# Transcriptome Analysis of the Accumulation of Astaxanthin in *Haematococcus pluvialis* Treated with White and Blue Lights as well as Salicylic Acid

**DOI:** 10.1155/2022/4827595

**Published:** 2022-07-14

**Authors:** Zuoxi Wei, Fengjie Sun, Chunxiao Meng, Wei Xing, Xiangyu Zhu, Chang Wang, Kai Cao, Chengsong Zhang, Bingkui Zhu, Ting Yao, Zhengquan Gao

**Affiliations:** ^1^School of Life Sciences and Medicine, Shandong University of Technology, Zibo 255049, China; ^2^School of Science and Technology, Georgia Gwinnett College, 1000 University Center Lane, Lawrenceville, GA 30043, USA; ^3^School of Pharmacy, Binzhou Medical University, Yantai 264003, China

## Abstract

*Haematococcus pluvialis* is the most commercially valuable microalga for the production of natural astaxanthin, showing enhanced production of astaxanthin with the treatments of high-intensity light and hormones. The molecular mechanisms regulating the biosynthesis of astaxanthin in *H*. *pluvialis* treated with white light, blue light, and blue light with salicylic acid (SA) were investigated based on the transcriptome analysis. Results showed that the combined treatment with both blue light and SA generated the highest production of astaxanthin. A total of 109,443 unigenes were identified to show that the genes involved in the tricarboxylic acid (TCA) cycle, the pentose phosphate pathway (PPP), and the astaxanthin biosynthesis were significantly upregulated to increase the production of the substrates for the synthesis of astaxanthin, i.e., pyruvate and glyceraldehyde-3-phosphate generated in the TCA cycle and PPP, respectively. Results of transcriptome analysis were further verified by the quantitative real-time PCR (qRT-PCR) analysis, showing that the highest content of astaxanthin was obtained with the expression of the *bkt* gene significantly increased. Our study provided the novel insights into the molecular mechanisms regulating the synthesis of astaxanthin and an innovative strategy combining the exogenous hormone and physical stress to increase the commercial production of astaxanthin by *H*. *pluvialis*.

## 1. Introduction

As a type of carotenoid, astaxanthin (3,3′-dihydroxy-4,4′-diketo-*β*-carotene, C_40_H_52_O_4_) is an efficient lutein, with the common functions and optimal performance as most carotenoids, showing red blood in color at room temperature [1]. There are a large number (up to 13) of conjugated double bonds in the molecule of astaxanthin, with a *β*-violet ketone ring in the head and tail of each molecule as well as both the hydroxyl and carbonyl groups on the ring. The prominent characteristics of the molecular structure determine the powerful and important functions of astaxanthin, such as scavenging free radicals, having antioxidant properties, and quenching the singlet oxygen [2]. As a type of health food, astaxanthin has received extensive attention in recent years. Due to its well-understood molecular structure, astaxanthin has been efficiently not only naturally produced in *Haematococcus pluvialis* but also synthesized through many chemical methods. To date, the chemically synthesized astaxanthin has not shown any known health risks to humans, while the natural product of astaxanthin has been approved for commercialization by the European Commission due to its high oxidation resistance, sustainable regeneration, and harmlessness to humans [3]. It is well known that natural astaxanthin has shown strong antioxidant capacity and excellent coloring effect. With the improvement of people's living standards worldwide, the natural product of astaxanthin is now in short supply in food, feed, cosmetics, medicine, and other industries [4]. In particular, astaxanthin has shown broad application prospects in the food industry. For example, astaxanthin has been widely used as a nutritional functional food and food additive due to its strong antioxidant capacity and unique coloring effect [5]. In order to further improve the chemical stability and the utilization of natural astaxanthin, the products of natural astaxanthin in the market are mainly made in the form of microcapsules and beverages [6].

A small amount of astaxanthin is detected in a variety of organisms and plays a key role in the life cycles of these organisms. Due to its low contents, the artificial extraction of astaxanthin is difficult in a group of microalgae, bacteria, fungi, yeasts, plants, and various marine animals. For example, both *Rhodococcus* and *Phaffia rhodozyma* are commercial astaxanthin-producing microorganisms [7], while the astaxanthin production by *H*. *pluvialis* has reached 5% dry cell weight (DCW) [8]. To date, the upstream and downstream processes, i.e., the cultivation of microorganisms and the extraction of astaxanthin, of *H*. *pluvialis* used for the large-scale production of natural astaxanthin are well established [2]. The life history of *H*. *pluvialis* is mainly divided into two stages, i.e., the green swimming cell stage showing rapid cell growth and large biomass accumulation, followed by the red inactive cell stage subjected to various stresses with cell growth nearly stopped and biomass accumulation significantly decreased. During the inactive cell stage, the astaxanthin is rapidly accumulated to protect the cells of *H*. *pluvialis* from environmental stresses [9]. Various types of biological regulators and stimulants have been used to increase the growth rate of *H*. *pluvialis* and its accumulation of astaxanthin. For example, studies have shown that the additions of salicylic acid (SA) and jasmonic acid (JA) have significantly promoted the production of astaxanthin, while the genes involved in the synthesis of astaxanthin are highly expressed at the transcriptional level [10, 11]. Furthermore, the treatment of additional precursors of ethylene (i.e., 1-aminocyclopropane-1-carboxylic acid) promotes the accumulation of both the green cell biomass and the astaxanthin in red cells of *H*. *pluvialis* [12]. Moreover, ethanol is used to induce the JA pathway to significantly promote the accumulation of astaxanthin and upregulate the key genes (i.e., *psy*, *bkt*, and *crtR*-*B*) involved in the synthetic pathway of astaxanthin [13]. Additionally, the exogenous *γ*-aminobutyric acid has significantly improved the photoprotection and resistance to stress in the cells of *H*. *pluvialis*, subsequently significantly improving the growth of algal cells, the biomass accumulation, and the astaxanthin production [14].

As a photosynthetic species, *H*. *pluvialis* converts light energy into biological energy. Therefore, light is an important factor in the growth and the accumulation of astaxanthin of *H*. *pluvialis*. The light-emitting diode (LED) has gradually replaced the traditional light source in the cultural process of algae. Studies have shown that the content of astaxanthin in *H*. *pluvialis* is significantly increased by the treatment of white light and blue light stresses with the highest content of astaxanthin achieved by blue light treatment [15]. The expression patterns of genes involved in the astaxanthin synthesis are slightly different under different light conditions; e.g., the expressions of genes *psy*, *lyc*, *crtO*, and *bkt2* are upregulated in *H*. *pluvialis* under blue light, while the expressions of genes *psy*, *crtO*, and *bkt2* are upregulated under white light [16]. Furthermore, the contents of astaxanthin are significantly different in *H*. *pluvialis* treated with different lights at different developmental stages. For example, the astaxanthin content of *H*. *pluvialis* treated with the combination of both the red and blue lights is significantly higher than that of only red light irradiation [17]. Additionally, the yield of astaxanthin is significantly increased by the combination of optimized white light and exogenous carbon sources [18]. Studies of lipid synthesis in *H*. *pluvialis* under different light combinations have shown that the white-blue light treatment is more efficient for the astaxanthin synthesis and the biomass accumulation of both glucose and protein than the white-red light treatment [19]. To date, the molecular mechanisms regulating the biosynthesis of astaxanthin in *H*. *pluvialis* treated with white light, blue light, and salicylic acid (SA) remain unclear.

Studies have revealed multiple genes involved in the biosynthesis of astaxanthin in *H*. *pluvialis*. First, the precursor, i.e., the isopentenyl diphosphate (IPP), of astaxanthin synthesis in *H*. *pluvialis* is synthesized in the nonmevalonate pathway (MEP). As the precursor of carotenoid synthesis, the IPP is involved in the biosynthetic pathway of the terpenoid skeleton with two genes (i.e., *ipi*-*1* and *ipi*-*2*) encoding the IPP isomerase identified. A molecule of dimethylallyl diphosphate (DMAPP) joins with three molecules of IPP to generate the geranylgeranyl diphosphate (GGPP) by extending the carbon chain. The genes encoding a group of enzymes, including the phytoene synthase (PSY), the phytoene desaturase (PDS), the zeta-carotene desaturase (ZDS), and the lycopene cyclase (LYC), involved in the synthesis of *β*-carotene have been identified and cloned. The synthesis of astaxanthin is completed by the catalysis of *β*-carotene, which is synthesized by the interaction of both *β*-carotene hydroxylase and *β*-carotene ketolase encoded by *crtZ* and *bkt*, respectively [4].

To date, the molecular mechanism of astaxanthin biosynthesis in *H*. *pluvialis* remains unclear. To further elucidate the molecular mechanism of astaxanthin biosynthesis in *H*. *pluvialis*, the transcriptome sequencing technology is applied to comprehensively analyze the gene expressions in algal cells treated with white light, blue light, and blue light with SA. The results have shown that the highest content of astaxanthin is achieved in *H*. *pluvialis* treated with the combination of both blue light and SA, with the key genes involved in the astaxanthin synthesis identified. Furthermore, both the tricarboxylic acid (TCA) cycle and the pentose phosphate pathway (PPP) provide the precursors and intermediates as well as both nicotinamide adenine dinucleotide (NADH) and nicotinamide adenine dinucleotide phosphate (NADPH) for the synthesis of astaxanthin. Our study provides (1) the novel insights into the molecular mechanism of the astaxanthin biosynthesis in *H*. *pluvialis* treated with white light, blue light, and blue light with SA and (2) a new method for the industrial production of astaxanthin in *H*. *pluvialis*.

## 2. Materials and Methods

### 2.1. Cultivation and Stress Treatment of *Haematococcus pluvialis*

*Haematococcus pluvialis* strain 712 (FACHB-712) was purchased from the Freshwater Algae Culture Collection at the Institute of Hydrobiology (FACHB-collection) and stored in the School of Life Sciences and Medicine, Shandong University of Technology. The algal cells of *H*. *pluvialis* were inoculated in Bold's Basal Medium (BBM) and cultured in an illumination chamber with 25 *μ*mol photons·m^−2^·s^−1^ at 23°C with a photoperiod cycle of 12 h light and 12 h dark. The cells of *H*. *pluvialis* during their logarithmic growth phase were subjected to three types of stress treatments: white light (150 *μ*mol photons·m^−2^·s^−1^), blue light (150 *μ*mol photons·m^−2^·s^−1^), and blue light (150 *μ*mol photons·m^−2^·s^−1^) with SA (2.5 mg·L^−1^) at 23°C with continuous light of 24 h. Samples were collected at 2 d and 7 d after the treatments, respectively, with each sample replicated three times. A total of 21 samples in seven groups were collected in this study, including the treatments of white light for 2 d (W02) and 7 d (W07), blue light for 2 d (B02) and 7 d (B07), and blue light and SA for 2 d (B252) and 7 d (B257), as well as the control group (N) with 0 h treatment. The samples were centrifuged at 8000 r/min for 5 min, then frozen in liquid nitrogen, and stored in a refrigerator at –80°C for subsequent experiments.

### 2.2. Extraction and Content Determination of Astaxanthin in *Haematococcus pluvialis*

A total of 10 mL algal sample solution was centrifuged at 8000 r/min for 10 min to remove the supernatant and collect the algae. Then, the sample was added with the methanol (30%) and KOH (5%) solution, with the algae evenly dispersed, placed in a water bath at constant temperature (65°C) for 15 min, and then centrifuged at 8000 r/min for 10 min to remove the supernatant and to precipitate. An appropriate amount of distilled water was added to centrifuge once to remove the residual alkali. A total of 4 mL dimethyl sulfoxide (DMSO) was added to remove the chlorophyll; then, the sample was treated with 200 W ultrasonic for 2 min and centrifuged, and the supernatant was collected, and the optical density (OD) value was measured at the wavelength of 490 nm. The content of astaxanthin in the sample of *H*. *pluvialis* per unit volume was determined by the Boussiba method [20] based on the following formula: *c* (mg L^–1^) = 4.5 × *A*_490_ × (*V*_a_/*V*_b_), where *c* is the content of astaxanthin, *A*_490_ is the absorbance value of astaxanthin at 490 nm, *V*_a_ is the volume of the extracted sample, and *V*_b_ is the total volume of the microalgal sample.

### 2.3. Total RNA Extraction and Transcriptome Sequencing

The total RNA was extracted with the TRIzol reagent (Invitrogen, CA, USA) following the procedure of the manufacturer. The purity and quantity of the total RNA were determined by the Bioanalyzer 2100 and RNA 1000 Nano LabChip Kit (Agilent, CA, USA) with the RIN larger than 7.0. The poly(A) RNA was purified from the total RNA (5 *μ*g) using the poly-T oligo-attached magnetic beads with two rounds of purification. Then, the purified mRNA was fragmented into small pieces with the divalent cations below elevated temperature. The cleaved RNAs were reverse-transcribed to generate the cDNA library following the protocol for the mRNA-Seq sample preparation kit (Illumina, San Diego, USA) with the mean insert size for the paired-end libraries of 300 ± 50 bp. The paired-end sequencing was performed on an Illumina HiSeq 4000 system (LC Sciences, Houston, USA) following the manufacturer's protocol.

### 2.4. De Novo Sequence Assembly and Unigene Annotation

Both Cutadapt and in-house Perl scripts were used to remove the reads containing junction contamination, low-quality bases, or undetermined bases. The sequence quality was detected by FastQC (http://www.bioinformatics.babraham.ac.uk/projects/fastqc/). All downstream analyses were based on high-quality clean data. The de novo assembly of the transcriptome was completed by Trinity 2.4.0.

All assembled unigenes were aligned against the nonredundant (Nr) protein (http://www.ncbi.nlm.nih.gov/), Gene Ontology (GO) (http://www.geneontology.org), Swiss-Prot (http://www.expasy.ch/sprot/), Kyoto Encyclopedia of Genes and Genomes (KEGG) (http://www.genome.jp/kegg/), Pfam (http://pfam.xfam.org/), and evolutionary genealogy of genes: Nonsupervised Orthologous Groups (eggNOG) (http://eggnogdb.embl.de/) databases using DIAMOND with a threshold of *E* value < 0.00001.

### 2.5. Analysis of Differentially Expressed Genes

Salmon was used to detect the expression levels of the unigenes by calculating the transcripts per million mapped reads (TPM). The differentially expressed genes (DEGs) were selected based on log_2_ (fold change) > 1 or <–1 with the statistical significance set at *p* value < 0.05 using the R package edgeR.

### 2.6. Quantitative Real-Time PCR Analysis

The expressions of a total of four genes (i.e., *bkt*, *crtZ*, *lyc*, and *psy*) involved in the biosynthetic pathway of astaxanthin were verified by the quantitative real-time PCR (qRT-PCR) analysis with *actin* as the reference gene [21]. The primers were designed using the Primer Premier 6.0 software (Premier, Toronto, Canada; Table 1). The qRT-PCR analysis was performed on an ABI 7500 Fast Real-Time PCR System (Applied Biosystems, USA) with the SYBR® Premix Ex Taq™ (TaKaRa, Beijing, China) following the manufacturer's instructions. Specifically, the qPCR procedures were as follows: 95°C for 30 s, followed by 40 cycles of 95°C for 5 s and 60°C for 34 s. Three biological replicates were set for each reaction of qRT-PCR analysis. The expression levels of genes were calculated and analyzed by the 2^*-ΔΔ*Ct^ method [22].

### 2.7. Statistical Analysis

Data were presented as mean ± standard deviation (SD) with three experimental replicates implemented in each group. Student's *t*-test (SPSS 17.0) was used to determine the statistical significance based on the *p* value < 0.05.

## 3. Results and Discussion

### 3.1. Accumulation of Astaxanthin in *Haematococcus pluvialis*

**Figure 1:**
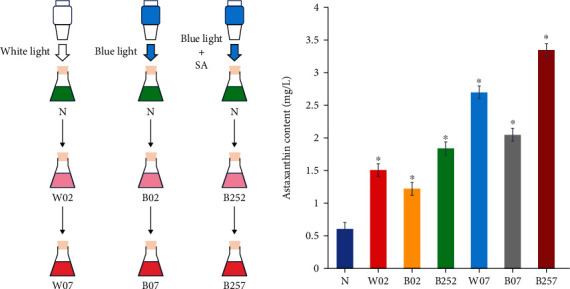
Experimental design of the determination of the astaxanthin accumulation in seven groups of *Haematococcus pluvialis*, i.e., the control group (N) and the six experimental groups, including the white light irradiation for two days (W02), the blue light irradiation for two days (B02), the blue light irradiation with salicylic acid (SA) for two days (B252), the white light irradiation for seven days (W07), the blue light irradiation for seven days (B07), and the blue light irradiation with SA for seven days (B257). Symbol “∗” indicates the significant difference in comparison to the control group.

The results showed that under the stress of blue light, white light, and blue light with SA for 2 d and 7 d, the contents of astaxanthin in *H*. *pluvialis* were significantly increased compared with that of the control group with the combined treatment of both blue light and SA achieving the highest content of astaxanthin, while the blue light generated the lowest production of astaxanthin (Figure 1). These results were consistent with those reported previously, showing the effect of SA as a plant growth regulator on promoting the accumulation of astaxanthin in *H*. *pluvialis* by providing additional carbon sources for the synthesis of astaxanthin [23]. Furthermore, studies showed that either blue light or white light promoted the accumulation of astaxanthin [24]. It was noted that in our study, the blue light caused less accumulation of astaxanthin than the white light, likely due to the stronger stress on *H*. *pluvialis* generated by the white light containing a mixture of different types of light, ultimately increasing the stimulation in *H*. *pluvialis* with increased generation of astaxanthin to protect the algal cells from damage.

### 3.2. Transcriptome Sequencing and Gene Annotation Analysis

The high-throughput transcriptome sequencing analysis was performed on the 21 samples of seven groups of *H*. *pluvialis* treated with blue light, white light, and blue light with SA for 0, 2, and 7 days [25]. The results of assembly based on Trinity showed that a total of 256,961 transcripts and 109,443 unigenes were obtained ranging from 201 bp to 13,626 bp in length with the medians of 588 bp and 429 bp, the GC contents of 55.92% and 53.09%, and the N50 values of 1392 bp and 1193 bp, respectively.

The total 109,443 genes identified were further annotated based on the GO, KEGG, Pfam, Swiss-Prot, eggNOG, and Nr databases with DIAMOND (Table 2).

**Figure 2:**
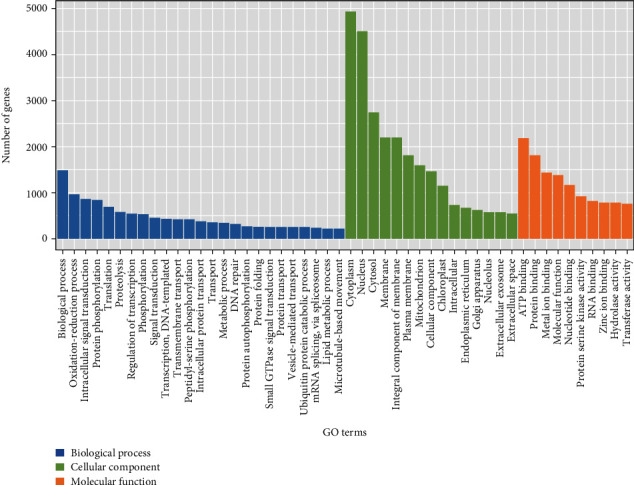
Annotation of a total of 24,798 unigenes in *Haematococcus pluvialis* based on the three categories (i.e., biological process, cellular component, and molecular function) of the Gene Ontology (GO) database.

The results of GO annotation showed that a total of 24,798 (~22.66%) unigenes were annotated into 50 subcategories of the three categories of GO terms, i.e., molecular function, biological process, and cellular component (Figure 2). In the category of the molecular function, the ATP binding (annotated with 2187 genes) and protein binding (1815 genes) were the top two GO terms with the highest number of genes annotated, while the biological process was annotated with the highest number of 1482 genes in the category of the biological process. The top three GO terms in the category of the cellular component with the highest number of genes annotated included the cytoplasm (4932 genes), nucleus (4507 genes), and cytosol (2750 genes).

**Figure 3:**
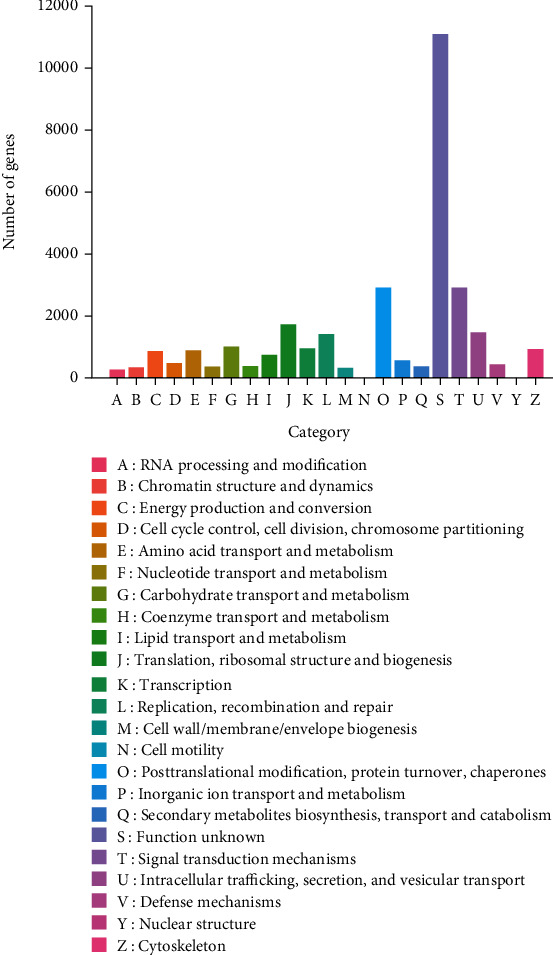
Annotation of a total of 31,590 unigenes in *Haematococcus pluvialis* based on the 23 categories (A-Z) in the evolutionary genealogy of genes: Nonsupervised Orthologous Groups (eggNOG) database.

A total of 31,590 (~28.86%) unigenes were annotated into 23 categories based on the eggNOG database (Figure 3). The category of the function unknown was annotated with the highest number of genes (11,146), followed by the signal transduction mechanisms (2950) and the posttranslational modification, protein turnover, and chaperones (2933), while the category of the nuclear structure was annotated by the least number of genes (4).

**Figure 4:**
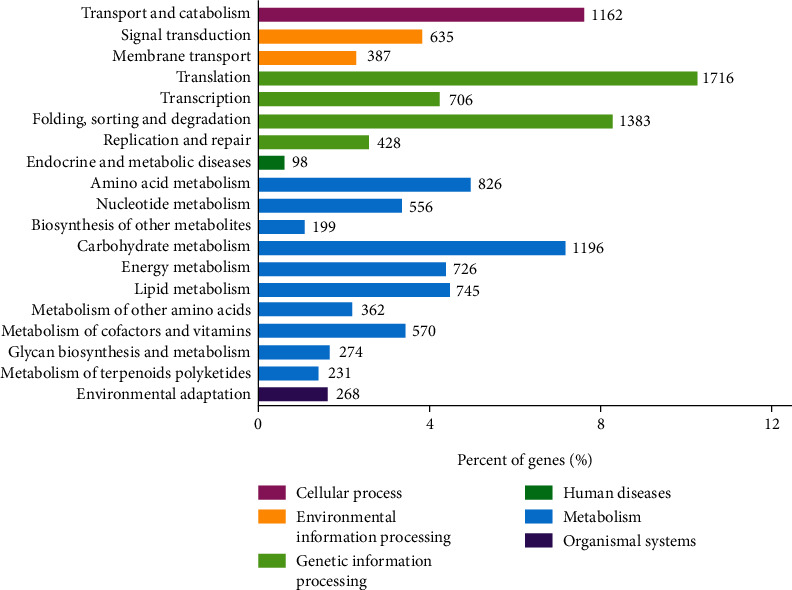
Annotation of a total of 19,648 unigenes in *Haematococcus pluvialis* based on six functional areas and 19 categories of the Kyoto Encyclopedia of Genes and Genomes (KEGG) database.

A total of 19,648 (~17.95%) unigenes were annotated into six functional areas in the first level and 19 categories in the second level based on the KEGG database (Figure 4). In the functional area of metabolism, the four categories annotated with the highest numbers of genes included the carbohydrate metabolism (1196 genes), the amino acid metabolism (826 genes), the lipid metabolism (745 genes), and the energy metabolism (726 genes). In the functional area of genetic information processing, the unigenes were mainly annotated in the categories of the replication and repair (428 genes), the folding, sorting, and degradation (1383 genes), the transcription (706 genes), and the translation (1716 genes). The functional area of environmental information processing included two categories, i.e., signal transduction (635 genes) and membrane transport (387 genes). There was only one category annotated in each of the three functional areas of the cellular processes (i.e., the transport and catabolism with 1162 genes), the human diseases (i.e., the endocrine and metabolic diseases with 98 genes), and the organismal systems (i.e., the environmental adaptation with 268 genes).

Additionally, a total of 21,504 unigenes were annotated based on the Swiss-Prot database and 1020 unigenes shared 100% similarity with genes annotated in the Swiss-Prot database (Table S1). A total of 27,965 unigenes were identified in the Nr protein database with 324 unigenes sharing 100% similarity with genes in the Nr database (Table S2), while a total of 26,248 unigenes were annotated based on the Pfam database with 475 unigenes sharing 100% similarity with genes in the Pfam database (Table S3).

### 3.3. Analysis of Differentially Expressed Genes in *Haematococcus pluvialis*

**Figure 5:**
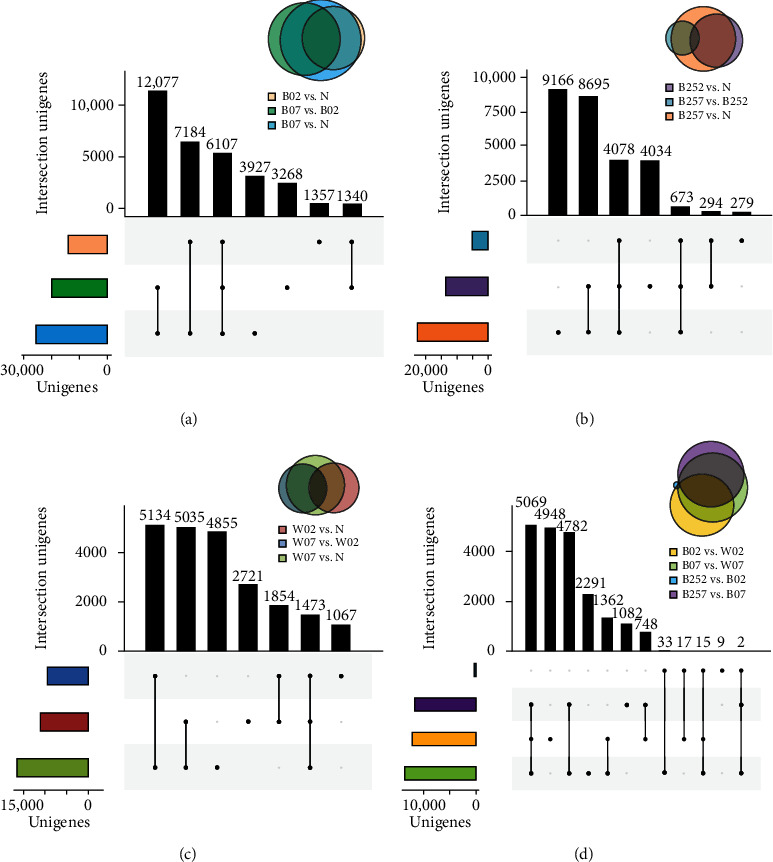
The distribution of differentially expressed genes (DEGs) in the comparison groups and their intersections of (a) blue light and the control group, (b) blue light with salicylic acid (SA) and the control group, (c) white light and the control group, and (d) blue light, white light, and blue light with SA of *Haematococcus pluvialis* treated with white light, blue light, and blue light with SA.

The DEGs were identified based on a total of 13 comparison groups of *H*. *pluvialis* treated with blue light, white light, and blue light with SA with samples collected 0, 2, and 7 days after the treatments (Figure 5). The results showed that the most DEGs of 29,295 (18,281 upregulated and 11,014 downregulated) and the least DEGs of 76 (5 upregulated and 71 downregulated) were detected in the comparison groups of B07 vs. N and B252 vs. B02, respectively (Figure S1). In the comparison groups treated with blue light, the B07 vs. N and B07 vs. B02 intersected with a total of 12,077 genes with significantly increased expression, while the intersection of B02 vs. N and B07 vs. N revealed 1340 genes (Figure 5(a)). In the comparison groups treated with blue light and SA, the intersection of B252 vs. N and B257 vs. N revealed 8695 genes showing significantly enhanced expression, while the intersection of B252 vs. N and B257 vs. N yielded 294 genes with insignificantly regulated expression (Figure 5(b)). In the comparison groups treated with white light, a total of 5134 genes were detected in the intersection of W07 vs. N and W07 vs. W02, while the intersection of W02 vs. N and W07 vs. W02 contained 1854 genes with insignificantly regulated expression (Figure 5(c)).

The DEGs were also identified based on four comparison groups between different treatments, e.g., B02 vs. W02, B07 vs. W07, B252 vs. B02, and B257 vs. B07 (Figure S1). A total of 4782 genes were detected in the intersection of the comparison groups of B07 vs. W07 and B257 vs. B07, while no intersection was detected between the comparison groups of B252 vs. B02 and B257 vs. B07 (Figure 5(d)). The results revealed similar expression patterns of DEGs in *H*. *pluvialis* treated with blue light and with both blue light and SA in comparison to the control group, with the numbers of DEGs identified in the experimental groups significantly higher than that in the control. Based on the changes in the expression patterns in the DEGs, it was clearly demonstrated that *H*. *pluvialis* showed the greatest response to blue light in comparison to either white light or blue light with SA. Future studies were necessary to illustrate the molecular mechanisms of these genes involved in the synthesis of astaxanthin in *H*. *pluvialis* treated with blue light.

The DEGs were further enriched based on the KEGG database to identify the metabolic pathways involved in the biosynthesis of astaxanthin of *H*. *pluvialis* under the treatments of white light, blue light, and blue light with SA (Table S4). These pathways identified under different stress treatments were discussed in the following sections.

### 3.4. KEGG Annotation of Differentially Expressed Genes in *Haematococcus pluvialis* Treated with Blue Light

**Figure 6:**
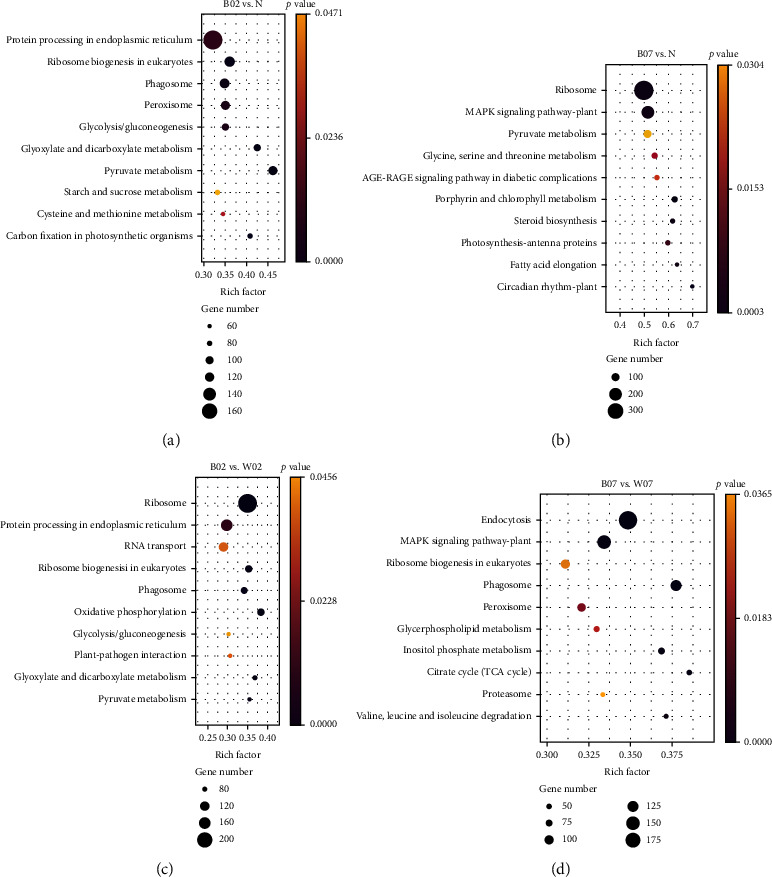
Enrichment analysis of the differentially expressed genes (DEGs) in the comparison groups of (a) B02 vs. control (N), (b) B07 vs. N, (c) B02 vs. W02, and (d) B07 vs. W07 of *Haematococcus pluvialis* treated with blue light based on the Kyoto Encyclopedia of Genes and Genomes (KEGG) database showing the top ten metabolic pathways.

In the comparison group of B02 vs. N, a total of 1922 genes (1339 upregulated and 583 downregulated) were significantly enriched in 44 KEGG metabolic pathways, e.g., the protein processing in the endoplasmic reticulum, the ribosome biogenesis in eukaryotes, the phagosome, the peroxisome, and the glycolysis/gluconeogenesis (Figure 6(a)). In the comparison group of B07 vs. N, a total of 982 genes (629 upregulated and 353 downregulated) were significantly enriched in 12 metabolic pathways, mainly including the ribosome, the MAPK signaling pathway-plant, the pyruvate metabolism, the glycine, serine, and threonine metabolism, and the AGE-RAGE signaling pathway in diabetic complications (Figure 6(b)). To investigate the effects of blue light on the accumulation of astaxanthin over time, the intersections of the comparison groups of B07 vs. N and B02 vs. N were enriched in the metabolic pathways of the KEGG database (Figure S2), with most genes enriched in the pyruvate metabolic pathway. These results were consistent with those previously reported, showing that the pyruvate metabolic pathway was enriched in *H*. *pluvialis* under stresses with similar gene expression patterns with subsequently largely the same gene expression patterns, suggesting that the pyruvate metabolism was closely related to the synthesis of astaxanthin, while the acetyl-CoA produced in the pyruvate metabolism was also essential for the lipid synthesis [23].

To further investigate the effect of blue light on the generation of astaxanthin in *H*. *pluvialis*, the DEGs identified between the blue light and white light treatments with the same time (i.e., 2 d or 7 d) were enriched into the metabolic pathways of the KEGG database. In the comparison group of B02 vs. W02, a total of 1980 genes (1115 upregulated and 865 downregulated) were significantly enriched in 38 KEGG pathways, mainly including the ribosome, the protein processing in the endoplasmic reticulum, the RNA transport, the ribosome biogenesis in eukaryotes, and the phagosome (Figure 6(c)). In the comparison group of B07 vs. W07, a total of 1152 genes (1127 upregulated and 25 downregulated) were significantly enriched in 22 KEGG pathways, e.g., the endocytosis, the MAPK signaling pathway-plant, the ribosome biogenesis in eukaryotes, the phagosome, and the peroxisome (Figure 6(d)). The genes detected in the intersection of the comparison groups of B02 vs. W02 and B07 vs. W07 were significantly enriched in 12 metabolic pathways in the KEGG database, mainly including the ribosome biogenesis in eukaryotes, the TCA cycle, and the biosynthesis of unsaturated fatty acids (Figure S2). These results were consistent with those reported previously, showing that the differential expressions of genes involved in the unsaturated fatty acid pathway accelerated the esterification of astaxanthin to promote the synthesis of astaxanthin and to improve its bioavailability [26], while the differential expressions of genes involved in the TCA cycle enhanced the substrate concentration for the synthesis of astaxanthin [27].

### 3.5. KEGG Annotation of Differentially Expressed Genes in *Haematococcus pluvialis* Treated with Both Blue Light and Salicylic Acid

Due to the induction effects of both simple blue light [15] and SA [27] on the accumulation of astaxanthin, *H*. *pluvialis* was treated with the combination of both blue light and SA in our study. The DEGs identified in *H*. *pluvialis* treated with both blue light and SA were enriched in the metabolic pathways of the KEGG database to investigate the effect of the combination of both physical stress (i.e., the blue light) and chemical induction (i.e., SA) on the accumulation of astaxanthin in *H*. *pluvialis*.

**Figure 7:**
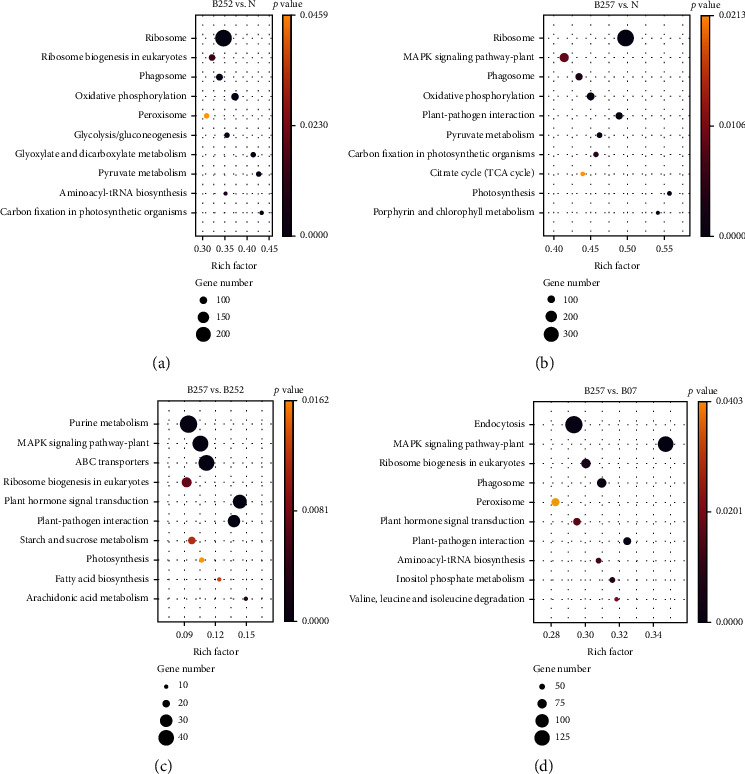
Enrichment analysis of the differentially expressed genes (DEGs) in the comparison groups of (a) B252 vs. control (N), (b) B257 vs. N, (c) B257 vs. B252, and (d) B257 vs. B07 of *Haematococcus pluvialis* treated with both blue light and salicylic acid (SA) based on the Kyoto Encyclopedia of Genes and Genomes (KEGG) database showing the top ten metabolic pathways.

In the comparison group of B252 vs. N, a total of 1847 DEGs (1145 upregulated and 702 downregulated) were significantly enriched in 39 KEGG pathways, e.g., the ribosome, the ribosome biogenesis in eukaryotes, the phagosome, the oxidative phosphorylation, and the peroxisome (Figure 7(a)), while in the comparison group of B257 vs. N, a total of 1503 DEGs (997 upregulated and 506 downregulated) were significantly enriched in 21 KEGG pathways, e.g., the ribosome, the MAPK signaling pathway-plant, the phagosome, the oxidative phosphorylation, and the plant-pathogen interaction (Figure 7(b)). The enrichment analysis of the DEGs detected in the comparison groups of B257 vs. N and B252 vs. N revealed a total of 12 KEGG pathways, e.g., the oxidative phosphorylation and the carbon fixation in photosynthetic organisms (Figure S2). The process of oxidative phosphorylation is essential to provide energy for various biological activities [28]. Studies showed that the pathway of oxidative phosphorylation was also enriched in *H*. *pluvialis* treated with *γ*-aminobutyric acid to enhance the production of astaxanthin [14]. The expressions of genes involved in the oxidative phosphorylation were significantly upregulated in *H*. *pluvialis* treated with blue light and SA, suggesting that the microalgal cells contained a sufficient amount of energy to resist the external stress and provided both ATP and coenzymes for their own redox reaction to promote the synthesis of astaxanthin and related substrates [29]. Furthermore, the metabolic pathway of carbon fixation in photosynthetic organisms was enriched in *H*. *pluvialis* treated with acetate and Fe^2+^ to enhance the production of astaxanthin [30]. These results suggested that the carbon flow and the distribution of carbon in *H*. *pluvialis* were regulated to ultimately enhance the synthesis of astaxanthin. Indeed, our study showed that the genes involved in the Calvin cycle were significantly enhanced, which was consistent with the previous study, showing the enhanced carbon metabolism in *H*. *pluvialis* treated with 15% CO_2_ [31].

A total of 299 DEGs (214 upregulated and 85 downregulated) identified in the comparison group of B257 vs. B252 were significantly enriched in 15 KEGG pathways, e.g., the purine metabolism, the MAPK signaling pathway-plant, the ABC transporters, the ribosome biogenesis in eukaryotes, and the plant hormone signal transduction (Figure 7(c)). Studies showed that as an important type of plant hormone, SA has caused the enrichment in the signal transduction pathway of plant hormones in the later stage of treatment of *H*. *pluvialis*, enhancing the accumulation of astaxanthin in *H*. *pluvialis* to resist the adverse effects caused by the environment [32]. The genes identified in the intersection of the comparison groups of B257 vs. N and B257 vs. B07 were significantly enriched in five KEGG pathways, i.e., the MAPK signaling pathway-plant, the phagosome, the plant-pathogen interaction, the steroidogenesis, and the fatty acid elongation (Figure S2).

Both steroids and fatty acids are necessary for energy and material metabolisms. In the comparison group of B257 vs. N, all of the DEGs enriched in the KEGG pathways of steroid biosynthesis and fatty acid elongation were upregulated, whereas most of the DEGs identified in the comparison group of B257 vs. B07 were downregulated (Figure 7(d)). Studies showed that with the enhanced production of astaxanthin and the significant upregulation of related genes, the expressions of genes in the competitive pathways were inhibited [33]. Therefore, compared with blue light, the addition of SA with blue light increased the content of astaxanthin. In the comparison groups B257 vs. N and B257 vs. B252, the DEGs were enriched in seven KEGG pathways, e.g., the fatty acid biosynthesis, the arachidonic acid metabolism, and the fatty acid elongation (Figure S2). It was noted that most of the genes enriched in these three pathways were significantly upregulated. Previous studies showed that astaxanthin existed largely in the form of astaxanthin esters in *H*. *pluvialis* [34]. Furthermore, the positive feedback regulation was identified between the astaxanthin synthesis and the fatty acid synthesis in *H*. *pluvialis* [35]. With the significantly promoted synthesis of astaxanthin by the blue light and the addition of SA as well as the significantly upregulated genes involved in the synthesis of fatty acids, it was evidently indicated that fatty acids were synthesized to further promote the synthesis of astaxanthin.

### 3.6. KEGG Annotation of Differentially Expressed Genes in *Haematococcus pluvialis* Treated with White Light

**Figure 8:**
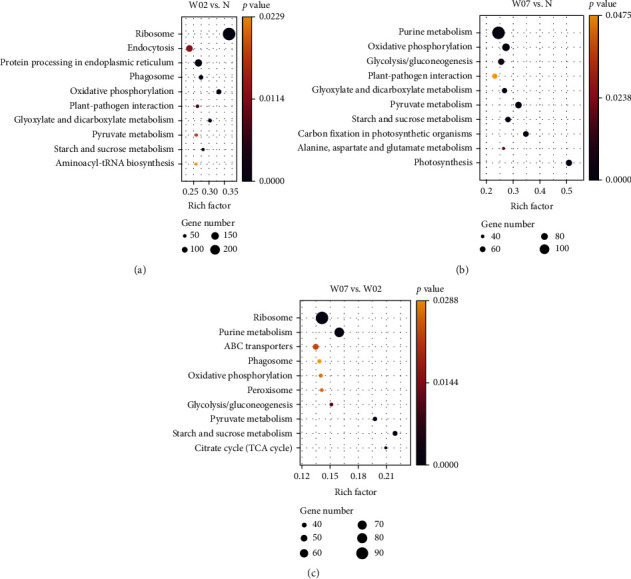
Enrichment analysis of the differentially expressed genes (DEGs) in the comparison groups of (a) W02 vs. control (N), (b) W07 vs. N, and (c) W07 vs. W02 of *Haematococcus pluvialis* treated with white light based on the Kyoto Encyclopedia of Genes and Genomes (KEGG) database showing the top ten metabolic pathways.

In the comparison group of W02 vs. N, a total of 1496 DEGs (1212 upregulated and 284 downregulated) were significantly enriched in 27 KEGG pathways, e.g., the ribosome, the endocytosis, the protein processing in the endoplasmic reticulum, the phagosome, and the oxidative phosphorylation (Figure 8(a)). The categories of enriched metabolic pathways varied greatly with the treatment time (i.e., 2 days or 7 days) of white light. A total of 937 DEGs (489 upregulated and 448 downregulated) identified in the comparison group of W07 vs. N were significantly enriched in a total of 24 KEGG pathways, e.g., the purine metabolism, the oxidative phosphorylation, the glycolysis/gluconeogenesis, the plant-pathogen interaction, and the glyoxylate and dicarboxylate metabolism (Figure 8(b)). A total of 714 DEGs (228 upregulated and 486 downregulated) identified in the comparison of W07 vs. W02 were significantly enriched in 26 KEGG pathways, e.g., the ribosome, the purine metabolism, the ABC transporters, the phagosome, and the oxidative phosphorylation (Figure 8(c)).

Studies compared the strong light to a “switch” of the astaxanthin accumulation [24]. To further explore the related synthetic pathways of astaxanthin in *H*. *pluvialis* under white light, the two comparison groups of W02 vs. N and W07 vs. N were intersected to identify 14 significantly enriched KEGG pathways (Figure S2). Most of the genes involved in the pathways of oxidative phosphorylation and pyruvate metabolism were upregulated, ultimately providing sufficient energy and coenzymes as well as substrates for the synthesis of astaxanthin in *H*. *pluvialis*. However, the genes involved in the pathway of carbon fixation in photosynthetic organisms were downregulated, suggesting that the photoprotection mechanism of *H*. *pluvialis* was triggered by the strong light, as reported previously [36]. Similarly, the genes revealed in the intersection of the comparison groups of W07 vs. W02 and W07 vs. N were also enriched in the pathways of oxidative degradation and pyruvate metabolism as well as photosynthesis, fatty acid biosynthesis, and carotenoid biosynthesis (Figure S2). Furthermore, our results showed that the genes related to photosynthesis were significantly downregulated in the later stage of the treatment of high-intensity white light irradiation, blocking the synthesis of photosynthetic pigment (i.e., chlorophyll), while the genes involved in the synthesis of carotenoids and fatty acids were significantly upregulated in the later stage of light stress. These results were consistent with those reported previously [23].

### 3.7. Transcriptome Analysis of the Tricarboxylic Acid Cycle in *Haematococcus pluvialis*

**Figure 9:**
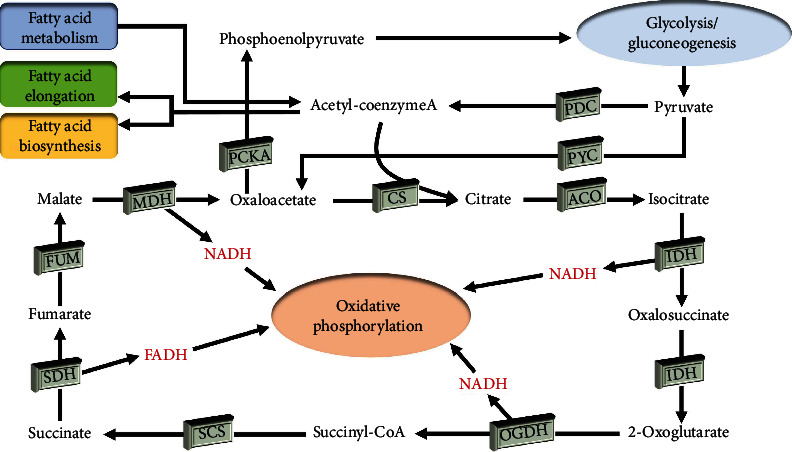
The tricarboxylic acid (TCA) cycle in *Haematococcus pluvialis*. MDH: malate dehydrogenase; CS: citrate synthase; ACO: aconitate hydratase; IDH: isocitrate dehydrogenase; OGDH: 2-oxoglutarate dehydrogenase E1 component; SCS: succinyl-CoA synthetase alpha subunit; SDH: succinate dehydrogenase (ubiquinone) flavoprotein subunit; FUM: fumarate hydratase; PYC: pyruvate carboxylase.

**Figure 10:**
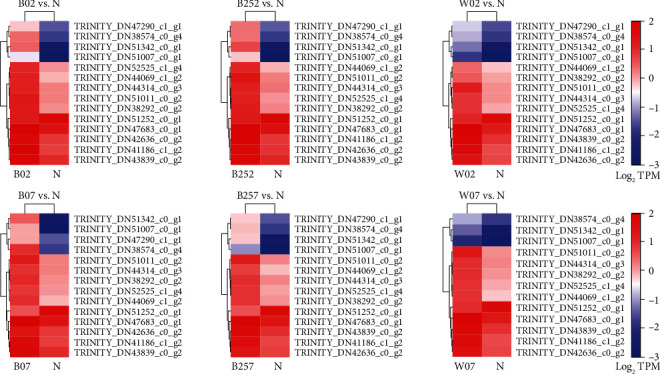
Cluster analysis of key genes involved in the tricarboxylic acid (TCA) cycle in *Haematococcus pluvialis* treated with white light, blue light, and blue light with the addition of salicylic acid (SA). The red and blue boxes represent the genes upregulated and downregulated, respectively.

The TCA cycle is a metabolic pathway largely involved in the decomposition and metabolism of pyruvate, fatty acids, amino acids, and other energy substances (Figure 9). Furthermore, many biosynthesis precursors are the intermediate products of the TCA cycle. The results of cluster analysis revealed the significant differences between the key genes involved in the TCA cycle in *H*. *pluvialis* treated with white light, blue light, and blue light with SA and those in the control group (Figure 10). The key genes involved in the TCA cycle with significantly regulated expressions in all the experimental groups of *H*. *pluvialis* in comparison to the control group were detected, including the genes encoding the malate dehydrogenase (MDH), the isocitrate dehydrogenase (IDH), the 2-oxoglutarate dehydrogenase E1 component (OGDH), the citrate synthase (CS), the succinate dehydrogenase (ubiquinone) flavoprotein subunit (SDH), the ATP citrate (pro-S)-lyase (ACLY), and the pyruvate carboxylase (PYC) (Table 3). Our results showed that most of these genes were upregulated in both 2 and 7 days after the treatments, with one gene encoding SDH downregulated in the experimental groups compared with the control group (Table 3).

Studies have shown that the TCA cycle is initiated with a preparatory stage; i.e., the pyruvate is used to generate the acetyl-CoA catalyzed by the pyruvate dehydrogenase complex [37]. Our results showed that during this preparatory stage, the expressions of genes involved in the pyruvate dehydrogenase complex were not significantly regulated by the treatments of white light, blue light, and blue light with the addition of SA, whereas the genes encoding PYC were significantly upregulated under these treatments. These results were consistent with those reported previously, showing the enhanced accumulation of pyruvate, which reacted with G3P to activate the MEP to generate IPP, leading to the large-scale synthesis of carotenoids [38]. Furthermore, the treatment of melatonin revealed the importance of succinate, isocitrate, *cis*-aconitate, and other intermediates in the astaxanthin synthesis in *H*. *pluvialis* [39]. Our study showed that the upregulation of genes involved in the TCA cycle strengthened the metabolism to enhance the production of the intermediates, ultimately increasing the synthesis of astaxanthin. Furthermore, our results showed that the genes encoding MDH, IDH, OGDH, and SDH were significantly upregulated in *H*. *pluvialis* of all the experimental groups to promote the metabolic pathways of related substances and to contribute to the production of both NADH and FADH, which entered the electron transfer chain of the oxidative phosphorylation to provide energy for various types of biological activities [40].

### 3.8. Transcriptome Analysis of the Pentose Phosphate Pathway in *Haematococcus pluvialis*

**Figure 11:**
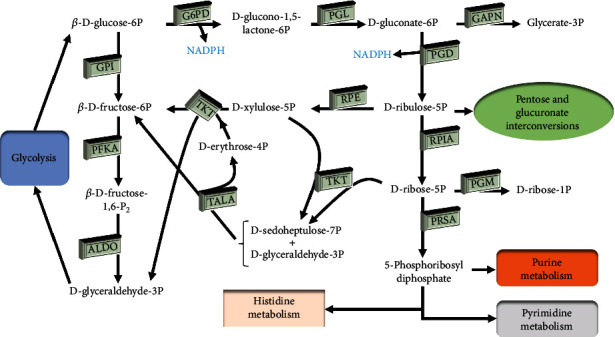
The pentose phosphate pathway (PPP) in *Haematococcus pluvialis*. G6PD: glucose-6-phosphate 1-dehydrogenase; PGL: 6-phosphogluconolactonase; GAPN: glyceraldehyde-3-phosphate dehydrogenase (NADP^+^); GPI: glucose-6-phosphate isomerase; PGD: 6-phosphogluconate dehydrogenase; RPE: ribulose-phosphate 3-epimerase; TKT: transketolase; TALA: transaldolase; PFKA: 6-phosphofructokinase; ALDO: fructose-bisphosphate aldolase; RPIA: ribose 5-phosphate isomerase; PRSA: ribose-phosphate pyrophosphokinase; PGM: phosphoglucomutase.

**Figure 12:**
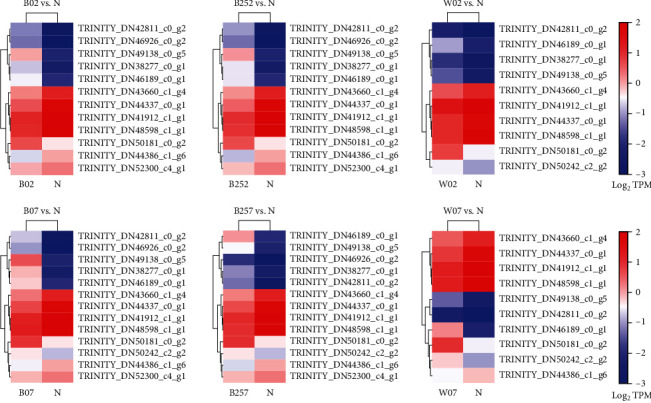
Cluster analysis of key genes involved in the pentose phosphate pathway (PPP) in *Haematococcus pluvialis* treated with white light, blue light, and blue light with the addition of salicylic acid (SA). The red and blue boxes represent the genes upregulated and downregulated, respectively.

The PPP is an important process for the generation of phosphorylated pentasaccharides from phosphorylated hexasaccharides (Figure 11). Among the many types of intermediates involved in the PPP, both NADPH and ribose 5-phosphate have shown their physiological significance. The results of cluster analysis revealed significant differences among the key genes involved in the PPP in *H*. *pluvialis* treated with white light, blue light, and blue light with SA and those in the control group (Figure 12). The key genes involved in the PPP with significantly regulated expressions in all the experimental groups of *H*. *pluvialis* in comparison to the control group were detected, including the genes encoding the 6-phosphogluconate dehydrogenase (PGD), the glucose-6-phosphate 1-dehydrogenase (G6PD), the glyceraldehyde-3-phosphate dehydrogenase (NADP^+^) (GAPN), the transketolase (TKT), the transaldolase (TALA), the fructose-bisphosphate aldolase (ALDO), the ribulose-phosphate 3-epimerase (RPE), the ribose 5-phosphate isomerase (RPIA), the glucose-6-phosphate isomerase (GPI), and the phosphoglucomutase (PGM) (Table 4). The results showed that genes encoding PGD, TALA, and GPI were significantly upregulated in *H*. *pluvialis* in the experimental groups compared with the control group. There were two copies of unigenes encoding G6PD, with one upregulated and the other downregulated, while the gene encoding ALDO showed a similar expression pattern to that of G6PD. The genes encoding GAPN, TKT, RPE, and RPIA were downregulated in the experimental groups in comparison to the control group (Table 4).

NADPH is catalyzed by two enzymes in the PPP, i.e., G6PD and PGD. Our results showed that most of the genes encoding these two enzymes were upregulated, with one gene encoding G6PD downregulated in *H*. *pluvialis* treated with white light, blue light, and blue light with SA. Studies showed that the overexpression of the G6PD gene in diatoms increased the levels of NADPH and lipid synthesis [41]. These results suggested that the increased expression of the G6PD gene promoted the lipid biosynthesis and facilitated the astaxanthin esterification in *H*. *pluvialis*. Furthermore, studies showed that the synthesis of *β*-carotene in *E*. *coli* was also completed through the MEP and the NADPH was used as a type of coenzyme for the synthesis of IPP in *E*. *coli* [42]. These results suggested that the significantly increased expression of G6PD and PGD genes in *H*. *pluvialis* promoted the synthesis of NADPH and indirectly enhanced the synthesis of *β*-carotene. Furthermore, as one of the intermediate products generated in the PPP, the ribose 5-phosphate is one of the important enzymes involved in the synthesis of ATP, ADP, AMP, cAMP, CoA, RNA, and DNA. Our results showed that the genes encoding the RPIA were significantly downregulated, suggesting that the synthesis of ribose 5-phosphate was reduced to indirectly inhibit the energy synthesis and nucleic acid synthesis in *H*. *pluvialis* treated with white light, blue light, and blue light with SA. Consequently, the cellular activities of *H*. *pluvialis* were generally decreased under these treatments. Moreover, our study showed that the gene encoding TALA was also significantly upregulated, suggesting the increased production of fructose-6-phosphate synthesized by both G3P and sedoheptulose-7-phosphate, ultimately enhancing the activities of the PPP and increasing the intermediate products of glycolysis.

### 3.9. Transcriptome Analysis and Verification of Keys Genes Involved in the Biosynthetic Pathway of Astaxanthin in *Haematococcus pluvialis*

**Figure 13:**
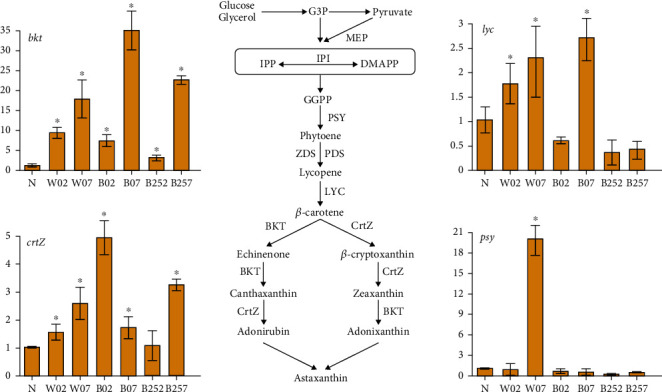
Expression patterns of four genes (i.e., *bkt*, *crtZ*, *lyc*, and *psy*) involved in the synthesis of astaxanthin in *Haematococcus pluvialis* treated with white light, blue light, and blue light with salicylic acid (SA). IPI: isopentenyl diphosphate isomerase; PSY: phytoene synthase; ZDS: zeta-carotene desaturase; PDS: phytoene desaturase; LYC: lycopene cyclase; CrtZ: *β*-carotene hydroxylase; BKT: *β*-carotene ketolase.

It is well known that both G3P and pyruvate are the precursors for the synthesis of astaxanthin in *H*. *pluvialis*. Studies have shown that the synthesis of the skeleton of the terpene compounds in the MEP is required for the biosynthesis of astaxanthin at the substrate level [43]. Furthermore, a sufficient amount of G3P and pyruvate is provided by both the PPP and the TCA cycle, which also provide a sufficient amount of NADPH for the MEP [42]. Our results of transcriptome analysis revealed different expression patterns of genes involved in the astaxanthin synthesis in *H*. *pluvialis* under different treatments of white light, blue light, and blue light with SA. Four genes related to the synthesis of astaxanthin were selected to verify their expression patterns based on the qRT-PCR analysis (Figure 13). The results of qRT-PCR were largely consistent with those revealed by the transcriptome analysis.

In 2 days, the highest accumulation of astaxanthin was achieved in *H*. *pluvialis* treated with blue light and SA compared with white light and blue light (Figure 1) with the expression of *bkt* significantly increased, while the expressions of both *bkt* and *crtZ* were significantly upregulated in the blue and white light groups (Figure 13). These results suggested that the expression of *bkt*played an important role in the early stage of astaxanthin accumulation. These results were consistent with those reported previously, showing the importance of the overexpression of *bkt* in the synthesis of astaxanthin [21, 44]. Furthermore, our results showed that the production of NADH was also promoted in the TCA cycle, suggesting that the significantly increased expression of *crtZ* enhanced the astaxanthin accumulation in *H*. *pluvialis*. These results were consistent with those reported previously, showing that the upregulation of *crtZ* was closely related to the enhanced production of NADH during the pyruvate metabolism [31]. Moreover, our results of qRT-PCR analysis showed that the expression of *psy* was significantly increased in *H*. *pluvialis* treated with white light for 7 days, while PSY played a key role in the extension of the carbon chain in the synthesis of astaxanthin [45]. Additionally, the expression of the *lyc* gene is directly related to the synthesis of *β*-carotene. For example, studies showed that the fulvic acid induced the expression of *lyc* in *H*. *pluvialis* to significantly increase the yield of *β*-carotene [46]. Our results of qRT-PCR analysis showed that the expression of *lyc* was upregulated in *H*. *pluvialis* treated with white light for 2 and 7 days and blue light for 7 days. Future studies are necessary to verify and further explore the molecular mechanisms of these genes regulating the synthesis of astaxanthin in *H*. *pluvialis* under both physical stress and chemical induction.

The expressions of these key genes involved in the synthetic pathway of astaxanthin were further investigated to reveal the molecular mechanism underlying the treatment of light affecting the astaxanthin synthesis. Our results showed that the treatments of blue light and blue light with SA did not cause significant changes in the expression of the *psy* gene, whereas the white light caused the significant change in the expression of the *psy* gene only during the later stage of treatment. Notably, the expression of the *lyc* gene was significantly upregulated in all stages of the treatment of white light and in the later stage of the treatment of blue light but was not affected by the treatment of blue light with SA. The expression of the *bkt* gene was significantly upregulated during all stages under the three groups of treatments, while the expression of the *crtZ* gene was significantly increased by the blue light and white light separately but not by the treatment of blue light with SA. Although the white light promoted the significant upregulation of these key genes, the highest content of astaxanthin was achieved by the treatment of blue light with SA, probably due to the addition of SA providing a sufficient carbon source for astaxanthin synthesis, ultimately resulting in the highest content of astaxanthin by the treatment of blue light with SA in comparison with the treatment of blue light and white light. Further studies are needed to clarify the molecular mechanisms regulating the astaxanthin synthesis by these key genes.

## 4. Conclusions

In this study, our results showed that the production of astaxanthin in *H*. *pluvialis* was significantly increased by the treatments of white light, blue light, and blue light with the addition of SA, with the highest contents of astaxanthin achieved by the treatment of the combination of both blue light and SA. The molecular mechanism regulating the astaxanthin accumulation in *H*. *pluvialis* was further explored at the genomic level based on the transcriptome analysis, with the key genes involved in the astaxanthin synthesis identified and confirmed at the transcriptional level based on the qRT-PCR analysis. Specifically, the genes involved in the TCA cycle, the PPP, and the astaxanthin biosynthesis were significantly upregulated to increase the production of the substrates for the synthesis of astaxanthin, i.e., the pyruvate and the G3P generated in the TCA cycle and the PPP, respectively. Our study provided a novel strategy by combining the exogenous hormone and physical stress to increase the commercial production of astaxanthin by *H*. *pluvialis*.

## Figures and Tables

**Table 1:** Primers used for the quantitative real-time PCR (qRT-PCR) analysis of four genes involved in the biosynthetic pathway of astaxanthin in *Haematococcus pluvialis*. Symbols “F” and “R” stand for upstream and downstream primers, respectively.

Gene (GenBank accession)	Primer and sequence
*actin* encoding actin of *H*. *lacustris* (GFH15240.1)	*actin*F: AGCGGGAGATAGTGCGGGACA *actin*R: ATGCCCACCGCCTCCATGC
*bkt* encoding beta-carotene ketolase (GU143688.1)	*bkt*F: AGCCACCACCTTCCGACACAA *bkt*R: ACGACGACGATGTGCATCAGAC
*crtZ* encoding carotenoid hydroxylase (AY187011.1)	*crtZ*F: CCAGCAGAGGCATCGACACATC *crtZ*R: AGGCGAGAGTGACCACTGAGAC
*lyc* encoding lycopene beta cyclase (KX424526.1)	*lyc*F: TCTGGACGCACGCCTCAAGT *lyc*R: GGCAGTTCAGGTGGCAGTTCAG
*psy* encoding phytoene synthase (AY835634.1)	*psy*F: CTCCCGCCATGCTTCACCAATC *psy*R: GGCAATCGGCGATGCAAGTCA

**Table 2:** Annotations of a total of 109,443 unigenes identified in *Haematococcus pluvialis* treated with white light, blue light, and blue light with salicylic acid based on six databases using DIAMOND.

Database	Number (percentage) of genes annotated
GO	24,798 (22.66%)
KEGG	19,648 (17.95%)
Pfam	26,248 (23.98%)
Swiss-Prot	21,504 (19.65%)
eggNOG	31,590 (28.86%)
Nr	27,965 (25.55%)
Total	109,443 (100%)

**Table 3:** Expression profile of the significantly regulated genes based on Trinity (showing the value of log_2_FC) annotated based on the Kyoto Encyclopedia of Genes and Genomes (KEGG) database involved in the tricarboxylic acid (TCA) cycle in *Haematococcus pluvialis* treated with blue light, white light, and blue light with salicylic acid (SA) for two and seven days.

Gene ID	EC no.	KEGG entry/name	2-day treatment			7-day treatment		
			B02 vs. N	B252 vs. N	W02 vs. N	B07 vs. N	B257 vs. N	W07 vs. N
DN44314_c0_g3	EC:1.1.1.37	K00026/MDH	1.90	1.84	1.88	1.37	1.69	1.89
DN47683_c0_g1	EC:1.1.1.37	K00025/MDH	1.18	1.23	1.91	1.21	1.42	1.59
DN47290_c1_g1	EC:1.1.1.41	K00030/IDH	2.86	4.08	2.14	3.55	2.79	NA
DN51011_c0_g2	EC:1.2.4.2	K00164/OGDH	2.21	2.27	2.58	2.11	2.35	2.58
DN41186_c1_g2	EC:1.3.5.1	K00235/SDH	2.53	2.53	2.58	1.90	2.00	2.29
DN38574_c0_g4	EC:1.3.5.1	K00235/SDH	4.44	4.37	2.12	5.84	3.49	1.84
DN51252_c0_g1	EC:1.3.5.1	K00234/SDH	–1.46	–1.55	–1.07	–2.87	–2.32	–1.60
DN42636_c0_g2	EC:1.3.5.1	K00234/SDH	1.78	1.91	1.93	1.15	1.30	1.77
DN52525_c1_g4	EC:2.3.1.61	K00658/DLST	2.28	2.40	2.57	1.87	2.17	2.37
DN44069_c1_g2	EC:2.3.3.1	K01647/CS	2.42	2.32	1.24	2.05	1.71	2.28
DN38292_c0_g2	EC:2.3.3.1	K01647/CS	2.21	2.08	1.04	1.68	1.59	1.95
DN51342_c0_g1	EC:2.3.3.8	K01648/ACLY	6.42	7.07	3.14	6.69	5.08	2.86
DN43839_c0_g2	EC:2.3.3.8	K01648/ACLY	1.66	1.57	2.22	1.39	1.44	1.75
DN51007_c0_g1	EC:6.4.1.1	K01958/PYC	5.11	6.19	2.80	6.54	4.19	2.28

**Table 4:** Expression profile of the significantly regulated genes based on Trinity (showing the value of log_2_FC) annotated based on the Kyoto Encyclopedia of Genes and Genomes (KEGG) database involved in the pentose phosphate pathway (PPP) in *Haematococcus pluvialis* treated with blue light, white light, and blue light with salicylic acid (SA) for two and seven days.

Gene ID	EC no.	KEGG entry/name	2-day treatment			7-day treatment		
			B02 vs. N	B252 vs. N	W02 vs. N	B07 vs. N	B257 vs. N	W07 vs. N
DN50181_c0_g2	EC:1.1.1.44 EC:1.1.1.343	K00033/PGD	2.34	2.43	3.10	3.03	3.04	3.53
DN38277_c0_g1	EC:1.1.1.49 EC:1.1.1.363	K00036/G6PD	3.74	4.24	2.05	5.56	2.82	NA
DN50242_c2_g2	EC:1.1.1.49 EC:1.1.1.363	K00036/G6PD	NA	NA	1.33	1.19	1.09	1.98
DN44386_c1_g6	EC:1.2.1.9	K00131/GAPN	–2.09	–2.36	NA	–1.72	–1.88	–1.09
DN44337_c0_g1	EC:2.2.1.1	K00615/TKT	–3.36	–3.75	–1.80	–2.96	–3.13	–2.29
DN46189_c0_g1	EC:2.2.1.2	K00616/TALA	3.22	3.21	3.08	4.30	5.04	5.82
DN49138_c0_g5	EC:4.1.2.13	K01623/ALDO	5.14	5.34	2.18	6.76	3.89	2.26
DN41912_c1_g1	EC:4.1.2.13	K01623/ALDO	–2.91	–3.15	–1.60	–2.73	–2.99	–2.23
DN48598_c1_g1	EC:5.1.3.1	K01783/RPE	–2.79	–2.91	–2.21	–2.16	–2.72	–1.81
DN43660_c1_g4	EC:5.3.1.6	K01807/RPIA	–2.72	–2.92	–1.20	–2.44	–2.67	–1.59
DN42811_c0_g2	EC:5.3.1.9	K01810/GPI	3.78	4.24	2.02	4.95	3.46	1.63
DN46926_c0_g2	EC:5.4.2.2	K01835/PGM	4.43	4.64	NA	5.28	3.08	NA
DN52300_c4_g1	EC:5.4.2.2	K01835/PGM	–1.26	–1.40	NA	–1.28	–1.19	NA

## Data Availability

The raw Illumina sequencing data are deposited in the Sequence Read Archive (SRA) at the NCBI (https://www.ncbi.nlm.nih.gov/sra/; accessed on 16 March 2020) database under accession number PRJNA766843.

## Supplementary Materials

Supplementary 1 Figure S1: differentially expressed genes (DEGs) identified by the pairwise comparisons in *Haematococcus pluvialis* treated with white light, blue light, and blue light with salicylic acid (SA).

Supplementary 2 Figure S2: the intersections of the pairwise comparisons of *Haematococcus pluvialis* treated with blue light (A), blue light with salicylic acid (SA) (B), and white light (C).

Supplementary 3 Table S1: enrichment analysis of differentially expressed genes (DEGs) identified in *Haematococcus pluvialis* treated with blue light, white light, and blue light with salicylic acid based on the Swiss-Prot database.

Supplementary 4 Table S2: enrichment analysis of differentially expressed genes (DEGs) identified in *Haematococcus pluvialis* treated with blue light, white light, and blue light with salicylic acid based on the nonredundant (Nr) protein database.

Supplementary 5 Table S3: enrichment analysis of differentially expressed genes (DEGs) identified in *Haematococcus pluvialis* treated with blue light, white light, and blue light with salicylic acid based on the Pfam database.

Supplementary 6 Table S4: enrichment analysis of differentially expressed genes (DEGs) identified in *Haematococcus pluvialis* treated with blue light, white light, and blue light with salicylic acid based on the Kyoto Encyclopedia of Genes and Genomes (KEGG) database.
