# Supplementary material for: Transcriptome Analysis of the Accumulation of Astaxanthin in Haematococcus pluvialis Treated with White and Blue Lights as well as Salicylic Acid
Source: Biomed Res Int. 2022 Jul 14;2022:4827595. doi: 10.1155/2022/4827595 (PMC9315456; doi:10.1155/2022/4827595)
Supplement: Supplementary 1 — Figure S1: differentially expressed genes (DEGs) identified by the pairwise comparisons in Haematococcus pluvialis treated with white light, blue light, and blue light with salicylic acid (SA). [file 4827595.f1.docx]

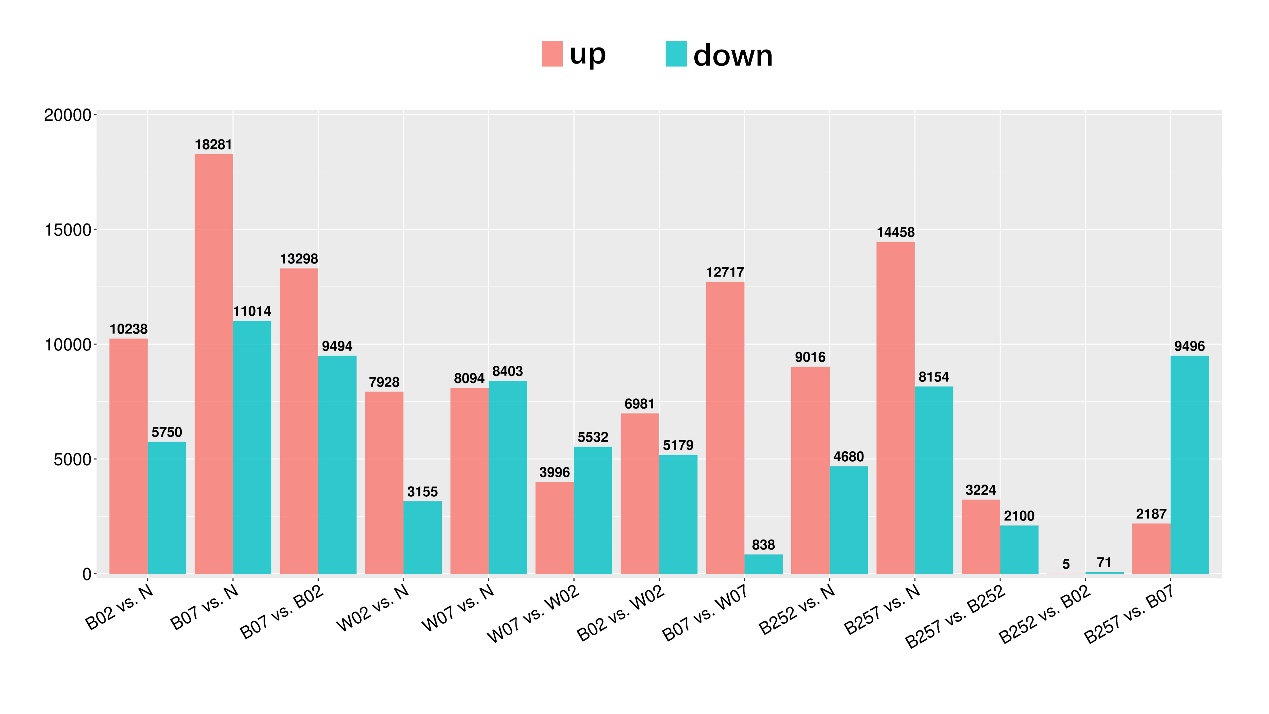


Figure S1. Differentially expressed genes (DEGs) showing up-regulated and down-regulated genes identified in the pairwise comparisons in *Haematococcus pluvialis* treated with white light, blue light, and blue light with salicylic acid (SA).
