# Supplementary material for: Transcriptome Analysis of the Accumulation of Astaxanthin in Haematococcus pluvialis Treated with White and Blue Lights as well as Salicylic Acid
Source: Biomed Res Int. 2022 Jul 14;2022:4827595. doi: 10.1155/2022/4827595 (PMC9315456; doi:10.1155/2022/4827595)
Supplement: Supplementary 2 — Figure S2: the intersections of the pairwise comparisons of Haematococcus pluvialis treated with blue light (A), blue light with salicylic acid (SA) (B), and white light (C). [file 4827595.f2.docx]

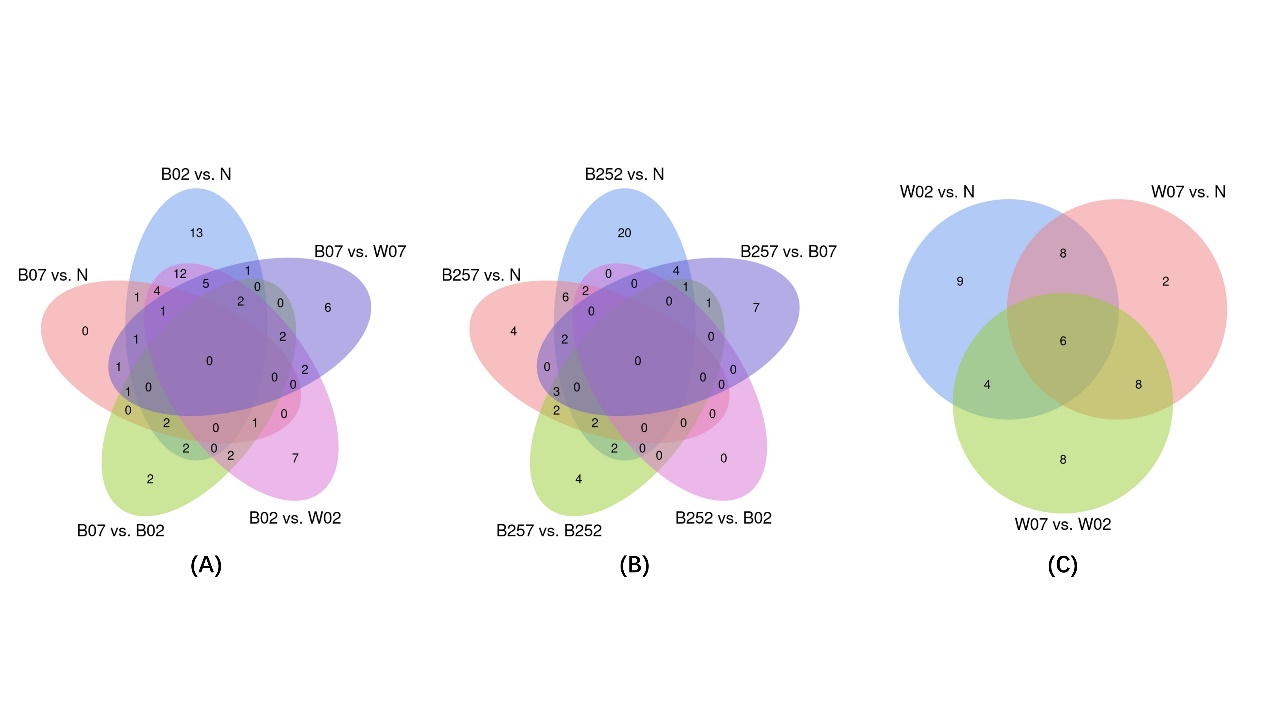


Figure S2. The intersections of the pairwise comparisons of *Haematococcus pluvialis* treated with blue light (A), blue light with salicylic acid (SA) (B), and white light (C).
