# Supplementary material for: Transcriptome Analysis of the Accumulation of Astaxanthin in Haematococcus pluvialis Treated with White and Blue Lights as well as Salicylic Acid
Source: Biomed Res Int. 2022 Jul 14;2022:4827595. doi: 10.1155/2022/4827595 (PMC9315456; doi:10.1155/2022/4827595)
Supplement: Supplementary 3 — Table S1: enrichment analysis of differentially expressed genes (DEGs) identified in Haematococcus pluvialis treated with blue light, white light, and blue light with salicylic acid based on the Swiss-Prot database. [file 4827595.f3.pdf]

Query	swisspro	swisspro	swissprot_descripti	swisspro	swissprot_evalue
TRINITY_	sp|Q8WVX	C4orf3	Uncharacterized pro	100.00	0.00
TRINITY_	sp|Q9252	H1FX	Histone H1x OS=Homo	100.00	0.00
TRINITY_	sp|Q9Y36	STARD10	PCTP-like protein O	100.00	0.00
TRINITY_	sp|O1551	ARPC5	Actin-related prote	100.00	0.00
TRINITY_	sp|Q1502	TNIP1	TNFAIP3-interacting	100.00	0.00
TRINITY_	sp|P1412	rpsA	30S ribosomal prote	100.00	0.00
TRINITY_	sp|Q9GZT	SLIRP	SRA stem-loop-inter	100.00	0.00
TRINITY_	sp|Q9NZ4	PSENEN	Gamma-secretase sub	100.00	0.00
TRINITY_	sp|P4863	PRRC2A	Protein PRRC2A OS=H	100.00	0.00
TRINITY_	sp|Q0542	FABP7	Fatty acid-binding	100.00	0.00
TRINITY_	sp|Q5M9E	ostc-b	Oligosaccharyltrans	100.00	0.00
TRINITY_	sp|Q9Y5V	MAGED1	Melanoma-associated	100.00	0.00
TRINITY_	sp|Q2797	CAPN2	Calpain-2 catalytic	100.00	0.00
TRINITY_	sp|Q95L4	EIF4G2	Eukaryotic translat	100.00	0.00
TRINITY_	sp|P1224	Apcs	Serum amyloid P-com	100.00	0.00
TRINITY_	sp|P0086	Odc1	Ornithine decarboxy	100.00	0.00
TRINITY_	sp|Q3UB7	Tbrg1	Transforming growth	100.00	0.00
TRINITY_	sp|P1517	GSPT1	Eukaryotic peptide	100.00	0.00
TRINITY_	sp|Q9H0U	MRPL18	39S ribosomal prote	100.00	0.00
TRINITY_	sp|Q3SZE	AAMP	Angio-associated mi	100.00	0.00
TRINITY_	sp|Q9UK4	VPS28	Vacuolar protein so	100.00	0.00
TRINITY_	sp|P4388	Plin2	Perilipin-2 OS=Mus	100.00	0.00
TRINITY_	sp|Q90WI	MXRA8	Matrix-remodeling-a	100.00	0.00
TRINITY_	sp|Q1686	ATP6V1F	V-type proton ATPas	100.00	0.00
TRINITY_	sp|Q5E95	DNAJA1	DnaJ homolog subfam	100.00	0.00
TRINITY_	sp|Q3SZC	ERH	Enhancer of rudimen	100.00	0.00
TRINITY_	sp|P1024	MYBL2	Myb-related protein	100.00	0.00
TRINITY_	sp|Q0P5I	RBM42	RNA-binding protein	100.00	0.00
TRINITY_	sp|Q9Y5M	SRPRB	Signal recognition	100.00	0.00
TRINITY_	sp|P0726	-	Serralysin OS=Serra	100.00	0.00
TRINITY_	sp|P2978	Vtn	Vitronectin OS=Mus	100.00	0.00
TRINITY_	sp|P5639	Cox6b1	Cytochrome c oxidas	100.00	0.00
TRINITY_	sp|P1485	COX6B1	Cytochrome c oxidas	100.00	0.00
TRINITY_	sp|Q0MQ0	NDUFAB1	Acyl carrier protei	100.00	0.00
TRINITY_	sp|P5046	Csrp3	Cysteine and glycin	100.00	0.00
TRINITY_	sp|P4125	GARS	Glycine--tRNA ligas	100.00	0.00
TRINITY_	sp|P1024	MYBL2	Myb-related protein	100.00	0.00
TRINITY_	sp|O0024	ATOX1	Copper transport pr	100.00	0.00
TRINITY_	sp|P0790	HSP90AA1	Heat shock protein	100.00	0.00
TRINITY_	sp|P6145	PCBD1	Pterin-4-alpha-carb	100.00	0.00
TRINITY_	sp|Q3T19	RPS23	40S ribosomal prote	100.00	0.00
TRINITY_	sp|Q3T19	RPS23	40S ribosomal prote	100.00	0.00
TRINITY_	sp|P1158	MTHFD1	C-1-tetrahydrofolat	100.00	0.00
TRINITY_	sp|Q6ZWV	Rpl35	60S ribosomal prote	100.00	0.00
TRINITY_	sp|P4276	RPL35	60S ribosomal prote	100.00	0.00
TRINITY_	sp|P6160	HSPE1	10 kDa heat shock p	100.00	0.00
TRINITY_	sp|P6148	rpl36a	60S ribosomal prote	100.00	0.00
TRINITY_	sp|Q6183	Pzp	Pregnancy zone prot	100.00	0.00
TRINITY_	sp|Q1515	NOMO1	Nodal modulator 1 O	100.00	0.00
TRINITY_	sp|Q96A3	MRPL24	39S ribosomal prote	100.00	0.00
TRINITY_	sp|O7043	Fhl2	Four and a half LIM	100.00	0.00
TRINITY_	sp|Q56JX	RPS13	40S ribosomal prote	100.00	0.00
TRINITY_	sp|Q9R06	Bcam	Basal cell adhesion	100.00	0.00
TRINITY_	sp|P0855	PDHA1	Pyruvate dehydrogen	100.00	0.00
TRINITY_	sp|Q9CQ0	Rnaset2	Ribonuclease T2 OS=	100.00	0.00

TRINITY_sp|Q1354	HDAC1	Histone deacetylase	100.00	0.00
TRINITY_sp|P2663	Sars	Serine--tRNA ligase	100.00	0.00
TRINITY_sp|P4173	Cd63	CD63 antigen OS=Mus	100.00	0.00
TRINITY_sp|Q3MHI	SMAP	Small acidic protei	100.00	0.00
TRINITY_sp|O7054	Tcap	Telethonin OS=Mus m	100.00	0.00
TRINITY_sp|Q9DBM	Ehhadh	Peroxisomal bifunct	100.00	0.00
TRINITY_sp|P7066	Sparcl1	SPARC-like protein	100.00	0.00
TRINITY_sp|P0512	Nppa	Natriuretic peptide	100.00	0.00
TRINITY_sp|P1517	GSPT1	Eukaryotic peptide	100.00	0.00
TRINITY_sp|Q29RI	THOC3	THO complex subunit	100.00	0.00
TRINITY_sp|Q1279	ASPH	Aspartyl/asparaginy	100.00	0.00
TRINITY_sp|Q8WUM	SLC20A1	Sodium-dependent ph	100.00	0.00
TRINITY_sp|P6330	SEPW1	Selenoprotein W OS=	100.00	0.00
TRINITY_sp|Q3T02	UBL5	Ubiquitin-like prot	100.00	0.00
TRINITY_sp|Q3T02	UBL5	Ubiquitin-like prot	100.00	0.00
TRINITY_sp|Q1677	GUK1	Guanylate kinase OS	100.00	0.00
TRINITY_sp|P3040	TM4SF1	Transmembrane 4 L6	100.00	0.00
TRINITY_sp|P3040	TM4SF1	Transmembrane 4 L6	100.00	0.00
TRINITY_sp|O7508	WDR1	WD repeat-containin	100.00	0.00
TRINITY_sp|O7564	SNRNP200	U5 small nuclear ri	100.00	0.00
TRINITY_sp|Q91VS	Mgst1	Microsomal glutathi	100.00	0.00
TRINITY_sp|Q0706	CKAP4	Cytoskeleton-associ	100.00	0.00
TRINITY_sp|P3062	SRI	Sorcin OS=Homo sapi	100.00	0.00
TRINITY_sp|Q5BIN	CNIH1	Protein cornichon h	100.00	0.00
TRINITY_sp|P3109	PSAN	Photosystem I react	100.00	0.00
TRINITY_sp|Q0745	Ambp	Protein AMBP OS=Mus	100.00	0.00
TRINITY_sp|Q0745	Ambp	Protein AMBP OS=Mus	100.00	0.00
TRINITY_sp|Q6NZJ	Eif4g1	Eukaryotic translat	100.00	0.00
TRINITY_sp|Q8VC1	Alas1	5-aminolevulinate s	100.00	0.00
TRINITY_sp|P9745	Atp5j	ATP synthase-coupli	100.00	0.00
TRINITY_sp|O7582	EIF3G	Eukaryotic translat	100.00	0.00
TRINITY_sp|P4900	POLD2	DNA polymerase delt	100.00	0.00
TRINITY_sp|O8883	Cyp4a10	Cytochrome P450 4A1	100.00	0.00
TRINITY_sp|Q7Z2W	MRPL21	39S ribosomal prote	100.00	0.00
TRINITY_sp|P3242	RPL7A	60S ribosomal prote	100.00	0.00
TRINITY_sp|Q2816	PABPN1	Polyadenylate-bindi	100.00	0.00
TRINITY_sp|Q9UK4	LSM7	U6 snRNA-associated	100.00	0.00
TRINITY_sp|P2167	SAT1	Diamine acetyltrans	100.00	0.00
TRINITY_sp|Q3T0Q	SAT1	Diamine acetyltrans	100.00	0.00
TRINITY_sp|Q9DCX	Atp5h	ATP synthase subuni	100.00	0.00
TRINITY_sp|Q9CZ1	Uqcrc1	Cytochrome b-c1 com	100.00	0.00
TRINITY_sp|Q1322	SELENBP1	Selenium-binding pr	100.00	0.00
TRINITY_sp|P1756	Selenbp1	Selenium-binding pr	100.00	0.00
TRINITY_sp|P5046	CSRP3	Cysteine and glycin	100.00	0.00
TRINITY_sp|P0775	Serpina1	Alpha-1-antitrypsin	100.00	0.00
TRINITY_sp|Q9CQ0	Tmem100	Transmembrane prote	100.00	0.00
TRINITY_sp|P3965	DDOST	Dolichyl-diphosphoo	100.00	0.00
TRINITY_sp|Q9H35	PRO1933	Putative uncharacte	100.00	0.00
TRINITY_sp|Q9QY0	Ndrg2	Protein NDRG2 OS=Mu	100.00	0.00
TRINITY_sp|P3840	GNAI2	Guanine nucleotide-	100.00	0.00
TRINITY_sp|P0261	-	Myosin regulatory l	100.00	0.00
TRINITY_sp|Q6142	Hadh	Hydroxyacyl-coenzym	100.00	0.00
TRINITY_sp|P7912	PPM1G	Protein phosphatase	100.00	0.00
TRINITY_sp|Q0MQH	NDUFC2	NADH dehydrogenase	100.00	0.00
TRINITY_sp|Q8BK3	Ndufv3	NADH dehydrogenase	100.00	0.00
TRINITY_sp|P0910	P4HB	Protein disulfide-i	100.00	0.00

TRINITY_sp|P2129	CSRP1	Cysteine and glycin	100.00	0.00
TRINITY_sp|P0758	-	Beta-galactoside-bi	100.00	0.00
TRINITY_sp|Q5E9B	CLIC1	Chloride intracellu	100.00	0.00
TRINITY_sp|Q0494	PLP2	Proteolipid protein	100.00	0.00
TRINITY_sp|Q9JJW	Myoz2	Myozenin-2 OS=Mus m	100.00	0.00
TRINITY_sp|P2659	PTBP1	Polypyrimidine trac	100.00	0.00
TRINITY_sp|Q3ZBI	PSMD7	26S proteasome non-	100.00	0.00
TRINITY_sp|Q96G2	CERS2	Ceramide synthase 2	100.00	0.00
TRINITY_sp|Q6P8J	Ckmt2	Creatine kinase S-t	100.00	0.00
TRINITY_sp|Q2825	SPCS2	Signal peptidase co	100.00	0.00
TRINITY_sp|P9743	Slpi	Antileukoproteinase	100.00	0.00
TRINITY_sp|P3362	Apoc3	Apolipoprotein C-II	100.00	0.00
TRINITY_sp|Q5JTY	CBWD3	COBW domain-contain	100.00	0.00
TRINITY_sp|Q32PI	RPS19	40S ribosomal prote	100.00	0.00
TRINITY_sp|Q1627	IGFBP7	Insulin-like growth	100.00	0.00
TRINITY_sp|P5516	CRYBA2	Beta-crystallin A2	100.00	0.00
TRINITY_sp|Q6114	Cp	Ceruloplasmin OS=Mu	100.00	0.00
TRINITY_sp|P4941	TUFM	Elongation factor T	100.00	0.00
TRINITY_sp|Q1558	TGFBI	Transforming growth	100.00	0.00
TRINITY_sp|O4151	MAP1LC3B	Microtubule-associa	100.00	0.00
TRINITY_sp|Q7L2H	EIF3M	Eukaryotic translat	100.00	0.00
TRINITY_sp|P2807	PSMB6	Proteasome subunit	100.00	0.00
TRINITY_sp|P2807	PSMB6	Proteasome subunit	100.00	0.00
TRINITY_sp|Q7L5N	COPS6	COP9 signalosome co	100.00	0.00
TRINITY_sp|Q8CE1	Bola3	BolA-like protein 3	100.00	0.00
TRINITY_sp|Q9690	SMARCE1	SWI/SNF-related mat	100.00	0.00
TRINITY_sp|Q9NPE	NGRN	Neugrin OS=Homo sap	100.00	0.00
TRINITY_sp|Q0811	AES	Amino-terminal enha	100.00	0.00
TRINITY_sp|Q0811	AES	Amino-terminal enha	100.00	0.00
TRINITY_sp|Q1651	PKN1	Serine/threonine-pr	100.00	0.00
TRINITY_sp|P1324	pac	Puromycin N-acetylt	100.00	0.00
TRINITY_sp|P3783	TALDO1	Transaldolase OS=Ho	100.00	0.00
TRINITY_sp|P6115	ACTR3	Actin-related prote	100.00	0.00
TRINITY_sp|P5196	UBE2E1	Ubiquitin-conjugati	100.00	0.00
TRINITY_sp|Q9BV5	ADI1	1,2-dihydroxy-3-ket	100.00	0.00
TRINITY_sp|Q9ER9	Ndufa13	NADH dehydrogenase	100.00	0.00
TRINITY_sp|P0952	ANXA4	Annexin A4 OS=Homo	100.00	0.00
TRINITY_sp|Q5BIN	SMARCB1	SWI/SNF-related mat	100.00	0.00
TRINITY_sp|P5278	SMS	Spermine synthase O	100.00	0.00
TRINITY_sp|P0862	SNRNP70	U1 small nuclear ri	100.00	0.00
TRINITY_sp|Q148J	ARPC4	Actin-related prote	100.00	0.00
TRINITY_sp|Q9D6U	Fam162a	Protein FAM162A OS=	100.00	0.00
TRINITY_sp|Q96A2	FAM162A	Protein FAM162A OS=	100.00	0.00
TRINITY_sp|P1090	CLU	Clusterin OS=Homo s	100.00	0.00
TRINITY_sp|Q6CZW	tuf1	Elongation factor T	100.00	0.00
TRINITY_sp|P0993	UCHL1	Ubiquitin carboxyl-	100.00	0.00
TRINITY_sp|P7837	CCT2	T-complex protein 1	100.00	0.00
TRINITY_sp|Q9JM7	Arpc3	Actin-related prote	100.00	0.00
TRINITY_sp|Q56JU	RPS24	40S ribosomal prote	100.00	0.00
TRINITY_sp|P6163	COX7C	Cytochrome c oxidas	100.00	0.00
TRINITY_sp|P1766	Cox7c	Cytochrome c oxidas	100.00	0.00
TRINITY_sp|P9902	Uqcrh	Cytochrome b-c1 com	100.00	0.00
TRINITY_sp|P8292	MRPS21	28S ribosomal prote	100.00	0.00
TRINITY_sp|P5099	CCT8	T-complex protein 1	100.00	0.00
TRINITY_sp|P2603	Tln1	Talin-1 OS=Mus musc	100.00	0.00
TRINITY_sp|P2603	Tln1	Talin-1 OS=Mus musc	100.00	0.00

TRINITY_sp|P2133	FLNA	Filamin-A OS=Homo s	100.00	0.00
TRINITY_sp|P0673	PYGL	Glycogen phosphoryl	100.00	0.00
TRINITY_sp|O3538	Phyh	Phytanoyl-CoA dioxy	100.00	0.00
TRINITY_sp|Q9UMY	NOL7	Nucleolar protein 7	100.00	0.00
TRINITY_sp|P0731	Ckm	Creatine kinase M-t	100.00	0.00
TRINITY_sp|P0731	Ckm	Creatine kinase M-t	100.00	0.00
TRINITY_sp|P2969	Ahsg	Alpha-2-HS-glycopro	100.00	0.00
TRINITY_sp|P4855	PSMD8	26S proteasome non-	100.00	0.00
TRINITY_sp|P1172	FN1	Fibronectin (Fragme	100.00	0.00
TRINITY_sp|Q9BRT	UQCC2	Ubiquinol-cytochrom	100.00	0.00
TRINITY_sp|P4855	PSMD8	26S proteasome non-	100.00	0.00
TRINITY_sp|Q32PH	LSM3	U6 snRNA-associated	100.00	0.00
TRINITY_sp|Q8QZT	Acat1	Acetyl-CoA acetyltr	100.00	0.00
TRINITY_sp|Q2M2T	WDR83OS	Protein Asterix OS=	100.00	0.00
TRINITY_sp|Q78IK	Usmg5	Up-regulated during	100.00	0.00
TRINITY_sp|P9902	Rplp2	60S acidic ribosoma	100.00	0.00
TRINITY_sp|P0538	RPLP2	60S acidic ribosoma	100.00	0.00
TRINITY_sp|Q9NZZ	CHMP5	Charged multivesicu	100.00	0.00
TRINITY_sp|P4982	NDUFV1	NADH dehydrogenase	100.00	0.00
TRINITY_sp|Q9D0M	Cyc1	Cytochrome c1, heme	100.00	0.00
TRINITY_sp|P2663	TARS	Threonine--tRNA lig	100.00	0.00
TRINITY_sp|P2663	TARS	Threonine--tRNA lig	100.00	0.00
TRINITY_sp|Q0446	IDH2	Isocitrate dehydrog	100.00	0.00
TRINITY_sp|Q9HB7	CACYBP	Calcyclin-binding p	100.00	0.00
TRINITY_sp|Q8BMS	Hadha	Trifunctional enzym	100.00	0.00
TRINITY_sp|Q9R0Y	Ak1	Adenylate kinase is	100.00	0.00
TRINITY_sp|Q9276	HDAC2	Histone deacetylase	100.00	0.00
TRINITY_sp|P2133	FLNA	Filamin-A OS=Homo s	100.00	0.00
TRINITY_sp|Q8MJJ	FBLN1	Fibulin-1 (Fragment	100.00	0.00
TRINITY_sp|Q1688	TXNRD1	Thioredoxin reducta	100.00	0.00
TRINITY_sp|Q1585	USF2	Upstream stimulator	100.00	0.00
TRINITY_sp|Q9W6H	ube2ia	SUMO-conjugating en	100.00	0.00
TRINITY_sp|P1150	HSP90AA1	Heat shock protein	100.00	0.00
TRINITY_sp|Q9NX1	NDUFB11	NADH dehydrogenase	100.00	0.00
TRINITY_sp|P5670	PSMD2	26S proteasome non-	100.00	0.00
TRINITY_sp|P7852	PRKDC	DNA-dependent prote	100.00	0.00
TRINITY_sp|Q9Z0M	Cd97	CD97 antigen OS=Mus	100.00	0.00
TRINITY_sp|P0035	ALDH1A1	Retinal dehydrogena	100.00	0.00
TRINITY_sp|Q56JU	RPS24	40S ribosomal prote	100.00	0.00
TRINITY_sp|P6219	PSMC5	26S protease regula	100.00	0.00
TRINITY_sp|P5190	PGK	Phosphoglycerate ki	100.00	0.00
TRINITY_sp|Q9NX2	NHP2	H/ACA ribonucleopro	100.00	0.00
TRINITY_sp|Q4234	RPL24A	60S ribosomal prote	100.00	0.00
TRINITY_sp|O7553	SF3B1	Splicing factor 3B	100.00	0.00
TRINITY_sp|Q1290	LMAN2	Vesicular integral-	100.00	0.00
TRINITY_sp|Q1558	TGFBI	Transforming growth	100.00	0.00
TRINITY_sp|Q0256	Myh6	Myosin-6 OS=Mus mus	100.00	0.00
TRINITY_sp|Q0256	Myh6	Myosin-6 OS=Mus mus	100.00	0.00
TRINITY_sp|Q3T0X	RPS16	40S ribosomal prote	100.00	0.00
TRINITY_sp|Q8BTM	Flna	Filamin-A OS=Mus mu	100.00	0.00
TRINITY_sp|P7027	Sepp1	Selenoprotein P OS=	100.00	0.00
TRINITY_sp|P2004	EIF2S2	Eukaryotic translat	100.00	0.00
TRINITY_sp|P2004	EIF2S2	Eukaryotic translat	100.00	0.00
TRINITY_sp|Q69BK	UQCRFS1	Cytochrome b-c1 com	100.00	0.00
TRINITY_sp|Q9NPE	NOP10	H/ACA ribonucleopro	100.00	0.00
TRINITY_sp|P6327	RPS17	40S ribosomal prote	100.00	0.00

<table>
<tr><td>TRINITY_sp|P087(</td><td>RPS17</td><td>40S ribosomal prote</td><td>100.00</td><td>0.00</td></tr>
<tr><td>TRINITY_sp|P231</td><td>CES1</td><td>Liver carboxylester</td><td>100.00</td><td>0.00</td></tr>
<tr><td>TRINITY_sp|Q1JP</td><td>ARPC1A</td><td>Actin-related prote</td><td>100.00</td><td>0.00</td></tr>
<tr><td>TRINITY_sp|P075</td><td>DCN</td><td>Decorin OS=Homo sap</td><td>100.00</td><td>0.00</td></tr>
<tr><td>TRINITY_sp|P075</td><td>DCN</td><td>Decorin OS=Homo sap</td><td>100.00</td><td>0.00</td></tr>
<tr><td>TRINITY_sp|Q9Y3</td><td>STRAP</td><td>Serine-threonine ki</td><td>100.00</td><td>0.00</td></tr>
<tr><td>TRINITY_sp|P497</td><td>PSMB2</td><td>Proteasome subunit</td><td>100.00</td><td>0.00</td></tr>
<tr><td>TRINITY_sp|O357</td><td>Cyp4a14</td><td>Cytochrome P450 4A1</td><td>100.00</td><td>0.00</td></tr>
<tr><td>TRINITY_sp|Q149</td><td>DRAP1</td><td>Dr1-associated core</td><td>100.00</td><td>0.00</td></tr>
<tr><td>TRINITY_sp|P460</td><td>RANGAP1</td><td>Ran GTPase-activati</td><td>100.00</td><td>0.00</td></tr>
<tr><td>TRINITY_sp|A3KN</td><td>UBE2M</td><td>NEDD8-conjugating e</td><td>100.00</td><td>0.00</td></tr>
<tr><td>TRINITY_sp|P117</td><td>FN1</td><td>Fibronectin (Fragme</td><td>100.00</td><td>0.00</td></tr>
<tr><td>TRINITY_sp|P117</td><td>FN1</td><td>Fibronectin (Fragme</td><td>100.00</td><td>0.00</td></tr>
<tr><td>TRINITY_sp|P179</td><td>TCP1</td><td>T-complex protein 1</td><td>100.00</td><td>0.00</td></tr>
<tr><td>TRINITY_sp|P190</td><td>Krt19</td><td>Keratin, type I cyt</td><td>100.00</td><td>0.00</td></tr>
<tr><td>TRINITY_sp|P070</td><td>S100a4</td><td>Protein S100-A4 OS=</td><td>100.00</td><td>0.00</td></tr>
<tr><td>TRINITY_sp|P264</td><td>S100A4</td><td>Protein S100-A4 OS=</td><td>100.00</td><td>0.00</td></tr>
<tr><td>TRINITY_sp|P186</td><td>NELFE</td><td>Negative elongation</td><td>100.00</td><td>0.00</td></tr>
<tr><td>TRINITY_sp|Q644</td><td>Cyp3a11</td><td>Cytochrome P450 3A1</td><td>100.00</td><td>0.00</td></tr>
<tr><td>TRINITY_sp|Q644</td><td>Cyp3a11</td><td>Cytochrome P450 3A1</td><td>100.00</td><td>0.00</td></tr>
<tr><td>TRINITY_sp|Q8K0</td><td>Fgb</td><td>Fibrinogen beta cha</td><td>100.00</td><td>0.00</td></tr>
<tr><td>TRINITY_sp|Q91X</td><td>Hpx</td><td>Hemopexin OS=Mus mu</td><td>100.00</td><td>0.00</td></tr>
<tr><td>TRINITY_sp|Q91X</td><td>Hpx</td><td>Hemopexin OS=Mus mu</td><td>100.00</td><td>0.00</td></tr>
<tr><td>TRINITY_sp|Q8K0</td><td>Fgb</td><td>Fibrinogen beta cha</td><td>100.00</td><td>0.00</td></tr>
<tr><td>TRINITY_sp|Q91X</td><td>Hpx</td><td>Hemopexin OS=Mus mu</td><td>100.00</td><td>0.00</td></tr>
<tr><td>TRINITY_sp|Q9JH</td><td>Cwc15</td><td>Spliceosome-associa</td><td>100.00</td><td>0.00</td></tr>
<tr><td>TRINITY_sp|P050</td><td>APP</td><td>Amyloid beta A4 pro</td><td>100.00</td><td>0.00</td></tr>
<tr><td>TRINITY_sp|O354</td><td>Ech1</td><td>Delta(3,5)-Delta(2,</td><td>100.00</td><td>0.00</td></tr>
<tr><td>TRINITY_sp|Q9UK</td><td>ACIN1</td><td>Apoptotic chromatin</td><td>100.00</td><td>0.00</td></tr>
<tr><td>TRINITY_sp|Q32K</td><td>-</td><td>Protein C10 OS=Bos</td><td>100.00</td><td>0.00</td></tr>
<tr><td>TRINITY_sp|Q32K</td><td>-</td><td>Protein C10 OS=Bos</td><td>100.00</td><td>0.00</td></tr>
<tr><td>TRINITY_sp|P180</td><td>RPL35A</td><td>60S ribosomal prote</td><td>100.00</td><td>0.00</td></tr>
<tr><td>TRINITY_sp|P678</td><td>CSNK2B</td><td>Casein kinase II su</td><td>100.00</td><td>0.00</td></tr>
<tr><td>TRINITY_sp|P149</td><td>UQCRB</td><td>Cytochrome b-c1 com</td><td>100.00</td><td>0.00</td></tr>
<tr><td>TRINITY_sp|Q56J</td><td>RPS13</td><td>40S ribosomal prote</td><td>100.00</td><td>0.00</td></tr>
<tr><td>TRINITY_sp|Q56J</td><td>RPS13</td><td>40S ribosomal prote</td><td>100.00</td><td>0.00</td></tr>
<tr><td>TRINITY_sp|Q56J</td><td>RPS13</td><td>40S ribosomal prote</td><td>100.00</td><td>0.00</td></tr>
<tr><td>TRINITY_sp|P005</td><td>PRKAR1A</td><td>cAMP-dependent prot</td><td>100.00</td><td>0.00</td></tr>
<tr><td>TRINITY_sp|A9JR</td><td>ppp4cb</td><td>Serine/threonine-pr</td><td>100.00</td><td>0.00</td></tr>
<tr><td>TRINITY_sp|O149</td><td>UQCR11</td><td>Cytochrome b-c1 com</td><td>100.00</td><td>0.00</td></tr>
<tr><td>TRINITY_sp|P565</td><td>EIF6</td><td>Eukaryotic translat</td><td>100.00</td><td>0.00</td></tr>
<tr><td>TRINITY_sp|P495</td><td>SARS</td><td>Serine--tRNA ligase</td><td>100.00</td><td>0.00</td></tr>
<tr><td>TRINITY_sp|Q622</td><td>Sptbn1</td><td>Spectrin beta chain</td><td>100.00</td><td>0.00</td></tr>
<tr><td>TRINITY_sp|P609</td><td>S100A10</td><td>Protein S100-A10 OS</td><td>100.00</td><td>0.00</td></tr>
<tr><td>TRINITY_sp|Q9QX</td><td>Egfl7</td><td>Epidermal growth fa</td><td>100.00</td><td>0.00</td></tr>
<tr><td>TRINITY_sp|P623</td><td>SNRPD3</td><td>Small nuclear ribon</td><td>100.00</td><td>0.00</td></tr>
<tr><td>TRINITY_sp|Q150</td><td>KARS</td><td>Lysine--tRNA ligase</td><td>100.00</td><td>0.00</td></tr>
<tr><td>TRINITY_sp|Q3MI</td><td>RPL37A</td><td>60S ribosomal prote</td><td>100.00</td><td>0.00</td></tr>
<tr><td>TRINITY_sp|Q3MI</td><td>RPL37A</td><td>60S ribosomal prote</td><td>100.00</td><td>0.00</td></tr>
<tr><td>TRINITY_sp|Q3T1</td><td>DYNLRB1</td><td>Dynein light chain</td><td>100.00</td><td>0.00</td></tr>
<tr><td>TRINITY_sp|P467</td><td>RPL28</td><td>60S ribosomal prote</td><td>100.00</td><td>0.00</td></tr>
<tr><td>TRINITY_sp|P411</td><td>Rpl28</td><td>60S ribosomal prote</td><td>100.00</td><td>0.00</td></tr>
<tr><td>TRINITY_sp|P042</td><td>H2-Eb1</td><td>H-2 class II histoc</td><td>100.00</td><td>0.00</td></tr>
<tr><td>TRINITY_sp|P591</td><td>HTR4</td><td>Histone H3.3 OS=Ara</td><td>100.00</td><td>0.00</td></tr>
<tr><td>TRINITY_sp|Q1RM</td><td>CTDNEP1</td><td>CTD nuclear envelop</td><td>100.00</td><td>0.00</td></tr>
<tr><td>TRINITY_sp|P494</td><td>Hpd</td><td>4-hydroxyphenylpyru</td><td>100.00</td><td>0.00</td></tr>
</table>

TRINITY_sp|Q32L/	H2AFV	Histone H2A.V OS=Bo	100.00	0.00
TRINITY_sp|Q56J	RPL31	60S ribosomal prote	100.00	0.00
TRINITY_sp|P990	Prdx5	Peroxiredoxin-5, mi	100.00	0.00
TRINITY_sp|Q144	RBM39	RNA-binding protein	100.00	0.00
TRINITY_sp|Q144	RBM39	RNA-binding protein	100.00	0.00
TRINITY_sp|Q3T0	RPL18A	60S ribosomal prote	100.00	0.00
TRINITY_sp|P627	Rpl18a	60S ribosomal prote	100.00	0.00
TRINITY_sp|Q9R0	Acox1	Peroxisomal acyl-co	100.00	0.00
TRINITY_sp|Q644	Cyp2c29	Cytochrome P450 2C2	100.00	0.00
TRINITY_sp|P091	P4hb	Protein disulfide-i	100.00	0.00
TRINITY_sp|Q9UN	STUB1	E3 ubiquitin-protei	100.00	0.00
TRINITY_sp|O436	PRC1	Protein regulator o	100.00	0.00
TRINITY_sp|P042	Mb	Myoglobin OS=Mus mu	100.00	0.00
TRINITY_sp|O753	NDUFS6	NADH dehydrogenase	100.00	0.00
TRINITY_sp|Q3SZ	SMS	Spermine synthase O	100.00	0.00
TRINITY_sp|Q9Y2	C14orf1	UPF0568 protein C14	100.00	0.00
TRINITY_sp|P622	RPS14	40S ribosomal prote	100.00	0.00
TRINITY_sp|O155	RGS5	Regulator of G-prot	100.00	0.00
TRINITY_sp|P0A9	cspC	Cold shock-like pro	100.00	0.00
TRINITY_sp|Q9UM	NENF	Neudesin OS=Homo sa	100.00	0.00
TRINITY_sp|P551	MANF	Mesencephalic astro	100.00	0.00
TRINITY_sp|Q082	DHX9	ATP-dependent RNA h	100.00	0.00
TRINITY_sp|Q3ZB	VCP	Transitional endopl	100.00	0.00
TRINITY_sp|Q140	CCNI	Cyclin-I OS=Homo sa	100.00	0.00
TRINITY_sp|Q9DC	Etfb	Electron transfer f	100.00	0.00
TRINITY_sp|P600	CD81	CD81 antigen OS=Hom	100.00	0.00
TRINITY_sp|P525	HNRNPF	Heterogeneous nucle	100.00	0.00
TRINITY_sp|Q9UB	RNF7	RING-box protein 2	100.00	0.00
TRINITY_sp|Q9BR	VPS25	Vacuolar protein-so	100.00	0.00
TRINITY_sp|Q1RM	RHOC	Rho-related GTP-bin	100.00	0.00
TRINITY_sp|Q3T0	PSMC4	26S protease regula	100.00	0.00
TRINITY_sp|A4FU	SNRPE	Small nuclear ribon	100.00	0.00
TRINITY_sp|P612	NEDD8	NEDD8 OS=Bos taurus	100.00	0.00
TRINITY_sp|Q32P	POLR2J	DNA-directed RNA po	100.00	0.00
TRINITY_sp|Q010	TIAL1	Nucleolysin TIAR OS	100.00	0.00
TRINITY_sp|Q2KI	PSMC6	26S protease regula	100.00	0.00
TRINITY_sp|Q91W	Ndufs2	NADH dehydrogenase	100.00	0.00
TRINITY_sp|Q3T0	RPL39	60S ribosomal prote	100.00	0.00
TRINITY_sp|P478	Igfbp4	Insulin-like growth	100.00	0.00
TRINITY_sp|P216	Gc	Vitamin D-binding p	100.00	0.00
TRINITY_sp|P291	CSDE1	Cold shock domain-c	100.00	0.00
TRINITY_sp|P052	Got2	Aspartate aminotran	100.00	0.00
TRINITY_sp|P052	Got2	Aspartate aminotran	100.00	0.00
TRINITY_sp|Q624	Ndrg1	Protein NDRG1 OS=Mu	100.00	0.00
TRINITY_sp|Q132	TRIM28	Transcription inter	100.00	0.00
TRINITY_sp|P146	CCNB1	G2/mitotic-specific	100.00	0.00
TRINITY_sp|P505	S100a11	Protein S100-A11 OS	100.00	0.00
TRINITY_sp|P319	S100A11	Protein S100-A11 OS	100.00	0.00
TRINITY_sp|P319	ATIC	Bifunctional purine	100.00	0.00
TRINITY_sp|P628	GNB1	Guanine nucleotide-	100.00	0.00
TRINITY_sp|P536	COPB1	Coatomer subunit be	100.00	0.00
TRINITY_sp|Q140	CIRBP	Cold-inducible RNA-	100.00	0.00
TRINITY_sp|P055	ITGB1	Integrin beta-1 OS=	100.00	0.00
TRINITY_sp|P840	ARF5	ADP-ribosylation fa	100.00	0.00
TRINITY_sp|Q9H6	RNF167	E3 ubiquitin-protei	100.00	0.00
TRINITY_sp|P073	CTSD	Cathepsin D OS=Homo	100.00	0.00

TRINITY_sp|Q2VI	EIF2S3L	Putative eukaryotic	100.00	0.00
TRINITY_sp|P148	HNRNPL	Heterogeneous nucle	100.00	0.00
TRINITY_sp|Q3ZB	RPS20	40S ribosomal prote	100.00	0.00
TRINITY_sp|Q3T0	VPS29	Vacuolar protein so	100.00	0.00
TRINITY_sp|Q8WU	BRK1	Protein BRICK1 OS=H	100.00	0.00
TRINITY_sp|Q5E9	RPL10A	60S ribosomal prote	100.00	0.00
TRINITY_sp|P628	RPS15	40S ribosomal prote	100.00	0.00
TRINITY_sp|P628	RPS15	40S ribosomal prote	100.00	0.00
TRINITY_sp|O003	EIF3F	Eukaryotic translat	100.00	0.00
TRINITY_sp|P612	RAP1B	Ras-related protein	100.00	0.00
TRINITY_sp|Q644	Cox8a	Cytochrome c oxidas	100.00	0.00
TRINITY_sp|Q9NZ	MTCH1	Mitochondrial carri	100.00	0.00
TRINITY_sp|Q9NZ	MTCH1	Mitochondrial carri	100.00	0.00
TRINITY_sp|O702	Eef1b	Elongation factor 1	100.00	0.00
TRINITY_sp|P245	EEF1B2	Elongation factor 1	100.00	0.00
TRINITY_sp|Q99L	Etfa	Electron transfer f	100.00	0.00
TRINITY_sp|O439	NDUFS5	NADH dehydrogenase	100.00	0.00
TRINITY_sp|Q56J	RPS24	40S ribosomal prote	100.00	0.00
TRINITY_sp|Q928	TAF15	TATA-binding protei	100.00	0.00
TRINITY_sp|Q945	-	Eukaryotic translat	100.00	0.00
TRINITY_sp|Q1RM	SNX3	Sorting nexin-3 OS=	100.00	0.00
TRINITY_sp|Q9Y6	AUP1	Ancient ubiquitous	100.00	0.00
TRINITY_sp|Q9H6	RNF167	E3 ubiquitin-protei	100.00	0.00
TRINITY_sp|P522	HNRNPM	Heterogeneous nucle	100.00	0.00
TRINITY_sp|Q96K	CLPTM1L	Cleft lip and palat	100.00	0.00
TRINITY_sp|O550	Copb2	Coatomer subunit be	100.00	0.00
TRINITY_sp|Q9Y6	NPTN	Neuroplastin OS=Hom	100.00	0.00
TRINITY_sp|Q8VC	Acaa1b	3-ketoacyl-CoA thio	100.00	0.00
TRINITY_sp|Q921	Acaa1a	3-ketoacyl-CoA thio	100.00	0.00
TRINITY_sp|P563	Atp5e	ATP synthase subuni	100.00	0.00
TRINITY_sp|Q3ZB	CKMT2	Creatine kinase S-t	100.00	0.00
TRINITY_sp|P507	Tnnt2	Troponin T, cardiac	100.00	0.00
TRINITY_sp|Q9D7	Mrpl30	39S ribosomal prote	100.00	0.00
TRINITY_sp|P118	CDK4	Cyclin-dependent ki	100.00	0.00
TRINITY_sp|P032	-	E1B protein, small	100.00	0.00
TRINITY_sp|P032	-	E1B 55 kDa protein	100.00	0.00
TRINITY_sp|Q8R1	Hopx	Homeodomain-only pr	100.00	0.00
TRINITY_sp|P458	VDAC1	Voltage-dependent a	100.00	0.00
TRINITY_sp|Q9UN	SSR3	Translocon-associat	100.00	0.00
TRINITY_sp|Q3SZ	SSR3	Translocon-associat	100.00	0.00
TRINITY_sp|P492	RPL34	60S ribosomal prote	100.00	0.00
TRINITY_sp|Q9D1	Rpl34	60S ribosomal prote	100.00	0.00
TRINITY_sp|P263	U2AF2	Splicing factor U2A	100.00	0.00
TRINITY_sp|P170	Hmga1	High mobility group	100.00	0.00
TRINITY_sp|P525	ARHGDIA	Rho GDP-dissociatio	100.00	0.00
TRINITY_sp|Q3T0	SNRPF	Small nuclear ribon	100.00	0.00
TRINITY_sp|Q862	RPL24	60S ribosomal prote	100.00	0.00
TRINITY_sp|Q862	RPL24	60S ribosomal prote	100.00	0.00
TRINITY_sp|P156	Gstm2	Glutathione S-trans	100.00	0.00
TRINITY_sp|Q3T0	PSMA1	Proteasome subunit	100.00	0.00
TRINITY_sp|P245	Aldh1a1	Retinal dehydrogena	100.00	0.00
TRINITY_sp|P245	Aldh1a1	Retinal dehydrogena	100.00	0.00
TRINITY_sp|Q9UN	NPIPA1	Nuclear pore comple	100.00	0.00
TRINITY_sp|P511	Acadl	Long-chain specific	100.00	0.00
TRINITY_sp|P511	Acadl	Long-chain specific	100.00	0.00
TRINITY_sp|P511	Acadl	Long-chain specific	100.00	0.00

TRINITY_sp|P025	ASL1	Delta-1 crystallin	100.00	0.00
TRINITY_sp|P106	Ctsb	Cathepsin B OS=Mus	100.00	0.00
TRINITY_sp|Q56K	RPLP1	60S acidic ribosoma	100.00	0.00
TRINITY_sp|Q9D6	Ndufv2	NADH dehydrogenase	100.00	0.00
TRINITY_sp|P617	B2M	Beta-2-microglobuli	100.00	0.00
TRINITY_sp|P352	RPL22	60S ribosomal prote	100.00	0.00
TRINITY_sp|Q4R5	RPL22	60S ribosomal prote	100.00	0.00
TRINITY_sp|O626	DES	Desmin OS=Bos tauru	100.00	0.00
TRINITY_sp|Q9CZ	Cs	Citrate synthase, m	100.00	0.00
TRINITY_sp|P516	Myl2	Myosin regulatory l	100.00	0.00
TRINITY_sp|P613	RPL27	60S ribosomal prote	100.00	0.00
TRINITY_sp|P613	RPL27	60S ribosomal prote	100.00	0.00
TRINITY_sp|P188	ATF4	Cyclic AMP-dependen	100.00	0.00
TRINITY_sp|P613	RPL27	60S ribosomal prote	100.00	0.00
TRINITY_sp|Q025	Myh6	Myosin-6 OS=Mus mus	100.00	0.00
TRINITY_sp|Q2KJ	CDC42	Cell division contr	100.00	0.00
TRINITY_sp|Q16Y	Cdc42	Cdc42 homolog OS=Ae	100.00	0.00
TRINITY_sp|O886	Psmc3	26S protease regula	100.00	0.00
TRINITY_sp|P412	IARS	Isoleucine--tRNA li	100.00	0.00
TRINITY_sp|P563	Cyb5a	Cytochrome b5 OS=Mu	100.00	0.00
TRINITY_sp|P206	COX5A	Cytochrome c oxidas	100.00	0.00
TRINITY_sp|Q3ZC	PAIP2	Polyadenylate-bindi	100.00	0.00
TRINITY_sp|P087	Gnai2	Guanine nucleotide-	100.00	0.00
TRINITY_sp|Q3T0	RPS10	40S ribosomal prote	100.00	0.00
TRINITY_sp|Q99P	Rtn4	Reticulon-4 OS=Mus	100.00	0.00
TRINITY_sp|P514	Rpl9	60S ribosomal prote	100.00	0.00
TRINITY_sp|P329	RPL9	60S ribosomal prote	100.00	0.00
TRINITY_sp|P319	UQCRC1	Cytochrome b-c1 com	100.00	0.00
TRINITY_sp|P319	UQCRC1	Cytochrome b-c1 com	100.00	0.00
TRINITY_sp|O884	Capns1	Calpain small subun	100.00	0.00
TRINITY_sp|Q3ZC	SKP1	S-phase kinase-asso	100.00	0.00
TRINITY_sp|P276	APEX1	DNA-(apurinic or ap	100.00	0.00
TRINITY_sp|Q9UN	RPL26L1	60S ribosomal prote	100.00	0.00
TRINITY_sp|P213	FLNA	Filamin-A OS=Homo s	100.00	0.00
TRINITY_sp|P223	NME2	Nucleoside diphosph	100.00	0.00
TRINITY_sp|P067	S100A6	Protein S100-A6 OS=	100.00	0.00
TRINITY_sp|Q3T0	RPL23	60S ribosomal prote	100.00	0.00
TRINITY_sp|Q3T0	RPL23	60S ribosomal prote	100.00	0.00
TRINITY_sp|P321	PRDX2	Peroxiredoxin-2 OS=	100.00	0.00
TRINITY_sp|P467	RPL21	60S ribosomal prote	100.00	0.00
TRINITY_sp|O436	PRC1	Protein regulator o	100.00	0.00
TRINITY_sp|O438	CALU	Calumenin OS=Homo s	100.00	0.00
TRINITY_sp|P048	RPN2	Dolichyl-diphosphoo	100.00	0.00
TRINITY_sp|P048	RPN2	Dolichyl-diphosphoo	100.00	0.00
TRINITY_sp|Q3T0	RPL23	60S ribosomal prote	100.00	0.00
TRINITY_sp|Q3T0	RPL23	60S ribosomal prote	100.00	0.00
TRINITY_sp|Q2KI	TCEB1	Transcription elong	100.00	0.00
TRINITY_sp|P612	RPL26	60S ribosomal prote	100.00	0.00
TRINITY_sp|P612	RPL26	60S ribosomal prote	100.00	0.00
TRINITY_sp|P612	RPL26	60S ribosomal prote	100.00	0.00
TRINITY_sp|P263	U2AF2	Splicing factor U2A	100.00	0.00
TRINITY_sp|Q3T0	RPS18	40S ribosomal prote	100.00	0.00
TRINITY_sp|Q3T0	RPS18	40S ribosomal prote	100.00	0.00
TRINITY_sp|P363	ARF1	ADP-ribosylation fa	100.00	0.00
TRINITY_sp|P180	ARF4	ADP-ribosylation fa	100.00	0.00
TRINITY_sp|Q3T0	RAN	GTP-binding nuclear	100.00	0.00

TRINITY_sp|Q2418	RpS5a	40S ribosomal prote	100.00	0.00
TRINITY_sp|Q5E98	RPS5	40S ribosomal prote	100.00	0.00
TRINITY_sp|P0578	KRT8	Keratin, type II cy	100.00	0.00
TRINITY_sp|P6066	MYL6	Myosin light polype	100.00	0.00
TRINITY_sp|Q1515	NOMO1	Nodal modulator 1 O	100.00	0.00
TRINITY_sp|P6128	PPP1CC	Serine/threonine-pr	100.00	0.00
TRINITY_sp|P6213	Ppp1ca	Serine/threonine-pr	100.00	0.00
TRINITY_sp|P0C09	H2AFZ	Histone H2A.Z OS=Bo	100.00	0.00
TRINITY_sp|Q27II	gag	Gag polyprotein OS=	100.00	0.00
TRINITY_sp|P0335	gag-pol	Gag-Pol polyprotein	100.00	0.00
TRINITY_sp|Q9690	RPL36AL	60S ribosomal prote	100.00	0.00
TRINITY_sp|Q3SZ5	RPL36A	60S ribosomal prote	100.00	0.00
TRINITY_sp|Q3ZCE	EIF3L	Eukaryotic translat	100.00	0.00
TRINITY_sp|Q3SZ5	RPL36A	60S ribosomal prote	100.00	0.00
TRINITY_sp|Q3ZC5	ACTN2	Alpha-actinin-2 OS=	100.00	0.00
TRINITY_sp|O4370	ACTN4	Alpha-actinin-4 OS=	100.00	0.00
TRINITY_sp|A5D7I	ACTN4	Alpha-actinin-4 OS=	100.00	0.00
TRINITY_sp|Q0184	EWSR1	RNA-binding protein	100.00	0.00
TRINITY_sp|Q3T10	SEC61G	Protein transport p	100.00	0.00
TRINITY_sp|P6242	RPL7A	60S ribosomal prote	100.00	0.00
TRINITY_sp|P1297	Rpl7a	60S ribosomal prote	100.00	0.00
TRINITY_sp|P1611	Lgals3	Galectin-3 OS=Mus m	100.00	0.00
TRINITY_sp|Q9Y30	RPL36	60S ribosomal prote	100.00	0.00
TRINITY_sp|Q3SZ6	PGAM1	Phosphoglycerate mu	100.00	0.00
TRINITY_sp|Q9Y30	RPL36	60S ribosomal prote	100.00	0.00
TRINITY_sp|Q3T0I	RPL30	60S ribosomal prote	100.00	0.00
TRINITY_sp|Q3T0I	RPL30	60S ribosomal prote	100.00	0.00
TRINITY_sp|Q3MHE	UBE2L3	Ubiquitin-conjugati	100.00	0.00
TRINITY_sp|Q5E93	EIF1	Eukaryotic translat	100.00	0.00
TRINITY_sp|Q9CP0	Atp5l	ATP synthase subuni	100.00	0.00
TRINITY_sp|Q2NK2	CCT7	T-complex protein 1	100.00	0.00
TRINITY_sp|Q6120	Psap	Prosaposin OS=Mus m	100.00	0.00
TRINITY_sp|Q3MHE	ARPC2	Actin-related prote	100.00	0.00
TRINITY_sp|Q9Y26	NUDC	Nuclear migration p	100.00	0.00
TRINITY_sp|Q9Z2E	Mbd2	Methyl-CpG-binding	100.00	0.00
TRINITY_sp|Q0683	PRDX1	Peroxiredoxin-1 OS=	100.00	0.00
TRINITY_sp|P0102	C3	Complement C3 OS=Mu	100.00	0.00
TRINITY_sp|Q0631	Scgb1a1	Uteroglobin OS=Mus	100.00	0.00
TRINITY_sp|P0102	C3	Complement C3 OS=Mu	100.00	0
TRINITY_sp|E9PV2	Fga	Fibrinogen alpha ch	100.00	0.00
TRINITY_sp|Q6164	Hp	Haptoglobin OS=Mus	100.00	0.00
TRINITY_sp|P0775	Serpina3	Serine protease inh	100.00	0.00
TRINITY_sp|Q0702	RPL18	60S ribosomal prote	100.00	0.00
TRINITY_sp|Q0062	Apoa1	Apolipoprotein A-I	100.00	0.00
TRINITY_sp|P0772	Alb	Serum albumin OS=Mu	100.00	0
TRINITY_sp|Q8VCM	Fgg	Fibrinogen gamma ch	100.00	0.00
TRINITY_sp|P0775	Serpina3	Serine protease inh	100.00	0.00
TRINITY_sp|P0822	Apoe	Apolipoprotein E OS	100.00	0.00
TRINITY_sp|P3598	Rpl18	60S ribosomal prote	100.00	0.00
TRINITY_sp|P0208	Hbb-b1	Hemoglobin subunit	100.00	0.00
TRINITY_sp|P5199	HNRNPA3	Heterogeneous nucle	100.00	0.00
TRINITY_sp|P7924	RPL37	60S ribosomal prote	100.00	0.00
TRINITY_sp|P3902	RPL3	60S ribosomal prote	100.00	0.00
TRINITY_sp|Q05AY	eif3e-a	Eukaryotic translat	100.00	0.00
TRINITY_sp|P7924	RPL37	60S ribosomal prote	100.00	0.00
TRINITY_sp|P0536	Saa1	Serum amyloid A-1 p	100.00	0.00

TRINITY_sp|P0491	Saa3	Serum amyloid A-3 p	100.00	0.00
TRINITY_sp|Q3SZ6	LAMTOR5	Ragulator complex p	100.00	0.00
TRINITY_sp|P6101	Pln	Cardiac phospholamb	100.00	0.00
TRINITY_sp|O5514	Atp2a2	Sarcoplasmic/endopl	100.00	0
TRINITY_sp|E2RKA	RPL32	60S ribosomal prote	100.00	0.00
TRINITY_sp|P0CG8	UBI11	Polyubiquitin OS=Ni	100.00	0.00
TRINITY_sp|P4820	ATP5G3	ATP synthase F(0) c	100.00	0.00
TRINITY_sp|P2133	FLNA	Filamin-A OS=Homo s	100.00	0.00
TRINITY_sp|P2352	CFL1	Cofilin-1 OS=Homo s	100.00	0.00
TRINITY_sp|P1876	Cfl1	Cofilin-1 OS=Mus mu	100.00	0.00
TRINITY_sp|P6107	UBE2D3	Ubiquitin-conjugati	100.00	0.00
TRINITY_sp|P1092	Spp1	Osteopontin OS=Mus	100.00	0.00
TRINITY_sp|P1092	Spp1	Osteopontin OS=Mus	100.00	0.00
TRINITY_sp|Q0133	Apoh	Beta-2-glycoprotein	100.00	0.00
TRINITY_sp|Q4296	-	Phosphoglycerate ki	100.00	0.00
TRINITY_sp|P5638	ATP5E	ATP synthase subuni	100.00	0.00
TRINITY_sp|Q5EAI	RPL15	60S ribosomal prote	100.00	0.00
TRINITY_sp|Q2NKW	BLOC1S1	Biogenesis of lysos	100.00	0.00
TRINITY_sp|P3657	RPL4	60S ribosomal prote	100.00	0.00
TRINITY_sp|P6317	DYNLT1	Dynein light chain	100.00	0.00
TRINITY_sp|Q3T16	RPS3	40S ribosomal prote	100.00	0.00
TRINITY_sp|E2RH4	RPS3	40S ribosomal prote	100.00	0.00
TRINITY_sp|P0189	HLA-A	HLA class I histoco	100.00	0.00
TRINITY_sp|Q76N2	RPS4X	40S ribosomal prote	100.00	0.00
TRINITY_sp|Q76N2	RPS4X	40S ribosomal prote	100.00	0.00
TRINITY_sp|Q32PH	EEF1A2	Elongation factor 1	100.00	0.00
TRINITY_sp|P2952	-	Elongation factor 1	100.00	0.00
TRINITY_sp|P1588	RPS2	40S ribosomal prote	100.00	0.00
TRINITY_sp|P1136	gag	Retrovirus-related	100.00	0.00
TRINITY_sp|P2544	Rps2	40S ribosomal prote	100.00	0.00
TRINITY_sp|P1136	gag	Retrovirus-related	100.00	0.00
TRINITY_sp|P1289	-	Probable Pol polypr	100.00	0.00
TRINITY_sp|P0397	Iap	IgE-binding protein	100.00	0.00
TRINITY_sp|P5648	Atp5b	ATP synthase subuni	100.00	0.00
TRINITY_sp|O7534	TBCA	Tubulin-specific ch	100.00	0.00
TRINITY_sp|P6293	PPIA	Peptidyl-prolyl cis	100.00	0.00
TRINITY_sp|P1774	Ppia	Peptidyl-prolyl cis	100.00	0.00
TRINITY_sp|P5347	-	Actin, cytoplasmic	100.00	0.00
TRINITY_sp|P5350	-	Actin, cytoplasmic	100.00	0.00
TRINITY_sp|A5D7I	ACTN4	Alpha-actinin-4 OS=	100.00	0.00
TRINITY_sp|P1363	EEF2	Elongation factor 2	100.00	0
TRINITY_sp|P0514	SLC25A5	ADP/ATP translocase	100.00	0.00
TRINITY_sp|P1411	Rpl27a	60S ribosomal prote	100.00	0.00
TRINITY_sp|P1462	HSP90B1	Endoplasmin OS=Homo	100.00	0.00
TRINITY_sp|P4677	RPL27A	60S ribosomal prote	100.00	0.00
TRINITY_sp|Q3T0W	RPL19	60S ribosomal prote	100.00	0.00
TRINITY_sp|Q3T0W	RPL19	60S ribosomal prote	100.00	0.00
TRINITY_sp|P4677	RPL5	60S ribosomal prote	100.00	0.00
TRINITY_sp|Q9R0H	Esd	S-formylglutathione	100.00	0.00
TRINITY_sp|P2763	RPL10	60S ribosomal prote	100.00	0.00
TRINITY_sp|P3104	SDHA	Succinate dehydroge	100.00	0.00
TRINITY_sp|Q8K2E	Sdha	Succinate dehydroge	100.00	0.00
TRINITY_sp|O1473	PDCD5	Programmed cell dea	100.00	0.00
TRINITY_sp|Q8K2E	Sdha	Succinate dehydroge	100.00	0.00
TRINITY_sp|P0044	SOD1	Superoxide dismutas	100.00	0.00
TRINITY_sp|P8417	RPS12	40S ribosomal prote	100.00	0.00

TRINITY_sp|P197	Cox4i1	Cytochrome c oxidas	100.00	0.00
TRINITY_sp|Q76I	RPS12	40S ribosomal prote	100.00	0.00
TRINITY_sp|P040	ALDOA	Fructose-bisphospha	100.00	0.00
TRINITY_sp|P082	Sod1	Superoxide dismutas	100.00	0.00
TRINITY_sp|Q76I	RPS12	40S ribosomal prote	100.00	0.00
TRINITY_sp|P116	Lcn2	Neutrophil gelatina	100.00	0.00
TRINITY_sp|P115	HSP90AA	Heat shock protein	100.00	0.00
TRINITY_sp|P0CG	ubi4	Polyubiquitin OS=Sc	100.00	0.00
TRINITY_sp|P548	Hmgcs2	Hydroxymethylglutar	100.00	0.00
TRINITY_sp|P682	rps27a	Ubiquitin-40S ribos	100.00	0.00
TRINITY_sp|P0CG	UBI11	Polyubiquitin OS=Ni	100.00	0.00
TRINITY_sp|P0CG	UBI11	Polyubiquitin OS=Ni	100.00	0.00
TRINITY_sp|Q165	RBBP7	Histone-binding pro	100.00	0.00
TRINITY_sp|P078	CTSB	Cathepsin B OS=Homo	100.00	0.00
TRINITY_sp|P129	XRCC6	X-ray repair cross-	100.00	0.00
TRINITY_sp|P073	Anxa2	Annexin A2 OS=Mus m	100.00	0.00
TRINITY_sp|P073	ANXA2	Annexin A2 OS=Homo	100.00	0.00
TRINITY_sp|Q3SZ	YWHAQ	14-3-3 protein thet	100.00	0.00
TRINITY_sp|P129	XRCC6	X-ray repair cross-	100.00	0.00
TRINITY_sp|Q3T0	RPS16	40S ribosomal prote	100.00	0.00
TRINITY_sp|P840	His4	Histone H4 OS=Acrol	100.00	0.00
TRINITY_sp|P082	-	Chlorophyll a-b bin	100.00	0.00
TRINITY_sp|P123	CAB1	Chlorophyll a-b bin	100.00	0.00
TRINITY_sp|A2XJ	OsI_012	Chlorophyll a-b bin	100.00	0.00
TRINITY_sp|Q371	rbcL	Ribulose bisphospha	100.00	0.00
TRINITY_sp|Q009	BEX3	Protein BEX3 OS=Hom	100.00	0.00
TRINITY_sp|P592	RPS27AA	Ubiquitin-40S ribos	100.00	0.00
TRINITY_sp|P0C2	crp-79	Ubiquitin-60S ribos	100.00	0.00
TRINITY_sp|P0CG	UBI11	Polyubiquitin OS=Ni	100.00	0.00
TRINITY_sp|P0CG	UBI11	Polyubiquitin OS=Ni	100.00	0.00
TRINITY_sp|P0CG	UBI11	Polyubiquitin OS=Ni	100.00	0.00
TRINITY_sp|Q060	ATP5G2	ATP synthase F(0) c	100.00	0.00
TRINITY_sp|Q9CR	Atp5g1	ATP synthase F(0) c	100.00	0.00
TRINITY_sp|P615	RHOA	Transforming protei	100.00	0.00
TRINITY_sp|Q9H1	NUCKS1	Nuclear ubiquitous	100.00	0.00
TRINITY_sp|Q5E9	H3F3A	Histone H3.3 OS=Bos	100.00	0.00
TRINITY_sp|P509	CCT4	T-complex protein 1	100.00	0.00
TRINITY_sp|O006	CYR61	Protein CYR61 OS=Ho	100.00	0.00
TRINITY_sp|Q143	FAM50A	Protein FAM50A OS=H	100.00	0.00
TRINITY_sp|P101	HMGB1	High mobility group	100.00	0.00
TRINITY_sp|P071	-	Catalase OS=Ipomoea	100.00	0.00
TRINITY_sp|P502	CRIP1	Cysteine-rich prote	100.00	0.00
TRINITY_sp|P027	FTL	Ferritin light chai	100.00	0.00
TRINITY_sp|P409	Inmt	Indolethylamine N-m	100.00	0.00
TRINITY_sp|P355	FAU	Ubiquitin-like prot	100.00	0.00
TRINITY_sp|P277	Pdia3	Protein disulfide-i	100.00	0.00
TRINITY_sp|P355	Fau	Ubiquitin-like prot	100.00	0.00
TRINITY_sp|Q8JG	PDIA3	Protein disulfide-i	100.00	0.00
TRINITY_sp|P277	Pdia3	Protein disulfide-i	100.00	0.00
TRINITY_sp|Q905	HSPA5	78 kDa glucose-regu	100.00	0.00
TRINITY_sp|P191	HSPA8	Heat shock cognate	100.00	0.00
TRINITY_sp|P200	Hspa5	78 kDa glucose-regu	100.00	0.00
TRINITY_sp|Q0VC	HSPA5	78 kDa glucose-regu	100.00	0.00
TRINITY_sp|P120	COX6A1	Cytochrome c oxidas	100.00	0.00
TRINITY_sp|P136	TPT1	Translationally-con	100.00	0.00
TRINITY_sp|P630	Tpt1	Translationally-con	100.00	0.00

TRINITY_sp|P0245	COL1A1	Collagen alpha-1(I)	100.00	0.00
TRINITY_sp|P5089	RPSA	40S ribosomal prote	100.00	0.00
TRINITY_sp|Q96C9	PPP1R14B	Protein phosphatase	100.00	0.00
TRINITY_sp|P1420	Rpsa	40S ribosomal prote	100.00	0.00
TRINITY_sp|Q4QY7	rpsa	40S ribosomal prote	100.00	0.00
TRINITY_sp|P1198	Tcp1	T-complex protein 1	100.00	0.00
TRINITY_sp|P0940	Ncl	Nucleolin OS=Mus mu	100.00	0.00
TRINITY_sp|P6298	Rps27a	Ubiquitin-40S ribos	100.00	0.00
TRINITY_sp|P0CG8	UBI11	Polyubiquitin OS=Ni	100.00	0.00
TRINITY_sp|P6299	RPS27A	Ubiquitin-40S ribos	100.00	0.00
TRINITY_sp|A2Q02	HSPA8	Heat shock cognate	100.00	0.00
TRINITY_sp|P1937	HSPA8	Heat shock cognate	100.00	0.00
TRINITY_sp|P3597	Rpl12	60S ribosomal prote	100.00	0.00
TRINITY_sp|P6128	RPL12	60S ribosomal prote	100.00	0.00
TRINITY_sp|P1784	DDX5	Probable ATP-depend	100.00	0.00
TRINITY_sp|Q95L4	EIF4G2	Eukaryotic translat	100.00	0.00
TRINITY_sp|Q95L4	EIF4G2	Eukaryotic translat	100.00	0.00
TRINITY_sp|Q95L4	EIF4G2	Eukaryotic translat	100.00	0.00
TRINITY_sp|Q95L4	EIF4G2	Eukaryotic translat	100.00	0.00
TRINITY_sp|Q6244	Eif4g2	Eukaryotic translat	100.00	0.00
TRINITY_sp|Q95L4	EIF4G2	Eukaryotic translat	100.00	0.00
TRINITY_sp|P5927	RPS27AA	Ubiquitin-40S ribos	100.00	0.00
TRINITY_sp|Q8VC1	Ces1d	Carboxylesterase 1D	100.00	0.00
TRINITY_sp|Q5DU5	Nlrc3	Protein NLRC3 OS=Mu	100.00	0.00
TRINITY_sp|P0807	Cbr2	Carbonyl reductase	100.00	0.00
TRINITY_sp|P2039	-	Actin, alpha cardia	100.00	0.00
TRINITY_sp|Q3ZC0	ACTC1	Actin, alpha cardia	100.00	0.00
TRINITY_sp|P0103	CST3	Cystatin-C OS=Homo	100.00	0.00
TRINITY_sp|P6273	ACTA2	Actin, aortic smoot	100.00	0.00
TRINITY_sp|P1271	Fabp1	Fatty acid-binding	100.00	0.00
TRINITY_sp|Q0072	Rbp4	Retinol-binding pro	100.00	0.00
TRINITY_sp|P5114	RAB7A	Ras-related protein	100.00	0.00
TRINITY_sp|P2358	EIF4B	Eukaryotic translat	100.00	0.00
TRINITY_sp|P0867	VIM	Vimentin OS=Homo sa	100.00	0.00
TRINITY_sp|Q9CR5	Rpl14	60S ribosomal prote	100.00	0.00
TRINITY_sp|P0986	HNRNPA1	Heterogeneous nucle	100.00	0.00
TRINITY_sp|Q2KH7	RPS27	40S ribosomal prote	100.00	0.00
TRINITY_sp|Q969X	ERGIC1	Endoplasmic reticul	100.00	0.00
TRINITY_sp|P1933	NCL	Nucleolin OS=Homo s	100.00	0.00
TRINITY_sp|Q9BQ6	C19orf43	Uncharacterized pro	100.00	0.00
TRINITY_sp|A1A4B	U2AF1	Splicing factor U2A	100.00	0.00
TRINITY_sp|Q1503	SNX17	Sorting nexin-17 OS	100.00	0.00
TRINITY_sp|P1383	Rlc-a	Myosin regulatory l	100.00	0.00
TRINITY_sp|P2403	-	Myosin regulatory l	100.00	0.00
TRINITY_sp|P0086	Odc1	Ornithine decarboxy	100.00	0.00
TRINITY_sp|P9735	Rps3a	40S ribosomal prote	100.00	0.00
TRINITY_sp|Q56JV	RPS3A	40S ribosomal prote	100.00	0.00
TRINITY_sp|P6017	TPI1	Triosephosphate iso	100.00	0.00
TRINITY_sp|Q9CQ6	Uqcrq	Cytochrome b-c1 com	100.00	0.00
TRINITY_sp|P1354	eef1as	Elongation factor 1	100.00	0.00
TRINITY_sp|P1354	eef1as	Elongation factor 1	100.00	0.00
TRINITY_sp|P2155	Eno3	Beta-enolase OS=Mus	100.00	0.00
TRINITY_sp|Q9CQ6	Uqcrq	Cytochrome b-c1 com	100.00	0.00
TRINITY_sp|Q1497	KPNB1	Importin subunit be	100.00	0.00
TRINITY_sp|P6287	RBX1	E3 ubiquitin-protei	100.00	0.00
TRINITY_sp|Q6242	Ndufa4	Cytochrome c oxidas	100.00	0.00

TRINITY_sp|P1812	RPL7	60S ribosomal prote	100.00	0.00
TRINITY_sp|Q2HJ(	HNRNPA2I	Heterogeneous nucle	100.00	0.00
TRINITY_sp|Q1654	CDC37	Hsp90 co-chaperone	100.00	0.00
TRINITY_sp|P1414	Rpl7	60S ribosomal prote	100.00	0.00
TRINITY_sp|Q2KJI	CKS2	Cyclin-dependent ki	100.00	0.00
TRINITY_sp|P094I	Pgk1	Phosphoglycerate ki	100.00	0.00
TRINITY_sp|Q921I	Tf	Serotransferrin OS=	100.00	0.00
TRINITY_sp|Q921I	Tf	Serotransferrin OS=	100.00	0.00
TRINITY_sp|Q921I	Tf	Serotransferrin OS=	100.00	0.00
TRINITY_sp|Q1334	EIF3I	Eukaryotic translat	100.00	0.00
TRINITY_sp|Q5E9(	EIF3I	Eukaryotic translat	100.00	0.00
TRINITY_sp|P0033	LDHA	L-lactate dehydroge	100.00	0.00
TRINITY_sp|Q3T0S	RPL8	60S ribosomal prote	100.00	0.00
TRINITY_sp|P0613	UROD	Uroporphyrinogen de	100.00	0.00
TRINITY_sp|Q3T0S	RPL8	60S ribosomal prote	100.00	0.00
TRINITY_sp|P7008	H4-VIII	Histone H4 type VII	100.00	0.00
TRINITY_sp|P7008	H4-VIII	Histone H4 type VII	100.00	0.00
TRINITY_sp|P7008	H4-VIII	Histone H4 type VII	100.00	0.00
TRINITY_sp|Q3T0\	RPS11	40S ribosomal prote	100.00	0.00
TRINITY_sp|Q3T0\	RPS11	40S ribosomal prote	100.00	0.00
TRINITY_sp|Q32PI	EEF1A2	Elongation factor 1	100.00	0.00
TRINITY_sp|P1868	HSPD1	60 kDa heat shock p	100.00	0.00
TRINITY_sp|P681(	EEF1A1	Elongation factor 1	100.00	0.00
TRINITY_sp|Q9083	EEF1A	Elongation factor 1	100.00	0.00
TRINITY_sp|P0773	PFN1	Profilin-1 OS=Homo	100.00	0.00
TRINITY_sp|O0054	PES1	Pescadillo homolog	100.00	0.00
TRINITY_sp|P1512	AKR1B1	Aldose reductase OS	100.00	0.00
TRINITY_sp|P0183	-	Ig kappa chain C re	100.00	0.00
TRINITY_sp|Q76I8	RPS15A	40S ribosomal prote	100.00	0.00
TRINITY_sp|Q76I8	RPS15A	40S ribosomal prote	100.00	0.00
TRINITY_sp|P0538	RPLP0	60S acidic ribosoma	100.00	0.00
TRINITY_sp|Q3SZ5	EIF4A1	Eukaryotic initiati	100.00	0.00
TRINITY_sp|P6325	ACTG1	Actin, cytoplasmic	100.00	0.00
TRINITY_sp|Q3T12	EIF3D	Eukaryotic translat	100.00	0.00
TRINITY_sp|P8194	-	Tubulin alpha-1B ch	100.00	0.00
TRINITY_sp|Q9Y2S	POLR1D	DNA-directed RNA po	100.00	0.00
TRINITY_sp|P8194	-	Tubulin alpha-1B ch	100.00	0.00
TRINITY_sp|P631(	YWHAZ	14-3-3 protein zeta	100.00	0.00
TRINITY_sp|Q3SZI	SNRPD2	Small nuclear ribon	100.00	0.00
TRINITY_sp|Q9D37	Ephx1	Epoxide hydrolase 1	100.00	0.00
TRINITY_sp|P0C67	OST4	Dolichyl-diphosphoo	100.00	0.00
TRINITY_sp|Q76L\	HSP90AB1	Heat shock protein	100.00	0.00
TRINITY_sp|Q8VDI	Atp1a1	Sodium/potassium-tr	100.00	0.00
TRINITY_sp|P0502	ATP1A1	Sodium/potassium-tr	100.00	0.00
TRINITY_sp|P0502	ATP1A1	Sodium/potassium-tr	100.00	0.00
TRINITY_sp|P1517	GSPT1	Eukaryotic peptide	100.00	0.00
TRINITY_sp|Q060(	YBX1	Nuclease-sensitive	100.00	0.00
TRINITY_sp|P678(	YBX1	Nuclease-sensitive	100.00	0.00
TRINITY_sp|Q9M6I	SAMDC	S-adenosylmethionin	100.00	0.00
TRINITY_sp|P1685	Gapdh	Glyceraldehyde-3-ph	100.00	0.00
TRINITY_sp|P0952	Fth1	Ferritin heavy chai	100.00	0.00
TRINITY_sp|P082(	FTH	Ferritin heavy chai	100.00	0.00
TRINITY_sp|P0279	FTH1	Ferritin heavy chai	100.00	0.00
TRINITY_sp|P044(	GAPDH	Glyceraldehyde-3-ph	100.00	0.00
TRINITY_sp|P089(	Lyz2	Lysozyme C-2 OS=Mus	100.00	0.00
TRINITY_sp|P0DN3	Ndufb1	NADH dehydrogenase	100.00	0.00

TRINITY_sp|P003	GAPDH	Glyceraldehyde-3-ph	100.00	0.00
TRINITY_sp|P192	hsp-3	Heat shock 70 kDa p	100.00	0.00
TRINITY_sp|P064	PTMA	Prothymosin alpha O	100.00	0.00
TRINITY_sp|P504	SERPINH	Serpin H1 OS=Homo s	100.00	0.00
TRINITY_sp|Q016	SLC7A5	Large neutral amino	100.00	0.00
TRINITY_sp|Q132	SRSF5	Serine/arginine-ric	100.00	0.00
TRINITY_sp|P511	RAB5C	Ras-related protein	100.00	0.00
TRINITY_sp|P511	RAB5C	Ras-related protein	100.00	0.00
TRINITY_sp|P176	CAPN2	Calpain-2 catalytic	100.00	0.00
TRINITY_sp|A6H7	RPS7	40S ribosomal prote	100.00	0.00
TRINITY_sp|Q8IV	MT-RNR2	Humanin OS=Homo sap	100.00	0.00
TRINITY_sp|A6H7	RPS7	40S ribosomal prote	100.00	0.00
TRINITY_sp|P619	SUMO2	Small ubiquitin-rel	100.00	0.00
TRINITY_sp|Q150	PDIA6	Protein disulfide-i	100.00	0.00
TRINITY_sp|Q9CX	Ndufs4	NADH dehydrogenase	100.00	0.00
TRINITY_sp|P024	COL1A1	Collagen alpha-1(I)	100.00	0.00
TRINITY_sp|P402	Cd9	CD9 antigen OS=Mus	100.00	0.00
TRINITY_sp|Q3T1	TMED9	Transmembrane emp24	100.00	0.00
TRINITY_sp|Q9CQ	Sdhb	Succinate dehydroge	100.00	0.00
TRINITY_sp|Q9CQ	Sdhb	Succinate dehydroge	100.00	0.00
TRINITY_sp|Q8JF	EIF4A2	Eukaryotic initiati	100.00	0.00
TRINITY_sp|Q3SZ	EIF4A2	Eukaryotic initiati	100.00	0.00
TRINITY_sp|P397	FEN1	Flap endonuclease 1	100.00	0.00
TRINITY_sp|P479	Rpl6	60S ribosomal prote	100.00	0.00
TRINITY_sp|Q66P	ARL6IP4	ADP-ribosylation fa	100.00	0.00
TRINITY_sp|O776	JUN	Transcription facto	100.00	0.00
TRINITY_sp|P128	ACTN1	Alpha-actinin-1 OS=	100.00	0.00
TRINITY_sp|P973	Psme1	Proteasome activato	100.00	0.00
TRINITY_sp|Q3V5	psbA	Photosystem II prot	100.00	0.00
TRINITY_sp|Q3V5	psbA	Photosystem II prot	100.00	0.00
TRINITY_sp|P114	Fabp3	Fatty acid-binding	100.00	0.00
TRINITY_sp|Q6EW	EIF5A	Eukaryotic translat	100.00	0.00
TRINITY_sp|Q3ZC	SNRPD1	Small nuclear ribon	100.00	0.00
TRINITY_sp|P453	Akr1b1	Aldose reductase OS	100.00	0.00
TRINITY_sp|P055	ITGB1	Integrin beta-1 OS=	100.00	0.00
TRINITY_sp|O975	GDI1	Rab GDP dissociatio	100.00	0.00
TRINITY_sp|P132	SPARC	SPARC OS=Bos taurus	100.00	0.00
TRINITY_sp|O043	TUBB	Tubulin beta chain	100.00	0.00
TRINITY_sp|P179	Pcna	Proliferating cell	100.00	0.00
TRINITY_sp|P263	RPL13	60S ribosomal prote	100.00	0.00
TRINITY_sp|P160	Lgals1	Galectin-1 OS=Mus m	100.00	0.00
TRINITY_sp|P093	LGALS1	Galectin-1 OS=Homo	100.00	0.00
TRINITY_sp|P612	PABPC1	Polyadenylate-bindi	100.00	0.00
TRINITY_sp|P263	RPL13	60S ribosomal prote	100.00	0.00
TRINITY_sp|Q4R7	GNB2L1	Guanine nucleotide-	100.00	0.00
TRINITY_sp|P612	PABPC1	Polyadenylate-bindi	100.00	0.00
TRINITY_sp|P122	CKB	Creatine kinase B-t	100.00	0.00
TRINITY_sp|A6QL	RPS9	40S ribosomal prote	100.00	0.00
TRINITY_sp|A6QL	RPS9	40S ribosomal prote	100.00	0.00
TRINITY_sp|A6QL	RPS9	40S ribosomal prote	100.00	0.00
TRINITY_sp|P032	-	Early E1A protein O	100.00	0.00
TRINITY_sp|Q3ZB	SHFM1	26S proteasome comp	100.00	0.00
TRINITY_sp|Q32P	SNRPC	U1 small nuclear ri	100.00	0.00
TRINITY_sp|P067	ENO1	Alpha-enolase OS=Ho	100.00	0.00
TRINITY_sp|P171	Eno1	Alpha-enolase OS=Mu	100.00	0.00
TRINITY_sp|P293	Pabpc1	Polyadenylate-bindi	100.00	0.00

TRINITY_sp|Q5E9[illegible]	RPS8	40S ribosomal prote	100.00	0.00
TRINITY_sp|Q5E9[illegible]	RPS8	40S ribosomal prote	100.00	0.00
TRINITY_sp|P433[illegible]	TPT1	Translationally-con	100.00	0.00
TRINITY_sp|Q99L[illegible]	Ndufa10	NADH dehydrogenase	100.00	0.00
TRINITY_sp|O952[illegible]	NDUFA10	NADH dehydrogenase	100.00	0.00
TRINITY_sp|P607[illegible]	ACTB	Actin, cytoplasmic	100.00	0.00
TRINITY_sp|P109[illegible]	Act5C	Actin-5C OS=Drosoph	100.00	0.00
TRINITY_sp|P184[illegible]	Cyr61	Protein CYR61 OS=Mu	100.00	0.00
TRINITY_sp|Q151[illegible]	NOMO1	Nodal modulator 1 O	100.00	0.00
TRINITY_sp|Q32P[illegible]	POLR2F	DNA-directed RNA po	100.00	0.00
TRINITY_sp|P509[illegible]	CCT4	T-complex protein 1	100.00	0.00
TRINITY_sp|P633[illegible]	TMSB10	Thymosin beta-10 OS	100.00	0.00
TRINITY_sp|Q3ZC[illegible]	METAP2	Methionine aminopep	100.00	0.00
TRINITY_sp|Q3ZC[illegible]	EIF3L	Eukaryotic translat	100.00	0.00
TRINITY_sp|Q058[illegible]	Fabp5	Fatty acid-binding	100.00	0.00
TRINITY_sp|P115[illegible]	Mup2	Major urinary prote	100.00	0.00
TRINITY_sp|Q9YH[illegible]	HMGB1	High mobility group	100.00	0.00
TRINITY_sp|Q98T[illegible]	RPL39	60S ribosomal prote	100.00	0.00
TRINITY_sp|Q3T0[illegible]	RPL39	60S ribosomal prote	100.00	0.00
TRINITY_sp|P141[illegible]	MIF	Macrophage migratio	100.00	0.00
TRINITY_sp|Q029[illegible]	MIF	Macrophage migratio	100.00	0.00
TRINITY_sp|P348[illegible]	Mif	Macrophage migratio	100.00	0.00
TRINITY_sp|P348[illegible]	SHMT2	Serine hydroxymethy	100.00	0.00
TRINITY_sp|P179[illegible]	PSMC3	26S protease regula	100.00	0.00
TRINITY_sp|P355[illegible]	MYH9	Myosin-9 OS=Homo sa	100.00	0.00
TRINITY_sp|P128[illegible]	-	Probable Pol polypr	100.00	0.00
TRINITY_sp|Q922[illegible]	Ubxn1	UBX domain-containi	100.00	0.00
TRINITY_sp|O149[illegible]	HNRNPDL	Heterogeneous nucle	100.00	0.00
TRINITY_sp|Q9DE[illegible]	-	Thymosin beta-15A h	100.00	0.00
TRINITY_sp|O551[illegible]	Sap18	Histone deacetylase	100.00	0.00
TRINITY_sp|Q994[illegible]	PFDN5	Prefoldin subunit 5	100.00	0.00
TRINITY_sp|P0CG[illegible]	UBB	Polyubiquitin-B OS=	100.00	0.00
TRINITY_sp|P0CG[illegible]	UBB	Polyubiquitin-B OS=	100.00	0.00
TRINITY_sp|Q6NZ[illegible]	MZT2B	Mitotic-spindle org	100.00	0.00
TRINITY_sp|Q9M4[illegible]	RPS10	30S ribosomal prote	100.00	0.00
TRINITY_sp|Q3ZB[illegible]	SNRPG	Small nuclear ribon	100.00	0.00
TRINITY_sp|P479[illegible]	RPL29	60S ribosomal prote	100.00	0.00
TRINITY_sp|P479[illegible]	Rpl29	60S ribosomal prote	100.00	0.00
TRINITY_sp|Q56J[illegible]	RPS25	40S ribosomal prote	100.00	0.00
TRINITY_sp|Q56J[illegible]	RPS25	40S ribosomal prote	100.00	0.00
TRINITY_sp|Q3SZ[illegible]	THOC7	THO complex subunit	100.00	0.00
TRINITY_sp|Q9CY[illegible]	Tecr	Very-long-chain eno	100.00	0.00
TRINITY_sp|A6X9[illegible]	Itih4	Inter alpha-trypsin	100.00	0.00
TRINITY_sp|Q9DB[illegible]	Ehhadh	Peroxisomal bifunct	100.00	0.00
TRINITY_sp|P704[illegible]	Ucp2	Mitochondrial uncou	100.00	0.00
TRINITY_sp|P332[illegible]	Cyp2f2	Cytochrome P450 2F2	100.00	0.00
TRINITY_sp|Q9JJ[illegible]	Rpl38	60S ribosomal prote	100.00	0.00
TRINITY_sp|P047[illegible]	PEP1	Phosphoenolpyruvate	100.00	0.00
TRINITY_sp|Q9WV[illegible]	Cxcl15	C-X-C motif chemoki	100.00	0.00
TRINITY_sp|Q71U[illegible]	RPS27L	40S ribosomal prote	100.00	0.00
TRINITY_sp|P025[illegible]	CRYAA	Alpha-crystallin A	100.00	0.00
TRINITY_sp|P235[illegible]	EIF4B	Eukaryotic translat	100.00	0.00
TRINITY_sp|P612[illegible]	DYNLL1	Dynein light chain	100.00	0.00
TRINITY_sp|Q2KJ[illegible]	TUBB5	Tubulin beta-5 chai	100.00	0.00
TRINITY_sp|P622[illegible]	RPS14	40S ribosomal prote	100.00	0.00
TRINITY_sp|Q2KJ[illegible]	TUBB5	Tubulin beta-5 chai	100.00	0.00

TRINITY_sp|P622(	RPS14	40S ribosomal prote	100.00	0.00
TRINITY_sp|P622(	RPS14	40S ribosomal prote	100.00	0.00
TRINITY_sp|Q9JJW	Myoz2	Myozenin-2 OS=Mus m	100.00	0.00
TRINITY_sp|Q0551	SRSF11	Serine/arginine-ric	100.00	0.00
TRINITY_sp|P567(	PSMD2	26S proteasome non-	100.00	0.00
TRINITY_sp|P4974	ACADVL	Very long-chain spe	100.00	0.00
TRINITY_sp|P1461	PKM	Pyruvate kinase PKM	100.00	0.00
TRINITY_sp|P0054	PKM	Pyruvate kinase PKM	100.00	0.00
TRINITY_sp|P5248	Pkm	Pyruvate kinase PKM	100.00	0.00
TRINITY_sp|A5D78	CSE1L	Exportin-2 OS=Bos t	100.00	0.00
TRINITY_sp|P683(	TUBA1A	Tubulin alpha-1A ch	100.00	0.00
TRINITY_sp|P683(	TUBA1A	Tubulin alpha-1A ch	100.00	0.00
TRINITY_sp|Q9CQ2	Ndufb3	NADH dehydrogenase	100.00	0.00
TRINITY_sp|P0CG7	ubi4	Polyubiquitin OS=Sc	100.00	0.00
TRINITY_sp|P0CG7	ubqD	Polyubiquitin-D OS=	100.00	0.00
TRINITY_sp|P0DM4	act-1	Actin-1 OS=Caenorha	100.00	0.00
TRINITY_sp|P5347	-	Actin, cytoplasmic	100.00	0.00
TRINITY_sp|P0DM4	act-1	Actin-1 OS=Caenorha	100.00	0.00
TRINITY_sp|P0674	NPM1	Nucleophosmin OS=Ho	100.00	0.00
TRINITY_sp|Q6193	Npm1	Nucleophosmin OS=Mu	100.00	0.00
TRINITY_sp|Q9H77	DCTPP1	dCTP pyrophosphatas	100.00	0.00
TRINITY_sp|P5588	EIF3B	Eukaryotic translat	100.00	0.00
TRINITY_sp|P1115	PPDK1	Pyruvate, phosphate	100.00	0.00
TRINITY_sp|P2664	EEF1G	Elongation factor 1	100.00	0.00
TRINITY_sp|Q9FF(	At5g2288	Histone H2B.10 OS=A	100.00	0.00
TRINITY_sp|Q8BJM	Tyw1	S-adenosyl-L-methio	100.00	0.00
TRINITY_sp|P1925	Rpl13a	60S ribosomal prote	100.00	0.00
TRINITY_sp|P4042	RPL13A	60S ribosomal prote	100.00	0.00
TRINITY_sp|P6814	acta1a	Actin, alpha skelet	100.00	0.00
TRINITY_sp|P3178	Dbi	Acyl-CoA-binding pr	100.00	0.00
TRINITY_sp|P0768	HEXB	Beta-hexosaminidase	100.00	0.00
TRINITY_sp|P0183	IGKC	Ig kappa chain C re	100.00	0.00
TRINITY_sp|Q0542	Cyp2e1	Cytochrome P450 2E1	100.00	0.00
TRINITY_sp|P622(	YWHAE	14-3-3 protein epsi	100.00	0.00
TRINITY_sp|P200(	Tmsb4x	Thymosin beta-4 OS=	100.00	0.00
TRINITY_sp|Q3T08	RPL11	60S ribosomal prote	100.00	0.00
TRINITY_sp|Q5ZLN	PGAM1	Phosphoglycerate mu	100.00	0.00
TRINITY_sp|P072(	GPX1	Glutathione peroxid	100.00	0.00
TRINITY_sp|P1135	Gpx1	Glutathione peroxid	100.00	0.00
TRINITY_sp|P567(	PSMD2	26S proteasome non-	100.00	0.00
TRINITY_sp|Q3ZC5	ACTN2	Alpha-actinin-2 OS=	100.00	0.00
TRINITY_sp|P2269	UQCRC2	Cytochrome b-c1 com	100.00	0.00
TRINITY_sp|P8031	Cct5	T-complex protein 1	100.00	0.00
TRINITY_sp|P209(	-	Actin OS=Volvox car	100.00	0.00
TRINITY_sp|P1978	Mgp	Matrix Gla protein	100.00	0.00
TRINITY_sp|P504(	Sftpb	Pulmonary surfactan	100.00	0.00
TRINITY_sp|P504(	Sftpb	Pulmonary surfactan	100.00	0.00
TRINITY_sp|P973(	Bpifa1	BPI fold-containing	100.00	0.00
TRINITY_sp|O1537	HDAC3	Histone deacetylase	100.00	0.00
TRINITY_sp|P0862	TXN	Thioredoxin OS=Gall	100.00	0.00
TRINITY_sp|P2081	CAST	Calpastatin OS=Homo	100.00	0.00
TRINITY_sp|Q2HJ2	MAP1LC3A	Microtubule-associa	100.00	0.00
TRINITY_sp|Q6PBI	rps28	40S ribosomal prote	100.00	0.00
TRINITY_sp|Q9BZ3	SLC25A39	Solute carrier fami	100.00	0.00
TRINITY_sp|P2015	Vim	Vimentin OS=Mus mus	100.00	0.00
TRINITY_sp|P1059	TXN	Thioredoxin OS=Homo	100.00	0.00

TRINITY_sp|Q5T65	MRPL2	39S ribosomal prote	100.00	0.00
TRINITY_sp|P6227	RPS29	40S ribosomal prote	100.00	0.00
TRINITY_sp|Q6139	Pcolce	Procollagen C-endop	100.00	0.00
TRINITY_sp|A5YK[illegible]	CNOT1	CCR4-NOT transcript	100.00	0.00
TRINITY_sp|Q0MQ[illegible]	NDUFB2	NADH dehydrogenase	100.00	0.00
TRINITY_sp|P5637	Mp68	6.8 kDa mitochondri	100.00	0.00
TRINITY_sp|Q0618	Atp5i	ATP synthase subuni	100.00	0.00
TRINITY_sp|Q0885	Cd36	Platelet glycoprote	100.00	0.00
TRINITY_sp|P3492	Apoc1	Apolipoprotein C-I	100.00	0.00
TRINITY_sp|Q9259	NDRG1	Protein NDRG1 OS=Ho	100.00	0.00
TRINITY_sp|A1L5A	ADRM1	Proteasomal ubiquit	100.00	0.00
TRINITY_sp|Q3ZC5	ACTN2	Alpha-actinin-2 OS=	100.00	0.00
TRINITY_sp|P4302	Cox6a2	Cytochrome c oxidas	100.00	0.00
TRINITY_sp|P0121	CGA	Glycoprotein hormon	100.00	0.00
TRINITY_sp|P3204	RPL37A	60S ribosomal prote	100.00	0.00
TRINITY_sp|Q56JV	RPS26	40S ribosomal prote	100.00	0.00
TRINITY_sp|Q32L5	GRINA	Protein lifeguard 1	100.00	0.00
TRINITY_sp|P9737	Psme2	Proteasome activato	100.00	0.00
TRINITY_sp|O8905	Coro1a	Coronin-1A OS=Mus m	100.00	0.00
TRINITY_sp|P0996	LTA4H	Leukotriene A-4 hyd	100.00	0.00
TRINITY_sp|P0771	CTSL	Cathepsin L1 OS=Hom	100.00	0.00
TRINITY_sp|P0726	-	Serralysin OS=Serra	100.00	0.00
TRINITY_sp|P3489	SHMT2	Serine hydroxymethy	100.00	0.00
TRINITY_sp|P2879	GRN	Granulins OS=Homo s	100.00	0.00
TRINITY_sp|Q56JV	RPS26	40S ribosomal prote	100.00	0.00
TRINITY_sp|Q9UB[illegible]	ATXN10	Ataxin-10 OS=Homo s	100.00	0.00
TRINITY_sp|O8883	Cyp4a10	Cytochrome P450 4A1	100.00	0.00
TRINITY_sp|O0048	NDUFA4	Cytochrome c oxidas	100.00	0.00
TRINITY_sp|Q920[illegible]	Scgb3a2	Secretoglobin famil	100.00	0.00
TRINITY_sp|Q32P[illegible]	RPL38	60S ribosomal prote	100.00	0.00
TRINITY_sp|Q0MQ[illegible]	NDUFA11	NADH dehydrogenase	100.00	0.00
TRINITY_sp|Q9UH[illegible]	SEPT9	Septin-9 OS=Homo sa	100.00	0.00
TRINITY_sp|P0185	-	T-cell receptor bet	100.00	0.00
TRINITY_sp|Q24JY	RPL23A	60S ribosomal prote	100.00	0.00
TRINITY_sp|O7579	RNASEH2[illegible]	Ribonuclease H2 sub	100.00	0.00
TRINITY_sp|P5639	Cox7a1	Cytochrome c oxidas	100.00	0.00
TRINITY_sp|Q9NX4	OCIAD1	OCIA domain-contain	100.00	0.00
TRINITY_sp|P3053	TSPO	Translocator protei	100.00	0.00
TRINITY_sp|P6227	RPS29	40S ribosomal prote	100.00	0.00
TRINITY_sp|P0730	Ttr	Transthyretin OS=Mu	100.00	0.00
TRINITY_sp|Q96A7	KIAA119[illegible]	Putative monooxygen	100.00	0.00
TRINITY_sp|P1239	Ctla2a	Protein CTLA-2-alph	100.00	0.00
TRINITY_sp|P1307	COX4I1	Cytochrome c oxidas	100.00	0.00
TRINITY_sp|Q1315	HNRNPA0	Heterogeneous nucle	100.00	0.00
TRINITY_sp|Q6PD[illegible]	Ppp2r5a	Serine/threonine-pr	100.00	0.00
TRINITY_sp|P4878	Tnni3	Troponin I, cardiac	100.00	0.00
TRINITY_sp|P1063	Txn	Thioredoxin OS=Mus	100.00	0.00
TRINITY_sp|O1523	NDUFA1	NADH dehydrogenase	100.00	0.00
TRINITY_sp|P3172	S100a9	Protein S100-A9 OS=	100.00	0.00
TRINITY_sp|P0DJ[illegible]	PET100	Protein PET100 homo	100.00	0.00
TRINITY_sp|Q5E9[illegible]	PSMC2	26S protease regula	100.00	0.00
TRINITY_sp|P1328	IFI30	Gamma-interferon-in	100.00	0.00
TRINITY_sp|Q8BW[illegible]	Acaa2	3-ketoacyl-CoA thio	100.00	0.00
TRINITY_sp|Q8BW[illegible]	Acaa2	3-ketoacyl-CoA thio	100.00	0.00
TRINITY_sp|P0423	CD74	HLA class II histoc	100.00	0.00
TRINITY_sp|Q9UB[illegible]	CKLF	Chemokine-like fact	100.00	0.00

TRINITY_sp|P561	ATP5J2	ATP synthase subuni	100.00	0.00
TRINITY_sp|P411	RPL13	60S ribosomal prote	100.00	0.00
TRINITY_sp|Q018	PFKP	ATP-dependent 6-pho	100.00	0.00
TRINITY_sp|Q9CX	Tmem50a	Transmembrane prote	100.00	0.00
TRINITY_sp|Q2KI	FHL2	Four and a half LIM	100.00	0.00
TRINITY_sp|Q135	DCTN2	Dynactin subunit 2	100.00	0.00
TRINITY_sp|Q99K	Aco2	Aconitate hydratase	100.00	0.00
TRINITY_sp|P162	MOD1	NADP-dependent mali	100.00	0.00
TRINITY_sp|P098	Apoa2	Apolipoprotein A-II	100.00	0.00
TRINITY_sp|Q56J	RPS28	40S ribosomal prote	100.00	0.00
TRINITY_sp|Q56J	RPS28	40S ribosomal prote	100.00	0.00
TRINITY_sp|Q9H4	CUEDC2	CUE domain-containi	100.00	0.00
TRINITY_sp|Q995	TIMM17A	Mitochondrial impor	100.00	0.00
TRINITY_sp|O958	AIFM1	Apoptosis-inducing	100.00	0.00
TRINITY_sp|P047	PEP1	Phosphoenolpyruvate	100.00	0.00
TRINITY_sp|P216	Gc	Vitamin D-binding p	100.00	0.00
TRINITY_sp|P109	CLU	Clusterin OS=Homo s	100.00	0.00
TRINITY_sp|Q32L	CNN3	Calponin-3 OS=Bos t	100.00	0.00
TRINITY_sp|Q9CQ	Rps21	40S ribosomal prote	100.00	0.00
TRINITY_sp|P287	Grn	Granulins OS=Mus mu	100.00	0.00
TRINITY_sp|Q32P	RPS21	40S ribosomal prote	100.00	0.00
TRINITY_sp|O086	Kng1	Kininogen-1 OS=Mus	100.00	0.00
TRINITY_sp|O551	Rpl35a	60S ribosomal prote	100.00	0.00
TRINITY_sp|O339	ompF	Outer membrane prot	100.00	0.00
TRINITY_sp|P561	Atp5j2	ATP synthase subuni	100.00	0.00
TRINITY_sp|A4QJ	psbA	Photosystem II prot	100.00	0.00
TRINITY_sp|P173	P	Protein P OS=Woodch	100.00	0.00
TRINITY_sp|Q86T	MRPL52	39S ribosomal prote	100.00	0.00
TRINITY_sp|Q9Y6	TACC3	Transforming acidic	100.00	0.00
TRINITY_sp|Q2HJ	OLA1	Obg-like ATPase 1 O	100.00	0.00
TRINITY_sp|P459	Acadm	Medium-chain specif	100.00	0.00
TRINITY_sp|Q71K	Fgl1	Fibrinogen-like pro	100.00	0.00
TRINITY_sp|Q2HJ	PHB2	Prohibitin-2 OS=Bos	100.00	0.00
TRINITY_sp|O947	MTA2	Metastasis-associat	100.00	0.00
TRINITY_sp|P106	MGST1	Microsomal glutathi	100.00	0.00
TRINITY_sp|Q068	Clu	Clusterin OS=Mus mu	100.00	0.00
TRINITY_sp|O351	Atpif1	ATPase inhibitor, m	100.00	0.00
TRINITY_sp|P153	FOLR1	Folate receptor alp	100.00	0.00
TRINITY_sp|Q9Y5	SNX5	Sorting nexin-5 OS=	100.00	0.00
TRINITY_sp|Q644	Cyp2c29	Cytochrome P450 2C2	100.00	0.00
TRINITY_sp|Q3T0	RPS16	40S ribosomal prote	100.00	0.00
TRINITY_sp|Q9QX	Egfl7	Epidermal growth fa	100.00	0.00
TRINITY_sp|P612	PABPC1	Polyadenylate-bindi	100.00	0.00
TRINITY_sp|Q129	ILF3	Interleukin enhance	100.00	0.00
TRINITY_sp|Q9Y3	UTP11	Probable U3 small n	100.00	0.00
TRINITY_sp|Q90Y	rps10	40S ribosomal prote	100.00	0.00
TRINITY_sp|Q057	CRYAB	Alpha-crystallin B	100.00	0.00
TRINITY_sp|P320	Scp2	Non-specific lipid-	100.00	0.00

TRINITY_sp|Q9H3Y	PPDPF	Pancreatic progenit	100.00	0.00
TRINITY_sp|Q3ZC	CCT8	T-complex protein 1	100.00	0.00
TRINITY_sp|P041	Fabp4	Fatty acid-binding	100.00	0.00
TRINITY_sp|Q025	Myh6	Myosin-6 OS=Mus mus	100.00	0.00
TRINITY_sp|P223	UBA1	Ubiquitin-like modi	100.00	0.00
TRINITY_sp|P493	NASP	Nuclear autoantigen	100.00	0.00
TRINITY_sp|P632	Crip1	Cysteine-rich prote	100.00	0.00
TRINITY_sp|Q128	CDC20	Cell division cycle	100.00	0.00
TRINITY_sp|Q96E	DAZAP1	DAZ-associated prot	100.00	0.00
TRINITY_sp|P378	TALDO1	Transaldolase OS=Ho	100.00	0.00
TRINITY_sp|A8YX	-	Uncharacterized pro	100.00	0.00
TRINITY_sp|Q9Y4	HYOU1	Hypoxia up-regulate	100.00	0.00
TRINITY_sp|P243	COX7B	Cytochrome c oxidas	100.00	0.00
TRINITY_sp|P352	RFC2	Replication factor	100.00	0.00
TRINITY_sp|Q3T0	PPP1CA	Serine/threonine-pr	100.00	0.00
TRINITY_sp|P611	Kap	Kidney androgen-reg	100.00	0.00
TRINITY_sp|P509	ANXA11	Annexin A11 OS=Homo	100.00	0.00
TRINITY_sp|Q604	APP	Amyloid beta A4 pro	100.00	0.00
TRINITY_sp|O704	Mybpc3	Myosin-binding prot	100.00	0.00
TRINITY_sp|P009	ASS1	Argininosuccinate s	100.00	0.00
TRINITY_sp|Q643	Tm4sf1	Transmembrane 4 L6	100.00	0.00
TRINITY_sp|P088	RPSA	40S ribosomal prote	99.70	0.00
TRINITY_sp|Q028	RPL6	60S ribosomal prote	99.70	0.00
TRINITY_sp|P024	COL3A1	Collagen alpha-1(II	99.60	0.00
TRINITY_sp|P148	Rplp0	60S acidic ribosoma	99.60	0.00
TRINITY_sp|P162	MOD1	NADP-dependent mali	99.60	0.00
TRINITY_sp|P519	ENO1	Alpha-enolase OS=Ga	99.50	0.00
TRINITY_sp|Q605	Orm1	Alpha-1-acid glycop	99.50	0.00
TRINITY_sp|P095	Myl3	Myosin light chain	99.50	0.00
TRINITY_sp|P974	Rps5	40S ribosomal prote	99.50	0.00
TRINITY_sp|P218	Sftpc	Pulmonary surfactan	99.50	0.00
TRINITY_sp|P276	RPL10	60S ribosomal prote	99.50	0.00
TRINITY_sp|P293	Ftl1	Ferritin light chai	99.50	0.00
TRINITY_sp|Q137	NACA	Nascent polypeptide	99.50	0.00
TRINITY_sp|P479	Rpl13	60S ribosomal prote	99.50	0.00
TRINITY_sp|E9PV	Fga	Fibrinogen alpha ch	99.40	0.00
TRINITY_sp|P543	OAZ1	Ornithine decarboxy	99.40	0.00
TRINITY_sp|Q3T0	RPL8	60S ribosomal prote	99.40	0.00
TRINITY_sp|Q24J	RPL23A	60S ribosomal prote	99.40	0.00
TRINITY_sp|Q24J	RPL23A	60S ribosomal prote	99.40	0.00
TRINITY_sp|P019	Hba	Hemoglobin subunit	99.30	0.00
TRINITY_sp|Q3BB	NBPF8	Putative neuroblast	99.30	0.00
TRINITY_sp|P183	DSTN	Destrin OS=Gallus g	99.30	0.00
TRINITY_sp|P422	GFP	Green fluorescent p	99.30	0.00
TRINITY_sp|P535	-	Actin, cytoplasmic	99.30	0.00
TRINITY_sp|P209	-	Actin OS=Volvox car	99.30	0.00
TRINITY_sp|Q90Y	rps23	40S ribosomal prote	99.30	0.00
TRINITY_sp|Q56J	RPS25	40S ribosomal prote	99.20	0.00
TRINITY_sp|P018	B2m	Beta-2-microglobuli	99.20	0.00
TRINITY_sp|P328	psaD	Photosystem I react	99.20	0.00
TRINITY_sp|Q76I	RPS15A	40S ribosomal prote	99.20	0.00
TRINITY_sp|Q0MQ	NDUFA6	NADH dehydrogenase	99.20	0.00
TRINITY_sp|Q5EA	RPL15	60S ribosomal prote	99.10	0.00
TRINITY_sp|Q56J	RPL31	60S ribosomal prote	99.10	0.00
TRINITY_sp|Q6UZ	rpl35	60S ribosomal prote	99.10	0.00
TRINITY_sp|P026	MYL6	Myosin light polype	99.10	0.00

TRINITY_sp|P097(	rplK	50S ribosomal prote	99.10	0.00
TRINITY_sp|Q9SPI	CRTZ	Beta-carotene 3-hyd	99.10	0.00
TRINITY_sp|Q008(	Serpina1	Alpha-1-antitrypsin	99.10	0.00
TRINITY_sp|P1112	CMD1	Calmodulin OS=Pleur	99.10	0.00
TRINITY_sp|O750(	WDR1	WD repeat-containin	99.00	0.00
TRINITY_sp|Q862I	RPL24	60S ribosomal prote	99.00	0.00
TRINITY_sp|Q3T0I	RPS10	40S ribosomal prote	99.00	0.00
TRINITY_sp|Q68F>	Idh3B	Isocitrate dehydrog	99.00	0.00
TRINITY_sp|Q5E9(	RPS6	40S ribosomal prote	99.00	0.00
TRINITY_sp|P534(	-	Actin OS=Chlamydomo	99.00	0.00
TRINITY_sp|Q54G>	act10	Actin-10 OS=Dictyos	99.00	0.00
TRINITY_sp|P483(	CAT1	Catalase isozyme 1	99.00	0.00
TRINITY_sp|P002(	PETE	Plastocyanin OS=Cuc	98.90	0.00
TRINITY_sp|Q1A7(	-	Dimethyladenosine t	98.90	0.00
TRINITY_sp|P079I	UQCRH	Cytochrome b-c1 com	98.90	0.00
TRINITY_sp|Q015I	CAP1	Adenylyl cyclase-as	98.90	0.00
TRINITY_sp|P114(	TUBB1	Tubulin beta chain	98.90	0.00
TRINITY_sp|Q3MI(	RPL37A	60S ribosomal prote	98.80	0.00
TRINITY_sp|P3242	RPL7A	60S ribosomal prote	98.80	0.00
TRINITY_sp|Q9NR>	MRPL17	39S ribosomal prote	98.80	0.00
TRINITY_sp|P048(	ompA	Outer membrane prot	98.80	0.00
TRINITY_sp|P611(	rps19	40S ribosomal prote	98.80	0.00
TRINITY_sp|P175(	Selenbp1	Selenium-binding pr	98.80	0.00
TRINITY_sp|Q410(	PSBQ1	Oxygen-evolving enh	98.80	0.00
TRINITY_sp|O223(	TUBA1	Tubulin alpha-1 cha	98.80	0.00
TRINITY_sp|Q9MI)	mt-co3	Cytochrome c oxidas	98.80	0.00
TRINITY_sp|P268(	gag	Gag polyprotein OS=	98.80	0.00
TRINITY_sp|P492(	PCK1	Phosphoenolpyruvate	98.80	0.00
TRINITY_sp|P684(	-	Histone H3.1 OS=Bos	98.80	0.00
TRINITY_sp|O043(	TUBB	Tubulin beta chain	98.80	0.00
TRINITY_sp|Q5E9(	RPS8	40S ribosomal prote	98.80	0.00
TRINITY_sp|Q7ZW(	rpl18a	60S ribosomal prote	98.80	0.00
TRINITY_sp|A8G8I	rplJ	50S ribosomal prote	98.80	0.00
TRINITY_sp|P033(	pol	Pol polyprotein OS=	98.70	0.00
TRINITY_sp|P025(	-	Calmodulin OS=Tetra	98.70	0.00
TRINITY_sp|Q9M6I	SAMDC	S-adenosylmethionin	98.70	0.00
TRINITY_sp|O043(	TUBB	Tubulin beta chain	98.70	0.00
TRINITY_sp|Q019(	TAGLN	Transgelin OS=Homo	98.70	0.00
TRINITY_sp|Q32S2	BRD2	Bromodomain-contain	98.60	0.00
TRINITY_sp|Q9CR(	Nsa2	Ribosome biogenesis	98.60	0.00
TRINITY_sp|O245(	SAMDC	S-adenosylmethionin	98.60	0.00
TRINITY_sp|Q9FG2	UBL5	Ubiquitin-like prot	98.60	0.00
TRINITY_sp|O187(	RPS2	40S ribosomal prote	98.60	0.00
TRINITY_sp|P612(	RPL12	60S ribosomal prote	98.60	0.00
TRINITY_sp|Q9M6I	SAMDC	S-adenosylmethionin	98.60	0.00
TRINITY_sp|P233(	-	Polyubiquitin (Frag	98.60	0.00
TRINITY_sp|Q2KH(	RPS27	40S ribosomal prote	98.60	0.00
TRINITY_sp|P563(	ATP5I	ATP synthase subuni	98.60	0.00
TRINITY_sp|A7Z0(	CLINT1	Clathrin interactor	98.60	0.00
TRINITY_sp|Q3SZ(	RPL13A	60S ribosomal prote	98.50	0.00
TRINITY_sp|P700(	rps15	40S ribosomal prote	98.50	0.00
TRINITY_sp|A8G8I	tuf1	Elongation factor T	98.50	0.00
TRINITY_sp|Q7ZV(	rpl27	60S ribosomal prote	98.50	0.00
TRINITY_sp|P319(	HNRNPH1	Heterogeneous nucle	98.50	0.00
TRINITY_sp|A2XJ(	OsI_012(	Chlorophyll a-b bin	98.50	0.00
TRINITY_sp|P047(	CAB25	Chlorophyll a-b bin	98.50	0.00

TRINITY_sp|P123	CAB1R	Chlorophyll a-b bin	98.50	0.00
TRINITY_sp|O738	rps3a	40S ribosomal prote	98.50	0.00
TRINITY_sp|P048	ompA	Outer membrane prot	98.40	0.00
TRINITY_sp|Q9LE	ENO1	Enolase 1 OS=Hevea	98.40	0.00
TRINITY_sp|P082	-	Chlorophyll a-b bin	98.40	0.00
TRINITY_sp|E2RH	RPS3	40S ribosomal prote	98.30	0.00
TRINITY_sp|P128	-	Probable Pol polypr	98.30	0.00
TRINITY_sp|P621	CALM	Calmodulin OS=Anas	98.30	0.00
TRINITY_sp|P628	FAU	40S ribosomal prote	98.30	0.00
TRINITY_sp|Q32P	RPL38	60S ribosomal prote	98.30	0.00
TRINITY_sp|P118	TUBB1	Tubulin beta chain	98.30	0.00
TRINITY_sp|P234	rps20	40S ribosomal prote	98.30	0.00
TRINITY_sp|Q127	ASPH	Aspartyl/asparaginy	98.20	0.00
TRINITY_sp|Q641	Btf3	Transcription facto	98.20	0.00
TRINITY_sp|P191	MYL12A	Myosin regulatory l	98.20	0.00
TRINITY_sp|Q76I	RPS15A	40S ribosomal prote	98.20	0.00
TRINITY_sp|A6QL	RPS9	40S ribosomal prote	98.20	0.00
TRINITY_sp|P208	-	Chlorophyll a-b bin	98.20	0.00
TRINITY_sp|O043	TUBB	Tubulin beta chain	98.20	0.00
TRINITY_sp|P092	TUBA1	Tubulin alpha-1 cha	98.20	0.00
TRINITY_sp|P583	rpl30	60S ribosomal prote	98.20	0.00
TRINITY_sp|P482	TM4SF4	Transmembrane 4 L6	98.20	0.00
TRINITY_sp|Q6Y2	rpl24	60S ribosomal prote	98.10	0.00
TRINITY_sp|P633	TNNC1	Troponin C, slow sk	98.10	0.00
TRINITY_sp|P109	GSPO-B1	Sporamin B OS=Ipomo	98.10	0.00
TRINITY_sp|P627	-	Histone H4 variant	98.10	0.00
TRINITY_sp|Q9SL	-	Phosphoenolpyruvate	98.10	0.00
TRINITY_sp|P066	alphaTub	Tubulin alpha-1 cha	98.00	0.00
TRINITY_sp|P828	-	Histone H4 OS=Olist	98.00	0.00
TRINITY_sp|P828	-	Histone H4 OS=Olist	98.00	0.00
TRINITY_sp|P628	-	Histone H4 OS=Bos t	98.00	0.00
TRINITY_sp|P627	-	Histone H4 variant	98.00	0.00
TRINITY_sp|P378	Tagln	Transgelin OS=Mus m	98.00	0.00
TRINITY_sp|P111	PPDK1	Pyruvate, phosphate	98.00	0.00
TRINITY_sp|Q130	MRPL28	39S ribosomal prote	97.90	0.00
TRINITY_sp|P0CG	UBB	Polyubiquitin-B OS=	97.90	0.00
TRINITY_sp|P627	-	Histone H4 variant	97.90	0.00
TRINITY_sp|P351	-	Ubiquitin-conjugati	97.80	0.00
TRINITY_sp|P082	-	Chlorophyll a-b bin	97.80	0.00
TRINITY_sp|P055	Ly6a	Lymphocyte antigen	97.80	0.00
TRINITY_sp|Q3BA	-	Uncharacterized pro	97.80	0.00
TRINITY_sp|Q008	Serpina1	Alpha-1-antitrypsin	97.80	0.00
TRINITY_sp|Q9CR	Ppid	Peptidyl-prolyl cis	97.80	0.00
TRINITY_sp|Q24J	RPL23A	60S ribosomal prote	97.70	0.00
TRINITY_sp|P792	RPL37	60S ribosomal prote	97.70	0.00
TRINITY_sp|P390	rps6	40S ribosomal prote	97.70	0.00
TRINITY_sp|O575	rpl7a	60S ribosomal prote	97.70	0.00
TRINITY_sp|P0CG	UBB	Polyubiquitin-B OS=	97.70	0.00
TRINITY_sp|Q9DD	Rarres2	Retinoic acid recep	97.70	0.00
TRINITY_sp|P148	Anxa6	Annexin A6 OS=Mus m	97.60	0.00
TRINITY_sp|Q7T3	rpl15	60S ribosomal prote	97.60	0.00
TRINITY_sp|P571	MDH2	Malate dehydrogenas	97.60	0.00
TRINITY_sp|P0CG	UBB	Polyubiquitin-B OS=	97.60	0.00
TRINITY_sp|P0CG	UBB	Polyubiquitin-B OS=	97.60	0.00
TRINITY_sp|Q99P	Arhgdia	Rho GDP-dissociatio	97.50	0.00
TRINITY_sp|P518	HDGF	Hepatoma-derived gr	97.40	0.00

TRINITY_sp|P0714	-	Catalase OS=Ipomoea	97.40	0.00
TRINITY_sp|P1136	pol	Putative Pol polypr	97.40	0.00
TRINITY_sp|P0CG5	UBB	Polyubiquitin-B OS=	97.40	0.00
TRINITY_sp|P2284	-	Histone H3 OS=Acrop	97.40	0.00
TRINITY_sp|P2284	-	Histone H3 OS=Acrop	97.40	0.00
TRINITY_sp|P3414	rac1A	Rho-related protein	97.40	0.00
TRINITY_sp|P6278	-	Histone H4 variant	97.40	0.00
TRINITY_sp|Q9033	-	Myosin heavy chain,	97.30	0.00
TRINITY_sp|P2760	DHAPS-1	Phospho-2-dehydro-3	97.30	0.00
TRINITY_sp|Q3ZBH	RPS20	40S ribosomal prote	97.30	0.00
TRINITY_sp|P2323	petD	Cytochrome b6-f com	97.30	0.00
TRINITY_sp|P4135	-	Tubulin alpha chain	97.30	0.00
TRINITY_sp|Q0571	CRYBB2	Beta-crystallin B2	97.30	0.00
TRINITY_sp|P6128	PABPC1	Polyadenylate-bindi	97.20	0.00
TRINITY_sp|P1807	RPL35A	60S ribosomal prote	97.20	0.00
TRINITY_sp|P1894	MT-ND6	NADH-ubiquinone oxi	97.20	0.00
TRINITY_sp|P3343	Mmp2	72 kDa type IV coll	97.20	0.00
TRINITY_sp|P0335	pol	Pol polyprotein OS=	97.10	0.00
TRINITY_sp|Q90Y9	rps2	40S ribosomal prote	97.10	0.00
TRINITY_sp|A6MZN	GOS2	Protein translation	97.10	0.00
TRINITY_sp|Q9FYV	-	Inositol-3-phosphat	97.10	0.00
TRINITY_sp|Q5E95	RPS8	40S ribosomal prote	97.10	0.00
TRINITY_sp|Q964E	-	Actin, cytoplasmic	97.10	0.00
TRINITY_sp|P6843	-	Histone H3.1 OS=Bos	97.10	0.00
TRINITY_sp|P8422	-	Histone H3.2 OS=Bos	97.10	0.00
TRINITY_sp|P0904	GAPB	Glyceraldehyde-3-ph	97.00	0.00
TRINITY_sp|Q8RX0	RPL37AC	60S ribosomal prote	97.00	0.00
TRINITY_sp|Q0162	IFITM2	Interferon-induced	97.00	0.00
TRINITY_sp|Q56K1	RPLP1	60S acidic ribosoma	97.00	0.00
TRINITY_sp|P4364	-	Elongation factor 1	97.00	0.00
TRINITY_sp|P5349	-	Actin OS=Chlamydomo	97.00	0.00
TRINITY_sp|P4221	GFP	Green fluorescent p	97.00	0.00
TRINITY_sp|Q56K1	RPLP1	60S acidic ribosoma	97.00	0.00
TRINITY_sp|P4233	AKR1C3	Aldo-keto reductase	96.90	0.00
TRINITY_sp|P4289	-	Enolase OS=Ricinus	96.90	0.00
TRINITY_sp|Q3965	PSAL	Photosystem I react	96.90	0.00
TRINITY_sp|Q6154	Ewsr1	RNA-binding protein	96.90	0.00
TRINITY_sp|Q9W6Y	hsc70	Heat shock cognate	96.90	0.00
TRINITY_sp|P1510	-	Glutamine synthetas	96.90	0.00
TRINITY_sp|P0CG5	UBB	Polyubiquitin-B OS=	96.90	0.00
TRINITY_sp|P0CG5	UBB	Polyubiquitin-B OS=	96.90	0.00
TRINITY_sp|P5349	-	Actin OS=Chlamydomo	96.90	0.00
TRINITY_sp|O4238	rps24	40S ribosomal prote	96.80	0.00
TRINITY_sp|Q3BA1	-	Uncharacterized pro	96.80	0.00
TRINITY_sp|P3511	UBC10	Ubiquitin-conjugati	96.70	0.00
TRINITY_sp|Q5E9E	RPL10A	60S ribosomal prote	96.70	0.00
TRINITY_sp|O0037	-	LINE-1 retrotranspo	96.70	0.00
TRINITY_sp|Q0MQI	NDUFB3	NADH dehydrogenase	96.70	0.00
TRINITY_sp|Q5EE0	CENPW	Centromere protein	96.70	0.00
TRINITY_sp|P9333	GAPN	NADP-dependent glyc	96.60	0.00
TRINITY_sp|P2492	EIF-5A2	Eukaryotic translat	96.60	0.00
TRINITY_sp|Q9SSD	PRP8A	Pre-mRNA-processing	96.60	0.00
TRINITY_sp|Q9YGE	rps6	40S ribosomal prote	96.60	0.00
TRINITY_sp|P0CG5	UBB	Polyubiquitin-B OS=	96.60	0.00
TRINITY_sp|P0CG5	UBB	Polyubiquitin-B OS=	96.60	0.00
TRINITY_sp|P6125	RPL26	60S ribosomal prote	96.50	0.00

TRINITY_sp|O491(	EF1	Elongation factor 1	96.50	0.00
TRINITY_sp|P060(	psbD	Photosystem II D2 p	96.50	0.00
TRINITY_sp|P301(	-	Actin-1 OS=Pisum sa	96.50	0.00
TRINITY_sp|P140(	MT-ATP6	ATP synthase subuni	96.40	0.00
TRINITY_sp|Q6PC(	rpl10a	60S ribosomal prote	96.40	0.00
TRINITY_sp|Q6P0\	rpl8	60S ribosomal prote	96.40	0.00
TRINITY_sp|Q90YI	rps7	40S ribosomal prote	96.40	0.00
TRINITY_sp|Q9FG	UBL5	Ubiquitin-like prot	96.40	0.00
TRINITY_sp|P690	EIF-5A1	Eukaryotic translat	96.30	0.00
TRINITY_sp|Q4UD(	TA12105	Heat shock protein	96.30	0.00
TRINITY_sp|P462	FBP	Fructose-1,6-bispho	96.30	0.00
TRINITY_sp|Q5XT	RPL17	60S ribosomal prote	96.20	0.00
TRINITY_sp|P627	-	Histone H4 variant	96.20	0.00
TRINITY_sp|P025	ARDA	Actin, plasmodial i	96.20	0.00
TRINITY_sp|P023	-	Histone H4, major O	96.20	0.00
TRINITY_sp|Q6PB	rps25	40S ribosomal prote	96.10	0.00
TRINITY_sp|Q1A7(	-	Dimethyladenosine t	96.10	0.00
TRINITY_sp|Q429(	-	Phosphoglycerate ki	96.10	0.00
TRINITY_sp|P030	-	Large T antigen OS=	96.10	0.00
TRINITY_sp|P0CG	UBB	Polyubiquitin-B OS=	96.10	0.00
TRINITY_sp|O491(	EF1	Elongation factor 1	96.00	0.00
TRINITY_sp|P0CG	UBB	Polyubiquitin-B OS=	95.90	0.00
TRINITY_sp|O237	-	Elongation factor 2	95.80	0.00
TRINITY_sp|Q9HZ	rpsA	30S ribosomal prote	95.80	0.00
TRINITY_sp|P033	gag-pol	Gag-Pol polyprotein	95.80	0.00
TRINITY_sp|Q9SH	RUB1	Ubiquitin-NEDD8-lik	95.80	0.00
TRINITY_sp|P492	RPL44	60S ribosomal prote	95.80	0.00
TRINITY_sp|Q54G	act10	Actin-10 OS=Dictyos	95.80	0.00
TRINITY_sp|Q9SB	PSBO	Oxygen-evolving enh	95.80	0.00
TRINITY_sp|P178	DDX5	Probable ATP-depend	95.70	0.00
TRINITY_sp|Q431	RPE	Ribulose-phosphate	95.70	0.00
TRINITY_sp|P690	rpl18	60S ribosomal prote	95.70	0.00
TRINITY_sp|P359	Rpl18	60S ribosomal prote	95.70	0.00
TRINITY_sp|P019(	H2-D1	H-2 class I histoco	95.70	0.00
TRINITY_sp|P0A9	cspC	Cold shock-like pro	95.70	0.00
TRINITY_sp|Q56J\	RPS26	40S ribosomal prote	95.70	0.00
TRINITY_sp|P033	gag-pol	Gag-Pol polyprotein	95.60	0.00
TRINITY_sp|P008	rbcL	Ribulose bisphospha	95.60	0.00
TRINITY_sp|Q90Y	rpl36	60S ribosomal prote	95.50	0.00
TRINITY_sp|P097	psbA	Photosystem II prot	95.50	0.00
TRINITY_sp|Q6R0	Gnas	Guanine nucleotide-	95.50	0.00
TRINITY_sp|P003	ALDH1A1	Retinal dehydrogena	95.40	0.00
TRINITY_sp|P824	RPL19	50S ribosomal prote	95.40	0.00
TRINITY_sp|Q90Y	rps3	40S ribosomal prote	95.40	0.00
TRINITY_sp|O575	rpl7a	60S ribosomal prote	95.40	0.00
TRINITY_sp|Q3SZ	RPL32	60S ribosomal prote	95.30	0.00
TRINITY_sp|P082	-	Chlorophyll a-b bin	95.30	0.00
TRINITY_sp|P413	BTU1	Tubulin beta chain	95.30	0.00
TRINITY_sp|P114	TUBB1	Tubulin beta chain	95.30	0.00
TRINITY_sp|P938	RPL10	60S ribosomal prote	95.20	0.00
TRINITY_sp|A0CD	CFAP20	Cilia- and flagella	95.20	0.00
TRINITY_sp|Q90Y(	rpl18a	60S ribosomal prote	95.20	0.00
TRINITY_sp|P258	GAPA1	Glyceraldehyde-3-ph	95.10	0.00
TRINITY_sp|Q3ZC	ACTN2	Alpha-actinin-2 OS=	95.10	0.00
TRINITY_sp|P508	rpl22	60S ribosomal prote	95.10	0.00
TRINITY_sp|P108	BETA-TT	Tubulin beta chain	95.10	0.00

TRINITY_sp|P2752	LHCA4	Chlorophyll a-b bin	95.00	0.00
TRINITY_sp|P134	HPR-A	Glycerate dehydroge	95.00	0.00
TRINITY_sp|P004	MT-CO2	Cytochrome c oxidas	95.00	0.00
TRINITY_sp|Q396	PSAL	Photosystem I react	94.90	0.00
TRINITY_sp|Q90Y	rpl9	60S ribosomal prote	94.90	0.00
TRINITY_sp|Q5Z9	FTSH1	ATP-dependent zinc	94.80	0.00
TRINITY_sp|P132	SPARC	SPARC OS=Bos taurus	94.80	0.00
TRINITY_sp|P121	psaA	Photosystem I P700	94.80	0
TRINITY_sp|Q9MI	mt-co1	Cytochrome c oxidas	94.70	0.00
TRINITY_sp|Q5R7	SLC25A3	Phosphate carrier p	94.70	0.00
TRINITY_sp|P390	RPL3	60S ribosomal prote	94.70	0.00
TRINITY_sp|A0CD	CFAP20	Cilia- and flagella	94.70	0.00
TRINITY_sp|P142	CAB4	Chlorophyll a-b bin	94.70	0.00
TRINITY_sp|Q1KV	tufA	Elongation factor T	94.70	0.00
TRINITY_sp|O237	-	Elongation factor 2	94.60	0.00
TRINITY_sp|Q3T0	RPL17	60S ribosomal prote	94.60	0.00
TRINITY_sp|Q9YI	eef1a	Elongation factor 1	94.60	0.00
TRINITY_sp|P123	PSAG	Photosystem I react	94.50	0.00
TRINITY_sp|P039	MT-ND5	NADH-ubiquinone oxi	94.50	0.00
TRINITY_sp|P298	Hsc70-3	Heat shock 70 kDa p	94.50	0.00
TRINITY_sp|Q9SN	UGE5	UDP-glucose 4-epime	94.50	0.00
TRINITY_sp|Q9FG	UBL5	Ubiquitin-like prot	94.50	0.00
TRINITY_sp|P120	Cox5b	Cytochrome c oxidas	94.40	0.00
TRINITY_sp|P138	-	Chlorophyll a-b bin	94.40	0.00
TRINITY_sp|O049	SODCP	Superoxide dismutas	94.40	0.00
TRINITY_sp|P099	ALDOC	Fructose-bisphospha	94.40	0.00
TRINITY_sp|P208	-	Chlorophyll a-b bin	94.40	0.00
TRINITY_sp|P128	GAPB	Glyceraldehyde-3-ph	94.30	0.00
TRINITY_sp|Q434	TUFA	Elongation factor T	94.30	0.00
TRINITY_sp|P079	HNRNPC	Heterogeneous nucle	94.30	0.00
TRINITY_sp|Q429	-	Phosphoglycerate ki	94.30	0.00
TRINITY_sp|Q1KV	petB	Cytochrome b6 OS=Ac	94.30	0.00
TRINITY_sp|Q8NF	ST13P5	Putative protein FA	94.20	0.00
TRINITY_sp|P226	-	Chlorophyll a-b bin	94.20	0.00
TRINITY_sp|Q9M4	TPI	Triosephosphate iso	94.20	0.00
TRINITY_sp|Q9XJ	RPS9	30S ribosomal prote	94.20	0.00
TRINITY_sp|Q9FP	PSBS	Photosystem II 22 k	94.10	0.00
TRINITY_sp|Q438	-	Ribulose-phosphate	94.10	0.00
TRINITY_sp|P108	psbC	Photosystem II CP43	94.10	0.00
TRINITY_sp|Q58D	SDC2	Syndecan-2 OS=Bos t	94.10	0.00
TRINITY_sp|P189	MT-ND1	NADH-ubiquinone oxi	94.00	0.00
TRINITY_sp|Q9LE	ENO1	Enolase 1 OS=Hevea	93.90	0.00
TRINITY_sp|O494	CCOAOMT	Caffeoyl-CoA O-meth	93.80	0.00
TRINITY_sp|Q134	SF3B2	Splicing factor 3B	93.80	0.00
TRINITY_sp|P189	MT-ND3	NADH-ubiquinone oxi	93.80	0.00
TRINITY_sp|P426	-	14-3-3-like protein	93.80	0.00
TRINITY_sp|P008	Mtatp6	ATP synthase subuni	93.80	0.00
TRINITY_sp|P122	SLC25A6	ADP/ATP translocase	93.70	0.00
TRINITY_sp|A0CD	CFAP20	Cilia- and flagella	93.70	0.00
TRINITY_sp|P792	ATP6V1G	V-type proton ATPas	93.70	0.00
TRINITY_sp|Q90Y	rps21	40S ribosomal prote	93.70	0.00
TRINITY_sp|Q9SQ	lhcA-P4	Chlorophyll a-b bin	93.60	0.00
TRINITY_sp|Q9ZT	RPE	Ribulose-phosphate	93.60	0.00
TRINITY_sp|Q9FK	LFNR1	Ferredoxin--NADP re	93.60	0.00
TRINITY_sp|P189	MT-CO2	Cytochrome c oxidas	93.60	0.00
TRINITY_sp|P792	RPL37	60S ribosomal prote	93.50	0.00

TRINITY_sp|Q9LRI	GLO1	Peroxisomal (S)-2-h	93.50	0.00
TRINITY_sp|Q9M3	RPL17	50S ribosomal prote	93.50	0.00
TRINITY_sp|Q913	eef1g-b	Elongation factor 1	93.50	0.00
TRINITY_sp|P004	MT-CO3	Cytochrome c oxidas	93.50	0.00
TRINITY_sp|Q2F7	gag-pol	Gag-Pol polyprotein	93.50	0.00
TRINITY_sp|Q080	CHSP70	Stromal 70 kDa heat	93.50	0.00
TRINITY_sp|Q9FU	-	Elongation factor 1	93.50	0.00
TRINITY_sp|Q914	rpl13a	60S ribosomal prote	93.40	0.00
TRINITY_sp|P328	psaD	Photosystem I react	93.30	0.00
TRINITY_sp|Q9XI	PBC1	Proteasome subunit	93.30	0.00
TRINITY_sp|P0CH	-	Ubiquitin-60S ribos	93.30	0.00
TRINITY_sp|Q8DR	radA	DNA repair protein	93.30	0.00
TRINITY_sp|Q412	PSAEA	Photosystem I react	93.30	0.00
TRINITY_sp|Q016	LHCA1	Chlorophyll a-b bin	93.20	0.00
TRINITY_sp|P275	CAB8	Chlorophyll a-b bin	93.20	0.00
TRINITY_sp|Q087	PIP1-3	Aquaporin PIP1-3 OS	93.20	0.00
TRINITY_sp|P033	gag-pol	Gag-Pol polyprotein	93.20	0.00
TRINITY_sp|Q5E9	RPS6	40S ribosomal prote	93.20	0.00
TRINITY_sp|A5JS	SERF2	Small EDRK-rich fac	93.20	0.00
TRINITY_sp|Q4VI	CALR	Calreticulin OS=Chl	93.20	0.00
TRINITY_sp|Q7ZW	chmp2a	Charged multivesicu	93.10	0.00
TRINITY_sp|Q9SD	LHC	Chlorophyll a-b bin	93.10	0.00
TRINITY_sp|Q961	mt-co3	Cytochrome c oxidas	93.10	0.00
TRINITY_sp|Q5E9	RPS6	40S ribosomal prote	93.10	0.00
TRINITY_sp|P372	psbB	Photosystem II CP47	93.10	0.00
TRINITY_sp|P499	-	Ferritin, heavy sub	93.10	0.00
TRINITY_sp|P629	PPIA	Peptidyl-prolyl cis	93.10	0.00
TRINITY_sp|P275	CAB215	Chlorophyll a-b bin	93.10	0.00
TRINITY_sp|P456	PA3623	Lipoprotein NlpD/Lp	93.10	0.00
TRINITY_sp|P938	-	Phosphoglucomutase,	93.00	0.00
TRINITY_sp|P274	CAB13	Chlorophyll a-b bin	92.90	0.00
TRINITY_sp|P249	MT-CO1	Cytochrome c oxidas	92.90	0.00
TRINITY_sp|Q431	LOX1.5	Probable linoleate	92.80	0.00
TRINITY_sp|P109	PETH	Ferredoxin--NADP re	92.80	0.00
TRINITY_sp|P478	RPS4	40S ribosomal prote	92.80	0.00
TRINITY_sp|P001	MT-CYB	Cytochrome b OS=Hom	92.80	0.00
TRINITY_sp|P199	-	40S ribosomal prote	92.80	0.00
TRINITY_sp|P089	HTA3	Histone H2A.Z OS=Te	92.80	0.00
TRINITY_sp|P091	psaB	Photosystem I P700	92.80	0
TRINITY_sp|P004	mt-Co3	Cytochrome c oxidas	92.70	0.00
TRINITY_sp|Q9FF	PIP2-4	Probable aquaporin	92.60	0.00
TRINITY_sp|P315	CD4B	ATP-dependent Clp p	92.60	0.00
TRINITY_sp|O485	RPL17	60S ribosomal prote	92.60	0.00
TRINITY_sp|Q020	PSBS	Photosystem II 22 k	92.60	0.00
TRINITY_sp|Q90Y	rps6	40S ribosomal prote	92.60	0.00
TRINITY_sp|P142	cabII-1	Chlorophyll a-b bin	92.60	0.00
TRINITY_sp|P497	petC	Cytochrome b6-f com	92.60	0.00
TRINITY_sp|P691	-	Histone H3 OS=Tetra	92.60	0.00
TRINITY_sp|Q9SI	HSP90-5	Heat shock protein	92.60	0.00
TRINITY_sp|P398	RPL3	60S ribosomal prote	92.50	0.00
TRINITY_sp|O486	ARF1	ADP-ribosylation fa	92.50	0.00
TRINITY_sp|Q1LZ	DDRGK1	DDRGK domain-contai	92.50	0.00
TRINITY_sp|Q395	YPTC4	GTP-binding protein	92.40	0.00
TRINITY_sp|P492	RPS18	40S ribosomal prote	92.30	0.00
TRINITY_sp|P142	cabII-1	Chlorophyll a-b bin	92.30	0.00
TRINITY_sp|Q9FW	HAC12	Histone acetyltrans	92.30	0.00

TRINITY_sp|O228(	RPL38A	60S ribosomal prote	92.30	0.00
TRINITY_sp|Q90Y	rpl32	60S ribosomal prote	92.20	0.00
TRINITY_sp|Q404	PSBO	Oxygen-evolving enh	92.20	0.00
TRINITY_sp|P039(	MT-ND4	NADH-ubiquinone oxi	92.20	0.00
TRINITY_sp|P265	atpA	ATP synthase subuni	92.20	0.00
TRINITY_sp|P003	MT-CO1	Cytochrome c oxidas	92.10	0.00
TRINITY_sp|P305	-	Metallothionein-lik	92.10	0.00
TRINITY_sp|Q8WY(	CHCHD10	Coiled-coil-helix-c	92.10	0.00
TRINITY_sp|Q085(	ACO1	1-aminocyclopropane	92.00	0.00
TRINITY_sp|P527(	trs-1	Threonine--tRNA lig	92.00	0.00
TRINITY_sp|Q080	CHSP70	Stromal 70 kDa heat	91.90	0.00
TRINITY_sp|Q95L	EIF4G2	Eukaryotic translat	91.90	0.00
TRINITY_sp|P258	HSP70	Heat shock 70 kDa p	91.90	0.00
TRINITY_sp|P212	CPN60B1	Chaperonin 60 subun	91.90	0.00
TRINITY_sp|Q6UN	RPL5	60S ribosomal prote	91.80	0.00
TRINITY_sp|Q426	TKT3	Transketolase, chlo	91.80	0.00
TRINITY_sp|Q7RT	PHF5A	PHD finger-like dom	91.80	0.00
TRINITY_sp|Q414	PCM3	Putative calmodulin	91.80	0.00
TRINITY_sp|P038	MT-ND1	NADH-ubiquinone oxi	91.80	0.00
TRINITY_sp|Q074	HSP70	Heat shock 70 kDa p	91.80	0.00
TRINITY_sp|P160	PSBP	Oxygen-evolving enh	91.80	0.00
TRINITY_sp|P003	Mtco1	Cytochrome c oxidas	91.70	0.00
TRINITY_sp|P004(	Mtco2	Cytochrome c oxidas	91.70	0.00
TRINITY_sp|O239	GDCST	Aminomethyltransfer	91.70	0.00
TRINITY_sp|Q9ZS	ABCI8	UPF0051 protein ABC	91.70	0.00
TRINITY_sp|P038	Mtnd3	NADH-ubiquinone oxi	91.70	0.00
TRINITY_sp|Q9FS	MD1	Malate dehydrogenas	91.70	0.00
TRINITY_sp|P038	Mtnd1	NADH-ubiquinone oxi	91.50	0.00
TRINITY_sp|Q396(	IDA4	28 kDa inner dynein	91.40	0.00
TRINITY_sp|P0C1	PETN	Cytochrome b6-f com	91.40	0.00
TRINITY_sp|P208(	-	Chlorophyll a-b bin	91.40	0.00
TRINITY_sp|Q400(	Amz2	Archaemetzincin-2 O	91.30	0.00
TRINITY_sp|P263(	-	Phosphoribulokinase	91.30	0.00
TRINITY_sp|P274	CAB40	Chlorophyll a-b bin	91.30	0.00
TRINITY_sp|Q9SA	CSP41B	Chloroplast stem-lo	91.20	0.00
TRINITY_sp|Q6K5(	GAPC3	Glyceraldehyde-3-ph	91.20	0.00
TRINITY_sp|Q395	-	Dynein 8 kDa light	91.20	0.00
TRINITY_sp|P413	HHT3	Histone H3.3 OS=Tet	91.20	0.00
TRINITY_sp|Q9SY	LHCA3	Photosystem I chlor	91.20	0.00
TRINITY_sp|Q3SY	RPL9	60S ribosomal prote	91.20	0.00
TRINITY_sp|A4XY(	smpB	SsrA-binding protei	91.10	0.00
TRINITY_sp|P194	-	Malate dehydrogenas	91.00	0.00
TRINITY_sp|B7EA	Os08g05(	Puromycin-sensitive	91.00	0.00
TRINITY_sp|O968(	Cdlc2	Dynein light chain	91.00	0.00
TRINITY_sp|P484	TPIP1	Triosephosphate iso	90.90	0.00
TRINITY_sp|P366	-	50S ribosomal prote	90.90	0.00
TRINITY_sp|Q9FJ	RPS3C	40S ribosomal prote	90.90	0.00
TRINITY_sp|P189	MT-ND4	NADH-ubiquinone oxi	90.80	0.00
TRINITY_sp|P463(	RPS28	40S ribosomal prote	90.80	0.00
TRINITY_sp|Q9LR	GLO2	Peroxisomal (S)-2-h	90.70	0.00
TRINITY_sp|Q414	PCM3	Putative calmodulin	90.70	0.00
TRINITY_sp|P038	MT-ND2	NADH-ubiquinone oxi	90.70	0.00
TRINITY_sp|Q412	FLBR	Leghemoglobin reduc	90.60	0.00
TRINITY_sp|Q4QY	rpsa	40S ribosomal prote	90.60	0.00
TRINITY_sp|P039	Mtnd5	NADH-ubiquinone oxi	90.60	0.00
TRINITY_sp|P147	ubqB	Ubiquitin-60S ribos	90.60	0.00

TRINITY_sp|P038	MT-ND3	NADH-ubiquinone oxi	90.60	0.00
TRINITY_sp|P483	CAT1	Catalase isozyme 1	90.60	0.00
TRINITY_sp|Q9SM	-	V-type proton ATPas	90.60	0.00
TRINITY_sp|Q031	PMA4	Plasma membrane ATP	90.50	0.00
TRINITY_sp|O486	GPX6	Probable phospholip	90.50	0.00
TRINITY_sp|P514	Rpl9	60S ribosomal prote	90.40	0.00
TRINITY_sp|P039	Mtnd4	NADH-ubiquinone oxi	90.40	0.00
TRINITY_sp|P529	-	14-3-3-like protein	90.40	0.00
TRINITY_sp|Q7XJ	GAS8	Growth arrest-speci	90.40	0.00
TRINITY_sp|P411	rps11	40S ribosomal prote	90.40	0.00
TRINITY_sp|Q7RT	PHF5A	PHD finger-like dom	90.30	0.00
TRINITY_sp|Q9SL	PSBP	Oxygen-evolving enh	90.20	0.00
TRINITY_sp|Q8LP	ABCE2	ABC transporter E f	90.20	0.00
TRINITY_sp|O202	-	Transketolase, chlo	90.20	0.00
TRINITY_sp|Q8W1	Cyp1	Peptidyl-prolyl cis	90.10	0.00
TRINITY_sp|P505	H2B	Histone H2B.1 OS=Ch	90.10	0.00
TRINITY_sp|P089	HTB1	Histone H2B.1 OS=Te	90.10	0.00
TRINITY_sp|Q3T0	RPL14	60S ribosomal prote	90.00	0.00
TRINITY_sp|P493	CAT1	Catalase isozyme 1	90.00	0.00
TRINITY_sp|P189	MT-ND2	NADH-ubiquinone oxi	90.00	0.00
TRINITY_sp|P235	-	2-methyl-6-phytyl-1	90.00	0.00
TRINITY_sp|Q7X9	RCA2	Ribulose bisphospha	90.00	0.00
TRINITY_sp|Q6ZD	SDH1	Succinate dehydroge	90.00	0.00
TRINITY_sp|A0CC	Ppx4	Serine/threonine-pr	90.00	0.00
TRINITY_sp|O817	IDH3	Isocitrate dehydrog	90.00	0.00
TRINITY_sp|Q9MB	DHC10	Dynein-1-beta heavy	90.00	0.00
TRINITY_sp|Q90Y	rpl35a	60S ribosomal prote	90.00	0.00
TRINITY_sp|P0DI	At2g300	PHD finger-like dom	89.90	0.00
TRINITY_sp|P478	rpl27a	60S ribosomal prote	89.90	0.00
TRINITY_sp|Q9XF	PIP1-2	Aquaporin PIP1-2 OS	89.90	0.00
TRINITY_sp|P265	GAPC	Glyceraldehyde-3-ph	89.90	0.00
TRINITY_sp|Q032	RBG7	Glycine-rich RNA-bi	89.80	0.00
TRINITY_sp|P505	H2A-II	Histone H2A OS=Chla	89.80	0.00
TRINITY_sp|P287	-	Formate--tetrahydro	89.70	0.00
TRINITY_sp|P294	TPT	Triose phosphate/ph	89.70	0.00
TRINITY_sp|P096	CTSL	Cathepsin L1 (Fragm	89.70	0.00
TRINITY_sp|Q426	TKT3	Transketolase, chlo	89.70	0.00
TRINITY_sp|O246	AS	Asparagine syntheta	89.70	0.00
TRINITY_sp|P933	GAPN	NADP-dependent glyc	89.60	0.00
TRINITY_sp|Q412	PSAEA	Photosystem I react	89.60	0.00
TRINITY_sp|Q9S7	GGAT2	Glutamate--glyoxyla	89.60	0.00
TRINITY_sp|Q9SL	PSBP	Oxygen-evolving enh	89.60	0.00
TRINITY_sp|P824	PSRP3	30S ribosomal prote	89.60	0.00
TRINITY_sp|P343	prp-8	Pre-mRNA-splicing f	89.50	0.00
TRINITY_sp|Q3T0	RPL17	60S ribosomal prote	89.50	0.00
TRINITY_sp|Q390	CO	Zinc finger protein	89.50	0.00
TRINITY_sp|P307	RPL9	60S ribosomal prote	89.40	0.00
TRINITY_sp|P485	APX1	L-ascorbate peroxid	89.40	0.00
TRINITY_sp|P365	RPL4	60S ribosomal prote	89.40	0.00
TRINITY_sp|Q9PV	c8b	Complement componen	89.40	0.00
TRINITY_sp|Q6VA	-	Tubulin alpha-4 cha	89.30	0.00
TRINITY_sp|P350	-	Histone H2B.1/H2B.2	89.30	0.00
TRINITY_sp|P503	GAPA	Glyceraldehyde-3-ph	89.30	0.00
TRINITY_sp|Q680	RPS29A	40S ribosomal prote	89.30	0.00
TRINITY_sp|P496	-	Ubiquitin-60S ribos	89.30	0.00
TRINITY_sp|Q8W1	PK	Pyridoxal kinase OS	89.30	0.00

TRINITY_sp|Q8VZ5	RPL35	50S ribosomal prote	89.20	0.00
TRINITY_sp|Q9ZSW	TCTP	Translationally-con	89.20	0.00
TRINITY_sp|Q9SA7	YchF1	Obg-like ATPase 1 O	89.10	0.00
TRINITY_sp|P4994	-	Ferritin, heavy sub	89.00	0.00
TRINITY_sp|Q944C	FBA2	Fructose-bisphospha	88.90	0.00
TRINITY_sp|O6481	At2g2309	Uncharacterized pro	88.90	0.00
TRINITY_sp|P1235	PSAG	Photosystem I react	88.90	0.00
TRINITY_sp|Q6EU9	PIP1-1	Aquaporin PIP1-1 OS	88.90	0.00
TRINITY_sp|Q3929	-	DNA-directed RNA po	88.90	0.00
TRINITY_sp|P0231	-	Histone H4, major O	88.90	0.00
TRINITY_sp|B7G81	PHATRDRA	Translation factor	88.90	0.00
TRINITY_sp|P3783	PFL	Formate acetyltrans	88.90	0.00
TRINITY_sp|Q5XJ1	gapdh	Glyceraldehyde-3-ph	88.80	0.00
TRINITY_sp|P1136	gag	Retrovirus-related	88.70	0.00
TRINITY_sp|P2752	CAP10A	Chlorophyll a-b bin	88.70	0.00
TRINITY_sp|P6914	-	Histone H3 OS=Tetra	88.70	0.00
TRINITY_sp|O7869	mt-cyb	Cytochrome b OS=Car	88.70	0.00
TRINITY_sp|O2216	At2g4486	Probable ribosome b	88.70	0.00
TRINITY_sp|Q9SV8	NIP5-1	Probable aquaporin	88.60	0.00
TRINITY_sp|Q9AXC	-	Eukaryotic translat	88.60	0.00
TRINITY_sp|Q3957	ODA2	Dynein gamma chain,	88.60	0.00
TRINITY_sp|Q9FNB	CHLH	Magnesium-chelatase	88.60	0.00
TRINITY_sp|Q58D1	RPL7	60S ribosomal prote	88.50	0.00
TRINITY_sp|Q56YA	AGT1	Serine--glyoxylate	88.50	0.00
TRINITY_sp|Q3868	-	V-type proton ATPas	88.50	0.00
TRINITY_sp|P5577	mt-co3	Cytochrome c oxidas	88.40	0.00
TRINITY_sp|P2752	CAP10A	Chlorophyll a-b bin	88.30	0.00
TRINITY_sp|P0CH2	-	Ubiquitin-60S ribos	88.30	0.00
TRINITY_sp|P6128	RPL12	60S ribosomal prote	88.30	0.00
TRINITY_sp|Q9S9N	HSP70-5	Heat shock 70 kDa p	88.30	0.00
TRINITY_sp|A8HS4	CHLREDRA	40S ribosomal prote	88.30	0.00
TRINITY_sp|P0C22	crp-79	Ubiquitin-60S ribos	88.30	0.00
TRINITY_sp|P1235	PSAF	Photosystem I react	88.20	0.00
TRINITY_sp|P4114	HSP90B1	Endoplasmin OS=Cani	88.20	0.00
TRINITY_sp|O8143	PAP1	Probable plastid-li	88.20	0.00
TRINITY_sp|Q5ADC	HHT3	Histone H3.3 OS=Can	88.20	0.00
TRINITY_sp|Q3961	ODA11	Dynein alpha chain,	88.20	0
TRINITY_sp|Q9SU1	PSAK	Photosystem I react	88.20	0.00
TRINITY_sp|A2XKU	OsI_0126	Costars family prot	88.10	0.00
TRINITY_sp|P5521	CGS1	Cystathionine gamma	88.10	0.00
TRINITY_sp|Q9AR2	CTH1	Magnesium-protoporp	88.10	0.00
TRINITY_sp|O9340	actb	Actin, cytoplasmic	88.00	0.00
TRINITY_sp|Q9CA6	CHLP	Geranylgeranyl diph	88.00	0.00
TRINITY_sp|P9754	Nptn	Neuroplastin OS=Rat	87.90	0.00
TRINITY_sp|P5932	Eif5	Eukaryotic translat	87.90	0.00
TRINITY_sp|O8908	Rbm3	RNA-binding protein	87.80	0.00
TRINITY_sp|Q3SZ1	RPN2	Dolichyl-diphosphoo	87.80	0.00
TRINITY_sp|Q9SSD	PRP8A	Pre-mRNA-processing	87.80	0.00
TRINITY_sp|Q9AR2	CTH1	Magnesium-protoporp	87.80	0.00
TRINITY_sp|Q7YKW	chlN	Light-independent p	87.80	0.00
TRINITY_sp|Q8TGM	ART2	Putative uncharacte	87.70	0.00
TRINITY_sp|Q3956	ODA4	Dynein beta chain,	87.70	0
TRINITY_sp|P3568	RPL3	60S ribosomal prote	87.70	0.00
TRINITY_sp|Q9SIE	CYP18-2	Peptidyl-prolyl cis	87.70	0.00
TRINITY_sp|P8387	TXNL4A	Thioredoxin-like pr	87.70	0.00
TRINITY_sp|Q8RXN	TYW1	S-adenosyl-L-methio	87.60	0.00

TRINITY_sp|Q9SZI	RPT2A	26S proteasome regu	87.60	0.00
TRINITY_sp|Q9LY/	CSP41A	Chloroplast stem-lo	87.50	0.00
TRINITY_sp|Q9FW(	HAC12	Histone acetyltrans	87.50	0.00
TRINITY_sp|Q9SL(	ATG8D	Autophagy-related p	87.50	0.00
TRINITY_sp|P505(	H2A-II	Histone H2A OS=Chla	87.50	0.00
TRINITY_sp|Q9H8I	METTL7A	Methyltransferase-l	87.50	0.00
TRINITY_sp|O809:	RPL36A	60S ribosomal prote	87.40	0.00
TRINITY_sp|Q56Y/	AGT1	Serine--glyoxylate	87.40	0.00
TRINITY_sp|Q54L\	smc4	Structural maintena	87.40	0.00
TRINITY_sp|P322(	CYSK	Cysteine synthase,	87.30	0.00
TRINITY_sp|Q8JJ(	fabp1	Fatty acid-binding	87.20	0.00
TRINITY_sp|P038!	Mtnd2	NADH-ubiquinone oxi	87.20	0.00
TRINITY_sp|Q396:	psaD	Photosystem I react	87.20	0.00
TRINITY_sp|C1F9I	thrS	Threonine--tRNA lig	87.10	0.00
TRINITY_sp|A2XJ:	OsI_012(	Chlorophyll a-b bin	87.10	0.00
TRINITY_sp|P485:	PPX2	Serine/threonine-pr	87.00	0.00
TRINITY_sp|Q9STI	-	Calreticulin OS=Chl	87.00	0.00
TRINITY_sp|Q9C9\	HPR	Glycerate dehydroge	87.00	0.00
TRINITY_sp|O655!	CIPK6	CBL-interacting ser	86.90	0.00
TRINITY_sp|P114!	TUBA1	Tubulin alpha-1/alp	86.90	0.00
TRINITY_sp|P0C2:	crp-79	Ubiquitin-60S ribos	86.90	0.00
TRINITY_sp|Q401	CYP-3	Cysteine proteinase	86.80	0.00
TRINITY_sp|P492:	RPL32A	60S ribosomal prote	86.80	0.00
TRINITY_sp|P493!	-	Serine hydroxymethy	86.80	0.00
TRINITY_sp|Q9MI\	mt-co1	Cytochrome c oxidas	86.80	0.00
TRINITY_sp|P257!	TRG-31	Probable aquaporin	86.70	0.00
TRINITY_sp|Q8LZ!	mt-cyb	Cytochrome b OS=Aeq	86.70	0.00
TRINITY_sp|O226(	RPL5	60S ribosomal prote	86.70	0.00
TRINITY_sp|Q86C(	tor	Target of rapamycin	86.70	0.00
TRINITY_sp|P168(	-	Histone H2B.3 OS=Vo	86.70	0.00
TRINITY_sp|P427:	RPS13	30S ribosomal prote	86.60	0.00
TRINITY_sp|P078:	act1	Major actin OS=Dict	86.60	0.00
TRINITY_sp|P263!	TFIID	TATA-box-binding pr	86.60	0.00
TRINITY_sp|Q94SI	mt-cyb	Cytochrome b OS=Dac	86.50	0.00
TRINITY_sp|P462!	RPS23	40S ribosomal prote	86.50	0.00
TRINITY_sp|P293	RPS1	30S ribosomal prote	86.40	0.00
TRINITY_sp|Q9AR:	CTH1	Magnesium-protoporp	86.30	0.00
TRINITY_sp|Q2QV(	CLPC2	Chaperone protein C	86.30	0.00
TRINITY_sp|P464(	TBP1	26S protease regula	86.20	0.00
TRINITY_sp|O225!	-	V-type proton ATPas	86.20	0.00
TRINITY_sp|P542(	GDCST	Aminomethyltransfer	86.20	0.00
TRINITY_sp|P481	MT-ND1	NADH-ubiquinone oxi	86.10	0.00
TRINITY_sp|F4J3(	CTPA3	Carboxyl-terminal-p	86.10	0.00
TRINITY_sp|F6H7I	THI1-2	Thiamine thiazole s	86.10	0.00
TRINITY_sp|P226!	-	Chlorophyll a-b bin	86.10	0.00
TRINITY_sp|P316!	ENO	Enolase (Fragment)	86.00	0.00
TRINITY_sp|Q8LG!	TDT	Tonoplast dicarboxy	85.90	0.00
TRINITY_sp|Q9SB!	PER42	Peroxidase 42 OS=Ar	85.90	0.00
TRINITY_sp|Q6K6(	Os02g07!	Leucine aminopeptid	85.90	0.00
TRINITY_sp|Q948I	-	Ferritin-3, chlorop	85.80	0.00
TRINITY_sp|P482	rps4	30S ribosomal prote	85.80	0.00
TRINITY_sp|Q9U7I	-	Histone H3 OS=Masti	85.80	0.00
TRINITY_sp|P325!	GLC7	Serine/threonine-pr	85.80	0.00
TRINITY_sp|A0CCI	Ppx4	Serine/threonine-pr	85.80	0.00
TRINITY_sp|P018!	H2-L	H-2 class I histoco	85.80	0.00
TRINITY_sp|P384!	ATP2	ATP synthase subuni	85.80	0.00

TRINITY_sp|Q7PXI	AGAP0013	Ubiquitin-fold modi	85.70	0.00
TRINITY_sp|Q5E9I	CHCHD3	MICOS complex subun	85.70	0.00
TRINITY_sp|B9DHA	RPL40A	Ubiquitin-60S ribos	85.70	0.00
TRINITY_sp|Q54RY	srfB	Serum response fact	85.70	0.00
TRINITY_sp|Q94A9	UBC35	Ubiquitin-conjugati	85.70	0.00
TRINITY_sp|P0538	RPLP2	60S acidic ribosoma	85.70	0.00
TRINITY_sp|Q8RX9	PNSB4	Photosynthetic NDH	85.70	0.00
TRINITY_sp|P4848	-	Serine/threonine-pr	85.60	0.00
TRINITY_sp|P0259	-	Calmodulin OS=Tetra	85.60	0.00
TRINITY_sp|P0CG8	TU20	Polyubiquitin OS=Te	85.60	0.00
TRINITY_sp|Q8LF3	NRPB10	DNA-directed RNA po	85.50	0.00
TRINITY_sp|O4851	RPL13	60S ribosomal prote	85.50	0.00
TRINITY_sp|Q3957	YPTC1	GTP-binding protein	85.50	0.00
TRINITY_sp|Q96CI	C8orf44	Putative uncharacte	85.40	0.00
TRINITY_sp|P2156	CYP	Peptidyl-prolyl cis	85.40	0.00
TRINITY_sp|P4191	RAN1	GTP-binding nuclear	85.40	0.00
TRINITY_sp|P5573	HSP90-2	Heat shock protein	85.40	0.00
TRINITY_sp|A4GF0	-	Profilin-4 OS=Olea	85.40	0.00
TRINITY_sp|O0461	CURT1A	Protein CURVATURE T	85.30	0.00
TRINITY_sp|P0909	GAPC	Glyceraldehyde-3-ph	85.30	0.00
TRINITY_sp|Q6369	Cdc37	Hsp90 co-chaperone	85.30	0.00
TRINITY_sp|Q9LR3	GGAT1	Glutamate--glyoxyla	85.20	0.00
TRINITY_sp|O0406	-	Acyl-CoA-binding pr	85.20	0.00
TRINITY_sp|Q8YZI	trpB1	Tryptophan synthase	85.20	0.00
TRINITY_sp|Q9Y0I	ppp4c	Serine/threonine-pr	85.20	0.00
TRINITY_sp|Q6P2Q	PRPF8	Pre-mRNA-processing	85.20	0.00
TRINITY_sp|O7419	UBC1	Ubiquitin-conjugati	85.10	0.00
TRINITY_sp|P2462	HSC-I	Heat shock cognate	85.10	0.00
TRINITY_sp|Q54YI	ints11	Integrator complex	85.10	0.00
TRINITY_sp|P2268	-	Chlorophyll a-b bin	85.10	0.00
TRINITY_sp|Q69TY	PRXIIE-1	Peroxiredoxin-2E-1,	85.10	0.00
TRINITY_sp|P4909	-	Asparagine syntheta	85.00	0.00
TRINITY_sp|P1686	-	Histone H2A-IV OS=V	85.00	0.00
TRINITY_sp|P0847	GAPC	Glyceraldehyde-3-ph	84.90	0.00
TRINITY_sp|Q9M2I	RPS27B	40S ribosomal prote	84.90	0.00
TRINITY_sp|Q9LZI	CDC48E	Cell division contr	84.90	0.00
TRINITY_sp|Q8BW9	Dnah3	Dynein heavy chain	84.80	0.00
TRINITY_sp|Q56Y8	MAP2B	Methionine aminopep	84.80	0.00
TRINITY_sp|O2301	KAB1	Probable voltage-ga	84.80	0.00
TRINITY_sp|Q9PVW	c8b	Complement componen	84.80	0.00
TRINITY_sp|O8266	HCF136	Photosystem II stab	84.70	0.00
TRINITY_sp|Q9HFQ	pphA	Serine/threonine-pr	84.70	0.00
TRINITY_sp|P4858	pph-1	Serine/threonine-pr	84.60	0.00
TRINITY_sp|O2438	-	Plastidic ATP/ADP-t	84.60	0.00
TRINITY_sp|Q8SEW	MT-CO3	Cytochrome c oxidas	84.60	0.00
TRINITY_sp|Q32LQ	bhmt	Betaine--homocystei	84.60	0.00
TRINITY_sp|Q3902	MPK7	Mitogen-activated p	84.60	0.00
TRINITY_sp|Q9SA5	CSP41B	Chloroplast stem-lo	84.50	0.00
TRINITY_sp|Q6553	FTSH2	ATP-dependent zinc	84.50	0.00
TRINITY_sp|O7419	UBC1	Ubiquitin-conjugati	84.50	0.00
TRINITY_sp|Q9STW	HSP70-6	Heat shock 70 kDa p	84.50	0.00
TRINITY_sp|P1686	-	Histone H2B.3 OS=Vo	84.50	0.00
TRINITY_sp|Q4JQI	MT-ND4	NADH-ubiquinone oxi	84.40	0.00
TRINITY_sp|P0853	tuba	Tubulin alpha chain	84.40	0.00
TRINITY_sp|Q9C6I	LSM3B	Sm-like protein LSM	84.40	0.00
TRINITY_sp|Q6553	FTSH2	ATP-dependent zinc	84.40	0.00

TRINITY_sp|Q94E\	PR46a	Ubiquitin-fold modi	84.40	0.00
TRINITY_sp|Q9M1\	LSM6A	Sm-like protein LSM	84.40	0.00
TRINITY_sp|Q9ZVI	NFYC3	Nuclear transcripti	84.40	0.00
TRINITY_sp|O650	RPS15	40S ribosomal prote	84.40	0.00
TRINITY_sp|P467	RPL5	60S ribosomal prote	84.30	0.00
TRINITY_sp|P208	-	Chlorophyll a-b bin	84.30	0.00
TRINITY_sp|P838	TXNL4A	Thioredoxin-like pr	84.30	0.00
TRINITY_sp|P547	Rad23a	UV excision repair	84.20	0.00
TRINITY_sp|Q3Y8I	DAW1	Dynein assembly fac	84.20	0.00
TRINITY_sp|O237	PBB1	Proteasome subunit	84.00	0.00
TRINITY_sp|Q56Y	AGT1	Serine--glyoxylate	84.00	0.00
TRINITY_sp|Q6KA	RPL39A	60S ribosomal prote	84.00	0.00
TRINITY_sp|P128	PSBO	Oxygen-evolving enh	84.00	0.00
TRINITY_sp|Q9ST\	HSP70-6	Heat shock 70 kDa p	84.00	0.00
TRINITY_sp|Q395	ODA4	Dynein beta chain,	83.90	0
TRINITY_sp|Q9SSI	PRP8A	Pre-mRNA-processing	83.90	0
TRINITY_sp|Q9STI	-	Calreticulin OS=Chl	83.80	0.00
TRINITY_sp|A2XF	TOP6A3	DNA topoisomerase 6	83.80	0.00
TRINITY_sp|Q9MI\	mt-co1	Cytochrome c oxidas	83.80	0.00
TRINITY_sp|P270	ABT	ADP,ATP carrier pro	83.80	0.00
TRINITY_sp|Q9MBI	DHC10	Dynein-1-beta heavy	83.80	0.00
TRINITY_sp|P557	HSP90-2	Heat shock protein	83.70	0.00
TRINITY_sp|A8JB	CCDC65	Coiled-coil domain-	83.70	0.00
TRINITY_sp|Q8W4I	CYP71	Peptidyl-prolyl cis	83.70	0.00
TRINITY_sp|P385	NIR1	Ferredoxin--nitrite	83.70	0.00
TRINITY_sp|Q406	ALDP	Fructose-bisphospha	83.60	0.00
TRINITY_sp|Q9LY	RPL1	50S ribosomal prote	83.60	0.00
TRINITY_sp|Q8AV	btd	Biotinidase OS=Taki	83.60	0.00
TRINITY_sp|O785	ftsH	ATP-dependent zinc	83.60	0.00
TRINITY_sp|Q9MBI	DHC10	Dynein-1-beta heavy	83.60	0
TRINITY_sp|P0DK	RPT1A	26S protease regula	83.50	0.00
TRINITY_sp|O934	actb	Actin, cytoplasmic	83.50	0.00
TRINITY_sp|Q9SJI	AGO5	Protein argonaute 5	83.30	0.00
TRINITY_sp|P057	KRT8	Keratin, type II cy	83.30	0.00
TRINITY_sp|P092	-	Parvalbumin alpha O	83.30	0.00
TRINITY_sp|Q9M7	RPL29A	60S ribosomal prote	83.30	0.00
TRINITY_sp|P462	-	Fructose-bisphospha	83.30	0.00
TRINITY_sp|Q9VL	Sirup	Succinate dehydroge	83.30	0.00
TRINITY_sp|Q7XS	PIP1-2	Probable aquaporin	83.30	0.00
TRINITY_sp|Q907	EEF2	Elongation factor 2	83.30	0.00
TRINITY_sp|Q396	PCKR1	Peptidyl-prolyl cis	83.30	0.00
TRINITY_sp|Q426	HEMB	Delta-aminolevulini	83.30	0.00
TRINITY_sp|Q436	-	Glutathione reducta	83.30	0.00
TRINITY_sp|P462	RPS13	40S ribosomal prote	83.20	0.00
TRINITY_sp|P368	ppp1cc-a	Serine/threonine-pr	83.20	0.00
TRINITY_sp|Q86C	tor	Target of rapamycin	83.10	0.00
TRINITY_sp|Q9AT	RPS15A	40S ribosomal prote	83.10	0.00
TRINITY_sp|Q395	ODA2	Dynein gamma chain,	83.10	0
TRINITY_sp|Q6YU	Os02g01	Putative multidrug	83.10	0.00
TRINITY_sp|Q9SSI	PRP8A	Pre-mRNA-processing	83.00	0.00
TRINITY_sp|P258	GAPA1	Glyceraldehyde-3-ph	83.00	0.00
TRINITY_sp|Q404	-	Ribulose bisphospha	83.00	0.00
TRINITY_sp|Q9MBI	DHC10	Dynein-1-beta heavy	83.00	0.00
TRINITY_sp|O772	catA	Catalase-A OS=Dicty	82.90	0.00
TRINITY_sp|Q74Z	DPH3	Diphthamide biosynt	82.90	0.00
TRINITY_sp|Q9VH	RpS29	40S ribosomal prote	82.90	0.00

TRINITY_sp|Q2NL1	CLPTM1	Cleft lip and palat	82.90	0.00
TRINITY_sp|Q074	LHCB4.1	Chlorophyll a-b bin	82.80	0.00
TRINITY_sp|Q8RWI	VTC2	GDP-L-galactose pho	82.80	0.00
TRINITY_sp|P0CG5	UBB	Polyubiquitin-B OS=	82.80	0.00
TRINITY_sp|Q926	DHX38	Pre-mRNA-splicing f	82.70	0.00
TRINITY_sp|Q9C5	RPT6A	26S protease regula	82.70	0.00
TRINITY_sp|Q9U7I	-	Histone H3 OS=Masti	82.70	0.00
TRINITY_sp|Q9FN	At5g085	NADH dehydrogenase	82.60	0.00
TRINITY_sp|Q9MA	RPT4B	26S protease regula	82.60	0.00
TRINITY_sp|O232	CKS1	Cyclin-dependent ki	82.50	0.00
TRINITY_sp|Q95V	RPL38	60S ribosomal prote	82.50	0.00
TRINITY_sp|Q0I5	rpsL	30S ribosomal prote	82.40	0.00
TRINITY_sp|P215	CYP	Peptidyl-prolyl cis	82.40	0.00
TRINITY_sp|P361	HSC80	Heat shock cognate	82.40	0.00
TRINITY_sp|Q9U9	ppp6c	Serine/threonine-pr	82.30	0.00
TRINITY_sp|Q048	CP31A	31 kDa ribonucleopr	82.20	0.00
TRINITY_sp|P128	GAPB	Glyceraldehyde-3-ph	82.20	0.00
TRINITY_sp|Q396	psaD	Photosystem I react	82.20	0.00
TRINITY_sp|Q54Y	dhkJ	Hybrid signal trans	82.20	0.00
TRINITY_sp|P361	HSC80	Heat shock cognate	82.20	0.00
TRINITY_sp|Q944	GLYK	D-glycerate 3-kinas	82.20	0.00
TRINITY_sp|Q9AS	PNSL5	Photosynthetic NDH	82.10	0.00
TRINITY_sp|Q9Y2	EIF3L	Eukaryotic translat	82.10	0.00
TRINITY_sp|O603	AQR	Intron-binding prot	82.10	0.00
TRINITY_sp|Q9M3	rps6	40S ribosomal prote	82.10	0.00
TRINITY_sp|P258	UBC2	Ubiquitin-conjugati	82.10	0.00
TRINITY_sp|P286	CLKR27	3-oxoacyl-[acyl-car	82.10	0.00
TRINITY_sp|Q426	SODA	Superoxide dismutas	82.10	0.00
TRINITY_sp|Q54Y	dhkJ	Hybrid signal trans	82.10	0.00
TRINITY_sp|Q9FG	UBL5	Ubiquitin-like prot	82.10	0.00
TRINITY_sp|P084	rpl4-a	60S ribosomal prote	82.00	0.00
TRINITY_sp|P929	KIN11	SNF1-related protei	82.00	0.00
TRINITY_sp|Q9MB	DHC10	Dynein-1-beta heavy	82.00	0
TRINITY_sp|Q9LY	RPL1	50S ribosomal prote	81.90	0.00
TRINITY_sp|Q395	TUBG	Tubulin gamma chain	81.90	0.00
TRINITY_sp|O494	AAC3	ADP,ATP carrier pro	81.80	0.00
TRINITY_sp|Q9ST	HSP70-6	Heat shock 70 kDa p	81.80	0.00
TRINITY_sp|Q5XX	MSH2	DNA mismatch repair	81.80	0.00
TRINITY_sp|O268	msrB	Peptide methionine	81.80	0.00
TRINITY_sp|P364	hspB	Heat shock cognate	81.80	0.00
TRINITY_sp|P160	GDCSH	Glycine cleavage sy	81.80	0.00
TRINITY_sp|P464	-	Malate dehydrogenas	81.80	0.00
TRINITY_sp|P832	hbb2	Hemoglobin subunit	81.70	0.00
TRINITY_sp|O805	RABH1B	Ras-related protein	81.70	0.00
TRINITY_sp|P234	-	Ribulose bisphospha	81.70	0.00
TRINITY_sp|P312	ACO	1-aminocyclopropane	81.60	0.00
TRINITY_sp|Q7X9	RCA1	Ribulose bisphospha	81.60	0.00
TRINITY_sp|A2XN	PP2A2	Serine/threonine-pr	81.60	0.00
TRINITY_sp|P281	TBP1	TATA-box-binding pr	81.60	0.00
TRINITY_sp|Q8LP	ABCE2	ABC transporter E f	81.60	0.00
TRINITY_sp|P084	RBCS	Ribulose bisphospha	81.60	0.00
TRINITY_sp|P061	-	40S ribosomal prote	81.60	0.00
TRINITY_sp|P411	Rpl28	60S ribosomal prote	81.50	0.00
TRINITY_sp|Q9XH	RPL37A	60S ribosomal prote	81.50	0.00
TRINITY_sp|P049	Fn1	Fibronectin OS=Ratt	81.50	0.00
TRINITY_sp|P592	RPS13B	40S ribosomal prote	81.50	0.00

TRINITY_sp|Q9C94	At1g5274	Probable histone H2	81.50	0.00
TRINITY_sp|Q9C81	At1g3342	PHD finger protein	81.50	0.00
TRINITY_sp|Q4051	PSBR	Photosystem II 10 k	81.50	0.00
TRINITY_sp|Q4308	-	Histone H4 OS=Pyren	81.40	0.00
TRINITY_sp|Q6ZR0	DNAH12	Dynein heavy chain	81.30	0.00
TRINITY_sp|Q4051	PSBR	Photosystem II 10 k	81.30	0.00
TRINITY_sp|Q9FR5	TOR	Serine/threonine-pr	81.30	0.00
TRINITY_sp|P3016	act-1a	Actin-1 OS=Onchocer	81.30	0.00
TRINITY_sp|P5036	GAPA	Glyceraldehyde-3-ph	81.30	0.00
TRINITY_sp|P3140	VHA55	V-type proton ATPas	81.30	0.00
TRINITY_sp|P2295	MED37D	Probable mediator o	81.20	0.00
TRINITY_sp|P0282	-	Heat shock 70 kDa p	81.20	0.00
TRINITY_sp|Q6559	FTSH2	ATP-dependent zinc	81.20	0.00
TRINITY_sp|Q9SUN	At4g3022	Probable small nucl	81.20	0.00
TRINITY_sp|P2267	ARG7	Argininosuccinate l	81.20	0.00
TRINITY_sp|P5710	MDH2	Malate dehydrogenas	81.20	0.00
TRINITY_sp|Q4R88	HSPA1L	Heat shock 70 kDa p	81.20	0.00
TRINITY_sp|Q0323	MYBL2	Myb-related protein	81.10	0.00
TRINITY_sp|Q3T0E	PPP1CA	Serine/threonine-pr	81.10	0.00
TRINITY_sp|Q86C6	tor	Target of rapamycin	81.10	0.00
TRINITY_sp|O2331	NFYB3	Nuclear transcripti	81.10	0.00
TRINITY_sp|P0554	Cd8b	T-cell surface glyc	81.00	0.00
TRINITY_sp|Q9LY6	RPL1	50S ribosomal prote	81.00	0.00
TRINITY_sp|Q2KH7	RPS27	40S ribosomal prote	81.00	0.00
TRINITY_sp|P1379	oprF	Outer membrane pori	81.00	0.00
TRINITY_sp|Q9SPE	CRTZ	Beta-carotene 3-hyd	81.00	0.00
TRINITY_sp|Q9SMH	DHC1	Dynein-1-alpha heav	81.00	0
TRINITY_sp|Q9Y24	POMP	Proteasome maturati	80.90	0.00
TRINITY_sp|Q8JH7	aldob	Fructose-bisphospha	80.90	0.00
TRINITY_sp|Q0806	RPS8	40S ribosomal prote	80.90	0.00
TRINITY_sp|P0743	BETA	Tubulin beta-1 chai	80.90	0.00
TRINITY_sp|P6777	PPP2CA	Serine/threonine-pr	80.90	0.00
TRINITY_sp|Q2156	M28.5	NHP2-like protein 1	80.80	0.00
TRINITY_sp|Q9ZRD	YKT61	VAMP-like protein Y	80.80	0.00
TRINITY_sp|P5481	-	26S protease regula	80.80	0.00
TRINITY_sp|P4969	RPL19C	60S ribosomal prote	80.70	0.00
TRINITY_sp|Q9FKE	LSM5	Sm-like protein LSM	80.70	0.00
TRINITY_sp|Q8TGM	ART2	Putative uncharacte	80.70	0.00
TRINITY_sp|Q3977	-	Acyl-CoA-binding pr	80.60	0.00
TRINITY_sp|P8088	petM	Cytochrome b6-f com	80.60	0.00
TRINITY_sp|P6920	UB-EP52	Ubiquitin-60S ribos	80.60	0.00
TRINITY_sp|Q54Y2	dhkJ	Hybrid signal trans	80.60	0.00
TRINITY_sp|Q3956	GSA	Glutamate-1-semiald	80.60	0.00
TRINITY_sp|Q852I	HD16	Casein kinase 1-lik	80.60	0.00
TRINITY_sp|Q9AR2	CTH1	Magnesium-protoporp	80.60	0.00
TRINITY_sp|Q9SJ1	VHA-a2	V-type proton ATPas	80.60	0.00
TRINITY_sp|Q9ZR0	FH	Frataxin, mitochond	80.60	0.00
TRINITY_sp|Q2RBN	Os11g010	Clathrin heavy chai	80.60	0.00
TRINITY_sp|A8JAN	DRC7	Dynein regulatory c	80.50	0.00
TRINITY_sp|Q7F91	CLPC1	Chaperone protein C	80.50	0.00
TRINITY_sp|P5476	RAN1A	GTP-binding nuclear	80.40	0.00
TRINITY_sp|Q54GN	nsa2	Ribosome biogenesis	80.40	0.00
TRINITY_sp|P5477	CDC48	Cell division cycle	80.40	0
TRINITY_sp|Q54Y2	dhkJ	Hybrid signal trans	80.40	0.00
TRINITY_sp|P1206	CAB37	Chlorophyll a-b bin	80.30	0.00
TRINITY_sp|P1507	env	Envelope glycoprote	80.30	0.00

TRINITY_sp|P492(	RPS18	40S ribosomal prote	80.30	0.00
TRINITY_sp|Q282	FN1	Fibronectin (Fragme	80.30	0.00
TRINITY_sp|Q426	ALDCHL	Fructose-bisphospha	80.30	0.00
TRINITY_sp|Q9UV	UBC1	Ubiquitin-conjugati	80.30	0.00
TRINITY_sp|Q9FJ	RPL26B	60S ribosomal prote	80.30	0.00
TRINITY_sp|Q9SM	DHC1	Dynein-1-alpha heav	80.30	0
TRINITY_sp|P0DI	At2g300(	PHD finger-like dom	80.20	0.00
TRINITY_sp|O040	FD1	Ferredoxin-1, chlor	80.20	0.00
TRINITY_sp|P507(	-	Osmotin-like protei	80.20	0.00
TRINITY_sp|Q7X7	ELP3	Elongator complex p	80.20	0.00
TRINITY_sp|Q69J	NFYB10	Nuclear transcripti	80.20	0.00
TRINITY_sp|F4IF	POL2B	DNA polymerase epsi	80.20	0.00
TRINITY_sp|Q9LT(	RPN11	26S proteasome non-	80.10	0.00
TRINITY_sp|Q9FN	At5g085	NADH dehydrogenase	80.10	0.00
TRINITY_sp|P108	sahA	Adenosylhomocystein	80.10	0.00
TRINITY_sp|Q682	RPT2	Root phototropism p	80.00	0.00
TRINITY_sp|O243	LOX2.1	Linoleate 13S-lipox	80.00	0.00
TRINITY_sp|Q8AV	btd	Biotinidase OS=Taki	80.00	0.00
TRINITY_sp|Q8L7	RH3	DEAD-box ATP-depend	80.00	0.00
TRINITY_sp|Q5F3	UGDH	UDP-glucose 6-dehyd	80.00	0.00
TRINITY_sp|Q9FN	At5g085	NADH dehydrogenase	80.00	0.00
TRINITY_sp|P364	hspB	Heat shock cognate	80.00	0.00
TRINITY_sp|Q5ZJ(	YARS	Tyrosine--tRNA liga	80.00	0.00
TRINITY_sp|P492(	RPS18	40S ribosomal prote	80.00	0.00
TRINITY_sp|P583	RpL30	60S ribosomal prote	80.00	0.00
TRINITY_sp|Q627	gsp-2	Serine/threonine-pr	80.00	0.00
TRINITY_sp|Q9FJ	SMC4	Structural maintena	80.00	0.00
TRINITY_sp|O498	FTRC	Ferredoxin-thioredo	80.00	0.00
TRINITY_sp|P206	bimG	Serine/threonine-pr	79.90	0.00
TRINITY_sp|P392(	NDK1	Nucleoside diphosph	79.90	0.00
TRINITY_sp|Q109(	Os10g05(	DEAD-box ATP-depend	79.90	0.00
TRINITY_sp|P492(	RPS18	40S ribosomal prote	79.80	0.00
TRINITY_sp|Q392	NTR2	Thioredoxin reducta	79.80	0.00
TRINITY_sp|P024(	Rplp2	60S acidic ribosoma	79.70	0.00
TRINITY_sp|P428	LDJ2	DnaJ protein homolo	79.70	0.00
TRINITY_sp|Q54C	mobB	MOB kinase activato	79.70	0.00
TRINITY_sp|Q9P2	DNAH1	Dynein heavy chain	79.70	0.00
TRINITY_sp|Q9JL	Spag6	Sperm-associated an	79.70	0.00
TRINITY_sp|Q9JL	Spag6	Sperm-associated an	79.70	0.00
TRINITY_sp|P564	Atp5b	ATP synthase subuni	79.70	0.00
TRINITY_sp|P479	Rpl29	60S ribosomal prote	79.70	0.00
TRINITY_sp|Q9ST	HSP70-6	Heat shock 70 kDa p	79.70	0.00
TRINITY_sp|P053	RPLP2	60S acidic ribosoma	79.70	0.00
TRINITY_sp|Q9ZV	CYP22	Peptidyl-prolyl cis	79.60	0.00
TRINITY_sp|Q8LP	ABCE2	ABC transporter E f	79.60	0.00
TRINITY_sp|P838	TXNL4A	Thioredoxin-like pr	79.60	0.00
TRINITY_sp|Q9AT	PDX1	Probable pyridoxal	79.60	0.00
TRINITY_sp|Q3ZB	RPS20	40S ribosomal prote	79.60	0.00
TRINITY_sp|Q9DE	ruvbl2	RuvB-like 2 OS=Xeno	79.60	0.00
TRINITY_sp|P264	S100A4	Protein S100-A4 OS=	79.60	0.00
TRINITY_sp|O820	CCH	Copper transport pr	79.50	0.00
TRINITY_sp|P086	COX1	Cytochrome c oxidas	79.50	0.00
TRINITY_sp|Q9FG	UBL5	Ubiquitin-like prot	79.50	0.00
TRINITY_sp|P486	FAD3	Acyl-lipid omega-3	79.50	0.00
TRINITY_sp|Q94B(	RPT6B	26S protease regula	79.50	0.00
TRINITY_sp|P505	H2A-II	Histone H2A OS=Chla	79.50	0.00

TRINITY_sp|Q6P2(	PRPF8	Pre-mRNA-processing	79.50	0
TRINITY_sp|Q8WX)	DNAH7	Dynein heavy chain	79.50	0.00
TRINITY_sp|Q9SZI	RPT2A	26S proteasome regu	79.40	0.00
TRINITY_sp|P297(	-	60S acidic ribosoma	79.40	0.00
TRINITY_sp|Q9S7I	TS1	Threonine synthase	79.40	0.00
TRINITY_sp|O826:	GATA9	GATA transcription	79.40	0.00
TRINITY_sp|O650(	SB62	60S ribosomal prote	79.40	0.00
TRINITY_sp|Q9Y2)	UBE2D4	Ubiquitin-conjugati	79.30	0.00
TRINITY_sp|P350(	SAHH	Adenosylhomocystein	79.30	0.00
TRINITY_sp|P329{	ATPD	ATP synthase delta	79.20	0.00
TRINITY_sp|P221'	PSAH	Photosystem I react	79.20	0.00
TRINITY_sp|Q8LP!	LACS6	Long chain acyl-CoA	79.20	0.00
TRINITY_sp|P123!	PSAE	Photosystem I react	79.20	0.00
TRINITY_sp|Q6PI\	FIGNL1	Fidgetin-like prote	79.10	0.00
TRINITY_sp|P111'	HSP70	Heat shock 70 kDa p	79.10	0.00
TRINITY_sp|O026'	mdh-2	Probable malate deh	79.10	0.00
TRINITY_sp|B9DGI	ACS	Acetyl-coenzyme A s	79.10	0.00
TRINITY_sp|P462{	CSBP	Sedoheptulose-1,7-b	79.00	0.00
TRINITY_sp|P563'	minD	Putative septum sit	79.00	0.00
TRINITY_sp|Q2QV(	CLPC2	Chaperone protein C	79.00	0.00
TRINITY_sp|P289!	-	Elongation factor 2	78.90	0.00
TRINITY_sp|P361{	HSC80	Heat shock cognate	78.90	0.00
TRINITY_sp|Q9UW{	pph-3	Serine/threonine-pr	78.90	0.00
TRINITY_sp|Q9MBI	DHC10	Dynein-1-beta heavy	78.90	0.00
TRINITY_sp|A1Z0I	hamp	Hepcidin OS=Larimic	78.80	0.00
TRINITY_sp|P344!	mog-1	Probable pre-mRNA-s	78.80	0.00
TRINITY_sp|O649{	RPT1	26S protease regula	78.80	0.00
TRINITY_sp|P0C8I	CCRP1	Probable serine/thr	78.80	0.00
TRINITY_sp|Q86A(	polr3a	DNA-directed RNA po	78.70	0.00
TRINITY_sp|Q9XI!	ELF5A-1	Eukaryotic translat	78.70	0.00
TRINITY_sp|P0C5I	PP2A4	Putative serine/thr	78.70	0.00
TRINITY_sp|Q9SHI	RUB1	Ubiquitin-NEDD8-lik	78.70	0.00
TRINITY_sp|P239!	PSAL	Photosystem I react	78.60	0.00
TRINITY_sp|P112'	Fn1	Fibronectin OS=Mus	78.60	0.00
TRINITY_sp|P350(	-	Histone H2A OS=Acro	78.60	0.00
TRINITY_sp|P350(	-	Histone H2A OS=Acro	78.60	0.00
TRINITY_sp|A8JF'	NCS6	Cytoplasmic tRNA 2-	78.60	0.00
TRINITY_sp|P022'	HTA1	Histone H2AX OS=Tet	78.60	0.00
TRINITY_sp|P206!	bimG	Serine/threonine-pr	78.60	0.00
TRINITY_sp|Q9SL(	PGR5	Protein PROTON GRAD	78.40	0.00
TRINITY_sp|P061{	PSBR	Photosystem II 10 k	78.40	0.00
TRINITY_sp|Q32PI	LSM3	U6 snRNA-associated	78.40	0.00
TRINITY_sp|Q93Y!	ppa1	Soluble inorganic p	78.40	0.00
TRINITY_sp|Q9LD!	CBF5	H/ACA ribonucleopro	78.40	0.00
TRINITY_sp|Q8LK!	DME	Transcriptional act	78.40	0.00
TRINITY_sp|Q377'	mt-co2	Cytochrome c oxidas	78.40	0.00
TRINITY_sp|A4IJ:	mns1	Meiosis-specific nu	78.40	0.00
TRINITY_sp|P277(	ODA6	Dynein, 70 kDa inte	78.40	0.00
TRINITY_sp|Q395(	ODA4	Dynein beta chain,	78.40	0
TRINITY_sp|Q6QJI	SULP2	Sulfate permease 2,	78.30	0.00
TRINITY_sp|O650{	SB62	60S ribosomal prote	78.30	0.00
TRINITY_sp|Q8LPI	ABCB2	ABC transporter B f	78.30	0.00
TRINITY_sp|Q9H6I	TASP1	Threonine aspartase	78.30	0.00
TRINITY_sp|Q6ZR(	DNAH12	Dynein heavy chain	78.30	0.00
TRINITY_sp|Q3T0(	RPL14	60S ribosomal prote	78.20	0.00
TRINITY_sp|A4QNI	nip7	60S ribosome subuni	78.20	0.00

TRINITY_sp|P484{	-	Serine/threonine-pr	78.20	0.00
TRINITY_sp|Q9UL(	INO80	DNA helicase INO80	78.10	0.00
TRINITY_sp|Q84U2	ERY2	50S ribosomal prote	78.10	0.00
TRINITY_sp|P175(	eef1ao	Elongation factor 1	78.10	0.00
TRINITY_sp|Q9MBI	DHC10	Dynein-1-beta heavy	78.10	0.00
TRINITY_sp|P099]	PETF	Ferredoxin-1, chlor	78.10	0.00
TRINITY_sp|O603(	AQR	Intron-binding prot	78.00	0.00
TRINITY_sp|P0DJ5	RPL23	60S ribosomal prote	78.00	0.00
TRINITY_sp|P078]	PETF	Ferredoxin, chlorop	78.00	0.00
TRINITY_sp|Q5ZIF	CPSF3L	Integrator complex	78.00	0.00
TRINITY_sp|P364]	rab7A	Ras-related protein	78.00	0.00
TRINITY_sp|P1285	PSBO	Oxygen-evolving enh	78.00	0.00
TRINITY_sp|Q7S6W	rps-28	40S ribosomal prote	78.00	0.00
TRINITY_sp|Q426{	-	Malate dehydrogenas	78.00	0.00
TRINITY_sp|Q395]	ODA9	Dynein, 78 kDa inte	78.00	0.00
TRINITY_sp|Q9Y0E	ppp4c	Serine/threonine-pr	78.00	0.00
TRINITY_sp|P4969	RPL23A	60S ribosomal prote	77.90	0.00
TRINITY_sp|P253{	GBLP	Guanine nucleotide-	77.90	0.00
TRINITY_sp|Q54QE	erkB	Extracellular signa	77.90	0.00
TRINITY_sp|P022]	HTA1	Histone H2AX OS=Tet	77.90	0.00
TRINITY_sp|P1545	CYC1	Cytochrome c OS=Chl	77.90	0.00
TRINITY_sp|Q9SE]	RPT4A	26S protease regula	77.80	0.00
TRINITY_sp|A8ISN	ARL3	ADP-ribosylation fa	77.80	0.00
TRINITY_sp|Q2KH(	EIF2S3	Eukaryotic translat	77.80	0.00
TRINITY_sp|P022]	HTA1	Histone H2AX OS=Tet	77.80	0.00
TRINITY_sp|I2CY2	CHLREDRA	Acyl-lipid (7-3)-de	77.80	0.00
TRINITY_sp|Q9CA]	CKL2	Casein kinase 1-lik	77.80	0.00
TRINITY_sp|Q8H1]	PGRL1A	PGR5-like protein 1	77.80	0.00
TRINITY_sp|P491]	-	Luminal-binding pro	77.70	0.00
TRINITY_sp|Q9C55	IPMS2	2-isopropylmalate s	77.70	0.00
TRINITY_sp|P464(	TBP1	26S protease regula	77.70	0.00
TRINITY_sp|P1114	-	Heat shock 70 kDa p	77.70	0.00
TRINITY_sp|Q1ZX(	elp3	Probable elongator	77.70	0.00
TRINITY_sp|Q4319	LOX1.5	Probable linoleate	77.60	0.00
TRINITY_sp|A9VE5	13033	Inosine triphosphat	77.60	0.00
TRINITY_sp|P1099	-	Actin, cytoplasmic	77.60	0.00
TRINITY_sp|A5EKI	gatA	Glutamyl-tRNA(Gln)	77.60	0.00
TRINITY_sp|O755]	SF3B1	Splicing factor 3B	77.60	0.00
TRINITY_sp|Q3895	At3g265(	Probable pre-mRNA-s	77.60	0
TRINITY_sp|P514]	RPL39A	60S ribosomal prote	77.60	0.00
TRINITY_sp|Q396]	ODA11	Dynein alpha chain,	77.60	0
TRINITY_sp|Q5F4E	AAT	Aspartate aminotran	77.50	0.00
TRINITY_sp|Q32L]	TRAPPC6E	Trafficking protein	77.50	0.00
TRINITY_sp|Q9FXE	GOLS2	Galactinol synthase	77.50	0.00
TRINITY_sp|Q3T0E	PPP1CA	Serine/threonine-pr	77.50	0.00
TRINITY_sp|Q9MAE	ISU2	Iron-sulfur cluster	77.50	0.00
TRINITY_sp|P524]	PAC1	Proteasome subunit	77.50	0.00
TRINITY_sp|Q9SP(	CYP20-1	Peptidyl-prolyl cis	77.50	0.00

TRINITY_sp|P505(	H2A-II	Histone H2A OS=Chla	77.50	0.00
TRINITY_sp|P427!	RPS11	40S ribosomal prote	77.50	0.00
TRINITY_sp|P341	racB	Rho-related protein	77.50	0.00
TRINITY_sp|Q426	ATPD	ATP synthase delta	77.50	0.00
TRINITY_sp|Q027(	RPL24	50S ribosomal prote	77.40	0.00
TRINITY_sp|Q9SM	DHC1	Dynein-1-alpha heav	77.40	0.00
TRINITY_sp|P286	-	28 kDa ribonucleopr	77.40	0.00
TRINITY_sp|F4KG	RVE1	Protein REVEILLE 1	77.40	0.00
TRINITY_sp|O500	AP-17	AP-2 complex subuni	77.40	0.00
TRINITY_sp|Q8H5	Os07g06	Manganese-dependent	77.40	0.00
TRINITY_sp|A2ZC	SC34	60S ribosomal prote	77.40	0.00
TRINITY_sp|Q631	Dnah7	Dynein heavy chain	77.40	0.00
TRINITY_sp|Q396	ODA11	Dynein alpha chain,	77.40	0.00
TRINITY_sp|Q9R0	Ctsf	Cathepsin F OS=Mus	77.30	0.00
TRINITY_sp|Q2S0(	pckA2	Phosphoenolpyruvate	77.30	0.00
TRINITY_sp|P341	cdk5	Cyclin-dependent ki	77.30	0.00
TRINITY_sp|A8HY	METM	S-adenosylmethionin	77.30	0.00
TRINITY_sp|Q9SE	RPT3	26S protease regula	77.30	0.00
TRINITY_sp|Q9SZ	SHM1	Serine hydroxymethy	77.30	0.00
TRINITY_sp|Q655	FTSH2	ATP-dependent zinc	77.30	0.00
TRINITY_sp|P0DL(	DRC1	Dynein regulatory c	77.20	0.00
TRINITY_sp|P492(	RPS20A	40S ribosomal prote	77.20	0.00
TRINITY_sp|Q9ST	-	Calreticulin OS=Chl	77.20	0.00
TRINITY_sp|Q9FM	RIN1	RuvB-like protein 1	77.20	0.00
TRINITY_sp|P215(	CYP	Peptidyl-prolyl cis	77.20	0.00
TRINITY_sp|P929	APR1	5'-adenylylsulfate	77.10	0.00
TRINITY_sp|Q3BA	-	Uncharacterized pro	77.10	0.00
TRINITY_sp|Q430	Os06g02(	Asparagine syntheta	77.10	0.00
TRINITY_sp|Q271	DHC-8	Dynein heavy chain,	77.10	0.00
TRINITY_sp|Q7F9	CLPC1	Chaperone protein C	77.00	0.00
TRINITY_sp|Q56Y	MAP2B	Methionine aminopep	77.00	0.00
TRINITY_sp|A8LP	hslU	ATP-dependent prote	77.00	0.00
TRINITY_sp|Q6ZD	SDH1	Succinate dehydroge	77.00	0.00
TRINITY_sp|P628	RAB1A	Ras-related protein	77.00	0.00
TRINITY_sp|Q76I	RPS15A	40S ribosomal prote	76.90	0.00
TRINITY_sp|Q963(	ARF1	ADP-ribosylation fa	76.90	0.00
TRINITY_sp|O650	SB62	60S ribosomal prote	76.90	0.00
TRINITY_sp|Q54U(	dnapkcs	DNA-dependent prote	76.90	0.00
TRINITY_sp|Q8LQ	FTSH5	ATP-dependent zinc	76.90	0.00
TRINITY_sp|P222	fbl	rRNA 2'-O-methyltra	76.90	0.00
TRINITY_sp|Q54J	pdx1	Probable pyridoxal	76.90	0.00
TRINITY_sp|P179	PSMC3	26S protease regula	76.90	0.00
TRINITY_sp|P539	PETH	Ferredoxin--NADP re	76.90	0.00
TRINITY_sp|Q9LH(	At3g123	Nascent polypeptide	76.90	0.00
TRINITY_sp|P462	RPL8A	60S ribosomal prote	76.90	0.00
TRINITY_sp|Q9VH	eIF4AII	Eukaryotic initiati	76.90	0.00
TRINITY_sp|P493	-	Glycine-rich RNA-bi	76.90	0.00
TRINITY_sp|Q9SF	PAH1	Phosphatidate phosp	76.90	0.00
TRINITY_sp|Q9CY	Serbp1	Plasminogen activat	76.80	0.00
TRINITY_sp|P838(	-	Histone H2B (Fragme	76.80	0.00
TRINITY_sp|P518	-	ADP-ribosylation fa	76.80	0.00
TRINITY_sp|P518	-	ADP-ribosylation fa	76.80	0.00
TRINITY_sp|P518	-	ADP-ribosylation fa	76.80	0.00
TRINITY_sp|Q9LX(	RPS9B	40S ribosomal prote	76.80	0.00
TRINITY_sp|P413	FTRC	Ferredoxin-thioredo	76.80	0.00
TRINITY_sp|Q9LR	RPL13AB	60S ribosomal prote	76.80	0.00

TRINITY_sp|P5141	RPL18AB	60S ribosomal prote	76.80	0.00
TRINITY_sp|P7938	PCCB	Propionyl-CoA carbo	76.80	0.00
TRINITY_sp|Q9VU(	SmD1	Probable small nucl	76.70	0.00
TRINITY_sp|P3783	PFL	Formate acetyltrans	76.70	0.00
TRINITY_sp|P4584	RPL31	60S ribosomal prote	76.70	0.00
TRINITY_sp|P4858	pph-1	Serine/threonine-pr	76.70	0.00
TRINITY_sp|Q9YGI	SUCLG1	Succinate--CoA liga	76.70	0.00
TRINITY_sp|Q5999	trpB	Tryptophan synthase	76.70	0.00
TRINITY_sp|Q9SI2	At2g4029	Eukaryotic translat	76.70	0.00
TRINITY_sp|O603(	AQR	Intron-binding prot	76.70	0.00
TRINITY_sp|P2641	HSP70	Heat shock 70 kDa p	76.70	0.00
TRINITY_sp|Q31K(	mnmG	tRNA uridine 5-carb	76.70	0.00
TRINITY_sp|P0CT1	HTB1	Histone H2B OS=Magn	76.60	0.00
TRINITY_sp|P0CY3	RPS23A	40S ribosomal prote	76.60	0.00
TRINITY_sp|Q3957	YPTC5	GTP-binding protein	76.60	0.00
TRINITY_sp|Q4G2	DER2.1	Derlin-2.1 OS=Zea m	76.60	0.00
TRINITY_sp|Q2W89	argG	Argininosuccinate s	76.60	0.00
TRINITY_sp|P4628	RPL8A	60S ribosomal prote	76.60	0.00
TRINITY_sp|Q8VZI	ASK5	Shaggy-related prot	76.60	0.00
TRINITY_sp|O6508	SB62	60S ribosomal prote	76.60	0.00
TRINITY_sp|Q3957	ODA2	Dynein gamma chain,	76.60	0
TRINITY_sp|Q9P2I	DNAH1	Dynein heavy chain	76.60	0.00
TRINITY_sp|P9WQ1	treS	Trehalose synthase/	76.50	0.00
TRINITY_sp|Q4268	GLN1	Glutamine synthetas	76.50	0.00
TRINITY_sp|Q9AUI	Os03g071	Coatomer subunit al	76.50	0.00
TRINITY_sp|Q9LD9	CBF5	H/ACA ribonucleopro	76.50	0.00
TRINITY_sp|P8433	ACTB	Actin, cytoplasmic	76.40	0.00
TRINITY_sp|Q4259	At1g7901	NADH dehydrogenase	76.40	0.00
TRINITY_sp|Q9TW3	cypB	Peptidyl-prolyl cis	76.40	0.00
TRINITY_sp|Q9FF5	RPL12C	60S ribosomal prote	76.40	0.00
TRINITY_sp|P4901	Os12g028	Protein mago nashi	76.40	0.00
TRINITY_sp|Q9ZW8	At2g4309	3-isopropylmalate d	76.40	0.00
TRINITY_sp|P4921	RPS17	40S ribosomal prote	76.40	0.00
TRINITY_sp|Q9LV(	BOB1	Protein BOBBER 1 OS	76.40	0.00
TRINITY_sp|P327(	RBCS-3	Ribulose bisphospha	76.40	0.00
TRINITY_sp|Q4259	At1g7901	NADH dehydrogenase	76.40	0.00
TRINITY_sp|Q4319	LOX1.5	Probable linoleate	76.40	0.00
TRINITY_sp|Q9SJ1	MBF1A	Multiprotein-bridgi	76.30	0.00
TRINITY_sp|Q94F7	CHLI	Magnesium-chelatase	76.30	0.00
TRINITY_sp|Q8GT(	SCOA	Succinate--CoA liga	76.30	0.00
TRINITY_sp|O2331	NFYB3	Nuclear transcripti	76.30	0.00
TRINITY_sp|Q32PI	FCF1	rRNA-processing pro	76.20	0.00
TRINITY_sp|Q32LA	H2AFV	Histone H2A.V OS=Bo	76.20	0.00
TRINITY_sp|P0CG5	UBB	Polyubiquitin-B OS=	76.20	0.00
TRINITY_sp|P484(	Pp1alpha	Serine/threonine-pr	76.20	0.00
TRINITY_sp|Q9NI(	cypE	Peptidyl-prolyl cis	76.10	0.00
TRINITY_sp|Q54II	ragA	Ras-related GTP-bin	76.10	0.00
TRINITY_sp|Q9SBN	PGK	Phosphoglycerate ki	76.10	0.00
TRINITY_sp|Q9UFI	DNAH17	Dynein heavy chain	76.10	0.00
TRINITY_sp|P0C8N	CCRP1	Probable serine/thr	76.10	0.00
TRINITY_sp|Q6C01	SNU13	13 kDa ribonucleopr	76.00	0.00
TRINITY_sp|Q59LN	CSE4	Histone H3-like cen	76.00	0.00
TRINITY_sp|P2295	MED37D	Probable mediator o	76.00	0.00
TRINITY_sp|C1MI9	MICPUCDI	tRNA-splicing ligas	76.00	0.00
TRINITY_sp|Q9AX(	DHS	Deoxyhypusine synth	76.00	0.00
TRINITY_sp|H3JU(	SGT1	Peptidyl serine alp	76.00	0.00

TRINITY_sp|Q9DE2	ruvbl2	RuvB-like 2 OS=Xeno	76.00	0.00
TRINITY_sp|Q94JV	At1g5429	Protein translation	75.90	0.00
TRINITY_sp|P817(	-	Lysozyme C, spleen	75.90	0.00
TRINITY_sp|Q3BAI	-	Uncharacterized pro	75.90	0.00
TRINITY_sp|Q9YH1	SDHB	Succinate dehydroge	75.90	0.00
TRINITY_sp|Q55CI	ascc3l	Activating signal c	75.90	0.00
TRINITY_sp|Q245I	EIF6	Eukaryotic translat	75.90	0.00
TRINITY_sp|O8034	ERF4	Ethylene-responsive	75.90	0.00
TRINITY_sp|Q3957	ODA2	Dynein gamma chain,	75.90	0
TRINITY_sp|A2Y6(	CAM2	Calmodulin-2 OS=Ory	75.80	0.00
TRINITY_sp|P0444	Cd74	H-2 class II histoc	75.80	0.00
TRINITY_sp|P325(	RPD3	Histone deacetylase	75.80	0.00
TRINITY_sp|Q54QI	erkB	Extracellular signa	75.80	0.00
TRINITY_sp|Q5E98	RPS5	40S ribosomal prote	75.80	0.00
TRINITY_sp|O5768	sf3b1	Splicing factor 3B	75.80	0
TRINITY_sp|P2065	bimG	Serine/threonine-pr	75.80	0.00
TRINITY_sp|Q4268	GLN2	Glutamine synthetas	75.80	0.00
TRINITY_sp|P4969	RPL23A	60S ribosomal prote	75.80	0.00
TRINITY_sp|Q3BAI	-	Uncharacterized pro	75.70	0.00
TRINITY_sp|Q0709	PP2A1	Serine/threonine-pr	75.70	0.00
TRINITY_sp|P798I	tf	Serotransferrin OS=	75.70	0.00
TRINITY_sp|Q9SU(	ALDH2B4	Aldehyde dehydrogen	75.70	0.00
TRINITY_sp|Q949X	ADS3	Palmitoyl-monogalac	75.70	0.00
TRINITY_sp|O7868	mt-atp6	ATP synthase subuni	75.60	0.00
TRINITY_sp|A2Y6(	CAM2	Calmodulin-2 OS=Ory	75.60	0.00
TRINITY_sp|Q0869	RPS14	40S ribosomal prote	75.60	0.00
TRINITY_sp|P6299	RAC1	Ras-related C3 botu	75.60	0.00
TRINITY_sp|Q8W2(	CSN2	COP9 signalosome co	75.60	0.00
TRINITY_sp|P3879	DYS1	Deoxyhypusine synth	75.60	0.00
TRINITY_sp|Q96CI	C8orf44	Putative uncharacte	75.60	0.00
TRINITY_sp|P5025	ALTA	Tubulin alpha-1A ch	75.60	0.00
TRINITY_sp|Q039(	L1818	Chlorophyll a-b bin	75.60	0.00
TRINITY_sp|Q9Y2(	RUVBL1	RuvB-like 1 OS=Homo	75.60	0.00
TRINITY_sp|Q84KI	SQD1	UDP-sulfoquinovose	75.60	0.00
TRINITY_sp|Q396I	ODA11	Dynein alpha chain,	75.60	0.00
TRINITY_sp|O8084	PMM	Phosphomannomutase	75.60	0.00
TRINITY_sp|P314I	AVP1	Pyrophosphate-energ	75.60	0.00
TRINITY_sp|P546I	fttB	14-3-3-like protein	75.50	0.00
TRINITY_sp|P364(	gpbA	Guanine nucleotide-	75.50	0.00
TRINITY_sp|Q2II8	rplL	50S ribosomal prote	75.50	0.00
TRINITY_sp|Q6LCV	ch3-II	Histone H3 type 2 O	75.50	0.00
TRINITY_sp|P2855	PDS	Phytoene dehydrogen	75.50	0.00
TRINITY_sp|Q6PFI	ppdpfa	Pancreatic progenit	75.40	0.00
TRINITY_sp|Q6PBI	rpl8	60S ribosomal prote	75.40	0.00
TRINITY_sp|A8HUA	CFAP58	Cilia- and flagella	75.40	0.00
TRINITY_sp|P301(	-	Actin-1 OS=Pisum sa	75.30	0.00
TRINITY_sp|P2577	Os04g065	Oryzain alpha chain	75.30	0.00
TRINITY_sp|O9662	arcD	Actin-related prote	75.30	0.00
TRINITY_sp|P258(	UBC1	Ubiquitin-conjugati	75.30	0.00
TRINITY_sp|Q137I	ATP1A4	Sodium/potassium-tr	75.30	0.00
TRINITY_sp|Q8LSN	FYPP	Phytochrome-associa	75.30	0.00
TRINITY_sp|Q8RV(	SULP1	Sulfate permease 1,	75.30	0.00
TRINITY_sp|Q9VHS	eIF4AIII	Eukaryotic initiati	75.20	0.00
TRINITY_sp|C4IZX	TATC	Sec-independent pro	75.20	0.00
TRINITY_sp|Q9SCN	RPS2D	40S ribosomal prote	75.20	0.00
TRINITY_sp|Q9SX(	PAD1	Proteasome subunit	75.20	0.00

TRINITY_sp|P677[illegible]	PPP2CA	Serine/threonine-pr	75.10	0.00
TRINITY_sp|P263[illegible]	TBP1	TATA-box-binding pr	75.10	0.00
TRINITY_sp|Q6MV[illegible]	dut-1	Deoxyuridine 5'-tri	75.00	0.00
TRINITY_sp|Q95J[illegible]	DNAJA1	DnaJ homolog subfam	75.00	0.00
TRINITY_sp|P340[illegible]	dhcA	Dynein heavy chain,	75.00	0.00
TRINITY_sp|Q9ST[illegible]	-	Calreticulin OS=Chl	75.00	0.00
TRINITY_sp|Q6P2[illegible]	PRPF8	Pre-mRNA-processing	75.00	0.00
TRINITY_sp|Q399[illegible]	-	Beta-carotene ketol	75.00	0.00
TRINITY_sp|Q5VR[illegible]	HGO	Homogentisate 1,2-d	75.00	0.00
TRINITY_sp|Q6DR[illegible]	nop10	H/ACA ribonucleopro	75.00	0.00
TRINITY_sp|Q8H1[illegible]	MED37B	Probable mediator o	75.00	0.00
TRINITY_sp|Q388[illegible]	DXS	1-deoxy-D-xylulose-	75.00	0.00
TRINITY_sp|Q94A[illegible]	IIL1	3-isopropylmalate d	75.00	0.00
TRINITY_sp|Q9C0[illegible]	rpl37	60S ribosomal prote	75.00	0.00
TRINITY_sp|Q0QH[illegible]	-	Probable citrate sy	75.00	0.00
TRINITY_sp|Q109[illegible]	Os10g05[illegible]	DEAD-box ATP-depend	74.90	0.00
TRINITY_sp|P364[illegible]	ARL2	ADP-ribosylation fa	74.90	0.00
TRINITY_sp|Q018[illegible]	-	Heat shock 70 kDa p	74.90	0.00
TRINITY_sp|P629[illegible]	RAC1	Ras-related C3 botu	74.90	0.00
TRINITY_sp|Q9DD[illegible]	-	Lysozyme C OS=Paral	74.80	0.00
TRINITY_sp|P258[illegible]	-	Cysteine proteinase	74.80	0.00
TRINITY_sp|P546[illegible]	fttB	14-3-3-like protein	74.80	0.00
TRINITY_sp|P281[illegible]	rps13	40S ribosomal prote	74.80	0.00
TRINITY_sp|Q86C[illegible]	atg8	Autophagy-related p	74.80	0.00
TRINITY_sp|Q395[illegible]	-	Dynein 11 kDa light	74.80	0.00
TRINITY_sp|Q426[illegible]	GLN2	Glutamine synthetas	74.80	0.00
TRINITY_sp|Q32L[illegible]	bhmt	Betaine--homocystei	74.80	0.00
TRINITY_sp|Q426[illegible]	-	RuBisCO large subun	74.80	0.00
TRINITY_sp|P258[illegible]	UBC1	Ubiquitin-conjugati	74.70	0.00
TRINITY_sp|Q9LD[illegible]	CBF5	H/ACA ribonucleopro	74.70	0.00
TRINITY_sp|O614[illegible]	RPL37A	60S ribosomal prote	74.70	0.00
TRINITY_sp|Q9MB[illegible]	DHC10	Dynein-1-beta heavy	74.70	0
TRINITY_sp|O808[illegible]	PEX11D	Peroxisomal membran	74.70	0.00
TRINITY_sp|Q84X[illegible]	PAPP5	Serine/threonine-pr	74.60	0.00
TRINITY_sp|O237[illegible]	PAB1	Proteasome subunit	74.60	0.00
TRINITY_sp|O141[illegible]	ino80	Putative DNA helica	74.60	0.00
TRINITY_sp|Q6DU[illegible]	Rab7b	Ras-related protein	74.60	0.00
TRINITY_sp|Q9AR[illegible]	HD6	Casein kinase II su	74.60	0.00
TRINITY_sp|P798[illegible]	tf	Serotransferrin OS=	74.60	0.00
TRINITY_sp|P700[illegible]	RAD51	DNA repair protein	74.60	0.00
TRINITY_sp|Q090[illegible]	TEF	Elongation factor 1	74.60	0.00
TRINITY_sp|P310[illegible]	RpS2	40S ribosomal prote	74.60	0.00
TRINITY_sp|P096[illegible]	LPD1	Dihydrolipoyl dehyd	74.60	0.00
TRINITY_sp|P832[illegible]	hba1	Hemoglobin subunit	74.60	0.00
TRINITY_sp|Q9XX[illegible]	H28O16.[illegible]	ATP synthase subuni	74.60	0.00
TRINITY_sp|Q9LE[illegible]	IRE	Probable serine/thr	74.60	0.00
TRINITY_sp|Q61L[illegible]	arf-1.2	ADP-ribosylation fa	74.60	0.00
TRINITY_sp|Q059[illegible]	RAB1A	Ras-related protein	74.60	0.00
TRINITY_sp|P408[illegible]	-	Carbonic anhydrase,	74.60	0.00
TRINITY_sp|Q004[illegible]	RPL27A	60S ribosomal prote	74.50	0.00
TRINITY_sp|Q9P2[illegible]	KIF17	Kinesin-like protei	74.50	0.00
TRINITY_sp|O485[illegible]	RPL17	60S ribosomal prote	74.50	0.00
TRINITY_sp|P123[illegible]	PSAF	Photosystem I react	74.50	0.00
TRINITY_sp|Q9AR[illegible]	HD6	Casein kinase II su	74.50	0.00
TRINITY_sp|Q8RW[illegible]	CHR11	ISWI chromatin-remo	74.50	0.00
TRINITY_sp|Q852[illegible]	HD16	Casein kinase 1-lik	74.50	0.00

TRINITY_sp|Q557[illegible]	fpaB-1	SCF ubiquitin ligas	74.40	0.00
TRINITY_sp|Q54F[illegible]	narfl	Probable cytosolic	74.40	0.00
TRINITY_sp|P629[illegible]	RAC1	Ras-related C3 botu	74.40	0.00
TRINITY_sp|P446[illegible]	mog	Molybdopterin adeny	74.40	0.00
TRINITY_sp|B0C0[illegible]	miaB	tRNA-2-methylthio-N	74.40	0.00
TRINITY_sp|A7MB[illegible]	RQCD1	Cell differentiatio	74.40	0.00
TRINITY_sp|Q54R[illegible]	arl8	ADP-ribosylation fa	74.40	0.00
TRINITY_sp|P520[illegible]	cyn-7	Peptidyl-prolyl cis	74.40	0.00
TRINITY_sp|O598[illegible]	SPCC550.	Putative ATP-depend	74.30	0.00
TRINITY_sp|Q54G[illegible]	ascc3	Activating signal c	74.30	0.00
TRINITY_sp|Q007[illegible]	-	Histone H2A type 4	74.30	0.00
TRINITY_sp|Q8LA[illegible]	GATA1	GATA transcription	74.30	0.00
TRINITY_sp|A8IE[illegible]	PRMT1	Protein arginine N-	74.30	0.00
TRINITY_sp|Q256[illegible]	VAPB	V-type proton ATPas	74.20	0.00
TRINITY_sp|P490[illegible]	-	Vacuolar-processing	74.20	0.00
TRINITY_sp|Q54Q[illegible]	dimt1	Probable dimethylad	74.20	0.00
TRINITY_sp|Q9LE[illegible]	ENO1	Enolase 1 OS=Hevea	74.20	0.00
TRINITY_sp|P094[illegible]	CLTA	Clathrin light chai	74.20	0.00
TRINITY_sp|Q9BZ[illegible]	DPH1	Diphthamide biosynt	74.20	0.00
TRINITY_sp|Q395[illegible]	ODA4	Dynein beta chain,	74.20	0
TRINITY_sp|Q8L3[illegible]	FZR2	Protein FIZZY-RELAT	74.20	0.00
TRINITY_sp|P049[illegible]	CLTB	Clathrin light chai	74.20	0.00
TRINITY_sp|Q9DC[illegible]	Ndufs7	NADH dehydrogenase	74.10	0.00
TRINITY_sp|Q9DC[illegible]	Ndufs7	NADH dehydrogenase	74.10	0.00
TRINITY_sp|Q3BA[illegible]	-	Uncharacterized pro	74.10	0.00
TRINITY_sp|P368[illegible]	YPTV3	GTP-binding protein	74.10	0.00
TRINITY_sp|Q9MA[illegible]	SDC	Serine decarboxylas	74.10	0.00
TRINITY_sp|O226[illegible]	RPS16	40S ribosomal prote	74.10	0.00
TRINITY_sp|P818[illegible]	Ppc1	Phosphoenolpyruvate	74.10	0.00
TRINITY_sp|Q9M0[illegible]	AUR1	Serine/threonine-pr	74.10	0.00
TRINITY_sp|Q6ZD[illegible]	SDH1	Succinate dehydroge	74.10	0.00
TRINITY_sp|Q425[illegible]	At5g117[illegible]	NADH dehydrogenase	74.10	0.00
TRINITY_sp|P559[illegible]	-	Probable cyclin-dep	74.00	0.00
TRINITY_sp|P340[illegible]	dhcA	Dynein heavy chain,	74.00	0.00
TRINITY_sp|P611[illegible]	RAB2A	Ras-related protein	74.00	0.00
TRINITY_sp|Q5R5[illegible]	ATP5A1	ATP synthase subuni	74.00	0.00
TRINITY_sp|O650[illegible]	SB62	60S ribosomal prote	74.00	0.00
TRINITY_sp|Q6H5[illegible]	Os09g02[illegible]	Enoyl-[acyl-carrier	74.00	0.00
TRINITY_sp|Q390[illegible]	CPK11	Calcium-dependent p	74.00	0.00
TRINITY_sp|O818[illegible]	PUMP1	Mitochondrial uncou	74.00	0.00
TRINITY_sp|Q9SE[illegible]	RPT4A	26S protease regula	74.00	0.00
TRINITY_sp|Q54X[illegible]	aco1	Probable cytoplasmi	74.00	0.00
TRINITY_sp|Q401[illegible]	HSF8	Heat shock factor p	74.00	0.00
TRINITY_sp|Q9CP[illegible]	Cox6c	Cytochrome c oxidas	74.00	0.00
TRINITY_sp|Q047[illegible]	-	Tubulin beta chain	73.90	0.00
TRINITY_sp|Q245[illegible]	EIF6	Eukaryotic translat	73.90	0.00
TRINITY_sp|Q945[illegible]	RPS15	40S ribosomal prote	73.90	0.00
TRINITY_sp|Q3T0[illegible]	RPL30	60S ribosomal prote	73.90	0.00
TRINITY_sp|Q9DE[illegible]	ruvbl1	RuvB-like 1 OS=Xeno	73.90	0.00
TRINITY_sp|Q940[illegible]	TMN2	Transmembrane 9 sup	73.90	0.00
TRINITY_sp|Q9I8[illegible]	fabp10a	Fatty acid-binding	73.80	0.00
TRINITY_sp|Q86C[illegible]	tor	Target of rapamycin	73.80	0.00
TRINITY_sp|Q54H[illegible]	mybQ	Myb-like protein Q	73.80	0.00
TRINITY_sp|Q1W3[illegible]	-	Phosphomannomutase	73.80	0.00
TRINITY_sp|P121[illegible]	ATPC	ATP synthase gamma	73.80	0.00
TRINITY_sp|Q9FM[illegible]	PI4KB1	Phosphatidylinosito	73.80	0.00

TRINITY_sp|P074(	CAM	Calmodulin OS=Param	73.80	0.00
TRINITY_sp|Q8H1(	NBP35	Cytosolic Fe-S clus	73.80	0.00
TRINITY_sp|Q2QM(	Os12g06:	2-methyl-6-phytyl-1	73.80	0.00
TRINITY_sp|A8ILI	CFAP52	Cilia- and flagella	73.80	0.00
TRINITY_sp|A8J9'	THI1	Thiamine thiazole s	73.80	0.00
TRINITY_sp|Q9SSI	PRP8A	Pre-mRNA-processing	73.80	0.00
TRINITY_sp|Q3BAI	-	Uncharacterized pro	73.70	0.00
TRINITY_sp|E9L7A	-	Bifunctional aspart	73.70	0.00
TRINITY_sp|A2YW(	HSP81-1	Heat shock protein	73.70	0.00
TRINITY_sp|O429'	fkh1	Peptidyl-prolyl cis	73.70	0.00
TRINITY_sp|O046:	THRRS	Threonine--tRNA lig	73.70	0.00
TRINITY_sp|Q946'	RAP1	Ras-related protein	73.70	0.00
TRINITY_sp|Q3BAI	-	Uncharacterized pro	73.70	0.00
TRINITY_sp|O154:	ABCC4	Multidrug resistanc	73.70	0.00
TRINITY_sp|Q390:	MPK6	Mitogen-activated p	73.60	0.00
TRINITY_sp|Q9SII	RPS25B	40S ribosomal prote	73.60	0.00
TRINITY_sp|F4K2I	CUV	Pre-mRNA-splicing f	73.60	0.00
TRINITY_sp|Q8IQI	Mocs1	Molybdenum cofactor	73.60	0.00
TRINITY_sp|Q064'	-	Cysteine proteinase	73.60	0.00
TRINITY_sp|Q5UAI	RpS4	40S ribosomal prote	73.50	0.00
TRINITY_sp|Q5E9(	RPS5	40S ribosomal prote	73.50	0.00
TRINITY_sp|O237(	PAB1	Proteasome subunit	73.50	0.00
TRINITY_sp|Q54MI	dhx16	Putative pre-mRNA-s	73.50	0.00
TRINITY_sp|Q54R'	srfB	Serum response fact	73.50	0.00
TRINITY_sp|Q9SL(	PGR5	Protein PROTON GRAD	73.50	0.00
TRINITY_sp|P805'	-	Alcohol dehydrogena	73.50	0.00
TRINITY_sp|P268!	ATPA	ATP synthase subuni	73.50	0.00
TRINITY_sp|A8I2\	LIP1P	Lipoyl synthase, ch	73.50	0.00
TRINITY_sp|Q3SZI	SNRPD2	Small nuclear ribon	73.40	0.00
TRINITY_sp|P229!	MED37E	Probable mediator o	73.40	0.00
TRINITY_sp|P170(	-	Carbonic anhydrase,	73.40	0.00
TRINITY_sp|Q8H8'	UAM1	UDP-arabinopyranose	73.40	0.00
TRINITY_sp|Q9FJI	ABCF1	ABC transporter F f	73.40	0.00
TRINITY_sp|Q3SZI	SNRPD2	Small nuclear ribon	73.40	0.00
TRINITY_sp|P123(	PSBQ	Oxygen-evolving enh	73.30	0.00
TRINITY_sp|P462'	RPS14	40S ribosomal prote	73.30	0.00
TRINITY_sp|O243(	-	Plastidic ATP/ADP-t	73.30	0.00
TRINITY_sp|Q54XI	sec61a	Protein transport p	73.30	0.00
TRINITY_sp|P496:	-	Ubiquitin-60S ribos	73.30	0.00
TRINITY_sp|Q426(	SODA	Superoxide dismutas	73.30	0.00
TRINITY_sp|Q8L7(	BSL1	Serine/threonine-pr	73.30	0.00
TRINITY_sp|Q9T0:	At4g388'	tRNA-dihydrouridine	73.30	0.00
TRINITY_sp|Q3B8I	katnal2	Katanin p60 ATPase-	73.30	0.00
TRINITY_sp|Q9C5:	ACLB-1	ATP-citrate synthas	73.30	0.00
TRINITY_sp|Q6BXI	RPS6	40S ribosomal prote	73.20	0.00
TRINITY_sp|Q950:	metK	S-adenosylmethionin	73.20	0.00
TRINITY_sp|Q9XXI	H28O16.:	ATP synthase subuni	73.20	0.00
TRINITY_sp|P364:	rab14	Ras-related protein	73.10	0.00
TRINITY_sp|Q757(	RPS29	40S ribosomal prote	73.10	0.00
TRINITY_sp|P529(	-	14-3-3-like protein	73.10	0.00
TRINITY_sp|Q9SF:	TIM14-2	Mitochondrial impor	73.10	0.00
TRINITY_sp|Q9SVA	DRG3	Developmentally-reg	73.10	0.00
TRINITY_sp|Q8CG(	Abcc1	Multidrug resistanc	73.10	0.00
TRINITY_sp|Q94A'	UBC35	Ubiquitin-conjugati	73.10	0.00
TRINITY_sp|Q9P3X	cyp41	41 kDa peptidyl-pro	73.10	0.00
TRINITY_sp|P383(	Os02g01'	Protein transport p	73.10	0.00

TRINITY_sp|P221[illegible]	ACTA	Actin-1 OS=Phytopht	73.10	0.00
TRINITY_sp|Q9M0[illegible]	EIF6-2	Eukaryotic translat	73.10	0.00
TRINITY_sp|Q4AE[illegible]	GPX4	Phospholipid hydrop	73.00	0.00
TRINITY_sp|Q7ZU[illegible]	snrpe	Small nuclear ribon	73.00	0.00
TRINITY_sp|P544[illegible]	-	T-complex protein 1	72.90	0.00
TRINITY_sp|P425[illegible]	arpC	Actin-related prote	72.90	0.00
TRINITY_sp|Q9LZ[illegible]	At3g623[illegible]	Probable pre-mRNA-s	72.90	0.00
TRINITY_sp|Q4JQ[illegible]	MT-ND2	NADH-ubiquinone oxi	72.90	0.00
TRINITY_sp|A2T2[illegible]	IFT46	Intraflagellar tran	72.80	0.00
TRINITY_sp|Q9ST[illegible]	DUT	Deoxyuridine 5'-tri	72.80	0.00
TRINITY_sp|O611[illegible]	svkA	Serine/threonine-pr	72.80	0.00
TRINITY_sp|Q395[illegible]	YPTC6	Ras-related protein	72.80	0.00
TRINITY_sp|Q408[illegible]	MPK1	Mitogen-activated p	72.80	0.00
TRINITY_sp|Q425[illegible]	FTSZ1	Cell division prote	72.80	0.00
TRINITY_sp|Q057[illegible]	IGFBP4	Insulin-like growth	72.70	0.00
TRINITY_sp|A8JB[illegible]	CHLREDR[illegible]	Pescadillo homolog	72.70	0.00
TRINITY_sp|Q3SZ[illegible]	RPL32	60S ribosomal prote	72.70	0.00
TRINITY_sp|Q9LR[illegible]	RPL27AB	60S ribosomal prote	72.70	0.00
TRINITY_sp|Q8LE[illegible]	CHMP1A	ESCRT-related prote	72.70	0.00
TRINITY_sp|P0C8[illegible]	CCRP1	Probable serine/thr	72.70	0.00
TRINITY_sp|Q6P2[illegible]	PRPF8	Pre-mRNA-processing	72.70	0.00
TRINITY_sp|Q9FL[illegible]	NRPB12	DNA-directed RNA po	72.70	0.00
TRINITY_sp|Q6WW[illegible]	UPL3	E3 ubiquitin-protei	72.70	0.00
TRINITY_sp|Q68E[illegible]	rab4b	Ras-related protein	72.70	0.00
TRINITY_sp|P479[illegible]	rps27	40S ribosomal prote	72.70	0.00
TRINITY_sp|P390[illegible]	-	Dynein beta chain,	72.70	0.00
TRINITY_sp|Q9BS[illegible]	NAA11	N-alpha-acetyltrans	72.70	0.00
TRINITY_sp|Q1ZX[illegible]	elp3	Probable elongator	72.60	0.00
TRINITY_sp|Q279[illegible]	HSPA1A	Heat shock 70 kDa p	72.60	0.00
TRINITY_sp|Q1KV[illegible]	infA	Translation initiat	72.60	0.00
TRINITY_sp|Q9Y3[illegible]	LSM2	U6 snRNA-associated	72.60	0.00
TRINITY_sp|P492[illegible]	PCK1	Phosphoenolpyruvate	72.60	0.00
TRINITY_sp|Q9P2[illegible]	DNAH1	Dynein heavy chain	72.50	0.00
TRINITY_sp|Q6F4[illegible]	RpL39	60S ribosomal prote	72.50	0.00
TRINITY_sp|Q6F4[illegible]	RpL39	60S ribosomal prote	72.50	0.00
TRINITY_sp|P420[illegible]	NDUFS7	NADH dehydrogenase	72.50	0.00
TRINITY_sp|P502[illegible]	ALTA	Tubulin alpha-1A ch	72.50	0.00
TRINITY_sp|Q2HJ[illegible]	LSM5	U6 snRNA-associated	72.50	0.00
TRINITY_sp|Q54K[illegible]	manG	Alpha-mannosidase G	72.50	0.00
TRINITY_sp|O044[illegible]	CCT5	T-complex protein 1	72.50	0.00
TRINITY_sp|Q56Y[illegible]	MAP2B	Methionine aminopep	72.50	0.00
TRINITY_sp|A5D8[illegible]	sbds	Ribosome maturation	72.50	0.00
TRINITY_sp|Q949[illegible]	MPC1	Mitochondrial pyruv	72.50	0.00
TRINITY_sp|Q75I[illegible]	Os05g01[illegible]	Isovaleryl-CoA dehy	72.50	0.00
TRINITY_sp|Q271[illegible]	EFA2	Elongation factor 1	72.50	0.00
TRINITY_sp|Q8L4[illegible]	CDKB1-1	Cyclin-dependent ki	72.50	0.00
TRINITY_sp|A8JF[illegible]	NCS6	Cytoplasmic tRNA 2-	72.50	0.00
TRINITY_sp|Q9ZS[illegible]	CHLP	Geranylgeranyl diph	72.50	0.00
TRINITY_sp|Q419[illegible]	PSBQ2	Oxygen-evolving enh	72.40	0.00
TRINITY_sp|Q9M0[illegible]	EIF6-2	Eukaryotic translat	72.40	0.00
TRINITY_sp|P419[illegible]	-	GTP-binding nuclear	72.40	0.00
TRINITY_sp|Q8LA[illegible]	GATA1	GATA transcription	72.40	0.00
TRINITY_sp|Q7X9[illegible]	PIE1	Protein PHOTOPERIOD	72.40	0.00
TRINITY_sp|P544[illegible]	-	T-complex protein 1	72.40	0.00
TRINITY_sp|O823[illegible]	THIC	Phosphomethylpyrimi	72.40	0.00
TRINITY_sp|Q9SI[illegible]	At2g299[illegible]	Presenilin-like pro	72.40	0.00

TRINITY_sp|P534{	arp2	Actin-related prote	72.40	0.00
TRINITY_sp|P677	PPP2CA	Serine/threonine-pr	72.30	0.00
TRINITY_sp|Q9XG	PRS2	Ribose-phosphate py	72.30	0.00
TRINITY_sp|Q631	Dnah1	Dynein heavy chain	72.30	0.00
TRINITY_sp|Q275	enol-1	Enolase OS=Caenorha	72.30	0.00
TRINITY_sp|Q9LU	RAP2-2	Ethylene-responsive	72.30	0.00
TRINITY_sp|P287	-	Formate--tetrahydro	72.30	0.00
TRINITY_sp|Q9LW	At3g233	Uncharacterized pro	72.30	0.00
TRINITY_sp|Q9ZP	SDH1-2	Succinate dehydroge	72.30	0.00
TRINITY_sp|Q8TD	DNAH3	Dynein heavy chain	72.30	0.00
TRINITY_sp|Q392	CAX1	Vacuolar cation/pro	72.20	0.00
TRINITY_sp|P502	TUBB4	Tubulin beta-4 chai	72.20	0.00
TRINITY_sp|P425	rnrB-1	Ribonucleoside-diph	72.20	0.00
TRINITY_sp|O860	bdhA	D-beta-hydroxybutyr	72.20	0.00
TRINITY_sp|Q54Q	rnrA	Ribonucleoside-diph	72.20	0.00
TRINITY_sp|Q54X	sec61a	Protein transport p	72.20	0.00
TRINITY_sp|Q9LT	RPN11	26S proteasome non-	72.20	0.00
TRINITY_sp|P542	CA1	Cation-transporting	72.20	0.00
TRINITY_sp|P078	act1	Major actin OS=Dict	72.20	0.00
TRINITY_sp|A8HT	tuf1	Elongation factor T	72.20	0.00
TRINITY_sp|Q84K	SQD1	UDP-sulfoquinovose	72.20	0.00
TRINITY_sp|Q76I	RPS15A	40S ribosomal prote	72.10	0.00
TRINITY_sp|Q419	PSBQ2	Oxygen-evolving enh	72.10	0.00
TRINITY_sp|P544	-	T-complex protein 1	72.10	0.00
TRINITY_sp|P341	myoD	Myosin ID heavy cha	72.10	0.00
TRINITY_sp|P629	RAC1	Ras-related C3 botu	72.10	0.00
TRINITY_sp|A2XC	CKS1	Cyclin-dependent ki	72.10	0.00
TRINITY_sp|Q5E9	SSNA1	Sjoegren syndrome n	72.10	0.00
TRINITY_sp|Q9JL	Spag6	Sperm-associated an	72.10	0.00
TRINITY_sp|A4SX	cynS	Cyanate hydratase O	72.10	0.00
TRINITY_sp|Q9SJ	VPS11	Vacuolar protein-so	72.10	0.00
TRINITY_sp|Q389	KIN10	SNF1-related protei	72.00	0.00
TRINITY_sp|O236	At1g021	Protein mago nashi	72.00	0.00
TRINITY_sp|O237	PBB1	Proteasome subunit	72.00	0.00
TRINITY_sp|Q86J	rab5A	Ras-related protein	72.00	0.00
TRINITY_sp|P0CT	rps3001	40S ribosomal prote	72.00	0.00
TRINITY_sp|Q3ZB	LSM4	U6 snRNA-associated	72.00	0.00
TRINITY_sp|Q9SJ	RNR1	Ribonucleoside-diph	72.00	0
TRINITY_sp|P105	SSA1	Heat shock protein	72.00	0.00
TRINITY_sp|Q9P2	DNAH1	Dynein heavy chain	72.00	0.00
TRINITY_sp|P229	let-60	Ras protein let-60	71.90	0.00
TRINITY_sp|Q54N	DDB_G02	Probable methylthio	71.90	0.00
TRINITY_sp|P128	PSBQ	Oxygen-evolving enh	71.90	0.00
TRINITY_sp|Q9FS	IVD	Isovaleryl-CoA dehy	71.90	0.00
TRINITY_sp|Q919	rad51-a	DNA repair protein	71.90	0.00
TRINITY_sp|P290	FER1	Ferritin-1, chlorop	71.90	0.00
TRINITY_sp|Q9XH	LC1	Dynein light chain	71.90	0.00
TRINITY_sp|P128	ANT2	ADP,ATP carrier pro	71.90	0.00
TRINITY_sp|P426	GRF5	14-3-3-like protein	71.90	0.00
TRINITY_sp|P318	-	RNA-directed DNA po	71.90	0.00
TRINITY_sp|P929	RABB1C	Ras-related protein	71.80	0.00
TRINITY_sp|P064	env	Envelope glycoprote	71.80	0.00
TRINITY_sp|Q6Z6	NCS6	Cytoplasmic tRNA 2-	71.80	0.00
TRINITY_sp|Q54R	gcdh	Glutaryl-CoA dehydr	71.80	0.00
TRINITY_sp|P519	NEK2	Serine/threonine-pr	71.80	0.00
TRINITY_sp|Q9SE	RPT3	26S protease regula	71.80	0.00

TRINITY_sp|Q09J1	RpL38	60S ribosomal prote	71.80	0.00
TRINITY_sp|Q919	rad51-a	DNA repair protein	71.80	0.00
TRINITY_sp|Q389	LWD2	WD repeat-containin	71.80	0.00
TRINITY_sp|Q395	-	5-methyltetrahydrop	71.80	0.00
TRINITY_sp|P341	racC	Rho-related protein	71.70	0.00
TRINITY_sp|P240	abpB	Calcium-regulated a	71.70	0.00
TRINITY_sp|Q9DE	ruvbl2	RuvB-like 2 OS=Xeno	71.70	0.00
TRINITY_sp|Q962	RpL37	60S ribosomal prote	71.70	0.00
TRINITY_sp|Q6ZD	SDH1	Succinate dehydroge	71.70	0.00
TRINITY_sp|Q9LX	RPS9B	40S ribosomal prote	71.70	0.00
TRINITY_sp|Q9ST	HOP3	Hsp70-Hsp90 organiz	71.70	0.00
TRINITY_sp|B9DG	ACS	Acetyl-coenzyme A s	71.70	0.00
TRINITY_sp|Q9W3	xit	Probable dolichyl p	71.60	0.00
TRINITY_sp|Q396	PCKR1	Peptidyl-prolyl cis	71.60	0.00
TRINITY_sp|Q294	HMGCL	Hydroxymethylglutar	71.60	0.00
TRINITY_sp|Q0UV	NSA2	Ribosome biogenesis	71.60	0.00
TRINITY_sp|Q54P	csn5	COP9 signalosome co	71.60	0.00
TRINITY_sp|Q9Z0	Eif2s3y	Eukaryotic translat	71.60	0.00
TRINITY_sp|Q9AU	UGD3	UDP-glucose 6-dehyd	71.60	0.00
TRINITY_sp|Q54M	dhx16	Putative pre-mRNA-s	71.50	0.00
TRINITY_sp|Q9LH	FYPP3	Phytochrome-associa	71.50	0.00
TRINITY_sp|Q9SA	CSP41B	Chloroplast stem-lo	71.50	0.00
TRINITY_sp|O004	PSMD14	26S proteasome non-	71.50	0.00
TRINITY_sp|Q9SY	NRPB10L	DNA-directed RNA po	71.40	0.00
TRINITY_sp|Q9UT	SPAC15E	Probable thymidylat	71.40	0.00
TRINITY_sp|O823	THIC	Phosphomethylpyrimi	71.40	0.00
TRINITY_sp|P419	-	GTP-binding nuclear	71.40	0.00
TRINITY_sp|P552	FUMR	Fumarate hydratase,	71.40	0.00
TRINITY_sp|P105	MIC	Myosin IC heavy cha	71.40	0.00
TRINITY_sp|Q54M	dhx16	Putative pre-mRNA-s	71.40	0.00
TRINITY_sp|P464	TBP2	26S protease regula	71.40	0.00
TRINITY_sp|Q9ZS	DER2.2	Derlin-2.2 OS=Arabi	71.40	0.00
TRINITY_sp|Q9W5	Roc1a	RING-box protein 1A	71.40	0.00
TRINITY_sp|P980	c3	Complement C3 (Frag	71.40	0.00
TRINITY_sp|P275	-	Chlorophyll a-b bin	71.40	0.00
TRINITY_sp|O822	At2g197	AP-4 complex subuni	71.40	0.00
TRINITY_sp|Q8IS	RPS18	40S ribosomal prote	71.40	0.00
TRINITY_sp|P071	DBI	Acyl-CoA-binding pr	71.40	0.00
TRINITY_sp|P128	PSBQ	Oxygen-evolving enh	71.40	0.00
TRINITY_sp|O498	-	Peptidyl-prolyl cis	71.40	0.00
TRINITY_sp|A2XF	PDC2	Pyruvate decarboxyl	71.40	0.00
TRINITY_sp|P565	HDAC1	Histone deacetylase	71.40	0.00
TRINITY_sp|Q8LP	ABCE2	ABC transporter E f	71.40	0.00
TRINITY_sp|Q2V9	-	Transcription facto	71.40	0.00
TRINITY_sp|Q17Q	TFDP1	Transcription facto	71.40	0.00
TRINITY_sp|Q9C5	SAC7	Phosphoinositide ph	71.40	0.00
TRINITY_sp|Q9C0	DNAH6	Dynein heavy chain	71.40	0.00
TRINITY_sp|P329	ATPD	ATP synthase delta	71.30	0.00
TRINITY_sp|P0DJ	RPL19	60S ribosomal prote	71.30	0.00
TRINITY_sp|Q9CA	ABCI6	ABC transporter I f	71.30	0.00
TRINITY_sp|P468	KLP1	Kinesin-like protei	71.30	0.00
TRINITY_sp|Q9AT	UVH3	DNA repair protein	71.30	0.00
TRINITY_sp|P128	bud31	Protein BUD31 homol	71.30	0.00
TRINITY_sp|P427	UBC4	Ubiquitin-conjugati	71.20	0.00
TRINITY_sp|Q9UV	UBC1	Ubiquitin-conjugati	71.20	0.00
TRINITY_sp|Q552	wdr68	DDB1- and CUL4-asso	71.20	0.00

TRINITY_sp|Q070[illegible]	HSP81-3	Heat shock protein	71.20	0.00
TRINITY_sp|B8HV[illegible]	rplK	50S ribosomal prote	71.20	0.00
TRINITY_sp|Q014[illegible]	SAR1B	GTP-binding protein	71.20	0.00
TRINITY_sp|P491[illegible]	RPS8	40S ribosomal prote	71.20	0.00
TRINITY_sp|A2XU[illegible]	CDKG-2	Cyclin-dependent ki	71.20	0.00
TRINITY_sp|P290[illegible]	-	Acidic endochitinas	71.10	0.00
TRINITY_sp|P351[illegible]	UBC2	Ubiquitin-conjugati	71.10	0.00
TRINITY_sp|Q3BA[illegible]	-	Uncharacterized pro	71.10	0.00
TRINITY_sp|Q3BA[illegible]	-	Uncharacterized pro	71.10	0.00
TRINITY_sp|Q9FJ[illegible]	ABCF1	ABC transporter F f	71.10	0.00
TRINITY_sp|B0C3[illegible]	dapF	Diaminopimelate epi	71.10	0.00
TRINITY_sp|Q8H6[illegible]	SPT16	FACT complex subuni	71.10	0.00
TRINITY_sp|P200[illegible]	pyr1-3	Protein PYR1-3 OS=D	71.10	0.00
TRINITY_sp|P733[illegible]	argB	Acetylglutamate kin	71.10	0.00
TRINITY_sp|Q9SL[illegible]	TCX6	Protein tesmin/TSO1	71.10	0.00
TRINITY_sp|Q9AR[illegible]	HD6	Casein kinase II su	71.10	0.00
TRINITY_sp|P287[illegible]	CCT1	T-complex protein 1	71.10	0.00
TRINITY_sp|Q6AV[illegible]	PPDK1	Pyruvate, phosphate	71.10	0.00
TRINITY_sp|P464[illegible]	-	Malate dehydrogenas	71.10	0.00
TRINITY_sp|Q54K[illegible]	rbx1	RING-box protein 1	71.10	0.00
TRINITY_sp|P322[illegible]	rasS	Ras-like protein ra	71.10	0.00
TRINITY_sp|Q949[illegible]	PSAO	Photosystem I subun	71.00	0.00
TRINITY_sp|P462[illegible]	RPS16	40S ribosomal prote	71.00	0.00
TRINITY_sp|Q9M8[illegible]	At1g742[illegible]	Probable phosphorib	71.00	0.00
TRINITY_sp|P581[illegible]	yhhW	Quercetin 2,3-dioxy	71.00	0.00
TRINITY_sp|Q2KI[illegible]	PSMC6	26S protease regula	71.00	0.00
TRINITY_sp|Q940[illegible]	RPL18C	60S ribosomal prote	71.00	0.00
TRINITY_sp|A9NU[illegible]	METK1	S-adenosylmethionin	71.00	0.00
TRINITY_sp|Q2KH[illegible]	GCDH	Glutaryl-CoA dehydr	71.00	0.00
TRINITY_sp|Q962[illegible]	RpS17	40S ribosomal prote	71.00	0.00
TRINITY_sp|A2XC[illegible]	CKS1	Cyclin-dependent ki	71.00	0.00
TRINITY_sp|Q944[illegible]	ctu1	Cytoplasmic tRNA 2-	71.00	0.00
TRINITY_sp|O756[illegible]	GCAT	2-amino-3-ketobutyr	71.00	0.00
TRINITY_sp|Q9SJ[illegible]	RNR1	Ribonucleoside-diph	71.00	0.00
TRINITY_sp|Q8LA[illegible]	GATA1	GATA transcription	71.00	0.00
TRINITY_sp|Q018[illegible]	-	Heat shock 70 kDa p	71.00	0.00
TRINITY_sp|Q050[illegible]	CPN60-2	Chaperonin CPN60-2,	71.00	0.00
TRINITY_sp|P535[illegible]	CYME_CM[illegible]	Actin OS=Cyanidiosc	71.00	0.00
TRINITY_sp|Q9P7[illegible]	nop10	H/ACA ribonucleopro	71.00	0.00
TRINITY_sp|O244[illegible]	-	Eukaryotic translat	71.00	0.00
TRINITY_sp|Q9LZ[illegible]	TOP6A	DNA topoisomerase 6	71.00	0.00
TRINITY_sp|Q94A[illegible]	MED36A	Mediator of RNA pol	70.90	0.00
TRINITY_sp|Q93W[illegible]	NIFU2	NifU-like protein 2	70.90	0.00
TRINITY_sp|Q9M7[illegible]	RPL29A	60S ribosomal prote	70.90	0.00
TRINITY_sp|P497[illegible]	Oat	Ornithine aminotran	70.90	0.00
TRINITY_sp|Q271[illegible]	DHC-8	Dynein heavy chain,	70.90	0.00
TRINITY_sp|Q54P[illegible]	prsA	Ribose-phosphate py	70.90	0.00
TRINITY_sp|Q84X[illegible]	PAPP5	Serine/threonine-pr	70.90	0.00
TRINITY_sp|Q9M0[illegible]	PFK1	ATP-dependent 6-pho	70.90	0.00
TRINITY_sp|Q9M3[illegible]	KPHMT2	3-methyl-2-oxobutan	70.90	0.00
TRINITY_sp|Q8LB[illegible]	PRP38	Pre-mRNA-splicing f	70.90	0.00
TRINITY_sp|P239[illegible]	KAS12	3-oxoacyl-[acyl-car	70.90	0.00
TRINITY_sp|Q9AR[illegible]	CTH1	Magnesium-protoporp	70.90	0.00
TRINITY_sp|Q8L7[illegible]	BSL1	Serine/threonine-pr	70.90	0.00
TRINITY_sp|P498[illegible]	GSK3A	Glycogen synthase k	70.80	0.00
TRINITY_sp|B8EQ[illegible]	mnmG	tRNA uridine 5-carb	70.80	0.00

TRINITY_sp|P797!	c9	Complement componen	70.80	0.00
TRINITY_sp|P905	-	Tubulin gamma-2 cha	70.80	0.00
TRINITY_sp|Q5QMI	Os01g01	DEAD-box ATP-depend	70.80	0.00
TRINITY_sp|Q8JFI	EIF4A2	Eukaryotic initiati	70.80	0.00
TRINITY_sp|Q3E8	YLR154C-	Uncharacterized pro	70.80	0.00
TRINITY_sp|P344!	mog-1	Probable pre-mRNA-s	70.80	0.00
TRINITY_sp|P483	FK506-b	12 kDa FK506-bindin	70.80	0.00
TRINITY_sp|P436!	drg1	Developmentally-reg	70.80	0.00
TRINITY_sp|O755	SF3B1	Splicing factor 3B	70.80	0
TRINITY_sp|Q9M3	NPF8.1	Protein NRT1/ PTR F	70.70	0.00
TRINITY_sp|P546	pikA	Phosphatidylinosito	70.70	0.00
TRINITY_sp|P496	RPL19C	60S ribosomal prote	70.70	0.00
TRINITY_sp|Q9SI	WRKY1	WRKY transcription	70.70	0.00
TRINITY_sp|Q388	CHLG	Chlorophyll synthas	70.70	0.00
TRINITY_sp|O426	smd1	Small nuclear ribon	70.70	0.00
TRINITY_sp|Q416	CDC2	Cell division contr	70.70	0.00
TRINITY_sp|Q2R1	GME-2	GDP-mannose 3,5-epi	70.70	0.00
TRINITY_sp|Q9FN	MBB1	PsbB mRNA maturatio	70.70	0.00
TRINITY_sp|Q54K	rbx1	RING-box protein 1	70.70	0.00
TRINITY_sp|P427	PAB2	Polyadenylate-bindi	70.70	0.00
TRINITY_sp|Q9SM	DHC1	Dynein-1-alpha heav	70.70	0
TRINITY_sp|Q3BA	-	Uncharacterized pro	70.70	0.00
TRINITY_sp|P168	-	Histone H2A-III OS=	70.70	0.00
TRINITY_sp|P222	fbl	rRNA 2'-O-methyltra	70.60	0.00
TRINITY_sp|P524	Ubc2	Ubiquitin-conjugati	70.60	0.00
TRINITY_sp|Q54I	acad8	Isobutyryl-CoA dehy	70.60	0.00
TRINITY_sp|Q426	SAMDC	S-adenosylmethionin	70.60	0.00
TRINITY_sp|O243	-	Plastidic ATP/ADP-t	70.60	0.00
TRINITY_sp|P736	mutS2	Endonuclease MutS2	70.60	0.00
TRINITY_sp|Q9SA	BCDH	2-oxoisovalerate de	70.60	0.00
TRINITY_sp|Q0E2	UVR3	(6-4)DNA photolyase	70.50	0.00
TRINITY_sp|Q55F	bkdB	2-oxoisovalerate de	70.50	0.00
TRINITY_sp|P491	RAB2A	Ras-related protein	70.50	0.00
TRINITY_sp|O157	vacA	Vacuolin-A OS=Dicty	70.50	0.00
TRINITY_sp|Q9AR	HD6	Casein kinase II su	70.50	0.00
TRINITY_sp|P800	GGPS1	Geranylgeranyl pyro	70.50	0.00
TRINITY_sp|Q8RY	PEX6	Peroxisome biogenes	70.50	0.00
TRINITY_sp|P804	PSBY	Photosystem II core	70.50	0.00
TRINITY_sp|B0G1	ap1s2	AP-1 complex subuni	70.50	0.00
TRINITY_sp|Q428	RPB2	DNA-directed RNA po	70.50	0
TRINITY_sp|P0C8	CCRP1	Probable serine/thr	70.50	0.00
TRINITY_sp|Q9HB	ATP6V0A	V-type proton ATPas	70.50	0.00
TRINITY_sp|P341	mvpA	Major vault protein	70.50	0.00
TRINITY_sp|Q9SL	RAD50	DNA repair protein	70.50	0.00
TRINITY_sp|Q84J	B''EPSI	Probable serine/thr	70.50	0.00
TRINITY_sp|A4FU	SNRPE	Small nuclear ribon	70.40	0.00
TRINITY_sp|Q9XG	PAA1	Proteasome subunit	70.40	0.00
TRINITY_sp|Q28I	poc1a	POC1 centriolar pro	70.40	0.00
TRINITY_sp|P207	MAK	Serine/threonine-pr	70.40	0.00
TRINITY_sp|Q922	Dyrk3	Dual specificity ty	70.40	0.00
TRINITY_sp|O225	CLA1	1-deoxy-D-xylulose-	70.40	0.00
TRINITY_sp|Q9C5	MSRB2	Peptide methionine	70.30	0.00
TRINITY_sp|Q182	rap-1	Ras-related protein	70.30	0.00
TRINITY_sp|P331	IDH2	Isocitrate dehydrog	70.30	0.00
TRINITY_sp|Q8H1	ABCI10	ABC transporter I f	70.30	0.00
TRINITY_sp|Q7ZX	rap1b	Ras-related protein	70.30	0.00

TRINITY_sp|P542(	CA1	Cation-transporting	70.30	0.00
TRINITY_sp|Q5Z9'	FTSH1	ATP-dependent zinc	70.30	0.00
TRINITY_sp|Q9T0!	At4g277‹	Protein yippee-like	70.30	0.00
TRINITY_sp|P068(	PLG	Plasminogen OS=Bos	70.20	0.00
TRINITY_sp|O228!	At2g472!	Probable pre-mRNA-s	70.20	0.00
TRINITY_sp|P506‹	RNR1	Ribonucleoside-diph	70.20	0.00
TRINITY_sp|A2XUV	CDKG-2	Cyclin-dependent ki	70.20	0.00
TRINITY_sp|P427!	RPL11B	60S ribosomal prote	70.20	0.00
TRINITY_sp|P557:	HSP90-2	Heat shock protein	70.20	0.00
TRINITY_sp|Q8LSI	FYPP	Phytochrome-associa	70.20	0.00
TRINITY_sp|Q8RW\	TKL-1	Transketolase-1, ch	70.20	0.00
TRINITY_sp|O822:	At2g239:	Probable small nucl	70.20	0.00
TRINITY_sp|P008'	RBCS-1	Ribulose bisphospha	70.20	0.00
TRINITY_sp|Q67E(	RAD51A	DNA repair protein	70.20	0.00
TRINITY_sp|Q9ZTI	ERD2	ER lumen protein-re	70.20	0.00
TRINITY_sp|A8XJ!	rpl-11.:	60S ribosomal prote	70.20	0.00
TRINITY_sp|O943{	psm1	Structural maintena	70.10	0.00
TRINITY_sp|P623:	LSM6	U6 snRNA-associated	70.10	0.00
TRINITY_sp|P088!	his-2	Histone H3 OS=Caeno	70.10	0.00
TRINITY_sp|Q9XF{	LHCB5	Chlorophyll a-b bin	70.10	0.00
TRINITY_sp|Q8T2I	nubp1	Cytosolic Fe-S clus	70.10	0.00
TRINITY_sp|Q148:	CHD4	Chromodomain-helica	70.10	0.00
TRINITY_sp|O813!	DRTS	Bifunctional dihydr	70.10	0.00
TRINITY_sp|P490:	Os12g02{	Protein mago nashi	70.10	0.00
TRINITY_sp|Q396:	PORA	Protochlorophyllide	70.10	0.00
TRINITY_sp|Q9LV\	At5g529'	Thylakoid lumenal 1	70.10	0.00
TRINITY_sp|Q55C/	vps26	Vacuolar protein so	70.10	0.00
TRINITY_sp|Q8LK(	GAPN	NADP-dependent glyc	70.10	0.00
TRINITY_sp|Q2JS‹	pyrH	Uridylate kinase OS	70.10	0.00
TRINITY_sp|P364:	hspB	Heat shock cognate	70.10	0.00
TRINITY_sp|P479(	rps27	40S ribosomal prote	70.10	0.00
TRINITY_sp|P806{	-	Ferredoxin-thioredo	70.00	0.00
TRINITY_sp|Q401!	MTB	Metallothionein-lik	70.00	0.00
TRINITY_sp|Q8VYI	CKL12	Casein kinase 1-lik	70.00	0.00
TRINITY_sp|P348!	CPN10	10 kDa chaperonin,	70.00	0.00
TRINITY_sp|P385!	TUBG2	Tubulin gamma-2 cha	70.00	0.00
TRINITY_sp|Q9LV:	CCT4	T-complex protein 1	70.00	0.00
TRINITY_sp|A7MB‹	RQCD1	Cell differentiatio	70.00	0.00
TRINITY_sp|Q9UFI	DNAH17	Dynein heavy chain	70.00	0.00
TRINITY_sp|Q437{	WAXY	Granule-bound starc	70.00	0.00
TRINITY_sp|Q9QXI	Trip4	Activating signal c	70.00	0.00
TRINITY_sp|Q154:	SF3B4	Splicing factor 3B	69.90	0.00
TRINITY_sp|Q9ZN:	GLU1	Ferredoxin-dependen	69.90	0.00
TRINITY_sp|Q93VI	At5g288‹	GDP-mannose 3,5-epi	69.90	0.00
TRINITY_sp|Q8AV{	btd	Biotinidase OS=Taki	69.80	0.00
TRINITY_sp|P0CG{	TU20	Polyubiquitin OS=Te	69.80	0.00
TRINITY_sp|P518!	PDC1	Pyruvate decarboxyl	69.80	0.00
TRINITY_sp|Q462(	pfl	Formate acetyltrans	69.80	0.00
TRINITY_sp|Q392!	SKP1A	SKP1-like protein 1	69.80	0.00
TRINITY_sp|B3QPI	gpmA	2,3-bisphosphoglyce	69.80	0.00
TRINITY_sp|A1JHI	HST	Homogentisate solan	69.80	0.00
TRINITY_sp|Q9SE:	RPT3	26S protease regula	69.80	0.00
TRINITY_sp|Q8WX:	DNAH7	Dynein heavy chain	69.80	0.00
TRINITY_sp|Q9LV:	PYD1	Dihydropyrimidine d	69.80	0.00
TRINITY_sp|Q8LSI	FYPP	Phytochrome-associa	69.80	0.00
TRINITY_sp|P296:	CDKA-1	Cyclin-dependent ki	69.80	0.00

TRINITY_sp|Q9STI	-	Calreticulin OS=Chl	69.80	0.00
TRINITY_sp|Q997S	ACO2	Aconitate hydratase	69.80	0.00
TRINITY_sp|Q0DHI	FTSH8	ATP-dependent zinc	69.80	0.00
TRINITY_sp|Q0E2Y	UVR3	(6-4)DNA photolyase	69.70	0.00
TRINITY_sp|Q54CW	rtpR	Probable adenosylco	69.70	0.00
TRINITY_sp|P677	PPP2CA	Serine/threonine-pr	69.70	0.00
TRINITY_sp|Q9ZPX	ARL2	ADP-ribosylation fa	69.70	0.00
TRINITY_sp|Q3E9A	ANTR6	Probable anion tran	69.70	0.00
TRINITY_sp|O2287	FKBP16-3	Peptidyl-prolyl cis	69.70	0.00
TRINITY_sp|P4973	-	Ribonucleoside-diph	69.70	0.00
TRINITY_sp|B3FHI	SCSa	Succinate--CoA liga	69.70	0.00
TRINITY_sp|Q8S2A	FTSH3	ATP-dependent zinc	69.70	0.00
TRINITY_sp|P4858	pph-1	Serine/threonine-pr	69.70	0.00
TRINITY_sp|Q8LCQ	LHCA6	Photosystem I chlor	69.70	0.00
TRINITY_sp|P4595	ARP	DNA-(apurinic or ap	69.70	0.00
TRINITY_sp|Q4256	ACO1	Aconitate hydratase	69.70	0.00
TRINITY_sp|P0082	ATP5B	ATP synthase subuni	69.70	0.00
TRINITY_sp|Q4372	APG1	Glucose-6-phosphate	69.70	0.00
TRINITY_sp|Q376I	ND4	NADH-ubiquinone oxi	69.70	0.00
TRINITY_sp|Q0368	BIP4	Luminal-binding pro	69.60	0.00
TRINITY_sp|Q6WV	-	Histone H4 OS=Mytil	69.60	0.00
TRINITY_sp|A7RWI	v1g22659	Probable cytosolic	69.60	0.00
TRINITY_sp|B6U15	GATA	Glutamyl-tRNA(Gln)	69.60	0.00
TRINITY_sp|Q54S3	DDB_G028	Probable myosin lig	69.60	0.00
TRINITY_sp|O6559	WRKY34	Probable WRKY trans	69.60	0.00
TRINITY_sp|P2227	RET1	DNA-directed RNA po	69.60	0.00
TRINITY_sp|Q9FJX	CYP65	Peptidyl-prolyl cis	69.60	0.00
TRINITY_sp|O1589	-	T-complex protein 1	69.60	0.00
TRINITY_sp|Q4HWQ	FGRRES_1	Putative heme-bindi	69.60	0.00
TRINITY_sp|Q8H0U	RH42	DEAD-box ATP-depend	69.60	0.00
TRINITY_sp|P6231	LSM6	U6 snRNA-associated	69.60	0.00
TRINITY_sp|Q55G3	psmB6	Proteasome subunit	69.50	0.00
TRINITY_sp|A8J22	FEN1	Flap endonuclease 1	69.50	0.00
TRINITY_sp|Q056C	CTR1	Serine/threonine-pr	69.50	0.00
TRINITY_sp|P1275	RSP3	Flagellar radial sp	69.50	0.00
TRINITY_sp|P1768	-	3-phosphoshikimate	69.50	0.00
TRINITY_sp|P3154	CD4B	ATP-dependent Clp p	69.50	0.00
TRINITY_sp|Q235N	RPL10	60S ribosomal prote	69.50	0.00
TRINITY_sp|P1806	PETE	Plastocyanin, chlor	69.50	0.00
TRINITY_sp|O2436	-	Proteasome subunit	69.50	0.00
TRINITY_sp|Q8VZC	ALDH12A1	Delta-1-pyrroline-5	69.50	0.00
TRINITY_sp|Q54MH	dhx16	Putative pre-mRNA-s	69.50	0.00
TRINITY_sp|Q9LHH	FOLD2	Bifunctional protei	69.50	0.00
TRINITY_sp|Q929C	UPF1	Regulator of nonsen	69.40	0.00
TRINITY_sp|O4995	GDCSP	Glycine dehydrogena	69.40	0.00
TRINITY_sp|Q8VXQ	GapA	Glyceraldehyde-3-ph	69.40	0.00
TRINITY_sp|P5196	Mybl1	Myb-related protein	69.40	0.00
TRINITY_sp|P2079	rab8A	Ras-related protein	69.40	0.00
TRINITY_sp|Q9SI7	CPEFG	Elongation factor G	69.40	0.00
TRINITY_sp|Q54JE	abcC3	ABC transporter C f	69.40	0.00
TRINITY_sp|Q3BAI	-	Uncharacterized pro	69.40	0.00
TRINITY_sp|P529C	-	14-3-3-like protein	69.30	0.00
TRINITY_sp|P9325	SAHH	Adenosylhomocystein	69.30	0.00
TRINITY_sp|P356I	ERF1-3	Eukaryotic peptide	69.30	0.00
TRINITY_sp|Q54E9	cct2	T-complex protein 1	69.30	0.00
TRINITY_sp|Q8W1I	SMU1	Suppressor of mec-8	69.30	0.00

TRINITY_sp|Q387(	PDH2	Pyruvate dehydrogen	69.30	0.00
TRINITY_sp|Q9LII	DHAD	Dihydroxy-acid dehy	69.30	0.00
TRINITY_sp|O039(	HSP90-4	Heat shock protein	69.30	0.00
TRINITY_sp|Q080(	-	Malate dehydrogenas	69.30	0.00
TRINITY_sp|O352(	Nsmaf	Protein FAN OS=Mus	69.20	0.00
TRINITY_sp|Q3B7N	CHD1L	Chromodomain-helica	69.20	0.00
TRINITY_sp|Q018(	-	Heat shock 70 kDa p	69.20	0.00
TRINITY_sp|Q7XR	RFC4	Replication factor	69.20	0.00
TRINITY_sp|O444	SmD3	Small nuclear ribon	69.20	0.00
TRINITY_sp|Q8L9(	At4g137	Inosine triphosphat	69.20	0.00
TRINITY_sp|O048(	NPP5	Serine/threonine-pr	69.20	0.00
TRINITY_sp|O824	At3g461(	Histidine--tRNA lig	69.20	0.00
TRINITY_sp|L7HU	ICL1	Isocitrate lyase OS	69.20	0.00
TRINITY_sp|Q6VNI	Wdfy3	WD repeat and FYVE	69.20	0.00
TRINITY_sp|Q8RY	DEGP7	Protease Do-like 7	69.20	0.00
TRINITY_sp|O860	bdhA	D-beta-hydroxybutyr	69.20	0.00
TRINITY_sp|O018(	acbp-1	Acyl-CoA-binding pr	69.20	0.00
TRINITY_sp|Q7XZ	SAC9	Probable phosphoino	69.20	0.00
TRINITY_sp|O422(	gnb2l1	Guanine nucleotide-	69.20	0.00
TRINITY_sp|Q631	Dnah7	Dynein heavy chain	69.20	0.00
TRINITY_sp|Q8TD	DNAH3	Dynein heavy chain	69.10	0.00
TRINITY_sp|Q17Q(	UBE2G2	Ubiquitin-conjugati	69.10	0.00
TRINITY_sp|Q54KI	cysS	Cysteine--tRNA liga	69.10	0.00
TRINITY_sp|P462(	RPL38	60S ribosomal prote	69.10	0.00
TRINITY_sp|O024	-	Dynein light chain	69.10	0.00
TRINITY_sp|P109(	-	Actin, macronuclear	69.10	0.00
TRINITY_sp|Q55F	pyd1	Dihydropyrimidine d	69.10	0
TRINITY_sp|Q9SS	RAP2-12	Ethylene-responsive	69.10	0.00
TRINITY_sp|B8ANI	CHLH	Magnesium-chelatase	69.10	0
TRINITY_sp|Q9D1(	Ube2c	Ubiquitin-conjugati	69.00	0.00
TRINITY_sp|O226	DRM1	Dormancy-associated	69.00	0.00
TRINITY_sp|Q9SSI	PRP8A	Pre-mRNA-processing	69.00	0.00
TRINITY_sp|B9L9I	rpmH	50S ribosomal prote	69.00	0.00
TRINITY_sp|Q8LGI	PEX4	Protein PEROXIN-4 O	69.00	0.00
TRINITY_sp|A8J7(	ATPG	ATP synthase subuni	69.00	0.00
TRINITY_sp|Q8H0	At1g081	Lactoylglutathione	69.00	0.00
TRINITY_sp|Q4P7	DED1	ATP-dependent RNA h	69.00	0.00
TRINITY_sp|Q6Z1	YchF1	Obg-like ATPase 1 O	69.00	0.00
TRINITY_sp|Q3BA	-	Uncharacterized pro	69.00	0.00
TRINITY_sp|Q9HZI	etfA	Electron transfer f	69.00	0.00
TRINITY_sp|P341	mvpA	Major vault protein	69.00	0.00
TRINITY_sp|Q9LJ	ABCB19	ABC transporter B f	68.90	0.00
TRINITY_sp|Q54X	mobA	MOB kinase activato	68.90	0.00
TRINITY_sp|Q86H	pdhB	Pyruvate dehydrogen	68.90	0.00
TRINITY_sp|Q8T2	mccb	Methylcrotonoyl-CoA	68.90	0.00
TRINITY_sp|Q9CAI	ABCI6	ABC transporter I f	68.90	0.00
TRINITY_sp|P368(	YPTV2	GTP-binding protein	68.90	0.00
TRINITY_sp|Q5DM	IFT172	Intraflagellar tran	68.90	0
TRINITY_sp|Q9FNI	PEX1	Peroxisome biogenes	68.90	0.00
TRINITY_sp|P0DJ	RPL37	60S ribosomal prote	68.90	0.00
TRINITY_sp|Q8WX	DNAH7	Dynein heavy chain	68.90	0.00
TRINITY_sp|Q0P5I	PPIH	Peptidyl-prolyl cis	68.80	0.00
TRINITY_sp|Q401	MTB	Metallothionein-lik	68.80	0.00
TRINITY_sp|P258	chcA	Clathrin heavy chai	68.80	0.00
TRINITY_sp|Q569I	selenbp	Selenium-binding pr	68.80	0.00
TRINITY_sp|Q8GX	CFIS2	Pre-mRNA cleavage f	68.80	0.00

TRINITY_sp|Q8W4	ML1	Protein MEI2-like 1	68.80	0.00
TRINITY_sp|Q54D	DDB_G02	Probable serine/thr	68.80	0.00
TRINITY_sp|Q229	TTHERM_(	POC1 centriolar pro	68.80	0.00
TRINITY_sp|Q9FL	DEGP9	Protease Do-like 9	68.80	0.00
TRINITY_sp|F4KD	NRPC2	DNA-directed RNA po	68.80	0.00
TRINITY_sp|Q9UW	pph-3	Serine/threonine-pr	68.80	0.00
TRINITY_sp|Q036	BIP4	Luminal-binding pro	68.80	0.00
TRINITY_sp|O495	NIFS1	Cysteine desulfuras	68.80	0.00
TRINITY_sp|P222	-	Pyruvate kinase, cy	68.80	0.00
TRINITY_sp|P520	cyn-7	Peptidyl-prolyl cis	68.80	0.00
TRINITY_sp|P614	rpl36a	60S ribosomal prote	68.80	0.00
TRINITY_sp|B5EZ	metE	5-methyltetrahydrop	68.80	0.00
TRINITY_sp|A2Q8	ncs6	Cytoplasmic tRNA 2-	68.80	0.00
TRINITY_sp|Q9C0	DNAH6	Dynein heavy chain	68.80	0.00
TRINITY_sp|Q8S8	TET8	Tetraspanin-8 OS=Ar	68.80	0.00
TRINITY_sp|A2XC	CKS1	Cyclin-dependent ki	68.70	0.00
TRINITY_sp|Q9LK	TATA	Sec-independent pro	68.70	0.00
TRINITY_sp|Q5NA	HDH	Histidinol dehydrog	68.70	0.00
TRINITY_sp|Q9CA	At1g777	Ubiquitin-fold modi	68.60	0.00
TRINITY_sp|P369	-	Protein AMBP (Fragm	68.60	0.00
TRINITY_sp|P171	-	Actin, non-muscle 6	68.60	0.00
TRINITY_sp|P816	-	Alcohol dehydrogena	68.60	0.00
TRINITY_sp|P869	TEF1	Elongation factor 1	68.60	0.00
TRINITY_sp|Q431	-	Proliferating cell	68.60	0.00
TRINITY_sp|A2YP	CARM1	Probable histone-ar	68.60	0.00
TRINITY_sp|Q8RY	PEX6	Peroxisome biogenes	68.60	0.00
TRINITY_sp|O045	FAD4L1	Fatty acid desatura	68.60	0.00
TRINITY_sp|Q550	stkA	Transcription facto	68.60	0.00
TRINITY_sp|O246	AS	Asparagine syntheta	68.60	0.00
TRINITY_sp|Q274	his-41	Probable histone H2	68.60	0.00
TRINITY_sp|Q86A	uroc1	Probable urocanate	68.60	0.00
TRINITY_sp|B1XK	lepA	Elongation factor 4	68.60	0.00
TRINITY_sp|F4K2	CUV	Pre-mRNA-splicing f	68.50	0.00
TRINITY_sp|Q102	rpc2	DNA-directed RNA po	68.50	0.00
TRINITY_sp|Q9M8	UGT80A2	Sterol 3-beta-gluco	68.50	0.00
TRINITY_sp|P114	PSBP	Oxygen-evolving enh	68.50	0.00
TRINITY_sp|P929	AATP2	ADP,ATP carrier pro	68.50	0.00
TRINITY_sp|Q9DG	tpt1	Translationally-con	68.50	0.00
TRINITY_sp|Q76N	vapA	V-type proton ATPas	68.50	0.00
TRINITY_sp|Q7S7	cpr2	Peptidyl-prolyl cis	68.50	0.00
TRINITY_sp|A4QN	nip7	60S ribosome subuni	68.50	0.00
TRINITY_sp|P0DJ	RPL37	60S ribosomal prote	68.50	0.00
TRINITY_sp|P0CZ	ACX3	Acyl-coenzyme A oxi	68.40	0.00
TRINITY_sp|P290	-	Acidic endochitinas	68.40	0.00
TRINITY_sp|Q091	rpl1001	60S ribosomal prote	68.40	0.00
TRINITY_sp|P130	acpA	F-actin-capping pro	68.40	0.00
TRINITY_sp|Q044	IDH2	Isocitrate dehydrog	68.40	0.00
TRINITY_sp|P540	Idh2	Isocitrate dehydrog	68.40	0.00
TRINITY_sp|P870	spg1	Septum-promoting GT	68.40	0.00
TRINITY_sp|Q395	YPTC4	GTP-binding protein	68.40	0.00
TRINITY_sp|F4HX	NUP155	Nuclear pore comple	68.40	0.00
TRINITY_sp|Q084	CKA2	Casein kinase II su	68.40	0.00
TRINITY_sp|Q7SD	rgt-1	tRNA(His) guanylylt	68.40	0.00
TRINITY_sp|Q395	ODA9	Dynein, 78 kDa inte	68.40	0.00
TRINITY_sp|P349	gap3	Glyceraldehyde-3-ph	68.40	0.00
TRINITY_sp|Q044	IDH2	Isocitrate dehydrog	68.30	0.00

TRINITY_sp|P611(	ACTR1A	Alpha-centractin OS	68.30	0.00
TRINITY_sp|Q9SB.	PDK	[Pyruvate dehydroge	68.30	0.00
TRINITY_sp|P147.	-	Alpha-galactosidase	68.30	0.00
TRINITY_sp|Q8LP.	ABCE2	ABC transporter E f	68.30	0.00
TRINITY_sp|Q964I	pyd3	Beta-ureidopropiona	68.30	0.00
TRINITY_sp|Q55G8	cnbA	Calcineurin subunit	68.30	0.00
TRINITY_sp|Q8699	scsB	Succinate--CoA liga	68.30	0.00
TRINITY_sp|P978(	Fh	Fumarate hydratase,	68.30	0.00
TRINITY_sp|P3281	gpd-3.1	Glyceraldehyde-3-ph	68.30	0.00
TRINITY_sp|P3352	ABCC1	Multidrug resistanc	68.30	0.00
TRINITY_sp|Q4288	CS1	Chorismate synthase	68.30	0.00
TRINITY_sp|Q9638	-	Metallothionein-lik	68.20	0.00
TRINITY_sp|P3411	cdk1	Cyclin-dependent ki	68.20	0.00
TRINITY_sp|P341(	myoD	Myosin ID heavy cha	68.20	0.00
TRINITY_sp|P9068	-	Actin OS=Brugia mal	68.20	0.00
TRINITY_sp|P0DKI	RNR2B	Ribonucleoside-diph	68.20	0.00
TRINITY_sp|P4234	MTOR	Serine/threonine-pr	68.20	0.00
TRINITY_sp|P5421	-	Caltractin OS=Dunal	68.20	0.00
TRINITY_sp|Q102(	ssa1	Probable heat shock	68.20	0.00
TRINITY_sp|Q9CAI	RFC2	Replication factor	68.20	0.00
TRINITY_sp|Q86JI	lvsB	BEACH domain-contai	68.10	0.00
TRINITY_sp|P2042	CSNK2A2	Casein kinase II su	68.10	0.00
TRINITY_sp|D4B0\	ARB_020	Probable glucan end	68.10	0.00
TRINITY_sp|P6304	UBA52	Ubiquitin-60S ribos	68.10	0.00
TRINITY_sp|P5464	vatD-1	V-type proton ATPas	68.10	0.00
TRINITY_sp|Q8W4I	XPD	DNA repair helicase	68.10	0.00
TRINITY_sp|Q9ZP>	ARL2	ADP-ribosylation fa	68.10	0.00
TRINITY_sp|P493(	GDCST	Aminomethyltransfer	68.10	0.00
TRINITY_sp|Q0DJ9	TOR	Serine/threonine-pr	68.10	0.00
TRINITY_sp|Q3ZB(	POLR2K	DNA-directed RNA po	68.10	0.00
TRINITY_sp|Q54NI	stt3	Dolichyl-diphosphoo	68.10	0.00
TRINITY_sp|P044(	-	Calmodulin OS=Triti	68.10	0.00
TRINITY_sp|B6SH(	-	tRNA (guanine-N(7)-	68.10	0.00
TRINITY_sp|Q9SR1	At3g046(	Tryptophan--tRNA li	68.10	0.00
TRINITY_sp|Q9C0(	DNAH6	Dynein heavy chain	68.10	0.00
TRINITY_sp|Q9JL1	Spag6	Sperm-associated an	68.10	0.00
TRINITY_sp|Q5BB\	has1	ATP-dependent RNA h	68.10	0.00
TRINITY_sp|Q9FFI	At5g416	6-phosphogluconate	68.10	0.00
TRINITY_sp|A8MS(	LPD1	Dihydrolipoyl dehyd	68.10	0.00
TRINITY_sp|Q4291	PPD	Pyruvate, phosphate	68.10	0.00
TRINITY_sp|Q9WV9	Mok	MAPK/MAK/MRK overla	68.00	0.00
TRINITY_sp|P0CT	rpl1101	60S ribosomal prote	68.00	0.00
TRINITY_sp|Q54C9	rad50	DNA repair protein	68.00	0.00
TRINITY_sp|Q2944	HMGCL	Hydroxymethylglutar	68.00	0.00
TRINITY_sp|Q54JI	oxct1	Probable succinyl-C	68.00	0.00
TRINITY_sp|O243(	TOP2	DNA topoisomerase 2	68.00	0.00
TRINITY_sp|Q54S(	ddx18	Probable ATP-depend	68.00	0.00
TRINITY_sp|O7603	CDKL5	Cyclin-dependent ki	68.00	0.00
TRINITY_sp|O0409	FD1	Ferredoxin-1, chlor	68.00	0.00
TRINITY_sp|Q9SZ.	SHM1	Serine hydroxymethy	68.00	0.00
TRINITY_sp|Q3761	NAD7	NADH-ubiquinone oxi	68.00	0.00
TRINITY_sp|Q70G5	Os07g065	Thioredoxin reducta	68.00	0.00
TRINITY_sp|P3251	-	Deoxyuridine 5'-tri	67.90	0.00
TRINITY_sp|Q54C9	pex6	Peroxisomal biogene	67.90	0.00
TRINITY_sp|O7722	catA	Catalase-A OS=Dicty	67.90	0.00
TRINITY_sp|Q54Y\	scai	Protein SCAI homolo	67.90	0.00

TRINITY_sp|Q9SUI	At4g302	Probable small nucl	67.90	0.00
TRINITY_sp|Q418	RPS21	40S ribosomal prote	67.90	0.00
TRINITY_sp|Q54K	pno1	RNA-binding protein	67.90	0.00
TRINITY_sp|Q9LP(	CYP18-1	Peptidyl-prolyl cis	67.90	0.00
TRINITY_sp|P737	slr1251	Peptidyl-prolyl cis	67.90	0.00
TRINITY_sp|Q9VCI	Ime4	Probable N6-adenosi	67.90	0.00
TRINITY_sp|Q9LJ	dVPE	Vacuolar-processing	67.90	0.00
TRINITY_sp|A9LNI	CPSF30	30-kDa cleavage and	67.90	0.00
TRINITY_sp|Q54CV	rtpR	Probable adenosylco	67.90	0.00
TRINITY_sp|Q8CDI	Agbl2	Cytosolic carboxype	67.90	0.00
TRINITY_sp|Q9LF	BOLA4	Protein BOLA4, chlo	67.90	0.00
TRINITY_sp|Q9M2I	RH52	DEAD-box ATP-depend	67.90	0.00
TRINITY_sp|Q8Y1(	msrA	Peptide methionine	67.90	0.00
TRINITY_sp|Q9CR(	Ufc1	Ubiquitin-fold modi	67.80	0.00
TRINITY_sp|P136	ATP1A3	Sodium/potassium-tr	67.80	0.00
TRINITY_sp|O939	pyrABCN	Protein pyrABCN OS=	67.80	0.00
TRINITY_sp|Q8HXI	SOD2	Superoxide dismutas	67.80	0.00
TRINITY_sp|O151	CETN3	Centrin-3 OS=Homo s	67.80	0.00
TRINITY_sp|Q8LE	AAP19-1	AP-1 complex subuni	67.80	0.00
TRINITY_sp|Q9C8	At1g523	Coatomer subunit be	67.80	0.00
TRINITY_sp|Q1W3	-	Phosphomannomutase	67.70	0.00
TRINITY_sp|P487	CDK1	Cyclin-dependent ki	67.70	0.00
TRINITY_sp|Q9T0	PCKA	Phosphoenolpyruvate	67.70	0.00
TRINITY_sp|B9DGI	ACS	Acetyl-coenzyme A s	67.70	0.00
TRINITY_sp|Q395	-	Dynein 16 kDa light	67.70	0.00
TRINITY_sp|Q425	SCE1	SUMO-conjugating en	67.70	0.00
TRINITY_sp|Q559(	ppsA	Phosphoenolpyruvate	67.70	0
TRINITY_sp|P341	psmA7	Proteasome subunit	67.70	0.00
TRINITY_sp|P008	RBCS-1	Ribulose bisphospha	67.70	0.00
TRINITY_sp|P341	mvpA	Major vault protein	67.70	0.00
TRINITY_sp|P479	zfs1	Zinc finger protein	67.70	0.00
TRINITY_sp|P479	zfs1	Zinc finger protein	67.70	0.00
TRINITY_sp|P462	ASP5	Aspartate aminotran	67.70	0.00
TRINITY_sp|P614	rpl36a	60S ribosomal prote	67.70	0.00
TRINITY_sp|Q5YLI	GYRA	DNA gyrase subunit	67.70	0.00
TRINITY_sp|Q8W4I	XPD	DNA repair helicase	67.70	0.00
TRINITY_sp|Q2W2I	tuf1	Elongation factor T	67.70	0.00
TRINITY_sp|Q631	Dnah7	Dynein heavy chain	67.70	0.00
TRINITY_sp|Q0P5	CKS1B	Cyclin-dependent ki	67.60	0.00
TRINITY_sp|O598	RPL44	60S ribosomal prote	67.60	0.00
TRINITY_sp|P491(	PSAN	Photosystem I react	67.60	0.00
TRINITY_sp|Q963	ARF1	ADP-ribosylation fa	67.60	0.00
TRINITY_sp|Q9BYI	ACTRT3	Actin-related prote	67.60	0.00
TRINITY_sp|Q425	At1g790	NADH dehydrogenase	67.60	0.00
TRINITY_sp|Q6F2	STT3A	Dolichyl-diphosphoo	67.60	0.00
TRINITY_sp|Q10I	Os03g05	DEAD-box ATP-depend	67.60	0.00
TRINITY_sp|Q9U9	ppp6c	Serine/threonine-pr	67.60	0.00
TRINITY_sp|Q9XF	RPL4	60S ribosomal prote	67.60	0.00
TRINITY_sp|Q9Z4I	ifcA	Fumarate reductase	67.60	0.00
TRINITY_sp|P427	UBC4	Ubiquitin-conjugati	67.60	0.00
TRINITY_sp|Q8DJ	clpB1	Chaperone protein C	67.60	0
TRINITY_sp|Q032	MYBL2	Myb-related protein	67.60	0.00
TRINITY_sp|A5UW	nfi	Endonuclease V OS=R	67.60	0.00
TRINITY_sp|O650	PBC1	Proteasome subunit	67.60	0.00
TRINITY_sp|P542	PMA1	Plasma membrane ATP	67.60	0
TRINITY_sp|Q9SQI	lhcA-P4	Chlorophyll a-b bin	67.60	0.00

TRINITY_sp|Q6NU4	chtf18	Chromosome transmis	67.50	0.00
TRINITY_sp|Q9QU(	Polk	DNA polymerase kapp	67.50	0.00
TRINITY_sp|Q9I67	dht	D-hydantoinase/dihy	67.50	0.00
TRINITY_sp|O946(	rpc1	DNA-directed RNA po	67.50	0.00
TRINITY_sp|B9KH2	clpP	ATP-dependent Clp p	67.50	0.00
TRINITY_sp|Q9NQI	RRAGD	Ras-related GTP-bin	67.50	0.00
TRINITY_sp|Q54CI	ddx17	Probable ATP-depend	67.50	0.00
TRINITY_sp|Q9SD(	At3g4712	Zinc finger CCCH do	67.50	0.00
TRINITY_sp|Q6H6I	HEMC	Porphobilinogen dea	67.50	0.00
TRINITY_sp|F4KG(	FBA4	Fructose-bisphospha	67.50	0.00
TRINITY_sp|Q56X5	AOX4	Ubiquinol oxidase 4	67.50	0.00
TRINITY_sp|Q9C0(	DNAH6	Dynein heavy chain	67.50	0.00
TRINITY_sp|Q95VI	-	Profilin-1B OS=Acan	67.50	0.00
TRINITY_sp|Q84KI	SQD1	UDP-sulfoquinovose	67.50	0.00
TRINITY_sp|Q9655	UGD1	UDP-glucose 6-dehyd	67.50	0.00
TRINITY_sp|Q9V6(	SEC61G1	Protein transport p	67.40	0.00
TRINITY_sp|A7SEI	v1g2447	U1 small nuclear ri	67.40	0.00
TRINITY_sp|Q1JQ9	DPM1	Dolichol-phosphate	67.40	0.00
TRINITY_sp|Q54VI	coq5	2-methoxy-6-polypre	67.40	0.00
TRINITY_sp|C1FI1	MICPUN_(	Inosine triphosphat	67.40	0.00
TRINITY_sp|P4034	YTA12	Mitochondrial respi	67.40	0.00
TRINITY_sp|Q462(	pfl	Formate acetyltrans	67.40	0.00
TRINITY_sp|O1552	OGG1	N-glycosylase/DNA l	67.40	0.00
TRINITY_sp|Q852(	OSK1	Serine/threonine pr	67.40	0.00
TRINITY_sp|F4HPI	DRP5A	Dynamin-related pro	67.40	0.00
TRINITY_sp|Q5WW2	purC	Phosphoribosylamino	67.40	0.00
TRINITY_sp|Q8S2I	Os01g072	Ribose-phosphate py	67.40	0.00
TRINITY_sp|Q86A)	uroc1	Probable urocanate	67.40	0.00
TRINITY_sp|Q014(	SBE1	1,4-alpha-glucan-br	67.40	0.00
TRINITY_sp|Q9SII	PHB6	Prohibitin-6, mitoc	67.40	0.00
TRINITY_sp|Q4234	RPS16C	40S ribosomal prote	67.40	0.00
TRINITY_sp|O5005	COL1	Zinc finger protein	67.40	0.00
TRINITY_sp|O8045	AMPD	AMP deaminase OS=Ar	67.40	0.00
TRINITY_sp|Q9S7(	MYB3R-1	Myb-related protein	67.30	0.00
TRINITY_sp|P044(	-	Calmodulin OS=Triti	67.30	0.00
TRINITY_sp|Q8TGI	ART2	Putative uncharacte	67.30	0.00
TRINITY_sp|P0DJ5	RPL30	60S ribosomal prote	67.30	0.00
TRINITY_sp|Q86A\	gltA	Citrate synthase OS	67.30	0.00
TRINITY_sp|Q1XDI	dnaK	Chaperone protein d	67.30	0.00
TRINITY_sp|Q9C62	PDH-E1	Pyruvate dehydrogen	67.30	0.00
TRINITY_sp|Q54QI	erkB	Extracellular signa	67.30	0.00
TRINITY_sp|P1861	NRPB1	DNA-directed RNA po	67.30	0.00
TRINITY_sp|Q6559	FTSH2	ATP-dependent zinc	67.30	0.00
TRINITY_sp|P5629	accD	Acetyl-coenzyme A c	67.30	0.00
TRINITY_sp|P3987	RPL3	60S ribosomal prote	67.30	0.00
TRINITY_sp|Q1542	SF3B4	Splicing factor 3B	67.20	0.00
TRINITY_sp|O1374	SPAC16E8	RING finger protein	67.20	0.00
TRINITY_sp|A4K43	RTEL1	Regulator of telome	67.20	0.00

TRINITY_sp|Q3BA	-	Uncharacterized pro	67.20	0.00
TRINITY_sp|Q8GY	RH10	DEAD-box ATP-depend	67.20	0.00
TRINITY_sp|O485	CAT4	Catalase-4 OS=Glyci	67.20	0.00
TRINITY_sp|Q9SM	DHC1B	Cytoplasmic dynein	67.20	0
TRINITY_sp|Q2QS	UGD4	UDP-glucose 6-dehyd	67.20	0.00
TRINITY_sp|Q9D0	Rfc5	Replication factor	67.10	0.00
TRINITY_sp|P225	-	Polyubiquitin OS=Ph	67.10	0.00
TRINITY_sp|P275	-	Chlorophyll a-b bin	67.10	0.00
TRINITY_sp|Q9VX	SNRPG	Probable small nucl	67.10	0.00
TRINITY_sp|Q5AG	HSL1	Serine/threonine-pr	67.10	0.00
TRINITY_sp|P025	Act79B	Actin, larval muscl	67.10	0.00
TRINITY_sp|Q425	SCE1	SUMO-conjugating en	67.10	0.00
TRINITY_sp|P534	CTN	Caltractin OS=Naegl	67.10	0.00
TRINITY_sp|P485	PPX2	Serine/threonine-pr	67.10	0.00
TRINITY_sp|Q9P2	IFT80	Intraflagellar tran	67.10	0.00
TRINITY_sp|P139	-	G2/mitotic-specific	67.10	0.00
TRINITY_sp|Q9Y3	SF3B6	Splicing factor 3B	67.00	0.00
TRINITY_sp|Q2HH	GAR1	H/ACA ribonucleopro	67.00	0.00
TRINITY_sp|Q9LH	FYPP3	Phytochrome-associa	67.00	0.00
TRINITY_sp|Q55F	bkdB	2-oxoisovalerate de	67.00	0.00
TRINITY_sp|O889	Yme1l1	ATP-dependent zinc	67.00	0.00
TRINITY_sp|P041	TK1	Thymidine kinase, c	66.90	0.00
TRINITY_sp|Q3SE	Icl1e	Caltractin ICL1e OS	66.90	0.00
TRINITY_sp|Q433	VAP	V-type proton ATPas	66.90	0.00
TRINITY_sp|Q9FN	BRR2C	DExH-box ATP-depend	66.90	0.00
TRINITY_sp|Q8NK	rpl3	60S ribosomal prote	66.90	0.00
TRINITY_sp|Q9U7	-	Histone H3 OS=Masti	66.90	0.00
TRINITY_sp|Q10G	MSI1	Histone-binding pro	66.90	0.00
TRINITY_sp|P546	vatA	V-type proton ATPas	66.90	0.00
TRINITY_sp|P519	UBE2E1	Ubiquitin-conjugati	66.90	0.00
TRINITY_sp|Q8VZ	SYP132	Syntaxin-132 OS=Ara	66.90	0.00
TRINITY_sp|O488	RPN2A	26S proteasome non-	66.90	0.00
TRINITY_sp|Q55G	uprt	Uracil phosphoribos	66.80	0.00
TRINITY_sp|Q54Q	dimt1	Probable dimethylad	66.80	0.00
TRINITY_sp|Q8S3	ALB3.1	Inner membrane ALBI	66.80	0.00
TRINITY_sp|P937	-	Acid beta-fructofur	66.80	0.00
TRINITY_sp|Q8RX	OVA4	Tryptophan--tRNA li	66.80	0.00
TRINITY_sp|Q54G	nsa2	Ribosome biogenesis	66.80	0.00
TRINITY_sp|Q9CA	CKL2	Casein kinase 1-lik	66.70	0.00
TRINITY_sp|Q86I	nat9	N-acetyltransferase	66.70	0.00
TRINITY_sp|A8IS	ARL3	ADP-ribosylation fa	66.70	0.00
TRINITY_sp|Q54R	cnrB	CLPTM1-like membran	66.70	0.00
TRINITY_sp|Q962	-	Protein BUD31 homol	66.70	0.00
TRINITY_sp|P427	UBC5	Ubiquitin-conjugati	66.70	0.00
TRINITY_sp|P546	fimA	Fimbrin OS=Dictyost	66.70	0.00
TRINITY_sp|Q54H	ap2s1	AP-2 complex subuni	66.70	0.00
TRINITY_sp|Q6UX	C16orf8	UPF0764 protein C16	66.70	0.00
TRINITY_sp|Q9NQ	KIF13B	Kinesin-like protei	66.70	0.00
TRINITY_sp|Q54Y	etfb	Electron transfer f	66.70	0.00
TRINITY_sp|Q9BM	erf1	Eukaryotic peptide	66.70	0.00
TRINITY_sp|Q56J	RPS26	40S ribosomal prote	66.70	0.00
TRINITY_sp|Q8HX	MUT	Methylmalonyl-CoA m	66.70	0.00
TRINITY_sp|Q8BJ	Chdh	Choline dehydrogena	66.70	0.00
TRINITY_sp|Q416	FKBP15	FK506-binding prote	66.70	0.00
TRINITY_sp|Q9M2	PGPS2	CDP-diacylglycerol-	66.70	0.00
TRINITY_sp|Q8RU	NLP3	Omega-amidase, chlo	66.70	0.00

TRINITY_sp|O038	MT-CO2	Cytochrome c oxidas	66.70	0.00
TRINITY_sp|Q9LF	VAMP713	Vesicle-associated	66.70	0.00
TRINITY_sp|Q9VI	SmD2	Probable small nucl	66.70	0.00
TRINITY_sp|O804	AMPD	AMP deaminase OS=Ar	66.70	0.00
TRINITY_sp|Q96N	ZMAT2	Zinc finger matrin-	66.70	0.00
TRINITY_sp|Q9DA	Pacrg	Parkin coregulated	66.70	0.00
TRINITY_sp|Q9K9	aceA	Isocitrate lyase OS	66.70	0.00
TRINITY_sp|P294	PGK1	Phosphoglycerate ki	66.70	0.00
TRINITY_sp|Q71U	RPS27L	40S ribosomal prote	66.70	0.00
TRINITY_sp|Q941	PMS1	DNA mismatch repair	66.70	0.00
TRINITY_sp|Q54U	kif3	Kinesin-related pro	66.70	0.00
TRINITY_sp|Q9I0	purB	Adenylosuccinate ly	66.70	0.00
TRINITY_sp|Q84T	CDKE-1	Cyclin-dependent ki	66.70	0.00
TRINITY_sp|Q8LP	At5g145	Isocitrate dehydrog	66.70	0.00
TRINITY_sp|Q2LR	rpmA	50S ribosomal prote	66.70	0.00
TRINITY_sp|B8ZR	proS	Proline--tRNA ligas	66.70	0.00
TRINITY_sp|A8ID	CHLREDR	Ribosome biogenesis	66.70	0.00
TRINITY_sp|P087	mhcA	Myosin-2 heavy chai	66.70	0.00
TRINITY_sp|Q7RY	cys-17	Cysteine synthase 1	66.60	0.00
TRINITY_sp|A2YI	OsI_250	Glutamine-dependent	66.60	0.00
TRINITY_sp|P331	IDH2	Isocitrate dehydrog	66.50	0.00
TRINITY_sp|P547	RAN1A	GTP-binding nuclear	66.50	0.00
TRINITY_sp|O772	catA	Catalase-A OS=Dicty	66.50	0.00
TRINITY_sp|O043	B'BETA	Serine/threonine pr	66.50	0.00
TRINITY_sp|Q54N	ino1	Inositol-3-phosphat	66.50	0.00
TRINITY_sp|Q015	Ot07g01	Probable alanine--t	66.50	0.00
TRINITY_sp|Q3SW	dnaK	Chaperone protein D	66.40	0.00
TRINITY_sp|Q86A	uroc1	Probable urocanate	66.40	0.00
TRINITY_sp|Q93V	RPS1	30S ribosomal prote	66.40	0.00
TRINITY_sp|Q9SV	DRG3	Developmentally-reg	66.40	0.00
TRINITY_sp|Q9VH	Gie	ADP-ribosylation fa	66.40	0.00
TRINITY_sp|Q555	trpC	Indole-3-glycerol p	66.40	0.00
TRINITY_sp|Q8DJ	clpB1	Chaperone protein C	66.40	0.00
TRINITY_sp|O811	-	Cysteine synthase,	66.40	0.00
TRINITY_sp|Q9FL	ABCA9	ABC transporter A f	66.30	0.00
TRINITY_sp|Q54W	abcB4	ABC transporter B f	66.30	0.00
TRINITY_sp|Q54M	dhps	Probable deoxyhypus	66.30	0.00
TRINITY_sp|Q9CA	CKL2	Casein kinase 1-lik	66.30	0.00
TRINITY_sp|P491	RAB2A	Ras-related protein	66.30	0.00
TRINITY_sp|Q9K9	aceA	Isocitrate lyase OS	66.30	0.00
TRINITY_sp|Q642	Serpind	Heparin cofactor 2	66.30	0.00
TRINITY_sp|P0C8	CCRP1	Probable serine/thr	66.30	0.00
TRINITY_sp|B9N8	POPTR_0	Biotin carboxylase	66.30	0.00
TRINITY_sp|A0CX	FEN1-1	Flap endonuclease 1	66.30	0.00
TRINITY_sp|Q7T2	csnk1da	Casein kinase I iso	66.30	0.00
TRINITY_sp|Q09J	ODA7	Leucine-rich repeat	66.30	0.00
TRINITY_sp|Q6DH	prpf38a	Pre-mRNA-splicing f	66.30	0.00
TRINITY_sp|Q55C	mekA	Dual specificity mi	66.30	0.00
TRINITY_sp|O426	smd1	Small nuclear ribon	66.30	0.00
TRINITY_sp|Q9FH	TMN4	Transmembrane 9 sup	66.30	0.00
TRINITY_sp|P275	CBR	Carotene biosynthes	66.30	0.00
TRINITY_sp|A4IH	ttc30a	Tetratricopeptide r	66.30	0.00
TRINITY_sp|P080	Mgst1	Microsomal glutathi	66.20	0.00
TRINITY_sp|Q3B8	katnal2	Katanin p60 ATPase-	66.20	0.00
TRINITY_sp|Q8IY	FANCM	Fanconi anemia grou	66.20	0.00
TRINITY_sp|Q86I	pex4	Ubiquitin-conjugati	66.20	0.00

TRINITY_sp|Q6F9)	msbA	Lipid A export ATP-	66.20	0.00
TRINITY_sp|Q9LSW	ETFB	Electron transfer f	66.20	0.00
TRINITY_sp|Q6P5I	hsdl2	Hydroxysteroid dehy	66.20	0.00
TRINITY_sp|G5EGI	let-92	Serine/threonine-pr	66.20	0.00
TRINITY_sp|Q8LGH	CUL4	Cullin-4 OS=Arabido	66.20	0.00
TRINITY_sp|Q8K22	Nat10	RNA cytidine acetyl	66.20	0.00
TRINITY_sp|Q9SUN	At4g3022	Probable small nucl	66.20	0.00
TRINITY_sp|O826(	At2g2042	Succinate--CoA liga	66.20	0.00
TRINITY_sp|P0DJ5	RPL8	60S ribosomal prote	66.20	0.00
TRINITY_sp|B7K9(	miaB	tRNA-2-methylthio-N	66.20	0.00
TRINITY_sp|Q237I	-	Elongation factor 2	66.20	0.00
TRINITY_sp|B0G18	ap1s2	AP-1 complex subuni	66.20	0.00
TRINITY_sp|Q0875	myb	Transcriptional act	66.10	0.00
TRINITY_sp|Q86C(	tor	Target of rapamycin	66.10	0.00
TRINITY_sp|P2338	WARS	Tryptophan--tRNA li	66.10	0.00
TRINITY_sp|Q57AI	dnaK	Chaperone protein D	66.10	0.00
TRINITY_sp|Q0J0H	Os09g05(	Pyruvate dehydrogen	66.10	0.00
TRINITY_sp|A0BD(	GSPATT0(	Adenylosuccinate sy	66.10	0.00
TRINITY_sp|P818(	Ppc1	Phosphoenolpyruvate	66.10	0.00
TRINITY_sp|Q8LPH	ABCB2	ABC transporter B f	66.10	0.00
TRINITY_sp|Q0848	ADK-B	Adenylate kinase 4	66.10	0.00
TRINITY_sp|A9NKI	-	Mitotic-spindle org	66.10	0.00
TRINITY_sp|Q6J9(	ERF086	Ethylene-responsive	66.10	0.00
TRINITY_sp|Q4CUN	Tc00.104	Major vault protein	66.10	0.00
TRINITY_sp|Q396I	ODA11	Dynein alpha chain,	66.10	0
TRINITY_sp|P1164	UOX	Uricase OS=Oryctola	66.00	0.00
TRINITY_sp|Q147)	NAA30	N-alpha-acetyltrans	66.00	0.00
TRINITY_sp|Q86K(	ube2n	Probable ubiquitin-	66.00	0.00
TRINITY_sp|A7RP(	pno1	RNA-binding protein	66.00	0.00
TRINITY_sp|Q54LN	acmsd	2-amino-3-carboxymu	66.00	0.00
TRINITY_sp|Q090(	Slc25a5	ADP/ATP translocase	66.00	0.00
TRINITY_sp|Q54E2	rfc2	Probable replicatio	66.00	0.00
TRINITY_sp|Q54QH	rab32A	Ras-related protein	66.00	0.00
TRINITY_sp|Q3HVN	CPA	N-carbamoylputresci	66.00	0.00
TRINITY_sp|Q94A(	STR6	Rhodanese-like doma	66.00	0.00
TRINITY_sp|P0CO2	HIS3	Imidazoleglycerol-p	66.00	0.00
TRINITY_sp|Q9C8(	NIFU5	NifU-like protein 5	66.00	0.00
TRINITY_sp|Q24J)	RPL23A	60S ribosomal prote	66.00	0.00
TRINITY_sp|P087(	mhcA	Myosin-2 heavy chai	66.00	0.00
TRINITY_sp|P0565	-	Myosin-2 heavy chai	66.00	0.00
TRINITY_sp|Q93ZN	DAP	LL-diaminopimelate	66.00	0.00
TRINITY_sp|P340(	manA	Lysosomal alpha-man	65.90	0.00
TRINITY_sp|A8GWH	iscS	Cysteine desulfuras	65.90	0.00
TRINITY_sp|Q86H4	malaS	Alanine--tRNA ligas	65.90	0.00
TRINITY_sp|Q23M(	Ttll6a	Probable beta-tubul	65.90	0.00
TRINITY_sp|P229(	MED37D	Probable mediator o	65.90	0.00
TRINITY_sp|Q9SX(	At1g275(	Ubiquitin-fold modi	65.90	0.00
TRINITY_sp|Q9ZVW	HEN2	DExH-box ATP-depend	65.90	0.00
TRINITY_sp|Q6E7I	DDB1	DNA damage-binding	65.90	0.00
TRINITY_sp|F4J3(	CTPA3	Carboxyl-terminal-p	65.90	0.00
TRINITY_sp|Q9LM(	MPK8	Mitogen-activated p	65.90	0.00
TRINITY_sp|Q9SGA	RPS19A	40S ribosomal prote	65.90	0.00
TRINITY_sp|Q9P6H	ppk15	Serine/threonine-pr	65.90	0.00
TRINITY_sp|Q6Z8(	Os02g01(	L-aspartate oxidase	65.90	0.00
TRINITY_sp|Q9RYH	folE	GTP cyclohydrolase	65.90	0.00
TRINITY_sp|Q9P42	B11B22.(	E3 ubiquitin-protei	65.90	0.00

TRINITY_sp|P008[illegible]	ACYP2	Acylphosphatase-2 O	65.80	0.00
TRINITY_sp|P410[illegible]	RPL34	60S ribosomal prote	65.80	0.00
TRINITY_sp|P360[illegible]	YNK1	Nucleoside diphosph	65.80	0.00
TRINITY_sp|O486[illegible]	RER1A	Protein RER1A OS=Ar	65.80	0.00
TRINITY_sp|Q9XF[illegible]	CBP20	Nuclear cap-binding	65.80	0.00
TRINITY_sp|P296[illegible]	eef-2	Elongation factor 2	65.80	0.00
TRINITY_sp|B8HU[illegible]	ndk	Nucleoside diphosph	65.80	0.00
TRINITY_sp|Q423[illegible]	PPDK2	Pyruvate, phosphate	65.80	0.00
TRINITY_sp|P204[illegible]	pyrK	UMP-CMP kinase OS=D	65.80	0.00
TRINITY_sp|P549[illegible]	CYPA	Peptidyl-prolyl cis	65.80	0.00
TRINITY_sp|Q388[illegible]	PP2AA1	Serine/threonine-pr	65.80	0.00
TRINITY_sp|Q746[illegible]	pckG	Phosphoenolpyruvate	65.80	0.00
TRINITY_sp|P088[illegible]	awd	Nucleoside diphosph	65.80	0.00
TRINITY_sp|P930[illegible]	RH21	DEAD-box ATP-depend	65.80	0.00
TRINITY_sp|Q8RX[illegible]	TYW1	S-adenosyl-L-methio	65.80	0.00
TRINITY_sp|A9RY[illegible]	PHYPADR[illegible]	ATP-dependent (S)-N	65.80	0.00
TRINITY_sp|P403[illegible]	RIC2	Ras-related protein	65.70	0.00
TRINITY_sp|P403[illegible]	RIC2	Ras-related protein	65.70	0.00
TRINITY_sp|Q9FI[illegible]	OXP1	5-oxoprolinase OS=A	65.70	0.00
TRINITY_sp|Q6AI[illegible]	aroC	Chorismate synthase	65.70	0.00
TRINITY_sp|Q3SE[illegible]	Icl1e	Caltractin ICL1e OS	65.70	0.00
TRINITY_sp|Q54Q[illegible]	erkB	Extracellular signa	65.70	0.00
TRINITY_sp|O496[illegible]	MPC4	Mitochondrial pyruv	65.70	0.00
TRINITY_sp|Q9M1[illegible]	ABCC9	ABC transporter C f	65.70	0.00
TRINITY_sp|P024[illegible]	Rplp2	60S acidic ribosoma	65.70	0.00
TRINITY_sp|Q9SJ[illegible]	RNR1	Ribonucleoside-diph	65.70	0.00
TRINITY_sp|Q073[illegible]	PDS	15-cis-phytoene des	65.70	0.00
TRINITY_sp|Q9LD[illegible]	CBF5	H/ACA ribonucleopro	65.70	0.00
TRINITY_sp|Q9FJ[illegible]	RPS3C	40S ribosomal prote	65.70	0.00
TRINITY_sp|P514[illegible]	RPL26A	60S ribosomal prote	65.70	0.00
TRINITY_sp|Q8VY[illegible]	ORLIKE	Protein ORANGE-LIKE	65.70	0.00
TRINITY_sp|Q9FE[illegible]	Os03g05[illegible]	Elongation factor G	65.70	0.00
TRINITY_sp|P530[illegible]	RFC2	Replication factor	65.70	0.00
TRINITY_sp|Q9LX[illegible]	RPS9B	40S ribosomal prote	65.60	0.00
TRINITY_sp|P483[illegible]	FK506-b[illegible]	12 kDa FK506-bindin	65.60	0.00
TRINITY_sp|P303[illegible]	pol3	DNA polymerase delt	65.60	0.00
TRINITY_sp|Q9SC[illegible]	RPS2D	40S ribosomal prote	65.60	0.00
TRINITY_sp|Q9LZ[illegible]	CDC48E	Cell division contr	65.60	0
TRINITY_sp|Q54Q[illegible]	urm1	Ubiquitin-related m	65.60	0.00
TRINITY_sp|P0C8[illegible]	CCRP1	Probable serine/thr	65.60	0.00
TRINITY_sp|P491[illegible]	PSBR	Photosystem II 10 k	65.60	0.00
TRINITY_sp|A6QP[illegible]	PIN4	Peptidyl-prolyl cis	65.60	0.00
TRINITY_sp|Q6QN[illegible]	-	Casein kinase I OS=	65.60	0.00
TRINITY_sp|Q9FS[illegible]	IVD	Isovaleryl-CoA dehy	65.60	0.00
TRINITY_sp|Q9FY[illegible]	OVA6	Proline--tRNA ligas	65.60	0.00
TRINITY_sp|P446[illegible]	ychF	Ribosome-binding AT	65.50	0.00
TRINITY_sp|Q9C8[illegible]	SUC5	Sucrose transport p	65.50	0.00
TRINITY_sp|P106[illegible]	SAM1	S-adenosylmethionin	65.50	0.00
TRINITY_sp|A7EQ[illegible]	INO80	Putative DNA helica	65.50	0.00
TRINITY_sp|P427[illegible]	UBC5	Ubiquitin-conjugati	65.50	0.00
TRINITY_sp|Q54P[illegible]	hbx10	Homeobox protein 10	65.50	0.00
TRINITY_sp|Q9Y8[illegible]	ubc15	Ubiquitin-conjugati	65.50	0.00
TRINITY_sp|Q66K[illegible]	clec3a	C-type lectin domai	65.50	0.00
TRINITY_sp|Q2QS[illegible]	UGD5	UDP-glucose 6-dehyd	65.50	0.00
TRINITY_sp|Q6C5[illegible]	YNG2	Chromatin modificat	65.50	0.00
TRINITY_sp|Q9S7[illegible]	TS1	Threonine synthase	65.50	0.00

TRINITY_sp|Q6Z4I	PL10B	DEAD-box ATP-depend	65.50	0.00
TRINITY_sp|Q9SPI	psbW	Photosystem II reac	65.50	0.00
TRINITY_sp|A4PBI	RAD54	DNA repair and reco	65.50	0.00
TRINITY_sp|P0DJI	RPL7	60S ribosomal prote	65.50	0.00
TRINITY_sp|Q397I	PPDK	Pyruvate, phosphate	65.50	0.00
TRINITY_sp|Q431S	LOX1.5	Probable linoleate	65.50	0.00
TRINITY_sp|Q70G5	Os07g065	Thioredoxin reducta	65.50	0.00
TRINITY_sp|A0JMA	katnal2	Katanin p60 ATPase-	65.40	0.00
TRINITY_sp|Q6PH(	lias	Lipoyl synthase, mi	65.40	0.00
TRINITY_sp|A8JJI	CHLREDRA	Molybdopterin synth	65.40	0.00
TRINITY_sp|Q8TGN	TAR1	Protein TAR1 OS=Sac	65.40	0.00
TRINITY_sp|Q9FJI	UPF1	Regulator of nonsen	65.40	0.00
TRINITY_sp|O246(	RPOT3	DNA-directed RNA po	65.40	0.00
TRINITY_sp|Q54CI	cct4	T-complex protein 1	65.40	0.00
TRINITY_sp|Q55BS	cpsf2	Cleavage and polyad	65.40	0.00
TRINITY_sp|B1LUN	nuoC	NADH-quinone oxidor	65.40	0.00
TRINITY_sp|Q9LQI	DRG1	Developmentally-reg	65.40	0.00
TRINITY_sp|Q570(	EFL4	Protein ELF4-LIKE 4	65.40	0.00
TRINITY_sp|Q9SW	RPL10A	60S ribosomal prote	65.40	0.00
TRINITY_sp|A7RX2	amdhd1	Probable imidazolon	65.40	0.00
TRINITY_sp|Q8S9	At2g338	Tyrosine--tRNA liga	65.40	0.00
TRINITY_sp|P435(	cpr-4	Cathepsin B-like cy	65.30	0.00
TRINITY_sp|P2688	FKBP2	Peptidyl-prolyl cis	65.30	0.00
TRINITY_sp|B9K8I	rpmA	50S ribosomal prote	65.30	0.00
TRINITY_sp|Q9M7I	SEP1	Stress enhanced pro	65.30	0.00
TRINITY_sp|Q0VD4	VPS4B	Vacuolar protein so	65.30	0.00
TRINITY_sp|Q3M69	lipB	Octanoyltransferase	65.30	0.00
TRINITY_sp|Q9UK2	CNOT11	CCR4-NOT transcript	65.30	0.00
TRINITY_sp|P341(	myoD	Myosin ID heavy cha	65.30	0.00
TRINITY_sp|P200(	ALDH2	Aldehyde dehydrogen	65.30	0.00
TRINITY_sp|Q4064	KOB1	Probable voltage-ga	65.30	0.00
TRINITY_sp|Q0875	myb	Transcriptional act	65.30	0.00
TRINITY_sp|P4664	ASP1	Aspartate aminotran	65.30	0.00
TRINITY_sp|Q9FHI	MOB1A	MOB kinase activato	65.30	0.00
TRINITY_sp|Q8LSN	FYPP	Phytochrome-associa	65.30	0.00
TRINITY_sp|P2685	ATP9	ATP synthase subuni	65.30	0.00
TRINITY_sp|P2685	ATP9	ATP synthase subuni	65.30	0.00
TRINITY_sp|Q93V(	RPS1	30S ribosomal prote	65.30	0.00
TRINITY_sp|P0565	-	Myosin-2 heavy chai	65.30	0.00
TRINITY_sp|P5344	CTN	Caltractin OS=Naegl	65.30	0.00
TRINITY_sp|Q0514	Rac2	Ras-related C3 botu	65.20	0.00
TRINITY_sp|Q4025	FIS1	Probable aldehyde d	65.20	0.00
TRINITY_sp|P5195	NEK2	Serine/threonine-pr	65.20	0.00
TRINITY_sp|Q54DU	febA	Eukaryotic translat	65.20	0.00
TRINITY_sp|Q6QNM	-	Casein kinase I OS=	65.20	0.00
TRINITY_sp|B8JHS	A2cp1_14	Maf-like protein A2	65.20	0.00
TRINITY_sp|P478I	-	Eukaryotic translat	65.20	0.00
TRINITY_sp|Q5RJQ	Sirt2	NAD-dependent prote	65.20	0.00
TRINITY_sp|Q54X(	nfs1	Probable cysteine d	65.20	0.00
TRINITY_sp|Q54TI	cct5	T-complex protein 1	65.20	0.00
TRINITY_sp|Q3MHI	RBBP4	Histone-binding pro	65.20	0.00
TRINITY_sp|P091I	ALS	Acetolactate syntha	65.20	0.00
TRINITY_sp|Q3JCN	pgi	Glucose-6-phosphate	65.20	0.00
TRINITY_sp|Q039(	L1818	Chlorophyll a-b bin	65.20	0.00
TRINITY_sp|O6099	culA	Cullin-1 OS=Dictyos	65.20	0.00
TRINITY_sp|Q23F	RPL26	60S ribosomal prote	65.20	0.00

TRINITY_sp|Q23F7	RPL26	60S ribosomal prote	65.20	0.00
TRINITY_sp|Q54DI	mrkA	Probable serine/thr	65.20	0.00
TRINITY_sp|P4963	-	Ubiquitin-60S ribos	65.20	0.00
TRINITY_sp|Q97UI	SSO2899	Uncharacterized pro	65.10	0.00
TRINITY_sp|Q9Y2X	UBE2D4	Ubiquitin-conjugati	65.10	0.00
TRINITY_sp|Q54QI	psmB7	Proteasome subunit	65.10	0.00
TRINITY_sp|O2264	CYTC	Cytochrome c OS=Fri	65.10	0.00
TRINITY_sp|P4234	MTOR	Serine/threonine-pr	65.10	0.00
TRINITY_sp|Q9SX2	CPLS1	ATP-dependent Clp p	65.10	0.00
TRINITY_sp|P146(	Echs1	Enoyl-CoA hydratase	65.10	0.00
TRINITY_sp|P340(	PAF1	Proteasome subunit	65.10	0.00
TRINITY_sp|Q8SSI	nat5	N-alpha-acetyltrans	65.10	0.00
TRINITY_sp|Q55C(	sdhB	Succinate dehydroge	65.10	0.00
TRINITY_sp|Q23M1	Ttll6a	Probable beta-tubul	65.10	0.00
TRINITY_sp|Q86A\	gltA	Citrate synthase OS	65.10	0.00
TRINITY_sp|Q9ZP9	TMN3	Transmembrane 9 sup	65.10	0.00
TRINITY_sp|Q9BGI	PRDX4	Peroxiredoxin-4 OS=	65.10	0.00
TRINITY_sp|Q86I2	fahd1	Acylpyruvase FAHD1,	65.10	0.00
TRINITY_sp|Q94J(	SHM3	Serine hydroxymethy	65.10	0.00
TRINITY_sp|Q9ZPI	At2g0369	Ubiquinone biosynth	65.10	0.00
TRINITY_sp|Q8L7I	RPL18AA	60S ribosomal prote	65.10	0.00
TRINITY_sp|P6327	RPS17	40S ribosomal prote	65.10	0.00
TRINITY_sp|Q99MI	Pccb	Propionyl-CoA carbo	65.10	0.00
TRINITY_sp|Q8MMI	paxB	Paxillin-B OS=Dicty	65.10	0.00
TRINITY_sp|P515(	AFC1	Serine/threonine-pr	65.10	0.00
TRINITY_sp|Q54T(	rio2	Serine/threonine-pr	65.00	0.00
TRINITY_sp|Q9W4I	rg	Neurobeachin OS=Dro	65.00	0.00
TRINITY_sp|Q9SN8	At3g4752	Malate dehydrogenas	65.00	0.00
TRINITY_sp|O8603	bdhA	D-beta-hydroxybutyr	65.00	0.00
TRINITY_sp|Q6ZII	VTE4	Probable tocopherol	65.00	0.00
TRINITY_sp|Q9FN4	CLPP2	ATP-dependent Clp p	65.00	0.00
TRINITY_sp|A4XKA	dnaJ	Chaperone protein D	65.00	0.00
TRINITY_sp|Q9LHI	FOLD2	Bifunctional protei	65.00	0.00
TRINITY_sp|Q4308	PYRB1	Aspartate carbamoyl	65.00	0.00
TRINITY_sp|P3787	KARS	Lysine--tRNA ligase	65.00	0.00
TRINITY_sp|P4608	NOP2	Probable 28S rRNA (	65.00	0.00
TRINITY_sp|P236(	MT-CYB	Cytochrome b OS=Chl	65.00	0.00
TRINITY_sp|Q031(	AAEL0061	Lysosomal aspartic	65.00	0.00
TRINITY_sp|Q68R	IFT81	Intraflagellar tran	65.00	0.00
TRINITY_sp|Q9LI(	NIFU4	NifU-like protein 4	64.90	0.00
TRINITY_sp|O1791	ran-1	GTP-binding nuclear	64.90	0.00
TRINITY_sp|B8B9I	RPL10A	60S ribosomal prote	64.90	0.00
TRINITY_sp|A4S6Y	OSTLU_41	Lon protease homolo	64.90	0.00
TRINITY_sp|Q9FJI	UPF1	Regulator of nonsen	64.90	0.00
TRINITY_sp|Q54D(	mdhB	Probable malate deh	64.90	0.00
TRINITY_sp|C6TBI	AKR1	Probable aldo-keto	64.90	0.00
TRINITY_sp|Q8E9\	hslV	ATP-dependent prote	64.90	0.00
TRINITY_sp|Q66L3	mak16-a	Protein MAK16 homol	64.90	0.00
TRINITY_sp|Q32PI	FCF1	rRNA-processing pro	64.90	0.00
TRINITY_sp|Q93ZY	STT3A	Dolichyl-diphosphoo	64.90	0.00
TRINITY_sp|Q7T31	ccdc25	Coiled-coil domain-	64.90	0.00
TRINITY_sp|Q9Y38	RBMX2	RNA-binding motif p	64.90	0.00
TRINITY_sp|Q8RW(	At4g3139	Uncharacterized aar	64.90	0.00
TRINITY_sp|Q5F3X	EFTUD2	116 kDa U5 small nu	64.90	0.00
TRINITY_sp|Q9ZV5	CCT1	Choline-phosphate c	64.90	0.00
TRINITY_sp|Q9SK2	CAF1-7	Probable CCR4-assoc	64.90	0.00

TRINITY_sp|Q9FJ[illegible]	UPF1	Regulator of nonsen	64.90	0
TRINITY_sp|Q54H[illegible]	apm1	AP-1 complex subuni	64.90	0.00
TRINITY_sp|P275[illegible]	LHCA4	Chlorophyll a-b bin	64.90	0.00
TRINITY_sp|Q8T1[illegible]	kif5	Kinesin-related pro	64.90	0.00
TRINITY_sp|Q9P8[illegible]	GPD1	Glyceraldehyde-3-ph	64.80	0.00
TRINITY_sp|Q23M[illegible]	Ttll6a	Probable beta-tubul	64.80	0.00
TRINITY_sp|Q3KQ[illegible]	nubp1-A	Cytosolic Fe-S clus	64.80	0.00
TRINITY_sp|Q9UE[illegible]	FTSJ1	Putative tRNA (cyti	64.80	0.00
TRINITY_sp|Q9FJ[illegible]	NRPB6A	DNA-directed RNA po	64.80	0.00
TRINITY_sp|P197[illegible]	MIB	Myosin heavy chain	64.80	0.00
TRINITY_sp|Q5RC[illegible]	ACOX1	Peroxisomal acyl-co	64.80	0.00
TRINITY_sp|Q9FJ[illegible]	ABCF1	ABC transporter F f	64.80	0.00
TRINITY_sp|Q25A[illegible]	H0410G0[illegible]	Double-strand break	64.80	0.00
TRINITY_sp|P264[illegible]	HSP70	Heat shock 70 kDa p	64.80	0.00
TRINITY_sp|O348[illegible]	yngF	Putative enoyl-CoA	64.80	0.00
TRINITY_sp|Q8WX[illegible]	DNAH7	Dynein heavy chain	64.80	0.00
TRINITY_sp|Q9BV[illegible]	EDEM2	ER degradation-enha	64.80	0.00
TRINITY_sp|A9IF[illegible]	gpmA	2,3-bisphosphoglyce	64.80	0.00
TRINITY_sp|Q413[illegible]	DIT1	Dicarboxylate trans	64.80	0.00
TRINITY_sp|P364[illegible]	ILSA	Isoleucine--tRNA li	64.80	0.00
TRINITY_sp|P537[illegible]	At3g570[illegible]	Cystathionine beta-	64.80	0.00
TRINITY_sp|B9EX[illegible]	CARB	Carbamoyl-phosphate	64.80	0
TRINITY_sp|Q964[illegible]	-	Actin, cytoplasmic	64.80	0.00
TRINITY_sp|Q5L6[illegible]	gatA	Glutamyl-tRNA(Gln)	64.70	0.00
TRINITY_sp|Q2NS[illegible]	ung	Uracil-DNA glycosyl	64.70	0.00
TRINITY_sp|P025[illegible]	-	Actin-1 OS=Acantham	64.70	0.00
TRINITY_sp|O600[illegible]	6-PGD	6-phosphogluconate	64.70	0.00
TRINITY_sp|Q425[illegible]	ACO1	Aconitate hydratase	64.70	0
TRINITY_sp|Q431[illegible]	APT1	Adenine phosphoribo	64.70	0.00
TRINITY_sp|Q631[illegible]	Dnah7	Dynein heavy chain	64.70	0.00
TRINITY_sp|P0C8[illegible]	CCRP1	Probable serine/thr	64.70	0.00
TRINITY_sp|Q9ZV[illegible]	CCT1	Choline-phosphate c	64.70	0.00
TRINITY_sp|Q9GP[illegible]	RpL31	60S ribosomal prote	64.70	0.00
TRINITY_sp|Q746[illegible]	pckG	Phosphoenolpyruvate	64.70	0.00
TRINITY_sp|Q9SH[illegible]	UBC34	Ubiquitin-conjugati	64.70	0.00
TRINITY_sp|P501[illegible]	Rdh2	Retinol dehydrogena	64.60	0.00
TRINITY_sp|Q54Q[illegible]	erkB	Extracellular signa	64.60	0.00
TRINITY_sp|O026[illegible]	ITIH2	Inter-alpha-trypsin	64.60	0.00
TRINITY_sp|Q6P8[illegible]	Alg8	Probable dolichyl p	64.60	0.00
TRINITY_sp|Q014[illegible]	SAR1B	GTP-binding protein	64.60	0.00
TRINITY_sp|B8EI[illegible]	dnaK	Chaperone protein D	64.60	0.00
TRINITY_sp|Q54F[illegible]	metK	S-adenosylmethionin	64.60	0.00
TRINITY_sp|Q4U4[illegible]	Xirp2	Xin actin-binding r	64.60	0.00
TRINITY_sp|Q8T6[illegible]	abcH2	ABC transporter H f	64.60	0.00
TRINITY_sp|Q9VW[illegible]	l(1)G01[illegible]	Probable isocitrate	64.60	0.00
TRINITY_sp|Q3KP[illegible]	tssc1	Protein TSSC1 OS=Xe	64.60	0.00
TRINITY_sp|P499[illegible]	SRP54-1	Signal recognition	64.60	0.00
TRINITY_sp|Q2QM[illegible]	BSL2	Serine/threonine-pr	64.60	0.00
TRINITY_sp|Q9SI[illegible]	LSM7	Sm-like protein LSM	64.60	0.00
TRINITY_sp|A8IW[illegible]	PURA	Adenylosuccinate sy	64.60	0.00
TRINITY_sp|Q7XT[illegible]	Os04g01[illegible]	tRNA-dihydrouridine	64.60	0.00
TRINITY_sp|O603[illegible]	AQR	Intron-binding prot	64.60	0.00
TRINITY_sp|Q921[illegible]	CAPN3	Calpain-3 OS=Gallus	64.60	0.00
TRINITY_sp|Q9S8[illegible]	At1g778[illegible]	Probable eukaryotic	64.60	0.00
TRINITY_sp|Q9M5[illegible]	LPD1	Dihydrolipoyl dehyd	64.60	0.00
TRINITY_sp|Q9SV[illegible]	At4g314[illegible]	Coatomer subunit be	64.60	0

TRINITY_sp|P383(	Os02g01'	Protein transport p	64.60	0.00
TRINITY_sp|Q553\	cs	Citrate synthase, m	64.50	0.00
TRINITY_sp|Q3ZC(	CNOT7	CCR4-NOT transcript	64.50	0.00
TRINITY_sp|Q94J(	RIDA	Reactive Intermedia	64.50	0.00
TRINITY_sp|Q54EV	shmt2	Serine hydroxymethy	64.50	0.00
TRINITY_sp|P234(	TRXM	Thioredoxin M-type,	64.50	0.00
TRINITY_sp|P545(	yqjY	Uncharacterized pro	64.50	0.00
TRINITY_sp|O226(	DEGP1	Protease Do-like 1,	64.50	0.00
TRINITY_sp|Q9ZVV	HEN2	DExH-box ATP-depend	64.50	0.00
TRINITY_sp|Q9SN5	UGE5	UDP-glucose 4-epime	64.50	0.00
TRINITY_sp|Q7G8\	Os01g03(	Probable chromatin-	64.50	0.00
TRINITY_sp|P2935	-	Fructose-bisphospha	64.50	0.00
TRINITY_sp|P403(	RIC2	Ras-related protein	64.50	0.00
TRINITY_sp|A8HS4	CHLREDRA	40S ribosomal prote	64.50	0.00
TRINITY_sp|P524I	AGPC	Glucose-1-phosphate	64.50	0.00
TRINITY_sp|Q0ABI	mdh	Malate dehydrogenas	64.50	0.00
TRINITY_sp|Q56WI	PED1	3-ketoacyl-CoA thio	64.50	0.00
TRINITY_sp|Q7XTJ	Os04g01I	Pyruvate dehydrogen	64.50	0.00
TRINITY_sp|Q9ZN1	SKD1	Protein SUPPRESSOR	64.50	0.00
TRINITY_sp|P546(	fimA	Fimbrin OS=Dictyost	64.50	0.00
TRINITY_sp|Q7ZV(	vps29	Vacuolar protein so	64.50	0.00
TRINITY_sp|O226(	DEGP1	Protease Do-like 1,	64.50	0.00
TRINITY_sp|Q9M0\	NSF	Vesicle-fusing ATPa	64.50	0.00
TRINITY_sp|Q215(	M28.5	NHP2-like protein 1	64.40	0.00
TRINITY_sp|P577'	EEFSEC	Selenocysteine-spec	64.40	0.00
TRINITY_sp|Q9SEI	-	Pirin-like protein	64.40	0.00
TRINITY_sp|O944(	tef3	Elongation factor 3	64.40	0.00
TRINITY_sp|F4K4I	LSM4	Sm-like protein LSM	64.40	0.00
TRINITY_sp|Q6MVI	dut-1	Deoxyuridine 5'-tri	64.40	0.00
TRINITY_sp|Q9Z1(	Vars	Valine--tRNA ligase	64.40	0.00
TRINITY_sp|O149(	BTAF1	TATA-binding protei	64.40	0.00
TRINITY_sp|O137I	smc5	Structural maintena	64.40	0.00
TRINITY_sp|Q3885	CRT2	Calreticulin-2 OS=A	64.40	0.00
TRINITY_sp|P4285	ZBP14	14 kDa zinc-binding	64.40	0.00
TRINITY_sp|Q9C5I	RPS7A	40S ribosomal prote	64.40	0.00
TRINITY_sp|Q55BI	raptor	Protein raptor homo	64.40	0.00
TRINITY_sp|Q9HG(	BiP	78 kDa glucose-regu	64.40	0.00
TRINITY_sp|P929(	APR3	5'-adenylylsulfate	64.40	0.00
TRINITY_sp|P305(	CAT2	Catalase isozyme 2	64.40	0.00
TRINITY_sp|Q7VA2	infB	Translation initiat	64.40	0.00
TRINITY_sp|P0AF>	rseA	Anti-sigma-E factor	64.30	0.00
TRINITY_sp|Q9UW(	pph-3	Serine/threonine-pr	64.30	0.00
TRINITY_sp|Q9HDV	gpi12	Probable N-acetylgl	64.30	0.00
TRINITY_sp|Q90Y(	rps4	40S ribosomal prote	64.30	0.00
TRINITY_sp|P4585	prpD	2-methylcitrate deh	64.30	0.00
TRINITY_sp|Q54W1	drg2	Developmentally-reg	64.30	0.00
TRINITY_sp|Q9MAI	NOP5-2	Probable nucleolar	64.30	0.00
TRINITY_sp|Q6H7(	Os02g064	DEAD-box ATP-depend	64.30	0.00
TRINITY_sp|Q437(	ROA1	DNA replication lic	64.30	0.00
TRINITY_sp|Q918:	aurka-a	Aurora kinase A-A O	64.30	0.00
TRINITY_sp|Q84W\	CCT3	T-complex protein 1	64.30	0.00
TRINITY_sp|Q275(	psmA1	Proteasome subunit	64.30	0.00
TRINITY_sp|A8J7(	ATPG	ATP synthase subuni	64.30	0.00
TRINITY_sp|Q141(	adc	Probable acetoaceta	64.30	0.00
TRINITY_sp|Q034(	-	Glutamate synthase	64.30	0
TRINITY_sp|Q9XII	PLT1	Putative polyol tra	64.30	0.00

TRINITY_sp|O235!	BAM3	Beta-amylase 3, chl	64.30	0.00
TRINITY_sp|A6GY)	dapF	Diaminopimelate epi	64.30	0.00
TRINITY_sp|F6QXV	dna2	DNA replication ATP	64.20	0.00
TRINITY_sp|P913(	F46F11.:	Inositol hexakispho	64.20	0.00
TRINITY_sp|P406:	ARL1	ADP-ribosylation fa	64.20	0.00
TRINITY_sp|Q4R5:	RPL22	60S ribosomal prote	64.20	0.00
TRINITY_sp|Q9VV4	CG4933	Probable tRNA N6-ad	64.20	0.00
TRINITY_sp|Q54E!	ppil3	Peptidyl-prolyl cis	64.20	0.00
TRINITY_sp|Q9DAI	Pacrg	Parkin coregulated	64.20	0.00
TRINITY_sp|B3RTI	TRIADDR/	Ubiquitin-fold modi	64.20	0.00
TRINITY_sp|O489(	MDH1	Malate dehydrogenas	64.20	0.00
TRINITY_sp|Q9M0N	ABCB9	ABC transporter B f	64.20	0.00
TRINITY_sp|O228!	At2g472!	Probable pre-mRNA-s	64.20	0.00
TRINITY_sp|Q54EV	lpd	Dihydrolipoyl dehyd	64.20	0.00
TRINITY_sp|B8AM/	OsI_140{	PHD finger protein	64.20	0.00
TRINITY_sp|Q96T(	inuE	Extracellular exo-i	64.20	0.00
TRINITY_sp|Q9LT(	RPN11	26S proteasome non-	64.20	0.00
TRINITY_sp|P611(	RAB2A	Ras-related protein	64.20	0.00
TRINITY_sp|Q9C8I	EMB3003	Dihydrolipoyllysine	64.20	0.00
TRINITY_sp|Q148:	TMEM167/	Protein kish-A OS=B	64.10	0.00
TRINITY_sp|Q5R6!	RBBP7	Histone-binding pro	64.10	0.00
TRINITY_sp|Q6UK(	pirA	Protein pirA OS=Dic	64.10	0.00
TRINITY_sp|Q1RM\	PEX5	Peroxisomal targeti	64.10	0.00
TRINITY_sp|Q0CBI	hta1	Histone H2A OS=Aspe	64.10	0.00
TRINITY_sp|F4IP\	SYCO	Cysteine--tRNA liga	64.10	0.00
TRINITY_sp|Q8IT!	RPS18	40S ribosomal prote	64.10	0.00
TRINITY_sp|Q9SJ/	RSZ22A	Serine/arginine-ric	64.10	0.00
TRINITY_sp|Q8LF:	At1g222'	Multifunctional met	64.10	0.00
TRINITY_sp|P386(	RPL24B	60S ribosomal prote	64.10	0.00
TRINITY_sp|P427!	RPS11	40S ribosomal prote	64.10	0.00
TRINITY_sp|Q207:	F53F4.1(	Probable NADH dehyd	64.10	0.00
TRINITY_sp|Q9HG(	BiP	78 kDa glucose-regu	64.10	0.00
TRINITY_sp|P479'	zfs1	Zinc finger protein	64.10	0.00
TRINITY_sp|Q86A)	uroc1	Probable urocanate	64.10	0.00
TRINITY_sp|Q9LNI	At1g344'	Probable magnesium	64.10	0.00
TRINITY_sp|Q9Y3I	SF3B6	Splicing factor 3B	64.10	0.00
TRINITY_sp|O236-	SBE2.1	1,4-alpha-glucan-br	64.10	0.00
TRINITY_sp|Q395'	YPTC6	Ras-related protein	64.00	0.00
TRINITY_sp|P257{	LCP2	Digestive cysteine	64.00	0.00
TRINITY_sp|Q8WX)	DNAH7	Dynein heavy chain	64.00	0.00
TRINITY_sp|Q965!	UGD1	UDP-glucose 6-dehyd	64.00	0.00
TRINITY_sp|P0C8N	CCRP1	Probable serine/thr	64.00	0.00
TRINITY_sp|Q940N	AGT2	Alanine--glyoxylate	64.00	0.00
TRINITY_sp|P243!	rps14	Plastid 30S ribosom	64.00	0.00
TRINITY_sp|Q396!	PSAL	Photosystem I react	64.00	0.00
TRINITY_sp|Q9FF!	RPS10B	40S ribosomal prote	64.00	0.00
TRINITY_sp|Q9MBI	DHC10	Dynein-1-beta heavy	64.00	0.00
TRINITY_sp|Q428'	RPB2	DNA-directed RNA po	64.00	0
TRINITY_sp|Q9CA:	CKL2	Casein kinase 1-lik	64.00	0.00
TRINITY_sp|Q5LI{	murQ	N-acetylmuramic aci	63.90	0.00
TRINITY_sp|O749'	SPCC182'	Probable choline-ph	63.90	0.00
TRINITY_sp|P117(	-	Probable pancreatic	63.90	0.00
TRINITY_sp|Q0P5I	PPIH	Peptidyl-prolyl cis	63.90	0.00
TRINITY_sp|O747'	mis3	KRR1 small subunit	63.90	0.00
TRINITY_sp|Q23M'	Ttll6a	Probable beta-tubul	63.90	0.00
TRINITY_sp|Q5NC(	Dph1	Diphthamide biosynt	63.90	0.00

TRINITY_sp|P5043	-	Serine hydroxymethy	63.90	0.00
TRINITY_sp|P4960	-	Aconitate hydratase	63.90	0
TRINITY_sp|O2334	HISN5B	Imidazoleglycerol-p	63.90	0.00
TRINITY_sp|Q9FNY	OGG1	N-glycosylase/DNA l	63.90	0.00
TRINITY_sp|Q55DV	cysA	Cystathionine gamma	63.90	0.00
TRINITY_sp|A8HN5	IFT27	Intraflagellar tran	63.90	0.00
TRINITY_sp|Q9LIE	GATA17	GATA transcription	63.90	0.00
TRINITY_sp|Q9M03	SFC1	Mitochondrial succi	63.90	0.00
TRINITY_sp|P4817	mt-atp6	ATP synthase subuni	63.80	0.00
TRINITY_sp|Q54S7	vps15	Probable serine/thr	63.80	0.00
TRINITY_sp|P0CQ5	RPL44	60S ribosomal prote	63.80	0.00
TRINITY_sp|Q9VK4	Tor	Target of rapamycin	63.80	0.00
TRINITY_sp|Q6BY8	RPS29	40S ribosomal prote	63.80	0.00
TRINITY_sp|O8253	PBF1	Proteasome subunit	63.80	0.00
TRINITY_sp|Q1542	SF3A2	Splicing factor 3A	63.80	0.00
TRINITY_sp|Q9SH3	HMA5	Probable copper-tra	63.80	0.00
TRINITY_sp|Q9M72	BCE2	Lipoamide acyltrans	63.80	0.00
TRINITY_sp|Q9D78	Ppil2	Peptidyl-prolyl cis	63.80	0.00
TRINITY_sp|P4061	F01G4.6	Phosphate carrier p	63.80	0.00
TRINITY_sp|Q54V7	ctps	CTP synthase OS=Dic	63.80	0.00
TRINITY_sp|A8IR4	CHLREDRA	Ribosome biogenesis	63.80	0.00
TRINITY_sp|H2L09	gck-1	Germinal center kin	63.80	0.00
TRINITY_sp|Q8LS1	WNK7	Probable serine/thr	63.80	0.00
TRINITY_sp|P5525	FUMR	Fumarate hydratase,	63.80	0.00
TRINITY_sp|Q08D6	abcb6	ATP-binding cassett	63.80	0.00
TRINITY_sp|A8IVX	DRC3	Dynein regulatory c	63.80	0.00
TRINITY_sp|Q9FV4	-	Zeta-carotene desat	63.80	0.00
TRINITY_sp|Q4007	PSBR	Photosystem II 10 k	63.80	0.00
TRINITY_sp|Q5FXM	acbA	Acyl-CoA-binding pr	63.80	0.00
TRINITY_sp|Q4VX0	PABPC1L	Polyadenylate-bindi	63.80	0.00
TRINITY_sp|Q4106	secA	Protein translocase	63.80	0
TRINITY_sp|A8ISM	ARL3	ADP-ribosylation fa	63.80	0.00
TRINITY_sp|P5156	AFC1	Serine/threonine-pr	63.80	0.00
TRINITY_sp|Q8WUI	RAB2B	Ras-related protein	63.80	0.00
TRINITY_sp|P9051	-	Proteasome subunit	63.70	0.00
TRINITY_sp|Q76I8	RPS12	40S ribosomal prote	63.70	0.00
TRINITY_sp|Q1ZXE	echs1	Probable enoyl-CoA	63.70	0.00
TRINITY_sp|Q940M	AGT2	Alanine--glyoxylate	63.70	0.00
TRINITY_sp|Q8W20	CSN2	COP9 signalosome co	63.70	0.00
TRINITY_sp|Q54J6	thrS1	Probable threonine-	63.70	0.00
TRINITY_sp|Q54Q1	hgd	Homogentisate 1,2-d	63.70	0.00
TRINITY_sp|P1344	HPR-A	Glycerate dehydroge	63.70	0.00
TRINITY_sp|P9383	IMDH2	3-isopropylmalate d	63.70	0.00
TRINITY_sp|P5364	sodB	Superoxide dismutas	63.70	0.00
TRINITY_sp|Q3919	PRL2	Protein pleiotropic	63.70	0.00
TRINITY_sp|Q8EJW	prpB	2-methylisocitrate	63.70	0.00
TRINITY_sp|P4686	FLA10	Kinesin-like protei	63.70	0.00
TRINITY_sp|P5376	uglA	Uracil-DNA glycosyl	63.70	0.00
TRINITY_sp|E1C1E	USP47	Ubiquitin carboxyl-	63.60	0.00
TRINITY_sp|Q6112	Cfi	Complement factor I	63.60	0.00
TRINITY_sp|Q4SUE	tma7	Translation machine	63.60	0.00
TRINITY_sp|O4962	ISU1	Iron-sulfur cluster	63.60	0.00
TRINITY_sp|A8IC4	URM1	Ubiquitin-related m	63.60	0.00
TRINITY_sp|Q9EPE	Atp13a1	Manganese-transport	63.60	0.00
TRINITY_sp|P8026	-	NADH dehydrogenase	63.60	0.00
TRINITY_sp|Q8K13	Sde2	Protein SDE2 homolo	63.60	0.00

TRINITY_sp|O811·	PAA2	Proteasome subunit	63.60	0.00
TRINITY_sp|P492(	RPL9B	60S ribosomal prote	63.60	0.00
TRINITY_sp|P123!	PSAE	Photosystem I react	63.60	0.00
TRINITY_sp|Q8LPI	ABCB2	ABC transporter B f	63.60	0.00
TRINITY_sp|Q54QI	erkB	Extracellular signa	63.60	0.00
TRINITY_sp|Q54QI	erkB	Extracellular signa	63.60	0.00
TRINITY_sp|Q9M3I	RPL35C	60S ribosomal prote	63.60	0.00
TRINITY_sp|Q9LP·	RPN6	26S proteasome non-	63.60	0.00
TRINITY_sp|Q54Y2	dhkJ	Hybrid signal trans	63.60	0.00
TRINITY_sp|Q9WV!	Mok	MAPK/MAK/MRK overla	63.60	0.00
TRINITY_sp|Q437:	OASC	Cysteine synthase,	63.60	0.00
TRINITY_sp|Q56Y8	MAP2B	Methionine aminopep	63.60	0.00
TRINITY_sp|Q9LYN	FER3	Ferritin-3, chlorop	63.60	0.00
TRINITY_sp|Q387	CDC2A	Cell division contr	63.60	0.00
TRINITY_sp|Q8LEI	ABCI11	ABC transporter I f	63.60	0.00
TRINITY_sp|Q6F2	CLPB1	Chaperone protein C	63.60	0.00
TRINITY_sp|Q84M2	ABCA1	ABC transporter A f	63.60	0.00
TRINITY_sp|Q940I	CCT2	T-complex protein 1	63.60	0.00
TRINITY_sp|P0DK(	PGLP1A	Phosphoglycolate ph	63.60	0.00
TRINITY_sp|A6BLV	NFYC2	Nuclear transcripti	63.50	0.00
TRINITY_sp|Q54RI	acadsb	Probable short/bran	63.50	0.00
TRINITY_sp|P335:	ABCC1	Multidrug resistanc	63.50	0.00
TRINITY_sp|O490(	-	Probable phospholip	63.50	0.00
TRINITY_sp|Q9BT(	DIDO1	Death-inducer oblit	63.50	0.00
TRINITY_sp|Q8I0I	lis1	Lissencephaly-1 hom	63.50	0.00
TRINITY_sp|Q9SHI	RUB1	Ubiquitin-NEDD8-lik	63.50	0.00
TRINITY_sp|P039(	rasD	Ras-like protein ra	63.50	0.00
TRINITY_sp|Q54EI	cct7	T-complex protein 1	63.50	0.00
TRINITY_sp|P517!	CLCN6	Chloride transport	63.50	0.00
TRINITY_sp|Q8K3	TSTA3	GDP-L-fucose syntha	63.50	0.00
TRINITY_sp|Q96A:	CPNE4	Copine-4 OS=Homo sa	63.50	0.00
TRINITY_sp|Q9LVI	ABCB25	ABC transporter B f	63.50	0.00
TRINITY_sp|Q389!	KIN10	SNF1-related protei	63.50	0.00
TRINITY_sp|A2Y4I	CDKD-1	Cyclin-dependent ki	63.50	0.00
TRINITY_sp|Q395	ODA2	Dynein gamma chain,	63.50	0.00
TRINITY_sp|F4K0.	KIN4C	Kinesin-like protei	63.50	0.00
TRINITY_sp|Q6CQI	TAR1-A	Protein TAR1 OS=Klu	63.50	0.00
TRINITY_sp|Q9UK(	ANGPTL2	Angiopoietin-relate	63.40	0.00
TRINITY_sp|Q8L8V	CYP21-2	Peptidyl-prolyl cis	63.40	0.00
TRINITY_sp|P327!	-	Alpha-1-antitrypsin	63.40	0.00
TRINITY_sp|Q9VD	Ino80	Putative DNA helica	63.40	0.00
TRINITY_sp|Q17Q(	AP2S1	AP-2 complex subuni	63.40	0.00
TRINITY_sp|Q54KI	ncbp2	Nuclear cap-binding	63.40	0.00
TRINITY_sp|Q7XA	ABCG21	ABC transporter G f	63.40	0.00
TRINITY_sp|Q059	RAB2	Ras-related protein	63.40	0.00
TRINITY_sp|O612:	daf-31	N-alpha-acetyltrans	63.40	0.00
TRINITY_sp|P340!	myoB	Myosin IB heavy cha	63.40	0.00
TRINITY_sp|Q950:	metK	S-adenosylmethionin	63.40	0.00
TRINITY_sp|Q94F(	IMPL1	Phosphatase IMPL1,	63.40	0.00
TRINITY_sp|P182(	Gsk3b	Glycogen synthase k	63.40	0.00
TRINITY_sp|Q6UK(	pirA	Protein pirA OS=Dic	63.40	0.00
TRINITY_sp|P0CO(	HHT1	Histone H3 OS=Crypt	63.40	0.00
TRINITY_sp|A0E7A	GSPATT0(	ATPase ASNA1 homolo	63.40	0.00
TRINITY_sp|P044(	-	Calmodulin OS=Triti	63.40	0.00
TRINITY_sp|P464:	GstS1	Glutathione S-trans	63.40	0.00
TRINITY_sp|A2ZV:	CPK1	Calcium-dependent p	63.40	0.00

TRINITY_sp|Q9LS\	GLYR1	Glyoxylate/succinic	63.40	0.00
TRINITY_sp|Q6F2\	CLPB1	Chaperone protein C	63.40	0.00
TRINITY_sp|Q169	PRKC1	Calcium-dependent p	63.40	0.00
TRINITY_sp|A4S6\	OSTLU_4	Lon protease homolo	63.40	0.00
TRINITY_sp|Q6YV	CARA	Carbamoyl-phosphate	63.40	0.00
TRINITY_sp|Q9M4	PGMP	Phosphoglucomutase,	63.40	0.00
TRINITY_sp|Q9ZV	CUL3A	Cullin-3A OS=Arabid	63.40	0.00
TRINITY_sp|Q8SS	nat5	N-alpha-acetyltrans	63.40	0.00
TRINITY_sp|Q9VT	CG7949	MIP18 family protei	63.40	0.00
TRINITY_sp|Q649	ORF1	RNA replication pol	63.40	0.00
TRINITY_sp|P207	MAK	Serine/threonine-pr	63.30	0.00
TRINITY_sp|Q8LD	CYP19-4	Peptidyl-prolyl cis	63.30	0.00
TRINITY_sp|F1Q4	atp9b	Probable phospholip	63.30	0.00
TRINITY_sp|Q9SB	PDK	[Pyruvate dehydroge	63.30	0.00
TRINITY_sp|A8XJ	cst-1	Serine/threonine-pr	63.30	0.00
TRINITY_sp|P133	PSAH	Photosystem I react	63.30	0.00
TRINITY_sp|Q9SC	KIN8B	Kinesin-like protei	63.30	0.00
TRINITY_sp|P469	dad1	Dolichyl-diphosphoo	63.30	0.00
TRINITY_sp|Q272	TFA	4-hydroxyphenylpyru	63.30	0.00
TRINITY_sp|Q55A	scsC	Succinate--CoA liga	63.30	0.00
TRINITY_sp|P242	yjiA	Uncharacterized GTP	63.30	0.00
TRINITY_sp|P222	-	Pyruvate kinase, cy	63.30	0.00
TRINITY_sp|Q9SS	ADT2	Arogenate dehydrata	63.30	0.00
TRINITY_sp|Q8RW	IBR3	Probable acyl-CoA d	63.30	0.00
TRINITY_sp|Q6QN	-	Casein kinase I OS=	63.30	0.00
TRINITY_sp|O826	HCF136	Photosystem II stab	63.30	0.00
TRINITY_sp|C5WZ	Sb01g02	Inosine triphosphat	63.20	0.00
TRINITY_sp|Q9Z0	Txnrd2	Thioredoxin reducta	63.20	0.00
TRINITY_sp|B8BK	OsI_361	DNA replication lic	63.20	0.00
TRINITY_sp|P0AB	elbB	Glyoxalase ElbB OS=	63.20	0.00
TRINITY_sp|Q8BW	Dnah3	Dynein heavy chain	63.20	0.00
TRINITY_sp|Q9FI	RPS8B	40S ribosomal prote	63.20	0.00
TRINITY_sp|Q23A	TTLL3E	Tubulin glycylase 3	63.20	0.00
TRINITY_sp|Q371	ACA1	Calcium-transportin	63.20	0.00
TRINITY_sp|P0CQ	PRP28	Pre-mRNA-splicing A	63.20	0.00
TRINITY_sp|Q8LB	PRP38	Pre-mRNA-splicing f	63.20	0.00
TRINITY_sp|O230	RPL6	50S ribosomal prote	63.20	0.00
TRINITY_sp|Q8GS	HCAR	7-hydroxymethyl chl	63.20	0.00
TRINITY_sp|Q9VV	CG4933	Probable tRNA N6-ad	63.20	0.00
TRINITY_sp|Q9LD	CAC3	Acetyl-coenzyme A c	63.20	0.00
TRINITY_sp|Q70G	Os07g06	Thioredoxin reducta	63.20	0.00
TRINITY_sp|P291	CPN60I	Chaperonin CPN60-1,	63.20	0.00
TRINITY_sp|Q9SR	At3g093	UPF0161 protein At3	63.20	0.00
TRINITY_sp|Q94K	At3g581	Phenylalanine--tRNA	63.20	0.00
TRINITY_sp|A0A0	OR	Protein ORANGE-GREE	63.20	0.00
TRINITY_sp|A4S6\	OSTLU_4	Lon protease homolo	63.20	0.00
TRINITY_sp|Q7G8	Os01g03	Probable chromatin-	63.20	0.00
TRINITY_sp|P937	TWN2	Valine--tRNA ligase	63.20	0.00
TRINITY_sp|Q66K	rbm42	RNA-binding protein	63.20	0.00
TRINITY_sp|Q2KI	NAA10	N-alpha-acetyltrans	63.10	0.00
TRINITY_sp|B4KC	Cbp20	Nuclear cap-binding	63.10	0.00
TRINITY_sp|Q9CA	CKL2	Casein kinase 1-lik	63.10	0.00
TRINITY_sp|Q556	mad2l1-	Mitotic spindle ass	63.10	0.00
TRINITY_sp|Q9GP	RpL31	60S ribosomal prote	63.10	0.00
TRINITY_sp|Q5B2	acpA	Acetate permease A	63.10	0.00
TRINITY_sp|P281	pkgB	Protein kinase 2 OS	63.10	0.00

TRINITY_sp|Q569I	selenbp1	Selenium-binding pr	63.10	0.00
TRINITY_sp|Q9FD2	RPL21E	60S ribosomal prote	63.10	0.00
TRINITY_sp|P2079	MAK	Serine/threonine-pr	63.10	0.00
TRINITY_sp|P5099	CCT4	T-complex protein 1	63.10	0.00
TRINITY_sp|O6157	gch	GTP cyclohydrolase	63.10	0.00
TRINITY_sp|O5003	OTC	Ornithine carbamoyl	63.10	0.00
TRINITY_sp|P2161	-	Pyrophosphate-energ	63.10	0.00
TRINITY_sp|Q9R0	Lgmn	Legumain OS=Rattus	63.10	0.00
TRINITY_sp|Q9VA	CG11837	Probable dimethylad	63.10	0.00
TRINITY_sp|Q9ZWE	CKL9	Casein kinase 1-lik	63.10	0.00
TRINITY_sp|C0SU1	JMJ16	Putative lysine-spe	63.10	0.00
TRINITY_sp|O8253	FTSZ2-1	Cell division prote	63.10	0.00
TRINITY_sp|Q9H0A	NAT10	RNA cytidine acetyl	63.10	0.00
TRINITY_sp|Q3MHI	RBBP4	Histone-binding pro	63.00	0.00
TRINITY_sp|P1187	-	High mobility group	63.00	0.00
TRINITY_sp|O7648	-	Casein kinase II su	63.00	0.00
TRINITY_sp|Q0913	tif213	Eukaryotic translat	63.00	0.00
TRINITY_sp|Q54H	auh	Methylglutaconyl-Co	63.00	0.00
TRINITY_sp|Q9SM	DHC1	Dynein-1-alpha heav	63.00	0.00
TRINITY_sp|Q8GX	CFIS2	Pre-mRNA cleavage f	63.00	0.00
TRINITY_sp|Q76N	hpd	4-hydroxyphenylpyru	63.00	0.00
TRINITY_sp|P9309	-	60S ribosomal prote	63.00	0.00
TRINITY_sp|Q9997	TEP1	Telomerase protein	63.00	0.00
TRINITY_sp|Q1542	SF3B4	Splicing factor 3B	63.00	0.00
TRINITY_sp|Q9BV	RNF126	E3 ubiquitin-protei	63.00	0.00
TRINITY_sp|Q0992	yakc	Aldo-keto reductase	63.00	0.00
TRINITY_sp|Q4309	-	Granule-bound starc	63.00	0.00
TRINITY_sp|Q8C3	Guf1	Translation factor	63.00	0.00
TRINITY_sp|Q9Y3	NAALAD2	N-acetylated-alpha-	63.00	0.00
TRINITY_sp|Q9C5	SMC2-1	Structural maintena	63.00	0.00
TRINITY_sp|Q9SY	PEX10	Peroxisome biogenes	63.00	0.00
TRINITY_sp|Q9LN	CLO	110 kDa U5 small nu	63.00	0
TRINITY_sp|P3204	RPL37A	60S ribosomal prote	63.00	0.00
TRINITY_sp|P3204	RPL37A	60S ribosomal prote	63.00	0.00
TRINITY_sp|P2311	CDC2	Cell division contr	63.00	0.00
TRINITY_sp|P2834	acuE	Malate synthase, gl	63.00	0.00
TRINITY_sp|P2090	aarA	Citrate synthase OS	62.90	0.00
TRINITY_sp|P3728	PIGA	Phosphatidylinosito	62.90	0.00
TRINITY_sp|Q9US	gpi10	GPI mannosyltransfe	62.90	0.00
TRINITY_sp|C1D67	pyrC	Dihydroorotase OS=L	62.90	0.00
TRINITY_sp|P0DK	RPS10-1	40S ribosomal prote	62.90	0.00
TRINITY_sp|Q8TF	DYNLRB2	Dynein light chain	62.90	0.00
TRINITY_sp|Q9C8	At1g6334	Putative flavin-con	62.90	0.00
TRINITY_sp|Q9LY8	RGLG2	E3 ubiquitin-protei	62.90	0.00
TRINITY_sp|Q9LR	CID9	Polyadenylate-bindi	62.90	0.00
TRINITY_sp|Q54W	pex7	Peroxisomal targeti	62.90	0.00
TRINITY_sp|O6030	AQR	Intron-binding prot	62.90	0.00
TRINITY_sp|Q84M2	ABCA1	ABC transporter A f	62.80	0.00
TRINITY_sp|P9310	CDC2	Cell division contr	62.80	0.00
TRINITY_sp|D4B0	ARB_0207	Probable glucan end	62.80	0.00
TRINITY_sp|Q7MA	clpX1	ATP-dependent Clp p	62.80	0.00
TRINITY_sp|Q55G	psmA5	Proteasome subunit	62.80	0.00
TRINITY_sp|Q86I	dst1	Serine/threonine-pr	62.80	0.00
TRINITY_sp|Q9LW	-	Inositol-3-phosphat	62.80	0.00
TRINITY_sp|Q9CA	DRG2	Developmentally-reg	62.80	0.00
TRINITY_sp|Q9BZ	CRNKL1	Crooked neck-like p	62.80	0.00

TRINITY_sp|Q2KJ:	PRPF6	Pre-mRNA-processing	62.80	0.00
TRINITY_sp|O0461	ADNT1	Mitochondrial adeni	62.80	0.00
TRINITY_sp|O2381	SIR	Sulfite reductase [	62.80	0.00
TRINITY_sp|P2348	-	Ribulose bisphospha	62.80	0.00
TRINITY_sp|Q8T6I	abcF2	ABC transporter F f	62.80	0.00
TRINITY_sp|P0AC:	fumA	Fumarate hydratase	62.80	0.00
TRINITY_sp|Q9LSI	UBC32	Ubiquitin-conjugati	62.70	0.00
TRINITY_sp|P024(	Rplp2	60S acidic ribosoma	62.70	0.00
TRINITY_sp|O2288	HEME2	Uroporphyrinogen de	62.70	0.00
TRINITY_sp|Q54I2	polr3b	DNA-directed RNA po	62.70	0.00
TRINITY_sp|Q6QNI	-	Casein kinase I OS=	62.70	0.00
TRINITY_sp|Q8GW(	At1g184	Peptidyl-tRNA hydro	62.70	0.00
TRINITY_sp|A8G8(	tatD	3'-5' ssDNA/RNA exo	62.70	0.00
TRINITY_sp|Q8LP:	ABCE2	ABC transporter E f	62.70	0.00
TRINITY_sp|O646	URT1	UTP:RNA uridylyltra	62.70	0.00
TRINITY_sp|Q9SEI	-	Pirin-like protein	62.70	0.00
TRINITY_sp|Q9SI2	At2g402	Eukaryotic translat	62.70	0.00
TRINITY_sp|Q655	FTSH2	ATP-dependent zinc	62.70	0.00
TRINITY_sp|O224	HDA19	Histone deacetylase	62.70	0.00
TRINITY_sp|P496(	-	Aconitate hydratase	62.70	0.00
TRINITY_sp|Q9SE	AAA1	Katanin p60 ATPase-	62.70	0.00
TRINITY_sp|Q9SMI	DHC1B	Cytoplasmic dynein	62.70	0.00
TRINITY_sp|Q652I	PHT4;4	Probable anion tran	62.70	0.00
TRINITY_sp|A5PJI	SF3A2	Splicing factor 3A	62.70	0.00
TRINITY_sp|C5D4	deoC	Deoxyribose-phospha	62.60	0.00
TRINITY_sp|P534	-	Actin OS=Chlamydomo	62.60	0.00
TRINITY_sp|Q55C	amdhd1	Probable imidazolon	62.60	0.00
TRINITY_sp|Q6QNI	-	Casein kinase I OS=	62.60	0.00
TRINITY_sp|P2641	HSP70	Heat shock 70 kDa p	62.60	0.00
TRINITY_sp|Q4142	PCM3	Putative calmodulin	62.60	0.00
TRINITY_sp|Q964	-	Meiotic recombinati	62.60	0.00
TRINITY_sp|Q9SVI	UEV1D	Ubiquitin-conjugati	62.60	0.00
TRINITY_sp|Q6AV:	Os03g06	Probable N-acetyl-g	62.60	0.00
TRINITY_sp|O005(	NOP56	Nucleolar protein 5	62.60	0.00
TRINITY_sp|O229(	RH24	DEAD-box ATP-depend	62.60	0.00
TRINITY_sp|Q9D2I	Dnaaf1	Dynein assembly fac	62.60	0.00
TRINITY_sp|Q9MBI	DHC10	Dynein-1-beta heavy	62.60	0.00
TRINITY_sp|Q9QYI	Abcc5	Multidrug resistanc	62.60	0.00
TRINITY_sp|P465	ubc4	Ubiquitin-conjugati	62.50	0.00
TRINITY_sp|Q7KWI	prp19	Pre-mRNA-processing	62.50	0.00
TRINITY_sp|E2RK	RPL32	60S ribosomal prote	62.50	0.00
TRINITY_sp|P306	cdc-25.	M-phase inducer pho	62.50	0.00
TRINITY_sp|Q54Y	mrpl33	Probable 39S riboso	62.50	0.00
TRINITY_sp|Q46EI	msrB	Peptide methionine	62.50	0.00
TRINITY_sp|Q2HJ8	PKNOX1	Homeobox protein PK	62.50	0.00
TRINITY_sp|P623	CPK1	Calcium-dependent p	62.50	0.00
TRINITY_sp|B8AMI	CHLD	Magnesium-chelatase	62.50	0.00
TRINITY_sp|O830	pip	Probable proline im	62.50	0.00
TRINITY_sp|Q2KI	TCEB1	Transcription elong	62.50	0.00
TRINITY_sp|Q54QI	erkB	Extracellular signa	62.50	0.00
TRINITY_sp|P316(	parp1	Poly [ADP-ribose] p	62.50	0.00
TRINITY_sp|Q9FV(	DHDPS2	4-hydroxy-tetrahydr	62.50	0.00
TRINITY_sp|Q56K	SF3B5	Splicing factor 3B	62.50	0.00
TRINITY_sp|Q922	Plrg1	Pleiotropic regulat	62.50	0.00
TRINITY_sp|Q8MQ	cshA	Citrate synthase, p	62.50	0.00
TRINITY_sp|A4K4	RTEL1	Regulator of telome	62.50	0.00

TRINITY_sp|Q388(	XPB1	DNA repair helicase	62.50	0.00
TRINITY_sp|O489;	CYP97B2	Cytochrome P450 97B	62.50	0.00
TRINITY_sp|B9DGI	ACS	Acetyl-coenzyme A s	62.50	0.00
TRINITY_sp|Q9Y3I	SF3B6	Splicing factor 3B	62.50	0.00
TRINITY_sp|A8J0.	CHLREDR/	1-acyl-sn-glycerol-	62.50	0.00
TRINITY_sp|Q8VYI	CKL12	Casein kinase 1-lik	62.50	0.00
TRINITY_sp|Q74ZI	AGR231C	Lipoyl synthase, mi	62.50	0.00
TRINITY_sp|Q5DM!	IFT172	Intraflagellar tran	62.50	0.00
TRINITY_sp|Q5ZJN	NGLY1	Peptide-N(4)-(N-ace	62.50	0.00
TRINITY_sp|Q1ZX]	DDB_G02	Probable serine/thr	62.50	0.00
TRINITY_sp|P027;	SLC25A4	ADP/ATP translocase	62.40	0.00
TRINITY_sp|P524;	PAC1	Proteasome subunit	62.40	0.00
TRINITY_sp|P168(	-	Histone H2A-III OS=	62.40	0.00
TRINITY_sp|A0L5	acsA	Acetyl-coenzyme A s	62.40	0.00
TRINITY_sp|Q54S;	nfyA	Nuclear transcripti	62.40	0.00
TRINITY_sp|P025	Act79B	Actin, larval muscl	62.40	0.00
TRINITY_sp|Q237]	-	Elongation factor 2	62.40	0
TRINITY_sp|Q54JI	oatA	Probable ornithine	62.40	0.00
TRINITY_sp|Q6YS:	Os07g03(	DEAD-box ATP-depend	62.40	0.00
TRINITY_sp|O827(	HISN3	1-(5-phosphoribosyl	62.40	0.00
TRINITY_sp|Q4KT]	RPL5	60S ribosomal prote	62.40	0.00
TRINITY_sp|P111	DLST	Dihydrolipoyllysine	62.40	0.00
TRINITY_sp|Q0WM;	ALDH6B2	Methylmalonate-semi	62.40	0.00
TRINITY_sp|A8IB;	CHLREDR/	40S ribosomal prote	62.40	0.00
TRINITY_sp|Q08D/	PANK3	Pantothenate kinase	62.30	0.00
TRINITY_sp|Q8GW\	GFA2	Chaperone protein d	62.30	0.00
TRINITY_sp|P0CC(	DDB_G02(	Enolase superfamily	62.30	0.00
TRINITY_sp|O240!	MT2	Metallothionein-lik	62.30	0.00
TRINITY_sp|Q86A;	psmB1	Proteasome subunit	62.30	0.00
TRINITY_sp|Q86K!	trappc3	Trafficking protein	62.30	0.00
TRINITY_sp|Q55D(	psmB3	Proteasome subunit	62.30	0.00
TRINITY_sp|Q9LHI	FYPP3	Phytochrome-associa	62.30	0.00
TRINITY_sp|Q75JI	pckA	Phosphoenolpyruvate	62.30	0.00
TRINITY_sp|Q059	RAB1A	Ras-related protein	62.30	0.00
TRINITY_sp|P423	MTOR	Serine/threonine-pr	62.30	0.00
TRINITY_sp|P460(	NOP2	Probable 28S rRNA (	62.30	0.00
TRINITY_sp|Q9XYI	fcpA	Probable C-terminal	62.30	0.00
TRINITY_sp|P067(	TOP2	DNA topoisomerase 2	62.30	0.00
TRINITY_sp|A8JA	DYF13	Intraflagellar tran	62.30	0.00
TRINITY_sp|Q3ED.	ABCI19	ABC transporter I f	62.30	0.00
TRINITY_sp|P503!	Gdi1	Rab GDP dissociatio	62.30	0.00
TRINITY_sp|O654!	XCP1	Cysteine protease X	62.30	0.00
TRINITY_sp|A1WZ(	rpmG	50S ribosomal prote	62.30	0.00
TRINITY_sp|Q243(	Iswi	Chromatin-remodelin	62.30	0.00
TRINITY_sp|Q8K0]	Oplah	5-oxoprolinase OS=M	62.30	0.00
TRINITY_sp|Q392	PP2AB2	Serine/threonine pr	62.30	0.00
TRINITY_sp|Q8RW(	RVE8	Protein REVEILLE 8	62.30	0.00
TRINITY_sp|A8HT\	tuf1	Elongation factor T	62.20	0.00
TRINITY_sp|Q2SJI	msrB	Peptide methionine	62.20	0.00
TRINITY_sp|P511:	MSK-2	Glycogen synthase k	62.20	0.00
TRINITY_sp|Q9UR!	ACT	Actin, gamma OS=Pen	62.20	0.00
TRINITY_sp|P297!	ACTB	Actin, cytoplasmic	62.20	0.00
TRINITY_sp|Q1JQI	MOCS1	Molybdenum cofactor	62.20	0.00
TRINITY_sp|Q9I0I	purB	Adenylosuccinate ly	62.20	0.00
TRINITY_sp|Q8DJ	clpB1	Chaperone protein C	62.20	0.00
TRINITY_sp|Q54QI	erkB	Extracellular signa	62.20	0.00

TRINITY_sp|Q59S(	IPL1	Spindle assembly ch	62.20	0.00
TRINITY_sp|Q6DB(	gmppb	Mannose-1-phosphate	62.20	0.00
TRINITY_sp|Q204'	kin-20	Casein kinase I iso	62.20	0.00
TRINITY_sp|Q8LA(	GATA1	GATA transcription	62.20	0.00
TRINITY_sp|Q54S)	nvl	Putative ribosome b	62.20	0.00
TRINITY_sp|P542	PGIC	Glucose-6-phosphate	62.20	0.00
TRINITY_sp|Q2RB	Os11g01(	Clathrin heavy chai	62.20	0
TRINITY_sp|P0DJ2	RPL35A	60S ribosomal prote	62.20	0.00
TRINITY_sp|Q10M	R40C1	Ricin B-like lectin	62.10	0.00
TRINITY_sp|Q391(	FTSH1	ATP-dependent zinc	62.10	0.00
TRINITY_sp|Q54V'	ctps	CTP synthase OS=Dic	62.10	0.00
TRINITY_sp|Q6FU	URA7	CTP synthase OS=Can	62.10	0.00
TRINITY_sp|A7MB'	SGK1	Serine/threonine-pr	62.10	0.00
TRINITY_sp|Q066	GDB1	Glycogen debranchin	62.10	0.00
TRINITY_sp|Q8L3)	KAS	3-oxoacyl-[acyl-car	62.10	0.00
TRINITY_sp|P233	WARS	Tryptophan--tRNA li	62.10	0.00
TRINITY_sp|Q9BP(	B9D2	B9 domain-containin	62.10	0.00
TRINITY_sp|Q568	gtdc1	Glycosyltransferase	62.10	0.00
TRINITY_sp|Q8TF	rpl1802	60S ribosomal prote	62.10	0.00
TRINITY_sp|P106	CTSA	Lysosomal protectiv	62.10	0.00
TRINITY_sp|Q9C6	At1g509	Nucleolar GTP-bindi	62.10	0.00
TRINITY_sp|Q9M8	At1g742	Probable phosphorib	62.10	0
TRINITY_sp|Q94F(	IMPL1	Phosphatase IMPL1,	62.10	0.00
TRINITY_sp|Q9LS(	ASF1B	Histone chaperone A	62.10	0.00
TRINITY_sp|Q7ZX)	kif19	Kinesin-like protei	62.10	0.00
TRINITY_sp|F4IS(	At2g320(	DNA topoisomerase 3	62.00	0.00
TRINITY_sp|Q9D7	Iscu	Iron-sulfur cluster	62.00	0.00
TRINITY_sp|Q8LB	At2g167	Iron-sulfur assembl	62.00	0.00
TRINITY_sp|Q402	FIS1	Probable aldehyde d	62.00	0.00
TRINITY_sp|Q6DE	epabp	Embryonic polyadeny	62.00	0.00
TRINITY_sp|Q9SI	DSK2B	Ubiquitin domain-co	62.00	0.00
TRINITY_sp|P323	DCTD	Deoxycytidylate dea	62.00	0.00
TRINITY_sp|Q560	prpC	2-methylcitrate syn	62.00	0.00
TRINITY_sp|Q8GW	AIH	Agmatine deiminase	62.00	0.00
TRINITY_sp|Q0A9(	Mlg_117	Putative 4-hydroxy-	62.00	0.00
TRINITY_sp|P558	RPS27	40S ribosomal prote	62.00	0.00
TRINITY_sp|O228	ETFQO	Electron transfer f	62.00	0.00
TRINITY_sp|Q8W3	MFDR	NADPH:adrenodoxin o	62.00	0.00
TRINITY_sp|Q29S	DDX47	Probable ATP-depend	62.00	0.00
TRINITY_sp|Q0WP	RMR4	Receptor homology r	62.00	0.00
TRINITY_sp|Q9C5(	SUF4	Protein SUPPRESSOR	62.00	0.00
TRINITY_sp|P742	ffh	Signal recognition	62.00	0.00
TRINITY_sp|A6QR	USP4	Ubiquitin carboxyl-	61.90	0.00
TRINITY_sp|Q014	AMPD2	AMP deaminase 2 OS=	61.90	0.00
TRINITY_sp|P464	Vps4b	Vacuolar protein so	61.90	0.00
TRINITY_sp|A3RL	-	40S ribosomal prote	61.90	0.00
TRINITY_sp|Q54B(	psmB5	Proteasome subunit	61.90	0.00
TRINITY_sp|Q78P(	Ccdc25	Coiled-coil domain-	61.90	0.00
TRINITY_sp|P227	ARP2	60S ribosomal prote	61.90	0.00
TRINITY_sp|P0C2	-	40S ribosomal prote	61.90	0.00
TRINITY_sp|Q9C5	NADK2	NAD kinase 2, chlor	61.90	0.00
TRINITY_sp|Q76N(	cct6	T-complex protein 1	61.90	0.00
TRINITY_sp|Q9ZV	PECT1	Ethanolamine-phosph	61.90	0.00
TRINITY_sp|Q8L6	RPOT1-S	DNA-directed RNA po	61.90	0.00
TRINITY_sp|O888	Sc5d	Lathosterol oxidase	61.80	0.00
TRINITY_sp|Q9UM	ANAPC10	Anaphase-promoting	61.80	0.00

TRINITY_sp|P107(	CAB7	Chlorophyll a-b bin	61.80	0.00
TRINITY_sp|Q86JI	lvsB	BEACH domain-contai	61.80	0.00
TRINITY_sp|Q9ZQI	ACX1.2	Putative peroxisoma	61.80	0.00
TRINITY_sp|Q59X(	RAS1	Ras-like protein 1	61.80	0.00
TRINITY_sp|P2431	-	Proliferating cell	61.80	0.00
TRINITY_sp|P823!	-	Non-specific lipid-	61.80	0.00
TRINITY_sp|Q31Q!	glgC	Glucose-1-phosphate	61.80	0.00
TRINITY_sp|Q9SZ1	LIP5	Protein HOMOLOG OF	61.80	0.00
TRINITY_sp|P0AF(	mog	Molybdopterin adeny	61.80	0.00
TRINITY_sp|Q065	-	Pyrophosphate-energ	61.80	0.00
TRINITY_sp|P197(	MIB	Myosin heavy chain	61.80	0.00
TRINITY_sp|Q9SUI	ATR2	NADPH--cytochrome P	61.80	0.00
TRINITY_sp|Q92MI	clpB	Chaperone protein C	61.80	0.00
TRINITY_sp|Q059	RAB1A	Ras-related protein	61.80	0.00
TRINITY_sp|Q059	UTP13	U3 small nucleolar	61.80	0.00
TRINITY_sp|O747	mis3	KRR1 small subunit	61.80	0.00
TRINITY_sp|O499!	GDCSP	Glycine dehydrogena	61.80	0.00
TRINITY_sp|P200	ATP2B1	Plasma membrane cal	61.80	0.00
TRINITY_sp|Q3ZBI	ASPN	Asporin OS=Bos taur	61.80	0.00
TRINITY_sp|P542	PGIC1	Glucose-6-phosphate	61.70	0.00
TRINITY_sp|Q8R3	Rfc3	Replication factor	61.70	0.00
TRINITY_sp|Q92J(	fdxB	2Fe-2S ferredoxin O	61.70	0.00
TRINITY_sp|Q6NS	cdkal1	Threonylcarbamoylad	61.70	0.00
TRINITY_sp|Q56X(	RH15	DEAD-box ATP-depend	61.70	0.00
TRINITY_sp|Q6TB)	CYP97C1	Carotene epsilon-mo	61.70	0.00
TRINITY_sp|Q9LJI	M3KE1	MAP3K epsilon prote	61.70	0.00
TRINITY_sp|Q9C9(	RPL6C	60S ribosomal prote	61.70	0.00
TRINITY_sp|P600	RPL7B	60S ribosomal prote	61.70	0.00
TRINITY_sp|Q3UT	Cdkl5	Cyclin-dependent ki	61.70	0.00
TRINITY_sp|Q9BW	CCDC94	Coiled-coil domain-	61.70	0.00
TRINITY_sp|Q45F	-	Serine/threonine-pr	61.70	0.00
TRINITY_sp|Q6YU(	Os02g01	Putative multidrug	61.70	0.00
TRINITY_sp|Q54DI	amdA	AMP deaminase OS=Di	61.70	0.00
TRINITY_sp|Q9C5(	At4g178	Acetylornithine dea	61.70	0.00
TRINITY_sp|O600	ubp12	Probable ubiquitin	61.70	0.00
TRINITY_sp|O747	mis3	KRR1 small subunit	61.70	0.00
TRINITY_sp|Q9AXI	Os01g06	Uroporphyrinogen de	61.70	0.00
TRINITY_sp|Q0021	DHS2	Phospho-2-dehydro-3	61.70	0.00
TRINITY_sp|Q9M1I	At3g621	Proline--tRNA ligas	61.70	0.00
TRINITY_sp|F4KE	EMB2247	Valine--tRNA ligase	61.70	0.00
TRINITY_sp|Q428!	WAXY	Granule-bound starc	61.70	0.00
TRINITY_sp|Q4V8I	Steap4	Metalloreductase ST	61.60	0.00
TRINITY_sp|Q9BG	PRDX2	Peroxiredoxin-2 OS=	61.60	0.00
TRINITY_sp|O655!	CIPK6	CBL-interacting ser	61.60	0.00
TRINITY_sp|P109!	-	Actin, macronuclear	61.60	0.00
TRINITY_sp|P357(	Prdx1	Peroxiredoxin-1 OS=	61.60	0.00
TRINITY_sp|P347	ARF	ADP-ribosylation fa	61.60	0.00
TRINITY_sp|Q86K(	valS1	Probable valine--tR	61.60	0.00
TRINITY_sp|P114	-	Actin OS=Entamoeba	61.60	0.00
TRINITY_sp|Q9ZWI	CAO	Chlorophyllide a ox	61.60	0.00
TRINITY_sp|Q6YZ)	Os08g01	Putative aconitate	61.60	0
TRINITY_sp|P0DJ1	RPL13A	60S ribosomal prote	61.60	0.00
TRINITY_sp|Q54P1	abcC8	ABC transporter C f	61.60	0.00
TRINITY_sp|P4041	-	T-complex protein 1	61.60	0.00
TRINITY_sp|Q9UL	RAB21	Ras-related protein	61.60	0.00
TRINITY_sp|Q9KN\	trpS	Tryptophan--tRNA li	61.60	0.00

TRINITY_sp|Q75IN	Os05g012	Isovaleryl-CoA dehy	61.60	0.00
TRINITY_sp|Q0396	L1818	Chlorophyll a-b bin	61.60	0.00
TRINITY_sp|O0757	yhdF	Uncharacterized oxi	61.60	0.00
TRINITY_sp|F4JLN	At4g1032	Isoleucine--tRNA li	61.60	0
TRINITY_sp|O2436	PAG1	Proteasome subunit	61.60	0.00
TRINITY_sp|Q9SYI	BRR2A	DExH-box ATP-depend	61.60	0.00
TRINITY_sp|O3200	yokD	SPBc2 prophage-deri	61.60	0.00
TRINITY_sp|E9L7A	-	Bifunctional aspart	61.60	0.00
TRINITY_sp|Q54U0	dnapkcs	DNA-dependent prote	61.60	0.00
TRINITY_sp|Q9XEA	EMB2369	Leucine--tRNA ligas	61.60	0
TRINITY_sp|Q94AN	OOP	Organellar oligopep	61.60	0.00
TRINITY_sp|Q6DJ9	cars2	Probable cysteine--	61.50	0.00
TRINITY_sp|Q8NKE	GLC3	1,4-alpha-glucan-br	61.50	0.00
TRINITY_sp|Q6NRN	mcm9	DNA helicase MCM9 O	61.50	0.00
TRINITY_sp|O1543	ABCC4	Multidrug resistanc	61.50	0.00
TRINITY_sp|P9054	-	Histone H3 OS=Euplo	61.50	0.00
TRINITY_sp|Q28H0	qtrt1	Queuine tRNA-ribosy	61.50	0.00
TRINITY_sp|Q9632	ACX4	Acyl-coenzyme A oxi	61.50	0.00
TRINITY_sp|A7SBN	v1g24415	Zinc finger CCCH-ty	61.50	0.00
TRINITY_sp|Q2HFE	TIF1	ATP-dependent RNA h	61.50	0.00
TRINITY_sp|B8AL3	OsI_1100	Arginine biosynthes	61.50	0.00
TRINITY_sp|P1506	rasG	Ras-like protein ra	61.50	0.00
TRINITY_sp|Q3957	YPTC6	Ras-related protein	61.50	0.00
TRINITY_sp|P3018	TOP2	DNA topoisomerase 2	61.50	0.00
TRINITY_sp|Q99MN	Kars	Lysine--tRNA ligase	61.50	0.00
TRINITY_sp|P6299	RAC1	Ras-related C3 botu	61.50	0.00
TRINITY_sp|Q9FE6	Os03g056	Elongation factor G	61.50	0.00
TRINITY_sp|O2204	ANP1	Mitogen-activated p	61.50	0.00
TRINITY_sp|Q6316	Dnah1	Dynein heavy chain	61.50	0.00
TRINITY_sp|Q9FN0	BRR2C	DExH-box ATP-depend	61.50	0
TRINITY_sp|P3328	RPS28	40S ribosomal prote	61.50	0.00
TRINITY_sp|Q2223	enpl-1	Endoplasmin homolog	61.40	0.00
TRINITY_sp|A9NKI	-	Mitotic-spindle org	61.40	0.00
TRINITY_sp|F4K2E	CUV	Pre-mRNA-splicing f	61.40	0.00
TRINITY_sp|Q9SLX	Os05g015	Importin subunit al	61.40	0.00
TRINITY_sp|P6295	HINT1	Histidine triad nuc	61.40	0.00
TRINITY_sp|Q4259	thrc	Threonine synthase	61.40	0.00
TRINITY_sp|Q55AE	vatF	V-type proton ATPas	61.40	0.00
TRINITY_sp|P3200	SLC25A6	ADP/ATP translocase	61.40	0.00
TRINITY_sp|Q8T2I	sepA	Serine/threonine-pr	61.40	0.00
TRINITY_sp|Q55BW	hdaB	Histone deacetylase	61.40	0.00
TRINITY_sp|Q6CQE	TAR1-A	Protein TAR1 OS=Klu	61.40	0.00
TRINITY_sp|Q9SW1	PUB35	U-box domain-contai	61.40	0.00
TRINITY_sp|P3568	RPL7A-1	60S ribosomal prote	61.40	0.00
TRINITY_sp|O2430	TOP2	DNA topoisomerase 2	61.40	0.00
TRINITY_sp|Q6T48	rbrA	Probable E3 ubiquit	61.40	0.00
TRINITY_sp|P1261	T-cp1	T-complex protein 1	61.40	0.00
TRINITY_sp|P4373	dnaJ	Chaperone protein D	61.40	0.00

TRINITY_sp|Q075!	Gyc32E	Guanylate cyclase 3	61.40	0.00
TRINITY_sp|Q6I5\	CDKC-1	Cyclin-dependent ki	61.40	0.00
TRINITY_sp|Q7XP.	Os04g06(	Kinesin-like calmod	61.40	0.00
TRINITY_sp|Q9LR.	GGAT1	Glutamate--glyoxyla	61.40	0.00
TRINITY_sp|Q9M4(	PGM1	Phosphoglucomutase,	61.40	0.00
TRINITY_sp|Q8T1:	kif5	Kinesin-related pro	61.40	0.00
TRINITY_sp|Q9C0(	DNAH6	Dynein heavy chain	61.40	0.00
TRINITY_sp|P277:	TTR	Transthyretin OS=Ga	61.30	0.00
TRINITY_sp|Q131:	PRKAA1	5'-AMP-activated pr	61.30	0.00
TRINITY_sp|Q54P\	ppa1	Inorganic pyrophosp	61.30	0.00
TRINITY_sp|Q9DAI	Pacrg	Parkin coregulated	61.30	0.00
TRINITY_sp|Q9C8I	EMB3003	Dihydrolipoyllysine	61.30	0.00
TRINITY_sp|Q949(	GLY1	Glycerol-3-phosphat	61.30	0.00
TRINITY_sp|Q5ZK!	SPAST	Spastin OS=Gallus g	61.30	0.00
TRINITY_sp|O489(	CMDH	Malate dehydrogenas	61.30	0.00
TRINITY_sp|P274{	GRP-2	Glycine-rich protei	61.30	0.00
TRINITY_sp|Q9WU{	DPM1	Dolichol-phosphate	61.30	0.00
TRINITY_sp|O743{	SPBC3H7.	Uncharacterized met	61.30	0.00
TRINITY_sp|B1XJI	prfA	Peptide chain relea	61.30	0.00
TRINITY_sp|B0G1:	pip5k3	1-phosphatidylinosi	61.30	0.00
TRINITY_sp|Q8W2I	SAT4	Serine acetyltransf	61.30	0.00
TRINITY_sp|P678:	SEC11A	Signal peptidase co	61.30	0.00
TRINITY_sp|B3VM(	BADH2	Betaine aldehyde de	61.30	0.00
TRINITY_sp|Q6DCI	selenbp:	Selenium-binding pr	61.30	0.00
TRINITY_sp|P484(	CNAG_00.	Actin OS=Cryptococc	61.30	0.00
TRINITY_sp|Q8W1:	SMU1	Suppressor of mec-8	61.20	0.00
TRINITY_sp|O429:	sec27	Probable coatomer s	61.20	0.00
TRINITY_sp|Q9W4I	rg	Neurobeachin OS=Dro	61.20	0.00
TRINITY_sp|P508{	RPL12A	60S ribosomal prote	61.20	0.00
TRINITY_sp|Q9HEI	2E4.130	Regulator of nonsen	61.20	0.00
TRINITY_sp|Q8IX:	ZC3H3	Zinc finger CCCH do	61.20	0.00
TRINITY_sp|Q9D8:	Dnajb4	DnaJ homolog subfam	61.20	0.00
TRINITY_sp|O820:	HPA	Histidinol-phosphat	61.20	0.00
TRINITY_sp|Q24JI	P4H5	Prolyl 4-hydroxylas	61.20	0.00
TRINITY_sp|A7YWI	HAL	Histidine ammonia-l	61.20	0.00
TRINITY_sp|Q9SB.	PDK	[Pyruvate dehydroge	61.20	0.00
TRINITY_sp|Q8C5I	Cwc22	Pre-mRNA-splicing f	61.20	0.00
TRINITY_sp|P323.	PPH3	Serine/threonine-pr	61.20	0.00
TRINITY_sp|Q75G:	CLPB2	Chaperone protein C	61.20	0.00
TRINITY_sp|B0C6:	prfC	Peptide chain relea	61.20	0.00
TRINITY_sp|Q623{	Atm	Serine-protein kina	61.20	0.00
TRINITY_sp|Q9SL:	At2g200!	Protein phosphatase	61.20	0.00
TRINITY_sp|B8AZ>	MCM6	DNA replication lic	61.20	0.00
TRINITY_sp|F4IF(	EMB2761	Threonine--tRNA lig	61.20	0.00
TRINITY_sp|Q6NYI	mak16	Protein MAK16 homol	61.10	0.00
TRINITY_sp|Q9TU:	RAC2	Ras-related C3 botu	61.10	0.00
TRINITY_sp|Q8S4\	AAT1	Acetyl-CoA acetyltr	61.10	0.00
TRINITY_sp|Q9BR>	WDR83	WD repeat domain-co	61.10	0.00
TRINITY_sp|Q8S9.	At2g338.	Tyrosine--tRNA liga	61.10	0.00
TRINITY_sp|P464(	Vps4b	Vacuolar protein so	61.10	0.00
TRINITY_sp|Q55F:	gabT	4-aminobutyrate ami	61.10	0.00
TRINITY_sp|A3KP:	ndufaf5	Arginine-hydroxylas	61.10	0.00
TRINITY_sp|Q029:	SLC25A1:	Mitochondrial 2-oxo	61.10	0.00
TRINITY_sp|J9RY:	DVR	Divinyl chlorophyll	61.10	0.00
TRINITY_sp|P549:	IMP2	Inositol monophosph	61.10	0.00
TRINITY_sp|Q9UHI	NDOR1	NADPH-dependent dif	61.10	0.00

TRINITY_sp|Q76P(	DDB_G02	Probable serine/thr	61.10	0.00
TRINITY_sp|C1F3	lipB	Octanoyltransferase	61.10	0.00
TRINITY_sp|P829	hamp	Hepcidin OS=Morone	61.10	0.00
TRINITY_sp|A0AU	wdsub1	WD repeat, SAM and	61.10	0.00
TRINITY_sp|P550	RPN10	26S proteasome non-	61.10	0.00
TRINITY_sp|Q9S7	RPI3	Probable ribose-5-p	61.10	0.00
TRINITY_sp|Q1MT	nip7	60S ribosome subuni	61.10	0.00
TRINITY_sp|Q9Z2	Mtm1	Myotubularin OS=Mus	61.10	0.00
TRINITY_sp|P486	FAD7	Omega-3 fatty acid	61.10	0.00
TRINITY_sp|Q7XZ	SAC1	Phosphoinositide ph	61.10	0.00
TRINITY_sp|P327	YME1	Mitochondrial inner	61.10	0.00
TRINITY_sp|Q688	GSH1-1	Glutamate--cysteine	61.10	0.00
TRINITY_sp|B8AP	MRS2-A	Magnesium transport	61.10	0.00
TRINITY_sp|Q54X	sec61a	Protein transport p	61.10	0.00
TRINITY_sp|Q9ZP	SDH1-2	Succinate dehydroge	61.10	0.00
TRINITY_sp|Q388	XPB1	DNA repair helicase	61.10	0.00
TRINITY_sp|P631	DYNLT1	Dynein light chain	61.10	0.00
TRINITY_sp|Q84M	ABCA1	ABC transporter A f	61.10	0.00
TRINITY_sp|P974	Bop1	Ribosome biogenesis	61.10	0.00
TRINITY_sp|O652	ACX1	Peroxisomal acyl-co	61.10	0.00
TRINITY_sp|Q929	SMARCC1	SWI/SNF complex sub	61.00	0.00
TRINITY_sp|Q154	SF3B4	Splicing factor 3B	61.00	0.00
TRINITY_sp|P550	Abcd3	ATP-binding cassett	61.00	0.00
TRINITY_sp|B9RA	RCOM_15	Probable aspartyl a	61.00	0.00
TRINITY_sp|Q099	has1	ATP-dependent RNA h	61.00	0.00
TRINITY_sp|P318	-	RNA-directed DNA po	61.00	0.00
TRINITY_sp|P491	Serpind	Heparin cofactor 2	61.00	0.00
TRINITY_sp|Q7WG	fumC	Fumarate hydratase	61.00	0.00
TRINITY_sp|O627	TXNRD1	Thioredoxin reducta	61.00	0.00
TRINITY_sp|Q2QM	UAH	Probable ureidoglyc	61.00	0.00
TRINITY_sp|P044	-	Calmodulin OS=Triti	61.00	0.00
TRINITY_sp|Q8VY	SQE3	Squalene epoxidase	61.00	0.00
TRINITY_sp|P364	ILSA	Isoleucine--tRNA li	61.00	0.00
TRINITY_sp|E9Q9	Rabl2	Rab-like protein 2A	61.00	0.00
TRINITY_sp|Q9V9	l(2)045	Probable methylcrot	61.00	0.00
TRINITY_sp|O246	MSH2	DNA mismatch repair	61.00	0.00
TRINITY_sp|Q93Y	MNS3	Mannosyl-oligosacch	61.00	0.00
TRINITY_sp|Q95K	NOP56	Nucleolar protein 5	61.00	0.00
TRINITY_sp|Q9UJ	DDX41	Probable ATP-depend	61.00	0.00
TRINITY_sp|A8N0	GET3	ATPase GET3 OS=Copr	61.00	0.00
TRINITY_sp|Q9C9	CDKD-1	Cyclin-dependent ki	61.00	0.00
TRINITY_sp|P585	chlI	Magnesium-chelatase	61.00	0.00
TRINITY_sp|Q2NK	DPY30	Protein dpy-30 homo	61.00	0.00
TRINITY_sp|P147	-	Alpha-galactosidase	60.90	0.00
TRINITY_sp|Q4P8	DPH3	Diphthamide biosynt	60.90	0.00
TRINITY_sp|P248	TSJT1	Stem-specific prote	60.90	0.00
TRINITY_sp|Q0E2	UVR3	(6-4)DNA photolyase	60.90	0.00
TRINITY_sp|Q097	pim1	Lon protease homolo	60.90	0.00
TRINITY_sp|Q9UK	LSM7	U6 snRNA-associated	60.90	0.00
TRINITY_sp|Q9ST	PH1	Pleckstrin homology	60.90	0.00
TRINITY_sp|A4IF	POLR3A	DNA-directed RNA po	60.90	0.00
TRINITY_sp|Q425	TSA1	Tryptophan synthase	60.90	0.00
TRINITY_sp|Q56Y	MAP2B	Methionine aminopep	60.90	0.00
TRINITY_sp|Q2T9	NIT2	Omega-amidase NIT2	60.90	0.00
TRINITY_sp|Q9SL	DGAT1	Diacylglycerol O-ac	60.90	0.00
TRINITY_sp|Q9FI	At5g477	Probable acetyl-CoA	60.90	0.00

TRINITY_sp|Q9SN5	UGE5	UDP-glucose 4-epime	60.90	0.00
TRINITY_sp|Q9C5M	DTC	Mitochondrial dicar	60.90	0.00
TRINITY_sp|Q9SJ2	RNR1	Ribonucleoside-diph	60.90	0.00
TRINITY_sp|Q6P5I	hsdl2	Hydroxysteroid dehy	60.90	0.00
TRINITY_sp|Q2XQY	IFT57	Intraflagellar tran	60.90	0.00
TRINITY_sp|Q2KI7	DNAJB4	DnaJ homolog subfam	60.90	0.00
TRINITY_sp|Q9M88	CCT6A	T-complex protein 1	60.90	0.00
TRINITY_sp|Q434I	LCY1	Lycopene beta cycla	60.90	0.00
TRINITY_sp|P2124	CPN60B1	Chaperonin 60 subun	60.90	0.00
TRINITY_sp|O8130	PAP11	Probable plastid-li	60.90	0.00
TRINITY_sp|P2269	celA	Endoglucanase OS=Di	60.90	0.00
TRINITY_sp|O8198	-	Serine--tRNA ligase	60.90	0.00
TRINITY_sp|Q54J2	nop10	H/ACA ribonucleopro	60.90	0.00
TRINITY_sp|P0743	BETA	Tubulin beta-1 chai	60.80	0.00
TRINITY_sp|Q9SJI	HIPP05	Heavy metal-associa	60.80	0.00
TRINITY_sp|O805I	CYCU4-1	Cyclin-U4-1 OS=Arab	60.80	0.00
TRINITY_sp|Q54NI	gtf2a2	Transcription initi	60.80	0.00
TRINITY_sp|P4098	hus5	SUMO-conjugating en	60.80	0.00
TRINITY_sp|Q557E	cblA-1	E3 ubiquitin-protei	60.80	0.00
TRINITY_sp|O0260	pp63-2	Phosphoglucomutase-	60.80	0.00
TRINITY_sp|P342I	TAL1	Transaldolase OS=Kl	60.80	0.00
TRINITY_sp|A7SG7	qprt	Nicotinate-nucleoti	60.80	0.00
TRINITY_sp|A1A4E	U2AF1	Splicing factor U2A	60.80	0.00
TRINITY_sp|Q6CQE	TAR1-A	Protein TAR1 OS=Klu	60.80	0.00
TRINITY_sp|P0257	-	Actin-1 OS=Acantham	60.80	0.00
TRINITY_sp|P3283	PPG1	Serine/threonine-pr	60.80	0.00
TRINITY_sp|Q6PFW	PPIP5K1	Inositol hexakispho	60.80	0.00
TRINITY_sp|Q9SB5	AP4M	AP-4 complex subuni	60.80	0.00
TRINITY_sp|A7RRJ	FEN1	Flap endonuclease 1	60.80	0.00
TRINITY_sp|M1V4Y	FAP73	Cilia- and flagella	60.80	0.00
TRINITY_sp|Q54J9	hspA	60 kDa heat shock p	60.80	0.00
TRINITY_sp|B4GZ2	Sgf11	SAGA-associated fac	60.80	0.00
TRINITY_sp|P2182	-	Triosephosphate iso	60.80	0.00
TRINITY_sp|P0C8M	CCRP1	Probable serine/thr	60.80	0.00
TRINITY_sp|Q9SRE	HIR3	Hypersensitive-indu	60.80	0.00
TRINITY_sp|Q6AX1	Sf3a2	Splicing factor 3A	60.80	0.00
TRINITY_sp|Q55E3	rab32B	Ras-related protein	60.80	0.00
TRINITY_sp|Q4359	-	Stearoyl-[acyl-carr	60.80	0.00
TRINITY_sp|H3JU0	SGT1	Peptidyl serine alp	60.80	0.00
TRINITY_sp|Q28EX	ublcp1	Ubiquitin-like doma	60.80	0.00
TRINITY_sp|Q7S80	pim1	Lon protease homolo	60.80	0.00
TRINITY_sp|P5435	dod	Putative peptidyl-p	60.70	0.00
TRINITY_sp|Q7SAE	msp-41	Pre-mRNA-splicing f	60.70	0.00
TRINITY_sp|Q9LXT	CALS3	Callose synthase 3	60.70	0.00
TRINITY_sp|P0044	SOD1	Superoxide dismutas	60.70	0.00
TRINITY_sp|P1760	ryh1	GTP-binding protein	60.70	0.00
TRINITY_sp|Q9SB5	AP4M	AP-4 complex subuni	60.70	0.00
TRINITY_sp|Q8TFM	-	60S acidic ribosoma	60.70	0.00
TRINITY_sp|Q9WV9	Mok	MAPK/MAK/MRK overla	60.70	0.00
TRINITY_sp|Q9FLE	HAM1	Histone acetyltrans	60.70	0.00
TRINITY_sp|O4236	apoeb	Apolipoprotein Eb O	60.70	0.00
TRINITY_sp|Q6B40	GLN1	Glutamine synthetas	60.70	0.00
TRINITY_sp|P5344	CTN	Caltractin OS=Naegl	60.70	0.00
TRINITY_sp|Q8R50	Mutyh	Adenine DNA glycosy	60.70	0.00
TRINITY_sp|Q19A2	F2	Prothrombin OS=Sus	60.70	0.00
TRINITY_sp|Q7XJJ	FAAH	Fatty acid amide hy	60.70	0.00

TRINITY_sp|Q8LDI	CYP23	Peptidyl-prolyl cis	60.70	0.00
TRINITY_sp|Q8N1I	LRRC75A-	Putative uncharacte	60.70	0.00
TRINITY_sp|Q9038	-	Peroxiredoxin OS=Cy	60.70	0.00
TRINITY_sp|Q8CXI	dnaJ	Chaperone protein D	60.70	0.00
TRINITY_sp|Q9LRI	POLD1	DNA polymerase delt	60.70	0
TRINITY_sp|Q9SA1	AKHSDH1	Bifunctional aspart	60.70	0.00
TRINITY_sp|P4233	At1g6049	Phosphatidylinosito	60.70	0.00
TRINITY_sp|Q94AI	CUL1	Cullin-1 OS=Arabido	60.70	0.00
TRINITY_sp|Q9SII	ACO2	Aconitate hydratase	60.60	0.00
TRINITY_sp|Q54NW	oplah	5-oxoprolinase OS=D	60.60	0.00
TRINITY_sp|Q0829	RD22	BURP domain protein	60.60	0.00
TRINITY_sp|Q86B0	rlp24	Probable ribosome b	60.60	0.00
TRINITY_sp|Q9WV9	Mok	MAPK/MAK/MRK overla	60.60	0.00
TRINITY_sp|P4837	FK506-bp	12 kDa FK506-bindin	60.60	0.00
TRINITY_sp|P2532	mlkA	Myosin light chain	60.60	0.00
TRINITY_sp|P0CG7	ubq-1	Polyubiquitin-A OS=	60.60	0.00
TRINITY_sp|Q5550	glgB	1,4-alpha-glucan-br	60.60	0.00
TRINITY_sp|O9431	SPBC215.	UPF0743 protein C21	60.60	0.00
TRINITY_sp|Q9M10	ABCB21	ABC transporter B f	60.60	0.00
TRINITY_sp|Q54N2	culC	Cullin-3 OS=Dictyos	60.60	0.00
TRINITY_sp|Q0WQ0	AGD15	Probable ADP-ribosy	60.60	0.00
TRINITY_sp|Q3Y8I	DAW1	Dynein assembly fac	60.60	0.00
TRINITY_sp|P2317	ABCB4	Phosphatidylcholine	60.60	0.00
TRINITY_sp|Q9C65	DCP5	Protein decapping 5	60.60	0.00
TRINITY_sp|P5628	tif211	Eukaryotic translat	60.60	0.00
TRINITY_sp|P5128	ycf45	Uncharacterized pro	60.60	0.00
TRINITY_sp|Q55E9	gfm1	Elongation factor G	60.60	0.00
TRINITY_sp|P4960	-	Aconitate hydratase	60.60	0.00
TRINITY_sp|Q5D01	rbm8a	RNA-binding protein	60.60	0.00
TRINITY_sp|Q9HEI	2E4.130	Regulator of nonsen	60.60	0.00
TRINITY_sp|Q9ZU1	At1g6007	AP-1 complex subuni	60.60	0.00
TRINITY_sp|Q8GWI	AIH	Agmatine deiminase	60.60	0.00
TRINITY_sp|Q94CI	EHD1	EH domain-containin	60.60	0.00
TRINITY_sp|Q9LZ0	At3g6231	Probable pre-mRNA-s	60.60	0.00
TRINITY_sp|Q9D61	Tube1	Tubulin epsilon cha	60.60	0.00
TRINITY_sp|Q9CA1	CKL2	Casein kinase 1-lik	60.50	0.00
TRINITY_sp|Q54F0	metK	S-adenosylmethionin	60.50	0.00
TRINITY_sp|Q9Y38	RBMX2	RNA-binding motif p	60.50	0.00
TRINITY_sp|Q9QY3	Abcb11	Bile salt export pu	60.50	0.00
TRINITY_sp|P5362	COPG1	Coatomer subunit ga	60.50	0.00
TRINITY_sp|Q8LA9	SFGH	S-formylglutathione	60.50	0.00
TRINITY_sp|Q6DW7	DGD1	Digalactosyldiacylg	60.50	0.00
TRINITY_sp|Q94JV	At1g5429	Protein translation	60.50	0.00
TRINITY_sp|B9DGI	ACS	Acetyl-coenzyme A s	60.50	0.00
TRINITY_sp|Q54H1	prmt2	Protein arginine N-	60.50	0.00
TRINITY_sp|Q5XHI	ppip5k2	Inositol hexakispho	60.50	0.00
TRINITY_sp|Q9SA2	RH36	DEAD-box ATP-depend	60.50	0.00
TRINITY_sp|Q9C77	RPN8B	26S proteasome non-	60.50	0.00
TRINITY_sp|Q9LTI	NMT1	Glycylpeptide N-tet	60.50	0.00
TRINITY_sp|Q9SMI	DHC1B	Cytoplasmic dynein	60.50	0
TRINITY_sp|Q6UB0	RSP2	Flagellar radial sp	60.50	0.00
TRINITY_sp|Q9FL0	At5g5507	Dihydrolipoyllysine	60.50	0.00
TRINITY_sp|A4IR3	dnaJ	Chaperone protein D	60.40	0.00
TRINITY_sp|Q9LSW	ETFB	Electron transfer f	60.40	0.00
TRINITY_sp|Q6QNM	-	Casein kinase I OS=	60.40	0.00
TRINITY_sp|O6490	NDPK2	Nucleoside diphosph	60.40	0.00

TRINITY_sp|Q5UP2	MIMI_R2(	DnaJ-like protein R	60.40	0.00
TRINITY_sp|Q916	aad-a	Alpha-aspartyl dipe	60.40	0.00
TRINITY_sp|B0BNI	Esd	S-formylglutathione	60.40	0.00
TRINITY_sp|Q941	Os01g08	Probable mannose-1-	60.40	0.00
TRINITY_sp|Q93WI	SBP2	Selenium-binding pr	60.40	0.00
TRINITY_sp|O142	mtr4	ATP-dependent RNA h	60.40	0.00
TRINITY_sp|Q93X	CPA	N-carbamoylputresci	60.40	0.00
TRINITY_sp|Q392	TOUSLED	Serine/threonine-pr	60.40	0.00
TRINITY_sp|O824	At3g461(	Histidine--tRNA lig	60.40	0.00
TRINITY_sp|O489(	CMDH	Malate dehydrogenas	60.40	0.00
TRINITY_sp|O232	SHM4	Serine hydroxymethy	60.40	0.00
TRINITY_sp|Q611(	Abcb7	ATP-binding cassett	60.40	0.00
TRINITY_sp|Q9SC	DHS1	Deoxyhypusine synth	60.40	0.00
TRINITY_sp|Q9SF	RPL4A	60S ribosomal prote	60.40	0.00
TRINITY_sp|B6D5	Dnaaf1	Dynein assembly fac	60.40	0.00
TRINITY_sp|Q5K5	Os07g06	DEAD-box ATP-depend	60.40	0.00
TRINITY_sp|Q9SR	FKBP16-	Peptidyl-prolyl cis	60.40	0.00
TRINITY_sp|Q9FI	RPS8B	40S ribosomal prote	60.40	0.00
TRINITY_sp|P073	ACYP1	Acylphosphatase-1 O	60.30	0.00
TRINITY_sp|P126	MLYCD	Malonyl-CoA decarbo	60.30	0.00
TRINITY_sp|P423	CNB1	Calcineurin subunit	60.30	0.00
TRINITY_sp|Q9SL	TCX6	Protein tesmin/TSO1	60.30	0.00
TRINITY_sp|Q0MQ	NDUFS6	NADH dehydrogenase	60.30	0.00
TRINITY_sp|Q9CW	Dph5	Diphthine methyl es	60.30	0.00
TRINITY_sp|Q97Y	msrA	Peptide methionine	60.30	0.00
TRINITY_sp|Q9FW	HAC12	Histone acetyltrans	60.30	0.00
TRINITY_sp|P515	AFC2	Serine/threonine-pr	60.30	0.00
TRINITY_sp|Q5X4	purC	Phosphoribosylamino	60.30	0.00
TRINITY_sp|Q3BA	-	Uncharacterized pro	60.30	0.00
TRINITY_sp|Q9FL	NLE1	Notchless protein h	60.30	0.00
TRINITY_sp|P348	-	Serine hydroxymethy	60.30	0.00
TRINITY_sp|Q5UP2	MIMI_R2(	DnaJ-like protein R	60.30	0.00
TRINITY_sp|P496	MAN2A2	Alpha-mannosidase 2	60.30	0.00
TRINITY_sp|Q6L4	DBB1	DNA damage-binding	60.30	0.00
TRINITY_sp|F4I9(	GLYR2	Glyoxylate/succinic	60.30	0.00
TRINITY_sp|Q9Y7	tor2	Serine/threonine-pr	60.30	0.00
TRINITY_sp|P352	Rab6a	Ras-related protein	60.30	0.00
TRINITY_sp|Q71U	RPS27L	40S ribosomal prote	60.30	0.00
TRINITY_sp|P056	sacC	Levanase OS=Bacillu	60.30	0.00
TRINITY_sp|Q9SP(	RRP41	Exosome complex com	60.30	0.00
TRINITY_sp|Q7KQ	PF10_00	Tubulin beta chain	60.30	0.00
TRINITY_sp|P114	PSBP	Oxygen-evolving enh	60.30	0.00
TRINITY_sp|Q54Q	erkB	Extracellular signa	60.30	0.00
TRINITY_sp|P493	CCT3	T-complex protein 1	60.30	0.00
TRINITY_sp|Q965	ADH2	Alcohol dehydrogena	60.30	0.00
TRINITY_sp|Q54K	gcvP	Glycine dehydrogena	60.30	0
TRINITY_sp|Q9MA	ERCC1	DNA excision repair	60.30	0.00
TRINITY_sp|B0R0	chd8	Chromodomain-helica	60.30	0.00
TRINITY_sp|Q9M8	At1g742	Probable phosphorib	60.20	0.00
TRINITY_sp|P217	rps-26	40S ribosomal prote	60.20	0.00
TRINITY_sp|P953	mls	Malate synthase OS=	60.20	0.00
TRINITY_sp|P462	-	Fructose-1,6-bispho	60.20	0.00
TRINITY_sp|P0DJ	RPL18	60S ribosomal prote	60.20	0.00
TRINITY_sp|P314	AVP1	Pyrophosphate-energ	60.20	0.00
TRINITY_sp|Q95U	Rab7a	Ras-related protein	60.20	0.00
TRINITY_sp|A7HW	recA	Protein RecA OS=Par	60.20	0.00

TRINITY_sp|Q8VX[illegible]	AGAL3	Alpha-galactosidase	60.20	0.00
TRINITY_sp|Q167[illegible]	MAN2A1	Alpha-mannosidase 2	60.20	0.00
TRINITY_sp|Q100[illegible]	SPAC3H1[illegible]	Glutathione gamma-g	60.20	0.00
TRINITY_sp|Q9ZQ[illegible]	U2AF35A	Splicing factor U2a	60.20	0.00
TRINITY_sp|Q86I[illegible]	dst1	Serine/threonine-pr	60.20	0.00
TRINITY_sp|Q6DF[illegible]	chmp2a	Charged multivesicu	60.20	0.00
TRINITY_sp|Q54X[illegible]	etfdh	Electron transfer f	60.20	0.00
TRINITY_sp|Q54M[illegible]	fhkD	Probable serine/thr	60.20	0.00
TRINITY_sp|Q9SR[illegible]	COG0212	5-formyltetrahydrof	60.20	0.00
TRINITY_sp|Q69T[illegible]	PRXIIE-[illegible]	Peroxiredoxin-2E-1,	60.20	0.00
TRINITY_sp|P368[illegible]	NITA	Nitrate reductase [	60.20	0
TRINITY_sp|P0C0[illegible]	NUDT24	Nudix hydrolase 24,	60.20	0.00
TRINITY_sp|Q425[illegible]	ASB1	Anthranilate syntha	60.20	0.00
TRINITY_sp|Q8LG[illegible]	CUL4	Cullin-4 OS=Arabido	60.20	0.00
TRINITY_sp|O222[illegible]	CYT1	Mannose-1-phosphate	60.20	0.00
TRINITY_sp|Q568[illegible]	gmd	GDP-mannose 4,6-deh	60.20	0.00
TRINITY_sp|Q54V[illegible]	ctps	CTP synthase OS=Dic	60.20	0.00
TRINITY_sp|P940[illegible]	HAL3B	Probable phosphopan	60.20	0.00
TRINITY_sp|Q8H1[illegible]	PGRL1A	PGR5-like protein 1	60.20	0.00
TRINITY_sp|Q54P[illegible]	mtr	Methionine synthase	60.20	0
TRINITY_sp|Q6AV[illegible]	PPDK1	Pyruvate, phosphate	60.20	0.00
TRINITY_sp|Q8H0[illegible]	ABCF3	ABC transporter F f	60.20	0.00
TRINITY_sp|Q965[illegible]	SAMDC	S-adenosylmethionin	60.20	0.00
TRINITY_sp|O966[illegible]	arcC	Actin-related prote	60.10	0.00
TRINITY_sp|Q93V[illegible]	At3g026[illegible]	WAT1-related protei	60.10	0.00
TRINITY_sp|Q8W4[illegible]	RH30	DEAD-box ATP-depend	60.10	0.00
TRINITY_sp|P481[illegible]	rpl27a	60S ribosomal prote	60.10	0.00
TRINITY_sp|O817[illegible]	At4g317[illegible]	Probable diphthine	60.10	0.00
TRINITY_sp|Q6S0[illegible]	kif6	Kinesin-related pro	60.10	0.00
TRINITY_sp|Q95Y[illegible]	cdk8	Probable cyclin-dep	60.10	0.00
TRINITY_sp|P0DJ[illegible]	RPL17	60S ribosomal prote	60.10	0.00
TRINITY_sp|Q86A[illegible]	gltA	Citrate synthase OS	60.10	0.00
TRINITY_sp|Q3Y8[illegible]	DAW1	Dynein assembly fac	60.10	0.00
TRINITY_sp|Q962[illegible]	GDI1	Guanosine nucleotid	60.10	0.00
TRINITY_sp|Q429[illegible]	Os12g06[illegible]	Malate dehydrogenas	60.10	0.00
TRINITY_sp|P178[illegible]	crn	Protein crooked nec	60.10	0.00
TRINITY_sp|Q2YD[illegible]	ARL1	ADP-ribosylation fa	60.10	0.00
TRINITY_sp|P177[illegible]	FBA1	Fructose-bisphospha	60.10	0.00
TRINITY_sp|A8IE[illegible]	PRMT1	Protein arginine N-	60.10	0.00
TRINITY_sp|Q5LA[illegible]	hisS	Histidine--tRNA lig	60.00	0.00
TRINITY_sp|Q8L7[illegible]	AGD11	Probable ADP-ribosy	60.00	0.00
TRINITY_sp|P176[illegible]	ypt3	GTP-binding protein	60.00	0.00
TRINITY_sp|B8AE[illegible]	MCM5	DNA replication lic	60.00	0.00
TRINITY_sp|Q217[illegible]	rskn-1	Putative ribosomal	60.00	0.00
TRINITY_sp|Q54J[illegible]	purL	Phosphoribosylformy	60.00	0.00
TRINITY_sp|P366[illegible]	rad8	DNA repair protein	60.00	0.00
TRINITY_sp|Q9VH[illegible]	CG11985	Probable splicing f	60.00	0.00
TRINITY_sp|Q93V[illegible]	GIF3	GRF1-interacting fa	60.00	0.00
TRINITY_sp|Q6ER[illegible]	CAD8B	Probable cinnamyl a	60.00	0.00
TRINITY_sp|Q8T6[illegible]	arsA	ATPase ASNA1 homolo	60.00	0.00
TRINITY_sp|Q4QQ[illegible]	Adhfe1	Hydroxyacid-oxoacid	60.00	0.00
TRINITY_sp|Q9VD[illegible]	Ino80	Putative DNA helica	60.00	0.00
TRINITY_sp|Q9LH[illegible]	At5g060[illegible]	Tropinone reductase	60.00	0.00
TRINITY_sp|Q29A[illegible]	GA17800	Leishmanolysin-like	60.00	0.00
TRINITY_sp|Q9UE[illegible]	FTSJ1	Putative tRNA (cyti	60.00	0.00
TRINITY_sp|Q84S[illegible]	Os07g05[illegible]	Zinc finger CCCH do	60.00	0.00

TRINITY_sp|O803(	RPL4	50S ribosomal prote	60.00	0.00
TRINITY_sp|Q6C0(	DPH3	Diphthamide biosynt	60.00	0.00
TRINITY_sp|Q8VX	ENT1	Equilibrative nucle	60.00	0.00
TRINITY_sp|Q6BJ	PNO1	Pre-rRNA-processing	60.00	0.00
TRINITY_sp|Q048	Mak	Serine/threonine-pr	60.00	0.00
TRINITY_sp|P094(	snrnp70	U1 small nuclear ri	60.00	0.00
TRINITY_sp|Q8VZ(	VPS25	Vacuolar protein so	60.00	0.00
TRINITY_sp|Q5BI	PIN1	Peptidyl-prolyl cis	60.00	0.00
TRINITY_sp|Q9M1	MPK10	Mitogen-activated p	60.00	0.00
TRINITY_sp|Q8VZ(	UXS1	UDP-glucuronic acid	60.00	0.00
TRINITY_sp|P275	-	Chlorophyll a-b bin	60.00	0.00
TRINITY_sp|P601	ELAC	Nuclear ribonucleas	60.00	0.00
TRINITY_sp|P200	pyr1-3	Protein PYR1-3 OS=D	60.00	0.00
TRINITY_sp|P592	RPS27AA	Ubiquitin-40S ribos	60.00	0.00
TRINITY_sp|Q9SZ	FAD4	Fatty acid desatura	60.00	0.00
TRINITY_sp|Q86I	DDB_G02	Uncharacterized pro	60.00	0.00
TRINITY_sp|Q7U6	dnaK1	Chaperone protein d	60.00	0.00
TRINITY_sp|O705	Srpk1	SRSF protein kinase	60.00	0.00
TRINITY_sp|Q286	ABCC2	Canalicular multisp	60.00	0.00
TRINITY_sp|P808	ydaD	General stress prot	60.00	0.00
TRINITY_sp|Q9LV	ABCB25	ABC transporter B f	60.00	0.00
TRINITY_sp|P108	PLCD1	1-phosphatidylinosi	60.00	0.00
TRINITY_sp|F4KE	FHIT	Bifunctional bis(5'	60.00	0.00
TRINITY_sp|Q5SP	chac1	Glutathione-specifi	60.00	0.00
TRINITY_sp|Q9SL(	RAD50	DNA repair protein	60.00	0.00
TRINITY_sp|Q9QY	Rdh11	Retinol dehydrogena	60.00	0.00
TRINITY_sp|Q9ZR	RH28	DEAD-box ATP-depend	60.00	0.00
TRINITY_sp|Q55G(	dst2	Serine/threonine-pr	60.00	0.00
TRINITY_sp|F4IL	P4H13	Prolyl 4-hydroxylas	60.00	0.00
TRINITY_sp|Q9XT	PREP	Prolyl endopeptidas	60.00	0.00
TRINITY_sp|Q2FK	pflB	Formate acetyltrans	60.00	0.00
TRINITY_sp|Q7ZZ	ran	GTP-binding nuclear	60.00	0.00
TRINITY_sp|Q9NP	NMRK2	Nicotinamide ribosi	60.00	0.00
TRINITY_sp|Q0MX	CSE4	Histone H3-like cen	60.00	0.00
TRINITY_sp|Q004	TPRP-F1	36.4 kDa proline-ri	60.00	0.00
TRINITY_sp|Q8CE	Rdh13	Retinol dehydrogena	60.00	0.00
TRINITY_sp|Q869	pakF	Serine/threonine-pr	59.90	0.00
TRINITY_sp|Q54Y	polr2h	DNA-directed RNA po	59.90	0.00
TRINITY_sp|O650	ECR1	NEDD8-activating en	59.90	0.00
TRINITY_sp|Q9FF	CKL7	Casein kinase 1-lik	59.90	0.00
TRINITY_sp|P524	PUR5	Phosphoribosylformy	59.90	0.00
TRINITY_sp|O598(	SPCC550.	Putative ATP-depend	59.90	0.00
TRINITY_sp|O499	ADK	Adenosine kinase OS	59.90	0.00
TRINITY_sp|Q8K2	Nat10	RNA cytidine acetyl	59.90	0.00
TRINITY_sp|Q9LU	PAP4	Probable plastid-li	59.90	0.00
TRINITY_sp|Q96K(	ARL5B	ADP-ribosylation fa	59.90	0.00
TRINITY_sp|Q9HC	GPN1	GPN-loop GTPase 1 O	59.90	0.00
TRINITY_sp|Q54H	drkA	Probable serine/thr	59.90	0.00
TRINITY_sp|Q392	SKP1A	SKP1-like protein 1	59.90	0.00
TRINITY_sp|O817	BETAC-AI	Beta-adaptin-like p	59.90	0.00
TRINITY_sp|P322	ARG2	Indole-3-acetic aci	59.80	0.00
TRINITY_sp|P136	Cp	Ceruloplasmin OS=Ra	59.80	0.00
TRINITY_sp|Q54Q	urm1	Ubiquitin-related m	59.80	0.00
TRINITY_sp|Q53N	MPK15	Mitogen-activated p	59.80	0.00
TRINITY_sp|Q6IV	UBA5	Ubiquitin-like modi	59.80	0.00
TRINITY_sp|Q2GH	rplT	50S ribosomal prote	59.80	0.00

TRINITY_sp|Q9JI1	Stk3	Serine/threonine-pr	59.80	0.00
TRINITY_sp|P872(	RPL34A	60S ribosomal prote	59.80	0.00
TRINITY_sp|Q8VY1	LSM8	Sm-like protein LSM	59.80	0.00
TRINITY_sp|Q9MB5	FKFBP	6-phosphofructo-2-k	59.80	0.00
TRINITY_sp|Q2TA(	KIF19	Kinesin-like protei	59.80	0.00
TRINITY_sp|P5831	ltaE	Low specificity L-t	59.80	0.00
TRINITY_sp|Q9C0(	DNAH6	Dynein heavy chain	59.80	0.00
TRINITY_sp|A2YGI	USP	UDP-sugar pyrophosp	59.70	0.00
TRINITY_sp|P217	rps-26	40S ribosomal prote	59.70	0.00
TRINITY_sp|O150	SYNJ2	Synaptojanin-2 OS=H	59.70	0.00
TRINITY_sp|Q9TU2	RAC2	Ras-related C3 botu	59.70	0.00
TRINITY_sp|Q9H52	DHX35	Probable ATP-depend	59.70	0.00
TRINITY_sp|Q9LXI	HAM2	Histone acetyltrans	59.70	0.00
TRINITY_sp|P127	RSP3	Flagellar radial sp	59.70	0.00
TRINITY_sp|Q940(	ZTP29	Zinc transporter ZT	59.70	0.00
TRINITY_sp|Q9ZUI	SPDSYN1	Spermidine synthase	59.70	0.00
TRINITY_sp|Q9AT(	TGD3	Protein TRIGALACTOS	59.70	0.00
TRINITY_sp|P372	PSY1	Phytoene synthase,	59.70	0.00
TRINITY_sp|Q1JPI	D2HGDH	D-2-hydroxyglutarat	59.70	0.00
TRINITY_sp|P468	KRP95	Kinesin-II 95 kDa s	59.70	0.00
TRINITY_sp|B9FM	KIN13A	Kinesin-like protei	59.70	0.00
TRINITY_sp|P0C8N	CCRP1	Probable serine/thr	59.70	0.00
TRINITY_sp|Q9FI	At5g478	Adenylate kinase 2,	59.70	0.00
TRINITY_sp|Q9FII	RPS8B	40S ribosomal prote	59.70	0.00
TRINITY_sp|P231	ABCB4	Phosphatidylcholine	59.70	0.00
TRINITY_sp|P187	pilA	Fimbrial protein OS	59.60	0.00
TRINITY_sp|Q9SG(	ALA5	Probable phospholip	59.60	0.00
TRINITY_sp|Q54II	cox17	Cytochrome c oxidas	59.60	0.00
TRINITY_sp|O822(	At2g197	AP-4 complex subuni	59.60	0.00
TRINITY_sp|Q5R4W	CPNE6	Copine-6 OS=Pongo a	59.60	0.00
TRINITY_sp|P828(	fdxr	NADPH:adrenodoxin o	59.60	0.00
TRINITY_sp|Q8BY	Usp47	Ubiquitin carboxyl-	59.60	0.00
TRINITY_sp|Q54G	act10	Actin-10 OS=Dictyos	59.60	0.00
TRINITY_sp|Q75H	SAPK10	Serine/threonine-pr	59.60	0.00
TRINITY_sp|Q54KN	cdh1	Anaphase-promoting	59.60	0.00
TRINITY_sp|Q9C7	ATE2	Arginyl-tRNA--prote	59.60	0.00
TRINITY_sp|A2XFI	PRR73	Two-component respo	59.60	0.00
TRINITY_sp|Q54J1	hal	Probable histidine	59.60	0.00
TRINITY_sp|P465	POL3	DNA polymerase delt	59.60	0.00
TRINITY_sp|Q54DI	DDB_G02	Protein unc-50 homo	59.60	0.00
TRINITY_sp|B6TRI	PYRD	Riboflavin biosynth	59.60	0.00
TRINITY_sp|Q54V	ddx47	Probable ATP-depend	59.60	0.00
TRINITY_sp|Q8S2	FTSH3	ATP-dependent zinc	59.60	0.00
TRINITY_sp|Q039	IM30	Membrane-associated	59.60	0.00
TRINITY_sp|P391(	YCF1	Metal resistance pr	59.60	0.00
TRINITY_sp|Q9C9I	KAS2	3-oxoacyl-[acyl-car	59.60	0.00
TRINITY_sp|Q9FT	RECQL3	ATP-dependent DNA h	59.60	0.00
TRINITY_sp|P356	RPS20	40S ribosomal prote	59.60	0.00
TRINITY_sp|P8031	Cct6a	T-complex protein 1	59.60	0.00
TRINITY_sp|P116	ND1	NADH-ubiquinone oxi	59.60	0.00
TRINITY_sp|Q145(	DHX8	ATP-dependent RNA h	59.60	0
TRINITY_sp|Q54X	DDB_G02	Probable serine/thr	59.60	0.00
TRINITY_sp|A8F1	prfA	Peptide chain relea	59.50	0.00
TRINITY_sp|Q93Z	At4g116	E3 ubiquitin-protei	59.50	0.00
TRINITY_sp|D4A5(	Hmgcll1	3-hydroxymethyl-3-m	59.50	0.00
TRINITY_sp|Q1PF	EDA2	Probable serine pro	59.50	0.00

TRINITY_sp|Q612/	vha-2	V-type proton ATPas	59.50	0.00
TRINITY_sp|P281	pkgB	Protein kinase 2 OS	59.50	0.00
TRINITY_sp|Q9LG\	CIPK1	CBL-interacting pro	59.50	0.00
TRINITY_sp|Q76N2	nubp2	Cytosolic Fe-S clus	59.50	0.00
TRINITY_sp|Q5RG	gnptab	N-acetylglucosamine	59.50	0.00
TRINITY_sp|Q395	YPTC6	Ras-related protein	59.50	0.00
TRINITY_sp|Q54C2	gpn1	GPN-loop GTPase 1 O	59.50	0.00
TRINITY_sp|O8102	HMGCL	Hydroxymethylglutar	59.50	0.00
TRINITY_sp|P3223	Cbs	Cystathionine beta-	59.50	0.00
TRINITY_sp|P5523	AGPS1	Glucose-1-phosphate	59.50	0.00
TRINITY_sp|O154	MPPED1	Metallophosphoester	59.50	0.00
TRINITY_sp|O8043	MAK3	N-alpha-acetyltrans	59.50	0.00
TRINITY_sp|P4921	RPS17	40S ribosomal prote	59.50	0.00
TRINITY_sp|P0DK(	PGLP1A	Phosphoglycolate ph	59.50	0.00
TRINITY_sp|Q9SJ	SAE2	SUMO-activating enz	59.50	0.00
TRINITY_sp|Q869	odhB	Dihydrolipoyllysine	59.50	0.00
TRINITY_sp|Q9SMI	NR1	Malate dehydrogenas	59.50	0.00
TRINITY_sp|P0432	pol	Retrovirus-related	59.50	0.00
TRINITY_sp|Q86B	anapc8	Anaphase-promoting	59.50	0.00
TRINITY_sp|P834(	DDB_G02	Putative aldehyde d	59.50	0.00
TRINITY_sp|Q9SZ3	HISN4	Imidazole glycerol	59.50	0.00
TRINITY_sp|F4K0I	ISPG	4-hydroxy-3-methylb	59.50	0.00
TRINITY_sp|Q6DH(	setd7	Histone-lysine N-me	59.40	0.00
TRINITY_sp|O745(	msh6	DNA mismatch repair	59.40	0.00
TRINITY_sp|P391(	YCF1	Metal resistance pr	59.40	0.00
TRINITY_sp|Q84M2	ABCA1	ABC transporter A f	59.40	0.00
TRINITY_sp|P0CM3	ATG7	Ubiquitin-like modi	59.40	0.00
TRINITY_sp|Q0III	GK	Glycerol kinase OS=	59.40	0.00
TRINITY_sp|P168	gpaA	Guanine nucleotide-	59.40	0.00
TRINITY_sp|A7Z01	SMARCA4	Transcription activ	59.40	0.00
TRINITY_sp|Q9PT	urod	Uroporphyrinogen de	59.40	0.00
TRINITY_sp|Q9LV(	TOE2	AP2-like ethylene-r	59.40	0.00
TRINITY_sp|P834(	DDB_G02	Putative aldehyde d	59.40	0.00
TRINITY_sp|P2333	TK	Thymidine kinase OS	59.40	0.00
TRINITY_sp|O009(	arfA	ADP-ribosylation fa	59.40	0.00
TRINITY_sp|P105	TXN	Thioredoxin OS=Homo	59.40	0.00
TRINITY_sp|O075	yheH	Probable multidrug	59.40	0.00
TRINITY_sp|Q2HJ	PRPF18	Pre-mRNA-splicing f	59.40	0.00
TRINITY_sp|Q437	-	Glutamate--tRNA lig	59.40	0.00
TRINITY_sp|P155	ade6	Phosphoribosylamino	59.40	0.00
TRINITY_sp|Q8H0	HDA9	Histone deacetylase	59.40	0.00
TRINITY_sp|Q5R6I	PIGK	GPI-anchor transami	59.40	0.00
TRINITY_sp|Q76P(	DDB_G02	Probable serine/thr	59.40	0.00
TRINITY_sp|Q9SI2	RKP	E3 ubiquitin-protei	59.40	0.00
TRINITY_sp|Q5J4	MPK2	Mitogen-activated p	59.40	0.00
TRINITY_sp|Q8LE	At3g022	E3 ubiquitin-protei	59.40	0.00
TRINITY_sp|P558	SUMO1	Small ubiquitin-rel	59.30	0.00
TRINITY_sp|Q9FH	MOB1A	MOB kinase activato	59.30	0.00
TRINITY_sp|O958	SDPR	Serum deprivation-r	59.30	0.00
TRINITY_sp|Q6UX	C16orf8	UPF0764 protein C16	59.30	0.00
TRINITY_sp|Q55A	snrpb	Small nuclear ribon	59.30	0.00
TRINITY_sp|Q5M7	PAP7	Probable plastid-li	59.30	0.00
TRINITY_sp|Q7YS	canA	Calcineurin subunit	59.30	0.00
TRINITY_sp|Q553	psmD4	26S proteasome non-	59.30	0.00
TRINITY_sp|Q8W1	SMU1	Suppressor of mec-8	59.30	0.00
TRINITY_sp|Q23F	TTLL3C	Tubulin glycylase 3	59.30	0.00

TRINITY_sp|P549(	BIO2	Biotin synthase OS=	59.30	0.00
TRINITY_sp|Q8LAI	UBP4	Ubiquitin carboxyl-	59.30	0.00
TRINITY_sp|A2XUV	CDKG-2	Cyclin-dependent ki	59.30	0.00
TRINITY_sp|Q392:	At5g274	Serine--tRNA ligase	59.30	0.00
TRINITY_sp|P361{	-	Endoplasmin homolog	59.30	0.00
TRINITY_sp|Q3B8:	SNU13	NHP2-like protein 1	59.30	0.00
TRINITY_sp|Q6P8I	snu13	NHP2-like protein 1	59.30	0.00
TRINITY_sp|Q9SJ:	NRPB6B	DNA-directed RNA po	59.30	0.00
TRINITY_sp|P078:	act18	Actin-18 OS=Dictyos	59.30	0.00
TRINITY_sp|Q9Y6I	STK24	Serine/threonine-pr	59.30	0.00
TRINITY_sp|Q8WU/	PPIL4	Peptidyl-prolyl cis	59.30	0.00
TRINITY_sp|Q9HCI	CHD8	Chromodomain-helica	59.30	0.00
TRINITY_sp|Q54WI	glnA3	Type-3 glutamine sy	59.20	0.00
TRINITY_sp|Q9I0I	purB	Adenylosuccinate ly	59.20	0.00
TRINITY_sp|Q394:	RAB1BV	Ras-related protein	59.20	0.00
TRINITY_sp|O266:	msrA	Peptide methionine	59.20	0.00
TRINITY_sp|A6VNI	infB	Translation initiat	59.20	0.00
TRINITY_sp|Q929:	BRF1	Transcription facto	59.20	0.00
TRINITY_sp|Q9XX\	myoK	Myosin-K heavy chai	59.20	0.00
TRINITY_sp|B7PXI	spas	Spastin OS=Ixodes s	59.20	0.00
TRINITY_sp|F1NTI	ascc3	Activating signal c	59.20	0.00
TRINITY_sp|Q24F!	RPL36	60S ribosomal prote	59.20	0.00
TRINITY_sp|O232	D2HGDH	D-2-hydroxyglutarat	59.20	0.00
TRINITY_sp|Q387	CDC2A	Cell division contr	59.20	0.00
TRINITY_sp|Q54PI	lvsF	BEACH domain-contai	59.20	0.00
TRINITY_sp|P119:	Scp2	Non-specific lipid-	59.20	0.00
TRINITY_sp|A2YVI	PAB1	Proteasome subunit	59.20	0.00
TRINITY_sp|P0C1:	cyp15	Peptidyl-prolyl cis	59.20	0.00
TRINITY_sp|Q9YGI	slbp2	Oocyte-specific his	59.20	0.00
TRINITY_sp|Q2VY:	hslV	ATP-dependent prote	59.20	0.00
TRINITY_sp|Q550I	DDB_G02	Probable serine/thr	59.20	0.00
TRINITY_sp|Q9H1/	ANAPC1	Anaphase-promoting	59.20	0.00
TRINITY_sp|P239(	VMA16	V-type proton ATPas	59.10	0.00
TRINITY_sp|P491(	RAB2B	Ras-related protein	59.10	0.00
TRINITY_sp|P403:	RIC2	Ras-related protein	59.10	0.00
TRINITY_sp|Q84NI	POLIB	DNA polymerase I B,	59.10	0.00
TRINITY_sp|Q54NV	oplah	5-oxoprolinase OS=D	59.10	0.00
TRINITY_sp|Q7SYI	qtrt1	Queuine tRNA-ribosy	59.10	0.00
TRINITY_sp|Q6Z1:	YchF1	Obg-like ATPase 1 O	59.10	0.00
TRINITY_sp|Q55F:	aatA	Aspartate aminotran	59.10	0.00
TRINITY_sp|Q86GI	Cys	Crustapain OS=Panda	59.10	0.00
TRINITY_sp|O648:	Acx	Acyl-coenzyme A oxi	59.10	0.00
TRINITY_sp|Q0P5I	GCAT	2-amino-3-ketobutyr	59.10	0.00
TRINITY_sp|P102	pol	Pol polyprotein OS=	59.10	0.00
TRINITY_sp|Q557I	g6pd-1	Glucose-6-phosphate	59.10	0.00
TRINITY_sp|Q5ZII	DOHH	Deoxyhypusine hydro	59.10	0.00
TRINITY_sp|Q7TMI	Prcp	Lysosomal Pro-X car	59.10	0.00
TRINITY_sp|Q9BY!	SEC11C	Signal peptidase co	59.10	0.00
TRINITY_sp|Q8H1:	RH14	DEAD-box ATP-depend	59.10	0.00
TRINITY_sp|Q8GWI	COX11	Cytochrome c oxidas	59.10	0.00
TRINITY_sp|Q9C5\	SMC2-1	Structural maintena	59.10	0.00
TRINITY_sp|A2CDI	rplA	50S ribosomal prote	59.10	0.00
TRINITY_sp|Q1JQI	BUB3	Mitotic checkpoint	59.10	0.00
TRINITY_sp|Q399{	-	Beta-carotene ketol	59.10	0.00
TRINITY_sp|A2XUI	OsI_163:	Pachytene checkpoin	59.10	0.00
TRINITY_sp|O226(	DEGP1	Protease Do-like 1,	59.10	0.00

TRINITY_sp|Q9LK3	UKL2	Uridine kinase-like	59.10	0.00
TRINITY_sp|Q9C8I	ABCC11	ABC transporter C f	59.10	0.00
TRINITY_sp|Q3867	-	V-type proton ATPas	59.10	0.00
TRINITY_sp|Q54QV	yakA	Probable serine/thr	59.10	0.00
TRINITY_sp|Q94JV	NLP2	Nitrilase-like prot	59.10	0.00
TRINITY_sp|Q4253	PIMT1	Protein-L-isoaspart	59.10	0.00
TRINITY_sp|P4964	GAPC	Glyceraldehyde-3-ph	59.10	0.00
TRINITY_sp|Q9M08	IBI1	Aspartate--tRNA lig	59.10	0.00
TRINITY_sp|Q94GI	ASA1	Anthranilate syntha	59.10	0.00
TRINITY_sp|Q8LI3	Os07g062	Putative MYST-like	59.10	0.00
TRINITY_sp|Q5BH8	spb1	AdoMet-dependent rR	59.10	0.00
TRINITY_sp|Q9NRI	UBQLN4	Ubiquilin-4 OS=Homo	59.10	0.00
TRINITY_sp|Q6UW6	PCSK4	Proprotein converta	59.10	0.00
TRINITY_sp|Q6CN6	ASF1	Histone chaperone A	59.00	0.00
TRINITY_sp|Q54D7	abcA9	ABC transporter A f	59.00	0.00
TRINITY_sp|P3641	rab11A	Ras-related protein	59.00	0.00
TRINITY_sp|Q23FI	TTLL3C	Tubulin glycylase 3	59.00	0.00
TRINITY_sp|Q6137	Ift88	Intraflagellar tran	59.00	0.00
TRINITY_sp|Q2JUI	rpmE	50S ribosomal prote	59.00	0.00
TRINITY_sp|P5242	PUR3	Phosphoribosylglyci	59.00	0.00
TRINITY_sp|Q54S9	rps11	40S ribosomal prote	59.00	0.00
TRINITY_sp|Q9ZQX	VHA-F	V-type proton ATPas	59.00	0.00
TRINITY_sp|Q8S21	GRIP	Protein GRIP OS=Ara	59.00	0.00
TRINITY_sp|P1444	FGA	Fibrinogen alpha ch	59.00	0.00
TRINITY_sp|P0774	Rrm1	Ribonucleoside-diph	59.00	0.00
TRINITY_sp|Q84K4	ABCA2	ABC transporter A f	59.00	0.00
TRINITY_sp|Q9NPI	EXOSC4	Exosome complex com	59.00	0.00
TRINITY_sp|Q54UI	serA	D-3-phosphoglycerat	59.00	0.00
TRINITY_sp|P9325	ELI3	Probable mannitol d	59.00	0.00
TRINITY_sp|Q9970	MTR	Methionine synthase	59.00	0.00
TRINITY_sp|Q8KA8	clpB2	Probable chaperone	59.00	0.00
TRINITY_sp|O7441	SPCC1223	Chorismate synthase	58.90	0.00
TRINITY_sp|Q56Y1	At5g5877	Dehydrodolichyl dip	58.90	0.00
TRINITY_sp|Q08DI	DNM1	Dynamin-1 OS=Bos ta	58.90	0.00
TRINITY_sp|P0236	rps7	40S ribosomal prote	58.90	0.00
TRINITY_sp|Q54U0	kif3	Kinesin-related pro	58.90	0.00
TRINITY_sp|Q84K4	ABCA2	ABC transporter A f	58.90	0.00
TRINITY_sp|Q9FN0	TOM9-2	Mitochondrial impor	58.90	0.00
TRINITY_sp|P1024	MYBL1	Myb-related protein	58.90	0.00
TRINITY_sp|Q75H7	SAPK10	Serine/threonine-pr	58.90	0.00
TRINITY_sp|P3260	FBP26	Fructose-2,6-bispho	58.90	0.00
TRINITY_sp|P0565	sacC	Levanase OS=Bacillu	58.90	0.00
TRINITY_sp|Q5B02	cwc15	Pre-mRNA-splicing f	58.90	0.00
TRINITY_sp|Q9LXI	RLF	Cytochrome b5 domai	58.90	0.00
TRINITY_sp|Q9FLI	At5g3990	Translation factor	58.90	0.00
TRINITY_sp|Q9LM9	SMD3B	Small nuclear ribon	58.90	0.00
TRINITY_sp|Q9S77	PKL	CHD3-type chromatin	58.90	0.00
TRINITY_sp|Q3V1I	Ampd1	AMP deaminase 1 OS=	58.90	0.00
TRINITY_sp|Q9313	-	Actin, muscle OS=Br	58.90	0.00
TRINITY_sp|P4627	-	DNA-directed RNA po	58.90	0.00
TRINITY_sp|Q9UK2	CNOT11	CCR4-NOT transcript	58.90	0.00
TRINITY_sp|P0787	Acaa1b	3-ketoacyl-CoA thio	58.90	0.00
TRINITY_sp|O6527	KEA2	K(+) efflux antipor	58.90	0.00
TRINITY_sp|Q55D1	DDB_G026	KIN17-like protein	58.90	0.00
TRINITY_sp|Q9LZI	PPCS2	Phosphopantothenate	58.90	0.00
TRINITY_sp|Q69RJ	GLU	Ferredoxin-dependen	58.90	0

TRINITY_sp|Q9SF	CCT7	T-complex protein 1	58.90	0.00
TRINITY_sp|Q9SM\	XPO1	Protein EXPORTIN 1A	58.90	0
TRINITY_sp|O823	THIC	Phosphomethylpyrimi	58.90	0.00
TRINITY_sp|Q9BY	SEC11C	Signal peptidase co	58.80	0.00
TRINITY_sp|Q4P2	UMAG_05	Tubulin beta chain	58.80	0.00
TRINITY_sp|Q9Z3	Top3b	DNA topoisomerase 3	58.80	0.00
TRINITY_sp|Q96R	BBS4	Bardet-Biedl syndro	58.80	0.00
TRINITY_sp|B6IP	gatB	Aspartyl/glutamyl-t	58.80	0.00
TRINITY_sp|O942	spt16	FACT complex subuni	58.80	0.00
TRINITY_sp|P492	RPL9	60S ribosomal prote	58.80	0.00
TRINITY_sp|Q8WV	SNRNP27	U4/U6.U5 small nucl	58.80	0.00
TRINITY_sp|P411	rplL	50S ribosomal prote	58.80	0.00
TRINITY_sp|Q86K	DDB_G02	Probable GH family	58.80	0.00
TRINITY_sp|P478	-	Eukaryotic translat	58.80	0.00
TRINITY_sp|P499	SRP-54C	Signal recognition	58.80	0.00
TRINITY_sp|Q8RX	OVA4	Tryptophan--tRNA li	58.80	0.00
TRINITY_sp|Q9P2	DNAH2	Dynein heavy chain	58.80	0.00
TRINITY_sp|Q55D	DDB_G02	Putative methylster	58.80	0.00
TRINITY_sp|A4IF	POLR3A	DNA-directed RNA po	58.80	0.00
TRINITY_sp|Q55G	glcS	Glycogen [starch] s	58.80	0.00
TRINITY_sp|Q059	mybl1	Myb-related protein	58.80	0.00
TRINITY_sp|O149	BTAF1	TATA-binding protei	58.80	0.00
TRINITY_sp|Q54M	asns	Probable asparagine	58.80	0.00
TRINITY_sp|Q55G	osgep	Probable tRNA N6-ad	58.80	0.00
TRINITY_sp|Q21C	dnaJ	Chaperone protein D	58.80	0.00
TRINITY_sp|Q8H0	PLSP1	Chloroplast process	58.80	0.00
TRINITY_sp|Q8MZ	-	Physarolisin OS=Phy	58.80	0.00
TRINITY_sp|Q8RH	lon	Lon protease OS=Fus	58.80	0.00
TRINITY_sp|Q9M1	ABCF4	ABC transporter F f	58.80	0.00
TRINITY_sp|Q8VX	RFC3	Replication factor	58.80	0.00
TRINITY_sp|Q9ZT	Os06g05	Probable methionine	58.80	0.00
TRINITY_sp|O231	HCF164	Thioredoxin-like pr	58.80	0.00
TRINITY_sp|P215	-	Malate dehydrogenas	58.80	0.00
TRINITY_sp|Q31K	tsf	Elongation factor T	58.80	0.00
TRINITY_sp|O153	PMM2	Phosphomannomutase	58.80	0.00
TRINITY_sp|P010	C4	Complement C4 (Frag	58.70	0.00
TRINITY_sp|Q55A	DDB_G02	PH and Rap-GAP doma	58.70	0.00
TRINITY_sp|P546	pikA	Phosphatidylinosito	58.70	0.00
TRINITY_sp|P399	DLD3	D-lactate dehydroge	58.70	0.00
TRINITY_sp|A1L2	d2hgdh	D-2-hydroxyglutarat	58.70	0.00
TRINITY_sp|Q6FL	ASF1	Histone chaperone A	58.70	0.00
TRINITY_sp|Q9BM	RPS6	40S ribosomal prote	58.70	0.00
TRINITY_sp|Q6ZI	ML2	Protein MEI2-like 2	58.70	0.00
TRINITY_sp|P0CN	CWC26	Pre-mRNA-splicing f	58.70	0.00
TRINITY_sp|Q3BA	-	Uncharacterized pro	58.70	0.00
TRINITY_sp|Q414	PCM3	Putative calmodulin	58.70	0.00
TRINITY_sp|P200	pyr1-3	Protein PYR1-3 OS=D	58.70	0.00
TRINITY_sp|Q60B	gloB	Hydroxyacylglutathi	58.70	0.00
TRINITY_sp|P265	hemA	5-aminolevulinate s	58.70	0.00
TRINITY_sp|Q8LF	IDH1	Isocitrate dehydrog	58.70	0.00
TRINITY_sp|Q8LG	CDC27B	Cell division cycle	58.70	0.00
TRINITY_sp|F4IR	ATG1C	Serine/threonine-pr	58.70	0.00
TRINITY_sp|Q6GP	dbr1-a	Lariat debranching	58.70	0.00
TRINITY_sp|Q8RX	WIN2	Probable protein ph	58.70	0.00
TRINITY_sp|Q8H8	UAM1	UDP-arabinopyranose	58.70	0.00
TRINITY_sp|P205	CAH1	Carbonic anhydrase	58.70	0.00

TRINITY_sp|Q9C7[illegible]	AERO1	Endoplasmic reticul	58.70	0.00
TRINITY_sp|Q9M8[illegible]	NSN1	Guanine nucleotide-	58.70	0.00
TRINITY_sp|Q54I[illegible]	lzic	Protein LZIC OS=Dic	58.70	0.00
TRINITY_sp|Q6P6[illegible]	Rsl24d1	Probable ribosome b	58.60	0.00
TRINITY_sp|P347[illegible]	pgp-1	Multidrug resistanc	58.60	0.00
TRINITY_sp|Q96D[illegible]	SNRNP40	U5 small nuclear ri	58.60	0.00
TRINITY_sp|A0AT[illegible]	-	Non-specific lipid-	58.60	0.00
TRINITY_sp|Q23M[illegible]	Ttll6a	Probable beta-tubul	58.60	0.00
TRINITY_sp|O942[illegible]	lys1	Homocitrate synthas	58.60	0.00
TRINITY_sp|Q1IQ[illegible]	rpsO	30S ribosomal prote	58.60	0.00
TRINITY_sp|Q8WX[illegible]	DNAH7	Dynein heavy chain	58.60	0.00
TRINITY_sp|Q150[illegible]	EFTUD2	116 kDa U5 small nu	58.60	0
TRINITY_sp|P085[illegible]	CYC1	Cytochrome c1, heme	58.60	0.00
TRINITY_sp|Q9VB[illegible]	CG6066	NKAP family protein	58.60	0.00
TRINITY_sp|O535[illegible]	ilvX	Putative acetolacta	58.60	0.00
TRINITY_sp|Q54Y[illegible]	shkC	Dual specificity pr	58.60	0.00
TRINITY_sp|O821[illegible]	GDH2	Glycine cleavage sy	58.60	0.00
TRINITY_sp|Q8VZ[illegible]	RIBF2	FAD synthetase 2, c	58.60	0.00
TRINITY_sp|Q86Z[illegible]	KLP1	Kinesin heavy chain	58.60	0.00
TRINITY_sp|Q075[illegible]	ALDH6A1	Methylmalonate-semi	58.60	0.00
TRINITY_sp|O224[illegible]	HDA19	Histone deacetylase	58.60	0.00
TRINITY_sp|Q9SI[illegible]	RPS25B	40S ribosomal prote	58.60	0.00
TRINITY_sp|Q631[illegible]	Dnah7	Dynein heavy chain	58.60	0.00
TRINITY_sp|P201[illegible]	CSY4	Citrate synthase 4,	58.50	0.00
TRINITY_sp|Q8LP[illegible]	ABCA8	ABC transporter A f	58.50	0.00
TRINITY_sp|Q9VR[illegible]	l(1)G01[illegible]	Inositol hexakispho	58.50	0.00
TRINITY_sp|Q6PF[illegible]	Cdc14b	Dual specificity pr	58.50	0.00
TRINITY_sp|Q7NB[illegible]	dnaJ	Chaperone protein D	58.50	0.00
TRINITY_sp|A2YM[illegible]	OsI_263[illegible]	Probable histone H2	58.50	0.00
TRINITY_sp|Q54P[illegible]	ucpC	Probable mitochondr	58.50	0.00
TRINITY_sp|Q7ZV[illegible]	vps29	Vacuolar protein so	58.50	0.00
TRINITY_sp|Q8TD[illegible]	HELQ	Helicase POLQ-like	58.50	0.00
TRINITY_sp|Q9VP[illegible]	Clp	Cleavage and polyad	58.50	0.00
TRINITY_sp|P207[illegible]	MAK	Serine/threonine-pr	58.50	0.00
TRINITY_sp|P525[illegible]	mybl2	Myb-related protein	58.50	0.00
TRINITY_sp|Q94C[illegible]	At4g271[illegible]	DDRGK domain-contai	58.50	0.00
TRINITY_sp|Q869[illegible]	mdn1	Midasin OS=Dictyost	58.50	0.00
TRINITY_sp|O492[illegible]	PPD4	PsbP domain-contain	58.50	0.00
TRINITY_sp|H9BW[illegible]	rab7	Ras-related protein	58.50	0.00
TRINITY_sp|B0KK[illegible]	cbpA	Curved DNA-binding	58.50	0.00
TRINITY_sp|A4SM[illegible]	fbp	Fructose-1,6-bispho	58.50	0.00
TRINITY_sp|Q9SH[illegible]	RRP44A	Exosome complex exo	58.50	0.00
TRINITY_sp|Q087[illegible]	myb	Transcriptional act	58.50	0.00
TRINITY_sp|Q9ZR[illegible]	ABCB1	ABC transporter B f	58.50	0.00
TRINITY_sp|Q6K6[illegible]	Os02g05[illegible]	Ubiquitin-like modi	58.50	0.00
TRINITY_sp|Q56A[illegible]	abcb8	ATP-binding cassett	58.50	0.00
TRINITY_sp|E8XY[illegible]	tatD	3'-5' ssDNA/RNA exo	58.50	0.00
TRINITY_sp|B2SA[illegible]	rlmE	Ribosomal RNA large	58.50	0.00
TRINITY_sp|P929[illegible]	APR2	5'-adenylylsulfate	58.50	0.00
TRINITY_sp|Q9ST[illegible]	At3g483[illegible]	Probable Ufm1-speci	58.50	0.00
TRINITY_sp|O805[illegible]	At1g228[illegible]	Putative methyltran	58.50	0.00
TRINITY_sp|Q59L[illegible]	CSE4	Histone H3-like cen	58.50	0.00
TRINITY_sp|P612[illegible]	DYNLL1	Dynein light chain	58.40	0.00
TRINITY_sp|P491[illegible]	PHI1	Glucose-6-phosphate	58.40	0.00
TRINITY_sp|Q9C9[illegible]	RPL6C	60S ribosomal prote	58.40	0.00
TRINITY_sp|Q4U4[illegible]	Xirp2	Xin actin-binding r	58.40	0.00

TRINITY_sp|Q9SK(	CYP19-2	Peptidyl-prolyl cis	58.40	0.00
TRINITY_sp|Q928:	KAT2A	Histone acetyltrans	58.40	0.00
TRINITY_sp|A0CX!	FEN1-1	Flap endonuclease 1	58.40	0.00
TRINITY_sp|Q4G2:	DER2.1	Derlin-2.1 OS=Zea m	58.40	0.00
TRINITY_sp|F4JL1	IMPA2	Importin subunit al	58.40	0.00
TRINITY_sp|Q9P3X	cyp41	41 kDa peptidyl-pro	58.40	0.00
TRINITY_sp|Q54C:	pex6	Peroxisomal biogene	58.40	0.00
TRINITY_sp|Q9XH:	RPS12	40S ribosomal prote	58.40	0.00
TRINITY_sp|O940	ANB1	Eukaryotic translat	58.40	0.00
TRINITY_sp|P341	glpD	Glycogen phosphoryl	58.40	0.00
TRINITY_sp|Q56X!	AOX4	Ubiquinol oxidase 4	58.40	0.00
TRINITY_sp|Q9FF(	UBC22	Ubiquitin-conjugati	58.40	0.00
TRINITY_sp|Q9FI(	BOLA2	Protein BOLA2 OS=Ar	58.40	0.00
TRINITY_sp|Q9SK(	CYP19-2	Peptidyl-prolyl cis	58.40	0.00
TRINITY_sp|P114:	-	Actin OS=Entamoeba	58.30	0.00
TRINITY_sp|Q8VX:	AGAL3	Alpha-galactosidase	58.30	0.00
TRINITY_sp|O657:	RPS5	40S ribosomal prote	58.30	0.00
TRINITY_sp|Q9LZI	CP12-2	Calvin cycle protei	58.30	0.00
TRINITY_sp|B4EU!	rbn	Ribonuclease BN OS=	58.30	0.00
TRINITY_sp|Q28C:	cyb5r4	Cytochrome b5 reduc	58.30	0.00
TRINITY_sp|Q803!	memo1	Protein MEMO1 OS=Da	58.30	0.00
TRINITY_sp|A3LP!	RKI1	Ribose-5-phosphate	58.30	0.00
TRINITY_sp|Q9DAI	Pacrg	Parkin coregulated	58.30	0.00
TRINITY_sp|P546	pkbA	RAC family serine/t	58.30	0.00
TRINITY_sp|Q54QI	rab32A	Ras-related protein	58.30	0.00
TRINITY_sp|Q86K!	ap2a1-1	AP-2 complex subuni	58.30	0.00
TRINITY_sp|Q8T2I	fam49	Protein FAM49 homol	58.30	0.00
TRINITY_sp|Q9SJ!	BLH1	BEL1-like homeodoma	58.30	0.00
TRINITY_sp|Q941(	HUA1	Zinc finger CCCH do	58.30	0.00
TRINITY_sp|Q75J(	dph5	Diphthine methyl es	58.30	0.00
TRINITY_sp|Q6PH!	trip13	Pachytene checkpoin	58.30	0.00
TRINITY_sp|A6Q0I	CP12	Calvin cycle protei	58.30	0.00
TRINITY_sp|Q8LFI	LSM1B	Sm-like protein LSM	58.30	0.00
TRINITY_sp|P546	pikE	Phosphatidylinosito	58.30	0.00
TRINITY_sp|Q173:	H24K24.:	Alcohol dehydrogena	58.30	0.00
TRINITY_sp|Q631	Dnah7	Dynein heavy chain	58.30	0.00
TRINITY_sp|Q2M3	KIAA103:	WASH complex subuni	58.30	0.00
TRINITY_sp|Q95RI	CG10038	UPF0528 protein CG1	58.30	0.00
TRINITY_sp|Q54P(	gcsA	Glutamate--cysteine	58.30	0.00
TRINITY_sp|P728	menG	2-phytyl-1,4-naphto	58.30	0.00
TRINITY_sp|Q54X:	DDB_G02	Probable serine/thr	58.30	0.00
TRINITY_sp|P536:	COPA	Coatomer subunit al	58.30	0.00
TRINITY_sp|Q8L4I	TGD1	Protein TRIGALACTOS	58.30	0.00
TRINITY_sp|Q8W4	At5g501(	Uncharacterized pro	58.30	0.00
TRINITY_sp|O646	At2g456	Histone deacetylase	58.30	0.00
TRINITY_sp|Q9SVI	UEV1D	Ubiquitin-conjugati	58.30	0.00
TRINITY_sp|Q9BUI	TUBB6	Tubulin beta-6 chai	58.30	0.00
TRINITY_sp|F8RP!	HOP	Hsp70-Hsp90 organiz	58.30	0.00
TRINITY_sp|Q9XGI	B''ALPH.	Serine/threonine pr	58.30	0.00
TRINITY_sp|Q9C8I	CID8	Polyadenylate-bindi	58.30	0.00
TRINITY_sp|Q7XQ	STT3B	Dolichyl-diphosphoo	58.30	0.00
TRINITY_sp|Q9LV!	ABCF5	ABC transporter F f	58.30	0.00
TRINITY_sp|Q6R2\	Ppc2	Phosphoenolpyruvate	58.30	0
TRINITY_sp|P289	-	Elongation factor 2	58.30	0.00
TRINITY_sp|P933	-	Actin-104 (Fragment	58.20	0.00
TRINITY_sp|P981	Atp9b	Probable phospholip	58.20	0.00

TRINITY_sp|P3352	ABCC1	Multidrug resistanc	58.20	0.00
TRINITY_sp|Q0CFY	ATEG_068	Leukotriene A-4 hyd	58.20	0.00
TRINITY_sp|Q8TD5	DNAH3	Dynein heavy chain	58.20	0.00
TRINITY_sp|P3787	KARS	Lysine--tRNA ligase	58.20	0.00
TRINITY_sp|P2086	-	Chlorophyll a-b bin	58.20	0.00
TRINITY_sp|Q55FE	rab6	Ras-related protein	58.20	0.00
TRINITY_sp|Q54B0	psmB5	Proteasome subunit	58.20	0.00
TRINITY_sp|B0G12	pip5k3	1-phosphatidylinosi	58.20	0.00
TRINITY_sp|B0JXE	fabH	3-oxoacyl-[acyl-car	58.20	0.00
TRINITY_sp|P5922	HTR2	Histone H3.2 OS=Ara	58.20	0.00
TRINITY_sp|Q9FN9	MBB1	PsbB mRNA maturatio	58.20	0.00
TRINITY_sp|Q9M8M	WIN1	Acetylornithine ami	58.20	0.00
TRINITY_sp|Q1491	DRAP1	Dr1-associated core	58.20	0.00
TRINITY_sp|Q9LHA	VHA-d2	V-type proton ATPas	58.20	0.00
TRINITY_sp|Q9UQ0	MOK	MAPK/MAK/MRK overla	58.20	0.00
TRINITY_sp|Q54Y2	ugpB	UTP--glucose-1-phos	58.20	0.00
TRINITY_sp|P2271	Gucy1b2	Guanylate cyclase s	58.20	0.00
TRINITY_sp|Q56X5	AOX4	Ubiquinol oxidase 4	58.20	0.00
TRINITY_sp|Q6I5Y	CDKC-1	Cyclin-dependent ki	58.20	0.00
TRINITY_sp|Q1H40	tuf1	Elongation factor T	58.20	0.00
TRINITY_sp|A2YX0	SPL15	Squamosa promoter-b	58.20	0.00
TRINITY_sp|Q54M4	pigC	Putative phosphatid	58.10	0.00
TRINITY_sp|P4175	PGK	Phosphoglycerate ki	58.10	0.00
TRINITY_sp|P5585	SUMO1	Small ubiquitin-rel	58.10	0.00
TRINITY_sp|P3513	UBC2	Ubiquitin-conjugati	58.10	0.00
TRINITY_sp|A2VE4	TTC30A	Tetratricopeptide r	58.10	0.00
TRINITY_sp|Q54KI	wdr5	WD repeat-containin	58.10	0.00
TRINITY_sp|P3854	Bm1_4472	GTP-binding nuclear	58.10	0.00
TRINITY_sp|P9099	djr-1.1	Glutathione-indepen	58.10	0.00
TRINITY_sp|A9UN0	bysl	Bystin OS=Monosiga	58.10	0.00
TRINITY_sp|Q64M7	ML4	Protein MEI2-like 4	58.10	0.00
TRINITY_sp|Q56K0	CRIP1	Cysteine-rich prote	58.10	0.00
TRINITY_sp|Q54N4	bcaA	Branched-chain-amin	58.10	0.00
TRINITY_sp|O4299	fkh1	Peptidyl-prolyl cis	58.10	0.00
TRINITY_sp|Q3884	PP2AA1	Serine/threonine-pr	58.10	0.00
TRINITY_sp|P1539	rpa1	DNA-directed RNA po	58.10	0.00
TRINITY_sp|Q9070	EEF2	Elongation factor 2	58.10	0.00
TRINITY_sp|Q9LM9	SMD3B	Small nuclear ribon	58.10	0.00
TRINITY_sp|Q8LD2	PBA1	Proteasome subunit	58.10	0.00
TRINITY_sp|Q54D0	lst8	Protein LST8 homolo	58.10	0.00
TRINITY_sp|O4948	PGDH1	D-3-phosphoglycerat	58.10	0.00
TRINITY_sp|O7703	trfA	General transcripti	58.10	0.00
TRINITY_sp|Q654V	Os06g024	Pyruvate dehydrogen	58.10	0.00
TRINITY_sp|O7060	Rsad2	Radical S-adenosyl	58.10	0.00
TRINITY_sp|Q6DW7	DGD1	Digalactosyldiacylg	58.10	0.00
TRINITY_sp|F4ICI	SEC31A	Protein transport p	58.10	0.00
TRINITY_sp|Q8BGE	Wdr92	WD repeat-containin	58.10	0.00
TRINITY_sp|B2RX1	Abcc3	Canalicular multisp	58.10	0.00
TRINITY_sp|F4IV4	CHR10	Probable helicase C	58.10	0.00
TRINITY_sp|Q5A59	NIK1	Histidine protein k	58.00	0.00
TRINITY_sp|Q9VCE	Ime4	Probable N6-adenosi	58.00	0.00
TRINITY_sp|Q55BM	nek2	Probable serine/thr	58.00	0.00
TRINITY_sp|A2VD9	ddx1	ATP-dependent RNA h	58.00	0.00
TRINITY_sp|Q8ST8	abcC10	ABC transporter C f	58.00	0.00
TRINITY_sp|Q6FL8	ASF1	Histone chaperone A	58.00	0.00
TRINITY_sp|Q60E6	Os05g038	Putative NAD kinase	58.00	0.00

TRINITY_sp|Q32LI	STOML2	Stomatin-like prote	58.00	0.00
TRINITY_sp|P4512	ettA	Energy-dependent tr	58.00	0.00
TRINITY_sp|Q9M9I	APC11	Anaphase-promoting	58.00	0.00
TRINITY_sp|Q148N	TAF13	Transcription initi	58.00	0.00
TRINITY_sp|P503I	PGK	Phosphoglycerate ki	58.00	0.00
TRINITY_sp|Q8IYI	FANCM	Fanconi anemia grou	58.00	0.00
TRINITY_sp|Q9FXI	PEX14	Peroxisomal membran	58.00	0.00
TRINITY_sp|Q54JI	dnmA	DNA (cytosine-5)-me	58.00	0.00
TRINITY_sp|Q54MI	nadsyn1	Glutamine-dependent	58.00	0.00
TRINITY_sp|Q9SH3	HMA5	Probable copper-tra	58.00	0.00
TRINITY_sp|P427	EMB2360	Glutathione reducta	58.00	0.00
TRINITY_sp|P6299	RAC1	Ras-related C3 botu	58.00	0.00
TRINITY_sp|P3860	CAS1	Cycloartenol syntha	58.00	0.00
TRINITY_sp|Q9LP9	At1g5059	Pirin-like protein	58.00	0.00
TRINITY_sp|Q6Z38	Os07g020	Coatomer subunit ga	58.00	0.00
TRINITY_sp|P5339	ACLY	ATP-citrate synthas	58.00	0
TRINITY_sp|Q9NU2	MDN1	Midasin OS=Homo sap	58.00	0.00
TRINITY_sp|P5855	rca	Ribulose bisphospha	58.00	0.00
TRINITY_sp|Q8WXX	DNAH7	Dynein heavy chain	58.00	0.00
TRINITY_sp|Q23T0	RPL21	60S ribosomal prote	58.00	0.00
TRINITY_sp|P0C8N	CCRP1	Probable serine/thr	58.00	0.00
TRINITY_sp|Q54SI	DDB_G028	Peroxiredoxin-like	57.90	0.00
TRINITY_sp|Q9C9N	CDKD-2	Cyclin-dependent ki	57.90	0.00
TRINITY_sp|O7052	ZNF330	Zinc finger protein	57.90	0.00
TRINITY_sp|Q2KJ8	AP1M1	AP-1 complex subuni	57.90	0.00
TRINITY_sp|Q2F63	14-3-3ze	14-3-3 protein zeta	57.90	0.00
TRINITY_sp|Q9JHI	Ide	Insulin-degrading e	57.90	0.00
TRINITY_sp|Q54M0	dhps	Probable deoxyhypus	57.90	0.00
TRINITY_sp|Q9FI6	UBC27	Ubiquitin-conjugati	57.90	0.00
TRINITY_sp|Q9LT0	PDR2	Probable manganese-	57.90	0.00
TRINITY_sp|Q9C7I	NTF2	Nuclear transport f	57.90	0.00
TRINITY_sp|O040	ctpA	C-terminal processi	57.90	0.00
TRINITY_sp|Q3903	ATPK2	Serine/threonine-pr	57.90	0.00
TRINITY_sp|F4JY3	RUK	Serine/threonine-pr	57.90	0.00
TRINITY_sp|Q55G9	cdk10	Probable cyclin-dep	57.90	0.00
TRINITY_sp|Q86I0	nek3	Probable serine/thr	57.90	0.00
TRINITY_sp|O8242	SPY	Probable UDP-N-acet	57.90	0.00
TRINITY_sp|P227I	Gucy1b2	Guanylate cyclase s	57.90	0.00
TRINITY_sp|Q6DDA	snrnp27	U4/U6.U5 small nucl	57.90	0.00
TRINITY_sp|Q5DU0	Cep164	Centrosomal protein	57.90	0.00
TRINITY_sp|P542I	-	SF-assemblin OS=Dun	57.90	0.00
TRINITY_sp|Q0VC	TTLL1	Probable tubulin po	57.90	0.00
TRINITY_sp|Q9T0A	LACS4	Long chain acyl-CoA	57.90	0.00
TRINITY_sp|Q1025	SPAC56F8	Eukaryotic translat	57.90	0.00
TRINITY_sp|Q56W0	PLC3	Phosphoinositide ph	57.90	0.00
TRINITY_sp|Q8S8Y	WNK2	Serine/threonine-pr	57.90	0.00
TRINITY_sp|Q28GI	tma7	Translation machine	57.80	0.00
TRINITY_sp|Q6ZQI	Ppip5k2	Inositol hexakispho	57.80	0.00
TRINITY_sp|P4984	GSK3B	Glycogen synthase k	57.80	0.00
TRINITY_sp|Q7KQN	PF14_036	DNA primase small s	57.80	0.00
TRINITY_sp|P973I	Csrp1	Cysteine and glycin	57.80	0.00
TRINITY_sp|Q5ZK9	SPAST	Spastin OS=Gallus g	57.80	0.00
TRINITY_sp|Q3ZC6	CRIPT	Cysteine-rich PDZ-b	57.80	0.00
TRINITY_sp|O3496	ytsJ	Probable NAD-depend	57.80	0.00
TRINITY_sp|Q9D2I	Ak7	Adenylate kinase 7	57.80	0.00
TRINITY_sp|Q8VXY	ENT1	Equilibrative nucle	57.80	0.00

TRINITY_sp|Q940	CCT2	T-complex protein 1	57.80	0.00
TRINITY_sp|P052	Su(var)	Heterochromatin pro	57.80	0.00
TRINITY_sp|O341	clpP2	ATP-dependent Clp p	57.80	0.00
TRINITY_sp|O226	CYN	Cyanate hydratase O	57.80	0.00
TRINITY_sp|Q54I	mmsdh	Probable methylmalo	57.80	0.00
TRINITY_sp|P0DL	DRC1	Dynein regulatory c	57.80	0.00
TRINITY_sp|Q8TE	DTD1	D-tyrosyl-tRNA(Tyr)	57.80	0.00
TRINITY_sp|Q474	pdxH	Pyridoxine/pyridoxa	57.80	0.00
TRINITY_sp|Q655	MCSU3	Molybdenum cofactor	57.80	0.00
TRINITY_sp|Q851	Os03g07	Probable GTP-bindin	57.80	0.00
TRINITY_sp|Q8H0	RH42	DEAD-box ATP-depend	57.80	0.00
TRINITY_sp|Q803	ergic3	Endoplasmic reticul	57.80	0.00
TRINITY_sp|Q8DG	gatB	Aspartyl/glutamyl-t	57.80	0.00
TRINITY_sp|O241	PPXI	Protoporphyrinogen	57.80	0.00
TRINITY_sp|B3CL	rplT	50S ribosomal prote	57.80	0.00
TRINITY_sp|Q1LZ	SNRNP35	U11/U12 small nucle	57.80	0.00
TRINITY_sp|Q72M	ppa	Inorganic pyrophosp	57.70	0.00
TRINITY_sp|Q0WN	CHC1	Clathrin heavy chai	57.70	0.00
TRINITY_sp|Q9UR	rmt1	Protein arginine N-	57.70	0.00
TRINITY_sp|Q6P6	dnajb6	DnaJ homolog subfam	57.70	0.00
TRINITY_sp|Q8LP	At3g199	E3 ubiquitin-protei	57.70	0.00
TRINITY_sp|Q55B	nek2	Probable serine/thr	57.70	0.00
TRINITY_sp|P086	R	Ras-like protein 3	57.70	0.00
TRINITY_sp|Q9VT	RpL10Ab	60S ribosomal prote	57.70	0.00
TRINITY_sp|Q152	RAB35	Ras-related protein	57.70	0.00
TRINITY_sp|Q54Q	erkB	Extracellular signa	57.70	0.00
TRINITY_sp|Q2W1	rpmG	50S ribosomal prote	57.70	0.00
TRINITY_sp|Q7U0	glpX	Fructose-1,6-bispho	57.70	0.00
TRINITY_sp|Q54I	ddx42	Probable ATP-depend	57.70	0.00
TRINITY_sp|Q6S0	kif6	Kinesin-related pro	57.70	0.00
TRINITY_sp|Q54G	DDB_G02	Coiled-coil domain-	57.70	0.00
TRINITY_sp|P551	galE	UDP-glucose 4-epime	57.70	0.00
TRINITY_sp|Q9W7	cpsf2	Cleavage and polyad	57.70	0.00
TRINITY_sp|Q962	PSAT1	Phosphoserine amino	57.70	0.00
TRINITY_sp|Q8L7	ALY2	THO complex subunit	57.70	0.00
TRINITY_sp|Q54J	oxct1	Probable succinyl-C	57.70	0.00
TRINITY_sp|Q5NV	TXNRD1	Thioredoxin reducta	57.70	0.00
TRINITY_sp|Q429	PGM1	2,3-bisphosphoglyce	57.70	0.00
TRINITY_sp|F4IA	SS3	Starch synthase 3,	57.70	0.00
TRINITY_sp|O429	eso1	N-acetyltransferase	57.70	0.00
TRINITY_sp|P934	Os05g01	Histidine--tRNA lig	57.70	0.00
TRINITY_sp|Q6K5	CDKG-1	Cyclin-dependent ki	57.70	0.00
TRINITY_sp|Q55B	pctA	Ethanolamine-phosph	57.70	0.00
TRINITY_sp|P629	RAC1	Ras-related C3 botu	57.70	0.00
TRINITY_sp|A8I9	CFAP45	Cilia- and flagella	57.70	0.00
TRINITY_sp|Q54P	DDB_G02	Stromal cell-derive	57.70	0.00
TRINITY_sp|Q54N	cyb5r1	NADH-cytochrome b5	57.60	0.00
TRINITY_sp|A6TU	mnmA	tRNA-specific 2-thi	57.60	0.00
TRINITY_sp|Q9VL	SmE	Probable small nucl	57.60	0.00
TRINITY_sp|Q9DG	RPLP0	60S acidic ribosoma	57.60	0.00
TRINITY_sp|Q9NU	MDN1	Midasin OS=Homo sap	57.60	0.00
TRINITY_sp|P464	Vps4b	Vacuolar protein so	57.60	0.00
TRINITY_sp|P318	-	RNA-directed DNA po	57.60	0.00
TRINITY_sp|Q54W	denr	Density-regulated p	57.60	0.00
TRINITY_sp|Q126	DNF2	Phospholipid-transp	57.60	0.00
TRINITY_sp|Q54F	xdh	Xanthine dehydrogen	57.60	0.00

TRINITY_sp|Q5ZJ[illegible]	KCTD7	BTB/POZ domain-cont	57.60	0.00
TRINITY_sp|Q8S1[illegible]	CML1	Calmodulin-like pro	57.60	0.00
TRINITY_sp|P297[illegible]	-	60S acidic ribosoma	57.60	0.00
TRINITY_sp|O655[illegible]	FBA5	Fructose-bisphospha	57.60	0.00
TRINITY_sp|O426[illegible]	nrc-2	Serine/threonine-pr	57.60	0.00
TRINITY_sp|B3DX[illegible]	aroC	Chorismate synthase	57.60	0.00
TRINITY_sp|Q9C6[illegible]	DCP5	Protein decapping 5	57.60	0.00
TRINITY_sp|Q94B[illegible]	PIGA	Phosphatidylinosito	57.60	0.00
TRINITY_sp|Q56Z[illegible]	AVPL1	Pyrophosphate-energ	57.60	0.00
TRINITY_sp|Q8DI[illegible]	fusA	Elongation factor G	57.50	0.00
TRINITY_sp|Q23M[illegible]	Ttll6a	Probable beta-tubul	57.50	0.00
TRINITY_sp|Q9VK[illegible]	Bug22	Cilia- and flagella	57.50	0.00
TRINITY_sp|P109[illegible]	-	Actin, cytoplasmic	57.50	0.00
TRINITY_sp|Q1ZX[illegible]	echs1	Probable enoyl-CoA	57.50	0.00
TRINITY_sp|Q99J[illegible]	Adi1	1,2-dihydroxy-3-ket	57.50	0.00
TRINITY_sp|Q91X[illegible]	Erlin1	Erlin-1 OS=Mus musc	57.50	0.00
TRINITY_sp|P168[illegible]	gpaA	Guanine nucleotide-	57.50	0.00
TRINITY_sp|Q54Q[illegible]	uba3	NEDD8-activating en	57.50	0.00
TRINITY_sp|O946[illegible]	tad2	tRNA-specific adeno	57.50	0.00
TRINITY_sp|Q8N1[illegible]	CFAP52	Cilia- and flagella	57.50	0.00
TRINITY_sp|P0DJ[illegible]	RPL4	60S ribosomal prote	57.50	0.00
TRINITY_sp|Q5XF[illegible]	Atp13a3	Probable cation-tra	57.50	0.00
TRINITY_sp|Q1LY[illegible]	setd1ba	Histone-lysine N-me	57.50	0.00
TRINITY_sp|Q84N[illegible]	POLIB	DNA polymerase I B,	57.50	0.00
TRINITY_sp|Q337[illegible]	Os10g05[illegible]	Electron transfer f	57.50	0.00
TRINITY_sp|F5A8[illegible]	DAB1	Dynein assembly fac	57.50	0.00
TRINITY_sp|Q8LP[illegible]	ABCB2	ABC transporter B f	57.50	0.00
TRINITY_sp|A4R0[illegible]	MSH3	DNA mismatch repair	57.50	0.00
TRINITY_sp|Q9Y4[illegible]	MYO5A	Unconventional myos	57.50	0.00
TRINITY_sp|Q93V[illegible]	CYP97A3	Protein LUTEIN DEFI	57.50	0.00
TRINITY_sp|O810[illegible]	NRPB5A	DNA-directed RNA po	57.50	0.00
TRINITY_sp|Q9AS[illegible]	PNSL5	Photosynthetic NDH	57.50	0.00
TRINITY_sp|Q9SZ[illegible]	At4g318[illegible]	Probable protein ph	57.50	0.00
TRINITY_sp|Q55G[illegible]	repD	TFIIH basal transcr	57.50	0.00
TRINITY_sp|Q54N[illegible]	pold1	DNA polymerase delt	57.40	0.00
TRINITY_sp|Q32P[illegible]	FCF1	rRNA-processing pro	57.40	0.00
TRINITY_sp|A6WV[illegible]	Oant_00[illegible]	Putative pterin-4-a	57.40	0.00
TRINITY_sp|Q9FK[illegible]	COX15	Cytochrome c oxidas	57.40	0.00
TRINITY_sp|Q9LR[illegible]	POLD1	DNA polymerase delt	57.40	0.00
TRINITY_sp|Q6GQ[illegible]	Cdc14a	Dual specificity pr	57.40	0.00
TRINITY_sp|A2Y5[illegible]	SNAT1	Serotonin N-acetylt	57.40	0.00
TRINITY_sp|Q54X[illegible]	pah	Phenylalanine-4-hyd	57.40	0.00
TRINITY_sp|D2K6[illegible]	SLT2	Sodium/sulfate cotr	57.40	0.00
TRINITY_sp|Q54Y[illegible]	DDB_G02[illegible]	Adenylate kinase is	57.40	0.00
TRINITY_sp|Q9FQ[illegible]	SPP1	Sucrose-phosphatase	57.40	0.00
TRINITY_sp|O813[illegible]	PAP11	Probable plastid-li	57.40	0.00
TRINITY_sp|Q9C9[illegible]	At1g527[illegible]	Probable histone H2	57.40	0.00
TRINITY_sp|P151[illegible]	efbA	Elongation factor 2	57.40	0.00

TRINITY_sp|Q500W	PIGM	GPI mannosyltransfe	57.40	0.00
TRINITY_sp|P0DJ1	RPL7A	60S ribosomal prote	57.40	0.00
TRINITY_sp|Q8CXI	dnaJ	Chaperone protein D	57.40	0.00
TRINITY_sp|Q9Y07	ctxB	Cortexillin-2 OS=Po	57.40	0.00
TRINITY_sp|Q6P8Y	Capsl	Calcyphosin-like pr	57.40	0.00
TRINITY_sp|O226(	DEGP1	Protease Do-like 1,	57.40	0.00
TRINITY_sp|Q9SRW	APK3	Adenylyl-sulfate ki	57.40	0.00
TRINITY_sp|P935S	CYP51	Obtusifoliol 14-alp	57.40	0.00
TRINITY_sp|P976(	Oplah	5-oxoprolinase OS=R	57.40	0.00
TRINITY_sp|Q9R0J	Lgmn	Legumain OS=Rattus	57.40	0.00
TRINITY_sp|P1457	Srp54	Signal recognition	57.40	0.00
TRINITY_sp|O7603	CDKL5	Cyclin-dependent ki	57.40	0.00
TRINITY_sp|P737(	slr1251	Peptidyl-prolyl cis	57.40	0.00
TRINITY_sp|Q5ZJI	DDX10	Probable ATP-depend	57.40	0.00
TRINITY_sp|Q6AZI	immp2l	Mitochondrial inner	57.40	0.00
TRINITY_sp|Q54MI	nadsyn1	Glutamine-dependent	57.40	0.00
TRINITY_sp|Q55F2	aatA	Aspartate aminotran	57.40	0.00
TRINITY_sp|P5521	CGS1	Cystathionine gamma	57.40	0.00
TRINITY_sp|Q84K4	ABCA2	ABC transporter A f	57.40	0.00
TRINITY_sp|A1A4J	ATP9B	Probable phospholip	57.40	0
TRINITY_sp|Q8TF(	ATP8B4	Probable phospholip	57.40	0.00
TRINITY_sp|O8083	At2g4587	UPF0187 protein At2	57.40	0.00
TRINITY_sp|O440(	PYK	Pyruvate kinase OS=	57.40	0.00
TRINITY_sp|P2752	CAB8	Chlorophyll a-b bin	57.40	0.00
TRINITY_sp|Q5UQ8	MIMI_R52	Probable ubiquitin-	57.30	0.00
TRINITY_sp|P4155	ICL1	Isocitrate lyase OS	57.30	0.00
TRINITY_sp|Q2392	copb	Coatomer subunit be	57.30	0.00
TRINITY_sp|Q9Y38	UBE2J1	Ubiquitin-conjugati	57.30	0.00
TRINITY_sp|Q54S3	rcl1	Probable RNA 3'-ter	57.30	0.00
TRINITY_sp|Q66GN	CLPX3	CLP protease regula	57.30	0.00
TRINITY_sp|Q4R5Y	NADSYN1	Glutamine-dependent	57.30	0.00
TRINITY_sp|Q9FMI	HDA6	Histone deacetylase	57.30	0.00
TRINITY_sp|Q9SQI	LTA2	Dihydrolipoyllysine	57.30	0.00
TRINITY_sp|Q9M5K	LPD1	Dihydrolipoyl dehyd	57.30	0.00
TRINITY_sp|P1295	PEPD	Xaa-Pro dipeptidase	57.30	0.00
TRINITY_sp|Q8T6(	abcH2	ABC transporter H f	57.30	0.00
TRINITY_sp|Q9LEN	NAC2	PsbD mRNA maturatio	57.30	0.00
TRINITY_sp|Q9FE(	OHP2	Light-harvesting co	57.30	0.00
TRINITY_sp|O658S	CDA1	Cytidine deaminase	57.30	0.00
TRINITY_sp|Q96MS	PACRG	Parkin coregulated	57.30	0.00
TRINITY_sp|P3411	mvpA	Major vault protein	57.30	0.00
TRINITY_sp|Q9LMR	TYRAAT2	Arogenate dehydroge	57.30	0.00
TRINITY_sp|P581(	coaE	Dephospho-CoA kinas	57.30	0.00
TRINITY_sp|Q940(	TMN1	Transmembrane 9 sup	57.30	0.00
TRINITY_sp|B1HM\	rpsM	30S ribosomal prote	57.30	0.00
TRINITY_sp|Q4285	DCUP	Uroporphyrinogen de	57.30	0.00
TRINITY_sp|Q54RI	DDB_G028	Probable serine/thr	57.30	0.00
TRINITY_sp|O2214	GCP1	Probable tRNA N6-ad	57.30	0.00
TRINITY_sp|P5467	apm2	AP-2 complex subuni	57.30	0.00
TRINITY_sp|P0C8N	CCRP1	Probable serine/thr	57.30	0.00
TRINITY_sp|Q55EI	DDB_G02(	NKAP family protein	57.30	0.00
TRINITY_sp|Q8RVI	SPY	Probable UDP-N-acet	57.30	0.00
TRINITY_sp|A8IQI	CCDC39	Coiled-coil domain-	57.30	0.00
TRINITY_sp|Q4277	-	Stearoyl-[acyl-carr	57.30	0.00
TRINITY_sp|Q9M9Y	CRTISO	Prolycopene isomera	57.30	0.00
TRINITY_sp|O6531	-	Actin OS=Coleochaet	57.30	0.00

TRINITY_sp|Q9LH	RHM3	Trifunctional UDP-g	57.20	0.00
TRINITY_sp|Q9FY	RPS15C	40S ribosomal prote	57.20	0.00
TRINITY_sp|Q8LD	CYP19-4	Peptidyl-prolyl cis	57.20	0.00
TRINITY_sp|Q55G	pakC	Serine/threonine-pr	57.20	0.00
TRINITY_sp|O598	gpx1	Glutathione peroxid	57.20	0.00
TRINITY_sp|Q9SL	MAP1A	Methionine aminopep	57.20	0.00
TRINITY_sp|Q9P2	CHD7	Chromodomain-helica	57.20	0.00
TRINITY_sp|Q238	racE	Rho-related protein	57.20	0.00
TRINITY_sp|P375	ychF	Ribosome-binding AT	57.20	0.00
TRINITY_sp|Q8BG	Wdr92	WD repeat-containin	57.20	0.00
TRINITY_sp|P0C8	CCRP1	Probable serine/thr	57.20	0.00
TRINITY_sp|F4I6	POLIA	DNA polymerase I A,	57.20	0.00
TRINITY_sp|O653	-	Actin OS=Coleochaet	57.20	0.00
TRINITY_sp|Q8LK	ALB3.2	Inner membrane ALBI	57.20	0.00
TRINITY_sp|Q9LZ	CP12-2	Calvin cycle protei	57.10	0.00
TRINITY_sp|P428	ZBP14	14 kDa zinc-binding	57.10	0.00
TRINITY_sp|Q633	coaE	Dephospho-CoA kinas	57.10	0.00
TRINITY_sp|B1AR	Cisd3	CDGSH iron-sulfur d	57.10	0.00
TRINITY_sp|O227	CB5LP	Cytochrome B5-like	57.10	0.00
TRINITY_sp|O652	FOLD4	Bifunctional protei	57.10	0.00
TRINITY_sp|P423	MTOR	Serine/threonine-pr	57.10	0.00
TRINITY_sp|P0CR	SLU7	Pre-mRNA-splicing f	57.10	0.00
TRINITY_sp|Q8BR	Man2a2	Alpha-mannosidase 2	57.10	0.00
TRINITY_sp|Q96R	IFT140	Intraflagellar tran	57.10	0.00
TRINITY_sp|P118	-	High mobility group	57.10	0.00
TRINITY_sp|Q94A	SEC22	25.3 kDa vesicle tr	57.10	0.00
TRINITY_sp|Q553	serp	Probable stress-ass	57.10	0.00
TRINITY_sp|Q3MH	PSMB6	Proteasome subunit	57.10	0.00
TRINITY_sp|Q3MH	PSMB6	Proteasome subunit	57.10	0.00
TRINITY_sp|Q9WU	Prodh	Proline dehydrogena	57.10	0.00
TRINITY_sp|O648	At2g230	Uncharacterized pro	57.10	0.00
TRINITY_sp|Q096	SPAC2F7.	Putative peptide ch	57.10	0.00
TRINITY_sp|P297	-	60S acidic ribosoma	57.10	0.00
TRINITY_sp|O825	RPL21	60S ribosomal prote	57.10	0.00
TRINITY_sp|Q422	At3g020	Probable mitochondr	57.10	0.00
TRINITY_sp|Q107	CCP2	Cysteine proteinase	57.10	0.00
TRINITY_sp|Q1JQ	AP3S2	AP-3 complex subuni	57.10	0.00
TRINITY_sp|P301	ybiA	N-glycosidase YbiA	57.10	0.00
TRINITY_sp|Q9WY	TM_0508	Uncharacterized pro	57.10	0.00
TRINITY_sp|Q9CZ	Skiv2l2	Superkiller viralic	57.10	0
TRINITY_sp|Q9XF	PEX7	Peroxisome biogenes	57.10	0.00
TRINITY_sp|O041	At4g189	Cyclic phosphodiest	57.10	0.00
TRINITY_sp|O671	gap	Glyceraldehyde-3-ph	57.10	0.00
TRINITY_sp|Q9M0	IBI1	Aspartate--tRNA lig	57.10	0.00
TRINITY_sp|Q1I5	ung	Uracil-DNA glycosyl	57.10	0.00
TRINITY_sp|O241	CHLD	Magnesium-chelatase	57.10	0.00
TRINITY_sp|P186	NRPB1	DNA-directed RNA po	57.10	0
TRINITY_sp|Q84M	ABCA1	ABC transporter A f	57.10	0.00
TRINITY_sp|Q080	CHSP70	Stromal 70 kDa heat	57.10	0.00
TRINITY_sp|Q8T1	kif5	Kinesin-related pro	57.10	0.00
TRINITY_sp|P801	-	Extracellular serin	57.00	0.00
TRINITY_sp|Q9M2	ABCG17	ABC transporter G f	57.00	0.00
TRINITY_sp|Q54G	mdhA	Probable malate deh	57.00	0.00
TRINITY_sp|Q9D1	Hddc3	Guanosine-3',5'-bis	57.00	0.00
TRINITY_sp|Q55E	pats1	Probable serine/thr	57.00	0.00
TRINITY_sp|Q9BW	NUDT9	ADP-ribose pyrophos	57.00	0.00

TRINITY_sp|Q9D0I	Rfc5	Replication factor	57.00	0.00
TRINITY_sp|O2432	-	Vacuolar-processing	57.00	0.00
TRINITY_sp|Q6Z1	YchF1	Obg-like ATPase 1 O	57.00	0.00
TRINITY_sp|Q54M2	cdc20	Anaphase-promoting	57.00	0.00
TRINITY_sp|Q8WU	PPIL4	Peptidyl-prolyl cis	57.00	0.00
TRINITY_sp|Q1KV	rpl12	50S ribosomal prote	57.00	0.00
TRINITY_sp|O756	GCAT	2-amino-3-ketobutyr	57.00	0.00
TRINITY_sp|Q6NR	mcts1-b	Malignant T-cell-am	57.00	0.00
TRINITY_sp|Q86C	tor	Target of rapamycin	57.00	0.00
TRINITY_sp|A7MB	RQCD1	Cell differentiatio	57.00	0.00
TRINITY_sp|Q9FK2	UBC33	Probable ubiquitin-	57.00	0.00
TRINITY_sp|Q32L	TRAPPC6	Trafficking protein	57.00	0.00
TRINITY_sp|P525	MYBL1	Myb-related protein	57.00	0.00
TRINITY_sp|P7452	slr1419	Uncharacterized Suf	57.00	0.00
TRINITY_sp|O024	-	Dynein light chain	57.00	0.00
TRINITY_sp|Q8LS	FYPP	Phytochrome-associa	57.00	0.00
TRINITY_sp|O153	NVL	Nuclear valosin-con	57.00	0.00
TRINITY_sp|Q66J	unc119b-	Protein unc-119 hom	57.00	0.00
TRINITY_sp|Q54Q	adkA	Adenylate kinase OS	57.00	0.00
TRINITY_sp|Q9SZ	GPX7	Putative glutathion	57.00	0.00
TRINITY_sp|Q8T1	kif5	Kinesin-related pro	57.00	0.00
TRINITY_sp|Q215	M28.5	NHP2-like protein 1	56.90	0.00
TRINITY_sp|Q294	LIPF	Gastric triacylglyc	56.90	0.00
TRINITY_sp|P000	-	Cytochrome c OS=Tet	56.90	0.00
TRINITY_sp|P9WQ	treY	Putative maltooligo	56.90	0.00
TRINITY_sp|O5992	SOD1	Superoxide dismutas	56.90	0.00
TRINITY_sp|Q1DR	CIMG_07	Mitotic-spindle org	56.90	0.00
TRINITY_sp|O440	PYK	Pyruvate kinase OS=	56.90	0.00
TRINITY_sp|Q54Y	gacA	Rho GTPase-activati	56.90	0.00
TRINITY_sp|Q86J	rab5A	Ras-related protein	56.90	0.00
TRINITY_sp|Q019	RAB2A	Ras-related protein	56.90	0.00
TRINITY_sp|Q8L7	BSL1	Serine/threonine-pr	56.90	0.00
TRINITY_sp|P427	ELI3	Mannitol dehydrogen	56.90	0.00
TRINITY_sp|A5DJ	HTA2	Histone H2A.2 OS=Me	56.90	0.00
TRINITY_sp|Q101	fep1	Iron-sensing transc	56.90	0.00
TRINITY_sp|Q9ZR	U2AF65B	Splicing factor U2a	56.90	0.00
TRINITY_sp|P318	tabA	Protein TabA OS=Pse	56.90	0.00
TRINITY_sp|B9G2	Os09g02	Auxin transport pro	56.90	0.00
TRINITY_sp|P519	Nek1	Serine/threonine-pr	56.90	0.00
TRINITY_sp|Q9SN	ATL7	RING-H2 finger prot	56.90	0.00
TRINITY_sp|A8HY	IFT43	Intraflagellar tran	56.90	0.00
TRINITY_sp|Q8G2	purD	Phosphoribosylamine	56.90	0.00
TRINITY_sp|Q220	pde-4	Probable 3',5'-cycl	56.90	0.00
TRINITY_sp|Q9MA	NOP5-2	Probable nucleolar	56.90	0.00
TRINITY_sp|Q392	SKP1A	SKP1-like protein 1	56.90	0.00
TRINITY_sp|P428	HEMA1	Glutamyl-tRNA reduc	56.90	0.00
TRINITY_sp|Q8L7	DIT2	Dicarboxylate trans	56.90	0.00
TRINITY_sp|A5VJ	dnaJ	Chaperone protein D	56.90	0.00
TRINITY_sp|Q9CA	HIR2	Hypersensitive-indu	56.90	0.00
TRINITY_sp|Q6H6	FTSH7	ATP-dependent zinc	56.90	0.00
TRINITY_sp|Q9FJ	CYP65	Peptidyl-prolyl cis	56.80	0.00
TRINITY_sp|O0062	PIR	Pirin OS=Homo sapie	56.80	0.00
TRINITY_sp|Q93W	SAG21	Protein SENESCENCE-	56.80	0.00
TRINITY_sp|Q9SZ	At4g291	Probable 3-hydroxyi	56.80	0.00
TRINITY_sp|P618	rpl-12	60S ribosomal prote	56.80	0.00
TRINITY_sp|Q2KJ	AFG3L2	AFG3-like protein 2	56.80	0.00

TRINITY_sp|P341(	myoD	Myosin ID heavy cha	56.80	0.00
TRINITY_sp|Q86AI	DDB_G02	Probable acetyl-CoA	56.80	0.00
TRINITY_sp|Q58DI	ALAD	Delta-aminolevulini	56.80	0.00
TRINITY_sp|P537(	GFA1	Glutamine--fructose	56.80	0.00
TRINITY_sp|Q10G8	MSI1	Histone-binding pro	56.80	0.00
TRINITY_sp|P258(	UBC1	Ubiquitin-conjugati	56.80	0.00
TRINITY_sp|Q944S	selD	Selenide, water dik	56.80	0.00
TRINITY_sp|Q8W58	FTSH8	ATP-dependent zinc	56.80	0.00
TRINITY_sp|Q551I	DDB_G02	WD repeat-containin	56.80	0.00
TRINITY_sp|B0TGY	secA	Protein translocase	56.80	0.00
TRINITY_sp|Q7XKI	TRX-X	Thioredoxin X, chlo	56.80	0.00
TRINITY_sp|Q58DI	GPN2	GPN-loop GTPase 2 O	56.80	0.00
TRINITY_sp|Q9FMA	PEX5	Peroxisome biogenes	56.80	0.00
TRINITY_sp|P239(	VMA16	V-type proton ATPas	56.80	0.00
TRINITY_sp|Q9FQ(	XRN3	5'-3' exoribonuclea	56.80	0.00
TRINITY_sp|D0NVI	PITG_16	Adenylosuccinate sy	56.80	0.00
TRINITY_sp|P109	-	Retrovirus-related	56.80	0.00
TRINITY_sp|P775	yfcG	Disulfide-bond oxid	56.80	0.00
TRINITY_sp|Q6PFY	Cdc14b	Dual specificity pr	56.70	0.00
TRINITY_sp|Q498I	smarcal	SWI/SNF-related mat	56.70	0.00
TRINITY_sp|O091	Hgd	Homogentisate 1,2-d	56.70	0.00
TRINITY_sp|A0LY	rplT	50S ribosomal prote	56.70	0.00
TRINITY_sp|Q54T(	DDB_G028	Maf-like protein DD	56.70	0.00
TRINITY_sp|Q551N	mettl1	tRNA (guanine-N(7)-	56.70	0.00
TRINITY_sp|Q54RV	trappc2	Trafficking protein	56.70	0.00
TRINITY_sp|Q5F3(	ABCC1	Multidrug resistanc	56.70	0.00
TRINITY_sp|Q86AI	DDB_G02	Probable myosin lig	56.70	0.00
TRINITY_sp|Q8ST8	abcC10	ABC transporter C f	56.70	0.00
TRINITY_sp|Q55GI	mcfT	Probable mitochondr	56.70	0.00
TRINITY_sp|P271	corA	Coronin-A OS=Dictyo	56.70	0.00
TRINITY_sp|O4878	HO1	Heme oxygenase 1, c	56.70	0.00
TRINITY_sp|E9Q9I	Rabl2	Rab-like protein 2A	56.70	0.00
TRINITY_sp|O574	fech	Ferrochelatase, mit	56.70	0.00
TRINITY_sp|Q9C5(	DPL1	Sphingosine-1-phosp	56.70	0.00
TRINITY_sp|O810	NRPB5A	DNA-directed RNA po	56.70	0.00
TRINITY_sp|Q869N	pakB	Serine/threonine-pr	56.70	0.00
TRINITY_sp|Q9FGS	MNS4	Alpha-mannosidase I	56.70	0.00
TRINITY_sp|Q9LV(	GLT1	Glutamate synthase	56.70	0.00
TRINITY_sp|Q3SZI	SNRPD2	Small nuclear ribon	56.70	0.00
TRINITY_sp|P304(	PPIF	Peptidyl-prolyl cis	56.70	0.00
TRINITY_sp|Q9ZW	TIM10	Mitochondrial impor	56.70	0.00
TRINITY_sp|A1CKN	get3	ATPase get3 OS=Aspe	56.70	0.00
TRINITY_sp|Q109I	CUTA1	Protein CutA 1, chl	56.70	0.00
TRINITY_sp|Q54N	hprT	Hypoxanthine-guanin	56.70	0.00
TRINITY_sp|O049	-	Alpha-glucosidase O	56.70	0.00
TRINITY_sp|P123	CAB1R	Chlorophyll a-b bin	56.70	0.00
TRINITY_sp|Q80VI	-	Probable peptide ch	56.60	0.00
TRINITY_sp|P469(	dad1	Dolichyl-diphosphoo	56.60	0.00
TRINITY_sp|Q54CV	rtpR	Probable adenosylco	56.60	0.00
TRINITY_sp|Q54L	anapc11	Anaphase-promoting	56.60	0.00
TRINITY_sp|Q2KJ	AFG3L2	AFG3-like protein 2	56.60	0.00
TRINITY_sp|Q6CQI	TAR1-A	Protein TAR1 OS=Klu	56.60	0.00
TRINITY_sp|Q554S	act17	Actin-17 OS=Dictyos	56.60	0.00
TRINITY_sp|Q23FI	TTLL3C	Tubulin glycylase 3	56.60	0.00
TRINITY_sp|Q0D5I	SAP16	Zinc finger AN1 and	56.60	0.00
TRINITY_sp|P391	citZ	Citrate synthase 2	56.60	0.00

TRINITY_sp|Q9SG(	MGL	Methionine gamma-ly	56.60	0.00
TRINITY_sp|Q9SSI	ADT2	Arogenate dehydrata	56.60	0.00
TRINITY_sp|Q3B8	sdhb	Succinate dehydroge	56.60	0.00
TRINITY_sp|Q9FMI	PYD2	Dihydropyrimidinase	56.60	0.00
TRINITY_sp|Q9SII	RPS3A	40S ribosomal prote	56.60	0.00
TRINITY_sp|P4968	RPS30A	40S ribosomal prote	56.60	0.00
TRINITY_sp|Q8T8I	abcD2	ABC transporter D f	56.60	0.00
TRINITY_sp|Q9QZN	Ubqln2	Ubiquilin-2 OS=Mus	56.60	0.00
TRINITY_sp|Q9JMI	Txnrd1	Thioredoxin reducta	56.60	0.00
TRINITY_sp|Q9SE4	Os09g05(	Ribulose-phosphate	56.60	0.00
TRINITY_sp|Q7SDN	rgt-1	tRNA(His) guanylylt	56.60	0.00
TRINITY_sp|P2752	CAB8	Chlorophyll a-b bin	56.60	0.00
TRINITY_sp|Q1KV\	psbK	Photosystem II reac	56.50	0.00
TRINITY_sp|Q6GL4	mcm4	DNA replication lic	56.50	0.00
TRINITY_sp|Q8T2I	masA	Malate synthase OS=	56.50	0.00
TRINITY_sp|Q395(	ODA4	Dynein beta chain,	56.50	0.00
TRINITY_sp|Q54YI	snfA	5'-AMP-activated se	56.50	0.00
TRINITY_sp|Q54J(	purL	Phosphoribosylformy	56.50	0.00
TRINITY_sp|Q9H0(	SLC25A3	ADP/ATP translocase	56.50	0.00
TRINITY_sp|Q9C8N	MSRB1	Peptide methionine	56.50	0.00
TRINITY_sp|Q7KQI	ARF1	ADP-ribosylation fa	56.50	0.00
TRINITY_sp|O239	GPXHA-1	Glutathione peroxid	56.50	0.00
TRINITY_sp|Q2462	ref(2)P	Protein ref(2)P OS=	56.50	0.00
TRINITY_sp|Q54QI	rab32A	Ras-related protein	56.50	0.00
TRINITY_sp|Q9SG	RPS19A	40S ribosomal prote	56.50	0.00
TRINITY_sp|Q6ME(	asnS	Asparagine--tRNA li	56.50	0.00
TRINITY_sp|P222	RAS2	Ras-like protein 2	56.50	0.00
TRINITY_sp|B8B9I	RPL10A	60S ribosomal prote	56.50	0.00
TRINITY_sp|O046	THRRS	Threonine--tRNA lig	56.50	0.00
TRINITY_sp|Q54T	ube2m	NEDD8-conjugating e	56.50	0.00
TRINITY_sp|O236	AK2	Aspartokinase 2, ch	56.50	0.00
TRINITY_sp|P072	URA2	Protein URA2 OS=Sac	56.50	0.00
TRINITY_sp|Q76NI	ERD2	ER lumen protein-re	56.50	0.00
TRINITY_sp|Q9C9	NUG2	Nuclear/nucleolar G	56.50	0.00
TRINITY_sp|P537(	GFA1	Glutamine--fructose	56.50	0.00
TRINITY_sp|Q4R6	AGBL2	Cytosolic carboxype	56.50	0.00
TRINITY_sp|Q86K	trappc3	Trafficking protein	56.40	0.00
TRINITY_sp|Q917	gsk3b	Glycogen synthase k	56.40	0.00
TRINITY_sp|O046	THRRS	Threonine--tRNA lig	56.40	0.00
TRINITY_sp|P4252	erkA	Extracellular signa	56.40	0.00
TRINITY_sp|Q553\	mcee	Methylmalonyl-CoA e	56.40	0.00
TRINITY_sp|Q6UX	C16orf8	UPF0764 protein C16	56.40	0.00
TRINITY_sp|P347	BMH2	Protein BMH2 OS=Sac	56.40	0.00
TRINITY_sp|Q29AI	GA17800	Leishmanolysin-like	56.40	0.00
TRINITY_sp|Q964I	-	Actin, cytoplasmic	56.40	0.00
TRINITY_sp|Q236	RPL32	60S ribosomal prote	56.40	0.00
TRINITY_sp|A9A5	guaB	Inosine-5'-monophos	56.40	0.00
TRINITY_sp|Q8I5I	proRS	Proline--tRNA ligas	56.40	0.00
TRINITY_sp|P207	MAK	Serine/threonine-pr	56.40	0.00
TRINITY_sp|P557	SEC13	Protein SEC13 homol	56.40	0.00
TRINITY_sp|F4JA	SKI2	DExH-box ATP-depend	56.40	0.00
TRINITY_sp|P318	-	RNA-directed DNA po	56.40	0.00
TRINITY_sp|O975(	TXN	Thioredoxin OS=Equu	56.40	0.00
TRINITY_sp|P558	mcm2	DNA replication lic	56.40	0.00
TRINITY_sp|Q6P5I	Uggt1	UDP-glucose:glycopr	56.40	0.00
TRINITY_sp|B9MQ	rplS	50S ribosomal prote	56.40	0.00

TRINITY_sp|Q9SK	At2g320	Folate-biopterin tr	56.40	0.00
TRINITY_sp|Q9CWI	Smyd3	Histone-lysine N-me	56.40	0.00
TRINITY_sp|Q9H8	MOB1A	MOB kinase activato	56.40	0.00
TRINITY_sp|P296	CYCL	Cytochrome c1-2, he	56.40	0.00
TRINITY_sp|Q7DL	PBB2	Proteasome subunit	56.40	0.00
TRINITY_sp|Q8N4	CARNMT1	Carnosine N-methylt	56.30	0.00
TRINITY_sp|Q7GB	ABCC5	ABC transporter C f	56.30	0.00
TRINITY_sp|Q8T1	udkB	Uridine-cytidine ki	56.30	0.00
TRINITY_sp|Q54X	metS	Probable methionine	56.30	0.00
TRINITY_sp|O141	lys12	Homoisocitrate dehy	56.30	0.00
TRINITY_sp|Q54I	ragA	Ras-related GTP-bin	56.30	0.00
TRINITY_sp|Q0JM	AIP1	DEAD-box ATP-depend	56.30	0.00
TRINITY_sp|P198	PRKA	Phosphoribulokinase	56.30	0.00
TRINITY_sp|O351	Emg1	Ribosomal RNA small	56.30	0.00
TRINITY_sp|B8B8	OsI_254	PHD finger protein	56.30	0.00
TRINITY_sp|Q051	Rac2	Ras-related C3 botu	56.30	0.00
TRINITY_sp|P563	-	Eukaryotic translat	56.30	0.00
TRINITY_sp|Q93Z	FKBP53	Peptidyl-prolyl cis	56.30	0.00
TRINITY_sp|Q54J	papA	Poly(A) polymerase	56.30	0.00
TRINITY_sp|O597	dph1	Diphthamide biosynt	56.30	0.00
TRINITY_sp|Q6YZ	RFC3	Replication factor	56.30	0.00
TRINITY_sp|Q91Z	Smarca5	SWI/SNF-related mat	56.30	0.00
TRINITY_sp|Q6R3	Poli	DNA polymerase iota	56.30	0.00
TRINITY_sp|Q087	myb	Transcriptional act	56.30	0.00
TRINITY_sp|O945	sua5	Threonylcarbamoyl-A	56.30	0.00
TRINITY_sp|Q631	Dnah1	Dynein heavy chain	56.30	0.00
TRINITY_sp|Q007	glpV	Glycogen phosphoryl	56.30	0.00
TRINITY_sp|P026	PMP2	Myelin P2 protein O	56.20	0.00
TRINITY_sp|P465	POL3	DNA polymerase delt	56.20	0.00
TRINITY_sp|O756	SNRNP20	U5 small nuclear ri	56.20	0.00
TRINITY_sp|Q9P4	SAR1	Small COPII coat GT	56.20	0.00
TRINITY_sp|Q96P	NEK1	Serine/threonine-pr	56.20	0.00
TRINITY_sp|Q54E	lpd	Dihydrolipoyl dehyd	56.20	0.00
TRINITY_sp|Q004	L41P1A	60S ribosomal prote	56.20	0.00
TRINITY_sp|Q9VL	SmE	Probable small nucl	56.20	0.00
TRINITY_sp|Q9FJ	PABN2	Polyadenylate-bindi	56.20	0.00
TRINITY_sp|Q54U	kif3	Kinesin-related pro	56.20	0.00
TRINITY_sp|A8EZ	hisS	Histidine--tRNA lig	56.20	0.00
TRINITY_sp|Q0IM	MT4A	Metallothionein-lik	56.20	0.00
TRINITY_sp|P546	pikC	Phosphatidylinosito	56.20	0.00
TRINITY_sp|Q55D	cysA	Cystathionine gamma	56.20	0.00
TRINITY_sp|Q8W1	SMU1	Suppressor of mec-8	56.20	0.00
TRINITY_sp|A2X2	OsI_006	Serine/threonine pr	56.20	0.00
TRINITY_sp|Q3ZC	OAT	Ornithine aminotran	56.20	0.00
TRINITY_sp|P735	sll0875	Uncharacterized mem	56.20	0.00
TRINITY_sp|Q01I	H0219H1	ATP-dependent RNA h	56.20	0.00
TRINITY_sp|Q997	ABCA3	ATP-binding cassett	56.20	0.00
TRINITY_sp|Q66I	aldh8a1	Aldehyde dehydrogen	56.20	0.00
TRINITY_sp|Q54S	sf3b3	Probable splicing f	56.20	0.00
TRINITY_sp|Q9SM	NFYC1	Nuclear transcripti	56.20	0.00
TRINITY_sp|Q9T0	At4g392	Phenylalanine--tRNA	56.20	0.00
TRINITY_sp|Q231	RPL5	60S ribosomal prote	56.20	0.00
TRINITY_sp|Q9CW	Cpsf3l	Integrator complex	56.20	0.00
TRINITY_sp|A8NE	TRP-1	Tryptophan synthase	56.20	0.00
TRINITY_sp|B2IZ	ileS	Isoleucine--tRNA li	56.20	0.00
TRINITY_sp|Q4WK	ppe1	Protein phosphatase	56.20	0.00

TRINITY_sp|Q84Q(	PPT2	Phosphoenolpyruvate	56.20	0.00
TRINITY_sp|Q141[illegible]	DHX34	Probable ATP-depend	56.20	0.00
TRINITY_sp|Q5PQ[illegible]	-	Uncharacterized pro	56.20	0.00
TRINITY_sp|Q56YI	SMC3	Structural maintena	56.20	0.00
TRINITY_sp|Q54F[illegible]	ndufv2	NADH dehydrogenase	56.20	0.00
TRINITY_sp|Q54P[illegible]	mtr	Methionine synthase	56.20	0.00
TRINITY_sp|Q704[illegible]	Abcb7	ATP-binding cassett	56.20	0.00
TRINITY_sp|Q8DJ[illegible]	clpB1	Chaperone protein C	56.20	0.00
TRINITY_sp|Q5F3[illegible]	ABCC1	Multidrug resistanc	56.20	0.00
TRINITY_sp|O757[illegible]	RNASEH2A	Ribonuclease H2 sub	56.10	0.00
TRINITY_sp|Q93V[illegible]	ATG18A	Autophagy-related p	56.10	0.00
TRINITY_sp|Q74Z(	YSH1	Endoribonuclease YS	56.10	0.00
TRINITY_sp|Q96B[illegible]	ARL8A	ADP-ribosylation fa	56.10	0.00
TRINITY_sp|A2ZB[illegible]	VATP-P1	V-type proton ATPas	56.10	0.00
TRINITY_sp|A5PK[illegible]	FAH	Fumarylacetoacetase	56.10	0.00
TRINITY_sp|P546[illegible]	fimA	Fimbrin OS=Dictyost	56.10	0.00
TRINITY_sp|Q9NX[illegible]	DUS2	tRNA-dihydrouridine	56.10	0.00
TRINITY_sp|Q6IN(	ttc30a	Tetratricopeptide r	56.10	0.00
TRINITY_sp|Q8VZ[illegible]	FZR1	Protein FIZZY-RELAT	56.10	0.00
TRINITY_sp|Q4WI(	rps21	40S ribosomal prote	56.10	0.00
TRINITY_sp|Q23F[illegible]	TTLL3C	Tubulin glycylase 3	56.10	0.00
TRINITY_sp|Q9XY[illegible]	fcpA	Probable C-terminal	56.10	0.00
TRINITY_sp|P341[illegible]	mvpA	Major vault protein	56.10	0.00
TRINITY_sp|P426[illegible]	yqjG	Glutathionyl-hydroq	56.10	0.00
TRINITY_sp|Q182[illegible]	dnaJ	Chaperone protein D	56.10	0.00
TRINITY_sp|Q9BL(	amtA	Ammonium transporte	56.10	0.00
TRINITY_sp|P341[illegible]	hmgB	3-hydroxy-3-methylg	56.10	0.00
TRINITY_sp|Q9SS[illegible]	RPS24A	40S ribosomal prote	56.10	0.00
TRINITY_sp|Q5A4[illegible]	GAT1	Transcriptional reg	56.10	0.00
TRINITY_sp|Q0WR[illegible]	FKBP20-2	Peptidyl-prolyl cis	56.10	0.00
TRINITY_sp|Q9S7[illegible]	HISN1A	ATP phosphoribosylt	56.10	0.00
TRINITY_sp|P468[illegible]	KIN14N	Kinesin-like protei	56.10	0.00
TRINITY_sp|P104[illegible]	-	Phosphatidylcholine	56.10	0.00
TRINITY_sp|Q9SJ[illegible]	UEV1C	Ubiquitin-conjugati	56.10	0.00
TRINITY_sp|P791(	CPSF3	Cleavage and polyad	56.10	0.00
TRINITY_sp|P114[illegible]	sod1	Superoxide dismutas	56.10	0.00
TRINITY_sp|Q9LZ[illegible]	RABE1D	Ras-related protein	56.10	0.00
TRINITY_sp|O756[illegible]	SNRNP20(	U5 small nuclear ri	56.00	0.00
TRINITY_sp|Q93W[illegible]	SAG21	Protein SENESCENCE-	56.00	0.00
TRINITY_sp|P565[illegible]	-	ES1 protein homolog	56.00	0.00
TRINITY_sp|Q6C4[illegible]	DBP2	ATP-dependent RNA h	56.00	0.00
TRINITY_sp|Q387(	THI1	Thiamine thiazole s	56.00	0.00
TRINITY_sp|Q3MI[illegible]	PUS10	Putative tRNA pseud	56.00	0.00
TRINITY_sp|B7T1[illegible]	ftsH	ATP-dependent zinc	56.00	0.00
TRINITY_sp|Q126[illegible]	DNF2	Phospholipid-transp	56.00	0.00
TRINITY_sp|O943[illegible]	psm1	Structural maintena	56.00	0.00
TRINITY_sp|Q9DA[illegible]	Pacrg	Parkin coregulated	56.00	0.00
TRINITY_sp|Q395[illegible]	YPTC1	GTP-binding protein	56.00	0.00
TRINITY_sp|Q75M[illegible]	ML3	Protein MEI2-like 3	56.00	0.00
TRINITY_sp|Q395[illegible]	-	5-methyltetrahydrop	56.00	0.00
TRINITY_sp|Q099[illegible]	yakc	Aldo-keto reductase	56.00	0.00
TRINITY_sp|Q6BV[illegible]	PMP3	Plasma membrane pro	56.00	0.00
TRINITY_sp|Q1ZX[illegible]	gxcDD	Guanine exchange fa	56.00	0.00
TRINITY_sp|Q54M[illegible]	bkdA	2-oxoisovalerate de	56.00	0.00
TRINITY_sp|Q8LH(	Os07g01(	DDRGK domain-contai	56.00	0.00
TRINITY_sp|O154[illegible]	ABCC4	Multidrug resistanc	56.00	0.00

TRINITY_sp|P529(	-	Pyruvate dehydrogen	56.00	0.00
TRINITY_sp|F4IYM	DEX1	Protein DEFECTIVE I	56.00	0.00
TRINITY_sp|Q0PGJ	AKR4C9	Aldo-keto reductase	56.00	0.00
TRINITY_sp|O489(	POLD1	DNA polymerase delt	56.00	0.00
TRINITY_sp|Q8RWM	NAPRT1	Nicotinate phosphor	56.00	0.00
TRINITY_sp|Q0564	dnaJ	Chaperone protein D	56.00	0.00
TRINITY_sp|O2214	GCP1	Probable tRNA N6-ad	56.00	0.00
TRINITY_sp|Q23FE	TTLL3C	Tubulin glycylase 3	56.00	0.00
TRINITY_sp|Q9SW9	SYNC1	Asparagine--tRNA li	56.00	0.00
TRINITY_sp|Q8W4I	CYP71	Peptidyl-prolyl cis	56.00	0.00
TRINITY_sp|A4QVE	TIF1	ATP-dependent RNA h	56.00	0.00
TRINITY_sp|F4K5X	RVE2	Protein REVEILLE 2	56.00	0.00
TRINITY_sp|Q5W91	USP	UDP-sugar pyrophosp	56.00	0.00
TRINITY_sp|P1629	F9	Coagulation factor	55.90	0.00
TRINITY_sp|Q6ZWY	Ube2d2b	Ubiquitin-conjugati	55.90	0.00
TRINITY_sp|Q9463	-	DNA polymerase alph	55.90	0.00
TRINITY_sp|Q54U8	dhkA	Hybrid signal trans	55.90	0.00
TRINITY_sp|Q86C6	tor	Target of rapamycin	55.90	0.00
TRINITY_sp|Q54RA	DDB_G028	Probable iron/ascor	55.90	0.00
TRINITY_sp|P5467	pikC	Phosphatidylinosito	55.90	0.00
TRINITY_sp|P6234	CPK4	Calcium-dependent p	55.90	0.00
TRINITY_sp|P252(	NFYB	Nuclear transcripti	55.90	0.00
TRINITY_sp|O6653	aq_141	Uncharacterized HIT	55.90	0.00
TRINITY_sp|Q9DAE	Pacrg	Parkin coregulated	55.90	0.00
TRINITY_sp|P484(	CNAG_004	Actin OS=Cryptococc	55.90	0.00
TRINITY_sp|P4123	Abca2	ATP-binding cassett	55.90	0.00
TRINITY_sp|Q86KI	ap2a1-1	AP-2 complex subuni	55.90	0.00
TRINITY_sp|Q0P5I	RBM42	RNA-binding protein	55.90	0.00
TRINITY_sp|Q4522	hbdA	3-hydroxybutyryl-Co	55.90	0.00
TRINITY_sp|Q653T	SPS1	Solanesyl-diphospha	55.90	0.00
TRINITY_sp|Q5572	sll0608	Ycf49-like protein	55.90	0.00
TRINITY_sp|Q54VI	rio1	Serine/threonine-pr	55.90	0.00
TRINITY_sp|Q94II	GGPS	Geranylgeranyl pyro	55.90	0.00
TRINITY_sp|Q2QPW	GK1	Guanylate kinase 1	55.90	0.00
TRINITY_sp|Q4057	AOX2	Ubiquinol oxidase 2	55.90	0.00
TRINITY_sp|P3289	IMP3	U3 small nucleolar	55.90	0.00
TRINITY_sp|P9323	-	Zeaxanthin epoxidas	55.90	0.00
TRINITY_sp|Q5R4I	XRN2	5'-3' exoribonuclea	55.90	0.00
TRINITY_sp|Q8PFE	dusA	tRNA-dihydrouridine	55.90	0.00
TRINITY_sp|Q9FM(	MPT3	Mitochondrial phosp	55.90	0.00
TRINITY_sp|O1815	ogt-1	UDP-N-acetylglucosa	55.90	0.00
TRINITY_sp|P4022	CKB1	Casein kinase II su	55.90	0.00
TRINITY_sp|Q93V(	RPS1	30S ribosomal prote	55.90	0.00
TRINITY_sp|Q54X8	ap1b1	AP-1 complex subuni	55.90	0.00
TRINITY_sp|Q8DIE	hemC	Porphobilinogen dea	55.90	0.00
TRINITY_sp|Q6P8Y	Capsl	Calcyphosin-like pr	55.90	0.00
TRINITY_sp|Q9FR3	AMI1	Amidase 1 OS=Arabid	55.90	0.00
TRINITY_sp|Q550(	rheb	GTP-binding protein	55.90	0.00
TRINITY_sp|Q5R63	RPF1	Ribosome production	55.90	0.00
TRINITY_sp|Q8H18	PYD3	Beta-ureidopropiona	55.90	0.00
TRINITY_sp|G0S8(	CTHT_002	Eukaryotic translat	55.90	0.00
TRINITY_sp|O0453	SNL4	Paired amphipathic	55.90	0.00
TRINITY_sp|Q336M	CDKE-1	Cyclin-dependent ki	55.90	0.00
TRINITY_sp|Q2QM4	BSL2	Serine/threonine-pr	55.90	0.00
TRINITY_sp|P2818	RABD2A	Ras-related protein	55.90	0.00
TRINITY_sp|Q9SHE	PSAF	Photosystem I react	55.80	0.00

TRINITY_sp|P409	Arf102F	ADP-ribosylation fa	55.80	0.00
TRINITY_sp|Q55G\	pakC	Serine/threonine-pr	55.80	0.00
TRINITY_sp|O741!	UBC1	Ubiquitin-conjugati	55.80	0.00
TRINITY_sp|Q6NP	HRD1B	ERAD-associated E3	55.80	0.00
TRINITY_sp|Q5TA(	DCAF8	DDB1- and CUL4-asso	55.80	0.00
TRINITY_sp|P519	Nek1	Serine/threonine-pr	55.80	0.00
TRINITY_sp|O744(	dps1	Aspartate--tRNA lig	55.80	0.00
TRINITY_sp|Q23H	CFAP206	Cilia- and flagella	55.80	0.00
TRINITY_sp|O611	svkA	Serine/threonine-pr	55.80	0.00
TRINITY_sp|Q9SY	BRR2A	DExH-box ATP-depend	55.80	0.00
TRINITY_sp|Q54K	mccA	Methylcrotonoyl-CoA	55.80	0.00
TRINITY_sp|P425	erkA	Extracellular signa	55.80	0.00
TRINITY_sp|Q8DJ	clpB1	Chaperone protein C	55.80	0.00
TRINITY_sp|P0C8	CCRP1	Probable serine/thr	55.80	0.00
TRINITY_sp|B0TE	efp	Elongation factor P	55.80	0.00
TRINITY_sp|P548	hgsA	Hydroxymethylglutar	55.80	0.00
TRINITY_sp|Q54J	purB	Adenylosuccinate ly	55.80	0.00
TRINITY_sp|Q8UV	brix1	Ribosome biogenesis	55.80	0.00
TRINITY_sp|A4S6	OSTLU_4	Lon protease homolo	55.80	0.00
TRINITY_sp|O221	GCP1	Probable tRNA N6-ad	55.80	0.00
TRINITY_sp|Q8VZ	GEP	Probable glutamyl e	55.80	0.00
TRINITY_sp|Q9C7	LACS3	Long chain acyl-CoA	55.80	0.00
TRINITY_sp|Q9UR\	plg7	Putative phospholip	55.80	0.00
TRINITY_sp|O760	CDKL5	Cyclin-dependent ki	55.80	0.00
TRINITY_sp|O230	ECA2	Calcium-transportin	55.80	0.00
TRINITY_sp|P527	-	Glutamine--tRNA lig	55.80	0.00
TRINITY_sp|Q631	Dnah1	Dynein heavy chain	55.80	0.00
TRINITY_sp|Q3E9(	CPK34	Calcium-dependent p	55.80	0.00
TRINITY_sp|Q557	dpp3-1	Dipeptidyl peptidas	55.80	0.00
TRINITY_sp|P426	-	14-3-3 protein 1 OS	55.70	0.00
TRINITY_sp|Q86C	tor	Target of rapamycin	55.70	0.00
TRINITY_sp|Q9VY	Coq5	2-methoxy-6-polypre	55.70	0.00
TRINITY_sp|Q6PB(	rpl35	60S ribosomal prote	55.70	0.00
TRINITY_sp|Q953	MANBA	Beta-mannosidase OS	55.70	0.00
TRINITY_sp|Q54N	pex12	Putative peroxisome	55.70	0.00
TRINITY_sp|Q54I	DDB_G02	WASH complex subuni	55.70	0.00
TRINITY_sp|O499	-	14-3-3-like protein	55.70	0.00
TRINITY_sp|Q8LG	ABCC8	ABC transporter C f	55.70	0.00
TRINITY_sp|Q9ZE	ogt	Methylated-DNA--pro	55.70	0.00
TRINITY_sp|B0TC	rplQ	50S ribosomal prote	55.70	0.00
TRINITY_sp|Q56Y	PLR1	Pyridoxal reductase	55.70	0.00
TRINITY_sp|Q9FX	RFC2	Replication factor	55.70	0.00
TRINITY_sp|Q3UM(	Spata5	Spermatogenesis-ass	55.70	0.00
TRINITY_sp|Q54E	sptB	Serine palmitoyltra	55.70	0.00
TRINITY_sp|Q6P8	Alg8	Probable dolichyl p	55.70	0.00
TRINITY_sp|O942	nop2	25S rRNA (cytosine-	55.70	0.00
TRINITY_sp|Q8RC	psuG	Pseudouridine-5'-ph	55.70	0.00
TRINITY_sp|Q9NW(	POLR3B	DNA-directed RNA po	55.70	0.00
TRINITY_sp|O154	MPPED1	Metallophosphoester	55.70	0.00
TRINITY_sp|Q021	PAT1	Anthranilate phosph	55.70	0.00
TRINITY_sp|Q94B	ISPH	4-hydroxy-3-methylb	55.70	0.00
TRINITY_sp|A0AV	UBA6	Ubiquitin-like modi	55.70	0.00
TRINITY_sp|Q54L	vps13A	Putative vacuolar p	55.70	0.00
TRINITY_sp|Q8H0	At5g234	Katanin p80 WD40 re	55.70	0.00
TRINITY_sp|Q6NV\	CDPF1	Cysteine-rich DPF m	55.70	0.00
TRINITY_sp|Q5XI(	Galk2	N-acetylgalactosami	55.70	0.00

TRINITY_sp|Q9FJ'	TOP1B	DNA topoisomerase 1	55.70	0.00
TRINITY_sp|Q86Z(	klp1	Kinesin heavy chain	55.70	0.00
TRINITY_sp|O860:	bdhA	D-beta-hydroxybutyr	55.70	0.00
TRINITY_sp|Q963(	SPY	Probable UDP-N-acet	55.70	0.00
TRINITY_sp|Q266!	-	Thioredoxin peroxid	55.60	0.00
TRINITY_sp|A5D7(	DDX52	Probable ATP-depend	55.60	0.00
TRINITY_sp|Q9C0(	DNAH6	Dynein heavy chain	55.60	0.00
TRINITY_sp|Q9ZQ!	MBR1	E3 ubiquitin-protei	55.60	0.00
TRINITY_sp|Q9LD(	DWF5	7-dehydrocholestero	55.60	0.00
TRINITY_sp|Q54X/	DDB_G02'	DEP domain-containi	55.60	0.00
TRINITY_sp|A8EX(	mnmG	tRNA uridine 5-carb	55.60	0.00
TRINITY_sp|Q94K'	At3g581	Phenylalanine--tRNA	55.60	0.00
TRINITY_sp|Q9FF(	UBC22	Ubiquitin-conjugati	55.60	0.00
TRINITY_sp|Q9SB!	AP4M	AP-4 complex subuni	55.60	0.00
TRINITY_sp|Q54DI	gcvT	Aminomethyltransfer	55.60	0.00
TRINITY_sp|Q944!	ubcB	Ubiquitin conjugati	55.60	0.00
TRINITY_sp|Q9NPI	UBE2T	Ubiquitin-conjugati	55.60	0.00
TRINITY_sp|Q9WV!	Mok	MAPK/MAK/MRK overla	55.60	0.00
TRINITY_sp|O141(	lys12	Homoisocitrate dehy	55.60	0.00
TRINITY_sp|Q5Z8'	Os06g05(	Probable inositol o	55.60	0.00
TRINITY_sp|P534'	CYIA	Actin, cytoskeletal	55.60	0.00
TRINITY_sp|Q54E!	rpc19	DNA-directed RNA po	55.60	0.00
TRINITY_sp|Q0VD4	VPS4B	Vacuolar protein so	55.60	0.00
TRINITY_sp|O753!	VPS4B	Vacuolar protein so	55.60	0.00
TRINITY_sp|Q9UQ(	MOK	MAPK/MAK/MRK overla	55.60	0.00
TRINITY_sp|Q926:	GCN1	eIF-2-alpha kinase	55.60	0.00
TRINITY_sp|P139!	-	G2/mitotic-specific	55.60	0.00
TRINITY_sp|P529(	-	14-3-3-like protein	55.60	0.00
TRINITY_sp|Q23M'	Ttll6a	Probable beta-tubul	55.60	0.00
TRINITY_sp|Q9SB!	AP4M	AP-4 complex subuni	55.60	0.00
TRINITY_sp|Q75M:	ML3	Protein MEI2-like 3	55.60	0.00
TRINITY_sp|P141!	AAC4	AAC-rich mRNA clone	55.60	0.00
TRINITY_sp|Q10M'	V2	Guanylate kinase 2,	55.60	0.00
TRINITY_sp|Q54GI	nsa2	Ribosome biogenesis	55.60	0.00
TRINITY_sp|Q54S/	sf3b3	Probable splicing f	55.60	0.00
TRINITY_sp|Q9SBI	ABC1	Protein ABC transpo	55.60	0.00
TRINITY_sp|Q275(	thyB	Thymidine kinase 1	55.60	0.00
TRINITY_sp|Q9SYI	RHM1	Trifunctional UDP-g	55.60	0.00
TRINITY_sp|Q9FLI	NRPB12	DNA-directed RNA po	55.60	0.00
TRINITY_sp|Q9P7)	rpa2	Probable DNA-direct	55.60	0.00
TRINITY_sp|Q9FSI	MD1	Malate dehydrogenas	55.60	0.00
TRINITY_sp|Q5VQ(	Os06g01	Translation factor	55.60	0.00
TRINITY_sp|P548(	P5CSB	Delta-1-pyrroline-5	55.60	0.00
TRINITY_sp|Q3V0(	Dnah12	Dynein heavy chain	55.60	0.00
TRINITY_sp|Q9ZQ!	MBR1	E3 ubiquitin-protei	55.60	0.00
TRINITY_sp|Q9D7I	Acad8	Isobutyryl-CoA dehy	55.60	0.00
TRINITY_sp|Q7X8:	GATA	Glutamyl-tRNA(Gln)	55.60	0.00
TRINITY_sp|Q54T(	rio2	Serine/threonine-pr	55.60	0.00
TRINITY_sp|O598!	gpx1	Glutathione peroxid	55.60	0.00
TRINITY_sp|Q9C6V	ABCG14	ABC transporter G f	55.60	0.00
TRINITY_sp|P427:	CLPB1	Chaperone protein C	55.60	0.00
TRINITY_sp|Q8NBI	RDH13	Retinol dehydrogena	55.50	0.00
TRINITY_sp|D4B0\	ARB_020'	Probable glucan end	55.50	0.00
TRINITY_sp|P039(	rasD	Ras-like protein ra	55.50	0.00
TRINITY_sp|Q631:	Abcc2	Canalicular multisp	55.50	0.00
TRINITY_sp|P372(	dis3	Exosome complex exo	55.50	0.00

TRINITY_sp|O226(	DEGP1	Protease Do-like 1,	55.50	0.00
TRINITY_sp|P499:	Ctsh	Pro-cathepsin H OS=	55.50	0.00
TRINITY_sp|Q8BW'	Kdm4a	Lysine-specific dem	55.50	0.00
TRINITY_sp|Q8BUI	Txnl4b	Thioredoxin-like pr	55.50	0.00
TRINITY_sp|Q6ZHI	Os02g07'	Putative D-cysteine	55.50	0.00
TRINITY_sp|P919:	-	ADP-ribosylation fa	55.50	0.00
TRINITY_sp|P328!	IMP3	U3 small nucleolar	55.50	0.00
TRINITY_sp|Q436:	-	Glutathione reducta	55.50	0.00
TRINITY_sp|Q007(	glpV	Glycogen phosphoryl	55.50	0.00
TRINITY_sp|Q7SYI	qtrt1	Queuine tRNA-ribosy	55.50	0.00
TRINITY_sp|P247!	ATP1A2	Sodium/potassium-tr	55.50	0.00
TRINITY_sp|A8IEI	PRMT1	Protein arginine N-	55.50	0.00
TRINITY_sp|P312!	UBA2	Ubiquitin-activatin	55.50	0
TRINITY_sp|O650{	PBC1	Proteasome subunit	55.50	0.00
TRINITY_sp|Q96I:	PYURF	Protein preY, mitoc	55.40	0.00
TRINITY_sp|P403!	RIC1	Ras-related protein	55.40	0.00
TRINITY_sp|P406:	F01G4.6	Phosphate carrier p	55.40	0.00
TRINITY_sp|P365!	rpb6	DNA-directed RNA po	55.40	0.00
TRINITY_sp|Q917!	gsk3b	Glycogen synthase k	55.40	0.00
TRINITY_sp|P132!	PCT1	Choline-phosphate c	55.40	0.00
TRINITY_sp|Q54X(	brk1	Protein BRICK1 OS=D	55.40	0.00
TRINITY_sp|Q552V	cchl	Probable cytochrome	55.40	0.00
TRINITY_sp|P551<	-	Glutaredoxin OS=Ric	55.40	0.00
TRINITY_sp|P176:	fgg	Fibrinogen gamma ch	55.40	0.00
TRINITY_sp|Q7T3:	ccdc25	Coiled-coil domain-	55.40	0.00
TRINITY_sp|P341:	mvpA	Major vault protein	55.40	0.00
TRINITY_sp|Q059(	GRP10	Glycine-rich RNA-bi	55.40	0.00
TRINITY_sp|Q5RG:	gnptab	N-acetylglucosamine	55.40	0.00
TRINITY_sp|P128(	-	Triosephosphate iso	55.40	0.00
TRINITY_sp|Q8LPI	FZR3	Protein FIZZY-RELAT	55.40	0.00
TRINITY_sp|P114'	PSBP	Oxygen-evolving enh	55.40	0.00
TRINITY_sp|B6JK>	acsA	Acetyl-coenzyme A s	55.40	0.00
TRINITY_sp|A4II:	ttc26	Intraflagellar tran	55.40	0.00
TRINITY_sp|A2XY\	PRMT6.1	Probable protein ar	55.40	0.00
TRINITY_sp|Q54RI	DDB_G02{	Delta-1-pyrroline-5	55.40	0.00
TRINITY_sp|B9RA:	RCOM_15(	Probable aspartyl a	55.40	0.00
TRINITY_sp|Q1JP>	farsa	Phenylalanine--tRNA	55.40	0.00
TRINITY_sp|P0AF(	mog	Molybdopterin adeny	55.40	0.00
TRINITY_sp|Q9LYI	FKBP19	Peptidyl-prolyl cis	55.40	0.00
TRINITY_sp|O677:	asd	Aspartate-semialdeh	55.40	0.00
TRINITY_sp|O008:	repB	TFIIH basal transcr	55.40	0.00
TRINITY_sp|Q134:	SF3B2	Splicing factor 3B	55.40	0.00
TRINITY_sp|Q5RF(	ALDH2	Aldehyde dehydrogen	55.40	0.00
TRINITY_sp|Q2QM(	MCCA	Methylcrotonoyl-CoA	55.40	0.00
TRINITY_sp|P334:	SOD1	Superoxide dismutas	55.40	0.00
TRINITY_sp|O044{	UCH2	Ubiquitin carboxyl-	55.40	0.00
TRINITY_sp|Q9ZR:	RH28	DEAD-box ATP-depend	55.40	0.00
TRINITY_sp|P459!	ARP	DNA-(apurinic or ap	55.40	0.00
TRINITY_sp|P0DJ:	RPL18A	60S ribosomal prote	55.30	0.00
TRINITY_sp|Q020!	rad3	Protein kinase rad3	55.30	0.00
TRINITY_sp|Q23M:	Ttll6a	Probable beta-tubul	55.30	0.00
TRINITY_sp|Q9LZI	RABE1D	Ras-related protein	55.30	0.00
TRINITY_sp|Q219<	R11F4.1	Probable glycerol k	55.30	0.00
TRINITY_sp|Q9US(	klp3	Kinesin-like protei	55.30	0.00
TRINITY_sp|Q9SZI	At4g291:	Probable 3-hydroxyi	55.30	0.00
TRINITY_sp|Q86A'	cpsf3	Cleavage and polyad	55.30	0.00

TRINITY_sp|P0C8N	CCRP1	Probable serine/thr	55.30	0.00
TRINITY_sp|Q940I	SRK2E	Serine/threonine-pr	55.30	0.00
TRINITY_sp|Q554{	slr0537	Uncharacterized sug	55.30	0.00
TRINITY_sp|Q54D(	comG	Aldehyde dehydrogen	55.30	0.00
TRINITY_sp|Q8WX)	DNAH7	Dynein heavy chain	55.30	0.00
TRINITY_sp|A5N6N	dnaJ	Chaperone protein D	55.30	0.00
TRINITY_sp|Q6S0(	kif6	Kinesin-related pro	55.30	0.00
TRINITY_sp|Q9LJI	M3KE1	MAP3K epsilon prote	55.30	0.00
TRINITY_sp|O2362	At1g298{	Glycine--tRNA ligas	55.30	0.00
TRINITY_sp|Q9FX2	STT3B	Dolichyl-diphosphoo	55.30	0.00
TRINITY_sp|P056{	CTSZ	Cathepsin Z OS=Bos	55.30	0.00
TRINITY_sp|Q55B.	sod2	Superoxide dismutas	55.30	0.00
TRINITY_sp|O353'	Abcc1	Multidrug resistanc	55.30	0.00
TRINITY_sp|Q39VI	mutS	DNA mismatch repair	55.30	0.00
TRINITY_sp|Q917!	gsk3b	Glycogen synthase k	55.30	0.00
TRINITY_sp|A2E3(	ALG5D	Dolichyl-phosphate	55.20	0.00
TRINITY_sp|Q9VD!	CG6353	Protein archease-li	55.20	0.00
TRINITY_sp|Q54NI	derl2	Probable derlin-2 h	55.20	0.00
TRINITY_sp|Q55G\	ChLim	Calponin homology a	55.20	0.00
TRINITY_sp|Q9SZ:	HISN4	Imidazole glycerol	55.20	0.00
TRINITY_sp|Q91VI	Ndufs1	NADH-ubiquinone oxi	55.20	0.00
TRINITY_sp|Q3MFI	rpsQ	30S ribosomal prote	55.20	0.00
TRINITY_sp|P3952	DRS2	Probable phospholip	55.20	0.00
TRINITY_sp|P265(	At2g3062	Histone H1.2 OS=Ara	55.20	0.00
TRINITY_sp|Q9FGI	PUB50	Putative U-box doma	55.20	0.00
TRINITY_sp|Q112(	PARP2	Poly [ADP-ribose] p	55.20	0.00
TRINITY_sp|Q54II	ndrB	Probable serine/thr	55.20	0.00
TRINITY_sp|F4IHS	SYD	Chromatin structure	55.20	0.00
TRINITY_sp|Q097!	pan2	PAB-dependent poly(	55.20	0.00
TRINITY_sp|Q9Y8:	ubc15	Ubiquitin-conjugati	55.20	0.00
TRINITY_sp|P266	VARS	Valine--tRNA ligase	55.20	0.00
TRINITY_sp|Q8W4I	CYP71	Peptidyl-prolyl cis	55.20	0.00
TRINITY_sp|Q27II	KIN12D	Kinesin-like protei	55.20	0.00
TRINITY_sp|P283	acuE	Malate synthase, gl	55.20	0.00
TRINITY_sp|Q54NI	gpn3	GPN-loop GTPase 3 O	55.20	0.00
TRINITY_sp|Q84W\	SHM7	Serine hydroxymethy	55.20	0.00
TRINITY_sp|Q52QU	AIL6	AP2-like ethylene-r	55.20	0.00
TRINITY_sp|Q6PB\	rps3a	40S ribosomal prote	55.20	0.00
TRINITY_sp|Q9LY(	AK1	Aspartokinase 1, ch	55.20	0.00
TRINITY_sp|P2432	al-3	Geranylgeranyl pyro	55.20	0.00
TRINITY_sp|Q9SZI	ABCG9	ABC transporter G f	55.20	0.00
TRINITY_sp|Q102!	SPAC56F{	Eukaryotic translat	55.20	0.00
TRINITY_sp|A2Y9N	OsI_021(	Soluble starch synt	55.20	0.00
TRINITY_sp|Q0VD(	IMP4	U3 small nucleolar	55.20	0.00
TRINITY_sp|P566!	KNOX1	Homeobox protein kn	55.20	0.00
TRINITY_sp|Q8VCV	Acsf2	Acyl-CoA synthetase	55.20	0.00
TRINITY_sp|F4HY.	At1g486!	DExH-box ATP-depend	55.20	0.00
TRINITY_sp|Q8WX)	DNAH7	Dynein heavy chain	55.20	0.00
TRINITY_sp|P872(	RPL34A	60S ribosomal prote	55.20	0.00
TRINITY_sp|O947:	ALG2	Alpha-1,3/1,6-manno	55.20	0.00
TRINITY_sp|Q9Y8(	TOP2	DNA topoisomerase 2	55.10	0.00
TRINITY_sp|A6Q4{	dnaJ	Chaperone protein D	55.10	0.00
TRINITY_sp|F4IG'	BCHC2	BEACH domain-contai	55.10	0.00
TRINITY_sp|F4IS(	At2g320(	DNA topoisomerase 3	55.10	0.00
TRINITY_sp|Q920I	Mlycd	Malonyl-CoA decarbo	55.10	0.00
TRINITY_sp|Q101(	SPAC26A:	Hydrolase C26A3.11	55.10	0.00

TRINITY_sp|Q86WA	SLC26A1	Sodium-independent	55.10	0.00
TRINITY_sp|Q92SI	ubiE	Ubiquinone/menaquin	55.10	0.00
TRINITY_sp|Q54WI	gna1	Glucosamine 6-phosp	55.10	0.00
TRINITY_sp|Q550I	DDB_G02	Probable serine/thr	55.10	0.00
TRINITY_sp|P4252	erkA	Extracellular signa	55.10	0.00
TRINITY_sp|Q54W	metap1	Methionine aminopep	55.10	0.00
TRINITY_sp|Q55E	gfm1	Elongation factor G	55.10	0.00
TRINITY_sp|Q9XI	ISCA	Iron-sulfur assembl	55.10	0.00
TRINITY_sp|Q54JI	abcC3	ABC transporter C f	55.10	0.00
TRINITY_sp|Q9FI	AGD9	Probable ADP-ribosy	55.10	0.00
TRINITY_sp|Q54NI	gpn3	GPN-loop GTPase 3 O	55.10	0.00
TRINITY_sp|Q54TI	DDB_G02	Coiled-coil domain-	55.10	0.00
TRINITY_sp|Q103	SPAC17G	Inositol phosphocer	55.10	0.00
TRINITY_sp|Q8WX	DNAH7	Dynein heavy chain	55.10	0.00
TRINITY_sp|Q54U	abcC12	ABC transporter C f	55.10	0.00
TRINITY_sp|Q1EC	tlk2	Serine/threonine-pr	55.10	0.00
TRINITY_sp|Q6TU	malA	NADP-dependent mali	55.10	0.00
TRINITY_sp|Q56Y	NADK1	NAD(H) kinase 1 OS=	55.10	0.00
TRINITY_sp|Q9C5	PFK7	ATP-dependent 6-pho	55.10	0.00
TRINITY_sp|Q5PN	AMSH3	AMSH-like ubiquitin	55.10	0.00
TRINITY_sp|A8IR	CHLREDR	Ribosome biogenesis	55.10	0.00
TRINITY_sp|Q445	nifS1	Cysteine desulfuras	55.10	0.00
TRINITY_sp|Q557	slr0074	UPF0051 protein slr	55.10	0.00
TRINITY_sp|Q9M0	ABCB9	ABC transporter B f	55.10	0.00
TRINITY_sp|Q9SG	At1g725	Phenylalanine--tRNA	55.10	0.00
TRINITY_sp|Q8C5	Cwc22	Pre-mRNA-splicing f	55.10	0.00
TRINITY_sp|P930	RH21	DEAD-box ATP-depend	55.10	0.00
TRINITY_sp|Q2QP	GK1	Guanylate kinase 1	55.10	0.00
TRINITY_sp|Q8RW	CCB3	Protein COFACTOR AS	55.10	0.00
TRINITY_sp|Q8BI	Dyrk4	Dual specificity ty	55.10	0.00
TRINITY_sp|P534	ACT7	Actin-7 OS=Arabidop	55.10	0.00
TRINITY_sp|Q9DB	Mcts1	Malignant T-cell-am	55.10	0.00
TRINITY_sp|Q071	Suox	Sulfite oxidase, mi	55.10	0.00
TRINITY_sp|Q94L	HOS66	Homeobox protein kn	55.10	0.00
TRINITY_sp|Q9VH	Coq2	4-hydroxybenzoate p	55.10	0.00
TRINITY_sp|Q55F	DDB_G02	Uncharacterized pro	55.00	0.00
TRINITY_sp|Q54R	mkkA	Mitogen-activated p	55.00	0.00
TRINITY_sp|Q55B	ddx49	Probable ATP-depend	55.00	0.00
TRINITY_sp|Q8L3	FZR2	Protein FIZZY-RELAT	55.00	0.00
TRINITY_sp|Q54Q	yipf5	Protein YIPF5 homol	55.00	0.00
TRINITY_sp|Q52J	VIP2	Probable NOT transc	55.00	0.00
TRINITY_sp|Q55F	rab6	Ras-related protein	55.00	0.00
TRINITY_sp|Q9Y2	UBE2D4	Ubiquitin-conjugati	55.00	0.00
TRINITY_sp|O421	apoa1	Apolipoprotein A-I	55.00	0.00
TRINITY_sp|P027	FN1	Fibronectin OS=Homo	55.00	0.00
TRINITY_sp|P341	myoD	Myosin ID heavy cha	55.00	0.00
TRINITY_sp|Q93W	NIFU1	NifU-like protein 1	55.00	0.00
TRINITY_sp|Q54P	pwp2	Periodic tryptophan	55.00	0.00
TRINITY_sp|P200	pyr1-3	Protein PYR1-3 OS=D	55.00	0.00
TRINITY_sp|Q6NL	ABCC2	ABC transporter D f	55.00	0.00
TRINITY_sp|Q54I	mycbp	C-Myc-binding prote	55.00	0.00
TRINITY_sp|Q2RA	CPK25	Calcium-dependent p	55.00	0.00
TRINITY_sp|Q554	aslB	Acyl-CoA synthetase	55.00	0.00
TRINITY_sp|O139	crn1	Coronin-like protei	55.00	0.00
TRINITY_sp|Q0DH	FTSH8	ATP-dependent zinc	55.00	0.00
TRINITY_sp|Q6ST	HCF101	Fe-S cluster assemb	55.00	0.00

TRINITY_sp|Q8L7I	HSK	Homoserine kinase O	55.00	0.00
TRINITY_sp|Q1LZ9	IDI1	Isopentenyl-diphosp	55.00	0.00
TRINITY_sp|F4KIA	At5g108(	Protein RRC1-like O	55.00	0.00
TRINITY_sp|Q429I	-	Glucose-6-phosphate	55.00	0.00
TRINITY_sp|A5D8N	sbds	Ribosome maturation	55.00	0.00
TRINITY_sp|O226(	DEGP1	Protease Do-like 1,	55.00	0.00
TRINITY_sp|Q54GI	ctdspl2	CTD small phosphata	55.00	0.00
TRINITY_sp|P025	-	Actin-1 OS=Acantham	55.00	0.00
TRINITY_sp|F4IUX	UPF2	Regulator of nonsen	55.00	0.00
TRINITY_sp|B0S6(	eral1	GTPase Era, mitocho	55.00	0.00
TRINITY_sp|Q4239	CPK12	Calcium-dependent p	55.00	0.00
TRINITY_sp|A8J63	SULTR2	Proton/sulfate cotr	55.00	0.00
TRINITY_sp|A5H03	DUR1,2	Urea amidolyase OS=	55.00	0.00
TRINITY_sp|P3642	ALATS	Alanine--tRNA ligas	55.00	0.00
TRINITY_sp|Q9SGU	MGL	Methionine gamma-ly	55.00	0.00
TRINITY_sp|Q9STI	VPS29	Vacuolar protein so	55.00	0.00
TRINITY_sp|A4FV	PPIE	Peptidyl-prolyl cis	55.00	0.00
TRINITY_sp|O049(	UMK3	UMP-CMP kinase 3 OS	54.90	0.00
TRINITY_sp|Q86J3	trmt61a	tRNA (adenine(58)-N	54.90	0.00
TRINITY_sp|Q8JI3	-	Type-4 ice-structur	54.90	0.00
TRINITY_sp|Q9KVI	lysA	Diaminopimelate dec	54.90	0.00
TRINITY_sp|Q66KY	cuta	Protein CutA homolo	54.90	0.00
TRINITY_sp|Q1339	TARBP1	Probable methyltran	54.90	0.00
TRINITY_sp|Q9FIV	DEGP10	Protease Do-like 10	54.90	0.00
TRINITY_sp|Q9C52	TIF3H1	Eukaryotic translat	54.90	0.00
TRINITY_sp|O2214	GCP1	Probable tRNA N6-ad	54.90	0.00
TRINITY_sp|B0R0I	si:dkey-	von Willebrand fact	54.90	0.00
TRINITY_sp|Q9TVI	cdk-9	Probable cyclin-dep	54.90	0.00
TRINITY_sp|Q7KWN	iunH	Inosine-uridine-pre	54.90	0.00
TRINITY_sp|Q7YZN	erf3	Eukaryotic peptide	54.90	0.00
TRINITY_sp|Q96N(	ZMAT2	Zinc finger matrin-	54.90	0.00
TRINITY_sp|Q0JCU	ZEP	Zeaxanthin epoxidas	54.90	0.00
TRINITY_sp|Q94BU	At1g718I	Uncharacterized aar	54.90	0.00
TRINITY_sp|P197(	MIB	Myosin heavy chain	54.90	0.00
TRINITY_sp|Q2KJ(	CDC5L	Cell division cycle	54.90	0.00
TRINITY_sp|Q9ZS9	OMR1	Threonine dehydrata	54.90	0.00
TRINITY_sp|Q32L9	CNN3	Calponin-3 OS=Bos t	54.90	0.00
TRINITY_sp|P0C03	RUB3	Ubiquitin-like prot	54.90	0.00
TRINITY_sp|Q9AT3	RPL23A	60S ribosomal prote	54.90	0.00
TRINITY_sp|Q8RCI	psuG	Pseudouridine-5'-ph	54.90	0.00
TRINITY_sp|Q7DMA	PAS1	Peptidyl-prolyl cis	54.90	0.00
TRINITY_sp|M1CZ(	EBP1	ERBB-3 BINDING PROT	54.90	0.00
TRINITY_sp|Q8W5I	KIN7D	Kinesin-like protei	54.90	0.00
TRINITY_sp|O8092	AGD7	ADP-ribosylation fa	54.90	0.00
TRINITY_sp|Q0VFV	kctd7	BTB/POZ domain-cont	54.90	0.00
TRINITY_sp|Q91V2	Abca7	ATP-binding cassett	54.90	0.00
TRINITY_sp|Q54JI	abcC3	ABC transporter C f	54.90	0.00
TRINITY_sp|P4228	Dbi	Acyl-CoA-binding pr	54.80	0.00
TRINITY_sp|Q95R(	CG11412	N-alpha-acetyltrans	54.80	0.00
TRINITY_sp|O8184	PUMP1	Mitochondrial uncou	54.80	0.00
TRINITY_sp|P3018	TOP2	DNA topoisomerase 2	54.80	0.00
TRINITY_sp|Q86BI	anapc8	Anaphase-promoting	54.80	0.00
TRINITY_sp|Q097(	pim1	Lon protease homolo	54.80	0.00
TRINITY_sp|Q2R2I	RFC1	Replication factor	54.80	0.00
TRINITY_sp|P527(	trs-1	Threonine--tRNA lig	54.80	0.00
TRINITY_sp|Q2PQI	AGL	Glycogen debranchin	54.80	0.00

TRINITY_sp|Q9ZQ\	U2AF35A	Splicing factor U2a	54.80	0.00
TRINITY_sp|Q54TI	gpn2	GPN-loop GTPase 2 h	54.80	0.00
TRINITY_sp|Q9SI2	RKP	E3 ubiquitin-protei	54.80	0.00
TRINITY_sp|P152	PCMT1	Protein-L-isoaspart	54.80	0.00
TRINITY_sp|Q9FMI	DIT2-1	Dicarboxylate trans	54.80	0.00
TRINITY_sp|Q6Q4	Atp2b4	Plasma membrane cal	54.80	0.00
TRINITY_sp|Q05B	shq1	Protein SHQ1 homolo	54.80	0.00
TRINITY_sp|Q6WW	UPL3	E3 ubiquitin-protei	54.80	0.00
TRINITY_sp|F4IV	CHR5	Protein CHROMATIN R	54.80	0.00
TRINITY_sp|Q631	Dnah1	Dynein heavy chain	54.80	0.00
TRINITY_sp|Q036	BIP4	Luminal-binding pro	54.80	0.00
TRINITY_sp|Q6NY	slc25a2	Calcium-binding mit	54.80	0.00
TRINITY_sp|B3PD	tal	Transaldolase OS=Ce	54.80	0.00
TRINITY_sp|P0C8	CCRP1	Probable serine/thr	54.80	0.00
TRINITY_sp|Q80U	Xpo1	Exportin-1 OS=Rattu	54.80	0
TRINITY_sp|P283	SMARCA1	Probable global tra	54.80	0.00
TRINITY_sp|Q5DM	IFT172	Intraflagellar tran	54.80	0.00
TRINITY_sp|Q56X	AOX4	Ubiquinol oxidase 4	54.80	0.00
TRINITY_sp|Q408	EMB8	Embryogenesis-assoc	54.80	0.00
TRINITY_sp|Q84S	NOL	Chlorophyll(ide) b	54.80	0.00
TRINITY_sp|Q7F2	CSB	DNA excision repair	54.80	0.00
TRINITY_sp|Q9BX	GTPBP2	GTP-binding protein	54.80	0.00
TRINITY_sp|O154	ABCC4	Multidrug resistanc	54.80	0.00
TRINITY_sp|Q28G	tma7	Translation machine	54.70	0.00
TRINITY_sp|Q9M5	PEAMT	Phosphoethanolamine	54.70	0.00
TRINITY_sp|Q9UP	CEP164	Centrosomal protein	54.70	0.00
TRINITY_sp|Q5SI	TTHA156	DNA base-flipping p	54.70	0.00
TRINITY_sp|Q54I	mycbp	C-Myc-binding prote	54.70	0.00
TRINITY_sp|Q015	facA	Acetyl-coenzyme A s	54.70	0.00
TRINITY_sp|Q54D	DDB_G02	Down syndrome criti	54.70	0.00
TRINITY_sp|Q86A	vamp7B	Vesicle-associated	54.70	0.00
TRINITY_sp|O819	-	Serine--tRNA ligase	54.70	0.00
TRINITY_sp|Q6K1	CAX3	Vacuolar cation/pro	54.70	0.00
TRINITY_sp|P321	PRDX2	Peroxiredoxin-2 OS=	54.70	0.00
TRINITY_sp|Q8H1	NBP35	Cytosolic Fe-S clus	54.70	0.00
TRINITY_sp|P312	pab1	Polyadenylate-bindi	54.70	0.00
TRINITY_sp|Q9P7	SPBC170	Probable mitochondr	54.70	0.00
TRINITY_sp|Q9UV	MRE11	Double-strand break	54.70	0.00
TRINITY_sp|P170	crtD	Hydroxyneurosporene	54.70	0.00
TRINITY_sp|Q940	CCT2	T-complex protein 1	54.70	0.00
TRINITY_sp|Q9C8	FAS4	ATP-dependent RNA h	54.70	0.00
TRINITY_sp|P078	Acaa1b	3-ketoacyl-CoA thio	54.70	0.00
TRINITY_sp|O351	Copb2	Coatomer subunit be	54.70	0.00
TRINITY_sp|Q9SY	TPS1	Alpha,alpha-trehalo	54.70	0.00
TRINITY_sp|Q54Y	ileS	Probable isoleucine	54.70	0
TRINITY_sp|P343	ced-7	ABC transporter ced	54.70	0.00
TRINITY_sp|P150	rasG	Ras-like protein ra	54.70	0.00
TRINITY_sp|Q67X	CBSDUF1	DUF21 domain-contai	54.70	0.00
TRINITY_sp|P402	hhp2	Casein kinase I hom	54.70	0.00
TRINITY_sp|O756	SNRNP20	U5 small nuclear ri	54.70	0
TRINITY_sp|P489	-	Beta-galactosidase	54.60	0.00
TRINITY_sp|Q141	MORC3	MORC family CW-type	54.60	0.00
TRINITY_sp|O809	PGPS1	CDP-diacylglycerol-	54.60	0.00
TRINITY_sp|Q929	BRF1	Transcription facto	54.60	0.00
TRINITY_sp|Q93Y	UBP9	Ubiquitin carboxyl-	54.60	0.00
TRINITY_sp|Q552	cct8	T-complex protein 1	54.60	0.00

TRINITY_sp|Q4P5(	DBP4	ATP-dependent RNA h	54.60	0.00
TRINITY_sp|Q9D3(	Pus10	Putative tRNA pseud	54.60	0.00
TRINITY_sp|Q8H1:	RH14	DEAD-box ATP-depend	54.60	0.00
TRINITY_sp|Q9LVI	TOP3A	DNA topoisomerase 3	54.60	0.00
TRINITY_sp|Q9CY2	Wbscr22	Probable 18S rRNA (	54.60	0.00
TRINITY_sp|P513!	ycf65	Probable 30S riboso	54.60	0.00
TRINITY_sp|Q2VY]	hslV	ATP-dependent prote	54.60	0.00
TRINITY_sp|O6012	SPBC16G!	Uncharacterized pro	54.60	0.00
TRINITY_sp|B4M3	GJ19011	NFU1 iron-sulfur cl	54.60	0.00
TRINITY_sp|Q949	EGY1	Probable zinc metal	54.60	0.00
TRINITY_sp|Q55EI	udkA	Uridine-cytidine ki	54.60	0.00
TRINITY_sp|Q9LUV	RH9	DEAD-box ATP-depend	54.60	0.00
TRINITY_sp|Q8W1	KIN14F	Kinesin-like protei	54.60	0.00
TRINITY_sp|Q9LD\	ALAAT2	Alanine aminotransf	54.60	0.00
TRINITY_sp|P2002	ATP2B1	Plasma membrane cal	54.50	0.00
TRINITY_sp|Q9EQI	Dpys	Dihydropyrimidinase	54.50	0.00
TRINITY_sp|Q8L6(	SIN2	Short integuments 2	54.50	0.00
TRINITY_sp|P531]	INO80	Putative DNA helica	54.50	0.00
TRINITY_sp|A0JN4	KIF3C	Kinesin-like protei	54.50	0.00
TRINITY_sp|P163	Galphao	G protein alpha o s	54.50	0.00
TRINITY_sp|P1302	acpB	F-actin-capping pro	54.50	0.00
TRINITY_sp|P433:	snf	U1 small nuclear ri	54.50	0.00
TRINITY_sp|P607(	RAC3	Ras-related C3 botu	54.50	0.00
TRINITY_sp|P026	FGB	Fibrinogen beta cha	54.50	0.00
TRINITY_sp|P4282	LDJ2	DnaJ protein homolo	54.50	0.00
TRINITY_sp|Q7ZVI	sirt2	NAD-dependent prote	54.50	0.00
TRINITY_sp|B1Y82	nuoA	NADH-quinone oxidor	54.50	0.00
TRINITY_sp|P0AF(	ribC	Riboflavin synthase	54.50	0.00
TRINITY_sp|Q54B(	idhB	Isocitrate dehydrog	54.50	0.00
TRINITY_sp|Q9W6I	ube2ia	SUMO-conjugating en	54.50	0.00
TRINITY_sp|Q9C8I	ABCC11	ABC transporter C f	54.50	0.00
TRINITY_sp|Q84V	FAD12	Delta(12)-acyl-lipi	54.50	0.00
TRINITY_sp|Q9WV:	Cpq	Carboxypeptidase Q	54.50	0.00
TRINITY_sp|Q8L3	KAS	3-oxoacyl-[acyl-car	54.50	0.00
TRINITY_sp|Q8YNI	gcvP	Glycine dehydrogena	54.50	0.00
TRINITY_sp|O803(	RPL3	50S ribosomal prote	54.50	0.00
TRINITY_sp|P3784	FES1	Cytochrome b-c1 com	54.50	0.00
TRINITY_sp|Q0992	yakc	Aldo-keto reductase	54.50	0.00
TRINITY_sp|Q9XZI	-	Peroxisomal catalas	54.50	0.00
TRINITY_sp|Q6RF(	rps-21	40S ribosomal prote	54.50	0.00
TRINITY_sp|Q243(	Iswi	Chromatin-remodelin	54.50	0.00
TRINITY_sp|B0U62	rpsI	30S ribosomal prote	54.50	0.00
TRINITY_sp|Q3KQI	nubp1-A	Cytosolic Fe-S clus	54.50	0.00
TRINITY_sp|P539	Rab2a	Ras-related protein	54.50	0.00
TRINITY_sp|A7YWI	HAL	Histidine ammonia-l	54.50	0.00
TRINITY_sp|Q86AV	guaD	Guanine deaminase O	54.50	0.00
TRINITY_sp|Q9CAI	YDA	Mitogen-activated p	54.50	0.00
TRINITY_sp|O7482	esf1	Pre-rRNA-processing	54.50	0.00
TRINITY_sp|Q149N	SHPRH	E3 ubiquitin-protei	54.50	0.00
TRINITY_sp|Q8RWI	At3g266	Probable magnesium	54.50	0.00
TRINITY_sp|Q407	-	Putative glucose-6-	54.50	0.00
TRINITY_sp|P340	pkaC	cAMP-dependent prot	54.50	0.00
TRINITY_sp|Q9SQI	AMT1-3	Ammonium transporte	54.50	0.00
TRINITY_sp|Q631(	Dnah1	Dynein heavy chain	54.50	0.00
TRINITY_sp|A2YQ(	OsI_276	Pre-mRNA-splicing f	54.50	0.00
TRINITY_sp|Q395	ODA2	Dynein gamma chain,	54.50	0.00

TRINITY_sp|A6GY	dapF	Diaminopimelate epi	54.50	0.00
TRINITY_sp|P0DJ	RPL27	60S ribosomal prote	54.40	0.00
TRINITY_sp|Q631	Abcc2	Canalicular multisp	54.40	0.00
TRINITY_sp|Q54H	drkA	Probable serine/thr	54.40	0.00
TRINITY_sp|O763	dco	Discs overgrown pro	54.40	0.00
TRINITY_sp|Q6DJ	cars2	Probable cysteine--	54.40	0.00
TRINITY_sp|Q182	rap-1	Ras-related protein	54.40	0.00
TRINITY_sp|Q86K	DDB_G02	Probable GH family	54.40	0.00
TRINITY_sp|Q390	CKL11	Casein kinase 1-lik	54.40	0.00
TRINITY_sp|Q993	TY3B-G	Transposon Ty3-G Ga	54.40	0.00
TRINITY_sp|O075	yhdW	Putative glyceropho	54.40	0.00
TRINITY_sp|P361	-	Endoplasmin homolog	54.40	0.00
TRINITY_sp|Q99J	Mlycd	Malonyl-CoA decarbo	54.40	0.00
TRINITY_sp|B2GU	Man1b1	Endoplasmic reticul	54.40	0.00
TRINITY_sp|P103	ND6	NADH-ubiquinone oxi	54.40	0.00
TRINITY_sp|P072	URA2	Protein URA2 OS=Sac	54.40	0.00
TRINITY_sp|Q8WX	DNAH7	Dynein heavy chain	54.40	0.00
TRINITY_sp|P068	Myb	Transcriptional act	54.40	0.00
TRINITY_sp|P0DO	PpSQ1_0	3-sulfolactaldehyde	54.40	0.00
TRINITY_sp|Q6FC	ACIAD12	Adenine deaminase O	54.40	0.00
TRINITY_sp|O750	mcm7	DNA replication lic	54.40	0.00
TRINITY_sp|Q55G	cpnD	Copine-D OS=Dictyos	54.40	0.00
TRINITY_sp|O500	PARP2	Poly [ADP-ribose] p	54.40	0.00
TRINITY_sp|Q8WX	DNAH7	Dynein heavy chain	54.40	0.00
TRINITY_sp|Q6CQ	TAR1-A	Protein TAR1 OS=Klu	54.40	0.00
TRINITY_sp|P558	sgaA	Serine--glyoxylate	54.40	0.00
TRINITY_sp|P118	-	High mobility group	54.40	0.00
TRINITY_sp|Q9HC	XAB2	Pre-mRNA-splicing f	54.40	0.00
TRINITY_sp|Q9UE	FTSJ1	Putative tRNA (cyti	54.40	0.00
TRINITY_sp|P048	-	Actin, cytoplasmic	54.40	0.00
TRINITY_sp|Q7NH	mnmE	tRNA modification G	54.40	0.00
TRINITY_sp|Q55F	acrA	Adenylate cyclase,	54.40	0.00
TRINITY_sp|Q5FW	Tmem2	Transmembrane prote	54.40	0.00
TRINITY_sp|A8JF	CFAP65	Cilia- and flagella	54.40	0.00
TRINITY_sp|Q7SD	ccp-1	Cytochrome c peroxi	54.40	0.00
TRINITY_sp|P519	Nek1	Serine/threonine-pr	54.40	0.00
TRINITY_sp|O489	TATB	Sec-independent pro	54.40	0.00
TRINITY_sp|P785	FCA1	Cytosine deaminase	54.40	0.00
TRINITY_sp|Q5X3	dnaJ	Chaperone protein D	54.40	0.00
TRINITY_sp|Q9VW	l(1)G01	Probable isocitrate	54.30	0.00
TRINITY_sp|P469	RPE1	Ribulose-phosphate	54.30	0.00
TRINITY_sp|Q131	PRKAA1	5'-AMP-activated pr	54.30	0.00
TRINITY_sp|Q54I	arpA	Centractin OS=Dicty	54.30	0.00
TRINITY_sp|Q32P	DYNLRB2	Dynein light chain	54.30	0.00
TRINITY_sp|P619	DCAF7	DDB1- and CUL4-asso	54.30	0.00
TRINITY_sp|Q007	glpV	Glycogen phosphoryl	54.30	0.00
TRINITY_sp|O308	HD_0322	RutC family protein	54.30	0.00
TRINITY_sp|O952	MBD4	Methyl-CpG-binding	54.30	0.00
TRINITY_sp|Q997	ABCA3	ATP-binding cassett	54.30	0.00
TRINITY_sp|Q9FM	RD21B	Probable cysteine p	54.30	0.00
TRINITY_sp|Q6C5	ERT1-1	Transcription activ	54.30	0.00
TRINITY_sp|P347	ARF	ADP-ribosylation fa	54.30	0.00
TRINITY_sp|P296	CDKA-2	Cyclin-dependent ki	54.30	0.00
TRINITY_sp|Q1ZX	fah	Fumarylacetoacetase	54.30	0.00
TRINITY_sp|O627	SCP2	Non-specific lipid-	54.30	0.00
TRINITY_sp|Q5ZK	METTL14	N6-adenosine-methyl	54.30	0.00

TRINITY_sp|D2GXI	AGBL5	Cytosolic carboxype	54.30	0.00
TRINITY_sp|Q8YPV	lysS	Lysine--tRNA ligase	54.30	0.00
TRINITY_sp|P340S	pkaC	cAMP-dependent prot	54.30	0.00
TRINITY_sp|Q338I	GCN5	Histone acetyltrans	54.30	0.00
TRINITY_sp|O232	D2HGDH	D-2-hydroxyglutarat	54.30	0.00
TRINITY_sp|Q8WX	DNAH7	Dynein heavy chain	54.30	0.00
TRINITY_sp|Q554	slr0537	Uncharacterized sug	54.20	0.00
TRINITY_sp|P341(	pkgC	Protein kinase 3 OS	54.20	0.00
TRINITY_sp|P011(	V-MYB	Transforming protei	54.20	0.00
TRINITY_sp|P056	sacC	Levanase OS=Bacillu	54.20	0.00
TRINITY_sp|Q8BV(	Dpp9	Dipeptidyl peptidas	54.20	0.00
TRINITY_sp|Q7ZV	pitrm1	Presequence proteas	54.20	0.00
TRINITY_sp|Q9LI	RBG4	Glycine-rich RNA-bi	54.20	0.00
TRINITY_sp|Q68J	LIPE	Hormone-sensitive l	54.20	0.00
TRINITY_sp|Q9UT	nnt1	Protein N-methyltra	54.20	0.00
TRINITY_sp|Q54U(	kif3	Kinesin-related pro	54.20	0.00
TRINITY_sp|O041	TAF10	Transcription initi	54.20	0.00
TRINITY_sp|P549(	PROC1	Pyrroline-5-carboxy	54.20	0.00
TRINITY_sp|A0BQI	GSPATT0(	Probable protein ph	54.20	0.00
TRINITY_sp|O046	NOP5-1	Probable nucleolar	54.20	0.00
TRINITY_sp|Q9XY	spsA	Spermidine synthase	54.20	0.00
TRINITY_sp|P428	ATPK1	Serine/threonine-pr	54.20	0.00
TRINITY_sp|A8XJ	cst-1	Serine/threonine-pr	54.20	0.00
TRINITY_sp|Q9LT	NMT1	Glycylpeptide N-tet	54.20	0.00
TRINITY_sp|P9WQ	treS	Trehalose synthase/	54.20	0.00
TRINITY_sp|Q54P	dph1	Diphthamide biosynt	54.20	0.00
TRINITY_sp|Q54M	gpt	Probable alanine am	54.20	0.00
TRINITY_sp|Q9NK	mfeA	Peroxisomal multifu	54.20	0.00
TRINITY_sp|Q9XF	LHCB5	Chlorophyll a-b bin	54.20	0.00
TRINITY_sp|Q556	rps30-1	40S ribosomal prote	54.20	0.00
TRINITY_sp|Q86JI	sec11	Signal peptidase co	54.20	0.00
TRINITY_sp|P546	pkbA	RAC family serine/t	54.20	0.00
TRINITY_sp|Q5VQ(	CPK2	Calcium-dependent p	54.20	0.00
TRINITY_sp|Q56J	RPL35A	60S ribosomal prote	54.20	0.00
TRINITY_sp|P407	Rac1	Ras-related protein	54.20	0.00
TRINITY_sp|Q67X(	GLB3	Two-on-two hemoglob	54.20	0.00
TRINITY_sp|Q430	Os06g02(	Asparagine syntheta	54.20	0.00
TRINITY_sp|Q9M2	OVA1	Methionine--tRNA li	54.20	0.00
TRINITY_sp|Q8VYI	PFK5	ATP-dependent 6-pho	54.20	0.00
TRINITY_sp|Q68R	IFT81	Intraflagellar tran	54.20	0.00
TRINITY_sp|Q8TA	RP9	Retinitis pigmentos	54.20	0.00
TRINITY_sp|Q9S7	SPL12	Squamosa promoter-b	54.20	0.00
TRINITY_sp|O498(	-	Glyoxysomal fatty a	54.20	0.00
TRINITY_sp|Q8L3	FZR2	Protein FIZZY-RELAT	54.20	0.00
TRINITY_sp|P727	typA	GTP-binding protein	54.20	0.00
TRINITY_sp|Q9LJI	PREP1	Presequence proteas	54.20	0.00
TRINITY_sp|W4VS	-	Acetylcholinesteras	54.20	0.00
TRINITY_sp|P109	Act87E	Actin-87E OS=Drosop	54.20	0.00

TRINITY_sp|Q6I5	SPL9	Squamosa promoter-b	54.20	0.00
TRINITY_sp|Q9I4	PA1195	N(G),N(G)-dimethyla	54.20	0.00
TRINITY_sp|Q5ZJ	FOPNL	LisH domain-contain	54.10	0.00
TRINITY_sp|Q54I	polr3b	DNA-directed RNA po	54.10	0.00
TRINITY_sp|A7HC	mutS	DNA mismatch repair	54.10	0.00
TRINITY_sp|Q8L3	FZR2	Protein FIZZY-RELAT	54.10	0.00
TRINITY_sp|Q9UD	DNAJB4	DnaJ homolog subfam	54.10	0.00
TRINITY_sp|F4HW	POL2A	DNA polymerase epsi	54.10	0.00
TRINITY_sp|P728	dus2	tRNA-dihydrouridine	54.10	0.00
TRINITY_sp|P919	ola-1	Obg-like ATPase 1 O	54.10	0.00
TRINITY_sp|Q84J	At2g392	Probable adenylate	54.10	0.00
TRINITY_sp|Q8TD	CHD6	Chromodomain-helica	54.10	0.00
TRINITY_sp|O608	EIF5B	Eukaryotic translat	54.10	0.00
TRINITY_sp|Q8W4	DDL	FHA domain-containi	54.10	0.00
TRINITY_sp|P002	-	Ferredoxin-1 OS=Des	54.10	0.00
TRINITY_sp|Q8CI	Copa	Coatomer subunit al	54.10	0.00
TRINITY_sp|Q949	PHL6	Myb family transcri	54.10	0.00
TRINITY_sp|P236	PREP	Prolyl endopeptidas	54.10	0.00
TRINITY_sp|Q1JP	farsa	Phenylalanine--tRNA	54.10	0.00
TRINITY_sp|Q9LN	TIM17-1	Mitochondrial impor	54.10	0.00
TRINITY_sp|Q54J	oxct1	Probable succinyl-C	54.10	0.00
TRINITY_sp|P286	CLKR27	3-oxoacyl-[acyl-car	54.10	0.00
TRINITY_sp|Q10R	Os03g01	DEAD-box ATP-depend	54.10	0.00
TRINITY_sp|B3Q1	thiM	Hydroxyethylthiazol	54.10	0.00
TRINITY_sp|Q0J0	Os09g05	Pantothenate kinase	54.10	0.00
TRINITY_sp|O605	EIF4E2	Eukaryotic translat	54.10	0.00
TRINITY_sp|Q54K	rab11B	Ras-related protein	54.10	0.00
TRINITY_sp|Q964	-	Actin, cytoplasmic	54.10	0.00
TRINITY_sp|P0C8	CCRP1	Probable serine/thr	54.10	0.00
TRINITY_sp|Q5XG	usp44-b	Ubiquitin carboxyl-	54.10	0.00
TRINITY_sp|Q057	THY-1	Bifunctional dihydr	54.10	0.00
TRINITY_sp|B6SF	MAA3	Probable helicase M	54.10	0.00
TRINITY_sp|Q9ZR	FH	Frataxin, mitochond	54.10	0.00
TRINITY_sp|Q680	At4g399	Haloacid dehalogena	54.10	0.00
TRINITY_sp|Q9XI	LACS2	Long chain acyl-CoA	54.00	0.00
TRINITY_sp|O949	TRIM37	E3 ubiquitin-protei	54.00	0.00
TRINITY_sp|P303	cdc25-1-	M-phase inducer pho	54.00	0.00
TRINITY_sp|Q8WU	PPIL4	Peptidyl-prolyl cis	54.00	0.00
TRINITY_sp|Q9H0	INTS2	Integrator complex	54.00	0.00
TRINITY_sp|Q9VF	CG5038	Transmembrane and T	54.00	0.00
TRINITY_sp|B3RM	TRIADDR	CDGSH iron-sulfur d	54.00	0.00
TRINITY_sp|P558	-	Probable 60S riboso	54.00	0.00
TRINITY_sp|F4JY	TCX7	Protein tesmin/TSO1	54.00	0.00
TRINITY_sp|Q803	sepsecs	O-phosphoseryl-tRNA	54.00	0.00
TRINITY_sp|Q6MD	tgt	Queuine tRNA-ribosy	54.00	0.00
TRINITY_sp|Q45F	-	Serine/threonine-pr	54.00	0.00
TRINITY_sp|O772	catA	Catalase-A OS=Dicty	54.00	0.00
TRINITY_sp|Q9FM	PI4KB1	Phosphatidylinosito	54.00	0.00
TRINITY_sp|P160	NIA1	Nitrate reductase [	54.00	0.00
TRINITY_sp|Q9W2	CG15309	Protein yippee-like	54.00	0.00
TRINITY_sp|Q088	H1-I	Histone H1-I OS=Vol	54.00	0.00
TRINITY_sp|Q7YS	canA	Calcineurin subunit	54.00	0.00
TRINITY_sp|Q552	pkgA	Probable serine/thr	54.00	0.00
TRINITY_sp|P546	patB	Probable plasma mem	54.00	0.00
TRINITY_sp|Q086	PUDP	Pseudouridine-5'-ph	54.00	0.00
TRINITY_sp|F1NT	ascc3	Activating signal c	54.00	0.00

TRINITY_sp|P462ː	-	Triosephosphate iso	54.00	0.00
TRINITY_sp|Q926ː	EDEM1	ER degradation-enha	54.00	0.00
TRINITY_sp|Q044ː	-	Glycine--tRNA ligas	54.00	0.00
TRINITY_sp|Q5KYː	GK2103	UPF0317 protein GK2	54.00	0.00
TRINITY_sp|P805ː	aro-8	Phospho-2-dehydro-3	54.00	0.00
TRINITY_sp|Q9C4ː	gyaR	Glyoxylate reductas	54.00	0.00
TRINITY_sp|Q146ː	BMS1	Ribosome biogenesis	54.00	0.00
TRINITY_sp|P286ː	SYNPCC7ː	Uncharacterized pro	54.00	0.00
TRINITY_sp|P936ː	LON1	Lon protease homolo	54.00	0.00
TRINITY_sp|P822ː	RRF	Ribosome-recycling	54.00	0.00
TRINITY_sp|Q9LFː	SMC5	Structural maintena	54.00	0.00
TRINITY_sp|Q6RCː	IFT74	Intraflagellar tran	54.00	0.00
TRINITY_sp|Q9BVː	KIFC3	Kinesin-like protei	54.00	0.00
TRINITY_sp|Q0WUː	SBT6.1	Subtilisin-like pro	54.00	0.00
TRINITY_sp|Q2HJː	DPH6	Diphthine--ammonia	54.00	0.00
TRINITY_sp|P275ː	LHCA4	Chlorophyll a-b bin	54.00	0.00
TRINITY_sp|P0DJː	RPL29	60S ribosomal prote	54.00	0.00
TRINITY_sp|O701ː	Abcb11	Bile salt export pu	53.90	0.00
TRINITY_sp|P207ː	rab8B	Ras-related protein	53.90	0.00
TRINITY_sp|P195ː	-	Probable reverse tr	53.90	0.00
TRINITY_sp|O818ː	PUMP1	Mitochondrial uncou	53.90	0.00
TRINITY_sp|Q9D8ː	Tex9	Testis-expressed se	53.90	0.00
TRINITY_sp|Q96Gː	IMP4	U3 small nucleolar	53.90	0.00
TRINITY_sp|P215ː	VPS1	Vacuolar protein so	53.90	0.00
TRINITY_sp|Q8VDː	Alg12	Dol-P-Man:Man(7)Glc	53.90	0.00
TRINITY_sp|P477ː	Gsr	Glutathione reducta	53.90	0.00
TRINITY_sp|Q54Cː	pdhA	Pyruvate dehydrogen	53.90	0.00
TRINITY_sp|Q405ː	NPK1	Mitogen-activated p	53.90	0.00
TRINITY_sp|Q3T0ː	PROSC	Proline synthase co	53.90	0.00
TRINITY_sp|Q5E9ː	EIF2S2	Eukaryotic translat	53.90	0.00
TRINITY_sp|O244ː	RPN8A	26S proteasome non-	53.90	0.00
TRINITY_sp|O611ː	svkA	Serine/threonine-pr	53.90	0.00
TRINITY_sp|P503ː	GDI2	Rab GDP dissociatio	53.90	0.00
TRINITY_sp|Q9C8ː	FAS4	ATP-dependent RNA h	53.90	0.00
TRINITY_sp|Q425ː	BCCP1	Biotin carboxyl car	53.90	0.00
TRINITY_sp|Q93Yː	At1g279ː	Probable pre-mRNA-s	53.90	0.00
TRINITY_sp|Q612ː	Abcd2	ATP-binding cassett	53.90	0.00
TRINITY_sp|Q74Hː	dnaJ	Chaperone protein D	53.90	0.00
TRINITY_sp|Q86Aː	DDB_G02ː	Probable myosin lig	53.90	0.00
TRINITY_sp|Q55Dː	dr1	Protein Dr1 homolog	53.90	0.00
TRINITY_sp|Q2PGː	SR	Serine racemase OS=	53.90	0.00
TRINITY_sp|Q9ZUː	QPT	Nicotinate-nucleoti	53.90	0.00
TRINITY_sp|Q9M8ː	SEC	Probable UDP-N-acet	53.90	0.00
TRINITY_sp|P713ː	HI_0568	Uncharacterized pro	53.90	0.00
TRINITY_sp|P176ː	PRKACA	cAMP-dependent prot	53.80	0.00
TRINITY_sp|P124ː	-	Actin A OS=Trypanos	53.80	0.00
TRINITY_sp|Q5PQː	-	Uncharacterized pro	53.80	0.00
TRINITY_sp|O154ː	ABCC4	Multidrug resistanc	53.80	0.00
TRINITY_sp|P546ː	pikB	Phosphatidylinosito	53.80	0.00
TRINITY_sp|P519ː	NEK3	Serine/threonine-pr	53.80	0.00
TRINITY_sp|Q0WQː	LTA3	Dihydrolipoyllysine	53.80	0.00
TRINITY_sp|Q3J5ː	rplC	50S ribosomal prote	53.80	0.00
TRINITY_sp|F4JAː	SKI2	DExH-box ATP-depend	53.80	0.00
TRINITY_sp|Q8R7ː	prs	Ribose-phosphate py	53.80	0.00
TRINITY_sp|Q9FHː	ABCB7	ABC transporter B f	53.80	0.00
TRINITY_sp|Q9P2ː	KIF17	Kinesin-like protei	53.80	0.00

TRINITY_sp|Q10P	ADIPOR3	Heptahelical transm	53.80	0.00
TRINITY_sp|Q9M8	FTSHI3	Probable inactive A	53.80	0.00
TRINITY_sp|Q2QP	GK1	Guanylate kinase 1	53.80	0.00
TRINITY_sp|Q3SZ	WDR18	WD repeat-containin	53.80	0.00
TRINITY_sp|Q6FR	GSP1	GTP-binding nuclear	53.80	0.00
TRINITY_sp|O747	SPBC170	4,5-DOPA dioxygenas	53.80	0.00
TRINITY_sp|P132	LCP1	Digestive cysteine	53.80	0.00
TRINITY_sp|Q54E	epnA	Epsin OS=Dictyostel	53.80	0.00
TRINITY_sp|P937	NUDT23	Nudix hydrolase 23,	53.80	0.00
TRINITY_sp|P548	cdc-48.	Transitional endopl	53.80	0.00
TRINITY_sp|Q420	ABCC2	ABC transporter C f	53.80	0.00
TRINITY_sp|O485	POLD2	DNA polymerase delt	53.80	0.00
TRINITY_sp|Q6ZR	DNAH12	Dynein heavy chain	53.80	0.00
TRINITY_sp|Q2TE	PCS2	Glutathione gamma-g	53.80	0.00
TRINITY_sp|A2RV	dph6	Diphthine--ammonia	53.80	0.00
TRINITY_sp|Q54W	metap1	Methionine aminopep	53.80	0.00
TRINITY_sp|Q8VY	ESP3	Pre-mRNA-splicing f	53.80	0.00
TRINITY_sp|Q1PD	SCL28	Serine/arginine-ric	53.80	0.00
TRINITY_sp|Q7XT	Os04g01	tRNA-dihydrouridine	53.80	0.00
TRINITY_sp|Q55E	nat10	RNA cytidine acetyl	53.80	0.00
TRINITY_sp|Q5F3	EFTUD2	116 kDa U5 small nu	53.80	0.00
TRINITY_sp|A8HM	FAP9	Intraflagellar tran	53.80	0.00
TRINITY_sp|P567	PSMD2	26S proteasome non-	53.80	0.00
TRINITY_sp|Q8NK	scon-3	E3 ubiquitin ligase	53.80	0.00
TRINITY_sp|Q7TM	Narfl	Cytosolic Fe-S clus	53.80	0.00
TRINITY_sp|Q2RB	Os11g01	Clathrin heavy chai	53.80	0.00
TRINITY_sp|A2XU	CDKG-2	Cyclin-dependent ki	53.70	0.00
TRINITY_sp|Q9SF	TIM14-2	Mitochondrial impor	53.70	0.00
TRINITY_sp|Q024	Sfri_36	Uncharacterized pro	53.70	0.00
TRINITY_sp|P0AB	elbB	Glyoxalase ElbB OS=	53.70	0.00
TRINITY_sp|Q9FK	LSM5	Sm-like protein LSM	53.70	0.00
TRINITY_sp|P092	-	Tubulin beta-1 chai	53.70	0.00
TRINITY_sp|P044	-	Calmodulin OS=Triti	53.70	0.00
TRINITY_sp|P322	rasS	Ras-like protein ra	53.70	0.00
TRINITY_sp|O808	MTACP2	Acyl carrier protei	53.70	0.00
TRINITY_sp|Q2RK	rsgA	Putative ribosome b	53.70	0.00
TRINITY_sp|Q6T3	Spata20	Spermatogenesis-ass	53.70	0.00
TRINITY_sp|Q9H4	WNK1	Serine/threonine-pr	53.70	0.00
TRINITY_sp|Q099	yakc	Aldo-keto reductase	53.70	0.00
TRINITY_sp|Q64J	PIMT2	Protein-L-isoaspart	53.70	0.00
TRINITY_sp|Q8HX	MUT	Methylmalonyl-CoA m	53.70	0.00
TRINITY_sp|Q479	-	L-sorbose 1-dehydro	53.70	0.00
TRINITY_sp|Q9C5	TIF3E1	Eukaryotic translat	53.70	0.00
TRINITY_sp|Q8EJ	prpC	2-methylcitrate syn	53.70	0.00
TRINITY_sp|P356	RPL7A-1	60S ribosomal prote	53.70	0.00
TRINITY_sp|Q9HC	CPNE5	Copine-5 OS=Homo sa	53.70	0.00
TRINITY_sp|P629	RAC1	Ras-related C3 botu	53.70	0.00
TRINITY_sp|Q395	-	Dynein 18 kDa light	53.70	0.00
TRINITY_sp|Q9Y6	ARL5A	ADP-ribosylation fa	53.70	0.00
TRINITY_sp|Q5N8	ISPD	2-C-methyl-D-erythr	53.70	0.00
TRINITY_sp|D3ZA	Chd6	Chromodomain-helica	53.70	0.00
TRINITY_sp|Q8S2	ISPE	4-diphosphocytidyl-	53.70	0.00
TRINITY_sp|B8NY	bxlB	Probable exo-1,4-be	53.70	0.00
TRINITY_sp|P087	ND5	NADH-ubiquinone oxi	53.70	0.00
TRINITY_sp|Q86I	dst1	Serine/threonine-pr	53.70	0.00
TRINITY_sp|Q150	EFTUD2	116 kDa U5 small nu	53.70	0.00

TRINITY_sp|P524:	GPDH	Glycerol-3-phosphat	53.70	0.00
TRINITY_sp|Q553:	dfa1	Diflavin flavoprote	53.70	0.00
TRINITY_sp|A2XVI	OsI_016(	DEAD-box ATP-depend	53.70	0.00
TRINITY_sp|C0SU1	JMJ16	Putative lysine-spe	53.70	0.00
TRINITY_sp|P871(	trz2	Ribonuclease Z 2, m	53.70	0.00
TRINITY_sp|Q94AI	OOP	Organellar oligopep	53.60	0.00
TRINITY_sp|Q6Q1!	CYP57	Peptidyl-prolyl cis	53.60	0.00
TRINITY_sp|Q99K:	Gak	Cyclin-G-associated	53.60	0.00
TRINITY_sp|Q993:	TY3B-G	Transposon Ty3-G Ga	53.60	0.00
TRINITY_sp|Q6DG(	supt4h1	Transcription elong	53.60	0.00
TRINITY_sp|Q9H5:	DHX35	Probable ATP-depend	53.60	0.00
TRINITY_sp|A7IP(	rplO	50S ribosomal prote	53.60	0.00
TRINITY_sp|O183:	Rab6	Ras-related protein	53.60	0.00
TRINITY_sp|Q032(	ced-10	Ras-related protein	53.60	0.00
TRINITY_sp|O049(	UMK3	UMP-CMP kinase 3 OS	53.60	0.00
TRINITY_sp|Q9I8I	MYO6	Unconventional myos	53.60	0.00
TRINITY_sp|Q84K:	PP5	Serine/threonine-pr	53.60	0.00
TRINITY_sp|Q91X\	DCXR	L-xylulose reductas	53.60	0.00
TRINITY_sp|Q9ZVI	PAHX	Phytanoyl-CoA dioxy	53.60	0.00
TRINITY_sp|P742!	gpx1	Hydroperoxy fatty a	53.60	0.00
TRINITY_sp|Q6I5:	CDKC-1	Cyclin-dependent ki	53.60	0.00
TRINITY_sp|Q7VG:	rlmN	Dual-specificity RN	53.60	0.00
TRINITY_sp|P600:	RPL7C	60S ribosomal prote	53.60	0.00
TRINITY_sp|O141:	SPAC31G!	Uncharacterized AAA	53.60	0.00
TRINITY_sp|Q9LZ.	ABCC14	ABC transporter C f	53.60	0.00
TRINITY_sp|Q54Q	DG1039	Probable ubiquitin	53.60	0.00
TRINITY_sp|Q9SJ(	GC1	Epimerase family pr	53.60	0.00
TRINITY_sp|Q9M8I	GLPK	Glycerol kinase OS=	53.60	0.00
TRINITY_sp|Q9M2I	RH52	DEAD-box ATP-depend	53.60	0.00
TRINITY_sp|Q9SC:	FKBP13	Peptidyl-prolyl cis	53.60	0.00
TRINITY_sp|Q9CAI	ERF013	Ethylene-responsive	53.60	0.00
TRINITY_sp|Q6V5I	Znf474	Zinc finger protein	53.60	0.00
TRINITY_sp|Q5F3:	EFTUD2	116 kDa U5 small nu	53.60	0.00
TRINITY_sp|Q6FPI	ICL1	Isocitrate lyase OS	53.60	0.00
TRINITY_sp|P283(	RPA2	DNA-directed RNA po	53.60	0
TRINITY_sp|Q9FP1	UBP12	Ubiquitin carboxyl-	53.60	0.00
TRINITY_sp|Q8W4!	VHA-a3	V-type proton ATPas	53.60	0.00
TRINITY_sp|Q0WVI	MCM4	DNA replication lic	53.60	0.00
TRINITY_sp|C6E5:	rnhB	Ribonuclease HII OS	53.60	0.00
TRINITY_sp|Q5VQI	Os01g01'	DEAD-box ATP-depend	53.60	0.00
TRINITY_sp|P519!	Nek1	Serine/threonine-pr	53.60	0.00
TRINITY_sp|A8JGI	LIP1	Lipoyl synthase, mi	53.60	0.00
TRINITY_sp|Q851:	GRXS7	Monothiol glutaredo	53.60	0.00
TRINITY_sp|Q9P2I	DNAH1	Dynein heavy chain	53.60	0.00
TRINITY_sp|Q9ZV.	At2g387:	Uncharacterized pro	53.50	0.00
TRINITY_sp|Q8BP'	Fra10ac:	Protein FRA10AC1 ho	53.50	0.00
TRINITY_sp|Q6CU!	SMP3	GPI mannosyltransfe	53.50	0.00
TRINITY_sp|Q9LP	RPN6	26S proteasome non-	53.50	0.00
TRINITY_sp|Q9UBI	MBD2	Methyl-CpG-binding	53.50	0.00
TRINITY_sp|B0T6I	mnmG	tRNA uridine 5-carb	53.50	0.00
TRINITY_sp|Q1PFI	CPK19	Calcium-dependent p	53.50	0.00
TRINITY_sp|Q68E:	agbl5	Cytosolic carboxype	53.50	0.00
TRINITY_sp|P051:	PRKACB	cAMP-dependent prot	53.50	0.00
TRINITY_sp|Q923'	trp1	Multifunctional try	53.50	0.00
TRINITY_sp|B8ARI	SRT1	NAD-dependent prote	53.50	0.00
TRINITY_sp|P409	Arf102F	ADP-ribosylation fa	53.50	0.00

TRINITY_sp|Q0PI7	NAXE	NAD(P)H-hydrate epi	53.50	0.00
TRINITY_sp|P3894	cat2	4-hydroxybutyrate c	53.50	0.00
TRINITY_sp|O8304	pip	Probable proline im	53.50	0.00
TRINITY_sp|P5467	patB	Probable plasma mem	53.50	0.00
TRINITY_sp|Q9SSN	At1g7290	Protein ROOT HAIR D	53.50	0.00
TRINITY_sp|Q80Z0	Arl5a	ADP-ribosylation fa	53.50	0.00
TRINITY_sp|Q9SX2	At1g6865	GDT1-like protein 5	53.50	0.00
TRINITY_sp|P4252	erkA	Extracellular signa	53.50	0.00
TRINITY_sp|Q5971	katA	Catalase OS=Pseudom	53.50	0.00
TRINITY_sp|Q4UJ1	TA09515	U1 small nuclear ri	53.50	0.00
TRINITY_sp|Q2462	ref(2)P	Protein ref(2)P OS=	53.50	0.00
TRINITY_sp|P3283	GSP1	GTP-binding nuclear	53.50	0.00
TRINITY_sp|Q9SF4	PAH1	Phosphatidate phosp	53.50	0.00
TRINITY_sp|P5464	pkbA	RAC family serine/t	53.50	0.00
TRINITY_sp|Q84V1	STT7	Serine/threonine-pr	53.50	0.00
TRINITY_sp|Q16K1	king-tub	Protein king tubby	53.50	0.00
TRINITY_sp|B1KU0	fabZ	3-hydroxyacyl-[acyl	53.50	0.00
TRINITY_sp|Q8BP7	Fra10ac1	Protein FRA10AC1 ho	53.50	0.00
TRINITY_sp|Q9SR7	TAF12	Transcription initi	53.50	0.00
TRINITY_sp|Q3960	IDA4	28 kDa inner dynein	53.50	0.00
TRINITY_sp|A9CR3	RAD25	Probable DNA repair	53.50	0.00
TRINITY_sp|Q55CA	vps26	Vacuolar protein so	53.40	0.00
TRINITY_sp|Q29RI	TBC1D31	TBC1 domain family	53.40	0.00
TRINITY_sp|Q9Y5K	PCYT1B	Choline-phosphate c	53.40	0.00
TRINITY_sp|Q8GWK	COX11	Cytochrome c oxidas	53.40	0.00
TRINITY_sp|Q9C50	At4g1783	Acetylornithine dea	53.40	0.00
TRINITY_sp|Q9FPR	EDR1	Serine/threonine-pr	53.40	0.00
TRINITY_sp|O6480	ACA7	Putative calcium-tr	53.40	0.00
TRINITY_sp|Q54S0	haao	3-hydroxyanthranila	53.40	0.00
TRINITY_sp|P4935	FPS	Farnesyl pyrophosph	53.40	0.00
TRINITY_sp|Q8WXX	DNAH7	Dynein heavy chain	53.40	0.00
TRINITY_sp|Q3TIX	Usp39	U4/U6.U5 tri-snRNP-	53.40	0.00
TRINITY_sp|P9809	c3	Complement C3 (Frag	53.40	0.00
TRINITY_sp|P4664	ASP2	Aspartate aminotran	53.40	0.00
TRINITY_sp|Q0992	yakc	Aldo-keto reductase	53.40	0.00
TRINITY_sp|Q8W1Y	SYN4	Sister chromatid co	53.40	0.00
TRINITY_sp|Q7Z07	-	Temptin OS=Aplysia	53.40	0.00
TRINITY_sp|Q5SN5	MPK8	Mitogen-activated p	53.40	0.00
TRINITY_sp|O6306	SECY	Preprotein transloc	53.40	0.00
TRINITY_sp|Q2717	DHC-8	Dynein heavy chain,	53.40	0.00
TRINITY_sp|O9600	MOCS2	Molybdopterin synth	53.40	0.00
TRINITY_sp|Q69TK	OPR3	Putative 12-oxophyt	53.40	0.00
TRINITY_sp|Q4054	NPK1	Mitogen-activated p	53.40	0.00
TRINITY_sp|Q54I5	smc6	Structural maintena	53.40	0.00
TRINITY_sp|P0545	SUP35	Eukaryotic peptide	53.40	0.00
TRINITY_sp|O2324	EMB1027	Arginine--tRNA liga	53.40	0.00
TRINITY_sp|Q8K0I	Acsm2	Acyl-coenzyme A syn	53.40	0.00
TRINITY_sp|Q2RAK	CCDA1	Cytochrome c-type b	53.40	0.00
TRINITY_sp|P4896	Slc25a4	ADP/ATP translocase	53.40	0.00
TRINITY_sp|Q6316	Dnah1	Dynein heavy chain	53.40	0.00
TRINITY_sp|Q0J03	Os09g053	Pantothenate kinase	53.40	0.00
TRINITY_sp|Q9C60	At1g5805	DExH-box ATP-depend	53.40	0.00
TRINITY_sp|Q29RI	CHORDC1	Cysteine and histid	53.30	0.00
TRINITY_sp|Q1456	DHX8	ATP-dependent RNA h	53.30	0.00
TRINITY_sp|Q9SSV	INPS1	Inositol-3-phosphat	53.30	0.00
TRINITY_sp|Q3SY3	DNAAF1	Dynein assembly fac	53.30	0.00

TRINITY_sp|Q9LY5	At3g5589	Protein yippee-like	53.30	0.00
TRINITY_sp|Q54JI	cnrN	Phosphatidylinosito	53.30	0.00
TRINITY_sp|A0A8N	lsm14a-a	Protein LSM14 homol	53.30	0.00
TRINITY_sp|P0705	NCU06606	Cytochrome b-c1 com	53.30	0.00
TRINITY_sp|Q54KN	ccbl	Kynurenine--oxoglut	53.30	0.00
TRINITY_sp|A2WNH	OsI_0138	Glucosidase 2 subun	53.30	0.00
TRINITY_sp|Q54MI	sun2	SUN domain-containi	53.30	0.00
TRINITY_sp|Q395T	YPTC6	Ras-related protein	53.30	0.00
TRINITY_sp|P3410	fhkC	Probable serine/thr	53.30	0.00
TRINITY_sp|P3202	Scp2	Non-specific lipid-	53.30	0.00
TRINITY_sp|Q32LI	STOML2	Stomatin-like prote	53.30	0.00
TRINITY_sp|Q2RK2	obg	GTPase Obg OS=Moore	53.30	0.00
TRINITY_sp|Q8VY8	LTD	Protein LHCP TRANSL	53.30	0.00
TRINITY_sp|Q9STY	PYRR	Riboflavin biosynth	53.30	0.00
TRINITY_sp|P3915	ywlC	Threonylcarbamoyl-A	53.30	0.00
TRINITY_sp|Q612B	pcca-1	Propionyl-CoA carbo	53.30	0.00
TRINITY_sp|Q9P6I	end4	Endocytosis protein	53.30	0.00
TRINITY_sp|P0783	PETF	Ferredoxin, chlorop	53.30	0.00
TRINITY_sp|Q75K2	ncsA	Calcium-binding pro	53.30	0.00
TRINITY_sp|P3783	Os01g076	Aspartate aminotran	53.30	0.00
TRINITY_sp|Q54RA	DDB_G028	Delta-1-pyrroline-5	53.30	0.00
TRINITY_sp|Q869N	pakB	Serine/threonine-pr	53.30	0.00
TRINITY_sp|Q9EQN	Ighmbp2	DNA-binding protein	53.30	0.00
TRINITY_sp|Q7KWQ	serS	Serine--tRNA ligase	53.30	0.00
TRINITY_sp|Q9LU4	RH35	DEAD-box ATP-depend	53.30	0.00
TRINITY_sp|Q9C7T	RPN8B	26S proteasome non-	53.30	0.00
TRINITY_sp|Q31LW	leuS	Leucine--tRNA ligas	53.30	0.00
TRINITY_sp|Q9XFH	SR30	Serine/arginine-ric	53.30	0.00
TRINITY_sp|P4245	cobA	Uroporphyrinogen-II	53.30	0.00
TRINITY_sp|Q9LY8	RGLG2	E3 ubiquitin-protei	53.30	0.00
TRINITY_sp|Q32P4	EML3	Echinoderm microtub	53.30	0.00
TRINITY_sp|Q6C3H	ALG13	UDP-N-acetylglucosa	53.30	0.00
TRINITY_sp|Q6NZH	Ptpdc1	Protein tyrosine ph	53.30	0.00
TRINITY_sp|Q2HJ9	FAHD1	Acylpyruvase FAHD1,	53.30	0.00
TRINITY_sp|Q5VYH	ECM29	Proteasome-associat	53.30	0.00
TRINITY_sp|Q86C6	tor	Target of rapamycin	53.20	0.00
TRINITY_sp|O8176	At4g3179	Probable diphthine	53.20	0.00
TRINITY_sp|A4IIN	zfp36l2	mRNA decay activato	53.20	0.00
TRINITY_sp|Q969Q	RAB24	Ras-related protein	53.20	0.00
TRINITY_sp|Q4162	ANT-G1	ADP,ATP carrier pro	53.20	0.00
TRINITY_sp|O2276	At4g0258	NADH dehydrogenase	53.20	0.00
TRINITY_sp|O1544	ABCC5	Multidrug resistanc	53.20	0.00
TRINITY_sp|Q9Y7R	set1	Histone-lysine N-me	53.20	0.00
TRINITY_sp|P1464	Pde4b	cAMP-specific 3',5'	53.20	0.00
TRINITY_sp|Q11EH	rlmN	Dual-specificity RN	53.20	0.00
TRINITY_sp|Q0AW3	purH	Bifunctional purine	53.20	0.00
TRINITY_sp|Q54EY	dtd	D-tyrosyl-tRNA(Tyr)	53.20	0.00
TRINITY_sp|Q9FIH	SPP	Stromal processing	53.20	0.00
TRINITY_sp|A2XUW	CDKG-2	Cyclin-dependent ki	53.20	0.00
TRINITY_sp|Q0P5H	ANKRD39	Ankyrin repeat doma	53.20	0.00
TRINITY_sp|Q5R8X	UBA5	Ubiquitin-like modi	53.20	0.00
TRINITY_sp|Q31L4	proB	Glutamate 5-kinase	53.20	0.00
TRINITY_sp|Q55F9	krsB	Serine/threonine-pr	53.20	0.00
TRINITY_sp|Q8VY2	ORTH2	E3 ubiquitin-protei	53.20	0.00
TRINITY_sp|O0448	UCH2	Ubiquitin carboxyl-	53.20	0.00
TRINITY_sp|Q99JX	Taf11	Transcription initi	53.20	0.00

TRINITY_sp|Q55AH	vatF	V-type proton ATPas	53.20	0.00
TRINITY_sp|A4IFE	KCTD7	BTB/POZ domain-cont	53.20	0.00
TRINITY_sp|P3124	RAD16	DNA repair protein	53.20	0.00
TRINITY_sp|Q5022	fmdA	Formamidase OS=Meth	53.20	0.00
TRINITY_sp|Q54KV	cdk5rap1	CDK5RAP1-like prote	53.20	0.00
TRINITY_sp|B3EM2	gatA	Glutamyl-tRNA(Gln)	53.20	0.00
TRINITY_sp|D2XNQ	FLOT2	Flotillin-like prot	53.20	0.00
TRINITY_sp|Q7KRW	CG1646	Pre-mRNA-processing	53.20	0.00
TRINITY_sp|P5099	ada1	AMP deaminase OS=Sc	53.20	0.00
TRINITY_sp|Q8GY8	SRX	Sulfiredoxin, chlor	53.20	0.00
TRINITY_sp|Q8YH2	ndvA	Beta-(1-->2)glucan	53.20	0.00
TRINITY_sp|P4937	XYL1	NAD(P)H-dependent D	53.10	0.00
TRINITY_sp|Q5XTY	RPL17	60S ribosomal prote	53.10	0.00
TRINITY_sp|P1366	HOM2	Aspartate-semialdeh	53.10	0.00
TRINITY_sp|Q94AX	DLD	D-lactate dehydroge	53.10	0.00
TRINITY_sp|Q54DA	drap1	Dr1-associated core	53.10	0.00
TRINITY_sp|Q54F2	mroh1	Maestro heat-like r	53.10	0.00
TRINITY_sp|Q8R75	prs	Ribose-phosphate py	53.10	0.00
TRINITY_sp|Q9ST7	ABCA7	ABC transporter A f	53.10	0.00
TRINITY_sp|Q8LPK	ABCA8	ABC transporter A f	53.10	0.00
TRINITY_sp|P4963	-	Ubiquitin-60S ribos	53.10	0.00
TRINITY_sp|P4121	Acsl1	Long-chain-fatty-ac	53.10	0.00
TRINITY_sp|Q72BE	gluQ	Glutamyl-Q tRNA(Asp	53.10	0.00
TRINITY_sp|P3421	rab-6.1	Ras-related protein	53.10	0.00
TRINITY_sp|O4931	At2g3322	NADH dehydrogenase	53.10	0.00
TRINITY_sp|P5195	Nek1	Serine/threonine-pr	53.10	0.00
TRINITY_sp|Q9C77	RPN8B	26S proteasome non-	53.10	0.00
TRINITY_sp|P4627	-	TATA-box-binding pr	53.10	0.00
TRINITY_sp|Q9SGY	ABCB10	ABC transporter B f	53.10	0.00
TRINITY_sp|Q6PIW	FIGNL1	Fidgetin-like prote	53.10	0.00
TRINITY_sp|P0DJ5	RPL24	60S ribosomal prote	53.10	0.00
TRINITY_sp|Q9FGE	RER1	Protein RETICULATA-	53.10	0.00
TRINITY_sp|Q8RWW	TMN10	Transmembrane 9 sup	53.10	0.00
TRINITY_sp|Q5M72	At1g5422	Dihydrolipoyllysine	53.10	0.00
TRINITY_sp|A3DJE	pckG	Phosphoenolpyruvate	53.10	0.00
TRINITY_sp|Q4R64	BBS5	Bardet-Biedl syndro	53.10	0.00
TRINITY_sp|Q55AX	dlpC	Dynamin-like protei	53.10	0.00
TRINITY_sp|Q9632	ACX4	Acyl-coenzyme A oxi	53.10	0.00
TRINITY_sp|Q54YS	DDB_G027	DNA-binding protein	53.10	0.00
TRINITY_sp|O8084	CPFTSY	Cell division prote	53.10	0.00
TRINITY_sp|D7SFE	LRK10L-2	Protein SUPPRESSOR	53.10	0.00
TRINITY_sp|Q7X2S	clpB	Chaperone protein C	53.10	0.00
TRINITY_sp|Q014C	SBE1	1,4-alpha-glucan-br	53.10	0.00
TRINITY_sp|Q9SU1	PSAK	Photosystem I react	53.10	0.00
TRINITY_sp|Q56Y1	At5g587	Dehydrodolichyl dip	53.00	0.00
TRINITY_sp|A8PTC	DUS3	tRNA-dihydrouridine	53.00	0.00
TRINITY_sp|Q1WCC	cript	Cysteine-rich PDZ-b	53.00	0.00
TRINITY_sp|P4548	ftsZ2	Cell division prote	53.00	0.00
TRINITY_sp|A8K2U	A2ML1	Alpha-2-macroglobul	53.00	0.00
TRINITY_sp|Q54IA	cox19	Cytochrome c oxidas	53.00	0.00
TRINITY_sp|Q6P96	hagh	Hydroxyacylglutathi	53.00	0.00
TRINITY_sp|Q4WVH	spb1	AdoMet-dependent rR	53.00	0.00
TRINITY_sp|Q8BMF	Dlat	Dihydrolipoyllysine	53.00	0.00
TRINITY_sp|Q9LZ5	RPL36C	60S ribosomal prote	53.00	0.00
TRINITY_sp|Q86IX	dst1	Serine/threonine-pr	53.00	0.00
TRINITY_sp|Q5ZIW	PEO1	Twinkle protein, mi	53.00	0.00

TRINITY_sp|P529:	lkhA	Leukotriene A-4 hyd	53.00	0.00
TRINITY_sp|Q9C5I	At5g062	2-Cys peroxiredoxin	53.00	0.00
TRINITY_sp|Q94A(	STR6	Rhodanese-like doma	53.00	0.00
TRINITY_sp|Q9HG>	-	Histone H2A OS=Agar	53.00	0.00
TRINITY_sp|A8J6>	CFAP221	Cilia- and flagella	53.00	0.00
TRINITY_sp|Q54DI	psenB	Presenilin-B OS=Dic	53.00	0.00
TRINITY_sp|P931(	PF20	Flagellar WD repeat	53.00	0.00
TRINITY_sp|O154:	ABCC4	Multidrug resistanc	53.00	0.00
TRINITY_sp|A8J1\	CFAP44	Cilia- and flagella	53.00	0
TRINITY_sp|Q101(	SPAC26A:	Hydrolase C26A3.11	53.00	0.00
TRINITY_sp|Q396(	IDA4	28 kDa inner dynein	53.00	0.00
TRINITY_sp|P0AF	yjbQ	UPF0047 protein Yjb	53.00	0.00
TRINITY_sp|Q6NYI	gpt2l	Alanine aminotransf	52.90	0.00
TRINITY_sp|Q059	RAB1A	Ras-related protein	52.90	0.00
TRINITY_sp|Q9D1	Fam96b	Mitotic spindle-ass	52.90	0.00
TRINITY_sp|Q8NCI	DYNC2H1	Cytoplasmic dynein	52.90	0.00
TRINITY_sp|P490	-	Cytochrome b5 OS=Ni	52.90	0.00
TRINITY_sp|Q428	GLX1	Lactoylglutathione	52.90	0.00
TRINITY_sp|Q5A5	NIK1	Histidine protein k	52.90	0.00
TRINITY_sp|Q9FFI	NFXL2	NF-X1-type zinc fin	52.90	0.00
TRINITY_sp|Q6CDI	BNA5	Kynureninase OS=Yar	52.90	0.00
TRINITY_sp|Q2KII	DNAL1	Dynein light chain	52.90	0.00
TRINITY_sp|Q820	serC	Phosphoserine amino	52.90	0.00
TRINITY_sp|Q9CP(	Mgst3	Microsomal glutathi	52.90	0.00
TRINITY_sp|B8G6	panC	Pantothenate synthe	52.90	0.00
TRINITY_sp|Q86H:	polr1a	DNA-directed RNA po	52.90	0.00
TRINITY_sp|Q6F9	ung	Uracil-DNA glycosyl	52.90	0.00
TRINITY_sp|O948	ATP10B	Probable phospholip	52.90	0.00
TRINITY_sp|O221	GCP1	Probable tRNA N6-ad	52.90	0.00
TRINITY_sp|P736	trpS	Tryptophan--tRNA li	52.90	0.00
TRINITY_sp|P435(	cpr-4	Cathepsin B-like cy	52.90	0.00
TRINITY_sp|Q1ED	nr2c2ap	Nuclear receptor 2C	52.90	0.00
TRINITY_sp|Q050	TRI1	Protein TRI1 OS=Sac	52.90	0.00
TRINITY_sp|A2YX(	SPL15	Squamosa promoter-b	52.90	0.00
TRINITY_sp|Q502	cyb5r4	Cytochrome b5 reduc	52.90	0.00
TRINITY_sp|C6DI	tatD	3'-5' ssDNA/RNA exo	52.90	0.00
TRINITY_sp|F4HT	SAMC2	Probable S-adenosyl	52.90	0.00
TRINITY_sp|Q7KWI	iunH	Inosine-uridine-pre	52.90	0.00
TRINITY_sp|Q9SB	AP4M	AP-4 complex subuni	52.90	0.00
TRINITY_sp|Q6L4	DBB1	DNA damage-binding	52.90	0.00
TRINITY_sp|P427	UBC5	Ubiquitin-conjugati	52.90	0.00
TRINITY_sp|Q940	KIN13B	Kinesin-like protei	52.90	0.00
TRINITY_sp|Q54Q(	impdh	Inosine-5'-monophos	52.90	0.00
TRINITY_sp|Q7G1	AAO1	Indole-3-acetaldehy	52.90	0.00
TRINITY_sp|B9MJ(	mutS	DNA mismatch repair	52.90	0.00
TRINITY_sp|Q9SAI	MRS2-10	Magnesium transport	52.90	0.00
TRINITY_sp|Q7DN	Cht2	Chitinase 2 OS=Oryz	52.90	0.00
TRINITY_sp|Q55CI	DDB_G02	Probable NADH dehyd	52.90	0.00
TRINITY_sp|Q275	cyp-13A	Putative cytochrome	52.90	0.00
TRINITY_sp|P614	thiDE	Thiamine biosynthes	52.90	0.00
TRINITY_sp|Q54V	abcC2	ABC transporter C f	52.90	0.00
TRINITY_sp|A0Q1I	dnaJ	Chaperone protein D	52.90	0.00
TRINITY_sp|P930	rps5	30S ribosomal prote	52.90	0.00
TRINITY_sp|Q7SY(	slc44a2	Choline transporter	52.90	0.00
TRINITY_sp|Q9SB(	PHR1	Deoxyribodipyrimidi	52.90	0.00
TRINITY_sp|Q8L6	SEC31B	Protein transport p	52.90	0.00

TRINITY_sp|Q54II	ragA	Ras-related GTP-bin	52.90	0.00
TRINITY_sp|Q9JJ5	Abcb9	ATP-binding cassett	52.90	0.00
TRINITY_sp|Q1339	TARBP1	Probable methyltran	52.90	0.00
TRINITY_sp|Q5SNI	Os06g017	Zinc finger CCCH do	52.90	0.00
TRINITY_sp|Q2756	psmA1	Proteasome subunit	52.90	0.00
TRINITY_sp|Q9Y39	DHRS7	Dehydrogenase/reduc	52.90	0.00
TRINITY_sp|Q4326	PCNA	Proliferating cell	52.90	0.00
TRINITY_sp|Q4054	NPK1	Mitogen-activated p	52.80	0.00
TRINITY_sp|Q5A59	NIK1	Histidine protein k	52.80	0.00
TRINITY_sp|P3245	GBP1	Guanylate-binding p	52.80	0.00
TRINITY_sp|Q3956	ODA4	Dynein beta chain,	52.80	0.00
TRINITY_sp|Q9NEW	rsp-3	Probable splicing f	52.80	0.00
TRINITY_sp|Q69K5	HAL3	Phosphopantothenoyl	52.80	0.00
TRINITY_sp|Q1703	Cdc42	Cdc42 homolog OS=An	52.80	0.00
TRINITY_sp|Q9FKI	ABCA11	ABC transporter A f	52.80	0.00
TRINITY_sp|Q9NR2	DYRK4	Dual specificity ty	52.80	0.00
TRINITY_sp|Q9SS9	RGLG1	E3 ubiquitin-protei	52.80	0.00
TRINITY_sp|A6QR4	RAB6B	Ras-related protein	52.80	0.00
TRINITY_sp|Q9C5	PFK7	ATP-dependent 6-pho	52.80	0.00
TRINITY_sp|Q9LT7	RD21C	Probable cysteine p	52.80	0.00
TRINITY_sp|P1511	efbA	Elongation factor 2	52.80	0.00
TRINITY_sp|Q0WW2	At4g3445	Coatomer subunit ga	52.80	0.00
TRINITY_sp|Q0825	CRYZ	Quinone oxidoreduct	52.80	0.00
TRINITY_sp|Q7DLI	PBG1	Proteasome subunit	52.80	0.00
TRINITY_sp|Q1414	DHX34	Probable ATP-depend	52.80	0.00
TRINITY_sp|P2361	WARS	Tryptophan--tRNA li	52.80	0.00
TRINITY_sp|Q95U	Rab7a	Ras-related protein	52.80	0.00
TRINITY_sp|B4FH	-	15-cis-zeta-caroten	52.80	0.00
TRINITY_sp|G0SE	RLI1	Translation initiat	52.80	0.00
TRINITY_sp|Q8L32	FZR2	Protein FIZZY-RELAT	52.80	0.00
TRINITY_sp|Q8CF	Aqr	Intron-binding prot	52.80	0.00
TRINITY_sp|F4IM8	At2g011	DExH-box ATP-depend	52.80	0.00
TRINITY_sp|Q9ZTI	ERD2	ER lumen protein-re	52.80	0.00
TRINITY_sp|Q7T3	rab13	Ras-related protein	52.80	0.00
TRINITY_sp|Q9SE9	-	Methylenetetrahydro	52.80	0.00
TRINITY_sp|Q54V9	isca1	Iron-sulfur cluster	52.80	0.00
TRINITY_sp|O9540	ZFYVE9	Zinc finger FYVE do	52.80	0.00
TRINITY_sp|Q1RJ	icd	Isocitrate dehydrog	52.80	0.00
TRINITY_sp|Q54S	nvl	Putative ribosome b	52.80	0.00
TRINITY_sp|Q9FMI	RD21B	Probable cysteine p	52.80	0.00
TRINITY_sp|Q9FY	BCAT5	Branched-chain-amin	52.80	0.00
TRINITY_sp|Q9LY	AK1	Aspartokinase 1, ch	52.80	0.00
TRINITY_sp|Q9WX3	rbpE	Putative RNA-bindin	52.80	0.00
TRINITY_sp|Q8H1	TL20.3	Thylakoid lumenal p	52.80	0.00
TRINITY_sp|P0565	-	Myosin-2 heavy chai	52.80	0.00
TRINITY_sp|P5368	-	Actin OS=Phaffia rh	52.80	0.00
TRINITY_sp|A8MS6	LPD1	Dihydrolipoyl dehyd	52.80	0.00
TRINITY_sp|Q9SF4	PAH1	Phosphatidate phosp	52.80	0.00
TRINITY_sp|Q8W1X	PK	Pyridoxal kinase OS	52.80	0.00
TRINITY_sp|Q9P22	DNAH2	Dynein heavy chain	52.80	0.00
TRINITY_sp|Q9FII	At5g4772	Probable acetyl-CoA	52.80	0.00
TRINITY_sp|Q6AU	XDH	Xanthine dehydrogen	52.80	0
TRINITY_sp|Q9M8I	DSPTP1B	Dual specificity pr	52.70	0.00
TRINITY_sp|P5337	NUDT6	Nucleoside diphosph	52.70	0.00
TRINITY_sp|Q9SKX	ABCC13	ABC transporter C f	52.70	0.00
TRINITY_sp|Q9ZRI	CAD	Probable mannitol d	52.70	0.00

TRINITY_sp|Q5U2!	usp47	Ubiquitin carboxyl-	52.70	0.00
TRINITY_sp|Q9M8I	DJ1D	Protein DJ-1 homolo	52.70	0.00
TRINITY_sp|Q10Q	Os03g02	Putative cysteine p	52.70	0.00
TRINITY_sp|Q8BYI	Rdh12	Retinol dehydrogena	52.70	0.00
TRINITY_sp|Q86KI	fhit	Bis(5'-adenosyl)-tr	52.70	0.00
TRINITY_sp|Q0VF\	kctd7	BTB/POZ domain-cont	52.70	0.00
TRINITY_sp|Q9NR	DDX21	Nucleolar RNA helic	52.70	0.00
TRINITY_sp|Q9LV(	BOB1	Protein BOBBER 1 OS	52.70	0.00
TRINITY_sp|O609!	limE	LIM domain-containi	52.70	0.00
TRINITY_sp|Q54Y(	trappc5	Trafficking protein	52.70	0.00
TRINITY_sp|Q32P	MPZL1	Myelin protein zero	52.70	0.00
TRINITY_sp|O356(	Abca4	Retinal-specific AT	52.70	0.00
TRINITY_sp|P094(	snrnp70	U1 small nuclear ri	52.70	0.00
TRINITY_sp|P047	-	Glutamine synthetas	52.70	0.00
TRINITY_sp|Q640\	tatdn1	Putative deoxyribon	52.70	0.00
TRINITY_sp|P790!	rhp16	ATP-dependent helic	52.70	0.00
TRINITY_sp|Q1DD(	kynU	Kynureninase OS=Myx	52.70	0.00
TRINITY_sp|Q9MBI	DHC10	Dynein-1-beta heavy	52.70	0.00
TRINITY_sp|Q3SZ	PMPCB	Mitochondrial-proce	52.70	0.00
TRINITY_sp|P407!	Rac1	Ras-related protein	52.70	0.00
TRINITY_sp|Q165(	CAMK4	Calcium/calmodulin-	52.70	0.00
TRINITY_sp|P297(	-	60S acidic ribosoma	52.70	0.00
TRINITY_sp|Q52R(	SPL	Sphingosine-1-phosp	52.70	0.00
TRINITY_sp|Q9C5!	RFC1	Replication factor	52.70	0.00
TRINITY_sp|P114!	TUBB1	Tubulin beta chain	52.70	0.00
TRINITY_sp|Q8GY!	At2g432	CMP-sialic acid tra	52.70	0.00
TRINITY_sp|O810!	NRPB8A	DNA-directed RNA po	52.70	0.00
TRINITY_sp|Q190	pho-1	Intestinal acid pho	52.70	0.00
TRINITY_sp|O959!	TOP3B	DNA topoisomerase 3	52.70	0.00
TRINITY_sp|Q9LUI	RAP2-2	Ethylene-responsive	52.70	0.00
TRINITY_sp|Q9NW	UCKL1	Uridine-cytidine ki	52.70	0.00
TRINITY_sp|P543!	eas	Ethanolamine kinase	52.70	0.00
TRINITY_sp|Q8VYI	HCC1	Protein SCO1 homolo	52.70	0.00
TRINITY_sp|O645	STR1	Thiosulfate/3-merca	52.70	0.00
TRINITY_sp|A7HT(	rpmA	50S ribosomal prote	52.70	0.00
TRINITY_sp|P965!	gtfB	Vancomycin aglycone	52.70	0.00
TRINITY_sp|Q027(	RPL24	50S ribosomal prote	52.70	0.00
TRINITY_sp|P0DJ!	RPL6	60S ribosomal prote	52.70	0.00
TRINITY_sp|Q2HJ!	FAHD1	Acylpyruvase FAHD1,	52.60	0.00
TRINITY_sp|O805	CYCU4-1	Cyclin-U4-1 OS=Arab	52.60	0.00
TRINITY_sp|Q9FV	E2FC	Transcription facto	52.60	0.00
TRINITY_sp|Q6PH(	hpx	Hemopexin OS=Danio	52.60	0.00
TRINITY_sp|Q54R	DDB_G02	Probable serine/thr	52.60	0.00
TRINITY_sp|Q210	kin-29	Serine/threonine-pr	52.60	0.00
TRINITY_sp|P518	PRKX	cAMP-dependent prot	52.60	0.00
TRINITY_sp|Q86A!	erh	Enhancer of rudimen	52.60	0.00
TRINITY_sp|Q9UFI	CNOT8	CCR4-NOT transcript	52.60	0.00
TRINITY_sp|Q710	spp2	Secreted phosphopro	52.60	0.00
TRINITY_sp|Q8RX	NDK4	Nucleoside diphosph	52.60	0.00
TRINITY_sp|Q5KW	dnaJ	Chaperone protein D	52.60	0.00
TRINITY_sp|Q23M	Ttll6a	Probable beta-tubul	52.60	0.00
TRINITY_sp|Q23FI	TTLL3C	Tubulin glycylase 3	52.60	0.00
TRINITY_sp|P386(	RPL24B	60S ribosomal prote	52.60	0.00
TRINITY_sp|Q86GI	Cys	Crustapain OS=Panda	52.60	0.00
TRINITY_sp|A9WS!	glpK	Glycerol kinase OS=	52.60	0.00
TRINITY_sp|Q99J(	Rfc4	Replication factor	52.60	0.00

TRINITY_sp|Q54HI	srr	Probable serine rac	52.60	0.00
TRINITY_sp|Q86I\	DDB_G02	PH domain-containin	52.60	0.00
TRINITY_sp|Q9R1S	B9d1	B9 domain-containin	52.60	0.00
TRINITY_sp|Q54FI	etfa	Electron transfer f	52.60	0.00
TRINITY_sp|P470	VTC4	Vacuolar transporte	52.60	0.00
TRINITY_sp|Q54E4	haox	Hydroxyacid oxidase	52.60	0.00
TRINITY_sp|Q6F3A	CPK10	Calcium-dependent p	52.60	0.00
TRINITY_sp|Q6EE3	CCT8	T-complex protein 1	52.60	0.00
TRINITY_sp|O2331	NFYB3	Nuclear transcripti	52.60	0.00
TRINITY_sp|Q869	pakF	Serine/threonine-pr	52.60	0.00
TRINITY_sp|Q9C6W	ABCG14	ABC transporter G f	52.60	0.00
TRINITY_sp|Q6EVK	BRM	ATP-dependent helic	52.60	0.00
TRINITY_sp|A2C40	rnz	Ribonuclease Z OS=P	52.60	0.00
TRINITY_sp|Q5KQN	CAX2	Vacuolar cation/pro	52.60	0.00
TRINITY_sp|P1824	-	Tubulin beta-1 chai	52.60	0.00
TRINITY_sp|H3JU0	SGT1	Peptidyl serine alp	52.60	0.00
TRINITY_sp|O2440	RABE1A	Ras-related protein	52.60	0.00
TRINITY_sp|O8188	LGALDH	L-galactose dehydro	52.60	0.00
TRINITY_sp|Q8IQ0	CG5021	Uncharacterized Gol	52.60	0.00
TRINITY_sp|Q9FG8	KCS20	3-ketoacyl-CoA synt	52.60	0.00
TRINITY_sp|B1WVY	hemE	Uroporphyrinogen de	52.60	0.00
TRINITY_sp|P2860	SYNPCC70	Uncharacterized pro	52.60	0.00
TRINITY_sp|Q6ZMV	KIF6	Kinesin-like protei	52.60	0.00
TRINITY_sp|Q7ZXK	nle1	Notchless protein h	52.60	0.00
TRINITY_sp|P1073	sevA	Severin OS=Dictyost	52.60	0.00
TRINITY_sp|Q6DI3	ublcp1	Ubiquitin-like doma	52.60	0.00
TRINITY_sp|Q6P7N	mak16	Protein MAK16 homol	52.60	0.00
TRINITY_sp|Q9FL6	AGD5	Probable ADP-ribosy	52.60	0.00
TRINITY_sp|Q0WL8	UGGT	UDP-glucose:glycopr	52.60	0.00
TRINITY_sp|Q9UT0	SPAC1093	Putative CCA tRNA n	52.50	0.00
TRINITY_sp|Q5ZIE	XRN2	5'-3' exoribonuclea	52.50	0.00
TRINITY_sp|Q9STI	CEP2	KDEL-tailed cystein	52.50	0.00
TRINITY_sp|Q5ZI8	TBCD	Tubulin-specific ch	52.50	0.00
TRINITY_sp|Q86C6	tor	Target of rapamycin	52.50	0.00
TRINITY_sp|Q54YS	DDB_G02	DNA-binding protein	52.50	0.00
TRINITY_sp|A8P5F	AMPP	Probable Xaa-Pro am	52.50	0.00
TRINITY_sp|Q9LTV	At3g1280	Peroxisomal 2,4-die	52.50	0.00
TRINITY_sp|Q0791	PAM18	Mitochondrial impor	52.50	0.00
TRINITY_sp|O0269	HSD17B10	3-hydroxyacyl-CoA d	52.50	0.00
TRINITY_sp|Q4IA5	CYM1	Mitochondrial prese	52.50	0.00
TRINITY_sp|Q54WE	ccdc94	Coiled-coil domain-	52.50	0.00
TRINITY_sp|Q86AV	mcfX	Mitochondrial subst	52.50	0.00
TRINITY_sp|B4JH1	GH18624	Lateral signaling t	52.50	0.00
TRINITY_sp|Q54QN	lig3	DNA ligase 3 OS=Dic	52.50	0.00
TRINITY_sp|B1LXV	apaG	Protein ApaG OS=Met	52.50	0.00
TRINITY_sp|Q5Z8I	Os06g07	RNA pseudouridine s	52.50	0.00
TRINITY_sp|Q6DR3	ric8b	Synembryn-B OS=Dani	52.50	0.00
TRINITY_sp|B7LS2	treF	Cytoplasmic trehala	52.50	0.00
TRINITY_sp|F4KG0	FBA4	Fructose-bisphospha	52.50	0.00
TRINITY_sp|Q4U48	Xirp2	Xin actin-binding r	52.50	0.00
TRINITY_sp|Q9FK6	RABA1C	Ras-related protein	52.50	0.00
TRINITY_sp|Q9NES	pph-5	Serine/threonine-pr	52.50	0.00
TRINITY_sp|P511	apeA	DNA-(apurinic or ap	52.50	0.00
TRINITY_sp|Q9SK2	At2g3204	Folate-biopterin tr	52.50	0.00
TRINITY_sp|O7842	ycf19	Uncharacterized pro	52.50	0.00
TRINITY_sp|P0C8N	CCRP1	Probable serine/thr	52.50	0.00

TRINITY_sp|P550(	RPN10	26S proteasome non-	52.50	0.00
TRINITY_sp|Q54DI	amdA	AMP deaminase OS=Di	52.50	0.00
TRINITY_sp|Q54H4	drkB	Probable serine/thr	52.50	0.00
TRINITY_sp|Q8VX\	UAH	Ureidoglycolate hyd	52.50	0.00
TRINITY_sp|Q5ZKI	DPH2	Diphthamide biosynt	52.50	0.00
TRINITY_sp|Q9C8(	LCV2	Protein LIKE COV 2	52.50	0.00
TRINITY_sp|O949(	PRPF6	Pre-mRNA-processing	52.50	0.00
TRINITY_sp|Q3TXS	Psmd1	26S proteasome non-	52.50	0.00
TRINITY_sp|Q53NI	Os11g01S	Probable NAD kinase	52.50	0.00
TRINITY_sp|Q5ZK(	SUB1	Activated RNA polym	52.50	0.00
TRINITY_sp|Q0074	Mdr65	Multidrug resistanc	52.50	0.00
TRINITY_sp|Q9DA/	Exosc1	Exosome complex com	52.50	0.00
TRINITY_sp|Q2HJI	USP15	Ubiquitin carboxyl-	52.50	0.00
TRINITY_sp|Q8DGS	rlmN	Probable dual-speci	52.50	0.00
TRINITY_sp|Q9LY8	RGLG2	E3 ubiquitin-protei	52.50	0.00
TRINITY_sp|O226(	DEGP1	Protease Do-like 1,	52.50	0.00
TRINITY_sp|P2082	pol	Retrovirus-related	52.50	0.00
TRINITY_sp|Q993I	TY3B-G	Transposon Ty3-G Ga	52.50	0.00
TRINITY_sp|P3412	psmA7	Proteasome subunit	52.50	0.00
TRINITY_sp|Q9C5\	TOP6B	DNA topoisomerase 6	52.50	0.00
TRINITY_sp|Q0VC/	SAMHD1	Deoxynucleoside tri	52.50	0.00
TRINITY_sp|Q9LFI	VAMP713	Vesicle-associated	52.50	0.00
TRINITY_sp|A5DQI	ACT1	Actin OS=Meyerozyma	52.50	0.00
TRINITY_sp|O2204	ANP3	Mitogen-activated p	52.40	0.00
TRINITY_sp|Q8ML2	pdeD	cGMP-dependent 3',5	52.40	0.00
TRINITY_sp|O3594	Nek2	Serine/threonine-pr	52.40	0.00
TRINITY_sp|Q7ZW2	alg11	GDP-Man:Man(3)GlcNA	52.40	0.00
TRINITY_sp|P189(	YPK2	Serine/threonine-pr	52.40	0.00
TRINITY_sp|Q5VV4	CDKAL1	Threonylcarbamoylad	52.40	0.00
TRINITY_sp|Q9MB5	FKFBP	6-phosphofructo-2-k	52.40	0.00
TRINITY_sp|Q9M8I	DSPTP1B	Dual specificity pr	52.40	0.00
TRINITY_sp|O8188	LGALDH	L-galactose dehydro	52.40	0.00
TRINITY_sp|Q0992	yakc	Aldo-keto reductase	52.40	0.00
TRINITY_sp|E9PUI	Abca17	ATP-binding cassett	52.40	0.00
TRINITY_sp|P280I	AAT-1	Aspartate aminotran	52.40	0.00
TRINITY_sp|Q1EB3	DBP4	ATP-dependent RNA h	52.40	0.00
TRINITY_sp|Q8LDI	CYP23	Peptidyl-prolyl cis	52.40	0.00
TRINITY_sp|Q4047	ERF4	Ethylene-responsive	52.40	0.00
TRINITY_sp|Q6PFI	ndor1	NADPH-dependent dif	52.40	0.00
TRINITY_sp|F4KF\	At5g3984	DExH-box ATP-depend	52.40	0.00
TRINITY_sp|B7KHI	PCC7424_	Nucleoid-associated	52.40	0.00
TRINITY_sp|A7YES	ttc30a	Tetratricopeptide r	52.40	0.00
TRINITY_sp|Q8SK(	TIC62	Protein TIC 62, chl	52.40	0.00
TRINITY_sp|Q9ZUI	CKL5	Casein kinase 1-lik	52.40	0.00
TRINITY_sp|P4333	At1g097(	U2 small nuclear ri	52.40	0.00
TRINITY_sp|A8ICS	CFAP46	Cilia- and flagella	52.40	0.00
TRINITY_sp|Q8WX)	DNAH7	Dynein heavy chain	52.40	0.00
TRINITY_sp|O0858	Gtpbp1	GTP-binding protein	52.40	0.00
TRINITY_sp|Q9SV(	ASAT1	Acyl-CoA--sterol O-	52.40	0.00
TRINITY_sp|Q7TXI	ppsB	Phthiocerol/phenolp	52.40	0.00
TRINITY_sp|Q8S6I	ACC1	Acetyl-CoA carboxyl	52.40	0.00
TRINITY_sp|Q0HA3	Ttc21b	Tetratricopeptide r	52.40	0.00
TRINITY_sp|Q5FG(	dnaJ	Chaperone protein D	52.40	0.00
TRINITY_sp|Q1K9(	SPAC167.	Probable E3 ubiquit	52.40	0.00
TRINITY_sp|Q8IU8	CAMK1D	Calcium/calmodulin-	52.40	0.00
TRINITY_sp|Q54YS	DDB_G02	DNA-binding protein	52.40	0.00

TRINITY_sp|B6SF/	MAA3	Probable helicase M	52.40	0.00
TRINITY_sp|Q7T3I	kyat3	Kynurenine--oxoglut	52.40	0.00
TRINITY_sp|Q9DB(	Selo	Selenoprotein O OS=	52.40	0.00
TRINITY_sp|A2Y4I	CDKD-1	Cyclin-dependent ki	52.40	0.00
TRINITY_sp|Q8MYI	DDB_G02'	Probable serine/thr	52.30	0.00
TRINITY_sp|P868(	-	Non-specific lipid-	52.30	0.00
TRINITY_sp|Q9UB!	DNAJB11	DnaJ homolog subfam	52.30	0.00
TRINITY_sp|P512{	ycf45	Uncharacterized pro	52.30	0.00
TRINITY_sp|Q93VI	PABN1	Polyadenylate-bindi	52.30	0.00
TRINITY_sp|F4HPI	IRE4	Probable serine/thr	52.30	0.00
TRINITY_sp|P465!	ubc4	Ubiquitin-conjugati	52.30	0.00
TRINITY_sp|A7RP'	v1g2284!	NAD(P)H-hydrate epi	52.30	0.00
TRINITY_sp|Q5ZJ\	GARNL3	GTPase-activating R	52.30	0.00
TRINITY_sp|P375!	iaaA	Isoaspartyl peptida	52.30	0.00
TRINITY_sp|Q99J\	Actr3	Actin-related prote	52.30	0.00
TRINITY_sp|P047'	-	Glutamine synthetas	52.30	0.00
TRINITY_sp|Q7ZV/	smu1	WD40 repeat-contain	52.30	0.00
TRINITY_sp|Q177.	trxr-1	Thioredoxin reducta	52.30	0.00
TRINITY_sp|P264.	PARP1	Poly [ADP-ribose] p	52.30	0.00
TRINITY_sp|Q8L5\	PANK2	Pantothenate kinase	52.30	0.00
TRINITY_sp|Q08CI	znrf2	E3 ubiquitin-protei	52.30	0.00
TRINITY_sp|B3EFI	infB	Translation initiat	52.30	0.00
TRINITY_sp|P078'	Acox1	Peroxisomal acyl-co	52.30	0.00
TRINITY_sp|Q8L9/	RPL21M	50S ribosomal prote	52.30	0.00
TRINITY_sp|P364(	rab2A	Ras-related protein	52.30	0.00
TRINITY_sp|P546'	patA	Calcium-transportin	52.30	0.00
TRINITY_sp|Q6GN(	adrm1-a	Proteasomal ubiquit	52.30	0.00
TRINITY_sp|Q96DI	HDAC11	Histone deacetylase	52.30	0.00
TRINITY_sp|Q55E\	ube2w	Probable ubiquitin-	52.30	0.00
TRINITY_sp|P538(	JJJ1	J protein JJJ1 OS=S	52.30	0.00
TRINITY_sp|P044(	-	Calmodulin OS=Triti	52.30	0.00
TRINITY_sp|P451.	ettA	Energy-dependent tr	52.30	0.00
TRINITY_sp|Q9SX(	ROA2	DNA replication lic	52.30	0.00
TRINITY_sp|O154.	MPPED1	Metallophosphoester	52.30	0.00
TRINITY_sp|B5X8I	brcc3	Lys-63-specific deu	52.30	0.00
TRINITY_sp|O947	ALG2	Alpha-1,3/1,6-manno	52.30	0.00
TRINITY_sp|P432.	Abcb1	Multidrug resistanc	52.30	0.00
TRINITY_sp|Q311(	panB	3-methyl-2-oxobutan	52.30	0.00
TRINITY_sp|O949(	PRPF6	Pre-mRNA-processing	52.30	0.00
TRINITY_sp|B2RW	Cfap58	Cilia- and flagella	52.30	0.00
TRINITY_sp|A9UN(	bysl	Bystin OS=Monosiga	52.30	0.00
TRINITY_sp|Q9ST!	ALDH10A!	Betaine aldehyde de	52.30	0.00
TRINITY_sp|P124	-	Vignain OS=Vigna mu	52.30	0.00
TRINITY_sp|Q23FI	TTLL3C	Tubulin glycylase 3	52.30	0.00
TRINITY_sp|P0CA.	TTLL3B	Tubulin glycylase 3	52.30	0.00
TRINITY_sp|Q6TEI	fam91a1	Protein FAM91A1 OS=	52.30	0.00
TRINITY_sp|Q8TAI	TTC8	Tetratricopeptide r	52.30	0.00
TRINITY_sp|C4LA(	hcp	Hydroxylamine reduc	52.30	0.00
TRINITY_sp|Q55D{	cas1	Cycloartenol syntha	52.30	0.00
TRINITY_sp|P598	RDH12	Retinol dehydrogena	52.30	0.00
TRINITY_sp|A7E3'	PTPDC1	Protein tyrosine ph	52.30	0.00
TRINITY_sp|P142.	PSAK	Photosystem I react	52.30	0.00
TRINITY_sp|Q1PFI	AIL1	AP2-like ethylene-r	52.30	0.00
TRINITY_sp|Q54JI	ogdh	2-oxoglutarate dehy	52.30	0.00
TRINITY_sp|Q9SS.	SDP6	Glycerol-3-phosphat	52.30	0.00
TRINITY_sp|Q755(	IPL1	Spindle assembly ch	52.30	0.00

TRINITY_sp|P2984	cysE	Serine acetyltransf	52.30	0.00
TRINITY_sp|O7580	CAPN15	Calpain-15 OS=Homo	52.20	0.00
TRINITY_sp|Q9C9P	PFP-ALPH	Pyrophosphate--fruc	52.20	0.00
TRINITY_sp|Q5XF8	Atp13a3	Probable cation-tra	52.20	0.00
TRINITY_sp|P0DJ5	RPL13	60S ribosomal prote	52.20	0.00
TRINITY_sp|Q9SII	DSK2B	Ubiquitin domain-co	52.20	0.00
TRINITY_sp|Q1729	unc-119	Protein unc-119 OS=	52.20	0.00
TRINITY_sp|O1415	SPAC4A8.	UPF0047 protein C4A	52.20	0.00
TRINITY_sp|O1415	SPAC4A8.	UPF0047 protein C4A	52.20	0.00
TRINITY_sp|Q6C0Y	COF1	Cofilin OS=Yarrowia	52.20	0.00
TRINITY_sp|B2ZX9	FSM	Chromatin assembly	52.20	0.00
TRINITY_sp|O3501	yfkJ	Low molecular weigh	52.20	0.00
TRINITY_sp|P5141	RPL26A	60S ribosomal prote	52.20	0.00
TRINITY_sp|Q54F1	nubpl	Iron-sulfur protein	52.20	0.00
TRINITY_sp|Q9Y68	ARL5A	ADP-ribosylation fa	52.20	0.00
TRINITY_sp|O0917	Amacr	Alpha-methylacyl-Co	52.20	0.00
TRINITY_sp|Q4L3I	SH2450	Uncharacterized epi	52.20	0.00
TRINITY_sp|Q0U3Y	DDI1	DNA damage-inducibl	52.20	0.00
TRINITY_sp|Q9FKB	ABCA11	ABC transporter A f	52.20	0.00
TRINITY_sp|Q55DY	mcfF	Mitoferrin OS=Dicty	52.20	0.00
TRINITY_sp|Q75SY	AFP1	Peroxiredoxin Q, ch	52.20	0.00
TRINITY_sp|Q9LM0	SMT1	Cycloartenol-C-24-m	52.20	0.00
TRINITY_sp|O4884	RPN2A	26S proteasome non-	52.20	0.00
TRINITY_sp|Q7MA0	ung	Uracil-DNA glycosyl	52.20	0.00
TRINITY_sp|P3411	mvpA	Major vault protein	52.20	0.00
TRINITY_sp|Q6T39	Spata20	Spermatogenesis-ass	52.20	0.00
TRINITY_sp|Q9D84	Tex9	Testis-expressed se	52.20	0.00
TRINITY_sp|Q8IZF	NALCN	Sodium leak channel	52.20	0.00
TRINITY_sp|Q0JL4	Os01g062	Neutral ceramidase	52.20	0.00
TRINITY_sp|Q3956	ODA4	Dynein beta chain,	52.20	0.00
TRINITY_sp|P0482	tpiA	Triosephosphate iso	52.20	0.00
TRINITY_sp|P4910	RAB2A	Ras-related protein	52.20	0.00
TRINITY_sp|C6E7B	gpsA	Glycerol-3-phosphat	52.20	0.00
TRINITY_sp|Q87AD	blh	Beta-lactamase hydr	52.20	0.00
TRINITY_sp|Q6NZ2	rbm22	Pre-mRNA-splicing f	52.20	0.00
TRINITY_sp|Q0PGJ	AKR4C9	Aldo-keto reductase	52.20	0.00
TRINITY_sp|Q9C55	RH5	DEAD-box ATP-depend	52.20	0.00
TRINITY_sp|Q5BP9	LOG2	Cytokinin riboside	52.20	0.00
TRINITY_sp|Q54SV	allB1	Probable allantoina	52.20	0.00
TRINITY_sp|P3184	-	RNA-directed DNA po	52.20	0.00
TRINITY_sp|B5XF1	kdm8	Lysine-specific dem	52.20	0.00
TRINITY_sp|Q52NJ	RAB1A	Ras-related protein	52.20	0.00
TRINITY_sp|O2220	NDT1	Nicotinamide adenin	52.20	0.00
TRINITY_sp|H9BFQ	-	Tropinone reductase	52.20	0.00
TRINITY_sp|Q08DH	-	Probable tRNA methy	52.20	0.00
TRINITY_sp|Q4315	RPE	Ribulose-phosphate	52.20	0.00
TRINITY_sp|Q949R	MPC1	Mitochondrial pyruv	52.20	0.00
TRINITY_sp|Q9NB7	hiw	E3 ubiquitin-protei	52.20	0.00
TRINITY_sp|Q9LST	PBD1	Proteasome subunit	52.20	0.00
TRINITY_sp|P9051	-	Elongation factor 1	52.20	0.00
TRINITY_sp|P4595	ARP	DNA-(apurinic or ap	52.10	0.00
TRINITY_sp|Q1ZXH	gxcDD	Guanine exchange fa	52.10	0.00
TRINITY_sp|B2RTY	MYO9A	Unconventional myos	52.10	0.00
TRINITY_sp|Q0654	GPN3	GPN-loop GTPase 3 O	52.10	0.00
TRINITY_sp|Q54JR	abcC3	ABC transporter C f	52.10	0.00
TRINITY_sp|Q99MN	Kars	Lysine--tRNA ligase	52.10	0.00

TRINITY_sp|Q55E]	atxn10	Ataxin-10 homolog O	52.10	0.00
TRINITY_sp|P342]	rab-6.1	Ras-related protein	52.10	0.00
TRINITY_sp|Q7T0[	poc1a	POC1 centriolar pro	52.10	0.00
TRINITY_sp|Q54V(	sepsecs	O-phosphoseryl-tRNA	52.10	0.00
TRINITY_sp|P412[	Abca1	ATP-binding cassett	52.10	0.00
TRINITY_sp|P402[	-	Histone H2A OS=Plas	52.10	0.00
TRINITY_sp|Q6ZM\	KIF6	Kinesin-like protei	52.10	0.00
TRINITY_sp|Q54J[	abcC3	ABC transporter C f	52.10	0.00
TRINITY_sp|Q9SF\	NLP5	Protein NLP5 OS=Ara	52.10	0.00
TRINITY_sp|Q0P5]	RBM42	RNA-binding protein	52.10	0.00
TRINITY_sp|Q54X(	nt5c2	Cytosolic purine 5'	52.10	0.00
TRINITY_sp|Q2KH(	MACROD1	O-acetyl-ADP-ribose	52.10	0.00
TRINITY_sp|Q54U(	kif3	Kinesin-related pro	52.10	0.00
TRINITY_sp|Q9C7(	BUB3.2	Mitotic checkpoint	52.10	0.00
TRINITY_sp|Q01J[	CYCB2-1	Cyclin-B2-1 OS=Oryz	52.10	0.00
TRINITY_sp|Q8TC[	POC1B	POC1 centriolar pro	52.10	0.00
TRINITY_sp|P0DJ[	RPL23A	60S ribosomal prote	52.10	0.00
TRINITY_sp|O360]	aap1	Aspartyl aminopepti	52.10	0.00
TRINITY_sp|Q1AR(	treT	Trehalose synthase	52.10	0.00
TRINITY_sp|Q7X9]	AERO2	Endoplasmic reticul	52.10	0.00
TRINITY_sp|P315[	RHN1	Ras-related protein	52.10	0.00
TRINITY_sp|P186]	NRPB1	DNA-directed RNA po	52.10	0.00
TRINITY_sp|Q6CL[	DED1	ATP-dependent RNA h	52.10	0.00
TRINITY_sp|Q58E[	armt1	Protein-glutamate O	52.10	0.00
TRINITY_sp|Q9S7]	COX6B-1	Cytochrome c oxidas	52.10	0.00
TRINITY_sp|Q395[	-	Dynein 14 kDa light	52.10	0.00
TRINITY_sp|Q5F3]	mcm8	DNA helicase MCM8 O	52.10	0.00
TRINITY_sp|Q1H5[	LSM2	Sm-like protein LSM	52.10	0.00
TRINITY_sp|Q8EJ\	prpC	2-methylcitrate syn	52.10	0.00
TRINITY_sp|Q6FL[	DBP2	ATP-dependent RNA h	52.10	0.00
TRINITY_sp|P116[	ypt1	GTP-binding protein	52.10	0.00
TRINITY_sp|Q8VY[	MED31	Mediator of RNA pol	52.10	0.00
TRINITY_sp|P239(	menB	1,4-dihydroxy-2-nap	52.10	0.00
TRINITY_sp|Q8L7[	EDD1	Glycine--tRNA ligas	52.10	0.00
TRINITY_sp|P208[	pol	Retrovirus-related	52.10	0.00
TRINITY_sp|B8BK]	OsI_361[	DNA replication lic	52.10	0.00
TRINITY_sp|O243(	SUS2	Sucrose synthase 2	52.10	0.00
TRINITY_sp|Q24J[	P4H5	Prolyl 4-hydroxylas	52.10	0.00
TRINITY_sp|Q8XY[	clpP	ATP-dependent Clp p	52.10	0.00
TRINITY_sp|P420[	NDUFS8	NADH dehydrogenase	52.00	0.00
TRINITY_sp|Q920[	DNMT1	DNA (cytosine-5)-me	52.00	0.00
TRINITY_sp|Q5DU(	Cep164	Centrosomal protein	52.00	0.00
TRINITY_sp|Q5XI[	Mccc2	Methylcrotonoyl-CoA	52.00	0.00
TRINITY_sp|Q9LR[	BSU1	Serine/threonine-pr	52.00	0.00
TRINITY_sp|Q9HF\	-	Cytochrome b5 OS=Rh	52.00	0.00
TRINITY_sp|B9DF(	ISE2	DExH-box ATP-depend	52.00	0.00
TRINITY_sp|Q9LR[	POLD1	DNA polymerase delt	52.00	0.00
TRINITY_sp|Q420[	ABCC2	ABC transporter C f	52.00	0.00
TRINITY_sp|Q94J]	HSFA4B	Heat stress transcr	52.00	0.00
TRINITY_sp|P0DJ[	RPL35	60S ribosomal prote	52.00	0.00
TRINITY_sp|Q643[	Sqstm1	Sequestosome-1 OS=M	52.00	0.00
TRINITY_sp|P546[	pkbA	RAC family serine/t	52.00	0.00
TRINITY_sp|Q017[	EXOSC10	Exosome component 1	52.00	0.00
TRINITY_sp|Q86A(	polr3a	DNA-directed RNA po	52.00	0.00
TRINITY_sp|Q8C5[	Cwc22	Pre-mRNA-splicing f	52.00	0.00
TRINITY_sp|Q54E[	exo1	Exonuclease 1 OS=Di	52.00	0.00

TRINITY_sp|Q9FII	RPN5A	26S proteasome non-	52.00	0.00
TRINITY_sp|Q9P38	SPBC13A2	Putative phosphatid	52.00	0.00
TRINITY_sp|Q9QY3	Abcb11	Bile salt export pu	52.00	0.00
TRINITY_sp|Q3957	YPTC6	Ras-related protein	52.00	0.00
TRINITY_sp|Q5T65	CFAP58	Cilia- and flagella	52.00	0.00
TRINITY_sp|Q6DG9	kctd6	BTB/POZ domain-cont	52.00	0.00
TRINITY_sp|Q8MYI	DDB_G027	Probable serine/thr	52.00	0.00
TRINITY_sp|Q93Y9	RPOT1	DNA-directed RNA po	52.00	0.00
TRINITY_sp|Q9ASX	At5g052(	Uncharacterized aar	52.00	0.00
TRINITY_sp|Q2371	-	Elongation factor 2	52.00	0.00
TRINITY_sp|B8BK8	OsI_3591	Protein KTI12 homol	52.00	0.00
TRINITY_sp|Q6WWW	UPL3	E3 ubiquitin-protei	52.00	0.00
TRINITY_sp|Q8LP9	LACS6	Long chain acyl-CoA	52.00	0.00
TRINITY_sp|Q9ZT9	Os06g05(	Probable methionine	52.00	0.00
TRINITY_sp|O7445	naa20	N-terminal acetyltr	52.00	0.00
TRINITY_sp|Q9ZT8	PEL1	Protein PELOTA 1 OS	52.00	0.00
TRINITY_sp|O9653	Ate1	Arginyl-tRNA--prote	52.00	0.00
TRINITY_sp|Q6413	rnf12-a	E3 ubiquitin-protei	52.00	0.00
TRINITY_sp|Q9ZU3	ABCG7	ABC transporter G f	52.00	0.00
TRINITY_sp|Q8H2I	POLH	DNA polymerase eta	52.00	0.00
TRINITY_sp|P0446	-	Calmodulin OS=Triti	52.00	0.00
TRINITY_sp|P4356	RIM15	Serine/threonine-pr	51.90	0.00
TRINITY_sp|Q8BVH	Mettl6	Methyltransferase-l	51.90	0.00
TRINITY_sp|Q54F0	dhx8	ATP-dependent RNA h	51.90	0.00
TRINITY_sp|Q5012	gtpbp3	tRNA modification G	51.90	0.00
TRINITY_sp|Q5R91	DARS	Aspartate--tRNA lig	51.90	0.00
TRINITY_sp|Q8LF(	IDH1	Isocitrate dehydrog	51.90	0.00
TRINITY_sp|Q9CAH	UEV1B	Ubiquitin-conjugati	51.90	0.00
TRINITY_sp|Q9FDW	CB5-A	Cytochrome b5 isofo	51.90	0.00
TRINITY_sp|P1056	MIC	Myosin IC heavy cha	51.90	0.00
TRINITY_sp|Q9SZ5	GPX7	Putative glutathion	51.90	0.00
TRINITY_sp|Q91Z5	Grhpr	Glyoxylate reductas	51.90	0.00
TRINITY_sp|Q9LGV	CIPK1	CBL-interacting pro	51.90	0.00
TRINITY_sp|Q9ZUI	ETL1	Protein CHROMATIN R	51.90	0.00
TRINITY_sp|Q9UL(	ACSL5	Long-chain-fatty-ac	51.90	0.00
TRINITY_sp|Q9SRH	LSF2	Phosphoglucan phosp	51.90	0.00
TRINITY_sp|Q2JS(	rpsP	30S ribosomal prote	51.90	0.00
TRINITY_sp|Q0992	yakc	Aldo-keto reductase	51.90	0.00
TRINITY_sp|P0A19	livF	High-affinity branc	51.90	0.00
TRINITY_sp|Q5F36	TBCK	TBC domain-containi	51.90	0.00
TRINITY_sp|Q9H7H	ATP13A3	Probable cation-tra	51.90	0.00
TRINITY_sp|Q9M1(	ABCB21	ABC transporter B f	51.90	0.00
TRINITY_sp|Q8P52	XCC3184	Macro domain-contai	51.90	0.00
TRINITY_sp|P1693	FAH	Fumarylacetoacetase	51.90	0.00
TRINITY_sp|Q8R3(	Rbm19	Probable RNA-bindin	51.90	0.00
TRINITY_sp|Q9VK4	Tor	Target of rapamycin	51.90	0.00
TRINITY_sp|Q9LE1	BRIX1-1	Ribosome biogenesis	51.90	0.00
TRINITY_sp|Q10S8	NLP1	Protein NLP1 OS=Ory	51.90	0.00
TRINITY_sp|Q9C5(	MSRB2	Peptide methionine	51.90	0.00
TRINITY_sp|Q9M0V	DDB1A	DNA damage-binding	51.90	0.00
TRINITY_sp|O049(	UMK3	UMP-CMP kinase 3 OS	51.90	0.00
TRINITY_sp|P511(	ANT18	Dihydroflavonol 4-r	51.90	0.00
TRINITY_sp|Q8I8U	ap1g1	AP-1 complex subuni	51.90	0.00
TRINITY_sp|Q9ZQH	ACX1.2	Putative peroxisoma	51.90	0.00
TRINITY_sp|Q54N4	asf1	Histone chaperone a	51.90	0.00
TRINITY_sp|Q2663	-	Cathepsin L OS=Sarc	51.90	0.00

TRINITY_sp|Q6L5(	Os05g05'	Zinc finger CCCH do	51.90	0.00
TRINITY_sp|P0C8N	CCRP1	Probable serine/thr	51.90	0.00
TRINITY_sp|P505:	smc4	Structural maintena	51.90	0.00
TRINITY_sp|Q9I8I	-	Heat shock 70 kDa p	51.90	0.00
TRINITY_sp|C0QT:	prfA	Peptide chain relea	51.90	0.00
TRINITY_sp|Q428'	RPB2	DNA-directed RNA po	51.90	0
TRINITY_sp|P406!	-	Aldo-keto reductase	51.90	0.00
TRINITY_sp|P0AG:	purF	Amidophosphoribosyl	51.90	0.00
TRINITY_sp|Q944(	dymA	Dynamin-A OS=Dictyo	51.90	0.00
TRINITY_sp|Q2N5I	groL2	60 kDa chaperonin 2	51.90	0.00
TRINITY_sp|Q9LDI	UMPS1	Uridine 5'-monophos	51.90	0.00
TRINITY_sp|Q4U2\	agtpbp1	Cytosolic carboxype	51.90	0.00
TRINITY_sp|Q9ZG:	pikAI	Narbonolide/10-deox	51.90	0.00
TRINITY_sp|Q025!	PK2	Probable serine/thr	51.90	0.00
TRINITY_sp|P086‹	R	Ras-like protein 3	51.90	0.00
TRINITY_sp|Q9M6‹	CPI1	Cycloeucalenol cycl	51.90	0.00
TRINITY_sp|O809{	FTSH4	ATP-dependent zinc	51.90	0.00
TRINITY_sp|A4FUI	RPL22L1	60S ribosomal prote	51.80	0.00
TRINITY_sp|Q55EI	bub2	Putative mitotic ch	51.80	0.00
TRINITY_sp|Q9LTI	NMT1	Glycylpeptide N-tet	51.80	0.00
TRINITY_sp|Q9P4>	utp7	Probable U3 small n	51.80	0.00
TRINITY_sp|Q8NEI	PIK3C3	Phosphatidylinosito	51.80	0.00
TRINITY_sp|Q54J‹	alg5	Dolichyl-phosphate	51.80	0.00
TRINITY_sp|P930(	RH21	DEAD-box ATP-depend	51.80	0.00
TRINITY_sp|Q424!	petM	Cytochrome b6-f com	51.80	0.00
TRINITY_sp|P562:	Gfer	FAD-linked sulfhydr	51.80	0.00
TRINITY_sp|P275:	CBR	Carotene biosynthes	51.80	0.00
TRINITY_sp|O134!	rca-1	Myb-like DNA-bindin	51.80	0.00
TRINITY_sp|Q93V:	PABN1	Polyadenylate-bindi	51.80	0.00
TRINITY_sp|P237(	ltxB	Leukotoxin export A	51.80	0.00
TRINITY_sp|P0AF‹	yjbQ	UPF0047 protein Yjb	51.80	0.00
TRINITY_sp|Q9M0:	KEA3	K(+) efflux antipor	51.80	0.00
TRINITY_sp|Q9SR(	DML2	DEMETER-like protei	51.80	0.00
TRINITY_sp|O827!	PSP	Phosphoserine phosp	51.80	0.00
TRINITY_sp|B2I6:	metE	5-methyltetrahydrop	51.80	0.00
TRINITY_sp|B9SQ:	RCOM_05!	Probable bifunction	51.80	0.00
TRINITY_sp|Q9ZU!	VAC14	Protein VAC14 homol	51.80	0.00
TRINITY_sp|P519!	Nek1	Serine/threonine-pr	51.80	0.00
TRINITY_sp|P490:	GPI8	GPI-anchor transami	51.80	0.00
TRINITY_sp|Q54VI	ddx56	Probable ATP-depend	51.80	0.00
TRINITY_sp|Q86G\	gcy-28	Receptor-type guany	51.80	0.00
TRINITY_sp|Q8LF(	AMY2	Probable alpha-amyl	51.80	0.00
TRINITY_sp|O491(	TIF3C1	Eukaryotic translat	51.80	0.00
TRINITY_sp|F4I1!	ELP2	Elongator complex p	51.80	0.00
TRINITY_sp|Q9T0I	LACS4	Long chain acyl-CoA	51.80	0.00
TRINITY_sp|Q3V0(	Dnah12	Dynein heavy chain	51.70	0.00
TRINITY_sp|Q9M7>	RPL29A	60S ribosomal prote	51.70	0.00
TRINITY_sp|Q7Z6:	HUWE1	E3 ubiquitin-protei	51.70	0.00
TRINITY_sp|Q6NW:	ypel3	Protein yippee-like	51.70	0.00
TRINITY_sp|Q9C7I	NTF2	Nuclear transport f	51.70	0.00
TRINITY_sp|Q9YH\	ncapd2	Condensin complex s	51.70	0.00
TRINITY_sp|E1BB(	ZRANB3	DNA annealing helic	51.70	0.00
TRINITY_sp|Q54MI	ku70	ATP-dependent DNA h	51.70	0.00
TRINITY_sp|Q410!	SBEI	1,4-alpha-glucan-br	51.70	0.00
TRINITY_sp|E1VB'	HELO_23:	Putative glutathion	51.70	0.00
TRINITY_sp|Q008!	MAPK	Mitogen-activated p	51.70	0.00

TRINITY_sp|Q0KIA	CG9801	PP2C-like domain-co	51.70	0.00
TRINITY_sp|Q9FH4	TAF14B	Transcription initi	51.70	0.00
TRINITY_sp|Q54S8	midA	Protein arginine me	51.70	0.00
TRINITY_sp|Q8BM1	Lipk	Lipase member K OS=	51.70	0.00
TRINITY_sp|P341(	pkgC	Protein kinase 3 OS	51.70	0.00
TRINITY_sp|Q8H16	ALEU	Thiol protease aleu	51.70	0.00
TRINITY_sp|P9819	Atp9b	Probable phospholip	51.70	0.00
TRINITY_sp|Q9SB(	PHR1	Deoxyribodipyrimidi	51.70	0.00
TRINITY_sp|Q556Y	rexo2-1	Probable oligoribon	51.70	0.00
TRINITY_sp|Q8VZ9	FZR1	Protein FIZZY-RELAT	51.70	0.00
TRINITY_sp|Q0085	MAPK	Mitogen-activated p	51.70	0.00
TRINITY_sp|A7HT6	rpmA	50S ribosomal prote	51.70	0.00
TRINITY_sp|Q9C0(	DNAH6	Dynein heavy chain	51.70	0.00
TRINITY_sp|Q942I	PDIL2-2	Protein disulfide i	51.70	0.00
TRINITY_sp|Q9JK8	Myg1	UPF0160 protein MYG	51.70	0.00
TRINITY_sp|Q6GLM	desi1	Desumoylating isope	51.70	0.00
TRINITY_sp|O6476	GLCNAC1I	UDP-N-acetylglucosa	51.70	0.00
TRINITY_sp|Q2074	atad-3	ATPase family AAA d	51.70	0.00
TRINITY_sp|Q8TGM	TAR1	Protein TAR1 OS=Sac	51.70	0.00
TRINITY_sp|Q6NQ	SDG40	Protein SET DOMAIN	51.70	0.00
TRINITY_sp|Q9Y68	ARL5A	ADP-ribosylation fa	51.70	0.00
TRINITY_sp|Q8RXM	PRP31	U4/U6 small nuclear	51.70	0.00
TRINITY_sp|Q8LG7	COL6	Zinc finger protein	51.70	0.00
TRINITY_sp|Q3232	ybiA	N-glycosidase YbiA	51.70	0.00
TRINITY_sp|Q9HZ(	PA3240	Putative quercetin	51.70	0.00
TRINITY_sp|Q8Y2I	RSc0270	UPF0225 protein RSc	51.70	0.00
TRINITY_sp|P0AG	trmH	tRNA (guanosine(18)	51.70	0.00
TRINITY_sp|Q6MD9	clpB	Chaperone protein C	51.70	0.00
TRINITY_sp|Q6PFM	jmjd6	Bifunctional argini	51.70	0.00
TRINITY_sp|Q8KAI	pckG	Phosphoenolpyruvate	51.70	0.00
TRINITY_sp|Q0WW5	ZEU1	Thymidylate kinase	51.70	0.00
TRINITY_sp|Q9C54	CM3	Chorismate mutase 3	51.70	0.00
TRINITY_sp|Q9261	EDEM1	ER degradation-enha	51.70	0.00
TRINITY_sp|Q3232	ybiA	N-glycosidase YbiA	51.70	0.00
TRINITY_sp|A8I4I	FAP100	Cilia- and flagella	51.70	0.00
TRINITY_sp|Q9UBN	HDAC6	Histone deacetylase	51.70	0.00
TRINITY_sp|O7485	caf1	Poly(A) ribonucleas	51.70	0.00
TRINITY_sp|Q7XK(	PYRD	Dihydroorotate dehy	51.70	0.00
TRINITY_sp|Q2868	ABCC2	Canalicular multisp	51.70	0.00
TRINITY_sp|Q711I	CCL4	C-C motif chemokine	51.70	0.00
TRINITY_sp|P4921	RPL9	60S ribosomal prote	51.60	0.00
TRINITY_sp|Q9LSU	PAE1	Proteasome subunit	51.60	0.00
TRINITY_sp|Q8I7	coq-2	4-hydroxybenzoate p	51.60	0.00
TRINITY_sp|Q6NV3	decr2	Peroxisomal 2,4-die	51.60	0.00
TRINITY_sp|Q9VHI	GstZ2	Probable maleylacet	51.60	0.00
TRINITY_sp|Q86A(	polr3a	DNA-directed RNA po	51.60	0.00
TRINITY_sp|Q7T3I	dbr1	Lariat debranching	51.60	0.00
TRINITY_sp|Q7YSW	canA	Calcineurin subunit	51.60	0.00

TRINITY_sp|O644(	BMY1	Beta-amylase OS=Vig	51.60	0.00
TRINITY_sp|Q9P7.	gad8	Serine/threonine-pr	51.60	0.00
TRINITY_sp|Q9C5(	ELF5A-3	Eukaryotic translat	51.60	0.00
TRINITY_sp|Q6Z5	SAP12	Zinc finger A20 and	51.60	0.00
TRINITY_sp|P231	ABCB4	Phosphatidylcholine	51.60	0.00
TRINITY_sp|Q9BU	TRMO	tRNA (adenine(37)-N	51.60	0.00
TRINITY_sp|Q9FZ	SMG7L	Protein SMG7L OS=Ar	51.60	0.00
TRINITY_sp|P485(	-	Cytochrome b-c1 com	51.60	0.00
TRINITY_sp|G5DB.	CPP1	Protein CHAPERONE-L	51.60	0.00
TRINITY_sp|O499	TLP40	Peptidyl-prolyl cis	51.60	0.00
TRINITY_sp|Q54W	lsm1	Probable U6 snRNA-a	51.60	0.00
TRINITY_sp|Q54GI	DDB_G02	PXMP2/4 family prot	51.60	0.00
TRINITY_sp|Q86A	purF	Amidophosphoribosyl	51.60	0.00
TRINITY_sp|Q54KI	mccA	Methylcrotonoyl-CoA	51.60	0.00
TRINITY_sp|Q7ZVI	sirt2	NAD-dependent prote	51.60	0.00
TRINITY_sp|Q9SH	UBC34	Ubiquitin-conjugati	51.60	0.00
TRINITY_sp|P078	SUOX	Sulfite oxidase OS=	51.60	0.00
TRINITY_sp|P524	PAC1	Proteasome subunit	51.60	0.00
TRINITY_sp|Q568I	gtdc1	Glycosyltransferase	51.60	0.00
TRINITY_sp|Q54S	sf3b3	Probable splicing f	51.60	0.00
TRINITY_sp|Q9FI	UBC27	Ubiquitin-conjugati	51.60	0.00
TRINITY_sp|Q018	YPT1	Ras-like GTP-bindin	51.60	0.00
TRINITY_sp|Q9BZI	GTPBP4	Nucleolar GTP-bindi	51.60	0.00
TRINITY_sp|Q9C9(	RPL6B	60S ribosomal prote	51.60	0.00
TRINITY_sp|Q2NC	ELI_029	Blue-light-activate	51.60	0.00
TRINITY_sp|F4IE	RID1	Pre-mRNA-splicing f	51.60	0.00
TRINITY_sp|Q8NDI	PAPD5	Non-canonical poly(	51.60	0.00
TRINITY_sp|Q099	yakc	Aldo-keto reductase	51.60	0.00
TRINITY_sp|F4KAI	MCM6	DNA replication lic	51.60	0.00
TRINITY_sp|F1Q4	atp9b	Probable phospholip	51.60	0.00
TRINITY_sp|P347	RAS1	Ras-like protein 1	51.60	0.00
TRINITY_sp|P153	RpS27A	Ubiquitin-40S ribos	51.60	0.00
TRINITY_sp|Q54R	mkkA	Mitogen-activated p	51.60	0.00
TRINITY_sp|Q9LX	At5g128	Zinc finger CCCH do	51.60	0.00
TRINITY_sp|Q9C5	SMC2-1	Structural maintena	51.60	0.00
TRINITY_sp|Q86A	spkA-1	Stress-activated pr	51.60	0.00
TRINITY_sp|P518	lig1	DNA ligase 1 OS=Xen	51.60	0.00
TRINITY_sp|Q551	purU	Formyltetrahydrofol	51.60	0.00
TRINITY_sp|A3QK	erlin2	Erlin-2 OS=Danio re	51.60	0.00
TRINITY_sp|B7ZC	KIF28P	Kinesin-like protei	51.50	0.00
TRINITY_sp|P051	CFI	Complement factor I	51.50	0.00
TRINITY_sp|Q3S4	AHK5	Histidine kinase 5	51.50	0.00
TRINITY_sp|F1Q4	atp9b	Probable phospholip	51.50	0.00
TRINITY_sp|Q9LEI	GPXMC1	Probable phospholip	51.50	0.00
TRINITY_sp|P521	kpna1	Importin subunit al	51.50	0.00
TRINITY_sp|Q9XGI	B''ALPH	Serine/threonine pr	51.50	0.00
TRINITY_sp|Q54SI	pemtA	Phosphatidylethanol	51.50	0.00
TRINITY_sp|Q55CI	rasU	Ras-like protein ra	51.50	0.00
TRINITY_sp|P981	ATP8B2	Phospholipid-transp	51.50	0.00
TRINITY_sp|Q5XJI	fip1l1	Pre-mRNA 3'-end-pro	51.50	0.00
TRINITY_sp|O230	ECA2	Calcium-transportin	51.50	0.00
TRINITY_sp|P229	let-60	Ras protein let-60	51.50	0.00
TRINITY_sp|A0Q3I	ribBA	Riboflavin biosynth	51.50	0.00
TRINITY_sp|Q8L7I	RPL18AA	60S ribosomal prote	51.50	0.00
TRINITY_sp|Q2KII	TDH	L-threonine 3-dehyd	51.50	0.00
TRINITY_sp|Q9FJI	UPF1	Regulator of nonsen	51.50	0.00

TRINITY_sp|Q082:	REX4	RNA exonuclease 4 O	51.50	0.00
TRINITY_sp|P525:	mybl2	Myb-related protein	51.50	0.00
TRINITY_sp|P142:	PSAG	Photosystem I react	51.50	0.00
TRINITY_sp|P519:	Nek1	Serine/threonine-pr	51.50	0.00
TRINITY_sp|Q8C0:	Agps	Alkyldihydroxyaceto	51.50	0.00
TRINITY_sp|P102:	MYB	Transcriptional act	51.50	0.00
TRINITY_sp|Q9PT:	kcnab2	Voltage-gated potas	51.50	0.00
TRINITY_sp|B0G1:	mcfC	Mitochondrial subst	51.50	0.00
TRINITY_sp|Q138:	EXOSC2	Exosome complex com	51.50	0.00
TRINITY_sp|P918:	Mo25	Protein Mo25 OS=Dro	51.50	0.00
TRINITY_sp|P351:	UBC11	Ubiquitin-conjugati	51.50	0.00
TRINITY_sp|Q8NG:	TTL	Tubulin--tyrosine l	51.50	0.00
TRINITY_sp|Q6DE:	etnppl	Ethanolamine-phosph	51.50	0.00
TRINITY_sp|B2RX:	Abcc3	Canalicular multisp	51.50	0.00
TRINITY_sp|Q9ST:	ABCA5	ABC transporter A f	51.50	0.00
TRINITY_sp|A8IB:	CFAP77	Cilia- and flagella	51.50	0.00
TRINITY_sp|Q389:	KIN10	SNF1-related protei	51.50	0.00
TRINITY_sp|Q6NM:	At5g597	UDP-galactose/UDP-g	51.50	0.00
TRINITY_sp|Q5ZL:	GPAT3	Glycerol-3-phosphat	51.50	0.00
TRINITY_sp|Q9SM:	DHC1	Dynein-1-alpha heav	51.50	0
TRINITY_sp|Q641:	ublcp1	Ubiquitin-like doma	51.50	0.00
TRINITY_sp|Q8NB:	RDH13	Retinol dehydrogena	51.50	0.00
TRINITY_sp|Q66K:	ube2z	Ubiquitin-conjugati	51.50	0.00
TRINITY_sp|Q8T1:	kif5	Kinesin-related pro	51.50	0.00
TRINITY_sp|P467:	cysB	Cystathionine beta-	51.50	0.00
TRINITY_sp|O860:	bdhA	D-beta-hydroxybutyr	51.50	0.00
TRINITY_sp|Q54P:	csnk2b	Casein kinase II su	51.50	0.00
TRINITY_sp|Q01J:	CYCB2-1	Cyclin-B2-1 OS=Oryz	51.50	0.00
TRINITY_sp|F4I1:	At1g096	Leucine--tRNA ligas	51.50	0
TRINITY_sp|Q9C5:	ATX1	Histone-lysine N-me	51.50	0.00
TRINITY_sp|F4JX:	NRPC1	DNA-directed RNA po	51.50	0.00
TRINITY_sp|P240:	-	Glu S.griseus prote	51.50	0.00
TRINITY_sp|Q9VQ:	CG3326	Fidgetin-like prote	51.40	0.00
TRINITY_sp|P529:	VPS4	Vacuolar protein so	51.40	0.00
TRINITY_sp|O322:	yvgN	Glyoxal reductase O	51.40	0.00
TRINITY_sp|Q55E:	vps13E	Putative vacuolar p	51.40	0.00
TRINITY_sp|Q5E9:	NFYA	Nuclear transcripti	51.40	0.00
TRINITY_sp|Q93Y:	RPN7	26S proteasome non-	51.40	0.00
TRINITY_sp|Q338:	GCN5	Histone acetyltrans	51.40	0.00
TRINITY_sp|Q9CB:	RABF1	Ras-related protein	51.40	0.00
TRINITY_sp|Q9SL:	TCX6	Protein tesmin/TSO1	51.40	0.00
TRINITY_sp|Q9UN:	CDC14A	Dual specificity pr	51.40	0.00
TRINITY_sp|O349:	ytsJ	Probable NAD-depend	51.40	0.00
TRINITY_sp|Q588:	TEB	Helicase and polyme	51.40	0.00
TRINITY_sp|P0C6:	Dnah2	Dynein heavy chain	51.40	0.00
TRINITY_sp|Q9NR:	HELLS	Lymphoid-specific h	51.40	0.00
TRINITY_sp|P241:	CDKA-1	Cyclin-dependent ki	51.40	0.00
TRINITY_sp|Q7ZU:	stk3	Serine/threonine-pr	51.40	0.00
TRINITY_sp|P832:	CBR2	NADH-cytochrome b5	51.40	0.00
TRINITY_sp|G5EG:	let-92	Serine/threonine-pr	51.40	0.00
TRINITY_sp|P777:	yajO	Uncharacterized oxi	51.40	0.00
TRINITY_sp|Q407:	RGP2	Ras-related protein	51.40	0.00
TRINITY_sp|Q9C5:	SUF4	Protein SUPPRESSOR	51.40	0.00
TRINITY_sp|Q0DJ:	Os05g03	Coatomer subunit de	51.40	0.00
TRINITY_sp|Q1LK:	gluQ	Glutamyl-Q tRNA(Asp	51.40	0.00
TRINITY_sp|B0F9:	GC6	Golgin candidate 6	51.40	0.00

TRINITY_sp|Q554{	slr0537	Uncharacterized sug	51.40	0.00
TRINITY_sp|O649{	RPT1	26S protease regula	51.40	0.00
TRINITY_sp|Q9UT{	SPAC343.	Uncharacterized WD	51.40	0.00
TRINITY_sp|Q6B8{	TKT	Transketolase OS=Bo	51.40	0.00
TRINITY_sp|P152{	AMD1	AMP deaminase OS=Sa	51.40	0.00
TRINITY_sp|B7NT{	ydiU	UPF0061 protein Ydi	51.40	0.00
TRINITY_sp|A8WK{	CBG2419{	Putative ATP-depend	51.40	0.00
TRINITY_sp|Q554{	aslB	Acyl-CoA synthetase	51.40	0.00
TRINITY_sp|Q9XY{	noxA	Superoxide-generati	51.40	0.00
TRINITY_sp|P428{	MTHFR	Methylenetetrahydro	51.40	0.00
TRINITY_sp|Q007{	glpV	Glycogen phosphoryl	51.40	0.00
TRINITY_sp|Q940{	RBX1A	RING-box protein 1a	51.40	0.00
TRINITY_sp|Q54B{	ggps1	Geranylgeranyl pyro	51.40	0.00
TRINITY_sp|Q8L8{	RECQL4A	ATP-dependent DNA h	51.40	0.00
TRINITY_sp|P0CR{	SEC23	Protein transport p	51.40	0.00
TRINITY_sp|Q9FL{	At5g645{	Probable sphingolip	51.40	0.00
TRINITY_sp|Q54U{	DDB_G02{	Bromodomain-contain	51.40	0.00
TRINITY_sp|P933{	UPP	Uracil phosphoribos	51.40	0.00
TRINITY_sp|Q7XP{	Os04g06{	Kinesin-like calmod	51.40	0.00
TRINITY_sp|D2K6{	SLT2	Sodium/sulfate cotr	51.40	0.00
TRINITY_sp|P0CM{	CNBB373{	Pentafunctional ARO	51.40	0.00
TRINITY_sp|Q642{	impact	Protein IMPACT OS=D	51.40	0.00
TRINITY_sp|Q3ZC{	ANAPC11	Anaphase-promoting	51.30	0.00
TRINITY_sp|O605{	CDC40	Pre-mRNA-processing	51.30	0.00
TRINITY_sp|Q8R1{	Phykpl	5-phosphohydroxy-L-	51.30	0.00
TRINITY_sp|P905{	-	66 kDa stress prote	51.30	0.00
TRINITY_sp|Q9FN{	At5g227{	Putative SWI/SNF-re	51.30	0.00
TRINITY_sp|Q498{	smarcal{	SWI/SNF-related mat	51.30	0.00
TRINITY_sp|Q8LF{	IDH1	Isocitrate dehydrog	51.30	0.00
TRINITY_sp|Q54D{	alg11	GDP-Man:Man(3)GlcNA	51.30	0.00
TRINITY_sp|Q7KW{	exosc3	Putative exosome co	51.30	0.00
TRINITY_sp|Q279{	COPA	Coatomer subunit al	51.30	0.00
TRINITY_sp|A4IQ{	namA	NADPH dehydrogenase	51.30	0.00
TRINITY_sp|Q102{	rrp8	25S rRNA (adenine(6	51.30	0.00
TRINITY_sp|Q55C{	DDB_G02{	Probable NADH dehyd	51.30	0.00
TRINITY_sp|Q920{	pcmt	Protein-L-isoaspart	51.30	0.00
TRINITY_sp|Q9FM{	RD21B	Probable cysteine p	51.30	0.00
TRINITY_sp|Q9QZ{	Eif2ak4	eIF-2-alpha kinase	51.30	0.00
TRINITY_sp|Q9M8{	EMB2768	Tyrosine--tRNA liga	51.30	0.00
TRINITY_sp|Q8GT{	CCS1	Cytochrome c biogen	51.30	0.00
TRINITY_sp|Q9Y2{	STK38L	Serine/threonine-pr	51.30	0.00
TRINITY_sp|Q84Q{	PPT2	Phosphoenolpyruvate	51.30	0.00
TRINITY_sp|P078{	SUOX	Sulfite oxidase OS=	51.30	0.00
TRINITY_sp|B9G2{	Os09g02{	Auxin transport pro	51.30	0.00
TRINITY_sp|Q339{	BGLU34	Beta-glucosidase 34	51.30	0.00
TRINITY_sp|Q89N{	guaA	GMP synthase [gluta	51.30	0.00
TRINITY_sp|Q75V{	dnaJ	Chaperone protein D	51.30	0.00
TRINITY_sp|F4J8{	RRP5	rRNA biogenesis pro	51.30	0.00
TRINITY_sp|F4I2{	CTN	Cactin OS=Arabidops	51.30	0.00
TRINITY_sp|Q55G{	cdk10	Probable cyclin-dep	51.30	0.00
TRINITY_sp|O748{	caf1	Poly(A) ribonucleas	51.30	0.00
TRINITY_sp|Q54P{	pwp2	Periodic tryptophan	51.30	0.00
TRINITY_sp|Q0WS{	ALDH22A{	Aldehyde dehydrogen	51.30	0.00
TRINITY_sp|Q9ZW{	PUMP2	Mitochondrial uncou	51.20	0.00
TRINITY_sp|P623{	CPK4	Calcium-dependent p	51.20	0.00
TRINITY_sp|Q9BS{	TUBGCP2	Gamma-tubulin compl	51.20	0.00

TRINITY_sp|Q4WP3	cym1	Mitochondrial prese	51.20	0.00
TRINITY_sp|Q55E5	pats1	Probable serine/thr	51.20	0.00
TRINITY_sp|P3560	COPZ1	Coatomer subunit ze	51.20	0.00
TRINITY_sp|Q0922	cpna-2	Copine family prote	51.20	0.00
TRINITY_sp|Q8BG0	Zadh2	Prostaglandin reduc	51.20	0.00
TRINITY_sp|P4079	Rac1	Ras-related protein	51.20	0.00
TRINITY_sp|O4309	-	Kinesin heavy chain	51.20	0.00
TRINITY_sp|Q9ZN1	SKD1	Protein SUPPRESSOR	51.20	0.00
TRINITY_sp|Q4254	UBC7	Ubiquitin-conjugati	51.20	0.00
TRINITY_sp|P5348	arp2	Actin-related prote	51.20	0.00
TRINITY_sp|Q9SU0	AUXI1	Auxilin-related pro	51.20	0.00
TRINITY_sp|Q5XIN	Traf3ip1	TRAF3-interacting p	51.20	0.00
TRINITY_sp|Q8H8U	Os03g028	Cardiolipin synthas	51.20	0.00
TRINITY_sp|Q8H0V	ABCF3	ABC transporter F f	51.20	0.00
TRINITY_sp|Q8VH2	Imp4	U3 small nucleolar	51.20	0.00
TRINITY_sp|Q8VZ1	CBSDUF6	DUF21 domain-contai	51.20	0.00
TRINITY_sp|Q6PC9	btf3l4	Transcription facto	51.20	0.00
TRINITY_sp|Q9C9F	APUM7	Putative pumilio ho	51.20	0.00
TRINITY_sp|Q6CF2	YAF9	Protein AF-9 homolo	51.20	0.00
TRINITY_sp|Q54EI	grwd1	Glutamate-rich WD r	51.20	0.00
TRINITY_sp|A9AF1	recA	Protein RecA OS=Bur	51.20	0.00
TRINITY_sp|Q8H10	At4g2691	Dihydrolipoyllysine	51.20	0.00
TRINITY_sp|P5464	pkbA	RAC family serine/t	51.20	0.00
TRINITY_sp|Q3921	NRPB3	DNA-directed RNA po	51.20	0.00
TRINITY_sp|Q9XY9	noxA	Superoxide-generati	51.20	0.00
TRINITY_sp|P5464	pkbA	RAC family serine/t	51.20	0.00
TRINITY_sp|Q557H	dpp3-1	Dipeptidyl peptidas	51.20	0.00
TRINITY_sp|P8391	CBX1	Chromobox protein h	51.20	0.00
TRINITY_sp|Q9ZV6	AXL1	NEDD8-activating en	51.20	0.00
TRINITY_sp|Q9SU7	PAO5	Probable polyamine	51.20	0.00
TRINITY_sp|Q8W4H	At4g0467	tRNA wybutosine-syn	51.20	0.00
TRINITY_sp|Q6K4N	RSZP21	Serine/arginine-ric	51.20	0.00
TRINITY_sp|O1543	ABCC4	Multidrug resistanc	51.20	0.00
TRINITY_sp|Q54U0	kif3	Kinesin-related pro	51.20	0.00
TRINITY_sp|A8IQ1	CCDC40	Coiled-coil domain-	51.20	0.00
TRINITY_sp|O0180	rab-11.1	Ras-related protein	51.20	0.00
TRINITY_sp|Q32PH	LSM3	U6 snRNA-associated	51.20	0.00
TRINITY_sp|Q9M82	UGT80A2	Sterol 3-beta-gluco	51.20	0.00
TRINITY_sp|O2289	At2g4725	Probable pre-mRNA-s	51.20	0.00
TRINITY_sp|Q0970	pld1	Phospholipase D1 OS	51.20	0.00
TRINITY_sp|O9443	SPBC660.	Uncharacterized RNA	51.10	0.00
TRINITY_sp|O0915	Man2b1	Lysosomal alpha-man	51.10	0.00
TRINITY_sp|Q3923	At5g2747	Serine--tRNA ligase	51.10	0.00
TRINITY_sp|Q7KQI	ARF1	ADP-ribosylation fa	51.10	0.00
TRINITY_sp|Q9LEY	At5g0818	H/ACA ribonucleopro	51.10	0.00
TRINITY_sp|Q3T00	PROSC	Proline synthase co	51.10	0.00
TRINITY_sp|Q69TY	PRXIIE-1	Peroxiredoxin-2E-1,	51.10	0.00
TRINITY_sp|Q1343	SF3B2	Splicing factor 3B	51.10	0.00
TRINITY_sp|Q6IP7	akt2-b	RAC-beta serine/thr	51.10	0.00
TRINITY_sp|Q54FY	cpnE	Copine-E OS=Dictyos	51.10	0.00
TRINITY_sp|Q9UQ0	MOK	MAPK/MAK/MRK overla	51.10	0.00
TRINITY_sp|Q54N0	sfxn	Sideroflexin OS=Dic	51.10	0.00
TRINITY_sp|P4123	Abca2	ATP-binding cassett	51.10	0.00
TRINITY_sp|P0CD6	alg7	UDP-N-acetylglucosa	51.10	0.00
TRINITY_sp|Q93Z1	CKL3	Casein kinase 1-lik	51.10	0.00
TRINITY_sp|Q1LZA	LIMD2	LIM domain-containi	51.10	0.00

TRINITY_sp|A8JF[illegible]	ODA1	Outer dynein arm pr	51.10	0.00
TRINITY_sp|Q54G[illegible]	DDB_G02[illegible]	Coiled-coil domain-	51.10	0.00
TRINITY_sp|Q869[illegible]	DDB_G02[illegible]	3-hydroxybutyryl-Co	51.10	0.00
TRINITY_sp|Q54R[illegible]	pyk	Pyruvate kinase OS=	51.10	0.00
TRINITY_sp|A2AR[illegible]	Ppip5k1	Inositol hexakispho	51.10	0.00
TRINITY_sp|Q9P2[illegible]	WDR35	WD repeat-containin	51.10	0.00
TRINITY_sp|Q86C[illegible]	tor	Target of rapamycin	51.10	0.00
TRINITY_sp|A5D9[illegible]	SPRTN	SprT-like domain-co	51.10	0.00
TRINITY_sp|Q9UJ[illegible]	TUBE1	Tubulin epsilon cha	51.10	0.00
TRINITY_sp|O049[illegible]	GDHA	Glutamate dehydroge	51.10	0.00
TRINITY_sp|Q54R[illegible]	yipf6	Protein YIPF6 homol	51.10	0.00
TRINITY_sp|O648[illegible]	COX10	Protoheme IX farnes	51.10	0.00
TRINITY_sp|Q9SI[illegible]	RPN1A	26S proteasome non-	51.10	0.00
TRINITY_sp|P290[illegible]	TIV1	Acid beta-fructofur	51.10	0.00
TRINITY_sp|Q3UT[illegible]	Cdkl5	Cyclin-dependent ki	51.10	0.00
TRINITY_sp|O751[illegible]	CNOT3	CCR4-NOT transcript	51.10	0.00
TRINITY_sp|Q9NV[illegible]	RLIM	E3 ubiquitin-protei	51.10	0.00
TRINITY_sp|Q5M7[illegible]	TH1	Thiamine biosynthet	51.10	0.00
TRINITY_sp|O823[illegible]	At2g258[illegible]	Probable transcript	51.10	0.00
TRINITY_sp|Q93Y[illegible]	RUS6	Protein root UVB se	51.10	0.00
TRINITY_sp|P109[illegible]	-	Retrovirus-related	51.10	0.00
TRINITY_sp|O647[illegible]	At2g348[illegible]	Coatomer subunit ep	51.10	0.00
TRINITY_sp|Q6DG[illegible]	kctd6	BTB/POZ domain-cont	51.10	0.00
TRINITY_sp|Q68E[illegible]	amdhd1	Probable imidazolon	51.10	0.00
TRINITY_sp|Q9BR[illegible]	RIOK1	Serine/threonine-pr	51.10	0.00
TRINITY_sp|P736[illegible]	sll1770	Uncharacterized pro	51.10	0.00
TRINITY_sp|O652[illegible]	ACX2	Acyl-coenzyme A oxi	51.10	0.00
TRINITY_sp|P565[illegible]	HDAC3	Histone deacetylase	51.10	0.00
TRINITY_sp|Q9C9[illegible]	SPPA	Serine protease SPP	51.10	0.00
TRINITY_sp|F4KD[illegible]	DUR3	Urea-proton symport	51.10	0.00
TRINITY_sp|Q9SM[illegible]	AAE3	Oxalate--CoA ligase	51.10	0.00
TRINITY_sp|P114[illegible]	CRYZ	Quinone oxidoreduct	51.00	0.00
TRINITY_sp|Q9C0[illegible]	DNAH6	Dynein heavy chain	51.00	0.00
TRINITY_sp|Q120[illegible]	TSR3	Ribosome biogenesis	51.00	0.00
TRINITY_sp|P509[illegible]	upp	Uracil phosphoribos	51.00	0.00
TRINITY_sp|Q9C5[illegible]	GRF12	14-3-3-like protein	51.00	0.00
TRINITY_sp|Q55E[illegible]	pats1	Probable serine/thr	51.00	0.00
TRINITY_sp|Q54Q[illegible]	phr2aB	Serine/threonine-pr	51.00	0.00
TRINITY_sp|Q9LE[illegible]	At5g081[illegible]	H/ACA ribonucleopro	51.00	0.00
TRINITY_sp|Q9JI[illegible]	Stk3	Serine/threonine-pr	51.00	0.00
TRINITY_sp|Q8RX[illegible]	RH8	DEAD-box ATP-depend	51.00	0.00
TRINITY_sp|Q038[illegible]	MSH6	DNA mismatch repair	51.00	0.00
TRINITY_sp|Q54R[illegible]	cnrB	CLPTM1-like membran	51.00	0.00
TRINITY_sp|Q9LX[illegible]	HAM2	Histone acetyltrans	51.00	0.00
TRINITY_sp|P264[illegible]	PARP1	Poly [ADP-ribose] p	51.00	0.00
TRINITY_sp|Q96P[illegible]	NSUN5	Probable 28S rRNA (	51.00	0.00
TRINITY_sp|Q9RH[illegible]	ssb2	Single-stranded DNA	51.00	0.00
TRINITY_sp|Q7ZX[illegible]	kif19	Kinesin-like protei	51.00	0.00
TRINITY_sp|B0DZ[illegible]	AMPP	Probable Xaa-Pro am	51.00	0.00
TRINITY_sp|Q8LR[illegible]	HDA18	Histone deacetylase	51.00	0.00
TRINITY_sp|Q8T2[illegible]	mccb	Methylcrotonoyl-CoA	51.00	0.00
TRINITY_sp|Q8AV[illegible]	syf2	Pre-mRNA-splicing f	51.00	0.00
TRINITY_sp|Q8BZ[illegible]	Slc25a2[illegible]	Mitochondrial 2-oxo	51.00	0.00
TRINITY_sp|B3PF[illegible]	dnaK	Chaperone protein D	51.00	0.00
TRINITY_sp|O046[illegible]	CURT1A	Protein CURVATURE T	51.00	0.00
TRINITY_sp|Q9LZ[illegible]	RPL36C	60S ribosomal prote	51.00	0.00

TRINITY_sp|P2752	LHCA4	Chlorophyll a-b bin	51.00	0.00
TRINITY_sp|Q9C0(	DNAH6	Dynein heavy chain	51.00	0.00
TRINITY_sp|Q6AU(	DBR1	Lariat debranching	51.00	0.00
TRINITY_sp|P279:	AMY1.2	Alpha-amylase isozy	51.00	0.00
TRINITY_sp|B4F1(	hcp	Hydroxylamine reduc	51.00	0.00
TRINITY_sp|P678(	CSNK2B	Casein kinase II su	51.00	0.00
TRINITY_sp|Q8MVI	gbpC	Cyclic GMP-binding	51.00	0.00
TRINITY_sp|A2XF1	PDC2	Pyruvate decarboxyl	51.00	0.00
TRINITY_sp|Q5SNI	Os06g01	Fanconi-associated	51.00	0.00
TRINITY_sp|O865(	sdaA	L-serine dehydratas	51.00	0.00
TRINITY_sp|O944:	SPBC660.	Uncharacterized RNA	50.90	0.00
TRINITY_sp|Q157:	INPP5J	Phosphatidylinosito	50.90	0.00
TRINITY_sp|Q7NMI	cry	Cryptochrome DASH O	50.90	0.00
TRINITY_sp|Q5FW4	aspdh	Putative L-aspartat	50.90	0.00
TRINITY_sp|P828(	Gfpt1	Glutamine--fructose	50.90	0.00
TRINITY_sp|O184'	mus308	DNA polymerase thet	50.90	0.00
TRINITY_sp|Q2SR	aguA	Putative agmatine d	50.90	0.00
TRINITY_sp|Q9H52	DHX35	Probable ATP-depend	50.90	0.00
TRINITY_sp|Q9SM1	AAE3	Oxalate--CoA ligase	50.90	0.00
TRINITY_sp|Q86A1	spkA-1	Stress-activated pr	50.90	0.00
TRINITY_sp|O516(	pfkA	ATP-dependent 6-pho	50.90	0.00
TRINITY_sp|Q103(	sck2	Serine/threonine-pr	50.90	0.00
TRINITY_sp|P258(	UBC1	Ubiquitin-conjugati	50.90	0.00
TRINITY_sp|Q9PW:	GPHN	Gephyrin OS=Gallus	50.90	0.00
TRINITY_sp|P5932	Ythdf1	YTH domain-containi	50.90	0.00
TRINITY_sp|Q0WR	TSC10A	3-dehydrosphinganin	50.90	0.00
TRINITY_sp|Q869!	scsB	Succinate--CoA liga	50.90	0.00
TRINITY_sp|Q54PI	csn5	COP9 signalosome co	50.90	0.00
TRINITY_sp|P2751	CBR	Carotene biosynthes	50.90	0.00
TRINITY_sp|Q8L6	RPOT1-T(	DNA-directed RNA po	50.90	0.00
TRINITY_sp|B0C0'	pdxH	Pyridoxine/pyridoxa	50.90	0.00
TRINITY_sp|O0754	yheH	Probable multidrug	50.90	0.00
TRINITY_sp|Q4KMI	cry-dasl	Cryptochrome DASH O	50.90	0.00
TRINITY_sp|O2204	ANP1	Mitogen-activated p	50.90	0.00
TRINITY_sp|Q8RWI	TMN10	Transmembrane 9 sup	50.90	0.00
TRINITY_sp|Q9XFI	DDM1	ATP-dependent DNA h	50.90	0.00
TRINITY_sp|P3404	gpaD	Guanine nucleotide-	50.90	0.00
TRINITY_sp|F8RP1	HOP	Hsp70-Hsp90 organiz	50.90	0.00
TRINITY_sp|P185!	Atp2a3	Sarcoplasmic/endopl	50.90	0.00
TRINITY_sp|Q86A1	spkA-1	Stress-activated pr	50.90	0.00
TRINITY_sp|Q5ZI!	TRAPPC3	Trafficking protein	50.90	0.00
TRINITY_sp|O548'	AIMP1	Aminoacyl tRNA synt	50.90	0.00
TRINITY_sp|H3JU(	SGT1	Peptidyl serine alp	50.90	0.00
TRINITY_sp|P5344	CTN	Caltractin OS=Naegl	50.90	0.00
TRINITY_sp|Q8LC	At5g039(	Iron-sulfur assembl	50.90	0.00
TRINITY_sp|F8WLI	Kif28p	Kinesin-like protei	50.80	0.00
TRINITY_sp|Q29A(	GA14342	Mitochondrial GTPas	50.80	0.00
TRINITY_sp|Q406(	RGB1	Guanine nucleotide-	50.80	0.00
TRINITY_sp|Q54N2	memo1	Protein MEMO1 homol	50.80	0.00
TRINITY_sp|Q9XW2	acdh-11	Acyl-CoA dehydrogen	50.80	0.00
TRINITY_sp|Q54V'	ctps	CTP synthase OS=Dic	50.80	0.00
TRINITY_sp|Q8UV	brix1	Ribosome biogenesis	50.80	0.00
TRINITY_sp|Q8R2	Ptrh2	Peptidyl-tRNA hydro	50.80	0.00
TRINITY_sp|Q54C!	csn6	COP9 signalosome co	50.80	0.00
TRINITY_sp|Q9M0\	MFDX1	Adrenodoxin-like pr	50.80	0.00
TRINITY_sp|Q9LK1	AMT1-5	Putative ammonium t	50.80	0.00

TRINITY_sp|Q91VI	Ddx1	ATP-dependent RNA h	50.80	0.00
TRINITY_sp|Q54EY	eif2b2	Translation initiat	50.80	0.00
TRINITY_sp|Q8K3F	Tdh	L-threonine 3-dehyd	50.80	0.00
TRINITY_sp|Q631	Dnah7	Dynein heavy chain	50.80	0
TRINITY_sp|Q1EBV	BPM5	BTB/POZ and MATH do	50.80	0.00
TRINITY_sp|Q1545	SF3A1	Splicing factor 3A	50.80	0.00
TRINITY_sp|Q9FK	COX15	Cytochrome c oxidas	50.80	0.00
TRINITY_sp|P3414	racA	Rho-related protein	50.80	0.00
TRINITY_sp|P216	-	Pyrophosphate-energ	50.80	0.00
TRINITY_sp|P417	Nolc1	Nucleolar and coile	50.80	0.00
TRINITY_sp|Q5QU	msbA	Lipid A export ATP-	50.80	0.00
TRINITY_sp|Q0922	cpna-2	Copine family prote	50.80	0.00
TRINITY_sp|Q3905	CNX1	Molybdopterin biosy	50.80	0.00
TRINITY_sp|Q8WX	DNAH7	Dynein heavy chain	50.80	0.00
TRINITY_sp|P546	patB	Probable plasma mem	50.80	0.00
TRINITY_sp|P3899	LYS5	Saccharopine dehydr	50.80	0.00
TRINITY_sp|Q9FV	NAP5	Probable non-intrin	50.80	0.00
TRINITY_sp|Q8T2	sepA	Serine/threonine-pr	50.80	0.00
TRINITY_sp|O155	POLR2D	DNA-directed RNA po	50.80	0.00
TRINITY_sp|P1512	AKR1B1	Aldose reductase OS	50.80	0.00
TRINITY_sp|Q3MG8	grpE	Protein GrpE OS=Ana	50.80	0.00
TRINITY_sp|Q9469	RAP1	Ras-related protein	50.80	0.00
TRINITY_sp|F4JN	P4H8	Probable prolyl 4-h	50.80	0.00
TRINITY_sp|Q9P95	alxA	Alternative oxidase	50.80	0.00
TRINITY_sp|Q9C0	DNAH6	Dynein heavy chain	50.80	0.00
TRINITY_sp|O8065	SKIP	SNW/SKI-interacting	50.80	0.00
TRINITY_sp|Q8X02	carRA	Bifunctional lycope	50.80	0.00
TRINITY_sp|P5498	carB	Phytoene desaturase	50.80	0.00
TRINITY_sp|Q56V9	FAD7	sn-2 acyl-lipid ome	50.80	0.00
TRINITY_sp|Q9C5I	At3g4742	Putative glycerol-3	50.80	0.00
TRINITY_sp|Q54IF	DDB_G028	WASH complex subuni	50.80	0.00
TRINITY_sp|O461	noi	Splicing factor 3A	50.80	0.00
TRINITY_sp|Q6C6	ATM1	Iron-sulfur cluster	50.80	0.00
TRINITY_sp|O600	ubp12	Probable ubiquitin	50.80	0.00
TRINITY_sp|Q86L	trap1	TNF receptor-associ	50.80	0.00
TRINITY_sp|O760	CDKL5	Cyclin-dependent ki	50.80	0.00
TRINITY_sp|Q9HC	XAB2	Pre-mRNA-splicing f	50.80	0.00
TRINITY_sp|Q2R0	U2AF65A	Splicing factor U2a	50.80	0.00
TRINITY_sp|Q8BM	Lars	Leucine--tRNA ligas	50.80	0
TRINITY_sp|Q9Z2	MBTPS1	Membrane-bound tran	50.80	0.00
TRINITY_sp|Q551	fray2	Serine/threonine-pr	50.80	0.00
TRINITY_sp|P025	-	Actin-1 OS=Acantham	50.80	0.00
TRINITY_sp|Q9Y3	COQ4	Ubiquinone biosynth	50.80	0.00
TRINITY_sp|P542	Pms2	Mismatch repair end	50.70	0.00
TRINITY_sp|Q8QZ	Gpt	Alanine aminotransf	50.70	0.00
TRINITY_sp|Q5ZM	PRPF19	Pre-mRNA-processing	50.70	0.00
TRINITY_sp|Q8RB	dnaJ	Chaperone protein D	50.70	0.00
TRINITY_sp|Q3S2	mokA	Lovastatin nonaketi	50.70	0.00
TRINITY_sp|Q86C	tor	Target of rapamycin	50.70	0.00
TRINITY_sp|Q6EM	TTLL5	Tubulin polyglutamy	50.70	0.00
TRINITY_sp|Q8VY	LSM8	Sm-like protein LSM	50.70	0.00
TRINITY_sp|Q7YS	canA	Calcineurin subunit	50.70	0.00
TRINITY_sp|Q9ZV	UBC23	Probable ubiquitin-	50.70	0.00
TRINITY_sp|Q6P3	NEK5	Serine/threonine-pr	50.70	0.00
TRINITY_sp|Q9V3	Dyrk2	Dual specificity ty	50.70	0.00
TRINITY_sp|Q52K	Srrm1	Serine/arginine rep	50.70	0.00

TRINITY_sp|Q8LB(	DAPB2	4-hydroxy-tetrahydr	50.70	0.00
TRINITY_sp|O816:	KIN14G	Kinesin-like protei	50.70	0.00
TRINITY_sp|Q235	dbt-1	Lipoamide acyltrans	50.70	0.00
TRINITY_sp|Q9P7N	vas2	AP-1 complex subuni	50.70	0.00
TRINITY_sp|Q54P!	cpnC	Copine-C OS=Dictyos	50.70	0.00
TRINITY_sp|Q54I:	smkA	Suppressor of Mek1	50.70	0.00
TRINITY_sp|Q9ATI	ADA2B	Transcriptional ada	50.70	0.00
TRINITY_sp|Q9ZTS	Os06g05(	Probable methionine	50.70	0.00
TRINITY_sp|Q558	argS1	Probable arginine--	50.70	0.00
TRINITY_sp|Q008!	MAPK	Mitogen-activated p	50.70	0.00
TRINITY_sp|Q5RJ(	Nol10	Nucleolar protein 1	50.70	0.00
TRINITY_sp|P551	PURKE	Phosphoribosylamino	50.70	0.00
TRINITY_sp|P432	MCM7	DNA replication lic	50.70	0.00
TRINITY_sp|P194(	Gclc	Glutamate--cysteine	50.70	0.00
TRINITY_sp|Q9SHI	RUB1	Ubiquitin-NEDD8-lik	50.70	0.00
TRINITY_sp|Q9ASU	PPAN	Peter Pan-like prot	50.70	0.00
TRINITY_sp|Q54U(	pgmB	Probable phosphoglu	50.70	0.00
TRINITY_sp|Q54Y	ndrA	Probable serine/thr	50.70	0.00
TRINITY_sp|P459	USP5	Ubiquitin carboxyl-	50.70	0.00
TRINITY_sp|Q6QNI	-	Casein kinase I OS=	50.70	0.00
TRINITY_sp|Q8LPI	ALPHA-AI	AP-2 complex subuni	50.70	0.00
TRINITY_sp|Q94C:	At3g521	SURP and G-patch do	50.70	0.00
TRINITY_sp|Q8T1:	kif5	Kinesin-related pro	50.70	0.00
TRINITY_sp|Q8VB	Clptm1	Cleft lip and palat	50.70	0.00
TRINITY_sp|Q631	Dnah7	Dynein heavy chain	50.70	0
TRINITY_sp|B4SD	xerC	Tyrosine recombinas	50.70	0.00
TRINITY_sp|Q9H5	HEATR1	HEAT repeat-contain	50.60	0.00
TRINITY_sp|P464	GDCSH	Glycine cleavage sy	50.60	0.00
TRINITY_sp|Q0VC	YTHDF2	YTH domain-containi	50.60	0.00
TRINITY_sp|Q5JLI	CIPK8	CBL-interacting pro	50.60	0.00
TRINITY_sp|I0IUI	MCM9	DNA helicase MCM9 O	50.60	0.00
TRINITY_sp|Q2PQI	AGL	Glycogen debranchin	50.60	0.00
TRINITY_sp|P348	pkc-1	Protein kinase C-li	50.60	0.00
TRINITY_sp|Q241:	TH1	Negative elongation	50.60	0.00
TRINITY_sp|Q54N	7tmk1	Seven transmembrane	50.60	0.00
TRINITY_sp|Q9ZPI	CDC48B	Cell division contr	50.60	0.00
TRINITY_sp|Q96S:	WDR24	WD repeat-containin	50.60	0.00
TRINITY_sp|Q86A	wars2	Tryptophan--tRNA li	50.60	0.00
TRINITY_sp|B3QWI	fbp	Fructose-1,6-bispho	50.60	0.00
TRINITY_sp|Q75L	Os03g06	U3 snoRNP-associate	50.60	0.00
TRINITY_sp|Q4P6	CYP3	Peptidyl-prolyl cis	50.60	0.00
TRINITY_sp|Q8INI	Akt1	RAC serine/threonin	50.60	0.00
TRINITY_sp|Q1ZX(	prlA	Proliferation-assoc	50.60	0.00
TRINITY_sp|F4HYI	DCD	Bifunctional D-cyst	50.60	0.00
TRINITY_sp|Q8LHI	Os07g06:	Transcription elong	50.60	0.00
TRINITY_sp|P114	PSBP	Oxygen-evolving enh	50.60	0.00
TRINITY_sp|Q8L7	DIT2	Dicarboxylate trans	50.60	0.00
TRINITY_sp|D2HX:	MASTL	Serine/threonine-pr	50.60	0.00
TRINITY_sp|Q54H	adprh	[Protein ADP-ribosy	50.60	0.00
TRINITY_sp|Q9R1	Abcc5	Multidrug resistanc	50.60	0.00
TRINITY_sp|Q8MZ	-	Physarolisin OS=Phy	50.60	0.00
TRINITY_sp|Q147:	PPP2R5D	Serine/threonine-pr	50.60	0.00
TRINITY_sp|Q9NWI	SMOX	Spermine oxidase OS	50.60	0.00
TRINITY_sp|P492	-	Citrate synthase, g	50.60	0.00
TRINITY_sp|Q057:	YPTM2	GTP-binding protein	50.60	0.00
TRINITY_sp|Q54P	ppa1	Inorganic pyrophosp	50.60	0.00

TRINITY_sp|Q0J0S	TOP3B	DNA topoisomerase 3	50.60	0.00
TRINITY_sp|Q963(	SPY	Probable UDP-N-acet	50.60	0.00
TRINITY_sp|Q54II	ndrB	Probable serine/thr	50.60	0.00
TRINITY_sp|Q0394	IM30	Membrane-associated	50.60	0.00
TRINITY_sp|Q9SS.	FAB1C	Putative 1-phosphat	50.60	0.00
TRINITY_sp|Q389!	At3g265(	Probable pre-mRNA-s	50.60	0.00
TRINITY_sp|Q94K(	CCT8	T-complex protein 1	50.60	0.00
TRINITY_sp|Q1DXI	PAB1	Polyadenylate-bindi	50.60	0.00
TRINITY_sp|Q54KI	elof1	Transcription elong	50.60	0.00
TRINITY_sp|Q7F8:	CYCA1-1	Cyclin-A1-1 OS=Oryz	50.60	0.00
TRINITY_sp|Q3894	RAE1	Protein RAE1 OS=Ara	50.60	0.00
TRINITY_sp|H2QL:	PDE9A	High affinity cGMP-	50.60	0.00
TRINITY_sp|Q390:	ATPK2	Serine/threonine-pr	50.60	0.00
TRINITY_sp|Q54PI	smc2	Structural maintena	50.50	0.00
TRINITY_sp|A6QL8	SACM1L	Phosphatidylinositi	50.50	0.00
TRINITY_sp|P6128	DYNLL1	Dynein light chain	50.50	0.00
TRINITY_sp|Q9LES	L2HGDH	L-2-hydroxyglutarat	50.50	0.00
TRINITY_sp|Q2NL2	TKTL1	Transketolase-like	50.50	0.00
TRINITY_sp|Q8RWI	STY17	Serine/threonine-pr	50.50	0.00
TRINITY_sp|P2788	ERV1	Mitochondrial FAD-l	50.50	0.00
TRINITY_sp|Q1ZX(	pXi	Probable serine/thr	50.50	0.00
TRINITY_sp|Q55FI	copa	Coatomer subunit al	50.50	0.00
TRINITY_sp|Q54X8	ap1b1	AP-1 complex subuni	50.50	0.00
TRINITY_sp|A8EX(	mnmG	tRNA uridine 5-carb	50.50	0.00
TRINITY_sp|P412:	Abca2	ATP-binding cassett	50.50	0.00
TRINITY_sp|Q9T0I	HSFB2B	Heat stress transcr	50.50	0.00
TRINITY_sp|Q9M8I	DJ1D	Protein DJ-1 homolo	50.50	0.00
TRINITY_sp|Q8BYI	Rdh12	Retinol dehydrogena	50.50	0.00
TRINITY_sp|P189(	YPK2	Serine/threonine-pr	50.50	0.00
TRINITY_sp|Q54QI	sir2A	NAD-dependent deace	50.50	0.00
TRINITY_sp|P312(	pab1	Polyadenylate-bindi	50.50	0.00
TRINITY_sp|O824]	At3g461(	Histidine--tRNA lig	50.50	0.00
TRINITY_sp|Q631(	Dnah1	Dynein heavy chain	50.50	0.00
TRINITY_sp|Q9Z2]	Letm1	LETM1 and EF-hand d	50.50	0.00
TRINITY_sp|Q5F3:	HCCS	Cytochrome c-type h	50.50	0.00
TRINITY_sp|P0C8I	CCRP1	Probable serine/thr	50.50	0.00
TRINITY_sp|O933(	smc3	Structural maintena	50.50	0.00
TRINITY_sp|Q016(	LHCA1	Chlorophyll a-b bin	50.50	0.00
TRINITY_sp|Q9SM(	SRP9	Signal recognition	50.50	0.00
TRINITY_sp|Q388!	NRPB11	DNA-directed RNA po	50.50	0.00
TRINITY_sp|Q8AXS	slc35b1	Solute carrier fami	50.50	0.00
TRINITY_sp|Q54EI	grwd1	Glutamate-rich WD r	50.50	0.00
TRINITY_sp|B0R0]	si:dkey-	von Willebrand fact	50.50	0.00
TRINITY_sp|Q9FY!	HPAT3	Hydroxyproline O-ar	50.50	0.00
TRINITY_sp|P775:	yfcG	Disulfide-bond oxid	50.50	0.00
TRINITY_sp|Q99JV	Acy1	Aminoacylase-1 OS=M	50.50	0.00
TRINITY_sp|O244!	UBP3	Ubiquitin carboxyl-	50.50	0.00
TRINITY_sp|F4JR.	At4g184(	Probable pre-mRNA-s	50.50	0.00
TRINITY_sp|Q8BWS	Dnah3	Dynein heavy chain	50.50	0.00
TRINITY_sp|P280(	RPC19	DNA-directed RNA po	50.50	0.00
TRINITY_sp|Q8LC\	PLP3B	Thioredoxin domain-	50.50	0.00
TRINITY_sp|Q8QZI	Tat	Tyrosine aminotrans	50.50	0.00
TRINITY_sp|O049"	-	Lon protease homolo	50.50	0.00
TRINITY_sp|Q53GS	USP39	U4/U6.U5 tri-snRNP-	50.50	0.00
TRINITY_sp|Q392·	NTR1	Thioredoxin reducta	50.50	0.00
TRINITY_sp|F4HY(	IRE3	Probable serine/thr	50.50	0.00

TRINITY_sp|Q9BW(	SLC4A1AI	Kanadaptin OS=Homo	50.50	0.00
TRINITY_sp|P3642	ILSA	Isoleucine--tRNA li	50.50	0.00
TRINITY_sp|B2V6I	hslU	ATP-dependent prote	50.50	0.00
TRINITY_sp|Q9FR5	TOR	Serine/threonine-pr	50.50	0.00
TRINITY_sp|P545(	yqjZ	Uncharacterized pro	50.50	0.00
TRINITY_sp|O042(	PXN	Peroxisomal nicotin	50.50	0.00
TRINITY_sp|O004(	MANBA	Beta-mannosidase OS	50.50	0.00
TRINITY_sp|Q9C0(	DNAH6	Dynein heavy chain	50.50	0.00
TRINITY_sp|Q9Y2Y	POLR3K	DNA-directed RNA po	50.50	0.00
TRINITY_sp|Q9SY9	LHCA3	Photosystem I chlor	50.50	0.00
TRINITY_sp|Q54BN	rfc3	Probable replicatio	50.40	0.00
TRINITY_sp|Q6443	Atp7a	Copper-transporting	50.40	0.00
TRINITY_sp|Q08E2	SARAF	Store-operated calc	50.40	0.00
TRINITY_sp|O3221	yvgN	Glyoxal reductase O	50.40	0.00
TRINITY_sp|Q54NI	DDB_G028	Protein UXT homolog	50.40	0.00
TRINITY_sp|Q1AU(	rplM	50S ribosomal prote	50.40	0.00
TRINITY_sp|Q86KI	cycC	Cyclin-C OS=Dictyos	50.40	0.00
TRINITY_sp|P4285	ZBP14	14 kDa zinc-binding	50.40	0.00
TRINITY_sp|Q54F(	mcfW	Mitochondrial subst	50.40	0.00
TRINITY_sp|Q86KJ	ap2a1-1	AP-2 complex subuni	50.40	0.00
TRINITY_sp|O8114	PAA1	Proteasome subunit	50.40	0.00
TRINITY_sp|Q10Q8	ERV1	FAD-linked sulfhydr	50.40	0.00
TRINITY_sp|P5112	ANP32A	Acidic leucine-rich	50.40	0.00
TRINITY_sp|Q9PV9	SNRPB	Small nuclear ribon	50.40	0.00
TRINITY_sp|Q8H0I	UEL-3	Probable UDP-arabin	50.40	0.00
TRINITY_sp|Q0II9	DNAJC21	DnaJ homolog subfam	50.40	0.00
TRINITY_sp|P5242	PAC1	Proteasome subunit	50.40	0.00
TRINITY_sp|Q5ZJ(	YARS	Tyrosine--tRNA liga	50.40	0.00
TRINITY_sp|O8873	Birc6	Baculoviral IAP rep	50.40	0.00
TRINITY_sp|P0678	CMD1	Calmodulin OS=Sacch	50.40	0.00
TRINITY_sp|Q9ES7	Nek7	Serine/threonine-pr	50.40	0.00
TRINITY_sp|Q9DBI	Alg2	Alpha-1,3/1,6-manno	50.40	0.00
TRINITY_sp|B0TE(	purH	Bifunctional purine	50.40	0.00
TRINITY_sp|O0403	At1g0894	Phosphoglycerate mu	50.40	0.00
TRINITY_sp|Q8W49	VHA-a3	V-type proton ATPas	50.40	0.00
TRINITY_sp|Q7662	REV3	DNA polymerase zeta	50.40	0.00
TRINITY_sp|Q9HZI	etfA	Electron transfer f	50.40	0.00
TRINITY_sp|Q6ZGI	SPP1	Signal peptide pept	50.40	0.00
TRINITY_sp|Q9C92	RER3	Protein RETICULATA-	50.40	0.00
TRINITY_sp|Q0E07	RIBA2	Probable bifunction	50.40	0.00
TRINITY_sp|P0568	CTSZ	Cathepsin Z OS=Bos	50.40	0.00
TRINITY_sp|P1424	RPS24	40S ribosomal prote	50.40	0.00
TRINITY_sp|Q91YI	Uckl1	Uridine-cytidine ki	50.30	0.00
TRINITY_sp|P0CD(	alg7	UDP-N-acetylglucosa	50.30	0.00
TRINITY_sp|Q47P(	pamO	Phenylacetone monoo	50.30	0.00
TRINITY_sp|Q9SHI	RRP44A	Exosome complex exo	50.30	0.00
TRINITY_sp|Q1824	rap-1	Ras-related protein	50.30	0.00
TRINITY_sp|Q9BW8	CCDC94	Coiled-coil domain-	50.30	0.00
TRINITY_sp|Q86AI	DDB_G027	Probable myosin lig	50.30	0.00
TRINITY_sp|P0111	RAS1	Ras-like protein 1	50.30	0.00
TRINITY_sp|Q6ZNI	AGMO	Alkylglycerol monoo	50.30	0.00
TRINITY_sp|Q99LJ	Hibadh	3-hydroxyisobutyrat	50.30	0.00
TRINITY_sp|Q9RX5	treZ	Malto-oligosyltreha	50.30	0.00
TRINITY_sp|B1M7(	prfA	Peptide chain relea	50.30	0.00
TRINITY_sp|Q17R(	RAB21	Ras-related protein	50.30	0.00
TRINITY_sp|Q80W.	Abcc12	Multidrug resistanc	50.30	0.00

TRINITY_sp|Q9FT	MED34	Mediator of RNA pol	50.30	0.00
TRINITY_sp|B0G1	ucpB	Mitochondrial subst	50.30	0.00
TRINITY_sp|Q63S	prmB	50S ribosomal prote	50.30	0.00
TRINITY_sp|P432	MSH2	DNA mismatch repair	50.30	0.00
TRINITY_sp|Q9LF	XPT	Xylulose 5-phosphat	50.30	0.00
TRINITY_sp|P374	hprT	Hypoxanthine-guanin	50.30	0.00
TRINITY_sp|O805	At2g440	6,7-dimethyl-8-ribi	50.30	0.00
TRINITY_sp|Q9XZ	culB	Cullin-2 OS=Dictyos	50.30	0.00
TRINITY_sp|Q84K	ABCA2	ABC transporter A f	50.30	0.00
TRINITY_sp|P524	UBA1	Ubiquitin-activatin	50.30	0.00
TRINITY_sp|Q438	SS3	Soluble starch synt	50.30	0.00
TRINITY_sp|P511	RAB22A	Ras-related protein	50.30	0.00
TRINITY_sp|P403	RIC1	Ras-related protein	50.30	0.00
TRINITY_sp|P403	RIC1	Ras-related protein	50.30	0.00
TRINITY_sp|Q9FG	QS	Quinolinate synthas	50.30	0.00
TRINITY_sp|Q965	CAT1	Catalase-1 OS=Arabi	50.30	0.00
TRINITY_sp|Q390	CNX1	Molybdopterin biosy	50.30	0.00
TRINITY_sp|Q631	Abcc2	Canalicular multisp	50.30	0.00
TRINITY_sp|A6N6	Wdr35	WD repeat-containin	50.30	0
TRINITY_sp|P537	GFA1	Glutamine--fructose	50.30	0.00
TRINITY_sp|Q5E9	BCS1L	Mitochondrial chape	50.20	0.00
TRINITY_sp|Q9VP	CG13690	Ribonuclease H2 sub	50.20	0.00
TRINITY_sp|Q54K	gluD	Glutamate dehydroge	50.20	0.00
TRINITY_sp|O322	yvgN	Glyoxal reductase O	50.20	0.00
TRINITY_sp|O154	ABCC4	Multidrug resistanc	50.20	0.00
TRINITY_sp|Q55E	pykA	Pyridoxal kinase OS	50.20	0.00
TRINITY_sp|Q8A0	asnS	Asparagine--tRNA li	50.20	0.00
TRINITY_sp|Q8BV	Mettl6	Methyltransferase-l	50.20	0.00
TRINITY_sp|Q7YS	canA	Calcineurin subunit	50.20	0.00
TRINITY_sp|O956	DUS4L	tRNA-dihydrouridine	50.20	0.00
TRINITY_sp|P159	acu-8	Acetyl-CoA hydrolas	50.20	0.00
TRINITY_sp|O824	At5g267	Glutamate--tRNA lig	50.20	0.00
TRINITY_sp|O611	svkA	Serine/threonine-pr	50.20	0.00
TRINITY_sp|P467	cysB	Cystathionine beta-	50.20	0.00
TRINITY_sp|Q73K	serS	Serine--tRNA ligase	50.20	0.00
TRINITY_sp|Q9C5	RH5	DEAD-box ATP-depend	50.20	0.00
TRINITY_sp|Q389	At3g265	Probable pre-mRNA-s	50.20	0.00
TRINITY_sp|Q8TD	DNAH3	Dynein heavy chain	50.20	0.00
TRINITY_sp|Q8XI	deoC	Deoxyribose-phospha	50.20	0.00
TRINITY_sp|Q8H3	ILL8	IAA-amino acid hydr	50.20	0.00
TRINITY_sp|P258	RPL15	50S ribosomal prote	50.20	0.00
TRINITY_sp|P546	pkbA	RAC family serine/t	50.20	0.00
TRINITY_sp|Q54T	rio2	Serine/threonine-pr	50.20	0.00
TRINITY_sp|Q55B	nmd3	60S ribosomal expor	50.20	0.00
TRINITY_sp|P155	ade6	Phosphoribosylamino	50.20	0.00
TRINITY_sp|Q563	TM_0019	Uncharacterized oxi	50.20	0.00
TRINITY_sp|Q9Y6	PARP3	Poly [ADP-ribose] p	50.20	0.00
TRINITY_sp|Q54S	wdr3	WD repeat-containin	50.20	0.00
TRINITY_sp|P736	mutS2	Endonuclease MutS2	50.20	0.00
TRINITY_sp|O041	ISA1	Isoamylase 1, chlor	50.20	0.00
TRINITY_sp|B9DH	ARIA	ARM REPEAT PROTEIN	50.20	0.00
TRINITY_sp|Q084	ADK-B	Adenylate kinase 4	50.20	0.00
TRINITY_sp|Q10M	Os03g03	DEAD-box ATP-depend	50.10	0.00
TRINITY_sp|Q103	gdi1	Probable secretory	50.10	0.00
TRINITY_sp|E1BN	ascc3	Activating signal c	50.10	0.00
TRINITY_sp|Q9Z0	Txnrd2	Thioredoxin reducta	50.10	0.00

TRINITY_sp|P047[illegible]	-	Glutamine synthetas	50.10	0.00
TRINITY_sp|Q54J[illegible]	ogdh	2-oxoglutarate dehy	50.10	0.00
TRINITY_sp|Q54G[illegible]	mdhA	Probable malate deh	50.00	0.00
TRINITY_sp|Q50E[illegible]	ODO1	Protein ODORANT1 OS	50.00	0.00
TRINITY_sp|Q9UR[illegible]	SPAC869.	Uncharacterized hem	50.00	0.00
TRINITY_sp|Q008[illegible]	MAPK	Mitogen-activated p	50.00	0.00
TRINITY_sp|Q4QT[illegible]	NCS1	S-norcoclaurine syn	50.00	0.00
TRINITY_sp|P407[illegible]	Rac1	Ras-related protein	50.00	0.00
TRINITY_sp|P631[illegible]	SNRPN	Small nuclear ribon	50.00	0.00
TRINITY_sp|Q93D[illegible]	sdcBA	Putative ABC transp	50.00	0.00
TRINITY_sp|P617[illegible]	aptx	Aprataxin OS=Danio	50.00	0.00
TRINITY_sp|O344[illegible]	pgl	6-phosphogluconolac	50.00	0.00
TRINITY_sp|Q3UD[illegible]	Wdr41	WD repeat-containin	50.00	0.00
TRINITY_sp|Q5E9[illegible]	ARF3	ADP-ribosylation fa	50.00	0.00
TRINITY_sp|P505[illegible]	vps34	Phosphatidylinosito	50.00	0.00
TRINITY_sp|Q8TA[illegible]	SNIP1	Smad nuclear-intera	50.00	0.00
TRINITY_sp|Q103[illegible]	SPBC582.	Putative alanine am	50.00	0.00
TRINITY_sp|Q212[illegible]	K07E3.7,	Probable cation-tra	50.00	0.00
TRINITY_sp|Q6XH[illegible]	roco4	Probable serine/thr	50.00	0.00
TRINITY_sp|Q9DE[illegible]	atr	Serine/threonine-pr	50.00	0.00
TRINITY_sp|Q9FP[illegible]	THA2	Probable low-specif	50.00	0.00
TRINITY_sp|Q55G[illegible]	gtaE	GATA zinc finger do	50.00	0.00
TRINITY_sp|P240[illegible]	-	Glu S.griseus prote	50.00	0.00
TRINITY_sp|P240[illegible]	-	Glu S.griseus prote	50.00	0.00
TRINITY_sp|P175[illegible]	RENBP	N-acylglucosamine 2	50.00	0.00
TRINITY_sp|Q220[illegible]	R166.3	Uncharacterized pro	50.00	0.00
TRINITY_sp|P0CM[illegible]	ARP6	Actin-like protein	50.00	0.00
TRINITY_sp|P176[illegible]	FMO2	Dimethylaniline mon	50.00	0.00
TRINITY_sp|Q6AX[illegible]	Sae1	SUMO-activating enz	50.00	0.00
TRINITY_sp|P0DJ[illegible]	STRA13	Centromere protein	50.00	0.00
TRINITY_sp|B1I1[illegible]	rpmD	50S ribosomal prote	50.00	0.00
TRINITY_sp|Q65N[illegible]	topB	DNA topoisomerase 3	50.00	0.00
TRINITY_sp|Q278[illegible]	DYH1B	Cytoplasmic dynein	50.00	0.00
TRINITY_sp|Q54U[illegible]	DDB_G02[illegible]	Glutathione S-trans	50.00	0.00
TRINITY_sp|Q8LF[illegible]	IDH1	Isocitrate dehydrog	50.00	0.00
TRINITY_sp|B2UX[illegible]	asnS	Asparagine--tRNA li	50.00	0.00
TRINITY_sp|Q9CZ[illegible]	Rab32	Ras-related protein	50.00	0.00
TRINITY_sp|Q5BM[illegible]	PLD	Phospholipase D OS=	50.00	0.00
TRINITY_sp|B9Q0[illegible]	TGVEG_2[illegible]	Anamorsin homolog O	50.00	0.00
TRINITY_sp|Q8WY[illegible]	SLC7A3	Cationic amino acid	50.00	0.00
TRINITY_sp|Q9XY[illegible]	fcpA	Probable C-terminal	50.00	0.00
TRINITY_sp|P454[illegible]	nudA	Dynein heavy chain,	50.00	0.00
TRINITY_sp|Q0V8[illegible]	ASNSD1	Asparagine syntheta	50.00	0.00
TRINITY_sp|Q53F[illegible]	TP53I3	Quinone oxidoreduct	50.00	0.00
TRINITY_sp|B8CX[illegible]	dnaJ	Chaperone protein D	50.00	0.00
TRINITY_sp|Q097[illegible]	SPBC12C[illegible]	Probable glutamine-	50.00	0.00
TRINITY_sp|Q59R[illegible]	PIF1	ATP-dependent DNA h	50.00	0.00
TRINITY_sp|Q9BV[illegible]	PBDC1	Protein PBDC1 OS=Ho	50.00	0.00
TRINITY_sp|Q5M9[illegible]	rnf181	E3 ubiquitin-protei	50.00	0.00
TRINITY_sp|Q09X[illegible]	Chd8	Chromodomain-helica	50.00	0.00
TRINITY_sp|Q9C7[illegible]	INT2	Probable inositol t	50.00	0.00
TRINITY_sp|Q401[illegible]	RAB11D	Ras-related protein	50.00	0.00
TRINITY_sp|Q4R5[illegible]	CTSB	Cathepsin B OS=Maca	50.00	0.00
TRINITY_sp|Q148[illegible]	LASP1	LIM and SH3 domain	50.00	0.00
TRINITY_sp|Q7YS[illegible]	canA	Calcineurin subunit	50.00	0.00
TRINITY_sp|Q1ZX[illegible]	gxcDD	Guanine exchange fa	50.00	0.00

TRINITY_sp|F4JY8	TCX7	Protein tesmin/TSO1	50.00	0.00
TRINITY_sp|Q9UKI	MRTO4	mRNA turnover prote	50.00	0.00
TRINITY_sp|Q1456	DHX8	ATP-dependent RNA h	50.00	0.00
TRINITY_sp|Q2945	MAN2B1	Lysosomal alpha-man	50.00	0.00
TRINITY_sp|Q9XFH	DDM1	ATP-dependent DNA h	50.00	0.00
TRINITY_sp|Q54WN	bzpF	Probable basic-leuc	50.00	0.00
TRINITY_sp|Q94K0	PAP2	Plastid lipid-assoc	50.00	0.00
TRINITY_sp|P2771	TRP1	Multifunctional try	50.00	0.00
TRINITY_sp|Q0990	nth1	Endonuclease III ho	50.00	0.00
TRINITY_sp|Q86C6	tor	Target of rapamycin	50.00	0.00
TRINITY_sp|Q99PI	Lpin3	Phosphatidate phosp	50.00	0.00
TRINITY_sp|Q9SN2	ATL59	E3 ubiquitin-protei	50.00	0.00
TRINITY_sp|A8I9E	CFAP45	Cilia- and flagella	50.00	0.00
TRINITY_sp|Q9D83	Dnajb4	DnaJ homolog subfam	50.00	0.00
TRINITY_sp|Q3ZBE	-	UPF0235 protein C15	50.00	0.00
TRINITY_sp|Q6DBE	At5g0582	Probable sugar phos	50.00	0.00
TRINITY_sp|Q0053	CDKL1	Cyclin-dependent ki	50.00	0.00
TRINITY_sp|Q93VE	ATG18A	Autophagy-related p	50.00	0.00
TRINITY_sp|P2676	bvgS	Virulence sensor pr	50.00	0.00
TRINITY_sp|P5127	ycf36	Uncharacterized pro	50.00	0.00
TRINITY_sp|Q54QU	DDB_G028	Probable zinc trans	50.00	0.00
TRINITY_sp|Q9FG3	GCP3	Gamma-tubulin compl	50.00	0.00
TRINITY_sp|A2YX0	SPL15	Squamosa promoter-b	50.00	0.00
TRINITY_sp|Q8VZ1	RMR2	Receptor homology r	50.00	0.00
TRINITY_sp|Q9NZJ	EIF2AK3	Eukaryotic translat	50.00	0.00
TRINITY_sp|Q9Y3E	EXOSC1	Exosome complex com	50.00	0.00
TRINITY_sp|Q1350	SQSTM1	Sequestosome-1 OS=H	50.00	0.00
TRINITY_sp|Q1045	gsk3	Protein kinase gsk3	50.00	0.00
TRINITY_sp|P3686	YPTV2	GTP-binding protein	50.00	0.00
TRINITY_sp|Q9NW1	RBM28	RNA-binding protein	50.00	0.00
TRINITY_sp|Q9262	NUP205	Nuclear pore comple	50.00	0.00
TRINITY_sp|Q2039	ppt-1	Palmitoyl-protein t	50.00	0.00
TRINITY_sp|B1XLE	glgC	Glucose-1-phosphate	50.00	0.00
TRINITY_sp|P9809	c3	Complement C3 (Frag	50.00	0.00
TRINITY_sp|O1543	ABCC4	Multidrug resistanc	50.00	0.00
TRINITY_sp|A5H0J	DUR1,2	Urea amidolyase OS=	50.00	0.00
TRINITY_sp|Q8BW9	Dnah3	Dynein heavy chain	50.00	0.00
TRINITY_sp|Q9HZE	etfB	Electron transfer f	50.00	0.00
TRINITY_sp|P9294	MDAR5	Monodehydroascorbat	50.00	0.00
TRINITY_sp|Q93VC	RPS1	30S ribosomal prote	50.00	0.00
TRINITY_sp|A3KMV	RANBP10	Ran-binding protein	50.00	0.00
TRINITY_sp|P9298	RANBP1C	Ran-binding protein	50.00	0.00
TRINITY_sp|P9438	ycgL	Uncharacterized pro	50.00	0.00
TRINITY_sp|Q3SZ8	PTRHD1	Putative peptidyl-t	50.00	0.00
TRINITY_sp|Q9ZRV	MLH1	DNA mismatch repair	50.00	0.00
TRINITY_sp|Q54FN	eif2b4	Translation initiat	50.00	0.00
TRINITY_sp|P0520	Su(var)2	Heterochromatin pro	50.00	0.00
TRINITY_sp|P9192	-	ADP-ribosylation fa	50.00	0.00
TRINITY_sp|Q9C5U	HISN8	Histidinol dehydrog	50.00	0.00
TRINITY_sp|Q7Z7H	TMED4	Transmembrane emp24	50.00	0.00
TRINITY_sp|O5973	gyp1	GTPase-activating p	50.00	0.00
TRINITY_sp|Q5RBU	PRCP	Lysosomal Pro-X car	50.00	0.00
TRINITY_sp|Q54S7	vps15	Probable serine/thr	50.00	0.00
TRINITY_sp|Q9NEW	rsp-3	Probable splicing f	50.00	0.00
TRINITY_sp|Q9NIV	PEK	Eukaryotic translat	50.00	0.00
TRINITY_sp|A2BDX	Mocs3	Adenylyltransferase	50.00	0.00

TRINITY_sp|Q839[illegible]	hpt	Hypoxanthine-guanin	50.00	0.00
TRINITY_sp|O597[illegible]	gyp1	GTPase-activating p	50.00	0.00
TRINITY_sp|Q395[illegible]	YPTC6	Ras-related protein	50.00	0.00
TRINITY_sp|O044[illegible]	SRP14	Signal recognition	50.00	0.00
TRINITY_sp|Q8GW[illegible]	BIOF	8-amino-7-oxononano	50.00	0.00
TRINITY_sp|O137[illegible]	ssr1	SWI/SNF and RSC com	50.00	0.00
TRINITY_sp|P762[illegible]	msrC	Free methionine-R-s	50.00	0.00
TRINITY_sp|Q3T0[illegible]	RANBP1	Ran-specific GTPase	50.00	0.00
TRINITY_sp|Q9JW[illegible]	rsmI	Ribosomal RNA small	50.00	0.00
TRINITY_sp|Q69Z[illegible]	Hbs1l	HBS1-like protein O	50.00	0.00
TRINITY_sp|Q9ZV[illegible]	RRP4	Exosome complex com	50.00	0.00
TRINITY_sp|O087[illegible]	Ampd3	AMP deaminase 3 OS=	50.00	0.00
TRINITY_sp|Q9C0[illegible]	DNAH6	Dynein heavy chain	50.00	0.00
TRINITY_sp|Q9P2[illegible]	IFT80	Intraflagellar tran	50.00	0.00
TRINITY_sp|O807[illegible]	MCM5	DNA replication lic	50.00	0.00
TRINITY_sp|P451[illegible]	can	Carbonic anhydrase	50.00	0.00
TRINITY_sp|Q0JG[illegible]	PSB28	Photosystem II reac	50.00	0.00
TRINITY_sp|Q93W[illegible]	Lhcb4	Chlorophyll a-b bin	50.00	0.00
TRINITY_sp|Q9FT[illegible]	TPT	Triose phosphate/ph	50.00	0.00
TRINITY_sp|Q8YM[illegible]	ftsH	ATP-dependent zinc	50.00	0.00
TRINITY_sp|Q6DD[illegible]	tbc1d31	TBC1 domain family	50.00	0.00
TRINITY_sp|Q27Y[illegible]	RSP5	Flagellar radial sp	50.00	0.00
TRINITY_sp|A7MV[illegible]	VIBHAR_[illegible]	UPF0061 protein VIB	50.00	0.00
TRINITY_sp|Q54X[illegible]	pah	Phenylalanine-4-hyd	50.00	0.00
TRINITY_sp|Q5U3[illegible]	katnal1	Katanin p60 ATPase-	50.00	0.00
TRINITY_sp|Q2KH[illegible]	MACROD1	O-acetyl-ADP-ribose	50.00	0.00
TRINITY_sp|Q2TB[illegible]	ZNHIT2	Zinc finger HIT dom	50.00	0.00
TRINITY_sp|Q027[illegible]	MAP3K10	Mitogen-activated p	50.00	0.00
TRINITY_sp|P628[illegible]	RAP1A	Ras-related protein	50.00	0.00
TRINITY_sp|Q9FF[illegible]	ABCG28	ABC transporter G f	50.00	0.00
TRINITY_sp|B8J8[illegible]	glgA	Glycogen synthase O	50.00	0.00
TRINITY_sp|Q7SC[illegible]	rps-25	40S ribosomal prote	50.00	0.00
TRINITY_sp|Q9VX[illegible]	CG6762	Putative sulfiredox	50.00	0.00
TRINITY_sp|A7E3[illegible]	PTPDC1	Protein tyrosine ph	50.00	0.00
TRINITY_sp|Q93V[illegible]	At3g026[illegible]	WAT1-related protei	50.00	0.00
TRINITY_sp|P623[illegible]	CPK4	Calcium-dependent p	50.00	0.00
TRINITY_sp|Q54F[illegible]	mcfW	Mitochondrial subst	50.00	0.00
TRINITY_sp|F4I3[illegible]	NRPA2	DNA-directed RNA po	50.00	0.00
TRINITY_sp|Q9H1[illegible]	KIF13A	Kinesin-like protei	50.00	0.00
TRINITY_sp|P0C7[illegible]	UMAG_11[illegible]	Putative DEAH-box A	50.00	0.00
TRINITY_sp|Q9ZQ[illegible]	ACX1.2	Putative peroxisoma	50.00	0.00
TRINITY_sp|O440[illegible]	PYK	Pyruvate kinase OS=	50.00	0.00
TRINITY_sp|Q653[illegible]	Os06g06[illegible]	Probable alpha-gluc	50.00	0.00
TRINITY_sp|Q9CQ[illegible]	-	Uncharacterized pro	50.00	0.00
TRINITY_sp|P078[illegible]	Cel	Bile salt-activated	50.00	0.00
TRINITY_sp|Q9FP[illegible]	UBP24	Ubiquitin carboxyl-	50.00	0.00
TRINITY_sp|Q213[illegible]	gst-4	Glutathione S-trans	50.00	0.00
TRINITY_sp|Q9S7[illegible]	ACL5	Thermospermine synt	50.00	0.00
TRINITY_sp|Q9DE[illegible]	BAZ2B	Bromodomain adjacen	50.00	0.00
TRINITY_sp|Q8ML[illegible]	pdeD	cGMP-dependent 3',5	50.00	0.00
TRINITY_sp|P505[illegible]	sck1	Serine/threonine-pr	50.00	0.00
TRINITY_sp|Q95P[illegible]	dhkC	Hybrid signal trans	50.00	0.00
TRINITY_sp|P126[illegible]	BCKDHA	2-oxoisovalerate de	49.90	0.00
TRINITY_sp|Q54P[illegible]	abcC8	ABC transporter C f	49.90	0.00
TRINITY_sp|Q9LR[illegible]	GLO1	Peroxisomal (S)-2-h	49.90	0.00
TRINITY_sp|Q9LI[illegible]	BAM1	Beta-amylase 1, chl	49.90	0.00

TRINITY_sp|B9F6	Os03g02	Probable glucan 1,3	49.90	0.00
TRINITY_sp|A0A0	tkt	Transketolase OS=Ge	49.90	0.00
TRINITY_sp|Q54Y2	alaS	Alanine--tRNA ligas	49.90	0.00
TRINITY_sp|Q6PFW	PPIP5K1	Inositol hexakispho	49.90	0.00
TRINITY_sp|O1413	elf1	mRNA export factor	49.90	0.00
TRINITY_sp|Q0508	UBE3A	Ubiquitin-protein l	49.80	0.00
TRINITY_sp|P4265	-	14-3-3-like protein	49.80	0.00
TRINITY_sp|Q86K0	DDB_G02	Probable GH family	49.80	0.00
TRINITY_sp|Q54N2	memo1	Protein MEMO1 homol	49.80	0.00
TRINITY_sp|Q9268	ALG3	Dol-P-Man:Man(5)Glc	49.80	0.00
TRINITY_sp|A5PLI	TMEM189	Transmembrane prote	49.80	0.00
TRINITY_sp|P5989	Nek6	Serine/threonine-pr	49.80	0.00
TRINITY_sp|Q9SA2	RH36	DEAD-box ATP-depend	49.80	0.00
TRINITY_sp|Q9C00	DNAH6	Dynein heavy chain	49.80	0.00
TRINITY_sp|Q54WW	DDB_G02	Probable serine/thr	49.80	0.00
TRINITY_sp|Q9LK0	SYP32	Syntaxin-32 OS=Arab	49.80	0.00
TRINITY_sp|Q86AI	DDB_G02	Probable myosin lig	49.80	0.00
TRINITY_sp|Q54MB	adk	Adenosine kinase OS	49.80	0.00
TRINITY_sp|Q8L32	FZR2	Protein FIZZY-RELAT	49.80	0.00
TRINITY_sp|P4333	At1g0976	U2 small nuclear ri	49.80	0.00
TRINITY_sp|Q7XK0	PYRD	Dihydroorotate dehy	49.80	0.00
TRINITY_sp|A2Q0U	rpsa	40S ribosomal prote	49.80	0.00
TRINITY_sp|Q84RR	ARI7	Probable E3 ubiquit	49.80	0.00
TRINITY_sp|Q3965	PSAL	Photosystem I react	49.80	0.00
TRINITY_sp|H2E71	TMT-1	Squalene methyltran	49.80	0.00
TRINITY_sp|A2YR1	ARP4	Actin-related prote	49.80	0.00
TRINITY_sp|Q9U64	cmfB	Conditioned medium	49.80	0.00
TRINITY_sp|P2818	RABD2A	Ras-related protein	49.80	0.00
TRINITY_sp|Q6WWW	UPL3	E3 ubiquitin-protei	49.80	0.00
TRINITY_sp|F4KD7	DUR3	Urea-proton symport	49.80	0.00
TRINITY_sp|P5181	PRKX	cAMP-dependent prot	49.80	0.00
TRINITY_sp|Q55GJ	nca2	Nuclear control of	49.70	0.00
TRINITY_sp|P0446	-	Calmodulin OS=Triti	49.70	0.00
TRINITY_sp|Q0573	YPTM2	GTP-binding protein	49.70	0.00
TRINITY_sp|P4039	RIC1	Ras-related protein	49.70	0.00
TRINITY_sp|Q6PC1	ssu72	RNA polymerase II s	49.70	0.00
TRINITY_sp|Q9D7B	Dus2	tRNA-dihydrouridine	49.70	0.00
TRINITY_sp|Q54IA	cpox	Oxygen-dependent co	49.70	0.00
TRINITY_sp|Q7YSW	canA	Calcineurin subunit	49.70	0.00
TRINITY_sp|F4HY0	IRE3	Probable serine/thr	49.70	0.00
TRINITY_sp|Q9UK5	CCNL1	Cyclin-L1 OS=Homo s	49.70	0.00
TRINITY_sp|Q3T0I	CTSH	Pro-cathepsin H OS=	49.70	0.00
TRINITY_sp|O9535	ATG7	Ubiquitin-like modi	49.70	0.00
TRINITY_sp|O6560	MSH3	DNA mismatch repair	49.70	0.00
TRINITY_sp|Q76NV	hpd	4-hydroxyphenylpyru	49.70	0.00
TRINITY_sp|A0JN5	SF3B3	Splicing factor 3B	49.70	0.00
TRINITY_sp|Q9FPH	THA2	Probable low-specif	49.70	0.00
TRINITY_sp|Q53GS	USP39	U4/U6.U5 tri-snRNP-	49.70	0.00
TRINITY_sp|O2457	RP-P0	60S acidic ribosoma	49.70	0.00
TRINITY_sp|Q0974	dbp5	ATP-dependent RNA h	49.70	0.00
TRINITY_sp|O4290	rti1	DNA repair and reco	49.70	0.00
TRINITY_sp|Q8BMF	Dlat	Dihydrolipoyllysine	49.70	0.00
TRINITY_sp|Q55D9	pakA	Serine/threonine-pr	49.70	0.00
TRINITY_sp|Q9P7B	SPAC186.	2-hydroxyacid dehyd	49.70	0.00
TRINITY_sp|Q5L07	psuG	Pseudouridine-5'-ph	49.70	0.00
TRINITY_sp|Q9UQW	spe1	Ornithine decarboxy	49.70	0.00

TRINITY_sp|Q91V2	Abca7	ATP-binding cassett	49.70	0.00
TRINITY_sp|Q86A(	polr3a	DNA-directed RNA po	49.70	0.00
TRINITY_sp|Q9QXI	Nme7	Nucleoside diphosph	49.70	0.00
TRINITY_sp|Q54BN	DDB_G02S	UPF0652 protein OS=	49.70	0.00
TRINITY_sp|Q8BU3	Ilvbl	Acetolactate syntha	49.70	0.00
TRINITY_sp|Q9MAE	SCPL44	Serine carboxypepti	49.70	0.00
TRINITY_sp|Q27Y(	RSP1	Flagellar radial sp	49.70	0.00
TRINITY_sp|Q6EVE	BRM	ATP-dependent helic	49.70	0.00
TRINITY_sp|Q9FNE	SS1	Starch synthase 1,	49.70	0.00
TRINITY_sp|Q9LF\	RPOT2	DNA-directed RNA po	49.70	0.00
TRINITY_sp|Q84K1	GAMMA-AI	AP-1 complex subuni	49.60	0.00
TRINITY_sp|O7584	SC5D	Lathosterol oxidase	49.60	0.00
TRINITY_sp|Q4312	-	Non-specific lipid-	49.60	0.00
TRINITY_sp|P136(	HOM2	Aspartate-semialdeh	49.60	0.00
TRINITY_sp|Q8VI4	Abcc2	Canalicular multisp	49.60	0.00
TRINITY_sp|O8603	bdhA	D-beta-hydroxybutyr	49.60	0.00
TRINITY_sp|A5G14	glpK	Glycerol kinase OS=	49.60	0.00
TRINITY_sp|Q91WA	Hdac11	Histone deacetylase	49.60	0.00
TRINITY_sp|Q86I(	nek3	Probable serine/thr	49.60	0.00
TRINITY_sp|Q7F83	CYCA1-1	Cyclin-A1-1 OS=Oryz	49.60	0.00
TRINITY_sp|Q9M39	NPF8.1	Protein NRT1/ PTR F	49.60	0.00
TRINITY_sp|Q323	ybiA	N-glycosidase YbiA	49.60	0.00
TRINITY_sp|Q9LX\	At5g128	Zinc finger CCCH do	49.60	0.00
TRINITY_sp|Q8WV(	CANT1	Soluble calcium-act	49.60	0.00
TRINITY_sp|Q54KE	gluD	Glutamate dehydroge	49.60	0.00
TRINITY_sp|Q94E(	At5g0224	Uncharacterized pro	49.60	0.00
TRINITY_sp|O1392	crn1	Coronin-like protei	49.60	0.00
TRINITY_sp|Q8RXN	TYW1	S-adenosyl-L-methio	49.60	0.00
TRINITY_sp|A8J78	ATPG	ATP synthase subuni	49.60	0.00
TRINITY_sp|O138(	SPAC1A6.	tRNA threonylcarbam	49.60	0.00
TRINITY_sp|P046	-	Uricase-2 isozyme 1	49.60	0.00
TRINITY_sp|P3472	RAS1	Ras-like protein 1	49.60	0.00
TRINITY_sp|Q9C0(	DNAH6	Dynein heavy chain	49.60	0.00
TRINITY_sp|A2YPE	OBGC1	Probable GTP-bindin	49.60	0.00
TRINITY_sp|Q091	krp1	Dibasic-processing	49.60	0.00
TRINITY_sp|A6NKE	SDR42E2	Putative short-chai	49.60	0.00
TRINITY_sp|Q556(	psmB4-1	Proteasome subunit	49.60	0.00
TRINITY_sp|Q8K1A	Mettl5	Methyltransferase-l	49.60	0.00
TRINITY_sp|Q8CG9	Elac2	Zinc phosphodiester	49.50	0.00
TRINITY_sp|P4981	DLD	Dihydrolipoyl dehyd	49.50	0.00
TRINITY_sp|P318(	XYL1	NAD(P)H-dependent D	49.50	0.00
TRINITY_sp|Q9ZWE	PCS2	Glutathione gamma-g	49.50	0.00
TRINITY_sp|Q68W(	grxC2	Probable monothiol	49.50	0.00
TRINITY_sp|P5025	TUBB1	Tubulin beta chain	49.50	0.00
TRINITY_sp|Q3T13	BOLA1	BolA-like protein 1	49.50	0.00
TRINITY_sp|Q8LD2	At1g5622	Dormancy-associated	49.50	0.00
TRINITY_sp|B1ZGW	glpK	Glycerol kinase OS=	49.50	0.00
TRINITY_sp|Q6123	Lcp1	Plastin-2 OS=Mus mu	49.50	0.00
TRINITY_sp|O9443	SPBC660.	Uncharacterized RNA	49.50	0.00
TRINITY_sp|P2391	DTYMK	Thymidylate kinase	49.50	0.00
TRINITY_sp|Q99JX	Gorasp2	Golgi reassembly-st	49.50	0.00
TRINITY_sp|Q54V(	hdaC	Type-2 histone deac	49.50	0.00
TRINITY_sp|Q201(	copb-2	Probable coatomer s	49.50	0.00
TRINITY_sp|Q8MXN	DDB_G02	Transmembrane prote	49.50	0.00
TRINITY_sp|Q941E	GONST1	GDP-mannose transpo	49.50	0.00
TRINITY_sp|Q9LEW	UBP22	Ubiquitin carboxyl-	49.50	0.00

TRINITY_sp|M0ZY‹	NDA1	Internal alternativ	49.50	0.00
TRINITY_sp|Q91YI	Mettl13	Methyltransferase-l	49.50	0.00
TRINITY_sp|Q9XVI	rpb-11	Probable DNA-direct	49.50	0.00
TRINITY_sp|Q54Q3	phbB	Prohibitin-2 OS=Dic	49.50	0.00
TRINITY_sp|O2301	KAB1	Probable voltage-ga	49.50	0.00
TRINITY_sp|Q0III	DUSP10	Dual specificity pr	49.50	0.00
TRINITY_sp|P4845	PPP3CC	Serine/threonine-pr	49.50	0.00
TRINITY_sp|Q8GXS	CFIS2	Pre-mRNA cleavage f	49.50	0.00
TRINITY_sp|D0NL(	PITG_128	tRNA (guanine(37)-N	49.50	0.00
TRINITY_sp|Q6BII	REX4	RNA exonuclease 4 O	49.50	0.00
TRINITY_sp|Q9UNI	CDC14A	Dual specificity pr	49.50	0.00
TRINITY_sp|Q127(	pab1	Protein phosphatase	49.50	0.00
TRINITY_sp|Q8VY(	DJ1C	Protein DJ-1 homolo	49.50	0.00
TRINITY_sp|O3555	Pfkfb3	6-phosphofructo-2-k	49.50	0.00
TRINITY_sp|Q165(	CAMK4	Calcium/calmodulin-	49.50	0.00
TRINITY_sp|Q9ES(	Ube4b	Ubiquitin conjugati	49.50	0.00
TRINITY_sp|Q5T0I	CFAP70	Cilia- and flagella	49.50	0.00
TRINITY_sp|P2951	TUBB7	Tubulin beta-7 chai	49.50	0.00
TRINITY_sp|Q3ZBI	PTRH2	Peptidyl-tRNA hydro	49.50	0.00
TRINITY_sp|P929(	RS31	Serine/arginine-ric	49.50	0.00
TRINITY_sp|Q9LT\	At3g128(	Peroxisomal 2,4-die	49.50	0.00
TRINITY_sp|O1511	NPC1	Niemann-Pick C1 pro	49.50	0.00
TRINITY_sp|Q6AY8	Alg14	UDP-N-acetylglucosa	49.50	0.00
TRINITY_sp|Q395(	ODA4	Dynein beta chain,	49.50	0.00
TRINITY_sp|Q9SYI	BRR2A	DExH-box ATP-depend	49.50	0.00
TRINITY_sp|Q1ZX(	pXi	Probable serine/thr	49.50	0.00
TRINITY_sp|Q9SD\	RCE1	NEDD8-conjugating e	49.50	0.00
TRINITY_sp|Q9C5I	DTC	Mitochondrial dicar	49.50	0.00
TRINITY_sp|Q9NKV	mfeA	Peroxisomal multifu	49.50	0.00
TRINITY_sp|Q3893	LCY1	Lycopene beta cycla	49.50	0.00
TRINITY_sp|Q425T	LIG1	DNA ligase 1 OS=Ara	49.50	0.00
TRINITY_sp|P320(	-	Stearoyl-[acyl-carr	49.50	0.00
TRINITY_sp|Q631(	Dnah1	Dynein heavy chain	49.50	0.00
TRINITY_sp|Q54DI	amdA	AMP deaminase OS=Di	49.50	0.00
TRINITY_sp|Q4378	WAXY	Granule-bound starc	49.50	0.00
TRINITY_sp|P5633	EIF5A4	Eukaryotic translat	49.50	0.00
TRINITY_sp|Q5RH\	dcun1d4	DCN1-like protein 4	49.50	0.00
TRINITY_sp|Q8INI	Gyc88E	Soluble guanylate c	49.50	0.00
TRINITY_sp|Q93Y2	At2g3181	Acetolactate syntha	49.50	0.00
TRINITY_sp|Q94B\	PIGA	Phosphatidylinosito	49.40	0.00
TRINITY_sp|Q8CHI	Ttll5	Tubulin polyglutamy	49.40	0.00
TRINITY_sp|Q54TA	drkC	Probable serine/thr	49.40	0.00
TRINITY_sp|Q2663	-	33 kDa inner dynein	49.40	0.00
TRINITY_sp|P1965	-	Non-specific lipid-	49.40	0.00
TRINITY_sp|Q9SRS	CHIP	E3 ubiquitin-protei	49.40	0.00
TRINITY_sp|A6LTI	apt	Adenine phosphoribo	49.40	0.00
TRINITY_sp|Q2VZ\	infB	Translation initiat	49.40	0.00
TRINITY_sp|P5184	pdcA	Pyruvate decarboxyl	49.40	0.00
TRINITY_sp|Q3SEI	Icl1e	Caltractin ICL1e OS	49.40	0.00
TRINITY_sp|Q0DHI	FTSH8	ATP-dependent zinc	49.40	0.00
TRINITY_sp|Q8YM5	clpB2	Chaperone protein C	49.40	0.00
TRINITY_sp|Q9GZI	REXO4	RNA exonuclease 4 O	49.40	0.00
TRINITY_sp|O9524	MBD4	Methyl-CpG-binding	49.40	0.00
TRINITY_sp|Q54SA	pldZ	Phospholipase D Z O	49.40	0.00
TRINITY_sp|Q8JI\	nifk	MKI67 FHA domain-in	49.40	0.00
TRINITY_sp|A7SL(	nat9	N-acetyltransferase	49.40	0.00

TRINITY_sp|Q8BRI	Man2a2	Alpha-mannosidase 2	49.40	0.00
TRINITY_sp|P167(	V-UBI	Ubiquitin-like prot	49.40	0.00
TRINITY_sp|A9V2(	37534	Probable nitrile hy	49.40	0.00
TRINITY_sp|O653!	GGH2	Gamma-glutamyl hydr	49.40	0.00
TRINITY_sp|B9F1(	LOGL2	Probable cytokinin	49.40	0.00
TRINITY_sp|Q9C8:	NUP50A	Nuclear pore comple	49.40	0.00
TRINITY_sp|Q9BR!	RIOK1	Serine/threonine-pr	49.40	0.00
TRINITY_sp|Q9SU\	At4g323!	Probable sugar phos	49.40	0.00
TRINITY_sp|Q5RK:	Sbds	Ribosome maturation	49.40	0.00
TRINITY_sp|P454:	CSN1	COP9 signalosome co	49.40	0.00
TRINITY_sp|A6LMI	alaS	Alanine--tRNA ligas	49.40	0.00
TRINITY_sp|Q8ST8	abcC10	ABC transporter C f	49.40	0.00
TRINITY_sp|Q0WQI	LTA3	Dihydrolipoyllysine	49.40	0.00
TRINITY_sp|Q056(	CTR1	Serine/threonine-pr	49.40	0.00
TRINITY_sp|A2X8N	PUT1	Polyamine transport	49.40	0.00
TRINITY_sp|O230:	KAB1	Probable voltage-ga	49.40	0.00
TRINITY_sp|Q7YSV	canA	Calcineurin subunit	49.40	0.00
TRINITY_sp|Q9M9!	UTR3	UDP-galactose/UDP-g	49.40	0.00
TRINITY_sp|Q8I8l	ap1g1	AP-1 complex subuni	49.40	0.00
TRINITY_sp|Q9CZ!	Fopnl	LisH domain-contain	49.40	0.00
TRINITY_sp|Q8L7N	SDN5	Small RNA degrading	49.40	0.00
TRINITY_sp|Q0WR)	PBL7	Probable serine/thr	49.40	0.00
TRINITY_sp|Q96RI	BBS4	Bardet-Biedl syndro	49.40	0.00
TRINITY_sp|Q86H:	polr1a	DNA-directed RNA po	49.40	0.00
TRINITY_sp|P109	-	Retrovirus-related	49.40	0.00
TRINITY_sp|Q9M3(	ATG6	Beclin-1-like prote	49.40	0.00
TRINITY_sp|P479:	AP2	Floral homeotic pro	49.40	0.00
TRINITY_sp|Q138:	GNL2	Nucleolar GTP-bindi	49.30	0.00
TRINITY_sp|Q9SBI	ABC1	Protein ABC transpo	49.30	0.00
TRINITY_sp|Q6DCI	hace1	E3 ubiquitin-protei	49.30	0.00
TRINITY_sp|Q39J(	dnaJ	Chaperone protein D	49.30	0.00
TRINITY_sp|Q54LI	ints4	Integrator complex	49.30	0.00
TRINITY_sp|Q54Cl	trappc2l	Trafficking protein	49.30	0.00
TRINITY_sp|A0PZI	NT01CX_1	Putative 3-methylad	49.30	0.00
TRINITY_sp|Q5MB:	ABCG2	ATP-binding cassett	49.30	0.00
TRINITY_sp|Q9BR	CBWD1	COBW domain-contain	49.30	0.00
TRINITY_sp|Q9SK	At2g202	Zinc finger CCCH do	49.30	0.00
TRINITY_sp|Q550V	slc35c1	GDP-fucose transpor	49.30	0.00
TRINITY_sp|Q8NCN	DYNC2H1	Cytoplasmic dynein	49.30	0.00
TRINITY_sp|Q8L4I	WEE1	Wee1-like protein k	49.30	0.00
TRINITY_sp|P396(	nirA	Ferredoxin--nitrite	49.30	0.00
TRINITY_sp|Q9M2I	RPS27B	40S ribosomal prote	49.30	0.00
TRINITY_sp|Q8IU8	CAMK1D	Calcium/calmodulin-	49.30	0.00
TRINITY_sp|Q940(	TMN1	Transmembrane 9 sup	49.30	0.00
TRINITY_sp|Q55EI	dhak	Probable dihydroxya	49.30	0.00
TRINITY_sp|Q75J(	aspS1	Aspartate--tRNA lig	49.30	0.00
TRINITY_sp|Q9LN:	P4H3	Probable prolyl 4-h	49.30	0.00
TRINITY_sp|Q4QEI	GMPR	GMP reductase OS=Le	49.30	0.00

TRINITY_sp|P011]	RAS1	Ras-like protein 1	49.30	0.00
TRINITY_sp|O810(	EMB2001	GTP-binding protein	49.30	0.00
TRINITY_sp|A8ILE	CFAP52	Cilia- and flagella	49.30	0.00
TRINITY_sp|D4AVI	ARB_001	Calnexin homolog AR	49.30	0.00
TRINITY_sp|A3GFA	SEC23	Protein transport p	49.30	0.00
TRINITY_sp|B0G1(	DDB_G02	Deoxynucleoside tri	49.30	0.00
TRINITY_sp|P275]	PHO91	Low-affinity phosph	49.30	0.00
TRINITY_sp|Q3SY2	XYLB	Xylulose kinase OS=	49.30	0.00
TRINITY_sp|Q136]	RAB32	Ras-related protein	49.30	0.00
TRINITY_sp|Q55DW	abcG1	ABC transporter G f	49.30	0.00
TRINITY_sp|Q8VZ	P4H9	Probable prolyl 4-h	49.30	0.00
TRINITY_sp|P138(	-	Chlorophyll a-b bin	49.30	0.00
TRINITY_sp|O137!	osm1	Fumarate reductase	49.30	0.00
TRINITY_sp|P581(	atp2b2	Plasma membrane cal	49.30	0.00
TRINITY_sp|Q803\	ppp2r3c	Serine/threonine-pr	49.30	0.00
TRINITY_sp|Q8IVH	MMAA	Methylmalonic acidu	49.30	0.00
TRINITY_sp|Q922!	SREP	GATA factor SREP OS	49.30	0.00
TRINITY_sp|A8J8E	CHLREDRA	Tektin OS=Chlamydom	49.30	0.00
TRINITY_sp|O753	GUCY1B2	Guanylate cyclase s	49.30	0.00
TRINITY_sp|P341]	mvpA	Major vault protein	49.30	0.00
TRINITY_sp|Q54D(	DDB_G02	Probable protein ty	49.30	0.00
TRINITY_sp|Q8H2I	POLH	DNA polymerase eta	49.30	0.00
TRINITY_sp|Q54ZW	phg1b	Putative phagocytic	49.30	0.00
TRINITY_sp|Q8C2I	Dus1l	tRNA-dihydrouridine	49.30	0.00
TRINITY_sp|Q9VL(	wol	Dolichyl-phosphate	49.30	0.00
TRINITY_sp|Q9FL]	DEGP9	Protease Do-like 9	49.30	0.00
TRINITY_sp|Q54E(	vatM	Vacuolar proton tra	49.30	0.00
TRINITY_sp|Q9VH]	Invadoly	Leishmanolysin-like	49.30	0.00
TRINITY_sp|Q82EI	acsA	Acetyl-coenzyme A s	49.30	0.00
TRINITY_sp|P551	-	Glutaredoxin OS=Ric	49.30	0.00
TRINITY_sp|P005]	PRKACA	cAMP-dependent prot	49.30	0.00
TRINITY_sp|F4IYE	At3g027	Histidine--tRNA lig	49.30	0.00
TRINITY_sp|Q9CA]	CKL2	Casein kinase 1-lik	49.30	0.00
TRINITY_sp|Q2KI	DNAJC5B	DnaJ homolog subfam	49.30	0.00
TRINITY_sp|P053(	Ef1alpha	Elongation factor 1	49.30	0.00
TRINITY_sp|Q54U(	trappc4	Trafficking protein	49.30	0.00
TRINITY_sp|Q7XQ]	AMT1-1	Ammonium transporte	49.30	0.00
TRINITY_sp|P402(	-	Histone H1 OS=Solan	49.30	0.00
TRINITY_sp|Q9JMA	Cyp3a41a	Cytochrome P450 3A4	49.30	0.00
TRINITY_sp|P395]	TIM17	Mitochondrial impor	49.20	0.00
TRINITY_sp|Q9ZWE	CKL9	Casein kinase 1-lik	49.20	0.00
TRINITY_sp|F4K4E	LSM4	Sm-like protein LSM	49.20	0.00
TRINITY_sp|Q9CR(	Golt1b	Vesicle transport p	49.20	0.00
TRINITY_sp|Q6YVX	Os02g05	Zinc finger CCCH do	49.20	0.00
TRINITY_sp|Q55E	mcfE	Mitochondrial subst	49.20	0.00
TRINITY_sp|P817]	-	Subtilisin inhibito	49.20	0.00
TRINITY_sp|Q9JJ(	Pigb	GPI mannosyltransfe	49.20	0.00
TRINITY_sp|Q150(	PSMD6	26S proteasome non-	49.20	0.00
TRINITY_sp|P629!	RAC1	Ras-related C3 botu	49.20	0.00
TRINITY_sp|Q9SC(	UPL7	E3 ubiquitin-protei	49.20	0.00
TRINITY_sp|Q5RAE	SEPSECS	O-phosphoseryl-tRNA	49.20	0.00
TRINITY_sp|Q9FGI	RKD3	Protein RKD3 OS=Ara	49.20	0.00
TRINITY_sp|Q1ZXI	psmD3	26S proteasome non-	49.20	0.00
TRINITY_sp|P175]	-	Acidic endochitinas	49.20	0.00
TRINITY_sp|Q6AJI	prs	Ribose-phosphate py	49.20	0.00
TRINITY_sp|Q7X9\	PIE1	Protein PHOTOPERIOD	49.20	0.00

TRINITY_sp|O947(	rpa12	DNA-directed RNA po	49.20	0.00
TRINITY_sp|Q9FN(	UVR8	Ultraviolet-B recep	49.20	0.00
TRINITY_sp|Q9LE5	SP1L2	Protein SPIRAL1-lik	49.20	0.00
TRINITY_sp|P258	chcA	Clathrin heavy chai	49.20	0.00
TRINITY_sp|P8714	SPBC25H2	Protein VAC14 homol	49.20	0.00
TRINITY_sp|P615	can	Carbonic anhydrase	49.20	0.00
TRINITY_sp|Q8LB	GRXS15	Monothiol glutaredo	49.20	0.00
TRINITY_sp|Q9NR(	BIRC6	Baculoviral IAP rep	49.20	0.00
TRINITY_sp|Q87L8	dusA	tRNA-dihydrouridine	49.20	0.00
TRINITY_sp|P1102	NNT	NAD(P) transhydroge	49.20	0.00
TRINITY_sp|Q54S	dhkM	Hybrid signal trans	49.20	0.00
TRINITY_sp|O226(	DEGP1	Protease Do-like 1,	49.20	0.00
TRINITY_sp|Q6ZT	ANKRD13	Ankyrin repeat doma	49.20	0.00
TRINITY_sp|Q8LG	CUL4	Cullin-4 OS=Arabido	49.20	0.00
TRINITY_sp|Q3895	PHT2-1	Inorganic phosphate	49.20	0.00
TRINITY_sp|Q8C7	Uba6	Ubiquitin-like modi	49.20	0.00
TRINITY_sp|Q56Z	At5g1615	Plastidic glucose t	49.20	0.00
TRINITY_sp|Q9NC	pitB	Phosphatidylinosito	49.20	0.00
TRINITY_sp|Q3T0	DHRS7B	Dehydrogenase/reduc	49.20	0.00
TRINITY_sp|Q9VH	Invadoly	Leishmanolysin-like	49.20	0.00
TRINITY_sp|Q8LB	At2g167	Iron-sulfur assembl	49.20	0.00
TRINITY_sp|Q9C0(	DNAH6	Dynein heavy chain	49.20	0.00
TRINITY_sp|Q8RY	ELI5	Tyrosine decarboxyl	49.20	0.00
TRINITY_sp|Q5XI(	Galk2	N-acetylgalactosami	49.20	0.00
TRINITY_sp|Q54X	etfdh	Electron transfer f	49.20	0.00
TRINITY_sp|P527(	Hrsp12	Ribonuclease UK114	49.20	0.00
TRINITY_sp|A2X6	CDKG-1	Cyclin-dependent ki	49.20	0.00
TRINITY_sp|Q6255	Ift52	Intraflagellar tran	49.20	0.00
TRINITY_sp|Q2KI2	FAM63B	Ubiquitin carboxyl-	49.20	0.00
TRINITY_sp|Q9Z2	Gfpt2	Glutamine--fructose	49.20	0.00
TRINITY_sp|Q8GY2	UPL1	E3 ubiquitin-protei	49.10	0.00
TRINITY_sp|Q9NB	hiw	E3 ubiquitin-protei	49.10	0.00
TRINITY_sp|Q6BV	PMP3	Plasma membrane pro	49.10	0.00
TRINITY_sp|P3225	rasC	Ras-like protein ra	49.10	0.00
TRINITY_sp|Q5JT2	RAB41	Ras-related protein	49.10	0.00
TRINITY_sp|P8408	ARF1	ADP-ribosylation fa	49.10	0.00
TRINITY_sp|Q9069	CDC42	Cell division contr	49.10	0.00
TRINITY_sp|A7MH	rbn	Ribonuclease BN OS=	49.10	0.00
TRINITY_sp|O944	SPBC660.	Uncharacterized RNA	49.10	0.00
TRINITY_sp|O0489	-	Alpha-glucosidase O	49.10	0.00
TRINITY_sp|Q23M	Ttll6a	Probable beta-tubul	49.10	0.00
TRINITY_sp|Q54G	uap1	Probable UDP-N-acet	49.10	0.00
TRINITY_sp|Q6UK(	pirA	Protein pirA OS=Dic	49.10	0.00
TRINITY_sp|P831(	Dyrk3	Putative dual speci	49.10	0.00
TRINITY_sp|Q7T3	rab13	Ras-related protein	49.10	0.00
TRINITY_sp|Q86A	DDB_G02	Probable myosin lig	49.10	0.00
TRINITY_sp|Q8H1(	MTPC4	Metal tolerance pro	49.10	0.00
TRINITY_sp|Q2845	CRYZ	Quinone oxidoreduct	49.10	0.00
TRINITY_sp|Q9LI	BAM1	Beta-amylase 1, chl	49.10	0.00
TRINITY_sp|P4908	AKHSDH2	Bifunctional aspart	49.10	0.00
TRINITY_sp|P0CR	SEC23	Protein transport p	49.10	0.00
TRINITY_sp|Q9CB	fbiC	FO synthase OS=Myco	49.10	0.00
TRINITY_sp|O7015	Dpm1	Dolichol-phosphate	49.10	0.00
TRINITY_sp|Q9T0	At4g1359	GDT1-like protein 2	49.10	0.00
TRINITY_sp|Q9M1(	ABCB21	ABC transporter B f	49.10	0.00
TRINITY_sp|Q9JI	Stk3	Serine/threonine-pr	49.10	0.00

TRINITY_sp|Q409[illegible]	-	Monodehydroascorbat	49.10	0.00
TRINITY_sp|P454[illegible]	CSN1	COP9 signalosome co	49.10	0.00
TRINITY_sp|Q54W[illegible]	glnA3	Type-3 glutamine sy	49.10	0.00
TRINITY_sp|Q54Y[illegible]	maf1	Repressor of RNA po	49.10	0.00
TRINITY_sp|Q6JQ[illegible]	ACAD10	Acyl-CoA dehydrogen	49.10	0.00
TRINITY_sp|Q10M[illegible]	Os03g02[illegible]	Asparagine syntheta	49.10	0.00
TRINITY_sp|P801[illegible]	-	Extracellular serin	49.10	0.00
TRINITY_sp|Q803[illegible]	ergic3	Endoplasmic reticul	49.10	0.00
TRINITY_sp|Q152[illegible]	PWP2	Periodic tryptophan	49.10	0.00
TRINITY_sp|Q9LE[illegible]	RANGAP1	RAN GTPase-activati	49.10	0.00
TRINITY_sp|P425[illegible]	-	Soma ferritin OS=Ly	49.10	0.00
TRINITY_sp|Q9C5[illegible]	HAC1	Histone acetyltrans	49.10	0.00
TRINITY_sp|Q389[illegible]	FTB	Protein farnesyltra	49.10	0.00
TRINITY_sp|Q963[illegible]	ACX4	Acyl-coenzyme A oxi	49.10	0.00
TRINITY_sp|P871[illegible]	not1	General negative re	49.10	0.00
TRINITY_sp|B0D6[illegible]	LACBIDR[illegible]	Pentafunctional ARO	49.10	0.00
TRINITY_sp|P258[illegible]	chcA	Clathrin heavy chai	49.10	0.00
TRINITY_sp|Q9SG[illegible]	ATL72	RING-H2 finger prot	49.00	0.00
TRINITY_sp|Q32P[illegible]	DYNLRB2	Dynein light chain	49.00	0.00
TRINITY_sp|C1FI[illegible]	MICPUN_[illegible]	Inosine triphosphat	49.00	0.00
TRINITY_sp|Q0WV[illegible]	RRP6L1	Protein RRP6-like 1	49.00	0.00
TRINITY_sp|Q66K[illegible]	TBC1D9B	TBC1 domain family	49.00	0.00
TRINITY_sp|O142[illegible]	trm10	tRNA (guanine(9)-N1	49.00	0.00
TRINITY_sp|Q9CQ[illegible]	Commd4	COMM domain-contain	49.00	0.00
TRINITY_sp|Q86A[illegible]	DDB_G02[illegible]	Probable myosin lig	49.00	0.00
TRINITY_sp|Q54I[illegible]	eif2b1	Translation initiat	49.00	0.00
TRINITY_sp|O158[illegible]	CPK2	Calcium-dependent p	49.00	0.00
TRINITY_sp|Q93Z[illegible]	SDF2	Stromal cell-derive	49.00	0.00
TRINITY_sp|Q58D[illegible]	SORD	Sorbitol dehydrogen	49.00	0.00
TRINITY_sp|F4HY[illegible]	IRE3	Probable serine/thr	49.00	0.00
TRINITY_sp|O227[illegible]	PXG3	Probable peroxygena	49.00	0.00
TRINITY_sp|Q86A[illegible]	spkA-1	Stress-activated pr	49.00	0.00
TRINITY_sp|Q5UP[illegible]	MIMI_R5[illegible]	Uncharacterized pro	49.00	0.00
TRINITY_sp|Q9Y5[illegible]	OARD1	O-acetyl-ADP-ribose	49.00	0.00
TRINITY_sp|Q8C9[illegible]	Spef2	Sperm flagellar pro	49.00	0.00
TRINITY_sp|A5GW[illegible]	rpsJ	30S ribosomal prote	49.00	0.00
TRINITY_sp|Q2M2[illegible]	ALKBH7	Alpha-ketoglutarate	49.00	0.00
TRINITY_sp|Q8LF[illegible]	DRP3B	Dynamin-related pro	49.00	0.00
TRINITY_sp|Q08D[illegible]	AP2B1	AP-1 complex subuni	49.00	0.00
TRINITY_sp|Q9XF[illegible]	LHCB5	Chlorophyll a-b bin	49.00	0.00
TRINITY_sp|P468[illegible]	KRP95	Kinesin-II 95 kDa s	49.00	0.00
TRINITY_sp|Q9XG[illegible]	VHA-D	V-type proton ATPas	49.00	0.00
TRINITY_sp|Q9XF[illegible]	DEE76	Degreening-related	49.00	0.00
TRINITY_sp|Q54J[illegible]	hal	Probable histidine	49.00	0.00
TRINITY_sp|F4J6[illegible]	IREH1	Probable serine/thr	49.00	0.00
TRINITY_sp|P223[illegible]	SODB	Superoxide dismutas	49.00	0.00
TRINITY_sp|A4S6[illegible]	OSTLU_4[illegible]	Lon protease homolo	49.00	0.00
TRINITY_sp|Q31V[illegible]	treF	Cytoplasmic trehala	49.00	0.00
TRINITY_sp|Q655[illegible]	MCSU3	Molybdenum cofactor	49.00	0.00
TRINITY_sp|Q86A[illegible]	DDB_G02[illegible]	Probable acetyl-CoA	49.00	0.00
TRINITY_sp|P272[illegible]	ahr	Aldehyde reductase	49.00	0.00
TRINITY_sp|Q993[illegible]	TY3B-G	Transposon Ty3-G Ga	49.00	0.00
TRINITY_sp|P0AB[illegible]	dedA	Protein DedA OS=Esc	49.00	0.00
TRINITY_sp|Q8GY[illegible]	PGM	Phosphoglycerate mu	49.00	0.00
TRINITY_sp|Q2NK[illegible]	DPY30	Protein dpy-30 homo	49.00	0.00
TRINITY_sp|Q9SF[illegible]	RABE1E	Ras-related protein	49.00	0.00

TRINITY_sp|P516	Akr1a1	Alcohol dehydrogena	49.00	0.00
TRINITY_sp|O154	ABCC4	Multidrug resistanc	49.00	0.00
TRINITY_sp|P129	PEPD	Xaa-Pro dipeptidase	49.00	0.00
TRINITY_sp|Q8LC	PLP3B	Thioredoxin domain-	49.00	0.00
TRINITY_sp|O350	yfkJ	Low molecular weigh	49.00	0.00
TRINITY_sp|Q8T6	abcH2	ABC transporter H f	48.90	0.00
TRINITY_sp|Q75W	OPLAH	5-oxoprolinase OS=B	48.90	0.00
TRINITY_sp|P118	-	High mobility group	48.90	0.00
TRINITY_sp|Q29S	OSCP1	Protein OSCP1 OS=Bo	48.90	0.00
TRINITY_sp|Q927	DPF3	Zinc finger protein	48.90	0.00
TRINITY_sp|P613	RPL27	60S ribosomal prote	48.90	0.00
TRINITY_sp|Q9LT	UKL5	Uridine kinase-like	48.90	0.00
TRINITY_sp|Q9SE	FIP2	FH protein interact	48.90	0.00
TRINITY_sp|Q54R	yipf6	Protein YIPF6 homol	48.90	0.00
TRINITY_sp|P222	RAS1	Ras-like protein 1	48.90	0.00
TRINITY_sp|Q54R	yipf6	Protein YIPF6 homol	48.90	0.00
TRINITY_sp|Q097	pld1	Phospholipase D1 OS	48.90	0.00
TRINITY_sp|Q54G	dgat2	Diacylglycerol O-ac	48.90	0.00
TRINITY_sp|Q54N	anapc1	Anaphase-promoting	48.90	0.00
TRINITY_sp|Q9Y6	SIRT4	NAD-dependent prote	48.90	0.00
TRINITY_sp|P307	Gstt2	Glutathione S-trans	48.90	0.00
TRINITY_sp|P422	SKIV2L2	Superkiller viralic	48.90	0.00
TRINITY_sp|Q55E	abcG23	ABC transporter G f	48.90	0.00
TRINITY_sp|P495	ycf45	Uncharacterized pro	48.90	0.00
TRINITY_sp|P551	galE	UDP-glucose 4-epime	48.90	0.00
TRINITY_sp|A0JM	celf5	CUGBP Elav-like fam	48.90	0.00
TRINITY_sp|P515	AFC1	Serine/threonine-pr	48.90	0.00
TRINITY_sp|Q044	Vars	Valine--tRNA ligase	48.90	0.00
TRINITY_sp|A4IF	KCTD7	BTB/POZ domain-cont	48.90	0.00
TRINITY_sp|Q084	PDE4D	cAMP-specific 3',5'	48.90	0.00
TRINITY_sp|A3DD	mutS	DNA mismatch repair	48.90	0.00
TRINITY_sp|Q9ZQ	CBSDUF3	DUF21 domain-contai	48.90	0.00
TRINITY_sp|Q6DI	rnf126	E3 ubiquitin-protei	48.90	0.00
TRINITY_sp|Q9FP	DJ1A	Protein DJ-1 homolo	48.90	0.00
TRINITY_sp|Q8W4	VHA-a3	V-type proton ATPas	48.90	0.00
TRINITY_sp|Q54F	etfa	Electron transfer f	48.90	0.00
TRINITY_sp|Q9C8	NIFU5	NifU-like protein 5	48.90	0.00
TRINITY_sp|O351	Emg1	Ribosomal RNA small	48.90	0.00
TRINITY_sp|P402	NDE1	External NADH-ubiqu	48.90	0.00
TRINITY_sp|Q9LF	CLPB3	Chaperone protein C	48.90	0.00
TRINITY_sp|Q061	NMA1	Nicotinamide/nicoti	48.90	0.00
TRINITY_sp|A7MB	ift46	Intraflagellar tran	48.90	0.00
TRINITY_sp|Q8LP	BTS	Zinc finger protein	48.90	0.00
TRINITY_sp|P143	glnS	Probable glutamine-	48.90	0.00
TRINITY_sp|Q5ZL	NSUN2	tRNA (cytosine(34)-	48.90	0.00
TRINITY_sp|Q96T	inuE	Extracellular exo-i	48.90	0.00
TRINITY_sp|P944	yciC	Putative metal chap	48.90	0.00
TRINITY_sp|Q0DI	Os05g03	Ferrochelatase-2, c	48.90	0.00
TRINITY_sp|Q8L7	RWA1	Protein REDUCED WAL	48.90	0.00
TRINITY_sp|Q9FI	CLPC1	Chaperone protein C	48.90	0.00
TRINITY_sp|Q8LP	ALPHA-A	AP-2 complex subuni	48.90	0.00
TRINITY_sp|Q9ZV	HEN2	DExH-box ATP-depend	48.90	0.00
TRINITY_sp|Q86G	gcy-28	Receptor-type guany	48.90	0.00
TRINITY_sp|Q9M1	ABCB21	ABC transporter B f	48.90	0.00
TRINITY_sp|Q1HF	Nsun2	tRNA (cytosine(34)-	48.90	0.00
TRINITY_sp|O202	-	Sedoheptulose-1,7-b	48.90	0.00

TRINITY_sp|Q08D2	PRPF4B	Serine/threonine-pr	48.90	0.00
TRINITY_sp|Q9FP1	UBP12	Ubiquitin carboxyl-	48.90	0.00
TRINITY_sp|Q9VHI	DppIII	Dipeptidyl peptidas	48.90	0.00
TRINITY_sp|P592	RPS27AA	Ubiquitin-40S ribos	48.80	0.00
TRINITY_sp|Q9FG(	EB1C	Microtubule-associa	48.80	0.00
TRINITY_sp|Q9P7	mit1	Chromatin remodelin	48.80	0.00
TRINITY_sp|Q86KI	fhit	Bis(5'-adenosyl)-tr	48.80	0.00
TRINITY_sp|B6EK	cutC	Copper homeostasis	48.80	0.00
TRINITY_sp|Q640	tatdn1	Putative deoxyribon	48.80	0.00
TRINITY_sp|A0DSI	GSPATT0(	Probable protein ph	48.80	0.00
TRINITY_sp|Q8BV(	Dpp9	Dipeptidyl peptidas	48.80	0.00
TRINITY_sp|Q2KH2	HBS1L	HBS1-like protein O	48.80	0.00
TRINITY_sp|Q059	Slc25a4	ADP/ATP translocase	48.80	0.00
TRINITY_sp|Q6EPI	FACE1	CAAX prenyl proteas	48.80	0.00
TRINITY_sp|Q9WX	rbpF	Putative RNA-bindin	48.80	0.00
TRINITY_sp|Q9C6I	GAMMACA2	Gamma carbonic anhy	48.80	0.00
TRINITY_sp|Q9FG	ARR18	Two-component respo	48.80	0.00
TRINITY_sp|Q869	pakF	Serine/threonine-pr	48.80	0.00
TRINITY_sp|Q54II	ndrB	Probable serine/thr	48.80	0.00
TRINITY_sp|O743	SPBC15D4	COBW domain-contain	48.80	0.00
TRINITY_sp|Q55G	DDB_G02(	Probable mitochondr	48.80	0.00
TRINITY_sp|Q7SZI	sec23a	Protein transport p	48.80	0.00
TRINITY_sp|F4JGI	KIN14D	Kinesin-like protei	48.80	0.00
TRINITY_sp|P2532	PRKACA	cAMP-dependent prot	48.80	0.00
TRINITY_sp|Q54DI	polr2c	DNA-directed RNA po	48.80	0.00
TRINITY_sp|F4HX	PLA1	Phospholipase A I O	48.80	0.00
TRINITY_sp|P046	tyrB	Aromatic-amino-acid	48.80	0.00
TRINITY_sp|P703(	Ufd1l	Ubiquitin fusion de	48.80	0.00
TRINITY_sp|Q29R2	PPWD1	Peptidylprolyl isom	48.80	0.00
TRINITY_sp|Q8H1	MNS2	Mannosyl-oligosacch	48.80	0.00
TRINITY_sp|B2RW	Cfap58	Cilia- and flagella	48.80	0.00
TRINITY_sp|Q8LF	At1g222	Multifunctional met	48.80	0.00
TRINITY_sp|Q8R1	Znfx1	NFX1-type zinc fing	48.80	0.00
TRINITY_sp|Q9AS	At1g324	Vacuolar protein so	48.80	0.00
TRINITY_sp|Q8AVI	rpf1	Ribosome production	48.80	0.00
TRINITY_sp|Q54JI	abcC3	ABC transporter C f	48.80	0.00
TRINITY_sp|O809	FTSH4	ATP-dependent zinc	48.80	0.00
TRINITY_sp|Q9SEI	GAL1	Galactokinase OS=Ar	48.80	0.00
TRINITY_sp|Q8JFV	adat3	Probable inactive t	48.80	0.00
TRINITY_sp|Q657	EX2	Protein EXECUTER 2,	48.80	0.00
TRINITY_sp|Q54X	DDB_G02	Glucose-induced deg	48.80	0.00
TRINITY_sp|Q617	Kif3b	Kinesin-like protei	48.80	0.00
TRINITY_sp|Q9SF(	At3g071(	Protein transport p	48.80	0.00
TRINITY_sp|Q6PC	btf3l4	Transcription facto	48.70	0.00
TRINITY_sp|O756	SNRNP20(	U5 small nuclear ri	48.70	0.00
TRINITY_sp|P727	typA	GTP-binding protein	48.70	0.00
TRINITY_sp|Q18C	rplM	50S ribosomal prote	48.70	0.00
TRINITY_sp|Q102	pla1	Poly(A) polymerase	48.70	0.00
TRINITY_sp|Q9QXI	Nme7	Nucleoside diphosph	48.70	0.00
TRINITY_sp|Q54Q	treh	Trehalase OS=Dictyo	48.70	0.00
TRINITY_sp|O246	PBD2	Proteasome subunit	48.70	0.00
TRINITY_sp|P281	pkgB	Protein kinase 2 OS	48.70	0.00
TRINITY_sp|C4YK	RAS1	Ras-like protein 1	48.70	0.00
TRINITY_sp|Q86AI	DDB_G02	Probable myosin lig	48.70	0.00
TRINITY_sp|Q55D	DDB_G02(	Probable 18S rRNA (	48.70	0.00
TRINITY_sp|Q0CC	gedE	Glutathione S-trans	48.70	0.00

TRINITY_sp|Q9C0(	DNAH6	Dynein heavy chain	48.70	0.00
TRINITY_sp|Q2S4]	SRU_076:	Maf-like protein SR	48.70	0.00
TRINITY_sp|Q681(	At1g039(	Uncharacterized pro	48.70	0.00
TRINITY_sp|Q9LRI	RPL3B	50S ribosomal prote	48.70	0.00
TRINITY_sp|Q9D5]	Ccdc130	Coiled-coil domain-	48.70	0.00
TRINITY_sp|Q54E\	dst3	Serine/threonine-pr	48.70	0.00
TRINITY_sp|P397:	Bx42	Puff-specific prote	48.70	0.00
TRINITY_sp|Q9LZ8	BTR1	Protein BTR1 OS=Ara	48.70	0.00
TRINITY_sp|Q1LVI	sf3b3	Splicing factor 3B	48.70	0.00
TRINITY_sp|Q10P\	Os03g02]	DEAD-box ATP-depend	48.70	0.00
TRINITY_sp|Q944]	GLYK	D-glycerate 3-kinas	48.70	0.00
TRINITY_sp|Q4I8)	TRM10	tRNA (guanine(9)-N1	48.70	0.00
TRINITY_sp|P629(	RAC1	Ras-related C3 botu	48.70	0.00
TRINITY_sp|Q9SQ2	At3g047(	PITH domain-contain	48.70	0.00
TRINITY_sp|Q54G]	phbA	Prohibitin-1, mitoc	48.70	0.00
TRINITY_sp|Q54SI	polr1c	DNA-directed RNA po	48.70	0.00
TRINITY_sp|Q54Y2	dhkJ	Hybrid signal trans	48.70	0.00
TRINITY_sp|O239(	GPXHA-2	Probable phospholip	48.70	0.00
TRINITY_sp|Q54Q/	trmt11	tRNA (guanine(10)-N	48.70	0.00
TRINITY_sp|Q197]	frs-2	Phenylalanine--tRNA	48.70	0.00
TRINITY_sp|Q1ZXI	gxcDD	Guanine exchange fa	48.60	0.00
TRINITY_sp|P966!	yddQ	Uncharacterized iso	48.60	0.00
TRINITY_sp|Q1LU]	hddc2	HD domain-containin	48.60	0.00
TRINITY_sp|Q6CE/	ALG10	Dol-P-Glc:Glc(2)Man	48.60	0.00
TRINITY_sp|Q8K4	Abca8b	ATP-binding cassett	48.60	0.00
TRINITY_sp|Q631	Dnah7	Dynein heavy chain	48.60	0.00
TRINITY_sp|Q8IU8	CAMK1D	Calcium/calmodulin-	48.60	0.00
TRINITY_sp|P245!	ptrB	Protease 2 OS=Esche	48.60	0.00
TRINITY_sp|Q9FT	MED34	Mediator of RNA pol	48.60	0.00
TRINITY_sp|P543]	Lipe	Hormone-sensitive l	48.60	0.00
TRINITY_sp|Q54WI	gna1	Glucosamine 6-phosp	48.60	0.00
TRINITY_sp|Q9P6I	brf1	Transcription facto	48.60	0.00
TRINITY_sp|P328:	BCS1	Mitochondrial chape	48.60	0.00
TRINITY_sp|Q9XT/	PREP	Prolyl endopeptidas	48.60	0.00
TRINITY_sp|A2YR]	ARP4	Actin-related prote	48.60	0.00
TRINITY_sp|O6978	betC	Choline-sulfatase O	48.60	0.00
TRINITY_sp|Q8VZ:	P4H9	Probable prolyl 4-h	48.60	0.00
TRINITY_sp|O652(	At4g005(	Putative threonine	48.60	0.00
TRINITY_sp|B4NJ\	Mocs2	Molybdopterin synth	48.60	0.00
TRINITY_sp|Q9SL)	Os05g01!	Importin subunit al	48.60	0.00
TRINITY_sp|P368(	YPTV3	GTP-binding protein	48.60	0.00
TRINITY_sp|Q6RSI	dnaJ	Chaperone protein D	48.60	0.00
TRINITY_sp|Q9GM\	PGA	Pepsin A OS=Sorex u	48.60	0.00
TRINITY_sp|Q55G]	dcd2B	Neutral ceramidase	48.60	0.00
TRINITY_sp|Q869(	DDB_G02	COBW domain-contain	48.60	0.00
TRINITY_sp|P436	-	DnaJ protein homolo	48.60	0.00
TRINITY_sp|Q22X2	TTHERM_(	Alpha-tubulin N-ace	48.60	0.00
TRINITY_sp|D2XV!	Gtpbp1	GTP-binding protein	48.60	0.00
TRINITY_sp|Q6I5\	CDKC-1	Cyclin-dependent ki	48.60	0.00
TRINITY_sp|Q9NSI	FARSB	Phenylalanine--tRNA	48.60	0.00
TRINITY_sp|P271]	ODC1	Ornithine decarboxy	48.60	0.00
TRINITY_sp|A0AU!	wdsub1	WD repeat, SAM and	48.60	0.00
TRINITY_sp|Q4V7I	Osgepl1	Probable tRNA N6-ad	48.60	0.00
TRINITY_sp|Q94A(	At1g066!	Uncharacterized oxi	48.60	0.00
TRINITY_sp|O230]	KAB1	Probable voltage-ga	48.60	0.00
TRINITY_sp|Q55G!	cdk10	Probable cyclin-dep	48.60	0.00

TRINITY_sp|P187(	NSF	Vesicle-fusing ATPa	48.60	0.00
TRINITY_sp|Q389	ACC1	Acetyl-CoA carboxyl	48.60	0.00
TRINITY_sp|Q8WX	DNAH7	Dynein heavy chain	48.60	0.00
TRINITY_sp|Q5M9	Dph6	Diphthine--ammonia	48.60	0.00
TRINITY_sp|Q165	MAP3K11	Mitogen-activated p	48.60	0.00
TRINITY_sp|O235	BAM3	Beta-amylase 3, chl	48.60	0.00
TRINITY_sp|Q9FL	RH18	DEAD-box ATP-depend	48.60	0.00
TRINITY_sp|Q8CG	Elac2	Zinc phosphodiester	48.60	0.00
TRINITY_sp|Q6P1	DUS1L	tRNA-dihydrouridine	48.60	0.00
TRINITY_sp|P496	-	Ubiquitin-60S ribos	48.60	0.00
TRINITY_sp|P982(	Atp8a2	Phospholipid-transp	48.60	0.00
TRINITY_sp|Q99M	Mccc1	Methylcrotonoyl-CoA	48.60	0.00
TRINITY_sp|P434	cdk2	Cyclin-dependent ki	48.60	0.00
TRINITY_sp|P525	mybl2	Myb-related protein	48.60	0.00
TRINITY_sp|Q075	Gyc32E	Guanylate cyclase 3	48.60	0.00
TRINITY_sp|Q9SN	NRAMP3	Metal transporter N	48.60	0.00
TRINITY_sp|B2GK(	mdh	Malate dehydrogenas	48.50	0.00
TRINITY_sp|O942	SPBC887.	Probable phospholip	48.50	0.00
TRINITY_sp|Q98E	mlr4350	Putative pterin-4-a	48.50	0.00
TRINITY_sp|F4JY	TCX7	Protein tesmin/TSO1	48.50	0.00
TRINITY_sp|P710	ygaD	Putative multidrug	48.50	0.00
TRINITY_sp|Q9Y3	MEMO1	Protein MEMO1 OS=Ho	48.50	0.00
TRINITY_sp|Q8RX	ABCG11	ABC transporter G f	48.50	0.00
TRINITY_sp|Q1ZX	gxcDD	Guanine exchange fa	48.50	0.00
TRINITY_sp|O975(	TXN	Thioredoxin OS=Equu	48.50	0.00
TRINITY_sp|Q84N	AMPD	Probable AMP deamin	48.50	0.00
TRINITY_sp|Q9H6	PIF1	ATP-dependent DNA h	48.50	0.00
TRINITY_sp|A2XD(	UBP26	Ubiquitin carboxyl-	48.50	0.00
TRINITY_sp|Q0DH	FTSH8	ATP-dependent zinc	48.50	0.00
TRINITY_sp|A7RX	amdhd1	Probable imidazolon	48.50	0.00
TRINITY_sp|P736	sll1773	Putative quercetin	48.50	0.00
TRINITY_sp|A7MK	mtnA	Methylthioribose-1-	48.50	0.00
TRINITY_sp|Q9XF	DDM1	ATP-dependent DNA h	48.50	0.00
TRINITY_sp|Q6P9(	CYB5D1	Cytochrome b5 domai	48.50	0.00
TRINITY_sp|P519	NEK2	Serine/threonine-pr	48.50	0.00
TRINITY_sp|Q8VI	Abcc2	Canalicular multisp	48.50	0.00
TRINITY_sp|Q93V	ATG18A	Autophagy-related p	48.50	0.00
TRINITY_sp|Q1ZX	gxcDD	Guanine exchange fa	48.50	0.00
TRINITY_sp|B1ZX	truB	tRNA pseudouridine	48.50	0.00
TRINITY_sp|Q54N	bcaA	Branched-chain-amin	48.50	0.00
TRINITY_sp|Q644	Cyp3a13	Cytochrome P450 3A1	48.50	0.00
TRINITY_sp|P466	GUS1	Glutamate--tRNA lig	48.50	0.00
TRINITY_sp|P251	Pfkfb4	6-phosphofructo-2-k	48.50	0.00
TRINITY_sp|Q9NV(	TBC1D13	TBC1 domain family	48.50	0.00
TRINITY_sp|Q67S	dnaJ	Chaperone protein D	48.50	0.00
TRINITY_sp|Q94K(	NAP1;3	Nucleosome assembly	48.50	0.00
TRINITY_sp|Q9FT	RECQL1	ATP-dependent DNA h	48.50	0.00
TRINITY_sp|Q8C0	Efl1	Elongation factor-l	48.50	0.00
TRINITY_sp|Q9PD	lipB	Octanoyltransferase	48.50	0.00
TRINITY_sp|Q8TB	DEPTOR	DEP domain-containi	48.50	0.00
TRINITY_sp|Q9SE	GAL1	Galactokinase OS=Ar	48.50	0.00
TRINITY_sp|P271	ODC1	Ornithine decarboxy	48.50	0.00
TRINITY_sp|Q7NB	dnaJ	Chaperone protein D	48.50	0.00
TRINITY_sp|Q9D6	Calml3	Calmodulin-like pro	48.50	0.00
TRINITY_sp|Q9SG	HMGB9	High mobility group	48.50	0.00
TRINITY_sp|Q9SZ	GPX7	Putative glutathion	48.50	0.00

TRINITY_sp|Q54NV	limB	LIM domain-containi	48.50	0.00
TRINITY_sp|Q0WWQ	ATG3	Autophagy-related p	48.50	0.00
TRINITY_sp|Q08J2	NSUN2	tRNA (cytosine(34)-	48.50	0.00
TRINITY_sp|P5314	TOS8	Homeobox protein TO	48.50	0.00
TRINITY_sp|Q5IFN	Ot01g064	Cryptochrome DASH,	48.50	0.00
TRINITY_sp|Q9NR0	BIRC6	Baculoviral IAP rep	48.50	0.00
TRINITY_sp|F4IA0	SS3	Starch synthase 3,	48.50	0.00
TRINITY_sp|F4IV9	CHR5	Protein CHROMATIN R	48.50	0.00
TRINITY_sp|Q6GN9	nt5dc3	5'-nucleotidase dom	48.50	0.00
TRINITY_sp|P3489	CPN10	10 kDa chaperonin,	48.50	0.00
TRINITY_sp|Q922R	Prkx	cAMP-dependent prot	48.40	0.00
TRINITY_sp|Q9M7M	ETR1	Ethylene receptor O	48.40	0.00
TRINITY_sp|O5483	Dusp5	Dual specificity pr	48.40	0.00
TRINITY_sp|P2563	PWP2	Periodic tryptophan	48.40	0.00
TRINITY_sp|Q47PU	pamO	Phenylacetone monoo	48.40	0.00
TRINITY_sp|Q6AYD	Pa2g4	Proliferation-assoc	48.40	0.00
TRINITY_sp|P4274	-	Polyubiquitin OS=Ag	48.40	0.00
TRINITY_sp|O4583	ndx-1	Putative nudix hydr	48.40	0.00
TRINITY_sp|Q9ZR3	DSPTP1	Dual specificity pr	48.40	0.00
TRINITY_sp|Q9SF3	TIM14-2	Mitochondrial impor	48.40	0.00
TRINITY_sp|Q0JI4	CIPK11	CBL-interacting pro	48.40	0.00
TRINITY_sp|Q9Z2A	Pdpk1	3-phosphoinositide-	48.40	0.00
TRINITY_sp|P0C58	mic-33	Putative mitochondr	48.40	0.00
TRINITY_sp|O6473	At2g3017	Probable protein ph	48.40	0.00
TRINITY_sp|Q8K13	Sde2	Protein SDE2 homolo	48.40	0.00
TRINITY_sp|Q54G1	agxt	Serine--pyruvate am	48.40	0.00
TRINITY_sp|P2817	pkgB	Protein kinase 2 OS	48.40	0.00
TRINITY_sp|Q1401	CAMK1	Calcium/calmodulin-	48.40	0.00
TRINITY_sp|O7453	qcr7	Cytochrome b-c1 com	48.40	0.00
TRINITY_sp|Q9SE9	FIP2	FH protein interact	48.40	0.00
TRINITY_sp|Q54DA	sti1	Protein STIP1 homol	48.40	0.00
TRINITY_sp|A7SDW	v1g16942	Eukaryotic translat	48.40	0.00
TRINITY_sp|Q5AIR	ENG1	Endo-1,3(4)-beta-gl	48.40	0.00
TRINITY_sp|Q1LUA	eif3eb	Eukaryotic translat	48.40	0.00
TRINITY_sp|A8JFU	CFAP65	Cilia- and flagella	48.40	0.00
TRINITY_sp|Q55C5	vps16	Vacuolar protein so	48.40	0.00
TRINITY_sp|P2532	mlkA	Myosin light chain	48.40	0.00
TRINITY_sp|Q9MB5	FKFBP	6-phosphofructo-2-k	48.40	0.00
TRINITY_sp|Q1RLD	Cpne9	Copine-9 OS=Mus mus	48.40	0.00
TRINITY_sp|Q9LE8	IRE	Probable serine/thr	48.40	0.00
TRINITY_sp|P2712	odc1-a	Ornithine decarboxy	48.40	0.00
TRINITY_sp|Q5T65	CFAP58	Cilia- and flagella	48.40	0.00
TRINITY_sp|Q9M82	UGT80A2	Sterol 3-beta-gluco	48.40	0.00
TRINITY_sp|P6179	APTX	Aprataxin (Fragment	48.40	0.00
TRINITY_sp|Q6177	Kif3b	Kinesin-like protei	48.40	0.00
TRINITY_sp|Q7XAB	THF1	Protein THYLAKOID F	48.40	0.00
TRINITY_sp|B8GFE	gpmA	2,3-bisphosphoglyce	48.40	0.00
TRINITY_sp|Q9FT7	RECQL3	ATP-dependent DNA h	48.40	0.00
TRINITY_sp|P1268	YPK1	Serine/threonine-pr	48.40	0.00
TRINITY_sp|Q54KE	cysS	Cysteine--tRNA liga	48.40	0.00
TRINITY_sp|A8AK2	hslU	ATP-dependent prote	48.40	0.00
TRINITY_sp|Q2291	C37C3.2	Eukaryotic translat	48.40	0.00
TRINITY_sp|O7440	dps1	Aspartate--tRNA lig	48.40	0.00
TRINITY_sp|P2233	HSF24	Heat shock factor p	48.40	0.00
TRINITY_sp|Q4522	hbdA	3-hydroxybutyryl-Co	48.40	0.00
TRINITY_sp|Q28FE	morn5	MORN repeat-contain	48.40	0.00

TRINITY_sp|A1Z9[illegible]	mip120	Protein lin-54 homo	48.40	0.00
TRINITY_sp|Q8GZ[illegible]	PLA2-BE[illegible]	Phospholipase A2-be	48.40	0.00
TRINITY_sp|Q9SE[illegible]	-	Pirin-like protein	48.40	0.00
TRINITY_sp|Q54Y[illegible]	dhkB	Hybrid signal trans	48.40	0.00
TRINITY_sp|C0QK[illegible]	groS	10 kDa chaperonin O	48.40	0.00
TRINITY_sp|P426[illegible]	-	14-3-3-like protein	48.30	0.00
TRINITY_sp|Q9XY[illegible]	fcpA	Probable C-terminal	48.30	0.00
TRINITY_sp|Q9U7[illegible]	xnp-1	Transcriptional reg	48.30	0.00
TRINITY_sp|Q86C[illegible]	tor	Target of rapamycin	48.30	0.00
TRINITY_sp|Q294[illegible]	MAN2B1	Lysosomal alpha-man	48.30	0.00
TRINITY_sp|Q991[illegible]	HIS1	ATP phosphoribosylt	48.30	0.00
TRINITY_sp|Q91W[illegible]	As3mt	Arsenite methyltran	48.30	0.00
TRINITY_sp|Q9LZ[illegible]	ABCC14	ABC transporter C f	48.30	0.00
TRINITY_sp|Q6DJ[illegible]	ccdc130	Coiled-coil domain-	48.30	0.00
TRINITY_sp|Q7XA[illegible]	MTN2	5'-methylthioadenos	48.30	0.00
TRINITY_sp|Q55C[illegible]	maspS	Aspartate--tRNA lig	48.30	0.00
TRINITY_sp|Q9H6[illegible]	PIF1	ATP-dependent DNA h	48.30	0.00
TRINITY_sp|P527[illegible]	-	Glutamine--tRNA lig	48.30	0.00
TRINITY_sp|P905[illegible]	-	66 kDa stress prote	48.30	0.00
TRINITY_sp|O027[illegible]	PARG	Poly(ADP-ribose) gl	48.30	0.00
TRINITY_sp|P044[illegible]	-	Calmodulin OS=Triti	48.30	0.00
TRINITY_sp|O494[illegible]	PHB1	Prohibitin-1, mitoc	48.30	0.00
TRINITY_sp|Q5RD[illegible]	PARP6	Poly [ADP-ribose] p	48.30	0.00
TRINITY_sp|P162[illegible]	MOD1	NADP-dependent mali	48.30	0.00
TRINITY_sp|P490[illegible]	POLD2	DNA polymerase delt	48.30	0.00
TRINITY_sp|Q5VR[illegible]	HGO	Homogentisate 1,2-d	48.30	0.00
TRINITY_sp|Q91Y[illegible]	Camk1	Calcium/calmodulin-	48.30	0.00
TRINITY_sp|P176[illegible]	ypt3	GTP-binding protein	48.30	0.00
TRINITY_sp|Q9C6[illegible]	LHCA5	Photosystem I chlor	48.30	0.00
TRINITY_sp|Q54T[illegible]	mrkC	Probable serine/thr	48.30	0.00
TRINITY_sp|A4IH[illegible]	bop1	Ribosome biogenesis	48.30	0.00
TRINITY_sp|Q9LN[illegible]	RRA3	Arabinosyltransfera	48.30	0.00
TRINITY_sp|Q6Z8[illegible]	Os02g01[illegible]	L-aspartate oxidase	48.30	0.00
TRINITY_sp|Q8LG[illegible]	IDH6	Isocitrate dehydrog	48.30	0.00
TRINITY_sp|Q5U2[illegible]	Fbxo9	F-box only protein	48.30	0.00
TRINITY_sp|P465[illegible]	pip	Proline iminopeptid	48.30	0.00
TRINITY_sp|Q053[illegible]	schC	Putative polyketide	48.30	0.00
TRINITY_sp|Q5XI[illegible]	Eci2	Enoyl-CoA delta iso	48.30	0.00
TRINITY_sp|Q168[illegible]	TXNRD1	Thioredoxin reducta	48.30	0.00
TRINITY_sp|A1A5[illegible]	Prpf6	Pre-mRNA-processing	48.30	0.00
TRINITY_sp|Q96J[illegible]	DNAH8	Dynein heavy chain	48.30	0.00
TRINITY_sp|Q9BU[illegible]	TRMO	tRNA (adenine(37)-N	48.30	0.00
TRINITY_sp|Q5M9[illegible]	arl6	ADP-ribosylation fa	48.30	0.00
TRINITY_sp|Q921[illegible]	Sf3b3	Splicing factor 3B	48.30	0
TRINITY_sp|Q125[illegible]	AO-I	Copper amine oxidas	48.30	0.00
TRINITY_sp|Q5U4[illegible]	meis3-a	Homeobox protein me	48.30	0.00
TRINITY_sp|Q9Y3[illegible]	AK6	Adenylate kinase is	48.20	0.00
TRINITY_sp|Q9SF[illegible]	RABE1E	Ras-related protein	48.20	0.00
TRINITY_sp|Q54X[illegible]	DDB_G02[illegible]	Glucose-induced deg	48.20	0.00
TRINITY_sp|B3DN[illegible]	APC6	Anaphase-promoting	48.20	0.00
TRINITY_sp|Q54F[illegible]	nubpl	Iron-sulfur protein	48.20	0.00
TRINITY_sp|O140[illegible]	mak2	Peroxide stress-act	48.20	0.00
TRINITY_sp|Q2KJ[illegible]	PGS1	CDP-diacylglycerol-	48.20	0.00
TRINITY_sp|Q8T1[illegible]	amd1	S-adenosylmethionin	48.20	0.00
TRINITY_sp|Q55F[illegible]	mef2A	Transcription facto	48.20	0.00
TRINITY_sp|Q0WW[illegible]	At4g344[illegible]	Coatomer subunit ga	48.20	0.00

TRINITY_sp|P049{	cprA	Cysteine proteinase	48.20	0.00
TRINITY_sp|Q9P2E	ZNFX1	NFX1-type zinc fing	48.20	0.00
TRINITY_sp|Q5ZIV	PEO1	Twinkle protein, mi	48.20	0.00
TRINITY_sp|B8BMI	MCM7	DNA replication lic	48.20	0.00
TRINITY_sp|Q8W1Y	KIN14F	Kinesin-like protei	48.20	0.00
TRINITY_sp|Q5ZI(	SLU7	Pre-mRNA-splicing f	48.20	0.00
TRINITY_sp|Q54Y2	ndrA	Probable serine/thr	48.20	0.00
TRINITY_sp|Q9LN(	RPN3A	26S proteasome non-	48.20	0.00
TRINITY_sp|P0432	pol	Retrovirus-related	48.20	0.00
TRINITY_sp|P2751	CBR	Carotene biosynthes	48.20	0.00
TRINITY_sp|P470:	PRY3	Cell wall protein P	48.20	0.00
TRINITY_sp|Q8R9V	acpP	Acyl carrier protei	48.20	0.00
TRINITY_sp|Q54B:	pakE	Serine/threonine-pr	48.20	0.00
TRINITY_sp|P546	pikD	Phosphatidylinosito	48.20	0.00
TRINITY_sp|P432{	SRK2G	Serine/threonine-pr	48.20	0.00
TRINITY_sp|Q8BW{	Camk1d	Calcium/calmodulin-	48.20	0.00
TRINITY_sp|Q920	Scpep1	Retinoid-inducible	48.20	0.00
TRINITY_sp|Q9FHI	KIN14E	Kinesin-like protei	48.20	0.00
TRINITY_sp|O0492	GPX2	Probable glutathion	48.20	0.00
TRINITY_sp|Q9FT	RECQL1	ATP-dependent DNA h	48.20	0.00
TRINITY_sp|Q043:	YMR196W	Uncharacterized pro	48.20	0.00
TRINITY_sp|Q9NR(	BIRC6	Baculoviral IAP rep	48.20	0.00
TRINITY_sp|Q9SC(	UPL7	E3 ubiquitin-protei	48.20	0.00
TRINITY_sp|Q9C0(	DNAH6	Dynein heavy chain	48.20	0.00
TRINITY_sp|Q8GY2	UPL1	E3 ubiquitin-protei	48.20	0.00
TRINITY_sp|Q52KI	Srrm1	Serine/arginine rep	48.20	0.00
TRINITY_sp|Q9LT(	PDR2	Probable manganese-	48.20	0.00
TRINITY_sp|Q54VE	hbx9	Homeobox protein 9	48.20	0.00
TRINITY_sp|Q3T0X	POLD4	DNA polymerase delt	48.20	0.00
TRINITY_sp|Q631	Dnah7	Dynein heavy chain	48.20	0
TRINITY_sp|Q93Z5	At3g161{	Probable inactive n	48.20	0.00
TRINITY_sp|Q9M35	AGD6	Probable ADP-ribosy	48.20	0.00
TRINITY_sp|Q0WVE	MCM4	DNA replication lic	48.20	0.00
TRINITY_sp|Q9UHI	DPP7	Dipeptidyl peptidas	48.10	0.00
TRINITY_sp|Q8X5X	ydiF	Acetate CoA-transfe	48.10	0.00
TRINITY_sp|Q9XIE	ALA3	Phospholipid-transp	48.10	0.00
TRINITY_sp|B3RY(	TRIADDR/	E3 UFM1-protein lig	48.10	0.00
TRINITY_sp|P0901	SNRPA	U1 small nuclear ri	48.10	0.00
TRINITY_sp|Q54YN	mai	Maleylacetoacetate	48.10	0.00
TRINITY_sp|A6QLV	TGDS	dTDP-D-glucose 4,6-	48.10	0.00
TRINITY_sp|P118	-	High mobility group	48.10	0.00
TRINITY_sp|Q212{	K07E3.7/	Probable cation-tra	48.10	0.00
TRINITY_sp|Q55G1	pomp	Proteasome maturati	48.10	0.00
TRINITY_sp|D5D8E	ftsH	ATP-dependent zinc	48.10	0.00
TRINITY_sp|Q9EP:	Arfgap1	ADP-ribosylation fa	48.10	0.00
TRINITY_sp|Q6GN1	dis3l	DIS3-like exonuclea	48.10	0.00
TRINITY_sp|Q9SF{	RABE1E	Ras-related protein	48.10	0.00
TRINITY_sp|Q54F2	mroh1	Maestro heat-like r	48.10	0.00
TRINITY_sp|Q9HDV	pgs1	CDP-diacylglycerol-	48.10	0.00
TRINITY_sp|P047	-	Glutamine synthetas	48.10	0.00
TRINITY_sp|O189{	DNASE1	Deoxyribonuclease-1	48.10	0.00
TRINITY_sp|Q4U2\	agtpbp1	Cytosolic carboxype	48.10	0.00
TRINITY_sp|Q9XTE	ZK262.3	Lipase ZK262.3 OS=C	48.10	0.00
TRINITY_sp|A8J6(	CFAP54	Cilia- and flagella	48.10	0.00
TRINITY_sp|P2744	MARK3	MAP/microtubule aff	48.10	0.00
TRINITY_sp|Q55BE	nmd3	60S ribosomal expor	48.10	0.00

TRINITY_sp|Q9FKI	At5g488	Pantoate--beta-alan	48.10	0.00
TRINITY_sp|P5353	SSU72	RNA polymerase II s	48.10	0.00
TRINITY_sp|O440(	PYK	Pyruvate kinase OS=	48.10	0.00
TRINITY_sp|Q9FYI	RS2Z32	Serine/arginine-ric	48.10	0.00
TRINITY_sp|Q141	DHX34	Probable ATP-depend	48.10	0.00
TRINITY_sp|Q2HJI	RAB8B	Ras-related protein	48.10	0.00
TRINITY_sp|A0Q3I	ribBA	Riboflavin biosynth	48.10	0.00
TRINITY_sp|Q9M3(	ATM	Serine/threonine-pr	48.10	0.00
TRINITY_sp|P3952	DRS2	Probable phospholip	48.10	0.00
TRINITY_sp|Q9P2I	DNAH1	Dynein heavy chain	48.10	0.00
TRINITY_sp|Q0VA(	usp16	Ubiquitin carboxyl-	48.10	0.00
TRINITY_sp|P157	Camk2d	Calcium/calmodulin-	48.10	0.00
TRINITY_sp|Q944I	HDA2	Histone deacetylase	48.10	0.00
TRINITY_sp|Q9LE8	IRE	Probable serine/thr	48.10	0.00
TRINITY_sp|P916(	l(2)0365	Probable multidrug	48.10	0.00
TRINITY_sp|F4JZ(	TERC	Thylakoid membrane	48.10	0.00
TRINITY_sp|P482	ycf39	Uncharacterized pro	48.10	0.00
TRINITY_sp|C1C4N	apip	Methylthioribulose-	48.10	0.00
TRINITY_sp|Q8GW(	At5g634	UPF0235 protein At5	48.10	0.00
TRINITY_sp|Q9D62	Nop56	Nucleolar protein 5	48.10	0.00
TRINITY_sp|A6LP	pth	Peptidyl-tRNA hydro	48.10	0.00
TRINITY_sp|F4HX	NUP155	Nuclear pore comple	48.10	0.00
TRINITY_sp|Q9LZI	NCS1	Purine-uracil perme	48.10	0.00
TRINITY_sp|Q9DE	BAZ2B	Bromodomain adjacen	48.10	0.00
TRINITY_sp|Q54B8	csn4	COP9 signalosome co	48.10	0.00
TRINITY_sp|A8GAI	mtnK	Methylthioribose ki	48.10	0.00
TRINITY_sp|Q8UA	blh	Beta-lactamase hydr	48.10	0.00
TRINITY_sp|Q54VI	glud2	Glutamate dehydroge	48.10	0.00
TRINITY_sp|P0CM	ATG7	Ubiquitin-like modi	48.10	0.00
TRINITY_sp|Q6NW	Ift122	Intraflagellar tran	48.10	0
TRINITY_sp|P0432	pol	Retrovirus-related	48.10	0.00
TRINITY_sp|Q54CN	cysK	Cysteine synthase O	48.10	0.00
TRINITY_sp|Q22X2	TTHERM_(	Alpha-tubulin N-ace	48.00	0.00
TRINITY_sp|P176(	ypt2	GTP-binding protein	48.00	0.00
TRINITY_sp|Q9ST	VPS29	Vacuolar protein so	48.00	0.00
TRINITY_sp|Q86CI	atg12	Ubiquitin-like prot	48.00	0.00
TRINITY_sp|Q2KII	OSTC	Oligosaccharyltrans	48.00	0.00
TRINITY_sp|P491(	Os05g01(	Cytochrome b5 OS=Or	48.00	0.00
TRINITY_sp|Q9SK	MP3	Probable steroid-bi	48.00	0.00
TRINITY_sp|Q810	Trim50	E3 ubiquitin-protei	48.00	0.00
TRINITY_sp|A9GFI	apt	Adenine phosphoribo	48.00	0.00
TRINITY_sp|O8072	ABCB4	ABC transporter B f	48.00	0.00
TRINITY_sp|Q5RB	PSMD12	26S proteasome non-	48.00	0.00
TRINITY_sp|Q1452	HABP2	Hyaluronan-binding	48.00	0.00
TRINITY_sp|Q54H	drkB	Probable serine/thr	48.00	0.00
TRINITY_sp|Q9BZ(	ABCA2	ATP-binding cassett	48.00	0.00
TRINITY_sp|Q52JI	VIP2	Probable NOT transc	48.00	0.00
TRINITY_sp|Q9FJ	EB1B	Microtubule-associa	48.00	0.00
TRINITY_sp|Q500	PIGM	GPI mannosyltransfe	48.00	0.00
TRINITY_sp|O6052	NEMF	Nuclear export medi	48.00	0.00
TRINITY_sp|Q5WL(	rplX	50S ribosomal prote	48.00	0.00
TRINITY_sp|Q9FE	SRT1	NAD-dependent prote	48.00	0.00
TRINITY_sp|A0T0(	rpl21	50S ribosomal prote	48.00	0.00
TRINITY_sp|Q9STI	CEP2	KDEL-tailed cystein	48.00	0.00
TRINITY_sp|Q22X2	TTHERM_(	Alpha-tubulin N-ace	48.00	0.00
TRINITY_sp|P527(	trs-1	Threonine--tRNA lig	48.00	0.00

TRINITY_sp|O599	pkaR	cAMP-dependent prot	48.00	0.00
TRINITY_sp|O001	AGPS	Alkyldihydroxyaceto	48.00	0.00
TRINITY_sp|Q7U2	Mb0150	Putative S-adenosyl	48.00	0.00
TRINITY_sp|P040	FUCA1	Tissue alpha-L-fuco	48.00	0.00
TRINITY_sp|Q9R1	Ctsz	Cathepsin Z OS=Ratt	48.00	0.00
TRINITY_sp|Q398	-	Calnexin homolog OS	48.00	0.00
TRINITY_sp|Q7L2	KCTD9	BTB/POZ domain-cont	48.00	0.00
TRINITY_sp|Q389	RABB1B	Ras-related protein	48.00	0.00
TRINITY_sp|Q6QN	-	Nascent polypeptide	48.00	0.00
TRINITY_sp|P288	PA3568	Uncharacterized pro	48.00	0.00
TRINITY_sp|O574	-	Voltage-dependent L	48.00	0.00
TRINITY_sp|E0X9	At3g635	Zinc finger CCCH do	48.00	0.00
TRINITY_sp|P127	RSP3	Flagellar radial sp	48.00	0.00
TRINITY_sp|O760	TM7SF2	Delta(14)-sterol re	48.00	0.00
TRINITY_sp|Q8W0	CCR4-1	Carbon catabolite r	48.00	0.00
TRINITY_sp|P541	MSR4	Peptide methionine	48.00	0.00
TRINITY_sp|B9DG	ACS	Acetyl-coenzyme A s	48.00	0.00
TRINITY_sp|Q54B	mcfG	Mitochondrial subst	48.00	0.00
TRINITY_sp|Q84R	ARI7	Probable E3 ubiquit	48.00	0.00
TRINITY_sp|B5FY	LAMTOR2	Ragulator complex p	48.00	0.00
TRINITY_sp|F5A8	DAB1	Dynein assembly fac	48.00	0.00
TRINITY_sp|Q2JM	aspS	Aspartate--tRNA(Asp	48.00	0.00
TRINITY_sp|Q3ZC	CNOT7	CCR4-NOT transcript	48.00	0.00
TRINITY_sp|P315	YPTV1	GTP-binding protein	48.00	0.00
TRINITY_sp|O808	ARASP	Membrane metallopro	48.00	0.00
TRINITY_sp|A2CE	cyb5d2	Neuferricin OS=Dani	48.00	0.00
TRINITY_sp|P534	-	Actin-2 OS=Schistos	48.00	0.00
TRINITY_sp|P227	Gucy1b2	Guanylate cyclase s	48.00	0.00
TRINITY_sp|Q6DF	papd4-b	Poly(A) RNA polymer	48.00	0.00
TRINITY_sp|Q617	Eif2b4	Translation initiat	48.00	0.00
TRINITY_sp|Q9C5	MKP1	Protein-tyrosine-ph	48.00	0.00
TRINITY_sp|Q603	MJ0079	Uncharacterized pro	48.00	0.00
TRINITY_sp|Q9T0	PCKA	Phosphoenolpyruvate	48.00	0.00
TRINITY_sp|P222	-	Pyruvate kinase, cy	48.00	0.00
TRINITY_sp|O944	tef3	Elongation factor 3	48.00	0.00
TRINITY_sp|Q15K	PHYLLO	Protein PHYLLO, chl	48.00	0.00
TRINITY_sp|P007	F2	Prothrombin OS=Homo	47.90	0.00
TRINITY_sp|Q9MB	DHC10	Dynein-1-beta heavy	47.90	0.00
TRINITY_sp|P501	Rdh3	Retinol dehydrogena	47.90	0.00
TRINITY_sp|O006	FCN1	Ficolin-1 OS=Homo s	47.90	0.00
TRINITY_sp|Q9FL	ABCA9	ABC transporter A f	47.90	0.00
TRINITY_sp|Q5VJ	gdt9	Probable serine/thr	47.90	0.00
TRINITY_sp|Q54T	mrpl17	Probable 39S riboso	47.90	0.00
TRINITY_sp|Q54Q	erkB	Extracellular signa	47.90	0.00
TRINITY_sp|Q9P9	alxA	Alternative oxidase	47.90	0.00
TRINITY_sp|Q9Z2	Mtmr2	Myotubularin-relate	47.90	0.00
TRINITY_sp|Q54X	DDB_G02	DEP domain-containi	47.90	0.00
TRINITY_sp|E9PV	Gcn1	eIF-2-alpha kinase	47.90	0.00
TRINITY_sp|Q54G	xpnpep1	Xaa-Pro aminopeptid	47.90	0.00
TRINITY_sp|Q96G	SDSL	Serine dehydratase-	47.90	0.00
TRINITY_sp|Q54W	bud32	EKC/KEOPS complex s	47.90	0.00
TRINITY_sp|Q5VJ	gdt9	Probable serine/thr	47.90	0.00
TRINITY_sp|Q54V	DDB_G02	Probable serine/thr	47.90	0.00
TRINITY_sp|Q4G3	-	Peptidyl-prolyl cis	47.90	0.00
TRINITY_sp|P736	sll1773	Putative quercetin	47.90	0.00
TRINITY_sp|Q76N	ERD2	ER lumen protein-re	47.90	0.00

TRINITY_sp|Q2KI2	TMEM256	Transmembrane prote	47.90	0.00
TRINITY_sp|Q62JH	alc1	Probable allantoica	47.90	0.00
TRINITY_sp|Q2556	UMP	Uridine 5'-monophos	47.90	0.00
TRINITY_sp|A7RZ8	bloc1s2	Biogenesis of lysos	47.90	0.00
TRINITY_sp|Q54H9	DDB_G028	CBS domain-containi	47.90	0.00
TRINITY_sp|A1A6H	NSRA	Nuclear speckle RNA	47.90	0.00
TRINITY_sp|Q6CR0	mge1	GrpE protein homolo	47.90	0.00
TRINITY_sp|Q54TW	pdkA	Probable serine/thr	47.90	0.00
TRINITY_sp|O6052	NEMF	Nuclear export medi	47.90	0.00
TRINITY_sp|P5467	apm2	AP-2 complex subuni	47.90	0.00
TRINITY_sp|Q3B40	zupT	Zinc transporter Zu	47.90	0.00
TRINITY_sp|Q9FIK	At5g4772	Probable acetyl-CoA	47.90	0.00
TRINITY_sp|Q8S0J	Os01g089	Probable membrane-a	47.90	0.00
TRINITY_sp|Q9M90	RKD1	Protein RKD1 OS=Ara	47.90	0.00
TRINITY_sp|Q4268	SODA	Superoxide dismutas	47.90	0.00
TRINITY_sp|Q7Z6E	RBBP6	E3 ubiquitin-protei	47.90	0.00
TRINITY_sp|A7SWH	v1g19425	Pescadillo homolog	47.90	0.00
TRINITY_sp|P4703	PRY3	Cell wall protein P	47.90	0.00
TRINITY_sp|P3209	mutT	8-oxo-dGTP diphosph	47.90	0.00
TRINITY_sp|Q66HE	Ift80	Intraflagellar tran	47.90	0.00
TRINITY_sp|Q94EY	PR46b	Coiled-coil domain-	47.90	0.00
TRINITY_sp|Q75LI	ADA2	Transcriptional ada	47.90	0.00
TRINITY_sp|Q6GQE	atg3	Ubiquitin-like-conj	47.90	0.00
TRINITY_sp|Q9Z9M	bsaA	Glutathione peroxid	47.90	0.00
TRINITY_sp|Q9FVN	AMT1-3	Ammonium transporte	47.90	0.00
TRINITY_sp|Q9WU0	Ctsz	Cathepsin Z OS=Mus	47.90	0.00
TRINITY_sp|Q9V3A	Catsup	Protein catecholami	47.90	0.00
TRINITY_sp|Q1IN7	gcvH	Glycine cleavage sy	47.90	0.00
TRINITY_sp|Q3919	PSBW	Photosystem II reac	47.80	0.00
TRINITY_sp|Q54LE	abcC5	ABC transporter C f	47.80	0.00
TRINITY_sp|O2356	RABB1A	Ras-related protein	47.80	0.00
TRINITY_sp|Q96P4	AGAP3	Arf-GAP with GTPase	47.80	0.00
TRINITY_sp|Q8WNE	CSTA	Cystatin-A OS=Felis	47.80	0.00
TRINITY_sp|Q9GLY	ITIH3	Inter-alpha-trypsin	47.80	0.00
TRINITY_sp|Q54RA	DDB_G028	Probable iron/ascor	47.80	0.00
TRINITY_sp|Q5PQ2	slc30a9	Zinc transporter 9	47.80	0.00
TRINITY_sp|Q9SRE	PEX13	Peroxisomal membran	47.80	0.00
TRINITY_sp|A7YT8	nmt2	Glycylpeptide N-tet	47.80	0.00
TRINITY_sp|Q54VE	coq7	5-demethoxyubiquino	47.80	0.00
TRINITY_sp|O9443	SPBC660.	Uncharacterized RNA	47.80	0.00
TRINITY_sp|Q58D4	MMAB	Cob(I)yrinic acid a	47.80	0.00
TRINITY_sp|C5CG7	glgA	Glycogen synthase O	47.80	0.00
TRINITY_sp|P3641	rab7A	Ras-related protein	47.80	0.00
TRINITY_sp|Q0VCA	OXSM	3-oxoacyl-[acyl-car	47.80	0.00
TRINITY_sp|Q2868	ABCC2	Canalicular multisp	47.80	0.00
TRINITY_sp|Q9Y4W	AFG3L2	AFG3-like protein 2	47.80	0.00
TRINITY_sp|C0QTM	rpsO	30S ribosomal prote	47.80	0.00
TRINITY_sp|Q54P0	gefA	Ras guanine nucleot	47.80	0.00
TRINITY_sp|Q8RWE	UPL6	E3 ubiquitin-protei	47.80	0.00
TRINITY_sp|P0CG5	UBB	Polyubiquitin-B OS=	47.80	0.00
TRINITY_sp|Q07G4	sde2	Protein SDE2 homolo	47.80	0.00
TRINITY_sp|Q54N3	uch2	Ubiquitin carboxyl-	47.80	0.00
TRINITY_sp|Q9M1I	At3g5446	F-box protein At3g5	47.80	0.00
TRINITY_sp|Q9CQ0	Immp1l	Mitochondrial inner	47.80	0.00
TRINITY_sp|O6146	-	60S acidic ribosoma	47.80	0.00
TRINITY_sp|P0846	Dlat	Dihydrolipoyllysine	47.80	0.00

TRINITY_sp|Q9SY〉	LIL3.1	Light-harvesting co	47.80	0.00
TRINITY_sp|O882(	Aspg	60 kDa lysophosphol	47.80	0.00
TRINITY_sp|Q9BII	B0464.9	Probable protein ph	47.80	0.00
TRINITY_sp|Q54PI	cwc2	Pre-mRNA-splicing f	47.80	0.00
TRINITY_sp|Q9AS	FREE1	Protein FREE1 OS=Ar	47.80	0.00
TRINITY_sp|Q5ZJI	NGLY1	Peptide-N(4)-(N-ace	47.80	0.00
TRINITY_sp|Q059	Dusp2	Dual specificity pr	47.80	0.00
TRINITY_sp|Q54Q(	tceb1	Transcription elong	47.80	0.00
TRINITY_sp|P005	V-YES	Tyrosine-protein ki	47.80	0.00
TRINITY_sp|Q9VN	CG12163	Putative cysteine p	47.80	0.00
TRINITY_sp|A2YH	OsI_023	Serine/threonine-pr	47.80	0.00
TRINITY_sp|Q8K2	Manba	Beta-mannosidase OS	47.80	0.00
TRINITY_sp|Q9TT(	pro-pol	Pro-Pol polyprotein	47.80	0.00
TRINITY_sp|Q2N2I	-	Probable phytol kin	47.80	0.00
TRINITY_sp|Q6ZR(	DNAH12	Dynein heavy chain	47.80	0.00
TRINITY_sp|P0A3	cspLA	Cold shock-like pro	47.80	0.00
TRINITY_sp|P459	ARP	DNA-(apurinic or ap	47.80	0.00
TRINITY_sp|Q641V	Ttll9	Probable tubulin po	47.80	0.00
TRINITY_sp|Q008	-	Cysteine synthase O	47.80	0.00
TRINITY_sp|Q099	yakc	Aldo-keto reductase	47.80	0.00
TRINITY_sp|Q9LK	UVH1	DNA repair endonucl	47.80	0.00
TRINITY_sp|P536	CLTCL1	Clathrin heavy chai	47.80	0.00
TRINITY_sp|Q9SH	RRP44A	Exosome complex exo	47.80	0.00
TRINITY_sp|Q9SNI	LUX	Transcription facto	47.80	0.00
TRINITY_sp|Q55C	uba1	Ubiquitin-like modi	47.80	0.00
TRINITY_sp|Q9LD	TIF3A1	Eukaryotic translat	47.80	0.00
TRINITY_sp|Q8IYI	DIS3L2	DIS3-like exonuclea	47.80	0.00
TRINITY_sp|Q395	-	5-methyltetrahydrop	47.80	0.00
TRINITY_sp|Q9C5(	DPL1	Sphingosine-1-phosp	47.80	0.00
TRINITY_sp|P258	chcA	Clathrin heavy chai	47.80	0
TRINITY_sp|Q9BX	GTPBP2	GTP-binding protein	47.80	0.00
TRINITY_sp|Q7Z7I	CENPV	Centromere protein	47.70	0.00
TRINITY_sp|Q9SA	AKHSDH1	Bifunctional aspart	47.70	0.00
TRINITY_sp|P706	Mapk14	Mitogen-activated p	47.70	0.00
TRINITY_sp|Q08B\	tyw5	tRNA wybutosine-syn	47.70	0.00
TRINITY_sp|Q54J	atg6B	Beclin-1-like prote	47.70	0.00
TRINITY_sp|Q6NLI	NRPB9A	DNA-directed RNA po	47.70	0.00
TRINITY_sp|Q5RI\	adat2	tRNA-specific adeno	47.70	0.00
TRINITY_sp|Q559(	slr0305	TVP38/TMEM64 family	47.70	0.00
TRINITY_sp|Q8L6	EXO1	Exonuclease 1 OS=Ar	47.70	0.00
TRINITY_sp|Q9UKI	PARP4	Poly [ADP-ribose] p	47.70	0.00
TRINITY_sp|P087	ND2	NADH-ubiquinone oxi	47.70	0.00
TRINITY_sp|Q1HD	CHX16	Cation/H(+) antipor	47.70	0.00
TRINITY_sp|O220	ANP3	Mitogen-activated p	47.70	0.00
TRINITY_sp|A5DN	YPI1	Type 1 phosphatases	47.70	0.00
TRINITY_sp|O154	MPPED1	Metallophosphoester	47.70	0.00
TRINITY_sp|Q5HKI	pflB	Formate acetyltrans	47.70	0.00
TRINITY_sp|Q94A(	MKK1	Mitogen-activated p	47.70	0.00
TRINITY_sp|Q6Z6V	MADS57	MADS-box transcript	47.70	0.00
TRINITY_sp|Q9AR	HAG1	Histone acetyltrans	47.70	0.00
TRINITY_sp|Q9SI	DSK2B	Ubiquitin domain-co	47.70	0.00
TRINITY_sp|P216	-	Pyrophosphate-energ	47.70	0.00
TRINITY_sp|Q6AE(	dnaJ	Chaperone protein D	47.70	0.00
TRINITY_sp|Q54SI	dhkD	Hybrid signal trans	47.70	0.00
TRINITY_sp|Q8BYI	Rdh12	Retinol dehydrogena	47.70	0.00
TRINITY_sp|B9RA	RCOM_15(	Probable aspartyl a	47.70	0.00

TRINITY_sp|Q9FKV	At5g6615	Probable alpha-mann	47.70	0.00
TRINITY_sp|B3N40	Trip1	Eukaryotic translat	47.70	0.00
TRINITY_sp|Q9GLV	PRDX5	Peroxiredoxin-5, mi	47.70	0.00
TRINITY_sp|Q9NRK	ABCB10	ATP-binding cassett	47.70	0.00
TRINITY_sp|Q84JI	At5g0930	2-oxoisovalerate de	47.70	0.00
TRINITY_sp|A5FN1	rpsK	30S ribosomal prote	47.70	0.00
TRINITY_sp|Q9M0E	PFK1	ATP-dependent 6-pho	47.70	0.00
TRINITY_sp|P5052	ssp1	Serine/threonine-pr	47.70	0.00
TRINITY_sp|Q9XEE	ALG11	GDP-Man:Man(3)GlcNA	47.70	0.00
TRINITY_sp|F4HP2	LIG6	DNA ligase 6 OS=Ara	47.70	0.00
TRINITY_sp|Q9XYI	fcpA	Probable C-terminal	47.70	0.00
TRINITY_sp|Q9FJI	KPNB1	Importin subunit be	47.70	0.00
TRINITY_sp|Q9LV9	DPE1	4-alpha-glucanotran	47.70	0.00
TRINITY_sp|O6638	sigA	RNA polymerase sigm	47.70	0.00
TRINITY_sp|Q9LSI	ARP2	Actin-related prote	47.70	0.00
TRINITY_sp|Q67XX	At5g0655	F-box protein At5g0	47.70	0.00
TRINITY_sp|Q9SM2	LKR/SDH	Alpha-aminoadipic s	47.70	0.00
TRINITY_sp|P2271	Gucy1b2	Guanylate cyclase s	47.70	0.00
TRINITY_sp|P3703	Por	NADPH--cytochrome P	47.70	0.00
TRINITY_sp|Q6454	Atp1a4	Sodium/potassium-tr	47.70	0.00
TRINITY_sp|P3905	-	Dynein beta chain,	47.70	0.00
TRINITY_sp|Q9M0E	RPL28C	60S ribosomal prote	47.70	0.00
TRINITY_sp|Q9C6I	MTK	Methylthioribose ki	47.70	0.00
TRINITY_sp|Q86SQ	DHDDS	Dehydrodolichyl dip	47.70	0.00
TRINITY_sp|Q8WXX	DNAH7	Dynein heavy chain	47.70	0.00
TRINITY_sp|Q9C5X	HAC1	Histone acetyltrans	47.70	0.00
TRINITY_sp|Q8WXX	DNAH7	Dynein heavy chain	47.70	0.00
TRINITY_sp|Q4142	PCM3	Putative calmodulin	47.60	0.00
TRINITY_sp|Q4551	-	D-hydantoinase OS=G	47.60	0.00
TRINITY_sp|F0NBE	SiRe_144	Protein-lysine N-me	47.60	0.00
TRINITY_sp|Q9188	hspa5	78 kDa glucose-regu	47.60	0.00
TRINITY_sp|Q55E5	pats1	Probable serine/thr	47.60	0.00
TRINITY_sp|P5707	NMA0194	Uncharacterized pro	47.60	0.00
TRINITY_sp|Q54NE	vps28	Vacuolar protein so	47.60	0.00
TRINITY_sp|Q05A1	Sdr16c6	Short-chain dehydro	47.60	0.00
TRINITY_sp|Q54BE	ggps1	Geranylgeranyl pyro	47.60	0.00
TRINITY_sp|P3862	PRE3	Proteasome subunit	47.60	0.00
TRINITY_sp|Q7RAV	CPK3	Calcium-dependent p	47.60	0.00
TRINITY_sp|Q9AT7	FAD2	Delta(12) fatty aci	47.60	0.00
TRINITY_sp|Q9P2E	KIF17	Kinesin-like protei	47.60	0.00
TRINITY_sp|Q9XT5	PDE6D	Retinal rod rhodops	47.60	0.00
TRINITY_sp|O2278	PRP19B	Pre-mRNA-processing	47.60	0.00
TRINITY_sp|Q9LP4	RPN6	26S proteasome non-	47.60	0.00
TRINITY_sp|O1544	MPPED1	Metallophosphoester	47.60	0.00
TRINITY_sp|Q86K2	cpnB-1	Copine-B OS=Dictyos	47.60	0.00
TRINITY_sp|P7070	Atp7a	Copper-transporting	47.60	0.00
TRINITY_sp|O1526	SPTLC1	Serine palmitoyltra	47.60	0.00
TRINITY_sp|P4512	ettA	Energy-dependent tr	47.60	0.00
TRINITY_sp|Q5Z8E	Os06g071	RNA pseudouridine s	47.60	0.00
TRINITY_sp|P0CQ4	REX4	RNA exonuclease 4 O	47.60	0.00
TRINITY_sp|Q6P3E	NEK5	Serine/threonine-pr	47.60	0.00
TRINITY_sp|C0QRE	gatA	Glutamyl-tRNA(Gln)	47.60	0.00
TRINITY_sp|O0090	-	Lysosomal acid alph	47.60	0.00
TRINITY_sp|Q8WZN	GCN5	Histone acetyltrans	47.60	0.00
TRINITY_sp|F1R34	ddx11	ATP-dependent DNA h	47.60	0.00
TRINITY_sp|A7MGV	pepB	Peptidase B OS=Cron	47.60	0.00

TRINITY_sp|Q54U	shkD	Dual specificity pr	47.60	0.00
TRINITY_sp|P067	Polb	DNA polymerase beta	47.60	0.00
TRINITY_sp|Q7Z4	HDDC2	HD domain-containin	47.60	0.00
TRINITY_sp|P136	Atpalpha	Sodium/potassium-tr	47.60	0.00
TRINITY_sp|Q96M	CFAP44	Cilia- and flagella	47.60	0.00
TRINITY_sp|P227	Gucy1b2	Guanylate cyclase s	47.60	0.00
TRINITY_sp|Q9AT	UVH3	DNA repair protein	47.60	0.00
TRINITY_sp|Q631	Dnah7	Dynein heavy chain	47.50	0.00
TRINITY_sp|P480	gacS	Sensor protein GacS	47.50	0.00
TRINITY_sp|O943	ppk31	Serine/threonine-pr	47.50	0.00
TRINITY_sp|Q54G	DDB_G02	Protein CLEC16A hom	47.50	0.00
TRINITY_sp|C1AB	engB	Probable GTP-bindin	47.50	0.00
TRINITY_sp|Q125	rasA	Ras-like protein OS	47.50	0.00
TRINITY_sp|P530	SLC25A1	Tricarboxylate tran	47.50	0.00
TRINITY_sp|A1CB	alg3	Dol-P-Man:Man(5)Glc	47.50	0.00
TRINITY_sp|O221	RHC1A	Probable E3 ubiquit	47.50	0.00
TRINITY_sp|Q418	-	ATP synthase subuni	47.50	0.00
TRINITY_sp|Q95U	rdiA	Putative rho GDP-di	47.50	0.00
TRINITY_sp|Q5T3	ABCC10	Multidrug resistanc	47.50	0.00
TRINITY_sp|O438	ATP9B	Probable phospholip	47.50	0.00
TRINITY_sp|Q246	ref(2)P	Protein ref(2)P OS=	47.50	0.00
TRINITY_sp|Q8RW	PUX4	Plant UBX domain-co	47.50	0.00
TRINITY_sp|Q91V	Memo1	Protein MEMO1 OS=Mu	47.50	0.00
TRINITY_sp|Q8BX	Ctdsp2	Carboxy-terminal do	47.50	0.00
TRINITY_sp|G0Y2	SSL-2	Botryococcus squale	47.50	0.00
TRINITY_sp|A8IZ	CHLREDR	Probable cytosolic	47.50	0.00
TRINITY_sp|Q3MG	rsgA	Putative ribosome b	47.50	0.00
TRINITY_sp|Q2UG	atg1	Serine/threonine-pr	47.50	0.00
TRINITY_sp|Q625	Ift52	Intraflagellar tran	47.50	0.00
TRINITY_sp|O087	Ube3a	Ubiquitin-protein l	47.50	0.00
TRINITY_sp|Q8TC	MARCH1	E3 ubiquitin-protei	47.50	0.00
TRINITY_sp|Q9AS	SBH2	Sphinganine C4-mono	47.50	0.00
TRINITY_sp|Q2RB	Os11g01	Clathrin heavy chai	47.50	0.00
TRINITY_sp|Q4V7	nsun2	tRNA (cytosine(34)-	47.50	0.00
TRINITY_sp|Q246	ref(2)P	Protein ref(2)P OS=	47.50	0.00
TRINITY_sp|Q6ZD	Os07g01	Phosphoacetylglucos	47.50	0.00
TRINITY_sp|Q9ST	HSP90-7	Endoplasmin homolog	47.50	0.00
TRINITY_sp|P308	Aldh3a2	Fatty aldehyde dehy	47.50	0.00
TRINITY_sp|O227	AP1M2	AP-1 complex subuni	47.50	0.00
TRINITY_sp|O043	PHB3	Prohibitin-3, mitoc	47.50	0.00
TRINITY_sp|Q6FS	DBP3	ATP-dependent RNA h	47.50	0.00
TRINITY_sp|Q9VX	Cyp4s3	Probable cytochrome	47.50	0.00
TRINITY_sp|A0AV	UBA6	Ubiquitin-like modi	47.50	0.00
TRINITY_sp|Q9FZ	PEX11A	Peroxisomal membran	47.50	0.00
TRINITY_sp|Q6EU	PUB4	U-box domain-contai	47.50	0.00
TRINITY_sp|Q7TX	ppsC	Phthiocerol/phenolp	47.50	0.00
TRINITY_sp|Q54T	gtaI	GATA zinc finger do	47.50	0.00
TRINITY_sp|Q7XR	YSL6	Probable metal-nico	47.50	0.00
TRINITY_sp|Q9D2	Mmab	Cob(I)yrinic acid a	47.50	0.00
TRINITY_sp|Q8L6	RPOT1-T	DNA-directed RNA po	47.50	0.00
TRINITY_sp|Q66K	phospho	Probable phosphatas	47.50	0.00
TRINITY_sp|Q3ZC	LSM8	U6 snRNA-associated	47.50	0.00
TRINITY_sp|Q69U	RMR1	Receptor homology r	47.50	0.00
TRINITY_sp|Q8GW	PMRT15	Protein arginine N-	47.50	0.00
TRINITY_sp|O944	tef3	Elongation factor 3	47.50	0.00
TRINITY_sp|Q5E9	ARF3	ADP-ribosylation fa	47.50	0.00

TRINITY_sp|Q945	Dlc90F	Dynein light chain	47.50	0.00
TRINITY_sp|P565	HDAC3	Histone deacetylase	47.40	0.00
TRINITY_sp|Q9LJ	M3KE1	MAP3K epsilon prote	47.40	0.00
TRINITY_sp|Q6BY	PSF3	DNA replication com	47.40	0.00
TRINITY_sp|Q6IM	alrA	Aldose reductase A	47.40	0.00
TRINITY_sp|Q9UI	MUTYH	Adenine DNA glycosy	47.40	0.00
TRINITY_sp|Q9P7	vas2	AP-1 complex subuni	47.40	0.00
TRINITY_sp|P823	scrK	Fructokinase OS=Lac	47.40	0.00
TRINITY_sp|Q8IB	CPK4	Calcium-dependent p	47.40	0.00
TRINITY_sp|P386	ro-4	Actin-like protein	47.40	0.00
TRINITY_sp|Q54K	talB	Talin-B OS=Dictyost	47.40	0.00
TRINITY_sp|P542	PMS1	PMS1 protein homolo	47.40	0.00
TRINITY_sp|B7F9	ADIPOR1	Heptahelical transm	47.40	0.00
TRINITY_sp|Q9SR	PEX19-1	Peroxisome biogenes	47.40	0.00
TRINITY_sp|P295	-	Eukaryotic translat	47.40	0.00
TRINITY_sp|O644	PBF1	Proteasome subunit	47.40	0.00
TRINITY_sp|Q2RR	msrA	Peptide methionine	47.40	0.00
TRINITY_sp|Q993	TY3B-G	Transposon Ty3-G Ga	47.40	0.00
TRINITY_sp|Q8T6	abcA6	ABC transporter A f	47.40	0.00
TRINITY_sp|Q101	mra1	Ribosomal RNA small	47.40	0.00
TRINITY_sp|Q54N	cyb5r1	NADH-cytochrome b5	47.40	0.00
TRINITY_sp|Q9LX	At5g106	Putative RNA methyl	47.40	0.00
TRINITY_sp|Q28I	glrx3	Glutaredoxin-3 OS=X	47.40	0.00
TRINITY_sp|Q0VG	Zgrf1	Protein ZGRF1 OS=Mu	47.40	0.00
TRINITY_sp|Q465	hndD	NADP-reducing hydro	47.40	0.00
TRINITY_sp|Q56X	RH15	DEAD-box ATP-depend	47.40	0.00
TRINITY_sp|A8X6	cmk-1	Calcium/calmodulin-	47.40	0.00
TRINITY_sp|O149	GAK	Cyclin-G-associated	47.40	0.00
TRINITY_sp|Q86I	omt6	Probable caffeoyl-C	47.40	0.00
TRINITY_sp|Q9FE	SRT1	NAD-dependent prote	47.40	0.00
TRINITY_sp|Q9M1	ABCF4	ABC transporter F f	47.40	0.00
TRINITY_sp|Q54Q	uba3	NEDD8-activating en	47.40	0.00
TRINITY_sp|Q9FK	COX15	Cytochrome c oxidas	47.40	0.00
TRINITY_sp|Q8LA	PDX2	Probable pyridoxal	47.40	0.00
TRINITY_sp|Q54L	DDB_G02	Uncharacterized pro	47.40	0.00
TRINITY_sp|Q5E9	DCAF11	DDB1- and CUL4-asso	47.40	0.00
TRINITY_sp|Q3T0	TRAPPC2	Trafficking protein	47.40	0.00
TRINITY_sp|Q6KC	NIPBL	Nipped-B-like prote	47.40	0.00
TRINITY_sp|Q016	RSP6	Flagellar radial sp	47.40	0.00
TRINITY_sp|Q165	MAP3K11	Mitogen-activated p	47.40	0.00
TRINITY_sp|Q9M1	ABCF4	ABC transporter F f	47.40	0.00
TRINITY_sp|Q9SS	GYRBC	DNA gyrase subunit	47.40	0.00
TRINITY_sp|P052	Su(var)	Heterochromatin pro	47.40	0.00
TRINITY_sp|Q928	gadB	Glutamate decarboxy	47.40	0.00
TRINITY_sp|Q8BI	Dyrk4	Dual specificity ty	47.40	0.00
TRINITY_sp|Q8UA	blh	Beta-lactamase hydr	47.40	0.00
TRINITY_sp|P0C7	UMAG_11	Putative DEAH-box A	47.40	0.00
TRINITY_sp|Q9UV	ACT	Actin, gamma OS=Acr	47.30	0.00
TRINITY_sp|O773	RPS10	40S ribosomal prote	47.30	0.00
TRINITY_sp|Q8L0	mgl	L-methionine gamma-	47.30	0.00
TRINITY_sp|Q67X	At5g065	F-box protein At5g0	47.30	0.00
TRINITY_sp|Q54T	ube2m	NEDD8-conjugating e	47.30	0.00
TRINITY_sp|Q54N	mybL	Myb-like protein L	47.30	0.00
TRINITY_sp|Q91V	Smap1	Stromal membrane-as	47.30	0.00
TRINITY_sp|O817	RKD5	Protein RKD5 OS=Ara	47.30	0.00
TRINITY_sp|Q9ZV	At2g387	Uncharacterized pro	47.30	0.00

TRINITY_sp|P477[illegible]	RAP1GAP	Rap1 GTPase-activat	47.30	0.00
TRINITY_sp|Q8L8[illegible]	NUDT3	Nudix hydrolase 3 O	47.30	0.00
TRINITY_sp|Q018[illegible]	YPT1	Ras-like GTP-bindin	47.30	0.00
TRINITY_sp|P549[illegible]	IMP2	Inositol monophosph	47.30	0.00
TRINITY_sp|A1CD[illegible]	ddi1	DNA damage-inducibl	47.30	0.00
TRINITY_sp|Q5XG[illegible]	rnps1-a	RNA-binding protein	47.30	0.00
TRINITY_sp|Q9FN[illegible]	E2FA	Transcription facto	47.30	0.00
TRINITY_sp|Q8TH[illegible]	dapA	4-hydroxy-tetrahydr	47.30	0.00
TRINITY_sp|P426[illegible]	yqjG	Glutathionyl-hydroq	47.30	0.00
TRINITY_sp|P258[illegible]	chcA	Clathrin heavy chai	47.30	0.00
TRINITY_sp|Q8RX[illegible]	FACE1	CAAX prenyl proteas	47.30	0.00
TRINITY_sp|P567[illegible]	PSMD2	26S proteasome non-	47.30	0.00
TRINITY_sp|P418[illegible]	rae1	Poly(A)+ RNA export	47.30	0.00
TRINITY_sp|B2GU[illegible]	kdm8	Lysine-specific dem	47.30	0.00
TRINITY_sp|B9GE[illegible]	KIN12F	Kinesin-like protei	47.30	0.00
TRINITY_sp|O888[illegible]	Arl6	ADP-ribosylation fa	47.30	0.00
TRINITY_sp|Q94A[illegible]	At1g641[illegible]	GDT1-like protein 1	47.30	0.00
TRINITY_sp|Q54S[illegible]	vps15	Probable serine/thr	47.30	0.00
TRINITY_sp|Q7Z6[illegible]	DNAJB7	DnaJ homolog subfam	47.30	0.00
TRINITY_sp|Q8L7[illegible]	BGLU6	Beta-glucosidase 6	47.30	0.00
TRINITY_sp|P268[illegible]	COX2	Cytochrome c oxidas	47.30	0.00
TRINITY_sp|Q9RU[illegible]	sodA	Superoxide dismutas	47.30	0.00
TRINITY_sp|Q9D8[illegible]	Bola1	BolA-like protein 1	47.30	0.00
TRINITY_sp|B8LI[illegible]	IFT25	Intraflagellar tran	47.30	0.00
TRINITY_sp|Q8VZ[illegible]	INT1	Inositol transporte	47.30	0.00
TRINITY_sp|Q6GQ[illegible]	Rab31	Ras-related protein	47.30	0.00
TRINITY_sp|P092[illegible]	-	Tubulin beta-6 chai	47.30	0.00
TRINITY_sp|O154[illegible]	ABCC4	Multidrug resistanc	47.30	0.00
TRINITY_sp|Q82W[illegible]	mdh	Malate dehydrogenas	47.20	0.00
TRINITY_sp|O044[illegible]	RABA2A	Ras-related protein	47.20	0.00
TRINITY_sp|Q158[illegible]	TBCC	Tubulin-specific ch	47.20	0.00
TRINITY_sp|Q55F[illegible]	hdaA	Type-1 histone deac	47.20	0.00
TRINITY_sp|Q54E[illegible]	eif2b2	Translation initiat	47.20	0.00
TRINITY_sp|O773[illegible]	MAL3P3.[illegible]	T-complex protein 1	47.20	0.00
TRINITY_sp|P9WH[illegible]	deaD	ATP-dependent RNA h	47.20	0.00
TRINITY_sp|Q9S7[illegible]	MYB98	Transcription facto	47.20	0.00
TRINITY_sp|P546[illegible]	pikB	Phosphatidylinosito	47.20	0.00
TRINITY_sp|Q9SX[illegible]	AHK1	Histidine kinase 1	47.20	0.00
TRINITY_sp|F4K5[illegible]	RVE2	Protein REVEILLE 2	47.20	0.00
TRINITY_sp|Q6Q1[illegible]	SMC1	Structural maintena	47.20	0.00
TRINITY_sp|Q1LZ[illegible]	ERCC1	DNA excision repair	47.20	0.00
TRINITY_sp|Q9V6[illegible]	Kdm4B	Probable lysine-spe	47.20	0.00
TRINITY_sp|Q0TM[illegible]	rsmA	Ribosomal RNA small	47.20	0.00
TRINITY_sp|Q6NV[illegible]	dcaf13	DDB1- and CUL4-asso	47.20	0.00
TRINITY_sp|Q9SG[illegible]	RPN12A	26S proteasome non-	47.20	0.00
TRINITY_sp|Q8IB[illegible]	CPK4	Calcium-dependent p	47.20	0.00
TRINITY_sp|P371[illegible]	-	NADPH--cytochrome P	47.20	0.00
TRINITY_sp|F4J2[illegible]	RVE7L	Protein REVEILLE 7-	47.20	0.00

TRINITY_sp|Q5ZJ(	HIBCH	3-hydroxyisobutyryl	47.20	0.00
TRINITY_sp|Q54L(	gatA	Glutamyl-tRNA(Gln)	47.20	0.00
TRINITY_sp|Q100:	grs-1	Glycine--tRNA ligas	47.20	0.00
TRINITY_sp|F4K4(	KIN14S	Kinesin-like protei	47.20	0.00
TRINITY_sp|Q1PE4	CDS3	Phosphatidate cytid	47.20	0.00
TRINITY_sp|Q9LE7	CIP111	Calmodulin-interact	47.20	0.00
TRINITY_sp|Q054(	LIPE	Hormone-sensitive l	47.20	0.00
TRINITY_sp|Q9SLI	DGAT1	Diacylglycerol O-ac	47.20	0.00
TRINITY_sp|Q9XFI	At3g027:	Thioredoxin F1, chl	47.20	0.00
TRINITY_sp|P493!	FNTB	Protein farnesyltra	47.20	0.00
TRINITY_sp|Q431:	CRY1	Cryptochrome-1 OS=A	47.20	0.00
TRINITY_sp|P176:	ypt3	GTP-binding protein	47.20	0.00
TRINITY_sp|Q8AB!	BT_0258	Putative glucosamin	47.20	0.00
TRINITY_sp|Q54L}	zntB	Protein zntB OS=Dic	47.20	0.00
TRINITY_sp|O646	At2g456	Histone deacetylase	47.20	0.00
TRINITY_sp|Q9ZT	STA1	Protein STABILIZED1	47.20	0.00
TRINITY_sp|A2YW!	PLP2	Patatin-like protei	47.20	0.00
TRINITY_sp|Q9UR}	SPAC195:	TBC domain-containi	47.20	0.00
TRINITY_sp|Q9M2!	CEF	Protein transport p	47.20	0.00
TRINITY_sp|P257(	RGP1	Ras-related protein	47.20	0.00
TRINITY_sp|Q9FG(	RAX1	Transcription facto	47.20	0.00
TRINITY_sp|O226(	DEGP1	Protease Do-like 1,	47.20	0.00
TRINITY_sp|P378!	CBP3	Serine carboxypepti	47.20	0.00
TRINITY_sp|Q54R(	top1	DNA topoisomerase 1	47.20	0.00
TRINITY_sp|Q9XI	PUMP3	Mitochondrial uncou	47.20	0.00
TRINITY_sp|Q945(	modA	Neutral alpha-gluco	47.20	0.00
TRINITY_sp|Q3URI	Acsf3	Acyl-CoA synthetase	47.20	0.00
TRINITY_sp|Q54WI	ap3d1	AP-3 complex subuni	47.20	0.00
TRINITY_sp|Q9FZ:	ANP2	Mitogen-activated p	47.20	0.00
TRINITY_sp|Q7G8}	Os01g03(	Probable chromatin-	47.20	0.00
TRINITY_sp|Q6P9(	CYB5D1	Cytochrome b5 domai	47.20	0.00
TRINITY_sp|Q9DB}	Nvl	Nuclear valosin-con	47.20	0.00
TRINITY_sp|P0DK(	NRT2.1	High-affinity nitra	47.20	0.00
TRINITY_sp|Q9MA:	At1g796(	Uncharacterized aar	47.20	0.00
TRINITY_sp|Q89Z(	cutC	Copper homeostasis	47.20	0.00
TRINITY_sp|Q54N(	cyb5r1	NADH-cytochrome b5	47.20	0.00
TRINITY_sp|Q9CF\	gpo	Glutathione peroxid	47.20	0.00
TRINITY_sp|Q126!	YPK9	Vacuolar cation-tra	47.10	0.00
TRINITY_sp|Q15R!	anmK	Anhydro-N-acetylmur	47.10	0.00
TRINITY_sp|P0AE(	barA	Signal transduction	47.10	0.00
TRINITY_sp|Q081:	-	Tubulin beta chain	47.10	0.00
TRINITY_sp|P345:	K11H3.3	Putative tricarboxy	47.10	0.00
TRINITY_sp|P303(	CDC25B	M-phase inducer pho	47.10	0.00
TRINITY_sp|Q018!	YPT1	Ras-like GTP-bindin	47.10	0.00
TRINITY_sp|Q9C0(	DNAH6	Dynein heavy chain	47.10	0.00
TRINITY_sp|P303:	cdc25-3	M-phase inducer pho	47.10	0.00
TRINITY_sp|Q55E!	pats1	Probable serine/thr	47.10	0.00
TRINITY_sp|F4JL:	IMPA2	Importin subunit al	47.10	0.00
TRINITY_sp|O147:	CDIPT	CDP-diacylglycerol-	47.10	0.00
TRINITY_sp|Q9FYI	RS2Z32	Serine/arginine-ric	47.10	0.00
TRINITY_sp|Q9SUI	COX6B-3	Cytochrome c oxidas	47.10	0.00
TRINITY_sp|B4SQI	pnp	Polyribonucleotide	47.10	0.00
TRINITY_sp|P0CD(	pan3	PAB-dependent poly(	47.10	0.00
TRINITY_sp|O042!	IMPA3	Importin subunit al	47.10	0.00
TRINITY_sp|Q9BZI	TBL1XR1	F-box-like/WD repea	47.10	0.00
TRINITY_sp|Q54J!	wdr24	WD repeat-containin	47.10	0.00

TRINITY_sp|Q54KI	DDB_G028	Uncharacterized pro	47.10	0.00
TRINITY_sp|Q7ZY(	acsbg2	Long-chain-fatty-ac	47.10	0.00
TRINITY_sp|Q7XHI	Os07g043	Tyrosine decarboxyl	47.10	0.00
TRINITY_sp|Q5EAI	PGGT1B	Geranylgeranyl tran	47.10	0.00
TRINITY_sp|Q503I	cpsf3l	Integrator complex	47.10	0.00
TRINITY_sp|Q7RAI	CPK1	Calcium-dependent p	47.10	0.00
TRINITY_sp|Q5ZKI	CEPT1	Choline/ethanolamin	47.10	0.00
TRINITY_sp|Q9MA(	PDIL2-2	Protein disulfide-i	47.10	0.00
TRINITY_sp|Q3SZI	MAPRE2	Microtubule-associa	47.10	0.00
TRINITY_sp|Q9FQ(	XRN3	5'-3' exoribonuclea	47.10	0.00
TRINITY_sp|Q5CCI	VAL2	B3 domain-containin	47.10	0.00
TRINITY_sp|Q8AB5	BT_0258	Putative glucosamin	47.10	0.00
TRINITY_sp|Q9XG>	TIM9	Mitochondrial impor	47.10	0.00
TRINITY_sp|Q9FPI	PSBS	Photosystem II 22 k	47.10	0.00
TRINITY_sp|Q9Z1(	Vars	Valine--tRNA ligase	47.10	0.00
TRINITY_sp|A6NNI	TTLL13P	Tubulin polyglutamy	47.10	0.00
TRINITY_sp|P1549	IDI1	Isopentenyl-diphosp	47.10	0.00
TRINITY_sp|Q6PD(	CTR9	RNA polymerase-asso	47.10	0.00
TRINITY_sp|Q9LZI	ERDJ3B	DnaJ protein ERDJ3B	47.10	0.00
TRINITY_sp|A1SP\	acyP	Acylphosphatase OS=	47.10	0.00
TRINITY_sp|Q7SH(	cys-12	Cysteine synthase 2	47.10	0.00
TRINITY_sp|A4FV.	rbm48	RNA-binding protein	47.10	0.00
TRINITY_sp|F4K2I	FPGS1	Folylpolyglutamate	47.10	0.00
TRINITY_sp|Q8K4(	Neil1	Endonuclease 8-like	47.10	0.00
TRINITY_sp|P3809	lamA	Putative urea carbo	47.10	0.00
TRINITY_sp|P099(	LTA4H	Leukotriene A-4 hyd	47.10	0.00
TRINITY_sp|Q5T9I	ATAD3B	ATPase family AAA d	47.10	0.00
TRINITY_sp|Q9EQ\	Tpp1	Tripeptidyl-peptida	47.10	0.00
TRINITY_sp|Q8MZS	-	Physarolisin OS=Phy	47.10	0.00
TRINITY_sp|Q7F0.	CML13	Probable calcium-bi	47.10	0.00
TRINITY_sp|O827(	HISN2	Histidine biosynthe	47.10	0.00
TRINITY_sp|A8JF(	CFAP65	Cilia- and flagella	47.10	0.00
TRINITY_sp|P0AG1	purF	Amidophosphoribosyl	47.10	0.00
TRINITY_sp|Q8BGI	Pan2	PAB-dependent poly(	47.10	0.00
TRINITY_sp|Q8K05	Ift80	Intraflagellar tran	47.10	0.00
TRINITY_sp|Q396I	ODA11	Dynein alpha chain,	47.10	0
TRINITY_sp|Q54R8	mkkA	Mitogen-activated p	47.10	0.00
TRINITY_sp|Q9FX(	BLH10	BEL1-like homeodoma	47.10	0.00
TRINITY_sp|Q1PFI	BLH11	BEL1-like homeodoma	47.10	0.00
TRINITY_sp|Q7XQI	SAPK7	Serine/threonine-pr	47.10	0.00
TRINITY_sp|P5264	ldhA	D-lactate dehydroge	47.10	0.00
TRINITY_sp|Q9SHI	At1g1722	Translation initiat	47.10	0.00
TRINITY_sp|Q6ZQ3	Cand1	Cullin-associated N	47.10	0.00
TRINITY_sp|F4K0I	VIII-2	Myosin-2 OS=Arabido	47.10	0.00
TRINITY_sp|Q54PS	osbI	Oxysterol-binding p	47.10	0.00
TRINITY_sp|Q92EI	rlmN	Probable dual-speci	47.10	0.00
TRINITY_sp|Q54JI	cnrN	Phosphatidylinosito	47.10	0.00
TRINITY_sp|P5179	CLCN7	H(+)/Cl(-) exchange	47.10	0.00
TRINITY_sp|Q0P44	dpcd	Protein DPCD OS=Dan	47.10	0.00
TRINITY_sp|Q95PI	dhkC	Hybrid signal trans	47.10	0.00
TRINITY_sp|Q7TMS	Abcg2	ATP-binding cassett	47.10	0.00
TRINITY_sp|P339(	bsr	Blasticidin-S deami	47.00	0.00
TRINITY_sp|Q54QI	sir2A	NAD-dependent deace	47.00	0.00
TRINITY_sp|Q9D8(	Actr6	Actin-related prote	47.00	0.00
TRINITY_sp|Q5RGI	mysm1	Histone H2A deubiqu	47.00	0.00
TRINITY_sp|P453(	Akr1b8	Aldose reductase-re	47.00	0.00

TRINITY_sp|P147	BPT1	Bile pigment transp	47.00	0.00
TRINITY_sp|P491	rps-12	40S ribosomal prote	47.00	0.00
TRINITY_sp|O704	Psma3	Proteasome subunit	47.00	0.00
TRINITY_sp|Q9C6	MTK	Methylthioribose ki	47.00	0.00
TRINITY_sp|B0JZ	agbl5	Cytosolic carboxype	47.00	0.00
TRINITY_sp|Q9M0	At5g014	LIMR family protein	47.00	0.00
TRINITY_sp|Q91V	Smap1	Stromal membrane-as	47.00	0.00
TRINITY_sp|Q9V4	Cyt-b5	Cytochrome b5 OS=Dr	47.00	0.00
TRINITY_sp|Q54E	ykt6	Putative synaptobre	47.00	0.00
TRINITY_sp|C5BS	mtaD	5-methylthioadenosi	47.00	0.00
TRINITY_sp|F4JW	QCR7-2	Cytochrome b-c1 com	47.00	0.00
TRINITY_sp|Q1NZ	F13E9.1	Uncharacterized pro	47.00	0.00
TRINITY_sp|Q9M4	AIP3	Probable prefoldin	47.00	0.00
TRINITY_sp|Q7XA	D27	Beta-carotene isome	47.00	0.00
TRINITY_sp|K7IM	-	Eukaryotic translat	47.00	0.00
TRINITY_sp|Q05D	CTDSPL2	CTD small phosphata	47.00	0.00
TRINITY_sp|Q157	NSDHL	Sterol-4-alpha-carb	47.00	0.00
TRINITY_sp|Q953	MANBA	Beta-mannosidase OS	47.00	0.00
TRINITY_sp|Q55D	sdad1	Protein SDA1 homolo	47.00	0.00
TRINITY_sp|Q928	GGH	Gamma-glutamyl hydr	47.00	0.00
TRINITY_sp|Q9SS	SDP6	Glycerol-3-phosphat	47.00	0.00
TRINITY_sp|O803	FPG1	Formamidopyrimidine	47.00	0.00
TRINITY_sp|Q54T	mrkC	Probable serine/thr	47.00	0.00
TRINITY_sp|Q9SX	TOM40-2	Probable mitochondr	47.00	0.00
TRINITY_sp|Q9BS	NOL10	Nucleolar protein 1	47.00	0.00
TRINITY_sp|O748	caf1	Poly(A) ribonucleas	47.00	0.00
TRINITY_sp|P372	PSY1	Phytoene synthase,	47.00	0.00
TRINITY_sp|Q631	Dnah1	Dynein heavy chain	47.00	0
TRINITY_sp|Q9H0	NAT10	RNA cytidine acetyl	47.00	0.00
TRINITY_sp|Q3V0	Dnah12	Dynein heavy chain	47.00	0.00
TRINITY_sp|P0C5	mic-33	Putative mitochondr	47.00	0.00
TRINITY_sp|P364	rab2A	Ras-related protein	47.00	0.00
TRINITY_sp|Q1EA	CCR4	Glucose-repressible	47.00	0.00
TRINITY_sp|P043	pol	Retrovirus-related	47.00	0.00
TRINITY_sp|Q9S7	MYB98	Transcription facto	46.90	0.00
TRINITY_sp|Q9ES	Abca2	ATP-binding cassett	46.90	0.00
TRINITY_sp|Q86K	fhit	Bis(5'-adenosyl)-tr	46.90	0.00
TRINITY_sp|Q8R2	Nabp2	SOSS complex subuni	46.90	0.00
TRINITY_sp|A1L1	ptpdc1	Protein tyrosine ph	46.90	0.00
TRINITY_sp|Q9V9	l(2)045	Probable methylcrot	46.90	0.00
TRINITY_sp|B9N1	POPTR_0	Probable bifunction	46.90	0.00
TRINITY_sp|P215	ALP1	Alpha-amylase OS=Sa	46.90	0.00
TRINITY_sp|O158	CPK2	Calcium-dependent p	46.90	0.00
TRINITY_sp|Q86H	gmppA	Mannose-1-phosphate	46.90	0.00
TRINITY_sp|Q652	PHT4;4	Probable anion tran	46.90	0.00
TRINITY_sp|O430	pre3	Probable proteasome	46.90	0.00
TRINITY_sp|O199	pbsA	Heme oxygenase OS=R	46.90	0.00
TRINITY_sp|A8IL	CFAP52	Cilia- and flagella	46.90	0.00
TRINITY_sp|Q55B	yod1	Ubiquitin thioester	46.90	0.00
TRINITY_sp|Q9LD	BETAA-A	Beta-adaptin-like p	46.90	0.00
TRINITY_sp|Q55C	ppp2r4A	Probable serine/thr	46.90	0.00
TRINITY_sp|Q9M2	RRP45B	Exosome complex com	46.90	0.00
TRINITY_sp|O597	gyp1	GTPase-activating p	46.90	0.00
TRINITY_sp|Q7SX	dnm1l	Dynamin-1-like prot	46.90	0.00
TRINITY_sp|Q9UH	SLC23A1	Solute carrier fami	46.90	0.00
TRINITY_sp|Q52T	PAT24	Protein S-acyltrans	46.90	0.00

TRINITY_sp|Q9M9H	SOL1	Carboxypeptidase SO	46.90	0.00
TRINITY_sp|Q8BW7	Kdm4a	Lysine-specific dem	46.90	0.00
TRINITY_sp|Q8XC0	cysQ	3'(2'),5'-bisphosph	46.90	0.00
TRINITY_sp|Q2RK2	obg	GTPase Obg OS=Moore	46.90	0.00
TRINITY_sp|Q8RY1	At5g435(	MATH domain-contain	46.90	0.00
TRINITY_sp|Q9SZV	MitHPPK/	Folate synthesis bi	46.90	0.00
TRINITY_sp|Q5EA9	MFAP1	Microfibrillar-asso	46.90	0.00
TRINITY_sp|Q7TN(	Ldhd	Probable D-lactate	46.90	0.00
TRINITY_sp|Q54H>	mybI	Myb-like protein I	46.90	0.00
TRINITY_sp|Q9SRI	ENGASE2	Cytosolic endo-beta	46.90	0.00
TRINITY_sp|A8IU(	CFAP99	Cilia- and flagella	46.90	0.00
TRINITY_sp|Q5D01	rbm8a	RNA-binding protein	46.90	0.00
TRINITY_sp|P1024	MYBL1	Myb-related protein	46.90	0.00
TRINITY_sp|P094(	mlcE	Myosin, essential l	46.90	0.00
TRINITY_sp|Q10QH	UROS	Uroporphyrinogen-II	46.90	0.00
TRINITY_sp|Q9Y6M	SLC30A1	Zinc transporter 1	46.90	0.00
TRINITY_sp|Q9ST1	ABCA4	ABC transporter A f	46.90	0.00
TRINITY_sp|Q8LC(	At3g457	Probable trans-2-en	46.90	0.00
TRINITY_sp|Q9SX3	ALA9	Putative phospholip	46.90	0.00
TRINITY_sp|Q54Y(	trappc5	Trafficking protein	46.90	0.00
TRINITY_sp|Q9SP(	TERT	Telomerase reverse	46.90	0.00
TRINITY_sp|P1097	-	Retrovirus-related	46.90	0.00
TRINITY_sp|Q1016	SPAC26A3	Hydrolase C26A3.11	46.90	0.00
TRINITY_sp|Q9SB4	PDK	[Pyruvate dehydroge	46.90	0.00
TRINITY_sp|P5344	CTN	Caltractin OS=Naegl	46.80	0.00
TRINITY_sp|Q278(	DYH1B	Cytoplasmic dynein	46.80	0.00
TRINITY_sp|O3498	yodQ	Uncharacterized met	46.80	0.00
TRINITY_sp|C6Y4I	SPCC16C4	Putative transcript	46.80	0.00
TRINITY_sp|D9HP2	CNR4	Cell number regulat	46.80	0.00
TRINITY_sp|Q54D9	DDB_G029	WD repeat-containin	46.80	0.00
TRINITY_sp|Q9CQ9	-	Uncharacterized pro	46.80	0.00
TRINITY_sp|Q6FL8	RPE1	Ribulose-phosphate	46.80	0.00
TRINITY_sp|Q0II3	NAA50	N-alpha-acetyltrans	46.80	0.00
TRINITY_sp|Q2TA1	PRCP	Lysosomal Pro-X car	46.80	0.00
TRINITY_sp|P160(	-	Subtilisin inhibito	46.80	0.00
TRINITY_sp|Q23K2	TTLL3D	Tubulin glycylase 3	46.80	0.00
TRINITY_sp|P0984	-	Hemoglobinase OS=Sc	46.80	0.00
TRINITY_sp|Q86AI	DDB_G027	Probable myosin lig	46.80	0.00
TRINITY_sp|O2837	rpl19e	50S ribosomal prote	46.80	0.00
TRINITY_sp|Q7XUH	Os04g05(	Lysine-specific his	46.80	0.00
TRINITY_sp|Q7Y0H	Os03g04(	Probable nucleoredo	46.80	0.00
TRINITY_sp|Q4244	PAI1	N-(5'-phosphoribosy	46.80	0.00
TRINITY_sp|Q9M82	NSN1	Guanine nucleotide-	46.80	0.00
TRINITY_sp|Q54RE	kmo	Kynurenine 3-monoox	46.80	0.00
TRINITY_sp|Q8IZY	ABCA7	ATP-binding cassett	46.80	0.00
TRINITY_sp|Q8KP(	mnmE	tRNA modification G	46.80	0.00
TRINITY_sp|Q424(	RNU1	U1 small nuclear ri	46.80	0.00
TRINITY_sp|F4JL2	EBS	Chromatin remodelin	46.80	0.00
TRINITY_sp|Q7S04	nhp-1	Non-histone chromos	46.80	0.00
TRINITY_sp|Q55G1	naprt	Nicotinate phosphor	46.80	0.00
TRINITY_sp|P3414	racA	Rho-related protein	46.80	0.00
TRINITY_sp|P2817	pkgB	Protein kinase 2 OS	46.80	0.00
TRINITY_sp|Q9NU2	MDN1	Midasin OS=Homo sap	46.80	0.00
TRINITY_sp|Q942I	PDIL2-2	Protein disulfide i	46.80	0.00
TRINITY_sp|Q8WWH	DNAJB3	DnaJ homolog subfam	46.80	0.00
TRINITY_sp|B0G15	mcfC	Mitochondrial subst	46.80	0.00

TRINITY_sp|Q06AI	CDC27A	Cell division cycle	46.80	0.00
TRINITY_sp|Q5B1(	atm1	Iron-sulfur cluster	46.80	0.00
TRINITY_sp|O231	AP2M	AP-2 complex subuni	46.80	0.00
TRINITY_sp|Q8C1	Thop1	Thimet oligopeptida	46.80	0.00
TRINITY_sp|F4K2	CUV	Pre-mRNA-splicing f	46.80	0.00
TRINITY_sp|Q9FH	DCI1	Delta(3,5)-Delta(2,	46.80	0.00
TRINITY_sp|Q812	Nadsyn1	Glutamine-dependent	46.80	0.00
TRINITY_sp|E4NK	PUB1	U-box domain-contai	46.80	0.00
TRINITY_sp|Q8L7	At1g317	AP-4 complex subuni	46.80	0.00
TRINITY_sp|F4HP	LIG6	DNA ligase 6 OS=Ara	46.80	0.00
TRINITY_sp|A4IF	METTL14	N6-adenosine-methyl	46.80	0.00
TRINITY_sp|Q2RF	rplA	50S ribosomal prote	46.80	0.00
TRINITY_sp|Q9FN	ATXR6	Histone-lysine N-me	46.80	0.00
TRINITY_sp|P227	Gucy1b2	Guanylate cyclase s	46.80	0.00
TRINITY_sp|P221	ACTB	Actin-2 OS=Phytopht	46.80	0.00
TRINITY_sp|Q617	Eif2b4	Translation initiat	46.80	0.00
TRINITY_sp|O244	-	LEC14B homolog OS=P	46.80	0.00
TRINITY_sp|O745	SPCC594.	Uncharacterized pro	46.80	0.00
TRINITY_sp|P146	Pde4b	cAMP-specific 3',5'	46.80	0.00
TRINITY_sp|Q9SA	SYP51	Syntaxin-51 OS=Arab	46.80	0.00
TRINITY_sp|Q9D8	Hm13	Minor histocompatib	46.80	0.00
TRINITY_sp|Q9UB	CTSZ	Cathepsin Z OS=Homo	46.80	0.00
TRINITY_sp|Q9FV	AMT1-3	Ammonium transporte	46.80	0.00
TRINITY_sp|Q949	ADS3	Palmitoyl-monogalac	46.80	0.00
TRINITY_sp|Q9SU	GLDH	L-galactono-1,4-lac	46.80	0.00
TRINITY_sp|Q9W4	CG3527	Ribosomal RNA small	46.80	0.00
TRINITY_sp|Q8R4	P4HB	Protein disulfide-i	46.80	0.00
TRINITY_sp|Q9M1	ABCB21	ABC transporter B f	46.70	0.00
TRINITY_sp|Q6AB	ileS	Isoleucine--tRNA li	46.70	0.00
TRINITY_sp|Q4JH	XBOS36	Probable E3 ubiquit	46.70	0.00
TRINITY_sp|P198	ITIH2	Inter-alpha-trypsin	46.70	0.00
TRINITY_sp|Q5TM	RPP21	Ribonuclease P prot	46.70	0.00
TRINITY_sp|Q9FL	MCM3	DNA replication lic	46.70	0.00
TRINITY_sp|Q8K4	Trib1	Tribbles homolog 1	46.70	0.00
TRINITY_sp|Q395	ODA2	Dynein gamma chain,	46.70	0.00
TRINITY_sp|Q104	alg8	Dolichyl pyrophosph	46.70	0.00
TRINITY_sp|Q08D	MRPL47	39S ribosomal prote	46.70	0.00
TRINITY_sp|A5DL	DBP8	ATP-dependent RNA h	46.70	0.00
TRINITY_sp|Q86H	coq9	Probable ubiquinone	46.70	0.00
TRINITY_sp|Q8MZ	-	Physarolisin OS=Phy	46.70	0.00
TRINITY_sp|O004	PSMD14	26S proteasome non-	46.70	0.00
TRINITY_sp|P511	RAB13	Ras-related protein	46.70	0.00
TRINITY_sp|O288	rtcA	RNA 3'-terminal pho	46.70	0.00
TRINITY_sp|A2BK	pcm	Protein-L-isoaspart	46.70	0.00
TRINITY_sp|Q6I5	CDKC-1	Cyclin-dependent ki	46.70	0.00
TRINITY_sp|Q9LJ	M3KE1	MAP3K epsilon prote	46.70	0.00
TRINITY_sp|P126	YPK1	Serine/threonine-pr	46.70	0.00
TRINITY_sp|Q8C6	Fam221b	Protein FAM221B OS=	46.70	0.00
TRINITY_sp|Q6GP	rnf181	E3 ubiquitin-protei	46.70	0.00
TRINITY_sp|Q9PI	lysA	Diaminopimelate dec	46.70	0.00
TRINITY_sp|Q9I1	treA	Periplasmic trehala	46.70	0.00
TRINITY_sp|Q9M0	ISA3	Isoamylase 3, chlor	46.70	0.00
TRINITY_sp|Q9UT	mrpl19	54S ribosomal prote	46.70	0.00
TRINITY_sp|Q8L7	NAD-ME2	NAD-dependent malic	46.70	0.00
TRINITY_sp|Q9M0	PUX10	Plant UBX domain-co	46.70	0.00
TRINITY_sp|Q3T0	SERP2	Stress-associated e	46.70	0.00

TRINITY_sp|P281	pkgB	Protein kinase 2 OS	46.70	0.00
TRINITY_sp|Q8GU	DGS1	Protein DGS1, mitoc	46.70	0.00
TRINITY_sp|Q9H0	TMEM222	Transmembrane prote	46.70	0.00
TRINITY_sp|Q9C1	SPBC713.	Periodic tryptophan	46.70	0.00
TRINITY_sp|Q84W	At5g121	Elongation factor 1	46.70	0.00
TRINITY_sp|Q9SD	VHA-C	V-type proton ATPas	46.70	0.00
TRINITY_sp|O485	POLD2	DNA polymerase delt	46.70	0.00
TRINITY_sp|Q0JL	Os01g06	Neutral ceramidase	46.70	0.00
TRINITY_sp|Q8MZ	-	Physarolisin OS=Phy	46.70	0.00
TRINITY_sp|P230	-	Dynein beta chain,	46.70	0.00
TRINITY_sp|Q9JM	Ap3b2	AP-3 complex subuni	46.70	0.00
TRINITY_sp|Q999	RNF5	E3 ubiquitin-protei	46.70	0.00
TRINITY_sp|Q6DC	desi2	Desumoylating isope	46.70	0.00
TRINITY_sp|Q69T	PRXIIE-	Peroxiredoxin-2E-1,	46.70	0.00
TRINITY_sp|C3Y4	BRAFLDR	DDRGK domain-contai	46.70	0.00
TRINITY_sp|Q94K	At3g484	Haloacid dehalogena	46.70	0.00
TRINITY_sp|P308	Aldh3a2	Fatty aldehyde dehy	46.70	0.00
TRINITY_sp|Q1H3	cysG	Siroheme synthase O	46.70	0.00
TRINITY_sp|Q5TY	lace1b	Lactation elevated	46.70	0.00
TRINITY_sp|Q9LR	RPL3B	50S ribosomal prote	46.70	0.00
TRINITY_sp|Q396	ODA11	Dynein alpha chain,	46.70	0.00
TRINITY_sp|B7GY	rlmE	Ribosomal RNA large	46.70	0.00
TRINITY_sp|O750	ABCB7	ATP-binding cassett	46.70	0.00
TRINITY_sp|Q9LE	SSRP1	FACT complex subuni	46.70	0.00
TRINITY_sp|O154	ABCC4	Multidrug resistanc	46.70	0.00
TRINITY_sp|Q9S7	PKL	CHD3-type chromatin	46.70	0.00
TRINITY_sp|Q641	Ttll9	Probable tubulin po	46.70	0.00
TRINITY_sp|Q9UT	vps29	Vacuolar protein so	46.70	0.00
TRINITY_sp|O701	Prep	Prolyl endopeptidas	46.70	0.00
TRINITY_sp|Q54H	drkB	Probable serine/thr	46.70	0.00
TRINITY_sp|Q949	ADS3	Palmitoyl-monogalac	46.70	0.00
TRINITY_sp|Q9C0	DNAH6	Dynein heavy chain	46.70	0.00
TRINITY_sp|P490	AS2	Asparagine syntheta	46.70	0.00
TRINITY_sp|B0BL	znf593	Zinc finger protein	46.60	0.00
TRINITY_sp|Q4WA	psoF	Dual-functional mon	46.60	0.00
TRINITY_sp|P480	gacS	Sensor protein GacS	46.60	0.00
TRINITY_sp|Q9YG	slbp2	Oocyte-specific his	46.60	0.00
TRINITY_sp|P249	rbr	Rubrerythrin OS=Des	46.60	0.00
TRINITY_sp|Q395	ODA2	Dynein gamma chain,	46.60	0.00
TRINITY_sp|Q9XF	MSBP1	Membrane steroid-bi	46.60	0.00
TRINITY_sp|Q9UQ	MOK	MAPK/MAK/MRK overla	46.60	0.00
TRINITY_sp|Q620	Ddx3y	ATP-dependent RNA h	46.60	0.00
TRINITY_sp|Q8R3	Cluap1	Clusterin-associate	46.60	0.00
TRINITY_sp|P546	pkbA	RAC family serine/t	46.60	0.00
TRINITY_sp|Q55C	DDB_G02	DDRGK domain-contai	46.60	0.00
TRINITY_sp|Q8TG	TAR1	Protein TAR1 OS=Sac	46.60	0.00
TRINITY_sp|Q55D	DDB_G02	Probable 18S rRNA (	46.60	0.00
TRINITY_sp|Q5U3	slc25a4	Solute carrier fami	46.60	0.00
TRINITY_sp|Q9CY	Dcp2	m7GpppN-mRNA hydrol	46.60	0.00
TRINITY_sp|Q55D	cdsA	Probable phosphatid	46.60	0.00
TRINITY_sp|Q8CH	Synj1	Synaptojanin-1 OS=M	46.60	0.00
TRINITY_sp|Q10R	Os03g01	Receptor homology r	46.60	0.00
TRINITY_sp|Q9C9	RRA2	Arabinosyltransfera	46.60	0.00
TRINITY_sp|Q54U	trappc4	Trafficking protein	46.60	0.00
TRINITY_sp|A1A5	cnot1	CCR4-NOT transcript	46.60	0.00
TRINITY_sp|Q8L7	BSL1	Serine/threonine-pr	46.60	0.00

TRINITY_sp|Q9I3	prmB	50S ribosomal prote	46.60	0.00
TRINITY_sp|Q9LU	EBP2	Probable rRNA-proce	46.60	0.00
TRINITY_sp|P043	pol	Retrovirus-related	46.60	0.00
TRINITY_sp|O086	Pi4ka	Phosphatidylinosito	46.60	0.00
TRINITY_sp|Q54R	pyk	Pyruvate kinase OS=	46.60	0.00
TRINITY_sp|E5R4	Lema_P0	Methionine aminopep	46.60	0.00
TRINITY_sp|O809	AGD7	ADP-ribosylation fa	46.60	0.00
TRINITY_sp|Q65J	cheB	Chemotaxis response	46.60	0.00
TRINITY_sp|Q54E	ddx55	Probable ATP-depend	46.60	0.00
TRINITY_sp|Q8I7	pol	Retrovirus-related	46.60	0.00
TRINITY_sp|Q8RW	OVA7	Serine--tRNA ligase	46.60	0.00
TRINITY_sp|Q5RJ	Nol10	Nucleolar protein 1	46.60	0.00
TRINITY_sp|Q9WV	Kif9	Kinesin-like protei	46.60	0.00
TRINITY_sp|O486	ARF1	ADP-ribosylation fa	46.60	0.00
TRINITY_sp|Q413	ETR1	Ethylene receptor 1	46.60	0.00
TRINITY_sp|Q9S7	ICS1	Isochorismate synth	46.60	0.00
TRINITY_sp|O221	RHC1A	Probable E3 ubiquit	46.60	0.00
TRINITY_sp|Q72J	lon2	Lon protease 2 OS=T	46.60	0.00
TRINITY_sp|Q9FJ	AAK6	Adenylate kinase is	46.60	0.00
TRINITY_sp|Q8LB	PIS1	CDP-diacylglycerol-	46.60	0.00
TRINITY_sp|Q2QM	BSL2	Serine/threonine-pr	46.60	0.00
TRINITY_sp|Q0P4	tmem14c	Transmembrane prote	46.60	0.00
TRINITY_sp|A2YB	OsI_223	tRNA (guanine-N(7)-	46.50	0.00
TRINITY_sp|F4JL	SFH1	Phosphatidylinosito	46.50	0.00
TRINITY_sp|Q7ZW	polr2g	DNA-directed RNA po	46.50	0.00
TRINITY_sp|Q323	ybiA	N-glycosidase YbiA	46.50	0.00
TRINITY_sp|Q556	DDB_G02	Probable rhodanese	46.50	0.00
TRINITY_sp|Q54N	pex12	Putative peroxisome	46.50	0.00
TRINITY_sp|P546	pkbA	RAC family serine/t	46.50	0.00
TRINITY_sp|P568	TIF3B1	Eukaryotic translat	46.50	0.00
TRINITY_sp|Q9SF	PAH1	Phosphatidate phosp	46.50	0.00
TRINITY_sp|Q3ZB	STIP1	Stress-induced-phos	46.50	0.00
TRINITY_sp|Q993	TY3B-G	Transposon Ty3-G Ga	46.50	0.00
TRINITY_sp|Q54I	tmem120	Transmembrane prote	46.50	0.00
TRINITY_sp|Q9FX	Os10g04	Alpha-galactosidase	46.50	0.00
TRINITY_sp|O355	Pfkfb3	6-phosphofructo-2-k	46.50	0.00
TRINITY_sp|Q54K	DDB_G02	Uncharacterized pro	46.50	0.00
TRINITY_sp|O647	EDA3	Protein EMBRYO SAC	46.50	0.00
TRINITY_sp|Q245	Xpc	DNA repair protein	46.50	0.00
TRINITY_sp|Q54S	wdr3	WD repeat-containin	46.50	0.00
TRINITY_sp|Q54T	DDB_G02	Probable zinc trans	46.50	0.00
TRINITY_sp|Q6P3	NEK5	Serine/threonine-pr	46.50	0.00
TRINITY_sp|P315	RABF2A	Ras-related protein	46.50	0.00
TRINITY_sp|Q55A	snrnp40	U5 small nuclear ri	46.50	0.00
TRINITY_sp|Q8VC	Mic1	Uncharacterized pro	46.50	0.00
TRINITY_sp|Q8CC	Vwa8	von Willebrand fact	46.50	0.00
TRINITY_sp|Q45F	-	Serine/threonine-pr	46.50	0.00
TRINITY_sp|B6IJ	mtp-18	Mitochondrial fissi	46.50	0.00
TRINITY_sp|Q9H5	ALG2	Alpha-1,3/1,6-manno	46.50	0.00
TRINITY_sp|P427	ctpA	Carboxyl-terminal-p	46.50	0.00
TRINITY_sp|A6RD	MRPL4	54S ribosomal prote	46.50	0.00
TRINITY_sp|P221	cta3	Calcium-transportin	46.50	0.00
TRINITY_sp|Q8BG	Zadh2	Prostaglandin reduc	46.50	0.00
TRINITY_sp|Q803	rnf8	E3 ubiquitin-protei	46.50	0.00
TRINITY_sp|A1A4	FAM188B	Probable ubiquitin	46.50	0.00
TRINITY_sp|P340	dhcA	Dynein heavy chain,	46.50	0.00

TRINITY_sp|C0SPI	ytcI	Uncharacterized acy	46.50	0.00
TRINITY_sp|Q9T0/	LACS4	Long chain acyl-CoA	46.50	0.00
TRINITY_sp|Q54F\	cpnE	Copine-E OS=Dictyos	46.50	0.00
TRINITY_sp|Q8I5I	proRS	Proline--tRNA ligas	46.50	0.00
TRINITY_sp|P109	-	Retrovirus-related	46.50	0.00
TRINITY_sp|Q75H	SAPK10	Serine/threonine-pr	46.50	0.00
TRINITY_sp|Q6QN	-	Casein kinase I OS=	46.50	0.00
TRINITY_sp|Q8RXI	BRCA1	Protein BREAST CANC	46.40	0.00
TRINITY_sp|Q9H0I	TMEM222	Transmembrane prote	46.40	0.00
TRINITY_sp|A0BD	Rpl7-2	60S ribosomal prote	46.40	0.00
TRINITY_sp|Q6UDI	ManS	Mannan synthase 1 O	46.40	0.00
TRINITY_sp|Q54L(	DDB_G02{	Probable LIM domain	46.40	0.00
TRINITY_sp|P458!	mmgB	Probable 3-hydroxyb	46.40	0.00
TRINITY_sp|Q8K4I	Nsun5	Probable 28S rRNA (	46.40	0.00
TRINITY_sp|Q96NI	RDH12	Retinol dehydrogena	46.40	0.00
TRINITY_sp|P0CP(	CPK1	Mitogen-activated p	46.40	0.00
TRINITY_sp|Q54PI	DDB_G02{	Probable myosin lig	46.40	0.00
TRINITY_sp|Q6Z3!	Os07g02{	Zinc finger CCCH do	46.40	0.00
TRINITY_sp|Q9LH(	ELC	Protein ELC OS=Arab	46.40	0.00
TRINITY_sp|Q9M8	UGT80A2	Sterol 3-beta-gluco	46.40	0.00
TRINITY_sp|Q8RW/	MEE4	NADH dehydrogenase	46.40	0.00
TRINITY_sp|Q5E9I	BCS1L	Mitochondrial chape	46.40	0.00
TRINITY_sp|Q54M	gpt	Probable alanine am	46.40	0.00
TRINITY_sp|A8WGI	ift122	Intraflagellar tran	46.40	0.00
TRINITY_sp|Q2RK(	rsgA	Putative ribosome b	46.40	0.00
TRINITY_sp|Q8W0	CCR4-1	Carbon catabolite r	46.40	0.00
TRINITY_sp|O222	ACA4	Calcium-transportin	46.40	0.00
TRINITY_sp|Q9P5(	tca-9	Succinate--CoA liga	46.40	0.00
TRINITY_sp|O761	Rab1C	Ras-related protein	46.40	0.00
TRINITY_sp|A2AP\	Ndufaf5	Arginine-hydroxylas	46.40	0.00
TRINITY_sp|Q5RB	DNPEP	Aspartyl aminopepti	46.40	0.00
TRINITY_sp|A2QC\	cbr1	NADH-cytochrome b5	46.40	0.00
TRINITY_sp|Q8H9(	NPC6	Non-specific phosph	46.40	0.00
TRINITY_sp|Q9XYI	fcpA	Probable C-terminal	46.40	0.00
TRINITY_sp|Q5XG	gtpbp1	GTP-binding protein	46.40	0.00
TRINITY_sp|Q54RI	shkA	Dual specificity pr	46.40	0.00
TRINITY_sp|A5D8(	xiap	E3 ubiquitin-protei	46.40	0.00
TRINITY_sp|O749	ria1	Ribosome assembly p	46.40	0.00
TRINITY_sp|P326(	FBP26	Fructose-2,6-bispho	46.40	0.00
TRINITY_sp|O803!	FPG1	Formamidopyrimidine	46.40	0.00
TRINITY_sp|Q3AC	ackA	Acetate kinase OS=C	46.40	0.00
TRINITY_sp|Q2HJ{	RTCA	RNA 3'-terminal pho	46.40	0.00
TRINITY_sp|Q8WU	SDR42E1	Short-chain dehydro	46.40	0.00
TRINITY_sp|Q9LU(	At3g236	Ribosome production	46.40	0.00
TRINITY_sp|P395	DRS2	Probable phospholip	46.40	0.00
TRINITY_sp|O045{	FAD4L1	Fatty acid desatura	46.40	0.00
TRINITY_sp|Q54W	ctns	Cystinosin homolog	46.40	0.00
TRINITY_sp|Q9SK	At2g320	Folate-biopterin tr	46.40	0.00
TRINITY_sp|P053{	PRKACB	cAMP-dependent prot	46.40	0.00
TRINITY_sp|Q555I	rnf160	E3 ubiquitin-protei	46.40	0.00
TRINITY_sp|Q9LKI	XI-2	Myosin-6 OS=Arabido	46.40	0.00
TRINITY_sp|P126{	YPK1	Serine/threonine-pr	46.40	0.00
TRINITY_sp|F1QB\	nipblb	Nipped-B-like prote	46.40	0.00
TRINITY_sp|Q54YI	vps11	Vacuolar protein so	46.40	0.00
TRINITY_sp|Q0UJ	NST1	Stress response pro	46.40	0.00
TRINITY_sp|Q54T/	mrkC	Probable serine/thr	46.40	0.00

TRINITY_sp|Q55E(	act23	Putative actin-23 O	46.40	0.00
TRINITY_sp|Q95YI	pefB	Penta-EF hand domai	46.40	0.00
TRINITY_sp|Q9LM	At1g180	Probable protein ph	46.40	0.00
TRINITY_sp|O154	ABCC5	Multidrug resistanc	46.40	0.00
TRINITY_sp|O239	-	4-hydroxyphenylpyru	46.40	0.00
TRINITY_sp|P499	SRP19	Signal recognition	46.40	0.00
TRINITY_sp|Q631	Dnah7	Dynein heavy chain	46.40	0
TRINITY_sp|P870	DODA	DOPA 4,5-dioxygenas	46.30	0.00
TRINITY_sp|Q4IL	INO80	Putative DNA helica	46.30	0.00
TRINITY_sp|Q917	ephb1-b	Ephrin type-B recep	46.30	0.00
TRINITY_sp|Q425	SCE1	SUMO-conjugating en	46.30	0.00
TRINITY_sp|F1MF	MICAL2	Protein-methionine	46.30	0.00
TRINITY_sp|Q5XJ	kctd15l	BTB/POZ domain-cont	46.30	0.00
TRINITY_sp|Q54YI	copb2	Coatomer subunit be	46.30	0.00
TRINITY_sp|O888	Lect2	Leukocyte cell-deri	46.30	0.00
TRINITY_sp|P0CH	UBC	Polyubiquitin-C OS=	46.30	0.00
TRINITY_sp|O496	CDS2	Phosphatidate cytid	46.30	0.00
TRINITY_sp|O654	XCP1	Cysteine protease X	46.30	0.00
TRINITY_sp|Q8VC	Cant1	Soluble calcium-act	46.30	0.00
TRINITY_sp|P426	yqjG	Glutathionyl-hydroq	46.30	0.00
TRINITY_sp|Q7TS	Nek5	Serine/threonine-pr	46.30	0.00
TRINITY_sp|F4JZ	TERC	Thylakoid membrane	46.30	0.00
TRINITY_sp|Q9UY	sua5	Threonylcarbamoyl-A	46.30	0.00
TRINITY_sp|Q25A	H0410G0	Double-strand break	46.30	0.00
TRINITY_sp|A1SW	lacZ	Beta-galactosidase	46.30	0.00
TRINITY_sp|Q116	tsf	Elongation factor T	46.30	0.00
TRINITY_sp|Q553	DDB_G02	Adenosine deaminase	46.30	0.00
TRINITY_sp|Q54N	bcaA	Branched-chain-amin	46.30	0.00
TRINITY_sp|Q075	sav	Protein SAV OS=Sulf	46.30	0.00
TRINITY_sp|Q5PP	At5g444	Alpha N-terminal pr	46.30	0.00
TRINITY_sp|Q390	ERF1-1	Eukaryotic peptide	46.30	0.00
TRINITY_sp|Q120	TSR3	Ribosome biogenesis	46.30	0.00
TRINITY_sp|Q098	SPAC16C	Putative general ne	46.30	0.00
TRINITY_sp|O952	TNKS	Tankyrase-1 OS=Homo	46.30	0.00
TRINITY_sp|Q9LJ	ABCB19	ABC transporter B f	46.30	0.00
TRINITY_sp|Q9SL	TCX6	Protein tesmin/TSO1	46.30	0.00
TRINITY_sp|O654	XCP1	Cysteine protease X	46.30	0.00
TRINITY_sp|Q9FL	MCM3	DNA replication lic	46.30	0.00
TRINITY_sp|Q9S7	MYB98	Transcription facto	46.30	0.00
TRINITY_sp|Q9HB	RDH14	Retinol dehydrogena	46.30	0.00
TRINITY_sp|F4J0	P4H6	Probable prolyl 4-h	46.30	0.00
TRINITY_sp|Q2TB	VBP1	Prefoldin subunit 3	46.30	0.00
TRINITY_sp|Q8GX	TGH	G patch domain-cont	46.30	0.00
TRINITY_sp|P201	BET2	Geranylgeranyl tran	46.30	0.00
TRINITY_sp|Q9HF	-	Cytochrome b5 OS=Rh	46.30	0.00
TRINITY_sp|Q6Y7	GIGYF2	PERQ amino acid-ric	46.30	0.00
TRINITY_sp|C5FP	sconB	Probable E3 ubiquit	46.30	0.00
TRINITY_sp|B0G1	DDB_G02	Deoxynucleoside tri	46.30	0.00
TRINITY_sp|Q9FJ	At5g607	P-loop NTPase domai	46.30	0.00
TRINITY_sp|Q8R1	Znfx1	NFX1-type zinc fing	46.30	0.00
TRINITY_sp|P459	CBX5	Chromobox protein h	46.30	0.00
TRINITY_sp|Q9C6	LHCA5	Photosystem I chlor	46.30	0.00
TRINITY_sp|O220	ANP3	Mitogen-activated p	46.30	0.00
TRINITY_sp|P693	-	Polyubiquitin (Frag	46.30	0.00
TRINITY_sp|Q610	Hars	Histidine--tRNA lig	46.30	0.00
TRINITY_sp|Q8L5	APRR9	Two-component respo	46.30	0.00

TRINITY_sp|C1C3I	ube2s	Ubiquitin-conjugati	46.30	0.00
TRINITY_sp|Q6NT\	nhp2	H/ACA ribonucleopro	46.30	0.00
TRINITY_sp|F4JII	TCX2	Protein tesmin/TSO1	46.30	0.00
TRINITY_sp|Q9Y3A	COQ4	Ubiquinone biosynth	46.30	0.00
TRINITY_sp|O756S	RP2	Protein XRP2 OS=Hom	46.30	0.00
TRINITY_sp|F4JTI	CHR7	CHD3-type chromatin	46.30	0.00
TRINITY_sp|Q9Y6I	ARFGEF2	Brefeldin A-inhibit	46.20	0.00
TRINITY_sp|Q9C72	At1g550S	Glutamine-dependent	46.20	0.00
TRINITY_sp|Q275(	cah-3	Putative carbonic a	46.20	0.00
TRINITY_sp|Q9FE3	SRT1	NAD-dependent prote	46.20	0.00
TRINITY_sp|Q2KII	EXOSC2	Exosome complex com	46.20	0.00
TRINITY_sp|Q54I4	slc44a2	Choline transporter	46.20	0.00
TRINITY_sp|Q042I	LGAS_134	Putative RNA (cytid	46.20	0.00
TRINITY_sp|Q9M8I	At1g742(	Probable phosphorib	46.20	0.00
TRINITY_sp|Q54KI	DDB_G028	Uncharacterized pro	46.20	0.00
TRINITY_sp|P528	sll0615	GDT1-like protein s	46.20	0.00
TRINITY_sp|Q9N2I	AHSG	Alpha-2-HS-glycopro	46.20	0.00
TRINITY_sp|Q0128	3PCR	Protochlorophyllide	46.20	0.00
TRINITY_sp|Q7F03	CML13	Probable calcium-bi	46.20	0.00
TRINITY_sp|O9662	arcC	Actin-related prote	46.20	0.00
TRINITY_sp|Q9UT(	paa1	Protein phosphatase	46.20	0.00
TRINITY_sp|Q2462	ref(2)P	Protein ref(2)P OS=	46.20	0.00
TRINITY_sp|Q5573	nit1-1	Nitrilase homolog 1	46.20	0.00
TRINITY_sp|Q2HH4	GAR1	H/ACA ribonucleopro	46.20	0.00
TRINITY_sp|Q1101	pepN	Aminopeptidase N OS	46.20	0.00
TRINITY_sp|O654S	XCP1	Cysteine protease X	46.20	0.00
TRINITY_sp|Q9M04	WLIM2B	LIM domain-containi	46.20	0.00
TRINITY_sp|Q54P4	isca2	Iron-sulfur cluster	46.20	0.00
TRINITY_sp|E1BP3	MMS19	MMS19 nucleotide ex	46.20	0.00
TRINITY_sp|O654S	XCP1	Cysteine protease X	46.20	0.00
TRINITY_sp|Q86H3	polr1a	DNA-directed RNA po	46.20	0.00
TRINITY_sp|Q9VII	Nbr	Exonuclease mut-7 h	46.20	0.00
TRINITY_sp|Q5TC8	OGFRL1	Opioid growth facto	46.20	0.00
TRINITY_sp|Q6WWW	UPL3	E3 ubiquitin-protei	46.20	0.00
TRINITY_sp|P344	hcp-3	Histone H3-like cen	46.20	0.00
TRINITY_sp|Q0WPI	ATG18D	Autophagy-related p	46.20	0.00
TRINITY_sp|Q1288	COX10	Protoheme IX farnes	46.20	0.00
TRINITY_sp|P581(	atp2b2	Plasma membrane cal	46.20	0.00
TRINITY_sp|Q4R7Y	GNB2L1	Guanine nucleotide-	46.20	0.00
TRINITY_sp|O704S	Clcn7	H(+)/Cl(-) exchange	46.20	0.00
TRINITY_sp|Q86AI	DDB_G02	Probable myosin lig	46.20	0.00
TRINITY_sp|Q32LS	SDR42E1	Short-chain dehydro	46.20	0.00
TRINITY_sp|Q94K(	ELIP2	Early light-induced	46.20	0.00
TRINITY_sp|Q9NXI	SIDT1	SID1 transmembrane	46.20	0.00
TRINITY_sp|Q7S5(	NCU02305	Probable hexaprenyl	46.20	0.00
TRINITY_sp|O138	not3	General negative re	46.20	0.00
TRINITY_sp|Q6P2(	PRPF8	Pre-mRNA-processing	46.20	0.00
TRINITY_sp|Q8LP3	At5g1398	Probable alpha-mann	46.20	0.00
TRINITY_sp|P1163	NHP6B	Non-histone chromos	46.20	0.00
TRINITY_sp|A7KAI	atg7	Ubiquitin-like modi	46.20	0.00
TRINITY_sp|Q1QZ>	mutS	DNA mismatch repair	46.20	0.00
TRINITY_sp|Q8LAI	PDX2	Probable pyridoxal	46.20	0.00
TRINITY_sp|Q8TC1	RDH11	Retinol dehydrogena	46.20	0.00
TRINITY_sp|Q5U52	AGBL2	Cytosolic carboxype	46.20	0.00
TRINITY_sp|G5E82	Atp2b1	Plasma membrane cal	46.20	0.00
TRINITY_sp|A5PKI	ube2d4	Ubiquitin-conjugati	46.20	0.00

TRINITY_sp|Q6CE1	TRM8	tRNA (guanine-N(7)-	46.20	0.00
TRINITY_sp|Q54HS	apm1	AP-1 complex subuni	46.20	0.00
TRINITY_sp|Q7U0C	fbiC	FO synthase OS=Myco	46.20	0.00
TRINITY_sp|Q0325	RBG8	Glycine-rich RNA-bi	46.20	0.00
TRINITY_sp|Q0WMI	ABCB27	ABC transporter B f	46.20	0.00
TRINITY_sp|B8ACH	ERH1	Phosphatidylinosito	46.20	0.00
TRINITY_sp|Q6DJ2	med10	Mediator of RNA pol	46.20	0.00
TRINITY_sp|A0RU1	proS	Proline--tRNA ligas	46.20	0.00
TRINITY_sp|Q9CR1	Ppid	Peptidyl-prolyl cis	46.20	0.00
TRINITY_sp|Q28Q0	der	GTPase Der OS=Janna	46.20	0.00
TRINITY_sp|Q0DW1	Os02g079	DEAD-box ATP-depend	46.20	0.00
TRINITY_sp|Q9M64	CPI1	Cycloeucalenol cycl	46.20	0.00
TRINITY_sp|Q8JZW	Cpne5	Copine-5 OS=Mus mus	46.20	0.00
TRINITY_sp|Q3961	ODA11	Dynein alpha chain,	46.20	0.00
TRINITY_sp|O0403	CCX5	Cation/calcium exch	46.20	0.00
TRINITY_sp|Q9450	modA	Neutral alpha-gluco	46.20	0.00
TRINITY_sp|Q75HV	CYCP3-1	Cyclin-P3-1 OS=Oryz	46.20	0.00
TRINITY_sp|Q3SX0	PUS3	tRNA pseudouridine(	46.20	0.00
TRINITY_sp|Q58WW	DCAF6	DDB1- and CUL4-asso	46.20	0.00
TRINITY_sp|Q1672	ATP2B3	Plasma membrane cal	46.20	0.00
TRINITY_sp|P5464	pkbA	RAC family serine/t	46.20	0.00
TRINITY_sp|O5496	Rnf13	E3 ubiquitin-protei	46.20	0.00
TRINITY_sp|Q54PW	dph1	Diphthamide biosynt	46.20	0.00
TRINITY_sp|Q1656	CAMK4	Calcium/calmodulin-	46.20	0.00
TRINITY_sp|Q7K0E	Mob4	MOB kinase activato	46.10	0.00
TRINITY_sp|Q9VLU	Wwox	WW domain-containin	46.10	0.00
TRINITY_sp|O949T	TRIM37	E3 ubiquitin-protei	46.10	0.00
TRINITY_sp|Q54S7	vps15	Probable serine/thr	46.10	0.00
TRINITY_sp|E1C65	HACE1	E3 ubiquitin-protei	46.10	0.00
TRINITY_sp|Q4019	RAB11A	Ras-related protein	46.10	0.00
TRINITY_sp|P6282	RAB1A	Ras-related protein	46.10	0.00
TRINITY_sp|P4910	RAB2A	Ras-related protein	46.10	0.00
TRINITY_sp|O2293	CIPK11	CBL-interacting ser	46.10	0.00
TRINITY_sp|P3238	YBT1	ATP-dependent bile	46.10	0.00
TRINITY_sp|P4252	cycB	G2/mitotic-specific	46.10	0.00
TRINITY_sp|P3410	myoD	Myosin ID heavy cha	46.10	0.00
TRINITY_sp|P7725	nemA	N-ethylmaleimide re	46.10	0.00
TRINITY_sp|P5585	smo-1	Small ubiquitin-rel	46.10	0.00
TRINITY_sp|Q5552	abcA4	ABC transporter A f	46.10	0.00
TRINITY_sp|P2664	eef1g-a	Elongation factor 1	46.10	0.00
TRINITY_sp|Q965T	Y57E12AM	Transmembrane prote	46.10	0.00
TRINITY_sp|O7049	Clcn7	H(+)/Cl(-) exchange	46.10	0.00
TRINITY_sp|Q61Y0	CBG03556	Leishmanolysin-like	46.10	0.00
TRINITY_sp|A6QLE	ERI3	ERI1 exoribonucleas	46.10	0.00
TRINITY_sp|Q86IV	ctnA	Countin-1 OS=Dictyo	46.10	0.00
TRINITY_sp|Q8W25	DXR	1-deoxy-D-xylulose	46.10	0.00
TRINITY_sp|Q9STI	CEP2	KDEL-tailed cystein	46.10	0.00
TRINITY_sp|O8051	CYCU4-1	Cyclin-U4-1 OS=Arab	46.10	0.00
TRINITY_sp|P4620	MYB	Transcriptional act	46.10	0.00
TRINITY_sp|Q54KN	cdh1	Anaphase-promoting	46.10	0.00
TRINITY_sp|Q54G7	agxt	Serine--pyruvate am	46.10	0.00
TRINITY_sp|O4885	VAMP725	Vesicle-associated	46.10	0.00
TRINITY_sp|Q7Z7E	CENPV	Centromere protein	46.10	0.00
TRINITY_sp|Q6NPM	HISN7	Bifunctional phosph	46.10	0.00
TRINITY_sp|Q68J4	LIPE	Hormone-sensitive l	46.10	0.00
TRINITY_sp|Q9P6E	ppk15	Serine/threonine-pr	46.10	0.00

TRINITY_sp|Q93WI	At5g193	Rhodanese-like/PpiC	46.10	0.00
TRINITY_sp|Q9VU	CG8745	Alanine--glyoxylate	46.10	0.00
TRINITY_sp|D9R4W	hflX	GTPase HflX OS=Clos	46.10	0.00
TRINITY_sp|Q9SI5	PI4KG7	Phosphatidylinosito	46.10	0.00
TRINITY_sp|A3EWI	REV1	DNA repair protein	46.10	0.00
TRINITY_sp|Q9C0	DNAH6	Dynein heavy chain	46.10	0.00
TRINITY_sp|Q149	Gspt2	Eukaryotic peptide	46.10	0.00
TRINITY_sp|C0LGN	LRR-RLK	Probable leucine-ri	46.10	0.00
TRINITY_sp|Q9FE	SRT1	NAD-dependent prote	46.00	0.00
TRINITY_sp|Q9UJ	TSR3	Ribosome biogenesis	46.00	0.00
TRINITY_sp|Q8LB	GPX8	Probable glutathion	46.00	0.00
TRINITY_sp|Q9LI	NIFU4	NifU-like protein 4	46.00	0.00
TRINITY_sp|P264	PARP1	Poly [ADP-ribose] p	46.00	0.00
TRINITY_sp|Q9M8	DSPTP1B	Dual specificity pr	46.00	0.00
TRINITY_sp|P425	EPS15	Epidermal growth fa	46.00	0.00
TRINITY_sp|Q9R0	Arpc1a	Actin-related prote	46.00	0.00
TRINITY_sp|A6Q4	dnaJ	Chaperone protein D	46.00	0.00
TRINITY_sp|A6QL	ERCC2	TFIIH basal transcr	46.00	0.00
TRINITY_sp|Q5E9	EIF2S2	Eukaryotic translat	46.00	0.00
TRINITY_sp|O075	yhdF	Uncharacterized oxi	46.00	0.00
TRINITY_sp|Q7YX	cpnA	Copine-A OS=Dictyos	46.00	0.00
TRINITY_sp|Q9M8	DJ1D	Protein DJ-1 homolo	46.00	0.00
TRINITY_sp|Q7KWI	wdsof1	DDB1- and CUL4-asso	46.00	0.00
TRINITY_sp|A8I9	CFAP45	Cilia- and flagella	46.00	0.00
TRINITY_sp|Q112	PARP2	Poly [ADP-ribose] p	46.00	0.00
TRINITY_sp|Q133	PPIL2	Peptidyl-prolyl cis	46.00	0.00
TRINITY_sp|P775	yfcG	Disulfide-bond oxid	46.00	0.00
TRINITY_sp|Q86Y	ZGRF1	Protein ZGRF1 OS=Ho	46.00	0.00
TRINITY_sp|Q9NW	RBM28	RNA-binding protein	46.00	0.00
TRINITY_sp|Q1RM	DCUN1D5	DCN1-like protein 5	46.00	0.00
TRINITY_sp|Q558	icmt-1	Protein-S-isoprenyl	46.00	0.00
TRINITY_sp|P285	-	Calcium-dependent p	46.00	0.00
TRINITY_sp|Q6X5	WRI1	Ethylene-responsive	46.00	0.00
TRINITY_sp|Q993	TY3B-G	Transposon Ty3-G Ga	46.00	0.00
TRINITY_sp|Q9EQ	Hsd17b1	Estradiol 17-beta-d	46.00	0.00
TRINITY_sp|Q8C7	Uba6	Ubiquitin-like modi	46.00	0.00
TRINITY_sp|F4IA	At1g059	ATPase family AAA d	46.00	0.00
TRINITY_sp|Q6M9	pc1998	Uncharacterized RNA	46.00	0.00
TRINITY_sp|O227	CB5LP	Cytochrome B5-like	46.00	0.00
TRINITY_sp|Q99M	Bbs2	Bardet-Biedl syndro	46.00	0.00
TRINITY_sp|Q9LE	At3g610	Protein-ribulosamin	46.00	0.00
TRINITY_sp|O810	NRPB5A	DNA-directed RNA po	46.00	0.00
TRINITY_sp|Q9Z3	Dynlt1	Dynein light chain	46.00	0.00
TRINITY_sp|F4K1	ATXR7	Histone-lysine N-me	46.00	0.00
TRINITY_sp|P743	sll1541	Apocarotenoid-15,15	46.00	0.00
TRINITY_sp|Q9C5	TIF3H1	Eukaryotic translat	46.00	0.00
TRINITY_sp|P281	pkgB	Protein kinase 2 OS	46.00	0.00
TRINITY_sp|Q567	dph1	Diphthamide biosynt	46.00	0.00
TRINITY_sp|Q9ND	dom	Helicase domino OS=	46.00	0.00
TRINITY_sp|P109	-	Retrovirus-related	46.00	0.00
TRINITY_sp|P526	Upp1	Uridine phosphoryla	46.00	0.00
TRINITY_sp|Q54Z	srpra	Signal recognition	46.00	0.00
TRINITY_sp|Q54C	hibA	Probable 3-hydroxyi	46.00	0.00
TRINITY_sp|Q94C	RER4	Protein RETICULATA-	46.00	0.00
TRINITY_sp|Q100	SPAC1F5.	Putative oxidoreduc	46.00	0.00
TRINITY_sp|Q8RV	DEK1	Calpain-type cystei	46.00	0.00

TRINITY_sp|Q32NW	leng8	Leukocyte receptor	46.00	0.00
TRINITY_sp|Q9ZQE	COAE	Dephospho-CoA kinas	46.00	0.00
TRINITY_sp|Q8LF2	DRP1C	Dynamin-related pro	46.00	0.00
TRINITY_sp|Q9P4X	utp7	Probable U3 small n	46.00	0.00
TRINITY_sp|Q9BZE	ASPSCR1	Tether containing U	46.00	0.00
TRINITY_sp|Q17QC	TAF9	Transcription initi	46.00	0.00
TRINITY_sp|Q8TFN	-	Thioredoxin-like pr	46.00	0.00
TRINITY_sp|Q6TPE	-	Cysteine proteinase	46.00	0.00
TRINITY_sp|D0N76	PITG_07(	Glutamyl-tRNA(Gln)	45.90	0.00
TRINITY_sp|P5164	arf4	ADP-ribosylation fa	45.90	0.00
TRINITY_sp|O5982	csl4	Exosome complex com	45.90	0.00
TRINITY_sp|F4IRW	ATG1C	Serine/threonine-pr	45.90	0.00
TRINITY_sp|O2292	U2B''	U2 small nuclear ri	45.90	0.00
TRINITY_sp|F4K68	NMNAT	Nicotinamide/nicoti	45.90	0.00
TRINITY_sp|P2817	pkgB	Protein kinase 2 OS	45.90	0.00
TRINITY_sp|Q54D0	mcfP	Mitochondrial subst	45.90	0.00
TRINITY_sp|G3MWE	MICAL3	Protein-methionine	45.90	0.00
TRINITY_sp|Q9MB5	FKFBP	6-phosphofructo-2-k	45.90	0.00
TRINITY_sp|Q0IIE	DDX1	ATP-dependent RNA h	45.90	0.00
TRINITY_sp|Q1565	TRIP4	Activating signal c	45.90	0.00
TRINITY_sp|Q7WHW	hmp	Flavohemoprotein OS	45.90	0.00
TRINITY_sp|Q5U4Y	seh1l	Nucleoporin seh1 OS	45.90	0.00
TRINITY_sp|Q2H2V	END3	Actin cytoskeleton-	45.90	0.00
TRINITY_sp|D0NL0	PITG_128	tRNA (guanine(37)-N	45.90	0.00
TRINITY_sp|A5D7E	DNAJC27	DnaJ homolog subfam	45.90	0.00
TRINITY_sp|Q6P2Y	dnajc2	DnaJ homolog subfam	45.90	0.00
TRINITY_sp|P4799	XDH	Xanthine dehydrogen	45.90	0.00
TRINITY_sp|Q86HN	PLK	Probable serine/thr	45.90	0.00
TRINITY_sp|Q1ENE	CytHPPK/	Folate synthesis bi	45.90	0.00
TRINITY_sp|Q8T66	abcH2	ABC transporter H f	45.90	0.00
TRINITY_sp|Q54IA	cox19	Cytochrome c oxidas	45.90	0.00
TRINITY_sp|Q5T65	CFAP58	Cilia- and flagella	45.90	0.00
TRINITY_sp|P2736	sbpA	Sulfate-binding pro	45.90	0.00
TRINITY_sp|Q9FZ3	ANP2	Mitogen-activated p	45.90	0.00
TRINITY_sp|Q9XGU	ARAC9	Rac-like GTP-bindin	45.90	0.00
TRINITY_sp|C6A38	ogt	Methylated-DNA--pro	45.90	0.00
TRINITY_sp|A6LM3	dnaK	Chaperone protein D	45.90	0.00
TRINITY_sp|P3677	LONP1	Lon protease homolo	45.90	0.00
TRINITY_sp|Q94AW	ATJ3	Chaperone protein d	45.90	0.00
TRINITY_sp|P4963	-	Ubiquitin-60S ribos	45.90	0.00
TRINITY_sp|A8HUA	CFAP58	Cilia- and flagella	45.90	0.00
TRINITY_sp|Q9Y6U	RNF215	RING finger protein	45.90	0.00
TRINITY_sp|B6JC1	grpE	Protein GrpE OS=Oli	45.90	0.00
TRINITY_sp|O4945	At4g284	Uncharacterized pro	45.90	0.00
TRINITY_sp|O3481	yfmJ	Putative NADP-depen	45.90	0.00
TRINITY_sp|P3516	TOR1	Serine/threonine-pr	45.90	0.00
TRINITY_sp|B1XHW	rpsT	30S ribosomal prote	45.90	0.00
TRINITY_sp|Q55BE	raptor	Protein raptor homo	45.90	0.00
TRINITY_sp|Q3899	KIN10	SNF1-related protei	45.90	0.00
TRINITY_sp|Q54ZE	srpra	Signal recognition	45.90	0.00
TRINITY_sp|P5437	sodA	Superoxide dismutas	45.90	0.00
TRINITY_sp|Q9C5N	DTC	Mitochondrial dicar	45.90	0.00
TRINITY_sp|Q9931	TY3B-G	Transposon Ty3-G Ga	45.90	0.00
TRINITY_sp|Q8H7E	GRXS16	Bifunctional monoth	45.90	0.00
TRINITY_sp|Q6K43	TRX1	Histone-lysine N-me	45.90	0.00
TRINITY_sp|Q54I1	DDB_G028	UPF0553 protein OS=	45.90	0.00

TRINITY_sp|P406	MLH1	DNA mismatch repair	45.90	0.00
TRINITY_sp|P410	cut3	Structural maintena	45.90	0.00
TRINITY_sp|Q294	ATP6V0A	V-type proton ATPas	45.90	0.00
TRINITY_sp|O154	ABCC4	Multidrug resistanc	45.90	0.00
TRINITY_sp|O487	SMU2	Suppressor of mec-8	45.90	0.00
TRINITY_sp|P227	Gucy1b2	Guanylate cyclase s	45.90	0.00
TRINITY_sp|Q993	TY3B-G	Transposon Ty3-G Ga	45.90	0.00
TRINITY_sp|Q55F	fam45	Protein FAM45 homol	45.90	0.00
TRINITY_sp|O430	-	Kinesin heavy chain	45.90	0.00
TRINITY_sp|A7GS	folD	Bifunctional protei	45.90	0.00
TRINITY_sp|Q287	PPP2R4	Serine/threonine-pr	45.90	0.00
TRINITY_sp|O607	EIF1B	Eukaryotic translat	45.90	0.00
TRINITY_sp|P710	fabD	Malonyl CoA-acyl ca	45.80	0.00
TRINITY_sp|Q323	ybiA	N-glycosidase YbiA	45.80	0.00
TRINITY_sp|P502	CRIP1	Cysteine-rich prote	45.80	0.00
TRINITY_sp|Q3UD	Ttll12	Tubulin--tyrosine l	45.80	0.00
TRINITY_sp|O955	MPC2	Mitochondrial pyruv	45.80	0.00
TRINITY_sp|O149	GAK	Cyclin-G-associated	45.80	0.00
TRINITY_sp|Q8L9	RPL21M	50S ribosomal prote	45.80	0.00
TRINITY_sp|Q6Y1	HACD2	Very-long-chain (3R	45.80	0.00
TRINITY_sp|Q9XW	acdh-11	Acyl-CoA dehydrogen	45.80	0.00
TRINITY_sp|A0KK	rlmI	Ribosomal RNA large	45.80	0.00
TRINITY_sp|Q5F3	PCMT1	Protein-L-isoaspart	45.80	0.00
TRINITY_sp|Q9FF	RPL13D	60S ribosomal prote	45.80	0.00
TRINITY_sp|P139	-	G2/mitotic-specific	45.80	0.00
TRINITY_sp|A7TS	TRM13	tRNA:m(4)X modifica	45.80	0.00
TRINITY_sp|P147	CRYL1	Lambda-crystallin O	45.80	0.00
TRINITY_sp|Q9P2	DNAH1	Dynein heavy chain	45.80	0.00
TRINITY_sp|P057	-	DNA endonuclease I-	45.80	0.00
TRINITY_sp|B5FZ	ENY2	Transcription and m	45.80	0.00
TRINITY_sp|Q55F	pyd1	Dihydropyrimidine d	45.80	0.00
TRINITY_sp|P546	pkbA	RAC family serine/t	45.80	0.00
TRINITY_sp|Q9P7	rpa2	Probable DNA-direct	45.80	0.00
TRINITY_sp|Q4R5	NOB1	RNA-binding protein	45.80	0.00
TRINITY_sp|Q7XK	BGLU12	Beta-glucosidase 12	45.80	0.00
TRINITY_sp|Q918	aurka-b	Aurora kinase A-B O	45.80	0.00
TRINITY_sp|P004	PAH	Phenylalanine-4-hyd	45.80	0.00
TRINITY_sp|Q9UK	PARP4	Poly [ADP-ribose] p	45.80	0.00
TRINITY_sp|O946	nog1	Probable nucleolar	45.80	0.00
TRINITY_sp|P0C8	CCRP1	Probable serine/thr	45.80	0.00
TRINITY_sp|Q944	dymA	Dynamin-A OS=Dictyo	45.80	0.00
TRINITY_sp|Q9MB	FKFBP	6-phosphofructo-2-k	45.80	0.00
TRINITY_sp|C6HQ	UTP25	U3 small nucleolar	45.80	0.00
TRINITY_sp|Q9ZR	RH28	DEAD-box ATP-depend	45.80	0.00
TRINITY_sp|Q929	CDS1	Phosphatidate cytid	45.80	0.00
TRINITY_sp|Q8TC	RDH11	Retinol dehydrogena	45.80	0.00
TRINITY_sp|Q99M	Pecr	Peroxisomal trans-2	45.80	0.00
TRINITY_sp|Q9NG	kif1	Kinesin-related pro	45.80	0.00
TRINITY_sp|P585	egl	Endoglucanase OS=Ra	45.80	0.00
TRINITY_sp|B3E5	hldE	Bifunctional protei	45.80	0.00
TRINITY_sp|Q993	TY3B-G	Transposon Ty3-G Ga	45.80	0.00
TRINITY_sp|F4IY	UGP3	UTP--glucose-1-phos	45.80	0.00
TRINITY_sp|Q54G	vps45	Vacuolar protein so	45.80	0.00
TRINITY_sp|Q5AT	apdG	Acyl-CoA dehydrogen	45.80	0.00
TRINITY_sp|Q8MV	gbpC	Cyclic GMP-binding	45.80	0.00
TRINITY_sp|Q993	TY3B-G	Transposon Ty3-G Ga	45.80	0.00

TRINITY_sp|P813[illegible]	-	Ferredoxin-A OS=Alo	45.80	0.00
TRINITY_sp|Q9XZ[illegible]	culB	Cullin-2 OS=Dictyos	45.80	0.00
TRINITY_sp|Q8LC[illegible]	At5g085[illegible]	Transcription elong	45.80	0.00
TRINITY_sp|Q69S[illegible]	PDIL5-4	Protein disulfide i	45.80	0.00
TRINITY_sp|Q101[illegible]	dph1	Deubiquitination-pr	45.80	0.00
TRINITY_sp|Q54H[illegible]	drkA	Probable serine/thr	45.80	0.00
TRINITY_sp|Q8LA[illegible]	P4H4	Probable prolyl 4-h	45.80	0.00
TRINITY_sp|Q9FJ[illegible]	CYP65	Peptidyl-prolyl cis	45.80	0.00
TRINITY_sp|Q5CC[illegible]	petC-1	Cytochrome b6-f com	45.80	0.00
TRINITY_sp|Q9M8[illegible]	DJ1D	Protein DJ-1 homolo	45.70	0.00
TRINITY_sp|Q042[illegible]	PDS5	Sister chromatid co	45.70	0.00
TRINITY_sp|P091[illegible]	P4HB	Protein disulfide-i	45.70	0.00
TRINITY_sp|P805[illegible]	TBCA	Tubulin-specific ch	45.70	0.00
TRINITY_sp|P137[illegible]	Msh3	DNA mismatch repair	45.70	0.00
TRINITY_sp|A0AU[illegible]	wdsub1	WD repeat, SAM and	45.70	0.00
TRINITY_sp|Q8WU[illegible]	RAB2B	Ras-related protein	45.70	0.00
TRINITY_sp|Q5RG[illegible]	coq8a	Atypical kinase COQ	45.70	0.00
TRINITY_sp|P132[illegible]	Camk4	Calcium/calmodulin-	45.70	0.00
TRINITY_sp|P546[illegible]	pikB	Phosphatidylinosito	45.70	0.00
TRINITY_sp|Q54T[illegible]	DDB_G02[illegible]	Probable dual speci	45.70	0.00
TRINITY_sp|Q133[illegible]	PLD1	Phospholipase D1 OS	45.70	0.00
TRINITY_sp|Q54T[illegible]	uch1	Probable ubiquitin	45.70	0.00
TRINITY_sp|P811[illegible]	NAPB	Beta-soluble NSF at	45.70	0.00
TRINITY_sp|Q7DM[illegible]	ABCC4	ABC transporter C f	45.70	0.00
TRINITY_sp|Q08B[illegible]	otud5a	OTU domain-containi	45.70	0.00
TRINITY_sp|A5HI[illegible]	-	Actinidain OS=Actin	45.70	0.00
TRINITY_sp|O755[illegible]	MYCBP2	E3 ubiquitin-protei	45.70	0.00
TRINITY_sp|Q54C[illegible]	xpo6	Exportin-6 OS=Dicty	45.70	0.00
TRINITY_sp|Q9NU[illegible]	MDN1	Midasin OS=Homo sap	45.70	0.00
TRINITY_sp|Q9FM[illegible]	PI4KB1	Phosphatidylinosito	45.70	0.00
TRINITY_sp|Q8NS[illegible]	prpB1	Probable 2-methylis	45.70	0.00
TRINITY_sp|P436[illegible]	-	DnaJ protein homolo	45.70	0.00
TRINITY_sp|Q54V[illegible]	DDB_G02[illegible]	Serine carboxypepti	45.70	0.00
TRINITY_sp|O229[illegible]	At2g417[illegible]	Protein N-terminal	45.70	0.00
TRINITY_sp|Q6GN[illegible]	-	UPF0454 protein C12	45.70	0.00
TRINITY_sp|Q0JL[illegible]	SGT1	Protein SGT1 homolo	45.70	0.00
TRINITY_sp|Q84X[illegible]	ILL1	IAA-amino acid hydr	45.70	0.00
TRINITY_sp|Q242[illegible]	eIF-2ga[illegible]	Eukaryotic translat	45.70	0.00
TRINITY_sp|Q201[illegible]	gcs-1	Glutamate--cysteine	45.70	0.00
TRINITY_sp|P423[illegible]	katA	Catalase OS=Proteus	45.70	0.00
TRINITY_sp|O587[illegible]	treT	Trehalose synthase	45.70	0.00
TRINITY_sp|P531[illegible]	ERV14	ER-derived vesicles	45.70	0.00
TRINITY_sp|Q9SC[illegible]	KINB2	SNF1-related protei	45.70	0.00
TRINITY_sp|Q396[illegible]	IDA4	28 kDa inner dynein	45.70	0.00
TRINITY_sp|Q9BX[illegible]	BRIP1	Fanconi anemia grou	45.70	0.00
TRINITY_sp|P109[illegible]	-	Retrovirus-related	45.70	0.00
TRINITY_sp|Q7XV[illegible]	BASS1	Probable sodium/met	45.70	0.00
TRINITY_sp|Q9M8[illegible]	UGT80A2	Sterol 3-beta-gluco	45.70	0.00
TRINITY_sp|Q9FN[illegible]	GRXC2	Glutaredoxin-C2 OS=	45.70	0.00
TRINITY_sp|Q54S[illegible]	rcl1	Probable RNA 3'-ter	45.70	0.00
TRINITY_sp|Q8GW[illegible]	UCH3	Ubiquitin carboxyl-	45.70	0.00
TRINITY_sp|Q75Q[illegible]	CFDP1	Craniofacial develo	45.70	0.00
TRINITY_sp|A1A4[illegible]	ATP9B	Probable phospholip	45.70	0.00
TRINITY_sp|Q8N1[illegible]	CYP4X1	Cytochrome P450 4X1	45.70	0.00
TRINITY_sp|Q8C1[illegible]	Cps1	Carbamoyl-phosphate	45.70	0.00
TRINITY_sp|Q0VA[illegible]	letm1	LETM1 and EF-hand d	45.70	0.00

TRINITY_sp|Q9MAI	DJ1B	Protein DJ-1 homolo	45.70	0.00
TRINITY_sp|Q8L9	P4H7	Probable prolyl 4-h	45.70	0.00
TRINITY_sp|Q0JJS	HCF101	Fe-S cluster assemb	45.70	0.00
TRINITY_sp|P2622	celD	Endoglucanase E-4 O	45.70	0.00
TRINITY_sp|Q9FJI	SMC4	Structural maintena	45.70	0.00
TRINITY_sp|Q9HGN	yta12	Mitochondrial respi	45.70	0.00
TRINITY_sp|Q54HI	csn2	COP9 signalosome co	45.70	0.00
TRINITY_sp|O440(	PYK	Pyruvate kinase OS=	45.70	0.00
TRINITY_sp|Q9931	TY3B-G	Transposon Ty3-G Ga	45.70	0.00
TRINITY_sp|Q22S\	RPL38	60S ribosomal prote	45.70	0.00
TRINITY_sp|P9252	AtMg008(	Uncharacterized mit	45.60	0.00
TRINITY_sp|P3513	UBC8	Ubiquitin-conjugati	45.60	0.00
TRINITY_sp|Q0593	mybl1	Myb-related protein	45.60	0.00
TRINITY_sp|Q0732	PIGF	Phosphatidylinosito	45.60	0.00
TRINITY_sp|Q9VR9	HERC2	Probable E3 ubiquit	45.60	0.00
TRINITY_sp|Q1269	YPK9	Vacuolar cation-tra	45.60	0.00
TRINITY_sp|Q6CGI	YALI0A18	Acyl-protein thioes	45.60	0.00
TRINITY_sp|Q3ZCI	ANAPC11	Anaphase-promoting	45.60	0.00
TRINITY_sp|A8AF	treA	Periplasmic trehala	45.60	0.00
TRINITY_sp|Q6Z8I	Os02g022	Probable protein ph	45.60	0.00
TRINITY_sp|P5361	ARCN1	Coatomer subunit de	45.60	0.00
TRINITY_sp|O601(	SPBC19F5	Uncharacterized pro	45.60	0.00
TRINITY_sp|Q7ZV(	bcs1l	Mitochondrial chape	45.60	0.00
TRINITY_sp|O486	RER1A	Protein RER1A OS=Ar	45.60	0.00
TRINITY_sp|Q7S45	rcf1	Respiratory superco	45.60	0.00
TRINITY_sp|Q2391	kinX	Probable serine/thr	45.60	0.00
TRINITY_sp|Q7RA\	CPK3	Calcium-dependent p	45.60	0.00
TRINITY_sp|Q9VR9	HERC2	Probable E3 ubiquit	45.60	0.00
TRINITY_sp|Q9FMJ	PI4KB1	Phosphatidylinosito	45.60	0.00
TRINITY_sp|P6299	RAC1	Ras-related C3 botu	45.60	0.00
TRINITY_sp|O2365	RABC1	Ras-related protein	45.60	0.00
TRINITY_sp|Q5VJI	gdt9	Probable serine/thr	45.60	0.00
TRINITY_sp|Q8LCU	At3g457	Probable trans-2-en	45.60	0.00
TRINITY_sp|Q54T	DDB_G028	Probable dual speci	45.60	0.00
TRINITY_sp|Q2GZ(	ESF2	Pre-rRNA-processing	45.60	0.00
TRINITY_sp|Q4254	UBC7	Ubiquitin-conjugati	45.60	0.00
TRINITY_sp|Q2R2I	KIN7L	Kinesin-like protei	45.60	0.00
TRINITY_sp|Q9H47	RBKS	Ribokinase OS=Homo	45.60	0.00
TRINITY_sp|Q388(	HSP22.0	22.0 kDa heat shock	45.60	0.00
TRINITY_sp|Q86YS	C2CD5	C2 domain-containin	45.60	0.00
TRINITY_sp|O2814	AF_2138	Phosphoserine phosp	45.60	0.00
TRINITY_sp|Q9SB5	AP4M	AP-4 complex subuni	45.60	0.00
TRINITY_sp|P2944	trxB	Thioredoxin-2 (Frag	45.60	0.00
TRINITY_sp|P5195	Nek1	Serine/threonine-pr	45.60	0.00
TRINITY_sp|Q9UT8	SPAC343.	Uncharacterized WD	45.60	0.00
TRINITY_sp|Q9411	PMS1	DNA mismatch repair	45.60	0.00
TRINITY_sp|A3QJU	coq8b	Atypical kinase COQ	45.60	0.00
TRINITY_sp|P5333	RNH70	RNA exonuclease 1 O	45.60	0.00
TRINITY_sp|Q9WX)	rbsA1	Ribose import ATP-b	45.60	0.00
TRINITY_sp|Q9LN2	P4H3	Probable prolyl 4-h	45.60	0.00
TRINITY_sp|P5487	hgsA	Hydroxymethylglutar	45.60	0.00
TRINITY_sp|O243(	TOP2	DNA topoisomerase 2	45.60	0.00
TRINITY_sp|A0JPI	Ccdc61	Coiled-coil domain-	45.60	0.00
TRINITY_sp|Q0314	Epha2	Ephrin type-A recep	45.60	0.00
TRINITY_sp|Q6GMI	prpf18	Pre-mRNA-splicing f	45.60	0.00
TRINITY_sp|Q9FJI	FKBP65	Peptidyl-prolyl cis	45.60	0.00

TRINITY_sp|Q8T1	fam91	Protein FAM91 homol	45.60	0.00
TRINITY_sp|Q9LZ	ORP3A	Oxysterol-binding p	45.60	0.00
TRINITY_sp|O824	At5g267	Glutamate--tRNA lig	45.60	0.00
TRINITY_sp|Q9AS	RFK1	Probable LRR recept	45.60	0.00
TRINITY_sp|Q993	VCX1	Vacuolar calcium io	45.60	0.00
TRINITY_sp|Q9FW	UBC16	Probable ubiquitin-	45.60	0.00
TRINITY_sp|P296	CDKA-1	Cyclin-dependent ki	45.60	0.00
TRINITY_sp|P332	ARC19	Actin-related prote	45.60	0.00
TRINITY_sp|Q55D	abcG22	ABC transporter G f	45.60	0.00
TRINITY_sp|O001	STXBP3	Syntaxin-binding pr	45.60	0.00
TRINITY_sp|A3EW	REV1	DNA repair protein	45.60	0.00
TRINITY_sp|P743	dfa3	Putative diflavin f	45.60	0.00
TRINITY_sp|Q5JV	EFHC1	EF-hand domain-cont	45.60	0.00
TRINITY_sp|Q86Y	CPNE8	Copine-8 OS=Homo sa	45.60	0.00
TRINITY_sp|O946	plh1	Phospholipid:diacyl	45.60	0.00
TRINITY_sp|Q9M8	UGT80A2	Sterol 3-beta-gluco	45.60	0.00
TRINITY_sp|Q10E	KIN8B	Kinesin-like protei	45.60	0.00
TRINITY_sp|Q10M	GEP	Probable glutamyl e	45.60	0.00
TRINITY_sp|O650	EAAC	Probable envelope A	45.60	0.00
TRINITY_sp|B9A1	SEP1	Thioredoxin reducta	45.50	0.00
TRINITY_sp|P976	Faah	Fatty-acid amide hy	45.50	0.00
TRINITY_sp|Q9UI	HERC5	E3 ISG15--protein l	45.50	0.00
TRINITY_sp|Q8BZ	Anapc2	Anaphase-promoting	45.50	0.00
TRINITY_sp|Q034	frk	Fructokinase OS=Zym	45.50	0.00
TRINITY_sp|O812	TK	Thymidine kinase OS	45.50	0.00
TRINITY_sp|Q390	ATJ13	Chaperone protein d	45.50	0.00
TRINITY_sp|P313	PKAR	cAMP-dependent prot	45.50	0.00
TRINITY_sp|O438	ATP9B	Probable phospholip	45.50	0.00
TRINITY_sp|P391	YCF1	Metal resistance pr	45.50	0.00
TRINITY_sp|Q286	ABCC2	Canalicular multisp	45.50	0.00
TRINITY_sp|Q9VH	Invadoly	Leishmanolysin-like	45.50	0.00
TRINITY_sp|P546	cap	Adenylyl cyclase-as	45.50	0.00
TRINITY_sp|Q9QX	Prss16	Thymus-specific ser	45.50	0.00
TRINITY_sp|Q755	DBP2	ATP-dependent RNA h	45.50	0.00
TRINITY_sp|F1Q4	atp9b	Probable phospholip	45.50	0.00
TRINITY_sp|P934	Os09g05	Cyclin-C1-1 OS=Oryz	45.50	0.00
TRINITY_sp|Q8W4	RH47	DEAD-box ATP-depend	45.50	0.00
TRINITY_sp|P486	pelo	Protein pelota OS=D	45.50	0.00
TRINITY_sp|O611	krsA	Serine/threonine-pr	45.50	0.00
TRINITY_sp|Q49G	pi4kb	Phosphatidylinosito	45.50	0.00
TRINITY_sp|E3QB	UTP25	U3 small nucleolar	45.50	0.00
TRINITY_sp|Q101	abc2	ATP-binding cassett	45.50	0.00
TRINITY_sp|P0CR	SEC23	Protein transport p	45.50	0.00
TRINITY_sp|Q9A8	CC_1201	Putative aldolase c	45.50	0.00
TRINITY_sp|Q8T2	fam49	Protein FAM49 homol	45.50	0.00
TRINITY_sp|Q94A	CDS4	Phosphatidate cytid	45.50	0.00
TRINITY_sp|P872	SPAC16A	Putative RNA polyme	45.50	0.00
TRINITY_sp|Q8WX	DNAH7	Dynein heavy chain	45.50	0
TRINITY_sp|Q8IY	GSPT2	Eukaryotic peptide	45.50	0.00
TRINITY_sp|Q9UI	EVL	Ena/VASP-like prote	45.50	0.00
TRINITY_sp|Q8RW	SD25	G-type lectin S-rec	45.50	0.00
TRINITY_sp|P743	sll0418	2-methyl-6-phytyl-1	45.50	0.00
TRINITY_sp|Q7L8	VASH1	Vasohibin-1 OS=Homo	45.50	0.00
TRINITY_sp|P257	Os04g06	Oryzain alpha chain	45.50	0.00
TRINITY_sp|P0A4	purU	Formyltetrahydrofol	45.50	0.00
TRINITY_sp|Q4V8	Lrrc56	Leucine-rich repeat	45.50	0.00

TRINITY_sp|Q54G5	ascc3	Activating signal c	45.50	0.00
TRINITY_sp|Q65XI	Os05g05	Ketol-acid reductoi	45.50	0.00
TRINITY_sp|Q0WW(	ATG3	Autophagy-related p	45.50	0.00
TRINITY_sp|Q9LS7	PBD1	Proteasome subunit	45.50	0.00
TRINITY_sp|U3KRI	DHQS	3-dehydroquinate sy	45.50	0.00
TRINITY_sp|Q3ZBI	TTC1	Tetratricopeptide r	45.50	0.00
TRINITY_sp|Q9FPI	EDR1	Serine/threonine-pr	45.50	0.00
TRINITY_sp|Q8BG(	Tmem184k	Transmembrane prote	45.50	0.00
TRINITY_sp|Q9FR5	TOR	Serine/threonine-pr	45.50	0.00
TRINITY_sp|Q8IVS	MCAT	Malonyl-CoA-acyl ca	45.50	0.00
TRINITY_sp|P4862	FAD8	Temperature-sensiti	45.50	0.00
TRINITY_sp|P4679	cysB	Cystathionine beta-	45.50	0.00
TRINITY_sp|Q7NDV	rnz	Ribonuclease Z OS=G	45.50	0.00
TRINITY_sp|F4J22	LPPE1	Lipid phosphate pho	45.50	0.00
TRINITY_sp|P1097	-	Retrovirus-related	45.50	0.00
TRINITY_sp|Q9SJI	At1g083	mRNA-decapping enzy	45.50	0.00
TRINITY_sp|B2V6N	lon	Lon protease OS=Sul	45.50	0.00
TRINITY_sp|Q9ZMN	mrp	Iron-sulfur cluster	45.50	0.00
TRINITY_sp|Q8VZ2	ABCC6	ABC transporter C f	45.50	0.00
TRINITY_sp|O4264	ubc6	Ubiquitin-conjugati	45.50	0.00
TRINITY_sp|Q8LFI	LPP3	Putative lipid phos	45.50	0.00
TRINITY_sp|Q6MAS	glgA	Glycogen synthase O	45.50	0.00
TRINITY_sp|Q96UI	radC	DNA repair and reco	45.50	0.00
TRINITY_sp|Q2787	pcm-1	Protein-L-isoaspart	45.50	0.00
TRINITY_sp|Q8LAN	P4H4	Probable prolyl 4-h	45.50	0.00
TRINITY_sp|Q8TD2	CHD6	Chromodomain-helica	45.50	0.00
TRINITY_sp|Q9ZVN	GTF2H2	General transcripti	45.50	0.00
TRINITY_sp|Q6K43	TRX1	Histone-lysine N-me	45.50	0.00
TRINITY_sp|O544(	relA	GTP pyrophosphokina	45.50	0.00
TRINITY_sp|P6101	RAB4B	Ras-related protein	45.50	0.00
TRINITY_sp|P4628	At3g558(	Sedoheptulose-1,7-b	45.50	0.00
TRINITY_sp|Q8W11	SMU1	Suppressor of mec-8	45.40	0.00
TRINITY_sp|P2227	RAS1	Ras-like protein 1	45.40	0.00
TRINITY_sp|Q53FA	TP53I3	Quinone oxidoreduct	45.40	0.00
TRINITY_sp|Q9JJI	No66	Bifunctional lysine	45.40	0.00
TRINITY_sp|P3413	ptpB	Tyrosine-protein ph	45.40	0.00
TRINITY_sp|P3282	SCPL49	Serine carboxypepti	45.40	0.00
TRINITY_sp|Q9P27	USP36	Ubiquitin carboxyl-	45.40	0.00
TRINITY_sp|Q9FMI	RD21B	Probable cysteine p	45.40	0.00
TRINITY_sp|Q54N4	bcaA	Branched-chain-amin	45.40	0.00
TRINITY_sp|Q7RAI	CPK1	Calcium-dependent p	45.40	0.00
TRINITY_sp|O5982	SPCC965.	Putative voltage-ga	45.40	0.00
TRINITY_sp|P2648	fixL	Sensor protein FixL	45.40	0.00
TRINITY_sp|Q76P(	DDB_G02	Probable serine/thr	45.40	0.00
TRINITY_sp|P136(	Atpalpha	Sodium/potassium-tr	45.40	0.00
TRINITY_sp|Q84U(	Os08g015	DEAD-box ATP-depend	45.40	0.00
TRINITY_sp|Q9UD\	DNAJB4	DnaJ homolog subfam	45.40	0.00
TRINITY_sp|Q8L72	GSTT2	Glutathione S-trans	45.40	0.00

TRINITY_sp|Q018	PFKP	ATP-dependent 6-pho	45.40	0.00
TRINITY_sp|Q9LT	NHD1	Sodium/proton antip	45.40	0.00
TRINITY_sp|Q5ZL	STRAP	Serine-threonine ki	45.40	0.00
TRINITY_sp|P230	-	Dynein beta chain,	45.40	0
TRINITY_sp|F4JJ	NDB3	External alternativ	45.40	0.00
TRINITY_sp|Q54D	DDB_G02	Probable serine/thr	45.40	0.00
TRINITY_sp|P546	cprD	Cysteine proteinase	45.40	0.00
TRINITY_sp|Q9FW	HAC12	Histone acetyltrans	45.40	0.00
TRINITY_sp|Q9FM	At5g564	Probable ADP,ATP ca	45.30	0.00
TRINITY_sp|Q3ZB	-	UPF0235 protein C15	45.30	0.00
TRINITY_sp|A0BD	Rpl7-2	60S ribosomal prote	45.30	0.00
TRINITY_sp|Q0IQ	CML3	Calmodulin-like pro	45.30	0.00
TRINITY_sp|Q000	tpsA	Alpha,alpha-trehalo	45.30	0.00
TRINITY_sp|Q6NR	chmp3	Charged multivesicu	45.30	0.00
TRINITY_sp|A0PJ	DHRS12	Dehydrogenase/reduc	45.30	0.00
TRINITY_sp|Q3TX	Trmt1	tRNA (guanine(26)-N	45.30	0.00
TRINITY_sp|P354	-	Stefin-C OS=Bos tau	45.30	0.00
TRINITY_sp|Q86A	atp6v1d	V-type proton ATPas	45.30	0.00
TRINITY_sp|Q58D	SDC2	Syndecan-2 OS=Bos t	45.30	0.00
TRINITY_sp|Q9LZ	BIG2	Brefeldin A-inhibit	45.30	0.00
TRINITY_sp|Q9ZR	CAD	Probable mannitol d	45.30	0.00
TRINITY_sp|Q9ZR	CAD	Probable mannitol d	45.30	0.00
TRINITY_sp|Q75J	aspS1	Aspartate--tRNA lig	45.30	0.00
TRINITY_sp|Q9HC	CPNE5	Copine-5 OS=Homo sa	45.30	0.00
TRINITY_sp|O654	XCP1	Cysteine protease X	45.30	0.00
TRINITY_sp|Q80X	Wnk3	Serine/threonine-pr	45.30	0.00
TRINITY_sp|Q7TS	Nek5	Serine/threonine-pr	45.30	0.00
TRINITY_sp|Q0VG	Zgrf1	Protein ZGRF1 OS=Mu	45.30	0.00
TRINITY_sp|Q4VB	metap1d	Methionine aminopep	45.30	0.00
TRINITY_sp|Q9C5	SMC2-1	Structural maintena	45.30	0.00
TRINITY_sp|A7Z0	PPIP5K1	Inositol hexakispho	45.30	0.00
TRINITY_sp|Q84L	AARE	Acylamino-acid-rele	45.30	0.00
TRINITY_sp|Q920	-	Nuclear factor 7, b	45.30	0.00
TRINITY_sp|P141	AAC4	AAC-rich mRNA clone	45.30	0.00
TRINITY_sp|Q54Q	DDB_G02	Probable E3 ubiquit	45.30	0.00
TRINITY_sp|P519	NEK4	Serine/threonine-pr	45.30	0.00
TRINITY_sp|Q9GR	efa1B	Elongation factor 1	45.30	0.00
TRINITY_sp|O822	At2g239	Probable small nucl	45.30	0.00
TRINITY_sp|Q54Q	dhkG	Hybrid signal trans	45.30	0.00
TRINITY_sp|Q9ER	Cars	Cysteine--tRNA liga	45.30	0.00
TRINITY_sp|A8IR	CFAP53	Cilia- and flagella	45.30	0.00
TRINITY_sp|P732	sll1917	Oxygen-independent	45.30	0.00
TRINITY_sp|Q5UN	MIMI_R7	Uncharacterized pro	45.30	0.00
TRINITY_sp|Q55D	fcsA	Fatty acyl-CoA synt	45.30	0.00
TRINITY_sp|O493	COQ3	Ubiquinone biosynth	45.30	0.00
TRINITY_sp|P118	Aldh3a1	Aldehyde dehydrogen	45.30	0.00
TRINITY_sp|Q9ZR	DSPTP1	Dual specificity pr	45.30	0.00
TRINITY_sp|O435	ATP8B1	Phospholipid-transp	45.30	0.00
TRINITY_sp|O040	At1g089	Phosphoglycerate mu	45.30	0.00
TRINITY_sp|Q9U5	Gtp-bp	Signal recognition	45.30	0.00
TRINITY_sp|Q627	Ddx46	Probable ATP-depend	45.30	0.00
TRINITY_sp|Q6GQ	Cdc14a	Dual specificity pr	45.30	0.00
TRINITY_sp|Q8IU	CBWD2	COBW domain-contain	45.30	0.00
TRINITY_sp|P087	Eip71CD	Peptide methionine	45.30	0.00
TRINITY_sp|Q9M1	CPK23	Calcium-dependent p	45.30	0.00
TRINITY_sp|Q9LD	RRP45A	Exosome complex com	45.30	0.00

TRINITY_sp|Q67PI	rnc	Ribonuclease 3 OS=S	45.30	0.00
TRINITY_sp|Q96HI	TMEM19	Transmembrane prote	45.30	0.00
TRINITY_sp|P9302	PHOT2	Phototropin-2 OS=Ar	45.30	0.00
TRINITY_sp|P5255	mybl2	Myb-related protein	45.30	0.00
TRINITY_sp|Q0DW	Os02g079	DEAD-box ATP-depend	45.30	0.00
TRINITY_sp|Q9NJU	CPK3	Calcium-dependent p	45.30	0.00
TRINITY_sp|P8088	AN1	Ananain OS=Ananas c	45.30	0.00
TRINITY_sp|Q8TV(	pth	Peptidyl-tRNA hydro	45.30	0.00
TRINITY_sp|Q8GWI	PGLP2	Phosphoglycolate ph	45.30	0.00
TRINITY_sp|Q94CI	NAR1	Protein NAR1 OS=Ara	45.30	0.00
TRINITY_sp|Q9NT(	SIRT3	NAD-dependent prote	45.30	0.00
TRINITY_sp|Q54TN	drkD	Probable serine/thr	45.30	0.00
TRINITY_sp|Q54E3	DDB_G029	Probable protein ki	45.30	0.00
TRINITY_sp|Q91Y(	Rab29	Ras-related protein	45.20	0.00
TRINITY_sp|Q4L7N	SH1032	Uncharacterized pro	45.20	0.00
TRINITY_sp|Q9C16	cat-1	Catalase-1 OS=Neuro	45.20	0.00
TRINITY_sp|Q1317	PAK2	Serine/threonine-pr	45.20	0.00
TRINITY_sp|P1370	fs(1)h	Homeotic protein fe	45.20	0.00
TRINITY_sp|P1103	NIA2	Nitrate reductase [	45.20	0.00
TRINITY_sp|Q54P1	abcC8	ABC transporter C f	45.20	0.00
TRINITY_sp|Q6DCI	slc30a9	Zinc transporter 9	45.20	0.00
TRINITY_sp|P2181	RYR1	Ryanodine receptor	45.20	0.00
TRINITY_sp|Q09J2	ODA7	Leucine-rich repeat	45.20	0.00
TRINITY_sp|P5195	NEK3	Serine/threonine-pr	45.20	0.00
TRINITY_sp|Q9C9I	At1g7818	Probable mitochondr	45.20	0.00
TRINITY_sp|Q6Q15	CYP57	Peptidyl-prolyl cis	45.20	0.00
TRINITY_sp|O9567	ASMTL	N-acetylserotonin O	45.20	0.00
TRINITY_sp|P4597	USP5	Ubiquitin carboxyl-	45.20	0.00
TRINITY_sp|Q1112	ofd1	Prolyl 3,4-dihydrox	45.20	0.00
TRINITY_sp|Q8C2I	Kiaa0196	WASH complex subuni	45.20	0.00
TRINITY_sp|Q5BJ2	Carnmt1	Carnosine N-methylt	45.20	0.00
TRINITY_sp|P0679	Ctsl	Cathepsin L1 OS=Mus	45.20	0.00
TRINITY_sp|Q8H0V	ABCF3	ABC transporter F f	45.20	0.00
TRINITY_sp|A2YFI	HK1	Probable histidine	45.20	0.00
TRINITY_sp|P0AD7	ygiC	Putative acid--amin	45.20	0.00
TRINITY_sp|Q6R56	RMA1H1	E3 ubiquitin-protei	45.20	0.00
TRINITY_sp|Q91WN	Rrp9	U3 small nucleolar	45.20	0.00
TRINITY_sp|O0862	Sqstm1	Sequestosome-1 OS=R	45.20	0.00
TRINITY_sp|O2340	PPD1	PsbP domain-contain	45.20	0.00
TRINITY_sp|Q5BKN	chmp3	Charged multivesicu	45.20	0.00
TRINITY_sp|B2S9N	der	GTPase Der OS=Bruce	45.20	0.00
TRINITY_sp|Q2JII	mnmE	tRNA modification G	45.20	0.00
TRINITY_sp|Q9VVN	Cyp312a1	Probable cytochrome	45.20	0.00
TRINITY_sp|P4232	CNB1	Calcineurin subunit	45.20	0.00
TRINITY_sp|Q55GV	pakC	Serine/threonine-pr	45.20	0.00
TRINITY_sp|A1L1I	dnlz	DNL-type zinc finge	45.20	0.00
TRINITY_sp|Q8RWI	UPL6	E3 ubiquitin-protei	45.20	0.00
TRINITY_sp|P8446	LSS	Lanosterol synthase	45.20	0.00
TRINITY_sp|Q2LAI	ASHH2	Histone-lysine N-me	45.20	0.00
TRINITY_sp|Q9XYI	fcpA	Probable C-terminal	45.20	0.00
TRINITY_sp|Q8H13	At1g0696	U2 small nuclear ri	45.20	0.00
TRINITY_sp|A2YFI	HK1	Probable histidine	45.20	0.00
TRINITY_sp|P0432	pol	Retrovirus-related	45.20	0.00
TRINITY_sp|P3192	SPS	Sucrose-phosphate s	45.20	0.00
TRINITY_sp|P9WP2	dapC	Probable N-succinyl	45.20	0.00
TRINITY_sp|Q7ZXI	mtmr4	Myotubularin-relate	45.20	0.00

TRINITY_sp|P3972	AIM1	Altered inheritance	45.20	0.00
TRINITY_sp|Q9P2E	ZNFX1	NFX1-type zinc fing	45.20	0.00
TRINITY_sp|Q2TA1	PRCP	Lysosomal Pro-X car	45.20	0.00
TRINITY_sp|Q54QJ	mcfR	Mitochondrial subst	45.10	0.00
TRINITY_sp|Q9VFE	rdx	Protein roadkill OS	45.10	0.00
TRINITY_sp|P4268	SRK1	Tyrosine-protein ki	45.10	0.00
TRINITY_sp|Q4Z1I	PB000323	Actin-1 OS=Plasmodi	45.10	0.00
TRINITY_sp|P5424	PGIC	Glucose-6-phosphate	45.10	0.00
TRINITY_sp|Q6G3I	ctaA	Heme A synthase OS=	45.10	0.00
TRINITY_sp|Q54GE	ctdspl2	CTD small phosphata	45.10	0.00
TRINITY_sp|Q32PI	PPP2R1A	Serine/threonine-pr	45.10	0.00
TRINITY_sp|O5992	pkaR	cAMP-dependent prot	45.10	0.00
TRINITY_sp|B5X0W	isoc1	Isochorismatase dom	45.10	0.00
TRINITY_sp|Q23M1	Ttll6a	Probable beta-tubul	45.10	0.00
TRINITY_sp|Q3TY0	Atp13a5	Probable cation-tra	45.10	0.00
TRINITY_sp|Q54FM	eif2b4	Translation initiat	45.10	0.00
TRINITY_sp|Q54Y1	mecr	Trans-2-enoyl-CoA r	45.10	0.00
TRINITY_sp|Q9LE8	IRE	Probable serine/thr	45.10	0.00
TRINITY_sp|B8J1A	rplA	50S ribosomal prote	45.10	0.00
TRINITY_sp|Q9P2E	SIPA1L2	Signal-induced prol	45.10	0.00
TRINITY_sp|Q8691	twfA	Twinfilin OS=Dictyo	45.10	0.00
TRINITY_sp|Q8CFE	Dnajc27	DnaJ homolog subfam	45.10	0.00
TRINITY_sp|B9DFX	PAA2	Copper-transporting	45.10	0.00
TRINITY_sp|Q5AT0	apdG	Acyl-CoA dehydrogen	45.10	0.00
TRINITY_sp|P0085	ATP6	ATP synthase subuni	45.10	0.00
TRINITY_sp|Q8H11	MNS2	Mannosyl-oligosacch	45.10	0.00
TRINITY_sp|Q60G0	EXO1	Exonuclease 1 OS=Or	45.10	0.00
TRINITY_sp|O1397	otu1	Putative ubiquitin	45.10	0.00
TRINITY_sp|O3182	yngI	Putative acyl-CoA s	45.10	0.00
TRINITY_sp|Q54X1	DDB_G027	Glucose-induced deg	45.10	0.00
TRINITY_sp|Q653V	NRAMP3	Metal transporter N	45.10	0.00
TRINITY_sp|Q8L77	CLPR3	ATP-dependent Clp p	45.10	0.00
TRINITY_sp|Q54IV	rft1	Protein RFT1 homolo	45.10	0.00
TRINITY_sp|Q29AE	GA17800	Leishmanolysin-like	45.10	0.00
TRINITY_sp|Q6NP0	At5g5113	Probable RNA methyl	45.10	0.00
TRINITY_sp|P5469	myoJ	Myosin-J heavy chai	45.10	0.00
TRINITY_sp|Q9KX7	xyoA	Xylitol oxidase OS=	45.10	0.00
TRINITY_sp|Q7XI1	IRE1	Serine/threonine-pr	45.10	0.00
TRINITY_sp|O7486	fmn1	Riboflavin kinase O	45.10	0.00
TRINITY_sp|O0410	-	Uricase-2 isozyme 2	45.10	0.00
TRINITY_sp|A3KMV	UBA1	Ubiquitin-like modi	45.10	0.00
TRINITY_sp|Q67XX	At5g0655	F-box protein At5g0	45.10	0.00
TRINITY_sp|Q3E90	CPK34	Calcium-dependent p	45.10	0.00
TRINITY_sp|Q67XQ	CBSDUF1	DUF21 domain-contai	45.10	0.00
TRINITY_sp|Q8K20	Alkbh6	Alpha-ketoglutarate	45.10	0.00
TRINITY_sp|Q0WVI	PRMT3	Probable protein ar	45.10	0.00
TRINITY_sp|Q9SE0	SCL33	Serine/arginine-ric	45.10	0.00
TRINITY_sp|Q5Z6X	CIPK25	CBL-interacting pro	45.10	0.00
TRINITY_sp|P5467	pikD	Phosphatidylinosito	45.10	0.00
TRINITY_sp|Q0939	hmg-1.2	High mobility group	45.10	0.00
TRINITY_sp|Q54S8	ech1	Delta(3,5)-Delta(2,	45.10	0.00
TRINITY_sp|Q8T21	top3	DNA topoisomerase 3	45.10	0.00
TRINITY_sp|O8185	IRT2	Fe(2+) transport pr	45.10	0.00
TRINITY_sp|Q9P79	SPBP35G2	Uncharacterized Nud	45.10	0.00
TRINITY_sp|P1658	proA	Alkaline serine exo	45.10	0.00
TRINITY_sp|O9423	mph1	Serine/threonine-pr	45.10	0.00

TRINITY_sp|O492	RH29	Putative DEAD-box A	45.10	0.00
TRINITY_sp|F1QC	rp2	Protein XRP2 OS=Dan	45.10	0.00
TRINITY_sp|Q96M	KCTD7	BTB/POZ domain-cont	45.10	0.00
TRINITY_sp|Q8CC	Nemf	Nuclear export medi	45.10	0.00
TRINITY_sp|A4II	sdad1	Protein SDA1 homolo	45.10	0.00
TRINITY_sp|Q4VB	ier3ip1	Immediate early res	45.10	0.00
TRINITY_sp|D2QZ	ftsH	ATP-dependent zinc	45.10	0.00
TRINITY_sp|Q8SS	gefR	Ras guanine nucleot	45.10	0.00
TRINITY_sp|P538	BOR1	Boron transporter 1	45.10	0.00
TRINITY_sp|P873	tma20	Translation machine	45.10	0.00
TRINITY_sp|Q9H0	NUAK2	NUAK family SNF1-li	45.00	0.00
TRINITY_sp|Q54I	pigm	GPI mannosyltransfe	45.00	0.00
TRINITY_sp|Q54K	talB	Talin-B OS=Dictyost	45.00	0.00
TRINITY_sp|Q9UK	LSM7	U6 snRNA-associated	45.00	0.00
TRINITY_sp|Q96N	ZMAT2	Zinc finger matrin-	45.00	0.00
TRINITY_sp|Q6GQ	tmem135	Transmembrane prote	45.00	0.00
TRINITY_sp|Q6TJ	RPS6KB1	Ribosomal protein S	45.00	0.00
TRINITY_sp|P168	gpaA	Guanine nucleotide-	45.00	0.00
TRINITY_sp|Q9UN	CDC14A	Dual specificity pr	45.00	0.00
TRINITY_sp|Q54K	talB	Talin-B OS=Dictyost	45.00	0.00
TRINITY_sp|Q9WY	TM_0508	Uncharacterized pro	45.00	0.00
TRINITY_sp|Q94A	MKK1	Mitogen-activated p	45.00	0.00
TRINITY_sp|P623	CPK1	Calcium-dependent p	45.00	0.00
TRINITY_sp|Q6S7	TAF5	Transcription initi	45.00	0.00
TRINITY_sp|Q9XY	noxA	Superoxide-generati	45.00	0.00
TRINITY_sp|P178	DDX5	Probable ATP-depend	45.00	0.00
TRINITY_sp|Q9FW	ABCB11	ABC transporter B f	45.00	0.00
TRINITY_sp|Q123	RIB2	Bifunctional protei	45.00	0.00
TRINITY_sp|O273	dnaJ	Chaperone protein D	45.00	0.00
TRINITY_sp|Q9LM	At1g137	Probable 6-phosphog	45.00	0.00
TRINITY_sp|Q9LX	PPD6	PsbP domain-contain	45.00	0.00
TRINITY_sp|Q9S9	VPS28-2	Vacuolar protein so	45.00	0.00
TRINITY_sp|Q54P	4cl1	Probable 4-coumarat	45.00	0.00
TRINITY_sp|F4J0	P4H6	Probable prolyl 4-h	45.00	0.00
TRINITY_sp|Q8T1	fam91	Protein FAM91 homol	45.00	0.00
TRINITY_sp|Q8VX	XEG113	Arabinosyltransfera	45.00	0.00
TRINITY_sp|P160	gpaB	Guanine nucleotide-	45.00	0.00
TRINITY_sp|Q8H0	ABCF3	ABC transporter F f	45.00	0.00
TRINITY_sp|P547	PSBS	Photosystem II 22 k	45.00	0.00
TRINITY_sp|Q631	Dnah1	Dynein heavy chain	45.00	0
TRINITY_sp|P109	-	Retrovirus-related	45.00	0.00
TRINITY_sp|O627	TXNRD1	Thioredoxin reducta	45.00	0.00
TRINITY_sp|Q5EA	MFAP1	Microfibrillar-asso	45.00	0.00
TRINITY_sp|Q8K5	Dus1l	tRNA-dihydrouridine	45.00	0.00
TRINITY_sp|O806	SKIP	SNW/SKI-interacting	45.00	0.00
TRINITY_sp|Q54B	nfx1	Transcriptional rep	45.00	0.00
TRINITY_sp|P546	pkbA	RAC family serine/t	45.00	0.00
TRINITY_sp|Q9P6	pcl1	Fe(2+)/Mn(2+) trans	45.00	0.00
TRINITY_sp|Q993	TY3B-G	Transposon Ty3-G Ga	45.00	0.00
TRINITY_sp|Q417	PFE2	Ferritin-2, chlorop	45.00	0.00
TRINITY_sp|Q9LY	NADP-ME	NADP-dependent mali	45.00	0.00
TRINITY_sp|A5PJ	HYKK	Hydroxylysine kinas	45.00	0.00
TRINITY_sp|Q418	-	ATP synthase subuni	45.00	0.00
TRINITY_sp|Q9FT	ABCG27	ABC transporter G f	45.00	0.00
TRINITY_sp|Q54S	dhkD	Hybrid signal trans	45.00	0.00
TRINITY_sp|Q9Z3	Top3b	DNA topoisomerase 3	44.90	0.00

TRINITY_sp|O489(	POLD1	DNA polymerase delt	44.90	0.00
TRINITY_sp|O009(	-	Lysosomal acid alph	44.90	0.00
TRINITY_sp|O009(	-	Lysosomal acid alph	44.90	0.00
TRINITY_sp|Q6DI5	nme6	Nucleoside diphosph	44.90	0.00
TRINITY_sp|F4IVI	GRV2	DnaJ homolog subfam	44.90	0.00
TRINITY_sp|Q9M0\	MFDX1	Adrenodoxin-like pr	44.90	0.00
TRINITY_sp|Q8VZ\	FIE2	Polycomb group prot	44.90	0.00
TRINITY_sp|Q54P1	abcC8	ABC transporter C f	44.90	0.00
TRINITY_sp|P0C8N	CCRP1	Probable serine/thr	44.90	0.00
TRINITY_sp|Q6365	Grk1	Rhodopsin kinase OS	44.90	0.00
TRINITY_sp|Q6265	Ech1	Delta(3,5)-Delta(2,	44.90	0.00
TRINITY_sp|Q1RM	TRNAU1AI	tRNA selenocysteine	44.90	0.00
TRINITY_sp|A9KH9	lon	Lon protease OS=Clo	44.90	0.00
TRINITY_sp|P552(	BRPF1	Peregrin OS=Homo sa	44.90	0.00
TRINITY_sp|O6549	XCP1	Cysteine protease X	44.90	0.00
TRINITY_sp|Q7G18	FIM1	Fimbrin-1 OS=Arabid	44.90	0.00
TRINITY_sp|P528	Tmem165	Transmembrane prote	44.90	0.00
TRINITY_sp|Q8L7N	TUN	UDP-glycosyltransfe	44.90	0.00
TRINITY_sp|Q9WTI	Lypla2	Acyl-protein thioes	44.90	0.00
TRINITY_sp|Q7ZY(	dcaf13	DDB1- and CUL4-asso	44.90	0.00
TRINITY_sp|Q6AY	Recql	ATP-dependent DNA h	44.90	0.00
TRINITY_sp|Q9532	MANBA	Beta-mannosidase OS	44.90	0.00
TRINITY_sp|Q9M2I	ACA11	Putative calcium-tr	44.90	0.00
TRINITY_sp|O9473	ALG2	Alpha-1,3/1,6-manno	44.90	0.00
TRINITY_sp|Q9LJI	M3KE1	MAP3K epsilon prote	44.90	0.00
TRINITY_sp|Q96F	TRMT61A	tRNA (adenine(58)-N	44.90	0.00
TRINITY_sp|F1QH	mical3a	Protein-methionine	44.90	0.00
TRINITY_sp|Q54N4	clp1	Protein CLP1 homolo	44.90	0.00
TRINITY_sp|O0471	MSH6	DNA mismatch repair	44.90	0.00
TRINITY_sp|Q55C5	glkA	Probable serine/thr	44.90	0.00
TRINITY_sp|Q9VCI	Dcr-1	Endoribonuclease Dc	44.90	0.00
TRINITY_sp|Q5AT(	apdG	Acyl-CoA dehydrogen	44.90	0.00
TRINITY_sp|Q54P5	cpnC	Copine-C OS=Dictyos	44.90	0.00
TRINITY_sp|O222(	CKI1	Histidine kinase CK	44.90	0.00
TRINITY_sp|Q9LXN	HIRA	Protein HIRA OS=Ara	44.90	0.00
TRINITY_sp|Q9P0I	MARK1	Serine/threonine-pr	44.90	0.00
TRINITY_sp|Q76N\	dyrk1	Probable serine/thr	44.90	0.00
TRINITY_sp|Q9VY(	Bap60	Brahma-associated p	44.90	0.00
TRINITY_sp|P7362	sll1770	Uncharacterized pro	44.90	0.00
TRINITY_sp|Q8BW	Kdm4a	Lysine-specific dem	44.90	0.00
TRINITY_sp|Q7TM\	Narfl	Cytosolic Fe-S clus	44.90	0.00
TRINITY_sp|Q94K(	ELIP2	Early light-induced	44.90	0.00
TRINITY_sp|P0432	pol	Retrovirus-related	44.90	0.00
TRINITY_sp|Q6AX(	Isg20l2	Interferon-stimulat	44.90	0.00
TRINITY_sp|Q9M2(	SPL15	Squamosa promoter-b	44.90	0.00
TRINITY_sp|P2332	-	Polyubiquitin OS=Eu	44.90	0.00
TRINITY_sp|Q9P6I	ppk15	Serine/threonine-pr	44.90	0.00
TRINITY_sp|Q9SJI	MEMB11	Membrin-11 OS=Arabi	44.90	0.00
TRINITY_sp|Q8WX	DNAH7	Dynein heavy chain	44.90	0.00
TRINITY_sp|Q9LM	TPS7	Probable alpha,alph	44.90	0.00
TRINITY_sp|O7445	scw1	Cell wall integrity	44.90	0.00
TRINITY_sp|Q9SR(	At3g0232	Probable tRNA (guan	44.90	0.00
TRINITY_sp|Q9ZGI	pikAIII	Narbonolide/10-deox	44.90	0.00
TRINITY_sp|Q76NN	ERD2	ER lumen protein-re	44.90	0.00
TRINITY_sp|Q9ZUI	TOM3	Tobamovirus multipl	44.90	0.00
TRINITY_sp|Q395	ODA2	Dynein gamma chain,	44.90	0.00

TRINITY_sp|A4QN	ctdspl2	CTD small phosphata	44.90	0.00
TRINITY_sp|P120	-	Cyclohexanone 1,2-m	44.80	0.00
TRINITY_sp|Q10G	NEK1	Serine/threonine-pr	44.80	0.00
TRINITY_sp|Q8R0	Trim31	E3 ubiquitin-protei	44.80	0.00
TRINITY_sp|Q9SF	FHA2	FHA domain-containi	44.80	0.00
TRINITY_sp|Q54G	phbA	Prohibitin-1, mitoc	44.80	0.00
TRINITY_sp|P341	pkgC	Protein kinase 3 OS	44.80	0.00
TRINITY_sp|Q5TJ	RING1	E3 ubiquitin-protei	44.80	0.00
TRINITY_sp|A6QR	USP4	Ubiquitin carboxyl-	44.80	0.00
TRINITY_sp|Q55D	gdt6	Probable inactive s	44.80	0.00
TRINITY_sp|Q01J	SPL7	Squamosa promoter-b	44.80	0.00
TRINITY_sp|B8AJ	MRS2-I	Magnesium transport	44.80	0.00
TRINITY_sp|Q9XY	noxA	Superoxide-generati	44.80	0.00
TRINITY_sp|P295	-	Eukaryotic translat	44.80	0.00
TRINITY_sp|Q54P	4cl2	Probable 4-coumarat	44.80	0.00
TRINITY_sp|Q8W2	DXR	1-deoxy-D-xylulose	44.80	0.00
TRINITY_sp|C0LG	LRR-RLK	Probable leucine-ri	44.80	0.00
TRINITY_sp|Q9NR	DDX21	Nucleolar RNA helic	44.80	0.00
TRINITY_sp|O230	KAB1	Probable voltage-ga	44.80	0.00
TRINITY_sp|Q8LP	DMT105	DNA (cytosine-5)-me	44.80	0.00
TRINITY_sp|Q7V6	uppS	Isoprenyl transfera	44.80	0.00
TRINITY_sp|A7SN	v1g2305	UPF0553 protein v1g	44.80	0.00
TRINITY_sp|Q99L	Usp16	Ubiquitin carboxyl-	44.80	0.00
TRINITY_sp|Q9FM	ICMTA	Protein-S-isoprenyl	44.80	0.00
TRINITY_sp|F4KB	AAE17	Probable acyl-activ	44.80	0.00
TRINITY_sp|Q54Y	mvd	Diphosphomevalonate	44.80	0.00
TRINITY_sp|Q9SL	Os05g01	Importin subunit al	44.80	0.00
TRINITY_sp|Q0VC	PQLC1	PQ-loop repeat-cont	44.80	0.00
TRINITY_sp|Q94J	WLIM1	LIM domain-containi	44.80	0.00
TRINITY_sp|Q8BK	Pdhx	Pyruvate dehydrogen	44.80	0.00
TRINITY_sp|Q7XR	YSL6	Probable metal-nico	44.80	0.00
TRINITY_sp|Q9X3	topA	DNA topoisomerase 1	44.80	0.00
TRINITY_sp|Q54T	drkD	Probable serine/thr	44.80	0.00
TRINITY_sp|Q6ZM	EML6	Echinoderm microtub	44.80	0.00
TRINITY_sp|Q9SH	RRP44A	Exosome complex exo	44.80	0.00
TRINITY_sp|Q9V9	CG30152	MIP18 family protei	44.80	0.00
TRINITY_sp|O652	CYP28	Peptidyl-prolyl cis	44.80	0.00
TRINITY_sp|Q0VC	TMEM65	Transmembrane prote	44.80	0.00
TRINITY_sp|Q69V	Os06g05	Probable protein ph	44.80	0.00
TRINITY_sp|P468	KLP1	Kinesin-like protei	44.80	0.00
TRINITY_sp|Q8IC	CPK2	Calcium-dependent p	44.80	0.00
TRINITY_sp|P412	Abca2	ATP-binding cassett	44.80	0.00
TRINITY_sp|P227	Gucy1b2	Guanylate cyclase s	44.80	0.00
TRINITY_sp|P0C6	Dnah2	Dynein heavy chain	44.80	0
TRINITY_sp|F4I7	ALAAT1	Alanine aminotransf	44.80	0.00
TRINITY_sp|Q010	PDE1B	Calcium/calmodulin-	44.80	0.00
TRINITY_sp|A1A5	usp47	Ubiquitin carboxyl-	44.70	0.00
TRINITY_sp|P490	-	Cytochrome b5 OS=Ni	44.70	0.00
TRINITY_sp|P462	At3g558	Sedoheptulose-1,7-b	44.70	0.00
TRINITY_sp|Q5UQ	MIMI_L5	Putative ADP-ribosy	44.70	0.00
TRINITY_sp|P426	DRT111	DNA-damage-repair/t	44.70	0.00
TRINITY_sp|Q3MI	PUS10	Putative tRNA pseud	44.70	0.00
TRINITY_sp|Q9ST	VPS29	Vacuolar protein so	44.70	0.00
TRINITY_sp|Q6PI	FIGNL1	Fidgetin-like prote	44.70	0.00
TRINITY_sp|Q550	DDB_G02	Protein TAPT1 homol	44.70	0.00
TRINITY_sp|A2BE	arid3a	AT-rich interactive	44.70	0.00

TRINITY_sp|Q9BII	B0464.9	Probable protein ph	44.70	0.00
TRINITY_sp|Q1362	CUL4B	Cullin-4B OS=Homo s	44.70	0.00
TRINITY_sp|Q9FG(	EB1C	Microtubule-associa	44.70	0.00
TRINITY_sp|Q394(	VH-PTP13	Dual specificity pr	44.70	0.00
TRINITY_sp|Q86H3	polr1a	DNA-directed RNA po	44.70	0.00
TRINITY_sp|Q3TUI	Yeats2	YEATS domain-contai	44.70	0.00
TRINITY_sp|Q5F44	GPR89	Golgi pH regulator	44.70	0.00
TRINITY_sp|F4NU.	TRM5	tRNA (guanine(37)-N	44.70	0.00
TRINITY_sp|Q8GW1	PMRT15	Protein arginine N-	44.70	0.00
TRINITY_sp|Q28B>	c2cd5	C2 domain-containin	44.70	0.00
TRINITY_sp|Q6UK(	pirA	Protein pirA OS=Dic	44.70	0.00
TRINITY_sp|Q8LP9	LACS6	Long chain acyl-CoA	44.70	0.00
TRINITY_sp|Q54TA	mrkC	Probable serine/thr	44.70	0.00
TRINITY_sp|P2577	Os04g065	Oryzain alpha chain	44.70	0.00
TRINITY_sp|Q8LP(	BTS	Zinc finger protein	44.70	0.00
TRINITY_sp|Q8VZI	PAS2	Very-long-chain (3R	44.70	0.00
TRINITY_sp|Q9SB(	At4g2473	Manganese-dependent	44.70	0.00
TRINITY_sp|Q2424	sw	Cytoplasmic dynein	44.70	0.00
TRINITY_sp|Q7T07	rhbg	Ammonium transporte	44.70	0.00
TRINITY_sp|Q9931	TY3B-G	Transposon Ty3-G Ga	44.70	0.00
TRINITY_sp|Q5NCI	Trappc1	Trafficking protein	44.70	0.00
TRINITY_sp|Q8BYI	Rdh12	Retinol dehydrogena	44.70	0.00
TRINITY_sp|Q3762	ND4L	NADH-ubiquinone oxi	44.70	0.00
TRINITY_sp|Q8WX>	DNAH7	Dynein heavy chain	44.70	0.00
TRINITY_sp|Q3957	ODA2	Dynein gamma chain,	44.70	0.00
TRINITY_sp|Q9WU(	Spint2	Kunitz-type proteas	44.70	0.00
TRINITY_sp|Q9LT2	MED35C	Pre-mRNA-processing	44.70	0.00
TRINITY_sp|P4115	HSFA1A	Heat stress transcr	44.70	0.00
TRINITY_sp|Q9ZPY	PPOX2	Pyridoxine/pyridoxa	44.70	0.00
TRINITY_sp|Q55G9	DDB_G02(	UPF0160 protein OS=	44.70	0.00
TRINITY_sp|Q54V(	odhA	Probable 2-oxogluta	44.70	0.00
TRINITY_sp|Q6DN1	MCTP1	Multiple C2 and tra	44.70	0.00
TRINITY_sp|Q8LB1	CLPR4	ATP-dependent Clp p	44.70	0.00
TRINITY_sp|A2ZV(	FTSH9	ATP-dependent zinc	44.70	0.00
TRINITY_sp|Q8GY9	APX6	Putative L-ascorbat	44.70	0.00
TRINITY_sp|Q8VYN	PFK5	ATP-dependent 6-pho	44.70	0.00
TRINITY_sp|Q3Y41	ckk-1	Calcium/calmodulin-	44.70	0.00
TRINITY_sp|Q9LM7	PAPS1	Nuclear poly(A) pol	44.70	0.00
TRINITY_sp|Q8L85	PIP5K9	Phosphatidylinosito	44.70	0.00
TRINITY_sp|P9251	AtMg0081	Uncharacterized mit	44.70	0.00
TRINITY_sp|P0432	pol	Retrovirus-related	44.70	0.00
TRINITY_sp|Q6DD>	cep76	Centrosomal protein	44.70	0.00
TRINITY_sp|P5271	CBP31	Serine carboxypepti	44.60	0.00
TRINITY_sp|Q6ZNI	ACP7	Acid phosphatase ty	44.60	0.00
TRINITY_sp|Q94A4	At1g6202	Coatomer subunit al	44.60	0.00
TRINITY_sp|Q9V85	lack	E3 ubiquitin-protei	44.60	0.00
TRINITY_sp|Q9Y6(	KPTN	Kaptin OS=Homo sapi	44.60	0.00
TRINITY_sp|Q02AI	thrB	Homoserine kinase O	44.60	0.00
TRINITY_sp|P3412	coaA	Coactosin OS=Dictyo	44.60	0.00
TRINITY_sp|Q9CB(	RABF1	Ras-related protein	44.60	0.00
TRINITY_sp|Q54RN	DDB_G028	NuA4 complex subuni	44.60	0.00
TRINITY_sp|Q54MI	fhkD	Probable serine/thr	44.60	0.00
TRINITY_sp|Q8R3I	Cluap1	Clusterin-associate	44.60	0.00
TRINITY_sp|Q5UP(	MIMI_L92	Putative ankyrin re	44.60	0.00
TRINITY_sp|Q9LJ9	At3g2248	Probable prefoldin	44.60	0.00
TRINITY_sp|Q9WY>	TM_0508	Uncharacterized pro	44.60	0.00

TRINITY_sp|Q5ZKI	ACSBG2	Long-chain-fatty-ac	44.60	0.00
TRINITY_sp|O650	PFC1	Ribosomal RNA small	44.60	0.00
TRINITY_sp|Q5HJ	dus	Probable tRNA-dihyd	44.60	0.00
TRINITY_sp|P427	CLPD	Chaperone protein C	44.60	0.00
TRINITY_sp|Q8DWI	pth	Peptidyl-tRNA hydro	44.60	0.00
TRINITY_sp|Q24C	RPL14	60S ribosomal prote	44.60	0.00
TRINITY_sp|Q54BV	cpras2	Circularly permutat	44.60	0.00
TRINITY_sp|P200	ATP2B1	Plasma membrane cal	44.60	0.00
TRINITY_sp|P0C8I	CCRP1	Probable serine/thr	44.60	0.00
TRINITY_sp|O810	EMB2001	GTP-binding protein	44.60	0.00
TRINITY_sp|Q8H8	Os03g02	Coatomer subunit ga	44.60	0.00
TRINITY_sp|Q89E	cobB2	NAD-dependent prote	44.60	0.00
TRINITY_sp|Q94J	At3g494	Nascent polypeptide	44.60	0.00
TRINITY_sp|P252	-	Cysteine proteinase	44.60	0.00
TRINITY_sp|O188	AOAH	Acyloxyacyl hydrola	44.60	0.00
TRINITY_sp|B4S4	menD	2-succinyl-5-enolpy	44.60	0.00
TRINITY_sp|A1RYI	proS	Proline--tRNA ligas	44.60	0.00
TRINITY_sp|Q2LAI	ASHH2	Histone-lysine N-me	44.60	0.00
TRINITY_sp|Q9S3	pcrA	ATP-dependent DNA h	44.60	0.00
TRINITY_sp|B8BD	LON1	Lon protease homolo	44.60	0.00
TRINITY_sp|Q54BI	DDB_G02	UPF0652 protein OS=	44.60	0.00
TRINITY_sp|P496	PRIM2	DNA primase large s	44.60	0.00
TRINITY_sp|Q9M8	DSPTP1B	Dual specificity pr	44.60	0.00
TRINITY_sp|Q7XJ	GAS8	Growth arrest-speci	44.60	0.00
TRINITY_sp|Q9Y7	set1	Histone-lysine N-me	44.60	0.00
TRINITY_sp|O426	prp22	Pre-mRNA-splicing f	44.60	0.00
TRINITY_sp|O503	bchH	Magnesium-chelatase	44.60	0.00
TRINITY_sp|Q54M	commd6	COMM domain-contain	44.60	0.00
TRINITY_sp|Q54T	mrkC	Probable serine/thr	44.60	0.00
TRINITY_sp|Q9FR	TOR	Serine/threonine-pr	44.60	0.00
TRINITY_sp|Q9Y2	CDYL	Chromodomain Y-like	44.60	0.00
TRINITY_sp|Q9Y2	ARL2BP	ADP-ribosylation fa	44.60	0.00
TRINITY_sp|Q54J	ogdh	2-oxoglutarate dehy	44.60	0.00
TRINITY_sp|Q225	dvc-1	SprT-like domain-co	44.60	0.00
TRINITY_sp|Q6IN	flad1	FAD synthase OS=Xen	44.60	0.00
TRINITY_sp|Q11H	rplP	50S ribosomal prote	44.60	0.00
TRINITY_sp|F4HX	PLA1	Phospholipase A I O	44.60	0.00
TRINITY_sp|Q9FJ	TOP1B	DNA topoisomerase 1	44.60	0.00
TRINITY_sp|Q9XF	SR30	Serine/arginine-ric	44.60	0.00
TRINITY_sp|P707	Atp8a1	Phospholipid-transp	44.60	0.00
TRINITY_sp|Q049	GTB1	Glucosidase 2 subun	44.60	0.00
TRINITY_sp|O622	F45G2.1	MIP18 family protei	44.60	0.00
TRINITY_sp|Q69Q	DPE2	4-alpha-glucanotran	44.60	0.00
TRINITY_sp|P497	VPS41	Vacuolar protein so	44.60	0.00
TRINITY_sp|Q54Z	srpra	Signal recognition	44.60	0.00
TRINITY_sp|O602	SMARCA5	SWI/SNF-related mat	44.60	0.00
TRINITY_sp|Q9BW	SLC4A1A	Kanadaptin OS=Homo	44.60	0.00
TRINITY_sp|Q993	TY3B-G	Transposon Ty3-G Ga	44.60	0.00
TRINITY_sp|B8BM	OsI_374	Pyruvate kinase 2,	44.60	0.00
TRINITY_sp|P044	-	Calmodulin OS=Triti	44.60	0.00
TRINITY_sp|Q909	RAB2A	Ras-related protein	44.50	0.00
TRINITY_sp|Q389	IPP1	Isopentenyl-diphosp	44.50	0.00
TRINITY_sp|O945	SPBC646.	Putative enoyl redu	44.50	0.00
TRINITY_sp|Q395	YPTC1	GTP-binding protein	44.50	0.00
TRINITY_sp|Q9LS	SAMDC3	S-adenosylmethionin	44.50	0.00
TRINITY_sp|Q54L	gghA	Gamma-glutamyl hydr	44.50	0.00

TRINITY_sp|B8G41	nfo	Probable endonuclea	44.50	0.00
TRINITY_sp|Q8L32	FZR2	Protein FIZZY-RELAT	44.50	0.00
TRINITY_sp|P5464	pkbA	RAC family serine/t	44.50	0.00
TRINITY_sp|Q1106	tag-320	Probable protein di	44.50	0.00
TRINITY_sp|P6234	CPK1	Calcium-dependent p	44.50	0.00
TRINITY_sp|P6234	CPK1	Calcium-dependent p	44.50	0.00
TRINITY_sp|Q9VX9	DENR	Density-regulated p	44.50	0.00
TRINITY_sp|P1268	YPK1	Serine/threonine-pr	44.50	0.00
TRINITY_sp|Q9W59	Ns3	Large subunit GTPas	44.50	0.00
TRINITY_sp|P0432	pol	Retrovirus-related	44.50	0.00
TRINITY_sp|Q9M3H	HMA1	Probable cadmium/zi	44.50	0.00
TRINITY_sp|Q5NTH	SK2	Shikimate kinase 2,	44.50	0.00
TRINITY_sp|Q96NR	RDH12	Retinol dehydrogena	44.50	0.00
TRINITY_sp|Q924H	Rad51c	DNA repair protein	44.50	0.00
TRINITY_sp|Q8N52	AADAT	Kynurenine/alpha-am	44.50	0.00
TRINITY_sp|Q8H0W	LPA3	Protein LOW PSII AC	44.50	0.00
TRINITY_sp|Q8L53	5FCL	5-formyltetrahydrof	44.50	0.00
TRINITY_sp|Q1XD0	ycf52	Uncharacterized N-a	44.50	0.00
TRINITY_sp|Q4PE3	SEC23	Protein transport p	44.50	0.00
TRINITY_sp|Q0JL7	Os01g061	DEAD-box ATP-depend	44.50	0.00
TRINITY_sp|Q7TX1	nudC	NADH pyrophosphatas	44.50	0.00
TRINITY_sp|Q9FM0	At5g1405	U3 small nucleolar	44.50	0.00
TRINITY_sp|Q9PUE	copg2	Coatomer subunit ga	44.50	0.00
TRINITY_sp|P5520	GUCY2C	Heat-stable enterot	44.50	0.00
TRINITY_sp|Q5VQ7	Os06g014	Coatomer subunit be	44.50	0.00
TRINITY_sp|O7482	esf1	Pre-rRNA-processing	44.50	0.00
TRINITY_sp|P6101	RAB4B	Ras-related protein	44.50	0.00
TRINITY_sp|O8039	MKK3	Mitogen-activated p	44.50	0.00
TRINITY_sp|Q7X65	VPS35A	Vacuolar protein so	44.50	0.00
TRINITY_sp|C4YKT	RAS1	Ras-like protein 1	44.40	0.00
TRINITY_sp|Q6T48	rbrA	Probable E3 ubiquit	44.40	0.00
TRINITY_sp|Q9ZVY	OBAP1A	Oil body-associated	44.40	0.00
TRINITY_sp|Q05A1	Sdr16c6	Short-chain dehydro	44.40	0.00
TRINITY_sp|Q86A0	polr3a	DNA-directed RNA po	44.40	0.00
TRINITY_sp|Q9XG2	At5g2601	Probable protein ph	44.40	0.00
TRINITY_sp|Q55C9	mpl1	MAP kinase phosphat	44.40	0.00
TRINITY_sp|Q6DFE	ttc37	Tetratricopeptide r	44.40	0.00
TRINITY_sp|Q9UB9	DNAJB9	DnaJ homolog subfam	44.40	0.00
TRINITY_sp|Q3943	RAB1BV	Ras-related protein	44.40	0.00
TRINITY_sp|Q9M35	AGD6	Probable ADP-ribosy	44.40	0.00
TRINITY_sp|Q9096	RAB2A	Ras-related protein	44.40	0.00
TRINITY_sp|Q9NU1	TBC1D22B	TBC1 domain family	44.40	0.00
TRINITY_sp|Q54Y3	mpl3	MAP kinase phosphat	44.40	0.00
TRINITY_sp|Q54TW	pdkA	Probable serine/thr	44.40	0.00
TRINITY_sp|Q0827	MED7	Mediator of RNA pol	44.40	0.00
TRINITY_sp|Q3957	ODA2	Dynein gamma chain,	44.40	0.00
TRINITY_sp|Q9WYX	TM_0508	Uncharacterized pro	44.40	0.00
TRINITY_sp|P9820	Atp8a2	Phospholipid-transp	44.40	0.00
TRINITY_sp|P3282	SCPL49	Serine carboxypepti	44.40	0.00
TRINITY_sp|Q9ZVF	PAHX	Phytanoyl-CoA dioxy	44.40	0.00
TRINITY_sp|Q3EA0	ZIFL2	Probable peptide/ni	44.40	0.00
TRINITY_sp|Q8MJJ	SPG21	Maspardin OS=Bos ta	44.40	0.00
TRINITY_sp|O7511	ATP9A	Probable phospholip	44.40	0.00
TRINITY_sp|Q6P3R	NEK5	Serine/threonine-pr	44.40	0.00
TRINITY_sp|Q8T67	abcG20	ABC transporter G f	44.40	0.00
TRINITY_sp|Q54RA	DDB_G028	Probable iron/ascor	44.40	0.00

TRINITY_sp|P593	Eif5	Eukaryotic translat	44.40	0.00
TRINITY_sp|Q5ZL	NSUN2	tRNA (cytosine(34)-	44.40	0.00
TRINITY_sp|Q6MA	rlmH	Ribosomal RNA large	44.40	0.00
TRINITY_sp|Q5M7	At3g522	RNA pseudouridine s	44.40	0.00
TRINITY_sp|Q96N	CAMK1G	Calcium/calmodulin-	44.40	0.00
TRINITY_sp|Q498	smarcal	SWI/SNF-related mat	44.40	0.00
TRINITY_sp|A2YF	HK1	Probable histidine	44.40	0.00
TRINITY_sp|P0AD	ygiC	Putative acid--amin	44.40	0.00
TRINITY_sp|Q9M7	CITRX	Thioredoxin-like pr	44.40	0.00
TRINITY_sp|Q6GQ	slc35b1	Solute carrier fami	44.40	0.00
TRINITY_sp|Q640	wdr82-a	WD repeat-containin	44.40	0.00
TRINITY_sp|Q631	Dnah7	Dynein heavy chain	44.40	0.00
TRINITY_sp|A8HN	RSP14	Radial spoke protei	44.40	0.00
TRINITY_sp|Q5D1	GBP1	Guanylate-binding p	44.40	0.00
TRINITY_sp|Q9CA	NUDT1	Nudix hydrolase 1 O	44.40	0.00
TRINITY_sp|Q54D	taf1	Transcription initi	44.40	0.00
TRINITY_sp|P153	rpa1	DNA-directed RNA po	44.40	0.00
TRINITY_sp|P470	PRY1	Protein PRY1 OS=Sac	44.40	0.00
TRINITY_sp|Q5RJ	snf8	Vacuolar-sorting pr	44.40	0.00
TRINITY_sp|Q031	Epha2	Ephrin type-A recep	44.40	0.00
TRINITY_sp|B7IF	dnaJ	Chaperone protein D	44.40	0.00
TRINITY_sp|Q9ZR	ABCB1	ABC transporter B f	44.40	0.00
TRINITY_sp|P098	PARP1	Poly [ADP-ribose] p	44.40	0.00
TRINITY_sp|Q921	Chmp1a	Charged multivesicu	44.40	0.00
TRINITY_sp|Q9SK	VPS2.1	Vacuolar protein so	44.40	0.00
TRINITY_sp|B1XJ	rplR	50S ribosomal prote	44.40	0.00
TRINITY_sp|Q9R1	LIPE	Hormone-sensitive l	44.40	0.00
TRINITY_sp|Q93Y	RPN7	26S proteasome non-	44.40	0.00
TRINITY_sp|Q9M5	PEAMT	Phosphoethanolamine	44.40	0.00
TRINITY_sp|Q138	GNL2	Nucleolar GTP-bindi	44.40	0.00
TRINITY_sp|Q9T0	HSFB2B	Heat stress transcr	44.40	0.00
TRINITY_sp|Q4R8	GNL1	Guanine nucleotide-	44.40	0.00
TRINITY_sp|P365	CPOX	Oxygen-dependent co	44.40	0.00
TRINITY_sp|Q9FL	VHA-e1	V-type proton ATPas	44.40	0.00
TRINITY_sp|Q9HE	2E4.130	Regulator of nonsen	44.40	0.00
TRINITY_sp|Q9LZ	ABCC14	ABC transporter C f	44.40	0.00
TRINITY_sp|Q55B	pctA	Ethanolamine-phosph	44.40	0.00
TRINITY_sp|P005	PRKAR1A	cAMP-dependent prot	44.40	0.00
TRINITY_sp|P519	NEK4	Serine/threonine-pr	44.40	0.00
TRINITY_sp|P519	NEK4	Serine/threonine-pr	44.40	0.00
TRINITY_sp|Q4FE	XBAT35	Putative E3 ubiquit	44.40	0.00
TRINITY_sp|Q86C	atg1	Serine/threonine-pr	44.40	0.00
TRINITY_sp|Q9SL	Os05g01	Importin subunit al	44.40	0.00
TRINITY_sp|Q104	pnu1	Nuclease 1, mitocho	44.40	0.00
TRINITY_sp|Q551	fray2	Serine/threonine-pr	44.40	0.00
TRINITY_sp|A6N6	Wdr35	WD repeat-containin	44.40	0.00
TRINITY_sp|Q9SF	CCT7	T-complex protein 1	44.40	0.00
TRINITY_sp|P829	-	Horcolin OS=Hordeum	44.40	0.00
TRINITY_sp|B8AR	SRT1	NAD-dependent prote	44.40	0.00
TRINITY_sp|P110	NIA2	Nitrate reductase [	44.30	0.00
TRINITY_sp|P373	rpiB	Ribose-5-phosphate	44.30	0.00
TRINITY_sp|Q9UP	B9D1	B9 domain-containin	44.30	0.00
TRINITY_sp|P472	ychF	Ribosome-binding AT	44.30	0.00
TRINITY_sp|C1DS	pyrG	CTP synthase OS=Azo	44.30	0.00
TRINITY_sp|O494	At4g284	Uncharacterized pro	44.30	0.00
TRINITY_sp|A6WX	ubiG	Ubiquinone biosynth	44.30	0.00

TRINITY_sp|Q8ICI	CPK2	Calcium-dependent p	44.30	0.00
TRINITY_sp|P0CS(	TRM61	tRNA (adenine(58)-N	44.30	0.00
TRINITY_sp|Q7RVI	NCU0834(	ADP-ribosylation fa	44.30	0.00
TRINITY_sp|A5N6I	dnaJ	Chaperone protein D	44.30	0.00
TRINITY_sp|Q56WI	SCPL48	Serine carboxypepti	44.30	0.00
TRINITY_sp|Q3UHI	Slc2a13	Proton myo-inositol	44.30	0.00
TRINITY_sp|Q140I	CAMK1	Calcium/calmodulin-	44.30	0.00
TRINITY_sp|Q9C0(	DNAH6	Dynein heavy chain	44.30	0.00
TRINITY_sp|P470I	PRY1	Protein PRY1 OS=Sac	44.30	0.00
TRINITY_sp|Q68E(	INTS3	Integrator complex	44.30	0.00
TRINITY_sp|Q043(	RPC11	DNA-directed RNA po	44.30	0.00
TRINITY_sp|Q86A(	DDB_G02	Probable helicase D	44.30	0.00
TRINITY_sp|Q9SEI	DEGP5	Protease Do-like 5,	44.30	0.00
TRINITY_sp|O139(	rrp40	Exosome complex com	44.30	0.00
TRINITY_sp|Q54NI	scrA	Protein SCAR OS=Dic	44.30	0.00
TRINITY_sp|Q85FI	dnaK	Chaperone protein d	44.30	0.00
TRINITY_sp|B5YF(	lon	Lon protease OS=Dic	44.30	0.00
TRINITY_sp|Q5F3I	HCCS	Cytochrome c-type h	44.30	0.00
TRINITY_sp|F4HP2	LIG6	DNA ligase 6 OS=Ara	44.30	0.00
TRINITY_sp|Q550I	ctxB	Cortexillin-2 OS=Di	44.30	0.00
TRINITY_sp|P360(	YKL069W	Free methionine-R-s	44.30	0.00
TRINITY_sp|P056(	sacC	Levanase OS=Bacillu	44.30	0.00
TRINITY_sp|Q9V5(	Cyp4p2	Probable cytochrome	44.30	0.00
TRINITY_sp|Q0J7I	Os08g01	Phosphopantothenate	44.30	0.00
TRINITY_sp|Q9FN	POLL	DNA polymerase lamb	44.30	0.00
TRINITY_sp|Q1LVI	selo	Selenoprotein O OS=	44.30	0.00
TRINITY_sp|P464I	GST1	Glutathione S-trans	44.30	0.00
TRINITY_sp|Q8BI(	Cstf2	Cleavage stimulatio	44.30	0.00
TRINITY_sp|Q54W(	DDB_G02	Probable protein ph	44.30	0.00
TRINITY_sp|Q9SJI	SAE2	SUMO-activating enz	44.30	0.00
TRINITY_sp|Q9FZ(	KINUA	Kinesin-like protei	44.30	0.00
TRINITY_sp|Q9FM(	At5g629	GDSL esterase/lipas	44.30	0.00
TRINITY_sp|Q9SL	At2g200	Protein phosphatase	44.30	0.00
TRINITY_sp|Q8S4I	SQD2	Sulfoquinovosyl tra	44.30	0.00
TRINITY_sp|Q9FWI	AVPL2	Pyrophosphate-energ	44.30	0.00
TRINITY_sp|Q54I(	upf1	Regulator of nonsen	44.30	0.00
TRINITY_sp|Q5W6(	MAN5	Putative mannan end	44.30	0.00
TRINITY_sp|F4HTI	GCS1	Mannosyl-oligosacch	44.30	0.00
TRINITY_sp|P936	LON1	Lon protease homolo	44.30	0.00
TRINITY_sp|Q28I	dnajb14	DnaJ homolog subfam	44.30	0.00
TRINITY_sp|Q8S92	ATG4A	Cysteine protease A	44.30	0.00
TRINITY_sp|Q9LF(	SMC5	Structural maintena	44.30	0.00
TRINITY_sp|Q9STI	CEP2	KDEL-tailed cystein	44.30	0.00
TRINITY_sp|Q7XA	ABCG21	ABC transporter G f	44.30	0.00
TRINITY_sp|Q9LY	CSP41A	Chloroplast stem-lo	44.30	0.00
TRINITY_sp|Q9P2I	IFT80	Intraflagellar tran	44.30	0.00
TRINITY_sp|B2B3I	Pa_6_65(	Very-long-chain 3-o	44.30	0.00
TRINITY_sp|P4052	NEO1	Probable phospholip	44.20	0.00
TRINITY_sp|Q58EI	hibch	3-hydroxyisobutyryl	44.20	0.00
TRINITY_sp|Q2TAI	PRCP	Lysosomal Pro-X car	44.20	0.00
TRINITY_sp|Q149I	Rpusd2	RNA pseudouridylate	44.20	0.00
TRINITY_sp|Q8LGI	ABCC8	ABC transporter C f	44.20	0.00
TRINITY_sp|P453	Akr1b8	Aldose reductase-re	44.20	0.00
TRINITY_sp|Q6GLI	desi1	Desumoylating isope	44.20	0.00
TRINITY_sp|Q2RA(	CYCT1-3	Cyclin-T1-3 OS=Oryz	44.20	0.00
TRINITY_sp|Q54U(	dhkA	Hybrid signal trans	44.20	0.00

TRINITY_sp|P3719	NUP62	Nuclear pore glycop	44.20	0.00
TRINITY_sp|Q8T10	gnt1	[Skp1-protein]-hydr	44.20	0.00
TRINITY_sp|Q1670	MAN2A1	Alpha-mannosidase 2	44.20	0.00
TRINITY_sp|P2230	SCP2	Non-specific lipid-	44.20	0.00
TRINITY_sp|Q86C6	tor	Target of rapamycin	44.20	0.00
TRINITY_sp|O0854	Sec22b	Vesicle-trafficking	44.20	0.00
TRINITY_sp|Q9H93	USP42	Ubiquitin carboxyl-	44.20	0.00
TRINITY_sp|Q54Y0	tcea1	Transcription elong	44.20	0.00
TRINITY_sp|Q941F	GONST1	GDP-mannose transpo	44.20	0.00
TRINITY_sp|Q5XIY	atp6v1c1	V-type proton ATPas	44.20	0.00
TRINITY_sp|Q54MV	ndufa12	NADH dehydrogenase	44.20	0.00
TRINITY_sp|Q9432	SRP19	Signal recognition	44.20	0.00
TRINITY_sp|Q9UG5	PISD	Phosphatidylserine	44.20	0.00
TRINITY_sp|C5PCN	CPC735_(	Putative dipeptidas	44.20	0.00
TRINITY_sp|P4252	erkA	Extracellular signa	44.20	0.00
TRINITY_sp|Q8LF9	GRXC4	Glutaredoxin-C4 OS=	44.20	0.00
TRINITY_sp|Q9LZI	RABE1D	Ras-related protein	44.20	0.00
TRINITY_sp|P6234	CPK4	Calcium-dependent p	44.20	0.00
TRINITY_sp|P3279	AFG2	ATPase family gene	44.20	0.00
TRINITY_sp|O0713	regX3	Sensory transductio	44.20	0.00
TRINITY_sp|Q5F45	PAN2	PAB-dependent poly(	44.20	0.00
TRINITY_sp|O6030	AQR	Intron-binding prot	44.20	0.00
TRINITY_sp|Q8S9F	DIVARICA	Transcription facto	44.20	0.00
TRINITY_sp|Q9VBX	Clbn	Nuclear export medi	44.20	0.00
TRINITY_sp|P4191	-	GTP-binding nuclear	44.20	0.00
TRINITY_sp|B8BM1	OsI_3745	Pyruvate kinase 2,	44.20	0.00
TRINITY_sp|Q2HJ9	FAHD1	Acylpyruvase FAHD1,	44.20	0.00
TRINITY_sp|Q8AWW	rnf128	E3 ubiquitin-protei	44.20	0.00
TRINITY_sp|Q70I5	hdaH	Histone deacetylase	44.20	0.00
TRINITY_sp|Q460N	PARP15	Poly [ADP-ribose] p	44.20	0.00
TRINITY_sp|A8JBE	CHLREDRA	Pescadillo homolog	44.20	0.00
TRINITY_sp|Q9D01	Wdsub1	WD repeat, SAM and	44.20	0.00
TRINITY_sp|Q9LTI	CBSDUF5	DUF21 domain-contai	44.20	0.00
TRINITY_sp|Q54B0	abcB1	ABC transporter B f	44.20	0.00
TRINITY_sp|Q94A9	SRT2	NAD-dependent prote	44.20	0.00
TRINITY_sp|Q1261	OLE1	Acyl-CoA desaturase	44.20	0.00
TRINITY_sp|Q4072	RGP2	Ras-related protein	44.20	0.00
TRINITY_sp|Q54N8	leuS	Leucine--tRNA ligas	44.20	0.00
TRINITY_sp|A2YH4	OsI_0236	Serine/threonine-pr	44.20	0.00
TRINITY_sp|Q8RWE	SRL1	Pre-mRNA splicing f	44.20	0.00
TRINITY_sp|B8DZ9	iscS	Cysteine desulfuras	44.20	0.00
TRINITY_sp|Q3SX0	PUS3	tRNA pseudouridine(	44.20	0.00
TRINITY_sp|Q9VWV	CG6179	Nitric oxide syntha	44.20	0.00
TRINITY_sp|Q86C9	atg1	Serine/threonine-pr	44.20	0.00
TRINITY_sp|Q96GF	RNF185	E3 ubiquitin-protei	44.20	0.00
TRINITY_sp|P5399	SUB1	Activated RNA polym	44.20	0.00
TRINITY_sp|Q7XA4	DODA	4,5-DOPA dioxygenas	44.20	0.00
TRINITY_sp|Q6C4I	DBP2	ATP-dependent RNA h	44.20	0.00
TRINITY_sp|P5431	Lipe	Hormone-sensitive l	44.20	0.00
TRINITY_sp|B9FDE	ALN	Probable allantoina	44.20	0.00
TRINITY_sp|Q69LE	PNP1	Probable polyribonu	44.20	0.00
TRINITY_sp|Q9M36	ATX3	Histone-lysine N-me	44.20	0.00
TRINITY_sp|Q54DE	fdfT	Squalene synthase O	44.20	0.00
TRINITY_sp|Q9KW0	pyc	Pyruvate carboxylas	44.20	0.00
TRINITY_sp|Q84M2	ABCA1	ABC transporter A f	44.20	0.00
TRINITY_sp|G5EE0	daf-18	Phosphatidylinosito	44.20	0.00

TRINITY_sp|P0C5A	-	3'(2'),5'-bisphosph	44.20	0.00
TRINITY_sp|P2587	chcA	Clathrin heavy chai	44.20	0
TRINITY_sp|O2446	RABE1A	Ras-related protein	44.10	0.00
TRINITY_sp|Q9VIE	CG2614	Methyltransferase-l	44.10	0.00
TRINITY_sp|Q8R1E	Hid1	Protein HID1 OS=Mus	44.10	0.00
TRINITY_sp|P5344	CTN	Caltractin OS=Naegl	44.10	0.00
TRINITY_sp|Q5AHA	CHK1	Histidine protein k	44.10	0.00
TRINITY_sp|Q54M4	pigC	Putative phosphatid	44.10	0.00
TRINITY_sp|Q9FR5	TOR	Serine/threonine-pr	44.10	0.00
TRINITY_sp|D3Z6E	Pdia2	Protein disulfide-i	44.10	0.00
TRINITY_sp|Q9US7	rnh1	Ribonuclease H OS=S	44.10	0.00
TRINITY_sp|Q0CCY	gedE	Glutathione S-trans	44.10	0.00
TRINITY_sp|B5XAN	mrpl58	Peptidyl-tRNA hydro	44.10	0.00
TRINITY_sp|P4778	THOP1	Thimet oligopeptida	44.10	0.00
TRINITY_sp|B9G3N	KIN7I	Kinesin-like protei	44.10	0.00
TRINITY_sp|Q9SF9	RABE1E	Ras-related protein	44.10	0.00
TRINITY_sp|P9166	l(2)0365	Probable multidrug	44.10	0.00
TRINITY_sp|Q95V2	carmil	Protein CARMIL OS=D	44.10	0.00
TRINITY_sp|Q8C1A	Abhd18	Protein ABHD18 OS=M	44.10	0.00
TRINITY_sp|B0SHE	def	Peptide deformylase	44.10	0.00
TRINITY_sp|Q8H85	Os03g022	Coatomer subunit ga	44.10	0.00
TRINITY_sp|Q54JE	abcC3	ABC transporter C f	44.10	0.00
TRINITY_sp|Q1502	EXOSC7	Exosome complex com	44.10	0.00
TRINITY_sp|Q6I62	PSS2	CDP-diacylglycerol-	44.10	0.00
TRINITY_sp|F4J7A	PPD7	PsbP domain-contain	44.10	0.00
TRINITY_sp|Q5842	MJ1023	Uncharacterized ABC	44.10	0.00
TRINITY_sp|P1134	Qdpr	Dihydropteridine re	44.10	0.00
TRINITY_sp|O2249	GSH2	Glutathione synthet	44.10	0.00
TRINITY_sp|Q1045	gsk3	Protein kinase gsk3	44.10	0.00
TRINITY_sp|Q8RWE	At5g1196	Probable magnesium	44.10	0.00
TRINITY_sp|P3508	polr2a	DNA-directed RNA po	44.10	0
TRINITY_sp|P0258	TNNC2	Troponin C, skeleta	44.10	0.00
TRINITY_sp|O6761	acpP	Acyl carrier protei	44.10	0.00
TRINITY_sp|O0091	dstA	Signal transducer a	44.10	0.00
TRINITY_sp|Q8VX2	AGAL3	Alpha-galactosidase	44.10	0.00
TRINITY_sp|Q96AJ	CLUAP1	Clusterin-associate	44.10	0.00
TRINITY_sp|A7SWE	v1g19425	Pescadillo homolog	44.10	0.00
TRINITY_sp|A0JPQ	Chid1	Chitinase domain-co	44.10	0.00
TRINITY_sp|Q9FLE	PRA1B3	PRA1 family protein	44.10	0.00
TRINITY_sp|Q9S7I	MYB98	Transcription facto	44.10	0.00
TRINITY_sp|Q54P4	isca2	Iron-sulfur cluster	44.10	0.00
TRINITY_sp|Q3T0X	POLD4	DNA polymerase delt	44.10	0.00
TRINITY_sp|F4JVA	SFH2	Phosphatidylinosito	44.10	0.00
TRINITY_sp|Q1261	OLE1	Acyl-CoA desaturase	44.10	0.00
TRINITY_sp|Q9U64	cmfB	Conditioned medium	44.10	0.00
TRINITY_sp|Q8LG9	At1g2812	Ubiquitin thioester	44.10	0.00
TRINITY_sp|A2QPQ	eglB	Probable endo-beta-	44.10	0.00
TRINITY_sp|Q8BYE	Rdh12	Retinol dehydrogena	44.10	0.00
TRINITY_sp|Q91V3	Atp6v0b	V-type proton ATPas	44.10	0.00
TRINITY_sp|Q0053	NAM8	Protein NAM8 OS=Sac	44.10	0.00
TRINITY_sp|P0922	-	25 kDa calcium-bind	44.10	0.00
TRINITY_sp|F4I93	STR8	Rhodanese-like doma	44.10	0.00
TRINITY_sp|P0432	pol	Retrovirus-related	44.10	0.00
TRINITY_sp|B8APE	MRS2-A	Magnesium transport	44.10	0.00
TRINITY_sp|Q6PD9	Sarm1	Sterile alpha and T	44.10	0.00
TRINITY_sp|Q3961	ODA11	Dynein alpha chain,	44.10	0.00

TRINITY_sp|Q54G(	dgat2	Diacylglycerol O-ac	44.10	0.00
TRINITY_sp|Q6NW	dnajc2	DnaJ homolog subfam	44.10	0.00
TRINITY_sp|Q1918	gcy-12	Receptor-type guany	44.10	0.00
TRINITY_sp|Q4UB	rab1	Ras-related protein	44.10	0.00
TRINITY_sp|P2969	eef-2	Elongation factor 2	44.10	0.00
TRINITY_sp|Q3SZ	POLE3	DNA polymerase epsi	44.00	0.00
TRINITY_sp|Q54Z(	xab2	Pre-mRNA-splicing f	44.00	0.00
TRINITY_sp|P201	GSTM2	Glutathione S-trans	44.00	0.00
TRINITY_sp|Q0209	rad3	Protein kinase rad3	44.00	0.00
TRINITY_sp|Q0178	EXOSC10	Exosome component 1	44.00	0.00
TRINITY_sp|Q9LE8	IRE	Probable serine/thr	44.00	0.00
TRINITY_sp|Q3958	-	Dynein 8 kDa light	44.00	0.00
TRINITY_sp|Q0DJ	TOR	Serine/threonine-pr	44.00	0.00
TRINITY_sp|Q86J	polr2j	DNA-directed RNA po	44.00	0.00
TRINITY_sp|Q8N4	CARNMT1	Carnosine N-methylt	44.00	0.00
TRINITY_sp|Q96M	HSPA12B	Heat shock 70 kDa p	44.00	0.00
TRINITY_sp|O087(	Vps45	Vacuolar protein so	44.00	0.00
TRINITY_sp|Q9LX	MBF1B	Multiprotein-bridgi	44.00	0.00
TRINITY_sp|P491(	-	Nitrate reductase [	44.00	0.00
TRINITY_sp|Q149	SHPRH	E3 ubiquitin-protei	44.00	0.00
TRINITY_sp|Q754	ERT1	Transcription activ	44.00	0.00
TRINITY_sp|Q103	p23fy	Translationally-con	44.00	0.00
TRINITY_sp|Q08D	MRPL47	39S ribosomal prote	44.00	0.00
TRINITY_sp|F4I2	SWI2	Switch 2 OS=Arabido	44.00	0.00
TRINITY_sp|P011(	MYB	Transcriptional act	44.00	0.00
TRINITY_sp|Q84M	LUL3	Probable E3 ubiquit	44.00	0.00
TRINITY_sp|O1625	sti-1	Stress-induced-phos	44.00	0.00
TRINITY_sp|Q9ZS	GLB1	Nitrogen regulatory	44.00	0.00
TRINITY_sp|P043	pol	Retrovirus-related	44.00	0.00
TRINITY_sp|Q6EE	TTLL5	Tubulin polyglutamy	44.00	0.00
TRINITY_sp|Q55A(	DDB_G02	Probable serine/thr	44.00	0.00
TRINITY_sp|O949(	PROSC	Proline synthase co	44.00	0.00
TRINITY_sp|Q5T0	CFAP70	Cilia- and flagella	44.00	0.00
TRINITY_sp|Q6QH	PAOX	Peroxisomal N(1)-ac	44.00	0.00
TRINITY_sp|A4II	tppp3	Tubulin polymerizat	44.00	0.00
TRINITY_sp|P1005	-	Caricain OS=Carica	44.00	0.00
TRINITY_sp|Q9P2	FAM135A	Protein FAM135A OS=	44.00	0.00
TRINITY_sp|Q9FG	MAG5	Protein transport p	44.00	0.00
TRINITY_sp|B6JE	rplD	50S ribosomal prote	44.00	0.00
TRINITY_sp|Q2HJ	USP15	Ubiquitin carboxyl-	44.00	0.00
TRINITY_sp|Q9UB	CTSZ	Cathepsin Z OS=Homo	44.00	0.00
TRINITY_sp|A3LU	FMP521	Protein FMP52-1, mi	44.00	0.00
TRINITY_sp|Q96P	LENG8	Leukocyte receptor	44.00	0.00
TRINITY_sp|Q650	Os09g05	DEAD-box ATP-depend	44.00	0.00
TRINITY_sp|Q9NG	mkcB	Probable serine/thr	44.00	0.00
TRINITY_sp|Q93Y	RUS6	Protein root UVB se	44.00	0.00
TRINITY_sp|O5509	Aurkb	Aurora kinase B OS=	44.00	0.00
TRINITY_sp|Q941	BON1	Protein BONZAI 1 OS	44.00	0.00
TRINITY_sp|O252	typA	GTP-binding protein	44.00	0.00
TRINITY_sp|Q3MH	NUBP2	Cytosolic Fe-S clus	44.00	0.00
TRINITY_sp|P231	ABCB4	Phosphatidylcholine	44.00	0.00
TRINITY_sp|Q9GR	aif	Apoptosis-inducing	44.00	0.00
TRINITY_sp|P250	CML12	Calmodulin-like pro	44.00	0.00
TRINITY_sp|P4039	RIC1	Ras-related protein	44.00	0.00
TRINITY_sp|Q9SU	KCS15	3-ketoacyl-CoA synt	44.00	0.00
TRINITY_sp|Q9LH	At5g352(	Putative clathrin a	44.00	0.00

TRINITY_sp|Q56X7	RH39	DEAD-box ATP-depend	44.00	0.00
TRINITY_sp|Q6C78	FIP1	Pre-mRNA polyadenyl	44.00	0.00
TRINITY_sp|Q94C4	PAT13	Probable protein S-	44.00	0.00
TRINITY_sp|Q54I1	DDB_G028	UPF0553 protein OS=	44.00	0.00
TRINITY_sp|Q6L49	DBB1	DNA damage-binding	44.00	0.00
TRINITY_sp|P2082	pol	Retrovirus-related	44.00	0.00
TRINITY_sp|Q3Y41	ckk-1	Calcium/calmodulin-	44.00	0.00
TRINITY_sp|P2873	MSP1	Protein MSP1 OS=Sac	44.00	0.00
TRINITY_sp|Q9S70	HSP70-14	Heat shock 70 kDa p	44.00	0.00
TRINITY_sp|A6WX0	ubiG	Ubiquinone biosynth	43.90	0.00
TRINITY_sp|Q8R0X	Sgpl1	Sphingosine-1-phosp	43.90	0.00
TRINITY_sp|Q5HR0	SERP0230	UPF0382 membrane pr	43.90	0.00
TRINITY_sp|Q18A8	speE	Polyamine aminoprop	43.90	0.00
TRINITY_sp|P3601	VPS21	Vacuolar protein so	43.90	0.00
TRINITY_sp|Q9M2U	ECR	Very-long-chain eno	43.90	0.00
TRINITY_sp|Q556Y	DDB_G027	Probable rhodanese	43.90	0.00
TRINITY_sp|Q5ZHW	RAB5B	Ras-related protein	43.90	0.00
TRINITY_sp|Q54IA	cox19	Cytochrome c oxidas	43.90	0.00
TRINITY_sp|P0DK0	PGLP1A	Phosphoglycolate ph	43.90	0.00
TRINITY_sp|Q9WV0	Kif9	Kinesin-like protei	43.90	0.00
TRINITY_sp|P4221	RAP	Aspartic proteinase	43.90	0.00
TRINITY_sp|P2126	CCA1	CCA tRNA nucleotidy	43.90	0.00
TRINITY_sp|Q8GWJ	OSB3	Protein OSB3, chlor	43.90	0.00
TRINITY_sp|Q9FW0	Os10g052	Probable trehalase	43.90	0.00
TRINITY_sp|Q8690	cpvl	Probable serine car	43.90	0.00
TRINITY_sp|Q6Z9U	Os08g018	mRNA cap guanine-N7	43.90	0.00
TRINITY_sp|Q9LU4	ACA9	Calcium-transportin	43.90	0.00
TRINITY_sp|O5994	fim1	Fimbrin OS=Schizosa	43.90	0.00
TRINITY_sp|P0A94	rimI	Ribosomal-protein-a	43.90	0.00
TRINITY_sp|Q2XQU	lacZ	Beta-galactosidase	43.90	0.00
TRINITY_sp|O6225	F45G2.10	MIP18 family protei	43.90	0.00
TRINITY_sp|Q8H96	NPC6	Non-specific phosph	43.90	0.00
TRINITY_sp|Q5W60	MAN5	Putative mannan end	43.90	0.00
TRINITY_sp|Q5GF2	Manea	Glycoprotein endo-a	43.90	0.00
TRINITY_sp|Q86AE	DDB_G027	Probable myosin lig	43.90	0.00
TRINITY_sp|Q9CZ4	Naxd	ATP-dependent (S)-N	43.90	0.00
TRINITY_sp|Q54TN	drkD	Probable serine/thr	43.90	0.00
TRINITY_sp|Q6317	Dnah7	Dynein heavy chain	43.90	0.00
TRINITY_sp|Q1LWI	dusp22a	Dual specificity pr	43.90	0.00
TRINITY_sp|Q5PP3	ATXR2	Histone-lysine N-me	43.90	0.00
TRINITY_sp|Q9M68	GK-2	Guanylate kinase 2	43.90	0.00
TRINITY_sp|O4928	RH29	Putative DEAD-box A	43.90	0.00
TRINITY_sp|P1097	-	Retrovirus-related	43.90	0.00
TRINITY_sp|Q54WA	pex7	Peroxisomal targeti	43.90	0.00
TRINITY_sp|P8705	SPAC57A1	Uncharacterized pro	43.90	0.00
TRINITY_sp|Q6113	Prpf4b	Serine/threonine-pr	43.90	0.00
TRINITY_sp|F2Z69	NDH2	External alternativ	43.90	0.00
TRINITY_sp|Q5VQ0	CPK2	Calcium-dependent p	43.90	0.00
TRINITY_sp|Q94F0	IMPL1	Phosphatase IMPL1,	43.90	0.00
TRINITY_sp|Q9ZBU	xyoA	Probable xylitol ox	43.90	0.00
TRINITY_sp|Q8IUE	CBWD2	COBW domain-contain	43.90	0.00
TRINITY_sp|P5431	Lipe	Hormone-sensitive l	43.90	0.00
TRINITY_sp|Q84ME	RBL11	Rhomboid-like prote	43.90	0.00
TRINITY_sp|Q2HJ4	PRPF18	Pre-mRNA-splicing f	43.90	0.00
TRINITY_sp|B1AQJ	Usp36	Ubiquitin carboxyl-	43.90	0.00
TRINITY_sp|Q5031	ngly1	Peptide-N(4)-(N-ace	43.90	0.00

TRINITY_sp|Q70I5	hdaH	Histone deacetylase	43.90	0.00
TRINITY_sp|Q5ZJI	ABHD13	Protein ABHD13 OS=G	43.90	0.00
TRINITY_sp|P5195	Nek1	Serine/threonine-pr	43.90	0.00
TRINITY_sp|Q6NV3	wdr82	WD repeat-containin	43.90	0.00
TRINITY_sp|Q9NRI	BAZ1A	Bromodomain adjacen	43.90	0.00
TRINITY_sp|O8837	Baz1a	Bromodomain adjacen	43.90	0.00
TRINITY_sp|Q0E2Y	UVR3	(6-4)DNA photolyase	43.90	0.00
TRINITY_sp|Q9SRF	AZG1	Adenine/guanine per	43.90	0.00
TRINITY_sp|Q8IS2	gefB	Ras guanine nucleot	43.90	0.00
TRINITY_sp|Q9R1(	LIPE	Hormone-sensitive l	43.90	0.00
TRINITY_sp|Q9ZWE	CKL9	Casein kinase 1-lik	43.90	0.00
TRINITY_sp|O8355	pfp	Pyrophosphate--fruc	43.80	0.00
TRINITY_sp|Q9FKW	At5g6615	Probable alpha-mann	43.80	0.00
TRINITY_sp|A8M52	rpsQ	30S ribosomal prote	43.80	0.00
TRINITY_sp|Q55C7	gtf2h5	General transcripti	43.80	0.00
TRINITY_sp|Q55C7	gtf2h5	General transcripti	43.80	0.00
TRINITY_sp|Q1007	SPAC3H1.	Glutathione gamma-g	43.80	0.00
TRINITY_sp|Q8BHY	Noc4l	Nucleolar complex p	43.80	0.00
TRINITY_sp|Q0WRE	PPC6-7	Probable protein ph	43.80	0.00
TRINITY_sp|P6857	hup2	SPBc2 prophage-deri	43.80	0.00
TRINITY_sp|Q2YDE	DRAP1	Dr1-associated core	43.80	0.00
TRINITY_sp|Q9H77	ELAC1	Zinc phosphodiester	43.80	0.00
TRINITY_sp|Q9H4E	PLK3	Serine/threonine-pr	43.80	0.00
TRINITY_sp|Q55E5	pats1	Probable serine/thr	43.80	0.00
TRINITY_sp|Q9632	ACX4	Acyl-coenzyme A oxi	43.80	0.00
TRINITY_sp|Q0297	CAD8	Cinnamyl alcohol de	43.80	0.00
TRINITY_sp|A1A69	HK5	Probable histidine	43.80	0.00
TRINITY_sp|Q9NJU	CPK3	Calcium-dependent p	43.80	0.00
TRINITY_sp|Q3ED6	At1g2295	Uncharacterized PKH	43.80	0.00
TRINITY_sp|A2Z3E	SGR	Protein STAY-GREEN,	43.80	0.00
TRINITY_sp|F4HXY	PSS1	CDP-diacylglycerol-	43.80	0.00
TRINITY_sp|P8415	TCTP	Translationally-con	43.80	0.00
TRINITY_sp|Q9JJ7	Dync2h1	Cytoplasmic dynein	43.80	0.00
TRINITY_sp|Q9X3X	topA	DNA topoisomerase 1	43.80	0.00
TRINITY_sp|Q9FL5	ATJ6	Chaperone protein d	43.80	0.00
TRINITY_sp|Q7TSI	Ppp6r1	Serine/threonine-pr	43.80	0.00
TRINITY_sp|Q9NGW	mkcB	Probable serine/thr	43.80	0.00
TRINITY_sp|Q8MZ9	-	Physarolisin OS=Phy	43.80	0.00
TRINITY_sp|Q8SS(	TCP1	T-complex protein 1	43.80	0.00
TRINITY_sp|P2271	Gucy1b2	Guanylate cyclase s	43.80	0.00
TRINITY_sp|Q9VVE	Rbp6	RNA-binding protein	43.80	0.00
TRINITY_sp|Q54U3	shkD	Dual specificity pr	43.80	0.00
TRINITY_sp|O8869	Stk16	Serine/threonine-pr	43.80	0.00
TRINITY_sp|Q8BNI	Kctd6	BTB/POZ domain-cont	43.80	0.00
TRINITY_sp|Q949E	At2g1734	Uncharacterized pro	43.80	0.00
TRINITY_sp|Q9SZE	ABCG9	ABC transporter G f	43.80	0.00
TRINITY_sp|P4215	CKL1	Casein kinase 1-lik	43.80	0.00
TRINITY_sp|Q8EI8	pepA1	Probable cytosol am	43.80	0.00
TRINITY_sp|Q9C42	MINE1	Cell division topol	43.80	0.00
TRINITY_sp|Q9NR3	DDX21	Nucleolar RNA helic	43.80	0.00
TRINITY_sp|Q6NY2	slc25a25	Calcium-binding mit	43.80	0.00
TRINITY_sp|A0JPE	Ccdc61	Coiled-coil domain-	43.80	0.00
TRINITY_sp|Q8LP9	LACS6	Long chain acyl-CoA	43.80	0.00
TRINITY_sp|Q68F3	vac14	Protein VAC14 homol	43.80	0.00
TRINITY_sp|P0CD6	alg7	UDP-N-acetylglucosa	43.80	0.00
TRINITY_sp|F4IV4	CHR10	Probable helicase C	43.80	0.00

TRINITY_sp|Q9ZU9	VAC14	Protein VAC14 homol	43.80	0.00
TRINITY_sp|Q9PWF	KCNAB1	Voltage-gated potas	43.80	0.00
TRINITY_sp|Q9I40	PA1210	Putative quercetin	43.80	0.00
TRINITY_sp|Q9LR7	CPX1	Coproporphyrinogen-	43.80	0.00
TRINITY_sp|Q3SZ2	RPP30	Ribonuclease P prot	43.80	0.00
TRINITY_sp|Q54Q6	dhkG	Hybrid signal trans	43.80	0.00
TRINITY_sp|P3905	-	Dynein beta chain,	43.80	0.00
TRINITY_sp|Q54Y5	shkC	Dual specificity pr	43.80	0.00
TRINITY_sp|Q54K3	rgaA	Ras GTPase-activati	43.80	0.00
TRINITY_sp|Q5VQ0	CPK2	Calcium-dependent p	43.80	0.00
TRINITY_sp|Q9632	HSFB1	Heat stress transcr	43.80	0.00
TRINITY_sp|Q2YDF	ANAPC10	Anaphase-promoting	43.80	0.00
TRINITY_sp|Q0560	CTR1	Serine/threonine-pr	43.80	0.00
TRINITY_sp|Q5F45	PAN2	PAB-dependent poly(	43.80	0.00
TRINITY_sp|Q3SZ2	RPP30	Ribonuclease P prot	43.80	0.00
TRINITY_sp|Q9C8F	ABCG12	ABC transporter G f	43.80	0.00
TRINITY_sp|O7495	SPCC736.	Uncharacterized oxi	43.80	0.00
TRINITY_sp|Q2302	unc-51	Serine/threonine-pr	43.80	0.00
TRINITY_sp|Q5597	polA	DNA polymerase I OS	43.80	0.00
TRINITY_sp|Q924F	Rad51c	DNA repair protein	43.80	0.00
TRINITY_sp|Q9ZR3	DSPTP1	Dual specificity pr	43.80	0.00
TRINITY_sp|Q1538	UBE3C	Ubiquitin-protein l	43.80	0.00
TRINITY_sp|P6099	-	Ervatamin-B OS=Tabe	43.80	0.00
TRINITY_sp|P0A90	adhE	Aldehyde-alcohol de	43.80	0.00
TRINITY_sp|Q9HEF	2E4.130	Regulator of nonsen	43.80	0.00
TRINITY_sp|Q9ZU5	BAT1	Amino-acid permease	43.80	0.00
TRINITY_sp|Q9ZSM	EZA1	Histone-lysine N-me	43.80	0.00
TRINITY_sp|Q9I4I	PA1204	NAD(P)H-dependent F	43.80	0.00
TRINITY_sp|Q0III	DYNC1I2	Cytoplasmic dynein	43.80	0.00
TRINITY_sp|Q55DF	DDB_G026	Alpha N-terminal pr	43.70	0.00
TRINITY_sp|F4JAA	SKI2	DExH-box ATP-depend	43.70	0.00
TRINITY_sp|P6110	RAB2A	Ras-related protein	43.70	0.00
TRINITY_sp|Q8VZ4	SAP11	Zinc finger AN1 and	43.70	0.00
TRINITY_sp|C3KJF	swi5	DNA repair protein	43.70	0.00
TRINITY_sp|Q6GN1	usp22-a	Ubiquitin carboxyl-	43.70	0.00
TRINITY_sp|Q9C1X	SPAP32A8	Uncharacterized RIN	43.70	0.00
TRINITY_sp|Q9MA0	ABCG24	ABC transporter G f	43.70	0.00
TRINITY_sp|A2XL3	ERS1	Probable ethylene r	43.70	0.00
TRINITY_sp|Q8VYA	RS2Z33	Serine/arginine-ric	43.70	0.00
TRINITY_sp|Q54F2	mroh1	Maestro heat-like r	43.70	0.00
TRINITY_sp|O4400	PYK	Pyruvate kinase OS=	43.70	0.00
TRINITY_sp|Q9HD4	SEPSECS	O-phosphoseryl-tRNA	43.70	0.00
TRINITY_sp|Q86X8	COMMD2	COMM domain-contain	43.70	0.00
TRINITY_sp|Q9FJF	UPF1	Regulator of nonsen	43.70	0.00
TRINITY_sp|Q8T19	ggtA	Probable UDP-glucos	43.70	0.00
TRINITY_sp|Q9C50	PUX13	Plant UBX domain-co	43.70	0.00
TRINITY_sp|Q9SSF	At1g5259	DCC family protein	43.70	0.00
TRINITY_sp|Q94AW	ATJ3	Chaperone protein d	43.70	0.00
TRINITY_sp|P5574	RAB21	Ras-related protein	43.70	0.00
TRINITY_sp|Q8L97	P4H7	Probable prolyl 4-h	43.70	0.00
TRINITY_sp|A0BQI	GSPATT00	Probable protein ph	43.70	0.00
TRINITY_sp|Q5U50	prpf31	U4/U6 small nuclear	43.70	0.00
TRINITY_sp|P4012	CAP2	Adenylyl cyclase-as	43.70	0.00
TRINITY_sp|Q3ZB7	TRA2B	Transformer-2 prote	43.70	0.00
TRINITY_sp|Q99K1	Aco2	Aconitate hydratase	43.70	0.00
TRINITY_sp|P4138	-	Eukaryotic initiati	43.70	0.00

TRINITY_sp|Q9SIK	At2g299(	Presenilin-like pro	43.70	0.00
TRINITY_sp|Q1359	TRA2A	Transformer-2 prote	43.70	0.00
TRINITY_sp|A5PJ9	SRD5A1	3-oxo-5-alpha-stero	43.70	0.00
TRINITY_sp|B8ARK	SRT1	NAD-dependent prote	43.70	0.00
TRINITY_sp|Q96NR	RDH12	Retinol dehydrogena	43.70	0.00
TRINITY_sp|B2T42	Bphyt_19	UPF0061 protein Bph	43.70	0.00
TRINITY_sp|Q54KE	mccA	Methylcrotonoyl-CoA	43.70	0.00
TRINITY_sp|Q75J9	rapgap1	RapA guanosine trip	43.70	0.00
TRINITY_sp|Q9HC2	TMBIM4	Protein lifeguard 4	43.70	0.00
TRINITY_sp|F4JA(	P4H2	Prolyl 4-hydroxylas	43.70	0.00
TRINITY_sp|P982(	ALA2	Phospholipid-transp	43.70	0.00
TRINITY_sp|Q3T01	GDE1	Glycerophosphodiest	43.70	0.00
TRINITY_sp|Q8LK9	LACS7	Long chain acyl-CoA	43.70	0.00
TRINITY_sp|Q86Y9	RAB43	Ras-related protein	43.70	0.00
TRINITY_sp|O822(	At2g4797	NPL4-like protein 2	43.70	0.00
TRINITY_sp|Q9XIE	ALA3	Phospholipid-transp	43.70	0.00
TRINITY_sp|O1543	ABCC4	Multidrug resistanc	43.70	0.00
TRINITY_sp|P499(	-	Arginase, hepatic O	43.70	0.00
TRINITY_sp|Q0III	DUSP10	Dual specificity pr	43.70	0.00
TRINITY_sp|O2445	UBP3	Ubiquitin carboxyl-	43.70	0.00
TRINITY_sp|P3259	SAC6	Fimbrin OS=Saccharo	43.70	0.00
TRINITY_sp|P0A91	tas	Protein tas OS=Esch	43.70	0.00
TRINITY_sp|Q9P4X	utp7	Probable U3 small n	43.70	0.00
TRINITY_sp|Q432(	PCM	Protein-L-isoaspart	43.70	0.00
TRINITY_sp|Q6238	Zpr1	Zinc finger protein	43.60	0.00
TRINITY_sp|B5DS7	GA24966	Adenylyltransferase	43.60	0.00
TRINITY_sp|Q96DI	SNRNP40	U5 small nuclear ri	43.60	0.00
TRINITY_sp|Q8VYE	MED31	Mediator of RNA pol	43.60	0.00
TRINITY_sp|Q291I	Aats-cys	Cysteine--tRNA liga	43.60	0.00
TRINITY_sp|G5ED(	mtm-6	Myotubularin-relate	43.60	0.00
TRINITY_sp|Q8HYI	PFDN5	Prefoldin subunit 5	43.60	0.00
TRINITY_sp|Q0P45	kti12	Protein KTI12 homol	43.60	0.00
TRINITY_sp|P1268	YPK1	Serine/threonine-pr	43.60	0.00
TRINITY_sp|Q54N4	ppan	Peter Pan-like prot	43.60	0.00
TRINITY_sp|F4JUI	ARPC4	Actin-related prote	43.60	0.00
TRINITY_sp|O1837	SelD	Selenide, water dik	43.60	0.00
TRINITY_sp|Q54I9	smt1	Probable cycloarten	43.60	0.00
TRINITY_sp|Q8WWV	RTN4IP1	Reticulon-4-interac	43.60	0.00
TRINITY_sp|Q4I8(	FGRRES_(	Acyl-protein thioes	43.60	0.00
TRINITY_sp|P1281	hsp16.9A	16.9 kDa class I he	43.60	0.00
TRINITY_sp|P1268	YPK1	Serine/threonine-pr	43.60	0.00
TRINITY_sp|Q6S4N	HSPA1B	Heat shock 70 kDa p	43.60	0.00
TRINITY_sp|Q9FWX	ABCB11	ABC transporter B f	43.60	0.00
TRINITY_sp|Q54NI	vmp1	Vacuole membrane pr	43.60	0.00
TRINITY_sp|Q9H39	TPK1	Thiamin pyrophospho	43.60	0.00
TRINITY_sp|Q680K	At4g3997	Haloacid dehalogena	43.60	0.00
TRINITY_sp|P5778	Snrpa1	U2 small nuclear ri	43.60	0.00
TRINITY_sp|P1922	adaB	Methylated-DNA--pro	43.60	0.00

TRINITY_sp|A1K5[illegible]	azo1574	UPF0061 protein azo	43.60	0.00
TRINITY_sp|Q94B[illegible]	NUDT2	Nudix hydrolase 2 O	43.60	0.00
TRINITY_sp|Q8H1[illegible]	RH14	DEAD-box ATP-depend	43.60	0.00
TRINITY_sp|Q8S2[illegible]	Os01g07[illegible]	Ribose-phosphate py	43.60	0.00
TRINITY_sp|Q9TV[illegible]	rab1	Ras-related protein	43.60	0.00
TRINITY_sp|Q9XY[illegible]	noxA	Superoxide-generati	43.60	0.00
TRINITY_sp|Q9VH[illegible]	Ada	Adenosine deaminase	43.60	0.00
TRINITY_sp|Q9SG[illegible]	ACLA-1	ATP-citrate synthas	43.60	0.00
TRINITY_sp|Q266[illegible]	-	cAMP-dependent prot	43.60	0.00
TRINITY_sp|B2V6[illegible]	gpsA	Glycerol-3-phosphat	43.60	0.00
TRINITY_sp|Q2QW[illegible]	Os12g01[illegible]	Probable thiamine b	43.60	0.00
TRINITY_sp|P929[illegible]	CLC-D	Chloride channel pr	43.60	0.00
TRINITY_sp|Q9UR[illegible]	ACT	Actin, gamma OS=Pen	43.60	0.00
TRINITY_sp|O654[illegible]	XCP1	Cysteine protease X	43.60	0.00
TRINITY_sp|Q869[illegible]	nramp1	Metal transporter n	43.60	0.00
TRINITY_sp|P136[illegible]	Atpalpha	Sodium/potassium-tr	43.60	0.00
TRINITY_sp|Q0II[illegible]	RAB5A	Ras-related protein	43.60	0.00
TRINITY_sp|O129[illegible]	RAD54L	DNA repair and reco	43.60	0.00
TRINITY_sp|P204[illegible]	pyrK	UMP-CMP kinase OS=D	43.60	0.00
TRINITY_sp|Q7ZX[illegible]	nle1	Notchless protein h	43.60	0.00
TRINITY_sp|Q24K[illegible]	IDE	Insulin-degrading e	43.60	0.00
TRINITY_sp|Q142[illegible]	DYNC1H1	Cytoplasmic dynein	43.60	0.00
TRINITY_sp|Q9H0[illegible]	TMEM222	Transmembrane prote	43.60	0.00
TRINITY_sp|Q963[illegible]	ACX4	Acyl-coenzyme A oxi	43.60	0.00
TRINITY_sp|P126[illegible]	RPL13	50S ribosomal prote	43.60	0.00
TRINITY_sp|Q073[illegible]	GAD	Glutamate decarboxy	43.60	0.00
TRINITY_sp|Q947[illegible]	GSPATT0[illegible]	Cathepsin L 1 OS=Pa	43.60	0.00
TRINITY_sp|Q8R9[illegible]	pckA	Phosphoenolpyruvate	43.60	0.00
TRINITY_sp|P401[illegible]	MMF1	Protein MMF1, mitoc	43.60	0.00
TRINITY_sp|Q5JK[illegible]	Os01g09[illegible]	Long chain base bio	43.60	0.00
TRINITY_sp|Q9FN[illegible]	BRR2C	DExH-box ATP-depend	43.60	0.00
TRINITY_sp|Q5RB[illegible]	PRCP	Lysosomal Pro-X car	43.60	0.00
TRINITY_sp|P546[illegible]	pkbA	RAC family serine/t	43.60	0.00
TRINITY_sp|Q8TC[illegible]	RDH11	Retinol dehydrogena	43.50	0.00
TRINITY_sp|Q86A[illegible]	atp6v1d	V-type proton ATPas	43.50	0.00
TRINITY_sp|A2WY[illegible]	HK3	Probable histidine	43.50	0.00
TRINITY_sp|Q9C5[illegible]	IRE1A	Serine/threonine-pr	43.50	0.00
TRINITY_sp|Q9MB[illegible]	DHC10	Dynein-1-beta heavy	43.50	0.00
TRINITY_sp|Q145[illegible]	ITPR2	Inositol 1,4,5-tris	43.50	0.00
TRINITY_sp|Q8RY[illegible]	NUP98A	Nuclear pore comple	43.50	0.00
TRINITY_sp|Q2GA[illegible]	engB	Probable GTP-bindin	43.50	0.00
TRINITY_sp|P163[illegible]	Pah	Phenylalanine-4-hyd	43.50	0.00
TRINITY_sp|Q55D[illegible]	mhisS	Probable histidine-	43.50	0.00
TRINITY_sp|P364[illegible]	rab2A	Ras-related protein	43.50	0.00
TRINITY_sp|Q8C1[illegible]	Thop1	Thimet oligopeptida	43.50	0.00
TRINITY_sp|Q6ZV[illegible]	TTLL10	Inactive polyglycyl	43.50	0.00
TRINITY_sp|P0C1[illegible]	amy3	Alpha-amylase A typ	43.50	0.00
TRINITY_sp|Q510[illegible]	-	Uncharacterized pro	43.50	0.00
TRINITY_sp|Q3ZB[illegible]	DEGS1	Sphingolipid delta(	43.50	0.00
TRINITY_sp|Q55G[illegible]	DDB_G02[illegible]	Probable serine/thr	43.50	0.00
TRINITY_sp|O823[illegible]	ANTR1	Sodium-dependent ph	43.50	0.00
TRINITY_sp|A7MB[illegible]	COMT	Catechol O-methyltr	43.50	0.00
TRINITY_sp|Q6H9[illegible]	alkB2	Alkane 1-monooxygen	43.50	0.00
TRINITY_sp|P516[illegible]	HSD17B4	Peroxisomal multifu	43.50	0.00
TRINITY_sp|Q5XM[illegible]	aprA	Autocrine prolifera	43.50	0.00
TRINITY_sp|Q54G[illegible]	ctdspl2	CTD small phosphata	43.50	0.00

TRINITY_sp|Q54TH	gacY	Rho GTPase-activati	43.50	0.00
TRINITY_sp|Q9MAX	COPZ1	Coatomer subunit ze	43.50	0.00
TRINITY_sp|P1535	RpS27A	Ubiquitin-40S ribos	43.50	0.00
TRINITY_sp|Q54U(	lvsC	BEACH domain-contai	43.50	0.00
TRINITY_sp|Q8N51	TBC1D19	TBC1 domain family	43.50	0.00
TRINITY_sp|Q9LX3	GUN4	Tetrapyrrole-bindin	43.50	0.00
TRINITY_sp|Q918I	aurka-b	Aurora kinase A-B O	43.50	0.00
TRINITY_sp|Q9ZVI	At1g5478	UPF0603 protein At1	43.50	0.00
TRINITY_sp|O807	WDR55	WD repeat-containin	43.50	0.00
TRINITY_sp|Q0WPT	At2g4104	Uncharacterized met	43.50	0.00
TRINITY_sp|P4269	DRT111	DNA-damage-repair/t	43.50	0.00
TRINITY_sp|O9571	HERC2	E3 ubiquitin-protei	43.50	0.00
TRINITY_sp|Q54G5	ascc3	Activating signal c	43.50	0.00
TRINITY_sp|Q9FWX	ABCB11	ABC transporter B f	43.50	0.00
TRINITY_sp|Q1RMH	LRRC6	Protein tilB homolo	43.50	0.00
TRINITY_sp|Q9VAN	Slbp	Histone RNA hairpin	43.50	0.00
TRINITY_sp|Q8LRH	HDA18	Histone deacetylase	43.50	0.00
TRINITY_sp|P2269	celA	Endoglucanase OS=Di	43.50	0.00
TRINITY_sp|A6QQN	ZNF474	Zinc finger protein	43.50	0.00
TRINITY_sp|P1372	hexa1	Beta-hexosaminidase	43.50	0.00
TRINITY_sp|P0509	abpA	Alpha-actinin A OS=	43.50	0.00
TRINITY_sp|Q93ZE	SFH3	Phosphatidylinosito	43.50	0.00
TRINITY_sp|Q7M62	Kif27	Kinesin-like protei	43.50	0.00
TRINITY_sp|Q9CVE	Arpc2	Actin-related prote	43.50	0.00
TRINITY_sp|A8CVX	ttll6	Tubulin polyglutamy	43.50	0.00
TRINITY_sp|F4I8H	GDPD4	Glycerophosphodiest	43.50	0.00
TRINITY_sp|Q5JVI	EFHC1	EF-hand domain-cont	43.50	0.00
TRINITY_sp|Q6K43	PAP2	Probable plastid-li	43.50	0.00
TRINITY_sp|Q1BK3	adc	Probable acetoaceta	43.50	0.00
TRINITY_sp|O4877	PDIL2-3	Protein disulfide-i	43.50	0.00
TRINITY_sp|Q3E9A	ANTR6	Probable anion tran	43.50	0.00
TRINITY_sp|Q1331	PABPC4	Polyadenylate-bindi	43.50	0.00
TRINITY_sp|C7EXE	ATP8A2	Phospholipid-transp	43.50	0.00
TRINITY_sp|P6175	Vbp1	Prefoldin subunit 3	43.50	0.00
TRINITY_sp|Q9XY9	noxA	Superoxide-generati	43.50	0.00
TRINITY_sp|O9465	ypt7	GTP-binding protein	43.50	0.00
TRINITY_sp|Q54W8	DDB_G027	Probable myosin lig	43.50	0.00
TRINITY_sp|P8286	CYP37	Peptidyl-prolyl cis	43.50	0.00
TRINITY_sp|Q9SRE	AZG1	Adenine/guanine per	43.50	0.00
TRINITY_sp|Q5AVE	ampp	Probable Xaa-Pro am	43.50	0.00
TRINITY_sp|Q3899	KIN10	SNF1-related protei	43.50	0.00
TRINITY_sp|Q6F3A	CPK10	Calcium-dependent p	43.50	0.00
TRINITY_sp|Q54SN	polr1c	DNA-directed RNA po	43.40	0.00
TRINITY_sp|P1370	fs(1)h	Homeotic protein fe	43.40	0.00
TRINITY_sp|Q6GPE	wdr70	WD repeat-containin	43.40	0.00
TRINITY_sp|B8B7X	D2HGDH	Probable D-2-hydrox	43.40	0.00
TRINITY_sp|Q55A0	DDB_G027	Probable serine/thr	43.40	0.00
TRINITY_sp|P8639	RPP14	Hydroxyacyl-thioest	43.40	0.00
TRINITY_sp|Q54VE	elmoB	ELMO domain-contain	43.40	0.00
TRINITY_sp|Q6GMI	tatdn1	Putative deoxyribon	43.40	0.00
TRINITY_sp|Q55C7	sec31	Protein transport p	43.40	0.00
TRINITY_sp|Q8BH0	-	UPF0587 protein C1o	43.40	0.00
TRINITY_sp|P6128	PSME3	Proteasome activato	43.40	0.00
TRINITY_sp|Q54F2	mroh1	Maestro heat-like r	43.40	0.00
TRINITY_sp|O5517	Pdpk1	3-phosphoinositide-	43.40	0.00
TRINITY_sp|Q86KI	ap2a1-1	AP-2 complex subuni	43.40	0.00

TRINITY_sp|Q551N	zfpl1	Zinc finger protein	43.40	0.00
TRINITY_sp|Q165(	CAMK4	Calcium/calmodulin-	43.40	0.00
TRINITY_sp|Q9C9	At3g0865	Putative zinc trans	43.40	0.00
TRINITY_sp|Q23FI	TTLL3C	Tubulin glycylase 3	43.40	0.00
TRINITY_sp|Q4KL\	chpt1	Cholinephosphotrans	43.40	0.00
TRINITY_sp|O159	SCGOA	Guanine nucleotide-	43.40	0.00
TRINITY_sp|P100	-	Caricain OS=Carica	43.40	0.00
TRINITY_sp|Q8H1I	DGP3	DAR GTPase 3, chlor	43.40	0.00
TRINITY_sp|Q9DE	BAZ2B	Bromodomain adjacen	43.40	0.00
TRINITY_sp|B1MGI	MAB_378	Putative S-adenosyl	43.40	0.00
TRINITY_sp|Q6NM	UTR5	UDP-galactose/UDP-g	43.40	0.00
TRINITY_sp|F4HX	PLA1	Phospholipase A I O	43.40	0.00
TRINITY_sp|P302	BpOF4_1(	Uncharacterized ami	43.40	0.00
TRINITY_sp|Q54I	eif2b1	Translation initiat	43.40	0.00
TRINITY_sp|Q0J0	Os09g05	Probable 1-acylglyc	43.40	0.00
TRINITY_sp|Q9HD	pmc1	Calcium-transportin	43.40	0.00
TRINITY_sp|Q9LT	VPS9A	Vacuolar protein so	43.40	0.00
TRINITY_sp|Q7XA(	D1BLIC	Cytoplasmic dynein	43.40	0.00
TRINITY_sp|Q9FGN	FTSH11	ATP-dependent zinc	43.40	0.00
TRINITY_sp|Q75A	ARF1	ADP-ribosylation fa	43.40	0.00
TRINITY_sp|Q9XYI	fcpA	Probable C-terminal	43.40	0.00
TRINITY_sp|Q1RJ	icd	Isocitrate dehydrog	43.40	0.00
TRINITY_sp|A1A4	ATP9B	Probable phospholip	43.40	0.00
TRINITY_sp|Q93V	PABN1	Polyadenylate-bindi	43.40	0.00
TRINITY_sp|Q90W	dusp1-b	Dual specificity pr	43.40	0.00
TRINITY_sp|Q7LH(	TY3B-I	Transposon Ty3-I Ga	43.40	0.00
TRINITY_sp|O154	ABCC4	Multidrug resistanc	43.40	0.00
TRINITY_sp|Q9SL(	RAD50	DNA repair protein	43.40	0.00
TRINITY_sp|O603(	AQR	Intron-binding prot	43.40	0.00
TRINITY_sp|O942	hmt2	Sulfide:quinone oxi	43.40	0.00
TRINITY_sp|Q6AYI	Isy1	Pre-mRNA-splicing f	43.40	0.00
TRINITY_sp|Q8L7I	NAD-ME2	NAD-dependent malic	43.40	0.00
TRINITY_sp|P226	-	Chlorophyll a-b bin	43.40	0.00
TRINITY_sp|Q141	WRN	Werner syndrome ATP	43.40	0.00
TRINITY_sp|Q9FW	HAC12	Histone acetyltrans	43.40	0.00
TRINITY_sp|Q8INI	Gyc88E	Soluble guanylate c	43.40	0.00
TRINITY_sp|P043	pol	Retrovirus-related	43.40	0.00
TRINITY_sp|Q9LS	ATL77	RING-H2 finger prot	43.30	0.00
TRINITY_sp|P299	Itp-r83	Inositol 1,4,5-tris	43.30	0.00
TRINITY_sp|O141	tps2	Trehalose-phosphata	43.30	0.00
TRINITY_sp|Q8NE(	DENND6B	Protein DENND6B OS=	43.30	0.00
TRINITY_sp|Q121	SGT2	Small glutamine-ric	43.30	0.00
TRINITY_sp|Q243(	Diap2	Death-associated in	43.30	0.00
TRINITY_sp|Q55G(	cpnD	Copine-D OS=Dictyos	43.30	0.00
TRINITY_sp|P840	RHOG	Rho-related GTP-bin	43.30	0.00
TRINITY_sp|F0NBI	SiRe_14	Protein-lysine N-me	43.30	0.00
TRINITY_sp|P350	cbpP	Calcium-binding pro	43.30	0.00
TRINITY_sp|Q4KLI	btf3l4	Transcription facto	43.30	0.00
TRINITY_sp|Q54TI	DDB_G02	Golgi to ER traffic	43.30	0.00
TRINITY_sp|Q613	Ift88	Intraflagellar tran	43.30	0.00
TRINITY_sp|Q9SF	RABE1E	Ras-related protein	43.30	0.00
TRINITY_sp|Q54P	abcC8	ABC transporter C f	43.30	0.00
TRINITY_sp|P049(	Gstm1	Glutathione S-trans	43.30	0.00
TRINITY_sp|Q54M	gpt	Probable alanine am	43.30	0.00
TRINITY_sp|Q923	SPAC6G9.	Uncharacterized pro	43.30	0.00
TRINITY_sp|Q94B\	NDB2	External alternativ	43.30	0.00

TRINITY_sp|O139:	trm4b	Multisite-specific	43.30	0.00
TRINITY_sp|Q0II:	NAA50	N-alpha-acetyltrans	43.30	0.00
TRINITY_sp|Q9D9(	Atg7	Ubiquitin-like modi	43.30	0.00
TRINITY_sp|P623	CPK1	Calcium-dependent p	43.30	0.00
TRINITY_sp|Q993	TY3B-G	Transposon Ty3-G Ga	43.30	0.00
TRINITY_sp|Q642	diexf	Digestive organ exp	43.30	0.00
TRINITY_sp|Q5SK	trmI	tRNA (adenine(58)-N	43.30	0.00
TRINITY_sp|Q9S7	LOG2	Probable E3 ubiquit	43.30	0.00
TRINITY_sp|Q941	BON1	Protein BONZAI 1 OS	43.30	0.00
TRINITY_sp|Q7X9	AERO2	Endoplasmic reticul	43.30	0.00
TRINITY_sp|Q06A	UCHL3	Ubiquitin carboxyl-	43.30	0.00
TRINITY_sp|P545	yqjY	Uncharacterized pro	43.30	0.00
TRINITY_sp|Q173	W	Protein white OS=Ce	43.30	0.00
TRINITY_sp|P080	top2	DNA topoisomerase 2	43.30	0.00
TRINITY_sp|O439	AIRE	Autoimmune regulato	43.30	0.00
TRINITY_sp|Q9SL	DGAT1	Diacylglycerol O-ac	43.30	0.00
TRINITY_sp|Q078	GPI13	GPI ethanolamine ph	43.30	0.00
TRINITY_sp|Q8RA	mutS	DNA mismatch repair	43.30	0.00
TRINITY_sp|Q84W	At5g045(	Glycosyltransferase	43.30	0.00
TRINITY_sp|Q9FH	TAF14B	Transcription initi	43.30	0.00
TRINITY_sp|P0CR	SEC23	Protein transport p	43.30	0.00
TRINITY_sp|Q469	tcdA	tRNA threonylcarbam	43.30	0.00
TRINITY_sp|Q1AU	rplM	50S ribosomal prote	43.30	0.00
TRINITY_sp|Q9NR	DDX21	Nucleolar RNA helic	43.30	0.00
TRINITY_sp|P550	Abcd3	ATP-binding cassett	43.30	0.00
TRINITY_sp|Q8LS	Os03g01	Probable signal rec	43.30	0.00
TRINITY_sp|Q54S	tmem104	Transmembrane prote	43.30	0.00
TRINITY_sp|F4KH	CTPA1	Carboxyl-terminal-p	43.30	0.00
TRINITY_sp|Q9XF	-	Profilin-3 OS=Malus	43.30	0.00
TRINITY_sp|Q86S	DHDDS	Dehydrodolichyl dip	43.30	0.00
TRINITY_sp|Q8LB	PHR2	Blue-light photorec	43.30	0.00
TRINITY_sp|Q949	PSAO	Photosystem I subun	43.30	0.00
TRINITY_sp|Q23M	Ttll6a	Probable beta-tubul	43.30	0.00
TRINITY_sp|Q7X9	PIE1	Protein PHOTOPERIOD	43.30	0.00
TRINITY_sp|Q5Z9	IMCEL1	Probable isoprenylc	43.30	0.00
TRINITY_sp|Q9SZ	ACA10	Calcium-transportin	43.30	0.00
TRINITY_sp|O235	BAM3	Beta-amylase 3, chl	43.30	0.00
TRINITY_sp|Q095	tag-124	Probable tRNA pseud	43.30	0.00
TRINITY_sp|Q7TM	Slc35f2	Solute carrier fami	43.30	0.00
TRINITY_sp|Q8VY	TIM50	Mitochondrial impor	43.30	0.00
TRINITY_sp|P222(	-	Pyruvate kinase, cy	43.30	0.00
TRINITY_sp|Q54W	mtyrS	Probable tyrosine--	43.30	0.00
TRINITY_sp|Q6GM	plaa	Phospholipase A-2-a	43.30	0.00
TRINITY_sp|O227	RPL28	50S ribosomal prote	43.30	0.00
TRINITY_sp|A5GE	rlmN	Dual-specificity RN	43.30	0.00
TRINITY_sp|C0LG	At4g361	Probable LRR recept	43.30	0.00
TRINITY_sp|P319	HNRNPH3	Heterogeneous nucle	43.30	0.00
TRINITY_sp|Q634	Prpsap1	Phosphoribosyl pyro	43.30	0.00
TRINITY_sp|Q54N	oplah	5-oxoprolinase OS=D	43.30	0.00
TRINITY_sp|Q7TX	pks15/1	Phenolphthiocerol s	43.20	0.00
TRINITY_sp|Q86Y	CPNE8	Copine-8 OS=Homo sa	43.20	0.00
TRINITY_sp|Q091	btl	Fibroblast growth f	43.20	0.00
TRINITY_sp|A1XD	stip-1	Septin and tuftelin	43.20	0.00
TRINITY_sp|A7SK	v1g1715	Eukaryotic translat	43.20	0.00
TRINITY_sp|P529	lacA	Galactoside O-acety	43.20	0.00
TRINITY_sp|P496	Kcnj10	ATP-sensitive inwar	43.20	0.00

TRINITY_sp|O430(	-	Kinesin heavy chain	43.20	0.00
TRINITY_sp|Q8BU\	Gpr107	Protein GPR107 OS=M	43.20	0.00
TRINITY_sp|P546(	mvpB	Major vault protein	43.20	0.00
TRINITY_sp|Q7ZTI	bcs1l	Mitochondrial chape	43.20	0.00
TRINITY_sp|Q5QNI	BZIP02	Basic leucine zippe	43.20	0.00
TRINITY_sp|P435(	AGX1	Alanine--glyoxylate	43.20	0.00
TRINITY_sp|Q863I	OXSR1	Serine/threonine-pr	43.20	0.00
TRINITY_sp|Q9FG3	GCP3	Gamma-tubulin compl	43.20	0.00
TRINITY_sp|Q9SR(	CLPS3	Protein CLP1 homolo	43.20	0.00
TRINITY_sp|Q962(	RpS7	40S ribosomal prote	43.20	0.00
TRINITY_sp|Q993I	TY3B-G	Transposon Ty3-G Ga	43.20	0.00
TRINITY_sp|Q1343	SF3B2	Splicing factor 3B	43.20	0.00
TRINITY_sp|Q3TT\	Plb1	Phospholipase B1, m	43.20	0.00
TRINITY_sp|Q9FN(	At5g0642	Zinc finger CCCH do	43.20	0.00
TRINITY_sp|Q6NXI	dennd5b	DENN domain-contain	43.20	0.00
TRINITY_sp|Q9FPI	EDR1	Serine/threonine-pr	43.20	0.00
TRINITY_sp|P612(	NEDD8	NEDD8 OS=Bos taurus	43.20	0.00
TRINITY_sp|Q9M2(	SPL15	Squamosa promoter-b	43.20	0.00
TRINITY_sp|Q9LD5	TIF3A1	Eukaryotic translat	43.20	0.00
TRINITY_sp|Q9LE(	IRE	Probable serine/thr	43.20	0.00
TRINITY_sp|Q906	THYN1	Thymocyte nuclear p	43.20	0.00
TRINITY_sp|Q5LR	dmdB	3-methylmercaptopro	43.20	0.00
TRINITY_sp|Q55DI	DDB_G02	Histidine protein m	43.20	0.00
TRINITY_sp|Q9FL	TSN2	Ribonuclease TUDOR	43.20	0.00
TRINITY_sp|Q554(	slr0537	Uncharacterized sug	43.10	0.00
TRINITY_sp|B7PXI	spas	Spastin OS=Ixodes s	43.10	0.00
TRINITY_sp|Q3V0I	Lrrc43	Leucine-rich repeat	43.10	0.00
TRINITY_sp|P476(	MG425	Probable ATP-depend	43.10	0.00
TRINITY_sp|Q91YI	Rnf126	E3 ubiquitin-protei	43.10	0.00
TRINITY_sp|P246(	PTP	Tyrosine-protein ph	43.10	0.00
TRINITY_sp|Q6GN	nrm	Nurim OS=Xenopus la	43.10	0.00
TRINITY_sp|Q197	F22B5.1(	Calcium load-activa	43.10	0.00
TRINITY_sp|Q54V(	DDB_G02(	Probable serine/thr	43.10	0.00
TRINITY_sp|Q5EB(	Wdr70	WD repeat-containin	43.10	0.00
TRINITY_sp|O827(	AGL24	MADS-box protein AG	43.10	0.00
TRINITY_sp|Q9WU	Akt3	RAC-gamma serine/th	43.10	0.00
TRINITY_sp|Q54QI	erkB	Extracellular signa	43.10	0.00
TRINITY_sp|Q9LZI	RABE1D	Ras-related protein	43.10	0.00
TRINITY_sp|F4J8I	RRP5	rRNA biogenesis pro	43.10	0.00
TRINITY_sp|Q8MVI	gbpC	Cyclic GMP-binding	43.10	0.00
TRINITY_sp|Q9JM(	Dnaja4	DnaJ homolog subfam	43.10	0.00
TRINITY_sp|Q84QI	OPR1	12-oxophytodienoate	43.10	0.00
TRINITY_sp|P198(	RABA5E	Ras-related protein	43.10	0.00
TRINITY_sp|P281(	RABA5C	Ras-related protein	43.10	0.00
TRINITY_sp|P025	-	Actin-1 OS=Acantham	43.10	0.00
TRINITY_sp|Q246	ref(2)P	Protein ref(2)P OS=	43.10	0.00
TRINITY_sp|Q9LT	RDL2	Probable cysteine p	43.10	0.00
TRINITY_sp|P905	alg1	Chitobiosyldiphosph	43.10	0.00
TRINITY_sp|P181(	splA	Dual specificity pr	43.10	0.00
TRINITY_sp|B5Y9	truA	tRNA pseudouridine	43.10	0.00
TRINITY_sp|P788(	SPBC16A	Probable lipase C16	43.10	0.00
TRINITY_sp|P043	pol	Retrovirus-related	43.10	0.00
TRINITY_sp|Q6P2	dnajc2	DnaJ homolog subfam	43.10	0.00
TRINITY_sp|Q8GY	RPN9B	26S proteasome non-	43.10	0.00
TRINITY_sp|Q9UGI	PARP2	Poly [ADP-ribose] p	43.10	0.00
TRINITY_sp|Q0P5	Vps8	Vacuolar protein so	43.10	0.00

TRINITY_sp|Q70F(	DODA	4,5-DOPA dioxygenas	43.10	0.00
TRINITY_sp|Q9MA(	TAF6	Transcription initi	43.10	0.00
TRINITY_sp|Q8TA(	RFESD	Rieske domain-conta	43.10	0.00
TRINITY_sp|Q6IN!	emc2-a	ER membrane protein	43.10	0.00
TRINITY_sp|Q3ED(	TPST	Protein-tyrosine su	43.10	0.00
TRINITY_sp|Q8H1]	XCT	Protein XAP5 CIRCAD	43.10	0.00
TRINITY_sp|P335]	RpS7	40S ribosomal prote	43.10	0.00
TRINITY_sp|Q54P(	abkD	Probable serine/thr	43.10	0.00
TRINITY_sp|Q96N(	ZMAT2	Zinc finger matrin-	43.10	0.00
TRINITY_sp|P470:	PRY3	Cell wall protein P	43.10	0.00
TRINITY_sp|Q54K:	rgaA	Ras GTPase-activati	43.10	0.00
TRINITY_sp|P146	Pde4c	cAMP-specific 3',5'	43.10	0.00
TRINITY_sp|P562!	pcrA	ATP-dependent DNA h	43.10	0.00
TRINITY_sp|Q9SL	SLY1	SEC1 family transpo	43.10	0.00
TRINITY_sp|Q7X9\	PIE1	Protein PHOTOPERIOD	43.10	0.00
TRINITY_sp|Q94A(	STR6	Rhodanese-like doma	43.10	0.00
TRINITY_sp|Q8GXI	NDC1	Alternative NAD(P)H	43.10	0.00
TRINITY_sp|C4L8(	adk	Adenylate kinase OS	43.10	0.00
TRINITY_sp|O225!	STY8	Serine/threonine-pr	43.10	0.00
TRINITY_sp|Q8W4]	BPG2	GTP-binding protein	43.10	0.00
TRINITY_sp|Q17R(	RAB21	Ras-related protein	43.10	0.00
TRINITY_sp|Q8LI:	Os07g06	Putative MYST-like	43.10	0.00
TRINITY_sp|Q8H6I	SPT16	FACT complex subuni	43.10	0.00
TRINITY_sp|Q9SC(	tubA	Tubulin alpha chain	43.10	0.00
TRINITY_sp|P109	-	Retrovirus-related	43.10	0.00
TRINITY_sp|Q8IY:	DHX37	Probable ATP-depend	43.10	0.00
TRINITY_sp|Q605(	IGHMBP2	DNA-binding protein	43.10	0.00
TRINITY_sp|Q54GI	ctdspl2	CTD small phosphata	43.10	0.00
TRINITY_sp|P252!	-	Cysteine proteinase	43.10	0.00
TRINITY_sp|Q9V1(	PYRAB06	tRNA (cytosine(49)-	43.00	0.00
TRINITY_sp|Q54P!	osbI	Oxysterol-binding p	43.00	0.00
TRINITY_sp|P469	GYG1	Glycogenin-1 OS=Hom	43.00	0.00
TRINITY_sp|Q8T1!	omt3	O-methyltransferase	43.00	0.00
TRINITY_sp|Q16QI	coq2	4-hydroxybenzoate p	43.00	0.00
TRINITY_sp|P533:	CAB4	Phosphopantetheine	43.00	0.00
TRINITY_sp|Q9SB!	AP4M	AP-4 complex subuni	43.00	0.00
TRINITY_sp|Q54VI	vps55	Vacuolar protein so	43.00	0.00
TRINITY_sp|Q9Z2	Tulp1	Tubby-related prote	43.00	0.00
TRINITY_sp|O142(	SPAC6B1	Uncharacterized ami	43.00	0.00
TRINITY_sp|P322!	rasS	Ras-like protein ra	43.00	0.00
TRINITY_sp|A1CV)	gar1	H/ACA ribonucleopro	43.00	0.00
TRINITY_sp|Q102	SPAC4G9.	Uncharacterized mit	43.00	0.00
TRINITY_sp|A6QR	RAB6B	Ras-related protein	43.00	0.00
TRINITY_sp|Q54S	wdr3	WD repeat-containin	43.00	0.00
TRINITY_sp|Q4AEI	DNASE1	Deoxyribonuclease-1	43.00	0.00
TRINITY_sp|Q5EA!	MFAP1	Microfibrillar-asso	43.00	0.00
TRINITY_sp|Q5U2I	Dis3l	DIS3-like exonuclea	43.00	0.00
TRINITY_sp|Q104	stm1	Seven transmembrane	43.00	0.00
TRINITY_sp|B0G1	rabggtb	Probable geranylger	43.00	0.00
TRINITY_sp|Q9BXI	PAPPA2	Pappalysin-2 OS=Hom	43.00	0.00
TRINITY_sp|Q8BRI	Man2a2	Alpha-mannosidase 2	43.00	0.00
TRINITY_sp|Q8RW(	NHX6	Sodium/hydrogen exc	43.00	0.00
TRINITY_sp|Q54RI	shkA	Dual specificity pr	43.00	0.00
TRINITY_sp|Q8GXI	VPS20.1	Vacuolar protein so	43.00	0.00
TRINITY_sp|Q6JQI	ACAD10	Acyl-CoA dehydrogen	43.00	0.00
TRINITY_sp|D4AZ!	PAC1	Nuclear distributio	43.00	0.00

TRINITY_sp|P2383	SCO1	Protein SCO1, mitoc	43.00	0.00
TRINITY_sp|P3905	-	Dynein beta chain,	43.00	0.00
TRINITY_sp|Q9UVN	MRE11	Double-strand break	43.00	0.00
TRINITY_sp|P4325	COP1	E3 ubiquitin-protei	43.00	0.00
TRINITY_sp|Q8TD0	BRSK1	Serine/threonine-pr	43.00	0.00
TRINITY_sp|O7446	taf6	Transcription initi	43.00	0.00
TRINITY_sp|Q9ZR0	FH	Frataxin, mitochond	43.00	0.00
TRINITY_sp|Q8L5Y	CAND1	Cullin-associated N	43.00	0.00
TRINITY_sp|Q9M1Q	ABCB21	ABC transporter B f	43.00	0.00
TRINITY_sp|Q9PL3	ribD	Riboflavin biosynth	43.00	0.00
TRINITY_sp|P5994	Sirt6	NAD-dependent prote	43.00	0.00
TRINITY_sp|P4861	pelo	Protein pelota OS=D	43.00	0.00
TRINITY_sp|Q9FL6	AGD5	Probable ADP-ribosy	43.00	0.00
TRINITY_sp|Q54V0	odhA	Probable 2-oxogluta	43.00	0.00
TRINITY_sp|O8076	At1g6042	Probable nucleoredo	43.00	0.00
TRINITY_sp|O7418	pop3	Target of rapamycin	43.00	0.00
TRINITY_sp|Q9FY0	PAO	Pheophorbide a oxyg	43.00	0.00
TRINITY_sp|O1543	ABCC4	Multidrug resistanc	43.00	0.00
TRINITY_sp|P1915	Gstp1	Glutathione S-trans	43.00	0.00
TRINITY_sp|Q9ATB	ADA2B	Transcriptional ada	43.00	0.00
TRINITY_sp|Q5A59	NIK1	Histidine protein k	43.00	0.00
TRINITY_sp|Q2H52	TMA22	Translation machine	43.00	0.00
TRINITY_sp|O9429	SPBC887.	Probable phospholip	43.00	0.00
TRINITY_sp|P0A9Q	adhE	Aldehyde-alcohol de	43.00	0.00
TRINITY_sp|Q9SHS	LSG1-1	GTPase LSG1-1 OS=Ar	43.00	0.00
TRINITY_sp|Q6T3U	Npc1l1	Niemann-Pick C1-lik	43.00	0.00
TRINITY_sp|P3079	ZMO1242	Uncharacterized pro	43.00	0.00
TRINITY_sp|O0493	-	Alpha-glucosidase O	43.00	0.00
TRINITY_sp|O7516	KDM4A	Lysine-specific dem	43.00	0.00
TRINITY_sp|Q7LH0	TY3B-I	Transposon Ty3-I Ga	43.00	0.00
TRINITY_sp|P9WQ2	treY	Putative maltooligo	42.90	0.00
TRINITY_sp|Q0VGB	Gdpd1	Glycerophosphodiest	42.90	0.00
TRINITY_sp|Q9UL1	HECTD1	E3 ubiquitin-protei	42.90	0.00
TRINITY_sp|Q5ZI8	TBCD	Tubulin-specific ch	42.90	0.00
TRINITY_sp|Q3S4A	AHK5	Histidine kinase 5	42.90	0.00
TRINITY_sp|Q9WYX	TM_0508	Uncharacterized pro	42.90	0.00
TRINITY_sp|Q9FN0	BGAL10	Beta-galactosidase	42.90	0.00
TRINITY_sp|A8WH1	narfl	Cytosolic Fe-S clus	42.90	0.00
TRINITY_sp|Q9XGU	ARAC9	Rac-like GTP-bindin	42.90	0.00
TRINITY_sp|Q9GZR	SENP6	Sentrin-specific pr	42.90	0.00
TRINITY_sp|Q769B	empA	Transmembrane emp24	42.90	0.00
TRINITY_sp|Q1241	TFC7	Transcription facto	42.90	0.00
TRINITY_sp|Q3TWB	Wdr70	WD repeat-containin	42.90	0.00
TRINITY_sp|Q96N4	TTC14	Tetratricopeptide r	42.90	0.00
TRINITY_sp|Q2MHB	HT1	Serine/threonine-pr	42.90	0.00
TRINITY_sp|Q8GX8	At1g0135	Zinc finger CCCH do	42.90	0.00
TRINITY_sp|Q0JL7	Os01g061	Probable protein ph	42.90	0.00
TRINITY_sp|P3120	pab1	Polyadenylate-bindi	42.90	0.00
TRINITY_sp|O1337	ded1	ATP-dependent RNA h	42.90	0.00
TRINITY_sp|P4325	COP1	E3 ubiquitin-protei	42.90	0.00
TRINITY_sp|Q8WTR	DUSP19	Dual specificity pr	42.90	0.00
TRINITY_sp|Q95YI	pefB	Penta-EF hand domai	42.90	0.00
TRINITY_sp|Q54Y2	dhkJ	Hybrid signal trans	42.90	0.00
TRINITY_sp|Q54WB	pldB	Phospholipase D B O	42.90	0.00
TRINITY_sp|Q9FIW	BGLU42	Beta-glucosidase 42	42.90	0.00
TRINITY_sp|Q6ZT9	TTLL7	Tubulin polyglutamy	42.90	0.00

TRINITY_sp|Q9H4	PIGU	Phosphatidylinosito	42.90	0.00
TRINITY_sp|A9CB	ZNF259	Zinc finger protein	42.90	0.00
TRINITY_sp|Q9BW	TPPP3	Tubulin polymerizat	42.90	0.00
TRINITY_sp|Q182	rap-1	Ras-related protein	42.90	0.00
TRINITY_sp|Q86J	rab5A	Ras-related protein	42.90	0.00
TRINITY_sp|Q29A	GA17800	Leishmanolysin-like	42.90	0.00
TRINITY_sp|Q8BX	Arfrp1	ADP-ribosylation fa	42.90	0.00
TRINITY_sp|P352	Rab23	Ras-related protein	42.90	0.00
TRINITY_sp|P468	yhhW	Quercetin 2,3-dioxy	42.90	0.00
TRINITY_sp|P220	bglA	Beta-glucosidase A	42.90	0.00
TRINITY_sp|Q236	gcy-5	Receptor-type guany	42.90	0.00
TRINITY_sp|P160	NIA1	Nitrate reductase [	42.90	0.00
TRINITY_sp|A2AR	Ppip5k1	Inositol hexakispho	42.90	0.00
TRINITY_sp|Q9W7	cpsf2	Cleavage and polyad	42.90	0.00
TRINITY_sp|Q9VQ	CG9662	Putative oligosacch	42.90	0.00
TRINITY_sp|O285	hmgA	3-hydroxy-3-methylg	42.90	0.00
TRINITY_sp|P678	SEC11A	Signal peptidase co	42.90	0.00
TRINITY_sp|Q557	DDB_G02	UPF0505 protein OS=	42.90	0.00
TRINITY_sp|Q9ER	SMC4	Structural maintena	42.90	0.00
TRINITY_sp|O221	GOS12	Golgi SNAP receptor	42.90	0.00
TRINITY_sp|Q323	ybiA	N-glycosidase YbiA	42.90	0.00
TRINITY_sp|O140	SPAC2C4.	Putative tRNA 2'-ph	42.90	0.00
TRINITY_sp|Q55C	DDB_G02	Probable NADH dehyd	42.90	0.00
TRINITY_sp|F4JL	IMPA2	Importin subunit al	42.90	0.00
TRINITY_sp|Q643	ATP12A	Potassium-transport	42.90	0.00
TRINITY_sp|P227	Gucy1b2	Guanylate cyclase s	42.90	0.00
TRINITY_sp|Q9C6	KCS5	3-ketoacyl-CoA synt	42.90	0.00
TRINITY_sp|P234	CYR1	Adenylate cyclase O	42.90	0.00
TRINITY_sp|Q8L7	TUN	UDP-glycosyltransfe	42.90	0.00
TRINITY_sp|P944	ynbB	Uncharacterized pro	42.90	0.00
TRINITY_sp|Q6DF	isoc2	Isochorismatase dom	42.90	0.00
TRINITY_sp|Q9UR	Tf2-11	Transposon Tf2-11 p	42.90	0.00
TRINITY_sp|Q9Y2	MTMR7	Myotubularin-relate	42.90	0.00
TRINITY_sp|P426	yqjG	Glutathionyl-hydroq	42.90	0.00
TRINITY_sp|Q4R5	CELF4	CUGBP Elav-like fam	42.90	0.00
TRINITY_sp|P408	USP8	Ubiquitin carboxyl-	42.90	0.00
TRINITY_sp|Q7PQ	AGAP002	ADP,ATP carrier pro	42.90	0.00
TRINITY_sp|Q9LK	UVH1	DNA repair endonucl	42.90	0.00
TRINITY_sp|Q8LP	ABCB28	ABC transporter B f	42.90	0.00
TRINITY_sp|Q9LM	HRD3A	ERAD-associated E3	42.90	0.00
TRINITY_sp|A4QN	leng8	Leukocyte receptor	42.90	0.00
TRINITY_sp|P487	uapC	Purine permease OS=	42.90	0.00
TRINITY_sp|Q5XI	Traf3ip	TRAF3-interacting p	42.90	0.00
TRINITY_sp|B5BU	pf13	Protein kintoun OS=	42.90	0.00
TRINITY_sp|Q9C7	AERO1	Endoplasmic reticul	42.90	0.00
TRINITY_sp|P109	-	Retrovirus-related	42.90	0.00
TRINITY_sp|P109	-	Retrovirus-related	42.90	0.00
TRINITY_sp|Q7LH	TY3B-I	Transposon Ty3-I Ga	42.90	0.00
TRINITY_sp|Q556	mad2l1-	Mitotic spindle ass	42.90	0.00
TRINITY_sp|P042	CD74	HLA class II histoc	42.90	0.00
TRINITY_sp|Q6NS	zgc:661	Serine/threonine-pr	42.90	0.00
TRINITY_sp|Q2YD	ANAPC10	Anaphase-promoting	42.90	0.00
TRINITY_sp|P339	bsr	Blasticidin-S deami	42.90	0.00
TRINITY_sp|A5DI	DHH1	ATP-dependent RNA h	42.90	0.00
TRINITY_sp|Q94A	DLD	D-lactate dehydroge	42.80	0.00
TRINITY_sp|Q9UJ	HACL1	2-hydroxyacyl-CoA l	42.80	0.00

TRINITY_sp|Q9USV	SPBC21B1	Uncharacterized pro	42.80	0.00
TRINITY_sp|Q9VH	Taf7	Transcription initi	42.80	0.00
TRINITY_sp|Q9SV2	At4g3148	Coatomer subunit be	42.80	0.00
TRINITY_sp|Q55E6	sys1	Protein SYS1 homolo	42.80	0.00
TRINITY_sp|F4IA1	THO2	THO complex subunit	42.80	0.00
TRINITY_sp|Q9DB8	Naa16	N-alpha-acetyltrans	42.80	0.00
TRINITY_sp|Q9P2I	FAM135A	Protein FAM135A OS=	42.80	0.00
TRINITY_sp|Q9938	VCX1	Vacuolar calcium io	42.80	0.00
TRINITY_sp|A6N63	Wdr35	WD repeat-containin	42.80	0.00
TRINITY_sp|Q6NR0	noc4l-a	Nucleolar complex p	42.80	0.00
TRINITY_sp|O5284	bgaM	Beta-galactosidase	42.80	0.00
TRINITY_sp|Q1469	BMS1	Ribosome biogenesis	42.80	0.00
TRINITY_sp|Q9LD8	BETAA-AD	Beta-adaptin-like p	42.80	0.00
TRINITY_sp|Q9C53	PFK7	ATP-dependent 6-pho	42.80	0.00
TRINITY_sp|Q9SD8	GDPD6	Glycerophosphodiest	42.80	0.00
TRINITY_sp|P0CQ7	TIF1	ATP-dependent RNA h	42.80	0.00
TRINITY_sp|Q7SXM	prpf31	U4/U6 small nuclear	42.80	0.00
TRINITY_sp|Q9LV0	At5g6672	Probable protein ph	42.80	0.00
TRINITY_sp|P6170	alaS	Alanine--tRNA ligas	42.80	0.00
TRINITY_sp|Q1101	Npepps	Puromycin-sensitive	42.80	0.00
TRINITY_sp|Q9FP3	RBP45A	Polyadenylate-bindi	42.80	0.00
TRINITY_sp|P1539	rpa1	DNA-directed RNA po	42.80	0.00
TRINITY_sp|Q7T3A	rab13	Ras-related protein	42.80	0.00
TRINITY_sp|Q8VC1	Plbd1	Phospholipase B-lik	42.80	0.00
TRINITY_sp|F4IYM	DEX1	Protein DEFECTIVE I	42.80	0.00
TRINITY_sp|Q8GTR	PU1	Pullulanase 1, chlo	42.80	0.00
TRINITY_sp|O5006	FIM2	Fimbrin-2 OS=Arabid	42.80	0.00
TRINITY_sp|Q9C6I	KCS5	3-ketoacyl-CoA synt	42.80	0.00
TRINITY_sp|Q9BW9	TARS2	Threonine--tRNA lig	42.70	0.00
TRINITY_sp|Q1RIM	RBE_0699	SCO2-like protein R	42.70	0.00
TRINITY_sp|Q0MQ0	NDUFAB1	Acyl carrier protei	42.70	0.00
TRINITY_sp|Q2780	DYH1B	Cytoplasmic dynein	42.70	0.00
TRINITY_sp|B0M0F	gefL	Ras guanine nucleot	42.70	0.00
TRINITY_sp|Q504M	Rab26	Ras-related protein	42.70	0.00
TRINITY_sp|Q68B3	TK1108	Phosphoglucomutase/	42.70	0.00
TRINITY_sp|Q7ZV2	abhd17c	Protein ABHD17C OS=	42.70	0.00
TRINITY_sp|O8092	AGD7	ADP-ribosylation fa	42.70	0.00
TRINITY_sp|Q8K21	Nmral1	NmrA-like family do	42.70	0.00
TRINITY_sp|Q1352	PIN1	Peptidyl-prolyl cis	42.70	0.00
TRINITY_sp|Q29F0	GA18292	Ubiquitin thioester	42.70	0.00
TRINITY_sp|F4JXE	NRPC1	DNA-directed RNA po	42.70	0.00
TRINITY_sp|O1496	RAB29	Ras-related protein	42.70	0.00
TRINITY_sp|Q8L70	BSL1	Serine/threonine-pr	42.70	0.00
TRINITY_sp|Q9FNA	NAGLU	Alpha-N-acetylgluco	42.70	0.00
TRINITY_sp|Q0169	-	Bacterial leucyl am	42.70	0.00
TRINITY_sp|Q54BW	ttc27	Tetratricopeptide r	42.70	0.00
TRINITY_sp|O8036	RPL17	50S ribosomal prote	42.70	0.00
TRINITY_sp|Q4105	SBEI	1,4-alpha-glucan-br	42.70	0.00
TRINITY_sp|Q8TB2	SPATA20	Spermatogenesis-ass	42.70	0.00
TRINITY_sp|Q75B4	MCA1	Metacaspase-1 OS=As	42.70	0.00
TRINITY_sp|Q54M2	dnajc3	DnaJ homolog subfam	42.70	0.00
TRINITY_sp|Q0554	HRQ1	ATP-dependent helic	42.70	0.00
TRINITY_sp|Q75B1	ADL233W	Leukotriene A-4 hyd	42.70	0.00
TRINITY_sp|Q7Z56	BRAP	BRCA1-associated pr	42.70	0.00
TRINITY_sp|Q9H8H	DDX31	Probable ATP-depend	42.70	0.00
TRINITY_sp|A1JK3	YE1008	tRNA1(Val) (adenine	42.70	0.00

TRINITY_sp|Q8WTI	DUSP19	Dual specificity pr	42.70	0.00
TRINITY_sp|Q9HD2	ATP13A1	Manganese-transport	42.70	0.00
TRINITY_sp|Q54Q6	dhkG	Hybrid signal trans	42.70	0.00
TRINITY_sp|Q55GU	DDB_G026	Probable serine/thr	42.70	0.00
TRINITY_sp|Q8C0I	Efl1	Elongation factor-l	42.70	0.00
TRINITY_sp|Q6MEI	truA1	tRNA pseudouridine	42.70	0.00
TRINITY_sp|Q8BQQ	Zdhhc14	Probable palmitoylt	42.70	0.00
TRINITY_sp|Q9ST2	PHOT2	Phototropin-2 OS=Or	42.70	0.00
TRINITY_sp|P3840	GNAI1	Guanine nucleotide-	42.70	0.00
TRINITY_sp|Q9SS4	APUM4	Pumilio homolog 4 O	42.70	0.00
TRINITY_sp|E9Q8I	Dnajc21	DnaJ homolog subfam	42.70	0.00
TRINITY_sp|Q9LS2	GTE12	Transcription facto	42.70	0.00
TRINITY_sp|P5639	Usp5	Ubiquitin carboxyl-	42.70	0.00
TRINITY_sp|F4KE6	EMB2247	Valine--tRNA ligase	42.70	0.00
TRINITY_sp|Q24UJ	rpsO	30S ribosomal prote	42.70	0.00
TRINITY_sp|P4959	PP2CA	Protein phosphatase	42.70	0.00
TRINITY_sp|Q6TGJ	ATG9	Autophagy-related p	42.70	0.00
TRINITY_sp|Q642I	Sf1	Splicing factor 1 O	42.70	0.00
TRINITY_sp|Q5VRI	BASS2	Probable sodium/met	42.70	0.00
TRINITY_sp|Q5BB4	tah18	NADPH-dependent dif	42.70	0.00
TRINITY_sp|Q0106	Pde1b	Calcium/calmodulin-	42.70	0.00
TRINITY_sp|Q9BW3	TPPP3	Tubulin polymerizat	42.70	0.00
TRINITY_sp|O6033	MAPKBP1	Mitogen-activated p	42.70	0.00
TRINITY_sp|P7614	sad	Succinate semialdeh	42.60	0.00
TRINITY_sp|H2L0I	chat-1	CDC50 family protei	42.60	0.00
TRINITY_sp|Q93VC	At4g1748	DeSI-like protein A	42.60	0.00
TRINITY_sp|Q86C6	tor	Target of rapamycin	42.60	0.00
TRINITY_sp|P2212	-	Ras-related protein	42.60	0.00
TRINITY_sp|Q72A1	acpS	Holo-[acyl-carrier-	42.60	0.00
TRINITY_sp|Q55BN	H3b	Histone H3.3 type b	42.60	0.00
TRINITY_sp|Q1971	F22B5.10	Calcium load-activa	42.60	0.00
TRINITY_sp|Q9LZI	ERDJ3B	DnaJ protein ERDJ3B	42.60	0.00
TRINITY_sp|Q54YI	fol1	Folic acid synthesi	42.60	0.00
TRINITY_sp|Q8RWA	NDT2	Nicotinamide adenin	42.60	0.00
TRINITY_sp|Q5AU6	nmrA	Nitrogen metabolite	42.60	0.00
TRINITY_sp|D4B0V	ARB_0207	Probable glucan end	42.60	0.00
TRINITY_sp|Q8K3I	Tdh	L-threonine 3-dehyd	42.60	0.00
TRINITY_sp|Q9NCI	pitB	Phosphatidylinosito	42.60	0.00
TRINITY_sp|O0090	-	Lysosomal acid alph	42.60	0.00
TRINITY_sp|Q9HC0	TMEM165	Transmembrane prote	42.60	0.00
TRINITY_sp|O1563	RPS19	40S ribosomal prote	42.60	0.00
TRINITY_sp|A1A4J	ATP9B	Probable phospholip	42.60	0.00
TRINITY_sp|P4412	ligA	DNA ligase OS=Haemo	42.60	0.00
TRINITY_sp|Q3UWA	Gucy2c	Heat-stable enterot	42.60	0.00
TRINITY_sp|O0199	efk-1	Eukaryotic elongati	42.60	0.00
TRINITY_sp|Q0712	maoI	Primary amine oxida	42.60	0.00
TRINITY_sp|F4JAA	SKI2	DExH-box ATP-depend	42.60	0.00
TRINITY_sp|O8073	TPS10	Probable alpha,alph	42.60	0.00
TRINITY_sp|Q93VI	PPCK2	Phosphoenolpyruvate	42.60	0.00
TRINITY_sp|Q9FE1	SRT1	NAD-dependent prote	42.60	0.00
TRINITY_sp|Q9FVV	UPF3	Regulator of nonsen	42.60	0.00
TRINITY_sp|Q9ZU9	VAC14	Protein VAC14 homol	42.60	0.00
TRINITY_sp|P3910	YCF1	Metal resistance pr	42.60	0.00
TRINITY_sp|A2ZMI	NEK2	Serine/threonine-pr	42.60	0.00
TRINITY_sp|Q9C70	PUB45	U-box domain-contai	42.60	0.00
TRINITY_sp|Q564I	CAPH	Condensin complex s	42.60	0.00

TRINITY_sp|P0AB8	nth	Endonuclease III OS	42.60	0.00
TRINITY_sp|P5153	SMARCA4	Transcription activ	42.60	0.00
TRINITY_sp|Q9ZNV	AHP1	Histidine-containin	42.60	0.00
TRINITY_sp|Q08DJ	AAR2	Protein AAR2 homolo	42.60	0.00
TRINITY_sp|Q9JIC	Dclre1a	DNA cross-link repa	42.60	0.00
TRINITY_sp|O7438	SPBC3H7.	Uncharacterized met	42.60	0.00
TRINITY_sp|Q55GU	DDB_G026	Probable serine/thr	42.60	0.00
TRINITY_sp|O6583	CRTL-E-1	Lycopene epsilon cy	42.60	0.00
TRINITY_sp|Q8LIE	CYP734A5	Cytochrome P450 734	42.60	0.00
TRINITY_sp|Q9239	spm1	Mitogen-activated p	42.60	0.00
TRINITY_sp|Q9VHF	Kdm2	JmjC domain-contain	42.60	0.00
TRINITY_sp|P0881	CBP2	Serine carboxypepti	42.60	0.00
TRINITY_sp|Q54XX	DDB_G027	Probable serine/thr	42.60	0.00
TRINITY_sp|Q94J2	PUX7	Plant UBX domain-co	42.60	0.00
TRINITY_sp|P5399	SEC24C	Protein transport p	42.60	0.00
TRINITY_sp|Q6BJW	INV	Invertase OS=Debary	42.60	0.00
TRINITY_sp|Q0981	nmd3	60S ribosomal expor	42.60	0.00
TRINITY_sp|P5520	GUCY2D	Retinal guanylyl cy	42.60	0.00
TRINITY_sp|Q5I02	slc30a8	Zinc transporter 8	42.60	0.00
TRINITY_sp|C0LGN	LRR-RLK	Probable leucine-ri	42.60	0.00
TRINITY_sp|Q3916	XI-1	Myosin-5 OS=Arabido	42.60	0.00
TRINITY_sp|Q5F38	PIGM	GPI mannosyltransfe	42.60	0.00
TRINITY_sp|P3820	NCL1	Multisite-specific	42.60	0.00
TRINITY_sp|Q54RV	trappc2	Trafficking protein	42.60	0.00
TRINITY_sp|Q54N2	DDB_G028	Isochorismatase fam	42.60	0.00
TRINITY_sp|P5463	cprD	Cysteine proteinase	42.50	0.00
TRINITY_sp|A5DN2	YPI1	Type 1 phosphatases	42.50	0.00
TRINITY_sp|A2XDC	UBP26	Ubiquitin carboxyl-	42.50	0.00
TRINITY_sp|P1401	ODC1	Ornithine decarboxy	42.50	0.00
TRINITY_sp|Q6P36	rnf121	RING finger protein	42.50	0.00
TRINITY_sp|Q6DFV	Ncoa7	Nuclear receptor co	42.50	0.00
TRINITY_sp|Q6FZ1	engB	Probable GTP-bindin	42.50	0.00
TRINITY_sp|P4080	Odc1	Ornithine decarboxy	42.50	0.00
TRINITY_sp|O2226	CKI1	Histidine kinase CK	42.50	0.00
TRINITY_sp|Q55BN	nek2	Probable serine/thr	42.50	0.00
TRINITY_sp|Q6116	Mapre1	Microtubule-associa	42.50	0.00
TRINITY_sp|A0NGI	AGAP0121	Transmembrane prote	42.50	0.00
TRINITY_sp|Q8GYE	CAT3	Cationic amino acid	42.50	0.00
TRINITY_sp|Q8N5X	EIF4E3	Eukaryotic translat	42.50	0.00
TRINITY_sp|Q9S7U	MKK2	Mitogen-activated p	42.50	0.00
TRINITY_sp|Q59PT	VMA2	V-type proton ATPas	42.50	0.00
TRINITY_sp|O1586	CPK2	Calcium-dependent p	42.50	0.00
TRINITY_sp|Q5ZM4	USP48	Ubiquitin carboxyl-	42.50	0.00
TRINITY_sp|O0876	Ogg1	N-glycosylase/DNA l	42.50	0.00
TRINITY_sp|Q6DDC	zc3h15	Zinc finger CCCH do	42.50	0.00
TRINITY_sp|Q0VFV	kctd7	BTB/POZ domain-cont	42.50	0.00
TRINITY_sp|Q55BE	ufd1	Ubiquitin fusion de	42.50	0.00
TRINITY_sp|Q8IBS	CPK4	Calcium-dependent p	42.50	0.00
TRINITY_sp|Q54P2	DDB_G028	Stromal cell-derive	42.50	0.00
TRINITY_sp|Q9FVU	CSN5A	COP9 signalosome co	42.50	0.00
TRINITY_sp|Q1ZXE	gxcDD	Guanine exchange fa	42.50	0.00
TRINITY_sp|P6048	PTEN	Phosphatidylinosito	42.50	0.00
TRINITY_sp|Q54LV	jcdC	JmjC domain-contain	42.50	0.00
TRINITY_sp|Q8RY2	NUP98A	Nuclear pore comple	42.50	0.00
TRINITY_sp|Q8WN9	ITPR2	Inositol 1,4,5-tris	42.50	0.00
TRINITY_sp|F4K5X	RVE2	Protein REVEILLE 2	42.50	0.00

TRINITY_sp|Q9SDI	HMT-1	Homocysteine S-meth	42.50	0.00
TRINITY_sp|Q075S	sav	Protein SAV OS=Sulf	42.50	0.00
TRINITY_sp|P0C1I	cyp5	Peptidyl-prolyl cis	42.50	0.00
TRINITY_sp|Q9TX3	cmk-1	Calcium/calmodulin-	42.50	0.00
TRINITY_sp|Q9SHS	BSL3	Serine/threonine-pr	42.50	0.00
TRINITY_sp|Q9M0Y	NSF	Vesicle-fusing ATPa	42.50	0.00
TRINITY_sp|P218	GART	Trifunctional purin	42.50	0.00
TRINITY_sp|F4JNY	APE2	DNA-(apurinic or ap	42.50	0.00
TRINITY_sp|A2WNE	ARP6	Actin-related prote	42.50	0.00
TRINITY_sp|P252	-	Cysteine proteinase	42.50	0.00
TRINITY_sp|P140	-	Chymopapain OS=Cari	42.50	0.00
TRINITY_sp|P529	VPS4	Vacuolar protein so	42.50	0.00
TRINITY_sp|P740	rsmI	Ribosomal RNA small	42.50	0.00
TRINITY_sp|Q097	trm112	Multifunctional met	42.50	0.00
TRINITY_sp|Q64J1	PIMT2	Protein-L-isoaspart	42.50	0.00
TRINITY_sp|P982	ALA2	Phospholipid-transp	42.50	0.00
TRINITY_sp|Q9BUE	MSTO1	Protein misato homo	42.50	0.00
TRINITY_sp|F4JA	P4H2	Prolyl 4-hydroxylas	42.50	0.00
TRINITY_sp|P577	EEFSEC	Selenocysteine-spec	42.50	0.00
TRINITY_sp|Q91WU	Slc22a7	Solute carrier fami	42.50	0.00
TRINITY_sp|P371	-	Actophorin OS=Acant	42.50	0.00
TRINITY_sp|Q08BW	zgc:154	Uncharacterized pro	42.50	0.00
TRINITY_sp|P109	-	Retrovirus-related	42.50	0.00
TRINITY_sp|Q87AI	PD_1892	UPF0394 membrane pr	42.50	0.00
TRINITY_sp|Q502	tmem41a	Transmembrane prote	42.50	0.00
TRINITY_sp|O597	ark1	Serine/threonine-pr	42.50	0.00
TRINITY_sp|Q8BH	Napepld	N-acyl-phosphatidyl	42.50	0.00
TRINITY_sp|Q651	Os09g05	P-loop NTPase domai	42.50	0.00
TRINITY_sp|Q9M2	CEF	Protein transport p	42.50	0.00
TRINITY_sp|P109	-	Retrovirus-related	42.50	0.00
TRINITY_sp|Q54X	mfeB	Probable enoyl-CoA	42.40	0.00
TRINITY_sp|Q4FZW	seh1l-a	Nucleoporin seh1-A	42.40	0.00
TRINITY_sp|Q54RE	dhkL	Hybrid signal trans	42.40	0.00
TRINITY_sp|Q5NC	Ttf2	Transcription termi	42.40	0.00
TRINITY_sp|P629	RAC1	Ras-related C3 botu	42.40	0.00
TRINITY_sp|Q8RUI	NLP3	Omega-amidase, chlo	42.40	0.00
TRINITY_sp|P206	Prim1	DNA primase small s	42.40	0.00
TRINITY_sp|Q2KJ	PPP2R4	Serine/threonine-pr	42.40	0.00
TRINITY_sp|Q3ABI	coaE	Dephospho-CoA kinas	42.40	0.00
TRINITY_sp|Q9LDU	DWF5	7-dehydrocholestero	42.40	0.00
TRINITY_sp|Q141	DOCK1	Dedicator of cytoki	42.40	0.00
TRINITY_sp|A4QQ	FMN1	Riboflavin kinase O	42.40	0.00
TRINITY_sp|P204	pyrK	UMP-CMP kinase OS=D	42.40	0.00
TRINITY_sp|Q3T0I	CTSH	Pro-cathepsin H OS=	42.40	0.00
TRINITY_sp|Q8H0W	HDA9	Histone deacetylase	42.40	0.00
TRINITY_sp|Q08B	alg11	GDP-Man:Man(3)GlcNA	42.40	0.00
TRINITY_sp|P077	SI	Sucrase-isomaltase,	42.40	0.00
TRINITY_sp|P502	ALTA	Tubulin alpha-1A ch	42.40	0.00
TRINITY_sp|Q925	GBF1	Golgi-specific bref	42.40	0.00
TRINITY_sp|Q6AI	HEATR6	HEAT repeat-contain	42.40	0.00
TRINITY_sp|Q930	TATDN2	Putative deoxyribon	42.40	0.00
TRINITY_sp|P534	CTN	Caltractin OS=Naegl	42.40	0.00
TRINITY_sp|Q6AY	Nudt5	ADP-sugar pyrophosp	42.40	0.00
TRINITY_sp|Q1ZXI	DDB_G02	Probable serine/thr	42.40	0.00
TRINITY_sp|F4JS2	SRFR1	Suppressor of RPS4-	42.40	0.00
TRINITY_sp|Q8IYI	TRMT44	Probable tRNA (urac	42.40	0.00

TRINITY_sp|Q54S	vps15	Probable serine/thr	42.40	0.00
TRINITY_sp|Q9SVI	RBG2	Glycine-rich RNA-bi	42.40	0.00
TRINITY_sp|O433	TXNL1	Thioredoxin-like pr	42.40	0.00
TRINITY_sp|Q9J5	FPV151	Probable deoxycytid	42.40	0.00
TRINITY_sp|Q54T	yipf1	Protein YIPF1 homol	42.40	0.00
TRINITY_sp|Q5RDI	TEX10	Testis-expressed se	42.40	0.00
TRINITY_sp|P196	yqxC	Putative rRNA methy	42.40	0.00
TRINITY_sp|Q54P	DDB_G02	OTU domain-containi	42.40	0.00
TRINITY_sp|Q86C	tor	Target of rapamycin	42.40	0.00
TRINITY_sp|Q8IN	Akt1	RAC serine/threonin	42.40	0.00
TRINITY_sp|Q54C	helA	ATP-dependent RNA h	42.40	0.00
TRINITY_sp|Q72Q	hppA	Putative K(+)-stimu	42.40	0.00
TRINITY_sp|A2YM	PDRP1	Probable pyruvate,	42.40	0.00
TRINITY_sp|P929	RS41	Serine/arginine-ric	42.40	0.00
TRINITY_sp|Q9M1	At3g544	F-box protein At3g5	42.40	0.00
TRINITY_sp|Q9SR	At3g089	LIMR family protein	42.40	0.00
TRINITY_sp|Q9FV	AMT1-3	Ammonium transporte	42.40	0.00
TRINITY_sp|Q54B	abcB2	ABC transporter B f	42.40	0.00
TRINITY_sp|P618	aptx	Aprataxin OS=Takifu	42.40	0.00
TRINITY_sp|P445	arcB	Aerobic respiration	42.40	0.00
TRINITY_sp|Q6GL	alyref-k	THO complex subunit	42.40	0.00
TRINITY_sp|Q123	RIB2	Bifunctional protei	42.40	0.00
TRINITY_sp|Q54Y	copb2	Coatomer subunit be	42.40	0.00
TRINITY_sp|O151	NPC1	Niemann-Pick C1 pro	42.40	0.00
TRINITY_sp|Q9AT	UVH3	DNA repair protein	42.40	0.00
TRINITY_sp|O047	MSH6	DNA mismatch repair	42.40	0.00
TRINITY_sp|P484	Ppp3cc	Serine/threonine-pr	42.40	0.00
TRINITY_sp|Q54B	fhkA	Probable serine/thr	42.40	0.00
TRINITY_sp|P546	patA	Calcium-transportin	42.40	0.00
TRINITY_sp|P491	CPK2	Calcium-dependent p	42.40	0.00
TRINITY_sp|Q5XI	Alkbh3	Alpha-ketoglutarate	42.40	0.00
TRINITY_sp|Q8GY	UPL1	E3 ubiquitin-protei	42.40	0.00
TRINITY_sp|Q9V5	Cyp4p2	Probable cytochrome	42.40	0.00
TRINITY_sp|Q9PW	DUSP4	Dual specificity pr	42.40	0.00
TRINITY_sp|O701	Tesk1	Dual specificity te	42.40	0.00
TRINITY_sp|P491	CPK2	Calcium-dependent p	42.40	0.00
TRINITY_sp|Q3UG	Wdr19	WD repeat-containin	42.30	0.00
TRINITY_sp|Q9FM	RHG1A	Probable E3 ubiquit	42.30	0.00
TRINITY_sp|Q69T	PRXIIE-1	Peroxiredoxin-2E-1,	42.30	0.00
TRINITY_sp|Q94A	At3g094	UNC93-like protein	42.30	0.00
TRINITY_sp|Q86H	abcG8	ABC transporter G f	42.30	0.00
TRINITY_sp|O520	arsM	Putative arsenite m	42.30	0.00
TRINITY_sp|Q1E7	TPC1	Mitochondrial thiam	42.30	0.00
TRINITY_sp|Q94B	ATL75	RING-H2 finger prot	42.30	0.00
TRINITY_sp|Q125	SET6	Potential protein l	42.30	0.00
TRINITY_sp|Q54R	DDB_G02	Probable tyrosine-p	42.30	0.00
TRINITY_sp|P0C6	Dnah2	Dynein heavy chain	42.30	0.00
TRINITY_sp|Q029	amyA	Alpha-amylase A OS=	42.30	0.00
TRINITY_sp|Q9SC	At3g505	Phosphoglycerate mu	42.30	0.00
TRINITY_sp|Q6U1	SGK1	Serine/threonine-pr	42.30	0.00
TRINITY_sp|D4AC	Recql5	ATP-dependent DNA h	42.30	0.00
TRINITY_sp|Q61Y	CBG0355	Leishmanolysin-like	42.30	0.00
TRINITY_sp|P393	iadA	Isoaspartyl dipepti	42.30	0.00
TRINITY_sp|Q4V8	Lrrc56	Leucine-rich repeat	42.30	0.00
TRINITY_sp|P105	MIC	Myosin IC heavy cha	42.30	0.00
TRINITY_sp|P053	p20	Uncharacterized N-a	42.30	0.00

TRINITY_sp|Q9FW(	HAC12	Histone acetyltrans	42.30	0.00
TRINITY_sp|Q949\	UBP6	Ubiquitin carboxyl-	42.30	0.00
TRINITY_sp|Q8GW	GFA2	Chaperone protein d	42.30	0.00
TRINITY_sp|Q9XYI	fcpA	Probable C-terminal	42.30	0.00
TRINITY_sp|Q54U(	dnapkcs	DNA-dependent prote	42.30	0.00
TRINITY_sp|Q9D8	Dnajb4	DnaJ homolog subfam	42.30	0.00
TRINITY_sp|Q388	XPB1	DNA repair helicase	42.30	0.00
TRINITY_sp|P583	atp1a3	Sodium/potassium-tr	42.30	0.00
TRINITY_sp|Q6AZI	immp2l	Mitochondrial inner	42.30	0.00
TRINITY_sp|F4ID\	At1g189	Zinc finger protein	42.30	0.00
TRINITY_sp|Q8GU	PIS2	Probable CDP-diacyl	42.30	0.00
TRINITY_sp|P324	pab	Para-aminobenzoate	42.30	0.00
TRINITY_sp|Q6BZ\	TIM22	Mitochondrial impor	42.30	0.00
TRINITY_sp|Q316	dnaJ	Chaperone protein D	42.30	0.00
TRINITY_sp|Q5D0	mettl10	Protein-lysine N-me	42.30	0.00
TRINITY_sp|Q553\	p2xB	P2X receptor B OS=D	42.30	0.00
TRINITY_sp|O654	XCP1	Cysteine protease X	42.30	0.00
TRINITY_sp|Q8LF(	RBL19	Rhomboid-like prote	42.30	0.00
TRINITY_sp|P485(	-	Cytochrome b-c1 com	42.30	0.00
TRINITY_sp|D3ZG	Ocrl	Inositol polyphosph	42.30	0.00
TRINITY_sp|P134	abpC	Gelation factor OS=	42.30	0.00
TRINITY_sp|Q9FM	PI4KB1	Phosphatidylinosito	42.30	0.00
TRINITY_sp|Q17QI	TMEM184(	Transmembrane prote	42.30	0.00
TRINITY_sp|Q8SS(	UGP1	UTP--glucose-1-phos	42.30	0.00
TRINITY_sp|Q9XHI	TIF3C1	Eukaryotic translat	42.30	0.00
TRINITY_sp|P704	Pld1	Phospholipase D1 OS	42.30	0.00
TRINITY_sp|P278	PDE4A	cAMP-specific 3',5'	42.30	0.00
TRINITY_sp|Q9FZI	MGD	Probable monogalact	42.30	0.00
TRINITY_sp|Q7KW	exosc3	Putative exosome co	42.30	0.00
TRINITY_sp|B2S7I	acsA	Acetyl-coenzyme A s	42.30	0.00
TRINITY_sp|P253	mlkA	Myosin light chain	42.30	0.00
TRINITY_sp|Q8H1	At2g344(	Uncharacterized pro	42.30	0.00
TRINITY_sp|Q6D7	lacZ	Beta-galactosidase	42.30	0.00
TRINITY_sp|Q8L8	PIP5K9	Phosphatidylinosito	42.30	0.00
TRINITY_sp|Q559	DDB_G02	Probable serine/thr	42.30	0.00
TRINITY_sp|P257	Os04g065	Oryzain alpha chain	42.30	0.00
TRINITY_sp|Q8VII	Dhrs4	Dehydrogenase/reduc	42.30	0.00
TRINITY_sp|Q8LP	R1	Alpha-glucan water	42.30	0.00
TRINITY_sp|P109	-	Retrovirus-related	42.30	0.00
TRINITY_sp|Q8WX	DNAH7	Dynein heavy chain	42.30	0.00
TRINITY_sp|O009(	arfA	ADP-ribosylation fa	42.30	0.00
TRINITY_sp|Q84M	ABCA1	ABC transporter A f	42.30	0.00
TRINITY_sp|O048	SAR1A	GTP-binding protein	42.30	0.00
TRINITY_sp|Q8VX	AGAL3	Alpha-galactosidase	42.20	0.00
TRINITY_sp|Q7PL	CG17528	Serine/threonine-pr	42.20	0.00
TRINITY_sp|O941	nudG	Dynein light chain,	42.20	0.00
TRINITY_sp|Q9UT	eng1	Endo-1,3(4)-beta-gl	42.20	0.00
TRINITY_sp|Q4E1	Tc00.10	Probable eukaryotic	42.20	0.00
TRINITY_sp|Q54Q	DDB_G02	Serine/threonine-pr	42.20	0.00
TRINITY_sp|A8ISI	ARL3	ADP-ribosylation fa	42.20	0.00
TRINITY_sp|Q54J	alg5	Dolichyl-phosphate	42.20	0.00
TRINITY_sp|Q9Y2	AKT3	RAC-gamma serine/th	42.20	0.00
TRINITY_sp|Q32L	ARFRP1	ADP-ribosylation fa	42.20	0.00
TRINITY_sp|Q5R9	VMP1	Vacuole membrane pr	42.20	0.00
TRINITY_sp|Q0J7I	Os08g01	Phosphopantothenate	42.20	0.00
TRINITY_sp|Q54R	fntA	Protein farnesyltra	42.20	0.00

TRINITY_sp|Q9H8S	MOB1A	MOB kinase activato	42.20	0.00
TRINITY_sp|O4400	HSP90	Heat shock protein	42.20	0.00
TRINITY_sp|F4HP2	LIG6	DNA ligase 6 OS=Ara	42.20	0.00
TRINITY_sp|Q9Y07	ctxA	Cortexillin-1 OS=Po	42.20	0.00
TRINITY_sp|P4802	gacS	Sensor protein GacS	42.20	0.00
TRINITY_sp|Q5UQ0	MIMI_R81	Putative serine/thr	42.20	0.00
TRINITY_sp|P4245	cobA	Uroporphyrinogen-II	42.20	0.00
TRINITY_sp|Q30W1	truB	tRNA pseudouridine	42.20	0.00
TRINITY_sp|Q9C58	RFC1	Replication factor	42.20	0.00
TRINITY_sp|O7445	scw1	Cell wall integrity	42.20	0.00
TRINITY_sp|Q9H7B	SMYD3	Histone-lysine N-me	42.20	0.00
TRINITY_sp|Q2NL8	TSR1	Pre-rRNA-processing	42.20	0.00
TRINITY_sp|Q9M90	RKD1	Protein RKD1 OS=Ara	42.20	0.00
TRINITY_sp|Q55B1	melA	Probable alpha-gala	42.20	0.00
TRINITY_sp|P4262	yqjG	Glutathionyl-hydroq	42.20	0.00
TRINITY_sp|Q9FWX	ABCB11	ABC transporter B f	42.20	0.00
TRINITY_sp|Q8IYI	GSPT2	Eukaryotic peptide	42.20	0.00
TRINITY_sp|Q9FIQ	AGD9	Probable ADP-ribosy	42.20	0.00
TRINITY_sp|O7035	Hsd17b10	3-hydroxyacyl-CoA d	42.20	0.00
TRINITY_sp|P0A97	tas	Protein tas OS=Esch	42.20	0.00
TRINITY_sp|Q6FV0	IPL1	Spindle assembly ch	42.20	0.00
TRINITY_sp|Q6AXQ	Mtm1	Myotubularin OS=Rat	42.20	0.00
TRINITY_sp|Q8WYH	ING5	Inhibitor of growth	42.20	0.00
TRINITY_sp|Q6DJN	tamm41	Phosphatidate cytid	42.20	0.00
TRINITY_sp|A6WYH	fmt	Methionyl-tRNA form	42.20	0.00
TRINITY_sp|P1346	abpC	Gelation factor OS=	42.20	0.00
TRINITY_sp|P5303	YIP1	Protein transport p	42.20	0.00
TRINITY_sp|P4973	-	Ribonucleoside-diph	42.20	0.00
TRINITY_sp|Q9D9R	Rnf125	E3 ubiquitin-protei	42.20	0.00
TRINITY_sp|Q0WU0	MTP10	Metal tolerance pro	42.20	0.00
TRINITY_sp|Q8W49	PTC52	Protochlorophyllide	42.20	0.00
TRINITY_sp|O6088	BRD4	Bromodomain-contain	42.20	0.00
TRINITY_sp|Q944K	OST48	Dolichyl-diphosphoo	42.20	0.00
TRINITY_sp|Q8VEH	Cbwd1	COBW domain-contain	42.20	0.00
TRINITY_sp|Q8LPK	ABCA8	ABC transporter A f	42.20	0.00
TRINITY_sp|F4KBI	AAE17	Probable acyl-activ	42.20	0.00
TRINITY_sp|Q9NZJ	SACS	Sacsin OS=Homo sapi	42.20	0.00
TRINITY_sp|Q8VYA	RS2Z33	Serine/arginine-ric	42.20	0.00
TRINITY_sp|Q9QXL	Kif21a	Kinesin-like protei	42.20	0.00
TRINITY_sp|Q54II	DDB_G028	Probable serine/thr	42.20	0.00
TRINITY_sp|Q9VTU	RhoGAP68	Rho GTPase-activati	42.20	0.00
TRINITY_sp|Q9LWM	CIPK5	CBL-interacting pro	42.10	0.00
TRINITY_sp|Q9CPP	Lipf	Gastric triacylglyc	42.10	0.00
TRINITY_sp|Q9P7Q	alg9	Alpha-1,2-mannosylt	42.10	0.00
TRINITY_sp|A2YS0	ATG8C	Autophagy-related p	42.10	0.00
TRINITY_sp|D9HP2	CNR11	Cell number regulat	42.10	0.00
TRINITY_sp|O0471	MSH6	DNA mismatch repair	42.10	0.00
TRINITY_sp|Q9Y2Y	POLR3K	DNA-directed RNA po	42.10	0.00
TRINITY_sp|P0DK0	HSD4	11-beta-hydroxyster	42.10	0.00
TRINITY_sp|P1160	NIA1	Nitrate reductase [	42.10	0.00
TRINITY_sp|Q9NZT	OGFR	Opioid growth facto	42.10	0.00
TRINITY_sp|Q9SB5	AP4M	AP-4 complex subuni	42.10	0.00
TRINITY_sp|P5407	F44G4.1	Brix domain-contain	42.10	0.00
TRINITY_sp|Q86I2	ddx52	Probable ATP-depend	42.10	0.00
TRINITY_sp|Q54C3	pgl	Probable 6-phosphog	42.10	0.00
TRINITY_sp|Q9283	INPP5D	Phosphatidylinosito	42.10	0.00

TRINITY_sp|Q9SL[illegible]	Os05g01[illegible]	Importin subunit al	42.10	0.00
TRINITY_sp|Q7L9[illegible]	MOB1B	MOB kinase activato	42.10	0.00
TRINITY_sp|P517[illegible]	USP11	Ubiquitin carboxyl-	42.10	0.00
TRINITY_sp|P323[illegible]	IRE1	Serine/threonine-pr	42.10	0.00
TRINITY_sp|O438[illegible]	ATP9B	Probable phospholip	42.10	0.00
TRINITY_sp|O944[illegible]	tef3	Elongation factor 3	42.10	0.00
TRINITY_sp|O652[illegible]	CPN20	20 kDa chaperonin,	42.10	0.00
TRINITY_sp|Q5ZL[illegible]	ZRANB2	Zinc finger Ran-bin	42.10	0.00
TRINITY_sp|Q9NZ[illegible]	SACS	Sacsin OS=Homo sapi	42.10	0.00
TRINITY_sp|Q8CH[illegible]	Ttll5	Tubulin polyglutamy	42.10	0.00
TRINITY_sp|O821[illegible]	VPS32.1	Vacuolar protein so	42.10	0.00
TRINITY_sp|O143[illegible]	ubl1	Ubiquitin-like prot	42.10	0.00
TRINITY_sp|A8QC[illegible]	Bm1_495[illegible]	Lateral signaling t	42.10	0.00
TRINITY_sp|Q9NZ[illegible]	OGFR	Opioid growth facto	42.10	0.00
TRINITY_sp|B0DZ[illegible]	AMPP	Probable Xaa-Pro am	42.10	0.00
TRINITY_sp|P252[illegible]	Rab3	Ras-related protein	42.10	0.00
TRINITY_sp|Q0P4[illegible]	dcakd	Dephospho-CoA kinas	42.10	0.00
TRINITY_sp|Q9LK[illegible]	ALA8	Probable phospholip	42.10	0.00
TRINITY_sp|P623[illegible]	CPK1	Calcium-dependent p	42.10	0.00
TRINITY_sp|Q9AS[illegible]	At5g274[illegible]	Branched-chain-amin	42.10	0.00
TRINITY_sp|O157[illegible]	vacA	Vacuolin-A OS=Dicty	42.10	0.00
TRINITY_sp|Q810[illegible]	Kiaa089[illegible]	Uncharacterized pro	42.10	0.00
TRINITY_sp|Q54G[illegible]	ctdspl2	CTD small phosphata	42.10	0.00
TRINITY_sp|Q24C[illegible]	RPL14	60S ribosomal prote	42.10	0.00
TRINITY_sp|Q9XI[illegible]	UGT80B1	Sterol 3-beta-gluco	42.10	0.00
TRINITY_sp|Q9TV[illegible]	DHDH	Trans-1,2-dihydrobe	42.10	0.00
TRINITY_sp|Q10S[illegible]	CIPK9	CBL-interacting pro	42.10	0.00
TRINITY_sp|Q88F[illegible]	nicT	Putative metabolite	42.10	0.00
TRINITY_sp|P134[illegible]	abpC	Gelation factor OS=	42.10	0.00
TRINITY_sp|Q86G[illegible]	gcy-28	Receptor-type guany	42.10	0.00
TRINITY_sp|Q8BH[illegible]	Rhbdd1	Rhomboid-related pr	42.10	0.00
TRINITY_sp|Q96M[illegible]	COG8	Conserved oligomeri	42.10	0.00
TRINITY_sp|Q2Y6[illegible]	rlmN	Dual-specificity RN	42.10	0.00
TRINITY_sp|O605[illegible]	CDC40	Pre-mRNA-processing	42.10	0.00
TRINITY_sp|Q8T8[illegible]	abcD2	ABC transporter D f	42.10	0.00
TRINITY_sp|P518[illegible]	lig1	DNA ligase 1 OS=Xen	42.10	0.00
TRINITY_sp|Q6ID[illegible]	At1g095[illegible]	Transmembrane emp24	42.10	0.00
TRINITY_sp|Q8RX[illegible]	At1g146[illegible]	Probable splicing f	42.10	0.00
TRINITY_sp|Q8RY[illegible]	ABCB26	ABC transporter B f	42.10	0.00
TRINITY_sp|Q272[illegible]	lap-2	Putative aminopepti	42.10	0.00
TRINITY_sp|Q00I[illegible]	CPL4	RNA polymerase II C	42.10	0.00
TRINITY_sp|Q96N[illegible]	AIFM3	Apoptosis-inducing	42.10	0.00
TRINITY_sp|P577[illegible]	EEFSEC	Selenocysteine-spec	42.10	0.00
TRINITY_sp|O432[illegible]	PSMD3	26S proteasome non-	42.10	0.00
TRINITY_sp|B9DG[illegible]	TAF7	Transcription initi	42.10	0.00
TRINITY_sp|B5DE[illegible]	Xpnpep3	Probable Xaa-Pro am	42.10	0.00
TRINITY_sp|P419[illegible]	YRB1	Ran-specific GTPase	42.10	0.00
TRINITY_sp|Q9FV[illegible]	At1g322[illegible]	Uncharacterized pro	42.10	0.00
TRINITY_sp|Q8ND[illegible]	TTC21A	Tetratricopeptide r	42.10	0.00
TRINITY_sp|Q9ZR[illegible]	YKT61	VAMP-like protein Y	42.10	0.00
TRINITY_sp|Q55C[illegible]	tpp1	Tripeptidyl-peptida	42.10	0.00
TRINITY_sp|Q0P5[illegible]	Tppp2	Tubulin polymerizat	42.10	0.00
TRINITY_sp|P484[illegible]	Tbca	Tubulin-specific ch	42.10	0.00
TRINITY_sp|A0AV[illegible]	UBA6	Ubiquitin-like modi	42.10	0.00
TRINITY_sp|O428[illegible]	ski6	Exosome complex com	42.10	0.00
TRINITY_sp|Q54P[illegible]	tmem184[illegible]	Transmembrane prote	42.00	0.00

TRINITY_sp|P908[illegible]	F56F10.[illegible]	Putative serine pro	42.00	0.00
TRINITY_sp|P973[illegible]	Nxn	Nucleoredoxin OS=Mu	42.00	0.00
TRINITY_sp|Q7TS[illegible]	Kif15	Kinesin-like protei	42.00	0.00
TRINITY_sp|Q6XH[illegible]	roco9	Probable serine/thr	42.00	0.00
TRINITY_sp|Q91Y[illegible]	Mettl13	Methyltransferase-l	42.00	0.00
TRINITY_sp|Q323[illegible]	ybiA	N-glycosidase YbiA	42.00	0.00
TRINITY_sp|Q9FI[illegible]	BOLA2	Protein BOLA2 OS=Ar	42.00	0.00
TRINITY_sp|Q8HY[illegible]	ACOX1	Peroxisomal acyl-co	42.00	0.00
TRINITY_sp|Q6BV[illegible]	IPL1	Spindle assembly ch	42.00	0.00
TRINITY_sp|Q86I[illegible]	ctnA	Countin-1 OS=Dictyo	42.00	0.00
TRINITY_sp|O460[illegible]	Cyp4ae1	Cytochrome P450 4ae	42.00	0.00
TRINITY_sp|P366[illegible]	bip1	78 kDa glucose-regu	42.00	0.00
TRINITY_sp|O289[illegible]	AF_1281	Uncharacterized pro	42.00	0.00
TRINITY_sp|A8Y1[illegible]	pfd-6	Probable prefoldin	42.00	0.00
TRINITY_sp|Q55E[illegible]	pats1	Probable serine/thr	42.00	0.00
TRINITY_sp|P047[illegible]	HSP17.5-[illegible]	17.5 kDa class I he	42.00	0.00
TRINITY_sp|A5DY[illegible]	DRS1	ATP-dependent RNA h	42.00	0.00
TRINITY_sp|Q7ZV[illegible]	zgc:559[illegible]	WASH complex subuni	42.00	0.00
TRINITY_sp|Q8C6[illegible]	-	Protein C21orf2 hom	42.00	0.00
TRINITY_sp|Q9XJ[illegible]	CLPR2	ATP-dependent Clp p	42.00	0.00
TRINITY_sp|P459[illegible]	USP5	Ubiquitin carboxyl-	42.00	0.00
TRINITY_sp|Q75L[illegible]	Os03g06[illegible]	U3 snoRNP-associate	42.00	0.00
TRINITY_sp|Q7TM[illegible]	Eri1	3'-5' exoribonuclea	42.00	0.00
TRINITY_sp|P340[illegible]	pkaC	cAMP-dependent prot	42.00	0.00
TRINITY_sp|Q86I[illegible]	commd7	COMM domain-contain	42.00	0.00
TRINITY_sp|Q9JJ[illegible]	Cdc20	Cell division cycle	42.00	0.00
TRINITY_sp|Q9U8[illegible]	DHFR	Dihydrofolate reduc	42.00	0.00
TRINITY_sp|Q9SJ[illegible]	ROS1	Protein ROS1 OS=Ara	42.00	0.00
TRINITY_sp|P235[illegible]	xynB	Beta-xylosidase OS=	42.00	0.00
TRINITY_sp|Q9VH[illegible]	CG8412	Probable Dol-P-Man:	42.00	0.00
TRINITY_sp|Q9SE[illegible]	At2g251[illegible]	Ribonuclease H2 sub	42.00	0.00
TRINITY_sp|Q91W[illegible]	-	RUS1 family protein	42.00	0.00
TRINITY_sp|Q9LS[illegible]	CASP	Protein CASP OS=Ara	42.00	0.00
TRINITY_sp|Q8H0[illegible]	ABCF3	ABC transporter F f	42.00	0.00
TRINITY_sp|B0TA[illegible]	ybeY	Endoribonuclease Yb	42.00	0.00
TRINITY_sp|Q8RD[illegible]	FN1419	L-methionine gamma-	42.00	0.00
TRINITY_sp|Q680[illegible]	GGT2	Gamma-glutamyltrans	42.00	0.00
TRINITY_sp|Q6L5[illegible]	Os05g05[illegible]	Probable protein ph	42.00	0.00
TRINITY_sp|P316[illegible]	-	ADP,ATP carrier pro	42.00	0.00
TRINITY_sp|Q9C7[illegible]	BUB3.2	Mitotic checkpoint	42.00	0.00
TRINITY_sp|O627[illegible]	CTNNBL1	Beta-catenin-like p	42.00	0.00
TRINITY_sp|Q4V7[illegible]	-	UPF0585 protein C16	42.00	0.00
TRINITY_sp|Q2TA[illegible]	KIF19	Kinesin-like protei	42.00	0.00
TRINITY_sp|P761[illegible]	ydcP	Uncharacterized pro	42.00	0.00
TRINITY_sp|Q9D0[illegible]	Ebpl	Emopamil-binding pr	42.00	0.00
TRINITY_sp|Q9S7[illegible]	MYB98	Transcription facto	42.00	0.00
TRINITY_sp|Q6PC[illegible]	dctn1	Dynactin subunit 1	42.00	0.00
TRINITY_sp|Q54I[illegible]	DDB_G02[illegible]	Probable serine/thr	42.00	0.00
TRINITY_sp|Q246[illegible]	ref(2)P	Protein ref(2)P OS=	42.00	0.00
TRINITY_sp|Q050[illegible]	UBE3A	Ubiquitin-protein l	42.00	0.00
TRINITY_sp|Q5YY[illegible]	idi	Isopentenyl-diphosp	42.00	0.00
TRINITY_sp|Q636[illegible]	Vps33a	Vacuolar protein so	42.00	0.00
TRINITY_sp|P319[illegible]	pac	Penicillin G acylas	42.00	0.00
TRINITY_sp|Q86U[illegible]	DTX2	Probable E3 ubiquit	42.00	0.00
TRINITY_sp|P0C0[illegible]	Chmp6	Charged multivesicu	42.00	0.00
TRINITY_sp|Q8L7[illegible]	SLP1	Shewanella-like pro	42.00	0.00

TRINITY_sp|Q037(	TAF12	Transcription initi	42.00	0.00
TRINITY_sp|A4QW4	TOA2	Transcription initi	42.00	0.00
TRINITY_sp|P352{	Rab22a	Ras-related protein	42.00	0.00
TRINITY_sp|Q8W03	CDC6B	Cell division contr	42.00	0.00
TRINITY_sp|Q8WYA	CTNNBL1	Beta-catenin-like p	42.00	0.00
TRINITY_sp|P091(	P4HB	Protein disulfide-i	42.00	0.00
TRINITY_sp|Q6R0I	LHY	Protein LHY OS=Arab	42.00	0.00
TRINITY_sp|Q9LIS	RBG4	Glycine-rich RNA-bi	42.00	0.00
TRINITY_sp|A8XW8	kin-1	cAMP-dependent prot	42.00	0.00
TRINITY_sp|P1097	-	Retrovirus-related	42.00	0.00
TRINITY_sp|Q6148	Pde1a	Calcium/calmodulin-	42.00	0.00
TRINITY_sp|Q84K4	ABCA2	ABC transporter A f	42.00	0.00
TRINITY_sp|Q8WX>	DNAH7	Dynein heavy chain	42.00	0.00
TRINITY_sp|F4IU>	UPF2	Regulator of nonsen	42.00	0.00
TRINITY_sp|Q962I	TUBA6	Tubulin alpha-6 cha	41.90	0.00
TRINITY_sp|Q54QY	yipf5	Protein YIPF5 homol	41.90	0.00
TRINITY_sp|Q9S84	SPL2	Squamosa promoter-b	41.90	0.00
TRINITY_sp|Q9LNW	HSP17.8	17.8 kDa class I he	41.90	0.00
TRINITY_sp|Q9UKI	DNAJC12	DnaJ homolog subfam	41.90	0.00
TRINITY_sp|Q4WI8	pam18	Mitochondrial impor	41.90	0.00
TRINITY_sp|Q4WT8	clf1	Pre-mRNA-splicing f	41.90	0.00
TRINITY_sp|Q9STV	CIPK8	CBL-interacting ser	41.90	0.00
TRINITY_sp|Q54Z2	pldC	Phospholipase D C O	41.90	0.00
TRINITY_sp|P3769	hetM	Polyketide synthase	41.90	0.00
TRINITY_sp|B9JVI	fusA	Elongation factor G	41.90	0.00
TRINITY_sp|P3952	DRS2	Probable phospholip	41.90	0.00
TRINITY_sp|A8GMI	rlmE	Ribosomal RNA large	41.90	0.00
TRINITY_sp|Q54BI	mcfN	Mitochondrial subst	41.90	0.00
TRINITY_sp|Q69VI	Os06g05(	Probable protein ph	41.90	0.00
TRINITY_sp|Q9XFA	PCK2	Phosphoenolpyruvate	41.90	0.00
TRINITY_sp|A3KPI	ttc38	Tetratricopeptide r	41.90	0.00
TRINITY_sp|Q9DBW	Cyp4v2	Cytochrome P450 4V2	41.90	0.00
TRINITY_sp|O807(	At1g6042	Probable nucleoredo	41.90	0.00
TRINITY_sp|Q54II	DDB_G028	WASH complex subuni	41.90	0.00
TRINITY_sp|Q1ZXI	gxcDD	Guanine exchange fa	41.90	0.00
TRINITY_sp|P388(	NMD3	60S ribosomal expor	41.90	0.00
TRINITY_sp|Q6Z82	WEE1	Wee1-like protein k	41.90	0.00
TRINITY_sp|B5YII	ligA	DNA ligase OS=Therm	41.90	0.00
TRINITY_sp|Q9C5I	At3g4742	Putative glycerol-3	41.90	0.00
TRINITY_sp|Q55C9	maspS	Aspartate--tRNA lig	41.90	0.00
TRINITY_sp|Q5RA7	FAM135A	Protein FAM135A OS=	41.90	0.00
TRINITY_sp|Q8IU8	CAMK1D	Calcium/calmodulin-	41.90	0.00
TRINITY_sp|A7HZ9	infB	Translation initiat	41.90	0.00
TRINITY_sp|Q55DI	DDB_G02(	Alpha N-terminal pr	41.90	0.00
TRINITY_sp|Q7TQI	Exosc3	Exosome complex com	41.90	0.00
TRINITY_sp|P1903	HSP18.1	18.1 kDa class I he	41.90	0.00
TRINITY_sp|Q9ZR3	DSPTP1	Dual specificity pr	41.90	0.00
TRINITY_sp|Q8T19	DDB_G027	Dehydrogenase/reduc	41.90	0.00

TRINITY_sp|F1RAX	coq6	Ubiquinone biosynth	41.90	0.00
TRINITY_sp|Q8CF9	Aqr	Intron-binding prot	41.90	0.00
TRINITY_sp|Q9LW5	PAP6	Probable plastid-li	41.90	0.00
TRINITY_sp|Q54V0	ascc2	Activating signal c	41.90	0.00
TRINITY_sp|Q8RD3	anmK	Anhydro-N-acetylmur	41.90	0.00
TRINITY_sp|Q54BN	sf1	Branchpoint-bridgin	41.90	0.00
TRINITY_sp|Q96HI	SH3YL1	SH3 domain-containi	41.90	0.00
TRINITY_sp|Q5RC8	RBM39	RNA-binding protein	41.90	0.00
TRINITY_sp|O2217	PAB4	Polyadenylate-bindi	41.90	0.00
TRINITY_sp|Q8L64	BB	E3 ubiquitin ligase	41.90	0.00
TRINITY_sp|Q3ZCH	EMC3	ER membrane protein	41.90	0.00
TRINITY_sp|P4851	TFIIB1	Transcription initi	41.90	0.00
TRINITY_sp|Q9UPU	USP24	Ubiquitin carboxyl-	41.90	0.00
TRINITY_sp|P4325	CSN8	COP9 signalosome co	41.90	0.00
TRINITY_sp|Q9GMY	PGA	Pepsin A OS=Rhinolo	41.90	0.00
TRINITY_sp|Q9LV0	At5g6672	Probable protein ph	41.90	0.00
TRINITY_sp|Q9XJ3	CLPR1	ATP-dependent Clp p	41.90	0.00
TRINITY_sp|Q68FA	march3	E3 ubiquitin-protei	41.90	0.00
TRINITY_sp|Q7TMR	Prcp	Lysosomal Pro-X car	41.90	0.00
TRINITY_sp|Q9FWY	IMPA6	Importin subunit al	41.90	0.00
TRINITY_sp|Q54PE	mrd1	Multiple RNA-bindin	41.90	0.00
TRINITY_sp|Q94JU	CSN7	COP9 signalosome co	41.90	0.00
TRINITY_sp|A6VCV	prmA	Ribosomal protein L	41.90	0.00
TRINITY_sp|P1097	-	Retrovirus-related	41.90	0.00
TRINITY_sp|A5GFW	RTFDC1	Protein RTF2 homolo	41.90	0.00
TRINITY_sp|O9443	SPBC660.	Uncharacterized RNA	41.90	0.00
TRINITY_sp|Q3896	SNM1	DNA cross-link repa	41.90	0.00
TRINITY_sp|Q47WE	lepA	Elongation factor 4	41.90	0.00
TRINITY_sp|A7SD8	cdc5l	Cell division cycle	41.90	0.00
TRINITY_sp|O2294	PXM16	Insulin-degrading e	41.90	0.00
TRINITY_sp|Q8LP2	CPK3	Calcium-dependent p	41.90	0.00
TRINITY_sp|P5467	pikA	Phosphatidylinosito	41.90	0.00
TRINITY_sp|Q2HIW	MAPR4	Membrane-associated	41.90	0.00
TRINITY_sp|Q0HA3	Ttc21b	Tetratricopeptide r	41.90	0.00
TRINITY_sp|P3233	YMC1	Carrier protein YMC	41.80	0.00
TRINITY_sp|Q9ZTP	EIN4	Protein EIN4 OS=Ara	41.80	0.00
TRINITY_sp|Q3SW2	WRAP53	Telomerase Cajal bo	41.80	0.00
TRINITY_sp|Q5RKI	Poll	DNA polymerase lamb	41.80	0.00
TRINITY_sp|Q7ZVE	esrp2	Epithelial splicing	41.80	0.00
TRINITY_sp|Q54S1	DDB_G028	DDT domain-containi	41.80	0.00
TRINITY_sp|Q9QZE	Dctn5	Dynactin subunit 5	41.80	0.00
TRINITY_sp|Q3958	-	Dynein 18 kDa light	41.80	0.00
TRINITY_sp|Q948R	DAD1	Phospholipase A(1)	41.80	0.00
TRINITY_sp|P4212	E(z)	Histone-lysine N-me	41.80	0.00
TRINITY_sp|Q3U48	Mfsd12	Major facilitator s	41.80	0.00
TRINITY_sp|Q8H18	PYD3	Beta-ureidopropiona	41.80	0.00
TRINITY_sp|Q58CU	MEAF6	Chromatin modificat	41.80	0.00
TRINITY_sp|P5038	SSO2706	Purine nucleoside p	41.80	0.00
TRINITY_sp|Q9LK9	ALA8	Probable phospholip	41.80	0.00
TRINITY_sp|P2780	Mgat1	Alpha-1,3-mannosyl-	41.80	0.00
TRINITY_sp|Q86VE	SLC25A42	Mitochondrial coenz	41.80	0.00
TRINITY_sp|Q8RXE	At1g1465	Probable splicing f	41.80	0.00
TRINITY_sp|P9295	KIN11	SNF1-related protei	41.80	0.00
TRINITY_sp|Q9FZ3	ANP2	Mitogen-activated p	41.80	0.00
TRINITY_sp|A7A1E	STE11	Serine/threonine-pr	41.80	0.00
TRINITY_sp|A7YW9	RARS	Arginine--tRNA liga	41.80	0.00

TRINITY_sp|Q9SG(	ALA5	Probable phospholip	41.80	0.00
TRINITY_sp|P749:	polA	DNA polymerase I OS	41.80	0.00
TRINITY_sp|Q54TI	gacY	Rho GTPase-activati	41.80	0.00
TRINITY_sp|A7MBI	pkn2	Serine/threonine-pr	41.80	0.00
TRINITY_sp|Q8RXI	At1g146!	Probable splicing f	41.80	0.00
TRINITY_sp|Q58D!	RAB5C	Ras-related protein	41.80	0.00
TRINITY_sp|Q6BC!	CYB5R2	NADH-cytochrome b5	41.80	0.00
TRINITY_sp|P049(	Gstm1	Glutathione S-trans	41.80	0.00
TRINITY_sp|Q9JH|	Eefsec	Selenocysteine-spec	41.80	0.00
TRINITY_sp|B4IB!	Mat89Ba	Nucleolar protein 6	41.80	0.00
TRINITY_sp|P435(	cpr-4	Cathepsin B-like cy	41.80	0.00
TRINITY_sp|Q6Z9I	KIN7H	Kinesin-like protei	41.80	0.00
TRINITY_sp|P539!	Anapc1	Anaphase-promoting	41.80	0.00
TRINITY_sp|Q5PP:	ATXR2	Histone-lysine N-me	41.80	0.00
TRINITY_sp|Q004:	Ptbp1	Polypyrimidine trac	41.80	0.00
TRINITY_sp|Q9ZW!	CYTB5-D	Cytochrome B5 isofo	41.80	0.00
TRINITY_sp|Q993!	TY3B-G	Transposon Ty3-G Ga	41.80	0.00
TRINITY_sp|P463!	bccA	Acetyl-/propionyl-c	41.80	0.00
TRINITY_sp|Q86Y(	CPNE8	Copine-8 OS=Homo sa	41.80	0.00
TRINITY_sp|Q86W!	METTL16	Methyltransferase-l	41.80	0.00
TRINITY_sp|Q056:	Cbei_02(	Uncharacterized pro	41.80	0.00
TRINITY_sp|Q11G(	rplY	50S ribosomal prote	41.80	0.00
TRINITY_sp|Q9UB!	DNAJB9	DnaJ homolog subfam	41.80	0.00
TRINITY_sp|Q8YH(	ssb	Single-stranded DNA	41.80	0.00
TRINITY_sp|Q54Z(	tbcb	Tubulin-specific ch	41.80	0.00
TRINITY_sp|Q9CS!	Ecd	Protein ecdysoneles	41.80	0.00
TRINITY_sp|Q6NS!	Pdcd11	Protein RRP5 homolo	41.80	0.00
TRINITY_sp|Q54BI	sf1	Branchpoint-bridgin	41.80	0.00
TRINITY_sp|Q945(	modA	Neutral alpha-gluco	41.80	0.00
TRINITY_sp|Q245(	U2af50	Splicing factor U2A	41.80	0.00
TRINITY_sp|Q9I6:	PA0142	8-oxoguanine deamin	41.80	0.00
TRINITY_sp|Q9LT:	VPS9A	Vacuolar protein so	41.80	0.00
TRINITY_sp|A2X6!	CDKG-1	Cyclin-dependent ki	41.80	0.00
TRINITY_sp|P118(	CDK4	Cyclin-dependent ki	41.80	0.00
TRINITY_sp|P973!	Nxn	Nucleoredoxin OS=Mu	41.80	0.00
TRINITY_sp|P278!	ERV1	Mitochondrial FAD-l	41.80	0.00
TRINITY_sp|Q9FE!	SRT1	NAD-dependent prote	41.80	0.00
TRINITY_sp|Q9KTI	truC	tRNA pseudouridine	41.80	0.00
TRINITY_sp|F4HN(	At1g069(	Nardilysin-like OS=	41.80	0.00
TRINITY_sp|O227!	PXG3	Probable peroxygena	41.80	0.00
TRINITY_sp|Q9C5!	PFK7	ATP-dependent 6-pho	41.80	0.00
TRINITY_sp|P378(	CNN1	Calponin-1 (Fragmen	41.80	0.00
TRINITY_sp|O246!	MSH2	DNA mismatch repair	41.80	0.00
TRINITY_sp|A4K2I	STK4	Serine/threonine-pr	41.80	0.00
TRINITY_sp|Q8L8!	RECQL4A	ATP-dependent DNA h	41.80	0.00
TRINITY_sp|Q9SJ!	SAE2	SUMO-activating enz	41.80	0.00
TRINITY_sp|Q9FFI	NFXL2	NF-X1-type zinc fin	41.80	0.00
TRINITY_sp|P422!	SKIV2L2	Superkiller viralic	41.80	0.00
TRINITY_sp|Q99K(	Aass	Alpha-aminoadipic s	41.80	0.00
TRINITY_sp|O229!	FTSHI1	Probable inactive A	41.80	0.00
TRINITY_sp|P221!	cta3	Calcium-transportin	41.80	0.00
TRINITY_sp|Q8KQI	cphE	Cyanophycinase OS=P	41.80	0.00
TRINITY_sp|P230!	-	Dynein beta chain,	41.80	0.00
TRINITY_sp|Q9ZP!	CAS	Exportin-2 OS=Arabi	41.80	0.00
TRINITY_sp|Q152(	PWP2	Periodic tryptophan	41.80	0.00
TRINITY_sp|P269!	-	ADP-ribosylation fa	41.80	0.00

TRINITY_sp|Q2TB(	UCHL3	Ubiquitin carboxyl-	41.80	0.00
TRINITY_sp|O078:	dapb1	Dipeptidyl aminopep	41.80	0.00
TRINITY_sp|Q9LW	HRD1A	ERAD-associated E3	41.80	0.00
TRINITY_sp|Q614	Pde1a	Calcium/calmodulin-	41.80	0.00
TRINITY_sp|Q8H1:	PAP3	Purple acid phospha	41.80	0.00
TRINITY_sp|Q9CQ:	Ndufa6	NADH dehydrogenase	41.70	0.00
TRINITY_sp|Q8RVI	DEK1	Calpain-type cystei	41.70	0.00
TRINITY_sp|Q54II	lzic	Protein LZIC OS=Dic	41.70	0.00
TRINITY_sp|P302	mik1	Mitosis inhibitor p	41.70	0.00
TRINITY_sp|Q54BI	psenen	Probable gamma-secr	41.70	0.00
TRINITY_sp|A8WU(	pkc-3	Protein kinase C-li	41.70	0.00
TRINITY_sp|O675(	aq_1546	Uncharacterized pho	41.70	0.00
TRINITY_sp|O942	atg8	Autophagy-related p	41.70	0.00
TRINITY_sp|Q96J	ABCC12	Multidrug resistanc	41.70	0.00
TRINITY_sp|Q395	ODA2	Dynein gamma chain,	41.70	0.00
TRINITY_sp|Q9H7I	SMYD3	Histone-lysine N-me	41.70	0.00
TRINITY_sp|P563	Cda	Cytidine deaminase	41.70	0.00
TRINITY_sp|B8AXI	OsI_182	E3 UFM1-protein lig	41.70	0.00
TRINITY_sp|O965	chmp1	Charged multivesicu	41.70	0.00
TRINITY_sp|Q54B	redA	NADPH oxidoreductas	41.70	0.00
TRINITY_sp|Q5M8I	Sdr39u1	Epimerase family pr	41.70	0.00
TRINITY_sp|Q294	LIPF	Gastric triacylglyc	41.70	0.00
TRINITY_sp|Q8BM	Lipk	Lipase member K OS=	41.70	0.00
TRINITY_sp|Q54M	mrkB	Probable serine/thr	41.70	0.00
TRINITY_sp|Q8L5	CAND1	Cullin-associated N	41.70	0.00
TRINITY_sp|Q8LPI	ALPHA-AI	AP-2 complex subuni	41.70	0.00
TRINITY_sp|O774(	-	Tryparedoxin OS=Try	41.70	0.00
TRINITY_sp|P497	Doa	Serine/threonine-pr	41.70	0.00
TRINITY_sp|P258(	cpr-1	Gut-specific cystei	41.70	0.00
TRINITY_sp|Q8L7	NUDT8	Nudix hydrolase 8 O	41.70	0.00
TRINITY_sp|A4FV	METTL21I	Protein-lysine meth	41.70	0.00
TRINITY_sp|P346	cap-2	F-actin-capping pro	41.70	0.00
TRINITY_sp|Q9JI	Utp3	Something about sil	41.70	0.00
TRINITY_sp|Q55C	DDB_G02	Mitochondrial inner	41.70	0.00
TRINITY_sp|Q7L2	KCTD9	BTB/POZ domain-cont	41.70	0.00
TRINITY_sp|Q96RI	CAMKK2	Calcium/calmodulin-	41.70	0.00
TRINITY_sp|O438:	C21orf2	Protein C21orf2 OS=	41.70	0.00
TRINITY_sp|Q505	fdhC	Probable formate tr	41.70	0.00
TRINITY_sp|Q5DU(	Cep164	Centrosomal protein	41.70	0.00
TRINITY_sp|Q54VI	rio1	Serine/threonine-pr	41.70	0.00
TRINITY_sp|Q9SG	HMGB9	High mobility group	41.70	0.00
TRINITY_sp|P558	EIF3B	Eukaryotic translat	41.70	0.00
TRINITY_sp|P109	-	Retrovirus-related	41.70	0.00
TRINITY_sp|Q271	DHC-8	Dynein heavy chain,	41.70	0.00
TRINITY_sp|P109	-	Actin, cytoplasmic	41.70	0.00
TRINITY_sp|P157	Camk2d	Calcium/calmodulin-	41.70	0.00
TRINITY_sp|Q9BXI	PAPPA2	Pappalysin-2 OS=Hom	41.70	0.00
TRINITY_sp|B2RW	Cfap58	Cilia- and flagella	41.70	0.00
TRINITY_sp|A6H7	PLS1	Plastin-1 OS=Bos ta	41.70	0.00
TRINITY_sp|Q8RWI	SRL1	Pre-mRNA splicing f	41.70	0.00
TRINITY_sp|B5FZ	EIF3I	Eukaryotic translat	41.70	0.00
TRINITY_sp|Q0WSI	ALDH22A	Aldehyde dehydrogen	41.70	0.00
TRINITY_sp|Q0P4	smarcal	SWI/SNF-related mat	41.70	0.00
TRINITY_sp|A1VE	selD	Selenide, water dik	41.70	0.00
TRINITY_sp|P227	Gucy1b2	Guanylate cyclase s	41.70	0.00
TRINITY_sp|P109	-	Retrovirus-related	41.70	0.00

TRINITY_sp|Q8VW:	HPT1	Homogentisate phyty	41.70	0.00
TRINITY_sp|P296	MPP	Mitochondrial-proce	41.70	0.00
TRINITY_sp|Q5U3I	Rabl6	Rab-like protein 6	41.70	0.00
TRINITY_sp|Q395(	ODA4	Dynein beta chain,	41.70	0.00
TRINITY_sp|B5F7:	rihC	Non-specific ribonu	41.70	0.00
TRINITY_sp|Q54II	tmem120	Transmembrane prote	41.70	0.00
TRINITY_sp|Q8GW:	At5g039(	Uncharacterized pro	41.70	0.00
TRINITY_sp|Q9LTI	TGD2	Protein TRIGALACTOS	41.70	0.00
TRINITY_sp|B7ZMI	Xpnpep3	Probable Xaa-Pro am	41.70	0.00
TRINITY_sp|Q5M8:	Pir	Pirin OS=Rattus nor	41.70	0.00
TRINITY_sp|Q9FMI	RD21B	Probable cysteine p	41.70	0.00
TRINITY_sp|Q55D/	abcG22	ABC transporter G f	41.70	0.00
TRINITY_sp|Q191{	gcy-12	Receptor-type guany	41.70	0.00
TRINITY_sp|Q9W4	dnc	cAMP-specific 3',5'	41.70	0.00
TRINITY_sp|A7RZV	dpp3	Dipeptidyl peptidas	41.70	0.00
TRINITY_sp|P167(	V-UBI	Ubiquitin-like prot	41.70	0.00
TRINITY_sp|P423:	CNB1	Calcineurin subunit	41.60	0.00
TRINITY_sp|Q9MBI	DHC10	Dynein-1-beta heavy	41.60	0.00
TRINITY_sp|Q54GI	dtymk	Thymidylate kinase	41.60	0.00
TRINITY_sp|Q6PBI	zgc:733:	Coenzyme Q-binding	41.60	0.00
TRINITY_sp|Q993	TY3B-G	Transposon Ty3-G Ga	41.60	0.00
TRINITY_sp|Q5ZKI	ACSBG2	Long-chain-fatty-ac	41.60	0.00
TRINITY_sp|Q9P7(	prp11	Pre-mRNA-processing	41.60	0.00
TRINITY_sp|A2XK(	OsI_012(	Costars family prot	41.60	0.00
TRINITY_sp|Q54P	tmem184/	Transmembrane prote	41.60	0.00
TRINITY_sp|Q9STI	WNK3	Probable serine/thr	41.60	0.00
TRINITY_sp|Q9SLI	ALIS3	ALA-interacting sub	41.60	0.00
TRINITY_sp|Q9C5)	ABA3	Molybdenum cofactor	41.60	0.00
TRINITY_sp|Q8ER/	OB1403	N-acetyldiaminopime	41.60	0.00
TRINITY_sp|Q104	mug157	Meiotically up-regu	41.60	0.00
TRINITY_sp|Q5FWI	pcid2	PCI domain-containi	41.60	0.00
TRINITY_sp|O809:	AGD7	ADP-ribosylation fa	41.60	0.00
TRINITY_sp|Q102	SPAC4H3.	Uncharacterized pro	41.60	0.00
TRINITY_sp|P253:	mlkA	Myosin light chain	41.60	0.00
TRINITY_sp|A8IN(	ARL13	ADP-ribosylation fa	41.60	0.00
TRINITY_sp|O008	cudA	Putative transcript	41.60	0.00
TRINITY_sp|P519!	Nek1	Serine/threonine-pr	41.60	0.00
TRINITY_sp|Q6ME(	rsmH	Ribosomal RNA small	41.60	0.00
TRINITY_sp|Q655I	MCSU3	Molybdenum cofactor	41.60	0.00
TRINITY_sp|Q9N9)	-	Ciliary WD repeat-c	41.60	0.00
TRINITY_sp|Q9H0	CCDC113	Coiled-coil domain-	41.60	0.00
TRINITY_sp|Q9M9I	At1g782{	F-box protein At1g7	41.60	0.00
TRINITY_sp|Q93Z/	NYC1	Probable chlorophyl	41.60	0.00
TRINITY_sp|Q050{	UBE3A	Ubiquitin-protein l	41.60	0.00
TRINITY_sp|P192:	adaB	Methylated-DNA--pro	41.60	0.00
TRINITY_sp|Q9FT	RECQL3	ATP-dependent DNA h	41.60	0.00
TRINITY_sp|Q54RI	shkA	Dual specificity pr	41.60	0.00
TRINITY_sp|O944{	bdp1	Transcription facto	41.60	0.00
TRINITY_sp|Q6NP:	ATR3	NADPH-dependent dif	41.60	0.00
TRINITY_sp|Q5D0	rbm8a	RNA-binding protein	41.60	0.00
TRINITY_sp|O226(	DEGP1	Protease Do-like 1,	41.60	0.00
TRINITY_sp|P739(	menA	2-carboxy-1,4-napht	41.60	0.00
TRINITY_sp|Q9P7	SPBC171	Uncharacterized WD	41.60	0.00
TRINITY_sp|Q5Y2(	HTAY	Histone H2A.Y OS=Te	41.60	0.00
TRINITY_sp|Q9P2I	IFT80	Intraflagellar tran	41.60	0.00
TRINITY_sp|P477:	Cbr1	Carbonyl reductase	41.60	0.00

TRINITY_sp|Q8GX	UGLYAH	(S)-ureidoglycine a	41.60	0.00
TRINITY_sp|Q503	ngly1	Peptide-N(4)-(N-ace	41.60	0.00
TRINITY_sp|P056	sacC	Levanase OS=Bacillu	41.60	0.00
TRINITY_sp|Q9SL	ALIS3	ALA-interacting sub	41.60	0.00
TRINITY_sp|Q9Y8	MET3	Sulfate adenylyltra	41.60	0.00
TRINITY_sp|P736	sll1770	Uncharacterized pro	41.60	0.00
TRINITY_sp|Q6NZ	Ddx31	Probable ATP-depend	41.60	0.00
TRINITY_sp|Q7ZZ	atad1a	ATPase family AAA d	41.60	0.00
TRINITY_sp|Q073	uapA	Uric acid-xanthine	41.60	0.00
TRINITY_sp|Q157	HERC1	Probable E3 ubiquit	41.60	0.00
TRINITY_sp|P109	-	Actin, cytoplasmic	41.60	0.00
TRINITY_sp|Q6PG	rnps1	RNA-binding protein	41.60	0.00
TRINITY_sp|Q5RF	PGM2	Phosphoglucomutase-	41.60	0.00
TRINITY_sp|P546	patA	Calcium-transportin	41.60	0.00
TRINITY_sp|Q2FI	saeS	Histidine protein k	41.50	0.00
TRINITY_sp|B2HL	MMAR_19	UPF0678 fatty acid-	41.50	0.00
TRINITY_sp|Q7Z4	TTC21B	Tetratricopeptide r	41.50	0.00
TRINITY_sp|P045	SPINK1	Serine protease inh	41.50	0.00
TRINITY_sp|P623	CPK1	Calcium-dependent p	41.50	0.00
TRINITY_sp|Q8LB	GRXS15	Monothiol glutaredo	41.50	0.00
TRINITY_sp|P930	BSH	Chromatin structure	41.50	0.00
TRINITY_sp|Q947	-	Probable dynein lig	41.50	0.00
TRINITY_sp|Q9NP	ALG13	Putative bifunction	41.50	0.00
TRINITY_sp|A2R5	tpc1	Mitochondrial thiam	41.50	0.00
TRINITY_sp|Q5N7	MOR1	Protein MOR1 OS=Ory	41.50	0.00
TRINITY_sp|Q9Y2	ATP8A1	Phospholipid-transp	41.50	0.00
TRINITY_sp|Q557	trappc1-	Trafficking protein	41.50	0.00
TRINITY_sp|Q55G	DDB_G02	UPF0160 protein OS=	41.50	0.00
TRINITY_sp|Q55E	pats1	Probable serine/thr	41.50	0.00
TRINITY_sp|Q041	HPT1	Hypoxanthine-guanin	41.50	0.00
TRINITY_sp|Q2SJ	msrB	Peptide methionine	41.50	0.00
TRINITY_sp|P277	ODA6	Dynein, 70 kDa inte	41.50	0.00
TRINITY_sp|P558	faf	Probable ubiquitin	41.50	0.00
TRINITY_sp|Q9UY	sua5	Threonylcarbamoyl-A	41.50	0.00
TRINITY_sp|Q929	BRF1	Transcription facto	41.50	0.00
TRINITY_sp|C0SU	JMJ16	Putative lysine-spe	41.50	0.00
TRINITY_sp|Q54D	DDB_G02	Serine carboxypepti	41.50	0.00
TRINITY_sp|Q54M	mcfB	Mitochondrial subst	41.50	0.00
TRINITY_sp|P140	-	Chymopapain OS=Cari	41.50	0.00
TRINITY_sp|P0C8	OsI_027	Uncharacterized pro	41.50	0.00
TRINITY_sp|Q55C	tpp1	Tripeptidyl-peptida	41.50	0.00
TRINITY_sp|Q3Z8	hisC	Histidinol-phosphat	41.50	0.00
TRINITY_sp|Q9VC	eIF-3p6	Eukaryotic translat	41.50	0.00
TRINITY_sp|P506	TUB	Tubby protein homol	41.50	0.00
TRINITY_sp|Q8L7	NPC1	Non-specific phosph	41.50	0.00
TRINITY_sp|P736	sppA	Protease 4 OS=Synec	41.50	0.00
TRINITY_sp|A0Q1	dnaJ	Chaperone protein D	41.50	0.00
TRINITY_sp|Q3MH	MSH2	DNA mismatch repair	41.50	0.00
TRINITY_sp|Q7XB	HGGT	Homogentisate geran	41.50	0.00
TRINITY_sp|Q9SV	At4g137	Methionine--tRNA li	41.50	0.00
TRINITY_sp|Q8WV	SCFD1	Sec1 family domain-	41.50	0.00
TRINITY_sp|Q8BG	Htatsf1	HIV Tat-specific fa	41.50	0.00
TRINITY_sp|Q86I	DDB_G02	Uncharacterized Gol	41.50	0.00
TRINITY_sp|Q8VE	Exd2	Exonuclease 3'-5' d	41.50	0.00
TRINITY_sp|Q32L	HMGB3	High mobility group	41.50	0.00
TRINITY_sp|O609	TBL1X	F-box-like/WD repea	41.50	0.00

TRINITY_sp|B9DF\	At5g474	E3 ubiquitin ligase	41.50	0.00
TRINITY_sp|F4J3(	CTPA3	Carboxyl-terminal-p	41.50	0.00
TRINITY_sp|P543	Lipe	Hormone-sensitive l	41.50	0.00
TRINITY_sp|Q476	mmuM	Homocysteine S-meth	41.50	0.00
TRINITY_sp|Q03AI	clpP	ATP-dependent Clp p	41.50	0.00
TRINITY_sp|O890	Tpp1	Tripeptidyl-peptida	41.50	0.00
TRINITY_sp|Q9Z1	DPM2	Dolichol phosphate-	41.50	0.00
TRINITY_sp|P495	Ppx1	Serine/threonine-pr	41.50	0.00
TRINITY_sp|O150	SYNJ2	Synaptojanin-2 OS=H	41.50	0.00
TRINITY_sp|O597	vcx1	Vacuolar calcium io	41.50	0.00
TRINITY_sp|E9PZ	Ascc3	Activating signal c	41.50	0.00
TRINITY_sp|Q145	NBR1	Next to BRCA1 gene	41.50	0.00
TRINITY_sp|Q9FZ	BGLU40	Beta-glucosidase 40	41.50	0.00
TRINITY_sp|P583	sseA	3-mercaptopyruvate	41.50	0.00
TRINITY_sp|Q9FN	E2FA	Transcription facto	41.50	0.00
TRINITY_sp|Q029	YRK	Proto-oncogene tyro	41.50	0.00
TRINITY_sp|Q8RX\	WIN2	Probable protein ph	41.50	0.00
TRINITY_sp|Q2TA(	AURKA	Aurora kinase A OS=	41.50	0.00
TRINITY_sp|P109	-	Retrovirus-related	41.50	0.00
TRINITY_sp|Q7X9	AERO2	Endoplasmic reticul	41.50	0.00
TRINITY_sp|Q704	xpo7-a	Exportin-7-A OS=Xen	41.50	0.00
TRINITY_sp|Q101	rtf2	Replication termina	41.50	0.00
TRINITY_sp|Q9BX	MAP1LC3	Microtubule-associa	41.40	0.00
TRINITY_sp|Q396	ODA11	Dynein alpha chain,	41.40	0.00
TRINITY_sp|O049	-	Alpha-glucosidase O	41.40	0.00
TRINITY_sp|Q5TH	VPS13D	Vacuolar protein so	41.40	0.00
TRINITY_sp|Q54G	vps45	Vacuolar protein so	41.40	0.00
TRINITY_sp|Q644	Atp7a	Copper-transporting	41.40	0.00
TRINITY_sp|P525	mybl2	Myb-related protein	41.40	0.00
TRINITY_sp|Q73W	MAP_283	UPF0678 fatty acid-	41.40	0.00
TRINITY_sp|Q54B	abcB2	ABC transporter B f	41.40	0.00
TRINITY_sp|Q54R	xpot	Exportin-T OS=Dicty	41.40	0.00
TRINITY_sp|Q502	eri2	ERI1 exoribonucleas	41.40	0.00
TRINITY_sp|Q96L	MARK4	MAP/microtubule aff	41.40	0.00
TRINITY_sp|Q8H8	Os03g02	Coatomer subunit ga	41.40	0.00
TRINITY_sp|Q6IR	abhd13	Protein ABHD13 OS=X	41.40	0.00
TRINITY_sp|Q5T3	ABCC10	Multidrug resistanc	41.40	0.00
TRINITY_sp|Q2GV	ADK1	Adenylate kinase OS	41.40	0.00
TRINITY_sp|Q9ZT	ATE1	Arginyl-tRNA--prote	41.40	0.00
TRINITY_sp|Q1JP	NDB1	External alternativ	41.40	0.00
TRINITY_sp|A7IS	QPCT	Glutaminyl-peptide	41.40	0.00
TRINITY_sp|D2XN	FLOT2	Flotillin-like prot	41.40	0.00
TRINITY_sp|Q9SB	AP4M	AP-4 complex subuni	41.40	0.00
TRINITY_sp|B3PG	agaA	Alpha-galactosidase	41.40	0.00
TRINITY_sp|Q9SH	HMA5	Probable copper-tra	41.40	0.00
TRINITY_sp|Q8W4	At4g046	tRNA wybutosine-syn	41.40	0.00
TRINITY_sp|Q9JM	Wrap73	WD repeat-containin	41.40	0.00
TRINITY_sp|Q9Z2\	Hdac6	Histone deacetylase	41.40	0.00
TRINITY_sp|Q90X	gpkow	G patch domain and	41.40	0.00
TRINITY_sp|Q9UN	A4GNT	Alpha-1,4-N-acetylg	41.40	0.00
TRINITY_sp|Q7TQ	Poln	DNA polymerase nu O	41.40	0.00
TRINITY_sp|P239	Eci1	Enoyl-CoA delta iso	41.40	0.00
TRINITY_sp|Q6NX	Rcbtb1	RCC1 and BTB domain	41.40	0.00
TRINITY_sp|Q9M2(	RRP45B	Exosome complex com	41.40	0.00
TRINITY_sp|Q55E	nat10	RNA cytidine acetyl	41.40	0.00
TRINITY_sp|P109	-	Retrovirus-related	41.40	0.00

TRINITY_sp|P128	hsp16.9	16.9 kDa class I he	41.40	0.00
TRINITY_sp|O671	aq_1088	Uncharacterized pro	41.40	0.00
TRINITY_sp|A5DE	FMP52	Protein FMP52, mito	41.40	0.00
TRINITY_sp|Q9UP	USP22	Ubiquitin carboxyl-	41.40	0.00
TRINITY_sp|B5DE	chd8	Chromodomain-helica	41.40	0.00
TRINITY_sp|Q9SY	ABCB3	ABC transporter B f	41.40	0.00
TRINITY_sp|Q554	bzpJ	Probable basic-leuc	41.40	0.00
TRINITY_sp|Q54C	DDB_G02	Probable myosin lig	41.40	0.00
TRINITY_sp|Q9Y7	AOX1	Alternative oxidase	41.40	0.00
TRINITY_sp|P0C8	Jmjd7	JmjC domain-contain	41.40	0.00
TRINITY_sp|Q54R	yipf6	Protein YIPF6 homol	41.40	0.00
TRINITY_sp|O749	POX2	Acyl-coenzyme A oxi	41.40	0.00
TRINITY_sp|Q54T	drkD	Probable serine/thr	41.40	0.00
TRINITY_sp|Q93V	VCL1	Protein VACUOLELESS	41.40	0.00
TRINITY_sp|Q9CR	Rchy1	RING finger and CHY	41.40	0.00
TRINITY_sp|P361	PRY2	Protein PRY2 OS=Sac	41.40	0.00
TRINITY_sp|P282	-	Betaine aldehyde de	41.40	0.00
TRINITY_sp|Q9ZV	At1g545	Acyltransferase-lik	41.40	0.00
TRINITY_sp|P0CH	adhc1	NADP-dependent alco	41.40	0.00
TRINITY_sp|P0AD	ygiC	Putative acid--amin	41.40	0.00
TRINITY_sp|P103	RRAS	Ras-related protein	41.40	0.00
TRINITY_sp|A8JI	CFAP157	Cilia- and flagella	41.40	0.00
TRINITY_sp|Q6ZY	GWD3	Phosphoglucan, wate	41.40	0.00
TRINITY_sp|P294	-	Thioredoxin H-type	41.40	0.00
TRINITY_sp|P109	-	Retrovirus-related	41.40	0.00
TRINITY_sp|Q3MH	RAB11B	Ras-related protein	41.40	0.00
TRINITY_sp|Q629	Slc30a2	Zinc transporter 2	41.30	0.00
TRINITY_sp|Q1ZX	gxcDD	Guanine exchange fa	41.30	0.00
TRINITY_sp|Q7Z3	C14orf1	UPF0317 protein C14	41.30	0.00
TRINITY_sp|P293	thrC	Threonine synthase	41.30	0.00
TRINITY_sp|Q911	-	Cystatin OS=Oncorhy	41.30	0.00
TRINITY_sp|P341	coaA	Coactosin OS=Dictyo	41.30	0.00
TRINITY_sp|O702	Atp9a	Probable phospholip	41.30	0.00
TRINITY_sp|Q869	mdn1	Midasin OS=Dictyost	41.30	0.00
TRINITY_sp|Q9M8	PMI1	Mannose-6-phosphate	41.30	0.00
TRINITY_sp|P546	pikD	Phosphatidylinosito	41.30	0.00
TRINITY_sp|Q54H	drkA	Probable serine/thr	41.30	0.00
TRINITY_sp|Q4V7	pigb	GPI mannosyltransfe	41.30	0.00
TRINITY_sp|P010	CSTA	Cystatin-A OS=Homo	41.30	0.00
TRINITY_sp|A2RV	TIC32	Short-chain dehydro	41.30	0.00
TRINITY_sp|P932	VPS41	Vacuolar protein so	41.30	0.00
TRINITY_sp|Q9SA	RH36	DEAD-box ATP-depend	41.30	0.00
TRINITY_sp|P306	-	Guanine nucleotide-	41.30	0.00
TRINITY_sp|P546	cap	Adenylyl cyclase-as	41.30	0.00
TRINITY_sp|Q949	ppa2	Soluble inorganic p	41.30	0.00
TRINITY_sp|Q8BT	Cpne3	Copine-3 OS=Mus mus	41.30	0.00
TRINITY_sp|P363	-	Endochitinase CH5B	41.30	0.00
TRINITY_sp|Q8W4	PI4KG6	Phosphatidylinosito	41.30	0.00
TRINITY_sp|P109	-	Retrovirus-related	41.30	0.00
TRINITY_sp|Q8R3	Ccdc12	Coiled-coil domain-	41.30	0.00
TRINITY_sp|P519	Nek1	Serine/threonine-pr	41.30	0.00
TRINITY_sp|Q8VC	Rbpms2	RNA-binding protein	41.30	0.00
TRINITY_sp|B6TV	-	Calcium sensing rec	41.30	0.00
TRINITY_sp|Q2RI	metX	Homoserine O-acetyl	41.30	0.00
TRINITY_sp|Q54K	DDB_G02	Uncharacterized pro	41.30	0.00
TRINITY_sp|Q9ZQ	PAP9	Probable inactive p	41.30	0.00

TRINITY_sp|Q5Z92	Os06g056	Solute carrier fami	41.30	0.00
TRINITY_sp|Q8L79	MIOX1	Inositol oxygenase	41.30	0.00
TRINITY_sp|Q9DG6	RAD54B	DNA repair and reco	41.30	0.00
TRINITY_sp|Q1ZXH	gxcDD	Guanine exchange fa	41.30	0.00
TRINITY_sp|P4136	BH0855	Thermostable alkali	41.30	0.00
TRINITY_sp|Q9632	SAC8	Phosphoinositide ph	41.30	0.00
TRINITY_sp|P1097	-	Retrovirus-related	41.30	0.00
TRINITY_sp|Q54KN	gemin2	Gem-associated prot	41.30	0.00
TRINITY_sp|Q9EQH	Vps35	Vacuolar protein so	41.30	0.00
TRINITY_sp|Q55BM	nek2	Probable serine/thr	41.30	0.00
TRINITY_sp|F4IRR	SAD2	Importin beta-like	41.30	0.00
TRINITY_sp|Q944R	ABCI12	Protein ABCI12, chl	41.30	0.00
TRINITY_sp|Q8LAN	P4H4	Probable prolyl 4-h	41.30	0.00
TRINITY_sp|P1097	-	Retrovirus-related	41.30	0.00
TRINITY_sp|Q652B	HSFB2C	Heat stress transcr	41.30	0.00
TRINITY_sp|P2379	RIT1	tRNA A64-2'-O-ribos	41.30	0.00
TRINITY_sp|P4820	dnaJ	Chaperone protein D	41.30	0.00
TRINITY_sp|Q9ZNW	MOCS3	Adenylyltransferase	41.30	0.00
TRINITY_sp|A6QP0	DHRS12	Dehydrogenase/reduc	41.30	0.00
TRINITY_sp|Q941R	GONST1	GDP-mannose transpo	41.30	0.00
TRINITY_sp|Q8TD5	DNAH3	Dynein heavy chain	41.30	0.00
TRINITY_sp|Q9HC0	GBA2	Non-lysosomal gluco	41.30	0.00
TRINITY_sp|P4601	KAPP	Protein phosphatase	41.30	0.00
TRINITY_sp|P0CQ2	ATP23	Mitochondrial inner	41.30	0.00
TRINITY_sp|F4ICH	RBL10	RHOMBOID-like prote	41.30	0.00
TRINITY_sp|O4604	Parg	Poly(ADP-ribose) gl	41.30	0.00
TRINITY_sp|Q8CG0	Abcc1	Multidrug resistanc	41.20	0.00
TRINITY_sp|Q9SCN	CYP71B31	Cytochrome P450 71B	41.20	0.00
TRINITY_sp|Q2TBB	PFDN4	Prefoldin subunit 4	41.20	0.00
TRINITY_sp|O3163	yjcK	Putative ribosomal-	41.20	0.00
TRINITY_sp|Q9Y24	GINS2	DNA replication com	41.20	0.00
TRINITY_sp|Q9QY2	Smok2b	Sperm motility kina	41.20	0.00
TRINITY_sp|Q9LK9	ALA8	Probable phospholip	41.20	0.00
TRINITY_sp|Q9LP0	RHM2	Trifunctional UDP-g	41.20	0.00
TRINITY_sp|Q3899	KIN10	SNF1-related protei	41.20	0.00
TRINITY_sp|Q5XM2	aprA	Autocrine prolifera	41.20	0.00
TRINITY_sp|Q9ZVT	At1g0337	C2 and GRAM domain-	41.20	0.00
TRINITY_sp|P9WLM	MT2089	Uncharacterized pro	41.20	0.00
TRINITY_sp|Q5ZKB	SLC25A36	Solute carrier fami	41.20	0.00
TRINITY_sp|Q54J9	wdr24	WD repeat-containin	41.20	0.00
TRINITY_sp|Q9ZTP	EIN4	Protein EIN4 OS=Ara	41.20	0.00
TRINITY_sp|Q9CZP	Bcs1l	Mitochondrial chape	41.20	0.00
TRINITY_sp|Q84WJ	At1g6732	Probable DNA primas	41.20	0.00
TRINITY_sp|Q5A4E	DED1	ATP-dependent RNA h	41.20	0.00
TRINITY_sp|D0PV9	laf-1	ATP-dependent RNA h	41.20	0.00
TRINITY_sp|Q9VJ3	Nedd8	NEDD8 OS=Drosophila	41.20	0.00
TRINITY_sp|Q6DC7	-	UPF0668 protein C10	41.20	0.00
TRINITY_sp|O9452	SPCC1281	ER membrane protein	41.20	0.00
TRINITY_sp|Q54P9	osbI	Oxysterol-binding p	41.20	0.00
TRINITY_sp|Q6GKX	FOLB3	Probable dihydroneo	41.20	0.00
TRINITY_sp|Q8K4F	Nsun5	Probable 28S rRNA (	41.20	0.00
TRINITY_sp|Q6PGP	TTC37	Tetratricopeptide r	41.20	0.00
TRINITY_sp|P0C8M	CCRP1	Probable serine/thr	41.20	0.00
TRINITY_sp|P1815	glpF	Glycerol uptake fac	41.20	0.00
TRINITY_sp|A2APX	Scn1a	Sodium channel prot	41.20	0.00
TRINITY_sp|Q6NUE	cwc15-a	Protein CWC15 homol	41.20	0.00

TRINITY_sp|Q584	pstS	Phosphate-binding p	41.20	0.00
TRINITY_sp|Q94K	VPS33	Vacuolar protein-so	41.20	0.00
TRINITY_sp|O755	MYCBP2	E3 ubiquitin-protei	41.20	0.00
TRINITY_sp|Q9D3	Pus10	Putative tRNA pseud	41.20	0.00
TRINITY_sp|Q401	RAB11A	Ras-related protein	41.20	0.00
TRINITY_sp|Q96D	DNAH11	Dynein heavy chain	41.20	0.00
TRINITY_sp|O354	Clcn6	Chloride transport	41.20	0.00
TRINITY_sp|Q9FP	UBP1	Ubiquitin carboxyl-	41.20	0.00
TRINITY_sp|Q48M	PSPPH_1	Renalase OS=Pseudom	41.20	0.00
TRINITY_sp|Q246	ref(2)P	Protein ref(2)P OS=	41.20	0.00
TRINITY_sp|Q9FN	PSL5	Probable glucan 1,3	41.20	0.00
TRINITY_sp|Q9FY	HPAT3	Hydroxyproline O-ar	41.20	0.00
TRINITY_sp|P817	-	Macrophage migratio	41.20	0.00
TRINITY_sp|Q67Z	TFCB	Tubulin-folding cof	41.20	0.00
TRINITY_sp|P049	GST3	Glutathione S-trans	41.20	0.00
TRINITY_sp|P925	AtMg008	Uncharacterized mit	41.20	0.00
TRINITY_sp|Q9EQ	Tpp1	Tripeptidyl-peptida	41.20	0.00
TRINITY_sp|P370	-	L-ascorbate oxidase	41.20	0.00
TRINITY_sp|Q124	SKM1	Serine/threonine-pr	41.20	0.00
TRINITY_sp|Q8VC	Rbpms2	RNA-binding protein	41.20	0.00
TRINITY_sp|Q9SI	HSP90-5	Heat shock protein	41.20	0.00
TRINITY_sp|Q67U	Os02g01	Probable protein ph	41.20	0.00
TRINITY_sp|Q6BU	RRD2	Serine/threonine-pr	41.20	0.00
TRINITY_sp|A2A5	Arfgef2	Brefeldin A-inhibit	41.20	0.00
TRINITY_sp|Q9FZ	At1g518	Probable LRR recept	41.20	0.00
TRINITY_sp|Q54N	atg9	Autophagy-related p	41.20	0.00
TRINITY_sp|P612	Arf79F	ADP-ribosylation fa	41.20	0.00
TRINITY_sp|Q9TT	RGN	Regucalcin OS=Oryct	41.20	0.00
TRINITY_sp|P0AE	barA	Signal transduction	41.20	0.00
TRINITY_sp|Q8N1	CCAR2	Cell cycle and apop	41.20	0.00
TRINITY_sp|O226	DEGP1	Protease Do-like 1,	41.20	0.00
TRINITY_sp|Q6R8	PHO1-H8	Phosphate transport	41.10	0.00
TRINITY_sp|Q9LP	BIG3	Brefeldin A-inhibit	41.10	0.00
TRINITY_sp|Q1ZX	echs1	Probable enoyl-CoA	41.10	0.00
TRINITY_sp|Q7XJ	GAS8	Growth arrest-speci	41.10	0.00
TRINITY_sp|Q8GY	SULTR4;2	Probable sulfate tr	41.10	0.00
TRINITY_sp|P126	YPK1	Serine/threonine-pr	41.10	0.00
TRINITY_sp|P056	sacC	Levanase OS=Bacillu	41.10	0.00
TRINITY_sp|Q9FE	EIF2A	Eukaryotic translat	41.10	0.00
TRINITY_sp|Q018	YPT1	Ras-like GTP-bindin	41.10	0.00
TRINITY_sp|Q72I	fusA	Elongation factor G	41.10	0.00
TRINITY_sp|O646	At2g456	Histone deacetylase	41.10	0.00
TRINITY_sp|O140	cta4	Manganese-transport	41.10	0.00
TRINITY_sp|Q8R4	Dusp15	Dual specificity pr	41.10	0.00
TRINITY_sp|Q9NA	iplA	Inositol 1,4,5-tris	41.10	0.00
TRINITY_sp|Q9M8	FTSHI3	Probable inactive A	41.10	0.00
TRINITY_sp|Q9FN	ATXR6	Histone-lysine N-me	41.10	0.00
TRINITY_sp|O784	ycf27	Probable transcript	41.10	0.00
TRINITY_sp|C7EX	ATP8A2	Phospholipid-transp	41.10	0.00
TRINITY_sp|Q9ST	CEP2	KDEL-tailed cystein	41.10	0.00
TRINITY_sp|Q54K	gtaN	GATA zinc finger do	41.10	0.00
TRINITY_sp|P328	GSP1	GTP-binding nuclear	41.10	0.00
TRINITY_sp|Q4IN	FGRRES_	Succinate dehydroge	41.10	0.00
TRINITY_sp|Q951	WASL	Neural Wiskott-Aldr	41.10	0.00
TRINITY_sp|Q9XY	fcpA	Probable C-terminal	41.10	0.00
TRINITY_sp|Q5A5	NIK1	Histidine protein k	41.10	0.00

TRINITY_sp|Q7Y21	GTE7	Transcription facto	41.10	0.00
TRINITY_sp|P1097	-	Retrovirus-related	41.10	0.00
TRINITY_sp|Q9WYX	TM_0508	Uncharacterized pro	41.10	0.00
TRINITY_sp|A6QLH	ERI3	ERI1 exoribonucleas	41.10	0.00
TRINITY_sp|Q54LE	vps13A	Putative vacuolar p	41.10	0.00
TRINITY_sp|Q9FN0	PP7	Serine/threonine-pr	41.10	0.00
TRINITY_sp|Q8RE3	adk	Adenylate kinase OS	41.10	0.00
TRINITY_sp|Q3MHI	RNPC3	RNA-binding protein	41.10	0.00
TRINITY_sp|Q8T8I	abcD2	ABC transporter D f	41.10	0.00
TRINITY_sp|P2585	FBP	Fructose-1,6-bispho	41.10	0.00
TRINITY_sp|Q86J0	mmgt	Membrane magnesium	41.10	0.00
TRINITY_sp|Q9C6I	MTK	Methylthioribose ki	41.10	0.00
TRINITY_sp|A0A0F	rnf213b	E3 ubiquitin-protei	41.10	0.00
TRINITY_sp|Q9C0(	DNAH6	Dynein heavy chain	41.10	0
TRINITY_sp|Q0UJ.	NST1	Stress response pro	41.10	0.00
TRINITY_sp|Q9S7I	TADA	tRNA(adenine(34)) d	41.10	0.00
TRINITY_sp|Q96PE	ARHGEF17	Rho guanine nucleot	41.10	0.00
TRINITY_sp|O0471	MSH6	DNA mismatch repair	41.10	0.00
TRINITY_sp|P2803	AOAH	Acyloxyacyl hydrola	41.10	0.00
TRINITY_sp|P3886	NMD3	60S ribosomal expor	41.10	0.00
TRINITY_sp|A3RF3	ALDH3A1	Aldehyde dehydrogen	41.10	0.00
TRINITY_sp|Q1569	MAPRE1	Microtubule-associa	41.10	0.00
TRINITY_sp|Q8WT1	NOC3L	Nucleolar complex p	41.10	0.00
TRINITY_sp|Q5BL5	atp8b1	Phospholipid-transp	41.10	0.00
TRINITY_sp|Q9SFU	At3g071(	Protein transport p	41.10	0.00
TRINITY_sp|Q9C50	PUX13	Plant UBX domain-co	41.10	0.00
TRINITY_sp|Q54YS	DDB_G027	DNA-binding protein	41.10	0.00
TRINITY_sp|A6R7X	PAN1	Actin cytoskeleton-	41.10	0.00
TRINITY_sp|Q5C92	NOM1	Nucleolar MIF4G dom	41.10	0.00
TRINITY_sp|Q8RWE	UPL6	E3 ubiquitin-protei	41.10	0.00
TRINITY_sp|B5XAM	mrpl58	Peptidyl-tRNA hydro	41.10	0.00
TRINITY_sp|Q9P7E	SPAC521.	NADP-dependent 3-hy	41.10	0.00
TRINITY_sp|O6549	XCP1	Cysteine protease X	41.10	0.00
TRINITY_sp|Q9FGE	CEP1	KDEL-tailed cystein	41.10	0.00
TRINITY_sp|Q6Q47	Atp2b4	Plasma membrane cal	41.10	0.00
TRINITY_sp|Q9LM1	PAPS1	Nuclear poly(A) pol	41.10	0.00
TRINITY_sp|P9819	Gnpat	Dihydroxyacetone ph	41.00	0.00
TRINITY_sp|Q54PE	pwp2	Periodic tryptophan	41.00	0.00
TRINITY_sp|Q7L21	ABHD13	Protein ABHD13 OS=H	41.00	0.00
TRINITY_sp|O4301	SPBC2G2.	Deoxycytidylate dea	41.00	0.00
TRINITY_sp|Q6ZR0	DNAH12	Dynein heavy chain	41.00	0.00
TRINITY_sp|Q0VC0	PQLC1	PQ-loop repeat-cont	41.00	0.00
TRINITY_sp|P0CQ2	SPT16	FACT complex subuni	41.00	0.00
TRINITY_sp|A1XD0	stip-1	Septin and tuftelin	41.00	0.00
TRINITY_sp|Q55F3	wdr91	WD repeat-containin	41.00	0.00
TRINITY_sp|O9571	HERC2	E3 ubiquitin-protei	41.00	0.00
TRINITY_sp|Q1RMH	ZNRD1	DNA-directed RNA po	41.00	0.00
TRINITY_sp|B0W37	CPIJ0016	Adenylyltransferase	41.00	0.00
TRINITY_sp|P2415	Thop1	Thimet oligopeptida	41.00	0.00
TRINITY_sp|Q9UBX	CTSF	Cathepsin F OS=Homo	41.00	0.00
TRINITY_sp|P1691	Pka-C3	Protein kinase DC2	41.00	0.00
TRINITY_sp|Q9M9H	SOL1	Carboxypeptidase SO	41.00	0.00
TRINITY_sp|P5439	Pdi	Protein disulfide-i	41.00	0.00
TRINITY_sp|P4412	ligA	DNA ligase OS=Haemo	41.00	0.00
TRINITY_sp|Q2KJ9	CDC42	Cell division contr	41.00	0.00
TRINITY_sp|Q5VQ7	Os06g014	Coatomer subunit be	41.00	0.00

TRINITY_sp|Q54GI	arv1	Protein arv1 homolo	41.00	0.00
TRINITY_sp|Q6NRI	ccnyl1-k	Cyclin-Y-like prote	41.00	0.00
TRINITY_sp|Q930(	USP9X	Probable ubiquitin	41.00	0.00
TRINITY_sp|Q5XM2	aprA	Autocrine prolifera	41.00	0.00
TRINITY_sp|P105(	MAL63	Maltose fermentatio	41.00	0.00
TRINITY_sp|O344{	yrrC	ATP-dependent RecD-	41.00	0.00
TRINITY_sp|P0C8N	CCRP1	Probable serine/thr	41.00	0.00
TRINITY_sp|Q7T03	rnf12-b	E3 ubiquitin-protei	41.00	0.00
TRINITY_sp|Q924(	pub1	E3 ubiquitin-protei	41.00	0.00
TRINITY_sp|F4I2I	SWI2	Switch 2 OS=Arabido	41.00	0.00
TRINITY_sp|Q8IS1	gefD	Ras guanine nucleot	41.00	0.00
TRINITY_sp|Q4PC(	HOG1	Mitogen-activated p	41.00	0.00
TRINITY_sp|O6052	NEMF	Nuclear export medi	41.00	0.00
TRINITY_sp|O596(	SPBC29A3	LisH domain-contain	41.00	0.00
TRINITY_sp|Q9NY5	STK32B	Serine/threonine-pr	41.00	0.00
TRINITY_sp|Q8GY2	UPL1	E3 ubiquitin-protei	41.00	0.00
TRINITY_sp|P487	uapC	Purine permease OS=	41.00	0.00
TRINITY_sp|Q9I8S	azin2	Antizyme inhibitor	41.00	0.00
TRINITY_sp|Q9TRY	FKBP4	Peptidyl-prolyl cis	41.00	0.00
TRINITY_sp|Q54RS	elmoC	ELMO domain-contain	41.00	0.00
TRINITY_sp|P494	GSPATT0(	Protein phosphatase	41.00	0.00
TRINITY_sp|O659(	CAP1	Cyclase-associated	41.00	0.00
TRINITY_sp|Q6INI	adck1	Uncharacterized aar	41.00	0.00
TRINITY_sp|P436	-	DnaJ protein homolo	41.00	0.00
TRINITY_sp|Q8INI	Gyc88E	Soluble guanylate c	41.00	0.00
TRINITY_sp|Q84WI	At5g045(	Glycosyltransferase	41.00	0.00
TRINITY_sp|P153	rpa1	DNA-directed RNA po	41.00	0.00
TRINITY_sp|O6782	guaB	Inosine-5'-monophos	41.00	0.00
TRINITY_sp|F4IE(	RID1	Pre-mRNA-splicing f	41.00	0.00
TRINITY_sp|O753{	ULK1	Serine/threonine-pr	41.00	0.00
TRINITY_sp|Q7T0V	bop1-a	Ribosome biogenesis	41.00	0.00
TRINITY_sp|Q9C95	SRK2B	Serine/threonine-pr	41.00	0.00
TRINITY_sp|Q8R3I	Mcat	Malonyl-CoA-acyl ca	41.00	0.00
TRINITY_sp|Q54FI	gefK	Ras guanine nucleot	41.00	0.00
TRINITY_sp|P270{	ABT	ADP,ATP carrier pro	41.00	0.00
TRINITY_sp|Q9FNY	E2FA	Transcription facto	41.00	0.00
TRINITY_sp|Q9LZV	STN8	Serine/threonine-pr	41.00	0.00
TRINITY_sp|P109	-	Retrovirus-related	41.00	0.00
TRINITY_sp|P623	CPK1	Calcium-dependent p	41.00	0.00
TRINITY_sp|E1BZI	TECPR1	Tectonin beta-prope	41.00	0.00
TRINITY_sp|Q8MR(	viaf	Viral IAP-associate	41.00	0.00
TRINITY_sp|Q994	CDC5L	Cell division cycle	41.00	0.00
TRINITY_sp|B9DF(	ISE2	DExH-box ATP-depend	41.00	0.00
TRINITY_sp|Q9XT(	tbh-1	Tyramine beta-hydro	41.00	0.00
TRINITY_sp|Q2XQY	IFT57	Intraflagellar tran	41.00	0.00
TRINITY_sp|P0CY3	SEC4	Ras-related protein	41.00	0.00
TRINITY_sp|O8102	EMB1187	Probable ethanolami	41.00	0.00
TRINITY_sp|P623	CPK4	Calcium-dependent p	41.00	0.00
TRINITY_sp|O433(	HSPA12A	Heat shock 70 kDa p	41.00	0.00
TRINITY_sp|Q7T0S	cfap36	Cilia- and flagella	41.00	0.00
TRINITY_sp|Q029	CAD8	Cinnamyl alcohol de	41.00	0.00
TRINITY_sp|Q5ZJY	GARNL3	GTPase-activating R	41.00	0.00
TRINITY_sp|Q5AVN	dbp5	ATP-dependent RNA h	41.00	0.00
TRINITY_sp|Q0JL	Os01g061	Probable protein ph	41.00	0.00
TRINITY_sp|Q9QX(	Dpf1	Zinc finger protein	41.00	0.00
TRINITY_sp|O3443	yloB	Calcium-transportin	41.00	0.00

TRINITY_sp|Q089	NEW1	[NU+] prion formati	41.00	0.00
TRINITY_sp|Q641	FAM21A	WASH complex subuni	41.00	0.00
TRINITY_sp|Q8RWI	STY17	Serine/threonine-pr	41.00	0.00
TRINITY_sp|Q6ZS	CAPN12	Calpain-12 OS=Homo	41.00	0.00
TRINITY_sp|Q158	STXBP2	Syntaxin-binding pr	41.00	0.00
TRINITY_sp|Q54E	dtd	D-tyrosyl-tRNA(Tyr)	40.90	0.00
TRINITY_sp|O596	SPBC29A	Uncharacterized ABC	40.90	0.00
TRINITY_sp|O966	arcE	Actin-related prote	40.90	0.00
TRINITY_sp|Q32P	TCTEX1D	Tctex1 domain-conta	40.90	0.00
TRINITY_sp|Q9X1	glpK2	Glycerol kinase 2 O	40.90	0.00
TRINITY_sp|Q098	rqh1	ATP-dependent DNA h	40.90	0.00
TRINITY_sp|Q54C	mobB	MOB kinase activato	40.90	0.00
TRINITY_sp|Q9LR	PLDZETA	Phospholipase D zet	40.90	0.00
TRINITY_sp|F1QC	rp2	Protein XRP2 OS=Dan	40.90	0.00
TRINITY_sp|Q84Z	Os07g05	Probable V-type pro	40.90	0.00
TRINITY_sp|Q9LD	MYB108	Transcription facto	40.90	0.00
TRINITY_sp|Q7XR	RMI1	RecQ-mediated genom	40.90	0.00
TRINITY_sp|Q55E	pats1	Probable serine/thr	40.90	0.00
TRINITY_sp|D7UQ	aur	Aurora kinase OS=Pa	40.90	0.00
TRINITY_sp|Q643	Sqstm1	Sequestosome-1 OS=M	40.90	0.00
TRINITY_sp|Q1DN	CIMG_08	Very-long-chain 3-o	40.90	0.00
TRINITY_sp|Q8RWI	STY17	Serine/threonine-pr	40.90	0.00
TRINITY_sp|Q141	MORC3	MORC family CW-type	40.90	0.00
TRINITY_sp|Q86X	NME9	Thioredoxin domain-	40.90	0.00
TRINITY_sp|Q5ZK	ARL2BP	ADP-ribosylation fa	40.90	0.00
TRINITY_sp|Q0QL	Dml	2,3-dimethylmalate	40.90	0.00
TRINITY_sp|Q100	shk2	Serine/threonine-pr	40.90	0.00
TRINITY_sp|Q7XZ	SAC1	Phosphoinositide ph	40.90	0.00
TRINITY_sp|Q55G	DDB_G02	Probable serine/thr	40.90	0.00
TRINITY_sp|Q4R8	RAB1B	Ras-related protein	40.90	0.00
TRINITY_sp|Q54Q	DDB_G02	Probable zinc trans	40.90	0.00
TRINITY_sp|P0CQ	DHH1	ATP-dependent RNA h	40.90	0.00
TRINITY_sp|Q103	SPBC582.	Uncharacterized ATP	40.90	0.00
TRINITY_sp|Q2YD	GOLT1B	Vesicle transport p	40.90	0.00
TRINITY_sp|Q86Y	CPNE8	Copine-8 OS=Homo sa	40.90	0.00
TRINITY_sp|O008	cudA	Putative transcript	40.90	0.00
TRINITY_sp|Q2MH	HT1	Serine/threonine-pr	40.90	0.00
TRINITY_sp|Q54B	mcfG	Mitochondrial subst	40.90	0.00
TRINITY_sp|Q56A	ccdc40	Coiled-coil domain-	40.90	0.00
TRINITY_sp|Q84L	RAD23B	Ubiquitin receptor	40.90	0.00
TRINITY_sp|Q5RJ	coq9	Ubiquinone biosynth	40.90	0.00
TRINITY_sp|Q94F	SDX1	Probable tocopherol	40.90	0.00
TRINITY_sp|Q9ZR	CAD	Probable mannitol d	40.90	0.00
TRINITY_sp|Q557	polr3h-	DNA-directed RNA po	40.90	0.00
TRINITY_sp|P837	GRP2	Putative NADPH-depe	40.90	0.00
TRINITY_sp|Q0D7	NRAMP1	Metal transporter N	40.90	0.00
TRINITY_sp|Q9C5	OEP80	Outer envelope prot	40.90	0.00
TRINITY_sp|Q69Z	Hbs1l	HBS1-like protein O	40.90	0.00
TRINITY_sp|Q9Y6	ARFGEF2	Brefeldin A-inhibit	40.90	0.00
TRINITY_sp|Q141	PDE1C	Calcium/calmodulin-	40.90	0.00
TRINITY_sp|O822	DEGP2	Protease Do-like 2,	40.90	0.00
TRINITY_sp|B5X4	NAGS2	Probable amino-acid	40.90	0.00
TRINITY_sp|O042	At2g400	Putative ABC1 prote	40.90	0.00
TRINITY_sp|Q8VY	CCR4-6	Carbon catabolite r	40.90	0.00
TRINITY_sp|P082	-	Pathogenesis-relate	40.90	0.00
TRINITY_sp|P281	pkgB	Protein kinase 2 OS	40.90	0.00

TRINITY_sp|Q84K	DDB2	DNA damage-binding	40.90	0.00
TRINITY_sp|Q8W5	At3g151	Uncharacterized exo	40.90	0.00
TRINITY_sp|P562	tif225	Probable translatio	40.90	0.00
TRINITY_sp|Q9UJ	TRMT6	tRNA (adenine(58)-N	40.90	0.00
TRINITY_sp|Q9M1	ABCB21	ABC transporter B f	40.90	0.00
TRINITY_sp|Q8UV	baz1a	Bromodomain adjacen	40.90	0.00
TRINITY_sp|Q7T6	MIMI_R8	Putative serine/thr	40.90	0.00
TRINITY_sp|Q294	Su(var)	Histone-lysine N-me	40.80	0.00
TRINITY_sp|P324	GBP2	Guanylate-binding p	40.80	0.00
TRINITY_sp|Q28F	mfsd7-A	Major facilitator s	40.80	0.00
TRINITY_sp|P347	RAS-2	Ras-like protein 2	40.80	0.00
TRINITY_sp|F4JZ	ENGASE1	Cytosolic endo-beta	40.80	0.00
TRINITY_sp|Q6Z8	Os02g02	Probable protein ph	40.80	0.00
TRINITY_sp|P201	GSTM2	Glutathione S-trans	40.80	0.00
TRINITY_sp|Q9LE	CAF1-10	Probable CCR4-assoc	40.80	0.00
TRINITY_sp|P412	Abca1	ATP-binding cassett	40.80	0.00
TRINITY_sp|O225	STY8	Serine/threonine-pr	40.80	0.00
TRINITY_sp|Q8GW	GFA2	Chaperone protein d	40.80	0.00
TRINITY_sp|Q6AX	Mtm1	Myotubularin OS=Rat	40.80	0.00
TRINITY_sp|Q8BL	Cpne4	Copine-4 OS=Mus mus	40.80	0.00
TRINITY_sp|P331	ACSL1	Long-chain-fatty-ac	40.80	0.00
TRINITY_sp|Q995	TSNAX	Translin-associated	40.80	0.00
TRINITY_sp|F4I2	SWI2	Switch 2 OS=Arabido	40.80	0.00
TRINITY_sp|O756	USP2	Ubiquitin carboxyl-	40.80	0.00
TRINITY_sp|O953	ATG7	Ubiquitin-like modi	40.80	0.00
TRINITY_sp|Q76F	NOL8	Nucleolar protein 8	40.80	0.00
TRINITY_sp|Q5TI	VWA5B1	von Willebrand fact	40.80	0.00
TRINITY_sp|Q9SU	PAO5	Probable polyamine	40.80	0.00
TRINITY_sp|Q9LV	APRR3	Two-component respo	40.80	0.00
TRINITY_sp|Q54D	rer1	Protein RER1 homolo	40.80	0.00
TRINITY_sp|O763	RACG	Rho-related protein	40.80	0.00
TRINITY_sp|O151	NPC1	Niemann-Pick C1 pro	40.80	0.00
TRINITY_sp|Q86Y	CPNE8	Copine-8 OS=Homo sa	40.80	0.00
TRINITY_sp|P975	Acox2	Peroxisomal acyl-co	40.80	0.00
TRINITY_sp|Q9FH	GSTT3	Glutathione S-trans	40.80	0.00
TRINITY_sp|O229	At2g341	Uncharacterized pro	40.80	0.00
TRINITY_sp|Q9HB	RRAGC	Ras-related GTP-bin	40.80	0.00
TRINITY_sp|Q9FF	EGY2	Probable zinc metal	40.80	0.00
TRINITY_sp|Q54W	gna1	Glucosamine 6-phosp	40.80	0.00
TRINITY_sp|Q94B	GCP1	Germination-specifi	40.80	0.00
TRINITY_sp|Q54U	abcC12	ABC transporter C f	40.80	0.00
TRINITY_sp|Q9VR	l(1)G01	Inositol hexakispho	40.80	0.00
TRINITY_sp|Q8BL	-	UPF0769 protein C21	40.80	0.00
TRINITY_sp|P270	Parp1	Poly [ADP-ribose] p	40.80	0.00
TRINITY_sp|Q80X	Usp6nl	USP6 N-terminal-lik	40.80	0.00
TRINITY_sp|P496	PRIM1	DNA primase small s	40.80	0.00
TRINITY_sp|O749	mug40	DNA polymerase kapp	40.80	0.00
TRINITY_sp|Q8RY	At2g332	Haloacid dehalogena	40.80	0.00
TRINITY_sp|Q9TT	pro-pol	Pro-Pol polyprotein	40.80	0.00
TRINITY_sp|P516	arf4	ADP-ribosylation fa	40.80	0.00
TRINITY_sp|Q0IW	GRXS11	Monothiol glutaredo	40.80	0.00
TRINITY_sp|Q9LP	RHM2	Trifunctional UDP-g	40.80	0.00
TRINITY_sp|O354	Folh1	Glutamate carboxype	40.80	0.00
TRINITY_sp|A1TF	hpxO	FAD-dependent urate	40.80	0.00
TRINITY_sp|Q9GR	efa1B	Elongation factor 1	40.80	0.00
TRINITY_sp|P0CT	UMAG_10	Small glutamine-ric	40.80	0.00

TRINITY_sp|O347	yvkC	Uncharacterized pho	40.80	0.00
TRINITY_sp|O753	VPS4B	Vacuolar protein so	40.80	0.00
TRINITY_sp|A8IC	CFAP46	Cilia- and flagella	40.80	0.00
TRINITY_sp|Q54W	bzpF	Probable basic-leuc	40.80	0.00
TRINITY_sp|Q6PG	traf3ip	TRAF3-interacting p	40.80	0.00
TRINITY_sp|Q66G	NOA1	NO-associated prote	40.80	0.00
TRINITY_sp|Q9LM	FKBP18	Peptidyl-prolyl cis	40.80	0.00
TRINITY_sp|P0CY	SEC4	Ras-related protein	40.80	0.00
TRINITY_sp|P109	-	Retrovirus-related	40.80	0.00
TRINITY_sp|Q7G1	AAO4	Benzaldehyde dehydr	40.80	0.00
TRINITY_sp|O597	ark1	Serine/threonine-pr	40.80	0.00
TRINITY_sp|Q9ST	At3g483	Probable Ufm1-speci	40.80	0.00
TRINITY_sp|Q7TX	ppsC	Phthiocerol/phenolp	40.80	0.00
TRINITY_sp|Q9FW	DHAR1	Glutathione S-trans	40.80	0.00
TRINITY_sp|Q391	At4g329	Vacuolar-processing	40.80	0.00
TRINITY_sp|Q99J	Taf11	Transcription initi	40.80	0.00
TRINITY_sp|Q7XM	Os04g05	DEAD-box ATP-depend	40.80	0.00
TRINITY_sp|Q54Q	DDB_G02	Probable zinc trans	40.70	0.00
TRINITY_sp|Q9Z2	Hdac6	Histone deacetylase	40.70	0.00
TRINITY_sp|P519	NEK3	Serine/threonine-pr	40.70	0.00
TRINITY_sp|Q96M	ZDHHC15	Palmitoyltransferas	40.70	0.00
TRINITY_sp|A5PK	ABHD17C	Protein ABHD17C OS=	40.70	0.00
TRINITY_sp|Q8MM	paxB	Paxillin-B OS=Dicty	40.70	0.00
TRINITY_sp|P0CG	UBI4	Polyubiquitin OS=Ca	40.70	0.00
TRINITY_sp|Q94K	At3g581	Phenylalanine--tRNA	40.70	0.00
TRINITY_sp|Q0J9	EME1	Crossover junction	40.70	0.00
TRINITY_sp|Q9SU	At4g165	Probable protein ph	40.70	0.00
TRINITY_sp|Q5VJ	gdt9	Probable serine/thr	40.70	0.00
TRINITY_sp|Q54Y	ngap	Probable Ras GTPase	40.70	0.00
TRINITY_sp|P520	proC	Pyrroline-5-carboxy	40.70	0.00
TRINITY_sp|Q8MI	TGIF2LX	Homeobox protein TG	40.70	0.00
TRINITY_sp|Q8WT	DUSP19	Dual specificity pr	40.70	0.00
TRINITY_sp|Q1ZX	DDB_G02	PH domain-containin	40.70	0.00
TRINITY_sp|Q9SJ	FIM4	Fimbrin-4 OS=Arabid	40.70	0.00
TRINITY_sp|Q9RS	pnp	Polyribonucleotide	40.70	0.00
TRINITY_sp|P086	CYP3A4	Cytochrome P450 3A4	40.70	0.00
TRINITY_sp|P085	pstI	Aqualysin-1 OS=Ther	40.70	0.00
TRINITY_sp|Q643	Sqstm1	Sequestosome-1 OS=M	40.70	0.00
TRINITY_sp|O087	Ogg1	N-glycosylase/DNA l	40.70	0.00
TRINITY_sp|Q551	fray2	Serine/threonine-pr	40.70	0.00
TRINITY_sp|Q869	dokA	Hybrid signal trans	40.70	0.00
TRINITY_sp|Q9D2	Vps33a	Vacuolar protein so	40.70	0.00
TRINITY_sp|Q6I5	CDKC-1	Cyclin-dependent ki	40.70	0.00
TRINITY_sp|P256	PWP2	Periodic tryptophan	40.70	0.00
TRINITY_sp|P169	CNJC	Conjugation stage-s	40.70	0.00
TRINITY_sp|O321	yuiD	Uncharacterized mem	40.70	0.00
TRINITY_sp|Q132	SRSF6	Serine/arginine-ric	40.70	0.00
TRINITY_sp|Q618	Rasl2-9	GTP-binding nuclear	40.70	0.00
TRINITY_sp|P629	RAC1	Ras-related C3 botu	40.70	0.00
TRINITY_sp|P0CS	AKR1	Palmitoyltransferas	40.70	0.00
TRINITY_sp|O155	PDPK1	3-phosphoinositide-	40.70	0.00
TRINITY_sp|Q022	hemB	Delta-aminolevulini	40.70	0.00
TRINITY_sp|A2XU	OsI_163	Pachytene checkpoin	40.70	0.00
TRINITY_sp|Q9UT	paa1	Protein phosphatase	40.70	0.00
TRINITY_sp|Q227	sym-2	RNA-binding protein	40.70	0.00
TRINITY_sp|Q019	CPP1	Cysteine proteinase	40.70	0.00

TRINITY_sp|Q097	dnm1	Dynamin-related pro	40.70	0.00
TRINITY_sp|Q646	Ptprf	Receptor-type tyros	40.70	0.00
TRINITY_sp|C0QT	rpsO	30S ribosomal prote	40.70	0.00
TRINITY_sp|Q9Y7	SPBC2A9.	Uncharacterized WD	40.70	0.00
TRINITY_sp|Q67Y	ATL65	RING-H2 finger prot	40.70	0.00
TRINITY_sp|Q3SZ	LTA4H	Leukotriene A-4 hyd	40.70	0.00
TRINITY_sp|P041	GIP	Copia protein OS=Dr	40.70	0.00
TRINITY_sp|Q9CW	Mcm8	DNA helicase MCM8 O	40.70	0.00
TRINITY_sp|P740	prmC	Release factor glut	40.70	0.00
TRINITY_sp|P043	pol	Retrovirus-related	40.70	0.00
TRINITY_sp|P291	isp	Intracellular serin	40.70	0.00
TRINITY_sp|Q6DI	ublcp1	Ubiquitin-like doma	40.70	0.00
TRINITY_sp|Q9FN	VIP4	Protein LEO1 homolo	40.70	0.00
TRINITY_sp|A7SK	dph2	Diphthamide biosynt	40.70	0.00
TRINITY_sp|Q2JN	ftsH	ATP-dependent zinc	40.70	0.00
TRINITY_sp|Q91V	Abca7	ATP-binding cassett	40.70	0.00
TRINITY_sp|O654	XCP1	Cysteine protease X	40.70	0.00
TRINITY_sp|P109	-	Retrovirus-related	40.70	0.00
TRINITY_sp|Q8GW	PGLP2	Phosphoglycolate ph	40.60	0.00
TRINITY_sp|Q053	PTK2	Focal adhesion kina	40.60	0.00
TRINITY_sp|Q9FK	SBT5.6	Subtilisin-like pro	40.60	0.00
TRINITY_sp|Q9SJ	WBC30	Putative white-brow	40.60	0.00
TRINITY_sp|Q9DA	Dynlrb2	Dynein light chain	40.60	0.00
TRINITY_sp|Q8K4	Abca5	ATP-binding cassett	40.60	0.00
TRINITY_sp|Q9ZR	DSPTP1	Dual specificity pr	40.60	0.00
TRINITY_sp|Q2FK	pflB	Formate acetyltrans	40.60	0.00
TRINITY_sp|Q84Q	OPR1	12-oxophytodienoate	40.60	0.00
TRINITY_sp|Q032	divJ	Histidine protein k	40.60	0.00
TRINITY_sp|O314	ydcI	Uncharacterized pro	40.60	0.00
TRINITY_sp|P040	-	Thermitase OS=Therm	40.60	0.00
TRINITY_sp|Q8ND	CCNY	Cyclin-Y OS=Homo sa	40.60	0.00
TRINITY_sp|Q149	SHPRH	E3 ubiquitin-protei	40.60	0.00
TRINITY_sp|B9KZ	der	GTPase Der OS=Therm	40.60	0.00
TRINITY_sp|Q9H0	RNF38	E3 ubiquitin-protei	40.60	0.00
TRINITY_sp|A2YE	SAP8	Zinc finger A20 and	40.60	0.00
TRINITY_sp|Q3Z6	trpD	Anthranilate phosph	40.60	0.00
TRINITY_sp|P435	RIM15	Serine/threonine-pr	40.60	0.00
TRINITY_sp|Q9ZP	AMT1-2	Ammonium transporte	40.60	0.00
TRINITY_sp|Q8N6	SIRT6	NAD-dependent prote	40.60	0.00
TRINITY_sp|Q4J6	PREPL	Prolyl endopeptidas	40.60	0.00
TRINITY_sp|Q90Y	ZNF622	Zinc finger protein	40.60	0.00
TRINITY_sp|O237	MVD1	Diphosphomevalonate	40.60	0.00
TRINITY_sp|Q9PK	malQ	4-alpha-glucanotran	40.60	0.00
TRINITY_sp|P495	ycf39	Uncharacterized pro	40.60	0.00
TRINITY_sp|Q9SD	GDPD6	Glycerophosphodiest	40.60	0.00
TRINITY_sp|Q965	Y57E12A	Transmembrane prote	40.60	0.00
TRINITY_sp|Q9CQ	Tmem216	Transmembrane prote	40.60	0.00
TRINITY_sp|Q8BT	Cpne3	Copine-3 OS=Mus mus	40.60	0.00
TRINITY_sp|Q948	THH1	Protein TOM THREE H	40.60	0.00
TRINITY_sp|Q74Z	PAB1	Polyadenylate-bindi	40.60	0.00
TRINITY_sp|Q9LT	At3g194	RNA pseudouridine s	40.60	0.00
TRINITY_sp|Q6PF	ndor1	NADPH-dependent dif	40.60	0.00
TRINITY_sp|O503	bchH	Magnesium-chelatase	40.60	0.00
TRINITY_sp|Q54D	mcfP	Mitochondrial subst	40.60	0.00
TRINITY_sp|Q54W	bzpF	Probable basic-leuc	40.60	0.00
TRINITY_sp|Q3E9	CPK34	Calcium-dependent p	40.60	0.00

TRINITY_sp|Q066(	OMS1	Methyltransferase O	40.60	0.00
TRINITY_sp|Q586	aroB'	3-dehydroquinate sy	40.60	0.00
TRINITY_sp|O944	bdp1	Transcription facto	40.60	0.00
TRINITY_sp|Q8L9	P4H7	Probable prolyl 4-h	40.60	0.00
TRINITY_sp|Q6IQ	sae1	SUMO-activating enz	40.60	0.00
TRINITY_sp|Q6P8(	arl8a	ADP-ribosylation fa	40.60	0.00
TRINITY_sp|Q5T2	C9orf11	Putative methyltran	40.60	0.00
TRINITY_sp|O794(	MT-CO3	Cytochrome c oxidas	40.60	0.00
TRINITY_sp|Q94A	NUDT19	Nudix hydrolase 19,	40.60	0.00
TRINITY_sp|B4I4	GM10981	Diacylglycerol kina	40.60	0.00
TRINITY_sp|Q758(	BTN1	Protein BTN1 OS=Ash	40.60	0.00
TRINITY_sp|Q929	BRF1	Transcription facto	40.60	0.00
TRINITY_sp|Q2KI	PRPF3	U4/U6 small nuclear	40.60	0.00
TRINITY_sp|Q9SS	FAB1C	Putative 1-phosphat	40.60	0.00
TRINITY_sp|Q9LY	FKBP19	Peptidyl-prolyl cis	40.60	0.00
TRINITY_sp|Q9P7	rpa2	Probable DNA-direct	40.60	0.00
TRINITY_sp|O738	HPGDS	Hematopoietic prost	40.60	0.00
TRINITY_sp|Q9JM(	Edf1	Endothelial differe	40.60	0.00
TRINITY_sp|O228	At2g436	Endochitinase At2g4	40.60	0.00
TRINITY_sp|A8XN	sgk-1	Serine/threonine-pr	40.50	0.00
TRINITY_sp|C7G0	DDB_G02	von Willebrand fact	40.50	0.00
TRINITY_sp|F4JM	At4g168(	Probable enoyl-CoA	40.50	0.00
TRINITY_sp|P509	INP52	Polyphosphatidylino	40.50	0.00
TRINITY_sp|Q0IX	Os10g04	Acylamino-acid-rele	40.50	0.00
TRINITY_sp|Q8N4	CBR4	Carbonyl reductase	40.50	0.00
TRINITY_sp|Q9JH(	Pigp	Phosphatidylinosito	40.50	0.00
TRINITY_sp|Q92J	tilS	tRNA(Ile)-lysidine	40.50	0.00
TRINITY_sp|Q569	selenbp	Selenium-binding pr	40.50	0.00
TRINITY_sp|Q8NI(	NCOA7	Nuclear receptor co	40.50	0.00
TRINITY_sp|P546	pkbA	RAC family serine/t	40.50	0.00
TRINITY_sp|O956	ASMTL	N-acetylserotonin O	40.50	0.00
TRINITY_sp|P819	-	Superoxide dismutas	40.50	0.00
TRINITY_sp|P828	fdxr	NADPH:adrenodoxin o	40.50	0.00
TRINITY_sp|Q9ZT	ATE1	Arginyl-tRNA--prote	40.50	0.00
TRINITY_sp|Q9D7	RtcA	RNA 3'-terminal pho	40.50	0.00
TRINITY_sp|Q6NV	wdr82	WD repeat-containin	40.50	0.00
TRINITY_sp|F4IP(	SWC2	SWR1 complex subuni	40.50	0.00
TRINITY_sp|P427	PAB2	Polyadenylate-bindi	40.50	0.00
TRINITY_sp|Q55B	csn7	COP9 signalosome co	40.50	0.00
TRINITY_sp|Q8CA	Iba57	Putative transferas	40.50	0.00
TRINITY_sp|Q7T6	MIMI_R8	Putative serine/thr	40.50	0.00
TRINITY_sp|Q54S	DDB_G02	Protein PIEZO homol	40.50	0.00
TRINITY_sp|D3YV	Ccdc13	Coiled-coil domain-	40.50	0.00
TRINITY_sp|P041	Cfb	Complement factor B	40.50	0.00
TRINITY_sp|Q9S7	MYB98	Transcription facto	40.50	0.00
TRINITY_sp|Q54B	mcfG	Mitochondrial subst	40.50	0.00
TRINITY_sp|P078	PETF	Ferredoxin, chlorop	40.50	0.00
TRINITY_sp|P623	CPK1	Calcium-dependent p	40.50	0.00
TRINITY_sp|Q5M7	nol6	Nucleolar protein 6	40.50	0.00
TRINITY_sp|Q99P(	Ogfr	Opioid growth facto	40.50	0.00
TRINITY_sp|Q9VB	Clbn	Nuclear export medi	40.50	0.00
TRINITY_sp|Q9JI(	Dclre1a	DNA cross-link repa	40.50	0.00
TRINITY_sp|Q6ZS	CAPN12	Calpain-12 OS=Homo	40.50	0.00
TRINITY_sp|P258(	-	Cysteine proteinase	40.50	0.00
TRINITY_sp|A7SN	v1g2464(	Serine/threonine-pr	40.50	0.00
TRINITY_sp|Q7TP	Pigv	GPI mannosyltransfe	40.50	0.00

TRINITY_sp|P2752	LHCA4	Chlorophyll a-b bin	40.50	0.00
TRINITY_sp|Q9LI(	TMN7	Transmembrane 9 sup	40.50	0.00
TRINITY_sp|A2YV8	OsI_0282	DEAD-box ATP-depend	40.50	0.00
TRINITY_sp|Q4R8I	GNL1	Guanine nucleotide-	40.50	0.00
TRINITY_sp|P1725	Mvk	Mevalonate kinase O	40.50	0.00
TRINITY_sp|Q8TF(	ATP8B4	Probable phospholip	40.50	0.00
TRINITY_sp|Q6DJ	pithd1	PITH domain-contain	40.50	0.00
TRINITY_sp|O7593	DNAJC8	DnaJ homolog subfam	40.50	0.00
TRINITY_sp|Q8RW\	SNRNP65	U11/U12 small nucle	40.50	0.00
TRINITY_sp|Q5562	snf12-1	SWI/SNF complex com	40.50	0.00
TRINITY_sp|Q4A1(	walR	Transcriptional reg	40.50	0.00
TRINITY_sp|Q58A4	DD3-3	Protein DD3-3 OS=Di	40.50	0.00
TRINITY_sp|Q67X2	At5g0655	F-box protein At5g0	40.50	0.00
TRINITY_sp|Q7TMI	Prcp	Lysosomal Pro-X car	40.50	0.00
TRINITY_sp|P2212	-	Ras-related protein	40.50	0.00
TRINITY_sp|D3ZN4	Ndufaf6	NADH dehydrogenase	40.50	0.00
TRINITY_sp|Q8GY2	UPL1	E3 ubiquitin-protei	40.50	0.00
TRINITY_sp|Q925(	FIG4	Polyphosphoinositid	40.50	0.00
TRINITY_sp|Q7YR(	PLCZ	1-phosphatidylinosi	40.50	0.00
TRINITY_sp|Q3958	-	Dynein 18 kDa light	40.50	0.00
TRINITY_sp|Q54HI	mybQ	Myb-like protein Q	40.50	0.00
TRINITY_sp|F4JBI	ATG1B	Serine/threonine-pr	40.50	0.00
TRINITY_sp|P4329	SRK2G	Serine/threonine-pr	40.50	0.00
TRINITY_sp|O1429	wis4	MAP kinase kinase k	40.50	0.00
TRINITY_sp|Q5RHI	ift172	Intraflagellar tran	40.50	0
TRINITY_sp|Q9SU(	ITPK3	Inositol-tetrakisph	40.50	0.00
TRINITY_sp|P1473	IDE	Insulin-degrading e	40.50	0.00
TRINITY_sp|Q7PNI	AGAP0058	Alpha-tubulin N-ace	40.50	0.00
TRINITY_sp|Q8LE\	At2g306(	BTB/POZ domain-cont	40.50	0.00
TRINITY_sp|Q9FFI	RLT2	Homeobox-DDT domain	40.50	0.00
TRINITY_sp|Q28D7	eed	Polycomb protein ee	40.50	0.00
TRINITY_sp|Q5FWI	ZRANB3	DNA annealing helic	40.50	0.00
TRINITY_sp|Q2663	-	Cathepsin L OS=Sarc	40.50	0.00
TRINITY_sp|O7534	GUCY1B2	Guanylate cyclase s	40.50	0.00
TRINITY_sp|P2577	Os04g065	Oryzain alpha chain	40.50	0.00
TRINITY_sp|Q54R5	DDB_G028	Probable tyrosine-p	40.50	0.00
TRINITY_sp|Q6DR	ddx51	ATP-dependent RNA h	40.40	0.00
TRINITY_sp|O5105	BB_0024	Uncharacterized pro	40.40	0.00
TRINITY_sp|P5399	Rab2a	Ras-related protein	40.40	0.00
TRINITY_sp|Q5ZJI	ABHD13	Protein ABHD13 OS=G	40.40	0.00
TRINITY_sp|Q9BXW	MAP1LC3(	Microtubule-associa	40.40	0.00
TRINITY_sp|Q6067	Sik1	Serine/threonine-pr	40.40	0.00
TRINITY_sp|P4031	CYB5	Cytochrome b5 OS=Sa	40.40	0.00
TRINITY_sp|P3245	APE2	Aminopeptidase 2, m	40.40	0.00
TRINITY_sp|Q9LHI	ABCB15	ABC transporter B f	40.40	0.00
TRINITY_sp|Q95Z9	tdc-1	Tyrosine decarboxyl	40.40	0.00
TRINITY_sp|Q9NZ7	OGFR	Opioid growth facto	40.40	0.00
TRINITY_sp|O8109	NRPB8A	DNA-directed RNA po	40.40	0.00
TRINITY_sp|Q9YDI	thsA	Thermosome subunit	40.40	0.00
TRINITY_sp|O1543	ABCC4	Multidrug resistanc	40.40	0.00
TRINITY_sp|Q2N2I	-	Probable phytol kin	40.40	0.00
TRINITY_sp|Q9FVI	AMT1-3	Ammonium transporte	40.40	0.00
TRINITY_sp|Q9NT	ATP8A2	Phospholipid-transp	40.40	0.00
TRINITY_sp|Q4VYA	hiuH	5-hydroxyisourate h	40.40	0.00
TRINITY_sp|Q8GW7	PMRT15	Protein arginine N-	40.40	0.00
TRINITY_sp|Q6CJ5	MDV1	Mitochondrial divis	40.40	0.00

TRINITY_sp|Q54R\	sglA	Sphingosine-1-phosp	40.40	0.00
TRINITY_sp|O697{	betC	Choline-sulfatase O	40.40	0.00
TRINITY_sp|Q54E{	rpc19	DNA-directed RNA po	40.40	0.00
TRINITY_sp|P220{	P100/11I	Prostaglandin F syn	40.40	0.00
TRINITY_sp|Q652I	PHT4;4	Probable anion tran	40.40	0.00
TRINITY_sp|Q925{	FIG4	Polyphosphoinositid	40.40	0.00
TRINITY_sp|P281	pkgB	Protein kinase 2 OS	40.40	0.00
TRINITY_sp|O751	CPNE3	Copine-3 OS=Homo sa	40.40	0.00
TRINITY_sp|B8AEI	RR23	Two-component respo	40.40	0.00
TRINITY_sp|Q4FE{	XBAT35	Putative E3 ubiquit	40.40	0.00
TRINITY_sp|Q9ST\	CIPK8	CBL-interacting ser	40.40	0.00
TRINITY_sp|Q8VYI	SQE3	Squalene epoxidase	40.40	0.00
TRINITY_sp|Q54I(	DDB_G02{	Vacuolar fusion pro	40.40	0.00
TRINITY_sp|Q54WI	tmem184(	Transmembrane prote	40.40	0.00
TRINITY_sp|Q247{	-	Myophilin OS=Echino	40.40	0.00
TRINITY_sp|Q169{	-	Dynein intermediate	40.40	0.00
TRINITY_sp|Q5ZJ	SUPV3L1	ATP-dependent RNA h	40.40	0.00
TRINITY_sp|O233	ATXR3	Histone-lysine N-me	40.40	0.00
TRINITY_sp|Q9PUI	smurf1	E3 ubiquitin-protei	40.40	0.00
TRINITY_sp|Q9GR{	aif	Apoptosis-inducing	40.40	0.00
TRINITY_sp|Q605{	Wrap53	Telomerase Cajal bo	40.40	0.00
TRINITY_sp|P0CB{	Ylpm1	YLP motif-containin	40.40	0.00
TRINITY_sp|Q9FII	RABA2D	Ras-related protein	40.40	0.00
TRINITY_sp|O597	SPBC3B8.	Uncharacterized tra	40.40	0.00
TRINITY_sp|Q8BH{	Dtd2	Probable D-tyrosyl-	40.40	0.00
TRINITY_sp|Q86A\	guaD	Guanine deaminase O	40.40	0.00
TRINITY_sp|Q9Y5I	N6AMT1	HemK methyltransfer	40.40	0.00
TRINITY_sp|Q84MI	LUL3	Probable E3 ubiquit	40.40	0.00
TRINITY_sp|Q148I	TAF13	Transcription initi	40.40	0.00
TRINITY_sp|Q9SV{	At4g314{	Coatomer subunit be	40.40	0.00
TRINITY_sp|O494{	AAH	Allantoate deiminas	40.40	0.00
TRINITY_sp|Q9FPI	EDR1	Serine/threonine-pr	40.40	0.00
TRINITY_sp|Q53F{	TP53I3	Quinone oxidoreduct	40.40	0.00
TRINITY_sp|Q5XG{	tsr1	Pre-rRNA-processing	40.40	0.00
TRINITY_sp|Q84J(	At5g405{	Ribosomal RNA-proce	40.40	0.00
TRINITY_sp|Q9SR{	CHIP	E3 ubiquitin-protei	40.40	0.00
TRINITY_sp|Q58D(	GTPBP1	GTP-binding protein	40.40	0.00
TRINITY_sp|P169	YPTM1	GTP-binding protein	40.40	0.00
TRINITY_sp|Q9FN(	UVR8	Ultraviolet-B recep	40.40	0.00
TRINITY_sp|Q395	ODA2	Dynein gamma chain,	40.40	0.00
TRINITY_sp|Q9SU(	FAH2	Dihydroceramide fat	40.40	0.00
TRINITY_sp|Q9LXI	RLF	Cytochrome b5 domai	40.40	0.00
TRINITY_sp|P283{	Gst1	Glutathione S-trans	40.40	0.00
TRINITY_sp|Q9LT{	PPOX1	Pyridoxine/pyridoxa	40.40	0.00
TRINITY_sp|Q9FY(	TMN11	Transmembrane 9 sup	40.40	0.00
TRINITY_sp|Q9M0{	NSF	Vesicle-fusing ATPa	40.40	0.00
TRINITY_sp|Q54W\	DDB_G02	Probable serine/thr	40.40	0.00
TRINITY_sp|Q9WT\	Slc23a1	Solute carrier fami	40.30	0.00

TRINITY_sp|Q8C6(	-	Protein C21orf2 hom	40.30	0.00
TRINITY_sp|Q6NP(	At5g511:	Probable RNA methyl	40.30	0.00
TRINITY_sp|Q8R5I	Utp11	Probable U3 small n	40.30	0.00
TRINITY_sp|Q7ZXI	mtmr4	Myotubularin-relate	40.30	0.00
TRINITY_sp|Q4JHI	XBOS36	Probable E3 ubiquit	40.30	0.00
TRINITY_sp|Q5ZMI	SETD3	Histone-lysine N-me	40.30	0.00
TRINITY_sp|P176:	FMO2	Dimethylaniline mon	40.30	0.00
TRINITY_sp|Q9DCI	Cyb5r3	NADH-cytochrome b5	40.30	0.00
TRINITY_sp|A8NU1	CC-RAS	24 kDa Ras-like pro	40.30	0.00
TRINITY_sp|Q9D6I	Ttc33	Tetratricopeptide r	40.30	0.00
TRINITY_sp|Q5M8:	Nudcd2	NudC domain-contain	40.30	0.00
TRINITY_sp|Q9LKV	NHX7	Sodium/hydrogen exc	40.30	0.00
TRINITY_sp|Q6DH(	mrpl24	Probable 39S riboso	40.30	0.00
TRINITY_sp|Q76P:	mcfQ	Mitochondrial subst	40.30	0.00
TRINITY_sp|B2GV1	Agbl5	Cytosolic carboxype	40.30	0.00
TRINITY_sp|Q08DI	TSN	Translin OS=Bos tau	40.30	0.00
TRINITY_sp|Q7DM!	ABCC4	ABC transporter C f	40.30	0.00
TRINITY_sp|Q0P5I	DNTTIP2	Deoxynucleotidyltra	40.30	0.00
TRINITY_sp|Q923V	Tgs1	Trimethylguanosine	40.30	0.00
TRINITY_sp|Q54C!	jcdE	JmjC domain-contain	40.30	0.00
TRINITY_sp|Q08BI	ctdspl2a	CTD small phosphata	40.30	0.00
TRINITY_sp|Q8VD1	Alg9	Alpha-1,2-mannosylt	40.30	0.00
TRINITY_sp|O550:	Copb2	Coatomer subunit be	40.30	0.00
TRINITY_sp|Q9H2V	MRPL46	39S ribosomal prote	40.30	0.00
TRINITY_sp|P929'	UBA2	Ubiquitin-activatin	40.30	0.00
TRINITY_sp|P208:	CYP3A5	Cytochrome P450 3A5	40.30	0.00
TRINITY_sp|Q102:	SPAC4H3.	Putative thiosulfat	40.30	0.00
TRINITY_sp|P0DM(	rcsC	Sensor histidine ki	40.30	0.00
TRINITY_sp|P323'	MVD1	Diphosphomevalonate	40.30	0.00
TRINITY_sp|Q9SKI	MON1	Vacuolar fusion pro	40.30	0.00
TRINITY_sp|Q8GX:	HDA15	Histone deacetylase	40.30	0.00
TRINITY_sp|P529:	lkhA	Leukotriene A-4 hyd	40.30	0.00
TRINITY_sp|P618(	rpl-12	60S ribosomal prote	40.30	0.00
TRINITY_sp|Q10S(	CIPK9	CBL-interacting pro	40.30	0.00
TRINITY_sp|Q86Y(	CPNE8	Copine-8 OS=Homo sa	40.30	0.00
TRINITY_sp|O745(	msh6	DNA mismatch repair	40.30	0.00
TRINITY_sp|Q8H1!	At1g069(	U2 small nuclear ri	40.30	0.00
TRINITY_sp|Q220(	R166.3	Uncharacterized pro	40.30	0.00
TRINITY_sp|P623	CPK1	Calcium-dependent p	40.30	0.00
TRINITY_sp|Q9CVI	Atxn3	Ataxin-3 OS=Mus mus	40.30	0.00
TRINITY_sp|Q3ED(	At1g229!	Uncharacterized PKH	40.30	0.00
TRINITY_sp|Q54PI	nagk	N-acetyl-D-glucosam	40.30	0.00
TRINITY_sp|Q9GPI	apm3	AP-3 complex subuni	40.30	0.00
TRINITY_sp|O225!	STY8	Serine/threonine-pr	40.30	0.00
TRINITY_sp|Q6D7:	lacZ	Beta-galactosidase	40.30	0.00
TRINITY_sp|Q9Y3I	SBDS	Ribosome maturation	40.30	0.00
TRINITY_sp|Q921I	Hemk1	HemK methyltransfer	40.30	0.00
TRINITY_sp|Q8IY:	CPNE9	Copine-9 OS=Homo sa	40.30	0.00
TRINITY_sp|Q102	SPAC4G9.	Uncharacterized mit	40.30	0.00
TRINITY_sp|Q203!	ppt-1	Palmitoyl-protein t	40.30	0.00
TRINITY_sp|F4JBI	ATG1B	Serine/threonine-pr	40.30	0.00
TRINITY_sp|O234(	ARA1	L-arabinokinase OS=	40.30	0.00
TRINITY_sp|Q54RI	eif2b5	Translation initiat	40.30	0.00
TRINITY_sp|Q3B7I	URI1	Unconventional pref	40.30	0.00
TRINITY_sp|Q54X	DDB_G02'	Probable serine/thr	40.30	0.00
TRINITY_sp|Q5ZKI	TRAPPC2	Trafficking protein	40.30	0.00

TRINITY_sp|O6469	CYP710A1	Cytochrome P450 710	40.30	0.00
TRINITY_sp|Q3YRE	folD	Bifunctional protei	40.30	0.00
TRINITY_sp|Q9FE[illegible]	TON2	Probable serine/thr	40.30	0.00
TRINITY_sp|P1163	NHP6A	Non-histone chromos	40.30	0.00
TRINITY_sp|P5191	CNN1	Calponin-1 OS=Homo	40.30	0.00
TRINITY_sp|Q4G0X	CCDC40	Coiled-coil domain-	40.30	0.00
TRINITY_sp|Q9M82	NSN1	Guanine nucleotide-	40.30	0.00
TRINITY_sp|Q940H	SRK2E	Serine/threonine-pr	40.30	0.00
TRINITY_sp|A7RK3	v1g23885	Quinone oxidoreduct	40.30	0.00
TRINITY_sp|P1048	-	Phosphatidylcholine	40.30	0.00
TRINITY_sp|Q9M8K	DSPTP1B	Dual specificity pr	40.30	0.00
TRINITY_sp|Q9LF4	PUB1	Probable ubiquitin	40.30	0.00
TRINITY_sp|P1097	-	Retrovirus-related	40.30	0.00
TRINITY_sp|Q804H	dio1	Type I iodothyronin	40.30	0.00
TRINITY_sp|Q9SLE	ALIS3	ALA-interacting sub	40.30	0.00
TRINITY_sp|Q9RSE	pnp	Polyribonucleotide	40.30	0.00
TRINITY_sp|P2115	csgA	C-factor OS=Myxococ	40.30	0.00
TRINITY_sp|Q7SY4	heatr1	HEAT repeat-contain	40.30	0.00
TRINITY_sp|P0770	LYS2	L-2-aminoadipate re	40.30	0.00
TRINITY_sp|Q7589	MRPL51	54S ribosomal prote	40.20	0.00
TRINITY_sp|Q5999	cyp120	Putative cytochrome	40.20	0.00
TRINITY_sp|Q8IYM	TRIM22	E3 ubiquitin-protei	40.20	0.00
TRINITY_sp|Q72M9	LIC_1329	Macro domain-contai	40.20	0.00
TRINITY_sp|D9HP2	CNR7	Cell number regulat	40.20	0.00
TRINITY_sp|P9761	Txn2	Thioredoxin, mitoch	40.20	0.00
TRINITY_sp|Q8IVD	NUDCD3	NudC domain-contain	40.20	0.00
TRINITY_sp|Q5ZKD	MOV10	Putative helicase M	40.20	0.00
TRINITY_sp|Q54RE	dhkL	Hybrid signal trans	40.20	0.00
TRINITY_sp|P5028	-	Isoaspartyl peptida	40.20	0.00
TRINITY_sp|O6549	XCP1	Cysteine protease X	40.20	0.00
TRINITY_sp|Q06A3	CHD7	Chromodomain-helica	40.20	0.00
TRINITY_sp|P2013	GSTM2	Glutathione S-trans	40.20	0.00
TRINITY_sp|Q9RSE	pnp	Polyribonucleotide	40.20	0.00
TRINITY_sp|F4HPN	IRE4	Probable serine/thr	40.20	0.00
TRINITY_sp|P0715	Ctsl	Cathepsin L1 OS=Rat	40.20	0.00
TRINITY_sp|Q94AW	ERF003	Ethylene-responsive	40.20	0.00
TRINITY_sp|B7ID0	THA_1434	UPF0145 protein THA	40.20	0.00
TRINITY_sp|Q7T2E	mx1	Interferon-induced	40.20	0.00
TRINITY_sp|Q1111	ubp14	Ubiquitin carboxyl-	40.20	0.00
TRINITY_sp|Q6NRE	agap1	Arf-GAP with GTPase	40.20	0.00
TRINITY_sp|Q2QE1	dio1	Type I iodothyronin	40.20	0.00
TRINITY_sp|P1689	gpaA	Guanine nucleotide-	40.20	0.00
TRINITY_sp|Q55EE	manC	Alpha-mannosidase C	40.20	0.00
TRINITY_sp|P4910	FMO5	Dimethylaniline mon	40.20	0.00
TRINITY_sp|Q9LZ9	ABCI20	ABC transporter I f	40.20	0.00
TRINITY_sp|Q9FH0	SCPL41	Serine carboxypepti	40.20	0.00
TRINITY_sp|E9PYY	Wdr78	WD repeat-containin	40.20	0.00
TRINITY_sp|Q9SEE	-	Pirin-like protein	40.20	0.00
TRINITY_sp|Q54P5	cpnC	Copine-C OS=Dictyos	40.20	0.00
TRINITY_sp|Q0WVE	MMS19	MMS19 nucleotide ex	40.20	0.00
TRINITY_sp|Q9VXE	CG9132	NECAP-like protein	40.20	0.00
TRINITY_sp|Q9C7E	NTF2	Nuclear transport f	40.20	0.00
TRINITY_sp|Q6CEW	MRD1	Multiple RNA-bindin	40.20	0.00
TRINITY_sp|P3303	yeiR	Zinc-binding GTPase	40.20	0.00
TRINITY_sp|P4123	Abca1	ATP-binding cassett	40.20	0.00
TRINITY_sp|Q24V7	htpG	Chaperone protein H	40.20	0.00

TRINITY_sp|Q102	SPAC4G9.	Uncharacterized mit	40.20	0.00
TRINITY_sp|Q9SX	PI4KA1	Phosphatidylinosito	40.20	0.00
TRINITY_sp|Q9UB	POLK	DNA polymerase kapp	40.20	0.00
TRINITY_sp|Q2V9	-	Transcription facto	40.20	0.00
TRINITY_sp|Q9SE	-	Pirin-like protein	40.20	0.00
TRINITY_sp|Q272	w	Protein white OS=An	40.20	0.00
TRINITY_sp|P455	-	Actin (Fragment) OS	40.20	0.00
TRINITY_sp|Q9LN	SKIP16	F-box protein SKIP1	40.20	0.00
TRINITY_sp|C0LG	LRR-RLK	Probable leucine-ri	40.20	0.00
TRINITY_sp|Q8TB	SPATA20	Spermatogenesis-ass	40.20	0.00
TRINITY_sp|O488	RPN2A	26S proteasome non-	40.20	0.00
TRINITY_sp|Q9NQ	UTP3	Something about sil	40.20	0.00
TRINITY_sp|Q9SP	APY2	Apyrase 2 OS=Arabid	40.20	0.00
TRINITY_sp|Q86C	atg1	Serine/threonine-pr	40.20	0.00
TRINITY_sp|P553	shc	Probable squalene--	40.20	0.00
TRINITY_sp|Q9HA	KIF9	Kinesin-like protei	40.20	0.00
TRINITY_sp|Q5JK	Os01g09	Probable NAD kinase	40.20	0.00
TRINITY_sp|P604	PTEN	Phosphatidylinosito	40.20	0.00
TRINITY_sp|Q8GW	At4g182	Uncharacterized pro	40.20	0.00
TRINITY_sp|P218	cryS	Crystal protein OS=	40.20	0.00
TRINITY_sp|Q2RJ	mtaD	5-methylthioadenosi	40.20	0.00
TRINITY_sp|O741	pop3	Target of rapamycin	40.20	0.00
TRINITY_sp|P432	RD21A	Cysteine proteinase	40.20	0.00
TRINITY_sp|Q55C	redB	NADPH--cytochrome P	40.20	0.00
TRINITY_sp|Q54M	mcfB	Mitochondrial subst	40.20	0.00
TRINITY_sp|Q4HX	CPR6	Peptidyl-prolyl cis	40.20	0.00
TRINITY_sp|Q8TD	CHD6	Chromodomain-helica	40.20	0.00
TRINITY_sp|Q96B	ALKBH8	Alkylated DNA repai	40.20	0.00
TRINITY_sp|P496	UAC1	Adenylate cyclase O	40.20	0.00
TRINITY_sp|P477	fdh	S-(hydroxymethyl)gl	40.20	0.00
TRINITY_sp|P271	odc1-a	Ornithine decarboxy	40.10	0.00
TRINITY_sp|Q68E	rab4b	Ras-related protein	40.10	0.00
TRINITY_sp|B7IF	dnaJ	Chaperone protein D	40.10	0.00
TRINITY_sp|Q395	ODA2	Dynein gamma chain,	40.10	0.00
TRINITY_sp|P0CC	DDB_G02	Enolase superfamily	40.10	0.00
TRINITY_sp|Q54S	rcl1	Probable RNA 3'-ter	40.10	0.00
TRINITY_sp|Q54S	midA	Protein arginine me	40.10	0.00
TRINITY_sp|O138	not3	General negative re	40.10	0.00
TRINITY_sp|Q9LU	CHX19	Cation/H(+) antipor	40.10	0.00
TRINITY_sp|Q54J	chmp5	Charged multivesicu	40.10	0.00
TRINITY_sp|P929	KIN11	SNF1-related protei	40.10	0.00
TRINITY_sp|Q54H	drkB	Probable serine/thr	40.10	0.00
TRINITY_sp|Q8L7	SPHK1	Sphingosine kinase	40.10	0.00
TRINITY_sp|Q6GL	nifk	MKI67 FHA domain-in	40.10	0.00
TRINITY_sp|O744	ubp16	Probable ubiquitin	40.10	0.00
TRINITY_sp|Q8RX	MIRO1	Mitochondrial Rho G	40.10	0.00
TRINITY_sp|P528	Tmem165	Transmembrane prote	40.10	0.00
TRINITY_sp|Q9D9	-	Leucine-rich repeat	40.10	0.00
TRINITY_sp|Q9LZ	SDP1	Triacylglycerol lip	40.10	0.00
TRINITY_sp|Q5XM	aprA	Autocrine prolifera	40.10	0.00
TRINITY_sp|Q8RW	STY17	Serine/threonine-pr	40.10	0.00
TRINITY_sp|Q136	MTMR1	Myotubularin-relate	40.10	0.00
TRINITY_sp|Q9M8	PEX12	Peroxisome biogenes	40.10	0.00
TRINITY_sp|D0NL	PITG_12	tRNA (guanine(37)-N	40.10	0.00
TRINITY_sp|P313	-	cAMP-dependent prot	40.10	0.00
TRINITY_sp|P129	PEPD	Xaa-Pro dipeptidase	40.10	0.00

TRINITY_sp|P519£	Nek1	Serine/threonine-pr	40.10	0.00
TRINITY_sp|Q61L/	arf-1.2	ADP-ribosylation fa	40.10	0.00
TRINITY_sp|Q126!	ENA5	Sodium transport AT	40.10	0.00
TRINITY_sp|Q7Y1V	SAP9	Zinc finger A20 and	40.10	0.00
TRINITY_sp|Q027!	FKBP4	Peptidyl-prolyl cis	40.10	0.00
TRINITY_sp|Q080{	CHSP70	Stromal 70 kDa heat	40.10	0.00
TRINITY_sp|Q4X0;	pim1	Lon protease homolo	40.00	0.00
TRINITY_sp|Q3V1I	Hephl1	Hephaestin-like pro	40.00	0.00
TRINITY_sp|A8WYI	par-1	Serine/threonine-pr	40.00	0.00
TRINITY_sp|Q426(	CYP89A2	Cytochrome P450 89A	40.00	0.00
TRINITY_sp|O603(	AQR	Intron-binding prot	40.00	0.00
TRINITY_sp|Q018!	YPT1	Ras-like GTP-bindin	40.00	0.00
TRINITY_sp|Q875!	AKR1	Palmitoyltransferas	40.00	0.00
TRINITY_sp|P468	KRP95	Kinesin-II 95 kDa s	40.00	0.00
TRINITY_sp|Q9Y7I	SPBC2A9.	Uncharacterized WD	40.00	0.00
TRINITY_sp|P981!	Atp11a	Probable phospholip	40.00	0.00
TRINITY_sp|Q1RMI	SNX3	Sorting nexin-3 OS=	40.00	0.00
TRINITY_sp|Q9D8\	Hm13	Minor histocompatib	40.00	0.00
TRINITY_sp|Q559I	sarA	GTP-binding protein	40.00	0.00
TRINITY_sp|G3MWI	MICAL3	Protein-methionine	40.00	0.00
TRINITY_sp|P435;	cya	Adenylate cyclase O	40.00	0.00
TRINITY_sp|O611;	krsA	Serine/threonine-pr	40.00	0.00
TRINITY_sp|P323	DAL1	Allantoinase OS=Sac	40.00	0.00
TRINITY_sp|P0CH:	RING1	E3 ubiquitin-protei	40.00	0.00
TRINITY_sp|F4JTI	STY46	Serine/threonine-pr	40.00	0.00
TRINITY_sp|Q9SM;	LKR/SDH	Alpha-aminoadipic s	40.00	0.00
TRINITY_sp|Q3URI	Kctd21	BTB/POZ domain-cont	40.00	0.00
TRINITY_sp|Q607:	Slc30a1	Zinc transporter 1	40.00	0.00
TRINITY_sp|Q6ID!	MKRN	E3 ubiquitin-protei	40.00	0.00
TRINITY_sp|P010!	-	Subtilisin-chymotry	40.00	0.00
TRINITY_sp|Q8WT!	ABHD5	1-acylglycerol-3-ph	40.00	0.00
TRINITY_sp|Q556I	cf50-1	Counting factor 50	40.00	0.00
TRINITY_sp|Q9M9I	At1g782{	F-box protein At1g7	40.00	0.00
TRINITY_sp|Q54M!	plbC	Phospholipase B-lik	40.00	0.00
TRINITY_sp|Q55A)	dotA	Histone-lysine N-me	40.00	0.00
TRINITY_sp|P433:	egl5	Endoglucanase-5 OS=	40.00	0.00
TRINITY_sp|O670{	pheA	P-protein OS=Aquife	40.00	0.00
TRINITY_sp|Q90Y:	timm8a	Mitochondrial impor	40.00	0.00
TRINITY_sp|A6W3(	mtnN	5'-methylthioadenos	40.00	0.00
TRINITY_sp|Q6K4:	TRX1	Histone-lysine N-me	40.00	0.00
TRINITY_sp|P146‹	Pde4c	cAMP-specific 3',5'	40.00	0.00
TRINITY_sp|P146‹	Pde4c	cAMP-specific 3',5'	40.00	0.00
TRINITY_sp|Q9ZN\	DES6	Acyl-lipid (9-3)-de	40.00	0.00
TRINITY_sp|Q51TI	CWC2	Pre-mRNA-splicing f	40.00	0.00
TRINITY_sp|Q94II	CCD1	Carotenoid 9,10(9',	40.00	0.00
TRINITY_sp|Q9FI‹	SDHAF2	Succinate dehydroge	40.00	0.00
TRINITY_sp|P0CT;	UMAG_12(	Glucosidase 2 subun	40.00	0.00
TRINITY_sp|P262;	celD	Endoglucanase E-4 O	40.00	0.00
TRINITY_sp|Q9P0I	VAPA	Vesicle-associated	40.00	0.00
TRINITY_sp|Q9N9)	-	Ciliary WD repeat-c	40.00	0.00
TRINITY_sp|O784;	ycf19	Uncharacterized pro	40.00	0.00
TRINITY_sp|Q17QI	SDR39U1	Epimerase family pr	40.00	0.00
TRINITY_sp|Q7XJI	AMC2	Metacaspase-2 OS=Ar	40.00	0.00
TRINITY_sp|P147	cyp102A:	Bifunctional cytoch	40.00	0.00
TRINITY_sp|Q1PD:	DGK4	Diacylglycerol kina	40.00	0.00
TRINITY_sp|Q556{	recG	ATP-dependent DNA h	40.00	0.00

TRINITY_sp|A7SD8	cdc5l	Cell division cycle	40.00	0.00
TRINITY_sp|Q9LQ\	RDR1	RNA-dependent RNA p	40.00	0.00
TRINITY_sp|Q54II	shkB	Dual specificity pr	40.00	0.00
TRINITY_sp|Q61M(	gpa-7	Guanine nucleotide-	40.00	0.00
TRINITY_sp|O496(	PAM68	Protein PAM68, chlo	40.00	0.00
TRINITY_sp|P629	RAC1	Ras-related C3 botu	40.00	0.00
TRINITY_sp|Q54Q	abcH4	ABC transporter H f	40.00	0.00
TRINITY_sp|Q9MU(	ycf66	Uncharacterized pro	40.00	0.00
TRINITY_sp|Q9LJ	At3g224	Probable prefoldin	40.00	0.00
TRINITY_sp|P797(	TSN	Translin OS=Gallus	40.00	0.00
TRINITY_sp|Q6NTV	nae1	NEDD8-activating en	40.00	0.00
TRINITY_sp|Q9C5(	AHK3	Histidine kinase 3	40.00	0.00
TRINITY_sp|A6UYV	bioC	Malonyl-[acyl-carri	40.00	0.00
TRINITY_sp|P268	ATPA	ATP synthase subuni	40.00	0.00
TRINITY_sp|P386(	-	Probable protein di	40.00	0.00
TRINITY_sp|Q5Z8(	PARP2-A	Poly [ADP-ribose] p	40.00	0.00
TRINITY_sp|Q9SX	FYPP1	Phytochrome-associa	40.00	0.00
TRINITY_sp|P487	EIF5	Eukaryotic translat	40.00	0.00
TRINITY_sp|O944	tef3	Elongation factor 3	40.00	0.00
TRINITY_sp|Q962	ATPC	ATP synthase subuni	40.00	0.00
TRINITY_sp|Q5R9(	ZFAND2A	AN1-type zinc finge	40.00	0.00
TRINITY_sp|O942	hmt2	Sulfide:quinone oxi	40.00	0.00
TRINITY_sp|Q9P2	SIPA1L2	Signal-induced prol	40.00	0.00
TRINITY_sp|O655	At4g309	BTB/POZ domain-cont	40.00	0.00
TRINITY_sp|O252	typA	GTP-binding protein	40.00	0.00
TRINITY_sp|O943	SPBC29A	Uncharacterized ATP	40.00	0.00
TRINITY_sp|Q84T	SR45A	Serine/arginine-ric	40.00	0.00
TRINITY_sp|Q618	Mns1	Meiosis-specific nu	40.00	0.00
TRINITY_sp|Q9SR(	PEX19-1	Peroxisome biogenes	40.00	0.00
TRINITY_sp|P109	-	Retrovirus-related	40.00	0.00
TRINITY_sp|P530	RfC4	Replication factor	40.00	0.00
TRINITY_sp|Q75V	SAPK9	Serine/threonine-pr	40.00	0.00
TRINITY_sp|P395	DRS2	Probable phospholip	40.00	0.00
TRINITY_sp|Q9UT	SPAC144	MIP18 family protei	40.00	0.00
TRINITY_sp|Q241	shark	Tyrosine-protein ki	40.00	0.00
TRINITY_sp|Q78JV	Ubfd1	Ubiquitin domain-co	40.00	0.00
TRINITY_sp|A5PK	METTL13	Methyltransferase-l	40.00	0.00
TRINITY_sp|O755	ECI2	Enoyl-CoA delta iso	40.00	0.00
TRINITY_sp|Q9HB	RDH14	Retinol dehydrogena	40.00	0.00
TRINITY_sp|Q6CP	TRM13	tRNA:m(4)X modifica	40.00	0.00
TRINITY_sp|Q86C(	tor	Target of rapamycin	40.00	0.00
TRINITY_sp|D0NL(	PITG_12	tRNA (guanine(37)-N	39.90	0.00
TRINITY_sp|Q8LE	ECH	Golgi apparatus mem	39.90	0.00
TRINITY_sp|P281	RABA5C	Ras-related protein	39.90	0.00
TRINITY_sp|P092	-	25 kDa calcium-bind	39.90	0.00
TRINITY_sp|Q8CF	Abca5	ATP-binding cassett	39.90	0.00
TRINITY_sp|Q9NT	ATP8A2	Phospholipid-transp	39.90	0.00
TRINITY_sp|Q93V	ATG18A	Autophagy-related p	39.90	0.00
TRINITY_sp|Q6IQ	alkbh6	Alpha-ketoglutarate	39.90	0.00
TRINITY_sp|O137	nop12	Nucleolar protein 1	39.90	0.00
TRINITY_sp|Q145	ITPR2	Inositol 1,4,5-tris	39.90	0.00
TRINITY_sp|Q927	SYN2	Synapsin-2 OS=Homo	39.90	0.00
TRINITY_sp|Q2KH\	USP2	Ubiquitin carboxyl-	39.90	0.00
TRINITY_sp|P215(	ALP1	Alpha-amylase OS=Sa	39.90	0.00
TRINITY_sp|O155	RNF113A	RING finger protein	39.90	0.00
TRINITY_sp|Q135(	TDG	G/T mismatch-specif	39.90	0.00

TRINITY_sp|Q54D.	bxdc1	Ribosome production	39.90	0.00
TRINITY_sp|P604	PTEN	Phosphatidylinosito	39.90	0.00
TRINITY_sp|Q9QW	Rad1	Cell cycle checkpoi	39.90	0.00
TRINITY_sp|Q6J4	SLC8B1	Sodium/potassium/ca	39.90	0.00
TRINITY_sp|A9RY	PHYPADR	ATP-dependent (S)-N	39.90	0.00
TRINITY_sp|Q6DE	slc25a3	Solute carrier fami	39.90	0.00
TRINITY_sp|Q5F4	PAN2	PAB-dependent poly(	39.90	0.00
TRINITY_sp|Q54M	mog1	Probable ran guanin	39.90	0.00
TRINITY_sp|P174	Ap2a2	AP-2 complex subuni	39.90	0.00
TRINITY_sp|Q54N	7tmk1	Seven transmembrane	39.90	0.00
TRINITY_sp|Q7RY	nmp-1	Nuclear distributio	39.90	0.00
TRINITY_sp|Q7KZ	SUPT6H	Transcription elong	39.90	0.00
TRINITY_sp|Q9SW	RH16	DEAD-box ATP-depend	39.90	0.00
TRINITY_sp|O157	spnA	Protein spalten OS=	39.90	0.00
TRINITY_sp|A5H0	DUR1,2	Urea amidolyase OS=	39.90	0.00
TRINITY_sp|P623	CPK1	Calcium-dependent p	39.90	0.00
TRINITY_sp|Q4V9	slc25a2	S-adenosylmethionin	39.90	0.00
TRINITY_sp|Q6NV	sdad1	Protein SDA1 homolo	39.90	0.00
TRINITY_sp|Q54Q	phr2aB	Serine/threonine-pr	39.90	0.00
TRINITY_sp|Q8VZ	RBL6	RHOMBOID-like prote	39.90	0.00
TRINITY_sp|Q8L5	CSN4	COP9 signalosome co	39.90	0.00
TRINITY_sp|Q5ZJ	NOC4L	Nucleolar complex p	39.90	0.00
TRINITY_sp|Q84W	IP5P15	Type II inositol po	39.90	0.00
TRINITY_sp|Q9SR	SUVR3	Histone-lysine N-me	39.90	0.00
TRINITY_sp|Q5JK	TPK1	Thiamine pyrophosph	39.90	0.00
TRINITY_sp|Q9M8	DSPTP1B	Dual specificity pr	39.90	0.00
TRINITY_sp|Q8WT	ABHD5	1-acylglycerol-3-ph	39.90	0.00
TRINITY_sp|Q07G	alkbh8	Alkylated DNA repai	39.90	0.00
TRINITY_sp|P081	ABCB1	Multidrug resistanc	39.90	0.00
TRINITY_sp|Q93Y	At1g279	Probable pre-mRNA-s	39.90	0.00
TRINITY_sp|Q54D	DDB_G02	Protein unc-50 homo	39.90	0.00
TRINITY_sp|A8J6	CFAP221	Cilia- and flagella	39.90	0.00
TRINITY_sp|Q9BY	YTHDF1	YTH domain-containi	39.90	0.00
TRINITY_sp|Q432	GDH1	Glutamate dehydroge	39.90	0.00
TRINITY_sp|Q9ZR	CAD	Probable mannitol d	39.90	0.00
TRINITY_sp|Q67Y	RUS4	Protein root UVB se	39.90	0.00
TRINITY_sp|Q9BX	GTPBP2	GTP-binding protein	39.90	0.00
TRINITY_sp|O954	SEC24B	Protein transport p	39.90	0.00
TRINITY_sp|O276	apgM1	2,3-bisphosphoglyce	39.90	0.00
TRINITY_sp|Q993	TY3B-G	Transposon Ty3-G Ga	39.90	0.00
TRINITY_sp|Q5A8	SLN1	Histidine protein k	39.80	0.00
TRINITY_sp|Q9VG	CG6345	CDK5RAP1-like prote	39.80	0.00
TRINITY_sp|Q54N	rab1D	Ras-related protein	39.80	0.00
TRINITY_sp|Q54C	mcm5	DNA replication lic	39.80	0.00
TRINITY_sp|P418	cut9	Anaphase-promoting	39.80	0.00
TRINITY_sp|Q8TC	RDH11	Retinol dehydrogena	39.80	0.00
TRINITY_sp|Q9V7	CG8152	G patch domain and	39.80	0.00
TRINITY_sp|Q56W	SCPL48	Serine carboxypepti	39.80	0.00
TRINITY_sp|P707	Atp8a1	Phospholipid-transp	39.80	0.00
TRINITY_sp|Q5EB	elp2	Elongator complex p	39.80	0.00
TRINITY_sp|Q54Y	dhkB	Hybrid signal trans	39.80	0.00
TRINITY_sp|Q9JH	Pigp	Phosphatidylinosito	39.80	0.00
TRINITY_sp|Q9LJ	FUT11	Glycoprotein 3-alph	39.80	0.00
TRINITY_sp|Q55G	pakC	Serine/threonine-pr	39.80	0.00
TRINITY_sp|Q9W0	NaCP60E	Sodium channel prot	39.80	0.00
TRINITY_sp|A4QN	ctdspl2	CTD small phosphata	39.80	0.00

TRINITY_sp|Q8VW2	SMO2-2	Methylsterol monoox	39.80	0.00
TRINITY_sp|Q54GE	ctdspl2	CTD small phosphata	39.80	0.00
TRINITY_sp|P341(	pkgC	Protein kinase 3 OS	39.80	0.00
TRINITY_sp|Q54EE	DDB_G02S	GTPase-activating p	39.80	0.00
TRINITY_sp|Q056(	CTR1	Serine/threonine-pr	39.80	0.00
TRINITY_sp|O496(	SAP9	Zinc finger A20 and	39.80	0.00
TRINITY_sp|Q9N4\	scpl-3	CTD small phosphata	39.80	0.00
TRINITY_sp|Q55A5	DDB_G027	Probable serine/thr	39.80	0.00
TRINITY_sp|Q8LPS	LACS6	Long chain acyl-CoA	39.80	0.00
TRINITY_sp|Q8WNS	ITPR2	Inositol 1,4,5-tris	39.80	0.00
TRINITY_sp|Q9SJ(	NUDT5	Nudix hydrolase 5 O	39.80	0.00
TRINITY_sp|Q9LV(	TOE2	AP2-like ethylene-r	39.80	0.00
TRINITY_sp|Q54E1	abpF	Actin-binding prote	39.80	0.00
TRINITY_sp|Q0V8E	TPP1	Tripeptidyl-peptida	39.80	0.00
TRINITY_sp|Q9JL\	Pnkp	Bifunctional polynu	39.80	0.00
TRINITY_sp|Q049S	HAT3.1	Homeobox protein HA	39.80	0.00
TRINITY_sp|Q86IE	gtf2h3	General transcripti	39.80	0.00
TRINITY_sp|Q8CXS	LA_3128	UPF0176 protein LA_	39.80	0.00
TRINITY_sp|Q7T6X	MIMI_R82	Putative serine/thr	39.80	0.00
TRINITY_sp|Q29S(	OSCP1	Protein OSCP1 OS=Bo	39.80	0.00
TRINITY_sp|B8AEE	MCM5	DNA replication lic	39.80	0.00
TRINITY_sp|P3414	racB	Rho-related protein	39.80	0.00
TRINITY_sp|Q2NK(	DPY30	Protein dpy-30 homo	39.80	0.00
TRINITY_sp|P0111	RAS1	Ras-like protein 1	39.80	0.00
TRINITY_sp|Q5ZI8	TBCD	Tubulin-specific ch	39.80	0.00
TRINITY_sp|Q9FNY	E2FA	Transcription facto	39.80	0.00
TRINITY_sp|Q86LS	gacHH	Rho GTPase-activati	39.80	0.00
TRINITY_sp|O8102	EMB1187	Probable ethanolami	39.80	0.00
TRINITY_sp|P2393	NDUFA5	NADH dehydrogenase	39.80	0.00
TRINITY_sp|P2392	metG	Methionine--tRNA li	39.80	0.00
TRINITY_sp|Q6DG\	mpv17l2	Mpv17-like protein	39.80	0.00
TRINITY_sp|O9452	iqw1	WD repeat protein i	39.80	0.00
TRINITY_sp|P9321	TFT7	14-3-3 protein 7 OS	39.80	0.00
TRINITY_sp|Q9BX(	NAA15	N-alpha-acetyltrans	39.80	0.00
TRINITY_sp|Q6317	Dnah7	Dynein heavy chain	39.80	0.00
TRINITY_sp|Q86AV	guaD	Guanine deaminase O	39.80	0.00
TRINITY_sp|P0288	psmD8-1	Probable 26S protea	39.80	0.00
TRINITY_sp|Q54JE	abcC3	ABC transporter C f	39.80	0.00
TRINITY_sp|D4B1E	ARB_023S	Probable glutamate	39.80	0.00
TRINITY_sp|O772(	CG4061	RNA 3'-terminal pho	39.80	0.00
TRINITY_sp|Q9W63	req-a	Zinc finger protein	39.80	0.00
TRINITY_sp|Q681Y	At1g609S	Putative transferas	39.80	0.00
TRINITY_sp|Q9UN3	VPS4A	Vacuolar protein so	39.80	0.00
TRINITY_sp|Q5RG(	ccdc53	WASH complex subuni	39.80	0.00
TRINITY_sp|Q912V	Rep	Replication-associa	39.80	0.00
TRINITY_sp|Q0P4E	dis3l	DIS3-like exonuclea	39.80	0.00
TRINITY_sp|Q9M2I	ACA11	Putative calcium-tr	39.80	0.00
TRINITY_sp|P1097	-	Retrovirus-related	39.80	0.00
TRINITY_sp|Q9FV(	PLGG1	Plastidal glycolate	39.80	0.00
TRINITY_sp|O9561	YEATS4	YEATS domain-contai	39.80	0.00
TRINITY_sp|Q929(	UPF1	Regulator of nonsen	39.80	0.00
TRINITY_sp|Q28GV	melk	Maternal embryonic	39.80	0.00
TRINITY_sp|Q944I	SLP2	Shewanella-like pro	39.80	0.00
TRINITY_sp|Q2ABE	pRB	Retinoblastoma-rela	39.80	0.00
TRINITY_sp|Q54HI	gghB	Gamma-glutamyl hydr	39.80	0.00
TRINITY_sp|Q5F47	RAB8A	Ras-related protein	39.80	0.00

TRINITY_sp|Q9FGY	PNG1	Peptide-N(4)-(N-ace	39.80	0.00
TRINITY_sp|Q75J3	CR4	Serine/threonine-pr	39.80	0.00
TRINITY_sp|Q54JH	abcC3	ABC transporter C f	39.80	0.00
TRINITY_sp|P7905	rhp16	ATP-dependent helic	39.80	0.00
TRINITY_sp|P2271	Gucy1b2	Guanylate cyclase s	39.80	0.00
TRINITY_sp|P1097	-	Retrovirus-related	39.80	0.00
TRINITY_sp|Q8CEQ	Cdkl1	Cyclin-dependent ki	39.70	0.00
TRINITY_sp|Q0IUU	CML2	Putative calmodulin	39.70	0.00
TRINITY_sp|Q7ZUN	ykt6	Synaptobrevin homol	39.70	0.00
TRINITY_sp|Q54W1	DDB_G027	U6 snRNA phosphodie	39.70	0.00
TRINITY_sp|Q9SLX	Os05g015	Importin subunit al	39.70	0.00
TRINITY_sp|P5352	ML1149	Cob(I)yrinic acid a	39.70	0.00
TRINITY_sp|Q5M9H	Rbm34	RNA-binding protein	39.70	0.00
TRINITY_sp|Q6P0X	Znf511	Zinc finger protein	39.70	0.00
TRINITY_sp|Q05AM	elp2	Elongator complex p	39.70	0.00
TRINITY_sp|Q6DCU	hikeshi	Protein Hikeshi OS=	39.70	0.00
TRINITY_sp|Q8642	RGS5	Regulator of G-prot	39.70	0.00
TRINITY_sp|Q9ZVJ	SGPP	Haloacid dehalogena	39.70	0.00
TRINITY_sp|Q9D11	Mthfs	5-formyltetrahydrof	39.70	0.00
TRINITY_sp|Q9NXH	TRMT1	tRNA (guanine(26)-N	39.70	0.00
TRINITY_sp|Q8S12	Os01g081	Probable U3 small n	39.70	0.00
TRINITY_sp|Q2KJA	INTS9	Integrator complex	39.70	0.00
TRINITY_sp|P3494	NDUFA9	NADH dehydrogenase	39.70	0.00
TRINITY_sp|P2668	B52	Serine-arginine pro	39.70	0.00
TRINITY_sp|Q5R82	OLA1	Obg-like ATPase 1 O	39.70	0.00
TRINITY_sp|P2440	RAB10	Ras-related protein	39.70	0.00
TRINITY_sp|Q8BYR	Rdh12	Retinol dehydrogena	39.70	0.00
TRINITY_sp|Q8R34	Ccdc12	Coiled-coil domain-	39.70	0.00
TRINITY_sp|O2255	STY8	Serine/threonine-pr	39.70	0.00
TRINITY_sp|Q84L0	GONST4	GDP-mannose transpo	39.70	0.00
TRINITY_sp|O6528	CPN20	20 kDa chaperonin,	39.70	0.00
TRINITY_sp|Q9DB9	Osbpl3	Oxysterol-binding p	39.70	0.00
TRINITY_sp|P9074	catp-8	Probable manganese-	39.70	0.00
TRINITY_sp|O0084	cudA	Putative transcript	39.70	0.00
TRINITY_sp|P6234	CPK4	Calcium-dependent p	39.70	0.00
TRINITY_sp|P1605	gpaB	Guanine nucleotide-	39.70	0.00
TRINITY_sp|Q9SUV	At4g3239	Probable sugar phos	39.70	0.00
TRINITY_sp|Q641K	Nuak1	NUAK family SNF1-li	39.70	0.00
TRINITY_sp|Q92JA	era	GTPase Era OS=Ricke	39.70	0.00
TRINITY_sp|Q54SP	dhkD	Hybrid signal trans	39.70	0.00
TRINITY_sp|Q5XM2	aprA	Autocrine prolifera	39.70	0.00
TRINITY_sp|P1097	-	Retrovirus-related	39.70	0.00
TRINITY_sp|Q7XA8	FOLT1	Folate transporter	39.70	0.00
TRINITY_sp|P3278	GTF2H1	General transcripti	39.70	0.00
TRINITY_sp|Q4W94	encD	2-oxoglutarate-Fe(I	39.70	0.00
TRINITY_sp|Q86I9	DDB_G027	Uncharacterized Gol	39.70	0.00
TRINITY_sp|P3430	C50C3.5	Uncharacterized cal	39.70	0.00
TRINITY_sp|Q7XRV	RMI1	RecQ-mediated genom	39.70	0.00
TRINITY_sp|Q9LJI	CRK2	CDPK-related kinase	39.70	0.00
TRINITY_sp|Q8C85	Myef2	Myelin expression f	39.70	0.00
TRINITY_sp|Q9QXI	Nme7	Nucleoside diphosph	39.70	0.00
TRINITY_sp|P5315	GUP1	Glycerol uptake pro	39.70	0.00
TRINITY_sp|O9450	SPBC12D1	Uncharacterized mit	39.70	0.00
TRINITY_sp|Q54FI	mybO	Myb-like protein O	39.70	0.00
TRINITY_sp|O0050	MPHOSPH1	U3 small nucleolar	39.70	0.00
TRINITY_sp|A8WYE	par-1	Serine/threonine-pr	39.70	0.00

TRINITY_sp|Q54JI	abcC3	ABC transporter C f	39.70	0.00
TRINITY_sp|Q556Y	DDB_G02	Probable rhodanese	39.70	0.00
TRINITY_sp|Q396	-	Glycerol-3-phosphat	39.70	0.00
TRINITY_sp|Q8RWI	STY17	Serine/threonine-pr	39.70	0.00
TRINITY_sp|Q3SX	LUC7L3	Luc7-like protein 3	39.70	0.00
TRINITY_sp|P259	MXAN_59	Uncharacterized oxi	39.70	0.00
TRINITY_sp|Q69Q	DPE2	4-alpha-glucanotran	39.70	0.00
TRINITY_sp|Q54H	drkB	Probable serine/thr	39.70	0.00
TRINITY_sp|Q56YN	SMC3	Structural maintena	39.70	0.00
TRINITY_sp|Q9LZ	SDP1	Triacylglycerol lip	39.70	0.00
TRINITY_sp|Q9FV	AGD12	ADP-ribosylation fa	39.70	0.00
TRINITY_sp|P329	CLB6	S-phase entry cycli	39.70	0.00
TRINITY_sp|Q96U	DGAT2A	Diacylglycerol O-ac	39.70	0.00
TRINITY_sp|Q246	ref(2)P	Protein ref(2)P OS=	39.70	0.00
TRINITY_sp|Q9C5	RFC1	Replication factor	39.70	0.00
TRINITY_sp|O942	SPBC887.	Probable phospholip	39.70	0.00
TRINITY_sp|Q5JW	DOPEY1	Protein dopey-1 OS=	39.70	0.00
TRINITY_sp|A2XQ	OsI_148	Senescence-specific	39.70	0.00
TRINITY_sp|Q0IQ	RAPTOR1	Regulatory-associat	39.70	0.00
TRINITY_sp|F4I9	At1g580	DExH-box ATP-depend	39.70	0.00
TRINITY_sp|Q54Y	dhkB	Hybrid signal trans	39.70	0.00
TRINITY_sp|Q9SL	NEK1	Serine/threonine-pr	39.70	0.00
TRINITY_sp|Q045	YDR109C	Uncharacterized sug	39.70	0.00
TRINITY_sp|Q6PB	grtp1a	Growth hormone-regu	39.60	0.00
TRINITY_sp|O550	Ralgapa	Ral GTPase-activati	39.60	0.00
TRINITY_sp|P303	cdc25-3	M-phase inducer pho	39.60	0.00
TRINITY_sp|Q9SE	-	Pirin-like protein	39.60	0.00
TRINITY_sp|Q9VC	CG17119	Cystinosin homolog	39.60	0.00
TRINITY_sp|Q632	Gmfb	Glia maturation fac	39.60	0.00
TRINITY_sp|Q2HV	MtrDRAF	Putative N6-adenosi	39.60	0.00
TRINITY_sp|Q9Y2	CHKB	Choline/ethanolamin	39.60	0.00
TRINITY_sp|O654	XCP1	Cysteine protease X	39.60	0.00
TRINITY_sp|Q9Y3	ACOT9	Acyl-coenzyme A thi	39.60	0.00
TRINITY_sp|Q6DC	mettl16	Methyltransferase-l	39.60	0.00
TRINITY_sp|O803	MKK3	Mitogen-activated p	39.60	0.00
TRINITY_sp|Q6UK	pirA	Protein pirA OS=Dic	39.60	0.00
TRINITY_sp|Q6NV	brd9	Bromodomain-contain	39.60	0.00
TRINITY_sp|O941	nudG	Dynein light chain,	39.60	0.00
TRINITY_sp|P341	rabC	Ras-related protein	39.60	0.00
TRINITY_sp|P300	PBLD	Phenazine biosynthe	39.60	0.00
TRINITY_sp|Q0WK	ULP2A	Probable ubiquitin-	39.60	0.00
TRINITY_sp|P351	PTC1	Protein phosphatase	39.60	0.00
TRINITY_sp|Q8JZ	Cpne5	Copine-5 OS=Mus mus	39.60	0.00
TRINITY_sp|P056	sacC	Levanase OS=Bacillu	39.60	0.00
TRINITY_sp|Q96A	ZUFSP	Zinc finger with UF	39.60	0.00
TRINITY_sp|Q9SL	Os05g01	Importin subunit al	39.60	0.00
TRINITY_sp|P623	CPK4	Calcium-dependent p	39.60	0.00
TRINITY_sp|Q5RB	SENP1	Sentrin-specific pr	39.60	0.00
TRINITY_sp|Q235	unc-22	Twitchin OS=Caenorh	39.60	0.00
TRINITY_sp|P214	ABCB4	Phosphatidylcholine	39.60	0.00
TRINITY_sp|Q55F	plbG	Phospholipase B-lik	39.60	0.00
TRINITY_sp|Q7ZV	otud6b	OTU domain-containi	39.60	0.00
TRINITY_sp|Q630	Add1	Alpha-adducin OS=Ra	39.60	0.00
TRINITY_sp|Q54R	pyk	Pyruvate kinase OS=	39.60	0.00
TRINITY_sp|B3DN	APC6	Anaphase-promoting	39.60	0.00
TRINITY_sp|Q9Z1	Nek4	Serine/threonine-pr	39.60	0.00

TRINITY_sp|Q8RYI	At2g3325	Haloacid dehalogena	39.60	0.00
TRINITY_sp|Q9FYI	RS2Z32	Serine/arginine-ric	39.60	0.00
TRINITY_sp|Q9BXV	FANCD2	Fanconi anemia grou	39.60	0.00
TRINITY_sp|Q9P7(	oma1	Mitochondrial metal	39.60	0.00
TRINITY_sp|Q9S83	CLPP5	ATP-dependent Clp p	39.60	0.00
TRINITY_sp|Q0593	mybl1	Myb-related protein	39.60	0.00
TRINITY_sp|A8XAI	rheb-1	GTP-binding protein	39.60	0.00
TRINITY_sp|Q54B7	abcB2	ABC transporter B f	39.60	0.00
TRINITY_sp|Q9BII	B0464.9	Probable protein ph	39.60	0.00
TRINITY_sp|Q5XI1	Grwd1	Glutamate-rich WD r	39.60	0.00
TRINITY_sp|Q9SJ4	VPS11	Vacuolar protein-so	39.60	0.00
TRINITY_sp|Q9LK3	CHY1	3-hydroxyisobutyryl	39.60	0.00
TRINITY_sp|P5828	Rnf216	E3 ubiquitin-protei	39.60	0.00
TRINITY_sp|Q5W6(	MAN5	Putative mannan end	39.60	0.00
TRINITY_sp|Q3891	ANT	AP2-like ethylene-r	39.60	0.00
TRINITY_sp|O7516	KDM4A	Lysine-specific dem	39.60	0.00
TRINITY_sp|Q5C9I	-	(S)-coclaurine N-me	39.60	0.00
TRINITY_sp|O4904	VPS45	Vacuolar protein so	39.60	0.00
TRINITY_sp|P0565	sacC	Levanase OS=Bacillu	39.60	0.00
TRINITY_sp|P3225	rasB	Ras-like protein ra	39.60	0.00
TRINITY_sp|Q6P7A	Gaa	Lysosomal alpha-glu	39.60	0.00
TRINITY_sp|E9Q49	Pde8b	High affinity cAMP-	39.60	0.00
TRINITY_sp|Q9BZI	OSBPL6	Oxysterol-binding p	39.60	0.00
TRINITY_sp|Q54N(	hus1	Checkpoint protein	39.60	0.00
TRINITY_sp|Q9446	dymA	Dynamin-A OS=Dictyo	39.60	0.00
TRINITY_sp|O0062	PIR	Pirin OS=Homo sapie	39.60	0.00
TRINITY_sp|O0647	yfmR	Uncharacterized ABC	39.60	0.00
TRINITY_sp|P0859	pstI	Aqualysin-1 OS=Ther	39.60	0.00
TRINITY_sp|Q54JI	abcC3	ABC transporter C f	39.60	0.00
TRINITY_sp|Q9SZ7	P4H11	Probable prolyl 4-h	39.60	0.00
TRINITY_sp|Q9M39	At3g5413	Ataxin-3 homolog OS	39.50	0.00
TRINITY_sp|A4FV7	PPIE	Peptidyl-prolyl cis	39.50	0.00
TRINITY_sp|Q8R1I	Hid1	Protein HID1 OS=Mus	39.50	0.00
TRINITY_sp|Q8BT6	Cpne3	Copine-3 OS=Mus mus	39.50	0.00
TRINITY_sp|U4PR8	pig-1	Maternal embryonic	39.50	0.00
TRINITY_sp|Q641I	acbd5	Acyl-CoA-binding do	39.50	0.00
TRINITY_sp|Q91YI	Rnf126	E3 ubiquitin-protei	39.50	0.00
TRINITY_sp|Q6056	IGHMBP2	DNA-binding protein	39.50	0.00
TRINITY_sp|Q54R3	cnrB	CLPTM1-like membran	39.50	0.00
TRINITY_sp|Q0999	K10B2.2	Uncharacterized ser	39.50	0.00
TRINITY_sp|A9UM(	lyrm1	LYR motif-containin	39.50	0.00
TRINITY_sp|P2533	mlkA	Myosin light chain	39.50	0.00
TRINITY_sp|Q2462	ref(2)P	Protein ref(2)P OS=	39.50	0.00
TRINITY_sp|B9DSI	pth	Peptidyl-tRNA hydro	39.50	0.00
TRINITY_sp|Q54L1	uox	Uricase OS=Dictyost	39.50	0.00
TRINITY_sp|Q5UP7	MIMI_R61	Putative band 7 fam	39.50	0.00
TRINITY_sp|Q5BL5	atp8b1	Phospholipid-transp	39.50	0.00
TRINITY_sp|O9562	CNOT4	CCR4-NOT transcript	39.50	0.00
TRINITY_sp|Q9AV8	PRP19	Pre-mRNA-processing	39.50	0.00
TRINITY_sp|Q8HY8	ELAC2	Zinc phosphodiester	39.50	0.00
TRINITY_sp|Q4U5I	RNF114	E3 ubiquitin-protei	39.50	0.00
TRINITY_sp|A7TMI	EFG1	rRNA-processing pro	39.50	0.00
TRINITY_sp|Q54BN	empB	Transmembrane emp24	39.50	0.00
TRINITY_sp|Q8WX)	DNAH7	Dynein heavy chain	39.50	0.00
TRINITY_sp|Q9995	DUSP9	Dual specificity pr	39.50	0.00
TRINITY_sp|Q4IEI	PPM2	tRNA wybutosine-syn	39.50	0.00

TRINITY_sp|P499(	SRP19	Signal recognition	39.50	0.00
TRINITY_sp|P524:	Polr1c	DNA-directed RNA po	39.50	0.00
TRINITY_sp|Q9SZ(	VIP3	WD repeat-containin	39.50	0.00
TRINITY_sp|A8HN\	RSP14	Radial spoke protei	39.50	0.00
TRINITY_sp|Q151:	CDK10	Cyclin-dependent ki	39.50	0.00
TRINITY_sp|Q8H1I	ABCI10	ABC transporter I f	39.50	0.00
TRINITY_sp|Q9H8!	TTC12	Tetratricopeptide r	39.50	0.00
TRINITY_sp|P220:	TUB1	Tubulin beta-1 chai	39.50	0.00
TRINITY_sp|O189(	SYNJ1	Synaptojanin-1 (Fra	39.50	0.00
TRINITY_sp|Q54DI	gcvT	Aminomethyltransfer	39.50	0.00
TRINITY_sp|Q013'	fox-2	Peroxisomal hydrata	39.50	0.00
TRINITY_sp|Q5BJ!	Cpne9	Copine-9 OS=Rattus	39.50	0.00
TRINITY_sp|O535!	ilvX	Putative acetolacta	39.50	0.00
TRINITY_sp|Q6ZM\	KIF6	Kinesin-like protei	39.50	0.00
TRINITY_sp|A9VS(	speE	Polyamine aminoprop	39.50	0.00
TRINITY_sp|Q6NMI	F8H	Probable glucuronox	39.50	0.00
TRINITY_sp|Q7PL:	Nipped-I	Nipped-B protein OS	39.50	0.00
TRINITY_sp|Q9WY)	TM_0508	Uncharacterized pro	39.50	0.00
TRINITY_sp|Q6P3(	rnf121	RING finger protein	39.50	0.00
TRINITY_sp|Q595:	ptrB	Protease 2 OS=Morax	39.50	0.00
TRINITY_sp|Q9UN:	VPS4A	Vacuolar protein so	39.50	0.00
TRINITY_sp|Q54BI	sf1	Branchpoint-bridgin	39.50	0.00
TRINITY_sp|Q9ER/	SMC4	Structural maintena	39.50	0.00
TRINITY_sp|Q9FR:	AMI1	Amidase 1 OS=Arabid	39.50	0.00
TRINITY_sp|Q3URI	Kctd21	BTB/POZ domain-cont	39.50	0.00
TRINITY_sp|P208(	PHYPADR/	Chlorophyll a-b bin	39.50	0.00
TRINITY_sp|Q9H8(	VCPKMT	Protein-lysine meth	39.50	0.00
TRINITY_sp|O430(	syj1	Inositol-1,4,5-tris	39.50	0.00
TRINITY_sp|F4J7!	SFH9	Phosphatidylinosito	39.50	0.00
TRINITY_sp|Q6AW(	HOL3	Probable thiol meth	39.50	0.00
TRINITY_sp|Q9LSI	UBC32	Ubiquitin-conjugati	39.50	0.00
TRINITY_sp|Q54X(	DDB_G02'	Uncharacterized G-p	39.50	0.00
TRINITY_sp|Q9FG(	ATO	Splicing factor SF3	39.50	0.00
TRINITY_sp|O826(	SDH1-1	Succinate dehydroge	39.50	0.00
TRINITY_sp|P305(	CAT2	Catalase isozyme 2	39.50	0.00
TRINITY_sp|Q55D/	abcG22	ABC transporter G f	39.50	0.00
TRINITY_sp|Q54X(	crop	Luc7-like protein O	39.50	0.00
TRINITY_sp|Q54JI	abcC3	ABC transporter C f	39.50	0.00
TRINITY_sp|Q8MVI	gbpC	Cyclic GMP-binding	39.50	0.00
TRINITY_sp|Q9N5I	parg-2	Poly(ADP-ribose) gl	39.40	0.00
TRINITY_sp|Q8BMI	Eif4e2	Eukaryotic translat	39.40	0.00
TRINITY_sp|Q55A\	sac1	Phosphatidylinositi	39.40	0.00
TRINITY_sp|Q7ZWI	bbs5	Bardet-Biedl syndro	39.40	0.00
TRINITY_sp|Q5E9/	VPS25	Vacuolar protein-so	39.40	0.00
TRINITY_sp|P161:	THR4	Threonine synthase	39.40	0.00
TRINITY_sp|Q54DI	amdA	AMP deaminase OS=Di	39.40	0.00
TRINITY_sp|Q9RP'	rhlG	Rhamnolipids biosyn	39.40	0.00
TRINITY_sp|Q9FV'	At1g577:	Probable elongation	39.40	0.00
TRINITY_sp|P436	-	DnaJ protein homolo	39.40	0.00
TRINITY_sp|P250'	CML12	Calmodulin-like pro	39.40	0.00
TRINITY_sp|Q9SC!	At3g505:	Phosphoglycerate mu	39.40	0.00
TRINITY_sp|Q9SY(	PEX10	Peroxisome biogenes	39.40	0.00
TRINITY_sp|Q8NG(	TTL	Tubulin--tyrosine l	39.40	0.00
TRINITY_sp|Q945(	modA	Neutral alpha-gluco	39.40	0.00
TRINITY_sp|Q31QI	rplJ	50S ribosomal prote	39.40	0.00
TRINITY_sp|Q6DE'	ogfod1	Prolyl 3-hydroxylas	39.40	0.00

TRINITY_sp|Q8BN[illegible]	Wdr35	WD repeat-containin	39.40	0.00
TRINITY_sp|P350[illegible]	cbpP	Calcium-binding pro	39.40	0.00
TRINITY_sp|Q9AS[illegible]	DGAT2	Diacylglycerol O-ac	39.40	0.00
TRINITY_sp|Q7KV[illegible]	Trf4-1	Non-canonical poly(	39.40	0.00
TRINITY_sp|Q8C1[illegible]	Ccz1	Vacuolar fusion pro	39.40	0.00
TRINITY_sp|Q028[illegible]	FMP30	N-acyl-phosphatidyl	39.40	0.00
TRINITY_sp|Q8L6[illegible]	BB	E3 ubiquitin ligase	39.40	0.00
TRINITY_sp|Q73P[illegible]	rpsS	30S ribosomal prote	39.40	0.00
TRINITY_sp|Q7ZX[illegible]	utp15	U3 small nucleolar	39.40	0.00
TRINITY_sp|Q124[illegible]	TRM11	tRNA (guanine(10)-N	39.40	0.00
TRINITY_sp|Q9VG[illegible]	CG17726	Protein arginine me	39.40	0.00
TRINITY_sp|Q630[illegible]	Gfer	FAD-linked sulfhydr	39.40	0.00
TRINITY_sp|F4JL[illegible]	LDL3	Lysine-specific his	39.40	0.00
TRINITY_sp|Q8IY[illegible]	PNPLA6	Neuropathy target e	39.40	0.00
TRINITY_sp|P412[illegible]	Cetn1	Centrin-1 OS=Mus mu	39.40	0.00
TRINITY_sp|Q946[illegible]	RAP1	Ras-related protein	39.40	0.00
TRINITY_sp|Q72L[illegible]	folE	GTP cyclohydrolase	39.40	0.00
TRINITY_sp|Q8TB[illegible]	KLHDC4	Kelch domain-contai	39.40	0.00
TRINITY_sp|Q8ZN[illegible]	pphA	Serine/threonine-pr	39.40	0.00
TRINITY_sp|P109[illegible]	-	Retrovirus-related	39.40	0.00
TRINITY_sp|Q9M8[illegible]	PMI1	Mannose-6-phosphate	39.40	0.00
TRINITY_sp|Q14C[illegible]	Mocos	Molybdenum cofactor	39.40	0.00
TRINITY_sp|Q9QY[illegible]	Smok3a	Sperm motility kina	39.40	0.00
TRINITY_sp|Q8H0[illegible]	MPA1	Puromycin-sensitive	39.40	0.00
TRINITY_sp|Q54P[illegible]	tmem184[illegible]	Transmembrane prote	39.40	0.00
TRINITY_sp|Q9VM[illegible]	TTLL3B	Tubulin glycylase 3	39.40	0.00
TRINITY_sp|Q5ZJ[illegible]	YARS	Tyrosine--tRNA liga	39.40	0.00
TRINITY_sp|Q8H1[illegible]	At1g064[illegible]	Probable sugar phos	39.40	0.00
TRINITY_sp|Q9KD[illegible]	era	GTPase Era OS=Bacil	39.40	0.00
TRINITY_sp|Q585[illegible]	nudF	ADP-ribose pyrophos	39.40	0.00
TRINITY_sp|Q8VZ[illegible]	SWC4	SWR1-complex protei	39.40	0.00
TRINITY_sp|Q9C0[illegible]	DNAH6	Dynein heavy chain	39.40	0.00
TRINITY_sp|Q54V[illegible]	DDB_G02[illegible]	Probable serine/thr	39.40	0.00
TRINITY_sp|P533[illegible]	Dbt	Lipoamide acyltrans	39.40	0.00
TRINITY_sp|Q726[illegible]	pta	Phosphate acetyltra	39.40	0.00
TRINITY_sp|B8BJ[illegible]	OsI_351[illegible]	Pyruvate kinase 1,	39.40	0.00
TRINITY_sp|P109[illegible]	-	Retrovirus-related	39.40	0.00
TRINITY_sp|Q9FI[illegible]	SPP	Stromal processing	39.40	0.00
TRINITY_sp|Q5A8[illegible]	SLN1	Histidine protein k	39.40	0.00
TRINITY_sp|Q8VE[illegible]	Ogfrl1	Opioid growth facto	39.40	0.00
TRINITY_sp|B4JV[illegible]	GH17388	E3 UFM1-protein lig	39.30	0.00
TRINITY_sp|P348[illegible]	-	Elongation factor 1	39.30	0.00
TRINITY_sp|Q9VL[illegible]	CG4968	Ubiquitin thioester	39.30	0.00
TRINITY_sp|Q944[illegible]	OST48	Dolichyl-diphosphoo	39.30	0.00
TRINITY_sp|P296[illegible]	eef-2	Elongation factor 2	39.30	0.00
TRINITY_sp|O490[illegible]	VPS45	Vacuolar protein so	39.30	0.00
TRINITY_sp|P384[illegible]	GNA14	Guanine nucleotide-	39.30	0.00
TRINITY_sp|F4JV[illegible]	SFH6	Phosphatidylinosito	39.30	0.00
TRINITY_sp|Q5E9[illegible]	EIF2S2	Eukaryotic translat	39.30	0.00
TRINITY_sp|Q3KP[illegible]	ANKRD55	Ankyrin repeat doma	39.30	0.00
TRINITY_sp|Q86K[illegible]	phyA	Prolyl 4-hydroxylas	39.30	0.00
TRINITY_sp|Q9CX[illegible]	Ndufs4	NADH dehydrogenase	39.30	0.00
TRINITY_sp|Q165[illegible]	MAP3K11	Mitogen-activated p	39.30	0.00
TRINITY_sp|Q9EP[illegible]	Acox3	Peroxisomal acyl-co	39.30	0.00
TRINITY_sp|Q4PC[illegible]	SEC13	Protein transport p	39.30	0.00
TRINITY_sp|Q2LV[illegible]	msbA	Lipid A export ATP-	39.30	0.00

TRINITY_sp|A7S7	v1g1672	Anamorsin homolog O	39.30	0.00
TRINITY_sp|A6UV	Maeo_10	Lactaldehyde dehydr	39.30	0.00
TRINITY_sp|Q6S7	TAF5	Transcription initi	39.30	0.00
TRINITY_sp|Q0P5	RPRD1A	Regulation of nucle	39.30	0.00
TRINITY_sp|Q54V	hdaC	Type-2 histone deac	39.30	0.00
TRINITY_sp|P353	-	Sodium/potassium-tr	39.30	0.00
TRINITY_sp|Q9P7	SPBP35G	ZZ-type zinc finger	39.30	0.00
TRINITY_sp|Q5BU	Eapp	E2F-associated phos	39.30	0.00
TRINITY_sp|Q75J	cdc123	Cell division cycle	39.30	0.00
TRINITY_sp|Q5E9	NECAP2	Adaptin ear-binding	39.30	0.00
TRINITY_sp|Q9VH	Invadol	Leishmanolysin-like	39.30	0.00
TRINITY_sp|Q095	F26C11.	Putative acid phosp	39.30	0.00
TRINITY_sp|O809	AGD7	ADP-ribosylation fa	39.30	0.00
TRINITY_sp|Q2RF	rsmG	Ribosomal RNA small	39.30	0.00
TRINITY_sp|O845	CT_544	Probable hexose pho	39.30	0.00
TRINITY_sp|O237	GSH2	Glutathione synthet	39.30	0.00
TRINITY_sp|Q090	-	Hibernation-specifi	39.30	0.00
TRINITY_sp|Q869	pakB	Serine/threonine-pr	39.30	0.00
TRINITY_sp|O188	AOAH	Acyloxyacyl hydrola	39.30	0.00
TRINITY_sp|Q7NM	cry	Cryptochrome DASH O	39.30	0.00
TRINITY_sp|Q9SL	ZIP3	Zinc transporter 3	39.30	0.00
TRINITY_sp|O624	Y43F4A.	Leishmanolysin-like	39.30	0.00
TRINITY_sp|Q9UD	DNAJB4	DnaJ homolog subfam	39.30	0.00
TRINITY_sp|Q9LE	IRE	Probable serine/thr	39.30	0.00
TRINITY_sp|Q54B	dstC	Signal transducer a	39.30	0.00
TRINITY_sp|Q54K	lmbrd1	Probable lysosomal	39.30	0.00
TRINITY_sp|Q53P	PHT4;6	Probable anion tran	39.30	0.00
TRINITY_sp|Q9UT	mrpl19	54S ribosomal prote	39.30	0.00
TRINITY_sp|Q9FM	At5g140	U3 small nucleolar	39.30	0.00
TRINITY_sp|Q8BW	Cmtr2	Cap-specific mRNA (	39.30	0.00
TRINITY_sp|Q556	snf12-1	SWI/SNF complex com	39.30	0.00
TRINITY_sp|Q582	MJ0812	Uncharacterized pro	39.30	0.00
TRINITY_sp|Q8MZ	-	Physarolisin OS=Phy	39.30	0.00
TRINITY_sp|Q134	MKRN4P	Putative E3 ubiquit	39.30	0.00
TRINITY_sp|P002	fd1	Ferredoxin-1 OS=Des	39.30	0.00
TRINITY_sp|O647	GLCNAC1	UDP-N-acetylglucosa	39.30	0.00
TRINITY_sp|P427	CLPB1	Chaperone protein C	39.30	0.00
TRINITY_sp|Q5E9	EMC2	ER membrane protein	39.30	0.00
TRINITY_sp|Q9CZ	Naalad2	N-acetylated-alpha-	39.30	0.00
TRINITY_sp|Q8LP	At3g199	E3 ubiquitin-protei	39.30	0.00
TRINITY_sp|Q8ND	EHBP1	EH domain-binding p	39.30	0.00
TRINITY_sp|Q555	gabD	Probable succinate-	39.30	0.00
TRINITY_sp|O140	SPAC2C4.	Putative tRNA 2'-ph	39.30	0.00
TRINITY_sp|Q93Y	GTE9	Transcription facto	39.30	0.00
TRINITY_sp|Q9C5	KEU	SNARE-interacting p	39.30	0.00
TRINITY_sp|O435	ATP8B1	Phospholipid-transp	39.30	0.00
TRINITY_sp|Q8R1	Znfx1	NFX1-type zinc fing	39.30	0.00
TRINITY_sp|B0D6	LACBIDR	Pentafunctional ARO	39.30	0.00
TRINITY_sp|Q54U	abcC12	ABC transporter C f	39.30	0.00
TRINITY_sp|A2XQ	OsI_148	Senescence-specific	39.30	0.00
TRINITY_sp|F4HW	XI-E	Myosin-11 OS=Arabid	39.30	0.00
TRINITY_sp|Q54J	abcC3	ABC transporter C f	39.30	0.00
TRINITY_sp|Q8AX	cyp3a56	Cytochrome P450 3A5	39.30	0.00
TRINITY_sp|Q2QY	CIPK14	CBL-interacting pro	39.30	0.00
TRINITY_sp|P213	pncA	Pyrazinamidase/nico	39.30	0.00
TRINITY_sp|Q2NL	GSTT1	Glutathione S-trans	39.20	0.00

TRINITY_sp|P937!	GK-1	Guanylate kinase 1	39.20	0.00
TRINITY_sp|P584	pepB	Putative peptidase	39.20	0.00
TRINITY_sp|O147	CDIPT	CDP-diacylglycerol-	39.20	0.00
TRINITY_sp|P011	V-MYB	Transforming protei	39.20	0.00
TRINITY_sp|Q9JM	Edf1	Endothelial differe	39.20	0.00
TRINITY_sp|Q9LD	CRK33	Putative cysteine-r	39.20	0.00
TRINITY_sp|Q636	Vps33a	Vacuolar protein so	39.20	0.00
TRINITY_sp|Q5M7	At3g522	RNA pseudouridine s	39.20	0.00
TRINITY_sp|C5H4	DBR2	Artemisinic aldehyd	39.20	0.00
TRINITY_sp|P487	Ppn1	Serine/threonine-pr	39.20	0.00
TRINITY_sp|O428	sco1	Protein sco1 OS=Sch	39.20	0.00
TRINITY_sp|Q4P6	COF1	Cofilin OS=Ustilago	39.20	0.00
TRINITY_sp|Q8CE	Trmt6	tRNA (adenine(58)-N	39.20	0.00
TRINITY_sp|Q239	kinX	Probable serine/thr	39.20	0.00
TRINITY_sp|O609	CTNS	Cystinosin OS=Homo	39.20	0.00
TRINITY_sp|Q05B	shq1	Protein SHQ1 homolo	39.20	0.00
TRINITY_sp|Q55G	DDB_G02	Probable serine/thr	39.20	0.00
TRINITY_sp|B8GQ	tsf	Elongation factor T	39.20	0.00
TRINITY_sp|Q55D	rabggta	Geranylgeranyl tran	39.20	0.00
TRINITY_sp|Q550	DDB_G02	Probable serine/thr	39.20	0.00
TRINITY_sp|O424	EPHA7	Ephrin type-A recep	39.20	0.00
TRINITY_sp|D4DH	LAP1	Probable leucine am	39.20	0.00
TRINITY_sp|P519	Nek1	Serine/threonine-pr	39.20	0.00
TRINITY_sp|Q0U3	SNOG_13	Very-long-chain 3-o	39.20	0.00
TRINITY_sp|Q8RV	DEK1	Calpain-type cystei	39.20	0.00
TRINITY_sp|P322	rasC	Ras-like protein ra	39.20	0.00
TRINITY_sp|Q8L7	At1g317	AP-4 complex subuni	39.20	0.00
TRINITY_sp|O597	lys9	Saccharopine dehydr	39.20	0.00
TRINITY_sp|Q6CT	FYV4	Protein FYV4, mitoc	39.20	0.00
TRINITY_sp|A6TW	gltX	Glutamate--tRNA lig	39.20	0.00
TRINITY_sp|P133	-	Actin OS=Phytophtho	39.20	0.00
TRINITY_sp|Q54C	DDB_G02	WAS protein family	39.20	0.00
TRINITY_sp|Q9XI	SERK2	Somatic embryogenes	39.20	0.00
TRINITY_sp|Q8T9	abcB3	ABC transporter B f	39.20	0.00
TRINITY_sp|F6Z5	usp15	Ubiquitin carboxyl-	39.20	0.00
TRINITY_sp|P383	PHO89	Phosphate permease	39.20	0.00
TRINITY_sp|Q5ZM	GK5	Putative glycerol k	39.20	0.00
TRINITY_sp|Q28I	hsd17b1	Very-long-chain 3-o	39.20	0.00
TRINITY_sp|Q9SR	UDP-GAL	UDP-galactose trans	39.20	0.00
TRINITY_sp|Q54T	yipf1	Protein YIPF1 homol	39.20	0.00
TRINITY_sp|Q5NA	MTP5	Metal tolerance pro	39.20	0.00
TRINITY_sp|P403	ptc1	Protein phosphatase	39.20	0.00
TRINITY_sp|Q96M	KREMEN1	Kremen protein 1 OS	39.20	0.00
TRINITY_sp|O040	DHBK	Putative 3,4-dihydr	39.20	0.00
TRINITY_sp|Q10Q	UROS	Uroporphyrinogen-II	39.20	0.00
TRINITY_sp|Q371	ACA1	Calcium-transportin	39.20	0.00
TRINITY_sp|Q9UT	SPAC227.	Putative uridine ki	39.20	0.00
TRINITY_sp|P200	Hexb	Beta-hexosaminidase	39.20	0.00
TRINITY_sp|Q8BG	Nudt15	Nucleotide triphosp	39.20	0.00
TRINITY_sp|A8IZ	CHLREDR	Probable cytosolic	39.20	0.00
TRINITY_sp|Q078	GPI13	GPI ethanolamine ph	39.20	0.00
TRINITY_sp|Q810	Mphosph	U3 small nucleolar	39.20	0.00
TRINITY_sp|P543	Lipe	Hormone-sensitive l	39.20	0.00
TRINITY_sp|Q9SR	SNL1	Paired amphipathic	39.20	0.00
TRINITY_sp|Q9SR	AZG1	Adenine/guanine per	39.20	0.00
TRINITY_sp|A7A2	ATG18	Autophagy-related p	39.20	0.00

TRINITY_sp|Q0WV)	SS4	Probable starch syn	39.20	0.00
TRINITY_sp|Q8W4I	CYP71	Peptidyl-prolyl cis	39.20	0.00
TRINITY_sp|Q54M\	mrkB	Probable serine/thr	39.20	0.00
TRINITY_sp|Q8L4.	CSTF50	Cleavage stimulatio	39.20	0.00
TRINITY_sp|Q7TN(	Eml2	Echinoderm microtub	39.20	0.00
TRINITY_sp|G5EB2	let-418	Protein let-418 OS=	39.20	0.00
TRINITY_sp|Q59W(	GIN4	Serine/threonine-pr	39.20	0.00
TRINITY_sp|Q9SKI	RPA1A	Replication protein	39.20	0.00
TRINITY_sp|D7UQN	aur	Aurora kinase OS=Pa	39.20	0.00
TRINITY_sp|Q747I	rimO	Ribosomal protein S	39.20	0.00
TRINITY_sp|Q084(	PDE4C	cAMP-specific 3',5'	39.20	0.00
TRINITY_sp|Q500\	DPB2	DNA polymerase epsi	39.20	0.00
TRINITY_sp|Q5E9N	BYSL	Bystin OS=Bos tauru	39.20	0.00
TRINITY_sp|Q141(	WRN	Werner syndrome ATP	39.20	0.00
TRINITY_sp|Q3ZCI	CCNC	Cyclin-C OS=Bos tau	39.20	0.00
TRINITY_sp|A0AV1	UBA6	Ubiquitin-like modi	39.20	0.00
TRINITY_sp|Q6RWA	-	Sodium/potassium-tr	39.20	0.00
TRINITY_sp|Q55D)	mcfF	Mitoferrin OS=Dicty	39.20	0.00
TRINITY_sp|P2622	celD	Endoglucanase E-4 O	39.20	0.00
TRINITY_sp|Q9SZ(	HHP4	Heptahelical transm	39.20	0.00
TRINITY_sp|Q3SY(	TNPO1	Transportin-1 OS=Bo	39.10	0.00
TRINITY_sp|P758(	yliI	Soluble aldose suga	39.10	0.00
TRINITY_sp|Q0WUI	FAB1A	1-phosphatidylinosi	39.10	0.00
TRINITY_sp|Q9P7I	clp1	Tyrosine-protein ph	39.10	0.00
TRINITY_sp|Q9U1I	Sras	CAAX prenyl proteas	39.10	0.00
TRINITY_sp|O494!	At4g284	Uncharacterized pro	39.10	0.00
TRINITY_sp|Q8NF(	SYNE1	Nesprin-1 OS=Homo s	39.10	0.00
TRINITY_sp|P981(	Atp8b2	Phospholipid-transp	39.10	0.00
TRINITY_sp|P0CH.	adhc1	NADP-dependent alco	39.10	0.00
TRINITY_sp|Q6EPN	ISPF	2-C-methyl-D-erythr	39.10	0.00
TRINITY_sp|P0C8N	CCRP1	Probable serine/thr	39.10	0.00
TRINITY_sp|Q9D3(	Snx29	Sorting nexin-29 OS	39.10	0.00
TRINITY_sp|Q55D!	DDB_G02	Putative methylster	39.10	0.00
TRINITY_sp|P0CD(	pan3	PAB-dependent poly(	39.10	0.00
TRINITY_sp|Q6TG(	CBK1	Serine/threonine-pr	39.10	0.00
TRINITY_sp|Q54SI	dhkM	Hybrid signal trans	39.10	0.00
TRINITY_sp|Q54B1	abcB2	ABC transporter B f	39.10	0.00
TRINITY_sp|Q8IQ1	CG31957	Probable RNA-bindin	39.10	0.00
TRINITY_sp|P366I	dsk1	Protein kinase dsk1	39.10	0.00
TRINITY_sp|F4J6I	IREH1	Probable serine/thr	39.10	0.00
TRINITY_sp|Q0D4I	CIPK21	CBL-interacting pro	39.10	0.00
TRINITY_sp|O422	kras1	GTPase KRas OS=Oryz	39.10	0.00
TRINITY_sp|Q55B.	plbB	Phospholipase B-lik	39.10	0.00
TRINITY_sp|P217(	EPHA1	Ephrin type-A recep	39.10	0.00
TRINITY_sp|Q55B2	dcd1A	Protein dcd1A OS=Di	39.10	0.00
TRINITY_sp|Q9QY2	Smok3a	Sperm motility kina	39.10	0.00
TRINITY_sp|Q8CF(	Aqr	Intron-binding prot	39.10	0.00
TRINITY_sp|O054(	yrpG	Uncharacterized oxi	39.10	0.00
TRINITY_sp|O704(	Prmt3	Protein arginine N-	39.10	0.00
TRINITY_sp|O154.	ABCC4	Multidrug resistanc	39.10	0.00
TRINITY_sp|P518(	lig1	DNA ligase 1 OS=Xen	39.10	0.00
TRINITY_sp|Q092!	ccdc-55	Nuclear speckle spl	39.10	0.00
TRINITY_sp|Q5T01	MARCH8	E3 ubiquitin-protei	39.10	0.00
TRINITY_sp|Q54G	ssrp1	FACT complex subuni	39.10	0.00
TRINITY_sp|Q84MA	CAT1	Cationic amino acid	39.10	0.00
TRINITY_sp|P0DK(	PGLP1A	Phosphoglycolate ph	39.10	0.00

TRINITY_sp|O009(	-	Lysosomal acid alph	39.10	0.00
TRINITY_sp|P707(	Atp8a1	Phospholipid-transp	39.10	0.00
TRINITY_sp|O890{	Pde4a	cAMP-specific 3',5'	39.10	0.00
TRINITY_sp|Q645{	Atp7b	Copper-transporting	39.10	0.00
TRINITY_sp|P186{	rapA	Ras-related protein	39.10	0.00
TRINITY_sp|F9VN	rnhA	Ribonuclease HI OS=	39.10	0.00
TRINITY_sp|B4SQ	pnp	Polyribonucleotide	39.10	0.00
TRINITY_sp|B9DF	PAA2	Copper-transporting	39.10	0.00
TRINITY_sp|Q91W(	Dgkg	Diacylglycerol kina	39.10	0.00
TRINITY_sp|Q8IW	DENND6A	Protein DENND6A OS=	39.10	0.00
TRINITY_sp|P623	CPK4	Calcium-dependent p	39.10	0.00
TRINITY_sp|Q5BJ	Carnmt1	Carnosine N-methylt	39.10	0.00
TRINITY_sp|Q9ZV	CHR8	Protein CHROMATIN R	39.10	0.00
TRINITY_sp|Q6N0	MFSD5	Molybdate-anion tra	39.10	0.00
TRINITY_sp|Q54GI	DDB_G02	PXMP2/4 family prot	39.10	0.00
TRINITY_sp|Q3V3	Lace1	Lactation elevated	39.10	0.00
TRINITY_sp|Q8N5I	RPP25L	Ribonuclease P prot	39.10	0.00
TRINITY_sp|Q9FN(	UVR8	Ultraviolet-B recep	39.10	0.00
TRINITY_sp|O805	CYCU4-1	Cyclin-U4-1 OS=Arab	39.10	0.00
TRINITY_sp|Q6Z9I	KIN7H	Kinesin-like protei	39.10	0.00
TRINITY_sp|Q9H0	WWP1	NEDD4-like E3 ubiqu	39.10	0.00
TRINITY_sp|O136	mrd1	Multiple RNA-bindin	39.10	0.00
TRINITY_sp|Q6UD	ManS	Mannan synthase 1 O	39.10	0.00
TRINITY_sp|O091	Man2b1	Lysosomal alpha-man	39.10	0.00
TRINITY_sp|P817	TL17	Thylakoid lumenal 1	39.10	0.00
TRINITY_sp|P623	CPK4	Calcium-dependent p	39.10	0.00
TRINITY_sp|Q8PN	glnS	Glutamine--tRNA lig	39.10	0.00
TRINITY_sp|O807	WDR55	WD repeat-containin	39.10	0.00
TRINITY_sp|Q9XG	B''ALPH	Serine/threonine pr	39.00	0.00
TRINITY_sp|A2YH	OsI_023	Serine/threonine-pr	39.00	0.00
TRINITY_sp|Q9FV	MAP1D	Methionine aminopep	39.00	0.00
TRINITY_sp|Q0AF	acsA	Acetyl-coenzyme A s	39.00	0.00
TRINITY_sp|Q0VA	wdr70	WD repeat-containin	39.00	0.00
TRINITY_sp|Q906	DPYSL2	Dihydropyrimidinase	39.00	0.00
TRINITY_sp|Q54G	vps45	Vacuolar protein so	39.00	0.00
TRINITY_sp|Q5XG	gtpbp1	GTP-binding protein	39.00	0.00
TRINITY_sp|Q6XH	roco4	Probable serine/thr	39.00	0.00
TRINITY_sp|Q96B	DOCK10	Dedicator of cytoki	39.00	0.00
TRINITY_sp|Q9NU	TBC1D22	TBC1 domain family	39.00	0.00
TRINITY_sp|Q6UE	nadA	NADH-dependent flav	39.00	0.00
TRINITY_sp|Q93V(	At4g174	DeSI-like protein A	39.00	0.00
TRINITY_sp|Q9FH	At5g051	Probable leucine-ri	39.00	0.00
TRINITY_sp|Q7KW	pgs1	Probable CDP-diacyl	39.00	0.00
TRINITY_sp|Q102	SPAC4G9.	Uncharacterized mit	39.00	0.00
TRINITY_sp|Q8IZ	TRMT2A	tRNA (uracil-5-)-me	39.00	0.00
TRINITY_sp|A1JS(	rsmJ	Ribosomal RNA small	39.00	0.00
TRINITY_sp|Q55F	DDB_G02	Probable carboxypep	39.00	0.00
TRINITY_sp|P497	RBM25	RNA-binding protein	39.00	0.00
TRINITY_sp|P805	aro-8	Phospho-2-dehydro-3	39.00	0.00
TRINITY_sp|Q0IU(	CML2	Putative calmodulin	39.00	0.00
TRINITY_sp|Q8VC	Zdhhc12	Probable palmitoylt	39.00	0.00
TRINITY_sp|Q23K	TTLL3D	Tubulin glycylase 3	39.00	0.00
TRINITY_sp|Q949	UBP6	Ubiquitin carboxyl-	39.00	0.00
TRINITY_sp|O139	pnk1	Bifunctional polynu	39.00	0.00
TRINITY_sp|Q7ZV	poc1a	POC1 centriolar pro	39.00	0.00
TRINITY_sp|Q8MY	DDB_G02	Probable serine/thr	39.00	0.00

TRINITY_sp|B0R01	chd8	Chromodomain-helica	39.00	0.00
TRINITY_sp|P4683	yhgF	Protein YhgF OS=Esc	39.00	0.00
TRINITY_sp|Q1269	YPK9	Vacuolar cation-tra	39.00	0.00
TRINITY_sp|Q9FV(	NUCL1	Nucleolin 1 OS=Arab	39.00	0.00
TRINITY_sp|Q6NV	HIBCH	3-hydroxyisobutyryl	39.00	0.00
TRINITY_sp|O6034	TBC1D12	TBC1 domain family	39.00	0.00
TRINITY_sp|Q95X2	laat-1	Lysosomal amino aci	39.00	0.00
TRINITY_sp|Q7TP(	Shprh	E3 ubiquitin-protei	39.00	0.00
TRINITY_sp|Q55B	plbB	Phospholipase B-lik	39.00	0.00
TRINITY_sp|Q962	MYB23	Transcription facto	39.00	0.00
TRINITY_sp|Q8RXI	AGD14	Probable ADP-ribosy	39.00	0.00
TRINITY_sp|Q8IU8	CAMK1D	Calcium/calmodulin-	39.00	0.00
TRINITY_sp|Q75JI	DDB_G02	SH3 and F-BAR domai	39.00	0.00
TRINITY_sp|O7491	rrp45	Exosome complex com	39.00	0.00
TRINITY_sp|P4703	PRY1	Protein PRY1 OS=Sac	39.00	0.00
TRINITY_sp|Q9UB	RNF14	E3 ubiquitin-protei	39.00	0.00
TRINITY_sp|Q4KWI	PLCH1	1-phosphatidylinosi	39.00	0.00
TRINITY_sp|Q927	SYN2	Synapsin-2 OS=Homo	39.00	0.00
TRINITY_sp|O1392	crn1	Coronin-like protei	39.00	0.00
TRINITY_sp|P5052	ssp1	Serine/threonine-pr	39.00	0.00
TRINITY_sp|P0513	PRKACB	cAMP-dependent prot	39.00	0.00
TRINITY_sp|Q941	PMS1	DNA mismatch repair	39.00	0.00
TRINITY_sp|O5489	Cacna1g	Voltage-dependent T	39.00	0.00
TRINITY_sp|C5FT2	LAP2	Leucine aminopeptid	39.00	0.00
TRINITY_sp|O9455	cut23	Anaphase-promoting	39.00	0.00
TRINITY_sp|Q6NXI	Rcbtb1	RCC1 and BTB domain	39.00	0.00
TRINITY_sp|Q6PC4	cdc123	Cell division cycle	39.00	0.00
TRINITY_sp|A4Q9I	Ttll2	Probable tubulin po	39.00	0.00
TRINITY_sp|Q5TC6	GRTP1	Growth hormone-regu	39.00	0.00
TRINITY_sp|Q7PC	SAT2	Diamine acetyltrans	39.00	0.00
TRINITY_sp|P109	-	Retrovirus-related	39.00	0.00
TRINITY_sp|Q6IR	tiprl	TIP41-like protein	39.00	0.00
TRINITY_sp|Q54WI	forA	Formin-A OS=Dictyos	39.00	0.00
TRINITY_sp|P5255	mybl2	Myb-related protein	39.00	0.00
TRINITY_sp|Q9XSI	TPP1	Tripeptidyl-peptida	39.00	0.00
TRINITY_sp|Q6148	Pde1a	Calcium/calmodulin-	39.00	0.00
TRINITY_sp|P2952	-	Elongation factor 1	39.00	0.00
TRINITY_sp|Q9BZI	PUS3	tRNA pseudouridine(	39.00	0.00
TRINITY_sp|O1496	AURKA	Aurora kinase A OS=	39.00	0.00
TRINITY_sp|Q9N5	cdc-73	Cell division cycle	39.00	0.00
TRINITY_sp|Q9SF4	PAH1	Phosphatidate phosp	39.00	0.00
TRINITY_sp|O0698	yvdB	Putative sulfate tr	39.00	0.00
TRINITY_sp|Q54YI	dhkB	Hybrid signal trans	39.00	0.00
TRINITY_sp|Q29RI	TBC1D31	TBC1 domain family	39.00	0.00
TRINITY_sp|D4P3I	CFAP74	Cilia- and flagella	39.00	0.00
TRINITY_sp|P2218	cta3	Calcium-transportin	39.00	0.00
TRINITY_sp|F4JY8	TCX7	Protein tesmin/TSO1	38.90	0.00
TRINITY_sp|Q9246	pub1	E3 ubiquitin-protei	38.90	0.00
TRINITY_sp|Q0651	TAZ1	Lysophosphatidylcho	38.90	0.00
TRINITY_sp|Q9PU	smurf1	E3 ubiquitin-protei	38.90	0.00
TRINITY_sp|O9706	CG1307	Probable peptidyl-t	38.90	0.00
TRINITY_sp|A6QL	MTMR2	Myotubularin-relate	38.90	0.00
TRINITY_sp|A0L5	rpmB	50S ribosomal prote	38.90	0.00
TRINITY_sp|Q6L5I	IMCE	Probable isoprenylc	38.90	0.00
TRINITY_sp|Q9SU(	CHX17	Cation/H(+) antipor	38.90	0.00
TRINITY_sp|A8WYI	par-1	Serine/threonine-pr	38.90	0.00

TRINITY_sp|A6H79	TRMT61A	tRNA (adenine(58)-N	38.90	0.00
TRINITY_sp|Q8BYR	Rdh12	Retinol dehydrogena	38.90	0.00
TRINITY_sp|Q6N06	NAA16	N-alpha-acetyltrans	38.90	0.00
TRINITY_sp|P8237	scrK	Fructokinase OS=Lac	38.90	0.00
TRINITY_sp|Q9SD3	At3g5113	UPF0183 protein At3	38.90	0.00
TRINITY_sp|P1101	isp	Major intracellular	38.90	0.00
TRINITY_sp|Q54BM	mcfG	Mitochondrial subst	38.90	0.00
TRINITY_sp|Q54B1	redA	NADPH oxidoreductas	38.90	0.00
TRINITY_sp|Q3924	PP2AB2	Serine/threonine pr	38.90	0.00
TRINITY_sp|A7SBR	ints9	Integrator complex	38.90	0.00
TRINITY_sp|Q5JTH	RRP12	RRP12-like protein	38.90	0.00
TRINITY_sp|Q7YXI	p80	Protein P80 OS=Dict	38.90	0.00
TRINITY_sp|Q8VHE	Dnah5	Dynein heavy chain	38.90	0.00
TRINITY_sp|Q5C9I	-	(S)-coclaurine N-me	38.90	0.00
TRINITY_sp|Q8LPT	ABCB6	ABC transporter B f	38.90	0.00
TRINITY_sp|P4969	pkwA	Probable serine/thr	38.90	0.00
TRINITY_sp|F4I2Y	RMR3	Receptor homology r	38.90	0.00
TRINITY_sp|Q3SZ1	HPRT1	Hypoxanthine-guanin	38.90	0.00
TRINITY_sp|P4123	Abca1	ATP-binding cassett	38.90	0.00
TRINITY_sp|O5830	alaXS	Alanyl-tRNA editing	38.90	0.00
TRINITY_sp|Q9VQ8	mio	WD repeat-containin	38.90	0.00
TRINITY_sp|Q67ZE	3BETAHSD	3beta-hydroxysteroi	38.90	0.00
TRINITY_sp|Q86G4	gefQ	Ras guanine nucleot	38.90	0.00
TRINITY_sp|Q8RWI	STY17	Serine/threonine-pr	38.90	0.00
TRINITY_sp|Q9SMH	DHC1B	Cytoplasmic dynein	38.90	0.00
TRINITY_sp|P2212	-	Ras-related protein	38.90	0.00
TRINITY_sp|Q4078	-	Putative glucose-6-	38.90	0.00
TRINITY_sp|Q7SCY	sym-1	Protein sym-1 OS=Ne	38.90	0.00
TRINITY_sp|Q9C52	UDP-GALT	UDP-galactose trans	38.90	0.00
TRINITY_sp|O6489	Acx	Acyl-coenzyme A oxi	38.90	0.00
TRINITY_sp|P1539	rpa1	DNA-directed RNA po	38.90	0.00
TRINITY_sp|O2289	LACS1	Long chain acyl-CoA	38.90	0.00
TRINITY_sp|Q1449	RBM39	RNA-binding protein	38.90	0.00
TRINITY_sp|B7KDE	glgC	Glucose-1-phosphate	38.90	0.00
TRINITY_sp|Q4PFE	ATG2	Autophagy-related p	38.90	0.00
TRINITY_sp|Q9FPS	UBP23	Ubiquitin carboxyl-	38.90	0.00
TRINITY_sp|Q9SR7	At3g1013	Heme-binding-like p	38.90	0.00
TRINITY_sp|P1464	Pde4c	cAMP-specific 3',5'	38.90	0.00
TRINITY_sp|Q88K2	pheT	Phenylalanine--tRNA	38.90	0.00
TRINITY_sp|Q08DI	PUS7	Pseudouridylate syn	38.90	0.00
TRINITY_sp|Q4V8I	Fam221a	Protein FAM221A OS=	38.90	0.00
TRINITY_sp|Q54DH	taf1	Transcription initi	38.90	0.00
TRINITY_sp|Q0DJ0	OST1A	Dolichyl-diphosphoo	38.90	0.00
TRINITY_sp|P1097	-	Retrovirus-related	38.90	0.00
TRINITY_sp|Q9FNI	At5g2275	Putative SWI/SNF-re	38.90	0.00
TRINITY_sp|Q9C6E	GAMMACA2	Gamma carbonic anhy	38.90	0.00
TRINITY_sp|Q47LE	rplX	50S ribosomal prote	38.90	0.00
TRINITY_sp|Q7580	BTN1	Protein BTN1 OS=Ash	38.90	0.00

TRINITY_sp|Q32KN	DNAL4	Dynein light chain	38.90	0.00
TRINITY_sp|Q5BL4	slc20a1	Sodium-dependent ph	38.90	0.00
TRINITY_sp|Q9BY1	SCAPER	S phase cyclin A-as	38.90	0.00
TRINITY_sp|Q0WW8	RBP47B	Polyadenylate-bindi	38.90	0.00
TRINITY_sp|Q758I	LAS21	GPI ethanolamine ph	38.90	0.00
TRINITY_sp|P2346	CYR1	Adenylate cyclase O	38.90	0.00
TRINITY_sp|Q1321	PAPPA	Pappalysin-1 OS=Hom	38.90	0.00
TRINITY_sp|Q2979	nero	Deoxyhypusine hydro	38.90	0.00
TRINITY_sp|Q54LI	abcC5	ABC transporter C f	38.90	0.00
TRINITY_sp|D7U60	ANR	Anthocyanidin reduc	38.90	0.00
TRINITY_sp|Q84W6	SUFE1	SufE-like protein 1	38.90	0.00
TRINITY_sp|Q9C83	RH34	DEAD-box ATP-depend	38.80	0.00
TRINITY_sp|Q557I	dpp3-1	Dipeptidyl peptidas	38.80	0.00
TRINITY_sp|A0A08	msl3	Mycolipanoate synth	38.80	0.00
TRINITY_sp|P4315	-	Microbial collagena	38.80	0.00
TRINITY_sp|Q1036	sck2	Serine/threonine-pr	38.80	0.00
TRINITY_sp|Q9Z32	Itpr2	Inositol 1,4,5-tris	38.80	0.00
TRINITY_sp|P3986	NIA2	Nitrate reductase [	38.80	0.00
TRINITY_sp|Q3T02	QDPR	Dihydropteridine re	38.80	0.00
TRINITY_sp|B8IS8	rplW	50S ribosomal prote	38.80	0.00
TRINITY_sp|Q2T9V	TRMT6	tRNA (adenine(58)-N	38.80	0.00
TRINITY_sp|Q1226	GIN4	Serine/threonine-pr	38.80	0.00
TRINITY_sp|Q9Y8N	APE_2601	Uncharacterized pro	38.80	0.00
TRINITY_sp|B4K61	fig	Protein phosphatase	38.80	0.00
TRINITY_sp|Q9QZ7	Dcun1d1	DCN1-like protein 1	38.80	0.00
TRINITY_sp|P5019	linX	2,5-dichloro-2,5-cy	38.80	0.00
TRINITY_sp|Q0587	NNT1	Protein N-methyltra	38.80	0.00
TRINITY_sp|Q9LT0	PDR2	Probable manganese-	38.80	0.00
TRINITY_sp|Q9VR9	HERC2	Probable E3 ubiquit	38.80	0.00
TRINITY_sp|Q55C9	mpl2	MAP kinase phosphat	38.80	0.00
TRINITY_sp|Q76PI	SPCC1223	E3 ubiquitin-protei	38.80	0.00
TRINITY_sp|Q2662	KAP115	Kinesin-associated	38.80	0.00
TRINITY_sp|Q3U29	Otud5	OTU domain-containi	38.80	0.00
TRINITY_sp|Q8GU0	RIE1	E3 ubiquitin protei	38.80	0.00
TRINITY_sp|Q9VR9	HERC2	Probable E3 ubiquit	38.80	0.00
TRINITY_sp|F4I23	CTN	Cactin OS=Arabidops	38.80	0.00
TRINITY_sp|O2446	RABE1A	Ras-related protein	38.80	0.00
TRINITY_sp|Q9Z12	Epn2	Epsin-2 OS=Rattus n	38.80	0.00
TRINITY_sp|O8124	RHL1	DNA-binding protein	38.80	0.00
TRINITY_sp|Q67Z0	LIP2	Triacylglycerol lip	38.80	0.00
TRINITY_sp|Q4V83	Gpat3	Glycerol-3-phosphat	38.80	0.00
TRINITY_sp|O8077	WDR55	WD repeat-containin	38.80	0.00
TRINITY_sp|Q9233	Dnah12	Dynein heavy chain	38.80	0.00
TRINITY_sp|O4339	TXNL1	Thioredoxin-like pr	38.80	0.00
TRINITY_sp|Q9NS8	KIF15	Kinesin-like protei	38.80	0.00
TRINITY_sp|Q9FL0	MRS2-2	Magnesium transport	38.80	0.00
TRINITY_sp|Q86H1	Pde4	cAMP-specific 3',5'	38.80	0.00
TRINITY_sp|A9V20	37534	Probable nitrile hy	38.80	0.00
TRINITY_sp|Q5E91	RBL	Protein RBL OS=Arab	38.80	0.00
TRINITY_sp|Q7K20	Rpn13	Proteasomal ubiquit	38.80	0.00
TRINITY_sp|Q8L7A	At1g3173	AP-4 complex subuni	38.80	0.00
TRINITY_sp|Q8GWV	GFA2	Chaperone protein d	38.80	0.00
TRINITY_sp|Q7Z62	HUWE1	E3 ubiquitin-protei	38.80	0.00
TRINITY_sp|Q9JIV	Polm	DNA-directed DNA/RN	38.80	0.00
TRINITY_sp|F4J71	EAF1A	Chromatin modificat	38.80	0.00
TRINITY_sp|Q80U9	Xpo1	Exportin-1 OS=Rattu	38.80	0.00

TRINITY_sp|Q8R5I	Usp33	Ubiquitin carboxyl-	38.80	0.00
TRINITY_sp|Q54MI	rbbE	Retinoblastoma-bind	38.80	0.00
TRINITY_sp|Q6Q1I	SMC1	Structural maintena	38.80	0.00
TRINITY_sp|Q54G(	slmo	Protein slowmo homo	38.80	0.00
TRINITY_sp|Q6AX(	Mtm1	Myotubularin OS=Rat	38.80	0.00
TRINITY_sp|Q6GLI	desi1	Desumoylating isope	38.80	0.00
TRINITY_sp|P361I	PRY2	Protein PRY2 OS=Sac	38.80	0.00
TRINITY_sp|Q7G8Y	Os01g03(	Probable chromatin-	38.80	0.00
TRINITY_sp|Q9ZNI	SAP3	Zinc finger A20 and	38.80	0.00
TRINITY_sp|A2XW(	BRE1A	E3 ubiquitin-protei	38.80	0.00
TRINITY_sp|Q8S94	DRP3A	Dynamin-related pro	38.80	0.00
TRINITY_sp|Q9FJI	KPNB1	Importin subunit be	38.80	0.00
TRINITY_sp|Q9M9(	SCPL50	Serine carboxypepti	38.80	0.00
TRINITY_sp|A2VEA	ARIH1	E3 ubiquitin-protei	38.80	0.00
TRINITY_sp|Q7XGI	Os10g014	Auxin transporter-l	38.80	0.00
TRINITY_sp|O268(	MTH_771	UPF0047 protein MTH	38.80	0.00
TRINITY_sp|Q9ZP\	At2g1822	Nucleolar complex p	38.80	0.00
TRINITY_sp|P025E	-	Actin, cytoplasmic	38.80	0.00
TRINITY_sp|Q5Z6	CML29	Probable calcium-bi	38.80	0.00
TRINITY_sp|Q032E	TOM1	E3 ubiquitin-protei	38.70	0.00
TRINITY_sp|Q54DA	rab4	Ras-related protein	38.70	0.00
TRINITY_sp|Q86VS	IQCH	IQ domain-containin	38.70	0.00
TRINITY_sp|Q631(	Dnah1	Dynein heavy chain	38.70	0.00
TRINITY_sp|Q0P45	kti12	Protein KTI12 homol	38.70	0.00
TRINITY_sp|P5315	GUP1	Glycerol uptake pro	38.70	0.00
TRINITY_sp|Q8MMI	paxB	Paxillin-B OS=Dicty	38.70	0.00
TRINITY_sp|Q5DU(	Cep164	Centrosomal protein	38.70	0.00
TRINITY_sp|O945E	trp3	Probable anthranila	38.70	0.00
TRINITY_sp|Q5XXI	MSH2	DNA mismatch repair	38.70	0.00
TRINITY_sp|Q54YS	DDB_G02	Probable serine/thr	38.70	0.00
TRINITY_sp|P4074	yuxG	Uncharacterized oxi	38.70	0.00
TRINITY_sp|Q54CJ	eloA	Elongation of fatty	38.70	0.00
TRINITY_sp|P391(	YCF1	Metal resistance pr	38.70	0.00
TRINITY_sp|Q0954	paa-1	Probable serine/thr	38.70	0.00
TRINITY_sp|F4KDA	MIP	Probable mitochondr	38.70	0.00
TRINITY_sp|Q6EWI	fyna	Tyrosine-protein ki	38.70	0.00
TRINITY_sp|F4I1I	ACC2	Acetyl-CoA carboxyl	38.70	0.00
TRINITY_sp|Q5U3\	slc25a43	Solute carrier fami	38.70	0.00
TRINITY_sp|Q653\	Os06g06	Probable alpha-gluc	38.70	0.00
TRINITY_sp|Q9ZVI	At1g033	C2 and GRAM domain-	38.70	0.00
TRINITY_sp|O1482	RASGRF2	Ras-specific guanin	38.70	0.00
TRINITY_sp|Q588\	TEB	Helicase and polyme	38.70	0.00
TRINITY_sp|Q1222	DIP2	U3 small nucleolar	38.70	0.00
TRINITY_sp|Q9BUI	DERL1	Derlin-1 OS=Homo sa	38.70	0.00
TRINITY_sp|Q99L4	St13	Hsc70-interacting p	38.70	0.00
TRINITY_sp|Q0CEI	bglL	Probable beta-gluco	38.70	0.00
TRINITY_sp|Q8LKS	LACS7	Long chain acyl-CoA	38.70	0.00
TRINITY_sp|Q068I	STAUR_42	Putative epoxide hy	38.70	0.00
TRINITY_sp|B9FJ(	SPP2	Signal peptide pept	38.70	0.00
TRINITY_sp|D2XNI	FLOT6	Flotillin-like prot	38.70	0.00
TRINITY_sp|P382(	NCL1	Multisite-specific	38.70	0.00
TRINITY_sp|P3283	GSP2	GTP-binding nuclear	38.70	0.00
TRINITY_sp|A1DGV	bna5-1	Kynureninase 1 OS=N	38.70	0.00
TRINITY_sp|Q8CH)	Mbtps2	Membrane-bound tran	38.70	0.00
TRINITY_sp|Q17Q2	TFDP1	Transcription facto	38.70	0.00
TRINITY_sp|O2814	AF_2138	Phosphoserine phosp	38.70	0.00

TRINITY_sp|Q9LP!	At1g505!	Pirin-like protein	38.70	0.00
TRINITY_sp|Q389!	KIN10	SNF1-related protei	38.70	0.00
TRINITY_sp|Q6DK8	kcnrg	Putative potassium	38.70	0.00
TRINITY_sp|Q9SAI	AKR2A	Ankyrin repeat doma	38.70	0.00
TRINITY_sp|B8ARV	OsI_180	Transportin-1 OS=Or	38.70	0.00
TRINITY_sp|O627(	CTNNBL1	Beta-catenin-like p	38.70	0.00
TRINITY_sp|Q9C6I	GAMMACA2	Gamma carbonic anhy	38.70	0.00
TRINITY_sp|Q9SH(	TIC21	Protein TIC 21, chl	38.70	0.00
TRINITY_sp|Q6NW\	Ift122	Intraflagellar tran	38.70	0.00
TRINITY_sp|P189(	ATP1A1	Sodium/potassium-tr	38.70	0.00
TRINITY_sp|O042(	TIF3F1	Eukaryotic translat	38.70	0.00
TRINITY_sp|Q9M0	NSF	Vesicle-fusing ATPa	38.70	0.00
TRINITY_sp|Q9STI	At3g483	Probable Ufm1-speci	38.70	0.00
TRINITY_sp|P459	USP5	Ubiquitin carboxyl-	38.70	0.00
TRINITY_sp|P0CB4	Ylpm1	YLP motif-containin	38.70	0.00
TRINITY_sp|Q5BPI	At4g066	Protein EI24 homolo	38.70	0.00
TRINITY_sp|Q8W3I	MFDR	NADPH:adrenodoxin o	38.70	0.00
TRINITY_sp|Q8J1I	TUBB2	Tubulin beta-2 chai	38.70	0.00
TRINITY_sp|Q54L5	DDB_G02	G8 domain-containin	38.70	0.00
TRINITY_sp|Q8LQ3	Os01g08	Putative ataxin-3 h	38.70	0.00
TRINITY_sp|Q8IYI	ZZZ3	ZZ-type zinc finger	38.70	0.00
TRINITY_sp|P2864	CLKR27	3-oxoacyl-[acyl-car	38.70	0.00
TRINITY_sp|Q7XT!	AGO2	Protein argonaute 2	38.70	0.00
TRINITY_sp|P3702	hrpB	ATP-dependent RNA h	38.70	0.00
TRINITY_sp|Q9SK2	At2g320	Folate-biopterin tr	38.70	0.00
TRINITY_sp|Q552N	DDB_G02	Putative ZDHHC-type	38.70	0.00
TRINITY_sp|P059!	-	Papaya proteinase 4	38.70	0.00
TRINITY_sp|O042I	At2g400	Putative ABC1 prote	38.70	0.00
TRINITY_sp|Q869!	dokA	Hybrid signal trans	38.70	0.00
TRINITY_sp|Q6QA3	hsd17b1	Very-long-chain 3-o	38.70	0.00
TRINITY_sp|Q395	ODA2	Dynein gamma chain,	38.70	0.00
TRINITY_sp|Q9H2	VPS11	Vacuolar protein so	38.70	0.00
TRINITY_sp|Q8GYN	RIN4	RPM1-interacting pr	38.70	0.00
TRINITY_sp|O598(	SPCC550.	Putative ATP-depend	38.60	0.00
TRINITY_sp|Q5572	slr0642	Folate-biopterin tr	38.60	0.00
TRINITY_sp|G5EC	csk-1	Tyrosine-protein ki	38.60	0.00
TRINITY_sp|Q241	Slh	Protein sly1 homolo	38.60	0.00
TRINITY_sp|O951!	RCBTB2	RCC1 and BTB domain	38.60	0.00
TRINITY_sp|Q55FI	zswim7	Zinc finger SWIM do	38.60	0.00
TRINITY_sp|Q56WI	SCPL48	Serine carboxypepti	38.60	0.00
TRINITY_sp|O803!	MKK3	Mitogen-activated p	38.60	0.00
TRINITY_sp|P497I	Hcls1	Hematopoietic linea	38.60	0.00
TRINITY_sp|Q96VI	HBN1	Putative nitroreduc	38.60	0.00
TRINITY_sp|Q9LXI	RLF	Cytochrome b5 domai	38.60	0.00
TRINITY_sp|Q8WYI	SSH1	Protein phosphatase	38.60	0.00
TRINITY_sp|Q603!	MJ0044	Isopentenyl phospha	38.60	0.00
TRINITY_sp|O942!	SPBC887.	Probable phospholip	38.60	0.00
TRINITY_sp|Q28BI	pitrm1	Presequence proteas	38.60	0.00
TRINITY_sp|O009(	-	Lysosomal acid alph	38.60	0.00
TRINITY_sp|C0SPA	adhA	Probable formaldehy	38.60	0.00
TRINITY_sp|Q9LK!	ALA8	Probable phospholip	38.60	0.00
TRINITY_sp|Q641I	fam135b	Protein FAM135B OS=	38.60	0.00
TRINITY_sp|Q94E	SPP1	Probable sucrose-ph	38.60	0.00
TRINITY_sp|Q55C!	vps16	Vacuolar protein so	38.60	0.00
TRINITY_sp|Q9FMI	VAMP714	Vesicle-associated	38.60	0.00
TRINITY_sp|A7YW(	GGH	Gamma-glutamyl hydr	38.60	0.00

TRINITY_sp|Q9WY(	TM_0225	Putative 1-aminocyc	38.60	0.00
TRINITY_sp|Q4PJ\	CYP51A1	Lanosterol 14-alpha	38.60	0.00
TRINITY_sp|P109	-	Retrovirus-related	38.60	0.00
TRINITY_sp|Q54E	grlE	Metabotropic glutam	38.60	0.00
TRINITY_sp|O149	BTAF1	TATA-binding protei	38.60	0.00
TRINITY_sp|Q8T2	DDB_G02	Putative ZDHHC-type	38.60	0.00
TRINITY_sp|A6TU	Amet_36	8-amino-7-oxononano	38.60	0.00
TRINITY_sp|O429	SPBC119.	Uncharacterized pro	38.60	0.00
TRINITY_sp|P187	RCC1	Regulator of chromo	38.60	0.00
TRINITY_sp|Q2XW	svop	Synaptic vesicle 2-	38.60	0.00
TRINITY_sp|Q2TA	KIF19	Kinesin-like protei	38.60	0.00
TRINITY_sp|O312	alkB	Alkane 1-monooxygen	38.60	0.00
TRINITY_sp|Q9FP	UBP23	Ubiquitin carboxyl-	38.60	0.00
TRINITY_sp|Q17R	DHX38	Pre-mRNA-splicing f	38.60	0.00
TRINITY_sp|O650	CYSEP	Vignain OS=Ricinus	38.60	0.00
TRINITY_sp|Q84K	CCD	Carotenoid 9,10(9',	38.60	0.00
TRINITY_sp|Q95Y	pefB	Penta-EF hand domai	38.60	0.00
TRINITY_sp|O646	At2g189	Thioredoxin domain-	38.60	0.00
TRINITY_sp|Q5U2	Arhgap2	Rho GTPase-activati	38.60	0.00
TRINITY_sp|Q6AX	Hexb	Beta-hexosaminidase	38.60	0.00
TRINITY_sp|Q55G	DDB_G02	Probable serine/thr	38.60	0.00
TRINITY_sp|Q9D2	Kif12	Kinesin-like protei	38.60	0.00
TRINITY_sp|O437	CYTH3	Cytohesin-3 OS=Homo	38.60	0.00
TRINITY_sp|Q84S	Os07g05	Zinc finger CCCH do	38.60	0.00
TRINITY_sp|Q3EA	At3g622	Probable protein ph	38.60	0.00
TRINITY_sp|Q9S7	At3g090	BTB/POZ domain-cont	38.60	0.00
TRINITY_sp|Q9LZ	STN8	Serine/threonine-pr	38.60	0.00
TRINITY_sp|Q54E	gacEE	Rho GTPase-activati	38.60	0.00
TRINITY_sp|Q9LW	HRD1A	ERAD-associated E3	38.60	0.00
TRINITY_sp|Q9SF	SYP71	Syntaxin-71 OS=Arab	38.60	0.00
TRINITY_sp|Q8R1	Bud13	BUD13 homolog OS=Mu	38.60	0.00
TRINITY_sp|Q9ZW	PRN2	Pirin-like protein	38.60	0.00
TRINITY_sp|Q8R3	Slf1	SMC5-SMC6 complex l	38.60	0.00
TRINITY_sp|Q54K	eps15	Epidermal growth fa	38.60	0.00
TRINITY_sp|Q2NU	cdd	Cytidine deaminase	38.60	0.00
TRINITY_sp|Q9SU	SYP43	Syntaxin-43 OS=Arab	38.60	0.00
TRINITY_sp|Q8LP	R1	Alpha-glucan water	38.60	0.00
TRINITY_sp|O159	CYCE	G1/S-specific cycli	38.60	0.00
TRINITY_sp|Q17Q	RAB30	Ras-related protein	38.50	0.00
TRINITY_sp|P599	DNAJB13	DnaJ homolog subfam	38.50	0.00
TRINITY_sp|Q552	pkgA	Probable serine/thr	38.50	0.00
TRINITY_sp|P341	ptpB	Tyrosine-protein ph	38.50	0.00
TRINITY_sp|Q0VA	wdr70	WD repeat-containin	38.50	0.00
TRINITY_sp|P623	CPK1	Calcium-dependent p	38.50	0.00
TRINITY_sp|A8WH	narfl	Cytosolic Fe-S clus	38.50	0.00
TRINITY_sp|P470	PRY1	Protein PRY1 OS=Sac	38.50	0.00
TRINITY_sp|Q0VC	PQLC1	PQ-loop repeat-cont	38.50	0.00
TRINITY_sp|O955	SFT2D2	Vesicle transport p	38.50	0.00
TRINITY_sp|Q9CQ	Lztr1	Leucine-zipper-like	38.50	0.00
TRINITY_sp|O823	At2g257	Probably inactive l	38.50	0.00
TRINITY_sp|Q5M8	Hsd17b1	17-beta-hydroxyster	38.50	0.00
TRINITY_sp|O582	gck	Glycerate 2-kinase	38.50	0.00
TRINITY_sp|D0PV	laf-1	ATP-dependent RNA h	38.50	0.00
TRINITY_sp|P217	EPHA1	Ephrin type-A recep	38.50	0.00
TRINITY_sp|P109	ugpQ	Glycerophosphoryl d	38.50	0.00
TRINITY_sp|Q91W	Txndc5	Thioredoxin domain-	38.50	0.00

TRINITY_sp|Q505(	fdhC	Probable formate tr	38.50	0.00
TRINITY_sp|Q9FG3	SNX1	Sorting nexin 1 OS=	38.50	0.00
TRINITY_sp|Q70GH	sed3	Tripeptidyl-peptida	38.50	0.00
TRINITY_sp|Q3S4A	AHK5	Histidine kinase 5	38.50	0.00
TRINITY_sp|Q86H3	polr1a	DNA-directed RNA po	38.50	0.00
TRINITY_sp|Q1968	F21D5.5	Uncharacterized pro	38.50	0.00
TRINITY_sp|Q54Y1	mecr	Trans-2-enoyl-CoA r	38.50	0.00
TRINITY_sp|A5DW1	PAB1	Polyadenylate-bindi	38.50	0.00
TRINITY_sp|Q4G0X	KCTD21	BTB/POZ domain-cont	38.50	0.00
TRINITY_sp|Q810A	Ddx42	ATP-dependent RNA h	38.50	0.00
TRINITY_sp|Q5HZN	sirt5-a	NAD-dependent prote	38.50	0.00
TRINITY_sp|Q9SLX	Os05g015	Importin subunit al	38.50	0.00
TRINITY_sp|P2577	Os04g065	Oryzain alpha chain	38.50	0.00
TRINITY_sp|Q557J	abpE-1	Drebrin-like protei	38.50	0.00
TRINITY_sp|Q59YI	ESF2	Pre-rRNA-processing	38.50	0.00
TRINITY_sp|Q9FFN	DGK2	Diacylglycerol kina	38.50	0.00
TRINITY_sp|Q3SZY	MED6	Mediator of RNA pol	38.50	0.00
TRINITY_sp|Q54LI	DDB_G028	Lipid droplet-assoc	38.50	0.00
TRINITY_sp|Q567X	ch25hl1.	Cholesterol 25-hydr	38.50	0.00
TRINITY_sp|Q1LVB	sf3b3	Splicing factor 3B	38.50	0.00
TRINITY_sp|Q9M3(	LCB2b	Long chain base bio	38.50	0.00
TRINITY_sp|Q7XI4	URA6	UMP-CMP kinase 3 OS	38.50	0.00
TRINITY_sp|Q3SWY	NOSIP	Nitric oxide syntha	38.50	0.00
TRINITY_sp|Q1ZXH	gxcDD	Guanine exchange fa	38.50	0.00
TRINITY_sp|Q8CHN	Sgpl1	Sphingosine-1-phosp	38.50	0.00
TRINITY_sp|Q8L7H	NUP62	Nuclear pore comple	38.50	0.00
TRINITY_sp|Q7PQV	AGAP0023	ADP,ATP carrier pro	38.50	0.00
TRINITY_sp|Q96AY	TTC28	Tetratricopeptide r	38.50	0.00
TRINITY_sp|Q54TN	drkD	Probable serine/thr	38.50	0.00
TRINITY_sp|P3311	spaR	Transcriptional reg	38.50	0.00
TRINITY_sp|Q9BZE	PUS3	tRNA pseudouridine(	38.50	0.00
TRINITY_sp|Q9XZV	-	Guanine nucleotide-	38.50	0.00
TRINITY_sp|O6144	p38b	Mitogen-activated p	38.50	0.00
TRINITY_sp|Q6318	Eif2ak2	Interferon-induced,	38.50	0.00
TRINITY_sp|Q5UQ(	MIMI_R81	Putative serine/thr	38.50	0.00
TRINITY_sp|Q1ZXH	gxcDD	Guanine exchange fa	38.50	0.00
TRINITY_sp|Q6TC4	SRY	Sex-determining reg	38.50	0.00
TRINITY_sp|Q6Q47	Atp2b4	Plasma membrane cal	38.50	0.00
TRINITY_sp|Q9ERY	ORF11	Probable ergosterol	38.50	0.00
TRINITY_sp|Q827H	soxA	Monomeric sarcosine	38.50	0.00
TRINITY_sp|Q7Y0B	SAPK8	Serine/threonine-pr	38.50	0.00
TRINITY_sp|Q9CQS	Riok2	Serine/threonine-pr	38.50	0.00
TRINITY_sp|A8IC9	CFAP46	Cilia- and flagella	38.50	0.00
TRINITY_sp|Q95PI	dhkC	Hybrid signal trans	38.50	0.00
TRINITY_sp|Q9499	MOS4	Pre-mRNA-splicing f	38.50	0.00
TRINITY_sp|Q9LZX	BIG2	Brefeldin A-inhibit	38.50	0.00
TRINITY_sp|Q6CX9	SET5	Potential protein l	38.50	0.00
TRINITY_sp|Q8LEM	CBL3	Calcineurin B-like	38.40	0.00
TRINITY_sp|Q3M8A	sasA	Adaptive-response s	38.40	0.00
TRINITY_sp|Q8ML2	pdeD	cGMP-dependent 3',5	38.40	0.00
TRINITY_sp|Q5FWY	mmcm6	Maternal DNA replic	38.40	0.00
TRINITY_sp|Q5RC5	GLRX2	Glutaredoxin-2, mit	38.40	0.00
TRINITY_sp|Q54YB	snfA	5'-AMP-activated se	38.40	0.00
TRINITY_sp|Q9D08	Alg14	UDP-N-acetylglucosa	38.40	0.00
TRINITY_sp|Q54YH	dhkB	Hybrid signal trans	38.40	0.00
TRINITY_sp|Q9JIM	Ercc6l2	DNA excision repair	38.40	0.00

TRINITY_sp|Q84LM	VPE1	Vacuolar-processing	38.40	0.00
TRINITY_sp|P5492	Napa	Alpha-soluble NSF a	38.40	0.00
TRINITY_sp|Q2M38	KIAA1033	WASH complex subuni	38.40	0.00
TRINITY_sp|P1423	Fgr	Tyrosine-protein ki	38.40	0.00
TRINITY_sp|Q9ZRF	CAD	Probable mannitol d	38.40	0.00
TRINITY_sp|Q54VV	DDB_G028	Probable serine/thr	38.40	0.00
TRINITY_sp|P3413	ptpB	Tyrosine-protein ph	38.40	0.00
TRINITY_sp|O1518	CETN3	Centrin-3 OS=Homo s	38.40	0.00
TRINITY_sp|Q6CGI	YALI0A18	Acyl-protein thioes	38.40	0.00
TRINITY_sp|Q8BUV	Gpr107	Protein GPR107 OS=M	38.40	0.00
TRINITY_sp|Q0QLF	mii	3-methylitaconate i	38.40	0.00
TRINITY_sp|O8035	FPG1	Formamidopyrimidine	38.40	0.00
TRINITY_sp|Q54MY	mkcA	Probable serine/thr	38.40	0.00
TRINITY_sp|Q0U6I	LAP1	Leucine aminopeptid	38.40	0.00
TRINITY_sp|Q7X6Y	MMD1	PHD finger protein	38.40	0.00
TRINITY_sp|Q8LPS	LACS6	Long chain acyl-CoA	38.40	0.00
TRINITY_sp|P0A2I	ucpA	Oxidoreductase UcpA	38.40	0.00
TRINITY_sp|Q66GI	PRORP1	Proteinaceous RNase	38.40	0.00
TRINITY_sp|Q9SJU	GC1	Epimerase family pr	38.40	0.00
TRINITY_sp|Q757I	HUL4	Probable E3 ubiquit	38.40	0.00
TRINITY_sp|P0CO7	GEM1	Mitochondrial Rho G	38.40	0.00
TRINITY_sp|P4480	HI_0658	Uncharacterized ABC	38.40	0.00
TRINITY_sp|P5779	ALA12	Probable phospholip	38.40	0.00
TRINITY_sp|O6050	CDC40	Pre-mRNA-processing	38.40	0.00
TRINITY_sp|P1890	ATP1A1	Sodium/potassium-tr	38.40	0.00
TRINITY_sp|B8AJ0	BASS4	Probable sodium/met	38.40	0.00
TRINITY_sp|Q9FL0	MRS2-2	Magnesium transport	38.40	0.00
TRINITY_sp|Q9VR9	HERC2	Probable E3 ubiquit	38.40	0.00
TRINITY_sp|Q6IA6	NADSYN1	Glutamine-dependent	38.40	0.00
TRINITY_sp|Q9P2H	IFT80	Intraflagellar tran	38.40	0.00
TRINITY_sp|Q9HDY	SPAPB1A1	Putative ATP-depend	38.40	0.00
TRINITY_sp|Q1ZXH	gxcDD	Guanine exchange fa	38.40	0.00
TRINITY_sp|Q08C0	rnf44	RING finger protein	38.40	0.00
TRINITY_sp|Q9ZU5	BAT1	Amino-acid permease	38.40	0.00
TRINITY_sp|Q54VV	DDB_G028	Probable serine/thr	38.40	0.00
TRINITY_sp|Q9FF8	VPS36	Vacuolar protein so	38.40	0.00
TRINITY_sp|B5BT1	BTAF1	TATA-binding protei	38.40	0.00
TRINITY_sp|Q2662	KAP115	Kinesin-associated	38.40	0.00
TRINITY_sp|Q9US0	mug71	Diphthine--ammonia	38.40	0.00
TRINITY_sp|Q4364	-	NADH dehydrogenase	38.40	0.00
TRINITY_sp|Q5SLM	TTHA0270	Thermostable carbox	38.40	0.00
TRINITY_sp|Q9SQT	EMB3004	Bifunctional 3-dehy	38.40	0.00
TRINITY_sp|Q9SYM	VTE6	Protein VTE6, chlor	38.40	0.00
TRINITY_sp|Q0IZ0	PHT4;5	Probable anion tran	38.40	0.00
TRINITY_sp|Q55E4	dhkE	Hybrid signal trans	38.30	0.00
TRINITY_sp|Q54YH	dhkB	Hybrid signal trans	38.30	0.00
TRINITY_sp|D9HP2	CNR11	Cell number regulat	38.30	0.00
TRINITY_sp|Q5BII	DUSP18	Dual specificity pr	38.30	0.00
TRINITY_sp|Q7T01	lamtor3-	Ragulator complex p	38.30	0.00
TRINITY_sp|P4471	HI_0454	Uncharacterized met	38.30	0.00
TRINITY_sp|Q7XA8	FOLT1	Folate transporter	38.30	0.00
TRINITY_sp|Q54V0	abcC15	ABC transporter C f	38.30	0.00
TRINITY_sp|Q0DA5	Os06g067	Zinc finger CCCH do	38.30	0.00
TRINITY_sp|Q54H4	drkB	Probable serine/thr	38.30	0.00
TRINITY_sp|F4JTI	STY46	Serine/threonine-pr	38.30	0.00
TRINITY_sp|Q1824	rap-1	Ras-related protein	38.30	0.00

TRINITY_sp|Q4R68	AMZ2	Archaemetzincin-2 O	38.30	0.00
TRINITY_sp|Q8TC1	RDH11	Retinol dehydrogena	38.30	0.00
TRINITY_sp|P2667	Sos	Protein son of seve	38.30	0.00
TRINITY_sp|E0X90	todS	Sensor histidine ki	38.30	0.00
TRINITY_sp|E1BFB	MASTL	Serine/threonine-pr	38.30	0.00
TRINITY_sp|A1A40	PRORSD1	Prolyl-tRNA synthet	38.30	0.00
TRINITY_sp|Q5RJ1	Ftsj3	pre-rRNA processing	38.30	0.00
TRINITY_sp|P3245	GBP2	Guanylate-binding p	38.30	0.00
TRINITY_sp|Q8L7B	SCPL20	Serine carboxypepti	38.30	0.00
TRINITY_sp|P3886	NMD3	60S ribosomal expor	38.30	0.00
TRINITY_sp|Q8BQ9	Adipor2	Adiponectin recepto	38.30	0.00
TRINITY_sp|A0LEA	dapL	LL-diaminopimelate	38.30	0.00
TRINITY_sp|O0761	yhfO	Uncharacterized N-a	38.30	0.00
TRINITY_sp|Q9LD4	BI-1	Bax inhibitor 1 OS=	38.30	0.00
TRINITY_sp|O4878	SPX2	SPX domain-containi	38.30	0.00
TRINITY_sp|Q9CAN	LPLAT2	Lysophospholipid ac	38.30	0.00
TRINITY_sp|Q9JLV	Pnkp	Bifunctional polynu	38.30	0.00
TRINITY_sp|Q54F0	gacJJ	Rho GTPase-activati	38.30	0.00
TRINITY_sp|Q86HB	cf45-1	Counting factor 45-	38.30	0.00
TRINITY_sp|Q55G8	abkC	Probable serine/thr	38.30	0.00
TRINITY_sp|Q9WTB	Cdyl	Chromodomain Y-like	38.30	0.00
TRINITY_sp|Q54WU	DDB_G027	Sestrin homolog OS=	38.30	0.00
TRINITY_sp|Q9Y59	GTF3C3	General transcripti	38.30	0.00
TRINITY_sp|Q54VW	DDB_G028	Serine carboxypepti	38.30	0.00
TRINITY_sp|Q96RB	CAMKK2	Calcium/calmodulin-	38.30	0.00
TRINITY_sp|Q5631	cheY	Chemotaxis protein	38.30	0.00
TRINITY_sp|Q5002	PAT15	Probable protein S-	38.30	0.00
TRINITY_sp|Q9SA9	At1g1178	Alpha-ketoglutarate	38.30	0.00
TRINITY_sp|Q8NDI	AGBL5	Cytosolic carboxype	38.30	0.00
TRINITY_sp|Q54GB	ctdspl2	CTD small phosphata	38.30	0.00
TRINITY_sp|F4IL5	KIN14I	Kinesin-like protei	38.30	0.00
TRINITY_sp|Q54GX	pex1	Peroxisome biogenes	38.30	0.00
TRINITY_sp|O9460	ptr3	Ubiquitin-activatin	38.30	0.00
TRINITY_sp|Q3UM1	Lsg1	Large subunit GTPas	38.30	0.00
TRINITY_sp|O0442	FCA	Flowering time cont	38.30	0.00
TRINITY_sp|Q9W1B	CG5532	Transmembrane prote	38.30	0.00
TRINITY_sp|Q6DH0	slc25a40	Solute carrier fami	38.30	0.00
TRINITY_sp|Q4N4M	TP02_060	Thioredoxin domain-	38.30	0.00
TRINITY_sp|P0DM4	Fer1l5	Fer-1-like protein	38.30	0.00
TRINITY_sp|O5505	Trdmt1	tRNA (cytosine(38)-	38.30	0.00
TRINITY_sp|Q5CC9	petC-1	Cytochrome b6-f com	38.30	0.00
TRINITY_sp|Q6A93	pgk	Phosphoglycerate ki	38.30	0.00
TRINITY_sp|Q9SJB	AGO5	Protein argonaute 5	38.30	0.00
TRINITY_sp|P1295	PEPD	Xaa-Pro dipeptidase	38.30	0.00
TRINITY_sp|Q16N9	Art7	Protein arginine N-	38.30	0.00
TRINITY_sp|Q8T7B	alxA	ALG-2 interacting p	38.30	0.00
TRINITY_sp|Q8BV7	Trank1	TPR and ankyrin rep	38.30	0.00
TRINITY_sp|O8049	At1g0916	Probable protein ph	38.30	0.00
TRINITY_sp|Q6P42	Lcmt1	Leucine carboxyl me	38.30	0.00
TRINITY_sp|P1890	ATP1A1	Sodium/potassium-tr	38.30	0.00
TRINITY_sp|Q8LL0	CPL3	RNA polymerase II C	38.30	0.00
TRINITY_sp|Q9961	DNAJC7	DnaJ homolog subfam	38.30	0.00
TRINITY_sp|Q88RB	davT	5-aminovalerate ami	38.30	0.00
TRINITY_sp|Q501V	pskh1	Serine/threonine-pr	38.30	0.00
TRINITY_sp|Q3E90	CPK34	Calcium-dependent p	38.30	0.00
TRINITY_sp|Q7XAB	RSH2	Probable GTP diphos	38.30	0.00

TRINITY_sp|Q993(	HRP1	Nuclear polyadenyla	38.30	0.00
TRINITY_sp|Q0WW	STR11	Rhodanese-like doma	38.30	0.00
TRINITY_sp|Q8I7I	pol	Retrovirus-related	38.30	0.00
TRINITY_sp|Q3T0	TPPP2	Tubulin polymerizat	38.30	0.00
TRINITY_sp|A2QCI	An02g03	Very-long-chain 3-o	38.30	0.00
TRINITY_sp|Q7Z6	HUWE1	E3 ubiquitin-protei	38.20	0.00
TRINITY_sp|P364(	rab2A	Ras-related protein	38.20	0.00
TRINITY_sp|O644	RMA1	E3 ubiquitin-protei	38.20	0.00
TRINITY_sp|Q903	FGFR4	Fibroblast growth f	38.20	0.00
TRINITY_sp|Q8CF(	Aqr	Intron-binding prot	38.20	0.00
TRINITY_sp|O018(	rab-11.	Ras-related protein	38.20	0.00
TRINITY_sp|Q9LT	RDL2	Probable cysteine p	38.20	0.00
TRINITY_sp|Q2HVI	MtrDRAF	Putative N6-adenosi	38.20	0.00
TRINITY_sp|Q916	epha4-b	Ephrin type-A recep	38.20	0.00
TRINITY_sp|Q0VC(	PQLC1	PQ-loop repeat-cont	38.20	0.00
TRINITY_sp|Q6NX(	ESRP1	Epithelial splicing	38.20	0.00
TRINITY_sp|Q54QI	commd3	COMM domain-contain	38.20	0.00
TRINITY_sp|P981	XIAP	E3 ubiquitin-protei	38.20	0.00
TRINITY_sp|P551	-	Hydrolase in pqqF 5	38.20	0.00
TRINITY_sp|P427(	GSTF10	Glutathione S-trans	38.20	0.00
TRINITY_sp|O954	PSMG1	Proteasome assembly	38.20	0.00
TRINITY_sp|P341(	SODB	Superoxide dismutas	38.20	0.00
TRINITY_sp|P283	acu-9	Malate synthase, gl	38.20	0.00
TRINITY_sp|P432	COP1	E3 ubiquitin-protei	38.20	0.00
TRINITY_sp|Q9M8	PMI1	Mannose-6-phosphate	38.20	0.00
TRINITY_sp|Q54Q	phr2aB	Serine/threonine-pr	38.20	0.00
TRINITY_sp|Q6X4	CIPK31	CBL-interacting pro	38.20	0.00
TRINITY_sp|Q94AI	CUL1	Cullin-1 OS=Arabido	38.20	0.00
TRINITY_sp|A8IW	CHLREDR	Mitochondrial cardi	38.20	0.00
TRINITY_sp|Q5VX	LIPK	Lipase member K OS=	38.20	0.00
TRINITY_sp|Q55G	pakC	Serine/threonine-pr	38.20	0.00
TRINITY_sp|Q55E	mcfE	Mitochondrial subst	38.20	0.00
TRINITY_sp|Q6IV	ssh	Protein phosphatase	38.20	0.00
TRINITY_sp|Q4U2I	Herc2	E3 ubiquitin-protei	38.20	0.00
TRINITY_sp|A8LVI	kmo	Kynurenine 3-monoox	38.20	0.00
TRINITY_sp|Q9LX	CSY2	Citrate synthase 2,	38.20	0.00
TRINITY_sp|Q9LE	IRE	Probable serine/thr	38.20	0.00
TRINITY_sp|P236	ATP2B4	Plasma membrane cal	38.20	0.00
TRINITY_sp|Q9NV(	TYW1	S-adenosyl-L-methio	38.20	0.00
TRINITY_sp|A2YR	ARP4	Actin-related prote	38.20	0.00
TRINITY_sp|Q6DF	coq6	Ubiquinone biosynth	38.20	0.00
TRINITY_sp|Q8BM	Lipk	Lipase member K OS=	38.20	0.00
TRINITY_sp|Q8RVI	DEK1	Calpain-type cystei	38.20	0.00
TRINITY_sp|Q9S7I	MYB98	Transcription facto	38.20	0.00
TRINITY_sp|Q9LDI	BETAA-AI	Beta-adaptin-like p	38.20	0.00
TRINITY_sp|Q9M9(	CSTF64	Cleavage stimulatin	38.20	0.00
TRINITY_sp|Q6VN	Ranbp10	Ran-binding protein	38.20	0.00
TRINITY_sp|Q9SK(	RAP2-7	Ethylene-responsive	38.20	0.00
TRINITY_sp|P143	Mp20	Muscle-specific pro	38.20	0.00
TRINITY_sp|P403	ptc1	Protein phosphatase	38.20	0.00
TRINITY_sp|Q3SY	TNPO1	Transportin-1 OS=Bo	38.20	0.00
TRINITY_sp|Q394	VH-PTP1	Dual specificity pr	38.20	0.00
TRINITY_sp|Q9XYI	fcpA	Probable C-terminal	38.20	0.00
TRINITY_sp|Q9H8(	VCPKMT	Protein-lysine meth	38.20	0.00
TRINITY_sp|P288	Rasgrf1	Ras-specific guanin	38.20	0.00
TRINITY_sp|P623	CPK1	Calcium-dependent p	38.20	0.00

TRINITY_sp|Q8R5I	Vps39	Vam6/Vps39-like pro	38.20	0.00
TRINITY_sp|Q9C63	LHCA5	Photosystem I chlor	38.20	0.00
TRINITY_sp|Q1033	SPBC582.	Uncharacterized ATP	38.20	0.00
TRINITY_sp|Q8RU9	ATG9	Autophagy-related p	38.20	0.00
TRINITY_sp|Q9CX1	Kdm8	Lysine-specific dem	38.20	0.00
TRINITY_sp|Q8IU8	CAMK1D	Calcium/calmodulin-	38.20	0.00
TRINITY_sp|P4643	GST1	Glutathione S-trans	38.20	0.00
TRINITY_sp|Q58D5	GLB1	Beta-galactosidase	38.20	0.00
TRINITY_sp|Q8CG0	Abcc1	Multidrug resistanc	38.20	0.00
TRINITY_sp|O7478	cho2	Phosphatidylethanol	38.20	0.00
TRINITY_sp|Q54S7	vps15	Probable serine/thr	38.20	0.00
TRINITY_sp|P5435	DNApol-d	DNA polymerase delt	38.20	0.00
TRINITY_sp|Q9KLK	luxQ	Autoinducer 2 senso	38.20	0.00
TRINITY_sp|Q8INE	Gyc88E	Soluble guanylate c	38.20	0.00
TRINITY_sp|Q5558	gabD	Probable succinate-	38.20	0.00
TRINITY_sp|Q8VEI	Mtmr14	Myotubularin-relate	38.20	0.00
TRINITY_sp|Q9P2D	DNAH1	Dynein heavy chain	38.20	0.00
TRINITY_sp|Q9FVJ	AGD12	ADP-ribosylation fa	38.20	0.00
TRINITY_sp|Q8RXX	ATL6	E3 ubiquitin-protei	38.20	0.00
TRINITY_sp|Q0VFV	kctd7	BTB/POZ domain-cont	38.20	0.00
TRINITY_sp|P9820	ALA1	Phospholipid-transp	38.20	0.00
TRINITY_sp|Q6ZKV	BIO3-BIO	Bifunctional dethio	38.20	0.00
TRINITY_sp|Q86XK	FBXO11	F-box only protein	38.20	0.00
TRINITY_sp|Q6K66	Os02g079	Leucine aminopeptid	38.20	0.00
TRINITY_sp|Q4R67	IQUB	IQ and ubiquitin-li	38.20	0.00
TRINITY_sp|Q1RMQ	SYS1	Protein SYS1 homolo	38.20	0.00
TRINITY_sp|Q59TN	TRM5	tRNA (guanine(37)-N	38.10	0.00
TRINITY_sp|P5710	-	Glutathione S-trans	38.10	0.00
TRINITY_sp|P3557	AGL	Glycogen debranchin	38.10	0.00
TRINITY_sp|Q4WG9	ddi1	DNA damage-inducibl	38.10	0.00
TRINITY_sp|P0406	GBA	Glucosylceramidase	38.10	0.00
TRINITY_sp|Q6I62	PSS2	CDP-diacylglycerol-	38.10	0.00
TRINITY_sp|Q84Y1	CXIP4	CAX-interacting pro	38.10	0.00
TRINITY_sp|C0SPA	adhA	Probable formaldehy	38.10	0.00
TRINITY_sp|Q9P80	trm1	tRNA (guanine(26)-N	38.10	0.00
TRINITY_sp|Q8VZ9	FZR1	Protein FIZZY-RELAT	38.10	0.00
TRINITY_sp|P6234	CPK1	Calcium-dependent p	38.10	0.00
TRINITY_sp|P8045	AOX1	Aldehyde oxidase 1	38.10	0.00
TRINITY_sp|O8826	Abcc6	Multidrug resistanc	38.10	0.00
TRINITY_sp|O1400	mak2	Peroxide stress-act	38.10	0.00
TRINITY_sp|Q9SD3	At3g5113	UPF0183 protein At3	38.10	0.00
TRINITY_sp|Q6NW5	spast	Spastin OS=Danio re	38.10	0.00
TRINITY_sp|Q6PG0	maf1	Repressor of RNA po	38.10	0.00
TRINITY_sp|Q55AE	snrnp40	U5 small nuclear ri	38.10	0.00
TRINITY_sp|Q9XIE	At1g5979	Putative cullin-lik	38.10	0.00
TRINITY_sp|Q2KJ1	PHKG2	Phosphorylase b kin	38.10	0.00
TRINITY_sp|Q96RY	IFT140	Intraflagellar tran	38.10	0.00
TRINITY_sp|Q28GH	uba2	SUMO-activating enz	38.10	0.00
TRINITY_sp|Q9Z0M	Lipa	Lysosomal acid lipa	38.10	0.00
TRINITY_sp|Q96BT	ALKBH8	Alkylated DNA repai	38.10	0.00
TRINITY_sp|Q9C6D	CPK33	Calcium-dependent p	38.10	0.00
TRINITY_sp|Q3899	KIN10	SNF1-related protei	38.10	0.00
TRINITY_sp|Q1057	CPSF1	Cleavage and polyad	38.10	0.00
TRINITY_sp|Q54TN	drkD	Probable serine/thr	38.10	0.00
TRINITY_sp|Q9M2E	RH52	DEAD-box ATP-depend	38.10	0.00
TRINITY_sp|Q9W0Y	NaCP60E	Sodium channel prot	38.10	0.00

TRINITY_sp|Q6IQ2	NAPEPLD	N-acyl-phosphatidyl	38.10	0.00
TRINITY_sp|Q6AX0	Rnf181	E3 ubiquitin-protei	38.10	0.00
TRINITY_sp|P3518	PTC1	Protein phosphatase	38.10	0.00
TRINITY_sp|P4773	RAP1GAP	Rap1 GTPase-activat	38.10	0.00
TRINITY_sp|O7053	CYP3A31	Cytochrome P450 3A3	38.10	0.00
TRINITY_sp|Q68J4	LIPE	Hormone-sensitive l	38.10	0.00
TRINITY_sp|Q5PRI	csnk1g1	Casein kinase I iso	38.10	0.00
TRINITY_sp|Q9D18	Cfap57	Cilia- and flagella	38.10	0.00
TRINITY_sp|Q6NZ0	Ddx31	Probable ATP-depend	38.10	0.00
TRINITY_sp|F4JC0	SUA	SUPPRESSOR OF ABI3-	38.10	0.00
TRINITY_sp|A7HZX	rpsM	30S ribosomal prote	38.10	0.00
TRINITY_sp|Q54SI	dhkD	Hybrid signal trans	38.10	0.00
TRINITY_sp|Q8RWI	STY17	Serine/threonine-pr	38.10	0.00
TRINITY_sp|Q5JJ1	TOM20	Probable mitochondr	38.10	0.00
TRINITY_sp|Q9SIE	ALKBH2	DNA oxidative demet	38.10	0.00
TRINITY_sp|Q8AV8	btd	Biotinidase OS=Taki	38.10	0.00
TRINITY_sp|P0CZ2	ACX3	Acyl-coenzyme A oxi	38.10	0.00
TRINITY_sp|Q9M3H	HMA1	Probable cadmium/zi	38.10	0.00
TRINITY_sp|P4781	-	Eukaryotic translat	38.10	0.00
TRINITY_sp|Q9C0H	ARHGAP39	Rho GTPase-activati	38.10	0.00
TRINITY_sp|P4268	SRK2	Tyrosine-protein ki	38.10	0.00
TRINITY_sp|Q54VV	DDB_G028	Probable serine/thr	38.10	0.00
TRINITY_sp|Q2RA0	CYCT1-3	Cyclin-T1-3 OS=Oryz	38.10	0.00
TRINITY_sp|Q9LZ2	CAT6	Cationic amino acid	38.10	0.00
TRINITY_sp|Q6NYH	tmem208	Transmembrane prote	38.10	0.00
TRINITY_sp|Q1255	agdA	Alpha-glucosidase O	38.00	0.00
TRINITY_sp|Q9XFX	cardA	Procardosin-A OS=Cy	38.00	0.00
TRINITY_sp|Q9P80	mis18	Kinetochore protein	38.00	0.00
TRINITY_sp|Q55GH	roco7	Probable serine/thr	38.00	0.00
TRINITY_sp|P2817	pkgB	Protein kinase 2 OS	38.00	0.00
TRINITY_sp|Q54VE	rsad1	Radical S-adenosyl	38.00	0.00
TRINITY_sp|Q54GE	ctdspl2	CTD small phosphata	38.00	0.00
TRINITY_sp|Q8K4F	Nsun5	Probable 28S rRNA (	38.00	0.00
TRINITY_sp|Q5854	nudF	ADP-ribose pyrophos	38.00	0.00
TRINITY_sp|Q84X1	ETN8	Equilibrative nucle	38.00	0.00
TRINITY_sp|B9G2A	Os09g024	Auxin transport pro	38.00	0.00
TRINITY_sp|Q5UP7	MIMI_R61	Putative band 7 fam	38.00	0.00
TRINITY_sp|P4620	usf	Protein usf OS=Aqui	38.00	0.00
TRINITY_sp|G5EC3	csk-1	Tyrosine-protein ki	38.00	0.00
TRINITY_sp|Q9278	STAM	Signal transducing	38.00	0.00
TRINITY_sp|Q0BU7	engB	Probable GTP-bindin	38.00	0.00
TRINITY_sp|Q5JK5	Os01g095	Probable NAD kinase	38.00	0.00
TRINITY_sp|C9WMM	-	Venom serine carbox	38.00	0.00
TRINITY_sp|P5156	AFC3	Serine/threonine-pr	38.00	0.00
TRINITY_sp|Q8BF9	Asnsd1	Asparagine syntheta	38.00	0.00
TRINITY_sp|P2873	MSP1	Protein MSP1 OS=Sac	38.00	0.00
TRINITY_sp|Q8IXY	PPIL6	Peptidyl-prolyl cis	38.00	0.00
TRINITY_sp|Q2R2E	KIN7L	Kinesin-like protei	38.00	0.00
TRINITY_sp|P1691	Pka-C3	Protein kinase DC2	38.00	0.00
TRINITY_sp|Q9173	ephb1-b	Ephrin type-B recep	38.00	0.00
TRINITY_sp|Q7TXH	Mb2977	Trans-acting enoyl	38.00	0.00
TRINITY_sp|Q8TEE	RHBDD1	Rhomboid-related pr	38.00	0.00
TRINITY_sp|Q9CA8	PEX2	Peroxisome biogenes	38.00	0.00
TRINITY_sp|P4620	MYB	Transcriptional act	38.00	0.00
TRINITY_sp|Q8R15	Znfx1	NFX1-type zinc fing	38.00	0.00
TRINITY_sp|Q641X	Hexa	Beta-hexosaminidase	38.00	0.00

TRINITY_sp|B1I1	rplD	50S ribosomal prote	38.00	0.00
TRINITY_sp|P3952	DRS2	Probable phospholip	38.00	0.00
TRINITY_sp|Q54II	shkB	Dual specificity pr	38.00	0.00
TRINITY_sp|O884	Aurkc	Aurora kinase C OS=	38.00	0.00
TRINITY_sp|A7SDV	v1g1694	Eukaryotic translat	38.00	0.00
TRINITY_sp|Q9SR	BIG	Auxin transport pro	38.00	0.00
TRINITY_sp|Q8H2	Os07g02	Glycerol-3-phosphat	38.00	0.00
TRINITY_sp|Q9SZ	PAA1	Copper-transporting	38.00	0.00
TRINITY_sp|P049	cprA	Cysteine proteinase	38.00	0.00
TRINITY_sp|Q207	atad-3	ATPase family AAA d	38.00	0.00
TRINITY_sp|Q96N	GUCD1	Protein GUCD1 OS=Ho	38.00	0.00
TRINITY_sp|Q55D	abcG22	ABC transporter G f	38.00	0.00
TRINITY_sp|P400	TPA1	Prolyl 3,4-dihydrox	38.00	0.00
TRINITY_sp|P123	GLRX	Glutaredoxin-1 OS=S	38.00	0.00
TRINITY_sp|P0C7	DA1	Protein DA1 OS=Arab	38.00	0.00
TRINITY_sp|O212	RPL14	60S ribosomal prote	38.00	0.00
TRINITY_sp|Q3MI	PUS10	Putative tRNA pseud	38.00	0.00
TRINITY_sp|Q8VZ	SUVH6	Histone-lysine N-me	38.00	0.00
TRINITY_sp|P041	Myb	Myb protein OS=Dros	38.00	0.00
TRINITY_sp|Q8LB	CYCU3-1	Cyclin-U3-1 OS=Arab	38.00	0.00
TRINITY_sp|Q9SI	TTM3	Triphosphate tunel	38.00	0.00
TRINITY_sp|P374	alkB	Alpha-ketoglutarate	38.00	0.00
TRINITY_sp|P3952	DRS2	Probable phospholip	38.00	0.00
TRINITY_sp|Q9FN	UVR8	Ultraviolet-B recep	38.00	0.00
TRINITY_sp|Q9C9	CEL3	Endoglucanase 9 OS=	38.00	0.00
TRINITY_sp|Q0W5	alaS	Alanine--tRNA ligas	38.00	0.00
TRINITY_sp|Q9M9	SRK2C	Serine/threonine-pr	38.00	0.00
TRINITY_sp|Q8LP	At3g199	E3 ubiquitin-protei	37.90	0.00
TRINITY_sp|Q9UT	pub2	E3 ubiquitin-protei	37.90	0.00
TRINITY_sp|P531	GUP1	Glycerol uptake pro	37.90	0.00
TRINITY_sp|O449	C34B2.1	Probable signal pep	37.90	0.00
TRINITY_sp|P357	-	Venom allergen 3 OS	37.90	0.00
TRINITY_sp|Q63Z	-	UPF0568 protein C14	37.90	0.00
TRINITY_sp|Q5XH	slc25a2	Calcium-binding mit	37.90	0.00
TRINITY_sp|Q4P9	CCR4	Glucose-repressible	37.90	0.00
TRINITY_sp|Q964	ORM1	Protein ORM1 OS=Sac	37.90	0.00
TRINITY_sp|Q99K	Usp11	Ubiquitin carboxyl-	37.90	0.00
TRINITY_sp|Q2TB	COG8	Conserved oligomeri	37.90	0.00
TRINITY_sp|A5PK	METTL13	Methyltransferase-l	37.90	0.00
TRINITY_sp|Q9QY	Dnaja2	DnaJ homolog subfam	37.90	0.00
TRINITY_sp|Q8BY	Trmt13	tRNA:m(4)X modifica	37.90	0.00
TRINITY_sp|Q420	ABCC2	ABC transporter C f	37.90	0.00
TRINITY_sp|P190	GNAT2	Guanine nucleotide-	37.90	0.00
TRINITY_sp|Q953	MANBA	Beta-mannosidase OS	37.90	0.00
TRINITY_sp|Q6V4	10HGO	8-hydroxygeraniol d	37.90	0.00
TRINITY_sp|Q004	pho2	4-nitrophenylphosph	37.90	0.00
TRINITY_sp|Q8W4	PTC52	Protochlorophyllide	37.90	0.00
TRINITY_sp|Q9N5	cdc-73	Cell division cycle	37.90	0.00
TRINITY_sp|Q8II	PF11_01	Probable cathepsin	37.90	0.00
TRINITY_sp|P176	ypt2	GTP-binding protein	37.90	0.00
TRINITY_sp|Q2QN	CYCA3-2	Cyclin-A3-2 OS=Oryz	37.90	0.00
TRINITY_sp|Q9LD	BETAA-AI	Beta-adaptin-like p	37.90	0.00
TRINITY_sp|Q9FP	UBP15	Ubiquitin carboxyl-	37.90	0.00
TRINITY_sp|A6W2	betB	NAD/NADP-dependent	37.90	0.00
TRINITY_sp|Q9S7	HSP70-1	Heat shock 70 kDa p	37.90	0.00
TRINITY_sp|Q9FE	SRT1	NAD-dependent prote	37.90	0.00

TRINITY_sp|Q111	wht-1	ABC transporter ATP	37.90	0.00
TRINITY_sp|A9Z1	Vwa5b1	von Willebrand fact	37.90	0.00
TRINITY_sp|Q087	myb	Transcriptional act	37.90	0.00
TRINITY_sp|Q9PK	fabG	3-oxoacyl-[acyl-car	37.90	0.00
TRINITY_sp|Q84N	CDSP32	Thioredoxin-like pr	37.90	0.00
TRINITY_sp|Q5TZ	mpv17	Protein Mpv17 OS=Da	37.90	0.00
TRINITY_sp|P109	-	Retrovirus-related	37.90	0.00
TRINITY_sp|P774	yqaB	Fructose-1-phosphat	37.90	0.00
TRINITY_sp|Q0WR	PPC6-7	Probable protein ph	37.90	0.00
TRINITY_sp|Q963	GSTF11	Glutathione S-trans	37.90	0.00
TRINITY_sp|A2RV	RRP41L	Exosome complex com	37.90	0.00
TRINITY_sp|B8AS	OsI_167	Vacuolar-processing	37.90	0.00
TRINITY_sp|Q9UT	och1	Initiation-specific	37.90	0.00
TRINITY_sp|Q559	slr0305	TVP38/TMEM64 family	37.90	0.00
TRINITY_sp|Q55F	mef2A	Transcription facto	37.90	0.00
TRINITY_sp|Q9VY	NnaD	Cytosolic carboxype	37.90	0.00
TRINITY_sp|Q1LZ	FAIM2	Protein lifeguard 2	37.90	0.00
TRINITY_sp|P671	yqfA	UPF0073 inner membr	37.90	0.00
TRINITY_sp|Q54G	pex1	Peroxisome biogenes	37.90	0.00
TRINITY_sp|Q9FV	At1g577	Probable elongation	37.90	0.00
TRINITY_sp|Q2LA	ASHH2	Histone-lysine N-me	37.90	0.00
TRINITY_sp|Q8JH	ddx55	ATP-dependent RNA h	37.90	0.00
TRINITY_sp|O652	ACX1	Peroxisomal acyl-co	37.90	0.00
TRINITY_sp|Q96F	KIF12	Kinesin-like protei	37.90	0.00
TRINITY_sp|Q246	ref(2)P	Protein ref(2)P OS=	37.90	0.00
TRINITY_sp|Q6L4	Os05g05	Probable protein ph	37.90	0.00
TRINITY_sp|O645	STR1	Thiosulfate/3-merca	37.90	0.00
TRINITY_sp|Q2QP	GK1	Guanylate kinase 1	37.90	0.00
TRINITY_sp|Q5UP	MIMI_R6	Putative band 7 fam	37.90	0.00
TRINITY_sp|O946	gyp10	GTPase-activating p	37.90	0.00
TRINITY_sp|Q6C6	ISN1	IMP-specific 5'-nuc	37.90	0.00
TRINITY_sp|Q3E6	DEGP14	Putative protease D	37.90	0.00
TRINITY_sp|Q9SL	ALIS3	ALA-interacting sub	37.90	0.00
TRINITY_sp|Q9FV	CYCA1-2	Cyclin-A1-2 OS=Arab	37.90	0.00
TRINITY_sp|Q851	ILL3	IAA-amino acid hydr	37.90	0.00
TRINITY_sp|O229	At2g303	Putative tRNA pseud	37.90	0.00
TRINITY_sp|Q9ER	Cars	Cysteine--tRNA liga	37.90	0.00
TRINITY_sp|Q9FY	RS2Z32	Serine/arginine-ric	37.90	0.00
TRINITY_sp|Q52K	NUDT13	Nudix hydrolase 13,	37.90	0.00
TRINITY_sp|Q8H1	AAE13	Malonate--CoA ligas	37.90	0.00
TRINITY_sp|Q6V4	10HGO	8-hydroxygeraniol d	37.90	0.00
TRINITY_sp|Q2T9	POLR3F	DNA-directed RNA po	37.90	0.00
TRINITY_sp|Q54S	spt16	FACT complex subuni	37.90	0.00
TRINITY_sp|Q941	PAP8	Probable plastid-li	37.90	0.00
TRINITY_sp|Q2RA	CYCT1-3	Cyclin-T1-3 OS=Oryz	37.90	0.00
TRINITY_sp|Q8VE	Tmem30a	Cell cycle control	37.90	0.00
TRINITY_sp|Q9SH	STIPL1	Septin and tuftelin	37.90	0.00
TRINITY_sp|O804	At1g091	Probable protein ph	37.90	0.00
TRINITY_sp|Q3E9	CPK34	Calcium-dependent p	37.90	0.00
TRINITY_sp|O221	GCP1	Probable tRNA N6-ad	37.90	0.00
TRINITY_sp|B1Q3	MET1B	DNA (cytosine-5)-me	37.90	0.00
TRINITY_sp|Q9V0	tuf	Elongation factor 1	37.90	0.00
TRINITY_sp|Q1LV	abhd14a	Protein ABHD14A OS=	37.90	0.00
TRINITY_sp|Q8DK	der	GTPase Der OS=Therm	37.90	0.00
TRINITY_sp|Q6C7	HAS1	ATP-dependent RNA h	37.90	0.00
TRINITY_sp|O577	rpl11	50S ribosomal prote	37.90	0.00

TRINITY_sp|P0A0N	SAOUHSC_	Putative acetyltran	37.90	0.00
TRINITY_sp|P1091	MCF2	Proto-oncogene DBL	37.80	0.00
TRINITY_sp|P137(	mvaA	3-hydroxy-3-methylg	37.80	0.00
TRINITY_sp|Q3958	-	Dynein 18 kDa light	37.80	0.00
TRINITY_sp|Q440S	-	L-sorbosone dehydro	37.80	0.00
TRINITY_sp|Q54J1	atg6B	Beclin-1-like prote	37.80	0.00
TRINITY_sp|Q6PQ	-	3-oxo-Delta(4,5)-st	37.80	0.00
TRINITY_sp|O8068	PAT02	Probable protein S-	37.80	0.00
TRINITY_sp|Q54RI	dhkL	Hybrid signal trans	37.80	0.00
TRINITY_sp|P2364	Rab27a	Ras-related protein	37.80	0.00
TRINITY_sp|Q54JI	chmp5	Charged multivesicu	37.80	0.00
TRINITY_sp|A8F0(	pepA	Probable cytosol am	37.80	0.00
TRINITY_sp|Q56X7	RH39	DEAD-box ATP-depend	37.80	0.00
TRINITY_sp|P1477	-	Soluble hydrogenase	37.80	0.00
TRINITY_sp|P3233	YMC1	Carrier protein YMC	37.80	0.00
TRINITY_sp|Q6PE(	Tbca	Tubulin-specific ch	37.80	0.00
TRINITY_sp|Q9NQ(	RPRD1B	Regulation of nucle	37.80	0.00
TRINITY_sp|Q9P42	B11B22.(	E3 ubiquitin-protei	37.80	0.00
TRINITY_sp|Q66HY	eif4e3	Eukaryotic translat	37.80	0.00
TRINITY_sp|Q9WUI	Nprl2	Nitrogen permease r	37.80	0.00
TRINITY_sp|F4JC(	At3g4783	Putative DNA glycos	37.80	0.00
TRINITY_sp|Q54IS	smt1	Probable cycloarten	37.80	0.00
TRINITY_sp|Q4015	-	LEC14B protein OS=L	37.80	0.00
TRINITY_sp|P6048	PTEN	Phosphatidylinosito	37.80	0.00
TRINITY_sp|P2251	CMK2	Calcium/calmodulin-	37.80	0.00
TRINITY_sp|Q54HI	copG	Coatomer subunit ga	37.80	0.00
TRINITY_sp|Q9ZU(	ADK	Adenylate kinase 1,	37.80	0.00
TRINITY_sp|A2Q8I	npl4	Nuclear protein loc	37.80	0.00
TRINITY_sp|Q8I4I	shk-1	Potassium voltage-g	37.80	0.00
TRINITY_sp|O226(	MSI4	WD-40 repeat-contai	37.80	0.00
TRINITY_sp|Q54I1	smkA	Suppressor of Mek1	37.80	0.00
TRINITY_sp|O0521	ywrD	Putative gamma-glut	37.80	0.00
TRINITY_sp|Q86W5	PARG	Poly(ADP-ribose) gl	37.80	0.00
TRINITY_sp|Q2125	gsnl-1	Gelsolin-like prote	37.80	0.00
TRINITY_sp|P2751	CBR	Carotene biosynthes	37.80	0.00
TRINITY_sp|Q0776	HVA22	Protein HVA22 OS=Ho	37.80	0.00
TRINITY_sp|P7438	sll0418	2-methyl-6-phytyl-1	37.80	0.00
TRINITY_sp|Q8GW(	TPPH	Probable trehalose-	37.80	0.00
TRINITY_sp|P2347	Xrcc6	X-ray repair cross-	37.80	0.00
TRINITY_sp|M4MRS	rnj	Ribonuclease J OS=S	37.80	0.00
TRINITY_sp|Q6NW2	RWDD4	RWD domain-containi	37.80	0.00
TRINITY_sp|Q54II	shkB	Dual specificity pr	37.80	0.00
TRINITY_sp|P7731	ybdR	Uncharacterized zin	37.80	0.00
TRINITY_sp|Q54B(	DDB_G02S	Probable GH family	37.80	0.00
TRINITY_sp|Q7XJS	GAS8	Growth arrest-speci	37.80	0.00
TRINITY_sp|Q8VEI	Tmem144	Transmembrane prote	37.80	0.00
TRINITY_sp|Q9NR2	DYRK4	Dual specificity ty	37.80	0.00
TRINITY_sp|Q1401	CAMK1	Calcium/calmodulin-	37.80	0.00
TRINITY_sp|O0448	At1g0964	Probable elongation	37.80	0.00
TRINITY_sp|B4JV(	GH22679	Eukaryotic translat	37.80	0.00
TRINITY_sp|Q69U4	RMR1	Receptor homology r	37.80	0.00
TRINITY_sp|Q0WW(	ATG3	Autophagy-related p	37.80	0.00
TRINITY_sp|Q852(	OSK1	Serine/threonine pr	37.80	0.00
TRINITY_sp|P1097	-	Retrovirus-related	37.80	0.00
TRINITY_sp|Q0GZS	USP	UDP-sugar pyrophosp	37.80	0.00
TRINITY_sp|P3172	OV16	OV-16 antigen OS=On	37.80	0.00

TRINITY_sp|P9WK8	nlhH	Carboxylesterase Nl	37.80	0.00
TRINITY_sp|Q54BI	ddx51	Probable ATP-depend	37.80	0.00
TRINITY_sp|Q0869	-	Pathogenesis-relate	37.80	0.00
TRINITY_sp|Q2RM6	hcp	Hydroxylamine reduc	37.80	0.00
TRINITY_sp|Q7K48	bin3	Probable RNA methyl	37.80	0.00
TRINITY_sp|A8J66	CFAP54	Cilia- and flagella	37.80	0.00
TRINITY_sp|Q993I	TY3B-G	Transposon Ty3-G Ga	37.80	0.00
TRINITY_sp|P7892	cof1	Cofilin OS=Schizosa	37.80	0.00
TRINITY_sp|Q9P2I	DNAH1	Dynein heavy chain	37.80	0.00
TRINITY_sp|Q8WVX	FAR1	Fatty acyl-CoA redu	37.80	0.00
TRINITY_sp|Q0210	-	Coat protein OS=Pop	37.70	0.00
TRINITY_sp|P0463	Lipf	Gastric triacylglyc	37.70	0.00
TRINITY_sp|Q5FWU	zmynd10	Zinc finger MYND do	37.70	0.00
TRINITY_sp|Q0917	cds1	Serine/threonine-pr	37.70	0.00
TRINITY_sp|Q940S	TMN2	Transmembrane 9 sup	37.70	0.00
TRINITY_sp|P7028	Vamp7	Vesicle-associated	37.70	0.00
TRINITY_sp|P2604	PTPN3	Tyrosine-protein ph	37.70	0.00
TRINITY_sp|O8222	SCPL23	Putative serine car	37.70	0.00
TRINITY_sp|Q7XQU	Os04g045	Probable protein ph	37.70	0.00
TRINITY_sp|Q5ZMV	RAP1GAP2	Rap1 GTPase-activat	37.70	0.00
TRINITY_sp|Q8GYI	MND1	Meiotic nuclear div	37.70	0.00
TRINITY_sp|P6300	AP2B1	AP-2 complex subuni	37.70	0.00
TRINITY_sp|P9586	treZ	Malto-oligosyltreha	37.70	0.00
TRINITY_sp|O9452	iqw1	WD repeat protein i	37.70	0.00
TRINITY_sp|O1543	ABCC4	Multidrug resistanc	37.70	0.00
TRINITY_sp|Q7SC4	cln3	Protein btn-1 OS=Ne	37.70	0.00
TRINITY_sp|Q8C9A	Endov	Endonuclease V OS=M	37.70	0.00
TRINITY_sp|Q6PDI	Fam63b	Ubiquitin carboxyl-	37.70	0.00
TRINITY_sp|P2082	pol	Retrovirus-related	37.70	0.00
TRINITY_sp|Q5EA5	ABHD4	Protein ABHD4 OS=Bo	37.70	0.00
TRINITY_sp|Q6GNE	coq10b	Coenzyme Q-binding	37.70	0.00
TRINITY_sp|Q9JHE	Ide	Insulin-degrading e	37.70	0.00
TRINITY_sp|A8QCE	Bm1_4952	Lateral signaling t	37.70	0.00
TRINITY_sp|P5380	Pctp	Phosphatidylcholine	37.70	0.00
TRINITY_sp|Q54WE	pldB	Phospholipase D B O	37.70	0.00
TRINITY_sp|Q94JV	At1g5429	Protein translation	37.70	0.00
TRINITY_sp|P2126	CCA1	CCA tRNA nucleotidy	37.70	0.00
TRINITY_sp|Q8R4E	Pappa	Pappalysin-1 OS=Mus	37.70	0.00
TRINITY_sp|Q54RJ	iksA	Probable serine/thr	37.70	0.00
TRINITY_sp|Q0278	Cacna1s	Voltage-dependent L	37.70	0.00
TRINITY_sp|A2XX5	COLD1	GPCR-type G protein	37.70	0.00
TRINITY_sp|F4I2H	SWI2	Switch 2 OS=Arabido	37.70	0.00
TRINITY_sp|Q9Z7Q	CPn_0654	Uncharacterized acy	37.70	0.00
TRINITY_sp|Q839E	hpt	Hypoxanthine-guanin	37.70	0.00
TRINITY_sp|Q1283	BPTF	Nucleosome-remodeli	37.70	0.00
TRINITY_sp|Q60EX	BSL1	Serine/threonine-pr	37.70	0.00
TRINITY_sp|Q2KHV	USP2	Ubiquitin carboxyl-	37.70	0.00
TRINITY_sp|Q9CWE	Smyd3	Histone-lysine N-me	37.70	0.00
TRINITY_sp|Q9LKW	NHX7	Sodium/hydrogen exc	37.70	0.00
TRINITY_sp|Q9288	ERCC4	DNA repair endonucl	37.70	0.00
TRINITY_sp|Q7T6X	MIMI_R82	Putative serine/thr	37.70	0.00
TRINITY_sp|Q8GRI	TFCE	Tubulin-folding cof	37.70	0.00
TRINITY_sp|O8102	EMB1187	Probable ethanolami	37.70	0.00
TRINITY_sp|A7EYW	dbp3	ATP-dependent RNA h	37.70	0.00
TRINITY_sp|Q5587	sll0103	Uncharacterized pro	37.70	0.00
TRINITY_sp|Q6F3A	CPK10	Calcium-dependent p	37.70	0.00

TRINITY_sp|P519!	NEK4	Serine/threonine-pr	37.70	0.00
TRINITY_sp|Q494(	HPAT2	Hydroxyproline O-ar	37.70	0.00
TRINITY_sp|B0F4(	BIO3-BI(	Bifunctional dethio	37.70	0.00
TRINITY_sp|D9HP2	CNR4	Cell number regulat	37.70	0.00
TRINITY_sp|Q5Y2(	HTAY	Histone H2A.Y OS=Te	37.70	0.00
TRINITY_sp|Q1QH2	thrB	Homoserine kinase O	37.70	0.00
TRINITY_sp|Q5UP	MIMI_R6	Putative band 7 fam	37.70	0.00
TRINITY_sp|P264(	fixL	Sensor protein FixL	37.70	0.00
TRINITY_sp|Q9C5	RRC1	Protein RRC1 OS=Ara	37.70	0.00
TRINITY_sp|B9DF(	ISE2	DExH-box ATP-depend	37.70	0.00
TRINITY_sp|Q8AY	cse1l	Exportin-2 OS=Oreoc	37.70	0.00
TRINITY_sp|Q6CB	RMT2	Protein arginine N-	37.70	0.00
TRINITY_sp|Q29R	PDIA4	Protein disulfide-i	37.70	0.00
TRINITY_sp|Q86U(	RBM23	Probable RNA-bindin	37.70	0.00
TRINITY_sp|Q9XF(	PDPK1	3-phosphoinositide-	37.70	0.00
TRINITY_sp|Q9ST(	NDB1	External alternativ	37.70	0.00
TRINITY_sp|Q96M	CFAP57	Cilia- and flagella	37.70	0.00
TRINITY_sp|Q54G	DDB_G02(	Protein CLEC16A hom	37.70	0.00
TRINITY_sp|O222	LIS	U4/U6 small nuclear	37.70	0.00
TRINITY_sp|P225(	-	Polyubiquitin OS=Ph	37.70	0.00
TRINITY_sp|Q8VD!	Sft2d2	Vesicle transport p	37.70	0.00
TRINITY_sp|Q54S(	midA	Protein arginine me	37.70	0.00
TRINITY_sp|Q9SJ	LACS8	Long chain acyl-CoA	37.60	0.00
TRINITY_sp|P112	UDA1	Lectin/endochitinas	37.60	0.00
TRINITY_sp|Q8RU	Os01g05(	Probable V-type pro	37.60	0.00
TRINITY_sp|Q9FI	At5g478!	NADH dehydrogenase	37.60	0.00
TRINITY_sp|Q338	GCN5	Histone acetyltrans	37.60	0.00
TRINITY_sp|Q2TB	COMMD9	COMM domain-contain	37.60	0.00
TRINITY_sp|Q54C	kxcB	Kinase and exchange	37.60	0.00
TRINITY_sp|Q95J	AK7	Adenylate kinase 7	37.60	0.00
TRINITY_sp|Q9ZU	At2g372	Thioredoxin-like pr	37.60	0.00
TRINITY_sp|Q9NR	ABCB10	ATP-binding cassett	37.60	0.00
TRINITY_sp|Q869	bpnt1	3'(2'),5'-bisphosph	37.60	0.00
TRINITY_sp|Q8AV(	syf2	Pre-mRNA-splicing f	37.60	0.00
TRINITY_sp|Q496	Elp2	Elongator complex p	37.60	0.00
TRINITY_sp|Q9LR(	PSB27-1	Photosystem II repa	37.60	0.00
TRINITY_sp|Q7XT	Os04g06	Nucleolin 2 OS=Oryz	37.60	0.00
TRINITY_sp|Q551	DDB_G02	WD repeat-containin	37.60	0.00
TRINITY_sp|O343!	ytcJ	Putative amidohydro	37.60	0.00
TRINITY_sp|P299(	CYP4C1	Cytochrome P450 4C1	37.60	0.00
TRINITY_sp|P496(	Os01g08	Coatomer subunit de	37.60	0.00
TRINITY_sp|Q063!	DER1	Derlin-1 OS=Oryza s	37.60	0.00
TRINITY_sp|Q9FG(	ATO	Splicing factor SF3	37.60	0.00
TRINITY_sp|O078	dapb1	Dipeptidyl aminopep	37.60	0.00
TRINITY_sp|Q225	chd-3	Chromodomain-helica	37.60	0.00
TRINITY_sp|K7WI	PYRR	Riboflavin biosynth	37.60	0.00
TRINITY_sp|O826	At4g329!	Probable protein ph	37.60	0.00
TRINITY_sp|Q389!	KIN10	SNF1-related protei	37.60	0.00
TRINITY_sp|Q67W!	RR25	Two-component respo	37.60	0.00
TRINITY_sp|O946	SPBC776.	Uncharacterized mem	37.60	0.00
TRINITY_sp|Q437	TOC75	Protein TOC75, chlo	37.60	0.00
TRINITY_sp|P407(	CYPRO4	Protein CYPRO4 OS=C	37.60	0.00
TRINITY_sp|Q54W	aurK	Aurora kinase OS=Di	37.60	0.00
TRINITY_sp|Q9FM(	TIC20-V	Protein TIC 20-v, c	37.60	0.00
TRINITY_sp|Q9EQ	Tpp1	Tripeptidyl-peptida	37.60	0.00
TRINITY_sp|A4QN	slc25a4	Solute carrier fami	37.60	0.00

TRINITY_sp|Q54LE	vps13A	Putative vacuolar p	37.60	0.00
TRINITY_sp|Q7TN(	Luc7l2	Putative RNA-bindin	37.60	0.00
TRINITY_sp|Q9FK1	THO3	THO complex subunit	37.60	0.00
TRINITY_sp|O7482	esf1	Pre-rRNA-processing	37.60	0.00
TRINITY_sp|O7425	GAM1	Glucoamylase 1 OS=C	37.60	0.00
TRINITY_sp|Q8VYU	CCR4-6	Carbon catabolite r	37.60	0.00
TRINITY_sp|O4331	PPIP5K2	Inositol hexakispho	37.60	0.00
TRINITY_sp|Q9NVE	EXD2	Exonuclease 3'-5' d	37.60	0.00
TRINITY_sp|P2532	mlkA	Myosin light chain	37.60	0.00
TRINITY_sp|Q54WW	DDB_G027	Probable serine/thr	37.60	0.00
TRINITY_sp|Q08DI	PUS7	Pseudouridylate syn	37.60	0.00
TRINITY_sp|Q9N9X	-	Ciliary WD repeat-c	37.60	0.00
TRINITY_sp|Q7Z47	MAATS1	Protein MAATS1 OS=H	37.60	0.00
TRINITY_sp|Q54K3	rgaA	Ras GTPase-activati	37.60	0.00
TRINITY_sp|Q54QE	erkB	Extracellular signa	37.60	0.00
TRINITY_sp|Q54TM	drkD	Probable serine/thr	37.60	0.00
TRINITY_sp|Q9MAM	KCS1	3-ketoacyl-CoA synt	37.60	0.00
TRINITY_sp|O0078	vatE	V-type proton ATPas	37.60	0.00
TRINITY_sp|Q0JI4	CIPK11	CBL-interacting pro	37.60	0.00
TRINITY_sp|P3225	rasC	Ras-like protein ra	37.60	0.00
TRINITY_sp|Q9US3	tum1	Probable 3-mercapto	37.60	0.00
TRINITY_sp|Q4Z8E	RanBPM	Ran-binding protein	37.60	0.00
TRINITY_sp|Q9625	At5g4703	ATP synthase subuni	37.60	0.00
TRINITY_sp|Q94F3	ESD4	Ubiquitin-like-spec	37.60	0.00
TRINITY_sp|Q6451	Tpp2	Tripeptidyl-peptida	37.60	0.00
TRINITY_sp|Q6137	Ift88	Intraflagellar tran	37.60	0.00
TRINITY_sp|A7Z91	uvsE	UV DNA damage endon	37.60	0.00
TRINITY_sp|Q9ATE	ADA2B	Transcriptional ada	37.60	0.00
TRINITY_sp|Q8GWU	PGLP2	Phosphoglycolate ph	37.60	0.00
TRINITY_sp|Q5TKI	Os05g02(	Thioredoxin-like 2,	37.60	0.00
TRINITY_sp|Q1RH3	grxC1	Glutaredoxin-1 OS=R	37.60	0.00
TRINITY_sp|Q86C2	dhkK	Hybrid signal trans	37.50	0.00
TRINITY_sp|Q9MBE	DHC10	Dynein-1-beta heavy	37.50	0.00
TRINITY_sp|Q6ZV1	TTLL10	Inactive polyglycyl	37.50	0.00
TRINITY_sp|Q9UUA	pfh1	ATP-dependent DNA h	37.50	0.00
TRINITY_sp|Q9W1E	CG5532	Transmembrane prote	37.50	0.00
TRINITY_sp|Q388(	HSP22.0	22.0 kDa heat shock	37.50	0.00
TRINITY_sp|Q8BYE	Rdh12	Retinol dehydrogena	37.50	0.00
TRINITY_sp|Q2481	RACB	Rho-related protein	37.50	0.00
TRINITY_sp|P0678	RHO2	GTP-binding protein	37.50	0.00
TRINITY_sp|Q99L(	Dhrs1	Dehydrogenase/reduc	37.50	0.00
TRINITY_sp|Q86C2	dhkK	Hybrid signal trans	37.50	0.00
TRINITY_sp|Q6IE5	Mug2	Murinoglobulin-2 OS	37.50	0.00
TRINITY_sp|P8032	-	Troponin C OS=Branc	37.50	0.00
TRINITY_sp|P1395	-	G2/mitotic-specific	37.50	0.00
TRINITY_sp|P1419	AAC4	AAC-rich mRNA clone	37.50	0.00
TRINITY_sp|Q8T19	DDB_G027	Dehydrogenase/reduc	37.50	0.00
TRINITY_sp|O6896	idhA	Inositol 2-dehydrog	37.50	0.00
TRINITY_sp|A1ZA1	CG8303	Putative fatty acyl	37.50	0.00
TRINITY_sp|Q9M8E	DSPTP1B	Dual specificity pr	37.50	0.00
TRINITY_sp|Q9LM6	ATL80	RING-H2 finger prot	37.50	0.00
TRINITY_sp|O1543	ABCC4	Multidrug resistanc	37.50	0.00
TRINITY_sp|Q2Y6E	rlmN	Dual-specificity RN	37.50	0.00
TRINITY_sp|Q54MA	ku70	ATP-dependent DNA h	37.50	0.00
TRINITY_sp|Q72NM	msrB	Peptide methionine	37.50	0.00
TRINITY_sp|Q6GV1	Kdsr	3-ketodihydrosphing	37.50	0.00

TRINITY_sp|O229:	CIPK11	CBL-interacting ser	37.50	0.00
TRINITY_sp|D0MV!	KEX1	Pheromone-processin	37.50	0.00
TRINITY_sp|O807(	At1g604:	Probable nucleoredo	37.50	0.00
TRINITY_sp|Q294!	LIPF	Gastric triacylglyc	37.50	0.00
TRINITY_sp|Q9LT!	BBR	E3 ubiquitin ligase	37.50	0.00
TRINITY_sp|Q9CAI	HIR2	Hypersensitive-indu	37.50	0.00
TRINITY_sp|P136	inaC	Protein kinase C, e	37.50	0.00
TRINITY_sp|Q54V\	DDB_G02	Probable serine/thr	37.50	0.00
TRINITY_sp|Q025!	PK2	Probable serine/thr	37.50	0.00
TRINITY_sp|Q7ZU	polr3c	DNA-directed RNA po	37.50	0.00
TRINITY_sp|Q6IN!	smek1	Serine/threonine-pr	37.50	0.00
TRINITY_sp|Q9SHI	RUB1	Ubiquitin-NEDD8-lik	37.50	0.00
TRINITY_sp|P044(	-	Calmodulin OS=Triti	37.50	0.00
TRINITY_sp|Q9N4I	pme-1	Poly(ADP-ribose) po	37.50	0.00
TRINITY_sp|Q555:	slr0328	Putative low molecu	37.50	0.00
TRINITY_sp|A6LLI	rpsG	30S ribosomal prote	37.50	0.00
TRINITY_sp|Q9M4I	AIP3	Probable prefoldin	37.50	0.00
TRINITY_sp|Q6P6I	Grina	Protein lifeguard 1	37.50	0.00
TRINITY_sp|Q38AI	MTR1	Cap-specific mRNA (	37.50	0.00
TRINITY_sp|Q102!	SPAC4H3.	MEMO1 family protei	37.50	0.00
TRINITY_sp|A6VD!	ubiB	Probable protein ki	37.50	0.00
TRINITY_sp|F4I6!	POLIA	DNA polymerase I A,	37.50	0.00
TRINITY_sp|Q869!	dokA	Hybrid signal trans	37.50	0.00
TRINITY_sp|Q9VCI	CG17119	Cystinosin homolog	37.50	0.00
TRINITY_sp|P0CN:	DUS3	tRNA-dihydrouridine	37.50	0.00
TRINITY_sp|Q54TI	gacY	Rho GTPase-activati	37.50	0.00
TRINITY_sp|Q8NF.	BBS1	Bardet-Biedl syndro	37.50	0.00
TRINITY_sp|F4JM.	HSP70-1	Heat shock 70 kDa p	37.50	0.00
TRINITY_sp|Q9SJI	LACS8	Long chain acyl-CoA	37.50	0.00
TRINITY_sp|P427	EMB2360	Glutathione reducta	37.50	0.00
TRINITY_sp|A5YKI	CNOT1	CCR4-NOT transcript	37.50	0.00
TRINITY_sp|Q55A	dlpC	Dynamin-like protei	37.50	0.00
TRINITY_sp|Q54M\	mrkB	Probable serine/thr	37.50	0.00
TRINITY_sp|P0AE(	barA	Signal transduction	37.50	0.00
TRINITY_sp|Q8RYI	At2g332!	Haloacid dehalogena	37.50	0.00
TRINITY_sp|P247!	ATP1A2	Sodium/potassium-tr	37.50	0.00
TRINITY_sp|Q1ZXI	roco5	Probable serine/thr	37.50	0.00
TRINITY_sp|Q3TZ/	Cdkl4	Cyclin-dependent ki	37.50	0.00
TRINITY_sp|Q4PH!	ATM1	Iron-sulfur cluster	37.50	0.00
TRINITY_sp|Q84M:	ABCA1	ABC transporter A f	37.50	0.00
TRINITY_sp|B0G1(	DDB_G02	Deoxynucleoside tri	37.50	0.00
TRINITY_sp|O149(	AURKA	Aurora kinase A OS=	37.50	0.00
TRINITY_sp|Q8S1	Os01g09:	Probable glucuronos	37.50	0.00
TRINITY_sp|Q3S4/	AHK5	Histidine kinase 5	37.50	0.00
TRINITY_sp|F4J1!	LSF1	Phosphoglucan phosp	37.40	0.00
TRINITY_sp|Q54Q!	DDB_G02	E3 UFM1-protein lig	37.40	0.00
TRINITY_sp|Q9UK:	CNOT11	CCR4-NOT transcript	37.40	0.00
TRINITY_sp|O946(	jmj4	JmjC domain-contain	37.40	0.00
TRINITY_sp|Q9M1!	At3g544(	F-box protein At3g5	37.40	0.00
TRINITY_sp|Q9UQ:	SRRM2	Serine/arginine rep	37.40	0.00
TRINITY_sp|Q3MQ:	ATG5	Autophagy protein 5	37.40	0.00
TRINITY_sp|Q5E9!	APEX2	DNA-(apurinic or ap	37.40	0.00
TRINITY_sp|Q0JI	CIPK11	CBL-interacting pro	37.40	0.00
TRINITY_sp|Q9JL(	Capn15	Calpain-15 OS=Mus m	37.40	0.00
TRINITY_sp|Q54HI	gghB	Gamma-glutamyl hydr	37.40	0.00
TRINITY_sp|Q5HM	SERP178!	Zinc-type alcohol d	37.40	0.00

TRINITY_sp|Q079!	-	NADPH--cytochrome P	37.40	0.00
TRINITY_sp|P418{	rbf-1	Rabphilin-1 OS=Caen	37.40	0.00
TRINITY_sp|Q9XFI	DDM1	ATP-dependent DNA h	37.40	0.00
TRINITY_sp|Q9BL(	amtB	Ammonium transporte	37.40	0.00
TRINITY_sp|D2GXI	AGBL5	Cytosolic carboxype	37.40	0.00
TRINITY_sp|P529]	VPS4	Vacuolar protein so	37.40	0.00
TRINITY_sp|Q993]	TY3B-G	Transposon Ty3-G Ga	37.40	0.00
TRINITY_sp|Q9ZS/	CPK27	Calcium-dependent p	37.40	0.00
TRINITY_sp|P617!	APTX	Aprataxin (Fragment	37.40	0.00
TRINITY_sp|Q8H0(	TRN1	Transportin-1 OS=Ar	37.40	0.00
TRINITY_sp|O152!	ACOX3	Peroxisomal acyl-co	37.40	0.00
TRINITY_sp|Q7ZVI	tigarb	Fructose-2,6-bispho	37.40	0.00
TRINITY_sp|P355:	CLNS1A	Methylosome subunit	37.40	0.00
TRINITY_sp|Q869:	DDB_G02	Putative protein di	37.40	0.00
TRINITY_sp|Q84M/	CAT1	Cationic amino acid	37.40	0.00
TRINITY_sp|Q9C5(	DPL1	Sphingosine-1-phosp	37.40	0.00
TRINITY_sp|B2X0!	MNR1	(+)-neomenthol dehy	37.40	0.00
TRINITY_sp|Q8S1:	Os01g08:	Probable U3 small n	37.40	0.00
TRINITY_sp|P432!	RD19A	Cysteine protease R	37.40	0.00
TRINITY_sp|Q8BN{	Slc17a5	Sialin OS=Mus muscu	37.40	0.00
TRINITY_sp|Q8K4]	Dusp19	Dual specificity pr	37.40	0.00
TRINITY_sp|Q91W(	As3mt	Arsenite methyltran	37.40	0.00
TRINITY_sp|A5PF4	garnl3	GTPase-activating R	37.40	0.00
TRINITY_sp|Q9NP(	HMG20A	High mobility group	37.40	0.00
TRINITY_sp|Q8DH(	secA	Protein translocase	37.40	0.00
TRINITY_sp|Q8VEI	Mtmr14	Myotubularin-relate	37.40	0.00
TRINITY_sp|Q96J(	VPS39	Vam6/Vps39-like pro	37.40	0.00
TRINITY_sp|Q614{	Pde1a	Calcium/calmodulin-	37.40	0.00
TRINITY_sp|Q54S!	DDB_G02{	Protein PIEZO homol	37.40	0.00
TRINITY_sp|O948:	LTN1	E3 ubiquitin-protei	37.40	0.00
TRINITY_sp|P509!	ANXA11	Annexin A11 OS=Homo	37.40	0.00
TRINITY_sp|P204{	stg	M-phase inducer pho	37.40	0.00
TRINITY_sp|Q9FKI	UBP17	Ubiquitin carboxyl-	37.40	0.00
TRINITY_sp|Q6GQ:	Anks6	Ankyrin repeat and	37.40	0.00
TRINITY_sp|Q9P2I	ZNFX1	NFX1-type zinc fing	37.40	0.00
TRINITY_sp|P375	radA	DNA repair protein	37.40	0.00
TRINITY_sp|Q9NZI	ITSN2	Intersectin-2 OS=Ho	37.40	0.00
TRINITY_sp|A2VD!	SCLY	Selenocysteine lyas	37.40	0.00
TRINITY_sp|O349	pbuO	Guanine/hypoxanthin	37.40	0.00
TRINITY_sp|Q54B]	abcB2	ABC transporter B f	37.40	0.00
TRINITY_sp|Q9P2!	RCC2	Protein RCC2 OS=Hom	37.40	0.00
TRINITY_sp|F4IF:	FGT1	Protein FORGETTER 1	37.40	0.00
TRINITY_sp|Q9HA(	KIF9	Kinesin-like protei	37.40	0.00
TRINITY_sp|Q7T6	MIMI_R8:	Putative serine/thr	37.40	0.00
TRINITY_sp|Q9C9(	RRA2	Arabinosyltransfera	37.40	0.00
TRINITY_sp|Q6TG:	ATG9	Autophagy-related p	37.40	0.00
TRINITY_sp|Q007]	mdmC	O-methyltransferase	37.40	0.00
TRINITY_sp|Q55A(	DDB_G02	Probable serine/thr	37.40	0.00

TRINITY_sp|Q55GI	vps25	Vacuolar protein-so	37.40	0.00
TRINITY_sp|Q55C8	DDB_G02	Bromodomain-contain	37.40	0.00
TRINITY_sp|P548	ymel-1	ATP-dependent zinc	37.40	0.00
TRINITY_sp|Q9BU	TRMO	tRNA (adenine(37)-N	37.40	0.00
TRINITY_sp|Q54W	aurK	Aurora kinase OS=Di	37.40	0.00
TRINITY_sp|Q69Z	Stk36	Serine/threonine-pr	37.40	0.00
TRINITY_sp|O602	SIPA1L3	Signal-induced prol	37.40	0.00
TRINITY_sp|Q54J	nae1	NEDD8-activating en	37.40	0.00
TRINITY_sp|Q2TB	PCID2	PCI domain-containi	37.40	0.00
TRINITY_sp|Q86G	gcy-28	Receptor-type guany	37.40	0.00
TRINITY_sp|Q150	HERC3	Probable E3 ubiquit	37.30	0.00
TRINITY_sp|A3PT	Mjls_04	UPF0678 fatty acid-	37.30	0.00
TRINITY_sp|P235	phoR	Alkaline phosphatas	37.30	0.00
TRINITY_sp|Q078	VPS13	Vacuolar protein so	37.30	0.00
TRINITY_sp|E1C6	HACE1	E3 ubiquitin-protei	37.30	0.00
TRINITY_sp|Q54T	tra1	Probable transcript	37.30	0.00
TRINITY_sp|Q8VY	At4g073	Mannose-P-dolichol	37.30	0.00
TRINITY_sp|O044	At1g096	Probable elongation	37.30	0.00
TRINITY_sp|Q168	PHKG1	Phosphorylase b kin	37.30	0.00
TRINITY_sp|Q155	SURF2	Surfeit locus prote	37.30	0.00
TRINITY_sp|P177	TDC	Aromatic-L-amino-ac	37.30	0.00
TRINITY_sp|P432	COP1	E3 ubiquitin-protei	37.30	0.00
TRINITY_sp|P604	PTEN	Phosphatidylinosito	37.30	0.00
TRINITY_sp|Q2A3	dnaJ	Chaperone protein D	37.30	0.00
TRINITY_sp|Q6ZS	NBEAL1	Neurobeachin-like p	37.30	0.00
TRINITY_sp|Q275	Cyp4d2	Cytochrome P450 4d2	37.30	0.00
TRINITY_sp|Q6P7	hmces	Embryonic stem cell	37.30	0.00
TRINITY_sp|Q000	SRP68	Signal recognition	37.30	0.00
TRINITY_sp|P840	RHOG	Rho-related GTP-bin	37.30	0.00
TRINITY_sp|P0C8	OsI_027	Uncharacterized pro	37.30	0.00
TRINITY_sp|Q9LH	OCT4	Organic cation/carn	37.30	0.00
TRINITY_sp|Q1MI	ctaA	Heme A synthase OS=	37.30	0.00
TRINITY_sp|Q9JJ	Rpf2	Ribosome production	37.30	0.00
TRINITY_sp|P143	-	Transposon TX1 unch	37.30	0.00
TRINITY_sp|Q5VV	GARNL3	GTPase-activating R	37.30	0.00
TRINITY_sp|Q9C5	MAPKKK5	Mitogen-activated p	37.30	0.00
TRINITY_sp|O959	NUDT3	Diphosphoinositol p	37.30	0.00
TRINITY_sp|O682	dusC	tRNA-dihydrouridine	37.30	0.00
TRINITY_sp|P281	pkgB	Protein kinase 2 OS	37.30	0.00
TRINITY_sp|Q3KQ	UAP1L1	UDP-N-acetylhexosam	37.30	0.00
TRINITY_sp|Q142	msbA	Lipid A export ATP-	37.30	0.00
TRINITY_sp|O811	PAE1	Proteasome subunit	37.30	0.00
TRINITY_sp|Q645	Atp2b3	Plasma membrane cal	37.30	0.00
TRINITY_sp|Q0WQ	RBL1	RHOMBOID-like prote	37.30	0.00
TRINITY_sp|Q556	DDB_G02	Probable rhodanese	37.30	0.00
TRINITY_sp|P404	HOS4	Protein HOS4 OS=Sac	37.30	0.00
TRINITY_sp|Q5ZP	SPO11-2	Meiotic recombinati	37.30	0.00
TRINITY_sp|Q5F3	WWOX	WW domain-containin	37.30	0.00
TRINITY_sp|A0A0	P58A	DnaJ protein P58IPK	37.30	0.00
TRINITY_sp|Q9SE	Os09g05	Ribulose-phosphate	37.30	0.00
TRINITY_sp|Q02P	lap	Aminopeptidase OS=P	37.30	0.00
TRINITY_sp|Q9D9	Efhc1	EF-hand domain-cont	37.30	0.00
TRINITY_sp|Q9ES	Sacm1l	Phosphatidylinositi	37.30	0.00
TRINITY_sp|Q8VZ	INT1	Inositol transporte	37.30	0.00
TRINITY_sp|Q9FK	KIN14B	Kinesin-like protei	37.30	0.00
TRINITY_sp|Q7ZT	gins2	DNA replication com	37.30	0.00

TRINITY_sp|Q9M8I	DSPTP1B	Dual specificity pr	37.30	0.00
TRINITY_sp|Q9CUI	Iqca1	IQ and AAA domain-c	37.30	0.00
TRINITY_sp|Q28GV	melk	Maternal embryonic	37.30	0.00
TRINITY_sp|Q6Z43	MPK3	Mitogen-activated p	37.30	0.00
TRINITY_sp|Q54G3	surf6	Surfeit locus prote	37.30	0.00
TRINITY_sp|Q8S95	NACK1	Kinesin-like protei	37.30	0.00
TRINITY_sp|Q5F35	NRAS	GTPase NRas OS=Gall	37.30	0.00
TRINITY_sp|A4KDI	D5Des	Acyl-lipid (8-3)-de	37.30	0.00
TRINITY_sp|Q9ESV	Ddx24	ATP-dependent RNA h	37.30	0.00
TRINITY_sp|B8B40	MCM9	Probable DNA helica	37.30	0.00
TRINITY_sp|Q9632	SAC8	Phosphoinositide ph	37.30	0.00
TRINITY_sp|Q9Y0A	itr-1	Inositol 1,4,5-tris	37.30	0.00
TRINITY_sp|Q9C80	MOS2	Protein MOS2 OS=Ara	37.30	0.00
TRINITY_sp|Q8TD5	DNAH3	Dynein heavy chain	37.30	0.00
TRINITY_sp|Q9FVX	CYCA1-2	Cyclin-A1-2 OS=Arab	37.30	0.00
TRINITY_sp|Q60G0	EXO1	Exonuclease 1 OS=Or	37.30	0.00
TRINITY_sp|Q8L7I	SPHK1	Sphingosine kinase	37.30	0.00
TRINITY_sp|E9PYY	Wdr78	WD repeat-containin	37.30	0.00
TRINITY_sp|Q9SQ2	CAT7	Cationic amino acid	37.30	0.00
TRINITY_sp|Q8IS1	gefI	Ras guanine nucleot	37.30	0.00
TRINITY_sp|Q2135	gst-4	Glutathione S-trans	37.30	0.00
TRINITY_sp|P1468	YAK1	Dual specificity pr	37.30	0.00
TRINITY_sp|Q91ZI	Nek8	Serine/threonine-pr	37.30	0.00
TRINITY_sp|Q9ZPX	ARL2	ADP-ribosylation fa	37.20	0.00
TRINITY_sp|Q5ZJV	MCMBP	Mini-chromosome mai	37.20	0.00
TRINITY_sp|Q9LU0	UBC25	Probable ubiquitin-	37.20	0.00
TRINITY_sp|O2373	GSH2	Glutathione synthet	37.20	0.00
TRINITY_sp|A1SSI	rlmF	Ribosomal RNA large	37.20	0.00
TRINITY_sp|B6EK7	cutC	Copper homeostasis	37.20	0.00
TRINITY_sp|O7484	fft2	ATP-dependent helic	37.20	0.00
TRINITY_sp|Q9UHI	SAP30BP	SAP30-binding prote	37.20	0.00
TRINITY_sp|Q5XJ3	kctd15l	BTB/POZ domain-cont	37.20	0.00
TRINITY_sp|Q6085	Ripk1	Receptor-interactin	37.20	0.00
TRINITY_sp|Q9ES0	Slc15a2	Solute carrier fami	37.20	0.00
TRINITY_sp|Q55C9	mpl1	MAP kinase phosphat	37.20	0.00
TRINITY_sp|Q54SI	sgmC	Sphingomyelinase ph	37.20	0.00
TRINITY_sp|Q1PF5	EDA2	Probable serine pro	37.20	0.00
TRINITY_sp|Q21L6	mgsA	Methylglyoxal synth	37.20	0.00
TRINITY_sp|Q9C77	At3g1123	Protein yippee-like	37.20	0.00
TRINITY_sp|Q9SB5	PAT08	Protein S-acyltrans	37.20	0.00
TRINITY_sp|Q8K20	Bbs7	Bardet-Biedl syndro	37.20	0.00
TRINITY_sp|Q556I	cf50-1	Counting factor 50	37.20	0.00
TRINITY_sp|Q2LTI	proS	Proline--tRNA ligas	37.20	0.00
TRINITY_sp|Q0373	YML018C	Uncharacterized vac	37.20	0.00
TRINITY_sp|Q6NLY	HVA22K	HVA22-like protein	37.20	0.00
TRINITY_sp|Q75J9	pex2	Peroxisome biogenes	37.20	0.00
TRINITY_sp|Q8WU0	STK32A	Serine/threonine-pr	37.20	0.00
TRINITY_sp|Q08E1	RNF10	RING finger protein	37.20	0.00
TRINITY_sp|Q9CXI	Tbc1d15	TBC1 domain family	37.20	0.00
TRINITY_sp|Q1552	SURF1	Surfeit locus prote	37.20	0.00
TRINITY_sp|A7SK3	dph2	Diphthamide biosynt	37.20	0.00
TRINITY_sp|Q9LY9	LAC13	Laccase-13 OS=Arabi	37.20	0.00
TRINITY_sp|C6E54	pepA	Probable cytosol am	37.20	0.00
TRINITY_sp|A0AV1	UBA6	Ubiquitin-like modi	37.20	0.00
TRINITY_sp|P7516	MPN_633	Uncharacterized pro	37.20	0.00
TRINITY_sp|P4433	rbsK	Ribokinase OS=Haemo	37.20	0.00

TRINITY_sp|P344(	gln-2	Probable glutamine	37.20	0.00
TRINITY_sp|Q6GL	grtp1	Growth hormone-regu	37.20	0.00
TRINITY_sp|Q8RXI	CLC-F	Chloride channel pr	37.20	0.00
TRINITY_sp|P777	yajO	Uncharacterized oxi	37.20	0.00
TRINITY_sp|Q0IH	spef1	Sperm flagellar pro	37.20	0.00
TRINITY_sp|Q54LI	vps13D	Putative vacuolar p	37.20	0.00
TRINITY_sp|O138	dcp2	mRNA decapping comp	37.20	0.00
TRINITY_sp|Q9VW(	l(3)76BI	E3 ubiquitin-protei	37.20	0.00
TRINITY_sp|Q9P2I	CHD7	Chromodomain-helica	37.20	0.00
TRINITY_sp|A1ZA	CG5065	Putative fatty acyl	37.20	0.00
TRINITY_sp|Q9JL(	Capn15	Calpain-15 OS=Mus m	37.20	0.00
TRINITY_sp|P399	SLN1	Osmosensing histidi	37.20	0.00
TRINITY_sp|P239(	glcA	Glucan endo-1,3-bet	37.20	0.00
TRINITY_sp|Q9LZ	PUB16	U-box domain-contai	37.20	0.00
TRINITY_sp|P411	CAPS	Calcyphosin OS=Oryc	37.20	0.00
TRINITY_sp|A4RF	PAN2	PAB-dependent poly(	37.20	0.00
TRINITY_sp|Q9SH	RRP44A	Exosome complex exo	37.20	0.00
TRINITY_sp|Q60H	TPP1	Tripeptidyl-peptida	37.20	0.00
TRINITY_sp|Q9UU	ppk34	Serine/threonine-pr	37.20	0.00
TRINITY_sp|Q8GU	MOS14	Transportin MOS14 O	37.20	0.00
TRINITY_sp|Q4P0	YOP1	Protein YOP1 OS=Ust	37.20	0.00
TRINITY_sp|Q6PC	ppt2-a	Lysosomal thioester	37.20	0.00
TRINITY_sp|Q54H	drkB	Probable serine/thr	37.20	0.00
TRINITY_sp|P109	-	Retrovirus-related	37.20	0.00
TRINITY_sp|P092	-	25 kDa calcium-bind	37.20	0.00
TRINITY_sp|Q5EB	Wdr70	WD repeat-containin	37.20	0.00
TRINITY_sp|Q94B(	At1g718	Uncharacterized aar	37.20	0.00
TRINITY_sp|Q9T0	LACS4	Long chain acyl-CoA	37.20	0.00
TRINITY_sp|Q641	tmem18	Transmembrane prote	37.20	0.00
TRINITY_sp|Q8IY	CCDC146	Coiled-coil domain-	37.20	0.00
TRINITY_sp|P398	EXO1	Exodeoxyribonucleas	37.20	0.00
TRINITY_sp|Q8S9	ATG8E	Autophagy-related p	37.20	0.00
TRINITY_sp|Q8H0	UPL2	E3 ubiquitin-protei	37.20	0.00
TRINITY_sp|Q54Y(	trappc5	Trafficking protein	37.10	0.00
TRINITY_sp|Q273	-	Gelsolin, cytoplasm	37.10	0.00
TRINITY_sp|Q9BZ(	ABCA2	ATP-binding cassett	37.10	0.00
TRINITY_sp|Q9QX	Trip4	Activating signal c	37.10	0.00
TRINITY_sp|A5PK	METTL13	Methyltransferase-l	37.10	0.00
TRINITY_sp|Q54B	abcB2	ABC transporter B f	37.10	0.00
TRINITY_sp|P067	CMD1	Calmodulin OS=Sacch	37.10	0.00
TRINITY_sp|Q921(	Tubgcp2	Gamma-tubulin compl	37.10	0.00
TRINITY_sp|D9HP	CNR4	Cell number regulat	37.10	0.00
TRINITY_sp|Q8IV(	HERC6	Probable E3 ubiquit	37.10	0.00
TRINITY_sp|P434	scrK	Fructokinase OS=Ped	37.10	0.00
TRINITY_sp|Q125	hxA	Xanthine dehydrogen	37.10	0.00
TRINITY_sp|Q136(	PEX6	Peroxisome assembly	37.10	0.00
TRINITY_sp|O315	yfhM	AB hydrolase superf	37.10	0.00
TRINITY_sp|Q392	PP2AB2	Serine/threonine pr	37.10	0.00
TRINITY_sp|Q9P2	USP36	Ubiquitin carboxyl-	37.10	0.00
TRINITY_sp|Q54E	gacEE	Rho GTPase-activati	37.10	0.00
TRINITY_sp|Q54S	spt16	FACT complex subuni	37.10	0.00
TRINITY_sp|Q54D(	mcfP	Mitochondrial subst	37.10	0.00
TRINITY_sp|P552(	BRPF1	Peregrin OS=Homo sa	37.10	0.00
TRINITY_sp|P9WK	nlhH	Carboxylesterase Nl	37.10	0.00
TRINITY_sp|Q5RJ	Mctp2	Multiple C2 and tra	37.10	0.00
TRINITY_sp|Q9WW	alkJ	Alcohol dehydrogena	37.10	0.00

TRINITY_sp|Q9ZVN	GTF2H2	General transcripti	37.10	0.00
TRINITY_sp|P912	gst-8	Probable glutathion	37.10	0.00
TRINITY_sp|Q2HJN	MEST	Mesoderm-specific t	37.10	0.00
TRINITY_sp|Q9NPI	ACP6	Lysophosphatidic ac	37.10	0.00
TRINITY_sp|Q0MQ8	NDUFA12	NADH dehydrogenase	37.10	0.00
TRINITY_sp|Q940I	SRK2E	Serine/threonine-pr	37.10	0.00
TRINITY_sp|Q10XN	infB	Translation initiat	37.10	0.00
TRINITY_sp|Q94F2	PAP1	Plastid lipid-assoc	37.10	0.00
TRINITY_sp|Q54KN	ccbl	Kynurenine--oxoglut	37.10	0.00
TRINITY_sp|G5ECI	gba-3	Putative glucosylce	37.10	0.00
TRINITY_sp|Q94A6	At4g277(	Rhodanese-like doma	37.10	0.00
TRINITY_sp|Q5ZI7	TAF2	Transcription initi	37.10	0.00
TRINITY_sp|P1295	PEPD	Xaa-Pro dipeptidase	37.10	0.00
TRINITY_sp|Q552N	gefY	Ras guanine nucleot	37.10	0.00
TRINITY_sp|P9782	Ctsc	Dipeptidyl peptidas	37.10	0.00
TRINITY_sp|Q55EI	cyp524A1	Probable cytochrome	37.10	0.00
TRINITY_sp|Q8AX(	kit	Mast/stem cell grow	37.10	0.00
TRINITY_sp|O4276	BMH1	14-3-3 protein homo	37.10	0.00
TRINITY_sp|P5861	glk	Glucokinase OS=Nost	37.10	0.00
TRINITY_sp|P5114	RAB5C	Ras-related protein	37.10	0.00
TRINITY_sp|Q8BZ3	Dcun1d2	DCN1-like protein 2	37.10	0.00
TRINITY_sp|Q5568	sll0005	Uncharacterized pro	37.10	0.00
TRINITY_sp|Q550W	captC	Uncharacterized CDP	37.10	0.00
TRINITY_sp|O2255	STY8	Serine/threonine-pr	37.10	0.00
TRINITY_sp|Q9ZTX	PSAM2	Secretory carrier-a	37.10	0.00
TRINITY_sp|Q9ZTN	ERD2	ER lumen protein-re	37.10	0.00
TRINITY_sp|Q9LF4	PUB1	Probable ubiquitin	37.10	0.00
TRINITY_sp|Q8L5I	TFCD	Tubulin-folding cof	37.10	0.00
TRINITY_sp|O1477	TCERG1	Transcription elong	37.10	0.00
TRINITY_sp|A8N53	EFG1	rRNA-processing pro	37.10	0.00
TRINITY_sp|Q8IB9	CPK4	Calcium-dependent p	37.10	0.00
TRINITY_sp|Q8049	mib1	E3 ubiquitin-protei	37.10	0.00
TRINITY_sp|Q6PC9	nmnat2	Nicotinamide/nicoti	37.10	0.00
TRINITY_sp|Q75K2	ncsA	Calcium-binding pro	37.10	0.00
TRINITY_sp|O5995	LIP	Lipase OS=Thermomyc	37.10	0.00
TRINITY_sp|Q9ZGI	pikAII	Narbonolide/10-deox	37.10	0.00
TRINITY_sp|Q9BX6	BRIP1	Fanconi anemia grou	37.10	0.00
TRINITY_sp|Q7YRA	EXOSC4	Exosome complex com	37.10	0.00
TRINITY_sp|Q9C53	PAT22	Probable protein S-	37.10	0.00
TRINITY_sp|Q9H89	CNTD2	Cyclin N-terminal d	37.10	0.00
TRINITY_sp|P0419	Myb	Myb protein OS=Dros	37.10	0.00
TRINITY_sp|Q3UGI	Wdr19	WD repeat-containin	37.10	0.00
TRINITY_sp|Q9M3(	PAT05	Probable protein S-	37.10	0.00
TRINITY_sp|Q8GWA	NDA1	Internal alternativ	37.10	0.00
TRINITY_sp|Q6VVW	Npr2	Atrial natriuretic	37.10	0.00
TRINITY_sp|Q54YI	dhkB	Hybrid signal trans	37.10	0.00
TRINITY_sp|Q96J(	ITCH	E3 ubiquitin-protei	37.00	0.00
TRINITY_sp|Q55FN	plbG	Phospholipase B-lik	37.00	0.00
TRINITY_sp|Q54YI	dhkB	Hybrid signal trans	37.00	0.00
TRINITY_sp|Q6Z82	WEE1	Wee1-like protein k	37.00	0.00
TRINITY_sp|Q06A(	RAB32	Ras-related protein	37.00	0.00
TRINITY_sp|O8902	Tpp1	Tripeptidyl-peptida	37.00	0.00
TRINITY_sp|P6276	VSNL1	Visinin-like protei	37.00	0.00
TRINITY_sp|P5836	arcB	Aerobic respiration	37.00	0.00
TRINITY_sp|Q941I	BON1	Protein BONZAI 1 OS	37.00	0.00
TRINITY_sp|Q6GR(	spopl	Speckle-type POZ pr	37.00	0.00

TRINITY_sp|O5852	taw2	tRNA(Phe) (4-demeth	37.00	0.00
TRINITY_sp|Q1PF5	EDA2	Probable serine pro	37.00	0.00
TRINITY_sp|Q0WUI	FAB1A	1-phosphatidylinosi	37.00	0.00
TRINITY_sp|Q9SZ1	LIP5	Protein HOMOLOG OF	37.00	0.00
TRINITY_sp|Q68EI	flad1	FAD synthase OS=Dan	37.00	0.00
TRINITY_sp|P3952	DRS2	Probable phospholip	37.00	0.00
TRINITY_sp|Q86Y0	CPNE8	Copine-8 OS=Homo sa	37.00	0.00
TRINITY_sp|Q059	RAB2	Ras-related protein	37.00	0.00
TRINITY_sp|P7378	slr1251	Peptidyl-prolyl cis	37.00	0.00
TRINITY_sp|Q9M9I	At1g7828	F-box protein At1g7	37.00	0.00
TRINITY_sp|Q69TI	OPR2	Putative 12-oxophyt	37.00	0.00
TRINITY_sp|Q9LPS	At1g5059	Pirin-like protein	37.00	0.00
TRINITY_sp|O3461	ytqB	Putative rRNA methy	37.00	0.00
TRINITY_sp|P5836	arcB	Aerobic respiration	37.00	0.00
TRINITY_sp|O1391	hpt1	Hypoxanthine-guanin	37.00	0.00
TRINITY_sp|Q8L7N	SDN5	Small RNA degrading	37.00	0.00
TRINITY_sp|Q9CQ0	Tmem216	Transmembrane prote	37.00	0.00
TRINITY_sp|Q9SI1	DSK2B	Ubiquitin domain-co	37.00	0.00
TRINITY_sp|B4LN4	Adam	Eukaryotic translat	37.00	0.00
TRINITY_sp|P0CE9	talA	Talin-A OS=Dictyost	37.00	0.00
TRINITY_sp|P7316	trmB	tRNA (guanine-N(7)-	37.00	0.00
TRINITY_sp|P501	Rdh2	Retinol dehydrogena	37.00	0.00
TRINITY_sp|Q9P30	dbl4	E3 ubiquitin-protei	37.00	0.00
TRINITY_sp|Q6NRI	snx33	Sorting nexin-33 OS	37.00	0.00
TRINITY_sp|Q9H49	PIGU	Phosphatidylinosito	37.00	0.00
TRINITY_sp|Q4KLV	chpt1	Cholinephosphotrans	37.00	0.00
TRINITY_sp|Q8WSI	IP3R	Inositol 1,4,5-tris	37.00	0.00
TRINITY_sp|P1059	SSA1	Heat shock protein	37.00	0.00
TRINITY_sp|Q17R0	DHX38	Pre-mRNA-splicing f	37.00	0.00
TRINITY_sp|Q86HI	cf45-1	Counting factor 45-	37.00	0.00
TRINITY_sp|Q7ZV2	abhd17c	Protein ABHD17C OS=	37.00	0.00
TRINITY_sp|Q9M08	At4g3264	Protein transport p	37.00	0.00
TRINITY_sp|Q9P2I	DNAH1	Dynein heavy chain	37.00	0.00
TRINITY_sp|Q9FJ1	At5g5673	Zinc protease PQQL-	37.00	0.00
TRINITY_sp|Q1ZXI	gxcDD	Guanine exchange fa	37.00	0.00
TRINITY_sp|P3414	racB	Rho-related protein	37.00	0.00
TRINITY_sp|Q027	MAP3K10	Mitogen-activated p	37.00	0.00
TRINITY_sp|F1RCI	trip12	E3 ubiquitin-protei	37.00	0.00
TRINITY_sp|Q7KQI	ARF1	ADP-ribosylation fa	37.00	0.00
TRINITY_sp|P5904	NLRP5	NACHT, LRR and PYD	37.00	0.00
TRINITY_sp|Q5ZKI	MOV10	Putative helicase M	37.00	0.00
TRINITY_sp|Q9W6V	PTPRJ	Receptor-type tyros	37.00	0.00
TRINITY_sp|O5253	cah	Carbonic anhydrase	36.90	0.00
TRINITY_sp|O6265	SI	Sucrase-isomaltase,	36.90	0.00
TRINITY_sp|Q9STV	CIPK8	CBL-interacting ser	36.90	0.00
TRINITY_sp|F4IUX	UPF2	Regulator of nonsen	36.90	0.00
TRINITY_sp|G5EF0	pak-2	Serine/threonine-pr	36.90	0.00
TRINITY_sp|Q28Y6	GA21225	Zinc finger CCCH do	36.90	0.00
TRINITY_sp|P3528	Rab12	Ras-related protein	36.90	0.00
TRINITY_sp|P618	-	Lipase OS=Rhizopus	36.90	0.00
TRINITY_sp|P9658	topB	DNA topoisomerase 3	36.90	0.00
TRINITY_sp|Q869W	DDB_G02	Probable myosin lig	36.90	0.00
TRINITY_sp|P2879	GRN	Granulins OS=Homo s	36.90	0.00
TRINITY_sp|Q94A4	At1g6202	Coatomer subunit al	36.90	0.00
TRINITY_sp|Q1698	-	Neurocalcin OS=Aply	36.90	0.00
TRINITY_sp|Q8N9V	WDSUB1	WD repeat, SAM and	36.90	0.00

TRINITY_sp|Q95N(	DIO1	Type I iodothyronin	36.90	0.00
TRINITY_sp|Q8R3I	Tbc1d13	TBC1 domain family	36.90	0.00
TRINITY_sp|P405(	YIR042C	Uncharacterized pro	36.90	0.00
TRINITY_sp|Q28I\	cops3	COP9 signalosome co	36.90	0.00
TRINITY_sp|F4IP\	SYCO	Cysteine--tRNA liga	36.90	0.00
TRINITY_sp|Q55DV	gacZ	Rho GTPase-activati	36.90	0.00
TRINITY_sp|Q3MII	Echdc3	Enoyl-CoA hydratase	36.90	0.00
TRINITY_sp|Q91V2	Abca7	ATP-binding cassett	36.90	0.00
TRINITY_sp|Q7K5!	Ttd14	TRPL translocation	36.90	0.00
TRINITY_sp|Q9QZ	Dcun1d1	DCN1-like protein 1	36.90	0.00
TRINITY_sp|P468	KIN14N	Kinesin-like protei	36.90	0.00
TRINITY_sp|Q993	TY3B-G	Transposon Ty3-G Ga	36.90	0.00
TRINITY_sp|Q9H1	ANAPC1	Anaphase-promoting	36.90	0.00
TRINITY_sp|A0BK(	GSPATT0(	Protein SEY1 homolo	36.90	0.00
TRINITY_sp|Q9ZVI	CUL3A	Cullin-3A OS=Arabid	36.90	0.00
TRINITY_sp|Q54P(	dlcA	Dynein light chain	36.90	0.00
TRINITY_sp|Q4V8(	Wdr78	WD repeat-containin	36.90	0.00
TRINITY_sp|Q8L72	GSTT2	Glutathione S-trans	36.90	0.00
TRINITY_sp|Q3MH(	SPTLC1	Serine palmitoyltra	36.90	0.00
TRINITY_sp|O757(	TRPA1	Transient receptor	36.90	0.00
TRINITY_sp|P478	Pdcd2	Programmed cell dea	36.90	0.00
TRINITY_sp|Q098!	ayr1	NADPH-dependent 1-a	36.90	0.00
TRINITY_sp|P239!	mpp	Mitochondrial-proce	36.90	0.00
TRINITY_sp|P492!	mao	Amine oxidase [flav	36.90	0.00
TRINITY_sp|Q1JPI	EIF4H	Eukaryotic translat	36.90	0.00
TRINITY_sp|P110	RAB3A	Ras-related protein	36.90	0.00
TRINITY_sp|Q9BQ(	NUDT12	Peroxisomal NADH py	36.90	0.00
TRINITY_sp|Q8TD!	DNAH3	Dynein heavy chain	36.90	0.00
TRINITY_sp|Q74FV	tdcB	L-threonine ammonia	36.90	0.00
TRINITY_sp|Q8K2\	Ipo11	Importin-11 OS=Mus	36.90	0.00
TRINITY_sp|Q111	wht-1	ABC transporter ATP	36.90	0.00
TRINITY_sp|P386(	PDIA6	Protein disulfide-i	36.90	0.00
TRINITY_sp|Q8N5	CAMKK1	Calcium/calmodulin-	36.90	0.00
TRINITY_sp|O043	B'BETA	Serine/threonine pr	36.90	0.00
TRINITY_sp|Q96HI	REEP6	Receptor expression	36.90	0.00
TRINITY_sp|P932	VPS41	Vacuolar protein so	36.90	0.00
TRINITY_sp|Q9FPI	EDR1	Serine/threonine-pr	36.90	0.00
TRINITY_sp|Q8TA(	JOSD2	Josephin-2 OS=Homo	36.90	0.00
TRINITY_sp|Q8IS	gefJ	Ras guanine nucleot	36.90	0.00
TRINITY_sp|Q5M7!	PAT12	Probable protein S-	36.90	0.00
TRINITY_sp|Q9QXI	Tbl1x	F-box-like/WD repea	36.90	0.00
TRINITY_sp|Q1ZXI	gxcDD	Guanine exchange fa	36.90	0.00
TRINITY_sp|Q5MB	ABCG2	ATP-binding cassett	36.90	0.00
TRINITY_sp|Q8RVI	DEK1	Calpain-type cystei	36.90	0.00
TRINITY_sp|O597	ark1	Serine/threonine-pr	36.90	0.00
TRINITY_sp|A6QR!	USP4	Ubiquitin carboxyl-	36.90	0.00
TRINITY_sp|Q408(	EMB8	Embryogenesis-assoc	36.90	0.00
TRINITY_sp|P395	DRS2	Probable phospholip	36.90	0.00
TRINITY_sp|P227	Gucy1b2	Guanylate cyclase s	36.90	0.00
TRINITY_sp|Q9S7	SYN1	Sister chromatid co	36.90	0.00
TRINITY_sp|Q0V9I	dis3l2	DIS3-like exonuclea	36.90	0.00
TRINITY_sp|Q6WN(	D4	Acyl-lipid (7-3)-de	36.90	0.00
TRINITY_sp|Q080	TEF1	Elongation factor 1	36.90	0.00
TRINITY_sp|Q54B(	dyrk2	Probable serine/thr	36.90	0.00
TRINITY_sp|P253	mlkA	Myosin light chain	36.90	0.00
TRINITY_sp|Q54KI	DDB_G02(	TPR repeat-containi	36.90	0.00

TRINITY_sp|O974	ancA	Mitochondrial subst	36.90	0.00
TRINITY_sp|Q8Z4N	yhfR	Uncharacterized pro	36.80	0.00
TRINITY_sp|P0979	CDC16	Anaphase-promoting	36.80	0.00
TRINITY_sp|Q141	VEZF1	Vascular endothelia	36.80	0.00
TRINITY_sp|Q9P25	RCC2	Protein RCC2 OS=Hom	36.80	0.00
TRINITY_sp|Q55F0	tipC	Putative vacuolar p	36.80	0.00
TRINITY_sp|Q8ND	PAPD5	Non-canonical poly(	36.80	0.00
TRINITY_sp|Q9FG0	ATO	Splicing factor SF3	36.80	0.00
TRINITY_sp|Q54C	rrp8	Ribosomal RNA-proce	36.80	0.00
TRINITY_sp|Q9M1	FAP1	Fatty-acid-binding	36.80	0.00
TRINITY_sp|O1436	naf1	H/ACA ribonucleopro	36.80	0.00
TRINITY_sp|P4902	PXN	Paxillin OS=Gallus	36.80	0.00
TRINITY_sp|Q5ZI	RAB10	Ras-related protein	36.80	0.00
TRINITY_sp|P4329	RD21A	Cysteine proteinase	36.80	0.00
TRINITY_sp|Q6UX0	DHRS13	Dehydrogenase/reduc	36.80	0.00
TRINITY_sp|Q7K55	Ttd14	TRPL translocation	36.80	0.00
TRINITY_sp|C6KT	SET1	Putative histone-ly	36.80	0.00
TRINITY_sp|Q9CA2	TKPR2	Tetraketide alpha-p	36.80	0.00
TRINITY_sp|Q68F0	-	Uncharacterized pro	36.80	0.00
TRINITY_sp|P9089	F56F10.1	Putative serine pro	36.80	0.00
TRINITY_sp|Q5JV	EFHC1	EF-hand domain-cont	36.80	0.00
TRINITY_sp|Q4990	Slc25a38	Solute carrier fami	36.80	0.00
TRINITY_sp|Q8NI3	WDR36	WD repeat-containin	36.80	0.00
TRINITY_sp|P7653	yfeX	Probable deferroche	36.80	0.00
TRINITY_sp|O3560	Abca4	Retinal-specific AT	36.80	0.00
TRINITY_sp|Q6IR	abhd13	Protein ABHD13 OS=X	36.80	0.00
TRINITY_sp|Q54W	DDB_G02	Probable serine/thr	36.80	0.00
TRINITY_sp|Q9T0	LACS5	Long chain acyl-CoA	36.80	0.00
TRINITY_sp|P5779	ALA12	Probable phospholip	36.80	0.00
TRINITY_sp|Q16X9	AAEL0089	Regulator of telome	36.80	0.00
TRINITY_sp|Q94E	At3g1441	Probable sugar phos	36.80	0.00
TRINITY_sp|Q554	bzpJ	Probable basic-leuc	36.80	0.00
TRINITY_sp|Q2HJ9	FAHD1	Acylpyruvase FAHD1,	36.80	0.00
TRINITY_sp|Q1ZX	gxcDD	Guanine exchange fa	36.80	0.00
TRINITY_sp|Q96FN	KIF12	Kinesin-like protei	36.80	0.00
TRINITY_sp|Q9210	Brwd1	Bromodomain and WD	36.80	0.00
TRINITY_sp|Q7X6	RUS1	Protein root UVB se	36.80	0.00
TRINITY_sp|Q4PK	CLCN7	H(+)/Cl(-) exchange	36.80	0.00
TRINITY_sp|Q4V3	MRG2	Protein MRG2 OS=Ara	36.80	0.00
TRINITY_sp|Q9CR2	Nudt8	Nucleoside diphosph	36.80	0.00
TRINITY_sp|Q75K	ATG4A	Cysteine protease A	36.80	0.00
TRINITY_sp|O3158	yfhM	AB hydrolase superf	36.80	0.00
TRINITY_sp|Q9C79	AERO1	Endoplasmic reticul	36.80	0.00
TRINITY_sp|P0C8N	CCRP1	Probable serine/thr	36.80	0.00
TRINITY_sp|Q8Z0	cysA	Sulfate/thiosulfate	36.80	0.00
TRINITY_sp|O0026	SUPT5H	Transcription elong	36.80	0.00
TRINITY_sp|Q6TM	poxA	Peroxinectin A OS=D	36.80	0.00
TRINITY_sp|O0023	PSMD9	26S proteasome non-	36.80	0.00
TRINITY_sp|Q5EA5	ABHD4	Protein ABHD4 OS=Bo	36.80	0.00
TRINITY_sp|B8BM1	OsI_3745	Pyruvate kinase 2,	36.80	0.00
TRINITY_sp|P4052	NEO1	Probable phospholip	36.80	0.00
TRINITY_sp|Q9928	GRESAG	Receptor-type adeny	36.80	0.00
TRINITY_sp|Q9R1	Sik1	Serine/threonine-pr	36.80	0.00
TRINITY_sp|B2GU2	Usp4	Ubiquitin carboxyl-	36.80	0.00
TRINITY_sp|Q99L2	Gstt3	Glutathione S-trans	36.80	0.00
TRINITY_sp|P1243	-	Actin A OS=Trypanos	36.70	0.00

TRINITY_sp|Q9SE	WER	Transcription facto	36.70	0.00
TRINITY_sp|Q636	Vps33a	Vacuolar protein so	36.70	0.00
TRINITY_sp|Q8NF	NUP37	Nucleoporin Nup37 O	36.70	0.00
TRINITY_sp|Q2KH	EXOSC8	Exosome complex com	36.70	0.00
TRINITY_sp|Q236	tgt-1	Queuine tRNA-ribosy	36.70	0.00
TRINITY_sp|P545	USP14	Ubiquitin carboxyl-	36.70	0.00
TRINITY_sp|Q8R7	rplD	50S ribosomal prote	36.70	0.00
TRINITY_sp|Q55A	captA	Uncharacterized CDP	36.70	0.00
TRINITY_sp|Q9T0	HMGB13	High mobility group	36.70	0.00
TRINITY_sp|Q95V	carmil	Protein CARMIL OS=D	36.70	0.00
TRINITY_sp|O826	VHA-G1	V-type proton ATPas	36.70	0.00
TRINITY_sp|P347	FAB1	1-phosphatidylinosi	36.70	0.00
TRINITY_sp|Q7ZV	pitrm1	Presequence proteas	36.70	0.00
TRINITY_sp|Q7SY	nt5c3	Cytosolic 5'-nucleo	36.70	0.00
TRINITY_sp|Q94B	FAO4B	Long-chain-alcohol	36.70	0.00
TRINITY_sp|P558	-	Probable 60S riboso	36.70	0.00
TRINITY_sp|Q9NV	UQCC1	Ubiquinol-cytochrom	36.70	0.00
TRINITY_sp|Q9TU	LMAN1	Protein ERGIC-53 OS	36.70	0.00
TRINITY_sp|Q023	ADA2	Transcriptional ada	36.70	0.00
TRINITY_sp|Q996	DNAJC7	DnaJ homolog subfam	36.70	0.00
TRINITY_sp|P495	ppm-2	Probable protein ph	36.70	0.00
TRINITY_sp|Q8NH	TTLL11	Tubulin polyglutamy	36.70	0.00
TRINITY_sp|Q9CP	Lipf	Gastric triacylglyc	36.70	0.00
TRINITY_sp|O642	10	Endolysin A OS=Myco	36.70	0.00
TRINITY_sp|Q9LJ	At3g224	Probable prefoldin	36.70	0.00
TRINITY_sp|O601	SPBC14F	Alkaline phosphatas	36.70	0.00
TRINITY_sp|Q146	ITIH4	Inter-alpha-trypsin	36.70	0.00
TRINITY_sp|Q9FJ	UPF1	Regulator of nonsen	36.70	0.00
TRINITY_sp|Q9SS	HIPL1	HIPL1 protein OS=Ar	36.70	0.00
TRINITY_sp|Q49U	sat	Sulfate adenylyltra	36.70	0.00
TRINITY_sp|Q584	coaD	Phosphopantetheine	36.70	0.00
TRINITY_sp|Q54E	DDB_G02	Protein TEX261 homo	36.70	0.00
TRINITY_sp|Q9NW	RBM28	RNA-binding protein	36.70	0.00
TRINITY_sp|P076	HEXB	Beta-hexosaminidase	36.70	0.00
TRINITY_sp|Q9LX	GCN2	eIF-2-alpha kinase	36.70	0.00
TRINITY_sp|A7UX	egt-2	Hercynylcysteine su	36.70	0.00
TRINITY_sp|O188	AOAH	Acyloxyacyl hydrola	36.70	0.00
TRINITY_sp|Q9M0	SCPL24	Serine carboxypepti	36.70	0.00
TRINITY_sp|Q8HY	ELAC2	Zinc phosphodiester	36.70	0.00
TRINITY_sp|Q035	Itk	Tyrosine-protein ki	36.70	0.00
TRINITY_sp|P211	P4HB	Protein disulfide-i	36.70	0.00
TRINITY_sp|Q425	FATA	Oleoyl-acyl carrier	36.70	0.00
TRINITY_sp|Q8BQ	Zdhhc14	Probable palmitoylt	36.70	0.00
TRINITY_sp|Q54D	DDB_G02	SET and MYND domain	36.70	0.00
TRINITY_sp|B7K2	truA	tRNA pseudouridine	36.70	0.00
TRINITY_sp|P485	eIF-4E	Eukaryotic translat	36.70	0.00
TRINITY_sp|Q8R3	Tcf25	Transcription facto	36.70	0.00
TRINITY_sp|Q888	PSPTO_1	Renalase OS=Pseudom	36.70	0.00
TRINITY_sp|Q995	MPHOSPH	M-phase phosphoprot	36.70	0.00
TRINITY_sp|Q1ZX	gxcDD	Guanine exchange fa	36.70	0.00
TRINITY_sp|Q9W0	bab2	Protein bric-a-brac	36.70	0.00
TRINITY_sp|Q9FL	PAT06	Probable protein S-	36.70	0.00
TRINITY_sp|O949	PCF11	Pre-mRNA cleavage c	36.70	0.00
TRINITY_sp|Q9UT	SPAC25B	Uncharacterized met	36.70	0.00
TRINITY_sp|Q7XJ	EB1A	Microtubule-associa	36.70	0.00
TRINITY_sp|P384	TIF5	Eukaryotic translat	36.70	0.00

TRINITY_sp|Q4FP	atpG	ATP synthase gamma	36.70	0.00
TRINITY_sp|Q54B	abcB2	ABC transporter B f	36.70	0.00
TRINITY_sp|F4IC	IWS1	Protein IWS1 homolo	36.70	0.00
TRINITY_sp|A8WX	CBG0456	Protein FAM50 homol	36.70	0.00
TRINITY_sp|Q7LH	TY3B-I	Transposon Ty3-I Ga	36.70	0.00
TRINITY_sp|Q6EU	Os02g06	Probable N6-adenosi	36.70	0.00
TRINITY_sp|Q54Y	ngap	Probable Ras GTPase	36.70	0.00
TRINITY_sp|Q54E	epnA	Epsin OS=Dictyostel	36.70	0.00
TRINITY_sp|Q8N1	CYP4X1	Cytochrome P450 4X1	36.70	0.00
TRINITY_sp|Q9ZG	pikAII	Narbonolide/10-deox	36.70	0.00
TRINITY_sp|Q56X	NHX2	Sodium/hydrogen exc	36.70	0.00
TRINITY_sp|Q96P	ACAP3	Arf-GAP with coiled	36.70	0.00
TRINITY_sp|Q2QQ	CYCT1-1	Cyclin-T1-4 OS=Oryz	36.70	0.00
TRINITY_sp|Q993	TY3B-G	Transposon Ty3-G Ga	36.70	0.00
TRINITY_sp|P623	CPK4	Calcium-dependent p	36.70	0.00
TRINITY_sp|Q9VY	Bap60	Brahma-associated p	36.70	0.00
TRINITY_sp|Q969	TMBIM1	Protein lifeguard 3	36.70	0.00
TRINITY_sp|Q640	slc37a3	Sugar phosphate exc	36.60	0.00
TRINITY_sp|O433	TXNL1	Thioredoxin-like pr	36.60	0.00
TRINITY_sp|Q962	MYB23	Transcription facto	36.60	0.00
TRINITY_sp|P623	CPK4	Calcium-dependent p	36.60	0.00
TRINITY_sp|Q3UJ	Armc7	Armadillo repeat-co	36.60	0.00
TRINITY_sp|O233	ECI3	Enoyl-CoA delta iso	36.60	0.00
TRINITY_sp|P259	MXAN_59	Uncharacterized oxi	36.60	0.00
TRINITY_sp|Q1ZX	gxcDD	Guanine exchange fa	36.60	0.00
TRINITY_sp|Q103	yaf9	Protein AF-9 homolo	36.60	0.00
TRINITY_sp|Q54S	mcfU	Mitochondrial subst	36.60	0.00
TRINITY_sp|B7GI	obg	GTPase Obg OS=Anoxy	36.60	0.00
TRINITY_sp|Q1ZX	gxcDD	Guanine exchange fa	36.60	0.00
TRINITY_sp|Q401	-	LEC14B protein OS=L	36.60	0.00
TRINITY_sp|Q1LX	ints3	Integrator complex	36.60	0.00
TRINITY_sp|Q0KI	CG9801	PP2C-like domain-co	36.60	0.00
TRINITY_sp|Q8LP	BTS	Zinc finger protein	36.60	0.00
TRINITY_sp|Q9CP	rsuA	Ribosomal small sub	36.60	0.00
TRINITY_sp|B8AA	CEST	Protein CHLOROPLAST	36.60	0.00
TRINITY_sp|Q9C1	SPBC713.	Periodic tryptophan	36.60	0.00
TRINITY_sp|Q9W5	CG43867	Uncharacterized pro	36.60	0.00
TRINITY_sp|Q056	CTR1	Serine/threonine-pr	36.60	0.00
TRINITY_sp|Q55F	plbG	Phospholipase B-lik	36.60	0.00
TRINITY_sp|Q944	dymA	Dynamin-A OS=Dictyo	36.60	0.00
TRINITY_sp|O946	bms1	Ribosome biogenesis	36.60	0.00
TRINITY_sp|Q5F3	SBNO1	Protein strawberry	36.60	0.00
TRINITY_sp|Q9LI	TMN7	Transmembrane 9 sup	36.60	0.00
TRINITY_sp|Q80W	Gadl1	Acidic amino acid d	36.60	0.00
TRINITY_sp|Q9FV	At1g577	Probable elongation	36.60	0.00
TRINITY_sp|Q8NC	MTMR14	Myotubularin-relate	36.60	0.00
TRINITY_sp|Q1E3	FES1	Hsp70 nucleotide ex	36.60	0.00
TRINITY_sp|O002	PSMD12	26S proteasome non-	36.60	0.00
TRINITY_sp|Q16A	rplO	50S ribosomal prote	36.60	0.00
TRINITY_sp|Q2SW	surE	5'-nucleotidase Sur	36.60	0.00
TRINITY_sp|A6QR	USP4	Ubiquitin carboxyl-	36.60	0.00
TRINITY_sp|Q7S7	NCU0673	Leukotriene A-4 hyd	36.60	0.00
TRINITY_sp|Q8L8	COG4	Conserved oligomeri	36.60	0.00
TRINITY_sp|Q8L9	P4H7	Probable prolyl 4-h	36.60	0.00
TRINITY_sp|Q9ZU	At2g372	Thioredoxin-like pr	36.60	0.00
TRINITY_sp|O158	CPK2	Calcium-dependent p	36.60	0.00

TRINITY_sp|O078	dapb1	Dipeptidyl aminopep	36.60	0.00
TRINITY_sp|Q8GY	UPL1	E3 ubiquitin-protei	36.60	0.00
TRINITY_sp|Q86K	cpnB-1	Copine-B OS=Dictyos	36.60	0.00
TRINITY_sp|Q8RW	STY17	Serine/threonine-pr	36.60	0.00
TRINITY_sp|Q435	CCAMK	Calcium and calcium	36.60	0.00
TRINITY_sp|Q9EP	Acox3	Peroxisomal acyl-co	36.60	0.00
TRINITY_sp|P328	STX2	Syntaxin-2 OS=Homo	36.50	0.00
TRINITY_sp|Q9W2	BTBD9	BTB/POZ domain-cont	36.50	0.00
TRINITY_sp|Q9AT	FAD2	Delta(12) fatty aci	36.50	0.00
TRINITY_sp|O138	its3	Phosphatidylinosito	36.50	0.00
TRINITY_sp|P445	arcB	Aerobic respiration	36.50	0.00
TRINITY_sp|Q08B	slc38a9	Sodium-coupled neut	36.50	0.00
TRINITY_sp|Q8RB	coaE	Dephospho-CoA kinas	36.50	0.00
TRINITY_sp|Q2MH	HT1	Serine/threonine-pr	36.50	0.00
TRINITY_sp|P981	ATP8B2	Phospholipid-transp	36.50	0.00
TRINITY_sp|Q54I	pyk3	Dual specificity pr	36.50	0.00
TRINITY_sp|Q135	CAMK2G	Calcium/calmodulin-	36.50	0.00
TRINITY_sp|Q7XJ	FAAH	Fatty acid amide hy	36.50	0.00
TRINITY_sp|Q101	lkh1	Dual specificity pr	36.50	0.00
TRINITY_sp|Q8X1	-	Actin OS=Exophiala	36.50	0.00
TRINITY_sp|Q4WX	pdcA	Pyruvate decarboxyl	36.50	0.00
TRINITY_sp|Q1PF	EDA2	Probable serine pro	36.50	0.00
TRINITY_sp|Q634	Mapk4	Mitogen-activated p	36.50	0.00
TRINITY_sp|P347	RAS-2	Ras-like protein 2	36.50	0.00
TRINITY_sp|Q54Q	yipf5	Protein YIPF5 homol	36.50	0.00
TRINITY_sp|Q5RC	NBR1	Next to BRCA1 gene	36.50	0.00
TRINITY_sp|P9WN	egtD	Histidine N-alpha-m	36.50	0.00
TRINITY_sp|Q5U2	Zfand2a	AN1-type zinc finge	36.50	0.00
TRINITY_sp|Q6P5	Uggt1	UDP-glucose:glycopr	36.50	0.00
TRINITY_sp|P435	RIM15	Serine/threonine-pr	36.50	0.00
TRINITY_sp|Q096	SPAC13C	Putative glutamine	36.50	0.00
TRINITY_sp|Q5YL	GYRA	DNA gyrase subunit	36.50	0.00
TRINITY_sp|Q4UM	RF_0314	Putative ankyrin re	36.50	0.00
TRINITY_sp|Q86C	dhkK	Hybrid signal trans	36.50	0.00
TRINITY_sp|Q9SF	At3g071	Protein transport p	36.50	0.00
TRINITY_sp|Q8N5	AADAT	Kynurenine/alpha-am	36.50	0.00
TRINITY_sp|Q9WW	alkJ	Alcohol dehydrogena	36.50	0.00
TRINITY_sp|Q146	TRIP12	E3 ubiquitin-protei	36.50	0.00
TRINITY_sp|O806	At2g393	Probable receptor-l	36.50	0.00
TRINITY_sp|Q8W2	CSN6A	COP9 signalosome co	36.50	0.00
TRINITY_sp|Q8LP	MYB86	Transcription facto	36.50	0.00
TRINITY_sp|Q8LP	MYB86	Transcription facto	36.50	0.00
TRINITY_sp|A1CQ	ACLA_02	Eukaryotic translat	36.50	0.00
TRINITY_sp|Q007	mdmC	O-methyltransferase	36.50	0.00
TRINITY_sp|Q9M9	At1g782	F-box protein At1g7	36.50	0.00
TRINITY_sp|Q940	ARID3	AT-rich interactive	36.50	0.00
TRINITY_sp|O236	TOC33	Translocase of chlo	36.50	0.00
TRINITY_sp|P837	GRP2	Putative NADPH-depe	36.50	0.00
TRINITY_sp|Q8L7	SCPL20	Serine carboxypepti	36.50	0.00
TRINITY_sp|Q55E	DDB_G02	Probable serine/thr	36.50	0.00
TRINITY_sp|P459	CBX5	Chromobox protein h	36.50	0.00
TRINITY_sp|Q5RD	CSTF3	Cleavage stimulatio	36.50	0.00
TRINITY_sp|Q55A	DDB_G02	Probable serine/thr	36.50	0.00
TRINITY_sp|Q9SJ	DJA6	Chaperone protein d	36.50	0.00
TRINITY_sp|Q54W	DDB_020	Protein SEY1 homolo	36.50	0.00
TRINITY_sp|Q555	vps13B	Putative vacuolar p	36.50	0.00

TRINITY_sp|B1AZ]	Thoc2	THO complex subunit	36.50	0.00
TRINITY_sp|Q95KI	RNF125	E3 ubiquitin-protei	36.50	0.00
TRINITY_sp|Q2JNI	ftsH	ATP-dependent zinc	36.50	0.00
TRINITY_sp|P388]	TRA1	Transcription-assoc	36.50	0.00
TRINITY_sp|Q93Y	ABCG22	ABC transporter G f	36.50	0.00
TRINITY_sp|Q993]	TY3B-G	Transposon Ty3-G Ga	36.50	0.00
TRINITY_sp|A8XN	sgk-1	Serine/threonine-pr	36.50	0.00
TRINITY_sp|P535(	Cebpz	CCAAT/enhancer-bind	36.50	0.00
TRINITY_sp|O956	FGFR1OP	FGFR1 oncogene part	36.50	0.00
TRINITY_sp|Q9SH	HMA5	Probable copper-tra	36.50	0.00
TRINITY_sp|Q55A(	DDB_G02	Probable serine/thr	36.50	0.00
TRINITY_sp|Q145	ITPR2	Inositol 1,4,5-tris	36.50	0.00
TRINITY_sp|Q9FGI	FTSH11	ATP-dependent zinc	36.50	0.00
TRINITY_sp|F4IV	CHR5	Protein CHROMATIN R	36.50	0.00
TRINITY_sp|O150	TRANK1	TPR and ankyrin rep	36.50	0.00
TRINITY_sp|Q7LH(	TY3B-I	Transposon Ty3-I Ga	36.50	0.00
TRINITY_sp|P341(	fhkC	Probable serine/thr	36.40	0.00
TRINITY_sp|Q9LZI	NCS1	Purine-uracil perme	36.40	0.00
TRINITY_sp|Q7SYI	arl2bp	ADP-ribosylation fa	36.40	0.00
TRINITY_sp|Q9FW	ABCB12	ABC transporter B f	36.40	0.00
TRINITY_sp|P117(	CYP3A6	Cytochrome P450 3A6	36.40	0.00
TRINITY_sp|Q86HI	mrt4	Ribosome assembly f	36.40	0.00
TRINITY_sp|P086	R	Ras-like protein 3	36.40	0.00
TRINITY_sp|Q99J	Def8	Differentially expr	36.40	0.00
TRINITY_sp|Q9USI	snx12	Sorting nexin-12 OS	36.40	0.00
TRINITY_sp|Q0CHI	sec13	Protein transport p	36.40	0.00
TRINITY_sp|Q8BWI	Ip6k3	Inositol hexakispho	36.40	0.00
TRINITY_sp|Q55BI	ufd1	Ubiquitin fusion de	36.40	0.00
TRINITY_sp|Q9LX]	PAP20	Probable purple aci	36.40	0.00
TRINITY_sp|P007	PGA	Pepsin A OS=Sus scr	36.40	0.00
TRINITY_sp|Q5XI	March7	E3 ubiquitin-protei	36.40	0.00
TRINITY_sp|A7SBI	ints9	Integrator complex	36.40	0.00
TRINITY_sp|P005	Src64B	Tyrosine-protein ki	36.40	0.00
TRINITY_sp|O537	ufaA1	Tuberculostearic ac	36.40	0.00
TRINITY_sp|Q54U(	lvsC	BEACH domain-contai	36.40	0.00
TRINITY_sp|Q8WN	ITPR3	Inositol 1,4,5-tris	36.40	0.00
TRINITY_sp|Q68W	ubiE	Ubiquinone/menaquin	36.40	0.00
TRINITY_sp|P538	DUG3	Probable glutamine	36.40	0.00
TRINITY_sp|Q8VY(	ESP3	Pre-mRNA-splicing f	36.40	0.00
TRINITY_sp|O745(	SPCC594.	Uncharacterized pro	36.40	0.00
TRINITY_sp|Q084(	CKA2	Casein kinase II su	36.40	0.00
TRINITY_sp|Q9VUI	Ran-like	GTP-binding nuclear	36.40	0.00
TRINITY_sp|P470	VTC4	Vacuolar transporte	36.40	0.00
TRINITY_sp|P199	Rplp0	60S acidic ribosoma	36.40	0.00
TRINITY_sp|Q3UJI	Usp19	Ubiquitin carboxyl-	36.40	0.00
TRINITY_sp|O138	its3	Phosphatidylinosito	36.40	0.00
TRINITY_sp|Q6K4I	Os09g03(	Aminopeptidase M1-D	36.40	0.00
TRINITY_sp|B6IS\	tsf	Elongation factor T	36.40	0.00
TRINITY_sp|P341	racD	Rho-related protein	36.40	0.00
TRINITY_sp|P0C8I	CCRP1	Probable serine/thr	36.40	0.00
TRINITY_sp|Q9EQ(	Enpp5	Ectonucleotide pyro	36.40	0.00
TRINITY_sp|F4HT	CYP26-2	Peptidyl-prolyl cis	36.40	0.00
TRINITY_sp|Q051(	-	Venom allergen 5 OS	36.40	0.00
TRINITY_sp|Q76L(	rbm24	RNA-binding protein	36.40	0.00
TRINITY_sp|Q7XWI	Os04g02	Probable cleavage a	36.40	0.00
TRINITY_sp|Q32KI	DNAL4	Dynein light chain	36.40	0.00

TRINITY_sp|Q7PC	PSD	Exportin-T OS=Arabi	36.40	0.00
TRINITY_sp|O805	CYCU4-1	Cyclin-U4-1 OS=Arab	36.40	0.00
TRINITY_sp|Q5A4	SKN7	Transcription facto	36.40	0.00
TRINITY_sp|Q9D1	Cfap57	Cilia- and flagella	36.40	0.00
TRINITY_sp|A1D3	sds23	Protein sds23 OS=Ne	36.40	0.00
TRINITY_sp|O744	sif2	Sad1-interacting fa	36.40	0.00
TRINITY_sp|Q54N	cyb5r1	NADH-cytochrome b5	36.40	0.00
TRINITY_sp|P495	ppm-2	Probable protein ph	36.40	0.00
TRINITY_sp|Q5ZL	TMEM230	Transmembrane prote	36.40	0.00
TRINITY_sp|G3X9	Sipa1l3	Signal-induced prol	36.40	0.00
TRINITY_sp|Q86H	cf45-1	Counting factor 45-	36.40	0.00
TRINITY_sp|O348	ykfA	Probable murein pep	36.40	0.00
TRINITY_sp|A0AU	wdsub1	WD repeat, SAM and	36.40	0.00
TRINITY_sp|Q8CH	Ttll5	Tubulin polyglutamy	36.40	0.00
TRINITY_sp|Q007	cmkA	Calcium/calmodulin-	36.40	0.00
TRINITY_sp|Q55F	dlpA	Dynamin-like protei	36.40	0.00
TRINITY_sp|O229	At2g341	Uncharacterized pro	36.40	0.00
TRINITY_sp|Q3SY	NOC2L	Nucleolar complex p	36.40	0.00
TRINITY_sp|F4JL	IMPA2	Importin subunit al	36.40	0.00
TRINITY_sp|P341	fhkC	Probable serine/thr	36.40	0.00
TRINITY_sp|Q6PD	Ecm29	Proteasome-associat	36.40	0.00
TRINITY_sp|Q075	YDL086W	Putative carboxymet	36.40	0.00
TRINITY_sp|Q54R	DDB_G02	Probable serine/thr	36.40	0.00
TRINITY_sp|Q684	RAP1GAP	Rap1 GTPase-activat	36.40	0.00
TRINITY_sp|Q86H	prodh	Proline dehydrogena	36.40	0.00
TRINITY_sp|Q9Z3	Itpr2	Inositol 1,4,5-tris	36.40	0.00
TRINITY_sp|Q616	Cbx5	Chromobox protein h	36.40	0.00
TRINITY_sp|Q7Z2	EFL1	Elongation factor-l	36.40	0.00
TRINITY_sp|B9FX	Os07g05	Eukaryotic translat	36.40	0.00
TRINITY_sp|P580	CYP71B1	Cytochrome P450 71B	36.40	0.00
TRINITY_sp|F4K0	KIN4C	Kinesin-like protei	36.40	0.00
TRINITY_sp|Q9N0	MOCOS	Molybdenum cofactor	36.40	0.00
TRINITY_sp|F4I8	ILA	Protein ILITYHIA OS	36.40	0.00
TRINITY_sp|Q15K	PHYLLO	Protein PHYLLO, chl	36.40	0.00
TRINITY_sp|P583	arcB	Aerobic respiration	36.30	0.00
TRINITY_sp|Q620	CBG0288	Mitoferrin OS=Caeno	36.30	0.00
TRINITY_sp|Q9LU	PAP28	Probable inactive p	36.30	0.00
TRINITY_sp|Q6IN	flad1	FAD synthase OS=Xen	36.30	0.00
TRINITY_sp|O433	MSI1	RNA-binding protein	36.30	0.00
TRINITY_sp|P352	Rab17	Ras-related protein	36.30	0.00
TRINITY_sp|Q54X	captB	Uncharacterized CDP	36.30	0.00
TRINITY_sp|Q9V4	Hcf	Host cell factor OS	36.30	0.00
TRINITY_sp|Q9D2	Polr3h	DNA-directed RNA po	36.30	0.00
TRINITY_sp|Q9FK	THO3	THO complex subunit	36.30	0.00
TRINITY_sp|Q9C9	VHA-E2	V-type proton ATPas	36.30	0.00
TRINITY_sp|Q8T6	abcA7	ABC transporter A f	36.30	0.00
TRINITY_sp|Q54S	dhkD	Hybrid signal trans	36.30	0.00
TRINITY_sp|P918	Mo25	Protein Mo25 OS=Dro	36.30	0.00
TRINITY_sp|P044	-	Calmodulin OS=Triti	36.30	0.00
TRINITY_sp|O157	spnA	Protein spalten OS=	36.30	0.00
TRINITY_sp|Q9ZQ	ACX1.2	Putative peroxisoma	36.30	0.00
TRINITY_sp|O044	RABA2A	Ras-related protein	36.30	0.00
TRINITY_sp|A6VX	rpsI	30S ribosomal prote	36.30	0.00
TRINITY_sp|Q8N5	FAM63A	Ubiquitin carboxyl-	36.30	0.00
TRINITY_sp|Q105	CPSF1	Cleavage and polyad	36.30	0.00
TRINITY_sp|Q54H	ctxA	Cortexillin-1 OS=Di	36.30	0.00

TRINITY_sp|Q6GR1	maea	Macrophage erythrob	36.30	0.00
TRINITY_sp|P3018	TOP2	DNA topoisomerase 2	36.30	0.00
TRINITY_sp|Q86UY	NAA40	N-alpha-acetyltrans	36.30	0.00
TRINITY_sp|Q86UC	RSPH3	Radial spoke head p	36.30	0.00
TRINITY_sp|Q5ZLE	SLC30A5	Zinc transporter 5	36.30	0.00
TRINITY_sp|O3560	Abca4	Retinal-specific AT	36.30	0.00
TRINITY_sp|Q86IC	gacH	Rho GTPase-activati	36.30	0.00
TRINITY_sp|Q8N5I	RPP25L	Ribonuclease P prot	36.30	0.00
TRINITY_sp|Q9C5U	AHK4	Histidine kinase 4	36.30	0.00
TRINITY_sp|P2803	AOAH	Acyloxyacyl hydrola	36.30	0.00
TRINITY_sp|Q9SGC	RDR6	RNA-dependent RNA p	36.30	0.00
TRINITY_sp|Q1575	HERC1	Probable E3 ubiquit	36.30	0.00
TRINITY_sp|Q9M7Z	BCE2	Lipoamide acyltrans	36.30	0.00
TRINITY_sp|Q9ZSE	UBP10	Ubiquitin carboxyl-	36.30	0.00
TRINITY_sp|Q7LHC	TY3B-I	Transposon Ty3-I Ga	36.30	0.00
TRINITY_sp|Q54CY	folC	Putative folylpolyg	36.30	0.00
TRINITY_sp|Q9JJ5	Abcb9	ATP-binding cassett	36.30	0.00
TRINITY_sp|Q9NXS	KLHL28	Kelch-like protein	36.30	0.00
TRINITY_sp|Q0GGW	STK11	Serine/threonine-pr	36.30	0.00
TRINITY_sp|B8AJ7	MRS2-I	Magnesium transport	36.30	0.00
TRINITY_sp|P5467	pikE	Phosphatidylinosito	36.30	0.00
TRINITY_sp|Q7TQC	Plekha6	Pleckstrin homology	36.30	0.00
TRINITY_sp|O4297	SPBC20F1	Uncharacterized mem	36.30	0.00
TRINITY_sp|Q9NJU	CPK3	Calcium-dependent p	36.30	0.00
TRINITY_sp|O5006	FIM2	Fimbrin-2 OS=Arabid	36.30	0.00
TRINITY_sp|Q6ZF2	ADL1	Calpain-type cystei	36.30	0.00
TRINITY_sp|Q9M9U	RKD1	Protein RKD1 OS=Ara	36.30	0.00
TRINITY_sp|Q2005	wdr-48	WD repeat-containin	36.30	0.00
TRINITY_sp|P1097	-	Retrovirus-related	36.30	0.00
TRINITY_sp|Q695U	ROM1	Rhomboid-like prote	36.30	0.00
TRINITY_sp|A8MXQ	-	Putative IQ motif a	36.30	0.00
TRINITY_sp|Q9X24	fabG	3-oxoacyl-[acyl-car	36.30	0.00
TRINITY_sp|P2319	Cbx3	Chromobox protein h	36.20	0.00
TRINITY_sp|Q9ZR7	ABCB1	ABC transporter B f	36.20	0.00
TRINITY_sp|Q8C00	Trim35	Tripartite motif-co	36.20	0.00
TRINITY_sp|Q8BH8	-	UPF0317 protein C14	36.20	0.00
TRINITY_sp|Q1DNC	CIMG_081	Very-long-chain 3-o	36.20	0.00
TRINITY_sp|Q54NI	abcC9	ABC transporter C f	36.20	0.00
TRINITY_sp|Q9UTC	SPAC227.	Putative uridine ki	36.20	0.00
TRINITY_sp|O7044	Syn3	Synapsin-3 OS=Rattu	36.20	0.00
TRINITY_sp|Q0VD1	SMPD1	Sphingomyelin phosp	36.20	0.00
TRINITY_sp|Q5F46	SLC38A2	Sodium-coupled neut	36.20	0.00
TRINITY_sp|Q8S1X	Os01g092	Probable glucuronos	36.20	0.00
TRINITY_sp|Q5T5U	ARHGAP21	Rho GTPase-activati	36.20	0.00
TRINITY_sp|P4859	eIF-4E	Eukaryotic translat	36.20	0.00
TRINITY_sp|Q5XGS	gtpbp1	GTP-binding protein	36.20	0.00
TRINITY_sp|Q9NVI	FANCI	Fanconi anemia grou	36.20	0.00
TRINITY_sp|Q6Q4C	LVRN	Aminopeptidase Q OS	36.20	0.00
TRINITY_sp|Q8BJ3	Marf1	Meiosis arrest fema	36.20	0.00
TRINITY_sp|Q5591	sll0286	Uncharacterized N-a	36.20	0.00
TRINITY_sp|Q8L64	BB	E3 ubiquitin ligase	36.20	0.00
TRINITY_sp|O5995	LIP	Lipase OS=Thermomyc	36.20	0.00
TRINITY_sp|Q9NI6	Myt1	Membrane-associated	36.20	0.00
TRINITY_sp|Q8HYI	ACOX1	Peroxisomal acyl-co	36.20	0.00
TRINITY_sp|Q8T63	abcA7	ABC transporter A f	36.20	0.00
TRINITY_sp|P6234	CPK1	Calcium-dependent p	36.20	0.00

TRINITY_sp|P134(	abpC	Gelation factor OS=	36.20	0.00
TRINITY_sp|A4W4(	pnp	Polyribonucleotide	36.20	0.00
TRINITY_sp|Q9C7S	AERO1	Endoplasmic reticul	36.20	0.00
TRINITY_sp|Q54I9	smt1	Probable cycloarten	36.20	0.00
TRINITY_sp|Q5589	pcyA	Phycocyanobilin:fer	36.20	0.00
TRINITY_sp|P3754	yabD	Uncharacterized met	36.20	0.00
TRINITY_sp|P1107	ARF1	ADP-ribosylation fa	36.20	0.00
TRINITY_sp|Q9UK3	NOCT	Nocturnin OS=Homo s	36.20	0.00
TRINITY_sp|Q54S4	gbpD	Cyclic GMP-binding	36.20	0.00
TRINITY_sp|F4IRW	ATG1C	Serine/threonine-pr	36.20	0.00
TRINITY_sp|P9817	RBM10	RNA-binding protein	36.20	0.00
TRINITY_sp|Q54II	shkB	Dual specificity pr	36.20	0.00
TRINITY_sp|Q9P7X	qcr1	Probable mitochondr	36.20	0.00
TRINITY_sp|P3552	CLNS1A	Methylosome subunit	36.20	0.00
TRINITY_sp|P5565	NGR_a015	Uncharacterized pep	36.20	0.00
TRINITY_sp|A2Y8I	PRMT7	Protein arginine N-	36.20	0.00
TRINITY_sp|D3YY2	Lonrf1	LON peptidase N-ter	36.20	0.00
TRINITY_sp|Q8CD(	Vcpip1	Deubiquitinating pr	36.20	0.00
TRINITY_sp|Q93Z3	SPPL1	Signal peptide pept	36.20	0.00
TRINITY_sp|A1L1\	loxl2b	Lysyl oxidase homol	36.20	0.00
TRINITY_sp|Q9DG(	RAD54B	DNA repair and reco	36.20	0.00
TRINITY_sp|Q869(	cpvl	Probable serine car	36.20	0.00
TRINITY_sp|Q8LEI	ABCI11	ABC transporter I f	36.20	0.00
TRINITY_sp|Q5TRI	AGAP0057	ATPase ASNA1 homolo	36.20	0.00
TRINITY_sp|Q55E4	mcfE	Mitochondrial subst	36.20	0.00
TRINITY_sp|Q9FG3	SNX1	Sorting nexin 1 OS=	36.20	0.00
TRINITY_sp|Q6RU(	Ptx4	Pentraxin-4 OS=Mus	36.20	0.00
TRINITY_sp|Q66H8	Ankzf1	Ankyrin repeat and	36.20	0.00
TRINITY_sp|Q9Z1N	ACOX1	Peroxisomal acyl-co	36.20	0.00
TRINITY_sp|O8248	MTA	N6-adenosine-methyl	36.20	0.00
TRINITY_sp|E1C1I	USP47	Ubiquitin carboxyl-	36.20	0.00
TRINITY_sp|Q9P2I	SIPA1L2	Signal-induced prol	36.20	0.00
TRINITY_sp|P3858	TTL	Tubulin--tyrosine l	36.20	0.00
TRINITY_sp|P0052	Abl1	Tyrosine-protein ki	36.20	0.00
TRINITY_sp|P1097	-	Retrovirus-related	36.20	0.00
TRINITY_sp|A5D7I	CDC25C	M-phase inducer pho	36.20	0.00
TRINITY_sp|P0432	pol	Retrovirus-related	36.20	0.00
TRINITY_sp|Q8NE7	ABCF1	ATP-binding cassett	36.20	0.00
TRINITY_sp|Q641X	Hexa	Beta-hexosaminidase	36.20	0.00
TRINITY_sp|Q9D8(	Tmem41a	Transmembrane prote	36.20	0.00
TRINITY_sp|Q9UKI	PARP4	Poly [ADP-ribose] p	36.20	0.00
TRINITY_sp|Q54RI	dhkL	Hybrid signal trans	36.20	0.00
TRINITY_sp|Q9SZ(	SAP7	Zinc finger A20 and	36.20	0.00
TRINITY_sp|Q8R75	prs	Ribose-phosphate py	36.10	0.00
TRINITY_sp|O0489	-	Alpha-glucosidase O	36.10	0.00
TRINITY_sp|Q9LTW	ALIS1	ALA-interacting sub	36.10	0.00
TRINITY_sp|Q9H87	RMND5A	Protein RMD5 homolo	36.10	0.00
TRINITY_sp|Q8TD1	NEK9	Serine/threonine-pr	36.10	0.00
TRINITY_sp|Q9VY9	Upf1	Regulator of nonsen	36.10	0.00
TRINITY_sp|Q54YI	dhkB	Hybrid signal trans	36.10	0.00
TRINITY_sp|Q550I	rab11C	Ras-related protein	36.10	0.00
TRINITY_sp|O6212	tag-175	Transmembrane prote	36.10	0.00
TRINITY_sp|Q8S92	ATG8E	Autophagy-related p	36.10	0.00
TRINITY_sp|O7482	esf1	Pre-rRNA-processing	36.10	0.00
TRINITY_sp|Q5R9(	ZFAND2A	AN1-type zinc finge	36.10	0.00
TRINITY_sp|G4RK4	tpsp	Bifunctional trehal	36.10	0.00

TRINITY_sp|Q8K0(	Eipr1	Protein TSSC1 OS=Mu	36.10	0.00
TRINITY_sp|P342	HCS1	DNA polymerase alph	36.10	0.00
TRINITY_sp|Q8ICI	CPK2	Calcium-dependent p	36.10	0.00
TRINITY_sp|O083	cypB	Bifunctional cytoch	36.10	0.00
TRINITY_sp|P702	Itpr3	Inositol 1,4,5-tris	36.10	0.00
TRINITY_sp|P038	AI2	Putative COX1/OXI3	36.10	0.00
TRINITY_sp|Q9SH	At2g379	Ribosome biogenesis	36.10	0.00
TRINITY_sp|P050	alkB	Alpha-ketoglutarate	36.10	0.00
TRINITY_sp|Q8T1	omt3	O-methyltransferase	36.10	0.00
TRINITY_sp|Q8L8	PIP5K9	Phosphatidylinosito	36.10	0.00
TRINITY_sp|Q056	Cbei_02(	Uncharacterized pro	36.10	0.00
TRINITY_sp|B4NP(	IntS3	Integrator complex	36.10	0.00
TRINITY_sp|Q8H6	PHT1-6	Inorganic phosphate	36.10	0.00
TRINITY_sp|Q3SZ(	NSFL1C	NSFL1 cofactor p47	36.10	0.00
TRINITY_sp|Q9BW	TPPP3	Tubulin polymerizat	36.10	0.00
TRINITY_sp|Q8VC	Plbd1	Phospholipase B-lik	36.10	0.00
TRINITY_sp|Q9LS	GTE12	Transcription facto	36.10	0.00
TRINITY_sp|Q9Y3(	ACOT9	Acyl-coenzyme A thi	36.10	0.00
TRINITY_sp|Q9SZ	ABCG9	ABC transporter G f	36.10	0.00
TRINITY_sp|Q9HF	B2J23.0	Acyl-protein thioes	36.10	0.00
TRINITY_sp|D4AY	ARB_013	ABC transporter G f	36.10	0.00
TRINITY_sp|Q9SF	OST1A	Dolichyl-diphosphoo	36.10	0.00
TRINITY_sp|O605	EIF4E2	Eukaryotic translat	36.10	0.00
TRINITY_sp|Q54R	pyk	Pyruvate kinase OS=	36.10	0.00
TRINITY_sp|Q687	npp	Nucleotide pyrophos	36.10	0.00
TRINITY_sp|Q863	OXSR1	Serine/threonine-pr	36.10	0.00
TRINITY_sp|Q067(	CSR1	Phosphatidylinosito	36.10	0.00
TRINITY_sp|Q9M9	RKD1	Protein RKD1 OS=Ara	36.10	0.00
TRINITY_sp|Q8CI(	Cog3	Conserved oligomeri	36.10	0.00
TRINITY_sp|Q8GY	MND1	Meiotic nuclear div	36.10	0.00
TRINITY_sp|Q928	ERCC4	DNA repair endonucl	36.10	0.00
TRINITY_sp|Q55E	gxcB	Rac guanine nucleot	36.10	0.00
TRINITY_sp|Q54J	abcC3	ABC transporter C f	36.10	0.00
TRINITY_sp|P785	ST5	Suppression of tumo	36.10	0.00
TRINITY_sp|Q7DM	PAS1	Peptidyl-prolyl cis	36.10	0.00
TRINITY_sp|A0RU	tuf	Elongation factor 1	36.10	0.00
TRINITY_sp|Q86C	tor	Target of rapamycin	36.10	0.00
TRINITY_sp|Q86G	gefQ	Ras guanine nucleot	36.10	0.00
TRINITY_sp|P548	mspn-1	Mitochondrial sorti	36.10	0.00
TRINITY_sp|O503	bchH	Magnesium-chelatase	36.10	0.00
TRINITY_sp|O158	CPK2	Calcium-dependent p	36.10	0.00
TRINITY_sp|Q390(	AATP1	ADP,ATP carrier pro	36.10	0.00
TRINITY_sp|O288	AF_1420	Uncharacterized pro	36.10	0.00
TRINITY_sp|Q7LH(	TY3B-I	Transposon Ty3-I Ga	36.10	0.00
TRINITY_sp|Q8L6	BB	E3 ubiquitin ligase	36.10	0.00
TRINITY_sp|Q6GP	rnf181	E3 ubiquitin-protei	36.00	0.00
TRINITY_sp|Q6NM	At5g459	GDSL esterase/lipas	36.00	0.00
TRINITY_sp|P341	coaA	Coactosin OS=Dictyo	36.00	0.00
TRINITY_sp|Q949	LIGB	Extradiol ring-clea	36.00	0.00
TRINITY_sp|Q930(	USP9X	Probable ubiquitin	36.00	0.00
TRINITY_sp|Q54S	pex10	Peroxisome biogenes	36.00	0.00
TRINITY_sp|Q0WP	SCPL34	Serine carboxypepti	36.00	0.00
TRINITY_sp|Q8J1(	KIP1	Kinesin-like protei	36.00	0.00
TRINITY_sp|Q8VZ	SWC4	SWR1-complex protei	36.00	0.00
TRINITY_sp|Q9C5	AHK4	Histidine kinase 4	36.00	0.00
TRINITY_sp|Q941	GONST1	GDP-mannose transpo	36.00	0.00

TRINITY_sp|Q54PI	lvsF	BEACH domain-contai	36.00	0.00
TRINITY_sp|Q32N(	rnf10	RING finger protein	36.00	0.00
TRINITY_sp|Q1RM3	cfap36	Cilia- and flagella	36.00	0.00
TRINITY_sp|Q6CR4	RRI1	COP9 signalosome co	36.00	0.00
TRINITY_sp|P4919	elo-3	Putative fatty acid	36.00	0.00
TRINITY_sp|Q9SI(	PPC3-1.2	Probable protein ph	36.00	0.00
TRINITY_sp|P7317	sll1290	Uncharacterized rib	36.00	0.00
TRINITY_sp|Q0896	LEA1	U2 small nuclear ri	36.00	0.00
TRINITY_sp|Q9DC5	Cpne8	Copine-8 OS=Mus mus	36.00	0.00
TRINITY_sp|Q9XA1	SCO3848	Probable serine/thr	36.00	0.00
TRINITY_sp|P341(	fhkC	Probable serine/thr	36.00	0.00
TRINITY_sp|Q54DI	DDB_G029	Probable serine/thr	36.00	0.00
TRINITY_sp|Q9FP9	UBP24	Ubiquitin carboxyl-	36.00	0.00
TRINITY_sp|Q9GZ1	NIF3L1	NIF3-like protein 1	36.00	0.00
TRINITY_sp|P0C8N	CCRP1	Probable serine/thr	36.00	0.00
TRINITY_sp|F4JBN	RBL4	RHOMBOID-like prote	36.00	0.00
TRINITY_sp|Q55C(	tnpo	Transportin OS=Dict	36.00	0.00
TRINITY_sp|Q810V	Mphosph1	U3 small nucleolar	36.00	0.00
TRINITY_sp|A1ZA1	CG5065	Putative fatty acyl	36.00	0.00
TRINITY_sp|Q2778	PYK	Pyruvate kinase OS=	36.00	0.00
TRINITY_sp|O9416	SNF1	Carbon catabolite-d	36.00	0.00
TRINITY_sp|Q9JM7	Arpc3	Actin-related prote	36.00	0.00
TRINITY_sp|O009(	-	Lysosomal acid alph	36.00	0.00
TRINITY_sp|O7418	pop3	Target of rapamycin	36.00	0.00
TRINITY_sp|Q75II	KU80	ATP-dependent DNA h	36.00	0.00
TRINITY_sp|P9376	-	Acid beta-fructofur	36.00	0.00
TRINITY_sp|Q9931	TY3B-G	Transposon Ty3-G Ga	36.00	0.00
TRINITY_sp|A2Z9W	MRS2-G	Putative magnesium	36.00	0.00
TRINITY_sp|O885(	Pde8a	High affinity cAMP-	36.00	0.00
TRINITY_sp|Q54F(	gacJJ	Rho GTPase-activati	36.00	0.00
TRINITY_sp|P6187	-	Lipase OS=Rhizopus	36.00	0.00
TRINITY_sp|Q2368	gcy-5	Receptor-type guany	36.00	0.00
TRINITY_sp|O745(	SPCC594.	Uncharacterized pro	36.00	0.00
TRINITY_sp|P3519	GCS1	ADP-ribosylation fa	36.00	0.00
TRINITY_sp|Q9SJ4	VPS11	Vacuolar protein-so	36.00	0.00
TRINITY_sp|P2622	cenB	Endoglucanase B OS=	36.00	0.00
TRINITY_sp|P8042	tlp	Trypsin-like protea	36.00	0.00
TRINITY_sp|O0262	aex-3	MAP kinase-activati	36.00	0.00
TRINITY_sp|Q9446	dymA	Dynamin-A OS=Dictyo	36.00	0.00
TRINITY_sp|Q0WUI	FAB1A	1-phosphatidylinosi	36.00	0.00
TRINITY_sp|P4221	RAP	Aspartic proteinase	36.00	0.00
TRINITY_sp|P137(	HMGCS1	Hydroxymethylglutar	36.00	0.00
TRINITY_sp|Q2HJI	USP15	Ubiquitin carboxyl-	36.00	0.00
TRINITY_sp|P6175	Vbp1	Prefoldin subunit 3	36.00	0.00
TRINITY_sp|Q9LX9	CALS3	Callose synthase 3	36.00	0.00
TRINITY_sp|F4JTI	STY46	Serine/threonine-pr	36.00	0.00
TRINITY_sp|P0509	abpA	Alpha-actinin A OS=	36.00	0.00
TRINITY_sp|Q9SH3	HMA5	Probable copper-tra	36.00	0.00
TRINITY_sp|Q8VZI	EDR2L	Protein ENHANCED DI	36.00	0.00
TRINITY_sp|O4604	Parg	Poly(ADP-ribose) gl	36.00	0.00
TRINITY_sp|O2124	CTAG	Cytochrome c oxidas	36.00	0.00
TRINITY_sp|P4035	NTA1	Protein N-terminal	35.90	0.00
TRINITY_sp|Q5SUI	Pfas	Phosphoribosylformy	35.90	0.00
TRINITY_sp|Q54KI	DDB_G028	Uncharacterized pro	35.90	0.00
TRINITY_sp|P5164	arf4	ADP-ribosylation fa	35.90	0.00
TRINITY_sp|P0CH3	adhc1	NADP-dependent alco	35.90	0.00

TRINITY_sp|Q196{	F21D5.5	Uncharacterized pro	35.90	0.00
TRINITY_sp|Q68F:	vac14	Protein VAC14 homol	35.90	0.00
TRINITY_sp|O646	URT1	UTP:RNA uridylyltra	35.90	0.00
TRINITY_sp|Q9D8	Arfgap3	ADP-ribosylation fa	35.90	0.00
TRINITY_sp|O642(	10	Endolysin A OS=Myco	35.90	0.00
TRINITY_sp|Q3V0I	Pls1	Plastin-1 OS=Mus mu	35.90	0.00
TRINITY_sp|Q550I	ctxB	Cortexillin-2 OS=Di	35.90	0.00
TRINITY_sp|Q5ZL(	GOLGA7	Golgin subfamily A	35.90	0.00
TRINITY_sp|Q227(	sym-2	RNA-binding protein	35.90	0.00
TRINITY_sp|Q54Q(	dhkG	Hybrid signal trans	35.90	0.00
TRINITY_sp|Q8H4	Os07g05(	Probable protein ph	35.90	0.00
TRINITY_sp|Q8T2	DDB_G02	Probable GH family	35.90	0.00
TRINITY_sp|Q294	LIPF	Gastric triacylglyc	35.90	0.00
TRINITY_sp|Q0V9	eif1ad	Probable RNA-bindin	35.90	0.00
TRINITY_sp|P435(	AGX1	Alanine--glyoxylate	35.90	0.00
TRINITY_sp|P0CC(	DDB_G02	Enolase superfamily	35.90	0.00
TRINITY_sp|O229	VSR2	Vacuolar-sorting re	35.90	0.00
TRINITY_sp|O944	bdp1	Transcription facto	35.90	0.00
TRINITY_sp|O597(	iki3	Elongator complex p	35.90	0.00
TRINITY_sp|Q8WTI	DUSP19	Dual specificity pr	35.90	0.00
TRINITY_sp|Q8H1I	TDP1	Tyrosyl-DNA phospho	35.90	0.00
TRINITY_sp|Q8ICI	CPK2	Calcium-dependent p	35.90	0.00
TRINITY_sp|Q8CBI	Wdr37	WD repeat-containin	35.90	0.00
TRINITY_sp|Q9WU	Pus1	tRNA pseudouridine	35.90	0.00
TRINITY_sp|O885	Cops4	COP9 signalosome co	35.90	0.00
TRINITY_sp|Q54RI	pyk	Pyruvate kinase OS=	35.90	0.00
TRINITY_sp|A6WS	Shew185_	Non-canonical purin	35.90	0.00
TRINITY_sp|O756	NUP155	Nuclear pore comple	35.90	0.00
TRINITY_sp|Q5BJI	Prune2	Protein prune homol	35.90	0.00
TRINITY_sp|Q0V8I	KRI1	Protein KRI1 homolo	35.90	0.00
TRINITY_sp|Q9ZW	PRN2	Pirin-like protein	35.90	0.00
TRINITY_sp|Q9VB	Clbn	Nuclear export medi	35.90	0.00
TRINITY_sp|Q9ZR\	MLH1	DNA mismatch repair	35.90	0.00
TRINITY_sp|Q8ICI	CPK2	Calcium-dependent p	35.90	0.00
TRINITY_sp|Q9SA	PGK3	Phosphoglycerate ki	35.90	0.00
TRINITY_sp|Q0WQI	VPS53	Vacuolar protein so	35.90	0.00
TRINITY_sp|O887	Birc6	Baculoviral IAP rep	35.90	0.00
TRINITY_sp|Q4DJ(	Tc00.10	Prostaglandin F syn	35.90	0.00
TRINITY_sp|O041	RABG3B	Ras-related protein	35.90	0.00
TRINITY_sp|Q4Z8I	RanBPM	Ran-binding protein	35.90	0.00
TRINITY_sp|Q9FHI	DTX16	Protein DETOXIFICAT	35.90	0.00
TRINITY_sp|Q1ZX(	fcsB	Fatty acyl-CoA synt	35.90	0.00
TRINITY_sp|Q8C0I	Efl1	Elongation factor-l	35.90	0.00
TRINITY_sp|P437	polA	DNA polymerase I OS	35.90	0.00
TRINITY_sp|Q5VQI	PPT3	Phosphoenolpyruvate	35.90	0.00
TRINITY_sp|Q641(	ncaph2	Condensin-2 complex	35.90	0.00
TRINITY_sp|Q55GI	mcfT	Probable mitochondr	35.90	0.00
TRINITY_sp|Q9FX	PEX14	Peroxisomal membran	35.90	0.00
TRINITY_sp|Q7S1	apg-12	Sterol 3-beta-gluco	35.90	0.00
TRINITY_sp|Q6DF	ppp4r3b	Serine/threonine-pr	35.90	0.00
TRINITY_sp|Q5VYI	ECM29	Proteasome-associat	35.90	0.00
TRINITY_sp|P128(	AMO	Peroxisomal primary	35.90	0.00
TRINITY_sp|Q925(	Slc8b1	Sodium/potassium/ca	35.90	0.00
TRINITY_sp|O186	pde-1	Probable 3',5'-cycl	35.90	0.00
TRINITY_sp|A6QQ	VPS51	Vacuolar protein so	35.90	0.00
TRINITY_sp|Q9UR(	Tf2-11	Transposon Tf2-11 p	35.80	0.00

TRINITY_sp|Q9K7\	BH3256	Probable non-canoni	35.80	0.00
TRINITY_sp|F4INI	GGP4	Gamma-glutamyl pept	35.80	0.00
TRINITY_sp|Q0974	SPBC12C2	Uncharacterized pro	35.80	0.00
TRINITY_sp|O1379	sgt2	Small glutamine-ric	35.80	0.00
TRINITY_sp|Q9FWI	GAMMACA1	Gamma carbonic anhy	35.80	0.00
TRINITY_sp|Q4PCI	SEC13	Protein transport p	35.80	0.00
TRINITY_sp|Q54PS	osbI	Oxysterol-binding p	35.80	0.00
TRINITY_sp|Q9HU1	aruS	Sensor histidine ki	35.80	0.00
TRINITY_sp|Q9GQI	XNP	Transcriptional reg	35.80	0.00
TRINITY_sp|Q55E1	ate1	Arginyl-tRNA--prote	35.80	0.00
TRINITY_sp|Q54KI	ccbl	Kynurenine--oxoglut	35.80	0.00
TRINITY_sp|Q9R05	Fhl3	Four and a half LIM	35.80	0.00
TRINITY_sp|Q1PF5	EDA2	Probable serine pro	35.80	0.00
TRINITY_sp|Q8T6	abcA7	ABC transporter A f	35.80	0.00
TRINITY_sp|Q5XH3	march8	E3 ubiquitin-protei	35.80	0.00
TRINITY_sp|Q0DLI	Os05g011	DEAD-box ATP-depend	35.80	0.00
TRINITY_sp|Q9NP8	PNPLA8	Calcium-independent	35.80	0.00
TRINITY_sp|P0505	alkB	Alpha-ketoglutarate	35.80	0.00
TRINITY_sp|Q9SI2	At2g4029	Eukaryotic translat	35.80	0.00
TRINITY_sp|Q5UP7	MIMI_R61	Putative band 7 fam	35.80	0.00
TRINITY_sp|Q5PP\	zc2hc1a	Zinc finger C2HC do	35.80	0.00
TRINITY_sp|P0463	Lipf	Gastric triacylglyc	35.80	0.00
TRINITY_sp|Q9LQ\	RDR1	RNA-dependent RNA p	35.80	0.00
TRINITY_sp|Q9FMI	PAP29	Probable inactive p	35.80	0.00
TRINITY_sp|O0277	PARG	Poly(ADP-ribose) gl	35.80	0.00
TRINITY_sp|P3333	SKN1	Beta-glucan synthes	35.80	0.00
TRINITY_sp|Q54GI	ctdspl2	CTD small phosphata	35.80	0.00
TRINITY_sp|Q926(	TBC1D5	TBC1 domain family	35.80	0.00
TRINITY_sp|P0A3U	ltrA	Group II intron-enc	35.80	0.00
TRINITY_sp|P0541	petA	Ubiquinol-cytochrom	35.80	0.00
TRINITY_sp|Q9KA6	frr	Ribosome-recycling	35.80	0.00
TRINITY_sp|C4L9I	mutL	DNA mismatch repair	35.80	0.00
TRINITY_sp|O806(	SUC3	Sucrose transport p	35.80	0.00
TRINITY_sp|Q9D91	Efhc1	EF-hand domain-cont	35.80	0.00
TRINITY_sp|O6016	SPBC19F5	Uncharacterized pro	35.80	0.00
TRINITY_sp|P3795	yusZ	Uncharacterized oxi	35.80	0.00
TRINITY_sp|O7632	abpD	Interaptin OS=Dicty	35.80	0.00
TRINITY_sp|P2227	RAS1	Ras-like protein 1	35.80	0.00
TRINITY_sp|Q5PP6	At2g3543	Zinc finger CCCH do	35.80	0.00
TRINITY_sp|Q8DJI	grpE	Protein GrpE OS=The	35.80	0.00
TRINITY_sp|Q9FLI	FKBP15-3	Peptidyl-prolyl cis	35.80	0.00
TRINITY_sp|A3D0(	rihA	Pyrimidine-specific	35.80	0.00
TRINITY_sp|Q7XU2	CCD7	Carotenoid cleavage	35.80	0.00
TRINITY_sp|Q9SSI	JMJ25	Lysine-specific dem	35.80	0.00
TRINITY_sp|B4ND(	GK24923	Regulator of telome	35.80	0.00
TRINITY_sp|B0W29	CPIJ0014	Clustered mitochond	35.80	0.00
TRINITY_sp|P2144	ABCB1	Multidrug resistanc	35.80	0.00
TRINITY_sp|B8AE(	OsI_0778	NAP1-related protei	35.80	0.00

TRINITY_sp|Q8TC1	RDH11	Retinol dehydrogena	35.80	0.00
TRINITY_sp|P1606	-	Speract receptor OS	35.80	0.00
TRINITY_sp|F4JTE	STY46	Serine/threonine-pr	35.80	0.00
TRINITY_sp|Q8MMI	vatH	V-type proton ATPas	35.80	0.00
TRINITY_sp|P1995	PSRP1	Ribosome-binding fa	35.80	0.00
TRINITY_sp|P2786	yigZ	IMPACT family membe	35.80	0.00
TRINITY_sp|Q9LJI	M3KE1	MAP3K epsilon prote	35.80	0.00
TRINITY_sp|Q7NBW	dnaJ	Chaperone protein D	35.80	0.00
TRINITY_sp|Q75EN	RAD18	Postreplication rep	35.80	0.00
TRINITY_sp|Q9FIV	DEGP10	Protease Do-like 10	35.80	0.00
TRINITY_sp|F4HY(	IRE3	Probable serine/thr	35.80	0.00
TRINITY_sp|Q9GKX	GALM	Aldose 1-epimerase	35.80	0.00
TRINITY_sp|A0FGE	ESYT3	Extended synaptotag	35.80	0.00
TRINITY_sp|P5475	PDE1A	Calcium/calmodulin-	35.80	0.00
TRINITY_sp|Q1101	pepN	Aminopeptidase N OS	35.80	0.00
TRINITY_sp|Q4209	ABCC2	ABC transporter C f	35.80	0.00
TRINITY_sp|Q7TPS	Rps6ka6	Ribosomal protein S	35.80	0.00
TRINITY_sp|Q9VJ3	Nedd8	NEDD8 OS=Drosophila	35.80	0.00
TRINITY_sp|Q32L5	GRINA	Protein lifeguard 1	35.80	0.00
TRINITY_sp|P4802	gacS	Sensor protein GacS	35.80	0.00
TRINITY_sp|Q9FPE	EDR1	Serine/threonine-pr	35.70	0.00
TRINITY_sp|Q8K9Y	dnaJ	Chaperone protein D	35.70	0.00
TRINITY_sp|Q9LSE	ARP2	Actin-related prote	35.70	0.00
TRINITY_sp|Q9SF9	RABE1E	Ras-related protein	35.70	0.00
TRINITY_sp|Q9LE8	IRE	Probable serine/thr	35.70	0.00
TRINITY_sp|Q1824	rap-1	Ras-related protein	35.70	0.00
TRINITY_sp|Q6C61	YALI0E09	Solute carrier fami	35.70	0.00
TRINITY_sp|Q9CA9	BAC2	Mitochondrial argin	35.70	0.00
TRINITY_sp|Q86AI	DDB_G027	Probable myosin lig	35.70	0.00
TRINITY_sp|Q55E4	dhkE	Hybrid signal trans	35.70	0.00
TRINITY_sp|Q94AH	CUL1	Cullin-1 OS=Arabido	35.70	0.00
TRINITY_sp|P3132	PKAR	cAMP-dependent prot	35.70	0.00
TRINITY_sp|Q55A(	DDB_G027	Probable serine/thr	35.70	0.00
TRINITY_sp|Q55F7	abiA	Abl interactor homo	35.70	0.00
TRINITY_sp|Q0252	Mgat3	Beta-1,4-mannosyl-g	35.70	0.00
TRINITY_sp|Q9LDE	FK	Delta(14)-sterol re	35.70	0.00
TRINITY_sp|P2126	CCA1	CCA tRNA nucleotidy	35.70	0.00
TRINITY_sp|Q9LK4	TMK4	Receptor-like kinas	35.70	0.00
TRINITY_sp|O5317	Rv2449c	Putative trans-acti	35.70	0.00
TRINITY_sp|Q8N35	SLC35F6	Solute carrier fami	35.70	0.00
TRINITY_sp|P5467	patA	Calcium-transportin	35.70	0.00
TRINITY_sp|Q7ZXE	nle1	Notchless protein h	35.70	0.00
TRINITY_sp|Q54P(	gefA	Ras guanine nucleot	35.70	0.00
TRINITY_sp|Q6Z8E	Os02g022	Probable protein ph	35.70	0.00
TRINITY_sp|Q8VZE	RAP	RAP domain-containi	35.70	0.00
TRINITY_sp|Q54X9	elp4	Probable elongator	35.70	0.00
TRINITY_sp|Q84L3	RAD23A	Probable ubiquitin	35.70	0.00
TRINITY_sp|Q54B9	med8	Putative mediator o	35.70	0.00
TRINITY_sp|Q5ZM9	NCOA7	Nuclear receptor co	35.70	0.00
TRINITY_sp|Q86B9	metl	Methyltransferase-l	35.70	0.00
TRINITY_sp|Q54Y5	shkC	Dual specificity pr	35.70	0.00
TRINITY_sp|Q1ZXE	gxcDD	Guanine exchange fa	35.70	0.00
TRINITY_sp|Q0591	iphP	Tyrosine-protein ph	35.70	0.00
TRINITY_sp|Q8LB6	CYCU3-1	Cyclin-U3-1 OS=Arab	35.70	0.00
TRINITY_sp|Q54U(	dnapkcs	DNA-dependent prote	35.70	0.00
TRINITY_sp|Q9P4E	GSP1	GTP-binding nuclear	35.70	0.00

TRINITY_sp|Q6MC	pepA	Probable cytosol am	35.70	0.00
TRINITY_sp|Q54M	DDB_G02	Coiled-coil domain-	35.70	0.00
TRINITY_sp|Q6VT	Vcath	Viral cathepsin OS=	35.70	0.00
TRINITY_sp|Q6ZU	ARHGAP2	Rho GTPase-activati	35.70	0.00
TRINITY_sp|O806	APY6	Probable apyrase 6	35.70	0.00
TRINITY_sp|Q81H	BC_0906	Delta(1)-pyrroline-	35.70	0.00
TRINITY_sp|Q9C5	IPT9	tRNA dimethylallylt	35.70	0.00
TRINITY_sp|Q2KN	HXK2	Hexokinase-2 OS=Ory	35.70	0.00
TRINITY_sp|Q9C0	WDR33	pre-mRNA 3' end pro	35.70	0.00
TRINITY_sp|Q3KT	GDPD5	Glycerophosphodiest	35.70	0.00
TRINITY_sp|P303	cadA	Probable cadmium-tr	35.70	0.00
TRINITY_sp|Q8L4	WEE1	Wee1-like protein k	35.70	0.00
TRINITY_sp|P221	-	Ras-related protein	35.70	0.00
TRINITY_sp|A5PK	ABHD17C	Protein ABHD17C OS=	35.70	0.00
TRINITY_sp|F4HV	VAD1	Protein VASCULAR AS	35.70	0.00
TRINITY_sp|Q6TE	fam91a1	Protein FAM91A1 OS=	35.70	0.00
TRINITY_sp|Q9LI	At3g213	Clavaminate synthas	35.70	0.00
TRINITY_sp|Q78J	Ubfd1	Ubiquitin domain-co	35.70	0.00
TRINITY_sp|B0F9	GC6	Golgin candidate 6	35.70	0.00
TRINITY_sp|Q097	akr1	Palmitoyltransferas	35.70	0.00
TRINITY_sp|P227	Gucy1b2	Guanylate cyclase s	35.70	0.00
TRINITY_sp|Q91Y	Mettl13	Methyltransferase-l	35.70	0.00
TRINITY_sp|Q6NL	ABCC2	ABC transporter D f	35.70	0.00
TRINITY_sp|Q993	TY3B-G	Transposon Ty3-G Ga	35.70	0.00
TRINITY_sp|Q9SH	TIC21	Protein TIC 21, chl	35.70	0.00
TRINITY_sp|P912	gst-6	Probable glutathion	35.70	0.00
TRINITY_sp|Q9FK	UBP17	Ubiquitin carboxyl-	35.70	0.00
TRINITY_sp|Q9SK	At2g203	NADH dehydrogenase	35.60	0.00
TRINITY_sp|B6ZJ	PNC1	Peroxisomal adenine	35.60	0.00
TRINITY_sp|G5EB	sma-5	Mitogen-activated p	35.60	0.00
TRINITY_sp|Q553	gxcJJ	Rac guanine nucleot	35.60	0.00
TRINITY_sp|Q6PE	Tbca	Tubulin-specific ch	35.60	0.00
TRINITY_sp|F4HP	IRE4	Probable serine/thr	35.60	0.00
TRINITY_sp|Q7ZU	ykt6	Synaptobrevin homol	35.60	0.00
TRINITY_sp|Q3T0	ABHD1	Protein ABHD1 OS=Bo	35.60	0.00
TRINITY_sp|F1QG	melk	Maternal embryonic	35.60	0.00
TRINITY_sp|Q088	SWA2	Alpha-amylase 2 OS=	35.60	0.00
TRINITY_sp|Q039	RUB1	NEDD8-like protein	35.60	0.00
TRINITY_sp|Q390	bVPE	Vacuolar-processing	35.60	0.00
TRINITY_sp|Q1PF	EDA2	Probable serine pro	35.60	0.00
TRINITY_sp|Q9SH	PVA12	Vesicle-associated	35.60	0.00
TRINITY_sp|O460	Parg	Poly(ADP-ribose) gl	35.60	0.00
TRINITY_sp|Q9W0	Cyp4d20	Probable cytochrome	35.60	0.00
TRINITY_sp|Q387	MTD	Mannitol dehydrogen	35.60	0.00
TRINITY_sp|Q274	mec-2	Mechanosensory prot	35.60	0.00
TRINITY_sp|Q54M	tiprl	TIP41-like protein	35.60	0.00
TRINITY_sp|O493	VAMP711	Vesicle-associated	35.60	0.00
TRINITY_sp|Q54P	gefA	Ras guanine nucleot	35.60	0.00
TRINITY_sp|Q86H	mrt4	Ribosome assembly f	35.60	0.00
TRINITY_sp|Q4WJ	mrd1	Multiple RNA-bindin	35.60	0.00
TRINITY_sp|Q9ZS	FIP37	FKBP12-interacting	35.60	0.00
TRINITY_sp|Q8MY	DDB_G02	Probable serine/thr	35.60	0.00
TRINITY_sp|Q556	psmB4-1	Proteasome subunit	35.60	0.00
TRINITY_sp|A2WY	HK3	Probable histidine	35.60	0.00
TRINITY_sp|O317	tdh	L-threonine 3-dehyd	35.60	0.00
TRINITY_sp|Q0WQ	LTA3	Dihydrolipoyllysine	35.60	0.00

TRINITY_sp|O608[illegible]	KIN	DNA/RNA-binding pro	35.60	0.00
TRINITY_sp|A7S8[illegible]	v1g1870[illegible]	Coiled-coil domain-	35.60	0.00
TRINITY_sp|A1A6[illegible]	HK4	Probable histidine	35.60	0.00
TRINITY_sp|Q86J[illegible]	DDB_G02[illegible]	Putative elongation	35.60	0.00
TRINITY_sp|P109[illegible]	-	Retrovirus-related	35.60	0.00
TRINITY_sp|Q54P[illegible]	exoc4	Exocyst complex com	35.60	0.00
TRINITY_sp|Q9ZQ[illegible]	CBSDUF3	DUF21 domain-contai	35.60	0.00
TRINITY_sp|Q5PP[illegible]	Eif2d	Eukaryotic translat	35.60	0.00
TRINITY_sp|Q1ZX[illegible]	gxcDD	Guanine exchange fa	35.60	0.00
TRINITY_sp|Q6C5[illegible]	YKT6	Synaptobrevin homol	35.60	0.00
TRINITY_sp|Q9C0[illegible]	WDR33	pre-mRNA 3' end pro	35.60	0.00
TRINITY_sp|Q54R[illegible]	dhkL	Hybrid signal trans	35.60	0.00
TRINITY_sp|P107[illegible]	-	Venom allergen 5.02	35.60	0.00
TRINITY_sp|Q940[illegible]	GLCNAC1[illegible]	UDP-N-acetylglucosa	35.60	0.00
TRINITY_sp|Q9FJ[illegible]	FKBP65	Peptidyl-prolyl cis	35.60	0.00
TRINITY_sp|Q8L4[illegible]	DAAT	D-amino-acid transa	35.60	0.00
TRINITY_sp|Q4U2[illegible]	Herc2	E3 ubiquitin-protei	35.60	0.00
TRINITY_sp|Q93Z[illegible]	LA1	La protein 1 OS=Ara	35.60	0.00
TRINITY_sp|P053[illegible]	p20	Uncharacterized N-a	35.60	0.00
TRINITY_sp|Q54T[illegible]	drkC	Probable serine/thr	35.60	0.00
TRINITY_sp|Q9M2[illegible]	SDR1	(+)-neomenthol dehy	35.60	0.00
TRINITY_sp|Q9US[illegible]	SPCC4B3.[illegible]	Uncharacterized tra	35.60	0.00
TRINITY_sp|Q6GP[illegible]	XAF1	XIAP-associated fac	35.60	0.00
TRINITY_sp|Q6FK[illegible]	RAT1	5'-3' exoribonuclea	35.60	0.00
TRINITY_sp|Q9CR[illegible]	Ccdc96	Coiled-coil domain-	35.60	0.00
TRINITY_sp|P054[illegible]	SSB	Lupus La protein OS	35.60	0.00
TRINITY_sp|O317[illegible]	cdsA	Phosphatidate cytid	35.60	0.00
TRINITY_sp|O537[illegible]	ufaA1	Tuberculostearic ac	35.60	0.00
TRINITY_sp|O088[illegible]	Rgs4	Regulator of G-prot	35.60	0.00
TRINITY_sp|P465[illegible]	cdk-12	Cyclin-dependent ki	35.60	0.00
TRINITY_sp|Q84K[illegible]	ABCA2	ABC transporter A f	35.60	0.00
TRINITY_sp|Q99J[illegible]	Mrm1	rRNA methyltransfer	35.60	0.00
TRINITY_sp|Q074[illegible]	EPHB5	Ephrin type-B recep	35.60	0.00
TRINITY_sp|D4AZ[illegible]	ARB_014[illegible]	Probable endo-1,3(4	35.60	0.00
TRINITY_sp|P171[illegible]	Cyp27a1	Sterol 26-hydroxyla	35.60	0.00
TRINITY_sp|Q993[illegible]	TY3B-G	Transposon Ty3-G Ga	35.60	0.00
TRINITY_sp|P071[illegible]	cyt-1	Cytochrome c1, heme	35.60	0.00
TRINITY_sp|Q9P7[illegible]	mak1	Peroxide stress-act	35.60	0.00
TRINITY_sp|P484[illegible]	RasGAP1	GTPase-activating p	35.50	0.00
TRINITY_sp|Q9SA[illegible]	At1g117[illegible]	Alpha-ketoglutarate	35.50	0.00
TRINITY_sp|Q8TE[illegible]	RAPGEF6	Rap guanine nucleot	35.50	0.00
TRINITY_sp|Q6AW[illegible]	At5g170[illegible]	D-xylose-proton sym	35.50	0.00
TRINITY_sp|Q54U[illegible]	trmt6	tRNA (adenine(58)-N	35.50	0.00
TRINITY_sp|Q9VT[illegible]	CG10681	KxDL motif-containi	35.50	0.00
TRINITY_sp|Q9XC[illegible]	tsf	Elongation factor T	35.50	0.00
TRINITY_sp|A0AU[illegible]	Gm7168	Sperm motility kina	35.50	0.00
TRINITY_sp|P623[illegible]	CPK4	Calcium-dependent p	35.50	0.00
TRINITY_sp|Q86H[illegible]	DDB_G02[illegible]	Probable serine/thr	35.50	0.00
TRINITY_sp|Q54Q[illegible]	dhkG	Hybrid signal trans	35.50	0.00
TRINITY_sp|Q9P3[illegible]	ptr2	Probable peptide tr	35.50	0.00
TRINITY_sp|F4K2[illegible]	ATG101	Autophagy-related p	35.50	0.00
TRINITY_sp|Q550[illegible]	captC	Uncharacterized CDP	35.50	0.00
TRINITY_sp|Q54M[illegible]	DDB_G02[illegible]	Serine hydrolase-li	35.50	0.00
TRINITY_sp|Q9ER[illegible]	Rdh14	Retinol dehydrogena	35.50	0.00
TRINITY_sp|Q4Z8[illegible]	RanBPM	Ran-binding protein	35.50	0.00
TRINITY_sp|Q96N[illegible]	RDH12	Retinol dehydrogena	35.50	0.00

TRINITY_sp|P3952	DRS2	Probable phospholip	35.50	0.00
TRINITY_sp|P385	LIPA	Lysosomal acid lipa	35.50	0.00
TRINITY_sp|Q9BT8	SOX7	Transcription facto	35.50	0.00
TRINITY_sp|Q2HVI	MtrDRAFT	Putative N6-adenosi	35.50	0.00
TRINITY_sp|Q08BE	ctdspl2a	CTD small phosphata	35.50	0.00
TRINITY_sp|Q9NJU	CPK3	Calcium-dependent p	35.50	0.00
TRINITY_sp|Q2896	CBR1	Carbonyl reductase	35.50	0.00
TRINITY_sp|Q7XU8	Os04g050	Probable protein ph	35.50	0.00
TRINITY_sp|B8ZXI	Qtrt2	Queuine tRNA-ribosy	35.50	0.00
TRINITY_sp|Q4V8Y	ergic1	Endoplasmic reticul	35.50	0.00
TRINITY_sp|P5406	bem46	Protein bem46 OS=Sc	35.50	0.00
TRINITY_sp|Q9FJW	FRO1	NADH dehydrogenase	35.50	0.00
TRINITY_sp|Q9LN0	At1g4812	Serine/threonine-pr	35.50	0.00
TRINITY_sp|Q84WV	QCT	Glutaminyl-peptide	35.50	0.00
TRINITY_sp|Q9I3Y	pmpM	Multidrug resistanc	35.50	0.00
TRINITY_sp|Q6NQI	At1g1358	LAG1 longevity assu	35.50	0.00
TRINITY_sp|Q5056	fdhC	Probable formate tr	35.50	0.00
TRINITY_sp|Q9M2Y	RAX3	Transcription facto	35.50	0.00
TRINITY_sp|Q8IX0	SLC35F2	Solute carrier fami	35.50	0.00
TRINITY_sp|P128I	hsp16.9A	16.9 kDa class I he	35.50	0.00
TRINITY_sp|Q70D0	ALDH3H1	Aldehyde dehydrogen	35.50	0.00
TRINITY_sp|O7493	POX2	Acyl-coenzyme A oxi	35.50	0.00
TRINITY_sp|Q3ZCE	EMC3	ER membrane protein	35.50	0.00
TRINITY_sp|Q6H70	CIPK26	CBL-interacting pro	35.50	0.00
TRINITY_sp|Q9VR5	l(1)G019	Inositol hexakispho	35.50	0.00
TRINITY_sp|Q502E	samhd1	Deoxynucleoside tri	35.50	0.00
TRINITY_sp|Q2898	-	Cystatin-A1 OS=Sus	35.50	0.00
TRINITY_sp|Q9S9N	CCR1	Cinnamoyl-CoA reduc	35.50	0.00
TRINITY_sp|Q8NF6	ZMIZ2	Zinc finger MIZ dom	35.50	0.00
TRINITY_sp|Q8RX3	At1g0716	Probable protein ph	35.50	0.00
TRINITY_sp|Q8VY0	CCR4-6	Carbon catabolite r	35.50	0.00
TRINITY_sp|Q55C7	tpp1	Tripeptidyl-peptida	35.50	0.00
TRINITY_sp|Q32L2	UBE2Q2	Ubiquitin-conjugati	35.50	0.00
TRINITY_sp|Q84JE	RUS3	Protein root UVB se	35.50	0.00
TRINITY_sp|Q55E5	pats1	Probable serine/thr	35.50	0.00
TRINITY_sp|A0A06	KIN4A	Kinesin-like protei	35.50	0.00
TRINITY_sp|Q0849	PDE4D	cAMP-specific 3',5'	35.50	0.00
TRINITY_sp|O7462	SPCC162.	Uncharacterized oxi	35.50	0.00
TRINITY_sp|Q6950	ROM1	Rhomboid-like prote	35.40	0.00
TRINITY_sp|Q8D52	luxQ	Autoinducer 2 senso	35.40	0.00
TRINITY_sp|Q4015	-	LEC14B protein OS=L	35.40	0.00
TRINITY_sp|Q5RB0	PRCP	Lysosomal Pro-X car	35.40	0.00
TRINITY_sp|O6010	snx41	Sorting nexin-41 OS	35.40	0.00
TRINITY_sp|O7580	ZMYND10	Zinc finger MYND do	35.40	0.00
TRINITY_sp|Q6IME	dnajc27	DnaJ homolog subfam	35.40	0.00
TRINITY_sp|Q9SV6	At4g1301	Putative quinone-ox	35.40	0.00
TRINITY_sp|Q9SL7	At2g2005	Protein phosphatase	35.40	0.00
TRINITY_sp|Q55BN	nek2	Probable serine/thr	35.40	0.00
TRINITY_sp|Q5RD6	SLC25A44	Solute carrier fami	35.40	0.00
TRINITY_sp|Q9XT6	RAD17	Cell cycle checkpoi	35.40	0.00
TRINITY_sp|Q0348	FBP1	Floral homeotic pro	35.40	0.00
TRINITY_sp|Q7Y0E	Os03g040	Probable nucleoredo	35.40	0.00
TRINITY_sp|Q54H4	drkB	Probable serine/thr	35.40	0.00
TRINITY_sp|Q2HCW	GWT1	GPI-anchored wall t	35.40	0.00
TRINITY_sp|P0419	Myb	Myb protein OS=Dros	35.40	0.00
TRINITY_sp|A4J47	hslO	33 kDa chaperonin O	35.40	0.00

TRINITY_sp|Q86C[illegible]	atg1	Serine/threonine-pr	35.40	0.00
TRINITY_sp|Q54R[illegible]	DDB_G02[illegible]	WASH complex subuni	35.40	0.00
TRINITY_sp|Q9LP[illegible]	ERF1-2	Eukaryotic peptide	35.40	0.00
TRINITY_sp|Q9SK[illegible]	NDB4	External alternativ	35.40	0.00
TRINITY_sp|Q803[illegible]	memo1	Protein MEMO1 OS=Da	35.40	0.00
TRINITY_sp|Q2KH[illegible]	CPNE6	Copine-6 OS=Bos tau	35.40	0.00
TRINITY_sp|P404[illegible]	PBX3	Pre-B-cell leukemia	35.40	0.00
TRINITY_sp|Q8CD[illegible]	Iqub	IQ and ubiquitin-li	35.40	0.00
TRINITY_sp|C5FW[illegible]	CPYA	Carboxypeptidase Y	35.40	0.00
TRINITY_sp|Q76P[illegible]	DDB_G02[illegible]	Probable serine/thr	35.40	0.00
TRINITY_sp|Q5ZJ[illegible]	TLDC1	TLD domain-containi	35.40	0.00
TRINITY_sp|A5FK[illegible]	rpsP	30S ribosomal prote	35.40	0.00
TRINITY_sp|Q55C[illegible]	wdr85	Diphthine methyltra	35.40	0.00
TRINITY_sp|P341[illegible]	pkgC	Protein kinase 3 OS	35.40	0.00
TRINITY_sp|Q01I[illegible]	H0219H1[illegible]	ATP-dependent RNA h	35.40	0.00
TRINITY_sp|Q652[illegible]	Os09g05[illegible]	Protein HIRA OS=Ory	35.40	0.00
TRINITY_sp|Q006[illegible]	gp63	Leishmanolysin OS=L	35.40	0.00
TRINITY_sp|Q8ST[illegible]	gefG	Ras guanine nucleot	35.40	0.00
TRINITY_sp|O944[illegible]	SPBC660.[illegible]	Uncharacterized RNA	35.40	0.00
TRINITY_sp|Q9ZR[illegible]	U2AF65A	Splicing factor U2a	35.40	0.00
TRINITY_sp|Q23F[illegible]	TTLL3C	Tubulin glycylase 3	35.40	0.00
TRINITY_sp|Q035[illegible]	Itk	Tyrosine-protein ki	35.40	0.00
TRINITY_sp|P239[illegible]	glcA	Glucan endo-1,3-bet	35.40	0.00
TRINITY_sp|Q9VR[illegible]	HERC2	Probable E3 ubiquit	35.40	0.00
TRINITY_sp|Q8MZ[illegible]	-	Physarolisin OS=Phy	35.40	0.00
TRINITY_sp|P364[illegible]	vilB	Protovillin OS=Dict	35.40	0.00
TRINITY_sp|Q407[illegible]	RGP2	Ras-related protein	35.40	0.00
TRINITY_sp|Q7F0[illegible]	CML13	Probable calcium-bi	35.40	0.00
TRINITY_sp|B1WA[illegible]	dvl3	Segment polarity pr	35.40	0.00
TRINITY_sp|Q6PN[illegible]	Dmxl1	DmX-like protein 1	35.40	0.00
TRINITY_sp|Q566[illegible]	Them4	Acyl-coenzyme A thi	35.40	0.00
TRINITY_sp|Q9MA[illegible]	At1g796[illegible]	Uncharacterized aar	35.40	0.00
TRINITY_sp|F4IU[illegible]	UPF2	Regulator of nonsen	35.40	0.00
TRINITY_sp|Q245[illegible]	Syx1A	Syntaxin-1A OS=Dros	35.30	0.00
TRINITY_sp|Q8WW[illegible]	TCTEX1D[illegible]	Tctex1 domain-conta	35.30	0.00
TRINITY_sp|Q9NP[illegible]	CABP5	Calcium-binding pro	35.30	0.00
TRINITY_sp|Q8IL[illegible]	PF14_03[illegible]	STI1-like protein O	35.30	0.00
TRINITY_sp|O133[illegible]	msh-2	DNA mismatch repair	35.30	0.00
TRINITY_sp|O138[illegible]	utp17	U3 small nucleolar	35.30	0.00
TRINITY_sp|Q9FH[illegible]	SWC6	SWR1 complex subuni	35.30	0.00
TRINITY_sp|Q6A0[illegible]	Fam179b	Crescerin-1 OS=Mus	35.30	0.00
TRINITY_sp|Q9LD[illegible]	MYB108	Transcription facto	35.30	0.00
TRINITY_sp|Q925[illegible]	GBF1	Golgi-specific bref	35.30	0.00
TRINITY_sp|P340[illegible]	manA	Lysosomal alpha-man	35.30	0.00
TRINITY_sp|A3KP[illegible]	ttc38	Tetratricopeptide r	35.30	0.00
TRINITY_sp|Q24K[illegible]	IDE	Insulin-degrading e	35.30	0.00
TRINITY_sp|Q9P2[illegible]	FAM135A	Protein FAM135A OS=	35.30	0.00
TRINITY_sp|Q58D[illegible]	ELMOD3	ELMO domain-contain	35.30	0.00
TRINITY_sp|Q125[illegible]	rasA	Ras-like protein OS	35.30	0.00
TRINITY_sp|Q5ZL[illegible]	LRRC40	Leucine-rich repeat	35.30	0.00
TRINITY_sp|Q9GY[illegible]	KIF15	Kinesin-like protei	35.30	0.00
TRINITY_sp|O646[illegible]	URT1	UTP:RNA uridylyltra	35.30	0.00
TRINITY_sp|O764[illegible]	nft-1	Nitrilase and fragi	35.30	0.00
TRINITY_sp|B4FG[illegible]	-	Spindle and kinetoc	35.30	0.00
TRINITY_sp|P232[illegible]	Mov10	Putative helicase M	35.30	0.00
TRINITY_sp|O170[illegible]	hpo-8	Very-long-chain (3R	35.30	0.00

TRINITY_sp|P3641	rab14	Ras-related protein	35.30	0.00
TRINITY_sp|P546	ptpC	Tyrosine-protein ph	35.30	0.00
TRINITY_sp|Q5ZJI	LSG1	Large subunit GTPas	35.30	0.00
TRINITY_sp|Q7S8V	esf-2	Pre-rRNA-processing	35.30	0.00
TRINITY_sp|P814	PGA	Pepsin A OS=Suncus	35.30	0.00
TRINITY_sp|Q7ZX	kif19	Kinesin-like protei	35.30	0.00
TRINITY_sp|Q54N	ppan	Peter Pan-like prot	35.30	0.00
TRINITY_sp|Q6IC	ASPHD2	Aspartate beta-hydr	35.30	0.00
TRINITY_sp|Q923	ssb1	Replication factor	35.30	0.00
TRINITY_sp|Q869	DDB_G02	COBW domain-contain	35.30	0.00
TRINITY_sp|Q9NA	tim-22	Mitochondrial impor	35.30	0.00
TRINITY_sp|Q851	Os03g07	Probable GTP-bindin	35.30	0.00
TRINITY_sp|P292	fol1	Folic acid synthesi	35.30	0.00
TRINITY_sp|O944	bdp1	Transcription facto	35.30	0.00
TRINITY_sp|Q2YD	ISG20L2	Interferon-stimulat	35.30	0.00
TRINITY_sp|Q86C	dhkK	Hybrid signal trans	35.30	0.00
TRINITY_sp|P251	rcc1	Regulator of chromo	35.30	0.00
TRINITY_sp|Q86Y	CPNE8	Copine-8 OS=Homo sa	35.30	0.00
TRINITY_sp|P533	tbg	Tubulin gamma chain	35.30	0.00
TRINITY_sp|Q945	CIB22	NADH dehydrogenase	35.30	0.00
TRINITY_sp|Q54C	pex6	Peroxisomal biogene	35.30	0.00
TRINITY_sp|P412	CSK	Tyrosine-protein ki	35.30	0.00
TRINITY_sp|Q9LT	ALIS1	ALA-interacting sub	35.30	0.00
TRINITY_sp|Q86I	dolpp1	Dolichyldiphosphata	35.30	0.00
TRINITY_sp|B0RZ	adk	Adenylate kinase OS	35.30	0.00
TRINITY_sp|Q680	GGT2	Gamma-glutamyltrans	35.30	0.00
TRINITY_sp|Q8IW	BBS7	Bardet-Biedl syndro	35.30	0.00
TRINITY_sp|Q6NS	trmt11	tRNA (guanine(10)-N	35.30	0.00
TRINITY_sp|Q86I	nek3	Probable serine/thr	35.30	0.00
TRINITY_sp|Q84K	ABCA2	ABC transporter A f	35.30	0.00
TRINITY_sp|Q96R	IFT140	Intraflagellar tran	35.30	0.00
TRINITY_sp|Q7T6	MIMI_R8	Putative serine/thr	35.30	0.00
TRINITY_sp|O601	lcf1	Long-chain-fatty-ac	35.20	0.00
TRINITY_sp|Q851	SLY1	SEC1 family transpo	35.20	0.00
TRINITY_sp|Q54R	dhkL	Hybrid signal trans	35.20	0.00
TRINITY_sp|Q7SD	msh-3	DNA mismatch repair	35.20	0.00
TRINITY_sp|A9I0	Bpet044	Nucleotide-binding	35.20	0.00
TRINITY_sp|Q75J	zakA	Dual specificity pr	35.20	0.00
TRINITY_sp|Q9BZ	WDR11	WD repeat-containin	35.20	0.00
TRINITY_sp|P213	NF1	Neurofibromin OS=Ho	35.20	0.00
TRINITY_sp|Q9NX	DUS2	tRNA-dihydrouridine	35.20	0.00
TRINITY_sp|Q54B	mcfG	Mitochondrial subst	35.20	0.00
TRINITY_sp|Q32P	C1D	Nuclear nucleic aci	35.20	0.00
TRINITY_sp|P412	Csk	Tyrosine-protein ki	35.20	0.00
TRINITY_sp|P426	SRK1	Tyrosine-protein ki	35.20	0.00
TRINITY_sp|Q9LK	ALA8	Probable phospholip	35.20	0.00
TRINITY_sp|Q0P5	Vps8	Vacuolar protein so	35.20	0.00
TRINITY_sp|Q90Z	lancl1	LanC-like protein 1	35.20	0.00
TRINITY_sp|Q588	TEB	Helicase and polyme	35.20	0.00
TRINITY_sp|Q0UU	TPC1	Mitochondrial thiam	35.20	0.00
TRINITY_sp|P308	evgS	Sensor protein EvgS	35.20	0.00
TRINITY_sp|Q84Y	CXIP4	CAX-interacting pro	35.20	0.00
TRINITY_sp|Q54F	eif2b3	Translation initiat	35.20	0.00
TRINITY_sp|Q55E	ctu2	Cytoplasmic tRNA 2-	35.20	0.00
TRINITY_sp|Q9FW	ABCB11	ABC transporter B f	35.20	0.00
TRINITY_sp|P518	fabG	3-oxoacyl-[acyl-car	35.20	0.00

TRINITY_sp|Q8K4I	Ddx54	ATP-dependent RNA h	35.20	0.00
TRINITY_sp|Q76NV	DDB_G02	Protein HGH1 homolo	35.20	0.00
TRINITY_sp|Q6C0	ATG18	Autophagy-related p	35.20	0.00
TRINITY_sp|Q96Q1	SMG1	Serine/threonine-pr	35.20	0.00
TRINITY_sp|Q198	F28H7.4	Putative selT-like	35.20	0.00
TRINITY_sp|Q96K(	EHMT2	Histone-lysine N-me	35.20	0.00
TRINITY_sp|Q8QZ	Tat	Tyrosine aminotrans	35.20	0.00
TRINITY_sp|Q2U6	caf17	Putative transferas	35.20	0.00
TRINITY_sp|Q8RW	CLASP	CLIP-associated pro	35.20	0.00
TRINITY_sp|F4JA	SKI2	DExH-box ATP-depend	35.20	0.00
TRINITY_sp|P418	rae1	Poly(A)+ RNA export	35.20	0.00
TRINITY_sp|P527	MSH6	DNA mismatch repair	35.20	0.00
TRINITY_sp|Q90Z	parn	Poly(A)-specific ri	35.20	0.00
TRINITY_sp|Q7T2	fam172a	Protein FAM172A OS=	35.20	0.00
TRINITY_sp|Q58D	MMTAG2	Multiple myeloma tu	35.20	0.00
TRINITY_sp|Q0II	ARL6	ADP-ribosylation fa	35.20	0.00
TRINITY_sp|Q5YD	CPL2	RNA polymerase II C	35.20	0.00
TRINITY_sp|Q8BY	Tbc1d4	TBC1 domain family	35.20	0.00
TRINITY_sp|Q54B	abcB2	ABC transporter B f	35.20	0.00
TRINITY_sp|Q5U2	Tbl3	Transducin beta-lik	35.20	0.00
TRINITY_sp|P0CM	CWC25	Pre-mRNA-splicing f	35.20	0.00
TRINITY_sp|Q9UI	HERC5	E3 ISG15--protein l	35.20	0.00
TRINITY_sp|Q000	SRP68	Signal recognition	35.20	0.00
TRINITY_sp|Q24K	GPATCH1	G patch domain-cont	35.20	0.00
TRINITY_sp|Q640	rmnd5a	Protein RMD5 homolo	35.20	0.00
TRINITY_sp|Q9SL	TOC132	Translocase of chlo	35.20	0.00
TRINITY_sp|Q6ZD	FAS2	Chromatin assembly	35.20	0.00
TRINITY_sp|Q128	MAP3K12	Mitogen-activated p	35.20	0.00
TRINITY_sp|Q1LU	fam206a	Protein Simiate OS=	35.20	0.00
TRINITY_sp|Q54L	vps13A	Putative vacuolar p	35.20	0.00
TRINITY_sp|Q9H2	HSD3B7	3 beta-hydroxystero	35.20	0.00
TRINITY_sp|P285	TRK2	Low-affinity potass	35.20	0.00
TRINITY_sp|Q8WU	PTPMT1	Phosphatidylglycero	35.20	0.00
TRINITY_sp|Q9M2	SDR1	(+)-neomenthol dehy	35.20	0.00
TRINITY_sp|Q9XI	SKIP32	F-box protein 7 OS=	35.20	0.00
TRINITY_sp|Q9FF	PPH	Pheophytinase, chlo	35.20	0.00
TRINITY_sp|O138	SPAC22A	Uncharacterized CDP	35.20	0.00
TRINITY_sp|Q7XJ	FAAH	Fatty acid amide hy	35.20	0.00
TRINITY_sp|A1ZA	CG5065	Putative fatty acyl	35.20	0.00
TRINITY_sp|Q96A	VPS33A	Vacuolar protein so	35.20	0.00
TRINITY_sp|X5M5	wnk-1	Serine/threonine-pr	35.20	0.00
TRINITY_sp|Q84Q	GGCT2;3	Gamma-glutamylcyclo	35.20	0.00
TRINITY_sp|Q3E9	ANTR6	Probable anion tran	35.20	0.00
TRINITY_sp|P385	HSF1	Heat shock factor p	35.20	0.00
TRINITY_sp|Q231	rsp-1	Probable splicing f	35.20	0.00
TRINITY_sp|Q8LP	CCD1	Carotenoid 9,10(9',	35.20	0.00
TRINITY_sp|A7RZ	dpp3	Dipeptidyl peptidas	35.20	0.00
TRINITY_sp|Q99K	Reep3	Receptor expression	35.10	0.00
TRINITY_sp|Q9UI	HERC5	E3 ISG15--protein l	35.10	0.00
TRINITY_sp|P341	rab1B	Ras-related protein	35.10	0.00
TRINITY_sp|Q6NW	arih1l	E3 ubiquitin-protei	35.10	0.00
TRINITY_sp|Q8WY	SSH1	Protein phosphatase	35.10	0.00
TRINITY_sp|Q2RA	CYCT1-3	Cyclin-T1-3 OS=Oryz	35.10	0.00
TRINITY_sp|P443	rbsK	Ribokinase OS=Haemo	35.10	0.00
TRINITY_sp|Q5AV	ampp	Probable Xaa-Pro am	35.10	0.00
TRINITY_sp|Q9P8	tcsA	Two-component syste	35.10	0.00

TRINITY_sp|F1Q4	atp9b	Probable phospholip	35.10	0.00
TRINITY_sp|A5PK	METTL13	Methyltransferase-l	35.10	0.00
TRINITY_sp|Q8UI	truA	tRNA pseudouridine	35.10	0.00
TRINITY_sp|Q54H	drkA	Probable serine/thr	35.10	0.00
TRINITY_sp|Q9P3	dbl4	E3 ubiquitin-protei	35.10	0.00
TRINITY_sp|P242	ARDD	Actin, spherule iso	35.10	0.00
TRINITY_sp|A8JB	CCDC65	Coiled-coil domain-	35.10	0.00
TRINITY_sp|Q3ED	At1g229	Uncharacterized PKH	35.10	0.00
TRINITY_sp|Q9CR	Rchy1	RING finger and CHY	35.10	0.00
TRINITY_sp|Q9DG	star	Steroidogenic acute	35.10	0.00
TRINITY_sp|Q69Z	Dnah17	Dynein heavy chain	35.10	0.00
TRINITY_sp|O648	CID7	Polyadenylate-bindi	35.10	0.00
TRINITY_sp|Q58E	parl	Presenilins-associa	35.10	0.00
TRINITY_sp|P200	PSAP	Saposin-C OS=Cavia	35.10	0.00
TRINITY_sp|Q54I	eif2b1	Translation initiat	35.10	0.00
TRINITY_sp|Q0VD	SMPD1	Sphingomyelin phosp	35.10	0.00
TRINITY_sp|Q9SJ	VPS11	Vacuolar protein-so	35.10	0.00
TRINITY_sp|Q869	napA	Nck-associated prot	35.10	0.00
TRINITY_sp|Q8N9	SCAI	Protein SCAI OS=Hom	35.10	0.00
TRINITY_sp|P608	Megf8	Multiple epidermal	35.10	0.00
TRINITY_sp|O510	trmH	tRNA (guanosine(18)	35.10	0.00
TRINITY_sp|P497	RBM25	RNA-binding protein	35.10	0.00
TRINITY_sp|P703	Usp9x	Probable ubiquitin	35.10	0.00
TRINITY_sp|P577	Rce1	CAAX prenyl proteas	35.10	0.00
TRINITY_sp|Q54M	DDB_G02	von Willebrand fact	35.10	0.00
TRINITY_sp|O086	Sqstm1	Sequestosome-1 OS=R	35.10	0.00
TRINITY_sp|Q8FM	dcd	dCTP deaminase OS=C	35.10	0.00
TRINITY_sp|Q4HW	RAT1	5'-3' exoribonuclea	35.10	0.00
TRINITY_sp|O138	not3	General negative re	35.10	0.00
TRINITY_sp|Q84R	CRSH	Probable GTP diphos	35.10	0.00
TRINITY_sp|Q8RV	SEC10	Exocyst complex com	35.10	0.00
TRINITY_sp|P048	Cyp3a1	Cytochrome P450 3A1	35.10	0.00
TRINITY_sp|Q8H1	AAE13	Malonate--CoA ligas	35.10	0.00
TRINITY_sp|Q9C5	GCP2	Gamma-tubulin compl	35.10	0.00
TRINITY_sp|Q6V5	Znf474	Zinc finger protein	35.10	0.00
TRINITY_sp|Q9P2	ZNFX1	NFX1-type zinc fing	35.10	0.00
TRINITY_sp|Q5ZJ	CREG1	Protein CREG1 OS=Ga	35.10	0.00
TRINITY_sp|Q9WV	March7	E3 ubiquitin-protei	35.10	0.00
TRINITY_sp|P313	PKAR	cAMP-dependent prot	35.10	0.00
TRINITY_sp|Q5BL	mettl21a	Protein N-lysine me	35.10	0.00
TRINITY_sp|Q8BL	Arhgap2	Rho GTPase-activati	35.10	0.00
TRINITY_sp|B4SD	Ppha_21	UPF0301 protein Pph	35.10	0.00
TRINITY_sp|Q54X	rckA	RGS domain-containi	35.10	0.00
TRINITY_sp|Q253	PKC1	Protein kinase C OS	35.10	0.00
TRINITY_sp|Q9HD	ypt71	GTP-binding protein	35.10	0.00
TRINITY_sp|Q050	UBE3A	Ubiquitin-protein l	35.10	0.00
TRINITY_sp|Q9VR	HERC2	Probable E3 ubiquit	35.10	0.00
TRINITY_sp|Q5BI	ERCC8	DNA excision repair	35.10	0.00
TRINITY_sp|Q294	ATP8A1	Probable phospholip	35.10	0.00
TRINITY_sp|F4IF	FGT1	Protein FORGETTER 1	35.10	0.00
TRINITY_sp|Q86I	DDB_G02	PH domain-containin	35.10	0.00
TRINITY_sp|Q6ZI	SAPK6	Serine/threonine-pr	35.10	0.00
TRINITY_sp|Q9SV	At4g217	RNA pseudouridine s	35.10	0.00
TRINITY_sp|Q0VF	bloc1s1	Biogenesis of lysos	35.10	0.00
TRINITY_sp|Q9P7	mak1	Peroxide stress-act	35.00	0.00
TRINITY_sp|Q90Z	parn	Poly(A)-specific ri	35.00	0.00

TRINITY_sp|Q179[illegible]	daf-36	Cholesterol 7-desat	35.00	0.00
TRINITY_sp|Q9UT[illegible]	paa1	Protein phosphatase	35.00	0.00
TRINITY_sp|Q84V[illegible]	PSD1	Phosphatidylserine	35.00	0.00
TRINITY_sp|Q9UK[illegible]	GPATCH8	G patch domain-cont	35.00	0.00
TRINITY_sp|O946[illegible]	SPCC622.[illegible]	Uncharacterized pro	35.00	0.00
TRINITY_sp|Q9M8[illegible]	DSPTP1B	Dual specificity pr	35.00	0.00
TRINITY_sp|Q54C[illegible]	DDB_G02[illegible]	Probable myosin lig	35.00	0.00
TRINITY_sp|Q5M8[illegible]	impact-[illegible]	Protein IMPACT-A OS	35.00	0.00
TRINITY_sp|Q9GL[illegible]	CTSL	Cathepsin L1 OS=Can	35.00	0.00
TRINITY_sp|Q8T6[illegible]	abcG20	ABC transporter G f	35.00	0.00
TRINITY_sp|Q9FG[illegible]	At5g068[illegible]	CDK5RAP3-like prote	35.00	0.00
TRINITY_sp|Q8QH[illegible]	dna2	DNA replication ATP	35.00	0.00
TRINITY_sp|P0CT[illegible]	UMAG_10[illegible]	Uncharacterized hyd	35.00	0.00
TRINITY_sp|Q9FP[illegible]	EDR1	Serine/threonine-pr	35.00	0.00
TRINITY_sp|O944[illegible]	bmt5	25S rRNA (uridine-N	35.00	0.00
TRINITY_sp|Q245[illegible]	U2af50	Splicing factor U2A	35.00	0.00
TRINITY_sp|O089[illegible]	Cyth3	Cytohesin-3 OS=Mus	35.00	0.00
TRINITY_sp|O646[illegible]	AUR3	Serine/threonine-pr	35.00	0.00
TRINITY_sp|Q6DE[illegible]	aurkb-a	Aurora kinase B-A O	35.00	0.00
TRINITY_sp|Q0JI[illegible]	CIPK11	CBL-interacting pro	35.00	0.00
TRINITY_sp|Q9ZP[illegible]	ARL2	ADP-ribosylation fa	35.00	0.00
TRINITY_sp|F4HP[illegible]	LIG6	DNA ligase 6 OS=Ara	35.00	0.00
TRINITY_sp|Q8ST[illegible]	gefG	Ras guanine nucleot	35.00	0.00
TRINITY_sp|Q8IZ[illegible]	DOCK3	Dedicator of cytoki	35.00	0.00
TRINITY_sp|A8MS[illegible]	SPT6	Transcription elong	35.00	0.00
TRINITY_sp|Q553[illegible]	DDB_G02[illegible]	Adenosine deaminase	35.00	0.00
TRINITY_sp|Q9LT[illegible]	PDR2	Probable manganese-	35.00	0.00
TRINITY_sp|O941[illegible]	SNF1	Carbon catabolite-d	35.00	0.00
TRINITY_sp|Q9UJ[illegible]	TUBD1	Tubulin delta chain	35.00	0.00
TRINITY_sp|Q9VB[illegible]	CG5521	Probable Rho GTPase	35.00	0.00
TRINITY_sp|O137[illegible]	SPAC16E[illegible]	Uncharacterized end	35.00	0.00
TRINITY_sp|Q4U2[illegible]	Herc2	E3 ubiquitin-protei	35.00	0.00
TRINITY_sp|Q9UR[illegible]	nrf1	Vacuolar transporte	35.00	0.00
TRINITY_sp|Q8CG[illegible]	Elac2	Zinc phosphodiester	35.00	0.00
TRINITY_sp|Q294[illegible]	LIPF	Gastric triacylglyc	35.00	0.00
TRINITY_sp|P418[illegible]	gar2	Protein gar2 OS=Sch	35.00	0.00
TRINITY_sp|O749[illegible]	nat1	N-terminal acetyltr	35.00	0.00
TRINITY_sp|Q86J[illegible]	polr3f	DNA-directed RNA po	35.00	0.00
TRINITY_sp|Q8L7[illegible]	SDN5	Small RNA degrading	35.00	0.00
TRINITY_sp|Q54E[illegible]	polr2e	DNA-directed RNA po	35.00	0.00
TRINITY_sp|Q8R3[illegible]	Rbm19	Probable RNA-bindin	35.00	0.00
TRINITY_sp|P360[illegible]	YPT52	GTP-binding protein	35.00	0.00
TRINITY_sp|Q949[illegible]	ARI1	Probable E3 ubiquit	35.00	0.00
TRINITY_sp|O225[illegible]	STY8	Serine/threonine-pr	35.00	0.00
TRINITY_sp|Q8N6[illegible]	ZFAND2A	AN1-type zinc finge	35.00	0.00
TRINITY_sp|Q75H[illegible]	SPS2	Solanesyl-diphospha	35.00	0.00
TRINITY_sp|Q389[illegible]	KIN10	SNF1-related protei	35.00	0.00
TRINITY_sp|Q5HN[illegible]	pcrA	ATP-dependent DNA h	35.00	0.00
TRINITY_sp|Q9FG[illegible]	SNX1	Sorting nexin 1 OS=	35.00	0.00
TRINITY_sp|Q93Y[illegible]	ABCG22	ABC transporter G f	35.00	0.00
TRINITY_sp|Q5AG[illegible]	HSL1	Serine/threonine-pr	35.00	0.00
TRINITY_sp|Q82W[illegible]	rluD	Ribosomal large sub	35.00	0.00
TRINITY_sp|Q8T8[illegible]	abcD2	ABC transporter D f	35.00	0.00
TRINITY_sp|O804[illegible]	At1g091[illegible]	Probable protein ph	35.00	0.00
TRINITY_sp|A4YI[illegible]	Msed_20[illegible]	3-hydroxypropionyl-	35.00	0.00
TRINITY_sp|P222[illegible]	RAS1	Ras-like protein 1	35.00	0.00

TRINITY_sp|Q9SR	At3g101	Heme-binding-like p	35.00	0.00
TRINITY_sp|Q5ZKI	ATP13A4	Probable cation-tra	34.90	0.00
TRINITY_sp|Q8L9	KCR1	Very-long-chain 3-o	34.90	0.00
TRINITY_sp|Q035	SCS7	Ceramide very long	34.90	0.00
TRINITY_sp|A1L2	slc29a4	Equilibrative nucle	34.90	0.00
TRINITY_sp|Q566	Them4	Acyl-coenzyme A thi	34.90	0.00
TRINITY_sp|P351	PTC1	Protein phosphatase	34.90	0.00
TRINITY_sp|Q59V	HHT1	Histone H3.1/H3.2 O	34.90	0.00
TRINITY_sp|Q28I	ngdn	Neuroguidin OS=Xeno	34.90	0.00
TRINITY_sp|Q041	-	Pathogenesis-relate	34.90	0.00
TRINITY_sp|Q96G	RMND5B	Protein RMD5 homolo	34.90	0.00
TRINITY_sp|Q634	Mapk4	Mitogen-activated p	34.90	0.00
TRINITY_sp|Q55F	atp5D	ATP synthase subuni	34.90	0.00
TRINITY_sp|Q8GY	RPN9B	26S proteasome non-	34.90	0.00
TRINITY_sp|Q401	-	LEC14B protein OS=L	34.90	0.00
TRINITY_sp|O944	SPBC660.	Uncharacterized RNA	34.90	0.00
TRINITY_sp|Q8LF	ASK21	SKP1-like protein 2	34.90	0.00
TRINITY_sp|Q9FE	RPL22C	60S ribosomal prote	34.90	0.00
TRINITY_sp|O188	AOAH	Acyloxyacyl hydrola	34.90	0.00
TRINITY_sp|Q9JK	Aatf	Protein AATF OS=Mus	34.90	0.00
TRINITY_sp|F4K2	ATG101	Autophagy-related p	34.90	0.00
TRINITY_sp|Q9P4	LYS3	Saccharopine dehydr	34.90	0.00
TRINITY_sp|O229	At2g303	Putative tRNA pseud	34.90	0.00
TRINITY_sp|O005	FAAH	Fatty-acid amide hy	34.90	0.00
TRINITY_sp|Q2QK	PCS3	Glutathione gamma-g	34.90	0.00
TRINITY_sp|Q9P4	AGM1	Phosphoacetylglucos	34.90	0.00
TRINITY_sp|Q554	argS	Arginine--tRNA liga	34.90	0.00
TRINITY_sp|Q9P7	mak1	Peroxide stress-act	34.90	0.00
TRINITY_sp|Q94D	PHT1-11	Inorganic phosphate	34.90	0.00
TRINITY_sp|Q0D9	STAR1	Protein STAR1 OS=Or	34.90	0.00
TRINITY_sp|Q94C	TIP1-2	Probable aquaporin	34.90	0.00
TRINITY_sp|B0G1	spcs3	Signal peptidase co	34.90	0.00
TRINITY_sp|Q8CG	Rbm28	RNA-binding protein	34.90	0.00
TRINITY_sp|P378	CBP1	Serine carboxypepti	34.90	0.00
TRINITY_sp|Q68C	HGSNAT	Heparan-alpha-gluco	34.90	0.00
TRINITY_sp|Q9LD	BETAA-AI	Beta-adaptin-like p	34.90	0.00
TRINITY_sp|Q6C7	PHO85	Negative regulator	34.90	0.00
TRINITY_sp|Q5KQ	CMT2	DNA (cytosine-5)-me	34.90	0.00
TRINITY_sp|Q500	TKPR1	Tetraketide alpha-p	34.90	0.00
TRINITY_sp|P546	pkbA	RAC family serine/t	34.90	0.00
TRINITY_sp|P004	COX5B	Cytochrome c oxidas	34.90	0.00
TRINITY_sp|Q8RW	CPK29	Calcium-dependent p	34.90	0.00
TRINITY_sp|Q0ZD	urad	2-oxo-4-hydroxy-4-c	34.90	0.00
TRINITY_sp|P148	yoxD	Uncharacterized oxi	34.90	0.00
TRINITY_sp|Q5M9	Rrp15	RRP15-like protein	34.90	0.00
TRINITY_sp|P323	Arp3	Actin-related prote	34.90	0.00
TRINITY_sp|Q9XT	tbh-1	Tyramine beta-hydro	34.90	0.00
TRINITY_sp|Q99J	Klhl22	Kelch-like protein	34.90	0.00
TRINITY_sp|Q9ZR	YKT61	VAMP-like protein Y	34.90	0.00
TRINITY_sp|Q5R9	ZFAND2A	AN1-type zinc finge	34.90	0.00
TRINITY_sp|P341	fhkC	Probable serine/thr	34.90	0.00
TRINITY_sp|Q095	C45G9.2	Uncharacterized tRN	34.90	0.00
TRINITY_sp|Q54N	vmp1	Vacuole membrane pr	34.90	0.00
TRINITY_sp|Q5F3	TTC27	Tetratricopeptide r	34.90	0.00
TRINITY_sp|Q220	pde-4	Probable 3',5'-cycl	34.90	0.00
TRINITY_sp|Q6NM	At5g656	Probable sphingolip	34.90	0.00

TRINITY_sp|P227[illegible]	Gucy1b2	Guanylate cyclase s	34.90	0.00
TRINITY_sp|Q9XW[illegible]	Y37D8A.[illegible]	Putative phospholip	34.80	0.00
TRINITY_sp|Q902[illegible]	es1	ES1 protein, mitoch	34.80	0.00
TRINITY_sp|Q9FP[illegible]	EDR1	Serine/threonine-pr	34.80	0.00
TRINITY_sp|P582[illegible]	LOXL3	Lysyl oxidase homol	34.80	0.00
TRINITY_sp|Q8WV[illegible]	NUDCD2	NudC domain-contain	34.80	0.00
TRINITY_sp|Q86C[illegible]	atg8	Autophagy-related p	34.80	0.00
TRINITY_sp|Q6MC[illegible]	pepA	Probable cytosol am	34.80	0.00
TRINITY_sp|Q0JI[illegible]	CIPK11	CBL-interacting pro	34.80	0.00
TRINITY_sp|Q9VR[illegible]	HERC2	Probable E3 ubiquit	34.80	0.00
TRINITY_sp|Q969[illegible]	POP5	Ribonuclease P/MRP	34.80	0.00
TRINITY_sp|Q652[illegible]	Os06g05[illegible]	Probable protein ph	34.80	0.00
TRINITY_sp|Q96Q[illegible]	ATAD5	ATPase family AAA d	34.80	0.00
TRINITY_sp|A8FH[illegible]	ppaX	Pyrophosphatase Ppa	34.80	0.00
TRINITY_sp|P276[illegible]	spoVK	Stage V sporulation	34.80	0.00
TRINITY_sp|Q86S[illegible]	C50D2.7	Probable ADP-depend	34.80	0.00
TRINITY_sp|Q55B[illegible]	dcd1A	Protein dcd1A OS=Di	34.80	0.00
TRINITY_sp|Q9VI[illegible]	RanGAP	Ran GTPase-activati	34.80	0.00
TRINITY_sp|Q556[illegible]	slr0021	Putative protease s	34.80	0.00
TRINITY_sp|Q54F[illegible]	mroh1	Maestro heat-like r	34.80	0.00
TRINITY_sp|O316[illegible]	yjcL	Uncharacterized mem	34.80	0.00
TRINITY_sp|Q6CF[illegible]	FIS1	Mitochondria fissio	34.80	0.00
TRINITY_sp|Q8YS[illegible]	alr2987	UPF0187 protein alr	34.80	0.00
TRINITY_sp|A2VD[illegible]	MAP3K7	Mitogen-activated p	34.80	0.00
TRINITY_sp|Q6GL[illegible]	alyref-[illegible]	THO complex subunit	34.80	0.00
TRINITY_sp|Q80L[illegible]	VCATH	Viral cathepsin OS=	34.80	0.00
TRINITY_sp|A1YV[illegible]	KDM5C	Lysine-specific dem	34.80	0.00
TRINITY_sp|Q8NE[illegible]	MCU	Calcium uniporter p	34.80	0.00
TRINITY_sp|Q7Z7[illegible]	YTHDF3	YTH domain-containi	34.80	0.00
TRINITY_sp|Q55C[illegible]	rasX	Ras-like protein ra	34.80	0.00
TRINITY_sp|Q6P0[illegible]	exosc6	Exosome complex com	34.80	0.00
TRINITY_sp|Q8L5[illegible]	LTO1	Thiol-disulfide oxi	34.80	0.00
TRINITY_sp|Q54B[illegible]	dstC	Signal transducer a	34.80	0.00
TRINITY_sp|Q181[illegible]	C25E10.[illegible]	UPF0046 protein C25	34.80	0.00
TRINITY_sp|Q6H7[illegible]	CIPK26	CBL-interacting pro	34.80	0.00
TRINITY_sp|P091[illegible]	P4HB	Protein disulfide-i	34.80	0.00
TRINITY_sp|Q93Y[illegible]	CLS	Cardiolipin synthas	34.80	0.00
TRINITY_sp|Q54D[illegible]	ergic3	Probable endoplasmi	34.80	0.00
TRINITY_sp|Q54N[illegible]	fsjC	Putative rRNA methy	34.80	0.00
TRINITY_sp|P742[illegible]	slr1673	Uncharacterized tRN	34.80	0.00
TRINITY_sp|Q8LF[illegible]	TFB4	RNA polymerase II t	34.80	0.00
TRINITY_sp|Q9QY[illegible]	Rdh11	Retinol dehydrogena	34.80	0.00
TRINITY_sp|B3MF[illegible]	Art7	Protein arginine N-	34.80	0.00
TRINITY_sp|Q86C[illegible]	dhkK	Hybrid signal trans	34.80	0.00
TRINITY_sp|Q6NZ[illegible]	Eif4g1	Eukaryotic translat	34.80	0.00
TRINITY_sp|A6RB[illegible]	HCAG_07[illegible]	Very-long-chain 3-o	34.80	0.00
TRINITY_sp|Q8GU[illegible]	RH50	DEAD-box ATP-depend	34.80	0.00
TRINITY_sp|P460[illegible]	RECQL	ATP-dependent DNA h	34.80	0.00
TRINITY_sp|Q9H1[illegible]	ANAPC1	Anaphase-promoting	34.80	0.00
TRINITY_sp|P356[illegible]	DEK	Protein DEK OS=Homo	34.80	0.00
TRINITY_sp|O958[illegible]	MLYCD	Malonyl-CoA decarbo	34.80	0.00
TRINITY_sp|Q9Y0[illegible]	itr-1	Inositol 1,4,5-tris	34.80	0.00
TRINITY_sp|Q75D[illegible]	FMN1	Riboflavin kinase O	34.80	0.00
TRINITY_sp|Q9LI[illegible]	UNG	Uracil-DNA glycosyl	34.80	0.00
TRINITY_sp|Q55C[illegible]	uba1	Ubiquitin-like modi	34.80	0.00
TRINITY_sp|Q54N[illegible]	kif4	Kinesin-related pro	34.80	0.00

TRINITY_sp|Q634[illegible]	Pcsk6	Proprotein converta	34.80	0.00
TRINITY_sp|Q9ZU[illegible]	BAT1	Amino-acid permease	34.80	0.00
TRINITY_sp|Q8BY[illegible]	Itpk1	Inositol-tetrakisph	34.80	0.00
TRINITY_sp|O220[illegible]	cfxQ	Protein CfxQ homolo	34.80	0.00
TRINITY_sp|Q6DF[illegible]	Ncapg2	Condensin-2 complex	34.80	0.00
TRINITY_sp|Q9S9[illegible]	SDRA	Short-chain dehydro	34.80	0.00
TRINITY_sp|Q5BP[illegible]	At4g066[illegible]	Protein EI24 homolo	34.80	0.00
TRINITY_sp|P435[illegible]	cya	Adenylate cyclase O	34.80	0.00
TRINITY_sp|Q6B9[illegible]	vwkA	Alpha-protein kinas	34.80	0.00
TRINITY_sp|Q94A[illegible]	SFH12	Phosphatidylinosito	34.70	0.00
TRINITY_sp|Q9FV[illegible]	At1g322[illegible]	Uncharacterized pro	34.70	0.00
TRINITY_sp|Q8VY[illegible]	RS2Z33	Serine/arginine-ric	34.70	0.00
TRINITY_sp|Q5P4[illegible]	gatA	Glutamyl-tRNA(Gln)	34.70	0.00
TRINITY_sp|Q9ES[illegible]	Pgpep1	Pyroglutamyl-peptid	34.70	0.00
TRINITY_sp|Q8BI[illegible]	Gpatch3	G patch domain-cont	34.70	0.00
TRINITY_sp|O827[illegible]	RBL7	RHOMBOID-like prote	34.70	0.00
TRINITY_sp|Q292[illegible]	CSTB	Cystatin-B OS=Sus s	34.70	0.00
TRINITY_sp|Q6IV[illegible]	ift57	Intraflagellar tran	34.70	0.00
TRINITY_sp|P237[illegible]	RIT1	tRNA A64-2'-O-ribos	34.70	0.00
TRINITY_sp|P633[illegible]	yheS	Uncharacterized ABC	34.70	0.00
TRINITY_sp|Q5ZI[illegible]	COPE	Coatomer subunit ep	34.70	0.00
TRINITY_sp|P308[illegible]	evgS	Sensor protein EvgS	34.70	0.00
TRINITY_sp|O346[illegible]	ytqB	Putative rRNA methy	34.70	0.00
TRINITY_sp|Q920[illegible]	Msi2	RNA-binding protein	34.70	0.00
TRINITY_sp|Q9FI[illegible]	ATL30	RING-H2 finger prot	34.70	0.00
TRINITY_sp|Q8VE[illegible]	Pla2g15	Group XV phospholip	34.70	0.00
TRINITY_sp|Q9AV[illegible]	PRP19	Pre-mRNA-processing	34.70	0.00
TRINITY_sp|Q2UN[illegible]	oct1	Mitochondrial inter	34.70	0.00
TRINITY_sp|Q9GV[illegible]	-	Endoglycoceramidase	34.70	0.00
TRINITY_sp|Q9M2[illegible]	At3g613[illegible]	UPF0187 protein At3	34.70	0.00
TRINITY_sp|Q9FV[illegible]	UPF3	Regulator of nonsen	34.70	0.00
TRINITY_sp|Q99L[illegible]	Abtb1	Ankyrin repeat and	34.70	0.00
TRINITY_sp|Q6TA[illegible]	ADCS	Aminodeoxychorismat	34.70	0.00
TRINITY_sp|Q54J[illegible]	DDB_G02[illegible]	Probable serine/thr	34.70	0.00
TRINITY_sp|Q5SS[illegible]	Sft2d1	Vesicle transport p	34.70	0.00
TRINITY_sp|Q9SC[illegible]	PAP17	Purple acid phospha	34.70	0.00
TRINITY_sp|P546[illegible]	pikD	Phosphatidylinosito	34.70	0.00
TRINITY_sp|Q54B[illegible]	redA	NADPH oxidoreductas	34.70	0.00
TRINITY_sp|Q54C[illegible]	cog6	Conserved oligomeri	34.70	0.00
TRINITY_sp|Q55G[illegible]	DDB_G02[illegible]	Probable phosphatid	34.70	0.00
TRINITY_sp|Q8CI[illegible]	Sidt2	SID1 transmembrane	34.70	0.00
TRINITY_sp|Q8BX[illegible]	Pxk	PX domain-containin	34.70	0.00
TRINITY_sp|Q91X[illegible]	Chia	Acidic mammalian ch	34.70	0.00
TRINITY_sp|Q54D[illegible]	DDB_G02[illegible]	Putative protein DD	34.70	0.00
TRINITY_sp|Q8LE[illegible]	NUDT11	Nudix hydrolase 11	34.70	0.00
TRINITY_sp|Q86U[illegible]	N4BP2	NEDD4-binding prote	34.70	0.00
TRINITY_sp|Q1HF[illegible]	Nsun2	tRNA (cytosine(34)-	34.70	0.00
TRINITY_sp|Q5SP[illegible]	wdr26	WD repeat-containin	34.70	0.00
TRINITY_sp|Q944[illegible]	SNF4	Sucrose nonfermenti	34.70	0.00
TRINITY_sp|Q54L[illegible]	cog7	Conserved oligomeri	34.70	0.00
TRINITY_sp|Q6GL[illegible]	brd9	Bromodomain-contain	34.70	0.00
TRINITY_sp|Q9TW[illegible]	myoM	Myosin-M heavy chai	34.70	0.00
TRINITY_sp|Q8W5[illegible]	CYCH1-1	Cyclin-H1-1 OS=Arab	34.70	0.00
TRINITY_sp|Q9ES[illegible]	Dusp10	Dual specificity pr	34.70	0.00
TRINITY_sp|Q8ND[illegible]	CCNY	Cyclin-Y OS=Homo sa	34.70	0.00
TRINITY_sp|Q54B[illegible]	nfx1	Transcriptional rep	34.70	0.00

TRINITY_sp|Q8NFI	FLAD1	FAD synthase OS=Hom	34.70	0.00
TRINITY_sp|Q61Y(	CBG0355(	Leishmanolysin-like	34.70	0.00
TRINITY_sp|Q8BL(	Mettl21(	Protein-lysine meth	34.70	0.00
TRINITY_sp|P480(	gacS	Sensor protein GacS	34.70	0.00
TRINITY_sp|P554(	NGR_a03(	Uncharacterized pro	34.70	0.00
TRINITY_sp|E9L7(	-	Bifunctional aspart	34.70	0.00
TRINITY_sp|Q8BT(	Rccd1	RCC1 domain-contain	34.70	0.00
TRINITY_sp|O806(	APY6	Probable apyrase 6	34.70	0.00
TRINITY_sp|Q9Y2(	TRAPPC4	Trafficking protein	34.70	0.00
TRINITY_sp|P590(	vps18	Vacuolar protein so	34.70	0.00
TRINITY_sp|O149(	PIP5K1B	Phosphatidylinosito	34.70	0.00
TRINITY_sp|Q5BU(	Eapp	E2F-associated phos	34.70	0.00
TRINITY_sp|Q8C0(	Slc25a1(	Graves disease carr	34.70	0.00
TRINITY_sp|P547(	pknA	Serine/threonine-pr	34.70	0.00
TRINITY_sp|Q8L5(	TFCD	Tubulin-folding cof	34.70	0.00
TRINITY_sp|Q80Y(	Alkbh8	Alkylated DNA repai	34.70	0.00
TRINITY_sp|O440(	PYK	Pyruvate kinase OS=	34.70	0.00
TRINITY_sp|A5PK(	OXR1	Oxidation resistanc	34.70	0.00
TRINITY_sp|O056(	vanA	Vanillate O-demethy	34.70	0.00
TRINITY_sp|Q86H(	cf45-1	Counting factor 45-	34.70	0.00
TRINITY_sp|Q6CN(	HSV2	SVP1-like protein 2	34.70	0.00
TRINITY_sp|Q54S(	allB1	Probable allantoina	34.60	0.00
TRINITY_sp|Q8RV(	DEK1	Calpain-type cystei	34.60	0.00
TRINITY_sp|P238(	sds21	Serine/threonine-pr	34.60	0.00
TRINITY_sp|Q8LA(	HCC2	Protein SCO1 homolo	34.60	0.00
TRINITY_sp|P372(	FRM2	Fatty acid repressi	34.60	0.00
TRINITY_sp|Q0BW(	mnmG	tRNA uridine 5-carb	34.60	0.00
TRINITY_sp|Q8RX(	HDA5	Histone deacetylase	34.60	0.00
TRINITY_sp|Q54R(	dhkL	Hybrid signal trans	34.60	0.00
TRINITY_sp|Q9UK(	GPATCH8	G patch domain-cont	34.60	0.00
TRINITY_sp|P684(	erg3	C-5 sterol desatura	34.60	0.00
TRINITY_sp|Q96Q(	ALKBH3	Alpha-ketoglutarate	34.60	0.00
TRINITY_sp|Q8WW(	RTN4IP1	Reticulon-4-interac	34.60	0.00
TRINITY_sp|Q5FV(	Wdsub1	WD repeat, SAM and	34.60	0.00
TRINITY_sp|Q9NB(	-	T-complex protein 1	34.60	0.00
TRINITY_sp|Q9SM(	LKR/SDH	Alpha-aminoadipic s	34.60	0.00
TRINITY_sp|Q6XP(	PLA2G15	Group XV phospholip	34.60	0.00
TRINITY_sp|P598(	RDH12	Retinol dehydrogena	34.60	0.00
TRINITY_sp|F4JT(	STY46	Serine/threonine-pr	34.60	0.00
TRINITY_sp|Q8LB(	RBL15	Rhomboid-like prote	34.60	0.00
TRINITY_sp|F4JY(	DHFS	Dihydrofolate synth	34.60	0.00
TRINITY_sp|O946(	rev1	DNA repair protein	34.60	0.00
TRINITY_sp|A0JP(	cnot1	CCR4-NOT transcript	34.60	0.00
TRINITY_sp|Q66J(	trmt10a	tRNA methyltransfer	34.60	0.00
TRINITY_sp|Q9UN(	ABCG2	ATP-binding cassett	34.60	0.00
TRINITY_sp|Q2KI(	SUGT1	Protein SGT1 homolo	34.60	0.00
TRINITY_sp|Q9P2(	VPS18	Vacuolar protein so	34.60	0.00
TRINITY_sp|Q9FY(	TRS120	Trafficking protein	34.60	0.00
TRINITY_sp|Q1EB(	BPM5	BTB/POZ and MATH do	34.60	0.00
TRINITY_sp|Q54T(	drkC	Probable serine/thr	34.60	0.00
TRINITY_sp|Q653(	Os09g05(	Probable protein ph	34.60	0.00
TRINITY_sp|Q000(	SLC25A5	ADP/ATP translocase	34.60	0.00
TRINITY_sp|P743(	sll1541	Apocarotenoid-15,15	34.60	0.00
TRINITY_sp|Q9JK(	Cyp39a1	24-hydroxycholester	34.60	0.00
TRINITY_sp|Q5JI(	TK1689	Subtilisin-like ser	34.60	0.00
TRINITY_sp|O154(	ABCC4	Multidrug resistanc	34.60	0.00

TRINITY_sp|Q6GPI	taok2	Serine/threonine-pr	34.60	0.00
TRINITY_sp|A3KM\	RANBP10	Ran-binding protein	34.60	0.00
TRINITY_sp|P800!	cpsA1	Thermostable carbox	34.60	0.00
TRINITY_sp|A6H7I	COPS3	COP9 signalosome co	34.60	0.00
TRINITY_sp|Q5VYI	ECM29	Proteasome-associat	34.60	0.00
TRINITY_sp|Q4PI8	RRP36	rRNA biogenesis pro	34.60	0.00
TRINITY_sp|A7Z0!	PPIP5K1	Inositol hexakispho	34.60	0.00
TRINITY_sp|A0JMI	mtmr2	Myotubularin-relate	34.60	0.00
TRINITY_sp|O138:	imp3	U3 small nucleolar	34.60	0.00
TRINITY_sp|Q8BX(	Pigt	GPI transamidase co	34.60	0.00
TRINITY_sp|P418!	gar2	Protein gar2 OS=Sch	34.60	0.00
TRINITY_sp|Q5BLI	mettl21a	Protein N-lysine me	34.60	0.00
TRINITY_sp|Q8N3!	SLC35F6	Solute carrier fami	34.60	0.00
TRINITY_sp|Q9UT	tif222	Probable translatio	34.60	0.00
TRINITY_sp|P621(	NCS1	Neuronal calcium se	34.60	0.00
TRINITY_sp|Q207:	tbcb-1	Tubulin-specific ch	34.60	0.00
TRINITY_sp|Q9ATI	ADA2B	Transcriptional ada	34.60	0.00
TRINITY_sp|Q9SK(	At2g203(	NADH dehydrogenase	34.60	0.00
TRINITY_sp|Q87AI	PD_1893	UPF0394 membrane pr	34.60	0.00
TRINITY_sp|Q55E:	gxcB	Rac guanine nucleot	34.60	0.00
TRINITY_sp|Q9SJ(	SPP2	Probable sucrose-ph	34.60	0.00
TRINITY_sp|A5YKI	CNOT1	CCR4-NOT transcript	34.60	0.00
TRINITY_sp|Q9ZUI	ETL1	Protein CHROMATIN R	34.60	0.00
TRINITY_sp|Q8Z0(	dfa5	Putative diflavin f	34.60	0.00
TRINITY_sp|P535(	Cebpz	CCAAT/enhancer-bind	34.60	0.00
TRINITY_sp|P109	-	Retrovirus-related	34.60	0.00
TRINITY_sp|Q54I8	upf1	Regulator of nonsen	34.60	0.00
TRINITY_sp|Q5U3:	-	UPF0769 protein C21	34.60	0.00
TRINITY_sp|D2Z0:	dcsG	Cycloserine biosynt	34.50	0.00
TRINITY_sp|Q6V5I	Znf474	Zinc finger protein	34.50	0.00
TRINITY_sp|Q91Y(	Slc13a3	Solute carrier fami	34.50	0.00
TRINITY_sp|A3B5:	CIPK28	CBL-interacting pro	34.50	0.00
TRINITY_sp|Q55B:	plbB	Phospholipase B-lik	34.50	0.00
TRINITY_sp|C9J7!	RASA4B	Ras GTPase-activati	34.50	0.00
TRINITY_sp|Q86J:	trmt61a	tRNA (adenine(58)-N	34.50	0.00
TRINITY_sp|Q4I0!	TIM50	Mitochondrial impor	34.50	0.00
TRINITY_sp|Q54N/	atg9	Autophagy-related p	34.50	0.00
TRINITY_sp|Q54BI	mcfG	Mitochondrial subst	34.50	0.00
TRINITY_sp|Q69Q	CIPK24	CBL-interacting pro	34.50	0.00
TRINITY_sp|P623	CPK4	Calcium-dependent p	34.50	0.00
TRINITY_sp|Q6C2I	NOP14	Probable nucleolar	34.50	0.00
TRINITY_sp|Q8EP)	ybeY	Endoribonuclease Yb	34.50	0.00
TRINITY_sp|Q136:	RAB32	Ras-related protein	34.50	0.00
TRINITY_sp|Q9LV:	ADS3.2	Probable lipid desa	34.50	0.00
TRINITY_sp|A2VE	ADPGK	ADP-dependent gluco	34.50	0.00
TRINITY_sp|Q9VY!	Upf1	Regulator of nonsen	34.50	0.00
TRINITY_sp|Q9VH1	Invadoly	Leishmanolysin-like	34.50	0.00
TRINITY_sp|P193:	NCL	Nucleolin OS=Homo s	34.50	0.00
TRINITY_sp|Q8W5I	KIN7D	Kinesin-like protei	34.50	0.00
TRINITY_sp|Q4V7I	nsun2	tRNA (cytosine(34)-	34.50	0.00
TRINITY_sp|Q55BI	dgat1	Diacylglycerol O-ac	34.50	0.00
TRINITY_sp|Q5AYI	pob3	FACT complex subuni	34.50	0.00
TRINITY_sp|Q5XI)	Dnajc14	DnaJ homolog subfam	34.50	0.00
TRINITY_sp|Q99L(	Ttc5	Tetratricopeptide r	34.50	0.00
TRINITY_sp|Q91ZI	Abhd3	Phospholipase ABHD3	34.50	0.00
TRINITY_sp|P966(	ydbI	UPF0118 membrane pr	34.50	0.00

TRINITY_sp|P3778	rfbC	dTDP-4-dehydrorhamn	34.50	0.00
TRINITY_sp|Q86JN	DDB_G027	Putative elongation	34.50	0.00
TRINITY_sp|Q8MYE	DDB_G027	Probable serine/thr	34.50	0.00
TRINITY_sp|Q755A	OXR1	Oxidation resistanc	34.50	0.00
TRINITY_sp|Q9632	IMPA1	Importin subunit al	34.50	0.00
TRINITY_sp|O4352	ATP8B1	Phospholipid-transp	34.50	0.00
TRINITY_sp|Q9UKK	PARP4	Poly [ADP-ribose] p	34.50	0.00
TRINITY_sp|Q9FPS	UBP24	Ubiquitin carboxyl-	34.50	0.00
TRINITY_sp|Q05B5	GTF2H3	General transcripti	34.50	0.00
TRINITY_sp|Q54B2	exoc6	Exocyst complex com	34.50	0.00
TRINITY_sp|Q9FG1	TIL	Temperature-induced	34.50	0.00
TRINITY_sp|Q9ZU5	BAT1	Amino-acid permease	34.50	0.00
TRINITY_sp|Q9S75	LOG2	Probable E3 ubiquit	34.50	0.00
TRINITY_sp|Q96E2	MCRS1	Microspherule prote	34.50	0.00
TRINITY_sp|Q8I7P	pol	Retrovirus-related	34.50	0.00
TRINITY_sp|O1294	RAD54L	DNA repair and reco	34.50	0.00
TRINITY_sp|Q8BG1	Tmtc4	Transmembrane and T	34.50	0.00
TRINITY_sp|Q9LQ8	ABCI7	Protein ABCI7, chlo	34.50	0.00
TRINITY_sp|Q8LB8	SPX1	SPX domain-containi	34.50	0.00
TRINITY_sp|A8ISN	ARL3	ADP-ribosylation fa	34.50	0.00
TRINITY_sp|Q8W4E	CAT4	Cationic amino acid	34.50	0.00
TRINITY_sp|Q0796	ARHGAP1	Rho GTPase-activati	34.50	0.00
TRINITY_sp|Q8C7X	Emc1	ER membrane protein	34.50	0.00
TRINITY_sp|Q55A5	DDB_G027	Probable serine/thr	34.50	0.00
TRINITY_sp|Q4P21	UMAG_058	ATP-dependent (S)-N	34.50	0.00
TRINITY_sp|Q8R3N	Nop14	Nucleolar protein 1	34.50	0.00
TRINITY_sp|O3433	ylbH	Putative rRNA methy	34.50	0.00
TRINITY_sp|Q0554	CHI17	Acidic 27 kDa endoc	34.50	0.00
TRINITY_sp|A7J11	map3k13-	Mitogen-activated p	34.50	0.00
TRINITY_sp|Q9S72	PPD3	PsbP domain-contain	34.50	0.00
TRINITY_sp|Q9P37	bud20	Zinc finger protein	34.50	0.00
TRINITY_sp|Q9ZU5	BAT1	Amino-acid permease	34.50	0.00
TRINITY_sp|Q9SW1	CHLM	Magnesium protoporp	34.50	0.00
TRINITY_sp|P3604	EBP2	rRNA-processing pro	34.50	0.00
TRINITY_sp|Q9M9W	At3g0564	Probable protein ph	34.50	0.00
TRINITY_sp|Q8HXQ	ABCC1	Multidrug resistanc	34.50	0.00
TRINITY_sp|P1097	-	Retrovirus-related	34.50	0.00
TRINITY_sp|Q0289	PNG1	Peptide-N(4)-(N-ace	34.50	0.00
TRINITY_sp|Q9P89	tcsA	Two-component syste	34.50	0.00
TRINITY_sp|Q7SXE	maea	Macrophage erythrob	34.50	0.00
TRINITY_sp|P6051	GABARAPL	Gamma-aminobutyric	34.40	0.00
TRINITY_sp|A5UXE	gatA	Glutamyl-tRNA(Gln)	34.40	0.00
TRINITY_sp|Q9VDE	meigo	Solute carrier fami	34.40	0.00
TRINITY_sp|Q02V9	mutS	DNA mismatch repair	34.40	0.00
TRINITY_sp|O1376	SPAC17A5	Uncharacterized pro	34.40	0.00
TRINITY_sp|Q499U	Tmco4	Transmembrane and c	34.40	0.00
TRINITY_sp|Q9UT6	SPAC513.	Probable phosphatas	34.40	0.00
TRINITY_sp|Q7G18	FIM1	Fimbrin-1 OS=Arabid	34.40	0.00
TRINITY_sp|A1A08	rpsE	30S ribosomal prote	34.40	0.00
TRINITY_sp|Q1MTE	vid27	Vacuolar import and	34.40	0.00
TRINITY_sp|P8002	-	Extracellular ribon	34.40	0.00
TRINITY_sp|Q2KIE	TDH	L-threonine 3-dehyd	34.40	0.00
TRINITY_sp|Q84M2	ABCA1	ABC transporter A f	34.40	0.00
TRINITY_sp|F4INN	GGP4	Gamma-glutamyl pept	34.40	0.00
TRINITY_sp|Q9LDN	CYCB2-3	Cyclin-B2-3 OS=Arab	34.40	0.00
TRINITY_sp|O5108	trmH	tRNA (guanosine(18)	34.40	0.00

TRINITY_sp|P0987	PARP1	Poly [ADP-ribose] p	34.40	0.00
TRINITY_sp|Q6444	Atp7b	Copper-transporting	34.40	0.00
TRINITY_sp|P0DO0	dkey-242	Protein C8orf37 hom	34.40	0.00
TRINITY_sp|P5198	-	G2/mitotic-specific	34.40	0.00
TRINITY_sp|P4917	IVR1	Beta-fructofuranosi	34.40	0.00
TRINITY_sp|Q3SZ8	BOLA3	BolA-like protein 3	34.40	0.00
TRINITY_sp|C7G04	DDB_G028	von Willebrand fact	34.40	0.00
TRINITY_sp|P1464	Pde4c	cAMP-specific 3',5'	34.40	0.00
TRINITY_sp|Q9C90	ENDO2	Endonuclease 2 OS=A	34.40	0.00
TRINITY_sp|P2817	pkgB	Protein kinase 2 OS	34.40	0.00
TRINITY_sp|Q6P3W	SCYL2	SCY1-like protein 2	34.40	0.00
TRINITY_sp|Q8RWI	STY17	Serine/threonine-pr	34.40	0.00
TRINITY_sp|Q149N	SHPRH	E3 ubiquitin-protei	34.40	0.00
TRINITY_sp|O8899	Marc2	Mitochondrial amido	34.40	0.00
TRINITY_sp|Q3ZC6	CFAP36	Cilia- and flagella	34.40	0.00
TRINITY_sp|Q52T3	PAT24	Protein S-acyltrans	34.40	0.00
TRINITY_sp|O7673	tupA	General transcripti	34.40	0.00
TRINITY_sp|Q6522	Os06g052	Probable protein ph	34.40	0.00
TRINITY_sp|Q9P77	SPBC1711	Dipeptidyl-peptidas	34.40	0.00
TRINITY_sp|Q9ULX	RNF112	RING finger protein	34.40	0.00
TRINITY_sp|Q5UP5	MIMI_R58	Uncharacterized pro	34.40	0.00
TRINITY_sp|Q54CE	ddx17	Probable ATP-depend	34.40	0.00
TRINITY_sp|P0509	abpA	Alpha-actinin A OS=	34.40	0.00
TRINITY_sp|P4189	gar2	Protein gar2 OS=Sch	34.40	0.00
TRINITY_sp|Q07G1	alkbh8	Alkylated DNA repai	34.40	0.00
TRINITY_sp|Q9UI0	LCMT1	Leucine carboxyl me	34.40	0.00
TRINITY_sp|Q1457	ITPR3	Inositol 1,4,5-tris	34.40	0.00
TRINITY_sp|Q8VZ6	At4g1919	Uncharacterized zin	34.40	0.00
TRINITY_sp|Q2XVE	scn4aa	Sodium channel prot	34.40	0.00
TRINITY_sp|Q8VY8	NUDT21	Nudix hydrolase 21,	34.40	0.00
TRINITY_sp|Q8LHH	SHL2	Probable RNA-depend	34.40	0.00
TRINITY_sp|O7580	CAPN15	Calpain-15 OS=Homo	34.40	0.00
TRINITY_sp|P7317	sll1290	Uncharacterized rib	34.40	0.00
TRINITY_sp|Q9QZ7	XPR1	Xenotropic and poly	34.40	0.00
TRINITY_sp|Q86TW	ADCK1	Uncharacterized aar	34.40	0.00
TRINITY_sp|Q54H4	drkA	Probable serine/thr	34.40	0.00
TRINITY_sp|P3858	TTL	Tubulin--tyrosine l	34.40	0.00
TRINITY_sp|Q0V9E	dis3l2	DIS3-like exonuclea	34.40	0.00
TRINITY_sp|Q9LU4	ACA9	Calcium-transportin	34.40	0.00
TRINITY_sp|Q2462	ref(2)P	Protein ref(2)P OS=	34.40	0.00
TRINITY_sp|Q96VE	HBN1	Putative nitroreduc	34.40	0.00
TRINITY_sp|Q3TZA	Cdkl4	Cyclin-dependent ki	34.40	0.00
TRINITY_sp|P2271	Gucy1b2	Guanylate cyclase s	34.40	0.00
TRINITY_sp|Q8IZE	XRN1	5'-3' exoribonuclea	34.40	0.00
TRINITY_sp|Q9BV8	EMC6	ER membrane protein	34.40	0.00
TRINITY_sp|P4977	aph1	Bis(5'-nucleosyl)-t	34.30	0.00
TRINITY_sp|Q6Z82	WEE1	Wee1-like protein k	34.30	0.00
TRINITY_sp|B6QM9	vps10	Vacuolar protein so	34.30	0.00
TRINITY_sp|Q6TBX	CYP97C1	Carotene epsilon-mo	34.30	0.00
TRINITY_sp|P3640	TRIM23	E3 ubiquitin-protei	34.30	0.00
TRINITY_sp|Q6X4A	CIPK31	CBL-interacting pro	34.30	0.00
TRINITY_sp|Q8IS1	gefI	Ras guanine nucleot	34.30	0.00
TRINITY_sp|P3245	GBP2	Guanylate-binding p	34.30	0.00
TRINITY_sp|Q2KI5	COPS7B	COP9 signalosome co	34.30	0.00
TRINITY_sp|Q9LET	RBL20	Rhomboid-like prote	34.30	0.00
TRINITY_sp|P4630	birA	Bifunctional ligase	34.30	0.00

TRINITY_sp|Q08D[illegible]	HSF1	Heat shock factor p	34.30	0.00
TRINITY_sp|Q54B[illegible]	ttc27	Tetratricopeptide r	34.30	0.00
TRINITY_sp|Q6L5[illegible]	Os05g05[illegible]	Probable protein ph	34.30	0.00
TRINITY_sp|P257[illegible]	-	Cruzipain OS=Trypan	34.30	0.00
TRINITY_sp|Q6NL[illegible]	CPK32	Calcium-dependent p	34.30	0.00
TRINITY_sp|P331[illegible]	yhxA	Uncharacterized ami	34.30	0.00
TRINITY_sp|Q9TW[illegible]	myoM	Myosin-M heavy chai	34.30	0.00
TRINITY_sp|Q54G[illegible]	ctdspl2	CTD small phosphata	34.30	0.00
TRINITY_sp|Q8VW[illegible]	SMO2-2	Methylsterol monoox	34.30	0.00
TRINITY_sp|E9Q4[illegible]	Pde8b	High affinity cAMP-	34.30	0.00
TRINITY_sp|Q6AY[illegible]	Mmadhc	Methylmalonic acidu	34.30	0.00
TRINITY_sp|Q80Z[illegible]	Nampt	Nicotinamide phosph	34.30	0.00
TRINITY_sp|Q9ZB[illegible]	lysA	Diaminopimelate dec	34.30	0.00
TRINITY_sp|Q9GS[illegible]	crtp1	Crt homolog 1 OS=Di	34.30	0.00
TRINITY_sp|Q552[illegible]	DDB_G02[illegible]	Putative ZDHHC-type	34.30	0.00
TRINITY_sp|Q9SF[illegible]	MCM8	Probable DNA helica	34.30	0.00
TRINITY_sp|Q54L[illegible]	DDB_G02[illegible]	Probable serine/thr	34.30	0.00
TRINITY_sp|Q6PC[illegible]	yif1a	Protein YIF1A OS=Da	34.30	0.00
TRINITY_sp|Q54H[illegible]	DDB_G02[illegible]	FAD-linked oxidored	34.30	0.00
TRINITY_sp|Q9VG[illegible]	CG17726	Protein arginine me	34.30	0.00
TRINITY_sp|Q98T[illegible]	rent1	Putative regulator	34.30	0.00
TRINITY_sp|Q9CP[illegible]	Glod4	Glyoxalase domain-c	34.30	0.00
TRINITY_sp|O751[illegible]	DNAJC13	DnaJ homolog subfam	34.30	0.00
TRINITY_sp|Q02P[illegible]	lap	Aminopeptidase OS=P	34.30	0.00
TRINITY_sp|Q123[illegible]	SFM1	Protein arginine N-	34.30	0.00
TRINITY_sp|Q6B9[illegible]	vwkA	Alpha-protein kinas	34.30	0.00
TRINITY_sp|P322[illegible]	rasS	Ras-like protein ra	34.30	0.00
TRINITY_sp|P428[illegible]	VNX1	Low affinity vacuol	34.30	0.00
TRINITY_sp|Q9SV[illegible]	GH9B16	Endoglucanase 22 OS	34.30	0.00
TRINITY_sp|Q9SM[illegible]	TFCC	Tubulin-folding cof	34.30	0.00
TRINITY_sp|O626[illegible]	CYP2C41	Cytochrome P450 2C4	34.30	0.00
TRINITY_sp|Q9SL[illegible]	HCS1	Biotin--protein lig	34.30	0.00
TRINITY_sp|A4W9[illegible]	selD	Selenide, water dik	34.30	0.00
TRINITY_sp|P824[illegible]	RPL19	50S ribosomal prote	34.30	0.00
TRINITY_sp|Q9ST[illegible]	At4g083[illegible]	Putative transcript	34.30	0.00
TRINITY_sp|O243[illegible]	TIC110	Protein TIC110, chl	34.30	0.00
TRINITY_sp|Q91Y[illegible]	Mettl13	Methyltransferase-l	34.30	0.00
TRINITY_sp|Q8N9[illegible]	GDPD1	Glycerophosphodiest	34.30	0.00
TRINITY_sp|Q6V4[illegible]	10HGO	8-hydroxygeraniol d	34.30	0.00
TRINITY_sp|P428[illegible]	ECE1	Endothelin-converti	34.30	0.00
TRINITY_sp|P357[illegible]	-	Venom allergen 5 OS	34.30	0.00
TRINITY_sp|Q9MA[illegible]	PRMT10	Protein arginine N-	34.30	0.00
TRINITY_sp|Q84J[illegible]	ZDP	Polynucleotide 3'-p	34.30	0.00
TRINITY_sp|Q9VG[illegible]	ClC-a	Chloride channel pr	34.30	0.00
TRINITY_sp|Q5BA[illegible]	dpp5	Probable dipeptidyl	34.30	0.00
TRINITY_sp|Q55C[illegible]	vps26	Vacuolar protein so	34.30	0.00
TRINITY_sp|Q55E[illegible]	DDB_G02[illegible]	Probable serine/thr	34.30	0.00
TRINITY_sp|Q8QZ[illegible]	Pde9a	High affinity cGMP-	34.30	0.00
TRINITY_sp|Q401[illegible]	-	LEC14B protein OS=L	34.30	0.00
TRINITY_sp|Q7LH[illegible]	TY3B-I	Transposon Ty3-I Ga	34.30	0.00
TRINITY_sp|Q94K[illegible]	VPS33	Vacuolar protein-so	34.20	0.00
TRINITY_sp|Q944[illegible]	OST48	Dolichyl-diphosphoo	34.20	0.00
TRINITY_sp|Q54W[illegible]	gcn1	eIF-2-alpha kinase	34.20	0.00
TRINITY_sp|Q074[illegible]	-	Ribonuclease OS=Aer	34.20	0.00
TRINITY_sp|Q82T[illegible]	def1	Peptide deformylase	34.20	0.00
TRINITY_sp|Q9XY[illegible]	noxA	Superoxide-generati	34.20	0.00

TRINITY_sp|P534(	-	Actin, larval muscl	34.20	0.00
TRINITY_sp|B1VG(	infB	Translation initiat	34.20	0.00
TRINITY_sp|P344(	polk-1	DNA polymerase kapp	34.20	0.00
TRINITY_sp|Q5U2!	usp47	Ubiquitin carboxyl-	34.20	0.00
TRINITY_sp|Q6AX:	mast3	Microtubule-associa	34.20	0.00
TRINITY_sp|P291.	PCSK6	Proprotein converta	34.20	0.00
TRINITY_sp|P0CM!	CWC21	Pre-mRNA-splicing f	34.20	0.00
TRINITY_sp|Q54UI	pldA	Phospholipase D A O	34.20	0.00
TRINITY_sp|P250	CML12	Calmodulin-like pro	34.20	0.00
TRINITY_sp|Q941.	rac-2	Ras-related protein	34.20	0.00
TRINITY_sp|F4IVI	GRV2	DnaJ homolog subfam	34.20	0.00
TRINITY_sp|Q8BG(	Zadh2	Prostaglandin reduc	34.20	0.00
TRINITY_sp|P418!	gar2	Protein gar2 OS=Sch	34.20	0.00
TRINITY_sp|Q9CX:	Utp23	rRNA-processing pro	34.20	0.00
TRINITY_sp|Q28B>	c2cd5	C2 domain-containin	34.20	0.00
TRINITY_sp|O140	SPAC2C4.	Putative tRNA 2'-ph	34.20	0.00
TRINITY_sp|Q9UR(	Tf2-11	Transposon Tf2-11 p	34.20	0.00
TRINITY_sp|P109	-	Retrovirus-related	34.20	0.00
TRINITY_sp|P280:	AOAH	Acyloxyacyl hydrola	34.20	0.00
TRINITY_sp|Q239:	kinX	Probable serine/thr	34.20	0.00
TRINITY_sp|Q9S7(	HSP70-1	Heat shock 70 kDa p	34.20	0.00
TRINITY_sp|Q86AI	DDB_G02	Probable myosin lig	34.20	0.00
TRINITY_sp|Q9SYI	HEXO2	Beta-hexosaminidase	34.20	0.00
TRINITY_sp|Q69Z(	Kiaa116:	Uncharacterized fam	34.20	0.00
TRINITY_sp|P209:	RASA1	Ras GTPase-activati	34.20	0.00
TRINITY_sp|F4IA:	THO2	THO complex subunit	34.20	0.00
TRINITY_sp|Q2KH\	USP2	Ubiquitin carboxyl-	34.20	0.00
TRINITY_sp|Q220(	pde-4	Probable 3',5'-cycl	34.20	0.00
TRINITY_sp|A5A6I	ASAH1	Acid ceramidase OS=	34.20	0.00
TRINITY_sp|O131	epha3	Ephrin type-A recep	34.20	0.00
TRINITY_sp|A8IU(	CFAP99	Cilia- and flagella	34.20	0.00
TRINITY_sp|B7J9.	rlmD	23S rRNA (uracil(19	34.20	0.00
TRINITY_sp|Q55E:	DDB_G02(	Probable serine/thr	34.20	0.00
TRINITY_sp|O826(	HCF136	Photosystem II stab	34.20	0.00
TRINITY_sp|Q96A>	RAB37	Ras-related protein	34.20	0.00
TRINITY_sp|Q9SLI	ALA6	Phospholipid-transp	34.20	0.00
TRINITY_sp|P831:	LACTB	Serine beta-lactama	34.20	0.00
TRINITY_sp|P436.	RMD8	Sporulation protein	34.20	0.00
TRINITY_sp|Q54JI	abcC3	ABC transporter C f	34.20	0.00
TRINITY_sp|Q7LH(	TY3B-I	Transposon Ty3-I Ga	34.20	0.00
TRINITY_sp|Q5MB:	ABCG2	ATP-binding cassett	34.20	0.00
TRINITY_sp|Q9LQ\	RDR1	RNA-dependent RNA p	34.20	0.00
TRINITY_sp|H2DH:	-	Cytochrome P450 CYP	34.20	0.00
TRINITY_sp|Q8L7I	LPEAT1	Lysophospholipid ac	34.20	0.00
TRINITY_sp|Q55E!	corB	Coronin-B OS=Dictyo	34.20	0.00
TRINITY_sp|Q4V3I	SPPL3	Signal peptide pept	34.20	0.00
TRINITY_sp|Q9ZU!	BAT1	Amino-acid permease	34.20	0.00
TRINITY_sp|Q8RY.	DEGP7	Protease Do-like 7	34.20	0.00

TRINITY_sp|Q9UT(	paa1	Protein phosphatase	34.20	0.00
TRINITY_sp|Q9VV]	Nedd4	E3 ubiquitin-protei	34.20	0.00
TRINITY_sp|Q1PFI	CPK19	Calcium-dependent p	34.20	0.00
TRINITY_sp|Q8K0]	Dync2li]	Cytoplasmic dynein	34.20	0.00
TRINITY_sp|Q55GV	DDB_G02(	AN1-type zinc finge	34.10	0.00
TRINITY_sp|A2YH4	OsI_023(	Serine/threonine-pr	34.10	0.00
TRINITY_sp|F4J5S	FMT	Clustered mitochond	34.10	0.00
TRINITY_sp|P1162	ypt1	GTP-binding protein	34.10	0.00
TRINITY_sp|Q9FJ(	IMPA5	Importin subunit al	34.10	0.00
TRINITY_sp|B2DBI	GGS-D	Geranylgeranyl pyro	34.10	0.00
TRINITY_sp|O004]	IPO5	Importin-5 OS=Homo	34.10	0.00
TRINITY_sp|Q2W7]	efp	Elongation factor P	34.10	0.00
TRINITY_sp|Q9M2I	SDR1	(+)-neomenthol dehy	34.10	0.00
TRINITY_sp|Q921Y	Mfsd5	Molybdate-anion tra	34.10	0.00
TRINITY_sp|Q5HZI	Slc25a2S	Mitochondrial basic	34.10	0.00
TRINITY_sp|P803(	-	Crustacean calcium-	34.10	0.00
TRINITY_sp|O4442	Es2	Protein DGCR14 homo	34.10	0.00
TRINITY_sp|Q643]	Syn2	Synapsin-2 OS=Mus m	34.10	0.00
TRINITY_sp|P2644	PARP1	Poly [ADP-ribose] p	34.10	0.00
TRINITY_sp|Q9UT(	SPAPYUK	Uncharacterized pro	34.10	0.00
TRINITY_sp|O677S	aspC	Aspartate aminotran	34.10	0.00
TRINITY_sp|Q86A]	spkA-1	Stress-activated pr	34.10	0.00
TRINITY_sp|Q53H(	KLHL26	Kelch-like protein	34.10	0.00
TRINITY_sp|O009(	-	Lysosomal acid alph	34.10	0.00
TRINITY_sp|B6EUA	PRP40A	Pre-mRNA-processing	34.10	0.00
TRINITY_sp|Q8QH	xpo6	Exportin-6 OS=Danio	34.10	0.00
TRINITY_sp|P357(	-	Venom allergen 5 OS	34.10	0.00
TRINITY_sp|B8FL]	rlmE	Ribosomal RNA large	34.10	0.00
TRINITY_sp|Q80XI	Bdh1	D-beta-hydroxybutyr	34.10	0.00
TRINITY_sp|P976]	Txn2	Thioredoxin, mitoch	34.10	0.00
TRINITY_sp|A3BN2	Os07g06(	RNA pseudouridine s	34.10	0.00
TRINITY_sp|Q1452	SCN5A	Sodium channel prot	34.10	0.00
TRINITY_sp|Q4R8I	STIP1	Stress-induced-phos	34.10	0.00
TRINITY_sp|Q050(	CPR	NADPH--cytochrome P	34.10	0.00
TRINITY_sp|Q8C1A	Mtrr	Methionine synthase	34.10	0.00
TRINITY_sp|P454(	yraR	Uncharacterized pro	34.10	0.00
TRINITY_sp|D4GP]	HVO_B002	D-xylose 1-dehydrog	34.10	0.00
TRINITY_sp|Q145]	ITPR3	Inositol 1,4,5-tris	34.10	0.00
TRINITY_sp|P2934	RPS1	30S ribosomal prote	34.10	0.00
TRINITY_sp|Q55DA	abcG22	ABC transporter G f	34.10	0.00
TRINITY_sp|Q55A(	DDB_G02	Probable serine/thr	34.10	0.00
TRINITY_sp|P352S	Rab24	Ras-related protein	34.10	0.00
TRINITY_sp|F4JTI	STY46	Serine/threonine-pr	34.10	0.00
TRINITY_sp|P0CS(	PFA4	Palmitoyltransferas	34.10	0.00
TRINITY_sp|Q9ZG]	pikAI	Narbonolide/10-deox	34.10	0.00
TRINITY_sp|Q9CXI	Tbc1d15	TBC1 domain family	34.10	0.00
TRINITY_sp|Q54DI	xpo7	Exportin-7 OS=Dicty	34.10	0.00
TRINITY_sp|Q54T(	sre1	Elongation of fatty	34.10	0.00
TRINITY_sp|Q3SWY	ZUFSP	Zinc finger with UF	34.10	0.00
TRINITY_sp|Q993]	TY3B-G	Transposon Ty3-G Ga	34.10	0.00
TRINITY_sp|Q626(	Frk	Tyrosine-protein ki	34.10	0.00
TRINITY_sp|Q55F]	cycH	Putative cyclin-H O	34.10	0.00
TRINITY_sp|C5DXI	AIM41	Altered inheritance	34.10	0.00
TRINITY_sp|P157]	NCL	Nucleolin OS=Gallus	34.10	0.00
TRINITY_sp|Q8W4S	VHA-a3	V-type proton ATPas	34.10	0.00
TRINITY_sp|Q7XZ(	SAC9	Probable phosphoino	34.10	0.00

TRINITY_sp|F4JY3	RUK	Serine/threonine-pr	34.10	0.00
TRINITY_sp|Q8HX9	ABCC1	Multidrug resistanc	34.10	0.00
TRINITY_sp|Q9CQ8	Rwdd1	RWD domain-containi	34.10	0.00
TRINITY_sp|Q95ZV	ddr-2	Discoidin domain-co	34.10	0.00
TRINITY_sp|Q6NNI	SAP1	Zinc finger A20 and	34.00	0.00
TRINITY_sp|Q54RI	dhkL	Hybrid signal trans	34.00	0.00
TRINITY_sp|Q71N5	LCAT4	Lecithin-cholestero	34.00	0.00
TRINITY_sp|Q8MSU	Sym	Symplekin OS=Drosop	34.00	0.00
TRINITY_sp|P0033	adh1	Alcohol dehydrogena	34.00	0.00
TRINITY_sp|Q7Z47	DHX29	ATP-dependent RNA h	34.00	0.00
TRINITY_sp|P3132	PKAR	cAMP-dependent prot	34.00	0.00
TRINITY_sp|B9DH1	ARIA	ARM REPEAT PROTEIN	34.00	0.00
TRINITY_sp|Q811I	Atpaf1	ATP synthase mitoch	34.00	0.00
TRINITY_sp|Q3904	bVPE	Vacuolar-processing	34.00	0.00
TRINITY_sp|Q93VI	ELF5A-2	Eukaryotic translat	34.00	0.00
TRINITY_sp|Q99PW	Kif17	Kinesin-like protei	34.00	0.00
TRINITY_sp|P8056	SGR_5809	Aminopeptidase S OS	34.00	0.00
TRINITY_sp|Q9FN0	UVR8	Ultraviolet-B recep	34.00	0.00
TRINITY_sp|Q17RE	LONRF1	LON peptidase N-ter	34.00	0.00
TRINITY_sp|Q8LDW	ENDO3	Endonuclease 3 OS=A	34.00	0.00
TRINITY_sp|Q08DE	STX5	Syntaxin-5 OS=Bos t	34.00	0.00
TRINITY_sp|Q54NI	abcC9	ABC transporter C f	34.00	0.00
TRINITY_sp|Q8C14	Dock8	Dedicator of cytoki	34.00	0.00
TRINITY_sp|Q149N	SHPRH	E3 ubiquitin-protei	34.00	0.00
TRINITY_sp|Q55C0	rsc5	Random slug protein	34.00	0.00
TRINITY_sp|Q1210	RTK1	Probable serine/thr	34.00	0.00
TRINITY_sp|Q9BZV	UBXN6	UBX domain-containi	34.00	0.00
TRINITY_sp|Q9FG7	EMB2731	ER membrane protein	34.00	0.00
TRINITY_sp|A7MBE	MAP3K13	Mitogen-activated p	34.00	0.00
TRINITY_sp|Q9GTW	GK1	Glucokinase 1 OS=Tr	34.00	0.00
TRINITY_sp|Q94A9	UBC35	Ubiquitin-conjugati	34.00	0.00
TRINITY_sp|P1697	YPTM1	GTP-binding protein	34.00	0.00
TRINITY_sp|Q9I3H	PA1538	Baeyer-Villiger mon	34.00	0.00
TRINITY_sp|Q1ZXI	DDB_G027	PH domain-containin	34.00	0.00
TRINITY_sp|Q4PC0	HOG1	Mitogen-activated p	34.00	0.00
TRINITY_sp|Q7M75	Abhd17b	Protein ABHD17B OS=	34.00	0.00
TRINITY_sp|Q9Z0M	Lipa	Lysosomal acid lipa	34.00	0.00
TRINITY_sp|P3660	rad8	DNA repair protein	34.00	0.00
TRINITY_sp|Q8ZGN	rsuA	Ribosomal small sub	34.00	0.00
TRINITY_sp|Q8SS9	gefR	Ras guanine nucleot	34.00	0.00
TRINITY_sp|Q5XF8	Atp13a3	Probable cation-tra	34.00	0.00
TRINITY_sp|Q0235	UTP6	U3 small nucleolar	34.00	0.00
TRINITY_sp|O8275	DTX49	Protein DETOXIFICAT	34.00	0.00
TRINITY_sp|O6057	EIF4E2	Eukaryotic translat	34.00	0.00
TRINITY_sp|P5416	ypgQ	Uncharacterized pro	34.00	0.00
TRINITY_sp|Q84JU	IBR5	Protein-tyrosine-ph	34.00	0.00
TRINITY_sp|Q9SFU	PAP15	Purple acid phospha	34.00	0.00
TRINITY_sp|B4JB4	Trip1	Eukaryotic translat	34.00	0.00
TRINITY_sp|Q8IL0	PF14_032	STI1-like protein O	34.00	0.00
TRINITY_sp|O1543	ABCC4	Multidrug resistanc	34.00	0.00
TRINITY_sp|Q9VGH	ClC-a	Chloride channel pr	34.00	0.00
TRINITY_sp|P9303	FUM1	Fumarate hydratase	34.00	0.00
TRINITY_sp|Q0259	PK2	Probable serine/thr	34.00	0.00
TRINITY_sp|Q6N1E	clpB	Chaperone protein C	34.00	0.00
TRINITY_sp|P2635	TBP1	TATA-box-binding pr	34.00	0.00
TRINITY_sp|Q54HI	csn2	COP9 signalosome co	34.00	0.00

TRINITY_sp|P4232	CNB1	Calcineurin subunit	34.00	0.00
TRINITY_sp|P3071	GSTT1	Glutathione S-trans	34.00	0.00
TRINITY_sp|Q8YXE	trpS	Tryptophan--tRNA li	34.00	0.00
TRINITY_sp|Q28I2	dtwd1	DTW domain-containi	34.00	0.00
TRINITY_sp|Q7XJ5	At3g0627	Probable protein ph	34.00	0.00
TRINITY_sp|Q8BG0	Htatsf1	HIV Tat-specific fa	34.00	0.00
TRINITY_sp|Q9DC0	Pbld1	Phenazine biosynthe	34.00	0.00
TRINITY_sp|Q7LFX	CHST15	Carbohydrate sulfot	34.00	0.00
TRINITY_sp|O4344	KCNAB3	Voltage-gated potas	34.00	0.00
TRINITY_sp|Q54BA	DDB_G029	Ankyrin repeat, bro	34.00	0.00
TRINITY_sp|Q0252	Mgat3	Beta-1,4-mannosyl-g	34.00	0.00
TRINITY_sp|Q4UKE	hspC2	Small heat shock pr	34.00	0.00
TRINITY_sp|Q08B7	pm20d1	N-fatty-acyl-amino	34.00	0.00
TRINITY_sp|Q7KZE	SND1	Staphylococcal nucl	34.00	0.00
TRINITY_sp|Q1457	ITPR2	Inositol 1,4,5-tris	34.00	0.00
TRINITY_sp|Q5577	sll0182	Uncharacterized ABC	34.00	0.00
TRINITY_sp|P8138	-	L-amino-acid oxidas	34.00	0.00
TRINITY_sp|Q54YE	dhkB	Hybrid signal trans	34.00	0.00
TRINITY_sp|P0432	pol	Retrovirus-related	34.00	0.00
TRINITY_sp|Q6312	Abcc2	Canalicular multisp	34.00	0.00
TRINITY_sp|Q9SH3	CALS7	Callose synthase 7	34.00	0.00
TRINITY_sp|Q3SZE	TTLL9	Probable tubulin po	34.00	0.00
TRINITY_sp|Q2203	rho-1	Ras-like GTP-bindin	33.90	0.00
TRINITY_sp|Q0740	htl	Fibroblast growth f	33.90	0.00
TRINITY_sp|Q7PC0	ANR	Anthocyanidin reduc	33.90	0.00
TRINITY_sp|Q0917	cds1	Serine/threonine-pr	33.90	0.00
TRINITY_sp|P5836	arcB	Aerobic respiration	33.90	0.00
TRINITY_sp|P2373	Si	Sucrase-isomaltase,	33.90	0.00
TRINITY_sp|P3695	RpII15	DNA-directed RNA po	33.90	0.00
TRINITY_sp|A9LL1	RRP6L3	Protein RRP6-like 3	33.90	0.00
TRINITY_sp|Q4WVE	caf17	Putative transferas	33.90	0.00
TRINITY_sp|P4882	cht60	Beta-hexosaminidase	33.90	0.00
TRINITY_sp|Q9Y7I	SPBC2A9.	Phosducin-like prot	33.90	0.00
TRINITY_sp|B0F9I	GC6	Golgin candidate 6	33.90	0.00
TRINITY_sp|Q54BM	mcfG	Mitochondrial subst	33.90	0.00
TRINITY_sp|Q6Q88	sirJ	Dipeptidase sirJ OS	33.90	0.00
TRINITY_sp|Q55D0	nosip	Nitric oxide syntha	33.90	0.00
TRINITY_sp|Q2M5E	RGS21	Regulator of G-prot	33.90	0.00
TRINITY_sp|Q5E99	DUSP11	RNA/RNP complex-1-i	33.90	0.00
TRINITY_sp|A4FV9	METTL21E	Protein-lysine meth	33.90	0.00
TRINITY_sp|Q5VST	ABHD17B	Protein ABHD17B OS=	33.90	0.00
TRINITY_sp|Q9S7E	KIN7N	Kinesin-like protei	33.90	0.00
TRINITY_sp|Q52L4	serpinb1	Leukocyte elastase	33.90	0.00
TRINITY_sp|P7270	slr0245	Uncharacterized pro	33.90	0.00
TRINITY_sp|P8723	mdj1	DnaJ homolog 1, mit	33.90	0.00
TRINITY_sp|Q5F3I	UTP15	U3 small nucleolar	33.90	0.00
TRINITY_sp|Q4P6E	COF1	Cofilin OS=Ustilago	33.90	0.00
TRINITY_sp|Q5XTS	PNPLA8	Calcium-independent	33.90	0.00
TRINITY_sp|Q6FN5	STE20	Serine/threonine-pr	33.90	0.00
TRINITY_sp|Q8QZE	Tat	Tyrosine aminotrans	33.90	0.00
TRINITY_sp|Q67UX	Os02g014	Probable protein ph	33.90	0.00
TRINITY_sp|P6048	PTEN	Phosphatidylinosito	33.90	0.00
TRINITY_sp|Q8LFA	At1g1934	Methyltransferase-l	33.90	0.00
TRINITY_sp|P4268	FRK	Tyrosine-protein ki	33.90	0.00
TRINITY_sp|Q54F0	gacJJ	Rho GTPase-activati	33.90	0.00
TRINITY_sp|Q9C5S	MKP1	Protein-tyrosine-ph	33.90	0.00

TRINITY_sp|P0C8N	CCRP1	Probable serine/thr	33.90	0.00
TRINITY_sp|O8068	PAT02	Probable protein S-	33.90	0.00
TRINITY_sp|P4364	-	DnaJ protein homolo	33.90	0.00
TRINITY_sp|Q6L73	kanJ	Kanamycin B dioxyge	33.90	0.00
TRINITY_sp|Q1KKV	mfsd5	Molybdate-anion tra	33.90	0.00
TRINITY_sp|A1C8(	manF	Mannan endo-1,4-bet	33.90	0.00
TRINITY_sp|Q6NV(	rhot1a	Mitochondrial Rho G	33.90	0.00
TRINITY_sp|Q8WNI	IKBKAP	Elongator complex p	33.90	0.00
TRINITY_sp|Q755(	IPL1	Spindle assembly ch	33.90	0.00
TRINITY_sp|Q54TE	gefP	Ras guanine nucleot	33.90	0.00
TRINITY_sp|Q566F	Them4	Acyl-coenzyme A thi	33.90	0.00
TRINITY_sp|P827I	PPD5	PsbP domain-contain	33.90	0.00
TRINITY_sp|Q3SX4	LUC7L3	Luc7-like protein 3	33.90	0.00
TRINITY_sp|A8JAE	CFAP43	Cilia- and flagella	33.90	0.00
TRINITY_sp|Q5ZM7	FAR1	Fatty acyl-CoA redu	33.90	0.00
TRINITY_sp|Q54L9	DDB_G028	Protein KRTCAP2 hom	33.90	0.00
TRINITY_sp|E0X9N	At3g6355	Zinc finger CCCH do	33.90	0.00
TRINITY_sp|F4IA1	LPXC1	Probable UDP-3-O-ac	33.90	0.00
TRINITY_sp|Q9ES5	Nploc4	Nuclear protein loc	33.90	0.00
TRINITY_sp|Q1KPV	FZL	Probable transmembr	33.90	0.00
TRINITY_sp|Q5T2I	OTUD3	OTU domain-containi	33.90	0.00
TRINITY_sp|Q9M2E	SDR1	(+)-neomenthol dehy	33.90	0.00
TRINITY_sp|D2SW9	COPB1	Coatomer subunit be	33.90	0.00
TRINITY_sp|A9F8E	infC	Translation initiat	33.90	0.00
TRINITY_sp|Q6DGV	mpv17l2	Mpv17-like protein	33.90	0.00
TRINITY_sp|Q9M0E	LAF1	Transcription facto	33.90	0.00
TRINITY_sp|Q8MYE	DDB_G027	Probable serine/thr	33.90	0.00
TRINITY_sp|O6034	TBC1D12	TBC1 domain family	33.90	0.00
TRINITY_sp|Q5RF9	ZNHIT6	Box C/D snoRNA prot	33.90	0.00
TRINITY_sp|H2QL3	PDE9A	High affinity cGMP-	33.90	0.00
TRINITY_sp|P428I	ATPK1	Serine/threonine-pr	33.90	0.00
TRINITY_sp|Q9FL1	DEGP9	Protease Do-like 9	33.90	0.00
TRINITY_sp|O1376	ercc3	Probable DNA repair	33.90	0.00
TRINITY_sp|O430(	syj1	Inositol-1,4,5-tris	33.80	0.00
TRINITY_sp|Q9C5V	GRF12	14-3-3-like protein	33.80	0.00
TRINITY_sp|Q8VI4	Abcc2	Canalicular multisp	33.80	0.00
TRINITY_sp|P6187	-	Lipase OS=Rhizopus	33.80	0.00
TRINITY_sp|Q6MC7	pepA	Probable cytosol am	33.80	0.00
TRINITY_sp|O9416	SNF1	Carbon catabolite-d	33.80	0.00
TRINITY_sp|O5983	SPCC965.	Probable cytosine d	33.80	0.00
TRINITY_sp|Q9H1I	NAPB	Beta-soluble NSF at	33.80	0.00
TRINITY_sp|Q9P38	ptr2	Probable peptide tr	33.80	0.00
TRINITY_sp|A7SG(	psmg2	Proteasome assembly	33.80	0.00
TRINITY_sp|D9N16	-	Inward rectifier po	33.80	0.00
TRINITY_sp|P707(	Atp7a	Copper-transporting	33.80	0.00
TRINITY_sp|Q9W0I	Cyp4d20	Probable cytochrome	33.80	0.00
TRINITY_sp|Q9UU9	SPCC23B6	CRAL-TRIO domain-co	33.80	0.00
TRINITY_sp|Q6NXI	dennd5b	DENN domain-contain	33.80	0.00
TRINITY_sp|Q9UR(	Tf2-11	Transposon Tf2-11 p	33.80	0.00
TRINITY_sp|Q1467	TTLL4	Tubulin polyglutamy	33.80	0.00
TRINITY_sp|P3852	HSF1	Heat shock factor p	33.80	0.00
TRINITY_sp|Q9SB6	At4g2473	Manganese-dependent	33.80	0.00
TRINITY_sp|Q9UT3	SPAC8F11	Putative ribosome b	33.80	0.00
TRINITY_sp|Q9Y26	PLAA	Phospholipase A-2-a	33.80	0.00
TRINITY_sp|Q2IME	proB	Glutamate 5-kinase	33.80	0.00
TRINITY_sp|Q9FHE	DTX16	Protein DETOXIFICAT	33.80	0.00

TRINITY_sp|Q9D5	Ubxn11	UBX domain-containi	33.80	0.00
TRINITY_sp|Q920	ACHE	Acetylcholinesteras	33.80	0.00
TRINITY_sp|Q8RWI	STY17	Serine/threonine-pr	33.80	0.00
TRINITY_sp|Q0IW	NDX1	Protein NEOXANTHIN-	33.80	0.00
TRINITY_sp|Q6H7	CIPK26	CBL-interacting pro	33.80	0.00
TRINITY_sp|Q55B	dgat1	Diacylglycerol O-ac	33.80	0.00
TRINITY_sp|Q9ST	CIPK8	CBL-interacting ser	33.80	0.00
TRINITY_sp|P212	GSTM3	Glutathione S-trans	33.80	0.00
TRINITY_sp|P445	arcB	Aerobic respiration	33.80	0.00
TRINITY_sp|O809	At2g376	Uncharacterized pro	33.80	0.00
TRINITY_sp|P181	splB	Dual specificity pr	33.80	0.00
TRINITY_sp|A6QN	NXT2	NTF2-related export	33.80	0.00
TRINITY_sp|B2HC	MMAR_10	Putative S-adenosyl	33.80	0.00
TRINITY_sp|Q9CR	Ccdc96	Coiled-coil domain-	33.80	0.00
TRINITY_sp|Q3SX	PUS3	tRNA pseudouridine(	33.80	0.00
TRINITY_sp|P431	CA7	Carbonic anhydrase	33.80	0.00
TRINITY_sp|Q2I6	inppl1b	Phosphatidylinosito	33.80	0.00
TRINITY_sp|Q055	CHI17	Acidic 27 kDa endoc	33.80	0.00
TRINITY_sp|Q124	OSH2	Oxysterol-binding p	33.80	0.00
TRINITY_sp|Q3A6	adk	Adenylate kinase OS	33.80	0.00
TRINITY_sp|Q420	ABCC2	ABC transporter C f	33.80	0.00
TRINITY_sp|Q0JI	CIPK11	CBL-interacting pro	33.80	0.00
TRINITY_sp|Q0WT	IP5P8	Type I inositol pol	33.80	0.00
TRINITY_sp|Q394	RAB1BV	Ras-related protein	33.70	0.00
TRINITY_sp|Q756	TOM1	Probable E3 ubiquit	33.70	0.00
TRINITY_sp|B4KY	SAK	Serine/threonine-pr	33.70	0.00
TRINITY_sp|P912	gst-6	Probable glutathion	33.70	0.00
TRINITY_sp|Q8H2	Os07g06	Probable protein ph	33.70	0.00
TRINITY_sp|Q6H6	HSFA3	Heat stress transcr	33.70	0.00
TRINITY_sp|Q9VC	lili	Protein Lilipod OS=	33.70	0.00
TRINITY_sp|Q9Y2	RNF24	RING finger protein	33.70	0.00
TRINITY_sp|Q122	PKH2	Serine/threonine-pr	33.70	0.00
TRINITY_sp|Q93Y	ppa1	Soluble inorganic p	33.70	0.00
TRINITY_sp|A1A4	ALKBH8	Alkylated DNA repai	33.70	0.00
TRINITY_sp|P059	cam1	Calmodulin OS=Schiz	33.70	0.00
TRINITY_sp|Q54T	gacY	Rho GTPase-activati	33.70	0.00
TRINITY_sp|Q5RE	SIK2	Serine/threonine-pr	33.70	0.00
TRINITY_sp|Q54H	drkA	Probable serine/thr	33.70	0.00
TRINITY_sp|Q9LK	UKL2	Uridine kinase-like	33.70	0.00
TRINITY_sp|Q0V8	ASNSD1	Asparagine syntheta	33.70	0.00
TRINITY_sp|Q9P8	tcsA	Two-component syste	33.70	0.00
TRINITY_sp|O289	AF_1297	Cell division cycle	33.70	0.00
TRINITY_sp|Q8H1	At2g344	Uncharacterized pro	33.70	0.00
TRINITY_sp|P175	chrA1	Chromate transport	33.70	0.00
TRINITY_sp|Q8BW	Nob1	RNA-binding protein	33.70	0.00
TRINITY_sp|P541	ypgQ	Uncharacterized pro	33.70	0.00
TRINITY_sp|Q2NL	TSR1	Pre-rRNA-processing	33.70	0.00
TRINITY_sp|Q8TD	HELQ	Helicase POLQ-like	33.70	0.00
TRINITY_sp|Q9U5	-	L-asparaginase OS=D	33.70	0.00
TRINITY_sp|B2RT	MYO9A	Unconventional myos	33.70	0.00
TRINITY_sp|Q9NV	FANCI	Fanconi anemia grou	33.70	0.00
TRINITY_sp|P981	ATP8B2	Phospholipid-transp	33.70	0.00
TRINITY_sp|Q9MA	ACBP4	Acyl-CoA-binding do	33.70	0.00
TRINITY_sp|C5DD	AIM32	Altered inheritance	33.70	0.00
TRINITY_sp|Q9P7	SPCC24B	Uncharacterized oxi	33.70	0.00
TRINITY_sp|Q8Y3	lmo2759	Macro domain-contai	33.70	0.00

TRINITY_sp|O745(	SPCC970.	Uncharacterized ino	33.70	0.00
TRINITY_sp|Q86H(	DDB_G02'	Probable serine/thr	33.70	0.00
TRINITY_sp|Q8CD!	Lrrc9	Leucine-rich repeat	33.70	0.00
TRINITY_sp|Q9SD(	At3g514'	Probable protein ph	33.70	0.00
TRINITY_sp|Q045!	CYP6B1	Cytochrome P450 6B1	33.70	0.00
TRINITY_sp|B4KB:	slx1	Structure-specific	33.70	0.00
TRINITY_sp|Q9ZU:	ABCG7	ABC transporter G f	33.70	0.00
TRINITY_sp|Q9FX!	FUT12	Putative fucosyltra	33.70	0.00
TRINITY_sp|Q8IV\	LOXHD1	Lipoxygenase homolo	33.70	0.00
TRINITY_sp|A9A3I	rlmN	Ribosomal RNA large	33.70	0.00
TRINITY_sp|P107:	PAM	Peptidyl-glycine al	33.70	0.00
TRINITY_sp|P127!	RSP3	Flagellar radial sp	33.70	0.00
TRINITY_sp|Q969(	TRIM63	E3 ubiquitin-protei	33.70	0.00
TRINITY_sp|Q2KN/	SPECC1L	Cytospin-A OS=Canis	33.70	0.00
TRINITY_sp|O756(	TIPRL	TIP41-like protein	33.70	0.00
TRINITY_sp|Q9FI)	At5g435:	Putative SWI/SNF-re	33.70	0.00
TRINITY_sp|Q6AX8	dcaf12-k	DDB1- and CUL4-asso	33.70	0.00
TRINITY_sp|O042(	PXN	Peroxisomal nicotin	33.70	0.00
TRINITY_sp|Q9H4I	DEF6	Differentially expr	33.70	0.00
TRINITY_sp|Q9C8:	TRO	Protein TRAUCO OS=A	33.70	0.00
TRINITY_sp|Q5XH:	hunk-a	Hormonally up-regul	33.70	0.00
TRINITY_sp|Q002:	melO	Tyrosinase OS=Asper	33.70	0.00
TRINITY_sp|A5PJ!	AIFM2	Apoptosis-inducing	33.70	0.00
TRINITY_sp|Q7XJ8	FRK2	Fructokinase-2 OS=S	33.70	0.00
TRINITY_sp|G5ED!	ceeh-2	Epoxide hydrolase 2	33.70	0.00
TRINITY_sp|Q5UQ8	MIMI_R52	Probable ubiquitin-	33.70	0.00
TRINITY_sp|Q54JI	abcC3	ABC transporter C f	33.70	0.00
TRINITY_sp|Q86L!	gacHH	Rho GTPase-activati	33.70	0.00
TRINITY_sp|Q6X4/	CIPK31	CBL-interacting pro	33.70	0.00
TRINITY_sp|Q5SN'	Os01g065	Probable protein ph	33.70	0.00
TRINITY_sp|Q8LP!	At3g199!	E3 ubiquitin-protei	33.70	0.00
TRINITY_sp|O670'	ftsH	ATP-dependent zinc	33.70	0.00
TRINITY_sp|Q6AX(	Iqca1l	IQ and AAA domain-c	33.70	0.00
TRINITY_sp|Q084	YOR059C	Putative lipase YOR	33.70	0.00
TRINITY_sp|Q9FM:	HIR1	Hypersensitive-indu	33.70	0.00
TRINITY_sp|Q9DD(	Rab13	Ras-related protein	33.70	0.00
TRINITY_sp|Q96N!	AIFM3	Apoptosis-inducing	33.70	0.00
TRINITY_sp|Q54M:	cdc20	Anaphase-promoting	33.70	0.00
TRINITY_sp|Q9VJ'	Pde11	Dual 3',5'-cyclic-A	33.70	0.00
TRINITY_sp|Q8RWI	CLT3	Protein CLT3, chlor	33.70	0.00
TRINITY_sp|Q54TI	ddx24	ATP-dependent RNA h	33.70	0.00
TRINITY_sp|Q05B(	TRMT11	tRNA (guanine(10)-N	33.70	0.00
TRINITY_sp|O758(	CAPN15	Calpain-15 OS=Homo	33.70	0.00
TRINITY_sp|Q755(	IPL1	Spindle assembly ch	33.70	0.00
TRINITY_sp|Q96F(	ELMOD3	ELMO domain-contain	33.70	0.00
TRINITY_sp|D7UQ!	aur	Aurora kinase OS=Pa	33.70	0.00
TRINITY_sp|O186!	pde-1	Probable 3',5'-cycl	33.70	0.00
TRINITY_sp|Q3E6!	CpNIFS3	Probable L-cysteine	33.70	0.00
TRINITY_sp|Q80Y8	Elac2	Zinc phosphodiester	33.70	0.00
TRINITY_sp|Q100!	seh1	Nucleoporin seh1 OS	33.60	0.00
TRINITY_sp|Q6INI	tollip-k	Toll-interacting pr	33.60	0.00
TRINITY_sp|P253:	mlkA	Myosin light chain	33.60	0.00
TRINITY_sp|Q9DA!	-	Uncharacterized pro	33.60	0.00
TRINITY_sp|Q5ZMI	SEPT2	Septin-2 OS=Gallus	33.60	0.00
TRINITY_sp|P470:	PRY1	Protein PRY1 OS=Sac	33.60	0.00
TRINITY_sp|Q2LVI	msbA	Lipid A export ATP-	33.60	0.00

TRINITY_sp|Q3ZC(	TPPP3	Tubulin polymerizat	33.60	0.00
TRINITY_sp|F4I0I	VPS35B	Vacuolar protein so	33.60	0.00
TRINITY_sp|Q041(	-	Pathogenesis-relate	33.60	0.00
TRINITY_sp|Q9C5(	CIPK10	CBL-interacting ser	33.60	0.00
TRINITY_sp|Q86W5	PARG	Poly(ADP-ribose) gl	33.60	0.00
TRINITY_sp|Q9GK7	FAXDC2	Fatty acid hydroxyl	33.60	0.00
TRINITY_sp|B5BSX	CYP88D6	Beta-amyrin 11-oxid	33.60	0.00
TRINITY_sp|Q9LZ3	ABCC14	ABC transporter C f	33.60	0.00
TRINITY_sp|Q0312	gtf2f2	General transcripti	33.60	0.00
TRINITY_sp|Q2M32	CCDC96	Coiled-coil domain-	33.60	0.00
TRINITY_sp|Q9DBW	Cyp4v2	Cytochrome P450 4V2	33.60	0.00
TRINITY_sp|Q9WVE	Pole	DNA polymerase epsi	33.60	0.00
TRINITY_sp|Q92EE	lin0452	Uncharacterized oxi	33.60	0.00
TRINITY_sp|Q9C82	At3g0653	Uncharacterized pro	33.60	0.00
TRINITY_sp|Q9VH1	Invadoly	Leishmanolysin-like	33.60	0.00
TRINITY_sp|Q54YH	dhkB	Hybrid signal trans	33.60	0.00
TRINITY_sp|O9458	SPCC1442	Ubiquitin and WLM d	33.60	0.00
TRINITY_sp|C9WMM	-	Venom serine carbox	33.60	0.00
TRINITY_sp|Q9UI1	RABAC1	Prenylated Rab acce	33.60	0.00
TRINITY_sp|C5D2E	deoD	Purine nucleoside p	33.60	0.00
TRINITY_sp|Q2312	W02B12.9	Mitoferrin OS=Caeno	33.60	0.00
TRINITY_sp|Q8QZE	Tat	Tyrosine aminotrans	33.60	0.00
TRINITY_sp|P2271	Gucy1b2	Guanylate cyclase s	33.60	0.00
TRINITY_sp|Q6444	Atp7b	Copper-transporting	33.60	0.00
TRINITY_sp|Q54Y(	ngap	Probable Ras GTPase	33.60	0.00
TRINITY_sp|Q869E	DDB_G027	CTL-like protein DD	33.60	0.00
TRINITY_sp|Q6CJ1	RSM10	37S ribosomal prote	33.60	0.00
TRINITY_sp|Q54Q(	alg6	Probable dolichyl p	33.60	0.00
TRINITY_sp|A6QL(	TRAPPC11	Trafficking protein	33.60	0.00
TRINITY_sp|P3763	yhiN	Uncharacterized pro	33.60	0.00
TRINITY_sp|O1392	crn1	Coronin-like protei	33.60	0.00
TRINITY_sp|Q9M11	At3g544(	F-box protein At3g5	33.60	0.00
TRINITY_sp|P2173	UBC1	Ubiquitin-conjugati	33.60	0.00
TRINITY_sp|Q9H44	RWDD1	RWD domain-containi	33.60	0.00
TRINITY_sp|Q0191	ACS5	1-aminocyclopropane	33.60	0.00
TRINITY_sp|Q7XR(	Os04g065	Probable protein ph	33.60	0.00
TRINITY_sp|O4327	PRODH	Proline dehydrogena	33.60	0.00
TRINITY_sp|Q339I	Os10g037	Probable protein ph	33.60	0.00
TRINITY_sp|Q8ICE	CPK2	Calcium-dependent p	33.60	0.00
TRINITY_sp|Q8G8Y	btrR	L-glutamine:2-deoxy	33.60	0.00
TRINITY_sp|Q8BX3	Acp7	Acid phosphatase ty	33.60	0.00
TRINITY_sp|P3992	SLN1	Osmosensing histidi	33.60	0.00
TRINITY_sp|Q0301	GSTM4	Glutathione S-trans	33.60	0.00
TRINITY_sp|Q8TD5	DNAH3	Dynein heavy chain	33.60	0.00
TRINITY_sp|Q3UDE	Ttll12	Tubulin--tyrosine l	33.60	0.00
TRINITY_sp|Q044(	Vars	Valine--tRNA ligase	33.60	0.00
TRINITY_sp|Q6P4W	fam175b	BRISC complex subun	33.60	0.00
TRINITY_sp|Q5BLI	mettl21a	Protein N-lysine me	33.60	0.00
TRINITY_sp|B4I1(	GM12339	Bifunctional lysine	33.60	0.00
TRINITY_sp|Q54U8	dhkA	Hybrid signal trans	33.60	0.00
TRINITY_sp|Q010(	Pde1b	Calcium/calmodulin-	33.60	0.00
TRINITY_sp|Q0J9(	Os04g06(	Probable carboxyles	33.60	0.00
TRINITY_sp|P1385	RSR1	Ras-related protein	33.50	0.00
TRINITY_sp|Q55B3	plbB	Phospholipase B-lik	33.50	0.00
TRINITY_sp|P0463	Lipf	Gastric triacylglyc	33.50	0.00
TRINITY_sp|O3581	Stip1	Stress-induced-phos	33.50	0.00

TRINITY_sp|Q8H12	At2g344(	Uncharacterized pro	33.50	0.00
TRINITY_sp|Q5TJ5	forD	Formin-D OS=Dictyos	33.50	0.00
TRINITY_sp|Q8L7E	LPEAT1	Lysophospholipid ac	33.50	0.00
TRINITY_sp|Q6FRX	TIM50	Mitochondrial impor	33.50	0.00
TRINITY_sp|P2803	AOAH	Acyloxyacyl hydrola	33.50	0.00
TRINITY_sp|B6E14	HP	Haptoglobin OS=Capr	33.50	0.00
TRINITY_sp|Q10L(	CIPK10	CBL-interacting pro	33.50	0.00
TRINITY_sp|Q6INS	emc2-a	ER membrane protein	33.50	0.00
TRINITY_sp|Q9D97	Efhc1	EF-hand domain-cont	33.50	0.00
TRINITY_sp|A9BG(	glgA	Glycogen synthase O	33.50	0.00
TRINITY_sp|Q7TXE	Mb2976	Phthiotriol/phenolp	33.50	0.00
TRINITY_sp|P3515	dacB	D-alanyl-D-alanine	33.50	0.00
TRINITY_sp|Q0373	YML018C	Uncharacterized vac	33.50	0.00
TRINITY_sp|O4418	F33D4.4	Putative sphingolip	33.50	0.00
TRINITY_sp|Q7MD1	luxQ	Autoinducer 2 senso	33.50	0.00
TRINITY_sp|P5485	TGL2	Lipase 2 OS=Sacchar	33.50	0.00
TRINITY_sp|Q9FWE	GAMMACA1	Gamma carbonic anhy	33.50	0.00
TRINITY_sp|Q9VS3	Srp68	Signal recognition	33.50	0.00
TRINITY_sp|Q9C77	ATE2	Arginyl-tRNA--prote	33.50	0.00
TRINITY_sp|Q9P30	dbl4	E3 ubiquitin-protei	33.50	0.00
TRINITY_sp|Q9LFE	DOT2	SART-1 family prote	33.50	0.00
TRINITY_sp|A7RX3	thoc7	THO complex subunit	33.50	0.00
TRINITY_sp|B0G12	pip5k3	1-phosphatidylinosi	33.50	0.00
TRINITY_sp|A4TE(	Mflv_502	Putative S-adenosyl	33.50	0.00
TRINITY_sp|Q96G2	CERS2	Ceramide synthase 2	33.50	0.00
TRINITY_sp|Q6CR(	mge1	GrpE protein homolo	33.50	0.00
TRINITY_sp|P3352	ABCC1	Multidrug resistanc	33.50	0.00
TRINITY_sp|Q9FZ4	ACR11	ACT domain-containi	33.50	0.00
TRINITY_sp|Q1L52	LONRF2	LON peptidase N-ter	33.50	0.00
TRINITY_sp|Q4G0E	HYDIN	Hydrocephalus-induc	33.50	0.00
TRINITY_sp|Q9V6(	Taz	Tafazzin homolog OS	33.50	0.00
TRINITY_sp|O2255	STY8	Serine/threonine-pr	33.50	0.00
TRINITY_sp|O1377	SPAC17A2	Putative metal ion	33.50	0.00
TRINITY_sp|Q9FV1	At1g5772	Probable elongation	33.50	0.00
TRINITY_sp|Q7TQ(	Poln	DNA polymerase nu O	33.50	0.00
TRINITY_sp|P6234	CPK4	Calcium-dependent p	33.50	0.00
TRINITY_sp|Q9LX1	PAP21	Purple acid phospha	33.50	0.00
TRINITY_sp|Q8VX2	XEG113	Arabinosyltransfera	33.50	0.00
TRINITY_sp|Q9LL8	LIG4	DNA ligase 4 OS=Ara	33.50	0.00
TRINITY_sp|P2183	cryS	Crystal protein OS=	33.50	0.00
TRINITY_sp|Q9FM(	At5g1405	U3 small nucleolar	33.50	0.00
TRINITY_sp|O1419	SPAC56E4	Aromatic amino acid	33.50	0.00
TRINITY_sp|O7388	HPGDS	Hematopoietic prost	33.50	0.00
TRINITY_sp|Q08DE	PDE12	2',5'-phosphodieste	33.50	0.00
TRINITY_sp|Q7TP(	Shprh	E3 ubiquitin-protei	33.50	0.00
TRINITY_sp|Q0597	-	Sodium channel prot	33.50	0.00
TRINITY_sp|Q0454	cbxXP	Protein CbxX, plasm	33.50	0.00
TRINITY_sp|Q9S9N	CCR1	Cinnamoyl-CoA reduc	33.40	0.00
TRINITY_sp|Q54WW	DDB_G027	Probable serine/thr	33.40	0.00
TRINITY_sp|O1543	ABCC4	Multidrug resistanc	33.40	0.00
TRINITY_sp|Q2TBE	CWF19L2	CWF19-like protein	33.40	0.00
TRINITY_sp|Q55G9	scy1	Probable inactive s	33.40	0.00
TRINITY_sp|Q9LQV	RDR1	RNA-dependent RNA p	33.40	0.00
TRINITY_sp|Q9P89	tcsA	Two-component syste	33.40	0.00
TRINITY_sp|P9WQE	MT1137	3 beta-hydroxystero	33.40	0.00
TRINITY_sp|Q1ZXE	DDB_G028	Sphingomyelinase DD	33.40	0.00

TRINITY_sp|Q96KN	CNDP1	Beta-Ala-His dipept	33.40	0.00
TRINITY_sp|Q8TAQ	SMARCC2	SWI/SNF complex sub	33.40	0.00
TRINITY_sp|O1375	osm1	Fumarate reductase	33.40	0.00
TRINITY_sp|F4IRR	SAD2	Importin beta-like	33.40	0.00
TRINITY_sp|Q9S7C	PNP2	Polyribonucleotide	33.40	0.00
TRINITY_sp|Q54PS	osbI	Oxysterol-binding p	33.40	0.00
TRINITY_sp|Q8W47	AAE15	Long-chain-fatty-ac	33.40	0.00
TRINITY_sp|O8406	tlcA	ADP,ATP carrier pro	33.40	0.00
TRINITY_sp|Q6Z6I	Os02g021	Aminopeptidase M1-A	33.40	0.00
TRINITY_sp|Q54S5	DDB_G028	Protein PIEZO homol	33.40	0.00
TRINITY_sp|O0041	IPO5	Importin-5 OS=Homo	33.40	0.00
TRINITY_sp|P4633	csbC	Probable metabolite	33.40	0.00
TRINITY_sp|A2XMR	ARP7	Actin-related prote	33.40	0.00
TRINITY_sp|O1581	clua	Clustered mitochond	33.40	0.00
TRINITY_sp|Q8WMR	PLA2G15	Group XV phospholip	33.30	0.00
TRINITY_sp|Q8L9T	ATL2	RING-H2 finger prot	33.30	0.00
TRINITY_sp|P3789	CBP1	Serine carboxypepti	33.30	0.00
TRINITY_sp|P1271	-	Actin, cytoplasmic	33.30	0.00
TRINITY_sp|Q8IBS	CPK4	Calcium-dependent p	33.30	0.00
TRINITY_sp|Q54R5	DDB_G028	Probable tyrosine-p	33.30	0.00
TRINITY_sp|Q0746	-	Ribonuclease OS=Aer	33.30	0.00
TRINITY_sp|Q95ZC	spc97	Spindle pole body c	33.30	0.00
TRINITY_sp|O1544	ABCC5	Multidrug resistanc	33.30	0.00
TRINITY_sp|P3999	GEA2	ARF guanine-nucleot	33.30	0.00
TRINITY_sp|Q869T	etnkA	Probable ethanolami	33.30	0.00
TRINITY_sp|Q5A87	SLN1	Histidine protein k	33.30	0.00
TRINITY_sp|P1877	est	Esterase OS=Acineto	33.30	0.00
TRINITY_sp|Q9532	MANBA	Beta-mannosidase OS	33.30	0.00
TRINITY_sp|P0939	strG	Streptomycin biosyn	33.30	0.00
TRINITY_sp|Q552N	DDB_G027	Putative ZDHHC-type	33.30	0.00
TRINITY_sp|P1163	NHP6B	Non-histone chromos	33.30	0.00
TRINITY_sp|Q54YH	dhkB	Hybrid signal trans	33.30	0.00
TRINITY_sp|Q5JLS	CIPK12	CBL-interacting pro	33.30	0.00
TRINITY_sp|Q54JR	abcC3	ABC transporter C f	33.30	0.00
TRINITY_sp|Q89W7	norM	Probable multidrug	33.30	0.00
TRINITY_sp|Q9M2I	At3g6132	UPF0187 protein At3	33.30	0.00
TRINITY_sp|P2018	-	Uncharacterized 37.	33.30	0.00
TRINITY_sp|Q87AR	infC	Translation initiat	33.30	0.00
TRINITY_sp|Q0451	Smpd1	Sphingomyelin phosp	33.30	0.00
TRINITY_sp|P2126	CCA1	CCA tRNA nucleotidy	33.30	0.00
TRINITY_sp|Q54TH	gacY	Rho GTPase-activati	33.30	0.00
TRINITY_sp|Q8S8S	PUB34	U-box domain-contai	33.30	0.00
TRINITY_sp|Q2KHV	USP2	Ubiquitin carboxyl-	33.30	0.00
TRINITY_sp|Q3839	37	Long tail fiber pro	33.30	0.00
TRINITY_sp|O4306	mot1	Probable helicase m	33.30	0.00
TRINITY_sp|Q6T41	CWC2	Pre-mRNA-splicing f	33.30	0.00
TRINITY_sp|P7934	RGS20	Regulator of G-prot	33.30	0.00
TRINITY_sp|Q9W4T	dnc	cAMP-specific 3',5'	33.30	0.00
TRINITY_sp|Q5M88	-	UPF0415 protein C7o	33.30	0.00
TRINITY_sp|Q6379	Map3k12	Mitogen-activated p	33.30	0.00
TRINITY_sp|A6ZU0	ATG1	Serine/threonine-pr	33.30	0.00
TRINITY_sp|Q1464	ITPR1	Inositol 1,4,5-tris	33.30	0.00
TRINITY_sp|Q54PS	osbI	Oxysterol-binding p	33.30	0.00
TRINITY_sp|Q9UR1	nrf1	Vacuolar transporte	33.30	0.00
TRINITY_sp|Q6NXM	Rcbtb1	RCC1 and BTB domain	33.30	0.00
TRINITY_sp|Q95YI	pefA	Penta-EF hand domai	33.30	0.00

TRINITY_sp|Q6P6?	csn8	COP9 signalosome co	33.30	0.00
TRINITY_sp|P317?	OV16	OV-16 antigen OS=On	33.30	0.00
TRINITY_sp|B0SD?	atpA	ATP synthase subuni	33.30	0.00
TRINITY_sp|Q5RH?	dtl	Denticleless protei	33.30	0.00
TRINITY_sp|Q9UR?	Tf2-11	Transposon Tf2-11 p	33.30	0.00
TRINITY_sp|Q8L7?	SYT5	Synaptotagmin-5 OS=	33.30	0.00
TRINITY_sp|Q9H2?	VPS16	Vacuolar protein so	33.30	0.00
TRINITY_sp|Q93Z?	NPF8.5	Protein NRT1/ PTR F	33.30	0.00
TRINITY_sp|Q08D?	PDE12	2',5'-phosphodieste	33.30	0.00
TRINITY_sp|Q550?	DDB_G02?	Probable serine/thr	33.30	0.00
TRINITY_sp|Q8BL?	Arhgap2?	Rho GTPase-activati	33.30	0.00
TRINITY_sp|Q28X?	Anp32a	Acidic leucine-rich	33.30	0.00
TRINITY_sp|Q6NR?	dcaf8	DDB1- and CUL4-asso	33.30	0.00
TRINITY_sp|P623?	CPK1	Calcium-dependent p	33.30	0.00
TRINITY_sp|Q9VJ?	Nnp-1	Ribosomal RNA proce	33.30	0.00
TRINITY_sp|Q54F?	gtf2b	Transcription initi	33.30	0.00
TRINITY_sp|Q6NR?	ppp6r3-?	Serine/threonine-pr	33.30	0.00
TRINITY_sp|Q6NQ?	At4g272?	Probable NAD(P)H de	33.30	0.00
TRINITY_sp|Q5BJ?	Ccdc93	Coiled-coil domain-	33.30	0.00
TRINITY_sp|Q17C?	Papst2	Adenosine 3'-phosph	33.30	0.00
TRINITY_sp|Q6P4?	Sik3	Serine/threonine-pr	33.30	0.00
TRINITY_sp|Q9SF?	OST1A	Dolichyl-diphosphoo	33.30	0.00
TRINITY_sp|O139?	hrq1	ATP-dependent helic	33.30	0.00
TRINITY_sp|Q8GU?	PUB33	U-box domain-contai	33.30	0.00
TRINITY_sp|Q6AX?	Utp3	Something about sil	33.30	0.00
TRINITY_sp|Q644?	Ptprj	Receptor-type tyros	33.30	0.00
TRINITY_sp|P144?	FGA	Fibrinogen alpha ch	33.30	0.00
TRINITY_sp|P641?	nei1	Endonuclease 8 1 OS	33.30	0.00
TRINITY_sp|Q9NS?	BRWD1	Bromodomain and WD	33.30	0.00
TRINITY_sp|O574?	-	Voltage-dependent L	33.30	0.00
TRINITY_sp|Q7XJ?	GAS8	Growth arrest-speci	33.30	0.00
TRINITY_sp|Q55A?	DDB_G02?	Probable serine/thr	33.30	0.00
TRINITY_sp|Q9Y5?	HEBP2	Heme-binding protei	33.30	0.00
TRINITY_sp|Q54R?	dhkL	Hybrid signal trans	33.30	0.00
TRINITY_sp|Q8BV?	Trank1	TPR and ankyrin rep	33.30	0.00
TRINITY_sp|Q9H2?	DHX36	ATP-dependent RNA h	33.30	0.00
TRINITY_sp|Q9R0?	Amfr	E3 ubiquitin-protei	33.30	0.00
TRINITY_sp|P528?	DGKQ	Diacylglycerol kina	33.30	0.00
TRINITY_sp|P527?	VAV2	Guanine nucleotide	33.30	0.00
TRINITY_sp|Q4R4?	IFT22	Intraflagellar tran	33.30	0.00
TRINITY_sp|P9WN?	MT2788	Uncharacterized pro	33.30	0.00
TRINITY_sp|C7YT?	RRP36	rRNA biogenesis pro	33.30	0.00
TRINITY_sp|Q5A5?	NIK1	Histidine protein k	33.30	0.00
TRINITY_sp|Q7LH?	TY3B-I	Transposon Ty3-I Ga	33.30	0.00
TRINITY_sp|Q6CB?	SAR1	Small COPII coat GT	33.30	0.00
TRINITY_sp|P235?	ACS1	1-aminocyclopropane	33.30	0.00
TRINITY_sp|Q54T?	drkD	Probable serine/thr	33.30	0.00
TRINITY_sp|P333?	PRP8	Pre-mRNA-splicing f	33.30	0.00
TRINITY_sp|P041?	Myb	Myb protein OS=Dros	33.30	0.00
TRINITY_sp|Q964?	CYP4C21	Cytochrome P450 4c2	33.30	0.00
TRINITY_sp|Q54D?	DDB_G02?	Putative potassium	33.30	0.00
TRINITY_sp|Q55G?	scy1	Probable inactive s	33.30	0.00
TRINITY_sp|Q0WT?	ERDJ2A	DnaJ protein ERDJ2A	33.30	0.00
TRINITY_sp|Q80U?	Sass6	Spindle assembly ab	33.30	0.00
TRINITY_sp|Q94B?	At1g718?	Uncharacterized aar	33.30	0.00
TRINITY_sp|Q944?	SNF4	Sucrose nonfermenti	33.30	0.00

TRINITY_sp|P146(	cyr1	Adenylate cyclase O	33.30	0.00
TRINITY_sp|A9CJ(	phrA	Deoxyribodipyrimidi	33.30	0.00
TRINITY_sp|Q039(	Eif2ak2	Interferon-induced,	33.30	0.00
TRINITY_sp|Q6ZI	ML2	Protein MEI2-like 2	33.30	0.00
TRINITY_sp|Q9ZUI	ETL1	Protein CHROMATIN R	33.30	0.00
TRINITY_sp|Q54MI	cnbB	Calcineurin subunit	33.30	0.00
TRINITY_sp|A1L1	Wdr55	WD repeat-containin	33.30	0.00
TRINITY_sp|P227	Gucy1b2	Guanylate cyclase s	33.30	0.00
TRINITY_sp|P041	GIP	Copia protein OS=Dr	33.30	0.00
TRINITY_sp|Q559(	slr0305	TVP38/TMEM64 family	33.30	0.00
TRINITY_sp|Q9ZP	TMN3	Transmembrane 9 sup	33.30	0.00
TRINITY_sp|Q9QY	Vapb	Vesicle-associated	33.30	0.00
TRINITY_sp|P411	hsf1	Heat shock factor p	33.20	0.00
TRINITY_sp|Q54N(	psmD13	26S proteasome non-	33.20	0.00
TRINITY_sp|Q9XZ	culB	Cullin-2 OS=Dictyos	33.20	0.00
TRINITY_sp|Q8R1(	Cog4	Conserved oligomeri	33.20	0.00
TRINITY_sp|Q8K2I	Trappc1	Trafficking protein	33.20	0.00
TRINITY_sp|Q1ZXI	DDB_G02	Sphingomyelinase DD	33.20	0.00
TRINITY_sp|Q6CS	ATG1	Serine/threonine-pr	33.20	0.00
TRINITY_sp|Q6H7(	CIPK26	CBL-interacting pro	33.20	0.00
TRINITY_sp|Q54C	dcd1B	Protein dcd1B OS=Di	33.20	0.00
TRINITY_sp|Q8PW	MM_1767	Geranylgeranyl diph	33.20	0.00
TRINITY_sp|Q5AG	HSL1	Serine/threonine-pr	33.20	0.00
TRINITY_sp|Q8RWI	STY17	Serine/threonine-pr	33.20	0.00
TRINITY_sp|Q8T2	prkag	5'-AMP-activated pr	33.20	0.00
TRINITY_sp|Q0IWI	GRXS11	Monothiol glutaredo	33.20	0.00
TRINITY_sp|P432	RD19B	Probable cysteine p	33.20	0.00
TRINITY_sp|Q0JI	CIPK11	CBL-interacting pro	33.20	0.00
TRINITY_sp|Q54EI	pdi2	Protein disulfide-i	33.20	0.00
TRINITY_sp|O429	ale1	Lysophospholipid ac	33.20	0.00
TRINITY_sp|Q8VY(	At4g073	Mannose-P-dolichol	33.20	0.00
TRINITY_sp|F4HTI	GCS1	Mannosyl-oligosacch	33.20	0.00
TRINITY_sp|A5PN	fam151a	Protein FAM151A OS=	33.20	0.00
TRINITY_sp|Q9CQI	Aasdhppt	L-aminoadipate-semi	33.20	0.00
TRINITY_sp|Q940	GLCNAC1	UDP-N-acetylglucosa	33.20	0.00
TRINITY_sp|Q5AF(	GLX3	Glyoxalase 3 OS=Can	33.20	0.00
TRINITY_sp|O239(	SF21	Pollen-specific pro	33.20	0.00
TRINITY_sp|A2RRI	WDR27	WD repeat-containin	33.20	0.00
TRINITY_sp|Q54W	abcD3	ABC transporter D f	33.20	0.00
TRINITY_sp|A6H8	sgsm3	Small G protein sig	33.20	0.00
TRINITY_sp|P141(	PDE1A	Calcium/calmodulin-	33.20	0.00
TRINITY_sp|P315	RHN1	Ras-related protein	33.20	0.00
TRINITY_sp|Q5JKI	Os01g05	Probable protein ph	33.20	0.00
TRINITY_sp|Q86VI	KIF27	Kinesin-like protei	33.20	0.00
TRINITY_sp|Q153	PUM3	Pumilio homolog 3 O	33.20	0.00
TRINITY_sp|Q9R1	PARP1	Poly [ADP-ribose] p	33.20	0.00
TRINITY_sp|O525	cah	Carbonic anhydrase	33.20	0.00
TRINITY_sp|P0C2	ddx20	Probable ATP-depend	33.20	0.00
TRINITY_sp|C5A1	gyaR	Glyoxylate reductas	33.20	0.00
TRINITY_sp|Q9M3I	RID3	Protein ROOT INITIA	33.20	0.00
TRINITY_sp|Q6UX(	DHRS13	Dehydrogenase/reduc	33.20	0.00
TRINITY_sp|Q102	SPAC4G9.	Uncharacterized mit	33.20	0.00
TRINITY_sp|P091(	P4HB	Protein disulfide-i	33.20	0.00
TRINITY_sp|Q5VV(	YOD1	Ubiquitin thioester	33.20	0.00
TRINITY_sp|P098	PARP1	Poly [ADP-ribose] p	33.20	0.00
TRINITY_sp|Q55G	abkC	Probable serine/thr	33.20	0.00

TRINITY_sp|Q5EB	nt5c2	Cytosolic purine 5'	33.20	0.00
TRINITY_sp|Q8CG	Abcc1	Multidrug resistanc	33.20	0.00
TRINITY_sp|Q9Y4	SPEF1	Sperm flagellar pro	33.20	0.00
TRINITY_sp|A7HQ	pyrD	Dihydroorotate dehy	33.10	0.00
TRINITY_sp|Q408	EMB8	Embryogenesis-assoc	33.10	0.00
TRINITY_sp|Q5TI	VWA5B1	von Willebrand fact	33.10	0.00
TRINITY_sp|O139	SPAC23C	UPF0665 family prot	33.10	0.00
TRINITY_sp|Q076	SPAG1	Sperm-associated an	33.10	0.00
TRINITY_sp|Q3SE	Icl1e	Caltractin ICL1e OS	33.10	0.00
TRINITY_sp|Q54D	abcA9	ABC transporter A f	33.10	0.00
TRINITY_sp|Q949	Reg-2	Rhythmically expres	33.10	0.00
TRINITY_sp|Q54P	xpo5	Exportin-5 OS=Dicty	33.10	0.00
TRINITY_sp|Q54C	sgmB	Sphingomyelin phosp	33.10	0.00
TRINITY_sp|Q55C	gdt2	Probable serine/thr	33.10	0.00
TRINITY_sp|Q9LT	BBR	E3 ubiquitin ligase	33.10	0.00
TRINITY_sp|Q9ZR	GSTT1	Glutathione S-trans	33.10	0.00
TRINITY_sp|Q1ZX	DDB_G02	PH domain-containin	33.10	0.00
TRINITY_sp|Q9W0	NaCP60E	Sodium channel prot	33.10	0.00
TRINITY_sp|Q92F	radA	DNA repair protein	33.10	0.00
TRINITY_sp|P153	rpa1	DNA-directed RNA po	33.10	0.00
TRINITY_sp|Q94A	STR6	Rhodanese-like doma	33.10	0.00
TRINITY_sp|O528	bgaM	Beta-galactosidase	33.10	0.00
TRINITY_sp|C0SV	RVE5	Protein REVEILLE 5	33.10	0.00
TRINITY_sp|O946	SPBC405.	Uncharacterized tra	33.10	0.00
TRINITY_sp|O957	HERC2	E3 ubiquitin-protei	33.10	0.00
TRINITY_sp|Q3UF	Nom1	Nucleolar MIF4G dom	33.10	0.00
TRINITY_sp|Q9X2	fabG	3-oxoacyl-[acyl-car	33.10	0.00
TRINITY_sp|G4SW	erg	Delta(14)-sterol re	33.10	0.00
TRINITY_sp|B1AZ	Otud3	OTU domain-containi	33.10	0.00
TRINITY_sp|Q9FJ	FKBP65	Peptidyl-prolyl cis	33.10	0.00
TRINITY_sp|Q7TN	Abca7	ATP-binding cassett	33.10	0.00
TRINITY_sp|Q9VR	HERC2	Probable E3 ubiquit	33.10	0.00
TRINITY_sp|P706	Siae	Sialate O-acetylest	33.10	0.00
TRINITY_sp|Q54P	gcsA	Glutamate--cysteine	33.10	0.00
TRINITY_sp|Q6NZ	ncl1	Nicalin-1 OS=Danio	33.10	0.00
TRINITY_sp|Q1L8	rnf19b	E3 ubiquitin-protei	33.10	0.00
TRINITY_sp|P242	yjiA	Uncharacterized GTP	33.10	0.00
TRINITY_sp|Q9DB	Cyp4v2	Cytochrome P450 4V2	33.10	0.00
TRINITY_sp|Q408	EMB8	Embryogenesis-assoc	33.10	0.00
TRINITY_sp|Q9DB	Osbpl3	Oxysterol-binding p	33.10	0.00
TRINITY_sp|Q994	PFDN5	Prefoldin subunit 5	33.10	0.00
TRINITY_sp|Q8RX	BRCA1	Protein BREAST CANC	33.10	0.00
TRINITY_sp|Q55F	tipC	Putative vacuolar p	33.10	0.00
TRINITY_sp|Q053	W	Protein white OS=Lu	33.10	0.00
TRINITY_sp|Q6P2	PNCK	Calcium/calmodulin-	33.10	0.00
TRINITY_sp|O805	CYCU4-1	Cyclin-U4-1 OS=Arab	33.10	0.00
TRINITY_sp|O147	TPP1	Tripeptidyl-peptida	33.10	0.00
TRINITY_sp|Q9SH	At2g379	Ribosome biogenesis	33.10	0.00
TRINITY_sp|Q54T	mybC	Myb-like protein C	33.10	0.00
TRINITY_sp|Q6DG	atg4b	Cysteine protease A	33.10	0.00
TRINITY_sp|Q91W	Pnpla3	Patatin-like phosph	33.10	0.00
TRINITY_sp|Q996	MGLL	Monoglyceride lipas	33.10	0.00
TRINITY_sp|O548	Gucy1b3	Guanylate cyclase s	33.10	0.00
TRINITY_sp|Q6J1	Usp19	Ubiquitin carboxyl-	33.10	0.00
TRINITY_sp|Q94K	VPS52	Vacuolar protein so	33.10	0.00
TRINITY_sp|B3E4	era	GTPase Era OS=Geoba	33.10	0.00

TRINITY_sp|O144	ssr2	SWI/SNF and RSC com	33.10	0.00
TRINITY_sp|Q9Y4	VPRBP	Protein VPRBP OS=Ho	33.10	0.00
TRINITY_sp|Q93W	At4g155	Uncharacterized pro	33.10	0.00
TRINITY_sp|Q9FM	CPK17	Calcium-dependent p	33.10	0.00
TRINITY_sp|A8I9	CFAP45	Cilia- and flagella	33.10	0.00
TRINITY_sp|P480	gacS	Sensor protein GacS	33.10	0.00
TRINITY_sp|O613	Dbp80	DEAD-box helicase D	33.10	0.00
TRINITY_sp|Q9Y1	fadA	Delta(5) fatty acid	33.10	0.00
TRINITY_sp|A8Y1	pfd-6	Probable prefoldin	33.10	0.00
TRINITY_sp|P532	NQM1	Transaldolase NQM1	33.10	0.00
TRINITY_sp|Q027	MAP3K10	Mitogen-activated p	33.10	0.00
TRINITY_sp|P788	vtc4	Vacuolar transporte	33.10	0.00
TRINITY_sp|G5CE	-	Eukaryotic translat	33.10	0.00
TRINITY_sp|Q54J	abcC3	ABC transporter C f	33.10	0.00
TRINITY_sp|Q3U4	Lipn	Lipase member N OS=	33.00	0.00
TRINITY_sp|P266	rad15	DNA repair helicase	33.00	0.00
TRINITY_sp|P459	Su(var)	Histone-lysine N-me	33.00	0.00
TRINITY_sp|Q0JI	CIPK11	CBL-interacting pro	33.00	0.00
TRINITY_sp|O089	Faah	Fatty-acid amide hy	33.00	0.00
TRINITY_sp|Q8VZ	At4g191	Uncharacterized zin	33.00	0.00
TRINITY_sp|Q8VE	Slc35f6	Solute carrier fami	33.00	0.00
TRINITY_sp|Q4U2	Herc2	E3 ubiquitin-protei	33.00	0.00
TRINITY_sp|Q9HC	TMBIM4	Protein lifeguard 4	33.00	0.00
TRINITY_sp|Q8RY	At4g176	Decapping nuclease	33.00	0.00
TRINITY_sp|Q647	Plk4	Serine/threonine-pr	33.00	0.00
TRINITY_sp|Q9VH	Invadol	Leishmanolysin-like	33.00	0.00
TRINITY_sp|Q9DB	Uqcrc2	Cytochrome b-c1 com	33.00	0.00
TRINITY_sp|Q5KU	COLEC12	Collectin-12 OS=Hom	33.00	0.00
TRINITY_sp|Q54D	DDB_G02	von Willebrand fact	33.00	0.00
TRINITY_sp|B0G0	mybAA	Myb-like protein AA	33.00	0.00
TRINITY_sp|Q9SS	RBL5	RHOMBOID-like prote	33.00	0.00
TRINITY_sp|P921	CP1	Cathepsin B-like CP	33.00	0.00
TRINITY_sp|P188	Dao	D-amino-acid oxidas	33.00	0.00
TRINITY_sp|Q9VN	Rheb	GTP-binding protein	33.00	0.00
TRINITY_sp|Q7M3	rngB	RING finger protein	33.00	0.00
TRINITY_sp|Q54J	papA	Poly(A) polymerase	33.00	0.00
TRINITY_sp|Q8Z0	gmk	Guanylate kinase OS	33.00	0.00
TRINITY_sp|Q643	Syn2	Synapsin-2 OS=Mus m	33.00	0.00
TRINITY_sp|Q9LD	BETAA-A	Beta-adaptin-like p	33.00	0.00
TRINITY_sp|Q8DI	tilS	tRNA(Ile)-lysidine	33.00	0.00
TRINITY_sp|Q94K	TPC1	Two pore calcium ch	33.00	0.00
TRINITY_sp|Q54T	drkD	Probable serine/thr	33.00	0.00
TRINITY_sp|Q191	gcy-12	Receptor-type guany	33.00	0.00
TRINITY_sp|P059	pkaR	cAMP-dependent prot	33.00	0.00
TRINITY_sp|Q5SM	TTHA106	Magnesium transport	33.00	0.00
TRINITY_sp|Q8IY	CCDC146	Coiled-coil domain-	33.00	0.00
TRINITY_sp|Q2QA	TIO	Serine/threonine-pr	33.00	0.00
TRINITY_sp|Q8IC	CPK2	Calcium-dependent p	33.00	0.00
TRINITY_sp|Q0VC	ABHD3	Phospholipase ABHD3	33.00	0.00
TRINITY_sp|P547	EPHA5	Ephrin type-A recep	33.00	0.00
TRINITY_sp|P104	pol	Retrovirus-related	33.00	0.00
TRINITY_sp|Q5UP	MIMI_L7	Uncharacterized pro	33.00	0.00
TRINITY_sp|Q8L7	LPEAT1	Lysophospholipid ac	33.00	0.00
TRINITY_sp|Q9HB	RDH14	Retinol dehydrogena	33.00	0.00
TRINITY_sp|Q86I	DDB_G02	Uncharacterized Gol	33.00	0.00
TRINITY_sp|O647	At2g301	Probable protein ph	33.00	0.00

TRINITY_sp|B8AJ(	BASS4	Probable sodium/met	33.00	0.00
TRINITY_sp|Q96B.	AIDA	Axin interactor, do	33.00	0.00
TRINITY_sp|O346{	ydiP	Probable BsuMI modi	33.00	0.00
TRINITY_sp|Q1PFI	CPK19	Calcium-dependent p	33.00	0.00
TRINITY_sp|Q9M0/	GGP3	Gamma-glutamyl pept	33.00	0.00
TRINITY_sp|Q95R	Orct2	Organic cation tran	33.00	0.00
TRINITY_sp|Q9M8V	SIP1-1	Aquaporin SIP1-1 OS	33.00	0.00
TRINITY_sp|Q177	nuc-1	Deoxyribonuclease-2	33.00	0.00
TRINITY_sp|P0CZ.	ACX3	Acyl-coenzyme A oxi	33.00	0.00
TRINITY_sp|Q10X/	obg	GTPase Obg OS=Trich	33.00	0.00
TRINITY_sp|Q9W4	dnc	cAMP-specific 3',5'	33.00	0.00
TRINITY_sp|O764{	GstD7	Glutathione S-trans	33.00	0.00
TRINITY_sp|O040	At1g089	Phosphoglycerate mu	33.00	0.00
TRINITY_sp|P340	manA	Lysosomal alpha-man	33.00	0.00
TRINITY_sp|Q75J	cpras1	Circularly permutat	33.00	0.00
TRINITY_sp|Q0V9	ccdc39	Coiled-coil domain-	33.00	0.00
TRINITY_sp|Q32P	Fubp1	Far upstream elemen	33.00	0.00
TRINITY_sp|Q6AX	bbs9	Protein PTHB1 OS=Xe	33.00	0.00
TRINITY_sp|Q8W4	UBP2	Ubiquitin carboxyl-	33.00	0.00
TRINITY_sp|P383	PHO89	Phosphate permease	33.00	0.00
TRINITY_sp|Q54R	dhkL	Hybrid signal trans	32.90	0.00
TRINITY_sp|Q1AU	fusA	Elongation factor G	32.90	0.00
TRINITY_sp|G5EF	gld-4	Poly(A) RNA polymer	32.90	0.00
TRINITY_sp|Q54F	arpin	Arpin OS=Dictyostel	32.90	0.00
TRINITY_sp|Q567	yod1	Ubiquitin thioester	32.90	0.00
TRINITY_sp|Q6PD	Ecm29	Proteasome-associat	32.90	0.00
TRINITY_sp|A0PQ	MUL_200	Probable phthiotrio	32.90	0.00
TRINITY_sp|Q9XT	ZK262.3	Lipase ZK262.3 OS=C	32.90	0.00
TRINITY_sp|B6MF	BRAFLDR	Protein Hook homolo	32.90	0.00
TRINITY_sp|Q9D1	Cfap57	Cilia- and flagella	32.90	0.00
TRINITY_sp|Q29A	GA17800	Leishmanolysin-like	32.90	0.00
TRINITY_sp|Q54H	gghB	Gamma-glutamyl hydr	32.90	0.00
TRINITY_sp|Q96G	DCUN1D1	DCN1-like protein 1	32.90	0.00
TRINITY_sp|O525	cah	Carbonic anhydrase	32.90	0.00
TRINITY_sp|Q8XJ	def2	Peptide deformylase	32.90	0.00
TRINITY_sp|Q3U4	Hectd3	E3 ubiquitin-protei	32.90	0.00
TRINITY_sp|Q54Y	dhkJ	Hybrid signal trans	32.90	0.00
TRINITY_sp|Q54H	drkA	Probable serine/thr	32.90	0.00
TRINITY_sp|Q84L	PAP14	Probable inactive p	32.90	0.00
TRINITY_sp|Q54Q	sir2A	NAD-dependent deace	32.90	0.00
TRINITY_sp|Q96R	CAMKK2	Calcium/calmodulin-	32.90	0.00
TRINITY_sp|Q3UG	Wdr19	WD repeat-containin	32.90	0.00
TRINITY_sp|Q9VY	Usp7	Ubiquitin carboxyl-	32.90	0.00
TRINITY_sp|Q0JI	CIPK11	CBL-interacting pro	32.90	0.00
TRINITY_sp|Q7XZ	APX5	L-ascorbate peroxid	32.90	0.00
TRINITY_sp|Q5XF	TIM44-2	Mitochondrial impor	32.90	0.00
TRINITY_sp|O087	Ube3a	Ubiquitin-protein l	32.90	0.00
TRINITY_sp|Q8RV	DEK1	Calpain-type cystei	32.90	0.00
TRINITY_sp|P404	rdgC	Serine/threonine-pr	32.90	0.00
TRINITY_sp|P484	Ppp3cc	Serine/threonine-pr	32.90	0.00
TRINITY_sp|Q7FG	RH1	DEAD-box ATP-depend	32.90	0.00
TRINITY_sp|Q7SY	slc44a2	Choline transporter	32.90	0.00
TRINITY_sp|O049	-	Alpha-glucosidase O	32.90	0.00
TRINITY_sp|Q6ZP	Ibtk	Inhibitor of Bruton	32.90	0.00
TRINITY_sp|Q8BV	Trank1	TPR and ankyrin rep	32.90	0.00
TRINITY_sp|Q54I	pyk3	Dual specificity pr	32.90	0.00

TRINITY_sp|Q6TY2	Pabpn1l-	Embryonic polyadeny	32.90	0.00
TRINITY_sp|Q5XG8	PAPD7	Non-canonical poly(	32.90	0.00
TRINITY_sp|A4IF8	GNPAT	Dihydroxyacetone ph	32.90	0.00
TRINITY_sp|A2QV\	creC	Probable catabolite	32.90	0.00
TRINITY_sp|A5PJ2	NUP93	Nuclear pore comple	32.90	0.00
TRINITY_sp|Q93VI	CCD8A	Carotenoid cleavage	32.90	0.00
TRINITY_sp|P5161	Xpc	DNA repair protein	32.90	0.00
TRINITY_sp|Q3UGN	Nek10	Serine/threonine-pr	32.90	0.00
TRINITY_sp|Q8I7I	pol	Retrovirus-related	32.90	0.00
TRINITY_sp|Q3905	CNX2	GTP 3',8-cyclase, m	32.90	0.00
TRINITY_sp|Q9ZUI	CKL5	Casein kinase 1-lik	32.90	0.00
TRINITY_sp|A5PJI	HSDL1	Inactive hydroxyste	32.90	0.00
TRINITY_sp|Q9902	act	Actin OS=Hypocrea j	32.90	0.00
TRINITY_sp|Q9LU4	PUB53	Putative U-box doma	32.90	0.00
TRINITY_sp|Q76L3	cpr-c1	NADPH-dependent con	32.90	0.00
TRINITY_sp|Q869I	bpnt1	3'(2'),5'-bisphosph	32.90	0.00
TRINITY_sp|A8JAI	CFAP43	Cilia- and flagella	32.90	0.00
TRINITY_sp|Q652N	PHT4;4	Probable anion tran	32.90	0.00
TRINITY_sp|Q4105	SBEI	1,4-alpha-glucan-br	32.90	0.00
TRINITY_sp|Q54RI	dhkL	Hybrid signal trans	32.80	0.00
TRINITY_sp|O9779	TBC1D1	TBC1 domain family	32.80	0.00
TRINITY_sp|Q96P5	ACAP3	Arf-GAP with coiled	32.80	0.00
TRINITY_sp|Q1ZXI	roco5	Probable serine/thr	32.80	0.00
TRINITY_sp|Q94A7	At5g5314	Probable protein ph	32.80	0.00
TRINITY_sp|O6763	aq_1758	Uncharacterized RNA	32.80	0.00
TRINITY_sp|Q1D92	deoD	Purine nucleoside p	32.80	0.00
TRINITY_sp|Q54DI	commd1	COMM domain-contain	32.80	0.00
TRINITY_sp|Q8RWI	STY17	Serine/threonine-pr	32.80	0.00
TRINITY_sp|O4263	pep2	Vacuolar protease A	32.80	0.00
TRINITY_sp|Q54P(	gefA	Ras guanine nucleot	32.80	0.00
TRINITY_sp|Q9UR(	Tf2-11	Transposon Tf2-11 p	32.80	0.00
TRINITY_sp|Q9ZVI	At1g5457	Acyltransferase-lik	32.80	0.00
TRINITY_sp|P4984	STK19	Serine/threonine-pr	32.80	0.00
TRINITY_sp|P2002	-	Myb-related protein	32.80	0.00
TRINITY_sp|Q9C8I	ABCC11	ABC transporter C f	32.80	0.00
TRINITY_sp|Q54S7	vps15	Probable serine/thr	32.80	0.00
TRINITY_sp|Q6IQ1	SPOPL	Speckle-type POZ pr	32.80	0.00
TRINITY_sp|O1882	AOAH	Acyloxyacyl hydrola	32.80	0.00
TRINITY_sp|Q54V\	DDB_G028	Probable serine/thr	32.80	0.00
TRINITY_sp|Q9P89	tcsA	Two-component syste	32.80	0.00
TRINITY_sp|P4081	USP8	Ubiquitin carboxyl-	32.80	0.00
TRINITY_sp|Q54DI	mrkA	Probable serine/thr	32.80	0.00
TRINITY_sp|Q9235	SPAC6G9.	Pumilio domain-cont	32.80	0.00
TRINITY_sp|Q54N(	alkB	Alpha-ketoglutarate	32.80	0.00
TRINITY_sp|O1394	avl9	Late secretory path	32.80	0.00
TRINITY_sp|Q5ZKU	DCUN1D1	DCN1-like protein 1	32.80	0.00
TRINITY_sp|Q54F(	gacJJ	Rho GTPase-activati	32.80	0.00
TRINITY_sp|O0431	NSP3	Nitrile-specifier p	32.80	0.00
TRINITY_sp|O0492	GPX2	Probable glutathion	32.80	0.00
TRINITY_sp|P0AF(	tsaE	tRNA threonylcarbam	32.80	0.00
TRINITY_sp|P5282	DGKQ	Diacylglycerol kina	32.80	0.00
TRINITY_sp|P5932	Eif5	Eukaryotic translat	32.80	0.00
TRINITY_sp|Q8RX\	WIN2	Probable protein ph	32.80	0.00
TRINITY_sp|Q0JI4	CIPK11	CBL-interacting pro	32.80	0.00
TRINITY_sp|Q8VHU	Ikbkap	Elongator complex p	32.80	0.00
TRINITY_sp|Q94C(	At5g4994	Uncharacterized pro	32.80	0.00

TRINITY_sp|Q6DH\	ADAL	Adenosine deaminase	32.80	0.00
TRINITY_sp|Q5ZM(	PEX5	Peroxisomal targeti	32.80	0.00
TRINITY_sp|B5FE(	rlmI	Ribosomal RNA large	32.80	0.00
TRINITY_sp|B5RM:	rplB	50S ribosomal prote	32.80	0.00
TRINITY_sp|Q54U:	shkD	Dual specificity pr	32.80	0.00
TRINITY_sp|Q9CA8	PEX2	Peroxisome biogenes	32.80	0.00
TRINITY_sp|Q80Z2	Nampt	Nicotinamide phosph	32.80	0.00
TRINITY_sp|O624	Y43F4A.1	Leishmanolysin-like	32.80	0.00
TRINITY_sp|P649(	stf3	PAPS-dependent sulf	32.80	0.00
TRINITY_sp|Q9LZ8	BTR1	Protein BTR1 OS=Ara	32.80	0.00
TRINITY_sp|Q54SI	dhkD	Hybrid signal trans	32.80	0.00
TRINITY_sp|F4ILI	At2g3592	DExH-box ATP-depend	32.80	0.00
TRINITY_sp|Q8GUI	PUB33	U-box domain-contai	32.80	0.00
TRINITY_sp|P3152	-	47 kDa protein OS=P	32.80	0.00
TRINITY_sp|Q9ZU5	BAT1	Amino-acid permease	32.80	0.00
TRINITY_sp|P6968	Necap1	Adaptin ear-binding	32.80	0.00
TRINITY_sp|Q1202	FSF1	Probable mitochondr	32.80	0.00
TRINITY_sp|Q9VY\	Evi5	Ecotropic viral int	32.70	0.00
TRINITY_sp|O1408	kap104	Importin subunit be	32.70	0.00
TRINITY_sp|Q8BUI	Usp43	Ubiquitin carboxyl-	32.70	0.00
TRINITY_sp|Q499\	xpo4	Exportin-4 OS=Xenop	32.70	0.00
TRINITY_sp|Q8VZ8	PLT5	Polyol transporter	32.70	0.00
TRINITY_sp|Q9CZ1	Polr3e	DNA-directed RNA po	32.70	0.00
TRINITY_sp|Q68D(	KCTD16	BTB/POZ domain-cont	32.70	0.00
TRINITY_sp|Q9VH1	Invadoly	Leishmanolysin-like	32.70	0.00
TRINITY_sp|Q5ZJI	ULK3	Serine/threonine-pr	32.70	0.00
TRINITY_sp|Q8N2(	ARHGAP24	Rho GTPase-activati	32.70	0.00
TRINITY_sp|Q8N9\	WDSUB1	WD repeat, SAM and	32.70	0.00
TRINITY_sp|O551	Pdpk1	3-phosphoinositide-	32.70	0.00
TRINITY_sp|Q6SP9	Enkur	Enkurin OS=Mus musc	32.70	0.00
TRINITY_sp|Q9YGI	sand	Protein SAND OS=Tak	32.70	0.00
TRINITY_sp|Q86L9	gacHH	Rho GTPase-activati	32.70	0.00
TRINITY_sp|Q54C1	dync1li1	Cytoplasmic dynein	32.70	0.00
TRINITY_sp|Q2TCI	tgfb1i1	Transforming growth	32.70	0.00
TRINITY_sp|Q5ZI:	KLHL7	Kelch-like protein	32.70	0.00
TRINITY_sp|O4312	csh3	Protein csh3 OS=Sch	32.70	0.00
TRINITY_sp|P2465	PTP	Tyrosine-protein ph	32.70	0.00
TRINITY_sp|Q1LW\	tldc1	TLD domain-containi	32.70	0.00
TRINITY_sp|Q94AI	TPS6	Alpha,alpha-trehalo	32.70	0.00
TRINITY_sp|O0881	Wasl	Neural Wiskott-Aldr	32.70	0.00
TRINITY_sp|P5249	UBA1	Ubiquitin-activatin	32.70	0.00
TRINITY_sp|Q0JC(	ZEP	Zeaxanthin epoxidas	32.70	0.00
TRINITY_sp|Q6ZR(	DNAH12	Dynein heavy chain	32.70	0.00
TRINITY_sp|Q105(	CPSF2	Cleavage and polyad	32.70	0.00
TRINITY_sp|Q9VI(	CG10166	Probable dolichol-p	32.70	0.00
TRINITY_sp|Q55A(	DDB_G02	Probable serine/thr	32.70	0.00
TRINITY_sp|Q5Y2(	HTAY	Histone H2A.Y OS=Te	32.70	0.00
TRINITY_sp|Q86C8	atg1	Serine/threonine-pr	32.70	0.00
TRINITY_sp|O702	Dgcr14	Protein DGCR14 OS=M	32.70	0.00
TRINITY_sp|P731	sll1290	Uncharacterized rib	32.70	0.00
TRINITY_sp|P8329	CBR2	NADH-cytochrome b5	32.70	0.00
TRINITY_sp|Q84U(	BAC1	Mitochondrial argin	32.70	0.00
TRINITY_sp|Q84MI	AL1	RAP domain-containi	32.70	0.00
TRINITY_sp|Q1ZXI	gxcDD	Guanine exchange fa	32.70	0.00
TRINITY_sp|Q9ZV1	RRP4	Exosome complex com	32.70	0.00
TRINITY_sp|Q8GT(	MUS81	Crossover junction	32.70	0.00

TRINITY_sp|O943⁷rex4		RNA exonuclease 4 O	32.70	0.00
TRINITY_sp|Q9LH(	OCT4	Organic cation/carn	32.70	0.00
TRINITY_sp|Q9VH1	Invadoly	Leishmanolysin-like	32.70	0.00
TRINITY_sp|Q8H2]	Os07g064	Probable protein ph	32.70	0.00
TRINITY_sp|Q6Z71	GLU14	Endoglucanase 4 OS=	32.70	0.00
TRINITY_sp|P554(	NGR_a03	Uncharacterized oxi	32.70	0.00
TRINITY_sp|Q6DRN	noc3l	Nucleolar complex p	32.70	0.00
TRINITY_sp|Q097(	akr1	Palmitoyltransferas	32.70	0.00
TRINITY_sp|Q54RN	osbG	Oxysterol-binding p	32.70	0.00
TRINITY_sp|Q9MA(	ABCG24	ABC transporter G f	32.70	0.00
TRINITY_sp|Q94A]	SEC6	Exocyst complex com	32.70	0.00
TRINITY_sp|P5775	DRT101	Phosphoacetylglucos	32.70	0.00
TRINITY_sp|Q220(	pde-4	Probable 3',5'-cycl	32.70	0.00
TRINITY_sp|P295(	-	Ubiquitin-40S ribos	32.70	0.00
TRINITY_sp|Q96W(	eglB	Probable endo-beta-	32.60	0.00
TRINITY_sp|Q6NXN	Rcbtb1	RCC1 and BTB domain	32.60	0.00
TRINITY_sp|P4055	YIA6	Mitochondrial nicot	32.60	0.00
TRINITY_sp|Q097]	apm4	AP-2 complex subuni	32.60	0.00
TRINITY_sp|P389	IGHMBP2	DNA-binding protein	32.60	0.00
TRINITY_sp|Q4Z8I	RanBPM	Ran-binding protein	32.60	0.00
TRINITY_sp|Q6PD]	Ecm29	Proteasome-associat	32.60	0.00
TRINITY_sp|O947]	SPCC125	Uncharacterized pro	32.60	0.00
TRINITY_sp|Q408(	EMB8	Embryogenesis-assoc	32.60	0.00
TRINITY_sp|Q7L5I	GDPD3	Glycerophosphodiest	32.60	0.00
TRINITY_sp|P111(	SLC2A2	Solute carrier fami	32.60	0.00
TRINITY_sp|Q923⁷	ssb1	Replication factor	32.60	0.00
TRINITY_sp|Q54E]	DDB_G02	Probable serine/thr	32.60	0.00
TRINITY_sp|Q6ZTI	CFAP47	Cilia- and flagella	32.60	0.00
TRINITY_sp|Q54M(	pigw	Phosphatidylinosito	32.60	0.00
TRINITY_sp|Q941]	EO	2-methylene-furan-3	32.60	0.00
TRINITY_sp|O549(	Kcnu1	Potassium channel s	32.60	0.00
TRINITY_sp|Q1ZXI	gxcDD	Guanine exchange fa	32.60	0.00
TRINITY_sp|Q54X	DDB_G02	Probable serine/thr	32.60	0.00
TRINITY_sp|Q093	Ugt	UDP-glucose:glycopr	32.60	0.00
TRINITY_sp|O757	RAD51D	DNA repair protein	32.60	0.00
TRINITY_sp|Q096(	tnc-2	Troponin C, isoform	32.60	0.00
TRINITY_sp|Q54B(	mcfZ	Mitochondrial subst	32.60	0.00
TRINITY_sp|O933]	rad21	Double-strand-break	32.60	0.00
TRINITY_sp|Q1EC(	SMO1-2	Methylsterol monoox	32.60	0.00
TRINITY_sp|Q08D(	abcb6	ATP-binding cassett	32.60	0.00
TRINITY_sp|Q9M35	GCP4	Gamma-tubulin compl	32.60	0.00
TRINITY_sp|Q8BT]	Srrm2	Serine/arginine rep	32.60	0.00
TRINITY_sp|Q59W(	GIN4	Serine/threonine-pr	32.60	0.00
TRINITY_sp|P0A8\	yidA	Sugar phosphatase Y	32.60	0.00
TRINITY_sp|Q54E	gacEE	Rho GTPase-activati	32.60	0.00
TRINITY_sp|Q84W(	UBP13	Ubiquitin carboxyl-	32.60	0.00
TRINITY_sp|Q7ZXI	far1	Fatty acyl-CoA redu	32.60	0.00
TRINITY_sp|Q54EI	abcC7	ABC transporter C f	32.60	0.00
TRINITY_sp|Q99M	Sec14l2	SEC14-like protein	32.60	0.00
TRINITY_sp|Q1DN(	CIMG_08	Very-long-chain 3-o	32.60	0.00
TRINITY_sp|Q54L\	DDB_G02	G8 domain-containin	32.60	0.00
TRINITY_sp|Q6F6\	hpxO	FAD-dependent urate	32.60	0.00
TRINITY_sp|Q9SR\	LPA1	Protein LOW PSII AC	32.60	0.00
TRINITY_sp|Q5UP5	MIMI_R5	Uncharacterized pro	32.60	0.00
TRINITY_sp|O7445	sfc4	Transcription facto	32.60	0.00
TRINITY_sp|Q9FL⁷	EXPA24	Expansin-A24 OS=Ara	32.60	0.00

TRINITY_sp|Q9BV(	SPATA5L1	Spermatogenesis-ass	32.60	0.00
TRINITY_sp|Q505I	iws1	Protein IWS1 homolo	32.60	0.00
TRINITY_sp|Q9KWI	proP	Putative proline/be	32.60	0.00
TRINITY_sp|Q612:	Lcp1	Plastin-2 OS=Mus mu	32.60	0.00
TRINITY_sp|P341(	pkgC	Protein kinase 3 OS	32.60	0.00
TRINITY_sp|P623	CPK1	Calcium-dependent p	32.60	0.00
TRINITY_sp|Q6PG	traf3ip1	TRAF3-interacting p	32.60	0.00
TRINITY_sp|Q923	SPAC6G9.	Pumilio domain-cont	32.60	0.00
TRINITY_sp|Q005	CDK17	Cyclin-dependent ki	32.60	0.00
TRINITY_sp|P740	rimM	Ribosome maturation	32.60	0.00
TRINITY_sp|Q3V1	Nt5c2	Cytosolic purine 5'	32.60	0.00
TRINITY_sp|Q027	MAP3K10	Mitogen-activated p	32.60	0.00
TRINITY_sp|Q4V9	dgat2	Diacylglycerol O-ac	32.60	0.00
TRINITY_sp|Q948	THH1	Protein TOM THREE H	32.60	0.00
TRINITY_sp|F4IXV	BIG5	Brefeldin A-inhibit	32.60	0.00
TRINITY_sp|F4IU	SAC3A	SAC3 family protein	32.60	0.00
TRINITY_sp|Q8BH	Rab9b	Ras-related protein	32.60	0.00
TRINITY_sp|Q28H	slc38a6	Probable sodium-cou	32.50	0.00
TRINITY_sp|Q66J	epc2	Enhancer of polycom	32.50	0.00
TRINITY_sp|Q9QX	Trip4	Activating signal c	32.50	0.00
TRINITY_sp|Q9NJ	CPK3	Calcium-dependent p	32.50	0.00
TRINITY_sp|Q552	DDB_G02	Putative ZDHHC-type	32.50	0.00
TRINITY_sp|Q9M9	IMPA7	Importin subunit al	32.50	0.00
TRINITY_sp|Q54X	syn7B	Probable syntaxin-7	32.50	0.00
TRINITY_sp|Q54W	roco6	Probable serine/thr	32.50	0.00
TRINITY_sp|P401	cyaA	Adenylate cyclase 1	32.50	0.00
TRINITY_sp|Q4UB	rab1	Ras-related protein	32.50	0.00
TRINITY_sp|Q8DE	hscB	Co-chaperone protei	32.50	0.00
TRINITY_sp|Q54E	abcC7	ABC transporter C f	32.50	0.00
TRINITY_sp|Q54HI	gghB	Gamma-glutamyl hydr	32.50	0.00
TRINITY_sp|Q54B	mcfG	Mitochondrial subst	32.50	0.00
TRINITY_sp|F1LR	Cd2ap	CD2-associated prot	32.50	0.00
TRINITY_sp|Q54D	atp5C1	ATP synthase subuni	32.50	0.00
TRINITY_sp|P434	CCNA2	Cyclin-A2 OS=Gallus	32.50	0.00
TRINITY_sp|Q5R6	DIRAS2	GTP-binding protein	32.50	0.00
TRINITY_sp|O805	LARP6B	La-related protein	32.50	0.00
TRINITY_sp|Q9NI	PEK	Eukaryotic translat	32.50	0.00
TRINITY_sp|Q70E	USP53	Inactive ubiquitin	32.50	0.00
TRINITY_sp|P871	not1	General negative re	32.50	0.00
TRINITY_sp|Q6NR	dcaf8	DDB1- and CUL4-asso	32.50	0.00
TRINITY_sp|Q54B	DDB_G02	LIMR family protein	32.50	0.00
TRINITY_sp|P187	RCC1	Regulator of chromo	32.50	0.00
TRINITY_sp|P406	HMGB3	High mobility group	32.50	0.00
TRINITY_sp|O810	SPP	Signal peptide pept	32.50	0.00
TRINITY_sp|Q5BG	mesA	Protein mesA OS=Eme	32.50	0.00
TRINITY_sp|Q5W7	STAR2	UPF0014 membrane pr	32.50	0.00
TRINITY_sp|Q6PF	Prorsd1	Prolyl-tRNA synthet	32.50	0.00
TRINITY_sp|P117	CYP3A6	Cytochrome P450 3A6	32.50	0.00

TRINITY_sp|Q5PQ(	nudt17	Nucleoside diphosph	32.50	0.00
TRINITY_sp|P945	araB	Ribulokinase OS=Bac	32.50	0.00
TRINITY_sp|Q5ZJ	PIP5K1B	Phosphatidylinosito	32.50	0.00
TRINITY_sp|O144	ssr2	SWI/SNF and RSC com	32.50	0.00
TRINITY_sp|Q3V0(	Garnl3	GTPase-activating R	32.50	0.00
TRINITY_sp|Q133	TARBP1	Probable methyltran	32.50	0.00
TRINITY_sp|O133	TRI11	Isotrichodermin C-1	32.50	0.00
TRINITY_sp|Q8RW	PUX4	Plant UBX domain-co	32.50	0.00
TRINITY_sp|Q8GU	At3g526	Nicastrin OS=Arabid	32.50	0.00
TRINITY_sp|Q9Y6	ARFGEF2	Brefeldin A-inhibit	32.50	0.00
TRINITY_sp|Q58D	DNAJB12	DnaJ homolog subfam	32.50	0.00
TRINITY_sp|Q7TX	fadD29	Long-chain-fatty-ac	32.50	0.00
TRINITY_sp|Q5ZK	RRP12	RRP12-like protein	32.50	0.00
TRINITY_sp|P940	RBCMT	Ribulose-1,5 bispho	32.50	0.00
TRINITY_sp|Q5SM	TTHA106	Magnesium transport	32.50	0.00
TRINITY_sp|Q496	Elp2	Elongator complex p	32.50	0.00
TRINITY_sp|Q9Y5	GTF3C3	General transcripti	32.50	0.00
TRINITY_sp|Q54G	acox1	Peroxisomal acyl-co	32.50	0.00
TRINITY_sp|Q9CR	Ccdc96	Coiled-coil domain-	32.50	0.00
TRINITY_sp|Q91W	Daglb	Sn1-specific diacyl	32.50	0.00
TRINITY_sp|Q9MA	ACBP4	Acyl-CoA-binding do	32.50	0.00
TRINITY_sp|Q9FW	GAMMACA	Gamma carbonic anhy	32.50	0.00
TRINITY_sp|Q020	-	Zinc-binding protei	32.50	0.00
TRINITY_sp|Q9Y7	SPCC645.	Putative CCA tRNA n	32.40	0.00
TRINITY_sp|P341(	pkaD	Developmentally-reg	32.40	0.00
TRINITY_sp|B8AS	OsI_167	Vacuolar-processing	32.40	0.00
TRINITY_sp|Q212	K07E3.7	Probable cation-tra	32.40	0.00
TRINITY_sp|P0CA	TTLL3B	Tubulin glycylase 3	32.40	0.00
TRINITY_sp|Q568	cfap161	Cilia- and flagella	32.40	0.00
TRINITY_sp|B2GV	Ufl1	E3 UFM1-protein lig	32.40	0.00
TRINITY_sp|O044	ECI1	Enoyl-CoA delta iso	32.40	0.00
TRINITY_sp|P195	-	Lipase OS=Rhizomuco	32.40	0.00
TRINITY_sp|Q6H7	CIPK26	CBL-interacting pro	32.40	0.00
TRINITY_sp|Q54C	vti1A	Vesicle transport t	32.40	0.00
TRINITY_sp|Q8CG	Rbm28	RNA-binding protein	32.40	0.00
TRINITY_sp|Q1QE	lplA	Lipoate-protein lig	32.40	0.00
TRINITY_sp|Q54D	DDB_G02	Serine carboxypepti	32.40	0.00
TRINITY_sp|O352	Aoah	Acyloxyacyl hydrola	32.40	0.00
TRINITY_sp|Q9C5	AHK4	Histidine kinase 4	32.40	0.00
TRINITY_sp|Q8RX	At1g068	Uncharacterized mem	32.40	0.00
TRINITY_sp|O605	BUB1B	Mitotic checkpoint	32.40	0.00
TRINITY_sp|Q86X	IQCA1	IQ and AAA domain-c	32.40	0.00
TRINITY_sp|Q5D1	GBP1	Guanylate-binding p	32.40	0.00
TRINITY_sp|Q76P	DDB_G02	Probable serine/thr	32.40	0.00
TRINITY_sp|Q9LR	BSU1	Serine/threonine-pr	32.40	0.00
TRINITY_sp|E1BV	HENMT1	Small RNA 2'-O-meth	32.40	0.00
TRINITY_sp|Q608	Akt2	RAC-beta serine/thr	32.40	0.00
TRINITY_sp|Q9N0	MX2	Interferon-induced	32.40	0.00
TRINITY_sp|Q9FM	At5g140	U3 small nucleolar	32.40	0.00
TRINITY_sp|O343	ytcJ	Putative amidohydro	32.40	0.00
TRINITY_sp|A3KM	RANBP10	Ran-binding protein	32.40	0.00
TRINITY_sp|A8IR	CHLREDR	Ribosome biogenesis	32.40	0.00
TRINITY_sp|Q756	TOM1	Probable E3 ubiquit	32.40	0.00
TRINITY_sp|Q9NP	PNPLA8	Calcium-independent	32.40	0.00
TRINITY_sp|Q920	ACHE	Acetylcholinesteras	32.40	0.00
TRINITY_sp|Q056	cna-1	Serine/threonine-pr	32.40	0.00

TRINITY_sp|Q5UR(	MIMI_L9(	Uncharacterized pro	32.40	0.00
TRINITY_sp|Q8CBI	March11	E3 ubiquitin-protei	32.40	0.00
TRINITY_sp|Q10L7	VLN2	Villin-2 OS=Oryza s	32.40	0.00
TRINITY_sp|Q9M3(	ATM	Serine/threonine-pr	32.40	0.00
TRINITY_sp|O9571	HERC2	E3 ubiquitin-protei	32.40	0.00
TRINITY_sp|Q6DRI	abhd11	Protein ABHD11 OS=D	32.40	0.00
TRINITY_sp|Q924V	Smc6	Structural maintena	32.40	0.00
TRINITY_sp|Q54P(	gefA	Ras guanine nucleot	32.40	0.00
TRINITY_sp|A8WYI	par-1	Serine/threonine-pr	32.40	0.00
TRINITY_sp|O2975	AF_0491	Ribosome maturation	32.40	0.00
TRINITY_sp|Q680(	TFB2	RNA polymerase II t	32.40	0.00
TRINITY_sp|P5628	tif225	Probable translatio	32.40	0.00
TRINITY_sp|Q55C7	sec31	Protein transport p	32.40	0.00
TRINITY_sp|Q5TC>	MLK4	Mitogen-activated p	32.40	0.00
TRINITY_sp|Q91WI	Ascc2	Activating signal c	32.40	0.00
TRINITY_sp|Q9FI2	NIP4-1	Putative aquaporin	32.40	0.00
TRINITY_sp|P4252	erkA	Extracellular signa	32.40	0.00
TRINITY_sp|Q54U(	kif3	Kinesin-related pro	32.40	0.00
TRINITY_sp|P803(	-	Crustacean calcium-	32.40	0.00
TRINITY_sp|Q5XG8	PAPD7	Non-canonical poly(	32.40	0.00
TRINITY_sp|Q70C(	USP34	Ubiquitin carboxyl-	32.40	0.00
TRINITY_sp|Q9NU2	MDN1	Midasin OS=Homo sap	32.40	0.00
TRINITY_sp|Q9VKI	ial	Aurora kinase B OS=	32.40	0.00
TRINITY_sp|B3EW2	-	Hephaestin-like pro	32.40	0.00
TRINITY_sp|Q9ZT(	RKS1	G-type lectin S-rec	32.40	0.00
TRINITY_sp|A4UM(	TFIP11	Tuftelin-interactin	32.40	0.00
TRINITY_sp|Q4PA5	CLU1	Clustered mitochond	32.30	0.00
TRINITY_sp|B0M0I	gefL	Ras guanine nucleot	32.30	0.00
TRINITY_sp|Q8N6I	METTL13	Methyltransferase-l	32.30	0.00
TRINITY_sp|P091(	P4HB	Protein disulfide-i	32.30	0.00
TRINITY_sp|P2533	YGL010W	Uncharacterized end	32.30	0.00
TRINITY_sp|P212(	GSTM3	Glutathione S-trans	32.30	0.00
TRINITY_sp|Q9ULI	PLEKHG1	Pleckstrin homology	32.30	0.00
TRINITY_sp|P2322	fixL	Sensor protein FixL	32.30	0.00
TRINITY_sp|P2739	sol	Calpain-D OS=Drosop	32.30	0.00
TRINITY_sp|O974(	-	Polygalacturonase O	32.30	0.00
TRINITY_sp|P583(	arcB	Aerobic respiration	32.30	0.00
TRINITY_sp|Q86A7	DDB_G027	Recoverin family pr	32.30	0.00
TRINITY_sp|Q55C4	gtaG	GATA zinc finger do	32.30	0.00
TRINITY_sp|Q7TP9	Rps6ka6	Ribosomal protein S	32.30	0.00
TRINITY_sp|Q54TI	mybC	Myb-like protein C	32.30	0.00
TRINITY_sp|Q1252	SET6	Potential protein l	32.30	0.00
TRINITY_sp|Q5W6(	MAN5	Putative mannan end	32.30	0.00
TRINITY_sp|Q5JJY	Os01g054	Protein kinase and	32.30	0.00
TRINITY_sp|Q6ZF2	ADL1	Calpain-type cystei	32.30	0.00
TRINITY_sp|Q6NY9	rngtt	mRNA-capping enzyme	32.30	0.00
TRINITY_sp|Q6DDV	gatsl2	GATS-like protein 2	32.30	0.00
TRINITY_sp|Q9SR1	RAF1.2	Rubisco accumulatio	32.30	0.00
TRINITY_sp|F4JTI	STY46	Serine/threonine-pr	32.30	0.00
TRINITY_sp|Q8H07	UPL2	E3 ubiquitin-protei	32.30	0.00
TRINITY_sp|Q4268	GLN1	Glutamine synthetas	32.30	0.00
TRINITY_sp|P0CE9	talA	Talin-A OS=Dictyost	32.30	0.00
TRINITY_sp|O758(	CAPN15	Calpain-15 OS=Homo	32.30	0.00
TRINITY_sp|A9AB3	rnz	Ribonuclease Z OS=M	32.30	0.00
TRINITY_sp|Q99PI	Arl4d	ADP-ribosylation fa	32.30	0.00
TRINITY_sp|Q1ZXI	DDB_G028	Sphingomyelinase DD	32.30	0.00

TRINITY_sp|Q9LD\	FKBP17-2	Peptidyl-prolyl cis	32.30	0.00
TRINITY_sp|Q2RBI	CIPK15	CBL-interacting pro	32.30	0.00
TRINITY_sp|Q9SVI	At4g1378	Methionine--tRNA li	32.30	0.00
TRINITY_sp|Q8IUI	CBWD2	COBW domain-contain	32.30	0.00
TRINITY_sp|Q8W4S	VHA-a3	V-type proton ATPas	32.30	0.00
TRINITY_sp|Q54Q9	DDB_G028	LMBR1 domain-contai	32.30	0.00
TRINITY_sp|Q8VX2	XEG113	Arabinosyltransfera	32.30	0.00
TRINITY_sp|Q6CF3	CBK1	Serine/threonine-pr	32.30	0.00
TRINITY_sp|Q6T48	rbrA	Probable E3 ubiquit	32.30	0.00
TRINITY_sp|P5178	CLCN2	Chloride channel pr	32.30	0.00
TRINITY_sp|Q54R2	DDB_G028	Probable serine/thr	32.30	0.00
TRINITY_sp|Q5UQ2	MIMI_R88	Uncharacterized pro	32.30	0.00
TRINITY_sp|Q5ZLI	CSNK1E	Casein kinase I iso	32.30	0.00
TRINITY_sp|O0760	yhfK	Uncharacterized sug	32.30	0.00
TRINITY_sp|P9WMY	MT3116	Glycogen synthase O	32.30	0.00
TRINITY_sp|Q55GU	DDB_G026	Probable serine/thr	32.30	0.00
TRINITY_sp|Q9M2I	At3g6132	UPF0187 protein At3	32.30	0.00
TRINITY_sp|Q9VMJ	lid	Lysine-specific dem	32.30	0.00
TRINITY_sp|Q554I	DDB_G027	Probable inactive s	32.30	0.00
TRINITY_sp|B7Q5I	ISCW0218	Queuine tRNA-ribosy	32.30	0.00
TRINITY_sp|Q9LYW	LUL1	Probable E3 ubiquit	32.30	0.00
TRINITY_sp|P3875	YHL008C	Uncharacterized tra	32.30	0.00
TRINITY_sp|P5398	YNL011C	Uncharacterized pro	32.30	0.00
TRINITY_sp|Q96ST	SIN3A	Paired amphipathic	32.30	0.00
TRINITY_sp|Q9KLI	luxQ	Autoinducer 2 senso	32.30	0.00
TRINITY_sp|B2GU\	Man1b1	Endoplasmic reticul	32.30	0.00
TRINITY_sp|Q017T	GLX	Aldehyde oxidase GL	32.30	0.00
TRINITY_sp|Q0304	Pkg21D	cGMP-dependent prot	32.30	0.00
TRINITY_sp|Q9NKW	mfeA	Peroxisomal multifu	32.30	0.00
TRINITY_sp|Q9975	ABCA3	ATP-binding cassett	32.30	0.00
TRINITY_sp|Q8BK0	Ipo5	Importin-5 OS=Mus m	32.30	0.00
TRINITY_sp|Q6L4\	MSH5	DNA mismatch repair	32.30	0.00
TRINITY_sp|P0096	glnA	Glutamine synthetas	32.30	0.00
TRINITY_sp|Q54LI	vps13A	Putative vacuolar p	32.30	0.00
TRINITY_sp|Q9SG0	RDR6	RNA-dependent RNA p	32.30	0.00
TRINITY_sp|Q54R\	sglA	Sphingosine-1-phosp	32.20	0.00
TRINITY_sp|Q9SLA	At2g2562	Probable protein ph	32.20	0.00
TRINITY_sp|P5972	hif1an	Hypoxia-inducible f	32.20	0.00
TRINITY_sp|Q54P\	eif2a	Eukaryotic translat	32.20	0.00
TRINITY_sp|P5972	hif1an	Hypoxia-inducible f	32.20	0.00
TRINITY_sp|P0709	LIPF	Gastric triacylglyc	32.20	0.00
TRINITY_sp|O1882	AOAH	Acyloxyacyl hydrola	32.20	0.00
TRINITY_sp|P3410	pkgC	Protein kinase 3 OS	32.20	0.00
TRINITY_sp|Q9Y0A	itr-1	Inositol 1,4,5-tris	32.20	0.00
TRINITY_sp|Q5ZJH	ULK3	Serine/threonine-pr	32.20	0.00
TRINITY_sp|P7582	hcr	NADH oxidoreductase	32.20	0.00
TRINITY_sp|O2832	AF_1954	Putative amidase AF	32.20	0.00
TRINITY_sp|O8050	At2g4466	Probable dolichyl p	32.20	0.00
TRINITY_sp|P8706	tea1	Tip elongation aber	32.20	0.00
TRINITY_sp|Q9SFU	PAP15	Purple acid phospha	32.20	0.00
TRINITY_sp|Q0502	TAF9	Transcription initi	32.20	0.00
TRINITY_sp|Q7TS\	Enkd1	Enkurin domain-cont	32.20	0.00
TRINITY_sp|Q9Z0I	Lipa	Lysosomal acid lipa	32.20	0.00
TRINITY_sp|D2H8\	DCLRE1B	5' exonuclease Apol	32.20	0.00
TRINITY_sp|A8I4I	FAP100	Cilia- and flagella	32.20	0.00
TRINITY_sp|Q8KB\	acsA	Acetyl-coenzyme A s	32.20	0.00

TRINITY_sp|Q9P2I	DISP3	Protein dispatched	32.20	0.00
TRINITY_sp|Q80X(	Usp6nl	USP6 N-terminal-lik	32.20	0.00
TRINITY_sp|O609(	culA	Cullin-1 OS=Dictyos	32.20	0.00
TRINITY_sp|Q3V3(	Lace1	Lactation elevated	32.20	0.00
TRINITY_sp|Q9SK(	ARI10	Probable E3 ubiquit	32.20	0.00
TRINITY_sp|P0AEI	ynaI	Low conductance mec	32.20	0.00
TRINITY_sp|Q9S7(	MKK2	Mitogen-activated p	32.20	0.00
TRINITY_sp|Q1ZXI	gacM	Rho GTPase-activati	32.20	0.00
TRINITY_sp|Q93V(	VCL1	Protein VACUOLELESS	32.20	0.00
TRINITY_sp|Q8BR(	Maats1	Protein MAATS1 OS=M	32.20	0.00
TRINITY_sp|O557(	IIV6-17(	Uncharacterized pro	32.20	0.00
TRINITY_sp|P109(	-	Retrovirus-related	32.20	0.00
TRINITY_sp|Q230(	unc-51	Serine/threonine-pr	32.20	0.00
TRINITY_sp|Q54P(	osbI	Oxysterol-binding p	32.20	0.00
TRINITY_sp|Q55A(	dlpC	Dynamin-like protei	32.20	0.00
TRINITY_sp|P831(	Dyrk3	Putative dual speci	32.20	0.00
TRINITY_sp|Q54P(	osbI	Oxysterol-binding p	32.20	0.00
TRINITY_sp|Q101(	trz1	Ribonuclease Z 1 OS	32.20	0.00
TRINITY_sp|P0C6(	Prkg1	cGMP-dependent prot	32.20	0.00
TRINITY_sp|Q094(	dpff-1	Zinc finger protein	32.20	0.00
TRINITY_sp|O488(	LUH	Transcriptional cor	32.20	0.00
TRINITY_sp|A8X7(	dlk-1	Mitogen-activated p	32.20	0.00
TRINITY_sp|O153(	INPPL1	Phosphatidylinosito	32.20	0.00
TRINITY_sp|Q02P(	PA14_24(	Probable lipid kina	32.20	0.00
TRINITY_sp|A8JA(	DRC7	Dynein regulatory c	32.20	0.00
TRINITY_sp|Q5TC(	GRTP1	Growth hormone-regu	32.20	0.00
TRINITY_sp|Q9SL(	At2g200(	Protein phosphatase	32.20	0.00
TRINITY_sp|P735(	slr0889	Uncharacterized pro	32.20	0.00
TRINITY_sp|Q182(	ula-1	NEDD8-activating en	32.20	0.00
TRINITY_sp|Q8GY(	ULP1A	Ubiquitin-like-spec	32.10	0.00
TRINITY_sp|Q9ZT(	ATE1	Arginyl-tRNA--prote	32.10	0.00
TRINITY_sp|A2CE(	piwil2	Piwi-like protein 2	32.10	0.00
TRINITY_sp|Q91X(	Osbpl1a	Oxysterol-binding p	32.10	0.00
TRINITY_sp|Q9DB(	Cyp4v2	Cytochrome P450 4V2	32.10	0.00
TRINITY_sp|Q9CR(	Exosc5	Exosome complex com	32.10	0.00
TRINITY_sp|O811(	ETR1	Ethylene receptor O	32.10	0.00
TRINITY_sp|G3XM(	azaE	Ketoreductase azaE	32.10	0.00
TRINITY_sp|Q9NR(	PRTFDC1	Phosphoribosyltrans	32.10	0.00
TRINITY_sp|Q0JI(	CIPK11	CBL-interacting pro	32.10	0.00
TRINITY_sp|Q095(	C45G9.2	Uncharacterized tRN	32.10	0.00
TRINITY_sp|Q54V(	fhkE	Probable serine/thr	32.10	0.00
TRINITY_sp|Q0JI(	CIPK11	CBL-interacting pro	32.10	0.00
TRINITY_sp|Q234(	asm-2	Sphingomyelin phosp	32.10	0.00
TRINITY_sp|Q9NE(	pph-5	Serine/threonine-pr	32.10	0.00
TRINITY_sp|B5X8(	brcc3	Lys-63-specific deu	32.10	0.00
TRINITY_sp|Q3SY(	PSMD8	26S proteasome non-	32.10	0.00
TRINITY_sp|Q920(	Ak5	Adenylate kinase is	32.10	0.00
TRINITY_sp|Q55C(	gdt2	Probable serine/thr	32.10	0.00
TRINITY_sp|B3FW(	hpm2	Glutathione S-trans	32.10	0.00
TRINITY_sp|B9DH(	ARIA	ARM REPEAT PROTEIN	32.10	0.00
TRINITY_sp|Q8C7(	Uba6	Ubiquitin-like modi	32.10	0.00
TRINITY_sp|Q9DB(	Cyp4v2	Cytochrome P450 4V2	32.10	0.00
TRINITY_sp|F4IN(	GGP4	Gamma-glutamyl pept	32.10	0.00
TRINITY_sp|Q54R(	dhkL	Hybrid signal trans	32.10	0.00
TRINITY_sp|O225(	STY8	Serine/threonine-pr	32.10	0.00
TRINITY_sp|P219(	glf	Glucose facilitated	32.10	0.00

TRINITY_sp|Q8CI3	Cwf19l1	CWF19-like protein	32.10	0.00
TRINITY_sp|Q6YY7	NEK6	Serine/threonine-pr	32.10	0.00
TRINITY_sp|Q74Z9	GPI13	GPI ethanolamine ph	32.10	0.00
TRINITY_sp|Q9CS7	Ecd	Protein ecdysoneles	32.10	0.00
TRINITY_sp|O7580	CAPN15	Calpain-15 OS=Homo	32.10	0.00
TRINITY_sp|D4GP3	HVO_B002	D-xylose 1-dehydrog	32.10	0.00
TRINITY_sp|P3901	MPT5	Suppressor protein	32.10	0.00
TRINITY_sp|Q9TW2	myoM	Myosin-M heavy chai	32.10	0.00
TRINITY_sp|Q0979	kri1	Protein kri1 OS=Sch	32.10	0.00
TRINITY_sp|Q8K0U	Hspa12a	Heat shock 70 kDa p	32.10	0.00
TRINITY_sp|Q9Y2W	KCNIP3	Calsenilin OS=Homo	32.10	0.00
TRINITY_sp|Q7KWM	iunH	Inosine-uridine-pre	32.10	0.00
TRINITY_sp|Q5953	ptrB	Protease 2 OS=Morax	32.10	0.00
TRINITY_sp|P4981	TSC2	Tuberin OS=Homo sap	32.10	0.00
TRINITY_sp|Q8R0F	Sec14l4	SEC14-like protein	32.10	0.00
TRINITY_sp|O2217	PAB4	Polyadenylate-bindi	32.10	0.00
TRINITY_sp|P2529	PHO84	Inorganic phosphate	32.10	0.00
TRINITY_sp|Q8NDN	RCBTB1	RCC1 and BTB domain	32.10	0.00
TRINITY_sp|Q10KY	CAMK1	Calcium/calmodulin-	32.10	0.00
TRINITY_sp|O6244	Y43F4A.1	Leishmanolysin-like	32.10	0.00
TRINITY_sp|Q54LF	abcC5	ABC transporter C f	32.10	0.00
TRINITY_sp|Q9RR1	menG	Demethylmenaquinone	32.10	0.00
TRINITY_sp|Q9FGI	RKD3	Protein RKD3 OS=Ara	32.10	0.00
TRINITY_sp|Q55GU	DDB_G026	Probable serine/thr	32.10	0.00
TRINITY_sp|Q0JI4	CIPK11	CBL-interacting pro	32.10	0.00
TRINITY_sp|F4HYJ	At1g4865	DExH-box ATP-depend	32.10	0.00
TRINITY_sp|Q8LPT	R1	Alpha-glucan water	32.10	0.00
TRINITY_sp|Q2KI0	PHOSPHO2	Pyridoxal phosphate	32.10	0.00
TRINITY_sp|B0TA5	rplI	50S ribosomal prote	32.10	0.00
TRINITY_sp|Q6ES1	HATB	Probable histone ac	32.10	0.00
TRINITY_sp|O0040	SLC33A1	Acetyl-coenzyme A t	32.10	0.00
TRINITY_sp|Q9JJK	Lancl2	LanC-like protein 2	32.10	0.00
TRINITY_sp|Q9XFM	MSBP1	Membrane steroid-bi	32.10	0.00
TRINITY_sp|Q54D1	abcA9	ABC transporter A f	32.10	0.00
TRINITY_sp|Q8S1X	Os01g092	Probable glucuronos	32.10	0.00
TRINITY_sp|Q54T9	yipf1	Protein YIPF1 homol	32.10	0.00
TRINITY_sp|O0274	GUCY2F	Retinal guanylyl cy	32.10	0.00
TRINITY_sp|O1917	UNC119	Protein unc-119 hom	32.10	0.00
TRINITY_sp|Q55A0	DDB_G027	Probable serine/thr	32.10	0.00
TRINITY_sp|Q0440	Adcy5	Adenylate cyclase t	32.10	0.00
TRINITY_sp|Q8LQ9	GLU8	Endoglucanase 3 OS=	32.10	0.00
TRINITY_sp|Q9H0E	TOLLIP	Toll-interacting pr	32.00	0.00
TRINITY_sp|Q6DE7	slc25a38	Solute carrier fami	32.00	0.00
TRINITY_sp|C4R19	VPS10	Vacuolar protein so	32.00	0.00
TRINITY_sp|O3501	yogA	Uncharacterized zin	32.00	0.00
TRINITY_sp|A0PQ2	MUL_2009	Probable phthiotrio	32.00	0.00
TRINITY_sp|Q6GP5	naa50	N-alpha-acetyltrans	32.00	0.00
TRINITY_sp|A8GVI	rplO	50S ribosomal prote	32.00	0.00
TRINITY_sp|Q1670	MAN2A1	Alpha-mannosidase 2	32.00	0.00
TRINITY_sp|P2967	SF3	Pollen-specific pro	32.00	0.00
TRINITY_sp|O7481	pet127	mRNA degradation pr	32.00	0.00
TRINITY_sp|O5253	cah	Carbonic anhydrase	32.00	0.00
TRINITY_sp|Q5F3A	YOD1	Ubiquitin thioester	32.00	0.00
TRINITY_sp|Q9VDI	meigo	Solute carrier fami	32.00	0.00
TRINITY_sp|Q0041	SEN1	Helicase SEN1 OS=Sa	32.00	0.00
TRINITY_sp|Q8K35	Pigu	Phosphatidylinosito	32.00	0.00

TRINITY_sp|Q8WU1	SLC25A43	Solute carrier fami	32.00	0.00
TRINITY_sp|Q9LQ(	RAD51D	DNA repair protein	32.00	0.00
TRINITY_sp|O745(	SPCC970.	Uncharacterized ino	32.00	0.00
TRINITY_sp|P546	pikD	Phosphatidylinosito	32.00	0.00
TRINITY_sp|Q9SJN	CML31	Probable calcium-bi	32.00	0.00
TRINITY_sp|Q5XI\	March10	Probable E3 ubiquit	32.00	0.00
TRINITY_sp|P231	todF	2-hydroxy-6-oxo-2,4	32.00	0.00
TRINITY_sp|Q9VC	CG7048	Probable prefoldin	32.00	0.00
TRINITY_sp|P0CM	CWC25	Pre-mRNA-splicing f	32.00	0.00
TRINITY_sp|P538	PDR17	Phosphatidylinosito	32.00	0.00
TRINITY_sp|Q1ZXI	DDB_G02	PH domain-containin	32.00	0.00
TRINITY_sp|Q54D(	DDB_G02	von Willebrand fact	32.00	0.00
TRINITY_sp|Q9SK(	ARI10	Probable E3 ubiquit	32.00	0.00
TRINITY_sp|O137	SPAC17A	Putative metal ion	32.00	0.00
TRINITY_sp|O647	At2g301	Probable protein ph	32.00	0.00
TRINITY_sp|P0CM	ATG2	Autophagy-related p	32.00	0.00
TRINITY_sp|Q163	PDCD2	Programmed cell dea	32.00	0.00
TRINITY_sp|P426(	VCP	Vitellogenic carbox	32.00	0.00
TRINITY_sp|Q86C	dhkK	Hybrid signal trans	32.00	0.00
TRINITY_sp|Q9C0(	DNAH6	Dynein heavy chain	32.00	0.00
TRINITY_sp|Q82D	SAV_512	UPF0301 protein SAV	32.00	0.00
TRINITY_sp|O044	At1g096	Probable elongation	32.00	0.00
TRINITY_sp|Q6ET	Os02g01	Probable protein ph	32.00	0.00
TRINITY_sp|Q80X	Ttc28	Tetratricopeptide r	32.00	0.00
TRINITY_sp|O758(	CAPN15	Calpain-15 OS=Homo	32.00	0.00
TRINITY_sp|Q8BL(	Slc25a2	Mitochondrial basic	32.00	0.00
TRINITY_sp|Q9ESN	Sel1l	Protein sel-1 homol	32.00	0.00
TRINITY_sp|Q96H(	SGSM3	Small G protein sig	32.00	0.00
TRINITY_sp|Q9SR	LPA1	Protein LOW PSII AC	32.00	0.00
TRINITY_sp|Q9H9	MARCH7	E3 ubiquitin-protei	32.00	0.00
TRINITY_sp|C9WM	-	Venom serine carbox	32.00	0.00
TRINITY_sp|Q8NF	BBS1	Bardet-Biedl syndro	32.00	0.00
TRINITY_sp|P358	BDF1	Bromodomain-contain	32.00	0.00
TRINITY_sp|Q7LH(	TY3B-I	Transposon Ty3-I Ga	32.00	0.00
TRINITY_sp|Q93W	NFS2	Cysteine desulfuras	32.00	0.00
TRINITY_sp|Q5ZJ	ULK3	Serine/threonine-pr	32.00	0.00
TRINITY_sp|Q6T3(	Npc1l1	Niemann-Pick C1-lik	32.00	0.00
TRINITY_sp|Q9FE	DSP4	Phosphoglucan phosp	32.00	0.00
TRINITY_sp|Q628	Pcsk7	Proprotein converta	32.00	0.00
TRINITY_sp|F4IVI	GRV2	DnaJ homolog subfam	32.00	0.00
TRINITY_sp|Q9W4	dnc	cAMP-specific 3',5'	32.00	0.00
TRINITY_sp|Q6U1	CMT1	Alpha-1,3-mannosylt	32.00	0.00
TRINITY_sp|P436	RMD8	Sporulation protein	32.00	0.00
TRINITY_sp|Q86U	N4BP2	NEDD4-binding prote	32.00	0.00
TRINITY_sp|O149	PLD2	Phospholipase D2 OS	31.90	0.00
TRINITY_sp|O749	toa2	Transcription initi	31.90	0.00
TRINITY_sp|Q3UR	Tgfbrap	Transforming growth	31.90	0.00
TRINITY_sp|Q54M\	mrkB	Probable serine/thr	31.90	0.00
TRINITY_sp|Q6IM	alrA	Aldose reductase A	31.90	0.00
TRINITY_sp|Q091	cds1	Serine/threonine-pr	31.90	0.00
TRINITY_sp|Q550	DDB_G02	Probable serine/thr	31.90	0.00
TRINITY_sp|O075	yhdW	Putative glyceropho	31.90	0.00
TRINITY_sp|Q9WU	Pus1	tRNA pseudouridine	31.90	0.00
TRINITY_sp|Q75W	Vwa5a	von Willebrand fact	31.90	0.00
TRINITY_sp|P774	yqaB	Fructose-1-phosphat	31.90	0.00
TRINITY_sp|Q9CQ(	Tctex1d	Tctex1 domain-conta	31.90	0.00

TRINITY_sp|P351(	resA	Thiol-disulfide oxi	31.90	0.00
TRINITY_sp|Q0JI4	CIPK11	CBL-interacting pro	31.90	0.00
TRINITY_sp|Q4R7I	TTLL10	Protein polyglycyla	31.90	0.00
TRINITY_sp|O888	Acsl5	Long-chain-fatty-ac	31.90	0.00
TRINITY_sp|Q54E	abpF	Actin-binding prote	31.90	0.00
TRINITY_sp|P906	-	Actin OS=Brugia mal	31.90	0.00
TRINITY_sp|B7U1	ABAP1	ARMADILLO BTB ARABI	31.90	0.00
TRINITY_sp|Q54E	grlE	Metabotropic glutam	31.90	0.00
TRINITY_sp|P168	gpaA	Guanine nucleotide-	31.90	0.00
TRINITY_sp|Q2G1	plc	1-phosphatidylinosi	31.90	0.00
TRINITY_sp|P427	dmpM	O-demethylpuromycin	31.90	0.00
TRINITY_sp|P428	ECE1	Endothelin-converti	31.90	0.00
TRINITY_sp|Q424	CPK3	Calcium-dependent p	31.90	0.00
TRINITY_sp|Q550	DDB_G02	Probable serine/thr	31.90	0.00
TRINITY_sp|Q8N5	DHRSX	Dehydrogenase/reduc	31.90	0.00
TRINITY_sp|O519	fes	Enterochelin estera	31.90	0.00
TRINITY_sp|P200	Hexb	Beta-hexosaminidase	31.90	0.00
TRINITY_sp|Q9K9	aceA	Isocitrate lyase OS	31.90	0.00
TRINITY_sp|A8I4	FAP100	Cilia- and flagella	31.90	0.00
TRINITY_sp|Q7JW	CG11007	Thioredoxin-related	31.90	0.00
TRINITY_sp|Q2XV	scn4ab	Sodium channel prot	31.90	0.00
TRINITY_sp|Q9JL	Capn15	Calpain-15 OS=Mus m	31.90	0.00
TRINITY_sp|Q556	bip1-1	Luminal-binding pro	31.90	0.00
TRINITY_sp|Q9DC	Ethe1	Persulfide dioxygen	31.90	0.00
TRINITY_sp|Q8BR	Maats1	Protein MAATS1 OS=M	31.90	0.00
TRINITY_sp|Q9JI	Sphk2	Sphingosine kinase	31.90	0.00
TRINITY_sp|Q564	CAPH	Condensin complex s	31.90	0.00
TRINITY_sp|Q9ZG	pikAI	Narbonolide/10-deox	31.90	0.00
TRINITY_sp|Q8VY	SWI3D	SWI/SNF complex sub	31.90	0.00
TRINITY_sp|O138	utp17	U3 small nucleolar	31.90	0.00
TRINITY_sp|P0C1	Os05g01	Endoglucanase 14 OS	31.90	0.00
TRINITY_sp|Q9LV	TTL	Uric acid degradati	31.90	0.00
TRINITY_sp|Q4PF	UMAG_01	Eukaryotic translat	31.90	0.00
TRINITY_sp|Q55G	abkC	Probable serine/thr	31.90	0.00
TRINITY_sp|Q8LL	REP	Rab escort protein	31.90	0.00
TRINITY_sp|O188	AOAH	Acyloxyacyl hydrola	31.90	0.00
TRINITY_sp|P182	Npr1	Atrial natriuretic	31.90	0.00
TRINITY_sp|Q9V0	tuf	Elongation factor 1	31.90	0.00
TRINITY_sp|F4IL	At2g359	DExH-box ATP-depend	31.90	0.00
TRINITY_sp|P044	-	Calmodulin OS=Triti	31.90	0.00
TRINITY_sp|O755	RPS6KA5	Ribosomal protein S	31.80	0.00
TRINITY_sp|O154	ABCC4	Multidrug resistanc	31.80	0.00
TRINITY_sp|Q6T9	fibpb	Acidic fibroblast g	31.80	0.00
TRINITY_sp|F4IP	SGR6	Protein SHOOT GRAVI	31.80	0.00
TRINITY_sp|Q5VQ	PPT3	Phosphoenolpyruvate	31.80	0.00
TRINITY_sp|Q8C4	Pex5l	PEX5-related protei	31.80	0.00
TRINITY_sp|Q9BS	ESYT1	Extended synaptotag	31.80	0.00
TRINITY_sp|P505	vps34	Phosphatidylinosito	31.80	0.00
TRINITY_sp|Q5JL	CIPK12	CBL-interacting pro	31.80	0.00
TRINITY_sp|Q6P6	nup35	Nucleoporin NUP53 O	31.80	0.00
TRINITY_sp|Q32P	RANGRF	Ran guanine nucleot	31.80	0.00
TRINITY_sp|P319	STIP1	Stress-induced-phos	31.80	0.00
TRINITY_sp|Q110	tmbi-4	Transmembrane BAX i	31.80	0.00
TRINITY_sp|Q9QY	Dnajb1	DnaJ homolog subfam	31.80	0.00
TRINITY_sp|O621	tag-175	Transmembrane prote	31.80	0.00
TRINITY_sp|Q61Y	CBG0355	Leishmanolysin-like	31.80	0.00

TRINITY_sp|O043[illegible]	NSP3	Nitrile-specifier p	31.80	0.00
TRINITY_sp|Q111[illegible]	ubp14	Ubiquitin carboxyl-	31.80	0.00
TRINITY_sp|Q7TM[illegible]	Eri1	3'-5' exoribonuclea	31.80	0.00
TRINITY_sp|P306[illegible]	svop-1	Putative transporte	31.80	0.00
TRINITY_sp|Q54D[illegible]	DDB_G02[illegible]	Serine carboxypepti	31.80	0.00
TRINITY_sp|Q559[illegible]	DDB_G02[illegible]	Probable serine/thr	31.80	0.00
TRINITY_sp|O428[illegible]	bub3	Mitotic checkpoint	31.80	0.00
TRINITY_sp|Q5R5[illegible]	SERINC3	Serine incorporator	31.80	0.00
TRINITY_sp|Q8RV[illegible]	DEK1	Calpain-type cystei	31.80	0.00
TRINITY_sp|Q9LV[illegible]	ARI4	Putative E3 ubiquit	31.80	0.00
TRINITY_sp|P581[illegible]	PRKAG1	5'-AMP-activated pr	31.80	0.00
TRINITY_sp|Q9SI[illegible]	TMK3	Receptor-like kinas	31.80	0.00
TRINITY_sp|Q5XF[illegible]	Atp13a3	Probable cation-tra	31.80	0.00
TRINITY_sp|Q7LH[illegible]	TY3B-I	Transposon Ty3-I Ga	31.80	0.00
TRINITY_sp|Q8LC[illegible]	At4g296[illegible]	Thioredoxin-like 2-	31.80	0.00
TRINITY_sp|Q0P4[illegible]	srd5a3	Polyprenol reductas	31.80	0.00
TRINITY_sp|Q54L[illegible]	vps13A	Putative vacuolar p	31.80	0.00
TRINITY_sp|Q75J[illegible]	cpras1	Circularly permutat	31.80	0.00
TRINITY_sp|Q9NW[illegible]	RBM28	RNA-binding protein	31.80	0.00
TRINITY_sp|Q9UT[illegible]	SPAC824.	Uncharacterized pro	31.80	0.00
TRINITY_sp|Q553[illegible]	gxcJJ	Rac guanine nucleot	31.80	0.00
TRINITY_sp|Q9US[illegible]	tim23	Mitochondrial impor	31.80	0.00
TRINITY_sp|P509[illegible]	AVT4	Vacuolar amino acid	31.80	0.00
TRINITY_sp|Q6FQ[illegible]	PKAR	cAMP-dependent prot	31.80	0.00
TRINITY_sp|Q6NX[illegible]	Rcbtb1	RCC1 and BTB domain	31.80	0.00
TRINITY_sp|Q9LU[illegible]	PNSB3	Photosynthetic NDH	31.80	0.00
TRINITY_sp|F4JJ[illegible]	ENDO4	Endonuclease 4 OS=A	31.80	0.00
TRINITY_sp|P531[illegible]	ROG1	Putative lipase ROG	31.80	0.00
TRINITY_sp|Q54T[illegible]	xpnpep3	Probable Xaa-Pro am	31.80	0.00
TRINITY_sp|Q9LH[illegible]	LPAT5	Probable 1-acyl-sn-	31.80	0.00
TRINITY_sp|P041[illegible]	GIP	Copia protein OS=Dr	31.80	0.00
TRINITY_sp|F4JE[illegible]	NSE4B	Non-structural main	31.80	0.00
TRINITY_sp|Q9UU[illegible]	gcn5	Histone acetyltrans	31.80	0.00
TRINITY_sp|Q9CX[illegible]	Dhrs7	Dehydrogenase/reduc	31.80	0.00
TRINITY_sp|Q9SY[illegible]	HEXO2	Beta-hexosaminidase	31.80	0.00
TRINITY_sp|Q9UN[illegible]	ABCG2	ATP-binding cassett	31.80	0.00
TRINITY_sp|Q55E[illegible]	gins4	DNA replication com	31.70	0.00
TRINITY_sp|Q06A[illegible]	RAB1B	Ras-related protein	31.70	0.00
TRINITY_sp|Q8N4[illegible]	RNF175	RING finger protein	31.70	0.00
TRINITY_sp|Q9VR[illegible]	HERC2	Probable E3 ubiquit	31.70	0.00
TRINITY_sp|Q9UM[illegible]	FZR1	Fizzy-related prote	31.70	0.00
TRINITY_sp|Q60D[illegible]	TPK3	Thiamine pyrophosph	31.70	0.00
TRINITY_sp|O316[illegible]	metC	Cystathionine beta-	31.70	0.00
TRINITY_sp|Q32L[illegible]	GRINA	Protein lifeguard 1	31.70	0.00
TRINITY_sp|Q70C[illegible]	USP34	Ubiquitin carboxyl-	31.70	0.00
TRINITY_sp|Q54Q[illegible]	wdr89	WD repeat-containin	31.70	0.00
TRINITY_sp|Q54Z[illegible]	tbcb	Tubulin-specific ch	31.70	0.00
TRINITY_sp|Q55G[illegible]	DDB_G02[illegible]	Probable phosphatid	31.70	0.00
TRINITY_sp|Q6FN[illegible]	STE20	Serine/threonine-pr	31.70	0.00
TRINITY_sp|Q9VY[illegible]	Cyp4g15	Cytochrome P450 4g1	31.70	0.00
TRINITY_sp|Q8L7[illegible]	At1g317[illegible]	AP-4 complex subuni	31.70	0.00
TRINITY_sp|Q96M[illegible]	AK7	Adenylate kinase 7	31.70	0.00
TRINITY_sp|Q54X[illegible]	captB	Uncharacterized CDP	31.70	0.00
TRINITY_sp|P583[illegible]	arcB	Aerobic respiration	31.70	0.00
TRINITY_sp|Q5BD[illegible]	sip5	Protein sip5 OS=Eme	31.70	0.00
TRINITY_sp|Q9FZ[illegible]	MAN1	Mannan endo-1,4-bet	31.70	0.00

TRINITY_sp|Q0WKV	ULP2A	Probable ubiquitin-	31.70	0.00
TRINITY_sp|F4JJI	ENDO4	Endonuclease 4 OS=A	31.70	0.00
TRINITY_sp|Q2TXA	sym1	Protein sym1 OS=Asp	31.70	0.00
TRINITY_sp|O1421	trm10	tRNA (guanine(9)-N1	31.70	0.00
TRINITY_sp|O7534	PDCD6	Programmed cell dea	31.70	0.00
TRINITY_sp|Q9HD4	PTPRH	Receptor-type tyros	31.70	0.00
TRINITY_sp|W5EP1	-	2-carboxy-D-arabini	31.70	0.00
TRINITY_sp|P5505	-	Protein disulfide-i	31.70	0.00
TRINITY_sp|O1498	BTAF1	TATA-binding protei	31.70	0.00
TRINITY_sp|O6562	CAD6	Probable cinnamyl a	31.70	0.00
TRINITY_sp|A8ILK	CFAP52	Cilia- and flagella	31.70	0.00
TRINITY_sp|Q54BI	DDB_G029	LIMR family protein	31.70	0.00
TRINITY_sp|P0053	Mos	Proto-oncogene seri	31.70	0.00
TRINITY_sp|Q0352	SCS7	Ceramide very long	31.70	0.00
TRINITY_sp|Q94EI	At3g1441	Probable sugar phos	31.70	0.00
TRINITY_sp|Q8N35	SLC35F6	Solute carrier fami	31.70	0.00
TRINITY_sp|Q9C77	ELP4	Elongator complex p	31.70	0.00
TRINITY_sp|Q0A79	rlmL	Ribosomal RNA large	31.70	0.00
TRINITY_sp|Q6ZTR	CFAP47	Cilia- and flagella	31.70	0.00
TRINITY_sp|Q75J9	cpras1	Circularly permutat	31.70	0.00
TRINITY_sp|Q8L70	SYT5	Synaptotagmin-5 OS=	31.70	0.00
TRINITY_sp|Q5YL0	eglA	Endoglucanase A OS=	31.70	0.00
TRINITY_sp|Q7FA2	LECRK1	G-type lectin S-rec	31.70	0.00
TRINITY_sp|Q55A0	DDB_G027	Probable serine/thr	31.70	0.00
TRINITY_sp|Q8I7R	pol	Retrovirus-related	31.70	0.00
TRINITY_sp|Q90Y0	eef1g	Elongation factor 1	31.70	0.00
TRINITY_sp|A8ITV	CFAP70	Cilia- and flagella	31.70	0.00
TRINITY_sp|Q9C72	TMN6	Transmembrane 9 sup	31.60	0.00
TRINITY_sp|O8856	Abcc3	Canalicular multisp	31.60	0.00
TRINITY_sp|Q9FY0	TMN11	Transmembrane 9 sup	31.60	0.00
TRINITY_sp|Q6H70	CIPK26	CBL-interacting pro	31.60	0.00
TRINITY_sp|Q9STV	CIPK8	CBL-interacting ser	31.60	0.00
TRINITY_sp|Q86TW	ADCK1	Uncharacterized aar	31.60	0.00
TRINITY_sp|P5242	DGKE	Diacylglycerol kina	31.60	0.00
TRINITY_sp|Q55G9	hibch	3-hydroxyisobutyryl	31.60	0.00
TRINITY_sp|Q8IZ6	TRMT2A	tRNA (uracil-5-)-me	31.60	0.00
TRINITY_sp|A1L1R	shq1	Protein SHQ1 homolo	31.60	0.00
TRINITY_sp|Q0VFI	bloc1s1	Biogenesis of lysos	31.60	0.00
TRINITY_sp|Q54P9	osbI	Oxysterol-binding p	31.60	0.00
TRINITY_sp|O0471	MSH6	DNA mismatch repair	31.60	0.00
TRINITY_sp|Q54L0	clcE	Chloride channel pr	31.60	0.00
TRINITY_sp|Q9QYI	Dnajb12	DnaJ homolog subfam	31.60	0.00
TRINITY_sp|P2588	rhlE	ATP-dependent RNA h	31.60	0.00
TRINITY_sp|Q2662	KAP115	Kinesin-associated	31.60	0.00
TRINITY_sp|F7AS2	BTBD3	BTB/POZ domain-cont	31.60	0.00
TRINITY_sp|Q9SC0	cbbX	Protein cbbX homolo	31.60	0.00
TRINITY_sp|Q4Z8R	RanBPM	Ran-binding protein	31.60	0.00
TRINITY_sp|O9467	SPBC776.	Uncharacterized mem	31.60	0.00
TRINITY_sp|Q0215	plc	1-phosphatidylinosi	31.60	0.00
TRINITY_sp|Q6PDJ	Fbxo42	F-box only protein	31.60	0.00
TRINITY_sp|Q9C5U	AHK4	Histidine kinase 4	31.60	0.00
TRINITY_sp|Q9Z10	Vars	Valine--tRNA ligase	31.60	0.00
TRINITY_sp|Q1742	trx-2	Probable thioredoxi	31.60	0.00
TRINITY_sp|Q8LPR	ALPHAC-A	AP-2 complex subuni	31.60	0.00
TRINITY_sp|Q96HR	DOCK6	Dedicator of cytoki	31.60	0.00
TRINITY_sp|O2602	HP_1491	Putative phosphate	31.60	0.00

TRINITY_sp|Q940(	FLU	Protein FLUORESCENT	31.60	0.00
TRINITY_sp|P235‹	-	Ribonuclease MC OS=	31.60	0.00
TRINITY_sp|P981‹	CYP4F8	Cytochrome P450 4F8	31.60	0.00
TRINITY_sp|Q54RN	osbG	Oxysterol-binding p	31.60	0.00
TRINITY_sp|Q9SK(	ARI10	Probable E3 ubiquit	31.60	0.00
TRINITY_sp|P521	NGTPT	Triose phosphate/ph	31.60	0.00
TRINITY_sp|Q6PHI	ULK3	Serine/threonine-pr	31.60	0.00
TRINITY_sp|Q54FI	gtf2b	Transcription initi	31.60	0.00
TRINITY_sp|Q6NNI	ATG16	Autophagy-related p	31.60	0.00
TRINITY_sp|Q5ZM	ADCK1	Uncharacterized aar	31.60	0.00
TRINITY_sp|Q4V8I	Rabepk	Rab9 effector prote	31.60	0.00
TRINITY_sp|P736‹	sppA	Protease 4 OS=Synec	31.60	0.00
TRINITY_sp|P0CD(	pan3	PAB-dependent poly(	31.60	0.00
TRINITY_sp|Q86JI	kdelr	ER lumen protein-re	31.60	0.00
TRINITY_sp|Q3U4‹	Mfsd12	Major facilitator s	31.60	0.00
TRINITY_sp|A4W8‹	rihA	Pyrimidine-specific	31.60	0.00
TRINITY_sp|Q7LH(	TY3B-I	Transposon Ty3-I Ga	31.60	0.00
TRINITY_sp|Q8K2\	Ppp4r1	Serine/threonine-pr	31.60	0.00
TRINITY_sp|Q9M2I	At3g613‹	UPF0187 protein At3	31.60	0.00
TRINITY_sp|Q8K2\	Ppp4r1	Serine/threonine-pr	31.60	0.00
TRINITY_sp|Q86G‹	gefQ	Ras guanine nucleot	31.60	0.00
TRINITY_sp|Q925(	RAPGEF5	Rap guanine nucleot	31.60	0.00
TRINITY_sp|Q499(	Tmco4	Transmembrane and c	31.60	0.00
TRINITY_sp|Q9Y5I	CTDP1	RNA polymerase II s	31.60	0.00
TRINITY_sp|Q227(	sym-2	RNA-binding protein	31.60	0.00
TRINITY_sp|O154‹	ABCC4	Multidrug resistanc	31.60	0.00
TRINITY_sp|P545(	yqkD	Uncharacterized pro	31.60	0.00
TRINITY_sp|Q0WP‹	GLE1	Protein GLE1 OS=Ara	31.60	0.00
TRINITY_sp|Q804‹	mib1	E3 ubiquitin-protei	31.60	0.00
TRINITY_sp|Q3E6‹	CpNIFS3	Probable L-cysteine	31.60	0.00
TRINITY_sp|Q55G(	DDB_G02(	Probable serine/thr	31.60	0.00
TRINITY_sp|Q5ZHI	CHMP2A	Charged multivesicu	31.60	0.00
TRINITY_sp|P108‹	PLCD1	1-phosphatidylinosi	31.60	0.00
TRINITY_sp|Q135	IQGAP2	Ras GTPase-activati	31.60	0.00
TRINITY_sp|Q7YXI	p80	Protein P80 OS=Dict	31.60	0.00
TRINITY_sp|Q5ZL\	NSUN2	tRNA (cytosine(34)-	31.60	0.00
TRINITY_sp|P403‹	exo2	5'-3' exoribonuclea	31.60	0.00
TRINITY_sp|Q9LX‹	GCN2	eIF-2-alpha kinase	31.50	0.00
TRINITY_sp|Q9C1X	SPAP32A‹	Uncharacterized RIN	31.50	0.00
TRINITY_sp|Q54ZI	plbE	Phospholipase B-lik	31.50	0.00
TRINITY_sp|Q99LI	Cdk5rap‹	CDK5 regulatory sub	31.50	0.00
TRINITY_sp|Q564I	CAPH	Condensin complex s	31.50	0.00
TRINITY_sp|A6QP‹	METTL21(	Protein-lysine meth	31.50	0.00
TRINITY_sp|Q6DCI	dym	Dymeclin OS=Xenopus	31.50	0.00
TRINITY_sp|B0F9I	GC6	Golgin candidate 6	31.50	0.00
TRINITY_sp|E9PZ‹	Ascc3	Activating signal c	31.50	0.00
TRINITY_sp|Q059	-	Sodium channel prot	31.50	0.00
TRINITY_sp|Q55DV	gacZ	Rho GTPase-activati	31.50	0.00
TRINITY_sp|Q7RJ(	CPK4	Calcium-dependent p	31.50	0.00
TRINITY_sp|Q9LSI	CCB1	Protein COFACTOR AS	31.50	0.00
TRINITY_sp|P623‹	CPK4	Calcium-dependent p	31.50	0.00
TRINITY_sp|P758(	ycbX	Uncharacterized pro	31.50	0.00
TRINITY_sp|Q5ACX	RAD5	DNA repair protein	31.50	0.00
TRINITY_sp|Q08DI	PUS7	Pseudouridylate syn	31.50	0.00
TRINITY_sp|Q54LY	ku80	X-ray repair cross-	31.50	0.00
TRINITY_sp|P302‹	WEE1	Wee1-like protein k	31.50	0.00

TRINITY_sp|Q58C\	FAM213B	Prostamide/prostagl	31.50	0.00
TRINITY_sp|Q8WZ	ibp1	Dual specificity ph	31.50	0.00
TRINITY_sp|P547	Tango2	Transport and Golgi	31.50	0.00
TRINITY_sp|O671	aq_1088	Uncharacterized pro	31.50	0.00
TRINITY_sp|O887	Alg10b	Putative Dol-P-Glc:	31.50	0.00
TRINITY_sp|Q559	slr0305	TVP38/TMEM64 family	31.50	0.00
TRINITY_sp|Q9SYI	GGH1	Gamma-glutamyl hydr	31.50	0.00
TRINITY_sp|Q5UP	MIMI_R2	Uncharacterized pro	31.50	0.00
TRINITY_sp|Q9FI	NIP4-1	Putative aquaporin	31.50	0.00
TRINITY_sp|Q56Y	PLR1	Pyridoxal reductase	31.50	0.00
TRINITY_sp|Q86C	dhkK	Hybrid signal trans	31.50	0.00
TRINITY_sp|Q55A	DDB_G02	Probable serine/thr	31.50	0.00
TRINITY_sp|P100	w	Protein white OS=Dr	31.50	0.00
TRINITY_sp|Q54M	mcfB	Mitochondrial subst	31.50	0.00
TRINITY_sp|P944	yciC	Putative metal chap	31.50	0.00
TRINITY_sp|O941	SNF1	Carbon catabolite-d	31.50	0.00
TRINITY_sp|B1AZ	Otud3	OTU domain-containi	31.50	0.00
TRINITY_sp|Q94KI	VPS52	Vacuolar protein so	31.50	0.00
TRINITY_sp|Q75J	cpras1	Circularly permutat	31.50	0.00
TRINITY_sp|Q9SZ	ACA10	Calcium-transportin	31.50	0.00
TRINITY_sp|Q201	gcs-1	Glutamate--cysteine	31.50	0.00
TRINITY_sp|P543	CkIalpha	Casein kinase I iso	31.50	0.00
TRINITY_sp|Q70L	lgrD	Linear gramicidin s	31.50	0.00
TRINITY_sp|Q923	ssb2	Replication factor	31.50	0.00
TRINITY_sp|Q0JI	CIPK11	CBL-interacting pro	31.50	0.00
TRINITY_sp|Q8VY	CCR4-6	Carbon catabolite r	31.50	0.00
TRINITY_sp|Q9M2	LPPD	Lipid phosphate pho	31.50	0.00
TRINITY_sp|Q2NL	PARP6	Poly [ADP-ribose] p	31.50	0.00
TRINITY_sp|Q8L7	BSL1	Serine/threonine-pr	31.50	0.00
TRINITY_sp|Q633	Nfkb1	Nuclear factor NF-k	31.50	0.00
TRINITY_sp|Q8CG	Smc5	Structural maintena	31.50	0.00
TRINITY_sp|Q8N6	OTUD6B	OTU domain-containi	31.50	0.00
TRINITY_sp|Q5TJ	forF	Formin-F OS=Dictyos	31.50	0.00
TRINITY_sp|Q54E	abpF	Actin-binding prote	31.50	0.00
TRINITY_sp|Q2MH	HT1	Serine/threonine-pr	31.50	0.00
TRINITY_sp|P370	lasT	Uncharacterized tRN	31.50	0.00
TRINITY_sp|Q460	PARP15	Poly [ADP-ribose] p	31.50	0.00
TRINITY_sp|Q7TS	Nsun6	Putative methyltran	31.50	0.00
TRINITY_sp|Q8BQ	Zdhhc14	Probable palmitoylt	31.50	0.00
TRINITY_sp|Q6Q2	MAOA	Amine oxidase [flav	31.50	0.00
TRINITY_sp|O759	CPD	Carboxypeptidase D	31.50	0.00
TRINITY_sp|O287	tspO	Tryptophan-rich pro	31.50	0.00
TRINITY_sp|P067	CMD1	Calmodulin OS=Sacch	31.50	0.00
TRINITY_sp|Q6ZF	ADL1	Calpain-type cystei	31.50	0.00
TRINITY_sp|O044	At1g096	Probable elongation	31.50	0.00
TRINITY_sp|P357	-	Pathogenesis-relate	31.40	0.00
TRINITY_sp|Q7ZW	Nt5c3b-a	7-methylguanosine p	31.40	0.00
TRINITY_sp|Q54FW	DDB_G02	WD repeat-containin	31.40	0.00
TRINITY_sp|Q9SL	Os05g01	Importin subunit al	31.40	0.00
TRINITY_sp|Q5PP	rnf170	E3 ubiquitin-protei	31.40	0.00
TRINITY_sp|P586	At2g399	Probable signal pep	31.40	0.00
TRINITY_sp|Q121	SPC3	Signal peptidase co	31.40	0.00
TRINITY_sp|P220	Pcmt1	Protein-L-isoaspart	31.40	0.00
TRINITY_sp|Q7LL	SPCC149	UPF0676 protein C14	31.40	0.00
TRINITY_sp|Q2TB	NSMCE4A	Non-structural main	31.40	0.00
TRINITY_sp|Q0JI	CIPK11	CBL-interacting pro	31.40	0.00

TRINITY_sp|Q86II	ppp4r2	Serine/threonine-pr	31.40	0.00
TRINITY_sp|Q6QA:	hsd17b12	Very-long-chain 3-o	31.40	0.00
TRINITY_sp|A0JN5	DGKA	Diacylglycerol kina	31.40	0.00
TRINITY_sp|Q54BW	ttc27	Tetratricopeptide r	31.40	0.00
TRINITY_sp|Q9VCI	CG17119	Cystinosin homolog	31.40	0.00
TRINITY_sp|Q9XZ4	5NUC	Protein 5NUC OS=Lut	31.40	0.00
TRINITY_sp|Q153	EPHA7	Ephrin type-A recep	31.40	0.00
TRINITY_sp|Q9M04	BHLH140	Transcription facto	31.40	0.00
TRINITY_sp|Q96B2	EXOSC8	Exosome complex com	31.40	0.00
TRINITY_sp|P0CD(	DDB_G028	Probable LIM domain	31.40	0.00
TRINITY_sp|B0VHI	kce	3-keto-5-aminohexan	31.40	0.00
TRINITY_sp|P0845	SPS1	Sporulation-specifi	31.40	0.00
TRINITY_sp|B9DF9	SNX2B	Sorting nexin 2B OS	31.40	0.00
TRINITY_sp|Q9P89	tcsA	Two-component syste	31.40	0.00
TRINITY_sp|Q8VI4	Abcc2	Canalicular multisp	31.40	0.00
TRINITY_sp|Q84X\	GTE1	Transcription facto	31.40	0.00
TRINITY_sp|Q55DW	gacZ	Rho GTPase-activati	31.40	0.00
TRINITY_sp|Q9N0\	ABCB11	Bile salt export pu	31.40	0.00
TRINITY_sp|Q9FN(	UVR8	Ultraviolet-B recep	31.40	0.00
TRINITY_sp|Q54N(	forH	Formin-H OS=Dictyos	31.40	0.00
TRINITY_sp|Q925(	RAPGEF5	Rap guanine nucleot	31.40	0.00
TRINITY_sp|Q8BT\	Scfd2	Sec1 family domain-	31.40	0.00
TRINITY_sp|F4IU)	UPF2	Regulator of nonsen	31.40	0.00
TRINITY_sp|Q7Z2W	ZC3HAV1	Zinc finger CCCH-ty	31.40	0.00
TRINITY_sp|G5EBI	ceeh-1	Epoxide hydrolase 1	31.40	0.00
TRINITY_sp|Q6NQ1	At1g1358	LAG1 longevity assu	31.40	0.00
TRINITY_sp|Q9LZ)	BIG2	Brefeldin A-inhibit	31.40	0.00
TRINITY_sp|Q9LHI	CHR27	Helicase-like trans	31.40	0.00
TRINITY_sp|Q9LH(	At3g2065	mRNA cap guanine-N7	31.40	0.00
TRINITY_sp|Q9K9I	speA	Arginine decarboxyl	31.40	0.00
TRINITY_sp|Q6AJW	msbA	Lipid A export ATP-	31.40	0.00
TRINITY_sp|Q940I	SRK2E	Serine/threonine-pr	31.40	0.00
TRINITY_sp|Q5MD8	flt4	Vascular endothelia	31.40	0.00
TRINITY_sp|Q3UZW	Eef2kmt	Protein-lysine N-me	31.40	0.00
TRINITY_sp|Q5ZLI	DCAF13	DDB1- and CUL4-asso	31.40	0.00
TRINITY_sp|Q9288	ABCC2	Canalicular multisp	31.40	0.00
TRINITY_sp|Q020(	PSBS	Photosystem II 22 k	31.40	0.00
TRINITY_sp|P4271	dmpM	O-demethylpuromycin	31.40	0.00
TRINITY_sp|Q2NK9	PSMG3	Proteasome assembly	31.40	0.00
TRINITY_sp|O0865	Tep1	Telomerase protein	31.40	0.00
TRINITY_sp|O430:	rpa43	DNA-directed RNA po	31.40	0.00
TRINITY_sp|Q9FG9	MNS4	Alpha-mannosidase I	31.40	0.00
TRINITY_sp|O9382	BET4	Geranylgeranyl tran	31.30	0.00
TRINITY_sp|Q9182	melk	Maternal embryonic	31.30	0.00
TRINITY_sp|Q084(	CKA2	Casein kinase II su	31.30	0.00
TRINITY_sp|O4288	SPAC8E11	Acyl-protein thioes	31.30	0.00
TRINITY_sp|E4US9	CPYA	Carboxypeptidase Y	31.30	0.00
TRINITY_sp|Q54KI	DDB_G028	TPR repeat-containi	31.30	0.00
TRINITY_sp|Q9SP(	TERT	Telomerase reverse	31.30	0.00
TRINITY_sp|Q54Z4	mybH	Myb-like protein H	31.30	0.00
TRINITY_sp|Q6UXI	FAM151B	Protein FAM151B OS=	31.30	0.00
TRINITY_sp|P3904	-	D-alanyl-D-alanine	31.30	0.00
TRINITY_sp|Q55B2	dcd1A	Protein dcd1A OS=Di	31.30	0.00
TRINITY_sp|O4302	fap1	L-pipecolate oxidas	31.30	0.00
TRINITY_sp|P511	Ppox	Protoporphyrinogen	31.30	0.00
TRINITY_sp|A0JPI	Slc39a4	Zinc transporter ZI	31.30	0.00

TRINITY_sp|Q9R1K	Fzr1	Fizzy-related prote	31.30	0.00
TRINITY_sp|O1377	SPAC17A2	Putative metal ion	31.30	0.00
TRINITY_sp|Q5UN9	MIMI_R66	Uncharacterized pro	31.30	0.00
TRINITY_sp|Q54MA	ku70	ATP-dependent DNA h	31.30	0.00
TRINITY_sp|Q94HF	TIF3K1	Eukaryotic translat	31.30	0.00
TRINITY_sp|P3515	rluB	Ribosomal large sub	31.30	0.00
TRINITY_sp|Q9D6J	Cers4	Ceramide synthase 4	31.30	0.00
TRINITY_sp|Q500U	TKPR1	Tetraketide alpha-p	31.30	0.00
TRINITY_sp|Q9FPF	EDR1	Serine/threonine-pr	31.30	0.00
TRINITY_sp|D4AYW	ARB_0137	ABC transporter G f	31.30	0.00
TRINITY_sp|Q653H	Os06g025	Potassium channel K	31.30	0.00
TRINITY_sp|Q6UVJ	SASS6	Spindle assembly ab	31.30	0.00
TRINITY_sp|Q54B2	DDB_G029	Uncharacterized mem	31.30	0.00
TRINITY_sp|Q6TN9	rabl3	Rab-like protein 3	31.30	0.00
TRINITY_sp|Q3MDY	pcxA	Proton extrusion pr	31.30	0.00
TRINITY_sp|Q6GLW	alyref-b	THO complex subunit	31.30	0.00
TRINITY_sp|P5194	MNAT1	CDK-activating kina	31.30	0.00
TRINITY_sp|Q8CGV	Thtpa	Thiamine-triphospha	31.30	0.00
TRINITY_sp|Q9FF4	ABCG28	ABC transporter G f	31.30	0.00
TRINITY_sp|Q925Q	Slc8b1	Sodium/potassium/ca	31.30	0.00
TRINITY_sp|Q9ST9	APC8	Anaphase-promoting	31.30	0.00
TRINITY_sp|Q81M9	speA	Arginine decarboxyl	31.30	0.00
TRINITY_sp|Q5L0Y	murE	UDP-N-acetylmuramoy	31.30	0.00
TRINITY_sp|Q5WEK	mutS2	Endonuclease MutS2	31.30	0.00
TRINITY_sp|Q7PC7	PSD	Exportin-T OS=Arabi	31.30	0.00
TRINITY_sp|Q7T2V	map3k10	Mitogen-activated p	31.30	0.00
TRINITY_sp|Q5UP2	MIMI_R25	Uncharacterized pro	31.20	0.00
TRINITY_sp|Q9LF4	PUB1	Probable ubiquitin	31.20	0.00
TRINITY_sp|Q54BN	mcfG	Mitochondrial subst	31.20	0.00
TRINITY_sp|Q5KTC	Naaa	N-acylethanolamine-	31.20	0.00
TRINITY_sp|Q54DI	DDB_G029	Thimet-like oligope	31.20	0.00
TRINITY_sp|P0DKC	HSD1	11-beta-hydroxyster	31.20	0.00
TRINITY_sp|A0DSF	GSPATT0(	Probable protein ph	31.20	0.00
TRINITY_sp|Q7T2I	sgsm3	Small G protein sig	31.20	0.00
TRINITY_sp|P4123	Abca2	ATP-binding cassett	31.20	0.00
TRINITY_sp|Q8RWF	UPL6	E3 ubiquitin-protei	31.20	0.00
TRINITY_sp|O9562	CNOT4	CCR4-NOT transcript	31.20	0.00
TRINITY_sp|P3071	Gstt2	Glutathione S-trans	31.20	0.00
TRINITY_sp|Q8BK6	Ahsa1	Activator of 90 kDa	31.20	0.00
TRINITY_sp|Q9VH1	Invadoly	Leishmanolysin-like	31.20	0.00
TRINITY_sp|P6126	STX1B	Syntaxin-1B OS=Bos	31.20	0.00
TRINITY_sp|Q8R43	Mgll	Monoglyceride lipas	31.20	0.00
TRINITY_sp|Q056C	CTR1	Serine/threonine-pr	31.20	0.00
TRINITY_sp|F4JL9	LDL3	Lysine-specific his	31.20	0.00
TRINITY_sp|Q8L7F	LPEAT1	Lysophospholipid ac	31.20	0.00
TRINITY_sp|Q7TQ(	Poln	DNA polymerase nu O	31.20	0.00
TRINITY_sp|Q84SI	Os07g056	Zinc finger CCCH do	31.20	0.00
TRINITY_sp|Q96P5	ACAP3	Arf-GAP with coiled	31.20	0.00
TRINITY_sp|Q55F9	krsB	Serine/threonine-pr	31.20	0.00
TRINITY_sp|Q96JC	VPS39	Vam6/Vps39-like pro	31.20	0.00
TRINITY_sp|A6H73	ACPP	Prostatic acid phos	31.20	0.00
TRINITY_sp|Q4254	UBC13	Ubiquitin-conjugati	31.20	0.00
TRINITY_sp|P3703	Por	NADPH--cytochrome P	31.20	0.00
TRINITY_sp|Q2905	ITIH1	Inter-alpha-trypsin	31.20	0.00
TRINITY_sp|O822C	At2g1979	AP-4 complex subuni	31.20	0.00
TRINITY_sp|Q559A	gacU	Rho GTPase-activati	31.20	0.00

TRINITY_sp|Q2QKI	U2AF65B	Splicing factor U2a	31.20	0.00
TRINITY_sp|Q5ZJ8	PRPF3	U4/U6 small nuclear	31.20	0.00
TRINITY_sp|P835(	ARHGAP35	Rho GTPase-activati	31.20	0.00
TRINITY_sp|O6535	SYP41	Syntaxin-41 OS=Arab	31.20	0.00
TRINITY_sp|Q8RXI	At1g0689	Uncharacterized mem	31.20	0.00
TRINITY_sp|O7493	POX2	Acyl-coenzyme A oxi	31.20	0.00
TRINITY_sp|O1408	imt1	Inositol phosphocer	31.20	0.00
TRINITY_sp|Q54TI	gacY	Rho GTPase-activati	31.20	0.00
TRINITY_sp|Q6BVA	IPL1	Spindle assembly ch	31.20	0.00
TRINITY_sp|Q94A(	RFS2	Probable galactinol	31.20	0.00
TRINITY_sp|F4J8I	RRP5	rRNA biogenesis pro	31.20	0.00
TRINITY_sp|Q9ZKV	vdlC	Probable short-chai	31.20	0.00
TRINITY_sp|Q8GYI	SULTR4;2	Probable sulfate tr	31.20	0.00
TRINITY_sp|P205(	CAH1	Carbonic anhydrase	31.20	0.00
TRINITY_sp|O6535	SYP41	Syntaxin-41 OS=Arab	31.20	0.00
TRINITY_sp|B6CZ(	Lrrc51	Leucine-rich repeat	31.20	0.00
TRINITY_sp|Q6L4I	SIZ1	E3 SUMO-protein lig	31.20	0.00
TRINITY_sp|Q84JI	PTAC14	Protein PLASTID TRA	31.20	0.00
TRINITY_sp|A7SNI	v1g2464(	Serine/threonine-pr	31.20	0.00
TRINITY_sp|Q75J9	rapgap1	RapA guanosine trip	31.20	0.00
TRINITY_sp|Q8W4I	HPAT1	Hydroxyproline O-ar	31.20	0.00
TRINITY_sp|Q8RXY	At3g6333	Probable inactive p	31.20	0.00
TRINITY_sp|P285(	DUSP1	Dual specificity pr	31.20	0.00
TRINITY_sp|Q54P(	gnt14	Glycosyltransferase	31.20	0.00
TRINITY_sp|Q86K8	DDB_G027	RUS1 family protein	31.20	0.00
TRINITY_sp|Q6C3V	NUF2	Probable kinetochor	31.20	0.00
TRINITY_sp|Q80V3	Cep104	Centrosomal protein	31.20	0.00
TRINITY_sp|Q54B7	abcB2	ABC transporter B f	31.20	0.00
TRINITY_sp|I7CT8	-	Protopanaxadiol 6-h	31.20	0.00
TRINITY_sp|Q6148	Pde1a	Calcium/calmodulin-	31.20	0.00
TRINITY_sp|Q9LV(	PVA21	Vesicle-associated	31.20	0.00
TRINITY_sp|Q5TJ5	forF	Formin-F OS=Dictyos	31.20	0.00
TRINITY_sp|Q337A	ADF10	Actin-depolymerizin	31.10	0.00
TRINITY_sp|P0DI1	SAE1B-1	SUMO-activating enz	31.10	0.00
TRINITY_sp|Q9FEI	DSP4	Phosphoglucan phosp	31.10	0.00
TRINITY_sp|P2264	dhlA	Haloalkane dehaloge	31.10	0.00
TRINITY_sp|A4II9	slc47a1	Multidrug and toxin	31.10	0.00
TRINITY_sp|Q7BWI	sasA	Adaptive-response s	31.10	0.00
TRINITY_sp|Q1227	INP53	Polyphosphatidylino	31.10	0.00
TRINITY_sp|Q9295	KCNB2	Potassium voltage-g	31.10	0.00
TRINITY_sp|Q96E2	MCRS1	Microspherule prote	31.10	0.00
TRINITY_sp|Q5852	MJ1123	Uncharacterized pro	31.10	0.00
TRINITY_sp|O1773	mpst-1	Putative thiosulfat	31.10	0.00
TRINITY_sp|F4JT7	VPS54	Vacuolar protein so	31.10	0.00
TRINITY_sp|P235(	MRS4	Mitochondrial RNA-s	31.10	0.00
TRINITY_sp|P3952	DRS2	Probable phospholip	31.10	0.00
TRINITY_sp|Q9BS3	TUBGCP2	Gamma-tubulin compl	31.10	0.00
TRINITY_sp|Q10L3	MTP2	Metal tolerance pro	31.10	0.00
TRINITY_sp|Q5K5I	Os07g064	DEAD-box ATP-depend	31.10	0.00
TRINITY_sp|P0A8Y	yidA	Sugar phosphatase Y	31.10	0.00
TRINITY_sp|Q9UP(	USP24	Ubiquitin carboxyl-	31.10	0.00
TRINITY_sp|P411(	Rpl28	60S ribosomal prote	31.10	0.00
TRINITY_sp|Q922Y	Trim59	Tripartite motif-co	31.10	0.00
TRINITY_sp|Q9SU\	At4g3239	Probable sugar phos	31.10	0.00
TRINITY_sp|F4JJ3	NDB3	External alternativ	31.10	0.00
TRINITY_sp|Q6539	Os09g055	Probable protein ph	31.10	0.00

TRINITY_sp|Q196X	IIV3-084	Putative ubiquitin	31.10	0.00
TRINITY_sp|Q555I	DDB_G027	SH3 and F-BAR domai	31.10	0.00
TRINITY_sp|Q8CQI	butA	Diacetyl reductase	31.10	0.00
TRINITY_sp|Q9FN9	At5g5968	Probable LRR recept	31.10	0.00
TRINITY_sp|Q2322	fat-3	Delta(6)-fatty-acid	31.10	0.00
TRINITY_sp|Q54U7	crlA	Cyclic AMP receptor	31.10	0.00
TRINITY_sp|Q6C29	NOP12	Nucleolar protein 1	31.10	0.00
TRINITY_sp|Q0023	melO	Tyrosinase OS=Asper	31.10	0.00
TRINITY_sp|Q75JI	DDB_G027	SH3 and F-BAR domai	31.10	0.00
TRINITY_sp|Q8GYI	SULTR4;2	Probable sulfate tr	31.10	0.00
TRINITY_sp|B9U3I	DISP3	Protein dispatched	31.10	0.00
TRINITY_sp|A7MQI	ubiB	Probable protein ki	31.10	0.00
TRINITY_sp|Q5H80	Vps13a	Vacuolar protein so	31.10	0.00
TRINITY_sp|Q1504	RAB3GAP1	Rab3 GTPase-activat	31.10	0.00
TRINITY_sp|Q9DB9	Osbpl3	Oxysterol-binding p	31.10	0.00
TRINITY_sp|P4055	YIA6	Mitochondrial nicot	31.10	0.00
TRINITY_sp|A9IV2	rplQ	50S ribosomal prote	31.10	0.00
TRINITY_sp|Q8R3N	Nop14	Nucleolar protein 1	31.10	0.00
TRINITY_sp|Q54HY	bcs1la	Probable mitochondr	31.10	0.00
TRINITY_sp|Q0041	SEN1	Helicase SEN1 OS=Sa	31.10	0.00
TRINITY_sp|Q0WQI	VPS53	Vacuolar protein so	31.10	0.00
TRINITY_sp|Q54XN	captB	Uncharacterized CDP	31.10	0.00
TRINITY_sp|Q1PF0	DTX7	Protein DETOXIFICAT	31.10	0.00
TRINITY_sp|Q9ZGI	pikAII	Narbonolide/10-deox	31.10	0.00
TRINITY_sp|O7489	ret3	Probable coatomer s	31.10	0.00
TRINITY_sp|Q8RY2	NUP98A	Nuclear pore comple	31.10	0.00
TRINITY_sp|Q7T2I	mx1	Interferon-induced	31.10	0.00
TRINITY_sp|P7392	glbN	Group 1 truncated h	31.10	0.00
TRINITY_sp|P4183	IME4	N6-adenosine-methyl	31.00	0.00
TRINITY_sp|Q0975	SPAC24H6	Putative membrane-b	31.00	0.00
TRINITY_sp|Q75A3	CDC123	Cell division cycle	31.00	0.00
TRINITY_sp|P3528	Rab12	Ras-related protein	31.00	0.00
TRINITY_sp|Q86W3	AMZ2	Archaemetzincin-2 O	31.00	0.00
TRINITY_sp|P1047	celE	Cellulase/esterase	31.00	0.00
TRINITY_sp|Q6DH0	hvcn1	Voltage-gated hydro	31.00	0.00
TRINITY_sp|O0713	echA8	Probable enoyl-CoA	31.00	0.00
TRINITY_sp|Q500U	TKPR1	Tetraketide alpha-p	31.00	0.00
TRINITY_sp|Q9S71	GTE3	Transcription facto	31.00	0.00
TRINITY_sp|Q9UU9	yop1	Protein yop1 OS=Sch	31.00	0.00
TRINITY_sp|Q54RI	dhkL	Hybrid signal trans	31.00	0.00
TRINITY_sp|Q1337	PDCL	Phosducin-like prot	31.00	0.00
TRINITY_sp|Q9963	RAD9A	Cell cycle checkpoi	31.00	0.00
TRINITY_sp|O8217	AGD10	ADP-ribosylation fa	31.00	0.00
TRINITY_sp|Q4R53	NOB1	RNA-binding protein	31.00	0.00
TRINITY_sp|Q9P89	tcsA	Two-component syste	31.00	0.00
TRINITY_sp|Q07G1	alkbh8	Alkylated DNA repai	31.00	0.00
TRINITY_sp|Q3V05	Slc47a2	Multidrug and toxin	31.00	0.00
TRINITY_sp|Q676U	ATG16L1	Autophagy-related p	31.00	0.00
TRINITY_sp|Q1581	ITSN1	Intersectin-1 OS=Ho	31.00	0.00
TRINITY_sp|Q5JJ6	TK1782	Uncharacterized ser	31.00	0.00
TRINITY_sp|Q8H1I	TDP1	Tyrosyl-DNA phospho	31.00	0.00
TRINITY_sp|P1318	KIN2	Serine/threonine-pr	31.00	0.00
TRINITY_sp|Q2KHY	CPNE6	Copine-6 OS=Bos tau	31.00	0.00
TRINITY_sp|Q68FI	Cog6	Conserved oligomeri	31.00	0.00
TRINITY_sp|A3LV4	DBP9	ATP-dependent RNA h	31.00	0.00
TRINITY_sp|P4786	Aqp4	Aquaporin-4 OS=Ratt	31.00	0.00

TRINITY_sp|P200(	Hexb	Beta-hexosaminidase	31.00	0.00
TRINITY_sp|Q494(	HPAT2	Hydroxyproline O-ar	31.00	0.00
TRINITY_sp|Q86Y(	KIF18B	Kinesin-like protei	31.00	0.00
TRINITY_sp|P477(	Srprb	Signal recognition	31.00	0.00
TRINITY_sp|Q9C5(	PAP6	Purple acid phospha	31.00	0.00
TRINITY_sp|Q5R7(	FAM213B	Prostamide/prostagl	31.00	0.00
TRINITY_sp|Q7M3(	rngB	RING finger protein	31.00	0.00
TRINITY_sp|Q4L7(	ilvA	L-threonine dehydra	31.00	0.00
TRINITY_sp|Q8NB(	RDH13	Retinol dehydrogena	31.00	0.00
TRINITY_sp|P981(	RBM10	RNA-binding protein	31.00	0.00
TRINITY_sp|Q9SH(	CYCU2-1	Cyclin-U2-1 OS=Arab	31.00	0.00
TRINITY_sp|Q124(	TRM10	tRNA (guanine(9)-N1	31.00	0.00
TRINITY_sp|O344(	yrrC	ATP-dependent RecD-	31.00	0.00
TRINITY_sp|Q8NI(	THOC2	THO complex subunit	31.00	0.00
TRINITY_sp|P940(	At1g149(	Putative clathrin a	31.00	0.00
TRINITY_sp|Q52Q(	CRTISO1	Prolycopene isomera	31.00	0.00
TRINITY_sp|Q8VW(	SSL3	Protein STRICTOSIDI	31.00	0.00
TRINITY_sp|Q9QY(	Smok3a	Sperm motility kina	31.00	0.00
TRINITY_sp|Q6NL(	LPPG	Lipid phosphate pho	31.00	0.00
TRINITY_sp|Q86I(	nek3	Probable serine/thr	31.00	0.00
TRINITY_sp|Q9LT(	MAD1	Mitotic spindle che	31.00	0.00
TRINITY_sp|Q9QY(	Dnajc7	DnaJ homolog subfam	31.00	0.00
TRINITY_sp|Q9C5(	TIF3E1	Eukaryotic translat	31.00	0.00
TRINITY_sp|B5X0(	VIP6	Protein CTR9 homolo	31.00	0.00
TRINITY_sp|Q8GU(	C50	Chaperone protein d	31.00	0.00
TRINITY_sp|Q9W4(	dnc	cAMP-specific 3',5'	31.00	0.00
TRINITY_sp|Q8H1(	RH14	DEAD-box ATP-depend	31.00	0.00
TRINITY_sp|Q145(	ITPR3	Inositol 1,4,5-tris	31.00	0.00
TRINITY_sp|Q9LY(	CALS6	Putative callose sy	31.00	0.00
TRINITY_sp|Q9AW(	R1	Alpha-glucan water	31.00	0.00
TRINITY_sp|Q54P(	osbI	Oxysterol-binding p	31.00	0.00
TRINITY_sp|P146(	Pde4c	cAMP-specific 3',5'	31.00	0.00
TRINITY_sp|A2WY(	HK3	Probable histidine	31.00	0.00
TRINITY_sp|Q5AF(	KEX1	Pheromone-processin	30.90	0.00
TRINITY_sp|O012(	vps-26	Vacuolar protein so	30.90	0.00
TRINITY_sp|Q8N5(	ATPAF2	ATP synthase mitoch	30.90	0.00
TRINITY_sp|Q389(	RABB1B	Ras-related protein	30.90	0.00
TRINITY_sp|O582(	gck	Glycerate 2-kinase	30.90	0.00
TRINITY_sp|O624(	Y43F4A.(	Leishmanolysin-like	30.90	0.00
TRINITY_sp|Q4R8(	DNAJC2	DnaJ homolog subfam	30.90	0.00
TRINITY_sp|Q55C(	gdt2	Probable serine/thr	30.90	0.00
TRINITY_sp|P218(	yaaD	Probable FKBP-type	30.90	0.00
TRINITY_sp|Q23F(	TTLL3C	Tubulin glycylase 3	30.90	0.00
TRINITY_sp|P328(	SSO1	Protein SSO1 OS=Sac	30.90	0.00
TRINITY_sp|Q9P7(	mak1	Peroxide stress-act	30.90	0.00
TRINITY_sp|P390(	MPT5	Suppressor protein	30.90	0.00
TRINITY_sp|Q9ZV(	TFIIS	Transcription elong	30.90	0.00
TRINITY_sp|Q55D(	pakD	Serine/threonine-pr	30.90	0.00
TRINITY_sp|Q149(	SHPRH	E3 ubiquitin-protei	30.90	0.00
TRINITY_sp|Q9SI(	RKP	E3 ubiquitin-protei	30.90	0.00
TRINITY_sp|Q5AX(	bcp1	Protein bcp1 OS=Eme	30.90	0.00
TRINITY_sp|Q0JI(	CIPK11	CBL-interacting pro	30.90	0.00
TRINITY_sp|Q8BK(	Ahsa1	Activator of 90 kDa	30.90	0.00
TRINITY_sp|Q5XG(	PAPD7	Non-canonical poly(	30.90	0.00
TRINITY_sp|Q8K2(	Ppp4r1	Serine/threonine-pr	30.90	0.00
TRINITY_sp|Q54P(	eif2a	Eukaryotic translat	30.90	0.00

TRINITY_sp|Q7ZUX	dohh	Deoxyhypusine hydro	30.90	0.00
TRINITY_sp|P2133	al-1	Phytoene desaturase	30.90	0.00
TRINITY_sp|Q54I8	shkB	Dual specificity pr	30.90	0.00
TRINITY_sp|Q93VI	CCD8A	Carotenoid cleavage	30.90	0.00
TRINITY_sp|Q4R9E	TCTN3	Tectonic-3 OS=Macac	30.90	0.00
TRINITY_sp|F4JMJ	HSP70-17	Heat shock 70 kDa p	30.90	0.00
TRINITY_sp|Q55C2	gdt2	Probable serine/thr	30.90	0.00
TRINITY_sp|Q1032	SPAC17G8	Inositol phosphocer	30.90	0.00
TRINITY_sp|F4HVJ	POD1	Protein POLLEN DEFE	30.90	0.00
TRINITY_sp|Q5VVW	GARNL3	GTPase-activating R	30.90	0.00
TRINITY_sp|P0414	GIP	Copia protein OS=Dr	30.90	0.00
TRINITY_sp|P6974	Trpv5	Transient receptor	30.90	0.00
TRINITY_sp|Q9FNX	DRP1E	Dynamin-related pro	30.90	0.00
TRINITY_sp|Q1ZXJ	clcD	Chloride channel pr	30.90	0.00
TRINITY_sp|O3296	ML0855	Uncharacterized zin	30.90	0.00
TRINITY_sp|B3QPW	dnaJ	Chaperone protein D	30.90	0.00
TRINITY_sp|Q9WTN	Tpcn1	Two pore calcium ch	30.90	0.00
TRINITY_sp|P9547	arbA	Extracellular exo-a	30.80	0.00
TRINITY_sp|F4IG7	BCHC2	BEACH domain-contai	30.80	0.00
TRINITY_sp|P5729	thrA	Bifunctional aspart	30.80	0.00
TRINITY_sp|Q9P2K	EIF2AK4	eIF-2-alpha kinase	30.80	0.00
TRINITY_sp|P5805	Nadk	NAD kinase OS=Mus m	30.80	0.00
TRINITY_sp|Q6361	Vps33a	Vacuolar protein so	30.80	0.00
TRINITY_sp|Q7ZVB	vps36	Vacuolar protein-so	30.80	0.00
TRINITY_sp|Q4251	GN	ARF guanine-nucleot	30.80	0.00
TRINITY_sp|F4JL2	SMO1-3	Methylsterol monoox	30.80	0.00
TRINITY_sp|Q5TGI	FAXC	Failed axon connect	30.80	0.00
TRINITY_sp|Q9VGI	Blm	Bloom syndrome prot	30.80	0.00
TRINITY_sp|Q8C14	Dock8	Dedicator of cytoki	30.80	0.00
TRINITY_sp|Q99L2	Gstt3	Glutathione S-trans	30.80	0.00
TRINITY_sp|Q2868	ABCC2	Canalicular multisp	30.80	0.00
TRINITY_sp|Q55E5	corB	Coronin-B OS=Dictyo	30.80	0.00
TRINITY_sp|P8219	Tgfbi	Transforming growth	30.80	0.00
TRINITY_sp|Q9FN0	UVR8	Ultraviolet-B recep	30.80	0.00
TRINITY_sp|Q0336	DNAJ1	DnaJ protein homolo	30.80	0.00
TRINITY_sp|Q9SR0	CLPS3	Protein CLP1 homolo	30.80	0.00
TRINITY_sp|Q9P30	dbl4	E3 ubiquitin-protei	30.80	0.00
TRINITY_sp|Q5UN9	MIMI_R66	Uncharacterized pro	30.80	0.00
TRINITY_sp|Q9632	IMPA1	Importin subunit al	30.80	0.00
TRINITY_sp|Q5500	DDB_G027	Protein TAPT1 homol	30.80	0.00
TRINITY_sp|Q8S89	LPEAT2	Lysophospholipid ac	30.80	0.00
TRINITY_sp|Q8N8V	GBP7	Guanylate-binding p	30.80	0.00
TRINITY_sp|Q641K	Nuak1	NUAK family SNF1-li	30.80	0.00
TRINITY_sp|Q54VJ	abcC2	ABC transporter C f	30.80	0.00
TRINITY_sp|Q6B51	sgkB	Sphingosine kinase	30.80	0.00
TRINITY_sp|Q9M0A	GGP2	Gamma-glutamyl pept	30.80	0.00
TRINITY_sp|Q55F9	dlpA	Dynamin-like protei	30.80	0.00
TRINITY_sp|Q552Y	psmD9	Probable 26S protea	30.80	0.00
TRINITY_sp|Q54G0	DDB_G029	SPX and EXS domain-	30.80	0.00
TRINITY_sp|Q5A32	CDG1	Cysteine dioxygenas	30.80	0.00
TRINITY_sp|Q8C43	Vcpkmt	Protein-lysine meth	30.80	0.00
TRINITY_sp|Q54VB	DDB_G028	TPR-containing prot	30.80	0.00
TRINITY_sp|Q8MIB	ABCG2	ATP-binding cassett	30.80	0.00
TRINITY_sp|A6QLB	ERI3	ERI1 exoribonucleas	30.80	0.00
TRINITY_sp|O6783	rplI	50S ribosomal prote	30.80	0.00
TRINITY_sp|Q54P0	abkD	Probable serine/thr	30.80	0.00

TRINITY_sp|P548!	TGL2	Lipase 2 OS=Sacchar	30.80	0.00
TRINITY_sp|Q93W2	At4g155	Uncharacterized pro	30.80	0.00
TRINITY_sp|Q9SY1	At4g029	CSC1-like protein A	30.80	0.00
TRINITY_sp|Q6GQ1	Nomo1	Nodal modulator 1 O	30.80	0.00
TRINITY_sp|Q5UR(	MIMI_L9(	Uncharacterized pro	30.80	0.00
TRINITY_sp|Q55GN	DDB_G02(	Probable phosphatid	30.80	0.00
TRINITY_sp|Q5XI/	Dzip1l	Zinc finger protein	30.80	0.00
TRINITY_sp|P0272	PCTP	Phosphatidylcholine	30.80	0.00
TRINITY_sp|P2271	Gucy1b2	Guanylate cyclase s	30.80	0.00
TRINITY_sp|Q6ZY5	GWD3	Phosphoglucan, wate	30.80	0.00
TRINITY_sp|O0078	vatE	V-type proton ATPas	30.70	0.00
TRINITY_sp|Q9M3(	PAT05	Probable protein S-	30.70	0.00
TRINITY_sp|Q2243	T12E12.1	Potential E3 ubiqui	30.70	0.00
TRINITY_sp|Q96M9	PACRG	Parkin coregulated	30.70	0.00
TRINITY_sp|Q55B(	abcA8	ABC transporter A f	30.70	0.00
TRINITY_sp|Q5F3(	C1GALT1	Glycoprotein-N-acet	30.70	0.00
TRINITY_sp|Q8VY/	RS2Z33	Serine/arginine-ric	30.70	0.00
TRINITY_sp|Q9FLN	PAT06	Probable protein S-	30.70	0.00
TRINITY_sp|Q6GL1	pip4k2c	Phosphatidylinosito	30.70	0.00
TRINITY_sp|Q54GY	rab18	Ras-related protein	30.70	0.00
TRINITY_sp|Q8LF(	RBL19	Rhomboid-like prote	30.70	0.00
TRINITY_sp|Q9VK8	CG6388	Probable tRNA (guan	30.70	0.00
TRINITY_sp|Q84M2	ABCA1	ABC transporter A f	30.70	0.00
TRINITY_sp|Q6P61	ak8	Adenylate kinase 8	30.70	0.00
TRINITY_sp|Q5AJ9	MRD1	Multiple RNA-bindin	30.70	0.00
TRINITY_sp|Q9VM7	Rat1	5'-3' exoribonuclea	30.70	0.00
TRINITY_sp|P5443	yrkH	Uncharacterized pro	30.70	0.00
TRINITY_sp|Q8L79	ULP2B	Probable ubiquitin-	30.70	0.00
TRINITY_sp|Q0992	tif224	Probable translatio	30.70	0.00
TRINITY_sp|O4853	BRR2B	DExH-box ATP-depend	30.70	0.00
TRINITY_sp|O1391	pnk1	Bifunctional polynu	30.70	0.00
TRINITY_sp|Q5UQF	MIMI_L4(	Putative alpha/beta	30.70	0.00
TRINITY_sp|Q8N6F	METTL13	Methyltransferase-l	30.70	0.00
TRINITY_sp|Q8BG4	Stk17b	Serine/threonine-pr	30.70	0.00
TRINITY_sp|A6UWF	hemL	Glutamate-1-semiald	30.70	0.00
TRINITY_sp|Q8C6(	-	Protein C21orf2 hom	30.70	0.00
TRINITY_sp|Q9Z1N	Ik	Protein Red OS=Mus	30.70	0.00
TRINITY_sp|B1ZW7	trpC	Indole-3-glycerol p	30.70	0.00
TRINITY_sp|P0414	GIP	Copia protein OS=Dr	30.70	0.00
TRINITY_sp|Q9LKW	NHX7	Sodium/hydrogen exc	30.70	0.00
TRINITY_sp|P5094	AVT4	Vacuolar amino acid	30.70	0.00
TRINITY_sp|P1027	pol	Pol polyprotein OS=	30.70	0.00
TRINITY_sp|Q9FV5	MAP1D	Methionine aminopep	30.70	0.00
TRINITY_sp|Q8H1I	APY4	Probable apyrase 4	30.70	0.00
TRINITY_sp|Q9SB	PDK	[Pyruvate dehydroge	30.70	0.00
TRINITY_sp|Q86U(	RSPH3	Radial spoke head p	30.70	0.00
TRINITY_sp|Q54B1	DDB_G029	LIMR family protein	30.70	0.00
TRINITY_sp|Q9LW2	At3g268	Acyltransferase-lik	30.70	0.00

TRINITY_sp|Q6DF(	ccdc77	Coiled-coil domain-	30.70	0.00
TRINITY_sp|Q7MG(	VVA0006	Riboflavin biosynth	30.70	0.00
TRINITY_sp|Q9P2(	ANKIB1	Ankyrin repeat and	30.70	0.00
TRINITY_sp|Q61Y(	CBG0355(	Leishmanolysin-like	30.70	0.00
TRINITY_sp|P443:	rbsK	Ribokinase OS=Haemo	30.70	0.00
TRINITY_sp|Q9W0	NaCP60E	Sodium channel prot	30.70	0.00
TRINITY_sp|Q558	sll0103	Uncharacterized pro	30.70	0.00
TRINITY_sp|Q9M0	At4g326	Protein transport p	30.70	0.00
TRINITY_sp|P181(	Acsl1	Long-chain-fatty-ac	30.70	0.00
TRINITY_sp|Q5XT	PNPLA8	Calcium-independent	30.70	0.00
TRINITY_sp|Q55G	abkC	Probable serine/thr	30.70	0.00
TRINITY_sp|Q8MI	ABCG2	ATP-binding cassett	30.70	0.00
TRINITY_sp|O818	IRT2	Fe(2+) transport pr	30.70	0.00
TRINITY_sp|Q9H8	CNTD2	Cyclin N-terminal d	30.70	0.00
TRINITY_sp|Q5FP	GOX2015	Glucose 1-dehydroge	30.60	0.00
TRINITY_sp|Q928	GGH	Gamma-glutamyl hydr	30.60	0.00
TRINITY_sp|Q55B(	abcA8	ABC transporter A f	30.60	0.00
TRINITY_sp|Q9FE	SULTR1;	Sulfate transporter	30.60	0.00
TRINITY_sp|Q8TD	NEK9	Serine/threonine-pr	30.60	0.00
TRINITY_sp|Q9Z1	ACOX1	Peroxisomal acyl-co	30.60	0.00
TRINITY_sp|P341(	pkgC	Protein kinase 3 OS	30.60	0.00
TRINITY_sp|Q8H1	TDP1	Tyrosyl-DNA phospho	30.60	0.00
TRINITY_sp|Q55G	l2hgdh	L-2-hydroxyglutarat	30.60	0.00
TRINITY_sp|Q8GY	CAT3	Cationic amino acid	30.60	0.00
TRINITY_sp|Q9D9	Zswim2	E3 ubiquitin-protei	30.60	0.00
TRINITY_sp|Q5A3	CDG1	Cysteine dioxygenas	30.60	0.00
TRINITY_sp|Q135	DGKZ	Diacylglycerol kina	30.60	0.00
TRINITY_sp|Q6PA	Klhdc10	Kelch domain-contai	30.60	0.00
TRINITY_sp|H3ZP	OCC_043	Aromatic-amino-acid	30.60	0.00
TRINITY_sp|Q6PG(	Dhx29	ATP-dependent RNA h	30.60	0.00
TRINITY_sp|D3ZV	Nat8l	N-acetylaspartate s	30.60	0.00
TRINITY_sp|O945	iqw1	WD repeat protein i	30.60	0.00
TRINITY_sp|Q679	PDLIM7	PDZ and LIM domain	30.60	0.00
TRINITY_sp|O821	VPS32.1	Vacuolar protein so	30.60	0.00
TRINITY_sp|Q3U1	Tmem64	Transmembrane prote	30.60	0.00
TRINITY_sp|Q6P5(	Mapk4	Mitogen-activated p	30.60	0.00
TRINITY_sp|F4I4	BARD1	BRCA1-associated RI	30.60	0.00
TRINITY_sp|O703	Nmt2	Glycylpeptide N-tet	30.60	0.00
TRINITY_sp|Q132	PRKG2	cGMP-dependent prot	30.60	0.00
TRINITY_sp|Q54T	yipf1	Protein YIPF1 homol	30.60	0.00
TRINITY_sp|F4JL	LDL3	Lysine-specific his	30.60	0.00
TRINITY_sp|Q9LR	APUM24	Pumilio homolog 24	30.60	0.00
TRINITY_sp|Q8LF	BRN1	RNA-binding protein	30.60	0.00
TRINITY_sp|Q0P4	acsf2	Acyl-CoA synthetase	30.60	0.00
TRINITY_sp|Q84M	AL1	RAP domain-containi	30.60	0.00
TRINITY_sp|Q9P4	CCA1	CCA tRNA nucleotidy	30.60	0.00
TRINITY_sp|Q0JI	CIPK11	CBL-interacting pro	30.60	0.00
TRINITY_sp|Q9NT	ATP8A2	Phospholipid-transp	30.60	0.00
TRINITY_sp|Q94B	CHR28	Helicase-like trans	30.60	0.00
TRINITY_sp|Q32T(	EFHC2	EF-hand domain-cont	30.60	0.00
TRINITY_sp|Q617	Kif3b	Kinesin-like protei	30.60	0.00
TRINITY_sp|P196	Gucy1a3	Guanylate cyclase s	30.60	0.00
TRINITY_sp|Q54Y	dhkJ	Hybrid signal trans	30.60	0.00
TRINITY_sp|Q8L5	PANK2	Pantothenate kinase	30.60	0.00
TRINITY_sp|Q54Y	dhkJ	Hybrid signal trans	30.60	0.00
TRINITY_sp|Q6IN(	ttc21b	Tetratricopeptide r	30.60	0.00

TRINITY_sp|Q5372	pcrA	ATP-dependent DNA h	30.60	0.00
TRINITY_sp|Q08BE	ctdspl2a	CTD small phosphata	30.60	0.00
TRINITY_sp|P0CH3	RING1	E3 ubiquitin-protei	30.50	0.00
TRINITY_sp|Q0II(	RAB18	Ras-related protein	30.50	0.00
TRINITY_sp|Q1DTI	FYV10	Protein FYV10 OS=Co	30.50	0.00
TRINITY_sp|E2RJI	MASTL	Serine/threonine-pr	30.50	0.00
TRINITY_sp|O1383	ptr1	E3 ubiquitin-protei	30.50	0.00
TRINITY_sp|Q9D09	Acer3	Alkaline ceramidase	30.50	0.00
TRINITY_sp|P0DKJ	SWEET15	Bidirectional sugar	30.50	0.00
TRINITY_sp|O138(	ebp2	Probable rRNA-proce	30.50	0.00
TRINITY_sp|Q8GUI	JMJ14	Probable lysine-spe	30.50	0.00
TRINITY_sp|Q5FVE	Wdr89	WD repeat-containin	30.50	0.00
TRINITY_sp|Q0917	cds1	Serine/threonine-pr	30.50	0.00
TRINITY_sp|Q555E	rnf160	E3 ubiquitin-protei	30.50	0.00
TRINITY_sp|P2805	gpa-3	Guanine nucleotide-	30.50	0.00
TRINITY_sp|B3FWE	hpm2	Glutathione S-trans	30.50	0.00
TRINITY_sp|Q9Y6E	PARP3	Poly [ADP-ribose] p	30.50	0.00
TRINITY_sp|Q9UUI	mok12	Cell wall alpha-1,3	30.50	0.00
TRINITY_sp|P3704	yaeI	Phosphodiesterase Y	30.50	0.00
TRINITY_sp|Q9FKE	CCX1	Cation/calcium exch	30.50	0.00
TRINITY_sp|Q3SX4	RRAGA	Ras-related GTP-bin	30.50	0.00
TRINITY_sp|Q6AY(	Mmadhc	Methylmalonic acidu	30.50	0.00
TRINITY_sp|Q9LG2	Os01g029	Putative L-cysteine	30.50	0.00
TRINITY_sp|O2279	GLTP1	Glycolipid transfer	30.50	0.00
TRINITY_sp|O444(	F37C4.5	Protein F37C4.5 OS=	30.50	0.00
TRINITY_sp|Q8Y2E	RSc0270	UPF0225 protein RSc	30.50	0.00
TRINITY_sp|Q6AXE	babam1	BRISC and BRCA1-A c	30.50	0.00
TRINITY_sp|Q8XRY	kynA2	Tryptophan 2,3-diox	30.50	0.00
TRINITY_sp|Q644(	Cyp3a13	Cytochrome P450 3A1	30.50	0.00
TRINITY_sp|A7IQW	C42C1.13	Protein-lysine meth	30.50	0.00
TRINITY_sp|Q7KZI	MARK2	Serine/threonine-pr	30.50	0.00
TRINITY_sp|Q96BE	SGK3	Serine/threonine-pr	30.50	0.00
TRINITY_sp|P2377	SERPINA(	Corticosteroid-bind	30.50	0.00
TRINITY_sp|O6649	adk	Adenylate kinase OS	30.50	0.00
TRINITY_sp|O1505	TRANK1	TPR and ankyrin rep	30.50	0.00
TRINITY_sp|O1328	xyrA	NAD(P)H-dependent D	30.50	0.00
TRINITY_sp|Q84KI	GAMMA-AI	AP-1 complex subuni	30.50	0.00
TRINITY_sp|Q9173	ephb1-b	Ephrin type-B recep	30.50	0.00
TRINITY_sp|Q9VM7	Cyp28d2	Probable cytochrome	30.50	0.00
TRINITY_sp|A7MB1	PDCD11	Protein RRP5 homolo	30.50	0.00
TRINITY_sp|Q9SX8	SEC3A	Exocyst complex com	30.50	0.00
TRINITY_sp|Q9FLE	VTC5	GDP-L-galactose pho	30.50	0.00
TRINITY_sp|B0M0E	gefL	Ras guanine nucleot	30.50	0.00
TRINITY_sp|Q55A(	DDB_G027	Probable serine/thr	30.50	0.00
TRINITY_sp|P2167	TAF1	Transcription initi	30.50	0.00
TRINITY_sp|Q9H0E	GBP3	Guanylate-binding p	30.50	0.00
TRINITY_sp|F4I6N	TOR1L1	TORTIFOLIA1-like pr	30.50	0.00
TRINITY_sp|Q2175	vdac-1	Probable voltage-de	30.50	0.00
TRINITY_sp|Q9VMW	CG3792	Mannose-P-dolichol	30.50	0.00
TRINITY_sp|Q552E	pkgA	Probable serine/thr	30.50	0.00
TRINITY_sp|Q4WXX	rna14	mRNA 3'-end-process	30.50	0.00
TRINITY_sp|Q6K64	Os02g061	Eukaryotic translat	30.50	0.00
TRINITY_sp|Q9UPU	USP24	Ubiquitin carboxyl-	30.50	0.00
TRINITY_sp|Q70C(	USP34	Ubiquitin carboxyl-	30.50	0.00
TRINITY_sp|Q9S9U	CALS11	Callose synthase 11	30.50	0.00
TRINITY_sp|Q1269	YPK9	Vacuolar cation-tra	30.40	0.00

TRINITY_sp|Q29RM	SLC25A19	Mitochondrial thiam	30.40	0.00
TRINITY_sp|O7487	ccr4	Glucose-repressible	30.40	0.00
TRINITY_sp|Q9C6B	GAMMACA2	Gamma carbonic anhy	30.40	0.00
TRINITY_sp|Q54LE	vps13A	Putative vacuolar p	30.40	0.00
TRINITY_sp|P0825	CHN48	Endochitinase A OS=	30.40	0.00
TRINITY_sp|P5204	crt	Short-chain-enoyl-C	30.40	0.00
TRINITY_sp|Q9FFI	RMV1	Polyamine transport	30.40	0.00
TRINITY_sp|F4KGU	At5g1037	ATP-dependent RNA h	30.40	0.00
TRINITY_sp|Q9ESB	Necab1	N-terminal EF-hand	30.40	0.00
TRINITY_sp|Q9JJ0	Plscr1	Phospholipid scramb	30.40	0.00
TRINITY_sp|Q96MB	CFAP57	Cilia- and flagella	30.40	0.00
TRINITY_sp|Q54W1	ctns	Cystinosin homolog	30.40	0.00
TRINITY_sp|Q4R7E	TTLL10	Protein polyglycyla	30.40	0.00
TRINITY_sp|F4I1S	ELP2	Elongator complex p	30.40	0.00
TRINITY_sp|Q9VR9	cactin	Cactin OS=Drosophil	30.40	0.00
TRINITY_sp|Q9SI0	MTP12	Metal tolerance pro	30.40	0.00
TRINITY_sp|A7MRY	luxN	Autoinducer 1 senso	30.40	0.00
TRINITY_sp|Q7TN3	Celf6	CUGBP Elav-like fam	30.40	0.00
TRINITY_sp|A8I9E	CFAP45	Cilia- and flagella	30.40	0.00
TRINITY_sp|A1Z8J	CG7741	CWF19-like protein	30.40	0.00
TRINITY_sp|P5874	Aaas	Aladin OS=Mus muscu	30.40	0.00
TRINITY_sp|A8WYE	par-1	Serine/threonine-pr	30.40	0.00
TRINITY_sp|Q6NU1	MFSD12	Major facilitator s	30.40	0.00
TRINITY_sp|Q86U3	NOP9	Nucleolar protein 9	30.40	0.00
TRINITY_sp|Q9SY2	TAF8	Transcription initi	30.40	0.00
TRINITY_sp|Q4R8E	EIF2AK1	Eukaryotic translat	30.40	0.00
TRINITY_sp|Q8LAY	At5g1074	Probable protein ph	30.40	0.00
TRINITY_sp|Q9H25	SPTBN4	Spectrin beta chain	30.40	0.00
TRINITY_sp|A8ILE	CFAP52	Cilia- and flagella	30.40	0.00
TRINITY_sp|P3796	pnbA	Para-nitrobenzyl es	30.40	0.00
TRINITY_sp|Q75J9	cpras1	Circularly permutat	30.40	0.00
TRINITY_sp|P2862	-	Endoglucanase 4 OS=	30.40	0.00
TRINITY_sp|Q54SW	grlF	Metabotropic glutam	30.40	0.00
TRINITY_sp|B2VF0	viaA	Protein ViaA OS=Erw	30.40	0.00
TRINITY_sp|Q9VR9	HERC2	Probable E3 ubiquit	30.40	0.00
TRINITY_sp|Q9MYE	HSD17B14	17-beta-hydroxyster	30.40	0.00
TRINITY_sp|Q9C82	At3g0653	Uncharacterized pro	30.40	0.00
TRINITY_sp|Q2905	ITIH1	Inter-alpha-trypsin	30.40	0.00
TRINITY_sp|Q14CX	NAA25	N-alpha-acetyltrans	30.40	0.00
TRINITY_sp|Q9235	SPAC6G9.	Pumilio domain-cont	30.40	0.00
TRINITY_sp|Q2I6J	inppl1a	Phosphatidylinosito	30.40	0.00
TRINITY_sp|Q0787	VPS13	Vacuolar protein so	30.40	0.00
TRINITY_sp|Q54Q0	sec1	Protein transport p	30.40	0.00
TRINITY_sp|Q4ZH9	GUCY1A3	Guanylate cyclase s	30.40	0.00
TRINITY_sp|P4006	GLE2	Nucleoporin GLE2 OS	30.40	0.00
TRINITY_sp|Q54GI	DDB_G029	PXMP2/4 family prot	30.40	0.00
TRINITY_sp|Q9M1E	SEN1	tRNA-splicing endon	30.40	0.00
TRINITY_sp|F4JTE	STY46	Serine/threonine-pr	30.40	0.00
TRINITY_sp|Q54B0	DDB_G029	Probable GH family	30.40	0.00
TRINITY_sp|O4339	HNRNPR	Heterogeneous nucle	30.40	0.00
TRINITY_sp|Q5A59	NIK1	Histidine protein k	30.40	0.00
TRINITY_sp|P5819	Plscr1	Phospholipid scramb	30.40	0.00
TRINITY_sp|Q9D10	Rrp7a	Ribosomal RNA-proce	30.40	0.00
TRINITY_sp|Q9SFU	PAP15	Purple acid phospha	30.40	0.00
TRINITY_sp|Q0225	YVH1	Tyrosine-protein ph	30.40	0.00
TRINITY_sp|Q0WVI	GC5	Golgin candidate 5	30.40	0.00

TRINITY_sp|Q3969	INV3	Beta-fructofuranosi	30.40	0.00
TRINITY_sp|Q32P4	EML3	Echinoderm microtub	30.40	0.00
TRINITY_sp|Q9CAE	FLD	Protein FLOWERING L	30.40	0.00
TRINITY_sp|Q54P1	abcC8	ABC transporter C f	30.40	0.00
TRINITY_sp|Q9931	TY3B-G	Transposon Ty3-G Ga	30.40	0.00
TRINITY_sp|Q9ATE	TIP2-1	Aquaporin TIP2-1 OS	30.30	0.00
TRINITY_sp|P2065	Ppm1a	Protein phosphatase	30.30	0.00
TRINITY_sp|Q5852	MJ1123	Uncharacterized pro	30.30	0.00
TRINITY_sp|Q8BG4	Stk17b	Serine/threonine-pr	30.30	0.00
TRINITY_sp|Q9D4W	-	Uncharacterized pro	30.30	0.00
TRINITY_sp|Q3URE	Kctd21	BTB/POZ domain-cont	30.30	0.00
TRINITY_sp|Q9NPI	NMRK2	Nicotinamide ribosi	30.30	0.00
TRINITY_sp|Q9C5U	AHK3	Histidine kinase 3	30.30	0.00
TRINITY_sp|Q66JI	acbd6	Acyl-CoA-binding do	30.30	0.00
TRINITY_sp|Q6H7U	CIPK26	CBL-interacting pro	30.30	0.00
TRINITY_sp|Q5ZIN	TSEN2	tRNA-splicing endon	30.30	0.00
TRINITY_sp|Q6312	Abcc2	Canalicular multisp	30.30	0.00
TRINITY_sp|Q96G0	EEF2KMT	Protein-lysine N-me	30.30	0.00
TRINITY_sp|Q96M9	FGD4	FYVE, RhoGEF and PH	30.30	0.00
TRINITY_sp|Q9699	ZNF622	Zinc finger protein	30.30	0.00
TRINITY_sp|P1464	Pde4c	cAMP-specific 3',5'	30.30	0.00
TRINITY_sp|Q8AX9	slc35b1	Solute carrier fami	30.30	0.00
TRINITY_sp|Q9M35	GCP4	Gamma-tubulin compl	30.30	0.00
TRINITY_sp|Q9V8W	Rep	Rab proteins gerany	30.30	0.00
TRINITY_sp|Q9ES0	Ube4b	Ubiquitin conjugati	30.30	0.00
TRINITY_sp|P1346	abpC	Gelation factor OS=	30.30	0.00
TRINITY_sp|Q9S70	HSP70-14	Heat shock 70 kDa p	30.30	0.00
TRINITY_sp|Q54TN	drkD	Probable serine/thr	30.30	0.00
TRINITY_sp|P5456	yqkD	Uncharacterized pro	30.30	0.00
TRINITY_sp|P3883	WSS1	DNA-dependent metal	30.30	0.00
TRINITY_sp|C4IY9	-	Ascorbate-specific	30.30	0.00
TRINITY_sp|P5094	AVT4	Vacuolar amino acid	30.30	0.00
TRINITY_sp|Q9SN3	RABA1D	Ras-related protein	30.30	0.00
TRINITY_sp|Q8BR0	Tmem209	Transmembrane prote	30.30	0.00
TRINITY_sp|O6005	afg2	ATPase family gene	30.30	0.00
TRINITY_sp|P9453	ysgA	Uncharacterized tRN	30.30	0.00
TRINITY_sp|Q7NSI	hmp	Flavohemoprotein OS	30.30	0.00
TRINITY_sp|Q54KX	vps13F	Putative vacuolar p	30.30	0.00
TRINITY_sp|B4JYN	fig	Protein phosphatase	30.30	0.00
TRINITY_sp|Q149N	SHPRH	E3 ubiquitin-protei	30.30	0.00
TRINITY_sp|A8IC9	CFAP46	Cilia- and flagella	30.30	0.00
TRINITY_sp|B2KI0	WDR76	WD repeat-containin	30.30	0.00
TRINITY_sp|Q55E4	dhkE	Hybrid signal trans	30.30	0.00
TRINITY_sp|Q8RWI	STY17	Serine/threonine-pr	30.30	0.00
TRINITY_sp|Q32LE	CORO2A	Coronin-2A OS=Bos t	30.30	0.00
TRINITY_sp|Q9JI9	Cacna1f	Voltage-dependent L	30.30	0.00
TRINITY_sp|A0A0I	FUB4	Hydrolase FUB4 OS=F	30.30	0.00
TRINITY_sp|Q9XTE	ZK262.3	Lipase ZK262.3 OS=C	30.30	0.00
TRINITY_sp|Q6ZV7	FGD6	FYVE, RhoGEF and PH	30.30	0.00
TRINITY_sp|Q7X91	SAC6	Phosphoinositide ph	30.30	0.00
TRINITY_sp|P4115	HSFA1A	Heat stress transcr	30.30	0.00
TRINITY_sp|P9294	CLC-D	Chloride channel pr	30.30	0.00
TRINITY_sp|Q5U4E	Wdr34	WD repeat-containin	30.30	0.00
TRINITY_sp|Q7T2E	slc44a4	Choline transporter	30.30	0.00
TRINITY_sp|O9683	fab1	Putative 1-phosphat	30.30	0.00
TRINITY_sp|B4F61	aifm2	Apoptosis-inducing	30.30	0.00

TRINITY_sp|A1L4)	CLT2	Protein CLT2, chlor	30.30	0.00
TRINITY_sp|O433(	HNRNPR	Heterogeneous nucle	30.30	0.00
TRINITY_sp|Q86G4	gefQ	Ras guanine nucleot	30.30	0.00
TRINITY_sp|P464(	Gst2	Glutathione S-trans	30.30	0.00
TRINITY_sp|Q9ZW(	CYP735A2	Cytokinin hydroxyla	30.30	0.00
TRINITY_sp|Q2QA\	TIO	Serine/threonine-pr	30.30	0.00
TRINITY_sp|A6WU(	tdh	L-threonine 3-dehyd	30.30	0.00
TRINITY_sp|Q5N74	MOR1	Protein MOR1 OS=Ory	30.30	0.00
TRINITY_sp|P053(	p20	Uncharacterized N-a	30.20	0.00
TRINITY_sp|Q96Q4	ALS2	Alsin OS=Homo sapie	30.20	0.00
TRINITY_sp|Q55CI	rasU	Ras-like protein ra	30.20	0.00
TRINITY_sp|Q9CX(	Ist1	IST1 homolog OS=Mus	30.20	0.00
TRINITY_sp|Q9NS\	DIAPH3	Protein diaphanous	30.20	0.00
TRINITY_sp|Q9T0I	MTN1	5'-methylthioadenos	30.20	0.00
TRINITY_sp|B1MZ(	frr	Ribosome-recycling	30.20	0.00
TRINITY_sp|Q86I/	pdi1	Protein disulfide-i	30.20	0.00
TRINITY_sp|Q0JI4	CIPK11	CBL-interacting pro	30.20	0.00
TRINITY_sp|Q9FL\	GNL1	ARF guanine-nucleot	30.20	0.00
TRINITY_sp|Q110I	pepN	Aminopeptidase N OS	30.20	0.00
TRINITY_sp|Q9U9I	snpC	Gamma-soluble NSF a	30.20	0.00
TRINITY_sp|Q9NG(	CenG1A	Centaurin-gamma-1A	30.20	0.00
TRINITY_sp|Q5F4	PPP6R3	Serine/threonine-pr	30.20	0.00
TRINITY_sp|Q54Y(	CRTF	CAR1 transcription	30.20	0.00
TRINITY_sp|Q9LE	RBL20	Rhomboid-like prote	30.20	0.00
TRINITY_sp|O668	bioF	Putative 8-amino-7-	30.20	0.00
TRINITY_sp|A2C5I	rpsF	30S ribosomal prote	30.20	0.00
TRINITY_sp|Q000(	PRMS	Pathogenesis-relate	30.20	0.00
TRINITY_sp|Q4U2I	Herc2	E3 ubiquitin-protei	30.20	0.00
TRINITY_sp|O944(	bdp1	Transcription facto	30.20	0.00
TRINITY_sp|Q037(	CEBPZ	CCAAT/enhancer-bind	30.20	0.00
TRINITY_sp|O822(	SKL2	Probable inactive s	30.20	0.00
TRINITY_sp|P408(	-	Crystallin J1C OS=T	30.20	0.00
TRINITY_sp|Q76P(	DDB_G02	Probable serine/thr	30.20	0.00
TRINITY_sp|Q6J4I	SLC8B1	Sodium/potassium/ca	30.20	0.00
TRINITY_sp|Q963(	IMPA1	Importin subunit al	30.20	0.00
TRINITY_sp|Q9V4\	GLE1	Nucleoporin GLE1 OS	30.20	0.00
TRINITY_sp|Q5A7(	CCR4	Glucose-repressible	30.20	0.00
TRINITY_sp|Q54I4	slc44a2	Choline transporter	30.20	0.00
TRINITY_sp|B3H5I	NEDD1	Protein NEDD1 OS=Ar	30.20	0.00
TRINITY_sp|Q6GR(	spopl	Speckle-type POZ pr	30.20	0.00
TRINITY_sp|Q9VR(	HERC2	Probable E3 ubiquit	30.20	0.00
TRINITY_sp|P561(	RRP1	Ribosomal RNA proce	30.20	0.00
TRINITY_sp|O548(	Dusp5	Dual specificity pr	30.20	0.00
TRINITY_sp|Q5RA	ARCN1	Coatomer subunit de	30.20	0.00
TRINITY_sp|Q54PI	mrd1	Multiple RNA-bindin	30.20	0.00
TRINITY_sp|Q8JI(	-	Cysteine-rich venom	30.20	0.00
TRINITY_sp|Q5VJI	gdt9	Probable serine/thr	30.20	0.00
TRINITY_sp|Q158	CACNA1E	Voltage-dependent R	30.20	0.00
TRINITY_sp|Q75J(	cpras1	Circularly permutat	30.20	0.00
TRINITY_sp|O830	rsmE	Ribosomal RNA small	30.20	0.00
TRINITY_sp|Q6S7I	TAF5	Transcription initi	30.20	0.00
TRINITY_sp|Q9HGI	gcn2	eIF-2-alpha kinase	30.20	0.00
TRINITY_sp|P340(	cmfA	Conditioned medium	30.20	0.00
TRINITY_sp|Q9XI(	WRKY4	Probable WRKY trans	30.20	0.00
TRINITY_sp|Q16TI	AAEL010I	Band 7 protein AAEL	30.20	0.00
TRINITY_sp|Q94A	At5g5314	Probable protein ph	30.20	0.00

TRINITY_sp|Q0WU(	MTP10	Metal tolerance pro	30.20	0.00
TRINITY_sp|P261S	ACT2	Actin-2 OS=Absidia	30.20	0.00
TRINITY_sp|Q9CT(	Atp13a2	Probable cation-tra	30.20	0.00
TRINITY_sp|O1372	SPAC15A1	Uncharacterized Na(	30.20	0.00
TRINITY_sp|P225(	bglB	Beta-glucosidase B	30.20	0.00
TRINITY_sp|F2Z4(	Herc6	E3 ISG15--protein l	30.20	0.00
TRINITY_sp|O243(	TIC110	Protein TIC110, chl	30.20	0.00
TRINITY_sp|Q8BRH	Kmt2c	Histone-lysine N-me	30.20	0.00
TRINITY_sp|O835(	rpoD	RNA polymerase sigm	30.20	0.00
TRINITY_sp|Q950l	ubc-9	SUMO-conjugating en	30.10	0.00
TRINITY_sp|F4IJK	KIN14R	Kinesin-like protei	30.10	0.00
TRINITY_sp|Q2TGJ	Zdhhc15	Palmitoyltransferas	30.10	0.00
TRINITY_sp|P395S	ywbO	Uncharacterized pro	30.10	0.00
TRINITY_sp|Q2LGH	TOLLIP	Toll-interacting pr	30.10	0.00
TRINITY_sp|Q8S91	UBC5B	Ubiquitin-conjugati	30.10	0.00
TRINITY_sp|P1382	-	Aspartic acid-rich	30.10	0.00
TRINITY_sp|Q9H8(	VCPKMT	Protein-lysine meth	30.10	0.00
TRINITY_sp|Q3952	HUP2	H(+)/hexose cotrans	30.10	0.00
TRINITY_sp|Q4R7H	TTLL10	Protein polyglycyla	30.10	0.00
TRINITY_sp|Q969X	TMBIM1	Protein lifeguard 3	30.10	0.00
TRINITY_sp|Q5ZK(	EIF2A	Eukaryotic translat	30.10	0.00
TRINITY_sp|Q6203	Cep131	Centrosomal protein	30.10	0.00
TRINITY_sp|P821S	Tgfbi	Transforming growth	30.10	0.00
TRINITY_sp|Q54PW	DDB_G028	Protein EI24 homolo	30.10	0.00
TRINITY_sp|Q5833	MJ0928	Putative protein me	30.10	0.00
TRINITY_sp|Q869W	DDB_G027	Probable myosin lig	30.10	0.00
TRINITY_sp|S4R2I	Slc8a3	Sodium/calcium exch	30.10	0.00
TRINITY_sp|Q9ZPI	CDC48B	Cell division contr	30.10	0.00
TRINITY_sp|Q9FF5	PDIL1-4	Protein disulfide i	30.10	0.00
TRINITY_sp|Q3905	CNX3	Cyclic pyranopterin	30.10	0.00
TRINITY_sp|Q79LY	hopD2	Effector protein ho	30.10	0.00
TRINITY_sp|Q28G(	mecr	Trans-2-enoyl-CoA r	30.10	0.00
TRINITY_sp|Q5A5S	NIK1	Histidine protein k	30.10	0.00
TRINITY_sp|Q8TBS	ITFG1	T-cell immunomodula	30.10	0.00
TRINITY_sp|A9US3	22789	Putative glycoside	30.10	0.00
TRINITY_sp|Q93VN	THO1	THO complex subunit	30.10	0.00
TRINITY_sp|P1464	Pde4c	cAMP-specific 3',5'	30.10	0.00
TRINITY_sp|Q8IY8	DRC7	Dynein regulatory c	30.10	0.00
TRINITY_sp|P750S	MPN_020	Uncharacterized ATP	30.10	0.00
TRINITY_sp|P7317	sll1290	Uncharacterized rib	30.10	0.00
TRINITY_sp|Q9NUJ	PLCXD1	PI-PLC X domain-con	30.10	0.00
TRINITY_sp|Q0068	gp63	Leishmanolysin OS=L	30.10	0.00
TRINITY_sp|Q9ZVJ	SGPP	Haloacid dehalogena	30.10	0.00
TRINITY_sp|Q4U2H	Herc2	E3 ubiquitin-protei	30.10	0.00
TRINITY_sp|A3EWI	REV1	DNA repair protein	30.10	0.00
TRINITY_sp|Q54Y5	shkC	Dual specificity pr	30.10	0.00
TRINITY_sp|Q8VY2	PAP8	Purple acid phospha	30.10	0.00
TRINITY_sp|Q9ZT2	-	Delta(7)-sterol-C5(	30.10	0.00
TRINITY_sp|C0ITI	-	Venom allergen 5 (F	30.10	0.00
TRINITY_sp|Q0554	CHI17	Acidic 27 kDa endoc	30.10	0.00
TRINITY_sp|Q9US3	SPAC1039	D-serine dehydratas	30.10	0.00
TRINITY_sp|Q7SY7	ncald	Neurocalcin-delta O	30.10	0.00
TRINITY_sp|Q7XSA	AGO1B	Protein argonaute 1	30.10	0.00
TRINITY_sp|P4671	Pdcd2	Programmed cell dea	30.10	0.00
TRINITY_sp|Q9LX(	PCO1	Plant cysteine oxid	30.10	0.00
TRINITY_sp|Q9P7(	oma1	Mitochondrial metal	30.10	0.00

TRINITY_sp|P788	tpp1	Trehalose-phosphata	30.00	0.00
TRINITY_sp|Q54U	abcC12	ABC transporter C f	30.00	0.00
TRINITY_sp|Q9LT	VPS9A	Vacuolar protein so	30.00	0.00
TRINITY_sp|Q9FN	UVR8	Ultraviolet-B recep	30.00	0.00
TRINITY_sp|P221	APA2	Diadenosine 5',5'''	30.00	0.00
TRINITY_sp|Q8LA	URH2	Probable uridine nu	30.00	0.00
TRINITY_sp|A4FV	RSL1D1	Ribosomal L1 domain	30.00	0.00
TRINITY_sp|A6QN	RRP36	Ribosomal RNA proce	30.00	0.00
TRINITY_sp|O137	cns1	Hsp70/Hsp90 co-chap	30.00	0.00
TRINITY_sp|Q0II	DNAJC21	DnaJ homolog subfam	30.00	0.00
TRINITY_sp|Q5EA	CHID1	Chitinase domain-co	30.00	0.00
TRINITY_sp|Q92K	afr	1,5-anhydro-D-fruct	30.00	0.00
TRINITY_sp|Q9LR	BSU1	Serine/threonine-pr	30.00	0.00
TRINITY_sp|Q6T4	rbrA	Probable E3 ubiquit	30.00	0.00
TRINITY_sp|Q9S9	CCR1	Cinnamoyl-CoA reduc	30.00	0.00
TRINITY_sp|Q9P3	dbl4	E3 ubiquitin-protei	30.00	0.00
TRINITY_sp|Q7TP	Apmap	Adipocyte plasma me	30.00	0.00
TRINITY_sp|Q9DB	Phkg2	Phosphorylase b kin	30.00	0.00
TRINITY_sp|P360	TUL1	Transmembrane E3 ub	30.00	0.00
TRINITY_sp|Q99J	Thumpd1	THUMP domain-contai	30.00	0.00
TRINITY_sp|Q94F	ABCC1	ABC transporter D f	30.00	0.00
TRINITY_sp|Q55A	DDB_G02	PH and Rap-GAP doma	30.00	0.00
TRINITY_sp|P533	YGR266W	Uncharacterized pro	30.00	0.00
TRINITY_sp|Q9M0	GGP3	Gamma-glutamyl pept	30.00	0.00
TRINITY_sp|Q84M	ABCA1	ABC transporter A f	30.00	0.00
TRINITY_sp|P0CU	CTHT_00	Protein-lysine N-me	30.00	0.00
TRINITY_sp|P511	A1	Dihydroflavonol 4-r	30.00	0.00
TRINITY_sp|Q54Y	dhkJ	Hybrid signal trans	30.00	0.00
TRINITY_sp|Q9Y5	POLR3H	DNA-directed RNA po	30.00	0.00
TRINITY_sp|A0QS	MSMEG_1	Putative S-adenosyl	30.00	0.00
TRINITY_sp|Q893	hutG	Formimidoylglutamas	30.00	0.00
TRINITY_sp|Q54R	dhkL	Hybrid signal trans	30.00	0.00
TRINITY_sp|P464	-	Glutathione S-trans	30.00	0.00
TRINITY_sp|Q6RG	RGS1	Regulator of G-prot	30.00	0.00
TRINITY_sp|P511	Taf1	Transcription initi	30.00	0.00
TRINITY_sp|P966	nap	Uncharacterized car	30.00	0.00
TRINITY_sp|P266	sigA	RNA polymerase sigm	30.00	0.00
TRINITY_sp|Q866	ADCY10	Adenylate cyclase t	30.00	0.00
TRINITY_sp|Q086	PUS7	Multisubstrate pseu	30.00	0.00
TRINITY_sp|Q9TW	myoM	Myosin-M heavy chai	30.00	0.00
TRINITY_sp|Q5SN	serac1	Protein SERAC1 OS=D	30.00	0.00
TRINITY_sp|P215	ALP1	Alpha-amylase OS=Sa	30.00	0.00
TRINITY_sp|Q054	pksL	Polyketide synthase	30.00	0.00
TRINITY_sp|Q29B	GA18864	Esterase GA18864 OS	30.00	0.00
TRINITY_sp|H3JU	SGT1	Peptidyl serine alp	30.00	0.00
TRINITY_sp|Q54Q	DDB_G02	Probable E3 ubiquit	30.00	0.00
TRINITY_sp|Q54G	gacW	Rho GTPase-activati	30.00	0.00
TRINITY_sp|Q5RA	ACOX3	Peroxisomal acyl-co	30.00	0.00
TRINITY_sp|Q9MA	ACBP4	Acyl-CoA-binding do	30.00	0.00
TRINITY_sp|Q079	PSR2	Probable phosphatas	30.00	0.00
TRINITY_sp|Q5A5	NIK1	Histidine protein k	30.00	0.00
TRINITY_sp|P388	TRA1	Transcription-assoc	30.00	0.00
TRINITY_sp|Q2QN	Os12g05	Probable protein ph	30.00	0.00
TRINITY_sp|Q9FF	At5g384	Probable dolichyl p	30.00	0.00
TRINITY_sp|Q944	FKBP16-	Peptidyl-prolyl cis	30.00	0.00
TRINITY_sp|Q55G	DDB_G02	Probable serine/thr	30.00	0.00

TRINITY_sp|Q9S3(	pcrA	ATP-dependent DNA h	30.00	0.00
TRINITY_sp|Q934!	rae-1	mRNA export factor	29.90	0.00
TRINITY_sp|Q28I(	ppp4r2	Serine/threonine-pr	29.90	0.00
TRINITY_sp|Q9H0I	TOLLIP	Toll-interacting pr	29.90	0.00
TRINITY_sp|Q54P!	osbI	Oxysterol-binding p	29.90	0.00
TRINITY_sp|A1JM>	rutB	Peroxyureidoacrylat	29.90	0.00
TRINITY_sp|O824:	ETR1	Ethylene receptor 1	29.90	0.00
TRINITY_sp|Q55C2	gdt2	Probable serine/thr	29.90	0.00
TRINITY_sp|Q2KJ]	AFG3L2	AFG3-like protein 2	29.90	0.00
TRINITY_sp|Q84M!	ARP4	Actin-related prote	29.90	0.00
TRINITY_sp|B3FWI	hpm2	Glutathione S-trans	29.90	0.00
TRINITY_sp|Q644]	PGC	Gastricsin OS=Cavia	29.90	0.00
TRINITY_sp|P306:	ZK637.14	Uncharacterized RIN	29.90	0.00
TRINITY_sp|P250(	puc1	Cyclin puc1 OS=Schi	29.90	0.00
TRINITY_sp|Q4UL!	RF_0863	Uncharacterized RNA	29.90	0.00
TRINITY_sp|P409	lys1	L-2-aminoadipate re	29.90	0.00
TRINITY_sp|Q5M7I	nol6	Nucleolar protein 6	29.90	0.00
TRINITY_sp|Q8BW(	Dclk3	Serine/threonine-pr	29.90	0.00
TRINITY_sp|Q54X8	ap1b1	AP-1 complex subuni	29.90	0.00
TRINITY_sp|Q920:	ACHE	Acetylcholinesteras	29.90	0.00
TRINITY_sp|A5DSI	FMP52	Protein FMP52, mito	29.90	0.00
TRINITY_sp|E1BY:	MSH6	DNA mismatch repair	29.90	0.00
TRINITY_sp|P312(	pab1	Polyadenylate-bindi	29.90	0.00
TRINITY_sp|O947]	SPCC1259	Uncharacterized pro	29.90	0.00
TRINITY_sp|Q10Q(	RPA1B	Replication protein	29.90	0.00
TRINITY_sp|Q9HE]	utp5	U3 small nucleolar	29.90	0.00
TRINITY_sp|Q6DBI	At4g0845	BTB/POZ domain-cont	29.90	0.00
TRINITY_sp|P499(	Taf12	Transcription initi	29.90	0.00
TRINITY_sp|Q86L!	gacHH	Rho GTPase-activati	29.90	0.00
TRINITY_sp|Q9Y8(	CYP505	Bifunctional cytoch	29.90	0.00
TRINITY_sp|Q9M04	BHLH140	Transcription facto	29.90	0.00
TRINITY_sp|Q8TB\	WDR66	WD repeat-containin	29.90	0.00
TRINITY_sp|P506]	CDK7	Cyclin-dependent ki	29.90	0.00
TRINITY_sp|Q2TH\	ZDHHC8	Probable palmitoylt	29.90	0.00
TRINITY_sp|Q6DG4	ubtd2	Ubiquitin domain-co	29.90	0.00
TRINITY_sp|Q5F1I	DNAJC21	DnaJ homolog subfam	29.90	0.00
TRINITY_sp|P3214	yihV	Sulfofructose kinas	29.90	0.00
TRINITY_sp|Q8IV!	DENND1C	DENN domain-contain	29.90	0.00
TRINITY_sp|Q9NV(	TBC1D13	TBC1 domain family	29.90	0.00
TRINITY_sp|O009]	dstA	Signal transducer a	29.90	0.00
TRINITY_sp|P262(	CD36	Platelet glycoprote	29.90	0.00
TRINITY_sp|Q9SA4	MED22A	Mediator of RNA pol	29.90	0.00
TRINITY_sp|Q3SZI	TTLL9	Probable tubulin po	29.90	0.00
TRINITY_sp|F4J1]	LSF1	Phosphoglucan phosp	29.90	0.00
TRINITY_sp|Q949>	LYSA1	Diaminopimelate dec	29.90	0.00
TRINITY_sp|Q9FW\	IMPA6	Importin subunit al	29.90	0.00
TRINITY_sp|Q54D]	DDB_G029	Down syndrome criti	29.90	0.00
TRINITY_sp|Q146	TTLL4	Tubulin polyglutamy	29.90	0.00
TRINITY_sp|Q9Y3(	ACOT9	Acyl-coenzyme A thi	29.90	0.00
TRINITY_sp|Q7M3!	rngB	RING finger protein	29.90	0.00
TRINITY_sp|Q54Y!	shkC	Dual specificity pr	29.90	0.00
TRINITY_sp|Q9UR(	Tf2-11	Transposon Tf2-11 p	29.90	0.00
TRINITY_sp|O947(	rxt3	Transcriptional reg	29.90	0.00
TRINITY_sp|Q4G2:	DER1.2	Derlin-1.2 OS=Zea m	29.90	0.00
TRINITY_sp|P179	Shaw	Potassium voltage-g	29.80	0.00
TRINITY_sp|P547	TYDC5	Tyrosine/DOPA decar	29.80	0.00

TRINITY_sp|Q54R4	gpaI	Guanine nucleotide-	29.80	0.00
TRINITY_sp|P3789	pleC	Non-motile and phag	29.80	0.00
TRINITY_sp|Q54ZI	DDB_G027	WD repeat-containin	29.80	0.00
TRINITY_sp|A4IH7	fignl1	Fidgetin-like prote	29.80	0.00
TRINITY_sp|Q5M72	RNFT1	RING finger and tra	29.80	0.00
TRINITY_sp|Q86A7	DDB_G027	Recoverin family pr	29.80	0.00
TRINITY_sp|P1926	AMY1	Alpha-amylase 1 OS=	29.80	0.00
TRINITY_sp|P3195	pac	Penicillin G acylas	29.80	0.00
TRINITY_sp|Q54Y2	dhkJ	Hybrid signal trans	29.80	0.00
TRINITY_sp|Q9LT7	VCS	Enhancer of mRNA-de	29.80	0.00
TRINITY_sp|A9UY9	serinc	Probable serine inc	29.80	0.00
TRINITY_sp|Q54Q0	pgtA	Bifunctional glycos	29.80	0.00
TRINITY_sp|F4ILY	At2g4200	Amino acid transpor	29.80	0.00
TRINITY_sp|Q9Y40	RBM19	Probable RNA-bindin	29.80	0.00
TRINITY_sp|A0LJ4	dnaJ	Chaperone protein D	29.80	0.00
TRINITY_sp|Q9436	gei-17	E3 SUMO-protein lig	29.80	0.00
TRINITY_sp|Q7XWI	Os04g025	Probable cleavage a	29.80	0.00
TRINITY_sp|O4440	eri-1	3'-5' exonuclease e	29.80	0.00
TRINITY_sp|Q7TS6	Nsun6	Putative methyltran	29.80	0.00
TRINITY_sp|Q5T50	STXBP5	Syntaxin-binding pr	29.80	0.00
TRINITY_sp|P1689	FUS3	Mitogen-activated p	29.80	0.00
TRINITY_sp|Q8L79	CID3	Polyadenylate-bindi	29.80	0.00
TRINITY_sp|Q9SMI	RABH1C	Ras-related protein	29.80	0.00
TRINITY_sp|Q9FPI	EDR1	Serine/threonine-pr	29.80	0.00
TRINITY_sp|Q05B1	shq1	Protein SHQ1 homolo	29.80	0.00
TRINITY_sp|Q553I	gxcJJ	Rac guanine nucleot	29.80	0.00
TRINITY_sp|Q9FXI	At1g6782	Probable protein ph	29.80	0.00
TRINITY_sp|Q9Y36	STARD10	PCTP-like protein O	29.80	0.00
TRINITY_sp|P1035	ry	Xanthine dehydrogen	29.80	0.00
TRINITY_sp|B0VX6	SETD3	Histone-lysine N-me	29.80	0.00
TRINITY_sp|Q54J9	gacX	Rho GTPase-activati	29.80	0.00
TRINITY_sp|O9519	RCBTB2	RCC1 and BTB domain	29.80	0.00
TRINITY_sp|O6034	TBC1D4	TBC1 domain family	29.80	0.00
TRINITY_sp|Q8VEM	Klhdc3	Kelch domain-contai	29.80	0.00
TRINITY_sp|B2RS9	Rrn3	RNA polymerase I-sp	29.80	0.00
TRINITY_sp|Q0304	Pkg21D	cGMP-dependent prot	29.80	0.00
TRINITY_sp|Q9VR9	HERC2	Probable E3 ubiquit	29.80	0.00
TRINITY_sp|Q6GLA	slc25a26	S-adenosylmethionin	29.80	0.00
TRINITY_sp|B5F0I	yfeW	UPF0214 protein Yfe	29.80	0.00
TRINITY_sp|Q55D0	rabggta	Geranylgeranyl tran	29.80	0.00
TRINITY_sp|A1L1I	slc47a1	Multidrug and toxin	29.80	0.00
TRINITY_sp|Q0077	cmkA	Calcium/calmodulin-	29.80	0.00
TRINITY_sp|Q9VR0	Npc1b	Niemann-Pick type p	29.80	0.00
TRINITY_sp|Q7M0I	Nudt19	Nucleoside diphosph	29.80	0.00
TRINITY_sp|O0783	dapb1	Dipeptidyl aminopep	29.80	0.00
TRINITY_sp|Q0349	STE20	Serine/threonine-pr	29.80	0.00
TRINITY_sp|Q9SCJ	UBP26	Ubiquitin carboxyl-	29.80	0.00
TRINITY_sp|Q9235	SPAC6G9.	Pumilio domain-cont	29.80	0.00
TRINITY_sp|A6SJW	BC1G_127	Eukaryotic translat	29.80	0.00
TRINITY_sp|O0053	VWA5A	von Willebrand fact	29.80	0.00
TRINITY_sp|Q55GU	DDB_G026	Probable serine/thr	29.80	0.00
TRINITY_sp|P3836	PHO89	Phosphate permease	29.80	0.00
TRINITY_sp|Q9JL0	Capn15	Calpain-15 OS=Mus m	29.80	0.00
TRINITY_sp|Q8C07	Mfsd9	Major facilitator s	29.70	0.00
TRINITY_sp|P6299	RAC1	Ras-related C3 botu	29.70	0.00
TRINITY_sp|Q6EEI	TTLL5	Tubulin polyglutamy	29.70	0.00

TRINITY_sp|Q9V9I	CG3107	Presequence proteas	29.70	0.00
TRINITY_sp|P3992	SLN1	Osmosensing histidi	29.70	0.00
TRINITY_sp|Q996I	DNAJC7	DnaJ homolog subfam	29.70	0.00
TRINITY_sp|Q6GNI	c1galt1	Glycoprotein-N-acet	29.70	0.00
TRINITY_sp|Q0JI4	CIPK11	CBL-interacting pro	29.70	0.00
TRINITY_sp|Q5XH9	ddx1	ATP-dependent RNA h	29.70	0.00
TRINITY_sp|Q94AI	CUL1	Cullin-1 OS=Arabido	29.70	0.00
TRINITY_sp|P2659	PTBP1	Polypyrimidine trac	29.70	0.00
TRINITY_sp|O947I	SPCC1259	Uncharacterized pro	29.70	0.00
TRINITY_sp|Q1457	ITPR2	Inositol 1,4,5-tris	29.70	0.00
TRINITY_sp|Q54WN	tmem184C	Transmembrane prote	29.70	0.00
TRINITY_sp|Q9UR0	Tf2-11	Transposon Tf2-11 p	29.70	0.00
TRINITY_sp|P3555	Ide	Insulin-degrading e	29.70	0.00
TRINITY_sp|P4657	PBL10	Probable serine/thr	29.70	0.00
TRINITY_sp|Q54DV	ergic3	Probable endoplasmi	29.70	0.00
TRINITY_sp|Q8BZI	Gucd1	Protein GUCD1 OS=Mu	29.70	0.00
TRINITY_sp|B9FMJ	KIN13A	Kinesin-like protei	29.70	0.00
TRINITY_sp|Q54PI	DDB_G028	Probable myosin lig	29.70	0.00
TRINITY_sp|Q0068	gp63	Leishmanolysin OS=L	29.70	0.00
TRINITY_sp|Q1048	tim13	Mitochondrial impor	29.70	0.00
TRINITY_sp|O9427	SPBP8B7.	Uncharacterized RIN	29.70	0.00
TRINITY_sp|Q68EI	wdr18	WD repeat-containin	29.70	0.00
TRINITY_sp|O7477	msh2	DNA mismatch repair	29.70	0.00
TRINITY_sp|Q6TN9	pak1ip1	p21-activated prote	29.70	0.00
TRINITY_sp|Q54E1	grlE	Metabotropic glutam	29.70	0.00
TRINITY_sp|B1ZX4	truB	tRNA pseudouridine	29.70	0.00
TRINITY_sp|P3117	-	Autolysin OS=Chlamy	29.70	0.00
TRINITY_sp|P222I	RAD53	Serine/threonine-pr	29.70	0.00
TRINITY_sp|Q0JL4	SGT1	Protein SGT1 homolo	29.70	0.00
TRINITY_sp|P463I	FAD6	Omega-6 fatty acid	29.70	0.00
TRINITY_sp|Q2648	FKBP46	46 kDa FK506-bindin	29.70	0.00
TRINITY_sp|P6187	-	Lipase OS=Rhizopus	29.70	0.00
TRINITY_sp|Q75EI	TPC1	Mitochondrial thiam	29.70	0.00
TRINITY_sp|O7648	GstD7	Glutathione S-trans	29.70	0.00
TRINITY_sp|Q86G4	gefQ	Ras guanine nucleot	29.70	0.00
TRINITY_sp|Q7K0I	PGAP3	Post-GPI attachment	29.70	0.00
TRINITY_sp|Q1760	cel-1	mRNA-capping enzyme	29.70	0.00
TRINITY_sp|Q0P5V	Vps8	Vacuolar protein so	29.70	0.00
TRINITY_sp|Q54E1	grlE	Metabotropic glutam	29.70	0.00
TRINITY_sp|Q5BCI	fmp52	Protein fmp52, mito	29.70	0.00
TRINITY_sp|P5179	CLCN7	H(+)/Cl(-) exchange	29.70	0.00
TRINITY_sp|B0SA2	adk	Adenylate kinase OS	29.70	0.00
TRINITY_sp|Q55G0	DDB_G026	Probable serine/thr	29.70	0.00
TRINITY_sp|Q54F2	cln3	Battenin OS=Dictyos	29.70	0.00
TRINITY_sp|Q9UKI	PARP4	Poly [ADP-ribose] p	29.70	0.00
TRINITY_sp|Q5TCX	MLK4	Mitogen-activated p	29.70	0.00
TRINITY_sp|Q9SE2	CIPK16	CBL-interacting ser	29.60	0.00
TRINITY_sp|P9407	At3g2672	Alpha-mannosidase A	29.60	0.00
TRINITY_sp|Q9P70	mak1	Peroxide stress-act	29.60	0.00
TRINITY_sp|Q54W1	DDB_G027	U6 snRNA phosphodie	29.60	0.00
TRINITY_sp|Q54P9	osbI	Oxysterol-binding p	29.60	0.00
TRINITY_sp|P2465	PTP	Tyrosine-protein ph	29.60	0.00
TRINITY_sp|Q9P70	mak1	Peroxide stress-act	29.60	0.00
TRINITY_sp|Q9VSI	CG7185	Cleavage and polyad	29.60	0.00
TRINITY_sp|Q8VY0	ESP3	Pre-mRNA-splicing f	29.60	0.00
TRINITY_sp|Q94EI	At3g1441	Probable sugar phos	29.60	0.00

TRINITY_sp|Q551	dhkF	Hybrid signal trans	29.60	0.00
TRINITY_sp|O157	tipD	Protein tipD OS=Dic	29.60	0.00
TRINITY_sp|Q9JL	Capn15	Calpain-15 OS=Mus m	29.60	0.00
TRINITY_sp|Q6AY	Sccpdh	Saccharopine dehydr	29.60	0.00
TRINITY_sp|Q9US	gaa1	GPI transamidase co	29.60	0.00
TRINITY_sp|A8MR	FMOGS-O	Flavin-containing m	29.60	0.00
TRINITY_sp|Q5UQ	MIMI_L4	Putative alpha/beta	29.60	0.00
TRINITY_sp|Q6NV	prpf31	U4/U6 small nuclear	29.60	0.00
TRINITY_sp|Q9H1	WDR13	WD repeat-containin	29.60	0.00
TRINITY_sp|Q4R5	SNX1	Sorting nexin-1 OS=	29.60	0.00
TRINITY_sp|Q9SZ	CYP81F4	Cytochrome P450 81F	29.60	0.00
TRINITY_sp|Q5Z9	Os06g05	Solute carrier fami	29.60	0.00
TRINITY_sp|Q5RC	RBM39	RNA-binding protein	29.60	0.00
TRINITY_sp|Q54Q	sec1	Protein transport p	29.60	0.00
TRINITY_sp|A0BK	GSPATT0	Protein SEY1 homolo	29.60	0.00
TRINITY_sp|Q5Z6	CIPK25	CBL-interacting pro	29.60	0.00
TRINITY_sp|Q287	-	Pregnancy-associate	29.60	0.00
TRINITY_sp|Q6TN	pak1ip1	p21-activated prote	29.60	0.00
TRINITY_sp|Q6PF	ngdn	Neuroguidin OS=Dani	29.60	0.00
TRINITY_sp|O890	Tpp1	Tripeptidyl-peptida	29.60	0.00
TRINITY_sp|Q9P3	dbl4	E3 ubiquitin-protei	29.60	0.00
TRINITY_sp|Q9D5	Zc2hc1b	Zinc finger C2HC do	29.60	0.00
TRINITY_sp|Q6JQ	ACAD10	Acyl-CoA dehydrogen	29.60	0.00
TRINITY_sp|Q54P	DDB_G02	Probable serine/thr	29.60	0.00
TRINITY_sp|P254	RAD51	DNA repair protein	29.60	0.00
TRINITY_sp|Q9KD	dnaJ	Chaperone protein D	29.60	0.00
TRINITY_sp|P407	recQ	ATP-dependent DNA h	29.60	0.00
TRINITY_sp|Q6NR	ppp6r3-a	Serine/threonine-pr	29.60	0.00
TRINITY_sp|A1L4	CLT2	Protein CLT2, chlor	29.60	0.00
TRINITY_sp|B3PJ	trmA	tRNA/tmRNA (uracil-	29.60	0.00
TRINITY_sp|O343	ytcJ	Putative amidohydro	29.60	0.00
TRINITY_sp|Q5SN	Os06g01	Fanconi-associated	29.60	0.00
TRINITY_sp|Q54N	mipp1	Multiple inositol p	29.60	0.00
TRINITY_sp|Q7YX	cpnA	Copine-A OS=Dictyos	29.60	0.00
TRINITY_sp|Q9FJ	CCB2	Protein COFACTOR AS	29.60	0.00
TRINITY_sp|Q9SV	ASAT1	Acyl-CoA--sterol O-	29.60	0.00
TRINITY_sp|Q573	HI_0036	Uncharacterized ABC	29.60	0.00
TRINITY_sp|Q9UQ	AURKC	Aurora kinase C OS=	29.60	0.00
TRINITY_sp|Q7S6	un-4	Mitochondrial impor	29.50	0.00
TRINITY_sp|O814	-	Eukaryotic translat	29.50	0.00
TRINITY_sp|Q9Y5	NUB1	NEDD8 ultimate bust	29.50	0.00
TRINITY_sp|P110	RAB3A	Ras-related protein	29.50	0.00
TRINITY_sp|Q8CH	Ttll5	Tubulin polyglutamy	29.50	0.00
TRINITY_sp|A6S3	ssh4	Protein ssh4 OS=Bot	29.50	0.00
TRINITY_sp|Q54E	cct2	T-complex protein 1	29.50	0.00
TRINITY_sp|P011	H-RAS	Transforming protei	29.50	0.00
TRINITY_sp|Q08J	NSUN2	tRNA (cytosine(34)-	29.50	0.00
TRINITY_sp|Q0VC	AP2A2	AP-2 complex subuni	29.50	0.00
TRINITY_sp|Q54W	mcfL	Mitochondrial subst	29.50	0.00
TRINITY_sp|Q3V1	Pnpla1	Patatin-like phosph	29.50	0.00
TRINITY_sp|Q54C	commd8	COMM domain-contain	29.50	0.00
TRINITY_sp|Q8T5	abcA1	ABC transporter A f	29.50	0.00
TRINITY_sp|P361	GPT2	Glycerol-3-phosphat	29.50	0.00
TRINITY_sp|Q93V	CYP97A3	Protein LUTEIN DEFI	29.50	0.00
TRINITY_sp|P837	GRP2	Putative NADPH-depe	29.50	0.00
TRINITY_sp|P748	fkbM	31-O-demethyl-FK506	29.50	0.00

TRINITY_sp|Q54L(	DDB_G028	Probable LIM domain	29.50	0.00
TRINITY_sp|A8WYI	par-1	Serine/threonine-pr	29.50	0.00
TRINITY_sp|Q8T1I	ltv1	Protein LTV1 homolo	29.50	0.00
TRINITY_sp|Q9P2I	CC2D2A	Coiled-coil and C2	29.50	0.00
TRINITY_sp|Q4E4I	Tc00.104	Phosphatidylinosito	29.50	0.00
TRINITY_sp|Q3LHI	wge	Protein winged eye	29.50	0.00
TRINITY_sp|Q7Z5(	POLN	DNA polymerase nu O	29.50	0.00
TRINITY_sp|A2WW\	ERG1	Elicitor-responsive	29.50	0.00
TRINITY_sp|Q557I	atg4-1	Cysteine protease a	29.50	0.00
TRINITY_sp|Q149N	SHPRH	E3 ubiquitin-protei	29.50	0.00
TRINITY_sp|Q9UI2	IPO11	Importin-11 OS=Homo	29.50	0.00
TRINITY_sp|Q1575	HERC1	Probable E3 ubiquit	29.50	0.00
TRINITY_sp|P5094	AVT4	Vacuolar amino acid	29.50	0.00
TRINITY_sp|Q55FI	gtf2h1	General transcripti	29.50	0.00
TRINITY_sp|O056I	vanA	Vanillate O-demethy	29.50	0.00
TRINITY_sp|Q54U3	shkD	Dual specificity pr	29.50	0.00
TRINITY_sp|A4IH(	ttc9c	Tetratricopeptide r	29.50	0.00
TRINITY_sp|Q3UYI	Rgl3	Ral guanine nucleot	29.50	0.00
TRINITY_sp|Q9VR9	HERC2	Probable E3 ubiquit	29.50	0.00
TRINITY_sp|P6187	-	Lipase OS=Rhizopus	29.40	0.00
TRINITY_sp|A4IIN	zfp36l2	mRNA decay activato	29.40	0.00
TRINITY_sp|Q5R97	GBP6	Guanylate-binding p	29.40	0.00
TRINITY_sp|Q8MP(	sno1	Senecionine N-oxyge	29.40	0.00
TRINITY_sp|Q5H7(	CDC20	Cell division cycle	29.40	0.00
TRINITY_sp|Q9SH(	STIPL1	Septin and tuftelin	29.40	0.00
TRINITY_sp|Q7XRI	PDIL1-2	Protein disulfide i	29.40	0.00
TRINITY_sp|P297(	AHSG	Alpha-2-HS-glycopro	29.40	0.00
TRINITY_sp|Q8DF(	nutA	5'-nucleotidase OS=	29.40	0.00
TRINITY_sp|F4J59	FMT	Clustered mitochond	29.40	0.00
TRINITY_sp|Q9LS4	CASP	Protein CASP OS=Ara	29.40	0.00
TRINITY_sp|P6126	STX1B	Syntaxin-1B OS=Homo	29.40	0.00
TRINITY_sp|Q2QV9	SEC10	Exocyst complex com	29.40	0.00
TRINITY_sp|Q0336	DNAJ1	DnaJ protein homolo	29.40	0.00
TRINITY_sp|Q3SZI	PIGS	GPI transamidase co	29.40	0.00
TRINITY_sp|Q0889	FAR2	Fatty acyl-CoA redu	29.40	0.00
TRINITY_sp|Q869(	napA	Nck-associated prot	29.40	0.00
TRINITY_sp|Q5I0S	serpinb1	Leukocyte elastase	29.40	0.00
TRINITY_sp|Q9Y4I	DAAM1	Disheveled-associat	29.40	0.00
TRINITY_sp|Q54M3	xpr1	SPX and EXS domain-	29.40	0.00
TRINITY_sp|O4317	PRPF4	U4/U6 small nuclear	29.40	0.00
TRINITY_sp|O749(	bcp1	Protein bcp1 OS=Sch	29.40	0.00
TRINITY_sp|A7RR(	v1g23216	Protein AKTIP homol	29.40	0.00
TRINITY_sp|Q9XI8	LSMT-L	[Fructose-bisphosph	29.40	0.00
TRINITY_sp|P5519	Sh3bp1	SH3 domain-binding	29.40	0.00
TRINITY_sp|Q018(	OTUD4	OTU domain-containi	29.40	0.00
TRINITY_sp|Q9XHN	TIF3C1	Eukaryotic translat	29.40	0.00
TRINITY_sp|Q9JHI	Adat1	tRNA-specific adeno	29.40	0.00
TRINITY_sp|Q91W(	Daglb	Sn1-specific diacyl	29.40	0.00
TRINITY_sp|C0HJI	-	Alpha-mannosidase O	29.40	0.00
TRINITY_sp|Q0II(	RAB18	Ras-related protein	29.40	0.00
TRINITY_sp|P1705	crtK	Protein CrtK OS=Rho	29.40	0.00
TRINITY_sp|Q95LI	TBC1D22I	TBC1 domain family	29.40	0.00
TRINITY_sp|B4ES7	gpmA	2,3-bisphosphoglyce	29.40	0.00
TRINITY_sp|Q84LI	WEX	Werner Syndrome-lik	29.40	0.00
TRINITY_sp|Q9C5I	At3g4742	Putative glycerol-3	29.40	0.00
TRINITY_sp|Q4VNI	SMT	Selenocysteine Se-m	29.40	0.00

TRINITY_sp|Q9XZ(	Cas	Exportin-2 OS=Droso	29.40	0.00
TRINITY_sp|P3711	Ache	Acetylcholinesteras	29.40	0.00
TRINITY_sp|Q944(	RH22	DEAD-box ATP-depend	29.40	0.00
TRINITY_sp|P743:	sll1541	Apocarotenoid-15,15	29.40	0.00
TRINITY_sp|Q9FN(	UVR8	Ultraviolet-B recep	29.40	0.00
TRINITY_sp|Q3UYI	Tbc1d9	TBC1 domain family	29.40	0.00
TRINITY_sp|Q7TP(	Shprh	E3 ubiquitin-protei	29.40	0.00
TRINITY_sp|Q066{	IRC3	Putative ATP-depend	29.40	0.00
TRINITY_sp|Q9C7I	PUB17	U-box domain-contai	29.40	0.00
TRINITY_sp|P075:	wee1	Mitosis inhibitor p	29.40	0.00
TRINITY_sp|Q9NQI	PIH1D3	Protein PIH1D3 OS=H	29.40	0.00
TRINITY_sp|Q045(	UTP14	U3 small nucleolar	29.40	0.00
TRINITY_sp|Q70C(	USP34	Ubiquitin carboxyl-	29.40	0.00
TRINITY_sp|Q54KI	DDB_G02{	TPR repeat-containi	29.40	0.00
TRINITY_sp|Q9GR(	mos	Serine/threonine-pr	29.40	0.00
TRINITY_sp|Q54LI	gghA	Gamma-glutamyl hydr	29.40	0.00
TRINITY_sp|Q8IY:	DHX37	Probable ATP-depend	29.30	0.00
TRINITY_sp|Q993{	VCX1	Vacuolar calcium io	29.30	0.00
TRINITY_sp|P343:	ncx-6	Putative sodium/cal	29.30	0.00
TRINITY_sp|Q640I	slc37a3	Sugar phosphate exc	29.30	0.00
TRINITY_sp|Q286{	ABCC2	Canalicular multisp	29.30	0.00
TRINITY_sp|Q076:	Slc2a3	Solute carrier fami	29.30	0.00
TRINITY_sp|Q9MAI	CIPK9	CBL-interacting ser	29.30	0.00
TRINITY_sp|Q9ES:	Sacm1l	Phosphatidylinositi	29.30	0.00
TRINITY_sp|Q9EP{	Wdr4	tRNA (guanine-N(7)-	29.30	0.00
TRINITY_sp|P428:	ATPK1	Serine/threonine-pr	29.30	0.00
TRINITY_sp|Q9SC)	KINB2	SNF1-related protei	29.30	0.00
TRINITY_sp|Q54MI	leo1	RNA polymerase-asso	29.30	0.00
TRINITY_sp|Q7RX:	egt-1	Ergothioneine biosy	29.30	0.00
TRINITY_sp|Q54Y(	DDB_G02'	Probable serine/thr	29.30	0.00
TRINITY_sp|Q6IN)	mlec-a	Malectin-A OS=Xenop	29.30	0.00
TRINITY_sp|Q9JI(	A4galt	Lactosylceramide 4-	29.30	0.00
TRINITY_sp|Q9LVI	DTX40	Protein DETOXIFICAT	29.30	0.00
TRINITY_sp|Q54FV	DDB_G02(	WD repeat-containin	29.30	0.00
TRINITY_sp|Q9Z0I	Gbp2	Guanylate-binding p	29.30	0.00
TRINITY_sp|Q9V6(	CG16935	Probable trans-2-en	29.30	0.00
TRINITY_sp|Q6LXI	rtcB	tRNA-splicing ligas	29.30	0.00
TRINITY_sp|Q29AI	GA17800	Leishmanolysin-like	29.30	0.00
TRINITY_sp|P210(	B4R	Ankyrin repeat prot	29.30	0.00
TRINITY_sp|P333:	SLA2	Protein SLA2 OS=Sac	29.30	0.00
TRINITY_sp|P333:	SLA2	Protein SLA2 OS=Sac	29.30	0.00
TRINITY_sp|Q9VK:	l(2)gd1	Coiled-coil and C2	29.30	0.00
TRINITY_sp|Q8RXI	At1g146!	Probable splicing f	29.30	0.00
TRINITY_sp|Q923!	SPAC6G9.	Pumilio domain-cont	29.30	0.00
TRINITY_sp|Q949{	ari-1	Potential E3 ubiqui	29.30	0.00
TRINITY_sp|Q54X:	gacR	Rho GTPase-activati	29.30	0.00
TRINITY_sp|A5YM'	CARNS1	Carnosine synthase	29.30	0.00
TRINITY_sp|Q3U1'	Dennd1b	DENN domain-contain	29.30	0.00
TRINITY_sp|Q9P3(	ubp2	Probable ubiquitin	29.30	0.00
TRINITY_sp|Q9DB(	Swt1	Transcriptional pro	29.30	0.00
TRINITY_sp|Q8BQ(	Zdhhc14	Probable palmitoylt	29.30	0.00
TRINITY_sp|Q8RWI	STY17	Serine/threonine-pr	29.30	0.00
TRINITY_sp|P937(	-	Acid beta-fructofur	29.30	0.00
TRINITY_sp|Q6P4I	lmbrd2b	LMBR1 domain-contai	29.30	0.00
TRINITY_sp|P743:	sll1541	Apocarotenoid-15,15	29.30	0.00
TRINITY_sp|Q8RY'	At4g176:	Decapping nuclease	29.30	0.00

TRINITY_sp|Q9FPT	UBP12	Ubiquitin carboxyl-	29.30	0.00
TRINITY_sp|P2862	-	Endoglucanase 4 OS=	29.30	0.00
TRINITY_sp|Q9FY0	PAO	Pheophorbide a oxyg	29.30	0.00
TRINITY_sp|P0C10	Pde11a	Dual 3',5'-cyclic-A	29.30	0.00
TRINITY_sp|Q8T1V	ndufs4	NADH dehydrogenase	29.20	0.00
TRINITY_sp|Q68EV	mrpl28	39S ribosomal prote	29.20	0.00
TRINITY_sp|O9473	MKP1	Mitogen-activated p	29.20	0.00
TRINITY_sp|Q54GI	DDB_G029	PXMP2/4 family prot	29.20	0.00
TRINITY_sp|Q8T2I	sepA	Serine/threonine-pr	29.20	0.00
TRINITY_sp|P5414	amt-1	Putative ammonium t	29.20	0.00
TRINITY_sp|Q9SIE	ALKBH2	DNA oxidative demet	29.20	0.00
TRINITY_sp|Q55FE	copa	Coatomer subunit al	29.20	0.00
TRINITY_sp|Q0991	SPAC30D1	Uncharacterized pro	29.20	0.00
TRINITY_sp|Q54LN	gghA	Gamma-glutamyl hydr	29.20	0.00
TRINITY_sp|O0429	IMPA3	Importin subunit al	29.20	0.00
TRINITY_sp|P2584	chic	Profilin OS=Drosoph	29.20	0.00
TRINITY_sp|Q923E	Steap4	Metalloreductase ST	29.20	0.00
TRINITY_sp|P7940	CYP3A29	Cytochrome P450 3A2	29.20	0.00
TRINITY_sp|Q8BJW	Eif2a	Eukaryotic translat	29.20	0.00
TRINITY_sp|Q86H2	prodh	Proline dehydrogena	29.20	0.00
TRINITY_sp|Q8K24	Uvrag	UV radiation resist	29.20	0.00
TRINITY_sp|O4374	PRKAB2	5'-AMP-activated pr	29.20	0.00
TRINITY_sp|O7053	CYP3A31	Cytochrome P450 3A3	29.20	0.00
TRINITY_sp|Q5597	DDB_G027	Probable serine/thr	29.20	0.00
TRINITY_sp|Q54AX	clcA	Chloride channel pr	29.20	0.00
TRINITY_sp|Q6H7J	RPA2B	Replication protein	29.20	0.00
TRINITY_sp|Q6NW0	prmt6	Protein arginine N-	29.20	0.00
TRINITY_sp|Q54CI	pex13	Probable peroxisoma	29.20	0.00
TRINITY_sp|Q9FN0	UVR8	Ultraviolet-B recep	29.20	0.00
TRINITY_sp|Q94A0	SAMC1	S-adenosylmethionin	29.20	0.00
TRINITY_sp|Q8BV9	Ppme1	Protein phosphatase	29.20	0.00
TRINITY_sp|Q75J9	cpras1	Circularly permutat	29.20	0.00
TRINITY_sp|Q54IE	shkB	Dual specificity pr	29.20	0.00
TRINITY_sp|Q9LVM	ABCB25	ABC transporter B f	29.20	0.00
TRINITY_sp|P1490	IDO1	Indoleamine 2,3-dio	29.20	0.00
TRINITY_sp|Q9FPJ	RBP45A	Polyadenylate-bindi	29.20	0.00
TRINITY_sp|B6EUA	PRP40A	Pre-mRNA-processing	29.20	0.00
TRINITY_sp|O9467	SPBC776.	Uncharacterized mem	29.20	0.00
TRINITY_sp|Q5789	MJ0457	Uncharacterized met	29.20	0.00
TRINITY_sp|F4JZ2	P4H10	Probable prolyl 4-h	29.20	0.00
TRINITY_sp|B0CA9	ycf3	Photosystem I assem	29.20	0.00
TRINITY_sp|Q9ZW9	CYP735A2	Cytokinin hydroxyla	29.20	0.00
TRINITY_sp|Q8BGA	Oxa1l	Mitochondrial inner	29.20	0.00
TRINITY_sp|O4944	AAC3	ADP,ATP carrier pro	29.20	0.00
TRINITY_sp|P5332	YGR266W	Uncharacterized pro	29.20	0.00
TRINITY_sp|Q91ZW	Tfam	Transcription facto	29.20	0.00
TRINITY_sp|Q5SS8	Dhrs13	Dehydrogenase/reduc	29.20	0.00
TRINITY_sp|Q55E5	pats1	Probable serine/thr	29.20	0.00
TRINITY_sp|Q54JE	abcC3	ABC transporter C f	29.20	0.00
TRINITY_sp|Q55GU	DDB_G026	Probable serine/thr	29.20	0.00
TRINITY_sp|Q7Z7H	TMED4	Transmembrane emp24	29.20	0.00
TRINITY_sp|D7UQM	aur	Aurora kinase OS=Pa	29.20	0.00
TRINITY_sp|Q9GR0	mos	Serine/threonine-pr	29.20	0.00
TRINITY_sp|P0C8M	CCRP1	Probable serine/thr	29.10	0.00
TRINITY_sp|Q3HRN	CBL10	Calcineurin B-like	29.10	0.00
TRINITY_sp|P2563	PWP2	Periodic tryptophan	29.10	0.00

TRINITY_sp|Q9SH(	At2g1531	Probable ADP-ribosy	29.10	0.00
TRINITY_sp|Q8VY(	CCR4-6	Carbon catabolite r	29.10	0.00
TRINITY_sp|O7594	RAD17	Cell cycle checkpoi	29.10	0.00
TRINITY_sp|Q500(	TKPR1	Tetraketide alpha-p	29.10	0.00
TRINITY_sp|Q5FVI	ACSBG2	Long-chain-fatty-ac	29.10	0.00
TRINITY_sp|Q9LYI	ML4	Protein MEI2-like 4	29.10	0.00
TRINITY_sp|Q6C7(	CGI121	EKC/KEOPS complex s	29.10	0.00
TRINITY_sp|Q1I61	plsB	Glycerol-3-phosphat	29.10	0.00
TRINITY_sp|Q8W4(	ARI8	Probable E3 ubiquit	29.10	0.00
TRINITY_sp|Q5TC!	AK9	Adenylate kinase 9	29.10	0.00
TRINITY_sp|Q7ZYI	ankrd13(	Ankyrin repeat doma	29.10	0.00
TRINITY_sp|Q6ZQ\	IDO2	Indoleamine 2,3-dio	29.10	0.00
TRINITY_sp|Q9Z2(	Rasal1	RasGAP-activating-l	29.10	0.00
TRINITY_sp|Q8W4{	RAD4	DNA repair protein	29.10	0.00
TRINITY_sp|Q87G(	luxQ	Autoinducer 2 senso	29.10	0.00
TRINITY_sp|Q9M8I	DSPTP1B	Dual specificity pr	29.10	0.00
TRINITY_sp|Q7TP(	Shprh	E3 ubiquitin-protei	29.10	0.00
TRINITY_sp|Q56Z(	VSR4	Vacuolar-sorting re	29.10	0.00
TRINITY_sp|Q55GI	DDB_G02(	Probable phosphatid	29.10	0.00
TRINITY_sp|A7MU4	hscB	Co-chaperone protei	29.10	0.00
TRINITY_sp|Q9VL7	FKBP59	FK506-binding prote	29.10	0.00
TRINITY_sp|O959(	ECD	Protein ecdysoneles	29.10	0.00
TRINITY_sp|C1DK2	rppH	RNA pyrophosphohydr	29.10	0.00
TRINITY_sp|P710(	epsO	Putative pyruvyl tr	29.10	0.00
TRINITY_sp|F4K43	THO5B	THO complex subunit	29.10	0.00
TRINITY_sp|Q8R5I	Usp33	Ubiquitin carboxyl-	29.10	0.00
TRINITY_sp|Q86I\	DDB_G027	PH domain-containin	29.10	0.00
TRINITY_sp|Q8GV4	PLC6	Phosphoinositide ph	29.10	0.00
TRINITY_sp|Q9LT2	MED35C	Pre-mRNA-processing	29.10	0.00
TRINITY_sp|Q9SL>	Os05g015	Importin subunit al	29.10	0.00
TRINITY_sp|Q84C(	vioD	Capreomycidine synt	29.10	0.00
TRINITY_sp|Q5ZII	kbp	KIF1-binding protei	29.10	0.00
TRINITY_sp|O6007	ykt6	Synaptobrevin homol	29.10	0.00
TRINITY_sp|Q9ZQ{	PAP9	Probable inactive p	29.10	0.00
TRINITY_sp|Q5XE7	HESO1	Protein HESO1 OS=Ar	29.10	0.00
TRINITY_sp|Q5F3I	DENND6A	Protein DENND6A OS=	29.10	0.00
TRINITY_sp|O1511	NPC1	Niemann-Pick C1 pro	29.10	0.00
TRINITY_sp|O883!	Avil	Advillin OS=Mus mus	29.10	0.00
TRINITY_sp|P340!	manA	Lysosomal alpha-man	29.10	0.00
TRINITY_sp|Q923!	SPAC6G9.	Pumilio domain-cont	29.10	0.00
TRINITY_sp|Q9C5(	SAC7	Phosphoinositide ph	29.10	0.00
TRINITY_sp|Q9CXI	Dhrs7	Dehydrogenase/reduc	29.10	0.00
TRINITY_sp|Q54SI	DDB_G02{	Beta-lactamase-like	29.10	0.00
TRINITY_sp|F1MH2	AAK1	AP2-associated prot	29.10	0.00
TRINITY_sp|Q96G4	DUS3L	tRNA-dihydrouridine	29.00	0.00
TRINITY_sp|B0G1(	exoc5	Exocyst complex com	29.00	0.00
TRINITY_sp|O758(	CAPN15	Calpain-15 OS=Homo	29.00	0.00
TRINITY_sp|P0AAI	tyrP	Tyrosine-specific t	29.00	0.00
TRINITY_sp|Q5R6!	ATG4A	Cysteine protease A	29.00	0.00
TRINITY_sp|P4712	BNA2	Indoleamine 2,3-dio	29.00	0.00
TRINITY_sp|F4HR(	THO5A	THO complex subunit	29.00	0.00
TRINITY_sp|O0044	RTCA	RNA 3'-terminal pho	29.00	0.00
TRINITY_sp|Q52K2	usp12-a	Ubiquitin carboxyl-	29.00	0.00
TRINITY_sp|Q7TT3	Ikbkap	Elongator complex p	29.00	0.00
TRINITY_sp|Q011(	bah	Acetyl-hydrolase OS	29.00	0.00
TRINITY_sp|P309!	RYR2	Ryanodine receptor	29.00	0.00

TRINITY_sp|P814(	GAPN	NADP-dependent glyc	29.00	0.00
TRINITY_sp|Q569	mfsd4b	Sodium-dependent gl	29.00	0.00
TRINITY_sp|Q4KL	rnf217	Probable E3 ubiquit	29.00	0.00
TRINITY_sp|Q84Y	CXIP4	CAX-interacting pro	29.00	0.00
TRINITY_sp|Q17Q	SCRN3	Secernin-3 OS=Bos t	29.00	0.00
TRINITY_sp|Q6C1	TIF32	Eukaryotic translat	29.00	0.00
TRINITY_sp|Q7T2	map3k10	Mitogen-activated p	29.00	0.00
TRINITY_sp|Q8GZ	BRN2	RNA-binding protein	29.00	0.00
TRINITY_sp|Q9FP	UBP12	Ubiquitin carboxyl-	29.00	0.00
TRINITY_sp|Q294	CYP3A24	Cytochrome P450 3A2	29.00	0.00
TRINITY_sp|Q67Z	TFCB	Tubulin-folding cof	29.00	0.00
TRINITY_sp|Q0JI	CIPK11	CBL-interacting pro	29.00	0.00
TRINITY_sp|Q430	GALE	UDP-glucose 4-epime	29.00	0.00
TRINITY_sp|Q129	ELAVL2	ELAV-like protein 2	29.00	0.00
TRINITY_sp|Q05B	EML5	Echinoderm microtub	29.00	0.00
TRINITY_sp|Q98T	PDCD4	Programmed cell dea	29.00	0.00
TRINITY_sp|Q55D	atxn2	Ataxin-2 homolog OS	29.00	0.00
TRINITY_sp|Q9SF	At3g071(	Protein transport p	29.00	0.00
TRINITY_sp|Q8H2	POLH	DNA polymerase eta	29.00	0.00
TRINITY_sp|O142	wis4	MAP kinase kinase k	29.00	0.00
TRINITY_sp|Q9LY	MED7A	Mediator of RNA pol	29.00	0.00
TRINITY_sp|P344	clu-1	Clustered mitochond	29.00	0.00
TRINITY_sp|Q9VA	aralar1	Calcium-binding mit	29.00	0.00
TRINITY_sp|Q6ZM	EML6	Echinoderm microtub	29.00	0.00
TRINITY_sp|Q54I	rft1	Protein RFT1 homolo	29.00	0.00
TRINITY_sp|Q15K	PHYLLO	Protein PHYLLO, chl	29.00	0.00
TRINITY_sp|Q232	fat-3	Delta(6)-fatty-acid	29.00	0.00
TRINITY_sp|O017	him-1	Structural maintena	28.90	0.00
TRINITY_sp|C0SP	ywtG	Putative metabolite	28.90	0.00
TRINITY_sp|Q5PP	rnf170	E3 ubiquitin-protei	28.90	0.00
TRINITY_sp|P418	rae1	Poly(A)+ RNA export	28.90	0.00
TRINITY_sp|P872	mus81	Crossover junction	28.90	0.00
TRINITY_sp|P041	GIP	Copia protein OS=Dr	28.90	0.00
TRINITY_sp|Q54W	DDB_G02	Putative actin-frag	28.90	0.00
TRINITY_sp|Q5ZL	VAMP7	Vesicle-associated	28.90	0.00
TRINITY_sp|Q54B	nsfl1c	NSFL1 cofactor p47	28.90	0.00
TRINITY_sp|Q122	NCR1	Niemann-Pick type C	28.90	0.00
TRINITY_sp|Q9DB	Cyp4v2	Cytochrome P450 4V2	28.90	0.00
TRINITY_sp|Q54B	DDB_G02	Probable GH family	28.90	0.00
TRINITY_sp|Q88F	nicC	6-hydroxynicotinate	28.90	0.00
TRINITY_sp|Q54N	abcC9	ABC transporter C f	28.90	0.00
TRINITY_sp|Q497	Kdm8	Lysine-specific dem	28.90	0.00
TRINITY_sp|A0A0	CMT1	DNA (cytosine-5)-me	28.90	0.00
TRINITY_sp|Q94I	OST3	Probable dolichyl-d	28.90	0.00
TRINITY_sp|Q9CA	At1g632	Transcription elong	28.90	0.00
TRINITY_sp|Q019	OCRL	Inositol polyphosph	28.90	0.00
TRINITY_sp|E1BY	MSH6	DNA mismatch repair	28.90	0.00
TRINITY_sp|Q11S	ctaB	Protoheme IX farnes	28.90	0.00
TRINITY_sp|Q9UT	cox1101	Rsm22-cox11 tandem	28.90	0.00
TRINITY_sp|Q917	gckr	Glucokinase regulat	28.90	0.00
TRINITY_sp|Q9JI	A4galt	Lactosylceramide 4-	28.90	0.00
TRINITY_sp|P041	GIP	Copia protein OS=Dr	28.90	0.00
TRINITY_sp|A4FI	iolG2	Inositol 2-dehydrog	28.90	0.00
TRINITY_sp|Q010	Pde4d	cAMP-specific 3',5'	28.90	0.00
TRINITY_sp|Q54X	gacP	Rho GTPase-activati	28.90	0.00
TRINITY_sp|A2VD	MAP3K7	Mitogen-activated p	28.90	0.00

TRINITY_sp|Q54D\	DDB_G02(	von Willebrand fact	28.90	0.00
TRINITY_sp|Q9LU(	CYP72A1:	Cytochrome P450 72A	28.90	0.00
TRINITY_sp|P164z	pol	Retrovirus-related	28.90	0.00
TRINITY_sp|O228(	At2g404:	Uncharacterized pro	28.90	0.00
TRINITY_sp|P586]	glk	Glucokinase OS=Nost	28.90	0.00
TRINITY_sp|Q9VZz	Pxn	Peroxidasin OS=Dros	28.90	0.00
TRINITY_sp|A3KM\	UBA1	Ubiquitin-like modi	28.90	0.00
TRINITY_sp|Q4V8:	tepsin	AP-4 complex access	28.90	0.00
TRINITY_sp|P290(	INVA	Acid beta-fructofur	28.90	0.00
TRINITY_sp|P393(	lgoT	Probable L-galacton	28.90	0.00
TRINITY_sp|Q9Y4{	DMXL1	DmX-like protein 1	28.90	0.00
TRINITY_sp|P311'	-	Autolysin OS=Chlamy	28.90	0.00
TRINITY_sp|Q5XF(	Atp13a4	Probable cation-tra	28.90	0.00
TRINITY_sp|Q7KV(	Trf4-1	Non-canonical poly(	28.90	0.00
TRINITY_sp|A7S4N	serinc	Probable serine inc	28.90	0.00
TRINITY_sp|Q54BI	exoc3	Exocyst complex com	28.90	0.00
TRINITY_sp|P360(	VPS24	Vacuolar protein-so	28.90	0.00
TRINITY_sp|Q092z	nrf-6	Nose resistant to f	28.90	0.00
TRINITY_sp|O154:	ABCC4	Multidrug resistanc	28.90	0.00
TRINITY_sp|Q4G0I	HYDIN	Hydrocephalus-induc	28.90	0.00
TRINITY_sp|Q1G3(	PCO3	Plant cysteine oxid	28.90	0.00
TRINITY_sp|Q54V(	DDB_G02{	Probable serine/thr	28.90	0.00
TRINITY_sp|A0JP(	heatr5a	HEAT repeat-contain	28.90	0.00
TRINITY_sp|Q94C(	ATG1A	Serine/threonine-pr	28.80	0.00
TRINITY_sp|P531(	MNP1	54S ribosomal prote	28.80	0.00
TRINITY_sp|Q6BM\	SYM1	Protein SYM1 OS=Deb	28.80	0.00
TRINITY_sp|Q4VB]	stx18	Syntaxin-18 OS=Dani	28.80	0.00
TRINITY_sp|Q916(	stk11	Serine/threonine-pr	28.80	0.00
TRINITY_sp|A5WW(	chfr	E3 ubiquitin-protei	28.80	0.00
TRINITY_sp|Q9SEz	CIPK16	CBL-interacting ser	28.80	0.00
TRINITY_sp|O168{	dhs-16	3 beta-hydroxystero	28.80	0.00
TRINITY_sp|Q8CN4	SE_2036	Uncharacterized oxi	28.80	0.00
TRINITY_sp|Q5LDI	engB	Probable GTP-bindin	28.80	0.00
TRINITY_sp|O547!	Hsd17b6	17-beta-hydroxyster	28.80	0.00
TRINITY_sp|Q9CQ(	Commd4	COMM domain-contain	28.80	0.00
TRINITY_sp|Q641I	Nuak1	NUAK family SNF1-li	28.80	0.00
TRINITY_sp|O189(	CYP3A21	Cytochrome P450 3A2	28.80	0.00
TRINITY_sp|O487{	CYP734A]	Cytochrome P450 734	28.80	0.00
TRINITY_sp|Q8LF4	RPA2B	Replication protein	28.80	0.00
TRINITY_sp|P341z	coaA	Coactosin OS=Dictyo	28.80	0.00
TRINITY_sp|Q5VS1	OBSCN	Obscurin OS=Homo sa	28.80	0.00
TRINITY_sp|Q6DE\	tbc1d31	TBC1 domain family	28.80	0.00
TRINITY_sp|Q55B(	scy2	Probable inactive s	28.80	0.00
TRINITY_sp|O229z	At2g303z	Putative tRNA pseud	28.80	0.00
TRINITY_sp|P825:	PPL1	PsbP-like protein 1	28.80	0.00
TRINITY_sp|Q100'	mdm31	Mitochondrial distr	28.80	0.00
TRINITY_sp|A2A5]	R3hdml	Peptidase inhibitor	28.80	0.00
TRINITY_sp|Q54Q(	sec1	Protein transport p	28.80	0.00
TRINITY_sp|P930(	PP2C27	Probable protein ph	28.80	0.00
TRINITY_sp|A8JF(	CFAP65	Cilia- and flagella	28.80	0.00
TRINITY_sp|P283(	qorA	Quinone oxidoreduct	28.80	0.00
TRINITY_sp|Q9WVI	Cyp46a1	Cholesterol 24-hydr	28.80	0.00
TRINITY_sp|Q944I	MTM1	Mitochondrial carri	28.80	0.00
TRINITY_sp|O231'	PLP1	Patatin-like protei	28.80	0.00
TRINITY_sp|A8MS{	SPT6	Transcription elong	28.80	0.00
TRINITY_sp|Q9LU(	CYP72A1:	Cytochrome P450 72A	28.80	0.00

TRINITY_sp|Q6DF(	wdr75	WD repeat-containin	28.80	0.00
TRINITY_sp|Q58C\	KLHDC3	Kelch domain-contai	28.80	0.00
TRINITY_sp|Q6C9(	BFR2	Protein BFR2 OS=Yar	28.80	0.00
TRINITY_sp|P445	arcB	Aerobic respiration	28.80	0.00
TRINITY_sp|Q9LV.	At3g178(	UV-B-induced protei	28.80	0.00
TRINITY_sp|O745	SPCC777.	Putative hydrolase	28.80	0.00
TRINITY_sp|A8LH/	Franean	Putative S-adenosyl	28.80	0.00
TRINITY_sp|Q54K	prmt5	Protein arginine N-	28.80	0.00
TRINITY_sp|P102	MYBL1	Myb-related protein	28.80	0.00
TRINITY_sp|D4AY\	ARB_013	ABC transporter G f	28.80	0.00
TRINITY_sp|Q84RI	ARI7	Probable E3 ubiquit	28.80	0.00
TRINITY_sp|B0E2(	VPS10	Vacuolar protein so	28.80	0.00
TRINITY_sp|Q6RF	WDR74	WD repeat-containin	28.80	0.00
TRINITY_sp|O155	PDPK1	3-phosphoinositide-	28.80	0.00
TRINITY_sp|O140	SPCC167	U3 small nucleolar	28.80	0.00
TRINITY_sp|Q8IS	gefJ	Ras guanine nucleot	28.80	0.00
TRINITY_sp|Q4G0	HYDIN	Hydrocephalus-induc	28.80	0.00
TRINITY_sp|Q8BU(	Cndp1	Beta-Ala-His dipept	28.80	0.00
TRINITY_sp|Q95YI	pefB	Penta-EF hand domai	28.80	0.00
TRINITY_sp|Q23T(	TTLL3A	Tubulin glycylase 3	28.70	0.00
TRINITY_sp|Q647(	Plk4	Serine/threonine-pr	28.70	0.00
TRINITY_sp|O429	ale1	Lysophospholipid ac	28.70	0.00
TRINITY_sp|Q5RA(	ACOX3	Peroxisomal acyl-co	28.70	0.00
TRINITY_sp|Q6P1	PLB1	Phospholipase B1, m	28.70	0.00
TRINITY_sp|Q9DG	star	Steroidogenic acute	28.70	0.00
TRINITY_sp|A2XD(	UBP26	Ubiquitin carboxyl-	28.70	0.00
TRINITY_sp|Q9HK	rhaD	L-rhamnose 1-dehydr	28.70	0.00
TRINITY_sp|O577	alaXL	Alanyl-tRNA editing	28.70	0.00
TRINITY_sp|Q54P	osbI	Oxysterol-binding p	28.70	0.00
TRINITY_sp|Q65G(	araB	Ribulokinase OS=Bac	28.70	0.00
TRINITY_sp|Q9ER	Capn12	Calpain-12 OS=Mus m	28.70	0.00
TRINITY_sp|Q0P5	ACP2	Lysosomal acid phos	28.70	0.00
TRINITY_sp|Q9DB\	Cyp4v2	Cytochrome P450 4V2	28.70	0.00
TRINITY_sp|P073	CTSD	Cathepsin D OS=Homo	28.70	0.00
TRINITY_sp|Q964	CYP4C21	Cytochrome P450 4c2	28.70	0.00
TRINITY_sp|Q7KV	Trf4-1	Non-canonical poly(	28.70	0.00
TRINITY_sp|P932	TFT9	14-3-3 protein 9 OS	28.70	0.00
TRINITY_sp|A2AP	Plch2	1-phosphatidylinosi	28.70	0.00
TRINITY_sp|Q94K	VPS52	Vacuolar protein so	28.70	0.00
TRINITY_sp|Q9Y6	CYP46A1	Cholesterol 24-hydr	28.70	0.00
TRINITY_sp|P981	CYP4F8	Cytochrome P450 4F8	28.70	0.00
TRINITY_sp|Q559	polA	DNA polymerase I OS	28.70	0.00
TRINITY_sp|Q072	-	Calcium/calmodulin-	28.70	0.00
TRINITY_sp|P618	-	Lipase OS=Rhizopus	28.70	0.00
TRINITY_sp|Q8BX	Arfrp1	ADP-ribosylation fa	28.70	0.00
TRINITY_sp|Q132	PRKG2	cGMP-dependent prot	28.70	0.00
TRINITY_sp|A8FD(	BPUM_16(	8-amino-7-oxononano	28.70	0.00
TRINITY_sp|Q96J	COG3	Conserved oligomeri	28.70	0.00

TRINITY_sp|Q2QMI	Os12g06(	Protein ROOT HAIR D	28.70	0.00
TRINITY_sp|Q8IS1	gefE	Ras guanine nucleot	28.70	0.00
TRINITY_sp|P715(	MexAM1_M	Putative carboxymet	28.70	0.00
TRINITY_sp|Q8GZ7	TIC20-I	Protein TIC 20-I, c	28.70	0.00
TRINITY_sp|P0957	nifA	Nif-specific regula	28.70	0.00
TRINITY_sp|A8MB(	cobB	NAD-dependent prote	28.70	0.00
TRINITY_sp|P2648	fixL	Sensor protein FixL	28.70	0.00
TRINITY_sp|Q8R15	Znfx1	NFX1-type zinc fing	28.70	0.00
TRINITY_sp|Q7SXW	emc3	ER membrane protein	28.60	0.00
TRINITY_sp|Q9SD1	At3g5137	Probable protein ph	28.60	0.00
TRINITY_sp|P5397	FAP1	FKBP12-associated p	28.60	0.00
TRINITY_sp|A0MEI	CYCA3-3	Cyclin-A3-3 OS=Arab	28.60	0.00
TRINITY_sp|Q8TA1	RHEBL1	GTPase RhebL1 OS=Ho	28.60	0.00
TRINITY_sp|Q1ZXI	emc6	ER membrane protein	28.60	0.00
TRINITY_sp|Q2QN(	TRS130	Trafficking protein	28.60	0.00
TRINITY_sp|Q5NC(	Ttf2	Transcription termi	28.60	0.00
TRINITY_sp|B2RX9	Plxnb2	Plexin-B2 OS=Mus mu	28.60	0.00
TRINITY_sp|Q6Z35	Os07g028	Putative aldehyde o	28.60	0.00
TRINITY_sp|O1394	ppk9	Protein kinase doma	28.60	0.00
TRINITY_sp|Q9LP5	CIPK18	CBL-interacting ser	28.60	0.00
TRINITY_sp|O1363	yvh1	Tyrosine-protein ph	28.60	0.00
TRINITY_sp|Q8RWI	STY17	Serine/threonine-pr	28.60	0.00
TRINITY_sp|Q61Y(	CBG03556	Leishmanolysin-like	28.60	0.00
TRINITY_sp|Q9Y0(	ripA	Ras-interacting pro	28.60	0.00
TRINITY_sp|Q1503	R3HDM1	R3H domain-containi	28.60	0.00
TRINITY_sp|Q75B4	NOP12	Nucleolar protein 1	28.60	0.00
TRINITY_sp|Q96S1	JMJD8	JmjC domain-contain	28.60	0.00
TRINITY_sp|Q9FR4	NMT1	Phosphoethanolamine	28.60	0.00
TRINITY_sp|Q9LF7	ACA8	Calcium-transportin	28.60	0.00
TRINITY_sp|Q2758	Cyp4d2	Cytochrome P450 4d2	28.60	0.00
TRINITY_sp|Q9Z16	Cog1	Conserved oligomeri	28.60	0.00
TRINITY_sp|Q6P8Y	Capsl	Calcyphosin-like pr	28.60	0.00
TRINITY_sp|Q5RF7	CHFR	E3 ubiquitin-protei	28.60	0.00
TRINITY_sp|Q8KIY	tmoS	Sensor histidine ki	28.60	0.00
TRINITY_sp|Q9H6I	NOL6	Nucleolar protein 6	28.60	0.00
TRINITY_sp|Q8HXI	LONRF3	LON peptidase N-ter	28.60	0.00
TRINITY_sp|Q9LRI	At3g1743	Probable sugar phos	28.60	0.00
TRINITY_sp|Q7PZI	AGAP0118	Clustered mitochond	28.60	0.00
TRINITY_sp|P0AG8	pstS	Phosphate-binding p	28.60	0.00
TRINITY_sp|Q9Z3(	Kcnj16	Inward rectifier po	28.60	0.00
TRINITY_sp|Q6EU1	AGO1A	Protein argonaute 1	28.60	0.00
TRINITY_sp|Q9SY1	At4g029(	CSC1-like protein A	28.60	0.00
TRINITY_sp|Q3E9(	CPK34	Calcium-dependent p	28.60	0.00
TRINITY_sp|Q8T63	abcA2	ABC transporter A f	28.60	0.00
TRINITY_sp|P3554	Ret	Proto-oncogene tyro	28.50	0.00
TRINITY_sp|Q93Z5	At3g1619	Probable inactive n	28.50	0.00
TRINITY_sp|P2507	CML12	Calmodulin-like pro	28.50	0.00
TRINITY_sp|O749(	bcp1	Protein bcp1 OS=Sch	28.50	0.00
TRINITY_sp|Q54DA	rab4	Ras-related protein	28.50	0.00
TRINITY_sp|Q8VX1	PDIL1-3	Protein disulfide i	28.50	0.00
TRINITY_sp|Q7ZY6	uba2-b	SUMO-activating enz	28.50	0.00
TRINITY_sp|O5748	-	Voltage-dependent L	28.50	0.00
TRINITY_sp|A2XH3	ATG4A	Cysteine protease A	28.50	0.00
TRINITY_sp|Q9P63	SPBC1683	Uncharacterized pro	28.50	0.00
TRINITY_sp|Q9ZQ1	REIL2	Cytoplasmic 60S sub	28.50	0.00
TRINITY_sp|P4088	-	Carbonic anhydrase,	28.50	0.00

TRINITY_sp|P0DKS	qmcA	Protein QmcA OS=Wig	28.50	0.00
TRINITY_sp|Q150(	POSTN	Periostin OS=Homo s	28.50	0.00
TRINITY_sp|Q929(	HPS1	Hermansky-Pudlak sy	28.50	0.00
TRINITY_sp|P270	Man2a1	Alpha-mannosidase 2	28.50	0.00
TRINITY_sp|O813(	-	Zeaxanthin epoxidas	28.50	0.00
TRINITY_sp|Q6BX	SLD5	DNA replication com	28.50	0.00
TRINITY_sp|Q96A	SYAP1	Synapse-associated	28.50	0.00
TRINITY_sp|Q9FJ	AT4	Probable long-chain	28.50	0.00
TRINITY_sp|Q54JI	DDB_G02	UPF0522 protein A O	28.50	0.00
TRINITY_sp|Q86Y	DNAAF5	Dynein assembly fac	28.50	0.00
TRINITY_sp|Q8BK	Tmem87b	Transmembrane prote	28.50	0.00
TRINITY_sp|Q6AZ	slc30a6-	Zinc transporter 6-	28.50	0.00
TRINITY_sp|Q8H2I	POLH	DNA polymerase eta	28.50	0.00
TRINITY_sp|Q4KL	wdr76	WD repeat-containin	28.50	0.00
TRINITY_sp|Q91Z	Fgd4	FYVE, RhoGEF and PH	28.50	0.00
TRINITY_sp|Q9SL	Os05g01	Importin subunit al	28.50	0.00
TRINITY_sp|Q8RX	RBL14	Rhomboid-like prote	28.50	0.00
TRINITY_sp|F4HX	NUP155	Nuclear pore comple	28.50	0.00
TRINITY_sp|Q5JJ	TK1782	Uncharacterized ser	28.50	0.00
TRINITY_sp|P435	cya	Adenylate cyclase O	28.50	0.00
TRINITY_sp|Q149	SHPRH	E3 ubiquitin-protei	28.50	0.00
TRINITY_sp|Q969	PIGT	GPI transamidase co	28.50	0.00
TRINITY_sp|Q145	ITPR2	Inositol 1,4,5-tris	28.50	0.00
TRINITY_sp|Q96E	ADAT3	Probable inactive t	28.50	0.00
TRINITY_sp|P311	-	Autolysin OS=Chlamy	28.50	0.00
TRINITY_sp|Q4KL	znrf3	E3 ubiquitin-protei	28.40	0.00
TRINITY_sp|P177	-	Group 1 truncated h	28.40	0.00
TRINITY_sp|B8NDI	abnA	Probable arabinan e	28.40	0.00
TRINITY_sp|Q8IL	PF14_02	Acidic leucine-rich	28.40	0.00
TRINITY_sp|A4FW	hisC	Histidinol-phosphat	28.40	0.00
TRINITY_sp|O091	Cyp3a25	Cytochrome P450 3A2	28.40	0.00
TRINITY_sp|Q6FI	YME2	Mitochondrial escap	28.40	0.00
TRINITY_sp|Q5NC	Ttf2	Transcription termi	28.40	0.00
TRINITY_sp|O139	erv25	Endoplasmic reticul	28.40	0.00
TRINITY_sp|Q9UT	SPAC8F1	Putative ribosome b	28.40	0.00
TRINITY_sp|Q644	Cyp3a16	Cytochrome P450 3A1	28.40	0.00
TRINITY_sp|A8WR	kin-29	Serine/threonine-pr	28.40	0.00
TRINITY_sp|Q9ZQ	REIL2	Cytoplasmic 60S sub	28.40	0.00
TRINITY_sp|Q54W	forA	Formin-A OS=Dictyos	28.40	0.00
TRINITY_sp|Q097	kri1	Protein kri1 OS=Sch	28.40	0.00
TRINITY_sp|Q9WZ	truB	tRNA pseudouridine	28.40	0.00
TRINITY_sp|Q9LT	VPS9A	Vacuolar protein so	28.40	0.00
TRINITY_sp|Q4L9	drp35	Lactonase drp35 OS=	28.40	0.00
TRINITY_sp|Q5JJ	Os01g05	Protein kinase and	28.40	0.00
TRINITY_sp|A4UH	SALR	Salutaridine reduct	28.40	0.00
TRINITY_sp|Q7SC	sym-1	Protein sym-1 OS=Ne	28.40	0.00
TRINITY_sp|Q5ZJI	ABHD13	Protein ABHD13 OS=G	28.40	0.00
TRINITY_sp|Q8R3	Tspan8	Tetraspanin-8 OS=Mu	28.40	0.00
TRINITY_sp|Q9UH	PGAP2	Post-GPI attachment	28.40	0.00
TRINITY_sp|Q8RX	AGD14	Probable ADP-ribosy	28.40	0.00
TRINITY_sp|Q6AU	QSOX1	Sulfhydryl oxidase	28.40	0.00
TRINITY_sp|P509	AVT4	Vacuolar amino acid	28.40	0.00
TRINITY_sp|P155	acyII	Penicillin acylase	28.40	0.00
TRINITY_sp|O235	At4g175	Putative glycerol-3	28.40	0.00
TRINITY_sp|Q70C	USP34	Ubiquitin carboxyl-	28.40	0.00
TRINITY_sp|Q992	CACNA1D	Voltage-dependent L	28.40	0.00

TRINITY_sp|Q7PZI	AGAP0118	Clustered mitochond	28.40	0.00
TRINITY_sp|P1789	recN	DNA repair protein	28.40	0.00
TRINITY_sp|P5819	Plscr1	Phospholipid scramb	28.40	0.00
TRINITY_sp|P3836	PHO89	Phosphate permease	28.40	0.00
TRINITY_sp|Q8L97	P4H7	Probable prolyl 4-h	28.40	0.00
TRINITY_sp|Q0157	Gstt1	Glutathione S-trans	28.40	0.00
TRINITY_sp|Q9USN	ubp1	Probable ubiquitin	28.40	0.00
TRINITY_sp|P2636	U2AF2	Splicing factor U2A	28.40	0.00
TRINITY_sp|A8JFU	CFAP65	Cilia- and flagella	28.40	0.00
TRINITY_sp|Q6NUE	PPP4R4	Serine/threonine-pr	28.40	0.00
TRINITY_sp|Q7LHG	TY3B-I	Transposon Ty3-I Ga	28.40	0.00
TRINITY_sp|Q75LX	OSH10	Homeobox protein kn	28.40	0.00
TRINITY_sp|Q8TB7	PUM2	Pumilio homolog 2 O	28.40	0.00
TRINITY_sp|P5775	At5g4067	Cystinosin homolog	28.30	0.00
TRINITY_sp|Q5VJI	gdt9	Probable serine/thr	28.30	0.00
TRINITY_sp|P8678	-	Gigasin-6 OS=Crasso	28.30	0.00
TRINITY_sp|Q6DEY	smndc1	Survival of motor n	28.30	0.00
TRINITY_sp|P4690	yccK	Uncharacterized oxi	28.30	0.00
TRINITY_sp|Q0C02	atpH	ATP synthase subuni	28.30	0.00
TRINITY_sp|P4232	CNB1	Calcineurin subunit	28.30	0.00
TRINITY_sp|P2047	-	23 kDa calcium-bind	28.30	0.00
TRINITY_sp|P1064	PRKAR1A	cAMP-dependent prot	28.30	0.00
TRINITY_sp|Q3EBC	PAT17	Probable protein S-	28.30	0.00
TRINITY_sp|Q9P27	USP36	Ubiquitin carboxyl-	28.30	0.00
TRINITY_sp|Q96BY	ATG2B	Autophagy-related p	28.30	0.00
TRINITY_sp|P5694	cphB	Cyanophycinase OS=G	28.30	0.00
TRINITY_sp|Q54I4	slc44a2	Choline transporter	28.30	0.00
TRINITY_sp|P5416	ypgQ	Uncharacterized pro	28.30	0.00
TRINITY_sp|P9348	BP80	Vacuolar-sorting re	28.30	0.00
TRINITY_sp|P3605	SRP102	Signal recognition	28.30	0.00
TRINITY_sp|O1519	VILL	Villin-like protein	28.30	0.00
TRINITY_sp|B5F0K	yfeW	UPF0214 protein Yfe	28.30	0.00
TRINITY_sp|Q9C95	VIP5	Protein RTF1 homolo	28.30	0.00
TRINITY_sp|Q8TWE	MK0970	Glyceraldehyde 3-ph	28.30	0.00
TRINITY_sp|Q9C6E	GAMMACA2	Gamma carbonic anhy	28.30	0.00
TRINITY_sp|Q7XWE	Os04g025	Probable cleavage a	28.30	0.00
TRINITY_sp|Q9263	NSMAF	Protein FAN OS=Homo	28.30	0.00
TRINITY_sp|Q9HD2	cwh43	Protein cwh43 OS=Sc	28.30	0.00
TRINITY_sp|Q6GL7	dscc1	Sister chromatid co	28.30	0.00
TRINITY_sp|Q08BY	jmjd4	JmjC domain-contain	28.30	0.00
TRINITY_sp|Q9NYI	ZAK	Mitogen-activated p	28.30	0.00
TRINITY_sp|Q54L1	DDB01869	Calcium uniporter p	28.30	0.00
TRINITY_sp|A4RDI	TIF34	Eukaryotic translat	28.30	0.00
TRINITY_sp|Q9UKE	PARP4	Poly [ADP-ribose] p	28.30	0.00
TRINITY_sp|O9424	mdn1	Midasin OS=Schizosa	28.30	0.00
TRINITY_sp|O1473	MAP2K7	Dual specificity mi	28.30	0.00
TRINITY_sp|Q8BRC	Maats1	Protein MAATS1 OS=M	28.30	0.00
TRINITY_sp|Q75WE	Vwa5a	von Willebrand fact	28.30	0.00
TRINITY_sp|Q84M9	MSL9	Mechanosensitive io	28.30	0.00
TRINITY_sp|Q86GV	gcy-28	Receptor-type guany	28.30	0.00
TRINITY_sp|B9DFV	At5g4743	E3 ubiquitin ligase	28.30	0.00
TRINITY_sp|Q8IV0	TBC1D10C	Carabin OS=Homo sap	28.30	0.00
TRINITY_sp|O7448	SPCC1840	Uncharacterized pro	28.20	0.00
TRINITY_sp|Q54EE	abcC7	ABC transporter C f	28.20	0.00
TRINITY_sp|P4232	Nca	Neurocalcin homolog	28.20	0.00
TRINITY_sp|Q9D2E	Iqch	IQ domain-containin	28.20	0.00

TRINITY_sp|Q91YI	Wasl	Neural Wiskott-Aldr	28.20	0.00
TRINITY_sp|Q54BI	DDB_G02	LIMR family protein	28.20	0.00
TRINITY_sp|Q9JL1	Bace2	Beta-secretase 2 OS	28.20	0.00
TRINITY_sp|Q1048	etr1	Probable trans-2-en	28.20	0.00
TRINITY_sp|P2047	-	23 kDa calcium-bind	28.20	0.00
TRINITY_sp|Q4R0W	rbmC	2-deoxy-scyllo-inos	28.20	0.00
TRINITY_sp|Q2NL6	PARP6	Poly [ADP-ribose] p	28.20	0.00
TRINITY_sp|Q54XQ	rckA	RGS domain-containi	28.20	0.00
TRINITY_sp|Q9BZE	GTPBP4	Nucleolar GTP-bindi	28.20	0.00
TRINITY_sp|Q9HCI	GPAM	Glycerol-3-phosphat	28.20	0.00
TRINITY_sp|P5186	Cyp4f4	Cytochrome P450 4F4	28.20	0.00
TRINITY_sp|Q0590	Ptprg	Receptor-type tyros	28.20	0.00
TRINITY_sp|O2330	BLUS1	Serine/threonine-pr	28.20	0.00
TRINITY_sp|Q9LS4	CASP	Protein CASP OS=Ara	28.20	0.00
TRINITY_sp|Q59K2	APE2	Aminopeptidase 2 OS	28.20	0.00
TRINITY_sp|Q54RE	agtA	UDP-galactose:fucos	28.20	0.00
TRINITY_sp|Q54PQ	gefA	Ras guanine nucleot	28.20	0.00
TRINITY_sp|Q54YW	elmoA	ELMO domain-contain	28.20	0.00
TRINITY_sp|C0LT2	CERK	Ceramide kinase OS=	28.20	0.00
TRINITY_sp|Q91WD	Kif18a	Kinesin-like protei	28.20	0.00
TRINITY_sp|P5213	yfjV	Putative arsenical	28.20	0.00
TRINITY_sp|P0C0E	rpfC	Sensory/regulatory	28.20	0.00
TRINITY_sp|Q5U2V	Plbd1	Phospholipase B-lik	28.20	0.00
TRINITY_sp|P5431	Lipe	Hormone-sensitive l	28.20	0.00
TRINITY_sp|Q1250	RKM4	Ribosomal lysine N-	28.20	0.00
TRINITY_sp|Q5QD0	SUVH3	Histone-lysine N-me	28.20	0.00
TRINITY_sp|B8MG7	vps10	Vacuolar protein so	28.20	0.00
TRINITY_sp|Q70CQ	USP34	Ubiquitin carboxyl-	28.20	0.00
TRINITY_sp|Q9JL1	Sart3	Squamous cell carci	28.20	0.00
TRINITY_sp|Q9U5N	-	V-type proton ATPas	28.20	0.00
TRINITY_sp|Q4ZPN	quiP	Acyl-homoserine lac	28.20	0.00
TRINITY_sp|Q8WUM	NUP133	Nuclear pore comple	28.20	0.00
TRINITY_sp|Q1JQE	FBLIM1	Filamin-binding LIM	28.10	0.00
TRINITY_sp|Q149N	SHPRH	E3 ubiquitin-protei	28.10	0.00
TRINITY_sp|P7394	hhoB	Putative serine pro	28.10	0.00
TRINITY_sp|Q9CR9	Ccdc96	Coiled-coil domain-	28.10	0.00
TRINITY_sp|Q0272	RKIN1	Carbon catabolite-d	28.10	0.00
TRINITY_sp|Q3EAQ	ZIFL2	Probable peptide/ni	28.10	0.00
TRINITY_sp|Q4WCV	mrs2	Mitochondrial inner	28.10	0.00
TRINITY_sp|Q54PE	lvsF	BEACH domain-contai	28.10	0.00
TRINITY_sp|Q6ZVT	TTLL10	Inactive polyglycyl	28.10	0.00
TRINITY_sp|P7940	CYP3A29	Cytochrome P450 3A2	28.10	0.00
TRINITY_sp|Q6443	Ptk6	Protein-tyrosine ki	28.10	0.00
TRINITY_sp|Q9D82	Ascc1	Activating signal c	28.10	0.00
TRINITY_sp|Q3T0D	WDYHV1	Protein N-terminal	28.10	0.00
TRINITY_sp|Q0IXE	Os10g041	Acylamino-acid-rele	28.10	0.00
TRINITY_sp|Q6ZF2	ADL1	Calpain-type cystei	28.10	0.00
TRINITY_sp|Q6DE8	chek1	Serine/threonine-pr	28.10	0.00
TRINITY_sp|P3285	STX2	Syntaxin-2 OS=Homo	28.10	0.00
TRINITY_sp|P2905	gtf2b	Transcription initi	28.10	0.00
TRINITY_sp|Q8NH9	MFSD8	Major facilitator s	28.10	0.00
TRINITY_sp|Q8BGE	Slc25a44	Solute carrier fami	28.10	0.00
TRINITY_sp|O4287	fcf2	rRNA-processing pro	28.10	0.00
TRINITY_sp|Q9BX6	BRIP1	Fanconi anemia grou	28.10	0.00
TRINITY_sp|P7150	MexAM1_M	Putative carboxymet	28.10	0.00
TRINITY_sp|A8WYE	par-1	Serine/threonine-pr	28.10	0.00

TRINITY_sp|Q5M7!	PAT12	Probable protein S-	28.10	0.00
TRINITY_sp|Q9M1I	BGLU27	Beta-glucosidase 27	28.10	0.00
TRINITY_sp|O091!	Man2b1	Lysosomal alpha-man	28.10	0.00
TRINITY_sp|Q08A7	PRMT6	Probable protein ar	28.10	0.00
TRINITY_sp|Q9XV1	rrbs-1	Ribosome biogenesis	28.10	0.00
TRINITY_sp|A5UW>	nfi	Endonuclease V OS=R	28.10	0.00
TRINITY_sp|Q9T01	SPA2	Protein SPA1-RELATE	28.10	0.00
TRINITY_sp|P332(	CYP3A8	Cytochrome P450 3A8	28.10	0.00
TRINITY_sp|Q262:	ku70	ATP-dependent DNA h	28.10	0.00
TRINITY_sp|A9B7I	Haur_17!	UPF0060 membrane pr	28.10	0.00
TRINITY_sp|Q057!	Dbh	Dopamine beta-hydro	28.10	0.00
TRINITY_sp|Q84M:	ABCA1	ABC transporter A f	28.10	0.00
TRINITY_sp|C8YR:	Loxhd1	Lipoxygenase homolo	28.10	0.00
TRINITY_sp|Q9C8I	ABCC11	ABC transporter C f	28.10	0.00
TRINITY_sp|Q54E1	abpF	Actin-binding prote	28.10	0.00
TRINITY_sp|Q9DBV	Cyp4v2	Cytochrome P450 4V2	28.10	0.00
TRINITY_sp|O430:	rpa43	DNA-directed RNA po	28.00	0.00
TRINITY_sp|Q29AI	GA17800	Leishmanolysin-like	28.00	0.00
TRINITY_sp|Q86K!	chmp7	Charged multivesicu	28.00	0.00
TRINITY_sp|Q9P2I	CHD7	Chromodomain-helica	28.00	0.00
TRINITY_sp|Q1MTI	SPBC16D1	Probable assembly c	28.00	0.00
TRINITY_sp|Q5TD!	RSPH4A	Radial spoke head p	28.00	0.00
TRINITY_sp|Q9ZR7	ABCB1	ABC transporter B f	28.00	0.00
TRINITY_sp|Q9WU4	CLCN2	Chloride channel pr	28.00	0.00
TRINITY_sp|Q89A1	bbp_296	Uncharacterized met	28.00	0.00
TRINITY_sp|Q1MTI	vid27	Vacuolar import and	28.00	0.00
TRINITY_sp|Q945I	DTX47	Protein DETOXIFICAT	28.00	0.00
TRINITY_sp|Q54T/	drkC	Probable serine/thr	28.00	0.00
TRINITY_sp|Q9BTV	TBCD	Tubulin-specific ch	28.00	0.00
TRINITY_sp|O243:	-	Vacuolar-processing	28.00	0.00
TRINITY_sp|P086{	CYP3A4	Cytochrome P450 3A4	28.00	0.00
TRINITY_sp|A2VD(	MAP3K7	Mitogen-activated p	28.00	0.00
TRINITY_sp|P471(	XPT1	Xanthine phosphorib	28.00	0.00
TRINITY_sp|Q80U(	Ankzf1	Ankyrin repeat and	28.00	0.00
TRINITY_sp|Q54P(	gefA	Ras guanine nucleot	28.00	0.00
TRINITY_sp|H3K2Y	MED12	Mediator of RNA pol	28.00	0.00
TRINITY_sp|Q80T{	Dcaf5	DDB1- and CUL4-asso	28.00	0.00
TRINITY_sp|Q54Y!	shkC	Dual specificity pr	28.00	0.00
TRINITY_sp|Q54B(	mcfZ	Mitochondrial subst	28.00	0.00
TRINITY_sp|Q54WI	forA	Formin-A OS=Dictyos	28.00	0.00
TRINITY_sp|Q5AD7	MNN24	Alpha-1,2-mannosylt	28.00	0.00
TRINITY_sp|Q8C0I	Cog5	Conserved oligomeri	28.00	0.00
TRINITY_sp|Q036	YMR210W	Putative esterase Y	28.00	0.00
TRINITY_sp|P277	acoC	Dihydrolipoyllysine	28.00	0.00
TRINITY_sp|A2RU(	TYW5	tRNA wybutosine-syn	28.00	0.00
TRINITY_sp|Q78Z/	Nap1l4	Nucleosome assembly	27.90	0.00
TRINITY_sp|Q215(	amt-3	Putative ammonium t	27.90	0.00
TRINITY_sp|F1MK(	RNF170	E3 ubiquitin-protei	27.90	0.00
TRINITY_sp|O042!	IMPA3	Importin subunit al	27.90	0.00
TRINITY_sp|Q69Q:	Os02g07!	Probable protein ph	27.90	0.00
TRINITY_sp|P400:	ERG28	Ergosterol biosynth	27.90	0.00
TRINITY_sp|Q96CI	EVI5L	EVI5-like protein O	27.90	0.00
TRINITY_sp|Q9C9I	CUL3B	Cullin-3B OS=Arabid	27.90	0.00
TRINITY_sp|Q8NC(	DAGLB	Sn1-specific diacyl	27.90	0.00
TRINITY_sp|P0AA!	qmcA	Protein QmcA OS=Esc	27.90	0.00
TRINITY_sp|O225!	STY8	Serine/threonine-pr	27.90	0.00

TRINITY_sp|Q9UT8	SPAC343.	Uncharacterized WD	27.90	0.00
TRINITY_sp|P4655	cdk-12	Cyclin-dependent ki	27.90	0.00
TRINITY_sp|Q7ZY0	cept1	Choline/ethanolamin	27.90	0.00
TRINITY_sp|P3881	TRA1	Transcription-assoc	27.90	0.00
TRINITY_sp|E1BY3	MSH6	DNA mismatch repair	27.90	0.00
TRINITY_sp|O6457	UBA1C	UBP1-associated pro	27.90	0.00
TRINITY_sp|P1555	acyII	Penicillin acylase	27.90	0.00
TRINITY_sp|Q5E98	SLC38A5	Sodium-coupled neut	27.90	0.00
TRINITY_sp|Q9FV0	At1g3209	CSC1-like protein A	27.90	0.00
TRINITY_sp|O7450	SPCC594.	Uncharacterized pro	27.90	0.00
TRINITY_sp|Q5BK1	Capn13	Calpain-13 OS=Rattu	27.90	0.00
TRINITY_sp|Q54V0	hdaC	Type-2 histone deac	27.90	0.00
TRINITY_sp|Q6NR1	ppp6r3-a	Serine/threonine-pr	27.90	0.00
TRINITY_sp|Q9UT2	pvg1	Pyruvyl transferase	27.90	0.00
TRINITY_sp|Q9FL7	ABCA12	ABC transporter A f	27.90	0.00
TRINITY_sp|Q6CL8	CDC123	Cell division cycle	27.90	0.00
TRINITY_sp|P3475	FAB1	1-phosphatidylinosi	27.80	0.00
TRINITY_sp|Q54RI	dhkL	Hybrid signal trans	27.80	0.00
TRINITY_sp|Q551A	DDB_G027	Protein DDB_G027668	27.80	0.00
TRINITY_sp|Q869N	pakB	Serine/threonine-pr	27.80	0.00
TRINITY_sp|Q1770	p	Hermansky-Pudlak sy	27.80	0.00
TRINITY_sp|P4284	YNL320W	Uncharacterized mem	27.80	0.00
TRINITY_sp|Q8CHE	Ttll5	Tubulin polyglutamy	27.80	0.00
TRINITY_sp|P3579	CHKA	Choline kinase alph	27.80	0.00
TRINITY_sp|P1275	RSP3	Flagellar radial sp	27.80	0.00
TRINITY_sp|Q5NC0	Ttf2	Transcription termi	27.80	0.00
TRINITY_sp|Q9MA0	At1g0497	Putative BPI/LBP fa	27.80	0.00
TRINITY_sp|P9WG0	MT1753.1	Uncharacterized oxi	27.80	0.00
TRINITY_sp|P7988	GUCA1B	Guanylyl cyclase-ac	27.80	0.00
TRINITY_sp|Q9HC6	SENP2	Sentrin-specific pr	27.80	0.00
TRINITY_sp|Q9XH5	ETR2	Ethylene receptor 2	27.80	0.00
TRINITY_sp|Q1LU9	fam206a	Protein Simiate OS=	27.80	0.00
TRINITY_sp|F4ICE	RBL10	RHOMBOID-like prote	27.80	0.00
TRINITY_sp|P9WK9	MT0656	Uncharacterized pro	27.80	0.00
TRINITY_sp|Q7LFX	CHST15	Carbohydrate sulfot	27.80	0.00
TRINITY_sp|O4375	STX6	Syntaxin-6 OS=Homo	27.80	0.00
TRINITY_sp|Q9FGE	MAG5	Protein transport p	27.80	0.00
TRINITY_sp|Q76PI	SPCC1223	E3 ubiquitin-protei	27.80	0.00
TRINITY_sp|Q8TD5	DNAH3	Dynein heavy chain	27.80	0.00
TRINITY_sp|Q99P2	Mutyh	Adenine DNA glycosy	27.80	0.00
TRINITY_sp|Q9NU2	MDN1	Midasin OS=Homo sap	27.80	0.00
TRINITY_sp|Q86WI	PKHD1L1	Fibrocystin-L OS=Ho	27.80	0.00
TRINITY_sp|Q9QY2	Smok2a	Sperm motility kina	27.70	0.00
TRINITY_sp|Q4PL6	HPCA	Neuron-specific cal	27.70	0.00
TRINITY_sp|Q95JE	AK7	Adenylate kinase 7	27.70	0.00
TRINITY_sp|Q9UT2	pvg1	Pyruvyl transferase	27.70	0.00
TRINITY_sp|Q9XGX	TIM9	Mitochondrial impor	27.70	0.00
TRINITY_sp|Q54Z2	nol9	Polynucleotide 5'-h	27.70	0.00
TRINITY_sp|Q6446	Cyp3a13	Cytochrome P450 3A1	27.70	0.00
TRINITY_sp|Q2397	Myo61F	Myosin-IB OS=Drosop	27.70	0.00
TRINITY_sp|Q6PD1	Ip6k1	Inositol hexakispho	27.70	0.00
TRINITY_sp|Q96M0	NSMCE3	Non-structural main	27.70	0.00
TRINITY_sp|Q8CFE	Scyl2	SCY1-like protein 2	27.70	0.00
TRINITY_sp|Q8BIW	Prune	Protein prune homol	27.70	0.00
TRINITY_sp|O4892	ETR1	Ethylene receptor O	27.70	0.00
TRINITY_sp|Q08D2	ELMOD2	ELMO domain-contain	27.70	0.00

TRINITY_sp|Q6YZ4	RPA1A	Replication protein	27.70	0.00
TRINITY_sp|Q55E7	frmB	FERM domain-contain	27.70	0.00
TRINITY_sp|Q9TUN	STAT5B	Signal transducer a	27.70	0.00
TRINITY_sp|O4440	F37C4.5	Protein F37C4.5 OS=	27.70	0.00
TRINITY_sp|O4264	SPAC10F6	RCC1 repeat-contain	27.70	0.00
TRINITY_sp|O1569	-	Trypanin OS=Trypano	27.70	0.00
TRINITY_sp|Q9SU0	CHX17	Cation/H(+) antipor	27.70	0.00
TRINITY_sp|Q0496	DNAJ1	DnaJ protein homolo	27.70	0.00
TRINITY_sp|Q0068	gp63	Leishmanolysin OS=L	27.70	0.00
TRINITY_sp|Q75J9	cpras1	Circularly permutat	27.70	0.00
TRINITY_sp|Q149N	NWD1	NACHT domain- and W	27.70	0.00
TRINITY_sp|Q9V60	CG16935	Probable trans-2-en	27.70	0.00
TRINITY_sp|Q0554	HRQ1	ATP-dependent helic	27.70	0.00
TRINITY_sp|P9752	Nf1	Neurofibromin OS=Ra	27.70	0.00
TRINITY_sp|P0678	CMD1	Calmodulin OS=Sacch	27.70	0.00
TRINITY_sp|Q23T0	TTLL3A	Tubulin glycylase 3	27.70	0.00
TRINITY_sp|Q6PD1	-	UPF0668 protein C10	27.70	0.00
TRINITY_sp|Q8BW7	Usp38	Ubiquitin carboxyl-	27.70	0.00
TRINITY_sp|P1149	PHO8	Repressible alkalin	27.70	0.00
TRINITY_sp|G5EFW	daf-10	Intraflagellar tran	27.70	0.00
TRINITY_sp|Q86G4	gefQ	Ras guanine nucleot	27.70	0.00
TRINITY_sp|O7442	SPCC162.	Uncharacterized pro	27.70	0.00
TRINITY_sp|Q9EP5	Tsc1	Hamartin OS=Mus mus	27.70	0.00
TRINITY_sp|Q5Z98	Os06g072	Serine/threonine-pr	27.70	0.00
TRINITY_sp|Q9Y11	NELF-B	Negative elongation	27.70	0.00
TRINITY_sp|Q99NI	Rwdd1	RWD domain-containi	27.70	0.00
TRINITY_sp|Q9300	USP9X	Probable ubiquitin	27.70	0.00
TRINITY_sp|P3471	-	Elongation factor 1	27.70	0.00
TRINITY_sp|Q6B9X	vwkA	Alpha-protein kinas	27.70	0.00
TRINITY_sp|Q56A4	ccdc40	Coiled-coil domain-	27.70	0.00
TRINITY_sp|O1416	SPAC4A8.	Putative lipase C4A	27.70	0.00
TRINITY_sp|Q6FM6	ATG18	Autophagy-related p	27.70	0.00
TRINITY_sp|Q6XHA	roco9	Probable serine/thr	27.70	0.00
TRINITY_sp|A7RF1	cdc123	Cell division cycle	27.70	0.00
TRINITY_sp|O3177	pbpX	Putative penicillin	27.70	0.00
TRINITY_sp|Q86H0	DDB_G027	Probable serine/thr	27.70	0.00
TRINITY_sp|F4KD7	DUR3	Urea-proton symport	27.70	0.00
TRINITY_sp|P3446	clu-1	Clustered mitochond	27.70	0.00
TRINITY_sp|Q9P2B	CC2D2A	Coiled-coil and C2	27.70	0.00
TRINITY_sp|Q8L61	SEC31B	Protein transport p	27.70	0.00
TRINITY_sp|Q8TBB	SLC25A40	Solute carrier fami	27.60	0.00
TRINITY_sp|A8ILB	CFAP52	Cilia- and flagella	27.60	0.00
TRINITY_sp|Q8C16	Cpne1	Copine-1 OS=Mus mus	27.60	0.00
TRINITY_sp|P2013	BET2	Geranylgeranyl tran	27.60	0.00
TRINITY_sp|P0C87	Jmjd7	JmjC domain-contain	27.60	0.00
TRINITY_sp|Q9BQ6	GRWD1	Glutamate-rich WD r	27.60	0.00
TRINITY_sp|Q8CHB	Ttll5	Tubulin polyglutamy	27.60	0.00
TRINITY_sp|Q9FF4	ABCG28	ABC transporter G f	27.60	0.00
TRINITY_sp|Q805B	ctse-b	Cathepsin E-B OS=Xe	27.60	0.00
TRINITY_sp|P5349	AC	Actin OS=Chondrus c	27.60	0.00
TRINITY_sp|Q2KJ7	USP21	Ubiquitin carboxyl-	27.60	0.00
TRINITY_sp|Q8VZ0	TSN1	Ribonuclease TUDOR	27.60	0.00
TRINITY_sp|Q8R0W	Ces4a	Carboxylesterase 4A	27.60	0.00
TRINITY_sp|A2X5B	SWEET15	Bidirectional sugar	27.60	0.00
TRINITY_sp|Q86CB	atg8	Autophagy-related p	27.60	0.00
TRINITY_sp|Q9FT5	GTE6	Transcription facto	27.60	0.00

TRINITY_sp|A4YI8	Msed_20(	3-hydroxypropionyl-	27.60	0.00
TRINITY_sp|O2217	PAB4	Polyadenylate-bindi	27.60	0.00
TRINITY_sp|Q8LLD	NUP96	Nuclear pore comple	27.60	0.00
TRINITY_sp|Q80ZE	Wdtc1	WD and tetratricope	27.60	0.00
TRINITY_sp|Q8TGX	rrp42	Exosome complex com	27.60	0.00
TRINITY_sp|Q0502	RRP5	rRNA biogenesis pro	27.60	0.00
TRINITY_sp|Q7ZX3	jmjd6-b	Bifunctional argini	27.60	0.00
TRINITY_sp|P7416	sll1374	Uncharacterized sym	27.60	0.00
TRINITY_sp|Q67BJ	A4galt	Lactosylceramide 4-	27.60	0.00
TRINITY_sp|Q9BV3	SLC25A23	Calcium-binding mit	27.60	0.00
TRINITY_sp|P3442	dpf-6	Dipeptidyl peptidas	27.60	0.00
TRINITY_sp|Q8CDN	Lrrc9	Leucine-rich repeat	27.60	0.00
TRINITY_sp|Q5U37	klhl12	Kelch-like protein	27.60	0.00
TRINITY_sp|Q2NL6	PARP6	Poly [ADP-ribose] p	27.60	0.00
TRINITY_sp|Q8LJW	ACR4	ACT domain-containi	27.60	0.00
TRINITY_sp|B5FY9	ARL3	ADP-ribosylation fa	27.60	0.00
TRINITY_sp|Q6B9X	vwkA	Alpha-protein kinas	27.50	0.00
TRINITY_sp|D4AP9	ARB_0622	Probable thioredoxi	27.50	0.00
TRINITY_sp|Q6NUT	MFSD12	Major facilitator s	27.50	0.00
TRINITY_sp|A1L4W	BPM6	BTB/POZ and MATH do	27.50	0.00
TRINITY_sp|Q54N5	dynB	Dynactin subunit 2	27.50	0.00
TRINITY_sp|Q9JI9	A4galt	Lactosylceramide 4-	27.50	0.00
TRINITY_sp|Q54PE	cf60	Counting factor 60	27.50	0.00
TRINITY_sp|Q84C0	vioD	Capreomycidine synt	27.50	0.00
TRINITY_sp|Q6C10	CDC123	Cell division cycle	27.50	0.00
TRINITY_sp|Q54JR	abcC3	ABC transporter C f	27.50	0.00
TRINITY_sp|Q56Z9	VSR4	Vacuolar-sorting re	27.50	0.00
TRINITY_sp|Q4Z8B	RanBPM	Ran-binding protein	27.50	0.00
TRINITY_sp|P4352	cya	Adenylate cyclase O	27.50	0.00
TRINITY_sp|Q9SKN	MON1	Vacuolar fusion pro	27.50	0.00
TRINITY_sp|Q99NE	Cep41	Centrosomal protein	27.50	0.00
TRINITY_sp|Q6AX0	Ddx11	ATP-dependent DNA h	27.50	0.00
TRINITY_sp|Q54P9	gefA	Ras guanine nucleot	27.50	0.00
TRINITY_sp|Q9QZ0	Abhd1	Protein ABHD1 OS=Mu	27.50	0.00
TRINITY_sp|B1XL1	tig	Trigger factor OS=S	27.50	0.00
TRINITY_sp|Q8VX2	XEG113	Arabinosyltransfera	27.50	0.00
TRINITY_sp|Q0023	melO	Tyrosinase OS=Asper	27.50	0.00
TRINITY_sp|Q7ZU9	parn	Poly(A)-specific ri	27.50	0.00
TRINITY_sp|B3E69	glmM	Phosphoglucosamine	27.50	0.00
TRINITY_sp|Q502J	lpcat2	Lysophosphatidylcho	27.50	0.00
TRINITY_sp|Q8GT6	TIC40	Protein TIC 40, chl	27.50	0.00
TRINITY_sp|Q9CA0	LOX6	Lipoxygenase 6, chl	27.50	0.00
TRINITY_sp|Q9RT4	sbcC	Nuclease SbcCD subu	27.50	0.00
TRINITY_sp|B6ET1	SYT2	Synaptotagmin-2 OS=	27.50	0.00
TRINITY_sp|Q9QYI	Dnajc7	DnaJ homolog subfam	27.50	0.00
TRINITY_sp|O1368	SPAC11E3	Uncharacterized RWD	27.50	0.00
TRINITY_sp|A8G77	ppc	Phosphoenolpyruvate	27.50	0.00
TRINITY_sp|Q8RWI	SCD2	Coiled-coil domain-	27.50	0.00
TRINITY_sp|Q9Y78	SPCC569.	Aromatic amino acid	27.50	0.00
TRINITY_sp|O3469	yloA	Uncharacterized pro	27.50	0.00
TRINITY_sp|O6073	PLA2G6	85/88 kDa calcium-i	27.50	0.00
TRINITY_sp|Q9SVE	DTX45	Protein DETOXIFICAT	27.50	0.00
TRINITY_sp|A6ZM0	PIF1	ATP-dependent DNA h	27.50	0.00
TRINITY_sp|Q9C8J	ABCG13	ABC transporter G f	27.40	0.00
TRINITY_sp|Q6D3E	idi	Isopentenyl-diphosp	27.40	0.00
TRINITY_sp|O0084	-	Uncharacterized 80	27.40	0.00

TRINITY_sp|D3ZEI	Micall2	MICAL-like protein	27.40	0.00
TRINITY_sp|Q8GWI	OS9	Protein OS-9 homolo	27.40	0.00
TRINITY_sp|Q964]	CYP4C21	Cytochrome P450 4c2	27.40	0.00
TRINITY_sp|O624	Y43F4A.1	Leishmanolysin-like	27.40	0.00
TRINITY_sp|Q01F(	Ogd	Prolyl 3,4-dihydrox	27.40	0.00
TRINITY_sp|Q17Q	TFDP1	Transcription facto	27.40	0.00
TRINITY_sp|Q5TFI	NT5DC1	5'-nucleotidase dom	27.40	0.00
TRINITY_sp|Q8W0	CCR4-1	Carbon catabolite r	27.40	0.00
TRINITY_sp|Q6UV	MOXD1	DBH-like monooxygen	27.40	0.00
TRINITY_sp|Q8IS	gefJ	Ras guanine nucleot	27.40	0.00
TRINITY_sp|Q1ZXI	DDB_G02	Sphingomyelinase DD	27.40	0.00
TRINITY_sp|Q8KEI	CT0663	UPF0301 protein CT0	27.40	0.00
TRINITY_sp|O548	Hmgb3	High mobility group	27.40	0.00
TRINITY_sp|P706	PLA2G7	Platelet-activating	27.40	0.00
TRINITY_sp|P389	MSS4	Probable phosphatid	27.40	0.00
TRINITY_sp|A4Q9	Ttll10	Protein polyglycyla	27.40	0.00
TRINITY_sp|B3DNI	APC6	Anaphase-promoting	27.40	0.00
TRINITY_sp|Q7ZVI	wdr75	WD repeat-containin	27.40	0.00
TRINITY_sp|Q8JH	arl13b	ADP-ribosylation fa	27.40	0.00
TRINITY_sp|Q9M0	ISA3	Isoamylase 3, chlor	27.40	0.00
TRINITY_sp|P418	scn1	Cut9-interacting pr	27.40	0.00
TRINITY_sp|Q94KI	VPS52	Vacuolar protein so	27.40	0.00
TRINITY_sp|Q969	UTP4	U3 small nucleolar	27.40	0.00
TRINITY_sp|Q084	PDE4D	cAMP-specific 3',5'	27.40	0.00
TRINITY_sp|Q9SA	CSP41B	Chloroplast stem-lo	27.40	0.00
TRINITY_sp|Q6TN	pak1ip1	p21-activated prote	27.40	0.00
TRINITY_sp|Q9VY	Evi5	Ecotropic viral int	27.40	0.00
TRINITY_sp|Q17R	GEN1	Flap endonuclease G	27.40	0.00
TRINITY_sp|Q6ZU(	-	Uncharacterized pro	27.40	0.00
TRINITY_sp|Q8S1	Os01g09	Probable glucuronos	27.40	0.00
TRINITY_sp|Q9NP	POLM	DNA-directed DNA/RN	27.40	0.00
TRINITY_sp|P335	ABCC1	Multidrug resistanc	27.30	0.00
TRINITY_sp|Q8CN	SE_2036	Uncharacterized oxi	27.30	0.00
TRINITY_sp|Q9MA	ACBP4	Acyl-CoA-binding do	27.30	0.00
TRINITY_sp|Q9VL	CCDC53	WASH complex subuni	27.30	0.00
TRINITY_sp|P345	epac-1	Rap guanine nucleot	27.30	0.00
TRINITY_sp|Q96S	JMJD8	JmjC domain-contain	27.30	0.00
TRINITY_sp|Q9Z1	Eif2ak3	Eukaryotic translat	27.30	0.00
TRINITY_sp|O960	CHEK2	Serine/threonine-pr	27.30	0.00
TRINITY_sp|Q8WV	KBTBD7	Kelch repeat and BT	27.30	0.00
TRINITY_sp|Q6G1(	truA	tRNA pseudouridine	27.30	0.00
TRINITY_sp|Q8BZI	Nuak2	NUAK family SNF1-li	27.30	0.00
TRINITY_sp|A2YW	PLP2	Patatin-like protei	27.30	0.00
TRINITY_sp|Q0CBI	faeA	Probable feruloyl e	27.30	0.00
TRINITY_sp|Q942	cif-1	COP9/Signalosome an	27.30	0.00
TRINITY_sp|Q54V	mcfJ	Mitochondrial subst	27.30	0.00
TRINITY_sp|Q7SX	slc25a3	Solute carrier fami	27.30	0.00
TRINITY_sp|Q9NXI	MKS1	Meckel syndrome typ	27.30	0.00
TRINITY_sp|P389	MSS4	Probable phosphatid	27.30	0.00
TRINITY_sp|Q002	melO	Tyrosinase OS=Asper	27.30	0.00
TRINITY_sp|Q6NK	At5g017(	Probable protein ph	27.30	0.00
TRINITY_sp|A6H8I	Kcnb2	Potassium voltage-g	27.30	0.00
TRINITY_sp|B1H1I	acp2	Lysosomal acid phos	27.30	0.00
TRINITY_sp|Q54K(	vps51	Vacuolar protein so	27.30	0.00
TRINITY_sp|P518	Cyp4f6	Cytochrome P450 4F6	27.30	0.00
TRINITY_sp|Q10S	Os03g01(	Probable glucuronos	27.30	0.00

TRINITY_sp|Q55D\	abcG1	ABC transporter G f	27.30	0.00
TRINITY_sp|Q6NM\	F8H	Probable glucuronox	27.30	0.00
TRINITY_sp|P344\	pat-2	Integrin alpha pat-	27.30	0.00
TRINITY_sp|Q016\	CACNA1D	Voltage-dependent L	27.30	0.00
TRINITY_sp|Q3S2\	mokB	Lovastatin diketide	27.30	0.00
TRINITY_sp|O493\	VAMP711	Vesicle-associated	27.20	0.00
TRINITY_sp|Q074\	-	Ribonuclease OS=Aer	27.20	0.00
TRINITY_sp|Q078\	SOS1	Son of sevenless ho	27.20	0.00
TRINITY_sp|Q86Y\	DZIP1	Zinc finger protein	27.20	0.00
TRINITY_sp|Q54R\	xpot	Exportin-T OS=Dicty	27.20	0.00
TRINITY_sp|Q54F\	mcfS	Mitochondrial subst	27.20	0.00
TRINITY_sp|O698\	SCO6023	Exopolysaccharide p	27.20	0.00
TRINITY_sp|Q075\	TRM3	tRNA (guanosine(18)	27.20	0.00
TRINITY_sp|O756\	PPM1B	Protein phosphatase	27.20	0.00
TRINITY_sp|Q8LP\	BTS	Zinc finger protein	27.20	0.00
TRINITY_sp|Q8BM\	Eif4e2	Eukaryotic translat	27.20	0.00
TRINITY_sp|Q9LX\	RRP46	Exosome complex exo	27.20	0.00
TRINITY_sp|Q8LE\	At2g306	BTB/POZ domain-cont	27.20	0.00
TRINITY_sp|Q653\	Os06g02	Potassium channel K	27.20	0.00
TRINITY_sp|A4TN\	betA	Oxygen-dependent ch	27.20	0.00
TRINITY_sp|Q8VY\	AAE14	2-succinylbenzoate-	27.20	0.00
TRINITY_sp|Q6L5\	IMCE	Probable isoprenylc	27.20	0.00
TRINITY_sp|Q8FT\	thiED	Thiamine biosynthes	27.20	0.00
TRINITY_sp|Q9FP\	EDR1	Serine/threonine-pr	27.20	0.00
TRINITY_sp|Q9WV\	Sept7	Septin-7 OS=Rattus	27.20	0.00
TRINITY_sp|Q969\	TMBIM1	Protein lifeguard 3	27.20	0.00
TRINITY_sp|Q8H1\	APC2	Anaphase-promoting	27.20	0.00
TRINITY_sp|Q6PD\	Ecm29	Proteasome-associat	27.20	0.00
TRINITY_sp|Q9Y2\	STK38L	Serine/threonine-pr	27.20	0.00
TRINITY_sp|Q91Z\	Fgd4	FYVE, RhoGEF and PH	27.20	0.00
TRINITY_sp|P209\	RASA1	Ras GTPase-activati	27.20	0.00
TRINITY_sp|Q0VC\	LSM12	Protein LSM12 homol	27.20	0.00
TRINITY_sp|C8YR\	Loxhd1	Lipoxygenase homolo	27.20	0.00
TRINITY_sp|Q54C\	gefF	Ras guanine nucleot	27.10	0.00
TRINITY_sp|Q9AW\	CML11	Probable calcium-bi	27.10	0.00
TRINITY_sp|Q8LE\	At2g306	BTB/POZ domain-cont	27.10	0.00
TRINITY_sp|Q254\	-	Muscle calcium chan	27.10	0.00
TRINITY_sp|Q4P9\	CCR4	Glucose-repressible	27.10	0.00
TRINITY_sp|Q9P3\	dbl4	E3 ubiquitin-protei	27.10	0.00
TRINITY_sp|P285\	TRK2	Low-affinity potass	27.10	0.00
TRINITY_sp|Q66H\	Klhl36	Kelch-like protein	27.10	0.00
TRINITY_sp|Q1ZX\	gxcDD	Guanine exchange fa	27.10	0.00
TRINITY_sp|Q869\	DDB_G02	Putative protein di	27.10	0.00
TRINITY_sp|Q9D7\	Pgc	Gastricsin OS=Mus m	27.10	0.00
TRINITY_sp|O751\	CPNE3	Copine-3 OS=Homo sa	27.10	0.00
TRINITY_sp|Q065\	dnrK	Carminomycin 4-O-me	27.10	0.00
TRINITY_sp|P109\	-	Retrovirus-related	27.10	0.00
TRINITY_sp|A6H7\	FBXO3	F-box only protein	27.10	0.00
TRINITY_sp|Q6F6\	dnaJ	Chaperone protein D	27.10	0.00
TRINITY_sp|Q7XJ\	GLR3.1	Glutamate receptor	27.10	0.00
TRINITY_sp|Q54G\	DDB_G02	PXMP2/4 family prot	27.10	0.00
TRINITY_sp|Q9FF\	At5g078	Flavin-containing m	27.10	0.00
TRINITY_sp|Q54E\	grlE	Metabotropic glutam	27.10	0.00
TRINITY_sp|Q9UI\	VPS51	Vacuolar protein so	27.10	0.00
TRINITY_sp|Q555\	DDB_G02	VID27-like protein	27.10	0.00
TRINITY_sp|Q8GU\	At3g526	Nicastrin OS=Arabid	27.10	0.00

TRINITY_sp|Q96N	KLHL32	Kelch-like protein	27.10	0.00
TRINITY_sp|Q5WY	kmo	Kynurenine 3-monoox	27.10	0.00
TRINITY_sp|O945	kap113	Importin beta-like	27.10	0.00
TRINITY_sp|Q2WG	FER1L6	Fer-1-like protein	27.10	0.00
TRINITY_sp|P800	LPO	Lactoperoxidase OS=	27.10	0.00
TRINITY_sp|Q54H	gins1	Probable DNA replic	27.10	0.00
TRINITY_sp|Q9SZ	HHP4	Heptahelical transm	27.10	0.00
TRINITY_sp|Q5JM	Os01g07	Potassium channel K	27.10	0.00
TRINITY_sp|Q99K	Smox	Spermine oxidase OS	27.10	0.00
TRINITY_sp|Q6P0	celf2	CUGBP Elav-like fam	27.10	0.00
TRINITY_sp|Q940	LARP1A	La-related protein	27.10	0.00
TRINITY_sp|Q9LT	VCR	Varicose-related pr	27.10	0.00
TRINITY_sp|Q8BW	Dnah3	Dynein heavy chain	27.10	0.00
TRINITY_sp|Q9P7	SPBC171	Uncharacterized WD	27.00	0.00
TRINITY_sp|Q6IC	At1g431	Polypyrimidine trac	27.00	0.00
TRINITY_sp|A2YF	HK1	Probable histidine	27.00	0.00
TRINITY_sp|Q38W	atpA	ATP synthase subuni	27.00	0.00
TRINITY_sp|Q6NW	arih1l	E3 ubiquitin-protei	27.00	0.00
TRINITY_sp|Q9C1	mcl1	Minichromosome loss	27.00	0.00
TRINITY_sp|Q96P	GBP4	Guanylate-binding p	27.00	0.00
TRINITY_sp|O546	Slc29a2	Equilibrative nucle	27.00	0.00
TRINITY_sp|Q4V8	brms1la	Breast cancer metas	27.00	0.00
TRINITY_sp|Q8H0	UPL2	E3 ubiquitin-protei	27.00	0.00
TRINITY_sp|Q84M	FHY	Bifunctional ribofl	27.00	0.00
TRINITY_sp|P871	SPBC3D6.	Uncharacterized WD	27.00	0.00
TRINITY_sp|Q6A3	SUC6	Putative sucrose tr	27.00	0.00
TRINITY_sp|O823	ATL67	RING-H2 finger prot	27.00	0.00
TRINITY_sp|A2VE	UNC93A	Protein unc-93 homo	27.00	0.00
TRINITY_sp|Q8LB	SWEET7	Bidirectional sugar	27.00	0.00
TRINITY_sp|Q0P5	NSUN3	Putative methyltran	27.00	0.00
TRINITY_sp|Q8MY	DDB_G02	Probable serine/thr	27.00	0.00
TRINITY_sp|Q9SR	SNL1	Paired amphipathic	27.00	0.00
TRINITY_sp|P0DL	DRC1	Dynein regulatory c	27.00	0.00
TRINITY_sp|P218	SRM1	Guanine nucleotide	27.00	0.00
TRINITY_sp|Q9Y2	CHKB	Choline/ethanolamin	27.00	0.00
TRINITY_sp|Q9CQ	Pcyox1	Prenylcysteine oxid	27.00	0.00
TRINITY_sp|Q5ZI	USP28	Ubiquitin carboxyl-	27.00	0.00
TRINITY_sp|Q641	Ttll9	Probable tubulin po	27.00	0.00
TRINITY_sp|P007	PGA	Pepsin A OS=Gallus	27.00	0.00
TRINITY_sp|Q9SL	TOC132	Translocase of chlo	27.00	0.00
TRINITY_sp|Q5UQ	MIMI_L5	Putative ADP-ribosy	27.00	0.00
TRINITY_sp|F4IP	MIP3	Sec1 family domain-	27.00	0.00
TRINITY_sp|Q126	YPK9	Vacuolar cation-tra	27.00	0.00
TRINITY_sp|Q86W	PKHD1L1	Fibrocystin-L OS=Ho	27.00	0.00
TRINITY_sp|Q0WT	CYP81F3	Cytochrome P450 81F	26.90	0.00
TRINITY_sp|Q9W0	Cyp4d20	Probable cytochrome	26.90	0.00
TRINITY_sp|O744	SPCC162.	Uncharacterized pro	26.90	0.00
TRINITY_sp|Q3U0	Tbc1d2b	TBC1 domain family	26.90	0.00
TRINITY_sp|Q08C	moxd2	DBH-like monooxygen	26.90	0.00
TRINITY_sp|Q129	BNIP1	Vesicle transport p	26.90	0.00
TRINITY_sp|P256	CDC39	General negative re	26.90	0.00
TRINITY_sp|Q68C	TNS3	Tensin-3 OS=Homo sa	26.90	0.00
TRINITY_sp|Q700	SPL16	Squamosa promoter-b	26.90	0.00
TRINITY_sp|Q9M2	SDIR1	E3 ubiquitin-protei	26.90	0.00
TRINITY_sp|Q6RC	IFT74	Intraflagellar tran	26.90	0.00
TRINITY_sp|Q86L	rapB	Ras-related protein	26.90	0.00

TRINITY_sp|Q80VI	Fam98b	Protein FAM98B OS=M	26.90	0.00
TRINITY_sp|P794(	CYP3A29	Cytochrome P450 3A2	26.90	0.00
TRINITY_sp|Q1ZXI	DDB_G028	Sphingomyelinase DD	26.90	0.00
TRINITY_sp|Q91X(	Pex16	Peroxisomal membran	26.90	0.00
TRINITY_sp|Q7LH(	TY3B-I	Transposon Ty3-I Ga	26.90	0.00
TRINITY_sp|Q99M!	Sec14l2	SEC14-like protein	26.90	0.00
TRINITY_sp|Q08D(	mael	Protein maelstrom h	26.90	0.00
TRINITY_sp|Q54I!	rtfdc1	Protein RTF2 homolo	26.90	0.00
TRINITY_sp|Q54E7	abpF	Actin-binding prote	26.90	0.00
TRINITY_sp|Q7RRN	PY00695	T-cell immunomodula	26.90	0.00
TRINITY_sp|Q61Y(	CBG0355(	Leishmanolysin-like	26.90	0.00
TRINITY_sp|B6K6N	mmm1	Maintenance of mito	26.90	0.00
TRINITY_sp|Q3T1!	YIF1A	Protein YIF1A OS=Bo	26.90	0.00
TRINITY_sp|Q9ST4	PH1	Pleckstrin homology	26.90	0.00
TRINITY_sp|B1AZA	Tmem245	Transmembrane prote	26.90	0.00
TRINITY_sp|B0U0(	rlmL	Ribosomal RNA large	26.90	0.00
TRINITY_sp|P2932	EPHA3	Ephrin type-A recep	26.90	0.00
TRINITY_sp|A6VY.	ddl	D-alanine--D-alanin	26.90	0.00
TRINITY_sp|Q7Z6.	FGD2	FYVE, RhoGEF and PH	26.90	0.00
TRINITY_sp|Q8VZI	DEG15	Glyoxysomal process	26.90	0.00
TRINITY_sp|O552(	Supt5h	Transcription elong	26.90	0.00
TRINITY_sp|Q1581	ITSN1	Intersectin-1 OS=Ho	26.90	0.00
TRINITY_sp|P5518	ybaR	Putative sulfate tr	26.90	0.00
TRINITY_sp|Q169.	-	Syntaxin OS=Aplysia	26.90	0.00
TRINITY_sp|Q4WZI	mvp1	Sorting nexin mvp1	26.90	0.00
TRINITY_sp|Q9FLY	GNL1	ARF guanine-nucleot	26.90	0.00
TRINITY_sp|Q08D.	AAR2	Protein AAR2 homolo	26.90	0.00
TRINITY_sp|Q9H3(	PABPC3	Polyadenylate-bindi	26.80	0.00
TRINITY_sp|Q5HRN	SERP0172	Putative TrmH famil	26.80	0.00
TRINITY_sp|Q0382	YMR166C	Uncharacterized mit	26.80	0.00
TRINITY_sp|Q5BLI	mettl21a	Protein N-lysine me	26.80	0.00
TRINITY_sp|Q5AHA	CHK1	Histidine protein k	26.80	0.00
TRINITY_sp|Q2VQ\	cecr1	Adenosine deaminase	26.80	0.00
TRINITY_sp|Q55A(	DDB_G027	Probable serine/thr	26.80	0.00
TRINITY_sp|Q551A	DDB_G027	Protein DDB_G027668	26.80	0.00
TRINITY_sp|P873(	tfb2	RNA polymerase II t	26.80	0.00
TRINITY_sp|Q29AI	GA17800	Leishmanolysin-like	26.80	0.00
TRINITY_sp|Q9M82	UGT80A2	Sterol 3-beta-gluco	26.80	0.00
TRINITY_sp|P8382	Ift81	Intraflagellar tran	26.80	0.00
TRINITY_sp|Q55A(	DDB_G027	Probable serine/thr	26.80	0.00
TRINITY_sp|Q8IYI	TEX11	Testis-expressed se	26.80	0.00
TRINITY_sp|Q08C!	moxd2	DBH-like monooxygen	26.80	0.00
TRINITY_sp|P0943	LOX1.2	Seed linoleate 9S-l	26.80	0.00
TRINITY_sp|Q6IN1	nol11	Nucleolar protein 1	26.80	0.00
TRINITY_sp|O3452	sppA	Putative signal pep	26.80	0.00
TRINITY_sp|O0403	CCX5	Cation/calcium exch	26.80	0.00
TRINITY_sp|Q14CW	ATXN7L3	Ataxin-7-like prote	26.80	0.00
TRINITY_sp|A4FII	iolG2	Inositol 2-dehydrog	26.80	0.00
TRINITY_sp|Q2TBI	RAB4A	Ras-related protein	26.80	0.00
TRINITY_sp|Q96J(	VPS50	Syndetin OS=Homo sa	26.80	0.00
TRINITY_sp|Q8N7A	KLHDC1	Kelch domain-contai	26.80	0.00
TRINITY_sp|P3043	Fur2	Furin-like protease	26.80	0.00
TRINITY_sp|Q28E!	ctu2	Cytoplasmic tRNA 2-	26.80	0.00
TRINITY_sp|Q8911	gatA	Glutamyl-tRNA(Gln)	26.80	0.00
TRINITY_sp|Q8IY2	DDX60	Probable ATP-depend	26.80	0.00
TRINITY_sp|Q9SP3	DCL1	Endoribonuclease Di	26.80	0.00

TRINITY_sp|P457(	MAN1A1	Mannosyl-oligosacch	26.80	0.00
TRINITY_sp|P085‹	-	LINE-1 reverse tran	26.80	0.00
TRINITY_sp|P157‹	-	Macronuclear solute	26.80	0.00
TRINITY_sp|B8H4‹	ftsH	ATP-dependent zinc	26.70	0.00
TRINITY_sp|P343‹	C14B9.2	Probable protein di	26.70	0.00
TRINITY_sp|P278‹	-	Pepsin F OS=Oryctol	26.70	0.00
TRINITY_sp|Q4P6‹	UMAG_04‹	Very-long-chain 3-o	26.70	0.00
TRINITY_sp|A1A6‹	HK5	Probable histidine	26.70	0.00
TRINITY_sp|P418‹	rae1	Poly(A)+ RNA export	26.70	0.00
TRINITY_sp|B0Y3‹	gmt1	GDP-mannose transpo	26.70	0.00
TRINITY_sp|P518‹	Cyp4f5	Cytochrome P450 4F5	26.70	0.00
TRINITY_sp|Q9VY‹	Cyp4g15	Cytochrome P450 4g1	26.70	0.00
TRINITY_sp|F4IR‹	SAD2	Importin beta-like	26.70	0.00
TRINITY_sp|Q9P9‹	xylS	Alpha-xylosidase OS	26.70	0.00
TRINITY_sp|A8IU‹	CFAP99	Cilia- and flagella	26.70	0.00
TRINITY_sp|Q9C5‹	PAP6	Purple acid phospha	26.70	0.00
TRINITY_sp|Q9FN‹	UVR8	Ultraviolet-B recep	26.70	0.00
TRINITY_sp|Q8R1‹	Kdelr3	ER lumen protein-re	26.70	0.00
TRINITY_sp|Q54W‹	DDB_G02‹	Putative actin-frag	26.70	0.00
TRINITY_sp|Q4U2‹	Herc2	E3 ubiquitin-protei	26.70	0.00
TRINITY_sp|Q4PE‹	SEC23	Protein transport p	26.70	0.00
TRINITY_sp|Q70C‹	USP34	Ubiquitin carboxyl-	26.70	0.00
TRINITY_sp|P339‹	shc	Squalene--hopene cy	26.70	0.00
TRINITY_sp|O433‹	HSPA12A	Heat shock 70 kDa p	26.70	0.00
TRINITY_sp|P312‹	DDO	D-aspartate oxidase	26.70	0.00
TRINITY_sp|Q6GY‹	Ralgapa‹	Ral GTPase-activati	26.70	0.00
TRINITY_sp|Q93V‹	CYP97A3	Protein LUTEIN DEFI	26.70	0.00
TRINITY_sp|O942‹	SPBC887.	Probable phospholip	26.70	0.00
TRINITY_sp|Q612‹	vha-11	V-type proton ATPas	26.60	0.00
TRINITY_sp|Q9LZ‹	EXO70A1	Exocyst complex com	26.60	0.00
TRINITY_sp|A7RF‹	cdc123	Cell division cycle	26.60	0.00
TRINITY_sp|Q224‹	T14G10.‹	Probable coatomer s	26.60	0.00
TRINITY_sp|P0CE‹	talA	Talin-A OS=Dictyost	26.60	0.00
TRINITY_sp|P221‹	XRN1	5'-3' exoribonuclea	26.60	0.00
TRINITY_sp|O596‹	ccc2	Copper-transporting	26.60	0.00
TRINITY_sp|Q3ZB‹	SIRT5	NAD-dependent prote	26.60	0.00
TRINITY_sp|Q91W‹	Ascc2	Activating signal c	26.60	0.00
TRINITY_sp|Q8K3‹	Dennd1a	DENN domain-contain	26.60	0.00
TRINITY_sp|Q9SY‹	IP5P13	Type I inositol pol	26.60	0.00
TRINITY_sp|Q0WV‹	RECQL5	ATP-dependent DNA h	26.60	0.00
TRINITY_sp|Q54C‹	gefF	Ras guanine nucleot	26.60	0.00
TRINITY_sp|Q6DF‹	ccnj	Cyclin-J OS=Xenopus	26.60	0.00
TRINITY_sp|Q6RK‹	car	Carboxylic acid red	26.60	0.00
TRINITY_sp|Q051‹	PAB5	Polyadenylate-bindi	26.60	0.00
TRINITY_sp|Q99L‹	Rcbtb2	RCC1 and BTB domain	26.60	0.00
TRINITY_sp|B7SI‹	xynC	Endo-1,4-beta-xylan	26.60	0.00
TRINITY_sp|Q5KZ‹	cobB2	NAD-dependent prote	26.60	0.00
TRINITY_sp|Q103‹	SPBC582.	Uncharacterized ATP	26.60	0.00
TRINITY_sp|O944‹	egt2	Hercynylcysteine su	26.60	0.00
TRINITY_sp|Q54P‹	gefA	Ras guanine nucleot	26.60	0.00
TRINITY_sp|Q54H‹	drkB	Probable serine/thr	26.60	0.00
TRINITY_sp|Q8IS‹	gefI	Ras guanine nucleot	26.60	0.00
TRINITY_sp|Q389‹	AKT1	Potassium channel A	26.60	0.00
TRINITY_sp|Q9AJ‹	treZ	Malto-oligosyltreha	26.60	0.00
TRINITY_sp|Q54B‹	DDB_G02‹	Succinate dehydroge	26.50	0.00
TRINITY_sp|Q189‹	cgp-1	GTP-binding protein	26.50	0.00

TRINITY_sp|B3TP(	SLC7A2	Cationic amino acid	26.50	0.00
TRINITY_sp|Q91W(	Setd3	Histone-lysine N-me	26.50	0.00
TRINITY_sp|Q7M3S	rngB	RING finger protein	26.50	0.00
TRINITY_sp|Q966Y	JNK	Stress-activated pr	26.50	0.00
TRINITY_sp|Q006S	gp63	Leishmanolysin OS=L	26.50	0.00
TRINITY_sp|Q8T6	abcA2	ABC transporter A f	26.50	0.00
TRINITY_sp|Q9LU(	CYP72A1!	Cytochrome P450 72A	26.50	0.00
TRINITY_sp|Q8ELS	OB3336	Putative modificati	26.50	0.00
TRINITY_sp|O960(	NAPSA	Napsin-A OS=Homo sa	26.50	0.00
TRINITY_sp|Q9D2I	Ak7	Adenylate kinase 7	26.50	0.00
TRINITY_sp|P4712	BNA2	Indoleamine 2,3-dio	26.50	0.00
TRINITY_sp|Q9CX(	Ist1	IST1 homolog OS=Mus	26.50	0.00
TRINITY_sp|P391	yeaD	Putative glucose-6-	26.50	0.00
TRINITY_sp|B0R0V	tbc1d8b	TBC1 domain family	26.50	0.00
TRINITY_sp|Q006S	gp63	Leishmanolysin OS=L	26.50	0.00
TRINITY_sp|Q8RWY	CLASP	CLIP-associated pro	26.50	0.00
TRINITY_sp|O743S	mak5	ATP-dependent RNA h	26.50	0.00
TRINITY_sp|Q54CI	dync1li1	Cytoplasmic dynein	26.50	0.00
TRINITY_sp|P273S	sol	Calpain-D OS=Drosop	26.50	0.00
TRINITY_sp|Q55E!	pats1	Probable serine/thr	26.50	0.00
TRINITY_sp|F4J5S	FMT	Clustered mitochond	26.50	0.00
TRINITY_sp|O6013	SPBC18H1	Uncharacterized WD	26.50	0.00
TRINITY_sp|Q9UTI	SPAC144.	Uncharacterized ATP	26.50	0.00
TRINITY_sp|Q55E1	zpr1	Zinc finger protein	26.50	0.00
TRINITY_sp|Q4G2	DER1.2	Derlin-1.2 OS=Zea m	26.50	0.00
TRINITY_sp|Q6NXN	Rcbtb1	RCC1 and BTB domain	26.50	0.00
TRINITY_sp|F4IQ	ELP5	Elongator complex p	26.50	0.00
TRINITY_sp|P041	GIP	Copia protein OS=Dr	26.50	0.00
TRINITY_sp|Q8K0I	Slc47a1	Multidrug and toxin	26.40	0.00
TRINITY_sp|P390!	-	Dynein beta chain,	26.40	0.00
TRINITY_sp|O942S	hmt2	Sulfide:quinone oxi	26.40	0.00
TRINITY_sp|P248(	Ccnb1	G2/mitotic-specific	26.40	0.00
TRINITY_sp|P2621	PG1	Exopolygalacturonas	26.40	0.00
TRINITY_sp|B4F6I	aifm2	Apoptosis-inducing	26.40	0.00
TRINITY_sp|A1D3\	sds23	Protein sds23 OS=Ne	26.40	0.00
TRINITY_sp|O666	pth	Peptidyl-tRNA hydro	26.40	0.00
TRINITY_sp|P332	EIF2AK1	Eukaryotic translat	26.40	0.00
TRINITY_sp|Q9P7V	SPBC1703	Probable ribonuclea	26.40	0.00
TRINITY_sp|Q9PTS	KCNH6	Potassium voltage-g	26.40	0.00
TRINITY_sp|Q9283	RPGR	X-linked retinitis	26.40	0.00
TRINITY_sp|P200!	HOP1	Meiosis-specific pr	26.40	0.00
TRINITY_sp|O285!	AF_1724	Uncharacterized pro	26.40	0.00
TRINITY_sp|P195S	-	Probable reverse tr	26.40	0.00
TRINITY_sp|Q91ZI	Abhd3	Phospholipase ABHD3	26.40	0.00
TRINITY_sp|A2RB	sds23	Protein sds23 OS=As	26.40	0.00
TRINITY_sp|Q9D6	Cers4	Ceramide synthase 4	26.40	0.00
TRINITY_sp|Q7F0	CML13	Probable calcium-bi	26.40	0.00
TRINITY_sp|P358I	BDF1	Bromodomain-contain	26.40	0.00
TRINITY_sp|Q9SV(	At4g382!	Amino acid transpor	26.40	0.00
TRINITY_sp|Q503(	slc38a6	Probable sodium-cou	26.40	0.00
TRINITY_sp|Q8CFI	Gbp5	Guanylate-binding p	26.40	0.00
TRINITY_sp|Q4UMI	RF_0338	Uncharacterized zin	26.40	0.00
TRINITY_sp|Q08B\	tyw5	tRNA wybutosine-syn	26.40	0.00
TRINITY_sp|Q2YDV	Msto1	Protein misato homo	26.40	0.00
TRINITY_sp|O600	CMD1	Calmodulin OS=Kluyv	26.30	0.00
TRINITY_sp|Q75JS	cpras1	Circularly permutat	26.30	0.00

TRINITY_sp|B4PR[illegible]	Mat89Ba	Nucleolar protein 6	26.30	0.00
TRINITY_sp|Q54S[illegible]	DDB_G02[illegible]	Protein PIEZO homol	26.30	0.00
TRINITY_sp|P213[illegible]	PWP1	Periodic tryptophan	26.30	0.00
TRINITY_sp|Q2TB[illegible]	POLR3C	DNA-directed RNA po	26.30	0.00
TRINITY_sp|Q9US[illegible]	toa1	Transcription initi	26.30	0.00
TRINITY_sp|Q9LF[illegible]	DOT2	SART-1 family prote	26.30	0.00
TRINITY_sp|Q75J[illegible]	cpras1	Circularly permutat	26.30	0.00
TRINITY_sp|Q5F3[illegible]	UTP15	U3 small nucleolar	26.30	0.00
TRINITY_sp|Q6ZU[illegible]	-	Uncharacterized pro	26.30	0.00
TRINITY_sp|F4I2[illegible]	TPX2	Protein TPX2 OS=Ara	26.30	0.00
TRINITY_sp|Q9Y6[illegible]	CEPT1	Choline/ethanolamin	26.30	0.00
TRINITY_sp|Q392[illegible]	SUC2	Sucrose transport p	26.30	0.00
TRINITY_sp|O573[illegible]	SLC1A3	Excitatory amino ac	26.30	0.00
TRINITY_sp|Q8BK[illegible]	Tmem87b	Transmembrane prote	26.30	0.00
TRINITY_sp|B3P3[illegible]	Pde6	cGMP-specific 3',5'	26.30	0.00
TRINITY_sp|Q6CS[illegible]	PAB1	Polyadenylate-bindi	26.30	0.00
TRINITY_sp|A7XD[illegible]	GALT2	Hydroxyproline O-ga	26.30	0.00
TRINITY_sp|Q8T4[illegible]	CG42684	Probable Ras GTPase	26.30	0.00
TRINITY_sp|Q54D[illegible]	psenB	Presenilin-B OS=Dic	26.30	0.00
TRINITY_sp|A0JM[illegible]	ccdc176	Basal body-orientat	26.30	0.00
TRINITY_sp|Q8BH[illegible]	Rab39a	Ras-related protein	26.30	0.00
TRINITY_sp|Q8UV[illegible]	supt6h	Transcription elong	26.30	0.00
TRINITY_sp|F4J1[illegible]	DTX24	Protein DETOXIFICAT	26.30	0.00
TRINITY_sp|Q207[illegible]	atad-3	ATPase family AAA d	26.30	0.00
TRINITY_sp|P276[illegible]	PGA	Pepsin A-4 OS=Macac	26.30	0.00
TRINITY_sp|Q017[illegible]	GLX	Aldehyde oxidase GL	26.30	0.00
TRINITY_sp|Q9C9[illegible]	CUL3B	Cullin-3B OS=Arabid	26.30	0.00
TRINITY_sp|P265[illegible]	PTBP1	Polypyrimidine trac	26.30	0.00
TRINITY_sp|B8M9[illegible]	tropI	Hydrolase tropI OS=	26.20	0.00
TRINITY_sp|B4EZ[illegible]	hscB	Co-chaperone protei	26.20	0.00
TRINITY_sp|Q75A[illegible]	AGC1	Mitochondrial aspar	26.20	0.00
TRINITY_sp|Q2YD[illegible]	impad1	Inositol monophosph	26.20	0.00
TRINITY_sp|A8JF[illegible]	ODA1	Outer dynein arm pr	26.20	0.00
TRINITY_sp|Q9SX[illegible]	FMOGS-O[illegible]	Flavin-containing m	26.20	0.00
TRINITY_sp|Q6GQ[illegible]	cep41	Centrosomal protein	26.20	0.00
TRINITY_sp|Q388[illegible]	CPK7	Calcium-dependent p	26.20	0.00
TRINITY_sp|Q155[illegible]	TGFBI	Transforming growth	26.20	0.00
TRINITY_sp|F4KB[illegible]	NUP205	Nuclear pore comple	26.20	0.00
TRINITY_sp|Q9W7[illegible]	clcnkb	Chloride channel pr	26.20	0.00
TRINITY_sp|O944[illegible]	bdp1	Transcription facto	26.20	0.00
TRINITY_sp|Q8QG[illegible]	ncor1	Nuclear receptor co	26.20	0.00
TRINITY_sp|Q239[illegible]	regA	3',5'-cyclic-nucleo	26.20	0.00
TRINITY_sp|P337[illegible]	SRP72	Signal recognition	26.20	0.00
TRINITY_sp|Q6DJ[illegible]	znf830	Zinc finger protein	26.20	0.00
TRINITY_sp|P513[illegible]	ycf21	Uncharacterized pro	26.20	0.00
TRINITY_sp|Q925[illegible]	Slc8b1	Sodium/potassium/ca	26.20	0.00
TRINITY_sp|Q54E[illegible]	grlE	Metabotropic glutam	26.20	0.00
TRINITY_sp|Q10M[illegible]	ERDJ3A	DnaJ protein ERDJ3A	26.20	0.00
TRINITY_sp|Q9NY[illegible]	RRN3	RNA polymerase I-sp	26.20	0.00
TRINITY_sp|Q438[illegible]	SS3	Soluble starch synt	26.20	0.00
TRINITY_sp|Q9ZV[illegible]	SE	Serrate RNA effecto	26.20	0.00
TRINITY_sp|Q922[illegible]	Ppp6r3	Serine/threonine-pr	26.20	0.00
TRINITY_sp|Q81Z[illegible]	ubiG	Ubiquinone biosynth	26.20	0.00
TRINITY_sp|C1F0[illegible]	pcp	Pyrrolidone-carboxy	26.20	0.00
TRINITY_sp|Q9FY[illegible]	GLOX1	Aldehyde oxidase GL	26.20	0.00
TRINITY_sp|Q194[illegible]	F14E5.2	Golgi apparatus pro	26.20	0.00

TRINITY_sp|Q9Y4I	RAD54L2	Helicase ARIP4 OS=H	26.20	0.00
TRINITY_sp|P945:	glcD	Glycolate oxidase s	26.20	0.00
TRINITY_sp|Q8CFI	Scyl2	SCY1-like protein 2	26.20	0.00
TRINITY_sp|Q7ZYA	lmbrd2	LMBR1 domain-contai	26.20	0.00
TRINITY_sp|Q8IW(	WDR63	WD repeat-containin	26.20	0.00
TRINITY_sp|P275{	gpa1	Guanine nucleotide-	26.10	0.00
TRINITY_sp|Q86X4	LRRC6	Protein tilB homolo	26.10	0.00
TRINITY_sp|Q9H2I	SLC38A1	Sodium-coupled neut	26.10	0.00
TRINITY_sp|Q8CI7	Rmnd1	Required for meioti	26.10	0.00
TRINITY_sp|P409!	ceg1	mRNA-capping enzyme	26.10	0.00
TRINITY_sp|Q9SR!	CHIP	E3 ubiquitin-protei	26.10	0.00
TRINITY_sp|Q29AI	GA17800	Leishmanolysin-like	26.10	0.00
TRINITY_sp|Q5ZH\	NAA35	N-alpha-acetyltrans	26.10	0.00
TRINITY_sp|A8ZN!	atpA2	ATP synthase subuni	26.10	0.00
TRINITY_sp|Q8LJ1	TKI1	TSL-kinase interact	26.10	0.00
TRINITY_sp|Q9UB(	NXF1	Nuclear RNA export	26.10	0.00
TRINITY_sp|Q6CUI	SDS23	Protein SDS23 OS=Kl	26.10	0.00
TRINITY_sp|P551!	ybbA	Uncharacterized pro	26.10	0.00
TRINITY_sp|Q8KGI	CT0009	Uncharacterized RNA	26.10	0.00
TRINITY_sp|P754:	MPN_348	5-formyltetrahydrof	26.10	0.00
TRINITY_sp|Q8IUI	ZDHHC17	Palmitoyltransferas	26.10	0.00
TRINITY_sp|Q94BI	SLK2	Probable transcript	26.10	0.00
TRINITY_sp|Q54L!	grlD	Metabotropic glutam	26.10	0.00
TRINITY_sp|Q3KP1	tssc1	Protein TSSC1 OS=Xe	26.10	0.00
TRINITY_sp|G4SLI	ttn-1	Titin homolog OS=Ca	26.10	0.00
TRINITY_sp|Q0297	GDE1	Glycerophosphodiest	26.10	0.00
TRINITY_sp|P0CM!	BST1	GPI inositol-deacyl	26.10	0.00
TRINITY_sp|P570!	SLC37A1	Glucose-6-phosphate	26.00	0.00
TRINITY_sp|P127!	RSP3	Flagellar radial sp	26.00	0.00
TRINITY_sp|Q81JI	yidC2	Membrane protein in	26.00	0.00
TRINITY_sp|Q9P7(	pmo25	Mo25-like protein O	26.00	0.00
TRINITY_sp|Q55E4	mcfE	Mitochondrial subst	26.00	0.00
TRINITY_sp|Q8BSA	Tyw3	tRNA wybutosine-syn	26.00	0.00
TRINITY_sp|O444(	kgb-1	GLH-binding kinase	26.00	0.00
TRINITY_sp|Q949:	At1g481(	Polygalacturonase A	26.00	0.00
TRINITY_sp|P710{	ygaD	Putative multidrug	26.00	0.00
TRINITY_sp|Q75J!	cpras1	Circularly permutat	26.00	0.00
TRINITY_sp|O345:	sppA	Putative signal pep	26.00	0.00
TRINITY_sp|Q96J(	VPS39	Vam6/Vps39-like pro	26.00	0.00
TRINITY_sp|O444(	F37C4.5	Protein F37C4.5 OS=	26.00	0.00
TRINITY_sp|Q7M3!	rngB	RING finger protein	26.00	0.00
TRINITY_sp|P2667	Sos	Protein son of seve	26.00	0.00
TRINITY_sp|Q95V:	carmil	Protein CARMIL OS=D	26.00	0.00
TRINITY_sp|Q68Y:	grid2	Glutamate receptor	26.00	0.00
TRINITY_sp|Q28FI	mfsd7-A	Major facilitator s	26.00	0.00
TRINITY_sp|P783:	IGBP1	Immunoglobulin-bind	26.00	0.00
TRINITY_sp|Q9SR1	FLK	Flowering locus K h	26.00	0.00
TRINITY_sp|Q8IS:	gefC	Ras guanine nucleot	26.00	0.00
TRINITY_sp|Q9TV(	rab1	Ras-related protein	26.00	0.00
TRINITY_sp|F4J5!	FMT	Clustered mitochond	26.00	0.00
TRINITY_sp|Q3KQ(	acpt	Testicular acid pho	26.00	0.00
TRINITY_sp|Q5JWI	DOPEY1	Protein dopey-1 OS=	26.00	0.00
TRINITY_sp|Q8LBI	SWEET7	Bidirectional sugar	26.00	0.00
TRINITY_sp|Q9RB(	absAa	2-aminobenzenesulfo	26.00	0.00
TRINITY_sp|Q9P2N	TBC1D14	TBC1 domain family	26.00	0.00
TRINITY_sp|O602:	SSSCA1	Sjoegren syndrome/s	26.00	0.00

TRINITY_sp|Q5VQ(	CPK2	Calcium-dependent p	26.00	0.00
TRINITY_sp|Q242	CngA	Cyclic nucleotide-g	26.00	0.00
TRINITY_sp|Q8L7(	At4g396	ACD11 homolog prote	26.00	0.00
TRINITY_sp|Q94A2	SAC2	Phosphoinositide ph	26.00	0.00
TRINITY_sp|Q8VD(	Mlk4	Mitogen-activated p	26.00	0.00
TRINITY_sp|Q126(	CYP51	Eburicol 14-alpha-d	26.00	0.00
TRINITY_sp|P598	slc39a1	Zinc transporter ZI	26.00	0.00
TRINITY_sp|P126	abg	Beta-glucosidase OS	26.00	0.00
TRINITY_sp|Q8PS	htpX2	Protease HtpX homol	26.00	0.00
TRINITY_sp|Q148	Kiaa146	LisH domain and HEA	26.00	0.00
TRINITY_sp|P278	Vav1	Proto-oncogene vav	26.00	0.00
TRINITY_sp|Q6TK	mx	Interferon-induced	26.00	0.00
TRINITY_sp|Q3MK	EMB2271	U3 snoRNP-associate	26.00	0.00
TRINITY_sp|B3EY	abnB	Intracellular endo-	25.90	0.00
TRINITY_sp|Q9BJ	fus	RNA-binding protein	25.90	0.00
TRINITY_sp|Q8GY	PGM	Phosphoglycerate mu	25.90	0.00
TRINITY_sp|Q9FN(	UVR8	Ultraviolet-B recep	25.90	0.00
TRINITY_sp|P612(	STX1B	Syntaxin-1B OS=Bos	25.90	0.00
TRINITY_sp|Q8VY	GC4	Golgin candidate 4	25.90	0.00
TRINITY_sp|Q96P	ACAP3	Arf-GAP with coiled	25.90	0.00
TRINITY_sp|Q923	SPAC6G9.	Pumilio domain-cont	25.90	0.00
TRINITY_sp|Q682	XRCC2	DNA repair protein	25.90	0.00
TRINITY_sp|Q54E(	nup98	Nuclear pore comple	25.90	0.00
TRINITY_sp|Q9UT	erd1	Protein ERD1 homolo	25.90	0.00
TRINITY_sp|Q8RW	ACBP5	Acyl-CoA-binding do	25.90	0.00
TRINITY_sp|Q54P(	gefA	Ras guanine nucleot	25.90	0.00
TRINITY_sp|Q9C4	paxM	FAD-dependent monoo	25.90	0.00
TRINITY_sp|Q59K	MNN21	Alpha-1,2-mannosylt	25.90	0.00
TRINITY_sp|Q5FW	gpr180	Integral membrane p	25.90	0.00
TRINITY_sp|Q9LG	PARN	Poly(A)-specific ri	25.90	0.00
TRINITY_sp|P276	Rasgrf1	Ras-specific guanin	25.90	0.00
TRINITY_sp|Q8MV	gbpC	Cyclic GMP-binding	25.90	0.00
TRINITY_sp|Q69X	CYP734A	Cytochrome P450 734	25.90	0.00
TRINITY_sp|Q8VY	KEA5	K(+) efflux antipor	25.90	0.00
TRINITY_sp|Q920	Ak5	Adenylate kinase is	25.90	0.00
TRINITY_sp|Q182	trpa-1	Transient receptor	25.90	0.00
TRINITY_sp|Q5JM(	Os01g07	Potassium channel K	25.90	0.00
TRINITY_sp|Q9SC	At3g505	Phosphoglycerate mu	25.80	0.00
TRINITY_sp|Q81V	prs	Ribose-phosphate py	25.80	0.00
TRINITY_sp|Q54E	abpF	Actin-binding prote	25.80	0.00
TRINITY_sp|P108	Prf1	Perforin-1 OS=Mus m	25.80	0.00
TRINITY_sp|Q99J	Thumpd1	THUMP domain-contai	25.80	0.00
TRINITY_sp|A1L0	rsph6a	Radial spoke head p	25.80	0.00
TRINITY_sp|Q700	BDG4	Probable lysophosph	25.80	0.00
TRINITY_sp|Q54B	dcd2A	Neutral ceramidase	25.80	0.00
TRINITY_sp|Q81M	tal2	Probable transaldol	25.80	0.00
TRINITY_sp|Q9SE	VTI11	Vesicle transport v	25.80	0.00
TRINITY_sp|Q8L5	CAND1	Cullin-associated N	25.80	0.00
TRINITY_sp|Q569	mfsd4b	Sodium-dependent gl	25.80	0.00
TRINITY_sp|Q94K	SYP72	Syntaxin-72 OS=Arab	25.80	0.00
TRINITY_sp|B8I7	era	GTPase Era OS=Clost	25.80	0.00
TRINITY_sp|Q995	SORT1	Sortilin OS=Homo sa	25.80	0.00
TRINITY_sp|Q94B	XBAT31	Putative E3 ubiquit	25.80	0.00
TRINITY_sp|O229	VSR2	Vacuolar-sorting re	25.80	0.00
TRINITY_sp|Q9HG	SPBC800.	Uncharacterized cal	25.80	0.00
TRINITY_sp|Q063	LOX1	Linoleate 9S-lipoxy	25.80	0.00

TRINITY_sp|O079:	yisT	Uncharacterized pro	25.80	0.00
TRINITY_sp|P397	mpr	Extracellular metal	25.80	0.00
TRINITY_sp|Q8VZ(	HSR4	Protein HYPER-SENSI	25.80	0.00
TRINITY_sp|Q9FG:	At5g068:	CDK5RAP3-like prote	25.80	0.00
TRINITY_sp|Q8VDI	Hnrnpul:	Heterogeneous nucle	25.80	0.00
TRINITY_sp|Q653I	Os06g02	Potassium channel K	25.80	0.00
TRINITY_sp|Q6TB	CYP97C1	Carotene epsilon-mo	25.80	0.00
TRINITY_sp|Q6FR	SEC62	Translocation prote	25.80	0.00
TRINITY_sp|Q125	aphA	Acid phosphatase OS	25.70	0.00
TRINITY_sp|Q32L	mfsd1	Major facilitator s	25.70	0.00
TRINITY_sp|Q86C	dhkK	Hybrid signal trans	25.70	0.00
TRINITY_sp|O243(	TIC110	Protein TIC110, chl	25.70	0.00
TRINITY_sp|P342	tc3a	Transposable elemen	25.70	0.00
TRINITY_sp|O624	Y43F4A.:	Leishmanolysin-like	25.70	0.00
TRINITY_sp|A2A4:	Cul4b	Cullin-4B OS=Mus mu	25.70	0.00
TRINITY_sp|Q8VEI	Klhdc3	Kelch domain-contai	25.70	0.00
TRINITY_sp|P871:	SPAC57A	Uncharacterized pro	25.70	0.00
TRINITY_sp|Q6WW	UPL3	E3 ubiquitin-protei	25.70	0.00
TRINITY_sp|Q95V	slo-1	Calcium-activated p	25.70	0.00
TRINITY_sp|Q9FF	At5g078(	Flavin-containing m	25.70	0.00
TRINITY_sp|Q8SS(	gefM	Ras guanine nucleot	25.70	0.00
TRINITY_sp|Q3SEI	Icl1e	Caltractin ICL1e OS	25.70	0.00
TRINITY_sp|Q8WNI	IKBKAP	Elongator complex p	25.70	0.00
TRINITY_sp|Q276(	Cyp4e2	Cytochrome P450 4e2	25.70	0.00
TRINITY_sp|Q9ZU:	ABCG7	ABC transporter G f	25.70	0.00
TRINITY_sp|Q145	ITPR2	Inositol 1,4,5-tris	25.70	0.00
TRINITY_sp|Q2FE:	SAUSA30(	Uncharacterized oxi	25.70	0.00
TRINITY_sp|Q54V	DDB_G02	Probable serine/thr	25.70	0.00
TRINITY_sp|Q28F	tmem147	Transmembrane prote	25.60	0.00
TRINITY_sp|Q86JI	DDB_G02	Recoverin family pr	25.60	0.00
TRINITY_sp|Q9NZI	GLTSCR2	Glioma tumor suppre	25.60	0.00
TRINITY_sp|Q54PI	DDB_G02	Probable myosin lig	25.60	0.00
TRINITY_sp|Q5S0(	Spag17	Sperm-associated an	25.60	0.00
TRINITY_sp|Q9PUI	smurf1	E3 ubiquitin-protei	25.60	0.00
TRINITY_sp|A5DSI	FMP52	Protein FMP52, mito	25.60	0.00
TRINITY_sp|P970	cobD	Threonine-phosphate	25.60	0.00
TRINITY_sp|P126	alkH	Aldehyde dehydrogen	25.60	0.00
TRINITY_sp|Q94A	EX2	Protein EXECUTER 2,	25.60	0.00
TRINITY_sp|Q75WI	Vwa5a	von Willebrand fact	25.60	0.00
TRINITY_sp|Q9FF	PPH	Pheophytinase, chlo	25.60	0.00
TRINITY_sp|Q5PP:	ATXR2	Histone-lysine N-me	25.60	0.00
TRINITY_sp|Q8LD(	TAP46	PP2A regulatory sub	25.60	0.00
TRINITY_sp|P209:	RASA1	Ras GTPase-activati	25.60	0.00
TRINITY_sp|Q75VI	TPC1B	Two pore calcium ch	25.60	0.00
TRINITY_sp|Q9FGI	RKD3	Protein RKD3 OS=Ara	25.60	0.00
TRINITY_sp|Q5RJI	Smg7	Protein SMG7 OS=Mus	25.60	0.00
TRINITY_sp|Q8NH(	CFAP61	Cilia- and flagella	25.60	0.00
TRINITY_sp|Q96EI	KIF1BP	KIF1-binding protei	25.60	0.00

TRINITY_sp|Q6DDW	kiaa1324	UPF0577 protein KIA	25.60	0.00
TRINITY_sp|Q2545	-	Muscle calcium chan	25.60	0.00
TRINITY_sp|Q29DY	Klp68D	Kinesin-like protei	25.60	0.00
TRINITY_sp|Q8NDN	RCBTB1	RCC1 and BTB domain	25.50	0.00
TRINITY_sp|P0800	Pdia4	Protein disulfide-i	25.50	0.00
TRINITY_sp|Q5RF7	CHFR	E3 ubiquitin-protei	25.50	0.00
TRINITY_sp|Q8WZ7	DNASE2B	Deoxyribonuclease-2	25.50	0.00
TRINITY_sp|Q6PG9	tmem56-b	Transmembrane prote	25.50	0.00
TRINITY_sp|Q9BV6	RNF126	E3 ubiquitin-protei	25.50	0.00
TRINITY_sp|P1468	KSS1	Mitogen-activated p	25.50	0.00
TRINITY_sp|Q68Y2	grid2	Glutamate receptor	25.50	0.00
TRINITY_sp|P2322	gp63	Leishmanolysin OS=L	25.50	0.00
TRINITY_sp|Q54Q0	wdr89	WD repeat-containin	25.50	0.00
TRINITY_sp|Q9VKY	Cand1	Cullin-associated N	25.50	0.00
TRINITY_sp|Q9CR4	Klhl28	Kelch-like protein	25.50	0.00
TRINITY_sp|Q0043	Ptbp1	Polypyrimidine trac	25.50	0.00
TRINITY_sp|Q0981	ppk5	Serine/threonine-pr	25.50	0.00
TRINITY_sp|F1MBI	KLHL3	Kelch-like protein	25.50	0.00
TRINITY_sp|Q9ULE	PALD1	Paladin OS=Homo sap	25.50	0.00
TRINITY_sp|A2VD2	PLK4	Serine/threonine-pr	25.50	0.00
TRINITY_sp|P1691	elav	Protein elav OS=Dro	25.50	0.00
TRINITY_sp|Q8RWI	STY17	Serine/threonine-pr	25.50	0.00
TRINITY_sp|P5468	tagB	Serine protease/ABC	25.50	0.00
TRINITY_sp|Q8690	DDB_G027	COBW domain-contain	25.50	0.00
TRINITY_sp|Q8R7X	adk	Adenylate kinase OS	25.40	0.00
TRINITY_sp|Q6ZJ0	URH1	Probable uridine nu	25.40	0.00
TRINITY_sp|Q2758	Cyp4d2	Cytochrome P450 4d2	25.40	0.00
TRINITY_sp|Q9NY2	SLC39A1	Zinc transporter ZI	25.40	0.00
TRINITY_sp|Q9VKH	CG6488	Conserved oligomeri	25.40	0.00
TRINITY_sp|B2AC4	Pa_2_141	Extracellular metal	25.40	0.00
TRINITY_sp|Q9975	ABCA3	ATP-binding cassett	25.40	0.00
TRINITY_sp|Q7Z3Z	PIWIL4	Piwi-like protein 4	25.40	0.00
TRINITY_sp|Q9C51	PAP6	Purple acid phospha	25.40	0.00
TRINITY_sp|P8310	Takl1	Putative mitogen-ac	25.40	0.00
TRINITY_sp|Q9C5H	At3g1627	VHS domain-containi	25.40	0.00
TRINITY_sp|Q9067	PLA2G7	Platelet-activating	25.40	0.00
TRINITY_sp|O4604	msta	Protein msta, isofo	25.40	0.00
TRINITY_sp|Q9V3H	heix	UbiA prenyltransfer	25.40	0.00
TRINITY_sp|Q0043	Ptbp1	Polypyrimidine trac	25.40	0.00
TRINITY_sp|F4JTE	STY46	Serine/threonine-pr	25.40	0.00
TRINITY_sp|O4440	F37C4.5	Protein F37C4.5 OS=	25.40	0.00
TRINITY_sp|O9445	SPAC1687	UPF0616 protein C16	25.40	0.00
TRINITY_sp|Q5U37	klhl12	Kelch-like protein	25.40	0.00
TRINITY_sp|Q5R40	SNX4	Sorting nexin-4 OS=	25.40	0.00
TRINITY_sp|P0864	ras1	Ras-like protein 1	25.40	0.00
TRINITY_sp|Q6NZ9	Ddx31	Probable ATP-depend	25.40	0.00
TRINITY_sp|Q7SCY	sym-1	Protein sym-1 OS=Ne	25.30	0.00
TRINITY_sp|P9085	F26E4.3	Uncharacterized pep	25.30	0.00
TRINITY_sp|Q9R11	Sqrdl	Sulfide:quinone oxi	25.30	0.00
TRINITY_sp|P5161	HCFC1	Host cell factor 1	25.30	0.00
TRINITY_sp|A7S4N	serinc	Probable serine inc	25.30	0.00
TRINITY_sp|Q9DG1	star	Steroidogenic acute	25.30	0.00
TRINITY_sp|Q6NRE	emc1	ER membrane protein	25.30	0.00
TRINITY_sp|Q2IBA	MET	Hepatocyte growth f	25.30	0.00
TRINITY_sp|A3LQ0	SDS23	Protein SDS23 OS=Sc	25.30	0.00
TRINITY_sp|Q8TEI	TRPC4AP	Short transient rec	25.30	0.00

TRINITY_sp|Q388	CPK7	Calcium-dependent p	25.30	0.00
TRINITY_sp|Q9HB	CYP4F11	Phylloquinone omega	25.30	0.00
TRINITY_sp|Q0UQ	BST1	GPI inositol-deacyl	25.30	0.00
TRINITY_sp|Q9UT	pvg1	Pyruvyl transferase	25.30	0.00
TRINITY_sp|P0CD	frmC	FERM domain-contain	25.30	0.00
TRINITY_sp|F4JM	PGSIP7	Putative glucuronos	25.30	0.00
TRINITY_sp|Q016	Pxd	Peroxidase OS=Droso	25.30	0.00
TRINITY_sp|Q9CT	Rhobtb3	Rho-related BTB dom	25.30	0.00
TRINITY_sp|Q9UB	ASH2L	Set1/Ash2 histone m	25.30	0.00
TRINITY_sp|P284	CAPZA2	F-actin-capping pro	25.30	0.00
TRINITY_sp|Q54E	grlE	Metabotropic glutam	25.30	0.00
TRINITY_sp|Q2UL	utp10	U3 small nucleolar	25.20	0.00
TRINITY_sp|Q6IM	dnajc27	DnaJ homolog subfam	25.20	0.00
TRINITY_sp|Q5RC	CYP4V2	Cytochrome P450 4V2	25.20	0.00
TRINITY_sp|Q28F	mfsd7-A	Major facilitator s	25.20	0.00
TRINITY_sp|O147	TPP1	Tripeptidyl-peptida	25.20	0.00
TRINITY_sp|Q0CR	atg5	Autophagy protein 5	25.20	0.00
TRINITY_sp|Q9SF	SYP71	Syntaxin-71 OS=Arab	25.20	0.00
TRINITY_sp|Q8LP	LACS6	Long chain acyl-CoA	25.20	0.00
TRINITY_sp|Q9SW	SYP42	Syntaxin-42 OS=Arab	25.20	0.00
TRINITY_sp|Q940	SRK2E	Serine/threonine-pr	25.20	0.00
TRINITY_sp|Q9YG	RPE65	Retinoid isomerohyd	25.20	0.00
TRINITY_sp|Q86A	mcfX	Mitochondrial subst	25.20	0.00
TRINITY_sp|Q9XT	tbh-1	Tyramine beta-hydro	25.20	0.00
TRINITY_sp|P432	npr	Bacillolysin OS=Bre	25.20	0.00
TRINITY_sp|Q023	GLG1	Golgi apparatus pro	25.20	0.00
TRINITY_sp|O434	EIF4G3	Eukaryotic translat	25.20	0.00
TRINITY_sp|Q5XG	Utp20	Small subunit proce	25.20	0.00
TRINITY_sp|P463	iolS	Protein IolS OS=Bac	25.10	0.00
TRINITY_sp|Q8DF	nutA	5'-nucleotidase OS=	25.10	0.00
TRINITY_sp|P515	BRCA2	Breast cancer type	25.10	0.00
TRINITY_sp|Q8CD	Ccdc38	Coiled-coil domain-	25.10	0.00
TRINITY_sp|Q8GU	HYP1	CSC1-like protein H	25.10	0.00
TRINITY_sp|Q9FI	ARC6	Protein ACCUMULATIO	25.10	0.00
TRINITY_sp|Q558	sll0103	Uncharacterized pro	25.10	0.00
TRINITY_sp|Q9NU	AGPAT5	1-acyl-sn-glycerol-	25.10	0.00
TRINITY_sp|Q8BI	Ccdc40	Coiled-coil domain-	25.10	0.00
TRINITY_sp|P511	GZMM	Granzyme M OS=Homo	25.10	0.00
TRINITY_sp|P891	CTR9	RNA polymerase-asso	25.10	0.00
TRINITY_sp|O285	AF_1724	Uncharacterized pro	25.10	0.00
TRINITY_sp|F4JT	STY46	Serine/threonine-pr	25.10	0.00
TRINITY_sp|Q5ZL	NSUN2	tRNA (cytosine(34)-	25.10	0.00
TRINITY_sp|P500	KEL2	Kelch repeat-contai	25.10	0.00
TRINITY_sp|Q9WU	Stxbp5	Syntaxin-binding pr	25.10	0.00
TRINITY_sp|Q86I	pscA	Penicillin-sensitiv	25.10	0.00
TRINITY_sp|C0Z9	mutS2	Endonuclease MutS2	25.10	0.00
TRINITY_sp|Q29K	GA19813	Ribosome biogenesis	25.10	0.00
TRINITY_sp|Q6GL	elavl1	ELAV-like protein 1	25.10	0.00
TRINITY_sp|O345	sppA	Putative signal pep	25.10	0.00
TRINITY_sp|Q9V3	Tak1	Mitogen-activated p	25.10	0.00
TRINITY_sp|P383	PHO89	Phosphate permease	25.10	0.00
TRINITY_sp|P175	SRV2	Adenylyl cyclase-as	25.10	0.00
TRINITY_sp|Q8N8	UBR7	Putative E3 ubiquit	25.00	0.00
TRINITY_sp|Q8BK	Smg1	Serine/threonine-pr	25.00	0.00
TRINITY_sp|O316	yjcL	Uncharacterized mem	25.00	0.00
TRINITY_sp|Q8IZ	CACNA2D	Voltage-dependent c	25.00	0.00

TRINITY_sp|Q9LKW	NHX7	Sodium/hydrogen exc	25.00	0.00
TRINITY_sp|O6017	SPBC23E6	Uncharacterized ATP	25.00	0.00
TRINITY_sp|Q4I32	BFR2	Protein BFR2 OS=Gib	25.00	0.00
TRINITY_sp|Q99JN	Klhl22	Kelch-like protein	25.00	0.00
TRINITY_sp|Q0WVE	RRP6L1	Protein RRP6-like 1	25.00	0.00
TRINITY_sp|Q94A6	At1g0669	Uncharacterized oxi	25.00	0.00
TRINITY_sp|Q9932	zip	Myosin heavy chain,	24.90	0.00
TRINITY_sp|P4099	ceg1	mRNA-capping enzyme	24.90	0.00
TRINITY_sp|Q9Z1N	ACOX1	Peroxisomal acyl-co	24.90	0.00
TRINITY_sp|Q9FE0	TOM1	Tobamovirus multipl	24.90	0.00
TRINITY_sp|Q23T0	TTLL3A	Tubulin glycylase 3	24.90	0.00
TRINITY_sp|Q6CSV	PAB1	Polyadenylate-bindi	24.90	0.00
TRINITY_sp|P3660	ncs-1	Neuronal calcium se	24.90	0.00
TRINITY_sp|Q7M39	rngB	RING finger protein	24.90	0.00
TRINITY_sp|Q6ICX	At1g4319	Polypyrimidine trac	24.90	0.00
TRINITY_sp|B1AU0	Arhgap36	Rho GTPase-activati	24.90	0.00
TRINITY_sp|Q54U7	crlA	Cyclic AMP receptor	24.90	0.00
TRINITY_sp|A8JF7	ODA1	Outer dynein arm pr	24.90	0.00
TRINITY_sp|Q9VRI	Membrin	Probable Golgi SNAP	24.90	0.00
TRINITY_sp|Q9LZX	BIG2	Brefeldin A-inhibit	24.90	0.00
TRINITY_sp|Q58DE	TRABD	TraB domain-contain	24.90	0.00
TRINITY_sp|Q7ZU0	ptges2	Prostaglandin E syn	24.90	0.00
TRINITY_sp|A2YFE	HK1	Probable histidine	24.90	0.00
TRINITY_sp|Q802E	smc6	Structural maintena	24.90	0.00
TRINITY_sp|Q0728	ADH1	Alcohol dehydrogena	24.80	0.00
TRINITY_sp|Q802V	abhd2a	Monoacylglycerol li	24.80	0.00
TRINITY_sp|P0A30	bp26	26 kDa periplasmic	24.80	0.00
TRINITY_sp|Q5U75	mx	Interferon-induced	24.80	0.00
TRINITY_sp|Q4P97	CCR4	Glucose-repressible	24.80	0.00
TRINITY_sp|A2X8A	OsI_0846	SPX domain-containi	24.80	0.00
TRINITY_sp|Q8RWI	CLT3	Protein CLT3, chlor	24.80	0.00
TRINITY_sp|Q6DF0	ccdc77	Coiled-coil domain-	24.80	0.00
TRINITY_sp|Q6F6Y	hpxO	FAD-dependent urate	24.80	0.00
TRINITY_sp|Q68AE	nfdA	N-substituted forma	24.80	0.00
TRINITY_sp|F4I11	At1g0957	Calcium uniporter p	24.80	0.00
TRINITY_sp|A8FW5	rlmL	Ribosomal RNA large	24.80	0.00
TRINITY_sp|Q6DFE	poc5	Centrosomal protein	24.80	0.00
TRINITY_sp|F4IA7	THO2	THO complex subunit	24.80	0.00
TRINITY_sp|Q4R8V	CFAP100	Cilia- and flagella	24.80	0.00
TRINITY_sp|A0JP8	cnot1	CCR4-NOT transcript	24.80	0.00
TRINITY_sp|Q1KPV	FZL	Probable transmembr	24.80	0.00
TRINITY_sp|A1XD9	TFIP11	Tuftelin-interactin	24.80	0.00
TRINITY_sp|P1438	-	Transposon TX1 unch	24.80	0.00
TRINITY_sp|Q54L5	grlD	Metabotropic glutam	24.80	0.00
TRINITY_sp|Q8RXE	MIRO1	Mitochondrial Rho G	24.80	0.00
TRINITY_sp|Q9LV8	At5g6497	Probable mitochondr	24.80	0.00
TRINITY_sp|Q54JA	sibE	Integrin beta-like	24.80	0.00
TRINITY_sp|Q9SI1	SFH10	Phosphatidylinosito	24.70	0.00
TRINITY_sp|Q6374	Dlc1	Rho GTPase-activati	24.70	0.00
TRINITY_sp|Q6P2E	TMEM53	Transmembrane prote	24.70	0.00
TRINITY_sp|Q7XB0	CNMT	(S)-coclaurine N-me	24.70	0.00
TRINITY_sp|O9495	FBXO21	F-box only protein	24.70	0.00
TRINITY_sp|P9818	CYP4F8	Cytochrome P450 4F8	24.70	0.00
TRINITY_sp|Q3SZI	LPCAT3	Lysophospholipid ac	24.70	0.00
TRINITY_sp|Q8CHE	Ttll5	Tubulin polyglutamy	24.70	0.00
TRINITY_sp|O1384	rsd1	RNA-binding protein	24.70	0.00

TRINITY_sp|Q8BV	Trank1	TPR and ankyrin rep	24.70	0.00
TRINITY_sp|Q098	SPAC2G1	Calcium permeable s	24.70	0.00
TRINITY_sp|Q8W0	CCR4-1	Carbon catabolite r	24.60	0.00
TRINITY_sp|Q3UR	Armc2	Armadillo repeat-co	24.60	0.00
TRINITY_sp|A8JF	ODA1	Outer dynein arm pr	24.60	0.00
TRINITY_sp|Q1XD	ycf37	Uncharacterized pro	24.60	0.00
TRINITY_sp|P516	HCFC1	Host cell factor 1	24.60	0.00
TRINITY_sp|O004	IPO5	Importin-5 OS=Homo	24.60	0.00
TRINITY_sp|Q9JH	Slc38a3	Sodium-coupled neut	24.60	0.00
TRINITY_sp|Q6PF	wdr48	WD repeat-containin	24.60	0.00
TRINITY_sp|Q6NZ	Dnajc8	DnaJ homolog subfam	24.60	0.00
TRINITY_sp|Q551	DDB_G02	Protein DDB_G027668	24.60	0.00
TRINITY_sp|P337	SRP72	Signal recognition	24.60	0.00
TRINITY_sp|A2AS	Ankrd16	Ankyrin repeat doma	24.60	0.00
TRINITY_sp|B1H1	acp2	Lysosomal acid phos	24.60	0.00
TRINITY_sp|O154	ABCC4	Multidrug resistanc	24.60	0.00
TRINITY_sp|P9WI	MT1821	L-gulono-1,4-lacton	24.50	0.00
TRINITY_sp|Q9ST	ABCA5	ABC transporter A f	24.50	0.00
TRINITY_sp|Q9AV	PRP19	Pre-mRNA-processing	24.50	0.00
TRINITY_sp|Q8BS	Ccdc151	Coiled-coil domain-	24.50	0.00
TRINITY_sp|Q8L7	ORP1C	Oxysterol-binding p	24.50	0.00
TRINITY_sp|Q7RR	PY00695	T-cell immunomodula	24.50	0.00
TRINITY_sp|P547	pkaA	Serine/threonine-pr	24.50	0.00
TRINITY_sp|Q54M	lrlA	Latrophilin recepto	24.50	0.00
TRINITY_sp|Q8BG	Zadh2	Prostaglandin reduc	24.50	0.00
TRINITY_sp|Q9LU	CYP72A1	Cytochrome P450 72A	24.50	0.00
TRINITY_sp|Q88N	quiP	Acyl-homoserine lac	24.50	0.00
TRINITY_sp|Q5TH	VPS13D	Vacuolar protein so	24.50	0.00
TRINITY_sp|Q54F	eif2b3	Translation initiat	24.50	0.00
TRINITY_sp|Q9Z0	Itsn2	Intersectin-2 OS=Mu	24.50	0.00
TRINITY_sp|P343	crn-7	Cell-death-related	24.50	0.00
TRINITY_sp|Q9PT	KCNH6	Potassium voltage-g	24.50	0.00
TRINITY_sp|Q5XI	Ttll10	Protein polyglycyla	24.50	0.00
TRINITY_sp|O141	pop2	WD repeat-containin	24.50	0.00
TRINITY_sp|A0R4	kshA	3-ketosteroid-9-alp	24.50	0.00
TRINITY_sp|Q54B	DDB_G02	LIMR family protein	24.40	0.00
TRINITY_sp|B4P5	eIF3-S9	Eukaryotic translat	24.40	0.00
TRINITY_sp|B3WC	cinA	Putative competence	24.40	0.00
TRINITY_sp|A2YW	PLP2	Patatin-like protei	24.40	0.00
TRINITY_sp|Q9NW	RNF216	E3 ubiquitin-protei	24.40	0.00
TRINITY_sp|Q9H7	NRDE2	Protein NRDE2 homol	24.40	0.00
TRINITY_sp|Q9FF	PPH	Pheophytinase, chlo	24.40	0.00
TRINITY_sp|Q9HF	tra1	Transcription-assoc	24.40	0.00
TRINITY_sp|Q08D	AP2B1	AP-1 complex subuni	24.40	0.00
TRINITY_sp|P779	secY	Protein translocase	24.40	0.00
TRINITY_sp|O749	ria1	Ribosome assembly p	24.40	0.00
TRINITY_sp|K7TQ	HIP	HSP-interacting pro	24.40	0.00
TRINITY_sp|P736	sppA	Protease 4 OS=Synec	24.40	0.00
TRINITY_sp|Q17Q	TFDP1	Transcription facto	24.40	0.00
TRINITY_sp|Q9V7	Cyp6a23	Probable cytochrome	24.40	0.00
TRINITY_sp|Q9SF	DTX43	Protein DETOXIFICAT	24.40	0.00
TRINITY_sp|Q6PD	AAGAB	Alpha- and gamma-ad	24.30	0.00
TRINITY_sp|Q8C1	Vps9d1	VPS9 domain-contain	24.30	0.00
TRINITY_sp|O185	Cyp4d10	Cytochrome P450 4d1	24.30	0.00
TRINITY_sp|Q9QX	Nufip1	Nuclear fragile X m	24.30	0.00
TRINITY_sp|Q6BZ	SNX4	Sorting nexin-4 OS=	24.30	0.00

TRINITY_sp|D3Z6	Pdia2	Protein disulfide-i	24.30	0.00
TRINITY_sp|Q8CH	Ttll5	Tubulin polyglutamy	24.30	0.00
TRINITY_sp|P532	MDR1	GTPase-activating p	24.30	0.00
TRINITY_sp|Q5XI	Sart1	U4/U6.U5 tri-snRNP-	24.30	0.00
TRINITY_sp|Q9SK	PAO2	Probable polyamine	24.20	0.00
TRINITY_sp|P516	HCFC1	Host cell factor 1	24.20	0.00
TRINITY_sp|Q8K3	Fanci	Fanconi anemia grou	24.20	0.00
TRINITY_sp|Q6CU	SDS23	Protein SDS23 OS=Kl	24.20	0.00
TRINITY_sp|Q8BS	Ccdc151	Coiled-coil domain-	24.20	0.00
TRINITY_sp|Q078	amdA	Acetamidase OS=Myco	24.20	0.00
TRINITY_sp|Q52K	rcc2	Protein RCC2 homolo	24.20	0.00
TRINITY_sp|Q9UB	RNF14	E3 ubiquitin-protei	24.20	0.00
TRINITY_sp|F4HX	PLA1	Phospholipase A I O	24.20	0.00
TRINITY_sp|Q9Z1	Cdc45	Cell division contr	24.20	0.00
TRINITY_sp|Q9SE	SURF1	Surfeit locus prote	24.20	0.00
TRINITY_sp|P0CB	med14	Putative mediator o	24.20	0.00
TRINITY_sp|Q54S	grlF	Metabotropic glutam	24.20	0.00
TRINITY_sp|Q80W	Slc29a3	Equilibrative nucle	24.20	0.00
TRINITY_sp|Q9SV	At4g382	Amino acid transpor	24.20	0.00
TRINITY_sp|Q802	xpo4	Exportin-4 OS=Danio	24.20	0.00
TRINITY_sp|A6VY	ddl	D-alanine--D-alanin	24.10	0.00
TRINITY_sp|Q085	ALE1	Lysophospholipid ac	24.10	0.00
TRINITY_sp|Q9C7	ATE2	Arginyl-tRNA--prote	24.10	0.00
TRINITY_sp|P908	F56F10.	Putative serine pro	24.10	0.00
TRINITY_sp|B4F6	aifm2	Apoptosis-inducing	24.10	0.00
TRINITY_sp|F4HP	DTX20	Protein DETOXIFICAT	24.10	0.00
TRINITY_sp|Q9LI	CHUP1	Protein CHUP1, chlo	24.10	0.00
TRINITY_sp|Q9LE	DTX54	Protein DETOXIFICAT	24.10	0.00
TRINITY_sp|Q9P3	fcp1	RNA polymerase II s	24.10	0.00
TRINITY_sp|Q672	Nox3	NADPH oxidase 3 OS=	24.10	0.00
TRINITY_sp|Q54I	slc44a2	Choline transporter	24.10	0.00
TRINITY_sp|Q7Z3	PIWIL3	Piwi-like protein 3	24.10	0.00
TRINITY_sp|P515	tetA	Tetracycline resist	24.00	0.00
TRINITY_sp|O349	yobE	Putative SOS respon	24.00	0.00
TRINITY_sp|Q8T2	prkag	5'-AMP-activated pr	24.00	0.00
TRINITY_sp|Q8IW	WDR63	WD repeat-containin	24.00	0.00
TRINITY_sp|Q5D1	TFAM	Transcription facto	24.00	0.00
TRINITY_sp|Q8S3	SEC5A	Exocyst complex com	24.00	0.00
TRINITY_sp|Q5RF	ZW10	Centromere/kinetoch	24.00	0.00
TRINITY_sp|Q5NC	Ttf2	Transcription termi	24.00	0.00
TRINITY_sp|Q9P8	tcsA	Two-component syste	24.00	0.00
TRINITY_sp|Q55F	dlpA	Dynamin-like protei	24.00	0.00
TRINITY_sp|Q556	DDB_G02	Probable rhodanese	24.00	0.00
TRINITY_sp|Q7MY	rsmB	Ribosomal RNA small	24.00	0.00
TRINITY_sp|Q6QB	Plscr3	Phospholipid scramb	24.00	0.00
TRINITY_sp|B0G1	ucpB	Mitochondrial subst	24.00	0.00
TRINITY_sp|Q275	cyp-13A	Putative cytochrome	24.00	0.00
TRINITY_sp|Q54W	gcn1	eIF-2-alpha kinase	23.90	0.00
TRINITY_sp|F4JT	VPS54	Vacuolar protein so	23.90	0.00
TRINITY_sp|P146	KSS1	Mitogen-activated p	23.90	0.00
TRINITY_sp|P400	AVT6	Vacuolar amino acid	23.90	0.00
TRINITY_sp|Q80V	Cfap100	Cilia- and flagella	23.90	0.00
TRINITY_sp|P395	ywbF	Uncharacterized tra	23.90	0.00
TRINITY_sp|P599	Sirt6	NAD-dependent prote	23.90	0.00
TRINITY_sp|Q29R	MAT2B	Methionine adenosyl	23.90	0.00
TRINITY_sp|Q103	SPBC582.	Uncharacterized ATP	23.90	0.00

TRINITY_sp|Q0912	Cyp24a1	1,25-dihydroxyvitam	23.90	0.00
TRINITY_sp|Q9V9	RhoGAP1(	Rho GTPase-activati	23.90	0.00
TRINITY_sp|Q54E	abpF	Actin-binding prote	23.90	0.00
TRINITY_sp|Q023	CACNA1E	Voltage-dependent R	23.90	0.00
TRINITY_sp|Q3KQ	acpt	Testicular acid pho	23.80	0.00
TRINITY_sp|Q54I	slc44a2	Choline transporter	23.80	0.00
TRINITY_sp|P278	PEP3	Vacuolar membrane p	23.80	0.00
TRINITY_sp|Q8BI	Ranbp6	Ran-binding protein	23.80	0.00
TRINITY_sp|Q54M	DDB_G02	von Willebrand fact	23.80	0.00
TRINITY_sp|Q3SZ	AAMP	Angio-associated mi	23.80	0.00
TRINITY_sp|Q2UK	clu1	Clustered mitochond	23.80	0.00
TRINITY_sp|O353	Phyh	Phytanoyl-CoA dioxy	23.70	0.00
TRINITY_sp|Q9LD	LAG1	LAG1 longevity assu	23.70	0.00
TRINITY_sp|Q5ZK	SETD6	N-lysine methyltran	23.70	0.00
TRINITY_sp|Q29L	mon2	Protein MON2 homolo	23.70	0.00
TRINITY_sp|Q017	EXOSC10	Exosome component 1	23.70	0.00
TRINITY_sp|Q103	SPBC582.	Uncharacterized ATP	23.70	0.00
TRINITY_sp|Q7Z4	NPHP3	Nephrocystin-3 OS=H	23.70	0.00
TRINITY_sp|Q17Q	SLC37A3	Sugar phosphate exc	23.70	0.00
TRINITY_sp|Q190	pho-1	Intestinal acid pho	23.70	0.00
TRINITY_sp|Q8LB	KING1	SNF1-related protei	23.70	0.00
TRINITY_sp|O152	ACOX3	Peroxisomal acyl-co	23.70	0.00
TRINITY_sp|Q55E	vps13E	Putative vacuolar p	23.70	0.00
TRINITY_sp|O042	IMPA3	Importin subunit al	23.60	0.00
TRINITY_sp|Q6EM	TTLL5	Tubulin polyglutamy	23.60	0.00
TRINITY_sp|Q9VR	HERC2	Probable E3 ubiquit	23.60	0.00
TRINITY_sp|Q54T	tbc1d5B	TBC1 domain family	23.60	0.00
TRINITY_sp|Q6FT	SFH1	Chromatin structure	23.60	0.00
TRINITY_sp|D3Z8	Slc38a1	Putative sodium-cou	23.60	0.00
TRINITY_sp|Q535	nahG	Salicylate hydroxyl	23.60	0.00
TRINITY_sp|Q9WZ	corA	Cobalt/magnesium tr	23.60	0.00
TRINITY_sp|P250	CML12	Calmodulin-like pro	23.60	0.00
TRINITY_sp|A7S4	serinc	Probable serine inc	23.60	0.00
TRINITY_sp|Q58D	DCAF4	DDB1- and CUL4-asso	23.50	0.00
TRINITY_sp|Q8UU	eed-a	Polycomb protein ee	23.50	0.00
TRINITY_sp|O805	ITPK4	Inositol 1,3,4-tris	23.50	0.00
TRINITY_sp|A6QP	TBC1D2	TBC1 domain family	23.50	0.00
TRINITY_sp|F2Z4	Herc6	E3 ISG15--protein l	23.50	0.00
TRINITY_sp|P400	FTR1	Plasma membrane iro	23.50	0.00
TRINITY_sp|Q4PI	UMAG_00	Acyl-protein thioes	23.50	0.00
TRINITY_sp|Q9LF	PIN8	Auxin efflux carrie	23.40	0.00
TRINITY_sp|Q8R3	Tcf25	Transcription facto	23.40	0.00
TRINITY_sp|Q6LL	pckA	Phosphoenolpyruvate	23.40	0.00
TRINITY_sp|Q6ZP	Ankib1	Ankyrin repeat and	23.40	0.00
TRINITY_sp|Q8NB	KDM1B	Lysine-specific his	23.40	0.00
TRINITY_sp|Q9VC	Cyp6d4	Probable cytochrome	23.40	0.00
TRINITY_sp|Q9LG	PARN	Poly(A)-specific ri	23.40	0.00
TRINITY_sp|Q9P4	tcsB	Two-component syste	23.40	0.00
TRINITY_sp|Q092	nrf-6	Nose resistant to f	23.40	0.00
TRINITY_sp|Q8SR	ECU08_1	Probable cell divis	23.40	0.00
TRINITY_sp|Q54E	gacEE	Rho GTPase-activati	23.40	0.00
TRINITY_sp|Q5B4	btgC	Glucan endo-1,3-bet	23.40	0.00
TRINITY_sp|Q4G0	CCDC40	Coiled-coil domain-	23.40	0.00
TRINITY_sp|P360	VPS24	Vacuolar protein-so	23.40	0.00
TRINITY_sp|Q8BV	Ifi44	Interferon-induced	23.40	0.00
TRINITY_sp|Q6DF	ccdc77	Coiled-coil domain-	23.30	0.00

TRINITY_sp|P207(	uvsW	ATP-dependent DNA h	23.30	0.00
TRINITY_sp|P345	K11H3.3	Putative tricarboxy	23.30	0.00
TRINITY_sp|Q9FK	PILS7	Protein PIN-LIKES 7	23.30	0.00
TRINITY_sp|Q0JJ	MAN2	Mannan endo-1,4-bet	23.30	0.00
TRINITY_sp|Q96Q	HS3ST6	Heparan sulfate glu	23.30	0.00
TRINITY_sp|Q66I	srrt	Serrate RNA effecto	23.30	0.00
TRINITY_sp|Q8CH	Mbtps2	Membrane-bound tran	23.30	0.00
TRINITY_sp|Q5XE	HESO1	Protein HESO1 OS=Ar	23.30	0.00
TRINITY_sp|P146(	cyr1	Adenylate cyclase O	23.30	0.00
TRINITY_sp|Q54K	talB	Talin-B OS=Dictyost	23.20	0.00
TRINITY_sp|Q4WL	utp10	U3 small nucleolar	23.20	0.00
TRINITY_sp|Q3KQ(	acpt	Testicular acid pho	23.20	0.00
TRINITY_sp|Q906	PLA2G7	Platelet-activating	23.20	0.00
TRINITY_sp|P973	Rhod	Rho-related GTP-bin	23.20	0.00
TRINITY_sp|P366(	rad8	DNA repair protein	23.20	0.00
TRINITY_sp|P343	ncx-6	Putative sodium/cal	23.20	0.00
TRINITY_sp|Q9SD(	GLR3.7	Glutamate receptor	23.20	0.00
TRINITY_sp|P292	pds	Phytoene dehydrogen	23.20	0.00
TRINITY_sp|A2VE	CG4495	Calcium uptake prot	23.20	0.00
TRINITY_sp|P196(	LTA4H	Leukotriene A-4 hyd	23.20	0.00
TRINITY_sp|Q6PE	Cfap206	Cilia- and flagella	23.20	0.00
TRINITY_sp|Q4GZ	ABCG2	ATP-binding cassett	23.10	0.00
TRINITY_sp|Q425	GN	ARF guanine-nucleot	23.10	0.00
TRINITY_sp|Q556	ctpA	Carboxyl-terminal-p	23.10	0.00
TRINITY_sp|Q9P7(	SPAC114	Uncharacterized NOC	23.10	0.00
TRINITY_sp|P509	AVT4	Vacuolar amino acid	23.10	0.00
TRINITY_sp|P355(	CSNK1B	Casein kinase I iso	23.10	0.00
TRINITY_sp|F4K3	KIN7L	Kinesin-like protei	23.10	0.00
TRINITY_sp|Q8VX	XEG113	Arabinosyltransfera	23.10	0.00
TRINITY_sp|Q75C(	MVP1	Sorting nexin MVP1	23.10	0.00
TRINITY_sp|Q9Y7	SPCC145(	PX domain-containin	23.10	0.00
TRINITY_sp|Q4PH	BRO1	Vacuolar protein-so	23.10	0.00
TRINITY_sp|P323	PSE1	Importin subunit be	23.10	0.00
TRINITY_sp|Q100	C28F5.4	Putative zinc prote	23.10	0.00
TRINITY_sp|Q425	GN	ARF guanine-nucleot	23.00	0.00
TRINITY_sp|A7S4	serinc	Probable serine inc	23.00	0.00
TRINITY_sp|O429	las1	Pre-rRNA-processing	23.00	0.00
TRINITY_sp|Q86K	phyA	Prolyl 4-hydroxylas	23.00	0.00
TRINITY_sp|Q8N8	TTLL6	Tubulin polyglutamy	23.00	0.00
TRINITY_sp|A0A0	M3KE1	MAP3K epsilon prote	23.00	0.00
TRINITY_sp|O758	GABBR2	Gamma-aminobutyric	23.00	0.00
TRINITY_sp|Q9LF	VAMP713	Vesicle-associated	23.00	0.00
TRINITY_sp|Q6P7	zc3hc1	NIPA-like protein O	22.90	0.00
TRINITY_sp|Q49A	FAM135B	Protein FAM135B OS=	22.90	0.00
TRINITY_sp|Q23M	Ttll6a	Probable beta-tubul	22.90	0.00
TRINITY_sp|Q7TS	Ppp6r1	Serine/threonine-pr	22.90	0.00
TRINITY_sp|Q93V	ATG18A	Autophagy-related p	22.90	0.00
TRINITY_sp|P400	AVT6	Vacuolar amino acid	22.90	0.00
TRINITY_sp|Q042	PDS5	Sister chromatid co	22.90	0.00
TRINITY_sp|Q5RD	KBTBD2	Kelch repeat and BT	22.90	0.00
TRINITY_sp|P343	ncx-6	Putative sodium/cal	22.90	0.00
TRINITY_sp|Q8R3	Dyx1c1	Dyslexia susceptibi	22.90	0.00
TRINITY_sp|Q6F4	AP25	Aspartyl protease 2	22.90	0.00
TRINITY_sp|Q54J	sibD	Integrin beta-like	22.80	0.00
TRINITY_sp|Q5F4	PPP6R3	Serine/threonine-pr	22.80	0.00
TRINITY_sp|Q9LE	DTX54	Protein DETOXIFICAT	22.80	0.00

TRINITY_sp|Q555(	vps13B	Putative vacuolar p	22.80	0.00
TRINITY_sp|P342	EDE1	EH domain-containin	22.80	0.00
TRINITY_sp|Q96M	CFAP44	Cilia- and flagella	22.80	0.00
TRINITY_sp|Q023	GLG1	Golgi apparatus pro	22.80	0.00
TRINITY_sp|Q9FL	ABCA10	ABC transporter A f	22.80	0.00
TRINITY_sp|Q9P2(	ANKIB1	Ankyrin repeat and	22.70	0.00
TRINITY_sp|P128	PEP5	E3 ubiquitin-protei	22.70	0.00
TRINITY_sp|Q222	ugt-50	Putative UDP-glucur	22.70	0.00
TRINITY_sp|Q58C	KLHDC3	Kelch domain-contai	22.70	0.00
TRINITY_sp|P029	tetA	Tetracycline resist	22.70	0.00
TRINITY_sp|Q9SY	IP5P13	Type I inositol pol	22.70	0.00
TRINITY_sp|Q6EM	TTLL5	Tubulin polyglutamy	22.70	0.00
TRINITY_sp|Q5EA	TMEM164	Transmembrane prote	22.70	0.00
TRINITY_sp|P189	IRA1	Inhibitory regulato	22.70	0.00
TRINITY_sp|Q6P6	ak8	Adenylate kinase 8	22.60	0.00
TRINITY_sp|Q8ND(	BBOF1	Basal body-orientat	22.60	0.00
TRINITY_sp|O046	GLR2.1	Glutamate receptor	22.60	0.00
TRINITY_sp|P870	pop1	WD repeat-containin	22.60	0.00
TRINITY_sp|Q8CG	Elac2	Zinc phosphodiester	22.60	0.00
TRINITY_sp|Q7T6	MIMI_R8	Putative serine/thr	22.60	0.00
TRINITY_sp|Q9LI	LDL2	Lysine-specific his	22.50	0.00
TRINITY_sp|Q5QN	Os01g02	Potassium channel K	22.50	0.00
TRINITY_sp|Q088	FAR2	Fatty acyl-CoA redu	22.50	0.00
TRINITY_sp|P741	sll1374	Uncharacterized sym	22.50	0.00
TRINITY_sp|Q5RD	CTC1	CST complex subunit	22.50	0.00
TRINITY_sp|O598(	SPCC550.	Probable importin c	22.50	0.00
TRINITY_sp|Q146	PDCD11	Protein RRP5 homolo	22.50	0.00
TRINITY_sp|B1H1	acp2	Lysosomal acid phos	22.50	0.00
TRINITY_sp|Q8CQ	drp35	Lactonase drp35 OS=	22.40	0.00
TRINITY_sp|Q67V	Os06g04	G patch domain-cont	22.40	0.00
TRINITY_sp|Q54P	DDB_G02	Probable acid phosp	22.40	0.00
TRINITY_sp|P871	SPAC57A	Uncharacterized pro	22.30	0.00
TRINITY_sp|Q6NX	Rcbtb1	RCC1 and BTB domain	22.30	0.00
TRINITY_sp|Q54L	vps13A	Putative vacuolar p	22.30	0.00
TRINITY_sp|Q1ZX(	grlK	Metabotropic glutam	22.30	0.00
TRINITY_sp|Q9P8	SNF4	Nuclear protein SNF	22.30	0.00
TRINITY_sp|Q1KK	lnpa	Protein lunapark-A	22.30	0.00
TRINITY_sp|Q0EE	Ptchd3	Patched domain-cont	22.30	0.00
TRINITY_sp|Q556	slr0021	Putative protease s	22.30	0.00
TRINITY_sp|P593	barA	Signal transduction	22.20	0.00
TRINITY_sp|P182	AAC3	ADP,ATP carrier pro	22.20	0.00
TRINITY_sp|P266	Sos	Protein son of seve	22.20	0.00
TRINITY_sp|P064(	RB1	Retinoblastoma-asso	22.20	0.00
TRINITY_sp|Q54H(	swp1	Dolichyl-diphosphoo	22.20	0.00
TRINITY_sp|Q7Z1	CYP51	Sterol 14-alpha dem	22.20	0.00
TRINITY_sp|Q3LX	RBR3	Retinoblastoma-rela	22.20	0.00
TRINITY_sp|O889(	Slc22a8	Solute carrier fami	22.20	0.00
TRINITY_sp|A8JF	ODA1	Outer dynein arm pr	22.20	0.00
TRINITY_sp|Q54F	gtf2b	Transcription initi	22.10	0.00
TRINITY_sp|O008	-	Uncharacterized 80	22.10	0.00
TRINITY_sp|Q4R5	ACP2	Lysosomal acid phos	22.10	0.00
TRINITY_sp|A6QL(	TRAPPC1	Trafficking protein	22.10	0.00
TRINITY_sp|Q8LB	KING1	SNF1-related protei	22.10	0.00
TRINITY_sp|O315	yesF	Uncharacterized oxi	22.10	0.00
TRINITY_sp|B2S1	mnmA	tRNA-specific 2-thi	22.10	0.00
TRINITY_sp|Q54U	sec24	Protein transport p	22.00	0.00

TRINITY_sp|Q9HF[illegible]	tra1	Transcription-assoc	22.00	0.00
TRINITY_sp|B3M1[illegible]	eIF3-S5-	Eukaryotic translat	22.00	0.00
TRINITY_sp|Q9M8[illegible]	SKOR	Potassium channel S	22.00	0.00
TRINITY_sp|Q54J[illegible]	sibC	Integrin beta-like	22.00	0.00
TRINITY_sp|Q10S[illegible]	NLP1	Protein NLP1 OS=Ory	22.00	0.00
TRINITY_sp|Q8RW[illegible]	CNGC5	Probable cyclic nuc	21.90	0.00
TRINITY_sp|Q54T[illegible]	drkD	Probable serine/thr	21.90	0.00
TRINITY_sp|Q9M9[illegible]	APCB1	Aspartyl protease A	21.90	0.00
TRINITY_sp|O076[illegible]	yhfW	Putative Rieske 2Fe	21.90	0.00
TRINITY_sp|Q54E[illegible]	grlE	Metabotropic glutam	21.90	0.00
TRINITY_sp|Q6DF[illegible]	poc5	Centrosomal protein	21.80	0.00
TRINITY_sp|Q078[illegible]	VPS13	Vacuolar protein so	21.80	0.00
TRINITY_sp|Q66K[illegible]	fam161a	Protein FAM161A OS=	21.80	0.00
TRINITY_sp|Q9BY[illegible]	SCAPER	S phase cyclin A-as	21.80	0.00
TRINITY_sp|P256[illegible]	CDC39	General negative re	21.80	0.00
TRINITY_sp|Q8WN[illegible]	KCNH2	Potassium voltage-g	21.80	0.00
TRINITY_sp|P366[illegible]	rad8	DNA repair protein	21.70	0.00
TRINITY_sp|A6ZU[illegible]	SDS23	Protein SDS23 OS=Sa	21.70	0.00
TRINITY_sp|Q5F3[illegible]	ABCC1	Multidrug resistanc	21.70	0.00
TRINITY_sp|Q9VF[illegible]	ldlCp	Conserved oligomeri	21.70	0.00
TRINITY_sp|Q5W2[illegible]	pigC	Prodigiosin synthes	21.70	0.00
TRINITY_sp|A9UY[illegible]	serinc	Probable serine inc	21.60	0.00
TRINITY_sp|Q6I5[illegible]	CPK16	Calcium-dependent p	21.60	0.00
TRINITY_sp|Q54J[illegible]	sibB	Integrin beta-like	21.60	0.00
TRINITY_sp|P540[illegible]	KTR6	Mannosyltransferase	21.50	0.00
TRINITY_sp|P556[illegible]	NGR_a01[illegible]	Uncharacterized zin	21.50	0.00
TRINITY_sp|F4JK[illegible]	SUD1	Probable E3 ubiquit	21.50	0.00
TRINITY_sp|Q5F3[illegible]	UTP15	U3 small nucleolar	21.40	0.00
TRINITY_sp|Q103[illegible]	cbs2	5'-AMP-activated pr	21.40	0.00
TRINITY_sp|Q9SZ[illegible]	PEP	RNA-binding KH doma	21.30	0.00
TRINITY_sp|Q4PS[illegible]	MAP65-9	65-kDa microtubule-	21.30	0.00
TRINITY_sp|O008[illegible]	-	Uncharacterized 80	21.30	0.00
TRINITY_sp|P549[illegible]	TLN1	Talin-1 OS=Gallus g	21.20	0.00
TRINITY_sp|Q9SD[illegible]	GLR3.7	Glutamate receptor	21.20	0.00
TRINITY_sp|Q640[illegible]	wdr82-a	WD repeat-containin	21.10	0.00
TRINITY_sp|Q56A[illegible]	ccdc40	Coiled-coil domain-	21.10	0.00
TRINITY_sp|Q54G[illegible]	crlF	Cyclic AMP receptor	21.10	0.00
TRINITY_sp|B7ZU[illegible]	setd3	Histone-lysine N-me	21.10	0.00
TRINITY_sp|Q5QJ[illegible]	TBCEL	Tubulin-specific ch	21.00	0.00
TRINITY_sp|Q74E[illegible]	ubiE	Ubiquinone/menaquin	21.00	0.00
TRINITY_sp|P111[illegible]	ACP2	Lysosomal acid phos	21.00	0.00
TRINITY_sp|P465[illegible]	ckb-3	Putative choline ki	20.90	0.00
TRINITY_sp|Q0J7[illegible]	TPR2	Protein TPR2 OS=Ory	20.80	0.00
TRINITY_sp|Q8AY[illegible]	CENPI	Centromere protein	20.80	0.00
TRINITY_sp|Q7SC[illegible]	sym-1	Protein sym-1 OS=Ne	20.70	0.00
TRINITY_sp|Q5FB[illegible]	tmcB	Tiny macrocysts pro	20.60	0.00
TRINITY_sp|Q9VS[illegible]	Rint1	RINT1-like protein	20.50	0.00
TRINITY_sp|Q5NV[illegible]	ACP2	Lysosomal acid phos	20.50	0.00
TRINITY_sp|O008[illegible]	-	Uncharacterized 80	20.40	0.00
TRINITY_sp|Q182[illegible]	sec-15	Exocyst complex com	20.40	0.00
TRINITY_sp|Q8ND[illegible]	CFAP43	Cilia- and flagella	20.20	0.00
TRINITY_sp|P345[illegible]	rfp-1	E3 ubiquitin-protei	20.20	0.00
TRINITY_sp|Q8IV[illegible]	LOXHD1	Lipoxygenase homolo	19.90	0.00
TRINITY_sp|Q50E[illegible]	CYP716B[illegible]	Cytochrome P450 716	19.60	0.00
TRINITY_sp|P400[illegible]	KAP123	Importin subunit be	19.50	0.00
TRINITY_sp|A8JF[illegible]	ODA1	Outer dynein arm pr	19.50	0.00

TRINITY_sp|Q922IPpp6r3	Serine/threonine-pr	19.30	0.00
