# Supplementary material for: Transcriptome Analysis of the Accumulation of Astaxanthin in Haematococcus pluvialis Treated with White and Blue Lights as well as Salicylic Acid
Source: Biomed Res Int. 2022 Jul 14;2022:4827595. doi: 10.1155/2022/4827595 (PMC9315456; doi:10.1155/2022/4827595)
Supplement: Supplementary 4 — Table S2: enrichment analysis of differentially expressed genes (DEGs) identified in Haematococcus pluvialis treated with blue light, white light, and blue light with salicylic acid based on the nonredundant (Nr) protein database. [file 4827595.f4.pdf]

Query	NR_hit	NR_descr	NR_species	NR_identity	NR_evalue
TRINITY_	XP_02294	root pho	Cucurbita_moschata	100.00	0.00
TRINITY_	XP_02292	50S ribo	Cucurbita_moschata	100.00	0.00
TRINITY_	XP_02295	photosys	Cucurbita_moschata	100.00	0.00
TRINITY_	XP_02293	30S ribo	Cucurbita_moschata	100.00	0.00
TRINITY_	XP_02352	photosys	Cucurbita_pepo	100.00	0.00
TRINITY_	XP_02352	uncharac	Cucurbita_pepo	100.00	0.00
TRINITY_	EEC80860	hypothet	Oryza_sativa	100.00	0.00
TRINITY_	XP_02296	chloropl	Cucurbita_moschata	100.00	0.00
TRINITY_	XP_02295	uncharac	Cucurbita_moschata	100.00	0.00
TRINITY_	XP_02296	chloropl	Cucurbita_moschata	100.00	0.00
TRINITY_	XP_02354	protein	Cucurbita_pepo	100.00	0.00
TRINITY_	XP_02295	photosyn	Cucurbita_moschata	100.00	0.00
TRINITY_	YP_00946	DNA-dire	Haematococcus_lacus	100.00	0.00
TRINITY_	NP_00134	uncharac	Zea_mays	100.00	0.00
TRINITY_	XP_02295	photosys	Cucurbita_moschata	100.00	0.00
TRINITY_	XP_02298	triosept	Cucurbita_maxima	100.00	0.00
TRINITY_	XP_02295	uncharac	Cucurbita_moschata	100.00	0.00
TRINITY_	XP_02353	ethylene	Cucurbita_pepo	100.00	0.00
TRINITY_	XP_02351	floral h	Cucurbita_pepo	100.00	0.00
TRINITY_	XP_02294	50S ribo	Cucurbita_moschata	100.00	0.00
TRINITY_	NP_00115	photosys	Zea_mays	100.00	0.00
TRINITY_	XP_01915	PREDICTE	Ipomoea_nil	100.00	0.00
TRINITY_	XP_01918	PREDICTE	Ipomoea_nil	100.00	0.00
TRINITY_	XP_02293	50S ribo	Cucurbita_moschata	100.00	0.00
TRINITY_	YP_00946	photosys	Haematococcus_lacus	100.00	0.00
TRINITY_	XP_01918	PREDICTE	Ipomoea_nil	100.00	0.00
TRINITY_	XP_02293	plastocy	Cucurbita_moschata	100.00	0.00
TRINITY_	XP_02296	photosys	Cucurbita_maxima	100.00	0.00
TRINITY_	XP_01916	PREDICTE	Ipomoea_nil	100.00	0.00
TRINITY_	XP_02292	proteaso	Cucurbita_moschata	100.00	0.00
TRINITY_	XP_02293	glyceral	Cucurbita_moschata	100.00	0.00
TRINITY_	XP_02353	glyceral	Cucurbita_pepo	100.00	0.00
TRINITY_	AFW90557	60S ribo	Solanum_tuberosum	100.00	0.00
TRINITY_	XP_02292	chloroph	Cucurbita_moschata	100.00	0.00
TRINITY_	XP_02213	chloroph	Momordica_charantia	100.00	0.00
TRINITY_	XP_02295	chloroph	Cucurbita_moschata	100.00	0.00
TRINITY_	AMS25549	catalase	Ipomoea_batatas	100.00	0.00
TRINITY_	XP_02299	photosys	Cucurbita_maxima	100.00	0.00
TRINITY_	QAY29594	enolase	Cucurbita_pepo	100.00	0.00
TRINITY_	XP_02292	costars	Cucurbita_moschata	100.00	0.00
TRINITY_	AAK69541	cathepsi	Ipomoea_batatas	100.00	0.00
TRINITY_	XP_02296	chloropl	Cucurbita_moschata	100.00	0.00
TRINITY_	XP_02294	metallot	Cucurbita_moschata	100.00	0.00
TRINITY_	XP_01917	PREDICTE	Ipomoea_nil	100.00	0.00
TRINITY_	XP_02293	uncharac	Cucurbita_moschata	100.00	0.00
TRINITY_	EMS46210	Elongati	Triticum_urartu	100.00	0.00
TRINITY_	XP_02292	triose p	Cucurbita_moschata	100.00	0.00
TRINITY_	XP_02294	phosphon	Cucurbita_moschata	100.00	0.00
TRINITY_	XP_02295	photosys	Cucurbita_moschata	100.00	0.00
TRINITY_	XP_00414	PREDICTE	Cucumis_sativus	100.00	0.00
TRINITY_	XP_02294	60S ribo	Cucurbita_moschata	100.00	0.00
TRINITY_	XP_02353	ATP-depe	Cucurbita_pepo	100.00	0.00
TRINITY_	XP_02353	ferritin	Cucurbita_pepo	100.00	0.00
TRINITY_	AMS24668	LOXA [Ip	Ipomoea_batatas	100.00	0.00
TRINITY_	XP_02354	60S ribo	Cucurbita_pepo	100.00	0.00
TRINITY_	NP_00129	ubiquiti	Jatropha_curcas	100.00	0.00
TRINITY_	ACF85961	unknown	Zea_mays	100.00	0.00
TRINITY_	NP_00113	photosys	Zea_mays	100.00	0.00
TRINITY_	XP_00414	PREDICTE	Cucumis_sativus	100.00	0.00
TRINITY_	YP_00946	RNA-depe	Haematococcus_lacus	100.00	0.00
TRINITY_	XP_01916	PREDICTE	Ipomoea_nil	100.00	0.00
TRINITY_	XP_02294	elongati	Cucurbita_moschata	100.00	0.00
TRINITY_	XP_02351	indole-3	Cucurbita_pepo	100.00	0.00
TRINITY_	XP_02294	transket	Cucurbita_moschata	100.00	0.00
TRINITY_	XP_00843	PREDICTE	Cucumis_melo	100.00	0.00
TRINITY_	XP_02298	uncharac	Cucurbita_maxima	100.00	0.00
TRINITY_	AAD22104	B12D pro	Ipomoea_batatas	100.00	0.00
TRINITY_	XP_02352	photosys	Cucurbita_pepo	100.00	0.00
TRINITY_	ALO21551	putative	Haematococcus_lacus	100.00	0.00
TRINITY_	XP_02354	uncharac	Cucurbita_pepo	100.00	0.00

TRINITY_XP_0279(	tubulin	Vigna_unguiculata	100.00	0.00
TRINITY_XP_02295	photosys	Cucurbita_moschata	100.00	0.00
TRINITY_XP_01918	PREDICTE	Ipomoea_nil	100.00	0.00
TRINITY_XP_02293	putative	Cucurbita_moschata	100.00	0.00
TRINITY_XP_00495	photosys	Setaria_italica	100.00	0.00
TRINITY_XP_02354	cysteine	Cucurbita_pepo	100.00	0.00
TRINITY_XP_00414	PREDICTE	Cucumis_sativus	100.00	0.00
TRINITY_XP_02292	enolase	Cucurbita_moschata	100.00	0.00
TRINITY_XP_02353	superoxi	Cucurbita_pepo	100.00	0.00
TRINITY_XP_02352	50S ribo	Cucurbita_pepo	100.00	0.00
TRINITY_XP_00413	PREDICTE	Cucumis_sativus	100.00	0.00
TRINITY_XP_02293	uncharac	Cucurbita_moschata	100.00	0.00
TRINITY_XP_02299	protein	Cucurbita_maxima	100.00	0.00
TRINITY_XP_02295	GTP-binc	Cucurbita_moschata	100.00	0.00
TRINITY_XP_00413	PREDICTE	Cucumis_sativus	100.00	0.00
TRINITY_XP_02355	photosys	Cucurbita_pepo	100.00	0.00
TRINITY_XP_00414	PREDICTE	Cucumis_sativus	100.00	0.00
TRINITY_XP_02352	ferredox	Cucurbita_pepo	100.00	0.00
TRINITY_XP_02354	malate c	Cucurbita_pepo	100.00	0.00
TRINITY_XP_02292	photosys	Cucurbita_moschata	100.00	0.00
TRINITY_XP_02297	ATP synt	Cucurbita_maxima	100.00	0.00
TRINITY_XP_02293	ATP synt	Cucurbita_moschata	100.00	0.00
TRINITY_XP_02352	ATP synt	Cucurbita_pepo	100.00	0.00
TRINITY_XP_02295	50S ribo	Cucurbita_moschata	100.00	0.00
TRINITY_XP_02292	chloroph	Cucurbita_moschata	100.00	0.00
TRINITY_XP_00244	S-adenos	Sorghum_bicolor	100.00	0.00
TRINITY_XP_02293	28 kDa r	Cucurbita_moschata	100.00	0.00
TRINITY_XP_02353	28 kDa r	Cucurbita_pepo	100.00	0.00
TRINITY_ADB03784	catalase	Ipomoea_batatas	100.00	0.00
TRINITY_XP_0230(	60S ribo	Cucurbita_maxima	100.00	0.00
TRINITY_NP_00114	chloroph	Zea_mays	100.00	0.00
TRINITY_XP_02352	thiamine	Cucurbita_pepo	100.00	0.00
TRINITY_XP_02352	thiamine	Cucurbita_pepo	100.00	0.00
TRINITY_XP_0114(	PREDICTE	Fragaria_vesca	100.00	0.00
TRINITY_XP_02293	eukaryot	Cucurbita_moschata	100.00	0.00
TRINITY_XP_02293	ATP-depe	Cucurbita_moschata	100.00	0.00
TRINITY_XP_02293	glyceral	Cucurbita_moschata	100.00	0.00
TRINITY_XP_02352	peptidyl	Cucurbita_pepo	100.00	0.00
TRINITY_XP_02294	60S ribo	Cucurbita_moschata	100.00	0.00
TRINITY_XP_02294	fructose	Cucurbita_moschata	100.00	0.00
TRINITY_XP_02293	calvin c	Cucurbita_moschata	100.00	0.00
TRINITY_NP_00123	ubiquiti	Solanum_lycopersicu	100.00	0.00
TRINITY_XP_02354	chloroph	Cucurbita_pepo	100.00	0.00
TRINITY_XP_02294	chloroph	Cucurbita_moschata	100.00	0.00
TRINITY_XP_02354	60S ribo	Cucurbita_pepo	100.00	0.00
TRINITY_XP_00844	PREDICTE	Cucumis_melo	100.00	0.00
TRINITY_XP_02355	chloroph	Cucurbita_pepo	100.00	0.00
TRINITY_XP_01918	PREDICTE	Ipomoea_nil	100.00	0.00
TRINITY_AAF69014	cysteine	Ipomoea_batatas	100.00	0.00
TRINITY_XP_02353	uncharac	Cucurbita_pepo	100.00	0.00
TRINITY_XP_02351	serine h	Cucurbita_pepo	100.00	0.00
TRINITY_XP_02298	glutamat	Cucurbita_maxima	100.00	0.00
TRINITY_XP_0230(	chloroph	Cucurbita_maxima	100.00	0.00
TRINITY_XP_02352	L-ascorb	Cucurbita_pepo	100.00	0.00
TRINITY_XP_02352	protein	Cucurbita_pepo	100.00	0.00
TRINITY_XP_02292	protein	Cucurbita_moschata	100.00	0.00
TRINITY_XP_02295	probable	Cucurbita_moschata	100.00	0.00
TRINITY_XP_02295	probable	Cucurbita_moschata	100.00	0.00
TRINITY_XP_0101(	malate c	Morus_notabilis	100.00	0.00
TRINITY_XP_0084(	PREDICTE	Cucumis_melo	100.00	0.00
TRINITY_XP_0191(	PREDICTE	Ipomoea_nil	100.00	0.00
TRINITY_XP_0191(	PREDICTE	Ipomoea_nil	100.00	0.00
TRINITY_XP_02299	photosys	Cucurbita_maxima	100.00	0.00
TRINITY_XP_02354	oxygen-e	Cucurbita_pepo	100.00	0.00
TRINITY_XP_0230(	oxygen-e	Cucurbita_maxima	100.00	0.00
TRINITY_XP_02293	50S ribo	Cucurbita_moschata	100.00	0.00
TRINITY_XP_02294	serine--	Cucurbita_moschata	100.00	0.00
TRINITY_XP_01915	PREDICTE	Ipomoea_nil	100.00	0.00
TRINITY_XP_02292	ribulose	Cucurbita_moschata	100.00	0.00
TRINITY_XP_0191(	PREDICTE	Ipomoea_nil	100.00	0.00
TRINITY_XP_02294	ribulose	Cucurbita_moschata	100.00	0.00

TRINITY_NP_00131	ADP-ribo	Arabidopsis_thalian	100.00	0.00
TRINITY_XP_02354	vacuola	Cucurbita_pepo	100.00	0.00
TRINITY_ACF85886	unknown	Zea_mays	100.00	0.00
TRINITY_XP_02294	oxygen-e	Cucurbita_moschata	100.00	0.00
TRINITY_XP_02352	oxygen-e	Cucurbita_pepo	100.00	0.00
TRINITY_XP_02294	ferredox	Cucurbita_moschata	100.00	0.00
TRINITY_XP_01917	PREDICTE	Ipomoea_nil	100.00	0.00
TRINITY_XP_02293	phosphor	Cucurbita_moschata	100.00	0.00
TRINITY_XP_02293	cystathi	Cucurbita_moschata	100.00	0.00
TRINITY_ACJ05910	putative	Ipomoea_batatas	100.00	0.00
TRINITY_ABP35528	cyclophi	Ipomoea_batatas	100.00	0.00
TRINITY_AAA33390	sporamin	Ipomoea_batatas	100.00	0.00
TRINITY_XP_00844	PREDICTE	Cucumis_melo	100.00	0.00
TRINITY_XP_02292	glyceral	Cucurbita_moschata	100.00	0.00
TRINITY_XP_02292	glyceral	Cucurbita_moschata	100.00	0.00
TRINITY_XP_02087	phosphog	Arabidopsis_lyrata	100.00	0.00
TRINITY_XP_01108	phosphog	Sesamum_indicum	100.00	0.00
TRINITY_XP_02294	phosphog	Cucurbita_moschata	100.00	0.00
TRINITY_XP_02355	photosys	Cucurbita_pepo	100.00	0.00
TRINITY_XP_02354	oxygen-e	Cucurbita_pepo	100.00	0.00
TRINITY_XP_02353	ribulose	Cucurbita_pepo	100.00	0.00
TRINITY_XP_02292	acyl-CoA	Cucurbita_moschata	100.00	0.00
TRINITY_XP_02295	elongati	Cucurbita_moschata	100.00	0.00
TRINITY_XP_01919	PREDICTE	Ipomoea_nil	100.00	0.00
TRINITY_YP_00946	30S ribo	Haematococcus_lacus	100.00	0.00
TRINITY_AIE89875	GFP (chl	Tetraselmis_subcord	100.00	0.00
TRINITY_AIE89875	GFP (chl	Tetraselmis_subcord	100.00	0.00
TRINITY_GAX81193	hypothet	Chlamydomonas_eusti	100.00	0.00
TRINITY_XP_02285	polyubic	Olea_europaea	100.00	0.00
TRINITY_XP_02285	polyubic	Olea_europaea	100.00	0.00
TRINITY_ALO21548	D1 react	Haematococcus_lacus	100.00	0.00
TRINITY_XP_00414	PREDICTE	Cucumis_sativus	100.00	0.00
TRINITY_XP_01564	chloroph	Oryza_sativa	100.00	0.00
TRINITY_AAR10886	chloroph	Trifolium_pratense	100.00	0.00
TRINITY_NP_00124	uncharac	Glycine_max	100.00	0.00
TRINITY_QBB20366	chloropl	Withania_somnifera	100.00	0.00
TRINITY_XP_02292	chloroph	Cucurbita_moschata	100.00	0.00
TRINITY_CAA48641	type II	Zea_mays	100.00	0.00
TRINITY_XP_02352	stress e	Cucurbita_pepo	100.00	0.00
TRINITY_NP_00127	ubiquiti	Solanum_tuberosum	100.00	0.00
TRINITY_XP_02374	polyubic	Lactuca_sativa	100.00	0.00
TRINITY_XP_02285	polyubic	Olea_europaea	100.00	0.00
TRINITY_BAD90809	histone	Conocephalum_conicu	100.00	0.00
TRINITY_P07145.1	RecName:	Ipomoea_batatas	100.00	0.00
TRINITY_AAQ10076	phosphoe	Megathyrsus_maximus	100.00	0.00
TRINITY_YP_00946	photosys	Haematococcus_lacus	100.00	0.00
TRINITY_AFU34347	70-kDa h	Ipomoea_batatas	100.00	0.00
TRINITY_RDY12638	Polyubic	Mucuna_pruriens	100.00	0.00
TRINITY_ACF88184	unknown	Zea_mays	100.00	0.00
TRINITY_ACF88184	unknown	Zea_mays	100.00	0.00
TRINITY_OVA00002	hypothet	Macleaya_cordata	100.00	0.00
TRINITY_YP_00946	homing e	Haematococcus_lacus	100.00	0.00
TRINITY_YP_00946	hypothet	Haematococcus_lacus	100.00	0.00
TRINITY_XP_02293	photosys	Cucurbita_moschata	100.00	0.00
TRINITY_XP_02351	chromopl	Cucurbita_pepo	100.00	0.00
TRINITY_XP_02295	cysteine	Cucurbita_moschata	100.00	0.00
TRINITY_XP_01389	hypothet	Monoraphidium_negle	100.00	0.00
TRINITY_YP_00946	cytochro	Haematococcus_lacus	100.00	0.00
TRINITY_ADN95182	oil glob	Haematococcus_lacus	100.00	0.00
TRINITY_XP_02294	50S ribo	Cucurbita_moschata	100.00	0.00
TRINITY_XP_02293	2-methyl	Cucurbita_moschata	100.00	0.00
TRINITY_AFU50381	chloropl	Ipomoea_batatas	100.00	0.00
TRINITY_XP_02354	galactin	Cucurbita_pepo	100.00	0.00
TRINITY_XP_02295	5'-adeny	Cucurbita_moschata	100.00	0.00
TRINITY_ADJ67213	hypothet	Jatropha_curcas	100.00	0.00
TRINITY_YP_00946	4-dihydr	Haematococcus_lacus	100.00	0.00
TRINITY_XP_02294	UDP-gluc	Cucurbita_moschata	100.00	0.00
TRINITY_AAZ15702	beta-car	Haematococcus_lacus	100.00	0.00
TRINITY_XP_02295	glutamin	Cucurbita_moschata	100.00	0.00
TRINITY_XP_02292	puromyci	Cucurbita_moschata	100.00	0.00
TRINITY_XP_02352	S-adenos	Cucurbita_pepo	100.00	0.00

TRINITY_AAN0349:	uORF [Ip	Ipomoea_batatas	100.00	0.00
TRINITY_Q9M6K1.1	RecName:	Ipomoea_batatas	100.00	0.00
TRINITY_XP_0229	photosys	Cucurbita_moschata	100.00	0.00
TRINITY_XP_0235	photosys	Cucurbita_pepo	100.00	0.00
TRINITY_ALO2155	D2 react	Haematococcus_lacus	100.00	0.00
TRINITY_EPS7450	hypothet	Genlisea_aurea	100.00	0.00
TRINITY_ABV7239	plastid	Haematococcus_lacus	100.00	0.00
TRINITY_XP_0235	60S ribo	Cucurbita_pepo	100.00	0.00
TRINITY_AAK1562	phytoene	Haematococcus_lacus	100.00	0.00
TRINITY_AAO7313	photosys	Bassia_scoparia	100.00	0.00
TRINITY_AAD3473	photosys	Poa_annua	100.00	0.00
TRINITY_XP_0235	uncharac	Cucurbita_pepo	100.00	0.00
TRINITY_AER5821	channelo	Haematococcus_lacus	100.00	0.00
TRINITY_AER5821	channelo	Haematococcus_lacus	100.00	0.00
TRINITY_BAL6108	polyubic	Dianthus_caryophyll	100.00	0.00
TRINITY_XP_0230	heat sho	Cucurbita_maxima	100.00	0.00
TRINITY_YP_0094	hypothet	Haematococcus_lacus	100.00	0.00
TRINITY_XP_0235	glycine	Cucurbita_pepo	100.00	0.00
TRINITY_XP_0016	beta tub	Chlamydomonas_reinh	100.00	0.00
TRINITY_ALO2152	CF1 alph	Haematococcus_lacus	100.00	0.00
TRINITY_ADP3695	hypothet	Asterochloris_sp._D	100.00	0.00
TRINITY_AJR2723	4-diphos	Haematococcus_lacus	100.00	0.00
TRINITY_ATU9014	WDR doma	Haematococcus_lacus	100.00	0.00
TRINITY_YP_0094	LAGLIDAD	Haematococcus_lacus	100.00	0.00
TRINITY_XP_0235	probable	Cucurbita_pepo	100.00	0.00
TRINITY_XP_0229	ferredox	Cucurbita_moschata	100.00	0.00
TRINITY_XP_0191	PREDICTE	Ipomoea_nil	100.00	0.00
TRINITY_AKT9517	chloropl	Haematococcus_lacus	100.00	0.00
TRINITY_ALO2154	P700 apo	Haematococcus_lacus	100.00	0
TRINITY_ADN9518	oil glob	Haematococcus_lacus	100.00	0.00
TRINITY_XP_0191	PREDICTE	Ipomoea_nil	100.00	0.00
TRINITY_XP_0041	PREDICTE	Cucumis_sativus	100.00	0.00
TRINITY_AJQ1948	1-deoxy-	Haematococcus_lacus	100.00	0.00
TRINITY_XP_0022	PREDICTE	Vitis_vinifera	100.00	0.00
TRINITY_AAW6929	mitochon	Haematococcus_lacus	100.00	0.00
TRINITY_CAD6055	phosphoe	Zea_mays	100.00	0.00
TRINITY_AAO6497	lycopene	Haematococcus_lacus	100.00	0.00
TRINITY_BAR9110	ribulose	Ipomoea_batatas	100.00	0.00
TRINITY_ABB1755	ribulose	Haematococcus_lacus	100.00	0.00
TRINITY_ABB1755	ribulose	Haematococcus_lacus	100.00	0.00
TRINITY_ABV7239	plastid	Haematococcus_lacus	100.00	0.00
TRINITY_XP_0041	PREDICTE	Cucumis_sativus	100.00	0.00
TRINITY_AAO6497	lycopene	Haematococcus_lacus	100.00	0.00
TRINITY_XP_0229	glycine-	Cucurbita_moschata	100.00	0.00
TRINITY_ADC4537	actin [C	Cleistogenes_songor	100.00	0.00
TRINITY_APO1583	actin 11	Sesuvium_portulacas	100.00	0.00
TRINITY_ADC4537	actin [C	Cleistogenes_songor	100.00	0.00
TRINITY_AAK3036	cytochro	Polytomella_sp._Pri	100.00	0.00
TRINITY_AAA3349	pyruvate	Zea_mays	100.00	0.00
TRINITY_XP_0029	S-adenos	Selaginella_moellen	100.00	0.00
TRINITY_XP_0016	predicte	Chlamydomonas_reinh	100.00	0.00
TRINITY_XP_0229	aminomet	Cucurbita_moschata	100.00	0.00
TRINITY_XP_0016	transmem	Chlamydomonas_reinh	100.00	0.00
TRINITY_ACF8588	unknown	Zea_mays	100.00	0.00
TRINITY_AAV3709	phytoene	Haematococcus_lacus	100.00	0.00
TRINITY_GAX7258	hypothet	Chlamydomonas_eusti	100.00	0.00
TRINITY_ABD3791	light-ha	Chlamydomonas_incer	100.00	0.00
TRINITY_XP_0084	PREDICTE	Cucumis_melo	100.00	0.00
TRINITY_XP_0235	S-adenos	Cucurbita_pepo	100.00	0.00
TRINITY_XP_0235	ferredox	Cucurbita_pepo	100.00	0.00
TRINITY_XP_0235	polyol t	Cucurbita_pepo	100.00	0.00
TRINITY_XP_0235	uncharac	Cucurbita_pepo	100.00	0.00
TRINITY_XP_0229	PGR5-lik	Cucurbita_moschata	100.00	0.00
TRINITY_XP_0235	MLO-like	Cucurbita_pepo	100.00	0.00
TRINITY_EOY0104	Phosphog	Theobroma_cacao	100.00	0.00
TRINITY_XP_0229	profilin	Cucurbita_moschata	100.00	0.00
TRINITY_YP_0094	30S ribo	Haematococcus_lacus	100.00	0.00
TRINITY_XP_0229	calmodul	Cucurbita_moschata	100.00	0.00
TRINITY_XP_0229	30S ribo	Cucurbita_moschata	100.00	0.00
TRINITY_NP_0011	PL3K2 [Z	Zea_mays	100.00	0.00
TRINITY_XP_0041	PREDICTE	Cucumis_sativus	100.00	0.00

TRINITY_XP_0235:	cysteine	Cucurbita_pepo	100.00	0.00
TRINITY_YP_0094(	Light-ir	Haematococcus_lacus	100.00	0.00
TRINITY_XP_02294	30S ribo	Cucurbita_moschata	100.00	0.00
TRINITY_QBB73041	ruBisCO	Cucurbita_pepo	100.00	0.00
TRINITY_XP_02294	peroxire	Cucurbita_moschata	100.00	0.00
TRINITY_EEC8086(	hypothet	Oryza_sativa	100.00	0.00
TRINITY_XP_02352	MLP-like	Cucurbita_pepo	100.00	0.00
TRINITY_RWR83888	60S ribo	Cinnamomum_micranth	100.00	0.00
TRINITY_XP_0235:	protein	Cucurbita_pepo	100.00	0.00
TRINITY_XP_0229:	cysteine	Cucurbita_moschata	100.00	0.00
TRINITY_XP_02292	magnesiu	Cucurbita_moschata	100.00	0.00
TRINITY_XP_02298	photosyr	Cucurbita_maxima	100.00	0.00
TRINITY_XP_02351	D-glycer	Cucurbita_pepo	100.00	0.00
TRINITY_XP_0229:	leucine	Cucurbita_moschata	100.00	0.00
TRINITY_XP_01918	PREDICTE	Ipomoea_nil	100.00	0.00
TRINITY_XP_02355	beta-amy	Cucurbita_pepo	100.00	0.00
TRINITY_XP_0235:	tetraspa	Cucurbita_pepo	100.00	0.00
TRINITY_XP_02292	peroxisc	Cucurbita_moschata	100.00	0.00
TRINITY_XP_0229:	catalase	Cucurbita_moschata	100.00	0.00
TRINITY_AKN08992	catalase	Luffa_aegyptiaca	100.00	0.00
TRINITY_ACF78489	unknown	Zea_mays	100.00	0.00
TRINITY_XP_00227	PREDICTE	Vitis_vinifera	100.00	0.00
TRINITY_NP_00114	chloroph	Zea_mays	100.00	0.00
TRINITY_NP_0011(	NADP-dep	Zea_mays	100.00	0.00
TRINITY_CAD60555	phosphoe	Zea_mays	100.00	0.00
TRINITY_NP_0013:	phosphoe	Zea_mays	100.00	0.00
TRINITY_XP_02292	ferredox	Cucurbita_moschata	100.00	0.00
TRINITY_XP_02294	probable	Cucurbita_moschata	100.00	0.00
TRINITY_ACG26704	photosys	Zea_mays	100.00	0.00
TRINITY_YP_0094(	cytochrc	Haematococcus_lacus	100.00	0.00
TRINITY_XP_02351	photosys	Cucurbita_pepo	100.00	0.00
TRINITY_XP_02351	photosys	Cucurbita_pepo	100.00	0.00
TRINITY_XP_0229:	30S ribo	Cucurbita_moschata	100.00	0.00
TRINITY_XP_0229:	fructose	Cucurbita_moschata	100.00	0.00
TRINITY_ACR3630(	unknown	Zea_mays	100.00	0.00
TRINITY_XP_0229:	ferredox	Cucurbita_moschata	100.00	0.00
TRINITY_XP_02295	V-type p	Cucurbita_moschata	100.00	0.00
TRINITY_XP_02295	malate c	Cucurbita_moschata	100.00	0.00
TRINITY_XP_02298	36.4 kDa	Cucurbita_maxima	100.00	0.00
TRINITY_XP_02287	oxygen-e	Olea_europaea	100.00	0.00
TRINITY_XP_02355	uncharac	Cucurbita_pepo	100.00	0.00
TRINITY_AML7765:	putative	Haematococcus_lacus	99.90	0
TRINITY_ALO2156(	CP43 chl	Haematococcus_lacus	99.80	0.00
TRINITY_AEY8002(	heat shc	Haematococcus_lacus	99.80	0.00
TRINITY_AJR27237	1-hydrox	Haematococcus_lacus	99.80	0.00
TRINITY_AKO7190(	phytoene	Haematococcus_lacus	99.80	0.00
TRINITY_AKR53702	chloropl	Haematococcus_lacus	99.80	0
TRINITY_XP_0230(	fructose	Cucurbita_maxima	99.70	0.00
TRINITY_ALO21554	hypothet	Haematococcus_lacus	99.70	0
TRINITY_ACR78535	cytosoli	Zea_mays	99.70	0.00
TRINITY_XP_02292	chloroph	Cucurbita_moschata	99.60	0.00
TRINITY_XP_02352	ribulose	Cucurbita_pepo	99.60	0.00
TRINITY_AKU47747	inositol	Cucurbita_moschata	99.60	0.00
TRINITY_AUT77175	glutamat	Haematococcus_lacus	99.60	0
TRINITY_AEF13159	beta-ket	Haematococcus_lacus	99.60	0.00
TRINITY_AAX86042	14-3-3 p	Haematococcus_lacus	99.50	0.00
TRINITY_AAB61311	htrA-lik	Haematococcus_lacus	99.40	0.00
TRINITY_AMK9782(	thioredc	Haematococcus_lacus	99.40	0.00
TRINITY_XP_02354	ribulose	Cucurbita_pepo	99.40	0.00
TRINITY_ABV72392	plastid	Haematococcus_lacus	99.40	0.00
TRINITY_AUT77175	glutamat	Haematococcus_lacus	99.40	0.00
TRINITY_XP_00295	hypothet	Volvox_carteri	99.30	0.00
TRINITY_XP_02298	glycerat	Cucurbita_maxima	99.20	0.00
TRINITY_XP_00168	splicing	Chlamydomonas_reinh	99.20	0.00
TRINITY_APX64487	geranylg	Haematococcus_lacus	99.20	0.00
TRINITY_YP_0094(	P-loop c	Haematococcus_lacus	99.20	0.00
TRINITY_AER58219	channelc	Haematococcus_lacus	99.20	0.00
TRINITY_BAK03519	predicte	Hordeum_vulgare	99.20	0.00
TRINITY_AUT7717:	glutamir	Haematococcus_lacus	99.10	0.00
TRINITY_OIV89924	hypothet	Lupinus_angustifoli	99.10	0.00
TRINITY_Q9SPK6.1	RecName:	Haematococcus_lacus	99.10	0.00

TRINITY_AYU5883{	alpha-tu	Haematococcus_lacus	99.10	0.00
TRINITY_XP_0191{	PREDICTE	Ipomoea_nil	99.00	0.00
TRINITY_AMS2466{	LOXA [Ip	Ipomoea_batatas	99.00	0.00
TRINITY_AAF6901{	cysteine	Ipomoea_batatas	99.00	0.00
TRINITY_AKT9517{	zeaxanth	Haematococcus_lacus	99.00	0.00
TRINITY_XP_0016{	actin [C	Chlamydomonas_reinh	99.00	0.00
TRINITY_XP_0229{	50S ribo	Cucurbita_maxima	98.90	0.00
TRINITY_XP_0191{	PREDICTE	Ipomoea_nil	98.90	0.00
TRINITY_XP_0191{	PREDICTE	Ipomoea_nil	98.90	0.00
TRINITY_XP_0235{	oxygen-e	Cucurbita_pepo	98.90	0.00
TRINITY_XP_0016{	beta tub	Chlamydomonas_reinh	98.90	0.00
TRINITY_AUI4110{	TAP38 [H	Haematococcus_lacus	98.90	0.00
TRINITY_AFR3690{	chloropl	Haematococcus_lacus	98.90	0.00
TRINITY_AAO6497{	lycopene	Haematococcus_lacus	98.90	0.00
TRINITY_XP_0093{	PREDICTE	Pyrus_x_bretschneid	98.80	0.00
TRINITY_ALO2158{	beta'' s	Haematococcus_lacus	98.80	0.00
TRINITY_XP_0191{	PREDICTE	Ipomoea_nil	98.80	0.00
TRINITY_ACG3675{	oxygen-e	Zea_mays	98.80	0.00
TRINITY_XP_0235{	non-spec	Cucurbita_pepo	98.80	0.00
TRINITY_XP_0191{	PREDICTE	Ipomoea_nil	98.80	0.00
TRINITY_AEI2916{	EF1a-lik	Vanilla_planifolia	98.80	0.00
TRINITY_AAK7200{	calmodul	Elaeis_oleifera	98.80	0.00
TRINITY_XP_0110{	PREDICTE	Populus_euphratica	98.80	0.00
TRINITY_O04386.1	RecName:	Chlamydomonas_incer	98.80	0.00
TRINITY_XP_0229{	copper t	Cucurbita_moschata	98.70	0.00
TRINITY_AFD6280{	3-deoxy-	Ipomoea_purpurea	98.70	0.00
TRINITY_XP_0229{	photosys	Cucurbita_moschata	98.70	0.00
TRINITY_BAD9558{	metallot	Ipomoea_batatas	98.70	0.00
TRINITY_Q9M6K1.1	RecName:	Ipomoea_batatas	98.70	0.00
TRINITY_XP_0016{	beta tub	Chlamydomonas_reinh	98.70	0.00
TRINITY_BAJ9821{	predicte	Hordeum_vulgare	98.70	0.00
TRINITY_XP_0229{	protein	Cucurbita_maxima	98.60	0.00
TRINITY_KCW5138{	hypothet	Eucalyptus_grandis	98.60	0.00
TRINITY_XP_0238{	cell div	Quercus_suber	98.60	0.00
TRINITY_Q9M6K1.1	RecName:	Ipomoea_batatas	98.60	0.00
TRINITY_AJQ1948{	4-diphos	Haematococcus_lacus	98.60	0.00
TRINITY_AJR2723{	4-diphos	Haematococcus_lacus	98.60	0.00
TRINITY_XP_0191{	PREDICTE	Ipomoea_nil	98.60	0.00
TRINITY_XP_0191{	PREDICTE	Ipomoea_nil	98.50	0.00
TRINITY_AWW1650{	stem-spe	Ipomoea_pes-caprae	98.50	0.00
TRINITY_BAO5727{	elongati	Ipomoea_nil	98.50	0.00
TRINITY_XP_0191{	PREDICTE	Ipomoea_nil	98.50	0.00
TRINITY_BAJ9369{	predicte	Hordeum_vulgare	98.50	0.00
TRINITY_ABV7239{	plastid	Haematococcus_lacus	98.50	0.00
TRINITY_YP_0094{	Light-in	Haematococcus_lacus	98.50	0.00
TRINITY_XP_0191{	PREDICTE	Ipomoea_nil	98.50	0.00
TRINITY_AAK8330{	calmodul	Capsicum_annuum	98.40	0.00
TRINITY_ACF8818{	unknown	Zea_mays	98.40	0.00
TRINITY_XP_0191{	PREDICTE	Ipomoea_nil	98.30	0.00
TRINITY_RWR9103{	polyubic	Cinnamomum_micranth	98.30	0.00
TRINITY_ABF8579{	plastid	Haematococcus_lacus	98.30	0.00
TRINITY_ANQ4519{	putative	Haematococcus_lacus	98.30	0.00
TRINITY_ABL6795{	1-aminoc	Ipomoea_nil	98.20	0.00
TRINITY_AFQ3161{	CYP97C [	Haematococcus_lacus	98.20	0.00
TRINITY_P20865.1	RecName:	Dunaliella_salina	98.20	0.00
TRINITY_XP_0016{	beta tub	Chlamydomonas_reinh	98.20	0.00
TRINITY_AFR3178{	cytochro	Haematococcus_lacus	98.20	0.00
TRINITY_XP_0271{	40S ribo	Coffea_arabica	98.20	0.00
TRINITY_OIV8972{	hypothet	Lupinus_angustifoli	98.10	0.00
TRINITY_ACG3053{	calmodul	Zea_mays	98.10	0.00
TRINITY_AFD6280{	GAPDH2 [	Ipomoea_purpurea	98.00	0.00
TRINITY_BAJ8900{	predicte	Hordeum_vulgare	98.00	0.00
TRINITY_AJY5897{	histone	Closterium_ehrenber	98.00	0.00
TRINITY_BAK0351{	predicte	Hordeum_vulgare	98.00	0.00
TRINITY_BAJ9006{	predicte	Hordeum_vulgare	98.00	0.00
TRINITY_AUI4110{	thylakoi	Haematococcus_lacus	98.00	0
TRINITY_XP_0191{	PREDICTE	Ipomoea_nil	97.90	0.00
TRINITY_XP_0191{	PREDICTE	Ipomoea_nil	97.90	0.00
TRINITY_XP_0191{	PREDICTE	Ipomoea_nil	97.90	0.00
TRINITY_XP_0235{	S-norcoc	Cucurbita_pepo	97.80	0.00
TRINITY_XP_0191{	PREDICTE	Ipomoea_nil	97.80	0.00

TRINITY_AUI4111(	violaxar	Haematococcus_lacus	97.80	0.00
TRINITY_ACN3155	unknown	Zea_mays	97.80	0.00
TRINITY_YP_3586	hypothet	Phalaenopsis_aphrod	97.80	0.00
TRINITY_AEF1316(	fatty ac	Haematococcus_lacus	97.80	0.00
TRINITY_XP_0235	aminomet	Cucurbita_pepo	97.80	0.00
TRINITY_XP_0191	PREDICTE	Ipomoea_nil	97.70	0.00
TRINITY_AUI4110	photosys	Haematococcus_lacus	97.70	0.00
TRINITY_AAK7150	soluble	Ipomoea_batatas	97.70	0.00
TRINITY_CAA5229(	polyubic	Volvox_carteri	97.70	0.00
TRINITY_AEP3960	heat shc	Haematococcus_lacus	97.70	0.00
TRINITY_XP_0191	PREDICTE	Ipomoea_nil	97.60	0.00
TRINITY_XP_0191	PREDICTE	Ipomoea_nil	97.60	0.00
TRINITY_AUI4110	LhcsR [	Haematococcus_lacus	97.60	0.00
TRINITY_CAI3483	ATP synt	Polytomella_sp._Pri	97.60	0.00
TRINITY_XP_0138	UDP-sul	Monoraphidium_negle	97.60	0.00
TRINITY_XP_0191	PREDICTE	Ipomoea_nil	97.50	0.00
TRINITY_XP_0191	PREDICTE	Ipomoea_nil	97.50	0.00
TRINITY_BAJ8510	predicte	Hordeum_vulgare	97.50	0.00
TRINITY_XP_0229	MLP-like	Cucurbita_moschata	97.40	0.00
TRINITY_XP_0191	PREDICTE	Ipomoea_nil	97.40	0.00
TRINITY_ANW7227	calmodul	Tradescantia_hirsut	97.40	0.00
TRINITY_GAX7401	hypothet	Chlamydomonas_eusti	97.40	0.00
TRINITY_XP_0058	hypothet	Chlorella_variabili	97.40	0.00
TRINITY_AEF1316	malonyl	Haematococcus_lacus	97.40	0.00
TRINITY_XP_0016	vacuolar	Chlamydomonas_reinh	97.40	0.00
TRINITY_AJR2723	1-hydrox	Haematococcus_lacus	97.40	0.00
TRINITY_AFK3361	unknown	Lotus_japonicus	97.30	0.00
TRINITY_KEH3115	polyubic	Medicago_truncatula	97.30	0.00
TRINITY_BAJ9006	predicte	Hordeum_vulgare	97.30	0.00
TRINITY_CAA1126	polyubic	Nicotiana_tabacum	97.30	0.00
TRINITY_XP_0191	PREDICTE	Ipomoea_nil	97.20	0.00
TRINITY_XP_0191	PREDICTE	Ipomoea_nil	97.20	0.00
TRINITY_KMS6506	hypothet	Beta_vulgaris	97.20	0.00
TRINITY_YP_0093	photosys	Chlamydomonas_leios	97.20	0
TRINITY_XP_0191	PREDICTE	Ipomoea_nil	97.20	0.00
TRINITY_XP_0191	PREDICTE	Ipomoea_nil	97.10	0.00
TRINITY_XP_0229	30S ribc	Cucurbita_maxima	97.10	0.00
TRINITY_AAK7290	proteina	Ipomoea_batatas	97.10	0.00
TRINITY_GAX8057	hypothet	Chlamydomonas_eusti	97.10	0.00
TRINITY_BAK0099	predicte	Hordeum_vulgare	97.10	0.00
TRINITY_OAE2671	hypothet	Marchantia_polymorp	97.00	0.00
TRINITY_XP_0016	actin [	Chlamydomonas_reinh	97.00	0.00
TRINITY_ACF8818	unknown	Zea_mays	97.00	0.00
TRINITY_GBG5920	Glycine	Chara_braunii	96.90	0.00
TRINITY_XP_0273	eukaryot	Abrus_precatorius	96.90	0.00
TRINITY_ALO6312	CP47 chl	Chloromonas_perfora	96.90	0.00
TRINITY_BAK0096(	predicte	Hordeum_vulgare	96.90	0.00
TRINITY_XP_0229	carbonic	Cucurbita_moschata	96.90	0.00
TRINITY_XP_0056	ribosoma	Coccomyxa_subellips	96.90	0.00
TRINITY_XP_0016	ribosoma	Chlamydomonas_reinh	96.90	0.00
TRINITY_AEP3960	heat shc	Haematococcus_lacus	96.90	0.00
TRINITY_GBG0034	hypothet	Raphidocelis_subcap	96.90	0.00
TRINITY_XP_0191	PREDICTE	Ipomoea_nil	96.80	0.00
TRINITY_XP_0228	histone	Olea_europaea	96.80	0.00
TRINITY_XP_0139(	Stromal	Monoraphidium_negle	96.80	0.00
TRINITY_AEP3960	heat shc	Haematococcus_lacus	96.80	0.00
TRINITY_PNH0370	PHD fing	Tetrabaena_socialis	96.80	0.00
TRINITY_KNA0614	hypothet	Spinacia_oleracea	96.70	0.00
TRINITY_ALO2159	hypothet	Haematococcus_lacus	96.70	0.00
TRINITY_ARN7958	low co2	Haematococcus_lacus	96.70	0.00
TRINITY_BAK0099	predicte	Hordeum_vulgare	96.70	0.00
TRINITY_XP_0139(	Stromal	Monoraphidium_negle	96.70	0.00
TRINITY_XP_0191	PREDICTE	Ipomoea_nil	96.60	0.00
TRINITY_XP_0191	PREDICTE	Ipomoea_nil	96.50	0.00
TRINITY_BAJ8510	predicte	Hordeum_vulgare	96.50	0.00
TRINITY_AAD4434	actin [	Dunaliella_salina	96.50	0.00
TRINITY_XP_0191	PREDICTE	Ipomoea_nil	96.40	0.00
TRINITY_BAJ8900	predicte	Hordeum_vulgare	96.40	0.00
TRINITY_ABC0018	chlorop	Dunaliella_viridis	96.40	0.00
TRINITY_AUI4110	LhcsR [	Haematococcus_lacus	96.40	0.00
TRINITY_XP_0016	naphthoa	Chlamydomonas_reinh	96.40	0.00

TRINITY_GAX7804	hypothet	Chlamydomonas_eusti	96.40	0.00
TRINITY_CDP0610	unnamed	Coffea_canephora	96.30	0.00
TRINITY_XP_0216	ankyrin	Hevea_brasiliensis	96.30	0.00
TRINITY_XP_0191	PREDICTE	Ipomoea_nil	96.30	0.00
TRINITY_EFJ0434	hypothet	Selaginella_moellen	96.30	0.00
TRINITY_ABC6796	heat sho	Triticum_aestivum	96.30	0.00
TRINITY_XP_0205	polyubiq	Phalaenopsis_equest	96.20	0.00
TRINITY_KMS6553	hypothet	Beta_vulgaris	96.20	0.00
TRINITY_AEY8002	1-deoxy-	Haematococcus_lacus	96.20	0.00
TRINITY_XP_0191	PREDICTE	Ipomoea_nil	96.20	0.00
TRINITY_BAK0099	predicte	Hordeum_vulgare	96.20	0.00
TRINITY_XP_0029	hypothet	Volvox_carteri	96.20	0.00
TRINITY_XP_0191	PREDICTE	Ipomoea_nil	96.10	0.00
TRINITY_XP_0191	PREDICTE	Ipomoea_nil	96.10	0.00
TRINITY_AKR7626	beta-1 t	Dendrobium_catenatu	96.10	0.00
TRINITY_XP_0191	PREDICTE	Ipomoea_nil	96.00	0.00
TRINITY_XP_0191	PREDICTE	Ipomoea_nil	96.00	0.00
TRINITY_AFD6280	GAPDH2 |	Ipomoea_purpurea	96.00	0.00
TRINITY_BAJ9006	predicte	Hordeum_vulgare	96.00	0.00
TRINITY_BAL6108	polyubiq	Dianthus_caryophyll	96.00	0.00
TRINITY_KZM8492	hypothet	Daucus_carota	96.00	0.00
TRINITY_ACG2745	60 ribos	Zea_mays	95.90	0.00
TRINITY_ACF8818	unknown	Zea_mays	95.90	0.00
TRINITY_BAF2013	Os06g065	Oryza_sativa	95.90	0.00
TRINITY_ACF8498	unknown	Zea_mays	95.80	0.00
TRINITY_KXZ5449	hypothet	Gonium_pectorale	95.80	0.00
TRINITY_XP_0017	predicte	Chlamydomonas_reinh	95.80	0.00
TRINITY_XP_0029	subunit	Volvox_carteri	95.80	0.00
TRINITY_XP_0191	PREDICTE	Ipomoea_nil	95.70	0.00
TRINITY_XP_0191	PREDICTE	Ipomoea_nil	95.70	0.00
TRINITY_AKR5370	chloropl	Haematococcus_lacus	95.70	0.00
TRINITY_ACN3153	unknown	Zea_mays	95.70	0.00
TRINITY_ADD5259	pyruvate	Dunaliella_salina	95.70	0.00
TRINITY_ACN3155	unknown	Zea_mays	95.60	0.00
TRINITY_XP_0029	hypothet	Volvox_carteri	95.60	0.00
TRINITY_XP_0128	PREDICTE	Erythranthe_guttata	95.50	0.00
TRINITY_XP_0191	PREDICTE	Ipomoea_nil	95.50	0.00
TRINITY_XP_0058	40S ribo	Chlorella_variabili	95.50	0.00
TRINITY_GAX8379	hypothet	Chlamydomonas_eusti	95.50	0.00
TRINITY_XP_0218	polyubiq	Prunus_avium	95.40	0.00
TRINITY_XP_0139	ribonucl	Monoraphidium_negle	95.30	0.00
TRINITY_XP_0084	PREDICTE	Cucumis_melo	95.30	0.00
TRINITY_XP_0191	PREDICTE	Ipomoea_nil	95.20	0.00
TRINITY_AMT9186	polyubiq	Apium_graveolens	95.20	0.00
TRINITY_GAX7938	hypothet	Chlamydomonas_eusti	95.20	0.00
TRINITY_XP_0239	uncharac	Quercus_suber	95.20	0.00
TRINITY_GAX8119	hypothet	Chlamydomonas_eusti	95.20	0.00
TRINITY_PNT6171	hypothet	Brachypodium_distac	95.20	0.00
TRINITY_GAX7827	hypothet	Chlamydomonas_eusti	95.20	0.00
TRINITY_BAJ9006	predicte	Hordeum_vulgare	95.10	0.00
TRINITY_ADQ0018	histone	Chlamydomonas_sp._I	95.10	0.00
TRINITY_CAA5229	polyubiq	Volvox_carteri	95.10	0.00
TRINITY_KZN0886	hypothet	Daucus_carota	95.10	0.00
TRINITY_CAA1126	polyubiq	Nicotiana_tabacum	95.10	0.00
TRINITY_ADV0277	lipid tr	Ipomoea_batatas	95.00	0.00
TRINITY_XP_0016	vacuolar	Chlamydomonas_reinh	95.00	0.00
TRINITY_XP_0029	p300/CBF	Volvox_carteri	95.00	0.00
TRINITY_CAI3483	ATP synt	Polytomella_sp._Pri	94.90	0.00
TRINITY_CAA1126	polyubiq	Nicotiana_tabacum	94.90	0.00
TRINITY_XP_0191	PREDICTE	Ipomoea_nil	94.90	0.00
TRINITY_GAX7873	hypothet	Chlamydomonas_eusti	94.80	0.00
TRINITY_XP_0191	PREDICTE	Ipomoea_nil	94.70	0.00
TRINITY_XP_0191	PREDICTE	Ipomoea_nil	94.70	0.00
TRINITY_GAX8437	hypothet	Chlamydomonas_eusti	94.70	0.00
TRINITY_GBF9010	hypothet	Raphidocelis_subcap	94.70	0.00
TRINITY_XP_0191	PREDICTE	Ipomoea_nil	94.70	0.00
TRINITY_XP_0017	MUT9-rel	Chlamydomonas_reinh	94.60	0.00
TRINITY_XP_0016	ran-like	Chlamydomonas_reinh	94.60	0.00
TRINITY_XP_0206	polyubiq	Dendrobium_catenatu	94.60	0.00
TRINITY_XP_0029	F1F0 ATF	Volvox_carteri	94.60	0.00
TRINITY_XP_0191	PREDICTE	Ipomoea_nil	94.50	0.00

TRINITY_KXZ5593(	hypothet	Gonium_pectorale	94.50	0.00
TRINITY_XP_0017(	dynein	Chlamydomonas_reinh	94.50	0.00
TRINITY_XP_0221(	translat	Momordica_charantia	94.40	0.00
TRINITY_XP_0200{	26S prot	Ananas_comosus	94.40	0.00
TRINITY_XP_0191!	PREDICTE	Ipomoea_nil	94.40	0.00
TRINITY_AAV5418{	chloropl	Haematococcus_lacus	94.40	0.00
TRINITY_XP_0056!	branchec	Coccomyxa_subellips	94.40	0.00
TRINITY_XP_0243!	histone	Physcomitrella_pate	94.40	0.00
TRINITY_XP_0093!	PREDICTE	Pyrus_x_bretschneid	94.30	0.00
TRINITY_XP_0139(	S-(hydrc	Monoraphidium_negle	94.30	0.00
TRINITY_ABL6795(	1-aminoc	Ipomoea_nil	94.20	0.00
TRINITY_XP_0191{	PREDICTE	Ipomoea_nil	94.20	0.00
TRINITY_XP_0029(	hypothet	Volvox_carteri	94.20	0.00
TRINITY_AUT7717(	glutamat	Haematococcus_lacus	94.20	0.00
TRINITY_GAX7460!	hypothet	Chlamydomonas_eusti	94.20	0.00
TRINITY_CAA5229(	polyubic	Volvox_carteri	94.20	0.00
TRINITY_XP_0139(	40S ribc	Monoraphidium_negle	94.20	0.00
TRINITY_CAA5229(	polyubic	Volvox_carteri	94.10	0.00
TRINITY_ABA0109!	40S ribc	Chlamydomonas_incer	94.10	0.00
TRINITY_ABV7239!	plastid	Haematococcus_lacus	94.10	0.00
TRINITY_XP_0016!	phosphoi	Chlamydomonas_reinh	94.10	0.00
TRINITY_XP_0029(	hypothet	Volvox_carteri	94.10	0.00
TRINITY_XP_0191!	PREDICTE	Ipomoea_nil	94.00	0.00
TRINITY_XP_0016!	predicte	Chlamydomonas_reinh	94.00	0.00
TRINITY_XP_0191!	PREDICTE	Ipomoea_nil	94.00	0.00
TRINITY_GAX8007!	hypothet	Chlamydomonas_eusti	94.00	0.00
TRINITY_XP_0191!	PREDICTE	Ipomoea_nil	94.00	0.00
TRINITY_XP_0191!	PREDICTE	Ipomoea_nil	93.90	0.00
TRINITY_XP_0029!	hypothet	Volvox_carteri	93.90	0.00
TRINITY_PSC7087!	nifU mit	Micractinium_conduc	93.90	0.00
TRINITY_BAD9564(	metallot	Ipomoea_batatas	93.90	0.00
TRINITY_KNA0614!	hypothet	Spinacia_oleracea	93.90	0.00
TRINITY_PON4418!	Ribosoma	Trema_orientale	93.80	0.00
TRINITY_XP_0191{	PREDICTE	Ipomoea_nil	93.80	0.00
TRINITY_KXZ4483{	hypothet	Gonium_pectorale	93.80	0.00
TRINITY_BAJ8900!	predicte	Hordeum_vulgare	93.80	0.00
TRINITY_XP_0109(	PREDICTE	Elaeis_guineensis	93.70	0.00
TRINITY_GAQ8928!	transcri	Klebsormidium_niten	93.60	0.00
TRINITY_GAX8510!	hypothet	Chlamydomonas_eusti	93.60	0.00
TRINITY_XP_0221(	photosys	Momordica_charantia	93.50	0.00
TRINITY_XP_0191{	PREDICTE	Ipomoea_nil	93.50	0.00
TRINITY_PNH0189(	hypothet	Tetrabaena_socialis	93.50	0.00
TRINITY_XP_0016!	imidazol	Chlamydomonas_reinh	93.50	0.00
TRINITY_PRW5641!	eukaryot	Chlorella_sorokinia	93.50	0.00
TRINITY_ABD3791!	light-ha	Chlamydomonas_incer	93.50	0.00
TRINITY_GAX7890!	hypothet	Chlamydomonas_eusti	93.40	0.00
TRINITY_GAX8057!	hypothet	Chlamydomonas_eusti	93.40	0.00
TRINITY_XP_0235!	uncharac	Cucurbita_pepo	93.30	0.00
TRINITY_KXZ4259!	hypothet	Gonium_pectorale	93.30	0.00
TRINITY_AMK9782(	thioredc	Haematococcus_lacus	93.30	0.00
TRINITY_KXZ5314{	hypothet	Gonium_pectorale	93.20	0.00
TRINITY_XP_0030(	predicte	Micromonas_pusilla	93.20	0.00
TRINITY_XP_0016!	26S prot	Chlamydomonas_reinh	93.20	0.00
TRINITY_OAE2671(	hypothet	Marchantia_polymorp	93.20	0.00
TRINITY_APO1583(	actin 1	Sesuvium_portulacas	93.20	0.00
TRINITY_XP_0029(	hypothet	Volvox_carteri	93.10	0.00
TRINITY_KXZ4400(	hypothet	Gonium_pectorale	93.10	0.00
TRINITY_AKR5370!	chloropl	Haematococcus_lacus	93.10	0.00
TRINITY_PNH1230(	Nucleosi	Tetrabaena_socialis	93.00	0.00
TRINITY_GAQ8928!	transcri	Klebsormidium_niten	93.00	0.00
TRINITY_RMZ5589(	hypothet	Auxenochlorella_pro	93.00	0.00
TRINITY_XP_0017(	U5 snRNE	Chlamydomonas_reinh	93.00	0.00
TRINITY_AAQ6369(	NADH:ubi	Chlamydomonas_reinh	93.00	0.00
TRINITY_PNH0118!	Peptidyl	Tetrabaena_socialis	93.00	0.00
TRINITY_XP_0093!	PREDICTE	Pyrus_x_bretschneid	92.90	0.00
TRINITY_XP_0192(	PREDICTE	Nicotiana_attenuata	92.90	0.00
TRINITY_XP_0230(	photosys	Cucurbita_maxima	92.90	0.00
TRINITY_GAX7262{	hypothet	Chlamydomonas_eusti	92.90	0.00
TRINITY_PNH0737(	GTP-binc	Tetrabaena_socialis	92.90	0.00
TRINITY_GAX8139!	hypothet	Chlamydomonas_eusti	92.90	0.00
TRINITY_ALM5498!	DEAD box	Chlamydomonas_sp._I	92.90	0.00

TRINITY_XP_0017(	flagella	Chlamydomonas_reinh	92.90	0.00
TRINITY_XP_0191S	PREDICTE	Ipomoea_nil	92.80	0.00
TRINITY_XP_0041S	PREDICTE	Cucumis_sativus	92.80	0.00
TRINITY_CAJ4537(	40S ribo	Chlamydomonas_sp._I	92.80	0.00
TRINITY_GAX7630S	hypothet	Chlamydomonas_eusti	92.80	0.00
TRINITY_ABA0113]	chloropl	Chlamydomonas_incer	92.60	0.00
TRINITY_ABA0114	chloropl	Chlamydomonas_incer	92.60	0.00
TRINITY_BAJ9019(	predicte	Hordeum_vulgare	92.50	0.00
TRINITY_XP_0056	ADP-ribo	Coccomyxa_subellips	92.50	0.00
TRINITY_GAX7921E	hypothet	Chlamydomonas_eusti	92.50	0.00
TRINITY_BAJ9928	predicte	Hordeum_vulgare	92.50	0.00
TRINITY_XP_0114(	Magnesiu	Auxenochlorella_pro	92.50	0.00
TRINITY_AQK3923	aluminum	Zea_mays	92.50	0.00
TRINITY_XP_0029	hypothet	Volvox_carteri	92.40	0.00
TRINITY_ABA0113]	chloropl	Chlamydomonas_incer	92.30	0.00
TRINITY_GAX7268]	hypothet	Chlamydomonas_eusti	92.30	0.00
TRINITY_PNH1214	Asparagi	Tetrabaena_socialis	92.30	0.00
TRINITY_XP_0029	hypothet	Volvox_carteri	92.30	0.00
TRINITY_XP_0017(	predicte	Chlamydomonas_reinh	92.30	0.00
TRINITY_GAX7910	hypothet	Chlamydomonas_eusti	92.30	0.00
TRINITY_AJR2723	1-hydrox	Haematococcus_lacus	92.30	0.00
TRINITY_XP_0016S	predicte	Chlamydomonas_reinh	92.30	0.00
TRINITY_XP_0191S	PREDICTE	Ipomoea_nil	92.10	0.00
TRINITY_KMS9327	hypothet	Beta_vulgaris	92.10	0.00
TRINITY_CBV7607	60S ribo	Polytomella_sp._Pri	92.10	0.00
TRINITY_RWW4353	hypothet	Ensete_ventricosum	92.10	0.00
TRINITY_GBF8774]	hypothet	Raphidocelis_subcap	92.10	0.00
TRINITY_XP_0025(	flagella	Micromonas_commoda	92.10	0.00
TRINITY_GAX7427	hypothet	Chlamydomonas_eusti	92.10	0
TRINITY_XP_0216E	60S ribo	Hevea_brasiliensis	92.00	0.00
TRINITY_XP_0191	PREDICTE	Ipomoea_nil	92.00	0.00
TRINITY_AAL8398S	NADH-ubi	Oryza_sativa	92.00	0.00
TRINITY_RVX0567	Polyubic	Vitis_vinifera	92.00	0.00
TRINITY_ACN3155	unknown	Zea_mays	92.00	0.00
TRINITY_KXZ5647	hypothet	Gonium_pectorale	92.00	0.00
TRINITY_GAX7304(	hypothet	Chlamydomonas_eusti	92.00	0.00
TRINITY_ACN3163]	unknown	Zea_mays	91.90	0.00
TRINITY_ASZ8517(	elongati	Hylocereus_polyrhiz	91.90	0.00
TRINITY_XP_0056	topoison	Coccomyxa_subellips	91.90	0.00
TRINITY_XP_0029	dynein h	Volvox_carteri	91.90	0.00
TRINITY_AFA5204(	PsbA (ch	Kirengeshoma_palmat	91.90	0.00
TRINITY_XP_0191E	PREDICTE	Ipomoea_nil	91.80	0.00
TRINITY_XP_0249(	ubiquiti	Cynara_cardunculus	91.80	0.00
TRINITY_GAX8490S	hypothet	Chlamydomonas_eusti	91.80	0
TRINITY_GBF9818E	ubiquiti	Raphidocelis_subcap	91.80	0.00
TRINITY_XP_0029	hypothet	Volvox_carteri	91.80	0.00
TRINITY_XP_0029	hypothet	Volvox_carteri	91.80	0.00
TRINITY_KMS6481(	hypothet	Beta_vulgaris	91.70	0.00
TRINITY_AAX7722	14-3-3 p	Dunaliella_salina	91.60	0.00
TRINITY_GAX7662(	hypothet	Chlamydomonas_eusti	91.60	0.00
TRINITY_KXZ5658E	hypothet	Gonium_pectorale	91.60	0.00
TRINITY_XP_0016S	eukaryot	Chlamydomonas_reinh	91.50	0.00
TRINITY_AUI4110	photosys	Haematococcus_lacus	91.50	0.00
TRINITY_XP_0056	ubiquiti	Coccomyxa_subellips	91.40	0.00
TRINITY_PNH0870(	Tyrosine	Tetrabaena_socialis	91.40	0.00
TRINITY_PNH0349	Ferredox	Tetrabaena_socialis	91.40	0.00
TRINITY_ABD3791]	light-ha	Chlamydomonas_incer	91.40	0.00
TRINITY_ABD3790S	light-ha	Chlamydomonas_incer	91.40	0.00
TRINITY_XP_0016E	predicte	Chlamydomonas_reinh	91.40	0.00
TRINITY_XP_0016S	ubiquino	Chlamydomonas_reinh	91.30	0.00
TRINITY_XP_0191(	PREDICTE	Ipomoea_nil	91.20	0.00
TRINITY_XP_0279	LOW QUAI	Vigna_unguiculata	91.20	0.00
TRINITY_XP_0017(	outer dy	Chlamydomonas_reinh	91.20	0.00
TRINITY_GAX7662	hypothet	Chlamydomonas_eusti	91.20	0.00
TRINITY_XP_0029	hypothet	Volvox_carteri	91.20	0.00
TRINITY_ADN9518	oil glob	Haematococcus_lacus	91.20	0.00
TRINITY_GBF9486	traffick	Raphidocelis_subcap	91.20	0.00
TRINITY_XP_0191S	PREDICTE	Ipomoea_nil	91.10	0.00
TRINITY_KDD7612	glycosyl	Helicosporidium_sp.	91.10	0.00
TRINITY_KXZ4829	hypothet	Gonium_pectorale	91.10	0.00
TRINITY_GAX8123	hypothet	Chlamydomonas_eusti	91.10	0.00

TRINITY_AAW6929	mitochor	Haematococcus_lacus	91.10	0.00
TRINITY_XP_0279	tubulin	Vigna_unguiculata	91.10	0.00
TRINITY_BAJ9160	predicte	Hordeum_vulgare	91.00	0.00
TRINITY_AFK3339	unknown	Lotus_japonicus	91.00	0.00
TRINITY_XP_0025	flagella	Micromonas_commoda	91.00	0.00
TRINITY_XP_0139	Tubulin	Monoraphidium_negle	91.00	0.00
TRINITY_XP_0029	serine/t	Volvox_carteri	91.00	0.00
TRINITY_XP_0184	PREDICTE	Raphanus_sativus	90.90	0.00
TRINITY_XP_0016	isopropy	Chlamydomonas_reinh	90.90	0.00
TRINITY_XP_0025	predicte	Micromonas_commoda	90.90	0.00
TRINITY_XP_0191	PREDICTE	Ipomoea_nil	90.80	0.00
TRINITY_CAB3861	splicing	Arabidopsis_thalian	90.80	0.00
TRINITY_PIN1445	Molecula	Handroanthus_impeti	90.80	0.00
TRINITY_KXZ4408	hypothet	Gonium_pectorale	90.80	0.00
TRINITY_KXZ4726	hypothet	Gonium_pectorale	90.80	0.00
TRINITY_XP_0016	predicte	Chlamydomonas_reinh	90.80	0.00
TRINITY_GAX7696	hypothet	Chlamydomonas_eusti	90.80	0.00
TRINITY_XP_0064	cell div	Citrus_clementina	90.70	0.00
TRINITY_GBF9432	40S ribo	Raphidocelis_subcap	90.70	0.00
TRINITY_PNH0555	Protein	Tetrabaena_socialis	90.70	0.00
TRINITY_XP_0029	adenylat	Volvox_carteri	90.70	0.00
TRINITY_XP_0191	PREDICTE	Ipomoea_nil	90.60	0.00
TRINITY_O82018.	RecName:	Mougeotia_scalaris	90.60	0.00
TRINITY_GAX7725	hypothet	Chlamydomonas_eusti	90.60	0.00
TRINITY_XP_0016	threonir	Chlamydomonas_reinh	90.60	0.00
TRINITY_PRW2110	malate c	Chlorella_sorokinia	90.60	0.00
TRINITY_GAX7423	hypothet	Chlamydomonas_eusti	90.60	0.00
TRINITY_CAH5940	hypothet	Plantago_major	90.50	0.00
TRINITY_XP_0029	hypothet	Volvox_carteri	90.50	0.00
TRINITY_GAX7420	hypothet	Chlamydomonas_eusti	90.50	0.00
TRINITY_GAX7474	hypothet	Chlamydomonas_eusti	90.50	0.00
TRINITY_GAX7732	hypothet	Chlamydomonas_eusti	90.50	0.00
TRINITY_ACG2906	bifuncti	Zea_mays	90.50	0
TRINITY_XP_0229	proline-	Cucurbita_moschata	90.40	0.00
TRINITY_XP_0029	hypothet	Volvox_carteri	90.40	0.00
TRINITY_XP_0165	PREDICTE	Nicotiana_tabacum	90.40	0.00
TRINITY_GAX7348	hypothet	Chlamydomonas_eusti	90.40	0.00
TRINITY_XP_0191	PREDICTE	Ipomoea_nil	90.30	0.00
TRINITY_XP_0093	PREDICTE	Pyrus_x_bretschneid	90.30	0.00
TRINITY_ACF7943	unknown	Zea_mays	90.30	0.00
TRINITY_KMS9468	hypothet	Beta_vulgaris	90.30	0.00
TRINITY_XP_0029	hypothet	Volvox_carteri	90.30	0.00
TRINITY_XP_0058	hypothet	Chlorella_variabili	90.30	0.00
TRINITY_GAX7256	hypothet	Chlamydomonas_eusti	90.30	0.00
TRINITY_GAX7265	hypothet	Chlamydomonas_eusti	90.30	0.00
TRINITY_AUT7717	glutamat	Haematococcus_lacus	90.30	0.00
TRINITY_XP_0056	small nu	Coccomyxa_subellips	90.20	0.00
TRINITY_ATU9014	WDR doma	Haematococcus_lacus	90.20	0.00
TRINITY_PNH0674	Outer ro	Tetrabaena_socialis	90.20	0.00
TRINITY_GAX7645	hypothet	Chlamydomonas_eusti	90.20	0.00
TRINITY_XP_0029	flagella	Volvox_carteri	90.20	0.00
TRINITY_XP_0029	hypothet	Volvox_carteri	90.20	0.00
TRINITY_GAX7528	hypothet	Chlamydomonas_eusti	90.20	0.00
TRINITY_BAJ9456	predicte	Hordeum_vulgare	90.10	0.00
TRINITY_BAJ9456	predicte	Hordeum_vulgare	90.10	0.00
TRINITY_XP_0029	dynein h	Volvox_carteri	90.10	0.00
TRINITY_GAX8020	hypothet	Chlamydomonas_eusti	90.10	0
TRINITY_GAX7562	hypothet	Chlamydomonas_eusti	90.10	0.00
TRINITY_XP_0029	hypothet	Volvox_carteri	90.00	0.00
TRINITY_OAE2140	hypothet	Marchantia_polymorp	90.00	0.00
TRINITY_XP_0030	predicte	Micromonas_pusilla	90.00	0.00
TRINITY_GAX7594	hypothet	Chlamydomonas_eusti	90.00	0.00
TRINITY_XP_0017	predicte	Chlamydomonas_reinh	90.00	0.00
TRINITY_XP_0102	PREDICTE	Nelumbo_nucifera	90.00	0.00
TRINITY_AAB6839	Rac-like	Arabidopsis_thalian	90.00	0.00
TRINITY_GAX7599	hypothet	Chlamydomonas_eusti	90.00	0.00
TRINITY_VDC7973	unnamed	Brassica_rapa	89.90	0.00
TRINITY_AKR5370	chloropl	Haematococcus_lacus	89.90	0.00
TRINITY_ADI4689	PR46af |	Volvox_carteri	89.90	0.00
TRINITY_GAX8040	hypothet	Chlamydomonas_eusti	89.90	0.00
TRINITY_BAU6157	heat sho	Gonium_pectorale	89.90	0.00

TRINITY_XP_0191(	PREDICTE	Ipomoea_nil	89.80	0.00
TRINITY_XP_0016(	histone	Chlamydomonas_reinh	89.80	0.00
TRINITY_GAX8428	hypothet	Chlamydomonas_eusti	89.80	0.00
TRINITY_XP_0245	tubulin	Selaginella_moellen	89.80	0.00
TRINITY_ADQ0018	histone	Chlamydomonas_sp._I	89.80	0.00
TRINITY_GAX8093(	hypothet	Chlamydomonas_eusti	89.80	0.00
TRINITY_PSR9157(	CBF1-int	Actinidia_chinensis	89.70	0.00
TRINITY_AEW4668	histone	Ulva_linza	89.70	0.00
TRINITY_XP_0029	hypothet	Volvox_carteri	89.70	0.00
TRINITY_BAK0000	predicte	Hordeum_vulgare	89.70	0.00
TRINITY_GAX8007	hypothet	Chlamydomonas_eusti	89.70	0.00
TRINITY_AFK3339	unknown	Lotus_japonicus	89.70	0.00
TRINITY_XP_0016(	molybdop	Chlamydomonas_reinh	89.70	0.00
TRINITY_XP_0239	ras-rela	Quercus_suber	89.70	0.00
TRINITY_XP_0017(	mitogen-	Chlamydomonas_reinh	89.70	0.00
TRINITY_XP_0029	hypothet	Volvox_carteri	89.50	0.00
TRINITY_GAX8172	hypothet	Chlamydomonas_eusti	89.50	0.00
TRINITY_BAE4524	GDP-mann	Oryza_sativa	89.50	0.00
TRINITY_GAX7434	hypothet	Chlamydomonas_eusti	89.40	0.00
TRINITY_XP_0016(	SEC61-ga	Chlamydomonas_reinh	89.40	0.00
TRINITY_XP_0066(	PREDICTE	Oryza_brachyantha	89.30	0.00
TRINITY_GBG0026	40S ribo	Raphidocelis_subcap	89.30	0.00
TRINITY_AKT9517	zeaxanth	Haematococcus_lacus	89.30	0.00
TRINITY_GBF8774	hypothet	Raphidocelis_subcap	89.30	0.00
TRINITY_GAX7814	hypothet	Chlamydomonas_eusti	89.30	0.00
TRINITY_XP_0155	ADP-ribo	Ricinus_communis	89.20	0.00
TRINITY_XP_0185(	PREDICTE	Brassica_rapa	89.20	0.00
TRINITY_GAX7842(	hypothet	Chlamydomonas_eusti	89.20	0.00
TRINITY_KXZ4543	hypothet	Gonium_pectorale	89.20	0.00
TRINITY_GBF8776	U6 snRNA	Raphidocelis_subcap	89.10	0.00
TRINITY_GAX8314	hypothet	Chlamydomonas_eusti	89.10	0.00
TRINITY_XP_0029	componen	Volvox_carteri	89.10	0.00
TRINITY_GAX8172	hypothet	Chlamydomonas_eusti	89.10	0.00
TRINITY_GAX7944	hypothet	Chlamydomonas_eusti	89.10	0.00
TRINITY_GAX7993	hypothet	Chlamydomonas_eusti	89.00	0.00
TRINITY_GAX7978(	hypothet	Chlamydomonas_eusti	89.00	0.00
TRINITY_XP_0102	PREDICTE	Nelumbo_nucifera	88.90	0.00
TRINITY_GAX7950	hypothet	Chlamydomonas_eusti	88.90	0.00
TRINITY_AFK3131	heat sho	Dunaliella_salina	88.90	0.00
TRINITY_XP_0016(	26S prot	Chlamydomonas_reinh	88.90	0.00
TRINITY_AQQ1175	clathrin	Chara_australis	88.90	0.00
TRINITY_CAF0412	pyruvate	Chlamydomonas_reinh	88.90	0.00
TRINITY_VDC7020	unnamed	Brassica_rapa	88.80	0.00
TRINITY_NP_0013	uncharac	Oryza_brachyantha	88.80	0.00
TRINITY_XP_0029	componen	Volvox_carteri	88.70	0.00
TRINITY_XP_0029	ribosoma	Volvox_carteri	88.70	0.00
TRINITY_KMS9401	hypothet	Beta_vulgaris	88.70	0.00
TRINITY_ABS4486	putative	Oryza_sativa	88.70	0.00
TRINITY_ABS4486	putative	Oryza_sativa	88.70	0.00
TRINITY_AYU5883	alpha-tu	Haematococcus_lacus	88.70	0.00
TRINITY_BAJ9456	predicte	Hordeum_vulgare	88.70	0.00
TRINITY_KXZ4882	hypothet	Gonium_pectorale	88.60	0.00
TRINITY_GBF9702	extracel	Raphidocelis_subcap	88.60	0.00
TRINITY_KXZ5695(	hypothet	Gonium_pectorale	88.60	0.00
TRINITY_XP_0016(	low CO2-	Chlamydomonas_reinh	88.60	0.00
TRINITY_BAU6157(	developm	Gonium_pectorale	88.60	0.00
TRINITY_GAX7596	hypothet	Chlamydomonas_eusti	88.60	0.00
TRINITY_GAX7350	hypothet	Chlamydomonas_eusti	88.60	0.00
TRINITY_ACG2691	hypothet	Zea_mays	88.50	0.00
TRINITY_KXZ4384	hypothet	Gonium_pectorale	88.50	0.00
TRINITY_XP_0058	hypothet	Chlorella_variabili	88.50	0.00
TRINITY_GAX8137	hypothet	Chlamydomonas_eusti	88.50	0.00
TRINITY_GAX8595	hypothet	Chlamydomonas_eusti	88.50	0.00
TRINITY_CAC8613	isocitra	Polytomella_sp._Pri	88.50	0.00
TRINITY_GBF9200	hypothet	Raphidocelis_subcap	88.50	0.00
TRINITY_XP_0016	deoxypus	Chlamydomonas_reinh	88.50	0.00
TRINITY_XP_0029	hypothet	Volvox_carteri	88.40	0.00
TRINITY_GAX7297(	hypothet	Chlamydomonas_eusti	88.40	0.00
TRINITY_GAX7732	hypothet	Chlamydomonas_eusti	88.30	0.00
TRINITY_XP_0029	componen	Volvox_carteri	88.30	0.00
TRINITY_KXZ4176	hypothet	Gonium_pectorale	88.30	0.00

TRINITY_BAK05232	predicte	Hordeum_vulgare	88.30	0.00
TRINITY_KXZ53469	hypothet	Gonium_pectorale	88.30	0.00
TRINITY_YP_00880	NADH deh	Monomastix_sp._OKE-	88.20	0.00
TRINITY_XP_00295	flagella	Volvox_carteri	88.20	0.00
TRINITY_PNH07089	Fumarate	Tetrabaena_socialis	88.20	0.00
TRINITY_XP_00295	hypothet	Volvox_carteri	88.20	0.00
TRINITY_ABD32351	Ubiquiti	Medicago_truncatula	88.20	0.00
TRINITY_OTG05221	putative	Helianthus_annuus	88.20	0.00
TRINITY_XP_00168	outer dy	Chlamydomonas_reinh	88.10	0.00
TRINITY_XP_00306	predicte	Micromonas_pusilla	88.10	0.00
TRINITY_XP_00169	RNA binc	Chlamydomonas_reinh	88.10	0.00
TRINITY_XP_00169	copper t	Chlamydomonas_reinh	88.10	0.00
TRINITY_XP_00295	hypothet	Volvox_carteri	88.10	0.00
TRINITY_AAT07657	putative	Oryza_sativa	88.00	0.00
TRINITY_XP_00584	ribosoma	Chlorella_variabili	88.00	0.00
TRINITY_XP_00294	hypothet	Volvox_carteri	88.00	0.00
TRINITY_GAX73492	hypothet	Chlamydomonas_eusti	88.00	0.00
TRINITY_GAX83668	hypothet	Chlamydomonas_eusti	88.00	0.00
TRINITY_XP_00657	remorin	Glycine_max	87.90	0.00
TRINITY_KMS94501	hypothet	Beta_vulgaris	87.90	0.00
TRINITY_GAX74804	hypothet	Chlamydomonas_eusti	87.90	0.00
TRINITY_PSC75502	N-alpha-	Micractinium_conduc	87.90	0.00
TRINITY_ACR35769	unknown	Zea_mays	87.90	0.00
TRINITY_GAX81565	hypothet	Chlamydomonas_eusti	87.90	0.00
TRINITY_AGZ19352	hypothet	Chlorella_sp._ArM00	87.80	0.00
TRINITY_GAX74018	hypothet	Chlamydomonas_eusti	87.80	0.00
TRINITY_KFK30470	hypothet	Arabis_alpina	87.80	0.00
TRINITY_KXZ54933	hypothet	Gonium_pectorale	87.80	0.00
TRINITY_KXZ55077	RABH1 pr	Gonium_pectorale	87.80	0.00
TRINITY_GAX84361	hypothet	Chlamydomonas_eusti	87.80	0.00
TRINITY_GAX72577	hypothet	Chlamydomonas_eusti	87.70	0.00
TRINITY_GAX77074	hypothet	Chlamydomonas_eusti	87.70	0.00
TRINITY_KMS94345	hypothet	Beta_vulgaris	87.70	0.00
TRINITY_AAK27970	metallot	Ipomoea_batatas	87.50	0.00
TRINITY_GAX86170	hypothet	Chlamydomonas_eusti	87.50	0.00
TRINITY_XP_01390	DNA-dire	Monoraphidium_negle	87.50	0.00
TRINITY_KMS93253	hypothet	Beta_vulgaris	87.50	0.00
TRINITY_XP_00564	ribosoma	Coccomyxa_subellips	87.50	0.00
TRINITY_PNH04881	Ubiquiti	Tetrabaena_socialis	87.50	0.00
TRINITY_GBF87832	hypothet	Raphidocelis_subcap	87.50	0.00
TRINITY_GAX74290	hypothet	Chlamydomonas_eusti	87.50	0.00
TRINITY_XP_02438	60S ribo	Physcomitrella_pate	87.40	0.00
TRINITY_GBF90140	diaminop	Raphidocelis_subcap	87.40	0.00
TRINITY_XP_00294	iron-sul	Volvox_carteri	87.40	0.00
TRINITY_XP_00294	2-isopro	Volvox_carteri	87.40	0.00
TRINITY_KXZ57012	hypothet	Gonium_pectorale	87.40	0.00
TRINITY_PNW86229	hypothet	Chlamydomonas_reinh	87.40	0.00
TRINITY_AHG05845	cold sho	Triticum_aestivum	87.30	0.00
TRINITY_PHT29099	hypothet	Capsicum_baccatum	87.30	0.00
TRINITY_KXZ45375	hypothet	Gonium_pectorale	87.30	0.00
TRINITY_KMS93274	hypothet	Beta_vulgaris	87.20	0.00
TRINITY_XP_00295	hypothet	Volvox_carteri	87.20	0.00
TRINITY_XP_02202	3-oxoacy	Helianthus_annuus	87.20	0.00
TRINITY_XP_02017	uncharac	Aegilops_tauschii	87.20	0.00
TRINITY_GAX76755	hypothet	Chlamydomonas_eusti	87.20	0.00
TRINITY_XP_00294	hypothet	Volvox_carteri	87.20	0.00
TRINITY_PNH10324	Pyrophos	Tetrabaena_socialis	87.20	0.00
TRINITY_XP_02387	heat sho	Quercus_suber	87.10	0.00
TRINITY_XP_02638	ubiquiti	Papaver_somniferum	87.10	0.00
TRINITY_XP_00295	hypothet	Volvox_carteri	87.10	0.00
TRINITY_XP_00305	magnesiu	Micromonas_pusilla	87.10	0.00
TRINITY_XP_02295	non-spec	Cucurbita_moschata	87.10	0.00
TRINITY_XP_01389	thylakoi	Monoraphidium_negle	87.10	0.00
TRINITY_KXZ55677	hypothet	Gonium_pectorale	87.10	0.00
TRINITY_GAX85578	hypothet	Chlamydomonas_eusti	87.10	0.00
TRINITY_XP_00305	predicte	Micromonas_pusilla	87.10	0.00
TRINITY_GAX80370	hypothet	Chlamydomonas_eusti	87.10	0.00
TRINITY_ONM39914	carbonic	Zea_mays	87.10	0.00
TRINITY_XP_02263	uncharac	Vigna_radiata	87.00	0.00
TRINITY_XP_01390	hypothet	Monoraphidium_negle	87.00	0.00
TRINITY_XP_00295	componer	Volvox_carteri	87.00	0.00

TRINITY_XP_0191	PREDICTE	Ipomoea_nil	86.90	0.00
TRINITY_GAX8015	hypothet	Chlamydomonas_eusti	86.90	0.00
TRINITY_KXZ4824	ODA2 pro	Gonium_pectorale	86.90	0
TRINITY_GAX8074	hypothet	Chlamydomonas_eusti	86.90	0
TRINITY_BAK0523	predicte	Hordeum_vulgare	86.90	0.00
TRINITY_XP_0087	actin, m	Phoenix_dactylifera	86.90	0.00
TRINITY_GAX8546	hypothet	Chlamydomonas_eusti	86.80	0.00
TRINITY_BAU6157	heat sho	Gonium_pectorale	86.80	0.00
TRINITY_XP_0016	R-SNARE	Chlamydomonas_reinh	86.80	0.00
TRINITY_KXZ5252	hypothet	Gonium_pectorale	86.80	0.00
TRINITY_BAJ9159	predicte	Hordeum_vulgare	86.80	0.00
TRINITY_XP_0017	predicte	Chlamydomonas_reinh	86.80	0.00
TRINITY_GAX8004	hypothet	Chlamydomonas_eusti	86.80	0.00
TRINITY_BAJ9928	predicte	Hordeum_vulgare	86.80	0.00
TRINITY_KMS6524	hypothet	Beta_vulgaris	86.80	0.00
TRINITY_AAN0409	S15 ribo	Dunaliella_tertiole	86.80	0.00
TRINITY_XP_0219	probable	Helianthus_annuus	86.70	0.00
TRINITY_XP_0029	hypothet	Volvox_carteri	86.70	0.00
TRINITY_O22608.1	RecName:	Dunaliella_salina	86.70	0.00
TRINITY_XP_0098	PREDICTE	Nicotiana_sylvestri	86.70	0.00
TRINITY_KMS6493	hypothet	Beta_vulgaris	86.70	0.00
TRINITY_PIN0321	Serine/t	Handroanthus_impeti	86.70	0.00
TRINITY_GAX7514	hypothet	Chlamydomonas_eusti	86.70	0.00
TRINITY_AAK8483	actin [E	Elaeis_oleifera	86.70	0.00
TRINITY_GBF9745	DEAD-box	Raphidocelis_subcap	86.70	0.00
TRINITY_XP_0070	PREDICTE	Theobroma_cacao	86.70	0.00
TRINITY_GBF9095	140S ribo	Raphidocelis_subcap	86.60	0.00
TRINITY_KXZ5055	hypothet	Gonium_pectorale	86.60	0.00
TRINITY_KXZ4869	hypothet	Gonium_pectorale	86.60	0.00
TRINITY_KXZ5327	hypothet	Gonium_pectorale	86.60	0.00
TRINITY_EAY8791	hypothet	Oryza_sativa	86.60	0.00
TRINITY_PNW7623	hypothet	Chlamydomonas_reinh	86.60	0.00
TRINITY_KJB1390	hypothet	Gossypium_raimondii	86.60	0.00
TRINITY_XP_0263	ubiquiti	Papaver_somniferum	86.50	0.00
TRINITY_PNH0979	hypothet	Tetrabaena_socialis	86.50	0.00
TRINITY_BAK0054	predicte	Hordeum_vulgare	86.50	0.00
TRINITY_GAX8325	hypothet	Chlamydomonas_eusti	86.50	0.00
TRINITY_KMS6493	hypothet	Beta_vulgaris	86.50	0.00
TRINITY_XP_0016	peptidyl	Chlamydomonas_reinh	86.50	0.00
TRINITY_PNW7928	hypothet	Chlamydomonas_reinh	86.50	0.00
TRINITY_GAX7709	hypothet	Chlamydomonas_eusti	86.50	0.00
TRINITY_KXZ5633	hypothet	Gonium_pectorale	86.50	0.00
TRINITY_GAX8287	hypothet	Chlamydomonas_eusti	86.50	0.00
TRINITY_PSC7066	mitochon	Micractinium_conduc	86.50	0.00
TRINITY_GAX8007	hypothet	Chlamydomonas_eusti	86.50	0.00
TRINITY_OAE3412	hypothet	Marchantia_polymorp	86.40	0.00
TRINITY_GBF8914	hypothet	Raphidocelis_subcap	86.40	0.00
TRINITY_GAX8278	hypothet	Chlamydomonas_eusti	86.40	0.00
TRINITY_XP_0029	hypothet	Volvox_carteri	86.40	0.00
TRINITY_XP_0243	60S ribo	Physcomitrella_pate	86.30	0.00
TRINITY_XP_0029	componen	Volvox_carteri	86.30	0.00
TRINITY_KMS6481	hypothet	Beta_vulgaris	86.30	0.00
TRINITY_BAS3051	histone	Polyblepharides_amy	86.30	0.00
TRINITY_KXZ5637	hypothet	Gonium_pectorale	86.30	0.00
TRINITY_XP_0228	Ubiquiti	Ostreococcus_tauri	86.20	0.00
TRINITY_XP_0243	60S ribo	Physcomitrella_pate	86.20	0.00
TRINITY_GAX7642	hypothet	Chlamydomonas_eusti	86.20	0.00
TRINITY_GAX8037	hypothet	Chlamydomonas_eusti	86.20	0.00
TRINITY_XP_0139	60S ribo	Monoraphidium_negle	86.20	0.00
TRINITY_KXZ4470	hypothet	Gonium_pectorale	86.20	0.00
TRINITY_KXZ5613	hypothet	Gonium_pectorale	86.20	0.00
TRINITY_GBG0026	aspartat	Raphidocelis_subcap	86.10	0.00
TRINITY_EPS7453	hypothet	Genlisea_aurea	86.10	0.00
TRINITY_GBF8957	hypothet	Raphidocelis_subcap	86.10	0.00
TRINITY_P22686.1	RecName:	Chlamydomonas_moewu	86.10	0.00
TRINITY_KXZ4839	hypothet	Gonium_pectorale	86.10	0.00
TRINITY_XP_0263	ubiquiti	Papaver_somniferum	86.00	0.00
TRINITY_GAX8025	hypothet	Chlamydomonas_eusti	86.00	0.00
TRINITY_XP_0029	componen	Volvox_carteri	86.00	0.00
TRINITY_PNH0793	AP-2 com	Tetrabaena_socialis	86.00	0.00
TRINITY_PNW8817	hypothet	Chlamydomonas_reinh	86.00	0.00

TRINITY_KXZ5195[illegible]	hypothet	Gonium_pectorale	86.00	0.00
TRINITY_GAX8249[illegible]	hypothet	Chlamydomonas_eusti	86.00	0.00
TRINITY_XP_0017[illegible]	ATP-NAD	Chlamydomonas_reinh	86.00	0.00
TRINITY_GAX8506[illegible]	hypothet	Chlamydomonas_eusti	86.00	0.00
TRINITY_XP_0104[illegible]	PREDICTE	Camelina_sativa	86.00	0.00
TRINITY_OAE2544[illegible]	hypothet	Marchantia_polymorp	85.90	0.00

TRINITY_XP_0017	elongat	Chlamydomonas_reinh	85.90	0.00
TRINITY_BAJ9608	predicte	Hordeum_vulgare	85.90	0.00
TRINITY_ADD5259	pyruvate	Dunaliella_salina	85.80	0.00
TRINITY_XP_0029	hypothet	Volvox_carteri	85.80	0.00
TRINITY_GAX8383	hypothet	Chlamydomonas_eusti	85.80	0.00
TRINITY_XP_0191	PREDICTI	Ipomoea_nil	85.70	0.00
TRINITY_XP_0264	DNA-dire	Papaver_somniferum	85.70	0.00
TRINITY_XP_0035	40S rib	Brachypodium_distac	85.70	0.00
TRINITY_KXZ4273	hypothet	Gonium_pectorale	85.70	0.00
TRINITY_XP_0138	40S rib	Monoraphidium_negle	85.70	0.00
TRINITY_PNH0711	Chloropl	Tetrabaena_socialis	85.70	0.00
TRINITY_XP_0136	PREDICTI	Brassica_oleracea	85.70	0.00
TRINITY_AEO8932	serine/t	Dunaliella_salina	85.70	0.00
TRINITY_GAX7502	hypothet	Chlamydomonas_eusti	85.70	0.00
TRINITY_XP_0016	predicte	Chlamydomonas_reinh	85.70	0.00
TRINITY_KXZ4903	hypothet	Gonium_pectorale	85.70	0.00
TRINITY_KXZ4630	hypothet	Gonium_pectorale	85.70	0.00
TRINITY_GBF9551	60S rib	Raphidocelis_subcap	85.70	0.00
TRINITY_XP_0029	phospho	Volvox_carteri	85.70	0.00
TRINITY_GBF9876	chaperon	Raphidocelis_subcap	85.70	0.00
TRINITY_XP_0016	flagella	Chlamydomonas_reinh	85.70	0.00
TRINITY_XP_0139	Stromal	Monoraphidium_negle	85.70	0.00
TRINITY_XP_0029	hypothet	Volvox_carteri	85.70	0.00
TRINITY_GBF9082	ADP,ATP	Raphidocelis_subcap	85.60	0.00
TRINITY_GAX7740	hypothet	Chlamydomonas_eusti	85.60	0.00
TRINITY_GAX8352	hypothet	Chlamydomonas_eusti	85.60	0.00
TRINITY_CAA7399	cytochro	Chlorogonium_elonga	85.50	0.00
TRINITY_XP_0016	predicte	Chlamydomonas_reinh	85.50	0.00
TRINITY_GAX7958	hypothet	Chlamydomonas_eusti	85.50	0.00
TRINITY_AFK3339	unknown	Lotus_japonicus	85.50	0.00
TRINITY_AFK3339	unknown	Lotus_japonicus	85.50	0.00
TRINITY_XP_0138	hypothet	Monoraphidium_negle	85.50	0.00
TRINITY_XP_0029	hypothet	Volvox_carteri	85.50	0.00
TRINITY_KXZ4685	hypothet	Gonium_pectorale	85.50	0.00
TRINITY_XP_0017	PP2A-tw	Chlamydomonas_reinh	85.50	0.00
TRINITY_XP_0243	T-comple	Physcomitrella_pate	85.40	0.00
TRINITY_EPS6896	hypothet	Genlisea_aurea	85.40	0.00
TRINITY_XP_0238	elongat	Quercus_suber	85.40	0.00
TRINITY_XP_0016	dynein	Chlamydomonas_reinh	85.40	0.00
TRINITY_AUI4110	LhcsR [	Haematococcus_lacus	85.40	0.00
TRINITY_GAX7354	hypothet	Chlamydomonas_eusti	85.40	0.00
TRINITY_XP_0029	mitochon	Volvox_carteri	85.40	0.00
TRINITY_PSC7038	SNF1-rel	Micractinium_conduc	85.40	0.00
TRINITY_GAX7586	hypothet	Chlamydomonas_eusti	85.40	0.00
TRINITY_AFK3971	unknown	Lotus_japonicus	85.30	0.00
TRINITY_XP_0191	PREDICTI	Ipomoea_nil	85.30	0.00
TRINITY_OMO5998	Ribosoma	Corchorus_capsulari	85.30	0.00
TRINITY_GBF9344	ribosoma	Raphidocelis_subcap	85.30	0.00
TRINITY_XP_0155	LOW QUAI	Ricinus_communis	85.30	0.00
TRINITY_XP_0238	serine/t	Quercus_suber	85.30	0.00
TRINITY_PSC7440	Diphthar	Micractinium_conduc	85.30	0.00
TRINITY_GAX8214	hypothet	Chlamydomonas_eusti	85.30	0.00
TRINITY_PIA3182	hypothet	Aquilegia_coerulea	85.30	0.00
TRINITY_PNH0366	Dynein	Tetrabaena_socialis	85.30	0.00
TRINITY_KCW4501	hypothet	Eucalyptus_grandis	85.20	0.00
TRINITY_GAQ8881	putaive	Klebsormidium_niten	85.20	0.00
TRINITY_XP_0227	ADP,ATP	Durio_zibethinus	85.20	0.00
TRINITY_PNH1242	hypothet	Tetrabaena_socialis	85.20	0.00
TRINITY_GAX7263	hypothet	Chlamydomonas_eusti	85.20	0.00
TRINITY_XP_0029	chaperon	Volvox_carteri	85.20	0.00
TRINITY_XP_0139	guanylat	Monoraphidium_negle	85.20	0.00
TRINITY_PRW5705	succinat	Chlorella_sorokinia	85.20	0.00
TRINITY_GAX7709	hypothet	Chlamydomonas_eusti	85.20	0.00
TRINITY_XP_0239	ubiquit	Quercus_suber	85.10	0.00
TRINITY_PSS0610	Serine/t	Actinidia_chinensis	85.10	0.00
TRINITY_KXZ5391	hypothet	Gonium_pectorale	85.10	0.00

TRINITY_BAJ9714(	predicte	Hordeum_vulgare	85.10	0.00
TRINITY_XP_0029!	hypothet	Volvox_carteri	85.10	0.00
TRINITY_ABP5742!	stearoyl	Haematococcus_lacus	85.10	0.00
TRINITY_P22686.]	RecName:	Chlamydomonas_moewu	85.10	0.00
TRINITY_XP_0016!	adenine	Chlamydomonas_reinh	85.00	0.00
TRINITY_XP_0191{	PREDICTI	Ipomoea_nil	85.00	0.00
TRINITY_XP_0029!	hypothet	Volvox_carteri	85.00	0.00
TRINITY_XP_0017(	arsenite	Chlamydomonas_reinh	85.00	0.00
TRINITY_XP_0029!	20S prot	Volvox_carteri	85.00	0.00
TRINITY_OAY8554!	ABC tran	Ananas_comosus	85.00	0.00
TRINITY_GAX7840(	hypothet	Chlamydomonas_eusti	85.00	0.00
TRINITY_XP_0029!	hypothet	Volvox_carteri	85.00	0.00
TRINITY_XP_0202(	thioredo	Cajanus_cajan	85.00	0.00
TRINITY_GBG6244	hypothet	Chara_braunii	84.90	0.00
TRINITY_KXZ5243(	hypothet	Gonium_pectorale	84.90	0.00
TRINITY_XP_0243{	serine/t	Physcomitrella_pate	84.90	0.00
TRINITY_KXZ4681(	hypothet	Gonium_pectorale	84.90	0.00
TRINITY_XP_0025(	predicte	Micromonas_commoda	84.80	0.00
TRINITY_RWR9034	stromal	Cinnamomum_micranth	84.80	0.00
TRINITY_KQK0586	hypothet	Brachypodium_distac	84.80	0.00
TRINITY_XP_0138!	40S ribo	Monoraphidium_negle	84.80	0.00
TRINITY_PNH0830	NFX1-typ	Tetrabaena_socialis	84.80	0.00
TRINITY_GAX7701	hypothet	Chlamydomonas_eusti	84.80	0.00
TRINITY_ABY6196(	chloropl	Dunaliella_salina	84.80	0.00
TRINITY_KXZ5310!	hypothet	Gonium_pectorale	84.80	0.00
TRINITY_GAX8386!	hypothet	Chlamydomonas_eusti	84.80	0.00
TRINITY_GAX8607	hypothet	Chlamydomonas_eusti	84.80	0.00
TRINITY_XP_0124	PREDICTI	Gossypium_raimondii	84.80	0.00
TRINITY_XP_0029!	flagella	Volvox_carteri	84.80	0.00
TRINITY_XP_0084!	PREDICTI	Cucumis_melo	84.70	0.00
TRINITY_XP_0201{	26S prot	Aegilops_tauschii	84.70	0.00
TRINITY_GAX8322(	hypothet	Chlamydomonas_eusti	84.70	0.00
TRINITY_AGZ1935	hypothet	Chlorella_sp._ArM00	84.70	0.00
TRINITY_GAX8316	hypothet	Chlamydomonas_eusti	84.70	0.00
TRINITY_XP_0016!	ribosoma	Chlamydomonas_reinh	84.70	0.00
TRINITY_GAX8105(	hypothet	Chlamydomonas_eusti	84.70	0.00
TRINITY_XP_0124(	PREDICTI	Gossypium_raimondii	84.70	0.00
TRINITY_XP_0016!	predicte	Chlamydomonas_reinh	84.70	0.00
TRINITY_GAQ8103	40S ribo	Klebsormidium_niten	84.60	0.00
TRINITY_KJB6925	hypothet	Gossypium_raimondii	84.60	0.00
TRINITY_GAX7925	hypothet	Chlamydomonas_eusti	84.60	0.00
TRINITY_XP_0016!	predicte	Chlamydomonas_reinh	84.60	0.00
TRINITY_XP_0191	PREDICTI	Ipomoea_nil	84.60	0.00
TRINITY_ABD3790{	light-ha	Chlamydomonas_incer	84.60	0.00
TRINITY_GAX7665!	hypothet	Chlamydomonas_eusti	84.60	0.00
TRINITY_XP_0205!	polyubiq	Phalaenopsis_equest	84.60	0.00
TRINITY_GAX8162!	hypothet	Chlamydomonas_eusti	84.60	0.00
TRINITY_ALL5522	NER endo	Chlamydomonas_reinh	84.60	0.00
TRINITY_KXZ4855	hypothet	Gonium_pectorale	84.60	0.00
TRINITY_XP_0239	ubiquiti	Quercus_suber	84.50	0.00
TRINITY_GAX7418{	hypothet	Chlamydomonas_eusti	84.50	0.00
TRINITY_XP_0139(	sec-inde	Monoraphidium_negle	84.50	0.00
TRINITY_KXZ5273	hypothet	Gonium_pectorale	84.50	0.00
TRINITY_KDP2046	hypothet	Jatropha_curcas	84.50	0.00
TRINITY_GAX8267(	hypothet	Chlamydomonas_eusti	84.50	0.00
TRINITY_PNH0209(	Sulfite	Tetrabaena_socialis	84.50	0.00
TRINITY_PNW7725	hypothet	Chlamydomonas_reinh	84.50	0.00
TRINITY_XP_0029!	hypothet	Volvox_carteri	84.50	0.00
TRINITY_XP_0029!	hypothet	Volvox_carteri	84.40	0.00
TRINITY_GAX8326	hypothet	Chlamydomonas_eusti	84.40	0.00
TRINITY_BAF3199	putative	Cryptomeria_japonic	84.40	0.00
TRINITY_XP_0016!	isomeras	Chlamydomonas_reinh	84.40	0.00
TRINITY_KXZ4661	hypothet	Gonium_pectorale	84.40	0.00
TRINITY_GBF9986!	hypothet	Raphidocelis_subcap	84.40	0.00
TRINITY_KXZ5601!	hypothet	Gonium_pectorale	84.40	0.00
TRINITY_KXZ4982	hypothet	Gonium_pectorale	84.40	0.00

TRINITY_XP_0029	hypothet	Volvox_carteri	84.40	0.00
TRINITY_XP_0016	predicte	Chlamydomonas_reinh	84.40	0.00
TRINITY_GAX7817	hypothet	Chlamydomonas_eusti	84.40	0.00
TRINITY_GAX7533	hypothet	Chlamydomonas_eusti	84.40	0.00
TRINITY_GAX7630	hypothet	Chlamydomonas_eusti	84.40	0
TRINITY_GAX7376	hypothet	Chlamydomonas_eusti	84.30	0.00
TRINITY_PNH0797	Magnesiu	Tetrabaena_socialis	84.30	0.00
TRINITY_ACN3155	unknown	Zea_mays	84.30	0.00
TRINITY_XP_0138	SufE-lik	Monoraphidium_negle	84.30	0.00
TRINITY_GAX7487	hypothet	Chlamydomonas_eusti	84.30	0.00
TRINITY_KMS6478	hypothet	Beta_vulgaris	84.20	0.00
TRINITY_PHT2871	hypothet	Capsicum_baccatum	84.20	0.00
TRINITY_PRW6026	Serine t	Chlorella_sorokinia	84.20	0.00
TRINITY_PNW8831	hypothet	Chlamydomonas_reinh	84.20	0.00
TRINITY_GAX8459	hypothet	Chlamydomonas_eusti	84.20	0.00
TRINITY_KXZ5043	VAM1/VAM	Gonium_pectorale	84.20	0.00
TRINITY_GAX8545	hypothet	Chlamydomonas_eusti	84.20	0.00
TRINITY_PNH0960	Frataxin	Tetrabaena_socialis	84.20	0.00
TRINITY_PNH0979	hypothet	Tetrabaena_socialis	84.20	0.00
TRINITY_GAX7423	hypothet	Chlamydomonas_eusti	84.20	0.00
TRINITY_PNH0567	Ubiquiti	Tetrabaena_socialis	84.10	0.00
TRINITY_XP_0155	ADP-ribo	Ricinus_communis	84.10	0.00
TRINITY_GAX8325	hypothet	Chlamydomonas_eusti	84.10	0.00
TRINITY_KXZ4194	hypothet	Gonium_pectorale	84.10	0.00
TRINITY_AAW2885	phytoene	Haematococcus_lacus	84.10	0.00
TRINITY_PNW8648	hypothet	Chlamydomonas_reinh	84.10	0.00
TRINITY_ADN3391	metallot	Cucumis_melo	84.00	0.00
TRINITY_PRW2081	Receptor	Chlorella_sorokinia	84.00	0.00
TRINITY_XP_0030	predicte	Micromonas_pusilla	84.00	0.00
TRINITY_XP_0238	transcri	Quercus_suber	84.00	0.00
TRINITY_GAX8054	hypothet	Chlamydomonas_eusti	84.00	0.00
TRINITY_XP_0029	hypothet	Volvox_carteri	84.00	0.00
TRINITY_KXZ4373	hypothet	Gonium_pectorale	84.00	0.00
TRINITY_AEU2929	ribulose	uncultured_Streptop	83.90	0.00
TRINITY_XP_0029	componen	Volvox_carteri	83.90	0.00
TRINITY_ACJ2424	RPN10 [D	Dunaliella_viridis	83.90	0.00
TRINITY_XP_0016	mitochon	Chlamydomonas_reinh	83.90	0.00
TRINITY_GBF9055	gamma tu	Raphidocelis_subcap	83.90	0.00
TRINITY_KXZ5290	hypothet	Gonium_pectorale	83.90	0.00
TRINITY_XP_0120	polyubiq	Jatropha_curcas	83.90	0.00
TRINITY_BAK0054	predicte	Hordeum_vulgare	83.90	0.00
TRINITY_XP_0016	calretic	Chlamydomonas_reinh	83.80	0.00
TRINITY_XP_0029	hypothet	Volvox_carteri	83.80	0.00
TRINITY_XP_0016	selenopr	Chlamydomonas_reinh	83.80	0.00
TRINITY_XP_0029	hypothet	Volvox_carteri	83.80	0.00
TRINITY_XP_0017	nuclear	Chlamydomonas_reinh	83.80	0.00
TRINITY_OAE2224	hypothet	Marchantia_polymorp	83.80	0.00
TRINITY_OAY5161	hypothet	Manihot_esculenta	83.80	0.00
TRINITY_GAX8322	hypothet	Chlamydomonas_eusti	83.80	0.00
TRINITY_GAX8138	hypothet	Chlamydomonas_eusti	83.80	0
TRINITY_KMS6493	hypothet	Beta_vulgaris	83.70	0.00
TRINITY_BAJ1178	dehydrat	Corchorus_olitorius	83.70	0.00
TRINITY_XP_0120	26S prot	Jatropha_curcas	83.70	0.00
TRINITY_XP_0212	histone	Herrania_umbratica	83.70	0.00
TRINITY_XP_0058	hypothet	Chlorella_variabili	83.70	0.00
TRINITY_AAT7272	putative	Cucurbita_maxima	83.60	0.00
TRINITY_XP_0228	acyl-CoA	Olea_europaea	83.60	0.00
TRINITY_RVW1687	NADH deh	Vitis_vinifera	83.60	0.00
TRINITY_XP_0194	PREDICTE	Lupinus_angustifoli	83.60	0.00
TRINITY_GAX7651	hypothet	Chlamydomonas_eusti	83.60	0.00
TRINITY_GAX8642	hypothet	Chlamydomonas_eusti	83.60	0.00
TRINITY_XP_0029	hypothet	Volvox_carteri	83.60	0.00
TRINITY_XP_0029	target o	Volvox_carteri	83.60	0
TRINITY_XP_0016	protein	Chlamydomonas_reinh	83.60	0.00
TRINITY_GAX8040	hypothet	Chlamydomonas_eusti	83.60	0.00
TRINITY_XP_0139	RING-box	Monoraphidium_negle	83.60	0.00

TRINITY_XP_0016‹	flagella	Chlamydomonas_reinh	83.60	0
TRINITY_XP_0155‹	LOW QUAI	Ricinus_communis	83.60	0.00
TRINITY_GAX7722‹	hypothet	Chlamydomonas_eusti	83.50	0.00
TRINITY_XP_0016‹	ketopant	Chlamydomonas_reinh	83.50	0.00
TRINITY_XP_0016‹	EMP/nona	Chlamydomonas_reinh	83.50	0.00
TRINITY_KXZ5541‹	hypothet	Gonium_pectorale	83.50	0.00
TRINITY_KXZ5563‹	hypothet	Gonium_pectorale	83.40	0.00
TRINITY_ALM5498‹	DEAD box	Chlamydomonas_sp._I	83.40	0.00
TRINITY_KXZ4490‹	hypothet	Gonium_pectorale	83.40	0.00
TRINITY_XP_0016‹	cytokine	Chlamydomonas_reinh	83.40	0.00
TRINITY_KXZ4956‹	hypothet	Gonium_pectorale	83.40	0.00
TRINITY_XP_0172‹	PREDICTI	Daucus_carota	83.30	0.00
TRINITY_PIA3575‹	hypothet	Aquilegia_coerulea	83.30	0.00
TRINITY_KXZ4925‹	hypothet	Gonium_pectorale	83.30	0.00
TRINITY_XP_0056‹	N-termin	Coccomyxa_subellips	83.30	0.00
TRINITY_KXZ5456‹	hypothet	Gonium_pectorale	83.30	0.00
TRINITY_GAQ7888‹	proteaso	Klebsormidium_niten	83.30	0.00
TRINITY_XP_0056‹	hypothet	Coccomyxa_subellips	83.30	0.00
TRINITY_GAX8509‹	hypothet	Chlamydomonas_eusti	83.30	0.00
TRINITY_XP_0016‹	inactive	Chlamydomonas_reinh	83.30	0.00
TRINITY_PWA4079‹	histone	Artemisia_annua	83.30	0.00
TRINITY_KXZ4635‹	hypothet	Gonium_pectorale	83.30	0.00
TRINITY_KXZ5310‹	hypothet	Gonium_pectorale	83.30	0.00
TRINITY_GBF8904‹	metal AI	Raphidocelis_subcap	83.30	0.00
TRINITY_GAX8025‹	hypothet	Chlamydomonas_eusti	83.30	0.00
TRINITY_KXZ4995‹	hypothet	Gonium_pectorale	83.30	0.00
TRINITY_GAX8510‹	hypothet	Chlamydomonas_eusti	83.30	0
TRINITY_XP_0093‹	PREDICTI	Pyrus_x_bretschneid	83.30	0.00
TRINITY_PKA6189‹	Heat sho	Apostasia_shenzheni	83.20	0.00
TRINITY_XP_0228‹	Structur	Ostreococcus_tauri	83.20	0.00
TRINITY_ABK4147‹	catalase	Haematococcus_lacus	83.20	0.00
TRINITY_GAX7429‹	hypothet	Chlamydomonas_eusti	83.20	0.00
TRINITY_XP_0029‹	magnesiu	Volvox_carteri	83.20	0
TRINITY_AAX4399‹	UDP-gluc	Dunaliella_salina	83.20	0.00
TRINITY_GAX7502‹	hypothet	Chlamydomonas_eusti	83.20	0.00
TRINITY_GAX8138‹	hypothet	Chlamydomonas_eusti	83.20	0
TRINITY_PNH0566‹	40S ribo	Tetrabaena_socialis	83.10	0.00
TRINITY_KXZ5106‹	hypothet	Gonium_pectorale	83.10	0.00
TRINITY_PIN0005‹	Molecula	Handroanthus_impeti	83.10	0.00
TRINITY_GAX7349‹	hypothet	Chlamydomonas_eusti	83.10	0.00
TRINITY_RMZ5358‹	hypothet	Auxenochlorella_pro	83.10	0.00
TRINITY_GBF8884‹	RNA-binc	Raphidocelis_subcap	83.10	0.00
TRINITY_GAQ9031‹	putative	Klebsormidium_niten	83.10	0.00
TRINITY_GAX8658‹	hypothet	Chlamydomonas_eusti	83.10	0.00
TRINITY_XP_0016‹	predicte	Chlamydomonas_reinh	83.10	0.00
TRINITY_ACG4404‹	60S ribo	Zea_mays	83.10	0.00
TRINITY_XP_0228‹	Ribosoma	Ostreococcus_tauri	83.00	0.00
TRINITY_XP_0228‹	Ribosoma	Ostreococcus_tauri	83.00	0.00
TRINITY_KXZ4476‹	hypothet	Gonium_pectorale	83.00	0.00
TRINITY_XP_0029‹	hypothet	Volvox_carteri	83.00	0.00
TRINITY_GAX8429‹	hypothet	Chlamydomonas_eusti	83.00	0.00
TRINITY_XP_0056‹	long-cha	Coccomyxa_subellips	83.00	0.00
TRINITY_PNT5216‹	hypothet	Populus_trichocarpa	83.00	0.00
TRINITY_GAX8247‹	hypothet	Chlamydomonas_eusti	83.00	0.00
TRINITY_XP_0094‹	PREDICTI	Musa_acuminata	83.00	0.00
TRINITY_KDD7707‹	hypothet	Helicosporidium_sp.	83.00	0.00
TRINITY_KXZ5241‹	hypothet	Gonium_pectorale	83.00	0.00
TRINITY_XP_0025‹	dynein l	Micromonas_commoda	83.00	0.00
TRINITY_XP_0025‹	dynein l	Micromonas_commoda	82.90	0.00
TRINITY_PRW5700‹	3-ketoac	Chlorella_sorokinia	82.90	0.00
TRINITY_PNW7366‹	hypothet	Chlamydomonas_reinh	82.90	0.00
TRINITY_GAX7828‹	hypothet	Chlamydomonas_eusti	82.90	0.00
TRINITY_GAX7777‹	hypothet	Chlamydomonas_eusti	82.90	0.00
TRINITY_VDD2476‹	unnamed	Brassica_rapa	82.90	0.00
TRINITY_XP_0016‹	mitogen-	Chlamydomonas_reinh	82.90	0.00
TRINITY_GAX8181‹	hypothet	Chlamydomonas_eusti	82.90	0.00

TRINITY_BAU3704(	choline-	Chlamydomonas_asymm	82.90	0.00
TRINITY_XP_0100;	PREDICTE	Eucalyptus_grandis	82.90	0.00
TRINITY_GAX8510(	hypothet	Chlamydomonas_eusti	82.90	0
TRINITY_XP_0109(	PREDICTE	Elaeis_guineensis	82.80	0.00
TRINITY_XP_0029!	hypothet	Volvox_carteri	82.80	0.00
TRINITY_GAX7556(	hypothet	Chlamydomonas_eusti	82.80	0.00
TRINITY_XP_0016(	ribose-p	Chlamydomonas_reinh	82.80	0.00
TRINITY_GAX7790(	hypothet	Chlamydomonas_eusti	82.80	0.00
TRINITY_GAX7711:	hypothet	Chlamydomonas_eusti	82.80	0.00
TRINITY_GAX8038(	hypothet	Chlamydomonas_eusti	82.80	0.00
TRINITY_GAX7834:	hypothet	Chlamydomonas_eusti	82.80	0.00
TRINITY_GAX7918'	hypothet	Chlamydomonas_eusti	82.80	0.00
TRINITY_PNW8004:	hypothet	Chlamydomonas_reinh	82.80	0.00
TRINITY_GAX8490(	hypothet	Chlamydomonas_eusti	82.80	0
TRINITY_ACL5390;	unknown	Zea_mays	82.70	0.00
TRINITY_BAJ9531!	predicte	Hordeum_vulgare	82.70	0.00
TRINITY_GAX7812'	hypothet	Chlamydomonas_eusti	82.70	0.00
TRINITY_KXZ5147(	hypothet	Gonium_pectorale	82.70	0.00
TRINITY_XP_0056(	ribosoma	Coccomyxa_subellips	82.70	0.00
TRINITY_PNW8777(	hypothet	Chlamydomonas_reinh	82.70	0.00
TRINITY_XP_0029!	hypothet	Volvox_carteri	82.70	0.00
TRINITY_XP_0191!	PREDICTE	Ipomoea_nil	82.70	0.00
TRINITY_GAX7992(	hypothet	Chlamydomonas_eusti	82.60	0.00
TRINITY_XP_0017(	PP2A-twi	Chlamydomonas_reinh	82.60	0.00
TRINITY_GAX8004(	hypothet	Chlamydomonas_eusti	82.60	0.00
TRINITY_GAQ9251'	F0F1-typ	Klebsormidium_niten	82.50	0.00
TRINITY_XP_0097(	PREDICTE	Nicotiana_sylvestri	82.50	0.00
TRINITY_PNH1275(	60S ribo	Tetrabaena_socialis	82.50	0.00
TRINITY_XP_0139(	Kinesin-	Monoraphidium_negle	82.50	0.00
TRINITY_XP_0029(	hypothet	Volvox_carteri	82.50	0.00
TRINITY_KXZ4196(	hypothet	Gonium_pectorale	82.50	0.00
TRINITY_GAQ7859(	Rab fami	Klebsormidium_niten	82.50	0.00
TRINITY_GAX7949(	hypothet	Chlamydomonas_eusti	82.50	0.00
TRINITY_KXZ5239(	hypothet	Gonium_pectorale	82.50	0.00
TRINITY_XP_0029(	hypothet	Volvox_carteri	82.50	0.00
TRINITY_GAX8461(	hypothet	Chlamydomonas_eusti	82.50	0.00
TRINITY_XP_0017(	fructose	Chlamydomonas_reinh	82.50	0.00
TRINITY_GAX7397(	hypothet	Chlamydomonas_eusti	82.50	0.00
TRINITY_AOO8541(	PII sign	Haematococcus_lacus	82.40	0.00
TRINITY_XP_0016(	26S prot	Chlamydomonas_reinh	82.40	0.00
TRINITY_XP_0029!	hypothet	Volvox_carteri	82.40	0.00
TRINITY_XP_0017(	predicte	Chlamydomonas_reinh	82.40	0.00
TRINITY_GAX8098:	hypothet	Chlamydomonas_eusti	82.40	0.00
TRINITY_PNH1269:	Translat	Tetrabaena_socialis	82.40	0.00
TRINITY_PNH0354(	Mitogen-	Tetrabaena_socialis	82.40	0.00
TRINITY_GAX7306(	hypothet	Chlamydomonas_eusti	82.40	0.00
TRINITY_XP_0029(	hypothet	Volvox_carteri	82.30	0
TRINITY_GAX8057:	hypothet	Chlamydomonas_eusti	82.30	0.00
TRINITY_XP_0029!	hypothet	Volvox_carteri	82.30	0.00
TRINITY_XP_0093(	PREDICTE	Pyrus_x_bretschneid	82.20	0.00
TRINITY_XP_0029(	componer	Volvox_carteri	82.20	0.00
TRINITY_XP_0029!	hypothet	Volvox_carteri	82.20	0.00
TRINITY_PTQ3630:	hypothet	Marchantia_polymorp	82.20	0.00
TRINITY_GBF9353;	peptidyl	Raphidocelis_subcap	82.20	0.00
TRINITY_KXZ5129(	hypothet	Gonium_pectorale	82.20	0
TRINITY_GAX7348;	hypothet	Chlamydomonas_eusti	82.20	0.00
TRINITY_KXZ4615(	DHC6 pro	Gonium_pectorale	82.20	0.00
TRINITY_GAX8138:	hypothet	Chlamydomonas_eusti	82.10	0.00
TRINITY_GBF9926:	hypothet	Raphidocelis_subcap	82.10	0.00
TRINITY_XP_0016(	predicte	Chlamydomonas_reinh	82.10	0.00
TRINITY_AFD6228(	mitochon	Chlamydomonas_reinh	82.10	0.00
TRINITY_XP_0264(	probable	Papaver_somniferum	82.10	0.00
TRINITY_GAX8037(	hypothet	Chlamydomonas_eusti	82.10	0.00
TRINITY_GAX7592:	hypothet	Chlamydomonas_eusti	82.10	0.00
TRINITY_PIA2624(	hypothet	Aquilegia_coerulea	82.10	0.00
TRINITY_ESQ4867(	hypothet	Eutrema_salsugineum	82.10	0.00

TRINITY_XP_0029	hypothet	Volvox_carteri	82.10	0.00
TRINITY_XP_0016	aminoac	Chlamydomonas_reinh	82.10	0.00
TRINITY_BAR8817	TAG acc	Chlamydomonas_reinh	82.10	0.00
TRINITY_GAX7798	hypothet	Chlamydomonas_eusti	82.10	0.00
TRINITY_GAX7519	hypothet	Chlamydomonas_eusti	82.10	0.00
TRINITY_AFD6228	mitocho	Chlamydomonas_reinh	82.10	0.00
TRINITY_XP_0056	WD40 re	Coccomyxa_subellips	82.10	0.00
TRINITY_XP_0245	pirin-l	Selaginella_moellen	82.00	0.00
TRINITY_ASZ8517	elongat	Hylocereus_polyrhiz	82.00	0.00
TRINITY_XP_0029	chapero	Volvox_carteri	82.00	0.00
TRINITY_GAX7768	hypothet	Chlamydomonas_eusti	82.00	0.00
TRINITY_GBF9344	ribosoma	Raphidocelis_subcap	82.00	0.00
TRINITY_XP_0243	cyclin-	Physcomitrella_pate	81.90	0.00
TRINITY_PNH0077	putative	Tetrabaena_socialis	81.90	0.00
TRINITY_GAX7363	hypothet	Chlamydomonas_eusti	81.90	0.00
TRINITY_BAJ9159	predicte	Hordeum_vulgare	81.90	0.00
TRINITY_GAX8154	hypothet	Chlamydomonas_eusti	81.90	0.00
TRINITY_XP_0016	flagella	Chlamydomonas_reinh	81.90	0.00
TRINITY_KXZ5188	hypothet	Gonium_pectorale	81.90	0.00
TRINITY_OAE2172	hypothet	Marchantia_polymorp	81.80	0.00
TRINITY_GAX7362	hypothet	Chlamydomonas_eusti	81.80	0.00
TRINITY_PNW8182	hypothet	Chlamydomonas_reinh	81.80	0.00
TRINITY_GAX7423	hypothet	Chlamydomonas_eusti	81.80	0.00
TRINITY_YP_0094	DNA-dir	Haematococcus_lacus	81.80	0.00
TRINITY_XP_0056	light-ha	Coccomyxa_subellips	81.80	0.00
TRINITY_GBF8932	magnesi	Raphidocelis_subcap	81.80	0.00
TRINITY_KXZ4483	hypothet	Gonium_pectorale	81.80	0.00
TRINITY_PNH1086	UDP-ara	Tetrabaena_socialis	81.80	0.00
TRINITY_GAX8546	hypothet	Chlamydomonas_eusti	81.80	0.00
TRINITY_XP_0016	20S pro	Chlamydomonas_reinh	81.70	0.00
TRINITY_XP_0205	polyubi	Phalaenopsis_equest	81.70	0.00
TRINITY_BAK0099	predicte	Hordeum_vulgare	81.70	0.00
TRINITY_GAX7991	hypothet	Chlamydomonas_eusti	81.70	0.00
TRINITY_GAX7305	hypothet	Chlamydomonas_eusti	81.70	0.00
TRINITY_KXZ5698	hypothet	Gonium_pectorale	81.70	0.00
TRINITY_XP_0029	hypothet	Volvox_carteri	81.70	0.00
TRINITY_KXZ5192	hypothet	Gonium_pectorale	81.70	0.00
TRINITY_GAX7874	hypothet	Chlamydomonas_eusti	81.70	0.00
TRINITY_XP_0029	hypothet	Volvox_carteri	81.70	0.00
TRINITY_XP_0016	nucleola	Chlamydomonas_reinh	81.70	0.00
TRINITY_PNW8037	hypothet	Chlamydomonas_reinh	81.70	0.00
TRINITY_XP_0270	sm-like	Coffea_arabica	81.60	0.00
TRINITY_XP_0016	flagella	Chlamydomonas_reinh	81.60	0.00
TRINITY_CAF0412	pyruvate	Chlamydomonas_reinh	81.60	0.00
TRINITY_XP_0016	ribosoma	Chlamydomonas_reinh	81.60	0.00
TRINITY_GAX7397	hypothet	Chlamydomonas_eusti	81.60	0.00
TRINITY_XP_0029	hypothet	Volvox_carteri	81.60	0.00
TRINITY_XP_0150	uncharac	Solanum_pennellii	81.60	0.00
TRINITY_GAX8410	hypothet	Chlamydomonas_eusti	81.60	0.00
TRINITY_PNG9983	hypothet	Tetrabaena_socialis	81.60	0.00
TRINITY_BAK0218	predicte	Hordeum_vulgare	81.50	0.00
TRINITY_AAX4399	UDP-glu	Dunaliella_salina	81.50	0.00
TRINITY_XP_0139	S-formyl	Monoraphidium_negle	81.50	0.00
TRINITY_XP_0029	hypothet	Volvox_carteri	81.50	0.00
TRINITY_XP_0029	hypothet	Volvox_carteri	81.50	0.00
TRINITY_GAX7262	hypothet	Chlamydomonas_eusti	81.50	0.00
TRINITY_KXZ5076	hypothet	Gonium_pectorale	81.50	0.00
TRINITY_AKA9537	heat sh	Bryum_argenteum	81.50	0.00
TRINITY_GAX7287	hypothet	Chlamydomonas_eusti	81.50	0.00
TRINITY_KMS6524	hypothet	Beta_vulgaris	81.50	0.00
TRINITY_XP_0016	Sigma1-	Chlamydomonas_reinh	81.50	0.00
TRINITY_GAQ9141	60S rib	Klebsormidium_niten	81.40	0.00
TRINITY_GAX7647	hypothet	Chlamydomonas_eusti	81.40	0.00
TRINITY_XP_0016	predicte	Chlamydomonas_reinh	81.40	0.00
TRINITY_GAX7777	hypothet	Chlamydomonas_eusti	81.40	0.00
TRINITY_KXZ4585	hypothet	Gonium_pectorale	81.40	0.00

TRINITY_GAX80541	hypothet	Chlamydomonas_eusti	81.40	0.00
TRINITY_GAX72586	hypothet	Chlamydomonas_eusti	81.40	0.00
TRINITY_PNW77846	hypothet	Chlamydomonas_reinh	81.40	0.00
TRINITY_XP_00294	dynein h	Volvox_carteri	81.40	0.00
TRINITY_XP_01165	PREDICTE	Cucumis_sativus	81.30	0.00
TRINITY_GAX77331	hypothet	Chlamydomonas_eusti	81.30	0.00
TRINITY_GAX78454	hypothet	Chlamydomonas_eusti	81.30	0.00
TRINITY_XP_00295	hypothet	Volvox_carteri	81.30	0.00
TRINITY_GAX83295	hypothet	Chlamydomonas_eusti	81.30	0.00
TRINITY_KXZ54647	hypothet	Gonium_pectorale	81.30	0.00
TRINITY_KXZ54401	hypothet	Gonium_pectorale	81.30	0.00
TRINITY_XP_00169	dihydrol	Chlamydomonas_reinh	81.30	0.00
TRINITY_XP_00229	PHD fing	Populus_trichocarpa	81.20	0.00
TRINITY_XP_02388	60S ribo	Quercus_suber	81.20	0.00
TRINITY_XP_00295	flagella	Volvox_carteri	81.20	0.00
TRINITY_PNH09822	Transcri	Tetrabaena_socialis	81.20	0.00
TRINITY_GAX83936	hypothet	Chlamydomonas_eusti	81.20	0.00
TRINITY_XP_00169	eukaryot	Chlamydomonas_reinh	81.20	0.00
TRINITY_KMS93251	hypothet	Beta_vulgaris	81.20	0.00
TRINITY_GBF88910	sodium:p	Raphidocelis_subcap	81.20	0.00
TRINITY_XP_01558	LOW QUAI	Ricinus_communis	81.20	0.00
TRINITY_XP_00170	threonin	Chlamydomonas_reinh	81.20	0.00
TRINITY_XP_00295	hypothet	Volvox_carteri	81.20	0.00
TRINITY_PNW84782	hypothet	Chlamydomonas_reinh	81.20	0.00
TRINITY_XP_00295	ubiquino	Volvox_carteri	81.20	0.00
TRINITY_XP_02437	60S ribo	Physcomitrella_pate	81.10	0.00
TRINITY_XP_00298	serine/t	Selaginella_moellen	81.10	0.00
TRINITY_XP_00295	hypothet	Volvox_carteri	81.10	0.00
TRINITY_KXZ46991	hypothet	Gonium_pectorale	81.10	0.00
TRINITY_GBF88998	plastid	Raphidocelis_subcap	81.10	0.00
TRINITY_XP_00170	predicte	Chlamydomonas_reinh	81.10	0.00
TRINITY_XP_00295	mitochon	Volvox_carteri	81.10	0.00
TRINITY_XP_00306	predicte	Micromonas_pusilla	81.10	0.00
TRINITY_BAJ11784	dehydrat	Corchorus_olitorius	81.10	0.00
TRINITY_Q9SPK6.1	RecName:	Haematococcus_lacus	81.00	0.00
TRINITY_XP_02213	uncharac	Momordica_charantia	81.00	0.00
TRINITY_ASZ85176	elongati	Hylocereus_polyrhiz	81.00	0.00
TRINITY_ACN31551	unknown	Zea_mays	81.00	0.00
TRINITY_ABR01232	heat sho	Chlorella_vulgaris	81.00	0.00
TRINITY_GAX73606	hypothet	Chlamydomonas_eusti	81.00	0.00
TRINITY_XP_00564	histone-	Coccomyxa_subellips	81.00	0.00
TRINITY_XP_00169	dynein h	Chlamydomonas_reinh	81.00	0.00
TRINITY_KXZ49012	DHC3 pro	Gonium_pectorale	81.00	0.00
TRINITY_GAX73078	hypothet	Chlamydomonas_eusti	81.00	0.00
TRINITY_PNH08819	Bifuncti	Tetrabaena_socialis	81.00	0.00
TRINITY_GBF88245	dihydrol	Raphidocelis_subcap	81.00	0.00
TRINITY_GAX83107	hypothet	Chlamydomonas_eusti	81.00	0.00
TRINITY_XP_00296	40S ribo	Selaginella_moellen	81.00	0.00
TRINITY_AML30861	axonemal	Marsilea_vestita	81.00	0.00
TRINITY_OAE34120	hypothet	Marchantia_polymorp	80.90	0.00
TRINITY_BAJ85454	predicte	Hordeum_vulgare	80.90	0.00
TRINITY_BAJ85454	predicte	Hordeum_vulgare	80.90	0.00
TRINITY_GAX80157	hypothet	Chlamydomonas_eusti	80.90	0.00
TRINITY_PNH05527	Chaperon	Tetrabaena_socialis	80.90	0.00
TRINITY_XP_01390	hypothet	Monoraphidium_negle	80.90	0.00
TRINITY_PNH05805	chaperon	Tetrabaena_socialis	80.90	0.00
TRINITY_GAX80657	hypothet	Chlamydomonas_eusti	80.90	0
TRINITY_XP_00169	vacuolar	Chlamydomonas_reinh	80.90	0.00
TRINITY_PNW80223	hypothet	Chlamydomonas_reinh	80.90	0.00
TRINITY_XP_00295	structur	Volvox_carteri	80.90	0.00
TRINITY_GAX73482	hypothet	Chlamydomonas_eusti	80.90	0.00
TRINITY_GAX72561	hypothet	Chlamydomonas_eusti	80.90	0.00
TRINITY_GAX82218	hypothet	Chlamydomonas_eusti	80.80	0.00
TRINITY_KXZ46360	hypothet	Gonium_pectorale	80.80	0.00
TRINITY_XP_00170	asparagi	Chlamydomonas_reinh	80.80	0.00
TRINITY_GAX77700	hypothet	Chlamydomonas_eusti	80.80	0

TRINITY_XP_0029	hypothet	Volvox_carteri	80.80	0.00
TRINITY_GAX8020	hypothet	Chlamydomonas_eusti	80.80	0
TRINITY_YP_0093	translat	Oedocladium_carolin	80.70	0.00
TRINITY_GAQ8159	ATP-depe	Klebsormidium_niten	80.70	0.00
TRINITY_OAY3900	hypothet	Manihot_esculenta	80.70	0.00
TRINITY_PNW8212	hypothet	Chlamydomonas_reinh	80.70	0.00
TRINITY_GAX7946	hypothet	Chlamydomonas_eusti	80.70	0.00
TRINITY_XP_0029	hypothet	Volvox_carteri	80.70	0.00
TRINITY_XP_0239	isocitra	Quercus_suber	80.60	0.00
TRINITY_XP_0270	chloropl	Coffea_arabica	80.60	0.00
TRINITY_BAJ9369	predicte	Hordeum_vulgare	80.60	0.00
TRINITY_GAQ8648	26S prot	Klebsormidium_niten	80.60	0.00
TRINITY_CAA6758	70 kD he	Medicago_sativa	80.60	0.00
TRINITY_XP_0029	hypothet	Volvox_carteri	80.60	0.00
TRINITY_PNH1237	hypothet	Tetrabaena_socialis	80.60	0.00
TRINITY_XP_0139	thioeste	Monoraphidium_negle	80.60	0.00
TRINITY_GBF9099	peptide-	Raphidocelis_subcap	80.60	0.00
TRINITY_XP_0016	glutamat	Chlamydomonas_reinh	80.60	0.00
TRINITY_KXZ5595	hypothet	Gonium_pectorale	80.60	0.00
TRINITY_GAX7514	hypothet	Chlamydomonas_eusti	80.60	0.00
TRINITY_XP_0016	acetyl-C	Chlamydomonas_reinh	80.60	0.00
TRINITY_PNW7163	hypothet	Chlamydomonas_reinh	80.60	0.00
TRINITY_GAX8397	hypothet	Chlamydomonas_eusti	80.60	0.00
TRINITY_XP_0056	ribosoma	Coccomyxa_subellips	80.60	0.00
TRINITY_XP_0104	PREDICTE	Camelina_sativa	80.60	0.00
TRINITY_GAX7938	hypothet	Chlamydomonas_eusti	80.60	0.00
TRINITY_GAX7258	hypothet	Chlamydomonas_eusti	80.60	0.00
TRINITY_PNW7037	hypothet	Chlamydomonas_reinh	80.50	0.00
TRINITY_PHT4683	hypothet	Capsicum_baccatum	80.50	0.00
TRINITY_GAX7533	hypothet	Chlamydomonas_eusti	80.50	0.00
TRINITY_PRW0594	nuclear	Chlorella_sorokinia	80.50	0.00
TRINITY_GAX8583	hypothet	Chlamydomonas_eusti	80.50	0.00
TRINITY_XP_0016	predicte	Chlamydomonas_reinh	80.50	0.00
TRINITY_GAX8371	hypothet	Chlamydomonas_eusti	80.50	0.00
TRINITY_XP_0029	mago nas	Volvox_carteri	80.50	0.00
TRINITY_XP_0017	ribosoma	Chlamydomonas_reinh	80.50	0.00
TRINITY_GAX8386	hypothet	Chlamydomonas_eusti	80.50	0.00
TRINITY_GAX8134	hypothet	Chlamydomonas_eusti	80.50	0.00
TRINITY_XP_0016	N-carbam	Chlamydomonas_reinh	80.50	0.00
TRINITY_GAX7771	hypothet	Chlamydomonas_eusti	80.50	0.00
TRINITY_XP_0056	hypothet	Coccomyxa_subellips	80.50	0.00
TRINITY_RWR9084	peptidyl	Cinnamomum_micranth	80.40	0.00
TRINITY_GBF9064	p-type a	Raphidocelis_subcap	80.40	0.00
TRINITY_XP_0249	40S ribo	Cynara_cardunculus	80.40	0.00
TRINITY_GAX8148	hypothet	Chlamydomonas_eusti	80.40	0.00
TRINITY_KFK2910	hypothet	Arabis_alpina	80.40	0.00
TRINITY_XP_0238	serine/t	Quercus_suber	80.40	0.00
TRINITY_KXZ4993	hypothet	Gonium_pectorale	80.40	0.00
TRINITY_XP_0016	ARF/SAR	Chlamydomonas_reinh	80.40	0.00
TRINITY_GAX7265	hypothet	Chlamydomonas_eusti	80.40	0.00
TRINITY_XP_0029	hypothet	Volvox_carteri	80.40	0.00
TRINITY_GBF9262	hypothet	Raphidocelis_subcap	80.40	0.00
TRINITY_XP_0030	predicte	Micromonas_pusilla	80.40	0.00
TRINITY_GAX7550	hypothet	Chlamydomonas_eusti	80.40	0.00
TRINITY_GAX8588	hypothet	Chlamydomonas_eusti	80.40	0.00
TRINITY_GAX8545	hypothet	Chlamydomonas_eusti	80.40	0.00
TRINITY_GAX7662	hypothet	Chlamydomonas_eusti	80.40	0.00
TRINITY_GAX8306	hypothet	Chlamydomonas_eusti	80.40	0.00
TRINITY_BAK0313	predicte	Hordeum_vulgare	80.30	0.00
TRINITY_ASL2477	mitochon	Allium_cepa	80.30	0.00
TRINITY_ESQ5038	hypothet	Eutrema_salsugineum	80.30	0.00
TRINITY_RVW9125	Serine/t	Vitis_vinifera	80.30	0.00
TRINITY_GAX7768	hypothet	Chlamydomonas_eusti	80.30	0.00
TRINITY_XP_0239	ATP-depe	Quercus_suber	80.30	0.00
TRINITY_KXZ4231	hypothet	Gonium_pectorale	80.30	0.00
TRINITY_XP_0029	hypothet	Volvox_carteri	80.30	0.00

TRINITY_GAX7581(	hypothet	Chlamydomonas_eusti	80.30	0
TRINITY_XP_0016(	aldehyde	Chlamydomonas_reinh	80.30	0.00
TRINITY_GAX7485(	hypothet	Chlamydomonas_eusti	80.30	0.00
TRINITY_GAX8379(	hypothet	Chlamydomonas_eusti	80.30	0.00
TRINITY_KXZ5118	hypothet	Gonium_pectorale	80.30	0.00
TRINITY_ADQ0018	histone	Chlamydomonas_sp._I	80.30	0.00
TRINITY_Q5PU89.1	RecName:	Chlamydomonas_incer	80.20	0.00
TRINITY_ASL2477(	mitochon	Allium_cepa	80.20	0.00
TRINITY_AAX6339	carotene	Haematococcus_lacus	80.20	0.00
TRINITY_XP_0209	uncharac	Arachis_ipaensis	80.20	0.00
TRINITY_KXZ5304(	hypothet	Gonium_pectorale	80.20	0.00
TRINITY_KXZ5576(	hypothet	Gonium_pectorale	80.20	0.00
TRINITY_BAJ9387(	predicte	Hordeum_vulgare	80.20	0.00
TRINITY_GAX8311(	hypothet	Chlamydomonas_eusti	80.20	0.00
TRINITY_AOP1244	RSH [Hae	Haematococcus_lacus	80.20	0.00
TRINITY_KXZ4194	hypothet	Gonium_pectorale	80.20	0.00
TRINITY_GAX7608	hypothet	Chlamydomonas_eusti	80.10	0.00
TRINITY_GAX8504	hypothet	Chlamydomonas_eusti	80.10	0.00
TRINITY_KXZ5471	hypothet	Gonium_pectorale	80.10	0.00
TRINITY_KXZ4274	hypothet	Gonium_pectorale	80.10	0.00
TRINITY_GAX7597	hypothet	Chlamydomonas_eusti	80.10	0.00
TRINITY_PNW8180	hypothet	Chlamydomonas_reinh	80.10	0.00
TRINITY_XP_0245	eukaryot	Selaginella_moellen	80.00	0.00
TRINITY_XP_0238	aldehyde	Quercus_suber	80.00	0.00
TRINITY_ABD7451	ribosoma	Pteris_vittata	80.00	0.00
TRINITY_AAK9564	proteina	Ipomoea_batatas	80.00	0.00
TRINITY_KXZ5636	hypothet	Gonium_pectorale	80.00	0.00
TRINITY_PNW7560	hypothet	Chlamydomonas_reinh	80.00	0.00
TRINITY_GAX8276	hypothet	Chlamydomonas_eusti	80.00	0.00
TRINITY_KXZ5171	hypothet	Gonium_pectorale	80.00	0.00
TRINITY_PRW5657	ADP-ribo	Chlorella_sorokinia	80.00	0.00
TRINITY_XP_0029	hypothet	Volvox_carteri	80.00	0.00
TRINITY_PNH1171	putative	Tetrabaena_socialis	80.00	0.00
TRINITY_GAX8637	hypothet	Chlamydomonas_eusti	80.00	0.00
TRINITY_XP_0029	hypothet	Volvox_carteri	80.00	0.00
TRINITY_XP_0029	1,4-alph	Volvox_carteri	80.00	0.00
TRINITY_AAZ1570	beta-car	Haematococcus_lacus	80.00	0.00
TRINITY_ALO2158	beta'' s	Haematococcus_lacus	80.00	0.00
TRINITY_PNH0382	COBW dom	Tetrabaena_socialis	80.00	0.00
TRINITY_GAX7877	hypothet	Chlamydomonas_eusti	80.00	0.00
TRINITY_XP_0029	chaperon	Volvox_carteri	80.00	0.00
TRINITY_GBF9054	hypothet	Raphidocelis_subcap	80.00	0.00
TRINITY_AIU4988	FMN-link	Acorus_calamus	80.00	0.00
TRINITY_XP_0029	hypothet	Volvox_carteri	80.00	0.00
TRINITY_XP_0058	hypothet	Chlorella_variabili	80.00	0.00
TRINITY_GAX7473	hypothet	Chlamydomonas_eusti	80.00	0
TRINITY_XP_0056	HAD-supe	Coccomyxa_subellips	80.00	0.00
TRINITY_XP_0139	GTP-bind	Monoraphidium_negle	80.00	0.00
TRINITY_XP_0029	hypothet	Volvox_carteri	79.90	0.00
TRINITY_XP_0025	predicte	Micromonas_commoda	79.90	0.00
TRINITY_XP_0016	UDP-gluc	Chlamydomonas_reinh	79.90	0.00
TRINITY_PNW7920	hypothet	Chlamydomonas_reinh	79.90	0.00
TRINITY_OWM7171	hypothet	Punica_granatum	79.90	0.00
TRINITY_XP_0138	prolyl-t	Monoraphidium_negle	79.90	0.00
TRINITY_XP_0030	predicte	Micromonas_pusilla	79.80	0.00
TRINITY_EFJ1119	hypothet	Selaginella_moellen	79.80	0.00
TRINITY_GAX8086	hypothet	Chlamydomonas_eusti	79.80	0.00
TRINITY_GAX7282	hypothet	Chlamydomonas_eusti	79.80	0.00
TRINITY_KXZ5140	hypothet	Gonium_pectorale	79.80	0.00
TRINITY_KXZ5567	hypothet	Gonium_pectorale	79.80	0.00
TRINITY_GAX7593	hypothet	Chlamydomonas_eusti	79.80	0.00
TRINITY_GAX8134	hypothet	Chlamydomonas_eusti	79.80	0.00
TRINITY_KXZ4575	hypothet	Gonium_pectorale	79.80	0.00
TRINITY_ABK2383	unknown	Picea_sitchensis	79.80	0.00
TRINITY_XP_0238	serine/t	Quercus_suber	79.80	0.00
TRINITY_AXF4155	CCP430 p	Chlorella_sp._ArM00	79.80	0.00

TRINITY_PNW7226(	hypothet	Chlamydomonas_reinh	79.80	0.00
TRINITY_KXZ5101	hypothet	Gonium_pectorale	79.80	0.00
TRINITY_XP_0029	chlorop	Volvox_carteri	79.80	0.00
TRINITY_GBG7007(	hypothet	Chara_braunii	79.80	0.00
TRINITY_ABA0110	mitochor	Chlamydomonas_incer	79.80	0.00
TRINITY_EES0224	hypothet	Sorghum_bicolor	79.70	0.00
TRINITY_EPS6218	hypothet	Genlisea_aurea	79.70	0.00
TRINITY_AIZ7433	eukaryot	Pinus_massoniana	79.70	0.00
TRINITY_GAX7572	hypothet	Chlamydomonas_eusti	79.70	0.00
TRINITY_GAX7698	hypothet	Chlamydomonas_eusti	79.70	0.00
TRINITY_KXZ4897(	hypothet	Gonium_pectorale	79.70	0.00
TRINITY_XP_0029	hypothet	Volvox_carteri	79.70	0.00
TRINITY_KXZ5546	hypothet	Gonium_pectorale	79.70	0.00
TRINITY_XP_0086	pre-mRN	Zea_mays	79.70	0
TRINITY_YP_0013	NADH del	Chlorokybus_atmophy	79.60	0.00
TRINITY_KMS6481(	hypothet	Beta_vulgaris	79.60	0.00
TRINITY_XP_0016	malate (	Chlamydomonas_reinh	79.60	0.00
TRINITY_OAE3144	hypothet	Marchantia_polymorp	79.60	0.00
TRINITY_KXZ4840(	hypothet	Gonium_pectorale	79.60	0.00
TRINITY_GAX7885	hypothet	Chlamydomonas_eusti	79.60	0.00
TRINITY_GBF9474	30S rib	Raphidocelis_subcap	79.60	0.00
TRINITY_GAX8301	hypothet	Chlamydomonas_eusti	79.60	0.00
TRINITY_XP_0029	hypothet	Volvox_carteri	79.60	0.00
TRINITY_GAX7593	hypothet	Chlamydomonas_eusti	79.60	0
TRINITY_GBG7101	hypothet	Chara_braunii	79.60	0.00
TRINITY_GAX8368	hypothet	Chlamydomonas_eusti	79.60	0.00
TRINITY_GAX8644	hypothet	Chlamydomonas_eusti	79.60	0.00
TRINITY_ACG2448	60S rib	Zea_mays	79.50	0.00
TRINITY_PIA3575(	hypothet	Aquilegia_coerulea	79.50	0.00
TRINITY_PIN2459	NADH del	Handroanthus_impeti	79.50	0.00
TRINITY_OAE2389	hypothet	Marchantia_polymorp	79.50	0.00
TRINITY_XP_0029	hypothet	Volvox_carteri	79.50	0.00
TRINITY_PSC7532	low-CO2-	Micractinium_conduc	79.50	0.00
TRINITY_BAJ9566	predicte	Hordeum_vulgare	79.50	0.00
TRINITY_GAX7634	hypothet	Chlamydomonas_eusti	79.50	0.00
TRINITY_GAX7878	hypothet	Chlamydomonas_eusti	79.50	0.00
TRINITY_KXZ4920	hypothet	Gonium_pectorale	79.50	0.00
TRINITY_XP_0029	hypothet	Volvox_carteri	79.50	0.00
TRINITY_KXZ5431	hypothet	Gonium_pectorale	79.50	0.00
TRINITY_PRW3925	SWR1 cor	Chlorella_sorokinia	79.50	0.00
TRINITY_XP_0216	nuclear	Hevea_brasiliensis	79.50	0.00
TRINITY_PIN2459	NADH del	Handroanthus_impeti	79.50	0.00
TRINITY_GAX8269	hypothet	Chlamydomonas_eusti	79.50	0.00
TRINITY_ABS8554	cycloph	Triticum_aestivum	79.50	0.00
TRINITY_KXZ4514	TTL8 pro	Gonium_pectorale	79.50	0.00
TRINITY_XP_0243	60S rib	Physcomitrella_pate	79.50	0.00
TRINITY_RWW9125	hypothet	Ensete_ventricosum	79.40	0.00
TRINITY_PNH1242	putative	Tetrabaena_socialis	79.40	0.00
TRINITY_XP_0243	40S rib	Physcomitrella_pate	79.40	0.00
TRINITY_OAE3483	hypothet	Marchantia_polymorp	79.40	0.00
TRINITY_XP_0029	DEAD-bo	Volvox_carteri	79.40	0.00
TRINITY_GAQ9220	Serine/t	Klebsormidium_niten	79.40	0.00
TRINITY_GAX7943	hypothet	Chlamydomonas_eusti	79.40	0.00
TRINITY_XP_0029	hypothet	Volvox_carteri	79.40	0.00
TRINITY_GAX8275	hypothet	Chlamydomonas_eusti	79.40	0.00
TRINITY_XP_0029	hypothet	Volvox_carteri	79.40	0.00
TRINITY_PNW8739	hypothet	Chlamydomonas_reinh	79.40	0.00
TRINITY_XP_0029	40S rib	Selaginella_moellen	79.40	0.00
TRINITY_XP_0029	40S rib	Selaginella_moellen	79.40	0.00
TRINITY_XP_0094	PREDICT	Musa_acuminata	79.30	0.00
TRINITY_XP_0029	hypothet	Volvox_carteri	79.30	0.00
TRINITY_KVH9279	NADH:ub	Cynara_cardunculus	79.30	0.00
TRINITY_KCW5391	hypothet	Eucalyptus_grandis	79.30	0.00
TRINITY_XP_0029	hypothet	Volvox_carteri	79.30	0.00
TRINITY_GAX8565	hypothet	Chlamydomonas_eusti	79.30	0.00
TRINITY_KXZ5680	METE pro	Gonium_pectorale	79.30	0.00

TRINITY_XP_0139(	Dynein I	Monoraphidium_negle	79.30	0.00
TRINITY_GAX7624{	hypothet	Chlamydomonas_eusti	79.20	0.00
TRINITY_GAX8305!	hypothet	Chlamydomonas_eusti	79.20	0.00
TRINITY_EPS6218!	hypothet	Genlisea_aurea	79.20	0.00
TRINITY_KXZ4792	hypothet	Gonium_pectorale	79.20	0.00
TRINITY_GBF9117	sulfur s	Raphidocelis_subcap	79.20	0.00
TRINITY_XP_0094	PREDICTI	Musa_acuminata	79.20	0.00
TRINITY_XP_0139(	50S ribo	Monoraphidium_negle	79.20	0.00
TRINITY_XP_0016!	predicte	Chlamydomonas_reinh	79.20	0.00
TRINITY_GAX7833(	hypothet	Chlamydomonas_eusti	79.20	0.00
TRINITY_KXZ5614	hypothet	Gonium_pectorale	79.20	0.00
TRINITY_XP_0235	60S ribo	Cucurbita_pepo	79.20	0.00
TRINITY_XP_0235	60S ribo	Cucurbita_pepo	79.20	0.00
TRINITY_GBG7844	hypothet	Chara_braunii	79.20	0.00
TRINITY_GAX8186	hypothet	Chlamydomonas_eusti	79.20	0.00
TRINITY_XP_0017(	GTP binc	Chlamydomonas_reinh	79.20	0.00
TRINITY_XP_0016!	peptidyl	Chlamydomonas_reinh	79.20	0.00
TRINITY_PIA3575(	hypothet	Aquilegia_coerulea	79.10	0.00
TRINITY_BAK0218{	predicte	Hordeum_vulgare	79.10	0
TRINITY_BAJ9566	predicte	Hordeum_vulgare	79.10	0.00
TRINITY_PNH0697	hypothet	Tetrabaena_socialis	79.10	0.00
TRINITY_XP_0029!	hypothet	Volvox_carteri	79.10	0.00
TRINITY_RYR6719	hypothet	Arachis_hypogaea	79.10	0.00
TRINITY_PNW8181(	hypothet	Chlamydomonas_reinh	79.10	0.00
TRINITY_XP_0016!	ribosoma	Chlamydomonas_reinh	79.10	0.00
TRINITY_GAX8007	hypothet	Chlamydomonas_eusti	79.10	0
TRINITY_GAX7360	hypothet	Chlamydomonas_eusti	79.10	0.00
TRINITY_XP_0111(	eukaryot	Sesamum_indicum	79.00	0.00
TRINITY_GAX8322(	hypothet	Chlamydomonas_eusti	79.00	0.00
TRINITY_GAX7839{	hypothet	Chlamydomonas_eusti	79.00	0.00
TRINITY_GAX7303	hypothet	Chlamydomonas_eusti	79.00	0.00
TRINITY_XP_0093	PREDICTI	Pyrus_x_bretschneid	79.00	0.00
TRINITY_XP_0016!	protein	Chlamydomonas_reinh	79.00	0.00
TRINITY_KMS6524!	hypothet	Beta_vulgaris	79.00	0.00
TRINITY_XP_0029	hypothet	Volvox_carteri	78.90	0.00
TRINITY_KXZ4730	hypothet	Gonium_pectorale	78.90	0.00
TRINITY_XP_0110	PREDICTI	Populus_euphratica	78.90	0.00
TRINITY_PNH0727	Threonir	Tetrabaena_socialis	78.90	0.00
TRINITY_GAX8636(	hypothet	Chlamydomonas_eusti	78.90	0.00
TRINITY_XP_0138!	cysteiny	Monoraphidium_negle	78.90	0.00
TRINITY_KXZ4794	hypothet	Gonium_pectorale	78.90	0.00
TRINITY_GAX7385	hypothet	Chlamydomonas_eusti	78.90	0.00
TRINITY_AEL2957!	chloropl	Auxenochlorella_pyr	78.90	0.00
TRINITY_PNH0216	Erythror	Tetrabaena_socialis	78.90	0.00
TRINITY_XP_0016!	flagella	Chlamydomonas_reinh	78.90	0.00
TRINITY_GAX7469	hypothet	Chlamydomonas_eusti	78.90	0.00
TRINITY_GBF9819!	hypothet	Raphidocelis_subcap	78.90	0.00
TRINITY_XP_0029!	hypothet	Volvox_carteri	78.90	0.00
TRINITY_KXZ4211	hypothet	Gonium_pectorale	78.90	0.00
TRINITY_GAX7771	hypothet	Chlamydomonas_eusti	78.90	0.00
TRINITY_KXZ4692	hypothet	Gonium_pectorale	78.90	0.00
TRINITY_GAX8223(	hypothet	Chlamydomonas_eusti	78.80	0.00
TRINITY_PIA3455!	hypothet	Aquilegia_coerulea	78.80	0.00
TRINITY_XP_0191{	PREDICTI	Ipomoea_nil	78.80	0.00
TRINITY_GAX7506	hypothet	Chlamydomonas_eusti	78.80	0.00
TRINITY_KXZ4464	hypothet	Gonium_pectorale	78.80	0.00
TRINITY_PRW3385	peptidyl	Chlorella_sorokinia	78.80	0.00
TRINITY_GAX7547{	hypothet	Chlamydomonas_eusti	78.80	0
TRINITY_YP_0094(	hypothet	Haematococcus_lacus	78.80	0.00
TRINITY_AAL8773{	chloropl	Chlamydomonas_reinh	78.80	0.00
TRINITY_XP_0030!	predicte	Micromonas_pusilla	78.80	0.00
TRINITY_XP_0075	60S ribo	Bathycoccus_prasinc	78.80	0.00
TRINITY_GAX8285	hypothet	Chlamydomonas_eusti	78.80	0.00
TRINITY_KXZ4331	hypothet	Gonium_pectorale	78.80	0.00
TRINITY_XP_0017(	aminotra	Chlamydomonas_reinh	78.80	0.00
TRINITY_XP_0029!	componer	Volvox_carteri	78.70	0.00

TRINITY_ACN3613	unknown	Zea_mays	78.70	0.00
TRINITY_ASL2477	mitochor	Allium_cepa	78.70	0.00
TRINITY_XP_0014	Heat Sh	Ostreococcus_sp._'l	78.70	0.00
TRINITY_XP_0029	hypothet	Volvox_carteri	78.70	0.00
TRINITY_BAJ8510	predicte	Hordeum_vulgare	78.70	0.00
TRINITY_XP_0042	PREDICTE	Fragaria_vesca	78.70	0.00
TRINITY_PNH0455	Ribofla	Tetrabaena_socialis	78.70	0.00
TRINITY_XP_0029	hypothet	Volvox_carteri	78.70	0.00
TRINITY_PNW7931	hypothet	Chlamydomonas_reinh	78.70	0.00
TRINITY_KXZ4385	hypothet	Gonium_pectorale	78.70	0.00
TRINITY_KXZ5591	hypothet	Gonium_pectorale	78.70	0.00
TRINITY_GAX8164	hypothet	Chlamydomonas_eusti	78.70	0.00
TRINITY_GAX8057	hypothet	Chlamydomonas_eusti	78.70	0.00
TRINITY_XP_0016	clp prot	Chlamydomonas_reinh	78.60	0.00
TRINITY_KRH1776	hypothet	Glycine_max	78.60	0.00
TRINITY_BAJ9606	predicte	Hordeum_vulgare	78.60	0.00
TRINITY_GBF9937	3-hydro	Raphidocelis_subcap	78.60	0.00
TRINITY_ABD3790	light-ha	Chlamydomonas_incer	78.60	0.00
TRINITY_GAX7540	hypothet	Chlamydomonas_eusti	78.60	0.00
TRINITY_AQK4420	Protein	Zea_mays	78.60	0.00
TRINITY_XP_0029	hypothet	Volvox_carteri	78.60	0.00
TRINITY_GAX7586	hypothet	Chlamydomonas_eusti	78.60	0.00
TRINITY_XP_0029	hypothet	Volvox_carteri	78.60	0.00
TRINITY_OEL3897	Cell di	Dichanthelium_oligo	78.60	0
TRINITY_XP_0029	hypothet	Volvox_carteri	78.60	0
TRINITY_XP_0029	adenylat	Volvox_carteri	78.60	0.00
TRINITY_XP_0016	oligoen	Chlamydomonas_reinh	78.60	0.00
TRINITY_XP_0025	predicte	Micromonas_commoda	78.60	0.00
TRINITY_GBG7101	hypothet	Chara_braunii	78.60	0.00
TRINITY_XP_0029	hypothet	Volvox_carteri	78.60	0
TRINITY_KXZ4746	hypothet	Gonium_pectorale	78.50	0.00
TRINITY_PNH0875	Carbon	Tetrabaena_socialis	78.50	0.00
TRINITY_KXZ5515	hypothet	Gonium_pectorale	78.50	0.00
TRINITY_XP_0016	succinat	Chlamydomonas_reinh	78.50	0.00
TRINITY_RLM7380	nudix h	Panicum_miliaceum	78.50	0.00
TRINITY_XP_0155	LOW QUAI	Ricinus_communis	78.50	0.00
TRINITY_XP_0029	ATP synt	Volvox_carteri	78.50	0.00
TRINITY_GAX8100	hypothet	Chlamydomonas_eusti	78.50	0.00
TRINITY_AAO8668	long fla	Chlamydomonas_reinh	78.40	0.00
TRINITY_GAX8334	hypothet	Chlamydomonas_eusti	78.40	0.00
TRINITY_GAX7991	hypothet	Chlamydomonas_eusti	78.40	0.00
TRINITY_KMS6524	hypothet	Beta_vulgaris	78.40	0.00
TRINITY_VDC6040	unnamed	Brassica_rapa	78.40	0.00
TRINITY_XP_0138	FK506-b	Monoraphidium_negle	78.40	0.00
TRINITY_GAX7490	hypothet	Chlamydomonas_eusti	78.40	0.00
TRINITY_XP_0029	componer	Volvox_carteri	78.40	0.00
TRINITY_GAX7943	hypothet	Chlamydomonas_eusti	78.40	0.00
TRINITY_XP_0030	predicte	Micromonas_pusilla	78.40	0.00
TRINITY_PNH0657	Succinat	Tetrabaena_socialis	78.40	0.00
TRINITY_GBG0016	hypothet	Raphidocelis_subcap	78.40	0.00
TRINITY_GAX7938	hypothet	Chlamydomonas_eusti	78.40	0.00
TRINITY_GAX8005	hypothet	Chlamydomonas_eusti	78.40	0.00
TRINITY_GAX8407	hypothet	Chlamydomonas_eusti	78.40	0.00
TRINITY_XP_0191	PREDICTE	Ipomoea_nil	78.30	0.00
TRINITY_GAX8575	hypothet	Chlamydomonas_eusti	78.30	0.00
TRINITY_KMS6524	hypothet	Beta_vulgaris	78.30	0.00
TRINITY_Q6QJE2.	RecName:	Chlamydomonas_reinh	78.30	0.00
TRINITY_XP_0017	dynein	Chlamydomonas_reinh	78.30	0.00
TRINITY_GBF9691	hypothet	Raphidocelis_subcap	78.30	0.00
TRINITY_GBF9958	glycera	Raphidocelis_subcap	78.30	0.00
TRINITY_RWR9524	26S prot	Cinnamomum_micranth	78.30	0.00
TRINITY_PSC7357	SRSF ki	Micractinium_conduc	78.30	0.00
TRINITY_XP_0016	mitochor	Chlamydomonas_reinh	78.30	0.00
TRINITY_XP_0030	predicte	Micromonas_pusilla	78.30	0.00
TRINITY_GAX7772	hypothet	Chlamydomonas_eusti	78.30	0.00
TRINITY_XP_0030	central	Micromonas_pusilla	78.30	0.00

TRINITY_GBF89622	ubiquiti	Raphidocelis_subcap	78.30	0.00
TRINITY_GAX74927	hypothet	Chlamydomonas_eusti	78.30	0.00
TRINITY_KMS64936	hypothet	Beta_vulgaris	78.30	0.00
TRINITY_XP_01946	PREDICTE	Lupinus_angustifoli	78.20	0.00
TRINITY_PNW78157	hypothet	Chlamydomonas_reinh	78.20	0.00
TRINITY_GBF93361	hypothet	Raphidocelis_subcap	78.20	0.00
TRINITY_OWM63623	hypothet	Punica_granatum	78.20	0
TRINITY_PNW70220	hypothet	Chlamydomonas_reinh	78.20	0.00
TRINITY_XP_00169	phosphog	Chlamydomonas_reinh	78.20	0.00
TRINITY_GAX72553	hypothet	Chlamydomonas_eusti	78.20	0.00
TRINITY_ADB65765	oligosac	Dunaliella_salina	78.20	0.00
TRINITY_GAX82698	hypothet	Chlamydomonas_eusti	78.20	0.00
TRINITY_GAX81185	hypothet	Chlamydomonas_eusti	78.20	0.00
TRINITY_PNW77006	hypothet	Chlamydomonas_reinh	78.20	0.00
TRINITY_XP_00170	fructose	Chlamydomonas_reinh	78.20	0.00
TRINITY_KXZ47681	hypothet	Gonium_pectorale	78.20	0.00
TRINITY_KXZ53516	hypothet	Gonium_pectorale	78.20	0.00
TRINITY_PNH08514	hypothet	Tetrabaena_socialis	78.20	0.00
TRINITY_XP_01389	Ribulose	Monoraphidium_negle	78.20	0.00
TRINITY_XP_00169	eukaryot	Chlamydomonas_reinh	78.20	0.00
TRINITY_XP_00564	topoisom	Coccomyxa_subellips	78.20	0.00
TRINITY_GAQ92572	catalase	Klebsormidium_niten	78.10	0.00
TRINITY_KMS64810	hypothet	Beta_vulgaris	78.10	0.00
TRINITY_XP_00294	R-SNARE,	Volvox_carteri	78.10	0.00
TRINITY_GAX78066	hypothet	Chlamydomonas_eusti	78.10	0.00
TRINITY_GAX85074	hypothet	Chlamydomonas_eusti	78.10	0.00
TRINITY_XP_00295	hypothet	Volvox_carteri	78.10	0.00
TRINITY_KXZ56723	hypothet	Gonium_pectorale	78.10	0.00
TRINITY_XP_02438	ferritin	Physcomitrella_pate	78.10	0.00
TRINITY_XP_01390	tyramine	Monoraphidium_negle	78.10	0.00
TRINITY_BAJ96199	predicte	Hordeum_vulgare	78.00	0.00
TRINITY_BAJ95967	predicte	Hordeum_vulgare	78.00	0.00
TRINITY_BAJ95967	predicte	Hordeum_vulgare	78.00	0.00
TRINITY_XP_02436	40S ribo	Physcomitrella_pate	78.00	0.00
TRINITY_XP_02436	40S ribo	Physcomitrella_pate	78.00	0.00
TRINITY_XP_00295	hypothet	Volvox_carteri	78.00	0.00
TRINITY_BAJ93795	predicte	Hordeum_vulgare	78.00	0.00
TRINITY_PRW57838	ubiquiti	Chlorella_sorokinia	78.00	0.00
TRINITY_XP_00170	intrafla	Chlamydomonas_reinh	78.00	0.00
TRINITY_GAX76941	hypothet	Chlamydomonas_eusti	78.00	0.00
TRINITY_GAX76713	hypothet	Chlamydomonas_eusti	78.00	0.00
TRINITY_XP_00169	COP9 sig	Chlamydomonas_reinh	78.00	0.00
TRINITY_XP_00170	malate d	Chlamydomonas_reinh	78.00	0.00
TRINITY_XP_00295	target o	Volvox_carteri	78.00	0.00
TRINITY_XP_01915	PREDICTE	Ipomoea_nil	78.00	0.00
TRINITY_GAX83720	hypothet	Chlamydomonas_eusti	78.00	0.00
TRINITY_XP_00169	ser/thr	Chlamydomonas_reinh	78.00	0.00
TRINITY_XP_00294	serine h	Volvox_carteri	78.00	0.00
TRINITY_XP_01107	heat sho	Sesamum_indicum	78.00	0.00
TRINITY_KXZ56136	hypothet	Gonium_pectorale	78.00	0.00
TRINITY_ACN31538	unknown	Zea_mays	77.90	0.00
TRINITY_GAX72816	hypothet	Chlamydomonas_eusti	77.90	0.00
TRINITY_PNW79053	hypothet	Chlamydomonas_reinh	77.90	0.00
TRINITY_GAX84862	hypothet	Chlamydomonas_eusti	77.90	0.00
TRINITY_XP_00169	predicte	Chlamydomonas_reinh	77.90	0.00
TRINITY_XP_00169	predicte	Chlamydomonas_reinh	77.90	0.00
TRINITY_GBF89693	PKHD-typ	Raphidocelis_subcap	77.90	0.00
TRINITY_GAX77355	hypothet	Chlamydomonas_eusti	77.90	0.00
TRINITY_XP_01389	ATP synt	Monoraphidium_negle	77.90	0.00
TRINITY_XP_00169	serine a	Chlamydomonas_reinh	77.90	0.00
TRINITY_KXZ56933	hypothet	Gonium_pectorale	77.90	0.00
TRINITY_XP_01390	Dynein h	Monoraphidium_negle	77.90	0.00
TRINITY_XP_02296	uncharac	Cucurbita_maxima	77.80	0.00
TRINITY_XP_00294	hypothet	Volvox_carteri	77.80	0.00
TRINITY_XP_01390	putative	Monoraphidium_negle	77.80	0.00
TRINITY_KXZ52281	hypothet	Gonium_pectorale	77.80	0.00

TRINITY_ERN0014	hypothet	Amborella_trichopod	77.80	0.00
TRINITY_XP_0030	predicte	Micromonas_pusilla	77.80	0.00
TRINITY_XP_0016	adenosir	Chlamydomonas_reinh	77.80	0.00
TRINITY_KXZ4941	hypothet	Gonium_pectorale	77.80	0.00
TRINITY_KXZ5382	hypothet	Gonium_pectorale	77.80	0.00
TRINITY_GAX7420	hypothet	Chlamydomonas_eusti	77.80	0.00
TRINITY_GAX7747	hypothet	Chlamydomonas_eusti	77.80	0.00
TRINITY_XP_0016	predicte	Chlamydomonas_reinh	77.80	0.00
TRINITY_XP_0029	hypothet	Volvox_carteri	77.80	0.00
TRINITY_XP_0029	hypothet	Volvox_carteri	77.80	0.00
TRINITY_XP_0025	predicte	Micromonas_commoda	77.80	0.00
TRINITY_XP_0202	serine/t	Asparagus_officinal	77.80	0.00
TRINITY_XP_0138	hypothet	Monoraphidium_negle	77.80	0.00
TRINITY_PNW7522	hypothet	Chlamydomonas_reinh	77.80	0
TRINITY_XP_0029	hypothet	Volvox_carteri	77.80	0.00
TRINITY_XP_0017	predicte	Chlamydomonas_reinh	77.80	0.00
TRINITY_PNW7080	hypothet	Chlamydomonas_reinh	77.80	0.00
TRINITY_PNW8632	hypothet	Chlamydomonas_reinh	77.70	0.00
TRINITY_XP_0238	serine/t	Quercus_suber	77.70	0.00
TRINITY_XP_0029	hypothet	Volvox_carteri	77.70	0.00
TRINITY_PNW8688	hypothet	Chlamydomonas_reinh	77.70	0.00
TRINITY_XP_0016	chlorop	Chlamydomonas_reinh	77.70	0.00
TRINITY_AGC1298	long fla	Chlamydomonas_reinh	77.70	0.00
TRINITY_XP_0017	flagella	Chlamydomonas_reinh	77.70	0
TRINITY_XP_0029	dynein h	Volvox_carteri	77.70	0.00
TRINITY_XP_0239	eukaryot	Quercus_suber	77.60	0.00
TRINITY_XP_0030	predicte	Micromonas_pusilla	77.60	0.00
TRINITY_GAV6443	ubiquiti	Cephalotus_follicul	77.60	0.00
TRINITY_GAX8399	hypothet	Chlamydomonas_eusti	77.60	0.00
TRINITY_XP_0188	PREDICTE	Juglans_regia	77.60	0.00
TRINITY_XP_0139	small ra	Monoraphidium_negle	77.60	0.00
TRINITY_GAX8119	hypothet	Chlamydomonas_eusti	77.60	0.00
TRINITY_XP_0029	hypothet	Volvox_carteri	77.60	0.00
TRINITY_GAX8508	hypothet	Chlamydomonas_eusti	77.60	0
TRINITY_ACO5073	NADH deh	Micromonas_pusilla	77.60	0.00
TRINITY_OIV9394	hypothet	Lupinus_angustifoli	77.50	0.00
TRINITY_XP_0062	60S ribo	Capsella_rubella	77.50	0.00
TRINITY_XP_0056	beta tub	Coccomyxa_subellips	77.50	0.00
TRINITY_BAJ9019	predicte	Hordeum_vulgare	77.50	0.00
TRINITY_OAE3225	hypothet	Marchantia_polymorp	77.50	0.00
TRINITY_XP_0030	central	Micromonas_pusilla	77.50	0.00
TRINITY_GAX8490	hypothet	Chlamydomonas_eusti	77.50	0
TRINITY_GAX8260	hypothet	Chlamydomonas_eusti	77.50	0.00
TRINITY_GAX8607	hypothet	Chlamydomonas_eusti	77.50	0.00
TRINITY_GAX7420	hypothet	Chlamydomonas_eusti	77.50	0
TRINITY_PUZ5074	hypothet	Panicum_hallii	77.50	0.00
TRINITY_XP_0016	dynein h	Chlamydomonas_reinh	77.50	0.00
TRINITY_KXZ4158	hypothet	Gonium_pectorale	77.40	0.00
TRINITY_GAX7729	hypothet	Chlamydomonas_eusti	77.40	0.00
TRINITY_XP_0113	DNA-dire	Auxenochlorella_pro	77.40	0.00
TRINITY_XP_0114	Alpha-ga	Auxenochlorella_pro	77.40	0.00
TRINITY_GAX7271	hypothet	Chlamydomonas_eusti	77.40	0.00
TRINITY_AXF4155	CCP430 p	Chlorella_sp._ArM00	77.40	0.00
TRINITY_GAX7481	hypothet	Chlamydomonas_eusti	77.40	0.00
TRINITY_AQZ3651	dehydin	Ipomoea_pes-caprae	77.40	0.00
TRINITY_KXZ5360	hypothet	Gonium_pectorale	77.40	0.00
TRINITY_XP_0016	glycine	Chlamydomonas_reinh	77.40	0.00
TRINITY_KXZ5182	hypothet	Gonium_pectorale	77.40	0.00
TRINITY_KXZ4583	hypothet	Gonium_pectorale	77.40	0.00
TRINITY_GAX7769	hypothet	Chlamydomonas_eusti	77.40	0.00
TRINITY_XP_0264	citrate	Papaver_somniferum	77.30	0.00
TRINITY_XP_0220	40S ribo	Helianthus_annuus	77.30	0.00
TRINITY_AAX4399	UDP-glu	Dunaliella_salina	77.30	0.00
TRINITY_PNH1023	Zinc fir	Tetrabaena_socialis	77.30	0.00
TRINITY_PNW7870	hypothet	Chlamydomonas_reinh	77.30	0.00
TRINITY_XP_0029	hypothet	Volvox_carteri	77.30	0.00

TRINITY_XP_00169	aquapori	Chlamydomonas_reinh	77.30	0.00
TRINITY_ADK36629	vacuolar	Dunaliella_viridis	77.30	0
TRINITY_KXZ44799	hypothet	Gonium_pectorale	77.30	0.00
TRINITY_XP_00170	gamma-gl	Chlamydomonas_reinh	77.30	0.00
TRINITY_GAX77461	hypothet	Chlamydomonas_eusti	77.30	0.00
TRINITY_GAQ79247	protein	Klebsormidium_niten	77.30	0.00
TRINITY_GAX85633	hypothet	Chlamydomonas_eusti	77.30	0.00
TRINITY_GAX80207	hypothet	Chlamydomonas_eusti	77.30	0.00
TRINITY_PSC72612	mRNA (2	Micractinium_conduc	77.20	0.00
TRINITY_BAJ98899	predicte	Hordeum_vulgare	77.20	0.00
TRINITY_XP_00169	predicte	Chlamydomonas_reinh	77.20	0.00
TRINITY_PRW59761	cytochro	Chlorella_sorokinia	77.20	0.00
TRINITY_PNH06203	Chloroph	Tetrabaena_socialis	77.20	0.00
TRINITY_GAX74099	hypothet	Chlamydomonas_eusti	77.20	0.00
TRINITY_PNH11270	putative	Tetrabaena_socialis	77.20	0.00
TRINITY_PSC72642	sar-type	Micractinium_conduc	77.20	0.00
TRINITY_KXZ46110	CDKA1 pr	Gonium_pectorale	77.20	0.00
TRINITY_XP_01389	uridylat	Monoraphidium_negle	77.20	0.00
TRINITY_XP_00169	predicte	Chlamydomonas_reinh	77.20	0.00
TRINITY_GAX80062	hypothet	Chlamydomonas_eusti	77.20	0.00
TRINITY_PNW73732	hypothet	Chlamydomonas_reinh	77.20	0.00
TRINITY_GAU39360	hypothet	Trifolium_subterran	77.10	0.00
TRINITY_XP_01389	formate	Monoraphidium_negle	77.10	0.00
TRINITY_KXZ46900	hypothet	Gonium_pectorale	77.10	0.00
TRINITY_GAX72626	hypothet	Chlamydomonas_eusti	77.10	0.00
TRINITY_XP_01596	60S ribo	Arachis_duranensis	77.10	0.00
TRINITY_GAX81643	hypothet	Chlamydomonas_eusti	77.10	0.00
TRINITY_GBF92721	hypothet	Raphidocelis_subcap	77.10	0.00
TRINITY_GAX72654	hypothet	Chlamydomonas_eusti	77.10	0.00
TRINITY_PNH09050	Peptidyl	Tetrabaena_socialis	77.10	0.00
TRINITY_XP_00294	hypothet	Volvox_carteri	77.10	0.00
TRINITY_XP_02436	phosphor	Physcomitrella_pate	77.10	0.00
TRINITY_GAX79183	hypothet	Chlamydomonas_eusti	77.10	0.00
TRINITY_XP_00294	trypsin	Volvox_carteri	77.10	0.00
TRINITY_XP_00169	transcri	Chlamydomonas_reinh	77.10	0.00
TRINITY_GAX81863	hypothet	Chlamydomonas_eusti	77.10	0.00
TRINITY_AFK49397	unknown	Lotus_japonicus	77.00	0.00
TRINITY_AFK49397	unknown	Lotus_japonicus	77.00	0.00
TRINITY_XP_02166	small nu	Hevea_brasiliensis	77.00	0.00
TRINITY_AFK49397	unknown	Lotus_japonicus	77.00	0.00
TRINITY_AFK49397	unknown	Lotus_japonicus	77.00	0.00
TRINITY_XP_02201	40S ribo	Helianthus_annuus	77.00	0.00
TRINITY_XP_00305	histone	Micromonas_pusilla	77.00	0.00
TRINITY_OIV89924	hypothet	Lupinus_angustifoli	77.00	0.00
TRINITY_GBF92034	hypothet	Raphidocelis_subcap	77.00	0.00
TRINITY_XP_00169	pyruvate	Chlamydomonas_reinh	77.00	0.00
TRINITY_PNW76230	hypothet	Chlamydomonas_reinh	77.00	0.00
TRINITY_KXZ43316	hypothet	Gonium_pectorale	77.00	0.00
TRINITY_XP_00169	flagella	Chlamydomonas_reinh	77.00	0.00
TRINITY_GAX73154	hypothet	Chlamydomonas_eusti	77.00	0.00
TRINITY_XP_00169	EMP/nona	Chlamydomonas_reinh	77.00	0.00
TRINITY_XP_00294	vitamin	Volvox_carteri	77.00	0.00
TRINITY_RRT59180	hypothet	Ensete_ventricosum	77.00	0.00
TRINITY_XP_00169	dUTP pyr	Chlamydomonas_reinh	76.90	0.00
TRINITY_XP_01943	PREDICTE	Lupinus_angustifoli	76.90	0.00
TRINITY_GBG70105	hypothet	Chara_braunii	76.90	0.00
TRINITY_XP_00295	iron-sul	Volvox_carteri	76.90	0.00
TRINITY_XP_01389	60S ribo	Monoraphidium_negle	76.90	0.00
TRINITY_GBF92418	small nu	Raphidocelis_subcap	76.90	0.00
TRINITY_KXZ44487	hypothet	Gonium_pectorale	76.90	0.00
TRINITY_GAX75405	hypothet	Chlamydomonas_eusti	76.90	0.00
TRINITY_XP_00169	protein	Chlamydomonas_reinh	76.90	0.00
TRINITY_XP_00169	lysyl-tr	Chlamydomonas_reinh	76.90	0.00
TRINITY_KXZ46531	hypothet	Gonium_pectorale	76.90	0.00
TRINITY_PNH08972	5-methyl	Tetrabaena_socialis	76.90	0.00
TRINITY_GAX72561	hypothet	Chlamydomonas_eusti	76.90	0.00

TRINITY_KMZ63289	Formate-	Zostera_marina	76.80	0.00
TRINITY_GBF91277	hypothet	Raphidocelis_subcap	76.80	0.00
TRINITY_EFJ09552	hypothet	Selaginella_moellen	76.80	0.00
TRINITY_BAJ96063	predicte	Hordeum_vulgare	76.80	0.00
TRINITY_OQU86657	hypothet	Sorghum_bicolor	76.80	0.00
TRINITY_KXZ51941	hypothet	Gonium_pectorale	76.80	0.00
TRINITY_GAX83443	hypothet	Chlamydomonas_eusti	76.80	0.00
TRINITY_KXZ51233	hypothet	Gonium_pectorale	76.80	0.00
TRINITY_GAX82163	hypothet	Chlamydomonas_eusti	76.80	0.00
TRINITY_GAX73618	hypothet	Chlamydomonas_eusti	76.80	0.00

TRINITY_XP_0094(	PREDICTE	Musa_acuminata	76.70	0.00
TRINITY_RZC7722	hypothet	Papaver_somniferum	76.70	0.00
TRINITY_PSC6755	hypothet	Micractinium_conduc	76.70	0.00
TRINITY_KXZ4990	hypothet	Gonium_pectorale	76.70	0.00
TRINITY_KMZ6153	Serine/t	Zostera_marina	76.70	0.00
TRINITY_XP_0239(	serine/t	Quercus_suber	76.70	0.00
TRINITY_XP_0029	hypothet	Volvox_carteri	76.70	0.00
TRINITY_XP_0016	acetylo	Chlamydomonas_reinh	76.70	0.00
TRINITY_KXZ4556	hypothet	Gonium_pectorale	76.70	0.00
TRINITY_XP_0029	hypothet	Volvox_carteri	76.70	0.00
TRINITY_XP_0016	phospho	Chlamydomonas_reinh	76.70	0.00
TRINITY_ADI8034	glutath	Chlamydomonas_sp._I	76.70	0.00
TRINITY_XP_0029	hypothet	Volvox_carteri	76.70	0.00
TRINITY_PNH0534	Palmito	Tetrabaena_socialis	76.70	0.00
TRINITY_PNH0754	Ubiquit	Tetrabaena_socialis	76.70	0.00
TRINITY_XP_0239(	histone	Quercus_suber	76.60	0.00
TRINITY_AFK3626	unknown	Medicago_truncatula	76.60	0.00
TRINITY_KXZ5497	hypothet	Gonium_pectorale	76.60	0.00
TRINITY_OTG2352	putative	Helianthus_annuus	76.60	0.00
TRINITY_GAX7455	hypothet	Chlamydomonas_eusti	76.60	0.00
TRINITY_KXZ4974	hypothet	Gonium_pectorale	76.60	0.00
TRINITY_GBF9551	60S rib	Raphidocelis_subcap	76.60	0.00
TRINITY_KXZ4732	hypothet	Gonium_pectorale	76.60	0.00
TRINITY_OIT2968	60s rib	Nicotiana_attenuata	76.60	0.00
TRINITY_GBF9032	aspartat	Raphidocelis_subcap	76.60	0.00
TRINITY_GAX7776	hypothet	Chlamydomonas_eusti	76.60	0.00
TRINITY_XP_0029	hypothet	Volvox_carteri	76.60	0.00
TRINITY_XP_0016	ribosoma	Chlamydomonas_reinh	76.60	0.00
TRINITY_XP_0029	hypothet	Volvox_carteri	76.60	0.00
TRINITY_PIA4570	hypothet	Aquilegia_coerulea	76.50	0.00
TRINITY_KXZ5004	hypothet	Gonium_pectorale	76.50	0.00
TRINITY_AAG5001	actin,	Helicosporidium_sp.	76.50	0.00
TRINITY_BAJ8768	predicte	Hordeum_vulgare	76.50	0.00
TRINITY_XP_0029	hypothet	Volvox_carteri	76.50	0.00
TRINITY_PNW8761(	hypothet	Chlamydomonas_reinh	76.50	0.00
TRINITY_XP_0138	60S rib	Monoraphidium_negle	76.50	0.00
TRINITY_PIN0931	60s rib	Handroanthus_impeti	76.50	0.00
TRINITY_XP_0016	aconitat	Chlamydomonas_reinh	76.50	0.00
TRINITY_XP_0016	nitrite	Chlamydomonas_reinh	76.50	0.00
TRINITY_RAL4756	hypothet	Cuscuta_australis	76.50	0.00
TRINITY_XP_0029	hypothet	Volvox_carteri	76.50	0.00
TRINITY_PRW5090(	Histone	Chlorella_sorokinia	76.50	0.00
TRINITY_XP_0029	hypothet	Volvox_carteri	76.50	0.00
TRINITY_XP_0138	hypothet	Monoraphidium_negle	76.50	0.00
TRINITY_GAX7649	hypothet	Chlamydomonas_eusti	76.50	0
TRINITY_GBF9631	cysteine	Raphidocelis_subcap	76.50	0.00
TRINITY_XP_0016	LOW QUA	Chlamydomonas_reinh	76.50	0.00
TRINITY_GAX7469	hypothet	Chlamydomonas_eusti	76.50	0.00
TRINITY_XP_0029	hypothet	Volvox_carteri	76.50	0.00
TRINITY_XP_0029	hypothet	Volvox_carteri	76.50	0.00
TRINITY_XP_0087	actin, r	Phoenix_dactylifera	76.40	0.00
TRINITY_OVA1488	Ribosoma	Macleaya_cordata	76.40	0.00
TRINITY_BAK0108	predicte	Hordeum_vulgare	76.40	0.00
TRINITY_XP_0245	serine/t	Selaginella_moellen	76.40	0.00
TRINITY_GAX7687(	hypothet	Chlamydomonas_eusti	76.40	0.00
TRINITY_GAX8619	hypothet	Chlamydomonas_eusti	76.40	0.00
TRINITY_PNW7750	hypothet	Chlamydomonas_reinh	76.40	0.00
TRINITY_GAX7398(	hypothet	Chlamydomonas_eusti	76.40	0.00
TRINITY_KXZ5060	hypothet	Gonium_pectorale	76.40	0.00
TRINITY_PNW8496(	hypothet	Chlamydomonas_reinh	76.40	0.00
TRINITY_GAX7344	hypothet	Chlamydomonas_eusti	76.40	0.00
TRINITY_XP_0017(	oxoglut	Chlamydomonas_reinh	76.40	0.00
TRINITY_GAX8373	hypothet	Chlamydomonas_eusti	76.40	0.00
TRINITY_XP_0016	protein	Chlamydomonas_reinh	76.40	0.00
TRINITY_GAX7363	hypothet	Chlamydomonas_eusti	76.30	0.00
TRINITY_XP_0029	hypothet	Volvox_carteri	76.30	0.00

TRINITY_RXH7611(	hypothet	Malus_domestica	76.30	0.00
TRINITY_GBF9420	cell di	Raphidocelis_subcap	76.30	0.00
TRINITY_XP_0029	hypothet	Volvox_carteri	76.30	0.00
TRINITY_GAX8043	hypothet	Chlamydomonas_eusti	76.30	0.00
TRINITY_GAX7978	hypothet	Chlamydomonas_eusti	76.30	0.00
TRINITY_GAX8370	hypothet	Chlamydomonas_eusti	76.30	0.00
TRINITY_PNW7080	hypothet	Chlamydomonas_reinh	76.30	0.00
TRINITY_GAX7506	hypothet	Chlamydomonas_eusti	76.30	0.00
TRINITY_XP_0017(	phospho	Chlamydomonas_reinh	76.30	0.00
TRINITY_KXZ4433	hypothet	Gonium_pectorale	76.30	0
TRINITY_OQU8625	hypothet	Sorghum_bicolor	76.30	0.00
TRINITY_XP_0228	40S rib	Olea_europaea	76.30	0.00
TRINITY_GAX8516	hypothet	Chlamydomonas_eusti	76.30	0.00
TRINITY_XP_0056	60S rib	Coccomyxa_subellips	76.20	0.00
TRINITY_XP_0094(	PREDICTI	Musa_acuminata	76.20	0.00
TRINITY_XP_0029	hypothet	Volvox_carteri	76.20	0.00
TRINITY_GBF9064	p-type a	Raphidocelis_subcap	76.20	0.00
TRINITY_EXC1088	hypothet	Morus_notabilis	76.20	0.00
TRINITY_PSC7357	SRSF kir	Micractinium_conduc	76.20	0.00
TRINITY_GAX7759	hypothet	Chlamydomonas_eusti	76.20	0.00
TRINITY_KMS6493	hypothet	Beta_vulgaris	76.20	0.00
TRINITY_XP_0229	luminal-	Cucurbita_moschata	76.20	0.00
TRINITY_GBF9508	glycine	Raphidocelis_subcap	76.20	0.00
TRINITY_PNW7667	hypothet	Chlamydomonas_reinh	76.20	0.00
TRINITY_PSC7244	fructose	Micractinium_conduc	76.20	0.00
TRINITY_XP_0029	hypothet	Volvox_carteri	76.20	0.00
TRINITY_KMS6524	hypothet	Beta_vulgaris	76.20	0.00
TRINITY_KXZ4299	hypothet	Gonium_pectorale	76.20	0.00
TRINITY_XP_0124	PREDICTI	Gossypium_raimondii	76.20	0.00
TRINITY_XP_0056	T-comple	Coccomyxa_subellips	76.20	0.00
TRINITY_XP_0096(	PREDICTI	Nicotiana_tomentosi	76.10	0.00
TRINITY_PNH1130	putative	Tetrabaena_socialis	76.10	0.00
TRINITY_GBF9448	proteas	Raphidocelis_subcap	76.10	0.00
TRINITY_PNW8629	hypothet	Chlamydomonas_reinh	76.10	0.00
TRINITY_GAX7801	hypothet	Chlamydomonas_eusti	76.10	0.00
TRINITY_KXZ5479(	hypothet	Gonium_pectorale	76.10	0.00
TRINITY_XP_0029	hypothet	Volvox_carteri	76.10	0.00
TRINITY_XP_0016	NADH nit	Chlamydomonas_reinh	76.10	0.00
TRINITY_PNW7351	hypothet	Chlamydomonas_reinh	76.10	0.00
TRINITY_PNW7598	hypothet	Chlamydomonas_reinh	76.10	0.00
TRINITY_XP_0017(	ATP-bin	Chlamydomonas_reinh	76.10	0.00
TRINITY_KXZ4996	hypothet	Gonium_pectorale	76.10	0.00
TRINITY_PNH0603	FACT cor	Tetrabaena_socialis	76.10	0.00
TRINITY_GAX8227	hypothet	Chlamydomonas_eusti	76.10	0.00
TRINITY_XP_0016	methylma	Chlamydomonas_reinh	76.10	0.00
TRINITY_KXZ4405(	hypothet	Gonium_pectorale	76.10	0.00
TRINITY_GAX8486(	hypothet	Chlamydomonas_eusti	76.10	0.00
TRINITY_GBG7721	hypothet	Chara_braunii	76.00	0.00
TRINITY_PTQ4241(	hypothet	Marchantia_polymorp	76.00	0.00
TRINITY_XP_0017(	predicte	Chlamydomonas_reinh	76.00	0.00
TRINITY_GAX8397	hypothet	Chlamydomonas_eusti	76.00	0.00
TRINITY_BAJ9708	predicte	Hordeum_vulgare	76.00	0.00
TRINITY_GAX7873	hypothet	Chlamydomonas_eusti	76.00	0.00
TRINITY_XP_0030	predicte	Micromonas_pusilla	76.00	0.00
TRINITY_GAX8374	hypothet	Chlamydomonas_eusti	76.00	0.00
TRINITY_PNW8621	hypothet	Chlamydomonas_reinh	76.00	0.00
TRINITY_GAQ8916	DEAD-bo	Klebsormidium_niten	75.90	0.00
TRINITY_KMS6524	hypothet	Beta_vulgaris	75.90	0.00
TRINITY_PNH0630	Calreti	Tetrabaena_socialis	75.90	0.00
TRINITY_XP_0243	cleavage	Physcomitrella_pate	75.90	0.00
TRINITY_PNH0895	Lysosoma	Tetrabaena_socialis	75.90	0.00
TRINITY_GAX7592	hypothet	Chlamydomonas_eusti	75.90	0.00
TRINITY_KXZ5200	hypothet	Gonium_pectorale	75.90	0.00
TRINITY_XP_0184	PREDICTI	Raphanus_sativus	75.90	0.00
TRINITY_GAX7805(	hypothet	Chlamydomonas_eusti	75.90	0.00
TRINITY_XP_0238	serine/t	Quercus_suber	75.90	0.00

TRINITY_KXZ49629	hypothet	Gonium_pectorale	75.90	0.00
TRINITY_KXZ54715	hypothet	Gonium_pectorale	75.90	0.00
TRINITY_XP_02172	LOW QUAI	Chenopodium_quinoa	75.90	0.00
TRINITY_GAX83076	hypothet	Chlamydomonas_eusti	75.90	0.00
TRINITY_KMS94501	hypothet	Beta_vulgaris	75.90	0.00
TRINITY_GAX77700	hypothet	Chlamydomonas_eusti	75.90	0
TRINITY_GAX78040	hypothet	Chlamydomonas_eusti	75.90	0.00
TRINITY_KXZ53642	hypothet	Gonium_pectorale	75.90	0.00
TRINITY_XP_02283	Ribosoma	Ostreococcus_tauri	75.80	0.00
TRINITY_GBG59037	hypothet	Chara_braunii	75.80	0.00
TRINITY_XP_00294	hypothet	Volvox_carteri	75.80	0.00
TRINITY_PIN17423	Casein k	Handroanthus_impeti	75.80	0.00
TRINITY_XP_01390	arogenat	Monoraphidium_negle	75.80	0.00
TRINITY_NP_00123	enolase	Solanum_lycopersicu	75.80	0.00
TRINITY_RWR72291	serine/t	Cinnamomum_micranth	75.80	0.00
TRINITY_KXZ56059	hypothet	Gonium_pectorale	75.80	0
TRINITY_GAX80571	hypothet	Chlamydomonas_eusti	75.80	0
TRINITY_XP_00170	magnesiu	Chlamydomonas_reinh	75.70	0.00
TRINITY_KXZ50506	hypothet	Gonium_pectorale	75.70	0.00
TRINITY_OAE32442	hypothet	Marchantia_polymorp	75.70	0.00
TRINITY_XP_02390	aldo-ket	Quercus_suber	75.70	0.00
TRINITY_PNW88460	hypothet	Chlamydomonas_reinh	75.70	0.00
TRINITY_GAX78260	hypothet	Chlamydomonas_eusti	75.70	0.00
TRINITY_GAX77457	hypothet	Chlamydomonas_eusti	75.70	0.00
TRINITY_XP_00169	minor ch	Chlamydomonas_reinh	75.70	0.00
TRINITY_GAX79634	hypothet	Chlamydomonas_eusti	75.70	0.00
TRINITY_GAX75900	hypothet	Chlamydomonas_eusti	75.70	0.00
TRINITY_XP_00169	predicte	Chlamydomonas_reinh	75.70	0.00
TRINITY_KXZ50847	hypothet	Gonium_pectorale	75.70	0.00
TRINITY_GAX73345	hypothet	Chlamydomonas_eusti	75.70	0.00
TRINITY_GAX76891	hypothet	Chlamydomonas_eusti	75.70	0.00
TRINITY_XP_00170	ClpB cha	Chlamydomonas_reinh	75.70	0.00
TRINITY_XP_00665	PREDICTE	Oryza_brachyantha	75.60	0.00
TRINITY_GAX84680	hypothet	Chlamydomonas_eusti	75.60	0.00
TRINITY_GAX84680	hypothet	Chlamydomonas_eusti	75.60	0.00
TRINITY_XP_00294	dynein h	Volvox_carteri	75.60	0.00
TRINITY_XP_00295	hypothet	Volvox_carteri	75.60	0.00
TRINITY_KMS64930	hypothet	Beta_vulgaris	75.60	0.00
TRINITY_GAX80430	hypothet	Chlamydomonas_eusti	75.60	0.00
TRINITY_GAX86074	hypothet	Chlamydomonas_eusti	75.60	0.00
TRINITY_GAX75401	hypothet	Chlamydomonas_eusti	75.60	0.00
TRINITY_PNW84441	hypothet	Chlamydomonas_reinh	75.60	0.00
TRINITY_XP_00169	ribosoma	Chlamydomonas_reinh	75.60	0.00
TRINITY_XP_00169	soluble	Chlamydomonas_reinh	75.60	0.00
TRINITY_PNW84425	hypothet	Chlamydomonas_reinh	75.60	0.00
TRINITY_GAX75524	hypothet	Chlamydomonas_eusti	75.60	0.00
TRINITY_XP_00295	branched	Volvox_carteri	75.60	0.00
TRINITY_XP_01674	PREDICTE	Gossypium_hirsutum	75.60	0.00
TRINITY_PSC73753	ruvB 1	Micractinium_conduc	75.60	0.00
TRINITY_XP_00294	heat sho	Volvox_carteri	75.50	0.00
TRINITY_GAX79758	hypothet	Chlamydomonas_eusti	75.50	0.00
TRINITY_GAX75729	hypothet	Chlamydomonas_eusti	75.50	0.00
TRINITY_GBF97760	peptidas	Raphidocelis_subcap	75.50	0.00
TRINITY_GAQ86818	Isovaler	Klebsormidium_niten	75.50	0.00
TRINITY_XP_00169	low-affi	Chlamydomonas_reinh	75.50	0.00
TRINITY_XP_00683	glutamyl	Amborella_trichopod	75.50	0.00
TRINITY_BAJ91591	predicte	Hordeum_vulgare	75.50	0.00
TRINITY_BAJ91591	predicte	Hordeum_vulgare	75.50	0.00
TRINITY_XP_00169	histone	Chlamydomonas_reinh	75.50	0.00
TRINITY_GAX77752	hypothet	Chlamydomonas_eusti	75.50	0.00
TRINITY_XP_01390	Ubiquili	Monoraphidium_negle	75.50	0.00
TRINITY_GAX80543	hypothet	Chlamydomonas_eusti	75.50	0.00
TRINITY_GAX73573	hypothet	Chlamydomonas_eusti	75.50	0.00
TRINITY_GAX77712	hypothet	Chlamydomonas_eusti	75.50	0.00
TRINITY_GAX78048	hypothet	Chlamydomonas_eusti	75.50	0.00
TRINITY_RDX63202	Casein k	Mucuna_pruriens	75.50	0.00

TRINITY_PNW7687(	hypothet	Chlamydomonas_reinh	75.50	0.00
TRINITY_XP_0029	hypothet	Volvox_carteri	75.50	0
TRINITY_GAX8322(	hypothet	Chlamydomonas_eusti	75.50	0.00
TRINITY_KXZ5513	hypothet	Gonium_pectorale	75.50	0
TRINITY_ACG2448	60S ribo	Zea_mays	75.40	0.00
TRINITY_ACG2448	60S ribo	Zea_mays	75.40	0.00
TRINITY_RLN0897	DEAD-bo	Panicum_miliaceum	75.40	0.00
TRINITY_PNH0118	Phospho	Tetrabaena_socialis	75.40	0.00
TRINITY_GAX8013	hypothet	Chlamydomonas_eusti	75.40	0.00
TRINITY_AFK3421	unknown	Lotus_japonicus	75.40	0.00
TRINITY_XP_0056	ribonucl	Coccomyxa_subellips	75.40	0.00
TRINITY_XP_0056	succinat	Coccomyxa_subellips	75.40	0.00
TRINITY_GAX8323	hypothet	Chlamydomonas_eusti	75.40	0.00
TRINITY_GBF9691	protopo	Raphidocelis_subcap	75.40	0.00
TRINITY_KXZ5585	hypothet	Gonium_pectorale	75.40	0.00
TRINITY_GAX8016	hypothet	Chlamydomonas_eusti	75.40	0.00
TRINITY_GAX8258	hypothet	Chlamydomonas_eusti	75.40	0.00
TRINITY_XP_0016	predicte	Chlamydomonas_reinh	75.40	0.00
TRINITY_XP_0029	hypothet	Volvox_carteri	75.40	0.00
TRINITY_KXZ5506	hypothet	Gonium_pectorale	75.40	0.00
TRINITY_ALM5499	DEAD bo	Chlamydomonas_sp._I	75.40	0.00
TRINITY_PRW6157	DNA gyr	Chlorella_sorokinia	75.40	0.00
TRINITY_KXZ5016	hypothet	Gonium_pectorale	75.40	0.00
TRINITY_KXZ4403	hypothet	Gonium_pectorale	75.40	0.00
TRINITY_PNH1222	Pirin-l	Tetrabaena_socialis	75.40	0.00
TRINITY_XP_0029	hypothet	Volvox_carteri	75.40	0.00
TRINITY_PNW7379	hypothet	Chlamydomonas_reinh	75.40	0.00
TRINITY_GAX7897	hypothet	Chlamydomonas_eusti	75.30	0.00
TRINITY_XP_0063	sm-like	Capsella_rubella	75.30	0.00
TRINITY_XP_0029	hypothet	Volvox_carteri	75.30	0.00
TRINITY_GBG9125	hypothet	Chara_braunii	75.30	0.00
TRINITY_XP_0124	PREDICTI	Gossypium_raimondii	75.30	0.00
TRINITY_GAQ8294	putative	Klebsormidium_niten	75.30	0.00
TRINITY_XP_0049	60S ribo	Setaria_italica	75.30	0.00
TRINITY_KDD7539	hypothet	Helicosporidium_sp.	75.30	0.00
TRINITY_XP_0014	predicte	Ostreococcus_sp._'l	75.30	0.00
TRINITY_GAX8134	hypothet	Chlamydomonas_eusti	75.30	0.00
TRINITY_XP_0017	predicte	Chlamydomonas_reinh	75.30	0.00
TRINITY_PNR4403	hypothet	Physcomitrella_pate	75.30	0.00
TRINITY_XP_0075	predicte	Bathycoccus_prasino	75.30	0.00
TRINITY_KXZ4438	hypothet	Gonium_pectorale	75.30	0.00
TRINITY_XP_0029	hypothet	Volvox_carteri	75.30	0
TRINITY_GBF9191	chloropl	Raphidocelis_subcap	75.30	0.00
TRINITY_XP_0016	fumarate	Chlamydomonas_reinh	75.30	0.00
TRINITY_XP_0238	60S ribo	Quercus_suber	75.20	0.00
TRINITY_BAK0038	predicte	Hordeum_vulgare	75.20	0.00
TRINITY_PIA5454	hypothet	Aquilegia_coerulea	75.20	0.00
TRINITY_XP_0056	ribosoma	Coccomyxa_subellips	75.20	0.00
TRINITY_XP_0017	componen	Chlamydomonas_reinh	75.20	0.00
TRINITY_GBF8734	photosys	Raphidocelis_subcap	75.20	0.00
TRINITY_KXZ5249	hypothet	Gonium_pectorale	75.20	0.00
TRINITY_GAX8343	hypothet	Chlamydomonas_eusti	75.20	0.00
TRINITY_KXZ5217	hypothet	Gonium_pectorale	75.20	0.00
TRINITY_GAX7437	hypothet	Chlamydomonas_eusti	75.20	0.00
TRINITY_GAX7693	hypothet	Chlamydomonas_eusti	75.20	0.00
TRINITY_GAX8212	hypothet	Chlamydomonas_eusti	75.20	0.00
TRINITY_GAX7627	hypothet	Chlamydomonas_eusti	75.20	0.00
TRINITY_GBF9460	hypothet	Raphidocelis_subcap	75.20	0.00
TRINITY_KXZ4473	hypothet	Gonium_pectorale	75.20	0.00
TRINITY_XP_0029	hypothet	Volvox_carteri	75.20	0.00
TRINITY_GAX8164	hypothet	Chlamydomonas_eusti	75.20	0.00
TRINITY_KXZ5600	hypothet	Gonium_pectorale	75.20	0.00
TRINITY_KXZ5312	hypothet	Gonium_pectorale	75.20	0
TRINITY_GAX8322	hypothet	Chlamydomonas_eusti	75.20	0.00
TRINITY_ADF4316	SPS1m [	Chlamydomonas_reinh	75.20	0.00
TRINITY_XP_0124	PREDICTI	Gossypium_raimondii	75.20	0.00

TRINITY_GAX8088	hypothet	Chlamydomonas_eusti	75.10	0.00
TRINITY_GAX7537	hypothet	Chlamydomonas_eusti	75.10	0.00
TRINITY_XP_0016	glycine	Chlamydomonas_reinh	75.10	0.00
TRINITY_KXZ4473	hypothet	Gonium_pectorale	75.10	0.00
TRINITY_XP_0239	60S rib	Quercus_suber	75.00	0.00
TRINITY_GBG7844	hypothet	Chara_braunii	75.00	0.00
TRINITY_XP_0029	hypothet	Volvox_carteri	75.00	0.00
TRINITY_XP_0064	serine/t	Eutrema_salsugineum	75.00	0.00
TRINITY_PRW5674	40S rib	Chlorella_sorokinia	75.00	0.00
TRINITY_GAX7570	hypothet	Chlamydomonas_eusti	75.00	0.00
TRINITY_XP_0056	ribosoma	Coccomyxa_subellips	75.00	0.00
TRINITY_PTQ3799	hypothet	Marchantia_polymorp	75.00	0.00
TRINITY_PNH0830	NFX1-typ	Tetrabaena_socialis	75.00	0.00
TRINITY_GAX8226	hypothet	Chlamydomonas_eusti	75.00	0.00
TRINITY_KDD7379	hypothet	Helicosporidium_sp.	75.00	0.00
TRINITY_XP_0029	hypothet	Volvox_carteri	75.00	0.00
TRINITY_ACG2692	glyceral	Zea_mays	75.00	0.00
TRINITY_GBF9954	hypothet	Raphidocelis_subcap	75.00	0.00
TRINITY_GAX7767	hypothet	Chlamydomonas_eusti	75.00	0.00
TRINITY_GAQ8291	putative	Klebsormidium_niten	75.00	0.00
TRINITY_GAX8571	hypothet	Chlamydomonas_eusti	75.00	0.00
TRINITY_ACL2816	hypothet	Dunaliella_viridis	75.00	0.00
TRINITY_KXZ4945	hypothet	Gonium_pectorale	75.00	0.00
TRINITY_PNW7263	hypothet	Chlamydomonas_reinh	75.00	0.00
TRINITY_PRW6121	actin-re	Chlorella_sorokinia	75.00	0.00
TRINITY_XP_0029	hypothet	Volvox_carteri	75.00	0.00
TRINITY_PNW7924	hypothet	Chlamydomonas_reinh	75.00	0.00
TRINITY_XP_0029	hypothet	Volvox_carteri	75.00	0.00
TRINITY_KXZ4701	hypothet	Gonium_pectorale	75.00	0.00
TRINITY_KXZ4275	hypothet	Gonium_pectorale	75.00	0.00
TRINITY_KDD7550	DUF933 I	Helicosporidium_sp.	75.00	0.00
TRINITY_GAX8236	hypothet	Chlamydomonas_eusti	75.00	0.00
TRINITY_XP_0029	hypothet	Volvox_carteri	75.00	0.00
TRINITY_XP_0029	hypothet	Volvox_carteri	75.00	0.00
TRINITY_GAX8164	hypothet	Chlamydomonas_eusti	75.00	0.00
TRINITY_XP_0139	40S rib	Monoraphidium_negle	75.00	0.00
TRINITY_XP_0058	hypothet	Chlorella_variabili	75.00	0.00
TRINITY_ALM5500	DEAD bo	Chlamydomonas_sp._I	75.00	0.00
TRINITY_OTG3562	putative	Helianthus_annuus	75.00	0
TRINITY_XP_0239	cytoplas	Quercus_suber	75.00	0.00
TRINITY_GAX7381	hypothet	Chlamydomonas_eusti	75.00	0.00
TRINITY_RLN2004	calreti	Panicum_miliaceum	75.00	0.00
TRINITY_GAX7348	hypothet	Chlamydomonas_eusti	74.90	0
TRINITY_XP_0016	deoxyhyp	Chlamydomonas_reinh	74.90	0.00
TRINITY_XP_0029	hypothet	Volvox_carteri	74.90	0.00
TRINITY_XP_0029	hypothet	Volvox_carteri	74.90	0.00
TRINITY_PNW8352	hypothet	Chlamydomonas_reinh	74.90	0.00
TRINITY_XP_0016	aspartat	Chlamydomonas_reinh	74.90	0.00
TRINITY_GAX7310	hypothet	Chlamydomonas_eusti	74.90	0.00
TRINITY_GAX7343	hypothet	Chlamydomonas_eusti	74.90	0.00
TRINITY_XP_0017	chaperon	Chlamydomonas_reinh	74.90	0.00
TRINITY_ACI2902	glucose-	Dunaliella_salina	74.90	0.00
TRINITY_XP_0239	putative	Quercus_suber	74.80	0.00
TRINITY_XP_0139	RNA-bin	Monoraphidium_negle	74.80	0.00
TRINITY_XP_0243	14-3-3 p	Physcomitrella_pate	74.80	0.00
TRINITY_XP_0029	hypothet	Volvox_carteri	74.80	0.00
TRINITY_GAX8461	hypothet	Chlamydomonas_eusti	74.80	0.00
TRINITY_XP_0139	hypothet	Monoraphidium_negle	74.80	0.00
TRINITY_GAX7498	hypothet	Chlamydomonas_eusti	74.80	0.00
TRINITY_GAX8428	hypothet	Chlamydomonas_eusti	74.80	0.00
TRINITY_GAX7742	hypothet	Chlamydomonas_eusti	74.80	0.00
TRINITY_XP_0029	flagella	Volvox_carteri	74.80	0
TRINITY_XP_0029	hypothet	Volvox_carteri	74.80	0.00
TRINITY_GAX8021	hypothet	Chlamydomonas_eusti	74.80	0.00
TRINITY_KXZ4092	hypothet	Gonium_pectorale	74.80	0.00
TRINITY_GAX7381	hypothet	Chlamydomonas_eusti	74.80	0

TRINITY_PNW77619	hypothet	Chlamydomonas_reinh	74.80	0.00
TRINITY_XP_00169	predicte	Chlamydomonas_reinh	74.80	0.00
TRINITY_XP_01020	PREDICTE	Nelumbo_nucifera	74.80	0.00
TRINITY_GBF92909	small nu	Raphidocelis_subcap	74.70	0.00
TRINITY_KXZ54843	hypothet	Gonium_pectorale	74.70	0.00
TRINITY_GAX84748	hypothet	Chlamydomonas_eusti	74.70	0.00
TRINITY_XP_00977	PREDICTE	Nicotiana_sylvestri	74.70	0.00
TRINITY_XP_00170	protein	Chlamydomonas_reinh	74.70	0.00
TRINITY_GAX74640	hypothet	Chlamydomonas_eusti	74.70	0.00
TRINITY_AGV54820	cell wal	Phaseolus_vulgaris	74.70	0.00
TRINITY_GAQ79973	bifuncti	Klebsormidium_niten	74.70	0.00
TRINITY_GAX83220	hypothet	Chlamydomonas_eusti	74.70	0
TRINITY_XP_01101	PREDICTE	Populus_euphratica	74.70	0.00
TRINITY_KMS65245	hypothet	Beta_vulgaris	74.70	0.00
TRINITY_GAX86358	hypothet	Chlamydomonas_eusti	74.70	0.00
TRINITY_XP_00295	hypothet	Volvox_carteri	74.70	0.00
TRINITY_PNW88810	hypothet	Chlamydomonas_reinh	74.70	0.00
TRINITY_PNW87408	hypothet	Chlamydomonas_reinh	74.70	0.00
TRINITY_GAX80610	hypothet	Chlamydomonas_eusti	74.70	0.00
TRINITY_GAX74230	hypothet	Chlamydomonas_eusti	74.70	0.00
TRINITY_KXZ44840	hypothet	Gonium_pectorale	74.70	0.00
TRINITY_GAX74989	hypothet	Chlamydomonas_eusti	74.70	0.00
TRINITY_GAX77905	hypothet	Chlamydomonas_eusti	74.70	0
TRINITY_PSC68270	DEAD-box	Micractinium_conduc	74.70	0.00
TRINITY_GAX84352	hypothet	Chlamydomonas_eusti	74.70	0.00
TRINITY_PNH06971	hypothet	Tetrabaena_socialis	74.70	0.00
TRINITY_GBF92909	small nu	Raphidocelis_subcap	74.70	0.00
TRINITY_ACN31538	unknown	Zea_mays	74.60	0.00
TRINITY_XP_00141	thioredo	Ostreococcus_sp._'l	74.60	0.00
TRINITY_P31692.1	RecName:	Parachlorella_kessl	74.60	0.00
TRINITY_EPS72024	proteaso	Genlisea_aurea	74.60	0.00
TRINITY_PNH05668	40S ribo	Tetrabaena_socialis	74.60	0.00
TRINITY_PNH05668	40S ribo	Tetrabaena_socialis	74.60	0.00
TRINITY_KMS64825	hypothet	Beta_vulgaris	74.60	0.00
TRINITY_KXZ51565	hypothet	Gonium_pectorale	74.60	0.00
TRINITY_KXZ46544	hypothet	Gonium_pectorale	74.60	0.00
TRINITY_PNH09628	hypothet	Tetrabaena_socialis	74.60	0.00
TRINITY_GAX84652	hypothet	Chlamydomonas_eusti	74.60	0.00
TRINITY_XP_00169	transcri	Chlamydomonas_reinh	74.60	0.00
TRINITY_AFI57622	Rpn8 [Du	Dunaliella_salina	74.60	0.00
TRINITY_PRW57197	Bromodom	Chlorella_sorokinia	74.60	0.00
TRINITY_XP_01389	Autophag	Monoraphidium_negle	74.60	0.00
TRINITY_GAX75459	hypothet	Chlamydomonas_eusti	74.60	0.00
TRINITY_XP_01561	eukaryot	Oryza_sativa	74.60	0.00
TRINITY_XP_00864	peptidyl	Zea_mays	74.60	0.00
TRINITY_ABN03944	mitogen-	Dunaliella_salina	74.60	0.00
TRINITY_XP_00294	DEAH-box	Volvox_carteri	74.60	0.00
TRINITY_GAX80055	hypothet	Chlamydomonas_eusti	74.60	0
TRINITY_XP_02390	histone	Quercus_suber	74.50	0.00
TRINITY_GAX83910	hypothet	Chlamydomonas_eusti	74.50	0.00
TRINITY_KMS64930	hypothet	Beta_vulgaris	74.50	0.00
TRINITY_GAX79622	hypothet	Chlamydomonas_eusti	74.50	0.00
TRINITY_PNH07524	Splicing	Tetrabaena_socialis	74.50	0.00
TRINITY_ACG26925	glyceral	Zea_mays	74.50	0.00
TRINITY_KXZ53902	hypothet	Gonium_pectorale	74.50	0.00
TRINITY_XP_00295	hypothet	Volvox_carteri	74.50	0.00
TRINITY_XP_00564	P-loop c	Coccomyxa_subellips	74.50	0.00
TRINITY_XP_00169	peptide	Chlamydomonas_reinh	74.50	0.00
TRINITY_PNW84615	hypothet	Chlamydomonas_reinh	74.50	0.00
TRINITY_GBG00117	RNA heli	Raphidocelis_subcap	74.50	0.00
TRINITY_XP_00294	hypothet	Volvox_carteri	74.50	0.00
TRINITY_GAX80600	hypothet	Chlamydomonas_eusti	74.50	0.00
TRINITY_XP_00300	predicte	Micromonas_pusilla	74.50	0.00
TRINITY_AJQ20789	acyl-CoA	Helianthus_annuus	74.40	0.00
TRINITY_RVW32220	hypothet	Vitis_vinifera	74.40	0.00
TRINITY_GAX77984	hypothet	Chlamydomonas_eusti	74.40	0.00

TRINITY_XP_0017(	adenylat	Chlamydomonas_reinh	74.40	0.00
TRINITY_XP_0016(	NADH-der	Chlamydomonas_reinh	74.40	0.00
TRINITY_ACG2448(	60S ribo	Zea_mays	74.40	0.00
TRINITY_PNH0643(	Vesicle	Tetrabaena_socialis	74.40	0.00
TRINITY_KXZ5302(	hypothet	Gonium_pectorale	74.40	0.00
TRINITY_XP_0016(	predicte	Chlamydomonas_reinh	74.40	0.00
TRINITY_XP_0017(	spermidi	Chlamydomonas_reinh	74.40	0.00
TRINITY_XP_0058(	hypothet	Chlorella_variabili	74.40	0.00
TRINITY_GBF9839(	hypothet	Raphidocelis_subcap	74.40	0.00
TRINITY_RZC6103(	hypothet	Papaver_somniferum	74.40	0.00
TRINITY_XP_0139(	hypothet	Monoraphidium_negle	74.40	0.00
TRINITY_KXZ4299(	hypothet	Gonium_pectorale	74.40	0.00
TRINITY_XP_0016(	SR prote	Chlamydomonas_reinh	74.40	0.00
TRINITY_GAX7636(	hypothet	Chlamydomonas_eusti	74.40	0.00
TRINITY_GBF9344(	ribosoma	Raphidocelis_subcap	74.40	0.00
TRINITY_GBF9621(	hypothet	Raphidocelis_subcap	74.40	0.00
TRINITY_VDC8615(	unnamed	Brassica_oleracea	74.40	0.00
TRINITY_GAX8155(	hypothet	Chlamydomonas_eusti	74.40	0.00
TRINITY_GBF9909(	peptidas	Raphidocelis_subcap	74.40	0.00
TRINITY_PNW7200(	hypothet	Chlamydomonas_reinh	74.40	0
TRINITY_KXZ4403(	hypothet	Gonium_pectorale	74.40	0.00
TRINITY_XP_0014(	predicte	Ostreococcus_sp._'l	74.40	0.00
TRINITY_XP_0017(	predicte	Chlamydomonas_reinh	74.40	0.00
TRINITY_XP_0029(	hypothet	Volvox_carteri	74.40	0.00
TRINITY_GAX7965(	hypothet	Chlamydomonas_eusti	74.40	0
TRINITY_XP_0016(	predicte	Chlamydomonas_reinh	74.40	0.00
TRINITY_XP_0030(	HAD-supe	Ostreococcus_tauri	74.40	0.00
TRINITY_XP_0016(	predicte	Chlamydomonas_reinh	74.40	0.00
TRINITY_XP_0113(	Peroxire	Auxenochlorella_pro	74.30	0.00
TRINITY_XP_0030(	predicte	Micromonas_pusilla	74.30	0.00
TRINITY_XP_0030(	predicte	Micromonas_pusilla	74.30	0.00
TRINITY_EPS7280(	hypothet	Genlisea_aurea	74.30	0.00
TRINITY_BAK0225(	predicte	Hordeum_vulgare	74.30	0.00
TRINITY_BAL4652(	hypothet	Gentiana_scabra_x_G	74.30	0.00
TRINITY_KXZ5320(	hypothet	Gonium_pectorale	74.30	0.00
TRINITY_GAX7418(	hypothet	Chlamydomonas_eusti	74.30	0.00
TRINITY_XP_0087(	protein	Phoenix_dactylifera	74.30	0.00
TRINITY_XP_0029(	hypothet	Volvox_carteri	74.30	0.00
TRINITY_KXZ5580(	hypothet	Gonium_pectorale	74.30	0.00
TRINITY_GAX7548(	hypothet	Chlamydomonas_eusti	74.30	0.00
TRINITY_GAX8155(	hypothet	Chlamydomonas_eusti	74.30	0.00
TRINITY_XP_0017(	protein	Chlamydomonas_reinh	74.30	0.00
TRINITY_GAQ8896(	Serine [	Klebsormidium_niten	74.30	0.00
TRINITY_GAX8483(	hypothet	Chlamydomonas_eusti	74.30	0.00
TRINITY_XP_0017(	predicte	Chlamydomonas_reinh	74.30	0.00
TRINITY_PNW8208(	hypothet	Chlamydomonas_reinh	74.30	0.00
TRINITY_XP_0106(	PREDICTI	Beta_vulgaris	74.30	0.00
TRINITY_XP_0029(	hypothet	Volvox_carteri	74.30	0.00
TRINITY_XP_0016(	oligsac(	Chlamydomonas_reinh	74.30	0.00
TRINITY_GAX7636(	hypothet	Chlamydomonas_eusti	74.30	0.00
TRINITY_GAX8068(	hypothet	Chlamydomonas_eusti	74.30	0.00
TRINITY_EPS7453(	hypothet	Genlisea_aurea	74.30	0.00
TRINITY_PIN0126(	60S ribo	Handroanthus_impeti	74.20	0.00
TRINITY_VDD0496(	unnamed	Brassica_rapa	74.20	0.00
TRINITY_GAX7713(	hypothet	Chlamydomonas_eusti	74.20	0.00
TRINITY_PNW8005(	hypothet	Chlamydomonas_reinh	74.20	0.00
TRINITY_XP_0029(	mitochon	Volvox_carteri	74.20	0.00
TRINITY_ERN0391(	hypothet	Amborella_trichopod	74.20	0.00
TRINITY_XP_0029(	hypothet	Volvox_carteri	74.20	0.00
TRINITY_XP_0017(	predicte	Chlamydomonas_reinh	74.20	0.00
TRINITY_KXZ4483(	hypothet	Gonium_pectorale	74.20	0.00
TRINITY_GBF8916(	phosphat	Raphidocelis_subcap	74.20	0.00
TRINITY_KXZ4352(	hypothet	Gonium_pectorale	74.20	0.00
TRINITY_XP_0016(	chlorop(	Chlamydomonas_reinh	74.20	0.00
TRINITY_PRW3917(	mismatch	Chlorella_sorokinia	74.20	0.00
TRINITY_KXZ4965(	hypothet	Gonium_pectorale	74.20	0.00

TRINITY_XP_0017(	predicte	Chlamydomonas_reinh	74.20	0.00
TRINITY_XP_0029	ABC trar	Volvox_carteri	74.20	0.00
TRINITY_KXZ4327	hypothet	Gonium_pectorale	74.20	0.00
TRINITY_BAK0096(	predicte	Hordeum_vulgare	74.20	0.00
TRINITY_KXZ4321	hypothet	Gonium_pectorale	74.20	0.00
TRINITY_GAX8040	hypothet	Chlamydomonas_eusti	74.20	0.00
TRINITY_AAX8604	14-3-3 p	Haematococcus_lacus	74.10	0.00
TRINITY_XP_0029	hypothet	Volvox_carteri	74.10	0.00
TRINITY_GAQ8639	Serine/t	Klebsormidium_niten	74.10	0.00
TRINITY_RWR7464	40S ribo	Cinnamomum_micranth	74.10	0.00
TRINITY_XP_0029	RabC/Rab	Volvox_carteri	74.10	0.00
TRINITY_XP_0016	catalyti	Chlamydomonas_reinh	74.10	0.00
TRINITY_GAX8223(	hypothet	Chlamydomonas_eusti	74.10	0.00
TRINITY_KXZ5055	hypothet	Gonium_pectorale	74.10	0.00
TRINITY_P81831.2	RecName:	Chlamydomonas_reinh	74.10	0.00
TRINITY_PNW7211	hypothet	Chlamydomonas_reinh	74.10	0.00
TRINITY_BBC2843	hypothet	Yamagishiella_unicc	74.10	0.00
TRINITY_BAJ9619	predicte	Hordeum_vulgare	74.10	0.00
TRINITY_GAX7856	hypothet	Chlamydomonas_eusti	74.10	0.00
TRINITY_KXZ4254	hypothet	Gonium_pectorale	74.10	0.00
TRINITY_KXZ5506	hypothet	Gonium_pectorale	74.10	0.00
TRINITY_XP_0029	hypothet	Volvox_carteri	74.10	0.00
TRINITY_KDD7549	hypothet	Helicosporidium_sp.	74.10	0.00
TRINITY_KDD7549	hypothet	Helicosporidium_sp.	74.10	0.00
TRINITY_XP_0016	gamma-gl	Chlamydomonas_reinh	74.10	0.00
TRINITY_GAX7900	hypothet	Chlamydomonas_eusti	74.10	0
TRINITY_ACN3153	unknown	Zea_mays	74.00	0.00
TRINITY_GAX7821	hypothet	Chlamydomonas_eusti	74.00	0.00
TRINITY_ACG2448	60S ribo	Zea_mays	74.00	0.00
TRINITY_GBF9901	hypothet	Raphidocelis_subcap	74.00	0.00
TRINITY_RLM6020	glyceral	Panicum_miliaceum	74.00	0.00
TRINITY_GBG6894	hypothet	Chara_braunii	74.00	0.00
TRINITY_KXZ4920	hypothet	Gonium_pectorale	74.00	0.00
TRINITY_GAX7996	hypothet	Chlamydomonas_eusti	74.00	0.00
TRINITY_GAX8438	hypothet	Chlamydomonas_eusti	74.00	0.00
TRINITY_PNH0198	DNA repa	Tetrabaena_socialis	74.00	0.00
TRINITY_P20364.2	RecName:	Daucus_carota	73.90	0.00
TRINITY_XP_0156	PREDICTE	Oryza_brachyantha	73.90	0.00
TRINITY_XP_0256	uncharac	Arachis_hypogaea	73.90	0.00
TRINITY_BAJ9379	predicte	Hordeum_vulgare	73.90	0.00
TRINITY_XP_0273	fumarate	Abrus_precatorius	73.90	0.00
TRINITY_GAX7976	hypothet	Chlamydomonas_eusti	73.90	0.00
TRINITY_GAX7832	hypothet	Chlamydomonas_eusti	73.90	0.00
TRINITY_KXZ5320	hypothet	Gonium_pectorale	73.90	0.00
TRINITY_KXZ5079	hypothet	Gonium_pectorale	73.90	0.00
TRINITY_KXZ4557	hypothet	Gonium_pectorale	73.90	0.00
TRINITY_GBF9381	hypothet	Raphidocelis_subcap	73.90	0.00
TRINITY_XP_0029	hypothet	Volvox_carteri	73.90	0.00
TRINITY_XP_0016	flagella	Chlamydomonas_reinh	73.90	0.00
TRINITY_XP_0016	mitochor	Chlamydomonas_reinh	73.90	0.00
TRINITY_XP_0017(	pyruvate	Chlamydomonas_reinh	73.90	0.00
TRINITY_XP_0029	hypothet	Volvox_carteri	73.90	0.00
TRINITY_XP_0228	ABC trar	Ostreococcus_tauri	73.90	0.00
TRINITY_GBG6513	hypothet	Chara_braunii	73.90	0.00
TRINITY_PNW8008	hypothet	Chlamydomonas_reinh	73.90	0.00
TRINITY_GAX7708	hypothet	Chlamydomonas_eusti	73.90	0.00
TRINITY_GAX8096	hypothet	Chlamydomonas_eusti	73.90	0.00
TRINITY_XP_0016	dynein h	Chlamydomonas_reinh	73.80	0.00
TRINITY_AFH8962	actin, p	Cymbidium_ensifoliu	73.80	0.00
TRINITY_XP_0096(	PREDICTE	Nicotiana_tomentosi	73.80	0.00
TRINITY_EFJ0748	hypothet	Selaginella_moellen	73.80	0.00
TRINITY_XP_0239(	40S ribo	Quercus_suber	73.80	0.00
TRINITY_KMS9401	hypothet	Beta_vulgaris	73.80	0.00
TRINITY_EPS7146	hypothet	Genlisea_aurea	73.80	0.00
TRINITY_GAX7513	hypothet	Chlamydomonas_eusti	73.80	0.00
TRINITY_PNW7570(	hypothet	Chlamydomonas_reinh	73.80	0.00

TRINITY_XP_0243	pre-mRN/	Physcomitrella_pate	73.80	0.00
TRINITY_XP_0029	hypothet	Volvox_carteri	73.80	0.00
TRINITY_GAX8243	hypothet	Chlamydomonas_eusti	73.80	0.00
TRINITY_XP_0110	PREDICTI	Populus_euphratica	73.80	0.00
TRINITY_XP_0200	tubulin	Ananas_comosus	73.80	0.00
TRINITY_XP_0238	DNA-dire	Quercus_suber	73.80	0.00
TRINITY_PNW8596	hypothet	Chlamydomonas_reinh	73.80	0.00
TRINITY_KXZ5409	hypothet	Gonium_pectorale	73.80	0.00
TRINITY_KXZ4672	hypothet	Gonium_pectorale	73.80	0.00
TRINITY_PRW0594	nuclear	Chlorella_sorokinia	73.80	0.00
TRINITY_BAK0734	predicte	Hordeum_vulgare	73.80	0.00
TRINITY_GAX8574	hypothet	Chlamydomonas_eusti	73.80	0.00
TRINITY_KXZ5475	hypothet	Gonium_pectorale	73.80	0.00
TRINITY_GAX8093	hypothet	Chlamydomonas_eusti	73.80	0.00
TRINITY_GAX7859	hypothet	Chlamydomonas_eusti	73.80	0
TRINITY_XP_0016	flagella	Chlamydomonas_reinh	73.80	0.00
TRINITY_PNW7169	hypothet	Chlamydomonas_reinh	73.80	0
TRINITY_GAX7605	hypothet	Chlamydomonas_eusti	73.80	0.00
TRINITY_GAX8376	hypothet	Chlamydomonas_eusti	73.80	0
TRINITY_XP_0207	60S ribo	Dendrobium_catenatu	73.70	0.00
TRINITY_XP_0156	DNA-dire	Oryza_sativa	73.70	0.00
TRINITY_GAX8248	hypothet	Chlamydomonas_eusti	73.70	0.00
TRINITY_KXZ5610	hypothet	Gonium_pectorale	73.70	0.00
TRINITY_ANA5703	ATP synt	Pyramimonas_parkeae	73.70	0.00
TRINITY_XP_0029	hypothet	Volvox_carteri	73.70	0.00
TRINITY_GAX7857	hypothet	Chlamydomonas_eusti	73.70	0.00
TRINITY_XP_0029	sterol 1	Volvox_carteri	73.70	0.00
TRINITY_XP_0138	Translat	Monoraphidium_negle	73.70	0.00
TRINITY_XP_0016	predicte	Chlamydomonas_reinh	73.70	0.00
TRINITY_AMY6315	sulfate	Tetraspora_sp._CU25	73.70	0.00
TRINITY_XP_0029	hypothet	Volvox_carteri	73.70	0.00
TRINITY_AEG7452	kinesin-	Dunaliella_salina	73.70	0.00
TRINITY_PNW8209	hypothet	Chlamydomonas_reinh	73.70	0.00
TRINITY_XP_0029	hypothet	Volvox_carteri	73.70	0.00
TRINITY_XP_0017	predicte	Chlamydomonas_reinh	73.70	0.00
TRINITY_AAO7355	reverse-	Chlamydomonas_reinh	73.70	0.00
TRINITY_KXZ4526	hypothet	Gonium_pectorale	73.70	0.00
TRINITY_GAX7662	hypothet	Chlamydomonas_eusti	73.70	0.00
TRINITY_GAX7864	hypothet	Chlamydomonas_eusti	73.70	0.00
TRINITY_GAX8146	hypothet	Chlamydomonas_eusti	73.70	0.00
TRINITY_KMS6461	hypothet	Beta_vulgaris	73.70	0.00
TRINITY_EFH5379	hypothet	Arabidopsis_lyrata	73.60	0.00
TRINITY_XP_0212	60S ribo	Herrania_umbratica	73.60	0.00
TRINITY_XP_0201	heat sho	Ananas_comosus	73.60	0.00
TRINITY_PNH0661	hypothet	Tetrabaena_socialis	73.60	0.00
TRINITY_XP_0016	iron-sul	Chlamydomonas_reinh	73.60	0.00
TRINITY_XP_0029	hypothet	Volvox_carteri	73.60	0.00
TRINITY_GAX8206	hypothet	Chlamydomonas_eusti	73.60	0.00
TRINITY_PNH1097	putative	Tetrabaena_socialis	73.60	0.00
TRINITY_BAK0046	predicte	Hordeum_vulgare	73.60	0.00
TRINITY_GAX7597	hypothet	Chlamydomonas_eusti	73.60	0.00
TRINITY_GAX8145	hypothet	Chlamydomonas_eusti	73.60	0.00
TRINITY_YP_0088	NADH deh	Monomastix_sp._OKE-	73.50	0.00
TRINITY_PHT3740	40S ribo	Capsicum_baccatum	73.50	0.00
TRINITY_PNW8807	hypothet	Chlamydomonas_reinh	73.50	0.00
TRINITY_PNW7165	hypothet	Chlamydomonas_reinh	73.50	0.00
TRINITY_GBF9704	histone	Raphidocelis_subcap	73.50	0.00
TRINITY_XP_0036	40S ribo	Medicago_truncatula	73.50	0.00
TRINITY_XP_0056	polyaden	Coccomyxa_subellips	73.50	0.00
TRINITY_XP_0029	hypothet	Volvox_carteri	73.50	0.00
TRINITY_KNA0305	hypothet	Spinacia_oleracea	73.50	0.00
TRINITY_GAX7877	hypothet	Chlamydomonas_eusti	73.50	0.00
TRINITY_ABR1809	unknown	Picea_sitchensis	73.50	0.00
TRINITY_GBF8735	CMGC RCK	Raphidocelis_subcap	73.50	0.00
TRINITY_CAI8489	putative	Medicago_sativa	73.50	0.00
TRINITY_GAX7280	hypothet	Chlamydomonas_eusti	73.50	0.00

TRINITY_XP_0279	probabl	Vigna_unguiculata	73.50	0.00
TRINITY_KFK3717	hypothet	Arabis_alpina	73.50	0.00
TRINITY_PRW5976	cytochr	Chlorella_sorokinia	73.50	0.00
TRINITY_ABU5296	hypothet	Dunaliella_viridis	73.50	0.00
TRINITY_XP_0016	mitochor	Chlamydomonas_reinh	73.50	0.00
TRINITY_XP_0212	60S rib	Herrania_umbratica	73.40	0.00
TRINITY_XP_0212	60S rib	Herrania_umbratica	73.40	0.00
TRINITY_XP_0016	eukaryot	Chlamydomonas_reinh	73.40	0.00
TRINITY_XP_0138	Stage V	Monoraphidium_negle	73.40	0.00
TRINITY_GBF9721	hypothet	Raphidocelis_subcap	73.40	0.00
TRINITY_GAX8352	hypothet	Chlamydomonas_eusti	73.40	0.00
TRINITY_GAX7557	hypothet	Chlamydomonas_eusti	73.40	0.00
TRINITY_GAX7451	hypothet	Chlamydomonas_eusti	73.40	0.00
TRINITY_GAX7978	hypothet	Chlamydomonas_eusti	73.40	0.00
TRINITY_PNH1037	Geranyl	Tetrabaena_socialis	73.40	0.00
TRINITY_ADI4711	nicotina	Chlamydomonas_reinh	73.40	0.00
TRINITY_XP_0045	peptidy	Cicer_arietinum	73.40	0.00
TRINITY_PHT5416	Ubiquit	Capsicum_baccatum	73.40	0.00
TRINITY_XP_0056	GDP-man	Coccomyxa_subellips	73.40	0.00
TRINITY_PNW7316	hypothet	Chlamydomonas_reinh	73.40	0.00
TRINITY_GAX8339	hypothet	Chlamydomonas_eusti	73.30	0.00
TRINITY_XP_0030	predict	Micromonas_pusilla	73.30	0.00
TRINITY_XP_0106	PREDICT	Beta_vulgaris	73.30	0.00
TRINITY_XP_0106	PREDICT	Beta_vulgaris	73.30	0.00
TRINITY_KMS6509	hypothet	Beta_vulgaris	73.30	0.00
TRINITY_PNG9987	NADH del	Tetrabaena_socialis	73.30	0.00
TRINITY_XP_0072	pyruvat	Prunus_persica	73.30	0.00
TRINITY_ABF8243	mitochor	Haematococcus_lacus	73.30	0.00
TRINITY_GAX7493	hypothet	Chlamydomonas_eusti	73.30	0.00
TRINITY_XP_0029	splicin	Volvox_carteri	73.30	0.00
TRINITY_XP_0023	40S rib	Populus_trichocarpa	73.30	0.00
TRINITY_CAA1126	polyubi	Nicotiana_tabacum	73.30	0.00
TRINITY_GAX8266	hypothet	Chlamydomonas_eusti	73.30	0.00
TRINITY_XP_0058	hypothet	Chlorella_variabili	73.30	0.00
TRINITY_GAX8049	hypothet	Chlamydomonas_eusti	73.30	0.00
TRINITY_GAX8376	hypothet	Chlamydomonas_eusti	73.30	0.00
TRINITY_XP_0029	RIO kin	Volvox_carteri	73.30	0.00
TRINITY_GAX8442	hypothet	Chlamydomonas_eusti	73.30	0.00
TRINITY_KZV4706	hypothet	Dorcoceras_hygromet	73.20	0.00
TRINITY_PNH0830	NFX1-ty	Tetrabaena_socialis	73.20	0.00
TRINITY_GBG7373	hypothet	Chara_braunii	73.20	0.00
TRINITY_XP_0029	hypothet	Volvox_carteri	73.20	0.00
TRINITY_GAX8512	hypothet	Chlamydomonas_eusti	73.20	0.00
TRINITY_XP_0220	histone	Helianthus_annuus	73.20	0.00
TRINITY_KXZ4760	hypothet	Gonium_pectorale	73.20	0.00
TRINITY_PNW7200	hypothet	Chlamydomonas_reinh	73.20	0.00
TRINITY_GAX7539	hypothet	Chlamydomonas_eusti	73.20	0.00
TRINITY_PNW7765	hypothet	Chlamydomonas_reinh	73.20	0.00
TRINITY_GAX8374	hypothet	Chlamydomonas_eusti	73.20	0.00
TRINITY_GAX7984	hypothet	Chlamydomonas_eusti	73.20	0.00
TRINITY_PNH0791	Chromos	Tetrabaena_socialis	73.20	0.00
TRINITY_PNW8742	hypothet	Chlamydomonas_reinh	73.20	0.00
TRINITY_KXZ4577	hypothet	Gonium_pectorale	73.20	0.00
TRINITY_GAX7821	hypothet	Chlamydomonas_eusti	73.20	0.00
TRINITY_XP_0016	coiled-	Chlamydomonas_reinh	73.20	0.00
TRINITY_GAX8123	hypothet	Chlamydomonas_eusti	73.20	0.00
TRINITY_GAX7624	hypothet	Chlamydomonas_eusti	73.20	0
TRINITY_OEL2077	Ras-rel	Dichanthelium_oligo	73.10	0.00
TRINITY_XP_0270	unchara	Coffea_arabica	73.10	0.00
TRINITY_XP_0229	60S rib	Cucurbita_moschata	73.10	0.00
TRINITY_XP_0029	hypothet	Volvox_carteri	73.10	0.00
TRINITY_XP_0229	serine/t	Cucurbita_maxima	73.10	0.00
TRINITY_GBF9312	diphthi	Raphidocelis_subcap	73.10	0.00
TRINITY_GBG6021	hypothet	Chara_braunii	73.10	0.00
TRINITY_KXZ5407	hypothet	Gonium_pectorale	73.10	0.00
TRINITY_GAQ8856	aconita	Klebsormidium_niten	73.10	0.00

TRINITY_GAX8418!	hypothet	Chlamydomonas_eusti	73.10	0.00
TRINITY_GAX7399	hypothet	Chlamydomonas_eusti	73.10	0.00
TRINITY_XP_0029	hypothet	Volvox_carteri	73.10	0.00
TRINITY_GAX8128	hypothet	Chlamydomonas_eusti	73.10	0.00
TRINITY_KXZ4631	hypothet	Gonium_pectorale	73.10	0.00
TRINITY_XP_0029	hypothet	Volvox_carteri	73.10	0.00
TRINITY_XP_0029	hypothet	Volvox_carteri	73.10	0.00
TRINITY_PNH0503	Histone	Tetrabaena_socialis	73.10	0.00
TRINITY_AFK0921	calcium-	Dunaliella_salina	73.10	0.00
TRINITY_XP_0238	unchara	Quercus_suber	73.00	0.00
TRINITY_XP_0072	SUMO-cor	Prunus_persica	73.00	0.00
TRINITY_XP_0029	componer	Volvox_carteri	73.00	0.00
TRINITY_AEB4001	cytochr	Funaria_hygrometric	73.00	0.00
TRINITY_XP_0138	hypothet	Monoraphidium_negle	73.00	0.00
TRINITY_XP_0017	predicte	Chlamydomonas_reinh	73.00	0.00
TRINITY_GAX7733	hypothet	Chlamydomonas_eusti	73.00	0.00
TRINITY_KFK3932	hypothet	Arabis_alpina	73.00	0.00
TRINITY_XP_0014	predicte	Ostreococcus_sp._'l	73.00	0.00
TRINITY_GAX8165	hypothet	Chlamydomonas_eusti	73.00	0.00
TRINITY_GAX7805	hypothet	Chlamydomonas_eusti	73.00	0.00
TRINITY_GAX7535	hypothet	Chlamydomonas_eusti	73.00	0.00
TRINITY_XP_0029	hypothet	Volvox_carteri	73.00	0.00
TRINITY_XP_0243	protein	Physcomitrella_pate	73.00	0.00
TRINITY_KXZ5099	RPT4 pr	Gonium_pectorale	73.00	0.00
TRINITY_GAX7922	hypothet	Chlamydomonas_eusti	73.00	0.00
TRINITY_XP_0016	low-CO2-	Chlamydomonas_reinh	73.00	0.00
TRINITY_XP_0029	hypothet	Volvox_carteri	73.00	0.00
TRINITY_KXZ5161	hypothet	Gonium_pectorale	73.00	0.00
TRINITY_GAX8658	hypothet	Chlamydomonas_eusti	73.00	0.00
TRINITY_GAX7297	hypothet	Chlamydomonas_eusti	73.00	0.00
TRINITY_XP_0029	hypothet	Volvox_carteri	73.00	0.00
TRINITY_KXZ5408	hypothet	Gonium_pectorale	73.00	0.00
TRINITY_XP_0058	hypothet	Chlorella_variabili	73.00	0.00
TRINITY_KDD7512	phosphat	Helicosporidium_sp.	72.90	0.00
TRINITY_YP_0088	NADH del	Monomastix_sp._OKE-	72.90	0.00
TRINITY_XP_0105	PREDICTI	Tarenaya_hasslerian	72.90	0.00
TRINITY_XP_0239	fimbrin-	Quercus_suber	72.90	0.00
TRINITY_BAK0218	predicte	Hordeum_vulgare	72.90	0.00
TRINITY_AFK4638	unknown	Lotus_japonicus	72.90	0.00
TRINITY_XP_0014	predicte	Ostreococcus_sp._'l	72.90	0.00
TRINITY_ABG6612	retrotra	Oryza_sativa	72.90	0.00
TRINITY_XP_0188	PREDICTI	Juglans_regia	72.90	0.00
TRINITY_KFK3651	hypothet	Arabis_alpina	72.90	0.00
TRINITY_GAX7756	hypothet	Chlamydomonas_eusti	72.90	0.00
TRINITY_XP_0139	60S ribo	Monoraphidium_negle	72.90	0.00
TRINITY_KXZ4588	hypothet	Gonium_pectorale	72.90	0.00
TRINITY_KXZ4708	hypothet	Gonium_pectorale	72.90	0.00
TRINITY_KXZ4636	hypothet	Gonium_pectorale	72.90	0.00
TRINITY_GAX8517	hypothet	Chlamydomonas_eusti	72.90	0.00
TRINITY_GAX8482	hypothet	Chlamydomonas_eusti	72.90	0.00
TRINITY_GAX7968	hypothet	Chlamydomonas_eusti	72.90	0.00
TRINITY_AWK6783	ACD [Dur	Dunaliella_tertiole	72.90	0.00
TRINITY_GAX7377	hypothet	Chlamydomonas_eusti	72.90	0.00
TRINITY_NP_0012	ripening	Solanum_lycopersicu	72.90	0.00
TRINITY_KXZ4468	hypothet	Gonium_pectorale	72.90	0.00
TRINITY_XP_0016	protopo	Chlamydomonas_reinh	72.90	0.00
TRINITY_GAX8045	hypothet	Chlamydomonas_eusti	72.90	0.00
TRINITY_XP_0029	hypothet	Volvox_carteri	72.90	0.00
TRINITY_KGN6251	hypothet	Cucumis_sativus	72.90	0.00
TRINITY_ACG2448	60S ribo	Zea_mays	72.80	0.00
TRINITY_XP_0063	PREDICTI	Solanum_tuberosum	72.80	0.00
TRINITY_PNH0179	Ras-rela	Tetrabaena_socialis	72.80	0.00
TRINITY_GAX7794	hypothet	Chlamydomonas_eusti	72.80	0.00
TRINITY_XP_0056	ribosoma	Coccomyxa_subellips	72.80	0.00
TRINITY_PNW8710	hypothet	Chlamydomonas_reinh	72.80	0.00
TRINITY_RRT7395	hypothet	Ensete_ventricosum	72.80	0.00

TRINITY_GBG72475	hypothet	Chara_braunii	72.80	0.00
TRINITY_A2T2X4.1	RecName:	Chlamydomonas_reinh	72.80	0.00
TRINITY_AML78291	putative	Pleurastrum_insigne	72.80	0.00
TRINITY_XP_00295	hypothet	Volvox_carteri	72.80	0.00
TRINITY_KXZ55695	hypothet	Gonium_pectorale	72.80	0.00
TRINITY_XP_00584	hypothet	Chlorella_variabili	72.80	0.00
TRINITY_GAX82654	hypothet	Chlamydomonas_eusti	72.80	0.00
TRINITY_PNW73109	hypothet	Chlamydomonas_reinh	72.80	0.00
TRINITY_XP_02644	RING-box	Papaver_somniferum	72.80	0.00
TRINITY_XP_02644	RING-box	Papaver_somniferum	72.80	0.00
TRINITY_GAX74406	hypothet	Chlamydomonas_eusti	72.80	0.00
TRINITY_PNW89001	hypothet	Chlamydomonas_reinh	72.80	0.00
TRINITY_OTG22836	putative	Helianthus_annuus	72.70	0.00
TRINITY_GAX83107	hypothet	Chlamydomonas_eusti	72.70	0.00
TRINITY_GBG71260	hypothet	Chara_braunii	72.70	0.00
TRINITY_XP_02390	40S ribo	Quercus_suber	72.70	0.00
TRINITY_KMS64494	hypothet	Beta_vulgaris	72.70	0.00
TRINITY_XP_00294	hypothet	Volvox_carteri	72.70	0.00
TRINITY_P0C8M8.1	RecName:	Zea_mays	72.70	0.00
TRINITY_PNW78506	hypothet	Chlamydomonas_reinh	72.70	0.00
TRINITY_GAX77312	hypothet	Chlamydomonas_eusti	72.70	0.00
TRINITY_PON97647	U5 small	Trema_orientale	72.70	0.00
TRINITY_GAX75458	hypothet	Chlamydomonas_eusti	72.70	0.00
TRINITY_XP_00295	hypothet	Volvox_carteri	72.70	0.00
TRINITY_KXZ45466	hypothet	Gonium_pectorale	72.70	0.00
TRINITY_RAL40391	hypothet	Cuscuta_australis	72.70	0.00
TRINITY_GAQ77748	hypothet	Klebsormidium_niten	72.70	0.00
TRINITY_KXZ51655	hypothet	Gonium_pectorale	72.70	0.00
TRINITY_XP_00250	DEAD/DEA	Micromonas_commoda	72.60	0.00
TRINITY_XP_00295	molecula	Volvox_carteri	72.60	0.00
TRINITY_VDD49652	unnamed	Brassica_oleracea	72.60	0.00
TRINITY_KMS64936	hypothet	Beta_vulgaris	72.60	0.00
TRINITY_XP_00169	predicte	Chlamydomonas_reinh	72.60	0.00
TRINITY_GAX82023	hypothet	Chlamydomonas_eusti	72.60	0.00
TRINITY_KXZ42331	hypothet	Gonium_pectorale	72.60	0.00
TRINITY_XP_00295	hypothet	Volvox_carteri	72.60	0.00
TRINITY_GAX79225	hypothet	Chlamydomonas_eusti	72.60	0.00
TRINITY_XP_00295	hypothet	Volvox_carteri	72.60	0.00
TRINITY_PSC74533	Protochl	Micractinium_conduc	72.60	0.00
TRINITY_GAX84230	hypothet	Chlamydomonas_eusti	72.60	0.00
TRINITY_KXZ52279	hypothet	Gonium_pectorale	72.60	0.00
TRINITY_XP_00294	mitochon	Volvox_carteri	72.60	0.00
TRINITY_PNW87875	hypothet	Chlamydomonas_reinh	72.60	0.00
TRINITY_GAX79372	hypothet	Chlamydomonas_eusti	72.60	0.00
TRINITY_CAA69144	apocytoc	Chlorogonium_elonga	72.60	0.00
TRINITY_GAX81367	hypothet	Chlamydomonas_eusti	72.60	0.00
TRINITY_GAX74230	hypothet	Chlamydomonas_eusti	72.60	0.00
TRINITY_GAX84030	hypothet	Chlamydomonas_eusti	72.60	0.00
TRINITY_XP_00294	hypothet	Volvox_carteri	72.60	0.00
TRINITY_XP_00295	histone	Volvox_carteri	72.60	0.00
TRINITY_XP_00564	ribosoma	Coccomyxa_subellips	72.50	0.00
TRINITY_EPS62326	hypothet	Genlisea_aurea	72.50	0.00
TRINITY_PNW84960	hypothet	Chlamydomonas_reinh	72.50	0.00
TRINITY_XP_01880	PREDICTE	Juglans_regia	72.50	0.00
TRINITY_XP_02389	60S ribo	Quercus_suber	72.50	0.00
TRINITY_ABK96400	unknown	Populus_trichocarpa	72.50	0.00
TRINITY_GAX85451	hypothet	Chlamydomonas_eusti	72.50	0.00
TRINITY_GBF95639	glutamin	Raphidocelis_subcap	72.50	0.00
TRINITY_GAX81867	hypothet	Chlamydomonas_eusti	72.50	0.00
TRINITY_PNW75700	hypothet	Chlamydomonas_reinh	72.50	0.00
TRINITY_GBF96066	chloroph	Raphidocelis_subcap	72.50	0.00
TRINITY_GAX84032	hypothet	Chlamydomonas_eusti	72.50	0.00
TRINITY_XP_00294	hypothet	Volvox_carteri	72.50	0.00
TRINITY_PNW88355	hypothet	Chlamydomonas_reinh	72.50	0.00
TRINITY_KXZ53533	hypothet	Gonium_pectorale	72.50	0.00
TRINITY_XP_02440	uncharac	Physcomitrella_pate	72.50	0.00

TRINITY_GAX7620	hypothet	Chlamydomonas_eusti	72.50	0.00
TRINITY_KMS6478	hypothet	Beta_vulgaris	72.50	0.00
TRINITY_PNW8630	hypothet	Chlamydomonas_reinh	72.50	0.00
TRINITY_PNW8614	hypothet	Chlamydomonas_reinh	72.50	0.00
TRINITY_XP_0138	ribonuc	Monoraphidium_negle	72.50	0.00
TRINITY_KXZ4323	hypothet	Gonium_pectorale	72.50	0.00
TRINITY_XP_0016	mixed l	Chlamydomonas_reinh	72.50	0.00
TRINITY_PSC7574	Amidoph	Micractinium_conduc	72.50	0.00
TRINITY_XP_0016	predict	Chlamydomonas_reinh	72.50	0.00
TRINITY_PIA3182	hypothet	Aquilegia_coerulea	72.50	0.00
TRINITY_KXZ4873	hypothet	Gonium_pectorale	72.50	0.00
TRINITY_PSC6927	Ribosom	Micractinium_conduc	72.50	0.00
TRINITY_XP_0029	alanine	Volvox_carteri	72.50	0.00
TRINITY_PNW7409	hypothet	Chlamydomonas_reinh	72.50	0.00
TRINITY_XP_0029	GTP-bin	Selaginella_moellen	72.50	0.00
TRINITY_KXZ4845	hypothet	Gonium_pectorale	72.50	0.00
TRINITY_PNW7427	hypothet	Chlamydomonas_reinh	72.50	0
TRINITY_GAX7420	hypothet	Chlamydomonas_eusti	72.50	0.00
TRINITY_XP_0139	ubiquit	Monoraphidium_negle	72.40	0.00
TRINITY_RXH6945	hypothet	Malus_domestica	72.40	0.00
TRINITY_XP_0029	hypothet	Volvox_carteri	72.40	0.00
TRINITY_XP_0063	PREDICT	Solanum_tuberosum	72.40	0.00
TRINITY_XP_0138	hypothet	Monoraphidium_negle	72.40	0.00
TRINITY_KXZ4921	hypothet	Gonium_pectorale	72.40	0.00
TRINITY_PHU0042	Polyubi	Capsicum_chinense	72.40	0.00
TRINITY_PNW7798	hypothet	Chlamydomonas_reinh	72.40	0.00
TRINITY_GAQ8332	sumo co	Klebsormidium_niten	72.40	0.00
TRINITY_GAX7738	hypothet	Chlamydomonas_eusti	72.40	0.00
TRINITY_GAX7448	hypothet	Chlamydomonas_eusti	72.40	0.00
TRINITY_RLM7435	60S rib	Panicum_miliaceum	72.30	0.00
TRINITY_XP_0202	cytochr	Asparagus_officinal	72.30	0.00
TRINITY_GAQ8693	hypothet	Klebsormidium_niten	72.30	0.00
TRINITY_EPS7280	hypothet	Genlisea_aurea	72.30	0.00
TRINITY_XP_0029	p300/CB	Volvox_carteri	72.30	0.00
TRINITY_XP_0029	isocitr	Volvox_carteri	72.30	0.00
TRINITY_BAT8558	hypothet	Vigna_angularis	72.30	0.00
TRINITY_PNW8183	hypothet	Chlamydomonas_reinh	72.30	0.00
TRINITY_XP_0243	26S prot	Physcomitrella_pate	72.30	0.00
TRINITY_GAX8001	hypothet	Chlamydomonas_eusti	72.30	0.00
TRINITY_XP_0016	predict	Chlamydomonas_reinh	72.30	0.00
TRINITY_XP_0243	ribosom	Physcomitrella_pate	72.30	0.00
TRINITY_KXZ4525	hypothet	Gonium_pectorale	72.30	0.00
TRINITY_GAX7866	hypothet	Chlamydomonas_eusti	72.30	0.00
TRINITY_XP_0016	Qb-SNAR	Chlamydomonas_reinh	72.30	0.00
TRINITY_PNW7891	hypothet	Chlamydomonas_reinh	72.30	0.00
TRINITY_XP_0016	O-linke	Chlamydomonas_reinh	72.30	0.00
TRINITY_KXZ5015	hypothet	Gonium_pectorale	72.30	0.00
TRINITY_GAX7260	hypothet	Chlamydomonas_eusti	72.30	0.00
TRINITY_OWM8129	hypothet	Punica_granatum	72.30	0.00
TRINITY_XP_0139	OTU dom	Monoraphidium_negle	72.30	0.00
TRINITY_PNW8257	hypothet	Chlamydomonas_reinh	72.30	0.00
TRINITY_PNW7423	hypothet	Chlamydomonas_reinh	72.20	0.00
TRINITY_XP_0243	ras-rel	Physcomitrella_pate	72.20	0.00
TRINITY_PNH0830	NFX1-ty	Tetrabaena_socialis	72.20	0.00
TRINITY_PRW2050	Pre-mRN	Chlorella_sorokinia	72.20	0.00
TRINITY_XP_0029	ribonuc	Selaginella_moellen	72.20	0.00
TRINITY_XP_0056	PLP-dep	Coccomyxa_subellips	72.20	0.00
TRINITY_GAX8277	hypothet	Chlamydomonas_eusti	72.20	0.00
TRINITY_KXZ5456	hypothet	Gonium_pectorale	72.20	0.00
TRINITY_PNW7672	hypothet	Chlamydomonas_reinh	72.20	0.00
TRINITY_GBF9115	hypothet	Raphidocelis_subcap	72.20	0.00
TRINITY_XP_0058	hypothet	Chlorella_variabili	72.20	0.00
TRINITY_KXZ4921	hypothet	Gonium_pectorale	72.20	0.00
TRINITY_XP_0056	FKBP-ty	Coccomyxa_subellips	72.20	0.00
TRINITY_GAX7876	hypothet	Chlamydomonas_eusti	72.20	0.00
TRINITY_PNW6989	hypothet	Chlamydomonas_reinh	72.20	0.00

TRINITY_KXZ5354	hypothet	Gonium_pectorale	72.20	0.00
TRINITY_XP_0194	PREDICTI	Lupinus_angustifoli	72.20	0.00
TRINITY_GAX8494	hypothet	Chlamydomonas_eusti	72.20	0.00
TRINITY_GAX7687	hypothet	Chlamydomonas_eusti	72.20	0.00
TRINITY_XP_0016	rubisco	Chlamydomonas_reinh	72.20	0.00
TRINITY_PNW7680	hypothet	Chlamydomonas_reinh	72.20	0.00
TRINITY_PNH0816	Splicing	Tetrabaena_socialis	72.20	0.00
TRINITY_XP_0029	succinat	Volvox_carteri	72.20	0.00
TRINITY_GAX7769	hypothet	Chlamydomonas_eusti	72.20	0
TRINITY_XP_0138	thiamine	Monoraphidium_negle	72.20	0.00
TRINITY_AWK6783	ACOX [Du	Dunaliella_tertiole	72.20	0.00
TRINITY_PNW8568	hypothet	Chlamydomonas_reinh	72.20	0.00
TRINITY_KXZ5371	hypothet	Gonium_pectorale	72.20	0.00
TRINITY_ABK2651	unknown	Picea_sitchensis	72.20	0.00
TRINITY_ABK2651	unknown	Picea_sitchensis	72.20	0.00
TRINITY_KMS9327	hypothet	Beta_vulgaris	72.10	0.00
TRINITY_PNH0737	GTP-binc	Tetrabaena_socialis	72.10	0.00
TRINITY_GAX7630	hypothet	Chlamydomonas_eusti	72.10	0.00
TRINITY_XP_0025	predicte	Micromonas_commoda	72.10	0.00
TRINITY_XP_0139	hypothet	Monoraphidium_negle	72.10	0.00
TRINITY_XP_0070	PREDICTI	Theobroma_cacao	72.10	0.00
TRINITY_KXZ5061	hypothet	Gonium_pectorale	72.10	0.00
TRINITY_XP_0016	sirohydı	Chlamydomonas_reinh	72.10	0.00
TRINITY_XP_0029	hypothet	Volvox_carteri	72.10	0.00
TRINITY_GAX8638	hypothet	Chlamydomonas_eusti	72.10	0.00
TRINITY_GAX8278	hypothet	Chlamydomonas_eusti	72.10	0.00
TRINITY_XP_0208	ATP-depe	Arabidopsis_lyrata	72.10	0.00
TRINITY_GBF8956	hypothet	Raphidocelis_subcap	72.10	0.00
TRINITY_KMZ5947	Cyclin-c	Zostera_marina	72.10	0.00
TRINITY_PNW8526	hypothet	Chlamydomonas_reinh	72.10	0.00
TRINITY_GAX8093	hypothet	Chlamydomonas_eusti	72.10	0.00
TRINITY_GAX8574	hypothet	Chlamydomonas_eusti	72.10	0.00
TRINITY_XP_0194	PREDICTI	Lupinus_angustifoli	72.10	0.00
TRINITY_KXZ4433	hypothet	Gonium_pectorale	72.10	0.00
TRINITY_KXZ4264	hypothet	Gonium_pectorale	72.10	0.00
TRINITY_PNH0552	Chaperor	Tetrabaena_socialis	72.10	0.00
TRINITY_XP_0230	ATP-depe	Cucurbita_maxima	72.10	0.00
TRINITY_XP_0106	PREDICTI	Beta_vulgaris	72.10	0.00
TRINITY_XP_0212	60S ribc	Herrania_umbratica	72.00	0.00
TRINITY_OAE3195	hypothet	Marchantia_polymorp	72.00	0.00
TRINITY_ACN9445	photosys	Dunaliella_salina	72.00	0.00
TRINITY_GAX7472	hypothet	Chlamydomonas_eusti	72.00	0.00
TRINITY_XP_0106	PREDICTI	Beta_vulgaris	72.00	0.00
TRINITY_XP_0097	PREDICTI	Nicotiana_sylvestri	72.00	0.00
TRINITY_XP_0029	hypothet	Volvox_carteri	72.00	0.00
TRINITY_ABD6459	ribosoma	Chlamydomonas_sp._I	72.00	0.00
TRINITY_XP_0058	hypothet	Chlorella_variabili	72.00	0.00
TRINITY_GAX8458	hypothet	Chlamydomonas_eusti	72.00	0.00
TRINITY_GAX8166	hypothet	Chlamydomonas_eusti	72.00	0.00
TRINITY_XP_0017	peroxisc	Chlamydomonas_reinh	72.00	0.00
TRINITY_XP_0243	26S prot	Physcomitrella_pate	72.00	0.00
TRINITY_XP_0016	splicing	Chlamydomonas_reinh	72.00	0.00
TRINITY_GBF9723	sec-inde	Raphidocelis_subcap	72.00	0.00
TRINITY_XP_0165	PREDICTI	Nicotiana_tabacum	71.90	0.00
TRINITY_XP_0016	ribosoma	Chlamydomonas_reinh	71.90	0.00
TRINITY_XP_0238	uncharac	Quercus_suber	71.90	0.00
TRINITY_XP_0016	mitochor	Chlamydomonas_reinh	71.90	0.00
TRINITY_PNH0210	hypothet	Tetrabaena_socialis	71.90	0.00
TRINITY_KXZ5155	hypothet	Gonium_pectorale	71.90	0.00
TRINITY_XP_0029	hypothet	Volvox_carteri	71.90	0.00
TRINITY_XP_0016	predicte	Chlamydomonas_reinh	71.90	0.00
TRINITY_XP_0058	hypothet	Chlorella_variabili	71.90	0.00
TRINITY_GAX7543	hypothet	Chlamydomonas_eusti	71.90	0.00
TRINITY_XP_0017	predicte	Chlamydomonas_reinh	71.90	0.00
TRINITY_GAX8324	hypothet	Chlamydomonas_eusti	71.90	0.00
TRINITY_GAX8024	hypothet	Chlamydomonas_eusti	71.90	0.00

TRINITY_GAX8072	hypothet	Chlamydomonas_eusti	71.90	0.00
TRINITY_XP_0106	PREDICTI	Beta_vulgaris	71.80	0.00
TRINITY_XP_0082	PREDICTI	Prunus_mume	71.80	0.00
TRINITY_XP_0082	PREDICTI	Prunus_mume	71.80	0.00
TRINITY_XP_0016	uroporph	Chlamydomonas_reinh	71.80	0.00
TRINITY_AAO0086	Unknown	Arabidopsis_thalian	71.80	0.00
TRINITY_XP_0201	cytoplas	Aegilops_tauschii	71.80	0.00
TRINITY_GAX7707	hypothet	Chlamydomonas_eusti	71.80	0.00
TRINITY_GAX7549	hypothet	Chlamydomonas_eusti	71.80	0.00
TRINITY_XP_0016	lipoate	Chlamydomonas_reinh	71.80	0.00
TRINITY_PNW8034	hypothet	Chlamydomonas_reinh	71.80	0.00
TRINITY_GBF9713	hypothet	Raphidocelis_subcap	71.80	0.00
TRINITY_GAX7665	hypothet	Chlamydomonas_eusti	71.80	0.00
TRINITY_PRW6078	Ribosoma	Chlorella_sorokinia	71.80	0.00
TRINITY_GBF9295	ubiquiti	Raphidocelis_subcap	71.80	0.00
TRINITY_GAX7926	hypothet	Chlamydomonas_eusti	71.80	0.00
TRINITY_XP_0016	predicte	Chlamydomonas_reinh	71.80	0.00
TRINITY_PNW8059	hypothet	Chlamydomonas_reinh	71.80	0.00
TRINITY_KMS6493	hypothet	Beta_vulgaris	71.80	0.00
TRINITY_GAX7423	hypothet	Chlamydomonas_eusti	71.80	0.00
TRINITY_XP_0243	NEDD8-co	Physcomitrella_pate	71.70	0.00
TRINITY_XP_0227	uncharac	Durio_zibethinus	71.70	0.00
TRINITY_GAX8019	hypothet	Chlamydomonas_eusti	71.70	0.00
TRINITY_XP_0029	hypothet	Volvox_carteri	71.70	0.00
TRINITY_GBG6292	hypothet	Chara_braunii	71.70	0.00
TRINITY_BAK0560	predicte	Hordeum_vulgare	71.70	0.00
TRINITY_XP_0017	peptidyl	Chlamydomonas_reinh	71.70	0.00
TRINITY_PNW8696	hypothet	Chlamydomonas_reinh	71.70	0.00
TRINITY_PNH0792	Ribokina	Tetrabaena_socialis	71.70	0.00
TRINITY_KXZ4713	hypothet	Gonium_pectorale	71.70	0.00
TRINITY_PRW6063	starch b	Chlorella_sorokinia	71.70	0.00
TRINITY_GAX8096	hypothet	Chlamydomonas_eusti	71.70	0.00
TRINITY_XP_0029	hypothet	Volvox_carteri	71.70	0.00
TRINITY_XP_0243	ruvB-lik	Physcomitrella_pate	71.70	0.00
TRINITY_GAX8214	hypothet	Chlamydomonas_eusti	71.70	0.00
TRINITY_GAX8601	hypothet	Chlamydomonas_eusti	71.70	0.00
TRINITY_KXZ5162	hypothet	Gonium_pectorale	71.70	0.00
TRINITY_KXZ4506	hypothet	Gonium_pectorale	71.70	0.00
TRINITY_GAX7312	hypothet	Chlamydomonas_eusti	71.70	0.00
TRINITY_XP_0228	heat sho	Olea_europaea	71.70	0.00
TRINITY_GAX7538	hypothet	Chlamydomonas_eusti	71.70	0.00
TRINITY_YP_0094	4-dihydr	Haematococcus_lacus	71.70	0.00
TRINITY_GAX7821	hypothet	Chlamydomonas_eusti	71.70	0.00
TRINITY_GAX7539	hypothet	Chlamydomonas_eusti	71.70	0.00
TRINITY_PNW6957	hypothet	Chlamydomonas_reinh	71.70	0.00
TRINITY_GAX8017	hypothet	Chlamydomonas_eusti	71.70	0.00
TRINITY_XP_0029	hypothet	Volvox_carteri	71.70	0.00
TRINITY_GAX7679	hypothet	Chlamydomonas_eusti	71.70	0
TRINITY_PNH0232	Histone	Tetrabaena_socialis	71.70	0.00
TRINITY_GAX8362	hypothet	Chlamydomonas_eusti	71.70	0.00
TRINITY_GAX8192	hypothet	Chlamydomonas_eusti	71.70	0.00
TRINITY_XP_0139	cytochro	Monoraphidium_negle	71.70	0.00
TRINITY_XP_0029	hypothet	Volvox_carteri	71.70	0.00
TRINITY_XP_0238	ras-rela	Quercus_suber	71.60	0.00
TRINITY_XP_0245	DEAD-box	Selaginella_moellen	71.60	0.00
TRINITY_XP_0156	protein	Oryza_sativa	71.60	0.00
TRINITY_XP_0205	60S ribo	Phalaenopsis_equest	71.60	0.00
TRINITY_BAK0024	predicte	Hordeum_vulgare	71.60	0
TRINITY_XP_0216	60S ribo	Hevea_brasiliensis	71.60	0.00
TRINITY_XP_0029	peptidyl	Selaginella_moellen	71.60	0.00
TRINITY_KXZ5092	hypothet	Gonium_pectorale	71.60	0.00
TRINITY_PNW7792	hypothet	Chlamydomonas_reinh	71.60	0.00
TRINITY_XP_0016	dihydrod	Chlamydomonas_reinh	71.60	0.00
TRINITY_KXZ5590	hypothet	Gonium_pectorale	71.60	0.00
TRINITY_PNW7301	hypothet	Chlamydomonas_reinh	71.60	0.00
TRINITY_XP_0017	peptidyl	Chlamydomonas_reinh	71.60	0.00

TRINITY_XP_0016(	predicte	Chlamydomonas_reinh	71.60	0.00
TRINITY_GAX7374	hypothet	Chlamydomonas_eusti	71.60	0.00
TRINITY_XP_0138	MAP kina	Monoraphidium_negle	71.60	0.00
TRINITY_KGN6252	hypothet	Cucumis_sativus	71.60	0.00
TRINITY_XP_0202	ubiquit	Cajanus_cajan	71.50	0.00
TRINITY_XP_0016(	sulfate	Chlamydomonas_reinh	71.50	0.00
TRINITY_KXZ5256	hypothet	Gonium_pectorale	71.50	0.00
TRINITY_GAX8134	hypothet	Chlamydomonas_eusti	71.50	0.00
TRINITY_KXZ5017	hypothet	Gonium_pectorale	71.50	0.00
TRINITY_BAK0118	predicte	Hordeum_vulgare	71.50	0.00

TRINITY_XP_00295	hypothet	Volvox_carteri	71.50	0.00
TRINITY_XP_00168	deoxypus	Chlamydomonas_reinh	71.50	0.00
TRINITY_PNW77591	hypothet	Chlamydomonas_reinh	71.50	0.00
TRINITY_GAX76784	hypothet	Chlamydomonas_eusti	71.50	0.00
TRINITY_GAX76470	hypothet	Chlamydomonas_eusti	71.50	0.00
TRINITY_XP_00295	meiotic	Volvox_carteri	71.50	0.00
TRINITY_PNW73926	hypothet	Chlamydomonas_reinh	71.50	0
TRINITY_XP_00305	predicte	Micromonas_pusilla	71.40	0.00
TRINITY_ACU24256	unknown	Glycine_max	71.40	0.00
TRINITY_XP_00305	predicte	Micromonas_pusilla	71.40	0.00
TRINITY_ACT33369	mago nas	Lycoris_radiata	71.40	0.00
TRINITY_BAK03491	predicte	Hordeum_vulgare	71.40	0.00
TRINITY_PNH12876	Mitochon	Tetrabaena_socialis	71.40	0.00
TRINITY_XP_01101	PREDICTE	Populus_euphratica	71.40	0.00
TRINITY_XP_01389	autophag	Monoraphidium_negle	71.40	0.00
TRINITY_XP_02439	60S ribo	Physcomitrella_pate	71.40	0.00
TRINITY_PNH04467	Methyltr	Tetrabaena_socialis	71.40	0.00
TRINITY_PIA58609	hypothet	Aquilegia_coerulea	71.40	0.00
TRINITY_CCC55422	S-adenos	Pinus_pinaster	71.40	0.00
TRINITY_GAX78892	hypothet	Chlamydomonas_eusti	71.40	0.00
TRINITY_XP_00170	ubiquino	Chlamydomonas_reinh	71.40	0.00
TRINITY_PNW79612	hypothet	Chlamydomonas_reinh	71.40	0.00
TRINITY_PNH03720	hypothet	Tetrabaena_socialis	71.40	0.00
TRINITY_PNW77491	hypothet	Chlamydomonas_reinh	71.40	0.00
TRINITY_PNH12731	hypothet	Tetrabaena_socialis	71.40	0.00
TRINITY_XP_00169	predicte	Chlamydomonas_reinh	71.40	0.00
TRINITY_GAX78639	hypothet	Chlamydomonas_eusti	71.40	0.00
TRINITY_GAQ85287	small nu	Klebsormidium_niten	71.40	0.00
TRINITY_GAX73482	hypothet	Chlamydomonas_eusti	71.40	0.00
TRINITY_GAX79289	hypothet	Chlamydomonas_eusti	71.40	0.00
TRINITY_KXZ43546	hypothet	Gonium_pectorale	71.40	0.00
TRINITY_KXZ51579	hypothet	Gonium_pectorale	71.40	0.00
TRINITY_XP_02454	putative	Selaginella_moellen	71.40	0.00
TRINITY_GAX74145	hypothet	Chlamydomonas_eusti	71.40	0.00
TRINITY_GAX82024	hypothet	Chlamydomonas_eusti	71.40	0.00
TRINITY_XP_02641	pyruvate	Papaver_somniferum	71.40	0.00
TRINITY_XP_00169	predicte	Chlamydomonas_reinh	71.40	0.00
TRINITY_GAX79141	hypothet	Chlamydomonas_eusti	71.40	0.00
TRINITY_GAX84985	hypothet	Chlamydomonas_eusti	71.40	0.00
TRINITY_GAX81139	hypothet	Chlamydomonas_eusti	71.40	0.00
TRINITY_ACG25031	60S ribo	Zea_mays	71.40	0.00
TRINITY_XP_00940	PREDICTE	Musa_acuminata	71.30	0.00
TRINITY_ADE77564	unknown	Picea_sitchensis	71.30	0.00
TRINITY_XP_02498	transcri	Cynara_cardunculus	71.30	0.00
TRINITY_AEG74028	alpha-tu	Apopellia_endiviifo	71.30	0.00
TRINITY_XP_02387	40S ribo	Quercus_suber	71.30	0.00
TRINITY_GAQ90682	DNA repa	Klebsormidium_niten	71.30	0.00
TRINITY_XP_01139	Tubulin	Auxenochlorella_pro	71.30	0.00
TRINITY_PNW80428	hypothet	Chlamydomonas_reinh	71.30	0.00
TRINITY_XP_00170	kinesin-	Chlamydomonas_reinh	71.30	0.00
TRINITY_XP_00170	myo-inos	Chlamydomonas_reinh	71.30	0.00
TRINITY_PNH02790	hypothet	Tetrabaena_socialis	71.30	0.00
TRINITY_XP_00564	formamid	Coccomyxa_subellips	71.30	0.00
TRINITY_ABA01140	chloropl	Chlamydomonas_incer	71.30	0.00
TRINITY_XP_01389	ribulose	Monoraphidium_negle	71.30	0.00
TRINITY_PNW78932	hypothet	Chlamydomonas_reinh	71.30	0.00
TRINITY_XP_00294	Kar3 mem	Volvox_carteri	71.30	0
TRINITY_GAX83470	hypothet	Chlamydomonas_eusti	71.30	0.00
TRINITY_GAX84287	hypothet	Chlamydomonas_eusti	71.30	0.00
TRINITY_GAX78677	hypothet	Chlamydomonas_eusti	71.30	0.00
TRINITY_GAQ90760	Inositol	Klebsormidium_niten	71.30	0.00
TRINITY_PNW76169	hypothet	Chlamydomonas_reinh	71.30	0
TRINITY_EFJ16334	hypothet	Selaginella_moellen	71.20	0.00
TRINITY_EMS68701	Inositol	Triticum_urartu	71.20	0.00
TRINITY_RLN24162	hypothet	Panicum_miliaceum	71.20	0.00
TRINITY_XP_00564	threonyl	Coccomyxa_subellips	71.20	0.00

TRINITY_RLM8674	hypothet	Panicum_miliaceum	71.20	0.00
TRINITY_XP_0238	40S rib	Quercus_suber	71.20	0.00
TRINITY_XP_0128	PREDICT	Erythranthe_guttata	71.20	0.00
TRINITY_XP_0238	60S rib	Quercus_suber	71.20	0.00
TRINITY_XP_0238	60S rib	Quercus_suber	71.20	0.00
TRINITY_XP_0029	hypothet	Volvox_carteri	71.20	0.00
TRINITY_XP_0029	hypothet	Volvox_carteri	71.20	0.00
TRINITY_XP_0029	hypothet	Volvox_carteri	71.20	0.00
TRINITY_XP_0016	predict	Chlamydomonas_reinh	71.20	0.00
TRINITY_OEL2224	Heat sh	Dichanthelium_oligo	71.20	0.00
TRINITY_PNH0830	NFX1-ty	Tetrabaena_socialis	71.20	0.00
TRINITY_XP_0029	hypothet	Volvox_carteri	71.20	0.00
TRINITY_PRW5738	FO synt	Chlorella_sorokinia	71.20	0.00
TRINITY_XP_0029	hypothet	Volvox_carteri	71.20	0.00
TRINITY_OUS4208	PsaL ph	Ostreococcus_tauri	71.20	0.00
TRINITY_KXZ5669	hypothet	Gonium_pectorale	71.20	0.00
TRINITY_KXZ5030	hypothet	Gonium_pectorale	71.20	0.00
TRINITY_XP_0029	hypothet	Volvox_carteri	71.20	0.00
TRINITY_PNW8460	hypothet	Chlamydomonas_reinh	71.20	0.00
TRINITY_XP_0139	DNA lig	Monoraphidium_negle	71.20	0.00
TRINITY_GAX7882	hypothet	Chlamydomonas_eusti	71.20	0
TRINITY_AAO7355	reverse-	Chlamydomonas_reinh	71.20	0.00
TRINITY_XP_0029	hypothet	Volvox_carteri	71.20	0.00
TRINITY_XP_0029	copper	Volvox_carteri	71.20	0.00
TRINITY_KRH1779	hypothet	Glycine_max	71.20	0.00
TRINITY_GBF8774	hypothet	Raphidocelis_subcap	71.20	0.00
TRINITY_GAX7633	hypothet	Chlamydomonas_eusti	71.10	0.00
TRINITY_XP_0279	ADP-rib	Vigna_unguiculata	71.10	0.00
TRINITY_XP_0029	isocitr	Volvox_carteri	71.10	0.00
TRINITY_XP_0016	predict	Chlamydomonas_reinh	71.10	0.00
TRINITY_XP_0029	hypothet	Volvox_carteri	71.10	0.00
TRINITY_XP_0017	predict	Chlamydomonas_reinh	71.10	0.00
TRINITY_GBF8997	FAD dep	Raphidocelis_subcap	71.10	0.00
TRINITY_XP_0014	predict	Ostreococcus_sp._'l	71.10	0.00
TRINITY_PWZ2408	ADP-rib	Zea_mays	71.10	0.00
TRINITY_XP_0218	eukaryo	Carica_papaya	71.10	0.00
TRINITY_KXZ4761	hypothet	Gonium_pectorale	71.10	0.00
TRINITY_GAX8283	hypothet	Chlamydomonas_eusti	71.10	0.00
TRINITY_KXZ5597	hypothet	Gonium_pectorale	71.10	0.00
TRINITY_GBF9929	hypothet	Raphidocelis_subcap	71.10	0.00
TRINITY_GAX8415	hypothet	Chlamydomonas_eusti	71.10	0.00
TRINITY_XP_0030	mRNA sp	Micromonas_pusilla	71.10	0.00
TRINITY_PNW7045	hypothet	Chlamydomonas_reinh	71.10	0.00
TRINITY_PNW7725	hypothet	Chlamydomonas_reinh	71.10	0.00
TRINITY_XP_0029	hypothet	Volvox_carteri	71.10	0.00
TRINITY_ADE7717	unknown	Picea_sitchensis	71.10	0.00
TRINITY_XP_0016	peptidy	Chlamydomonas_reinh	71.10	0.00
TRINITY_XP_0016	N-acety	Chlamydomonas_reinh	71.10	0.00
TRINITY_XP_0016	peptidy	Chlamydomonas_reinh	71.10	0.00
TRINITY_PNH0908	Prolyl	Tetrabaena_socialis	71.10	0.00
TRINITY_PNH1122	Peptidy	Tetrabaena_socialis	71.10	0.00
TRINITY_GAX7890	hypothet	Chlamydomonas_eusti	71.10	0.00
TRINITY_GAX8597	hypothet	Chlamydomonas_eusti	71.10	0.00
TRINITY_ABU9501	intrafl	Chlamydomonas_reinh	71.10	0.00
TRINITY_XP_0056	hypothet	Coccomyxa_subellips	71.00	0.00
TRINITY_XP_0240	H/ACA r	Morus_notabilis	71.00	0.00
TRINITY_BAJ9387	predict	Hordeum_vulgare	71.00	0.00
TRINITY_XP_0030	predict	Micromonas_pusilla	71.00	0.00
TRINITY_GAX7899	hypothet	Chlamydomonas_eusti	71.00	0.00
TRINITY_PRW5806	ribonuc	Chlorella_sorokinia	71.00	0.00
TRINITY_XP_0239	ras-rel	Quercus_suber	71.00	0.00
TRINITY_GAX7492	hypothet	Chlamydomonas_eusti	71.00	0.00
TRINITY_XP_0016	light-h	Chlamydomonas_reinh	71.00	0.00
TRINITY_XP_0016	R-SNARE	Chlamydomonas_reinh	71.00	0.00
TRINITY_XP_0058	hypothet	Chlorella_variabili	71.00	0.00
TRINITY_XP_0016	inactiv	Chlamydomonas_reinh	71.00	0.00

TRINITY_PSC7223(	FO synth	Micractinium_conduc	71.00	0.00
TRINITY_PNW8627	hypothet	Chlamydomonas_reinh	71.00	0.00
TRINITY_XP_0029	hypothet	Volvox_carteri	71.00	0.00
TRINITY_XP_0016	protein	Chlamydomonas_reinh	71.00	0.00
TRINITY_KXZ4350	hypothet	Gonium_pectorale	71.00	0.00
TRINITY_XP_0029	flagella	Volvox_carteri	71.00	0.00
TRINITY_PNW7415	hypothet	Chlamydomonas_reinh	71.00	0.00
TRINITY_GAX8227	hypothet	Chlamydomonas_eusti	71.00	0.00
TRINITY_GAX7863	hypothet	Chlamydomonas_eusti	71.00	0.00
TRINITY_PNW8529	hypothet	Chlamydomonas_reinh	71.00	0.00
TRINITY_KMS9434	hypothet	Beta_vulgaris	71.00	0.00
TRINITY_XP_0167	PREDICTE	Gossypium_hirsutum	71.00	0.00
TRINITY_XP_0058	hypothet	Chlorella_variabili	71.00	0.00
TRINITY_XP_0058	hypothet	Chlorella_variabili	70.90	0.00
TRINITY_RLN4024	ubiquiti	Panicum_miliaceum	70.90	0.00
TRINITY_XP_0139	hypothet	Monoraphidium_negle	70.90	0.00
TRINITY_XP_0256	60S ribo	Arachis_hypogaea	70.90	0.00
TRINITY_XP_0029	hypothet	Volvox_carteri	70.90	0.00
TRINITY_OEL2224	Heat sho	Dichanthelium_oligo	70.90	0.00
TRINITY_PNW7109	hypothet	Chlamydomonas_reinh	70.90	0.00
TRINITY_GAX8627	hypothet	Chlamydomonas_eusti	70.90	0.00
TRINITY_KXZ5582	hypothet	Gonium_pectorale	70.90	0.00
TRINITY_GAX7279	hypothet	Chlamydomonas_eusti	70.90	0.00
TRINITY_KXZ4937	hypothet	Gonium_pectorale	70.90	0.00
TRINITY_PNH0368	Poly(rC)	Tetrabaena_socialis	70.90	0.00
TRINITY_XP_0243	protein	Physcomitrella_pate	70.90	0.00
TRINITY_XP_0016	mitochon	Chlamydomonas_reinh	70.90	0.00
TRINITY_RAL4797	hypothet	Cuscuta_australis	70.90	0.00
TRINITY_XP_0240	DNA-dire	Morus_notabilis	70.90	0.00
TRINITY_ERM9372	hypothet	Amborella_trichopod	70.90	0.00
TRINITY_KXZ4766	hypothet	Gonium_pectorale	70.90	0.00
TRINITY_XP_0025	histone	Micromonas_commoda	70.90	0.00
TRINITY_GBF9061	cadmium	Raphidocelis_subcap	70.90	0.00
TRINITY_ACF7902	unknown	Zea_mays	70.90	0.00
TRINITY_GBF9937	3-hydrox	Raphidocelis_subcap	70.90	0.00
TRINITY_XP_0029	ATP-depe	Volvox_carteri	70.90	0.00
TRINITY_PNW8861	hypothet	Chlamydomonas_reinh	70.90	0.00
TRINITY_GAX7694	hypothet	Chlamydomonas_eusti	70.90	0.00
TRINITY_KXZ4493	hypothet	Gonium_pectorale	70.90	0.00
TRINITY_XP_0016	mitogen-	Chlamydomonas_reinh	70.90	0.00
TRINITY_KXZ5394	hypothet	Gonium_pectorale	70.90	0.00
TRINITY_PTQ4421	hypothet	Marchantia_polymorp	70.90	0.00
TRINITY_XP_0029	peroxire	Volvox_carteri	70.80	0.00
TRINITY_XP_0029	tubulin	Selaginella_moellen	70.80	0.00
TRINITY_PKA5716	hypothet	Apostasia_shenzheni	70.80	0.00
TRINITY_KXZ5199	hypothet	Gonium_pectorale	70.80	0.00
TRINITY_XP_0105	PREDICTE	Tarenaya_hasslerian	70.80	0.00
TRINITY_XP_0238	succinat	Quercus_suber	70.80	0.00
TRINITY_GAX8022	hypothet	Chlamydomonas_eusti	70.80	0.00
TRINITY_GAX7532	hypothet	Chlamydomonas_eusti	70.80	0.00
TRINITY_GAX7493	hypothet	Chlamydomonas_eusti	70.80	0.00
TRINITY_KXZ4193	hypothet	Gonium_pectorale	70.80	0.00
TRINITY_GAX7747	hypothet	Chlamydomonas_eusti	70.80	0.00
TRINITY_ONL9857	P-loop c	Zea_mays	70.80	0.00
TRINITY_PSC7303	asparagi	Micractinium_conduc	70.80	0.00
TRINITY_GAX8316	hypothet	Chlamydomonas_eusti	70.80	0.00
TRINITY_XP_0016	DegP-typ	Chlamydomonas_reinh	70.80	0.00
TRINITY_KXZ5593	hypothet	Gonium_pectorale	70.80	0.00
TRINITY_GAX7544	hypothet	Chlamydomonas_eusti	70.80	0.00
TRINITY_KXZ4970	hypothet	Gonium_pectorale	70.80	0.00
TRINITY_XP_0029	hypothet	Volvox_carteri	70.80	0.00
TRINITY_PTQ4421	hypothet	Marchantia_polymorp	70.80	0.00
TRINITY_XP_0058	hypothet	Chlorella_variabili	70.70	0.00
TRINITY_XP_0109	PREDICTE	Elaeis_guineensis	70.70	0.00
TRINITY_XP_0191	PREDICTE	Ipomoea_nil	70.70	0.00
TRINITY_XP_0016	DNA-dire	Chlamydomonas_reinh	70.70	0.00

TRINITY_XP_0029	hypothet	Volvox_carteri	70.70	0.00
TRINITY_PNH0464	putative	Tetrabaena_socialis	70.70	0.00
TRINITY_GAX7326	hypothet	Chlamydomonas_eusti	70.70	0.00
TRINITY_XP_0205	AP-4 cor	Phalaenopsis_equest	70.70	0.00
TRINITY_XP_0016	nickel (	Chlamydomonas_reinh	70.70	0.00
TRINITY_PTQ4266	hypothet	Marchantia_polymorp	70.70	0.00
TRINITY_GAX7487	hypothet	Chlamydomonas_eusti	70.70	0.00
TRINITY_GAX7913	hypothet	Chlamydomonas_eusti	70.70	0.00
TRINITY_GAX7272	hypothet	Chlamydomonas_eusti	70.70	0.00
TRINITY_PNW8249	hypothet	Chlamydomonas_reinh	70.70	0.00
TRINITY_KXZ5280	hypothet	Gonium_pectorale	70.70	0.00
TRINITY_PNW7200	hypothet	Chlamydomonas_reinh	70.70	0.00
TRINITY_GAX7397	hypothet	Chlamydomonas_eusti	70.70	0.00
TRINITY_XP_0016	glutath	Chlamydomonas_reinh	70.70	0.00
TRINITY_GAX7482	hypothet	Chlamydomonas_eusti	70.70	0.00
TRINITY_XP_0239	26S prot	Quercus_suber	70.60	0.00
TRINITY_BAK0244	predicte	Hordeum_vulgare	70.60	0.00
TRINITY_PPD6687	hypothet	Gossypium_barbadens	70.60	0.00
TRINITY_GAX7689	hypothet	Chlamydomonas_eusti	70.60	0.00
TRINITY_EFJ2312	hypothet	Selaginella_moellen	70.60	0.00
TRINITY_XP_0087	diphthar	Phoenix_dactylifera	70.60	0.00
TRINITY_GAX7601	hypothet	Chlamydomonas_eusti	70.60	0.00
TRINITY_GAX8334	hypothet	Chlamydomonas_eusti	70.60	0.00
TRINITY_XP_0237	histone	Lactuca_sativa	70.60	0.00
TRINITY_GBF9053	20S prot	Raphidocelis_subcap	70.60	0.00
TRINITY_GAX8271	hypothet	Chlamydomonas_eusti	70.60	0.00
TRINITY_XP_0029	hypothet	Volvox_carteri	70.60	0.00
TRINITY_XP_0016	isocitra	Chlamydomonas_reinh	70.60	0.00
TRINITY_GAX7779	hypothet	Chlamydomonas_eusti	70.60	0.00
TRINITY_GAQ7892	Dynein I	Klebsormidium_niten	70.60	0.00
TRINITY_GAX7754	hypothet	Chlamydomonas_eusti	70.60	0.00
TRINITY_BAK0075	predicte	Hordeum_vulgare	70.60	0.00
TRINITY_GAX8654	hypothet	Chlamydomonas_eusti	70.60	0.00
TRINITY_XP_0188	PREDICTE	Juglans_regia	70.60	0.00
TRINITY_XP_0029	hypothet	Volvox_carteri	70.60	0.00
TRINITY_XP_0016	predicte	Chlamydomonas_reinh	70.60	0.00
TRINITY_BAJ9387	predicte	Hordeum_vulgare	70.50	0.00
TRINITY_XP_0093	PREDICTE	Musa_acuminata	70.50	0.00
TRINITY_PNW8311	hypothet	Chlamydomonas_reinh	70.50	0.00
TRINITY_XP_0016	riboflav	Chlamydomonas_reinh	70.50	0.00
TRINITY_PNW7116	hypothet	Chlamydomonas_reinh	70.50	0.00
TRINITY_XP_0194	PREDICTE	Lupinus_angustifoli	70.50	0.00
TRINITY_GAX8035	hypothet	Chlamydomonas_eusti	70.50	0.00
TRINITY_GAX8152	hypothet	Chlamydomonas_eusti	70.50	0.00
TRINITY_GAX8252	hypothet	Chlamydomonas_eusti	70.50	0.00
TRINITY_XP_0029	hypothet	Volvox_carteri	70.50	0.00
TRINITY_GAX8142	hypothet	Chlamydomonas_eusti	70.50	0.00
TRINITY_XP_0139	arogenat	Monoraphidium_negle	70.50	0.00
TRINITY_BAU7113	cysteine	Parachlorella_kessl	70.50	0.00
TRINITY_PNH0880	V-type p	Tetrabaena_socialis	70.50	0.00
TRINITY_GAX7908	hypothet	Chlamydomonas_eusti	70.50	0.00
TRINITY_GAX7906	hypothet	Chlamydomonas_eusti	70.50	0.00
TRINITY_GAX8390	hypothet	Chlamydomonas_eusti	70.50	0.00
TRINITY_KXZ4281	hypothet	Gonium_pectorale	70.50	0.00
TRINITY_P0C8M8.1	RecName:	Zea_mays	70.50	0.00
TRINITY_XP_0029	L-aspart	Volvox_carteri	70.50	0.00
TRINITY_XP_0017	mitochor	Chlamydomonas_reinh	70.50	0.00
TRINITY_GAX7514	hypothet	Chlamydomonas_eusti	70.50	0
TRINITY_RRT3535	hypothet	Ensete_ventricosum	70.50	0.00
TRINITY_GAX7676	hypothet	Chlamydomonas_eusti	70.50	0.00
TRINITY_GAX8138	hypothet	Chlamydomonas_eusti	70.50	0
TRINITY_XP_0016	triacylg	Chlamydomonas_reinh	70.50	0.00
TRINITY_XP_0029	light-ha	Volvox_carteri	70.50	0.00
TRINITY_XP_0113	60S ribo	Auxenochlorella_pro	70.40	0.00
TRINITY_PRW6096	pre-mRNA	Chlorella_sorokinia	70.40	0.00
TRINITY_PRW6143	Proteaso	Chlorella_sorokinia	70.40	0.00

TRINITY_PNH0562	ribonucl	Tetrabaena_socialis	70.40	0.00
TRINITY_XP_0016	methion	Chlamydomonas_reinh	70.40	0.00
TRINITY_GAX7376	hypothet	Chlamydomonas_eusti	70.40	0.00
TRINITY_GAX8155	hypothet	Chlamydomonas_eusti	70.40	0.00
TRINITY_PNH1217	hypothet	Tetrabaena_socialis	70.40	0.00
TRINITY_XP_0016	predicte	Chlamydomonas_reinh	70.40	0.00
TRINITY_XP_0029	hypothet	Volvox_carteri	70.40	0.00
TRINITY_AAB6131	htrA-lil	Haematococcus_lacus	70.40	0.00
TRINITY_GAQ8961	nucleot	Klebsormidium_niten	70.40	0.00
TRINITY_PRW5975	Propion	Chlorella_sorokinia	70.40	0.00
TRINITY_GBG7929	hypothet	Chara_braunii	70.40	0.00
TRINITY_GAX7899	hypothet	Chlamydomonas_eusti	70.40	0.00
TRINITY_PNH1163	Two-comp	Tetrabaena_socialis	70.40	0.00
TRINITY_GAX7906	hypothet	Chlamydomonas_eusti	70.40	0.00
TRINITY_GAX7356	hypothet	Chlamydomonas_eusti	70.40	0.00
TRINITY_GBF9373	hypothet	Raphidocelis_subcap	70.40	0.00
TRINITY_PSC7280	replicat	Micractinium_conduc	70.40	0.00
TRINITY_BAK0038	predicte	Hordeum_vulgare	70.30	0.00
TRINITY_XP_0056	nucleic	Coccomyxa_subellips	70.30	0.00
TRINITY_XP_0243	ubiquit	Physcomitrella_pate	70.30	0.00
TRINITY_PRW3284	S-adeno	Chlorella_sorokinia	70.30	0.00
TRINITY_XP_0025	predicte	Micromonas_commoda	70.30	0.00
TRINITY_KJB0726	hypothet	Gossypium_raimondii	70.30	0.00
TRINITY_KFK2736	hypothet	Arabis_alpina	70.30	0.00
TRINITY_GAX7598	hypothet	Chlamydomonas_eusti	70.30	0.00
TRINITY_XP_0156	DNA-dire	Oryza_sativa	70.30	0.00
TRINITY_XP_0217	cytopla	Chenopodium_quinoa	70.30	0.00
TRINITY_XP_0029	hypothet	Volvox_carteri	70.30	0
TRINITY_P54209.1	RecName:	Dunaliella_bioculat	70.30	0.00
TRINITY_XP_0016	predicte	Chlamydomonas_reinh	70.30	0.00
TRINITY_XP_0228	Zinc fir	Ostreococcus_tauri	70.30	0.00
TRINITY_XP_0017	predicte	Chlamydomonas_reinh	70.30	0.00
TRINITY_GAX7694	hypothet	Chlamydomonas_eusti	70.30	0.00
TRINITY_ACG2503	60S ribo	Zea_mays	70.30	0.00
TRINITY_XP_0029	flagella	Volvox_carteri	70.30	0.00
TRINITY_RAL4084	hypothet	Cuscuta_australis	70.30	0.00
TRINITY_XP_0023	DNA poly	Populus_trichocarpa	70.20	0.00
TRINITY_XP_0194	PREDICTE	Lupinus_angustifoli	70.20	0.00
TRINITY_GBG8739	hypothet	Chara_braunii	70.20	0.00
TRINITY_GAX7512	hypothet	Chlamydomonas_eusti	70.20	0.00
TRINITY_XP_0016	predicte	Chlamydomonas_reinh	70.20	0.00
TRINITY_PIA3906	hypothet	Aquilegia_coerulea	70.20	0.00
TRINITY_PNW8883	hypothet	Chlamydomonas_reinh	70.20	0.00
TRINITY_OAE3244	hypothet	Marchantia_polymorp	70.20	0.00
TRINITY_EXC0191	hypothet	Morus_notabilis	70.20	0.00
TRINITY_KXZ5123	hypothet	Gonium_pectorale	70.20	0.00
TRINITY_PNH0503	Histone	Tetrabaena_socialis	70.20	0.00
TRINITY_KXZ4838	hypothet	Gonium_pectorale	70.20	0.00
TRINITY_XP_0110	AP-1 com	Sesamum_indicum	70.20	0.00
TRINITY_PNW8367	hypothet	Chlamydomonas_reinh	70.20	0.00
TRINITY_GAX7698	hypothet	Chlamydomonas_eusti	70.20	0.00
TRINITY_XP_0217	ATP synt	Chenopodium_quinoa	70.20	0.00
TRINITY_GAX7273	hypothet	Chlamydomonas_eusti	70.20	0.00
TRINITY_GAX7359	hypothet	Chlamydomonas_eusti	70.20	0.00
TRINITY_PNW7202	hypothet	Chlamydomonas_reinh	70.20	0
TRINITY_GAX7745	hypothet	Chlamydomonas_eusti	70.20	0.00
TRINITY_GAQ8823	putative	Klebsormidium_niten	70.20	0.00
TRINITY_GAX7426	hypothet	Chlamydomonas_eusti	70.20	0.00
TRINITY_ACN9445	photosys	Dunaliella_salina	70.20	0.00
TRINITY_XP_0064	vacuola	Eutrema_salsugineum	70.10	0.00
TRINITY_GBF9447	hypothet	Raphidocelis_subcap	70.10	0.00
TRINITY_GAX7607	hypothet	Chlamydomonas_eusti	70.10	0.00
TRINITY_XP_0056	Metallo-	Coccomyxa_subellips	70.10	0.00
TRINITY_KZV4340	hypothet	Dorcoceras_hygromet	70.10	0.00
TRINITY_GAX7689	hypothet	Chlamydomonas_eusti	70.10	0.00
TRINITY_XP_0058	hypothet	Chlorella_variabili	70.10	0.00

TRINITY_XP_0105:	PREDICTE	Tarenaya_hasslerian	70.10	0.00
TRINITY_AAC3220	isopente	Haematococcus_lacus	70.10	0.00
TRINITY_RAL5163	hypothet	Cuscuta_australis	70.10	0.00
TRINITY_OWM6883	hypothet	Punica_granatum	70.10	0.00
TRINITY_XP_0058	hypothet	Chlorella_variabili	70.10	0.00
TRINITY_XP_0017(	TspO/MBE	Chlamydomonas_reinh	70.10	0.00
TRINITY_GAX8230	hypothet	Chlamydomonas_eusti	70.10	0.00
TRINITY_GAX8568	hypothet	Chlamydomonas_eusti	70.10	0.00
TRINITY_PNH0981(	hypothet	Tetrabaena_socialis	70.10	0.00
TRINITY_XP_0017(	mitochor	Chlamydomonas_reinh	70.10	0.00
TRINITY_GAX8052	hypothet	Chlamydomonas_eusti	70.10	0.00
TRINITY_PON8203	Ribosoma	Trema_orientale	70.10	0.00
TRINITY_GBF9358	hypothet	Raphidocelis_subcap	70.10	0.00
TRINITY_GAX7383	hypothet	Chlamydomonas_eusti	70.10	0.00
TRINITY_GAX8490	hypothet	Chlamydomonas_eusti	70.10	0.00
TRINITY_XP_0014	predicte	Ostreococcus_sp._'l	70.00	0.00
TRINITY_XP_0205	ras-rela	Phalaenopsis_equest	70.00	0.00
TRINITY_GBG6683	hypothet	Chara_braunii	70.00	0.00
TRINITY_XP_0239	60S ribo	Quercus_suber	70.00	0.00
TRINITY_PUZ5074	hypothet	Panicum_hallii	70.00	0.00
TRINITY_GAX7425	hypothet	Chlamydomonas_eusti	70.00	0.00
TRINITY_GAX8229	hypothet	Chlamydomonas_eusti	70.00	0.00
TRINITY_GAX7360	hypothet	Chlamydomonas_eusti	70.00	0.00
TRINITY_XP_0056	hypothet	Coccomyxa_subellips	70.00	0.00
TRINITY_GAX7671	hypothet	Chlamydomonas_eusti	70.00	0.00
TRINITY_XP_0016	predicte	Chlamydomonas_reinh	70.00	0.00
TRINITY_XP_0188	PREDICTE	Juglans_regia	70.00	0.00
TRINITY_GAX7986	hypothet	Chlamydomonas_eusti	70.00	0.00
TRINITY_EFJ3775	ATP-binc	Selaginella_moellen	70.00	0.00
TRINITY_KMZ6812	tubulin	Zostera_marina	70.00	0.00
TRINITY_KJB3932	hypothet	Gossypium_raimondii	70.00	0.00
TRINITY_XP_0029	hypothet	Volvox_carteri	70.00	0.00
TRINITY_PNW7369	hypothet	Chlamydomonas_reinh	70.00	0.00
TRINITY_XP_0029	hypothet	Volvox_carteri	70.00	0.00
TRINITY_XP_0029	serine	Volvox_carteri	70.00	0.00
TRINITY_PRW5842	Peptidy	Chlorella_sorokinia	70.00	0.00
TRINITY_XP_0016	CDP-Etha	Chlamydomonas_reinh	70.00	0.00
TRINITY_PNW8128	hypothet	Chlamydomonas_reinh	70.00	0.00
TRINITY_KXZ5506	hypothet	Gonium_pectorale	70.00	0.00
TRINITY_GAX7530	hypothet	Chlamydomonas_eusti	70.00	0.00
TRINITY_XP_0016	hybrid-c	Chlamydomonas_reinh	70.00	0.00
TRINITY_PNW8186	hypothet	Chlamydomonas_reinh	70.00	0.00
TRINITY_PNH1060(	E3 ubiqu	Tetrabaena_socialis	70.00	0.00
TRINITY_KMS6449	hypothet	Beta_vulgaris	70.00	0.00
TRINITY_KYP7875	SsrA-bir	Cajanus_cajan	70.00	0.00
TRINITY_XP_0109	PREDICTE	Elaeis_guineensis	69.90	0.00
TRINITY_GAX8010	hypothet	Chlamydomonas_eusti	69.90	0.00
TRINITY_GAX7967	hypothet	Chlamydomonas_eusti	69.90	0.00
TRINITY_XP_0075	predicte	Bathycoccus_prasinc	69.90	0
TRINITY_PNH0194	Diphthar	Tetrabaena_socialis	69.90	0.00
TRINITY_XP_0016	mitochor	Chlamydomonas_reinh	69.90	0.00
TRINITY_CAI8489	putative	Medicago_sativa	69.90	0.00
TRINITY_XP_0016	predicte	Chlamydomonas_reinh	69.90	0.00
TRINITY_XP_0029	hypothet	Volvox_carteri	69.90	0.00
TRINITY_XP_0056	MT-A70-c	Coccomyxa_subellips	69.90	0.00
TRINITY_PNW7111	hypothet	Chlamydomonas_reinh	69.90	0.00
TRINITY_XP_0206	tRNA (cy	Dendrobium_catenatu	69.90	0.00
TRINITY_KXZ5668	hypothet	Gonium_pectorale	69.90	0.00
TRINITY_KXZ5011	hypothet	Gonium_pectorale	69.90	0.00
TRINITY_XP_0029	cobalt t	Volvox_carteri	69.90	0.00
TRINITY_PNH1058	Vacuola	Tetrabaena_socialis	69.90	0.00
TRINITY_PKA5170	60S ribo	Apostasia_shenzheni	69.80	0.00
TRINITY_GBG9125	hypothet	Chara_braunii	69.80	0.00
TRINITY_ABK2279(	unknown	Picea_sitchensis	69.80	0.00
TRINITY_PNH0786	Mitogen-	Tetrabaena_socialis	69.80	0.00
TRINITY_XP_0205	60S ribo	Phalaenopsis_equest	69.80	0.00

TRINITY_PNR2653	hypothet	Physcomitrella_pate	69.80	0.00
TRINITY_XP_0029	hypothet	Volvox_carteri	69.80	0.00
TRINITY_GAX8428	hypothet	Chlamydomonas_eusti	69.80	0.00
TRINITY_A1JHN0.	RecName:	Chlamydomonas_reinh	69.80	0.00
TRINITY_XP_0029	hypothet	Volvox_carteri	69.80	0.00
TRINITY_PRW5739	5-oxopro	Chlorella_sorokinia	69.80	0.00
TRINITY_GBF8937	dihydrop	Raphidocelis_subcap	69.80	0.00
TRINITY_PNG8930	Pyruvate	Tetrabaena_socialis	69.80	0.00
TRINITY_GAX7747	hypothet	Chlamydomonas_eusti	69.80	0.00
TRINITY_XP_0243	ATP-depe	Physcomitrella_pate	69.80	0.00
TRINITY_GBG0009	COP9 sig	Raphidocelis_subcap	69.80	0.00
TRINITY_XP_0116	40S ribo	Amborella_trichopod	69.80	0.00
TRINITY_AJR2723	4-diphos	Haematococcus_lacus	69.80	0.00
TRINITY_KXZ4503	hypothet	Gonium_pectorale	69.80	0.00
TRINITY_EOX9902	Calcineu	Theobroma_cacao	69.80	0.00
TRINITY_KMS6449	hypothet	Beta_vulgaris	69.80	0.00
TRINITY_GAX8266	hypothet	Chlamydomonas_eusti	69.80	0.00
TRINITY_KZV1720	luminal-	Dorcoceras_hygromet	69.80	0.00
TRINITY_XP_0087	heat sho	Phoenix_dactylifera	69.80	0.00
TRINITY_GAQ8318	Protein-	Klebsormidium_niten	69.80	0.00
TRINITY_GAX8299	hypothet	Chlamydomonas_eusti	69.80	0.00
TRINITY_PNW7708	hypothet	Chlamydomonas_reinh	69.80	0.00
TRINITY_XP_0063	PREDICTE	Solanum_tuberosum	69.80	0.00
TRINITY_XP_0139	hypothet	Monoraphidium_negle	69.80	0.00
TRINITY_AGT1609	cyclophi	Saccharum_hybrid_cu	69.80	0.00
TRINITY_PNH0956	C-facto	Tetrabaena_socialis	69.80	0.00
TRINITY_XP_0075	H/ACA ri	Bathycoccus_prasino	69.80	0.00
TRINITY_GAX7402	hypothet	Chlamydomonas_eusti	69.80	0.00
TRINITY_PSC7160	hypothet	Micractinium_conduc	69.80	0.00
TRINITY_KXZ5610	hypothet	Gonium_pectorale	69.80	0.00
TRINITY_BAJ9055	predicte	Hordeum_vulgare	69.70	0.00
TRINITY_NP_0011	uncharac	Zea_mays	69.70	0.00
TRINITY_EPS6413	hypothet	Genlisea_aurea	69.70	0.00
TRINITY_GBF9186	hypothet	Raphidocelis_subcap	69.70	0.00
TRINITY_KXZ4944	RSP3 pro	Gonium_pectorale	69.70	0.00
TRINITY_XP_0029	hypothet	Volvox_carteri	69.70	0.00
TRINITY_XP_0025	predicte	Micromonas_commoda	69.70	0.00
TRINITY_XP_0016	flagella	Chlamydomonas_reinh	69.70	0.00
TRINITY_GBF9892	appr-1-p	Raphidocelis_subcap	69.70	0.00
TRINITY_PNW8495	hypothet	Chlamydomonas_reinh	69.70	0.00
TRINITY_Q9FPL6.	RecName:	Suaeda_salsa	69.70	0.00
TRINITY_XP_0017	radial s	Chlamydomonas_reinh	69.70	0.00
TRINITY_XP_0017	predicte	Chlamydomonas_reinh	69.70	0.00
TRINITY_GAX7771	hypothet	Chlamydomonas_eusti	69.70	0.00
TRINITY_GAX7284	hypothet	Chlamydomonas_eusti	69.70	0.00
TRINITY_GAX8222	hypothet	Chlamydomonas_eusti	69.70	0.00
TRINITY_PTQ3795	hypothet	Marchantia_polymorp	69.70	0.00
TRINITY_GBF8946	GTP-bind	Raphidocelis_subcap	69.70	0.00
TRINITY_GAX8324	hypothet	Chlamydomonas_eusti	69.70	0.00
TRINITY_GAX8222	hypothet	Chlamydomonas_eusti	69.70	0.00
TRINITY_GAX8574	hypothet	Chlamydomonas_eusti	69.70	0.00
TRINITY_KXZ5229	hypothet	Gonium_pectorale	69.70	0.00
TRINITY_GAX7596	hypothet	Chlamydomonas_eusti	69.70	0.00
TRINITY_OAY6886	Heat sho	Ananas_comosus	69.70	0.00
TRINITY_GAX8164	hypothet	Chlamydomonas_eusti	69.70	0.00
TRINITY_XP_0056	DUF92-do	Coccomyxa_subellips	69.70	0.00
TRINITY_XP_0216	protein	Hevea_brasiliensis	69.60	0.00
TRINITY_KXZ5157	hypothet	Gonium_pectorale	69.60	0.00
TRINITY_RZC9232	hypothet	Papaver_somniferum	69.60	0.00
TRINITY_KXZ4403	FTT1 14	Gonium_pectorale	69.60	0.00
TRINITY_XP_0016	serine/t	Chlamydomonas_reinh	69.60	0.00
TRINITY_XP_0029	Selenopr	Volvox_carteri	69.60	0.00
TRINITY_XP_0029	membrane	Volvox_carteri	69.60	0.00
TRINITY_XP_0016	deoxypus	Chlamydomonas_reinh	69.60	0.00
TRINITY_AUE2188	GADPH [	Uncaria_rhynchophyl	69.60	0.00
TRINITY_GAX7520	hypothet	Chlamydomonas_eusti	69.60	0.00

TRINITY_KXZ4697(	hypothet	Gonium_pectorale	69.60	0.00
TRINITY_KZV1890!	hypothet	Dorcoceras_hygromet	69.60	0.00
TRINITY_KXZ4875!	hypothet	Gonium_pectorale	69.60	0.00
TRINITY_OAE1910'	hypothet	Marchantia_polymorp	69.60	0.00
TRINITY_OAE3310!	hypothet	Marchantia_polymorp	69.60	0.00
TRINITY_XP_0016!	predicte	Chlamydomonas_reinh	69.60	0.00
TRINITY_PNW8027(	hypothet	Chlamydomonas_reinh	69.60	0.00
TRINITY_XP_0029(	hypothet	Volvox_carteri	69.60	0.00
TRINITY_XP_0029!	hypothet	Volvox_carteri	69.60	0.00
TRINITY_KXZ5690(	hypothet	Gonium_pectorale	69.60	0.00
TRINITY_XP_0029(	hypothet	Volvox_carteri	69.60	0.00
TRINITY_XP_0016!	predicte	Chlamydomonas_reinh	69.60	0.00
TRINITY_PNW8408!	hypothet	Chlamydomonas_reinh	69.60	0.00
TRINITY_XP_0016!	lysine (	Chlamydomonas_reinh	69.60	0.00
TRINITY_KXZ5150!	hypothet	Gonium_pectorale	69.60	0.00
TRINITY_KXZ4194(	hypothet	Gonium_pectorale	69.60	0.00
TRINITY_GAX8002!	hypothet	Chlamydomonas_eusti	69.60	0.00
TRINITY_GAX7266(	hypothet	Chlamydomonas_eusti	69.60	0.00
TRINITY_XP_0102(	PREDICTI	Nelumbo_nucifera	69.50	0.00
TRINITY_NP_0011(	uncharac	Zea_mays	69.50	0.00
TRINITY_BAJ8540(	predicte	Hordeum_vulgare	69.50	0.00
TRINITY_KVH9112!	Protein	Cynara_cardunculus	69.50	0.00
TRINITY_O65353.!	RecName:	Helianthus_annuus	69.50	0.00
TRINITY_XP_0016!	nuclease	Chlamydomonas_reinh	69.50	0.00
TRINITY_KMS9434!	hypothet	Beta_vulgaris	69.50	0.00
TRINITY_PNH0895'	Transcri	Tetrabaena_socialis	69.50	0.00
TRINITY_PNH0830!	NFX1-typ	Tetrabaena_socialis	69.50	0.00
TRINITY_KXZ5199!	hypothet	Gonium_pectorale	69.50	0.00
TRINITY_GAX7279(	hypothet	Chlamydomonas_eusti	69.50	0.00
TRINITY_GAX7361(	hypothet	Chlamydomonas_eusti	69.50	0.00
TRINITY_KXZ4646'	hypothet	Gonium_pectorale	69.50	0.00
TRINITY_XP_0017(	5,10-met	Chlamydomonas_reinh	69.50	0.00
TRINITY_GAX8339!	hypothet	Chlamydomonas_eusti	69.50	0.00
TRINITY_XP_0058(	hypothet	Chlorella_variabili	69.50	0.00
TRINITY_XP_0058(	hypothet	Chlorella_variabili	69.50	0.00
TRINITY_GAX7403'	hypothet	Chlamydomonas_eusti	69.50	0.00
TRINITY_XP_0029!	hypothet	Volvox_carteri	69.50	0.00
TRINITY_GAX7382'	hypothet	Chlamydomonas_eusti	69.50	0.00
TRINITY_GAX8503!	hypothet	Chlamydomonas_eusti	69.50	0.00
TRINITY_KXZ5422(	hypothet	Gonium_pectorale	69.50	0.00
TRINITY_XP_0016!	DNA repl	Chlamydomonas_reinh	69.50	0.00
TRINITY_XP_0029(	hypothet	Volvox_carteri	69.50	0.00
TRINITY_XP_0016!	predicte	Chlamydomonas_reinh	69.50	0.00
TRINITY_GAX8602!	hypothet	Chlamydomonas_eusti	69.50	0
TRINITY_PNW8496(	hypothet	Chlamydomonas_reinh	69.50	0
TRINITY_XP_0016(	predicte	Chlamydomonas_reinh	69.40	0.00
TRINITY_OVA1238(	Proteas(	Macleaya_cordata	69.40	0.00
TRINITY_XP_0025!	60S rib(	Ricinus_communis	69.40	0.00
TRINITY_XP_0025!	60S rib(	Ricinus_communis	69.40	0.00
TRINITY_XP_0029!	hypothet	Volvox_carteri	69.40	0.00
TRINITY_AAB8649(	calmodul	Zea_mays	69.40	0.00
TRINITY_NP_0013!	ribosoma	Solanum_lycopersicu	69.40	0.00
TRINITY_KDD7497(	hypothet	Helicosporidium_sp.	69.40	0.00
TRINITY_XP_0029!	hypothet	Volvox_carteri	69.40	0.00
TRINITY_RWR7682!	Ribosoma	Cinnamomum_micranth	69.40	0.00
TRINITY_RWR7682!	Ribosoma	Cinnamomum_micranth	69.40	0.00
TRINITY_GAQ8502!	RNAse L	Klebsormidium_niten	69.40	0.00
TRINITY_GAX8410!	hypothet	Chlamydomonas_eusti	69.40	0.00
TRINITY_XP_0016!	predicte	Chlamydomonas_reinh	69.40	0.00
TRINITY_XP_0029!	hypothet	Volvox_carteri	69.40	0.00
TRINITY_AIJ0088!	multi-fu	Dunaliella_salina	69.40	0.00
TRINITY_XP_0016!	predicte	Chlamydomonas_reinh	69.40	0.00
TRINITY_XP_0100!	PREDICTI	Eucalyptus_grandis	69.40	0.00
TRINITY_PNW8815!	hypothet	Chlamydomonas_reinh	69.40	0.00
TRINITY_GAX7977(	hypothet	Chlamydomonas_eusti	69.40	0.00
TRINITY_PNW8655(	hypothet	Chlamydomonas_reinh	69.40	0.00

TRINITY_BAJ9500!	predicte	Hordeum_vulgare	69.40	0.00
TRINITY_KXZ5567	hypothet	Gonium_pectorale	69.40	0
TRINITY_GAX8056!	hypothet	Chlamydomonas_eusti	69.40	0.00
TRINITY_GAX7328(	hypothet	Chlamydomonas_eusti	69.40	0.00
TRINITY_XP_0058	hypothet	Chlorella_variabili	69.40	0.00
TRINITY_BAJ9973	predicte	Hordeum_vulgare	69.30	0.00
TRINITY_XP_0212	histone	Herrania_umbratica	69.30	0.00
TRINITY_RAL4661(	hypothet	Cuscuta_australis	69.30	0.00
TRINITY_RAL4661(	hypothet	Cuscuta_australis	69.30	0.00
TRINITY_RAL4661(	hypothet	Cuscuta_australis	69.30	0.00
TRINITY_PNW8267	hypothet	Chlamydomonas_reinh	69.30	0.00
TRINITY_XP_0029	plastid/	Volvox_carteri	69.30	0.00
TRINITY_GAX7695	hypothet	Chlamydomonas_eusti	69.30	0.00
TRINITY_KXZ4753	hypothet	Gonium_pectorale	69.30	0.00
TRINITY_PRW6045(	aldehyde	Chlorella_sorokinia	69.30	0.00
TRINITY_XP_0138	hypothet	Monoraphidium_negle	69.30	0.00
TRINITY_PNW8609	hypothet	Chlamydomonas_reinh	69.30	0.00
TRINITY_XP_0029!	DEAH-box	Volvox_carteri	69.30	0.00
TRINITY_GAX7463	hypothet	Chlamydomonas_eusti	69.30	0.00
TRINITY_GAQ8909	GTP-binc	Klebsormidium_niten	69.30	0.00
TRINITY_GAX7766	hypothet	Chlamydomonas_eusti	69.30	0.00
TRINITY_GAX7884!	hypothet	Chlamydomonas_eusti	69.30	0
TRINITY_GAX7967	hypothet	Chlamydomonas_eusti	69.30	0.00
TRINITY_KXZ4473!	hypothet	Gonium_pectorale	69.30	0.00
TRINITY_XP_0219(	uncharac	Carica_papaya	69.20	0.00
TRINITY_XP_0239(	serine/t	Quercus_suber	69.20	0.00
TRINITY_XP_0016	triacylg	Chlamydomonas_reinh	69.20	0.00
TRINITY_XP_0243	uncharac	Physcomitrella_pate	69.20	0.00
TRINITY_GAX8315	hypothet	Chlamydomonas_eusti	69.20	0.00
TRINITY_KMS9327	hypothet	Beta_vulgaris	69.20	0.00
TRINITY_GBG7247!	hypothet	Chara_braunii	69.20	0.00
TRINITY_GAX7667(	hypothet	Chlamydomonas_eusti	69.20	0.00
TRINITY_XP_0138	hypothet	Monoraphidium_negle	69.20	0.00
TRINITY_XP_0113	Deoxycyt	Auxenochlorella_prc	69.20	0.00
TRINITY_GBF9841	hypothet	Raphidocelis_subcap	69.20	0.00
TRINITY_GAX7899	hypothet	Chlamydomonas_eusti	69.20	0.00
TRINITY_GBF9326	U6 snRNA	Raphidocelis_subcap	69.20	0.00
TRINITY_XP_0016	nucleic	Chlamydomonas_reinh	69.20	0.00
TRINITY_GAX7306	hypothet	Chlamydomonas_eusti	69.20	0.00
TRINITY_XP_0029!	hypothet	Volvox_carteri	69.20	0.00
TRINITY_XP_0188(	PREDICTE	Juglans_regia	69.20	0.00
TRINITY_XP_0029!	hypothet	Volvox_carteri	69.20	0.00
TRINITY_KXZ4090	hypothet	Gonium_pectorale	69.20	0.00
TRINITY_GAX8188	hypothet	Chlamydomonas_eusti	69.20	0.00
TRINITY_XP_0016	AP2-doma	Chlamydomonas_reinh	69.20	0.00
TRINITY_KXZ4821	DHC-9 pı	Gonium_pectorale	69.20	0.00
TRINITY_XP_0056	hypothet	Coccomyxa_subellips	69.20	0.00
TRINITY_GAX8255(	hypothet	Chlamydomonas_eusti	69.20	0.00
TRINITY_GAX8525	hypothet	Chlamydomonas_eusti	69.20	0.00
TRINITY_GAX7468	hypothet	Chlamydomonas_eusti	69.20	0.00
TRINITY_PNH0967	DNA-binc	Tetrabaena_socialis	69.20	0.00
TRINITY_XP_0228	Proteas	Ostreococcus_tauri	69.20	0.00
TRINITY_GAX7844	hypothet	Chlamydomonas_eusti	69.20	0.00
TRINITY_GBF9261	chloropl	Raphidocelis_subcap	69.20	0.00
TRINITY_XP_0025(	predicte	Micromonas_commoda	69.10	0.00
TRINITY_XP_0238	40S ribo	Quercus_suber	69.10	0.00
TRINITY_PSC7669	60S acic	Micractinium_conduc	69.10	0.00
TRINITY_XP_0105	PREDICTE	Tarenaya_hasslerian	69.10	0.00
TRINITY_GAX8654!	hypothet	Chlamydomonas_eusti	69.10	0.00
TRINITY_KMS6493	hypothet	Beta_vulgaris	69.10	0.00
TRINITY_GAX7752	hypothet	Chlamydomonas_eusti	69.10	0.00
TRINITY_BAK0289	predicte	Hordeum_vulgare	69.10	0.00
TRINITY_CAA1126	polyubic	Nicotiana_tabacum	69.10	0.00
TRINITY_PNH0440	Glutathi	Tetrabaena_socialis	69.10	0.00
TRINITY_XP_0029!	hypothet	Volvox_carteri	69.10	0.00
TRINITY_XP_0094(	PREDICTE	Musa_acuminata	69.10	0.00

TRINITY_PRW6027	glyoxyla	Chlorella_sorokinia	69.10	0.00
TRINITY_GAX8020	hypothet	Chlamydomonas_eusti	69.10	0.00
TRINITY_XP_0029	photosys	Volvox_carteri	69.10	0.00
TRINITY_GBF9844	DNA top	Raphidocelis_subcap	69.10	0.00
TRINITY_PNH0074	Serine/t	Tetrabaena_socialis	69.10	0.00
TRINITY_XP_0029	hypothet	Volvox_carteri	69.10	0.00
TRINITY_PNW7042	hypothet	Chlamydomonas_reinh	69.10	0.00
TRINITY_GAX8294	hypothet	Chlamydomonas_eusti	69.10	0.00
TRINITY_GAX8546	hypothet	Chlamydomonas_eusti	69.10	0.00
TRINITY_KXZ4944	hypothet	Gonium_pectorale	69.10	0.00
TRINITY_XP_0016	UDP-glu	Chlamydomonas_reinh	69.10	0.00
TRINITY_KXZ5395	hypothet	Gonium_pectorale	69.10	0.00
TRINITY_XP_0029	hypothet	Volvox_carteri	69.10	0.00
TRINITY_XP_0029	hypothet	Volvox_carteri	69.10	0.00
TRINITY_XP_0014	predicte	Ostreococcus_sp._'l	69.10	0.00
TRINITY_GAX7885	hypothet	Chlamydomonas_eusti	69.00	0.00
TRINITY_GAQ9307	mitochor	Klebsormidium_niten	69.00	0.00
TRINITY_GAQ8393	Ubiquit	Klebsormidium_niten	69.00	0.00
TRINITY_PSC7262	DEAD-bo	Micractinium_conduc	69.00	0.00
TRINITY_GAX8060	hypothet	Chlamydomonas_eusti	69.00	0.00
TRINITY_XP_0029	hypothet	Volvox_carteri	69.00	0.00
TRINITY_GAX7714	hypothet	Chlamydomonas_eusti	69.00	0.00
TRINITY_XP_0029	DEAD/DE	Volvox_carteri	69.00	0.00
TRINITY_XP_0243	double-	Physcomitrella_pate	69.00	0.00
TRINITY_KXZ5505	hypothet	Gonium_pectorale	69.00	0.00
TRINITY_XP_0139	ESCRT-I	Monoraphidium_negle	69.00	0.00
TRINITY_GAX8293	hypothet	Chlamydomonas_eusti	69.00	0.00
TRINITY_GAX8362	hypothet	Chlamydomonas_eusti	69.00	0.00
TRINITY_KXZ5133	hypothet	Gonium_pectorale	69.00	0.00
TRINITY_GAX7651	hypothet	Chlamydomonas_eusti	69.00	0.00
TRINITY_XP_0279	developr	Vigna_unguiculata	69.00	0.00
TRINITY_PNW7776	hypothet	Chlamydomonas_reinh	69.00	0.00
TRINITY_PNW7423	hypothet	Chlamydomonas_reinh	69.00	0.00
TRINITY_XP_0016	predicte	Chlamydomonas_reinh	69.00	0.00
TRINITY_BAJ9606	predicte	Hordeum_vulgare	69.00	0.00
TRINITY_PNW8028	hypothet	Chlamydomonas_reinh	69.00	0.00
TRINITY_XP_0029	hypothet	Volvox_carteri	69.00	0.00
TRINITY_GAX8570	hypothet	Chlamydomonas_eusti	69.00	0.00
TRINITY_KXZ4194	hypothet	Gonium_pectorale	69.00	0.00
TRINITY_XP_0017	predicte	Chlamydomonas_reinh	69.00	0.00
TRINITY_XP_0024	predicte	Micromonas_commoda	68.90	0.00
TRINITY_ADI4689	PR46af	Volvox_carteri	68.90	0.00
TRINITY_BAJ2156	cytochr	Prasinococcus_capsu	68.90	0.00
TRINITY_GAX7826	hypothet	Chlamydomonas_eusti	68.90	0.00
TRINITY_KXZ4480	hypothet	Gonium_pectorale	68.90	0.00
TRINITY_GAX8335	hypothet	Chlamydomonas_eusti	68.90	0.00
TRINITY_OWM8809	hypothet	Punica_granatum	68.90	0.00
TRINITY_RWW9078	hypothet	Ensete_ventricosum	68.90	0.00
TRINITY_PSC7532	low-CO2-	Micractinium_conduc	68.90	0.00
TRINITY_XP_0029	hypothet	Volvox_carteri	68.90	0.00
TRINITY_XP_0165	PREDICTE	Capsicum_annuum	68.90	0.00
TRINITY_PTQ4311	hypothet	Marchantia_polymorp	68.90	0.00
TRINITY_GAQ8681	Isovale	Klebsormidium_niten	68.90	0.00
TRINITY_GAX7582	hypothet	Chlamydomonas_eusti	68.90	0.00
TRINITY_PNH0809	NADH del	Tetrabaena_socialis	68.90	0.00
TRINITY_PNH1046	Leucine-	Tetrabaena_socialis	68.90	0.00
TRINITY_XP_0277	uncharac	Solanum_pennellii	68.90	0.00
TRINITY_XP_0029	hypothet	Volvox_carteri	68.90	0.00
TRINITY_PNW8830	hypothet	Chlamydomonas_reinh	68.90	0.00
TRINITY_BAJ9943	predicte	Hordeum_vulgare	68.90	0.00
TRINITY_PRW2097	phosphor	Chlorella_sorokinia	68.90	0.00
TRINITY_PNW8438	hypothet	Chlamydomonas_reinh	68.90	0.00
TRINITY_XP_0056	hypothet	Coccomyxa_subellips	68.90	0.00
TRINITY_OMP1412	hypothet	Corchorus_olitorius	68.90	0.00
TRINITY_XP_0264	ubiquit	Papaver_somniferum	68.80	0.00
TRINITY_XP_0202	nifU-li	Asparagus_officinal	68.80	0.00

TRINITY_XP_0238(	eukaryot	Quercus_suber	68.80	0.00
TRINITY_XP_0243(	uncharac	Physcomitrella_pate	68.80	0.00
TRINITY_VDC8380	unnamed	Brassica_rapa	68.80	0.00
TRINITY_GAQ9167	methion	Klebsormidium_niten	68.80	0.00
TRINITY_KMS6478	hypothet	Beta_vulgaris	68.80	0.00
TRINITY_GAX8505	hypothet	Chlamydomonas_eusti	68.80	0.00
TRINITY_GAX7649	hypothet	Chlamydomonas_eusti	68.80	0.00
TRINITY_GAX7546	hypothet	Chlamydomonas_eusti	68.80	0.00
TRINITY_XP_0029	hypothet	Volvox_carteri	68.80	0.00
TRINITY_KMS6493	hypothet	Beta_vulgaris	68.80	0.00
TRINITY_XP_0128	PREDICTE	Erythranthe_guttata	68.80	0.00
TRINITY_GAX8417	hypothet	Chlamydomonas_eusti	68.80	0.00
TRINITY_PRW5606	beta-ure	Chlorella_sorokinia	68.80	0.00
TRINITY_GAX7495	hypothet	Chlamydomonas_eusti	68.80	0.00
TRINITY_GAX8440	hypothet	Chlamydomonas_eusti	68.80	0.00
TRINITY_GAX8349	hypothet	Chlamydomonas_eusti	68.80	0.00
TRINITY_PNW8651	hypothet	Chlamydomonas_reinh	68.80	0.00
TRINITY_GAX8345	hypothet	Chlamydomonas_eusti	68.80	0.00
TRINITY_GAX7289	hypothet	Chlamydomonas_eusti	68.80	0.00
TRINITY_PNW8173	hypothet	Chlamydomonas_reinh	68.80	0.00
TRINITY_GAX8277	hypothet	Chlamydomonas_eusti	68.80	0.00
TRINITY_GAX8566	hypothet	Chlamydomonas_eusti	68.80	0.00
TRINITY_XP_0029	hypothet	Volvox_carteri	68.80	0.00
TRINITY_XP_0016	hydroxy	Chlamydomonas_reinh	68.80	0.00
TRINITY_XP_0029	hypothet	Volvox_carteri	68.80	0.00
TRINITY_XP_0029	hypothet	Volvox_carteri	68.80	0.00
TRINITY_GAX7401	hypothet	Chlamydomonas_eusti	68.80	0.00
TRINITY_KXZ5538	hypothet	Gonium_pectorale	68.80	0.00
TRINITY_PNW7569	hypothet	Chlamydomonas_reinh	68.80	0.00
TRINITY_PNW8033	hypothet	Chlamydomonas_reinh	68.80	0.00
TRINITY_XP_0056	hypothet	Coccomyxa_subellips	68.70	0.00
TRINITY_OWM7406	hypothet	Punica_granatum	68.70	0.00
TRINITY_XP_0273	isocitra	Abrus_precatorius	68.70	0.00
TRINITY_GAX8505	hypothet	Chlamydomonas_eusti	68.70	0.00
TRINITY_GAX7442	hypothet	Chlamydomonas_eusti	68.70	0.00
TRINITY_GBF8937	dihydrop	Raphidocelis_subcap	68.70	0.00
TRINITY_GAX7637	hypothet	Chlamydomonas_eusti	68.70	0.00
TRINITY_KXZ4834	hypothet	Gonium_pectorale	68.70	0.00
TRINITY_XP_0139	RAN GTPa	Monoraphidium_negle	68.70	0.00
TRINITY_PNH0656	Chaperor	Tetrabaena_socialis	68.70	0.00
TRINITY_XP_0029	hypothet	Volvox_carteri	68.70	0.00
TRINITY_KXZ4402	hypothet	Gonium_pectorale	68.70	0.00
TRINITY_XP_0016	NADP-dep	Chlamydomonas_reinh	68.70	0.00
TRINITY_AAY4238	soluble	Chlamydomonas_reinh	68.70	0.00
TRINITY_PNH1016	Serine a	Tetrabaena_socialis	68.70	0.00
TRINITY_KYP5700	Putative	Cajanus_cajan	68.60	0.00
TRINITY_KDD7573	hypothet	Helicosporidium_sp.	68.60	0.00
TRINITY_XP_0016	predicte	Chlamydomonas_reinh	68.60	0.00
TRINITY_XP_0016	predicte	Chlamydomonas_reinh	68.60	0.00
TRINITY_BAK0099	predicte	Hordeum_vulgare	68.60	0.00
TRINITY_PNH1196	hypothet	Tetrabaena_socialis	68.60	0.00
TRINITY_PNW8355	hypothet	Chlamydomonas_reinh	68.60	0.00
TRINITY_ACL9736	elongat	Ignatius_tetrasporu	68.60	0.00
TRINITY_PIN0863	Chaperor	Handroanthus_impeti	68.60	0.00
TRINITY_KXZ4766	hypothet	Gonium_pectorale	68.60	0.00
TRINITY_XP_0029	hypothet	Volvox_carteri	68.60	0.00
TRINITY_XP_0058	hypothet	Chlorella_variabili	68.60	0.00
TRINITY_XP_0139	NudC dor	Monoraphidium_negle	68.60	0.00
TRINITY_GAX7525	hypothet	Chlamydomonas_eusti	68.60	0.00
TRINITY_OUS4300	hypothet	Ostreococcus_tauri	68.60	0.00
TRINITY_GAX8298	hypothet	Chlamydomonas_eusti	68.60	0.00
TRINITY_PNH0191	Pantothe	Tetrabaena_socialis	68.60	0.00
TRINITY_GAX7468	hypothet	Chlamydomonas_eusti	68.60	0.00
TRINITY_XP_0029	hypothet	Volvox_carteri	68.60	0.00
TRINITY_XP_0017	predicte	Chlamydomonas_reinh	68.60	0.00
TRINITY_KXZ4331	hypothet	Gonium_pectorale	68.60	0.00

TRINITY_GAX7423(	hypothet	Chlamydomonas_eusti	68.60	0
TRINITY_KXZ4194(	hypothet	Gonium_pectorale	68.60	0.00
TRINITY_GAX7998	hypothet	Chlamydomonas_eusti	68.60	0.00
TRINITY_KXZ4781	hypothet	Gonium_pectorale	68.60	0
TRINITY_PRW5849	secondar	Chlorella_sorokinia	68.60	0.00
TRINITY_GAX8020	hypothet	Chlamydomonas_eusti	68.60	0
TRINITY_XP_0017(	ribosoma	Chlamydomonas_reinh	68.50	0.00
TRINITY_BAK0046(	predicte	Hordeum_vulgare	68.50	0.00
TRINITY_BAK0046(	predicte	Hordeum_vulgare	68.50	0.00
TRINITY_PON5752	ATPase,	Trema_orientale	68.50	0.00
TRINITY_KXZ5668	hypothet	Gonium_pectorale	68.50	0.00
TRINITY_XP_0016	predicte	Chlamydomonas_reinh	68.50	0.00
TRINITY_XP_0016	predicte	Chlamydomonas_reinh	68.50	0.00
TRINITY_KXZ5079	hypothet	Gonium_pectorale	68.50	0.00
TRINITY_XP_0016	esterase	Chlamydomonas_reinh	68.50	0.00
TRINITY_XP_0029	hypothet	Volvox_carteri	68.50	0.00
TRINITY_PNH0816	Conserve	Tetrabaena_socialis	68.50	0.00
TRINITY_XP_0045(	DNA repa	Cicer_arietinum	68.50	0.00
TRINITY_OAE2032(	hypothet	Marchantia_polymorp	68.50	0.00
TRINITY_KXZ4901	hypothet	Gonium_pectorale	68.50	0.00
TRINITY_GAX7634	hypothet	Chlamydomonas_eusti	68.50	0
TRINITY_PNW8870	hypothet	Chlamydomonas_reinh	68.50	0.00
TRINITY_XP_0025(	predicte	Micromonas_commoda	68.50	0.00
TRINITY_PNW8761	hypothet	Chlamydomonas_reinh	68.50	0.00
TRINITY_GBG6063	hypothet	Chara_braunii	68.50	0.00
TRINITY_ACH8758	trehalos	Dunaliella_viridis	68.50	0.00
TRINITY_GAX7448	hypothet	Chlamydomonas_eusti	68.50	0.00
TRINITY_ADW0902(	ammoniur	Dunaliella_viridis	68.50	0.00
TRINITY_XP_0017(	sterol s	Chlamydomonas_reinh	68.50	0.00
TRINITY_XP_0016	dynein h	Chlamydomonas_reinh	68.50	0.00
TRINITY_BAK0046(	predicte	Hordeum_vulgare	68.50	0.00
TRINITY_OAE3336	hypothet	Marchantia_polymorp	68.50	0.00
TRINITY_GBG5883	hypothet	Chara_braunii	68.50	0.00
TRINITY_GAQ8798(	Phosphat	Klebsormidium_niten	68.40	0.00
TRINITY_KZN0100	hypothet	Daucus_carota	68.40	0.00
TRINITY_XP_0029	calmodul	Selaginella_moellen	68.40	0.00
TRINITY_RWR7259	Ribosoma	Cinnamomum_micranth	68.40	0.00
TRINITY_XP_0072(	60S ribo	Prunus_persica	68.40	0.00
TRINITY_XP_0016	small ra	Chlamydomonas_reinh	68.40	0.00
TRINITY_GBG5915	hypothet	Chara_braunii	68.40	0.00
TRINITY_KCW5592	hypothet	Eucalyptus_grandis	68.40	0.00
TRINITY_XP_0238	protein	Quercus_suber	68.40	0.00
TRINITY_GAX7472	hypothet	Chlamydomonas_eusti	68.40	0.00
TRINITY_XP_0243	probable	Physcomitrella_pate	68.40	0.00
TRINITY_XP_0029	hypothet	Volvox_carteri	68.40	0.00
TRINITY_GBF9474	photosys	Raphidocelis_subcap	68.40	0.00
TRINITY_GAX8321	hypothet	Chlamydomonas_eusti	68.40	0.00
TRINITY_XP_0139(	Ribosome	Monoraphidium_negle	68.40	0.00
TRINITY_XP_0029	hypothet	Volvox_carteri	68.40	0.00
TRINITY_GAX7574	hypothet	Chlamydomonas_eusti	68.40	0.00
TRINITY_XP_0016	PsaB RNA	Chlamydomonas_reinh	68.40	0.00
TRINITY_XP_0016	predicte	Chlamydomonas_reinh	68.40	0.00
TRINITY_GAX8027	hypothet	Chlamydomonas_eusti	68.40	0.00
TRINITY_RYR5149	hypothet	Arachis_hypogaea	68.30	0.00
TRINITY_XP_0029	hypothet	Volvox_carteri	68.30	0.00
TRINITY_ACV8343	QM-like	Zea_mays	68.30	0.00
TRINITY_XP_0056	Metallo-	Coccomyxa_subellips	68.30	0.00
TRINITY_XP_0017(	DNA repa	Chlamydomonas_reinh	68.30	0.00
TRINITY_XP_0082	PREDICTE	Prunus_mume	68.30	0.00
TRINITY_GAX7680	hypothet	Chlamydomonas_eusti	68.30	0.00
TRINITY_XP_0139(	Uncharac	Monoraphidium_negle	68.30	0.00
TRINITY_GAX8602	hypothet	Chlamydomonas_eusti	68.30	0.00
TRINITY_PNW8691	hypothet	Chlamydomonas_reinh	68.30	0.00
TRINITY_XP_0016	pyruvate	Chlamydomonas_reinh	68.30	0.00
TRINITY_XP_0056	small nu	Coccomyxa_subellips	68.30	0.00
TRINITY_PNW7003	hypothet	Chlamydomonas_reinh	68.30	0.00

TRINITY_GBF8787(	hypothet	Raphidocelis_subcap	68.30	0.00
TRINITY_PNW7573(	hypothet	Chlamydomonas_reinh	68.30	0.00
TRINITY_GAX7504(	hypothet	Chlamydomonas_eusti	68.30	0.00
TRINITY_PNW7632(	hypothet	Chlamydomonas_reinh	68.30	0.00
TRINITY_GBF9588(	hypothet	Raphidocelis_subcap	68.30	0.00
TRINITY_AAT0251(	unknown	Chlamydomonas_reinh	68.30	0.00
TRINITY_GAX8065(	hypothet	Chlamydomonas_eusti	68.30	0.00
TRINITY_PRW5718(	Proteas(	Chlorella_sorokinia	68.30	0.00
TRINITY_XP_0029(	hypothet	Volvox_carteri	68.30	0.00
TRINITY_PNW8123(	hypothet	Chlamydomonas_reinh	68.30	0.00
TRINITY_GAX8256(	hypothet	Chlamydomonas_eusti	68.30	0.00
TRINITY_GAX8368(	hypothet	Chlamydomonas_eusti	68.30	0.00
TRINITY_XP_0016(	EMP/nona	Chlamydomonas_reinh	68.30	0.00
TRINITY_PNW8902(	hypothet	Chlamydomonas_reinh	68.30	0.00
TRINITY_GAX7309(	hypothet	Chlamydomonas_eusti	68.30	0.00
TRINITY_PNW8885(	hypothet	Chlamydomonas_reinh	68.30	0.00
TRINITY_KXZ4466(	hypothet	Gonium_pectorale	68.30	0.00
TRINITY_ACF9853(	delta-9	Chlamydomonas_reinh	68.30	0.00
TRINITY_XP_0016(	pyruvate	Chlamydomonas_reinh	68.30	0.00
TRINITY_CAJ3481(	methion:	Plantago_major	68.20	0.00
TRINITY_XP_0091(	PREDICTE	Brassica_rapa	68.20	0.00
TRINITY_KMS6524(	hypothet	Beta_vulgaris	68.20	0.00
TRINITY_XP_0029(	hypothet	Volvox_carteri	68.20	0.00
TRINITY_XP_0016(	flagella	Chlamydomonas_reinh	68.20	0.00
TRINITY_PNW7713(	hypothet	Chlamydomonas_reinh	68.20	0.00
TRINITY_PNW6958(	hypothet	Chlamydomonas_reinh	68.20	0.00
TRINITY_XP_0058(	hypothet	Chlorella_variabili	68.20	0.00
TRINITY_XP_0263(	serine/t	Papaver_somniferum	68.20	0.00
TRINITY_XP_0139(	hypothet	Monoraphidium_negle	68.20	0.00
TRINITY_PNW8734(	hypothet	Chlamydomonas_reinh	68.20	0.00
TRINITY_XP_0264(	AMP dear	Papaver_somniferum	68.20	0.00
TRINITY_KXZ4329(	hypothet	Gonium_pectorale	68.20	0.00
TRINITY_XP_0016(	predicte	Chlamydomonas_reinh	68.20	0.00
TRINITY_GAX7910(	hypothet	Chlamydomonas_eusti	68.20	0.00
TRINITY_RWR9150(	mitochor	Cinnamomum_micranth	68.20	0.00
TRINITY_KXZ5106(	hypothet	Gonium_pectorale	68.20	0.00
TRINITY_GAX8565(	hypothet	Chlamydomonas_eusti	68.20	0.00
TRINITY_P54213.(	RecName:	Dunaliella_salina	68.20	0.00
TRINITY_XP_0029(	dynein (	Volvox_carteri	68.20	0.00
TRINITY_GAX8035(	hypothet	Chlamydomonas_eusti	68.20	0.00
TRINITY_GBF9955(	ABC trar	Raphidocelis_subcap	68.20	0.00
TRINITY_KXZ4889(	hypothet	Gonium_pectorale	68.20	0.00
TRINITY_PSC7085(	G8 doma:	Micractinium_conduc	68.20	0.00
TRINITY_XP_0017(	seleniur	Chlamydomonas_reinh	68.20	0.00
TRINITY_XP_0025(	predicte	Micromonas_commoda	68.10	0.00
TRINITY_XP_0238(	probable	Quercus_suber	68.10	0.00
TRINITY_XP_0017(	pyruvate	Chlamydomonas_reinh	68.10	0.00
TRINITY_OAE1877(	hypothet	Marchantia_polymorp	68.10	0.00
TRINITY_PHT2541(	hypothet	Capsicum_baccatum	68.10	0.00
TRINITY_XP_0016(	ribosoma	Chlamydomonas_reinh	68.10	0.00
TRINITY_XP_0218(	pre-mRNA	Spinacia_oleracea	68.10	0.00
TRINITY_GAX7694(	hypothet	Chlamydomonas_eusti	68.10	0.00
TRINITY_PNH0504(	putative	Tetrabaena_socialis	68.10	0.00
TRINITY_XP_0024(	heat sho	Sorghum_bicolor	68.10	0.00
TRINITY_CDP1826(	unnamed	Coffea_canephora	68.10	0.00
TRINITY_AAO4575(	pol prot	Cucumis_melo	68.10	0.00
TRINITY_GAX8185(	hypothet	Chlamydomonas_eusti	68.10	0.00
TRINITY_GAX7828(	hypothet	Chlamydomonas_eusti	68.10	0.00
TRINITY_GBF9035(	tRNA mod	Raphidocelis_subcap	68.10	0.00
TRINITY_XP_0029(	hypothet	Volvox_carteri	68.10	0.00
TRINITY_GAX7595(	hypothet	Chlamydomonas_eusti	68.10	0.00
TRINITY_XP_0017(	predicte	Chlamydomonas_reinh	68.10	0.00
TRINITY_P04464.(	RecName:	Triticum_aestivum	68.10	0.00
TRINITY_GAX8041(	hypothet	Chlamydomonas_eusti	68.10	0.00
TRINITY_GAX8006(	hypothet	Chlamydomonas_eusti	68.10	0.00
TRINITY_XP_0058(	hypothet	Chlorella_variabili	68.10	0.00

TRINITY_AAG2983	4-alpha-	Chlamydomonas_reinh	68.10	0.00
TRINITY_GBF8857	hypothet	Raphidocelis_subcap	68.10	0.00
TRINITY_PNW7362	hypothet	Chlamydomonas_reinh	68.10	0.00
TRINITY_PNH0356	Heat sh	Tetrabaena_socialis	68.10	0.00
TRINITY_PNW7062	hypothet	Chlamydomonas_reinh	68.10	0.00
TRINITY_GAX7480	hypothet	Chlamydomonas_eusti	68.10	0.00
TRINITY_XP_0216	14-3-3-	Hevea_brasiliensis	68.10	0.00
TRINITY_PNW7497	hypothet	Chlamydomonas_reinh	68.10	0.00
TRINITY_PNW7683	hypothet	Chlamydomonas_reinh	68.10	0.00
TRINITY_GAX8059	hypothet	Chlamydomonas_eusti	68.10	0.00
TRINITY_XP_0241	actin-r	Rosa_chinensis	68.00	0.00
TRINITY_XP_0016	casein	Chlamydomonas_reinh	68.00	0.00
TRINITY_NP_0012	uncharac	Glycine_max	68.00	0.00
TRINITY_PNW8907	hypothet	Chlamydomonas_reinh	68.00	0.00
TRINITY_AAO7355	reverse-	Chlamydomonas_reinh	68.00	0.00
TRINITY_GAX8356	hypothet	Chlamydomonas_eusti	68.00	0.00
TRINITY_XP_0029	hypothet	Volvox_carteri	68.00	0.00
TRINITY_PNW7834	hypothet	Chlamydomonas_reinh	68.00	0.00
TRINITY_KXZ5075	hypothet	Gonium_pectorale	68.00	0.00
TRINITY_XP_0029	hypothet	Volvox_carteri	68.00	0.00
TRINITY_GAX7411	hypothet	Chlamydomonas_eusti	68.00	0.00
TRINITY_KXZ4274	hypothet	Gonium_pectorale	68.00	0
TRINITY_KXZ4938	hypothet	Gonium_pectorale	68.00	0.00
TRINITY_XP_0139	hypothet	Monoraphidium_negle	68.00	0.00
TRINITY_GAX7605	hypothet	Chlamydomonas_eusti	68.00	0.00
TRINITY_PNW8236	hypothet	Chlamydomonas_reinh	68.00	0.00
TRINITY_XP_0017	predict	Chlamydomonas_reinh	68.00	0.00
TRINITY_KXZ5299	hypothet	Gonium_pectorale	68.00	0.00
TRINITY_GAX7271	hypothet	Chlamydomonas_eusti	68.00	0.00
TRINITY_PNW7849	hypothet	Chlamydomonas_reinh	68.00	0.00
TRINITY_GAX7323	hypothet	Chlamydomonas_eusti	68.00	0.00
TRINITY_PNW8498	hypothet	Chlamydomonas_reinh	68.00	0.00
TRINITY_KXZ5537	hypothet	Gonium_pectorale	68.00	0.00
TRINITY_XP_0056	hypothet	Coccomyxa_subellips	68.00	0.00
TRINITY_OWM7932	hypothet	Punica_granatum	68.00	0.00
TRINITY_GAX7902	hypothet	Chlamydomonas_eusti	68.00	0.00
TRINITY_XP_0239	myosin	Quercus_suber	68.00	0.00
TRINITY_NP_0011	uncharac	Zea_mays	68.00	0.00
TRINITY_GAX8642	hypothet	Chlamydomonas_eusti	67.90	0.00
TRINITY_PNW7793	hypothet	Chlamydomonas_reinh	67.90	0.00
TRINITY_YP_0090	cytochr	Buxbaumia_aphylla	67.90	0.00
TRINITY_PNW7548	hypothet	Chlamydomonas_reinh	67.90	0.00
TRINITY_XP_0016	mitocho	Chlamydomonas_reinh	67.90	0.00
TRINITY_XP_0228	ABC tra	Olea_europaea	67.90	0.00
TRINITY_XP_0113	Oxygen-	Auxenochlorella_prc	67.90	0.00
TRINITY_XP_0029	hypothet	Volvox_carteri	67.90	0.00
TRINITY_RMZ5223	hypothet	Auxenochlorella_prc	67.90	0.00
TRINITY_GAX7372	hypothet	Chlamydomonas_eusti	67.90	0.00
TRINITY_XP_0029	hypothet	Volvox_carteri	67.90	0.00
TRINITY_GAX7694	hypothet	Chlamydomonas_eusti	67.90	0.00
TRINITY_GAX8166	hypothet	Chlamydomonas_eusti	67.90	0.00
TRINITY_XP_0029	hypothet	Volvox_carteri	67.90	0.00
TRINITY_GAX7638	hypothet	Chlamydomonas_eusti	67.90	0.00
TRINITY_KXZ4578	hypothet	Gonium_pectorale	67.90	0.00
TRINITY_PRW5757	ornithi	Chlorella_sorokinia	67.90	0.00
TRINITY_PNW8461	hypothet	Chlamydomonas_reinh	67.90	0.00
TRINITY_AFC8884	putativ	Tetragonia_tetragon	67.90	0.00
TRINITY_XP_0029	hypothet	Volvox_carteri	67.90	0.00
TRINITY_KXZ4433	hypothet	Gonium_pectorale	67.90	0.00
TRINITY_PSC6821	GPI man	Micractinium_conduc	67.90	0.00
TRINITY_XP_0029	hypothet	Volvox_carteri	67.90	0.00
TRINITY_PNW7170	hypothet	Chlamydomonas_reinh	67.90	0.00
TRINITY_XP_0063	PREDICT	Solanum_tuberosum	67.90	0.00
TRINITY_XP_0029	hypothet	Volvox_carteri	67.90	0.00
TRINITY_GAX7517	hypothet	Chlamydomonas_eusti	67.90	0.00
TRINITY_KXZ4978	hypothet	Gonium_pectorale	67.90	0.00

TRINITY_GAX8007	hypothet	Chlamydomonas_eusti	67.90	0.00
TRINITY_KXZ4823	hypothet	Gonium_pectorale	67.90	0.00
TRINITY_GBG6695	hypothet	Chara_braunii	67.90	0.00
TRINITY_PNW7082	hypothet	Chlamydomonas_reinh	67.90	0.00
TRINITY_XP_0172	PREDICT	Daucus_carota	67.80	0.00
TRINITY_XP_0016	ribosom	Chlamydomonas_reinh	67.80	0.00
TRINITY_XP_0017	plastid	Chlamydomonas_reinh	67.80	0.00
TRINITY_GBG8957	hypothet	Chara_braunii	67.80	0.00
TRINITY_XP_0238	DNA rep	Quercus_suber	67.80	0.00
TRINITY_XP_0016	mitocho	Chlamydomonas_reinh	67.80	0.00
TRINITY_GAX8481	hypothet	Chlamydomonas_eusti	67.80	0.00
TRINITY_XP_0239	ras-rel	Quercus_suber	67.80	0.00
TRINITY_XP_0218	prolife	Carica_papaya	67.80	0.00
TRINITY_GBG7769	hypothet	Chara_braunii	67.80	0.00
TRINITY_GAX7431	hypothet	Chlamydomonas_eusti	67.80	0.00
TRINITY_ABK2475	unknown	Picea_sitchensis	67.80	0.00
TRINITY_PNH0842	Retrovi	Tetrabaena_socialis	67.80	0.00
TRINITY_GAX7937	hypothet	Chlamydomonas_eusti	67.80	0.00
TRINITY_GAX8026	hypothet	Chlamydomonas_eusti	67.80	0.00
TRINITY_GAX7264	hypothet	Chlamydomonas_eusti	67.80	0.00
TRINITY_KXZ4686	hypothet	Gonium_pectorale	67.80	0.00
TRINITY_KXZ4730	hypothet	Gonium_pectorale	67.80	0.00
TRINITY_GAX7923	hypothet	Chlamydomonas_eusti	67.80	0
TRINITY_ONK7573	uncharac	Asparagus_officinal	67.70	0.00
TRINITY_XP_0056	nucleos	Coccomyxa_subellips	67.70	0.00
TRINITY_KXZ5546	hypothet	Gonium_pectorale	67.70	0.00
TRINITY_XP_0016	regulat	Chlamydomonas_reinh	67.70	0.00
TRINITY_GAX7923	hypothet	Chlamydomonas_eusti	67.70	0.00
TRINITY_XP_0016	predict	Chlamydomonas_reinh	67.70	0.00
TRINITY_GAX7702	hypothet	Chlamydomonas_eusti	67.70	0.00
TRINITY_XP_0029	fumarat	Volvox_carteri	67.70	0.00
TRINITY_PNW8863	hypothet	Chlamydomonas_reinh	67.70	0.00
TRINITY_XP_0017	predict	Chlamydomonas_reinh	67.70	0.00
TRINITY_GAX7286	hypothet	Chlamydomonas_eusti	67.70	0.00
TRINITY_RAL5292	hypothet	Cuscuta_australis	67.70	0.00
TRINITY_PNW8013	hypothet	Chlamydomonas_reinh	67.70	0.00
TRINITY_XP_0016	ser/thr	Chlamydomonas_reinh	67.70	0.00
TRINITY_PNW7167	hypothet	Chlamydomonas_reinh	67.70	0.00
TRINITY_XP_0029	compone	Volvox_carteri	67.70	0.00
TRINITY_XP_0228	Zinc fi	Ostreococcus_tauri	67.70	0.00
TRINITY_XP_0228	Zinc fi	Ostreococcus_tauri	67.70	0.00
TRINITY_XP_0029	hypothet	Volvox_carteri	67.70	0.00
TRINITY_XP_0029	hypothet	Volvox_carteri	67.70	0.00
TRINITY_PNW7777	hypothet	Chlamydomonas_reinh	67.70	0.00
TRINITY_XP_0029	hypothet	Volvox_carteri	67.70	0.00
TRINITY_PNH1083	Helicas	Tetrabaena_socialis	67.70	0.00
TRINITY_KXZ5032	hypothet	Gonium_pectorale	67.70	0
TRINITY_PNW6969	hypothet	Chlamydomonas_reinh	67.70	0.00
TRINITY_GAX7770	hypothet	Chlamydomonas_eusti	67.70	0.00
TRINITY_XP_0016	N-ethyl	Chlamydomonas_reinh	67.70	0.00
TRINITY_PSC7669	60S aci	Micractinium_conduc	67.60	0.00
TRINITY_GAQ8150	Selenoc	Klebsormidium_niten	67.60	0.00
TRINITY_XP_0030	predict	Micromonas_pusilla	67.60	0.00
TRINITY_RMZ5585	hypothet	Auxenochlorella_pro	67.60	0.00
TRINITY_XP_0029	isocitr	Volvox_carteri	67.60	0.00
TRINITY_KXZ4193	hypothet	Gonium_pectorale	67.60	0.00
TRINITY_GAX8186	hypothet	Chlamydomonas_eusti	67.60	0.00
TRINITY_XP_0022	PREDICT	Vitis_vinifera	67.60	0.00
TRINITY_GBF9256	hypothet	Raphidocelis_subcap	67.60	0.00
TRINITY_XP_0029	hypothet	Volvox_carteri	67.60	0.00
TRINITY_XP_0017	molybdo	Chlamydomonas_reinh	67.60	0.00
TRINITY_KXZ5309	hypothet	Gonium_pectorale	67.60	0.00
TRINITY_XP_0030	predict	Micromonas_pusilla	67.60	0.00
TRINITY_GAX8041	hypothet	Chlamydomonas_eusti	67.60	0.00
TRINITY_KNA0305	hypothet	Spinacia_oleracea	67.60	0.00
TRINITY_GAX7405	hypothet	Chlamydomonas_eusti	67.60	0.00

TRINITY_AFI57622	Rpn8 [Du	Dunaliella_salina	67.60	0.00
TRINITY_XP_00666	PREDICTE	Oryza_brachyantha	67.60	0.00
TRINITY_GAX80861	hypothet	Chlamydomonas_eusti	67.60	0.00
TRINITY_GAX79640	hypothet	Chlamydomonas_eusti	67.60	0.00
TRINITY_KXZ53964	hypothet	Gonium_pectorale	67.60	0.00
TRINITY_PNH07195	Elicitor	Tetrabaena_socialis	67.60	0.00
TRINITY_XP_00169	peptidyl	Chlamydomonas_reinh	67.60	0.00
TRINITY_PNW69788	hypothet	Chlamydomonas_reinh	67.60	0.00
TRINITY_XP_00230	heat sho	Populus_trichocarpa	67.60	0.00
TRINITY_GAX85297	hypothet	Chlamydomonas_eusti	67.60	0.00

TRINITY_GBG8506(	hypothet	Chara_braunii	67.60	0.00
TRINITY_GBG7545{	hypothet	Chara_braunii	67.60	0.00
TRINITY_PNW8452	hypothet	Chlamydomonas_reinh	67.60	0.00
TRINITY_XP_0056	ribosoma	Coccomyxa_subellips	67.60	0.00
TRINITY_PNW8180	hypothet	Chlamydomonas_reinh	67.60	0.00
TRINITY_PSC7629	integral	Micractinium_conduc	67.60	0.00
TRINITY_XP_0029	hypothet	Volvox_carteri	67.60	0
TRINITY_GAQ8971	GTP-binc	Klebsormidium_niten	67.60	0.00
TRINITY_GAX7746	hypothet	Chlamydomonas_eusti	67.60	0.00
TRINITY_PNW7964	hypothet	Chlamydomonas_reinh	67.60	0.00
TRINITY_PNW7067	hypothet	Chlamydomonas_reinh	67.60	0.00
TRINITY_XP_0094	PREDICTE	Musa_acuminata	67.50	0.00
TRINITY_XP_0029	mitochor	Volvox_carteri	67.50	0.00
TRINITY_XP_0216	60S ribc	Hevea_brasiliensis	67.50	0.00
TRINITY_KZV1520	hypothet	Dorcoceras_hygromet	67.50	0.00
TRINITY_KXZ5701(	hypothet	Gonium_pectorale	67.50	0.00
TRINITY_ANE1053	gamma ca	Dunaliella_salina	67.50	0.00
TRINITY_XP_0029	hypothet	Volvox_carteri	67.50	0.00
TRINITY_AAB6131	htrA-lik	Haematococcus_lacus	67.50	0.00
TRINITY_GAX7883	hypothet	Chlamydomonas_eusti	67.50	0.00
TRINITY_KXZ4637	hypothet	Gonium_pectorale	67.50	0.00
TRINITY_XP_0056	WD40 rep	Coccomyxa_subellips	67.50	0.00
TRINITY_XP_0056	polyader	Coccomyxa_subellips	67.50	0.00
TRINITY_GAX8018	hypothet	Chlamydomonas_eusti	67.50	0.00
TRINITY_KXZ5673	hypothet	Gonium_pectorale	67.50	0.00
TRINITY_GAX7778	hypothet	Chlamydomonas_eusti	67.50	0.00
TRINITY_XP_0016	predicte	Chlamydomonas_reinh	67.50	0.00
TRINITY_GAX7281(	hypothet	Chlamydomonas_eusti	67.50	0.00
TRINITY_XP_0016	predicte	Chlamydomonas_reinh	67.50	0.00
TRINITY_GAX7477	hypothet	Chlamydomonas_eusti	67.50	0.00
TRINITY_XP_0029	hypothet	Volvox_carteri	67.50	0.00
TRINITY_KXZ4748(	hypothet	Gonium_pectorale	67.50	0.00
TRINITY_KXZ5469	hypothet	Gonium_pectorale	67.50	0.00
TRINITY_KXZ4498	hypothet	Gonium_pectorale	67.50	0.00
TRINITY_XP_0029	NimA-rel	Volvox_carteri	67.50	0.00
TRINITY_GAX7909	hypothet	Chlamydomonas_eusti	67.50	0.00
TRINITY_XP_0145	(6-4)DNA	Vigna_radiata	67.40	0.00
TRINITY_BAJ9379	predicte	Hordeum_vulgare	67.40	0.00
TRINITY_XP_0025(	predicte	Micromonas_commoda	67.40	0.00
TRINITY_GBG6826	hypothet	Chara_braunii	67.40	0.00
TRINITY_CBI2297	unnamed	Vitis_vinifera	67.40	0.00
TRINITY_BAJ8923	predicte	Hordeum_vulgare	67.40	0.00
TRINITY_XP_0139(	glycine	Monoraphidium_negle	67.40	0.00
TRINITY_XP_0238	cysteine	Quercus_suber	67.40	0.00
TRINITY_PHT8317	hypothet	Capsicum_annuum	67.40	0.00
TRINITY_XP_0016	predicte	Chlamydomonas_reinh	67.40	0.00
TRINITY_XP_0016	predicte	Chlamydomonas_reinh	67.40	0.00
TRINITY_GAX7634(	hypothet	Chlamydomonas_eusti	67.40	0.00
TRINITY_XP_0029	hypothet	Volvox_carteri	67.40	0.00
TRINITY_XP_0229	ras-rela	Cucurbita_maxima	67.40	0.00
TRINITY_GAX7937	hypothet	Chlamydomonas_eusti	67.40	0.00
TRINITY_GAX8363(	hypothet	Chlamydomonas_eusti	67.40	0.00
TRINITY_GBF9555	hypothet	Raphidocelis_subcap	67.40	0.00
TRINITY_XP_0029	hypothet	Volvox_carteri	67.40	0.00
TRINITY_KXZ4349	hypothet	Gonium_pectorale	67.40	0.00
TRINITY_GAX8118	hypothet	Chlamydomonas_eusti	67.40	0.00
TRINITY_GAX8349	hypothet	Chlamydomonas_eusti	67.40	0.00
TRINITY_XP_0029	hypothet	Volvox_carteri	67.40	0.00
TRINITY_KXZ4606	hypothet	Gonium_pectorale	67.40	0.00
TRINITY_GAX8091(	hypothet	Chlamydomonas_eusti	67.40	0.00
TRINITY_GAX7398	hypothet	Chlamydomonas_eusti	67.40	0.00
TRINITY_KXZ4703	hypothet	Gonium_pectorale	67.40	0.00
TRINITY_XP_0029	hypothet	Volvox_carteri	67.40	0
TRINITY_PTQ4603	hypothet	Marchantia_polymorp	67.40	0.00
TRINITY_GAX7925	hypothet	Chlamydomonas_eusti	67.40	0.00
TRINITY_BAJ9409	predicte	Hordeum_vulgare	67.30	0.00

TRINITY_XP_0110	PREDICTE	Populus_euphratica	67.30	0.00
TRINITY_GAQ8302	rotamase	Klebsormidium_niten	67.30	0.00
TRINITY_PIN0453	Molecula	Handroanthus_impeti	67.30	0.00
TRINITY_KXZ5273	hypothet	Gonium_pectorale	67.30	0.00
TRINITY_GAX8393	hypothet	Chlamydomonas_eusti	67.30	0.00
TRINITY_GAX8481	hypothet	Chlamydomonas_eusti	67.30	0.00
TRINITY_XP_0016	ubiquino	Chlamydomonas_reinh	67.30	0.00
TRINITY_XP_0243	phytoch	Physcomitrella_pate	67.30	0.00
TRINITY_GAX7985	hypothet	Chlamydomonas_eusti	67.30	0.00
TRINITY_GAX8285	hypothet	Chlamydomonas_eusti	67.30	0.00
TRINITY_KXZ5583	hypothet	Gonium_pectorale	67.30	0.00
TRINITY_GAX7412	hypothet	Chlamydomonas_eusti	67.30	0.00
TRINITY_GAX8219	hypothet	Chlamydomonas_eusti	67.30	0.00
TRINITY_KXZ4092	hypothet	Gonium_pectorale	67.30	0.00
TRINITY_KXZ4824	hypothet	Gonium_pectorale	67.30	0.00
TRINITY_PNW8634	hypothet	Chlamydomonas_reinh	67.30	0.00
TRINITY_GAX8467	hypothet	Chlamydomonas_eusti	67.30	0.00
TRINITY_OWM8305	hypothet	Punica_granatum	67.30	0.00
TRINITY_XP_0017	intrafla	Chlamydomonas_reinh	67.30	0.00
TRINITY_XP_0016	isocitra	Chlamydomonas_reinh	67.30	0.00
TRINITY_KMS6524	hypothet	Beta_vulgaris	67.30	0.00
TRINITY_XP_0138	20S prot	Monoraphidium_negle	67.30	0.00
TRINITY_GAX8446	hypothet	Chlamydomonas_eusti	67.30	0.00
TRINITY_PNW7001	hypothet	Chlamydomonas_reinh	67.30	0.00
TRINITY_GAX7950	hypothet	Chlamydomonas_eusti	67.30	0.00
TRINITY_GAX8266	hypothet	Chlamydomonas_eusti	67.30	0.00
TRINITY_GAX8015	hypothet	Chlamydomonas_eusti	67.30	0.00
TRINITY_GAX7423	hypothet	Chlamydomonas_eusti	67.30	0.00
TRINITY_KXZ4262	hypothet	Gonium_pectorale	67.20	0.00
TRINITY_XP_0058	hypothet	Chlorella_variabili	67.20	0.00
TRINITY_CBI2297	unnamed	Vitis_vinifera	67.20	0.00
TRINITY_ALM5501	DEAD box	Chlamydomonas_sp._I	67.20	0.00
TRINITY_PNH0894	hypothet	Tetrabaena_socialis	67.20	0.00
TRINITY_PNW7759	hypothet	Chlamydomonas_reinh	67.20	0.00
TRINITY_PNH0830	NFX1-typ	Tetrabaena_socialis	67.20	0.00
TRINITY_XP_0029	hypothet	Volvox_carteri	67.20	0.00
TRINITY_KXZ5702	hypothet	Gonium_pectorale	67.20	0.00
TRINITY_GAX7414	hypothet	Chlamydomonas_eusti	67.20	0.00
TRINITY_XP_0029	hypothet	Volvox_carteri	67.20	0.00
TRINITY_XP_0029	Hsp70 pr	Volvox_carteri	67.20	0.00
TRINITY_GAX8166	hypothet	Chlamydomonas_eusti	67.20	0.00
TRINITY_GAX7882	hypothet	Chlamydomonas_eusti	67.20	0.00
TRINITY_GAX8165	hypothet	Chlamydomonas_eusti	67.20	0.00
TRINITY_XP_0184	PREDICTE	Raphanus_sativus	67.20	0.00
TRINITY_GAX7983	hypothet	Chlamydomonas_eusti	67.20	0.00
TRINITY_AEF7996	phosphos	Dunaliella_salina	67.20	0.00
TRINITY_PRW5994	pfkB far	Chlorella_sorokinia	67.20	0.00
TRINITY_KXZ4155	hypothet	Gonium_pectorale	67.20	0.00
TRINITY_XP_0029	hypothet	Volvox_carteri	67.20	0.00
TRINITY_XP_0029	hypothet	Volvox_carteri	67.20	0.00
TRINITY_GAX7606	hypothet	Chlamydomonas_eusti	67.20	0.00
TRINITY_ABL0948	omega-3	Chlamydomonas_reinh	67.20	0.00
TRINITY_GAX7406	hypothet	Chlamydomonas_eusti	67.20	0.00
TRINITY_GAX8006	hypothet	Chlamydomonas_eusti	67.20	0.00
TRINITY_QBG3806	NADH deh	Chlorosarcinopsis_e	67.20	0.00
TRINITY_GAX7667	hypothet	Chlamydomonas_eusti	67.20	0.00
TRINITY_GAX8204	hypothet	Chlamydomonas_eusti	67.20	0.00
TRINITY_XP_0029	2-oxoglu	Volvox_carteri	67.20	0.00
TRINITY_GAX8460	hypothet	Chlamydomonas_eusti	67.20	0.00
TRINITY_ABS1121	protein	Oldenlandia_affinis	67.10	0.00
TRINITY_XP_0219	uracil-D	Helianthus_annuus	67.10	0.00
TRINITY_RWR9412	ISWI chr	Cinnamomum_micranth	67.10	0.00
TRINITY_EPS6264	hypothet	Genlisea_aurea	67.10	0.00
TRINITY_PTQ3691	hypothet	Marchantia_polymorp	67.10	0.00
TRINITY_BAK0277	predicte	Hordeum_vulgare	67.10	0.00
TRINITY_RWW9078	hypothet	Ensete_ventricosum	67.10	0.00

TRINITY_XP_0239(	aldo-ket	Quercus_suber	67.10	0.00
TRINITY_XP_0029	hypothet	Volvox_carteri	67.10	0.00
TRINITY_KXZ5206	hypothet	Gonium_pectorale	67.10	0.00
TRINITY_KMS6524	hypothet	Beta_vulgaris	67.10	0.00
TRINITY_XP_0029	hypothet	Volvox_carteri	67.10	0.00
TRINITY_GAX7786	hypothet	Chlamydomonas_eusti	67.10	0.00
TRINITY_GAX8288	hypothet	Chlamydomonas_eusti	67.10	0.00
TRINITY_XP_0029	hypothet	Volvox_carteri	67.10	0.00
TRINITY_GAX7317	hypothet	Chlamydomonas_eusti	67.10	0.00
TRINITY_KXZ5353	hypothet	Gonium_pectorale	67.10	0.00
TRINITY_GAX8277	hypothet	Chlamydomonas_eusti	67.10	0.00
TRINITY_KXZ4402	hypothet	Gonium_pectorale	67.10	0.00
TRINITY_XP_0029	hypothet	Volvox_carteri	67.10	0.00
TRINITY_GAX8425	hypothet	Chlamydomonas_eusti	67.10	0.00
TRINITY_GAX7400	hypothet	Chlamydomonas_eusti	67.10	0
TRINITY_GAX8257	hypothet	Chlamydomonas_eusti	67.10	0.00
TRINITY_XP_0105	PREDICTE	Tarenaya_hasslerian	67.10	0.00
TRINITY_KXZ4731	hypothet	Gonium_pectorale	67.10	0.00
TRINITY_PSC7395	flagella	Micractinium_conduc	67.10	0.00
TRINITY_GAX7358	hypothet	Chlamydomonas_eusti	67.10	0
TRINITY_OVA1233	K Homolo	Macleaya_cordata	67.00	0.00
TRINITY_XP_0228	ABC tran	Olea_europaea	67.00	0.00
TRINITY_KMS6493	hypothet	Beta_vulgaris	67.00	0.00
TRINITY_XP_0239(	septum-p	Quercus_suber	67.00	0.00
TRINITY_XP_0029	hypothet	Volvox_carteri	67.00	0.00
TRINITY_XP_0176	PREDICTE	Gossypium_arboreum	67.00	0.00
TRINITY_GAX7780	hypothet	Chlamydomonas_eusti	67.00	0.00
TRINITY_KXZ4891(	hypothet	Gonium_pectorale	67.00	0.00
TRINITY_XP_0138	hypothet	Monoraphidium_negle	67.00	0.00
TRINITY_GAX7711	hypothet	Chlamydomonas_eusti	67.00	0.00
TRINITY_XP_0263	agmatine	Papaver_somniferum	67.00	0.00
TRINITY_GAX8351	hypothet	Chlamydomonas_eusti	67.00	0.00
TRINITY_PNW7336	hypothet	Chlamydomonas_reinh	67.00	0.00
TRINITY_KXZ5520	hypothet	Gonium_pectorale	67.00	0.00
TRINITY_KXZ5683	hypothet	Gonium_pectorale	67.00	0.00
TRINITY_PNW8005	hypothet	Chlamydomonas_reinh	67.00	0.00
TRINITY_KXZ4661	hypothet	Gonium_pectorale	67.00	0.00
TRINITY_GAX8007	hypothet	Chlamydomonas_eusti	67.00	0.00
TRINITY_GBG7832	hypothet	Chara_braunii	67.00	0.00
TRINITY_XP_0029	hypothet	Volvox_carteri	67.00	0.00
TRINITY_KXZ4527	hypothet	Gonium_pectorale	67.00	0.00
TRINITY_XP_0016	predicte	Chlamydomonas_reinh	67.00	0.00
TRINITY_XP_0138	hypothet	Monoraphidium_negle	67.00	0.00
TRINITY_XP_0029	hypothet	Volvox_carteri	67.00	0.00
TRINITY_GAX8310	hypothet	Chlamydomonas_eusti	67.00	0.00
TRINITY_GAX7527	hypothet	Chlamydomonas_eusti	67.00	0.00
TRINITY_XP_0238	nucleosi	Quercus_suber	66.90	0.00
TRINITY_AWW1652	phosphor	Ipomoea_pes-caprae	66.90	0.00
TRINITY_KXZ5046	hypothet	Gonium_pectorale	66.90	0.00
TRINITY_YP_0060	cox1 gen	Nitella_hyalina	66.90	0.00
TRINITY_PSC7350	cytochro	Micractinium_conduc	66.90	0.00
TRINITY_XP_0016	mitogen	Chlamydomonas_reinh	66.90	0.00
TRINITY_GAX7755	hypothet	Chlamydomonas_eusti	66.90	0.00
TRINITY_XP_0029	hypothet	Volvox_carteri	66.90	0.00
TRINITY_XP_0016	T-comple	Chlamydomonas_reinh	66.90	0.00
TRINITY_GAX8383	hypothet	Chlamydomonas_eusti	66.90	0.00
TRINITY_KXZ4612	hypothet	Gonium_pectorale	66.90	0.00
TRINITY_XP_0029	hypothet	Volvox_carteri	66.90	0.00
TRINITY_GAX8183	hypothet	Chlamydomonas_eusti	66.90	0.00
TRINITY_XP_0016	predicte	Chlamydomonas_reinh	66.90	0.00
TRINITY_GAX7547	hypothet	Chlamydomonas_eusti	66.90	0.00
TRINITY_XP_0016	phosphat	Chlamydomonas_reinh	66.90	0.00
TRINITY_XP_0029	hypothet	Volvox_carteri	66.90	0.00
TRINITY_KXZ4638	hypothet	Gonium_pectorale	66.90	0.00
TRINITY_KXZ5128	hypothet	Gonium_pectorale	66.90	0.00
TRINITY_GAX7460	hypothet	Chlamydomonas_eusti	66.90	0.00

TRINITY_PNW7618	hypothet	Chlamydomonas_reinh	66.90	0.00
TRINITY_KXZ5426	hypothet	Gonium_pectorale	66.90	0.00
TRINITY_PNW8406	hypothet	Chlamydomonas_reinh	66.90	0.00
TRINITY_PNW8182	hypothet	Chlamydomonas_reinh	66.90	0.00
TRINITY_KXZ5239	hypothet	Gonium_pectorale	66.90	0.00
TRINITY_OMO7846	Pyruvate	Corchorus_olitorius	66.80	0.00
TRINITY_GAQ8062	mitochor	Klebsormidium_niten	66.80	0.00
TRINITY_XP_0029	hypothet	Volvox_carteri	66.80	0.00
TRINITY_GBF9745	DEAD-bo	Raphidocelis_subcap	66.80	0.00
TRINITY_PNW7514	hypothet	Chlamydomonas_reinh	66.80	0.00
TRINITY_GAX7632	hypothet	Chlamydomonas_eusti	66.80	0.00
TRINITY_OEL3389	Calreti	Dichanthelium_oligo	66.80	0.00
TRINITY_GAX7815	hypothet	Chlamydomonas_eusti	66.80	0.00
TRINITY_XP_0029	hypothet	Volvox_carteri	66.80	0.00
TRINITY_GAX8342	hypothet	Chlamydomonas_eusti	66.80	0.00
TRINITY_GAX8311	hypothet	Chlamydomonas_eusti	66.80	0.00
TRINITY_GAX7905	hypothet	Chlamydomonas_eusti	66.80	0.00
TRINITY_GAX8540	hypothet	Chlamydomonas_eusti	66.80	0.00
TRINITY_GAX7490	hypothet	Chlamydomonas_eusti	66.80	0.00
TRINITY_XP_0243	angio-a	Physcomitrella_pate	66.80	0.00
TRINITY_KXZ4213	hypothet	Gonium_pectorale	66.80	0.00
TRINITY_XP_0029	hypothet	Volvox_carteri	66.80	0
TRINITY_KXZ5614	hypothet	Gonium_pectorale	66.80	0.00
TRINITY_EFJ0733	hypothet	Selaginella_moellen	66.70	0.00
TRINITY_PSS1447	26S prot	Actinidia_chinensis	66.70	0.00
TRINITY_XP_0064	ras-rel	Citrus_sinensis	66.70	0.00
TRINITY_XP_0184	PREDICT	Raphanus_sativus	66.70	0.00
TRINITY_A8ISN6.2	RecName:	Chlamydomonas_reinh	66.70	0.00
TRINITY_YP_0091	cytochr	Lobosphaera_incisa	66.70	0.00
TRINITY_XP_0184	PREDICT	Raphanus_sativus	66.70	0.00
TRINITY_RXI0691	hypothet	Malus_domestica	66.70	0.00
TRINITY_XP_0030	predict	Micromonas_pusilla	66.70	0.00
TRINITY_XP_0030	Pyridox	Ostreococcus_tauri	66.70	0.00
TRINITY_XP_0228	Cytochr	Ostreococcus_tauri	66.70	0.00
TRINITY_XP_0238	ubiquit	Quercus_suber	66.70	0.00
TRINITY_XP_0216	ribonuc	Manihot_esculenta	66.70	0.00
TRINITY_NP_0011	cell di	Zea_mays	66.70	0.00
TRINITY_PNW8183	hypothet	Chlamydomonas_reinh	66.70	0.00
TRINITY_GBF9660	hypothet	Raphidocelis_subcap	66.70	0.00
TRINITY_XP_0029	hypothet	Volvox_carteri	66.70	0.00
TRINITY_XP_0029	hypothet	Volvox_carteri	66.70	0.00
TRINITY_QBB2000	heat sh	Ettlia_sp._YC001	66.70	0.00
TRINITY_KXZ4193	hypothet	Gonium_pectorale	66.70	0.00
TRINITY_GAX7993	hypothet	Chlamydomonas_eusti	66.70	0.00
TRINITY_ADC4537	actin [	Cleistogenes_songor	66.70	0.00
TRINITY_PNH0363	Phosphat	Tetrabaena_socialis	66.70	0.00
TRINITY_XP_0029	hypothet	Volvox_carteri	66.70	0.00
TRINITY_GBF9697	hypothet	Raphidocelis_subcap	66.70	0.00
TRINITY_KZV5869	hypothet	Dorcoceras_hygromet	66.70	0.00
TRINITY_AKR1403	translat	Tengia_sp._MEN-2015	66.70	0.00
TRINITY_XP_0016	hypothet	Chlamydomonas_reinh	66.70	0.00
TRINITY_KXZ5134	hypothet	Gonium_pectorale	66.70	0.00
TRINITY_PNW7095	hypothet	Chlamydomonas_reinh	66.70	0.00
TRINITY_PRW6027	sm LSM8	Chlorella_sorokinia	66.70	0.00
TRINITY_XP_0029	luminal	Volvox_carteri	66.70	0.00
TRINITY_XP_0029	hypothet	Volvox_carteri	66.70	0.00
TRINITY_XP_0029	hypothet	Volvox_carteri	66.70	0.00
TRINITY_XP_0217	LOW QUA	Chenopodium_quinoa	66.70	0.00
TRINITY_AGH5599	acyl Co	Tetraena_mongolica	66.70	0.00
TRINITY_PNW7386	hypothet	Chlamydomonas_reinh	66.70	0.00
TRINITY_PNH1001	hypothet	Tetrabaena_socialis	66.70	0.00
TRINITY_KXZ4092	hypothet	Gonium_pectorale	66.70	0.00
TRINITY_GAX7262	hypothet	Chlamydomonas_eusti	66.70	0.00
TRINITY_PNW7138	hypothet	Chlamydomonas_reinh	66.70	0.00
TRINITY_KXZ4579	hypothet	Gonium_pectorale	66.70	0.00
TRINITY_GAX7560	hypothet	Chlamydomonas_eusti	66.70	0.00

TRINITY_XP_00169	predicte	Chlamydomonas_reinh	66.70	0.00
TRINITY_GAX72908	hypothet	Chlamydomonas_eusti	66.70	0.00
TRINITY_GAX84843	hypothet	Chlamydomonas_eusti	66.70	0.00
TRINITY_KMZ65423	Phytochi	Zostera_marina	66.70	0.00
TRINITY_XP_00584	hypothet	Chlorella_variabili	66.70	0.00
TRINITY_GAX82617	hypothet	Chlamydomonas_eusti	66.70	0.00
TRINITY_KMS91954	hypothet	Beta_vulgaris	66.70	0.00
TRINITY_PNH06819	hypothet	Tetrabaena_socialis	66.70	0.00
TRINITY_XP_00294	hypothet	Volvox_carteri	66.70	0.00
TRINITY_ONM18921	ABC tran	Zea_mays	66.70	0.00
TRINITY_GAX82432	hypothet	Chlamydomonas_eusti	66.70	0.00
TRINITY_XP_00751	predicte	Bathycoccus_prasino	66.70	0.00
TRINITY_PRW44853	tRNA-spe	Chlorella_sorokinia	66.70	0.00
TRINITY_XP_00564	hypothet	Coccomyxa_subellips	66.70	0.00
TRINITY_XP_00294	hypothet	Volvox_carteri	66.70	0.00
TRINITY_XP_01389	hypothet	Monoraphidium_negle	66.70	0.00
TRINITY_GAX80939	hypothet	Chlamydomonas_eusti	66.70	0.00
TRINITY_KXZ41310	hypothet	Gonium_pectorale	66.70	0.00
TRINITY_PNW88858	hypothet	Chlamydomonas_reinh	66.70	0.00
TRINITY_PNW76800	hypothet	Chlamydomonas_reinh	66.70	0.00
TRINITY_PRW44928	nuclear	Chlorella_sorokinia	66.70	0.00
TRINITY_GBF88376	hypothet	Raphidocelis_subcap	66.70	0.00
TRINITY_XP_00564	Aldo/ket	Coccomyxa_subellips	66.70	0.00
TRINITY_GAX82949	hypothet	Chlamydomonas_eusti	66.70	0.00
TRINITY_KXZ41819	hypothet	Gonium_pectorale	66.70	0.00
TRINITY_PNW72964	hypothet	Chlamydomonas_reinh	66.70	0.00
TRINITY_XP_00564	glutathi	Coccomyxa_subellips	66.70	0.00
TRINITY_GAX84161	hypothet	Chlamydomonas_eusti	66.70	0.00
TRINITY_KZV15020	ATP-depe	Dorcoceras_hygromet	66.70	0.00
TRINITY_XP_02215	peroxiso	Momordica_charantia	66.70	0.00
TRINITY_XP_02452	U3 small	Selaginella_moellen	66.60	0.00
TRINITY_XP_02389	phosphog	Quercus_suber	66.60	0.00
TRINITY_OMO78468	Pyruvate	Corchorus_olitorius	66.60	0.00
TRINITY_XP_00295	Deoxyrib	Volvox_carteri	66.60	0.00
TRINITY_KXZ54712	hypothet	Gonium_pectorale	66.60	0.00
TRINITY_GAX84353	hypothet	Chlamydomonas_eusti	66.60	0.00
TRINITY_GAX73802	hypothet	Chlamydomonas_eusti	66.60	0.00
TRINITY_GAX73201	hypothet	Chlamydomonas_eusti	66.60	0.00
TRINITY_PNW79674	hypothet	Chlamydomonas_reinh	66.60	0.00
TRINITY_GAX84732	hypothet	Chlamydomonas_eusti	66.60	0.00
TRINITY_GAX79512	hypothet	Chlamydomonas_eusti	66.60	0.00
TRINITY_XP_01594	60S ribo	Arachis_duranensis	66.50	0.00
TRINITY_GAQ91252	oxoproli	Klebsormidium_niten	66.50	0.00
TRINITY_XP_00170	SM/Sec1-	Chlamydomonas_reinh	66.50	0.00
TRINITY_BAK02361	predicte	Hordeum_vulgare	66.50	0.00
TRINITY_GAQ83528	Alanine-	Klebsormidium_niten	66.50	0.00
TRINITY_KXZ53925	hypothet	Gonium_pectorale	66.50	0.00
TRINITY_GBF93536	hypothet	Raphidocelis_subcap	66.50	0.00
TRINITY_XP_00295	hypothet	Volvox_carteri	66.50	0.00
TRINITY_KXZ49319	hypothet	Gonium_pectorale	66.50	0.00
TRINITY_KMS64936	hypothet	Beta_vulgaris	66.50	0.00
TRINITY_RRT33576	hypothet	Ensete_ventricosum	66.50	0.00
TRINITY_GAX79081	hypothet	Chlamydomonas_eusti	66.50	0.00
TRINITY_GAX82090	hypothet	Chlamydomonas_eusti	66.50	0.00
TRINITY_XP_01946	PREDICTE	Lupinus_angustifoli	66.50	0.00
TRINITY_PNH07830	NADPH--c	Tetrabaena_socialis	66.50	0.00
TRINITY_PNH07466	Dihydrol	Tetrabaena_socialis	66.50	0.00
TRINITY_PNW84989	hypothet	Chlamydomonas_reinh	66.50	0.00
TRINITY_XP_00295	hypothet	Volvox_carteri	66.50	0.00
TRINITY_XP_02186	fumarate	Spinacia_oleracea	66.50	0.00
TRINITY_ADE92943	mitochon	Polytomella_sp._Pri	66.50	0.00
TRINITY_GAX81765	hypothet	Chlamydomonas_eusti	66.50	0
TRINITY_GAX78813	hypothet	Chlamydomonas_eusti	66.50	0.00
TRINITY_PNW71496	hypothet	Chlamydomonas_reinh	66.50	0.00
TRINITY_OIV93733	hypothet	Lupinus_angustifoli	66.40	0.00
TRINITY_AAT73619	calmodul	Daucus_carota	66.40	0.00

TRINITY_YP_00912	NADH deh	Dunaliella_viridis	66.40	0.00
TRINITY_XP_00834	PREDICTE	Malus_domestica	66.40	0.00
TRINITY_EPS74505	hypothet	Genlisea_aurea	66.40	0.00
TRINITY_PNW77485	hypothet	Chlamydomonas_reinh	66.40	0.00
TRINITY_GAX75461	hypothet	Chlamydomonas_eusti	66.40	0.00
TRINITY_GBF87921	superoxi	Raphidocelis_subcap	66.40	0.00
TRINITY_KXZ49062	hypothet	Gonium_pectorale	66.40	0.00
TRINITY_AAL79356	assimila	Dunaliella_tertiole	66.40	0
TRINITY_GBG00631	ATP-depe	Raphidocelis_subcap	66.40	0.00
TRINITY_XP_01761	PREDICTE	Gossypium_arboreum	66.40	0.00
TRINITY_KXZ51346	hypothet	Gonium_pectorale	66.40	0.00
TRINITY_GAX82322	hypothet	Chlamydomonas_eusti	66.40	0.00
TRINITY_XP_02452	bifuncti	Selaginella_moellen	66.40	0.00
TRINITY_XP_02199	40S ribo	Helianthus_annuus	66.40	0.00
TRINITY_GAX80865	hypothet	Chlamydomonas_eusti	66.40	0.00
TRINITY_PNW75925	hypothet	Chlamydomonas_reinh	66.40	0.00
TRINITY_GAX73999	hypothet	Chlamydomonas_eusti	66.40	0.00
TRINITY_GAX82621	hypothet	Chlamydomonas_eusti	66.40	0.00
TRINITY_ABD37904	light-ha	Chlamydomonas_incer	66.40	0.00
TRINITY_BAJ99430	predicte	Hordeum_vulgare	66.40	0.00
TRINITY_XP_00564	prokaryo	Coccomyxa_subellips	66.30	0.00
TRINITY_OTG04903	putative	Helianthus_annuus	66.30	0.00
TRINITY_PRW58972	transmem	Chlorella_sorokinia	66.30	0.00
TRINITY_XP_02437	protein	Physcomitrella_pate	66.30	0.00
TRINITY_KXZ45013	hypothet	Gonium_pectorale	66.30	0.00
TRINITY_PNR37154	hypothet	Physcomitrella_pate	66.30	0.00
TRINITY_XP_00295	hypothet	Volvox_carteri	66.30	0.00
TRINITY_GAQ82917	Acyl-CoA	Klebsormidium_niten	66.30	0.00
TRINITY_BAK02778	predicte	Hordeum_vulgare	66.30	0.00
TRINITY_KXZ44026	hypothet	Gonium_pectorale	66.30	0.00
TRINITY_XP_01048	PREDICTE	Camelina_sativa	66.30	0.00
TRINITY_XP_01389	hypothet	Monoraphidium_negle	66.30	0.00
TRINITY_XP_00564	NAD(P)-b	Coccomyxa_subellips	66.30	0.00
TRINITY_XP_00295	hypothet	Volvox_carteri	66.30	0.00
TRINITY_EFJ24224	hypothet	Selaginella_moellen	66.30	0.00
TRINITY_P0C8M8.1	RecName:	Zea_mays	66.30	0.00
TRINITY_XP_00643	peptidyl	Citrus_clementina	66.30	0.00
TRINITY_GAX83661	hypothet	Chlamydomonas_eusti	66.30	0.00
TRINITY_KXZ50885	hypothet	Gonium_pectorale	66.30	0.00
TRINITY_GAX86224	hypothet	Chlamydomonas_eusti	66.30	0.00
TRINITY_PNW79570	hypothet	Chlamydomonas_reinh	66.30	0.00
TRINITY_XP_00170	zinc fin	Chlamydomonas_reinh	66.30	0.00
TRINITY_XP_00564	PRP38-do	Coccomyxa_subellips	66.30	0.00
TRINITY_XP_02283	Ribosoma	Ostreococcus_tauri	66.30	0.00
TRINITY_PRW45173	Malonyl-	Chlorella_sorokinia	66.30	0.00
TRINITY_XP_00170	ubiquino	Chlamydomonas_reinh	66.30	0.00
TRINITY_XP_01596	regulato	Arachis_duranensis	66.30	0
TRINITY_KXZ52444	hypothet	Gonium_pectorale	66.30	0.00
TRINITY_GBF90642	p-type a	Raphidocelis_subcap	66.30	0.00
TRINITY_KXZ47073	hypothet	Gonium_pectorale	66.30	0.00
TRINITY_GAQ81168	hypothet	Klebsormidium_niten	66.30	0.00
TRINITY_XP_02402	1,2-dihy	Morus_notabilis	66.20	0.00
TRINITY_GBG89525	hypothet	Chara_braunii	66.20	0.00
TRINITY_XP_00636	DNA repl	Populus_trichocarpa	66.20	0.00
TRINITY_XP_01946	PREDICTE	Lupinus_angustifoli	66.20	0.00
TRINITY_GAQ78449	Coatomer	Klebsormidium_niten	66.20	0.00
TRINITY_GAX78992	hypothet	Chlamydomonas_eusti	66.20	0.00
TRINITY_XP_01723	PREDICTE	Daucus_carota	66.20	0.00
TRINITY_EFJ21806	hypothet	Selaginella_moellen	66.20	0.00
TRINITY_XP_00170	alpha-SN	Chlamydomonas_reinh	66.20	0.00
TRINITY_XP_00295	hypothet	Volvox_carteri	66.20	0.00
TRINITY_GAQ79025	Regulato	Klebsormidium_niten	66.20	0.00
TRINITY_PIN07335	hypothet	Handroanthus_impeti	66.20	0.00
TRINITY_PKI33291	hypothet	Punica_granatum	66.20	0.00
TRINITY_GAX73857	hypothet	Chlamydomonas_eusti	66.20	0.00
TRINITY_XP_00169	plasma m	Chlamydomonas_reinh	66.20	0.00

TRINITY_GBF89762	hypothet	Raphidocelis_subcap	66.20	0.00
TRINITY_XP_00295	hypothet	Volvox_carteri	66.20	0.00
TRINITY_GAQ84757	putative	Klebsormidium_niten	66.20	0.00
TRINITY_KXZ46309	hypothet	Gonium_pectorale	66.20	0.00
TRINITY_KXZ49072	hypothet	Gonium_pectorale	66.20	0.00
TRINITY_XP_00295	flagella	Volvox_carteri	66.20	0.00
TRINITY_ABR21759	calmodul	Actinidia_deliciosa	66.20	0.00
TRINITY_PNW84728	hypothet	Chlamydomonas_reinh	66.20	0.00
TRINITY_GAX72783	hypothet	Chlamydomonas_eusti	66.20	0.00
TRINITY_PNH08115	Peroxire	Tetrabaena_socialis	66.20	0.00
TRINITY_GAX82630	hypothet	Chlamydomonas_eusti	66.20	0.00
TRINITY_KXZ48399	hypothet	Gonium_pectorale	66.20	0.00
TRINITY_KXZ56275	hypothet	Gonium_pectorale	66.20	0.00
TRINITY_GAX78445	hypothet	Chlamydomonas_eusti	66.20	0.00
TRINITY_XP_00295	CF0 ATP	Volvox_carteri	66.20	0.00
TRINITY_GBF92560	hypothet	Raphidocelis_subcap	66.20	0.00
TRINITY_GBF99462	hypothet	Raphidocelis_subcap	66.20	0.00
TRINITY_GAX79708	hypothet	Chlamydomonas_eusti	66.20	0.00
TRINITY_XP_01564	ras-rela	Oryza_sativa	66.20	0.00
TRINITY_PNW71230	hypothet	Chlamydomonas_reinh	66.20	0.00
TRINITY_ACF87913	unknown	Zea_mays	66.20	0.00
TRINITY_PNW70131	hypothet	Chlamydomonas_reinh	66.20	0.00
TRINITY_PNW69797	hypothet	Chlamydomonas_reinh	66.20	0.00
TRINITY_KXZ46408	hypothet	Gonium_pectorale	66.20	0.00
TRINITY_XP_00295	hypothet	Volvox_carteri	66.20	0.00
TRINITY_GAX78238	hypothet	Chlamydomonas_eusti	66.20	0.00
TRINITY_XP_00879	kinesin-	Phoenix_dactylifera	66.20	0.00
TRINITY_XP_00250	predicte	Micromonas_commoda	66.10	0.00
TRINITY_PNW84602	hypothet	Chlamydomonas_reinh	66.10	0.00
TRINITY_GAX85351	hypothet	Chlamydomonas_eusti	66.10	0.00
TRINITY_XP_01741	PREDICTE	Vigna_angularis	66.10	0.00
TRINITY_XP_00295	transcri	Volvox_carteri	66.10	0.00
TRINITY_XP_02452	aconitat	Selaginella_moellen	66.10	0
TRINITY_PNH08913	Vacuolar	Tetrabaena_socialis	66.10	0.00
TRINITY_XP_00450	DNA repa	Cicer_arietinum	66.10	0.00
TRINITY_XP_02389	floccula	Quercus_suber	66.10	0.00
TRINITY_KXZ48819	hypothet	Gonium_pectorale	66.10	0.00
TRINITY_GAX78060	hypothet	Chlamydomonas_eusti	66.10	0.00
TRINITY_GAX84826	hypothet	Chlamydomonas_eusti	66.10	0.00
TRINITY_PRW55889	alpha-1,	Chlorella_sorokinia	66.10	0.00
TRINITY_AAZ31184	central	Chlamydomonas_reinh	66.10	0.00
TRINITY_P81831.2	RecName:	Chlamydomonas_reinh	66.10	0.00
TRINITY_XP_00628	inositol	Capsella_rubella	66.10	0.00
TRINITY_PNW81070	hypothet	Chlamydomonas_reinh	66.10	0.00
TRINITY_GBF98872	hypothet	Raphidocelis_subcap	66.10	0.00
TRINITY_GAQ90432	Histone	Klebsormidium_niten	66.10	0.00
TRINITY_PSC76139	nucleosi	Micractinium_conduc	66.10	0.00
TRINITY_GAX78150	hypothet	Chlamydomonas_eusti	66.10	0
TRINITY_ALM55004	DEAD box	Chlamydomonas_sp._I	66.10	0.00
TRINITY_XP_00295	hypothet	Volvox_carteri	66.10	0.00
TRINITY_KXZ49584	hypothet	Gonium_pectorale	66.10	0.00
TRINITY_GAX77344	hypothet	Chlamydomonas_eusti	66.10	0.00
TRINITY_PNW74821	hypothet	Chlamydomonas_reinh	66.10	0.00
TRINITY_GAX82457	hypothet	Chlamydomonas_eusti	66.10	0.00
TRINITY_GBF92304	hypothet	Raphidocelis_subcap	66.10	0.00
TRINITY_XP_00865	uncharac	Zea_mays	66.00	0.00
TRINITY_XP_00250	long fla	Micromonas_commoda	66.00	0.00
TRINITY_GAX85084	hypothet	Chlamydomonas_eusti	66.00	0.00
TRINITY_KXZ54656	hypothet	Gonium_pectorale	66.00	0.00
TRINITY_PNW73325	hypothet	Chlamydomonas_reinh	66.00	0.00
TRINITY_GAX77127	hypothet	Chlamydomonas_eusti	66.00	0.00
TRINITY_XP_00294	componen	Volvox_carteri	66.00	0.00
TRINITY_XP_00295	p300/CBP	Volvox_carteri	66.00	0.00
TRINITY_PNW85495	hypothet	Chlamydomonas_reinh	66.00	0.00
TRINITY_XP_01053	PREDICTE	Tarenaya_hasslerian	66.00	0.00
TRINITY_PNW73456	hypothet	Chlamydomonas_reinh	66.00	0.00

TRINITY_XP_0016(	proline	Chlamydomonas_reinh	66.00	0.00
TRINITY_XP_0017(	predicte	Chlamydomonas_reinh	66.00	0.00
TRINITY_BAJ9323	predicte	Hordeum_vulgare	66.00	0.00
TRINITY_XP_0139(	pyruvate	Monoraphidium_negle	66.00	0.00
TRINITY_AAB8649	calmodul	Zea_mays	66.00	0.00
TRINITY_XP_0029!	hypothet	Volvox_carteri	66.00	0.00
TRINITY_GBF9312	alpha be	Raphidocelis_subcap	66.00	0.00
TRINITY_KXZ4565	hypothet	Gonium_pectorale	66.00	0.00
TRINITY_XP_0017(	predicte	Chlamydomonas_reinh	66.00	0.00
TRINITY_GBF8863	hypothet	Raphidocelis_subcap	66.00	0.00
TRINITY_XP_0029	hypothet	Volvox_carteri	66.00	0.00
TRINITY_XP_0029	hypothet	Volvox_carteri	66.00	0.00
TRINITY_GAX7428	hypothet	Chlamydomonas_eusti	66.00	0.00
TRINITY_XP_0016	caroten	Chlamydomonas_reinh	66.00	0.00
TRINITY_XP_0029!	hypothet	Volvox_carteri	66.00	0.00
TRINITY_GBF9226	hypothet	Raphidocelis_subcap	66.00	0.00
TRINITY_XP_0243	60S rib	Physcomitrella_pate	66.00	0.00
TRINITY_GAQ8349	propion	Klebsormidium_niten	66.00	0.00
TRINITY_XP_0016	predicte	Chlamydomonas_reinh	66.00	0.00
TRINITY_GAX7642	hypothet	Chlamydomonas_eusti	66.00	0.00
TRINITY_XP_0029!	hypothet	Volvox_carteri	66.00	0.00
TRINITY_KXZ5601	hypothet	Gonium_pectorale	66.00	0
TRINITY_GAX8253	hypothet	Chlamydomonas_eusti	66.00	0.00
TRINITY_EXC2676(	hypothet	Morus_notabilis	66.00	0.00
TRINITY_EXC2676(	hypothet	Morus_notabilis	66.00	0.00
TRINITY_GAX8123	hypothet	Chlamydomonas_eusti	66.00	0.00
TRINITY_XP_0029!	hypothet	Volvox_carteri	66.00	0.00
TRINITY_GAX7675(	hypothet	Chlamydomonas_eusti	66.00	0.00
TRINITY_XP_0238	60S rib	Quercus_suber	65.90	0.00
TRINITY_XP_0165	PREDICTE	Capsicum_annuum	65.90	0.00
TRINITY_KXZ5045	hypothet	Gonium_pectorale	65.90	0.00
TRINITY_XP_0016	SEC61-ga	Chlamydomonas_reinh	65.90	0.00
TRINITY_XP_0238	probable	Quercus_suber	65.90	0.00
TRINITY_XP_0104	PREDICTE	Camelina_sativa	65.90	0.00
TRINITY_XP_0093!	PREDICTE	Pyrus_x_bretschneid	65.90	0.00
TRINITY_XP_0029!	hypothet	Volvox_carteri	65.90	0.00
TRINITY_PNH1014(	hypothet	Tetrabaena_socialis	65.90	0.00
TRINITY_XP_0029!	hypothet	Volvox_carteri	65.90	0.00
TRINITY_AKI3238!	DNA mei	Watanabea_reniformi	65.90	0.00
TRINITY_KXZ5206	hypothet	Gonium_pectorale	65.90	0.00
TRINITY_BAJ9371	predicte	Hordeum_vulgare	65.90	0.00
TRINITY_XP_0017(	cycloph	Chlamydomonas_reinh	65.90	0.00
TRINITY_KXZ5597!	hypothet	Gonium_pectorale	65.90	0.00
TRINITY_GAX8285	hypothet	Chlamydomonas_eusti	65.90	0.00
TRINITY_GAX8080	hypothet	Chlamydomonas_eusti	65.90	0.00
TRINITY_GAU1829	hypothet	Trifolium_subterran	65.90	0.00
TRINITY_KXZ4924	hypothet	Gonium_pectorale	65.90	0.00
TRINITY_GAX8182	hypothet	Chlamydomonas_eusti	65.90	0.00
TRINITY_GAX7937	hypothet	Chlamydomonas_eusti	65.90	0.00
TRINITY_XP_0218	fructose	Spinacia_oleracea	65.90	0.00
TRINITY_XP_0113	V-type	Auxenochlorella_pro	65.90	0.00
TRINITY_XP_0017(	sterol	Chlamydomonas_reinh	65.90	0.00
TRINITY_GAX7988	hypothet	Chlamydomonas_eusti	65.90	0.00
TRINITY_XP_0058	hypothet	Chlorella_variabili	65.90	0.00
TRINITY_XP_0016	chlorop	Chlamydomonas_reinh	65.90	0.00
TRINITY_GAX7363	hypothet	Chlamydomonas_eusti	65.90	0.00
TRINITY_GAX8570	hypothet	Chlamydomonas_eusti	65.90	0.00
TRINITY_XP_0016	predicte	Chlamydomonas_reinh	65.90	0.00
TRINITY_XP_0029!	hypothet	Volvox_carteri	65.90	0.00
TRINITY_XP_0058	hypothet	Chlorella_variabili	65.90	0.00
TRINITY_XP_0017(	predicte	Chlamydomonas_reinh	65.90	0.00
TRINITY_GAX7589	hypothet	Chlamydomonas_eusti	65.90	0.00
TRINITY_XP_0014	predicte	Ostreococcus_sp._'l	65.80	0.00
TRINITY_CAA1126	polyubi	Nicotiana_tabacum	65.80	0.00
TRINITY_XP_0029	componer	Volvox_carteri	65.80	0.00
TRINITY_KXZ5010	hypothet	Gonium_pectorale	65.80	0.00

TRINITY_XP_01389	aquapori	Monoraphidium_negle	65.80	0.00
TRINITY_XP_00294	hypothet	Volvox_carteri	65.80	0.00
TRINITY_CAE75981	B1160F02	Oryza_sativa	65.80	0.00
TRINITY_GAX80591	hypothet	Chlamydomonas_eusti	65.80	0.00
TRINITY_XP_00294	hypothet	Volvox_carteri	65.80	0.00
TRINITY_XP_00295	hypothet	Volvox_carteri	65.80	0.00
TRINITY_XP_02736	UDP-gluc	Abrus_precatorius	65.80	0.00
TRINITY_KXZ42325	hypothet	Gonium_pectorale	65.80	0.00
TRINITY_PNW73794	hypothet	Chlamydomonas_reinh	65.80	0.00
TRINITY_XP_02392	ras-rela	Quercus_suber	65.80	0.00
TRINITY_PNH12751	F-box-li	Tetrabaena_socialis	65.80	0.00
TRINITY_GAX82581	hypothet	Chlamydomonas_eusti	65.80	0.00
TRINITY_PNW74497	hypothet	Chlamydomonas_reinh	65.80	0.00
TRINITY_GAX86246	hypothet	Chlamydomonas_eusti	65.80	0.00
TRINITY_XP_00169	bifuncti	Chlamydomonas_reinh	65.80	0.00
TRINITY_KXZ55547	hypothet	Gonium_pectorale	65.80	0.00
TRINITY_XP_00169	componen	Chlamydomonas_reinh	65.80	0.00
TRINITY_GAX77228	hypothet	Chlamydomonas_eusti	65.80	0.00
TRINITY_PNH03318	SUN doma	Tetrabaena_socialis	65.80	0.00
TRINITY_GAX76615	hypothet	Chlamydomonas_eusti	65.80	0.00
TRINITY_GBF87462	quinolin	Raphidocelis_subcap	65.80	0.00
TRINITY_XP_00565	galactos	Coccomyxa_subellips	65.80	0.00
TRINITY_XP_02388	nucleosi	Quercus_suber	65.80	0.00
TRINITY_GAX84816	hypothet	Chlamydomonas_eusti	65.80	0.00
TRINITY_KXZ42459	hypothet	Gonium_pectorale	65.80	0.00
TRINITY_KXZ49298	hypothet	Gonium_pectorale	65.80	0
TRINITY_PNR45218	hypothet	Physcomitrella_pate	65.70	0.00
TRINITY_RVW69187	Zinc fin	Vitis_vinifera	65.70	0.00
TRINITY_GAX78858	hypothet	Chlamydomonas_eusti	65.70	0.00
TRINITY_GAQ80419	40S ribo	Klebsormidium_niten	65.70	0.00
TRINITY_YP_00881	cytochro	Roya_obtusa	65.70	0.00
TRINITY_GAQ78943	methylma	Klebsormidium_niten	65.70	0.00
TRINITY_KXZ50930	hypothet	Gonium_pectorale	65.70	0.00
TRINITY_GAX75759	hypothet	Chlamydomonas_eusti	65.70	0.00
TRINITY_GBF97375	hypothet	Raphidocelis_subcap	65.70	0.00
TRINITY_KNA09350	hypothet	Spinacia_oleracea	65.70	0.00
TRINITY_AER58219	channelo	Haematococcus_lacus	65.70	0.00
TRINITY_GAX84249	hypothet	Chlamydomonas_eusti	65.70	0.00
TRINITY_KDD74537	protein	Helicosporidium_sp.	65.70	0.00
TRINITY_GAX82716	hypothet	Chlamydomonas_eusti	65.70	0.00
TRINITY_GAX79250	hypothet	Chlamydomonas_eusti	65.70	0.00
TRINITY_PSC68273	diphthar	Micractinium_conduc	65.70	0.00
TRINITY_PNW71615	hypothet	Chlamydomonas_reinh	65.70	0.00
TRINITY_GAX74581	hypothet	Chlamydomonas_eusti	65.70	0.00
TRINITY_KXZ52148	hypothet	Gonium_pectorale	65.70	0.00
TRINITY_GAX77615	hypothet	Chlamydomonas_eusti	65.70	0.00
TRINITY_XP_00295	hypothet	Volvox_carteri	65.70	0.00
TRINITY_KXZ52857	hypothet	Gonium_pectorale	65.70	0.00
TRINITY_XP_00294	hypothet	Volvox_carteri	65.70	0.00
TRINITY_GAQ93561	hypothet	Klebsormidium_niten	65.70	0.00
TRINITY_NP_19127	homologu	Arabidopsis_thalian	65.70	0.00
TRINITY_PNW81009	hypothet	Chlamydomonas_reinh	65.70	0.00
TRINITY_KXZ50900	hypothet	Gonium_pectorale	65.70	0.00
TRINITY_PNW73634	hypothet	Chlamydomonas_reinh	65.70	0.00
TRINITY_PNW88553	hypothet	Chlamydomonas_reinh	65.70	0.00
TRINITY_PNW84782	hypothet	Chlamydomonas_reinh	65.70	0.00
TRINITY_PNW86443	hypothet	Chlamydomonas_reinh	65.70	0.00
TRINITY_XP_02389	GTP-bind	Quercus_suber	65.70	0.00
TRINITY_XP_02436	40S ribo	Physcomitrella_pate	65.70	0.00
TRINITY_XP_02390	ras-like	Quercus_suber	65.60	0.00
TRINITY_ACJ85890	unknown	Medicago_truncatula	65.60	0.00
TRINITY_BAK02493	predicte	Hordeum_vulgare	65.60	0.00
TRINITY_BAK02363	predicte	Hordeum_vulgare	65.60	0.00
TRINITY_PNW77176	hypothet	Chlamydomonas_reinh	65.60	0.00
TRINITY_XP_00169	predicte	Chlamydomonas_reinh	65.60	0.00
TRINITY_PNW83618	hypothet	Chlamydomonas_reinh	65.60	0.00

TRINITY_P0C8M8.1	RecName:	Zea_mays	65.60	0.00
TRINITY_GAX74404	hypothet	Chlamydomonas_eusti	65.60	0.00
TRINITY_PNW75091	hypothet	Chlamydomonas_reinh	65.60	0.00
TRINITY_XP_00295	hypothet	Volvox_carteri	65.60	0.00
TRINITY_KXZ56469	hypothet	Gonium_pectorale	65.60	0.00
TRINITY_GAX75992	hypothet	Chlamydomonas_eusti	65.60	0.00
TRINITY_KXZ47132	hypothet	Gonium_pectorale	65.60	0.00
TRINITY_GAX77008	hypothet	Chlamydomonas_eusti	65.60	0.00
TRINITY_KXZ44082	hypothet	Gonium_pectorale	65.60	0.00
TRINITY_XP_00170	photosys	Chlamydomonas_reinh	65.60	0.00
TRINITY_GAX80936	hypothet	Chlamydomonas_eusti	65.60	0.00
TRINITY_GBF95525	hypothet	Raphidocelis_subcap	65.60	0.00
TRINITY_GAX84621	hypothet	Chlamydomonas_eusti	65.60	0.00
TRINITY_XP_00169	predicte	Chlamydomonas_reinh	65.60	0.00
TRINITY_PNW78539	hypothet	Chlamydomonas_reinh	65.60	0.00
TRINITY_PSC69495	Altered	Micractinium_conduc	65.60	0.00
TRINITY_PSC74501	Serine t	Micractinium_conduc	65.60	0.00
TRINITY_XP_00298	26S prot	Selaginella_moellen	65.60	0.00
TRINITY_KDD76551	GTP-bind	Helicosporidium_sp.	65.60	0.00
TRINITY_PNW79609	hypothet	Chlamydomonas_reinh	65.60	0.00
TRINITY_XP_00299	5-oxopro	Selaginella_moellen	65.50	0.00
TRINITY_KZN03552	hypothet	Daucus_carota	65.50	0.00
TRINITY_XP_00142	predicte	Ostreococcus_sp._'l	65.50	0.00
TRINITY_XP_00429	PREDICTE	Fragaria_vesca	65.50	0.00
TRINITY_GBF88604	cytosoli	Raphidocelis_subcap	65.50	0.00
TRINITY_GAX78491	hypothet	Chlamydomonas_eusti	65.50	0.00
TRINITY_XP_02175	ubiquiti	Chenopodium_quinoa	65.50	0.00
TRINITY_GAX77956	hypothet	Chlamydomonas_eusti	65.50	0.00
TRINITY_XP_00585	hypothet	Chlorella_variabili	65.50	0.00
TRINITY_KXZ45928	hypothet	Gonium_pectorale	65.50	0.00
TRINITY_BAK05241	predicte	Hordeum_vulgare	65.50	0.00
TRINITY_PNW70906	hypothet	Chlamydomonas_reinh	65.50	0.00
TRINITY_GBF93690	hypothet	Raphidocelis_subcap	65.50	0.00
TRINITY_BAG12189	Parkin-c	Chlamydomonas_reinh	65.50	0.00
TRINITY_GAX82949	hypothet	Chlamydomonas_eusti	65.50	0.00
TRINITY_GAQ84546	3'-5'-ex	Klebsormidium_niten	65.50	0.00
TRINITY_XP_02437	RNA cyti	Physcomitrella_pate	65.50	0.00
TRINITY_GAX85242	hypothet	Chlamydomonas_eusti	65.50	0.00
TRINITY_GAX74338	hypothet	Chlamydomonas_eusti	65.50	0.00
TRINITY_KXZ53682	hypothet	Gonium_pectorale	65.50	0.00
TRINITY_XP_02793	actin-re	Vigna_unguiculata	65.50	0.00
TRINITY_XP_00169	predicte	Chlamydomonas_reinh	65.50	0.00
TRINITY_KXZ51190	hypothet	Gonium_pectorale	65.50	0.00
TRINITY_GAX73592	hypothet	Chlamydomonas_eusti	65.50	0.00
TRINITY_XP_00565	beach-do	Coccomyxa_subellips	65.40	0.00
TRINITY_BAJ93074	predicte	Hordeum_vulgare	65.40	0.00
TRINITY_RID78408	hypothet	Brassica_rapa	65.40	0.00
TRINITY_XP_01847	PREDICTE	Raphanus_sativus	65.40	0.00
TRINITY_PSC72161	carbohyd	Micractinium_conduc	65.40	0.00
TRINITY_XP_00141	predicte	Ostreococcus_sp._'l	65.40	0.00
TRINITY_GAX82822	hypothet	Chlamydomonas_eusti	65.40	0.00
TRINITY_GAX74910	hypothet	Chlamydomonas_eusti	65.40	0.00
TRINITY_EFJ11196	hypothet	Selaginella_moellen	65.40	0.00
TRINITY_GAX86170	hypothet	Chlamydomonas_eusti	65.40	0.00
TRINITY_GBF88109	hypothet	Raphidocelis_subcap	65.40	0.00
TRINITY_XP_00295	hypothet	Volvox_carteri	65.40	0.00
TRINITY_XP_00295	hypothet	Volvox_carteri	65.40	0.00
TRINITY_PNW77038	hypothet	Chlamydomonas_reinh	65.40	0.00
TRINITY_XP_00170	predicte	Chlamydomonas_reinh	65.40	0.00
TRINITY_GBF88298	hypothet	Raphidocelis_subcap	65.40	0.00
TRINITY_GAX82119	hypothet	Chlamydomonas_eusti	65.40	0.00
TRINITY_XP_00170	cyclin	Chlamydomonas_reinh	65.40	0.00
TRINITY_XP_00294	hypothet	Volvox_carteri	65.40	0.00
TRINITY_XP_00250	predicte	Micromonas_commoda	65.40	0.00
TRINITY_GAX73229	hypothet	Chlamydomonas_eusti	65.40	0.00
TRINITY_GBF99877	hypothet	Raphidocelis_subcap	65.40	0.00

TRINITY_KXZ5697(	hypothet	Gonium_pectorale	65.40	0.00
TRINITY_KXZ4274!	hypothet	Gonium_pectorale	65.40	0.00
TRINITY_KXZ5402!	hypothet	Gonium_pectorale	65.40	0.00
TRINITY_GAX7659(	hypothet	Chlamydomonas_eusti	65.40	0.00
TRINITY_PNW8122!	hypothet	Chlamydomonas_reinh	65.40	0.00
TRINITY_GAX7291!	hypothet	Chlamydomonas_eusti	65.40	0.00
TRINITY_ABM7438(	major l:	Dunaliella_salina	65.40	0.00
TRINITY_XP_0029	hypothet	Volvox_carteri	65.40	0.00
TRINITY_GAQ9125	oxoprol:	Klebsormidium_niten	65.30	0.00
TRINITY_XP_0219	casein }	Helianthus_annuus	65.30	0.00
TRINITY_ESQ5399	hypothet	Eutrema_salsugineum	65.30	0.00
TRINITY_EPS7450!	hypothet	Genlisea_aurea	65.30	0.00
TRINITY_KDD7549!	hypothet	Helicosporidium_sp.	65.30	0.00
TRINITY_BAJ9637!	predicte	Hordeum_vulgare	65.30	0.00
TRINITY_GBG7567	hypothet	Chara_braunii	65.30	0.00
TRINITY_XP_0036	casein }	Medicago_truncatula	65.30	0.00
TRINITY_XP_0238	uncharac	Quercus_suber	65.30	0.00
TRINITY_PNW7579(	hypothet	Chlamydomonas_reinh	65.30	0.00
TRINITY_GAQ9066	Cleavage	Klebsormidium_niten	65.30	0.00
TRINITY_XP_0205	V-type p	Phalaenopsis_equest	65.30	0.00
TRINITY_XP_0257	casein }	Panicum_hallii	65.30	0.00
TRINITY_PNW7107	hypothet	Chlamydomonas_reinh	65.30	0.00
TRINITY_XP_0016	predicte	Chlamydomonas_reinh	65.30	0.00
TRINITY_GAX8175	hypothet	Chlamydomonas_eusti	65.30	0.00
TRINITY_PRW5951	Rab fami	Chlorella_sorokinia	65.30	0.00
TRINITY_GAX7423(	hypothet	Chlamydomonas_eusti	65.30	0.00
TRINITY_GBF9566	hypothet	Raphidocelis_subcap	65.30	0.00
TRINITY_KXZ5455	hypothet	Gonium_pectorale	65.30	0.00
TRINITY_EFJ1726	hypothet	Selaginella_moellen	65.30	0.00
TRINITY_XP_0271	T-comple	Coffea_arabica	65.30	0.00
TRINITY_GAX7356(	hypothet	Chlamydomonas_eusti	65.30	0.00
TRINITY_KXZ5438	hypothet	Gonium_pectorale	65.30	0.00
TRINITY_GBF9640(	malate s	Raphidocelis_subcap	65.30	0.00
TRINITY_XP_0029	anaphase	Selaginella_moellen	65.30	0.00
TRINITY_XP_0029	chaperor	Volvox_carteri	65.30	0.00
TRINITY_XP_0029	hypothet	Volvox_carteri	65.30	0.00
TRINITY_XP_0176	PREDICTE	Gossypium_arboreum	65.30	0.00
TRINITY_GAX8322	hypothet	Chlamydomonas_eusti	65.30	0
TRINITY_ONM0351	60S ribo	Zea_mays	65.30	0.00
TRINITY_XP_0030	flagella	Micromonas_pusilla	65.20	0.00
TRINITY_KNA0305!	hypothet	Spinacia_oleracea	65.20	0.00
TRINITY_GAQ8768!	AAA-type	Klebsormidium_niten	65.20	0.00
TRINITY_XP_0243	pre-mRNA	Physcomitrella_pate	65.20	0.00
TRINITY_KXZ4326	hypothet	Gonium_pectorale	65.20	0.00
TRINITY_KXZ5407	hypothet	Gonium_pectorale	65.20	0.00
TRINITY_PNW8517!	hypothet	Chlamydomonas_reinh	65.20	0.00
TRINITY_PNW8634(	hypothet	Chlamydomonas_reinh	65.20	0.00
TRINITY_XP_0016	predicte	Chlamydomonas_reinh	65.20	0.00
TRINITY_XP_0243	spliceos	Physcomitrella_pate	65.20	0.00
TRINITY_KXZ4316!	hypothet	Gonium_pectorale	65.20	0.00
TRINITY_PNW8461!	hypothet	Chlamydomonas_reinh	65.20	0.00
TRINITY_KXZ5029	hypothet	Gonium_pectorale	65.20	0.00
TRINITY_XP_0058	hypothet	Chlorella_variabili	65.20	0.00
TRINITY_XP_0016	predicte	Chlamydomonas_reinh	65.20	0.00
TRINITY_GBG0025	60S acic	Raphidocelis_subcap	65.20	0.00
TRINITY_GAX8224	hypothet	Chlamydomonas_eusti	65.20	0.00
TRINITY_XP_0273	U1 small	Abrus_precatorius	65.20	0.00
TRINITY_XP_0237	ras-rela	Lactuca_sativa	65.20	0.00
TRINITY_PNH1164	hypothet	Tetrabaena_socialis	65.20	0.00
TRINITY_RIA0560!	hypothet	Brassica_rapa	65.20	0.00
TRINITY_XP_0138	amidase	Monoraphidium_negle	65.20	0.00
TRINITY_GAX7690(	hypothet	Chlamydomonas_eusti	65.20	0.00
TRINITY_GBF9530	mitochor	Raphidocelis_subcap	65.20	0.00
TRINITY_GAX7609(	hypothet	Chlamydomonas_eusti	65.20	0.00
TRINITY_GAX8624(	hypothet	Chlamydomonas_eusti	65.20	0.00
TRINITY_KCW5023!	hypothet	Eucalyptus_grandis	65.20	0.00

TRINITY_XP_00584	hypothet	Chlorella_variabili	65.10	0.00
TRINITY_XP_02436	DExH-box	Physcomitrella_pate	65.10	0.00
TRINITY_PPD70748	hypothet	Gossypium_barbadens	65.10	0.00
TRINITY_XP_01389	hypothet	Monoraphidium_negle	65.10	0.00
TRINITY_XP_00295	hypothet	Volvox_carteri	65.10	0.00
TRINITY_GAX82413	hypothet	Chlamydomonas_eusti	65.10	0.00
TRINITY_GAX77204	hypothet	Chlamydomonas_eusti	65.10	0.00
TRINITY_AEY80027	1-deoxy-	Haematococcus_lacus	65.10	0.00
TRINITY_KXZ56460	hypothet	Gonium_pectorale	65.10	0.00
TRINITY_XP_00295	hypothet	Volvox_carteri	65.10	0.00
TRINITY_XP_00295	componen	Volvox_carteri	65.10	0.00
TRINITY_PNG99855	Nuclear	Tetrabaena_socialis	65.10	0.00
TRINITY_ALM55011	DEAD box	Chlamydomonas_sp._I	65.10	0.00
TRINITY_PNW78717	hypothet	Chlamydomonas_reinh	65.10	0.00
TRINITY_XP_01390	Vacuolar	Monoraphidium_negle	65.10	0.00
TRINITY_XP_01944	PREDICTE	Lupinus_angustifoli	65.10	0.00
TRINITY_XP_01390	aldo-ket	Monoraphidium_negle	65.10	0.00
TRINITY_XP_00169	inositol	Chlamydomonas_reinh	65.10	0.00
TRINITY_PNH03908	hypothet	Tetrabaena_socialis	65.10	0.00
TRINITY_XP_00170	glycosyl	Chlamydomonas_reinh	65.10	0.00
TRINITY_PWA70694	DNA poly	Artemisia_annua	65.10	0.00
TRINITY_XP_02437	110 kDa	Physcomitrella_pate	65.10	0.00
TRINITY_XP_00169	predicte	Chlamydomonas_reinh	65.10	0.00
TRINITY_PNH00507	hypothet	Tetrabaena_socialis	65.10	0.00
TRINITY_PNH10380	Intrafla	Tetrabaena_socialis	65.10	0.00
TRINITY_KXZ45586	hypothet	Gonium_pectorale	65.10	0.00
TRINITY_GAX73160	hypothet	Chlamydomonas_eusti	65.10	0.00
TRINITY_GAX73618	hypothet	Chlamydomonas_eusti	65.10	0
TRINITY_GAX84207	hypothet	Chlamydomonas_eusti	65.10	0.00
TRINITY_GAX78346	hypothet	Chlamydomonas_eusti	65.10	0.00
TRINITY_KXZ56965	hypothet	Gonium_pectorale	65.10	0.00
TRINITY_PNW86026	hypothet	Chlamydomonas_reinh	65.10	0.00
TRINITY_KXZ45175	hypothet	Gonium_pectorale	65.10	0.00
TRINITY_AKI32383	DNA mism	Watanabea_reniformi	65.10	0.00
TRINITY_XP_02390	phospho	Quercus_suber	65.00	0.00
TRINITY_RZC93243	hypothet	Papaver_somniferum	65.00	0.00
TRINITY_XP_00430	PREDICTE	Fragaria_vesca	65.00	0.00
TRINITY_EXC35991	Ribulose	Morus_notabilis	65.00	0.00
TRINITY_XP_00306	predicte	Micromonas_pusilla	65.00	0.00
TRINITY_YP_00607	cox1 gen	Nitella_hyalina	65.00	0.00
TRINITY_XP_00294	hypothet	Volvox_carteri	65.00	0.00
TRINITY_XP_01449	60S ribo	Vigna_radiata	65.00	0.00
TRINITY_KXZ56690	hypothet	Gonium_pectorale	65.00	0.00
TRINITY_OAE21011	hypothet	Marchantia_polymorp	65.00	0.00
TRINITY_GAX77887	hypothet	Chlamydomonas_eusti	65.00	0.00
TRINITY_GBF95537	hypothet	Raphidocelis_subcap	65.00	0.00
TRINITY_GAX80285	hypothet	Chlamydomonas_eusti	65.00	0.00
TRINITY_GBF90772	hypothet	Raphidocelis_subcap	65.00	0.00
TRINITY_XP_00564	hypothet	Coccomyxa_subellips	65.00	0.00
TRINITY_PNW86627	hypothet	Chlamydomonas_reinh	65.00	0.00
TRINITY_GAX83989	hypothet	Chlamydomonas_eusti	65.00	0.00
TRINITY_AAO73551	reverse-	Chlamydomonas_reinh	65.00	0.00
TRINITY_XP_00170	predicte	Chlamydomonas_reinh	65.00	0.00
TRINITY_XP_00169	isovaler	Chlamydomonas_reinh	65.00	0.00
TRINITY_PNW72802	hypothet	Chlamydomonas_reinh	65.00	0.00
TRINITY_GAX73330	hypothet	Chlamydomonas_eusti	65.00	0.00
TRINITY_XP_00294	hypothet	Volvox_carteri	65.00	0.00
TRINITY_XP_00169	predicte	Chlamydomonas_reinh	65.00	0.00
TRINITY_GAX72950	hypothet	Chlamydomonas_eusti	65.00	0.00
TRINITY_GBF89852	proline	Raphidocelis_subcap	65.00	0.00
TRINITY_XP_00295	hypothet	Volvox_carteri	65.00	0.00
TRINITY_RYQ82032	hypothet	Arachis_hypogaea	65.00	0.00
TRINITY_XP_00295	hypothet	Volvox_carteri	65.00	0.00
TRINITY_XP_02070	protein	Dendrobium_catenatu	65.00	0.00
TRINITY_PNW87924	hypothet	Chlamydomonas_reinh	65.00	0.00
TRINITY_XP_02276	unchara	Durio_zibethinus	64.90	0.00

TRINITY_AFK3783 unknown	Medicago_truncatula	64.90	0.00
TRINITY_XP_0105 PREDICTE	Tarenaya_hasslerian	64.90	0.00
TRINITY_NP_0011 putative	Zea_mays	64.90	0.00
TRINITY_XP_0062 regulato	Capsella_rubella	64.90	0.00
TRINITY_PRW3390 Ubiquiti	Chlorella_sorokinia	64.90	0.00
TRINITY_GAX7425 hypothet	Chlamydomonas_eusti	64.90	0.00
TRINITY_GBF9495 hypothet	Raphidocelis_subcap	64.90	0.00
TRINITY_CAC1491 triosep	Triticum_aestivum	64.90	0.00
TRINITY_KXZ5006 hypothet	Gonium_pectorale	64.90	0.00
TRINITY_KXZ4740 hypothet	Gonium_pectorale	64.90	0.00
TRINITY_XP_0017 cyclophi	Chlamydomonas_reinh	64.90	0.00
TRINITY_XP_0029 hypothet	Volvox_carteri	64.90	0.00
TRINITY_KMS6524 hypothet	Beta_vulgaris	64.90	0.00
TRINITY_XP_0016 peptidyl	Chlamydomonas_reinh	64.90	0.00
TRINITY_RDX8343 T-comple	Mucuna_pruriens	64.90	0.00
TRINITY_XP_0056 SHMT-dor	Coccomyxa_subellips	64.90	0.00
TRINITY_PNW8844 hypothet	Chlamydomonas_reinh	64.90	0.00
TRINITY_XP_0243 probable	Physcomitrella_pate	64.90	0.00
TRINITY_XP_0017 soluble	Chlamydomonas_reinh	64.90	0.00
TRINITY_PNH0504 Cyclin-c	Tetrabaena_socialis	64.90	0.00
TRINITY_GAQ8649 mitochor	Klebsormidium_niten	64.90	0.00
TRINITY_PNW8373 hypothet	Chlamydomonas_reinh	64.90	0.00
TRINITY_XP_0029 hypothet	Volvox_carteri	64.90	0.00
TRINITY_KXZ4910 hypothet	Gonium_pectorale	64.90	0.00
TRINITY_GAX8315 hypothet	Chlamydomonas_eusti	64.90	0.00
TRINITY_PNW8223 hypothet	Chlamydomonas_reinh	64.90	0.00
TRINITY_XP_0029 hypothet	Volvox_carteri	64.90	0.00
TRINITY_GAX8221 hypothet	Chlamydomonas_eusti	64.90	0.00
TRINITY_GBG6524 hypothet	Chara_braunii	64.80	0.00
TRINITY_PNH1072 hypothet	Tetrabaena_socialis	64.80	0.00
TRINITY_GAQ8150 hypothet	Klebsormidium_niten	64.80	0.00
TRINITY_XP_0106 PREDICTE	Beta_vulgaris	64.80	0.00
TRINITY_OAE2075 hypothet	Marchantia_polymorp	64.80	0.00
TRINITY_PNW7722 hypothet	Chlamydomonas_reinh	64.80	0.00
TRINITY_XP_0017 5'-methy	Chlamydomonas_reinh	64.80	0.00
TRINITY_XP_0240 peroxiso	Morus_notabilis	64.80	0.00
TRINITY_KXZ4275 hypothet	Gonium_pectorale	64.80	0.00
TRINITY_GBG7375 hypothet	Chara_braunii	64.80	0.00
TRINITY_XP_0029 small A	Volvox_carteri	64.80	0.00
TRINITY_KMS6461 hypothet	Beta_vulgaris	64.80	0.00
TRINITY_XP_0139 Uncharac	Monoraphidium_negle	64.80	0.00
TRINITY_XP_0138 hypothet	Monoraphidium_negle	64.80	0.00
TRINITY_GAX7383 hypothet	Chlamydomonas_eusti	64.80	0.00
TRINITY_GAX7672 hypothet	Chlamydomonas_eusti	64.80	0.00
TRINITY_PNW8556 hypothet	Chlamydomonas_reinh	64.80	0.00
TRINITY_GAX8346 hypothet	Chlamydomonas_eusti	64.80	0.00
TRINITY_XP_0016 predicte	Chlamydomonas_reinh	64.80	0.00
TRINITY_XP_0016 predicte	Chlamydomonas_reinh	64.80	0.00
TRINITY_XP_0029 mitochor	Volvox_carteri	64.80	0.00
TRINITY_XP_0029 componer	Volvox_carteri	64.80	0.00
TRINITY_PNW8561 hypothet	Chlamydomonas_reinh	64.80	0.00
TRINITY_KXZ5057 hypothet	Gonium_pectorale	64.80	0.00
TRINITY_XP_0029 hypothet	Volvox_carteri	64.80	0.00
TRINITY_XP_0017 F1F0 ATI	Chlamydomonas_reinh	64.80	0.00
TRINITY_XP_0016 haloacid	Chlamydomonas_reinh	64.80	0.00
TRINITY_PNW7256 hypothet	Chlamydomonas_reinh	64.80	0.00
TRINITY_PNW7464 hypothet	Chlamydomonas_reinh	64.80	0.00
TRINITY_PNW7464 hypothet	Chlamydomonas_reinh	64.80	0.00
TRINITY_RYR0140 hypothet	Arachis_hypogaea	64.80	0.00
TRINITY_PNW7546 hypothet	Chlamydomonas_reinh	64.80	0.00
TRINITY_KXZ4756 hypothet	Gonium_pectorale	64.80	0.00
TRINITY_CAA1126 polyubic	Nicotiana_tabacum	64.80	0.00
TRINITY_XP_0138 U6 snRNA	Monoraphidium_negle	64.70	0.00
TRINITY_KVH9698 Ran GTPa	Cynara_cardunculus	64.70	0.00
TRINITY_GBF9487 polynucl	Raphidocelis_subcap	64.70	0.00
TRINITY_BAK0267 predicte	Hordeum_vulgare	64.70	0.00

TRINITY_AMP8292!	ABC trar	Catalpa_bungei	64.70	0.00
TRINITY_AML3086(	axonemal	Marsilea_vestita	64.70	0.00
TRINITY_XP_0243(	14 kDa z	Physcomitrella_pate	64.70	0.00
TRINITY_XP_0218!	aldehyde	Spinacia_oleracea	64.70	0.00
TRINITY_XP_0243(	40S ribo	Physcomitrella_pate	64.70	0.00
TRINITY_XP_0218!	ubiquiti	Spinacia_oleracea	64.70	0.00
TRINITY_XP_0030!	predicte	Micromonas_pusilla	64.70	0.00
TRINITY_XP_0240:	peroxiso	Morus_notabilis	64.70	0.00
TRINITY_GAX7606:	hypothet	Chlamydomonas_eusti	64.70	0.00
TRINITY_GBG7247!	hypothet	Chara_braunii	64.70	0.00
TRINITY_GBF8847!	hypothet	Raphidocelis_subcap	64.70	0.00
TRINITY_EFJ2940(	inner ar	Selaginella_moellen	64.70	0.00
TRINITY_PNH0754:	Ubiquiti	Tetrabaena_socialis	64.70	0.00
TRINITY_XP_0139(	hypothet	Monoraphidium_negle	64.70	0.00
TRINITY_XP_0016!	subunit	Chlamydomonas_reinh	64.70	0.00
TRINITY_PNW8574(	hypothet	Chlamydomonas_reinh	64.70	0.00
TRINITY_P0C8M8.:	RecName:	Zea_mays	64.70	0.00
TRINITY_XP_0016!	lysyl-tr	Chlamydomonas_reinh	64.70	0.00
TRINITY_KXZ5616	hypothet	Gonium_pectorale	64.70	0.00
TRINITY_QBG3806(	NADH del	Chlorosarcinopsis_e	64.70	0.00
TRINITY_GAX7540:	hypothet	Chlamydomonas_eusti	64.70	0.00
TRINITY_XP_0109:	PREDICTE	Elaeis_guineensis	64.70	0.00
TRINITY_XP_0029	20S prot	Volvox_carteri	64.70	0.00
TRINITY_GAX8041!	hypothet	Chlamydomonas_eusti	64.70	0.00
TRINITY_GAX8350(	hypothet	Chlamydomonas_eusti	64.70	0.00
TRINITY_XP_0029	componer	Volvox_carteri	64.70	0.00
TRINITY_XP_0016!	predicte	Chlamydomonas_reinh	64.70	0.00
TRINITY_XP_0138!	pyridoxa	Monoraphidium_negle	64.70	0.00
TRINITY_XP_0016!	glycerol	Chlamydomonas_reinh	64.70	0.00
TRINITY_XP_0029!	hypothet	Volvox_carteri	64.70	0.00
TRINITY_GAX7690(	hypothet	Chlamydomonas_eusti	64.70	0.00
TRINITY_XP_0016!	predicte	Chlamydomonas_reinh	64.70	0.00
TRINITY_KXZ5076!	hypothet	Gonium_pectorale	64.70	0.00
TRINITY_AAO7355:	reverse-	Chlamydomonas_reinh	64.70	0.00
TRINITY_XP_0024!	predicte	Micromonas_commoda	64.70	0.00
TRINITY_XP_0016!	Qa-SNARE	Chlamydomonas_reinh	64.70	0.00
TRINITY_PNW8493(	hypothet	Chlamydomonas_reinh	64.70	0.00
TRINITY_XP_0016!	dynein h	Chlamydomonas_reinh	64.70	0
TRINITY_ADQ0018:	histone	Chlamydomonas_sp._I	64.70	0.00
TRINITY_PNH0922(	Granule-	Tetrabaena_socialis	64.70	0.00
TRINITY_XP_0120(	acyl car	Jatropha_curcas	64.60	0.00
TRINITY_XP_0056	UBC-like	Coccomyxa_subellips	64.60	0.00
TRINITY_BAJ9379!	predicte	Hordeum_vulgare	64.60	0.00
TRINITY_EMS6305	Nucleosi	Triticum_urartu	64.60	0.00
TRINITY_ABK2279(	unknown	Picea_sitchensis	64.60	0.00
TRINITY_ABK2279(	unknown	Picea_sitchensis	64.60	0.00
TRINITY_XP_0029!	hypothet	Volvox_carteri	64.60	0.00
TRINITY_GAX8027!	hypothet	Chlamydomonas_eusti	64.60	0.00
TRINITY_OVA1018!	Cation-t	Macleaya_cordata	64.60	0.00
TRINITY_YP_0060	cox1 ger	Nitella_hyalina	64.60	0.00
TRINITY_PRW6149!	tryptopl	Chlorella_sorokinia	64.60	0.00
TRINITY_XP_0029!	molecula	Volvox_carteri	64.60	0.00
TRINITY_PSC7632:	Phosphor	Micractinium_conduc	64.60	0.00
TRINITY_GAX7574(	hypothet	Chlamydomonas_eusti	64.60	0.00
TRINITY_XP_0016!	NADPH-de	Chlamydomonas_reinh	64.60	0.00
TRINITY_XP_0016!	glyoxyla	Chlamydomonas_reinh	64.60	0.00
TRINITY_KXZ4767(	hypothet	Gonium_pectorale	64.60	0.00
TRINITY_KXZ5017	hypothet	Gonium_pectorale	64.60	0.00
TRINITY_GAQ8682!	dynein h	Klebsormidium_niten	64.60	0.00
TRINITY_GAX8178:	hypothet	Chlamydomonas_eusti	64.60	0.00
TRINITY_GBF9172(	isoleucy	Raphidocelis_subcap	64.60	0.00
TRINITY_XP_0138!	pyridoxa	Monoraphidium_negle	64.60	0.00
TRINITY_PNW8420:	hypothet	Chlamydomonas_reinh	64.60	0.00
TRINITY_XP_0029	hypothet	Volvox_carteri	64.60	0.00
TRINITY_KXZ4369!	hypothet	Gonium_pectorale	64.60	0.00
TRINITY_XP_0096:	PREDICTE	Nicotiana_tomentosi	64.60	0.00

TRINITY_PNW8779	hypothet	Chlamydomonas_reinh	64.60	0.00
TRINITY_PNY1131	casein	Trifolium_pratense	64.60	0.00
TRINITY_PNR3183	hypothet	Physcomitrella_pate	64.60	0.00
TRINITY_GAX7649	hypothet	Chlamydomonas_eusti	64.60	0.00
TRINITY_KXZ4818	hypothet	Gonium_pectorale	64.60	0.00
TRINITY_GBF9953	hypothet	Raphidocelis_subcap	64.60	0.00
TRINITY_PNW7252	hypothet	Chlamydomonas_reinh	64.60	0.00
TRINITY_EFH6841	predicte	Arabidopsis_lyrata	64.60	0.00
TRINITY_GBG0007	hypothet	Raphidocelis_subcap	64.60	0.00
TRINITY_KXZ5431	hypothet	Gonium_pectorale	64.60	0.00
TRINITY_GAX7304	hypothet	Chlamydomonas_eusti	64.60	0.00
TRINITY_GAX8624	hypothet	Chlamydomonas_eusti	64.60	0.00
TRINITY_GAX7503	hypothet	Chlamydomonas_eusti	64.60	0.00
TRINITY_XP_0110	electror	Sesamum_indicum	64.50	0.00
TRINITY_XP_0091	PREDICTE	Brassica_rapa	64.50	0.00
TRINITY_PTQ3809	hypothet	Marchantia_polymorp	64.50	0.00
TRINITY_XP_0243	ubiquit	Physcomitrella_pate	64.50	0.00
TRINITY_GAX8579	hypothet	Chlamydomonas_eusti	64.50	0.00
TRINITY_KNA1220	hypothet	Spinacia_oleracea	64.50	0.00
TRINITY_KXZ5170	hypothet	Gonium_pectorale	64.50	0.00
TRINITY_KXZ4756	hypothet	Gonium_pectorale	64.50	0.00
TRINITY_XP_0029	peptide	Volvox_carteri	64.50	0.00
TRINITY_PNW7885	hypothet	Chlamydomonas_reinh	64.50	0.00
TRINITY_PNW7230	hypothet	Chlamydomonas_reinh	64.50	0.00
TRINITY_XP_0139	50S rib	Monoraphidium_negle	64.50	0.00
TRINITY_GAX8352	hypothet	Chlamydomonas_eusti	64.50	0.00
TRINITY_GAX8253	hypothet	Chlamydomonas_eusti	64.50	0.00
TRINITY_GAX8104	hypothet	Chlamydomonas_eusti	64.50	0.00
TRINITY_XP_0083	PREDICTE	Malus_domestica	64.50	0.00
TRINITY_PNW8901	hypothet	Chlamydomonas_reinh	64.50	0.00
TRINITY_XP_0029	transcri	Volvox_carteri	64.50	0.00
TRINITY_XP_0158	casein	Ziziphus_jujuba	64.50	0.00
TRINITY_XP_0016	ribosoma	Chlamydomonas_reinh	64.50	0.00
TRINITY_XP_0016	tail-spe	Chlamydomonas_reinh	64.50	0.00
TRINITY_GAX8236	hypothet	Chlamydomonas_eusti	64.50	0.00
TRINITY_GAQ8362	HEAT rep	Klebsormidium_niten	64.50	0.00
TRINITY_KXZ5334	hypothet	Gonium_pectorale	64.50	0.00
TRINITY_XP_0095	PREDICTE	Nicotiana_tomentosi	64.50	0.00
TRINITY_GAX7948	hypothet	Chlamydomonas_eusti	64.50	0.00
TRINITY_XP_0014	predicte	Ostreococcus_sp._'l	64.50	0.00
TRINITY_GAX8624	hypothet	Chlamydomonas_eusti	64.50	0.00
TRINITY_GAX7300	hypothet	Chlamydomonas_eusti	64.50	0.00
TRINITY_GAX7942	hypothet	Chlamydomonas_eusti	64.50	0.00
TRINITY_KXZ4701	hypothet	Gonium_pectorale	64.50	0.00
TRINITY_XP_0014	predicte	Ostreococcus_sp._'l	64.50	0.00
TRINITY_XP_0029	hypothet	Volvox_carteri	64.50	0
TRINITY_XP_0139	peptide-	Monoraphidium_negle	64.50	0.00
TRINITY_GBF9995	chorisma	Raphidocelis_subcap	64.40	0.00
TRINITY_BAK0268	predicte	Hordeum_vulgare	64.40	0.00
TRINITY_RVW3358	hypothet	Vitis_vinifera	64.40	0.00
TRINITY_KZV4215	proline	Dorcoceras_hygromet	64.40	0.00
TRINITY_PNW7226	hypothet	Chlamydomonas_reinh	64.40	0.00
TRINITY_XP_0029	photosys	Volvox_carteri	64.40	0.00
TRINITY_GAX7928	hypothet	Chlamydomonas_eusti	64.40	0.00
TRINITY_PNW8323	hypothet	Chlamydomonas_reinh	64.40	0.00
TRINITY_GAX7873	hypothet	Chlamydomonas_eusti	64.40	0.00
TRINITY_XP_0016	predicte	Chlamydomonas_reinh	64.40	0.00
TRINITY_KXZ5396	hypothet	Gonium_pectorale	64.40	0.00
TRINITY_XP_0228	Cation-t	Ostreococcus_tauri	64.40	0.00
TRINITY_XP_0191	PREDICTE	Ipomoea_nil	64.40	0.00
TRINITY_GAX8403	hypothet	Chlamydomonas_eusti	64.40	0.00
TRINITY_XP_0056	hypothet	Coccomyxa_subellips	64.40	0.00
TRINITY_XP_0156	methion	Oryza_sativa	64.40	0.00
TRINITY_XP_0056	mitochor	Coccomyxa_subellips	64.40	0.00
TRINITY_XP_0095	PREDICTE	Nicotiana_tomentosi	64.40	0.00
TRINITY_XP_0016	DNA dama	Chlamydomonas_reinh	64.40	0.00

TRINITY_XP_00295	hypothet	Volvox_carteri	64.40	0.00
TRINITY_GAX75546	hypothet	Chlamydomonas_eusti	64.40	0.00
TRINITY_XP_00169	sodium/p	Chlamydomonas_reinh	64.40	0.00
TRINITY_GAQ90652	Alpha-L	Klebsormidium_niten	64.40	0.00
TRINITY_PNH01280	Bifuncti	Tetrabaena_socialis	64.40	0.00
TRINITY_XP_00294	hypothet	Volvox_carteri	64.40	0.00
TRINITY_KXZ50689	hypothet	Gonium_pectorale	64.40	0.00
TRINITY_PNW71553	hypothet	Chlamydomonas_reinh	64.40	0.00
TRINITY_KXZ44021	hypothet	Gonium_pectorale	64.40	0.00
TRINITY_GAX81853	hypothet	Chlamydomonas_eusti	64.40	0.00

TRINITY_KXZ42022	VAM5/VAM	Gonium_pectorale	64.40	0.00
TRINITY_GAX83107	hypothet	Chlamydomonas_eusti	64.40	0
TRINITY_KGN62516	hypothet	Cucumis_sativus	64.40	0.00
TRINITY_XP_02438	transcri	Physcomitrella_pate	64.30	0.00
TRINITY_XP_00306	target (	Micromonas_pusilla	64.30	0.00
TRINITY_GAX81039	hypothet	Chlamydomonas_eusti	64.30	0.00
TRINITY_XP_02389	guanine	Quercus_suber	64.30	0.00
TRINITY_XP_01884	PREDICTE	Juglans_regia	64.30	0.00
TRINITY_XP_01067	PREDICTE	Beta_vulgaris	64.30	0.00
TRINITY_XP_02387	probable	Quercus_suber	64.30	0.00
TRINITY_XP_00295	hypothet	Volvox_carteri	64.30	0.00
TRINITY_XP_01390	anaphase	Monoraphidium_negle	64.30	0.00
TRINITY_GAX74519	hypothet	Chlamydomonas_eusti	64.30	0.00
TRINITY_EPS74717	hypothet	Genlisea_aurea	64.30	0.00
TRINITY_KXZ46836	hypothet	Gonium_pectorale	64.30	0.00
TRINITY_XP_02288	splicing	Olea_europaea	64.30	0.00
TRINITY_XP_02452	queuine	Selaginella_moellen	64.30	0.00
TRINITY_PNH07835	GATA tra	Tetrabaena_socialis	64.30	0.00
TRINITY_XP_00294	hypothet	Volvox_carteri	64.30	0.00
TRINITY_GBG72475	hypothet	Chara_braunii	64.30	0.00
TRINITY_XP_00169	major ir	Chlamydomonas_reinh	64.30	0.00
TRINITY_BAU03701	hypothet	Vigna_angularis	64.30	0.00
TRINITY_GAX76021	hypothet	Chlamydomonas_eusti	64.30	0.00
TRINITY_XP_00564	ALG6, AI	Coccomyxa_subellips	64.30	0.00
TRINITY_XP_00169	predicte	Chlamydomonas_reinh	64.30	0.00
TRINITY_XP_00168	DEAD/DEA	Chlamydomonas_reinh	64.30	0.00
TRINITY_KXZ41814	hypothet	Gonium_pectorale	64.30	0.00
TRINITY_XP_00294	hypothet	Volvox_carteri	64.30	0.00
TRINITY_GAX73308	hypothet	Chlamydomonas_eusti	64.30	0.00
TRINITY_PNH03285	Uroporph	Tetrabaena_socialis	64.30	0.00
TRINITY_XP_02174	dolichyl	Chenopodium_quinoa	64.30	0.00
TRINITY_GAX84321	hypothet	Chlamydomonas_eusti	64.30	0.00
TRINITY_XP_00230	40S ribo	Populus_trichocarpa	64.30	0.00
TRINITY_GAX79473	hypothet	Chlamydomonas_eusti	64.30	0.00
TRINITY_XP_00294	hypothet	Volvox_carteri	64.30	0.00
TRINITY_KXZ42994	hypothet	Gonium_pectorale	64.30	0.00
TRINITY_PRW05739	Gag-Pol	Chlorella_sorokinia	64.30	0.00
TRINITY_GAX82247	hypothet	Chlamydomonas_eusti	64.30	0.00
TRINITY_PNW73765	hypothet	Chlamydomonas_reinh	64.30	0.00
TRINITY_GAX82417	hypothet	Chlamydomonas_eusti	64.30	0.00
TRINITY_GAX77417	hypothet	Chlamydomonas_eusti	64.30	0.00
TRINITY_KXZ53206	hypothet	Gonium_pectorale	64.30	0.00
TRINITY_GAX82406	hypothet	Chlamydomonas_eusti	64.30	0.00
TRINITY_GAX77541	hypothet	Chlamydomonas_eusti	64.30	0.00
TRINITY_XP_00169	O-acetyl	Chlamydomonas_reinh	64.30	0.00
TRINITY_XP_00169	predicte	Chlamydomonas_reinh	64.30	0.00
TRINITY_XP_00295	hypothet	Volvox_carteri	64.30	0.00
TRINITY_AAL35381	putative	Chlamydomonas_reinh	64.30	0.00
TRINITY_KXZ42158	hypothet	Gonium_pectorale	64.30	0.00
TRINITY_GAX84605	hypothet	Chlamydomonas_eusti	64.30	0.00
TRINITY_XP_00294	hypothet	Volvox_carteri	64.30	0.00
TRINITY_GAX84180	hypothet	Chlamydomonas_eusti	64.30	0.00
TRINITY_KXZ49298	hypothet	Gonium_pectorale	64.30	0.00
TRINITY_PNW76114	hypothet	Chlamydomonas_reinh	64.30	0.00
TRINITY_EPS74717	hypothet	Genlisea_aurea	64.30	0.00
TRINITY_PRQ36381	putative	Rosa_chinensis	64.30	0.00
TRINITY_BAJ98301	predicte	Hordeum_vulgare	64.20	0.00
TRINITY_XP_00305	predicte	Micromonas_pusilla	64.20	0.00
TRINITY_NP_04225	cytochro	Prototheca_wickerha	64.20	0.00
TRINITY_XP_01041	PREDICTE	Camelina_sativa	64.20	0.00
TRINITY_GBF92513	eukaryot	Raphidocelis_subcap	64.20	0.00
TRINITY_XP_02024	cell div	Asparagus_officinal	64.20	0.00
TRINITY_BAK00999	predicte	Hordeum_vulgare	64.20	0.00
TRINITY_PNW73801	hypothet	Chlamydomonas_reinh	64.20	0.00
TRINITY_XP_00295	mitogen-	Volvox_carteri	64.20	0.00
TRINITY_XP_01389	heat sho	Monoraphidium_negle	64.20	0.00

TRINITY_GAX8160	hypothet	Chlamydomonas_eusti	64.20	0.00
TRINITY_XP_0216	putative	Manihot_esculenta	64.20	0.00
TRINITY_PNW7075	hypothet	Chlamydomonas_reinh	64.20	0.00
TRINITY_PNW8816	hypothet	Chlamydomonas_reinh	64.20	0.00
TRINITY_GAX8115	hypothet	Chlamydomonas_eusti	64.20	0.00
TRINITY_KXZ4775	hypothet	Gonium_pectorale	64.20	0.00
TRINITY_ABD9689	hypothet	Tarenaya_spinosa	64.20	0.00
TRINITY_XP_0238	serine/t	Quercus_suber	64.20	0.00
TRINITY_XP_0029	hypothet	Volvox_carteri	64.20	0.00
TRINITY_XP_0025	predicte	Micromonas_commoda	64.20	0.00
TRINITY_XP_0029	hypothet	Volvox_carteri	64.20	0.00
TRINITY_PWA4164	rab GDP	Artemisia_annua	64.20	0.00
TRINITY_XP_0138	DNA pol	Monoraphidium_negle	64.20	0.00
TRINITY_GAX8259	hypothet	Chlamydomonas_eusti	64.20	0.00
TRINITY_PON7848	Isocitra	Trema_orientale	64.20	0.00
TRINITY_GAQ8856	aconitat	Klebsormidium_niten	64.20	0
TRINITY_XP_0016	predicte	Chlamydomonas_reinh	64.20	0.00
TRINITY_ALM5501	DEAD bo	Chlamydomonas_sp._I	64.20	0.00
TRINITY_PNW7403	hypothet	Chlamydomonas_reinh	64.20	0.00
TRINITY_ABK2516	unknown	Picea_sitchensis	64.20	0.00
TRINITY_OAE3091	hypothet	Marchantia_polymorp	64.20	0.00
TRINITY_KXZ4092	hypothet	Gonium_pectorale	64.20	0.00
TRINITY_XP_0238	dynein	Quercus_suber	64.20	0.00
TRINITY_XP_0144	60S rib	Vigna_radiata	64.20	0.00
TRINITY_XP_0228	cell di	Olea_europaea	64.20	0.00
TRINITY_XP_0016	O-linke	Chlamydomonas_reinh	64.20	0.00
TRINITY_GAX8624	hypothet	Chlamydomonas_eusti	64.20	0.00
TRINITY_XP_0017	glycera	Chlamydomonas_reinh	64.10	0.00
TRINITY_GAQ8958	alpha-1,	Klebsormidium_niten	64.10	0.00
TRINITY_GBG8021	hypothet	Chara_braunii	64.10	0.00
TRINITY_XP_0165	PREDICTE	Capsicum_annuum	64.10	0.00
TRINITY_XP_0139	60S aci	Monoraphidium_negle	64.10	0.00
TRINITY_XP_0139	60S aci	Monoraphidium_negle	64.10	0.00
TRINITY_XP_0097	PREDICTE	Nicotiana_sylvestri	64.10	0.00
TRINITY_XP_0238	threoni	Quercus_suber	64.10	0.00
TRINITY_PSC7673	E3 ubiq	Micractinium_conduc	64.10	0.00
TRINITY_ACQ8347	CBL-int	Physcomitrella_pate	64.10	0.00
TRINITY_RVW9974	Serine	Vitis_vinifera	64.10	0.00
TRINITY_XP_0138	ethanol	Monoraphidium_negle	64.10	0.00
TRINITY_PNH0405	CCR4-NO	Tetrabaena_socialis	64.10	0.00
TRINITY_GAX8210	hypothet	Chlamydomonas_eusti	64.10	0.00
TRINITY_XP_0016	peptidy	Chlamydomonas_reinh	64.10	0.00
TRINITY_GBF9473	IAA-ami	Raphidocelis_subcap	64.10	0.00
TRINITY_KXZ4571	hypothet	Gonium_pectorale	64.10	0.00
TRINITY_XP_0017	peroxir	Chlamydomonas_reinh	64.10	0.00
TRINITY_GAX7987	hypothet	Chlamydomonas_eusti	64.10	0.00
TRINITY_XP_0029	hypothet	Volvox_carteri	64.10	0.00
TRINITY_GAX8524	hypothet	Chlamydomonas_eusti	64.10	0.00
TRINITY_PNR6202	hypothet	Physcomitrella_pate	64.10	0.00
TRINITY_PNW7328	hypothet	Chlamydomonas_reinh	64.10	0.00
TRINITY_GAQ8698	Serine	Klebsormidium_niten	64.10	0.00
TRINITY_XP_0243	uncharac	Physcomitrella_pate	64.10	0.00
TRINITY_XP_0128	PREDICTE	Erythranthe_guttata	64.10	0.00
TRINITY_XP_0139	copropo	Monoraphidium_negle	64.10	0.00
TRINITY_XP_0016	bardet-	Chlamydomonas_reinh	64.10	0.00
TRINITY_XP_0016	mitocho	Chlamydomonas_reinh	64.10	0.00
TRINITY_KXZ5438	hypothet	Gonium_pectorale	64.10	0.00
TRINITY_GAX7537	hypothet	Chlamydomonas_eusti	64.10	0.00
TRINITY_GAX7419	hypothet	Chlamydomonas_eusti	64.10	0.00
TRINITY_KXZ5506	hypothet	Gonium_pectorale	64.10	0.00
TRINITY_XP_0105	PREDICTE	Tarenaya_hasslerian	64.00	0.00
TRINITY_GBG6437	hypothet	Chara_braunii	64.00	0.00
TRINITY_KMZ5989	putative	Zostera_marina	64.00	0.00
TRINITY_BAJ9317	predicte	Hordeum_vulgare	64.00	0.00
TRINITY_PSC7447	60S rib	Micractinium_conduc	64.00	0.00
TRINITY_KXZ5655	hypothet	Gonium_pectorale	64.00	0.00

TRINITY_XP_00294	hypothet	Volvox_carteri	64.00	0.00
TRINITY_KXZ43041	hypothet	Gonium_pectorale	64.00	0.00
TRINITY_GAX82981	hypothet	Chlamydomonas_eusti	64.00	0.00
TRINITY_GAX80829	hypothet	Chlamydomonas_eusti	64.00	0.00
TRINITY_XP_00170	predicte	Chlamydomonas_reinh	64.00	0.00
TRINITY_XP_00295	diacylgl	Volvox_carteri	64.00	0.00
TRINITY_XP_00295	hypothet	Volvox_carteri	64.00	0.00
TRINITY_P0C8M8.1	RecName:	Zea_mays	64.00	0.00
TRINITY_KXZ51597	hypothet	Gonium_pectorale	64.00	0.00
TRINITY_KXZ41940	hypothet	Gonium_pectorale	64.00	0.00
TRINITY_GAX77364	hypothet	Chlamydomonas_eusti	64.00	0.00
TRINITY_GBF93615	zeaxanth	Raphidocelis_subcap	64.00	0.00
TRINITY_XP_02181	flavin-c	Prunus_avium	64.00	0.00
TRINITY_GAX74989	hypothet	Chlamydomonas_eusti	64.00	0.00
TRINITY_GAX79819	hypothet	Chlamydomonas_eusti	64.00	0.00
TRINITY_GAX75810	hypothet	Chlamydomonas_eusti	64.00	0.00
TRINITY_GAX81341	hypothet	Chlamydomonas_eusti	64.00	0.00
TRINITY_XP_00295	hypothet	Volvox_carteri	64.00	0.00
TRINITY_XP_00295	hypothet	Volvox_carteri	64.00	0.00
TRINITY_GAX73288	hypothet	Chlamydomonas_eusti	64.00	0.00
TRINITY_GAX72737	hypothet	Chlamydomonas_eusti	64.00	0
TRINITY_XP_00565	TPR-like	Coccomyxa_subellips	64.00	0.00
TRINITY_PNW70419	hypothet	Chlamydomonas_reinh	64.00	0.00
TRINITY_GAX76466	hypothet	Chlamydomonas_eusti	64.00	0.00
TRINITY_XP_02451	DNA-dire	Selaginella_moellen	64.00	0
TRINITY_PNH12171	Golgi ap	Tetrabaena_socialis	64.00	0.00
TRINITY_XP_00295	hypothet	Volvox_carteri	64.00	0.00
TRINITY_XP_00294	hypothet	Volvox_carteri	64.00	0.00
TRINITY_XP_00294	hypothet	Volvox_carteri	64.00	0.00
TRINITY_KXZ49758	hypothet	Gonium_pectorale	64.00	0.00
TRINITY_GBF97031	amino ac	Raphidocelis_subcap	64.00	0.00
TRINITY_GAX77489	hypothet	Chlamydomonas_eusti	64.00	0.00
TRINITY_KXZ51618	hypothet	Gonium_pectorale	64.00	0.00
TRINITY_GBF87381	N-acetyl	Raphidocelis_subcap	64.00	0.00
TRINITY_AFK36347	unknown	Lotus_japonicus	63.90	0.00
TRINITY_GBG84961	hypothet	Chara_braunii	63.90	0.00
TRINITY_GAQ77648	Dyneins	Klebsormidium_niten	63.90	0.00
TRINITY_GAX80604	hypothet	Chlamydomonas_eusti	63.90	0.00
TRINITY_GAX72792	hypothet	Chlamydomonas_eusti	63.90	0.00
TRINITY_GAX77149	hypothet	Chlamydomonas_eusti	63.90	0.00
TRINITY_GAX73284	hypothet	Chlamydomonas_eusti	63.90	0.00
TRINITY_XP_00250	predicte	Micromonas_commoda	63.90	0.00
TRINITY_PRW56270	Deoxyhyp	Chlorella_sorokinia	63.90	0.00
TRINITY_GAX82062	hypothet	Chlamydomonas_eusti	63.90	0.00
TRINITY_PRW60116	3-oxoacy	Chlorella_sorokinia	63.90	0.00
TRINITY_PNW71355	hypothet	Chlamydomonas_reinh	63.90	0.00
TRINITY_GAX83348	hypothet	Chlamydomonas_eusti	63.90	0.00
TRINITY_XP_00360	WD-40 re	Medicago_truncatula	63.90	0.00
TRINITY_GAX82477	hypothet	Chlamydomonas_eusti	63.90	0.00
TRINITY_BAJ90795	predicte	Hordeum_vulgare	63.90	0.00
TRINITY_XP_01389	hypothet	Monoraphidium_negle	63.90	0.00
TRINITY_GAX84579	hypothet	Chlamydomonas_eusti	63.90	0.00
TRINITY_KXZ52799	hypothet	Gonium_pectorale	63.90	0.00
TRINITY_GAX76959	hypothet	Chlamydomonas_eusti	63.90	0.00
TRINITY_XP_00170	predicte	Chlamydomonas_reinh	63.90	0.00
TRINITY_XP_00565	putative	Coccomyxa_subellips	63.90	0.00
TRINITY_XP_00295	hypothet	Volvox_carteri	63.90	0.00
TRINITY_PNH09542	Vacuolar	Tetrabaena_socialis	63.90	0.00
TRINITY_XP_00169	ARF-rela	Chlamydomonas_reinh	63.90	0.00
TRINITY_GAX83216	hypothet	Chlamydomonas_eusti	63.90	0.00
TRINITY_GAX76630	hypothet	Chlamydomonas_eusti	63.90	0.00
TRINITY_XP_00169	peptidyl	Chlamydomonas_reinh	63.90	0.00
TRINITY_GAX83903	hypothet	Chlamydomonas_eusti	63.90	0
TRINITY_AAO73551	reverse-	Chlamydomonas_reinh	63.90	0.00
TRINITY_XP_00878	actin, r	Phoenix_dactylifera	63.90	0.00
TRINITY_AAP85534	isoamyla	Chlamydomonas_reinh	63.90	0

TRINITY_XP_0206	splicing	Dendrobium_catenatu	63.90	0.00
TRINITY_GAX7330	hypothet	Chlamydomonas_eusti	63.90	0
TRINITY_PNW7037	hypothet	Chlamydomonas_reinh	63.90	0.00
TRINITY_XP_0029	light-ha	Volvox_carteri	63.90	0.00
TRINITY_GAQ8357	RNA liga	Klebsormidium_niten	63.80	0.00
TRINITY_GAQ8098	Anaphase	Klebsormidium_niten	63.80	0.00
TRINITY_GAQ9156	clathrin	Klebsormidium_niten	63.80	0.00
TRINITY_PRW6083	chitinas	Chlorella_sorokinia	63.80	0.00
TRINITY_XP_0159	BEL1-lik	Arachis_duranensis	63.80	0.00
TRINITY_KMS6524	hypothet	Beta_vulgaris	63.80	0.00
TRINITY_XP_0100	PREDICTE	Eucalyptus_grandis	63.80	0.00
TRINITY_GBG0043	hypothet	Raphidocelis_subcap	63.80	0.00
TRINITY_PRW6062	ATPase A	Chlorella_sorokinia	63.80	0.00
TRINITY_XP_0017	predicte	Chlamydomonas_reinh	63.80	0.00
TRINITY_GAQ7902	Regulato	Klebsormidium_niten	63.80	0.00
TRINITY_XP_0201	DNA poly	Aegilops_tauschii	63.80	0.00
TRINITY_XP_0113	Serine/t	Auxenochlorella_pro	63.80	0.00
TRINITY_XP_0024	40S ribo	Sorghum_bicolor	63.80	0.00
TRINITY_XP_0139	Beclin-1	Monoraphidium_negle	63.80	0.00
TRINITY_XP_0184	PREDICTE	Raphanus_sativus	63.80	0.00
TRINITY_PNW8710	hypothet	Chlamydomonas_reinh	63.80	0.00
TRINITY_PNW7411	hypothet	Chlamydomonas_reinh	63.80	0.00
TRINITY_XP_0029	60S ribo	Selaginella_moellen	63.80	0.00
TRINITY_XP_0138	ATP-depe	Monoraphidium_negle	63.80	0.00
TRINITY_XP_0056	oxygenas	Coccomyxa_subellips	63.80	0.00
TRINITY_XP_0058	hypothet	Chlorella_variabili	63.80	0.00
TRINITY_GAX8386	hypothet	Chlamydomonas_eusti	63.80	0.00
TRINITY_GAX7341	hypothet	Chlamydomonas_eusti	63.80	0.00
TRINITY_GAX8470	hypothet	Chlamydomonas_eusti	63.80	0.00
TRINITY_XP_0016	guanylat	Chlamydomonas_reinh	63.80	0.00
TRINITY_GAX7808	hypothet	Chlamydomonas_eusti	63.80	0.00
TRINITY_KXZ4941	hypothet	Gonium_pectorale	63.80	0.00
TRINITY_KMS6493	hypothet	Beta_vulgaris	63.80	0.00
TRINITY_KXZ4141	hypothet	Gonium_pectorale	63.80	0.00
TRINITY_GAX7437	hypothet	Chlamydomonas_eusti	63.80	0.00
TRINITY_KXZ5555	hypothet	Gonium_pectorale	63.80	0.00
TRINITY_XP_0017	mitochon	Chlamydomonas_reinh	63.80	0.00
TRINITY_GBF8875	hypothet	Raphidocelis_subcap	63.80	0.00
TRINITY_GAX8007	hypothet	Chlamydomonas_eusti	63.80	0
TRINITY_GAX7855	hypothet	Chlamydomonas_eusti	63.80	0.00
TRINITY_XP_0138	UDP-sulf	Monoraphidium_negle	63.80	0.00
TRINITY_XP_0029	hypothet	Volvox_carteri	63.80	0.00
TRINITY_ADW0902	ammonium	Dunaliella_viridis	63.80	0.00
TRINITY_KXZ4815	hypothet	Gonium_pectorale	63.80	0.00
TRINITY_GAX8208	hypothet	Chlamydomonas_eusti	63.80	0.00
TRINITY_RID8060	hypothet	Brassica_rapa	63.80	0.00
TRINITY_RDY1294	Splicing	Mucuna_pruriens	63.70	0.00
TRINITY_PNW7813	hypothet	Chlamydomonas_reinh	63.70	0.00
TRINITY_RMZ5614	hypothet	Auxenochlorella_pro	63.70	0.00
TRINITY_GAX7311	hypothet	Chlamydomonas_eusti	63.70	0.00
TRINITY_XP_0202	prolifer	Asparagus_officinal	63.70	0.00
TRINITY_KZV1535	hypothet	Dorcoceras_hygromet	63.70	0.00
TRINITY_XP_0188	PREDICTE	Juglans_regia	63.70	0.00
TRINITY_GAX7278	hypothet	Chlamydomonas_eusti	63.70	0.00
TRINITY_XP_0056	Metallo-	Coccomyxa_subellips	63.70	0.00
TRINITY_KXZ5049	hypothet	Gonium_pectorale	63.70	0.00
TRINITY_GBF8899	plastid	Raphidocelis_subcap	63.70	0.00
TRINITY_GAX8352	hypothet	Chlamydomonas_eusti	63.70	0.00
TRINITY_GAX8022	hypothet	Chlamydomonas_eusti	63.70	0.00
TRINITY_GAX7857	hypothet	Chlamydomonas_eusti	63.70	0.00
TRINITY_GAX7570	hypothet	Chlamydomonas_eusti	63.70	0.00
TRINITY_KXZ4425	hypothet	Gonium_pectorale	63.70	0.00
TRINITY_XP_0017	kinesin-	Chlamydomonas_reinh	63.70	0.00
TRINITY_KHN0718	60S ribo	Glycine_soja	63.70	0.00
TRINITY_RYR0140	hypothet	Arachis_hypogaea	63.70	0.00
TRINITY_KXZ4955	hypothet	Gonium_pectorale	63.70	0.00

TRINITY_GAX7447	hypothet	Chlamydomonas_eusti	63.70	0.00
TRINITY_PSC7605	parvulir	Micractinium_conduc	63.60	0.00
TRINITY_KXZ4460	hypothet	Gonium_pectorale	63.60	0.00
TRINITY_XP_0029	hypothet	Volvox_carteri	63.60	0.00
TRINITY_NP_0012	uncharac	Glycine_max	63.60	0.00
TRINITY_XP_0139	vacuolar	Monoraphidium_negle	63.60	0.00
TRINITY_XP_0110	protein	Sesamum_indicum	63.60	0.00
TRINITY_RMZ5451	hypothet	Auxenochlorella_prc	63.60	0.00
TRINITY_GAQ8749	RNA recc	Klebsormidium_niten	63.60	0.00
TRINITY_GAQ8183	20S prot	Klebsormidium_niten	63.60	0.00
TRINITY_PSC7568	Equilibr	Micractinium_conduc	63.60	0.00
TRINITY_PTQ2678	hypothet	Marchantia_polymorp	63.60	0.00
TRINITY_XP_0017	ubiquiti	Chlamydomonas_reinh	63.60	0.00
TRINITY_OIW1000	hypothet	Lupinus_angustifoli	63.60	0.00
TRINITY_PWA4413	cofactor	Artemisia_annua	63.60	0.00
TRINITY_ADK7912	astaxant	Haematococcus_lacus	63.60	0.00
TRINITY_XP_0200	protein	Ananas_comosus	63.60	0.00
TRINITY_XP_0029	hypothet	Volvox_carteri	63.60	0.00
TRINITY_GAX8167	hypothet	Chlamydomonas_eusti	63.60	0.00
TRINITY_XP_0017	photosys	Chlamydomonas_reinh	63.60	0.00
TRINITY_OAE3244	hypothet	Marchantia_polymorp	63.60	0.00
TRINITY_GAX8282	hypothet	Chlamydomonas_eusti	63.60	0.00
TRINITY_PNW8133	hypothet	Chlamydomonas_reinh	63.60	0.00
TRINITY_PNW8848	hypothet	Chlamydomonas_reinh	63.60	0.00
TRINITY_PNH0854	hypothet	Tetrabaena_socialis	63.60	0.00
TRINITY_BAJ1178	dehydrat	Corchorus_olitorius	63.60	0.00
TRINITY_KXZ4195	hypothet	Gonium_pectorale	63.60	0.00
TRINITY_XP_0238	uncharac	Quercus_suber	63.60	0.00
TRINITY_XP_0016	mitogen-	Chlamydomonas_reinh	63.60	0.00
TRINITY_XP_0029	RabC/Rak	Volvox_carteri	63.60	0.00
TRINITY_GAX7284	hypothet	Chlamydomonas_eusti	63.60	0.00
TRINITY_XP_0109	PREDICTE	Elaeis_guineensis	63.60	0.00
TRINITY_XP_0202	40S ribc	Asparagus_officinal	63.60	0.00
TRINITY_PNW8252	hypothet	Chlamydomonas_reinh	63.60	0.00
TRINITY_XP_0243	CTP synt	Physcomitrella_pate	63.60	0.00
TRINITY_GAX8464	hypothet	Chlamydomonas_eusti	63.60	0.00
TRINITY_XP_0016	serine/t	Chlamydomonas_reinh	63.60	0.00
TRINITY_XP_0064	cysteine	Eutrema_salsugineum	63.60	0.00
TRINITY_GBF8778	hypothet	Raphidocelis_subcap	63.60	0.00
TRINITY_PNW7094	hypothet	Chlamydomonas_reinh	63.60	0.00
TRINITY_OAE3542	hypothet	Marchantia_polymorp	63.60	0.00
TRINITY_PNW7713	hypothet	Chlamydomonas_reinh	63.60	0.00
TRINITY_XP_0220	chaperor	Helianthus_annuus	63.60	0.00
TRINITY_XP_0243	protein	Physcomitrella_pate	63.60	0.00
TRINITY_XP_0017	predicte	Chlamydomonas_reinh	63.60	0.00
TRINITY_GAX7951	hypothet	Chlamydomonas_eusti	63.60	0.00
TRINITY_PNW8499	hypothet	Chlamydomonas_reinh	63.60	0.00
TRINITY_GAX7834	hypothet	Chlamydomonas_eusti	63.60	0.00
TRINITY_GBF9570	aspartat	Raphidocelis_subcap	63.60	0.00
TRINITY_PNH0898	Selenocy	Tetrabaena_socialis	63.60	0.00
TRINITY_GAX8597	hypothet	Chlamydomonas_eusti	63.60	0.00
TRINITY_GAX8344	hypothet	Chlamydomonas_eusti	63.60	0.00
TRINITY_GAX8152	hypothet	Chlamydomonas_eusti	63.60	0.00
TRINITY_GBG6903	hypothet	Chara_braunii	63.60	0.00
TRINITY_GAX7361	hypothet	Chlamydomonas_eusti	63.60	0
TRINITY_GAX7541	hypothet	Chlamydomonas_eusti	63.60	0.00
TRINITY_GAX8249	hypothet	Chlamydomonas_eusti	63.60	0.00
TRINITY_KXZ5381	hypothet	Gonium_pectorale	63.60	0.00
TRINITY_GAX7722	hypothet	Chlamydomonas_eusti	63.60	0.00
TRINITY_KXZ5550	hypothet	Gonium_pectorale	63.60	0.00
TRINITY_GBF9340	serine h	Raphidocelis_subcap	63.60	0.00
TRINITY_GAQ7945	DNA topc	Klebsormidium_niten	63.50	0.00
TRINITY_XP_0058	hypothet	Chlorella_variabili	63.50	0.00
TRINITY_PLY6869	hypothet	Lactuca_sativa	63.50	0.00
TRINITY_GAX7401	hypothet	Chlamydomonas_eusti	63.50	0.00
TRINITY_PNW8488	hypothet	Chlamydomonas_reinh	63.50	0.00

TRINITY_PNH0200(	RNA pseı	Tetrabaena_socialis	63.50	0.00
TRINITY_XP_0029!	hypothet	Volvox_carteri	63.50	0.00
TRINITY_XP_0029!	hypothet	Volvox_carteri	63.50	0.00
TRINITY_XP_0029(	hypothet	Volvox_carteri	63.50	0.00
TRINITY_XP_0058(	hypothet	Chlorella_variabili	63.50	0.00
TRINITY_PNR6252(	hypothet	Physcomitrella_pate	63.50	0.00
TRINITY_KXZ4325!	hypothet	Gonium_pectorale	63.50	0.00
TRINITY_BAJ9577(	predicte	Hordeum_vulgare	63.50	0.00
TRINITY_GAX7361(	hypothet	Chlamydomonas_eusti	63.50	0.00
TRINITY_XP_0206(	poly [AI	Dendrobium_catenatu	63.50	0.00
TRINITY_PNH0571(	hypothet	Tetrabaena_socialis	63.50	0.00
TRINITY_BAK0277(	predicte	Hordeum_vulgare	63.50	0.00
TRINITY_OAE3283(	hypothet	Marchantia_polymorp	63.50	0
TRINITY_PSC6754'	hypothet	Micractinium_conduc	63.50	0.00
TRINITY_XP_0191(	PREDICTI	Ipomoea_nil	63.50	0.00
TRINITY_PNH1000(	hypothet	Tetrabaena_socialis	63.50	0.00
TRINITY_XP_0029!	hypothet	Volvox_carteri	63.50	0.00
TRINITY_GAX8111(	hypothet	Chlamydomonas_eusti	63.50	0.00
TRINITY_XP_0150(	SUMO-act	Solanum_pennellii	63.50	0.00
TRINITY_XP_0029(	acetylgl	Volvox_carteri	63.50	0.00
TRINITY_GAX8302(	hypothet	Chlamydomonas_eusti	63.50	0.00
TRINITY_ADI4685(	DHC1bf	Volvox_carteri	63.50	0.00
TRINITY_GAX7598(	hypothet	Chlamydomonas_eusti	63.50	0.00
TRINITY_XP_0030(	Ribosoma	Ostreococcus_tauri	63.50	0.00
TRINITY_KMS6524!	hypothet	Beta_vulgaris	63.50	0.00
TRINITY_PNW8197(	hypothet	Chlamydomonas_reinh	63.50	0.00
TRINITY_XP_0029!	hypothet	Volvox_carteri	63.40	0.00
TRINITY_PSC7261(	mRNA (2	Micractinium_conduc	63.40	0.00
TRINITY_BAJ9887!	predicte	Hordeum_vulgare	63.40	0.00
TRINITY_AAO7355(	reverse-	Chlamydomonas_reinh	63.40	0.00
TRINITY_XP_0029!	hypothet	Volvox_carteri	63.40	0.00
TRINITY_GAQ7859(	Rab fami	Klebsormidium_niten	63.40	0.00
TRINITY_GBG7247!	hypothet	Chara_braunii	63.40	0.00
TRINITY_PNW8180(	hypothet	Chlamydomonas_reinh	63.40	0.00
TRINITY_XP_0016(	predicte	Chlamydomonas_reinh	63.40	0.00
TRINITY_GAX8242(	hypothet	Chlamydomonas_eusti	63.40	0.00
TRINITY_XP_0016(	subunit	Chlamydomonas_reinh	63.40	0.00
TRINITY_KXZ4753(	hypothet	Gonium_pectorale	63.40	0.00
TRINITY_XP_0016(	mitochor	Chlamydomonas_reinh	63.40	0.00
TRINITY_XP_0139(	heme per	Monoraphidium_negle	63.40	0.00
TRINITY_XP_0058(	hypothet	Chlorella_variabili	63.40	0.00
TRINITY_GAQ8276(	Actin-re	Klebsormidium_niten	63.40	0.00
TRINITY_XP_0017(	flagella	Chlamydomonas_reinh	63.40	0.00
TRINITY_XP_0029!	hypothet	Volvox_carteri	63.40	0.00
TRINITY_ONK7023!	unchara(	Asparagus_officinal	63.40	0.00
TRINITY_XP_0271(	60S ribo	Coffea_arabica	63.40	0.00
TRINITY_KXZ4803(	hypothet	Gonium_pectorale	63.40	0.00
TRINITY_PNW7909'	hypothet	Chlamydomonas_reinh	63.40	0.00
TRINITY_GAX8190(	hypothet	Chlamydomonas_eusti	63.40	0.00
TRINITY_PNW7806(	hypothet	Chlamydomonas_reinh	63.40	0.00
TRINITY_GAX7811(	hypothet	Chlamydomonas_eusti	63.40	0.00
TRINITY_XP_0029(	hypothet	Volvox_carteri	63.40	0.00
TRINITY_GAX8297(	hypothet	Chlamydomonas_eusti	63.40	0.00
TRINITY_GAX8558(	hypothet	Chlamydomonas_eusti	63.40	0.00
TRINITY_GAX7348(	hypothet	Chlamydomonas_eusti	63.40	0.00
TRINITY_XP_0134!	mitochor	Medicago_truncatula	63.40	0.00
TRINITY_XP_0070(	PREDICTI	Theobroma_cacao	63.30	0.00
TRINITY_GAQ8831(	hypothet	Klebsormidium_niten	63.30	0.00
TRINITY_GBF9126(	ABC trar	Raphidocelis_subcap	63.30	0.00
TRINITY_AAL3264(	fructose	Arabidopsis_thalian	63.30	0.00
TRINITY_GAX7899(	hypothet	Chlamydomonas_eusti	63.30	0.00
TRINITY_XP_0109(	PREDICTI	Elaeis_guineensis	63.30	0.00
TRINITY_PNW7482(	hypothet	Chlamydomonas_reinh	63.30	0.00
TRINITY_XP_0176(	PREDICTI	Gossypium_arboreum	63.30	0.00
TRINITY_OAE3212(	hypothet	Marchantia_polymorp	63.30	0.00
TRINITY_EXC3489(	Metal tı	Morus_notabilis	63.30	0.00

TRINITY_GBF9373	hypothet	Raphidocelis_subcap	63.30	0.00
TRINITY_GAX7498	hypothet	Chlamydomonas_eusti	63.30	0.00
TRINITY_GBG9239	hypothet	Chara_braunii	63.30	0.00
TRINITY_PNH0961	putative	Tetrabaena_socialis	63.30	0.00
TRINITY_GBF9073	nitrile	Raphidocelis_subcap	63.30	0.00
TRINITY_GBF9521	hypothet	Raphidocelis_subcap	63.30	0.00
TRINITY_XP_0017	cyclin	Chlamydomonas_reinh	63.30	0.00
TRINITY_KXZ5489	hypothet	Gonium_pectorale	63.30	0.00
TRINITY_GBF9418	tRNA (g	Raphidocelis_subcap	63.30	0.00
TRINITY_GAX7898	hypothet	Chlamydomonas_eusti	63.30	0.00
TRINITY_GAX7621	hypothet	Chlamydomonas_eusti	63.30	0.00
TRINITY_XP_0029	hypothet	Volvox_carteri	63.30	0.00
TRINITY_XP_0239	eukaryot	Quercus_suber	63.30	0.00
TRINITY_GAX7840	hypothet	Chlamydomonas_eusti	63.30	0.00
TRINITY_XP_0016	predicte	Chlamydomonas_reinh	63.30	0.00
TRINITY_XP_0016	vacuola	Chlamydomonas_reinh	63.30	0.00
TRINITY_GAX8248	hypothet	Chlamydomonas_eusti	63.30	0.00
TRINITY_XP_0017	predicte	Chlamydomonas_reinh	63.30	0.00
TRINITY_CAA6623	cyclin-	Antirrhinum_majus	63.30	0.00
TRINITY_PNW7920	hypothet	Chlamydomonas_reinh	63.30	0.00
TRINITY_PNW7736	hypothet	Chlamydomonas_reinh	63.30	0.00
TRINITY_GAQ7865	Ribosome	Klebsormidium_niten	63.30	0.00
TRINITY_GAQ8311	Fumarase	Klebsormidium_niten	63.30	0.00
TRINITY_KXZ4419	hypothet	Gonium_pectorale	63.30	0.00
TRINITY_PNW7583	hypothet	Chlamydomonas_reinh	63.30	0.00
TRINITY_GAX7941	hypothet	Chlamydomonas_eusti	63.30	0.00
TRINITY_KXZ4782	hypothet	Gonium_pectorale	63.30	0.00
TRINITY_XP_0016	predicte	Chlamydomonas_reinh	63.30	0.00
TRINITY_XP_0029	hypothet	Volvox_carteri	63.30	0.00
TRINITY_PNW8514	hypothet	Chlamydomonas_reinh	63.30	0.00
TRINITY_XP_0029	hypothet	Volvox_carteri	63.30	0.00
TRINITY_GAX8315	hypothet	Chlamydomonas_eusti	63.30	0.00
TRINITY_PNW7447	hypothet	Chlamydomonas_reinh	63.30	0.00
TRINITY_BAK0267	predicte	Hordeum_vulgare	63.30	0.00
TRINITY_XP_0025	hypothet	Micromonas_commoda	63.20	0.00
TRINITY_XP_0229	pirin-l	Cucurbita_moschata	63.20	0.00
TRINITY_XP_0238	uncharac	Quercus_suber	63.20	0.00
TRINITY_RWR9250	AP-4 cor	Cinnamomum_micranth	63.20	0.00
TRINITY_BAK0096	predicte	Hordeum_vulgare	63.20	0.00
TRINITY_XP_0029	hypothet	Volvox_carteri	63.20	0.00
TRINITY_XP_0066	PREDICTE	Oryza_brachyantha	63.20	0.00
TRINITY_A9NUH8.	RecName:	Picea_sitchensis	63.20	0.00
TRINITY_PNH1155	hypothet	Tetrabaena_socialis	63.20	0.00
TRINITY_XP_0017	actin-l	Chlamydomonas_reinh	63.20	0.00
TRINITY_PRW5993	RNA 3 -t	Chlorella_sorokinia	63.20	0.00
TRINITY_XP_0029	hypothet	Volvox_carteri	63.20	0.00
TRINITY_XP_0114	hypothet	Auxenochlorella_prc	63.20	0.00
TRINITY_OAE2113	hypothet	Marchantia_polymorp	63.20	0.00
TRINITY_OVA1982	WD40 re	Macleaya_cordata	63.20	0.00
TRINITY_GAX7351	hypothet	Chlamydomonas_eusti	63.20	0.00
TRINITY_GAX8564	hypothet	Chlamydomonas_eusti	63.20	0.00
TRINITY_PNW8680	hypothet	Chlamydomonas_reinh	63.20	0.00
TRINITY_GAX8630	hypothet	Chlamydomonas_eusti	63.20	0.00
TRINITY_GAX7509	hypothet	Chlamydomonas_eusti	63.20	0.00
TRINITY_GAX8282	hypothet	Chlamydomonas_eusti	63.20	0.00
TRINITY_KXZ5342	hypothet	Gonium_pectorale	63.20	0.00
TRINITY_PNW6989	hypothet	Chlamydomonas_reinh	63.20	0.00
TRINITY_GAX8168	hypothet	Chlamydomonas_eusti	63.20	0.00
TRINITY_KXZ4694	hypothet	Gonium_pectorale	63.20	0.00
TRINITY_XP_0016	peptidy	Chlamydomonas_reinh	63.20	0.00
TRINITY_XP_0022	PREDICTE	Vitis_vinifera	63.20	0.00
TRINITY_XP_0029	hypothet	Volvox_carteri	63.20	0.00
TRINITY_XP_0029	hypothet	Volvox_carteri	63.20	0.00
TRINITY_GBG7247	hypothet	Chara_braunii	63.20	0.00
TRINITY_GAX7558	hypothet	Chlamydomonas_eusti	63.20	0.00
TRINITY_GAX7522	hypothet	Chlamydomonas_eusti	63.20	0.00

TRINITY_KXZ4330	hypothet	Gonium_pectorale	63.20	0.00
TRINITY_XP_0016	predicte	Chlamydomonas_reinh	63.20	0.00
TRINITY_KXZ5670	hypothet	Gonium_pectorale	63.20	0.00
TRINITY_AAO7355	reverse-	Chlamydomonas_reinh	63.20	0.00
TRINITY_GBF9761	hypothet	Raphidocelis_subcap	63.20	0.00
TRINITY_OAE2519	hypothet	Marchantia_polymorp	63.10	0.00
TRINITY_RDY1253	ADP-ribo	Mucuna_pruriens	63.10	0.00
TRINITY_GAX7264	hypothet	Chlamydomonas_eusti	63.10	0.00
TRINITY_PRW4449	translat	Chlorella_sorokinia	63.10	0.00
TRINITY_XP_0245	isocitra	Selaginella_moellen	63.10	0.00
TRINITY_XP_0243	eukaryot	Physcomitrella_pate	63.10	0.00
TRINITY_GAX7961	hypothet	Chlamydomonas_eusti	63.10	0.00
TRINITY_XP_0029	hypothet	Volvox_carteri	63.10	0.00
TRINITY_PNW7464	hypothet	Chlamydomonas_reinh	63.10	0.00
TRINITY_GAX7671	hypothet	Chlamydomonas_eusti	63.10	0.00
TRINITY_XP_0239	ras-like	Quercus_suber	63.10	0.00
TRINITY_PNW8326	hypothet	Chlamydomonas_reinh	63.10	0.00
TRINITY_AIU8018	acyl-CoA	Chlorella_sp._KQ-20	63.10	0.00
TRINITY_NP_5636	dual spe	Arabidopsis_thalian	63.10	0.00
TRINITY_AGC1298	long fla	Chlamydomonas_reinh	63.10	0.00
TRINITY_XP_0138	pyridoxa	Monoraphidium_negle	63.10	0.00
TRINITY_XP_0016	CAX fami	Chlamydomonas_reinh	63.10	0.00
TRINITY_KXZ4975	hypothet	Gonium_pectorale	63.10	0.00
TRINITY_XP_0030	FERM/acy	Ostreococcus_tauri	63.10	0.00
TRINITY_XP_0025	predicte	Micromonas_commoda	63.10	0.00
TRINITY_BAK0046	predicte	Hordeum_vulgare	63.00	0.00
TRINITY_XP_0243	seleniur	Physcomitrella_pate	63.00	0.00
TRINITY_XP_0239	dolichol	Quercus_suber	63.00	0.00
TRINITY_RWR8170	ubiquiti	Cinnamomum_micranth	63.00	0.00
TRINITY_KXZ4424	hypothet	Gonium_pectorale	63.00	0.00
TRINITY_XP_0088	uncharac	Phoenix_dactylifera	63.00	0.00
TRINITY_XP_0228	Ribosoma	Ostreococcus_tauri	63.00	0.00
TRINITY_XP_0029	hypothet	Volvox_carteri	63.00	0.00
TRINITY_XP_0138	hypothet	Monoraphidium_negle	63.00	0.00
TRINITY_ABF9356	Cell div	Oryza_sativa	63.00	0.00
TRINITY_AXU9887	hypothet	Brassica_juncea	63.00	0.00
TRINITY_KXZ4742	hypothet	Gonium_pectorale	63.00	0.00
TRINITY_GAX7401	hypothet	Chlamydomonas_eusti	63.00	0.00
TRINITY_XP_0029	hypothet	Volvox_carteri	63.00	0.00
TRINITY_XP_0058	hypothet	Chlorella_variabili	63.00	0.00
TRINITY_XP_0016	predicte	Chlamydomonas_reinh	63.00	0.00
TRINITY_XP_0110	PREDICTE	Populus_euphratica	63.00	0.00
TRINITY_XP_0029	flagella	Volvox_carteri	63.00	0.00
TRINITY_XP_0138	myo-inos	Monoraphidium_negle	63.00	0.00
TRINITY_GAX8310	hypothet	Chlamydomonas_eusti	63.00	0.00
TRINITY_PNW7620	hypothet	Chlamydomonas_reinh	63.00	0.00
TRINITY_XP_0014	predicte	Ostreococcus_sp._'l	63.00	0.00
TRINITY_GBF9354	hypothet	Raphidocelis_subcap	63.00	0.00
TRINITY_GAX8165	hypothet	Chlamydomonas_eusti	63.00	0.00
TRINITY_PNW8311	hypothet	Chlamydomonas_reinh	63.00	0.00
TRINITY_PNW8148	hypothet	Chlamydomonas_reinh	63.00	0.00
TRINITY_XP_0207	uncharac	Dendrobium_catenatu	63.00	0.00
TRINITY_PTQ3695	hypothet	Marchantia_polymorp	63.00	0.00
TRINITY_GAX8642	hypothet	Chlamydomonas_eusti	63.00	0.00
TRINITY_PNH1097	Interfer	Tetrabaena_socialis	63.00	0.00
TRINITY_XP_0229	intron-b	Cucurbita_moschata	63.00	0.00
TRINITY_GBF8935	hypothet	Raphidocelis_subcap	63.00	0.00
TRINITY_XP_0056	hypothet	Coccomyxa_subellips	63.00	0.00
TRINITY_XP_0139	Uncharac	Monoraphidium_negle	63.00	0.00
TRINITY_XP_0229	ubiquiti	Cucurbita_moschata	63.00	0.00
TRINITY_PNW7182	hypothet	Chlamydomonas_reinh	63.00	0.00
TRINITY_XP_0138	type I p	Monoraphidium_negle	63.00	0.00
TRINITY_XP_0167	PREDICTE	Gossypium_hirsutum	63.00	0.00
TRINITY_PNW8272	hypothet	Chlamydomonas_reinh	63.00	0.00
TRINITY_PNR3110	hypothet	Physcomitrella_pate	63.00	0.00
TRINITY_XP_0016	beta-ure	Chlamydomonas_reinh	63.00	0.00

TRINITY_GAX7898	hypothet	Chlamydomonas_eusti	63.00	0.00
TRINITY_XP_0075	predict	Bathycoccus_prasinc	63.00	0.00
TRINITY_GAX7381	hypothet	Chlamydomonas_eusti	63.00	0.00
TRINITY_GBF8866	diacylg	Raphidocelis_subcap	63.00	0.00
TRINITY_KXZ4482	hypothet	Gonium_pectorale	63.00	0.00
TRINITY_PRW4526	apurini	Chlorella_sorokinia	63.00	0.00
TRINITY_GBF9197	methylt	Raphidocelis_subcap	63.00	0.00
TRINITY_GAX7648	hypothet	Chlamydomonas_eusti	63.00	0.00
TRINITY_KXZ5189	hypothet	Gonium_pectorale	63.00	0.00
TRINITY_XP_0113	Tubulin	Auxenochlorella_prc	62.90	0.00
TRINITY_XP_0016	hydin-l	Chlamydomonas_reinh	62.90	0.00
TRINITY_XP_0029	hypothet	Volvox_carteri	62.90	0.00
TRINITY_XP_0228	Histidi	Ostreococcus_tauri	62.90	0.00
TRINITY_XP_0017	ubiquin	Chlamydomonas_reinh	62.90	0.00
TRINITY_PNH0153	actin [	Tetrabaena_socialis	62.90	0.00
TRINITY_XP_0258	V-type	Panicum_hallii	62.90	0.00
TRINITY_XP_0238	uncharac	Quercus_suber	62.90	0.00
TRINITY_XP_0029	Mg2+ tr	Volvox_carteri	62.90	0.00
TRINITY_XP_0029	hypothet	Volvox_carteri	62.90	0.00
TRINITY_GBF8839	WD repe	Raphidocelis_subcap	62.90	0.00
TRINITY_GBF9263	hypothet	Raphidocelis_subcap	62.90	0.00
TRINITY_PSC7438	ethylen	Micractinium_conduc	62.90	0.00
TRINITY_GAX8359	hypothet	Chlamydomonas_eusti	62.90	0.00
TRINITY_XP_0016	NAD-dep	Chlamydomonas_reinh	62.90	0.00
TRINITY_PRW5625	20S prot	Chlorella_sorokinia	62.90	0.00
TRINITY_KXZ4838	hypothet	Gonium_pectorale	62.90	0.00
TRINITY_XP_0029	hypothet	Volvox_carteri	62.90	0.00
TRINITY_PNH1174	hypothet	Tetrabaena_socialis	62.90	0.00
TRINITY_XP_0139	baculov	Monoraphidium_negle	62.90	0.00
TRINITY_XP_0138	hypothet	Monoraphidium_negle	62.90	0.00
TRINITY_VDC7218	unnamed	Brassica_rapa	62.90	0.00
TRINITY_XP_0238	phospho	Quercus_suber	62.90	0.00
TRINITY_XP_0029	hypothet	Volvox_carteri	62.90	0.00
TRINITY_XP_0243	pre-mRN	Physcomitrella_pate	62.90	0.00
TRINITY_GBG0052	phospho	Raphidocelis_subcap	62.90	0.00
TRINITY_GAX8344	hypothet	Chlamydomonas_eusti	62.90	0.00
TRINITY_RMZ5415	hypothet	Auxenochlorella_prc	62.90	0.00
TRINITY_GAX7539	hypothet	Chlamydomonas_eusti	62.90	0.00
TRINITY_GAX8138	hypothet	Chlamydomonas_eusti	62.90	0.00
TRINITY_GAX7894	hypothet	Chlamydomonas_eusti	62.90	0.00
TRINITY_XP_0139	aurora	Monoraphidium_negle	62.90	0.00
TRINITY_PRW2067	40S rib	Chlorella_sorokinia	62.90	0.00
TRINITY_XP_0029	p300/CB	Volvox_carteri	62.90	0.00
TRINITY_GAX7575	hypothet	Chlamydomonas_eusti	62.90	0
TRINITY_XP_0063	PREDICT	Solanum_tuberosum	62.90	0.00
TRINITY_XP_0016	predict	Chlamydomonas_reinh	62.80	0.00
TRINITY_XP_0167	PREDICT	Gossypium_hirsutum	62.80	0.00
TRINITY_KXZ5680	METE pr	Gonium_pectorale	62.80	0.00
TRINITY_GAX7820	hypothet	Chlamydomonas_eusti	62.80	0.00
TRINITY_XP_0138	hypothet	Monoraphidium_negle	62.80	0.00
TRINITY_XP_0017	serine	Chlamydomonas_reinh	62.80	0.00
TRINITY_PNW8336	hypothet	Chlamydomonas_reinh	62.80	0.00
TRINITY_KXZ4200	hypothet	Gonium_pectorale	62.80	0.00
TRINITY_XP_0239	aldo-ket	Quercus_suber	62.80	0.00
TRINITY_GAX8214	hypothet	Chlamydomonas_eusti	62.80	0.00
TRINITY_XP_0017	SM/Sec1-	Chlamydomonas_reinh	62.80	0.00
TRINITY_PNH1070	Zeaxant	Tetrabaena_socialis	62.80	0.00
TRINITY_GAX8518	hypothet	Chlamydomonas_eusti	62.80	0.00
TRINITY_GAQ8244	Membran	Klebsormidium_niten	62.80	0.00
TRINITY_XP_0029	hypothet	Volvox_carteri	62.80	0.00
TRINITY_EOY3025	Cystein	Theobroma_cacao	62.80	0.00
TRINITY_GAX7981	hypothet	Chlamydomonas_eusti	62.80	0.00
TRINITY_PNH1171	L-galac	Tetrabaena_socialis	62.80	0.00
TRINITY_GAX8322	hypothet	Chlamydomonas_eusti	62.80	0.00
TRINITY_KZV1503	NAD-dep	Dorcoceras_hygromet	62.80	0.00
TRINITY_XP_0238	uncharac	Quercus_suber	62.80	0.00

TRINITY_XP_0094(	PREDICTI	Musa_acuminata	62.80	0.00
TRINITY_GAX8320	hypothet	Chlamydomonas_eusti	62.80	0.00
TRINITY_GAX7401	hypothet	Chlamydomonas_eusti	62.80	0
TRINITY_GAQ9213	putative	Klebsormidium_niten	62.80	0.00
TRINITY_KXZ5506	hypothet	Gonium_pectorale	62.80	0.00
TRINITY_GAX8127	hypothet	Chlamydomonas_eusti	62.80	0.00
TRINITY_GAX8025	hypothet	Chlamydomonas_eusti	62.80	0.00
TRINITY_PTQ4226	hypothet	Marchantia_polymorp	62.80	0.00
TRINITY_XP_0139(	prolyl	Monoraphidium_negle	62.80	0.00
TRINITY_GAX7544	hypothet	Chlamydomonas_eusti	62.80	0.00
TRINITY_XP_0056	threonyl	Coccomyxa_subellips	62.70	0.00
TRINITY_XP_0068	heat sh	Amborella_trichopod	62.70	0.00
TRINITY_BAJ9943(	predicte	Hordeum_vulgare	62.70	0.00
TRINITY_XP_0124	PREDICTI	Gossypium_raimondii	62.70	0.00
TRINITY_XP_0017(	predicte	Chlamydomonas_reinh	62.70	0.00
TRINITY_XP_0243	alpha-ma	Physcomitrella_pate	62.70	0.00
TRINITY_PRQ4599	putative	Rosa_chinensis	62.70	0.00
TRINITY_GBF9186	hypothet	Raphidocelis_subcap	62.70	0.00
TRINITY_OAE3367	hypothet	Marchantia_polymorp	62.70	0.00
TRINITY_GAX7941	hypothet	Chlamydomonas_eusti	62.70	0.00
TRINITY_KXZ4194(	hypothet	Gonium_pectorale	62.70	0.00
TRINITY_PNH0794	putative	Tetrabaena_socialis	62.70	0.00
TRINITY_GBF9114	hypothet	Raphidocelis_subcap	62.70	0.00
TRINITY_XP_0139(	hypothet	Monoraphidium_negle	62.70	0.00
TRINITY_XP_0191	PREDICTI	Ipomoea_nil	62.70	0.00
TRINITY_XP_0017(	NADH:ub	Chlamydomonas_reinh	62.70	0.00
TRINITY_XP_0029	hypothet	Volvox_carteri	62.70	0.00
TRINITY_GAX8344	hypothet	Chlamydomonas_eusti	62.70	0.00
TRINITY_GAX8188	hypothet	Chlamydomonas_eusti	62.70	0.00
TRINITY_GAX7980	hypothet	Chlamydomonas_eusti	62.70	0.00
TRINITY_XP_0017(	inositol	Chlamydomonas_reinh	62.70	0.00
TRINITY_ALM5500	DEAD box	Chlamydomonas_sp._I	62.70	0.00
TRINITY_GAX7622	hypothet	Chlamydomonas_eusti	62.70	0.00
TRINITY_GBG8742	hypothet	Chara_braunii	62.70	0.00
TRINITY_KXZ5134	hypothet	Gonium_pectorale	62.70	0.00
TRINITY_XP_0029	hypothet	Volvox_carteri	62.70	0.00
TRINITY_CAD7125	asparagi	Lotus_japonicus	62.70	0.00
TRINITY_GAX8030	hypothet	Chlamydomonas_eusti	62.70	0.00
TRINITY_GAX8607(	hypothet	Chlamydomonas_eusti	62.70	0.00
TRINITY_XP_0218	eukaryot	Spinacia_oleracea	62.70	0.00
TRINITY_RID6206	hypothet	Brassica_rapa	62.70	0.00
TRINITY_KXZ5398	hypothet	Gonium_pectorale	62.70	0.00
TRINITY_GAX7530	hypothet	Chlamydomonas_eusti	62.70	0.00
TRINITY_GBF9253	DNA repa	Raphidocelis_subcap	62.70	0.00
TRINITY_GAX7352	hypothet	Chlamydomonas_eusti	62.70	0.00
TRINITY_XP_0016	predicte	Chlamydomonas_reinh	62.70	0.00
TRINITY_PNW8026	hypothet	Chlamydomonas_reinh	62.70	0.00
TRINITY_PNH0453	E3 ubiqu	Tetrabaena_socialis	62.70	0.00
TRINITY_GAX8246	hypothet	Chlamydomonas_eusti	62.70	0.00
TRINITY_GAX7596	hypothet	Chlamydomonas_eusti	62.70	0.00
TRINITY_VDD1037	unnamed	Brassica_rapa	62.70	0.00
TRINITY_PNW7893	hypothet	Chlamydomonas_reinh	62.70	0.00
TRINITY_XP_0109	PREDICTI	Elaeis_guineensis	62.60	0.00
TRINITY_XP_0056	Mak16 p	Coccomyxa_subellips	62.60	0.00
TRINITY_XP_0093	PREDICTI	Pyrus_x_bretschneid	62.60	0.00
TRINITY_GAX8155	hypothet	Chlamydomonas_eusti	62.60	0.00
TRINITY_EFJ2312	hypothet	Selaginella_moellen	62.60	0.00
TRINITY_KCW7583	hypothet	Eucalyptus_grandis	62.60	0.00
TRINITY_ACG3645	histone	Zea_mays	62.60	0.00
TRINITY_XP_0191	PREDICTI	Ipomoea_nil	62.60	0.00
TRINITY_AGC8213	phytoche	Suaeda_salsa	62.60	0.00
TRINITY_GAX8205	hypothet	Chlamydomonas_eusti	62.60	0.00
TRINITY_GBG0057	hypothet	Raphidocelis_subcap	62.60	0.00
TRINITY_GBF9885	hypothet	Raphidocelis_subcap	62.60	0.00
TRINITY_XP_0029	hypothet	Volvox_carteri	62.60	0.00
TRINITY_XP_0016	low CO2-	Chlamydomonas_reinh	62.60	0.00

TRINITY_GAX7351	hypothet	Chlamydomonas_eusti	62.60	0.00
TRINITY_GAX7588	hypothet	Chlamydomonas_eusti	62.60	0.00
TRINITY_GAX8126	hypothet	Chlamydomonas_eusti	62.60	0.00
TRINITY_PNW8390	hypothet	Chlamydomonas_reinh	62.60	0.00
TRINITY_XP_0228	Chaperon	Ostreococcus_tauri	62.60	0.00
TRINITY_KXZ5102	hypothet	Gonium_pectorale	62.60	0.00
TRINITY_XP_0113	60S ribo	Auxenochlorella_pro	62.60	0.00
TRINITY_XP_0029	hypothet	Volvox_carteri	62.60	0.00
TRINITY_B9NJI2.	RecName:	Populus_trichocarpa	62.60	0.00
TRINITY_KXZ5104	hypothet	Gonium_pectorale	62.60	0.00
TRINITY_KXZ4750	hypothet	Gonium_pectorale	62.60	0.00
TRINITY_KXZ4327	hypothet	Gonium_pectorale	62.60	0.00
TRINITY_GAX7329	hypothet	Chlamydomonas_eusti	62.60	0.00
TRINITY_GAX7904	hypothet	Chlamydomonas_eusti	62.60	0.00
TRINITY_XP_0105	PREDICTE	Tarenaya_hasslerian	62.60	0.00
TRINITY_KXZ4269	hypothet	Gonium_pectorale	62.60	0.00
TRINITY_PNW7785	hypothet	Chlamydomonas_reinh	62.60	0.00
TRINITY_GAX8247	hypothet	Chlamydomonas_eusti	62.60	0.00
TRINITY_XP_0016	sterol c	Chlamydomonas_reinh	62.60	0.00
TRINITY_XP_0029	hypothet	Volvox_carteri	62.60	0.00
TRINITY_XP_0273	peptidyl	Abrus_precatorius	62.60	0.00
TRINITY_XP_0030	UTP--glu	Ostreococcus_tauri	62.50	0.00
TRINITY_XP_0025	probable	Ricinus_communis	62.50	0.00
TRINITY_XP_0110	2-methox	Sesamum_indicum	62.50	0.00
TRINITY_XP_0100	PREDICTE	Eucalyptus_grandis	62.50	0.00
TRINITY_GAX8557	hypothet	Chlamydomonas_eusti	62.50	0.00
TRINITY_GAX7997	hypothet	Chlamydomonas_eusti	62.50	0.00
TRINITY_ABH0932	leucine	Arachis_hypogaea	62.50	0.00
TRINITY_XP_0056	protein	Coccomyxa_subellips	62.50	0.00
TRINITY_XP_0029	hypothet	Volvox_carteri	62.50	0.00
TRINITY_OMO8227	Band 7 p	Corchorus_olitorius	62.50	0.00
TRINITY_OAE3439	hypothet	Marchantia_polymorp	62.50	0.00
TRINITY_XP_0058	hypothet	Chlorella_variabili	62.50	0.00
TRINITY_GAX7733	hypothet	Chlamydomonas_eusti	62.50	0.00
TRINITY_GAQ8559	Calossin	Klebsormidium_niten	62.50	0.00
TRINITY_GAX7919	hypothet	Chlamydomonas_eusti	62.50	0.00
TRINITY_GAX7565	hypothet	Chlamydomonas_eusti	62.50	0.00
TRINITY_OTG1849	putative	Helianthus_annuus	62.50	0.00
TRINITY_GAX8508	hypothet	Chlamydomonas_eusti	62.50	0.00
TRINITY_GAX8435	hypothet	Chlamydomonas_eusti	62.50	0.00
TRINITY_XP_0029	hypothet	Volvox_carteri	62.50	0.00
TRINITY_XP_0016	MutT/NUD	Chlamydomonas_reinh	62.50	0.00
TRINITY_GAX8240	hypothet	Chlamydomonas_eusti	62.50	0.00
TRINITY_GBG0036	hypothet	Raphidocelis_subcap	62.50	0.00
TRINITY_KXZ5325	hypothet	Gonium_pectorale	62.50	0.00
TRINITY_PNH1115	Vegetati	Tetrabaena_socialis	62.50	0.00
TRINITY_GAX8177	hypothet	Chlamydomonas_eusti	62.50	0.00
TRINITY_PNW7909	hypothet	Chlamydomonas_reinh	62.50	0.00
TRINITY_BAK0075	predicte	Hordeum_vulgare	62.50	0.00
TRINITY_XP_0139	Protein	Monoraphidium_negle	62.50	0.00
TRINITY_GAX7765	hypothet	Chlamydomonas_eusti	62.50	0.00
TRINITY_GBF8823	alpha be	Raphidocelis_subcap	62.50	0.00
TRINITY_KXZ5391	hypothet	Gonium_pectorale	62.50	0.00
TRINITY_GAX7732	hypothet	Chlamydomonas_eusti	62.50	0.00
TRINITY_GAX7892	hypothet	Chlamydomonas_eusti	62.50	0.00
TRINITY_GAX8406	hypothet	Chlamydomonas_eusti	62.50	0.00
TRINITY_KXZ5013	hypothet	Gonium_pectorale	62.50	0.00
TRINITY_GAX7358	hypothet	Chlamydomonas_eusti	62.50	0
TRINITY_GAX7352	hypothet	Chlamydomonas_eusti	62.50	0.00
TRINITY_PNW8452	hypothet	Chlamydomonas_reinh	62.50	0.00
TRINITY_GAX8263	hypothet	Chlamydomonas_eusti	62.50	0.00
TRINITY_XP_0035	ras-rela	Brachypodium_distac	62.40	0.00
TRINITY_BAK0103	predicte	Hordeum_vulgare	62.40	0.00
TRINITY_BAK0103	predicte	Hordeum_vulgare	62.40	0.00
TRINITY_XP_0029	hypothet	Volvox_carteri	62.40	0.00
TRINITY_GAQ8463	hypothet	Klebsormidium_niten	62.40	0.00

TRINITY_XP_0094	PREDICTE	Musa_acuminata	62.40	0.00
TRINITY_XP_0094	PREDICTE	Musa_acuminata	62.40	0.00
TRINITY_GBG8278	hypothet	Chara_braunii	62.40	0.00
TRINITY_KXZ5143	hypothet	Gonium_pectorale	62.40	0.00
TRINITY_PNW6997	hypothet	Chlamydomonas_reinh	62.40	0.00
TRINITY_XP_0114	ATP-depe	Auxenochlorella_pro	62.40	0.00
TRINITY_PNH0656	putative	Tetrabaena_socialis	62.40	0.00
TRINITY_BAK0077	predicte	Hordeum_vulgare	62.40	0.00
TRINITY_XP_0029	hypothet	Volvox_carteri	62.40	0.00
TRINITY_XP_0016	predicte	Chlamydomonas_reinh	62.40	0.00
TRINITY_KXZ5199	hypothet	Gonium_pectorale	62.40	0.00
TRINITY_XP_0029	hypothet	Volvox_carteri	62.40	0.00
TRINITY_PNH1190	Nuclear	Tetrabaena_socialis	62.40	0.00
TRINITY_GAX8142	hypothet	Chlamydomonas_eusti	62.40	0.00
TRINITY_GBF9528	hypothet	Raphidocelis_subcap	62.40	0.00
TRINITY_XP_0243	nascent	Physcomitrella_pate	62.40	0.00
TRINITY_XP_0016	serine/t	Chlamydomonas_reinh	62.40	0.00
TRINITY_XP_0017	predicte	Chlamydomonas_reinh	62.40	0.00
TRINITY_GAX8251	hypothet	Chlamydomonas_eusti	62.40	0.00
TRINITY_KXZ4879	hypothet	Gonium_pectorale	62.40	0.00
TRINITY_PNW8875	hypothet	Chlamydomonas_reinh	62.40	0.00
TRINITY_GAX7422	hypothet	Chlamydomonas_eusti	62.40	0.00
TRINITY_XP_0264	ras-rela	Papaver_somniferum	62.40	0.00
TRINITY_XP_0017	ribosoma	Chlamydomonas_reinh	62.40	0.00
TRINITY_GAX8251	hypothet	Chlamydomonas_eusti	62.40	0.00
TRINITY_GBF9656	ATP-depe	Raphidocelis_subcap	62.40	0.00
TRINITY_GAX8280	hypothet	Chlamydomonas_eusti	62.40	0.00
TRINITY_PNW7906	hypothet	Chlamydomonas_reinh	62.40	0.00
TRINITY_GAX7520	hypothet	Chlamydomonas_eusti	62.40	0.00
TRINITY_PNW8578	hypothet	Chlamydomonas_reinh	62.40	0.00
TRINITY_GAX8164	hypothet	Chlamydomonas_eusti	62.40	0
TRINITY_GAX8525	hypothet	Chlamydomonas_eusti	62.40	0.00
TRINITY_GBF8904	hypothet	Raphidocelis_subcap	62.40	0.00
TRINITY_XP_0064	glucose-	Eutrema_salsugineum	62.30	0.00
TRINITY_XP_0138	hypothet	Monoraphidium_negle	62.30	0.00
TRINITY_GAX8223	hypothet	Chlamydomonas_eusti	62.30	0.00
TRINITY_XP_0097	PREDICTE	Nicotiana_sylvestri	62.30	0.00
TRINITY_BAK0289	predicte	Hordeum_vulgare	62.30	0.00
TRINITY_XP_0243	spermato	Physcomitrella_pate	62.30	0.00
TRINITY_XP_0016	26S prot	Chlamydomonas_reinh	62.30	0.00
TRINITY_GAX7865	hypothet	Chlamydomonas_eusti	62.30	0.00
TRINITY_GAX7591	hypothet	Chlamydomonas_eusti	62.30	0.00
TRINITY_KXZ4092	hypothet	Gonium_pectorale	62.30	0.00
TRINITY_XP_0239	ras-rela	Quercus_suber	62.30	0.00
TRINITY_GBF8805	hypothet	Raphidocelis_subcap	62.30	0.00
TRINITY_GAX8496	hypothet	Chlamydomonas_eusti	62.30	0.00
TRINITY_GAX7286	hypothet	Chlamydomonas_eusti	62.30	0.00
TRINITY_GBG0021	hypothet	Raphidocelis_subcap	62.30	0.00
TRINITY_PNH1137	Gramicid	Tetrabaena_socialis	62.30	0.00
TRINITY_PNH1147	hypothet	Tetrabaena_socialis	62.30	0.00
TRINITY_KXZ4861	hypothet	Gonium_pectorale	62.30	0.00
TRINITY_PNH1158	Serine/t	Tetrabaena_socialis	62.30	0.00
TRINITY_PNW7380	hypothet	Chlamydomonas_reinh	62.30	0.00
TRINITY_XP_0058	hypothet	Chlorella_variabili	62.30	0.00
TRINITY_XP_0139	Serine/t	Monoraphidium_negle	62.30	0.00
TRINITY_XP_0016	amino ac	Chlamydomonas_reinh	62.30	0.00
TRINITY_GAX8563	hypothet	Chlamydomonas_eusti	62.30	0
TRINITY_PNW7586	hypothet	Chlamydomonas_reinh	62.30	0.00
TRINITY_XP_0029	hypothet	Volvox_carteri	62.30	0.00
TRINITY_XP_0017	predicte	Chlamydomonas_reinh	62.30	0.00
TRINITY_GBF9576	hypothet	Raphidocelis_subcap	62.30	0.00
TRINITY_KXZ4464	hypothet	Gonium_pectorale	62.30	0.00
TRINITY_KXZ4407	hypothet	Gonium_pectorale	62.30	0.00
TRINITY_ESQ4444	hypothet	Eutrema_salsugineum	62.30	0.00
TRINITY_GAX8525	hypothet	Chlamydomonas_eusti	62.20	0.00
TRINITY_GAX7562	hypothet	Chlamydomonas_eusti	62.20	0.00

TRINITY_GAX85959	hypothet	Chlamydomonas_eusti	62.20	0.00
TRINITY_PNW88793	hypothet	Chlamydomonas_reinh	62.20	0.00
TRINITY_KXZ56339	hypothet	Gonium_pectorale	62.20	0.00
TRINITY_XP_00295	hypothet	Volvox_carteri	62.20	0.00
TRINITY_GAQ87080	2-amino-	Klebsormidium_niten	62.20	0.00
TRINITY_GAQ87528	GSK3/SHA	Klebsormidium_niten	62.20	0.00
TRINITY_XP_00141	predicte	Ostreococcus_sp._'l	62.20	0.00
TRINITY_KDD72157	hypothet	Helicosporidium_sp.	62.20	0.00
TRINITY_XP_00295	hypothet	Volvox_carteri	62.20	0.00
TRINITY_XP_02390	ATP-binc	Quercus_suber	62.20	0.00
TRINITY_ABU52967	hypothet	Dunaliella_viridis	62.20	0.00
TRINITY_PNW81248	hypothet	Chlamydomonas_reinh	62.20	0.00
TRINITY_ABR18116	unknown	Picea_sitchensis	62.20	0.00
TRINITY_PNH02689	Phosphol	Tetrabaena_socialis	62.20	0.00
TRINITY_XP_00170	presenil	Chlamydomonas_reinh	62.20	0.00
TRINITY_XP_01389	Transcri	Monoraphidium_negle	62.20	0.00
TRINITY_XP_00295	hypothet	Volvox_carteri	62.20	0.00
TRINITY_GAX73629	hypothet	Chlamydomonas_eusti	62.20	0.00
TRINITY_PTQ47427	hypothet	Marchantia_polymorp	62.20	0.00
TRINITY_GAX73144	hypothet	Chlamydomonas_eusti	62.20	0.00
TRINITY_XP_00169	low CO2-	Chlamydomonas_reinh	62.20	0.00
TRINITY_XP_00294	hypothet	Volvox_carteri	62.20	0.00
TRINITY_GBF97319	folylpol	Raphidocelis_subcap	62.20	0.00
TRINITY_XP_00295	hypothet	Volvox_carteri	62.20	0.00
TRINITY_XP_00169	2-cys pe	Chlamydomonas_reinh	62.20	0.00
TRINITY_PRW05739	Gag-Pol	Chlorella_sorokinia	62.20	0.00
TRINITY_PNW85112	hypothet	Chlamydomonas_reinh	62.20	0.00
TRINITY_KXZ55454	hypothet	Gonium_pectorale	62.20	0.00
TRINITY_GAX79250	hypothet	Chlamydomonas_eusti	62.20	0.00
TRINITY_KXZ49629	hypothet	Gonium_pectorale	62.20	0.00
TRINITY_GAX73891	hypothet	Chlamydomonas_eusti	62.20	0.00
TRINITY_KXZ51924	hypothet	Gonium_pectorale	62.20	0.00
TRINITY_GBG87683	hypothet	Chara_braunii	62.20	0.00
TRINITY_XP_00169	gamma-gl	Chlamydomonas_reinh	62.20	0.00
TRINITY_PNH02069	Histone	Tetrabaena_socialis	62.20	0.00
TRINITY_GAX83626	hypothet	Chlamydomonas_eusti	62.20	0.00
TRINITY_XP_01067	PREDICTE	Beta_vulgaris	62.20	0.00
TRINITY_RXI04586	hypothet	Malus_domestica	62.20	0.00
TRINITY_GAQ91504	Translat	Klebsormidium_niten	62.10	0.00
TRINITY_XP_02389	guanine	Quercus_suber	62.10	0.00
TRINITY_XP_01068	PREDICTE	Beta_vulgaris	62.10	0.00
TRINITY_XP_01562	protein	Oryza_sativa	62.10	0.00
TRINITY_OAE18627	hypothet	Marchantia_polymorp	62.10	0.00
TRINITY_XP_02180	probable	Prunus_avium	62.10	0.00
TRINITY_XP_01389	hypothet	Monoraphidium_negle	62.10	0.00
TRINITY_XP_00170	thioredc	Chlamydomonas_reinh	62.10	0.00
TRINITY_XP_01674	PREDICTE	Gossypium_hirsutum	62.10	0.00
TRINITY_KXZ43502	hypothet	Gonium_pectorale	62.10	0.00
TRINITY_GBG80403	hypothet	Chara_braunii	62.10	0.00
TRINITY_XP_02436	40S ribc	Physcomitrella_pate	62.10	0.00
TRINITY_XP_02436	40S ribc	Physcomitrella_pate	62.10	0.00
TRINITY_XP_00169	DnaJ-lik	Chlamydomonas_reinh	62.10	0.00
TRINITY_GAX79606	hypothet	Chlamydomonas_eusti	62.10	0.00
TRINITY_VDD46802	unnamed	Brassica_oleracea	62.10	0.00
TRINITY_PNW86049	hypothet	Chlamydomonas_reinh	62.10	0.00
TRINITY_GAX76491	hypothet	Chlamydomonas_eusti	62.10	0.00
TRINITY_GAX83843	hypothet	Chlamydomonas_eusti	62.10	0.00
TRINITY_PNW80361	hypothet	Chlamydomonas_reinh	62.10	0.00
TRINITY_GAX80571	hypothet	Chlamydomonas_eusti	62.10	0
TRINITY_XP_00169	predicte	Chlamydomonas_reinh	62.10	0.00
TRINITY_KXZ42000	hypothet	Gonium_pectorale	62.10	0.00
TRINITY_GAX76295	hypothet	Chlamydomonas_eusti	62.10	0.00
TRINITY_XP_00170	predicte	Chlamydomonas_reinh	62.10	0.00
TRINITY_KXZ55968	hypothet	Gonium_pectorale	62.10	0.00
TRINITY_PNW71344	hypothet	Chlamydomonas_reinh	62.10	0.00
TRINITY_XP_00294	molecula	Volvox_carteri	62.10	0.00

TRINITY_GBF8771!	hypothet	Raphidocelis_subcap	62.10	0.00
TRINITY_GAX7728!	hypothet	Chlamydomonas_eusti	62.10	0
TRINITY_PNW7792(	hypothet	Chlamydomonas_reinh	62.10	0.00
TRINITY_GAX7736	hypothet	Chlamydomonas_eusti	62.10	0.00
TRINITY_XP_0017(	sulfite	Chlamydomonas_reinh	62.10	0.00
TRINITY_GBF9091!	calcium-	Raphidocelis_subcap	62.10	0.00
TRINITY_XP_0243!	40S ribo	Physcomitrella_pate	62.00	0.00
TRINITY_OTG1836(	putative	Helianthus_annuus	62.00	0.00
TRINITY_XP_0238!	4-hydro>	Quercus_suber	62.00	0.00
TRINITY_GAX8588(	hypothet	Chlamydomonas_eusti	62.00	0.00
TRINITY_XP_0125	probable	Cicer_arietinum	62.00	0.00
TRINITY_GAX7482	hypothet	Chlamydomonas_eusti	62.00	0.00
TRINITY_XP_0159	clathrir	Arachis_duranensis	62.00	0.00
TRINITY_XP_0156	ADP-ribo	Oryza_sativa	62.00	0.00
TRINITY_XP_0136(	PREDICTE	Brassica_oleracea	62.00	0.00
TRINITY_PNR5240(	hypothet	Physcomitrella_pate	62.00	0.00
TRINITY_GAQ8924(	phosphat	Klebsormidium_niten	62.00	0.00
TRINITY_XP_0058	hypothet	Chlorella_variabili	62.00	0.00
TRINITY_XP_0134!	proteaso	Medicago_truncatula	62.00	0.00
TRINITY_PLY9692!	hypothet	Lactuca_sativa	62.00	0.00
TRINITY_OTG2711	putative	Helianthus_annuus	62.00	0.00
TRINITY_XP_0219(	threonir	Carica_papaya	62.00	0.00
TRINITY_XP_0017(	DNA rep	Chlamydomonas_reinh	62.00	0.00
TRINITY_PRW6118!	Glutare	Chlorella_sorokinia	62.00	0.00
TRINITY_GAX7436	hypothet	Chlamydomonas_eusti	62.00	0.00
TRINITY_XP_0058	hypothet	Chlorella_variabili	62.00	0.00
TRINITY_XP_0056!	RNA-binc	Coccomyxa_subellips	62.00	0.00
TRINITY_XP_0245	transcri	Selaginella_moellen	62.00	0.00
TRINITY_XP_0025(	heat sho	Micromonas_commoda	62.00	0.00
TRINITY_PNW7346	hypothet	Chlamydomonas_reinh	62.00	0.00
TRINITY_XP_0025(	predicte	Micromonas_commoda	62.00	0.00
TRINITY_OMO7607	Aminoacy	Corchorus_capsulari	62.00	0.00
TRINITY_XP_0139(	hypothet	Monoraphidium_negle	62.00	0.00
TRINITY_BAJ9457!	predicte	Hordeum_vulgare	62.00	0.00
TRINITY_PNW7567!	hypothet	Chlamydomonas_reinh	62.00	0.00
TRINITY_XP_0094	PREDICTE	Musa_acuminata	62.00	0.00
TRINITY_PNH1139	ASC1-lik	Tetrabaena_socialis	62.00	0.00
TRINITY_XP_0029	hypothet	Volvox_carteri	62.00	0.00
TRINITY_GAQ8040	Mitochor	Klebsormidium_niten	62.00	0.00
TRINITY_XP_0017(	histidir	Chlamydomonas_reinh	62.00	0.00
TRINITY_GBF9075	ubiquiti	Raphidocelis_subcap	62.00	0.00
TRINITY_GBF8803	uridine	Raphidocelis_subcap	62.00	0.00
TRINITY_KXZ5501(	hypothet	Gonium_pectorale	62.00	0.00
TRINITY_XP_0156	small nu	Oryza_sativa	62.00	0.00
TRINITY_KXZ4421!	hypothet	Gonium_pectorale	62.00	0.00
TRINITY_GAX7625!	hypothet	Chlamydomonas_eusti	62.00	0.00
TRINITY_KXZ5014	hypothet	Gonium_pectorale	62.00	0.00
TRINITY_KXZ5504	hypothet	Gonium_pectorale	62.00	0.00
TRINITY_GAQ8928!	TCP-1/cp	Klebsormidium_niten	62.00	0.00
TRINITY_GAX7884	hypothet	Chlamydomonas_eusti	62.00	0.00
TRINITY_RMZ5271(	hypothet	Auxenochlorella_pro	62.00	0.00
TRINITY_KXZ4455	hypothet	Gonium_pectorale	62.00	0.00
TRINITY_ACF7929	unknown	Zea_mays	62.00	0.00
TRINITY_XP_0016!	DNA-dire	Chlamydomonas_reinh	62.00	0.00
TRINITY_KXZ4245!	hypothet	Gonium_pectorale	62.00	0.00
TRINITY_XP_0017(	sodium/p	Chlamydomonas_reinh	62.00	0.00
TRINITY_KVH9478	AAA+ ATI	Cynara_cardunculus	62.00	0.00
TRINITY_XP_0238!	40S ribo	Quercus_suber	62.00	0.00
TRINITY_XP_0139(	beta-ket	Monoraphidium_negle	61.90	0.00
TRINITY_AAK2610	SKP1-lik	Arabidopsis_thalian	61.90	0.00
TRINITY_GAX7714!	hypothet	Chlamydomonas_eusti	61.90	0.00
TRINITY_XP_0110	PREDICTE	Populus_euphratica	61.90	0.00
TRINITY_GAX7908	hypothet	Chlamydomonas_eusti	61.90	0.00
TRINITY_XP_0017(	sulfite	Chlamydomonas_reinh	61.90	0.00
TRINITY_RWW0186!	hypothet	Ensete_ventricosum	61.90	0.00
TRINITY_KMS9450	hypothet	Beta_vulgaris	61.90	0.00

TRINITY_PNW7239	hypothet	Chlamydomonas_reinh	61.90	0.00
TRINITY_KMS6493	hypothet	Beta_vulgaris	61.90	0.00
TRINITY_PNW7532	hypothet	Chlamydomonas_reinh	61.90	0.00
TRINITY_KXZ4092	hypothet	Gonium_pectorale	61.90	0.00
TRINITY_PNH0047	hypothet	Tetrabaena_socialis	61.90	0.00
TRINITY_GBG5920	Glycine	Chara_braunii	61.90	0.00
TRINITY_PNW7654	hypothet	Chlamydomonas_reinh	61.90	0.00
TRINITY_GBF9284	hypothet	Raphidocelis_subcap	61.90	0.00
TRINITY_GAX7384	hypothet	Chlamydomonas_eusti	61.90	0.00
TRINITY_PRW4433	DNA ann	Chlorella_sorokinia	61.90	0.00
TRINITY_XP_0017	vacuola	Chlamydomonas_reinh	61.90	0.00
TRINITY_KXZ5285	hypothet	Gonium_pectorale	61.90	0.00
TRINITY_GAX8285	hypothet	Chlamydomonas_eusti	61.90	0.00
TRINITY_KXZ5275	hypothet	Gonium_pectorale	61.90	0.00
TRINITY_GAX7334	hypothet	Chlamydomonas_eusti	61.90	0.00
TRINITY_XP_0016	mitochon	Chlamydomonas_reinh	61.90	0.00
TRINITY_GAX8589	hypothet	Chlamydomonas_eusti	61.90	0.00
TRINITY_GAX8341	hypothet	Chlamydomonas_eusti	61.90	0
TRINITY_XP_0056	exostos	Coccomyxa_subellips	61.90	0.00
TRINITY_XP_0138	Chaperon	Monoraphidium_negle	61.90	0.00
TRINITY_GBF9916	aconitat	Raphidocelis_subcap	61.90	0.00
TRINITY_XP_0017	F1F0 AT	Chlamydomonas_reinh	61.90	0.00
TRINITY_GAX7525	hypothet	Chlamydomonas_eusti	61.90	0.00
TRINITY_PNW7402	hypothet	Chlamydomonas_reinh	61.90	0.00
TRINITY_BAK0075	predict	Hordeum_vulgare	61.90	0.00
TRINITY_GBF9140	hypothet	Raphidocelis_subcap	61.90	0.00
TRINITY_XP_0056	polyade	Coccomyxa_subellips	61.90	0.00
TRINITY_PNW8032	hypothet	Chlamydomonas_reinh	61.90	0.00
TRINITY_GAX7722	hypothet	Chlamydomonas_eusti	61.90	0.00
TRINITY_GAX7912	hypothet	Chlamydomonas_eusti	61.90	0
TRINITY_GAX8285	hypothet	Chlamydomonas_eusti	61.90	0.00
TRINITY_GAX8038	hypothet	Chlamydomonas_eusti	61.90	0.00
TRINITY_XP_0191	PREDICT	Ipomoea_nil	61.80	0.00
TRINITY_PRW2046	lysine--	Chlorella_sorokinia	61.80	0.00
TRINITY_XP_0029	compone	Volvox_carteri	61.80	0.00
TRINITY_XP_0165	PREDICT	Capsicum_annuum	61.80	0.00
TRINITY_ESQ5180	hypothet	Eutrema_salsugineu	61.80	0.00
TRINITY_ABI4895	putativ	Viola_baoshanensis	61.80	0.00
TRINITY_XP_0238	cytosol	Quercus_suber	61.80	0.00
TRINITY_PNW8780	hypothet	Chlamydomonas_reinh	61.80	0.00
TRINITY_GBF9967	hypothet	Raphidocelis_subcap	61.80	0.00
TRINITY_GAQ9257	catalas	Klebsormidium_niten	61.80	0.00
TRINITY_AAK3873	nucleos	Dunaliella_tertiole	61.80	0.00
TRINITY_KXZ5512	hypothet	Gonium_pectorale	61.80	0.00
TRINITY_KMZ5965	Elongat	Zostera_marina	61.80	0.00
TRINITY_PNW8304	hypothet	Chlamydomonas_reinh	61.80	0.00
TRINITY_AAV5418	chlorop	Haematococcus_lacus	61.80	0.00
TRINITY_PNW8403	hypothet	Chlamydomonas_reinh	61.80	0.00
TRINITY_PNW8809	hypothet	Chlamydomonas_reinh	61.80	0.00
TRINITY_GAX7356	hypothet	Chlamydomonas_eusti	61.80	0.00
TRINITY_AAD2338	gamete-	Chlamydomonas_reinh	61.80	0.00
TRINITY_PNH1240	hypothet	Tetrabaena_socialis	61.80	0.00
TRINITY_XP_0029	hypothet	Volvox_carteri	61.80	0.00
TRINITY_XP_0239	aldo-ket	Quercus_suber	61.80	0.00
TRINITY_GAX7938	hypothet	Chlamydomonas_eusti	61.80	0.00
TRINITY_GAX8351	hypothet	Chlamydomonas_eusti	61.80	0.00
TRINITY_GAV6882	Ribosom	Cephalotus_follicul	61.80	0.00
TRINITY_XP_0227	NHP2-li	Durio_zibethinus	61.80	0.00
TRINITY_KXZ5336	hypothet	Gonium_pectorale	61.80	0.00
TRINITY_XP_0058	hypothet	Chlorella_variabili	61.80	0.00
TRINITY_GAX8631	hypothet	Chlamydomonas_eusti	61.80	0.00
TRINITY_GAX8624	hypothet	Chlamydomonas_eusti	61.80	0.00
TRINITY_GAQ8928	TCP-1/c	Klebsormidium_niten	61.80	0.00
TRINITY_KXZ5513	hypothet	Gonium_pectorale	61.80	0.00
TRINITY_BAX3899	phospho	Chlamydomonas_appla	61.80	0.00
TRINITY_GAQ8931	ABC tra	Klebsormidium_niten	61.80	0.00

TRINITY_PRW6021[illegible]	Transcri[illegible]	Chlorella_sorokinia	61.80	0.00
TRINITY_KXZ5001[illegible]	hypothet	Gonium_pectorale	61.80	0.00
TRINITY_KXZ4092[illegible]	hypothet	Gonium_pectorale	61.80	0.00
TRINITY_GAX7315[illegible]	hypothet	Chlamydomonas_eusti	61.80	0.00
TRINITY_KXZ4918[illegible]	hypothet	Gonium_pectorale	61.80	0.00
TRINITY_GAX8121[illegible]	hypothet	Chlamydomonas_eusti	61.80	0.00
TRINITY_KXZ4422[illegible]	hypothet	Gonium_pectorale	61.80	0.00
TRINITY_PNW7292[illegible]	hypothet	Chlamydomonas_reinh	61.80	0
TRINITY_GAX7907[illegible]	hypothet	Chlamydomonas_eusti	61.80	0.00
TRINITY_PNW6990[illegible]	hypothet	Chlamydomonas_reinh	61.80	0.00

TRINITY_GAX7511	hypothet	Chlamydomonas_eusti	61.80	0.00
TRINITY_XP_0017(	predicte	Chlamydomonas_reinh	61.80	0.00
TRINITY_PNH0296	Monothi	Tetrabaena_socialis	61.80	0.00
TRINITY_XP_0184	PREDICTI	Raphanus_sativus	61.70	0.00
TRINITY_GAQ7958	DNA rep	Klebsormidium_niten	61.70	0.00
TRINITY_EFJ2629	hypothet	Selaginella_moellen	61.70	0.00
TRINITY_XP_0042	small n	Solanum_lycopersicu	61.70	0.00
TRINITY_XP_0042	small n	Solanum_lycopersicu	61.70	0.00
TRINITY_NP_0011	unchara	Zea_mays	61.70	0.00
TRINITY_GAQ9035	copper/	Klebsormidium_niten	61.70	0.00
TRINITY_YP_0013	putative	Chlorokybus_atmophy	61.70	0.00
TRINITY_XP_0014	predicte	Ostreococcus_sp._'l	61.70	0.00
TRINITY_XP_0219	T-comple	Helianthus_annuus	61.70	0.00
TRINITY_PNW7786	hypothet	Chlamydomonas_reinh	61.70	0.00
TRINITY_GAX7393	hypothet	Chlamydomonas_eusti	61.70	0.00
TRINITY_XP_0139	hypothet	Monoraphidium_negle	61.70	0.00
TRINITY_GAX8298	hypothet	Chlamydomonas_eusti	61.70	0.00
TRINITY_PNH1101	Mitochor	Tetrabaena_socialis	61.70	0.00
TRINITY_GAX7458	hypothet	Chlamydomonas_eusti	61.70	0.00
TRINITY_PNH1025	Outer r	Tetrabaena_socialis	61.70	0.00
TRINITY_XP_0029	hypothet	Volvox_carteri	61.70	0.00
TRINITY_GAX7269	hypothet	Chlamydomonas_eusti	61.70	0.00
TRINITY_PNW7158	hypothet	Chlamydomonas_reinh	61.70	0.00
TRINITY_GAX7424	hypothet	Chlamydomonas_eusti	61.70	0.00
TRINITY_PNW7313	hypothet	Chlamydomonas_reinh	61.70	0.00
TRINITY_GBG6764	hypothet	Chara_braunii	61.70	0.00
TRINITY_GAX8150	hypothet	Chlamydomonas_eusti	61.70	0.00
TRINITY_KXZ5665	hypothet	Gonium_pectorale	61.70	0.00
TRINITY_PNW7348	hypothet	Chlamydomonas_reinh	61.70	0.00
TRINITY_GAX7807	hypothet	Chlamydomonas_eusti	61.70	0.00
TRINITY_XP_0139	hypothet	Monoraphidium_negle	61.70	0.00
TRINITY_GAX8580	hypothet	Chlamydomonas_eusti	61.70	0.00
TRINITY_XP_0139	dynein	Monoraphidium_negle	61.70	0.00
TRINITY_GAX8148	hypothet	Chlamydomonas_eusti	61.70	0.00
TRINITY_XP_0016	signal	Chlamydomonas_reinh	61.70	0.00
TRINITY_XP_0029	hypothet	Volvox_carteri	61.70	0.00
TRINITY_XP_0239	ras-like	Quercus_suber	61.70	0.00
TRINITY_GAQ8892	hypothet	Klebsormidium_niten	61.70	0.00
TRINITY_GAX8624	hypothet	Chlamydomonas_eusti	61.70	0.00
TRINITY_XP_0029	hypothet	Volvox_carteri	61.70	0.00
TRINITY_GAX7398	hypothet	Chlamydomonas_eusti	61.70	0.00
TRINITY_RAL4803	hypothet	Cuscuta_australis	61.70	0.00
TRINITY_GAX8205	hypothet	Chlamydomonas_eusti	61.70	0.00
TRINITY_GAX7678	hypothet	Chlamydomonas_eusti	61.70	0.00
TRINITY_XP_0238	probable	Quercus_suber	61.60	0.00
TRINITY_XP_0271	V-type	Coffea_arabica	61.60	0.00
TRINITY_PNH0920	Peroxir	Tetrabaena_socialis	61.60	0.00
TRINITY_OVA1319	Tetrapy	Macleaya_cordata	61.60	0.00
TRINITY_XP_0058	hypothet	Chlorella_variabili	61.60	0.00
TRINITY_XP_0238	acetate	Quercus_suber	61.60	0.00
TRINITY_XP_0228	Tetrahy	Ostreococcus_tauri	61.60	0.00
TRINITY_BAJ8540	predicte	Hordeum_vulgare	61.60	0.00
TRINITY_XP_0124	PREDICTI	Gossypium_raimondii	61.60	0.00
TRINITY_XP_0016	predicte	Chlamydomonas_reinh	61.60	0.00
TRINITY_OWM7047	hypothet	Punica_granatum	61.60	0.00
TRINITY_XP_0066	PREDICTI	Oryza_brachyantha	61.60	0.00
TRINITY_PSC7229	Yippee	Micractinium_conduc	61.60	0.00
TRINITY_XP_0258	DEAD-bo	Panicum_hallii	61.60	0.00
TRINITY_GBF9841	hypothet	Raphidocelis_subcap	61.60	0.00
TRINITY_NP_0012	unchara	Glycine_max	61.60	0.00
TRINITY_EPS7106	hypothet	Genlisea_aurea	61.60	0.00
TRINITY_GAX7742	hypothet	Chlamydomonas_eusti	61.60	0.00
TRINITY_XP_0056	polyade	Coccomyxa_subellips	61.60	0.00
TRINITY_KXZ5121	hypothet	Gonium_pectorale	61.60	0.00
TRINITY_XP_0029	hypothet	Volvox_carteri	61.60	0.00
TRINITY_EFJ2312	hypothet	Selaginella_moellen	61.60	0.00

TRINITY_EFJ08658	hypothet	Selaginella_moellen	61.60	0.00
TRINITY_PNW86229	hypothet	Chlamydomonas_reinh	61.60	0.00
TRINITY_XP_00294	mitochor	Volvox_carteri	61.60	0.00
TRINITY_KXZ50996	hypothet	Gonium_pectorale	61.60	0.00
TRINITY_XP_00169	predicte	Chlamydomonas_reinh	61.60	0.00
TRINITY_XP_00169	serine/t	Chlamydomonas_reinh	61.60	0.00
TRINITY_GAX85902	hypothet	Chlamydomonas_eusti	61.60	0.00
TRINITY_XP_00169	predicte	Chlamydomonas_reinh	61.60	0.00
TRINITY_KXZ51382	hypothet	Gonium_pectorale	61.60	0.00
TRINITY_GAX85753	hypothet	Chlamydomonas_eusti	61.60	0.00
TRINITY_GAX79138	hypothet	Chlamydomonas_eusti	61.60	0.00
TRINITY_GBF94202	cell div	Raphidocelis_subcap	61.60	0.00
TRINITY_KXZ42459	hypothet	Gonium_pectorale	61.60	0.00
TRINITY_PNW86256	hypothet	Chlamydomonas_reinh	61.60	0.00
TRINITY_PNW85730	hypothet	Chlamydomonas_reinh	61.60	0.00
TRINITY_PNW74877	hypothet	Chlamydomonas_reinh	61.60	0.00
TRINITY_GAX72618	hypothet	Chlamydomonas_eusti	61.60	0
TRINITY_XP_00564	anti-sil	Coccomyxa_subellips	61.60	0.00
TRINITY_GAX72717	hypothet	Chlamydomonas_eusti	61.60	0.00
TRINITY_KXZ54643	hypothet	Gonium_pectorale	61.60	0.00
TRINITY_OAE29611	hypothet	Marchantia_polymorp	61.60	0.00
TRINITY_PNX55116	glutamyl	Trifolium_pratense	61.50	0.00
TRINITY_XP_02389	unchara	Quercus_suber	61.50	0.00
TRINITY_GBG63342	hypothet	Chara_braunii	61.50	0.00
TRINITY_XP_02098	GATA tra	Arachis_duranensis	61.50	0.00
TRINITY_XP_02437	unchara	Physcomitrella_pate	61.50	0.00
TRINITY_XP_00564	Actin/ac	Coccomyxa_subellips	61.50	0.00
TRINITY_GAQ88609	hypothet	Klebsormidium_niten	61.50	0.00
TRINITY_XP_00295	eukaryot	Volvox_carteri	61.50	0.00
TRINITY_XP_02283	V-ATPase	Ostreococcus_tauri	61.50	0.00
TRINITY_XP_00978	PREDICTE	Nicotiana_sylvestri	61.50	0.00
TRINITY_KXZ53691	hypothet	Gonium_pectorale	61.50	0.00
TRINITY_XP_00250	predicte	Micromonas_commoda	61.50	0.00
TRINITY_XP_02024	ankyrin-	Asparagus_officinal	61.50	0.00
TRINITY_RHN40324	putative	Medicago_truncatula	61.50	0.00
TRINITY_XP_02389	homocit	Quercus_suber	61.50	0.00
TRINITY_GAX72933	hypothet	Chlamydomonas_eusti	61.50	0.00
TRINITY_GAX77539	hypothet	Chlamydomonas_eusti	61.50	0.00
TRINITY_XP_00169	predicte	Chlamydomonas_reinh	61.50	0.00
TRINITY_ERM96741	hypothet	Amborella_trichopod	61.50	0.00
TRINITY_BAK02186	predicte	Hordeum_vulgare	61.50	0.00
TRINITY_PNH07594	Solute c	Tetrabaena_socialis	61.50	0.00
TRINITY_XP_02639	T-comple	Papaver_somniferum	61.50	0.00
TRINITY_XP_00169	small ra	Chlamydomonas_reinh	61.50	0.00
TRINITY_ONL95843	Small nu	Zea_mays	61.50	0.00
TRINITY_KXZ44386	hypothet	Gonium_pectorale	61.50	0.00
TRINITY_KXZ41940	hypothet	Gonium_pectorale	61.50	0.00
TRINITY_KXZ52275	hypothet	Gonium_pectorale	61.50	0.00
TRINITY_PNW83113	hypothet	Chlamydomonas_reinh	61.50	0.00
TRINITY_PNW84898	hypothet	Chlamydomonas_reinh	61.50	0.00
TRINITY_KXZ44045	hypothet	Gonium_pectorale	61.50	0.00
TRINITY_XP_02352	transcri	Cucurbita_pepo	61.50	0.00
TRINITY_XP_00294	hypothet	Volvox_carteri	61.50	0.00
TRINITY_GAX85700	hypothet	Chlamydomonas_eusti	61.50	0.00
TRINITY_GAX81797	hypothet	Chlamydomonas_eusti	61.50	0.00
TRINITY_GAX78872	hypothet	Chlamydomonas_eusti	61.50	0.00
TRINITY_XP_00170	predicte	Chlamydomonas_reinh	61.50	0.00
TRINITY_XP_00294	hypothet	Volvox_carteri	61.50	0.00
TRINITY_PNW83046	hypothet	Chlamydomonas_reinh	61.50	0.00
TRINITY_XP_00702	PREDICTE	Theobroma_cacao	61.50	0.00
TRINITY_GAX77698	hypothet	Chlamydomonas_eusti	61.50	0.00
TRINITY_PNW77316	hypothet	Chlamydomonas_reinh	61.50	0.00
TRINITY_GAQ78769	hypothet	Klebsormidium_niten	61.50	0.00
TRINITY_GAX84206	hypothet	Chlamydomonas_eusti	61.50	0.00
TRINITY_XP_00169	cytochro	Chlamydomonas_reinh	61.50	0.00
TRINITY_XP_01563	importir	Oryza_sativa	61.40	0.00

TRINITY_XP_0279(	casein }	Vigna_unguiculata	61.40	0.00
TRINITY_XP_0016!	flagella	Chlamydomonas_reinh	61.40	0.00
TRINITY_GBG9125‹	hypothet	Chara_braunii	61.40	0.00
TRINITY_XP_0075]	PREDICTI	Bathycoccus_prasinc	61.40	0.00
TRINITY_PNW7082!	hypothet	Chlamydomonas_reinh	61.40	0.00
TRINITY_XP_0217]	DEAD-bo›	Chenopodium_quinoa	61.40	0.00
TRINITY_XP_0024‹	probable	Sorghum_bicolor	61.40	0.00
TRINITY_GBG7003‹	hypothet	Chara_braunii	61.40	0.00
TRINITY_PNW7321]	hypothet	Chlamydomonas_reinh	61.40	0.00
TRINITY_XP_0235‹	succinat	Cucurbita_pepo	61.40	0.00
TRINITY_PNW7496!	hypothet	Chlamydomonas_reinh	61.40	0.00
TRINITY_GAX7975‹	hypothet	Chlamydomonas_eusti	61.40	0.00
TRINITY_O65315.]	RecName:	Coleochaete_scutata	61.40	0.00
TRINITY_ONK5940(	uncharac	Asparagus_officinal	61.40	0.00
TRINITY_KXZ4887(	hypothet	Gonium_pectorale	61.40	0.00
TRINITY_XP_0029!	hypothet	Volvox_carteri	61.40	0.00
TRINITY_XP_0146(	flap enc	Setaria_italica	61.40	0.00
TRINITY_KXZ5549(	hypothet	Gonium_pectorale	61.40	0.00
TRINITY_GAX7411]	hypothet	Chlamydomonas_eusti	61.40	0.00
TRINITY_XP_0016!	signal p	Chlamydomonas_reinh	61.40	0.00
TRINITY_XP_0029‹	3'5'-cy(	Volvox_carteri	61.40	0.00
TRINITY_GAX7600(	hypothet	Chlamydomonas_eusti	61.40	0.00
TRINITY_XP_0176‹	PREDICTI	Gossypium_arboreum	61.40	0.00
TRINITY_AGC1298'	long fla	Chlamydomonas_reinh	61.40	0.00
TRINITY_XP_0029!	light-ha	Volvox_carteri	61.40	0.00
TRINITY_KZV1751]	dolichol	Dorcoceras_hygromet	61.40	0.00
TRINITY_OAP0894'	RNR1 [A	Arabidopsis_thalian	61.40	0.00
TRINITY_KXZ5541]	hypothet	Gonium_pectorale	61.40	0.00
TRINITY_XP_0029!	hypothet	Volvox_carteri	61.40	0.00
TRINITY_PNW7716]	hypothet	Chlamydomonas_reinh	61.40	0.00
TRINITY_XP_0270'	proteas(	Coffea_arabica	61.40	0.00
TRINITY_PRW4573(	lysine-s	Chlorella_sorokinia	61.40	0.00
TRINITY_PNW7253!	hypothet	Chlamydomonas_reinh	61.40	0.00
TRINITY_XP_0029‹	hypothet	Volvox_carteri	61.40	0.00
TRINITY_XP_0016!	predicte	Chlamydomonas_reinh	61.40	0.00
TRINITY_XP_0029!	hypothet	Volvox_carteri	61.40	0.00
TRINITY_AML7829]	putative	Pleurastrum_insigne	61.40	0.00
TRINITY_XP_0017(	predicte	Chlamydomonas_reinh	61.40	0.00
TRINITY_KXZ4646'	hypothet	Gonium_pectorale	61.40	0.00
TRINITY_XP_0029!	tRNA pse	Volvox_carteri	61.40	0.00
TRINITY_GAX8091]	hypothet	Chlamydomonas_eusti	61.40	0.00
TRINITY_XP_0228‹	Ribosoma	Ostreococcus_tauri	61.40	0.00
TRINITY_PNW7059]	hypothet	Chlamydomonas_reinh	61.40	0.00
TRINITY_XP_0029!	hypothet	Volvox_carteri	61.40	0.00
TRINITY_GAX8446‹	hypothet	Chlamydomonas_eusti	61.40	0.00
TRINITY_XP_0029‹	hypothet	Volvox_carteri	61.40	0.00
TRINITY_KMZ6147]	Protein	Zostera_marina	61.40	0.00
TRINITY_GAX7994'	hypothet	Chlamydomonas_eusti	61.40	0.00
TRINITY_XP_0025(	predicte	Micromonas_commoda	61.30	0.00
TRINITY_EFJ3361(	hypothet	Selaginella_moellen	61.30	0.00
TRINITY_ACF8016(	unknown	Zea_mays	61.30	0.00
TRINITY_PTQ3616!	hypothet	Marchantia_polymorp	61.30	0.00
TRINITY_XP_0029‹	hypothet	Volvox_carteri	61.30	0.00
TRINITY_GAX7949!	hypothet	Chlamydomonas_eusti	61.30	0.00
TRINITY_GBG5920!	Glycine	Chara_braunii	61.30	0.00
TRINITY_OAY8091'	Serine/t	Ananas_comosus	61.30	0.00
TRINITY_GAQ8802(	Ribosome	Klebsormidium_niten	61.30	0.00
TRINITY_XP_0056‹	eukaryot	Coccomyxa_subellips	61.30	0.00
TRINITY_KVI0579'	AAA+ ATI	Cynara_cardunculus	61.30	0.00
TRINITY_XP_0029!	hypothet	Volvox_carteri	61.30	0.00
TRINITY_XP_0056‹	polyader	Coccomyxa_subellips	61.30	0.00
TRINITY_XP_0277(	uncharac	Solanum_pennellii	61.30	0.00
TRINITY_KXZ5285‹	hypothet	Gonium_pectorale	61.30	0.00
TRINITY_PIA6133‹	hypothet	Aquilegia_coerulea	61.30	0.00
TRINITY_GAX8181(	hypothet	Chlamydomonas_eusti	61.30	0.00
TRINITY_XP_0029!	hypothet	Volvox_carteri	61.30	0.00

TRINITY_GAX7725	hypothet	Chlamydomonas_eusti	61.30	0.00
TRINITY_KXZ5439	hypothet	Gonium_pectorale	61.30	0.00
TRINITY_PNH0892	Protoher	Tetrabaena_socialis	61.30	0.00
TRINITY_GAX8305	hypothet	Chlamydomonas_eusti	61.30	0.00
TRINITY_PNW8817	hypothet	Chlamydomonas_reinh	61.30	0.00
TRINITY_XP_0029	hypothet	Volvox_carteri	61.30	0.00
TRINITY_GBG7157	hypothet	Chara_braunii	61.30	0.00
TRINITY_XP_0220	ubiquit	Helianthus_annuus	61.30	0.00
TRINITY_XP_0029	hypothet	Volvox_carteri	61.30	0.00
TRINITY_XP_0194	PREDICT	Lupinus_angustifoli	61.30	0.00
TRINITY_XP_0016	predict	Chlamydomonas_reinh	61.30	0.00
TRINITY_PNW7735	hypothet	Chlamydomonas_reinh	61.30	0.00
TRINITY_GAX8001	hypothet	Chlamydomonas_eusti	61.30	0.00
TRINITY_KXZ5409	hypothet	Gonium_pectorale	61.30	0.00
TRINITY_XP_0243	phospho	Physcomitrella_pate	61.30	0.00
TRINITY_XP_0017	predict	Chlamydomonas_reinh	61.30	0.00
TRINITY_BAG5542	hypothet	Trebouxiophyceae_sp	61.20	0.00
TRINITY_PIA6434	hypothet	Aquilegia_coerulea	61.20	0.00
TRINITY_RWR9801	LOW QUA	Cinnamomum_micranth	61.20	0.00
TRINITY_GAX7572	hypothet	Chlamydomonas_eusti	61.20	0.00
TRINITY_XP_0194	PREDICT	Lupinus_angustifoli	61.20	0.00
TRINITY_PNW8229	hypothet	Chlamydomonas_reinh	61.20	0.00
TRINITY_XP_0024	methion	Sorghum_bicolor	61.20	0.00
TRINITY_XP_0075	ABC tra	Bathycoccus_prasino	61.20	0.00
TRINITY_XP_0138	hypothet	Monoraphidium_negle	61.20	0.00
TRINITY_XP_0029	rab-rel	Volvox_carteri	61.20	0.00
TRINITY_GAX7306	hypothet	Chlamydomonas_eusti	61.20	0.00
TRINITY_PNW7392	hypothet	Chlamydomonas_reinh	61.20	0.00
TRINITY_XP_0201	cyclin-	Ananas_comosus	61.20	0.00
TRINITY_GAX8456	hypothet	Chlamydomonas_eusti	61.20	0.00
TRINITY_GAX8181	hypothet	Chlamydomonas_eusti	61.20	0.00
TRINITY_XP_0049	DNA dam	Setaria_italica	61.20	0.00
TRINITY_XP_0128	PREDICT	Erythranthe_guttata	61.20	0.00
TRINITY_XP_0017	3-methy	Chlamydomonas_reinh	61.20	0.00
TRINITY_KXZ4306	hypothet	Gonium_pectorale	61.20	0.00
TRINITY_XP_0029	ferropo	Volvox_carteri	61.20	0.00
TRINITY_PNH1240	Signal	Tetrabaena_socialis	61.20	0.00
TRINITY_GAX7627	hypothet	Chlamydomonas_eusti	61.20	0.00
TRINITY_PNW8038	hypothet	Chlamydomonas_reinh	61.20	0.00
TRINITY_GAX7725	hypothet	Chlamydomonas_eusti	61.20	0.00
TRINITY_GBF9542	hypothet	Raphidocelis_subcap	61.20	0.00
TRINITY_GBF9572	hypothet	Raphidocelis_subcap	61.20	0.00
TRINITY_RVW5219	Multidr	Vitis_vinifera	61.20	0.00
TRINITY_XP_0139	purine	Monoraphidium_negle	61.20	0.00
TRINITY_XP_0025	dihydro	Micromonas_commoda	61.20	0.00
TRINITY_PNH0528	ATP-dep	Tetrabaena_socialis	61.20	0.00
TRINITY_GAX8026	hypothet	Chlamydomonas_eusti	61.20	0.00
TRINITY_PNW7948	hypothet	Chlamydomonas_reinh	61.20	0.00
TRINITY_XP_0056	DUF292-	Coccomyxa_subellips	61.20	0.00
TRINITY_XP_0016	predict	Chlamydomonas_reinh	61.20	0.00
TRINITY_XP_0202	casein	Asparagus_officinal	61.20	0.00
TRINITY_XP_0016	dynein	Chlamydomonas_reinh	61.20	0.00
TRINITY_XP_0229	MOB kin	Cucurbita_maxima	61.10	0.00
TRINITY_XP_0158	unchara	Ziziphus_jujuba	61.10	0.00
TRINITY_OAE2663	hypothet	Marchantia_polymorp	61.10	0.00
TRINITY_GAX8032	hypothet	Chlamydomonas_eusti	61.10	0.00
TRINITY_CAI8489	putativ	Medicago_sativa	61.10	0.00
TRINITY_GAQ8481	ubiquit	Klebsormidium_niten	61.10	0.00
TRINITY_ESR3877	hypothet	Citrus_clementina	61.10	0.00
TRINITY_GAX8242	hypothet	Chlamydomonas_eusti	61.10	0.00
TRINITY_GAU2165	hypothet	Trifolium_subterran	61.10	0.00
TRINITY_RAL4980	hypothet	Cuscuta_australis	61.10	0.00
TRINITY_GAX7922	hypothet	Chlamydomonas_eusti	61.10	0.00
TRINITY_XP_0058	hypothet	Chlorella_variabili	61.10	0.00
TRINITY_PNW8438	hypothet	Chlamydomonas_reinh	61.10	0.00
TRINITY_PRW2113	RING fi	Chlorella_sorokinia	61.10	0.00

TRINITY_PSC7516	DEAD-dor	Micractinium_conduc	61.10	0.00
TRINITY_GAX8127	hypothet	Chlamydomonas_eusti	61.10	0.00
TRINITY_XP_0029	tubulin	Volvox_carteri	61.10	0.00
TRINITY_XP_0029	hypothet	Volvox_carteri	61.10	0.00
TRINITY_KXZ4624	hypothet	Gonium_pectorale	61.10	0.00
TRINITY_GAX8200	hypothet	Chlamydomonas_eusti	61.10	0.00
TRINITY_GAX7563	hypothet	Chlamydomonas_eusti	61.10	0.00
TRINITY_GAQ8017	homogent	Klebsormidium_niten	61.10	0.00
TRINITY_XP_0056	hypothet	Coccomyxa_subellips	61.10	0.00
TRINITY_XP_0110	PREDICTI	Populus_euphratica	61.10	0.00
TRINITY_GAX7597	hypothet	Chlamydomonas_eusti	61.10	0.00
TRINITY_XP_0017	acetyl (	Chlamydomonas_reinh	61.10	0.00
TRINITY_XP_0017	predicte	Chlamydomonas_reinh	61.10	0.00
TRINITY_XP_0029	hypothet	Volvox_carteri	61.10	0.00
TRINITY_GAX8134	hypothet	Chlamydomonas_eusti	61.10	0.00
TRINITY_GAX7615	hypothet	Chlamydomonas_eusti	61.10	0.00
TRINITY_RZB6834	ATP-depe	Glycine_soja	61.10	0.00
TRINITY_GAQ9356	hypothet	Klebsormidium_niten	61.10	0.00
TRINITY_XP_0202	putative	Cajanus_cajan	61.10	0.00
TRINITY_GAX8174	hypothet	Chlamydomonas_eusti	61.10	0.00
TRINITY_PHT6190	Phosphat	Capsicum_annuum	61.10	0.00
TRINITY_PSC7669	60S aci	Micractinium_conduc	61.10	0.00
TRINITY_KXZ4771	hypothet	Gonium_pectorale	61.10	0.00
TRINITY_EFJ1747	hypothet	Selaginella_moellen	61.00	0.00
TRINITY_PRW4538	ATP-depe	Chlorella_sorokinia	61.00	0.00
TRINITY_XP_0029	vesicle	Volvox_carteri	61.00	0.00
TRINITY_XP_0106	PREDICTI	Beta_vulgaris	61.00	0.00
TRINITY_XP_0136	PREDICTI	Brassica_oleracea	61.00	0.00
TRINITY_XP_0194	PREDICTI	Lupinus_angustifoli	61.00	0.00
TRINITY_BAU7155	parkin (	Marchantia_polymorp	61.00	0.00
TRINITY_GBF8793	ATP synt	Raphidocelis_subcap	61.00	0.00
TRINITY_RMZ5518	hypothet	Auxenochlorella_prc	61.00	0.00
TRINITY_GAX8002	hypothet	Chlamydomonas_eusti	61.00	0.00
TRINITY_GBF9088	phosphat	Raphidocelis_subcap	61.00	0.00
TRINITY_KXZ5075	hypothet	Gonium_pectorale	61.00	0.00
TRINITY_PRW6038	glyoxal	Chlorella_sorokinia	61.00	0.00
TRINITY_XP_0239	ATP-depe	Quercus_suber	61.00	0.00
TRINITY_GAX8155	hypothet	Chlamydomonas_eusti	61.00	0.00
TRINITY_PNH1083	Desiccat	Tetrabaena_socialis	61.00	0.00
TRINITY_XP_0035	malate (	Medicago_truncatula	61.00	0.00
TRINITY_XP_0029	hypothet	Volvox_carteri	61.00	0.00
TRINITY_XP_0093	PREDICTI	Musa_acuminata	61.00	0.00
TRINITY_GAQ9343	hypothet	Klebsormidium_niten	61.00	0.00
TRINITY_XP_0239	dynein h	Quercus_suber	61.00	0.00
TRINITY_GAX7394	hypothet	Chlamydomonas_eusti	61.00	0.00
TRINITY_PNW8126	hypothet	Chlamydomonas_reinh	61.00	0.00
TRINITY_GAX7965	hypothet	Chlamydomonas_eusti	61.00	0.00
TRINITY_GAX7636	hypothet	Chlamydomonas_eusti	61.00	0.00
TRINITY_XP_0029	chloropl	Volvox_carteri	61.00	0.00
TRINITY_XP_0084	PREDICTI	Cucumis_melo	61.00	0.00
TRINITY_PRW6156	Retrovi	Chlorella_sorokinia	61.00	0.00
TRINITY_GAX7891	hypothet	Chlamydomonas_eusti	61.00	0.00
TRINITY_GAX7714	hypothet	Chlamydomonas_eusti	61.00	0.00
TRINITY_GAX7276	hypothet	Chlamydomonas_eusti	61.00	0.00
TRINITY_GBF9776	peptidas	Raphidocelis_subcap	61.00	0.00
TRINITY_PKA6203	60S ribo	Apostasia_shenzheni	61.00	0.00
TRINITY_XP_0056	exonucle	Coccomyxa_subellips	61.00	0.00
TRINITY_GBF9417	hypothet	Raphidocelis_subcap	61.00	0.00
TRINITY_GAX8520	hypothet	Chlamydomonas_eusti	61.00	0.00
TRINITY_GAX8034	hypothet	Chlamydomonas_eusti	61.00	0.00
TRINITY_GAX7753	hypothet	Chlamydomonas_eusti	61.00	0.00
TRINITY_GBF9846	hypothet	Raphidocelis_subcap	61.00	0.00
TRINITY_PNW8404	hypothet	Chlamydomonas_reinh	61.00	0.00
TRINITY_CAX2743	Na P-typ	Marchantia_polymorp	61.00	0.00
TRINITY_XP_0113	60S aci	Auxenochlorella_prc	60.90	0.00
TRINITY_XP_0068	ubiquit	Amborella_trichopod	60.90	0.00

TRINITY_ABK2260(	unknown	Picea_sitchensis	60.90	0.00
TRINITY_KZV1532	hypothet	Dorcoceras_hygromet	60.90	0.00
TRINITY_KQK0232	hypothet	Brachypodium_distac	60.90	0.00
TRINITY_PIN0705(	Molecula	Handroanthus_impeti	60.90	0.00
TRINITY_KXZ5123	hypothet	Gonium_pectorale	60.90	0.00
TRINITY_XP_0270	iron-su	Coffea_arabica	60.90	0.00
TRINITY_EXB3704	Coatome	Morus_notabilis	60.90	0.00
TRINITY_XP_0191	PREDICTI	Ipomoea_nil	60.90	0.00
TRINITY_XP_0106	PREDICTI	Beta_vulgaris	60.90	0.00
TRINITY_XP_0029	hypothet	Volvox_carteri	60.90	0.00
TRINITY_XP_0029	hypothet	Volvox_carteri	60.90	0.00
TRINITY_XP_0044	protein	Cicer_arietinum	60.90	0.00
TRINITY_XP_0016	predicte	Chlamydomonas_reinh	60.90	0.00
TRINITY_XP_0083	PREDICTI	Malus_domestica	60.90	0.00
TRINITY_XP_0056	hypothet	Coccomyxa_subellips	60.90	0.00
TRINITY_GBF9399	calcium	Raphidocelis_subcap	60.90	0.00
TRINITY_XP_0029	hypothet	Volvox_carteri	60.90	0.00
TRINITY_XP_0016	predicte	Chlamydomonas_reinh	60.90	0.00
TRINITY_KXZ5309	hypothet	Gonium_pectorale	60.90	0.00
TRINITY_XP_0058	GTP bin	Chlorella_variabili	60.90	0.00
TRINITY_GAX7275	hypothet	Chlamydomonas_eusti	60.90	0.00
TRINITY_GAX7710	hypothet	Chlamydomonas_eusti	60.90	0.00
TRINITY_XP_0016	monooxy	Chlamydomonas_reinh	60.90	0.00
TRINITY_XP_0029	luminal	Volvox_carteri	60.90	0.00
TRINITY_XP_0017	rhomboi	Chlamydomonas_reinh	60.90	0.00
TRINITY_KXZ4617	hypothet	Gonium_pectorale	60.90	0.00
TRINITY_PNW7140	hypothet	Chlamydomonas_reinh	60.90	0.00
TRINITY_GAX7773	hypothet	Chlamydomonas_eusti	60.90	0.00
TRINITY_GAX8239	hypothet	Chlamydomonas_eusti	60.90	0.00
TRINITY_XP_0029	hypothet	Volvox_carteri	60.90	0.00
TRINITY_PNW7502	hypothet	Chlamydomonas_reinh	60.90	0.00
TRINITY_GAQ8407	fructose	Klebsormidium_niten	60.90	0.00
TRINITY_XP_0029	hypothet	Volvox_carteri	60.90	0.00
TRINITY_GAX8150	hypothet	Chlamydomonas_eusti	60.90	0.00
TRINITY_XP_0245	glycine	Selaginella_moellen	60.90	0
TRINITY_XP_0056	lipoic a	Coccomyxa_subellips	60.90	0.00
TRINITY_XP_0016	cryptoc	Chlamydomonas_reinh	60.90	0.00
TRINITY_XP_0016	predicte	Chlamydomonas_reinh	60.90	0.00
TRINITY_GAX7265	hypothet	Chlamydomonas_eusti	60.90	0
TRINITY_KXZ4915	hypothet	Gonium_pectorale	60.90	0.00
TRINITY_XP_0075	H/ACA r	Bathycoccus_prasinc	60.90	0.00
TRINITY_BAJ8597	predicte	Hordeum_vulgare	60.80	0.00
TRINITY_GAQ7856	putative	Klebsormidium_niten	60.80	0.00
TRINITY_NP_0011	unchara	Zea_mays	60.80	0.00
TRINITY_OAE2751	hypothet	Marchantia_polymorp	60.80	0.00
TRINITY_XP_0016	sugar 1,	Chlamydomonas_reinh	60.80	0.00
TRINITY_GAX7645	hypothet	Chlamydomonas_eusti	60.80	0.00
TRINITY_PSS3171	Fructose	Actinidia_chinensis	60.80	0.00
TRINITY_GAX8494	hypothet	Chlamydomonas_eusti	60.80	0.00
TRINITY_GBF9099	hypothet	Raphidocelis_subcap	60.80	0.00
TRINITY_GAX8204	hypothet	Chlamydomonas_eusti	60.80	0.00
TRINITY_XP_0094	PREDICTI	Musa_acuminata	60.80	0.00
TRINITY_GAQ8591	3-hydro	Klebsormidium_niten	60.80	0.00
TRINITY_BAK0099	predicte	Hordeum_vulgare	60.80	0.00
TRINITY_GBF8936	adenylat	Raphidocelis_subcap	60.80	0.00
TRINITY_PNR6053	hypothet	Physcomitrella_pate	60.80	0.00
TRINITY_XP_0016	predicte	Chlamydomonas_reinh	60.80	0.00
TRINITY_KXZ5683	hypothet	Gonium_pectorale	60.80	0.00
TRINITY_GAX7941	hypothet	Chlamydomonas_eusti	60.80	0.00
TRINITY_GAX7559	hypothet	Chlamydomonas_eusti	60.80	0.00
TRINITY_OAE3095	hypothet	Marchantia_polymorp	60.80	0.00
TRINITY_PSC7583	Sterol-4	Micractinium_conduc	60.80	0.00
TRINITY_GBG7678	hypothet	Chara_braunii	60.80	0.00
TRINITY_XP_0058	hypothet	Chlorella_variabili	60.80	0.00
TRINITY_GAX8116	hypothet	Chlamydomonas_eusti	60.80	0.00
TRINITY_XP_0058	hypothet	Chlorella_variabili	60.80	0.00

TRINITY_XP_0029!	hypothet	Volvox_carteri	60.80	0.00
TRINITY_BAF4422	malate (	Iris_x_hollandica	60.80	0.00
TRINITY_RMZ5257	hypothet	Auxenochlorella_prc	60.80	0.00
TRINITY_P0C8M8.1	RecName:	Zea_mays	60.80	0.00
TRINITY_XP_0200	ATP-depe	Ananas_comosus	60.80	0.00
TRINITY_KXZ5377	hypothet	Gonium_pectorale	60.80	0.00
TRINITY_PNH0983	Polyubic	Tetrabaena_socialis	60.80	0.00
TRINITY_GAX7814	hypothet	Chlamydomonas_eusti	60.80	0.00
TRINITY_XP_0172	PREDICTI	Daucus_carota	60.80	0.00
TRINITY_XP_0221	malignar	Momordica_charantia	60.80	0.00
TRINITY_XP_0029	molecula	Volvox_carteri	60.80	0.00
TRINITY_KXZ5546	hypothet	Gonium_pectorale	60.80	0
TRINITY_KXZ5446	hypothet	Gonium_pectorale	60.80	0.00
TRINITY_GAX7839	hypothet	Chlamydomonas_eusti	60.80	0.00
TRINITY_GAX8627	hypothet	Chlamydomonas_eusti	60.80	0.00
TRINITY_PNH0960	Catheps:	Tetrabaena_socialis	60.80	0.00
TRINITY_GAX7667	hypothet	Chlamydomonas_eusti	60.80	0.00
TRINITY_XP_0025	predicte	Micromonas_commoda	60.80	0.00
TRINITY_GAX8549	hypothet	Chlamydomonas_eusti	60.80	0.00
TRINITY_XP_0030	predicte	Micromonas_pusilla	60.80	0.00
TRINITY_XP_0238	40S ribo	Quercus_suber	60.80	0.00
TRINITY_XP_0243	uncharac	Physcomitrella_pate	60.70	0.00
TRINITY_XP_0139	aldehyde	Monoraphidium_negle	60.70	0.00
TRINITY_XP_0104	PREDICTI	Camelina_sativa	60.70	0.00
TRINITY_XP_0221	acetyl-c	Momordica_charantia	60.70	0.00
TRINITY_GAX8562	hypothet	Chlamydomonas_eusti	60.70	0.00
TRINITY_XP_0084	PREDICTI	Cucumis_melo	60.70	0.00
TRINITY_XP_0184	PREDICTI	Raphanus_sativus	60.70	0.00
TRINITY_XP_0029	hypothet	Volvox_carteri	60.70	0.00
TRINITY_PNW7419	hypothet	Chlamydomonas_reinh	60.70	0.00
TRINITY_PNW7234	hypothet	Chlamydomonas_reinh	60.70	0.00
TRINITY_GBG8057	hypothet	Chara_braunii	60.70	0.00
TRINITY_KXZ5317	hypothet	Gonium_pectorale	60.70	0.00
TRINITY_AMB1969	3-hydrox	Taraxacum_kok-saghy	60.70	0.00
TRINITY_XP_0017	DnaJ-lik	Chlamydomonas_reinh	60.70	0.00
TRINITY_GAX7882	hypothet	Chlamydomonas_eusti	60.70	0.00
TRINITY_XP_0086	uncharac	Zea_mays	60.70	0.00
TRINITY_PSC7112	serine t	Micractinium_conduc	60.70	0.00
TRINITY_XP_0139	Pentatri	Monoraphidium_negle	60.70	0.00
TRINITY_GAX7320	hypothet	Chlamydomonas_eusti	60.70	0.00
TRINITY_BAU3704	ethanola	Chlamydomonas_asymm	60.70	0.00
TRINITY_XP_0138	hypothet	Monoraphidium_negle	60.70	0.00
TRINITY_GAX7452	hypothet	Chlamydomonas_eusti	60.70	0.00
TRINITY_XP_0042	40S ribo	Solanum_lycopersicu	60.70	0.00
TRINITY_PNH1099	hypothet	Tetrabaena_socialis	60.70	0.00
TRINITY_XP_0029	hypothet	Volvox_carteri	60.70	0.00
TRINITY_PNW7922	hypothet	Chlamydomonas_reinh	60.70	0.00
TRINITY_XP_0218	aldehyde	Spinacia_oleracea	60.70	0.00
TRINITY_GAX7579	hypothet	Chlamydomonas_eusti	60.70	0.00
TRINITY_GAX8507	hypothet	Chlamydomonas_eusti	60.70	0.00
TRINITY_GAX7273	hypothet	Chlamydomonas_eusti	60.70	0.00
TRINITY_KXZ5678	hypothet	Gonium_pectorale	60.70	0.00
TRINITY_PNW8528	hypothet	Chlamydomonas_reinh	60.70	0.00
TRINITY_KXZ4189	hypothet	Gonium_pectorale	60.70	0.00
TRINITY_GAX7502	hypothet	Chlamydomonas_eusti	60.70	0.00
TRINITY_XP_0172	PREDICTI	Daucus_carota	60.70	0.00
TRINITY_KDD7389	seleniur	Helicosporidium_sp.	60.60	0.00
TRINITY_XP_0238	isocitra	Quercus_suber	60.60	0.00
TRINITY_XP_0239	60S ribo	Quercus_suber	60.60	0.00
TRINITY_XP_0030	predicte	Micromonas_pusilla	60.60	0.00
TRINITY_XP_0238	GTP-bind	Quercus_suber	60.60	0.00
TRINITY_EMS4581	ABC trar	Triticum_urartu	60.60	0.00
TRINITY_AAV4181	pyruvate	Chlamydomonas_incer	60.60	0.00
TRINITY_KXZ4495	hypothet	Gonium_pectorale	60.60	0.00
TRINITY_XP_0029	hypothet	Volvox_carteri	60.60	0.00
TRINITY_XP_0056	2-amino-	Coccomyxa_subellips	60.60	0.00

TRINITY_CAA11269	polyubiq	Nicotiana_tabacum	60.60	0.00
TRINITY_GAX76395	hypothet	Chlamydomonas_eusti	60.60	0.00
TRINITY_PNH08037	Glutathi	Tetrabaena_socialis	60.60	0.00
TRINITY_XP_00170	predicte	Chlamydomonas_reinh	60.60	0.00
TRINITY_XP_00169	predicte	Chlamydomonas_reinh	60.60	0.00
TRINITY_GBF91731	hypothet	Raphidocelis_subcap	60.60	0.00
TRINITY_KZV15216	citrate	Dorcoceras_hygromet	60.60	0.00
TRINITY_GAX74763	hypothet	Chlamydomonas_eusti	60.60	0.00
TRINITY_XP_00584	hypothet	Chlorella_variabili	60.60	0.00
TRINITY_GAQ77581	ABC tran	Klebsormidium_niten	60.60	0.00
TRINITY_KMS64810	hypothet	Beta_vulgaris	60.60	0.00
TRINITY_KXZ51385	hypothet	Gonium_pectorale	60.60	0.00
TRINITY_GAX83459	hypothet	Chlamydomonas_eusti	60.60	0.00
TRINITY_RXH70648	hypothet	Malus_domestica	60.60	0.00
TRINITY_YP_00131	putative	Chlorokybus_atmophy	60.60	0.00
TRINITY_XP_00449	ADP-ribo	Cicer_arietinum	60.60	0.00
TRINITY_GAX78791	hypothet	Chlamydomonas_eusti	60.60	0.00
TRINITY_PTQ47551	hypothet	Marchantia_polymorp	60.60	0.00
TRINITY_XP_00424	serine/a	Solanum_lycopersicu	60.60	0.00
TRINITY_KXZ47291	hypothet	Gonium_pectorale	60.60	0.00
TRINITY_GBF96246	acyl-coe	Raphidocelis_subcap	60.60	0.00
TRINITY_KXZ53546	hypothet	Gonium_pectorale	60.60	0.00
TRINITY_XP_00169	U2 snRNP	Chlamydomonas_reinh	60.60	0.00
TRINITY_GAX75127	hypothet	Chlamydomonas_eusti	60.60	0.00
TRINITY_GAX75128	hypothet	Chlamydomonas_eusti	60.60	0.00
TRINITY_PNW70426	hypothet	Chlamydomonas_reinh	60.60	0.00
TRINITY_PNW78463	hypothet	Chlamydomonas_reinh	60.60	0.00
TRINITY_PNH05717	Regulato	Tetrabaena_socialis	60.60	0.00
TRINITY_KXZ50034	hypothet	Gonium_pectorale	60.60	0.00
TRINITY_KXZ45540	hypothet	Gonium_pectorale	60.60	0.00
TRINITY_KXZ49376	hypothet	Gonium_pectorale	60.60	0.00
TRINITY_GAX79695	hypothet	Chlamydomonas_eusti	60.60	0.00
TRINITY_PNW79678	hypothet	Chlamydomonas_reinh	60.60	0
TRINITY_EFJ04860	hypothet	Selaginella_moellen	60.60	0.00
TRINITY_RAL51299	hypothet	Cuscuta_australis	60.60	0.00
TRINITY_RIA05220	hypothet	Brassica_rapa	60.50	0.00
TRINITY_PRW60491	apoptoti	Chlorella_sorokinia	60.50	0.00
TRINITY_XP_00305	predicte	Micromonas_pusilla	60.50	0.00
TRINITY_GBF96403	glycosyl	Raphidocelis_subcap	60.50	0.00
TRINITY_XP_02392	ras-rela	Quercus_suber	60.50	0.00
TRINITY_GAX80193	hypothet	Chlamydomonas_eusti	60.50	0.00
TRINITY_GAQ92572	catalase	Klebsormidium_niten	60.50	0.00
TRINITY_XP_00169	fructose	Chlamydomonas_reinh	60.50	0.00
TRINITY_XP_00294	hypothet	Volvox_carteri	60.50	0.00
TRINITY_BAK02778	predicte	Hordeum_vulgare	60.50	0.00
TRINITY_PNW78548	hypothet	Chlamydomonas_reinh	60.50	0.00
TRINITY_EEC68421	hypothet	Oryza_sativa	60.50	0.00
TRINITY_XP_01390	Bifuncti	Monoraphidium_negle	60.50	0.00
TRINITY_GBF89866	hypothet	Raphidocelis_subcap	60.50	0.00
TRINITY_XP_00294	hypothet	Volvox_carteri	60.50	0.00
TRINITY_XP_00170	Emp24/gp	Chlamydomonas_reinh	60.50	0.00
TRINITY_GAX74707	hypothet	Chlamydomonas_eusti	60.50	0.00
TRINITY_XP_00168	mitochon	Chlamydomonas_reinh	60.50	0.00
TRINITY_XP_00141	predicte	Ostreococcus_sp._'l	60.50	0.00
TRINITY_KXZ51420	hypothet	Gonium_pectorale	60.50	0.00
TRINITY_GAX84295	hypothet	Chlamydomonas_eusti	60.50	0.00
TRINITY_GAQ85173	polyaden	Klebsormidium_niten	60.50	0.00
TRINITY_PNW70675	hypothet	Chlamydomonas_reinh	60.50	0.00
TRINITY_XP_00168	metal io	Chlamydomonas_reinh	60.50	0.00
TRINITY_PNW69900	hypothet	Chlamydomonas_reinh	60.50	0.00
TRINITY_KXZ53085	hypothet	Gonium_pectorale	60.50	0.00
TRINITY_XP_00169	NADH:ubi	Chlamydomonas_reinh	60.50	0.00
TRINITY_XP_00295	histone	Volvox_carteri	60.50	0.00
TRINITY_GAX73330	hypothet	Chlamydomonas_eusti	60.50	0.00
TRINITY_XP_00721	DNA-dire	Prunus_persica	60.50	0.00
TRINITY_XP_00295	adenylat	Volvox_carteri	60.50	0.00

TRINITY_GAX8257(	hypothet	Chlamydomonas_eusti	60.50	0.00
TRINITY_GAX7745(	hypothet	Chlamydomonas_eusti	60.50	0.00
TRINITY_KXZ5138(	hypothet	Gonium_pectorale	60.50	0.00
TRINITY_PNW8660(	hypothet	Chlamydomonas_reinh	60.50	0.00
TRINITY_XP_0156(	AP-1 cor	Oryza_sativa	60.50	0.00
TRINITY_GAX8400(	hypothet	Chlamydomonas_eusti	60.50	0.00
TRINITY_XP_0017(	radial s	Chlamydomonas_reinh	60.50	0.00
TRINITY_XP_0271(	uncharac	Coffea_eugenioides	60.50	0.00
TRINITY_XP_0227(	uncharac	Durio_zibethinus	60.50	0.00
TRINITY_BAG5542(	hypothet	Trebouxiophyceae_sp	60.40	0.00
TRINITY_GAQ8420(	ubiquiti	Klebsormidium_niten	60.40	0.00
TRINITY_XP_0228(	GPI manr	Ostreococcus_tauri	60.40	0.00
TRINITY_XP_0243(	superoxi	Physcomitrella_pate	60.40	0.00
TRINITY_XP_0016(	hypothet	Chlamydomonas_reinh	60.40	0.00
TRINITY_XP_0024(	O-phosph	Micromonas_commoda	60.40	0.00
TRINITY_GAX8351(	hypothet	Chlamydomonas_eusti	60.40	0.00
TRINITY_XP_0029(	hypothet	Volvox_carteri	60.40	0.00
TRINITY_XP_0029(	hypothet	Volvox_carteri	60.40	0.00
TRINITY_PNW7860(	hypothet	Chlamydomonas_reinh	60.40	0.00
TRINITY_XP_0271(	omega-ar	Coffea_eugenioides	60.40	0.00
TRINITY_GAX8470(	hypothet	Chlamydomonas_eusti	60.40	0.00
TRINITY_PNH0762(	Chaperor	Tetrabaena_socialis	60.40	0.00
TRINITY_PNW8706(	hypothet	Chlamydomonas_reinh	60.40	0.00
TRINITY_GAX8291(	hypothet	Chlamydomonas_eusti	60.40	0.00
TRINITY_XP_0029(	hypothet	Volvox_carteri	60.40	0.00
TRINITY_GBG5917(	hypothet	Chara_braunii	60.40	0.00
TRINITY_PNW8185(	hypothet	Chlamydomonas_reinh	60.40	0.00
TRINITY_AEA9262(	peroxire	Dunaliella_viridis	60.40	0.00
TRINITY_XP_0029(	hypothet	Volvox_carteri	60.40	0.00
TRINITY_XP_0014(	predicte	Ostreococcus_sp._'l	60.40	0.00
TRINITY_GAX7943(	hypothet	Chlamydomonas_eusti	60.40	0.00
TRINITY_GAX7385(	hypothet	Chlamydomonas_eusti	60.40	0.00
TRINITY_XP_0104(	PREDICTE	Camelina_sativa	60.40	0.00
TRINITY_GAX7691(	hypothet	Chlamydomonas_eusti	60.40	0.00
TRINITY_PRW5844(	isoleuci	Chlorella_sorokinia	60.40	0.00
TRINITY_XP_0029(	hypothet	Volvox_carteri	60.40	0.00
TRINITY_KXZ4703(	hypothet	Gonium_pectorale	60.40	0.00
TRINITY_XP_0029(	hypothet	Volvox_carteri	60.40	0.00
TRINITY_KXZ5586(	hypothet	Gonium_pectorale	60.40	0.00
TRINITY_PRW6055(	LOW PSI	Chlorella_sorokinia	60.40	0.00
TRINITY_KXZ4347(	hypothet	Gonium_pectorale	60.40	0.00
TRINITY_GAX7834(	hypothet	Chlamydomonas_eusti	60.40	0.00
TRINITY_PNW7066(	hypothet	Chlamydomonas_reinh	60.40	0.00
TRINITY_GAX7539(	hypothet	Chlamydomonas_eusti	60.40	0
TRINITY_XP_0094(	PREDICTE	Musa_acuminata	60.30	0.00
TRINITY_XP_0030(	flagella	Micromonas_pusilla	60.30	0.00
TRINITY_BAJ9524(	predicte	Hordeum_vulgare	60.30	0.00
TRINITY_XP_0201(	AP-4 cor	Ananas_comosus	60.30	0.00
TRINITY_GAX8595(	hypothet	Chlamydomonas_eusti	60.30	0.00
TRINITY_XP_0243(	chaperor	Physcomitrella_pate	60.30	0.00
TRINITY_XP_0239(	6-phosph	Quercus_suber	60.30	0.00
TRINITY_PSC6843(	Ubiquiti	Micractinium_conduc	60.30	0.00
TRINITY_PWA4602(	microtub	Artemisia_annua	60.30	0.00
TRINITY_XP_0227(	developr	Durio_zibethinus	60.30	0.00
TRINITY_GBF9342(	DNA-dire	Raphidocelis_subcap	60.30	0.00
TRINITY_XP_0056(	hypothet	Coccomyxa_subellips	60.30	0.00
TRINITY_KXZ4731(	hypothet	Gonium_pectorale	60.30	0.00
TRINITY_XP_0139(	UreA car	Monoraphidium_negle	60.30	0.00
TRINITY_XP_0128(	PREDICTE	Erythranthe_guttata	60.30	0.00
TRINITY_XP_0139(	putative	Monoraphidium_negle	60.30	0.00
TRINITY_XP_0063(	60S aci	Eutrema_salsugineum	60.30	0.00
TRINITY_XP_0029(	hypothet	Volvox_carteri	60.30	0.00
TRINITY_XP_0029(	hypothet	Volvox_carteri	60.30	0.00
TRINITY_GAX8503(	hypothet	Chlamydomonas_eusti	60.30	0.00
TRINITY_XP_0029(	hypothet	Volvox_carteri	60.30	0.00
TRINITY_GAX8157(	hypothet	Chlamydomonas_eusti	60.30	0.00

TRINITY_PNH1143	hypothet	Tetrabaena_socialis	60.30	0.00
TRINITY_PNW7373	hypothet	Chlamydomonas_reinh	60.30	0.00
TRINITY_GAQ9194	Cytochr	Klebsormidium_niten	60.30	0.00
TRINITY_GAX7262	hypothet	Chlamydomonas_eusti	60.30	0.00
TRINITY_XP_0058	hypothet	Chlorella_variabili	60.30	0.00
TRINITY_XP_0138	hypothet	Monoraphidium_negle	60.30	0.00
TRINITY_PSC7345	dephosp	Micractinium_conduc	60.30	0.00
TRINITY_XP_0139	Unchara	Monoraphidium_negle	60.30	0.00
TRINITY_XP_0101	RNA-bin	Morus_notabilis	60.30	0.00
TRINITY_GAX8054	hypothet	Chlamydomonas_eusti	60.30	0.00
TRINITY_PNW8278	hypothet	Chlamydomonas_reinh	60.30	0.00
TRINITY_PNW7069	hypothet	Chlamydomonas_reinh	60.30	0.00
TRINITY_PNW7867	hypothet	Chlamydomonas_reinh	60.30	0.00
TRINITY_KXZ4651	hypothet	Gonium_pectorale	60.30	0.00
TRINITY_XP_0056	cation	Coccomyxa_subellips	60.30	0.00
TRINITY_XP_0238	structu	Quercus_suber	60.30	0.00
TRINITY_BAK0075	predict	Hordeum_vulgare	60.30	0.00
TRINITY_XP_0029	hypothet	Volvox_carteri	60.30	0.00
TRINITY_PNW8535	hypothet	Chlamydomonas_reinh	60.30	0.00
TRINITY_GAX7808	hypothet	Chlamydomonas_eusti	60.30	0.00
TRINITY_GAQ9247	EF-Hand	Klebsormidium_niten	60.30	0.00
TRINITY_GAX8216	hypothet	Chlamydomonas_eusti	60.30	0.00
TRINITY_GAX8396	hypothet	Chlamydomonas_eusti	60.30	0.00
TRINITY_XP_0087	ABC tra	Phoenix_dactylifera	60.30	0.00
TRINITY_BAK0277	predict	Hordeum_vulgare	60.30	0.00
TRINITY_KXZ5419	hypothet	Gonium_pectorale	60.30	0.00
TRINITY_XP_0016	sodium/	Chlamydomonas_reinh	60.30	0.00
TRINITY_PNH0890	Elongat	Tetrabaena_socialis	60.30	0.00
TRINITY_GAV8298	RRM_1 d	Cephalotus_follicul	60.30	0.00
TRINITY_XP_0087	60S rib	Phoenix_dactylifera	60.20	0.00
TRINITY_GAQ8089	hypothet	Klebsormidium_niten	60.20	0.00
TRINITY_XP_0179	PREDICT	Theobroma_cacao	60.20	0.00
TRINITY_YP_6656	cytochr	Nephroselmis_olivac	60.20	0.00
TRINITY_RZC6065	hypothet	Papaver_somniferum	60.20	0.00
TRINITY_PKU6286	60S rib	Dendrobium_catenatu	60.20	0.00
TRINITY_XP_0238	dynein	Quercus_suber	60.20	0.00
TRINITY_RWR9572	4Fe-4S	Cinnamomum_micranth	60.20	0.00
TRINITY_XP_0128	PREDICT	Erythranthe_guttata	60.20	0.00
TRINITY_GAX7441	hypothet	Chlamydomonas_eusti	60.20	0.00
TRINITY_EFJ0945	hypothet	Selaginella_moellen	60.20	0.00
TRINITY_KXZ5036	hypothet	Gonium_pectorale	60.20	0.00
TRINITY_GAX7284	hypothet	Chlamydomonas_eusti	60.20	0.00
TRINITY_GAX7450	hypothet	Chlamydomonas_eusti	60.20	0.00
TRINITY_ABK2455	unknown	Picea_sitchensis	60.20	0.00
TRINITY_ONK5610	unchara	Asparagus_officinal	60.20	0.00
TRINITY_GAX7569	hypothet	Chlamydomonas_eusti	60.20	0.00
TRINITY_PNW7976	hypothet	Chlamydomonas_reinh	60.20	0.00
TRINITY_XP_0228	DNA-dir	Ostreococcus_tauri	60.20	0.00
TRINITY_KXZ5552	hypothet	Gonium_pectorale	60.20	0.00
TRINITY_EMS4635	Serine/	Triticum_urartu	60.20	0.00
TRINITY_XP_0056	hypothet	Coccomyxa_subellips	60.20	0.00
TRINITY_PPS1612	hypothet	Gossypium_barbadens	60.20	0.00
TRINITY_GBF9777	hypothet	Raphidocelis_subcap	60.20	0.00
TRINITY_PNW7883	hypothet	Chlamydomonas_reinh	60.20	0.00
TRINITY_GAX7643	hypothet	Chlamydomonas_eusti	60.20	0.00
TRINITY_RMZ5214	hypothet	Auxenochlorella_pro	60.20	0.00
TRINITY_EPS6935	hypothet	Genlisea_aurea	60.20	0.00
TRINITY_GAX7285	hypothet	Chlamydomonas_eusti	60.20	0.00
TRINITY_PNW7710	hypothet	Chlamydomonas_reinh	60.20	0.00
TRINITY_GAX7774	hypothet	Chlamydomonas_eusti	60.20	0.00
TRINITY_KXZ5377	hypothet	Gonium_pectorale	60.20	0.00
TRINITY_XP_0139	DNA pol	Monoraphidium_negle	60.20	0.00
TRINITY_GAX7550	hypothet	Chlamydomonas_eusti	60.20	0.00
TRINITY_GAX7942	hypothet	Chlamydomonas_eusti	60.20	0.00
TRINITY_KXZ4938	hypothet	Gonium_pectorale	60.20	0.00
TRINITY_PNH0886	Protein	Tetrabaena_socialis	60.20	0.00

TRINITY_XP_00169	phosphat	Chlamydomonas_reinh	60.20	0.00
TRINITY_KXZ56965	hypothet	Gonium_pectorale	60.20	0.00
TRINITY_PNW87181	hypothet	Chlamydomonas_reinh	60.20	0.00
TRINITY_XP_02390	dipeptic	Quercus_suber	60.20	0.00
TRINITY_AST08679	cytochrc	Chlorella_heliozoae	60.10	0.00
TRINITY_XP_00295	hypothet	Volvox_carteri	60.10	0.00
TRINITY_XP_00250	predicte	Micromonas_commoda	60.10	0.00
TRINITY_PSC69805	phosphol	Micractinium_conduc	60.10	0.00
TRINITY_PNW74128	hypothet	Chlamydomonas_reinh	60.10	0.00
TRINITY_GAX76342	hypothet	Chlamydomonas_eusti	60.10	0.00
TRINITY_XP_01024	PREDICTE	Nelumbo_nucifera	60.10	0.00
TRINITY_XP_00295	hypothet	Volvox_carteri	60.10	0.00
TRINITY_KXZ51727	hypothet	Gonium_pectorale	60.10	0.00
TRINITY_PSC68120	Methylti	Micractinium_conduc	60.10	0.00
TRINITY_PSC67729	putative	Micractinium_conduc	60.10	0.00
TRINITY_GAX73367	hypothet	Chlamydomonas_eusti	60.10	0.00
TRINITY_GAX77716	hypothet	Chlamydomonas_eusti	60.10	0.00
TRINITY_GAX82117	hypothet	Chlamydomonas_eusti	60.10	0.00
TRINITY_PNH01078	Retrovii	Tetrabaena_socialis	60.10	0.00
TRINITY_XP_00170	protein	Chlamydomonas_reinh	60.10	0.00
TRINITY_RWW91067	hypothet	Ensete_ventricosum	60.10	0.00
TRINITY_GAX76897	hypothet	Chlamydomonas_eusti	60.10	0.00
TRINITY_RID64137	hypothet	Brassica_rapa	60.10	0.00
TRINITY_PNW77156	hypothet	Chlamydomonas_reinh	60.10	0.00
TRINITY_PPR93764	hypothet	Gossypium_barbadens	60.10	0
TRINITY_PRW33706	low-CO2	Chlorella_sorokinia	60.10	0.00
TRINITY_GAX81666	hypothet	Chlamydomonas_eusti	60.10	0.00
TRINITY_XP_02131	uncharac	Sorghum_bicolor	60.00	0.00
TRINITY_BAF22198	Os07g061	Oryza_sativa	60.00	0.00
TRINITY_EOX98560	Dolichyl	Theobroma_cacao	60.00	0.00
TRINITY_PNH11946	BBSome-i	Tetrabaena_socialis	60.00	0.00
TRINITY_KDD76647	hypothet	Helicosporidium_sp.	60.00	0.00
TRINITY_GAQ78813	Dynein-a	Klebsormidium_niten	60.00	0.00
TRINITY_XP_01051	PREDICTE	Camelina_sativa	60.00	0.00
TRINITY_PIN08707	hypothet	Handroanthus_impeti	60.00	0.00
TRINITY_BAJ99260	predicte	Hordeum_vulgare	60.00	0.00
TRINITY_GAQ85240	hypothet	Klebsormidium_niten	60.00	0.00
TRINITY_XP_00306	predicte	Micromonas_pusilla	60.00	0.00
TRINITY_XP_02175	peptidyl	Chenopodium_quinoa	60.00	0.00
TRINITY_PSC67348	ATP:cob	Micractinium_conduc	60.00	0.00
TRINITY_ARQ87992	ABC tran	Helianthus_annuus	60.00	0.00
TRINITY_PKA54619	hypothet	Apostasia_shenzheni	60.00	0.00
TRINITY_GAQ82529	hypothet	Klebsormidium_niten	60.00	0.00
TRINITY_GAU17963	hypothet	Trifolium_subterran	60.00	0.00
TRINITY_OEL25669	S-adenos	Dichanthelium_oligo	60.00	0.00
TRINITY_XP_02582	obg-like	Panicum_hallii	60.00	0.00
TRINITY_XP_00685	zinc fir	Amborella_trichopod	60.00	0.00
TRINITY_GAX72786	hypothet	Chlamydomonas_eusti	60.00	0.00
TRINITY_XP_02288	D-2-hydi	Olea_europaea	60.00	0.00
TRINITY_GAX82150	hypothet	Chlamydomonas_eusti	60.00	0.00
TRINITY_GAX74302	hypothet	Chlamydomonas_eusti	60.00	0.00
TRINITY_GBF93626	hypothet	Raphidocelis_subcap	60.00	0.00
TRINITY_XP_00565	P-loop c	Coccomyxa_subellips	60.00	0.00
TRINITY_KXZ55116	hypothet	Gonium_pectorale	60.00	0.00
TRINITY_XP_00295	hypothet	Volvox_carteri	60.00	0.00
TRINITY_XP_02058	calmodul	Phalaenopsis_equest	60.00	0.00
TRINITY_EPS74505	hypothet	Genlisea_aurea	60.00	0.00
TRINITY_PNH12936	hypothet	Tetrabaena_socialis	60.00	0.00
TRINITY_XP_00298	cinnamoy	Selaginella_moellen	60.00	0.00
TRINITY_BAJ98875	predicte	Hordeum_vulgare	60.00	0.00
TRINITY_GAX84550	hypothet	Chlamydomonas_eusti	60.00	0.00
TRINITY_KXZ48733	hypothet	Gonium_pectorale	60.00	0.00
TRINITY_PNW84070	hypothet	Chlamydomonas_reinh	60.00	0.00
TRINITY_XP_01139	GAF doma	Auxenochlorella_pro	60.00	0.00
TRINITY_PNW77434	hypothet	Chlamydomonas_reinh	60.00	0.00
TRINITY_PNG99777	hypothet	Tetrabaena_socialis	60.00	0.00

TRINITY_XP_0058	hypothet	Chlorella_variabili	60.00	0.00
TRINITY_ABS1106	reverse	Malus_domestica	60.00	0.00
TRINITY_PSC7237	acetola	Micractinium_conduc	60.00	0.00
TRINITY_GAX7890	hypothet	Chlamydomonas_eusti	60.00	0.00
TRINITY_PNW7513	hypothet	Chlamydomonas_reinh	60.00	0.00
TRINITY_XP_0029	hypothet	Volvox_carteri	60.00	0.00
TRINITY_AGC7972	glycero	Dunaliella_tertiole	60.00	0.00
TRINITY_GAX8336	hypothet	Chlamydomonas_eusti	60.00	0.00
TRINITY_PNW8018	hypothet	Chlamydomonas_reinh	60.00	0.00
TRINITY_XP_0029	hypothet	Volvox_carteri	60.00	0.00
TRINITY_GAX8322	hypothet	Chlamydomonas_eusti	60.00	0.00
TRINITY_KXZ5100	hypothet	Gonium_pectorale	60.00	0.00
TRINITY_XP_0273	ABC tra	Abrus_precatorius	60.00	0.00
TRINITY_PNH0982	F-box/W	Tetrabaena_socialis	60.00	0.00
TRINITY_XP_0139	hypothet	Monoraphidium_negle	60.00	0.00
TRINITY_PNW7466	hypothet	Chlamydomonas_reinh	60.00	0.00
TRINITY_PRW6039	UPF0420	Chlorella_sorokinia	60.00	0.00
TRINITY_XP_0017	Qa-SNAR	Chlamydomonas_reinh	60.00	0.00
TRINITY_GAX7288	hypothet	Chlamydomonas_eusti	60.00	0.00
TRINITY_GAX7594	hypothet	Chlamydomonas_eusti	60.00	0.00
TRINITY_GAX8126	hypothet	Chlamydomonas_eusti	60.00	0.00
TRINITY_XP_0201	mitogen-	Aegilops_tauschii	60.00	0.00
TRINITY_GAX8576	hypothet	Chlamydomonas_eusti	60.00	0.00
TRINITY_XP_0138	putative	Monoraphidium_negle	60.00	0.00
TRINITY_PNW8206	hypothet	Chlamydomonas_reinh	60.00	0.00
TRINITY_PNW7936	hypothet	Chlamydomonas_reinh	60.00	0.00
TRINITY_KMS6460	hypothet	Beta_vulgaris	60.00	0.00
TRINITY_GAX7840	hypothet	Chlamydomonas_eusti	60.00	0.00
TRINITY_PNW7832	hypothet	Chlamydomonas_reinh	60.00	0.00
TRINITY_PNH0382	General	Tetrabaena_socialis	60.00	0.00
TRINITY_XP_0138	Cation t	Monoraphidium_negle	60.00	0.00
TRINITY_KNA2370	hypothet	Spinacia_oleracea	60.00	0.00
TRINITY_GAX8569	hypothet	Chlamydomonas_eusti	60.00	0.00
TRINITY_GAX7320	hypothet	Chlamydomonas_eusti	60.00	0.00
TRINITY_XP_0068	60S rib	Amborella_trichopod	59.90	0.00
TRINITY_XP_0058	hypothet	Chlorella_variabili	59.90	0.00
TRINITY_GAX7573	hypothet	Chlamydomonas_eusti	59.90	0.00
TRINITY_KXZ5189	hypothet	Gonium_pectorale	59.90	0.00
TRINITY_XP_0029	pseudou	Volvox_carteri	59.90	0.00
TRINITY_XP_0035	aldehyd	Brachypodium_distac	59.90	0.00
TRINITY_GAX7354	hypothet	Chlamydomonas_eusti	59.90	0.00
TRINITY_PNW8340	hypothet	Chlamydomonas_reinh	59.90	0.00
TRINITY_GBG7162	hypothet	Chara_braunii	59.90	0.00
TRINITY_GAX7667	hypothet	Chlamydomonas_eusti	59.90	0.00
TRINITY_OAE2211	hypothet	Marchantia_polymorp	59.90	0.00
TRINITY_XP_0016	flavin-	Chlamydomonas_reinh	59.90	0.00
TRINITY_GBF8845	hypothet	Raphidocelis_subcap	59.90	0.00
TRINITY_BAK0277	predict	Hordeum_vulgare	59.90	0.00
TRINITY_XP_0029	hypothet	Volvox_carteri	59.90	0.00
TRINITY_PNH1201	hypothet	Tetrabaena_socialis	59.90	0.00
TRINITY_XP_0237	DEAD-bo	Lactuca_sativa	59.90	0.00
TRINITY_KZV1535	hypothet	Dorcoceras_hygromet	59.90	0.00
TRINITY_PSC7348	tubulin	Micractinium_conduc	59.90	0.00
TRINITY_GAX8634	hypothet	Chlamydomonas_eusti	59.90	0.00
TRINITY_XP_0016	esteras	Chlamydomonas_reinh	59.90	0.00
TRINITY_PKA6203	60S rib	Apostasia_shenzheni	59.90	0.00
TRINITY_XP_0243	T-compl	Physcomitrella_pate	59.90	0.00
TRINITY_XP_0016	Qc-SNAR	Chlamydomonas_reinh	59.90	0.00
TRINITY_XP_0017	predict	Chlamydomonas_reinh	59.90	0.00
TRINITY_KXZ5558	hypothet	Gonium_pectorale	59.90	0.00
TRINITY_PRW6113	N-acyl-	Chlorella_sorokinia	59.90	0.00
TRINITY_PNW7482	hypothet	Chlamydomonas_reinh	59.90	0.00
TRINITY_EFJ3272	hypothet	Selaginella_moellen	59.80	0.00
TRINITY_XP_0025	predict	Micromonas_commoda	59.80	0.00
TRINITY_XP_0238	lon prot	Quercus_suber	59.80	0.00
TRINITY_GAX7488	hypothet	Chlamydomonas_eusti	59.80	0.00

TRINITY_PRW1829	methylma	Chlorella_sorokinia	59.80	0.00
TRINITY_XP_0100	PREDICTE	Eucalyptus_grandis	59.80	0.00
TRINITY_XP_0216	erlin-2-	Hevea_brasiliensis	59.80	0.00
TRINITY_XP_0017	leucine-	Chlamydomonas_reinh	59.80	0.00
TRINITY_XP_0016	peptidy	Chlamydomonas_reinh	59.80	0.00
TRINITY_PNH0340	hypothet	Tetrabaena_socialis	59.80	0.00
TRINITY_XP_0029	intrafla	Volvox_carteri	59.80	0.00
TRINITY_GAX8650	hypothet	Chlamydomonas_eusti	59.80	0.00
TRINITY_GAX8491	hypothet	Chlamydomonas_eusti	59.80	0.00
TRINITY_PNW8779	hypothet	Chlamydomonas_reinh	59.80	0.00
TRINITY_XP_0016	cytochro	Chlamydomonas_reinh	59.80	0.00
TRINITY_GAX8418	hypothet	Chlamydomonas_eusti	59.80	0.00
TRINITY_PNW8405	hypothet	Chlamydomonas_reinh	59.80	0.00
TRINITY_PNW7294	hypothet	Chlamydomonas_reinh	59.80	0.00
TRINITY_XP_0029	hypothet	Volvox_carteri	59.80	0.00
TRINITY_PNW7076	hypothet	Chlamydomonas_reinh	59.80	0.00
TRINITY_KXZ4394	hypothet	Gonium_pectorale	59.80	0.00
TRINITY_XP_0029	hypothet	Volvox_carteri	59.80	0.00
TRINITY_PNW7221	hypothet	Chlamydomonas_reinh	59.80	0.00
TRINITY_GAX7385	hypothet	Chlamydomonas_eusti	59.80	0.00
TRINITY_XP_0016	predicte	Chlamydomonas_reinh	59.80	0.00
TRINITY_XP_0029	glutamir	Selaginella_moellen	59.80	0.00
TRINITY_POO0413	ATP-binc	Trema_orientale	59.80	0.00
TRINITY_GAX7376	hypothet	Chlamydomonas_eusti	59.80	0.00
TRINITY_PNH0693	Sorting	Tetrabaena_socialis	59.80	0.00
TRINITY_KMS6524	hypothet	Beta_vulgaris	59.80	0.00
TRINITY_KXZ4192	hypothet	Gonium_pectorale	59.80	0.00
TRINITY_PNW8499	hypothet	Chlamydomonas_reinh	59.80	0.00
TRINITY_BAJ9943	predicte	Hordeum_vulgare	59.80	0.00
TRINITY_PNW7922	hypothet	Chlamydomonas_reinh	59.70	0.00
TRINITY_XP_0063	PREDICTE	Solanum_tuberosum	59.70	0.00
TRINITY_XP_0238	bifuncti	Quercus_suber	59.70	0.00
TRINITY_AII2055	cytosol	Festuca_ovina	59.70	0.00
TRINITY_BAB0874	nucleoti	Arabidopsis_thalian	59.70	0.00
TRINITY_XP_0218	40S ribo	Spinacia_oleracea	59.70	0.00
TRINITY_XP_0058	hypothet	Chlorella_variabili	59.70	0.00
TRINITY_ABK2444	unknown	Picea_sitchensis	59.70	0.00
TRINITY_KXZ5125	hypothet	Gonium_pectorale	59.70	0.00
TRINITY_EFJ2649	hypothet	Selaginella_moellen	59.70	0.00
TRINITY_XP_0016	radial s	Chlamydomonas_reinh	59.70	0.00
TRINITY_BAK0219	predicte	Hordeum_vulgare	59.70	0.00
TRINITY_XP_0030	Guanylat	Ostreococcus_tauri	59.70	0.00
TRINITY_GAX7606	hypothet	Chlamydomonas_eusti	59.70	0.00
TRINITY_PNW7806	hypothet	Chlamydomonas_reinh	59.70	0.00
TRINITY_XP_0029	hypothet	Volvox_carteri	59.70	0.00
TRINITY_XP_0138	hypothet	Monoraphidium_negle	59.70	0.00
TRINITY_XP_0058	hypothet	Chlorella_variabili	59.70	0.00
TRINITY_XP_0029	hypothet	Volvox_carteri	59.70	0.00
TRINITY_XP_0017	COP-II c	Chlamydomonas_reinh	59.70	0.00
TRINITY_XP_0029	hypothet	Volvox_carteri	59.70	0.00
TRINITY_PNW7431	hypothet	Chlamydomonas_reinh	59.70	0.00
TRINITY_GAX7680	hypothet	Chlamydomonas_eusti	59.70	0.00
TRINITY_ESR6354	hypothet	Citrus_clementina	59.70	0.00
TRINITY_XP_0029	hypothet	Volvox_carteri	59.70	0.00
TRINITY_XP_0016	flagella	Chlamydomonas_reinh	59.70	0.00
TRINITY_XP_0075	40S ribo	Bathycoccus_prasino	59.70	0.00
TRINITY_GAX8170	hypothet	Chlamydomonas_eusti	59.70	0.00
TRINITY_XP_0138	hypothet	Monoraphidium_negle	59.70	0.00
TRINITY_GAX8487	hypothet	Chlamydomonas_eusti	59.70	0.00
TRINITY_GBF9805	histone-	Raphidocelis_subcap	59.70	0.00
TRINITY_GAX7901	hypothet	Chlamydomonas_eusti	59.70	0.00
TRINITY_P0C8M8.	RecName:	Zea_mays	59.70	0.00
TRINITY_KXZ4221	hypothet	Gonium_pectorale	59.70	0.00
TRINITY_KXZ5094	hypothet	Gonium_pectorale	59.70	0.00
TRINITY_GAX8378	hypothet	Chlamydomonas_eusti	59.70	0.00
TRINITY_KXZ5665	hypothet	Gonium_pectorale	59.70	0.00

TRINITY_XP_0138	hypothet	Monoraphidium_negle	59.70	0.00
TRINITY_XP_0029	amine o	Volvox_carteri	59.70	0.00
TRINITY_GAX8243	hypothet	Chlamydomonas_eusti	59.70	0.00
TRINITY_XP_0110	40S rib	Sesamum_indicum	59.70	0.00
TRINITY_XP_0025	predict	Micromonas_commoda	59.60	0.00
TRINITY_AGN5202	MYB-rel	Salvia_miltiorrhiza	59.60	0.00
TRINITY_XP_0029	dr1-ass	Selaginella_moellen	59.60	0.00
TRINITY_XP_0271	pre-mRN	Coffea_arabica	59.60	0.00
TRINITY_XP_0238	SUMO-co	Quercus_suber	59.60	0.00
TRINITY_PNY1170	phospho	Trifolium_pratense	59.60	0.00
TRINITY_GAQ8921	hypothet	Klebsormidium_niten	59.60	0.00
TRINITY_XP_0056	U6 snRN	Coccomyxa_subellips	59.60	0.00
TRINITY_XP_0029	SET and	Volvox_carteri	59.60	0.00
TRINITY_XP_0239	ras-rel	Quercus_suber	59.60	0.00
TRINITY_GAX7271	hypothet	Chlamydomonas_eusti	59.60	0.00
TRINITY_AGL0947	TBP-ass	Piper_nigrum	59.60	0.00
TRINITY_KXZ4827	hypothet	Gonium_pectorale	59.60	0.00
TRINITY_OAE2260	hypothet	Marchantia_polymorp	59.60	0.00
TRINITY_XP_0271	actin-1	Coffea_arabica	59.60	0.00
TRINITY_GAX7731	hypothet	Chlamydomonas_eusti	59.60	0.00
TRINITY_OAE3359	hypothet	Marchantia_polymorp	59.60	0.00
TRINITY_KXZ5455	hypothet	Gonium_pectorale	59.60	0.00
TRINITY_GAX7389	hypothet	Chlamydomonas_eusti	59.60	0.00
TRINITY_PSC7629	integra	Micractinium_conduc	59.60	0.00
TRINITY_XP_0056	alcohol	Coccomyxa_subellips	59.60	0.00
TRINITY_PKA5985	60S rib	Apostasia_shenzheni	59.60	0.00
TRINITY_XP_0058	hypothet	Chlorella_variabili	59.60	0.00
TRINITY_KXZ4427	hypothet	Gonium_pectorale	59.60	0.00
TRINITY_GAQ8936	putativ	Klebsormidium_niten	59.60	0.00
TRINITY_KXZ5285	hypothet	Gonium_pectorale	59.60	0.00
TRINITY_XP_0138	hypothet	Monoraphidium_negle	59.60	0.00
TRINITY_KXZ4794	hypothet	Gonium_pectorale	59.60	0.00
TRINITY_XP_0016	DRAP1-l	Chlamydomonas_reinh	59.60	0.00
TRINITY_GAX8513	hypothet	Chlamydomonas_eusti	59.60	0.00
TRINITY_GAX7396	hypothet	Chlamydomonas_eusti	59.60	0.00
TRINITY_BAK0218	predict	Hordeum_vulgare	59.60	0.00
TRINITY_EAY8744	hypothet	Oryza_sativa	59.60	0.00
TRINITY_GAX8090	hypothet	Chlamydomonas_eusti	59.60	0.00
TRINITY_XP_0016	N6-DNA-	Chlamydomonas_reinh	59.60	0.00
TRINITY_GAX8370	hypothet	Chlamydomonas_eusti	59.60	0.00
TRINITY_KMS9434	hypothet	Beta_vulgaris	59.60	0.00
TRINITY_GAX8355	hypothet	Chlamydomonas_eusti	59.60	0.00
TRINITY_GAX8072	hypothet	Chlamydomonas_eusti	59.60	0.00
TRINITY_XP_0017	ribosom	Chlamydomonas_reinh	59.60	0.00
TRINITY_XP_0239	heat sh	Quercus_suber	59.60	0.00
TRINITY_KXZ4643	hypothet	Gonium_pectorale	59.60	0.00
TRINITY_PNW7593	hypothet	Chlamydomonas_reinh	59.60	0.00
TRINITY_GBF9812	ABC tra	Raphidocelis_subcap	59.60	0.00
TRINITY_GAX7634	hypothet	Chlamydomonas_eusti	59.60	0.00
TRINITY_GAX7975	hypothet	Chlamydomonas_eusti	59.60	0.00
TRINITY_GAX8503	hypothet	Chlamydomonas_eusti	59.60	0.00
TRINITY_GAX8594	hypothet	Chlamydomonas_eusti	59.60	0.00
TRINITY_GAX8569	hypothet	Chlamydomonas_eusti	59.60	0.00
TRINITY_XP_0029	hypothet	Volvox_carteri	59.60	0.00
TRINITY_PSC6774	Gag-Pol	Micractinium_conduc	59.60	0.00
TRINITY_XP_0029	hypothet	Volvox_carteri	59.60	0.00
TRINITY_XP_0029	hypothet	Volvox_carteri	59.60	0.00
TRINITY_XP_0239	lon pro	Quercus_suber	59.60	0.00
TRINITY_PSC7135	RCC2-li	Micractinium_conduc	59.50	0.00
TRINITY_XP_0016	flagell	Chlamydomonas_reinh	59.50	0.00
TRINITY_PWA7013	pigment	Artemisia_annua	59.50	0.00
TRINITY_BAK0051	predict	Hordeum_vulgare	59.50	0.00
TRINITY_XP_0217	spastin-	Chenopodium_quinoa	59.50	0.00
TRINITY_EMS5789	hypothet	Triticum_urartu	59.50	0.00
TRINITY_XP_0238	ras-rel	Quercus_suber	59.50	0.00
TRINITY_XP_0238	40S rib	Quercus_suber	59.50	0.00

TRINITY_XP_0270	tryptop	Coffea_arabica	59.50	0.00
TRINITY_KDD7516	hypothet	Helicosporidium_sp.	59.50	0.00
TRINITY_RMZ5369	hypothet	Auxenochlorella_pro	59.50	0.00
TRINITY_PNW7193	hypothet	Chlamydomonas_reinh	59.50	0.00
TRINITY_RLM7453	hypothet	Panicum_miliaceum	59.50	0.00
TRINITY_PNW7586	hypothet	Chlamydomonas_reinh	59.50	0.00
TRINITY_XP_0016	small r	Chlamydomonas_reinh	59.50	0.00
TRINITY_XP_0029	hypothet	Volvox_carteri	59.50	0.00
TRINITY_GAX8390	hypothet	Chlamydomonas_eusti	59.50	0.00
TRINITY_GAX7756	hypothet	Chlamydomonas_eusti	59.50	0.00
TRINITY_GAX8404	hypothet	Chlamydomonas_eusti	59.50	0.00
TRINITY_PRW3917	mismatc	Chlorella_sorokinia	59.50	0.00
TRINITY_PSC7492	ubiquit	Micractinium_conduc	59.50	0.00
TRINITY_ADN4307	beta-ca	Haematococcus_lacus	59.50	0.00
TRINITY_GAX8495	hypothet	Chlamydomonas_eusti	59.50	0.00
TRINITY_GBF9034	hypothet	Raphidocelis_subcap	59.50	0.00
TRINITY_GBF9563	hypothet	Raphidocelis_subcap	59.50	0.00
TRINITY_XP_0016	predict	Chlamydomonas_reinh	59.50	0.00
TRINITY_XP_0029	hypothet	Volvox_carteri	59.50	0.00
TRINITY_XP_0239	serine/t	Quercus_suber	59.50	0.00
TRINITY_KXZ5425	hypothet	Gonium_pectorale	59.50	0.00
TRINITY_XP_0035	chapero	Brachypodium_distac	59.50	0.00
TRINITY_GAX7934	hypothet	Chlamydomonas_eusti	59.50	0.00
TRINITY_XP_0016	allanto	Chlamydomonas_reinh	59.50	0.00
TRINITY_RYQ8594	hypothet	Arachis_hypogaea	59.50	0.00
TRINITY_XP_0029	3'5'-cy	Volvox_carteri	59.50	0.00
TRINITY_PRW5789	RING fi	Chlorella_sorokinia	59.50	0.00
TRINITY_GAX8346	hypothet	Chlamydomonas_eusti	59.50	0.00
TRINITY_PNW7881	hypothet	Chlamydomonas_reinh	59.50	0.00
TRINITY_OUS4300	uracil-I	Ostreococcus_tauri	59.50	0.00
TRINITY_PNR4546	hypothet	Physcomitrella_pate	59.50	0.00
TRINITY_XP_0029	hypothet	Volvox_carteri	59.50	0.00
TRINITY_GBF9838	DNA rep	Raphidocelis_subcap	59.50	0.00
TRINITY_RZB6735	Cyclin-	Glycine_soja	59.50	0.00
TRINITY_PNW8396	hypothet	Chlamydomonas_reinh	59.50	0.00
TRINITY_GAX8410	hypothet	Chlamydomonas_eusti	59.50	0.00
TRINITY_GAX8164	hypothet	Chlamydomonas_eusti	59.50	0.00
TRINITY_GAX7714	hypothet	Chlamydomonas_eusti	59.50	0.00
TRINITY_XP_0116	PREDICT	Cucumis_sativus	59.50	0.00
TRINITY_XP_0238	40S rib	Quercus_suber	59.50	0.00
TRINITY_OUS4464	5-amino	Ostreococcus_tauri	59.40	0.00
TRINITY_XP_0138	citrate	Monoraphidium_negle	59.40	0.00
TRINITY_RZB9585	Program	Glycine_soja	59.40	0.00
TRINITY_XP_0216	probabl	Manihot_esculenta	59.40	0.00
TRINITY_OAY8236	ABC tra	Ananas_comosus	59.40	0.00
TRINITY_XP_0014	predict	Ostreococcus_sp._'l	59.40	0.00
TRINITY_GAX8105	hypothet	Chlamydomonas_eusti	59.40	0.00
TRINITY_XP_0110	PREDICT	Populus_euphratica	59.40	0.00
TRINITY_BAK0268	predict	Hordeum_vulgare	59.40	0.00
TRINITY_KXZ4863	hypothet	Gonium_pectorale	59.40	0.00
TRINITY_PNW7183	hypothet	Chlamydomonas_reinh	59.40	0.00
TRINITY_PNH0961	hypothet	Tetrabaena_socialis	59.40	0.00
TRINITY_KXZ5025	hypothet	Gonium_pectorale	59.40	0.00
TRINITY_XP_0058	hypothet	Chlorella_variabili	59.40	0.00
TRINITY_GAX7287	hypothet	Chlamydomonas_eusti	59.40	0.00
TRINITY_GAX8370	hypothet	Chlamydomonas_eusti	59.40	0.00
TRINITY_KXZ5481	hypothet	Gonium_pectorale	59.40	0.00
TRINITY_GAX7672	hypothet	Chlamydomonas_eusti	59.40	0.00
TRINITY_GAX7985	hypothet	Chlamydomonas_eusti	59.40	0.00
TRINITY_GAX7269	hypothet	Chlamydomonas_eusti	59.40	0.00
TRINITY_PSC6940	Lysosom	Micractinium_conduc	59.40	0.00
TRINITY_GAX7965	hypothet	Chlamydomonas_eusti	59.40	0.00
TRINITY_PSC7431	epimera	Micractinium_conduc	59.40	0.00
TRINITY_PNW8806	hypothet	Chlamydomonas_reinh	59.40	0.00
TRINITY_PNW7536	hypothet	Chlamydomonas_reinh	59.40	0.00
TRINITY_XP_0016	predict	Chlamydomonas_reinh	59.40	0.00

TRINITY_KXZ5645(	hypothet	Gonium_pectorale	59.40	0.00
TRINITY_GBF9460!	hypothet	Raphidocelis_subcap	59.40	0.00
TRINITY_XP_0016!	methylc:	Chlamydomonas_reinh	59.40	0.00
TRINITY_KZV1528{	cystath:	Dorcoceras_hygromet	59.40	0.00
TRINITY_PNW7374(	hypothet	Chlamydomonas_reinh	59.40	0.00
TRINITY_GAX7712(	hypothet	Chlamydomonas_eusti	59.40	0.00
TRINITY_KXZ4688:	hypothet	Gonium_pectorale	59.40	0.00
TRINITY_GAX7451!	hypothet	Chlamydomonas_eusti	59.40	0.00
TRINITY_AER5821{	channel(	Chlamydomonas_raude	59.40	0.00
TRINITY_GAX8238!	hypothet	Chlamydomonas_eusti	59.40	0

TRINITY_XP_0228	Ribosoma	Ostreococcus_tauri	59.40	0.00
TRINITY_XP_0016	predicte	Chlamydomonas_reinh	59.40	0.00
TRINITY_XP_0029	hypothet	Volvox_carteri	59.40	0.00
TRINITY_PNW8187	hypothet	Chlamydomonas_reinh	59.40	0.00
TRINITY_GAX8026	hypothet	Chlamydomonas_eusti	59.40	0.00
TRINITY_GAX8190	hypothet	Chlamydomonas_eusti	59.40	0
TRINITY_GAX7986	hypothet	Chlamydomonas_eusti	59.40	0.00
TRINITY_PNW7037	hypothet	Chlamydomonas_reinh	59.40	0.00
TRINITY_EPS7450	hypothet	Genlisea_aurea	59.40	0.00
TRINITY_GAX7598	hypothet	Chlamydomonas_eusti	59.40	0.00
TRINITY_GAX8017	hypothet	Chlamydomonas_eusti	59.40	0.00
TRINITY_XP_0191	PREDICTE	Beta_vulgaris	59.40	0.00
TRINITY_BAK0286	predicte	Hordeum_vulgare	59.30	0.00
TRINITY_XP_0239	transalc	Quercus_suber	59.30	0.00
TRINITY_XP_0191	PREDICTE	Ipomoea_nil	59.30	0.00
TRINITY_XP_0030	ligase,	Micromonas_pusilla	59.30	0.00
TRINITY_BAK0051	predicte	Hordeum_vulgare	59.30	0.00
TRINITY_BAK0051	predicte	Hordeum_vulgare	59.30	0.00
TRINITY_PNH0792	Methylat	Tetrabaena_socialis	59.30	0.00
TRINITY_XP_0094	PREDICTE	Musa_acuminata	59.30	0.00
TRINITY_XP_0138	Mitotic	Monoraphidium_negle	59.30	0.00
TRINITY_XP_0238	1,4-alph	Quercus_suber	59.30	0.00
TRINITY_EMS6852	hypothet	Triticum_urartu	59.30	0.00
TRINITY_XP_0194	PREDICTE	Lupinus_angustifoli	59.30	0.00
TRINITY_XP_0243	probable	Physcomitrella_pate	59.30	0.00
TRINITY_KMS6481	hypothet	Beta_vulgaris	59.30	0.00
TRINITY_PNW8279	hypothet	Chlamydomonas_reinh	59.30	0.00
TRINITY_KXZ5111	hypothet	Gonium_pectorale	59.30	0.00
TRINITY_XP_0139	putative	Monoraphidium_negle	59.30	0.00
TRINITY_XP_0139	PKHD-typ	Monoraphidium_negle	59.30	0.00
TRINITY_GAX7259	hypothet	Chlamydomonas_eusti	59.30	0.00
TRINITY_XP_0158	uncharac	Ziziphus_jujuba	59.30	0.00
TRINITY_KXZ4304	hypothet	Gonium_pectorale	59.30	0.00
TRINITY_XP_0134	uncharac	Medicago_truncatula	59.30	0.00
TRINITY_XP_0058	hypothet	Chlorella_variabili	59.30	0.00
TRINITY_XP_0145	vacuolar	Vigna_radiata	59.30	0.00
TRINITY_EOY2032	Small GT	Theobroma_cacao	59.30	0.00
TRINITY_KXZ4484	hypothet	Gonium_pectorale	59.30	0.00
TRINITY_OAE2964	hypothet	Marchantia_polymorp	59.30	0.00
TRINITY_ABR1682	unknown	Picea_sitchensis	59.30	0.00
TRINITY_AWK6783	MME2 [Du	Dunaliella_tertiole	59.30	0.00
TRINITY_XP_0102	PREDICTE	Nelumbo_nucifera	59.30	0.00
TRINITY_GAX7923	hypothet	Chlamydomonas_eusti	59.30	0.00
TRINITY_GAX8248	hypothet	Chlamydomonas_eusti	59.30	0.00
TRINITY_XP_0219	protein	Carica_papaya	59.30	0.00
TRINITY_XP_0029	hypothet	Volvox_carteri	59.30	0.00
TRINITY_GAX7821	hypothet	Chlamydomonas_eusti	59.30	0.00
TRINITY_PSC6977	Peroxiso	Micractinium_conduc	59.30	0.00
TRINITY_GAQ8486	peroxin	Klebsormidium_niten	59.30	0.00
TRINITY_KXZ4671	hypothet	Gonium_pectorale	59.30	0.00
TRINITY_KXZ5471	hypothet	Gonium_pectorale	59.30	0.00
TRINITY_XP_0029	hypothet	Volvox_carteri	59.30	0.00
TRINITY_XP_0029	hypothet	Volvox_carteri	59.30	0.00
TRINITY_PNW7777	hypothet	Chlamydomonas_reinh	59.30	0.00
TRINITY_GAQ8899	MEKK and	Klebsormidium_niten	59.30	0.00
TRINITY_GBF9055	hypothet	Raphidocelis_subcap	59.30	0.00
TRINITY_RMZ5233	hypothet	Auxenochlorella_pro	59.30	0.00
TRINITY_XP_0016	predicte	Chlamydomonas_reinh	59.30	0.00
TRINITY_XP_0084	PREDICTE	Cucumis_melo	59.30	0.00
TRINITY_GAX7305	hypothet	Chlamydomonas_eusti	59.30	0.00
TRINITY_XP_0124	PREDICTE	Gossypium_raimondii	59.20	0.00
TRINITY_XP_0030	CTP synt	Micromonas_pusilla	59.20	0.00
TRINITY_PSC6734	ATP:cob	Micractinium_conduc	59.20	0.00
TRINITY_XP_0239	60S ribo	Quercus_suber	59.20	0.00
TRINITY_XP_0279	probable	Vigna_unguiculata	59.20	0.00
TRINITY_XP_0058	hypothet	Chlorella_variabili	59.20	0.00

TRINITY_GAX8086(	hypothet	Chlamydomonas_eusti	59.20	0.00
TRINITY_PNH0396!	putative	Tetrabaena_socialis	59.20	0.00
TRINITY_PNW8676'	hypothet	Chlamydomonas_reinh	59.20	0.00
TRINITY_KXZ5247'	hypothet	Gonium_pectorale	59.20	0.00
TRINITY_GAX7670	hypothet	Chlamydomonas_eusti	59.20	0.00
TRINITY_XP_0138!	ribosome	Monoraphidium_negle	59.20	0.00
TRINITY_XP_0029	iron-sul	Volvox_carteri	59.20	0.00
TRINITY_XP_0016!	plastid	Chlamydomonas_reinh	59.20	0.00
TRINITY_PRW5676!	valine--	Chlorella_sorokinia	59.20	0.00
TRINITY_GAX7549	hypothet	Chlamydomonas_eusti	59.20	0.00
TRINITY_KXZ4680!	hypothet	Gonium_pectorale	59.20	0.00
TRINITY_PNH1289!	ATP-depe	Tetrabaena_socialis	59.20	0.00
TRINITY_GAX7867	hypothet	Chlamydomonas_eusti	59.20	0.00
TRINITY_GAQ7894	methylma	Klebsormidium_niten	59.20	0.00
TRINITY_XP_0216!	mannose-	Hevea_brasiliensis	59.20	0.00
TRINITY_XP_0016!	gamma tu	Chlamydomonas_reinh	59.20	0.00
TRINITY_AWK6783	ACD [Dur	Dunaliella_tertiole	59.20	0.00
TRINITY_XP_0172	PREDICTE	Daucus_carota	59.20	0.00
TRINITY_KXZ4332	hypothet	Gonium_pectorale	59.20	0.00
TRINITY_KXZ5084	hypothet	Gonium_pectorale	59.20	0.00
TRINITY_RMZ5438	hypothet	Auxenochlorella_prc	59.20	0.00
TRINITY_PNH1061	hypothet	Tetrabaena_socialis	59.20	0.00
TRINITY_GAQ9143!	Kinesin-	Klebsormidium_niten	59.20	0.00
TRINITY_CAI8489	putative	Medicago_sativa	59.20	0.00
TRINITY_XP_0036	ras-rela	Medicago_truncatula	59.20	0.00
TRINITY_PNW8270	hypothet	Chlamydomonas_reinh	59.20	0.00
TRINITY_KXZ5281	hypothet	Gonium_pectorale	59.20	0.00
TRINITY_GAX8046	hypothet	Chlamydomonas_eusti	59.20	0.00
TRINITY_GAX8127(	hypothet	Chlamydomonas_eusti	59.20	0.00
TRINITY_GAX7875	hypothet	Chlamydomonas_eusti	59.20	0.00
TRINITY_GAX7937'	hypothet	Chlamydomonas_eusti	59.20	0.00
TRINITY_XP_0102(	PREDICTE	Nelumbo_nucifera	59.20	0.00
TRINITY_PNW8276!	hypothet	Chlamydomonas_reinh	59.20	0.00
TRINITY_XP_0029	histone	Volvox_carteri	59.20	0.00
TRINITY_GAQ8291'	Acyl-CoA	Klebsormidium_niten	59.20	0.00
TRINITY_XP_0138!	NFX1-typ	Monoraphidium_negle	59.20	0.00
TRINITY_GAX7945'	hypothet	Chlamydomonas_eusti	59.20	0
TRINITY_XP_0029!	hypothet	Volvox_carteri	59.20	0.00
TRINITY_EEF2623!	conserve	Ricinus_communis	59.10	0.00
TRINITY_XP_0229(	unchara	Cucurbita_maxima	59.10	0.00
TRINITY_XP_0106(	PREDICTE	Beta_vulgaris	59.10	0.00
TRINITY_XP_0029(	superoxi	Selaginella_moellen	59.10	0.00
TRINITY_XP_0114(	Actin [A	Auxenochlorella_prc	59.10	0.00
TRINITY_GBG7924!	hypothet	Chara_braunii	59.10	0.00
TRINITY_RMZ5448(	hypothet	Auxenochlorella_prc	59.10	0.00
TRINITY_PNW7573!	hypothet	Chlamydomonas_reinh	59.10	0.00
TRINITY_XP_0093!	PREDICTE	Pyrus_x_bretschneid	59.10	0.00
TRINITY_XP_0243'	threonyl	Physcomitrella_pate	59.10	0.00
TRINITY_GAX7347!	hypothet	Chlamydomonas_eusti	59.10	0.00
TRINITY_XP_0239(	aldo-ket	Quercus_suber	59.10	0.00
TRINITY_PNH1293	Mitogen-	Tetrabaena_socialis	59.10	0.00
TRINITY_XP_0029!	hypothet	Volvox_carteri	59.10	0.00
TRINITY_XP_0217	protein	Chenopodium_quinoa	59.10	0.00
TRINITY_GAX7412	hypothet	Chlamydomonas_eusti	59.10	0.00
TRINITY_KXZ5525(	hypothet	Gonium_pectorale	59.10	0.00
TRINITY_XP_0045(	signal r	Cicer_arietinum	59.10	0.00
TRINITY_GAX7609'	hypothet	Chlamydomonas_eusti	59.10	0.00
TRINITY_PSC6969(	ATP-depe	Micractinium_conduc	59.10	0.00
TRINITY_XP_0029!	hypothet	Volvox_carteri	59.10	0.00
TRINITY_XP_0016!	triose p	Chlamydomonas_reinh	59.10	0.00
TRINITY_PNW7528	hypothet	Chlamydomonas_reinh	59.10	0.00
TRINITY_XP_0058	hypothet	Chlorella_variabili	59.10	0.00
TRINITY_PNW8679	hypothet	Chlamydomonas_reinh	59.10	0.00
TRINITY_YP_6656	cytochro	Nephroselmis_olivac	59.10	0.00
TRINITY_PRW5607(	GATA tra	Chlorella_sorokinia	59.10	0.00
TRINITY_XP_0029	hypothet	Volvox_carteri	59.10	0.00

TRINITY_XP_0017(	acetyl-(	Chlamydomonas_reinh	59.10	0.00
TRINITY_XP_0029⁄	hypothet	Volvox_carteri	59.10	0.00
TRINITY_XP_0029!	hypothet	Volvox_carteri	59.10	0.00
TRINITY_PNH1056!	Reticul(	Tetrabaena_socialis	59.10	0.00
TRINITY_PRW4564(	L-ascorl	Chlorella_sorokinia	59.10	0.00
TRINITY_XP_0016!	hypothet	Chlamydomonas_reinh	59.10	0.00
TRINITY_KZV1521!	petal de	Dorcoceras_hygromet	59.10	0.00
TRINITY_ABW8726(	ferritir	Chlamydomonas_reinh	59.10	0.00
TRINITY_GBF9365!	hypothet	Raphidocelis_subcap	59.10	0.00
TRINITY_XP_0029!	hypothet	Volvox_carteri	59.10	0.00
TRINITY_ACB2847:	polyprot	Ananas_comosus	59.10	0.00
TRINITY_ABH1167:	actin, p	Chara_contraria	59.10	0.00
TRINITY_XP_0220:	NAD-depe	Helianthus_annuus	59.10	0.00
TRINITY_XP_0041⁄	PREDICTI	Cucumis_sativus	59.00	0.00
TRINITY_XP_0238!	guanine	Quercus_suber	59.00	0.00
TRINITY_BAJ9764:	predicte	Hordeum_vulgare	59.00	0.00
TRINITY_XP_0264:	queuine	Papaver_somniferum	59.00	0.00
TRINITY_GBG6683:	hypothet	Chara_braunii	59.00	0.00
TRINITY_XP_0239:	E3 ubiqu	Quercus_suber	59.00	0.00
TRINITY_XP_0243{	transcri	Physcomitrella_pate	59.00	0.00
TRINITY_XP_0016!	predicte	Chlamydomonas_reinh	59.00	0.00
TRINITY_KMS6481(	hypothet	Beta_vulgaris	59.00	0.00
TRINITY_XP_0029⁄	hypothet	Volvox_carteri	59.00	0.00
TRINITY_XP_0017(	ribosoma	Chlamydomonas_reinh	59.00	0.00
TRINITY_KXZ4589:	hypothet	Gonium_pectorale	59.00	0.00
TRINITY_XP_0192(	PREDICTI	Nicotiana_attenuata	59.00	0.00
TRINITY_PNW7681'	hypothet	Chlamydomonas_reinh	59.00	0.00
TRINITY_KXZ5284'	hypothet	Gonium_pectorale	59.00	0.00
TRINITY_GAX8245{	hypothet	Chlamydomonas_eusti	59.00	0.00
TRINITY_KXZ5434{	hypothet	Gonium_pectorale	59.00	0.00
TRINITY_XP_0058⁄	hypothet	Chlorella_variabili	59.00	0.00
TRINITY_GAX7732:	hypothet	Chlamydomonas_eusti	59.00	0.00
TRINITY_KXZ4269⁄	hypothet	Gonium_pectorale	59.00	0.00
TRINITY_XP_0217!	unchara(	Chenopodium_quinoa	59.00	0.00
TRINITY_XP_0093!	PREDICTI	Pyrus_x_bretschneid	59.00	0.00
TRINITY_KXZ4336(	hypothet	Gonium_pectorale	59.00	0.00
TRINITY_XP_0029!	hypothet	Volvox_carteri	59.00	0.00
TRINITY_KXZ5400'	hypothet	Gonium_pectorale	59.00	0.00
TRINITY_AXF4155!	HLA3 pro	Chlorella_sp._ArM00	59.00	0.00
TRINITY_BAJ9461!	predicte	Hordeum_vulgare	59.00	0.00
TRINITY_GAX7383!	hypothet	Chlamydomonas_eusti	59.00	0.00
TRINITY_XP_0016{	predicte	Chlamydomonas_reinh	59.00	0.00
TRINITY_KXZ5193⁄	hypothet	Gonium_pectorale	59.00	0.00
TRINITY_GAQ8105!	Nuclear	Klebsormidium_niten	59.00	0.00
TRINITY_GAX7645{	hypothet	Chlamydomonas_eusti	59.00	0.00
TRINITY_KXZ5174!	hypothet	Gonium_pectorale	59.00	0.00
TRINITY_XP_0056⁄	FAD/NAD	Coccomyxa_subellips	59.00	0.00
TRINITY_CDP0464'	unnamed	Coffea_canephora	59.00	0.00
TRINITY_XP_0058!	hypothet	Chlorella_variabili	59.00	0.00
TRINITY_XP_0029!	hypothet	Volvox_carteri	59.00	0.00
TRINITY_GAX8224'	hypothet	Chlamydomonas_eusti	59.00	0.00
TRINITY_GAX8519⁄	hypothet	Chlamydomonas_eusti	59.00	0.00
TRINITY_ALM5499:	DEAD box	Chlamydomonas_sp._I	59.00	0.00
TRINITY_XP_0016!	subunit	Chlamydomonas_reinh	59.00	0.00
TRINITY_XP_0128!	PREDICTI	Erythranthe_guttata	59.00	0.00
TRINITY_PNW8531!	hypothet	Chlamydomonas_reinh	59.00	0.00
TRINITY_PNW7496'	hypothet	Chlamydomonas_reinh	59.00	0.00
TRINITY_KXZ4519'	hypothet	Gonium_pectorale	59.00	0.00
TRINITY_GAX8092!	hypothet	Chlamydomonas_eusti	59.00	0.00
TRINITY_XP_0016{	predicte	Chlamydomonas_reinh	59.00	0.00
TRINITY_KDO5298!	hypothet	Citrus_sinensis	59.00	0.00
TRINITY_PNH0752'	hypothet	Tetrabaena_socialis	59.00	0.00
TRINITY_PWA7909(	MOB kina	Artemisia_annua	59.00	0.00
TRINITY_XP_0104:	PREDICTI	Camelina_sativa	59.00	0.00
TRINITY_PNW8332:	hypothet	Chlamydomonas_reinh	59.00	0.00
TRINITY_GAX8317!	hypothet	Chlamydomonas_eusti	59.00	0.00

TRINITY_XP_0028(	ABC trar	Arabidopsis_lyrata	58.90	0.00
TRINITY_GAX8035(	hypothet	Chlamydomonas_eusti	58.90	0.00
TRINITY_XP_0016(	flavoher	Chlamydomonas_reinh	58.90	0.00
TRINITY_PTQ3358(	hypothet	Marchantia_polymorp	58.90	0.00
TRINITY_XP_0202(	40S ribo	Asparagus_officinal	58.90	0.00
TRINITY_KXZ5600(	hypothet	Gonium_pectorale	58.90	0.00
TRINITY_XP_0025(	predicte	Micromonas_commoda	58.90	0.00
TRINITY_PNW8117(	hypothet	Chlamydomonas_reinh	58.90	0.00
TRINITY_XP_0014(	predicte	Ostreococcus_sp._'l	58.90	0.00
TRINITY_GAX8254(	hypothet	Chlamydomonas_eusti	58.90	0.00
TRINITY_PRW5951(	inositol	Chlorella_sorokinia	58.90	0.00
TRINITY_PNW8220(	hypothet	Chlamydomonas_reinh	58.90	0.00
TRINITY_XP_0097(	PREDICTE	Nicotiana_sylvestri	58.90	0.00
TRINITY_GAX7800(	hypothet	Chlamydomonas_eusti	58.90	0.00
TRINITY_XP_0029(	hypothet	Volvox_carteri	58.90	0.00
TRINITY_GAX7262(	hypothet	Chlamydomonas_eusti	58.90	0.00
TRINITY_XP_0139(	putative	Monoraphidium_negle	58.90	0.00
TRINITY_GAX7329(	hypothet	Chlamydomonas_eusti	58.90	0.00
TRINITY_PNH0844(	hypothet	Tetrabaena_socialis	58.90	0.00
TRINITY_XP_0029(	actin-7	Selaginella_moellen	58.90	0.00
TRINITY_GAX7494(	hypothet	Chlamydomonas_eusti	58.90	0.00
TRINITY_GAX7982(	hypothet	Chlamydomonas_eusti	58.90	0.00
TRINITY_PNW8046(	hypothet	Chlamydomonas_reinh	58.90	0.00
TRINITY_PSC6700(	Gag-Pol	Micractinium_conduc	58.90	0.00
TRINITY_OAE2101(	hypothet	Marchantia_polymorp	58.80	0.00
TRINITY_XP_0156(	glycine-	Oryza_sativa	58.80	0.00
TRINITY_XP_0028(	SWI/SNF-	Arabidopsis_lyrata	58.80	0.00
TRINITY_XP_0138(	Casein k	Monoraphidium_negle	58.80	0.00
TRINITY_XP_0025(	predicte	Micromonas_commoda	58.80	0.00
TRINITY_XP_0238(	dynein h	Quercus_suber	58.80	0.00
TRINITY_RAL4789(	hypothet	Cuscuta_australis	58.80	0.00
TRINITY_PHT2909(	hypothet	Capsicum_baccatum	58.80	0.00
TRINITY_GBG7777(	hypothet	Chara_braunii	58.80	0.00
TRINITY_GAX7534(	hypothet	Chlamydomonas_eusti	58.80	0.00
TRINITY_GAX8512(	hypothet	Chlamydomonas_eusti	58.80	0.00
TRINITY_GBF8819(	hypothet	Raphidocelis_subcap	58.80	0.00
TRINITY_PNH1105(	Protein	Tetrabaena_socialis	58.80	0.00
TRINITY_XP_0029(	hypothet	Volvox_carteri	58.80	0.00
TRINITY_XP_0156(	RNA-bind	Oryza_sativa	58.80	0.00
TRINITY_XP_0017(	predicte	Chlamydomonas_reinh	58.80	0.00
TRINITY_KXZ4473(	hypothet	Gonium_pectorale	58.80	0.00
TRINITY_KXZ5168(	hypothet	Gonium_pectorale	58.80	0.00
TRINITY_XP_0017(	predicte	Chlamydomonas_reinh	58.80	0.00
TRINITY_KXZ5356(	hypothet	Gonium_pectorale	58.80	0.00
TRINITY_KXZ4796(	hypothet	Gonium_pectorale	58.80	0.00
TRINITY_XP_0029(	hypothet	Volvox_carteri	58.80	0.00
TRINITY_GAX7357(	hypothet	Chlamydomonas_eusti	58.80	0.00
TRINITY_XP_0056(	putative	Coccomyxa_subellips	58.80	0.00
TRINITY_GAX8351(	hypothet	Chlamydomonas_eusti	58.80	0.00
TRINITY_GAX7551(	hypothet	Chlamydomonas_eusti	58.80	0.00
TRINITY_GBF9218(	exonucle	Raphidocelis_subcap	58.80	0.00
TRINITY_XP_0205(	histone	Phalaenopsis_equest	58.80	0.00
TRINITY_GAX7373(	hypothet	Chlamydomonas_eusti	58.80	0.00
TRINITY_GAX7418(	hypothet	Chlamydomonas_eusti	58.80	0.00
TRINITY_GAX7994(	hypothet	Chlamydomonas_eusti	58.80	0.00
TRINITY_XP_0017(	predicte	Chlamydomonas_reinh	58.80	0.00
TRINITY_XP_0056(	NLI inte	Coccomyxa_subellips	58.80	0.00
TRINITY_XP_0221(	serine/t	Momordica_charantia	58.80	0.00
TRINITY_KZV4753(	hypothet	Dorcoceras_hygromet	58.80	0.00
TRINITY_XP_0096(	PREDICTE	Nicotiana_tomentosi	58.80	0.00
TRINITY_GAX7949(	hypothet	Chlamydomonas_eusti	58.80	0
TRINITY_XP_0239(	myosin t	Quercus_suber	58.80	0.00
TRINITY_PNW8242(	hypothet	Chlamydomonas_reinh	58.80	0.00
TRINITY_GAX8368(	hypothet	Chlamydomonas_eusti	58.80	0.00
TRINITY_XP_0029(	1,4-alph	Volvox_carteri	58.80	0.00
TRINITY_PRW5867(	Intron-b	Chlorella_sorokinia	58.80	0.00

TRINITY_XP_0216(	tRNAse Z	Hevea_brasiliensis	58.80	0.00
TRINITY_PSC7361:	type I p	Micractinium_conduc	58.80	0.00
TRINITY_GAX7749(	hypothet	Chlamydomonas_eusti	58.80	0.00
TRINITY_XP_0238(	ubiquiti	Quercus_suber	58.80	0.00
TRINITY_XP_0071:	hypothet	Phaseolus_vulgaris	58.70	0.00
TRINITY_XP_0201!	small ub	Aegilops_tauschii	58.70	0.00
TRINITY_XP_0201'	GTP-binc	Aegilops_tauschii	58.70	0.00
TRINITY_XP_0158'	ATP-depe	Ziziphus_jujuba	58.70	0.00
TRINITY_PTQ4268'	hypothet	Marchantia_polymorp	58.70	0.00
TRINITY_OAE2408(	hypothet	Marchantia_polymorp	58.70	0.00
TRINITY_XP_0238(	alpha-ac	Quercus_suber	58.70	0.00
TRINITY_BAJ9999(	predicte	Hordeum_vulgare	58.70	0.00
TRINITY_XP_0239(	myosin-1	Quercus_suber	58.70	0.00
TRINITY_GAX8175:	hypothet	Chlamydomonas_eusti	58.70	0.00
TRINITY_XP_0161'	ubiquiti	Arachis_ipaensis	58.70	0.00
TRINITY_PNH0289(	hypothet	Tetrabaena_socialis	58.70	0.00
TRINITY_XP_0109'	PREDICTE	Elaeis_guineensis	58.70	0.00
TRINITY_XP_0139(	phosphat	Monoraphidium_negle	58.70	0.00
TRINITY_XP_0229(	cyclin-c	Cucurbita_maxima	58.70	0.00
TRINITY_GAX7647:	hypothet	Chlamydomonas_eusti	58.70	0.00
TRINITY_GAX7279:	hypothet	Chlamydomonas_eusti	58.70	0.00
TRINITY_PNH0579!	2,3-bisp	Tetrabaena_socialis	58.70	0.00
TRINITY_XP_0139(	hypothet	Monoraphidium_negle	58.70	0.00
TRINITY_GAX8315'	hypothet	Chlamydomonas_eusti	58.70	0.00
TRINITY_GAX7651:	hypothet	Chlamydomonas_eusti	58.70	0.00
TRINITY_PNW7123(	hypothet	Chlamydomonas_reinh	58.70	0.00
TRINITY_GBG8742'	hypothet	Chara_braunii	58.70	0.00
TRINITY_PNH0198:	Pre-mRNA	Tetrabaena_socialis	58.70	0.00
TRINITY_GAX7858(	hypothet	Chlamydomonas_eusti	58.70	0.00
TRINITY_XP_0017(	predicte	Chlamydomonas_reinh	58.70	0.00
TRINITY_XP_0029!	hypothet	Volvox_carteri	58.70	0.00
TRINITY_XP_0058'	protein	Chlorella_variabili	58.70	0.00
TRINITY_XP_0093(	PREDICTE	Musa_acuminata	58.70	0.00
TRINITY_KXZ5452:	hypothet	Gonium_pectorale	58.70	0.00
TRINITY_GAX8334:	hypothet	Chlamydomonas_eusti	58.70	0.00
TRINITY_XP_0017(	predicte	Chlamydomonas_reinh	58.70	0.00
TRINITY_GAX8624(	hypothet	Chlamydomonas_eusti	58.70	0.00
TRINITY_GAX8386!	hypothet	Chlamydomonas_eusti	58.70	0.00
TRINITY_GAX7607(	hypothet	Chlamydomonas_eusti	58.70	0.00
TRINITY_PNG9395(	hypothet	Tetrabaena_socialis	58.70	0.00
TRINITY_XP_0243'	cAMP-dep	Physcomitrella_pate	58.60	0.00
TRINITY_XP_0270(	short ir	Coffea_arabica	58.60	0.00
TRINITY_XP_0138(	hypothet	Monoraphidium_negle	58.60	0.00
TRINITY_EEF2554(	beta-mar	Ricinus_communis	58.60	0.00
TRINITY_OEL1377'	Myb-rela	Dichanthelium_oligo	58.60	0.00
TRINITY_XP_0188:	PREDICTE	Juglans_regia	58.60	0.00
TRINITY_GAX7378(	hypothet	Chlamydomonas_eusti	58.60	0.00
TRINITY_XP_0124'	PREDICTE	Gossypium_raimondii	58.60	0.00
TRINITY_XP_0277(	unchara(	Solanum_pennellii	58.60	0.00
TRINITY_GAX7884:	hypothet	Chlamydomonas_eusti	58.60	0.00
TRINITY_PNH0449(	Transmer	Tetrabaena_socialis	58.60	0.00
TRINITY_GAX7351'	hypothet	Chlamydomonas_eusti	58.60	0.00
TRINITY_RWW9113(	hypothet	Ensete_ventricosum	58.60	0.00
TRINITY_XP_0016(	predicte	Chlamydomonas_reinh	58.60	0.00
TRINITY_GAX8165:	hypothet	Chlamydomonas_eusti	58.60	0.00
TRINITY_GAX8305:	hypothet	Chlamydomonas_eusti	58.60	0.00
TRINITY_XP_0237'	ADP-ribo	Lactuca_sativa	58.60	0.00
TRINITY_GAX7265!	hypothet	Chlamydomonas_eusti	58.60	0.00
TRINITY_XP_0188'	PREDICTE	Juglans_regia	58.60	0.00
TRINITY_XP_0029!	hypothet	Volvox_carteri	58.60	0.00
TRINITY_XP_0016(	5-Formyl	Chlamydomonas_reinh	58.60	0.00
TRINITY_XP_0016(	RNA binc	Chlamydomonas_reinh	58.60	0.00
TRINITY_XP_0029'	hypothet	Volvox_carteri	58.60	0.00
TRINITY_KXZ4516:	hypothet	Gonium_pectorale	58.60	0.00
TRINITY_XP_0029!	hypothet	Volvox_carteri	58.60	0.00
TRINITY_XP_0029'	hypothet	Volvox_carteri	58.60	0.00

TRINITY_PNW8336	hypothet	Chlamydomonas_reinh	58.60	0.00
TRINITY_PSC7288	E3 ubiq	Micractinium_conduc	58.60	0.00
TRINITY_GAX7309	hypothet	Chlamydomonas_eusti	58.60	0.00
TRINITY_GAX8220	hypothet	Chlamydomonas_eusti	58.60	0.00
TRINITY_PNW7556	hypothet	Chlamydomonas_reinh	58.60	0.00
TRINITY_XP_0239	ras-like	Quercus_suber	58.60	0.00
TRINITY_GAX7551	hypothet	Chlamydomonas_eusti	58.60	0.00
TRINITY_PNW8344	hypothet	Chlamydomonas_reinh	58.60	0.00
TRINITY_PNW8487	hypothet	Chlamydomonas_reinh	58.60	0.00
TRINITY_PNW7509	hypothet	Chlamydomonas_reinh	58.60	0.00
TRINITY_XP_0229	transcri	Cucurbita_moschata	58.60	0.00
TRINITY_GBF9628	SNF2 far	Raphidocelis_subcap	58.60	0.00
TRINITY_AFK3634	unknown	Lotus_japonicus	58.50	0.00
TRINITY_XP_0116	inosine	Amborella_trichopod	58.50	0.00
TRINITY_XP_0105	PREDICTE	Camelina_sativa	58.50	0.00
TRINITY_XP_0029	nascent	Selaginella_moellen	58.50	0.00
TRINITY_XP_0087	diphthar	Phoenix_dactylifera	58.50	0.00
TRINITY_KDD7549	hypothet	Helicosporidium_sp.	58.50	0.00
TRINITY_AAT7361	calmodul	Daucus_carota	58.50	0.00
TRINITY_XP_0016	cell di	Chlamydomonas_reinh	58.50	0.00
TRINITY_XP_0017	predicte	Chlamydomonas_reinh	58.50	0.00
TRINITY_KXZ4180	hypothet	Gonium_pectorale	58.50	0.00
TRINITY_GBF9755	hypothet	Raphidocelis_subcap	58.50	0.00
TRINITY_XP_0139	trans-2-	Monoraphidium_negle	58.50	0.00
TRINITY_BAK0277	predicte	Hordeum_vulgare	58.50	0.00
TRINITY_ACJ8550	unknown	Medicago_truncatula	58.50	0.00
TRINITY_PRW5605	starch b	Chlorella_sorokinia	58.50	0.00
TRINITY_GBF9941	peptidyl	Raphidocelis_subcap	58.50	0.00
TRINITY_XP_0191	PREDICTE	Ipomoea_nil	58.50	0.00
TRINITY_KXZ5526	hypothet	Gonium_pectorale	58.50	0.00
TRINITY_GBF8793	ATP synt	Raphidocelis_subcap	58.50	0.00
TRINITY_XP_0058	hypothet	Chlorella_variabili	58.50	0.00
TRINITY_PNW6960	hypothet	Chlamydomonas_reinh	58.50	0.00
TRINITY_XP_0029	hypothet	Volvox_carteri	58.50	0.00
TRINITY_PTQ4733	hypothet	Marchantia_polymorp	58.50	0
TRINITY_XP_0029	hypothet	Volvox_carteri	58.50	0.00
TRINITY_PNW7646	hypothet	Chlamydomonas_reinh	58.50	0.00
TRINITY_PRW5869	DNA-dire	Chlorella_sorokinia	58.50	0.00
TRINITY_XP_0016	predicte	Chlamydomonas_reinh	58.50	0.00
TRINITY_GAX8459	hypothet	Chlamydomonas_eusti	58.50	0.00
TRINITY_XP_0029	hypothet	Volvox_carteri	58.50	0.00
TRINITY_GAX8301	hypothet	Chlamydomonas_eusti	58.50	0.00
TRINITY_GAX7948	hypothet	Chlamydomonas_eusti	58.50	0.00
TRINITY_XP_0029	metacasp	Volvox_carteri	58.50	0.00
TRINITY_XP_0217	ABC tran	Chenopodium_quinoa	58.50	0.00
TRINITY_GAX7305	hypothet	Chlamydomonas_eusti	58.50	0.00
TRINITY_XP_0030	predicte	Micromonas_pusilla	58.50	0.00
TRINITY_AFK3980	unknown	Medicago_truncatula	58.50	0.00
TRINITY_PNH0731	putative	Tetrabaena_socialis	58.50	0.00
TRINITY_XP_0239	nicotina	Quercus_suber	58.50	0.00
TRINITY_GAX7854	hypothet	Chlamydomonas_eusti	58.50	0.00
TRINITY_GAX7634	hypothet	Chlamydomonas_eusti	58.50	0.00
TRINITY_EFJ1746	hypothet	Selaginella_moellen	58.50	0.00
TRINITY_RLM6636	hypothet	Panicum_miliaceum	58.50	0.00
TRINITY_KXZ4736	hypothet	Gonium_pectorale	58.50	0.00
TRINITY_KXZ4677	hypothet	Gonium_pectorale	58.50	0.00
TRINITY_XP_0029	hypothet	Volvox_carteri	58.50	0.00
TRINITY_PNW7421	hypothet	Chlamydomonas_reinh	58.50	0.00
TRINITY_PNW8806	hypothet	Chlamydomonas_reinh	58.50	0.00
TRINITY_XP_0017	Fe-S ox	Chlamydomonas_reinh	58.50	0.00
TRINITY_KXZ4738	hypothet	Gonium_pectorale	58.50	0.00
TRINITY_EEC7090	hypothet	Oryza_sativa	58.50	0.00
TRINITY_GAX8420	hypothet	Chlamydomonas_eusti	58.50	0.00
TRINITY_XP_0029	hypothet	Volvox_carteri	58.50	0.00
TRINITY_XP_0128	PREDICTE	Erythranthe_guttata	58.40	0.00
TRINITY_XP_0042	PREDICTE	Fragaria_vesca	58.40	0.00

TRINITY_GAQ7892(	Dynein]	Klebsormidium_niten	58.40	0.00
TRINITY_BAJ9317(	predicte	Hordeum_vulgare	58.40	0.00
TRINITY_XP_0106(	PREDICTI	Beta_vulgaris	58.40	0.00
TRINITY_XP_0030'	P-loop (	Ostreococcus_tauri	58.40	0.00
TRINITY_XP_0056!	UBC-like	Coccomyxa_subellips	58.40	0.00
TRINITY_GBG9251	hypothet	Chara_braunii	58.40	0.00
TRINITY_YP_0088]	apocyto(	Entransia_fimbriata	58.40	0.00
TRINITY_XP_0093(	PREDICTI	Musa_acuminata	58.40	0.00
TRINITY_AFC8882(	actin 5-	Miscanthus_sinensis	58.40	0.00
TRINITY_GAX7359!	hypothet	Chlamydomonas_eusti	58.40	0.00
TRINITY_BAK0277(	predicte	Hordeum_vulgare	58.40	0.00
TRINITY_XP_0016!	ATP-sul	Chlamydomonas_reinh	58.40	0.00
TRINITY_GAX7390]	hypothet	Chlamydomonas_eusti	58.40	0.00
TRINITY_PNW8875!	hypothet	Chlamydomonas_reinh	58.40	0.00
TRINITY_XP_0228(	glycine-	Olea_europaea	58.40	0.00
TRINITY_XP_0029	hypothet	Volvox_carteri	58.40	0.00
TRINITY_KXZ4928	hypothet	Gonium_pectorale	58.40	0.00
TRINITY_BAJ9959(	predicte	Hordeum_vulgare	58.40	0.00
TRINITY_PNW8577(	hypothet	Chlamydomonas_reinh	58.40	0.00
TRINITY_GAX7496	hypothet	Chlamydomonas_eusti	58.40	0.00
TRINITY_GAX7888	hypothet	Chlamydomonas_eusti	58.40	0.00
TRINITY_KXZ5100!	hypothet	Gonium_pectorale	58.40	0.00
TRINITY_GAX8405'	hypothet	Chlamydomonas_eusti	58.40	0.00
TRINITY_GAX7870	hypothet	Chlamydomonas_eusti	58.40	0.00
TRINITY_KZV1480(	hypothet	Dorcoceras_hygromet	58.40	0.00
TRINITY_GAX7916(	hypothet	Chlamydomonas_eusti	58.40	0.00
TRINITY_XP_0124!	PREDICTI	Gossypium_raimondii	58.40	0.00
TRINITY_PNW8775	hypothet	Chlamydomonas_reinh	58.40	0.00
TRINITY_GAQ8389	Iron-sul	Klebsormidium_niten	58.40	0.00
TRINITY_KXZ4316!	hypothet	Gonium_pectorale	58.40	0.00
TRINITY_PNW8635!	hypothet	Chlamydomonas_reinh	58.40	0.00
TRINITY_XP_0017(	Qc-SNARI	Chlamydomonas_reinh	58.40	0.00
TRINITY_GAX8091	hypothet	Chlamydomonas_eusti	58.40	0.00
TRINITY_GAX7933(	hypothet	Chlamydomonas_eusti	58.40	0.00
TRINITY_GAX8012!	hypothet	Chlamydomonas_eusti	58.40	0.00
TRINITY_XP_0016!	heat sh(	Chlamydomonas_reinh	58.40	0.00
TRINITY_PNW8510(	hypothet	Chlamydomonas_reinh	58.40	0
TRINITY_XP_0139(	hypothet	Monoraphidium_negle	58.40	0.00
TRINITY_GAQ9356]	hypothet	Klebsormidium_niten	58.40	0.00
TRINITY_PNW8404(	hypothet	Chlamydomonas_reinh	58.40	0.00
TRINITY_ABK2446(	unknown	Picea_sitchensis	58.30	0.00
TRINITY_XP_0238!	ubiquit:	Quercus_suber	58.30	0.00
TRINITY_XP_0096(	PREDICTI	Nicotiana_tomentosi	58.30	0.00
TRINITY_GAX7707	hypothet	Chlamydomonas_eusti	58.30	0.00
TRINITY_RZC1093]	Protein	Glycine_soja	58.30	0.00
TRINITY_XP_0017(	predicte	Chlamydomonas_reinh	58.30	0.00
TRINITY_KXZ4973(	hypothet	Gonium_pectorale	58.30	0.00
TRINITY_XP_0062(	60S ribo	Capsella_rubella	58.30	0.00
TRINITY_XP_0056	cytosol:	Coccomyxa_subellips	58.30	0.00
TRINITY_GAQ8058!	RIO-like	Klebsormidium_niten	58.30	0.00
TRINITY_CAA8775]	Apocyto(	Tetraselmis_subcord	58.30	0.00
TRINITY_GAQ8895	Putative	Klebsormidium_niten	58.30	0.00
TRINITY_XP_0029	hypothet	Volvox_carteri	58.30	0.00
TRINITY_XP_0139(	Prestal}	Monoraphidium_negle	58.30	0.00
TRINITY_XP_0216!	histone	Hevea_brasiliensis	58.30	0.00
TRINITY_OAE3542!	hypothet	Marchantia_polymorp	58.30	0.00
TRINITY_GAX7575!	hypothet	Chlamydomonas_eusti	58.30	0.00
TRINITY_PNH1166	La-relat	Tetrabaena_socialis	58.30	0.00
TRINITY_AAU0098(	trehalo:	Dunaliella_viridis	58.30	0.00
TRINITY_GAX7906]	hypothet	Chlamydomonas_eusti	58.30	0.00
TRINITY_XP_0139(	hypothet	Monoraphidium_negle	58.30	0.00
TRINITY_GBG0041'	hypothet	Raphidocelis_subcap	58.30	0.00
TRINITY_KXZ5129	hypothet	Gonium_pectorale	58.30	0.00
TRINITY_PNH0831(	Nucleol:	Tetrabaena_socialis	58.30	0.00
TRINITY_PNW7460	hypothet	Chlamydomonas_reinh	58.30	0.00
TRINITY_KCW6637(	hypothet	Eucalyptus_grandis	58.30	0.00

TRINITY_GAX8111	hypothet	Chlamydomonas_eusti	58.30	0.00
TRINITY_XP_0228	structu	Olea_europaea	58.30	0.00
TRINITY_AKS2968	UV-B ph	Chlamydomonas_reinh	58.30	0.00
TRINITY_GAX8418	hypothet	Chlamydomonas_eusti	58.30	0.00
TRINITY_XP_0138	TPR rep	Monoraphidium_negle	58.30	0.00
TRINITY_GAX7831	hypothet	Chlamydomonas_eusti	58.30	0.00
TRINITY_PKA6512	60S rib	Apostasia_shenzheni	58.30	0.00
TRINITY_GAX7859	hypothet	Chlamydomonas_eusti	58.30	0.00
TRINITY_XP_0029	60S rib	Selaginella_moellen	58.30	0.00
TRINITY_PNH1056	Exonucl	Tetrabaena_socialis	58.30	0.00
TRINITY_KXZ5290	hypothet	Gonium_pectorale	58.30	0.00
TRINITY_GAX7964	hypothet	Chlamydomonas_eusti	58.30	0.00
TRINITY_PNW8834	hypothet	Chlamydomonas_reinh	58.30	0.00
TRINITY_XP_0016	predict	Chlamydomonas_reinh	58.30	0.00
TRINITY_XP_0029	hypothet	Volvox_carteri	58.30	0.00
TRINITY_XP_0017	predict	Chlamydomonas_reinh	58.30	0.00
TRINITY_XP_0245	beta-ad	Selaginella_moellen	58.30	0.00
TRINITY_XP_0188	PREDICT	Juglans_regia	58.30	0.00
TRINITY_XP_0029	hypothet	Volvox_carteri	58.30	0.00
TRINITY_GAX7700	hypothet	Chlamydomonas_eusti	58.30	0.00
TRINITY_GAX8108	hypothet	Chlamydomonas_eusti	58.30	0.00
TRINITY_XP_0104	PREDICT	Camelina_sativa	58.30	0.00
TRINITY_XP_0238	serine/t	Quercus_suber	58.30	0.00
TRINITY_GAQ9047	KRAB-A	Klebsormidium_niten	58.30	0.00
TRINITY_GAX8592	hypothet	Chlamydomonas_eusti	58.30	0.00
TRINITY_KXZ5495	hypothet	Gonium_pectorale	58.30	0.00
TRINITY_GBF9334	hypothet	Raphidocelis_subcap	58.30	0.00
TRINITY_KXZ4919	hypothet	Gonium_pectorale	58.30	0.00
TRINITY_XP_0017	NADH:ub	Chlamydomonas_reinh	58.30	0.00
TRINITY_PNH0658	Cytosol	Tetrabaena_socialis	58.30	0.00
TRINITY_GBG8952	hypothet	Chara_braunii	58.30	0.00
TRINITY_OAE2900	hypothet	Marchantia_polymorp	58.30	0.00
TRINITY_XP_0029	hypothet	Volvox_carteri	58.30	0.00
TRINITY_GBF9701	hypothet	Raphidocelis_subcap	58.30	0.00
TRINITY_PNW8753	hypothet	Chlamydomonas_reinh	58.30	0.00
TRINITY_XP_0139	beta-ket	Monoraphidium_negle	58.20	0.00
TRINITY_PSC7558	family t	Micractinium_conduc	58.20	0.00
TRINITY_RWW9034	hypothet	Ensete_ventricosum	58.20	0.00
TRINITY_XP_0221	inositol	Momordica_charantia	58.20	0.00
TRINITY_PTQ3022	hypothet	Marchantia_polymorp	58.20	0.00
TRINITY_BAK0043	predict	Hordeum_vulgare	58.20	0.00
TRINITY_ACF7929	unknown	Zea_mays	58.20	0.00
TRINITY_XP_0202	40S rib	Asparagus_officinal	58.20	0.00
TRINITY_XP_0227	ras-rel	Durio_zibethinus	58.20	0.00
TRINITY_KDO6612	hypothet	Citrus_sinensis	58.20	0.00
TRINITY_GAX8189	hypothet	Chlamydomonas_eusti	58.20	0.00
TRINITY_XP_0243	tRNase	Physcomitrella_pate	58.20	0.00
TRINITY_XP_0088	WD-40 r	Phoenix_dactylifera	58.20	0.00
TRINITY_XP_0058	hypothet	Chlorella_variabili	58.20	0.00
TRINITY_XP_0097	PREDICT	Nicotiana_sylvestri	58.20	0.00
TRINITY_XP_0029	hypothet	Volvox_carteri	58.20	0.00
TRINITY_PNW8650	hypothet	Chlamydomonas_reinh	58.20	0.00
TRINITY_XP_0029	hypothet	Volvox_carteri	58.20	0.00
TRINITY_XP_0029	hypothet	Volvox_carteri	58.20	0.00
TRINITY_XP_0016	PsbB mR	Chlamydomonas_reinh	58.20	0.00
TRINITY_PNW7232	hypothet	Chlamydomonas_reinh	58.20	0.00
TRINITY_GAX8463	hypothet	Chlamydomonas_eusti	58.20	0.00
TRINITY_XP_0058	hypothet	Chlorella_variabili	58.20	0.00
TRINITY_XP_0056	eukaryot	Coccomyxa_subellips	58.20	0.00
TRINITY_GAX7626	hypothet	Chlamydomonas_eusti	58.20	0.00
TRINITY_XP_0029	hypothet	Volvox_carteri	58.20	0.00
TRINITY_KXZ4986	hypothet	Gonium_pectorale	58.20	0.00
TRINITY_XP_0029	isocitr	Volvox_carteri	58.20	0.00
TRINITY_XP_0100	PREDICT	Eucalyptus_grandis	58.20	0.00
TRINITY_KXZ5325	hypothet	Gonium_pectorale	58.20	0.00
TRINITY_GAX7420	hypothet	Chlamydomonas_eusti	58.20	0

TRINITY_GAX7809	hypothet	Chlamydomonas_eusti	58.20	0.00
TRINITY_PNW7808	hypothet	Chlamydomonas_reinh	58.20	0.00
TRINITY_PNW8858	hypothet	Chlamydomonas_reinh	58.20	0.00
TRINITY_XP_0030	predict	Micromonas_pusilla	58.20	0.00
TRINITY_PNW8868	hypothet	Chlamydomonas_reinh	58.20	0.00
TRINITY_GBF8956	hypothet	Raphidocelis_subcap	58.20	0.00
TRINITY_GAX7471	hypothet	Chlamydomonas_eusti	58.20	0.00
TRINITY_XP_0056	short-c	Coccomyxa_subellips	58.20	0.00
TRINITY_XP_0244	ABC tra	Physcomitrella_pate	58.20	0.00
TRINITY_CBI2687	unnamed	Vitis_vinifera	58.10	0.00
TRINITY_XP_0029	hypothet	Volvox_carteri	58.10	0.00
TRINITY_GAX7771	hypothet	Chlamydomonas_eusti	58.10	0.00
TRINITY_XP_0114	PREDICT	Fragaria_vesca	58.10	0.00
TRINITY_OTG3652	putativ	Helianthus_annuus	58.10	0.00
TRINITY_XP_0029	hypothet	Volvox_carteri	58.10	0.00
TRINITY_XP_0215	obg-lik	Manihot_esculenta	58.10	0.00
TRINITY_GAX7670	hypothet	Chlamydomonas_eusti	58.10	0.00
TRINITY_XP_0024	predict	Micromonas_commoda	58.10	0.00
TRINITY_GAX7994	hypothet	Chlamydomonas_eusti	58.10	0.00
TRINITY_RYR2709	hypothet	Arachis_hypogaea	58.10	0.00
TRINITY_PSC7496	60S rib	Micractinium_conduc	58.10	0.00
TRINITY_XP_0016	predict	Chlamydomonas_reinh	58.10	0.00
TRINITY_ABR1781	unknown	Picea_sitchensis	58.10	0.00
TRINITY_XP_0058	hypothet	Chlorella_variabili	58.10	0.00
TRINITY_PNW7663	hypothet	Chlamydomonas_reinh	58.10	0.00
TRINITY_OMO5606	hypothet	Corchorus_capsulari	58.10	0.00
TRINITY_XP_0029	hypothet	Volvox_carteri	58.10	0.00
TRINITY_XP_0101	splicin	Morus_notabilis	58.10	0.00
TRINITY_GAX7529	hypothet	Chlamydomonas_eusti	58.10	0.00
TRINITY_PRW6026	Serine	Chlorella_sorokinia	58.10	0.00
TRINITY_GAX8538	hypothet	Chlamydomonas_eusti	58.10	0.00
TRINITY_PNW8248	hypothet	Chlamydomonas_reinh	58.10	0.00
TRINITY_XP_0139	hypothet	Monoraphidium_negle	58.10	0.00
TRINITY_PNH0588	Dehydro	Tetrabaena_socialis	58.10	0.00
TRINITY_PNW8876	hypothet	Chlamydomonas_reinh	58.10	0.00
TRINITY_XP_0229	ABC tra	Cucurbita_moschata	58.10	0.00
TRINITY_ALL5522	NER end	Chlamydomonas_reinh	58.10	0.00
TRINITY_GAX8624	hypothet	Chlamydomonas_eusti	58.10	0.00
TRINITY_PNW8521	hypothet	Chlamydomonas_reinh	58.10	0.00
TRINITY_XP_0056	Pkinase-	Coccomyxa_subellips	58.10	0.00
TRINITY_XP_0029	hypothet	Volvox_carteri	58.10	0.00
TRINITY_KHF9972	E3 ubiq	Gossypium_arboreum	58.00	0.00
TRINITY_XP_0217	40S rib	Chenopodium_quinoa	58.00	0.00
TRINITY_XP_0056	hypothet	Coccomyxa_subellips	58.00	0.00
TRINITY_XP_0056	MT-A70-	Coccomyxa_subellips	58.00	0.00
TRINITY_XP_0106	PREDICT	Beta_vulgaris	58.00	0.00
TRINITY_XP_0128	PREDICT	Erythranthe_guttata	58.00	0.00
TRINITY_KXZ5223	hypothet	Gonium_pectorale	58.00	0.00
TRINITY_GBG6683	hypothet	Chara_braunii	58.00	0.00
TRINITY_GAX8049	hypothet	Chlamydomonas_eusti	58.00	0.00
TRINITY_GAX8310	hypothet	Chlamydomonas_eusti	58.00	0.00
TRINITY_PNW7084	hypothet	Chlamydomonas_reinh	58.00	0.00
TRINITY_PNW8317	hypothet	Chlamydomonas_reinh	58.00	0.00
TRINITY_PTQ3894	hypothet	Marchantia_polymorp	58.00	0.00
TRINITY_KXZ5424	hypothet	Gonium_pectorale	58.00	0.00
TRINITY_GAX7634	hypothet	Chlamydomonas_eusti	58.00	0.00
TRINITY_XP_0017	cytochr	Chlamydomonas_reinh	58.00	0.00
TRINITY_PSC7670	RNA exo	Micractinium_conduc	58.00	0.00
TRINITY_GAX7826	hypothet	Chlamydomonas_eusti	58.00	0.00
TRINITY_PNH1140	hypothet	Tetrabaena_socialis	58.00	0.00
TRINITY_PNW7719	hypothet	Chlamydomonas_reinh	58.00	0.00
TRINITY_XP_0239	calcium	Quercus_suber	58.00	0.00
TRINITY_XP_0243	citrate	Physcomitrella_pate	58.00	0.00
TRINITY_XP_0017	ER DnaJ-	Chlamydomonas_reinh	58.00	0.00
TRINITY_KXZ5667	hypothet	Gonium_pectorale	58.00	0.00
TRINITY_GAX8524	hypothet	Chlamydomonas_eusti	58.00	0.00

TRINITY_KXZ5442	hypothet	Gonium_pectorale	58.00	0.00
TRINITY_XP_0016	delta t	Chlamydomonas_reinh	58.00	0.00
TRINITY_GAX8148	hypothet	Chlamydomonas_eusti	58.00	0.00
TRINITY_XP_0029	hypothet	Volvox_carteri	58.00	0.00
TRINITY_GBG6119	hypothet	Chara_braunii	58.00	0.00
TRINITY_XP_0114	PREDICTI	Fragaria_vesca	58.00	0.00
TRINITY_BAA2591	actin [N	Nannochloris_bacill	58.00	0.00
TRINITY_KXZ4747	hypothet	Gonium_pectorale	58.00	0.00
TRINITY_GAX8108	hypothet	Chlamydomonas_eusti	58.00	0.00
TRINITY_GAX7571	hypothet	Chlamydomonas_eusti	58.00	0.00
TRINITY_GBG7983	hypothet	Chara_braunii	58.00	0.00
TRINITY_XP_0058	hypothet	Chlorella_variabili	58.00	0.00
TRINITY_GAQ9184	cobalam	Klebsormidium_niten	58.00	0
TRINITY_PNW8797	hypothet	Chlamydomonas_reinh	58.00	0.00
TRINITY_PNW7757	hypothet	Chlamydomonas_reinh	58.00	0.00
TRINITY_GAX8357	hypothet	Chlamydomonas_eusti	58.00	0.00
TRINITY_CAB7113	heat sh	Dunaliella_salina	58.00	0.00
TRINITY_XP_0243	DExH-bo	Physcomitrella_pate	58.00	0.00
TRINITY_XP_0114	40S rib	Auxenochlorella_pro	58.00	0.00
TRINITY_GAQ7987	Vacuola	Klebsormidium_niten	57.90	0.00
TRINITY_GAQ9273	Putative	Klebsormidium_niten	57.90	0.00
TRINITY_NP_6893	cytochr	Chaetosphaeridium_g	57.90	0.00
TRINITY_RAL5291	hypothet	Cuscuta_australis	57.90	0.00
TRINITY_XP_0164	PREDICTI	Nicotiana_tabacum	57.90	0.00
TRINITY_XP_0106	PREDICTI	Beta_vulgaris	57.90	0.00
TRINITY_XP_0239	probable	Quercus_suber	57.90	0.00
TRINITY_XP_0243	40S rib	Physcomitrella_pate	57.90	0.00
TRINITY_XP_0056	Rab5a G	Coccomyxa_subellips	57.90	0.00
TRINITY_XP_0201	V-type p	Aegilops_tauschii	57.90	0.00
TRINITY_GAQ9196	hypothet	Klebsormidium_niten	57.90	0.00
TRINITY_XP_0238	lipoyl s	Quercus_suber	57.90	0.00
TRINITY_GBG6683	hypothet	Chara_braunii	57.90	0.00
TRINITY_XP_0237	geranylg	Lactuca_sativa	57.90	0.00
TRINITY_PSC6975	60S rib	Micractinium_conduc	57.90	0.00
TRINITY_OIW1138	hypothet	Lupinus_angustifoli	57.90	0.00
TRINITY_XP_0243	cAMP-dep	Physcomitrella_pate	57.90	0.00
TRINITY_PPR8338	hypothet	Gossypium_barbadens	57.90	0.00
TRINITY_GAX7459	hypothet	Chlamydomonas_eusti	57.90	0.00
TRINITY_XP_0029	hypothet	Volvox_carteri	57.90	0.00
TRINITY_GAX8462	hypothet	Chlamydomonas_eusti	57.90	0.00
TRINITY_GBG0007	hypothet	Raphidocelis_subcap	57.90	0.00
TRINITY_PTQ3041	hypothet	Marchantia_polymorp	57.90	0.00
TRINITY_XP_0017	predicte	Chlamydomonas_reinh	57.90	0.00
TRINITY_XP_0058	hypothet	Chlorella_variabili	57.90	0.00
TRINITY_ABR1688	unknown	Picea_sitchensis	57.90	0.00
TRINITY_O04073.1	RecName:	Tetradesmus_obliquu	57.90	0.00
TRINITY_GBF9937	hypothet	Raphidocelis_subcap	57.90	0.00
TRINITY_GAX8145	hypothet	Chlamydomonas_eusti	57.90	0.00
TRINITY_PRW5955	ATPase f	Chlorella_sorokinia	57.90	0.00
TRINITY_XP_0239	aldo-ket	Quercus_suber	57.90	0.00
TRINITY_GBF8936	hypothet	Raphidocelis_subcap	57.90	0.00
TRINITY_XP_0207	MAP3K e	Dendrobium_catenatu	57.90	0.00
TRINITY_GAX7886	hypothet	Chlamydomonas_eusti	57.90	0.00
TRINITY_KXZ4607	hypothet	Gonium_pectorale	57.90	0.00
TRINITY_PNW8843	hypothet	Chlamydomonas_reinh	57.90	0.00
TRINITY_GAX7982	hypothet	Chlamydomonas_eusti	57.90	0.00
TRINITY_GAX7982	hypothet	Chlamydomonas_eusti	57.90	0.00
TRINITY_P54214.1	RecName:	Dunaliella_bioculat	57.90	0.00
TRINITY_XP_0238	geranylg	Quercus_suber	57.90	0.00
TRINITY_KXZ5284	hypothet	Gonium_pectorale	57.90	0.00
TRINITY_GAX7986	hypothet	Chlamydomonas_eusti	57.90	0
TRINITY_XP_0029	hypothet	Volvox_carteri	57.90	0.00
TRINITY_PNW8187	hypothet	Chlamydomonas_reinh	57.90	0.00
TRINITY_PNW8775	hypothet	Chlamydomonas_reinh	57.90	0.00
TRINITY_RYR6371	hypothet	Arachis_hypogaea	57.90	0.00
TRINITY_GAX7994	hypothet	Chlamydomonas_eusti	57.90	0.00

TRINITY_GAU1160	hypothet	Trifolium_subterran	57.90	0.00
TRINITY_GAX7968	hypothet	Chlamydomonas_eusti	57.90	0.00
TRINITY_EFJ0486	hypothet	Selaginella_moellen	57.90	0.00
TRINITY_GAQ8931	Dyneins	Klebsormidium_niten	57.90	0.00
TRINITY_CDP0417	unnamed	Coffea_canephora	57.90	0.00
TRINITY_ONK8191	uncharac	Asparagus_officinal	57.80	0.00
TRINITY_KXZ5551	hypothet	Gonium_pectorale	57.80	0.00
TRINITY_GAX7716	hypothet	Chlamydomonas_eusti	57.80	0.00
TRINITY_GAX7596	hypothet	Chlamydomonas_eusti	57.80	0.00
TRINITY_PNH1057	Retrovi	Tetrabaena_socialis	57.80	0.00
TRINITY_XP_0264	WD repe	Papaver_somniferum	57.80	0.00
TRINITY_GAX7392	hypothet	Chlamydomonas_eusti	57.80	0.00
TRINITY_XP_0229	putative	Cucurbita_moschata	57.80	0.00
TRINITY_GAX7317	hypothet	Chlamydomonas_eusti	57.80	0.00
TRINITY_XP_0239	myosin-	Quercus_suber	57.80	0.00
TRINITY_GBF8763	hypothet	Raphidocelis_subcap	57.80	0.00
TRINITY_PNH0769	Methyl-	Tetrabaena_socialis	57.80	0.00
TRINITY_XP_0058	hypothet	Chlorella_variabili	57.80	0.00
TRINITY_XP_0056	hypothet	Coccomyxa_subellips	57.80	0.00
TRINITY_XP_0138	hypothet	Monoraphidium_negle	57.80	0.00
TRINITY_KXZ4923	hypothet	Gonium_pectorale	57.80	0.00
TRINITY_KXZ4550	hypothet	Gonium_pectorale	57.80	0.00
TRINITY_GAX7759	hypothet	Chlamydomonas_eusti	57.80	0.00
TRINITY_KXZ4949	hypothet	Gonium_pectorale	57.80	0.00
TRINITY_PNW7425	hypothet	Chlamydomonas_reinh	57.80	0.00
TRINITY_PNW7811	hypothet	Chlamydomonas_reinh	57.80	0.00
TRINITY_PNW7350	hypothet	Chlamydomonas_reinh	57.80	0.00
TRINITY_XP_0029	hypothet	Volvox_carteri	57.80	0.00
TRINITY_GAX7590	hypothet	Chlamydomonas_eusti	57.80	0.00
TRINITY_KZN0886	hypothet	Daucus_carota	57.80	0.00
TRINITY_PSC7267	Aldo ket	Micractinium_conduc	57.80	0.00
TRINITY_XP_0056	kinesin-	Coccomyxa_subellips	57.80	0.00
TRINITY_KXZ4189	hypothet	Gonium_pectorale	57.80	0.00
TRINITY_GAX7558	hypothet	Chlamydomonas_eusti	57.80	0.00
TRINITY_PIA4696	hypothet	Aquilegia_coerulea	57.80	0.00
TRINITY_XP_0017	peptidy	Chlamydomonas_reinh	57.80	0.00
TRINITY_XP_0029	hypothet	Volvox_carteri	57.80	0.00
TRINITY_XP_0188	PREDICT	Juglans_regia	57.80	0.00
TRINITY_GBF9903	hypothet	Raphidocelis_subcap	57.80	0.00
TRINITY_GAQ7854	Cytidin	Klebsormidium_niten	57.80	0.00
TRINITY_GAX7854	hypothet	Chlamydomonas_eusti	57.80	0.00
TRINITY_XP_0064	U11/U12	Citrus_clementina	57.80	0.00
TRINITY_GAQ9059	DNA-dir	Klebsormidium_niten	57.80	0.00
TRINITY_XP_0139	hypothet	Monoraphidium_negle	57.80	0.00
TRINITY_GAX7422	hypothet	Chlamydomonas_eusti	57.80	0.00
TRINITY_GAX8343	hypothet	Chlamydomonas_eusti	57.80	0.00
TRINITY_AAB8649	calmodu	Zea_mays	57.70	0.00
TRINITY_RMZ5553	hypothet	Auxenochlorella_pro	57.70	0.00
TRINITY_XP_0064	zinc fi	Citrus_sinensis	57.70	0.00
TRINITY_XP_0208	tubulin	Arabidopsis_lyrata	57.70	0.00
TRINITY_OAE2770	hypothet	Marchantia_polymorp	57.70	0.00
TRINITY_XP_0025	SNF2 su	Micromonas_commoda	57.70	0.00
TRINITY_XP_0110	ABC tra	Sesamum_indicum	57.70	0.00
TRINITY_XP_0075	nucleot	Bathycoccus_prasino	57.70	0.00
TRINITY_OUS4456	alas pr	Ostreococcus_tauri	57.70	0.00
TRINITY_GBF9432	hypothet	Raphidocelis_subcap	57.70	0.00
TRINITY_GAX7690	hypothet	Chlamydomonas_eusti	57.70	0.00
TRINITY_EMS6059	40S rib	Triticum_urartu	57.70	0.00
TRINITY_XP_0239	myosin-	Quercus_suber	57.70	0.00
TRINITY_XP_0056	hypothet	Coccomyxa_subellips	57.70	0.00
TRINITY_GAX8512	hypothet	Chlamydomonas_eusti	57.70	0.00
TRINITY_XP_0016	predict	Chlamydomonas_reinh	57.70	0.00
TRINITY_XP_0063	PREDICT	Solanum_tuberosum	57.70	0.00
TRINITY_KXZ5246	hypothet	Gonium_pectorale	57.70	0.00
TRINITY_XP_0029	prohibit	Volvox_carteri	57.70	0.00
TRINITY_XP_0100	PREDICT	Eucalyptus_grandis	57.70	0.00

TRINITY_XP_0243(	uncharac	Physcomitrella_pate	57.70	0.00
TRINITY_PNW8449(	hypothet	Chlamydomonas_reinh	57.70	0.00
TRINITY_XP_0017(	predicte	Chlamydomonas_reinh	57.70	0.00
TRINITY_KXZ5325(	hypothet	Gonium_pectorale	57.70	0.00
TRINITY_XP_0017(	phosphat	Chlamydomonas_reinh	57.70	0.00
TRINITY_XP_0093(	PREDICTI	Musa_acuminata	57.70	0.00
TRINITY_GAX7743(	hypothet	Chlamydomonas_eusti	57.70	0.00
TRINITY_GAQ8497(	Aspartyl	Klebsormidium_niten	57.70	0.00
TRINITY_GAX7287(	hypothet	Chlamydomonas_eusti	57.70	0.00
TRINITY_XP_0016(	R53.5-re	Chlamydomonas_reinh	57.70	0.00
TRINITY_XP_0029(	pyruvate	Volvox_carteri	57.70	0.00
TRINITY_GBF9028(	kar3 typ	Raphidocelis_subcap	57.70	0.00
TRINITY_GAX8569(	hypothet	Chlamydomonas_eusti	57.70	0.00
TRINITY_XP_0056(	DEAD-dom	Coccomyxa_subellips	57.70	0.00
TRINITY_GBF8970(	hypothet	Raphidocelis_subcap	57.70	0.00
TRINITY_GAX7263(	hypothet	Chlamydomonas_eusti	57.70	0.00
TRINITY_XP_0093(	PREDICTI	Pyrus_x_bretschneid	57.70	0.00
TRINITY_GAQ8131(	Molybdop	Klebsormidium_niten	57.70	0.00
TRINITY_GAX8386(	hypothet	Chlamydomonas_eusti	57.70	0.00
TRINITY_XP_0029(	hypothet	Volvox_carteri	57.70	0.00
TRINITY_GAX8431(	hypothet	Chlamydomonas_eusti	57.70	0.00
TRINITY_PNW7402(	hypothet	Chlamydomonas_reinh	57.70	0.00
TRINITY_PNW8046(	hypothet	Chlamydomonas_reinh	57.70	0.00
TRINITY_XP_0016(	predicte	Chlamydomonas_reinh	57.70	0.00
TRINITY_XP_0016(	acid pho	Chlamydomonas_reinh	57.70	0.00
TRINITY_PNW8904(	hypothet	Chlamydomonas_reinh	57.70	0.00
TRINITY_GAX7989(	hypothet	Chlamydomonas_eusti	57.70	0.00
TRINITY_GAX8588(	hypothet	Chlamydomonas_eusti	57.70	0
TRINITY_XP_0017(	flagella	Chlamydomonas_reinh	57.70	0.00
TRINITY_BAK0277(	predicte	Hordeum_vulgare	57.70	0.00
TRINITY_GAX7707(	hypothet	Chlamydomonas_eusti	57.70	0
TRINITY_CAA4928(	gag,prot	Volvox_carteri	57.70	0.00
TRINITY_ABU5296(	hypothet	Dunaliella_viridis	57.70	0.00
TRINITY_BAK0244(	predicte	Hordeum_vulgare	57.60	0.00
TRINITY_PTQ2993(	hypothet	Marchantia_polymorp	57.60	0.00
TRINITY_XP_0240(	mitogen-	Morus_notabilis	57.60	0.00
TRINITY_KFK4262(	hypothet	Arabis_alpina	57.60	0.00
TRINITY_XP_0016(	flagella	Chlamydomonas_reinh	57.60	0.00
TRINITY_XP_0093(	PREDICTI	Pyrus_x_bretschneid	57.60	0.00
TRINITY_XP_0239(	hydroxya	Quercus_suber	57.60	0.00
TRINITY_XP_0243(	ras-rela	Physcomitrella_pate	57.60	0.00
TRINITY_GAQ8088(	adaptor	Klebsormidium_niten	57.60	0.00
TRINITY_XP_0202(	probable	Asparagus_officinal	57.60	0.00
TRINITY_PRQ2606(	putative	Rosa_chinensis	57.60	0.00
TRINITY_XP_0029(	hypothet	Volvox_carteri	57.60	0.00
TRINITY_PNH0290(	Tubulin	Tetrabaena_socialis	57.60	0.00
TRINITY_XP_0056(	hypothet	Coccomyxa_subellips	57.60	0.00
TRINITY_PNW8848(	hypothet	Chlamydomonas_reinh	57.60	0.00
TRINITY_XP_0016(	coiled-c	Chlamydomonas_reinh	57.60	0.00
TRINITY_PNW8385(	hypothet	Chlamydomonas_reinh	57.60	0.00
TRINITY_KDD7640(	electron	Helicosporidium_sp.	57.60	0.00
TRINITY_XP_0104(	PREDICTI	Camelina_sativa	57.60	0.00
TRINITY_GAX8003(	hypothet	Chlamydomonas_eusti	57.60	0.00
TRINITY_GAU2503(	hypothet	Trifolium_subterran	57.60	0.00
TRINITY_GAX7314(	hypothet	Chlamydomonas_eusti	57.60	0.00
TRINITY_XP_0156(	calmodul	Oryza_sativa	57.60	0.00
TRINITY_XP_0095(	PREDICTI	Nicotiana_tomentosi	57.60	0.00
TRINITY_PNW7157(	hypothet	Chlamydomonas_reinh	57.60	0.00
TRINITY_PNW8245(	hypothet	Chlamydomonas_reinh	57.60	0.00
TRINITY_KXZ5626(	hypothet	Gonium_pectorale	57.60	0.00
TRINITY_GAX7934(	hypothet	Chlamydomonas_eusti	57.60	0.00
TRINITY_XP_0029(	hypothet	Volvox_carteri	57.60	0.00
TRINITY_PNW7394(	hypothet	Chlamydomonas_reinh	57.60	0.00
TRINITY_GAX8370(	hypothet	Chlamydomonas_eusti	57.60	0.00
TRINITY_GBG6774(	hypothet	Chara_braunii	57.60	0.00
TRINITY_XP_0045(	pyrophos	Cicer_arietinum	57.60	0.00

TRINITY_PNW7437(	hypothet	Chlamydomonas_reinh	57.60	0.00
TRINITY_GAX7818'	hypothet	Chlamydomonas_eusti	57.60	0.00
TRINITY_KXZ4801	hypothet	Gonium_pectorale	57.60	0.00
TRINITY_GAX8060(	hypothet	Chlamydomonas_eusti	57.60	0.00
TRINITY_GAX8230'	hypothet	Chlamydomonas_eusti	57.60	0
TRINITY_GAX8624(	hypothet	Chlamydomonas_eusti	57.60	0.00
TRINITY_XP_0025(	predicte	Micromonas_commoda	57.60	0.00
TRINITY_XP_0029	hypothet	Volvox_carteri	57.60	0.00
TRINITY_GAQ9120	glutath	Klebsormidium_niten	57.60	0.00
TRINITY_EPS7223	hypothet	Genlisea_aurea	57.50	0.00
TRINITY_XP_0263	14-3-3	Papaver_somniferum	57.50	0.00
TRINITY_RMZ5446(	hypothet	Auxenochlorella_pro	57.50	0.00
TRINITY_XP_0029	DNA rep	Volvox_carteri	57.50	0.00
TRINITY_XP_0030	predicte	Micromonas_pusilla	57.50	0.00
TRINITY_PRW3303'	RNA-bin	Chlorella_sorokinia	57.50	0.00
TRINITY_XP_0238	cytosol	Quercus_suber	57.50	0.00
TRINITY_XP_0029	hypothet	Volvox_carteri	57.50	0.00
TRINITY_XP_0029	hypothet	Volvox_carteri	57.50	0.00
TRINITY_GAX7995	hypothet	Chlamydomonas_eusti	57.50	0.00
TRINITY_GAX8055	hypothet	Chlamydomonas_eusti	57.50	0.00
TRINITY_PNW8901	hypothet	Chlamydomonas_reinh	57.50	0.00
TRINITY_GAX8065	hypothet	Chlamydomonas_eusti	57.50	0.00
TRINITY_GAX8443	hypothet	Chlamydomonas_eusti	57.50	0.00
TRINITY_KDD7281	hypothet	Helicosporidium_sp.	57.50	0.00
TRINITY_GAX8081	hypothet	Chlamydomonas_eusti	57.50	0.00
TRINITY_GAX8439(	hypothet	Chlamydomonas_eusti	57.50	0.00
TRINITY_XP_0110'	signal	Sesamum_indicum	57.50	0.00
TRINITY_GAX8419(	hypothet	Chlamydomonas_eusti	57.50	0.00
TRINITY_KXZ4861	hypothet	Gonium_pectorale	57.50	0.00
TRINITY_XP_0029	hypothet	Volvox_carteri	57.50	0.00
TRINITY_GAX7294	hypothet	Chlamydomonas_eusti	57.50	0.00
TRINITY_XP_0017(	predicte	Chlamydomonas_reinh	57.50	0.00
TRINITY_PNW8018'	hypothet	Chlamydomonas_reinh	57.50	0.00
TRINITY_XP_0138	putative	Monoraphidium_negle	57.50	0.00
TRINITY_GAX8009	hypothet	Chlamydomonas_eusti	57.50	0.00
TRINITY_GAX7357	hypothet	Chlamydomonas_eusti	57.50	0.00
TRINITY_PRW5667	decarbo	Chlorella_sorokinia	57.50	0.00
TRINITY_GBF9111	water d	Raphidocelis_subcap	57.50	0.00
TRINITY_GAX8058	hypothet	Chlamydomonas_eusti	57.50	0.00
TRINITY_GAX7804(	hypothet	Chlamydomonas_eusti	57.50	0.00
TRINITY_XP_0024(	actin-3	Sorghum_bicolor	57.50	0.00
TRINITY_XP_0016	ADP-rib	Chlamydomonas_reinh	57.50	0.00
TRINITY_XP_0094	PREDICT	Musa_acuminata	57.50	0.00
TRINITY_XP_0174	PREDICT	Vigna_angularis	57.50	0.00
TRINITY_KXZ5621'	hypothet	Gonium_pectorale	57.50	0.00
TRINITY_GAX7415	hypothet	Chlamydomonas_eusti	57.50	0.00
TRINITY_XP_0139(	Signal	Monoraphidium_negle	57.50	0.00
TRINITY_PRW3930(	zinc ca	Chlorella_sorokinia	57.50	0.00
TRINITY_XP_0238'	COBW do	Quercus_suber	57.40	0.00
TRINITY_XP_0206'	ubiquit	Dendrobium_catenatu	57.40	0.00
TRINITY_GAQ8798(	Phosphat	Klebsormidium_niten	57.40	0.00
TRINITY_XP_0110	probable	Sesamum_indicum	57.40	0.00
TRINITY_GBF9899	cytochr	Raphidocelis_subcap	57.40	0.00
TRINITY_XP_0243	probable	Physcomitrella_pate	57.40	0.00
TRINITY_GBF9931	hypothet	Raphidocelis_subcap	57.40	0.00
TRINITY_XP_0016	predicte	Chlamydomonas_reinh	57.40	0.00
TRINITY_KXZ4906	hypothet	Gonium_pectorale	57.40	0.00
TRINITY_XP_0138	ERD4-re	Monoraphidium_negle	57.40	0.00
TRINITY_XP_0029	hypothet	Volvox_carteri	57.40	0.00
TRINITY_PNW8460	hypothet	Chlamydomonas_reinh	57.40	0.00
TRINITY_XP_0243(	U3 smal	Physcomitrella_pate	57.40	0.00
TRINITY_GAX7513	hypothet	Chlamydomonas_eusti	57.40	0.00
TRINITY_PRW5797	ATP-dep	Chlorella_sorokinia	57.40	0.00
TRINITY_XP_0025(	elongat	Micromonas_commoda	57.40	0.00
TRINITY_PRW6069'	DNA dam	Chlorella_sorokinia	57.40	0.00
TRINITY_KXZ4334	hypothet	Gonium_pectorale	57.40	0.00

TRINITY_XP_0239(	aldo-ket	Quercus_suber	57.40	0.00
TRINITY_XP_0016(	FAD-depe	Chlamydomonas_reinh	57.40	0.00
TRINITY_KXZ4230	hypothet	Gonium_pectorale	57.40	0.00
TRINITY_KVI0348	Gelsolir	Cynara_cardunculus	57.40	0.00
TRINITY_OWM6501	hypothet	Punica_granatum	57.40	0.00
TRINITY_KXZ5255	hypothet	Gonium_pectorale	57.40	0.00
TRINITY_GBG9044	hypothet	Chara_braunii	57.40	0.00
TRINITY_GBF9744	importir	Raphidocelis_subcap	57.40	0.00
TRINITY_GBF8911	hypothet	Raphidocelis_subcap	57.40	0.00
TRINITY_XP_0016	predicte	Chlamydomonas_reinh	57.40	0.00
TRINITY_XP_0172	PREDICTE	Daucus_carota	57.40	0.00
TRINITY_PRW5987	GDP-Man:	Chlorella_sorokinia	57.40	0.00
TRINITY_GAX7306	hypothet	Chlamydomonas_eusti	57.40	0.00
TRINITY_GAX7921	hypothet	Chlamydomonas_eusti	57.40	0.00
TRINITY_XP_0043(	PREDICTE	Fragaria_vesca	57.40	0.00
TRINITY_XP_0017(	lipid-b	Chlamydomonas_reinh	57.40	0.00
TRINITY_GAX7638	hypothet	Chlamydomonas_eusti	57.40	0.00
TRINITY_ERN1059	hypothet	Amborella_trichopod	57.40	0.00
TRINITY_GAX8006	hypothet	Chlamydomonas_eusti	57.40	0.00
TRINITY_XP_0192	PREDICTE	Nicotiana_attenuata	57.40	0.00
TRINITY_GAX7540	hypothet	Chlamydomonas_eusti	57.40	0.00
TRINITY_XP_0029	hypothet	Volvox_carteri	57.40	0.00
TRINITY_GAX7626	hypothet	Chlamydomonas_eusti	57.40	0.00
TRINITY_PNW8155	hypothet	Chlamydomonas_reinh	57.40	0.00
TRINITY_XP_0139(	hypothet	Monoraphidium_negle	57.40	0.00
TRINITY_GBG8457	hypothet	Chara_braunii	57.40	0.00
TRINITY_KXZ4245	hypothet	Gonium_pectorale	57.40	0.00
TRINITY_GAX8211(	hypothet	Chlamydomonas_eusti	57.40	0.00
TRINITY_XP_0016	sar-type	Chlamydomonas_reinh	57.30	0.00
TRINITY_RLM5484	shaggy-r	Panicum_miliaceum	57.30	0.00
TRINITY_KXZ5181	hypothet	Gonium_pectorale	57.30	0.00
TRINITY_XP_0025(	predicte	Micromonas_commoda	57.30	0.00
TRINITY_PWA5060	COX15/Ct	Artemisia_annua	57.30	0.00
TRINITY_XP_0239(	chorisma	Quercus_suber	57.30	0.00
TRINITY_EFJ3744	hypothet	Selaginella_moellen	57.30	0.00
TRINITY_XP_0167	PREDICTE	Gossypium_hirsutum	57.30	0.00
TRINITY_OMO6756	hypothet	Corchorus_capsulari	57.30	0.00
TRINITY_PKU6731	hypothet	Dendrobium_catenatu	57.30	0.00
TRINITY_XP_0243	40S ribo	Physcomitrella_pate	57.30	0.00
TRINITY_GAX8082	hypothet	Chlamydomonas_eusti	57.30	0.00
TRINITY_PSC7221	50S ribo	Micractinium_conduc	57.30	0.00
TRINITY_XP_0041	PREDICTE	Cucumis_sativus	57.30	0.00
TRINITY_XP_0029	hypothet	Volvox_carteri	57.30	0.00
TRINITY_GBF9041	hypothet	Raphidocelis_subcap	57.30	0.00
TRINITY_XP_0139(	6-phosph	Monoraphidium_negle	57.30	0.00
TRINITY_XP_0128	PREDICTE	Erythranthe_guttata	57.30	0.00
TRINITY_XP_0025(	predicte	Micromonas_commoda	57.30	0.00
TRINITY_XP_0063	aldehyde	Eutrema_salsugineum	57.30	0.00
TRINITY_PNW7923	hypothet	Chlamydomonas_reinh	57.30	0.00
TRINITY_XP_0138	NifU-lik	Monoraphidium_negle	57.30	0.00
TRINITY_XP_0016	predicte	Chlamydomonas_reinh	57.30	0.00
TRINITY_XP_0029	hypothet	Volvox_carteri	57.30	0.00
TRINITY_XP_0016	predicte	Chlamydomonas_reinh	57.30	0.00
TRINITY_KFK3120	hypothet	Arabis_alpina	57.30	0.00
TRINITY_GBF9559	hypothet	Raphidocelis_subcap	57.30	0.00
TRINITY_AXU9887	hypothet	Brassica_juncea	57.30	0.00
TRINITY_GBF9458	hypothet	Raphidocelis_subcap	57.30	0.00
TRINITY_XP_0138	ethanola	Monoraphidium_negle	57.30	0.00
TRINITY_GAX7408	hypothet	Chlamydomonas_eusti	57.30	0.00
TRINITY_GAX7283	hypothet	Chlamydomonas_eusti	57.30	0.00
TRINITY_XP_0016	predicte	Chlamydomonas_reinh	57.30	0.00
TRINITY_GAX8396(	hypothet	Chlamydomonas_eusti	57.30	0.00
TRINITY_XP_0243	uncharac	Physcomitrella_pate	57.30	0.00
TRINITY_P0C8M8.	RecName:	Zea_mays	57.30	0.00
TRINITY_PNW8001	hypothet	Chlamydomonas_reinh	57.30	0.00
TRINITY_RMZ5401	hypothet	Auxenochlorella_prc	57.30	0.00

TRINITY_XP_0243(	general	Physcomitrella_pate	57.30	0.00
TRINITY_GAX7823(	hypothet	Chlamydomonas_eusti	57.30	0.00
TRINITY_PNW8067(	hypothet	Chlamydomonas_reinh	57.30	0.00
TRINITY_GAQ8081(	Seryl-tF	Klebsormidium_niten	57.30	0.00
TRINITY_GAX7818	hypothet	Chlamydomonas_eusti	57.30	0.00
TRINITY_GAX8400	hypothet	Chlamydomonas_eusti	57.30	0.00
TRINITY_XP_0191	PREDICTE	Ipomoea_nil	57.30	0.00
TRINITY_PNW8315	hypothet	Chlamydomonas_reinh	57.30	0.00
TRINITY_GAX8067	hypothet	Chlamydomonas_eusti	57.30	0.00
TRINITY_XP_0139(	superox	Monoraphidium_negle	57.30	0.00
TRINITY_PNW7717(	hypothet	Chlamydomonas_reinh	57.30	0.00
TRINITY_O65315.	RecName:	Coleochaete_scutata	57.30	0.00
TRINITY_XP_0228	60S rib	Olea_europaea	57.30	0.00
TRINITY_XP_0025(	predicte	Micromonas_commoda	57.30	0.00
TRINITY_GAQ8867	20S prot	Klebsormidium_niten	57.20	0.00
TRINITY_XP_0028	40S rib	Arabidopsis_lyrata	57.20	0.00
TRINITY_GAQ9260	Hypothet	Klebsormidium_niten	57.20	0.00
TRINITY_XP_0016	phospho	Chlamydomonas_reinh	57.20	0.00
TRINITY_PSC7066	Solute (	Micractinium_conduc	57.20	0.00
TRINITY_GAX8387	hypothet	Chlamydomonas_eusti	57.20	0.00
TRINITY_XP_0016	predicte	Chlamydomonas_reinh	57.20	0.00
TRINITY_XP_0029	hypothet	Volvox_carteri	57.20	0.00
TRINITY_GAQ7947(	ER lumen	Klebsormidium_niten	57.20	0.00
TRINITY_P0C8M8.	RecName:	Zea_mays	57.20	0.00
TRINITY_PNH0198	Ankyrin-	Tetrabaena_socialis	57.20	0.00
TRINITY_ABI9400	mitochon	Dunaliella_salina	57.20	0.00
TRINITY_KXZ5660	hypothet	Gonium_pectorale	57.20	0.00
TRINITY_XP_0239	adenylat	Quercus_suber	57.20	0.00
TRINITY_XP_0029	hypothet	Volvox_carteri	57.20	0.00
TRINITY_O65315.	RecName:	Coleochaete_scutata	57.20	0.00
TRINITY_PNW7759	hypothet	Chlamydomonas_reinh	57.20	0.00
TRINITY_XP_0104	PREDICTE	Camelina_sativa	57.10	0.00
TRINITY_XP_0056	RNA hel	Coccomyxa_subellips	57.10	0.00
TRINITY_GBG7650	hypothet	Chara_braunii	57.10	0.00
TRINITY_KDD7443(	hypothet	Helicosporidium_sp.	57.10	0.00
TRINITY_PNW8357	hypothet	Chlamydomonas_reinh	57.10	0.00
TRINITY_XP_0072(	cyclin-U	Prunus_persica	57.10	0.00
TRINITY_XP_0113	Peroxire	Auxenochlorella_pro	57.10	0.00
TRINITY_XP_0218	zinc fir	Carica_papaya	57.10	0.00
TRINITY_XP_0172	PREDICTE	Daucus_carota	57.10	0.00
TRINITY_XP_0030	predicte	Micromonas_pusilla	57.10	0.00
TRINITY_KXZ4533	hypothet	Gonium_pectorale	57.10	0.00
TRINITY_PRW3289	WD40 rep	Chlorella_sorokinia	57.10	0.00
TRINITY_XP_0257(	cyclin-c	Arachis_hypogaea	57.10	0.00
TRINITY_PSC7231	DNA poly	Micractinium_conduc	57.10	0.00
TRINITY_KXZ4203	hypothet	Gonium_pectorale	57.10	0.00
TRINITY_XP_0029	hypothet	Volvox_carteri	57.10	0.00
TRINITY_KXZ5064	hypothet	Gonium_pectorale	57.10	0.00
TRINITY_KNA1554	hypothet	Spinacia_oleracea	57.10	0.00
TRINITY_NP_0013	14-3-3 p	Nicotiana_tabacum	57.10	0.00
TRINITY_GBF9119	hypothet	Raphidocelis_subcap	57.10	0.00
TRINITY_XP_0029	hypothet	Volvox_carteri	57.10	0.00
TRINITY_XP_0216	ABC tran	Manihot_esculenta	57.10	0.00
TRINITY_XP_0029	hypothet	Volvox_carteri	57.10	0.00
TRINITY_PNW7856(	hypothet	Chlamydomonas_reinh	57.10	0.00
TRINITY_ONK7211	uncharac	Asparagus_officinal	57.10	0.00
TRINITY_XP_0029	hypothet	Volvox_carteri	57.10	0.00
TRINITY_PIA4991	hypothet	Aquilegia_coerulea	57.10	0.00
TRINITY_GAX8286	hypothet	Chlamydomonas_eusti	57.10	0.00
TRINITY_BAK0277	predicte	Hordeum_vulgare	57.10	0.00
TRINITY_GAX8313	hypothet	Chlamydomonas_eusti	57.10	0.00
TRINITY_PPS1685	hypothet	Gossypium_barbadens	57.10	0.00
TRINITY_KXZ5292(	hypothet	Gonium_pectorale	57.10	0.00
TRINITY_PNW7121	hypothet	Chlamydomonas_reinh	57.10	0.00
TRINITY_XP_0014	predicte	Ostreococcus_sp._'l	57.10	0.00
TRINITY_XP_0017(	histone	Chlamydomonas_reinh	57.10	0.00

TRINITY_GBG8174[illegible]	hypothet	Chara_braunii	57.10	0.00
TRINITY_GAX7686[illegible]	hypothet	Chlamydomonas_eusti	57.10	0.00
TRINITY_ONI0023[illegible]	hypothet	Prunus_persica	57.10	0.00
TRINITY_XP_0029[illegible]	hypothet	Volvox_carteri	57.10	0.00
TRINITY_PNH0403[illegible]	hypothet	Tetrabaena_socialis	57.10	0.00
TRINITY_GAQ8367[illegible]	homeobo	Klebsormidium_niten	57.10	0.00
TRINITY_KCW8530[illegible]	hypothet	Eucalyptus_grandis	57.10	0.00
TRINITY_GAX8569[illegible]	hypothet	Chlamydomonas_eusti	57.10	0.00
TRINITY_GBF9350[illegible]	hypothet	Raphidocelis_subcap	57.10	0.00
TRINITY_XP_0239[illegible]	AMP dear	Quercus_suber	57.10	0.00

TRINITY_PNW8323	hypothet	Chlamydomonas_reinh	57.10	0.00
TRINITY_GAX7528	hypothet	Chlamydomonas_eusti	57.10	0.00
TRINITY_GBF9425	110 kDa	Raphidocelis_subcap	57.10	0.00
TRINITY_GAX8112	hypothet	Chlamydomonas_eusti	57.10	0.00
TRINITY_XP_0063	PREDICTE	Solanum_tuberosum	57.10	0.00
TRINITY_XP_0030	Tetratri	Ostreococcus_tauri	57.10	0.00
TRINITY_GAX7496	hypothet	Chlamydomonas_eusti	57.10	0.00
TRINITY_GBF9031	transcri	Raphidocelis_subcap	57.10	0.00
TRINITY_KXZ5594	hypothet	Gonium_pectorale	57.10	0.00
TRINITY_GBF9342	type I	Raphidocelis_subcap	57.10	0.00
TRINITY_XP_0238	chorisma	Quercus_suber	57.10	0.00
TRINITY_XP_0016	predicte	Chlamydomonas_reinh	57.10	0.00
TRINITY_GAX7949	hypothet	Chlamydomonas_eusti	57.10	0.00
TRINITY_RWR9279	tRNA-bir	Cinnamomum_micranth	57.10	0.00
TRINITY_PNH0234	Tubulin-	Tetrabaena_socialis	57.10	0.00
TRINITY_KXZ5592	hypothet	Gonium_pectorale	57.10	0.00
TRINITY_XP_0016	predicte	Chlamydomonas_reinh	57.10	0.00
TRINITY_GBG7447	hypothet	Chara_braunii	57.10	0.00
TRINITY_OAE2772	hypothet	Marchantia_polymorp	57.10	0.00
TRINITY_GAQ8884	diaminop	Klebsormidium_niten	57.10	0.00
TRINITY_GAQ7919	DNA-dire	Klebsormidium_niten	57.00	0.00
TRINITY_XP_0219	probable	Helianthus_annuus	57.00	0.00
TRINITY_XP_0102	PREDICTE	Nelumbo_nucifera	57.00	0.00
TRINITY_OWM6961	hypothet	Punica_granatum	57.00	0.00
TRINITY_KDD7218	hypothet	Helicosporidium_sp.	57.00	0.00
TRINITY_GAX7881	hypothet	Chlamydomonas_eusti	57.00	0.00
TRINITY_GBG6532	hypothet	Chara_braunii	57.00	0.00
TRINITY_XP_0155	uncharac	Ricinus_communis	57.00	0.00
TRINITY_GBG7574	hypothet	Chara_braunii	57.00	0.00
TRINITY_ACQ8348	CBL-inte	Selaginella_moellen	57.00	0.00
TRINITY_XP_0120	heparan-	Jatropha_curcas	57.00	0.00
TRINITY_ARO5009	indolear	Yamagishiella_unicc	57.00	0.00
TRINITY_XP_0029	hypothet	Volvox_carteri	57.00	0.00
TRINITY_KXZ4404	hypothet	Gonium_pectorale	57.00	0.00
TRINITY_GAV7609	UCH doma	Cephalotus_follicul	57.00	0.00
TRINITY_XP_0029	hypothet	Volvox_carteri	57.00	0.00
TRINITY_KXZ5059	hypothet	Gonium_pectorale	57.00	0.00
TRINITY_XP_0218	succinat	Spinacia_oleracea	57.00	0.00
TRINITY_GAX7727	hypothet	Chlamydomonas_eusti	57.00	0.00
TRINITY_PNW8314	hypothet	Chlamydomonas_reinh	57.00	0.00
TRINITY_XP_0029	hypothet	Volvox_carteri	57.00	0.00
TRINITY_KXZ5226	hypothet	Gonium_pectorale	57.00	0.00
TRINITY_NP_0012	uncharac	Glycine_max	57.00	0.00
TRINITY_KXZ4258	hypothet	Gonium_pectorale	57.00	0.00
TRINITY_XP_0029	hypothet	Volvox_carteri	57.00	0.00
TRINITY_AML7829	putative	Pleurastrum_insigne	57.00	0.00
TRINITY_PNW8488	hypothet	Chlamydomonas_reinh	57.00	0.00
TRINITY_XP_0016	predicte	Chlamydomonas_reinh	57.00	0.00
TRINITY_PSC7349	rRNA met	Micractinium_conduc	57.00	0.00
TRINITY_PNW8212	hypothet	Chlamydomonas_reinh	57.00	0.00
TRINITY_NP_0012	malate (	Glycine_max	57.00	0.00
TRINITY_PNW8531	hypothet	Chlamydomonas_reinh	57.00	0.00
TRINITY_XP_0016	general	Chlamydomonas_reinh	57.00	0.00
TRINITY_PNH0682	Carbonyl	Tetrabaena_socialis	57.00	0.00
TRINITY_XP_0016	predicte	Chlamydomonas_reinh	57.00	0.00
TRINITY_GAX7255	hypothet	Chlamydomonas_eusti	57.00	0.00
TRINITY_XP_0029	hypothet	Volvox_carteri	57.00	0.00
TRINITY_GBF9588	hypothet	Raphidocelis_subcap	57.00	0.00
TRINITY_KXZ4546	hypothet	Gonium_pectorale	57.00	0.00
TRINITY_GAX7423	hypothet	Chlamydomonas_eusti	57.00	0.00
TRINITY_GBF9925	polypren	Raphidocelis_subcap	57.00	0.00
TRINITY_ABF5767	chloropl	Chlamydomonas_reinh	57.00	0.00
TRINITY_GAX8192	hypothet	Chlamydomonas_eusti	57.00	0.00
TRINITY_EFJ0498	hypothet	Selaginella_moellen	57.00	0.00
TRINITY_XP_0056	ABC fami	Coccomyxa_subellips	57.00	0.00
TRINITY_PNW7785	hypothet	Chlamydomonas_reinh	57.00	0.00

TRINITY_KXZ52654	hypothet	Gonium_pectorale	57.00	0.00
TRINITY_XP_01390	Centroso	Monoraphidium_negle	56.90	0.00
TRINITY_XP_01004	PREDICTE	Eucalyptus_grandis	56.90	0.00
TRINITY_ADE77455	unknown	Picea_sitchensis	56.90	0.00
TRINITY_RLN35216	hypothet	Panicum_miliaceum	56.90	0.00
TRINITY_PTQ50103	hypothet	Marchantia_polymorp	56.90	0.00
TRINITY_ABK23787	unknown	Picea_sitchensis	56.90	0.00
TRINITY_XP_00751	predicte	Bathycoccus_prasino	56.90	0.00
TRINITY_KXZ46637	hypothet	Gonium_pectorale	56.90	0.00
TRINITY_XP_02438	putative	Physcomitrella_pate	56.90	0.00
TRINITY_GAX84594	hypothet	Chlamydomonas_eusti	56.90	0.00
TRINITY_OAY82958	NADH-cyt	Ananas_comosus	56.90	0.00
TRINITY_XP_01146	PREDICTE	Fragaria_vesca	56.90	0.00
TRINITY_XP_01282	PREDICTE	Erythranthe_guttata	56.90	0.00
TRINITY_XP_00169	predicte	Chlamydomonas_reinh	56.90	0.00
TRINITY_XP_00294	hypothet	Volvox_carteri	56.90	0.00
TRINITY_KXZ43041	hypothet	Gonium_pectorale	56.90	0.00
TRINITY_GBG87308	hypothet	Chara_braunii	56.90	0.00
TRINITY_GBF89375	guanylat	Raphidocelis_subcap	56.90	0.00
TRINITY_BAK02778	predicte	Hordeum_vulgare	56.90	0.00
TRINITY_PNH11885	hypothet	Tetrabaena_socialis	56.90	0.00
TRINITY_GAQ92474	Electron	Klebsormidium_niten	56.90	0.00
TRINITY_XP_00564	cytochro	Coccomyxa_subellips	56.90	0.00
TRINITY_GAX77679	hypothet	Chlamydomonas_eusti	56.90	0.00
TRINITY_RYR23351	hypothet	Arachis_hypogaea	56.90	0.00
TRINITY_GBF90443	hypothet	Raphidocelis_subcap	56.90	0.00
TRINITY_PNH10384	hypothet	Tetrabaena_socialis	56.90	0.00
TRINITY_GBF90935	voltage-	Raphidocelis_subcap	56.90	0.00
TRINITY_PNW72452	hypothet	Chlamydomonas_reinh	56.90	0.00
TRINITY_XP_00169	cytochro	Chlamydomonas_reinh	56.90	0.00
TRINITY_RID81070	hypothet	Brassica_rapa	56.90	0.00
TRINITY_XP_00295	hypothet	Volvox_carteri	56.90	0.00
TRINITY_XP_00665	PREDICTE	Oryza_brachyantha	56.90	0.00
TRINITY_GBF91498	hypothet	Raphidocelis_subcap	56.90	0.00
TRINITY_XP_00751	major fa	Bathycoccus_prasino	56.90	0.00
TRINITY_GBF92377	disulfic	Raphidocelis_subcap	56.90	0.00
TRINITY_PNW78438	hypothet	Chlamydomonas_reinh	56.90	0.00
TRINITY_RXH69554	hypothet	Malus_domestica	56.90	0.00
TRINITY_KXZ51833	hypothet	Gonium_pectorale	56.90	0.00
TRINITY_XP_00295	hypothet	Volvox_carteri	56.90	0.00
TRINITY_GAX83927	hypothet	Chlamydomonas_eusti	56.90	0.00
TRINITY_GAX76162	hypothet	Chlamydomonas_eusti	56.90	0.00
TRINITY_GAX79612	hypothet	Chlamydomonas_eusti	56.90	0.00
TRINITY_GBF90169	galactol	Raphidocelis_subcap	56.90	0.00
TRINITY_PRW39388	DNA gyra	Chlorella_sorokinia	56.90	0.00
TRINITY_PNW78545	hypothet	Chlamydomonas_reinh	56.90	0.00
TRINITY_GAX78046	hypothet	Chlamydomonas_eusti	56.90	0.00
TRINITY_KXZ51758	hypothet	Gonium_pectorale	56.90	0.00
TRINITY_XP_00564	eukaryot	Coccomyxa_subellips	56.90	0.00
TRINITY_GBF87741	hypothet	Raphidocelis_subcap	56.90	0.00
TRINITY_PNW72222	hypothet	Chlamydomonas_reinh	56.90	0.00
TRINITY_KXZ42934	hypothet	Gonium_pectorale	56.90	0.00
TRINITY_PRW44843	prolyl e	Chlorella_sorokinia	56.90	0.00
TRINITY_EEF24733	pterin-4	Ricinus_communis	56.80	0.00
TRINITY_XP_01655	PREDICTE	Capsicum_annuum	56.80	0.00
TRINITY_GBG66199	hypothet	Chara_braunii	56.80	0.00
TRINITY_XP_02389	kinesin	Quercus_suber	56.80	0.00
TRINITY_GAQ92868	polyader	Klebsormidium_niten	56.80	0.00
TRINITY_NP_19464	6-phosph	Arabidopsis_thalian	56.80	0.00
TRINITY_RLN39760	hypothet	Panicum_miliaceum	56.80	0.00
TRINITY_PRW60832	chitinas	Chlorella_sorokinia	56.80	0.00
TRINITY_XP_00294	hypothet	Volvox_carteri	56.80	0.00
TRINITY_BAK00960	predicte	Hordeum_vulgare	56.80	0.00
TRINITY_GAQ86828	NADH:fla	Klebsormidium_niten	56.80	0.00
TRINITY_XP_00297	L-galact	Selaginella_moellen	56.80	0.00
TRINITY_XP_00295	hypothet	Volvox_carteri	56.80	0.00

TRINITY_XP_0016(	predicte	Chlamydomonas_reinh	56.80	0.00
TRINITY_GAQ7787(	U6 snRNA	Klebsormidium_niten	56.80	0.00
TRINITY_GAX7471	hypothet	Chlamydomonas_eusti	56.80	0.00
TRINITY_KXZ5015	hypothet	Gonium_pectorale	56.80	0.00
TRINITY_XP_0017(	histone	Chlamydomonas_reinh	56.80	0.00
TRINITY_XP_0200	probable	Ananas_comosus	56.80	0.00
TRINITY_PNH0525	hypothet	Tetrabaena_socialis	56.80	0.00
TRINITY_XP_0029	hypothet	Volvox_carteri	56.80	0.00
TRINITY_PNW8189	hypothet	Chlamydomonas_reinh	56.80	0.00
TRINITY_GBF9657	hypothet	Raphidocelis_subcap	56.80	0.00
TRINITY_PNH1223	Protein	Tetrabaena_socialis	56.80	0.00
TRINITY_GAX8372	hypothet	Chlamydomonas_eusti	56.80	0.00
TRINITY_XP_0029	hypothet	Volvox_carteri	56.80	0.00
TRINITY_KXZ5218	hypothet	Gonium_pectorale	56.80	0.00
TRINITY_XP_0110	isoleuc	Sesamum_indicum	56.80	0.00
TRINITY_XP_0104	PREDICTE	Camelina_sativa	56.80	0.00
TRINITY_OWM7166	hypothet	Punica_granatum	56.80	0.00
TRINITY_GAX7387	hypothet	Chlamydomonas_eusti	56.80	0.00
TRINITY_XP_0139(	Putative	Monoraphidium_negle	56.80	0.00
TRINITY_GBG6087(	hypothet	Chara_braunii	56.80	0.00
TRINITY_GAX7665	hypothet	Chlamydomonas_eusti	56.80	0.00
TRINITY_GAX7381	hypothet	Chlamydomonas_eusti	56.80	0.00
TRINITY_PNW8725	hypothet	Chlamydomonas_reinh	56.80	0.00
TRINITY_XP_0017(	predicte	Chlamydomonas_reinh	56.80	0.00
TRINITY_PNW8515	hypothet	Chlamydomonas_reinh	56.80	0
TRINITY_PNW7358	hypothet	Chlamydomonas_reinh	56.80	0
TRINITY_GAX8086	hypothet	Chlamydomonas_eusti	56.80	0.00
TRINITY_PNH1283	Calcium-	Tetrabaena_socialis	56.80	0
TRINITY_XP_0016	predicte	Chlamydomonas_reinh	56.80	0.00
TRINITY_PNW8718	hypothet	Chlamydomonas_reinh	56.80	0.00
TRINITY_EOA3705	hypothet	Capsella_rubella	56.80	0.00
TRINITY_XP_0030	predicte	Micromonas_pusilla	56.70	0.00
TRINITY_XP_0068	ubiquit	Amborella_trichopod	56.70	0.00
TRINITY_GBG6681	hypothet	Chara_braunii	56.70	0.00
TRINITY_XP_0249	probable	Ziziphus_jujuba	56.70	0.00
TRINITY_XP_0244(	uncharac	Physcomitrella_pate	56.70	0.00
TRINITY_PWA4778	NAD(P)-	Artemisia_annua	56.70	0.00
TRINITY_XP_0042	PREDICTE	Fragaria_vesca	56.70	0.00
TRINITY_AWK2409	mitochon	Chlamydomonas_sp._U	56.70	0.00
TRINITY_KMZ6229	Replicat	Zostera_marina	56.70	0.00
TRINITY_XP_0217	threonyl	Chenopodium_quinoa	56.70	0.00
TRINITY_KXZ5244	hypothet	Gonium_pectorale	56.70	0.00
TRINITY_KZV1503	NAD-depe	Dorcoceras_hygromet	56.70	0.00
TRINITY_GAX8579	hypothet	Chlamydomonas_eusti	56.70	0.00
TRINITY_GBG7203	hypothet	Chara_braunii	56.70	0.00
TRINITY_XP_0056	P-loop (	Coccomyxa_subellips	56.70	0.00
TRINITY_PNH1208	hypothet	Tetrabaena_socialis	56.70	0.00
TRINITY_GAX7693	hypothet	Chlamydomonas_eusti	56.70	0.00
TRINITY_GAX8146	hypothet	Chlamydomonas_eusti	56.70	0.00
TRINITY_PRW4433	Kanadapt	Chlorella_sorokinia	56.70	0.00
TRINITY_GAX7868	hypothet	Chlamydomonas_eusti	56.70	0.00
TRINITY_XP_0097	PREDICTE	Nicotiana_sylvestri	56.70	0.00
TRINITY_GAX7305	hypothet	Chlamydomonas_eusti	56.70	0.00
TRINITY_XP_0101(	protein	Morus_notabilis	56.70	0.00
TRINITY_XP_0029	hypothet	Volvox_carteri	56.70	0.00
TRINITY_XP_0139(	IMPACT f	Monoraphidium_negle	56.70	0.00
TRINITY_GAX7522	hypothet	Chlamydomonas_eusti	56.70	0.00
TRINITY_XP_0029	hypothet	Volvox_carteri	56.70	0.00
TRINITY_GAX7411	hypothet	Chlamydomonas_eusti	56.70	0.00
TRINITY_XP_0239	ras-rela	Quercus_suber	56.70	0.00
TRINITY_BAK0697	predicte	Hordeum_vulgare	56.70	0.00
TRINITY_KXZ4806	hypothet	Gonium_pectorale	56.70	0.00
TRINITY_KXZ4560	hypothet	Gonium_pectorale	56.70	0.00
TRINITY_PNH1194	JmjC dor	Tetrabaena_socialis	56.70	0.00
TRINITY_PNW8539	hypothet	Chlamydomonas_reinh	56.70	0.00
TRINITY_PNH1155	30S ribo	Tetrabaena_socialis	56.70	0.00

TRINITY_XP_0029	hypothet	Volvox_carteri	56.70	0.00
TRINITY_PNH0614	hypothet	Tetrabaena_socialis	56.70	0.00
TRINITY_PNW7788	hypothet	Chlamydomonas_reinh	56.70	0.00
TRINITY_XP_0030	central	Micromonas_pusilla	56.70	0.00
TRINITY_XP_0243	eukaryot	Physcomitrella_pate	56.70	0.00
TRINITY_XP_0029	hypothet	Volvox_carteri	56.70	0.00
TRINITY_XP_0063	DExH-bo	Capsella_rubella	56.70	0.00
TRINITY_GAX7707	hypothet	Chlamydomonas_eusti	56.70	0.00
TRINITY_GBF9777	hypothet	Raphidocelis_subcap	56.70	0.00
TRINITY_PNW8849	hypothet	Chlamydomonas_reinh	56.70	0.00
TRINITY_AML7796	putative	Dunaliella_salina	56.70	0.00
TRINITY_KXZ4577	hypothet	Gonium_pectorale	56.70	0.00
TRINITY_PNH0920	Tiny ma	Tetrabaena_socialis	56.70	0.00
TRINITY_XP_0238	septin	Quercus_suber	56.70	0.00
TRINITY_XP_0113	Translo	Auxenochlorella_pro	56.70	0.00
TRINITY_ONK6448	uncharac	Asparagus_officinal	56.60	0.00
TRINITY_XP_0237	myosin-	Lactuca_sativa	56.60	0.00
TRINITY_XP_0088	T-compl	Phoenix_dactylifera	56.60	0.00
TRINITY_OWM8856	hypothet	Punica_granatum	56.60	0.00
TRINITY_XP_0238	actin-l	Quercus_suber	56.60	0.00
TRINITY_XP_0100	PREDICT	Eucalyptus_grandis	56.60	0.00
TRINITY_XP_0102	coiled-	Brachypodium_distac	56.60	0.00
TRINITY_XP_0220	imidazo	Helianthus_annuus	56.60	0.00
TRINITY_OVA0402	ABC tra	Macleaya_cordata	56.60	0.00
TRINITY_XP_0128	PREDICT	Erythranthe_guttata	56.60	0.00
TRINITY_ALM5500	DEAD bo	Chlamydomonas_sp._I	56.60	0.00
TRINITY_XP_0016	hypothet	Chlamydomonas_reinh	56.60	0.00
TRINITY_PNW8713	hypothet	Chlamydomonas_reinh	56.60	0.00
TRINITY_CAA1126	polyubi	Nicotiana_tabacum	56.60	0.00
TRINITY_KXZ5582	hypothet	Gonium_pectorale	56.60	0.00
TRINITY_GAX7625	hypothet	Chlamydomonas_eusti	56.60	0.00
TRINITY_GAQ8167	hypothet	Klebsormidium_niten	56.60	0.00
TRINITY_KXZ5624	hypothet	Gonium_pectorale	56.60	0.00
TRINITY_XP_0238	non-spe	Quercus_suber	56.60	0.00
TRINITY_XP_0238	kinesin-	Quercus_suber	56.60	0.00
TRINITY_VDD4191	unnamed	Brassica_oleracea	56.60	0.00
TRINITY_GAX7924	hypothet	Chlamydomonas_eusti	56.60	0.00
TRINITY_PNW8166	hypothet	Chlamydomonas_reinh	56.60	0.00
TRINITY_PNW8578	hypothet	Chlamydomonas_reinh	56.60	0.00
TRINITY_XP_0016	tail-sp	Chlamydomonas_reinh	56.60	0.00
TRINITY_XP_0016	predict	Chlamydomonas_reinh	56.60	0.00
TRINITY_GAX7299	hypothet	Chlamydomonas_eusti	56.60	0.00
TRINITY_GAX7314	hypothet	Chlamydomonas_eusti	56.60	0.00
TRINITY_GAX8522	hypothet	Chlamydomonas_eusti	56.60	0.00
TRINITY_XP_0017	predict	Chlamydomonas_reinh	56.60	0.00
TRINITY_GAX8202	hypothet	Chlamydomonas_eusti	56.60	0.00
TRINITY_GAX7554	hypothet	Chlamydomonas_eusti	56.60	0.00
TRINITY_GAX8256	hypothet	Chlamydomonas_eusti	56.60	0.00
TRINITY_GBF8972	ubiquit	Raphidocelis_subcap	56.60	0.00
TRINITY_PNW8875	hypothet	Chlamydomonas_reinh	56.60	0.00
TRINITY_XP_0016	uncoupl	Chlamydomonas_reinh	56.60	0.00
TRINITY_XP_0238	kinesin	Quercus_suber	56.60	0.00
TRINITY_XP_0056	glutamat	Coccomyxa_subellips	56.60	0.00
TRINITY_KXZ5550	hypothet	Gonium_pectorale	56.60	0.00
TRINITY_RZC2446	Eukaryot	Glycine_soja	56.60	0.00
TRINITY_XP_0058	hypothet	Chlorella_variabili	56.50	0.00
TRINITY_XP_0264	cystein	Papaver_somniferum	56.50	0.00
TRINITY_XP_0016	predict	Chlamydomonas_reinh	56.50	0.00
TRINITY_XP_0014	predict	Ostreococcus_sp._'l	56.50	0.00
TRINITY_XP_0029	hypothet	Volvox_carteri	56.50	0.00
TRINITY_OMO6271	hypothet	Corchorus_capsulari	56.50	0.00
TRINITY_XP_0028	peptide	Arabidopsis_lyrata	56.50	0.00
TRINITY_XP_0029	40S rib	Selaginella_moellen	56.50	0.00
TRINITY_ACQ8348	CBL-int	Selaginella_moellen	56.50	0.00
TRINITY_GBG7885	hypothet	Chara_braunii	56.50	0.00
TRINITY_XP_0068	S-formyl	Amborella_trichopod	56.50	0.00

TRINITY_XP_0016(	LOW QUAl	Chlamydomonas_reinh	56.50	0.00
TRINITY_XP_0029!	hypothet	Volvox_carteri	56.50	0.00
TRINITY_XP_0058	hypothet	Chlorella_variabili	56.50	0.00
TRINITY_PNW8543	hypothet	Chlamydomonas_reinh	56.50	0.00
TRINITY_EFJ1351	hypothet	Selaginella_moellen	56.50	0.00
TRINITY_GAQ8178	Rab fam	Klebsormidium_niten	56.50	0.00
TRINITY_XP_0066!	PREDICTI	Oryza_brachyantha	56.50	0.00
TRINITY_PNW7070	hypothet	Chlamydomonas_reinh	56.50	0.00
TRINITY_RLM7333	uncharac	Panicum_miliaceum	56.50	0.00
TRINITY_XP_0138	insulysi	Monoraphidium_negle	56.50	0.00
TRINITY_PNW7291	hypothet	Chlamydomonas_reinh	56.50	0.00
TRINITY_PNW8736	hypothet	Chlamydomonas_reinh	56.50	0.00
TRINITY_GAQ8169	Long-Cha	Klebsormidium_niten	56.50	0.00
TRINITY_XP_0243	ABC tran	Physcomitrella_pate	56.50	0.00
TRINITY_GAX7563	hypothet	Chlamydomonas_eusti	56.50	0.00
TRINITY_XP_0172	PREDICTI	Daucus_carota	56.50	0.00
TRINITY_XP_0017(	fructose	Chlamydomonas_reinh	56.50	0.00
TRINITY_ABR2175	calmodul	Actinidia_deliciosa	56.50	0.00
TRINITY_XP_0062	mitotic	Capsella_rubella	56.50	0.00
TRINITY_XP_0139(	hypothet	Monoraphidium_negle	56.50	0.00
TRINITY_XP_0056!	mitochor	Coccomyxa_subellips	56.50	0.00
TRINITY_KXZ4931	hypothet	Gonium_pectorale	56.50	0.00
TRINITY_GAQ8199	fragment	Klebsormidium_niten	56.50	0.00
TRINITY_KXZ4189	hypothet	Gonium_pectorale	56.50	0.00
TRINITY_XP_0016	predicte	Chlamydomonas_reinh	56.50	0.00
TRINITY_XP_0056	DNA misr	Coccomyxa_subellips	56.50	0.00
TRINITY_GAQ7819	ATP-depe	Klebsormidium_niten	56.50	0.00
TRINITY_XP_0056	D-3-phos	Coccomyxa_subellips	56.50	0.00
TRINITY_VDC7403	unnamed	Brassica_rapa	56.50	0.00
TRINITY_KXZ4498	hypothet	Gonium_pectorale	56.50	0.00
TRINITY_KXZ5371	hypothet	Gonium_pectorale	56.50	0.00
TRINITY_GAX8319(	hypothet	Chlamydomonas_eusti	56.50	0.00
TRINITY_KXZ4761(	hypothet	Gonium_pectorale	56.50	0.00
TRINITY_VDC8973	unnamed	Brassica_oleracea	56.50	0.00
TRINITY_XP_0029!	programm	Volvox_carteri	56.50	0.00
TRINITY_GAX8400	hypothet	Chlamydomonas_eusti	56.50	0.00
TRINITY_GAX8165	hypothet	Chlamydomonas_eusti	56.50	0.00
TRINITY_XP_0029(	vacuolai	Selaginella_moellen	56.50	0.00
TRINITY_GBF8833	hypothet	Raphidocelis_subcap	56.50	0.00
TRINITY_GAX7726	hypothet	Chlamydomonas_eusti	56.50	0.00
TRINITY_PON6243	ER lumen	Trema_orientale	56.50	0.00
TRINITY_XP_0025(	intrafla	Micromonas_commoda	56.50	0.00
TRINITY_PNH0830	NFX1-typ	Tetrabaena_socialis	56.50	0.00
TRINITY_KXZ5000	hypothet	Gonium_pectorale	56.50	0.00
TRINITY_PNH0921	Tiny mac	Tetrabaena_socialis	56.50	0.00
TRINITY_GAQ7904	isocitra	Klebsormidium_niten	56.40	0.00
TRINITY_XP_0105	PREDICTI	Tarenaya_hasslerian	56.40	0.00
TRINITY_XP_0238	aspartat	Quercus_suber	56.40	0.00
TRINITY_XP_0083	PREDICTI	Malus_domestica	56.40	0.00
TRINITY_GBG6057	hypothet	Chara_braunii	56.40	0.00
TRINITY_ACN3155	unknown	Zea_mays	56.40	0.00
TRINITY_XP_0056	RNA-binc	Coccomyxa_subellips	56.40	0.00
TRINITY_XP_0238	glucose-	Quercus_suber	56.40	0.00
TRINITY_GAX7594	hypothet	Chlamydomonas_eusti	56.40	0.00
TRINITY_PNW8447	hypothet	Chlamydomonas_reinh	56.40	0.00
TRINITY_XP_0016	predicte	Chlamydomonas_reinh	56.40	0.00
TRINITY_GAX8039	hypothet	Chlamydomonas_eusti	56.40	0.00
TRINITY_GAQ9247	60S acic	Klebsormidium_niten	56.40	0.00
TRINITY_XP_0058	hypothet	Chlorella_variabili	56.40	0.00
TRINITY_PNW8625	hypothet	Chlamydomonas_reinh	56.40	0.00
TRINITY_XP_0216	citrate	Hevea_brasiliensis	56.40	0.00
TRINITY_PNH0712	AP-1 cor	Tetrabaena_socialis	56.40	0.00
TRINITY_GAQ8463	hypothet	Klebsormidium_niten	56.40	0.00
TRINITY_PNW7423(	hypothet	Chlamydomonas_reinh	56.40	0.00
TRINITY_OAE2398	hypothet	Marchantia_polymorp	56.40	0.00
TRINITY_GBF9316	hypothet	Raphidocelis_subcap	56.40	0.00

TRINITY_PNW7069	hypothet	Chlamydomonas_reinh	56.40	0.00
TRINITY_KXZ5012	hypothet	Gonium_pectorale	56.40	0.00
TRINITY_GAX7363	hypothet	Chlamydomonas_eusti	56.40	0.00
TRINITY_GAX8373	hypothet	Chlamydomonas_eusti	56.40	0.00
TRINITY_XP_0029	hypothet	Volvox_carteri	56.40	0.00
TRINITY_XP_0058	hypothet	Chlorella_variabili	56.40	0.00
TRINITY_XP_0035	eukaryot	Medicago_truncatula	56.40	0.00
TRINITY_PNR2788	hypothet	Physcomitrella_pate	56.40	0.00
TRINITY_GAX7544	hypothet	Chlamydomonas_eusti	56.40	0.00
TRINITY_GAX8106	hypothet	Chlamydomonas_eusti	56.40	0.00
TRINITY_XP_0016	predicte	Chlamydomonas_reinh	56.40	0.00
TRINITY_GAX8486	hypothet	Chlamydomonas_eusti	56.40	0.00
TRINITY_XP_0172	PREDICTE	Daucus_carota	56.40	0.00
TRINITY_GAX8061	hypothet	Chlamydomonas_eusti	56.40	0
TRINITY_OAE1992	hypothet	Marchantia_polymorp	56.40	0
TRINITY_KMZ6167	3-ketoac	Zostera_marina	56.40	0.00
TRINITY_KXZ5561	hypothet	Gonium_pectorale	56.30	0.00
TRINITY_GBF8822	ATP-depe	Raphidocelis_subcap	56.30	0.00
TRINITY_GAX8054	hypothet	Chlamydomonas_eusti	56.30	0.00
TRINITY_XP_0208	calcium-	Arabidopsis_lyrata	56.30	0.00
TRINITY_YP_0088	apocytoc	Entransia_fimbriata	56.30	0.00
TRINITY_XP_0025	predicte	Micromonas_commoda	56.30	0.00
TRINITY_XP_0239	aldo-ket	Quercus_suber	56.30	0.00
TRINITY_XP_0239	aldo-ket	Quercus_suber	56.30	0.00
TRINITY_BAJ1178	dehydrat	Corchorus_olitorius	56.30	0.00
TRINITY_XP_0138	putative	Monoraphidium_negle	56.30	0.00
TRINITY_PNW7310	hypothet	Chlamydomonas_reinh	56.30	0.00
TRINITY_XP_0023	probable	Populus_trichocarpa	56.30	0.00
TRINITY_XP_0016	DnaJ-lik	Chlamydomonas_reinh	56.30	0.00
TRINITY_PNH0292	Transmem	Tetrabaena_socialis	56.30	0.00
TRINITY_KXZ4576	hypothet	Gonium_pectorale	56.30	0.00
TRINITY_XP_0029	ABC tran	Volvox_carteri	56.30	0.00
TRINITY_PNH0920	Peroxire	Tetrabaena_socialis	56.30	0.00
TRINITY_KXZ4086	hypothet	Gonium_pectorale	56.30	0.00
TRINITY_KXZ5409	hypothet	Gonium_pectorale	56.30	0.00
TRINITY_KXZ5652	hypothet	Gonium_pectorale	56.30	0.00
TRINITY_KXZ5409	hypothet	Gonium_pectorale	56.30	0.00
TRINITY_XP_0063	60S acid	Eutrema_salsugineum	56.30	0.00
TRINITY_PNW8434	hypothet	Chlamydomonas_reinh	56.30	0.00
TRINITY_XP_0139	hypothet	Monoraphidium_negle	56.30	0.00
TRINITY_PNW8244	hypothet	Chlamydomonas_reinh	56.30	0.00
TRINITY_GAX7919	hypothet	Chlamydomonas_eusti	56.30	0.00
TRINITY_GAQ8091	putative	Klebsormidium_niten	56.30	0.00
TRINITY_KXZ5021	hypothet	Gonium_pectorale	56.30	0.00
TRINITY_XP_0017	SM/Sec1-	Chlamydomonas_reinh	56.30	0.00
TRINITY_XP_0207	structur	Dendrobium_catenatu	56.30	0.00
TRINITY_GAX7725	hypothet	Chlamydomonas_eusti	56.30	0.00
TRINITY_PNW8176	hypothet	Chlamydomonas_reinh	56.30	0.00
TRINITY_PNH0378	Phosphat	Tetrabaena_socialis	56.30	0.00
TRINITY_XP_0029	hypothet	Volvox_carteri	56.30	0.00
TRINITY_ACL2815	hypothet	Dunaliella_viridis	56.30	0.00
TRINITY_GAQ8002	histone	Klebsormidium_niten	56.30	0.00
TRINITY_KXZ5127	hypothet	Gonium_pectorale	56.30	0.00
TRINITY_KXZ4685	hypothet	Gonium_pectorale	56.30	0.00
TRINITY_XP_0017	high int	Chlamydomonas_reinh	56.30	0.00
TRINITY_APW8374	caroteno	Dunaliella_salina	56.30	0.00
TRINITY_KXZ4676	hypothet	Gonium_pectorale	56.30	0.00
TRINITY_XP_0206	BEACH do	Dendrobium_catenatu	56.20	0.00
TRINITY_GAX7633	hypothet	Chlamydomonas_eusti	56.20	0.00
TRINITY_AES8227	peptide	Medicago_truncatula	56.20	0.00
TRINITY_GBF9839	hypothet	Raphidocelis_subcap	56.20	0.00
TRINITY_XP_0238	GTP-bind	Quercus_suber	56.20	0.00
TRINITY_GAX8175	hypothet	Chlamydomonas_eusti	56.20	0.00
TRINITY_GBG8478	hypothet	Chara_braunii	56.20	0.00
TRINITY_GAQ8874	adenine	Klebsormidium_niten	56.20	0.00
TRINITY_XP_0043	PREDICTE	Fragaria_vesca	56.20	0.00

TRINITY_XP_0238	transal	Quercus_suber	56.20	0.00
TRINITY_OEL2901	Dynamin-	Dichanthelium_oligo	56.20	0.00
TRINITY_XP_0017	vacuola	Chlamydomonas_reinh	56.20	0.00
TRINITY_XP_0029	hypothet	Volvox_carteri	56.20	0.00
TRINITY_ESR6269	hypothet	Citrus_clementina	56.20	0.00
TRINITY_XP_0139	dual-sp	Monoraphidium_negle	56.20	0.00
TRINITY_GBF9241	RNA pol	Raphidocelis_subcap	56.20	0.00
TRINITY_GAX8084	hypothet	Chlamydomonas_eusti	56.20	0.00
TRINITY_GAQ9018	lysosom	Klebsormidium_niten	56.20	0.00
TRINITY_XP_0056	ornithi	Coccomyxa_subellips	56.20	0.00
TRINITY_XP_0058	hypothet	Chlorella_variabili	56.20	0.00
TRINITY_PNW8093	hypothet	Chlamydomonas_reinh	56.20	0.00
TRINITY_OMO7877	hypothet	Corchorus_capsulari	56.20	0.00
TRINITY_GAX8055	hypothet	Chlamydomonas_eusti	56.20	0.00
TRINITY_XP_0139	hydroxy	Monoraphidium_negle	56.20	0.00
TRINITY_GBF8855	hypothet	Raphidocelis_subcap	56.20	0.00
TRINITY_KXZ4738	hypothet	Gonium_pectorale	56.20	0.00
TRINITY_KMZ6641	putativ	Zostera_marina	56.20	0.00
TRINITY_VDC9191	unnamed	Brassica_oleracea	56.20	0.00
TRINITY_KXZ5314	hypothet	Gonium_pectorale	56.20	0.00
TRINITY_GAX7395	hypothet	Chlamydomonas_eusti	56.20	0.00
TRINITY_XP_0105	PREDICT	Tarenaya_hasslerian	56.20	0.00
TRINITY_PNW7291	hypothet	Chlamydomonas_reinh	56.20	0.00
TRINITY_PNW7926	hypothet	Chlamydomonas_reinh	56.20	0.00
TRINITY_GAX7977	hypothet	Chlamydomonas_eusti	56.20	0.00
TRINITY_XP_0138	hypothet	Monoraphidium_negle	56.20	0.00
TRINITY_XP_0238	probabl	Quercus_suber	56.20	0.00
TRINITY_XP_0016	flagell	Chlamydomonas_reinh	56.20	0.00
TRINITY_XP_0084	PREDICT	Cucumis_melo	56.20	0.00
TRINITY_XP_0030	predict	Micromonas_pusilla	56.20	0.00
TRINITY_XP_0128	PREDICT	Erythranthe_guttata	56.20	0.00
TRINITY_XP_0029	hypothet	Volvox_carteri	56.20	0.00
TRINITY_GAQ8654	TCP-1/c	Klebsormidium_niten	56.20	0.00
TRINITY_GAX8139	hypothet	Chlamydomonas_eusti	56.20	0.00
TRINITY_GBF9579	hypothet	Raphidocelis_subcap	56.20	0.00
TRINITY_PSC7186	DEAD-bo	Micractinium_conduc	56.20	0.00
TRINITY_GAX7995	hypothet	Chlamydomonas_eusti	56.20	0.00
TRINITY_XP_0271	uncharac	Coffea_eugenioides	56.20	0.00
TRINITY_PRW3394	acyl- d	Chlorella_sorokinia	56.20	0.00
TRINITY_XP_0238	acyl-Co	Quercus_suber	56.20	0.00
TRINITY_GAX8296	hypothet	Chlamydomonas_eusti	56.20	0.00
TRINITY_BAK0000	predict	Hordeum_vulgare	56.20	0.00
TRINITY_ONK6835	uncharac	Asparagus_officinal	56.20	0.00
TRINITY_BAJ9926	predict	Hordeum_vulgare	56.20	0.00
TRINITY_GAX8335	hypothet	Chlamydomonas_eusti	56.20	0.00
TRINITY_PNW8864	hypothet	Chlamydomonas_reinh	56.20	0.00
TRINITY_XP_0016	predict	Chlamydomonas_reinh	56.20	0.00
TRINITY_PRW6114	mitogen-	Chlorella_sorokinia	56.20	0.00
TRINITY_XP_0029	hypothet	Volvox_carteri	56.20	0.00
TRINITY_KXZ4988	hypothet	Gonium_pectorale	56.20	0.00
TRINITY_GAX7416	hypothet	Chlamydomonas_eusti	56.20	0.00
TRINITY_KXZ5401	hypothet	Gonium_pectorale	56.20	0.00
TRINITY_XP_0017	glyoxal	Chlamydomonas_reinh	56.20	0.00
TRINITY_KXZ5000	hypothet	Gonium_pectorale	56.20	0.00
TRINITY_PNW8531	hypothet	Chlamydomonas_reinh	56.20	0.00
TRINITY_GAX8076	hypothet	Chlamydomonas_eusti	56.20	0.00
TRINITY_PSC7271	IMPACT	Micractinium_conduc	56.20	0.00
TRINITY_GAX8216	hypothet	Chlamydomonas_eusti	56.20	0.00
TRINITY_XP_0202	DExH-bo	Asparagus_officinal	56.20	0.00
TRINITY_GAX8100	hypothet	Chlamydomonas_eusti	56.20	0.00
TRINITY_GAX7849	hypothet	Chlamydomonas_eusti	56.20	0.00
TRINITY_XP_0139	hypothet	Monoraphidium_negle	56.10	0.00
TRINITY_AHB0888	HD doma	Suaeda_glauca	56.10	0.00
TRINITY_XP_0238	lactoba	Quercus_suber	56.10	0.00
TRINITY_EFJ1951	hypothet	Selaginella_moellen	56.10	0.00
TRINITY_OTF9730	putativ	Helianthus_annuus	56.10	0.00

TRINITY_PWA49914	Nascent	Artemisia_annua	56.10	0.00
TRINITY_XP_02438	probable	Physcomitrella_pate	56.10	0.00
TRINITY_XP_02387	homoiso	Quercus_suber	56.10	0.00
TRINITY_GAX83579	hypothet	Chlamydomonas_eusti	56.10	0.00
TRINITY_XP_00295	hypothet	Volvox_carteri	56.10	0.00
TRINITY_GAX79311	hypothet	Chlamydomonas_eusti	56.10	0.00
TRINITY_GAX81461	hypothet	Chlamydomonas_eusti	56.10	0.00
TRINITY_GAQ81411	Metallop	Klebsormidium_niten	56.10	0.00
TRINITY_GAX78157	hypothet	Chlamydomonas_eusti	56.10	0.00
TRINITY_XP_00295	hypothet	Volvox_carteri	56.10	0.00
TRINITY_XP_01140	Calvin c	Auxenochlorella_pro	56.10	0.00
TRINITY_KXZ53775	hypothet	Gonium_pectorale	56.10	0.00
TRINITY_GAQ79684	peroxiso	Klebsormidium_niten	56.10	0.00
TRINITY_BAJ93795	predicte	Hordeum_vulgare	56.10	0.00
TRINITY_XP_00295	hypothet	Volvox_carteri	56.10	0.00
TRINITY_KXZ55138	hypothet	Gonium_pectorale	56.10	0.00
TRINITY_XP_00250	predicte	Micromonas_commoda	56.10	0.00
TRINITY_KXZ53139	hypothet	Gonium_pectorale	56.10	0.00
TRINITY_XP_00168	DNA-dire	Chlamydomonas_reinh	56.10	0.00
TRINITY_GAX75591	hypothet	Chlamydomonas_eusti	56.10	0.00
TRINITY_XP_02010	aspartat	Ananas_comosus	56.10	0.00
TRINITY_XP_00169	predicte	Chlamydomonas_reinh	56.10	0.00
TRINITY_XP_00295	hypothet	Volvox_carteri	56.10	0.00
TRINITY_KXZ51825	hypothet	Gonium_pectorale	56.10	0.00
TRINITY_PRW57963	Thioredo	Chlorella_sorokinia	56.10	0.00
TRINITY_XP_00169	2-cys pe	Chlamydomonas_reinh	56.10	0.00
TRINITY_KXZ44955	hypothet	Gonium_pectorale	56.10	0.00
TRINITY_GAX75066	hypothet	Chlamydomonas_eusti	56.10	0.00
TRINITY_XP_02439	40S ribo	Physcomitrella_pate	56.10	0.00
TRINITY_KXZ51733	hypothet	Gonium_pectorale	56.10	0.00
TRINITY_GAX76258	hypothet	Chlamydomonas_eusti	56.10	0.00
TRINITY_XP_00169	tRNA (ur	Chlamydomonas_reinh	56.10	0.00
TRINITY_AEF13162	acyl car	Haematococcus_lacus	56.10	0.00
TRINITY_GAX75389	hypothet	Chlamydomonas_eusti	56.10	0.00
TRINITY_GAX83245	hypothet	Chlamydomonas_eusti	56.10	0.00
TRINITY_GAX82200	hypothet	Chlamydomonas_eusti	56.10	0.00
TRINITY_PNW79029	hypothet	Chlamydomonas_reinh	56.10	0.00
TRINITY_XP_01140	hypothet	Auxenochlorella_pro	56.10	0.00
TRINITY_XP_00169	thiosulf	Chlamydomonas_reinh	56.10	0.00
TRINITY_GAX81923	hypothet	Chlamydomonas_eusti	56.10	0.00
TRINITY_PNW86359	hypothet	Chlamydomonas_reinh	56.10	0.00
TRINITY_XP_00295	hypothet	Volvox_carteri	56.10	0.00
TRINITY_BAK02254	predicte	Hordeum_vulgare	56.10	0.00
TRINITY_PSC73672	ferredox	Micractinium_conduc	56.10	0.00
TRINITY_GBF99614	hypothet	Raphidocelis_subcap	56.10	0.00
TRINITY_KXZ56762	hypothet	Gonium_pectorale	56.10	0.00
TRINITY_RWR95274	Ribosoma	Cinnamomum_micranth	56.00	0.00
TRINITY_XP_00564	acyl car	Coccomyxa_subellips	56.00	0.00
TRINITY_XP_02439	phosphat	Physcomitrella_pate	56.00	0.00
TRINITY_XP_00564	acetyl-C	Coccomyxa_subellips	56.00	0.00
TRINITY_RWR95530	DNA-dire	Cinnamomum_micranth	56.00	0.00
TRINITY_BAJ94294	predicte	Hordeum_vulgare	56.00	0.00
TRINITY_XP_01725	PREDICTE	Daucus_carota	56.00	0.00
TRINITY_XP_01109	malate d	Sesamum_indicum	56.00	0.00
TRINITY_PNH03558	Armadill	Tetrabaena_socialis	56.00	0.00
TRINITY_AJQ19483	2-C-meth	Haematococcus_lacus	56.00	0.00
TRINITY_OAE20355	hypothet	Marchantia_polymorp	56.00	0.00
TRINITY_XP_00295	hypothet	Volvox_carteri	56.00	0.00
TRINITY_XP_02439	1,4-alph	Physcomitrella_pate	56.00	0.00
TRINITY_AGH30310	nitrate	Chromochloris_zofin	56.00	0.00
TRINITY_GBG80885	hypothet	Chara_braunii	56.00	0.00
TRINITY_KXZ46788	hypothet	Gonium_pectorale	56.00	0.00
TRINITY_KXZ41650	hypothet	Gonium_pectorale	56.00	0.00
TRINITY_PWA73941	duplicat	Artemisia_annua	56.00	0.00
TRINITY_GAX76835	hypothet	Chlamydomonas_eusti	56.00	0.00
TRINITY_GBF97643	hypothet	Raphidocelis_subcap	56.00	0.00

TRINITY_PNW7074	hypothet	Chlamydomonas_reinh	56.00	0.00
TRINITY_XP_0239	ras-rela	Quercus_suber	56.00	0.00
TRINITY_GAX7348	hypothet	Chlamydomonas_eusti	56.00	0.00
TRINITY_GAX8427	hypothet	Chlamydomonas_eusti	56.00	0.00
TRINITY_BAK0524	predicte	Hordeum_vulgare	56.00	0.00
TRINITY_XP_0017	predicte	Chlamydomonas_reinh	56.00	0.00
TRINITY_XP_0029	hypothet	Volvox_carteri	56.00	0.00
TRINITY_GAX7764	hypothet	Chlamydomonas_eusti	56.00	0.00
TRINITY_GBF8788	hypothet	Raphidocelis_subcap	56.00	0.00
TRINITY_PNW7649	hypothet	Chlamydomonas_reinh	56.00	0.00
TRINITY_OMO5658	Small G	Corchorus_capsulari	56.00	0.00
TRINITY_GAQ8325	nucleot	Klebsormidium_niten	56.00	0.00
TRINITY_XP_0029	hypothet	Volvox_carteri	56.00	0.00
TRINITY_KXZ4607	hypothet	Gonium_pectorale	56.00	0.00
TRINITY_GAX8134	hypothet	Chlamydomonas_eusti	56.00	0.00
TRINITY_GAX8260	hypothet	Chlamydomonas_eusti	56.00	0.00
TRINITY_GAX8338	hypothet	Chlamydomonas_eusti	56.00	0.00
TRINITY_XP_0101	transcr	Morus_notabilis	56.00	0.00
TRINITY_XP_0241	DNA top	Rosa_chinensis	56.00	0.00
TRINITY_GAX7897	hypothet	Chlamydomonas_eusti	56.00	0
TRINITY_XP_0029	hypothet	Volvox_carteri	56.00	0.00
TRINITY_GAX7277	hypothet	Chlamydomonas_eusti	56.00	0.00
TRINITY_KXZ4730	hypothet	Gonium_pectorale	56.00	0.00
TRINITY_ASQ4164	lipase	Dunaliella_parva	56.00	0.00
TRINITY_XP_0029	hypothet	Volvox_carteri	56.00	0.00
TRINITY_XP_0243	ubiquit	Physcomitrella_pate	56.00	0.00
TRINITY_GAX8323	hypothet	Chlamydomonas_eusti	56.00	0.00
TRINITY_XP_0016	starch	Chlamydomonas_reinh	56.00	0.00
TRINITY_XP_0239	ATP-depe	Quercus_suber	56.00	0.00
TRINITY_GAX8220	hypothet	Chlamydomonas_eusti	56.00	0.00
TRINITY_PNW8102	hypothet	Chlamydomonas_reinh	56.00	0.00
TRINITY_RWR7571	transcr	Cinnamomum_micranth	56.00	0.00
TRINITY_GAX8153	hypothet	Chlamydomonas_eusti	56.00	0.00
TRINITY_GBG9232	hypothet	Chara_braunii	56.00	0.00
TRINITY_BAK0277	predicte	Hordeum_vulgare	56.00	0.00
TRINITY_PNW7042	hypothet	Chlamydomonas_reinh	56.00	0.00
TRINITY_KXZ4659	hypothet	Gonium_pectorale	56.00	0.00
TRINITY_EPS7450	hypothet	Genlisea_aurea	56.00	0.00
TRINITY_KXZ5618	hypothet	Gonium_pectorale	56.00	0.00
TRINITY_XP_0029	20S prot	Volvox_carteri	56.00	0.00
TRINITY_XP_0058	hypothet	Chlorella_variabili	56.00	0.00
TRINITY_XP_0024	E3 ubiq	Sorghum_bicolor	55.90	0.00
TRINITY_XP_0056	low-CO2-	Coccomyxa_subellips	55.90	0.00
TRINITY_PIN0892	Diphthi	Handroanthus_impeti	55.90	0.00
TRINITY_XP_0025	KH doma	Micromonas_commoda	55.90	0.00
TRINITY_XP_0016	flagell	Chlamydomonas_reinh	55.90	0.00
TRINITY_XP_0266	probabl	Phoenix_dactylifera	55.90	0.00
TRINITY_XP_0217	E3 ubiq	Chenopodium_quinoa	55.90	0.00
TRINITY_KMS6524	hypothet	Beta_vulgaris	55.90	0.00
TRINITY_GBF8822	ATP-depe	Raphidocelis_subcap	55.90	0.00
TRINITY_AEM8927	actin [	Narcissus_tazetta	55.90	0.00
TRINITY_PSC7583	glutami	Micractinium_conduc	55.90	0.00
TRINITY_GAX7588	hypothet	Chlamydomonas_eusti	55.90	0.00
TRINITY_KXZ5627	hypothet	Gonium_pectorale	55.90	0.00
TRINITY_XP_0029	26S prot	Volvox_carteri	55.90	0.00
TRINITY_XP_0029	hypothet	Volvox_carteri	55.90	0.00
TRINITY_PON4516	ABC tra	Parasponia_anderson	55.90	0.00
TRINITY_GAX7446	hypothet	Chlamydomonas_eusti	55.90	0.00
TRINITY_XP_0029	hypothet	Volvox_carteri	55.90	0.00
TRINITY_XP_0029	hypothet	Volvox_carteri	55.90	0.00
TRINITY_XP_0017	SM/Sec1-	Chlamydomonas_reinh	55.90	0.00
TRINITY_OWM7818	hypothet	Punica_granatum	55.90	0.00
TRINITY_KXZ4488	hypothet	Gonium_pectorale	55.90	0.00
TRINITY_KXZ5259	hypothet	Gonium_pectorale	55.90	0.00
TRINITY_PNH0747	hypothet	Tetrabaena_socialis	55.90	0.00
TRINITY_GAX8363	hypothet	Chlamydomonas_eusti	55.90	0.00

TRINITY_XP_0245	asparag	Selaginella_moellen	55.90	0.00
TRINITY_XP_0243	serine/	Physcomitrella_pate	55.90	0.00
TRINITY_KXZ5351	hypothet	Gonium_pectorale	55.90	0.00
TRINITY_XP_0138	hypothet	Monoraphidium_negle	55.90	0.00
TRINITY_GAQ9123	hypothet	Klebsormidium_niten	55.90	0.00
TRINITY_XP_0029	hypothet	Volvox_carteri	55.90	0.00
TRINITY_XP_0029	hypothet	Volvox_carteri	55.90	0.00
TRINITY_PNW8549	hypothet	Chlamydomonas_reinh	55.90	0.00
TRINITY_XP_0056	Isochor	Coccomyxa_subellips	55.90	0.00
TRINITY_GAX7819	hypothet	Chlamydomonas_eusti	55.90	0.00
TRINITY_XP_0029	hypothet	Volvox_carteri	55.90	0.00
TRINITY_KXZ4196	hypothet	Gonium_pectorale	55.90	0.00
TRINITY_GAX8401	hypothet	Chlamydomonas_eusti	55.90	0.00
TRINITY_XP_0029	hypothet	Volvox_carteri	55.90	0.00
TRINITY_GAX8220	hypothet	Chlamydomonas_eusti	55.90	0.00
TRINITY_XP_0016	dephosp	Chlamydomonas_reinh	55.90	0.00
TRINITY_ADQ0018	glutath	Chlamydomonas_sp._I	55.90	0.00
TRINITY_XP_0017	DnaJ-li	Chlamydomonas_reinh	55.90	0.00
TRINITY_KXZ5329	hypothet	Gonium_pectorale	55.90	0.00
TRINITY_XP_0016	predict	Chlamydomonas_reinh	55.90	0.00
TRINITY_GAX7551	hypothet	Chlamydomonas_eusti	55.90	0.00
TRINITY_XP_0217	ervatam	Chenopodium_quinoa	55.90	0.00
TRINITY_PNH0346	Transmem	Tetrabaena_socialis	55.90	0.00
TRINITY_PRW3374	transcr	Chlorella_sorokinia	55.80	0.00
TRINITY_GAQ7945	DNA top	Klebsormidium_niten	55.80	0.00
TRINITY_GAQ7836	hypothet	Klebsormidium_niten	55.80	0.00
TRINITY_XP_0043	PREDICT	Fragaria_vesca	55.80	0.00
TRINITY_GAX7699	hypothet	Chlamydomonas_eusti	55.80	0.00
TRINITY_GAX7788	hypothet	Chlamydomonas_eusti	55.80	0.00
TRINITY_PIN2131	Fumaryl	Handroanthus_impeti	55.80	0.00
TRINITY_BAA2591	actin [	Nannochloris_bacill	55.80	0.00
TRINITY_XP_0139	dual-sp	Monoraphidium_negle	55.80	0.00
TRINITY_KMZ7128	Multidr	Zostera_marina	55.80	0.00
TRINITY_XP_0056	pyruvat	Coccomyxa_subellips	55.80	0.00
TRINITY_XP_0016	Sec14p-	Chlamydomonas_reinh	55.80	0.00
TRINITY_O65353.	RecName	Helianthus_annuus	55.80	0.00
TRINITY_XP_0217	glycero	Chenopodium_quinoa	55.80	0.00
TRINITY_PSC7653	NAD-dep	Micractinium_conduc	55.80	0.00
TRINITY_BAJ9738	predict	Hordeum_vulgare	55.80	0.00
TRINITY_GAQ7758	ABC tra	Klebsormidium_niten	55.80	0.00
TRINITY_GAX7406	hypothet	Chlamydomonas_eusti	55.80	0.00
TRINITY_XP_0243	protein	Physcomitrella_pate	55.80	0.00
TRINITY_XP_0029	hypothet	Volvox_carteri	55.80	0.00
TRINITY_XP_0016	molybde	Chlamydomonas_reinh	55.80	0.00
TRINITY_XP_0029	hypothet	Volvox_carteri	55.80	0.00
TRINITY_GAX8509	hypothet	Chlamydomonas_eusti	55.80	0.00
TRINITY_PNH1056	Zinc fi	Tetrabaena_socialis	55.80	0.00
TRINITY_P0C8M8.	RecName	Zea_mays	55.80	0.00
TRINITY_GAQ8903	DNA rep	Klebsormidium_niten	55.80	0.00
TRINITY_GAX7997	hypothet	Chlamydomonas_eusti	55.80	0.00
TRINITY_XP_0243	AP-1 co	Physcomitrella_pate	55.80	0.00
TRINITY_PRW5855	flagell	Chlorella_sorokinia	55.80	0.00
TRINITY_XP_0191	PREDICT	Ipomoea_nil	55.80	0.00
TRINITY_CAN6267	hypothet	Vitis_vinifera	55.80	0.00
TRINITY_GAX8455	hypothet	Chlamydomonas_eusti	55.80	0.00
TRINITY_PNW7890	hypothet	Chlamydomonas_reinh	55.80	0.00
TRINITY_XP_0113	Desicca	Auxenochlorella_pro	55.80	0.00
TRINITY_GAX7709	hypothet	Chlamydomonas_eusti	55.80	0.00
TRINITY_XP_0014	predict	Ostreococcus_sp._'l	55.80	0.00
TRINITY_XP_0016	NADH:ub	Chlamydomonas_reinh	55.80	0.00
TRINITY_XP_0191	PREDICT	Ipomoea_nil	55.80	0.00
TRINITY_PNW8260	hypothet	Chlamydomonas_reinh	55.80	0.00
TRINITY_XP_0016	predict	Chlamydomonas_reinh	55.80	0.00
TRINITY_XP_0016	ferroch	Chlamydomonas_reinh	55.80	0.00
TRINITY_XP_0086	cyclin-	Zea_mays	55.80	0.00
TRINITY_XP_0138	hypothet	Monoraphidium_negle	55.80	0.00

TRINITY_GAX8265	hypothet	Chlamydomonas_eusti	55.80	0.00
TRINITY_XP_0029	3'5'-cy	Volvox_carteri	55.80	0.00
TRINITY_XP_0093	PREDICTI	Musa_acuminata	55.80	0.00
TRINITY_XP_0029	hypothet	Volvox_carteri	55.80	0.00
TRINITY_XP_0016	predicte	Chlamydomonas_reinh	55.80	0.00
TRINITY_XP_0017	predicte	Chlamydomonas_reinh	55.80	0.00
TRINITY_KXZ5147	hypothet	Gonium_pectorale	55.80	0.00
TRINITY_GAX7491	hypothet	Chlamydomonas_eusti	55.80	0.00
TRINITY_BAJ9847	predicte	Hordeum_vulgare	55.80	0.00
TRINITY_GAX7490	hypothet	Chlamydomonas_eusti	55.80	0.00
TRINITY_KXZ5075	hypothet	Gonium_pectorale	55.80	0.00
TRINITY_XP_0192	PREDICTI	Nicotiana_attenuata	55.80	0.00
TRINITY_XP_0102	PREDICTI	Nelumbo_nucifera	55.80	0.00
TRINITY_BAJ9260	predicte	Hordeum_vulgare	55.70	0.00
TRINITY_XP_0022	PREDICTI	Vitis_vinifera	55.70	0.00
TRINITY_GBF9051	hypothet	Raphidocelis_subcap	55.70	0.00
TRINITY_RMZ5698	hypothet	Auxenochlorella_pro	55.70	0.00
TRINITY_XP_0237	actin-7-	Lactuca_sativa	55.70	0.00
TRINITY_XP_0093	PREDICTI	Musa_acuminata	55.70	0.00
TRINITY_GBG6361	hypothet	Chara_braunii	55.70	0.00
TRINITY_XP_0071	hypothet	Phaseolus_vulgaris	55.70	0.00
TRINITY_EEC7870	hypothet	Oryza_sativa	55.70	0.00
TRINITY_GBF9845	hypothet	Raphidocelis_subcap	55.70	0.00
TRINITY_GAQ7978	hypothet	Klebsormidium_niten	55.70	0.00
TRINITY_GAX8158	hypothet	Chlamydomonas_eusti	55.70	0.00
TRINITY_XP_0104	PREDICTI	Camelina_sativa	55.70	0.00
TRINITY_GAX7488	hypothet	Chlamydomonas_eusti	55.70	0.00
TRINITY_RYR0255	hypothet	Arachis_hypogaea	55.70	0.00
TRINITY_PTQ3303	hypothet	Marchantia_polymorp	55.70	0.00
TRINITY_KZV1534	hypothet	Dorcoceras_hygromet	55.70	0.00
TRINITY_GAQ8403	Anaphase	Klebsormidium_niten	55.70	0.00
TRINITY_KXZ5493	hypothet	Gonium_pectorale	55.70	0.00
TRINITY_XP_0036	alpha-ma	Medicago_truncatula	55.70	0.00
TRINITY_XP_0056	trypsin-	Coccomyxa_subellips	55.70	0.00
TRINITY_XP_0068	chaperon	Amborella_trichopod	55.70	0.00
TRINITY_GAX8565	hypothet	Chlamydomonas_eusti	55.70	0.00
TRINITY_PON4836	hypothet	Parasponia_anderson	55.70	0.00
TRINITY_KXZ4950	hypothet	Gonium_pectorale	55.70	0.00
TRINITY_XP_0016	dihydrox	Chlamydomonas_reinh	55.70	0.00
TRINITY_XP_0064	ubiquiti	Citrus_clementina	55.70	0.00
TRINITY_XP_0029	hypothet	Volvox_carteri	55.70	0.00
TRINITY_XP_0016	predicte	Chlamydomonas_reinh	55.70	0.00
TRINITY_XP_0029	hypothet	Volvox_carteri	55.70	0.00
TRINITY_PNH0278	Ankycork	Tetrabaena_socialis	55.70	0.00
TRINITY_GAX7257	hypothet	Chlamydomonas_eusti	55.70	0.00
TRINITY_GAX7478	hypothet	Chlamydomonas_eusti	55.70	0.00
TRINITY_PNH0109	Ankyrin	Tetrabaena_socialis	55.70	0.00
TRINITY_PNW8008	hypothet	Chlamydomonas_reinh	55.70	0.00
TRINITY_GAX8464	hypothet	Chlamydomonas_eusti	55.70	0.00
TRINITY_XP_0016	predicte	Chlamydomonas_reinh	55.70	0.00
TRINITY_GAX7580	hypothet	Chlamydomonas_eusti	55.70	0.00
TRINITY_OUS4464	5-aminol	Ostreococcus_tauri	55.70	0.00
TRINITY_GAX7378	hypothet	Chlamydomonas_eusti	55.70	0.00
TRINITY_GAX8078	hypothet	Chlamydomonas_eusti	55.70	0.00
TRINITY_AFK4295	unknown	Lotus_japonicus	55.70	0.00
TRINITY_GAX7381	hypothet	Chlamydomonas_eusti	55.70	0.00
TRINITY_GAX7926	hypothet	Chlamydomonas_eusti	55.70	0.00
TRINITY_GAX7860	hypothet	Chlamydomonas_eusti	55.70	0.00
TRINITY_PNH1189	Patatin-	Tetrabaena_socialis	55.70	0.00
TRINITY_PNH0630	Omega-3	Tetrabaena_socialis	55.70	0.00
TRINITY_XP_0016	flagella	Chlamydomonas_reinh	55.70	0.00
TRINITY_XP_0172	PREDICTI	Daucus_carota	55.70	0.00
TRINITY_PRW5592	translat	Chlorella_sorokinia	55.70	0.00
TRINITY_PNW8895	hypothet	Chlamydomonas_reinh	55.70	0.00
TRINITY_XP_0075	ABC tra	Bathycoccus_prasino	55.70	0.00
TRINITY_KXZ4790	hypothet	Gonium_pectorale	55.70	0.00

TRINITY_PNW81055	hypothet	Chlamydomonas_reinh	55.70	0.00
TRINITY_GAX80439	hypothet	Chlamydomonas_eusti	55.70	0.00
TRINITY_GAX76918	hypothet	Chlamydomonas_eusti	55.70	0.00
TRINITY_XP_01027	PREDICTE	Nelumbo_nucifera	55.60	0.00
TRINITY_GAQ84171	Ubiquiti	Klebsormidium_niten	55.60	0.00
TRINITY_PKI40656	hypothet	Punica_granatum	55.60	0.00
TRINITY_BAJ96688	predicte	Hordeum_vulgare	55.60	0.00
TRINITY_XP_00305	dynein h	Micromonas_pusilla	55.60	0.00
TRINITY_GAQ87980	Phosphat	Klebsormidium_niten	55.60	0.00
TRINITY_XP_00565	sterol c	Coccomyxa_subellips	55.60	0.00
TRINITY_GAQ91618	alpha-1,	Klebsormidium_niten	55.60	0.00
TRINITY_XP_00295	hypothet	Volvox_carteri	55.60	0.00
TRINITY_XP_01908	PREDICTE	Camelina_sativa	55.60	0.00
TRINITY_XP_02174	tRNA-dih	Chenopodium_quinoa	55.60	0.00
TRINITY_GAQ79025	Regulato	Klebsormidium_niten	55.60	0.00
TRINITY_XP_02201	ABC tran	Helianthus_annuus	55.60	0.00
TRINITY_XP_00250	predicte	Micromonas_commoda	55.60	0.00
TRINITY_GAQ91151	hypothet	Klebsormidium_niten	55.60	0.00
TRINITY_OTG33006	putative	Helianthus_annuus	55.60	0.00
TRINITY_OWM74552	hypothet	Punica_granatum	55.60	0.00
TRINITY_XP_00683	ras-rela	Amborella_trichopod	55.60	0.00
TRINITY_XP_00250	glycosyl	Micromonas_commoda	55.60	0.00
TRINITY_XP_00295	hypothet	Volvox_carteri	55.60	0.00
TRINITY_KZV54876	wall-ass	Dorcoceras_hygromet	55.60	0.00
TRINITY_XP_00294	hypothet	Volvox_carteri	55.60	0.00
TRINITY_GAX79861	hypothet	Chlamydomonas_eusti	55.60	0.00
TRINITY_PIA46551	hypothet	Aquilegia_coerulea	55.60	0.00
TRINITY_XP_00170	mitogen-	Chlamydomonas_reinh	55.60	0.00
TRINITY_GAX74379	hypothet	Chlamydomonas_eusti	55.60	0.00
TRINITY_PNW77428	hypothet	Chlamydomonas_reinh	55.60	0.00
TRINITY_KXZ53401	hypothet	Gonium_pectorale	55.60	0.00
TRINITY_GAX77908	hypothet	Chlamydomonas_eusti	55.60	0.00
TRINITY_PNH09476	Purple a	Tetrabaena_socialis	55.60	0.00
TRINITY_KXZ48414	hypothet	Gonium_pectorale	55.60	0.00
TRINITY_PNH07661	Serine/t	Tetrabaena_socialis	55.60	0.00
TRINITY_XP_00564	proteaso	Coccomyxa_subellips	55.60	0.00
TRINITY_XP_00295	hypothet	Volvox_carteri	55.60	0.00
TRINITY_GAX84660	hypothet	Chlamydomonas_eusti	55.60	0.00
TRINITY_PNW79691	hypothet	Chlamydomonas_reinh	55.60	0.00
TRINITY_PNW74811	hypothet	Chlamydomonas_reinh	55.60	0.00
TRINITY_KXZ45439	hypothet	Gonium_pectorale	55.60	0.00
TRINITY_XP_02387	ribose-p	Quercus_suber	55.60	0.00
TRINITY_XP_00169	26S prot	Chlamydomonas_reinh	55.60	0.00
TRINITY_GAX79801	hypothet	Chlamydomonas_eusti	55.60	0.00
TRINITY_KXZ54104	hypothet	Gonium_pectorale	55.60	0.00
TRINITY_PNW81569	hypothet	Chlamydomonas_reinh	55.60	0.00
TRINITY_PNH05351	hypothet	Tetrabaena_socialis	55.60	0.00
TRINITY_PRQ55656	putative	Rosa_chinensis	55.60	0.00
TRINITY_PNH08069	hypothet	Tetrabaena_socialis	55.60	0.00
TRINITY_GAX73793	hypothet	Chlamydomonas_eusti	55.60	0.00
TRINITY_RWR84593	actin [C	Cinnamomum_micranth	55.60	0.00
TRINITY_GAX74929	hypothet	Chlamydomonas_eusti	55.60	0.00
TRINITY_GAX80960	hypothet	Chlamydomonas_eusti	55.60	0.00
TRINITY_XP_02021	DExH-box	Cajanus_cajan	55.60	0.00
TRINITY_GAX81533	hypothet	Chlamydomonas_eusti	55.60	0.00
TRINITY_XP_00169	transloc	Chlamydomonas_reinh	55.60	0.00
TRINITY_GAX81441	hypothet	Chlamydomonas_eusti	55.60	0.00
TRINITY_XP_00305	predicte	Micromonas_pusilla	55.60	0.00
TRINITY_KXZ54517	hypothet	Gonium_pectorale	55.60	0.00
TRINITY_XP_00169	transcri	Chlamydomonas_reinh	55.60	0.00
TRINITY_GBG91680	hypothet	Chara_braunii	55.60	0.00
TRINITY_XP_00451	serine c	Cicer_arietinum	55.60	0.00
TRINITY_EOY26390	Uncharac	Theobroma_cacao	55.60	0.00
TRINITY_XP_00295	hypothet	Volvox_carteri	55.60	0.00
TRINITY_XP_00295	hypothet	Volvox_carteri	55.60	0.00
TRINITY_XP_00305	thioredo	Micromonas_pusilla	55.60	0.00

TRINITY_XP_0064	DEAD-bo›	Eutrema_salsugineum	55.60	0.00
TRINITY_GAX8390	hypothet	Chlamydomonas_eusti	55.60	0.00
TRINITY_KDO4039	hypothet	Citrus_sinensis	55.60	0.00
TRINITY_XP_0029	hypothet	Volvox_carteri	55.60	0.00
TRINITY_PSC7581	BRCA1-a	Micractinium_conduc	55.60	0.00
TRINITY_ABF9495	retrotra	Oryza_sativa	55.60	0.00
TRINITY_XP_0016	SM/Sec1-	Chlamydomonas_reinh	55.60	0.00
TRINITY_GAX8138	hypothet	Chlamydomonas_eusti	55.60	0.00
TRINITY_PNH0497	hypothet	Tetrabaena_socialis	55.60	0.00
TRINITY_GAX8470	hypothet	Chlamydomonas_eusti	55.60	0.00
TRINITY_PNW7682	hypothet	Chlamydomonas_reinh	55.60	0.00
TRINITY_GAX7531	hypothet	Chlamydomonas_eusti	55.60	0
TRINITY_GAQ7760	haloaci	Klebsormidium_niten	55.60	0.00
TRINITY_BAK0277	predicte	Hordeum_vulgare	55.50	0.00
TRINITY_XP_0029	ribulose	Selaginella_moellen	55.50	0.00
TRINITY_XP_0237	S-formyl	Lactuca_sativa	55.50	0.00
TRINITY_AAO7355	reverse-	Chlamydomonas_reinh	55.50	0.00
TRINITY_BAU6158	eukaryot	Gonium_pectorale	55.50	0.00
TRINITY_GAX8107	hypothet	Chlamydomonas_eusti	55.50	0.00
TRINITY_OAE2398	hypothet	Marchantia_polymorp	55.50	0.00
TRINITY_PNH0483	LL-diam	Tetrabaena_socialis	55.50	0.00
TRINITY_PNH1105	hypothet	Tetrabaena_socialis	55.50	0.00
TRINITY_KXZ4534	hypothet	Gonium_pectorale	55.50	0.00
TRINITY_XP_0016	maltose	Chlamydomonas_reinh	55.50	0.00
TRINITY_GAX7757	hypothet	Chlamydomonas_eusti	55.50	0.00
TRINITY_GAX7948	hypothet	Chlamydomonas_eusti	55.50	0.00
TRINITY_KXZ5562	hypothet	Gonium_pectorale	55.50	0.00
TRINITY_PNW7222	hypothet	Chlamydomonas_reinh	55.50	0.00
TRINITY_XP_0239	ras-like	Quercus_suber	55.50	0.00
TRINITY_RMZ5563	hypothet	Auxenochlorella_prc	55.50	0.00
TRINITY_GAX7445	hypothet	Chlamydomonas_eusti	55.50	0.00
TRINITY_GAX8240	hypothet	Chlamydomonas_eusti	55.50	0.00
TRINITY_XP_0056	hypothet	Coccomyxa_subellips	55.50	0.00
TRINITY_XP_0228	E3 ubiq	Olea_europaea	55.50	0.00
TRINITY_XP_0024	acyl-co	Sorghum_bicolor	55.50	0.00
TRINITY_GAX8611	hypothet	Chlamydomonas_eusti	55.50	0.00
TRINITY_XP_0017	3-phosp	Chlamydomonas_reinh	55.50	0.00
TRINITY_GAX8383	hypothet	Chlamydomonas_eusti	55.50	0.00
TRINITY_XP_0239	ras-like	Quercus_suber	55.50	0.00
TRINITY_PNW7723	hypothet	Chlamydomonas_reinh	55.50	0.00
TRINITY_GAQ9031	20S prot	Klebsormidium_niten	55.50	0.00
TRINITY_XP_0016	voltage-	Chlamydomonas_reinh	55.50	0.00
TRINITY_GAX8043	hypothet	Chlamydomonas_eusti	55.50	0.00
TRINITY_O65084.	RecName:	Picea_mariana	55.50	0.00
TRINITY_XP_0273	eukaryot	Abrus_precatorius	55.40	0.00
TRINITY_XP_0238	probable	Quercus_suber	55.40	0.00
TRINITY_GAQ8347	ubiquin	Klebsormidium_niten	55.40	0.00
TRINITY_GAQ8713	Ribonuc	Klebsormidium_niten	55.40	0.00
TRINITY_AIU4943	translat	Schrenkiella_parvul	55.40	0.00
TRINITY_PSC7496	60S rib	Micractinium_conduc	55.40	0.00
TRINITY_BAJ8923	predicte	Hordeum_vulgare	55.40	0.00
TRINITY_GAX7502	hypothet	Chlamydomonas_eusti	55.40	0.00
TRINITY_XP_0056	AAA-ATPa	Coccomyxa_subellips	55.40	0.00
TRINITY_PRW5758	coatome	Chlorella_sorokinia	55.40	0.00
TRINITY_KXZ4177	hypothet	Gonium_pectorale	55.40	0.00
TRINITY_GAX7954	hypothet	Chlamydomonas_eusti	55.40	0.00
TRINITY_ADI4692	MTM0417	Volvox_carteri	55.40	0.00
TRINITY_PNH0520	Protein	Tetrabaena_socialis	55.40	0.00
TRINITY_RVW7594	Nucleola	Vitis_vinifera	55.40	0.00
TRINITY_XP_0029	hypothet	Volvox_carteri	55.40	0.00
TRINITY_KHN2807	Serine/t	Glycine_soja	55.40	0.00
TRINITY_XP_0075	predicte	Bathycoccus_prasinc	55.40	0.00
TRINITY_VDD1348	unnamed	Brassica_oleracea	55.40	0.00
TRINITY_PNW7412	hypothet	Chlamydomonas_reinh	55.40	0.00
TRINITY_XP_0238	probable	Quercus_suber	55.40	0.00
TRINITY_PNW8620	hypothet	Chlamydomonas_reinh	55.40	0.00

TRINITY_XP_0243(	exonucle	Physcomitrella_pate	55.40	0.00
TRINITY_KXZ4626!	hypothet	Gonium_pectorale	55.40	0.00
TRINITY_XP_0017(	sulfite	Chlamydomonas_reinh	55.40	0.00
TRINITY_KXZ4702!	hypothet	Gonium_pectorale	55.40	0.00
TRINITY_XP_0014:	predicte	Ostreococcus_sp._'l	55.40	0.00
TRINITY_XP_0096:	PREDICTI	Nicotiana_tomentosi	55.40	0.00
TRINITY_PSR9345:	Cleavage	Actinidia_chinensis	55.40	0.00
TRINITY_XP_0146:	hydroxyr	Glycine_max	55.40	0.00
TRINITY_XP_0110	ATP-depe	Sesamum_indicum	55.40	0.00
TRINITY_OAE2473	hypothet	Marchantia_polymorp	55.40	0.00
TRINITY_PNH0146:	hypothet	Tetrabaena_socialis	55.40	0.00
TRINITY_XP_0138	2-oxodi	Monoraphidium_negle	55.40	0.00
TRINITY_XP_0098(	PREDICTI	Nicotiana_sylvestri	55.40	0.00
TRINITY_XP_0043(	PREDICTI	Fragaria_vesca	55.40	0.00
TRINITY_XP_0016	DNA dama	Chlamydomonas_reinh	55.40	0.00
TRINITY_PRW5708	N-alpha-	Chlorella_sorokinia	55.40	0.00
TRINITY_XP_0139(	hypothet	Monoraphidium_negle	55.40	0.00
TRINITY_GAX7818	hypothet	Chlamydomonas_eusti	55.40	0.00
TRINITY_PRQ2958	putative	Rosa_chinensis	55.40	0.00
TRINITY_AHA1536:	isopente	Taraxacum_kok-saghy	55.40	0.00
TRINITY_GAX7530(	hypothet	Chlamydomonas_eusti	55.40	0.00
TRINITY_XP_0016	predicte	Chlamydomonas_reinh	55.40	0.00
TRINITY_GAX8121	hypothet	Chlamydomonas_eusti	55.40	0.00
TRINITY_PRW4436!	DNA repa	Chlorella_sorokinia	55.40	0.00
TRINITY_KXZ5212	ARPC4 p	Gonium_pectorale	55.40	0.00
TRINITY_PNH0230:	hypothet	Tetrabaena_socialis	55.40	0.00
TRINITY_XP_0219	calcium-	Helianthus_annuus	55.40	0.00
TRINITY_GAX8387(	hypothet	Chlamydomonas_eusti	55.40	0.00
TRINITY_KXZ5146	hypothet	Gonium_pectorale	55.40	0.00
TRINITY_KVH9034:	Histone-	Cynara_cardunculus	55.40	0.00
TRINITY_GAX7658	hypothet	Chlamydomonas_eusti	55.40	0.00
TRINITY_XP_0016	transcr:	Chlamydomonas_reinh	55.40	0.00
TRINITY_XP_0029	hypothet	Volvox_carteri	55.40	0.00
TRINITY_GAX7788:	hypothet	Chlamydomonas_eusti	55.40	0.00
TRINITY_GAQ7758:	ABC tra	Klebsormidium_niten	55.40	0.00
TRINITY_XP_0238	calcine	Quercus_suber	55.30	0.00
TRINITY_XP_0243(	putative	Physcomitrella_pate	55.30	0.00
TRINITY_PNR2956:	hypothet	Physcomitrella_pate	55.30	0.00
TRINITY_GAX7492(	hypothet	Chlamydomonas_eusti	55.30	0.00
TRINITY_OAE3085!	hypothet	Marchantia_polymorp	55.30	0.00
TRINITY_XP_0194!	PREDICTI	Lupinus_angustifoli	55.30	0.00
TRINITY_XP_0102!	PREDICTI	Nelumbo_nucifera	55.30	0.00
TRINITY_XP_0215	39S rib	Manihot_esculenta	55.30	0.00
TRINITY_XP_0014:	predicte	Ostreococcus_sp._'l	55.30	0.00
TRINITY_OAE3102	hypothet	Marchantia_polymorp	55.30	0.00
TRINITY_XP_0016	predicte	Chlamydomonas_reinh	55.30	0.00
TRINITY_GAX8411	hypothet	Chlamydomonas_eusti	55.30	0.00
TRINITY_P0C8M8.:	RecName:	Zea_mays	55.30	0.00
TRINITY_KXZ5060:	hypothet	Gonium_pectorale	55.30	0.00
TRINITY_GAX8315	hypothet	Chlamydomonas_eusti	55.30	0.00
TRINITY_KXZ4473!	hypothet	Gonium_pectorale	55.30	0.00
TRINITY_ERN1059:	hypothet	Amborella_trichopod	55.30	0.00
TRINITY_PRW6062:	ATPase	Chlorella_sorokinia	55.30	0.00
TRINITY_XP_0056	pre-rRN	Coccomyxa_subellips	55.30	0.00
TRINITY_RMZ5313	hypothet	Auxenochlorella_pro	55.30	0.00
TRINITY_GBG5896:	hypothet	Chara_braunii	55.30	0.00
TRINITY_XP_0029	hypothet	Volvox_carteri	55.30	0.00
TRINITY_PNW8841	hypothet	Chlamydomonas_reinh	55.30	0.00
TRINITY_XP_0238	glucose-	Quercus_suber	55.30	0.00
TRINITY_GAQ9356:	hypothet	Klebsormidium_niten	55.30	0.00
TRINITY_GBF8870!	hypothet	Raphidocelis_subcap	55.30	0.00
TRINITY_XP_0049(	GTP-bin	Setaria_italica	55.30	0.00
TRINITY_XP_0206	DNA rep:	Dendrobium_catenatu	55.30	0.00
TRINITY_XP_0016	predicte	Chlamydomonas_reinh	55.30	0.00
TRINITY_GAX7529	hypothet	Chlamydomonas_eusti	55.30	0.00
TRINITY_XP_0138	hypothet	Monoraphidium_negle	55.30	0.00

TRINITY_PNW7745!	hypothet	Chlamydomonas_reinh	55.30	0.00
TRINITY_GAX8055	hypothet	Chlamydomonas_eusti	55.30	0.00
TRINITY_GAX7904	hypothet	Chlamydomonas_eusti	55.30	0.00
TRINITY_XP_0161	lon prot	Arachis_ipaensis	55.30	0.00
TRINITY_XP_0279	DEAD-bo	Vigna_unguiculata	55.30	0.00
TRINITY_PON9199	RidA fa	Trema_orientale	55.30	0.00
TRINITY_PNW7276	hypothet	Chlamydomonas_reinh	55.30	0.00
TRINITY_GAX7456	hypothet	Chlamydomonas_eusti	55.30	0.00
TRINITY_KCW7237	hypothet	Eucalyptus_grandis	55.30	0.00
TRINITY_XP_0029	3'5'-cy	Volvox_carteri	55.30	0.00
TRINITY_XP_0243	serine/t	Physcomitrella_pate	55.20	0.00
TRINITY_GAX7492	hypothet	Chlamydomonas_eusti	55.20	0.00
TRINITY_XP_0202	phosphat	Asparagus_officinal	55.20	0.00
TRINITY_XP_0113	Cystein	Auxenochlorella_pro	55.20	0.00
TRINITY_XP_0017	protein	Chlamydomonas_reinh	55.20	0.00
TRINITY_XP_0058	hypothet	Chlorella_variabili	55.20	0.00
TRINITY_RAL4800	hypothet	Cuscuta_australis	55.20	0.00
TRINITY_XP_0238	guanine	Quercus_suber	55.20	0.00
TRINITY_XP_0106	PREDICT	Beta_vulgaris	55.20	0.00
TRINITY_OAY3289	hypothet	Manihot_esculenta	55.20	0.00
TRINITY_GAQ9158	RNA pol	Klebsormidium_niten	55.20	0.00
TRINITY_RVX1487	ABC tra	Vitis_vinifera	55.20	0.00
TRINITY_XP_0239	ras-rel	Quercus_suber	55.20	0.00
TRINITY_XP_0049	methion	Setaria_italica	55.20	0.00
TRINITY_GBG7022	hypothet	Chara_braunii	55.20	0.00
TRINITY_XP_0249	60S rib	Cynara_cardunculus	55.20	0.00
TRINITY_PNW8334	hypothet	Chlamydomonas_reinh	55.20	0.00
TRINITY_OAE3233	hypothet	Marchantia_polymorp	55.20	0.00
TRINITY_XP_0056	carbon-r	Coccomyxa_subellips	55.20	0.00
TRINITY_PHU2846	hypothet	Capsicum_chinense	55.20	0.00
TRINITY_PNW8827	hypothet	Chlamydomonas_reinh	55.20	0.00
TRINITY_PNH0580	Metallo	Tetrabaena_socialis	55.20	0.00
TRINITY_GAX8069	hypothet	Chlamydomonas_eusti	55.20	0.00
TRINITY_BAJ9323	predict	Hordeum_vulgare	55.20	0.00
TRINITY_ALM5498	DEAD bo	Chlamydomonas_sp._I	55.20	0.00
TRINITY_PSC7599	2-oxois	Micractinium_conduc	55.20	0.00
TRINITY_GBF8915	hypothet	Raphidocelis_subcap	55.20	0.00
TRINITY_XP_0239	ras-lik	Quercus_suber	55.20	0.00
TRINITY_KXZ4640	hypothet	Gonium_pectorale	55.20	0.00
TRINITY_PNH0808	BTB/POZ	Tetrabaena_socialis	55.20	0.00
TRINITY_GAX7570	hypothet	Chlamydomonas_eusti	55.20	0.00
TRINITY_PNH0419	hypothet	Tetrabaena_socialis	55.20	0.00
TRINITY_XP_0029	hypothet	Volvox_carteri	55.20	0.00
TRINITY_GAX8011	hypothet	Chlamydomonas_eusti	55.20	0.00
TRINITY_XP_0106	PREDICT	Beta_vulgaris	55.20	0.00
TRINITY_GAX7273	hypothet	Chlamydomonas_eusti	55.20	0.00
TRINITY_RMZ5666	hypothet	Auxenochlorella_pro	55.20	0.00
TRINITY_GAX7735	hypothet	Chlamydomonas_eusti	55.20	0.00
TRINITY_GAX7900	hypothet	Chlamydomonas_eusti	55.20	0.00
TRINITY_KXZ5597	hypothet	Gonium_pectorale	55.20	0.00
TRINITY_KXZ5672	hypothet	Gonium_pectorale	55.20	0.00
TRINITY_PNW8261	hypothet	Chlamydomonas_reinh	55.20	0.00
TRINITY_XP_0016	predict	Chlamydomonas_reinh	55.20	0.00
TRINITY_GBF9439	importi	Raphidocelis_subcap	55.20	0.00
TRINITY_GAQ8229	hypothet	Klebsormidium_niten	55.20	0.00
TRINITY_PNH1116	Ankyrin	Tetrabaena_socialis	55.20	0.00
TRINITY_XP_0271	replicat	Coffea_eugenioides	55.20	0.00
TRINITY_GAX8054	hypothet	Chlamydomonas_eusti	55.20	0.00
TRINITY_GAX7339	hypothet	Chlamydomonas_eusti	55.20	0.00
TRINITY_KXZ4596	hypothet	Gonium_pectorale	55.20	0.00
TRINITY_PNH1200	Arsenic	Tetrabaena_socialis	55.20	0.00
TRINITY_XP_0128	PREDICT	Erythranthe_guttata	55.20	0.00
TRINITY_PNH0732	Cell di	Tetrabaena_socialis	55.20	0.00
TRINITY_XP_0264	DNA pol	Papaver_somniferum	55.10	0.00
TRINITY_GAQ8591	3-hydro	Klebsormidium_niten	55.10	0.00
TRINITY_XP_0025	predict	Micromonas_commoda	55.10	0.00

TRINITY_XP_0017(	predicte	Chlamydomonas_reinh	55.10	0.00
TRINITY_PNH0937(	Cytochro	Tetrabaena_socialis	55.10	0.00
TRINITY_XP_00685	exosome	Amborella_trichopod	55.10	0.00
TRINITY_ERN12399	hypothet	Amborella_trichopod	55.10	0.00
TRINITY_GAQ82295	hypothet	Klebsormidium_niten	55.10	0.00
TRINITY_XP_01284	PREDICTE	Erythranthe_guttata	55.10	0.00
TRINITY_PIN07962	Protein	Handroanthus_impeti	55.10	0.00
TRINITY_XP_0271(	actin-11	Coffea_arabica	55.10	0.00
TRINITY_XP_0124(	PREDICTE	Gossypium_raimondii	55.10	0.00
TRINITY_XP_00628	casein }	Capsella_rubella	55.10	0.00

TRINITY_XP_0083	PREDICT	Malus_domestica	55.10	0.00
TRINITY_BAK0228	predict	Hordeum_vulgare	55.10	0.00
TRINITY_XP_0109	PREDICT	Elaeis_guineensis	55.10	0.00
TRINITY_XP_0191	PREDICT	Ipomoea_nil	55.10	0.00
TRINITY_GAX7488	hypothet	Chlamydomonas_eusti	55.10	0.00
TRINITY_GAX7898	hypothet	Chlamydomonas_eusti	55.10	0.00
TRINITY_GAX8605	hypothet	Chlamydomonas_eusti	55.10	0.00
TRINITY_XP_0139	hypothet	Monoraphidium_negle	55.10	0.00
TRINITY_XP_0075	thymidi	Bathycoccus_prasin	55.10	0.00
TRINITY_GAX7834	hypothet	Chlamydomonas_eusti	55.10	0.00
TRINITY_GAX8172	hypothet	Chlamydomonas_eusti	55.10	0.00
TRINITY_AAZ3107	peroxis	Medicago_sativa	55.10	0.00
TRINITY_GAX7359	hypothet	Chlamydomonas_eusti	55.10	0.00
TRINITY_GAQ8101	glycyl-	Klebsormidium_niten	55.10	0.00
TRINITY_GAX8395	hypothet	Chlamydomonas_eusti	55.10	0.00
TRINITY_RAL4529	hypothet	Cuscuta_australis	55.10	0.00
TRINITY_XP_0029	hypothet	Volvox_carteri	55.10	0.00
TRINITY_XP_0016	predict	Chlamydomonas_reinh	55.10	0.00
TRINITY_GAX7415	hypothet	Chlamydomonas_eusti	55.10	0.00
TRINITY_GAX7760	hypothet	Chlamydomonas_eusti	55.10	0.00
TRINITY_KXZ4651	hypothet	Gonium_pectorale	55.10	0.00
TRINITY_AFK4521	unknown	Lotus_japonicus	55.10	0.00
TRINITY_XP_0029	ATP-ene	Volvox_carteri	55.10	0.00
TRINITY_PNH0961	hypothet	Tetrabaena_socialis	55.10	0.00
TRINITY_XP_0110	PREDICT	Populus_euphratica	55.10	0.00
TRINITY_KXZ5345	hypothet	Gonium_pectorale	55.10	0.00
TRINITY_GAX7375	hypothet	Chlamydomonas_eusti	55.10	0.00
TRINITY_GBF9344	hypothet	Raphidocelis_subcap	55.10	0.00
TRINITY_GAX8019	hypothet	Chlamydomonas_eusti	55.10	0.00
TRINITY_PNH0634	Protein	Tetrabaena_socialis	55.10	0.00
TRINITY_GAX8245	hypothet	Chlamydomonas_eusti	55.10	0.00
TRINITY_PRW5674	chromat	Chlorella_sorokinia	55.10	0.00
TRINITY_GBF9456	aldo ket	Raphidocelis_subcap	55.10	0.00
TRINITY_XP_0087	SURP an	Phoenix_dactylifera	55.10	0.00
TRINITY_XP_0239	kinesin	Quercus_suber	55.10	0.00
TRINITY_KXZ4986	hypothet	Gonium_pectorale	55.10	0.00
TRINITY_XP_0016	ribofla	Chlamydomonas_reinh	55.10	0.00
TRINITY_XP_0240	protein	Morus_notabilis	55.10	0.00
TRINITY_KXZ4442	hypothet	Gonium_pectorale	55.10	0.00
TRINITY_PNW8806	hypothet	Chlamydomonas_reinh	55.10	0.00
TRINITY_XP_0016	predict	Chlamydomonas_reinh	55.10	0.00
TRINITY_PNR6194	hypothet	Physcomitrella_pate	55.10	0.00
TRINITY_XP_0238	kinesin	Quercus_suber	55.10	0.00
TRINITY_GAQ7909	26S Prot	Klebsormidium_niten	55.10	0.00
TRINITY_RMZ5446	hypothet	Auxenochlorella_pr	55.00	0.00
TRINITY_XP_0035	inosito	Brachypodium_distac	55.00	0.00
TRINITY_XP_0029	hypothet	Volvox_carteri	55.00	0.00
TRINITY_GBG7935	hypothet	Chara_braunii	55.00	0.00
TRINITY_PHT9878	putativ	Capsicum_chinense	55.00	0.00
TRINITY_GAX8357	hypothet	Chlamydomonas_eusti	55.00	0.00
TRINITY_BAK0108	predict	Hordeum_vulgare	55.00	0.00
TRINITY_GAX8379	hypothet	Chlamydomonas_eusti	55.00	0.00
TRINITY_XP_0029	ATM/ATR-	Volvox_carteri	55.00	0.00
TRINITY_XP_0029	hypothet	Volvox_carteri	55.00	0.00
TRINITY_GAX7714	hypothet	Chlamydomonas_eusti	55.00	0.00
TRINITY_KNA0987	hypothet	Spinacia_oleracea	55.00	0.00
TRINITY_PNW7579	hypothet	Chlamydomonas_reinh	55.00	0.00
TRINITY_XP_0056	Metallo-	Coccomyxa_subellips	55.00	0.00
TRINITY_XP_0243	nuclear	Physcomitrella_pate	55.00	0.00
TRINITY_XP_0161	60S rib	Arachis_ipaensis	55.00	0.00
TRINITY_GAV8731	UQ_con	Cephalotus_follicul	55.00	0.00
TRINITY_XP_0029	thylako	Volvox_carteri	55.00	0.00
TRINITY_GAX7998	hypothet	Chlamydomonas_eusti	55.00	0.00
TRINITY_XP_0017	mitocho	Chlamydomonas_reinh	55.00	0.00
TRINITY_GBG6743	hypothet	Chara_braunii	55.00	0.00
TRINITY_GAX8098	hypothet	Chlamydomonas_eusti	55.00	0.00

TRINITY_XP_0270	protein-	Coffea_arabica	55.00	0.00
TRINITY_XP_0017(	predicte	Chlamydomonas_reinh	55.00	0.00
TRINITY_PNW7810	hypothet	Chlamydomonas_reinh	55.00	0.00
TRINITY_XP_0016	methion:	Chlamydomonas_reinh	55.00	0.00
TRINITY_PSC6705	Retrovi	Micractinium_conduc	55.00	0.00
TRINITY_XP_0016	predicte	Chlamydomonas_reinh	55.00	0.00
TRINITY_XP_0167	PREDICTE	Gossypium_hirsutum	55.00	0.00
TRINITY_ONM4123	Cytochr	Zea_mays	55.00	0.00
TRINITY_GAX7530	hypothet	Chlamydomonas_eusti	55.00	0.00
TRINITY_PNW8566	hypothet	Chlamydomonas_reinh	55.00	0.00
TRINITY_PRW2090	Oxidore	Chlorella_sorokinia	55.00	0.00
TRINITY_GAX8227	hypothet	Chlamydomonas_eusti	55.00	0.00
TRINITY_PSC6774	E3 ubiq	Micractinium_conduc	55.00	0.00
TRINITY_RMZ5565	hypothet	Auxenochlorella_pro	55.00	0.00
TRINITY_GAX7813	hypothet	Chlamydomonas_eusti	55.00	0.00
TRINITY_PRW2065	DNA mism	Chlorella_sorokinia	55.00	0.00
TRINITY_KXZ5037	hypothet	Gonium_pectorale	55.00	0.00
TRINITY_XP_0243	probable	Physcomitrella_pate	55.00	0.00
TRINITY_PRW5844	isoleuc:	Chlorella_sorokinia	55.00	0
TRINITY_GAX8496	hypothet	Chlamydomonas_eusti	55.00	0.00
TRINITY_KXZ5409	hypothet	Gonium_pectorale	55.00	0.00
TRINITY_GAX8339	hypothet	Chlamydomonas_eusti	55.00	0.00
TRINITY_AAD4434	actin [I	Dunaliella_salina	55.00	0.00
TRINITY_XP_0016	sulfate	Chlamydomonas_reinh	55.00	0.00
TRINITY_GAX8163	hypothet	Chlamydomonas_eusti	55.00	0.00
TRINITY_KXZ5105	hypothet	Gonium_pectorale	55.00	0.00
TRINITY_GAX7287	hypothet	Chlamydomonas_eusti	55.00	0
TRINITY_GAX8166	hypothet	Chlamydomonas_eusti	55.00	0.00
TRINITY_GAX8047	hypothet	Chlamydomonas_eusti	55.00	0.00
TRINITY_XP_0097	PREDICTE	Nicotiana_sylvestri	54.90	0.00
TRINITY_XP_0029	hypothet	Volvox_carteri	54.90	0.00
TRINITY_KEH1554	acyl-Co	Medicago_truncatula	54.90	0.00
TRINITY_XP_0104	PREDICTE	Camelina_sativa	54.90	0.00
TRINITY_XP_0093	PREDICTE	Pyrus_x_bretschneid	54.90	0.00
TRINITY_PWZ3258	hypothet	Zea_mays	54.90	0.00
TRINITY_XP_0239	nicotina	Quercus_suber	54.90	0.00
TRINITY_XP_0205	GPI mann	Amborella_trichopod	54.90	0.00
TRINITY_ABK2377	unknown	Picea_sitchensis	54.90	0.00
TRINITY_GBG8802	hypothet	Chara_braunii	54.90	0.00
TRINITY_XP_0025	predicte	Micromonas_commoda	54.90	0.00
TRINITY_KXZ4685	hypothet	Gonium_pectorale	54.90	0.00
TRINITY_KXZ4319	hypothet	Gonium_pectorale	54.90	0.00
TRINITY_XP_0192	PREDICTE	Nicotiana_attenuata	54.90	0.00
TRINITY_XP_0139	40S rib	Monoraphidium_negle	54.90	0.00
TRINITY_KXZ5512	hypothet	Gonium_pectorale	54.90	0.00
TRINITY_XP_0029	hypothet	Volvox_carteri	54.90	0.00
TRINITY_GAX7595	hypothet	Chlamydomonas_eusti	54.90	0.00
TRINITY_GAX7506	hypothet	Chlamydomonas_eusti	54.90	0.00
TRINITY_KXZ4463	hypothet	Gonium_pectorale	54.90	0.00
TRINITY_GAX7493	hypothet	Chlamydomonas_eusti	54.90	0.00
TRINITY_GBF9726	hypothet	Raphidocelis_subcap	54.90	0.00
TRINITY_PNH0329	hypothet	Tetrabaena_socialis	54.90	0.00
TRINITY_XP_0150	DNA mism	Solanum_pennellii	54.90	0.00
TRINITY_KXZ5544	hypothet	Gonium_pectorale	54.90	0.00
TRINITY_KDD7222	G10-like	Helicosporidium_sp.	54.90	0.00
TRINITY_GAQ8636	6-phosph	Klebsormidium_niten	54.90	0.00
TRINITY_GAX8519	hypothet	Chlamydomonas_eusti	54.90	0.00
TRINITY_GAX8098	hypothet	Chlamydomonas_eusti	54.90	0.00
TRINITY_GAX7391	hypothet	Chlamydomonas_eusti	54.90	0.00
TRINITY_XP_0029	hypothet	Volvox_carteri	54.90	0.00
TRINITY_BAK0047	predicte	Hordeum_vulgare	54.90	0.00
TRINITY_GAQ7953	DNA dama	Klebsormidium_niten	54.90	0.00
TRINITY_GBF9903	hypothet	Raphidocelis_subcap	54.90	0.00
TRINITY_XP_0016	predicte	Chlamydomonas_reinh	54.90	0.00
TRINITY_XP_0139	peptide-	Monoraphidium_negle	54.90	0.00
TRINITY_CAA9816	RAB5A [I	Lotus_japonicus	54.90	0.00

TRINITY_XP_0024	vacuola	Micromonas_commoda	54.90	0.00
TRINITY_PTQ5007	hypothet	Marchantia_polymorp	54.90	0.00
TRINITY_PNW8487	hypothet	Chlamydomonas_reinh	54.90	0.00
TRINITY_XP_0029	betaine	Selaginella_moellen	54.90	0.00
TRINITY_PNW7366	hypothet	Chlamydomonas_reinh	54.90	0.00
TRINITY_GBF9082	hypothet	Raphidocelis_subcap	54.90	0.00
TRINITY_PNH0685	GATA tra	Tetrabaena_socialis	54.90	0.00
TRINITY_KXZ4331	hypothet	Gonium_pectorale	54.90	0.00
TRINITY_PNW8273	hypothet	Chlamydomonas_reinh	54.90	0.00
TRINITY_GAQ7874	Putative	Klebsormidium_niten	54.90	0.00
TRINITY_KXZ5324	hypothet	Gonium_pectorale	54.90	0.00
TRINITY_XP_0016	guanylat	Chlamydomonas_reinh	54.90	0.00
TRINITY_GAX7274	hypothet	Chlamydomonas_eusti	54.90	0.00
TRINITY_XP_0239	ras-like	Quercus_suber	54.90	0.00
TRINITY_GAX7326	hypothet	Chlamydomonas_eusti	54.90	0.00
TRINITY_GAX7995	hypothet	Chlamydomonas_eusti	54.90	0.00
TRINITY_GAX8083	hypothet	Chlamydomonas_eusti	54.90	0.00
TRINITY_NP_0013	Cyclin A	Arabidopsis_thalian	54.90	0.00
TRINITY_XP_0201	anaphase	Aegilops_tauschii	54.80	0.00
TRINITY_XP_0156	PREDICTE	Oryza_brachyantha	54.80	0.00
TRINITY_P51824.2	RecName:	Solanum_tuberosum	54.80	0.00
TRINITY_XP_0243	cAMP-dep	Physcomitrella_pate	54.80	0.00
TRINITY_KMZ7611	Ras-rela	Zostera_marina	54.80	0.00
TRINITY_XP_0244	type IV	Physcomitrella_pate	54.80	0.00
TRINITY_XP_0243	xylulose	Physcomitrella_pate	54.80	0.00
TRINITY_XP_0025	predicte	Micromonas_commoda	54.80	0.00
TRINITY_XP_0030	predicte	Micromonas_pusilla	54.80	0.00
TRINITY_OUS4725	P-loop c	Ostreococcus_tauri	54.80	0.00
TRINITY_GAX7898	hypothet	Chlamydomonas_eusti	54.80	0.00
TRINITY_GAX7263	hypothet	Chlamydomonas_eusti	54.80	0.00
TRINITY_GAX7359	hypothet	Chlamydomonas_eusti	54.80	0.00
TRINITY_XP_0029	hypothet	Volvox_carteri	54.80	0.00
TRINITY_KXZ4545	hypothet	Gonium_pectorale	54.80	0.00
TRINITY_XP_0243	ATP-bind	Physcomitrella_pate	54.80	0.00
TRINITY_XP_0279	LOW QUAL	Vigna_unguiculata	54.80	0.00
TRINITY_PRW5759	Diphthar	Chlorella_sorokinia	54.80	0.00
TRINITY_GAX8059	hypothet	Chlamydomonas_eusti	54.80	0.00
TRINITY_XP_0186	PREDICTE	Musa_acuminata	54.80	0.00
TRINITY_GAQ8908	Electron	Klebsormidium_niten	54.80	0.00
TRINITY_XP_0029	hypothet	Volvox_carteri	54.80	0.00
TRINITY_PTQ4930	hypothet	Marchantia_polymorp	54.80	0.00
TRINITY_XP_0056	PTPA-dom	Coccomyxa_subellips	54.80	0.00
TRINITY_PNH0483	Cyclin-d	Tetrabaena_socialis	54.80	0.00
TRINITY_PNW8898	hypothet	Chlamydomonas_reinh	54.80	0.00
TRINITY_PSR9281	Cell div	Actinidia_chinensis	54.80	0.00
TRINITY_KXZ4449	hypothet	Gonium_pectorale	54.80	0.00
TRINITY_PNW7715	hypothet	Chlamydomonas_reinh	54.80	0.00
TRINITY_P0C8M8.1	RecName:	Zea_mays	54.80	0.00
TRINITY_GBG8658	hypothet	Chara_braunii	54.80	0.00
TRINITY_XP_0016	predicte	Chlamydomonas_reinh	54.80	0.00
TRINITY_GAX8569	hypothet	Chlamydomonas_eusti	54.80	0.00
TRINITY_XP_0029	hypothet	Volvox_carteri	54.80	0.00
TRINITY_ANE1053	carbonic	Dunaliella_salina	54.80	0.00
TRINITY_GAX7769	hypothet	Chlamydomonas_eusti	54.80	0.00
TRINITY_GAX8569	hypothet	Chlamydomonas_eusti	54.80	0.00
TRINITY_XP_0102	CBL-inte	Brachypodium_distac	54.80	0.00
TRINITY_XP_0029	hypothet	Volvox_carteri	54.80	0.00
TRINITY_GAX7354	hypothet	Chlamydomonas_eusti	54.80	0.00
TRINITY_GAX8194	hypothet	Chlamydomonas_eusti	54.80	0.00
TRINITY_GBF9990	hypothet	Raphidocelis_subcap	54.80	0.00
TRINITY_GAX7415	hypothet	Chlamydomonas_eusti	54.80	0.00
TRINITY_GAX7322	hypothet	Chlamydomonas_eusti	54.80	0.00
TRINITY_PNW7322	hypothet	Chlamydomonas_reinh	54.70	0.00
TRINITY_XP_0191	PREDICTE	Ipomoea_nil	54.70	0.00
TRINITY_ESR5330	hypothet	Citrus_clementina	54.70	0.00
TRINITY_GAQ7932	malonyl-	Klebsormidium_niten	54.70	0.00

TRINITY_PON6631(	Coatome:	Trema_orientale	54.70	0.00
TRINITY_XP_0058	hypothet	Chlorella_variabili	54.70	0.00
TRINITY_XP_0239	uncharac	Quercus_suber	54.70	0.00
TRINITY_XP_0238	SWI/SNF	Quercus_suber	54.70	0.00
TRINITY_EEF2473	pterin-4	Ricinus_communis	54.70	0.00
TRINITY_GBF9918	DNA mis	Raphidocelis_subcap	54.70	0.00
TRINITY_PWA3889	dual spe	Artemisia_annua	54.70	0.00
TRINITY_PNW7118	hypothet	Chlamydomonas_reinh	54.70	0.00
TRINITY_GAQ8167	OTU-like	Klebsormidium_niten	54.70	0.00
TRINITY_GAX7402	hypothet	Chlamydomonas_eusti	54.70	0.00
TRINITY_XP_0243	probable	Physcomitrella_pate	54.70	0.00
TRINITY_ABK2632	unknown	Picea_sitchensis	54.70	0.00
TRINITY_XP_0056	AAA-ATPa	Coccomyxa_subellips	54.70	0.00
TRINITY_XP_0016	predicte	Chlamydomonas_reinh	54.70	0.00
TRINITY_CCF1754	RabE GT	Micrasterias_dentic	54.70	0.00
TRINITY_EPS7450	hypothet	Genlisea_aurea	54.70	0.00
TRINITY_OWM6704	hypothet	Punica_granatum	54.70	0.00
TRINITY_GBF9326	hypothet	Raphidocelis_subcap	54.70	0.00
TRINITY_PRW4501	serine t	Chlorella_sorokinia	54.70	0.00
TRINITY_XP_0029	hypothet	Volvox_carteri	54.70	0.00
TRINITY_XP_0165	PREDICTE	Capsicum_annuum	54.70	0.00
TRINITY_PNW8100	hypothet	Chlamydomonas_reinh	54.70	0.00
TRINITY_PNW7724	hypothet	Chlamydomonas_reinh	54.70	0.00
TRINITY_XP_0138	hypothet	Monoraphidium_negle	54.70	0.00
TRINITY_XP_0145	ribosoma	Vigna_radiata	54.70	0.00
TRINITY_XP_0184	PREDICTE	Raphanus_sativus	54.70	0.00
TRINITY_PRW3306	glycolat	Chlorella_sorokinia	54.70	0.00
TRINITY_GAX8083	hypothet	Chlamydomonas_eusti	54.70	0.00
TRINITY_AXU9887	hypothet	Brassica_juncea	54.70	0.00
TRINITY_XP_0017	predicte	Chlamydomonas_reinh	54.70	0.00
TRINITY_GAX7763	hypothet	Chlamydomonas_eusti	54.70	0.00
TRINITY_GAX7823	hypothet	Chlamydomonas_eusti	54.70	0.00
TRINITY_KXZ5345	hypothet	Gonium_pectorale	54.70	0.00
TRINITY_GAX8270	hypothet	Chlamydomonas_eusti	54.70	0.00
TRINITY_PNW7020	hypothet	Chlamydomonas_reinh	54.70	0.00
TRINITY_PSC6797	nucleola	Micractinium_conduc	54.70	0.00
TRINITY_KXZ5207	hypothet	Gonium_pectorale	54.70	0.00
TRINITY_PNW8278	hypothet	Chlamydomonas_reinh	54.70	0.00
TRINITY_PNH0007	hypothet	Tetrabaena_socialis	54.70	0.00
TRINITY_XP_0102	phosphat	Brachypodium_distac	54.70	0.00
TRINITY_PSC6706	Retrovi	Micractinium_conduc	54.70	0.00
TRINITY_PNH1104	Thiol-d	Tetrabaena_socialis	54.70	0.00
TRINITY_GAQ8462	putative	Klebsormidium_niten	54.70	0.00
TRINITY_XP_0029	hypothet	Volvox_carteri	54.70	0.00
TRINITY_KZV1521	citrate	Dorcoceras_hygromet	54.70	0.00
TRINITY_GAX7558	hypothet	Chlamydomonas_eusti	54.70	0.00
TRINITY_PNW7553	hypothet	Chlamydomonas_reinh	54.70	0.00
TRINITY_GAQ8177	26S prot	Klebsormidium_niten	54.70	0.00
TRINITY_KXZ5598	hypothet	Gonium_pectorale	54.70	0.00
TRINITY_XP_0016	cytochr	Chlamydomonas_reinh	54.70	0.00
TRINITY_PNW8624	hypothet	Chlamydomonas_reinh	54.70	0.00
TRINITY_GAX8057	hypothet	Chlamydomonas_eusti	54.70	0.00
TRINITY_OAY6630	Oryzain	Ananas_comosus	54.70	0.00
TRINITY_GBG8367	hypothet	Chara_braunii	54.70	0.00
TRINITY_GAQ8270	Dyneins	Klebsormidium_niten	54.70	0.00
TRINITY_ACB2847	polyprot	Ananas_comosus	54.70	0.00
TRINITY_GAX8461	hypothet	Chlamydomonas_eusti	54.70	0.00
TRINITY_XP_0205	heteroge	Phalaenopsis_equest	54.70	0.00
TRINITY_KMZ6268	Phospho	Zostera_marina	54.70	0.00
TRINITY_RLM7909	transcr	Panicum_miliaceum	54.60	0.00
TRINITY_XP_0100	PREDICTE	Eucalyptus_grandis	54.60	0.00
TRINITY_XP_0205	polyubi	Phalaenopsis_equest	54.60	0.00
TRINITY_XP_0192	PREDICTE	Nicotiana_attenuata	54.60	0.00
TRINITY_XP_0083	PREDICTE	Malus_domestica	54.60	0.00
TRINITY_PNH1016	Serine a	Tetrabaena_socialis	54.60	0.00
TRINITY_XP_0017	expansi	Chlamydomonas_reinh	54.60	0.00

TRINITY_GAX8369(	hypothet	Chlamydomonas_eusti	54.60	0.00
TRINITY_GBG5935	hypothet	Chara_braunii	54.60	0.00
TRINITY_XP_0238	acyl-CoA	Quercus_suber	54.60	0.00
TRINITY_XP_0238	GTP cycl	Quercus_suber	54.60	0.00
TRINITY_XP_0016	homoseri	Chlamydomonas_reinh	54.60	0.00
TRINITY_KXZ5162	hypothet	Gonium_pectorale	54.60	0.00
TRINITY_GAX8536	hypothet	Chlamydomonas_eusti	54.60	0.00
TRINITY_KXZ5645	hypothet	Gonium_pectorale	54.60	0.00
TRINITY_PNW7354	hypothet	Chlamydomonas_reinh	54.60	0.00
TRINITY_PNW8071	hypothet	Chlamydomonas_reinh	54.60	0.00
TRINITY_XP_0016	cytochro	Chlamydomonas_reinh	54.60	0.00
TRINITY_GAX7355	hypothet	Chlamydomonas_eusti	54.60	0.00
TRINITY_PNW7220	hypothet	Chlamydomonas_reinh	54.60	0.00
TRINITY_PNH0962	Regulato	Tetrabaena_socialis	54.60	0.00
TRINITY_OIW1138	hypothet	Lupinus_angustifoli	54.60	0.00
TRINITY_XP_0041	PREDICTE	Cucumis_sativus	54.60	0.00
TRINITY_KXZ5386	hypothet	Gonium_pectorale	54.60	0.00
TRINITY_XP_0068	DEAD-box	Amborella_trichopod	54.60	0.00
TRINITY_GBF9091	hypothet	Raphidocelis_subcap	54.60	0.00
TRINITY_GAX7906	hypothet	Chlamydomonas_eusti	54.60	0.00
TRINITY_KXZ5424	hypothet	Gonium_pectorale	54.60	0.00
TRINITY_KXZ5686	hypothet	Gonium_pectorale	54.60	0.00
TRINITY_BAK0524	predicte	Hordeum_vulgare	54.60	0.00
TRINITY_XP_0138	hypothet	Monoraphidium_negle	54.60	0.00
TRINITY_PNW8318	hypothet	Chlamydomonas_reinh	54.60	0.00
TRINITY_OAE2517	hypothet	Marchantia_polymorp	54.60	0.00
TRINITY_XP_0042	PREDICTE	Fragaria_vesca	54.50	0.00
TRINITY_GAQ8323	hypothet	Klebsormidium_niten	54.50	0.00
TRINITY_PIN2279	Cyclin	Handroanthus_impeti	54.50	0.00
TRINITY_EPS7415	hypothet	Genlisea_aurea	54.50	0.00
TRINITY_OVA0416	Mitochor	Macleaya_cordata	54.50	0.00
TRINITY_GBG6150	hypothet	Chara_braunii	54.50	0.00
TRINITY_XP_0016	ATP-depe	Chlamydomonas_reinh	54.50	0.00
TRINITY_XP_0105	PREDICTE	Tarenaya_hasslerian	54.50	0.00
TRINITY_XP_0263	probable	Papaver_somniferum	54.50	0.00
TRINITY_RWW9113	hypothet	Ensete_ventricosum	54.50	0.00
TRINITY_GAX7396	hypothet	Chlamydomonas_eusti	54.50	0.00
TRINITY_GAX7784	hypothet	Chlamydomonas_eusti	54.50	0.00
TRINITY_GAX7750	hypothet	Chlamydomonas_eusti	54.50	0.00
TRINITY_XP_0016	predicte	Chlamydomonas_reinh	54.50	0.00
TRINITY_GBF9660	hypothet	Raphidocelis_subcap	54.50	0.00
TRINITY_GAX8431	hypothet	Chlamydomonas_eusti	54.50	0.00
TRINITY_KDO6676	hypothet	Citrus_sinensis	54.50	0.00
TRINITY_XP_0016	voltage-	Chlamydomonas_reinh	54.50	0.00
TRINITY_XP_0023	xylulose	Populus_trichocarpa	54.50	0.00
TRINITY_KXZ5025	hypothet	Gonium_pectorale	54.50	0.00
TRINITY_GBF9253	hypothet	Raphidocelis_subcap	54.50	0.00
TRINITY_XP_0191	PREDICTE	Ipomoea_nil	54.50	0.00
TRINITY_KMS6524	hypothet	Beta_vulgaris	54.50	0.00
TRINITY_GAX8091	hypothet	Chlamydomonas_eusti	54.50	0.00
TRINITY_KXZ4130	hypothet	Gonium_pectorale	54.50	0.00
TRINITY_XP_0029	hypothet	Volvox_carteri	54.50	0.00
TRINITY_XP_0238	40S ribo	Quercus_suber	54.50	0.00
TRINITY_XP_0245	ubiquiti	Selaginella_moellen	54.50	0.00
TRINITY_XP_0029	hypothet	Volvox_carteri	54.50	0.00
TRINITY_XP_0058	hypothet	Chlorella_variabili	54.50	0.00
TRINITY_XP_0029	hypothet	Volvox_carteri	54.50	0.00
TRINITY_XP_0058	hypothet	Chlorella_variabili	54.50	0.00
TRINITY_PNW7064	hypothet	Chlamydomonas_reinh	54.50	0.00
TRINITY_GAX7817	hypothet	Chlamydomonas_eusti	54.50	0.00
TRINITY_GAX7326	hypothet	Chlamydomonas_eusti	54.50	0.00
TRINITY_PNW8879	hypothet	Chlamydomonas_reinh	54.50	0.00
TRINITY_GAX7362	hypothet	Chlamydomonas_eusti	54.50	0.00
TRINITY_GAX7674	hypothet	Chlamydomonas_eusti	54.50	0.00
TRINITY_GAX8226	hypothet	Chlamydomonas_eusti	54.50	0.00
TRINITY_GAX7290	hypothet	Chlamydomonas_eusti	54.50	0.00

TRINITY_RLM5796	E3 ubiq	Panicum_miliaceum	54.50	0.00
TRINITY_XP_0056	nucleot	Coccomyxa_subellips	54.50	0.00
TRINITY_KXZ4750	hypothet	Gonium_pectorale	54.50	0.00
TRINITY_PNW7210	hypothet	Chlamydomonas_reinh	54.50	0.00
TRINITY_RWR7368	putativ	Cinnamomum_micranth	54.50	0.00
TRINITY_GAX8500	hypothet	Chlamydomonas_eusti	54.50	0.00
TRINITY_RQL8146	hypothet	Brassica_cretica	54.50	0.00
TRINITY_XP_0056	hypothet	Coccomyxa_subellips	54.50	0.00
TRINITY_CAI8489	putativ	Medicago_sativa	54.50	0.00
TRINITY_XP_0258	nifU-li	Panicum_hallii	54.50	0.00
TRINITY_PNW7395	hypothet	Chlamydomonas_reinh	54.50	0.00
TRINITY_GAX8213	hypothet	Chlamydomonas_eusti	54.50	0.00
TRINITY_GAX7667	hypothet	Chlamydomonas_eusti	54.50	0.00
TRINITY_PNH0476	Protein	Tetrabaena_socialis	54.50	0.00
TRINITY_XP_0029	hypothet	Volvox_carteri	54.50	0.00
TRINITY_GAX8021	hypothet	Chlamydomonas_eusti	54.50	0.00
TRINITY_PNW8443	hypothet	Chlamydomonas_reinh	54.50	0.00
TRINITY_GAX7988	hypothet	Chlamydomonas_eusti	54.50	0
TRINITY_AGB8503	hypothet	Auxenochlorella_prc	54.50	0.00
TRINITY_EFJ3493	hypothet	Selaginella_moellen	54.40	0.00
TRINITY_XP_0023	CBL-int	Populus_trichocarpa	54.40	0.00
TRINITY_VDC6048	unnamed	Brassica_rapa	54.40	0.00
TRINITY_GBF9163	alanine	Raphidocelis_subcap	54.40	0.00
TRINITY_GAQ7875	NADH-cy	Klebsormidium_niten	54.40	0.00
TRINITY_XP_0239	ATP-dep	Quercus_suber	54.40	0.00
TRINITY_GBG8312	hypothet	Chara_braunii	54.40	0.00
TRINITY_XP_0245	kinesin-	Selaginella_moellen	54.40	0.00
TRINITY_XP_0025	predict	Micromonas_commoda	54.40	0.00
TRINITY_KMS9450	hypothet	Beta_vulgaris	54.40	0.00
TRINITY_GAQ8747	P-type	Klebsormidium_niten	54.40	0.00
TRINITY_GAX8351	hypothet	Chlamydomonas_eusti	54.40	0.00
TRINITY_KYP5101	ABC tra	Cajanus_cajan	54.40	0.00
TRINITY_BAJ9317	predict	Hordeum_vulgare	54.40	0.00
TRINITY_GAX8361	hypothet	Chlamydomonas_eusti	54.40	0.00
TRINITY_XP_0029	probabl	Selaginella_moellen	54.40	0.00
TRINITY_KXZ5116	hypothet	Gonium_pectorale	54.40	0.00
TRINITY_XP_0227	probabl	Durio_zibethinus	54.40	0.00
TRINITY_XP_0239	spermid	Quercus_suber	54.40	0.00
TRINITY_PNW8467	hypothet	Chlamydomonas_reinh	54.40	0.00
TRINITY_GAX7686	hypothet	Chlamydomonas_eusti	54.40	0.00
TRINITY_GAX7258	hypothet	Chlamydomonas_eusti	54.40	0.00
TRINITY_XP_0029	hypothet	Volvox_carteri	54.40	0.00
TRINITY_GAX7882	hypothet	Chlamydomonas_eusti	54.40	0.00
TRINITY_XP_0139	hypothet	Monoraphidium_negle	54.40	0.00
TRINITY_XP_0029	hypothet	Volvox_carteri	54.40	0.00
TRINITY_KXZ4316	hypothet	Gonium_pectorale	54.40	0.00
TRINITY_PSC6889	RNA met	Micractinium_conduc	54.40	0.00
TRINITY_GAX7606	hypothet	Chlamydomonas_eusti	54.40	0.00
TRINITY_PSC7284	40S rib	Micractinium_conduc	54.40	0.00
TRINITY_KXZ4215	hypothet	Gonium_pectorale	54.40	0.00
TRINITY_XP_0016	predict	Chlamydomonas_reinh	54.40	0.00
TRINITY_XP_0016	predict	Chlamydomonas_reinh	54.40	0.00
TRINITY_PRW5725	rae1 [C	Chlorella_sorokinia	54.40	0.00
TRINITY_GAQ8502	RNAse L	Klebsormidium_niten	54.40	0.00
TRINITY_XP_0016	dynein	Chlamydomonas_reinh	54.40	0.00
TRINITY_XP_0029	heat sh	Volvox_carteri	54.40	0.00
TRINITY_KXZ4776	hypothet	Gonium_pectorale	54.40	0.00
TRINITY_GAX7502	hypothet	Chlamydomonas_eusti	54.40	0.00
TRINITY_KXZ5670	hypothet	Gonium_pectorale	54.40	0.00
TRINITY_XP_0228	Alterna	Ostreococcus_tauri	54.40	0.00
TRINITY_PNH0687	hypothet	Tetrabaena_socialis	54.40	0.00
TRINITY_XP_0016	predict	Chlamydomonas_reinh	54.40	0.00
TRINITY_GBF9736	hypothet	Raphidocelis_subcap	54.40	0.00
TRINITY_PNW7594	hypothet	Chlamydomonas_reinh	54.40	0.00
TRINITY_GAX8116	hypothet	Chlamydomonas_eusti	54.40	0.00
TRINITY_GAX8386	hypothet	Chlamydomonas_eusti	54.40	0.00

TRINITY_PNR55449	hypothet	Physcomitrella_pate	54.40	0.00
TRINITY_PSC76227	UPF0759	Micractinium_conduc	54.40	0.00
TRINITY_EPS74717	hypothet	Genlisea_aurea	54.40	0.00
TRINITY_XP_00584	hypothet	Chlorella_variabili	54.40	0.00
TRINITY_XP_00169	predicte	Chlamydomonas_reinh	54.40	0.00
TRINITY_XP_00295	hypothet	Volvox_carteri	54.40	0.00
TRINITY_GAX83125	hypothet	Chlamydomonas_eusti	54.40	0.00
TRINITY_GAX77819	hypothet	Chlamydomonas_eusti	54.40	0.00
TRINITY_KXZ51223	hypothet	Gonium_pectorale	54.40	0.00
TRINITY_PRQ29559	putative	Rosa_chinensis	54.40	0.00
TRINITY_GAX73796	hypothet	Chlamydomonas_eusti	54.40	0.00
TRINITY_XP_00294	3'5'-cyc	Volvox_carteri	54.40	0.00
TRINITY_GAX73826	hypothet	Chlamydomonas_eusti	54.40	0.00
TRINITY_XP_00141	predicte	Ostreococcus_sp._'l	54.40	0.00
TRINITY_XP_02711	proteaso	Coffea_arabica	54.30	0.00
TRINITY_XP_00295	hypothet	Volvox_carteri	54.30	0.00
TRINITY_XP_02097	ABC tran	Arachis_ipaensis	54.30	0.00
TRINITY_XP_02389	phosphog	Quercus_suber	54.30	0.00
TRINITY_PIN08707	hypothet	Handroanthus_impeti	54.30	0.00
TRINITY_XP_01284	PREDICTE	Erythranthe_guttata	54.30	0.00
TRINITY_XP_01041	PREDICTE	Camelina_sativa	54.30	0.00
TRINITY_XP_02392	ubiquiti	Quercus_suber	54.30	0.00
TRINITY_PRW18407	glucosam	Chlorella_sorokinia	54.30	0.00
TRINITY_XP_00665	PREDICTE	Oryza_brachyantha	54.30	0.00
TRINITY_CDY63600	BnaUnng(	Brassica_napus	54.30	0.00
TRINITY_KDD75175	SNF2 fam	Helicosporidium_sp.	54.30	0.00
TRINITY_AXU98877	hypothet	Brassica_juncea	54.30	0.00
TRINITY_ACF79371	unknown	Zea_mays	54.30	0.00
TRINITY_XP_01053	PREDICTE	Tarenaya_hasslerian	54.30	0.00
TRINITY_KZN04010	hypothet	Daucus_carota	54.30	0.00
TRINITY_GBF98720	cytochro	Raphidocelis_subcap	54.30	0.00
TRINITY_KRH17780	hypothet	Glycine_max	54.30	0.00
TRINITY_KXZ42000	hypothet	Gonium_pectorale	54.30	0.00
TRINITY_GAX81374	hypothet	Chlamydomonas_eusti	54.30	0.00
TRINITY_XP_00564	P25-alph	Coccomyxa_subellips	54.30	0.00
TRINITY_PNW73826	hypothet	Chlamydomonas_reinh	54.30	0.00
TRINITY_KXZ51336	hypothet	Gonium_pectorale	54.30	0.00
TRINITY_AAO73551	reverse-	Chlamydomonas_reinh	54.30	0.00
TRINITY_PRW61391	dnaJ-lik	Chlorella_sorokinia	54.30	0.00
TRINITY_PRW59412	RNA exon	Chlorella_sorokinia	54.30	0.00
TRINITY_PNW79709	hypothet	Chlamydomonas_reinh	54.30	0.00
TRINITY_ACF81765	unknown	Zea_mays	54.30	0.00
TRINITY_BAK02778	predicte	Hordeum_vulgare	54.30	0.00
TRINITY_XP_02053	serine/t	Amborella_trichopod	54.30	0.00
TRINITY_PNW70189	hypothet	Chlamydomonas_reinh	54.30	0.00
TRINITY_YP_00946	hypothet	Haematococcus_lacus	54.30	0.00
TRINITY_KXZ55440	ACK1 pro	Gonium_pectorale	54.30	0.00
TRINITY_XP_02274	ras-rela	Durio_zibethinus	54.30	0.00
TRINITY_GBF87693	hypothet	Raphidocelis_subcap	54.30	0.00
TRINITY_EOY26781	ATP binc	Theobroma_cacao	54.30	0.00
TRINITY_XP_02203	ABC tran	Helianthus_annuus	54.30	0.00
TRINITY_XP_00295	hypothet	Volvox_carteri	54.30	0.00
TRINITY_PNW84668	hypothet	Chlamydomonas_reinh	54.30	0.00
TRINITY_XP_00295	hypothet	Volvox_carteri	54.30	0.00
TRINITY_GAX73708	hypothet	Chlamydomonas_eusti	54.30	0.00
TRINITY_KXZ50280	hypothet	Gonium_pectorale	54.30	0.00
TRINITY_GAX77396	hypothet	Chlamydomonas_eusti	54.30	0.00
TRINITY_GAQ91802	ATP-depe	Klebsormidium_niten	54.30	0.00
TRINITY_XP_02388	ubiquiti	Quercus_suber	54.30	0.00
TRINITY_GAX75423	hypothet	Chlamydomonas_eusti	54.30	0.00
TRINITY_XP_00980	PREDICTE	Nicotiana_sylvestri	54.30	0.00
TRINITY_PNW76094	hypothet	Chlamydomonas_reinh	54.30	0.00
TRINITY_GAX85640	hypothet	Chlamydomonas_eusti	54.30	0.00
TRINITY_KXZ51612	hypothet	Gonium_pectorale	54.30	0.00
TRINITY_GAX81530	hypothet	Chlamydomonas_eusti	54.30	0.00
TRINITY_GAX77871	hypothet	Chlamydomonas_eusti	54.30	0.00

TRINITY_XP_0029	hypothet	Volvox_carteri	54.30	0.00
TRINITY_GAX8460	hypothet	Chlamydomonas_eusti	54.30	0.00
TRINITY_GAX7700	hypothet	Chlamydomonas_eusti	54.30	0.00
TRINITY_GBF9213	kinase	Raphidocelis_subcap	54.30	0.00
TRINITY_GAQ8538	clathrin	Klebsormidium_niten	54.30	0.00
TRINITY_GAQ9358	putative	Klebsormidium_niten	54.30	0.00
TRINITY_PKA5591	60S rib	Apostasia_shenzheni	54.30	0.00
TRINITY_GAX7581	hypothet	Chlamydomonas_eusti	54.20	0.00
TRINITY_XP_0206	peroxis	Dendrobium_catenatu	54.20	0.00
TRINITY_XP_0083	PREDICTI	Malus_domestica	54.20	0.00
TRINITY_XP_0025	predicte	Micromonas_commoda	54.20	0.00
TRINITY_PVH6638	hypothet	Panicum_hallii	54.20	0.00
TRINITY_OAE2596	hypothet	Marchantia_polymorp	54.20	0.00
TRINITY_XP_0029	hypothet	Volvox_carteri	54.20	0.00
TRINITY_NP_0013	ABC tran	Zea_mays	54.20	0.00
TRINITY_GAQ7899	Replicat	Klebsormidium_niten	54.20	0.00
TRINITY_KXZ4863	hypothet	Gonium_pectorale	54.20	0.00
TRINITY_OWM7638	hypothet	Punica_granatum	54.20	0.00
TRINITY_GAQ8727	ABC tran	Klebsormidium_niten	54.20	0.00
TRINITY_GAX7350	hypothet	Chlamydomonas_eusti	54.20	0.00
TRINITY_XP_0138	hypothet	Monoraphidium_negle	54.20	0.00
TRINITY_BAK0277	predicte	Hordeum_vulgare	54.20	0.00
TRINITY_XP_0016	predicte	Chlamydomonas_reinh	54.20	0.00
TRINITY_XP_0243	DNA dama	Physcomitrella_pate	54.20	0.00
TRINITY_XP_0239	probable	Quercus_suber	54.20	0.00
TRINITY_XP_0029	hypothet	Volvox_carteri	54.20	0.00
TRINITY_XP_0218	60S rib	Spinacia_oleracea	54.20	0.00
TRINITY_XP_0239	probable	Quercus_suber	54.20	0.00
TRINITY_GBF9517	hypothet	Raphidocelis_subcap	54.20	0.00
TRINITY_XP_0264	ABC tran	Papaver_somniferum	54.20	0.00
TRINITY_XP_0139	hypothet	Monoraphidium_negle	54.20	0.00
TRINITY_XP_0029	hypothet	Volvox_carteri	54.20	0.00
TRINITY_EFJ2128	hypothet	Selaginella_moellen	54.20	0.00
TRINITY_KXZ5208	hypothet	Gonium_pectorale	54.20	0.00
TRINITY_XP_0245	nascent	Selaginella_moellen	54.20	0.00
TRINITY_XP_0056	hypothet	Coccomyxa_subellips	54.20	0.00
TRINITY_XP_0029	hypothet	Volvox_carteri	54.20	0.00
TRINITY_GBF9316	hypothet	Raphidocelis_subcap	54.20	0.00
TRINITY_GAX7764	hypothet	Chlamydomonas_eusti	54.20	0.00
TRINITY_XP_0016	adenylyl	Chlamydomonas_reinh	54.20	0.00
TRINITY_XP_0017	ATM/ATR-	Chlamydomonas_reinh	54.20	0.00
TRINITY_KQJ9597	hypothet	Brachypodium_distac	54.20	0.00
TRINITY_GAX8007	hypothet	Chlamydomonas_eusti	54.20	0.00
TRINITY_KXZ5111	hypothet	Gonium_pectorale	54.20	0.00
TRINITY_XP_0245	tRNA pse	Selaginella_moellen	54.20	0.00
TRINITY_GAX7809	hypothet	Chlamydomonas_eusti	54.20	0.00
TRINITY_XP_0016	guanylat	Chlamydomonas_reinh	54.20	0.00
TRINITY_GAX7787	hypothet	Chlamydomonas_eusti	54.20	0.00
TRINITY_XP_0016	intrafla	Chlamydomonas_reinh	54.20	0.00
TRINITY_GAX7817	hypothet	Chlamydomonas_eusti	54.20	0.00
TRINITY_PSC7653	NAD-depe	Micractinium_conduc	54.20	0.00
TRINITY_XP_0277	uncharac	Solanum_pennellii	54.20	0.00
TRINITY_KXZ5552	hypothet	Gonium_pectorale	54.20	0.00
TRINITY_EEF2660	tRNA (5-	Ricinus_communis	54.10	0.00
TRINITY_EFJ3302	hypothet	Selaginella_moellen	54.10	0.00
TRINITY_EFH7016	hypothet	Arabidopsis_lyrata	54.10	0.00
TRINITY_GBG6475	hypothet	Chara_braunii	54.10	0.00
TRINITY_OEL1670	1,4-alph	Dichanthelium_oligo	54.10	0.00
TRINITY_XP_0035	isocitra	Brachypodium_distac	54.10	0.00
TRINITY_XP_0025	predicte	Micromonas_commoda	54.10	0.00
TRINITY_EFJ2160	hypothet	Selaginella_moellen	54.10	0.00
TRINITY_PRQ4151	putative	Rosa_chinensis	54.10	0.00
TRINITY_GBF9620	hypothet	Raphidocelis_subcap	54.10	0.00
TRINITY_XP_0094	PREDICTI	Musa_acuminata	54.10	0.00
TRINITY_XP_0238	short/b	Quercus_suber	54.10	0.00
TRINITY_PNW7003	hypothet	Chlamydomonas_reinh	54.10	0.00

TRINITY_XP_0249	ubiquit	Cynara_cardunculus	54.10	0.00
TRINITY_BAK0277	predict	Hordeum_vulgare	54.10	0.00
TRINITY_KXZ4207	hypothet	Gonium_pectorale	54.10	0.00
TRINITY_XP_0029	hypothet	Volvox_carteri	54.10	0.00
TRINITY_PNW7260	hypothet	Chlamydomonas_reinh	54.10	0.00
TRINITY_XP_0239	4-amino	Quercus_suber	54.10	0.00
TRINITY_XP_0017	sodium/	Chlamydomonas_reinh	54.10	0.00
TRINITY_KMZ6395	GTP-bin	Zostera_marina	54.10	0.00
TRINITY_XP_0056	hypothet	Coccomyxa_subellips	54.10	0.00
TRINITY_GAX7659	hypothet	Chlamydomonas_eusti	54.10	0.00
TRINITY_AKT9517	zeaxant	Haematococcus_lacus	54.10	0.00
TRINITY_GAX8558	hypothet	Chlamydomonas_eusti	54.10	0.00
TRINITY_PNW7547	hypothet	Chlamydomonas_reinh	54.10	0.00
TRINITY_PNH1217	hypothet	Tetrabaena_socialis	54.10	0.00
TRINITY_GBG7546	hypothet	Chara_braunii	54.10	0.00
TRINITY_XP_0029	hypothet	Volvox_carteri	54.10	0.00
TRINITY_KXZ5172	hypothet	Gonium_pectorale	54.10	0.00
TRINITY_GAX8263	hypothet	Chlamydomonas_eusti	54.10	0.00
TRINITY_XP_0029	hypothet	Volvox_carteri	54.10	0.00
TRINITY_XP_0029	flagell	Volvox_carteri	54.10	0.00
TRINITY_XP_0029	hypothet	Volvox_carteri	54.10	0.00
TRINITY_GBF9056	mitocho	Raphidocelis_subcap	54.10	0.00
TRINITY_KXZ4584	hypothet	Gonium_pectorale	54.10	0.00
TRINITY_GBF9760	26S prot	Raphidocelis_subcap	54.10	0.00
TRINITY_GAX7803	hypothet	Chlamydomonas_eusti	54.10	0.00
TRINITY_PNH0571	hypothet	Tetrabaena_socialis	54.10	0.00
TRINITY_P0C8M8.	RecName:	Zea_mays	54.10	0.00
TRINITY_XP_0016	predict	Chlamydomonas_reinh	54.10	0.00
TRINITY_PNH0456	Ethylen	Tetrabaena_socialis	54.10	0.00
TRINITY_GAX8439	hypothet	Chlamydomonas_eusti	54.10	0.00
TRINITY_GAX7648	hypothet	Chlamydomonas_eusti	54.10	0.00
TRINITY_XP_0238	kinesin	Quercus_suber	54.10	0.00
TRINITY_GAX8091	hypothet	Chlamydomonas_eusti	54.10	0.00
TRINITY_KJB3686	hypothet	Gossypium_raimondii	54.10	0.00
TRINITY_PNW7766	hypothet	Chlamydomonas_reinh	54.10	0.00
TRINITY_GAX7423	hypothet	Chlamydomonas_eusti	54.10	0.00
TRINITY_XP_0264	casein	Papaver_somniferum	54.10	0.00
TRINITY_GAX7381	hypothet	Chlamydomonas_eusti	54.10	0.00
TRINITY_XP_0110	2-oxogl	Sesamum_indicum	54.10	0
TRINITY_XP_0029	compone	Volvox_carteri	54.10	0.00
TRINITY_PNH0815	Protein	Tetrabaena_socialis	54.10	0.00
TRINITY_GAX7995	hypothet	Chlamydomonas_eusti	54.10	0.00
TRINITY_PNW8711	hypothet	Chlamydomonas_reinh	54.10	0.00
TRINITY_KXZ5381	hypothet	Gonium_pectorale	54.10	0.00
TRINITY_XP_0016	hypothet	Chlamydomonas_reinh	54.10	0.00
TRINITY_PRW5862	chlorop	Chlorella_sorokinia	54.10	0.00
TRINITY_ART6685	ATP bin	Phyllostachys_sp._K	54.00	0.00
TRINITY_OAY8266	Mitocho	Ananas_comosus	54.00	0.00
TRINITY_XP_0084	PREDICT	Cucumis_melo	54.00	0.00
TRINITY_EFJ3215	hypothet	Selaginella_moellen	54.00	0.00
TRINITY_XP_0063	tRNA (g	Eutrema_salsugineu	54.00	0.00
TRINITY_XP_0029	p300/CB	Volvox_carteri	54.00	0.00
TRINITY_GAV6363	UCH dom	Cephalotus_follicul	54.00	0.00
TRINITY_OAE2926	hypothet	Marchantia_polymorp	54.00	0.00
TRINITY_GBF9420	hypothet	Raphidocelis_subcap	54.00	0.00
TRINITY_XP_0025	radial	Micromonas_commoda	54.00	0.00
TRINITY_GAQ7976	Tryptop	Klebsormidium_niten	54.00	0.00
TRINITY_GAX8266	hypothet	Chlamydomonas_eusti	54.00	0.00
TRINITY_XP_0017	predict	Chlamydomonas_reinh	54.00	0.00
TRINITY_KXZ5258	hypothet	Gonium_pectorale	54.00	0.00
TRINITY_XP_0025	radial	Micromonas_commoda	54.00	0.00
TRINITY_GAX8650	hypothet	Chlamydomonas_eusti	54.00	0.00
TRINITY_PRW3370	hypothet	Chlorella_sorokinia	54.00	0.00
TRINITY_XP_0029	hypothet	Volvox_carteri	54.00	0.00
TRINITY_GAX7815	hypothet	Chlamydomonas_eusti	54.00	0.00
TRINITY_PVH3228	hypothet	Panicum_hallii	54.00	0.00

TRINITY_XP_00295	hypothet	Volvox_carteri	54.00	0.00
TRINITY_GAX78651	hypothet	Chlamydomonas_eusti	54.00	0.00
TRINITY_KXZ44943	hypothet	Gonium_pectorale	54.00	0.00
TRINITY_GBG61308	hypothet	Chara_braunii	54.00	0.00
TRINITY_GAX82673	hypothet	Chlamydomonas_eusti	54.00	0.00
TRINITY_KXZ44277	hypothet	Gonium_pectorale	54.00	0.00
TRINITY_PNW80720	hypothet	Chlamydomonas_reinh	54.00	0.00
TRINITY_XP_00295	hypothet	Volvox_carteri	54.00	0.00
TRINITY_GAX73544	hypothet	Chlamydomonas_eusti	54.00	0.00
TRINITY_GBF88979	hypothet	Raphidocelis_subcap	54.00	0.00
TRINITY_PNH07589	LYR moti	Tetrabaena_socialis	54.00	0.00
TRINITY_GAQ93561	hypothet	Klebsormidium_niten	54.00	0.00
TRINITY_XP_02390	aldo-ket	Quercus_suber	54.00	0.00
TRINITY_GAX85577	hypothet	Chlamydomonas_eusti	54.00	0.00
TRINITY_XP_02438	DNA topo	Physcomitrella_pate	54.00	0.00
TRINITY_KXZ56876	hypothet	Gonium_pectorale	54.00	0.00
TRINITY_KXZ52475	hypothet	Gonium_pectorale	54.00	0.00
TRINITY_GAX81164	hypothet	Chlamydomonas_eusti	54.00	0.00
TRINITY_GAX80212	hypothet	Chlamydomonas_eusti	54.00	0.00
TRINITY_PNW69613	hypothet	Chlamydomonas_reinh	54.00	0.00
TRINITY_GAX84389	hypothet	Chlamydomonas_eusti	54.00	0.00
TRINITY_XP_00294	hypothet	Volvox_carteri	54.00	0.00
TRINITY_XP_00168	intrafla	Chlamydomonas_reinh	54.00	0.00
TRINITY_KXZ50024	hypothet	Gonium_pectorale	54.00	0.00
TRINITY_XP_00295	molecula	Volvox_carteri	54.00	0.00
TRINITY_XP_00565	cobalami	Coccomyxa_subellips	54.00	0.00
TRINITY_KXZ53253	hypothet	Gonium_pectorale	54.00	0.00
TRINITY_XP_00295	hypothet	Volvox_carteri	54.00	0.00
TRINITY_PNW79521	hypothet	Chlamydomonas_reinh	54.00	0.00
TRINITY_XP_00295	hypothet	Volvox_carteri	54.00	0.00
TRINITY_GAX78363	hypothet	Chlamydomonas_eusti	54.00	0.00
TRINITY_GAX80495	hypothet	Chlamydomonas_eusti	54.00	0.00
TRINITY_XP_01918	PREDICTE	Ipomoea_nil	54.00	0.00
TRINITY_KXZ44832	hypothet	Gonium_pectorale	54.00	0.00
TRINITY_GAX77376	hypothet	Chlamydomonas_eusti	54.00	0.00
TRINITY_ABR17680	unknown	Picea_sitchensis	53.90	0.00
TRINITY_XP_02499	60S ribo	Cynara_cardunculus	53.90	0.00
TRINITY_AAG15839	NADPH-de	Phelipanche_ramosa	53.90	0.00
TRINITY_XP_01506	dynein I	Solanum_pennellii	53.90	0.00
TRINITY_PSC69898	sulfate	Micractinium_conduc	53.90	0.00
TRINITY_XP_00564	hypothet	Coccomyxa_subellips	53.90	0.00
TRINITY_XP_02026	ADP-ribo	Asparagus_officinal	53.90	0.00
TRINITY_ADA67931	putative	Wolffia_arrhiza	53.90	0.00
TRINITY_XP_00682	glutamin	Amborella_trichopod	53.90	0.00
TRINITY_XP_01942	PREDICTE	Lupinus_angustifoli	53.90	0.00
TRINITY_KXZ56224	hypothet	Gonium_pectorale	53.90	0.00
TRINITY_KXZ56385	hypothet	Gonium_pectorale	53.90	0.00
TRINITY_GAX72664	hypothet	Chlamydomonas_eusti	53.90	0.00
TRINITY_XP_00295	hypothet	Volvox_carteri	53.90	0.00
TRINITY_GBG72803	hypothet	Chara_braunii	53.90	0.00
TRINITY_XP_00959	PREDICTE	Nicotiana_tomentosi	53.90	0.00
TRINITY_OWM63713	hypothet	Punica_granatum	53.90	0.00
TRINITY_KXZ53629	hypothet	Gonium_pectorale	53.90	0.00
TRINITY_GAX82713	hypothet	Chlamydomonas_eusti	53.90	0.00
TRINITY_KXZ56257	hypothet	Gonium_pectorale	53.90	0.00
TRINITY_XP_01944	PREDICTE	Lupinus_angustifoli	53.90	0.00
TRINITY_KXZ45237	hypothet	Gonium_pectorale	53.90	0.00
TRINITY_XP_01162	protein	Amborella_trichopod	53.90	0.00
TRINITY_GAX81362	hypothet	Chlamydomonas_eusti	53.90	0.00
TRINITY_PNW74677	hypothet	Chlamydomonas_reinh	53.90	0.00
TRINITY_XP_00564	APG9-dom	Coccomyxa_subellips	53.90	0.00
TRINITY_XP_02389	peroxiso	Quercus_suber	53.90	0.00
TRINITY_GAX72672	hypothet	Chlamydomonas_eusti	53.90	0.00
TRINITY_GBF89736	pheophor	Raphidocelis_subcap	53.90	0.00
TRINITY_XP_00564	purine 5	Coccomyxa_subellips	53.90	0.00
TRINITY_GAX79630	hypothet	Chlamydomonas_eusti	53.90	0.00

TRINITY_GBF9752!	hypothet	Raphidocelis_subcap	53.90	0.00
TRINITY_GAX7953	hypothet	Chlamydomonas_eusti	53.90	0.00
TRINITY_KXZ5305(	hypothet	Gonium_pectorale	53.90	0.00
TRINITY_GAX8130(	hypothet	Chlamydomonas_eusti	53.90	0.00
TRINITY_XP_0244(	AP-1 cor	Physcomitrella_pate	53.80	0.00
TRINITY_BAA2591	actin [N	Nannochloris_bacill	53.80	0.00
TRINITY_EPS7341(	hypothet	Genlisea_aurea	53.80	0.00
TRINITY_XP_0082	PREDICTI	Prunus_mume	53.80	0.00
TRINITY_PNW7850	hypothet	Chlamydomonas_reinh	53.80	0.00
TRINITY_ESR5665	hypothet	Citrus_clementina	53.80	0.00
TRINITY_XP_0239(	serine/t	Quercus_suber	53.80	0.00
TRINITY_XP_0072(	vacuolar	Prunus_persica	53.80	0.00
TRINITY_XP_0101(	probable	Morus_notabilis	53.80	0.00
TRINITY_PNH1124!	Chaperor	Tetrabaena_socialis	53.80	0.00
TRINITY_XP_0241(	WD40 rep	Rosa_chinensis	53.80	0.00
TRINITY_ALD8431	acetyl-C	Morus_alba	53.80	0.00
TRINITY_PNW8343!	hypothet	Chlamydomonas_reinh	53.80	0.00
TRINITY_GAX8094(	hypothet	Chlamydomonas_eusti	53.80	0.00
TRINITY_GAX7319	hypothet	Chlamydomonas_eusti	53.80	0.00
TRINITY_EOY2772	Ribosoma	Theobroma_cacao	53.80	0.00
TRINITY_XP_0245	histone	Selaginella_moellen	53.80	0.00
TRINITY_XP_0029	hypothet	Volvox_carteri	53.80	0.00
TRINITY_PRW3329(	cytochro	Chlorella_sorokinia	53.80	0.00
TRINITY_KXZ5239	hypothet	Gonium_pectorale	53.80	0.00
TRINITY_GAX8075	hypothet	Chlamydomonas_eusti	53.80	0.00
TRINITY_GAX8003	hypothet	Chlamydomonas_eusti	53.80	0.00
TRINITY_KXZ4092	hypothet	Gonium_pectorale	53.80	0.00
TRINITY_PNW8883	hypothet	Chlamydomonas_reinh	53.80	0.00
TRINITY_XP_0238	uncharac	Quercus_suber	53.80	0.00
TRINITY_GAX8221	hypothet	Chlamydomonas_eusti	53.80	0.00
TRINITY_KXZ5191	hypothet	Gonium_pectorale	53.80	0.00
TRINITY_OUS4255	hypothet	Ostreococcus_tauri	53.80	0.00
TRINITY_XP_0194	PREDICTI	Lupinus_angustifoli	53.80	0.00
TRINITY_PNW8013	hypothet	Chlamydomonas_reinh	53.80	0.00
TRINITY_XP_0017(	predicte	Chlamydomonas_reinh	53.80	0.00
TRINITY_GAX8050	hypothet	Chlamydomonas_eusti	53.80	0.00
TRINITY_XP_0029	hypothet	Volvox_carteri	53.80	0.00
TRINITY_PTQ3503	hypothet	Marchantia_polymorp	53.80	0.00
TRINITY_RMZ5608(	hypothet	Auxenochlorella_pro	53.80	0.00
TRINITY_XP_0016	predicte	Chlamydomonas_reinh	53.80	0.00
TRINITY_PNW7075!	hypothet	Chlamydomonas_reinh	53.80	0.00
TRINITY_GAX8265	hypothet	Chlamydomonas_eusti	53.80	0.00
TRINITY_XP_0029	E3 ubiqu	Selaginella_moellen	53.80	0.00
TRINITY_GAQ7762	hypothet	Klebsormidium_niten	53.80	0.00
TRINITY_PNW7434	hypothet	Chlamydomonas_reinh	53.80	0.00
TRINITY_XP_0016	predicte	Chlamydomonas_reinh	53.80	0.00
TRINITY_GAQ8120	DNA repl	Klebsormidium_niten	53.80	0.00
TRINITY_GAX8217!	hypothet	Chlamydomonas_eusti	53.80	0.00
TRINITY_KXZ4567	hypothet	Gonium_pectorale	53.80	0.00
TRINITY_XP_0056!	DUF1313-	Coccomyxa_subellips	53.80	0.00
TRINITY_PNW8324	hypothet	Chlamydomonas_reinh	53.80	0.00
TRINITY_GAX8410	hypothet	Chlamydomonas_eusti	53.80	0.00
TRINITY_GAX7667(	hypothet	Chlamydomonas_eusti	53.80	0.00
TRINITY_GAX7591	hypothet	Chlamydomonas_eusti	53.80	0.00
TRINITY_PNW7227	hypothet	Chlamydomonas_reinh	53.80	0.00
TRINITY_XP_0239(	uncharac	Quercus_suber	53.80	0.00
TRINITY_PNW8048	hypothet	Chlamydomonas_reinh	53.80	0.00
TRINITY_XP_0058	hypothet	Chlorella_variabili	53.80	0.00
TRINITY_PNW7435(	hypothet	Chlamydomonas_reinh	53.80	0.00
TRINITY_GAX7399	hypothet	Chlamydomonas_eusti	53.80	0.00
TRINITY_PNH1158(	Serine/t	Tetrabaena_socialis	53.80	0.00
TRINITY_KXZ5129(	hypothet	Gonium_pectorale	53.80	0.00
TRINITY_XP_0041!	PREDICTI	Cucumis_sativus	53.80	0.00
TRINITY_GAX7970!	hypothet	Chlamydomonas_eusti	53.80	0.00
TRINITY_KXZ5313	hypothet	Gonium_pectorale	53.80	0.00
TRINITY_KDD7549	hypothet	Helicosporidium_sp.	53.80	0.00

TRINITY_XP_00169	major fa	Chlamydomonas_reinh	53.80	0.00
TRINITY_GAX78701	hypothet	Chlamydomonas_eusti	53.80	0.00
TRINITY_GBF99286	hypothet	Raphidocelis_subcap	53.80	0.00
TRINITY_PNW84061	hypothet	Chlamydomonas_reinh	53.80	0.00
TRINITY_XP_01670	PREDICTE	Gossypium_hirsutum	53.80	0.00
TRINITY_GBF88898	isochori	Raphidocelis_subcap	53.80	0.00
TRINITY_GAX79790	hypothet	Chlamydomonas_eusti	53.80	0.00
TRINITY_RYR06578	hypothet	Arachis_hypogaea	53.80	0.00
TRINITY_XP_00250	predicte	Micromonas_commoda	53.70	0.00
TRINITY_XP_02199	mitogen-	Helianthus_annuus	53.70	0.00
TRINITY_XP_00935	PREDICTE	Pyrus_x_bretschneid	53.70	0.00
TRINITY_XP_02185	mitochon	Spinacia_oleracea	53.70	0.00
TRINITY_EFJ19475	hypothet	Selaginella_moellen	53.70	0.00
TRINITY_XP_00584	hypothet	Chlorella_variabili	53.70	0.00
TRINITY_XP_02284	Fatty ac	Ostreococcus_tauri	53.70	0.00
TRINITY_XP_00305	predicte	Micromonas_pusilla	53.70	0.00
TRINITY_RLN04554	hypothet	Panicum_miliaceum	53.70	0.00
TRINITY_XP_00564	hypothet	Coccomyxa_subellips	53.70	0.00
TRINITY_PNW75024	hypothet	Chlamydomonas_reinh	53.70	0.00
TRINITY_XP_00169	trehalas	Chlamydomonas_reinh	53.70	0.00
TRINITY_XP_01724	PREDICTE	Daucus_carota	53.70	0.00
TRINITY_KXZ46597	hypothet	Gonium_pectorale	53.70	0.00
TRINITY_XP_02010	serine--	Ananas_comosus	53.70	0.00
TRINITY_GAQ78262	putative	Klebsormidium_niten	53.70	0.00
TRINITY_GAX78569	hypothet	Chlamydomonas_eusti	53.70	0.00
TRINITY_CAC95163	RNA poly	Physcomitrella_pate	53.70	0.00
TRINITY_RWR81965	proteaso	Cinnamomum_micranth	53.70	0.00
TRINITY_XP_00564	hypothet	Coccomyxa_subellips	53.70	0.00
TRINITY_GAX79746	hypothet	Chlamydomonas_eusti	53.70	0.00
TRINITY_XP_02373	ubiquiti	Lactuca_sativa	53.70	0.00
TRINITY_GAX83157	hypothet	Chlamydomonas_eusti	53.70	0.00
TRINITY_GAX82166	hypothet	Chlamydomonas_eusti	53.70	0.00
TRINITY_PSC75091	SAM-depe	Micractinium_conduc	53.70	0.00
TRINITY_XP_00169	flagella	Chlamydomonas_reinh	53.70	0.00
TRINITY_OVA00162	WD40 rep	Macleaya_cordata	53.70	0.00
TRINITY_ACL13981	NADPH de	Dunaliella_salina	53.70	0.00
TRINITY_XP_00169	predicte	Chlamydomonas_reinh	53.70	0.00
TRINITY_XP_01475	serine/t	Brachypodium_distac	53.70	0.00
TRINITY_KXZ43985	hypothet	Gonium_pectorale	53.70	0.00
TRINITY_GAX75021	hypothet	Chlamydomonas_eusti	53.70	0.00
TRINITY_XP_00295	hypothet	Volvox_carteri	53.70	0.00
TRINITY_GAX80391	hypothet	Chlamydomonas_eusti	53.70	0.00
TRINITY_XP_00250	dynein h	Micromonas_commoda	53.70	0.00
TRINITY_GBF90820	hypothet	Raphidocelis_subcap	53.70	0.00
TRINITY_PNW75147	hypothet	Chlamydomonas_reinh	53.70	0.00
TRINITY_BBC28473	serine/t	Yamagishiella_unicc	53.70	0.00
TRINITY_GBF89436	hypothet	Raphidocelis_subcap	53.70	0.00
TRINITY_KXZ54440	hypothet	Gonium_pectorale	53.70	0.00
TRINITY_PNH05133	3-ketoac	Tetrabaena_socialis	53.70	0.00
TRINITY_GAX77522	hypothet	Chlamydomonas_eusti	53.70	0.00
TRINITY_PNW80796	hypothet	Chlamydomonas_reinh	53.70	0.00
TRINITY_XP_01390	cytochro	Monoraphidium_negle	53.70	0.00
TRINITY_XP_00169	aconitat	Chlamydomonas_reinh	53.70	0.00
TRINITY_KDD73785	hypothet	Helicosporidium_sp.	53.70	0.00
TRINITY_XP_00664	PREDICTE	Oryza_brachyantha	53.60	0.00
TRINITY_XP_01002	PREDICTE	Eucalyptus_grandis	53.60	0.00
TRINITY_KXZ49012	DHC3 pro	Gonium_pectorale	53.60	0.00
TRINITY_OAE25820	hypothet	Marchantia_polymorp	53.60	0.00
TRINITY_XP_00414	PREDICTE	Cucumis_sativus	53.60	0.00
TRINITY_GAQ89333	transpor	Klebsormidium_niten	53.60	0.00
TRINITY_XP_00628	CTD smal	Capsella_rubella	53.60	0.00
TRINITY_GAQ79025	Regulato	Klebsormidium_niten	53.60	0.00
TRINITY_KMS65245	hypothet	Beta_vulgaris	53.60	0.00
TRINITY_XP_00288	cytochro	Arabidopsis_lyrata	53.60	0.00
TRINITY_GAQ88954	Putative	Klebsormidium_niten	53.60	0.00
TRINITY_KMS65245	hypothet	Beta_vulgaris	53.60	0.00

TRINITY_GBF92665	mitochor	Raphidocelis_subcap	53.60	0.00
TRINITY_AEW90637	KU80-lik	Pinus_sylvestris	53.60	0.00
TRINITY_XP_0243(	unchara(	Physcomitrella_pate	53.60	0.00
TRINITY_XP_0220Z	serine/t	Helianthus_annuus	53.60	0.00
TRINITY_XP_00169	flavin-c	Chlamydomonas_reinh	53.60	0.00
TRINITY_XP_00169	predicte	Chlamydomonas_reinh	53.60	0.00
TRINITY_XP_0139(	hypothet	Monoraphidium_negle	53.60	0.00
TRINITY_KZM84921	hypothet	Daucus_carota	53.60	0.00
TRINITY_KYP53097	Retrovir	Cajanus_cajan	53.60	0.00
TRINITY_GAX77995	hypothet	Chlamydomonas_eusti	53.60	0.00
TRINITY_BAJ86872	predicte	Hordeum_vulgare	53.60	0.00
TRINITY_GAX84207	hypothet	Chlamydomonas_eusti	53.60	0.00
TRINITY_GAX8574(	hypothet	Chlamydomonas_eusti	53.60	0.00
TRINITY_XP_00294	hypothet	Volvox_carteri	53.60	0.00
TRINITY_XP_00294	hypothet	Volvox_carteri	53.60	0.00
TRINITY_PNH07117	Chloroph	Tetrabaena_socialis	53.60	0.00
TRINITY_BAK00435	predicte	Hordeum_vulgare	53.60	0.00
TRINITY_GAX72769	hypothet	Chlamydomonas_eusti	53.60	0.00
TRINITY_XP_0017(	predicte	Chlamydomonas_reinh	53.60	0.00
TRINITY_XP_02098	DNA-dire	Arachis_duranensis	53.60	0.00
TRINITY_XP_0017(	response	Chlamydomonas_reinh	53.60	0.00
TRINITY_GAQ80193	serine/t	Klebsormidium_niten	53.60	0.00
TRINITY_XP_00294	hypothet	Volvox_carteri	53.60	0.00
TRINITY_PRW45077	HBS1 is(	Chlorella_sorokinia	53.60	0.00
TRINITY_XP_0017(	predicte	Chlamydomonas_reinh	53.60	0.00
TRINITY_PNW73618	hypothet	Chlamydomonas_reinh	53.60	0.00
TRINITY_PRW59514	Rab fami	Chlorella_sorokinia	53.60	0.00
TRINITY_GAX73633	hypothet	Chlamydomonas_eusti	53.60	0.00
TRINITY_PNW77802	hypothet	Chlamydomonas_reinh	53.60	0.00
TRINITY_GAX81364	hypothet	Chlamydomonas_eusti	53.60	0.00
TRINITY_XP_0030(	predicte	Micromonas_pusilla	53.60	0.00
TRINITY_PRW59003	thymidyl	Chlorella_sorokinia	53.60	0.00
TRINITY_XP_00295	hypothet	Volvox_carteri	53.60	0.00
TRINITY_KXZ54063	hypothet	Gonium_pectorale	53.60	0.00
TRINITY_GAX74557	hypothet	Chlamydomonas_eusti	53.60	0.00
TRINITY_XP_00424	ethanola	Solanum_lycopersicu	53.60	0.00
TRINITY_GAX7818(	hypothet	Chlamydomonas_eusti	53.60	0.00
TRINITY_GAX8447(	hypothet	Chlamydomonas_eusti	53.60	0.00
TRINITY_PNW87485	hypothet	Chlamydomonas_reinh	53.60	0.00
TRINITY_XP_01389	hypothet	Monoraphidium_negle	53.60	0.00
TRINITY_XP_0017(	F1F0 ATI	Chlamydomonas_reinh	53.60	0.00
TRINITY_PNH05468	hypothet	Tetrabaena_socialis	53.60	0.00
TRINITY_GAX75297	hypothet	Chlamydomonas_eusti	53.60	0.00
TRINITY_PNW70441	hypothet	Chlamydomonas_reinh	53.60	0
TRINITY_PRW05739	Gag-Pol	Chlorella_sorokinia	53.60	0.00
TRINITY_GAQ89367	putative	Klebsormidium_niten	53.60	0.00
TRINITY_GAX82607	hypothet	Chlamydomonas_eusti	53.60	0.00
TRINITY_PNW7602(	hypothet	Chlamydomonas_reinh	53.60	0.00
TRINITY_GAX74733	hypothet	Chlamydomonas_eusti	53.60	0.00
TRINITY_AXF41555	HLA3 pro	Chlorella_sp._ArM00	53.60	0.00
TRINITY_GAQ80911	putative	Klebsormidium_niten	53.60	0.00
TRINITY_GAQ84757	putative	Klebsormidium_niten	53.50	0.00
TRINITY_XP_0208(	unchara(	Arabidopsis_lyrata	53.50	0.00
TRINITY_GAQ8723(	Dyneins	Klebsormidium_niten	53.50	0.00
TRINITY_OAE2659(	hypothet	Marchantia_polymorp	53.50	0.00
TRINITY_GAX7423(	hypothet	Chlamydomonas_eusti	53.50	0.00
TRINITY_GAX84715	hypothet	Chlamydomonas_eusti	53.50	0.00
TRINITY_XP_00685	ubiquiti	Amborella_trichopod	53.50	0.00
TRINITY_XP_00169	predicte	Chlamydomonas_reinh	53.50	0.00
TRINITY_PNW83435	hypothet	Chlamydomonas_reinh	53.50	0.00
TRINITY_GBG7023(	hypothet	Chara_braunii	53.50	0.00
TRINITY_GAX75625	hypothet	Chlamydomonas_eusti	53.50	0.00
TRINITY_XP_0150(	E2F-ass(	Solanum_pennellii	53.50	0.00
TRINITY_KZV1498(	hypothet	Dorcoceras_hygromet	53.50	0.00
TRINITY_XP_00751	chaperor	Bathycoccus_prasinc	53.50	0.00
TRINITY_PNH01184	Nif-spe(	Tetrabaena_socialis	53.50	0.00

TRINITY_XP_0025(	predicte	Micromonas_commoda	53.50	0.00
TRINITY_GAX7413(	hypothet	Chlamydomonas_eusti	53.50	0.00
TRINITY_GBF9486(	hypothet	Raphidocelis_subcap	53.50	0.00
TRINITY_XP_0238(	non-spec	Quercus_suber	53.50	0.00
TRINITY_XP_0138(	hypothet	Monoraphidium_negle	53.50	0.00
TRINITY_GAX7993(	hypothet	Chlamydomonas_eusti	53.50	0.00
TRINITY_XP_0029(	hypothet	Volvox_carteri	53.50	0.00
TRINITY_GAX7856(	hypothet	Chlamydomonas_eusti	53.50	0.00
TRINITY_XP_0238(	uncharac	Quercus_suber	53.50	0.00
TRINITY_XP_0029(	hypothet	Volvox_carteri	53.50	0.00
TRINITY_PPS1631(	hypothet	Gossypium_barbadens	53.50	0.00
TRINITY_XP_0029(	hypothet	Volvox_carteri	53.50	0.00
TRINITY_GAX7526(	hypothet	Chlamydomonas_eusti	53.50	0.00
TRINITY_GAX7290(	hypothet	Chlamydomonas_eusti	53.50	0.00
TRINITY_PNH1207(	Protein	Tetrabaena_socialis	53.50	0.00
TRINITY_XP_0017(	predicte	Chlamydomonas_reinh	53.50	0.00
TRINITY_AEZ4987(	FK506 bi	Dunaliella_salina	53.50	0.00
TRINITY_GAX8585(	hypothet	Chlamydomonas_eusti	53.50	0.00
TRINITY_GAX8592(	hypothet	Chlamydomonas_eusti	53.50	0.00
TRINITY_GAX7567(	hypothet	Chlamydomonas_eusti	53.50	0.00
TRINITY_QBB2001(	E3 ubiqu	Ettlia_sp._YC001	53.50	0.00
TRINITY_XP_0029(	hypothet	Volvox_carteri	53.50	0.00
TRINITY_PSC7355(	DUF1295	Micractinium_conduc	53.50	0.00
TRINITY_XP_0017(	formate	Chlamydomonas_reinh	53.50	0.00
TRINITY_PNH0915(	Transcri	Tetrabaena_socialis	53.50	0.00
TRINITY_GAQ9356(	hypothet	Klebsormidium_niten	53.50	0.00
TRINITY_XP_0128(	PREDICTE	Erythranthe_guttata	53.50	0.00
TRINITY_OWM6547(	hypothet	Punica_granatum	53.50	0.00
TRINITY_GAX7649(	hypothet	Chlamydomonas_eusti	53.50	0.00
TRINITY_GAX8238(	hypothet	Chlamydomonas_eusti	53.50	0.00
TRINITY_GAX8049(	hypothet	Chlamydomonas_eusti	53.50	0.00
TRINITY_XP_0120(	tRNAse Z	Jatropha_curcas	53.40	0.00
TRINITY_GAQ9199(	mitochon	Klebsormidium_niten	53.40	0.00
TRINITY_XP_0025(	predicte	Micromonas_commoda	53.40	0.00
TRINITY_OIW1599(	hypothet	Lupinus_angustifoli	53.40	0.00
TRINITY_XP_0202(	probable	Asparagus_officinal	53.40	0.00
TRINITY_PLY8260(	hypothet	Lactuca_sativa	53.40	0.00
TRINITY_XP_0029(	hypothet	Volvox_carteri	53.40	0.00
TRINITY_XP_0102(	PREDICTE	Nelumbo_nucifera	53.40	0.00
TRINITY_XP_0106(	PREDICTE	Beta_vulgaris	53.40	0.00
TRINITY_PHT3869(	Long cha	Capsicum_baccatum	53.40	0.00
TRINITY_XP_0237(	serine/t	Lactuca_sativa	53.40	0.00
TRINITY_XP_0093(	PREDICTE	Musa_acuminata	53.40	0.00
TRINITY_XP_0240(	uncharac	Eutrema_salsugineum	53.40	0.00
TRINITY_KXZ4833(	hypothet	Gonium_pectorale	53.40	0.00
TRINITY_PSC7208(	P-type A	Micractinium_conduc	53.40	0.00
TRINITY_GAX8065(	hypothet	Chlamydomonas_eusti	53.40	0.00
TRINITY_GAX7834(	hypothet	Chlamydomonas_eusti	53.40	0.00
TRINITY_GAX7378(	hypothet	Chlamydomonas_eusti	53.40	0.00
TRINITY_XP_0029(	hypothet	Volvox_carteri	53.40	0.00
TRINITY_GAX7397(	hypothet	Chlamydomonas_eusti	53.40	0.00
TRINITY_GBF9816(	hypothet	Raphidocelis_subcap	53.40	0.00
TRINITY_XP_0029(	hypothet	Volvox_carteri	53.40	0.00
TRINITY_XP_0238(	triosepł	Quercus_suber	53.40	0.00
TRINITY_GAX7392(	hypothet	Chlamydomonas_eusti	53.40	0.00
TRINITY_GAX8418(	hypothet	Chlamydomonas_eusti	53.40	0.00
TRINITY_XP_0243(	galactol	Physcomitrella_pate	53.40	0.00
TRINITY_GAX8127(	hypothet	Chlamydomonas_eusti	53.40	0.00
TRINITY_APT6933(	ATP-citr	Dunaliella_tertiole	53.40	0.00
TRINITY_KXZ4676(	hypothet	Gonium_pectorale	53.40	0.00
TRINITY_PSC6780(	ketopant	Micractinium_conduc	53.40	0.00
TRINITY_XP_0029(	hypothet	Volvox_carteri	53.40	0.00
TRINITY_GBF9630(	hypothet	Raphidocelis_subcap	53.40	0.00
TRINITY_XP_0239(	RNA cyti	Quercus_suber	53.40	0.00
TRINITY_PNH1022(	GATA tra	Tetrabaena_socialis	53.40	0.00
TRINITY_EEC6696(	hypothet	Oryza_sativa	53.40	0.00

TRINITY_GAX7807	hypothet	Chlamydomonas_eusti	53.40	0.00
TRINITY_GAX8373	hypothet	Chlamydomonas_eusti	53.40	0.00
TRINITY_GAX7337	hypothet	Chlamydomonas_eusti	53.40	0.00
TRINITY_PNW7041	hypothet	Chlamydomonas_reinh	53.40	0.00
TRINITY_GAX7311	hypothet	Chlamydomonas_eusti	53.40	0.00
TRINITY_GAX7354	hypothet	Chlamydomonas_eusti	53.40	0.00
TRINITY_PRW4456	ribosoma	Chlorella_sorokinia	53.40	0.00
TRINITY_XP_0058	hypothet	Chlorella_variabili	53.40	0.00
TRINITY_XP_0016	predicte	Chlamydomonas_reinh	53.40	0.00
TRINITY_GAX8534	hypothet	Chlamydomonas_eusti	53.40	0.00
TRINITY_GAX8403	hypothet	Chlamydomonas_eusti	53.40	0.00
TRINITY_RYQ8995	hypothet	Arachis_hypogaea	53.40	0.00
TRINITY_GAX7624	hypothet	Chlamydomonas_eusti	53.40	0.00
TRINITY_KXZ4534	hypothet	Gonium_pectorale	53.40	0.00
TRINITY_GAX8125	hypothet	Chlamydomonas_eusti	53.40	0.00
TRINITY_XP_0029	hypothet	Volvox_carteri	53.40	0.00
TRINITY_XP_0238	cytochro	Quercus_suber	53.30	0.00
TRINITY_ABR1770	unknown	Picea_sitchensis	53.30	0.00
TRINITY_RVX0571	Polyubio	Vitis_vinifera	53.30	0.00
TRINITY_PKI6880	hypothet	Punica_granatum	53.30	0.00
TRINITY_XP_0138	DNA (cyt	Monoraphidium_negle	53.30	0.00
TRINITY_GAX7995	hypothet	Chlamydomonas_eusti	53.30	0.00
TRINITY_XP_0243	probable	Physcomitrella_pate	53.30	0.00
TRINITY_GAQ8447	NAD(P)-I	Klebsormidium_niten	53.30	0.00
TRINITY_GAQ8798	Phosphat	Klebsormidium_niten	53.30	0.00
TRINITY_KXZ4635	hypothet	Gonium_pectorale	53.30	0.00
TRINITY_XP_0216	uncharac	Hevea_brasiliensis	53.30	0.00
TRINITY_XP_0206	thymidyl	Dendrobium_catenatu	53.30	0.00
TRINITY_XP_0218	activati	Spinacia_oleracea	53.30	0.00
TRINITY_XP_0192	PREDICTE	Nicotiana_attenuata	53.30	0.00
TRINITY_PNH1077	ERI1 exo	Tetrabaena_socialis	53.30	0.00
TRINITY_XP_0229	coatomer	Cucurbita_moschata	53.30	0.00
TRINITY_BAK0277	predicte	Hordeum_vulgare	53.30	0.00
TRINITY_KXZ4567	hypothet	Gonium_pectorale	53.30	0.00
TRINITY_OEL1407	Heptahel	Dichanthelium_oligo	53.30	0.00
TRINITY_OMO5688	SNF2-rel	Corchorus_capsulari	53.30	0.00
TRINITY_XP_0194	PREDICTE	Lupinus_angustifoli	53.30	0.00
TRINITY_GAQ8155	Xanthine	Klebsormidium_niten	53.30	0.00
TRINITY_XP_0016	small ra	Chlamydomonas_reinh	53.30	0.00
TRINITY_GAX7297	hypothet	Chlamydomonas_eusti	53.30	0.00
TRINITY_XP_0091	PREDICTE	Brassica_rapa	53.30	0.00
TRINITY_XP_0016	methylt	Chlamydomonas_reinh	53.30	0.00
TRINITY_GAX8223	hypothet	Chlamydomonas_eusti	53.30	0.00
TRINITY_XP_0094	PREDICTE	Musa_acuminata	53.30	0.00
TRINITY_XP_0029	hypothet	Volvox_carteri	53.30	0.00
TRINITY_XP_0029	hypothet	Volvox_carteri	53.30	0.00
TRINITY_GAX8554	hypothet	Chlamydomonas_eusti	53.30	0.00
TRINITY_GAX7320	hypothet	Chlamydomonas_eusti	53.30	0.00
TRINITY_GAQ7868	Kinesin-	Klebsormidium_niten	53.30	0.00
TRINITY_KXZ5670	hypothet	Gonium_pectorale	53.30	0.00
TRINITY_KXZ4401	hypothet	Gonium_pectorale	53.30	0.00
TRINITY_GAX7532	hypothet	Chlamydomonas_eusti	53.30	0.00
TRINITY_KXZ4319	hypothet	Gonium_pectorale	53.30	0.00
TRINITY_AAK7087	pyruvate	Chlamydomonas_reinh	53.30	0.00
TRINITY_KXZ4936	hypothet	Gonium_pectorale	53.30	0.00
TRINITY_XP_0184	PREDICTE	Raphanus_sativus	53.30	0.00
TRINITY_XP_0029	hypothet	Volvox_carteri	53.30	0.00
TRINITY_XP_0243	superoxi	Physcomitrella_pate	53.30	0.00
TRINITY_XP_0029	hypothet	Volvox_carteri	53.30	0.00
TRINITY_XP_0058	hypothet	Chlorella_variabili	53.30	0.00
TRINITY_GAX8119	hypothet	Chlamydomonas_eusti	53.30	0.00
TRINITY_XP_0024	casein k	Sorghum_bicolor	53.30	0.00
TRINITY_PNW8499	hypothet	Chlamydomonas_reinh	53.30	0.00
TRINITY_GBG8176	hypothet	Chara_braunii	53.30	0.00
TRINITY_PNW8655	hypothet	Chlamydomonas_reinh	53.30	0.00
TRINITY_GAX7743	hypothet	Chlamydomonas_eusti	53.30	0.00

TRINITY_PNW83142	hypothet	Chlamydomonas_reinh	53.30	0.00
TRINITY_XP_00295	hypothet	Volvox_carteri	53.30	0.00
TRINITY_GAX83899	hypothet	Chlamydomonas_eusti	53.30	0.00
TRINITY_KXZ51622	hypothet	Gonium_pectorale	53.30	0.00
TRINITY_GAX80387	hypothet	Chlamydomonas_eusti	53.30	0
TRINITY_XP_00564	MT-A70	Coccomyxa_subellips	53.30	0.00
TRINITY_PTQ34569	hypothet	Marchantia_polymorp	53.20	0.00
TRINITY_XP_02733	dual spe	Abrus_precatorius	53.20	0.00
TRINITY_GAX77102	hypothet	Chlamydomonas_eusti	53.20	0.00
TRINITY_YP_00931	cytochro	Pyramimonas_parkeae	53.20	0.00

TRINITY_KDD7610	MT-A70	Helicosporidium_sp.	53.20	0.00
TRINITY_XP_0270	nuclear	Coffea_arabica	53.20	0.00
TRINITY_GBG6110	hypothet	Chara_braunii	53.20	0.00
TRINITY_XP_0030	predict	Micromonas_pusilla	53.20	0.00
TRINITY_XP_0201	protein	Aegilops_tauschii	53.20	0.00
TRINITY_XP_0030	dihydro	Micromonas_pusilla	53.20	0.00
TRINITY_XP_0239	ras-rel	Quercus_suber	53.20	0.00
TRINITY_GBG6826	hypothet	Chara_braunii	53.20	0.00
TRINITY_XP_0016	predict	Chlamydomonas_reinh	53.20	0.00
TRINITY_KXZ4973	hypothet	Gonium_pectorale	53.20	0.00
TRINITY_KXZ5612	hypothet	Gonium_pectorale	53.20	0.00
TRINITY_CAN6267	hypothet	Vitis_vinifera	53.20	0.00
TRINITY_GAQ8145	hypothet	Klebsormidium_niten	53.20	0.00
TRINITY_PNW8696	hypothet	Chlamydomonas_reinh	53.20	0.00
TRINITY_GAX7625	hypothet	Chlamydomonas_eusti	53.20	0.00
TRINITY_GAX8053	hypothet	Chlamydomonas_eusti	53.20	0.00
TRINITY_PNW7865	hypothet	Chlamydomonas_reinh	53.20	0.00
TRINITY_PNW7677	hypothet	Chlamydomonas_reinh	53.20	0.00
TRINITY_GAX7581	hypothet	Chlamydomonas_eusti	53.20	0.00
TRINITY_GAQ7991	ABC tra	Klebsormidium_niten	53.20	0.00
TRINITY_GAX7531	hypothet	Chlamydomonas_eusti	53.20	0.00
TRINITY_XP_0241	plasma	Rosa_chinensis	53.20	0.00
TRINITY_PNH1206	hypothet	Tetrabaena_socialis	53.20	0.00
TRINITY_GAX8072	hypothet	Chlamydomonas_eusti	53.20	0.00
TRINITY_GAX7852	hypothet	Chlamydomonas_eusti	53.20	0.00
TRINITY_KXZ5311	hypothet	Gonium_pectorale	53.20	0.00
TRINITY_GAQ8791	S-adeno	Klebsormidium_niten	53.20	0.00
TRINITY_GAX8154	hypothet	Chlamydomonas_eusti	53.20	0.00
TRINITY_GAX7834	hypothet	Chlamydomonas_eusti	53.20	0.00
TRINITY_GAX7605	hypothet	Chlamydomonas_eusti	53.20	0.00
TRINITY_XP_0091	PREDICT	Brassica_rapa	53.20	0.00
TRINITY_PSC6782	40S rib	Micractinium_conduc	53.20	0.00
TRINITY_GAV7858	RNA_pol_	Cephalotus_follicul	53.20	0.00
TRINITY_RWW9113	hypothet	Ensete_ventricosum	53.20	0.00
TRINITY_XP_0014	predict	Ostreococcus_sp._'l	53.20	0.00
TRINITY_GAX7947	hypothet	Chlamydomonas_eusti	53.20	0.00
TRINITY_GAX8316	hypothet	Chlamydomonas_eusti	53.20	0.00
TRINITY_KXZ5465	hypothet	Gonium_pectorale	53.20	0.00
TRINITY_GAX7274	hypothet	Chlamydomonas_eusti	53.20	0.00
TRINITY_PNH1006	Anaphas	Tetrabaena_socialis	53.20	0.00
TRINITY_XP_0238	probabl	Quercus_suber	53.20	0.00
TRINITY_KMZ6310	putativ	Zostera_marina	53.20	0.00
TRINITY_XP_0238	1-phosp	Quercus_suber	53.20	0.00
TRINITY_GAX8245	hypothet	Chlamydomonas_eusti	53.20	0.00
TRINITY_GAX8515	hypothet	Chlamydomonas_eusti	53.20	0.00
TRINITY_PRW4564	OTU dom	Chlorella_sorokinia	53.20	0.00
TRINITY_XP_0016	guanyla	Chlamydomonas_reinh	53.20	0.00
TRINITY_XP_0029	hypothet	Volvox_carteri	53.20	0.00
TRINITY_GAX7918	hypothet	Chlamydomonas_eusti	53.20	0.00
TRINITY_GAX7466	hypothet	Chlamydomonas_eusti	53.20	0.00
TRINITY_XP_0016	eyespot	Chlamydomonas_reinh	53.20	0.00
TRINITY_GAX8125	hypothet	Chlamydomonas_eusti	53.20	0.00
TRINITY_PNW7075	hypothet	Chlamydomonas_reinh	53.20	0.00
TRINITY_GAX8221	hypothet	Chlamydomonas_eusti	53.20	0.00
TRINITY_PRW5919	thiored	Chlorella_sorokinia	53.20	0.00
TRINITY_OVA0901	Ribosom	Macleaya_cordata	53.20	0.00
TRINITY_GAQ8270	Dyneins	Klebsormidium_niten	53.10	0.00
TRINITY_XP_0029	hypothet	Volvox_carteri	53.10	0.00
TRINITY_GBG6526	Glycine	Chara_braunii	53.10	0.00
TRINITY_XP_0156	60S rib	Oryza_sativa	53.10	0.00
TRINITY_XP_0025	predict	Micromonas_commoda	53.10	0.00
TRINITY_GAX8482	hypothet	Chlamydomonas_eusti	53.10	0.00
TRINITY_XP_0264	cyclin-	Papaver_somniferum	53.10	0.00
TRINITY_PNW8004	hypothet	Chlamydomonas_reinh	53.10	0.00
TRINITY_GAX8503	hypothet	Chlamydomonas_eusti	53.10	0.00
TRINITY_PNW8884	hypothet	Chlamydomonas_reinh	53.10	0.00

TRINITY_GAX7948	hypothet	Chlamydomonas_eusti	53.10	0.00
TRINITY_BAK0228	predicte	Hordeum_vulgare	53.10	0.00
TRINITY_PRW5088	intrace	Chlorella_sorokinia	53.10	0.00
TRINITY_GBG5927	hypothet	Chara_braunii	53.10	0.00
TRINITY_XP_0016	predicte	Chlamydomonas_reinh	53.10	0.00
TRINITY_KNA1004	hypothet	Spinacia_oleracea	53.10	0.00
TRINITY_PNW7631	hypothet	Chlamydomonas_reinh	53.10	0.00
TRINITY_XP_0056	acyl-Co	Coccomyxa_subellips	53.10	0.00
TRINITY_GAX7617	hypothet	Chlamydomonas_eusti	53.10	0.00
TRINITY_GAX7615	hypothet	Chlamydomonas_eusti	53.10	0.00
TRINITY_PSC6941	anaphas	Micractinium_conduc	53.10	0.00
TRINITY_XP_0016	WD40 re	Chlamydomonas_reinh	53.10	0.00
TRINITY_XP_0029	hypothet	Volvox_carteri	53.10	0.00
TRINITY_BAK0302	predicte	Hordeum_vulgare	53.10	0.00
TRINITY_XP_0030	flagell	Micromonas_pusilla	53.10	0.00
TRINITY_XP_0058	hypothet	Chlorella_variabili	53.10	0.00
TRINITY_XP_0138	hypothet	Monoraphidium_negle	53.10	0.00
TRINITY_XP_0016	predicte	Chlamydomonas_reinh	53.10	0.00
TRINITY_XP_0238	serine/t	Quercus_suber	53.10	0.00
TRINITY_XP_0016	phenyla	Chlamydomonas_reinh	53.10	0.00
TRINITY_XP_0029	hypothet	Volvox_carteri	53.10	0.00
TRINITY_PSC7395	26S prot	Micractinium_conduc	53.10	0.00
TRINITY_BAK0277	predicte	Hordeum_vulgare	53.10	0.00
TRINITY_AAO7355	reverse-	Chlamydomonas_reinh	53.10	0.00
TRINITY_PWA5899	phenyla	Artemisia_annua	53.10	0.00
TRINITY_GBF8823	metal-n	Raphidocelis_subcap	53.10	0.00
TRINITY_KXZ4786	hypothet	Gonium_pectorale	53.10	0.00
TRINITY_PNW7783	hypothet	Chlamydomonas_reinh	53.10	0.00
TRINITY_XP_0016	chromod	Chlamydomonas_reinh	53.10	0.00
TRINITY_GBF9080	hypothet	Raphidocelis_subcap	53.10	0.00
TRINITY_XP_0029	hypothet	Volvox_carteri	53.10	0.00
TRINITY_GAX7552	hypothet	Chlamydomonas_eusti	53.10	0.00
TRINITY_PNH0697	hypothet	Tetrabaena_socialis	53.10	0.00
TRINITY_GBF9986	hypothet	Raphidocelis_subcap	53.10	0.00
TRINITY_XP_0270	SNF1-re	Coffea_arabica	53.10	0.00
TRINITY_GAQ8464	hypothet	Klebsormidium_niten	53.10	0.00
TRINITY_KXZ5388	hypothet	Gonium_pectorale	53.10	0.00
TRINITY_GAX7623	hypothet	Chlamydomonas_eusti	53.10	0.00
TRINITY_PNW7006	hypothet	Chlamydomonas_reinh	53.10	0.00
TRINITY_XP_0016	actin-re	Chlamydomonas_reinh	53.10	0.00
TRINITY_PNW7133	hypothet	Chlamydomonas_reinh	53.10	0.00
TRINITY_KXZ4215	hypothet	Gonium_pectorale	53.10	0.00
TRINITY_KXZ5313	hypothet	Gonium_pectorale	53.10	0.00
TRINITY_BAJ9847	predicte	Hordeum_vulgare	53.10	0
TRINITY_GAX8296	hypothet	Chlamydomonas_eusti	53.10	0.00
TRINITY_GAX8261	hypothet	Chlamydomonas_eusti	53.10	0.00
TRINITY_KXZ5146	hypothet	Gonium_pectorale	53.10	0.00
TRINITY_KXZ4389	hypothet	Gonium_pectorale	53.10	0.00
TRINITY_PNW8139	hypothet	Chlamydomonas_reinh	53.10	0.00
TRINITY_GAX7720	hypothet	Chlamydomonas_eusti	53.10	0.00
TRINITY_GAX7741	hypothet	Chlamydomonas_eusti	53.10	0.00
TRINITY_GAQ8026	thiored	Klebsormidium_niten	53.10	0.00
TRINITY_RID5177	hypothet	Brassica_rapa	53.10	0.00
TRINITY_XP_0239	arginin	Quercus_suber	53.10	0.00
TRINITY_KMZ7358	Serine--	Zostera_marina	53.00	0.00
TRINITY_XP_0093	PREDICT	Pyrus_x_bretschneid	53.00	0.00
TRINITY_XP_0243	NAD-dep	Physcomitrella_pate	53.00	0.00
TRINITY_GAQ8211	hypothet	Klebsormidium_niten	53.00	0.00
TRINITY_XP_0113	Proteas	Auxenochlorella_prc	53.00	0.00
TRINITY_XP_0249	unchara	Cynara_cardunculus	53.00	0.00
TRINITY_XP_0245	39S rib	Selaginella_moellen	53.00	0.00
TRINITY_XP_0029	proteas	Selaginella_moellen	53.00	0.00
TRINITY_AAU9394	thiored	Helicosporidium_sp.	53.00	0.00
TRINITY_GAQ7818	20S prot	Klebsormidium_niten	53.00	0.00
TRINITY_XP_0058	hypothet	Chlorella_variabili	53.00	0.00
TRINITY_GAX8505	hypothet	Chlamydomonas_eusti	53.00	0.00

TRINITY_XP_00295	hypothet	Volvox_carteri	53.00	0.00
TRINITY_XP_02389	4-hydrox	Quercus_suber	53.00	0.00
TRINITY_XP_02440	methylc	Physcomitrella_pate	53.00	0.00
TRINITY_XP_01361	PREDICTE	Brassica_oleracea	53.00	0.00
TRINITY_KXZ41741	hypothet	Gonium_pectorale	53.00	0.00
TRINITY_XP_00565	pyruvate	Coccomyxa_subellips	53.00	0.00
TRINITY_GAX75635	hypothet	Chlamydomonas_eusti	53.00	0.00
TRINITY_GAX81005	hypothet	Chlamydomonas_eusti	53.00	0.00
TRINITY_GAX74273	hypothet	Chlamydomonas_eusti	53.00	0.00
TRINITY_GBF90454	hypothet	Raphidocelis_subcap	53.00	0.00
TRINITY_XP_00584	hypothet	Chlorella_variabili	53.00	0.00
TRINITY_KXZ50975	hypothet	Gonium_pectorale	53.00	0.00
TRINITY_GBF87867	beta-ada	Raphidocelis_subcap	53.00	0.00
TRINITY_PNW74676	hypothet	Chlamydomonas_reinh	53.00	0.00
TRINITY_XP_00564	RCC1/BLI	Coccomyxa_subellips	53.00	0.00
TRINITY_XP_00295	hypothet	Volvox_carteri	53.00	0.00
TRINITY_GAX85082	hypothet	Chlamydomonas_eusti	53.00	0.00
TRINITY_APW83744	caroteno	Dunaliella_salina	53.00	0.00
TRINITY_KXZ46522	hypothet	Gonium_pectorale	53.00	0.00
TRINITY_XP_02438	PAN2-PAN	Physcomitrella_pate	53.00	0.00
TRINITY_XP_00305	predicte	Micromonas_pusilla	53.00	0.00
TRINITY_GAX73140	hypothet	Chlamydomonas_eusti	53.00	0.00
TRINITY_PNW72916	hypothet	Chlamydomonas_reinh	53.00	0.00
TRINITY_PNW70485	hypothet	Chlamydomonas_reinh	53.00	0.00
TRINITY_XP_00169	predicte	Chlamydomonas_reinh	53.00	0.00
TRINITY_XP_00294	hypothet	Volvox_carteri	53.00	0.00
TRINITY_GBF87502	50S ribo	Raphidocelis_subcap	53.00	0.00
TRINITY_PNW80525	hypothet	Chlamydomonas_reinh	53.00	0.00
TRINITY_GAX79718	hypothet	Chlamydomonas_eusti	53.00	0.00
TRINITY_GBF99162	hypothet	Raphidocelis_subcap	53.00	0.00
TRINITY_GBF88408	hypothet	Raphidocelis_subcap	53.00	0.00
TRINITY_GAX78230	hypothet	Chlamydomonas_eusti	53.00	0.00
TRINITY_GAX74906	hypothet	Chlamydomonas_eusti	53.00	0.00
TRINITY_GAX78694	hypothet	Chlamydomonas_eusti	53.00	0.00
TRINITY_XP_00169	flagella	Chlamydomonas_reinh	53.00	0
TRINITY_XP_00305	dpy-30 I	Micromonas_pusilla	53.00	0.00
TRINITY_XP_02067	calmodul	Dendrobium_catenatu	53.00	0.00
TRINITY_XP_00565	hypothet	Coccomyxa_subellips	52.90	0.00
TRINITY_XP_01104	PREDICTE	Populus_euphratica	52.90	0.00
TRINITY_XP_00296	60S ribo	Selaginella_moellen	52.90	0.00
TRINITY_XP_02388	putative	Quercus_suber	52.90	0.00
TRINITY_GBG59821	hypothet	Chara_braunii	52.90	0.00
TRINITY_XP_01587	probable	Ziziphus_jujuba	52.90	0.00
TRINITY_XP_00168	peroxire	Chlamydomonas_reinh	52.90	0.00
TRINITY_KCW55564	hypothet	Eucalyptus_grandis	52.90	0.00
TRINITY_PNR31432	hypothet	Physcomitrella_pate	52.90	0.00
TRINITY_ESQ53580	hypothet	Eutrema_salsugineum	52.90	0.00
TRINITY_KZV56239	ATP synt	Dorcoceras_hygromet	52.90	0.00
TRINITY_OVA04022	ABC tran	Macleaya_cordata	52.90	0.00
TRINITY_XP_02735	casein k	Abrus_precatorius	52.90	0.00
TRINITY_GAQ87713	alpha-2-	Klebsormidium_niten	52.90	0.00
TRINITY_GAQ86106	Zinc fin	Klebsormidium_niten	52.90	0.00
TRINITY_CAI84893	putative	Medicago_sativa	52.90	0.00
TRINITY_KXZ55702	hypothet	Gonium_pectorale	52.90	0.00
TRINITY_XP_00639	eukaryot	Eutrema_salsugineum	52.90	0.00
TRINITY_XP_01918	PREDICTE	Ipomoea_nil	52.90	0.00
TRINITY_PRW20803	60S ribo	Chlorella_sorokinia	52.90	0.00
TRINITY_GAX74132	hypothet	Chlamydomonas_eusti	52.90	0.00
TRINITY_GBF89823	hypothet	Raphidocelis_subcap	52.90	0.00
TRINITY_XP_00168	LOW QUAL	Chlamydomonas_reinh	52.90	0.00
TRINITY_XP_00297	GPN-loop	Selaginella_moellen	52.90	0.00
TRINITY_GAX72657	hypothet	Chlamydomonas_eusti	52.90	0.00
TRINITY_XP_00565	hypothet	Coccomyxa_subellips	52.90	0.00
TRINITY_GAQ81454	hypothet	Klebsormidium_niten	52.90	0.00
TRINITY_XP_00250	bah-phd	Micromonas_commoda	52.90	0.00
TRINITY_XP_02454	glycine	Selaginella_moellen	52.90	0.00

TRINITY_GBF8818	phosphat	Raphidocelis_subcap	52.90	0.00
TRINITY_GAX8202	hypothet	Chlamydomonas_eusti	52.90	0.00
TRINITY_XP_0030	Inosine,	Ostreococcus_tauri	52.90	0.00
TRINITY_PNW7805	hypothet	Chlamydomonas_reinh	52.90	0.00
TRINITY_GAX7898	hypothet	Chlamydomonas_eusti	52.90	0.00
TRINITY_XP_0016	aminoacy	Chlamydomonas_reinh	52.90	0.00
TRINITY_EOY1656	Polynucl	Theobroma_cacao	52.90	0.00
TRINITY_XP_0063	PREDICTE	Solanum_tuberosum	52.90	0.00
TRINITY_GAX7674	hypothet	Chlamydomonas_eusti	52.90	0.00
TRINITY_PNH1226	Tiny mac	Tetrabaena_socialis	52.90	0.00
TRINITY_RMZ5476	hypothet	Auxenochlorella_pro	52.90	0.00
TRINITY_XP_0016	predicte	Chlamydomonas_reinh	52.90	0.00
TRINITY_GBF9404	hypothet	Raphidocelis_subcap	52.90	0.00
TRINITY_GAX8465	hypothet	Chlamydomonas_eusti	52.90	0.00
TRINITY_XP_0056	monodehy	Coccomyxa_subellips	52.90	0.00
TRINITY_XP_0239	ras-rela	Quercus_suber	52.90	0.00
TRINITY_GAX7675	hypothet	Chlamydomonas_eusti	52.90	0.00
TRINITY_XP_0029	hypothet	Volvox_carteri	52.90	0.00
TRINITY_XP_0243	protein	Physcomitrella_pate	52.90	0
TRINITY_XP_0029	hypothet	Volvox_carteri	52.90	0.00
TRINITY_GAX7767	hypothet	Chlamydomonas_eusti	52.90	0.00
TRINITY_PNH0540	hypothet	Tetrabaena_socialis	52.90	0.00
TRINITY_XP_0016	predicte	Chlamydomonas_reinh	52.90	0.00
TRINITY_PNW7727	hypothet	Chlamydomonas_reinh	52.90	0.00
TRINITY_XP_0016	cyclin c	Chlamydomonas_reinh	52.90	0.00
TRINITY_XP_0017	MaoC-lik	Chlamydomonas_reinh	52.90	0.00
TRINITY_GAQ9305	hypothet	Klebsormidium_niten	52.90	0.00
TRINITY_GBF9296	hypothet	Raphidocelis_subcap	52.90	0.00
TRINITY_PNW8723	hypothet	Chlamydomonas_reinh	52.90	0.00
TRINITY_GAX7683	hypothet	Chlamydomonas_eusti	52.90	0.00
TRINITY_GAX7416	hypothet	Chlamydomonas_eusti	52.90	0.00
TRINITY_XP_0029	4-hydrox	Volvox_carteri	52.90	0.00
TRINITY_GAX8438	hypothet	Chlamydomonas_eusti	52.90	0.00
TRINITY_ERN0063	hypothet	Amborella_trichopod	52.90	0.00
TRINITY_XP_0017	predicte	Chlamydomonas_reinh	52.90	0.00
TRINITY_GAX8408	hypothet	Chlamydomonas_eusti	52.90	0.00
TRINITY_GAX8163	hypothet	Chlamydomonas_eusti	52.90	0.00
TRINITY_GAX8028	hypothet	Chlamydomonas_eusti	52.90	0.00
TRINITY_GAX7798	hypothet	Chlamydomonas_eusti	52.90	0.00
TRINITY_KXZ4540	hypothet	Gonium_pectorale	52.90	0.00
TRINITY_GAV8585	Pkinase	Cephalotus_follicul	52.80	0.00
TRINITY_ACX4395	cysteine	Brachiaria_hybrid_c	52.80	0.00
TRINITY_GAQ8416	hypothet	Klebsormidium_niten	52.80	0.00
TRINITY_PQM3956	splicing	Prunus_yedoensis	52.80	0.00
TRINITY_RVX0872	Transcri	Vitis_vinifera	52.80	0.00
TRINITY_PAN1819	hypothet	Panicum_hallii	52.80	0.00
TRINITY_GAX8220	hypothet	Chlamydomonas_eusti	52.80	0.00
TRINITY_BAK0043	predicte	Hordeum_vulgare	52.80	0.00
TRINITY_OIW1138	hypothet	Lupinus_angustifoli	52.80	0.00
TRINITY_XP_0016	NADH:ubi	Chlamydomonas_reinh	52.80	0.00
TRINITY_BAK0277	predicte	Hordeum_vulgare	52.80	0.00
TRINITY_XP_0239	ras-rela	Quercus_suber	52.80	0.00
TRINITY_OAE3367	hypothet	Marchantia_polymorp	52.80	0.00
TRINITY_RMZ5592	hypothet	Auxenochlorella_pro	52.80	0.00
TRINITY_XP_0238	protein	Quercus_suber	52.80	0.00
TRINITY_PNW8411	hypothet	Chlamydomonas_reinh	52.80	0.00
TRINITY_XP_0238	guanine	Quercus_suber	52.80	0.00
TRINITY_XP_0029	hypothet	Volvox_carteri	52.80	0.00
TRINITY_PNW7326	hypothet	Chlamydomonas_reinh	52.80	0.00
TRINITY_GAX7742	hypothet	Chlamydomonas_eusti	52.80	0.00
TRINITY_GAX7393	hypothet	Chlamydomonas_eusti	52.80	0.00
TRINITY_XP_0017	predicte	Chlamydomonas_reinh	52.80	0.00
TRINITY_PKA5193	hypothet	Apostasia_shenzheni	52.80	0.00
TRINITY_GAX7797	hypothet	Chlamydomonas_eusti	52.80	0.00
TRINITY_PNW7865	hypothet	Chlamydomonas_reinh	52.80	0.00
TRINITY_XP_0042	alpha-ma	Solanum_lycopersicu	52.80	0.00

TRINITY_PNW75107	hypothet	Chlamydomonas_reinh	52.80	0.00
TRINITY_XP_00305	predicte	Micromonas_pusilla	52.80	0.00
TRINITY_GAX82152	hypothet	Chlamydomonas_eusti	52.80	0.00
TRINITY_XP_00170	mitochon	Chlamydomonas_reinh	52.80	0.00
TRINITY_PTQ30844	hypothet	Marchantia_polymorp	52.80	0.00
TRINITY_GAQ88060	methylcr	Klebsormidium_niten	52.80	0.00
TRINITY_GAX77631	hypothet	Chlamydomonas_eusti	52.80	0.00
TRINITY_EPS74505	hypothet	Genlisea_aurea	52.80	0.00
TRINITY_PNH06431	Transmem	Tetrabaena_socialis	52.80	0.00
TRINITY_XP_02389	probable	Quercus_suber	52.80	0.00
TRINITY_XP_02199	uncharac	Helianthus_annuus	52.80	0.00
TRINITY_XP_00585	hypothet	Chlorella_variabili	52.80	0.00
TRINITY_PNW78269	hypothet	Chlamydomonas_reinh	52.80	0.00
TRINITY_PNW81821	hypothet	Chlamydomonas_reinh	52.80	0.00
TRINITY_PRW60832	chitinas	Chlorella_sorokinia	52.80	0.00
TRINITY_XP_00295	hypothet	Volvox_carteri	52.80	0.00
TRINITY_XP_00295	eukaryot	Volvox_carteri	52.80	0.00
TRINITY_XP_01389	hypothet	Monoraphidium_negle	52.80	0.00
TRINITY_GBF92499	major fa	Raphidocelis_subcap	52.80	0.00
TRINITY_GAQ90142	Putative	Klebsormidium_niten	52.80	0.00
TRINITY_KXZ48051	hypothet	Gonium_pectorale	52.80	0.00
TRINITY_XP_02387	serine/t	Quercus_suber	52.80	0.00
TRINITY_GAQ79718	alpha-tu	Klebsormidium_niten	52.70	0.00
TRINITY_GBG69603	hypothet	Chara_braunii	52.70	0.00
TRINITY_XP_02716	ABC tran	Coffea_eugenioides	52.70	0.00
TRINITY_OAE24696	hypothet	Marchantia_polymorp	52.70	0.00
TRINITY_EFH56348	hypothet	Arabidopsis_lyrata	52.70	0.00
TRINITY_GAX77888	hypothet	Chlamydomonas_eusti	52.70	0.00
TRINITY_BAJ99035	predicte	Hordeum_vulgare	52.70	0.00
TRINITY_XP_02438	mediator	Physcomitrella_pate	52.70	0.00
TRINITY_XP_02189	60S acid	Carica_papaya	52.70	0.00
TRINITY_KZV21680	ABC tran	Dorcoceras_hygromet	52.70	0.00
TRINITY_XP_00295	hypothet	Volvox_carteri	52.70	0.00
TRINITY_XP_02390	myosin-1	Quercus_suber	52.70	0.00
TRINITY_XP_02438	OTU doma	Physcomitrella_pate	52.70	0.00
TRINITY_XP_02791	DNA poly	Vigna_unguiculata	52.70	0.00
TRINITY_KDD72661	hypothet	Helicosporidium_sp.	52.70	0.00
TRINITY_QBG38065	NADH deh	Chlorosarcinopsis_e	52.70	0.00
TRINITY_GAX80914	hypothet	Chlamydomonas_eusti	52.70	0.00
TRINITY_PNH05624	hypothet	Tetrabaena_socialis	52.70	0.00
TRINITY_XP_00295	hypothet	Volvox_carteri	52.70	0.00
TRINITY_ACN40777	unknown	Picea_sitchensis	52.70	0.00
TRINITY_BAK02778	predicte	Hordeum_vulgare	52.70	0.00
TRINITY_XP_00564	RBP11-li	Coccomyxa_subellips	52.70	0.00
TRINITY_XP_00295	hypothet	Volvox_carteri	52.70	0.00
TRINITY_PSC71663	Pre-mRNA	Micractinium_conduc	52.70	0.00
TRINITY_PRW34018	trans-2-	Chlorella_sorokinia	52.70	0.00
TRINITY_GAX75731	hypothet	Chlamydomonas_eusti	52.70	0.00
TRINITY_XP_02710	probable	Coffea_arabica	52.70	0.00
TRINITY_XP_01053	PREDICTE	Tarenaya_hasslerian	52.70	0.00
TRINITY_GAX85168	hypothet	Chlamydomonas_eusti	52.70	0.00
TRINITY_XP_00231	6-phosph	Populus_trichocarpa	52.70	0.00
TRINITY_XP_02388	ras-rela	Quercus_suber	52.70	0.00
TRINITY_GAX77983	hypothet	Chlamydomonas_eusti	52.70	0.00
TRINITY_GAX83343	hypothet	Chlamydomonas_eusti	52.70	0.00
TRINITY_XP_00169	beta tub	Chlamydomonas_reinh	52.70	0.00
TRINITY_KXZ55513	hypothet	Gonium_pectorale	52.70	0.00
TRINITY_PRW61141	mitogen-	Chlorella_sorokinia	52.70	0.00
TRINITY_RZC14159	Tubulin	Glycine_soja	52.70	0.00
TRINITY_XP_00170	diacylgl	Chlamydomonas_reinh	52.70	0.00
TRINITY_GAQ88245	hypothet	Klebsormidium_niten	52.70	0.00
TRINITY_KXZ42056	hypothet	Gonium_pectorale	52.70	0.00
TRINITY_GAX74129	hypothet	Chlamydomonas_eusti	52.70	0.00
TRINITY_GAX76031	hypothet	Chlamydomonas_eusti	52.70	0.00
TRINITY_KXZ44688	hypothet	Gonium_pectorale	52.70	0.00
TRINITY_GAX73182	hypothet	Chlamydomonas_eusti	52.70	0.00

TRINITY_GAQ7782	Nucleos	Klebsormidium_niten	52.70	0.00
TRINITY_XP_0064	notchles	Citrus_clementina	52.70	0.00
TRINITY_GAQ8826	Ca2+ tra	Klebsormidium_niten	52.70	0.00
TRINITY_GAX8027	hypothet	Chlamydomonas_eusti	52.70	0.00
TRINITY_KXZ4092	hypothet	Gonium_pectorale	52.70	0.00
TRINITY_XP_0058	hypothet	Chlorella_variabili	52.70	0.00
TRINITY_GAX8256	hypothet	Chlamydomonas_eusti	52.70	0.00
TRINITY_GBF9276	hypothet	Raphidocelis_subcap	52.70	0.00
TRINITY_GAX8270	hypothet	Chlamydomonas_eusti	52.70	0.00
TRINITY_KXZ4993	hypothet	Gonium_pectorale	52.70	0.00
TRINITY_GAX8557	hypothet	Chlamydomonas_eusti	52.70	0.00
TRINITY_GAX8091	hypothet	Chlamydomonas_eusti	52.70	0.00
TRINITY_XP_0017	predicte	Chlamydomonas_reinh	52.70	0.00
TRINITY_PNW7815	hypothet	Chlamydomonas_reinh	52.70	0.00
TRINITY_ERN0058	hypothet	Amborella_trichopod	52.60	0.00
TRINITY_XP_0138	DNA (cyt	Monoraphidium_negle	52.60	0.00
TRINITY_RDX6977	Actin-in	Mucuna_pruriens	52.60	0.00
TRINITY_XP_0239	inorgani	Quercus_suber	52.60	0.00
TRINITY_XP_0124	PREDICTE	Gossypium_raimondii	52.60	0.00
TRINITY_XP_0138	H/ACA ri	Monoraphidium_negle	52.60	0.00
TRINITY_XP_0156	probable	Oryza_sativa	52.60	0.00
TRINITY_KMS6524	hypothet	Beta_vulgaris	52.60	0.00
TRINITY_XP_0083	PREDICTE	Malus_domestica	52.60	0.00
TRINITY_XP_0058	hypothet	Chlorella_variabili	52.60	0.00
TRINITY_PNW7423	hypothet	Chlamydomonas_reinh	52.60	0.00
TRINITY_GAQ7846	cyclophi	Klebsormidium_niten	52.60	0.00
TRINITY_XP_0201	ribose-p	Aegilops_tauschii	52.60	0.00
TRINITY_RZC1381	ATP-depe	Glycine_soja	52.60	0.00
TRINITY_XP_0106	PREDICTE	Beta_vulgaris	52.60	0.00
TRINITY_GBG9231	hypothet	Chara_braunii	52.60	0.00
TRINITY_GAQ8895	Putative	Klebsormidium_niten	52.60	0.00
TRINITY_GAX7953	hypothet	Chlamydomonas_eusti	52.60	0.00
TRINITY_ABD9816	2-succin	Chlamydomonas_reinh	52.60	0.00
TRINITY_GAX7966	hypothet	Chlamydomonas_eusti	52.60	0.00
TRINITY_XP_0217	ubiquino	Chenopodium_quinoa	52.60	0.00
TRINITY_EPS7450	hypothet	Genlisea_aurea	52.60	0.00
TRINITY_KXZ4910	hypothet	Gonium_pectorale	52.60	0.00
TRINITY_GAX7411	hypothet	Chlamydomonas_eusti	52.60	0.00
TRINITY_GAX8224	hypothet	Chlamydomonas_eusti	52.60	0.00
TRINITY_XP_0093	PREDICTE	Musa_acuminata	52.60	0.00
TRINITY_XP_0016	thylakoi	Chlamydomonas_reinh	52.60	0.00
TRINITY_GAX7497	hypothet	Chlamydomonas_eusti	52.60	0.00
TRINITY_PNW7358	hypothet	Chlamydomonas_reinh	52.60	0.00
TRINITY_XP_0075	predicte	Bathycoccus_prasino	52.60	0.00
TRINITY_GAX8004	hypothet	Chlamydomonas_eusti	52.60	0.00
TRINITY_RYR2251	hypothet	Arachis_hypogaea	52.60	0.00
TRINITY_GBF9347	hypothet	Raphidocelis_subcap	52.60	0.00
TRINITY_XP_0094	PREDICTE	Musa_acuminata	52.60	0.00
TRINITY_PON4897	Splicing	Parasponia_anderson	52.60	0.00
TRINITY_XP_0184	PREDICTE	Raphanus_sativus	52.60	0.00
TRINITY_XP_0227	GTP-bind	Durio_zibethinus	52.60	0.00
TRINITY_XP_0139	hypothet	Monoraphidium_negle	52.60	0.00
TRINITY_GAX8535	hypothet	Chlamydomonas_eusti	52.60	0.00
TRINITY_XP_0058	hypothet	Chlorella_variabili	52.60	0.00
TRINITY_GBG8134	hypothet	Chara_braunii	52.60	0.00
TRINITY_GAX7615	hypothet	Chlamydomonas_eusti	52.60	0.00
TRINITY_GAX7702	hypothet	Chlamydomonas_eusti	52.60	0.00
TRINITY_PNW8124	hypothet	Chlamydomonas_reinh	52.60	0.00
TRINITY_XP_0058	hypothet	Chlorella_variabili	52.60	0.00
TRINITY_PSC7221	50S ribo	Micractinium_conduc	52.60	0.00
TRINITY_XP_0249	glutamin	Cynara_cardunculus	52.60	0.00
TRINITY_PSC7166	Phosphol	Micractinium_conduc	52.60	0.00
TRINITY_PNH0009	putative	Tetrabaena_socialis	52.60	0.00
TRINITY_GBF9755	hypothet	Raphidocelis_subcap	52.60	0.00
TRINITY_GAX8338	hypothet	Chlamydomonas_eusti	52.60	0.00
TRINITY_GAX7449	hypothet	Chlamydomonas_eusti	52.60	0.00

TRINITY_XP_0191	PREDICTE	Ipomoea_nil	52.60	0.00
TRINITY_XP_0058	hypothet	Chlorella_variabili	52.60	0.00
TRINITY_KXZ5190	hypothet	Gonium_pectorale	52.60	0.00
TRINITY_EFJ2667	hypothet	Selaginella_moellen	52.50	0.00
TRINITY_XP_0217	5'-3' e	Chenopodium_quinoa	52.50	0.00
TRINITY_XP_0201	ribonucl	Aegilops_tauschii	52.50	0.00
TRINITY_XP_0056	NAD(P)-k	Coccomyxa_subellips	52.50	0.00
TRINITY_ABK2291	unknown	Picea_sitchensis	52.50	0.00
TRINITY_KXZ4661	hypothet	Gonium_pectorale	52.50	0.00
TRINITY_XP_0238	histidir	Quercus_suber	52.50	0.00
TRINITY_PNR2715	hypothet	Physcomitrella_pate	52.50	0.00
TRINITY_VDD1262	unnamed	Brassica_rapa	52.50	0.00
TRINITY_BAJ9114	predicte	Hordeum_vulgare	52.50	0.00
TRINITY_XP_0239	mitochor	Quercus_suber	52.50	0.00
TRINITY_XP_0029	1,2-dihy	Selaginella_moellen	52.50	0.00
TRINITY_XP_0172	PREDICTE	Daucus_carota	52.50	0.00
TRINITY_GAX7369	hypothet	Chlamydomonas_eusti	52.50	0.00
TRINITY_PRW5899	carboxy-	Chlorella_sorokinia	52.50	0.00
TRINITY_PRW5609	phosphat	Chlorella_sorokinia	52.50	0.00
TRINITY_PNH0973	Traffick	Tetrabaena_socialis	52.50	0.00
TRINITY_XP_0241	microsor	Rosa_chinensis	52.50	0.00
TRINITY_XP_0030	predicte	Micromonas_pusilla	52.50	0.00
TRINITY_XP_0239	exosome	Quercus_suber	52.50	0.00
TRINITY_PNW7200	hypothet	Chlamydomonas_reinh	52.50	0.00
TRINITY_PNH1106	hypothet	Tetrabaena_socialis	52.50	0.00
TRINITY_PNH0228	Guanylat	Tetrabaena_socialis	52.50	0.00
TRINITY_OMO5487	hypothet	Corchorus_capsulari	52.50	0.00
TRINITY_PNH1070	hypothet	Tetrabaena_socialis	52.50	0.00
TRINITY_GAX8646	hypothet	Chlamydomonas_eusti	52.50	0.00
TRINITY_PWA9765	periodic	Artemisia_annua	52.50	0.00
TRINITY_XP_0058	hypothet	Chlorella_variabili	52.50	0.00
TRINITY_PRW5665	Exodeoxy	Chlorella_sorokinia	52.50	0.00
TRINITY_P0C8M8.	RecName:	Zea_mays	52.50	0.00
TRINITY_ABI7907	chloropl	Dunaliella_salina	52.50	0.00
TRINITY_PRW6063	isoform	Chlorella_sorokinia	52.50	0.00
TRINITY_PNH1008	hypothet	Tetrabaena_socialis	52.50	0.00
TRINITY_PNW6998	hypothet	Chlamydomonas_reinh	52.50	0.00
TRINITY_GAX7723	hypothet	Chlamydomonas_eusti	52.50	0.00
TRINITY_GBG8768	hypothet	Chara_braunii	52.50	0.00
TRINITY_XP_0228	Thioredo	Ostreococcus_tauri	52.50	0.00
TRINITY_GBF9978	hypothet	Raphidocelis_subcap	52.50	0.00
TRINITY_XP_0016	peptidyl	Chlamydomonas_reinh	52.50	0.00
TRINITY_GAQ7868	ubiquiti	Klebsormidium_niten	52.50	0.00
TRINITY_GAX7257	hypothet	Chlamydomonas_eusti	52.50	0.00
TRINITY_GBF8970	hypothet	Raphidocelis_subcap	52.50	0.00
TRINITY_GAX7459	hypothet	Chlamydomonas_eusti	52.50	0.00
TRINITY_XP_0243	unchara	Physcomitrella_pate	52.50	0.00
TRINITY_KXZ5357	hypothet	Gonium_pectorale	52.50	0.00
TRINITY_XP_0062	arginyl-	Capsella_rubella	52.50	0.00
TRINITY_BAK0058	predicte	Hordeum_vulgare	52.50	0.00
TRINITY_GAQ9358	putative	Klebsormidium_niten	52.50	0.00
TRINITY_XP_0029	hypothet	Volvox_carteri	52.50	0.00
TRINITY_GAX8569	hypothet	Chlamydomonas_eusti	52.50	0.00
TRINITY_AAV8379	putative	Chorispora_bungeana	52.50	0.00
TRINITY_XP_0022	PREDICTE	Vitis_vinifera	52.40	0.00
TRINITY_XP_0203	heat sh	Zea_mays	52.40	0.00
TRINITY_XP_0279	trifunct	Vigna_unguiculata	52.40	0.00
TRINITY_XP_0029	probable	Selaginella_moellen	52.40	0.00
TRINITY_GAQ8872	putative	Klebsormidium_niten	52.40	0.00
TRINITY_XP_0138	hypothet	Monoraphidium_negle	52.40	0.00
TRINITY_XP_0042	14 kDa	Solanum_lycopersicu	52.40	0.00
TRINITY_GAQ8098	Aldo/ket	Klebsormidium_niten	52.40	0.00
TRINITY_XP_0058	hypothet	Chlorella_variabili	52.40	0.00
TRINITY_XP_0058	hypothet	Chlorella_variabili	52.40	0.00
TRINITY_XP_0206	6-phosph	Dendrobium_catenatu	52.40	0.00
TRINITY_XP_0030	predicte	Micromonas_pusilla	52.40	0.00

TRINITY_YP_7172	cytochr	Ostreococcus_tauri	52.40	0.00
TRINITY_ONK6944	unchara	Asparagus_officinal	52.40	0.00
TRINITY_XP_0124	PREDICTI	Gossypium_raimondii	52.40	0.00
TRINITY_KXZ5280	hypothet	Gonium_pectorale	52.40	0.00
TRINITY_KZV1670	hypothet	Dorcoceras_hygromet	52.40	0.00
TRINITY_XP_0102	PREDICTI	Nelumbo_nucifera	52.40	0.00
TRINITY_XP_0029	hypothet	Volvox_carteri	52.40	0.00
TRINITY_PSC6924	aminotra	Micractinium_conduc	52.40	0.00
TRINITY_GAX8388	hypothet	Chlamydomonas_eusti	52.40	0.00
TRINITY_XP_0056	alcohol	Coccomyxa_subellips	52.40	0.00
TRINITY_XP_0102	PREDICTI	Nelumbo_nucifera	52.40	0.00
TRINITY_XP_0075	coatome	Bathycoccus_prasinc	52.40	0.00
TRINITY_PNW8723	hypothet	Chlamydomonas_reinh	52.40	0.00
TRINITY_GAX8321	hypothet	Chlamydomonas_eusti	52.40	0.00
TRINITY_EMS5930	Plasma r	Triticum_urartu	52.40	0.00
TRINITY_XP_0270	proteas	Coffea_arabica	52.40	0.00
TRINITY_BAK0524	predicte	Hordeum_vulgare	52.40	0.00
TRINITY_PRW5839	PAB-depe	Chlorella_sorokinia	52.40	0.00
TRINITY_XP_0239	DNA repa	Quercus_suber	52.40	0.00
TRINITY_XP_0101	putative	Morus_notabilis	52.40	0.00
TRINITY_PRW5942	acyl- N-	Chlorella_sorokinia	52.40	0.00
TRINITY_GAX8274	hypothet	Chlamydomonas_eusti	52.40	0.00
TRINITY_XP_0114	PREDICTI	Fragaria_vesca	52.40	0.00
TRINITY_GAX7753	hypothet	Chlamydomonas_eusti	52.40	0.00
TRINITY_XP_0017	iron-su	Chlamydomonas_reinh	52.40	0.00
TRINITY_PTQ4731	hypothet	Marchantia_polymorp	52.40	0.00
TRINITY_KXZ5444	hypothet	Gonium_pectorale	52.40	0.00
TRINITY_XP_0093	PREDICTI	Pyrus_x_bretschneid	52.40	0.00
TRINITY_GBF9045	choline-	Raphidocelis_subcap	52.40	0.00
TRINITY_PNW7738	hypothet	Chlamydomonas_reinh	52.40	0.00
TRINITY_KDD7527	hypothet	Helicosporidium_sp.	52.40	0.00
TRINITY_XP_0029	hypothet	Volvox_carteri	52.40	0.00
TRINITY_GAX7517	hypothet	Chlamydomonas_eusti	52.40	0.00
TRINITY_XP_0139	hypothet	Monoraphidium_negle	52.40	0.00
TRINITY_XP_0029	hypothet	Volvox_carteri	52.40	0.00
TRINITY_PNW8407	hypothet	Chlamydomonas_reinh	52.40	0.00
TRINITY_GAQ9356	hypothet	Klebsormidium_niten	52.40	0.00
TRINITY_KXZ4736	hypothet	Gonium_pectorale	52.40	0.00
TRINITY_XP_0016	mixed l	Chlamydomonas_reinh	52.40	0.00
TRINITY_XP_0025	protein	Micromonas_commoda	52.40	0.00
TRINITY_GAX8034	hypothet	Chlamydomonas_eusti	52.40	0.00
TRINITY_XP_0016	predicte	Chlamydomonas_reinh	52.40	0.00
TRINITY_XP_0029	hypothet	Volvox_carteri	52.40	0.00
TRINITY_GAX8154	hypothet	Chlamydomonas_eusti	52.40	0.00
TRINITY_PNW7878	hypothet	Chlamydomonas_reinh	52.40	0.00
TRINITY_XP_0245	sucrose	Selaginella_moellen	52.40	0.00
TRINITY_GAX7339	hypothet	Chlamydomonas_eusti	52.40	0.00
TRINITY_AAM6103	contains	Arabidopsis_thalian	52.30	0.00
TRINITY_XP_0030	Adenylyl	Ostreococcus_tauri	52.30	0.00
TRINITY_XP_0106	PREDICTI	Beta_vulgaris	52.30	0.00
TRINITY_AAB8649	calmodul	Zea_mays	52.30	0.00
TRINITY_EEF2664	conserve	Ricinus_communis	52.30	0.00
TRINITY_GAX7723	hypothet	Chlamydomonas_eusti	52.30	0.00
TRINITY_GBF9667	T-comple	Raphidocelis_subcap	52.30	0.00
TRINITY_GAX7885	hypothet	Chlamydomonas_eusti	52.30	0.00
TRINITY_XP_0030	predicte	Micromonas_pusilla	52.30	0.00
TRINITY_XP_0065	tip elor	Glycine_max	52.30	0.00
TRINITY_XP_0029	hypothet	Volvox_carteri	52.30	0.00
TRINITY_XP_0029	hypothet	Volvox_carteri	52.30	0.00
TRINITY_XP_0201	protein	Aegilops_tauschii	52.30	0.00
TRINITY_GBF9732	hypothet	Raphidocelis_subcap	52.30	0.00
TRINITY_XP_0194	PREDICTI	Lupinus_angustifoli	52.30	0.00
TRINITY_XP_0017	predicte	Chlamydomonas_reinh	52.30	0.00
TRINITY_PNW7856	hypothet	Chlamydomonas_reinh	52.30	0.00
TRINITY_XP_0029	hypothet	Volvox_carteri	52.30	0.00
TRINITY_GAQ8145	hypothet	Klebsormidium_niten	52.30	0.00

TRINITY_XP_0056	ATP-depe	Coccomyxa_subellips	52.30	0.00
TRINITY_PSC7546	very-lo	Micractinium_conduc	52.30	0.00
TRINITY_XP_0135	PREDICT	Brassica_oleracea	52.30	0.00
TRINITY_NP_5649	Protein	Arabidopsis_thalian	52.30	0.00
TRINITY_XP_0243	spliceo	Physcomitrella_pate	52.30	0.00
TRINITY_XP_0056	SPRY-dor	Coccomyxa_subellips	52.30	0.00
TRINITY_PNW7802	hypothet	Chlamydomonas_reinh	52.30	0.00
TRINITY_XP_0029	hypothet	Volvox_carteri	52.30	0.00
TRINITY_PNH1077	hypothet	Tetrabaena_socialis	52.30	0.00
TRINITY_GAX8470	hypothet	Chlamydomonas_eusti	52.30	0.00
TRINITY_XP_0016	predicte	Chlamydomonas_reinh	52.30	0.00
TRINITY_KXZ4409	hypothet	Gonium_pectorale	52.30	0.00
TRINITY_GAX7417	hypothet	Chlamydomonas_eusti	52.30	0.00
TRINITY_GBF8897	hypothet	Raphidocelis_subcap	52.30	0.00
TRINITY_GAX7489	hypothet	Chlamydomonas_eusti	52.30	0.00
TRINITY_XP_0029	hypothet	Volvox_carteri	52.30	0.00
TRINITY_GAX7415	hypothet	Chlamydomonas_eusti	52.30	0.00
TRINITY_XP_0139	hypothet	Monoraphidium_negle	52.30	0.00
TRINITY_XP_0023	iron-su	Populus_trichocarpa	52.30	0.00
TRINITY_PNW7654	hypothet	Chlamydomonas_reinh	52.30	0.00
TRINITY_KXZ4329	hypothet	Gonium_pectorale	52.30	0.00
TRINITY_GAX8417	hypothet	Chlamydomonas_eusti	52.30	0.00
TRINITY_GAX8592	hypothet	Chlamydomonas_eusti	52.30	0.00
TRINITY_XP_0029	hypothet	Volvox_carteri	52.30	0.00
TRINITY_GAX7260	hypothet	Chlamydomonas_eusti	52.30	0.00
TRINITY_BAK0145	predicte	Hordeum_vulgare	52.30	0.00
TRINITY_PNH1228	WW doma	Tetrabaena_socialis	52.30	0.00
TRINITY_XP_0243	uncharac	Physcomitrella_pate	52.30	0.00
TRINITY_GAX7829	hypothet	Chlamydomonas_eusti	52.30	0.00
TRINITY_PNW7716	hypothet	Chlamydomonas_reinh	52.30	0.00
TRINITY_XP_0029	hypothet	Volvox_carteri	52.30	0.00
TRINITY_PNW7375	hypothet	Chlamydomonas_reinh	52.30	0.00
TRINITY_PNH0305	hypothet	Tetrabaena_socialis	52.30	0.00
TRINITY_GAX7949	hypothet	Chlamydomonas_eusti	52.30	0.00
TRINITY_KXZ4486	hypothet	Gonium_pectorale	52.30	0
TRINITY_PRW4461	macropha	Chlorella_sorokinia	52.30	0.00
TRINITY_XP_0029	hypothet	Volvox_carteri	52.30	0.00
TRINITY_XP_0218	intron-k	Spinacia_oleracea	52.30	0.00
TRINITY_XP_0239	uncharac	Quercus_suber	52.30	0.00
TRINITY_GAX7907	hypothet	Chlamydomonas_eusti	52.30	0.00
TRINITY_KXZ4823	hypothet	Gonium_pectorale	52.30	0.00
TRINITY_PTQ3209	hypothet	Marchantia_polymorp	52.20	0.00
TRINITY_PTQ3141	hypothet	Marchantia_polymorp	52.20	0.00
TRINITY_RID5623	hypothet	Brassica_rapa	52.20	0.00
TRINITY_GAX7512	hypothet	Chlamydomonas_eusti	52.20	0.00
TRINITY_XP_0227	60S ribo	Durio_zibethinus	52.20	0.00
TRINITY_XP_0104	PREDICT	Camelina_sativa	52.20	0.00
TRINITY_XP_0014	predicte	Ostreococcus_sp._'l	52.20	0.00
TRINITY_XP_0056	DER1-dor	Coccomyxa_subellips	52.20	0.00
TRINITY_GAQ8892	hypothet	Klebsormidium_niten	52.20	0.00
TRINITY_AHI4990	membrane	Lavandula_angustifo	52.20	0.00
TRINITY_PNW8816	hypothet	Chlamydomonas_reinh	52.20	0.00
TRINITY_XP_0217	cytochro	Chenopodium_quinoa	52.20	0.00
TRINITY_XP_0218	phosphat	Carica_papaya	52.20	0.00
TRINITY_XP_0216	putative	Hevea_brasiliensis	52.20	0.00
TRINITY_XP_0029	hypothet	Volvox_carteri	52.20	0.00
TRINITY_PUZ4445	hypothet	Panicum_hallii	52.20	0.00
TRINITY_XP_0084	PREDICT	Cucumis_melo	52.20	0.00
TRINITY_PNH0132	hypothet	Tetrabaena_socialis	52.20	0.00
TRINITY_XP_0016	predicte	Chlamydomonas_reinh	52.20	0.00
TRINITY_XP_0014	predicte	Ostreococcus_sp._'l	52.20	0.00
TRINITY_PNW8676	hypothet	Chlamydomonas_reinh	52.20	0.00
TRINITY_XP_0016	flagella	Chlamydomonas_reinh	52.20	0.00
TRINITY_GAX8636	hypothet	Chlamydomonas_eusti	52.20	0.00
TRINITY_XP_0105	PREDICT	Tarenaya_hasslerian	52.20	0.00
TRINITY_GAX7504	hypothet	Chlamydomonas_eusti	52.20	0.00

TRINITY_XP_0202(	putative	Asparagus_officinal	52.20	0.00
TRINITY_PNW7865:	hypothet	Chlamydomonas_reinh	52.20	0.00
TRINITY_XP_0217	ras-rela	Chenopodium_quinoa	52.20	0.00
TRINITY_PNW8523:	hypothet	Chlamydomonas_reinh	52.20	0.00
TRINITY_XP_0016!	flagella	Chlamydomonas_reinh	52.20	0.00
TRINITY_PNW7151!	hypothet	Chlamydomonas_reinh	52.20	0.00
TRINITY_GBF9274!	carbohy	Raphidocelis_subcap	52.20	0.00
TRINITY_GAX7487!	hypothet	Chlamydomonas_eusti	52.20	0.00
TRINITY_XP_0029!	dynein I	Selaginella_moellen	52.20	0.00
TRINITY_GAX7510!	hypothet	Chlamydomonas_eusti	52.20	0.00
TRINITY_PRW0592!	methioni	Chlorella_sorokinia	52.20	0.00
TRINITY_PNW8805!	hypothet	Chlamydomonas_reinh	52.20	0.00
TRINITY_XP_0110!	DNA-dire	Sesamum_indicum	52.20	0
TRINITY_XP_0113!	Putative	Auxenochlorella_prc	52.20	0.00
TRINITY_XP_0219!	FH prote	Helianthus_annuus	52.20	0.00
TRINITY_PSC7424:	mitochor	Micractinium_conduc	52.20	0.00
TRINITY_GAX7538!	hypothet	Chlamydomonas_eusti	52.20	0.00
TRINITY_GAQ9305:	hypothet	Klebsormidium_niten	52.20	0.00
TRINITY_PNW7352:	hypothet	Chlamydomonas_reinh	52.20	0.00
TRINITY_GAX8000(	hypothet	Chlamydomonas_eusti	52.20	0.00
TRINITY_PSC7278!	hypothet	Micractinium_conduc	52.20	0.00
TRINITY_XP_0029!	hypothet	Volvox_carteri	52.20	0.00
TRINITY_GAX7291(	hypothet	Chlamydomonas_eusti	52.20	0.00
TRINITY_KXZ4193!	hypothet	Gonium_pectorale	52.20	0.00
TRINITY_XP_0105	PREDICTI	Tarenaya_hasslerian	52.20	0.00
TRINITY_ALM5498	DEAD bo	Chlamydomonas_sp._I	52.20	0.00
TRINITY_GAX7426!	hypothet	Chlamydomonas_eusti	52.20	0.00
TRINITY_AAO7355:	reverse-	Chlamydomonas_reinh	52.20	0.00
TRINITY_PNH0830:	NFX1-typ	Tetrabaena_socialis	52.20	0.00
TRINITY_BAK0354:	predicte	Hordeum_vulgare	52.20	0.00
TRINITY_XP_0239:	myosin t	Quercus_suber	52.10	0.00
TRINITY_XP_0120(	chaperor	Jatropha_curcas	52.10	0.00
TRINITY_AFK3634	unknown	Lotus_japonicus	52.10	0.00
TRINITY_XP_0238!	transcri	Quercus_suber	52.10	0.00
TRINITY_XP_0016!	predicte	Chlamydomonas_reinh	52.10	0.00
TRINITY_GAX7537!	hypothet	Chlamydomonas_eusti	52.10	0.00
TRINITY_OMO6619:	hypothet	Corchorus_capsulari	52.10	0.00
TRINITY_XP_0094:	PREDICTI	Musa_acuminata	52.10	0.00
TRINITY_GBF9683:	archease	Raphidocelis_subcap	52.10	0.00
TRINITY_XP_0100:	PREDICTI	Eucalyptus_grandis	52.10	0.00
TRINITY_XP_0025(	predicte	Micromonas_commoda	52.10	0.00
TRINITY_XP_0030(	predicte	Micromonas_pusilla	52.10	0.00
TRINITY_GAQ7991(	ABC trar	Klebsormidium_niten	52.10	0.00
TRINITY_XP_0202:	agmatine	Cajanus_cajan	52.10	0.00
TRINITY_XP_0101(	calcium-	Morus_notabilis	52.10	0.00
TRINITY_XP_0017(	NimA-rel	Chlamydomonas_reinh	52.10	0.00
TRINITY_GBF9937(	3-hydro	Raphidocelis_subcap	52.10	0.00
TRINITY_GAQ7974!	ATP-depe	Klebsormidium_niten	52.10	0.00
TRINITY_GAX8422	hypothet	Chlamydomonas_eusti	52.10	0.00
TRINITY_GAX7831!	hypothet	Chlamydomonas_eusti	52.10	0.00
TRINITY_XP_0239(	NADPH o	Quercus_suber	52.10	0.00
TRINITY_XP_0239(	delta-ar	Quercus_suber	52.10	0.00
TRINITY_GAX7413(	hypothet	Chlamydomonas_eusti	52.10	0.00
TRINITY_GBF9576!	hypothet	Raphidocelis_subcap	52.10	0.00
TRINITY_GAX7807!	hypothet	Chlamydomonas_eusti	52.10	0.00
TRINITY_XP_0243!	probable	Physcomitrella_pate	52.10	0.00
TRINITY_XP_0029	hypothet	Volvox_carteri	52.10	0.00
TRINITY_XP_0029	hypothet	Volvox_carteri	52.10	0.00
TRINITY_GAX8021:	hypothet	Chlamydomonas_eusti	52.10	0.00
TRINITY_XP_0245:	pre-mRN	Selaginella_moellen	52.10	0.00
TRINITY_XP_0058!	hypothet	Chlorella_variabili	52.10	0.00
TRINITY_XP_0139(	beta-am	Monoraphidium_negle	52.10	0.00
TRINITY_XP_0128	PREDICTI	Erythranthe_guttata	52.10	0.00
TRINITY_KXZ5323	hypothet	Gonium_pectorale	52.10	0.00
TRINITY_XP_0016!	predicte	Chlamydomonas_reinh	52.10	0.00
TRINITY_GAQ9078!	hypothet	Klebsormidium_niten	52.10	0.00

TRINITY_PNW7423	hypothet	Chlamydomonas_reinh	52.10	0.00
TRINITY_PNW7768	hypothet	Chlamydomonas_reinh	52.10	0.00
TRINITY_XP_0239	ras-rel	Quercus_suber	52.10	0.00
TRINITY_XP_0128	PREDICTE	Erythranthe_guttata	52.10	0.00
TRINITY_GAX8553	hypothet	Chlamydomonas_eusti	52.10	0.00
TRINITY_KXZ5237	hypothet	Gonium_pectorale	52.10	0.00
TRINITY_GAQ8903	hypothet	Klebsormidium_niten	52.10	0.00
TRINITY_XP_0091	PREDICTE	Brassica_rapa	52.10	0.00
TRINITY_GAX8459	hypothet	Chlamydomonas_eusti	52.10	0.00
TRINITY_XP_0104	PREDICTE	Camelina_sativa	52.10	0.00
TRINITY_EAZ0162	hypothet	Oryza_sativa	52.10	0.00
TRINITY_BAK0277	predicte	Hordeum_vulgare	52.10	0.00
TRINITY_GAX8063	hypothet	Chlamydomonas_eusti	52.10	0.00
TRINITY_GAX7533	hypothet	Chlamydomonas_eusti	52.10	0.00
TRINITY_XP_0016	superox	Chlamydomonas_reinh	52.10	0.00
TRINITY_PNW8233	hypothet	Chlamydomonas_reinh	52.10	0.00
TRINITY_GAX8098	hypothet	Chlamydomonas_eusti	52.10	0.00
TRINITY_GAX7525	hypothet	Chlamydomonas_eusti	52.10	0.00
TRINITY_PNW7261	hypothet	Chlamydomonas_reinh	52.10	0.00
TRINITY_PNW7530	hypothet	Chlamydomonas_reinh	52.10	0.00
TRINITY_XP_0017	expansin	Chlamydomonas_reinh	52.10	0.00
TRINITY_XP_0029	hypothet	Volvox_carteri	52.10	0.00
TRINITY_XP_0229	calcium-	Cucurbita_moschata	52.10	0.00
TRINITY_BAK0218	predicte	Hordeum_vulgare	52.10	0.00
TRINITY_XP_0029	hypothet	Volvox_carteri	52.10	0.00
TRINITY_XP_0056	hypothet	Coccomyxa_subellips	52.10	0.00
TRINITY_XP_0016	predicte	Chlamydomonas_reinh	52.10	0.00
TRINITY_PNW7735	hypothet	Chlamydomonas_reinh	52.10	0.00
TRINITY_GAX8166	hypothet	Chlamydomonas_eusti	52.10	0.00
TRINITY_PNH1000	hypothet	Tetrabaena_socialis	52.10	0.00
TRINITY_XP_0016	predicte	Chlamydomonas_reinh	52.10	0.00
TRINITY_XP_0029	60S rib	Selaginella_moellen	52.00	0.00
TRINITY_GBG7262	hypothet	Chara_braunii	52.00	0.00
TRINITY_RDY0919	DNA mis	Mucuna_pruriens	52.00	0.00
TRINITY_PNW7844	hypothet	Chlamydomonas_reinh	52.00	0.00
TRINITY_KMZ7087	DNA-dir	Zostera_marina	52.00	0.00
TRINITY_PKA5674	Putative	Apostasia_shenzheni	52.00	0.00
TRINITY_PTQ2770	hypothet	Marchantia_polymorp	52.00	0.00
TRINITY_XP_0194	PREDICTE	Lupinus_angustifoli	52.00	0.00
TRINITY_XP_0029	hypothet	Volvox_carteri	52.00	0.00
TRINITY_XP_0209	uncharac	Arachis_ipaensis	52.00	0.00
TRINITY_RWR8897	Cytopla	Cinnamomum_micranth	52.00	0.00
TRINITY_GAX7498	hypothet	Chlamydomonas_eusti	52.00	0.00
TRINITY_BAK0277	predicte	Hordeum_vulgare	52.00	0.00
TRINITY_XP_0238	ferroche	Quercus_suber	52.00	0.00
TRINITY_XP_0273	putative	Abrus_precatorius	52.00	0.00
TRINITY_BAK0277	predicte	Hordeum_vulgare	52.00	0.00
TRINITY_RXH6756	hypothet	Malus_domestica	52.00	0.00
TRINITY_GAQ8942	ankyrin	Klebsormidium_niten	52.00	0.00
TRINITY_KXZ4516	hypothet	Gonium_pectorale	52.00	0.00
TRINITY_KXZ4785	hypothet	Gonium_pectorale	52.00	0.00
TRINITY_XP_0217	E3 ubiq	Chenopodium_quinoa	52.00	0.00
TRINITY_GAQ8430	Histone	Klebsormidium_niten	52.00	0.00
TRINITY_GAX8558	hypothet	Chlamydomonas_eusti	52.00	0.00
TRINITY_GAX7285	hypothet	Chlamydomonas_eusti	52.00	0.00
TRINITY_ABK2108	unknown	Picea_sitchensis	52.00	0.00
TRINITY_PNW7506	hypothet	Chlamydomonas_reinh	52.00	0.00
TRINITY_XP_0100	DEAD-bo	Morus_notabilis	52.00	0.00
TRINITY_KEH1699	hypothet	Medicago_truncatula	52.00	0.00
TRINITY_PNW7332	hypothet	Chlamydomonas_reinh	52.00	0.00
TRINITY_PRW4447	Peptide-	Chlorella_sorokinia	52.00	0.00
TRINITY_ADE7606	unknown	Picea_sitchensis	52.00	0.00
TRINITY_XP_0016	predicte	Chlamydomonas_reinh	52.00	0.00
TRINITY_KXZ5446	hypothet	Gonium_pectorale	52.00	0.00
TRINITY_GAX8270	hypothet	Chlamydomonas_eusti	52.00	0.00
TRINITY_PNH1081	putative	Tetrabaena_socialis	52.00	0.00

TRINITY_GAX7916	hypothet	Chlamydomonas_eusti	52.00	0.00
TRINITY_XP_0056	RCC1/BL	Coccomyxa_subellips	52.00	0.00
TRINITY_GAX8325	hypothet	Chlamydomonas_eusti	52.00	0.00
TRINITY_GAX7622	hypothet	Chlamydomonas_eusti	52.00	0.00
TRINITY_XP_0029	hypothet	Volvox_carteri	52.00	0.00
TRINITY_GAX7427	hypothet	Chlamydomonas_eusti	52.00	0.00
TRINITY_GAQ8633	lysine-k	Klebsormidium_niten	52.00	0.00
TRINITY_GAX7763	hypothet	Chlamydomonas_eusti	52.00	0.00
TRINITY_XP_0138	hypothet	Monoraphidium_negle	52.00	0.00
TRINITY_GAX8346	hypothet	Chlamydomonas_eusti	52.00	0.00
TRINITY_XP_0016	predicte	Chlamydomonas_reinh	52.00	0.00
TRINITY_XP_0058	hypothet	Chlorella_variabili	51.90	0.00
TRINITY_PWA5381	mitochor	Artemisia_annua	51.90	0.00
TRINITY_GAQ7782	Nucleos	Klebsormidium_niten	51.90	0.00
TRINITY_XP_0063	proteas	Populus_trichocarpa	51.90	0.00
TRINITY_OAE3360	hypothet	Marchantia_polymorp	51.90	0.00
TRINITY_RXH8901	hypothet	Malus_domestica	51.90	0.00
TRINITY_PNW7916	hypothet	Chlamydomonas_reinh	51.90	0.00
TRINITY_PNH1134	Protein	Tetrabaena_socialis	51.90	0.00
TRINITY_GBF9127	2-phosph	Raphidocelis_subcap	51.90	0.00
TRINITY_KXZ4889	hypothet	Gonium_pectorale	51.90	0.00
TRINITY_NP_1949	glutath	Arabidopsis_thalian	51.90	0.00
TRINITY_GAX7826	hypothet	Chlamydomonas_eusti	51.90	0.00
TRINITY_XP_0264	importin	Papaver_somniferum	51.90	0.00
TRINITY_OAE2799	hypothet	Marchantia_polymorp	51.90	0.00
TRINITY_XP_0093	PREDICTE	Musa_acuminata	51.90	0.00
TRINITY_ONK7545	uncharac	Asparagus_officinal	51.90	0.00
TRINITY_OAE2135	hypothet	Marchantia_polymorp	51.90	0.00
TRINITY_GAX8314	hypothet	Chlamydomonas_eusti	51.90	0.00
TRINITY_XP_0238	protein	Quercus_suber	51.90	0.00
TRINITY_XP_0056	hypothet	Coccomyxa_subellips	51.90	0.00
TRINITY_GAX8021	hypothet	Chlamydomonas_eusti	51.90	0.00
TRINITY_GBF8997	FAD depe	Raphidocelis_subcap	51.90	0.00
TRINITY_XP_0238	4-hydrox	Quercus_suber	51.90	0.00
TRINITY_OAE2601	hypothet	Marchantia_polymorp	51.90	0.00
TRINITY_KXZ4263	hypothet	Gonium_pectorale	51.90	0.00
TRINITY_GBF9408	hypothet	Raphidocelis_subcap	51.90	0.00
TRINITY_GAX8128	hypothet	Chlamydomonas_eusti	51.90	0.00
TRINITY_GAX7998	hypothet	Chlamydomonas_eusti	51.90	0.00
TRINITY_XP_0194	PREDICTE	Lupinus_angustifoli	51.90	0.00
TRINITY_PNH1274	3-oxo-5-	Tetrabaena_socialis	51.90	0.00
TRINITY_XP_0238	acyl-CoA	Quercus_suber	51.90	0.00
TRINITY_OAE3509	hypothet	Marchantia_polymorp	51.90	0.00
TRINITY_GAX8535	hypothet	Chlamydomonas_eusti	51.90	0.00
TRINITY_GBF9499	hypothet	Raphidocelis_subcap	51.90	0.00
TRINITY_XP_0016	ammonium	Chlamydomonas_reinh	51.90	0.00
TRINITY_KXZ4449	hypothet	Gonium_pectorale	51.90	0.00
TRINITY_GBG5935	hypothet	Chara_braunii	51.90	0.00
TRINITY_PSC7056	Glycosyl	Micractinium_conduc	51.90	0.00
TRINITY_GAX8507	hypothet	Chlamydomonas_eusti	51.90	0.00
TRINITY_GAX8458	hypothet	Chlamydomonas_eusti	51.90	0.00
TRINITY_XP_0058	hypothet	Chlorella_variabili	51.90	0.00
TRINITY_KXZ4277	hypothet	Gonium_pectorale	51.90	0.00
TRINITY_GAX7726	hypothet	Chlamydomonas_eusti	51.90	0.00
TRINITY_PTQ3989	hypothet	Marchantia_polymorp	51.90	0.00
TRINITY_PNW7357	hypothet	Chlamydomonas_reinh	51.90	0.00
TRINITY_P0C8M8.	RecName:	Zea_mays	51.90	0.00
TRINITY_PNH0781	putative	Tetrabaena_socialis	51.90	0.00
TRINITY_XP_0058	hypothet	Chlorella_variabili	51.90	0.00
TRINITY_GBG8581	hypothet	Chara_braunii	51.90	0.00
TRINITY_KXZ4748	hypothet	Gonium_pectorale	51.90	0.00
TRINITY_BAJ8687	predicte	Hordeum_vulgare	51.90	0.00
TRINITY_VDD4660	unnamed	Brassica_oleracea	51.90	0.00
TRINITY_GAX8134	hypothet	Chlamydomonas_eusti	51.90	0.00
TRINITY_KXZ5598	hypothet	Gonium_pectorale	51.90	0.00
TRINITY_PNW8098	hypothet	Chlamydomonas_reinh	51.90	0.00

TRINITY_XP_0017(	low-CO2-	Chlamydomonas_reinh	51.90	0.00
TRINITY_GAX7836(	hypothet	Chlamydomonas_eusti	51.90	0.00
TRINITY_GAX8329:	hypothet	Chlamydomonas_eusti	51.90	0.00
TRINITY_XP_0029!	hypothet	Volvox_carteri	51.90	0.00
TRINITY_XP_0016!	predicte	Chlamydomonas_reinh	51.90	0.00
TRINITY_XP_0138!	hypothet	Monoraphidium_negle	51.90	0.00
TRINITY_PNW7730{	hypothet	Chlamydomonas_reinh	51.90	0.00
TRINITY_PNW8488:	hypothet	Chlamydomonas_reinh	51.90	0.00
TRINITY_KXZ4189{	hypothet	Gonium_pectorale	51.90	0.00
TRINITY_PNH1144:	Granule-	Tetrabaena_socialis	51.90	0.00
TRINITY_GAX7608(	hypothet	Chlamydomonas_eusti	51.90	0.00
TRINITY_XP_0030(	dynein l	Micromonas_pusilla	51.90	0.00
TRINITY_PKU8508	putative	Dendrobium_catenatu	51.90	0.00
TRINITY_XP_0217(	cytochro	Chenopodium_quinoa	51.90	0.00
TRINITY_XP_0158	uncharac	Ziziphus_jujuba	51.90	0.00
TRINITY_XP_0244(	uncharac	Physcomitrella_pate	51.80	0.00
TRINITY_GBG8668(	hypothet	Chara_braunii	51.80	0.00
TRINITY_GAQ8965	NADP-dep	Klebsormidium_niten	51.80	0.00
TRINITY_GAX8133	hypothet	Chlamydomonas_eusti	51.80	0.00
TRINITY_KMZ6206!	E3 ubiqu	Zostera_marina	51.80	0.00
TRINITY_KXZ5072!	hypothet	Gonium_pectorale	51.80	0.00
TRINITY_GAQ8805!	putative	Klebsormidium_niten	51.80	0.00
TRINITY_XP_0093!	PREDICTE	Pyrus_x_bretschneid	51.80	0.00
TRINITY_GBG7721:	hypothet	Chara_braunii	51.80	0.00
TRINITY_PSC7553!	SUPPRESS	Micractinium_conduc	51.80	0.00
TRINITY_GBG6143!	hypothet	Chara_braunii	51.80	0.00
TRINITY_GBF9066:	hypothet	Raphidocelis_subcap	51.80	0.00
TRINITY_KMS9327	hypothet	Beta_vulgaris	51.80	0.00
TRINITY_OAY7668	E3 ubiqu	Ananas_comosus	51.80	0.00
TRINITY_PNW8734:	hypothet	Chlamydomonas_reinh	51.80	0.00
TRINITY_XP_0029!	hypothet	Volvox_carteri	51.80	0.00
TRINITY_RAL3746:	hypothet	Cuscuta_australis	51.80	0.00
TRINITY_KXZ5374{	hypothet	Gonium_pectorale	51.80	0.00
TRINITY_GAX7726!	hypothet	Chlamydomonas_eusti	51.80	0.00
TRINITY_BAJ9830:	predicte	Hordeum_vulgare	51.80	0.00
TRINITY_GAX8569{	hypothet	Chlamydomonas_eusti	51.80	0.00
TRINITY_XP_0030!	predicte	Micromonas_pusilla	51.80	0.00
TRINITY_OAP1605!	DCD [Ara	Arabidopsis_thalian	51.80	0.00
TRINITY_GAX8584:	hypothet	Chlamydomonas_eusti	51.80	0.00
TRINITY_PNW8620:	hypothet	Chlamydomonas_reinh	51.80	0.00
TRINITY_KXZ4684:	hypothet	Gonium_pectorale	51.80	0.00
TRINITY_PKU8666!	RNA-dire	Dendrobium_catenatu	51.80	0.00
TRINITY_XP_0056	TPR-like	Coccomyxa_subellips	51.80	0.00
TRINITY_GAX7982!	hypothet	Chlamydomonas_eusti	51.80	0.00
TRINITY_PSC7645	nuclear	Micractinium_conduc	51.80	0.00
TRINITY_PNW8310:	hypothet	Chlamydomonas_reinh	51.80	0.00
TRINITY_GAX8383:	hypothet	Chlamydomonas_eusti	51.80	0.00
TRINITY_XP_0138!	Protein	Monoraphidium_negle	51.80	0.00
TRINITY_GAX7741	hypothet	Chlamydomonas_eusti	51.80	0.00
TRINITY_KXZ4949(	hypothet	Gonium_pectorale	51.80	0.00
TRINITY_GAX7558{	hypothet	Chlamydomonas_eusti	51.80	0.00
TRINITY_PNR4137{	hypothet	Physcomitrella_pate	51.80	0.00
TRINITY_GAX8226:	hypothet	Chlamydomonas_eusti	51.80	0.00
TRINITY_GAX8350:	hypothet	Chlamydomonas_eusti	51.80	0.00
TRINITY_PNW8322(	hypothet	Chlamydomonas_reinh	51.80	0.00
TRINITY_GAX8282!	hypothet	Chlamydomonas_eusti	51.80	0.00
TRINITY_GAX7995	hypothet	Chlamydomonas_eusti	51.80	0.00
TRINITY_XP_0016!	flagella	Chlamydomonas_reinh	51.80	0.00
TRINITY_GAX8469!	hypothet	Chlamydomonas_eusti	51.80	0.00
TRINITY_XP_0056	aminotra	Coccomyxa_subellips	51.80	0.00
TRINITY_XP_0068:	actin-re	Amborella_trichopod	51.80	0.00
TRINITY_XP_0238	uncharac	Quercus_suber	51.80	0.00
TRINITY_XP_0138!	Uncharac	Monoraphidium_negle	51.80	0.00
TRINITY_PNW8668:	hypothet	Chlamydomonas_reinh	51.80	0
TRINITY_ESR3845{	hypothet	Citrus_clementina	51.80	0.00
TRINITY_PNW8169:	hypothet	Chlamydomonas_reinh	51.80	0.00

TRINITY_PNH0939	Purple a	Tetrabaena_socialis	51.80	0.00
TRINITY_KXZ5151	hypothet	Gonium_pectorale	51.80	0.00
TRINITY_XP_0128	PREDICTE	Erythranthe_guttata	51.80	0.00
TRINITY_KXZ4132	hypothet	Gonium_pectorale	51.80	0.00
TRINITY_XP_0029	hypothet	Volvox_carteri	51.80	0.00
TRINITY_GAX8183	hypothet	Chlamydomonas_eusti	51.80	0.00
TRINITY_GAX8439	hypothet	Chlamydomonas_eusti	51.80	0.00
TRINITY_XP_0025	histone	Micromonas_commoda	51.80	0.00
TRINITY_GAX7923	hypothet	Chlamydomonas_eusti	51.80	0.00
TRINITY_GAX8338	hypothet	Chlamydomonas_eusti	51.80	0.00
TRINITY_GAQ8753	dynein	Klebsormidium_niten	51.80	0.00
TRINITY_KXZ5161	hypothet	Gonium_pectorale	51.80	0.00
TRINITY_XP_0025	predicte	Micromonas_commoda	51.80	0.00
TRINITY_GAX8306	hypothet	Chlamydomonas_eusti	51.80	0.00
TRINITY_XP_0243	RNA cyt	Physcomitrella_pate	51.80	0.00
TRINITY_GAX8046	hypothet	Chlamydomonas_eusti	51.80	0.00
TRINITY_GAX7415	hypothet	Chlamydomonas_eusti	51.80	0.00
TRINITY_RAL4803	hypothet	Cuscuta_australis	51.80	0.00
TRINITY_PNH0563	ADP,ATP	Tetrabaena_socialis	51.80	0.00
TRINITY_PNH0956	C-facto	Tetrabaena_socialis	51.80	0.00
TRINITY_XP_0165	PREDICTE	Capsicum_annuum	51.70	0.00
TRINITY_OWM7870	hypothet	Punica_granatum	51.70	0.00
TRINITY_XP_0030	Exosome	Ostreococcus_tauri	51.70	0.00
TRINITY_XP_0230	dual spe	Cucurbita_maxima	51.70	0.00
TRINITY_ABK2339	unknown	Picea_sitchensis	51.70	0.00
TRINITY_XP_0238	UDP-N-ac	Quercus_suber	51.70	0.00
TRINITY_AES8227	peptide	Medicago_truncatula	51.70	0.00
TRINITY_XP_0102	PREDICTE	Nelumbo_nucifera	51.70	0.00
TRINITY_XP_0110	serine	Sesamum_indicum	51.70	0.00
TRINITY_PNH0421	hypothet	Tetrabaena_socialis	51.70	0.00
TRINITY_PNW8534	hypothet	Chlamydomonas_reinh	51.70	0.00
TRINITY_XP_0138	Dual spe	Monoraphidium_negle	51.70	0.00
TRINITY_XP_0238	branche	Quercus_suber	51.70	0.00
TRINITY_XP_0014	predicte	Ostreococcus_sp._'l	51.70	0.00
TRINITY_XP_0030	flagella	Micromonas_pusilla	51.70	0.00
TRINITY_XP_0029	dynein h	Volvox_carteri	51.70	0.00
TRINITY_GBF9666	ABC tran	Raphidocelis_subcap	51.70	0.00
TRINITY_XP_0158	dnaJ hom	Ziziphus_jujuba	51.70	0.00
TRINITY_XP_0191	PREDICTE	Ipomoea_nil	51.70	0.00
TRINITY_PNW8100	hypothet	Chlamydomonas_reinh	51.70	0.00
TRINITY_XP_0056	nucleic	Coccomyxa_subellips	51.70	0.00
TRINITY_GAX8316	hypothet	Chlamydomonas_eusti	51.70	0.00
TRINITY_GAX7497	hypothet	Chlamydomonas_eusti	51.70	0.00
TRINITY_PNW8117	hypothet	Chlamydomonas_reinh	51.70	0.00
TRINITY_GBG7185	hypothet	Chara_braunii	51.70	0.00
TRINITY_XP_0016	predicte	Chlamydomonas_reinh	51.70	0.00
TRINITY_KGN5865	hypothet	Cucumis_sativus	51.70	0.00
TRINITY_GAX7950	hypothet	Chlamydomonas_eusti	51.70	0.00
TRINITY_GAX7792	hypothet	Chlamydomonas_eusti	51.70	0.00
TRINITY_XP_0017	high int	Chlamydomonas_reinh	51.70	0.00
TRINITY_XP_0016	predicte	Chlamydomonas_reinh	51.70	0.00
TRINITY_XP_0029	hypothet	Volvox_carteri	51.70	0.00
TRINITY_GAX8612	hypothet	Chlamydomonas_eusti	51.70	0.00
TRINITY_PNH0365	Tiny mac	Tetrabaena_socialis	51.70	0.00
TRINITY_KXZ4695	hypothet	Gonium_pectorale	51.70	0.00
TRINITY_XP_0192	PREDICTE	Nicotiana_attenuata	51.70	0.00
TRINITY_XP_0056	bifuncti	Coccomyxa_subellips	51.60	0.00
TRINITY_XP_0075	seryl-tR	Bathycoccus_prasino	51.60	0.00
TRINITY_GBG8367	hypothet	Chara_braunii	51.60	0.00
TRINITY_GAX7598	hypothet	Chlamydomonas_eusti	51.60	0.00
TRINITY_XP_0264	nucleola	Papaver_somniferum	51.60	0.00
TRINITY_GAQ8564	Fragile	Klebsormidium_niten	51.60	0.00
TRINITY_RMZ5586	hypothet	Auxenochlorella_pro	51.60	0.00
TRINITY_GAX7909	hypothet	Chlamydomonas_eusti	51.60	0.00
TRINITY_XP_0120	26S prot	Jatropha_curcas	51.60	0.00
TRINITY_GAQ7937	glutathi	Klebsormidium_niten	51.60	0.00

TRINITY_PNW7483	hypothet	Chlamydomonas_reinh	51.60	0.00
TRINITY_GAX7496	hypothet	Chlamydomonas_eusti	51.60	0.00
TRINITY_XP_0138	Coiled-	Monoraphidium_negle	51.60	0.00
TRINITY_PTQ4279	hypothet	Marchantia_polymorp	51.60	0.00
TRINITY_GBF9986	kinesin-	Raphidocelis_subcap	51.60	0.00
TRINITY_KXZ5455	hypothet	Gonium_pectorale	51.60	0.00
TRINITY_EFJ2695	hypothet	Selaginella_moellen	51.60	0.00
TRINITY_RRT5634	hypothet	Ensete_ventricosum	51.60	0.00
TRINITY_GAX7528	hypothet	Chlamydomonas_eusti	51.60	0.00
TRINITY_PNW8637	hypothet	Chlamydomonas_reinh	51.60	0.00
TRINITY_XP_0029	hypothet	Volvox_carteri	51.60	0.00
TRINITY_OAE2513	hypothet	Marchantia_polymorp	51.60	0.00
TRINITY_XP_0105	PREDICTE	Tarenaya_hasslerian	51.60	0.00
TRINITY_XP_0238	cystath	Quercus_suber	51.60	0.00
TRINITY_GAX7763	hypothet	Chlamydomonas_eusti	51.60	0.00
TRINITY_KDP4623	hypothet	Jatropha_curcas	51.60	0.00
TRINITY_PNW7163	hypothet	Chlamydomonas_reinh	51.60	0.00
TRINITY_XP_0029	mitogen-	Selaginella_moellen	51.60	0.00
TRINITY_GAX7674	hypothet	Chlamydomonas_eusti	51.60	0.00
TRINITY_XP_0124	PREDICTE	Gossypium_raimondii	51.60	0.00
TRINITY_GAQ9120	glutath	Klebsormidium_niten	51.60	0.00
TRINITY_PNW8226	hypothet	Chlamydomonas_reinh	51.60	0.00
TRINITY_PRW5887	P-ATPase	Chlorella_sorokinia	51.60	0.00
TRINITY_PNW7222	hypothet	Chlamydomonas_reinh	51.60	0.00
TRINITY_XP_0029	hypothet	Volvox_carteri	51.60	0.00
TRINITY_XP_0056	hypothet	Coccomyxa_subellips	51.60	0.00
TRINITY_GAX8509	hypothet	Chlamydomonas_eusti	51.60	0.00
TRINITY_XP_0035	glutamyl	Glycine_max	51.60	0.00
TRINITY_GAX7579	hypothet	Chlamydomonas_eusti	51.60	0.00
TRINITY_PWA5250	Ubiquit	Artemisia_annua	51.60	0.00
TRINITY_GAX7856	hypothet	Chlamydomonas_eusti	51.60	0.00
TRINITY_PNH1069	hypothet	Tetrabaena_socialis	51.60	0.00
TRINITY_GBF9082	transien	Raphidocelis_subcap	51.60	0.00
TRINITY_XP_0016	predicte	Chlamydomonas_reinh	51.60	0.00
TRINITY_GAX7675	hypothet	Chlamydomonas_eusti	51.60	0.00
TRINITY_XP_0025	glutath	Micromonas_commoda	51.60	0.00
TRINITY_XP_0016	cationic	Chlamydomonas_reinh	51.60	0.00
TRINITY_XP_0201	40S rib	Aegilops_tauschii	51.60	0.00
TRINITY_XP_0030	Helicase	Ostreococcus_tauri	51.60	0.00
TRINITY_XP_0058	hypothet	Chlorella_variabili	51.50	0.00
TRINITY_XP_0241	type IV	Rosa_chinensis	51.50	0.00
TRINITY_BAB6121	P0460E0	Oryza_sativa	51.50	0.00
TRINITY_OMO7888	Ubiquit	Corchorus_capsulari	51.50	0.00
TRINITY_XP_0056	proteas	Coccomyxa_subellips	51.50	0.00
TRINITY_GBF9013	hypothet	Raphidocelis_subcap	51.50	0.00
TRINITY_GAQ8017	homogent	Klebsormidium_niten	51.50	0.00
TRINITY_PTQ4674	hypothet	Marchantia_polymorp	51.50	0.00
TRINITY_KOM4028	hypothet	Vigna_angularis	51.50	0.00
TRINITY_PHT2792	hypothet	Capsicum_baccatum	51.50	0.00
TRINITY_PON5916	Glycerop	Parasponia_anderson	51.50	0.00
TRINITY_OAE2211	hypothet	Marchantia_polymorp	51.50	0.00
TRINITY_XP_0201	putative	Aegilops_tauschii	51.50	0.00
TRINITY_PSC7100	Glycopro	Micractinium_conduc	51.50	0.00
TRINITY_KZV3090	transmem	Dorcoceras_hygromet	51.50	0.00
TRINITY_GAX8149	hypothet	Chlamydomonas_eusti	51.50	0.00
TRINITY_EFJ2239	hypothet	Selaginella_moellen	51.50	0.00
TRINITY_XP_0029	hypothet	Volvox_carteri	51.50	0.00
TRINITY_PNH1293	hypothet	Tetrabaena_socialis	51.50	0.00
TRINITY_XP_0116	LOW QUAL	Amborella_trichopod	51.50	0.00
TRINITY_PRW5920	oxidored	Chlorella_sorokinia	51.50	0.00
TRINITY_PNW8850	hypothet	Chlamydomonas_reinh	51.50	0.00
TRINITY_XP_0016	NADH:ub	Chlamydomonas_reinh	51.50	0.00
TRINITY_GAX8346	hypothet	Chlamydomonas_eusti	51.50	0.00
TRINITY_GBG8253	hypothet	Chara_braunii	51.50	0.00
TRINITY_GAX7637	hypothet	Chlamydomonas_eusti	51.50	0.00
TRINITY_XP_0239	eukaryot	Quercus_suber	51.50	0.00

TRINITY_GAX7521(	hypothet	Chlamydomonas_eusti	51.50	0.00
TRINITY_XP_0016(	low CO2	Chlamydomonas_reinh	51.50	0.00
TRINITY_XP_0017(	flagella	Chlamydomonas_reinh	51.50	0.00
TRINITY_PNW8047(	hypothet	Chlamydomonas_reinh	51.50	0.00
TRINITY_KXZ4358(	hypothet	Gonium_pectorale	51.50	0.00
TRINITY_NP_0013(	uncharac	Zea_mays	51.50	0.00
TRINITY_XP_0106(	PREDICTE	Beta_vulgaris	51.50	0.00
TRINITY_PIA4675(	hypothet	Aquilegia_coerulea	51.50	0.00
TRINITY_GBG0043(	hypothet	Raphidocelis_subcap	51.50	0.00
TRINITY_ALM5499(	DEAD box	Chlamydomonas_sp._I	51.50	0.00

TRINITY_XP_02024	SNF1-rel	Asparagus_officinal	51.50	0.00
TRINITY_KXZ49276	hypothet	Gonium_pectorale	51.50	0.00
TRINITY_XP_01139	40S ribo	Auxenochlorella_prc	51.50	0.00
TRINITY_XP_01104	PREDICTE	Populus_euphratica	51.50	0.00
TRINITY_PRW58906	selenocy	Chlorella_sorokinia	51.50	0.00
TRINITY_GBG93046	hypothet	Chara_braunii	51.50	0.00
TRINITY_XP_00294	acetyl-c	Volvox_carteri	51.50	0.00
TRINITY_GAX84397	hypothet	Chlamydomonas_eusti	51.50	0.00
TRINITY_PNH06959	Proline	Tetrabaena_socialis	51.50	0.00
TRINITY_XP_00295	hypothet	Volvox_carteri	51.50	0.00
TRINITY_XP_00170	plastid	Chlamydomonas_reinh	51.50	0.00
TRINITY_GAX79008	hypothet	Chlamydomonas_eusti	51.50	0.00
TRINITY_KXZ43508	hypothet	Gonium_pectorale	51.50	0.00
TRINITY_XP_02390	profilin	Quercus_suber	51.50	0.00
TRINITY_GBG75211	hypothet	Chara_braunii	51.50	0.00
TRINITY_GAX79027	hypothet	Chlamydomonas_eusti	51.50	0.00
TRINITY_XP_00308	Domain c	Ostreococcus_tauri	51.50	0.00
TRINITY_GAX84568	hypothet	Chlamydomonas_eusti	51.40	0.00
TRINITY_RMZ52881	hypothet	Auxenochlorella_prc	51.40	0.00
TRINITY_XP_00295	hypothet	Volvox_carteri	51.40	0.00
TRINITY_XP_01941	PREDICTE	Lupinus_angustifoli	51.40	0.00
TRINITY_GBF88570	glyoxal	Raphidocelis_subcap	51.40	0.00
TRINITY_XP_01139	Malate s	Auxenochlorella_prc	51.40	0.00
TRINITY_VDC72836	unnamed	Brassica_rapa	51.40	0.00
TRINITY_XP_00299	anaphase	Selaginella_moellen	51.40	0.00
TRINITY_XP_02403	mediator	Morus_notabilis	51.40	0.00
TRINITY_PWA53816	mitochon	Artemisia_annua	51.40	0.00
TRINITY_EEF27463	Tartrate	Ricinus_communis	51.40	0.00
TRINITY_XP_00250	hypothet	Micromonas_commoda	51.40	0.00
TRINITY_GBG66831	hypothet	Chara_braunii	51.40	0.00
TRINITY_XP_01027	PREDICTE	Nelumbo_nucifera	51.40	0.00
TRINITY_GAX76358	hypothet	Chlamydomonas_eusti	51.40	0.00
TRINITY_PNW72739	hypothet	Chlamydomonas_reinh	51.40	0.00
TRINITY_PRW58375	putative	Chlorella_sorokinia	51.40	0.00
TRINITY_ACG27456	60 ribos	Zea_mays	51.40	0.00
TRINITY_RAL46865	hypothet	Cuscuta_australis	51.40	0.00
TRINITY_GBF91148	hypothet	Raphidocelis_subcap	51.40	0.00
TRINITY_PIA63301	hypothet	Aquilegia_coerulea	51.40	0.00
TRINITY_PIA42118	hypothet	Aquilegia_coerulea	51.40	0.00
TRINITY_KXZ55166	hypothet	Gonium_pectorale	51.40	0.00
TRINITY_XP_00565	putative	Coccomyxa_subellips	51.40	0.00
TRINITY_XP_00935	PREDICTE	Pyrus_x_bretschneid	51.40	0.00
TRINITY_XP_01918	PREDICTE	Ipomoea_nil	51.40	0.00
TRINITY_GAX85837	hypothet	Chlamydomonas_eusti	51.40	0.00
TRINITY_KCW75837	hypothet	Eucalyptus_grandis	51.40	0.00
TRINITY_PVH31962	hypothet	Panicum_hallii	51.40	0.00
TRINITY_XP_02188	transcri	Carica_papaya	51.40	0.00
TRINITY_PNH09922	Random s	Tetrabaena_socialis	51.40	0.00
TRINITY_XP_02452	methyltr	Selaginella_moellen	51.40	0.00
TRINITY_GBF88550	tRNA pse	Raphidocelis_subcap	51.40	0.00
TRINITY_ABR16494	unknown	Picea_sitchensis	51.40	0.00
TRINITY_GAX82008	hypothet	Chlamydomonas_eusti	51.40	0.00
TRINITY_PNW88593	hypothet	Chlamydomonas_reinh	51.40	0.00
TRINITY_BAK01125	predicte	Hordeum_vulgare	51.40	0.00
TRINITY_GBF94818	hypothet	Raphidocelis_subcap	51.40	0.00
TRINITY_GAX76876	hypothet	Chlamydomonas_eusti	51.40	0.00
TRINITY_CAA49281	gag,prot	Volvox_carteri	51.40	0.00
TRINITY_PNW80715	hypothet	Chlamydomonas_reinh	51.40	0.00
TRINITY_RWW90253	hypothet	Ensete_ventricosum	51.40	0.00
TRINITY_XP_01649	PREDICTE	Nicotiana_tabacum	51.40	0.00
TRINITY_XP_00294	hypothet	Volvox_carteri	51.40	0.00
TRINITY_GBF94444	flagella	Raphidocelis_subcap	51.40	0.00
TRINITY_PNW76408	hypothet	Chlamydomonas_reinh	51.40	0.00
TRINITY_KXZ56411	hypothet	Gonium_pectorale	51.40	0.00
TRINITY_GBF88261	rab3 GTE	Raphidocelis_subcap	51.40	0.00
TRINITY_PNW80471	hypothet	Chlamydomonas_reinh	51.40	0.00

TRINITY_GAX7747	hypothet	Chlamydomonas_eusti	51.40	0.00
TRINITY_XP_0029	hypothet	Volvox_carteri	51.40	0.00
TRINITY_XP_0083	PREDICTI	Malus_domestica	51.40	0.00
TRINITY_GAX8550	hypothet	Chlamydomonas_eusti	51.40	0.00
TRINITY_GAX7708	hypothet	Chlamydomonas_eusti	51.40	0.00
TRINITY_PNH1228	WW domai	Tetrabaena_socialis	51.40	0.00
TRINITY_XP_0017	predicte	Chlamydomonas_reinh	51.40	0.00
TRINITY_GBF8886	chitinas	Raphidocelis_subcap	51.40	0.00
TRINITY_GAX8100	hypothet	Chlamydomonas_eusti	51.40	0.00
TRINITY_KDD7528	AMP-bin	Helicosporidium_sp.	51.40	0.00
TRINITY_XP_0113	hypothet	Auxenochlorella_pro	51.40	0.00
TRINITY_KXZ5280	hypothet	Gonium_pectorale	51.40	0.00
TRINITY_ABH0932	leucine	Arachis_hypogaea	51.40	0.00
TRINITY_KXZ4181	hypothet	Gonium_pectorale	51.40	0.00
TRINITY_GAQ9356	hypothet	Klebsormidium_niten	51.40	0.00
TRINITY_PNH0415	hypothet	Tetrabaena_socialis	51.40	0.00
TRINITY_GAQ8492	histone	Klebsormidium_niten	51.40	0.00
TRINITY_XP_0239	ATP-depe	Quercus_suber	51.40	0.00
TRINITY_OUS4916	NUDIX hy	Ostreococcus_tauri	51.30	0.00
TRINITY_XP_0104	PREDICTI	Camelina_sativa	51.30	0.00
TRINITY_RWW9025	hypothet	Ensete_ventricosum	51.30	0.00
TRINITY_BAJ9390	predicte	Hordeum_vulgare	51.30	0.00
TRINITY_XP_0014	predicte	Ostreococcus_sp._'l	51.30	0.00
TRINITY_GAX8225	hypothet	Chlamydomonas_eusti	51.30	0.00
TRINITY_GBF9862	beta-ada	Raphidocelis_subcap	51.30	0.00
TRINITY_XP_0239	AP-2 cor	Quercus_suber	51.30	0.00
TRINITY_XP_0016	predicte	Chlamydomonas_reinh	51.30	0.00
TRINITY_PRW3298	erythror	Chlorella_sorokinia	51.30	0.00
TRINITY_PTQ4262	hypothet	Marchantia_polymorp	51.30	0.00
TRINITY_GAX8322	hypothet	Chlamydomonas_eusti	51.30	0.00
TRINITY_XP_0016	ammoniur	Chlamydomonas_reinh	51.30	0.00
TRINITY_XP_0151	PREDICTI	Solanum_tuberosum	51.30	0.00
TRINITY_XP_0106	PREDICTI	Beta_vulgaris	51.30	0.00
TRINITY_XP_0025	predicte	Micromonas_commoda	51.30	0.00
TRINITY_XP_0023	imidazol	Populus_trichocarpa	51.30	0.00
TRINITY_GAX8150	hypothet	Chlamydomonas_eusti	51.30	0.00
TRINITY_PNH0853	Methylt	Tetrabaena_socialis	51.30	0.00
TRINITY_XP_0029	hypothet	Volvox_carteri	51.30	0.00
TRINITY_XP_0022	PREDICTI	Vitis_vinifera	51.30	0.00
TRINITY_XP_0188	PREDICTI	Juglans_regia	51.30	0.00
TRINITY_XP_0029	exosome	Selaginella_moellen	51.30	0.00
TRINITY_GBF9284	hypothet	Raphidocelis_subcap	51.30	0.00
TRINITY_KDD7669	hypothet	Helicosporidium_sp.	51.30	0.00
TRINITY_XP_0016	predicte	Chlamydomonas_reinh	51.30	0.00
TRINITY_XP_0235	DExH-box	Cucurbita_pepo	51.30	0.00
TRINITY_XP_0202	protein	Cajanus_cajan	51.30	0.00
TRINITY_GBG8840	hypothet	Chara_braunii	51.30	0.00
TRINITY_GAX7312	hypothet	Chlamydomonas_eusti	51.30	0.00
TRINITY_XP_0030	predicte	Micromonas_pusilla	51.30	0.00
TRINITY_RDY1237	Serine/t	Mucuna_pruriens	51.30	0.00
TRINITY_PNW7924	hypothet	Chlamydomonas_reinh	51.30	0.00
TRINITY_GBF8911	hypothet	Raphidocelis_subcap	51.30	0.00
TRINITY_GAX8635	hypothet	Chlamydomonas_eusti	51.30	0.00
TRINITY_XP_0191	PREDICTI	Ipomoea_nil	51.30	0.00
TRINITY_GAX8312	hypothet	Chlamydomonas_eusti	51.30	0.00
TRINITY_XP_0219	dihydrol	Helianthus_annuus	51.30	0.00
TRINITY_KXZ4812	hypothet	Gonium_pectorale	51.30	0.00
TRINITY_KXZ5691	hypothet	Gonium_pectorale	51.30	0.00
TRINITY_XP_0016	peroxire	Chlamydomonas_reinh	51.30	0.00
TRINITY_KXZ4582	hypothet	Gonium_pectorale	51.30	0.00
TRINITY_GAX8266	hypothet	Chlamydomonas_eusti	51.30	0.00
TRINITY_XP_0030	predicte	Micromonas_pusilla	51.30	0.00
TRINITY_XP_0029	hypothet	Volvox_carteri	51.30	0.00
TRINITY_GAX7427	hypothet	Chlamydomonas_eusti	51.30	0.00
TRINITY_GAX7302	hypothet	Chlamydomonas_eusti	51.30	0.00
TRINITY_KXZ5425	hypothet	Gonium_pectorale	51.30	0.00

TRINITY_OTG2090(	putative	Helianthus_annuus	51.30	0.00
TRINITY_GAX8483:	hypothet	Chlamydomonas_eusti	51.30	0.00
TRINITY_KXZ5420:	hypothet	Gonium_pectorale	51.30	0.00
TRINITY_GAX7320(	hypothet	Chlamydomonas_eusti	51.30	0.00
TRINITY_PNW7310:	hypothet	Chlamydomonas_reinh	51.30	0.00
TRINITY_GBF9572:	hypothet	Raphidocelis_subcap	51.30	0.00
TRINITY_PNH1289!	hypothet	Tetrabaena_socialis	51.30	0.00
TRINITY_PNW7983	hypothet	Chlamydomonas_reinh	51.30	0.00
TRINITY_GAX7353(	hypothet	Chlamydomonas_eusti	51.30	0.00
TRINITY_KDD7530(	fumble,	Helicosporidium_sp.	51.20	0.00
TRINITY_GAQ7822	Serine/t	Klebsormidium_niten	51.20	0.00
TRINITY_GBF9117	hypothet	Raphidocelis_subcap	51.20	0.00
TRINITY_ABK2587	unknown	Picea_sitchensis	51.20	0.00
TRINITY_GAQ7758	ABC tra	Klebsormidium_niten	51.20	0.00
TRINITY_XP_0228(	ras-rel	Olea_europaea	51.20	0.00
TRINITY_GAQ8276	Actin-re	Klebsormidium_niten	51.20	0.00
TRINITY_GAQ8787(	Aromati	Klebsormidium_niten	51.20	0.00
TRINITY_XP_0056	putative	Coccomyxa_subellips	51.20	0.00
TRINITY_GAX8503	hypothet	Chlamydomonas_eusti	51.20	0.00
TRINITY_EPS7450!	hypothet	Genlisea_aurea	51.20	0.00
TRINITY_PNW8777!	hypothet	Chlamydomonas_reinh	51.20	0.00
TRINITY_GAQ8762(	Dyneins	Klebsormidium_niten	51.20	0
TRINITY_XP_0114(	Glycerol	Auxenochlorella_pro	51.20	0.00
TRINITY_XP_0219	probable	Helianthus_annuus	51.20	0.00
TRINITY_OAE2772	hypothet	Marchantia_polymorp	51.20	0.00
TRINITY_XP_0087	uncharac	Phoenix_dactylifera	51.20	0.00
TRINITY_PNH1039!	Beta-fru	Tetrabaena_socialis	51.20	0.00
TRINITY_XP_0205!	probable	Phalaenopsis_equest	51.20	0.00
TRINITY_AEU1103	flagella	Dunaliella_salina	51.20	0.00
TRINITY_PNW7419!	hypothet	Chlamydomonas_reinh	51.20	0.00
TRINITY_KZV1521(	citrate	Dorcoceras_hygromet	51.20	0.00
TRINITY_XP_0016!	predicte	Chlamydomonas_reinh	51.20	0.00
TRINITY_GAX8418	hypothet	Chlamydomonas_eusti	51.20	0.00
TRINITY_GAX8021:	hypothet	Chlamydomonas_eusti	51.20	0.00
TRINITY_CCF1754(	RabE GT	Micrasterias_dentic	51.20	0.00
TRINITY_XP_0029	hypothet	Volvox_carteri	51.20	0.00
TRINITY_XP_0075	predicte	Bathycoccus_prasinc	51.20	0.00
TRINITY_PNW7264(	hypothet	Chlamydomonas_reinh	51.20	0.00
TRINITY_PSC7691	cupin su	Micractinium_conduc	51.20	0.00
TRINITY_PNW7817	hypothet	Chlamydomonas_reinh	51.20	0.00
TRINITY_PSC7135!	arsenite	Micractinium_conduc	51.20	0.00
TRINITY_XP_0279	zinc fir	Vigna_unguiculata	51.20	0.00
TRINITY_XP_0029(	traffick	Selaginella_moellen	51.20	0.00
TRINITY_PNW7206(	hypothet	Chlamydomonas_reinh	51.20	0.00
TRINITY_GAQ9067!	hypothet	Klebsormidium_niten	51.20	0.00
TRINITY_XP_0029	hypothet	Volvox_carteri	51.20	0.00
TRINITY_BAJ9946(	predicte	Hordeum_vulgare	51.20	0.00
TRINITY_XP_0058	hypothet	Chlorella_variabili	51.20	0.00
TRINITY_XP_0263!	glycerol	Papaver_somniferum	51.20	0.00
TRINITY_GAX8346:	hypothet	Chlamydomonas_eusti	51.20	0.00
TRINITY_PNH1213:	hypothet	Tetrabaena_socialis	51.20	0.00
TRINITY_XP_0041	PREDICTE	Cucumis_sativus	51.20	0.00
TRINITY_GAX8505	hypothet	Chlamydomonas_eusti	51.20	0.00
TRINITY_XP_0029	hypothet	Volvox_carteri	51.20	0.00
TRINITY_XP_0029!	CF0 ATP	Volvox_carteri	51.20	0.00
TRINITY_PRW5921!	tRNA (cy	Chlorella_sorokinia	51.20	0.00
TRINITY_XP_0056!	DNA/RNA	Coccomyxa_subellips	51.20	0.00
TRINITY_KJB2409	hypothet	Gossypium_raimondii	51.20	0.00
TRINITY_GAX7961	hypothet	Chlamydomonas_eusti	51.20	0.00
TRINITY_PRW2081	Altered	Chlorella_sorokinia	51.20	0.00
TRINITY_GBF9444:	hypothet	Raphidocelis_subcap	51.20	0.00
TRINITY_XP_0174:	PREDICTE	Vigna_angularis	51.20	0.00
TRINITY_GAX8386!	hypothet	Chlamydomonas_eusti	51.20	0.00
TRINITY_GAX7420	hypothet	Chlamydomonas_eusti	51.20	0.00
TRINITY_BAJ9372	predicte	Hordeum_vulgare	51.20	0.00
TRINITY_GAX8056!	hypothet	Chlamydomonas_eusti	51.20	0.00

TRINITY_XP_00294	hypothet	Volvox_carteri	51.20	0.00
TRINITY_GAX72670	hypothet	Chlamydomonas_eusti	51.20	0.00
TRINITY_PNW81213	hypothet	Chlamydomonas_reinh	51.20	0.00
TRINITY_XP_01139	hypothet	Auxenochlorella_pro	51.20	0.00
TRINITY_GAX82301	hypothet	Chlamydomonas_eusti	51.20	0.00
TRINITY_XP_00249	predicte	Micromonas_commoda	51.20	0.00
TRINITY_KMS65245	hypothet	Beta_vulgaris	51.20	0.00
TRINITY_PNH06133	Adenylat	Tetrabaena_socialis	51.20	0.00
TRINITY_KXZ55099	hypothet	Gonium_pectorale	51.20	0.00
TRINITY_XP_00250	dynein g	Micromonas_commoda	51.20	0.00
TRINITY_KXZ51016	hypothet	Gonium_pectorale	51.20	0
TRINITY_PNH12254	hypothet	Tetrabaena_socialis	51.20	0.00
TRINITY_GAX73324	hypothet	Chlamydomonas_eusti	51.20	0.00
TRINITY_GAX82592	hypothet	Chlamydomonas_eusti	51.20	0.00
TRINITY_GBG88409	hypothet	Chara_braunii	51.20	0.00
TRINITY_GAX72841	hypothet	Chlamydomonas_eusti	51.20	0.00
TRINITY_GBG68577	Glutamat	Chara_braunii	51.20	0.00
TRINITY_GBF91428	cytochro	Raphidocelis_subcap	51.20	0.00
TRINITY_XP_02389	kinesin	Quercus_suber	51.20	0.00
TRINITY_XP_02390	replicat	Quercus_suber	51.10	0.00
TRINITY_XP_00751	ribulose	Bathycoccus_prasino	51.10	0.00
TRINITY_XP_01107	UDP-N-ac	Sesamum_indicum	51.10	0.00
TRINITY_XP_02166	RNA poly	Hevea_brasiliensis	51.10	0.00
TRINITY_KFK25052	d-lactat	Arabis_alpina	51.10	0.00
TRINITY_PNY14246	cationic	Trifolium_pratense	51.10	0.00
TRINITY_GBF99792	26S prot	Raphidocelis_subcap	51.10	0.00
TRINITY_RID69577	hypothet	Brassica_rapa	51.10	0.00
TRINITY_GAX80188	hypothet	Chlamydomonas_eusti	51.10	0.00
TRINITY_PNW78266	hypothet	Chlamydomonas_reinh	51.10	0.00
TRINITY_ARO50091	indolear	Yamagishiella_unico	51.10	0.00
TRINITY_GBF93659	hypothet	Raphidocelis_subcap	51.10	0.00
TRINITY_PNH05873	Inositol	Tetrabaena_socialis	51.10	0.00
TRINITY_GAQ81917	heat sho	Klebsormidium_niten	51.10	0.00
TRINITY_GAX73014	hypothet	Chlamydomonas_eusti	51.10	0.00
TRINITY_XP_02387	transcri	Quercus_suber	51.10	0.00
TRINITY_GAX77611	hypothet	Chlamydomonas_eusti	51.10	0.00
TRINITY_GAX80391	hypothet	Chlamydomonas_eusti	51.10	0.00
TRINITY_XP_00564	PhyH-dom	Coccomyxa_subellips	51.10	0.00
TRINITY_PNW73807	hypothet	Chlamydomonas_reinh	51.10	0.00
TRINITY_XP_01093	PREDICTE	Elaeis_guineensis	51.10	0.00
TRINITY_XP_00939	PREDICTE	Musa_acuminata	51.10	0.00
TRINITY_GAX83533	hypothet	Chlamydomonas_eusti	51.10	0.00
TRINITY_GAX84165	hypothet	Chlamydomonas_eusti	51.10	0.00
TRINITY_AQA29589	retrovir	Zea_mays	51.10	0.00
TRINITY_GAX81168	hypothet	Chlamydomonas_eusti	51.10	0.00
TRINITY_GAX80199	hypothet	Chlamydomonas_eusti	51.10	0.00
TRINITY_KDO51447	hypothet	Citrus_sinensis	51.10	0.00
TRINITY_GAX75807	hypothet	Chlamydomonas_eusti	51.10	0.00
TRINITY_XP_00295	hypothet	Volvox_carteri	51.10	0.00
TRINITY_PNW71306	hypothet	Chlamydomonas_reinh	51.10	0.00
TRINITY_XP_00295	hypothet	Volvox_carteri	51.10	0.00
TRINITY_GAX76255	hypothet	Chlamydomonas_eusti	51.10	0.00
TRINITY_GBF88513	ABC tran	Raphidocelis_subcap	51.10	0.00
TRINITY_GBF88513	ABC tran	Raphidocelis_subcap	51.10	0.00
TRINITY_XP_00498	heat sho	Setaria_italica	51.10	0.00
TRINITY_XP_02710	putative	Coffea_arabica	51.10	0.00
TRINITY_GAX77485	hypothet	Chlamydomonas_eusti	51.10	0.00
TRINITY_GAX85323	hypothet	Chlamydomonas_eusti	51.10	0.00
TRINITY_PNH10576	Retrovir	Tetrabaena_socialis	51.10	0.00
TRINITY_XP_01102	PREDICTE	Populus_euphratica	51.10	0.00
TRINITY_PNW77923	hypothet	Chlamydomonas_reinh	51.10	0.00
TRINITY_GAX77211	hypothet	Chlamydomonas_eusti	51.10	0.00
TRINITY_GAU21959	hypothet	Trifolium_subterran	51.10	0.00
TRINITY_GBF87879	sucrose	Raphidocelis_subcap	51.10	0.00
TRINITY_XP_00170	glyoxal	Chlamydomonas_reinh	51.10	0.00
TRINITY_BAK00999	predicte	Hordeum_vulgare	51.10	0.00

TRINITY_XP_00865	cytochro	Zea_mays	51.10	0.00
TRINITY_PSC73882	ABC tran	Micractinium_conduc	51.10	0.00
TRINITY_GAX85698	hypothet	Chlamydomonas_eusti	51.10	0.00
TRINITY_GAQ88658	Dyneins	Klebsormidium_niten	51.10	0.00
TRINITY_KXZ48269	hypothet	Gonium_pectorale	51.10	0.00
TRINITY_KXZ40924	hypothet	Gonium_pectorale	51.10	0.00
TRINITY_PNH07816	putative	Tetrabaena_socialis	51.10	0.00
TRINITY_OMO73136	reverse	Corchorus_capsulari	51.10	0.00
TRINITY_XP_01847	PREDICTE	Raphanus_sativus	51.10	0.00
TRINITY_XP_01389	nucleosi	Monoraphidium_negle	51.00	0.00
TRINITY_XP_00141	predicte	Ostreococcus_sp._'l	51.00	0.00
TRINITY_XP_02440	uncharac	Physcomitrella_pate	51.00	0.00
TRINITY_GBG62284	hypothet	Chara_braunii	51.00	0.00
TRINITY_XP_00297	calmodul	Selaginella_moellen	51.00	0.00
TRINITY_XP_00305	entriole	Micromonas_pusilla	51.00	0.00
TRINITY_GBF88231	alpha be	Raphidocelis_subcap	51.00	0.00
TRINITY_XP_02390	ras-like	Quercus_suber	51.00	0.00
TRINITY_XP_02712	tRNAse Z	Coffea_arabica	51.00	0.00
TRINITY_XP_02390	LETM1 do	Quercus_suber	51.00	0.00
TRINITY_GBG80331	hypothet	Chara_braunii	51.00	0.00
TRINITY_XP_02173	carnosin	Chenopodium_quinoa	51.00	0.00
TRINITY_XP_00424	putative	Solanum_lycopersicu	51.00	0.00
TRINITY_KXZ45964	hypothet	Gonium_pectorale	51.00	0.00
TRINITY_XP_02435	mitochon	Physcomitrella_pate	51.00	0.00
TRINITY_NP_00134	ABC tran	Zea_mays	51.00	0.00
TRINITY_GAX77779	hypothet	Chlamydomonas_eusti	51.00	0.00
TRINITY_XP_00169	predicte	Chlamydomonas_reinh	51.00	0.00
TRINITY_PNH05981	Ubiquino	Tetrabaena_socialis	51.00	0.00
TRINITY_KXZ42805	hypothet	Gonium_pectorale	51.00	0.00
TRINITY_XP_01657	PREDICTE	Capsicum_annuum	51.00	0.00
TRINITY_GAX74027	hypothet	Chlamydomonas_eusti	51.00	0.00
TRINITY_GBF95504	transcri	Raphidocelis_subcap	51.00	0.00
TRINITY_PNW87886	hypothet	Chlamydomonas_reinh	51.00	0.00
TRINITY_XP_00169	predicte	Chlamydomonas_reinh	51.00	0.00
TRINITY_XP_00169	predicte	Chlamydomonas_reinh	51.00	0.00
TRINITY_XP_00294	hypothet	Volvox_carteri	51.00	0.00
TRINITY_KXZ52752	hypothet	Gonium_pectorale	51.00	0.00
TRINITY_XP_02402	LOW QUAL	Morus_notabilis	51.00	0.00
TRINITY_PNW87389	hypothet	Chlamydomonas_reinh	51.00	0.00
TRINITY_KXZ45775	hypothet	Gonium_pectorale	51.00	0.00
TRINITY_KXZ49323	hypothet	Gonium_pectorale	51.00	0.00
TRINITY_XP_01721	PREDICTE	Daucus_carota	51.00	0.00
TRINITY_GAX79874	hypothet	Chlamydomonas_eusti	51.00	0.00
TRINITY_KXZ49585	hypothet	Gonium_pectorale	51.00	0.00
TRINITY_KXZ53403	hypothet	Gonium_pectorale	51.00	0.00
TRINITY_GAX73530	hypothet	Chlamydomonas_eusti	51.00	0.00
TRINITY_GAX80482	hypothet	Chlamydomonas_eusti	51.00	0.00
TRINITY_GAX80271	hypothet	Chlamydomonas_eusti	51.00	0.00
TRINITY_RWR97491	ras-rela	Cinnamomum_micranth	51.00	0.00
TRINITY_GAX79596	hypothet	Chlamydomonas_eusti	51.00	0.00
TRINITY_GAX82619	hypothet	Chlamydomonas_eusti	51.00	0.00
TRINITY_XP_00170	predicte	Chlamydomonas_reinh	51.00	0.00
TRINITY_GAX73447	hypothet	Chlamydomonas_eusti	51.00	0.00
TRINITY_XP_00251	RNA-bind	Ricinus_communis	51.00	0.00
TRINITY_RWR84219	DNA heli	Cinnamomum_micranth	50.90	0.00
TRINITY_XP_00168	predicte	Chlamydomonas_reinh	50.90	0.00
TRINITY_GAX85410	hypothet	Chlamydomonas_eusti	50.90	0.00
TRINITY_XP_02644	ras-rela	Papaver_somniferum	50.90	0.00
TRINITY_KZV42179	hypothet	Dorcoceras_hygromet	50.90	0.00
TRINITY_XP_00564	peptide	Coccomyxa_subellips	50.90	0.00
TRINITY_XP_02284	Ribosoma	Ostreococcus_tauri	50.90	0.00
TRINITY_XP_01945	PREDICTE	Lupinus_angustifoli	50.90	0.00
TRINITY_XP_02168	E3 ubiqu	Hevea_brasiliensis	50.90	0.00
TRINITY_AET02413	hypothet	Medicago_truncatula	50.90	0.00
TRINITY_ONK65918	uncharac	Asparagus_officinal	50.90	0.00
TRINITY_XP_00169	regulato	Chlamydomonas_reinh	50.90	0.00

TRINITY_BAK0277{	predicte	Hordeum_vulgare	50.90	0.00
TRINITY_XP_0191	PREDICTE	Ipomoea_nil	50.90	0.00
TRINITY_XP_0064	ubiquiti	Citrus_clementina	50.90	0.00
TRINITY_OAE2573	hypothet	Marchantia_polymorp	50.90	0.00
TRINITY_PNW7739	hypothet	Chlamydomonas_reinh	50.90	0.00
TRINITY_GAX8431	hypothet	Chlamydomonas_eusti	50.90	0.00
TRINITY_KZV1520	hypothet	Dorcoceras_hygromet	50.90	0.00
TRINITY_BAK0277	predicte	Hordeum_vulgare	50.90	0.00
TRINITY_XP_0238	glycoger	Quercus_suber	50.90	0.00
TRINITY_GAX7571	hypothet	Chlamydomonas_eusti	50.90	0.00
TRINITY_KZV2800	putative	Dorcoceras_hygromet	50.90	0.00
TRINITY_GAX7306	hypothet	Chlamydomonas_eusti	50.90	0.00
TRINITY_XP_0093	PREDICTE	Musa_acuminata	50.90	0.00
TRINITY_PNH0769	Initiati	Tetrabaena_socialis	50.90	0.00
TRINITY_XP_0058	hypothet	Chlorella_variabili	50.90	0.00
TRINITY_GBG7830	hypothet	Chara_braunii	50.90	0.00
TRINITY_XP_0238	serine/t	Quercus_suber	50.90	0.00
TRINITY_GAX8092	hypothet	Chlamydomonas_eusti	50.90	0.00
TRINITY_GAX8260	hypothet	Chlamydomonas_eusti	50.90	0.00
TRINITY_GAX7743	hypothet	Chlamydomonas_eusti	50.90	0.00
TRINITY_GAX7312	hypothet	Chlamydomonas_eusti	50.90	0.00
TRINITY_PRW4496	cysteine	Chlorella_sorokinia	50.90	0.00
TRINITY_XP_0219	26S prot	Helianthus_annuus	50.90	0.00
TRINITY_OAE2308	hypothet	Marchantia_polymorp	50.90	0.00
TRINITY_PRW2019	formamic	Chlorella_sorokinia	50.90	0.00
TRINITY_GBG0012	thioredc	Raphidocelis_subcap	50.90	0.00
TRINITY_PNW8717	hypothet	Chlamydomonas_reinh	50.90	0.00
TRINITY_XP_0017	mitotic	Chlamydomonas_reinh	50.90	0.00
TRINITY_GAX7272	hypothet	Chlamydomonas_eusti	50.90	0.00
TRINITY_XP_0058	hypothet	Chlorella_variabili	50.90	0.00
TRINITY_PTQ3636	hypothet	Marchantia_polymorp	50.90	0.00
TRINITY_EOX9140	Heat shc	Theobroma_cacao	50.90	0.00
TRINITY_XP_0029	guanylyl	Volvox_carteri	50.90	0.00
TRINITY_XP_0017	predicte	Chlamydomonas_reinh	50.90	0.00
TRINITY_KXZ5334	hypothet	Gonium_pectorale	50.90	0.00
TRINITY_XP_0238	kinesin	Quercus_suber	50.90	0.00
TRINITY_H3JU05.1	RecName:	Chlamydomonas_reinh	50.90	0.00
TRINITY_PNW7813	hypothet	Chlamydomonas_reinh	50.90	0.00
TRINITY_EOX9790	DNA repl	Theobroma_cacao	50.90	0.00
TRINITY_GAQ8319	Galactos	Klebsormidium_niten	50.80	0.00
TRINITY_GBF9848	hypothet	Raphidocelis_subcap	50.80	0.00
TRINITY_PWA6087	CID doma	Artemisia_annua	50.80	0.00
TRINITY_KXZ4590	DHC7 prc	Gonium_pectorale	50.80	0.00
TRINITY_GAQ8380	Protein	Klebsormidium_niten	50.80	0.00
TRINITY_GAX7606	hypothet	Chlamydomonas_eusti	50.80	0.00
TRINITY_XP_0116	protein	Amborella_trichopod	50.80	0.00
TRINITY_XP_0014	predicte	Ostreococcus_sp._'l	50.80	0.00
TRINITY_XP_0237	CAAX pre	Lactuca_sativa	50.80	0.00
TRINITY_PNR4808	hypothet	Physcomitrella_pate	50.80	0.00
TRINITY_GAX8113	hypothet	Chlamydomonas_eusti	50.80	0.00
TRINITY_XP_0100	PREDICTE	Eucalyptus_grandis	50.80	0.00
TRINITY_PIN2169	Transcri	Handroanthus_impeti	50.80	0.00
TRINITY_XP_0202	LOW QUAI	Asparagus_officinal	50.80	0.00
TRINITY_OAE2074	hypothet	Marchantia_polymorp	50.80	0.00
TRINITY_XP_0017	expansir	Chlamydomonas_reinh	50.80	0.00
TRINITY_GAX7374	hypothet	Chlamydomonas_eusti	50.80	0.00
TRINITY_GAX7703	hypothet	Chlamydomonas_eusti	50.80	0.00
TRINITY_KXZ5158	hypothet	Gonium_pectorale	50.80	0.00
TRINITY_XP_0049	alpha-1,	Setaria_italica	50.80	0.00
TRINITY_GAX8444	hypothet	Chlamydomonas_eusti	50.80	0.00
TRINITY_XP_0243	chromodc	Physcomitrella_pate	50.80	0.00
TRINITY_XP_0016	predicte	Chlamydomonas_reinh	50.80	0.00
TRINITY_XP_0139	putative	Monoraphidium_negle	50.80	0.00
TRINITY_XP_0239	protein	Quercus_suber	50.80	0.00
TRINITY_GAX8605	hypothet	Chlamydomonas_eusti	50.80	0.00
TRINITY_GBF8774	hypothet	Raphidocelis_subcap	50.80	0.00

TRINITY_XP_0058	hypothet	Chlorella_variabili	50.80	0.00
TRINITY_XP_0138	hypothet	Monoraphidium_negle	50.80	0.00
TRINITY_PNW7678	hypothet	Chlamydomonas_reinh	50.80	0.00
TRINITY_PNW7589	hypothet	Chlamydomonas_reinh	50.80	0.00
TRINITY_XP_0024	DExH-bo	Sorghum_bicolor	50.80	0.00
TRINITY_GAX7547	hypothet	Chlamydomonas_eusti	50.80	0.00
TRINITY_XP_0139	Protein	Monoraphidium_negle	50.80	0.00
TRINITY_GAX8315	hypothet	Chlamydomonas_eusti	50.80	0.00
TRINITY_PNW8152	hypothet	Chlamydomonas_reinh	50.80	0.00
TRINITY_GAX8398	hypothet	Chlamydomonas_eusti	50.80	0.00
TRINITY_KXZ5169	hypothet	Gonium_pectorale	50.80	0.00
TRINITY_PNW7439	hypothet	Chlamydomonas_reinh	50.80	0.00
TRINITY_NP_0011	dihydro	Zea_mays	50.80	0.00
TRINITY_XP_0017	ARF-lik	Chlamydomonas_reinh	50.80	0.00
TRINITY_OUS4521	FAD-cont	Ostreococcus_tauri	50.80	0.00
TRINITY_RID6288	hypothet	Brassica_rapa	50.80	0.00
TRINITY_XP_0029	glutamat	Selaginella_moellen	50.80	0.00
TRINITY_PHU2332	hypothet	Capsicum_chinense	50.80	0.00
TRINITY_GAX7888	hypothet	Chlamydomonas_eusti	50.80	0.00
TRINITY_XP_0029	hypothet	Volvox_carteri	50.80	0.00
TRINITY_XP_0239	ras-like	Quercus_suber	50.80	0.00
TRINITY_GAX7542	hypothet	Chlamydomonas_eusti	50.80	0.00
TRINITY_GAQ8781	sodium p	Klebsormidium_niten	50.80	0.00
TRINITY_KXZ5395	FAP91 p	Gonium_pectorale	50.80	0.00
TRINITY_GAX7300	hypothet	Chlamydomonas_eusti	50.80	0.00
TRINITY_XP_0218	nucleola	Carica_papaya	50.80	0.00
TRINITY_GBF8915	Xaa-Pro	Raphidocelis_subcap	50.80	0.00
TRINITY_GAX8236	hypothet	Chlamydomonas_eusti	50.80	0.00
TRINITY_RLN0878	protein	Panicum_miliaceum	50.80	0.00
TRINITY_XP_0194	PREDICTE	Lupinus_angustifoli	50.80	0.00
TRINITY_PRW3935	phospho	Chlorella_sorokinia	50.80	0.00
TRINITY_XP_0029	hypothet	Volvox_carteri	50.80	0.00
TRINITY_PNH0628	hypothet	Tetrabaena_socialis	50.80	0.00
TRINITY_PSC6764	MAK16-li	Micractinium_conduc	50.80	0.00
TRINITY_GAX7564	hypothet	Chlamydomonas_eusti	50.80	0.00
TRINITY_GBF9127	hypothet	Raphidocelis_subcap	50.80	0.00
TRINITY_XP_0017	copper h	Chlamydomonas_reinh	50.80	0.00
TRINITY_KXZ5519	hypothet	Gonium_pectorale	50.80	0.00
TRINITY_GAX8529	hypothet	Chlamydomonas_eusti	50.80	0.00
TRINITY_AAO7355	reverse-	Chlamydomonas_reinh	50.80	0.00
TRINITY_KFK2583	hypothet	Arabis_alpina	50.80	0.00
TRINITY_PTQ3141	hypothet	Marchantia_polymorp	50.70	0.00
TRINITY_XP_0228	histone	Olea_europaea	50.70	0.00
TRINITY_PPS0837	hypothet	Gossypium_barbadens	50.70	0.00
TRINITY_XP_0208	UPF0235	Arabidopsis_lyrata	50.70	0.00
TRINITY_GAX7900	hypothet	Chlamydomonas_eusti	50.70	0.00
TRINITY_XP_0056	prolife	Coccomyxa_subellips	50.70	0.00
TRINITY_XP_0041	PREDICTE	Cucumis_sativus	50.70	0.00
TRINITY_GAQ8382	M-phase	Klebsormidium_niten	50.70	0.00
TRINITY_XP_0110	PREDICTE	Populus_euphratica	50.70	0.00
TRINITY_XP_0063	nitrate	Eutrema_salsugineum	50.70	0.00
TRINITY_AAQ0725	putative	Oryza_sativa	50.70	0.00
TRINITY_GBG7247	hypothet	Chara_braunii	50.70	0.00
TRINITY_XP_0228	D-2-hyd	Olea_europaea	50.70	0.00
TRINITY_XP_0229	histidi	Cucurbita_moschata	50.70	0.00
TRINITY_PRQ3492	putative	Rosa_chinensis	50.70	0.00
TRINITY_KVH9905	hypothet	Cynara_cardunculus	50.70	0.00
TRINITY_XP_0030	flagella	Micromonas_pusilla	50.70	0.00
TRINITY_PNH0365	Tiny ma	Tetrabaena_socialis	50.70	0.00
TRINITY_GAQ8676	hypothet	Klebsormidium_niten	50.70	0.00
TRINITY_EFJ1061	hypothet	Selaginella_moellen	50.70	0.00
TRINITY_KYP6674	U1 smal	Cajanus_cajan	50.70	0.00
TRINITY_GAQ9121	Calcium	Klebsormidium_niten	50.70	0.00
TRINITY_XP_0029	hypothet	Volvox_carteri	50.70	0.00
TRINITY_XP_0016	flagella	Chlamydomonas_reinh	50.70	0.00
TRINITY_XP_0016	predicte	Chlamydomonas_reinh	50.70	0.00

TRINITY_AID2386	vanillir	Glechoma_hederacea	50.70	0.00
TRINITY_PSS1130	UDP-gluc	Actinidia_chinensis	50.70	0.00
TRINITY_XP_0109	PREDICTE	Elaeis_guineensis	50.70	0.00
TRINITY_ACF9852	delta-12	Chlorella_vulgaris	50.70	0.00
TRINITY_PWA4367	autophag	Artemisia_annua	50.70	0.00
TRINITY_XP_0188	PREDICTE	Juglans_regia	50.70	0.00
TRINITY_XP_0202	serine/t	Asparagus_officinal	50.70	0.00
TRINITY_GAX8277	hypothet	Chlamydomonas_eusti	50.70	0.00
TRINITY_KXZ5051	hypothet	Gonium_pectorale	50.70	0.00
TRINITY_GAX8054	hypothet	Chlamydomonas_eusti	50.70	0.00
TRINITY_XP_0194	PREDICTE	Lupinus_angustifoli	50.70	0.00
TRINITY_KXZ4988	hypothet	Gonium_pectorale	50.70	0.00
TRINITY_XP_0139	Pre-rRNA	Monoraphidium_negle	50.70	0.00
TRINITY_GAQ8199	Amino ac	Klebsormidium_niten	50.70	0.00
TRINITY_XP_0111	splicing	Sesamum_indicum	50.70	0.00
TRINITY_XP_0184	PREDICTE	Raphanus_sativus	50.70	0.00
TRINITY_GAQ8559	DNA-dire	Klebsormidium_niten	50.70	0.00
TRINITY_KXZ5273	hypothet	Gonium_pectorale	50.70	0.00
TRINITY_XP_0029	hypothet	Volvox_carteri	50.70	0.00
TRINITY_RZC2607	NADPH--c	Glycine_soja	50.70	0.00
TRINITY_GAX8458	hypothet	Chlamydomonas_eusti	50.70	0.00
TRINITY_OUS4480	putative	Ostreococcus_tauri	50.70	0.00
TRINITY_BAV5834	GTP-bind	Ulva_partita	50.70	0.00
TRINITY_BAK0112	predicte	Hordeum_vulgare	50.70	0.00
TRINITY_XP_0239	delta-1-	Quercus_suber	50.70	0.00
TRINITY_KXZ4687	hypothet	Gonium_pectorale	50.70	0.00
TRINITY_XP_0238	tryptoph	Quercus_suber	50.70	0.00
TRINITY_NP_0012	seed mat	Glycine_max	50.70	0.00
TRINITY_GBG7449	hypothet	Chara_braunii	50.70	0.00
TRINITY_XP_0029	hypothet	Volvox_carteri	50.70	0.00
TRINITY_GBF9907	hypothet	Raphidocelis_subcap	50.70	0.00
TRINITY_XP_0056	TPR-like	Coccomyxa_subellips	50.70	0.00
TRINITY_XP_0029	hypothet	Volvox_carteri	50.70	0.00
TRINITY_GAX8122	hypothet	Chlamydomonas_eusti	50.70	0.00
TRINITY_PNH1145	EF-hand	Tetrabaena_socialis	50.70	0.00
TRINITY_GAX8469	hypothet	Chlamydomonas_eusti	50.70	0.00
TRINITY_YP_6656	cytochro	Nephroselmis_olivac	50.70	0.00
TRINITY_EFJ0645	hypothet	Selaginella_moellen	50.70	0.00
TRINITY_PNW7434	hypothet	Chlamydomonas_reinh	50.70	0.00
TRINITY_GAX7602	hypothet	Chlamydomonas_eusti	50.70	0.00
TRINITY_GAX7903	hypothet	Chlamydomonas_eusti	50.70	0.00
TRINITY_XP_0028	putative	Arabidopsis_lyrata	50.70	0.00
TRINITY_XP_0113	Uridine	Auxenochlorella_pro	50.70	0.00
TRINITY_PTQ4327	hypothet	Marchantia_polymorp	50.70	0.00
TRINITY_XP_0097	PREDICTE	Nicotiana_sylvestri	50.70	0.00
TRINITY_PNH0794	hypothet	Tetrabaena_socialis	50.70	0.00
TRINITY_XP_0045	ATP-depe	Cicer_arietinum	50.70	0.00
TRINITY_PNH0877	NEDD8 ul	Tetrabaena_socialis	50.70	0.00
TRINITY_GAQ8926	hypothet	Klebsormidium_niten	50.70	0.00
TRINITY_GAX7662	hypothet	Chlamydomonas_eusti	50.70	0.00
TRINITY_OMO5699	Glutared	Corchorus_capsulari	50.70	0.00
TRINITY_GAX7840	hypothet	Chlamydomonas_eusti	50.70	0.00
TRINITY_XP_0030	P-loop c	Ostreococcus_tauri	50.70	0.00
TRINITY_XP_0138	hypothet	Monoraphidium_negle	50.70	0.00
TRINITY_PNW7490	hypothet	Chlamydomonas_reinh	50.70	0.00
TRINITY_GAQ8798	Phosphat	Klebsormidium_niten	50.70	0.00
TRINITY_GAX7827	hypothet	Chlamydomonas_eusti	50.70	0.00
TRINITY_GAX7370	hypothet	Chlamydomonas_eusti	50.70	0.00
TRINITY_PNH0830	NFX1-typ	Tetrabaena_socialis	50.70	0.00
TRINITY_XP_0161	ADP-ribo	Arachis_ipaensis	50.70	0.00
TRINITY_EFJ2714	hypothet	Selaginella_moellen	50.60	0.00
TRINITY_GAQ8673	triparti	Klebsormidium_niten	50.60	0.00
TRINITY_XP_0220	anaphase	Helianthus_annuus	50.60	0.00
TRINITY_XP_0016	predicte	Chlamydomonas_reinh	50.60	0.00
TRINITY_XP_0164	PREDICTE	Nicotiana_tabacum	50.60	0.00
TRINITY_XP_0104	PREDICTE	Camelina_sativa	50.60	0.00

TRINITY_XP_0101(	succinat	Morus_notabilis	50.60	0.00
TRINITY_XP_0264:	nudC dor	Papaver_somniferum	50.60	0.00
TRINITY_XP_0139(	DEAD-bo:	Monoraphidium_negle	50.60	0.00
TRINITY_XP_0243(	eukaryot	Physcomitrella_pate	50.60	0.00
TRINITY_GAQ8308:	Maltose	Klebsormidium_niten	50.60	0.00
TRINITY_XP_0238'	pyruvate	Quercus_suber	50.60	0.00
TRINITY_PKA6430(	Glycine-	Apostasia_shenzheni	50.60	0.00
TRINITY_XP_0029!	hypothet	Volvox_carteri	50.60	0.00
TRINITY_KXZ4903(	hypothet	Gonium_pectorale	50.60	0.00
TRINITY_KXZ5155(	hypothet	Gonium_pectorale	50.60	0.00
TRINITY_EEF2551:	conserve	Ricinus_communis	50.60	0.00
TRINITY_PNW7810:	hypothet	Chlamydomonas_reinh	50.60	0.00
TRINITY_XP_0029(	hypothet	Volvox_carteri	50.60	0.00
TRINITY_XP_0239:	ras-rela	Quercus_suber	50.60	0.00
TRINITY_XP_0016(	predicte	Chlamydomonas_reinh	50.60	0.00
TRINITY_XP_0239(	myosin-1	Quercus_suber	50.60	0.00
TRINITY_XP_0205:	mitogen-	Amborella_trichopod	50.60	0.00
TRINITY_PNH1098(	hypothet	Tetrabaena_socialis	50.60	0.00
TRINITY_XP_0219(	autopha(	Carica_papaya	50.60	0.00
TRINITY_PNH1041!	WD repea	Tetrabaena_socialis	50.60	0.00
TRINITY_AFK3379'	unknown	Lotus_japonicus	50.60	0.00
TRINITY_RLN3604!	hypothet	Panicum_miliaceum	50.60	0.00
TRINITY_XP_0134(	ras-rela	Medicago_truncatula	50.60	0.00
TRINITY_XP_0029!	hypothet	Volvox_carteri	50.60	0.00
TRINITY_PNW8524(	hypothet	Chlamydomonas_reinh	50.60	0.00
TRINITY_PNW8270'	hypothet	Chlamydomonas_reinh	50.60	0.00
TRINITY_GAX7758(	hypothet	Chlamydomonas_eusti	50.60	0.00
TRINITY_NP_0013(	uncharac	Zea_mays	50.60	0.00
TRINITY_XP_0029!	hypothet	Volvox_carteri	50.60	0.00
TRINITY_KXZ5097!	hypothet	Gonium_pectorale	50.60	0.00
TRINITY_XP_0145:	DExH-bo:	Vigna_radiata	50.60	0.00
TRINITY_GBF9228(	hypothet	Raphidocelis_subcap	50.60	0.00
TRINITY_XP_0258(	uncharac	Oryza_sativa	50.60	0.00
TRINITY_GAX7622:	hypothet	Chlamydomonas_eusti	50.60	0.00
TRINITY_GAX8431(	hypothet	Chlamydomonas_eusti	50.60	0.00
TRINITY_KQJ9067!	hypothet	Brachypodium_distac	50.60	0.00
TRINITY_PRW5835:	ATP-binc	Chlorella_sorokinia	50.60	0.00
TRINITY_XP_0029!	hypothet	Volvox_carteri	50.60	0.00
TRINITY_XP_0191!	PREDICTI	Ipomoea_nil	50.60	0.00
TRINITY_XP_0016(	predicte	Chlamydomonas_reinh	50.60	0.00
TRINITY_XP_0016(	hypothet	Chlamydomonas_reinh	50.60	0.00
TRINITY_XP_0139(	Activat(	Monoraphidium_negle	50.60	0.00
TRINITY_XP_0228:	Rudiment	Ostreococcus_tauri	50.60	0.00
TRINITY_XP_0219'	probable	Helianthus_annuus	50.60	0.00
TRINITY_AHA9309(	PLC prot	Dunaliella_salina	50.60	0.00
TRINITY_PNH0807!	hypothet	Tetrabaena_socialis	50.60	0.00
TRINITY_GAX8138(	hypothet	Chlamydomonas_eusti	50.60	0.00
TRINITY_PNH0405(	putative	Tetrabaena_socialis	50.60	0.00
TRINITY_GAX8511(	hypothet	Chlamydomonas_eusti	50.60	0.00
TRINITY_XP_0218(	serine/t	Spinacia_oleracea	50.60	0.00
TRINITY_GAX8270:	hypothet	Chlamydomonas_eusti	50.60	0.00
TRINITY_GAX7868(	hypothet	Chlamydomonas_eusti	50.60	0.00
TRINITY_GAX8157(	hypothet	Chlamydomonas_eusti	50.60	0.00
TRINITY_XP_0229:	ras-rela	Cucurbita_moschata	50.60	0.00
TRINITY_XP_0165(	PREDICTI	Capsicum_annuum	50.60	0.00
TRINITY_XP_0138!	hypothet	Monoraphidium_negle	50.60	0.00
TRINITY_PRW5953!	hypothet	Chlorella_sorokinia	50.60	0.00
TRINITY_PNW8062!	hypothet	Chlamydomonas_reinh	50.60	0.00
TRINITY_GAX7568(	hypothet	Chlamydomonas_eusti	50.60	0.00
TRINITY_KXZ4316!	hypothet	Gonium_pectorale	50.60	0.00
TRINITY_XP_0139(	Inositol	Monoraphidium_negle	50.60	0.00
TRINITY_PNW8221!	hypothet	Chlamydomonas_reinh	50.60	0.00
TRINITY_PNW8053(	hypothet	Chlamydomonas_reinh	50.60	0.00
TRINITY_XP_0075(	glutath:	Bathycoccus_prasinc	50.60	0.00
TRINITY_OVA0044(	DnaJ dor	Macleaya_cordata	50.60	0.00
TRINITY_XP_0201(	uncharac	Ananas_comosus	50.60	0.00

TRINITY_GAX7573(	hypothet	Chlamydomonas_eusti	50.60	0.00
TRINITY_XP_0025(	protein	Micromonas_commoda	50.60	0.00
TRINITY_XP_0239(	myosin t	Quercus_suber	50.50	0.00
TRINITY_BAK0236	predicte	Hordeum_vulgare	50.50	0.00
TRINITY_VDC9824	unnamed	Brassica_oleracea	50.50	0.00
TRINITY_XP_0017(	YCII-re	Chlamydomonas_reinh	50.50	0.00
TRINITY_XP_0100	cell nur	Morus_notabilis	50.50	0.00
TRINITY_AIU4948	RNA hel	Dioscorea_oppositif	50.50	0.00
TRINITY_XP_0120	methylt	Jatropha_curcas	50.50	0.00
TRINITY_XP_0191	PREDICT	Ipomoea_nil	50.50	0.00
TRINITY_RAL4648	hypothet	Cuscuta_australis	50.50	0.00
TRINITY_GAX7471	hypothet	Chlamydomonas_eusti	50.50	0.00
TRINITY_XP_0083	PREDICT	Malus_domestica	50.50	0.00
TRINITY_XP_0041	PREDICT	Cucumis_sativus	50.50	0.00
TRINITY_PNH1286	Microsor	Tetrabaena_socialis	50.50	0.00
TRINITY_P0C8M8.	RecName:	Zea_mays	50.50	0.00
TRINITY_XP_0145	zingipa	Vigna_radiata	50.50	0.00
TRINITY_GBF9091	hypothet	Raphidocelis_subcap	50.50	0.00
TRINITY_PNH1250(	hypothet	Tetrabaena_socialis	50.50	0.00
TRINITY_GBF9379	hypothet	Raphidocelis_subcap	50.50	0.00
TRINITY_PNW7973	hypothet	Chlamydomonas_reinh	50.50	0.00
TRINITY_GBG0010	hypothet	Raphidocelis_subcap	50.50	0.00
TRINITY_XP_0016	predicte	Chlamydomonas_reinh	50.50	0.00
TRINITY_GAX7456	hypothet	Chlamydomonas_eusti	50.50	0.00
TRINITY_GBF9529	hypothet	Raphidocelis_subcap	50.50	0.00
TRINITY_PNW8397	hypothet	Chlamydomonas_reinh	50.50	0.00
TRINITY_GAX7411	hypothet	Chlamydomonas_eusti	50.50	0.00
TRINITY_PNH1169	hypothet	Tetrabaena_socialis	50.50	0.00
TRINITY_PNW7788	hypothet	Chlamydomonas_reinh	50.50	0.00
TRINITY_GAX7758	hypothet	Chlamydomonas_eusti	50.50	0.00
TRINITY_KXZ5488	hypothet	Gonium_pectorale	50.50	0.00
TRINITY_PNW7889	hypothet	Chlamydomonas_reinh	50.50	0.00
TRINITY_XP_0184	PREDICT	Raphanus_sativus	50.50	0.00
TRINITY_GAX7517	hypothet	Chlamydomonas_eusti	50.50	0.00
TRINITY_KZV4271	mediato	Dorcoceras_hygromet	50.50	0.00
TRINITY_GAX7385	hypothet	Chlamydomonas_eusti	50.50	0.00
TRINITY_BAG1297	RHYTHM (	Chlamydomonas_reinh	50.50	0.00
TRINITY_GBG8251	hypothet	Chara_braunii	50.50	0.00
TRINITY_OAE2263	hypothet	Marchantia_polymorp	50.50	0.00
TRINITY_XP_0071	hypothet	Phaseolus_vulgaris	50.50	0.00
TRINITY_GAX8217	hypothet	Chlamydomonas_eusti	50.50	0.00
TRINITY_PRW5973	Acyl-coe	Chlorella_sorokinia	50.50	0.00
TRINITY_GAX7450	hypothet	Chlamydomonas_eusti	50.50	0.00
TRINITY_KXZ5559(	hypothet	Gonium_pectorale	50.50	0.00
TRINITY_XP_0139(	hypothet	Monoraphidium_negle	50.50	0.00
TRINITY_XP_0016	predicte	Chlamydomonas_reinh	50.50	0.00
TRINITY_XP_0063(	protein	Capsella_rubella	50.50	0.00
TRINITY_GBF9787	hypothet	Raphidocelis_subcap	50.50	0.00
TRINITY_KXZ4547	hypothet	Gonium_pectorale	50.50	0.00
TRINITY_GAX7487	hypothet	Chlamydomonas_eusti	50.50	0.00
TRINITY_GAX8554	hypothet	Chlamydomonas_eusti	50.50	0.00
TRINITY_GAX8220(	hypothet	Chlamydomonas_eusti	50.50	0.00
TRINITY_KXZ5199	hypothet	Gonium_pectorale	50.50	0.00
TRINITY_XP_0138	alpha-1,	Monoraphidium_negle	50.50	0.00
TRINITY_AMN1485	putative	Nepenthes_mirabilis	50.40	0.00
TRINITY_XP_0056	protein	Coccomyxa_subellips	50.40	0.00
TRINITY_XP_0239(	mitochor	Quercus_suber	50.40	0.00
TRINITY_GBF9918	DNA mism	Raphidocelis_subcap	50.40	0.00
TRINITY_XP_0128	PREDICT	Erythranthe_guttata	50.40	0.00
TRINITY_BAG1218	Parkin-(	Chlamydomonas_reinh	50.40	0.00
TRINITY_KDD7549	hypothet	Helicosporidium_sp.	50.40	0.00
TRINITY_XP_0106	PREDICT	Vitis_vinifera	50.40	0.00
TRINITY_XP_0237	zinc fir	Lactuca_sativa	50.40	0.00
TRINITY_GBF9934	hypothet	Raphidocelis_subcap	50.40	0.00
TRINITY_KXZ5668	hypothet	Gonium_pectorale	50.40	0.00
TRINITY_XP_0030	predicte	Micromonas_pusilla	50.40	0.00

TRINITY_XP_0245	O-acety	Selaginella_moellen	50.40	0.00
TRINITY_XP_0016	predict	Chlamydomonas_reinh	50.40	0.00
TRINITY_GAX8085	hypothet	Chlamydomonas_eusti	50.40	0.00
TRINITY_PNW8702	hypothet	Chlamydomonas_reinh	50.40	0.00
TRINITY_XP_0030	Protein-	Ostreococcus_tauri	50.40	0.00
TRINITY_XP_0139	Protein	Monoraphidium_negle	50.40	0.00
TRINITY_XP_0058	hypothet	Chlorella_variabili	50.40	0.00
TRINITY_KXZ4854	hypothet	Gonium_pectorale	50.40	0.00
TRINITY_GAX7667	hypothet	Chlamydomonas_eusti	50.40	0.00
TRINITY_KXZ4636	hypothet	Gonium_pectorale	50.40	0.00
TRINITY_XP_0029	hypothet	Volvox_carteri	50.40	0.00
TRINITY_XP_0029	hypothet	Volvox_carteri	50.40	0.00
TRINITY_XP_0068	endopla	Amborella_trichopod	50.40	0.00
TRINITY_GBF9029	hypothet	Raphidocelis_subcap	50.40	0.00
TRINITY_GAX7755	hypothet	Chlamydomonas_eusti	50.40	0.00
TRINITY_KXZ5603	hypothet	Gonium_pectorale	50.40	0.00
TRINITY_PNW7172	hypothet	Chlamydomonas_reinh	50.40	0.00
TRINITY_XP_0014	predict	Ostreococcus_sp._'l	50.40	0.00
TRINITY_GAX7403	hypothet	Chlamydomonas_eusti	50.40	0.00
TRINITY_GAX8500	hypothet	Chlamydomonas_eusti	50.40	0.00
TRINITY_PNW8186	hypothet	Chlamydomonas_reinh	50.40	0.00
TRINITY_PNW8801	hypothet	Chlamydomonas_reinh	50.40	0.00
TRINITY_XP_0029	hypothet	Volvox_carteri	50.40	0.00
TRINITY_KXZ5187	hypothet	Gonium_pectorale	50.40	0.00
TRINITY_XP_0058	hypothet	Chlorella_variabili	50.40	0.00
TRINITY_XP_0156	PREDICT	Oryza_brachyantha	50.40	0.00
TRINITY_GAX8138	hypothet	Chlamydomonas_eusti	50.40	0
TRINITY_GAX8501	hypothet	Chlamydomonas_eusti	50.40	0.00
TRINITY_GAX8321	hypothet	Chlamydomonas_eusti	50.40	0.00
TRINITY_AAV7115	PR46b [	Chlamydomonas_incer	50.40	0.00
TRINITY_XP_0029	hypothet	Volvox_carteri	50.40	0.00
TRINITY_GAX8374	hypothet	Chlamydomonas_eusti	50.40	0.00
TRINITY_XP_0029	hypothet	Volvox_carteri	50.40	0.00
TRINITY_KZV1512	hypothet	Dorcoceras_hygromet	50.40	0.00
TRINITY_GAX8061	hypothet	Chlamydomonas_eusti	50.40	0.00
TRINITY_GBF9349	hypothet	Raphidocelis_subcap	50.40	0.00
TRINITY_XP_0200	leucine-	Ananas_comosus	50.40	0
TRINITY_XP_0029	DEAD-bo	Selaginella_moellen	50.40	0.00
TRINITY_XP_0270	serine/	Coffea_arabica	50.40	0.00
TRINITY_BAK0277	predict	Hordeum_vulgare	50.40	0.00
TRINITY_GAX8561	hypothet	Chlamydomonas_eusti	50.40	0.00
TRINITY_XP_0239	cytosin	Quercus_suber	50.40	0.00
TRINITY_XP_0017	COP-II	Chlamydomonas_reinh	50.40	0.00
TRINITY_PIA6167	hypothet	Aquilegia_coerulea	50.30	0.00
TRINITY_EFJ3209	hypothet	Selaginella_moellen	50.30	0.00
TRINITY_EFJ3376	hypothet	Selaginella_moellen	50.30	0.00
TRINITY_GAQ8016	hypothet	Klebsormidium_niten	50.30	0.00
TRINITY_XP_0239	ras-lik	Quercus_suber	50.30	0.00
TRINITY_XP_0238	guanine	Quercus_suber	50.30	0.00
TRINITY_RWW9085	hypothet	Ensete_ventricosum	50.30	0.00
TRINITY_AAB8649	calmodu	Zea_mays	50.30	0.00
TRINITY_KXZ4481	hypothet	Gonium_pectorale	50.30	0.00
TRINITY_PNW8830	hypothet	Chlamydomonas_reinh	50.30	0.00
TRINITY_XP_0239	uncharac	Quercus_suber	50.30	0.00
TRINITY_XP_0029	hypothet	Volvox_carteri	50.30	0.00
TRINITY_XP_0217	U4/U6.U	Chenopodium_quinoa	50.30	0.00
TRINITY_OAE3509	hypothet	Marchantia_polymorp	50.30	0.00
TRINITY_GBF9459	metallo-	Raphidocelis_subcap	50.30	0.00
TRINITY_KFK3243	hypothet	Arabis_alpina	50.30	0.00
TRINITY_KXZ5103	hypothet	Gonium_pectorale	50.30	0.00
TRINITY_GBF9153	hypothet	Raphidocelis_subcap	50.30	0.00
TRINITY_GAX8048	hypothet	Chlamydomonas_eusti	50.30	0.00
TRINITY_GAX7550	hypothet	Chlamydomonas_eusti	50.30	0.00
TRINITY_XP_0029	hypothet	Volvox_carteri	50.30	0.00
TRINITY_PNW8866	hypothet	Chlamydomonas_reinh	50.30	0.00
TRINITY_BAK0317	predict	Hordeum_vulgare	50.30	0.00

TRINITY_PTQ35039	hypothet	Marchantia_polymorp	50.30	0.00
TRINITY_XP_00584	hypothet	Chlorella_variabili	50.30	0.00
TRINITY_XP_00565	nucleoti	Coccomyxa_subellips	50.30	0.00
TRINITY_XP_00169	predicte	Chlamydomonas_reinh	50.30	0.00
TRINITY_XP_00295	hypothet	Volvox_carteri	50.30	0.00
TRINITY_PNW82922	hypothet	Chlamydomonas_reinh	50.30	0.00
TRINITY_KXZ51382	hypothet	Gonium_pectorale	50.30	0.00
TRINITY_XP_00295	hypothet	Volvox_carteri	50.30	0.00
TRINITY_XP_02451	casein k	Selaginella_moellen	50.30	0.00
TRINITY_KMS65245	hypothet	Beta_vulgaris	50.30	0.00
TRINITY_PTQ35287	hypothet	Marchantia_polymorp	50.30	0.00
TRINITY_PLY76581	hypothet	Lactuca_sativa	50.30	0.00
TRINITY_GAX84455	hypothet	Chlamydomonas_eusti	50.30	0.00
TRINITY_PSC67553	hypothet	Micractinium_conduc	50.30	0.00
TRINITY_GAX85565	hypothet	Chlamydomonas_eusti	50.30	0.00
TRINITY_KXZ43500	hypothet	Gonium_pectorale	50.30	0.00
TRINITY_GAX82138	hypothet	Chlamydomonas_eusti	50.30	0.00
TRINITY_GAX82380	hypothet	Chlamydomonas_eusti	50.30	0.00
TRINITY_EPS69920	hypothet	Genlisea_aurea	50.20	0.00
TRINITY_XP_00564	pyruvate	Coccomyxa_subellips	50.20	0.00
TRINITY_XP_00350	COBW dor	Brachypodium_distac	50.20	0.00
TRINITY_GAQ88954	Putative	Klebsormidium_niten	50.20	0.00
TRINITY_GAX74335	hypothet	Chlamydomonas_eusti	50.20	0.00
TRINITY_GAX78602	hypothet	Chlamydomonas_eusti	50.20	0.00
TRINITY_GAX79708	hypothet	Chlamydomonas_eusti	50.20	0.00
TRINITY_PIA37640	hypothet	Aquilegia_coerulea	50.20	0.00
TRINITY_PNH07369	hypothet	Tetrabaena_socialis	50.20	0.00
TRINITY_GAQ86540	TCP-1/cp	Klebsormidium_niten	50.20	0.00
TRINITY_BAK02778	predicte	Hordeum_vulgare	50.20	0.00
TRINITY_BAJ98902	predicte	Hordeum_vulgare	50.20	0.00
TRINITY_XP_00294	hypothet	Volvox_carteri	50.20	0.00
TRINITY_XP_00169	predicte	Chlamydomonas_reinh	50.20	0.00
TRINITY_GAQ83887	CCR4-NOT	Klebsormidium_niten	50.20	0.00
TRINITY_XP_00250	protein	Micromonas_commoda	50.20	0.00
TRINITY_PNW80458	hypothet	Chlamydomonas_reinh	50.20	0.00
TRINITY_GAX83594	hypothet	Chlamydomonas_eusti	50.20	0.00
TRINITY_GAX73683	hypothet	Chlamydomonas_eusti	50.20	0.00
TRINITY_PNH12987	hypothet	Tetrabaena_socialis	50.20	0.00
TRINITY_KXZ49820	hypothet	Gonium_pectorale	50.20	0.00
TRINITY_PNW69788	hypothet	Chlamydomonas_reinh	50.20	0.00
TRINITY_XP_02058	serine/t	Phalaenopsis_equest	50.20	0.00
TRINITY_GAX84084	hypothet	Chlamydomonas_eusti	50.20	0.00
TRINITY_GAX79457	hypothet	Chlamydomonas_eusti	50.20	0.00
TRINITY_GAQ88070	adenylos	Klebsormidium_niten	50.20	0.00
TRINITY_XP_00295	hexokina	Volvox_carteri	50.20	0.00
TRINITY_GBF91987	hypothet	Raphidocelis_subcap	50.20	0.00
TRINITY_GAX84618	hypothet	Chlamydomonas_eusti	50.20	0.00
TRINITY_GBF88042	histone	Raphidocelis_subcap	50.20	0.00
TRINITY_XP_00169	aldose-1	Chlamydomonas_reinh	50.20	0.00
TRINITY_XP_02390	delta-1-	Quercus_suber	50.20	0.00
TRINITY_XP_00169	predicte	Chlamydomonas_reinh	50.20	0.00
TRINITY_XP_02390	protein	Quercus_suber	50.20	0.00
TRINITY_KXZ50888	hypothet	Gonium_pectorale	50.20	0.00
TRINITY_KXZ50183	NTR1 pro	Gonium_pectorale	50.20	0.00
TRINITY_GBF91240	hypothet	Raphidocelis_subcap	50.20	0.00
TRINITY_PNW88100	hypothet	Chlamydomonas_reinh	50.20	0.00
TRINITY_GAX76895	hypothet	Chlamydomonas_eusti	50.20	0.00
TRINITY_GAX84165	hypothet	Chlamydomonas_eusti	50.20	0
TRINITY_GAX74020	hypothet	Chlamydomonas_eusti	50.20	0.00
TRINITY_GAX81660	hypothet	Chlamydomonas_eusti	50.20	0.00
TRINITY_PNW76018	hypothet	Chlamydomonas_reinh	50.20	0.00
TRINITY_GAX82325	hypothet	Chlamydomonas_eusti	50.20	0.00
TRINITY_GAX73064	hypothet	Chlamydomonas_eusti	50.20	0.00
TRINITY_XP_00357	isocitra	Brachypodium_distac	50.10	0.00
TRINITY_XP_02390	uroporph	Quercus_suber	50.10	0.00
TRINITY_XP_02389	uncharac	Quercus_suber	50.10	0.00

TRINITY_XP_0194	PREDICTI	Lupinus_angustifoli	50.10	0.00
TRINITY_KXZ4839	hypothet	Gonium_pectorale	50.10	0.00
TRINITY_GAX8426	hypothet	Chlamydomonas_eusti	50.10	0.00
TRINITY_KXZ4447	hypothet	Gonium_pectorale	50.10	0.00
TRINITY_PNW7610	hypothet	Chlamydomonas_reinh	50.10	0.00
TRINITY_XP_0016	subunit	Chlamydomonas_reinh	50.10	0.00
TRINITY_GAX7731	hypothet	Chlamydomonas_eusti	50.10	0.00
TRINITY_PNW7420	hypothet	Chlamydomonas_reinh	50.10	0.00
TRINITY_XP_0238	malate (	Quercus_suber	50.00	0.00
TRINITY_XP_0238	cytochr	Quercus_suber	50.00	0.00
TRINITY_GAQ7922	hypothet	Klebsormidium_niten	50.00	0.00
TRINITY_KDD7659	ABC tra	Helicosporidium_sp.	50.00	0.00
TRINITY_XP_0017	predicte	Chlamydomonas_reinh	50.00	0.00
TRINITY_XP_0165	PREDICTI	Capsicum_annuum	50.00	0.00
TRINITY_GAX7770	hypothet	Chlamydomonas_eusti	50.00	0.00
TRINITY_XP_0125	ABC tra	Cicer_arietinum	50.00	0.00
TRINITY_EMS5875	Dihydrop	Triticum_urartu	50.00	0.00
TRINITY_XP_0172	PREDICTI	Daucus_carota	50.00	0.00
TRINITY_OAE2668	hypothet	Marchantia_polymorp	50.00	0.00
TRINITY_KZM9572	hypothet	Daucus_carota	50.00	0.00
TRINITY_EEC6955	hypothet	Oryza_sativa	50.00	0.00
TRINITY_RMZ5364	hypothet	Auxenochlorella_pro	50.00	0.00
TRINITY_BAK0550	predicte	Hordeum_vulgare	50.00	0.00
TRINITY_XP_0056	flagella	Coccomyxa_subellips	50.00	0.00
TRINITY_GAX7451	hypothet	Chlamydomonas_eusti	50.00	0.00
TRINITY_GAU4795	hypothet	Trifolium_subterran	50.00	0.00
TRINITY_XP_0075	ATP-depe	Bathycoccus_prasino	50.00	0.00
TRINITY_XP_0243	probable	Physcomitrella_pate	50.00	0.00
TRINITY_XP_0106	PREDICTI	Beta_vulgaris	50.00	0.00
TRINITY_OEL1295	Tubulin	Dichanthelium_oligo	50.00	0.00
TRINITY_PNW7013	hypothet	Chlamydomonas_reinh	50.00	0.00
TRINITY_P51824.	RecName:	Solanum_tuberosum	50.00	0.00
TRINITY_XP_0190	PREDICTI	Nelumbo_nucifera	50.00	0.00
TRINITY_ABF0007	chloropl	Capsicum_annuum	50.00	0.00
TRINITY_XP_0156	PREDICTI	Oryza_brachyantha	50.00	0.00
TRINITY_XP_0093	PREDICTI	Pyrus_x_bretschneid	50.00	0.00
TRINITY_PSC7428	DNA-dire	Micractinium_conduc	50.00	0.00
TRINITY_XP_0075	predicte	Bathycoccus_prasino	50.00	0.00
TRINITY_BAJ9317	predicte	Hordeum_vulgare	50.00	0.00
TRINITY_XP_0162	protein	Arachis_ipaensis	50.00	0.00
TRINITY_GBF9672	hypothet	Raphidocelis_subcap	50.00	0.00
TRINITY_XP_0116	PREDICTI	Cucumis_sativus	50.00	0.00
TRINITY_XP_0093	PREDICTI	Pyrus_x_bretschneid	50.00	0.00
TRINITY_GBG8644	hypothet	Chara_braunii	50.00	0.00
TRINITY_AIA8853	FGE-sul	uncultured_Ostreocc	50.00	0.00
TRINITY_GAX7573	hypothet	Chlamydomonas_eusti	50.00	0.00
TRINITY_OWM8259	hypothet	Punica_granatum	50.00	0.00
TRINITY_XP_0029	uncharac	Selaginella_moellen	50.00	0.00
TRINITY_XP_0093	PREDICTI	Pyrus_x_bretschneid	50.00	0.00
TRINITY_BAJ9884	predicte	Hordeum_vulgare	50.00	0.00
TRINITY_XP_0035	2-oxoiso	Glycine_max	50.00	0.00
TRINITY_XP_0016	predicte	Chlamydomonas_reinh	50.00	0.00
TRINITY_CDP1137	unnamed	Coffea_canephora	50.00	0.00
TRINITY_XP_0243	mitochon	Physcomitrella_pate	50.00	0.00
TRINITY_XP_0279	U1 small	Vigna_unguiculata	50.00	0.00
TRINITY_KXZ4929	hypothet	Gonium_pectorale	50.00	0.00
TRINITY_XP_0064	aminomet	Citrus_clementina	50.00	0.00
TRINITY_GBF9502	hypothet	Raphidocelis_subcap	50.00	0.00
TRINITY_XP_0239	probable	Quercus_suber	50.00	0.00
TRINITY_XP_0201	serine/t	Ananas_comosus	50.00	0.00
TRINITY_GAX8203	hypothet	Chlamydomonas_eusti	50.00	0.00
TRINITY_GBF9085	hypothet	Raphidocelis_subcap	50.00	0.00
TRINITY_GAQ8372	vesicle-	Klebsormidium_niten	50.00	0.00
TRINITY_GBG7996	hypothet	Chara_braunii	50.00	0.00
TRINITY_ACN3155	unknown	Zea_mays	50.00	0.00
TRINITY_XP_0228	Tetratri	Ostreococcus_tauri	50.00	0.00

TRINITY_GAX79377	hypothet	Chlamydomonas_eusti	50.00	0.00
TRINITY_PLY73027	hypothet	Lactuca_sativa	50.00	0.00
TRINITY_XP_01389	Phosphat	Monoraphidium_negle	50.00	0.00
TRINITY_GAX75760	hypothet	Chlamydomonas_eusti	50.00	0.00
TRINITY_XP_00305	predicte	Micromonas_pusilla	50.00	0.00
TRINITY_XP_00169	predicte	Chlamydomonas_reinh	50.00	0.00
TRINITY_XP_00295	hypothet	Volvox_carteri	50.00	0.00
TRINITY_XP_00306	flagella	Micromonas_pusilla	50.00	0.00
TRINITY_XP_02022	probable	Cajanus_cajan	50.00	0.00
TRINITY_KXZ53116	hypothet	Gonium_pectorale	50.00	0.00
TRINITY_PNW76637	hypothet	Chlamydomonas_reinh	50.00	0.00
TRINITY_ABR16144	unknown	Picea_sitchensis	50.00	0.00
TRINITY_BAK00435	predicte	Hordeum_vulgare	50.00	0.00
TRINITY_PSC76533	NAD-depe	Micractinium_conduc	50.00	0.00
TRINITY_GAQ84468	histidin	Klebsormidium_niten	50.00	0.00
TRINITY_XP_02389	histone-	Quercus_suber	50.00	0.00
TRINITY_XP_00169	predicte	Chlamydomonas_reinh	50.00	0.00
TRINITY_GAQ85748	Sof1-lik	Klebsormidium_niten	50.00	0.00
TRINITY_GAX78453	hypothet	Chlamydomonas_eusti	50.00	0.00
TRINITY_XP_00700	PREDICTE	Theobroma_cacao	50.00	0.00
TRINITY_XP_02389	eukaryot	Quercus_suber	50.00	0.00
TRINITY_GAX75303	hypothet	Chlamydomonas_eusti	50.00	0.00
TRINITY_XP_00564	isocitra	Coccomyxa_subellips	50.00	0.00
TRINITY_KXZ52415	hypothet	Gonium_pectorale	50.00	0.00
TRINITY_XP_01389	Peroxiso	Monoraphidium_negle	50.00	0.00
TRINITY_GAQ92802	hypothet	Klebsormidium_niten	50.00	0.00
TRINITY_XP_00584	hypothet	Chlorella_variabili	50.00	0.00
TRINITY_XP_02199	serine/t	Helianthus_annuus	50.00	0.00
TRINITY_XP_01390	abc tran	Monoraphidium_negle	50.00	0.00
TRINITY_GAX75087	hypothet	Chlamydomonas_eusti	50.00	0.00
TRINITY_PNH07672	hypothet	Tetrabaena_socialis	50.00	0.00
TRINITY_KXZ51363	hypothet	Gonium_pectorale	50.00	0.00
TRINITY_PNW80243	hypothet	Chlamydomonas_reinh	50.00	0.00
TRINITY_XP_02016	prohibit	Aegilops_tauschii	50.00	0.00
TRINITY_GAQ80175	hypothet	Klebsormidium_niten	50.00	0.00
TRINITY_VDD44417	unnamed	Brassica_oleracea	50.00	0.00
TRINITY_GAX78192	hypothet	Chlamydomonas_eusti	50.00	0.00
TRINITY_EFJ07398	hypothet	Selaginella_moellen	50.00	0.00
TRINITY_XP_00295	hypothet	Volvox_carteri	50.00	0.00
TRINITY_GAX73042	hypothet	Chlamydomonas_eusti	50.00	0.00
TRINITY_XP_02162	ABC tran	Manihot_esculenta	50.00	0.00
TRINITY_GAX82869	hypothet	Chlamydomonas_eusti	50.00	0.00
TRINITY_PNH03055	Spindle	Tetrabaena_socialis	50.00	0.00
TRINITY_KXZ46446	hypothet	Gonium_pectorale	50.00	0.00
TRINITY_XP_01389	glutathi	Monoraphidium_negle	50.00	0.00
TRINITY_KZV51417	hypothet	Dorcoceras_hygromet	50.00	0.00
TRINITY_KDD75357	hypothet	Helicosporidium_sp.	50.00	0.00
TRINITY_XP_00295	hypothet	Volvox_carteri	50.00	0.00
TRINITY_KXZ46159	DHC6 pro	Gonium_pectorale	50.00	0.00
TRINITY_XP_00244	serine/t	Sorghum_bicolor	50.00	0.00
TRINITY_GAX83075	hypothet	Chlamydomonas_eusti	50.00	0.00
TRINITY_XP_00294	hypothet	Volvox_carteri	50.00	0.00
TRINITY_KXZ44733	hypothet	Gonium_pectorale	50.00	0.00
TRINITY_KXZ44336	hypothet	Gonium_pectorale	50.00	0.00
TRINITY_KXZ46433	hypothet	Gonium_pectorale	50.00	0.00
TRINITY_GAX80288	hypothet	Chlamydomonas_eusti	50.00	0.00
TRINITY_KXZ52827	hypothet	Gonium_pectorale	50.00	0.00
TRINITY_KMZ59834	putative	Zostera_marina	50.00	0.00
TRINITY_XP_02453	dynein h	Selaginella_moellen	50.00	0.00
TRINITY_GAX72554	hypothet	Chlamydomonas_eusti	50.00	0.00
TRINITY_XP_00295	RWP-RK d	Volvox_carteri	50.00	0.00
TRINITY_KXZ47561	hypothet	Gonium_pectorale	50.00	0.00
TRINITY_XP_00295	SET and	Volvox_carteri	50.00	0.00
TRINITY_XP_01390	hypothet	Monoraphidium_negle	50.00	0.00
TRINITY_ANA57061	cytochro	Pyramimonas_parkeae	50.00	0.00
TRINITY_PRW55925	translat	Chlorella_sorokinia	50.00	0.00

TRINITY_XP_00295	hypothet	Volvox_carteri	50.00	0.00
TRINITY_XP_00751	predicte	Bathycoccus_prasino	50.00	0.00
TRINITY_PKA63295	Histidin	Apostasia_shenzheni	50.00	0.00
TRINITY_AHF81484	small GT	Mangifera_indica	50.00	0.00
TRINITY_OVA19138	Tetratri	Macleaya_cordata	50.00	0.00
TRINITY_PRW45536	light-ha	Chlorella_sorokinia	50.00	0.00
TRINITY_GAX74857	hypothet	Chlamydomonas_eusti	50.00	0.00
TRINITY_PNH06745	putative	Tetrabaena_socialis	50.00	0.00
TRINITY_XP_01389	Golgi SN	Monoraphidium_negle	50.00	0.00
TRINITY_PNW83135	hypothet	Chlamydomonas_reinh	50.00	0.00
TRINITY_XP_01915	PREDICTE	Ipomoea_nil	50.00	0.00
TRINITY_OAE35245	hypothet	Marchantia_polymorp	50.00	0.00
TRINITY_PNW85663	hypothet	Chlamydomonas_reinh	50.00	0.00
TRINITY_XP_00169	predicte	Chlamydomonas_reinh	50.00	0.00
TRINITY_PNH03432	hypothet	Tetrabaena_socialis	50.00	0.00
TRINITY_XP_01389	Peroxiso	Monoraphidium_negle	50.00	0.00
TRINITY_PNW73331	hypothet	Chlamydomonas_reinh	50.00	0.00
TRINITY_GAX83237	hypothet	Chlamydomonas_eusti	50.00	0.00
TRINITY_GBF88218	CTD smal	Raphidocelis_subcap	50.00	0.00
TRINITY_GBG89007	hypothet	Chara_braunii	50.00	0.00
TRINITY_GAX72678	hypothet	Chlamydomonas_eusti	50.00	0.00
TRINITY_XP_02439	huntingt	Physcomitrella_pate	50.00	0.00
TRINITY_KFK30433	hypothet	Arabis_alpina	50.00	0.00
TRINITY_RID53662	hypothet	Brassica_rapa	50.00	0.00
TRINITY_GAX84581	hypothet	Chlamydomonas_eusti	50.00	0.00
TRINITY_XP_00168	prenylat	Chlamydomonas_reinh	50.00	0.00
TRINITY_XP_01617	tRNA pse	Arachis_ipaensis	50.00	0.00
TRINITY_GAX73059	hypothet	Chlamydomonas_eusti	50.00	0.00
TRINITY_XP_01884	PREDICTE	Juglans_regia	50.00	0.00
TRINITY_GAX73558	hypothet	Chlamydomonas_eusti	50.00	0.00
TRINITY_XP_02354	30S ribo	Cucurbita_pepo	50.00	0.00
TRINITY_GAX81717	hypothet	Chlamydomonas_eusti	50.00	0.00
TRINITY_XP_00250	H+-or Na	Micromonas_commoda	50.00	0.00
TRINITY_XP_02437	DNA-dire	Physcomitrella_pate	50.00	0.00
TRINITY_PKA66764	putative	Apostasia_shenzheni	50.00	0.00
TRINITY_GAX76291	hypothet	Chlamydomonas_eusti	50.00	0.00
TRINITY_GBF93626	hypothet	Raphidocelis_subcap	50.00	0.00
TRINITY_KXZ54967	hypothet	Gonium_pectorale	50.00	0.00
TRINITY_KXZ55858	hypothet	Gonium_pectorale	50.00	0.00
TRINITY_KFK36178	hypothet	Arabis_alpina	50.00	0.00
TRINITY_XP_00294	hypothet	Volvox_carteri	50.00	0.00
TRINITY_GBG00283	hypothet	Raphidocelis_subcap	50.00	0.00
TRINITY_XP_01915	PREDICTE	Ipomoea_nil	50.00	0.00
TRINITY_XP_01389	enolase-	Monoraphidium_negle	50.00	0.00
TRINITY_GAX83104	hypothet	Chlamydomonas_eusti	50.00	0.00
TRINITY_KXZ49577	hypothet	Gonium_pectorale	50.00	0.00
TRINITY_XP_00245	isocitra	Sorghum_bicolor	50.00	0.00
TRINITY_KXZ50765	hypothet	Gonium_pectorale	50.00	0.00
TRINITY_ONK60784	uncharac	Asparagus_officinal	50.00	0.00
TRINITY_PNW69850	hypothet	Chlamydomonas_reinh	50.00	0.00
TRINITY_RWR73664	chaperon	Cinnamomum_micranth	50.00	0.00
TRINITY_XP_02404	histone-	Citrus_clementina	50.00	0.00
TRINITY_KXZ57020	MYO5 pro	Gonium_pectorale	50.00	0.00
TRINITY_GAX78929	hypothet	Chlamydomonas_eusti	50.00	0.00
TRINITY_GBF99171	hypothet	Raphidocelis_subcap	50.00	0.00
TRINITY_GAX74060	hypothet	Chlamydomonas_eusti	50.00	0.00
TRINITY_GBF97659	carbonic	Raphidocelis_subcap	50.00	0.00
TRINITY_GAX82609	hypothet	Chlamydomonas_eusti	50.00	0.00
TRINITY_XP_02390	aldo-ket	Quercus_suber	50.00	0.00
TRINITY_XP_00169	predicte	Chlamydomonas_reinh	50.00	0.00
TRINITY_GAX75503	hypothet	Chlamydomonas_eusti	50.00	0.00
TRINITY_XP_00565	hypothet	Coccomyxa_subellips	50.00	0.00
TRINITY_XP_00169	predicte	Chlamydomonas_reinh	50.00	0.00
TRINITY_XP_02440	galactol	Physcomitrella_pate	50.00	0.00
TRINITY_PNH08460	dTDP-3-a	Tetrabaena_socialis	50.00	0.00
TRINITY_PNW86160	hypothet	Chlamydomonas_reinh	50.00	0.00

TRINITY_GAX7353	hypothet	Chlamydomonas_eusti	50.00	0.00
TRINITY_GAX7287	hypothet	Chlamydomonas_eusti	50.00	0.00
TRINITY_PNH1057	Retrovi	Tetrabaena_socialis	50.00	0.00
TRINITY_XP_0238	uncharac	Quercus_suber	50.00	0.00
TRINITY_GAX7605	hypothet	Chlamydomonas_eusti	50.00	0.00
TRINITY_GAX7389	hypothet	Chlamydomonas_eusti	50.00	0.00
TRINITY_PRW5095	DNA cros	Chlorella_sorokinia	50.00	0.00
TRINITY_GAQ8270	Dyneins	Klebsormidium_niten	50.00	0.00
TRINITY_BAK0218	predicte	Hordeum_vulgare	50.00	0.00
TRINITY_XP_0139	Dynein	Monoraphidium_negle	50.00	0.00

TRINITY_XP_0191(	PREDICTI	Ipomoea_nil	50.00	0.00
TRINITY_KXZ4963]	hypothet	Gonium_pectorale	50.00	0.00
TRINITY_XP_0239(	ras-like	Quercus_suber	50.00	0.00
TRINITY_XP_0102]	lysine-s	Brachypodium_distac	50.00	0.00
TRINITY_GAX8202(	hypothet	Chlamydomonas_eusti	50.00	0.00
TRINITY_XP_0128!	PREDICTI	Erythranthe_guttata	50.00	0.00
TRINITY_GAX7631!	hypothet	Chlamydomonas_eusti	50.00	0.00
TRINITY_XP_0029!	hypothet	Volvox_carteri	50.00	0.00
TRINITY_XP_0064'	sm-like	Citrus_sinensis	50.00	0.00
TRINITY_XP_0029(	hypothet	Volvox_carteri	50.00	0.00
TRINITY_XP_0093(	PREDICTI	Pyrus_x_bretschneid	50.00	0.00
TRINITY_XP_0016!	predicte	Chlamydomonas_reinh	50.00	0.00
TRINITY_XP_0016!	predicte	Chlamydomonas_reinh	50.00	0.00
TRINITY_KXZ4219(	hypothet	Gonium_pectorale	50.00	0.00
TRINITY_GAX8484!	hypothet	Chlamydomonas_eusti	50.00	0.00
TRINITY_GBF9260(	SAL1 ph(	Raphidocelis_subcap	50.00	0.00
TRINITY_XP_0113!	DNA pol	Auxenochlorella_pro	50.00	0.00
TRINITY_GAX8592!	hypothet	Chlamydomonas_eusti	50.00	0.00
TRINITY_GAQ9305]	hypothet	Klebsormidium_niten	50.00	0.00
TRINITY_PNH0629!	Alpha-ke	Tetrabaena_socialis	50.00	0.00
TRINITY_XP_0017(	DNA meth	Chlamydomonas_reinh	50.00	0.00
TRINITY_PNW7902(	hypothet	Chlamydomonas_reinh	50.00	0.00
TRINITY_XP_0016!	protein	Chlamydomonas_reinh	50.00	0.00
TRINITY_XP_0029!	hypothet	Volvox_carteri	50.00	0.00
TRINITY_XP_0243!	omega-ar	Physcomitrella_pate	50.00	0.00
TRINITY_PNW7759!	hypothet	Chlamydomonas_reinh	50.00	0.00
TRINITY_GAX8530]	hypothet	Chlamydomonas_eusti	50.00	0.00
TRINITY_OAE3474'	hypothet	Marchantia_polymorp	50.00	0.00
TRINITY_PTQ3233]	hypothet	Marchantia_polymorp	50.00	0.00
TRINITY_XP_0110'	probable	Sesamum_indicum	50.00	0.00
TRINITY_GAQ8185!	DNA-dire	Klebsormidium_niten	49.90	0.00
TRINITY_XP_0238!	glutathi	Quercus_suber	49.90	0.00
TRINITY_ONI2156]	hypothet	Prunus_persica	49.90	0.00
TRINITY_BAJ8696!	predicte	Hordeum_vulgare	49.90	0.00
TRINITY_XP_0025(	predicte	Micromonas_commoda	49.90	0.00
TRINITY_XP_0029(	hypothet	Volvox_carteri	49.90	0.00
TRINITY_KXZ4855(	hypothet	Gonium_pectorale	49.90	0.00
TRINITY_KXZ5589!	hypothet	Gonium_pectorale	49.90	0.00
TRINITY_XP_0029!	26S prot	Selaginella_moellen	49.90	0.00
TRINITY_XP_0029!	Kif19 t	Volvox_carteri	49.90	0.00
TRINITY_GAX8443(	hypothet	Chlamydomonas_eusti	49.90	0.00
TRINITY_KXZ5000(	hypothet	Gonium_pectorale	49.90	0.00
TRINITY_PSC7248'	Nucleola	Micractinium_conduc	49.90	0.00
TRINITY_KXZ5687(	hypothet	Gonium_pectorale	49.90	0.00
TRINITY_PTQ4576!	hypothet	Marchantia_polymorp	49.90	0.00
TRINITY_GAX7921(	hypothet	Chlamydomonas_eusti	49.90	0.00
TRINITY_PSC7326(	L-arabir	Micractinium_conduc	49.90	0.00
TRINITY_PNW8838(	hypothet	Chlamydomonas_reinh	49.90	0.00
TRINITY_GAX7578(	hypothet	Chlamydomonas_eusti	49.90	0.00
TRINITY_XP_0017(	predicte	Chlamydomonas_reinh	49.90	0.00
TRINITY_GAX7361!	hypothet	Chlamydomonas_eusti	49.90	0.00
TRINITY_PNW8499!	hypothet	Chlamydomonas_reinh	49.90	0.00
TRINITY_XP_0014]	predicte	Ostreococcus_sp._'l	49.90	0.00
TRINITY_GBF8757(	NAD-depe	Raphidocelis_subcap	49.90	0.00
TRINITY_GAX7423!	hypothet	Chlamydomonas_eusti	49.90	0.00
TRINITY_XP_0029!	hypothet	Volvox_carteri	49.90	0.00
TRINITY_OAE2502(	hypothet	Marchantia_polymorp	49.90	0.00
TRINITY_GAQ8447(	NAD(P)-]	Klebsormidium_niten	49.80	0.00
TRINITY_XP_0041(	PREDICTI	Cucumis_sativus	49.80	0.00
TRINITY_XP_0236]	TP53-reg	Capsella_rubella	49.80	0.00
TRINITY_XP_0124!	PREDICTI	Gossypium_raimondii	49.80	0.00
TRINITY_AXF4155!	HLA3 pro	Chlorella_sp._ArM00	49.80	0.00
TRINITY_XP_0217]	gamma ca	Chenopodium_quinoa	49.80	0.00
TRINITY_PNH0733!	Mitochor	Tetrabaena_socialis	49.80	0.00
TRINITY_XP_0138!	hypothet	Monoraphidium_negle	49.80	0.00
TRINITY_PNW7348(	hypothet	Chlamydomonas_reinh	49.80	0.00

TRINITY_XP_02439	protein	Physcomitrella_pate	49.80	0.00
TRINITY_XP_01723	PREDICTE	Daucus_carota	49.80	0.00
TRINITY_GAX75134	hypothet	Chlamydomonas_eusti	49.80	0.00
TRINITY_XP_00294	eukaryot	Volvox_carteri	49.80	0.00
TRINITY_AID51435	3-hydrox	Astragalus_membrana	49.80	0.00
TRINITY_XP_00250	protein	Micromonas_commoda	49.80	0.00
TRINITY_XP_00295	hypothet	Volvox_carteri	49.80	0.00
TRINITY_GAX75134	hypothet	Chlamydomonas_eusti	49.80	0.00
TRINITY_XP_00295	hypothet	Volvox_carteri	49.80	0.00
TRINITY_GAX82412	hypothet	Chlamydomonas_eusti	49.80	0.00
TRINITY_PNW80578	hypothet	Chlamydomonas_reinh	49.80	0.00
TRINITY_GAX84826	hypothet	Chlamydomonas_eusti	49.80	0.00
TRINITY_PSC73715	caroteno	Micractinium_conduc	49.80	0.00
TRINITY_XP_00295	hypothet	Volvox_carteri	49.80	0.00
TRINITY_GAX73935	hypothet	Chlamydomonas_eusti	49.80	0.00
TRINITY_XP_00295	hypothet	Volvox_carteri	49.80	0.00
TRINITY_PNW73259	hypothet	Chlamydomonas_reinh	49.80	0.00
TRINITY_GAX76119	hypothet	Chlamydomonas_eusti	49.80	0.00
TRINITY_EPS71732	hypothet	Genlisea_aurea	49.80	0.00
TRINITY_XP_01009	senescen	Morus_notabilis	49.80	0.00
TRINITY_XP_00294	hypothet	Volvox_carteri	49.80	0.00
TRINITY_PNW85268	hypothet	Chlamydomonas_reinh	49.80	0.00
TRINITY_BAK00435	predicte	Hordeum_vulgare	49.80	0.00
TRINITY_GAX80085	hypothet	Chlamydomonas_eusti	49.80	0.00
TRINITY_GAX79451	hypothet	Chlamydomonas_eusti	49.80	0.00
TRINITY_GAX81666	hypothet	Chlamydomonas_eusti	49.80	0.00
TRINITY_OMO82479	Calcineu	Corchorus_olitorius	49.70	0.00
TRINITY_XP_02439	coatomer	Physcomitrella_pate	49.70	0.00
TRINITY_EFJ33766	hypothet	Selaginella_moellen	49.70	0.00
TRINITY_GBG62941	hypothet	Chara_braunii	49.70	0.00
TRINITY_GBG66199	hypothet	Chara_braunii	49.70	0.00
TRINITY_BAK03491	predicte	Hordeum_vulgare	49.70	0.00
TRINITY_XP_02389	eukaryot	Quercus_suber	49.70	0.00
TRINITY_PNW85746	hypothet	Chlamydomonas_reinh	49.70	0.00
TRINITY_XP_02390	mitochon	Quercus_suber	49.70	0.00
TRINITY_KXZ46407	hypothet	Gonium_pectorale	49.70	0.00
TRINITY_RLM78911	putative	Panicum_miliaceum	49.70	0.00
TRINITY_GAQ82709	Dyneins	Klebsormidium_niten	49.70	0.00
TRINITY_XP_00564	hypothet	Coccomyxa_subellips	49.70	0.00
TRINITY_XP_01092	PREDICTE	Elaeis_guineensis	49.70	0.00
TRINITY_PNW74264	hypothet	Chlamydomonas_reinh	49.70	0.00
TRINITY_ABR18228	unknown	Picea_sitchensis	49.70	0.00
TRINITY_BAV58347	GTP-bind	Ulva_partita	49.70	0.00
TRINITY_RMZ52073	hypothet	Auxenochlorella_pro	49.70	0.00
TRINITY_PNW69996	hypothet	Chlamydomonas_reinh	49.70	0.00
TRINITY_XP_00565	HAD-like	Coccomyxa_subellips	49.70	0.00
TRINITY_PNW86249	hypothet	Chlamydomonas_reinh	49.70	0.00
TRINITY_XP_00295	hypothet	Volvox_carteri	49.70	0.00
TRINITY_XP_00824	PREDICTE	Prunus_mume	49.70	0.00
TRINITY_XP_00720	eukaryot	Prunus_persica	49.70	0.00
TRINITY_XP_00169	predicte	Chlamydomonas_reinh	49.70	0.00
TRINITY_KXZ56834	hypothet	Gonium_pectorale	49.70	0.00
TRINITY_XP_00359	DNA topo	Medicago_truncatula	49.70	0.00
TRINITY_XP_01107	signal p	Sesamum_indicum	49.70	0.00
TRINITY_PRW59581	MYST-lik	Chlorella_sorokinia	49.70	0.00
TRINITY_GAX74416	hypothet	Chlamydomonas_eusti	49.70	0.00
TRINITY_GAX85636	hypothet	Chlamydomonas_eusti	49.70	0.00
TRINITY_XP_00584	hypothet	Chlorella_variabili	49.70	0.00
TRINITY_GAX72794	hypothet	Chlamydomonas_eusti	49.70	0.00
TRINITY_KXZ52829	hypothet	Gonium_pectorale	49.70	0.00
TRINITY_XP_00169	guanylat	Chlamydomonas_reinh	49.70	0.00
TRINITY_KXZ52725	hypothet	Gonium_pectorale	49.70	0.00
TRINITY_ACG27162	charged	Zea_mays	49.70	0.00
TRINITY_GAX74993	hypothet	Chlamydomonas_eusti	49.70	0.00
TRINITY_BAJ93905	predicte	Hordeum_vulgare	49.70	0.00
TRINITY_XP_00169	predicte	Chlamydomonas_reinh	49.70	0.00

TRINITY_XP_02392	uncharac	Quercus_suber	49.70	0.00
TRINITY_XP_02067	probable	Dendrobium_catenatu	49.70	0.00
TRINITY_GAX84726	hypothet	Chlamydomonas_eusti	49.70	0.00
TRINITY_XP_02392	uncharac	Quercus_suber	49.70	0.00
TRINITY_GAX81106	hypothet	Chlamydomonas_eusti	49.70	0.00
TRINITY_XP_00168	predicte	Chlamydomonas_reinh	49.70	0.00
TRINITY_GAQ82849	Adaptor	Klebsormidium_niten	49.70	0.00
TRINITY_XP_00941	PREDICTE	Musa_acuminata	49.70	0.00
TRINITY_KXZ43333	hypothet	Gonium_pectorale	49.70	0.00
TRINITY_XP_01847	PREDICTE	Raphanus_sativus	49.70	0.00
TRINITY_XP_00295	hypothet	Volvox_carteri	49.70	0.00
TRINITY_GAX75027	hypothet	Chlamydomonas_eusti	49.70	0.00
TRINITY_XP_01390	hypothet	Monoraphidium_negle	49.70	0.00
TRINITY_GAX83458	hypothet	Chlamydomonas_eusti	49.70	0.00
TRINITY_XP_01640	PREDICTE	Nicotiana_tabacum	49.60	0.00
TRINITY_XP_00498	telomera	Setaria_italica	49.60	0.00
TRINITY_GAQ86969	eukaryot	Klebsormidium_niten	49.60	0.00
TRINITY_PWA39469	ribosoma	Artemisia_annua	49.60	0.00
TRINITY_XP_00362	prohibit	Medicago_truncatula	49.60	0.00
TRINITY_GAQ83213	Aminotra	Klebsormidium_niten	49.60	0.00
TRINITY_EEF27536	hydroxya	Ricinus_communis	49.60	0.00
TRINITY_PNW84466	hypothet	Chlamydomonas_reinh	49.60	0.00
TRINITY_GBF99812	hypothet	Raphidocelis_subcap	49.60	0.00
TRINITY_XP_00295	hypothet	Volvox_carteri	49.60	0.00
TRINITY_XP_00295	hypothet	Volvox_carteri	49.60	0.00
TRINITY_GAQ88088	Heme A s	Klebsormidium_niten	49.60	0.00
TRINITY_GAX78802	hypothet	Chlamydomonas_eusti	49.60	0.00
TRINITY_KXZ51995	hypothet	Gonium_pectorale	49.60	0.00
TRINITY_PNR35353	hypothet	Physcomitrella_pate	49.60	0.00
TRINITY_GAX79803	hypothet	Chlamydomonas_eusti	49.60	0.00
TRINITY_GAX78698	hypothet	Chlamydomonas_eusti	49.60	0.00
TRINITY_CAA49283	gag,prot	Volvox_carteri	49.60	0.00
TRINITY_GAX79409	hypothet	Chlamydomonas_eusti	49.60	0.00
TRINITY_GAX74229	hypothet	Chlamydomonas_eusti	49.60	0.00
TRINITY_XP_00584	hypothet	Chlorella_variabili	49.60	0.00
TRINITY_XP_02430	probable	Physcomitrella_pate	49.60	0.00
TRINITY_XP_02435	chromodo	Physcomitrella_pate	49.60	0.00
TRINITY_ESQ35112	hypothet	Eutrema_salsugineum	49.60	0.00
TRINITY_GAX79289	hypothet	Chlamydomonas_eusti	49.60	0.00
TRINITY_GAX77392	hypothet	Chlamydomonas_eusti	49.60	0.00
TRINITY_GAX85222	hypothet	Chlamydomonas_eusti	49.60	0.00
TRINITY_PNH10090	Ran-bind	Tetrabaena_socialis	49.60	0.00
TRINITY_GAX73644	hypothet	Chlamydomonas_eusti	49.60	0.00
TRINITY_GBG74762	hypothet	Chara_braunii	49.60	0.00
TRINITY_GAX84397	hypothet	Chlamydomonas_eusti	49.60	0.00
TRINITY_XP_00564	hypothet	Coccomyxa_subellips	49.60	0.00
TRINITY_XP_02638	structur	Papaver_somniferum	49.60	0.00
TRINITY_KXZ52452	hypothet	Gonium_pectorale	49.60	0.00
TRINITY_XP_00168	predicte	Chlamydomonas_reinh	49.60	0.00
TRINITY_PNW74048	hypothet	Chlamydomonas_reinh	49.60	0.00
TRINITY_KZV15034	acetate-	Dorcoceras_hygromet	49.60	0.00
TRINITY_KXZ54091	hypothet	Gonium_pectorale	49.60	0.00
TRINITY_GBF88897	hypothet	Raphidocelis_subcap	49.60	0.00
TRINITY_GAX85524	hypothet	Chlamydomonas_eusti	49.60	0.00
TRINITY_GAX73309	hypothet	Chlamydomonas_eusti	49.60	0.00
TRINITY_GAX84316	hypothet	Chlamydomonas_eusti	49.60	0.00
TRINITY_XP_01045	PREDICTE	Camelina_sativa	49.50	0.00
TRINITY_GBG66199	hypothet	Chara_braunii	49.50	0.00
TRINITY_BAJ94097	predicte	Hordeum_vulgare	49.50	0.00
TRINITY_XP_02735	CTP synt	Abrus_precatorius	49.50	0.00
TRINITY_XP_00294	hypothet	Volvox_carteri	49.50	0.00
TRINITY_XP_00564	hypothet	Coccomyxa_subellips	49.50	0.00
TRINITY_XP_02042	probable	Prunus_persica	49.50	0.00
TRINITY_XP_01919	PREDICTE	Ipomoea_nil	49.50	0.00
TRINITY_GAQ91622	RNA-bind	Klebsormidium_niten	49.50	0.00
TRINITY_XP_01248	PREDICTE	Gossypium_raimondii	49.50	0.00

TRINITY_XP_0062(	RNA-binc	Capsella_rubella	49.50	0.00
TRINITY_XP_0120(	ER lumer	Jatropha_curcas	49.50	0.00
TRINITY_XP_0029(	hypothet	Volvox_carteri	49.50	0.00
TRINITY_GAX7423(	hypothet	Chlamydomonas_eusti	49.50	0
TRINITY_XP_0058(	hypothet	Chlorella_variabili	49.50	0.00
TRINITY_OMO6071(	Uracil-I	Corchorus_capsulari	49.50	0.00
TRINITY_BAK0277(	predicte	Hordeum_vulgare	49.50	0.00
TRINITY_GAX7949(	hypothet	Chlamydomonas_eusti	49.50	0.00
TRINITY_GAX8429(	hypothet	Chlamydomonas_eusti	49.50	0.00
TRINITY_XP_0058(	hypothet	Chlorella_variabili	49.50	0.00
TRINITY_KMZ7088(	Phospho:	Zostera_marina	49.50	0.00
TRINITY_GAX7788(	hypothet	Chlamydomonas_eusti	49.50	0.00
TRINITY_GAX8080(	hypothet	Chlamydomonas_eusti	49.50	0.00
TRINITY_XP_0271(	serine/a	Coffea_arabica	49.50	0.00
TRINITY_XP_0025(	predicte	Micromonas_commoda	49.50	0.00
TRINITY_XP_0066(	PREDICTI	Oryza_brachyantha	49.50	0.00
TRINITY_XP_0014(	predicte	Ostreococcus_sp._'l	49.50	0.00
TRINITY_XP_0016(	protein	Chlamydomonas_reinh	49.50	0.00
TRINITY_GAX7454(	hypothet	Chlamydomonas_eusti	49.50	0.00
TRINITY_GAQ9157(	protein	Klebsormidium_niten	49.50	0.00
TRINITY_PNH0800(	Soluble	Tetrabaena_socialis	49.50	0.00
TRINITY_GAX8363(	hypothet	Chlamydomonas_eusti	49.50	0.00
TRINITY_XP_0029(	hypothet	Volvox_carteri	49.50	0.00
TRINITY_GAX8218(	hypothet	Chlamydomonas_eusti	49.50	0.00
TRINITY_PNW8863(	hypothet	Chlamydomonas_reinh	49.50	0.00
TRINITY_GAX8395(	hypothet	Chlamydomonas_eusti	49.50	0.00
TRINITY_PRW2057(	E3 ubiqu	Chlorella_sorokinia	49.50	0.00
TRINITY_PNH0786(	hypothet	Tetrabaena_socialis	49.50	0.00
TRINITY_PRW4569(	glucosar	Chlorella_sorokinia	49.50	0.00
TRINITY_BBC2842(	phospho:	Chlamydomonas_appla	49.50	0.00
TRINITY_XP_0017(	predicte	Chlamydomonas_reinh	49.50	0.00
TRINITY_PRW1831(	NADPH-de	Chlorella_sorokinia	49.50	0.00
TRINITY_GAX7260(	hypothet	Chlamydomonas_eusti	49.50	0.00
TRINITY_XP_0029(	hypothet	Volvox_carteri	49.50	0.00
TRINITY_KXZ5258(	hypothet	Gonium_pectorale	49.50	0.00
TRINITY_OAE3087(	hypothet	Marchantia_polymorp	49.50	0.00
TRINITY_GAX7501(	hypothet	Chlamydomonas_eusti	49.50	0.00
TRINITY_PRW5750(	transket	Chlorella_sorokinia	49.50	0.00
TRINITY_PKA6682(	putative	Apostasia_shenzheni	49.50	0.00
TRINITY_PSC7476(	notchles	Micractinium_conduc	49.50	0.00
TRINITY_GAX7990(	hypothet	Chlamydomonas_eusti	49.50	0.00
TRINITY_XP_0029(	hypothet	Volvox_carteri	49.50	0.00
TRINITY_XP_0159(	transcri	Arachis_duranensis	49.50	0.00
TRINITY_GAX8384(	hypothet	Chlamydomonas_eusti	49.50	0.00
TRINITY_GAX7500(	hypothet	Chlamydomonas_eusti	49.50	0
TRINITY_XP_0128(	PREDICTI	Erythranthe_guttata	49.50	0.00
TRINITY_XP_0029(	pyruvate	Volvox_carteri	49.50	0.00
TRINITY_KXZ4189(	hypothet	Gonium_pectorale	49.50	0.00
TRINITY_PNW7964(	hypothet	Chlamydomonas_reinh	49.50	0.00
TRINITY_GAX7298(	hypothet	Chlamydomonas_eusti	49.50	0.00
TRINITY_RWW9108(	hypothet	Ensete_ventricosum	49.50	0.00
TRINITY_XP_0243(	probable	Physcomitrella_pate	49.40	0.00
TRINITY_XP_0165(	PREDICTI	Capsicum_annuum	49.40	0.00
TRINITY_PRW5722(	60S ribo	Chlorella_sorokinia	49.40	0.00
TRINITY_XP_0113(	Oxystero	Auxenochlorella_pro	49.40	0.00
TRINITY_XP_0030(	ligase,	Micromonas_pusilla	49.40	0.00
TRINITY_PON5956(	Lysine r	Trema_orientale	49.40	0.00
TRINITY_KDD7677(	hypothet	Helicosporidium_sp.	49.40	0.00
TRINITY_RXH8504(	hypothet	Malus_domestica	49.40	0.00
TRINITY_GAX7303(	hypothet	Chlamydomonas_eusti	49.40	0.00
TRINITY_KXZ4949(	hypothet	Gonium_pectorale	49.40	0.00
TRINITY_XP_0102(	PREDICTI	Nelumbo_nucifera	49.40	0.00
TRINITY_XP_0158(	probable	Ziziphus_jujuba	49.40	0.00
TRINITY_XP_0029(	probable	Selaginella_moellen	49.40	0.00
TRINITY_RCV2007(	hypothet	Setaria_italica	49.40	0.00
TRINITY_XP_0235(	DNA misr	Cucurbita_pepo	49.40	0.00

TRINITY_GAQ8888	NAD-dep	Klebsormidium_niten	49.40	0.00
TRINITY_XP_0243	LOW QUA	Brachypodium_distac	49.40	0.00
TRINITY_GAX7433	hypothet	Chlamydomonas_eusti	49.40	0.00
TRINITY_GAX8422	hypothet	Chlamydomonas_eusti	49.40	0.00
TRINITY_KXZ4327	hypothet	Gonium_pectorale	49.40	0.00
TRINITY_XP_0139	hypothet	Monoraphidium_negle	49.40	0.00
TRINITY_XP_0029	hypothet	Volvox_carteri	49.40	0.00
TRINITY_OVA1013	NUDIX h	Macleaya_cordata	49.40	0.00
TRINITY_GBF9019	hypothet	Raphidocelis_subcap	49.40	0.00
TRINITY_XP_0070	PREDICT	Theobroma_cacao	49.40	0.00
TRINITY_GBG6937	hypothet	Chara_braunii	49.40	0.00
TRINITY_KXZ4341	hypothet	Gonium_pectorale	49.40	0.00
TRINITY_PNW8142	hypothet	Chlamydomonas_reinh	49.40	0.00
TRINITY_XP_0056	Pkinase-	Coccomyxa_subellips	49.40	0.00
TRINITY_GAX8297	hypothet	Chlamydomonas_eusti	49.40	0.00
TRINITY_XP_0243	methion	Physcomitrella_pate	49.40	0.00
TRINITY_XP_0017	predict	Chlamydomonas_reinh	49.40	0.00
TRINITY_GAX7984	hypothet	Chlamydomonas_eusti	49.40	0.00
TRINITY_GAX7599	hypothet	Chlamydomonas_eusti	49.40	0.00
TRINITY_ERN1918	hypothet	Amborella_trichopod	49.40	0.00
TRINITY_PNW7115	hypothet	Chlamydomonas_reinh	49.40	0.00
TRINITY_GBF8772	hypothet	Raphidocelis_subcap	49.40	0.00
TRINITY_XP_0029	hypothet	Volvox_carteri	49.40	0.00
TRINITY_XP_0056	P-loop	Coccomyxa_subellips	49.40	0.00
TRINITY_KXZ5609	hypothet	Gonium_pectorale	49.40	0.00
TRINITY_XP_0116	PREDICT	Cucumis_sativus	49.40	0.00
TRINITY_KXZ4280	hypothet	Gonium_pectorale	49.40	0.00
TRINITY_XP_0270	serine/t	Coffea_arabica	49.40	0.00
TRINITY_RRT4380	hypothet	Ensete_ventricosum	49.40	0.00
TRINITY_PRW4444	catheps	Chlorella_sorokinia	49.40	0.00
TRINITY_KXZ4254	hypothet	Gonium_pectorale	49.40	0.00
TRINITY_BAK0302	predict	Hordeum_vulgare	49.40	0.00
TRINITY_KZV1541	phenyla	Dorcoceras_hygromet	49.40	0.00
TRINITY_XP_0239	probabl	Quercus_suber	49.40	0.00
TRINITY_GAX8394	hypothet	Chlamydomonas_eusti	49.40	0.00
TRINITY_GAX8260	hypothet	Chlamydomonas_eusti	49.40	0.00
TRINITY_GAX7554	hypothet	Chlamydomonas_eusti	49.40	0.00
TRINITY_XP_0056	S-adeno	Coccomyxa_subellips	49.40	0.00
TRINITY_XP_0029	uncharac	Selaginella_moellen	49.40	0.00
TRINITY_GAX7314	hypothet	Chlamydomonas_eusti	49.40	0.00
TRINITY_GAX7271	hypothet	Chlamydomonas_eusti	49.40	0.00
TRINITY_PNW7146	hypothet	Chlamydomonas_reinh	49.40	0.00
TRINITY_XP_0025	protein	Micromonas_commoda	49.40	0.00
TRINITY_GAX7408	hypothet	Chlamydomonas_eusti	49.40	0.00
TRINITY_PNW7092	hypothet	Chlamydomonas_reinh	49.40	0.00
TRINITY_PRW5825	cytochr	Chlorella_sorokinia	49.40	0.00
TRINITY_XP_0064	UPF0160	Citrus_clementina	49.40	0.00
TRINITY_GAQ8089	WD doma	Klebsormidium_niten	49.40	0.00
TRINITY_RQM0438	hypothet	Brassica_cretica	49.40	0.00
TRINITY_XP_0137	ras-rel	Brassica_napus	49.40	0.00
TRINITY_GAX8634	hypothet	Chlamydomonas_eusti	49.40	0.00
TRINITY_PNW8687	hypothet	Chlamydomonas_reinh	49.40	0.00
TRINITY_GAX7677	hypothet	Chlamydomonas_eusti	49.40	0.00
TRINITY_GAX7490	hypothet	Chlamydomonas_eusti	49.40	0.00
TRINITY_PNH0499	Cycloart	Tetrabaena_socialis	49.40	0.00
TRINITY_PSC6846	Transme	Micractinium_conduc	49.40	0.00
TRINITY_GAX7502	hypothet	Chlamydomonas_eusti	49.40	0.00
TRINITY_PNH1093	Protein	Tetrabaena_socialis	49.40	0.00
TRINITY_XP_0086	ABC tra	Zea_mays	49.40	0.00
TRINITY_XP_0217	lysosom	Chenopodium_quinoa	49.40	0.00
TRINITY_GBG7398	hypothet	Chara_braunii	49.40	0.00
TRINITY_XP_0241	zinc fi	Rosa_chinensis	49.30	0.00
TRINITY_ALC7885	MTP2h (	Sedum_alfredii	49.30	0.00
TRINITY_OAE3203	hypothet	Marchantia_polymorp	49.30	0.00
TRINITY_XP_0188	PREDICT	Juglans_regia	49.30	0.00
TRINITY_RLN3067	E3 ubiq	Panicum_miliaceum	49.30	0.00

TRINITY_PPD6781	hypothet	Gossypium_barbadens	49.30	0.00
TRINITY_XP_0205	actin-re	Phalaenopsis_equest	49.30	0.00
TRINITY_XP_0216	serine/t	Manihot_esculenta	49.30	0.00
TRINITY_CAI8489	putative	Medicago_sativa	49.30	0.00
TRINITY_XP_0205	metal to	Phalaenopsis_equest	49.30	0.00
TRINITY_XP_0270	RNA-bin	Coffea_arabica	49.30	0.00
TRINITY_GAQ8098	Aldo/ket	Klebsormidium_niten	49.30	0.00
TRINITY_AFK4814	unknown	Medicago_truncatula	49.30	0.00
TRINITY_XP_0042	mitochor	Solanum_lycopersicu	49.30	0.00
TRINITY_XP_0049	ras-rela	Setaria_italica	49.30	0.00
TRINITY_XP_0243	60S ribo	Physcomitrella_pate	49.30	0.00
TRINITY_XP_0093	PREDICTI	Pyrus_x_bretschneid	49.30	0.00
TRINITY_GBF9578	hypothet	Raphidocelis_subcap	49.30	0.00
TRINITY_GAX8357	hypothet	Chlamydomonas_eusti	49.30	0.00
TRINITY_XP_0243	ATP-bin	Physcomitrella_pate	49.30	0.00
TRINITY_OAE2772	hypothet	Marchantia_polymorp	49.30	0.00
TRINITY_PNW7682	hypothet	Chlamydomonas_reinh	49.30	0.00
TRINITY_XP_0029	SET doma	Volvox_carteri	49.30	0.00
TRINITY_GBG7812	hypothet	Chara_braunii	49.30	0.00
TRINITY_BAU0370	hypothet	Vigna_angularis	49.30	0.00
TRINITY_KXZ4215	hypothet	Gonium_pectorale	49.30	0.00
TRINITY_GAX7580	hypothet	Chlamydomonas_eusti	49.30	0.00
TRINITY_GAX7572	hypothet	Chlamydomonas_eusti	49.30	0.00
TRINITY_PNW8202	hypothet	Chlamydomonas_reinh	49.30	0.00
TRINITY_XP_0017	C-type	Chlamydomonas_reinh	49.30	0.00
TRINITY_XP_0165	PREDICTI	Capsicum_annuum	49.30	0.00
TRINITY_OAE2735	hypothet	Marchantia_polymorp	49.30	0.00
TRINITY_ONM0319	Proteas	Zea_mays	49.30	0.00
TRINITY_XP_0029	hypothet	Volvox_carteri	49.30	0.00
TRINITY_PNH1104	R3H and	Tetrabaena_socialis	49.30	0.00
TRINITY_XP_0016	predicte	Chlamydomonas_reinh	49.30	0.00
TRINITY_XP_0238	probable	Quercus_suber	49.30	0.00
TRINITY_XP_0016	cryptoch	Chlamydomonas_reinh	49.30	0.00
TRINITY_XP_0217	probable	Chenopodium_quinoa	49.30	0.00
TRINITY_XP_0138	hypothet	Monoraphidium_negle	49.30	0.00
TRINITY_GAX8666	hypothet	Chlamydomonas_eusti	49.30	0.00
TRINITY_XP_0138	hypothet	Monoraphidium_negle	49.30	0.00
TRINITY_XP_0056	hypothet	Coccomyxa_subellips	49.30	0.00
TRINITY_XP_0029	hypothet	Volvox_carteri	49.30	0.00
TRINITY_BAJ9390	predicte	Hordeum_vulgare	49.30	0.00
TRINITY_GBF9453	DNA repl	Raphidocelis_subcap	49.30	0.00
TRINITY_GAX7599	hypothet	Chlamydomonas_eusti	49.30	0.00
TRINITY_PNW7909	hypothet	Chlamydomonas_reinh	49.30	0.00
TRINITY_GBF9814	hypothet	Raphidocelis_subcap	49.30	0.00
TRINITY_XP_0056	hypothet	Coccomyxa_subellips	49.30	0.00
TRINITY_PNW8247	hypothet	Chlamydomonas_reinh	49.30	0.00
TRINITY_GAX7391	hypothet	Chlamydomonas_eusti	49.30	0.00
TRINITY_GAQ8284	Prolyl	Klebsormidium_niten	49.30	0.00
TRINITY_GBF9323	insulin-	Raphidocelis_subcap	49.30	0.00
TRINITY_PSC7129	ATP-bin	Micractinium_conduc	49.30	0.00
TRINITY_GAX8522	hypothet	Chlamydomonas_eusti	49.30	0.00
TRINITY_NP_0013	uncharac	Zea_mays	49.30	0.00
TRINITY_XP_0239	structu	Quercus_suber	49.30	0.00
TRINITY_OAE3223	hypothet	Marchantia_polymorp	49.30	0.00
TRINITY_GAX7916	hypothet	Chlamydomonas_eusti	49.30	0.00
TRINITY_EFJ2871	hypothet	Selaginella_moellen	49.30	0.00
TRINITY_OAE2104	hypothet	Marchantia_polymorp	49.30	0.00
TRINITY_GAX7355	hypothet	Chlamydomonas_eusti	49.30	0.00
TRINITY_GAX7777	hypothet	Chlamydomonas_eusti	49.30	0.00
TRINITY_GAX7834	hypothet	Chlamydomonas_eusti	49.30	0.00
TRINITY_XP_0264	calcium-	Papaver_somniferum	49.30	0.00
TRINITY_GAX7288	hypothet	Chlamydomonas_eusti	49.30	0.00
TRINITY_XP_0206	homeobo	Dendrobium_catenatu	49.30	0.00
TRINITY_PNW8898	hypothet	Chlamydomonas_reinh	49.30	0.00
TRINITY_KDO4394	hypothet	Citrus_sinensis	49.30	0.00
TRINITY_XP_0023	probable	Populus_trichocarpa	49.30	0.00

TRINITY_OUS4836	hypothet	Ostreococcus_tauri	49.30	0.00
TRINITY_XP_0098	PREDICT	Nicotiana_sylvestri	49.30	0.00
TRINITY_OTG2048	putativ	Helianthus_annuus	49.20	0.00
TRINITY_KHN2058	COP9 si	Glycine_soja	49.20	0.00
TRINITY_XP_0191	PREDICT	Ipomoea_nil	49.20	0.00
TRINITY_GBG7296	hypothet	Chara_braunii	49.20	0.00
TRINITY_XP_0238	RNA-bin	Quercus_suber	49.20	0.00
TRINITY_KFK4273	hypothet	Arabis_alpina	49.20	0.00
TRINITY_PRQ5311	putativ	Rosa_chinensis	49.20	0.00
TRINITY_GAX7978	hypothet	Chlamydomonas_eusti	49.20	0.00
TRINITY_RLM6448	50S rib	Panicum_miliaceum	49.20	0.00
TRINITY_XP_0205	dol-P-M	Phalaenopsis_equest	49.20	0.00
TRINITY_XP_0238	glycoge	Quercus_suber	49.20	0.00
TRINITY_XP_0016	predict	Chlamydomonas_reinh	49.20	0.00
TRINITY_GAX7479	hypothet	Chlamydomonas_eusti	49.20	0.00
TRINITY_PON4723	Ubiquit	Parasponia_anderson	49.20	0.00
TRINITY_GAX7974	hypothet	Chlamydomonas_eusti	49.20	0.00
TRINITY_KDD7659	hypothet	Helicosporidium_sp.	49.20	0.00
TRINITY_XP_0014	predict	Ostreococcus_sp._'l	49.20	0.00
TRINITY_XP_0094	PREDICT	Musa_acuminata	49.20	0.00
TRINITY_XP_0056	GRIM-19	Coccomyxa_subellips	49.20	0.00
TRINITY_OAY4714	hypothet	Manihot_esculenta	49.20	0.00
TRINITY_GBG6961	hypothet	Chara_braunii	49.20	0.00
TRINITY_GAX7948	hypothet	Chlamydomonas_eusti	49.20	0.00
TRINITY_GAX7814	hypothet	Chlamydomonas_eusti	49.20	0.00
TRINITY_KXZ4202	hypothet	Gonium_pectorale	49.20	0.00
TRINITY_ONK7360	uncharac	Asparagus_officinal	49.20	0.00
TRINITY_GAX7412	hypothet	Chlamydomonas_eusti	49.20	0.00
TRINITY_XP_0201	calcium-	Aegilops_tauschii	49.20	0.00
TRINITY_XP_0029	cytochr	Volvox_carteri	49.20	0.00
TRINITY_XP_0016	predict	Chlamydomonas_reinh	49.20	0.00
TRINITY_PNW8574	hypothet	Chlamydomonas_reinh	49.20	0.00
TRINITY_XP_0056	WD40 re	Coccomyxa_subellips	49.20	0.00
TRINITY_XP_0206	ubiquit	Dendrobium_catenatu	49.20	0.00
TRINITY_XP_0139	hypothet	Monoraphidium_negle	49.20	0.00
TRINITY_GAX7510	hypothet	Chlamydomonas_eusti	49.20	0.00
TRINITY_KXZ4998	hypothet	Gonium_pectorale	49.20	0.00
TRINITY_XP_0016	predict	Chlamydomonas_reinh	49.20	0.00
TRINITY_PNW8190	hypothet	Chlamydomonas_reinh	49.20	0.00
TRINITY_GAX7860	hypothet	Chlamydomonas_eusti	49.20	0.00
TRINITY_GAX8166	hypothet	Chlamydomonas_eusti	49.20	0.00
TRINITY_CBU3041	asparag	Polytomella_sp._Pri	49.20	0.00
TRINITY_KZV1503	hypothet	Dorcoceras_hygromet	49.20	0.00
TRINITY_RWW3170	hypothet	Ensete_ventricosum	49.20	0.00
TRINITY_PNW8410	hypothet	Chlamydomonas_reinh	49.20	0.00
TRINITY_GAX7413	hypothet	Chlamydomonas_eusti	49.20	0.00
TRINITY_KXZ5151	hypothet	Gonium_pectorale	49.20	0.00
TRINITY_XP_0082	PREDICT	Prunus_mume	49.20	0.00
TRINITY_GAX8540	hypothet	Chlamydomonas_eusti	49.20	0.00
TRINITY_PNH0286	hypothet	Tetrabaena_socialis	49.20	0.00
TRINITY_PNW7423	hypothet	Chlamydomonas_reinh	49.20	0.00
TRINITY_GAX8021	hypothet	Chlamydomonas_eusti	49.20	0.00
TRINITY_KXZ5110	hypothet	Gonium_pectorale	49.20	0.00
TRINITY_PRW4505	E3 ubiq	Chlorella_sorokinia	49.20	0.00
TRINITY_CDY2302	BnaC07g	Brassica_napus	49.20	0.00
TRINITY_GAX8268	hypothet	Chlamydomonas_eusti	49.20	0.00
TRINITY_PNW8485	hypothet	Chlamydomonas_reinh	49.20	0.00
TRINITY_GAX7493	hypothet	Chlamydomonas_eusti	49.20	0.00
TRINITY_KXZ4840	hypothet	Gonium_pectorale	49.20	0.00
TRINITY_XP_0058	hypothet	Chlorella_variabili	49.10	0.00
TRINITY_PSC7466	Serine	Micractinium_conduc	49.10	0.00
TRINITY_KFK3834	hypothet	Arabis_alpina	49.10	0.00
TRINITY_OMO6070	hypothet	Corchorus_capsulari	49.10	0.00
TRINITY_XP_0161	DNA-dir	Arachis_ipaensis	49.10	0.00
TRINITY_XP_0030	unnamed	Ostreococcus_tauri	49.10	0.00
TRINITY_OUS4468	glucose	Ostreococcus_tauri	49.10	0.00

TRINITY_PRW0585(	decappir	Chlorella_sorokinia	49.10	0.00
TRINITY_OVA0582(	Glutath:	Macleaya_cordata	49.10	0.00
TRINITY_XP_0239(	ras-like	Quercus_suber	49.10	0.00
TRINITY_XP_0202'	40S ribo	Asparagus_officinal	49.10	0.00
TRINITY_XP_0139(	dual-spe	Monoraphidium_negle	49.10	0.00
TRINITY_GAX8065!	hypothet	Chlamydomonas_eusti	49.10	0.00
TRINITY_XP_0029‹	hypothet	Volvox_carteri	49.10	0.00
TRINITY_KXZ5320(	hypothet	Gonium_pectorale	49.10	0.00
TRINITY_GBG7673(	hypothet	Chara_braunii	49.10	0.00
TRINITY_PRW5825‹	cytochro	Chlorella_sorokinia	49.10	0.00
TRINITY_GAX7809!	hypothet	Chlamydomonas_eusti	49.10	0.00
TRINITY_XP_0229!	thioredo	Cucurbita_moschata	49.10	0.00
TRINITY_XP_0138!	Mannan e	Monoraphidium_negle	49.10	0.00
TRINITY_XP_0056‹	WD40 rep	Coccomyxa_subellips	49.10	0.00
TRINITY_PLY8179:	hypothet	Lactuca_sativa	49.10	0.00
TRINITY_XP_0016!	predicte	Chlamydomonas_reinh	49.10	0.00
TRINITY_GAX8151(	hypothet	Chlamydomonas_eusti	49.10	0.00
TRINITY_GAX7395!	hypothet	Chlamydomonas_eusti	49.10	0.00
TRINITY_XP_0029!	hypothet	Volvox_carteri	49.10	0.00
TRINITY_GAX7830(	hypothet	Chlamydomonas_eusti	49.10	0.00
TRINITY_CAB7187!	P-glycop	Arabidopsis_thalian	49.10	0.00
TRINITY_PNH0415'	hypothet	Tetrabaena_socialis	49.10	0.00
TRINITY_XP_0082‹	PREDICTE	Prunus_mume	49.10	0.00
TRINITY_XP_0239‹	probable	Quercus_suber	49.10	0.00
TRINITY_KXZ4490!	hypothet	Gonium_pectorale	49.10	0.00
TRINITY_GAX7382‹	hypothet	Chlamydomonas_eusti	49.10	0.00
TRINITY_PWA8090!	Phospho:	Artemisia_annua	49.10	0.00
TRINITY_PNH0285'	Adenine/	Tetrabaena_socialis	49.10	0.00
TRINITY_GBG7303(	hypothet	Chara_braunii	49.10	0.00
TRINITY_KXZ5422‹	hypothet	Gonium_pectorale	49.10	0.00
TRINITY_BAK0193(	predicte	Hordeum_vulgare	49.10	0.00
TRINITY_GAQ8187:	26S prot	Klebsormidium_niten	49.10	0.00
TRINITY_RXI0558(	hypothet	Malus_domestica	49.10	0.00
TRINITY_GAX7836(	hypothet	Chlamydomonas_eusti	49.10	0.00
TRINITY_PNW8504!	hypothet	Chlamydomonas_reinh	49.10	0.00
TRINITY_PNW7744(	hypothet	Chlamydomonas_reinh	49.10	0.00
TRINITY_GAX7413!	hypothet	Chlamydomonas_eusti	49.10	0.00
TRINITY_XP_0029!	alpha-1,	Volvox_carteri	49.10	0.00
TRINITY_KXZ4350‹	hypothet	Gonium_pectorale	49.10	0.00
TRINITY_PNW7883!	hypothet	Chlamydomonas_reinh	49.10	0.00
TRINITY_PNW7530(	hypothet	Chlamydomonas_reinh	49.10	0.00
TRINITY_PNW7382:	hypothet	Chlamydomonas_reinh	49.10	0.00
TRINITY_GAX7949‹	hypothet	Chlamydomonas_eusti	49.10	0.00
TRINITY_GAX8400!	hypothet	Chlamydomonas_eusti	49.10	0.00
TRINITY_GAX7624(	hypothet	Chlamydomonas_eusti	49.10	0.00
TRINITY_GAX7787‹	hypothet	Chlamydomonas_eusti	49.10	0
TRINITY_EPS5921(	hypothet	Genlisea_aurea	49.10	0.00
TRINITY_KHG0862!	Chaperor	Gossypium_arboreum	49.00	0.00
TRINITY_XP_0102!	PREDICTE	Nelumbo_nucifera	49.00	0.00
TRINITY_RYR4286:	hypothet	Arachis_hypogaea	49.00	0.00
TRINITY_AGV5438!	tubulin	Phaseolus_vulgaris	49.00	0.00
TRINITY_XP_0217‹	squamosa	Chenopodium_quinoa	49.00	0.00
TRINITY_XP_0056!	flavin-c	Coccomyxa_subellips	49.00	0.00
TRINITY_GAX8071(	hypothet	Chlamydomonas_eusti	49.00	0.00
TRINITY_XP_0036‹	histone	Medicago_truncatula	49.00	0.00
TRINITY_XP_0227‹	E3 ubiqu	Durio_zibethinus	49.00	0.00
TRINITY_GAQ8888!	NAD-depe	Klebsormidium_niten	49.00	0.00
TRINITY_XP_0029!	hypothet	Volvox_carteri	49.00	0.00
TRINITY_XP_0264(	sorbitol	Papaver_somniferum	49.00	0.00
TRINITY_XP_0218!	prolyl e	Spinacia_oleracea	49.00	0.00
TRINITY_XP_0029‹	hypothet	Volvox_carteri	49.00	0.00
TRINITY_GAQ7956'	valyl-tE	Klebsormidium_niten	49.00	0.00
TRINITY_PNH0312(	hypothet	Tetrabaena_socialis	49.00	0.00
TRINITY_GAX8640:	hypothet	Chlamydomonas_eusti	49.00	0.00
TRINITY_GAQ8456!	ATP citr	Klebsormidium_niten	49.00	0.00
TRINITY_GBF9073(	nitrile	Raphidocelis_subcap	49.00	0.00

TRINITY_GAX8403	hypothet	Chlamydomonas_eusti	49.00	0.00
TRINITY_PNW7059	hypothet	Chlamydomonas_reinh	49.00	0.00
TRINITY_GAQ8101	glycyl-	Klebsormidium_niten	49.00	0.00
TRINITY_EFJ3836	hypothet	Selaginella_moellen	49.00	0.00
TRINITY_PNW8553	hypothet	Chlamydomonas_reinh	49.00	0.00
TRINITY_OAE2496	hypothet	Marchantia_polymorp	49.00	0.00
TRINITY_GAQ8065	hypothet	Klebsormidium_niten	49.00	0.00
TRINITY_XP_0219	pyropho	Helianthus_annuus	49.00	0.00
TRINITY_XP_0239	probabl	Quercus_suber	49.00	0.00
TRINITY_XP_0029	hypothet	Volvox_carteri	49.00	0.00
TRINITY_XP_0016	predict	Chlamydomonas_reinh	49.00	0.00
TRINITY_XP_0083	PREDICT	Malus_domestica	49.00	0.00
TRINITY_GAX8258	hypothet	Chlamydomonas_eusti	49.00	0.00
TRINITY_XP_0029	hypothet	Volvox_carteri	49.00	0.00
TRINITY_GAX8038	hypothet	Chlamydomonas_eusti	49.00	0.00
TRINITY_XP_0228	DNA-dir	Olea_europaea	49.00	0.00
TRINITY_GAX8063	hypothet	Chlamydomonas_eusti	49.00	0.00
TRINITY_XP_0138	nicotin	Monoraphidium_negle	49.00	0.00
TRINITY_GBG8134	hypothet	Chara_braunii	49.00	0.00
TRINITY_XP_0063	protein	Eutrema_salsugineum	49.00	0.00
TRINITY_PNW7279	hypothet	Chlamydomonas_reinh	49.00	0.00
TRINITY_GAX7303	hypothet	Chlamydomonas_eusti	49.00	0.00
TRINITY_XP_0109	PREDICT	Elaeis_guineensis	49.00	0.00
TRINITY_KDD7266	hypothet	Helicosporidium_sp.	49.00	0.00
TRINITY_PIN0134	Glutami	Handroanthus_impeti	49.00	0.00
TRINITY_PNR3038	hypothet	Physcomitrella_pate	49.00	0.00
TRINITY_KXZ4427	hypothet	Gonium_pectorale	49.00	0.00
TRINITY_XP_0029	hybrid-	Volvox_carteri	49.00	0.00
TRINITY_PNW8080	hypothet	Chlamydomonas_reinh	49.00	0.00
TRINITY_GAX7357	hypothet	Chlamydomonas_eusti	49.00	0.00
TRINITY_GAX7257	hypothet	Chlamydomonas_eusti	49.00	0.00
TRINITY_PNW7645	hypothet	Chlamydomonas_reinh	49.00	0.00
TRINITY_GAQ7758	ABC tra	Klebsormidium_niten	49.00	0.00
TRINITY_GAX7398	hypothet	Chlamydomonas_eusti	49.00	0.00
TRINITY_AMK9782	thiored	Haematococcus_lacus	49.00	0.00
TRINITY_GAX8162	hypothet	Chlamydomonas_eusti	49.00	0.00
TRINITY_GAX8439	hypothet	Chlamydomonas_eusti	49.00	0.00
TRINITY_GBF9827	hypothet	Raphidocelis_subcap	49.00	0.00
TRINITY_GAX7918	hypothet	Chlamydomonas_eusti	49.00	0.00
TRINITY_PNW8291	hypothet	Chlamydomonas_reinh	49.00	0.00
TRINITY_PNW8004	hypothet	Chlamydomonas_reinh	49.00	0.00
TRINITY_GAX7276	hypothet	Chlamydomonas_eusti	49.00	0.00
TRINITY_GAX7361	hypothet	Chlamydomonas_eusti	49.00	0.00
TRINITY_XP_0036	tRNA (c	Medicago_truncatula	49.00	0.00
TRINITY_KXZ4771	hypothet	Gonium_pectorale	49.00	0.00
TRINITY_BAD2510	unknown	Oryza_sativa	48.90	0.00
TRINITY_XP_0238	protein	Quercus_suber	48.90	0.00
TRINITY_GAX7677	hypothet	Chlamydomonas_eusti	48.90	0.00
TRINITY_PRW3394	acyl- d	Chlorella_sorokinia	48.90	0.00
TRINITY_XP_0257	multipr	Arachis_hypogaea	48.90	0.00
TRINITY_KZV1545	hypothet	Dorcoceras_hygromet	48.90	0.00
TRINITY_BAJ9608	predict	Hordeum_vulgare	48.90	0.00
TRINITY_XP_0095	PREDICT	Nicotiana_tomentosi	48.90	0.00
TRINITY_XP_0243	WD repe	Brachypodium_distac	48.90	0.00
TRINITY_XP_0105	PREDICT	Tarenaya_hasslerian	48.90	0.00
TRINITY_GAQ8974	Inward	Klebsormidium_niten	48.90	0.00
TRINITY_KXZ4695	hypothet	Gonium_pectorale	48.90	0.00
TRINITY_BAK0277	predict	Hordeum_vulgare	48.90	0.00
TRINITY_GAX7258	hypothet	Chlamydomonas_eusti	48.90	0.00
TRINITY_XP_0206	probabl	Dendrobium_catenatu	48.90	0.00
TRINITY_XP_0114	G2/mito	Auxenochlorella_pro	48.90	0.00
TRINITY_KXZ5187	hypothet	Gonium_pectorale	48.90	0.00
TRINITY_GAX8131	hypothet	Chlamydomonas_eusti	48.90	0.00
TRINITY_GAQ9228	E3 ubiq	Klebsormidium_niten	48.90	0.00
TRINITY_PNW8586	hypothet	Chlamydomonas_reinh	48.90	0.00
TRINITY_GBF8972	hypothet	Raphidocelis_subcap	48.90	0.00

TRINITY_GAX8076(	hypothet	Chlamydomonas_eusti	48.90	0.00
TRINITY_PSC6828	Bifuncti	Micractinium_conduc	48.90	0.00
TRINITY_GAX7847	hypothet	Chlamydomonas_eusti	48.90	0.00
TRINITY_XP_0029	hypothet	Volvox_carteri	48.90	0.00
TRINITY_GAX8383(	hypothet	Chlamydomonas_eusti	48.90	0.00
TRINITY_XP_0238	histidir	Quercus_suber	48.90	0.00
TRINITY_PIN2703	Cysteine	Handroanthus_impeti	48.90	0.00
TRINITY_XP_0029	hypothet	Volvox_carteri	48.90	0.00
TRINITY_XP_0025(	predicte	Micromonas_commoda	48.90	0.00
TRINITY_XP_0041	PREDICTI	Cucumis_sativus	48.90	0.00
TRINITY_GAX7678	hypothet	Chlamydomonas_eusti	48.90	0.00
TRINITY_XP_0238	lid2 cor	Quercus_suber	48.90	0.00
TRINITY_GAX7909(	hypothet	Chlamydomonas_eusti	48.90	0.00
TRINITY_GAX7965	hypothet	Chlamydomonas_eusti	48.90	0.00
TRINITY_EEF2726	nhl repe	Ricinus_communis	48.90	0.00
TRINITY_XP_0056	Yip1-dor	Coccomyxa_subellips	48.90	0.00
TRINITY_GAX7580	hypothet	Chlamydomonas_eusti	48.90	0.00
TRINITY_GAX8053	hypothet	Chlamydomonas_eusti	48.90	0.00
TRINITY_EFJ1726	hypothet	Selaginella_moellen	48.90	0.00
TRINITY_XP_0138	Uncharac	Monoraphidium_negle	48.90	0.00
TRINITY_GBF9903	sec-inde	Raphidocelis_subcap	48.90	0.00
TRINITY_EFJ1485	hypothet	Selaginella_moellen	48.90	0.00
TRINITY_XP_0029	hypothet	Volvox_carteri	48.90	0.00
TRINITY_XP_0029	hypothet	Volvox_carteri	48.90	0.00
TRINITY_GAQ8807	NADH det	Klebsormidium_niten	48.90	0.00
TRINITY_BAF9891	subunit	Chlamydomonas_reinh	48.90	0.00
TRINITY_XP_0029	hypothet	Volvox_carteri	48.90	0.00
TRINITY_XP_0056	hypothet	Coccomyxa_subellips	48.90	0.00
TRINITY_PNW8566	hypothet	Chlamydomonas_reinh	48.90	0.00
TRINITY_PKA5195	Myb-rela	Apostasia_shenzheni	48.90	0.00
TRINITY_XP_0075(	unnamed	Bathycoccus_prasinc	48.90	0.00
TRINITY_GAQ9105	mannosy	Klebsormidium_niten	48.90	0.00
TRINITY_GAX7309	hypothet	Chlamydomonas_eusti	48.90	0.00
TRINITY_XP_0220	traffick	Helianthus_annuus	48.90	0.00
TRINITY_GBF9999	hypothet	Raphidocelis_subcap	48.90	0.00
TRINITY_GAX7608	hypothet	Chlamydomonas_eusti	48.90	0.00
TRINITY_GAX8431	hypothet	Chlamydomonas_eusti	48.90	0.00
TRINITY_XP_0068	NADH det	Amborella_trichopod	48.90	0.00
TRINITY_RZB8566	ADP-ribc	Glycine_soja	48.90	0.00
TRINITY_OMP1396	hypothet	Corchorus_olitorius	48.80	0.00
TRINITY_XP_0063	PREDICTI	Solanum_tuberosum	48.80	0.00
TRINITY_XP_0238	uncharac	Quercus_suber	48.80	0.00
TRINITY_XP_0263	probable	Papaver_somniferum	48.80	0.00
TRINITY_XP_0063(	CWF19-li	Capsella_rubella	48.80	0.00
TRINITY_XP_0091	PREDICTI	Brassica_rapa	48.80	0.00
TRINITY_XP_0270	protein	Coffea_arabica	48.80	0.00
TRINITY_XP_0014	predicte	Ostreococcus_sp._'l	48.80	0.00
TRINITY_XP_0239	putative	Quercus_suber	48.80	0.00
TRINITY_XP_0243	probable	Physcomitrella_pate	48.80	0.00
TRINITY_GAQ8088	mitochor	Klebsormidium_niten	48.80	0.00
TRINITY_KXZ5658	hypothet	Gonium_pectorale	48.80	0.00
TRINITY_XP_0056	SMAD/FHA	Coccomyxa_subellips	48.80	0.00
TRINITY_BAK0193	predicte	Hordeum_vulgare	48.80	0.00
TRINITY_XP_0219	protein	Helianthus_annuus	48.80	0.00
TRINITY_PKA6484	hypothet	Apostasia_shenzheni	48.80	0.00
TRINITY_XP_0165	PREDICTI	Capsicum_annuum	48.80	0.00
TRINITY_PQQ2121(	ABC trar	Prunus_yedoensis	48.80	0.00
TRINITY_XP_0056	ras-doma	Coccomyxa_subellips	48.80	0.00
TRINITY_OMO5504	hypothet	Corchorus_capsulari	48.80	0.00
TRINITY_KXZ4568	hypothet	Gonium_pectorale	48.80	0.00
TRINITY_KXZ5396	hypothet	Gonium_pectorale	48.80	0.00
TRINITY_XP_0016	predicte	Chlamydomonas_reinh	48.80	0.00
TRINITY_GAX7413	hypothet	Chlamydomonas_eusti	48.80	0.00
TRINITY_GAX8223	hypothet	Chlamydomonas_eusti	48.80	0.00
TRINITY_PRW4564(	L-ascorb	Chlorella_sorokinia	48.80	0.00
TRINITY_RVW2642	Histone-	Vitis_vinifera	48.80	0.00

TRINITY_PNH1287	Serine/t	Tetrabaena_socialis	48.80	0.00
TRINITY_GAX7751	hypothet	Chlamydomonas_eusti	48.80	0.00
TRINITY_XP_0068	protein	Amborella_trichopod	48.80	0.00
TRINITY_XP_0016	componen	Chlamydomonas_reinh	48.80	0.00
TRINITY_XP_0238	sacchar	Quercus_suber	48.80	0.00
TRINITY_GBF9014	hypothet	Raphidocelis_subcap	48.80	0.00
TRINITY_XP_0016	predicte	Chlamydomonas_reinh	48.80	0.00
TRINITY_OAE2503	hypothet	Marchantia_polymorp	48.80	0.00
TRINITY_XP_0138	peptidas	Monoraphidium_negle	48.80	0.00
TRINITY_XP_0139	hypothet	Monoraphidium_negle	48.80	0.00
TRINITY_KXZ4333	hypothet	Gonium_pectorale	48.80	0.00
TRINITY_XP_0016	predicte	Chlamydomonas_reinh	48.80	0.00
TRINITY_GBF8839	hypothet	Raphidocelis_subcap	48.80	0.00
TRINITY_GAX8428	hypothet	Chlamydomonas_eusti	48.80	0.00
TRINITY_PRW5856	cereblon	Chlorella_sorokinia	48.80	0.00
TRINITY_XP_0016	predicte	Chlamydomonas_reinh	48.80	0.00
TRINITY_XP_0184	PREDICTE	Raphanus_sativus	48.80	0.00
TRINITY_XP_0025	predicte	Micromonas_commoda	48.80	0.00
TRINITY_GAX8237	hypothet	Chlamydomonas_eusti	48.80	0.00
TRINITY_GAQ8931	Dyneins	Klebsormidium_niten	48.80	0.00
TRINITY_XP_0207	dolichol	Dendrobium_catenatu	48.80	0.00
TRINITY_GAX7906	hypothet	Chlamydomonas_eusti	48.80	0.00
TRINITY_GAX7897	hypothet	Chlamydomonas_eusti	48.80	0.00
TRINITY_PTQ4121	hypothet	Marchantia_polymorp	48.80	0.00
TRINITY_XP_0056	EMP/nona	Coccomyxa_subellips	48.80	0.00
TRINITY_CDL9305	cytosoli	Micrasterias_rotata	48.80	0.00
TRINITY_PNH1013	hypothet	Tetrabaena_socialis	48.80	0.00
TRINITY_XP_0029	alpha-ma	Selaginella_moellen	48.80	0.00
TRINITY_GAQ8268	Serine T	Klebsormidium_niten	48.80	0.00
TRINITY_XP_0238	geranylg	Quercus_suber	48.80	0.00
TRINITY_XP_0016	organic	Chlamydomonas_reinh	48.80	0.00
TRINITY_GAX7865	hypothet	Chlamydomonas_eusti	48.80	0.00
TRINITY_XP_0016	predicte	Chlamydomonas_reinh	48.80	0.00
TRINITY_PNW7481	hypothet	Chlamydomonas_reinh	48.80	0.00
TRINITY_BAK0223	predicte	Hordeum_vulgare	48.80	0.00
TRINITY_GAQ7762	Myosin f	Klebsormidium_niten	48.80	0.00
TRINITY_GAX7865	hypothet	Chlamydomonas_eusti	48.80	0.00
TRINITY_OVA2034	Peptidas	Macleaya_cordata	48.80	0.00
TRINITY_ACX4396	cysteine	Brachiaria_hybrid_c	48.80	0.00
TRINITY_PRW4440	translat	Chlorella_sorokinia	48.80	0.00
TRINITY_KXZ4690	hypothet	Gonium_pectorale	48.80	0.00
TRINITY_XP_0201	MAP3K ep	Aegilops_tauschii	48.70	0.00
TRINITY_XP_0229	oil body	Cucurbita_moschata	48.70	0.00
TRINITY_PRW0585	decappin	Chlorella_sorokinia	48.70	0.00
TRINITY_GAQ7838	dephosph	Klebsormidium_niten	48.70	0.00
TRINITY_XP_0139	hypothet	Monoraphidium_negle	48.70	0.00
TRINITY_XP_0056	glycosyl	Coccomyxa_subellips	48.70	0.00
TRINITY_GAQ8300	hypothet	Klebsormidium_niten	48.70	0.00
TRINITY_GBG7296	hypothet	Chara_braunii	48.70	0.00
TRINITY_PNR3152	hypothet	Physcomitrella_pate	48.70	0.00
TRINITY_PRW5901	molybdat	Chlorella_sorokinia	48.70	0.00
TRINITY_XP_0016	triacylg	Chlamydomonas_reinh	48.70	0.00
TRINITY_XP_0016	predicte	Chlamydomonas_reinh	48.70	0.00
TRINITY_GBF9419	calcium	Raphidocelis_subcap	48.70	0.00
TRINITY_GAX7890	hypothet	Chlamydomonas_eusti	48.70	0.00
TRINITY_PHT7887	Peroxiso	Capsicum_annuum	48.70	0.00
TRINITY_XP_0016	flagella	Chlamydomonas_reinh	48.70	0.00
TRINITY_GAX7880	hypothet	Chlamydomonas_eusti	48.70	0.00
TRINITY_GBG8205	hypothet	Chara_braunii	48.70	0.00
TRINITY_GAX7389	hypothet	Chlamydomonas_eusti	48.70	0.00
TRINITY_XP_0238	U3 small	Quercus_suber	48.70	0.00
TRINITY_XP_0029	hypothet	Volvox_carteri	48.70	0.00
TRINITY_GAX7495	hypothet	Chlamydomonas_eusti	48.70	0.00
TRINITY_XP_0058	hypothet	Chlorella_variabili	48.70	0.00
TRINITY_GAX8552	hypothet	Chlamydomonas_eusti	48.70	0.00
TRINITY_PSC7017	zinc fin	Micractinium_conduc	48.70	0.00

TRINITY_RWR8703(	non-spe(	Cinnamomum_micranth	48.70	0.00
TRINITY_XP_0029(	hypothet	Volvox_carteri	48.70	0.00
TRINITY_GBG0045(	hypothet	Raphidocelis_subcap	48.70	0.00
TRINITY_XP_0238(	bifuncti	Quercus_suber	48.70	0.00
TRINITY_PNH0625(	Meiotic	Tetrabaena_socialis	48.70	0.00
TRINITY_PNH0576(	30S ribo	Tetrabaena_socialis	48.70	0.00
TRINITY_PNW7067(	hypothet	Chlamydomonas_reinh	48.70	0.00
TRINITY_GAX8389(	hypothet	Chlamydomonas_eusti	48.70	0.00
TRINITY_GAX7519(	hypothet	Chlamydomonas_eusti	48.70	0.00
TRINITY_GAQ8019(	serine/t	Klebsormidium_niten	48.70	0.00
TRINITY_PNW8734(	hypothet	Chlamydomonas_reinh	48.70	0.00
TRINITY_RMZ5514(	hypothet	Auxenochlorella_pro	48.70	0.00
TRINITY_PNH0920(	Tiny mac	Tetrabaena_socialis	48.70	0.00
TRINITY_PNH0920(	Tiny mac	Tetrabaena_socialis	48.70	0.00
TRINITY_OAE2542(	hypothet	Marchantia_polymorp	48.70	0.00
TRINITY_XP_0239(	ras-like	Quercus_suber	48.70	0.00
TRINITY_XP_0138(	hypothet	Monoraphidium_negle	48.70	0.00
TRINITY_KXZ5143(	hypothet	Gonium_pectorale	48.70	0.00
TRINITY_GAX8527(	hypothet	Chlamydomonas_eusti	48.70	0.00
TRINITY_XP_0239(	pentafur	Quercus_suber	48.70	0.00
TRINITY_GAX7628(	hypothet	Chlamydomonas_eusti	48.70	0.00
TRINITY_AAQ1627(	histidir	Chlamydomonas_reinh	48.70	0.00
TRINITY_GAQ8538(	clathrir	Klebsormidium_niten	48.70	0.00
TRINITY_PNW7766(	hypothet	Chlamydomonas_reinh	48.70	0.00
TRINITY_XP_0017(	predicte	Chlamydomonas_reinh	48.70	0.00
TRINITY_XP_0029(	mitochor	Selaginella_moellen	48.60	0.00
TRINITY_EFJ3127(	hypothet	Selaginella_moellen	48.60	0.00
TRINITY_XP_0030(	tRNA wyk	Ostreococcus_tauri	48.60	0.00
TRINITY_OAE2940(	hypothet	Marchantia_polymorp	48.60	0.00
TRINITY_XP_0165(	PREDICTE	Capsicum_annuum	48.60	0.00
TRINITY_EFJ1875(	hypothet	Selaginella_moellen	48.60	0.00
TRINITY_XP_0124(	PREDICTE	Gossypium_raimondii	48.60	0.00
TRINITY_PSC7366(	dual spe	Micractinium_conduc	48.60	0.00
TRINITY_CBI3445(	unnamed	Vitis_vinifera	48.60	0.00
TRINITY_XP_0084(	PREDICTE	Cucumis_melo	48.60	0.00
TRINITY_OWM7752(	hypothet	Punica_granatum	48.60	0.00
TRINITY_XP_0056(	phosphoi	Coccomyxa_subellips	48.60	0.00
TRINITY_XP_0014(	predicte	Ostreococcus_sp._'l	48.60	0.00
TRINITY_XP_0056(	Sec7-dor	Coccomyxa_subellips	48.60	0.00
TRINITY_RLN3121(	ATP-depe	Panicum_miliaceum	48.60	0.00
TRINITY_PPS0868(	hypothet	Gossypium_barbadens	48.60	0.00
TRINITY_PHU1012(	hypothet	Capsicum_chinense	48.60	0.00
TRINITY_PHT2561(	Protein	Capsicum_baccatum	48.60	0.00
TRINITY_XP_0239(	cystathi	Quercus_suber	48.60	0.00
TRINITY_VDC8627(	unnamed	Brassica_rapa	48.60	0.00
TRINITY_PNW8169(	hypothet	Chlamydomonas_reinh	48.60	0.00
TRINITY_PNH0950(	hypothet	Tetrabaena_socialis	48.60	0.00
TRINITY_XP_0156(	actin-re	Oryza_sativa	48.60	0.00
TRINITY_XP_0213(	molybdop	Sorghum_bicolor	48.60	0.00
TRINITY_XP_0030(	RNA binc	Micromonas_pusilla	48.60	0.00
TRINITY_XP_0029(	hypothet	Volvox_carteri	48.60	0.00
TRINITY_XP_0029(	coatomer	Selaginella_moellen	48.60	0.00
TRINITY_XP_0049(	mitochor	Setaria_italica	48.60	0.00
TRINITY_PNH0947(	Purple a	Tetrabaena_socialis	48.60	0.00
TRINITY_GAX8321(	hypothet	Chlamydomonas_eusti	48.60	0.00
TRINITY_GAX8403(	hypothet	Chlamydomonas_eusti	48.60	0.00
TRINITY_GAX8561(	hypothet	Chlamydomonas_eusti	48.60	0.00
TRINITY_AGT2960(	poly [AI	Solanum_tuberosum	48.60	0.00
TRINITY_GAQ7843(	ubiquiti	Klebsormidium_niten	48.60	0.00
TRINITY_PNH0421(	hypothet	Tetrabaena_socialis	48.60	0.00
TRINITY_KXZ5664(	hypothet	Gonium_pectorale	48.60	0.00
TRINITY_GAQ8091(	hypothet	Klebsormidium_niten	48.60	0.00
TRINITY_PNW7617(	hypothet	Chlamydomonas_reinh	48.60	0.00
TRINITY_PSC7047(	tetratri	Micractinium_conduc	48.60	0.00
TRINITY_GAX7923(	hypothet	Chlamydomonas_eusti	48.60	0.00
TRINITY_PSC7639(	F-box SI	Micractinium_conduc	48.60	0.00

TRINITY_XP_0243	exosome	Physcomitrella_pate	48.60	0.00
TRINITY_XP_0016	predicte	Chlamydomonas_reinh	48.60	0.00
TRINITY_XP_0239	probable	Quercus_suber	48.60	0.00
TRINITY_GAX7677	hypothet	Chlamydomonas_eusti	48.60	0.00
TRINITY_KXZ4303	hypothet	Gonium_pectorale	48.60	0.00
TRINITY_RLM9773	myb-rela	Panicum_miliaceum	48.60	0.00
TRINITY_XP_0041	PREDICTE	Cucumis_sativus	48.60	0.00
TRINITY_EFJ2421	hypothet	Selaginella_moellen	48.60	0.00
TRINITY_KXZ4473	hypothet	Gonium_pectorale	48.60	0.00
TRINITY_XP_0029	uncharac	Selaginella_moellen	48.60	0.00
TRINITY_PNW8444	hypothet	Chlamydomonas_reinh	48.60	0.00
TRINITY_GAX8242	hypothet	Chlamydomonas_eusti	48.60	0.00
TRINITY_GAX8370	hypothet	Chlamydomonas_eusti	48.60	0.00
TRINITY_XP_0029	hypothet	Volvox_carteri	48.60	0.00
TRINITY_GBF9767	aminoacy	Raphidocelis_subcap	48.60	0.00
TRINITY_GAX8020	hypothet	Chlamydomonas_eusti	48.60	0.00
TRINITY_PNW8140	hypothet	Chlamydomonas_reinh	48.60	0.00
TRINITY_XP_0206	protein	Dendrobium_catenatu	48.60	0.00
TRINITY_GAX8621	hypothet	Chlamydomonas_eusti	48.60	0.00
TRINITY_XP_0029	hypothet	Volvox_carteri	48.60	0.00
TRINITY_XP_0029	hypothet	Volvox_carteri	48.60	0.00
TRINITY_GAQ7843	ubiquit	Klebsormidium_niten	48.60	0.00
TRINITY_GAX8569	hypothet	Chlamydomonas_eusti	48.60	0.00
TRINITY_XP_0191	PREDICTE	Ipomoea_nil	48.60	0.00
TRINITY_BAK0223	predicte	Hordeum_vulgare	48.60	0.00
TRINITY_PSC7196	acetyl-c	Micractinium_conduc	48.60	0.00
TRINITY_PNW8029	hypothet	Chlamydomonas_reinh	48.60	0.00
TRINITY_PNW8124	hypothet	Chlamydomonas_reinh	48.60	0.00
TRINITY_GAX8049	hypothet	Chlamydomonas_eusti	48.60	0.00
TRINITY_OAE2597	hypothet	Marchantia_polymorp	48.60	0.00
TRINITY_KMT0153	hypothet	Beta_vulgaris	48.60	0.00
TRINITY_XP_0120	phosphoe	Jatropha_curcas	48.50	0.00
TRINITY_XP_0056	acyl-CoA	Coccomyxa_subellips	48.50	0.00
TRINITY_PRW4534	serine p	Chlorella_sorokinia	48.50	0.00
TRINITY_XP_0017	predicte	Chlamydomonas_reinh	48.50	0.00
TRINITY_PNH1205	hypothet	Tetrabaena_socialis	48.50	0.00
TRINITY_EFJ1494	hypothet	Selaginella_moellen	48.50	0.00
TRINITY_GAY4284	hypothet	Citrus_unshiu	48.50	0.00
TRINITY_KDD7715	hypothet	Helicosporidium_sp.	48.50	0.00
TRINITY_P51824.2	RecName:	Solanum_tuberosum	48.50	0.00
TRINITY_XP_0029	flagella	Volvox_carteri	48.50	0.00
TRINITY_XP_0206	chaperon	Dendrobium_catenatu	48.50	0.00
TRINITY_XP_0218	ABC tran	Carica_papaya	48.50	0.00
TRINITY_XP_0028	coatomer	Arabidopsis_lyrata	48.50	0.00
TRINITY_XP_0014	predicte	Ostreococcus_sp._'l	48.50	0.00
TRINITY_XP_0106	PREDICTE	Beta_vulgaris	48.50	0.00
TRINITY_XP_0172	PREDICTE	Daucus_carota	48.50	0.00
TRINITY_KDO6747	hypothet	Citrus_sinensis	48.50	0.00
TRINITY_GAX7284	hypothet	Chlamydomonas_eusti	48.50	0.00
TRINITY_GAX7383	hypothet	Chlamydomonas_eusti	48.50	0.00
TRINITY_XP_0239	ras-like	Quercus_suber	48.50	0.00
TRINITY_GBG6252	hypothet	Chara_braunii	48.50	0.00
TRINITY_KJB7773	hypothet	Gossypium_raimondii	48.50	0.00
TRINITY_GAX7975	hypothet	Chlamydomonas_eusti	48.50	0.00
TRINITY_XP_0104	PREDICTE	Camelina_sativa	48.50	0.00
TRINITY_GAX8210	hypothet	Chlamydomonas_eusti	48.50	0.00
TRINITY_XP_0017	flagella	Chlamydomonas_reinh	48.50	0.00
TRINITY_GAX7546	hypothet	Chlamydomonas_eusti	48.50	0.00
TRINITY_CAA4928	gag,prot	Volvox_carteri	48.50	0.00
TRINITY_XP_0029	hypothet	Volvox_carteri	48.50	0.00
TRINITY_GAX8400	hypothet	Chlamydomonas_eusti	48.50	0.00
TRINITY_XP_0243	mannosyl	Physcomitrella_pate	48.50	0.00
TRINITY_XP_0243	serine/t	Physcomitrella_pate	48.50	0.00
TRINITY_GAQ8908	Electron	Klebsormidium_niten	48.50	0.00
TRINITY_XP_0016	predicte	Chlamydomonas_reinh	48.50	0.00
TRINITY_GAX8529	hypothet	Chlamydomonas_eusti	48.50	0.00

TRINITY_XP_0071!	hypothet	Phaseolus_vulgaris	48.50	0.00
TRINITY_PNW7455!	hypothet	Chlamydomonas_reinh	48.50	0.00
TRINITY_PNW8423!	hypothet	Chlamydomonas_reinh	48.50	0.00
TRINITY_VDD2290!	unnamed	Brassica_oleracea	48.50	0.00
TRINITY_GAX7546!	hypothet	Chlamydomonas_eusti	48.50	0.00
TRINITY_GAX7474!	hypothet	Chlamydomonas_eusti	48.50	0.00
TRINITY_XP_0058!	hypothet	Chlorella_variabili	48.50	0.00
TRINITY_XP_0136!	PREDICTI	Brassica_oleracea	48.50	0.00
TRINITY_GAX7828!	hypothet	Chlamydomonas_eusti	48.50	0.00
TRINITY_GAX7387!	hypothet	Chlamydomonas_eusti	48.50	0.00
TRINITY_PNW8859!	hypothet	Chlamydomonas_reinh	48.50	0.00
TRINITY_PSC7456!	DNA repa	Micractinium_conduc	48.50	0.00
TRINITY_XP_0064!	eukaryot	Citrus_clementina	48.50	0.00
TRINITY_PNW7793!	hypothet	Chlamydomonas_reinh	48.50	0.00
TRINITY_PNW8013!	hypothet	Chlamydomonas_reinh	48.50	0.00
TRINITY_GAX7788!	hypothet	Chlamydomonas_eusti	48.50	0.00
TRINITY_GAX8181!	hypothet	Chlamydomonas_eusti	48.50	0.00
TRINITY_GAX8166!	hypothet	Chlamydomonas_eusti	48.50	0.00
TRINITY_GAX7737!	hypothet	Chlamydomonas_eusti	48.50	0.00
TRINITY_XP_0238!	cAMP-dep	Quercus_suber	48.50	0.00
TRINITY_XP_0029!	hypothet	Volvox_carteri	48.50	0.00
TRINITY_OAE1961!	hypothet	Marchantia_polymorp	48.50	0.00
TRINITY_XP_0042!	subtilis	Solanum_lycopersicu	48.50	0.00
TRINITY_GBF9059!	hypothet	Raphidocelis_subcap	48.50	0.00
TRINITY_XP_0071!	hypothet	Phaseolus_vulgaris	48.50	0.00
TRINITY_ABK2221!	unknown	Picea_sitchensis	48.50	0.00
TRINITY_XP_0063!	PREDICTI	Solanum_tuberosum	48.40	0.00
TRINITY_YP_0088!	NADH deh	Entransia_fimbriata	48.40	0.00
TRINITY_XP_0221!	bifuncti	Momordica_charantia	48.40	0.00
TRINITY_GBG6364!	hypothet	Chara_braunii	48.40	0.00
TRINITY_XP_0056!	hypothet	Coccomyxa_subellips	48.40	0.00
TRINITY_XP_0238!	probable	Quercus_suber	48.40	0.00
TRINITY_XP_0075!	predicte	Bathycoccus_prasinc	48.40	0.00
TRINITY_XP_0208!	uncharac	Arabidopsis_lyrata	48.40	0.00
TRINITY_GAX7609!	hypothet	Chlamydomonas_eusti	48.40	0.00
TRINITY_KXZ4360!	hypothet	Gonium_pectorale	48.40	0.00
TRINITY_XP_0066!	PREDICTI	Oryza_brachyantha	48.40	0.00
TRINITY_XP_0056!	hypothet	Coccomyxa_subellips	48.40	0.00
TRINITY_AAQ6468!	NIMA-rel	Chlamydomonas_reinh	48.40	0.00
TRINITY_XP_0058!	hypothet	Chlorella_variabili	48.40	0.00
TRINITY_XP_0029!	ADC synt	Volvox_carteri	48.40	0.00
TRINITY_XP_0075!	predicte	Bathycoccus_prasinc	48.40	0.00
TRINITY_XP_0029!	hypothet	Volvox_carteri	48.40	0.00
TRINITY_XP_0017!	flagella	Chlamydomonas_reinh	48.40	0.00
TRINITY_GAX8186!	hypothet	Chlamydomonas_eusti	48.40	0.00
TRINITY_OMP0290!	ABC tran	Corchorus_olitorius	48.40	0.00
TRINITY_GAQ8812!	Putative	Klebsormidium_niten	48.40	0.00
TRINITY_PSC7474!	Methylc	Micractinium_conduc	48.40	0.00
TRINITY_KHN1382!	Putative	Glycine_soja	48.40	0.00
TRINITY_EEF2455!	3-oxoacy	Ricinus_communis	48.40	0.00
TRINITY_PTQ5024!	hypothet	Marchantia_polymorp	48.40	0.00
TRINITY_GBG6182!	hypothet	Chara_braunii	48.40	0.00
TRINITY_XP_0029!	hypothet	Volvox_carteri	48.40	0.00
TRINITY_XP_0016!	global t	Chlamydomonas_reinh	48.40	0.00
TRINITY_PIA3512!	hypothet	Aquilegia_coerulea	48.40	0.00
TRINITY_KMZ7001!	Zinc fir	Zostera_marina	48.40	0.00
TRINITY_XP_0091!	PREDICTI	Brassica_rapa	48.40	0.00
TRINITY_PNW7475!	hypothet	Chlamydomonas_reinh	48.40	0.00
TRINITY_XP_0229!	serine/t	Cucurbita_moschata	48.40	0.00
TRINITY_KXZ4811!	hypothet	Gonium_pectorale	48.40	0.00
TRINITY_CDO9996!	unnamed	Coffea_canephora	48.40	0.00
TRINITY_RID4819!	hypothet	Brassica_rapa	48.40	0.00
TRINITY_KXZ5625!	hypothet	Gonium_pectorale	48.40	0.00
TRINITY_PNW8637!	hypothet	Chlamydomonas_reinh	48.40	0.00
TRINITY_GAQ8511!	hypothet	Klebsormidium_niten	48.40	0.00
TRINITY_GAX7753!	hypothet	Chlamydomonas_eusti	48.40	0.00

TRINITY_KHG2079	Peroxis	Gossypium_arboreum	48.40	0.00
TRINITY_XP_0025	dynein	Micromonas_commoda	48.40	0.00
TRINITY_XP_0243	uncharac	Physcomitrella_pate	48.40	0.00
TRINITY_KXZ4792	hypothet	Gonium_pectorale	48.40	0.00
TRINITY_GAX7309	hypothet	Chlamydomonas_eusti	48.40	0.00
TRINITY_KXZ5371	hypothet	Gonium_pectorale	48.40	0.00
TRINITY_XP_0097	PREDICTE	Nicotiana_sylvestri	48.40	0.00
TRINITY_XP_0219	uncharac	Helianthus_annuus	48.40	0.00
TRINITY_GAX7308	hypothet	Chlamydomonas_eusti	48.40	0.00
TRINITY_XP_0029	hypothet	Volvox_carteri	48.40	0.00
TRINITY_PNH0939	Purple a	Tetrabaena_socialis	48.40	0.00
TRINITY_XP_0029	RWP-RK c	Volvox_carteri	48.40	0.00
TRINITY_EPS7352	hypothet	Genlisea_aurea	48.40	0.00
TRINITY_XP_0030	predicte	Micromonas_pusilla	48.40	0.00
TRINITY_XP_0138	hypothet	Monoraphidium_negle	48.40	0.00
TRINITY_BBC2847	serine/t	Yamagishiella_unicc	48.40	0.00
TRINITY_XP_0017	flagella	Chlamydomonas_reinh	48.40	0.00
TRINITY_GAX8139	hypothet	Chlamydomonas_eusti	48.40	0.00
TRINITY_PNW8784	hypothet	Chlamydomonas_reinh	48.40	0.00
TRINITY_PNH0492	hypothet	Tetrabaena_socialis	48.40	0.00
TRINITY_KXZ5653	hypothet	Gonium_pectorale	48.40	0.00
TRINITY_PNH1181	CDP-diac	Tetrabaena_socialis	48.40	0.00
TRINITY_XP_0029	hypothet	Volvox_carteri	48.40	0.00
TRINITY_XP_0202	ubiquiti	Asparagus_officinal	48.40	0.00
TRINITY_PNW7009	hypothet	Chlamydomonas_reinh	48.40	0.00
TRINITY_KXZ5174	hypothet	Gonium_pectorale	48.40	0.00
TRINITY_PNW7970	hypothet	Chlamydomonas_reinh	48.40	0.00
TRINITY_XP_0029	hypothet	Volvox_carteri	48.40	0.00
TRINITY_RWW8425	hypothet	Ensete_ventricosum	48.40	0.00
TRINITY_GBG8422	hypothet	Chara_braunii	48.30	0.00
TRINITY_XP_0200	heavy me	Ananas_comosus	48.30	0.00
TRINITY_XP_0063	PREDICTE	Solanum_tuberosum	48.30	0.00
TRINITY_XP_0029	hypothet	Volvox_carteri	48.30	0.00
TRINITY_XP_0239	probable	Quercus_suber	48.30	0.00
TRINITY_XP_0029	probable	Selaginella_moellen	48.30	0.00
TRINITY_XP_0043	PREDICTE	Fragaria_vesca	48.30	0.00
TRINITY_XP_0093	PREDICTE	Musa_acuminata	48.30	0.00
TRINITY_GBG6365	hypothet	Chara_braunii	48.30	0.00
TRINITY_XP_0158	zinc fin	Ziziphus_jujuba	48.30	0.00
TRINITY_XP_0056	hypothet	Coccomyxa_subellips	48.30	0.00
TRINITY_PON6944	Fructose	Parasponia_anderson	48.30	0.00
TRINITY_PNW7937	hypothet	Chlamydomonas_reinh	48.30	0.00
TRINITY_XP_0029	hypothet	Volvox_carteri	48.30	0.00
TRINITY_ERN0391	hypothet	Amborella_trichopod	48.30	0.00
TRINITY_GAQ8021	Band 7/S	Klebsormidium_niten	48.30	0.00
TRINITY_KXZ4988	hypothet	Gonium_pectorale	48.30	0.00
TRINITY_XP_0016	predicte	Chlamydomonas_reinh	48.30	0.00
TRINITY_PSC7687	Alpha-ga	Micractinium_conduc	48.30	0.00
TRINITY_XP_0243	beta-glu	Physcomitrella_pate	48.30	0.00
TRINITY_XP_0237	serine/t	Lactuca_sativa	48.30	0.00
TRINITY_XP_0029	methylst	Selaginella_moellen	48.30	0.00
TRINITY_XP_0016	puf prot	Chlamydomonas_reinh	48.30	0.00
TRINITY_GAX8216	hypothet	Chlamydomonas_eusti	48.30	0.00
TRINITY_XP_0110	PREDICTE	Populus_euphratica	48.30	0.00
TRINITY_XP_0029	hypothet	Volvox_carteri	48.30	0.00
TRINITY_XP_0205	54S ribo	Phalaenopsis_equest	48.30	0.00
TRINITY_EFJ1460	hypothet	Selaginella_moellen	48.30	0.00
TRINITY_GAX8305	hypothet	Chlamydomonas_eusti	48.30	0.00
TRINITY_GAX8554	hypothet	Chlamydomonas_eusti	48.30	0.00
TRINITY_BAK0735	predicte	Hordeum_vulgare	48.30	0.00
TRINITY_XP_0227	LIM doma	Durio_zibethinus	48.30	0.00
TRINITY_PTQ3482	hypothet	Marchantia_polymorp	48.30	0.00
TRINITY_GAX7418	hypothet	Chlamydomonas_eusti	48.30	0.00
TRINITY_PNH0393	Expansin	Tetrabaena_socialis	48.30	0.00
TRINITY_GAX8254	hypothet	Chlamydomonas_eusti	48.30	0.00
TRINITY_PRW5690	fumarate	Chlorella_sorokinia	48.30	0.00

TRINITY_GAQ8270[illegible]	Dyneins	Klebsormidium_niten	48.30	0
TRINITY_EPS6286[illegible]	hypothet	Genlisea_aurea	48.30	0.00
TRINITY_PNW6970[illegible]	hypothet	Chlamydomonas_reinh	48.30	0.00
TRINITY_PNW7625[illegible]	hypothet	Chlamydomonas_reinh	48.30	0.00
TRINITY_GAX7350[illegible]	hypothet	Chlamydomonas_eusti	48.30	0.00
TRINITY_BAJ9497[illegible]	predicte	Hordeum_vulgare	48.30	0.00
TRINITY_XP_0016[illegible]	predicte	Chlamydomonas_reinh	48.30	0.00
TRINITY_PRW4548[illegible]	ABC trar	Chlorella_sorokinia	48.30	0.00
TRINITY_GBF9588[illegible]	hypothet	Raphidocelis_subcap	48.30	0.00
TRINITY_XP_0029[illegible]	hypothet	Volvox_carteri	48.30	0.00

TRINITY_XP_0226	ABC tra	Vigna_radiata	48.30	0.00
TRINITY_GAX8339	hypothet	Chlamydomonas_eusti	48.30	0.00
TRINITY_XP_0138	hypothet	Monoraphidium_negle	48.30	0.00
TRINITY_GAX8063	hypothet	Chlamydomonas_eusti	48.30	0.00
TRINITY_XP_0029	hypothet	Volvox_carteri	48.30	0.00
TRINITY_XP_0029	hypothet	Volvox_carteri	48.30	0.00
TRINITY_GBG6886	Chlorid	Chara_braunii	48.30	0.00
TRINITY_GAX7285	hypothet	Chlamydomonas_eusti	48.30	0.00
TRINITY_XP_0017	protein	Chlamydomonas_reinh	48.30	0
TRINITY_XP_0167	PREDICT	Gossypium_hirsutum	48.30	0.00
TRINITY_GAX7432	hypothet	Chlamydomonas_eusti	48.30	0.00
TRINITY_GAQ8019	serine/	Klebsormidium_niten	48.30	0.00
TRINITY_EFJ0860	hypothet	Selaginella_moellen	48.20	0.00
TRINITY_RZC2385	Adenylat	Glycine_soja	48.20	0.00
TRINITY_GAQ8759	G-patch	Klebsormidium_niten	48.20	0.00
TRINITY_PNR4433	hypothet	Physcomitrella_pate	48.20	0.00
TRINITY_XP_0025	predict	Micromonas_commoda	48.20	0.00
TRINITY_XP_0239	probabl	Quercus_suber	48.20	0.00
TRINITY_KXZ5173	hypothet	Gonium_pectorale	48.20	0.00
TRINITY_XP_0024	vacuola	Sorghum_bicolor	48.20	0.00
TRINITY_PRW0585	decappi	Chlorella_sorokinia	48.20	0.00
TRINITY_XP_0238	ATP pho	Quercus_suber	48.20	0.00
TRINITY_XP_0215	cyclin-	Manihot_esculenta	48.20	0.00
TRINITY_GBG8367	hypothet	Chara_braunii	48.20	0.00
TRINITY_XP_0192	PREDICT	Nicotiana_attenuata	48.20	0.00
TRINITY_KXZ5127	hypothet	Gonium_pectorale	48.20	0.00
TRINITY_XP_0245	tetratr	Selaginella_moellen	48.20	0.00
TRINITY_XP_0056	DUF185-	Coccomyxa_subellips	48.20	0.00
TRINITY_PTQ3255	hypothet	Marchantia_polymorp	48.20	0.00
TRINITY_XP_0167	PREDICT	Gossypium_hirsutum	48.20	0.00
TRINITY_APX6448	geranyl	Haematococcus_lacus	48.20	0.00
TRINITY_PSC7142	PKHD-ty	Micractinium_conduc	48.20	0.00
TRINITY_XP_0120	DDB1- a	Jatropha_curcas	48.20	0.00
TRINITY_GAX8343	hypothet	Chlamydomonas_eusti	48.20	0.00
TRINITY_GAX8605	hypothet	Chlamydomonas_eusti	48.20	0.00
TRINITY_XP_0016	predict	Chlamydomonas_reinh	48.20	0.00
TRINITY_XP_0273	phosphat	Abrus_precatorius	48.20	0.00
TRINITY_XP_0105	PREDICT	Tarenaya_hasslerian	48.20	0.00
TRINITY_GAX7887	hypothet	Chlamydomonas_eusti	48.20	0.00
TRINITY_OAY4928	hypothet	Manihot_esculenta	48.20	0.00
TRINITY_KXZ5051	hypothet	Gonium_pectorale	48.20	0.00
TRINITY_GAX8036	hypothet	Chlamydomonas_eusti	48.20	0.00
TRINITY_GBF9708	hypothet	Raphidocelis_subcap	48.20	0.00
TRINITY_KHG1634	Lon pro	Gossypium_arboreum	48.20	0.00
TRINITY_GAX7959	hypothet	Chlamydomonas_eusti	48.20	0.00
TRINITY_XP_0237	zinc fi	Lactuca_sativa	48.20	0.00
TRINITY_XP_0075	unnamed	Bathycoccus_prasino	48.20	0.00
TRINITY_GBF9466	hypothet	Raphidocelis_subcap	48.20	0.00
TRINITY_GAX7783	hypothet	Chlamydomonas_eusti	48.20	0.00
TRINITY_ABH0932	leucine	Arachis_hypogaea	48.20	0.00
TRINITY_GAX7644	hypothet	Chlamydomonas_eusti	48.20	0.00
TRINITY_BAK0277	predict	Hordeum_vulgare	48.20	0.00
TRINITY_ARO5009	hypothet	Yamagishiella_unico	48.20	0.00
TRINITY_XP_0029	hypothet	Volvox_carteri	48.20	0.00
TRINITY_KXZ5653	hypothet	Gonium_pectorale	48.20	0.00
TRINITY_AAD2215	polyprot	Sorghum_bicolor	48.20	0.00
TRINITY_GAQ8429	alpha-m	Klebsormidium_niten	48.20	0.00
TRINITY_PNW7478	hypothet	Chlamydomonas_reinh	48.20	0.00
TRINITY_GAX7910	hypothet	Chlamydomonas_eusti	48.20	0.00
TRINITY_KXZ4333	hypothet	Gonium_pectorale	48.20	0.00
TRINITY_KXZ4839	hypothet	Gonium_pectorale	48.20	0.00
TRINITY_GAX7389	hypothet	Chlamydomonas_eusti	48.20	0.00
TRINITY_XP_0241	phospho	Rosa_chinensis	48.20	0.00
TRINITY_PNH0363	hypothet	Tetrabaena_socialis	48.20	0.00
TRINITY_PNW8903	hypothet	Chlamydomonas_reinh	48.20	0.00
TRINITY_PNW8041	hypothet	Chlamydomonas_reinh	48.20	0.00

TRINITY_XP_0239(	kinesin	Quercus_suber	48.20	0.00
TRINITY_KXZ4748	hypothet	Gonium_pectorale	48.20	0.00
TRINITY_XP_0056	hypothet	Coccomyxa_subellips	48.20	0.00
TRINITY_KXZ4131(	hypothet	Gonium_pectorale	48.20	0.00
TRINITY_GAX8097	hypothet	Chlamydomonas_eusti	48.20	0.00
TRINITY_GAX8235	hypothet	Chlamydomonas_eusti	48.20	0.00
TRINITY_XP_0139(	hypothet	Monoraphidium_negle	48.20	0.00
TRINITY_ERM9673	hypothet	Amborella_trichopod	48.20	0.00
TRINITY_XP_0202(	exosome	Cajanus_cajan	48.20	0.00
TRINITY_GAX8090	hypothet	Chlamydomonas_eusti	48.20	0.00
TRINITY_XP_0184	PREDICTI	Raphanus_sativus	48.20	0.00
TRINITY_PNW7865	hypothet	Chlamydomonas_reinh	48.20	0.00
TRINITY_KXZ4926(	hypothet	Gonium_pectorale	48.20	0.00
TRINITY_GBF9588	hypothet	Raphidocelis_subcap	48.20	0.00
TRINITY_PNW7003(	hypothet	Chlamydomonas_reinh	48.20	0.00
TRINITY_XP_0217	NEP1-int	Chenopodium_quinoa	48.10	0.00
TRINITY_XP_0136	uridine	Brassica_napus	48.10	0.00
TRINITY_GAQ8223	Actin-re	Klebsormidium_niten	48.10	0.00
TRINITY_XP_0029	hypothet	Volvox_carteri	48.10	0.00
TRINITY_GAX8647	hypothet	Chlamydomonas_eusti	48.10	0.00
TRINITY_PLY7631	hypothet	Lactuca_sativa	48.10	0.00
TRINITY_GAX7970	hypothet	Chlamydomonas_eusti	48.10	0.00
TRINITY_GAQ8019	serine/t	Klebsormidium_niten	48.10	0.00
TRINITY_GBF9357	vacuola	Raphidocelis_subcap	48.10	0.00
TRINITY_GAX8510	hypothet	Chlamydomonas_eusti	48.10	0.00
TRINITY_EFJ1402	hypothet	Selaginella_moellen	48.10	0.00
TRINITY_XP_0056	JmjC-dor	Coccomyxa_subellips	48.10	0.00
TRINITY_KXZ5473	hypothet	Gonium_pectorale	48.10	0.00
TRINITY_XP_0029	mitochor	Volvox_carteri	48.10	0.00
TRINITY_XP_0030	predicte	Micromonas_pusilla	48.10	0.00
TRINITY_GAX7270(	hypothet	Chlamydomonas_eusti	48.10	0.00
TRINITY_XP_0239(	guanine	Quercus_suber	48.10	0.00
TRINITY_XP_0191	PREDICTI	Ipomoea_nil	48.10	0.00
TRINITY_PNW7438	hypothet	Chlamydomonas_reinh	48.10	0.00
TRINITY_XP_0030	flagella	Micromonas_pusilla	48.10	0.00
TRINITY_XP_0239(	succinat	Quercus_suber	48.10	0.00
TRINITY_PRW5724	acetyl-	Chlorella_sorokinia	48.10	0.00
TRINITY_RAL5251	hypothet	Cuscuta_australis	48.10	0.00
TRINITY_KXZ5009	hypothet	Gonium_pectorale	48.10	0.00
TRINITY_XP_0029	hypothet	Volvox_carteri	48.10	0.00
TRINITY_GBF8748	hypothet	Raphidocelis_subcap	48.10	0.00
TRINITY_GAX7841	hypothet	Chlamydomonas_eusti	48.10	0.00
TRINITY_GAX8457	hypothet	Chlamydomonas_eusti	48.10	0.00
TRINITY_GBF8863(	cystath	Raphidocelis_subcap	48.10	0.00
TRINITY_EFJ0486(	hypothet	Selaginella_moellen	48.10	0.00
TRINITY_XP_0068	ribosoma	Amborella_trichopod	48.10	0.00
TRINITY_XP_0016	predicte	Chlamydomonas_reinh	48.10	0.00
TRINITY_RQO9868	hypothet	Populus_trichocarpa	48.10	0.00
TRINITY_XP_0158	unchara	Ziziphus_jujuba	48.10	0.00
TRINITY_GBG6003	hypothet	Chara_braunii	48.10	0.00
TRINITY_XP_0238	serine/t	Quercus_suber	48.10	0.00
TRINITY_XP_0056	aspartat	Coccomyxa_subellips	48.10	0.00
TRINITY_XP_0238	cytosol	Quercus_suber	48.10	0.00
TRINITY_BAA7858	hypothet	Chlamydomonas_sp._H	48.10	0.00
TRINITY_XP_0245	DEAD-bo	Selaginella_moellen	48.10	0.00
TRINITY_RWR7465	class I	Cinnamomum_micranth	48.10	0.00
TRINITY_GAX8323	hypothet	Chlamydomonas_eusti	48.10	0.00
TRINITY_XP_0138	Serine/t	Monoraphidium_negle	48.10	0.00
TRINITY_PNW8018	hypothet	Chlamydomonas_reinh	48.10	0.00
TRINITY_XP_0238	phospho	Quercus_suber	48.10	0.00
TRINITY_XP_0093	PREDICTI	Musa_acuminata	48.10	0.00
TRINITY_XP_0030	dp-like	Micromonas_pusilla	48.10	0.00
TRINITY_XP_0245	zinc fi	Selaginella_moellen	48.10	0.00
TRINITY_GAX8238	hypothet	Chlamydomonas_eusti	48.10	0.00
TRINITY_PNW8874	hypothet	Chlamydomonas_reinh	48.10	0.00
TRINITY_PNW6993	hypothet	Chlamydomonas_reinh	48.10	0.00

TRINITY_GAX8217	hypothet	Chlamydomonas_eusti	48.10	0.00
TRINITY_XP_0017	mitogen-	Chlamydomonas_reinh	48.10	0.00
TRINITY_APW8374	caroten	Dunaliella_salina	48.10	0.00
TRINITY_XP_0105	PREDICTI	Tarenaya_hasslerian	48.10	0.00
TRINITY_GAX8072	hypothet	Chlamydomonas_eusti	48.10	0.00
TRINITY_GAX8386	hypothet	Chlamydomonas_eusti	48.10	0.00
TRINITY_XP_0029	hypothet	Volvox_carteri	48.10	0.00
TRINITY_GAX8005	hypothet	Chlamydomonas_eusti	48.10	0.00
TRINITY_GAX7458	hypothet	Chlamydomonas_eusti	48.10	0.00
TRINITY_KXZ4199	hypothet	Gonium_pectorale	48.10	0.00
TRINITY_KXZ4403	hypothet	Gonium_pectorale	48.10	0.00
TRINITY_XP_0138	hypothet	Monoraphidium_negle	48.10	0.00
TRINITY_XP_0239	AMP dear	Quercus_suber	48.10	0.00
TRINITY_XP_0139	ATP synt	Monoraphidium_negle	48.10	0.00
TRINITY_PNW7159	hypothet	Chlamydomonas_reinh	48.10	0.00
TRINITY_GAX8422	hypothet	Chlamydomonas_eusti	48.10	0.00
TRINITY_XP_0191	PREDICTI	Ipomoea_nil	48.00	0.00
TRINITY_XP_0139	Dynein-1	Monoraphidium_negle	48.00	0.00
TRINITY_XP_0120	probable	Jatropha_curcas	48.00	0.00
TRINITY_PNW7471	hypothet	Chlamydomonas_reinh	48.00	0.00
TRINITY_PNR5083	hypothet	Physcomitrella_pate	48.00	0.00
TRINITY_GAQ7922	hypothet	Klebsormidium_niten	48.00	0.00
TRINITY_PNH0920	Tiny mac	Tetrabaena_socialis	48.00	0.00
TRINITY_PSC7078	mitogen-	Micractinium_conduc	48.00	0.00
TRINITY_XP_0237	lariat c	Lactuca_sativa	48.00	0.00
TRINITY_XP_0245	striatec	Selaginella_moellen	48.00	0.00
TRINITY_XP_0238	zinc hor	Quercus_suber	48.00	0.00
TRINITY_GAX8194	hypothet	Chlamydomonas_eusti	48.00	0.00
TRINITY_PNH1118	Mitochor	Tetrabaena_socialis	48.00	0.00
TRINITY_XP_0243	eukaryot	Physcomitrella_pate	48.00	0.00
TRINITY_PNW8849	hypothet	Chlamydomonas_reinh	48.00	0.00
TRINITY_XP_0124	PREDICTI	Gossypium_raimondii	48.00	0.00
TRINITY_XP_0029	hypothet	Volvox_carteri	48.00	0.00
TRINITY_XP_0036	ras-rela	Medicago_truncatula	48.00	0.00
TRINITY_XP_0029	hypothet	Volvox_carteri	48.00	0.00
TRINITY_PSC7305	ATP-depe	Micractinium_conduc	48.00	0.00
TRINITY_XP_0036	nucleola	Medicago_truncatula	48.00	0.00
TRINITY_XP_0016	DNA dama	Chlamydomonas_reinh	48.00	0.00
TRINITY_BAK0043	predicte	Hordeum_vulgare	48.00	0.00
TRINITY_KXZ4785	hypothet	Gonium_pectorale	48.00	0.00
TRINITY_KZV1521	citrate	Dorcoceras_hygromet	48.00	0.00
TRINITY_XP_0164	PREDICTI	Nicotiana_tabacum	48.00	0.00
TRINITY_GAQ8393	hypothet	Klebsormidium_niten	48.00	0.00
TRINITY_XP_0139	oligopep	Monoraphidium_negle	48.00	0.00
TRINITY_XP_0029	hypothet	Volvox_carteri	48.00	0.00
TRINITY_GBF9382	pre trar	Raphidocelis_subcap	48.00	0.00
TRINITY_KXZ5130	hypothet	Gonium_pectorale	48.00	0.00
TRINITY_GAX8351	hypothet	Chlamydomonas_eusti	48.00	0.00
TRINITY_XP_0082	PREDICTI	Prunus_mume	48.00	0.00
TRINITY_KXZ5408	hypothet	Gonium_pectorale	48.00	0.00
TRINITY_XP_0102	PREDICTI	Nelumbo_nucifera	48.00	0.00
TRINITY_GAX7572	hypothet	Chlamydomonas_eusti	48.00	0.00
TRINITY_KXZ5571	hypothet	Gonium_pectorale	48.00	0.00
TRINITY_XP_0239	glutamat	Quercus_suber	48.00	0.00
TRINITY_XP_0029	fructose	Volvox_carteri	48.00	0.00
TRINITY_GAX7593	hypothet	Chlamydomonas_eusti	48.00	0.00
TRINITY_XP_0056	Shwachma	Coccomyxa_subellips	48.00	0.00
TRINITY_XP_0221	cycloeu	Momordica_charantia	48.00	0.00
TRINITY_XP_0029	kinesin-	Volvox_carteri	48.00	0.00
TRINITY_GAX8010	hypothet	Chlamydomonas_eusti	48.00	0.00
TRINITY_XP_0273	ethanola	Abrus_precatorius	48.00	0.00
TRINITY_XP_0238	kinesin	Quercus_suber	48.00	0.00
TRINITY_GAX7916	hypothet	Chlamydomonas_eusti	48.00	0.00
TRINITY_GAX7401	hypothet	Chlamydomonas_eusti	48.00	0.00
TRINITY_AMB1903	actin, p	Panax_ginseng	48.00	0.00
TRINITY_KQK1529	hypothet	Brachypodium_distac	48.00	0.00

TRINITY_GAX8371{	hypothet	Chlamydomonas_eusti	48.00	0.00
TRINITY_GAX7350{	hypothet	Chlamydomonas_eusti	48.00	0.00
TRINITY_XP_0017(	flagella	Chlamydomonas_reinh	48.00	0.00
TRINITY_XP_0105;	PREDICTI	Tarenaya_hasslerian	48.00	0.00
TRINITY_GAX7984{	hypothet	Chlamydomonas_eusti	48.00	0.00
TRINITY_CAA4928:	gag,prot	Volvox_carteri	48.00	0.00
TRINITY_PNR4792;	hypothet	Physcomitrella_pate	47.90	0.00
TRINITY_OAY7023{	ABC trar	Ananas_comosus	47.90	0.00
TRINITY_XP_0106(	PREDICTI	Beta_vulgaris	47.90	0.00
TRINITY_XP_0206{	60S ribc	Dendrobium_catenatu	47.90	0.00
TRINITY_XP_0058<	hypothet	Chlorella_variabili	47.90	0.00
TRINITY_XP_0109:	PREDICTI	Elaeis_guineensis	47.90	0.00
TRINITY_XP_0110:	PREDICTI	Populus_euphratica	47.90	0.00
TRINITY_GAX7418;	hypothet	Chlamydomonas_eusti	47.90	0.00
TRINITY_GAX7797:	hypothet	Chlamydomonas_eusti	47.90	0.00
TRINITY_GAQ8839(	Prefold:	Klebsormidium_niten	47.90	0.00
TRINITY_BAK0043!	predicte	Hordeum_vulgare	47.90	0.00
TRINITY_XP_0024!	ATP-depe	Sorghum_bicolor	47.90	0.00
TRINITY_XP_0030{	Pyridoxa	Ostreococcus_tauri	47.90	0.00
TRINITY_GAQ8798(	Phosphat	Klebsormidium_niten	47.90	0.00
TRINITY_XP_0243{	eukaryot	Physcomitrella_pate	47.90	0.00
TRINITY_GAX7633{	hypothet	Chlamydomonas_eusti	47.90	0.00
TRINITY_GAQ9065{	GroES-l:	Klebsormidium_niten	47.90	0.00
TRINITY_GAQ8253	Transcr:	Klebsormidium_niten	47.90	0.00
TRINITY_PNW8784{	hypothet	Chlamydomonas_reinh	47.90	0.00
TRINITY_KXZ4807:	hypothet	Gonium_pectorale	47.90	0.00
TRINITY_GAX8256	hypothet	Chlamydomonas_eusti	47.90	0.00
TRINITY_PNW7334:	hypothet	Chlamydomonas_reinh	47.90	0.00
TRINITY_GAX7975:	hypothet	Chlamydomonas_eusti	47.90	0.00
TRINITY_EPS7228{	hypothet	Genlisea_aurea	47.90	0.00
TRINITY_GAQ8538:	Putative	Klebsormidium_niten	47.90	0.00
TRINITY_XP_0056<	hypothet	Coccomyxa_subellips	47.90	0.00
TRINITY_GAX8355;	hypothet	Chlamydomonas_eusti	47.90	0.00
TRINITY_XP_0238	protein	Quercus_suber	47.90	0.00
TRINITY_KXZ5282{	hypothet	Gonium_pectorale	47.90	0.00
TRINITY_PNW7138<	hypothet	Chlamydomonas_reinh	47.90	0.00
TRINITY_PNW7022{	hypothet	Chlamydomonas_reinh	47.90	0.00
TRINITY_PNH1075;	putative	Tetrabaena_socialis	47.90	0.00
TRINITY_XP_0238	phospho-	Quercus_suber	47.90	0.00
TRINITY_XP_0014;	predicte	Ostreococcus_sp._'l	47.90	0.00
TRINITY_GAX8021;	hypothet	Chlamydomonas_eusti	47.90	0.00
TRINITY_RAL5137;	hypothet	Cuscuta_australis	47.90	0.00
TRINITY_XP_0025(	methiony	Micromonas_commoda	47.90	0.00
TRINITY_XP_0017(	superox:	Chlamydomonas_reinh	47.90	0.00
TRINITY_BAJ9390!	predicte	Hordeum_vulgare	47.90	0.00
TRINITY_XP_0029!	hypothet	Volvox_carteri	47.90	0.00
TRINITY_KXZ4303;	hypothet	Gonium_pectorale	47.90	0.00
TRINITY_XP_0197(	PREDICTI	Elaeis_guineensis	47.90	0.00
TRINITY_GAX8007:	hypothet	Chlamydomonas_eusti	47.90	0.00
TRINITY_GAX8220(	hypothet	Chlamydomonas_eusti	47.90	0.00
TRINITY_XP_0243{	PAN2-PAI	Physcomitrella_pate	47.90	0.00
TRINITY_PNH0505<	hypothet	Tetrabaena_socialis	47.90	0.00
TRINITY_PNW7096:	hypothet	Chlamydomonas_reinh	47.90	0.00
TRINITY_GBF8951{	multidrı	Raphidocelis_subcap	47.90	0.00
TRINITY_XP_0139(	dual-spe	Monoraphidium_negle	47.90	0.00
TRINITY_AFK3975:	unknown	Lotus_japonicus	47.90	0.00
TRINITY_XP_0029!	hypothet	Volvox_carteri	47.90	0.00
TRINITY_GAX7905:	hypothet	Chlamydomonas_eusti	47.90	0.00
TRINITY_GAX7465(	hypothet	Chlamydomonas_eusti	47.90	0.00
TRINITY_GAX7369	hypothet	Chlamydomonas_eusti	47.90	0.00
TRINITY_KXZ4678{	hypothet	Gonium_pectorale	47.90	0.00
TRINITY_KXZ5320{	hypothet	Gonium_pectorale	47.90	0.00
TRINITY_PNW7082{	hypothet	Chlamydomonas_reinh	47.90	0.00
TRINITY_PNW8847	hypothet	Chlamydomonas_reinh	47.90	0.00
TRINITY_BAK0277{	predicte	Hordeum_vulgare	47.90	0.00
TRINITY_BAK0277{	predicte	Hordeum_vulgare	47.90	0.00

TRINITY_KMZ7564	Serine/t	Zostera_marina	47.90	0.00
TRINITY_GAX8074	hypothet	Chlamydomonas_eusti	47.90	0.00
TRINITY_GAX8484	hypothet	Chlamydomonas_eusti	47.90	0.00
TRINITY_KNA0858	hypothet	Spinacia_oleracea	47.90	0.00
TRINITY_GAX7579	hypothet	Chlamydomonas_eusti	47.90	0.00
TRINITY_GAQ9305	hypothet	Klebsormidium_niten	47.90	0.00
TRINITY_XP_0139	Putative	Monoraphidium_negle	47.90	0.00
TRINITY_GAX8570	hypothet	Chlamydomonas_eusti	47.80	0.00
TRINITY_GAV8360	Response	Cephalotus_follicul	47.80	0.00
TRINITY_PNH0231	Leucine	Tetrabaena_socialis	47.80	0.00
TRINITY_XP_0120	serine/t	Jatropha_curcas	47.80	0.00
TRINITY_XP_0202	glycerol	Cajanus_cajan	47.80	0.00
TRINITY_EPS6995	hypothet	Genlisea_aurea	47.80	0.00
TRINITY_AFK3519	unknown	Lotus_japonicus	47.80	0.00
TRINITY_XP_0165	PREDICTE	Capsicum_annuum	47.80	0.00
TRINITY_GBG0049	formate	Raphidocelis_subcap	47.80	0.00
TRINITY_XP_0098	PREDICTE	Nicotiana_sylvestri	47.80	0.00
TRINITY_XP_0239	putative	Quercus_suber	47.80	0.00
TRINITY_GBG8033	hypothet	Chara_braunii	47.80	0.00
TRINITY_XP_0241	LOW QUAI	Rosa_chinensis	47.80	0.00
TRINITY_KNA1649	hypothet	Spinacia_oleracea	47.80	0.00
TRINITY_XP_0239	ras-like	Quercus_suber	47.80	0.00
TRINITY_XP_0238	uncharac	Quercus_suber	47.80	0.00
TRINITY_PHT9950	Protein	Capsicum_chinense	47.80	0.00
TRINITY_XP_0105	PREDICTE	Tarenaya_hasslerian	47.80	0.00
TRINITY_PHT2702	Protein	Capsicum_baccatum	47.80	0.00
TRINITY_XP_0273	uncharac	Abrus_precatorius	47.80	0.00
TRINITY_GAX7593	hypothet	Chlamydomonas_eusti	47.80	0.00
TRINITY_RDX6862	ABC tran	Mucuna_pruriens	47.80	0.00
TRINITY_PNR4128	hypothet	Physcomitrella_pate	47.80	0.00
TRINITY_GAX8607	hypothet	Chlamydomonas_eusti	47.80	0.00
TRINITY_GAX8491	hypothet	Chlamydomonas_eusti	47.80	0.00
TRINITY_XP_0239	myosin-1	Quercus_suber	47.80	0.00
TRINITY_PNW8185	hypothet	Chlamydomonas_reinh	47.80	0.00
TRINITY_GBF9104	hypothet	Raphidocelis_subcap	47.80	0.00
TRINITY_PSC7127	exodeoxy	Micractinium_conduc	47.80	0.00
TRINITY_PIA5906	hypothet	Aquilegia_coerulea	47.80	0.00
TRINITY_XP_0241	transpo	Rosa_chinensis	47.80	0.00
TRINITY_XP_0029	hypothet	Volvox_carteri	47.80	0.00
TRINITY_XP_0029	hypothet	Volvox_carteri	47.80	0.00
TRINITY_GAX8371	hypothet	Chlamydomonas_eusti	47.80	0.00
TRINITY_GAX8075	hypothet	Chlamydomonas_eusti	47.80	0.00
TRINITY_GBF9395	signal p	Raphidocelis_subcap	47.80	0.00
TRINITY_XP_0016	amino ac	Chlamydomonas_reinh	47.80	0.00
TRINITY_GBF9419	calcium	Raphidocelis_subcap	47.80	0.00
TRINITY_XP_0016	low-CO2-	Chlamydomonas_reinh	47.80	0.00
TRINITY_GAX7837	hypothet	Chlamydomonas_eusti	47.80	0.00
TRINITY_KXZ4506	hypothet	Gonium_pectorale	47.80	0.00
TRINITY_GAX8436	hypothet	Chlamydomonas_eusti	47.80	0.00
TRINITY_KXZ5159	hypothet	Gonium_pectorale	47.80	0.00
TRINITY_XP_0029	histone	Volvox_carteri	47.80	0.00
TRINITY_KXZ5576	hypothet	Gonium_pectorale	47.80	0.00
TRINITY_XP_0029	hypothet	Volvox_carteri	47.80	0.00
TRINITY_OAE3450	hypothet	Marchantia_polymorp	47.80	0.00
TRINITY_XP_0056	hypothet	Coccomyxa_subellips	47.80	0.00
TRINITY_PKA5227	Metal tr	Apostasia_shenzheni	47.80	0.00
TRINITY_XP_0028	elongati	Arabidopsis_lyrata	47.80	0.00
TRINITY_PRW5075	Formylte	Chlorella_sorokinia	47.80	0.00
TRINITY_XP_0017	chloropl	Chlamydomonas_reinh	47.80	0.00
TRINITY_XP_0239	ER-deriv	Quercus_suber	47.80	0.00
TRINITY_GAQ8006	oligosac	Klebsormidium_niten	47.80	0.00
TRINITY_KXZ4748	hypothet	Gonium_pectorale	47.80	0.00
TRINITY_XP_0044	uncharac	Cicer_arietinum	47.80	0.00
TRINITY_XP_0030	Major fa	Ostreococcus_tauri	47.80	0.00
TRINITY_GAX8171	hypothet	Chlamydomonas_eusti	47.80	0.00
TRINITY_PRW5955	FAD synt	Chlorella_sorokinia	47.80	0.00

TRINITY_PNW7730(	hypothet	Chlamydomonas_reinh	47.80	0.00
TRINITY_PNW7244!	hypothet	Chlamydomonas_reinh	47.80	0.00
TRINITY_GAX7493!	hypothet	Chlamydomonas_eusti	47.80	0.00
TRINITY_XP_0029!	hypothet	Volvox_carteri	47.80	0.00
TRINITY_GAX8374(	hypothet	Chlamydomonas_eusti	47.80	0.00
TRINITY_XP_0110!	PREDICTI	Populus_euphratica	47.80	0.00
TRINITY_XP_0029	hypothet	Volvox_carteri	47.80	0.00
TRINITY_GAX7389	hypothet	Chlamydomonas_eusti	47.80	0.00
TRINITY_PRW6083	chitinas	Chlorella_sorokinia	47.80	0.00
TRINITY_KXZ4791	hypothet	Gonium_pectorale	47.80	0.00
TRINITY_GAX8472	hypothet	Chlamydomonas_eusti	47.80	0.00
TRINITY_GAX8439(	hypothet	Chlamydomonas_eusti	47.80	0.00
TRINITY_OWM7045	hypothet	Punica_granatum	47.80	0.00
TRINITY_KXZ4788	hypothet	Gonium_pectorale	47.80	0.00
TRINITY_GBF9558	hypothet	Raphidocelis_subcap	47.80	0.00
TRINITY_PSC6926	snurport	Micractinium_conduc	47.80	0.00
TRINITY_GAQ8495	hypothet	Klebsormidium_niten	47.80	0.00
TRINITY_XP_0016	predicte	Chlamydomonas_reinh	47.80	0.00
TRINITY_PNW8423	hypothet	Chlamydomonas_reinh	47.80	0.00
TRINITY_XP_0029	hypothet	Volvox_carteri	47.80	0.00
TRINITY_GAX7888	hypothet	Chlamydomonas_eusti	47.80	0.00
TRINITY_PNW7968	hypothet	Chlamydomonas_reinh	47.80	0.00
TRINITY_ESR6345	hypothet	Citrus_clementina	47.80	0.00
TRINITY_GAX7940	hypothet	Chlamydomonas_eusti	47.80	0.00
TRINITY_XP_0071	hypothet	Phaseolus_vulgaris	47.80	0.00
TRINITY_XP_0116	PREDICTI	Cucumis_sativus	47.80	0.00
TRINITY_XP_0134	glutamir	Medicago_truncatula	47.70	0.00
TRINITY_KDD7187	acyl-Co	Helicosporidium_sp.	47.70	0.00
TRINITY_KGN5787	hypothet	Cucumis_sativus	47.70	0.00
TRINITY_XP_0167	PREDICTI	Gossypium_hirsutum	47.70	0.00
TRINITY_PRW5102	ADP-ribo	Chlorella_sorokinia	47.70	0.00
TRINITY_AYK2756	agamous-	Morus_alba	47.70	0.00
TRINITY_XP_0239	ras-like	Quercus_suber	47.70	0.00
TRINITY_XP_0228	protein-	Olea_europaea	47.70	0.00
TRINITY_XP_0100	PREDICTI	Eucalyptus_grandis	47.70	0.00
TRINITY_KXZ4519	hypothet	Gonium_pectorale	47.70	0.00
TRINITY_XP_0084	PREDICTI	Cucumis_melo	47.70	0.00
TRINITY_PHT2702	Protein	Capsicum_baccatum	47.70	0.00
TRINITY_KXZ5456	hypothet	Gonium_pectorale	47.70	0.00
TRINITY_KXZ5518	hypothet	Gonium_pectorale	47.70	0.00
TRINITY_XP_0066	PREDICTI	Oryza_brachyantha	47.70	0.00
TRINITY_BAJ9866	predicte	Hordeum_vulgare	47.70	0.00
TRINITY_GAX7909	hypothet	Chlamydomonas_eusti	47.70	0.00
TRINITY_XP_0213	kinesin-	Sorghum_bicolor	47.70	0.00
TRINITY_PNH0688	Transmer	Tetrabaena_socialis	47.70	0.00
TRINITY_XP_0029	hypothet	Volvox_carteri	47.70	0.00
TRINITY_XP_0056	pyruvate	Coccomyxa_subellips	47.70	0.00
TRINITY_XP_0228	Rieske	Ostreococcus_tauri	47.70	0.00
TRINITY_RWW9036(	hypothet	Ensete_ventricosum	47.70	0.00
TRINITY_GAX7733	hypothet	Chlamydomonas_eusti	47.70	0.00
TRINITY_XP_0238	kinesin	Quercus_suber	47.70	0.00
TRINITY_GAX7955	hypothet	Chlamydomonas_eusti	47.70	0.00
TRINITY_GAX7621	hypothet	Chlamydomonas_eusti	47.70	0.00
TRINITY_PRW6143	pre-rRN	Chlorella_sorokinia	47.70	0.00
TRINITY_XP_0029	hypothet	Volvox_carteri	47.70	0.00
TRINITY_XP_0029	hypothet	Volvox_carteri	47.70	0.00
TRINITY_KHG2386	NADP-dep	Gossypium_arboreum	47.70	0.00
TRINITY_GAX8379	hypothet	Chlamydomonas_eusti	47.70	0.00
TRINITY_OAE2923	hypothet	Marchantia_polymorp	47.70	0.00
TRINITY_XP_0240	unchara	Citrus_clementina	47.70	0.00
TRINITY_XP_0139(	Nodulat	Monoraphidium_negle	47.70	0.00
TRINITY_GBF9000	short-ch	Raphidocelis_subcap	47.70	0.00
TRINITY_KXZ5546	hypothet	Gonium_pectorale	47.70	0.00
TRINITY_PNW7526	hypothet	Chlamydomonas_reinh	47.70	0.00
TRINITY_GAQ9254	hypothet	Klebsormidium_niten	47.70	0.00
TRINITY_GAX7292	hypothet	Chlamydomonas_eusti	47.70	0.00

TRINITY_VDC9663	unnamed	Brassica_rapa	47.70	0.00
TRINITY_GAX8288	hypothet	Chlamydomonas_eusti	47.70	0.00
TRINITY_GAQ8218	jmjc dor	Klebsormidium_niten	47.70	0.00
TRINITY_XP_0029	F-box p	Selaginella_moellen	47.70	0.00
TRINITY_PSC7399	heme oxy	Micractinium_conduc	47.70	0.00
TRINITY_EEF2383	hypothet	Ricinus_communis	47.70	0.00
TRINITY_GBF8909	clavamir	Raphidocelis_subcap	47.70	0.00
TRINITY_KXZ5302	hypothet	Gonium_pectorale	47.70	0.00
TRINITY_XP_0029	hypothet	Volvox_carteri	47.70	0.00
TRINITY_PNH0593	hypothet	Tetrabaena_socialis	47.70	0.00
TRINITY_BAK0302	predicte	Hordeum_vulgare	47.70	0.00
TRINITY_GAX8614	hypothet	Chlamydomonas_eusti	47.70	0.00
TRINITY_PRW5819	acetyl-	Chlorella_sorokinia	47.70	0.00
TRINITY_XP_0238	uncharac	Quercus_suber	47.70	0.00
TRINITY_RYR2416	hypothet	Arachis_hypogaea	47.70	0.00
TRINITY_RID8114	hypothet	Brassica_rapa	47.70	0.00
TRINITY_GAX7688	hypothet	Chlamydomonas_eusti	47.70	0.00
TRINITY_GAX8199	hypothet	Chlamydomonas_eusti	47.70	0.00
TRINITY_GAX7273	hypothet	Chlamydomonas_eusti	47.70	0.00
TRINITY_XP_0029	hypothet	Volvox_carteri	47.70	0.00
TRINITY_GAX7983	hypothet	Chlamydomonas_eusti	47.70	0.00
TRINITY_GAX7314	hypothet	Chlamydomonas_eusti	47.70	0.00
TRINITY_XP_0216	casein	Manihot_esculenta	47.70	0.00
TRINITY_XP_0156	PREDICTI	Oryza_brachyantha	47.60	0.00
TRINITY_XP_0243	isoleuc	Physcomitrella_pate	47.60	0.00
TRINITY_XP_0201	cell nur	Aegilops_tauschii	47.60	0.00
TRINITY_GAQ8814	Oxidatio	Klebsormidium_niten	47.60	0.00
TRINITY_XP_0200	ubiquinc	Ananas_comosus	47.60	0.00
TRINITY_XP_0227	GRF1-int	Durio_zibethinus	47.60	0.00
TRINITY_NP_1970	glucose-	Arabidopsis_thalian	47.60	0.00
TRINITY_XP_0036	PREDICTI	Vitis_vinifera	47.60	0.00
TRINITY_GAX8389	hypothet	Chlamydomonas_eusti	47.60	0.00
TRINITY_XP_0201	choline-	Ananas_comosus	47.60	0.00
TRINITY_XP_0176	PREDICTI	Gossypium_arboreum	47.60	0.00
TRINITY_OAE2747	hypothet	Marchantia_polymorp	47.60	0.00
TRINITY_XP_0064	ras-rela	Citrus_clementina	47.60	0.00
TRINITY_XP_0064	putative	Citrus_sinensis	47.60	0.00
TRINITY_GBG7211	hypothet	Chara_braunii	47.60	0.00
TRINITY_XP_0217	fimbrin-	Chenopodium_quinoa	47.60	0.00
TRINITY_GBG4810	hypothet	Chara_braunii	47.60	0.00
TRINITY_GBG6183	hypothet	Chara_braunii	47.60	0.00
TRINITY_RLN1279	uncharac	Panicum_miliaceum	47.60	0.00
TRINITY_XP_0241	DCC fam	Rosa_chinensis	47.60	0.00
TRINITY_OVA1868	Clathrir	Macleaya_cordata	47.60	0.00
TRINITY_GAX7770	hypothet	Chlamydomonas_eusti	47.60	0.00
TRINITY_GAX8120	hypothet	Chlamydomonas_eusti	47.60	0.00
TRINITY_KXZ5043	hypothet	Gonium_pectorale	47.60	0.00
TRINITY_PNW7673	hypothet	Chlamydomonas_reinh	47.60	0.00
TRINITY_XP_0029	hypothet	Volvox_carteri	47.60	0.00
TRINITY_GAX7381	hypothet	Chlamydomonas_eusti	47.60	0.00
TRINITY_XP_0192	PREDICTI	Nicotiana_attenuata	47.60	0.00
TRINITY_XP_0016	oxygen-e	Chlamydomonas_reinh	47.60	0.00
TRINITY_PNW8487	hypothet	Chlamydomonas_reinh	47.60	0.00
TRINITY_GBG7546	hypothet	Chara_braunii	47.60	0.00
TRINITY_PNW6985	hypothet	Chlamydomonas_reinh	47.60	0.00
TRINITY_XP_0206	protein	Dendrobium_catenatu	47.60	0.00
TRINITY_GAQ7864	4-couma	Klebsormidium_niten	47.60	0.00
TRINITY_PNH0354	hypothet	Tetrabaena_socialis	47.60	0.00
TRINITY_GAX7938	hypothet	Chlamydomonas_eusti	47.60	0.00
TRINITY_RMZ5531	hypothet	Auxenochlorella_prc	47.60	0.00
TRINITY_GAX8461	hypothet	Chlamydomonas_eusti	47.60	0.00
TRINITY_GAX8013	hypothet	Chlamydomonas_eusti	47.60	0.00
TRINITY_PNW7755	hypothet	Chlamydomonas_reinh	47.60	0.00
TRINITY_KXZ5342	hypothet	Gonium_pectorale	47.60	0.00
TRINITY_XP_0257	proteas	Panicum_hallii	47.60	0.00
TRINITY_GAX7886	hypothet	Chlamydomonas_eusti	47.60	0.00

TRINITY_XP_00295	hypothet	Volvox_carteri	47.60	0.00
TRINITY_GAX74293	hypothet	Chlamydomonas_eusti	47.60	0.00
TRINITY_XP_00449	serine/t	Cicer_arietinum	47.60	0.00
TRINITY_GAQ90877	Dipeptic	Klebsormidium_niten	47.60	0.00
TRINITY_GAX80960	hypothet	Chlamydomonas_eusti	47.60	0.00
TRINITY_XP_00295	hypothet	Volvox_carteri	47.60	0.00
TRINITY_PNW81173	hypothet	Chlamydomonas_reinh	47.60	0.00
TRINITY_PNW74998	hypothet	Chlamydomonas_reinh	47.60	0.00
TRINITY_GAX76248	hypothet	Chlamydomonas_eusti	47.60	0.00
TRINITY_PNW78299	hypothet	Chlamydomonas_reinh	47.60	0.00
TRINITY_XP_01915	PREDICTE	Ipomoea_nil	47.60	0.00
TRINITY_XP_00169	predicte	Chlamydomonas_reinh	47.60	0.00
TRINITY_XP_00294	hypothet	Volvox_carteri	47.60	0.00
TRINITY_AFL70161	actin [A	Agastache_rugosa	47.60	0.00
TRINITY_GAX72998	hypothet	Chlamydomonas_eusti	47.60	0.00
TRINITY_XP_02439	translat	Physcomitrella_pate	47.60	0.00
TRINITY_XP_00751	GTP-binc	Bathycoccus_prasino	47.60	0.00
TRINITY_ABK24372	unknown	Picea_sitchensis	47.60	0.00
TRINITY_KXZ51275	hypothet	Gonium_pectorale	47.60	0.00
TRINITY_GAX72557	hypothet	Chlamydomonas_eusti	47.60	0.00
TRINITY_GAX79566	hypothet	Chlamydomonas_eusti	47.60	0.00
TRINITY_PRW57440	senescer	Chlorella_sorokinia	47.60	0.00
TRINITY_XP_01667	PREDICTE	Gossypium_hirsutum	47.60	0.00
TRINITY_GBG73999	hypothet	Chara_braunii	47.60	0.00
TRINITY_GAX75038	hypothet	Chlamydomonas_eusti	47.60	0.00
TRINITY_PKA57201	Potassiu	Apostasia_shenzheni	47.50	0.00
TRINITY_GAX78150	hypothet	Chlamydomonas_eusti	47.50	0.00
TRINITY_RWR90155	nitrate	Cinnamomum_micranth	47.50	0.00
TRINITY_PNW74711	hypothet	Chlamydomonas_reinh	47.50	0.00
TRINITY_XP_02388	elongati	Quercus_suber	47.50	0.00
TRINITY_BAJ90002	predicte	Hordeum_vulgare	47.50	0.00
TRINITY_PNY15863	60S ribc	Trifolium_pratense	47.50	0.00
TRINITY_XP_02025	tyrosyl-	Asparagus_officinal	47.50	0.00
TRINITY_PRW05850	decappir	Chlorella_sorokinia	47.50	0.00
TRINITY_ABK24554	unknown	Picea_sitchensis	47.50	0.00
TRINITY_XP_01558	2-amino-	Ricinus_communis	47.50	0.00
TRINITY_EEF27535	two-comp	Ricinus_communis	47.50	0.00
TRINITY_XP_00250	predicte	Micromonas_commoda	47.50	0.00
TRINITY_GAQ77581	ABC trar	Klebsormidium_niten	47.50	0.00
TRINITY_GBF94409	hypothet	Raphidocelis_subcap	47.50	0.00
TRINITY_EPS70048	hypothet	Genlisea_aurea	47.50	0.00
TRINITY_KXZ47022	hypothet	Gonium_pectorale	47.50	0.00
TRINITY_GAQ88954	Putative	Klebsormidium_niten	47.50	0.00
TRINITY_GBF94547	dual spe	Raphidocelis_subcap	47.50	0.00
TRINITY_GAX74887	hypothet	Chlamydomonas_eusti	47.50	0.00
TRINITY_BAK00750	predicte	Hordeum_vulgare	47.50	0.00
TRINITY_GAQ85240	hypothet	Klebsormidium_niten	47.50	0.00
TRINITY_GAX73709	hypothet	Chlamydomonas_eusti	47.50	0.00
TRINITY_VDD42655	unnamed	Brassica_oleracea	47.50	0.00
TRINITY_XP_02389	serine/t	Quercus_suber	47.50	0.00
TRINITY_PTQ34642	hypothet	Marchantia_polymorp	47.50	0.00
TRINITY_PRW59519	diphthir	Chlorella_sorokinia	47.50	0.00
TRINITY_KXZ42898	hypothet	Gonium_pectorale	47.50	0.00
TRINITY_XP_01920	PREDICTE	Ipomoea_nil	47.50	0.00
TRINITY_KXZ54860	hypothet	Gonium_pectorale	47.50	0.00
TRINITY_XP_02439	protein	Physcomitrella_pate	47.50	0.00
TRINITY_GBF94829	hypothet	Raphidocelis_subcap	47.50	0.00
TRINITY_GAX77541	hypothet	Chlamydomonas_eusti	47.50	0.00
TRINITY_XP_02438	NAD-depe	Physcomitrella_pate	47.50	0.00
TRINITY_XP_00169	predicte	Chlamydomonas_reinh	47.50	0.00
TRINITY_AZI70899	acyl-CoA	Lobosphaera_incisa	47.50	0.00
TRINITY_XP_00169	predicte	Chlamydomonas_reinh	47.50	0.00
TRINITY_XP_02160	DNA-(apu	Hevea_brasiliensis	47.50	0.00
TRINITY_XP_01093	PREDICTE	Elaeis_guineensis	47.50	0.00
TRINITY_GAX72877	hypothet	Chlamydomonas_eusti	47.50	0.00
TRINITY_RDY02236	Dual spe	Mucuna_pruriens	47.50	0.00

TRINITY_PRW5843	mitotic	Chlorella_sorokinia	47.50	0.00
TRINITY_XP_0216	ubiquit	Hevea_brasiliensis	47.50	0.00
TRINITY_GAX7722	hypothet	Chlamydomonas_eusti	47.50	0.00
TRINITY_XP_0106	PREDICT	Beta_vulgaris	47.50	0.00
TRINITY_XP_0239	externa	Quercus_suber	47.50	0.00
TRINITY_XP_0239	ras-lik	Quercus_suber	47.50	0.00
TRINITY_XP_0029	hypothet	Volvox_carteri	47.50	0.00
TRINITY_GAX8594	hypothet	Chlamydomonas_eusti	47.50	0.00
TRINITY_GAX8225	hypothet	Chlamydomonas_eusti	47.50	0.00
TRINITY_BAK0286	predict	Hordeum_vulgare	47.50	0.00
TRINITY_BAK0043	predict	Hordeum_vulgare	47.50	0.00
TRINITY_GAQ8123	2-oxogl	Klebsormidium_niten	47.50	0.00
TRINITY_GBF8752	hypothet	Raphidocelis_subcap	47.50	0.00
TRINITY_PNW8750	hypothet	Chlamydomonas_reinh	47.50	0.00
TRINITY_PNW8311	hypothet	Chlamydomonas_reinh	47.50	0.00
TRINITY_XP_0029	hypothet	Volvox_carteri	47.50	0.00
TRINITY_AHA9309	PLC prot	Dunaliella_salina	47.50	0.00
TRINITY_XP_0158	kanadapt	Ziziphus_jujuba	47.50	0.00
TRINITY_GBF9323	insulin-	Raphidocelis_subcap	47.50	0.00
TRINITY_GAX7459	hypothet	Chlamydomonas_eusti	47.50	0.00
TRINITY_OAE3131	hypothet	Marchantia_polymorp	47.50	0.00
TRINITY_XP_0139	Vacuola	Monoraphidium_negle	47.50	0.00
TRINITY_KXZ5431	hypothet	Gonium_pectorale	47.50	0.00
TRINITY_XP_0029	hypothet	Volvox_carteri	47.50	0.00
TRINITY_GAQ8985	putative	Klebsormidium_niten	47.50	0.00
TRINITY_GAX7314	hypothet	Chlamydomonas_eusti	47.50	0.00
TRINITY_PRW3939	hypothet	Chlorella_sorokinia	47.50	0.00
TRINITY_XP_0025	predict	Micromonas_commoda	47.40	0.00
TRINITY_XP_0058	hypothet	Chlorella_variabili	47.40	0.00
TRINITY_PTQ4273	hypothet	Marchantia_polymorp	47.40	0.00
TRINITY_XP_0056	thiored	Coccomyxa_subellips	47.40	0.00
TRINITY_XP_0105	PREDICT	Tarenaya_hasslerian	47.40	0.00
TRINITY_AML3085	axonema	Marsilea_vestita	47.40	0.00
TRINITY_XP_0029	flagell	Volvox_carteri	47.40	0.00
TRINITY_BAD9069	sigma1	Botryococcus_brauni	47.40	0.00
TRINITY_RLM8676	nuclear	Panicum_miliaceum	47.40	0.00
TRINITY_EPS6592	hypothet	Genlisea_aurea	47.40	0.00
TRINITY_BAK0277	predict	Hordeum_vulgare	47.40	0.00
TRINITY_GAX8162	hypothet	Chlamydomonas_eusti	47.40	0.00
TRINITY_XP_0058	hypothet	Chlorella_variabili	47.40	0.00
TRINITY_XP_0014	thiored	Ostreococcus_sp._'l	47.40	0.00
TRINITY_XP_0238	multifu	Quercus_suber	47.40	0.00
TRINITY_XP_0139	calcium	Monoraphidium_negle	47.40	0.00
TRINITY_PSC6853	3,4-dih	Micractinium_conduc	47.40	0.00
TRINITY_PWA6522	long-ch	Artemisia_annua	47.40	0.00
TRINITY_GAQ8293	chorism	Klebsormidium_niten	47.40	0.00
TRINITY_GAX8095	hypothet	Chlamydomonas_eusti	47.40	0.00
TRINITY_GAX7486	hypothet	Chlamydomonas_eusti	47.40	0.00
TRINITY_BAK0075	predict	Hordeum_vulgare	47.40	0.00
TRINITY_GAX7972	hypothet	Chlamydomonas_eusti	47.40	0.00
TRINITY_GAX8093	hypothet	Chlamydomonas_eusti	47.40	0.00
TRINITY_GBG6619	hypothet	Chara_braunii	47.40	0.00
TRINITY_GBG6475	hypothet	Chara_braunii	47.40	0.00
TRINITY_GAX7910	hypothet	Chlamydomonas_eusti	47.40	0.00
TRINITY_XP_0029	hypothet	Volvox_carteri	47.40	0.00
TRINITY_GAX8087	hypothet	Chlamydomonas_eusti	47.40	0.00
TRINITY_XP_0016	predict	Chlamydomonas_reinh	47.40	0.00
TRINITY_XP_0172	PREDICT	Daucus_carota	47.40	0.00
TRINITY_RWW3434	hypothet	Ensete_ventricosum	47.40	0.00
TRINITY_GAX7502	hypothet	Chlamydomonas_eusti	47.40	0.00
TRINITY_XP_0058	hypothet	Chlorella_variabili	47.40	0.00
TRINITY_XP_0016	predict	Chlamydomonas_reinh	47.40	0.00
TRINITY_KHG0244	hypothet	Gossypium_arboreum	47.40	0.00
TRINITY_XP_0102	PREDICT	Nelumbo_nucifera	47.40	0.00
TRINITY_XP_0029	hypothet	Volvox_carteri	47.40	0.00
TRINITY_XP_0029	hypothet	Volvox_carteri	47.40	0.00

TRINITY_PWZ0764(	YlmG 2,	Zea_mays	47.40	0.00
TRINITY_KXZ5609(	hypothet	Gonium_pectorale	47.40	0.00
TRINITY_XP_0029(	hypothet	Volvox_carteri	47.40	0.00
TRINITY_KXZ5550(	hypothet	Gonium_pectorale	47.40	0.00
TRINITY_XP_0016(	predicte	Chlamydomonas_reinh	47.40	0.00
TRINITY_GAX7947(	hypothet	Chlamydomonas_eusti	47.40	0.00
TRINITY_PNH0386(	hypothet	Tetrabaena_socialis	47.40	0.00
TRINITY_XP_0056(	prohibit	Coccomyxa_subellips	47.40	0.00
TRINITY_KXZ5539(	hypothet	Gonium_pectorale	47.40	0.00
TRINITY_GAX7262(	hypothet	Chlamydomonas_eusti	47.40	0.00
TRINITY_XP_0030(	vacuola	Micromonas_pusilla	47.40	0.00
TRINITY_GAQ8723(	Dyneins	Klebsormidium_niten	47.40	0.00
TRINITY_XP_0258(	protein-	Panicum_hallii	47.40	0.00
TRINITY_ESQ3540(	hypothet	Eutrema_salsugineum	47.40	0.00
TRINITY_PRW6001(	protopo	Chlorella_sorokinia	47.40	0.00
TRINITY_PRW5699(	DNA-3-me	Chlorella_sorokinia	47.40	0.00
TRINITY_XP_0279(	hyperse	Vigna_unguiculata	47.40	0.00
TRINITY_KXZ5647(	hypothet	Gonium_pectorale	47.40	0.00
TRINITY_KXZ5252(	hypothet	Gonium_pectorale	47.40	0.00
TRINITY_GAX7847(	hypothet	Chlamydomonas_eusti	47.40	0.00
TRINITY_GAX7987(	hypothet	Chlamydomonas_eusti	47.40	0.00
TRINITY_GAX7279(	hypothet	Chlamydomonas_eusti	47.40	0.00
TRINITY_XP_0273(	probable	Abrus_precatorius	47.40	0.00
TRINITY_XP_0239(	activat	Quercus_suber	47.40	0.00
TRINITY_XP_0243(	uncharac	Physcomitrella_pate	47.30	0.00
TRINITY_AFK4559(	unknown	Medicago_truncatula	47.30	0.00
TRINITY_ADC4537(	actin [(	Cleistogenes_songor	47.30	0.00
TRINITY_PON6201(	1,4-alph	Parasponia_anderson	47.30	0.00
TRINITY_CAB4076(	putative	Arabidopsis_thalian	47.30	0.00
TRINITY_XP_0238(	polyubi	Quercus_suber	47.30	0.00
TRINITY_XP_0243(	uncharac	Physcomitrella_pate	47.30	0.00
TRINITY_XP_0093(	PREDICTI	Musa_acuminata	47.30	0.00
TRINITY_XP_0056(	sterol (	Coccomyxa_subellips	47.30	0.00
TRINITY_XP_0239(	ras-like	Quercus_suber	47.30	0.00
TRINITY_KDD7467(	hypothet	Helicosporidium_sp.	47.30	0.00
TRINITY_PTQ2950(	hypothet	Marchantia_polymorp	47.30	0.00
TRINITY_PTQ3208(	hypothet	Marchantia_polymorp	47.30	0.00
TRINITY_XP_0094(	PREDICTI	Musa_acuminata	47.30	0.00
TRINITY_XP_0025(	predicte	Micromonas_commoda	47.30	0.00
TRINITY_GBF9099(	ubiquit	Raphidocelis_subcap	47.30	0.00
TRINITY_XP_0238(	uncharac	Quercus_suber	47.30	0.00
TRINITY_XP_0030(	predicte	Micromonas_pusilla	47.30	0.00
TRINITY_XP_0206(	probable	Dendrobium_catenatu	47.30	0.00
TRINITY_XP_0066(	PREDICTI	Oryza_brachyantha	47.30	0.00
TRINITY_XP_0035(	cyclin-(	Brachypodium_distac	47.30	0.00
TRINITY_XP_0165(	PREDICTI	Capsicum_annuum	47.30	0.00
TRINITY_XP_0094(	PREDICTI	Musa_acuminata	47.30	0.00
TRINITY_PLY9094(	hypothet	Lactuca_sativa	47.30	0.00
TRINITY_XP_0250(	protein	Ricinus_communis	47.30	0.00
TRINITY_GAQ8019(	serine/t	Klebsormidium_niten	47.30	0.00
TRINITY_XP_0029(	hypothet	Volvox_carteri	47.30	0.00
TRINITY_ALM5498(	DEAD bo	Chlamydomonas_sp._I	47.30	0.00
TRINITY_PNW7501(	hypothet	Chlamydomonas_reinh	47.30	0.00
TRINITY_BAK0236(	predicte	Hordeum_vulgare	47.30	0.00
TRINITY_XP_0016(	flagella	Chlamydomonas_reinh	47.30	0.00
TRINITY_KXZ5482(	hypothet	Gonium_pectorale	47.30	0.00
TRINITY_XP_0243(	probable	Physcomitrella_pate	47.30	0.00
TRINITY_GAX7411(	hypothet	Chlamydomonas_eusti	47.30	0.00
TRINITY_GAX7261(	hypothet	Chlamydomonas_eusti	47.30	0.00
TRINITY_GAX8389(	hypothet	Chlamydomonas_eusti	47.30	0.00
TRINITY_GAQ7784(	Kinesin-	Klebsormidium_niten	47.30	0.00
TRINITY_PNW8402(	hypothet	Chlamydomonas_reinh	47.30	0.00
TRINITY_XP_0016(	flagella	Chlamydomonas_reinh	47.30	0.00
TRINITY_PNW7159(	hypothet	Chlamydomonas_reinh	47.30	0.00
TRINITY_PNW7963(	hypothet	Chlamydomonas_reinh	47.30	0.00
TRINITY_XP_0270(	THO comp	Coffea_arabica	47.30	0.00

TRINITY_XP_0075	predict	Bathycoccus_prasinc	47.30	0.00
TRINITY_GAQ8437	helicas	Klebsormidium_niten	47.30	0.00
TRINITY_XP_0024	predict	Micromonas_commoda	47.30	0.00
TRINITY_BAK0075	predict	Hordeum_vulgare	47.30	0.00
TRINITY_GBF9872	hypothet	Raphidocelis_subcap	47.30	0.00
TRINITY_GAX8261	hypothet	Chlamydomonas_eusti	47.30	0.00
TRINITY_XP_0016	predict	Chlamydomonas_reinh	47.30	0.00
TRINITY_GAX8569	hypothet	Chlamydomonas_eusti	47.30	0.00
TRINITY_GAX7266	hypothet	Chlamydomonas_eusti	47.30	0.00
TRINITY_XP_0016	DnaJ-li	Chlamydomonas_reinh	47.30	0.00
TRINITY_RMZ5489	hypothet	Auxenochlorella_prc	47.30	0.00
TRINITY_GAX7990	hypothet	Chlamydomonas_eusti	47.30	0.00
TRINITY_PRW3370	low-CO2	Chlorella_sorokinia	47.30	0.00
TRINITY_XP_0016	predict	Chlamydomonas_reinh	47.30	0.00
TRINITY_GAX8066	hypothet	Chlamydomonas_eusti	47.30	0.00
TRINITY_XP_0014	P-ATPas	Ostreococcus_sp._'l	47.30	0.00
TRINITY_GAQ9033	hypothet	Klebsormidium_niten	47.30	0.00
TRINITY_PNW7688	hypothet	Chlamydomonas_reinh	47.30	0.00
TRINITY_XP_0017	predict	Chlamydomonas_reinh	47.30	0.00
TRINITY_GAX7372	hypothet	Chlamydomonas_eusti	47.30	0.00
TRINITY_XP_0030	predict	Micromonas_pusilla	47.30	0.00
TRINITY_OAE3359	hypothet	Marchantia_polymorp	47.30	0.00
TRINITY_GAQ8106	ATP-dep	Klebsormidium_niten	47.30	0.00
TRINITY_GAX7320	hypothet	Chlamydomonas_eusti	47.30	0.00
TRINITY_GAX7424	hypothet	Chlamydomonas_eusti	47.30	0.00
TRINITY_GAQ8317	hypothet	Klebsormidium_niten	47.30	0.00
TRINITY_PRW5859	ADP-sug	Chlorella_sorokinia	47.30	0.00
TRINITY_GAX7958	hypothet	Chlamydomonas_eusti	47.30	0.00
TRINITY_GAX7625	hypothet	Chlamydomonas_eusti	47.30	0.00
TRINITY_PNW6993	hypothet	Chlamydomonas_reinh	47.30	0.00
TRINITY_GAX7540	hypothet	Chlamydomonas_eusti	47.30	0.00
TRINITY_XP_0103	troponi	Solanum_lycopersicu	47.30	0.00
TRINITY_PNH1156	Cationi	Tetrabaena_socialis	47.30	0.00
TRINITY_XP_0029	hypothet	Volvox_carteri	47.30	0.00
TRINITY_YP_6656	cytochr	Nephroselmis_olivac	47.30	0.00
TRINITY_AAB8649	calmodu	Zea_mays	47.30	0.00
TRINITY_PWA6526	P-glyco	Artemisia_annua	47.30	0.00
TRINITY_XP_0228	casein	Olea_europaea	47.30	0.00
TRINITY_RAL4461	hypothet	Cuscuta_australis	47.20	0.00
TRINITY_XP_0191	PREDICT	Ipomoea_nil	47.20	0.00
TRINITY_XP_0014	F-ATPas	Ostreococcus_sp._'l	47.20	0.00
TRINITY_XP_0093	PREDICT	Musa_acuminata	47.20	0.00
TRINITY_XP_0184	PREDICT	Pyrus_x_bretschneid	47.20	0.00
TRINITY_XP_0028	uncharac	Arabidopsis_lyrata	47.20	0.00
TRINITY_XP_0270	pyridox	Coffea_arabica	47.20	0.00
TRINITY_XP_0191	PREDICT	Ipomoea_nil	47.20	0.00
TRINITY_OMO5108	Sugar/i	Corchorus_capsulari	47.20	0.00
TRINITY_PSC7499	mannose-	Micractinium_conduc	47.20	0.00
TRINITY_GBF9359	hypothet	Raphidocelis_subcap	47.20	0.00
TRINITY_XP_0116	regulat	Amborella_trichopod	47.20	0.00
TRINITY_XP_0238	thiosul	Quercus_suber	47.20	0.00
TRINITY_AFK4603	unknown	Lotus_japonicus	47.20	0.00
TRINITY_AAT7361	calmodu	Daucus_carota	47.20	0.00
TRINITY_PNH1270	HEAT re	Tetrabaena_socialis	47.20	0.00
TRINITY_PNW8446	hypothet	Chlamydomonas_reinh	47.20	0.00
TRINITY_GAX8374	hypothet	Chlamydomonas_eusti	47.20	0.00
TRINITY_XP_0207	dual sp	Dendrobium_catenatu	47.20	0.00
TRINITY_GBG7935	hypothet	Chara_braunii	47.20	0.00
TRINITY_GAX7646	hypothet	Chlamydomonas_eusti	47.20	0.00
TRINITY_PNW7601	hypothet	Chlamydomonas_reinh	47.20	0.00
TRINITY_GBF9465	hypothet	Raphidocelis_subcap	47.20	0.00
TRINITY_XP_0025	protein	Micromonas_commoda	47.20	0.00
TRINITY_PNW7774	hypothet	Chlamydomonas_reinh	47.20	0.00
TRINITY_PWA5459	MUTS-li	Artemisia_annua	47.20	0.00
TRINITY_GAX7939	hypothet	Chlamydomonas_eusti	47.20	0.00
TRINITY_XP_0238	putativ	Quercus_suber	47.20	0.00

TRINITY_XP_00294	hypothet	Volvox_carteri	47.20	0.00
TRINITY_GAX80008	hypothet	Chlamydomonas_eusti	47.20	0.00
TRINITY_XP_01055	PREDICTE	Tarenaya_hasslerian	47.20	0.00
TRINITY_PNW87033	hypothet	Chlamydomonas_reinh	47.20	0.00
TRINITY_GBF99846	hypothet	Raphidocelis_subcap	47.20	0.00
TRINITY_GAX75633	hypothet	Chlamydomonas_eusti	47.20	0.00
TRINITY_GAX83777	hypothet	Chlamydomonas_eusti	47.20	0.00
TRINITY_PSC75979	alpha-ke	Micractinium_conduc	47.20	0.00
TRINITY_OAE35699	hypothet	Marchantia_polymorp	47.20	0.00
TRINITY_GAX73391	hypothet	Chlamydomonas_eusti	47.20	0.00
TRINITY_BAJ94736	predicte	Hordeum_vulgare	47.20	0.00
TRINITY_KXZ54521	hypothet	Gonium_pectorale	47.20	0.00
TRINITY_GAX79859	hypothet	Chlamydomonas_eusti	47.20	0.00
TRINITY_KXZ47900	hypothet	Gonium_pectorale	47.20	0.00
TRINITY_PNW70517	hypothet	Chlamydomonas_reinh	47.20	0.00
TRINITY_PNW71453	hypothet	Chlamydomonas_reinh	47.20	0.00
TRINITY_XP_01910	PREDICTE	Camelina_sativa	47.20	0.00
TRINITY_PRW57278	Nucleola	Chlorella_sorokinia	47.20	0.00
TRINITY_GAX79184	hypothet	Chlamydomonas_eusti	47.20	0.00
TRINITY_PSC76384	tubulin	Micractinium_conduc	47.20	0.00
TRINITY_PNW80809	hypothet	Chlamydomonas_reinh	47.20	0.00
TRINITY_GAX76173	hypothet	Chlamydomonas_eusti	47.20	0.00
TRINITY_XP_00169	flagella	Chlamydomonas_reinh	47.20	0
TRINITY_GAX80207	hypothet	Chlamydomonas_eusti	47.20	0.00
TRINITY_OAE35055	hypothet	Marchantia_polymorp	47.20	0.00
TRINITY_KXZ47616	hypothet	Gonium_pectorale	47.20	0.00
TRINITY_PNW74817	hypothet	Chlamydomonas_reinh	47.20	0.00
TRINITY_XP_01643	PREDICTE	Nicotiana_tabacum	47.20	0.00
TRINITY_GAX82802	hypothet	Chlamydomonas_eusti	47.20	0.00
TRINITY_GAX80647	hypothet	Chlamydomonas_eusti	47.20	0.00
TRINITY_PNW77278	hypothet	Chlamydomonas_reinh	47.20	0.00
TRINITY_GAX83280	hypothet	Chlamydomonas_eusti	47.20	0.00
TRINITY_GBF91241	hypothet	Raphidocelis_subcap	47.20	0.00
TRINITY_GAX74891	hypothet	Chlamydomonas_eusti	47.20	0.00
TRINITY_KXZ46245	hypothet	Gonium_pectorale	47.20	0.00
TRINITY_GAX74015	hypothet	Chlamydomonas_eusti	47.20	0.00
TRINITY_PNG99754	hypothet	Tetrabaena_socialis	47.20	0.00
TRINITY_GBG83671	hypothet	Chara_braunii	47.20	0.00
TRINITY_XP_02199	histone-	Helianthus_annuus	47.10	0.00
TRINITY_XP_00169	LOW QUAL	Chlamydomonas_reinh	47.10	0.00
TRINITY_XP_02372	ultravio	Lactuca_sativa	47.10	0.00
TRINITY_XP_01207	coatomer	Jatropha_curcas	47.10	0.00
TRINITY_RWR94650	NADH deh	Cinnamomum_micranth	47.10	0.00
TRINITY_XP_02164	probable	Hevea_brasiliensis	47.10	0.00
TRINITY_GAQ81440	Hypothet	Klebsormidium_niten	47.10	0.00
TRINITY_XP_01048	PREDICTE	Camelina_sativa	47.10	0.00
TRINITY_XP_00294	hypothet	Volvox_carteri	47.10	0.00
TRINITY_GBG66485	hypothet	Chara_braunii	47.10	0.00
TRINITY_XP_02439	uncharac	Physcomitrella_pate	47.10	0.00
TRINITY_XP_00250	cytoplas	Micromonas_commoda	47.10	0.00
TRINITY_PIN09288	Thioredo	Handroanthus_impeti	47.10	0.00
TRINITY_XP_02375	uncharac	Lactuca_sativa	47.10	0.00
TRINITY_XP_00306	dynein h	Micromonas_pusilla	47.10	0.00
TRINITY_XP_02172	transcri	Chenopodium_quinoa	47.10	0.00
TRINITY_XP_01724	PREDICTE	Daucus_carota	47.10	0.00
TRINITY_EFJ13296	hypothet	Selaginella_moellen	47.10	0.00
TRINITY_KZM92183	hypothet	Daucus_carota	47.10	0.00
TRINITY_XP_00250	FYVE zin	Micromonas_commoda	47.10	0.00
TRINITY_EFJ27143	hypothet	Selaginella_moellen	47.10	0.00
TRINITY_KXZ45538	hypothet	Gonium_pectorale	47.10	0.00
TRINITY_KZV36364	calcium-	Dorcoceras_hygromet	47.10	0.00
TRINITY_KXZ41194	hypothet	Gonium_pectorale	47.10	0.00
TRINITY_XP_02294	serine/a	Cucurbita_moschata	47.10	0.00
TRINITY_XP_01054	PREDICTE	Tarenaya_hasslerian	47.10	0.00
TRINITY_ONK62624	uncharac	Asparagus_officinal	47.10	0.00
TRINITY_PTQ41104	hypothet	Marchantia_polymorp	47.10	0.00

TRINITY_PRW20789	Mitochon	Chlorella_sorokinia	47.10	0.00
TRINITY_XP_00169	predicte	Chlamydomonas_reinh	47.10	0.00
TRINITY_PNW88549	hypothet	Chlamydomonas_reinh	47.10	0.00
TRINITY_PNH09585	Protein	Tetrabaena_socialis	47.10	0.00
TRINITY_GAX75276	hypothet	Chlamydomonas_eusti	47.10	0.00
TRINITY_XP_01848	PREDICTE	Raphanus_sativus	47.10	0.00
TRINITY_GAX74811	hypothet	Chlamydomonas_eusti	47.10	0.00
TRINITY_OMO87578	Ribosoma	Corchorus_capsulari	47.10	0.00
TRINITY_GAX78747	hypothet	Chlamydomonas_eusti	47.10	0.00
TRINITY_KXZ53138	hypothet	Gonium_pectorale	47.10	0.00
TRINITY_GAX73268	hypothet	Chlamydomonas_eusti	47.10	0.00
TRINITY_XP_00565	hypothet	Coccomyxa_subellips	47.10	0.00
TRINITY_XP_01847	PREDICTE	Raphanus_sativus	47.10	0.00
TRINITY_XP_00295	hypothet	Volvox_carteri	47.10	0.00
TRINITY_KXZ51149	hypothet	Gonium_pectorale	47.10	0.00
TRINITY_XP_00295	hypothet	Volvox_carteri	47.10	0.00
TRINITY_PSC76276	thiamine	Micractinium_conduc	47.10	0.00
TRINITY_GAX84012	hypothet	Chlamydomonas_eusti	47.10	0.00
TRINITY_XP_00564	RNI-like	Coccomyxa_subellips	47.10	0.00
TRINITY_GAQ82553	hypothet	Klebsormidium_niten	47.10	0.00
TRINITY_GAX84697	hypothet	Chlamydomonas_eusti	47.10	0.00
TRINITY_XP_01389	ZIP fami	Monoraphidium_negle	47.10	0.00
TRINITY_XP_02130	uncharac	Sorghum_bicolor	47.10	0.00
TRINITY_BAJ98473	predicte	Hordeum_vulgare	47.10	0.00
TRINITY_GAX78499	hypothet	Chlamydomonas_eusti	47.10	0.00
TRINITY_XP_00296	probable	Selaginella_moellen	47.10	0.00
TRINITY_XP_00294	hypothet	Volvox_carteri	47.10	0.00
TRINITY_GBF94645	hypothet	Raphidocelis_subcap	47.10	0.00
TRINITY_GBF89518	multidru	Raphidocelis_subcap	47.10	0.00
TRINITY_PNW80969	hypothet	Chlamydomonas_reinh	47.10	0.00
TRINITY_EFJ13284	hypothet	Selaginella_moellen	47.10	0.00
TRINITY_GAX81353	hypothet	Chlamydomonas_eusti	47.10	0.00
TRINITY_KZV15019	hypothet	Dorcoceras_hygromet	47.10	0.00
TRINITY_GAX80361	hypothet	Chlamydomonas_eusti	47.10	0.00
TRINITY_GAX85886	hypothet	Chlamydomonas_eusti	47.10	0.00
TRINITY_XP_02642	betaine	Papaver_somniferum	47.10	0.00
TRINITY_PNW71032	hypothet	Chlamydomonas_reinh	47.10	0.00
TRINITY_ALD84318	acetyl-C	Morus_alba	47.10	0.00
TRINITY_GAX78806	hypothet	Chlamydomonas_eusti	47.10	0.00
TRINITY_GAX78155	hypothet	Chlamydomonas_eusti	47.10	0.00
TRINITY_PNW71871	hypothet	Chlamydomonas_reinh	47.10	0.00
TRINITY_KXZ48350	hypothet	Gonium_pectorale	47.10	0.00
TRINITY_GAX75270	hypothet	Chlamydomonas_eusti	47.10	0.00
TRINITY_XP_00634	PREDICTE	Solanum_tuberosum	47.10	0.00
TRINITY_NP_00134	uncharac	Zea_mays	47.10	0.00
TRINITY_XP_00169	phosphoa	Chlamydomonas_reinh	47.10	0.00
TRINITY_GBG83673	hypothet	Chara_braunii	47.10	0.00
TRINITY_XP_01207	uncharac	Jatropha_curcas	47.10	0.00
TRINITY_PNG99787	hypothet	Tetrabaena_socialis	47.10	0.00
TRINITY_GAQ79529	hypothet	Klebsormidium_niten	47.10	0.00
TRINITY_GAQ80294	calcium-	Klebsormidium_niten	47.10	0.00
TRINITY_ONK60287	uncharac	Asparagus_officinal	47.00	0.00
TRINITY_EMS57267	Serine (	Triticum_urartu	47.00	0.00
TRINITY_XP_00751	predicte	Bathycoccus_prasino	47.00	0.00
TRINITY_XP_01285	PREDICTE	Erythranthe_guttata	47.00	0.00
TRINITY_XP_00297	calmodul	Selaginella_moellen	47.00	0.00
TRINITY_GAQ81809	Anaphase	Klebsormidium_niten	47.00	0.00
TRINITY_XP_02172	DEAD-box	Chenopodium_quinoa	47.00	0.00
TRINITY_XP_01109	probable	Sesamum_indicum	47.00	0.00
TRINITY_VDD29745	unnamed	Brassica_oleracea	47.00	0.00
TRINITY_XP_00684	protein	Amborella_trichopod	47.00	0.00
TRINITY_XP_00684	probable	Amborella_trichopod	47.00	0.00
TRINITY_BAK00461	predicte	Hordeum_vulgare	47.00	0.00
TRINITY_XP_01905	PREDICTE	Tarenaya_hasslerian	47.00	0.00
TRINITY_PTQ36733	hypothet	Marchantia_polymorp	47.00	0.00
TRINITY_GBG62536	hypothet	Chara_braunii	47.00	0.00

TRINITY_RXI0963	hypothet	Malus_domestica	47.00	0.00
TRINITY_EPS6592	hypothet	Genlisea_aurea	47.00	0.00
TRINITY_XP_0138	malate (	Monoraphidium_negle	47.00	0.00
TRINITY_XP_0239	myosin-1	Quercus_suber	47.00	0.00
TRINITY_PKI3164	hypothet	Punica_granatum	47.00	0.00
TRINITY_XP_0243	GDT1-lil	Physcomitrella_pate	47.00	0.00
TRINITY_GAX8297	hypothet	Chlamydomonas_eusti	47.00	0.00
TRINITY_BAK0302	predicte	Hordeum_vulgare	47.00	0.00
TRINITY_PNH0830	NFX1-typ	Tetrabaena_socialis	47.00	0.00
TRINITY_GAX7560	hypothet	Chlamydomonas_eusti	47.00	0.00
TRINITY_PSC7653	NAD-depe	Micractinium_conduc	47.00	0.00
TRINITY_XP_0043	PREDICTI	Fragaria_vesca	47.00	0.00
TRINITY_PLY8328	hypothet	Lactuca_sativa	47.00	0.00
TRINITY_XP_0030	predicte	Micromonas_pusilla	47.00	0.00
TRINITY_GAX8089	hypothet	Chlamydomonas_eusti	47.00	0.00
TRINITY_GAQ8460	inosine-	Klebsormidium_niten	47.00	0.00
TRINITY_KXZ4925	hypothet	Gonium_pectorale	47.00	0.00
TRINITY_XP_0056	hypothet	Coccomyxa_subellips	47.00	0.00
TRINITY_PNH1057	Retrovi	Tetrabaena_socialis	47.00	0.00
TRINITY_KXZ4962	hypothet	Gonium_pectorale	47.00	0.00
TRINITY_PNW7101	hypothet	Chlamydomonas_reinh	47.00	0.00
TRINITY_KZV5296	calcium-	Dorcoceras_hygromet	47.00	0.00
TRINITY_GAX8292	hypothet	Chlamydomonas_eusti	47.00	0.00
TRINITY_GAX8223	hypothet	Chlamydomonas_eusti	47.00	0.00
TRINITY_XP_0056	ras-rela	Coccomyxa_subellips	47.00	0.00
TRINITY_EOA3845	hypothet	Capsella_rubella	47.00	0.00
TRINITY_XP_0271	probable	Cicer_arietinum	47.00	0.00
TRINITY_XP_0139	hypothet	Monoraphidium_negle	47.00	0.00
TRINITY_GAX7302	hypothet	Chlamydomonas_eusti	47.00	0.00
TRINITY_PNH0383	Retinol	Tetrabaena_socialis	47.00	0.00
TRINITY_GAQ8759	WD40 rep	Klebsormidium_niten	47.00	0.00
TRINITY_GAX7925	hypothet	Chlamydomonas_eusti	47.00	0.00
TRINITY_GAX7639	hypothet	Chlamydomonas_eusti	47.00	0.00
TRINITY_XP_0239	SWI/SNF	Quercus_suber	47.00	0.00
TRINITY_XP_0237	ATP-depe	Lactuca_sativa	47.00	0.00
TRINITY_GAX7573	hypothet	Chlamydomonas_eusti	47.00	0.00
TRINITY_XP_0075	major fa	Bathycoccus_prasino	47.00	0.00
TRINITY_PNW8826	hypothet	Chlamydomonas_reinh	47.00	0.00
TRINITY_XP_0249	short-ch	Cynara_cardunculus	47.00	0.00
TRINITY_PNW7087	hypothet	Chlamydomonas_reinh	47.00	0.00
TRINITY_GAX7505	hypothet	Chlamydomonas_eusti	47.00	0.00
TRINITY_GAX7313	hypothet	Chlamydomonas_eusti	47.00	0.00
TRINITY_XP_0064	actin-2	Eutrema_salsugineum	47.00	0.00
TRINITY_PHT3450	hypothet	Capsicum_baccatum	47.00	0.00
TRINITY_PNW8334	hypothet	Chlamydomonas_reinh	47.00	0.00
TRINITY_XP_0126	putative	Setaria_italica	47.00	0.00
TRINITY_EFJ3204	hypothet	Selaginella_moellen	47.00	0.00
TRINITY_RAL3859	hypothet	Cuscuta_australis	47.00	0.00
TRINITY_GAX7798	hypothet	Chlamydomonas_eusti	47.00	0.00
TRINITY_GBG7788	hypothet	Chara_braunii	47.00	0.00
TRINITY_XP_0172	PREDICTI	Daucus_carota	47.00	0.00
TRINITY_GAX8523	hypothet	Chlamydomonas_eusti	47.00	0.00
TRINITY_PNW7067	hypothet	Chlamydomonas_reinh	47.00	0.00
TRINITY_KXZ5000	hypothet	Gonium_pectorale	47.00	0.00
TRINITY_GAX7636	hypothet	Chlamydomonas_eusti	47.00	0.00
TRINITY_XP_0128	PREDICTI	Erythranthe_guttata	47.00	0.00
TRINITY_PNH0875	Dynein (	Tetrabaena_socialis	47.00	0.00
TRINITY_GBF9480	hypothet	Raphidocelis_subcap	47.00	0.00
TRINITY_XP_0104	PREDICTI	Camelina_sativa	46.90	0.00
TRINITY_XP_0239	ras-like	Quercus_suber	46.90	0.00
TRINITY_XP_0174	PREDICTI	Vigna_angularis	46.90	0.00
TRINITY_KMS9346	hypothet	Beta_vulgaris	46.90	0.00
TRINITY_GAX7701	hypothet	Chlamydomonas_eusti	46.90	0.00
TRINITY_ABK9524	unknown	Populus_trichocarpa	46.90	0.00
TRINITY_PKA4842	hypothet	Apostasia_shenzheni	46.90	0.00
TRINITY_GAQ7994	Putative	Klebsormidium_niten	46.90	0.00

TRINITY_PNT6132	hypothet	Brachypodium_distac	46.90	0.00
TRINITY_XP_0190	PREDICTI	Camelina_sativa	46.90	0.00
TRINITY_BAJ9457	predicte	Hordeum_vulgare	46.90	0.00
TRINITY_GBF9321	ABC tra	Raphidocelis_subcap	46.90	0.00
TRINITY_GAQ8438	hypothet	Klebsormidium_niten	46.90	0.00
TRINITY_XP_0016	mitochor	Chlamydomonas_reinh	46.90	0.00
TRINITY_PKA4936	Phospho	Apostasia_shenzheni	46.90	0.00
TRINITY_PRW5845	tRNA (g	Chlorella_sorokinia	46.90	0.00
TRINITY_XP_0239	ras-like	Quercus_suber	46.90	0.00
TRINITY_GBG9235	hypothet	Chara_braunii	46.90	0.00
TRINITY_XP_0029	hypothet	Volvox_carteri	46.90	0.00
TRINITY_PNY0167	ras-rela	Trifolium_pratense	46.90	0.00
TRINITY_PNW7526	hypothet	Chlamydomonas_reinh	46.90	0.00
TRINITY_GAX8205	hypothet	Chlamydomonas_eusti	46.90	0.00
TRINITY_XP_0029	DNA-dire	Selaginella_moellen	46.90	0.00
TRINITY_GAQ8123	2-oxoglu	Klebsormidium_niten	46.90	0.00
TRINITY_ANF0469	omega-6	Chlamydomonas_sp._I	46.90	0.00
TRINITY_XP_0209	cell di	Arachis_ipaensis	46.90	0.00
TRINITY_KXZ5605	hypothet	Gonium_pectorale	46.90	0.00
TRINITY_XP_0194	PREDICTI	Lupinus_angustifoli	46.90	0.00
TRINITY_XP_0110	PREDICTI	Populus_euphratica	46.90	0.00
TRINITY_XP_0227	dehydro	Durio_zibethinus	46.90	0.00
TRINITY_GAX8389	hypothet	Chlamydomonas_eusti	46.90	0.00
TRINITY_GAX7918	hypothet	Chlamydomonas_eusti	46.90	0.00
TRINITY_RYR3709	hypothet	Arachis_hypogaea	46.90	0.00
TRINITY_XP_0056	arad-li	Coccomyxa_subellips	46.90	0.00
TRINITY_XP_0029	hypothet	Volvox_carteri	46.90	0.00
TRINITY_GAX8130	hypothet	Chlamydomonas_eusti	46.90	0.00
TRINITY_PNW7290	hypothet	Chlamydomonas_reinh	46.90	0.00
TRINITY_XP_0095	PREDICTI	Nicotiana_tomentosi	46.90	0.00
TRINITY_GAX8209	hypothet	Chlamydomonas_eusti	46.90	0.00
TRINITY_PRW3926	acetola	Chlorella_sorokinia	46.90	0.00
TRINITY_XP_0029	hypothet	Volvox_carteri	46.90	0.00
TRINITY_PRW5787	Transpo	Chlorella_sorokinia	46.90	0.00
TRINITY_ACH8758	hypothet	Dunaliella_viridis	46.90	0.00
TRINITY_XP_0029	hypothet	Volvox_carteri	46.90	0.00
TRINITY_OAY6262	Importi	Ananas_comosus	46.90	0.00
TRINITY_GAX8062	hypothet	Chlamydomonas_eusti	46.90	0.00
TRINITY_GAX7888	hypothet	Chlamydomonas_eusti	46.90	0.00
TRINITY_PLY8557	hypothet	Lactuca_sativa	46.90	0.00
TRINITY_GBG8576	hypothet	Chara_braunii	46.90	0.00
TRINITY_GAX7285	hypothet	Chlamydomonas_eusti	46.90	0.00
TRINITY_XP_0226	DEAD-bo	Vigna_radiata	46.90	0.00
TRINITY_XP_0238	radical	Quercus_suber	46.90	0.00
TRINITY_PRW5844	isoleuc	Chlorella_sorokinia	46.90	0.00
TRINITY_GAX7910	hypothet	Chlamydomonas_eusti	46.90	0.00
TRINITY_XP_0110	protein	Sesamum_indicum	46.90	0.00
TRINITY_XP_0056	hypothet	Coccomyxa_subellips	46.90	0.00
TRINITY_KXZ5382	hypothet	Gonium_pectorale	46.90	0.00
TRINITY_GAX7575	hypothet	Chlamydomonas_eusti	46.90	0.00
TRINITY_GAX8141	hypothet	Chlamydomonas_eusti	46.90	0.00
TRINITY_PNW7206	hypothet	Chlamydomonas_reinh	46.90	0.00
TRINITY_XP_0029	hypothet	Volvox_carteri	46.90	0.00
TRINITY_XP_0017	transcr	Chlamydomonas_reinh	46.90	0.00
TRINITY_NP_0013	ras-rela	Solanum_lycopersicu	46.90	0.00
TRINITY_GAX7745	hypothet	Chlamydomonas_eusti	46.90	0.00
TRINITY_GAX8150	hypothet	Chlamydomonas_eusti	46.90	0.00
TRINITY_XP_0239	uncharac	Quercus_suber	46.90	0.00
TRINITY_OAE2605	hypothet	Marchantia_polymorp	46.90	0.00
TRINITY_KXZ5683	hypothet	Gonium_pectorale	46.90	0.00
TRINITY_PNW8443	hypothet	Chlamydomonas_reinh	46.90	0.00
TRINITY_RID4452	hypothet	Brassica_rapa	46.90	0.00
TRINITY_XP_0014	predicte	Ostreococcus_sp._'l	46.90	0.00
TRINITY_XP_0138	dehydro	Monoraphidium_negle	46.90	0.00
TRINITY_XP_0138	Protein	Monoraphidium_negle	46.90	0.00
TRINITY_XP_0056	thiored	Coccomyxa_subellips	46.90	0.00

TRINITY_GAX7440[illegible]	hypothet	Chlamydomonas_eusti	46.90	0.00
TRINITY_XP_0239[illegible]	cAMP-dep	Quercus_suber	46.80	0.00
TRINITY_PTQ4621[illegible]	hypothet	Marchantia_polymorp	46.80	0.00
TRINITY_KDD7455[illegible]	hypothet	Helicosporidium_sp.	46.80	0.00
TRINITY_PIN1605[illegible]	GTPase [illegible]	Handroanthus_impeti	46.80	0.00
TRINITY_GAQ8206[illegible]	phosphat	Klebsormidium_niten	46.80	0.00
TRINITY_BAJ9541[illegible]	predicte	Hordeum_vulgare	46.80	0.00
TRINITY_GAQ9059[illegible]	DNA-dire	Klebsormidium_niten	46.80	0.00
TRINITY_XP_0238[illegible]	unchara	Quercus_suber	46.80	0.00
TRINITY_PKA4966[illegible]	Ras-rela	Apostasia_shenzheni	46.80	0.00

TRINITY_GAX7836	hypothet	Chlamydomonas_eusti	46.80	0.00
TRINITY_XP_0058	hypothet	Chlorella_variabili	46.80	0.00
TRINITY_XP_0205	probable	Amborella_trichopod	46.80	0.00
TRINITY_XP_0138	malate (	Monoraphidium_negle	46.80	0.00
TRINITY_GBG6037	hypothet	Chara_braunii	46.80	0.00
TRINITY_XP_0238	store-op	Quercus_suber	46.80	0.00
TRINITY_XP_0097	PREDICTE	Nicotiana_sylvestri	46.80	0.00
TRINITY_XP_0025	serine/t	Ricinus_communis	46.80	0.00
TRINITY_XP_0237	nuclear	Lactuca_sativa	46.80	0.00
TRINITY_PNW8394	hypothet	Chlamydomonas_reinh	46.80	0.00
TRINITY_XP_0016	eukaryot	Chlamydomonas_reinh	46.80	0.00
TRINITY_GAX7314	hypothet	Chlamydomonas_eusti	46.80	0.00
TRINITY_BAK0075	predicte	Hordeum_vulgare	46.80	0.00
TRINITY_RYR1150	hypothet	Arachis_hypogaea	46.80	0.00
TRINITY_XP_0238	probable	Quercus_suber	46.80	0.00
TRINITY_XP_0056	myocyte	Coccomyxa_subellips	46.80	0.00
TRINITY_XP_0165	PREDICTE	Capsicum_annuum	46.80	0.00
TRINITY_XP_0058	hypothet	Chlorella_variabili	46.80	0.00
TRINITY_PSC7355	DUF1295	Micractinium_conduc	46.80	0.00
TRINITY_XP_0042	PREDICTE	Fragaria_vesca	46.80	0.00
TRINITY_ABK2332	unknown	Picea_sitchensis	46.80	0.00
TRINITY_PSC7639	charged	Micractinium_conduc	46.80	0.00
TRINITY_XP_0139	hypothet	Monoraphidium_negle	46.80	0.00
TRINITY_PNW7793	hypothet	Chlamydomonas_reinh	46.80	0.00
TRINITY_GAX7798	hypothet	Chlamydomonas_eusti	46.80	0.00
TRINITY_EPS6830	hypothet	Genlisea_aurea	46.80	0.00
TRINITY_PWA5442	DNA repa	Artemisia_annua	46.80	0.00
TRINITY_XP_0058	hypothet	Chlorella_variabili	46.80	0.00
TRINITY_XP_0241	dual spe	Rosa_chinensis	46.80	0.00
TRINITY_XP_0066	PREDICTE	Oryza_brachyantha	46.80	0.00
TRINITY_XP_0105	PREDICTE	Tarenaya_hasslerian	46.80	0.00
TRINITY_GAQ8561	Nucleola	Klebsormidium_niten	46.80	0.00
TRINITY_VDD1162	unnamed	Brassica_oleracea	46.80	0.00
TRINITY_GAX7543	hypothet	Chlamydomonas_eusti	46.80	0.00
TRINITY_GAX7313	hypothet	Chlamydomonas_eusti	46.80	0.00
TRINITY_XP_0017	predicte	Chlamydomonas_reinh	46.80	0.00
TRINITY_XP_0014	predicte	Ostreococcus_sp._'l	46.80	0.00
TRINITY_XP_0228	Zinc fin	Ostreococcus_tauri	46.80	0.00
TRINITY_GAX7334	hypothet	Chlamydomonas_eusti	46.80	0.00
TRINITY_KXZ4474	hypothet	Gonium_pectorale	46.80	0.00
TRINITY_GAX8116	hypothet	Chlamydomonas_eusti	46.80	0.00
TRINITY_PNH1199	hypothet	Tetrabaena_socialis	46.80	0.00
TRINITY_XP_0025	uncharac	Ricinus_communis	46.80	0.00
TRINITY_ABU5296	hypothet	Dunaliella_viridis	46.80	0.00
TRINITY_GAX7725	hypothet	Chlamydomonas_eusti	46.80	0.00
TRINITY_XP_0124	PREDICTE	Gossypium_raimondii	46.80	0.00
TRINITY_KXZ5167	hypothet	Gonium_pectorale	46.80	0.00
TRINITY_GAX8227	hypothet	Chlamydomonas_eusti	46.80	0.00
TRINITY_PRW3917	mismatch	Chlorella_sorokinia	46.80	0.00
TRINITY_KXZ4579	hypothet	Gonium_pectorale	46.80	0.00
TRINITY_GAX8404	hypothet	Chlamydomonas_eusti	46.80	0.00
TRINITY_BAK0286	predicte	Hordeum_vulgare	46.80	0.00
TRINITY_GAX7609	hypothet	Chlamydomonas_eusti	46.80	0.00
TRINITY_KXZ5493	hypothet	Gonium_pectorale	46.80	0.00
TRINITY_GAX8031	hypothet	Chlamydomonas_eusti	46.80	0.00
TRINITY_KMZ6314	Ras-rela	Zostera_marina	46.80	0.00
TRINITY_XP_0213	SAC3 fam	Sorghum_bicolor	46.80	0.00
TRINITY_XP_0138	Histone-	Monoraphidium_negle	46.80	0.00
TRINITY_GAX7771	hypothet	Chlamydomonas_eusti	46.80	0.00
TRINITY_GAX8228	hypothet	Chlamydomonas_eusti	46.80	0.00
TRINITY_GAX8446	hypothet	Chlamydomonas_eusti	46.80	0.00
TRINITY_GAQ8166	ABC tran	Klebsormidium_niten	46.80	0.00
TRINITY_GAX8173	hypothet	Chlamydomonas_eusti	46.80	0.00
TRINITY_XP_0191	PREDICTE	Ipomoea_nil	46.80	0.00
TRINITY_AML7930	putative	Prasinococcus_capsu	46.80	0.00
TRINITY_PRW3287	Isochori	Chlorella_sorokinia	46.80	0.00

TRINITY_XP_0056	putative	Coccomyxa_subellips	46.70	0.00
TRINITY_XP_0075	DNA poly	Bathycoccus_prasinc	46.70	0.00
TRINITY_XP_0243	U6 small	Physcomitrella_pate	46.70	0.00
TRINITY_GBG6380	hypothet	Chara_braunii	46.70	0.00
TRINITY_XP_0201	kinesin-	Aegilops_tauschii	46.70	0.00
TRINITY_XP_0239	phosphat	Quercus_suber	46.70	0.00
TRINITY_GAX7271	hypothet	Chlamydomonas_eusti	46.70	0.00
TRINITY_XP_0229	rRNA bio	Cucurbita_maxima	46.70	0.00
TRINITY_XP_0238	ATP-depe	Quercus_suber	46.70	0.00
TRINITY_ABK2491	unknown	Picea_sitchensis	46.70	0.00
TRINITY_PKI7493	hypothet	Punica_granatum	46.70	0.00
TRINITY_KXZ5089	hypothet	Gonium_pectorale	46.70	0.00
TRINITY_XP_0243	ABC tran	Physcomitrella_pate	46.70	0.00
TRINITY_XP_0239	myosin-1	Quercus_suber	46.70	0.00
TRINITY_XP_0279	FH prote	Vigna_unguiculata	46.70	0.00
TRINITY_KFK3842	hypothet	Arabis_alpina	46.70	0.00
TRINITY_XP_0024	26S prot	Sorghum_bicolor	46.70	0.00
TRINITY_XP_0217	ubiquiti	Chenopodium_quinoa	46.70	0.00
TRINITY_XP_0243	protein	Physcomitrella_pate	46.70	0.00
TRINITY_GBG8205	hypothet	Chara_braunii	46.70	0.00
TRINITY_PTQ2836	hypothet	Marchantia_polymorp	46.70	0.00
TRINITY_PRW5760	phosphat	Chlorella_sorokinia	46.70	0.00
TRINITY_GAX7553	hypothet	Chlamydomonas_eusti	46.70	0.00
TRINITY_GBF9293	hypothet	Raphidocelis_subcap	46.70	0.00
TRINITY_XP_0014	P-ATPase	Ostreococcus_sp._'l	46.70	0.00
TRINITY_PRW4569	glucosam	Chlorella_sorokinia	46.70	0.00
TRINITY_XP_0138	Importin	Monoraphidium_negle	46.70	0.00
TRINITY_KXZ5639	hypothet	Gonium_pectorale	46.70	0.00
TRINITY_GAX8155	hypothet	Chlamydomonas_eusti	46.70	0.00
TRINITY_XP_0058	hypothet	Chlorella_variabili	46.70	0.00
TRINITY_GAQ8924	hypothet	Klebsormidium_niten	46.70	0.00
TRINITY_XP_0025	ATP-bind	Micromonas_commoda	46.70	0.00
TRINITY_GAX8289	hypothet	Chlamydomonas_eusti	46.70	0.00
TRINITY_GAX8440	hypothet	Chlamydomonas_eusti	46.70	0.00
TRINITY_KXZ5565	hypothet	Gonium_pectorale	46.70	0.00
TRINITY_CBI2312	unnamed	Vitis_vinifera	46.70	0.00
TRINITY_GAX8488	hypothet	Chlamydomonas_eusti	46.70	0.00
TRINITY_XP_0025	ABC tran	Ricinus_communis	46.70	0.00
TRINITY_ABK2414	unknown	Picea_sitchensis	46.70	0.00
TRINITY_KXZ4690	hypothet	Gonium_pectorale	46.70	0.00
TRINITY_XP_0016	predicte	Chlamydomonas_reinh	46.70	0.00
TRINITY_XP_0016	plastid	Chlamydomonas_reinh	46.70	0.00
TRINITY_PNW7843	hypothet	Chlamydomonas_reinh	46.70	0.00
TRINITY_PNW7058	hypothet	Chlamydomonas_reinh	46.70	0.00
TRINITY_GAX7930	hypothet	Chlamydomonas_eusti	46.70	0.00
TRINITY_XP_0016	predicte	Chlamydomonas_reinh	46.70	0.00
TRINITY_GAX7844	hypothet	Chlamydomonas_eusti	46.70	0.00
TRINITY_GAQ8899	Rab fami	Klebsormidium_niten	46.70	0.00
TRINITY_GAX7616	hypothet	Chlamydomonas_eusti	46.70	0.00
TRINITY_XP_0273	ADP-ribo	Abrus_precatorius	46.70	0.00
TRINITY_PNH1242	hypothet	Tetrabaena_socialis	46.70	0.00
TRINITY_PNW8794	hypothet	Chlamydomonas_reinh	46.70	0.00
TRINITY_PIN0673	putative	Handroanthus_impeti	46.70	0.00
TRINITY_GAQ8514	hypothet	Klebsormidium_niten	46.70	0.00
TRINITY_PNH0696	Transpos	Tetrabaena_socialis	46.70	0.00
TRINITY_CBL4300	Na+/K+ I	Riccia_fluitans	46.70	0.00
TRINITY_XP_0245	cAMP-dep	Selaginella_moellen	46.70	0.00
TRINITY_XP_0238	ras-rela	Quercus_suber	46.70	0.00
TRINITY_GAX7560	hypothet	Chlamydomonas_eusti	46.70	0.00
TRINITY_KXZ4919	hypothet	Gonium_pectorale	46.70	0.00
TRINITY_GAX7837	hypothet	Chlamydomonas_eusti	46.70	0.00
TRINITY_XP_0221	multipro	Momordica_charantia	46.70	0.00
TRINITY_XP_0029	pathogen	Volvox_carteri	46.70	0.00
TRINITY_EFJ3341	hypothet	Selaginella_moellen	46.70	0.00
TRINITY_XP_0220	fe-S clu	Helianthus_annuus	46.70	0.00
TRINITY_XP_0243	probable	Physcomitrella_pate	46.70	0.00

TRINITY_KXZ4923	hypothet	Gonium_pectorale	46.70	0.00
TRINITY_PNH0981	hypothet	Tetrabaena_socialis	46.70	0.00
TRINITY_XP_0245	protein	Selaginella_moellen	46.70	0.00
TRINITY_KXZ4863	hypothet	Gonium_pectorale	46.70	0.00
TRINITY_PNW8854	hypothet	Chlamydomonas_reinh	46.70	0.00
TRINITY_VDD4722	unnamed	Brassica_oleracea	46.60	0.00
TRINITY_NP_0011	uncharac	Zea_mays	46.60	0.00
TRINITY_BAS9358	Os05g03	Oryza_sativa	46.60	0.00
TRINITY_XP_0113	Malate	Auxenochlorella_pro	46.60	0.00
TRINITY_XP_0201	SUMO-act	Aegilops_tauschii	46.60	0.00
TRINITY_XP_0029	spoke s	Volvox_carteri	46.60	0.00
TRINITY_GAX8509	hypothet	Chlamydomonas_eusti	46.60	0.00
TRINITY_XP_0128	PREDICT	Erythranthe_guttata	46.60	0.00
TRINITY_XP_0025	predicte	Micromonas_commoda	46.60	0.00
TRINITY_PIN1149	Sensory	Handroanthus_impeti	46.60	0.00
TRINITY_XP_0271	nicotina	Coffea_arabica	46.60	0.00
TRINITY_XP_0016	predicte	Chlamydomonas_reinh	46.60	0.00
TRINITY_GAQ8238	delta(3)	Klebsormidium_niten	46.60	0.00
TRINITY_GAX7920	hypothet	Chlamydomonas_eusti	46.60	0.00
TRINITY_XP_0238	UDP-N-a	Quercus_suber	46.60	0.00
TRINITY_KMS6524	hypothet	Beta_vulgaris	46.60	0.00
TRINITY_GAX8316	hypothet	Chlamydomonas_eusti	46.60	0.00
TRINITY_XP_0029	hypothet	Volvox_carteri	46.60	0.00
TRINITY_BAK0274	predicte	Hordeum_vulgare	46.60	0.00
TRINITY_CAA9652	G prote	Nicotiana_plumbagin	46.60	0.00
TRINITY_GAX8127	hypothet	Chlamydomonas_eusti	46.60	0.00
TRINITY_XP_0016	MATE ef	Chlamydomonas_reinh	46.60	0.00
TRINITY_GAX8036	hypothet	Chlamydomonas_eusti	46.60	0.00
TRINITY_GBF9312	3-dehyd	Raphidocelis_subcap	46.60	0.00
TRINITY_PNW7525	hypothet	Chlamydomonas_reinh	46.60	0.00
TRINITY_XP_0238	metal r	Quercus_suber	46.60	0.00
TRINITY_XP_0029	hypothet	Volvox_carteri	46.60	0.00
TRINITY_KXZ5204	hypothet	Gonium_pectorale	46.60	0.00
TRINITY_XP_0109	PREDICT	Elaeis_guineensis	46.60	0.00
TRINITY_XP_0174	PREDICT	Vigna_angularis	46.60	0.00
TRINITY_BAK0277	predicte	Hordeum_vulgare	46.60	0.00
TRINITY_GBF9064	p-type	Raphidocelis_subcap	46.60	0.00
TRINITY_XP_0217	nicotina	Chenopodium_quinoa	46.60	0.00
TRINITY_XP_0029	uncharac	Selaginella_moellen	46.60	0.00
TRINITY_XP_0239	ribosome	Quercus_suber	46.60	0.00
TRINITY_KXZ5320	hypothet	Gonium_pectorale	46.60	0.00
TRINITY_PNH0747	hypothet	Tetrabaena_socialis	46.60	0.00
TRINITY_XP_0063	PREDICT	Solanum_tuberosum	46.60	0.00
TRINITY_GAQ8191	N-acyl-	Klebsormidium_niten	46.60	0.00
TRINITY_GAQ9104	calcium-	Klebsormidium_niten	46.60	0.00
TRINITY_XP_0056	calcium-	Coccomyxa_subellips	46.60	0.00
TRINITY_KXZ5360	hypothet	Gonium_pectorale	46.60	0.00
TRINITY_XP_0030	flagella	Micromonas_pusilla	46.60	0.00
TRINITY_GBF8978	hypothet	Raphidocelis_subcap	46.60	0.00
TRINITY_PNW7708	hypothet	Chlamydomonas_reinh	46.60	0.00
TRINITY_GAQ7922	hypothet	Klebsormidium_niten	46.60	0.00
TRINITY_PSC6964	putative	Micractinium_conduc	46.60	0.00
TRINITY_GAX8270	hypothet	Chlamydomonas_eusti	46.60	0.00
TRINITY_GAX7763	hypothet	Chlamydomonas_eusti	46.60	0.00
TRINITY_PNH1103	Endoglu	Tetrabaena_socialis	46.60	0.00
TRINITY_XP_0022	PREDICT	Vitis_vinifera	46.60	0.00
TRINITY_XP_0239	ras-rel	Quercus_suber	46.60	0.00
TRINITY_GBG8383	hypothet	Chara_braunii	46.60	0.00
TRINITY_GBF9837	hypothet	Raphidocelis_subcap	46.60	0.00
TRINITY_GAX7295	hypothet	Chlamydomonas_eusti	46.60	0.00
TRINITY_GBG8088	hypothet	Chara_braunii	46.60	0.00
TRINITY_XP_0082	PREDICT	Prunus_mume	46.60	0.00
TRINITY_XP_0029	hypothet	Volvox_carteri	46.60	0.00
TRINITY_GAX7486	hypothet	Chlamydomonas_eusti	46.60	0.00
TRINITY_XP_0030	flagella	Micromonas_pusilla	46.60	0.00
TRINITY_XP_0243	aminoac	Physcomitrella_pate	46.60	0.00

TRINITY_PNW7878(	hypothet	Chlamydomonas_reinh	46.60	0.00
TRINITY_PNW7744	hypothet	Chlamydomonas_reinh	46.60	0.00
TRINITY_PNW7374	hypothet	Chlamydomonas_reinh	46.60	0.00
TRINITY_GAQ8167	cAMP cGI	Klebsormidium_niten	46.60	0.00
TRINITY_XP_0063	PREDICTI	Solanum_tuberosum	46.60	0.00
TRINITY_GAQ9162	RNA-binc	Klebsormidium_niten	46.50	0.00
TRINITY_XP_0104	PREDICTI	Camelina_sativa	46.50	0.00
TRINITY_OTF9515	putative	Helianthus_annuus	46.50	0.00
TRINITY_XP_0016	calcium-	Chlamydomonas_reinh	46.50	0.00
TRINITY_XP_0128	PREDICTI	Erythranthe_guttata	46.50	0.00
TRINITY_KNA1020(	hypothet	Spinacia_oleracea	46.50	0.00
TRINITY_XP_0056	allanto:	Coccomyxa_subellips	46.50	0.00
TRINITY_PSS3324	E3 ubiq	Actinidia_chinensis	46.50	0.00
TRINITY_PNR4343	hypothet	Physcomitrella_pate	46.50	0.00
TRINITY_XP_0014	predicte	Ostreococcus_sp._'l	46.50	0.00
TRINITY_GAX7616(	hypothet	Chlamydomonas_eusti	46.50	0.00
TRINITY_GAX7752	hypothet	Chlamydomonas_eusti	46.50	0.00
TRINITY_PSS3567	Actin-re	Actinidia_chinensis	46.50	0.00
TRINITY_PNW7044(	hypothet	Chlamydomonas_reinh	46.50	0.00
TRINITY_XP_0016	ATP synt	Chlamydomonas_reinh	46.50	0.00
TRINITY_XP_0216	8-hydro:	Hevea_brasiliensis	46.50	0.00
TRINITY_XP_0068	glycero:	Amborella_trichopod	46.50	0.00
TRINITY_GAX7875(	hypothet	Chlamydomonas_eusti	46.50	0.00
TRINITY_XP_0016	plastid	Chlamydomonas_reinh	46.50	0.00
TRINITY_GAX7784(	hypothet	Chlamydomonas_eusti	46.50	0.00
TRINITY_XP_0241	chaperor	Rosa_chinensis	46.50	0.00
TRINITY_XP_0217	DNA-dire	Chenopodium_quinoa	46.50	0.00
TRINITY_XP_0243	glutamir	Physcomitrella_pate	46.50	0.00
TRINITY_GAX7658	hypothet	Chlamydomonas_eusti	46.50	0.00
TRINITY_GAX8115	hypothet	Chlamydomonas_eusti	46.50	0.00
TRINITY_GAX7402	hypothet	Chlamydomonas_eusti	46.50	0.00
TRINITY_KXZ4216	hypothet	Gonium_pectorale	46.50	0.00
TRINITY_XP_0139(	subunit	Monoraphidium_negle	46.50	0.00
TRINITY_GAX8386	hypothet	Chlamydomonas_eusti	46.50	0.00
TRINITY_GBF9439	rRNA met	Raphidocelis_subcap	46.50	0.00
TRINITY_AEI6192	MOSC dor	Chlamydomonas_reinh	46.50	0.00
TRINITY_GAX8002	hypothet	Chlamydomonas_eusti	46.50	0.00
TRINITY_PKA6538	Beta-fr	Apostasia_shenzheni	46.50	0.00
TRINITY_GAQ8116	hypothet	Klebsormidium_niten	46.50	0.00
TRINITY_GBF9795	hypothet	Raphidocelis_subcap	46.50	0.00
TRINITY_GAX8393	hypothet	Chlamydomonas_eusti	46.50	0.00
TRINITY_GAX7359	hypothet	Chlamydomonas_eusti	46.50	0.00
TRINITY_KXZ5187(	hypothet	Gonium_pectorale	46.50	0.00
TRINITY_XP_0113	hypothet	Auxenochlorella_prc	46.50	0.00
TRINITY_GBF8871	hypothet	Raphidocelis_subcap	46.50	0.00
TRINITY_PWA7999	sirtuin	Artemisia_annua	46.50	0.00
TRINITY_XP_0029	hypothet	Volvox_carteri	46.50	0.00
TRINITY_PKA5221	Serine/t	Apostasia_shenzheni	46.50	0.00
TRINITY_GAX8279	hypothet	Chlamydomonas_eusti	46.50	0.00
TRINITY_GAQ8328	autopha	Klebsormidium_niten	46.50	0.00
TRINITY_GAQ8753	dynein	Klebsormidium_niten	46.50	0.00
TRINITY_XP_0243	ras-rela	Physcomitrella_pate	46.50	0.00
TRINITY_XP_0058	hypothet	Chlorella_variabili	46.50	0.00
TRINITY_GAX7553	hypothet	Chlamydomonas_eusti	46.50	0.00
TRINITY_ABU5296	hypothet	Dunaliella_viridis	46.50	0.00
TRINITY_GAX7590	hypothet	Chlamydomonas_eusti	46.50	0.00
TRINITY_XP_0016	predicte	Chlamydomonas_reinh	46.50	0.00
TRINITY_GAQ8488	heat sh	Klebsormidium_niten	46.50	0.00
TRINITY_XP_0239(	aorsin-	Quercus_suber	46.50	0.00
TRINITY_KXZ4406	hypothet	Gonium_pectorale	46.50	0.00
TRINITY_KXZ5199	hypothet	Gonium_pectorale	46.50	0.00
TRINITY_GAX8220(	hypothet	Chlamydomonas_eusti	46.50	0.00
TRINITY_PNW7149	hypothet	Chlamydomonas_reinh	46.50	0.00
TRINITY_GAQ8075	DNA top	Klebsormidium_niten	46.50	0.00
TRINITY_PNH0612	Thiored	Tetrabaena_socialis	46.50	0.00
TRINITY_PNW8014	hypothet	Chlamydomonas_reinh	46.50	0.00

TRINITY_GAX8086	hypothet	Chlamydomonas_eusti	46.50	0.00
TRINITY_GAX8212	hypothet	Chlamydomonas_eusti	46.50	0.00
TRINITY_GAX7763	hypothet	Chlamydomonas_eusti	46.50	0.00
TRINITY_GAX8306	hypothet	Chlamydomonas_eusti	46.50	0.00
TRINITY_PNH1179	Ubiquit	Tetrabaena_socialis	46.50	0.00
TRINITY_XP_0138	hypothet	Monoraphidium_negle	46.50	0.00
TRINITY_XP_0016	predict	Chlamydomonas_reinh	46.50	0.00
TRINITY_XP_0016	T-compl	Chlamydomonas_reinh	46.50	0.00
TRINITY_GAX7859	hypothet	Chlamydomonas_eusti	46.50	0.00
TRINITY_GBF9870	mitogen-	Raphidocelis_subcap	46.50	0.00
TRINITY_OAE2953	hypothet	Marchantia_polymorp	46.40	0.00
TRINITY_XP_0212	FGGY ca	Herrania_umbratica	46.40	0.00
TRINITY_KNA0612	hypothet	Spinacia_oleracea	46.40	0.00
TRINITY_XP_0058	GTP-bin	Chlorella_variabili	46.40	0.00
TRINITY_XP_0158	uncharac	Ziziphus_jujuba	46.40	0.00
TRINITY_PNH0496	hypothet	Tetrabaena_socialis	46.40	0.00
TRINITY_EFJ1092	hypothet	Selaginella_moellen	46.40	0.00
TRINITY_PKA5220	Serine/t	Apostasia_shenzheni	46.40	0.00
TRINITY_XP_0030	predict	Micromonas_pusilla	46.40	0.00
TRINITY_EYU4153	hypothet	Erythranthe_guttata	46.40	0.00
TRINITY_GAQ7941	Na+/H+	Klebsormidium_niten	46.40	0.00
TRINITY_XP_0172	PREDICT	Daucus_carota	46.40	0.00
TRINITY_XP_0075	predict	Bathycoccus_prasino	46.40	0.00
TRINITY_KJB1825	hypothet	Gossypium_raimondii	46.40	0.00
TRINITY_XP_0217	ras-rel	Chenopodium_quinoa	46.40	0.00
TRINITY_XP_0238	probabl	Quercus_suber	46.40	0.00
TRINITY_XP_0025	short c	Micromonas_commoda	46.40	0.00
TRINITY_PNH0074	hypothet	Tetrabaena_socialis	46.40	0.00
TRINITY_GAQ9227	hypothet	Klebsormidium_niten	46.40	0.00
TRINITY_GBG6254	hypothet	Chara_braunii	46.40	0.00
TRINITY_XP_0243	BEACH d	Physcomitrella_pate	46.40	0.00
TRINITY_GBG6619	hypothet	Chara_braunii	46.40	0.00
TRINITY_CAN7088	hypothet	Vitis_vinifera	46.40	0.00
TRINITY_XP_0238	probabl	Quercus_suber	46.40	0.00
TRINITY_GBG6857	Glutama	Chara_braunii	46.40	0.00
TRINITY_PNH1104	Thiol-d	Tetrabaena_socialis	46.40	0.00
TRINITY_XP_0056	xanthin	Coccomyxa_subellips	46.40	0.00
TRINITY_GAQ9137	diphosp	Klebsormidium_niten	46.40	0.00
TRINITY_KXZ4729	hypothet	Gonium_pectorale	46.40	0.00
TRINITY_XP_0075	unnamed	Bathycoccus_prasino	46.40	0.00
TRINITY_GAQ7802	Six-hai	Klebsormidium_niten	46.40	0.00
TRINITY_GAQ8828	Tubulin-	Klebsormidium_niten	46.40	0.00
TRINITY_XP_0029	hypothet	Volvox_carteri	46.40	0.00
TRINITY_XP_0109	PREDICT	Elaeis_guineensis	46.40	0.00
TRINITY_BAJ9779	predict	Hordeum_vulgare	46.40	0.00
TRINITY_GAX8423	hypothet	Chlamydomonas_eusti	46.40	0.00
TRINITY_OUS4456	putativ	Ostreococcus_tauri	46.40	0.00
TRINITY_GAX7302	hypothet	Chlamydomonas_eusti	46.40	0.00
TRINITY_KXZ5275	hypothet	Gonium_pectorale	46.40	0.00
TRINITY_XP_0220	probabl	Helianthus_annuus	46.40	0.00
TRINITY_PRW5611	Nuclear	Chlorella_sorokinia	46.40	0.00
TRINITY_GAX8462	hypothet	Chlamydomonas_eusti	46.40	0.00
TRINITY_GAX7271	hypothet	Chlamydomonas_eusti	46.40	0.00
TRINITY_XP_0029	NimA-re	Volvox_carteri	46.40	0.00
TRINITY_XP_0239	tyrosin	Quercus_suber	46.40	0.00
TRINITY_XP_0029	hypothet	Volvox_carteri	46.40	0.00
TRINITY_XP_0244	protein	Populus_trichocarpa	46.40	0.00
TRINITY_GAX7548	hypothet	Chlamydomonas_eusti	46.40	0.00
TRINITY_PNW7718	hypothet	Chlamydomonas_reinh	46.40	0.00
TRINITY_KXZ4973	hypothet	Gonium_pectorale	46.40	0.00
TRINITY_XP_0263	uncharac	Papaver_somniferum	46.40	0.00
TRINITY_KXZ5266	ARPC2 p	Gonium_pectorale	46.40	0.00
TRINITY_KXZ4998	hypothet	Gonium_pectorale	46.40	0.00
TRINITY_EPS7066	hypothet	Genlisea_aurea	46.40	0.00
TRINITY_GAX8521	hypothet	Chlamydomonas_eusti	46.40	0.00
TRINITY_GAX8636	hypothet	Chlamydomonas_eusti	46.40	0.00

TRINITY_XP_00305	predicte	Micromonas_pusilla	46.40	0.00
TRINITY_PNW76485	hypothet	Chlamydomonas_reinh	46.40	0.00
TRINITY_XP_00564	hypothet	Coccomyxa_subellips	46.40	0.00
TRINITY_XP_00935	PREDICTE	Pyrus_x_bretschneid	46.40	0.00
TRINITY_GAX75428	hypothet	Chlamydomonas_eusti	46.40	0.00
TRINITY_AHA42268	putative	Chlamydomonas_reinh	46.40	0.00
TRINITY_XP_01722	PREDICTE	Daucus_carota	46.40	0.00
TRINITY_PNW74393	hypothet	Chlamydomonas_reinh	46.40	0.00
TRINITY_PNW75650	hypothet	Chlamydomonas_reinh	46.40	0.00
TRINITY_RAL41559	hypothet	Cuscuta_australis	46.40	0.00
TRINITY_XP_01108	UPF0678	Sesamum_indicum	46.30	0.00
TRINITY_XP_02498	tubulin	Cynara_cardunculus	46.30	0.00
TRINITY_XP_01002	PREDICTE	Eucalyptus_grandis	46.30	0.00
TRINITY_BAK00506	predicte	Hordeum_vulgare	46.30	0.00
TRINITY_PIN25923	hypothet	Handroanthus_impeti	46.30	0.00
TRINITY_AIU48358	pseudour	Dioscorea_oppositif	46.30	0.00
TRINITY_EFJ16368	hypothet	Selaginella_moellen	46.30	0.00
TRINITY_XP_00297	calmodul	Selaginella_moellen	46.30	0.00
TRINITY_BAK02238	predicte	Hordeum_vulgare	46.30	0.00
TRINITY_XP_00249	predicte	Micromonas_commoda	46.30	0.00
TRINITY_XP_02387	isopenic	Quercus_suber	46.30	0.00
TRINITY_XP_02716	serine (	Coffea_eugenioides	46.30	0.00
TRINITY_XP_02643	ras-rela	Papaver_somniferum	46.30	0.00
TRINITY_PSC73998	ammonium	Micractinium_conduc	46.30	0.00
TRINITY_XP_02167	beta-glu	Hevea_brasiliensis	46.30	0.00
TRINITY_GAX82963	hypothet	Chlamydomonas_eusti	46.30	0.00
TRINITY_XP_01654	PREDICTE	Capsicum_annuum	46.30	0.00
TRINITY_PNW86118	hypothet	Chlamydomonas_reinh	46.30	0.00
TRINITY_PNW78541	hypothet	Chlamydomonas_reinh	46.30	0.00
TRINITY_XP_00295	hypothet	Volvox_carteri	46.30	0.00
TRINITY_GBF91043	hypothet	Raphidocelis_subcap	46.30	0.00
TRINITY_XP_00295	hypothet	Volvox_carteri	46.30	0.00
TRINITY_GAX81279	hypothet	Chlamydomonas_eusti	46.30	0.00
TRINITY_XP_00294	hypothet	Volvox_carteri	46.30	0.00
TRINITY_KXZ47851	hypothet	Gonium_pectorale	46.30	0.00
TRINITY_XP_02172	splicing	Chenopodium_quinoa	46.30	0.00
TRINITY_GAX78220	hypothet	Chlamydomonas_eusti	46.30	0.00
TRINITY_XP_00584	hypothet	Chlorella_variabili	46.30	0.00
TRINITY_GAX85453	hypothet	Chlamydomonas_eusti	46.30	0.00
TRINITY_ACF79297	unknown	Zea_mays	46.30	0.00
TRINITY_PNW70261	hypothet	Chlamydomonas_reinh	46.30	0.00
TRINITY_GAX73646	hypothet	Chlamydomonas_eusti	46.30	0.00
TRINITY_XP_01918	PREDICTE	Ipomoea_nil	46.30	0.00
TRINITY_XP_02026	ABC tran	Asparagus_officinal	46.30	0.00
TRINITY_AES59818	ACT-like	Medicago_truncatula	46.30	0.00
TRINITY_GAX76789	hypothet	Chlamydomonas_eusti	46.30	0.00
TRINITY_GAX77074	hypothet	Chlamydomonas_eusti	46.30	0.00
TRINITY_XP_00685	ABC tran	Amborella_trichopod	46.30	0.00
TRINITY_PNH11695	hypothet	Tetrabaena_socialis	46.30	0.00
TRINITY_XP_00294	hypothet	Volvox_carteri	46.30	0.00
TRINITY_PNW74616	hypothet	Chlamydomonas_reinh	46.30	0.00
TRINITY_GAX77362	hypothet	Chlamydomonas_eusti	46.30	0.00
TRINITY_XP_01390	Protein	Monoraphidium_negle	46.30	0.00
TRINITY_GAX84222	hypothet	Chlamydomonas_eusti	46.30	0.00
TRINITY_GBF91515	hypothet	Raphidocelis_subcap	46.30	0.00
TRINITY_XP_01390	DNA repa	Monoraphidium_negle	46.30	0.00
TRINITY_BAJ86552	predicte	Hordeum_vulgare	46.30	0.00
TRINITY_GBF98918	hypothet	Raphidocelis_subcap	46.30	0.00
TRINITY_XP_00170	predicte	Chlamydomonas_reinh	46.30	0.00
TRINITY_GAX83419	hypothet	Chlamydomonas_eusti	46.30	0.00
TRINITY_PNW70694	hypothet	Chlamydomonas_reinh	46.30	0.00
TRINITY_XP_02024	probable	Asparagus_officinal	46.30	0.00
TRINITY_PNW79818	hypothet	Chlamydomonas_reinh	46.30	0.00
TRINITY_GAX81946	hypothet	Chlamydomonas_eusti	46.30	0.00
TRINITY_XP_00170	low-CO2-	Chlamydomonas_reinh	46.30	0.00
TRINITY_XP_02389	lipase 1	Quercus_suber	46.30	0.00

TRINITY_GAQ8985(	putative	Klebsormidium_niten	46.30	0.00
TRINITY_XP_0029(	protein	Selaginella_moellen	46.30	0.00
TRINITY_GAQ8019:	serine/t	Klebsormidium_niten	46.30	0.00
TRINITY_NP_0011:	chromati	Arabidopsis_thalian	46.30	0.00
TRINITY_KRH0048:	hypothet	Glycine_max	46.20	0.00
TRINITY_XP_0084!	PREDICTI	Cucumis_melo	46.20	0.00
TRINITY_XP_0056	putative	Coccomyxa_subellips	46.20	0.00
TRINITY_XP_0156!	PREDICTI	Oryza_brachyantha	46.20	0.00
TRINITY_XP_0128!	PREDICTI	Erythranthe_guttata	46.20	0.00
TRINITY_PSC7305:	ATP-depe	Micractinium_conduc	46.20	0.00
TRINITY_XP_0014:	predicte	Ostreococcus_sp._'l	46.20	0.00
TRINITY_KDP2666(	hypothet	Jatropha_curcas	46.20	0.00
TRINITY_PNH0875:	Dynein (	Tetrabaena_socialis	46.20	0.00
TRINITY_XP_0100!	tubulin-	Morus_notabilis	46.20	0.00
TRINITY_XP_0016!	predicte	Chlamydomonas_reinh	46.20	0.00
TRINITY_GAQ7822	Serine/t	Klebsormidium_niten	46.20	0.00
TRINITY_XP_0239(	ras-like	Quercus_suber	46.20	0.00
TRINITY_XP_0241!	dual spe	Rosa_chinensis	46.20	0.00
TRINITY_CDL6798(	putative	Olea_europaea	46.20	0.00
TRINITY_XP_0030(	Domain (	Ostreococcus_tauri	46.20	0.00
TRINITY_PNR4563(	hypothet	Physcomitrella_pate	46.20	0.00
TRINITY_XP_0029	hypothet	Volvox_carteri	46.20	0.00
TRINITY_XP_0238	translat	Quercus_suber	46.20	0.00
TRINITY_XP_0056	hypothet	Coccomyxa_subellips	46.20	0.00
TRINITY_VDC6575(	unnamed	Brassica_rapa	46.20	0.00
TRINITY_XP_0063:	PREDICTI	Solanum_tuberosum	46.20	0.00
TRINITY_PNH0167(	hypothet	Tetrabaena_socialis	46.20	0.00
TRINITY_XP_0029!	hypothet	Volvox_carteri	46.20	0.00
TRINITY_GAX7423(	hypothet	Chlamydomonas_eusti	46.20	0.00
TRINITY_XP_0109:	PREDICTI	Elaeis_guineensis	46.20	0.00
TRINITY_XP_0017(	TPR prot	Chlamydomonas_reinh	46.20	0.00
TRINITY_RWW9121(	hypothet	Ensete_ventricosum	46.20	0.00
TRINITY_XP_0128	PREDICTI	Erythranthe_guttata	46.20	0.00
TRINITY_XP_0174(	PREDICTI	Vigna_angularis	46.20	0.00
TRINITY_XP_0200(	serine/t	Ananas_comosus	46.20	0.00
TRINITY_XP_0238	ADP-ribo	Quercus_suber	46.20	0.00
TRINITY_KXZ5628:	hypothet	Gonium_pectorale	46.20	0.00
TRINITY_XP_0270!	poly [AI	Coffea_arabica	46.20	0.00
TRINITY_XP_0228(	MAP3K ep	Olea_europaea	46.20	0.00
TRINITY_GBF9594!	hypothet	Raphidocelis_subcap	46.20	0.00
TRINITY_PNW7335!	hypothet	Chlamydomonas_reinh	46.20	0.00
TRINITY_BAL4652:	hypothet	Gentiana_scabra_x_G	46.20	0.00
TRINITY_XP_0016!	ran-like	Chlamydomonas_reinh	46.20	0.00
TRINITY_XP_0058!	hypothet	Chlorella_variabili	46.20	0.00
TRINITY_PNW7671(	hypothet	Chlamydomonas_reinh	46.20	0.00
TRINITY_XP_0042!	PREDICTI	Fragaria_vesca	46.20	0.00
TRINITY_GAX8507	hypothet	Chlamydomonas_eusti	46.20	0.00
TRINITY_GAX8061:	hypothet	Chlamydomonas_eusti	46.20	0.00
TRINITY_GAX8328:	hypothet	Chlamydomonas_eusti	46.20	0.00
TRINITY_GBF9067:	hypothet	Raphidocelis_subcap	46.20	0.00
TRINITY_XP_0270	probable	Coffea_arabica	46.20	0.00
TRINITY_GAQ8369	putative	Klebsormidium_niten	46.20	0.00
TRINITY_XP_0264:	1-phosph	Papaver_somniferum	46.20	0.00
TRINITY_XP_0029!	hypothet	Volvox_carteri	46.20	0.00
TRINITY_PRW6143!	pre-rRNA	Chlorella_sorokinia	46.20	0.00
TRINITY_GAX8430:	hypothet	Chlamydomonas_eusti	46.20	0.00
TRINITY_KXZ5310(	hypothet	Gonium_pectorale	46.20	0.00
TRINITY_KYP5182:	Protein	Cajanus_cajan	46.20	0.00
TRINITY_PRW5604(	multidru	Chlorella_sorokinia	46.20	0.00
TRINITY_XP_0217	probable	Chenopodium_quinoa	46.20	0.00
TRINITY_OAE2250!	hypothet	Marchantia_polymorp	46.20	0.00
TRINITY_BAK0075(	predicte	Hordeum_vulgare	46.20	0.00
TRINITY_GAX7304:	hypothet	Chlamydomonas_eusti	46.20	0.00
TRINITY_KXZ4438	hypothet	Gonium_pectorale	46.20	0.00
TRINITY_XP_0139(	Aquapori	Monoraphidium_negle	46.20	0.00
TRINITY_XP_0030(	predicte	Micromonas_pusilla	46.20	0.00

TRINITY_XP_0191	PREDICTE	Ipomoea_nil	46.20	0.00
TRINITY_PNW8631	hypothet	Chlamydomonas_reinh	46.20	0.00
TRINITY_KXZ4562	hypothet	Gonium_pectorale	46.20	0.00
TRINITY_GAX7344	hypothet	Chlamydomonas_eusti	46.20	0.00
TRINITY_GAX7928	hypothet	Chlamydomonas_eusti	46.20	0.00
TRINITY_GAX7289	hypothet	Chlamydomonas_eusti	46.20	0.00
TRINITY_GAX8471	hypothet	Chlamydomonas_eusti	46.20	0.00
TRINITY_KXZ4786	hypothet	Gonium_pectorale	46.20	0.00
TRINITY_XP_0016	predicte	Chlamydomonas_reinh	46.20	0.00
TRINITY_XP_0244	cyclin-c	Physcomitrella_pate	46.20	0.00
TRINITY_XP_0041	PREDICTE	Cucumis_sativus	46.20	0.00
TRINITY_XP_0258	ubiquiti	Panicum_hallii	46.20	0.00
TRINITY_PSC7384	ABC tran	Micractinium_conduc	46.20	0.00
TRINITY_PRW1834	putative	Chlorella_sorokinia	46.20	0.00
TRINITY_XP_0245	uncharac	Selaginella_moellen	46.20	0.00
TRINITY_KXZ4188	hypothet	Gonium_pectorale	46.20	0.00
TRINITY_RMZ5514	hypothet	Auxenochlorella_pro	46.20	0.00
TRINITY_KXZ4683	hypothet	Gonium_pectorale	46.20	0.00
TRINITY_OAE3294	hypothet	Marchantia_polymorp	46.20	0.00
TRINITY_PNH1226	Tiny mac	Tetrabaena_socialis	46.20	0.00
TRINITY_XP_0062	NADPH:qu	Capsella_rubella	46.20	0.00
TRINITY_GAX7722	hypothet	Chlamydomonas_eusti	46.10	0.00
TRINITY_KHG1450	Histidir	Gossypium_arboreum	46.10	0.00
TRINITY_Q40191.1	RecName:	Lotus_japonicus	46.10	0.00
TRINITY_XP_0093	PREDICTE	Musa_acuminata	46.10	0.00
TRINITY_XP_0082	PREDICTE	Prunus_mume	46.10	0.00
TRINITY_XP_0158	auxin tr	Ziziphus_jujuba	46.10	0.00
TRINITY_XP_0205	LOW QUAL	Phalaenopsis_equest	46.10	0.00
TRINITY_GAX7377	hypothet	Chlamydomonas_eusti	46.10	0.00
TRINITY_XP_0029	hypothet	Volvox_carteri	46.10	0.00
TRINITY_GAX8407	hypothet	Chlamydomonas_eusti	46.10	0.00
TRINITY_XP_0100	PREDICTE	Eucalyptus_grandis	46.10	0.00
TRINITY_XP_0116	ubiquiti	Amborella_trichopod	46.10	0.00
TRINITY_GAX7603	hypothet	Chlamydomonas_eusti	46.10	0.00
TRINITY_XP_0058	hypothet	Chlorella_variabili	46.10	0.00
TRINITY_GAX8153	hypothet	Chlamydomonas_eusti	46.10	0.00
TRINITY_XP_0025	predicte	Micromonas_commoda	46.10	0.00
TRINITY_XP_0029	hypothet	Volvox_carteri	46.10	0.00
TRINITY_PNW8842	hypothet	Chlamydomonas_reinh	46.10	0.00
TRINITY_GAQ7967	geranyl	Klebsormidium_niten	46.10	0.00
TRINITY_GAX8097	hypothet	Chlamydomonas_eusti	46.10	0.00
TRINITY_KXZ4675	hypothet	Gonium_pectorale	46.10	0.00
TRINITY_PRW5903	nudix hy	Chlorella_sorokinia	46.10	0.00
TRINITY_PSC6759	Gag-Pol	Micractinium_conduc	46.10	0.00
TRINITY_XP_0029	hypothet	Volvox_carteri	46.10	0.00
TRINITY_ABK2643	unknown	Picea_sitchensis	46.10	0.00
TRINITY_GAX8481	hypothet	Chlamydomonas_eusti	46.10	0.00
TRINITY_KZM9376	hypothet	Daucus_carota	46.10	0.00
TRINITY_GAQ8985	putative	Klebsormidium_niten	46.10	0.00
TRINITY_XP_0238	probable	Quercus_suber	46.10	0.00
TRINITY_XP_0194	PREDICTE	Lupinus_angustifoli	46.10	0.00
TRINITY_GAX7432	hypothet	Chlamydomonas_eusti	46.10	0.00
TRINITY_XP_0029	hypothet	Volvox_carteri	46.10	0.00
TRINITY_GAX7487	hypothet	Chlamydomonas_eusti	46.10	0.00
TRINITY_GAX8241	hypothet	Chlamydomonas_eusti	46.10	0.00
TRINITY_RWW0672	hypothet	Ensete_ventricosum	46.10	0.00
TRINITY_GBG7104	hypothet	Chara_braunii	46.10	0.00
TRINITY_GAX7942	hypothet	Chlamydomonas_eusti	46.10	0.00
TRINITY_XP_0205	myosin-(	Sesamum_indicum	46.10	0.00
TRINITY_XP_0087	1-phosph	Phoenix_dactylifera	46.10	0.00
TRINITY_XP_0238	ubiquiti	Quercus_suber	46.10	0.00
TRINITY_XP_0029	hypothet	Volvox_carteri	46.10	0.00
TRINITY_XP_0029	hypothet	Volvox_carteri	46.10	0.00
TRINITY_PNW8559	hypothet	Chlamydomonas_reinh	46.10	0.00
TRINITY_GAX7411	hypothet	Chlamydomonas_eusti	46.10	0.00
TRINITY_XP_0016	predicte	Chlamydomonas_reinh	46.10	0.00

TRINITY_OTG04014	putative	Helianthus_annuus	46.10	0.00
TRINITY_XP_00565	DNA/RNA	Coccomyxa_subellips	46.10	0.00
TRINITY_XP_00250	pyruvate	Micromonas_commoda	46.10	0.00
TRINITY_PNH04805	hypothet	Tetrabaena_socialis	46.10	0.00
TRINITY_DAA34891	TPA_exp:	Ostreococcus_sp._'l	46.10	0.00
TRINITY_GAX82427	hypothet	Chlamydomonas_eusti	46.10	0.00
TRINITY_GAX76608	hypothet	Chlamydomonas_eusti	46.10	0.00
TRINITY_GAX81366	hypothet	Chlamydomonas_eusti	46.10	0.00
TRINITY_PUZ55071	hypothet	Panicum_hallii	46.10	0.00
TRINITY_GAX77010	hypothet	Chlamydomonas_eusti	46.10	0.00
TRINITY_ACL28159	hypothet	Dunaliella_viridis	46.10	0.00
TRINITY_XP_02438	serine/t	Physcomitrella_pate	46.10	0.00
TRINITY_XP_02284	UBA-like	Ostreococcus_tauri	46.10	0.00
TRINITY_KXZ48999	hypothet	Gonium_pectorale	46.10	0.00
TRINITY_OAE27724	hypothet	Marchantia_polymorp	46.10	0.00
TRINITY_EPS65101	hypothet	Genlisea_aurea	46.10	0.00
TRINITY_GAX75484	hypothet	Chlamydomonas_eusti	46.10	0.00
TRINITY_GAX76218	hypothet	Chlamydomonas_eusti	46.10	0.00
TRINITY_XP_00448	transcri	Cicer_arietinum	46.00	0.00
TRINITY_OIW07290	hypothet	Lupinus_angustifoli	46.00	0.00
TRINITY_ERM95185	hypothet	Amborella_trichopod	46.00	0.00
TRINITY_PRW57533	Ras-rela	Chlorella_sorokinia	46.00	0.00
TRINITY_XP_01561	glutathi	Oryza_sativa	46.00	0.00
TRINITY_OVA14754	Small G	Macleaya_cordata	46.00	0.00
TRINITY_XP_01654	PREDICTE	Capsicum_annuum	46.00	0.00
TRINITY_XP_02201	serine/t	Helianthus_annuus	46.00	0.00
TRINITY_PVH47236	hypothet	Panicum_hallii	46.00	0.00
TRINITY_OAY80294	Heat sho	Ananas_comosus	46.00	0.00
TRINITY_XP_00935	PREDICTE	Pyrus_x_bretschneid	46.00	0.00
TRINITY_RMZ56992	hypothet	Auxenochlorella_pro	46.00	0.00
TRINITY_GAQ91212	Calcium	Klebsormidium_niten	46.00	0.00
TRINITY_PNH07996	hypothet	Tetrabaena_socialis	46.00	0.00
TRINITY_XP_02437	NPC intr	Physcomitrella_pate	46.00	0.00
TRINITY_OAY77860	Signal r	Ananas_comosus	46.00	0.00
TRINITY_GAQ78644	4-coumar	Klebsormidium_niten	46.00	0.00
TRINITY_GAX75814	hypothet	Chlamydomonas_eusti	46.00	0.00
TRINITY_KXZ54859	hypothet	Gonium_pectorale	46.00	0.00
TRINITY_GAX84565	hypothet	Chlamydomonas_eusti	46.00	0.00
TRINITY_XP_02451	calmodul	Selaginella_moellen	46.00	0.00
TRINITY_GAX73943	hypothet	Chlamydomonas_eusti	46.00	0.00
TRINITY_GAX73779	hypothet	Chlamydomonas_eusti	46.00	0.00
TRINITY_XP_00938	PREDICTE	Musa_acuminata	46.00	0.00
TRINITY_KXZ46283	hypothet	Gonium_pectorale	46.00	0.00
TRINITY_RWW34630	hypothet	Ensete_ventricosum	46.00	0.00
TRINITY_XP_00647	bifuncti	Citrus_sinensis	46.00	0.00
TRINITY_XP_00297	cleft li	Selaginella_moellen	46.00	0.00
TRINITY_GBF87526	hypothet	Raphidocelis_subcap	46.00	0.00
TRINITY_XP_00295	hypothet	Volvox_carteri	46.00	0.00
TRINITY_XP_00170	predicte	Chlamydomonas_reinh	46.00	0.00
TRINITY_PNW73818	hypothet	Chlamydomonas_reinh	46.00	0.00
TRINITY_XP_01761	PREDICTE	Gossypium_arboreum	46.00	0.00
TRINITY_GAX83997	hypothet	Chlamydomonas_eusti	46.00	0.00
TRINITY_PRW21061	AMP-bind	Chlorella_sorokinia	46.00	0.00
TRINITY_RZB43048	Flap end	Glycine_soja	46.00	0.00
TRINITY_XP_00226	PREDICTE	Vitis_vinifera	46.00	0.00
TRINITY_KXZ42499	hypothet	Gonium_pectorale	46.00	0.00
TRINITY_OWM90366	hypothet	Punica_granatum	46.00	0.00
TRINITY_GAQ83112	pescadil	Klebsormidium_niten	46.00	0.00
TRINITY_GAX82821	hypothet	Chlamydomonas_eusti	46.00	0.00
TRINITY_ABF95791	Serine/t	Oryza_sativa	46.00	0.00
TRINITY_GAQ93189	hypothet	Klebsormidium_niten	46.00	0.00
TRINITY_KXZ43536	hypothet	Gonium_pectorale	46.00	0.00
TRINITY_KXZ48346	hypothet	Gonium_pectorale	46.00	0.00
TRINITY_GAX74691	hypothet	Chlamydomonas_eusti	46.00	0.00
TRINITY_XP_00294	hypothet	Volvox_carteri	46.00	0.00
TRINITY_XP_02392	fimbrin-	Quercus_suber	46.00	0.00

TRINITY_GBG8498(	hypothet	Chara_braunii	46.00	0.00
TRINITY_GAQ9215:	hypothet	Klebsormidium_niten	46.00	0.00
TRINITY_PNH1180	hypothet	Tetrabaena_socialis	46.00	0.00
TRINITY_GAX8666:	hypothet	Chlamydomonas_eusti	46.00	0.00
TRINITY_XP_0058	hypothet	Chlorella_variabili	46.00	0.00
TRINITY_XP_0029	hypothet	Volvox_carteri	46.00	0.00
TRINITY_XP_0029	trypsin	Volvox_carteri	46.00	0.00
TRINITY_GAX7534(	hypothet	Chlamydomonas_eusti	46.00	0.00
TRINITY_XP_0030(	predicte	Micromonas_pusilla	46.00	0.00
TRINITY_XP_0025(	predicte	Micromonas_commoda	45.90	0.00
TRINITY_PNR2974	hypothet	Physcomitrella_pate	45.90	0.00
TRINITY_PNH0810	hypothet	Tetrabaena_socialis	45.90	0.00
TRINITY_XP_0220	ras-rela	Helianthus_annuus	45.90	0.00
TRINITY_XP_0273	tubulin	Abrus_precatorius	45.90	0.00
TRINITY_XP_0239	putative	Quercus_suber	45.90	0.00
TRINITY_XP_0025(	predicte	Micromonas_commoda	45.90	0.00
TRINITY_XP_0114(	PREDICTE	Fragaria_vesca	45.90	0.00
TRINITY_PRW5682	oxidored	Chlorella_sorokinia	45.90	0.00
TRINITY_ESQ3075	hypothet	Eutrema_salsugineum	45.90	0.00
TRINITY_GBG7565	hypothet	Chara_braunii	45.90	0.00
TRINITY_GBG8866	hypothet	Chara_braunii	45.90	0.00
TRINITY_XP_0014	predicte	Ostreococcus_sp._'l	45.90	0.00
TRINITY_GBF9419(	calcium	Raphidocelis_subcap	45.90	0.00
TRINITY_XP_0068	ubiquiti	Amborella_trichopod	45.90	0.00
TRINITY_GAQ7873	alpha-ar	Klebsormidium_niten	45.90	0.00
TRINITY_XP_0221	switch 2	Momordica_charantia	45.90	0.00
TRINITY_XP_0068	protein	Amborella_trichopod	45.90	0.00
TRINITY_GAQ8393	glycerop	Klebsormidium_niten	45.90	0.00
TRINITY_XP_0029	hypothet	Volvox_carteri	45.90	0.00
TRINITY_EFJ2081	hypothet	Selaginella_moellen	45.90	0.00
TRINITY_PRW5882	Pol [Ch]	Chlorella_sorokinia	45.90	0.00
TRINITY_VDD1023	unnamed	Brassica_rapa	45.90	0.00
TRINITY_XP_0083	PREDICTE	Malus_domestica	45.90	0.00
TRINITY_XP_0100	PREDICTE	Eucalyptus_grandis	45.90	0.00
TRINITY_XP_0016	diacylgl	Chlamydomonas_reinh	45.90	0.00
TRINITY_KZV4647(	protein	Dorcoceras_hygromet	45.90	0.00
TRINITY_GBF9327	hypothet	Raphidocelis_subcap	45.90	0.00
TRINITY_PSC7124(	WD repea	Micractinium_conduc	45.90	0.00
TRINITY_GAX7880	hypothet	Chlamydomonas_eusti	45.90	0.00
TRINITY_XP_0056	hypothet	Coccomyxa_subellips	45.90	0.00
TRINITY_XP_0220	N-glycos	Helianthus_annuus	45.90	0.00
TRINITY_PTQ3750	hypothet	Marchantia_polymorp	45.90	0.00
TRINITY_XP_0022	PREDICTE	Vitis_vinifera	45.90	0.00
TRINITY_GAX7811	hypothet	Chlamydomonas_eusti	45.90	0.00
TRINITY_GAX7282	hypothet	Chlamydomonas_eusti	45.90	0.00
TRINITY_PNW7878	hypothet	Chlamydomonas_reinh	45.90	0.00
TRINITY_XP_0091	PREDICTE	Brassica_rapa	45.90	0.00
TRINITY_XP_0029	hypothet	Volvox_carteri	45.90	0.00
TRINITY_XP_0238	protein	Quercus_suber	45.90	0.00
TRINITY_GAX7348	hypothet	Chlamydomonas_eusti	45.90	0.00
TRINITY_XP_0238	uncharac	Quercus_suber	45.90	0.00
TRINITY_GAX7457	hypothet	Chlamydomonas_eusti	45.90	0.00
TRINITY_RID4227	hypothet	Brassica_rapa	45.90	0.00
TRINITY_XP_0202(	uncharac	Cajanus_cajan	45.90	0.00
TRINITY_GAQ8752	cupin-do	Klebsormidium_niten	45.90	0.00
TRINITY_XP_0016	predicte	Chlamydomonas_reinh	45.90	0.00
TRINITY_PNW8263	hypothet	Chlamydomonas_reinh	45.90	0.00
TRINITY_XP_0156	histone	Oryza_sativa	45.90	0.00
TRINITY_XP_0204	ribosome	Prunus_persica	45.90	0.00
TRINITY_XP_0014	predicte	Ostreococcus_sp._'l	45.90	0.00
TRINITY_GAX7883	hypothet	Chlamydomonas_eusti	45.90	0.00
TRINITY_XP_0029	hypothet	Volvox_carteri	45.90	0.00
TRINITY_GAX7761	hypothet	Chlamydomonas_eusti	45.90	0.00
TRINITY_GBF8992	hypothet	Raphidocelis_subcap	45.90	0.00
TRINITY_GAQ8400	intrafla	Klebsormidium_niten	45.90	0.00
TRINITY_XP_0120	long cha	Jatropha_curcas	45.90	0.00

TRINITY_GAQ8109	hypothet	Klebsormidium_niten	45.90	0.00
TRINITY_OAE2722	hypothet	Marchantia_polymorp	45.90	0.00
TRINITY_PNW8431	hypothet	Chlamydomonas_reinh	45.90	0.00
TRINITY_CAN7043	hypothet	Vitis_vinifera	45.90	0.00
TRINITY_GAX7268	hypothet	Chlamydomonas_eusti	45.90	0.00
TRINITY_GAX8215	hypothet	Chlamydomonas_eusti	45.90	0.00
TRINITY_GBG6548	hypothet	Chara_braunii	45.90	0.00
TRINITY_GAX8558	hypothet	Chlamydomonas_eusti	45.90	0.00
TRINITY_PSC7702	NUDIX h	Micractinium_conduc	45.90	0.00
TRINITY_PNW8654	hypothet	Chlamydomonas_reinh	45.90	0.00
TRINITY_PNH1237	Ferredo	Tetrabaena_socialis	45.90	0.00
TRINITY_GAX7981	hypothet	Chlamydomonas_eusti	45.90	0.00
TRINITY_GAX7919	hypothet	Chlamydomonas_eusti	45.90	0.00
TRINITY_PSC6759	Gag-Pol	Micractinium_conduc	45.90	0.00
TRINITY_RMZ5254	hypothet	Auxenochlorella_pro	45.90	0.00
TRINITY_GAX7408	hypothet	Chlamydomonas_eusti	45.90	0.00
TRINITY_XP_0029	small A	Volvox_carteri	45.90	0.00
TRINITY_XP_0029	hypothet	Volvox_carteri	45.90	0.00
TRINITY_GBG6463	hypothet	Chara_braunii	45.90	0.00
TRINITY_RHN3997	putative	Medicago_truncatula	45.90	0.00
TRINITY_KXZ4430	hypothet	Gonium_pectorale	45.90	0.00
TRINITY_GAU2656	hypothet	Trifolium_subterran	45.90	0.00
TRINITY_RZC8237	hypothet	Papaver_somniferum	45.90	0.00
TRINITY_XP_0191	PREDICT	Ipomoea_nil	45.80	0.00
TRINITY_XP_0045	ubiquit	Cicer_arietinum	45.80	0.00
TRINITY_XP_0100	PREDICT	Eucalyptus_grandis	45.80	0.00
TRINITY_KJB8180	hypothet	Gossypium_raimondii	45.80	0.00
TRINITY_XP_0030	SET dom	Ostreococcus_tauri	45.80	0.00
TRINITY_BAK0277	predict	Hordeum_vulgare	45.80	0.00
TRINITY_RVX0265	Retrovi	Vitis_vinifera	45.80	0.00
TRINITY_GAQ8602	Serine	Klebsormidium_niten	45.80	0.00
TRINITY_XP_0025	histone	Ricinus_communis	45.80	0.00
TRINITY_XP_0139	hypothet	Monoraphidium_negle	45.80	0.00
TRINITY_XP_0192	PREDICT	Nicotiana_attenuata	45.80	0.00
TRINITY_GAU2745	hypothet	Trifolium_subterran	45.80	0.00
TRINITY_GBG7399	hypothet	Chara_braunii	45.80	0.00
TRINITY_GAQ8889	N-acety	Klebsormidium_niten	45.80	0.00
TRINITY_AIN3982	hypothet	Zoysia_matrella	45.80	0.00
TRINITY_OAY8040	Glycine-	Ananas_comosus	45.80	0.00
TRINITY_EFH6780	predict	Arabidopsis_lyrata	45.80	0.00
TRINITY_GAQ8774	Adenylat	Klebsormidium_niten	45.80	0.00
TRINITY_KZV1511	ATP bin	Dorcoceras_hygromet	45.80	0.00
TRINITY_PNW7161	hypothet	Chlamydomonas_reinh	45.80	0.00
TRINITY_CAD1167	putative	Physcomitrella_pate	45.80	0.00
TRINITY_GAX7401	hypothet	Chlamydomonas_eusti	45.80	0.00
TRINITY_XP_0029	nudix h	Selaginella_moellen	45.80	0.00
TRINITY_PNW7873	hypothet	Chlamydomonas_reinh	45.80	0.00
TRINITY_KXZ5398	hypothet	Gonium_pectorale	45.80	0.00
TRINITY_CAI8489	putative	Medicago_sativa	45.80	0.00
TRINITY_XP_0158	protein	Ziziphus_jujuba	45.80	0.00
TRINITY_XP_0056	palmito	Coccomyxa_subellips	45.80	0.00
TRINITY_GAX7841	hypothet	Chlamydomonas_eusti	45.80	0.00
TRINITY_GBF8834	hypothet	Raphidocelis_subcap	45.80	0.00
TRINITY_P0C8M8.	RecName:	Zea_mays	45.80	0.00
TRINITY_GBG7546	hypothet	Chara_braunii	45.80	0.00
TRINITY_XP_0064	dynamin-	Eutrema_salsugineum	45.80	0.00
TRINITY_XP_0256	serine/t	Arachis_hypogaea	45.80	0.00
TRINITY_XP_0093	PREDICT	Pyrus_x_bretschneid	45.80	0.00
TRINITY_XP_0216	DEAD-bo	Manihot_esculenta	45.80	0.00
TRINITY_XP_0029	hypothet	Volvox_carteri	45.80	0.00
TRINITY_PNW7373	hypothet	Chlamydomonas_reinh	45.80	0.00
TRINITY_XP_0155	condens	Ricinus_communis	45.80	0.00
TRINITY_KXZ4681	hypothet	Gonium_pectorale	45.80	0.00
TRINITY_PSC7436	nucleot	Micractinium_conduc	45.80	0.00
TRINITY_GBF9757	hypothet	Raphidocelis_subcap	45.80	0.00
TRINITY_ERN1372	hypothet	Amborella_trichopod	45.80	0.00

TRINITY_GBF8945	30S rib	Raphidocelis_subcap	45.80	0.00
TRINITY_GAX8192	hypothet	Chlamydomonas_eusti	45.80	0.00
TRINITY_KXZ5286	hypothet	Gonium_pectorale	45.80	0.00
TRINITY_RRT8057	hypothet	Ensete_ventricosum	45.80	0.00
TRINITY_GAQ8199	fragment	Klebsormidium_niten	45.80	0.00
TRINITY_XP_0245	cycloart	Selaginella_moellen	45.80	0.00
TRINITY_GAX7641	hypothet	Chlamydomonas_eusti	45.80	0.00
TRINITY_XP_0227	serine	Durio_zibethinus	45.80	0.00
TRINITY_PNW8840	hypothet	Chlamydomonas_reinh	45.80	0.00
TRINITY_XP_0075	SNF2 su	Bathycoccus_prasinc	45.80	0.00
TRINITY_XP_0164	PREDICT	Nicotiana_tabacum	45.80	0.00
TRINITY_PNW8209	hypothet	Chlamydomonas_reinh	45.80	0.00
TRINITY_RID7422	hypothet	Brassica_rapa	45.80	0.00
TRINITY_XP_0017	predict	Chlamydomonas_reinh	45.80	0.00
TRINITY_PNW7192	hypothet	Chlamydomonas_reinh	45.80	0.00
TRINITY_XP_0138	hypothet	Monoraphidium_negle	45.80	0.00
TRINITY_XP_0029	hypothet	Volvox_carteri	45.80	0.00
TRINITY_BAJ9457	predict	Hordeum_vulgare	45.80	0.00
TRINITY_XP_0114	Adenyly	Auxenochlorella_prc	45.80	0.00
TRINITY_XP_0029	hypothet	Volvox_carteri	45.80	0.00
TRINITY_KXZ5626	hypothet	Gonium_pectorale	45.80	0.00
TRINITY_XP_0136	ubiquit	Brassica_napus	45.80	0.00
TRINITY_XP_0063	PREDICT	Solanum_tuberosum	45.80	0.00
TRINITY_ABB9055	putativ	Triticum_aestivum	45.80	0.00
TRINITY_GAX8095	hypothet	Chlamydomonas_eusti	45.80	0.00
TRINITY_PNW8703	hypothet	Chlamydomonas_reinh	45.80	0.00
TRINITY_PNW8572	hypothet	Chlamydomonas_reinh	45.80	0.00
TRINITY_GAX8008	hypothet	Chlamydomonas_eusti	45.80	0.00
TRINITY_GBF8910	hypothet	Raphidocelis_subcap	45.80	0.00
TRINITY_GAX8210	hypothet	Chlamydomonas_eusti	45.80	0.00
TRINITY_XP_0102	unchara	Brachypodium_distac	45.80	0.00
TRINITY_XP_0017	RNA met	Chlamydomonas_reinh	45.80	0.00
TRINITY_KXZ4361	hypothet	Gonium_pectorale	45.80	0.00
TRINITY_PSS3064	Kinesin-	Actinidia_chinensis	45.80	0.00
TRINITY_RAL3909	hypothet	Cuscuta_australis	45.70	0.00
TRINITY_PRW5901	deoxyri	Chlorella_sorokinia	45.70	0.00
TRINITY_OAE2128	hypothet	Marchantia_polymorp	45.70	0.00
TRINITY_XP_0172	PREDICT	Daucus_carota	45.70	0.00
TRINITY_GBF8951	multidr	Raphidocelis_subcap	45.70	0.00
TRINITY_GBG7193	hypothet	Chara_braunii	45.70	0.00
TRINITY_XP_0105	PREDICT	Tarenaya_hasslerian	45.70	0.00
TRINITY_XP_0273	autopha	Abrus_precatorius	45.70	0.00
TRINITY_XP_0220	AP-4 co	Helianthus_annuus	45.70	0.00
TRINITY_GAQ8088	adaptor	Klebsormidium_niten	45.70	0.00
TRINITY_XP_0243	probabl	Physcomitrella_pate	45.70	0.00
TRINITY_GAQ8891	peroxin	Klebsormidium_niten	45.70	0.00
TRINITY_XP_0029	hypothet	Volvox_carteri	45.70	0.00
TRINITY_GBF8774	hypothet	Raphidocelis_subcap	45.70	0.00
TRINITY_XP_0243	RHOMBOI	Physcomitrella_pate	45.70	0.00
TRINITY_XP_0041	PREDICT	Cucumis_sativus	45.70	0.00
TRINITY_XP_0241	chaperor	Rosa_chinensis	45.70	0.00
TRINITY_GAQ8333	17 beta-	Klebsormidium_niten	45.70	0.00
TRINITY_XP_0238	translat	Quercus_suber	45.70	0.00
TRINITY_XP_0159	coiled-	Arachis_duranensis	45.70	0.00
TRINITY_XP_0056	CheY-li	Coccomyxa_subellips	45.70	0.00
TRINITY_KDP3931	hypothet	Jatropha_curcas	45.70	0.00
TRINITY_GAX8540	hypothet	Chlamydomonas_eusti	45.70	0.00
TRINITY_XP_0025	unchara	Ricinus_communis	45.70	0.00
TRINITY_RQL7928	hypothet	Brassica_cretica	45.70	0.00
TRINITY_KXZ4254	hypothet	Gonium_pectorale	45.70	0.00
TRINITY_PSC7624	multidr	Micractinium_conduc	45.70	0.00
TRINITY_BAK0043	predict	Hordeum_vulgare	45.70	0.00
TRINITY_XP_0094	PREDICT	Musa_acuminata	45.70	0.00
TRINITY_GAX8483	hypothet	Chlamydomonas_eusti	45.70	0.00
TRINITY_XP_0138	hypothet	Monoraphidium_negle	45.70	0.00
TRINITY_AAT5883	hypothet	Oryza_sativa	45.70	0.00

TRINITY_XP_00702	PREDICTE	Theobroma_cacao	45.70	0.00
TRINITY_GAQ78227	Serine/t	Klebsormidium_niten	45.70	0.00
TRINITY_XP_01245	PREDICTE	Gossypium_raimondii	45.70	0.00
TRINITY_XP_02199	probable	Helianthus_annuus	45.70	0.00
TRINITY_GBG68748	hypothet	Chara_braunii	45.70	0.00
TRINITY_GAX84407	hypothet	Chlamydomonas_eusti	45.70	0.00
TRINITY_GAQ88061	Protein	Klebsormidium_niten	45.70	0.00
TRINITY_XP_00294	hypothet	Volvox_carteri	45.70	0.00
TRINITY_PTQ35570	hypothet	Marchantia_polymorp	45.70	0.00
TRINITY_BAK02869	predicte	Hordeum_vulgare	45.70	0.00
TRINITY_XP_00294	hypothet	Volvox_carteri	45.70	0.00
TRINITY_PNW81070	hypothet	Chlamydomonas_reinh	45.70	0.00
TRINITY_XP_00295	hypothet	Volvox_carteri	45.70	0.00
TRINITY_XP_01208	calcium-	Jatropha_curcas	45.70	0.00
TRINITY_GAX77031	hypothet	Chlamydomonas_eusti	45.70	0.00
TRINITY_XP_00834	PREDICTE	Malus_domestica	45.70	0.00
TRINITY_RXH89823	hypothet	Malus_domestica	45.70	0.00
TRINITY_XP_00253	uncharac	Ricinus_communis	45.70	0.00
TRINITY_GAX78492	hypothet	Chlamydomonas_eusti	45.70	0.00
TRINITY_XP_01847	PREDICTE	Raphanus_sativus	45.70	0.00
TRINITY_XP_00564	hypothet	Coccomyxa_subellips	45.70	0.00
TRINITY_GAX73639	hypothet	Chlamydomonas_eusti	45.70	0.00
TRINITY_KXZ54201	hypothet	Gonium_pectorale	45.70	0.00
TRINITY_GAX73216	hypothet	Chlamydomonas_eusti	45.70	0.00
TRINITY_GAQ78675	tRNA-dih	Klebsormidium_niten	45.70	0.00
TRINITY_GAX83270	hypothet	Chlamydomonas_eusti	45.70	0.00
TRINITY_GAQ91546	Zn2+ tra	Klebsormidium_niten	45.70	0.00
TRINITY_GAX75733	hypothet	Chlamydomonas_eusti	45.70	0.00
TRINITY_GAX74506	hypothet	Chlamydomonas_eusti	45.70	0.00
TRINITY_PNW79117	hypothet	Chlamydomonas_reinh	45.70	0.00
TRINITY_KXZ55378	hypothet	Gonium_pectorale	45.70	0.00
TRINITY_GBF90761	ABC tran	Raphidocelis_subcap	45.70	0.00
TRINITY_XP_00170	predicte	Chlamydomonas_reinh	45.70	0.00
TRINITY_KXZ48271	hypothet	Gonium_pectorale	45.70	0.00
TRINITY_CAA49283	gag,prot	Volvox_carteri	45.70	0.00
TRINITY_KXZ51147	MYO2 pro	Gonium_pectorale	45.70	0.00
TRINITY_XP_00450	traffick	Cicer_arietinum	45.70	0.00
TRINITY_XP_00722	N-alpha-	Prunus_persica	45.60	0.00
TRINITY_PON54885	Zinc fin	Trema_orientale	45.60	0.00
TRINITY_OEL25606	putative	Dichanthelium_oligo	45.60	0.00
TRINITY_BAK02837	predicte	Hordeum_vulgare	45.60	0.00
TRINITY_XP_00429	PREDICTE	Fragaria_vesca	45.60	0.00
TRINITY_XP_00250	predicte	Micromonas_commoda	45.60	0.00
TRINITY_PSS24219	Beclin-1	Actinidia_chinensis	45.60	0.00
TRINITY_NP_00133	NADH-ubi	Solanum_lycopersicu	45.60	0.00
TRINITY_XP_02443	FH prote	Populus_trichocarpa	45.60	0.00
TRINITY_XP_00429	PREDICTE	Fragaria_vesca	45.60	0.00
TRINITY_EFJ26425	hypothet	Selaginella_moellen	45.60	0.00
TRINITY_OAE18745	hypothet	Marchantia_polymorp	45.60	0.00
TRINITY_XP_00305	predicte	Micromonas_pusilla	45.60	0.00
TRINITY_XP_02387	peroxiso	Quercus_suber	45.60	0.00
TRINITY_XP_02188	cationic	Carica_papaya	45.60	0.00
TRINITY_XP_00564	molybden	Coccomyxa_subellips	45.60	0.00
TRINITY_XP_02437	DNA-dire	Physcomitrella_pate	45.60	0.00
TRINITY_PKA65890	putative	Apostasia_shenzheni	45.60	0.00
TRINITY_EPS64916	hypothet	Genlisea_aurea	45.60	0.00
TRINITY_OAE32172	hypothet	Marchantia_polymorp	45.60	0.00
TRINITY_XP_02580	ATP-depe	Panicum_hallii	45.60	0.00
TRINITY_PSC69611	MYST-lik	Micractinium_conduc	45.60	0.00
TRINITY_GAQ81424	NLI inte	Klebsormidium_niten	45.60	0.00
TRINITY_GBF88438	hypothet	Raphidocelis_subcap	45.60	0.00
TRINITY_GAQ81991	fragment	Klebsormidium_niten	45.60	0.00
TRINITY_OAE20068	hypothet	Marchantia_polymorp	45.60	0.00
TRINITY_BAK00750	predicte	Hordeum_vulgare	45.60	0.00
TRINITY_GAX82861	hypothet	Chlamydomonas_eusti	45.60	0.00
TRINITY_XP_00294	hypothet	Volvox_carteri	45.60	0.00

TRINITY_XP_0102	serine/t	Brachypodium_distac	45.60	0.00
TRINITY_XP_0016	Hsp70 es	Chlamydomonas_reinh	45.60	0.00
TRINITY_PTQ4097	hypothet	Marchantia_polymorp	45.60	0.00
TRINITY_XP_0014	predicte	Ostreococcus_sp._'l	45.60	0.00
TRINITY_GAX8238	hypothet	Chlamydomonas_eusti	45.60	0.00
TRINITY_ERN0391	hypothet	Amborella_trichopod	45.60	0.00
TRINITY_GAX7960	hypothet	Chlamydomonas_eusti	45.60	0.00
TRINITY_XP_0029	hypothet	Volvox_carteri	45.60	0.00
TRINITY_PNW7453	hypothet	Chlamydomonas_reinh	45.60	0.00
TRINITY_XP_0029	hypothet	Volvox_carteri	45.60	0.00
TRINITY_XP_0191	PREDICTE	Ipomoea_nil	45.60	0.00
TRINITY_GAQ9257	catalase	Klebsormidium_niten	45.60	0.00
TRINITY_XP_0016	predicte	Chlamydomonas_reinh	45.60	0.00
TRINITY_GAX7429	hypothet	Chlamydomonas_eusti	45.60	0.00
TRINITY_EFJ2159	hypothet	Selaginella_moellen	45.60	0.00
TRINITY_XP_0216	polyader	Hevea_brasiliensis	45.60	0.00
TRINITY_XP_0194	PREDICTE	Lupinus_angustifoli	45.60	0.00
TRINITY_GAQ8897	cycloph	Klebsormidium_niten	45.60	0.00
TRINITY_XP_0029	componer	Volvox_carteri	45.60	0.00
TRINITY_XP_0029	hypothet	Volvox_carteri	45.60	0.00
TRINITY_XP_0029	hypothet	Volvox_carteri	45.60	0.00
TRINITY_XP_0228	NAD/GMP	Ostreococcus_tauri	45.60	0.00
TRINITY_PNW7244	hypothet	Chlamydomonas_reinh	45.60	0.00
TRINITY_PKA6128	Protein-	Apostasia_shenzheni	45.60	0.00
TRINITY_XP_0029	hypothet	Volvox_carteri	45.60	0.00
TRINITY_GAQ9356	hypothet	Klebsormidium_niten	45.60	0.00
TRINITY_XP_0072	transcri	Prunus_persica	45.60	0.00
TRINITY_XP_0165	PREDICTE	Capsicum_annuum	45.60	0.00
TRINITY_GAX8111	hypothet	Chlamydomonas_eusti	45.60	0.00
TRINITY_GAQ8102	beta-cat	Klebsormidium_niten	45.60	0.00
TRINITY_GBF9433	hypothet	Raphidocelis_subcap	45.60	0.00
TRINITY_GAX8166	hypothet	Chlamydomonas_eusti	45.60	0.00
TRINITY_XP_0029	hypothet	Volvox_carteri	45.60	0.00
TRINITY_GAX7940	hypothet	Chlamydomonas_eusti	45.60	0.00
TRINITY_PRW2046	lysine--	Chlorella_sorokinia	45.60	0.00
TRINITY_XP_0049	uncharac	Setaria_italica	45.60	0.00
TRINITY_XP_0239	calcium/	Quercus_suber	45.60	0.00
TRINITY_XP_0218	protein	Spinacia_oleracea	45.60	0.00
TRINITY_XP_0243	transpor	Physcomitrella_pate	45.50	0.00
TRINITY_XP_0098	PREDICTE	Nicotiana_sylvestri	45.50	0.00
TRINITY_XP_0249	deSI-lik	Cynara_cardunculus	45.50	0.00
TRINITY_EEF2267	two-comp	Ricinus_communis	45.50	0.00
TRINITY_GAV8394	MCM doma	Cephalotus_follicul	45.50	0.00
TRINITY_XP_0238	probable	Quercus_suber	45.50	0.00
TRINITY_XP_0156	PREDICTE	Oryza_brachyantha	45.50	0.00
TRINITY_XP_0220	(+)-neor	Helianthus_annuus	45.50	0.00
TRINITY_XP_0244	adenine	Physcomitrella_pate	45.50	0.00
TRINITY_XP_0068	putative	Amborella_trichopod	45.50	0.00
TRINITY_VDD2060	unnamed	Brassica_oleracea	45.50	0.00
TRINITY_XP_0088	regulato	Phoenix_dactylifera	45.50	0.00
TRINITY_GBF9551	cytoplas	Raphidocelis_subcap	45.50	0.00
TRINITY_GAQ8672	Nuclear	Klebsormidium_niten	45.50	0.00
TRINITY_GAQ8635	GTPase-a	Klebsormidium_niten	45.50	0.00
TRINITY_GAQ8278	phosphop	Klebsormidium_niten	45.50	0.00
TRINITY_GAQ8225	hypothet	Klebsormidium_niten	45.50	0.00
TRINITY_XP_0238	glycoger	Quercus_suber	45.50	0.00
TRINITY_GBG6848	hypothet	Chara_braunii	45.50	0.00
TRINITY_BAM1109	ABC prot	Coptis_japonica	45.50	0.00
TRINITY_XP_0029	hypothet	Volvox_carteri	45.50	0.00
TRINITY_XP_0056	hypothet	Coccomyxa_subellips	45.50	0.00
TRINITY_OAY7081	Ubiquiti	Ananas_comosus	45.50	0.00
TRINITY_GAX7502	hypothet	Chlamydomonas_eusti	45.50	0.00
TRINITY_XP_0191	PREDICTE	Ipomoea_nil	45.50	0.00
TRINITY_XP_0093	PREDICTE	Pyrus_x_bretschneid	45.50	0.00
TRINITY_XP_0221	probable	Momordica_charantia	45.50	0.00
TRINITY_YP_0088	NADH deh	Roya_obtusa	45.50	0.00

TRINITY_PIA43382	hypothet	Aquilegia_coerulea	45.50	0.00
TRINITY_GAX85978	hypothet	Chlamydomonas_eusti	45.50	0.00
TRINITY_XP_00564	P25-alph	Coccomyxa_subellips	45.50	0.00
TRINITY_PNH04301	putative	Tetrabaena_socialis	45.50	0.00
TRINITY_RWR83301	Nop doma	Cinnamomum_micranth	45.50	0.00
TRINITY_XP_00169	shikimat	Chlamydomonas_reinh	45.50	0.00
TRINITY_XP_02257	ubiquiti	Brassica_napus	45.50	0.00
TRINITY_PNW75532	hypothet	Chlamydomonas_reinh	45.50	0.00
TRINITY_XP_02452	protein	Selaginella_moellen	45.50	0.00
TRINITY_PNH01704	Digalact	Tetrabaena_socialis	45.50	0.00
TRINITY_EFJ10528	hypothet	Selaginella_moellen	45.50	0.00
TRINITY_PNH06934	Dynein l	Tetrabaena_socialis	45.50	0.00
TRINITY_EOY33035	Geminivi	Theobroma_cacao	45.50	0.00
TRINITY_XP_01650	PREDICTE	Nicotiana_tabacum	45.50	0.00
TRINITY_KXZ55953	hypothet	Gonium_pectorale	45.50	0.00
TRINITY_AGW18228	axonemal	Chlamydomonas_reinh	45.50	0.00
TRINITY_GAX86225	hypothet	Chlamydomonas_eusti	45.50	0.00
TRINITY_GAQ91878	Diacylgl	Klebsormidium_niten	45.50	0.00
TRINITY_XP_02351	FH prote	Cucurbita_pepo	45.50	0.00
TRINITY_PSC72887	E3 ubiqu	Micractinium_conduc	45.50	0.00
TRINITY_GAX80121	hypothet	Chlamydomonas_eusti	45.50	0.00
TRINITY_PNW78592	hypothet	Chlamydomonas_reinh	45.50	0.00
TRINITY_XP_00170	ABC tran	Chlamydomonas_reinh	45.50	0.00
TRINITY_XP_02171	protein	Chenopodium_quinoa	45.50	0.00
TRINITY_OAY79583	JmjC dom	Ananas_comosus	45.50	0.00
TRINITY_XP_01284	PREDICTE	Erythranthe_guttata	45.50	0.00
TRINITY_GAX74972	hypothet	Chlamydomonas_eusti	45.50	0.00
TRINITY_GAX73468	hypothet	Chlamydomonas_eusti	45.50	0.00
TRINITY_XP_00414	PREDICTE	Cucumis_sativus	45.50	0.00
TRINITY_XP_02642	ABC tran	Papaver_somniferum	45.50	0.00
TRINITY_XP_01919	PREDICTE	Ipomoea_nil	45.50	0.00
TRINITY_GAX84813	hypothet	Chlamydomonas_eusti	45.50	0.00
TRINITY_XP_02376	probable	Lactuca_sativa	45.50	0.00
TRINITY_GAX80368	hypothet	Chlamydomonas_eusti	45.50	0.00
TRINITY_RMZ56974	hypothet	Auxenochlorella_pro	45.50	0.00
TRINITY_GAQ92204	hypothet	Klebsormidium_niten	45.50	0.00
TRINITY_AHG05844	cold sho	Triticum_aestivum	45.50	0.00
TRINITY_XP_00295	hypothet	Volvox_carteri	45.50	0.00
TRINITY_XP_02454	coilin-l	Selaginella_moellen	45.50	0.00
TRINITY_XP_02440	deoxynuc	Physcomitrella_pate	45.50	0.00
TRINITY_PNH04986	hypothet	Tetrabaena_socialis	45.50	0.00
TRINITY_PNW75220	hypothet	Chlamydomonas_reinh	45.50	0.00
TRINITY_XP_01596	nucleola	Arachis_duranensis	45.50	0.00
TRINITY_PNW85362	hypothet	Chlamydomonas_reinh	45.50	0.00
TRINITY_KXZ51085	hypothet	Gonium_pectorale	45.50	0.00
TRINITY_KXZ54006	hypothet	Gonium_pectorale	45.50	0.00
TRINITY_GAX80195	hypothet	Chlamydomonas_eusti	45.50	0.00
TRINITY_PNW87768	hypothet	Chlamydomonas_reinh	45.50	0.00
TRINITY_GBF91936	thioredo	Raphidocelis_subcap	45.50	0.00
TRINITY_GAX77318	hypothet	Chlamydomonas_eusti	45.50	0.00
TRINITY_GAQ89088	ATP-depe	Klebsormidium_niten	45.50	0.00
TRINITY_EFJ34372	hypothet	Selaginella_moellen	45.50	0.00
TRINITY_GAX77182	hypothet	Chlamydomonas_eusti	45.50	0.00
TRINITY_XP_00565	tRNA gua	Coccomyxa_subellips	45.50	0.00
TRINITY_XP_00250	predicte	Micromonas_commoda	45.50	0.00
TRINITY_PIN00081	Anion ex	Handroanthus_impeti	45.50	0.00
TRINITY_PNW80534	hypothet	Chlamydomonas_reinh	45.50	0.00
TRINITY_XP_01764	PREDICTE	Gossypium_arboreum	45.50	0.00
TRINITY_GAX82620	hypothet	Chlamydomonas_eusti	45.50	0.00
TRINITY_OAE35542	hypothet	Marchantia_polymorp	45.50	0.00
TRINITY_NP_00114	signal r	Zea_mays	45.50	0.00
TRINITY_GAX77328	hypothet	Chlamydomonas_eusti	45.50	0.00
TRINITY_GAQ81501	hypothet	Klebsormidium_niten	45.50	0.00
TRINITY_XP_00170	SIN3 cor	Chlamydomonas_reinh	45.50	0.00
TRINITY_XP_02440	NADP-dep	Physcomitrella_pate	45.40	0.00
TRINITY_AFK09219	calcium-	Dunaliella_salina	45.40	0.00

TRINITY_GAQ7839	HSP70/H	Klebsormidium_niten	45.40	0.00
TRINITY_KFK2954	hypothet	Arabis_alpina	45.40	0.00
TRINITY_BAK0223	predict	Hordeum_vulgare	45.40	0.00
TRINITY_GAQ8983	hypothet	Klebsormidium_niten	45.40	0.00
TRINITY_KXZ4276	hypothet	Gonium_pectorale	45.40	0.00
TRINITY_GAX8519	hypothet	Chlamydomonas_eusti	45.40	0.00
TRINITY_XP_0093	PREDICT	Pyrus_x_bretschneid	45.40	0.00
TRINITY_ABU5296	hypothet	Dunaliella_viridis	45.40	0.00
TRINITY_GAX7348	hypothet	Chlamydomonas_eusti	45.40	0.00
TRINITY_PHT2702	Protein	Capsicum_baccatum	45.40	0.00

TRINITY_GAX8212(	hypothet	Chlamydomonas_eusti	45.40	0.00
TRINITY_GAX8142(	hypothet	Chlamydomonas_eusti	45.40	0.00
TRINITY_PNH1221'	Beta-gal	Tetrabaena_socialis	45.40	0.00
TRINITY_KMZ6412:	IMP cycl	Zostera_marina	45.40	0.00
TRINITY_XP_0139(	Prestalk	Monoraphidium_negle	45.40	0.00
TRINITY_GAX7956'	hypothet	Chlamydomonas_eusti	45.40	0.00
TRINITY_PNW8460:	hypothet	Chlamydomonas_reinh	45.40	0.00
TRINITY_XP_0243(	BEACH do	Physcomitrella_pate	45.40	0.00
TRINITY_PNW7393(	hypothet	Chlamydomonas_reinh	45.40	0.00
TRINITY_GAX8465!	hypothet	Chlamydomonas_eusti	45.40	0.00
TRINITY_PNH0310!	Calmodul	Tetrabaena_socialis	45.40	0.00
TRINITY_GAX7855(	hypothet	Chlamydomonas_eusti	45.40	0.00
TRINITY_GAQ9101:	gamma-gl	Klebsormidium_niten	45.40	0.00
TRINITY_GAX7398(	hypothet	Chlamydomonas_eusti	45.40	0.00
TRINITY_XP_0097!	PREDICTE	Nicotiana_sylvestri	45.40	0.00
TRINITY_XP_0124!	PREDICTE	Gossypium_raimondii	45.40	0.00
TRINITY_GAQ7857:	Transcri	Klebsormidium_niten	45.40	0.00
TRINITY_GAQ8093(	short ch	Klebsormidium_niten	45.40	0.00
TRINITY_KXZ5615!	hypothet	Gonium_pectorale	45.40	0.00
TRINITY_XP_0102(	PREDICTE	Nelumbo_nucifera	45.40	0.00
TRINITY_GAX8389!	hypothet	Chlamydomonas_eusti	45.40	0.00
TRINITY_GAX7632!	hypothet	Chlamydomonas_eusti	45.40	0.00
TRINITY_KXZ5306!	hypothet	Gonium_pectorale	45.40	0.00
TRINITY_XP_0244!	protein	Populus_trichocarpa	45.40	0.00
TRINITY_GAX8116!	hypothet	Chlamydomonas_eusti	45.40	0.00
TRINITY_PNW8254!	hypothet	Chlamydomonas_reinh	45.40	0.00
TRINITY_PNH0858(	Adenine	Tetrabaena_socialis	45.40	0.00
TRINITY_GAX7341(	hypothet	Chlamydomonas_eusti	45.40	0.00
TRINITY_PNH1240!	putative	Tetrabaena_socialis	45.40	0.00
TRINITY_OAE2180:	hypothet	Marchantia_polymorp	45.40	0.00
TRINITY_GAX7684!	hypothet	Chlamydomonas_eusti	45.40	0.00
TRINITY_KXZ4165(	hypothet	Gonium_pectorale	45.40	0.00
TRINITY_PNW7942'	hypothet	Chlamydomonas_reinh	45.40	0.00
TRINITY_XP_0029!	hybrid-c	Volvox_carteri	45.40	0.00
TRINITY_XP_0245:	casein k	Selaginella_moellen	45.40	0.00
TRINITY_XP_0030(	predicte	Micromonas_pusilla	45.30	0.00
TRINITY_GAX8386:	hypothet	Chlamydomonas_eusti	45.30	0.00
TRINITY_RLN1717!	hypothet	Panicum_miliaceum	45.30	0.00
TRINITY_RMZ5555!	hypothet	Auxenochlorella_pro	45.30	0.00
TRINITY_GAX7350!	hypothet	Chlamydomonas_eusti	45.30	0.00
TRINITY_XP_0279:	ankyrin	Vigna_unguiculata	45.30	0.00
TRINITY_XP_0056!	hypothet	Coccomyxa_subellips	45.30	0.00
TRINITY_GAQ9143!	Kinesin-	Klebsormidium_niten	45.30	0.00
TRINITY_XP_0239:	spindle	Quercus_suber	45.30	0.00
TRINITY_GAX8058!	hypothet	Chlamydomonas_eusti	45.30	0.00
TRINITY_XP_0066!	PREDICTE	Oryza_brachyantha	45.30	0.00
TRINITY_XP_0167(	PREDICTE	Gossypium_hirsutum	45.30	0.00
TRINITY_XP_0245:	protein	Selaginella_moellen	45.30	0.00
TRINITY_XP_0243!	calcium	Physcomitrella_pate	45.30	0.00
TRINITY_XP_0202(	peptidyl	Cajanus_cajan	45.30	0.00
TRINITY_PSR9343!	Transcri	Actinidia_chinensis	45.30	0.00
TRINITY_OTF9970!	putative	Helianthus_annuus	45.30	0.00
TRINITY_GAQ7978!	Signal t	Klebsormidium_niten	45.30	0.00
TRINITY_XP_0238!	vacuolar	Quercus_suber	45.30	0.00
TRINITY_XP_0245:	geranylg	Selaginella_moellen	45.30	0.00
TRINITY_XP_0025(	predicte	Micromonas_commoda	45.30	0.00
TRINITY_XP_0273!	ABC tran	Abrus_precatorius	45.30	0.00
TRINITY_GAU3377(	hypothet	Trifolium_subterran	45.30	0.00
TRINITY_XP_0029(	26S prot	Selaginella_moellen	45.30	0.00
TRINITY_GAQ8089(	WD domai	Klebsormidium_niten	45.30	0.00
TRINITY_XP_0243'	phyteps:	Physcomitrella_pate	45.30	0.00
TRINITY_BAJ9390!	predicte	Hordeum_vulgare	45.30	0.00
TRINITY_KFK3659:	hypothet	Arabis_alpina	45.30	0.00
TRINITY_XP_0188:	PREDICTE	Juglans_regia	45.30	0.00
TRINITY_GBG9050:	hypothet	Chara_braunii	45.30	0.00
TRINITY_OVA1664'	WD40 rep	Macleaya_cordata	45.30	0.00

TRINITY_XP_0029!	hypothet	Volvox_carteri	45.30	0.00
TRINITY_AML7843(	putative	Botryococcus_territ	45.30	0.00
TRINITY_GBF8945:	hypothet	Raphidocelis_subcap	45.30	0.00
TRINITY_PNW7735{	hypothet	Chlamydomonas_reinh	45.30	0.00
TRINITY_XP_0200!	60S ribc	Ananas_comosus	45.30	0.00
TRINITY_XP_0205'	SNW/SKI-	Phalaenopsis_equest	45.30	0.00
TRINITY_GBF9550:	hypothet	Raphidocelis_subcap	45.30	0.00
TRINITY_XP_0245:	PITH dor	Selaginella_moellen	45.30	0.00
TRINITY_XP_0159!	serine/t	Arachis_duranensis	45.30	0.00
TRINITY_PIA5108!	hypothet	Aquilegia_coerulea	45.30	0.00
TRINITY_XP_0105:	PREDICTI	Tarenaya_hasslerian	45.30	0.00
TRINITY_XP_0029!	hypothet	Volvox_carteri	45.30	0.00
TRINITY_GAQ9149!	acyl-act	Klebsormidium_niten	45.30	0.00
TRINITY_GAX7510:	hypothet	Chlamydomonas_eusti	45.30	0.00
TRINITY_XP_0243{	uncharac	Physcomitrella_pate	45.30	0.00
TRINITY_KXZ4484{	hypothet	Gonium_pectorale	45.30	0.00
TRINITY_PNH0141!	Attracti	Tetrabaena_socialis	45.30	0.00
TRINITY_XP_0016!	flagella	Chlamydomonas_reinh	45.30	0.00
TRINITY_GAQ8678{	ARM repe	Klebsormidium_niten	45.30	0.00
TRINITY_KXZ5670'	hypothet	Gonium_pectorale	45.30	0.00
TRINITY_XP_0102(	PREDICTI	Nelumbo_nucifera	45.30	0.00
TRINITY_KFK3216:	hypothet	Arabis_alpina	45.30	0.00
TRINITY_GAX7486(	hypothet	Chlamydomonas_eusti	45.30	0.00
TRINITY_XP_0029<	hypothet	Volvox_carteri	45.30	0.00
TRINITY_PSR9296:	RING-H2	Actinidia_chinensis	45.30	0.00
TRINITY_GAX7874<	hypothet	Chlamydomonas_eusti	45.30	0.00
TRINITY_XP_0239(	guanine	Quercus_suber	45.30	0.00
TRINITY_GAQ8899{	Rab fami	Klebsormidium_niten	45.30	0.00
TRINITY_PNH0873<	Pre-mRNA	Tetrabaena_socialis	45.30	0.00
TRINITY_XP_0029!	hypothet	Volvox_carteri	45.30	0.00
TRINITY_KXZ4391!	hypothet	Gonium_pectorale	45.30	0.00
TRINITY_XP_0016!	predicte	Chlamydomonas_reinh	45.30	0.00
TRINITY_XP_0030{	Small GT	Ostreococcus_tauri	45.30	0.00
TRINITY_XP_0029!	hypothet	Volvox_carteri	45.30	0.00
TRINITY_GBF9891{	hypothet	Raphidocelis_subcap	45.30	0.00
TRINITY_XP_0029{	ultravic	Selaginella_moellen	45.30	0.00
TRINITY_GAX8220!	hypothet	Chlamydomonas_eusti	45.30	0.00
TRINITY_XP_0087!	ABC trar	Phoenix_dactylifera	45.30	0.00
TRINITY_PNW7759!	hypothet	Chlamydomonas_reinh	45.30	0.00
TRINITY_XP_0094(	PREDICTI	Musa_acuminata	45.30	0
TRINITY_XP_0138!	hypothet	Monoraphidium_negle	45.30	0.00
TRINITY_PNW8586(	hypothet	Chlamydomonas_reinh	45.30	0.00
TRINITY_XP_0110<	PREDICTI	Populus_euphratica	45.30	0.00
TRINITY_RYQ9455(	hypothet	Arachis_hypogaea	45.30	0.00
TRINITY_XP_0114(	5-oxoprc	Auxenochlorella_prc	45.20	0.00
TRINITY_XP_0239:	myosin t	Quercus_suber	45.20	0.00
TRINITY_KFK4011(	hypothet	Arabis_alpina	45.20	0.00
TRINITY_XP_0014:	predicte	Ostreococcus_sp._'l	45.20	0.00
TRINITY_XP_0249!	serine/a	Citrus_sinensis	45.20	0.00
TRINITY_OAY7709!	Protein	Ananas_comosus	45.20	0.00
TRINITY_XP_0270'	histone	Coffea_arabica	45.20	0.00
TRINITY_XP_0202(	CBL-inte	Asparagus_officinal	45.20	0.00
TRINITY_XP_0145:	dnaJ prc	Vigna_radiata	45.20	0.00
TRINITY_GAQ8025<	Serine/T	Klebsormidium_niten	45.20	0.00
TRINITY_GAQ8787(	Aromatic	Klebsormidium_niten	45.20	0.00
TRINITY_XP_0030'	HCaRG [(	Ostreococcus_tauri	45.20	0.00
TRINITY_XP_0056<	L30e-lik	Coccomyxa_subellips	45.20	0.00
TRINITY_XP_0216!	subtilis	Hevea_brasiliensis	45.20	0.00
TRINITY_XP_0191!	PREDICTI	Ipomoea_nil	45.20	0.00
TRINITY_XP_0264<	phosphoc	Papaver_somniferum	45.20	0.00
TRINITY_XP_0243{	mitogen-	Physcomitrella_pate	45.20	0.00
TRINITY_PNW8519'	hypothet	Chlamydomonas_reinh	45.20	0.00
TRINITY_GBF9648'	phosphoe	Raphidocelis_subcap	45.20	0.00
TRINITY_XP_0243{	mitogen-	Physcomitrella_pate	45.20	0.00
TRINITY_XP_0229!	FH prote	Cucurbita_moschata	45.20	0.00
TRINITY_OAE2344(	hypothet	Marchantia_polymorp	45.20	0.00

TRINITY_KMS9433	hypothet	Beta_vulgaris	45.20	0.00
TRINITY_BAK0277	predict	Hordeum_vulgare	45.20	0.00
TRINITY_GAQ8919	hypothet	Klebsormidium_niten	45.20	0.00
TRINITY_GAX8307	hypothet	Chlamydomonas_eusti	45.20	0.00
TRINITY_AII8486	NADH del	Jungermannia_exsert	45.20	0.00
TRINITY_PNY1051	ABC tra	Trifolium_pratense	45.20	0.00
TRINITY_XP_0264	probabl	Papaver_somniferum	45.20	0.00
TRINITY_XP_0056	uroporph	Coccomyxa_subellips	45.20	0.00
TRINITY_PNH1172	tRNA wyk	Tetrabaena_socialis	45.20	0.00
TRINITY_GAX8437	hypothet	Chlamydomonas_eusti	45.20	0.00
TRINITY_OAE3414	hypothet	Marchantia_polymorp	45.20	0.00
TRINITY_GAX8322	hypothet	Chlamydomonas_eusti	45.20	0.00
TRINITY_KXZ5415	hypothet	Gonium_pectorale	45.20	0.00
TRINITY_XP_0016	Mg2+ tra	Chlamydomonas_reinh	45.20	0.00
TRINITY_GAX8050	hypothet	Chlamydomonas_eusti	45.20	0.00
TRINITY_XP_0029	hypothet	Volvox_carteri	45.20	0.00
TRINITY_PNH0495	putative	Tetrabaena_socialis	45.20	0.00
TRINITY_GAX8176	hypothet	Chlamydomonas_eusti	45.20	0.00
TRINITY_XP_0244	OTU doma	Physcomitrella_pate	45.20	0.00
TRINITY_GAX8384	hypothet	Chlamydomonas_eusti	45.20	0.00
TRINITY_GAQ8739	Serine	Klebsormidium_niten	45.20	0.00
TRINITY_PNW7118	hypothet	Chlamydomonas_reinh	45.20	0.00
TRINITY_KXZ5173	hypothet	Gonium_pectorale	45.20	0.00
TRINITY_XP_0029	hypothet	Volvox_carteri	45.20	0.00
TRINITY_RLM9338	hypothet	Panicum_miliaceum	45.20	0.00
TRINITY_PNW7634	hypothet	Chlamydomonas_reinh	45.20	0.00
TRINITY_GAX8140	hypothet	Chlamydomonas_eusti	45.20	0.00
TRINITY_XP_0273	pirin-l	Abrus_precatorius	45.20	0.00
TRINITY_XP_0238	translat	Quercus_suber	45.20	0.00
TRINITY_XP_0029	hypothet	Volvox_carteri	45.20	0.00
TRINITY_XP_0029	hypothet	Volvox_carteri	45.20	0.00
TRINITY_PON4323	ABC tra	Parasponia_anderson	45.20	0.00
TRINITY_XP_0056	hypothet	Coccomyxa_subellips	45.20	0.00
TRINITY_XP_0029	hypothet	Volvox_carteri	45.20	0.00
TRINITY_XP_0217	probabl	Chenopodium_quinoa	45.20	0.00
TRINITY_XP_0016	predict	Chlamydomonas_reinh	45.20	0.00
TRINITY_XP_0029	hypothet	Volvox_carteri	45.20	0.00
TRINITY_GAX8388	hypothet	Chlamydomonas_eusti	45.20	0.00
TRINITY_NP_0011	dual sp	Arabidopsis_thalian	45.20	0.00
TRINITY_XP_0016	poly(a)	Chlamydomonas_reinh	45.20	0.00
TRINITY_KXZ4999	hypothet	Gonium_pectorale	45.20	0.00
TRINITY_PNW8047	hypothet	Chlamydomonas_reinh	45.20	0.00
TRINITY_RWR9581	pre-mRN	Cinnamomum_micranth	45.20	0.00
TRINITY_GAX8343	hypothet	Chlamydomonas_eusti	45.20	0.00
TRINITY_XP_0016	KDEL re	Chlamydomonas_reinh	45.20	0.00
TRINITY_ARO5010	20S prot	Yamagishiella_unicc	45.20	0.00
TRINITY_XP_0056	NUC173-	Coccomyxa_subellips	45.20	0.00
TRINITY_AAG4409	cycloart	Abies_magnifica	45.20	0.00
TRINITY_KXZ4931	hypothet	Gonium_pectorale	45.20	0.00
TRINITY_GAQ8068	Serine	Klebsormidium_niten	45.20	0.00
TRINITY_XP_0239	dynamin-	Quercus_suber	45.20	0.00
TRINITY_XP_0025	histone	Micromonas_commoda	45.20	0.00
TRINITY_PNW8749	hypothet	Chlamydomonas_reinh	45.20	0.00
TRINITY_GAX8184	hypothet	Chlamydomonas_eusti	45.20	0.00
TRINITY_XP_0109	PREDICT	Elaeis_guineensis	45.20	0.00
TRINITY_KXZ5666	hypothet	Gonium_pectorale	45.20	0.00
TRINITY_PNH0281	hypothet	Tetrabaena_socialis	45.20	0.00
TRINITY_XP_0045	pyropho	Cicer_arietinum	45.20	0.00
TRINITY_XP_0029	hypothet	Volvox_carteri	45.20	0.00
TRINITY_XP_0075	predict	Bathycoccus_prasinc	45.20	0.00
TRINITY_PRW4546	pseudou	Chlorella_sorokinia	45.20	0.00
TRINITY_XP_0016	signal	Chlamydomonas_reinh	45.20	0.00
TRINITY_GAX8011	hypothet	Chlamydomonas_eusti	45.20	0.00
TRINITY_KXZ4595	hypothet	Gonium_pectorale	45.20	0.00
TRINITY_GAX8375	hypothet	Chlamydomonas_eusti	45.20	0.00
TRINITY_XP_0029	hypothet	Volvox_carteri	45.20	0.00

TRINITY_XP_0056	hypothet	Coccomyxa_subellips	45.20	0.00
TRINITY_PNW7973	hypothet	Chlamydomonas_reinh	45.20	0.00
TRINITY_PNW8423	hypothet	Chlamydomonas_reinh	45.20	0.00
TRINITY_PNW8053	hypothet	Chlamydomonas_reinh	45.20	0.00
TRINITY_XP_0030	predicte	Micromonas_pusilla	45.20	0.00
TRINITY_XP_0116	PREDICTE	Cucumis_sativus	45.20	0.00
TRINITY_PRW5644	ADP-ribo	Chlorella_sorokinia	45.20	0.00
TRINITY_XP_0239	myosin t	Quercus_suber	45.20	0.00
TRINITY_EMS5000	Enhance	Triticum_urartu	45.10	0.00
TRINITY_XP_0228	Cytochro	Ostreococcus_tauri	45.10	0.00
TRINITY_OAE1992	hypothet	Marchantia_polymorp	45.10	0.00
TRINITY_XP_0058	hypothet	Chlorella_variabili	45.10	0.00
TRINITY_PSC6998	ubiquiti	Micractinium_conduc	45.10	0.00
TRINITY_EPS7121	glucose-	Genlisea_aurea	45.10	0.00
TRINITY_GAQ7777	Nicotina	Klebsormidium_niten	45.10	0.00
TRINITY_XP_0238	phosphol	Quercus_suber	45.10	0.00
TRINITY_GAQ8835	alpha/be	Klebsormidium_niten	45.10	0.00
TRINITY_XP_0239	ras-like	Quercus_suber	45.10	0.00
TRINITY_XP_0202	glutamin	Cajanus_cajan	45.10	0.00
TRINITY_XP_0066	PREDICTE	Oryza_brachyantha	45.10	0.00
TRINITY_GAX7891	hypothet	Chlamydomonas_eusti	45.10	0.00
TRINITY_GAQ7785	GTP-bind	Klebsormidium_niten	45.10	0.00
TRINITY_PNH1198	hypothet	Tetrabaena_socialis	45.10	0.00
TRINITY_XP_0227	MOB kina	Durio_zibethinus	45.10	0.00
TRINITY_KXZ5553	hypothet	Gonium_pectorale	45.10	0.00
TRINITY_XP_0030	predicte	Micromonas_pusilla	45.10	0.00
TRINITY_XP_0029	hypothet	Volvox_carteri	45.10	0.00
TRINITY_KEH4009	metallo	Medicago_truncatula	45.10	0.00
TRINITY_PNH0313	Alpha-p	Tetrabaena_socialis	45.10	0.00
TRINITY_XP_0016	predicte	Chlamydomonas_reinh	45.10	0.00
TRINITY_GAX7386	hypothet	Chlamydomonas_eusti	45.10	0.00
TRINITY_AYU7094	sucrose	Ginkgo_biloba	45.10	0.00
TRINITY_XP_0017	predicte	Chlamydomonas_reinh	45.10	0.00
TRINITY_XP_0240	transcri	Morus_notabilis	45.10	0.00
TRINITY_BAG7097	epsin N-	Musa_balbisiana	45.10	0.00
TRINITY_PRW5739	eukaryot	Chlorella_sorokinia	45.10	0.00
TRINITY_ERN0563	hypothet	Amborella_trichopod	45.10	0.00
TRINITY_XP_0205	serine/t	Phalaenopsis_equest	45.10	0.00
TRINITY_CDM8391	unnamed	Triticum_aestivum	45.10	0.00
TRINITY_XP_0202	ribose-p	Cajanus_cajan	45.10	0.00
TRINITY_GBF9208	hypothet	Raphidocelis_subcap	45.10	0.00
TRINITY_BAK0112	predicte	Hordeum_vulgare	45.10	0.00
TRINITY_XP_0243	sensor p	Physcomitrella_pate	45.10	0.00
TRINITY_XP_0056	Clc chlo	Coccomyxa_subellips	45.10	0.00
TRINITY_RDX6039	Senescen	Mucuna_pruriens	45.10	0.00
TRINITY_GAX7335	hypothet	Chlamydomonas_eusti	45.10	0.00
TRINITY_XP_0091	PREDICTE	Brassica_rapa	45.10	0.00
TRINITY_XP_0239	uncharac	Quercus_suber	45.10	0.00
TRINITY_GAX7971	hypothet	Chlamydomonas_eusti	45.10	0.00
TRINITY_XP_0029	phosphat	Selaginella_moellen	45.10	0.00
TRINITY_RQL8169	hypothet	Brassica_cretica	45.10	0.00
TRINITY_XP_0238	structu	Quercus_suber	45.10	0.00
TRINITY_PNW7813	hypothet	Chlamydomonas_reinh	45.10	0.00
TRINITY_XP_0016	p53-indu	Chlamydomonas_reinh	45.10	0.00
TRINITY_PRW5902	arginine	Chlorella_sorokinia	45.10	0.00
TRINITY_XP_0016	potassiu	Chlamydomonas_reinh	45.10	0.00
TRINITY_GAX7875	hypothet	Chlamydomonas_eusti	45.10	0.00
TRINITY_XP_0014	predicte	Ostreococcus_sp._'l	45.10	0.00
TRINITY_GBF9373	hypothet	Raphidocelis_subcap	45.10	0.00
TRINITY_KXZ5329	hypothet	Gonium_pectorale	45.10	0.00
TRINITY_XP_0158	galacto	Ziziphus_jujuba	45.10	0.00
TRINITY_GAQ7874	Putative	Klebsormidium_niten	45.10	0.00
TRINITY_PNH0919	hypothet	Tetrabaena_socialis	45.10	0.00
TRINITY_AAV5418	chloropl	Haematococcus_lacus	45.10	0.00
TRINITY_XP_0139	hypothet	Monoraphidium_negle	45.10	0.00
TRINITY_XP_0239	kinesin	Quercus_suber	45.10	0.00

TRINITY_XP_00295	hypothet	Volvox_carteri	45.10	0.00
TRINITY_PNW75161	hypothet	Chlamydomonas_reinh	45.10	0.00
TRINITY_EPS64534	hypothet	Genlisea_aurea	45.10	0.00
TRINITY_XP_00750	predicte	Bathycoccus_prasino	45.10	0.00
TRINITY_XP_02374	autophag	Lactuca_sativa	45.10	0.00
TRINITY_XP_00628	ras-rela	Capsella_rubella	45.00	0.00
TRINITY_XP_00250	fatty ac	Micromonas_commoda	45.00	0.00
TRINITY_XP_02438	H/ACA ri	Physcomitrella_pate	45.00	0.00
TRINITY_XP_02387	acetamid	Quercus_suber	45.00	0.00
TRINITY_KXZ45555	hypothet	Gonium_pectorale	45.00	0.00
TRINITY_XP_02389	LOW QUAL	Quercus_suber	45.00	0.00
TRINITY_ONM33382	CTP synt	Zea_mays	45.00	0.00
TRINITY_GAX77180	hypothet	Chlamydomonas_eusti	45.00	0.00
TRINITY_XP_02439	serine/t	Physcomitrella_pate	45.00	0.00
TRINITY_GAQ88885	NAD-depe	Klebsormidium_niten	45.00	0.00
TRINITY_XP_00227	PREDICTE	Vitis_vinifera	45.00	0.00
TRINITY_GAQ85015	ATP-depe	Klebsormidium_niten	45.00	0.00
TRINITY_XP_02389	probable	Quercus_suber	45.00	0.00
TRINITY_ONK66039	uncharac	Asparagus_officinal	45.00	0.00
TRINITY_OAO94309	TAF5 [Ar	Arabidopsis_thalian	45.00	0.00
TRINITY_KXZ55513	hypothet	Gonium_pectorale	45.00	0.00
TRINITY_XP_01284	PREDICTE	Erythranthe_guttata	45.00	0.00
TRINITY_PNH06372	histone	Tetrabaena_socialis	45.00	0.00
TRINITY_XP_00664	PREDICTE	Oryza_brachyantha	45.00	0.00
TRINITY_XP_01882	PREDICTE	Juglans_regia	45.00	0.00
TRINITY_KXZ51810	hypothet	Gonium_pectorale	45.00	0.00
TRINITY_PTQ31328	hypothet	Marchantia_polymorp	45.00	0.00
TRINITY_XP_00584	expresse	Chlorella_variabili	45.00	0.00
TRINITY_GBF91241	hypothet	Raphidocelis_subcap	45.00	0.00
TRINITY_GAQ77818	branched	Klebsormidium_niten	45.00	0.00
TRINITY_GAX76876	hypothet	Chlamydomonas_eusti	45.00	0.00
TRINITY_XP_01026	PREDICTE	Nelumbo_nucifera	45.00	0.00
TRINITY_XP_02388	alpha-ac	Quercus_suber	45.00	0.00
TRINITY_GBG61046	hypothet	Chara_braunii	45.00	0.00
TRINITY_XP_01390	hypothet	Monoraphidium_negle	45.00	0.00
TRINITY_GBF90545	zinc pho	Raphidocelis_subcap	45.00	0.00
TRINITY_XP_00564	GBP-doma	Coccomyxa_subellips	45.00	0.00
TRINITY_XP_02437	ATP-bind	Physcomitrella_pate	45.00	0.00
TRINITY_KXZ54443	hypothet	Gonium_pectorale	45.00	0.00
TRINITY_PNH02844	hypothet	Tetrabaena_socialis	45.00	0.00
TRINITY_GBF90810	ubiquiti	Raphidocelis_subcap	45.00	0.00
TRINITY_GAX75949	hypothet	Chlamydomonas_eusti	45.00	0.00
TRINITY_KXZ49339	hypothet	Gonium_pectorale	45.00	0.00
TRINITY_PNH06295	Apocarot	Tetrabaena_socialis	45.00	0.00
TRINITY_GAX81212	hypothet	Chlamydomonas_eusti	45.00	0.00
TRINITY_GAQ83960	hypothet	Klebsormidium_niten	45.00	0.00
TRINITY_GAQ85616	VPS45 va	Klebsormidium_niten	45.00	0.00
TRINITY_XP_00169	plastid	Chlamydomonas_reinh	45.00	0.00
TRINITY_XP_02168	GTP-bind	Hevea_brasiliensis	45.00	0.00
TRINITY_XP_00960	PREDICTE	Nicotiana_tomentosi	45.00	0.00
TRINITY_GAX72981	hypothet	Chlamydomonas_eusti	45.00	0.00
TRINITY_XP_01027	PREDICTE	Nelumbo_nucifera	45.00	0.00
TRINITY_GAX86128	hypothet	Chlamydomonas_eusti	45.00	0.00
TRINITY_GAQ86264	dehydrog	Klebsormidium_niten	45.00	0.00
TRINITY_PNW72245	hypothet	Chlamydomonas_reinh	45.00	0.00
TRINITY_XP_01389	hypothet	Monoraphidium_negle	45.00	0.00
TRINITY_GBF94173	hypothet	Raphidocelis_subcap	45.00	0.00
TRINITY_PNW82359	hypothet	Chlamydomonas_reinh	45.00	0.00
TRINITY_GAQ80925	solute s	Klebsormidium_niten	45.00	0.00
TRINITY_XP_00294	hypothet	Volvox_carteri	45.00	0.00
TRINITY_GAQ80621	hypothet	Klebsormidium_niten	45.00	0.00
TRINITY_GAX83351	hypothet	Chlamydomonas_eusti	45.00	0.00
TRINITY_XP_01023	transcri	Brachypodium_distac	45.00	0.00
TRINITY_XP_02387	probable	Quercus_suber	45.00	0.00
TRINITY_KXZ40924	hypothet	Gonium_pectorale	45.00	0.00
TRINITY_XP_02387	DNA repa	Quercus_suber	45.00	0.00

TRINITY_GAX7259	hypothet	Chlamydomonas_eusti	45.00	0.00
TRINITY_XP_0029	hypothet	Volvox_carteri	45.00	0.00
TRINITY_GAX7578	hypothet	Chlamydomonas_eusti	45.00	0.00
TRINITY_XP_0258	probable	Panicum_hallii	45.00	0.00
TRINITY_GAQ8865	Dyneins	Klebsormidium_niten	45.00	0
TRINITY_KXZ5105	hypothet	Gonium_pectorale	45.00	0.00
TRINITY_OAE2406	hypothet	Marchantia_polymorp	45.00	0
TRINITY_XP_0029	hypothet	Volvox_carteri	45.00	0.00
TRINITY_XP_0029	hypothet	Volvox_carteri	44.90	0.00
TRINITY_PIN1149	Sensory	Handroanthus_impeti	44.90	0.00
TRINITY_AMD8267	long cha	Vernicia_fordii	44.90	0.00
TRINITY_GAQ8635	AAA-type	Klebsormidium_niten	44.90	0.00
TRINITY_PNW8612	hypothet	Chlamydomonas_reinh	44.90	0.00
TRINITY_BAK0050	predicte	Hordeum_vulgare	44.90	0.00
TRINITY_XP_0264	EEF1A ly	Papaver_somniferum	44.90	0.00
TRINITY_XP_0158	uncharac	Ziziphus_jujuba	44.90	0.00
TRINITY_XP_0239	serine/t	Quercus_suber	44.90	0.00
TRINITY_XP_0100	PREDICTE	Eucalyptus_grandis	44.90	0.00
TRINITY_XP_0218	probable	Prunus_avium	44.90	0.00
TRINITY_XP_0191	PREDICTE	Ipomoea_nil	44.90	0.00
TRINITY_XP_0241	uncharac	Rosa_chinensis	44.90	0.00
TRINITY_RWW9025	hypothet	Ensete_ventricosum	44.90	0.00
TRINITY_OUS4467	putative	Ostreococcus_tauri	44.90	0.00
TRINITY_XP_0239	alpha,al	Quercus_suber	44.90	0.00
TRINITY_XP_0016	flavohem	Chlamydomonas_reinh	44.90	0.00
TRINITY_P0C8M8.	RecName:	Zea_mays	44.90	0.00
TRINITY_XP_0240	uncharac	Morus_notabilis	44.90	0.00
TRINITY_XP_0137	ras-rela	Brassica_napus	44.90	0.00
TRINITY_PSC7114	coatomer	Micractinium_conduc	44.90	0.00
TRINITY_XP_0270	very-lon	Coffea_arabica	44.90	0.00
TRINITY_XP_0016	plastid	Chlamydomonas_reinh	44.90	0.00
TRINITY_XP_0138	hypothet	Monoraphidium_negle	44.90	0.00
TRINITY_XP_0017	nucleore	Chlamydomonas_reinh	44.90	0.00
TRINITY_GBG6857	Glutamat	Chara_braunii	44.90	0.00
TRINITY_XP_0243	uncharac	Physcomitrella_pate	44.90	0.00
TRINITY_PNW7793	hypothet	Chlamydomonas_reinh	44.90	0.00
TRINITY_XP_0238	ras-rela	Quercus_suber	44.90	0.00
TRINITY_XP_0017	predicte	Chlamydomonas_reinh	44.90	0.00
TRINITY_GBF9190	gamma-tu	Raphidocelis_subcap	44.90	0.00
TRINITY_XP_0243	protein	Physcomitrella_pate	44.90	0.00
TRINITY_GAX7605	hypothet	Chlamydomonas_eusti	44.90	0.00
TRINITY_PON4323	ABC tran	Parasponia_anderson	44.90	0.00
TRINITY_KXZ5530	hypothet	Gonium_pectorale	44.90	0.00
TRINITY_PNW8596	hypothet	Chlamydomonas_reinh	44.90	0.00
TRINITY_XP_0056	RabGAP/T	Coccomyxa_subellips	44.90	0.00
TRINITY_KXZ4950	hypothet	Gonium_pectorale	44.90	0.00
TRINITY_OAE3007	hypothet	Marchantia_polymorp	44.90	0.00
TRINITY_GAX8397	hypothet	Chlamydomonas_eusti	44.90	0.00
TRINITY_XP_0238	ubiquiti	Quercus_suber	44.90	0.00
TRINITY_PNW8884	hypothet	Chlamydomonas_reinh	44.90	0.00
TRINITY_KXZ5275	hypothet	Gonium_pectorale	44.90	0.00
TRINITY_PWA7601	Arf GTPa	Artemisia_annua	44.90	0.00
TRINITY_XP_0238	glutamat	Quercus_suber	44.90	0.00
TRINITY_EEF2536	3-hydrox	Ricinus_communis	44.90	0.00
TRINITY_GAX7750	hypothet	Chlamydomonas_eusti	44.90	0.00
TRINITY_GAQ8247	hypothet	Klebsormidium_niten	44.90	0.00
TRINITY_XP_0029	hypothet	Volvox_carteri	44.90	0.00
TRINITY_XP_0025	uncharac	Ricinus_communis	44.90	0.00
TRINITY_XP_0120	probable	Jatropha_curcas	44.90	0.00
TRINITY_GAQ8311	pescadil	Klebsormidium_niten	44.90	0.00
TRINITY_XP_0106	PREDICTE	Beta_vulgaris	44.90	0.00
TRINITY_GAX8485	hypothet	Chlamydomonas_eusti	44.90	0.00
TRINITY_GAX8592	hypothet	Chlamydomonas_eusti	44.90	0.00
TRINITY_GBF8939	hypothet	Raphidocelis_subcap	44.90	0.00
TRINITY_PNW8156	hypothet	Chlamydomonas_reinh	44.90	0.00
TRINITY_XP_0165	PREDICTE	Capsicum_annuum	44.90	0.00

TRINITY_GBG6548(	hypothet	Chara_braunii	44.90	0.00
TRINITY_GAX7578(	hypothet	Chlamydomonas_eusti	44.90	0.00
TRINITY_GAX8574(	hypothet	Chlamydomonas_eusti	44.90	0.00
TRINITY_EFJ2446(	hypothet	Selaginella_moellen	44.90	0.00
TRINITY_PNW7499(	hypothet	Chlamydomonas_reinh	44.90	0.00
TRINITY_XP_0201(	uncharac	Ananas_comosus	44.90	0.00
TRINITY_KXZ4446(	hypothet	Gonium_pectorale	44.90	0.00
TRINITY_XP_0056(	urea act	Coccomyxa_subellips	44.90	0.00
TRINITY_XP_0188(	PREDICTE	Juglans_regia	44.90	0.00
TRINITY_XP_0036(	uncharac	Medicago_truncatula	44.90	0.00
TRINITY_PNW8850(	hypothet	Chlamydomonas_reinh	44.90	0.00
TRINITY_ABU5296(	hypothet	Dunaliella_viridis	44.90	0.00
TRINITY_PNW7786(	hypothet	Chlamydomonas_reinh	44.90	0.00
TRINITY_KXG2977(	hypothet	Sorghum_bicolor	44.90	0.00
TRINITY_XP_0228(	ER lumen	Olea_europaea	44.90	0.00
TRINITY_XP_0030(	dpy-30	Micromonas_pusilla	44.90	0.00
TRINITY_KXZ4165(	hypothet	Gonium_pectorale	44.90	0.00
TRINITY_GAX7367(	hypothet	Chlamydomonas_eusti	44.90	0.00
TRINITY_XP_0068(	protein	Amborella_trichopod	44.90	0.00
TRINITY_PNH0912(	hypothet	Tetrabaena_socialis	44.90	0.00
TRINITY_XP_0100(	PREDICTE	Eucalyptus_grandis	44.80	0.00
TRINITY_PNR3798(	hypothet	Physcomitrella_pate	44.80	0.00
TRINITY_XP_0239(	uncharac	Quercus_suber	44.80	0.00
TRINITY_GAQ8578(	Cysteiny	Klebsormidium_niten	44.80	0.00
TRINITY_XP_0188(	PREDICTE	Juglans_regia	44.80	0.00
TRINITY_GBG6494(	hypothet	Chara_braunii	44.80	0.00
TRINITY_XP_0029(	hypothet	Volvox_carteri	44.80	0.00
TRINITY_XP_0014(	predicte	Ostreococcus_sp._'l	44.80	0.00
TRINITY_GAV5948(	ADH_zinc	Cephalotus_follicul	44.80	0.00
TRINITY_OAE2839(	hypothet	Marchantia_polymorp	44.80	0.00
TRINITY_PIA3329(	hypothet	Aquilegia_coerulea	44.80	0.00
TRINITY_XP_0058(	hypothet	Chlorella_variabili	44.80	0.00
TRINITY_XP_0237(	serine/t	Lactuca_sativa	44.80	0.00
TRINITY_XP_0110(	ABC tran	Sesamum_indicum	44.80	0.00
TRINITY_XP_0239(	fimbrin-	Quercus_suber	44.80	0.00
TRINITY_XP_0075(	predicte	Bathycoccus_prasino	44.80	0.00
TRINITY_XP_0109(	PREDICTE	Elaeis_guineensis	44.80	0.00
TRINITY_BAJ9385(	predicte	Hordeum_vulgare	44.80	0.00
TRINITY_GAX8364(	hypothet	Chlamydomonas_eusti	44.80	0.00
TRINITY_VDD0534(	unnamed	Brassica_oleracea	44.80	0.00
TRINITY_XP_0243(	N-alpha-	Physcomitrella_pate	44.80	0.00
TRINITY_XP_0058(	hypothet	Chlorella_variabili	44.80	0.00
TRINITY_XP_0029(	hypothet	Volvox_carteri	44.80	0.00
TRINITY_XP_0273(	uncharac	Abrus_precatorius	44.80	0.00
TRINITY_PNW7973(	hypothet	Chlamydomonas_reinh	44.80	0.00
TRINITY_XP_0208(	importin	Arabidopsis_lyrata	44.80	0.00
TRINITY_GAX8022(	hypothet	Chlamydomonas_eusti	44.80	0.00
TRINITY_GAX7714(	hypothet	Chlamydomonas_eusti	44.80	0.00
TRINITY_GAV8311(	K_tetra	Cephalotus_follicul	44.80	0.00
TRINITY_OAE2889(	hypothet	Marchantia_polymorp	44.80	0.00
TRINITY_GAX7562(	hypothet	Chlamydomonas_eusti	44.80	0.00
TRINITY_PRW5890(	selenocy	Chlorella_sorokinia	44.80	0.00
TRINITY_XP_0186(	PREDICTE	Musa_acuminata	44.80	0.00
TRINITY_GAX8459(	hypothet	Chlamydomonas_eusti	44.80	0.00
TRINITY_KXZ5330(	hypothet	Gonium_pectorale	44.80	0.00
TRINITY_KHG2359(	Serine/t	Gossypium_arboreum	44.80	0.00
TRINITY_GAX8114(	hypothet	Chlamydomonas_eusti	44.80	0.00
TRINITY_XP_0279(	probable	Vigna_unguiculata	44.80	0.00
TRINITY_GBG5939(	hypothet	Chara_braunii	44.80	0.00
TRINITY_GAX8549(	hypothet	Chlamydomonas_eusti	44.80	0.00
TRINITY_PNH0870(	hypothet	Tetrabaena_socialis	44.80	0.00
TRINITY_GAX8498(	hypothet	Chlamydomonas_eusti	44.80	0.00
TRINITY_GBF8939(	membrane	Raphidocelis_subcap	44.80	0.00
TRINITY_XP_0139(	hypothet	Monoraphidium_negle	44.80	0.00
TRINITY_XP_0093(	PREDICTE	Musa_acuminata	44.80	0.00
TRINITY_XP_0239(	phytoene	Quercus_suber	44.80	0.00

TRINITY_GBG6437	hypothet	Chara_braunii	44.80	0.00
TRINITY_XP_0239	LOW QUAI	Quercus_suber	44.80	0.00
TRINITY_PNW8114	hypothet	Chlamydomonas_reinh	44.80	0.00
TRINITY_GAX8057	hypothet	Chlamydomonas_eusti	44.80	0.00
TRINITY_GAX7454	hypothet	Chlamydomonas_eusti	44.80	0.00
TRINITY_GAX8624	hypothet	Chlamydomonas_eusti	44.80	0.00
TRINITY_XP_0167	PREDICTE	Gossypium_hirsutum	44.80	0.00
TRINITY_GAX8326	hypothet	Chlamydomonas_eusti	44.80	0.00
TRINITY_KFK3803	hypothet	Arabis_alpina	44.80	0.00
TRINITY_XP_0093	PREDICTE	Musa_acuminata	44.80	0.00
TRINITY_XP_0239	probable	Quercus_suber	44.80	0.00
TRINITY_XP_0124	PREDICTE	Gossypium_raimondii	44.80	0.00
TRINITY_PNW8278	hypothet	Chlamydomonas_reinh	44.80	0.00
TRINITY_PNW7393	hypothet	Chlamydomonas_reinh	44.80	0.00
TRINITY_XP_0029	hypothet	Volvox_carteri	44.80	0.00
TRINITY_PNW7069	hypothet	Chlamydomonas_reinh	44.80	0.00
TRINITY_XP_0114	Sedohept	Auxenochlorella_pro	44.80	0.00
TRINITY_XP_0014	predicte	Ostreococcus_sp._'l	44.80	0.00
TRINITY_XP_0072	GTP-binc	Prunus_persica	44.80	0.00
TRINITY_XP_0165	PREDICTE	Capsicum_annuum	44.70	0.00
TRINITY_XP_0094	PREDICTE	Musa_acuminata	44.70	0.00
TRINITY_XP_0194	PREDICTE	Lupinus_angustifoli	44.70	0.00
TRINITY_XP_0045	ras-rela	Cicer_arietinum	44.70	0.00
TRINITY_XP_0184	PREDICTE	Raphanus_sativus	44.70	0.00
TRINITY_RZC2051	Short ir	Glycine_soja	44.70	0.00
TRINITY_GAX7760	hypothet	Chlamydomonas_eusti	44.70	0.00
TRINITY_XP_0075	predicte	Bathycoccus_prasinc	44.70	0.00
TRINITY_XP_0227	GTP-binc	Durio_zibethinus	44.70	0.00
TRINITY_GAX7567	hypothet	Chlamydomonas_eusti	44.70	0.00
TRINITY_XP_0219	E3 ubiqu	Helianthus_annuus	44.70	0.00
TRINITY_GAQ8491	hypothet	Klebsormidium_niten	44.70	0.00
TRINITY_GAQ8315	hypothet	Klebsormidium_niten	44.70	0.00
TRINITY_PIN1005	Tyrosine	Handroanthus_impeti	44.70	0.00
TRINITY_XP_0029	hypothet	Volvox_carteri	44.70	0.00
TRINITY_GAX7848	hypothet	Chlamydomonas_eusti	44.70	0.00
TRINITY_XP_0243	N-alpha-	Physcomitrella_pate	44.70	0.00
TRINITY_GBG7712	hypothet	Chara_braunii	44.70	0.00
TRINITY_KXZ4954	hypothet	Gonium_pectorale	44.70	0.00
TRINITY_XP_0156	sphingol	Oryza_sativa	44.70	0.00
TRINITY_OAE2670	hypothet	Marchantia_polymorp	44.70	0.00
TRINITY_PNW8856	hypothet	Chlamydomonas_reinh	44.70	0.00
TRINITY_GAQ9196	hypothet	Klebsormidium_niten	44.70	0.00
TRINITY_XP_0068	methylth	Amborella_trichopod	44.70	0.00
TRINITY_PWA8309	pantoate	Artemisia_annua	44.70	0.00
TRINITY_KNA1888	hypothet	Spinacia_oleracea	44.70	0.00
TRINITY_XP_0158	bZIP tra	Ziziphus_jujuba	44.70	0.00
TRINITY_GBF8862	hypothet	Raphidocelis_subcap	44.70	0.00
TRINITY_XP_0058	hypothet	Chlorella_variabili	44.70	0.00
TRINITY_KOM4859	hypothet	Vigna_angularis	44.70	0.00
TRINITY_XP_0218	nudix hy	Carica_papaya	44.70	0.00
TRINITY_PNW8866	hypothet	Chlamydomonas_reinh	44.70	0.00
TRINITY_PNW8303	hypothet	Chlamydomonas_reinh	44.70	0.00
TRINITY_GAX7318	hypothet	Chlamydomonas_eusti	44.70	0.00
TRINITY_GAX7789	hypothet	Chlamydomonas_eusti	44.70	0.00
TRINITY_PNH1178	Heat shc	Tetrabaena_socialis	44.70	0.00
TRINITY_EOY1998	Geminivi	Theobroma_cacao	44.70	0.00
TRINITY_PRW5816	transfer	Chlorella_sorokinia	44.70	0.00
TRINITY_XP_0239	ATP-depe	Quercus_suber	44.70	0.00
TRINITY_PNW7711	hypothet	Chlamydomonas_reinh	44.70	0.00
TRINITY_XP_0243	putative	Physcomitrella_pate	44.70	0.00
TRINITY_XP_0221	DNA poly	Momordica_charantia	44.70	0.00
TRINITY_PNH0892	hypothet	Tetrabaena_socialis	44.70	0.00
TRINITY_PTQ5061	hypothet	Marchantia_polymorp	44.70	0.00
TRINITY_XP_0138	hypothet	Monoraphidium_negle	44.70	0.00
TRINITY_PNW8268	hypothet	Chlamydomonas_reinh	44.70	0.00
TRINITY_XP_0124	PREDICTE	Gossypium_raimondii	44.70	0.00

TRINITY_XP_02387	acyl-CoA	Quercus_suber	44.70	0.00
TRINITY_EFJ18487	hypothet	Selaginella_moellen	44.70	0.00
TRINITY_GAX77499	hypothet	Chlamydomonas_eusti	44.70	0.00
TRINITY_PNW77180	hypothet	Chlamydomonas_reinh	44.70	0.00
TRINITY_KZN09756	hypothet	Daucus_carota	44.70	0.00
TRINITY_XP_00565	hypothet	Coccomyxa_subellips	44.70	0.00
TRINITY_GAX78086	hypothet	Chlamydomonas_eusti	44.70	0.00
TRINITY_GBG87191	hypothet	Chara_braunii	44.70	0.00
TRINITY_GAQ85880	tyrosine	Klebsormidium_niten	44.70	0.00
TRINITY_XP_00169	predicte	Chlamydomonas_reinh	44.70	0.00
TRINITY_XP_02022	YLP moti	Cajanus_cajan	44.70	0.00
TRINITY_KXZ48818	hypothet	Gonium_pectorale	44.70	0.00
TRINITY_GAX84324	hypothet	Chlamydomonas_eusti	44.70	0.00
TRINITY_GAX80853	hypothet	Chlamydomonas_eusti	44.70	0.00
TRINITY_BAK02199	predicte	Hordeum_vulgare	44.70	0.00
TRINITY_OAE32643	hypothet	Marchantia_polymorp	44.70	0.00
TRINITY_XP_01941	PREDICTE	Lupinus_angustifoli	44.70	0.00
TRINITY_GAX79453	hypothet	Chlamydomonas_eusti	44.70	0.00
TRINITY_GAX72727	hypothet	Chlamydomonas_eusti	44.70	0.00
TRINITY_PNW69702	hypothet	Chlamydomonas_reinh	44.70	0.00
TRINITY_GAX77149	hypothet	Chlamydomonas_eusti	44.70	0.00
TRINITY_GAX84028	hypothet	Chlamydomonas_eusti	44.70	0.00
TRINITY_PNH07860	hypothet	Tetrabaena_socialis	44.70	0.00
TRINITY_PNW73085	hypothet	Chlamydomonas_reinh	44.70	0.00
TRINITY_GAX72985	hypothet	Chlamydomonas_eusti	44.70	0.00
TRINITY_PNW81774	hypothet	Chlamydomonas_reinh	44.70	0.00
TRINITY_XP_00169	predicte	Chlamydomonas_reinh	44.70	0.00
TRINITY_GAX83220	hypothet	Chlamydomonas_eusti	44.60	0.00
TRINITY_XP_00565	pyrophos	Coccomyxa_subellips	44.60	0.00
TRINITY_PSS13870	Actin-re	Actinidia_chinensis	44.60	0.00
TRINITY_XP_02716	kinesin-	Coffea_eugenioides	44.60	0.00
TRINITY_PLY95837	hypothet	Lactuca_sativa	44.60	0.00
TRINITY_XP_02439	protein	Physcomitrella_pate	44.60	0.00
TRINITY_XP_01763	PREDICTE	Gossypium_arboreum	44.60	0.00
TRINITY_XP_02388	elongati	Quercus_suber	44.60	0.00
TRINITY_OWM71838	hypothet	Punica_granatum	44.60	0.00
TRINITY_PTQ37914	hypothet	Marchantia_polymorp	44.60	0.00
TRINITY_EEC73387	hypothet	Oryza_sativa	44.60	0.00
TRINITY_XP_00250	predicte	Micromonas_commoda	44.60	0.00
TRINITY_XP_00564	metal-de	Coccomyxa_subellips	44.60	0.00
TRINITY_GAQ82327	phosphat	Klebsormidium_niten	44.60	0.00
TRINITY_XP_00564	hypothet	Coccomyxa_subellips	44.60	0.00
TRINITY_XP_02388	ras-rela	Quercus_suber	44.60	0.00
TRINITY_XP_02387	cell div	Quercus_suber	44.60	0.00
TRINITY_PIN04432	Cystathi	Handroanthus_impeti	44.60	0.00
TRINITY_GAQ85273	WD-40 re	Klebsormidium_niten	44.60	0.00
TRINITY_OAE29545	hypothet	Marchantia_polymorp	44.60	0.00
TRINITY_XP_00751	unnamed	Bathycoccus_prasino	44.60	0.00
TRINITY_GAQ78735	alpha-an	Klebsormidium_niten	44.60	0.00
TRINITY_XP_01046	PREDICTE	Camelina_sativa	44.60	0.00
TRINITY_KDD74340	hypothet	Helicosporidium_sp.	44.60	0.00
TRINITY_XP_01055	PREDICTE	Tarenaya_hasslerian	44.60	0.00
TRINITY_AKD28017	translat	Morus_alba	44.60	0.00
TRINITY_GAQ84241	Class I	Klebsormidium_niten	44.60	0.00
TRINITY_PHT59984	Dual spe	Capsicum_baccatum	44.60	0.00
TRINITY_XP_01918	PREDICTE	Ipomoea_nil	44.60	0.00
TRINITY_XP_00170	CDK inhi	Chlamydomonas_reinh	44.60	0.00
TRINITY_XP_00564	zf-AN1-c	Coccomyxa_subellips	44.60	0.00
TRINITY_XP_01067	PREDICTE	Beta_vulgaris	44.60	0.00
TRINITY_XP_01390	Cell cyc	Monoraphidium_negle	44.60	0.00
TRINITY_GAX79006	hypothet	Chlamydomonas_eusti	44.60	0.00
TRINITY_EFJ22602	hypothet	Selaginella_moellen	44.60	0.00
TRINITY_P0C8M8.1	RecName:	Zea_mays	44.60	0.00
TRINITY_KXZ47621	hypothet	Gonium_pectorale	44.60	0.00
TRINITY_GAX74117	hypothet	Chlamydomonas_eusti	44.60	0.00
TRINITY_PNW86709	hypothet	Chlamydomonas_reinh	44.60	0.00

TRINITY_XP_00295	hypothet	Volvox_carteri	44.60	0.00
TRINITY_GAX77809	hypothet	Chlamydomonas_eusti	44.60	0.00
TRINITY_XP_00880	DNA-dire	Phoenix_dactylifera	44.60	0
TRINITY_KXZ44418	hypothet	Gonium_pectorale	44.60	0.00
TRINITY_XP_01101	PREDICTE	Populus_euphratica	44.60	0.00
TRINITY_GAX79067	hypothet	Chlamydomonas_eusti	44.60	0.00
TRINITY_GBG73474	hypothet	Chara_braunii	44.60	0.00
TRINITY_NP_85052	MAPK pho	Arabidopsis_thalian	44.60	0.00
TRINITY_Q7XJ96.1	RecName:	Chlamydomonas_reinh	44.60	0.00
TRINITY_XP_01040	PREDICTE	Camelina_sativa	44.60	0.00
TRINITY_XP_00564	Sec63-do	Coccomyxa_subellips	44.60	0.00
TRINITY_XP_01452	ervatami	Vigna_radiata	44.60	0.00
TRINITY_GBF94188	hypothet	Raphidocelis_subcap	44.60	0.00
TRINITY_XP_00294	hypothet	Volvox_carteri	44.60	0.00
TRINITY_GAX83358	hypothet	Chlamydomonas_eusti	44.60	0.00
TRINITY_GAX81923	hypothet	Chlamydomonas_eusti	44.60	0.00
TRINITY_GAX84289	hypothet	Chlamydomonas_eusti	44.60	0.00
TRINITY_PRW33549	thiosulf	Chlorella_sorokinia	44.60	0.00
TRINITY_KXZ56862	hypothet	Gonium_pectorale	44.60	0.00
TRINITY_OEL38809	Serine/t	Dichanthelium_oligo	44.60	0.00
TRINITY_ADF43165	97782m	Chlamydomonas_reinh	44.60	0.00
TRINITY_PIN17324	S-methyl	Handroanthus_impeti	44.60	0.00
TRINITY_XP_00300	predicte	Micromonas_pusilla	44.60	0.00
TRINITY_XP_02390	inorgani	Quercus_suber	44.60	0.00
TRINITY_XP_00295	proline	Volvox_carteri	44.60	0.00
TRINITY_XP_02452	phosphat	Selaginella_moellen	44.60	0.00
TRINITY_XP_02640	mitochon	Papaver_somniferum	44.60	0.00
TRINITY_PNH11052	Phosphog	Tetrabaena_socialis	44.60	0.00
TRINITY_PNH10793	Bifuncti	Tetrabaena_socialis	44.60	0.00
TRINITY_XP_00170	RabGAP/T	Chlamydomonas_reinh	44.60	0.00
TRINITY_PNH06283	putative	Tetrabaena_socialis	44.60	0.00
TRINITY_XP_02387	uncharac	Quercus_suber	44.60	0.00
TRINITY_XP_00295	hypothet	Volvox_carteri	44.60	0.00
TRINITY_XP_02389	sorting	Quercus_suber	44.60	0.00
TRINITY_XP_01722	PREDICTE	Daucus_carota	44.60	0.00
TRINITY_KXZ48718	hypothet	Gonium_pectorale	44.60	0.00
TRINITY_XP_00170	predicte	Chlamydomonas_reinh	44.60	0.00
TRINITY_OAE27810	hypothet	Marchantia_polymorp	44.60	0.00
TRINITY_XP_00935	PREDICTE	Pyrus_x_bretschneid	44.60	0.00
TRINITY_XP_00295	hypothet	Volvox_carteri	44.60	0.00
TRINITY_XP_01390	calcium/	Monoraphidium_negle	44.60	0.00
TRINITY_PNW80254	hypothet	Chlamydomonas_reinh	44.60	0.00
TRINITY_PPS08138	hypothet	Gossypium_barbadens	44.60	0.00
TRINITY_GAX79335	hypothet	Chlamydomonas_eusti	44.60	0.00
TRINITY_XP_00307	Translat	Ostreococcus_tauri	44.60	0.00
TRINITY_PNH05203	Endo-1,3	Tetrabaena_socialis	44.60	0.00
TRINITY_XP_01724	PREDICTE	Daucus_carota	44.60	0.00
TRINITY_GAX78078	hypothet	Chlamydomonas_eusti	44.60	0.00
TRINITY_RVW11860	Transpos	Vitis_vinifera	44.60	0.00
TRINITY_GBG91496	hypothet	Chara_braunii	44.60	0.00
TRINITY_EYU45971	hypothet	Erythranthe_guttata	44.50	0.00
TRINITY_XP_02437	metal to	Physcomitrella_pate	44.50	0.00
TRINITY_GBF90328	hypothet	Raphidocelis_subcap	44.50	0.00
TRINITY_RRT65770	hypothet	Ensete_ventricosum	44.50	0.00
TRINITY_XP_02640	haloacid	Papaver_somniferum	44.50	0.00
TRINITY_GAQ89310	Dyneins	Klebsormidium_niten	44.50	0.00
TRINITY_XP_02392	eukaryot	Quercus_suber	44.50	0.00
TRINITY_GBF95304	mitochon	Raphidocelis_subcap	44.50	0.00
TRINITY_XP_01843	PREDICTE	Raphanus_sativus	44.50	0.00
TRINITY_XP_00879	AP-2 com	Phoenix_dactylifera	44.50	0.00
TRINITY_GAX75433	hypothet	Chlamydomonas_eusti	44.50	0.00
TRINITY_GBF89851	hypothet	Raphidocelis_subcap	44.50	0.00
TRINITY_GAX73106	hypothet	Chlamydomonas_eusti	44.50	0.00
TRINITY_XP_02389	4-hydrox	Quercus_suber	44.50	0.00
TRINITY_GAX73655	hypothet	Chlamydomonas_eusti	44.50	0.00
TRINITY_GAX77309	hypothet	Chlamydomonas_eusti	44.50	0.00

TRINITY_XP_0245	protein	Selaginella_moellen	44.50	0.00
TRINITY_OMO9557	Crotona	Corchorus_capsulari	44.50	0.00
TRINITY_XP_0138	hypothet	Monoraphidium_negle	44.50	0.00
TRINITY_PNW8217	hypothet	Chlamydomonas_reinh	44.50	0.00
TRINITY_XP_0243	pre-mRN	Physcomitrella_pate	44.50	0.00
TRINITY_GAX7377	hypothet	Chlamydomonas_eusti	44.50	0.00
TRINITY_GAX8151	hypothet	Chlamydomonas_eusti	44.50	0.00
TRINITY_GAX7767	hypothet	Chlamydomonas_eusti	44.50	0.00
TRINITY_GAQ8357	RNA lig	Klebsormidium_niten	44.50	0.00
TRINITY_PNW7793	hypothet	Chlamydomonas_reinh	44.50	0.00
TRINITY_GBG5950	hypothet	Chara_braunii	44.50	0.00
TRINITY_PTQ3559	hypothet	Marchantia_polymorp	44.50	0.00
TRINITY_KEH2974	signal	Medicago_truncatula	44.50	0.00
TRINITY_EFH6567	hypothet	Arabidopsis_lyrata	44.50	0.00
TRINITY_KXZ5519	hypothet	Gonium_pectorale	44.50	0.00
TRINITY_XP_0042	PREDICT	Fragaria_vesca	44.50	0.00
TRINITY_CAD4169	OSJNBb0	Oryza_sativa	44.50	0.00
TRINITY_XP_0056	hypothet	Coccomyxa_subellips	44.50	0.00
TRINITY_PNW7569	hypothet	Chlamydomonas_reinh	44.50	0.00
TRINITY_PNW8508	hypothet	Chlamydomonas_reinh	44.50	0.00
TRINITY_XP_0082	PREDICT	Prunus_mume	44.50	0.00
TRINITY_XP_0243	pirin-l	Physcomitrella_pate	44.50	0.00
TRINITY_XP_0243	uncharac	Physcomitrella_pate	44.50	0.00
TRINITY_KXZ5188	hypothet	Gonium_pectorale	44.50	0.00
TRINITY_XP_0029	hypothet	Volvox_carteri	44.50	0.00
TRINITY_XP_0243	DCN1-li	Physcomitrella_pate	44.50	0.00
TRINITY_XP_0058	hypothet	Chlorella_variabili	44.50	0.00
TRINITY_PNH0281	Vam6/Vp	Tetrabaena_socialis	44.40	0.00
TRINITY_RLN4056	hypothet	Panicum_miliaceum	44.40	0.00
TRINITY_BAJ9779	predict	Hordeum_vulgare	44.40	0.00
TRINITY_XP_0206	phosphat	Dendrobium_catenatu	44.40	0.00
TRINITY_BAK0274	predict	Hordeum_vulgare	44.40	0.00
TRINITY_XP_0162	beta-ga	Arachis_ipaensis	44.40	0.00
TRINITY_XP_0104	PREDICT	Camelina_sativa	44.40	0.00
TRINITY_XP_0043	PREDICT	Fragaria_vesca	44.40	0.00
TRINITY_OMO8536	hypothet	Corchorus_olitorius	44.40	0.00
TRINITY_PNH1226	Tiny ma	Tetrabaena_socialis	44.40	0.00
TRINITY_XP_0208	peroxis	Arabidopsis_lyrata	44.40	0.00
TRINITY_XP_0243	serine/	Physcomitrella_pate	44.40	0.00
TRINITY_XP_0114	Adenyly	Auxenochlorella_pro	44.40	0.00
TRINITY_XP_0218	importi	Spinacia_oleracea	44.40	0.00
TRINITY_XP_0106	PREDICT	Beta_vulgaris	44.40	0.00
TRINITY_RWW9084	hypothet	Ensete_ventricosum	44.40	0.00
TRINITY_NP_0011	caltract	Zea_mays	44.40	0.00
TRINITY_XP_0221	calcium-	Momordica_charantia	44.40	0.00
TRINITY_XP_0096	PREDICT	Nicotiana_tomentosi	44.40	0.00
TRINITY_OMO6670	hypothet	Corchorus_capsulari	44.40	0.00
TRINITY_XP_0229	glutamat	Cucurbita_maxima	44.40	0.00
TRINITY_GAQ9121	Calcium	Klebsormidium_niten	44.40	0.00
TRINITY_PNH1240	putativ	Tetrabaena_socialis	44.40	0.00
TRINITY_BAK0075	predict	Hordeum_vulgare	44.40	0.00
TRINITY_XP_0218	GTP-bin	Spinacia_oleracea	44.40	0.00
TRINITY_XP_0016	predict	Chlamydomonas_reinh	44.40	0.00
TRINITY_KNA0354	hypothet	Spinacia_oleracea	44.40	0.00
TRINITY_GAQ7880	Transcr	Klebsormidium_niten	44.40	0.00
TRINITY_XP_0056	calcium-	Coccomyxa_subellips	44.40	0.00
TRINITY_XP_0094	PREDICT	Musa_acuminata	44.40	0.00
TRINITY_XP_0016	phospho	Chlamydomonas_reinh	44.40	0.00
TRINITY_GAX7466	hypothet	Chlamydomonas_eusti	44.40	0.00
TRINITY_GAQ9177	mitocho	Klebsormidium_niten	44.40	0.00
TRINITY_KZV1531	hypothet	Dorcoceras_hygromet	44.40	0.00
TRINITY_XP_0124	PREDICT	Gossypium_raimondii	44.40	0.00
TRINITY_GAX8597	hypothet	Chlamydomonas_eusti	44.40	0.00
TRINITY_XP_0075	predict	Bathycoccus_prasin	44.40	0.00
TRINITY_RXH8537	hypothet	Malus_domestica	44.40	0.00
TRINITY_KXZ4776	hypothet	Gonium_pectorale	44.40	0.00

TRINITY_XP_01102	PREDICTE	Populus_euphratica	44.40	0.00
TRINITY_XP_0239(	serine/t	Quercus_suber	44.40	0.00
TRINITY_KXZ5536!	hypothet	Gonium_pectorale	44.40	0.00
TRINITY_XP_0239(	probable	Quercus_suber	44.40	0.00
TRINITY_XP_0243(	uncharac	Physcomitrella_pate	44.40	0.00
TRINITY_GAX7543(	hypothet	Chlamydomonas_eusti	44.40	0.00
TRINITY_XP_02392	ubiquiti	Quercus_suber	44.40	0.00
TRINITY_XP_0219'	probable	Helianthus_annuus	44.40	0.00
TRINITY_KXZ4322	hypothet	Gonium_pectorale	44.40	0.00
TRINITY_XP_0238'	translat	Quercus_suber	44.40	0.00
TRINITY_GAX7829(	hypothet	Chlamydomonas_eusti	44.40	0.00
TRINITY_PSC7504(	apoptoti	Micractinium_conduc	44.40	0.00
TRINITY_KXZ4838!	hypothet	Gonium_pectorale	44.40	0.00
TRINITY_XP_0056	snare-dc	Coccomyxa_subellips	44.40	0.00
TRINITY_XP_0016!	early l	Chlamydomonas_reinh	44.40	0.00
TRINITY_GAX8033'	hypothet	Chlamydomonas_eusti	44.40	0.00
TRINITY_XP_0029!	hypothet	Volvox_carteri	44.40	0.00
TRINITY_PNH1023	DEAD-box	Tetrabaena_socialis	44.40	0.00
TRINITY_GAX8260'	hypothet	Chlamydomonas_eusti	44.40	0.00
TRINITY_GAX7725(	hypothet	Chlamydomonas_eusti	44.40	0.00
TRINITY_PNH00992	Phytochr	Tetrabaena_socialis	44.40	0.00
TRINITY_KXZ4756(	hypothet	Gonium_pectorale	44.40	0.00
TRINITY_XP_0016!	DEAD/DEA	Chlamydomonas_reinh	44.40	0.00
TRINITY_PNW8836(	hypothet	Chlamydomonas_reinh	44.40	0.00
TRINITY_GBG8982(	hypothet	Chara_braunii	44.40	0.00
TRINITY_GAX8569(	hypothet	Chlamydomonas_eusti	44.40	0.00
TRINITY_GAX8612	hypothet	Chlamydomonas_eusti	44.40	0.00
TRINITY_XP_0025	uncharac	Ricinus_communis	44.40	0.00
TRINITY_PNW8689(	hypothet	Chlamydomonas_reinh	44.40	0.00
TRINITY_GAX8317!	hypothet	Chlamydomonas_eusti	44.40	0.00
TRINITY_XP_0156	importir	Oryza_sativa	44.40	0.00
TRINITY_GAX7382(	hypothet	Chlamydomonas_eusti	44.40	0.00
TRINITY_GAQ8074!	phosphoe	Klebsormidium_niten	44.40	0.00
TRINITY_GAX7752!	hypothet	Chlamydomonas_eusti	44.40	0.00
TRINITY_PNW8086!	hypothet	Chlamydomonas_reinh	44.40	0.00
TRINITY_GAX7682	hypothet	Chlamydomonas_eusti	44.40	0.00
TRINITY_XP_0201(	ADP-ribc	Aegilops_tauschii	44.40	0.00
TRINITY_OMO8837!	hypothet	Corchorus_capsulari	44.40	0.00
TRINITY_XP_0202(	RING-H2	Asparagus_officinal	44.30	0.00
TRINITY_XP_0071	hypothet	Phaseolus_vulgaris	44.30	0.00
TRINITY_XP_0093	PREDICTE	Pyrus_x_bretschneid	44.30	0.00
TRINITY_GAX7454(	hypothet	Chlamydomonas_eusti	44.30	0.00
TRINITY_GAQ89612	enoyl-Cc	Klebsormidium_niten	44.30	0.00
TRINITY_OAE3410	hypothet	Marchantia_polymorp	44.30	0.00
TRINITY_GAQ8201(	Serine-t	Klebsormidium_niten	44.30	0.00
TRINITY_RAL4527'	hypothet	Cuscuta_australis	44.30	0.00
TRINITY_XP_0238(	threonir	Quercus_suber	44.30	0.00
TRINITY_GBG8776(	hypothet	Chara_braunii	44.30	0.00
TRINITY_XP_0063	PREDICTE	Solanum_tuberosum	44.30	0.00
TRINITY_XP_0238'	cell div	Quercus_suber	44.30	0.00
TRINITY_XP_0029!	hypothet	Volvox_carteri	44.30	0.00
TRINITY_XP_0029(	mitochor	Selaginella_moellen	44.30	0.00
TRINITY_XP_0024!	predicte	Micromonas_commoda	44.30	0.00
TRINITY_XP_0161(	serine (	Arachis_ipaensis	44.30	0.00
TRINITY_XP_0029	hypothet	Volvox_carteri	44.30	0.00
TRINITY_GBG7769'	hypothet	Chara_braunii	44.30	0.00
TRINITY_XP_0245	coatomer	Selaginella_moellen	44.30	0.00
TRINITY_PUZ7737	hypothet	Panicum_hallii	44.30	0.00
TRINITY_GAQ8019	serine/t	Klebsormidium_niten	44.30	0.00
TRINITY_PNH0460!	hypothet	Tetrabaena_socialis	44.30	0.00
TRINITY_XP_0056!	hypothet	Coccomyxa_subellips	44.30	0.00
TRINITY_XP_0082	PREDICTE	Prunus_mume	44.30	0.00
TRINITY_GAX75992	hypothet	Chlamydomonas_eusti	44.30	0.00
TRINITY_GAX77212	hypothet	Chlamydomonas_eusti	44.30	0.00
TRINITY_GAX7522	hypothet	Chlamydomonas_eusti	44.30	0.00
TRINITY_XP_0041	PREDICTE	Cucumis_sativus	44.30	0.00

TRINITY_GAX7480	hypothet	Chlamydomonas_eusti	44.30	0.00
TRINITY_GAX8378	hypothet	Chlamydomonas_eusti	44.30	0.00
TRINITY_XP_0238	probable	Quercus_suber	44.30	0.00
TRINITY_XP_0035	serpin-Z	Brachypodium_distac	44.30	0.00
TRINITY_GAX7725	hypothet	Chlamydomonas_eusti	44.30	0.00
TRINITY_GAX7950	hypothet	Chlamydomonas_eusti	44.30	0.00
TRINITY_PHT7881	Aldehyde	Capsicum_annuum	44.30	0.00
TRINITY_XP_0014	CDF fami	Ostreococcus_sp._'l	44.30	0.00
TRINITY_PNW7343	hypothet	Chlamydomonas_reinh	44.30	0.00
TRINITY_XP_0138	hypothet	Monoraphidium_negle	44.30	0.00
TRINITY_XP_0066	PREDICTE	Oryza_brachyantha	44.30	0.00
TRINITY_XP_0029	hypothet	Volvox_carteri	44.30	0.00
TRINITY_GAX8266	hypothet	Chlamydomonas_eusti	44.30	0.00
TRINITY_XP_0058	hypothet	Chlorella_variabili	44.30	0.00
TRINITY_XP_0144	coatomer	Vigna_radiata	44.30	0.00
TRINITY_XP_0139	heme per	Monoraphidium_negle	44.30	0.00
TRINITY_XP_0264	cyclin-d	Papaver_somniferum	44.30	0.00
TRINITY_PNG9983	hypothet	Tetrabaena_socialis	44.30	0.00
TRINITY_GAX7745	hypothet	Chlamydomonas_eusti	44.30	0.00
TRINITY_PNH0311	Peptidyl	Tetrabaena_socialis	44.30	0.00
TRINITY_GAX7856	hypothet	Chlamydomonas_eusti	44.30	0.00
TRINITY_GAX7593	hypothet	Chlamydomonas_eusti	44.30	0.00
TRINITY_OAO8909	hypothet	Arabidopsis_thalian	44.30	0.00
TRINITY_KXZ4370	hypothet	Gonium_pectorale	44.30	0.00
TRINITY_XP_0238	acyl-CoA	Quercus_suber	44.30	0.00
TRINITY_PNH0762	hypothet	Tetrabaena_socialis	44.30	0.00
TRINITY_XP_0029	hypothet	Volvox_carteri	44.30	0.00
TRINITY_XP_0102	PREDICTE	Nelumbo_nucifera	44.30	0.00
TRINITY_RAL4052	hypothet	Cuscuta_australis	44.30	0.00
TRINITY_XP_0270	phosphol	Coffea_arabica	44.30	0.00
TRINITY_PNH1265	putative	Tetrabaena_socialis	44.30	0.00
TRINITY_RWR7294	dnaJ sub	Cinnamomum_micranth	44.30	0.00
TRINITY_PSC6940	symplek	Micractinium_conduc	44.30	0.00
TRINITY_KXZ5474	hypothet	Gonium_pectorale	44.30	0.00
TRINITY_XP_0120	ras-rela	Jatropha_curcas	44.30	0.00
TRINITY_GAX8576	hypothet	Chlamydomonas_eusti	44.30	0.00
TRINITY_XP_0029	hypothet	Volvox_carteri	44.30	0.00
TRINITY_KXZ4907	hypothet	Gonium_pectorale	44.30	0.00
TRINITY_PNW8411	hypothet	Chlamydomonas_reinh	44.30	0.00
TRINITY_PNW8195	hypothet	Chlamydomonas_reinh	44.30	0.00
TRINITY_ADE7569	unknown	Picea_sitchensis	44.30	0.00
TRINITY_KXZ4901	hypothet	Gonium_pectorale	44.30	0.00
TRINITY_XP_0245	cAMP-dep	Selaginella_moellen	44.30	0.00
TRINITY_PNH1127	Mitochon	Tetrabaena_socialis	44.30	0.00
TRINITY_KXZ5409	hypothet	Gonium_pectorale	44.30	0.00
TRINITY_XP_0014	predicte	Ostreococcus_sp._'l	44.30	0.00
TRINITY_XP_0029	hypothet	Volvox_carteri	44.30	0.00
TRINITY_GAX8182	hypothet	Chlamydomonas_eusti	44.30	0.00
TRINITY_GAX8150	hypothet	Chlamydomonas_eusti	44.30	0.00
TRINITY_XP_0016	mitogen	Chlamydomonas_reinh	44.30	0.00
TRINITY_KXZ5471	hypothet	Gonium_pectorale	44.30	0.00
TRINITY_GBF9415	cytochro	Raphidocelis_subcap	44.30	0.00
TRINITY_KZV1511	ATP bind	Dorcoceras_hygromet	44.20	0.00
TRINITY_XP_0064	ABC tran	Eutrema_salsugineum	44.20	0.00
TRINITY_OIW0110	hypothet	Lupinus_angustifoli	44.20	0.00
TRINITY_XP_0245	uncharac	Selaginella_moellen	44.20	0.00
TRINITY_XP_0279	probable	Vigna_unguiculata	44.20	0.00
TRINITY_KVH9006	Glutathi	Cynara_cardunculus	44.20	0.00
TRINITY_XP_0243	ubiquiti	Physcomitrella_pate	44.20	0.00
TRINITY_XP_0043	PREDICTE	Fragaria_vesca	44.20	0.00
TRINITY_XP_0243	serine/t	Physcomitrella_pate	44.20	0.00
TRINITY_XP_0064	very-lon	Eutrema_salsugineum	44.20	0.00
TRINITY_XP_0245	protein	Selaginella_moellen	44.20	0.00
TRINITY_BAK0356	predicte	Hordeum_vulgare	44.20	0.00
TRINITY_KYP7736	Translat	Cajanus_cajan	44.20	0.00
TRINITY_GAX8281	hypothet	Chlamydomonas_eusti	44.20	0.00

TRINITY_BAJ94575	predicte	Hordeum_vulgare	44.20	0.00
TRINITY_GAQ90048	embryo s	Klebsormidium_niten	44.20	0.00
TRINITY_XP_00295	hypothet	Volvox_carteri	44.20	0.00
TRINITY_KMS65186	hypothet	Beta_vulgaris	44.20	0.00
TRINITY_XP_02438	ras-rela	Physcomitrella_pate	44.20	0.00
TRINITY_XP_00245	ubiquiti	Sorghum_bicolor	44.20	0.00
TRINITY_NP_00114	uncharac	Zea_mays	44.20	0.00
TRINITY_XP_02059	ABC tran	Phalaenopsis_equest	44.20	0.00
TRINITY_PNW85676	hypothet	Chlamydomonas_reinh	44.20	0.00
TRINITY_XP_00295	eukaryot	Volvox_carteri	44.20	0.00
TRINITY_XP_02582	probable	Panicum_hallii	44.20	0.00
TRINITY_XP_02452	17.4 kDa	Selaginella_moellen	44.20	0.00
TRINITY_GAX79371	hypothet	Chlamydomonas_eusti	44.20	0.00
TRINITY_KXZ42004	hypothet	Gonium_pectorale	44.20	0.00
TRINITY_GAX81164	hypothet	Chlamydomonas_eusti	44.20	0.00
TRINITY_XP_02288	cyclin-c	Olea_europaea	44.20	0.00
TRINITY_XP_00252	two-comp	Ricinus_communis	44.20	0.00
TRINITY_GAQ87645	Ribonucl	Klebsormidium_niten	44.20	0.00
TRINITY_KXZ46814	hypothet	Gonium_pectorale	44.20	0.00
TRINITY_GAX75720	hypothet	Chlamydomonas_eusti	44.20	0.00
TRINITY_GAX83015	hypothet	Chlamydomonas_eusti	44.20	0.00
TRINITY_XP_00305	hypothet	Micromonas_pusilla	44.20	0.00
TRINITY_PNW77507	hypothet	Chlamydomonas_reinh	44.20	0.00
TRINITY_GAX84705	hypothet	Chlamydomonas_eusti	44.20	0.00
TRINITY_EFJ25003	hypothet	Selaginella_moellen	44.20	0.00
TRINITY_GAX77365	hypothet	Chlamydomonas_eusti	44.20	0.00
TRINITY_KXZ44209	hypothet	Gonium_pectorale	44.20	0.00
TRINITY_GAX79855	hypothet	Chlamydomonas_eusti	44.20	0.00
TRINITY_KXZ52928	hypothet	Gonium_pectorale	44.20	0.00
TRINITY_XP_02014	putative	Aegilops_tauschii	44.20	0.00
TRINITY_XP_02435	cAMP-dep	Physcomitrella_pate	44.20	0.00
TRINITY_OWM90727	hypothet	Punica_granatum	44.20	0.00
TRINITY_GAX83199	hypothet	Chlamydomonas_eusti	44.20	0.00
TRINITY_GAX76371	hypothet	Chlamydomonas_eusti	44.20	0.00
TRINITY_XP_00295	cytoplas	Volvox_carteri	44.20	0.00
TRINITY_XP_00565	threonyl	Coccomyxa_subellips	44.20	0.00
TRINITY_KXZ53872	hypothet	Gonium_pectorale	44.20	0.00
TRINITY_XP_00833	PREDICTE	Malus_domestica	44.20	0.00
TRINITY_XP_02289	SH3 doma	Olea_europaea	44.20	0.00
TRINITY_XP_02390	ATP-binc	Quercus_suber	44.20	0.00
TRINITY_PNW88060	hypothet	Chlamydomonas_reinh	44.20	0.00
TRINITY_GAX79811	hypothet	Chlamydomonas_eusti	44.20	0.00
TRINITY_XP_00169	predicte	Chlamydomonas_reinh	44.20	0.00
TRINITY_GAX80028	hypothet	Chlamydomonas_eusti	44.20	0.00
TRINITY_PRW57036	glycosyl	Chlorella_sorokinia	44.20	0.00
TRINITY_GAX73032	hypothet	Chlamydomonas_eusti	44.20	0.00
TRINITY_GBG00009	hypothet	Raphidocelis_subcap	44.20	0.00
TRINITY_AKP06209	glutathi	Eurya_emarginata	44.20	0.00
TRINITY_GAX74653	hypothet	Chlamydomonas_eusti	44.20	0.00
TRINITY_XP_01390	hypothet	Monoraphidium_negle	44.20	0.00
TRINITY_GAX85084	hypothet	Chlamydomonas_eusti	44.20	0.00
TRINITY_GAX81240	hypothet	Chlamydomonas_eusti	44.20	0.00
TRINITY_GAX73771	hypothet	Chlamydomonas_eusti	44.20	0.00
TRINITY_PNH09631	Protein	Tetrabaena_socialis	44.20	0.00
TRINITY_GAQ93053	hypothet	Klebsormidium_niten	44.20	0.00
TRINITY_GAQ85347	Ca2+-bin	Klebsormidium_niten	44.20	0.00
TRINITY_XP_00295	hypothet	Volvox_carteri	44.20	0.00
TRINITY_GBF92854	hypothet	Raphidocelis_subcap	44.20	0.00
TRINITY_PRW45071	family r	Chlorella_sorokinia	44.20	0.00
TRINITY_RID59711	hypothet	Brassica_rapa	44.20	0.00
TRINITY_GAX82633	hypothet	Chlamydomonas_eusti	44.20	0.00
TRINITY_KDD73154	E1-E2 AT	Helicosporidium_sp.	44.20	0.00
TRINITY_GAX85193	hypothet	Chlamydomonas_eusti	44.10	0.00
TRINITY_XP_00685	transcri	Amborella_trichopod	44.10	0.00
TRINITY_ONK79391	uncharac	Asparagus_officinal	44.10	0.00
TRINITY_ERM95197	hypothet	Amborella_trichopod	44.10	0.00

TRINITY_XP_02287	acid bet	Olea_europaea	44.10	0.00
TRINITY_XP_02438	putative	Physcomitrella_pate	44.10	0.00
TRINITY_OAE31192	hypothet	Marchantia_polymorp	44.10	0.00
TRINITY_XP_01925	PREDICTE	Nicotiana_attenuata	44.10	0.00
TRINITY_XP_00823	PREDICTE	Prunus_mume	44.10	0.00
TRINITY_XP_02388	serine/t	Quercus_suber	44.10	0.00
TRINITY_GAQ78449	Coatomer	Klebsormidium_niten	44.10	0.00
TRINITY_XP_02712	uncharac	Coffea_arabica	44.10	0.00
TRINITY_GAQ81348	mitochon	Klebsormidium_niten	44.10	0.00
TRINITY_PIN05633	5'-AMP-a	Handroanthus_impeti	44.10	0.00
TRINITY_KXZ45909	DHC7 pro	Gonium_pectorale	44.10	0.00
TRINITY_GAQ89783	hypothet	Klebsormidium_niten	44.10	0.00
TRINITY_PTQ47112	hypothet	Marchantia_polymorp	44.10	0.00
TRINITY_XP_02436	uncharac	Physcomitrella_pate	44.10	0.00
TRINITY_PSC68578	NUDIX do	Micractinium_conduc	44.10	0.00
TRINITY_XP_02214	protein	Momordica_charantia	44.10	0.00
TRINITY_XP_00751	predicte	Bathycoccus_prasino	44.10	0.00
TRINITY_XP_01764	PREDICTE	Gossypium_arboreum	44.10	0.00
TRINITY_PNW80581	hypothet	Chlamydomonas_reinh	44.10	0.00
TRINITY_XP_01943	PREDICTE	Lupinus_angustifoli	44.10	0.00
TRINITY_XP_00665	PREDICTE	Oryza_brachyantha	44.10	0.00
TRINITY_KZV15033	hypothet	Dorcoceras_hygromet	44.10	0.00
TRINITY_XP_01389	hypothet	Monoraphidium_negle	44.10	0.00
TRINITY_XP_02068	guanylat	Dendrobium_catenatu	44.10	0.00
TRINITY_XP_00295	hypothet	Volvox_carteri	44.10	0.00
TRINITY_GAX75620	hypothet	Chlamydomonas_eusti	44.10	0.00
TRINITY_XP_02439	monothio	Physcomitrella_pate	44.10	0.00
TRINITY_PNW75001	hypothet	Chlamydomonas_reinh	44.10	0.00
TRINITY_XP_02453	probable	Selaginella_moellen	44.10	0.00
TRINITY_XP_00169	predicte	Chlamydomonas_reinh	44.10	0.00
TRINITY_PNW82627	hypothet	Chlamydomonas_reinh	44.10	0.00
TRINITY_KXZ52413	hypothet	Gonium_pectorale	44.10	0.00
TRINITY_KXZ45697	hypothet	Gonium_pectorale	44.10	0.00
TRINITY_GAX75635	hypothet	Chlamydomonas_eusti	44.10	0.00
TRINITY_XP_02719	cysteine	Cicer_arietinum	44.10	0.00
TRINITY_PNW84734	hypothet	Chlamydomonas_reinh	44.10	0.00
TRINITY_GAX82546	hypothet	Chlamydomonas_eusti	44.10	0.00
TRINITY_KXZ42029	hypothet	Gonium_pectorale	44.10	0.00
TRINITY_XP_00308	WD40 rep	Ostreococcus_tauri	44.10	0.00
TRINITY_RWR93988	protein	Cinnamomum_micranth	44.10	0.00
TRINITY_XP_00169	predicte	Chlamydomonas_reinh	44.10	0.00
TRINITY_OMO90340	Peptidas	Corchorus_olitorius	44.10	0.00
TRINITY_PNH05756	putative	Tetrabaena_socialis	44.10	0.00
TRINITY_XP_00169	flagella	Chlamydomonas_reinh	44.10	0.00
TRINITY_GAX72887	hypothet	Chlamydomonas_eusti	44.10	0.00
TRINITY_GAX76977	hypothet	Chlamydomonas_eusti	44.10	0.00
TRINITY_PWA96904	Protein	Artemisia_annua	44.10	0.00
TRINITY_GAX74552	hypothet	Chlamydomonas_eusti	44.10	0.00
TRINITY_XP_02285	alpha-gl	Olea_europaea	44.10	0.00
TRINITY_XP_01284	PREDICTE	Erythranthe_guttata	44.10	0.00
TRINITY_OIW09435	hypothet	Lupinus_angustifoli	44.10	0.00
TRINITY_XP_00305	thioredo	Micromonas_pusilla	44.00	0.00
TRINITY_KXZ56792	hypothet	Gonium_pectorale	44.00	0.00
TRINITY_VDD24307	unnamed	Brassica_oleracea	44.00	0.00
TRINITY_EEF27264	nhl repe	Ricinus_communis	44.00	0.00
TRINITY_XP_01094	PREDICTE	Elaeis_guineensis	44.00	0.00
TRINITY_XP_00294	hypothet	Volvox_carteri	44.00	0.00
TRINITY_EMS63369	Nitrate	Triticum_urartu	44.00	0.00
TRINITY_EOY04722	Carrot E	Theobroma_cacao	44.00	0.00
TRINITY_GAQ79830	dipeptid	Klebsormidium_niten	44.00	0.00
TRINITY_GAQ85561	hypothet	Klebsormidium_niten	44.00	0.00
TRINITY_XP_02435	cAMP-dep	Physcomitrella_pate	44.00	0.00
TRINITY_XP_02164	ADP-ribo	Hevea_brasiliensis	44.00	0.00
TRINITY_XP_02164	indole-3	Hevea_brasiliensis	44.00	0.00
TRINITY_RAL45204	hypothet	Cuscuta_australis	44.00	0.00
TRINITY_PSC69139	spindle	Micractinium_conduc	44.00	0.00

TRINITY_XP_0238	probable	Quercus_suber	44.00	0.00
TRINITY_KXZ4895	hypothet	Gonium_pectorale	44.00	0.00
TRINITY_XP_0017	sulfite	Chlamydomonas_reinh	44.00	0.00
TRINITY_ABK2476	unknown	Picea_sitchensis	44.00	0.00
TRINITY_XP_0165	PREDICTE	Capsicum_annuum	44.00	0.00
TRINITY_GAX8393	hypothet	Chlamydomonas_eusti	44.00	0.00
TRINITY_XP_0017	predicte	Chlamydomonas_reinh	44.00	0.00
TRINITY_XP_0016	rhodanes	Chlamydomonas_reinh	44.00	0.00
TRINITY_ONK5916	uncharac	Asparagus_officinal	44.00	0.00
TRINITY_PNW8559	hypothet	Chlamydomonas_reinh	44.00	0.00

TRINITY_KXZ54718	hypothet	Gonium_pectorale	44.00	0.00
TRINITY_GAX75460	hypothet	Chlamydomonas_eusti	44.00	0.00
TRINITY_PNW78396	hypothet	Chlamydomonas_reinh	44.00	0.00
TRINITY_GAQ87770	Membrane	Klebsormidium_niten	44.00	0.00
TRINITY_GAX73064	hypothet	Chlamydomonas_eusti	44.00	0.00
TRINITY_GAX84988	hypothet	Chlamydomonas_eusti	44.00	0.00
TRINITY_XP_00169	predicte	Chlamydomonas_reinh	44.00	0.00
TRINITY_GAQ79816	Sec23 p	Klebsormidium_niten	44.00	0.00
TRINITY_XP_01069	PREDICTE	Beta_vulgaris	44.00	0.00
TRINITY_XP_01649	PREDICTE	Nicotiana_tabacum	44.00	0.00
TRINITY_RAL54334	hypothet	Cuscuta_australis	44.00	0.00
TRINITY_GAX72866	hypothet	Chlamydomonas_eusti	44.00	0.00
TRINITY_XP_00170	flagella	Chlamydomonas_reinh	44.00	0.00
TRINITY_XP_00295	hypothet	Volvox_carteri	44.00	0.00
TRINITY_RMZ53779	hypothet	Auxenochlorella_pro	44.00	0.00
TRINITY_XP_00168	predicte	Chlamydomonas_reinh	44.00	0.00
TRINITY_XP_01023	E3 ubiqu	Brachypodium_distac	44.00	0.00
TRINITY_GAX84594	hypothet	Chlamydomonas_eusti	44.00	0.00
TRINITY_AAK98797	BONZAI1	Arabidopsis_thalian	44.00	0.00
TRINITY_XP_00638	40S ribo	Populus_trichocarpa	44.00	0.00
TRINITY_XP_00169	glycosyl	Chlamydomonas_reinh	44.00	0.00
TRINITY_XP_01206	chloride	Jatropha_curcas	44.00	0.00
TRINITY_XP_00295	hypothet	Volvox_carteri	44.00	0.00
TRINITY_XP_02438	cysteine	Physcomitrella_pate	44.00	0.00
TRINITY_XP_01589	cysteine	Ziziphus_jujuba	44.00	0.00
TRINITY_GBG68282	hypothet	Chara_braunii	44.00	0.00
TRINITY_XP_01108	GPN-loop	Sesamum_indicum	44.00	0.00
TRINITY_GAX76469	hypothet	Chlamydomonas_eusti	44.00	0.00
TRINITY_XP_02435	riboflav	Physcomitrella_pate	44.00	0.00
TRINITY_GAX75028	hypothet	Chlamydomonas_eusti	44.00	0.00
TRINITY_PSC76703	hypothet	Micractinium_conduc	44.00	0.00
TRINITY_XP_00564	hypothet	Coccomyxa_subellips	44.00	0.00
TRINITY_XP_00169	predicte	Chlamydomonas_reinh	44.00	0.00
TRINITY_GBF96595	hypothet	Raphidocelis_subcap	44.00	0.00
TRINITY_GAQ80193	serine/t	Klebsormidium_niten	44.00	0.00
TRINITY_GAX79898	hypothet	Chlamydomonas_eusti	44.00	0.00
TRINITY_GBG87427	hypothet	Chara_braunii	44.00	0.00
TRINITY_PSC73152	aldehyde	Micractinium_conduc	44.00	0.00
TRINITY_GAX78628	hypothet	Chlamydomonas_eusti	44.00	0.00
TRINITY_GAQ88892	aminopep	Klebsormidium_niten	44.00	0.00
TRINITY_XP_00720	uncharac	Prunus_persica	44.00	0.00
TRINITY_XP_00294	basal bo	Volvox_carteri	44.00	0.00
TRINITY_GAX80378	hypothet	Chlamydomonas_eusti	44.00	0.00
TRINITY_XP_02390	kinesin	Quercus_suber	44.00	0.00
TRINITY_RID71494	hypothet	Brassica_rapa	44.00	0.00
TRINITY_PNW87342	hypothet	Chlamydomonas_reinh	44.00	0.00
TRINITY_XP_01743	PREDICTE	Vigna_angularis	44.00	0.00
TRINITY_BBA27223	DYX1C1-l	Chlamydomonas_reinh	44.00	0.00
TRINITY_XP_00295	hypothet	Volvox_carteri	44.00	0.00
TRINITY_GAX73373	hypothet	Chlamydomonas_eusti	44.00	0.00
TRINITY_XP_00564	hypothet	Coccomyxa_subellips	44.00	0.00
TRINITY_XP_00294	hypothet	Volvox_carteri	44.00	0.00
TRINITY_XP_01389	lysine N	Monoraphidium_negle	44.00	0.00
TRINITY_GAX75744	hypothet	Chlamydomonas_eusti	44.00	0.00
TRINITY_OAE18600	hypothet	Marchantia_polymorp	44.00	0.00
TRINITY_XP_00294	RWP-RK c	Volvox_carteri	44.00	0.00
TRINITY_GAX75027	hypothet	Chlamydomonas_eusti	44.00	0.00
TRINITY_KXZ48405	hypothet	Gonium_pectorale	44.00	0.00
TRINITY_XP_00564	pseudour	Coccomyxa_subellips	44.00	0.00
TRINITY_GAX72920	hypothet	Chlamydomonas_eusti	44.00	0.00
TRINITY_KMS94413	hypothet	Beta_vulgaris	44.00	0.00
TRINITY_EEF22619	conserve	Ricinus_communis	44.00	0.00
TRINITY_XP_00250	dynein-1	Micromonas_commoda	44.00	0.00
TRINITY_PSC74229	ADP-ribo	Micractinium_conduc	43.90	0.00
TRINITY_PNW72281	hypothet	Chlamydomonas_reinh	43.90	0.00
TRINITY_EOA39665	hypothet	Capsella_rubella	43.90	0.00

TRINITY_XP_0102	VAMP-li	Brachypodium_distac	43.90	0.00
TRINITY_RXH8695	hypothet	Malus_domestica	43.90	0.00
TRINITY_RMZ5547	hypothet	Auxenochlorella_pro	43.90	0.00
TRINITY_XP_0220	cell nu	Helianthus_annuus	43.90	0.00
TRINITY_XP_0239	hypoxant	Quercus_suber	43.90	0.00
TRINITY_XP_0017	small r	Chlamydomonas_reinh	43.90	0.00
TRINITY_XP_0086	uncharac	Zea_mays	43.90	0.00
TRINITY_XP_0190	PREDICT	Nelumbo_nucifera	43.90	0.00
TRINITY_RQL7594	hypothet	Brassica_cretica	43.90	0.00
TRINITY_RWR8891	Methylt	Cinnamomum_micranth	43.90	0.00
TRINITY_PSC6925	ammoniu	Micractinium_conduc	43.90	0.00
TRINITY_PNR2930	hypothet	Physcomitrella_pate	43.90	0.00
TRINITY_PTQ3293	hypothet	Marchantia_polymorp	43.90	0.00
TRINITY_OVA0016	WD40 re	Macleaya_cordata	43.90	0.00
TRINITY_GBF9921	hypothet	Raphidocelis_subcap	43.90	0.00
TRINITY_XP_0058	hypothet	Chlorella_variabili	43.90	0.00
TRINITY_RWW9034	hypothet	Ensete_ventricosum	43.90	0.00
TRINITY_XP_0239	histone-	Quercus_suber	43.90	0.00
TRINITY_GAX7822	hypothet	Chlamydomonas_eusti	43.90	0.00
TRINITY_PNH1190	Phospho	Tetrabaena_socialis	43.90	0.00
TRINITY_XP_0216	non-spe	Manihot_esculenta	43.90	0.00
TRINITY_PSC7639	Jumonji	Micractinium_conduc	43.90	0.00
TRINITY_OMO5302	BTB/POZ-	Corchorus_olitorius	43.90	0.00
TRINITY_XP_0136	dual sp	Brassica_napus	43.90	0.00
TRINITY_KXZ5504	hypothet	Gonium_pectorale	43.90	0.00
TRINITY_PTQ3502	hypothet	Marchantia_polymorp	43.90	0.00
TRINITY_GAX8519	hypothet	Chlamydomonas_eusti	43.90	0.00
TRINITY_XP_0237	protein	Lactuca_sativa	43.90	0.00
TRINITY_XP_0029	hypothet	Volvox_carteri	43.90	0.00
TRINITY_GAX8154	hypothet	Chlamydomonas_eusti	43.90	0.00
TRINITY_XP_0029	hypothet	Volvox_carteri	43.90	0.00
TRINITY_XP_0239	glutamat	Quercus_suber	43.90	0.00
TRINITY_PTQ3200	hypothet	Marchantia_polymorp	43.90	0.00
TRINITY_XP_0029	hypothet	Volvox_carteri	43.90	0.00
TRINITY_OVA1673	Galacto	Macleaya_cordata	43.90	0.00
TRINITY_XP_0016	EMP/non	Chlamydomonas_reinh	43.90	0.00
TRINITY_KXZ4698	hypothet	Gonium_pectorale	43.90	0.00
TRINITY_XP_0029	probabl	Selaginella_moellen	43.90	0.00
TRINITY_GAX8263	hypothet	Chlamydomonas_eusti	43.90	0.00
TRINITY_XP_0029	hypothet	Volvox_carteri	43.90	0.00
TRINITY_EFJ3330	hypothet	Selaginella_moellen	43.90	0.00
TRINITY_PHT4237	hypothet	Capsicum_baccatum	43.90	0.00
TRINITY_XP_0218	26S pro	Carica_papaya	43.90	0.00
TRINITY_KXZ5147	hypothet	Gonium_pectorale	43.90	0.00
TRINITY_PNH0880	ADP-rib	Tetrabaena_socialis	43.90	0.00
TRINITY_XP_0056	hypothet	Coccomyxa_subellips	43.90	0.00
TRINITY_XP_0110	PREDICT	Populus_euphratica	43.90	0.00
TRINITY_XP_0056	calcium-	Coccomyxa_subellips	43.90	0.00
TRINITY_GAX7717	hypothet	Chlamydomonas_eusti	43.90	0.00
TRINITY_GAX8338	hypothet	Chlamydomonas_eusti	43.90	0.00
TRINITY_XP_0017	Zeta2-C	Chlamydomonas_reinh	43.90	0.00
TRINITY_GBF8753	hypothet	Raphidocelis_subcap	43.90	0.00
TRINITY_PNH1178	Heat sh	Tetrabaena_socialis	43.90	0.00
TRINITY_XP_0014	predict	Ostreococcus_sp._'l	43.90	0.00
TRINITY_GAX7412	hypothet	Chlamydomonas_eusti	43.90	0.00
TRINITY_GAX8389	hypothet	Chlamydomonas_eusti	43.90	0.00
TRINITY_PNW8534	hypothet	Chlamydomonas_reinh	43.90	0.00
TRINITY_RYR6719	hypothet	Arachis_hypogaea	43.90	0.00
TRINITY_XP_0035	60S rib	Brachypodium_distac	43.90	0.00
TRINITY_BAJ9981	predict	Hordeum_vulgare	43.90	0.00
TRINITY_EFH6827	predict	Arabidopsis_lyrata	43.80	0.00
TRINITY_EFH4134	glycosy	Arabidopsis_lyrata	43.80	0.00
TRINITY_XP_0245	zinc fi	Selaginella_moellen	43.80	0.00
TRINITY_RZB7894	Phospho	Glycine_soja	43.80	0.00
TRINITY_EFJ2158	hypothet	Selaginella_moellen	43.80	0.00
TRINITY_XP_0217	CBL-int	Chenopodium_quinoa	43.80	0.00

TRINITY_XP_0270{	uncharac	Coffea_arabica	43.80	0.00
TRINITY_GAX7530{	hypothet	Chlamydomonas_eusti	43.80	0.00
TRINITY_XP_0094(	PREDICTE	Musa_acuminata	43.80	0.00
TRINITY_GAQ8477	Sensory	Klebsormidium_niten	43.80	0.00
TRINITY_XP_0028{	basic t	Arabidopsis_lyrata	43.80	0.00
TRINITY_XP_0058{	hypothet	Chlorella_variabili	43.80	0.00
TRINITY_XP_0029	hypothet	Volvox_carteri	43.80	0.00
TRINITY_KXZ4165(	hypothet	Gonium_pectorale	43.80	0.00
TRINITY_KXZ4353(	hypothet	Gonium_pectorale	43.80	0.00
TRINITY_PNW8839	hypothet	Chlamydomonas_reinh	43.80	0.00
TRINITY_PNH1169	hypothet	Tetrabaena_socialis	43.80	0.00
TRINITY_XP_0229	26S prot	Cucurbita_maxima	43.80	0.00
TRINITY_GAQ9194(	hypothet	Klebsormidium_niten	43.80	0.00
TRINITY_GAQ8185{	DNA-dire	Klebsormidium_niten	43.80	0.00
TRINITY_KXZ5613	hypothet	Gonium_pectorale	43.80	0.00
TRINITY_PSC7207(	L-isoasp	Micractinium_conduc	43.80	0.00
TRINITY_PNW8407	hypothet	Chlamydomonas_reinh	43.80	0.00
TRINITY_XP_0227	type IV	Durio_zibethinus	43.80	0.00
TRINITY_XP_0137	ras-rela	Brassica_napus	43.80	0.00
TRINITY_XP_0239	kynureni	Quercus_suber	43.80	0.00
TRINITY_BAX2519	centroso	Chlamydomonas_reinh	43.80	0.00
TRINITY_XP_0016	histone	Chlamydomonas_reinh	43.80	0.00
TRINITY_XP_0100{	17.5 kDa	Morus_notabilis	43.80	0.00
TRINITY_RWW7997{	hypothet	Ensete_ventricosum	43.80	0.00
TRINITY_EOY0582	Autoinhi	Theobroma_cacao	43.80	0.00
TRINITY_XP_0213	regulato	Sorghum_bicolor	43.80	0.00
TRINITY_XP_0016	predicte	Chlamydomonas_reinh	43.80	0.00
TRINITY_XP_0194	PREDICTE	Lupinus_angustifoli	43.80	0.00
TRINITY_OTG2965	putative	Helianthus_annuus	43.80	0.00
TRINITY_PNH0484	Peroxiso	Tetrabaena_socialis	43.80	0.00
TRINITY_KXZ5060	hypothet	Gonium_pectorale	43.80	0.00
TRINITY_GAX8071	hypothet	Chlamydomonas_eusti	43.80	0.00
TRINITY_XP_0245	U3 snoRN	Selaginella_moellen	43.80	0.00
TRINITY_PNW8040	hypothet	Chlamydomonas_reinh	43.80	0.00
TRINITY_KXZ5696	hypothet	Gonium_pectorale	43.80	0.00
TRINITY_XP_0238{	protein	Quercus_suber	43.80	0.00
TRINITY_XP_0017(	componen	Chlamydomonas_reinh	43.80	0.00
TRINITY_XP_0084	PREDICTE	Cucumis_melo	43.80	0.00
TRINITY_PWA9380	alcohol	Artemisia_annua	43.80	0.00
TRINITY_PSC6759	Gag-Pol	Micractinium_conduc	43.80	0.00
TRINITY_GAX7778	hypothet	Chlamydomonas_eusti	43.80	0.00
TRINITY_XP_0029	hypothet	Volvox_carteri	43.80	0.00
TRINITY_XP_0042	PREDICTE	Fragaria_vesca	43.80	0.00
TRINITY_GAX8645	hypothet	Chlamydomonas_eusti	43.80	0.00
TRINITY_GAQ8093	short ch	Klebsormidium_niten	43.80	0.00
TRINITY_GAX7314	hypothet	Chlamydomonas_eusti	43.80	0.00
TRINITY_GAX7401(	hypothet	Chlamydomonas_eusti	43.80	0.00
TRINITY_KMZ5923	Regulato	Zostera_marina	43.80	0.00
TRINITY_GAX8156	hypothet	Chlamydomonas_eusti	43.80	0.00
TRINITY_AES7312	SNF1-rel	Medicago_truncatula	43.80	0.00
TRINITY_PNW7067	hypothet	Chlamydomonas_reinh	43.80	0.00
TRINITY_XP_0139(	hypothet	Monoraphidium_negle	43.80	0.00
TRINITY_KMZ6047	DNA cros	Zostera_marina	43.80	0.00
TRINITY_GAX8388(	hypothet	Chlamydomonas_eusti	43.80	0.00
TRINITY_XP_0029	hypothet	Volvox_carteri	43.80	0.00
TRINITY_PNH1129	NifU-lik	Tetrabaena_socialis	43.80	0.00
TRINITY_GAQ8280	Sec1 fam	Klebsormidium_niten	43.80	0.00
TRINITY_KDD7145{	protein	Helicosporidium_sp.	43.80	0.00
TRINITY_XP_0058	hypothet	Chlorella_variabili	43.80	0.00
TRINITY_XP_0025(	predicte	Micromonas_commoda	43.80	0.00
TRINITY_PTQ3451{	hypothet	Marchantia_polymorp	43.80	0.00
TRINITY_GAX7773	hypothet	Chlamydomonas_eusti	43.80	0.00
TRINITY_XP_0228(	phospho	Olea_europaea	43.80	0.00
TRINITY_PNH1283(	hypothet	Tetrabaena_socialis	43.80	0.00
TRINITY_GAX7544	hypothet	Chlamydomonas_eusti	43.80	0.00
TRINITY_GAX8337	hypothet	Chlamydomonas_eusti	43.80	0.00

TRINITY_KXZ4215(	hypothet	Gonium_pectorale	43.80	0.00
TRINITY_KXZ4653(	hypothet	Gonium_pectorale	43.80	0.00
TRINITY_XP_0114(	PREDICTI	Fragaria_vesca	43.80	0.00
TRINITY_XP_0191(	PREDICTI	Ipomoea_nil	43.80	0.00
TRINITY_KXZ4595(	hypothet	Gonium_pectorale	43.80	0.00
TRINITY_XP_0186(	PREDICTI	Nicotiana_tomentosi	43.80	0.00
TRINITY_OVA0205(	Alcohol	Macleaya_cordata	43.80	0.00
TRINITY_PNW8716(	hypothet	Chlamydomonas_reinh	43.80	0.00
TRINITY_XP_0029(	RWP-RK (	Volvox_carteri	43.80	0.00
TRINITY_GAX7483(	hypothet	Chlamydomonas_eusti	43.80	0.00
TRINITY_GAX7513(	hypothet	Chlamydomonas_eusti	43.80	0.00
TRINITY_GAX7670(	hypothet	Chlamydomonas_eusti	43.80	0.00
TRINITY_GAX7726(	hypothet	Chlamydomonas_eusti	43.80	0.00
TRINITY_XP_0029(	flagella	Volvox_carteri	43.80	0.00
TRINITY_KGN5877(	hypothet	Cucumis_sativus	43.80	0.00
TRINITY_GAX8265(	hypothet	Chlamydomonas_eusti	43.80	0.00
TRINITY_BAJ9847(	predicte	Hordeum_vulgare	43.80	0.00
TRINITY_GAX8241(	hypothet	Chlamydomonas_eusti	43.80	0.00
TRINITY_GBF9460(	hypothet	Raphidocelis_subcap	43.80	0.00
TRINITY_XP_0016(	predicte	Chlamydomonas_reinh	43.80	0.00
TRINITY_GAX7735(	hypothet	Chlamydomonas_eusti	43.80	0
TRINITY_KXZ5144(	hypothet	Gonium_pectorale	43.80	0.00
TRINITY_GAX7886(	hypothet	Chlamydomonas_eusti	43.80	0.00
TRINITY_GBF8774(	hypothet	Raphidocelis_subcap	43.80	0.00
TRINITY_XP_0158(	NADH--cy	Ziziphus_jujuba	43.80	0.00
TRINITY_XP_0029(	wee1 kir	Volvox_carteri	43.70	0.00
TRINITY_ESR3242(	hypothet	Citrus_clementina	43.70	0.00
TRINITY_GAX7455(	hypothet	Chlamydomonas_eusti	43.70	0.00
TRINITY_GAQ9000(	Serine (	Klebsormidium_niten	43.70	0.00
TRINITY_ABR1774(	unknown	Picea_sitchensis	43.70	0.00
TRINITY_EFJ2455(	hypothet	Selaginella_moellen	43.70	0.00
TRINITY_GAQ8265(	RNA-binc	Klebsormidium_niten	43.70	0.00
TRINITY_EEF2267(	two-comp	Ricinus_communis	43.70	0.00
TRINITY_KMZ5846(	Cysteine	Zostera_marina	43.70	0.00
TRINITY_GAX7711(	hypothet	Chlamydomonas_eusti	43.70	0.00
TRINITY_XP_0029(	annexin	Selaginella_moellen	43.70	0.00
TRINITY_XP_0238(	cytochrc	Quercus_suber	43.70	0.00
TRINITY_KXZ5438(	hypothet	Gonium_pectorale	43.70	0.00
TRINITY_XP_0029(	hypothet	Volvox_carteri	43.70	0.00
TRINITY_XP_0064(	ubiquit:	Eutrema_salsugineum	43.70	0.00
TRINITY_GBG7841(	hypothet	Chara_braunii	43.70	0.00
TRINITY_XP_0172(	PREDICTI	Daucus_carota	43.70	0.00
TRINITY_XP_0056(	hypothet	Coccomyxa_subellips	43.70	0.00
TRINITY_XP_0139(	hypothet	Monoraphidium_negle	43.70	0.00
TRINITY_PNW7758(	hypothet	Chlamydomonas_reinh	43.70	0.00
TRINITY_KXZ4178(	hypothet	Gonium_pectorale	43.70	0.00
TRINITY_XP_0239(	metacasp	Quercus_suber	43.70	0.00
TRINITY_GBF9482(	hypothet	Raphidocelis_subcap	43.70	0.00
TRINITY_GAV8244(	WD40 dor	Cephalotus_follicul	43.70	0.00
TRINITY_GAX8538(	hypothet	Chlamydomonas_eusti	43.70	0.00
TRINITY_XP_0104(	PREDICTI	Camelina_sativa	43.70	0.00
TRINITY_GBF9889(	hypothet	Raphidocelis_subcap	43.70	0.00
TRINITY_XP_0030(	predicte	Micromonas_pusilla	43.70	0.00
TRINITY_OUS4464(	aldolase	Ostreococcus_tauri	43.70	0.00
TRINITY_BAK0277(	predicte	Hordeum_vulgare	43.70	0.00
TRINITY_GAX8269(	hypothet	Chlamydomonas_eusti	43.70	0.00
TRINITY_XP_0063(	probable	Capsella_rubella	43.70	0.00
TRINITY_PNW7228(	hypothet	Chlamydomonas_reinh	43.70	0.00
TRINITY_PNW8199(	hypothet	Chlamydomonas_reinh	43.70	0.00
TRINITY_PNH0891(	E3 ubiqu	Tetrabaena_socialis	43.70	0.00
TRINITY_XP_0124(	PREDICTI	Gossypium_raimondii	43.70	0.00
TRINITY_XP_0016(	predicte	Chlamydomonas_reinh	43.70	0.00
TRINITY_PNH1272(	Glycosyl	Tetrabaena_socialis	43.70	0.00
TRINITY_GAX8297(	hypothet	Chlamydomonas_eusti	43.70	0.00
TRINITY_GAX7600(	hypothet	Chlamydomonas_eusti	43.70	0.00
TRINITY_PNH1269(	Protein	Tetrabaena_socialis	43.70	0.00

TRINITY_GAX7688	hypothet	Chlamydomonas_eusti	43.70	0.00
TRINITY_PTQ4850	hypothet	Marchantia_polymorp	43.70	0.00
TRINITY_XP_0029	mitochon	Volvox_carteri	43.70	0.00
TRINITY_GBF9160	hypothet	Raphidocelis_subcap	43.70	0.00
TRINITY_GAX7633	hypothet	Chlamydomonas_eusti	43.70	0.00
TRINITY_PTQ5032	hypothet	Marchantia_polymorp	43.70	0.00
TRINITY_XP_0029	hypothet	Volvox_carteri	43.70	0.00
TRINITY_GAX7625	hypothet	Chlamydomonas_eusti	43.70	0.00
TRINITY_XP_0201	probable	Ananas_comosus	43.70	0.00
TRINITY_GAX8549	hypothet	Chlamydomonas_eusti	43.70	0.00
TRINITY_KXZ4432	hypothet	Gonium_pectorale	43.70	0.00
TRINITY_XP_0239	116 kDa	Quercus_suber	43.70	0.00
TRINITY_GBG0053	splicing	Raphidocelis_subcap	43.70	0.00
TRINITY_EFJ1207	hypothet	Selaginella_moellen	43.70	0
TRINITY_PNW8324	hypothet	Chlamydomonas_reinh	43.70	0.00
TRINITY_GAX7877	hypothet	Chlamydomonas_eusti	43.70	0.00
TRINITY_GAX8447	hypothet	Chlamydomonas_eusti	43.70	0.00
TRINITY_XP_0029	hypothet	Volvox_carteri	43.70	0.00
TRINITY_XP_0109	PREDICTE	Elaeis_guineensis	43.70	0.00
TRINITY_RRT6141	hypothet	Ensete_ventricosum	43.70	0.00
TRINITY_EFH6442	hypothet	Arabidopsis_lyrata	43.70	0.00
TRINITY_PON6686	P-type	Parasponia_anderson	43.70	0.00
TRINITY_GAX8457	hypothet	Chlamydomonas_eusti	43.70	0.00
TRINITY_BAJ9668	predicte	Hordeum_vulgare	43.70	0.00
TRINITY_KXZ4331	hypothet	Gonium_pectorale	43.70	0.00
TRINITY_XP_0056	acyl-CoA	Coccomyxa_subellips	43.70	0.00
TRINITY_GAX8124	hypothet	Chlamydomonas_eusti	43.70	0.00
TRINITY_XP_0016	predicte	Chlamydomonas_reinh	43.70	0.00
TRINITY_XP_0202	unchara	Cajanus_cajan	43.70	0.00
TRINITY_GAX8367	hypothet	Chlamydomonas_eusti	43.70	0.00
TRINITY_GAX7453	hypothet	Chlamydomonas_eusti	43.70	0.00
TRINITY_GAX7334	hypothet	Chlamydomonas_eusti	43.70	0.00
TRINITY_XP_0239	cytochro	Quercus_suber	43.60	0.00
TRINITY_XP_0194	PREDICTE	Lupinus_angustifoli	43.60	0.00
TRINITY_EEF2444	Outer me	Ricinus_communis	43.60	0.00
TRINITY_BAJ9317	predicte	Hordeum_vulgare	43.60	0.00
TRINITY_PSC7675	general	Micractinium_conduc	43.60	0.00
TRINITY_XP_0113	Protein	Auxenochlorella_pro	43.60	0.00
TRINITY_GAQ7789	photolya	Klebsormidium_niten	43.60	0.00
TRINITY_GAX7386	hypothet	Chlamydomonas_eusti	43.60	0.00
TRINITY_XP_0238	probable	Quercus_suber	43.60	0.00
TRINITY_XP_0139	hypothet	Monoraphidium_negle	43.60	0.00
TRINITY_PRW5914	nad-depe	Chlorella_sorokinia	43.60	0.00
TRINITY_XP_0035	serine/t	Glycine_max	43.60	0.00
TRINITY_RID6792	hypothet	Brassica_rapa	43.60	0.00
TRINITY_GAX8518	hypothet	Chlamydomonas_eusti	43.60	0.00
TRINITY_XP_0139	hypothet	Monoraphidium_negle	43.60	0.00
TRINITY_XP_0016	nucleola	Chlamydomonas_reinh	43.60	0.00
TRINITY_XP_0239	multifun	Quercus_suber	43.60	0.00
TRINITY_AAC0426	(S)-aden	Zea_mays	43.60	0.00
TRINITY_XP_0029	hypothet	Volvox_carteri	43.60	0.00
TRINITY_OVA0373	RNA reco	Macleaya_cordata	43.60	0.00
TRINITY_XP_0270	unchara	Coffea_arabica	43.60	0.00
TRINITY_GBG0018	ATP-depe	Raphidocelis_subcap	43.60	0.00
TRINITY_GAX7642	hypothet	Chlamydomonas_eusti	43.60	0.00
TRINITY_GBG7421	hypothet	Chara_braunii	43.60	0.00
TRINITY_GBF9277	hypothet	Raphidocelis_subcap	43.60	0.00
TRINITY_PTQ3292	hypothet	Marchantia_polymorp	43.60	0.00
TRINITY_XP_0066	PREDICTE	Oryza_brachyantha	43.60	0.00
TRINITY_XP_0017	subunit	Chlamydomonas_reinh	43.60	0.00
TRINITY_XP_0025	predicte	Micromonas_commoda	43.60	0.00
TRINITY_GAQ8413	hypothet	Klebsormidium_niten	43.60	0.00
TRINITY_OAY6262	Importin	Ananas_comosus	43.60	0.00
TRINITY_GAQ8719	hypothet	Klebsormidium_niten	43.60	0.00
TRINITY_GAQ8153	hypothet	Klebsormidium_niten	43.60	0.00
TRINITY_KFK3422	hypothet	Arabis_alpina	43.60	0.00

TRINITY_KXZ5515]	hypothet	Gonium_pectorale	43.60	0.00
TRINITY_GAX7281	hypothet	Chlamydomonas_eusti	43.60	0.00
TRINITY_PRW5832	c trans	Chlorella_sorokinia	43.60	0.00
TRINITY_XP_0245	dynein	Selaginella_moellen	43.60	0.00
TRINITY_GAQ8635	GTPase-a	Klebsormidium_niten	43.60	0.00
TRINITY_PNH0256	Protein	Tetrabaena_socialis	43.60	0.00
TRINITY_KMZ7496	DNA poly	Zostera_marina	43.60	0.00
TRINITY_GAQ8744	Rhomboid	Klebsormidium_niten	43.60	0.00
TRINITY_GAX7742	hypothet	Chlamydomonas_eusti	43.60	0.00
TRINITY_GAX8392	hypothet	Chlamydomonas_eusti	43.60	0.00
TRINITY_XP_0017	tryptoph	Chlamydomonas_reinh	43.60	0.00
TRINITY_PRW5753	Ras-rela	Chlorella_sorokinia	43.60	0.00
TRINITY_PTQ3454	hypothet	Marchantia_polymorp	43.60	0.00
TRINITY_KXZ4679	hypothet	Gonium_pectorale	43.60	0.00
TRINITY_XP_0017	predicte	Chlamydomonas_reinh	43.60	0.00
TRINITY_XP_0016	caroten	Chlamydomonas_reinh	43.60	0.00
TRINITY_GBF8961	hypothet	Raphidocelis_subcap	43.60	0.00
TRINITY_XP_0029	hypothet	Volvox_carteri	43.60	0.00
TRINITY_XP_0264	protein	Papaver_somniferum	43.60	0.00
TRINITY_PSC7334	tRNA wyb	Micractinium_conduc	43.60	0.00
TRINITY_GAX7644	hypothet	Chlamydomonas_eusti	43.60	0.00
TRINITY_GAQ7908	hypothet	Klebsormidium_niten	43.60	0.00
TRINITY_XP_0029	hypothet	Volvox_carteri	43.60	0.00
TRINITY_XP_0104	PREDICTE	Camelina_sativa	43.60	0.00
TRINITY_OAE3096	hypothet	Marchantia_polymorp	43.60	0.00
TRINITY_KXZ5278	hypothet	Gonium_pectorale	43.60	0.00
TRINITY_GAX8179	hypothet	Chlamydomonas_eusti	43.60	0.00
TRINITY_KXZ5302	hypothet	Gonium_pectorale	43.60	0.00
TRINITY_GAX8484	hypothet	Chlamydomonas_eusti	43.60	0.00
TRINITY_GAX8279	hypothet	Chlamydomonas_eusti	43.60	0.00
TRINITY_PNW8526	hypothet	Chlamydomonas_reinh	43.60	0.00
TRINITY_EFJ2069	hypothet	Selaginella_moellen	43.60	0.00
TRINITY_OAE1901	hypothet	Marchantia_polymorp	43.60	0.00
TRINITY_RXH8938	hypothet	Malus_domestica	43.60	0.00
TRINITY_PNW8405	hypothet	Chlamydomonas_reinh	43.60	0.00
TRINITY_GAX8407	hypothet	Chlamydomonas_eusti	43.60	0.00
TRINITY_GAQ8357	RNA liga	Klebsormidium_niten	43.60	0.00
TRINITY_GBF9427	dual-spe	Raphidocelis_subcap	43.60	0.00
TRINITY_XP_0025	predicte	Micromonas_commoda	43.60	0.00
TRINITY_NP_1943	chloropl	Arabidopsis_thalian	43.60	0.00
TRINITY_GAX7490	hypothet	Chlamydomonas_eusti	43.60	0.00
TRINITY_PRW0585	copine-3	Chlorella_sorokinia	43.60	0.00
TRINITY_BAK0236	predicte	Hordeum_vulgare	43.60	0.00
TRINITY_XP_0029	hypothet	Volvox_carteri	43.60	0.00
TRINITY_XP_0104	PREDICTE	Camelina_sativa	43.60	0.00
TRINITY_XP_0014	predicte	Ostreococcus_sp._'l	43.60	0.00
TRINITY_XP_0024	ras-rela	Micromonas_commoda	43.50	0.00
TRINITY_XP_0216	potassiu	Hevea_brasiliensis	43.50	0.00
TRINITY_PIN1220	hypothet	Handroanthus_impeti	43.50	0.00
TRINITY_XP_0191	PREDICTE	Ipomoea_nil	43.50	0.00
TRINITY_XP_0205	uncharac	Phalaenopsis_equest	43.50	0.00
TRINITY_GAQ8842	Putative	Klebsormidium_niten	43.50	0.00
TRINITY_KCW5078	hypothet	Eucalyptus_grandis	43.50	0.00
TRINITY_GBG9229	hypothet	Chara_braunii	43.50	0.00
TRINITY_XP_0239	probable	Quercus_suber	43.50	0.00
TRINITY_XP_0042	major fa	Solanum_lycopersicu	43.50	0.00
TRINITY_RWW9047	hypothet	Ensete_ventricosum	43.50	0.00
TRINITY_XP_0243	MAPK/MAK	Physcomitrella_pate	43.50	0.00
TRINITY_XP_0161	serine/t	Arachis_ipaensis	43.50	0.00
TRINITY_PRW6043	ATP-depe	Chlorella_sorokinia	43.50	0.00
TRINITY_XP_0238	actin cy	Quercus_suber	43.50	0.00
TRINITY_GBF9624	adenylat	Raphidocelis_subcap	43.50	0.00
TRINITY_XP_0105	PREDICTE	Tarenaya_hasslerian	43.50	0.00
TRINITY_GAX7555	hypothet	Chlamydomonas_eusti	43.50	0.00
TRINITY_XP_0228	ATPase,	Ostreococcus_tauri	43.50	0.00
TRINITY_OEL3581	Actin-1	Dichanthelium_oligo	43.50	0.00

TRINITY_XP_00564	ARM repe	Coccomyxa_subellips	43.50	0.00
TRINITY_XP_00169	predicte	Chlamydomonas_reinh	43.50	0.00
TRINITY_GBF99583	hypothet	Raphidocelis_subcap	43.50	0.00
TRINITY_XP_02643	RNA heli	Papaver_somniferum	43.50	0.00
TRINITY_GAQ88406	RNA heli	Klebsormidium_niten	43.50	0.00
TRINITY_XP_00666	PREDICTE	Oryza_brachyantha	43.50	0.00
TRINITY_XP_01389	hypothet	Monoraphidium_negle	43.50	0.00
TRINITY_ABK22565	unknown	Picea_sitchensis	43.50	0.00
TRINITY_XP_02439	auxin-in	Physcomitrella_pate	43.50	0.00
TRINITY_XP_00584	hypothet	Chlorella_variabili	43.50	0.00
TRINITY_XP_00720	DNA repl	Prunus_persica	43.50	0.00
TRINITY_XP_00169	glycosid	Chlamydomonas_reinh	43.50	0.00
TRINITY_GAX80452	hypothet	Chlamydomonas_eusti	43.50	0.00
TRINITY_KXZ56979	hypothet	Gonium_pectorale	43.50	0.00
TRINITY_PSR85402	NAD-depe	Actinidia_chinensis	43.50	0.00
TRINITY_GAX77778	hypothet	Chlamydomonas_eusti	43.50	0.00
TRINITY_PTQ43879	hypothet	Marchantia_polymorp	43.50	0.00
TRINITY_PIN14056	hypothet	Handroanthus_impeti	43.50	0.00
TRINITY_XP_00295	hypothet	Volvox_carteri	43.50	0.00
TRINITY_GAX78849	hypothet	Chlamydomonas_eusti	43.50	0.00
TRINITY_GAX85463	hypothet	Chlamydomonas_eusti	43.50	0.00
TRINITY_XP_02184	vacuolar	Spinacia_oleracea	43.50	0.00
TRINITY_XP_02453	N-termin	Selaginella_moellen	43.50	0.00
TRINITY_PNW75897	hypothet	Chlamydomonas_reinh	43.50	0.00
TRINITY_XP_00295	hypothet	Volvox_carteri	43.50	0.00
TRINITY_XP_00584	hypothet	Chlorella_variabili	43.50	0.00
TRINITY_KDD71253	hypothet	Helicosporidium_sp.	43.50	0.00
TRINITY_XP_01109	polyaden	Sesamum_indicum	43.50	0.00
TRINITY_PNH08278	putative	Tetrabaena_socialis	43.50	0.00
TRINITY_XP_00979	PREDICTE	Nicotiana_sylvestri	43.50	0.00
TRINITY_XP_02394	erythroc	Quercus_suber	43.50	0.00
TRINITY_OAE20668	hypothet	Marchantia_polymorp	43.50	0.00
TRINITY_KXZ55461	hypothet	Gonium_pectorale	43.50	0.00
TRINITY_GAX83532	hypothet	Chlamydomonas_eusti	43.50	0.00
TRINITY_GAX78062	hypothet	Chlamydomonas_eusti	43.50	0.00
TRINITY_XP_00564	APG9-dom	Coccomyxa_subellips	43.50	0.00
TRINITY_GAX81940	hypothet	Chlamydomonas_eusti	43.50	0.00
TRINITY_GAX84995	hypothet	Chlamydomonas_eusti	43.50	0.00
TRINITY_PPR84446	hypothet	Gossypium_barbadens	43.50	0.00
TRINITY_GAX73618	hypothet	Chlamydomonas_eusti	43.50	0.00
TRINITY_GAX75719	hypothet	Chlamydomonas_eusti	43.50	0.00
TRINITY_GAX77871	hypothet	Chlamydomonas_eusti	43.50	0.00
TRINITY_XP_02293	regulato	Cucurbita_moschata	43.50	0.00
TRINITY_RMZ52372	hypothet	Auxenochlorella_pro	43.50	0.00
TRINITY_KVI00993	D-tyrosy	Cynara_cardunculus	43.40	0.00
TRINITY_KXZ42025	hypothet	Gonium_pectorale	43.40	0.00
TRINITY_XP_00246	DNA mism	Sorghum_bicolor	43.40	0.00
TRINITY_PRW57410	Adenylyl	Chlorella_sorokinia	43.40	0.00
TRINITY_XP_02376	cysteine	Lactuca_sativa	43.40	0.00
TRINITY_KNA17937	hypothet	Spinacia_oleracea	43.40	0.00
TRINITY_OVA15926	hypothet	Macleaya_cordata	43.40	0.00
TRINITY_EOX99153	4-hydrox	Theobroma_cacao	43.40	0.00
TRINITY_B8B7X6.1	RecName:	Oryza_sativa	43.40	0.00
TRINITY_XP_01023	ubiquiti	Brachypodium_distac	43.40	0.00
TRINITY_XP_02792	chaperon	Vigna_unguiculata	43.40	0.00
TRINITY_XP_01389	Serine/t	Monoraphidium_negle	43.40	0.00
TRINITY_XP_02638	isopente	Papaver_somniferum	43.40	0.00
TRINITY_KVH88882	Bacteria	Cynara_cardunculus	43.40	0.00
TRINITY_XP_02392	phosphol	Quercus_suber	43.40	0.00
TRINITY_XP_02197	cullin-3	Helianthus_annuus	43.40	0.00
TRINITY_GBF95268	inosine	Raphidocelis_subcap	43.40	0.00
TRINITY_XP_00298	translin	Selaginella_moellen	43.40	0.00
TRINITY_ONK63418	uncharac	Asparagus_officinal	43.40	0.00
TRINITY_XP_02436	3-phosph	Physcomitrella_pate	43.40	0.00
TRINITY_XP_00636	PREDICTE	Solanum_tuberosum	43.40	0.00
TRINITY_PNW70693	hypothet	Chlamydomonas_reinh	43.40	0.00

TRINITY_PNW82662	hypothet	Chlamydomonas_reinh	43.40	0.00
TRINITY_XP_02058	translat	Phalaenopsis_equest	43.40	0.00
TRINITY_XP_02711	uncharac	Coffea_arabica	43.40	0.00
TRINITY_GAQ87329	stearoyl	Klebsormidium_niten	43.40	0.00
TRINITY_GAX75820	hypothet	Chlamydomonas_eusti	43.40	0.00
TRINITY_GBG82332	hypothet	Chara_braunii	43.40	0.00
TRINITY_XP_00169	predicte	Chlamydomonas_reinh	43.40	0.00
TRINITY_GBG77697	hypothet	Chara_braunii	43.40	0.00
TRINITY_XP_01390	hypothet	Monoraphidium_negle	43.40	0.00
TRINITY_KXZ49643	hypothet	Gonium_pectorale	43.40	0.00
TRINITY_BAJ99217	predicte	Hordeum_vulgare	43.40	0.00
TRINITY_XP_00751	predicte	Bathycoccus_prasino	43.40	0.00
TRINITY_XP_00294	hypothet	Volvox_carteri	43.40	0.00
TRINITY_XP_00295	hypothet	Volvox_carteri	43.40	0.00
TRINITY_KMZ69303	CBL-inte	Zostera_marina	43.40	0.00
TRINITY_XP_00584	hypothet	Chlorella_variabili	43.40	0.00
TRINITY_GBG60178	hypothet	Chara_braunii	43.40	0.00
TRINITY_GAX84471	hypothet	Chlamydomonas_eusti	43.40	0.00
TRINITY_RHN56561	putative	Medicago_truncatula	43.40	0.00
TRINITY_XP_02436	ABC tran	Physcomitrella_pate	43.40	0.00
TRINITY_KXZ47642	hypothet	Gonium_pectorale	43.40	0.00
TRINITY_GAX83638	hypothet	Chlamydomonas_eusti	43.40	0.00
TRINITY_XP_00305	predicte	Micromonas_pusilla	43.40	0.00
TRINITY_PNW81941	hypothet	Chlamydomonas_reinh	43.40	0.00
TRINITY_XP_00497	chaperon	Setaria_italica	43.40	0.00
TRINITY_XP_00714	hypothet	Phaseolus_vulgaris	43.40	0.00
TRINITY_GBG74592	hypothet	Chara_braunii	43.40	0.00
TRINITY_XP_02388	J domain	Quercus_suber	43.40	0.00
TRINITY_XP_01023	transcri	Brachypodium_distac	43.40	0.00
TRINITY_XP_00295	hypothet	Volvox_carteri	43.40	0.00
TRINITY_XP_02059	bifuncti	Phalaenopsis_equest	43.40	0.00
TRINITY_GAX76007	hypothet	Chlamydomonas_eusti	43.40	0.00
TRINITY_KFK30497	hypothet	Arabis_alpina	43.40	0.00
TRINITY_XP_02389	serine/t	Quercus_suber	43.40	0.00
TRINITY_GAX84713	hypothet	Chlamydomonas_eusti	43.40	0.00
TRINITY_XP_02392	erythroc	Quercus_suber	43.40	0.00
TRINITY_XP_02160	glutathi	Manihot_esculenta	43.40	0.00
TRINITY_GAX84615	hypothet	Chlamydomonas_eusti	43.40	0.00
TRINITY_GAX84322	hypothet	Chlamydomonas_eusti	43.40	0.00
TRINITY_XP_00294	hypothet	Volvox_carteri	43.40	0.00
TRINITY_GAX84989	hypothet	Chlamydomonas_eusti	43.40	0.00
TRINITY_XP_00294	hypothet	Volvox_carteri	43.40	0.00
TRINITY_GAX78374	hypothet	Chlamydomonas_eusti	43.40	0.00
TRINITY_GAX76114	hypothet	Chlamydomonas_eusti	43.40	0.00
TRINITY_XP_02351	protein	Cucurbita_pepo	43.40	0.00
TRINITY_GAX82333	hypothet	Chlamydomonas_eusti	43.40	0.00
TRINITY_KXZ54730	hypothet	Gonium_pectorale	43.40	0.00
TRINITY_XP_01052	PREDICTE	Tarenaya_hasslerian	43.40	0.00
TRINITY_OAE24627	hypothet	Marchantia_polymorp	43.40	0.00
TRINITY_XP_01944	PREDICTE	Lupinus_angustifoli	43.40	0.00
TRINITY_KXZ53282	hypothet	Gonium_pectorale	43.40	0.00
TRINITY_GAX83285	hypothet	Chlamydomonas_eusti	43.40	0.00
TRINITY_KXZ55127	hypothet	Gonium_pectorale	43.40	0.00
TRINITY_BAJ11784	dehydrat	Corchorus_olitorius	43.40	0.00
TRINITY_EMS49126	Dihydrof	Triticum_urartu	43.40	0.00
TRINITY_XP_01594	potassiu	Arachis_duranensis	43.30	0.00
TRINITY_GAQ81758	hypothet	Klebsormidium_niten	43.30	0.00
TRINITY_GBG74265	hypothet	Chara_braunii	43.30	0.00
TRINITY_XP_00169	predicte	Chlamydomonas_reinh	43.30	0.00
TRINITY_XP_02389	cytochrc	Quercus_suber	43.30	0.00
TRINITY_XP_01616	serine/t	Arachis_ipaensis	43.30	0.00
TRINITY_GBF97343	hypothet	Raphidocelis_subcap	43.30	0.00
TRINITY_KZM89369	hypothet	Daucus_carota	43.30	0.00
TRINITY_PNW87730	hypothet	Chlamydomonas_reinh	43.30	0.00
TRINITY_XP_00250	predicte	Micromonas_commoda	43.30	0.00
TRINITY_GAQ78227	Serine/t	Klebsormidium_niten	43.30	0.00

TRINITY_PSC7240	M-phase	Micractinium_conduc	43.30	0.00
TRINITY_XP_0191	PREDICTI	Ipomoea_nil	43.30	0.00
TRINITY_NP_0013	uncharac	Zea_mays	43.30	0.00
TRINITY_PNR3024	hypothet	Physcomitrella_pate	43.30	0.00
TRINITY_XP_0238	probable	Quercus_suber	43.30	0.00
TRINITY_XP_0184	PREDICTI	Raphanus_sativus	43.30	0.00
TRINITY_XP_0071	hypothet	Phaseolus_vulgaris	43.30	0.00
TRINITY_GAQ8175	hypothet	Klebsormidium_niten	43.30	0.00
TRINITY_XP_0238	protein	Quercus_suber	43.30	0.00
TRINITY_XP_0243	uncharac	Physcomitrella_pate	43.30	0.00
TRINITY_GAX7492	hypothet	Chlamydomonas_eusti	43.30	0.00
TRINITY_GBG5901	hypothet	Chara_braunii	43.30	0.00
TRINITY_KXZ5196	hypothet	Gonium_pectorale	43.30	0.00
TRINITY_GAX8363	hypothet	Chlamydomonas_eusti	43.30	0.00
TRINITY_PHT2654	hypothet	Capsicum_baccatum	43.30	0.00
TRINITY_PNH0274	Ferredox	Tetrabaena_socialis	43.30	0.00
TRINITY_GAX7900	hypothet	Chlamydomonas_eusti	43.30	0.00
TRINITY_XP_0243	coatomer	Physcomitrella_pate	43.30	0.00
TRINITY_XP_0097	PREDICTI	Nicotiana_sylvestri	43.30	0.00
TRINITY_GAX7260	hypothet	Chlamydomonas_eusti	43.30	0.00
TRINITY_KCW8752	hypothet	Eucalyptus_grandis	43.30	0.00
TRINITY_NP_0013	copper r	Malus_domestica	43.30	0.00
TRINITY_GAX8193	hypothet	Chlamydomonas_eusti	43.30	0.00
TRINITY_GAX7579	hypothet	Chlamydomonas_eusti	43.30	0.00
TRINITY_GBG8531	hypothet	Chara_braunii	43.30	0.00
TRINITY_XP_0258	nitrate	Panicum_hallii	43.30	0.00
TRINITY_PNW7952	hypothet	Chlamydomonas_reinh	43.30	0.00
TRINITY_KXZ5552	hypothet	Gonium_pectorale	43.30	0.00
TRINITY_XP_0217	ABC tran	Chenopodium_quinoa	43.30	0.00
TRINITY_XP_0159	lysosoma	Arachis_duranensis	43.30	0.00
TRINITY_RZC1411	Eukaryot	Glycine_soja	43.30	0.00
TRINITY_XP_0235	dynamin-	Cucurbita_pepo	43.30	0.00
TRINITY_XP_0138	hypothet	Monoraphidium_negle	43.30	0.00
TRINITY_XP_0139	hypothet	Monoraphidium_negle	43.30	0.00
TRINITY_PNW8145	hypothet	Chlamydomonas_reinh	43.30	0.00
TRINITY_GBF9111	hypothet	Raphidocelis_subcap	43.30	0.00
TRINITY_GAX8493	hypothet	Chlamydomonas_eusti	43.30	0.00
TRINITY_XP_0237	serine/t	Lactuca_sativa	43.30	0.00
TRINITY_PUZ5898	hypothet	Panicum_hallii	43.30	0.00
TRINITY_PTQ3167	hypothet	Marchantia_polymorp	43.30	0.00
TRINITY_GAX8511	hypothet	Chlamydomonas_eusti	43.30	0.00
TRINITY_PNW8642	hypothet	Chlamydomonas_reinh	43.30	0.00
TRINITY_GAX7786	hypothet	Chlamydomonas_eusti	43.30	0.00
TRINITY_RAL4773	hypothet	Cuscuta_australis	43.30	0.00
TRINITY_KXZ5065	hypothet	Gonium_pectorale	43.30	0.00
TRINITY_GAX8251	hypothet	Chlamydomonas_eusti	43.30	0.00
TRINITY_GAX8102	hypothet	Chlamydomonas_eusti	43.30	0.00
TRINITY_GAX8157	hypothet	Chlamydomonas_eusti	43.30	0.00
TRINITY_GAX8461	hypothet	Chlamydomonas_eusti	43.30	0.00
TRINITY_XP_0139	hypothet	Monoraphidium_negle	43.30	0.00
TRINITY_XP_0029	hypothet	Volvox_carteri	43.30	0.00
TRINITY_GAX7421	hypothet	Chlamydomonas_eusti	43.30	0.00
TRINITY_KXZ5390	hypothet	Gonium_pectorale	43.30	0.00
TRINITY_AAM4588	multicop	Chlamydomonas_reinh	43.30	0.00
TRINITY_GAX7499	hypothet	Chlamydomonas_eusti	43.30	0
TRINITY_GBG6548	hypothet	Chara_braunii	43.30	0.00
TRINITY_PNH0696	Sperm fl	Tetrabaena_socialis	43.30	0.00
TRINITY_XP_0016	fibrocys	Chlamydomonas_reinh	43.20	0.00
TRINITY_XP_0030	methylt	Micromonas_pusilla	43.20	0.00
TRINITY_PPD9720	hypothet	Gossypium_barbadens	43.20	0.00
TRINITY_OWM6362	hypothet	Punica_granatum	43.20	0.00
TRINITY_XP_0258	recQ-med	Panicum_hallii	43.20	0.00
TRINITY_OMO6606	hypothet	Corchorus_olitorius	43.20	0.00
TRINITY_GBG7247	hypothet	Chara_braunii	43.20	0.00
TRINITY_XP_0104	PREDICTI	Camelina_sativa	43.20	0.00
TRINITY_XP_0094	PREDICTI	Musa_acuminata	43.20	0.00

TRINITY_KXZ5013	hypothet	Gonium_pectorale	43.20	0.00
TRINITY_GAV8363	E1-E2_A	Cephalotus_follicul	43.20	0.00
TRINITY_OAE2506	hypothet	Marchantia_polymorp	43.20	0.00
TRINITY_AGZ1935	hypothet	Chlorella_sp._ArM00	43.20	0.00
TRINITY_GAX8376	hypothet	Chlamydomonas_eusti	43.20	0.00
TRINITY_XP_0138	hypothet	Monoraphidium_negle	43.20	0.00
TRINITY_XP_0030	predicte	Micromonas_pusilla	43.20	0.00
TRINITY_EXC3489	Metal t	Morus_notabilis	43.20	0.00
TRINITY_XP_0194	PREDICT	Lupinus_angustifoli	43.20	0.00
TRINITY_GAQ7935	Arginyl-	Klebsormidium_niten	43.20	0.00
TRINITY_PTQ4668	hypothet	Marchantia_polymorp	43.20	0.00
TRINITY_XP_0030	predicte	Micromonas_pusilla	43.20	0.00
TRINITY_GAX7599	hypothet	Chlamydomonas_eusti	43.20	0.00
TRINITY_XP_0264	ras-rela	Papaver_somniferum	43.20	0.00
TRINITY_KFK4029	hypothet	Arabis_alpina	43.20	0.00
TRINITY_PTQ4810	hypothet	Marchantia_polymorp	43.20	0.00
TRINITY_XP_0063	PREDICT	Solanum_tuberosum	43.20	0.00
TRINITY_CAI8489	putative	Medicago_sativa	43.20	0.00
TRINITY_PSC6996	metal t	Micractinium_conduc	43.20	0.00
TRINITY_XP_0138	hypothet	Monoraphidium_negle	43.20	0.00
TRINITY_GAX8307	hypothet	Chlamydomonas_eusti	43.20	0.00
TRINITY_XP_0064	pre-mRN	Citrus_clementina	43.20	0.00
TRINITY_XP_0016	predicte	Chlamydomonas_reinh	43.20	0.00
TRINITY_XP_0029	unchara	Selaginella_moellen	43.20	0.00
TRINITY_XP_0238	non-spe	Quercus_suber	43.20	0.00
TRINITY_XP_0029	hypothet	Volvox_carteri	43.20	0.00
TRINITY_XP_0100	PREDICT	Eucalyptus_grandis	43.20	0.00
TRINITY_GAX7399	hypothet	Chlamydomonas_eusti	43.20	0.00
TRINITY_XP_0243	Fanconi	Physcomitrella_pate	43.20	0.00
TRINITY_PNW8313	hypothet	Chlamydomonas_reinh	43.20	0.00
TRINITY_GBF9042	prolyl e	Raphidocelis_subcap	43.20	0.00
TRINITY_GAX7634	hypothet	Chlamydomonas_eusti	43.20	0.00
TRINITY_GAX7934	hypothet	Chlamydomonas_eusti	43.20	0.00
TRINITY_XP_0238	FYVE-typ	Quercus_suber	43.20	0.00
TRINITY_EEF2753	hydroxya	Ricinus_communis	43.20	0.00
TRINITY_PKA6449	putative	Apostasia_shenzheni	43.20	0.00
TRINITY_XP_0056	hypothet	Coccomyxa_subellips	43.20	0.00
TRINITY_XP_0016	glycerol	Chlamydomonas_reinh	43.20	0.00
TRINITY_PTQ4682	hypothet	Marchantia_polymorp	43.20	0.00
TRINITY_RMZ5533	hypothet	Auxenochlorella_pro	43.20	0.00
TRINITY_GAX8159	hypothet	Chlamydomonas_eusti	43.20	0.00
TRINITY_GAX7564	hypothet	Chlamydomonas_eusti	43.20	0.00
TRINITY_PTQ2901	hypothet	Marchantia_polymorp	43.20	0.00
TRINITY_KXZ4596	hypothet	Gonium_pectorale	43.20	0.00
TRINITY_AEY8245	FAP133	Chlamydomonas_reinh	43.20	0.00
TRINITY_XP_0109	PREDICT	Elaeis_guineensis	43.20	0.00
TRINITY_XP_0238	floccula	Quercus_suber	43.20	0.00
TRINITY_XP_0239	protein	Quercus_suber	43.20	0.00
TRINITY_XP_0035	putative	Brachypodium_distac	43.20	0.00
TRINITY_GAX7789	hypothet	Chlamydomonas_eusti	43.20	0.00
TRINITY_OAE3251	hypothet	Marchantia_polymorp	43.20	0.00
TRINITY_XP_0014	predicte	Ostreococcus_sp._'l	43.20	0.00
TRINITY_GAX7404	hypothet	Chlamydomonas_eusti	43.20	0.00
TRINITY_XP_0029	hypothet	Volvox_carteri	43.20	0.00
TRINITY_GAQ7881	hypothet	Klebsormidium_niten	43.20	0.00
TRINITY_PNW7368	hypothet	Chlamydomonas_reinh	43.20	0.00
TRINITY_XP_0017	predicte	Chlamydomonas_reinh	43.20	0.00
TRINITY_XP_0056	Di-trans	Coccomyxa_subellips	43.20	0.00
TRINITY_GAX8508	hypothet	Chlamydomonas_eusti	43.20	0.00
TRINITY_PNW8816	hypothet	Chlamydomonas_reinh	43.20	0.00
TRINITY_XP_0016	predicte	Chlamydomonas_reinh	43.20	0.00
TRINITY_GBF9195	hypothet	Raphidocelis_subcap	43.20	0.00
TRINITY_XP_0172	PREDICT	Daucus_carota	43.10	0.00
TRINITY_PON4130	Tubulin-	Parasponia_anderson	43.10	0.00
TRINITY_XP_0271	DNA (cyt	Coffea_arabica	43.10	0.00
TRINITY_KXZ4746	hypothet	Gonium_pectorale	43.10	0.00

TRINITY_XP_0138	FKBP12-i	Monoraphidium_negle	43.10	0.00
TRINITY_XP_0075	predicte	Bathycoccus_prasinc	43.10	0.00
TRINITY_XP_0109	PREDICTE	Elaeis_guineensis	43.10	0.00
TRINITY_GAQ8393	hypothet	Klebsormidium_niten	43.10	0.00
TRINITY_XP_0192	PREDICTE	Nicotiana_attenuata	43.10	0.00
TRINITY_XP_0093	PREDICTE	Pyrus_x_bretschneid	43.10	0.00
TRINITY_PWA8614	NAF doma	Artemisia_annua	43.10	0.00
TRINITY_XP_0197	PREDICTE	Elaeis_guineensis	43.10	0.00
TRINITY_XP_0056	hypothet	Coccomyxa_subellips	43.10	0.00
TRINITY_XP_0134	serine/t	Medicago_truncatula	43.10	0.00
TRINITY_GBF9162	hypothet	Raphidocelis_subcap	43.10	0.00
TRINITY_EFH6806	predicte	Arabidopsis_lyrata	43.10	0.00
TRINITY_XP_0227	calcium-	Durio_zibethinus	43.10	0.00
TRINITY_GAQ8123	2-oxoglu	Klebsormidium_niten	43.10	0.00
TRINITY_XP_0264	ras-rela	Papaver_somniferum	43.10	0.00
TRINITY_XP_0202	MMS19 nu	Asparagus_officinal	43.10	0.00
TRINITY_GAX7498	hypothet	Chlamydomonas_eusti	43.10	0.00
TRINITY_XP_0172	PREDICTE	Daucus_carota	43.10	0.00
TRINITY_EPS6891	hypothet	Genlisea_aurea	43.10	0.00
TRINITY_XP_0029	hypothet	Volvox_carteri	43.10	0.00
TRINITY_GAQ8020	protein	Klebsormidium_niten	43.10	0.00
TRINITY_EFJ3369	hypothet	Selaginella_moellen	43.10	0.00
TRINITY_GAX8107	hypothet	Chlamydomonas_eusti	43.10	0.00
TRINITY_OAE3174	hypothet	Marchantia_polymorp	43.10	0.00
TRINITY_XP_0238	uncharac	Quercus_suber	43.10	0.00
TRINITY_XP_0237	traB dor	Lactuca_sativa	43.10	0.00
TRINITY_PSC7143	serine t	Micractinium_conduc	43.10	0.00
TRINITY_ACH6322	O-linked	Rheum_australe	43.10	0.00
TRINITY_XP_0017	predicte	Chlamydomonas_reinh	43.10	0.00
TRINITY_GAX7448	hypothet	Chlamydomonas_eusti	43.10	0.00
TRINITY_XP_0245	zinc fir	Selaginella_moellen	43.10	0.00
TRINITY_GAX8503	hypothet	Chlamydomonas_eusti	43.10	0.00
TRINITY_GAX8552	hypothet	Chlamydomonas_eusti	43.10	0.00
TRINITY_XP_0243	uncharac	Physcomitrella_pate	43.10	0.00
TRINITY_XP_0029	RWP-RK d	Volvox_carteri	43.10	0.00
TRINITY_KXZ5510	hypothet	Gonium_pectorale	43.10	0.00
TRINITY_GAX7263	hypothet	Chlamydomonas_eusti	43.10	0.00
TRINITY_XP_0030	monovale	Micromonas_pusilla	43.10	0.00
TRINITY_XP_0068	thioredo	Amborella_trichopod	43.10	0.00
TRINITY_KHG0557	Protein	Gossypium_arboreum	43.10	0.00
TRINITY_GAX7787	hypothet	Chlamydomonas_eusti	43.10	0.00
TRINITY_XP_0014	predicte	Ostreococcus_sp._'l	43.10	0.00
TRINITY_GAX8053	hypothet	Chlamydomonas_eusti	43.10	0.00
TRINITY_KXZ4407	hypothet	Gonium_pectorale	43.10	0.00
TRINITY_XP_0194	PREDICTE	Lupinus_angustifoli	43.10	0.00
TRINITY_XP_0239	myosin t	Quercus_suber	43.10	0.00
TRINITY_GBG5927	hypothet	Chara_braunii	43.10	0.00
TRINITY_GAX7602	hypothet	Chlamydomonas_eusti	43.10	0.00
TRINITY_GAQ8141	Metallop	Klebsormidium_niten	43.10	0.00
TRINITY_PNH0921	Tiny mac	Tetrabaena_socialis	43.10	0.00
TRINITY_XP_0024	uncharac	Sorghum_bicolor	43.10	0.00
TRINITY_XP_0029	hypothet	Volvox_carteri	43.10	0.00
TRINITY_KXZ5546	hypothet	Gonium_pectorale	43.10	0.00
TRINITY_PNH0255	hypothet	Tetrabaena_socialis	43.10	0.00
TRINITY_GAQ8279	thiol me	Klebsormidium_niten	43.10	0.00
TRINITY_XP_0238	uncharac	Quercus_suber	43.10	0.00
TRINITY_GAX7748	hypothet	Chlamydomonas_eusti	43.10	0.00
TRINITY_GAX8504	hypothet	Chlamydomonas_eusti	43.10	0.00
TRINITY_PTQ3028	hypothet	Marchantia_polymorp	43.10	0.00
TRINITY_XP_0016	peptidyl	Chlamydomonas_reinh	43.10	0.00
TRINITY_GBG5905	hypothet	Chara_braunii	43.10	0.00
TRINITY_GAX7930	hypothet	Chlamydomonas_eusti	43.10	0.00
TRINITY_XP_0202	lipid ph	Aegilops_tauschii	43.10	0.00
TRINITY_XP_0056	hypothet	Coccomyxa_subellips	43.10	0.00
TRINITY_XP_0056	hypothet	Coccomyxa_subellips	43.10	0.00
TRINITY_GAX7334	hypothet	Chlamydomonas_eusti	43.10	0.00

TRINITY_GAX81208	hypothet	Chlamydomonas_eusti	43.10	0.00
TRINITY_XP_01362	PREDICTE	Brassica_oleracea	43.10	0.00
TRINITY_XP_01844	PREDICTE	Raphanus_sativus	43.10	0.00
TRINITY_XP_02391	uncharac	Quercus_suber	43.10	0.00
TRINITY_KMS93400	hypothet	Beta_vulgaris	43.00	0.00
TRINITY_RWR86501	Guanylat	Cinnamomum_micranth	43.00	0.00
TRINITY_XP_01908	PREDICTE	Camelina_sativa	43.00	0.00
TRINITY_XP_02388	catalase	Quercus_suber	43.00	0.00
TRINITY_XP_01055	PREDICTE	Tarenaya_hasslerian	43.00	0.00
TRINITY_XP_01004	PREDICTE	Eucalyptus_grandis	43.00	0.00
TRINITY_ACY00391	calmodul	Knorringia_sibirica	43.00	0.00
TRINITY_XP_01051	PREDICTE	Camelina_sativa	43.00	0.00
TRINITY_XP_00244	carnosin	Sorghum_bicolor	43.00	0.00
TRINITY_GAQ81917	heat sho	Klebsormidium_niten	43.00	0.00
TRINITY_BAJ93729	predicte	Hordeum_vulgare	43.00	0.00
TRINITY_XP_02015	probable	Aegilops_tauschii	43.00	0.00
TRINITY_XP_01054	PREDICTE	Tarenaya_hasslerian	43.00	0.00
TRINITY_XP_02197	U2 small	Helianthus_annuus	43.00	0.00
TRINITY_XP_02272	cinnamoy	Durio_zibethinus	43.00	0.00
TRINITY_XP_01508	lysosoma	Solanum_pennellii	43.00	0.00
TRINITY_OAE30073	hypothet	Marchantia_polymorp	43.00	0.00
TRINITY_XP_02162	17.3 kDa	Manihot_esculenta	43.00	0.00
TRINITY_GAX78668	hypothet	Chlamydomonas_eusti	43.00	0.00
TRINITY_XP_00297	annexin	Selaginella_moellen	43.00	0.00
TRINITY_XP_00294	hypothet	Volvox_carteri	43.00	0.00
TRINITY_NP_00135	4-methyl	Solanum_lycopersicu	43.00	0.00
TRINITY_GBF88513	ABC tran	Raphidocelis_subcap	43.00	0.00
TRINITY_XP_00287	SNF1-rel	Arabidopsis_lyrata	43.00	0.00
TRINITY_XP_02452	uncharac	Selaginella_moellen	43.00	0.00
TRINITY_XP_01139	Ribosome	Auxenochlorella_pro	43.00	0.00
TRINITY_XP_02069	putative	Dendrobium_catenatu	43.00	0.00
TRINITY_XP_00664	PREDICTE	Oryza_brachyantha	43.00	0.00
TRINITY_XP_00295	hypothet	Volvox_carteri	43.00	0.00
TRINITY_XP_02438	ras-rela	Physcomitrella_pate	43.00	0.00
TRINITY_PNW70451	hypothet	Chlamydomonas_reinh	43.00	0.00
TRINITY_BAK00461	predicte	Hordeum_vulgare	43.00	0.00
TRINITY_GAQ85740	Galactos	Klebsormidium_niten	43.00	0.00
TRINITY_XP_02390	myosin-1	Quercus_suber	43.00	0.00
TRINITY_XP_00141	predicte	Ostreococcus_sp._'l	43.00	0.00
TRINITY_XP_02440	elongati	Physcomitrella_pate	43.00	0.00
TRINITY_XP_02390	kinesin-	Quercus_suber	43.00	0.00
TRINITY_XP_00935	PREDICTE	Pyrus_x_bretschneid	43.00	0.00
TRINITY_GAQ87963	Ubiquiti	Klebsormidium_niten	43.00	0.00
TRINITY_GAX73780	hypothet	Chlamydomonas_eusti	43.00	0.00
TRINITY_GBF93759	polyribo	Raphidocelis_subcap	43.00	0.00
TRINITY_XP_00250	cysteine	Micromonas_commoda	43.00	0.00
TRINITY_ADE77788	unknown	Picea_sitchensis	43.00	0.00
TRINITY_GAX73543	hypothet	Chlamydomonas_eusti	43.00	0.00
TRINITY_GAX74097	hypothet	Chlamydomonas_eusti	43.00	0.00
TRINITY_GBG92316	hypothet	Chara_braunii	43.00	0.00
TRINITY_PNH09146	hypothet	Tetrabaena_socialis	43.00	0.00
TRINITY_XP_01589	mitochon	Ziziphus_jujuba	43.00	0.00
TRINITY_XP_00294	hypothet	Volvox_carteri	43.00	0.00
TRINITY_GBF88744	iron-sul	Raphidocelis_subcap	43.00	0.00
TRINITY_CAI84893	putative	Medicago_sativa	43.00	0.00
TRINITY_GAQ82894	ThiJ/Pfp	Klebsormidium_niten	43.00	0.00
TRINITY_GAQ77855	S1 RNA b	Klebsormidium_niten	43.00	0.00
TRINITY_PNH00836	hypothet	Tetrabaena_socialis	43.00	0.00
TRINITY_GAX80339	hypothet	Chlamydomonas_eusti	43.00	0.00
TRINITY_XP_02708	hsp70-Hs	Coffea_arabica	43.00	0.00
TRINITY_GAX77168	hypothet	Chlamydomonas_eusti	43.00	0.00
TRINITY_PNW80082	hypothet	Chlamydomonas_reinh	43.00	0.00
TRINITY_GAX80954	hypothet	Chlamydomonas_eusti	43.00	0.00
TRINITY_GAX75930	hypothet	Chlamydomonas_eusti	43.00	0.00
TRINITY_KXZ54324	hypothet	Gonium_pectorale	43.00	0.00
TRINITY_XP_00685	probable	Amborella_trichopod	43.00	0.00

TRINITY_XP_01048	PREDICTE	Camelina_sativa	43.00	0.00
TRINITY_RZB70070	Pre-mRNA	Glycine_soja	43.00	0.00
TRINITY_GAX83731	hypothet	Chlamydomonas_eusti	43.00	0.00
TRINITY_KXZ41675	hypothet	Gonium_pectorale	43.00	0.00
TRINITY_XP_02390	ras-like	Quercus_suber	43.00	0.00
TRINITY_GBF99171	hypothet	Raphidocelis_subcap	43.00	0.00
TRINITY_XP_02289	poly [AD	Olea_europaea	43.00	0.00
TRINITY_XP_00295	hypothet	Volvox_carteri	43.00	0.00
TRINITY_GAX78287	hypothet	Chlamydomonas_eusti	43.00	0.00
TRINITY_GAQ77938	NMD prot	Klebsormidium_niten	43.00	0.00
TRINITY_PNW80327	hypothet	Chlamydomonas_reinh	43.00	0.00
TRINITY_XP_00169	predicte	Chlamydomonas_reinh	43.00	0.00
TRINITY_OWM71614	hypothet	Punica_granatum	43.00	0.00
TRINITY_KXZ43850	hypothet	Gonium_pectorale	43.00	0.00
TRINITY_KXZ49197	hypothet	Gonium_pectorale	43.00	0.00
TRINITY_GAX85230	hypothet	Chlamydomonas_eusti	43.00	0.00
TRINITY_PSC74106	ubiquiti	Micractinium_conduc	43.00	0.00
TRINITY_GAX76051	hypothet	Chlamydomonas_eusti	43.00	0.00
TRINITY_XP_00169	uric aci	Chlamydomonas_reinh	43.00	0.00
TRINITY_GAX86251	hypothet	Chlamydomonas_eusti	43.00	0.00
TRINITY_XP_00641	cysteine	Eutrema_salsugineum	43.00	0.00
TRINITY_KXZ41310	hypothet	Gonium_pectorale	43.00	0.00
TRINITY_RXH87698	hypothet	Malus_domestica	43.00	0.00
TRINITY_XP_00564	JmjC-dom	Coccomyxa_subellips	43.00	0.00
TRINITY_PNH12291	putative	Tetrabaena_socialis	43.00	0.00
TRINITY_GAX73064	hypothet	Chlamydomonas_eusti	43.00	0.00
TRINITY_PSC70392	Leukotri	Micractinium_conduc	43.00	0.00
TRINITY_PIN09740	RNA poly	Handroanthus_impeti	42.90	0.00
TRINITY_XP_00305	predicte	Micromonas_pusilla	42.90	0.00
TRINITY_GAX85110	hypothet	Chlamydomonas_eusti	42.90	0.00
TRINITY_XP_00298	brefeldi	Selaginella_moellen	42.90	0.00
TRINITY_XP_01069	PREDICTE	Beta_vulgaris	42.90	0.00
TRINITY_XP_01589	LAG1 lon	Ziziphus_jujuba	42.90	0.00
TRINITY_KXZ46834	DHC5 pro	Gonium_pectorale	42.90	0.00
TRINITY_XP_02437	adenylat	Physcomitrella_pate	42.90	0.00
TRINITY_GAQ89808	Sensory	Klebsormidium_niten	42.90	0.00
TRINITY_GAX73580	hypothet	Chlamydomonas_eusti	42.90	0.00
TRINITY_PRW57531	Ras-rela	Chlorella_sorokinia	42.90	0.00
TRINITY_XP_00357	E3 ubiqu	Brachypodium_distac	42.90	0.00
TRINITY_GBG66199	hypothet	Chara_braunii	42.90	0.00
TRINITY_XP_02280	putative	Olea_europaea	42.90	0.00
TRINITY_OAE18299	hypothet	Marchantia_polymorp	42.90	0.00
TRINITY_GAQ92515	hypothet	Klebsormidium_niten	42.90	0.00
TRINITY_NP_00110	uncharac	Zea_mays	42.90	0.00
TRINITY_XP_00660	PREDICTE	Oryza_brachyantha	42.90	0.00
TRINITY_RHN70169	putative	Medicago_truncatula	42.90	0.00
TRINITY_GAQ89977	two-comp	Klebsormidium_niten	42.90	0.00
TRINITY_XP_02387	serine/t	Quercus_suber	42.90	0.00
TRINITY_XP_02392	hydroxym	Quercus_suber	42.90	0.00
TRINITY_XP_00865	COMPASS-	Zea_mays	42.90	0.00
TRINITY_XP_00940	PREDICTE	Musa_acuminata	42.90	0.00
TRINITY_KDD74838	hypothet	Helicosporidium_sp.	42.90	0.00
TRINITY_GAQ88357	alpha-sc	Klebsormidium_niten	42.90	0.00
TRINITY_XP_02277	serine/t	Durio_zibethinus	42.90	0.00
TRINITY_XP_02197	stromal	Helianthus_annuus	42.90	0.00
TRINITY_XP_02022	probable	Cajanus_cajan	42.90	0.00
TRINITY_PRW58678	Tetratri	Chlorella_sorokinia	42.90	0.00
TRINITY_OAE23185	hypothet	Marchantia_polymorp	42.90	0.00
TRINITY_XP_00169	peroxiso	Chlamydomonas_reinh	42.90	0.00
TRINITY_GAQ85789	hypothet	Klebsormidium_niten	42.90	0.00
TRINITY_XP_01762	PREDICTE	Gossypium_arboreum	42.90	0.00
TRINITY_XP_00300	predicte	Micromonas_pusilla	42.90	0.00
TRINITY_GAQ84402	Serine T	Klebsormidium_niten	42.90	0.00
TRINITY_RYR40254	hypothet	Arachis_hypogaea	42.90	0.00
TRINITY_GBF99850	hypothet	Raphidocelis_subcap	42.90	0.00
TRINITY_PNW71712	hypothet	Chlamydomonas_reinh	42.90	0.00

TRINITY_XP_0029⁴	hypothet	Volvox_carteri	42.90	0.00
TRINITY_KXZ4568⁴	hypothet	Gonium_pectorale	42.90	0.00
TRINITY_EMS5945	Periodic	Triticum_urartu	42.90	0.00
TRINITY_GAX7752	hypothet	Chlamydomonas_eusti	42.90	0.00
TRINITY_GAQ8369	putative	Klebsormidium_niten	42.90	0.00
TRINITY_RDX9731	CBL-inte	Mucuna_pruriens	42.90	0.00
TRINITY_XP_0017	predicte	Chlamydomonas_reinh	42.90	0.00
TRINITY_XP_0064	splicing	Citrus_sinensis	42.90	0.00
TRINITY_KDP3422	hypothet	Jatropha_curcas	42.90	0.00
TRINITY_XP_0245	GPI manr	Selaginella_moellen	42.90	0.00
TRINITY_GAX7727	hypothet	Chlamydomonas_eusti	42.90	0.00
TRINITY_KXZ4689	hypothet	Gonium_pectorale	42.90	0.00
TRINITY_XP_0110	PREDICTE	Populus_euphratica	42.90	0.00
TRINITY_KXZ5048	hypothet	Gonium_pectorale	42.90	0.00
TRINITY_GAX8108	hypothet	Chlamydomonas_eusti	42.90	0.00
TRINITY_XP_0239	ATP-depe	Quercus_suber	42.90	0.00
TRINITY_POO0179	Guanine	Trema_orientale	42.90	0.00
TRINITY_XP_0087	SEC1 far	Phoenix_dactylifera	42.90	0.00
TRINITY_XP_0029	hypothet	Volvox_carteri	42.90	0.00
TRINITY_GAQ8238	delta(3)	Klebsormidium_niten	42.90	0.00
TRINITY_PNR4639	hypothet	Physcomitrella_pate	42.90	0.00
TRINITY_GAX8500	hypothet	Chlamydomonas_eusti	42.90	0.00
TRINITY_XP_0113	Aminomet	Auxenochlorella_prc	42.90	0.00
TRINITY_XP_0138	hypothet	Monoraphidium_negle	42.90	0.00
TRINITY_XP_0218	diphospl	Spinacia_oleracea	42.90	0.00
TRINITY_GAX8379	hypothet	Chlamydomonas_eusti	42.90	0.00
TRINITY_XP_0014	predicte	Ostreococcus_sp._'l	42.90	0.00
TRINITY_KXZ4763	hypothet	Gonium_pectorale	42.90	0.00
TRINITY_XP_0239	ATP-depe	Quercus_suber	42.90	0.00
TRINITY_GAX7978	hypothet	Chlamydomonas_eusti	42.90	0.00
TRINITY_GAQ8517	Dihydroc	Klebsormidium_niten	42.90	0.00
TRINITY_PRW5720	metallop	Chlorella_sorokinia	42.90	0.00
TRINITY_GAX8422	hypothet	Chlamydomonas_eusti	42.90	0.00
TRINITY_GAX7631	hypothet	Chlamydomonas_eusti	42.90	0.00
TRINITY_XP_0056	hypothet	Coccomyxa_subellips	42.90	0.00
TRINITY_XP_0124	PREDICTE	Gossypium_raimondii	42.90	0.00
TRINITY_XP_0238	bifuncti	Quercus_suber	42.90	0.00
TRINITY_KXZ5147	hypothet	Gonium_pectorale	42.90	0.00
TRINITY_GAX7487	hypothet	Chlamydomonas_eusti	42.90	0.00
TRINITY_PNH0778	Represso	Tetrabaena_socialis	42.90	0.00
TRINITY_PNW8555	hypothet	Chlamydomonas_reinh	42.90	0.00
TRINITY_XP_0024	CBL-inte	Sorghum_bicolor	42.90	0.00
TRINITY_KXZ5324	hypothet	Gonium_pectorale	42.90	0.00
TRINITY_XP_0105	PREDICTE	Tarenaya_hasslerian	42.90	0.00
TRINITY_PTQ4992	hypothet	Marchantia_polymorp	42.90	0.00
TRINITY_GBF8951	hypothet	Raphidocelis_subcap	42.90	0.00
TRINITY_GAX8439	hypothet	Chlamydomonas_eusti	42.90	0.00
TRINITY_GBF8951	multidru	Raphidocelis_subcap	42.90	0.00
TRINITY_XP_0138	hypothet	Monoraphidium_negle	42.90	0.00
TRINITY_GBF9440	hypothet	Raphidocelis_subcap	42.90	0.00
TRINITY_PNH0281	Vam6/Vps	Tetrabaena_socialis	42.90	0.00
TRINITY_KXZ5251	hypothet	Gonium_pectorale	42.90	0.00
TRINITY_XP_0016	predicte	Chlamydomonas_reinh	42.90	0.00
TRINITY_PON9027	Transcri	Trema_orientale	42.90	0.00
TRINITY_GAX8223	hypothet	Chlamydomonas_eusti	42.90	0.00
TRINITY_KXZ4946	hypothet	Gonium_pectorale	42.90	0.00
TRINITY_XP_0238	cystathi	Quercus_suber	42.90	0.00
TRINITY_XP_0191	PREDICTE	Ipomoea_nil	42.90	0.00
TRINITY_PNH0747	Leucine	Tetrabaena_socialis	42.90	0.00
TRINITY_GAX8146	hypothet	Chlamydomonas_eusti	42.90	0.00
TRINITY_KXZ4781	hypothet	Gonium_pectorale	42.90	0.00
TRINITY_GAX7459	hypothet	Chlamydomonas_eusti	42.90	0.00
TRINITY_PNH1226	Tiny mac	Tetrabaena_socialis	42.90	0.00
TRINITY_GAX7822	hypothet	Chlamydomonas_eusti	42.90	0.00
TRINITY_GAX7682	hypothet	Chlamydomonas_eusti	42.90	0.00
TRINITY_GAX7710	hypothet	Chlamydomonas_eusti	42.90	0.00

TRINITY_GAX7739(	hypothet	Chlamydomonas_eusti	42.90	0.00
TRINITY_PRW4518(	importir	Chlorella_sorokinia	42.90	0.00
TRINITY_XP_0243(	unchara(	Physcomitrella_pate	42.90	0.00
TRINITY_PNW8235(	hypothet	Chlamydomonas_reinh	42.90	0.00
TRINITY_XP_0016(	glutath:	Chlamydomonas_reinh	42.90	0.00
TRINITY_XP_0014(	predicte	Ostreococcus_sp._'l	42.90	0.00
TRINITY_XP_0238(	coronin-	Quercus_suber	42.90	0.00
TRINITY_XP_0093(	PREDICTI	Musa_acuminata	42.90	0.00
TRINITY_ONM3180(	Suppress	Zea_mays	42.80	0.00
TRINITY_ONM1167(	ADP-rib(	Zea_mays	42.80	0.00
TRINITY_XP_0220(	NADPH:a(	Helianthus_annuus	42.80	0.00
TRINITY_XP_0030(	predicte	Micromonas_pusilla	42.80	0.00
TRINITY_XP_0237(	carboxyp	Lactuca_sativa	42.80	0.00
TRINITY_XP_0238(	calcium-	Quercus_suber	42.80	0.00
TRINITY_XP_0228(	cation/I	Olea_europaea	42.80	0.00
TRINITY_GAX7742(	hypothet	Chlamydomonas_eusti	42.80	0.00
TRINITY_KZV4358(	serine/t	Dorcoceras_hygromet	42.80	0.00
TRINITY_XP_0139(	3-methyl	Monoraphidium_negle	42.80	0.00
TRINITY_GAX7598(	hypothet	Chlamydomonas_eusti	42.80	0.00
TRINITY_PRW5892(	esterase	Chlorella_sorokinia	42.80	0.00
TRINITY_GBG0030(	hypothet	Raphidocelis_subcap	42.80	0.00
TRINITY_PNW7889(	hypothet	Chlamydomonas_reinh	42.80	0.00
TRINITY_XP_0075(	riboflav	Bathycoccus_prasinc	42.80	0.00
TRINITY_GAX7423(	hypothet	Chlamydomonas_eusti	42.80	0.00
TRINITY_PIN2510(	GTP-binc	Handroanthus_impeti	42.80	0.00
TRINITY_XP_0016(	predicte	Chlamydomonas_reinh	42.80	0.00
TRINITY_GAX7938(	hypothet	Chlamydomonas_eusti	42.80	0.00
TRINITY_GAX8215(	hypothet	Chlamydomonas_eusti	42.80	0.00
TRINITY_XP_0029(	probable	Selaginella_moellen	42.80	0.00
TRINITY_OIV9525(	hypothet	Lupinus_angustifoli	42.80	0.00
TRINITY_PNW8599(	hypothet	Chlamydomonas_reinh	42.80	0.00
TRINITY_OAE3354(	hypothet	Marchantia_polymorp	42.80	0.00
TRINITY_ALE1528(	CDK-like	Chlamydomonas_reinh	42.80	0.00
TRINITY_GAX8255(	hypothet	Chlamydomonas_eusti	42.80	0.00
TRINITY_GAX8357(	hypothet	Chlamydomonas_eusti	42.80	0.00
TRINITY_GAX7809(	hypothet	Chlamydomonas_eusti	42.80	0.00
TRINITY_ATY3519(	actin [	Ixeridium_dentatum	42.80	0.00
TRINITY_XP_0056(	threonyl	Coccomyxa_subellips	42.70	0.00
TRINITY_GBG7153(	hypothet	Chara_braunii	42.70	0.00
TRINITY_XP_0244(	histidir	Physcomitrella_pate	42.70	0.00
TRINITY_XP_0124(	PREDICTI	Gossypium_raimondii	42.70	0.00
TRINITY_XP_0029(	dynein l	Selaginella_moellen	42.70	0.00
TRINITY_GAQ8857(	enoyl-C(	Klebsormidium_niten	42.70	0.00
TRINITY_AFN8820(	haloaci(	Phaseolus_vulgaris	42.70	0.00
TRINITY_GBF9708(	hypothet	Raphidocelis_subcap	42.70	0.00
TRINITY_GBG8414(	hypothet	Chara_braunii	42.70	0.00
TRINITY_PNW8079(	hypothet	Chlamydomonas_reinh	42.70	0.00
TRINITY_GAQ7922(	hypothet	Klebsormidium_niten	42.70	0.00
TRINITY_GBG6500(	hypothet	Chara_braunii	42.70	0.00
TRINITY_PIA3664(	hypothet	Aquilegia_coerulea	42.70	0.00
TRINITY_GAQ7815(	hypothet	Klebsormidium_niten	42.70	0.00
TRINITY_XP_0244(	thimet (	Physcomitrella_pate	42.70	0.00
TRINITY_XP_0228(	MT-A70-l	Ostreococcus_tauri	42.70	0.00
TRINITY_XP_0264(	EEF1A ly	Papaver_somniferum	42.70	0.00
TRINITY_XP_0216(	serine/t	Hevea_brasiliensis	42.70	0.00
TRINITY_GAQ9054(	Ubiquit:	Klebsormidium_niten	42.70	0.00
TRINITY_RXI0063(	hypothet	Malus_domestica	42.70	0.00
TRINITY_XP_0221(	calcium-	Momordica_charantia	42.70	0.00
TRINITY_AES8925(	alpha-ma	Medicago_truncatula	42.70	0.00
TRINITY_XP_0084(	PREDICTI	Cucumis_melo	42.70	0.00
TRINITY_XP_0228(	Helicase	Ostreococcus_tauri	42.70	0.00
TRINITY_OAE3250(	hypothet	Marchantia_polymorp	42.70	0.00
TRINITY_RHN3848(	hypothet	Medicago_truncatula	42.70	0.00
TRINITY_GBG6960(	hypothet	Chara_braunii	42.70	0.00
TRINITY_APO1583(	actin 1	Sesuvium_portulacas	42.70	0.00
TRINITY_XP_0029(	ras-rela	Selaginella_moellen	42.70	0.00

TRINITY_XP_02272	COP9 si	Durio_zibethinus	42.70	0.00
TRINITY_GAX8170	hypothet	Chlamydomonas_eusti	42.70	0.00
TRINITY_PSC7339	ribosoma	Micractinium_conduc	42.70	0.00
TRINITY_GAX8130	hypothet	Chlamydomonas_eusti	42.70	0.00
TRINITY_GAX7340	hypothet	Chlamydomonas_eusti	42.70	0.00
TRINITY_XP_0029	NimA-re	Volvox_carteri	42.70	0.00
TRINITY_KXZ4644	hypothet	Gonium_pectorale	42.70	0.00
TRINITY_PWA6780	serine	Artemisia_annua	42.70	0.00
TRINITY_XP_0243	60S rib	Physcomitrella_pate	42.70	0.00
TRINITY_GAQ8180	glycopr	Klebsormidium_niten	42.70	0.00

TRINITY_OEL32079	Glycerop	Dichanthelium_oligo	42.70	0.00
TRINITY_KXZ47865	hypothet	Gonium_pectorale	42.70	0.00
TRINITY_XP_00294	hypothet	Volvox_carteri	42.70	0.00
TRINITY_GBF92723	hypothet	Raphidocelis_subcap	42.70	0.00
TRINITY_XP_01655	PREDICTE	Capsicum_annuum	42.70	0.00
TRINITY_GBF89232	DNA repa	Raphidocelis_subcap	42.70	0.00
TRINITY_XP_00564	protein	Coccomyxa_subellips	42.70	0.00
TRINITY_XP_01108	squalene	Sesamum_indicum	42.70	0.00
TRINITY_PNW70068	hypothet	Chlamydomonas_reinh	42.70	0.00
TRINITY_PNW89000	hypothet	Chlamydomonas_reinh	42.70	0.00
TRINITY_XP_02391	N-acyl-p	Quercus_suber	42.70	0.00
TRINITY_XP_00715	hypothet	Phaseolus_vulgaris	42.70	0.00
TRINITY_AQK71573	Transcri	Zea_mays	42.70	0.00
TRINITY_GAX84118	hypothet	Chlamydomonas_eusti	42.70	0.00
TRINITY_VDC60350	unnamed	Brassica_rapa	42.70	0.00
TRINITY_RWR87709	ABC tran	Cinnamomum_micranth	42.70	0.00
TRINITY_RLM56339	hypothet	Panicum_miliaceum	42.70	0.00
TRINITY_XP_00169	predicte	Chlamydomonas_reinh	42.70	0.00
TRINITY_BAF14329	Os04g030	Oryza_sativa	42.70	0.00
TRINITY_KXZ45198	hypothet	Gonium_pectorale	42.70	0.00
TRINITY_GAX72932	hypothet	Chlamydomonas_eusti	42.70	0.00
TRINITY_GAX73181	hypothet	Chlamydomonas_eusti	42.70	0.00
TRINITY_ERM99023	hypothet	Amborella_trichopod	42.70	0.00
TRINITY_XP_01390	hypothet	Monoraphidium_negle	42.70	0.00
TRINITY_RAL53155	hypothet	Cuscuta_australis	42.70	0.00
TRINITY_XP_00294	hypothet	Volvox_carteri	42.70	0.00
TRINITY_KXZ44782	SNAP34 p	Gonium_pectorale	42.70	0.00
TRINITY_XP_01668	PREDICTE	Gossypium_hirsutum	42.70	0.00
TRINITY_XP_00565	P-loop c	Coccomyxa_subellips	42.70	0.00
TRINITY_XP_00295	hypothet	Volvox_carteri	42.70	0.00
TRINITY_XP_00250	predicte	Micromonas_commoda	42.70	0.00
TRINITY_PSC69635	Myosin-1	Micractinium_conduc	42.70	0.00
TRINITY_KXZ48376	hypothet	Gonium_pectorale	42.70	0.00
TRINITY_XP_01390	Molybdop	Monoraphidium_negle	42.70	0.00
TRINITY_BAE98683	hypothet	Arabidopsis_thalian	42.70	0.00
TRINITY_PNH06268	ALA-inte	Tetrabaena_socialis	42.70	0.00
TRINITY_XP_00685	SNW/SKI-	Amborella_trichopod	42.70	0.00
TRINITY_GBF94605	hypothet	Raphidocelis_subcap	42.70	0.00
TRINITY_XP_00305	predicte	Micromonas_pusilla	42.70	0.00
TRINITY_OMO74329	hypothet	Corchorus_capsulari	42.70	0.00
TRINITY_XP_00168	predicte	Chlamydomonas_reinh	42.70	0.00
TRINITY_GAX77225	hypothet	Chlamydomonas_eusti	42.70	0.00
TRINITY_KXZ53363	hypothet	Gonium_pectorale	42.70	0.00
TRINITY_KMS94413	hypothet	Beta_vulgaris	42.70	0.00
TRINITY_PNH05717	Regulato	Tetrabaena_socialis	42.70	0.00
TRINITY_ADB85273	putative	Phyllostachys_eduli	42.70	0.00
TRINITY_RAL51222	hypothet	Cuscuta_australis	42.70	0.00
TRINITY_XP_00298	uricase-	Selaginella_moellen	42.60	0.00
TRINITY_PHT48811	hypothet	Capsicum_baccatum	42.60	0.00
TRINITY_XP_02213	probable	Momordica_charantia	42.60	0.00
TRINITY_XP_02390	NAD-depe	Quercus_suber	42.60	0.00
TRINITY_RWW90255	hypothet	Ensete_ventricosum	42.60	0.00
TRINITY_EFJ09289	hypothet	Selaginella_moellen	42.60	0.00
TRINITY_XP_02388	M-phase	Quercus_suber	42.60	0.00
TRINITY_XP_02438	heteroge	Physcomitrella_pate	42.60	0.00
TRINITY_XP_00564	iron hyd	Coccomyxa_subellips	42.60	0.00
TRINITY_XP_00296	tRNA (ad	Selaginella_moellen	42.60	0.00
TRINITY_GAX79165	hypothet	Chlamydomonas_eusti	42.60	0.00
TRINITY_XP_02167	delta(3,	Hevea_brasiliensis	42.60	0.00
TRINITY_XP_02391	LOW QUAL	Quercus_suber	42.60	0.00
TRINITY_XP_00584	hypothet	Chlorella_variabili	42.60	0.00
TRINITY_XP_02644	coatomer	Papaver_somniferum	42.60	0.00
TRINITY_XP_00142	predicte	Ostreococcus_sp._'l	42.60	0.00
TRINITY_PRW59438	40S ribo	Chlorella_sorokinia	42.60	0.00
TRINITY_PNW81867	hypothet	Chlamydomonas_reinh	42.60	0.00
TRINITY_XP_00141	predicte	Ostreococcus_sp._'l	42.60	0.00

TRINITY_PRW6157	DNA gyra	Chlorella_sorokinia	42.60	0.00
TRINITY_XP_0176	PREDICTI	Gossypium_arboreum	42.60	0.00
TRINITY_XP_0056	hypothet	Coccomyxa_subellips	42.60	0.00
TRINITY_PIM9944	Thiored	Handroanthus_impeti	42.60	0.00
TRINITY_XP_0217	alpha N-	Chenopodium_quinoa	42.60	0.00
TRINITY_KXZ4287	hypothet	Gonium_pectorale	42.60	0.00
TRINITY_XP_0114	PREDICTI	Fragaria_vesca	42.60	0.00
TRINITY_XP_0016	predicte	Chlamydomonas_reinh	42.60	0.00
TRINITY_XP_0058	hypothet	Chlorella_variabili	42.60	0.00
TRINITY_XP_0206	beta-he	Dendrobium_catenatu	42.60	0.00
TRINITY_GAX7265	hypothet	Chlamydomonas_eusti	42.60	0.00
TRINITY_PTQ3920	hypothet	Marchantia_polymorp	42.60	0.00
TRINITY_GAX7299	hypothet	Chlamydomonas_eusti	42.60	0.00
TRINITY_KXZ4322	hypothet	Gonium_pectorale	42.60	0.00
TRINITY_XP_0029	hypothet	Volvox_carteri	42.60	0.00
TRINITY_XP_0166	PREDICTI	Gossypium_hirsutum	42.60	0.00
TRINITY_XP_0029	hypothet	Volvox_carteri	42.60	0.00
TRINITY_XP_0017	predicte	Chlamydomonas_reinh	42.60	0.00
TRINITY_PNW7875	hypothet	Chlamydomonas_reinh	42.60	0.00
TRINITY_XP_0029	hypothet	Volvox_carteri	42.60	0.00
TRINITY_PRW3285	putative	Chlorella_sorokinia	42.60	0.00
TRINITY_OAE3071	hypothet	Marchantia_polymorp	42.60	0.00
TRINITY_XP_0245	glutathi	Selaginella_moellen	42.60	0.00
TRINITY_KXZ4205	hypothet	Gonium_pectorale	42.60	0.00
TRINITY_XP_0058	hypothet	Chlorella_variabili	42.60	0.00
TRINITY_XP_0030	Armadil	Ostreococcus_tauri	42.60	0.00
TRINITY_PNW7222	hypothet	Chlamydomonas_reinh	42.60	0.00
TRINITY_AFK4640	unknown	Lotus_japonicus	42.60	0.00
TRINITY_PNW8521	hypothet	Chlamydomonas_reinh	42.60	0.00
TRINITY_PNW8311	hypothet	Chlamydomonas_reinh	42.60	0.00
TRINITY_GAX7522	hypothet	Chlamydomonas_eusti	42.60	0.00
TRINITY_RIA0405	hypothet	Brassica_rapa	42.60	0.00
TRINITY_PNW8440	hypothet	Chlamydomonas_reinh	42.60	0.00
TRINITY_ADE7774	unknown	Picea_sitchensis	42.60	0.00
TRINITY_GBF8877	hypothet	Raphidocelis_subcap	42.60	0.00
TRINITY_XP_0239	craniofa	Quercus_suber	42.60	0.00
TRINITY_PKA5975	Zinc fi	Apostasia_shenzheni	42.60	0.00
TRINITY_XP_0139	hypothet	Monoraphidium_negle	42.60	0.00
TRINITY_PSC6994	Zinc fi	Micractinium_conduc	42.60	0.00
TRINITY_KDD7216	hypothet	Helicosporidium_sp.	42.60	0.00
TRINITY_KXZ4678	hypothet	Gonium_pectorale	42.60	0.00
TRINITY_XP_0241	calcium-	Rosa_chinensis	42.60	0.00
TRINITY_XP_0239	NAD-spe	Quercus_suber	42.60	0.00
TRINITY_XP_0093	PREDICTI	Pyrus_x_bretschneid	42.60	0.00
TRINITY_GAX7405	hypothet	Chlamydomonas_eusti	42.60	0.00
TRINITY_OAY8355	Pumilio	Ananas_comosus	42.60	0.00
TRINITY_XP_0014	predicte	Ostreococcus_sp._'l	42.50	0.00
TRINITY_XP_0270	multipr	Coffea_arabica	42.50	0.00
TRINITY_XP_0243	tRNA (g	Physcomitrella_pate	42.50	0.00
TRINITY_XP_0191	PREDICTI	Ipomoea_nil	42.50	0.00
TRINITY_XP_0216	COX ass	Hevea_brasiliensis	42.50	0.00
TRINITY_RID7651	hypothet	Brassica_rapa	42.50	0.00
TRINITY_XP_0217	ubiquit	Chenopodium_quinoa	42.50	0.00
TRINITY_PRW2065	SWI SNF-	Chlorella_sorokinia	42.50	0.00
TRINITY_CAJ2752	beclin	Gossypium_raimondii	42.50	0.00
TRINITY_GAQ8578	Cystein	Klebsormidium_niten	42.50	0.00
TRINITY_RLN1844	histone	Panicum_miliaceum	42.50	0.00
TRINITY_OMO6888	hypothet	Corchorus_olitorius	42.50	0.00
TRINITY_XP_0105	PREDICTI	Camelina_sativa	42.50	0.00
TRINITY_XP_0025	predicte	Micromonas_commoda	42.50	0.00
TRINITY_EEF2554	beta-ma	Ricinus_communis	42.50	0.00
TRINITY_XP_0270	meiotic	Coffea_arabica	42.50	0.00
TRINITY_GAX7770	hypothet	Chlamydomonas_eusti	42.50	0.00
TRINITY_XP_0093	PREDICTI	Musa_acuminata	42.50	0.00
TRINITY_PWA8241	leucine-	Artemisia_annua	42.50	0.00
TRINITY_KXZ5220	hypothet	Gonium_pectorale	42.50	0.00

TRINITY_XP_0239	protein	Quercus_suber	42.50	0.00
TRINITY_XP_0030	Pyridoxa	Ostreococcus_tauri	42.50	0.00
TRINITY_GAX7527	hypothet	Chlamydomonas_eusti	42.50	0.00
TRINITY_PHT2654	hypothet	Capsicum_baccatum	42.50	0.00
TRINITY_GAX7985	hypothet	Chlamydomonas_eusti	42.50	0.00
TRINITY_XP_0030	AMP-depe	Ostreococcus_tauri	42.50	0.00
TRINITY_PNW8639	hypothet	Chlamydomonas_reinh	42.50	0.00
TRINITY_XP_0128	PREDICTE	Erythranthe_guttata	42.50	0.00
TRINITY_PNH0927	Acidic I	Tetrabaena_socialis	42.50	0.00
TRINITY_XP_0243	guanylat	Physcomitrella_pate	42.50	0.00
TRINITY_PSC7398	CAMK CAM	Micractinium_conduc	42.50	0.00
TRINITY_ACJ3819	chitinas	Malus_hupehensis	42.50	0.00
TRINITY_GAQ8787	Aromatic	Klebsormidium_niten	42.50	0.00
TRINITY_XP_0017	predicte	Chlamydomonas_reinh	42.50	0.00
TRINITY_XP_0227	U6 small	Durio_zibethinus	42.50	0.00
TRINITY_GAX7610	hypothet	Chlamydomonas_eusti	42.50	0.00
TRINITY_GAX7528	hypothet	Chlamydomonas_eusti	42.50	0.00
TRINITY_KXZ4239	hypothet	Gonium_pectorale	42.50	0.00
TRINITY_XP_0030	predicte	Micromonas_pusilla	42.50	0.00
TRINITY_KMS6516	hypothet	Beta_vulgaris	42.50	0.00
TRINITY_NP_6893	NADH deh	Chaetosphaeridium_g	42.50	0.00
TRINITY_GAX7872	hypothet	Chlamydomonas_eusti	42.50	0.00
TRINITY_GAX7872	hypothet	Chlamydomonas_eusti	42.50	0.00
TRINITY_XP_0086	uncharac	Zea_mays	42.50	0.00
TRINITY_PNH0853	hypothet	Tetrabaena_socialis	42.50	0.00
TRINITY_XP_0042	ubiquiti	Solanum_lycopersicu	42.50	0.00
TRINITY_PKA5061	Putative	Apostasia_shenzheni	42.50	0.00
TRINITY_XP_0128	PREDICTE	Erythranthe_guttata	42.50	0.00
TRINITY_XP_0096	PREDICTE	Nicotiana_tomentosi	42.50	0.00
TRINITY_XP_0227	SNF1-rel	Durio_zibethinus	42.50	0.00
TRINITY_XP_0016	mitogen	Chlamydomonas_reinh	42.50	0.00
TRINITY_XP_0136	PREDICTE	Brassica_oleracea	42.50	0.00
TRINITY_XP_0238	putative	Quercus_suber	42.50	0.00
TRINITY_PNH0396	Transcri	Tetrabaena_socialis	42.50	0.00
TRINITY_GAX8321	hypothet	Chlamydomonas_eusti	42.50	0.00
TRINITY_XP_0138	threonin	Monoraphidium_negle	42.50	0.00
TRINITY_CAM9653	16.5 kDa	Aegilops_longissima	42.50	0.00
TRINITY_GAX7286	hypothet	Chlamydomonas_eusti	42.50	0.00
TRINITY_GAX8377	hypothet	Chlamydomonas_eusti	42.50	0.00
TRINITY_XP_0216	serine/t	Hevea_brasiliensis	42.50	0.00
TRINITY_XP_0239	probable	Quercus_suber	42.50	0.00
TRINITY_XP_0264	ABC tran	Papaver_somniferum	42.50	0.00
TRINITY_PRW5943	Apocarot	Chlorella_sorokinia	42.50	0.00
TRINITY_XP_0075	predicte	Bathycoccus_prasino	42.50	0.00
TRINITY_KXZ4322	hypothet	Gonium_pectorale	42.50	0.00
TRINITY_XP_0029	hypothet	Volvox_carteri	42.50	0.00
TRINITY_GAX7444	hypothet	Chlamydomonas_eusti	42.50	0.00
TRINITY_KXZ4823	hypothet	Gonium_pectorale	42.50	0.00
TRINITY_OVA1147	Synaptot	Macleaya_cordata	42.50	0.00
TRINITY_XP_0014	P-ATPase	Ostreococcus_sp._'l	42.50	0.00
TRINITY_XP_0029	hypothet	Volvox_carteri	42.50	0.00
TRINITY_PNW8206	hypothet	Chlamydomonas_reinh	42.50	0.00
TRINITY_PNW7501	hypothet	Chlamydomonas_reinh	42.50	0.00
TRINITY_XP_0104	PREDICTE	Camelina_sativa	42.50	0.00
TRINITY_XP_0165	PREDICTE	Capsicum_annuum	42.50	0.00
TRINITY_XP_0243	delta(3,	Physcomitrella_pate	42.50	0.00
TRINITY_OAE3206	hypothet	Marchantia_polymorp	42.50	0.00
TRINITY_KXZ4802	TTL4 pro	Gonium_pectorale	42.50	0.00
TRINITY_XP_0188	PREDICTE	Juglans_regia	42.50	0.00
TRINITY_PNW7418	hypothet	Chlamydomonas_reinh	42.50	0.00
TRINITY_PNW7056	hypothet	Chlamydomonas_reinh	42.50	0.00
TRINITY_XP_0139	peptidas	Monoraphidium_negle	42.50	0.00
TRINITY_GBF9557	hypothet	Raphidocelis_subcap	42.50	0.00
TRINITY_PNW7046	hypothet	Chlamydomonas_reinh	42.50	0.00
TRINITY_XP_0016	predicte	Chlamydomonas_reinh	42.50	0.00
TRINITY_GAX7475	hypothet	Chlamydomonas_eusti	42.50	0.00

TRINITY_XP_0139	hypothet	Monoraphidium_negle	42.50	0.00
TRINITY_KXZ5472	hypothet	Gonium_pectorale	42.50	0
TRINITY_GBF9843	polyubi	Raphidocelis_subcap	42.50	0.00
TRINITY_XP_0197	PREDICT	Elaeis_guineensis	42.50	0.00
TRINITY_XP_0134	DExH-bo	Medicago_truncatula	42.40	0.00
TRINITY_XP_0063	phospho	Eutrema_salsugineum	42.40	0.00
TRINITY_RYR6375	hypothet	Arachis_hypogaea	42.40	0.00
TRINITY_XP_0243	protein	Physcomitrella_pate	42.40	0.00
TRINITY_PNR5077	hypothet	Physcomitrella_pate	42.40	0.00
TRINITY_GAQ8988	Transcr	Klebsormidium_niten	42.40	0.00
TRINITY_KXZ5384	hypothet	Gonium_pectorale	42.40	0.00
TRINITY_XP_0243	nucleol	Physcomitrella_pate	42.40	0.00
TRINITY_XP_0191	PREDICT	Ipomoea_nil	42.40	0.00
TRINITY_CBI3370	unnamed	Vitis_vinifera	42.40	0.00
TRINITY_KXZ5547	hypothet	Gonium_pectorale	42.40	0.00
TRINITY_RWW3330	hypothet	Ensete_ventricosum	42.40	0.00
TRINITY_EYU3836	hypothet	Erythranthe_guttata	42.40	0.00
TRINITY_AFK4638	unknown	Lotus_japonicus	42.40	0.00
TRINITY_XP_0161	high mo	Arachis_ipaensis	42.40	0.00
TRINITY_AAT3804	putative	Oryza_sativa	42.40	0.00
TRINITY_XP_0239	phospho	Quercus_suber	42.40	0.00
TRINITY_GAQ8894	Cytopla	Klebsormidium_niten	42.40	0.00
TRINITY_RWW9047	hypothet	Ensete_ventricosum	42.40	0.00
TRINITY_XP_0056	translat	Coccomyxa_subellips	42.40	0.00
TRINITY_AET0473	phosphat	Medicago_truncatula	42.40	0.00
TRINITY_GBG7003	hypothet	Chara_braunii	42.40	0.00
TRINITY_PSC7078	mitogen-	Micractinium_conduc	42.40	0.00
TRINITY_XP_0207	AP-2 co	Dendrobium_catenatu	42.40	0.00
TRINITY_XP_0128	PREDICT	Erythranthe_guttata	42.40	0.00
TRINITY_PNW7275	hypothet	Chlamydomonas_reinh	42.40	0.00
TRINITY_XP_0029	hypothet	Volvox_carteri	42.40	0.00
TRINITY_PSC7632	interfe	Micractinium_conduc	42.40	0.00
TRINITY_BAE5369	CW80Cd4	Chlamydomonas_sp._W	42.40	0.00
TRINITY_XP_0114	Upstrea	Auxenochlorella_pro	42.40	0.00
TRINITY_VDD1622	unnamed	Brassica_rapa	42.40	0.00
TRINITY_PNW7037	hypothet	Chlamydomonas_reinh	42.40	0.00
TRINITY_GAX8105	hypothet	Chlamydomonas_eusti	42.40	0.00
TRINITY_PNH0892	hypothet	Tetrabaena_socialis	42.40	0.00
TRINITY_EFJ1566	hypothet	Selaginella_moellen	42.40	0.00
TRINITY_XP_0029	zinc tr	Selaginella_moellen	42.40	0.00
TRINITY_KJB7631	hypothet	Gossypium_raimondii	42.40	0.00
TRINITY_XP_0228	casein	Olea_europaea	42.40	0.00
TRINITY_XP_0238	multidr	Quercus_suber	42.40	0.00
TRINITY_KFK2777	hypothet	Arabis_alpina	42.40	0.00
TRINITY_XP_0045	probabl	Cicer_arietinum	42.40	0.00
TRINITY_GAX8372	hypothet	Chlamydomonas_eusti	42.40	0.00
TRINITY_XP_0205	short-c	Phalaenopsis_equest	42.40	0.00
TRINITY_GAX8056	hypothet	Chlamydomonas_eusti	42.40	0.00
TRINITY_GAX8277	hypothet	Chlamydomonas_eusti	42.40	0.00
TRINITY_XP_0100	PREDICT	Eucalyptus_grandis	42.40	0.00
TRINITY_GAX8186	hypothet	Chlamydomonas_eusti	42.40	0.00
TRINITY_GAX7976	hypothet	Chlamydomonas_eusti	42.40	0.00
TRINITY_XP_0239	non-hist	Quercus_suber	42.40	0.00
TRINITY_XP_0273	probabl	Abrus_precatorius	42.40	0.00
TRINITY_PNH0540	hypothet	Tetrabaena_socialis	42.40	0.00
TRINITY_GBG8855	hypothet	Chara_braunii	42.40	0.00
TRINITY_GBF9123	hypothet	Raphidocelis_subcap	42.40	0.00
TRINITY_XP_0029	hypothet	Volvox_carteri	42.40	0.00
TRINITY_XP_0239	polyade	Quercus_suber	42.40	0.00
TRINITY_GAQ7774	Translat	Klebsormidium_niten	42.40	0.00
TRINITY_GAX7611	hypothet	Chlamydomonas_eusti	42.40	0.00
TRINITY_XP_0029	hypothet	Volvox_carteri	42.40	0.00
TRINITY_PNW8832	hypothet	Chlamydomonas_reinh	42.40	0.00
TRINITY_KXZ5253	hypothet	Gonium_pectorale	42.40	0.00
TRINITY_GAX8086	hypothet	Chlamydomonas_eusti	42.40	0.00
TRINITY_PNW7885	hypothet	Chlamydomonas_reinh	42.40	0.00

TRINITY_PRW5877	ABC trar	Chlorella_sorokinia	42.40	0.00
TRINITY_PNW7303	hypothet	Chlamydomonas_reinh	42.40	0.00
TRINITY_KXZ5426	hypothet	Gonium_pectorale	42.40	0.00
TRINITY_XP_0239	ATP-depe	Quercus_suber	42.40	0.00
TRINITY_PNW8878	hypothet	Chlamydomonas_reinh	42.40	0.00
TRINITY_GAX7884	hypothet	Chlamydomonas_eusti	42.40	0.00
TRINITY_XP_0029	hypothet	Volvox_carteri	42.40	0.00
TRINITY_PNH0356	hypothet	Tetrabaena_socialis	42.40	0.00
TRINITY_KFK4493	hypothet	Arabis_alpina	42.40	0.00
TRINITY_KQK0957	hypothet	Brachypodium_distac	42.40	0.00
TRINITY_VDC9323	unnamed	Brassica_rapa	42.40	0.00
TRINITY_GAX7486	hypothet	Chlamydomonas_eusti	42.40	0.00
TRINITY_BAJ9628	predicte	Hordeum_vulgare	42.40	0.00
TRINITY_GAX8206	hypothet	Chlamydomonas_eusti	42.40	0.00
TRINITY_XP_0106	PREDICTE	Beta_vulgaris	42.40	0.00
TRINITY_PRW3936	senescer	Chlorella_sorokinia	42.40	0.00
TRINITY_PNH0721	hypothet	Tetrabaena_socialis	42.30	0.00
TRINITY_XP_0025	predicte	Micromonas_commoda	42.30	0.00
TRINITY_XP_0056	Phospho	Coccomyxa_subellips	42.30	0.00
TRINITY_PWA8898	protein	Artemisia_annua	42.30	0.00
TRINITY_GBF9496	hypothet	Raphidocelis_subcap	42.30	0.00
TRINITY_GAX7829	hypothet	Chlamydomonas_eusti	42.30	0.00
TRINITY_EFJ0545	hypothet	Selaginella_moellen	42.30	0.00
TRINITY_XP_0239	phosphat	Quercus_suber	42.30	0.00
TRINITY_XP_0029	hypothet	Volvox_carteri	42.30	0.00
TRINITY_XP_0201	uncharac	Ananas_comosus	42.30	0.00
TRINITY_XP_0217	probable	Chenopodium_quinoa	42.30	0.00
TRINITY_BAK0043	predicte	Hordeum_vulgare	42.30	0.00
TRINITY_XP_0145	protein	Vigna_radiata	42.30	0.00
TRINITY_OAE2028	hypothet	Marchantia_polymorp	42.30	0.00
TRINITY_KFK2634	hypothet	Arabis_alpina	42.30	0.00
TRINITY_XP_0030	predicte	Micromonas_pusilla	42.30	0.00
TRINITY_KXZ4437	hypothet	Gonium_pectorale	42.30	0.00
TRINITY_GAQ8282	TATA-box	Klebsormidium_niten	42.30	0.00
TRINITY_GAX7948	hypothet	Chlamydomonas_eusti	42.30	0.00
TRINITY_XP_0056	epocide	Coccomyxa_subellips	42.30	0.00
TRINITY_XP_0106	PREDICTE	Beta_vulgaris	42.30	0.00
TRINITY_GAQ9014	hypothet	Klebsormidium_niten	42.30	0.00
TRINITY_GAX7260	hypothet	Chlamydomonas_eusti	42.30	0.00
TRINITY_GAX8041	hypothet	Chlamydomonas_eusti	42.30	0.00
TRINITY_XP_0024	uncharac	Sorghum_bicolor	42.30	0.00
TRINITY_GAQ8493	3'-5'-ex	Klebsormidium_niten	42.30	0.00
TRINITY_XP_0017	predicte	Chlamydomonas_reinh	42.30	0.00
TRINITY_GAX8012	hypothet	Chlamydomonas_eusti	42.30	0.00
TRINITY_XP_0216	sodium/h	Manihot_esculenta	42.30	0.00
TRINITY_XP_0200	LOW QUAI	Ananas_comosus	42.30	0.00
TRINITY_XP_0029	hypothet	Volvox_carteri	42.30	0.00
TRINITY_PWA5849	chromati	Artemisia_annua	42.30	0.00
TRINITY_PNG9977	hypothet	Tetrabaena_socialis	42.30	0.00
TRINITY_XP_0105	PREDICTE	Tarenaya_hasslerian	42.30	0.00
TRINITY_GAX7864	hypothet	Chlamydomonas_eusti	42.30	0.00
TRINITY_KXZ5362	hypothet	Gonium_pectorale	42.30	0.00
TRINITY_XP_0229	E3 ubiqu	Cucurbita_maxima	42.30	0.00
TRINITY_PNW8543	hypothet	Chlamydomonas_reinh	42.30	0.00
TRINITY_GBG8422	hypothet	Chara_braunii	42.30	0.00
TRINITY_GAX7785	hypothet	Chlamydomonas_eusti	42.30	0.00
TRINITY_GAX8203	hypothet	Chlamydomonas_eusti	42.30	0.00
TRINITY_NP_0012	U1 small	Solanum_lycopersicu	42.30	0.00
TRINITY_PNH0432	GTPase (	Tetrabaena_socialis	42.30	0.00
TRINITY_GAX7682	hypothet	Chlamydomonas_eusti	42.30	0.00
TRINITY_GAX7686	hypothet	Chlamydomonas_eusti	42.30	0.00
TRINITY_GAX8258	hypothet	Chlamydomonas_eusti	42.30	0.00
TRINITY_PNW8330	hypothet	Chlamydomonas_reinh	42.30	0.00
TRINITY_XP_0030	predicte	Micromonas_pusilla	42.30	0.00
TRINITY_XP_0029	hypothet	Volvox_carteri	42.30	0.00
TRINITY_GAQ8672	ABC trar	Klebsormidium_niten	42.30	0.00

TRINITY_XP_0218(	thiosul	Spinacia_oleracea	42.30	0.00
TRINITY_PWA4428	Zinc fir	Artemisia_annua	42.30	0.00
TRINITY_XP_0029	kinesin-	Volvox_carteri	42.30	0.00
TRINITY_KEH2996	serine (	Medicago_truncatula	42.30	0.00
TRINITY_KXZ4933	hypothet	Gonium_pectorale	42.30	0.00
TRINITY_XP_0245	probable	Selaginella_moellen	42.30	0.00
TRINITY_GAQ9280	hypothet	Klebsormidium_niten	42.30	0.00
TRINITY_KXZ4748	hypothet	Gonium_pectorale	42.30	0.00
TRINITY_GAX8606	hypothet	Chlamydomonas_eusti	42.30	0.00
TRINITY_PNH0831	Dolichol	Tetrabaena_socialis	42.30	0.00
TRINITY_KCW8343	hypothet	Eucalyptus_grandis	42.30	0.00
TRINITY_XP_0239	60S rib	Quercus_suber	42.30	0.00
TRINITY_GBF9046	hypothet	Raphidocelis_subcap	42.30	0.00
TRINITY_GAX7257	hypothet	Chlamydomonas_eusti	42.30	0.00
TRINITY_KVH9064	Alpha tu	Cynara_cardunculus	42.30	0.00
TRINITY_XP_0029	hypothet	Volvox_carteri	42.30	0.00
TRINITY_PNH0148	putative	Tetrabaena_socialis	42.30	0.00
TRINITY_PNW7139	hypothet	Chlamydomonas_reinh	42.30	0.00
TRINITY_GBF8849	hypothet	Raphidocelis_subcap	42.30	0.00
TRINITY_PRW5870	multidru	Chlorella_sorokinia	42.30	0.00
TRINITY_XP_0029	hypothet	Volvox_carteri	42.30	0.00
TRINITY_GAX7688	hypothet	Chlamydomonas_eusti	42.30	0.00
TRINITY_PNW8898	hypothet	Chlamydomonas_reinh	42.30	0
TRINITY_PNW8523	hypothet	Chlamydomonas_reinh	42.30	0.00
TRINITY_XP_0056	calcium-	Coccomyxa_subellips	42.20	0.00
TRINITY_XP_0102	E3 UFM1-	Brachypodium_distac	42.20	0.00
TRINITY_RMZ5769	hypothet	Auxenochlorella_pro	42.20	0.00
TRINITY_KMS9368	hypothet	Beta_vulgaris	42.20	0.00
TRINITY_XP_0127	serine/t	Setaria_italica	42.20	0.00
TRINITY_GAX7560	hypothet	Chlamydomonas_eusti	42.20	0.00
TRINITY_XP_0243	E3 UFM1-	Physcomitrella_pate	42.20	0.00
TRINITY_A8ISN6.2	RecName:	Chlamydomonas_reinh	42.20	0.00
TRINITY_RXH8872	hypothet	Malus_domestica	42.20	0.00
TRINITY_XP_0116	PREDICTE	Cucumis_sativus	42.20	0.00
TRINITY_XP_0243	transcri	Physcomitrella_pate	42.20	0.00
TRINITY_PNR5300	hypothet	Physcomitrella_pate	42.20	0.00
TRINITY_XP_0058	hypothet	Chlorella_variabili	42.20	0.00
TRINITY_XP_0062	vacuolar	Capsella_rubella	42.20	0.00
TRINITY_GAX8188	hypothet	Chlamydomonas_eusti	42.20	0.00
TRINITY_GAX7772	hypothet	Chlamydomonas_eusti	42.20	0.00
TRINITY_KXZ4752	hypothet	Gonium_pectorale	42.20	0.00
TRINITY_GBG4227	hypothet	Chara_braunii	42.20	0.00
TRINITY_XP_0245	phosphat	Selaginella_moellen	42.20	0.00
TRINITY_XP_0227	external	Durio_zibethinus	42.20	0.00
TRINITY_XP_0070	PREDICTE	Theobroma_cacao	42.20	0.00
TRINITY_GAX8306	hypothet	Chlamydomonas_eusti	42.20	0.00
TRINITY_GAQ8019	serine/t	Klebsormidium_niten	42.20	0.00
TRINITY_XP_0124	PREDICTE	Gossypium_raimondii	42.20	0.00
TRINITY_GAX8158	hypothet	Chlamydomonas_eusti	42.20	0.00
TRINITY_GAQ8088	adaptor	Klebsormidium_niten	42.20	0.00
TRINITY_OAY7436	Geranylg	Ananas_comosus	42.20	0.00
TRINITY_PON3591	GPCR kin	Parasponia_anderson	42.20	0.00
TRINITY_GAQ8458	histidin	Klebsormidium_niten	42.20	0.00
TRINITY_OAE2310	hypothet	Marchantia_polymorp	42.20	0.00
TRINITY_XP_0239	probable	Quercus_suber	42.20	0.00
TRINITY_GAQ8964	transcri	Klebsormidium_niten	42.20	0.00
TRINITY_XP_0116	PREDICTE	Cucumis_sativus	42.20	0.00
TRINITY_PSC6950	Oxidored	Micractinium_conduc	42.20	0.00
TRINITY_GAX8495	hypothet	Chlamydomonas_eusti	42.20	0.00
TRINITY_GBF9811	hypothet	Raphidocelis_subcap	42.20	0.00
TRINITY_PNW8338	hypothet	Chlamydomonas_reinh	42.20	0.00
TRINITY_RWR9417	Peptidas	Cinnamomum_micranth	42.20	0.00
TRINITY_XP_0068	ABC tran	Amborella_trichopod	42.20	0.00
TRINITY_GBF9399	1-phosph	Raphidocelis_subcap	42.20	0.00
TRINITY_KXZ4690	hypothet	Gonium_pectorale	42.20	0.00
TRINITY_GBG8399	hypothet	Chara_braunii	42.20	0.00

TRINITY_KHG1242	hydrola	Gossypium_arboreum	42.20	0.00
TRINITY_XP_0241	WD and	Rosa_chinensis	42.20	0.00
TRINITY_PNH0146	hypothet	Tetrabaena_socialis	42.20	0.00
TRINITY_XP_0029	hypothet	Volvox_carteri	42.20	0.00
TRINITY_KXZ5078	hypothet	Gonium_pectorale	42.20	0.00
TRINITY_KXZ4998	hypothet	Gonium_pectorale	42.20	0.00
TRINITY_GBG8855	hypothet	Chara_braunii	42.20	0.00
TRINITY_XP_0029	hypothet	Volvox_carteri	42.20	0.00
TRINITY_KXZ5640	hypothet	Gonium_pectorale	42.20	0.00
TRINITY_XP_0243	DNA mis	Physcomitrella_pate	42.20	0.00
TRINITY_PLY6959	hypothet	Lactuca_sativa	42.20	0.00
TRINITY_XP_0243	probabl	Physcomitrella_pate	42.20	0.00
TRINITY_RXH6957	hypothet	Malus_domestica	42.20	0.00
TRINITY_XP_0270	WD40 re	Coffea_arabica	42.20	0.00
TRINITY_XP_0228	Aminoac	Ostreococcus_tauri	42.20	0.00
TRINITY_OAE2722	hypothet	Marchantia_polymorp	42.20	0.00
TRINITY_XP_0017	predict	Chlamydomonas_reinh	42.20	0.00
TRINITY_XP_0029	hypothet	Volvox_carteri	42.20	0.00
TRINITY_PNH1268	hypothet	Tetrabaena_socialis	42.20	0.00
TRINITY_GAX7865	hypothet	Chlamydomonas_eusti	42.20	0.00
TRINITY_GBG7917	hypothet	Chara_braunii	42.20	0.00
TRINITY_XP_0095	PREDICT	Nicotiana_tomentosi	42.20	0.00
TRINITY_PNW8470	hypothet	Chlamydomonas_reinh	42.20	0.00
TRINITY_RWW9025	hypothet	Ensete_ventricosum	42.20	0.00
TRINITY_GAX7493	hypothet	Chlamydomonas_eusti	42.20	0.00
TRINITY_PNW7911	hypothet	Chlamydomonas_reinh	42.20	0.00
TRINITY_PWA7999	sirtuin	Artemisia_annua	42.10	0.00
TRINITY_XP_0062	putativ	Capsella_rubella	42.10	0.00
TRINITY_OUS4248	glutami	Ostreococcus_tauri	42.10	0.00
TRINITY_XP_0066	PREDICT	Oryza_brachyantha	42.10	0.00
TRINITY_XP_0239	ras-lik	Quercus_suber	42.10	0.00
TRINITY_XP_0201	BTB/POZ	Aegilops_tauschii	42.10	0.00
TRINITY_OVA0083	Cytochr	Macleaya_cordata	42.10	0.00
TRINITY_RWW9100	hypothet	Ensete_ventricosum	42.10	0.00
TRINITY_XP_0194	PREDICT	Lupinus_angustifoli	42.10	0.00
TRINITY_ADE7785	unknown	Picea_sitchensis	42.10	0.00
TRINITY_XP_0172	PREDICT	Daucus_carota	42.10	0.00
TRINITY_RYQ8186	hypothet	Arachis_hypogaea	42.10	0.00
TRINITY_XP_0029	hypothet	Volvox_carteri	42.10	0.00
TRINITY_KMS6524	hypothet	Beta_vulgaris	42.10	0.00
TRINITY_XP_0243	short-c	Physcomitrella_pate	42.10	0.00
TRINITY_XP_0049	NADH de	Setaria_italica	42.10	0.00
TRINITY_XP_0058	hypothet	Chlorella_variabili	42.10	0.00
TRINITY_GBF9841	hypothet	Raphidocelis_subcap	42.10	0.00
TRINITY_XP_0102	lanC-li	Brachypodium_distac	42.10	0.00
TRINITY_OAE3180	hypothet	Marchantia_polymorp	42.10	0.00
TRINITY_EOY3460	Mitocho	Theobroma_cacao	42.10	0.00
TRINITY_XP_0030	Transcr	Ostreococcus_tauri	42.10	0.00
TRINITY_AET0241	hypothet	Medicago_truncatula	42.10	0.00
TRINITY_XP_0249	long ch	Cynara_cardunculus	42.10	0.00
TRINITY_RCU6142	hypothet	Setaria_italica	42.10	0.00
TRINITY_GAX7314	hypothet	Chlamydomonas_eusti	42.10	0.00
TRINITY_XP_0056	peptida	Coccomyxa_subellips	42.10	0.00
TRINITY_XP_0249	heterog	Cynara_cardunculus	42.10	0.00
TRINITY_XP_0144	ABC tra	Vigna_radiata	42.10	0.00
TRINITY_GAX8086	hypothet	Chlamydomonas_eusti	42.10	0.00
TRINITY_GAX7635	hypothet	Chlamydomonas_eusti	42.10	0.00
TRINITY_PTQ2884	hypothet	Marchantia_polymorp	42.10	0.00
TRINITY_OAE2879	hypothet	Marchantia_polymorp	42.10	0.00
TRINITY_XP_0239	lon pro	Quercus_suber	42.10	0.00
TRINITY_GAX7736	hypothet	Chlamydomonas_eusti	42.10	0.00
TRINITY_KXZ5556	hypothet	Gonium_pectorale	42.10	0.00
TRINITY_XP_0273	probabl	Abrus_precatorius	42.10	0.00
TRINITY_XP_0239	protein	Quercus_suber	42.10	0.00
TRINITY_KXZ5459	hypothet	Gonium_pectorale	42.10	0.00
TRINITY_XP_0016	mitogen	Chlamydomonas_reinh	42.10	0.00

TRINITY_RLM69424	hypothet	Panicum_miliaceum	42.10	0.00
TRINITY_GBF93029	hypothet	Raphidocelis_subcap	42.10	0.00
TRINITY_XP_01390	hypothet	Monoraphidium_negle	42.10	0.00
TRINITY_GBF92257	thioredo	Raphidocelis_subcap	42.10	0.00
TRINITY_XP_02131	phosphol	Sorghum_bicolor	42.10	0.00
TRINITY_GBF99949	hypothet	Raphidocelis_subcap	42.10	0.00
TRINITY_KXZ44063	hypothet	Gonium_pectorale	42.10	0.00
TRINITY_XP_01390	Sodium (	Monoraphidium_negle	42.10	0.00
TRINITY_XP_01918	PREDICTE	Ipomoea_nil	42.10	0.00
TRINITY_GAX73207	hypothet	Chlamydomonas_eusti	42.10	0.00
TRINITY_GAX72786	hypothet	Chlamydomonas_eusti	42.10	0.00
TRINITY_GAX84054	hypothet	Chlamydomonas_eusti	42.10	0.00
TRINITY_GAX75212	hypothet	Chlamydomonas_eusti	42.10	0.00
TRINITY_GAX82503	hypothet	Chlamydomonas_eusti	42.10	0.00
TRINITY_ADE76381	unknown	Picea_sitchensis	42.10	0.00
TRINITY_GAX81523	hypothet	Chlamydomonas_eusti	42.10	0.00
TRINITY_GAX75618	hypothet	Chlamydomonas_eusti	42.10	0.00
TRINITY_XP_00564	P-loop (	Coccomyxa_subellips	42.10	0.00
TRINITY_BBC28459	predicte	Yamagishiella_unico	42.10	0.00
TRINITY_GBF88516	hypothet	Raphidocelis_subcap	42.10	0.00
TRINITY_XP_00169	predicte	Chlamydomonas_reinh	42.10	0.00
TRINITY_GAX77748	hypothet	Chlamydomonas_eusti	42.10	0.00
TRINITY_GAX77105	hypothet	Chlamydomonas_eusti	42.10	0.00
TRINITY_GAX83671	hypothet	Chlamydomonas_eusti	42.10	0.00
TRINITY_XP_00141	predicte	Ostreococcus_sp._'l	42.10	0.00
TRINITY_XP_02059	serine/a	Phalaenopsis_equest	42.10	0.00
TRINITY_PNW78927	hypothet	Chlamydomonas_reinh	42.10	0.00
TRINITY_KXZ49410	hypothet	Gonium_pectorale	42.10	0.00
TRINITY_XP_02390	dipeptic	Quercus_suber	42.10	0.00
TRINITY_XP_02390	GTP-binc	Quercus_suber	42.00	0.00
TRINITY_XP_00962	PREDICTE	Nicotiana_tomentosi	42.00	0.00
TRINITY_XP_00250	predicte	Micromonas_commoda	42.00	0.00
TRINITY_RWW90255	hypothet	Ensete_ventricosum	42.00	0.00
TRINITY_XP_00226	PREDICTE	Vitis_vinifera	42.00	0.00
TRINITY_XP_02644	cell div	Papaver_somniferum	42.00	0.00
TRINITY_NP_00123	uncharac	Glycine_max	42.00	0.00
TRINITY_XP_00879	flavin-c	Phoenix_dactylifera	42.00	0.00
TRINITY_XP_01363	PREDICTE	Brassica_oleracea	42.00	0.00
TRINITY_XP_01245	PREDICTE	Gossypium_raimondii	42.00	0.00
TRINITY_XP_02008	uncharac	Ananas_comosus	42.00	0.00
TRINITY_XP_00935	PREDICTE	Pyrus_x_bretschneid	42.00	0.00
TRINITY_ONM09725	Serine/t	Zea_mays	42.00	0.00
TRINITY_EFJ38356	hypothet	Selaginella_moellen	42.00	0.00
TRINITY_XP_01140	Alkylgly	Auxenochlorella_pro	42.00	0.00
TRINITY_OAE29107	hypothet	Marchantia_polymorp	42.00	0.00
TRINITY_GBG84318	hypothet	Chara_braunii	42.00	0.00
TRINITY_ABR21719	calmodul	Actinidia_kolomikta	42.00	0.00
TRINITY_GBG89628	hypothet	Chara_braunii	42.00	0.00
TRINITY_XP_02375	protein	Lactuca_sativa	42.00	0.00
TRINITY_XP_00294	hypothet	Volvox_carteri	42.00	0.00
TRINITY_XP_00751	aminopho	Bathycoccus_prasino	42.00	0.00
TRINITY_GAX76816	hypothet	Chlamydomonas_eusti	42.00	0.00
TRINITY_XP_01564	ras-rela	Oryza_sativa	42.00	0.00
TRINITY_XP_01052	PREDICTE	Tarenaya_hasslerian	42.00	0.00
TRINITY_KXZ48315	hypothet	Gonium_pectorale	42.00	0.00
TRINITY_GAQ80700	lactosyl	Klebsormidium_niten	42.00	0.00
TRINITY_GAX85788	hypothet	Chlamydomonas_eusti	42.00	0.00
TRINITY_ERN18092	hypothet	Amborella_trichopod	42.00	0.00
TRINITY_KXZ56905	hypothet	Gonium_pectorale	42.00	0.00
TRINITY_XP_00975	PREDICTE	Nicotiana_sylvestri	42.00	0.00
TRINITY_XP_02436	double-s	Physcomitrella_pate	42.00	0.00
TRINITY_GAX76004	hypothet	Chlamydomonas_eusti	42.00	0.00
TRINITY_XP_00295	hypothet	Volvox_carteri	42.00	0.00
TRINITY_PTQ45236	hypothet	Marchantia_polymorp	42.00	0.00
TRINITY_XP_00565	ADP,ATP	Coccomyxa_subellips	42.00	0.00
TRINITY_XP_00713	hypothet	Phaseolus_vulgaris	42.00	0.00

TRINITY_XP_0025(	predicte	Micromonas_commoda	42.00	0.00
TRINITY_XP_0217(	conserve	Chenopodium_quinoa	42.00	0.00
TRINITY_XP_0239(	probable	Quercus_suber	42.00	0.00
TRINITY_GAX8008(	hypothet	Chlamydomonas_eusti	42.00	0.00
TRINITY_XP_0136:	PREDICTI	Brassica_oleracea	42.00	0.00
TRINITY_XP_0029:	hypothet	Volvox_carteri	42.00	0.00
TRINITY_XP_0029!	hypothet	Volvox_carteri	42.00	0.00
TRINITY_XP_0016!	predicte	Chlamydomonas_reinh	42.00	0.00
TRINITY_XP_0221:	serine/t	Momordica_charantia	42.00	0.00
TRINITY_GAX8058(	hypothet	Chlamydomonas_eusti	42.00	0.00
TRINITY_OAE2715:	hypothet	Marchantia_polymorp	42.00	0.00
TRINITY_KXZ4385:	hypothet	Gonium_pectorale	42.00	0.00
TRINITY_EFH5167'	predicte	Arabidopsis_lyrata	42.00	0.00
TRINITY_GAX7344:	hypothet	Chlamydomonas_eusti	42.00	0.00
TRINITY_XP_0138!	hypothet	Monoraphidium_negle	42.00	0.00
TRINITY_GAX7653:	hypothet	Chlamydomonas_eusti	42.00	0.00
TRINITY_GAX7499:	hypothet	Chlamydomonas_eusti	42.00	0.00
TRINITY_PNG9953:	hypothet	Tetrabaena_socialis	42.00	0.00
TRINITY_XP_0087!	probable	Phoenix_dactylifera	42.00	0.00
TRINITY_PNW8044:	hypothet	Chlamydomonas_reinh	42.00	0.00
TRINITY_GAX7695(	hypothet	Chlamydomonas_eusti	42.00	0.00
TRINITY_PNW8683(	hypothet	Chlamydomonas_reinh	42.00	0.00
TRINITY_XP_0114(	Cob(I)y:	Auxenochlorella_prc	42.00	0.00
TRINITY_GAX7935:	hypothet	Chlamydomonas_eusti	42.00	0.00
TRINITY_XP_0241{	nodal m	Rosa_chinensis	42.00	0.00
TRINITY_PNW8061:	hypothet	Chlamydomonas_reinh	42.00	0.00
TRINITY_XP_0056:	hypothet	Coccomyxa_subellips	42.00	0.00
TRINITY_PNW7900:	hypothet	Chlamydomonas_reinh	42.00	0.00
TRINITY_GAX8080'	hypothet	Chlamydomonas_eusti	42.00	0.00
TRINITY_PNW8810(	hypothet	Chlamydomonas_reinh	42.00	0.00
TRINITY_GAX7734:	hypothet	Chlamydomonas_eusti	42.00	0.00
TRINITY_GAX8012{	hypothet	Chlamydomonas_eusti	42.00	0.00
TRINITY_PNW7834{	hypothet	Chlamydomonas_reinh	42.00	0.00
TRINITY_GAX7636'	hypothet	Chlamydomonas_eusti	42.00	0.00
TRINITY_GAX8489!	hypothet	Chlamydomonas_eusti	42.00	0.00
TRINITY_XP_0239(	uncharac	Quercus_suber	42.00	0.00
TRINITY_XP_0188:	PREDICTI	Juglans_regia	42.00	0.00
TRINITY_NP_0011:	uncharac	Zea_mays	41.90	0.00
TRINITY_XP_0014:	predicte	Ostreococcus_sp._'l	41.90	0.00
TRINITY_ERN1242(	hypothet	Amborella_trichopod	41.90	0.00
TRINITY_XP_0165(	PREDICTI	Capsicum_annuum	41.90	0.00
TRINITY_XP_0172!	PREDICTI	Daucus_carota	41.90	0.00
TRINITY_GAX7672:	hypothet	Chlamydomonas_eusti	41.90	0.00
TRINITY_PKA5124:	E3 ubiq	Apostasia_shenzheni	41.90	0.00
TRINITY_XP_0220:	ras-rela	Helianthus_annuus	41.90	0.00
TRINITY_XP_0279:	GDSL est	Vigna_unguiculata	41.90	0.00
TRINITY_XP_0017(	seleniur	Chlamydomonas_reinh	41.90	0.00
TRINITY_XP_0114(	PREDICTI	Fragaria_vesca	41.90	0.00
TRINITY_XP_0194:	PREDICTI	Lupinus_angustifoli	41.90	0.00
TRINITY_XP_0240:	CBL-inte	Citrus_clementina	41.90	0.00
TRINITY_AFK4375!	unknown	Lotus_japonicus	41.90	0.00
TRINITY_RAL5060:	hypothet	Cuscuta_australis	41.90	0.00
TRINITY_XP_0030(	predicte	Micromonas_pusilla	41.90	0.00
TRINITY_GBG8022!	hypothet	Chara_braunii	41.90	0.00
TRINITY_XP_0172:	PREDICTI	Daucus_carota	41.90	0.00
TRINITY_XP_0014:	predicte	Ostreococcus_sp._'l	41.90	0.00
TRINITY_XP_0245:	tRNA (gu	Selaginella_moellen	41.90	0.00
TRINITY_XP_0016!	copper :	Chlamydomonas_reinh	41.90	0.00
TRINITY_XP_0058!	hypothet	Chlorella_variabili	41.90	0.00
TRINITY_GAQ9171'	hypothet	Klebsormidium_niten	41.90	0.00
TRINITY_GAX8100:	hypothet	Chlamydomonas_eusti	41.90	0.00
TRINITY_XP_0030(	predicte	Micromonas_pusilla	41.90	0.00
TRINITY_XP_0128:	PREDICTI	Erythranthe_guttata	41.90	0.00
TRINITY_AGC1314!	theta c:	Pinus_tabuliformis	41.90	0.00
TRINITY_VDD4901!	unnamed	Brassica_oleracea	41.90	0.00
TRINITY_KXZ5702:	hypothet	Gonium_pectorale	41.90	0.00

TRINITY_XP_0243	beta-ada	Physcomitrella_pate	41.90	0.00
TRINITY_GAQ7781	branche	Klebsormidium_niten	41.90	0.00
TRINITY_XP_0258	uncharac	Panicum_hallii	41.90	0.00
TRINITY_GAX8557	hypothet	Chlamydomonas_eusti	41.90	0.00
TRINITY_PNW8533	hypothet	Chlamydomonas_reinh	41.90	0.00
TRINITY_PNH0820	hypothet	Tetrabaena_socialis	41.90	0.00
TRINITY_EYU2887	hypothet	Erythranthe_guttata	41.90	0.00
TRINITY_GAQ8231	protein-	Klebsormidium_niten	41.90	0.00
TRINITY_GAX7959	hypothet	Chlamydomonas_eusti	41.90	0.00
TRINITY_XP_0016	uric ac	Chlamydomonas_reinh	41.90	0.00
TRINITY_XP_0029	subunit	Volvox_carteri	41.90	0.00
TRINITY_XP_0228	serine/t	Olea_europaea	41.90	0.00
TRINITY_KZV0671	single-s	Dorcoceras_hygromet	41.90	0.00
TRINITY_XP_0025	predicte	Micromonas_commoda	41.90	0.00
TRINITY_PSC6921	FAD-link	Micractinium_conduc	41.90	0.00
TRINITY_XP_0239	arginine	Quercus_suber	41.90	0.00
TRINITY_PNW8427	hypothet	Chlamydomonas_reinh	41.90	0.00
TRINITY_XP_0100	PREDICTE	Eucalyptus_grandis	41.90	0.00
TRINITY_GAX7522	hypothet	Chlamydomonas_eusti	41.90	0.00
TRINITY_XP_0228	uncharac	Olea_europaea	41.90	0.00
TRINITY_GAX7561	hypothet	Chlamydomonas_eusti	41.90	0.00
TRINITY_XP_0138	Esterase	Monoraphidium_negle	41.90	0.00
TRINITY_GAX7342	hypothet	Chlamydomonas_eusti	41.90	0.00
TRINITY_ABU5296	hypothet	Dunaliella_viridis	41.90	0.00
TRINITY_XP_0156	pirin-li	Oryza_sativa	41.90	0.00
TRINITY_XP_0066	PREDICTE	Oryza_brachyantha	41.90	0.00
TRINITY_PTQ3075	hypothet	Marchantia_polymorp	41.90	0.00
TRINITY_XP_0056	Metallo-	Coccomyxa_subellips	41.90	0.00
TRINITY_GAX8388	hypothet	Chlamydomonas_eusti	41.90	0.00
TRINITY_XP_0029	hypothet	Volvox_carteri	41.90	0.00
TRINITY_GAX7924	hypothet	Chlamydomonas_eusti	41.90	0.00
TRINITY_GAX8099	hypothet	Chlamydomonas_eusti	41.90	0.00
TRINITY_GAX8206	hypothet	Chlamydomonas_eusti	41.90	0.00
TRINITY_PKA6194	Ras-rela	Apostasia_shenzheni	41.90	0.00
TRINITY_XP_0270	bifuncti	Coffea_arabica	41.90	0.00
TRINITY_GBG7703	hypothet	Chara_braunii	41.90	0.00
TRINITY_XP_0056	hypothet	Coccomyxa_subellips	41.90	0.00
TRINITY_XP_0216	serine/t	Hevea_brasiliensis	41.90	0.00
TRINITY_EPS5921	hypothet	Genlisea_aurea	41.90	0.00
TRINITY_XP_0017	predicte	Chlamydomonas_reinh	41.90	0.00
TRINITY_ABK2543	unknown	Picea_sitchensis	41.80	0.00
TRINITY_XP_0110	uncharac	Sesamum_indicum	41.80	0.00
TRINITY_XP_0239	vacuola	Quercus_suber	41.80	0.00
TRINITY_XP_0106	PREDICTE	Beta_vulgaris	41.80	0.00
TRINITY_GBF8979	hypothet	Raphidocelis_subcap	41.80	0.00
TRINITY_XP_0237	glycerol	Lactuca_sativa	41.80	0.00
TRINITY_GAQ8386	hypothet	Klebsormidium_niten	41.80	0.00
TRINITY_OAE2508	hypothet	Marchantia_polymorp	41.80	0.00
TRINITY_XP_0106	PREDICTE	Beta_vulgaris	41.80	0.00
TRINITY_PQQ1034	uncharac	Prunus_yedoensis	41.80	0.00
TRINITY_GAX8177	hypothet	Chlamydomonas_eusti	41.80	0.00
TRINITY_KMZ6112	putative	Zostera_marina	41.80	0.00
TRINITY_XP_0219	autophag	Carica_papaya	41.80	0.00
TRINITY_PKA6239	WD-40 re	Apostasia_shenzheni	41.80	0.00
TRINITY_PTQ3691	hypothet	Marchantia_polymorp	41.80	0.00
TRINITY_GBF9760	acidstab	Raphidocelis_subcap	41.80	0.00
TRINITY_PRW5818	manganes	Chlorella_sorokinia	41.80	0.00
TRINITY_XP_0158	isoaspa	Ziziphus_jujuba	41.80	0.00
TRINITY_AZS6417	tubulin	Hypericum_perforatu	41.80	0.00
TRINITY_GAQ8841	Phosphat	Klebsormidium_niten	41.80	0.00
TRINITY_XP_0084	PREDICTE	Cucumis_melo	41.80	0.00
TRINITY_XP_0263	probable	Papaver_somniferum	41.80	0.00
TRINITY_XP_0109	PREDICTE	Elaeis_guineensis	41.80	0.00
TRINITY_XP_0238	ras-rela	Quercus_suber	41.80	0.00
TRINITY_GAX7272	hypothet	Chlamydomonas_eusti	41.80	0.00
TRINITY_RZB7823	Regulato	Glycine_soja	41.80	0.00

TRINITY_KXZ4165(	hypothet	Gonium_pectorale	41.80	0.00
TRINITY_PLY7766(	hypothet	Lactuca_sativa	41.80	0.00
TRINITY_XP_0219(	serine/t	Helianthus_annuus	41.80	0.00
TRINITY_GAQ9054(	Ubiquiti	Klebsormidium_niten	41.80	0.00
TRINITY_EFJ2488(	hypothet	Selaginella_moellen	41.80	0.00
TRINITY_XP_0194(	PREDICTE	Lupinus_angustifoli	41.80	0.00
TRINITY_XP_0191(	PREDICTE	Ipomoea_nil	41.80	0.00
TRINITY_PRW3317(	acyl- th	Chlorella_sorokinia	41.80	0.00
TRINITY_RWW9036(	hypothet	Ensete_ventricosum	41.80	0.00
TRINITY_XP_0030(	predicte	Micromonas_pusilla	41.80	0.00
TRINITY_XP_0029(	homeodor	Volvox_carteri	41.80	0.00
TRINITY_PNW8805(	hypothet	Chlamydomonas_reinh	41.80	0.00
TRINITY_XP_0166(	PREDICTE	Gossypium_hirsutum	41.80	0.00
TRINITY_XP_0016(	CCR4-NOT	Chlamydomonas_reinh	41.80	0.00
TRINITY_XP_0139(	FkbM fam	Monoraphidium_negle	41.80	0.00
TRINITY_XP_0029(	tRNA 2'-	Selaginella_moellen	41.80	0.00
TRINITY_XP_0025(	p-type A	Micromonas_commoda	41.80	0.00
TRINITY_XP_0029(	hypothet	Volvox_carteri	41.80	0.00
TRINITY_PSC7036(	BTB POZ	Micractinium_conduc	41.80	0.00
TRINITY_GBF9414(	hypothet	Raphidocelis_subcap	41.80	0.00
TRINITY_GAX8057(	hypothet	Chlamydomonas_eusti	41.80	0.00
TRINITY_XP_0228(	UBA-like	Ostreococcus_tauri	41.80	0.00
TRINITY_XP_0263(	short-ch	Papaver_somniferum	41.80	0.00
TRINITY_KZV1512(	oxidorec	Dorcoceras_hygromet	41.80	0.00
TRINITY_PNW7570(	hypothet	Chlamydomonas_reinh	41.80	0.00
TRINITY_PKA5595(	hypothet	Apostasia_shenzheni	41.80	0.00
TRINITY_XP_0219(	cinnamoy	Helianthus_annuus	41.80	0.00
TRINITY_GAX8575(	hypothet	Chlamydomonas_eusti	41.80	0.00
TRINITY_XP_0243(	epiderma	Physcomitrella_pate	41.80	0.00
TRINITY_PNW8400(	hypothet	Chlamydomonas_reinh	41.80	0.00
TRINITY_PNW7525(	hypothet	Chlamydomonas_reinh	41.80	0.00
TRINITY_GAQ8941(	Kinesin-	Klebsormidium_niten	41.80	0.00
TRINITY_XP_0025(	predicte	Micromonas_commoda	41.80	0.00
TRINITY_XP_0136(	PREDICTE	Brassica_oleracea	41.80	0.00
TRINITY_XP_0279(	small gl	Vigna_unguiculata	41.80	0.00
TRINITY_XP_0243(	PHD fing	Physcomitrella_pate	41.80	0.00
TRINITY_PNW7053(	hypothet	Chlamydomonas_reinh	41.80	0.00
TRINITY_OAO8919(	hypothet	Arabidopsis_thalian	41.80	0.00
TRINITY_PNW7906(	hypothet	Chlamydomonas_reinh	41.80	0.00
TRINITY_PNW7174(	hypothet	Chlamydomonas_reinh	41.80	0.00
TRINITY_XP_0104(	PREDICTE	Camelina_sativa	41.80	0.00
TRINITY_XP_0029(	hypothet	Volvox_carteri	41.80	0.00
TRINITY_GAQ8152(	Vesicle	Klebsormidium_niten	41.80	0.00
TRINITY_XP_0016(	SNF2 sup	Chlamydomonas_reinh	41.80	0.00
TRINITY_PNW8868(	hypothet	Chlamydomonas_reinh	41.80	0.00
TRINITY_PNW8327(	hypothet	Chlamydomonas_reinh	41.80	0.00
TRINITY_BAJ9689(	predicte	Hordeum_vulgare	41.80	0.00
TRINITY_BAV5831(	hypothet	Ulva_partita	41.80	0.00
TRINITY_GAX7990(	hypothet	Chlamydomonas_eusti	41.80	0.00
TRINITY_XP_0056(	alpha/be	Coccomyxa_subellips	41.80	0.00
TRINITY_XP_0017(	predicte	Chlamydomonas_reinh	41.80	0.00
TRINITY_XP_0016(	predicte	Chlamydomonas_reinh	41.80	0.00
TRINITY_GAX7505(	hypothet	Chlamydomonas_eusti	41.80	0.00
TRINITY_GAX7950(	hypothet	Chlamydomonas_eusti	41.80	0.00
TRINITY_GBG6475(	hypothet	Chara_braunii	41.80	0.00
TRINITY_EFJ1467(	hypothet	Selaginella_moellen	41.80	0.00
TRINITY_GAX8643(	hypothet	Chlamydomonas_eusti	41.80	0.00
TRINITY_OMO8630(	hypothet	Corchorus_capsulari	41.80	0.00
TRINITY_EEF2267(	two-comp	Ricinus_communis	41.80	0.00
TRINITY_GBF9178(	hypothet	Raphidocelis_subcap	41.70	0.00
TRINITY_PIA3303(	hypothet	Aquilegia_coerulea	41.70	0.00
TRINITY_XP_0016(	predicte	Chlamydomonas_reinh	41.70	0.00
TRINITY_PWA4260(	Regulato	Artemisia_annua	41.70	0.00
TRINITY_XP_0217(	serine/t	Chenopodium_quinoa	41.70	0.00
TRINITY_GAX7682(	hypothet	Chlamydomonas_eusti	41.70	0.00
TRINITY_EEF2487(	acyl-CoA	Ricinus_communis	41.70	0.00

TRINITY_GBF8955	canalic	Raphidocelis_subcap	41.70	0.00
TRINITY_GBF9591	hypothet	Raphidocelis_subcap	41.70	0.00
TRINITY_XP_0176	PREDICT	Gossypium_arboreum	41.70	0.00
TRINITY_XP_0238	protein	Quercus_suber	41.70	0.00
TRINITY_XP_0024	replicat	Sorghum_bicolor	41.70	0.00
TRINITY_XP_0239	ras-lik	Quercus_suber	41.70	0.00
TRINITY_XP_0238	G2-spec	Quercus_suber	41.70	0.00
TRINITY_XP_0239	kinesin	Quercus_suber	41.70	0.00
TRINITY_XP_0245	probabl	Selaginella_moellen	41.70	0.00
TRINITY_BAJ9794	predict	Hordeum_vulgare	41.70	0.00
TRINITY_RLN0070	hypothet	Panicum_miliaceum	41.70	0.00
TRINITY_GAX8391	hypothet	Chlamydomonas_eusti	41.70	0.00
TRINITY_XP_0016	predict	Chlamydomonas_reinh	41.70	0.00
TRINITY_XP_0093	PREDICT	Pyrus_x_bretschneid	41.70	0.00
TRINITY_GAQ8519	hypothet	Klebsormidium_niten	41.70	0.00
TRINITY_KXZ4771	hypothet	Gonium_pectorale	41.70	0.00
TRINITY_GAX7660	hypothet	Chlamydomonas_eusti	41.70	0.00
TRINITY_GAQ8019	serine/t	Klebsormidium_niten	41.70	0.00
TRINITY_XP_0024	dynein	Sorghum_bicolor	41.70	0.00
TRINITY_XP_0029	hypothet	Volvox_carteri	41.70	0.00
TRINITY_PKI6159	hypothet	Punica_granatum	41.70	0.00
TRINITY_XP_0271	cell di	Coffea_arabica	41.70	0.00
TRINITY_PHT2617	Protein	Capsicum_baccatum	41.70	0.00
TRINITY_PRW5662	denticl	Chlorella_sorokinia	41.70	0.00
TRINITY_GBG7003	hypothet	Chara_braunii	41.70	0.00
TRINITY_PIA4113	hypothet	Aquilegia_coerulea	41.70	0.00
TRINITY_PRW6140	nifU ch	Chlorella_sorokinia	41.70	0.00
TRINITY_GAX7505	hypothet	Chlamydomonas_eusti	41.70	0.00
TRINITY_XP_0082	PREDICT	Prunus_mume	41.70	0.00
TRINITY_GBF9506	hypothet	Raphidocelis_subcap	41.70	0.00
TRINITY_PRW5618	replicat	Chlorella_sorokinia	41.70	0.00
TRINITY_XP_0139	Phosphat	Monoraphidium_negle	41.70	0.00
TRINITY_XP_0243	transme	Physcomitrella_pate	41.70	0.00
TRINITY_XP_0219	unchara	Helianthus_annuus	41.70	0.00
TRINITY_GBF9940	DNA rep	Raphidocelis_subcap	41.70	0.00
TRINITY_XP_0016	predict	Chlamydomonas_reinh	41.70	0.00
TRINITY_XP_0029	hypothet	Volvox_carteri	41.70	0.00
TRINITY_XP_0043	PREDICT	Fragaria_vesca	41.70	0.00
TRINITY_EFJ0749	hypothet	Selaginella_moellen	41.70	0.00
TRINITY_ABK2481	unknown	Picea_sitchensis	41.70	0.00
TRINITY_XP_0216	transme	Hevea_brasiliensis	41.70	0.00
TRINITY_XP_0238	unchara	Quercus_suber	41.70	0.00
TRINITY_XP_0239	LOW QUA	Quercus_suber	41.70	0.00
TRINITY_GAV8916	UCH dom	Cephalotus_follicul	41.70	0.00
TRINITY_KXZ4624	hypothet	Gonium_pectorale	41.70	0.00
TRINITY_GAX8625	hypothet	Chlamydomonas_eusti	41.70	0.00
TRINITY_PON9520	GPCR ki	Trema_orientale	41.70	0.00
TRINITY_GAX7926	hypothet	Chlamydomonas_eusti	41.70	0.00
TRINITY_GAX8113	hypothet	Chlamydomonas_eusti	41.70	0.00
TRINITY_KMS9362	hypothet	Beta_vulgaris	41.70	0.00
TRINITY_GBG0036	hypothet	Raphidocelis_subcap	41.70	0.00
TRINITY_PNW8348	hypothet	Chlamydomonas_reinh	41.70	0.00
TRINITY_GAX8178	hypothet	Chlamydomonas_eusti	41.70	0.00
TRINITY_GAX7650	hypothet	Chlamydomonas_eusti	41.70	0.00
TRINITY_EMS6747	hypothet	Triticum_urartu	41.70	0.00
TRINITY_KZN0975	hypothet	Daucus_carota	41.70	0.00
TRINITY_PNH0442	Heme-bi	Tetrabaena_socialis	41.70	0.00
TRINITY_RWR7759	ADP-rib	Cinnamomum_micranth	41.70	0.00
TRINITY_GAX7629	hypothet	Chlamydomonas_eusti	41.70	0.00
TRINITY_GBF8735	K-stimu	Raphidocelis_subcap	41.70	0.00
TRINITY_GAX7566	hypothet	Chlamydomonas_eusti	41.70	0.00
TRINITY_GAX7578	hypothet	Chlamydomonas_eusti	41.70	0.00
TRINITY_XP_0243	vacuola	Physcomitrella_pate	41.70	0.00
TRINITY_XP_0229	eukaryo	Cucurbita_maxima	41.70	0.00
TRINITY_XP_0239	replicat	Quercus_suber	41.70	0.00
TRINITY_XP_0025	predict	Micromonas_commoda	41.70	0.00

TRINITY_XP_0238	probable	Quercus_suber	41.70	0.00
TRINITY_XP_0139	LanC-lik	Monoraphidium_negle	41.70	0.00
TRINITY_PNH0393	Condensi	Tetrabaena_socialis	41.70	0.00
TRINITY_XP_0239	dipeptic	Quercus_suber	41.70	0.00
TRINITY_XP_0220	uncharac	Helianthus_annuus	41.70	0.00
TRINITY_XP_0014	Ribosoma	Ostreococcus_sp._'l	41.70	0.00
TRINITY_XP_0016	predicte	Chlamydomonas_reinh	41.70	0.00
TRINITY_YP_0090	NADH deh	Chlorotetraedron_in	41.70	0.00
TRINITY_XP_0158	uncharac	Ziziphus_jujuba	41.70	0.00
TRINITY_BAD9589	Ser/Thr	Lotus_japonicus	41.70	0.00
TRINITY_XP_0075	riboflav	Bathycoccus_prasino	41.60	0.00
TRINITY_GAQ9115	Hypothet	Klebsormidium_niten	41.60	0.00
TRINITY_GBG6934	hypothet	Chara_braunii	41.60	0.00
TRINITY_RDX8694	hypothet	Mucuna_pruriens	41.60	0.00
TRINITY_XP_0238	trehalos	Quercus_suber	41.60	0.00
TRINITY_GBG8507	hypothet	Chara_braunii	41.60	0.00
TRINITY_EFJ1044	hypothet	Selaginella_moellen	41.60	0.00
TRINITY_XP_0206	signal p	Dendrobium_catenatu	41.60	0.00
TRINITY_PRW6087	Calcium	Chlorella_sorokinia	41.60	0.00
TRINITY_XP_0024	predicte	Micromonas_commoda	41.60	0.00
TRINITY_XP_0205	ABC tran	Phalaenopsis_equest	41.60	0.00
TRINITY_XP_0094	PREDICTE	Musa_acuminata	41.60	0.00
TRINITY_XP_0176	PREDICTE	Gossypium_arboreum	41.60	0.00
TRINITY_XP_0064	E3 ubiqu	Eutrema_salsugineum	41.60	0.00
TRINITY_PRW5703	UPF0544	Chlorella_sorokinia	41.60	0.00
TRINITY_XP_0147	eukaryot	Brachypodium_distac	41.60	0.00
TRINITY_GAX7303	hypothet	Chlamydomonas_eusti	41.60	0.00
TRINITY_PNW8719	hypothet	Chlamydomonas_reinh	41.60	0.00
TRINITY_XP_0156	costars	Oryza_sativa	41.60	0.00
TRINITY_KXZ4165	hypothet	Gonium_pectorale	41.60	0.00
TRINITY_GAX8562	hypothet	Chlamydomonas_eusti	41.60	0.00
TRINITY_GAX7398	hypothet	Chlamydomonas_eusti	41.60	0.00
TRINITY_XP_0075	predicte	Bathycoccus_prasino	41.60	0.00
TRINITY_XP_0238	guanine	Quercus_suber	41.60	0.00
TRINITY_KXZ4841	hypothet	Gonium_pectorale	41.60	0.00
TRINITY_XP_0016	predicte	Chlamydomonas_reinh	41.60	0.00
TRINITY_GBG7734	hypothet	Chara_braunii	41.60	0.00
TRINITY_XP_0102	PREDICTE	Nelumbo_nucifera	41.60	0.00
TRINITY_GAX7562	hypothet	Chlamydomonas_eusti	41.60	0.00
TRINITY_GAX8002	hypothet	Chlamydomonas_eusti	41.60	0.00
TRINITY_GAX7468	hypothet	Chlamydomonas_eusti	41.60	0.00
TRINITY_GAX7939	hypothet	Chlamydomonas_eusti	41.60	0.00
TRINITY_XP_0029	hypothet	Volvox_carteri	41.60	0.00
TRINITY_XP_0042	probable	Solanum_lycopersicu	41.60	0.00
TRINITY_GAQ7981	Sec23 pr	Klebsormidium_niten	41.60	0.00
TRINITY_XP_0238	LOW QUAL	Quercus_suber	41.60	0.00
TRINITY_BAB9039	ADP-ribo	Oryza_sativa	41.60	0.00
TRINITY_EEF2734	acylamin	Ricinus_communis	41.60	0.00
TRINITY_GAQ8815	Catalase	Klebsormidium_niten	41.60	0.00
TRINITY_EFH4694	PQ-loop	Arabidopsis_lyrata	41.60	0.00
TRINITY_CBI3729	unnamed	Vitis_vinifera	41.60	0.00
TRINITY_XP_0206	uncharac	Dendrobium_catenatu	41.60	0.00
TRINITY_XP_0138	putative	Monoraphidium_negle	41.60	0.00
TRINITY_PNW8634	hypothet	Chlamydomonas_reinh	41.60	0.00
TRINITY_CBN2077	mitochon	Polytomella_sp._Pri	41.60	0.00
TRINITY_RWW9025	hypothet	Ensete_ventricosum	41.60	0.00
TRINITY_BAJ9390	predicte	Hordeum_vulgare	41.60	0.00
TRINITY_XP_0029	hypothet	Volvox_carteri	41.60	0.00
TRINITY_XP_0201	ras-rela	Ananas_comosus	41.60	0.00
TRINITY_XP_0139	hypothet	Monoraphidium_negle	41.60	0.00
TRINITY_XP_0064	RING fin	Eutrema_salsugineum	41.60	0.00
TRINITY_XP_0110	PREDICTE	Populus_euphratica	41.60	0.00
TRINITY_XP_0042	eukaryot	Solanum_lycopersicu	41.60	0.00
TRINITY_XP_0056	calcium-	Coccomyxa_subellips	41.60	0.00
TRINITY_XP_0030	flagella	Micromonas_pusilla	41.60	0.00
TRINITY_PNH1099	hypothet	Tetrabaena_socialis	41.60	0.00

TRINITY_XP_0016	predicte	Chlamydomonas_reinh	41.60	0.00
TRINITY_XP_0217	TBC1 dor	Chenopodium_quinoa	41.60	0.00
TRINITY_RVX0256	Beta-glu	Vitis_vinifera	41.60	0.00
TRINITY_GAX8324	hypothet	Chlamydomonas_eusti	41.60	0.00
TRINITY_XP_0191	PREDICTE	Ipomoea_nil	41.60	0.00
TRINITY_GAQ7959	Insulina	Klebsormidium_niten	41.60	0.00
TRINITY_PNW8868	hypothet	Chlamydomonas_reinh	41.60	0.00
TRINITY_XP_0029	hypothet	Volvox_carteri	41.60	0.00
TRINITY_XP_0271	uncharac	Coffea_arabica	41.60	0.00
TRINITY_KXZ5297	DHC1 pro	Gonium_pectorale	41.60	0
TRINITY_PRW5990	histidyl	Chlorella_sorokinia	41.60	0.00
TRINITY_XP_0029	hypothet	Volvox_carteri	41.60	0.00
TRINITY_XP_0016	predicte	Chlamydomonas_reinh	41.60	0.00
TRINITY_XP_0063	serine/a	Populus_trichocarpa	41.60	0.00
TRINITY_OWM8583	hypothet	Punica_granatum	41.60	0.00
TRINITY_PNH1057	Retrovir	Tetrabaena_socialis	41.60	0.00
TRINITY_XP_0191	PREDICTE	Ipomoea_nil	41.50	0.00
TRINITY_EFJ2323	hypothet	Selaginella_moellen	41.50	0.00
TRINITY_OMO7601	hypothet	Corchorus_olitorius	41.50	0.00
TRINITY_XP_0138	protein	Monoraphidium_negle	41.50	0.00
TRINITY_GAQ8900	hypothet	Klebsormidium_niten	41.50	0.00
TRINITY_OTF8781	putative	Helianthus_annuus	41.50	0.00
TRINITY_XP_0058	hypothet	Chlorella_variabili	41.50	0.00
TRINITY_GAQ7905	ATP-depe	Klebsormidium_niten	41.50	0.00
TRINITY_XP_0016	voltage-	Chlamydomonas_reinh	41.50	0.00
TRINITY_GAX7871	hypothet	Chlamydomonas_eusti	41.50	0.00
TRINITY_XP_0200	serine/t	Ananas_comosus	41.50	0.00
TRINITY_OUS4782	adenylyl	Ostreococcus_tauri	41.50	0.00
TRINITY_GAQ7937	glutathi	Klebsormidium_niten	41.50	0.00
TRINITY_XP_0245	uncharac	Selaginella_moellen	41.50	0.00
TRINITY_XP_0058	hypothet	Chlorella_variabili	41.50	0.00
TRINITY_XP_0243	PAN2-PAN	Physcomitrella_pate	41.50	0.00
TRINITY_GAX8146	hypothet	Chlamydomonas_eusti	41.50	0.00
TRINITY_PTQ4957	hypothet	Marchantia_polymorp	41.50	0.00
TRINITY_EFJ1247	hypothet	Selaginella_moellen	41.50	0.00
TRINITY_GAX7759	hypothet	Chlamydomonas_eusti	41.50	0.00
TRINITY_XP_0137	uncharac	Brassica_napus	41.50	0.00
TRINITY_PNW8273	hypothet	Chlamydomonas_reinh	41.50	0.00
TRINITY_ERN1614	hypothet	Amborella_trichopod	41.50	0.00
TRINITY_XP_0110	vignain-	Sesamum_indicum	41.50	0.00
TRINITY_GAX7765	hypothet	Chlamydomonas_eusti	41.50	0.00
TRINITY_GBF9317	histone-	Raphidocelis_subcap	41.50	0.00
TRINITY_XP_0042	PREDICTE	Fragaria_vesca	41.50	0.00
TRINITY_PNH0879	hypothet	Tetrabaena_socialis	41.50	0.00
TRINITY_XP_0105	PREDICTE	Camelina_sativa	41.50	0.00
TRINITY_GAQ9228	hypothet	Klebsormidium_niten	41.50	0.00
TRINITY_GAX7347	hypothet	Chlamydomonas_eusti	41.50	0.00
TRINITY_GAX7318	hypothet	Chlamydomonas_eusti	41.50	0.00
TRINITY_PNW8781	hypothet	Chlamydomonas_reinh	41.50	0.00
TRINITY_XP_0105	PREDICTE	Tarenaya_hasslerian	41.50	0.00
TRINITY_GAQ8585	cytosoli	Klebsormidium_niten	41.50	0.00
TRINITY_XP_0172	PREDICTE	Daucus_carota	41.50	0.00
TRINITY_XP_0016	predicte	Chlamydomonas_reinh	41.50	0.00
TRINITY_XP_0238	eukaryot	Quercus_suber	41.50	0.00
TRINITY_GAX7418	hypothet	Chlamydomonas_eusti	41.50	0.00
TRINITY_VDD3240	unnamed	Brassica_oleracea	41.50	0.00
TRINITY_GAX7540	hypothet	Chlamydomonas_eusti	41.50	0.00
TRINITY_GAQ8369	putative	Klebsormidium_niten	41.50	0.00
TRINITY_OUS4587	hypothet	Ostreococcus_tauri	41.50	0.00
TRINITY_GAX7733	hypothet	Chlamydomonas_eusti	41.50	0.00
TRINITY_KXZ4664	hypothet	Gonium_pectorale	41.50	0.00
TRINITY_GAQ8172	Protein	Klebsormidium_niten	41.50	0.00
TRINITY_XP_0017	predicte	Chlamydomonas_reinh	41.50	0.00
TRINITY_KXZ4943	hypothet	Gonium_pectorale	41.50	0.00
TRINITY_XP_0071	hypothet	Phaseolus_vulgaris	41.50	0.00
TRINITY_XP_0105	PREDICTE	Tarenaya_hasslerian	41.50	0.00

TRINITY_XP_01003	PREDICTE	Eucalyptus_grandis	41.50	0.00
TRINITY_GAX73773	hypothet	Chlamydomonas_eusti	41.50	0.00
TRINITY_XP_02436	cAMP-dep	Physcomitrella_pate	41.50	0.00
TRINITY_GAX75058	hypothet	Chlamydomonas_eusti	41.50	0.00
TRINITY_KXZ51992	hypothet	Gonium_pectorale	41.50	0.00
TRINITY_OAE31210	hypothet	Marchantia_polymorp	41.50	0.00
TRINITY_XP_00564	protein	Coccomyxa_subellips	41.50	0.00
TRINITY_GAX77385	hypothet	Chlamydomonas_eusti	41.50	0.00
TRINITY_GBF94583	hypothet	Raphidocelis_subcap	41.50	0.00
TRINITY_GAX82655	hypothet	Chlamydomonas_eusti	41.50	0.00
TRINITY_KXZ54040	hypothet	Gonium_pectorale	41.50	0.00
TRINITY_KXZ49666	hypothet	Gonium_pectorale	41.50	0.00
TRINITY_XP_00295	flagella	Volvox_carteri	41.50	0.00
TRINITY_XP_00169	3'-5' ex	Chlamydomonas_reinh	41.50	0.00
TRINITY_GBF91333	hypothet	Raphidocelis_subcap	41.50	0.00
TRINITY_GAX84289	hypothet	Chlamydomonas_eusti	41.50	0.00
TRINITY_GAX82053	hypothet	Chlamydomonas_eusti	41.50	0.00
TRINITY_XP_01002	PREDICTE	Eucalyptus_grandis	41.50	0.00
TRINITY_KXZ51993	hypothet	Gonium_pectorale	41.50	0.00
TRINITY_OMO92046	Diphthar	Corchorus_olitorius	41.50	0.00
TRINITY_XP_00295	Selenopr	Volvox_carteri	41.40	0.00
TRINITY_BAJ98303	predicte	Hordeum_vulgare	41.40	0.00
TRINITY_XP_01389	Guanylat	Monoraphidium_negle	41.40	0.00
TRINITY_XP_00564	Metallo-	Coccomyxa_subellips	41.40	0.00
TRINITY_XP_02271	importin	Durio_zibethinus	41.40	0.00
TRINITY_RLM80357	NADP-dep	Panicum_miliaceum	41.40	0.00
TRINITY_XP_02452	multidru	Selaginella_moellen	41.40	0.00
TRINITY_XP_02387	mitochon	Quercus_suber	41.40	0.00
TRINITY_XP_01917	PREDICTE	Ipomoea_nil	41.40	0.00
TRINITY_XP_02710	F-box pr	Coffea_arabica	41.40	0.00
TRINITY_XP_02392	phosphol	Quercus_suber	41.40	0.00
TRINITY_XP_02087	2-hydrox	Arabidopsis_lyrata	41.40	0.00
TRINITY_XP_00713	hypothet	Phaseolus_vulgaris	41.40	0.00
TRINITY_OTG02532	putative	Helianthus_annuus	41.40	0.00
TRINITY_XP_02057	ubiquiti	Phalaenopsis_equest	41.40	0.00
TRINITY_XP_02436	uncharac	Physcomitrella_pate	41.40	0.00
TRINITY_XP_01027	PREDICTE	Nelumbo_nucifera	41.40	0.00
TRINITY_BAK00435	predicte	Hordeum_vulgare	41.40	0.00
TRINITY_XP_00565	hypothet	Coccomyxa_subellips	41.40	0.00
TRINITY_EFJ07355	hypothet	Selaginella_moellen	41.40	0.00
TRINITY_KXZ48406	hypothet	Gonium_pectorale	41.40	0.00
TRINITY_XP_01723	PREDICTE	Daucus_carota	41.40	0.00
TRINITY_XP_02026	uncharac	Asparagus_officinal	41.40	0.00
TRINITY_XP_01052	PREDICTE	Tarenaya_hasslerian	41.40	0.00
TRINITY_XP_00423	protein	Solanum_lycopersicu	41.40	0.00
TRINITY_KXZ55614	hypothet	Gonium_pectorale	41.40	0.00
TRINITY_BAK06483	predicte	Hordeum_vulgare	41.40	0.00
TRINITY_GBG84821	hypothet	Chara_braunii	41.40	0.00
TRINITY_PNR31549	hypothet	Physcomitrella_pate	41.40	0.00
TRINITY_XP_02202	adenylat	Helianthus_annuus	41.40	0.00
TRINITY_GAX81862	hypothet	Chlamydomonas_eusti	41.40	0.00
TRINITY_PUZ46870	hypothet	Panicum_hallii	41.40	0.00
TRINITY_OAE22049	hypothet	Marchantia_polymorp	41.40	0.00
TRINITY_GBF90554	hypothet	Raphidocelis_subcap	41.40	0.00
TRINITY_GBF91112	hypothet	Raphidocelis_subcap	41.40	0.00
TRINITY_PNW76055	hypothet	Chlamydomonas_reinh	41.40	0.00
TRINITY_KXZ48222	hypothet	Gonium_pectorale	41.40	0.00
TRINITY_GAQ89245	hypothet	Klebsormidium_niten	41.40	0.00
TRINITY_XP_02439	uncharac	Physcomitrella_pate	41.40	0.00
TRINITY_EFJ16549	hypothet	Selaginella_moellen	41.40	0.00
TRINITY_PNW72749	hypothet	Chlamydomonas_reinh	41.40	0.00
TRINITY_XP_02199	protein	Helianthus_annuus	41.40	0.00
TRINITY_PRW39149	dnaJ-lik	Chlorella_sorokinia	41.40	0.00
TRINITY_XP_00294	hypothet	Volvox_carteri	41.40	0.00
TRINITY_KXZ49330	hypothet	Gonium_pectorale	41.40	0.00
TRINITY_XP_02389	inositol	Quercus_suber	41.40	0.00

TRINITY_GBG7975(	hypothet	Chara_braunii	41.40	0.00
TRINITY_ADB7781	actin, p	Ulva_intestinalis	41.40	0.00
TRINITY_XP_0217	RHOMBOI	Chenopodium_quinoa	41.40	0.00
TRINITY_GAX8069	hypothet	Chlamydomonas_eusti	41.40	0.00
TRINITY_GAX7798	hypothet	Chlamydomonas_eusti	41.40	0.00
TRINITY_NP_0011(	uncharac	Zea_mays	41.40	0.00
TRINITY_OWM8374	hypothet	Punica_granatum	41.40	0.00
TRINITY_GAX8482	hypothet	Chlamydomonas_eusti	41.40	0.00
TRINITY_GAX7578	hypothet	Chlamydomonas_eusti	41.40	0.00
TRINITY_XP_0029	hypothet	Volvox_carteri	41.40	0.00

TRINITY_XP_00169	puf prot	Chlamydomonas_reinh	41.40	0.00
TRINITY_KXZ53360	hypothet	Gonium_pectorale	41.40	0.00
TRINITY_EFJ21271	hypothet	Selaginella_moellen	41.40	0.00
TRINITY_XP_00295	componer	Volvox_carteri	41.40	0.00
TRINITY_GAX74444	hypothet	Chlamydomonas_eusti	41.40	0.00
TRINITY_KJB70446	hypothet	Gossypium_raimondii	41.40	0.00
TRINITY_GAQ84304	prefold:	Klebsormidium_niten	41.40	0.00
TRINITY_XP_00565	hydroxyr	Coccomyxa_subellips	41.40	0.00
TRINITY_PSC70463	putative	Micractinium_conduc	41.40	0.00
TRINITY_KXZ45276	hypothet	Gonium_pectorale	41.40	0.00
TRINITY_GAX86553	hypothet	Chlamydomonas_eusti	41.40	0.00
TRINITY_GAX75027	hypothet	Chlamydomonas_eusti	41.40	0.00
TRINITY_XP_02127	acyl-CoA	Herrania_umbratica	41.40	0.00
TRINITY_KXZ44149	hypothet	Gonium_pectorale	41.40	0.00
TRINITY_GAX77102	hypothet	Chlamydomonas_eusti	41.40	0.00
TRINITY_XP_00305	predicte	Micromonas_pusilla	41.30	0.00
TRINITY_XP_00750	predicte	Bathycoccus_prasino	41.30	0.00
TRINITY_KDD76369	spermine	Helicosporidium_sp.	41.30	0.00
TRINITY_XP_02058	lipase	Phalaenopsis_equest	41.30	0.00
TRINITY_GAV67379	Ribosoma	Cephalotus_follicul	41.30	0.00
TRINITY_XP_00584	hypothet	Chlorella_variabili	41.30	0.00
TRINITY_EEC77874	hypothet	Oryza_sativa	41.30	0.00
TRINITY_KXZ44006	hypothet	Gonium_pectorale	41.30	0.00
TRINITY_PNW76533	hypothet	Chlamydomonas_reinh	41.30	0.00
TRINITY_XP_00751	predicte	Bathycoccus_prasino	41.30	0.00
TRINITY_GAX77638	hypothet	Chlamydomonas_eusti	41.30	0.00
TRINITY_XP_02452	enoyl-[a	Selaginella_moellen	41.30	0.00
TRINITY_PNH07767	hypothet	Tetrabaena_socialis	41.30	0.00
TRINITY_GAQ80394	phosphat	Klebsormidium_niten	41.30	0.00
TRINITY_RAL54451	hypothet	Cuscuta_australis	41.30	0.00
TRINITY_ABW71683	chymotry	Hordeum_vulgare	41.30	0.00
TRINITY_XP_02392	erythroc	Quercus_suber	41.30	0.00
TRINITY_XP_01915	PREDICTE	Ipomoea_nil	41.30	0.00
TRINITY_PNH04210	hypothet	Tetrabaena_socialis	41.30	0.00
TRINITY_KZV15311	beta-mar	Dorcoceras_hygromet	41.30	0.00
TRINITY_PNW75845	hypothet	Chlamydomonas_reinh	41.30	0.00
TRINITY_KMZ59522	Exosome	Zostera_marina	41.30	0.00
TRINITY_XP_00169	soluble	Chlamydomonas_reinh	41.30	0.00
TRINITY_XP_02453	DNA topo	Selaginella_moellen	41.30	0.00
TRINITY_PNH07400	hypothet	Tetrabaena_socialis	41.30	0.00
TRINITY_GAX84659	hypothet	Chlamydomonas_eusti	41.30	0.00
TRINITY_ESQ28921	hypothet	Eutrema_salsugineum	41.30	0.00
TRINITY_RMZ57701	hypothet	Auxenochlorella_pro	41.30	0.00
TRINITY_GAX76711	hypothet	Chlamydomonas_eusti	41.30	0.00
TRINITY_XP_01594	GTP-binc	Arachis_duranensis	41.30	0.00
TRINITY_PRW45322	copine-8	Chlorella_sorokinia	41.30	0.00
TRINITY_PNH07697	Methyl-C	Tetrabaena_socialis	41.30	0.00
TRINITY_GAX76632	hypothet	Chlamydomonas_eusti	41.30	0.00
TRINITY_XP_02284	serine/a	Olea_europaea	41.30	0.00
TRINITY_XP_02436	peter Pa	Physcomitrella_pate	41.30	0.00
TRINITY_XP_02299	E3 ubiqu	Cucurbita_maxima	41.30	0.00
TRINITY_PRW59177	hypothet	Chlorella_sorokinia	41.30	0.00
TRINITY_GAX82582	hypothet	Chlamydomonas_eusti	41.30	0.00
TRINITY_XP_00246	actin-3	Sorghum_bicolor	41.30	0.00
TRINITY_PNH01161	hypothet	Tetrabaena_socialis	41.30	0.00
TRINITY_XP_01389	Dual spe	Monoraphidium_negle	41.30	0.00
TRINITY_XP_00170	predicte	Chlamydomonas_reinh	41.30	0.00
TRINITY_XP_00141	predicte	Ostreococcus_sp._'l	41.30	0.00
TRINITY_XP_02388	fimbrin-	Quercus_suber	41.30	0.00
TRINITY_PIA54622	hypothet	Aquilegia_coerulea	41.30	0.00
TRINITY_XP_00245	probable	Sorghum_bicolor	41.30	0.00
TRINITY_GAQ87734	Ribosoma	Klebsormidium_niten	41.30	0.00
TRINITY_GAX81533	hypothet	Chlamydomonas_eusti	41.30	0.00
TRINITY_GAX85201	hypothet	Chlamydomonas_eusti	41.30	0.00
TRINITY_XP_00141	TRAP1, H	Ostreococcus_sp._'l	41.30	0.00
TRINITY_XP_00169	protein	Chlamydomonas_reinh	41.30	0.00

TRINITY_GAQ78227	Serine/t	Klebsormidium_niten	41.30	0.00
TRINITY_BAJ96894	predicte	Hordeum_vulgare	41.30	0.00
TRINITY_GAX73992	hypothet	Chlamydomonas_eusti	41.30	0.00
TRINITY_XP_00306	predicte	Micromonas_pusilla	41.30	0.00
TRINITY_GAX74807	hypothet	Chlamydomonas_eusti	41.30	0.00
TRINITY_XP_02582	protein	Panicum_hallii	41.30	0.00
TRINITY_GAX79893	hypothet	Chlamydomonas_eusti	41.30	0.00
TRINITY_PNH12872	Serine/t	Tetrabaena_socialis	41.30	0.00
TRINITY_XP_02374	protein	Lactuca_sativa	41.30	0.00
TRINITY_XP_02057	phosphat	Phalaenopsis_equest	41.20	0.00
TRINITY_XP_01282	PREDICTE	Erythranthe_guttata	41.20	0.00
TRINITY_GAQ83028	hypothet	Klebsormidium_niten	41.20	0.00
TRINITY_PSC69984	ubiquiti	Micractinium_conduc	41.20	0.00
TRINITY_RID42037	hypothet	Brassica_rapa	41.20	0.00
TRINITY_XP_01024	PREDICTE	Nelumbo_nucifera	41.20	0.00
TRINITY_XP_02640	ras-rela	Papaver_somniferum	41.20	0.00
TRINITY_GBG00183	hypothet	Raphidocelis_subcap	41.20	0.00
TRINITY_XP_00429	PREDICTE	Fragaria_vesca	41.20	0.00
TRINITY_RWW84243	hypothet	Ensete_ventricosum	41.20	0.00
TRINITY_XP_01207	alpha-ke	Jatropha_curcas	41.20	0.00
TRINITY_GAQ78747	Putative	Klebsormidium_niten	41.20	0.00
TRINITY_XP_01884	PREDICTE	Juglans_regia	41.20	0.00
TRINITY_PRW20868	cinnamyl	Chlorella_sorokinia	41.20	0.00
TRINITY_XP_00305	calcium-	Micromonas_pusilla	41.20	0.00
TRINITY_PNW84630	hypothet	Chlamydomonas_reinh	41.20	0.00
TRINITY_XP_02498	calcium-	Cynara_cardunculus	41.20	0.00
TRINITY_KRH35821	hypothet	Glycine_max	41.20	0.00
TRINITY_RLN15429	calcium-	Panicum_miliaceum	41.20	0.00
TRINITY_XP_01762	PREDICTE	Gossypium_arboreum	41.20	0.00
TRINITY_PNW79299	hypothet	Chlamydomonas_reinh	41.20	0.00
TRINITY_XP_00307	G-protei	Ostreococcus_tauri	41.20	0.00
TRINITY_PNW75763	hypothet	Chlamydomonas_reinh	41.20	0.00
TRINITY_ESR57544	hypothet	Citrus_clementina	41.20	0.00
TRINITY_ABR21719	calmodul	Actinidia_kolomikta	41.20	0.00
TRINITY_GAX79409	hypothet	Chlamydomonas_eusti	41.20	0.00
TRINITY_XP_01919	PREDICTE	Ipomoea_nil	41.20	0.00
TRINITY_XP_01093	PREDICTE	Elaeis_guineensis	41.20	0.00
TRINITY_GAX83510	hypothet	Chlamydomonas_eusti	41.20	0.00
TRINITY_XP_01146	PREDICTE	Fragaria_vesca	41.20	0.00
TRINITY_XP_01653	PREDICTE	Capsicum_annuum	41.20	0.00
TRINITY_XP_02275	calcium-	Durio_zibethinus	41.20	0.00
TRINITY_GAX74833	hypothet	Chlamydomonas_eusti	41.20	0.00
TRINITY_KZV42214	pseudour	Dorcoceras_hygromet	41.20	0.00
TRINITY_PNW87472	hypothet	Chlamydomonas_reinh	41.20	0.00
TRINITY_XP_02070	uncharac	Dendrobium_catenatu	41.20	0.00
TRINITY_PNW75717	hypothet	Chlamydomonas_reinh	41.20	0.00
TRINITY_GAX82228	hypothet	Chlamydomonas_eusti	41.20	0.00
TRINITY_XP_01245	PREDICTE	Gossypium_raimondii	41.20	0.00
TRINITY_XP_02165	RNA-bind	Hevea_brasiliensis	41.20	0.00
TRINITY_XP_00287	uncharac	Arabidopsis_lyrata	41.20	0.00
TRINITY_P0C8M8.1	RecName:	Zea_mays	41.20	0.00
TRINITY_KXZ43877	hypothet	Gonium_pectorale	41.20	0.00
TRINITY_OMO98802	hypothet	Corchorus_olitorius	41.20	0.00
TRINITY_GAX85017	hypothet	Chlamydomonas_eusti	41.20	0.00
TRINITY_PNW74551	hypothet	Chlamydomonas_reinh	41.20	0.00
TRINITY_GAX77536	hypothet	Chlamydomonas_eusti	41.20	0.00
TRINITY_OIT02836	kinesin	Nicotiana_attenuata	41.20	0.00
TRINITY_XP_01844	PREDICTE	Raphanus_sativus	41.20	0.00
TRINITY_XP_02453	UDP-gala	Selaginella_moellen	41.20	0.00
TRINITY_GAQ86524	hypothet	Klebsormidium_niten	41.20	0.00
TRINITY_XP_01162	uncharac	Amborella_trichopod	41.20	0.00
TRINITY_OAE26586	hypothet	Marchantia_polymorp	41.20	0.00
TRINITY_XP_02498	protein	Cynara_cardunculus	41.20	0.00
TRINITY_XP_00295	hypothet	Volvox_carteri	41.20	0.00
TRINITY_PRW45049	jmjC dor	Chlorella_sorokinia	41.20	0.00
TRINITY_GAX84182	hypothet	Chlamydomonas_eusti	41.20	0.00

TRINITY_GAX8540	hypothet	Chlamydomonas_eusti	41.20	0.00
TRINITY_NP_0011	ubiquit	Zea_mays	41.20	0.00
TRINITY_XP_0243	luc7-lik	Physcomitrella_pate	41.20	0.00
TRINITY_GBF9415	cytochr	Raphidocelis_subcap	41.20	0.00
TRINITY_PON4064	Protein	Parasponia_anderson	41.20	0.00
TRINITY_BAK0043	predicte	Hordeum_vulgare	41.20	0.00
TRINITY_XP_0217	ras-rela	Chenopodium_quinoa	41.20	0.00
TRINITY_EFJ0777	hypothet	Selaginella_moellen	41.20	0.00
TRINITY_XP_0016	amino ac	Chlamydomonas_reinh	41.20	0.00
TRINITY_XP_0245	NFX1-typ	Selaginella_moellen	41.20	0.00
TRINITY_KYP7862	hypothet	Cajanus_cajan	41.20	0.00
TRINITY_GAX8188	hypothet	Chlamydomonas_eusti	41.20	0.00
TRINITY_GAX8400	hypothet	Chlamydomonas_eusti	41.20	0.00
TRINITY_PRW4566	MKI67 FH	Chlorella_sorokinia	41.20	0.00
TRINITY_XP_0264	cyclin-I	Papaver_somniferum	41.20	0.00
TRINITY_GBF8759	hypothet	Raphidocelis_subcap	41.20	0.00
TRINITY_XP_0184	PREDICTE	Raphanus_sativus	41.20	0.00
TRINITY_XP_0238	alpha-ac	Quercus_suber	41.20	0.00
TRINITY_GAX8099	hypothet	Chlamydomonas_eusti	41.20	0.00
TRINITY_GAX8498	hypothet	Chlamydomonas_eusti	41.20	0.00
TRINITY_XP_0029	hypothet	Volvox_carteri	41.20	0.00
TRINITY_XP_0113	Alkylate	Auxenochlorella_pro	41.20	0.00
TRINITY_GAX7534	hypothet	Chlamydomonas_eusti	41.20	0.00
TRINITY_XP_0279	serine/a	Vigna_unguiculata	41.20	0.00
TRINITY_GAQ8483	Alpha-ki	Klebsormidium_niten	41.20	0.00
TRINITY_KZV3928	actin-7-	Dorcoceras_hygromet	41.20	0.00
TRINITY_XP_0138	retinol	Monoraphidium_negle	41.10	0.00
TRINITY_OWM8641	hypothet	Punica_granatum	41.10	0.00
TRINITY_XP_0025	predicte	Micromonas_commoda	41.10	0.00
TRINITY_PKA5802	DNA misr	Apostasia_shenzheni	41.10	0.00
TRINITY_XP_0016	predicte	Chlamydomonas_reinh	41.10	0.00
TRINITY_GAQ8580	Zinc fir	Klebsormidium_niten	41.10	0.00
TRINITY_XP_0110	carbon c	Sesamum_indicum	41.10	0.00
TRINITY_XP_0025	glycosyl	Micromonas_commoda	41.10	0.00
TRINITY_BAJ9984	predicte	Hordeum_vulgare	41.10	0.00
TRINITY_XP_0056	ATP-depe	Coccomyxa_subellips	41.10	0.00
TRINITY_XP_0217	uncharac	Chenopodium_quinoa	41.10	0.00
TRINITY_GAX7675	hypothet	Chlamydomonas_eusti	41.10	0.00
TRINITY_NP_5663	ubiquit	Arabidopsis_thalian	41.10	0.00
TRINITY_XP_0243	ER membr	Physcomitrella_pate	41.10	0.00
TRINITY_XP_0207	tyrosine	Dendrobium_catenatu	41.10	0.00
TRINITY_RWW9025	hypothet	Ensete_ventricosum	41.10	0.00
TRINITY_GAQ8166	ABC tran	Klebsormidium_niten	41.10	0.00
TRINITY_XP_0165	PREDICTE	Capsicum_annuum	41.10	0.00
TRINITY_OAE2770	hypothet	Marchantia_polymorp	41.10	0.00
TRINITY_GAX8094	hypothet	Chlamydomonas_eusti	41.10	0.00
TRINITY_XP_0056	DNA glyc	Coccomyxa_subellips	41.10	0.00
TRINITY_XP_0188	PREDICTE	Juglans_regia	41.10	0.00
TRINITY_XP_0086	probable	Zea_mays	41.10	0.00
TRINITY_XP_0217	tRNA wyk	Chenopodium_quinoa	41.10	0.00
TRINITY_PNW8189	hypothet	Chlamydomonas_reinh	41.10	0.00
TRINITY_GAX7463	hypothet	Chlamydomonas_eusti	41.10	0.00
TRINITY_GAX7358	hypothet	Chlamydomonas_eusti	41.10	0.00
TRINITY_KXZ5399	hypothet	Gonium_pectorale	41.10	0.00
TRINITY_XP_0106	PREDICTE	Beta_vulgaris	41.10	0.00
TRINITY_XP_0241	uncharac	Rosa_chinensis	41.10	0.00
TRINITY_PNG9923	60S ribo	Tetrabaena_socialis	41.10	0.00
TRINITY_PNW8843	hypothet	Chlamydomonas_reinh	41.10	0.00
TRINITY_KXZ5329	hypothet	Gonium_pectorale	41.10	0.00
TRINITY_XP_0138	hypothet	Monoraphidium_negle	41.10	0.00
TRINITY_RID6036	hypothet	Brassica_rapa	41.10	0.00
TRINITY_XP_0029	peptidyl	Selaginella_moellen	41.10	0.00
TRINITY_GAX7953	hypothet	Chlamydomonas_eusti	41.10	0.00
TRINITY_PRW5962	tyrosyl-	Chlorella_sorokinia	41.10	0.00
TRINITY_PNW7033	hypothet	Chlamydomonas_reinh	41.10	0.00
TRINITY_GAX8635	hypothet	Chlamydomonas_eusti	41.10	0.00

TRINITY_GAX8059	hypothet	Chlamydomonas_eusti	41.10	0.00
TRINITY_XP_0200	temperat	Ananas_comosus	41.10	0.00
TRINITY_XP_0138	Uncharac	Monoraphidium_negle	41.10	0.00
TRINITY_XP_0029	hypothet	Volvox_carteri	41.10	0.00
TRINITY_XP_0066	PREDICTE	Oryza_brachyantha	41.10	0.00
TRINITY_GAX8211	hypothet	Chlamydomonas_eusti	41.10	0.00
TRINITY_GAQ7836	transcri	Klebsormidium_niten	41.10	0.00
TRINITY_XP_0243	somethin	Physcomitrella_pate	41.10	0.00
TRINITY_XP_0016	predicte	Chlamydomonas_reinh	41.10	0.00
TRINITY_XP_0029	Mg2+ tra	Volvox_carteri	41.10	0.00
TRINITY_AXR8531	EF1.278	Sanionia_uncinata	41.10	0.00
TRINITY_OMO8801	hypothet	Corchorus_capsulari	41.10	0.00
TRINITY_XP_0029	hypothet	Volvox_carteri	41.10	0.00
TRINITY_XP_0109	PREDICTE	Elaeis_guineensis	41.10	0.00
TRINITY_PWA6583	Armadil	Artemisia_annua	41.10	0.00
TRINITY_XP_0071	hypothet	Phaseolus_vulgaris	41.10	0.00
TRINITY_GAX8210	hypothet	Chlamydomonas_eusti	41.10	0.00
TRINITY_GAQ8995	hypothet	Klebsormidium_niten	41.10	0.00
TRINITY_RQL7862	hypothet	Brassica_cretica	41.10	0.00
TRINITY_BAK0043	predicte	Hordeum_vulgare	41.10	0.00
TRINITY_BAK0219	predicte	Hordeum_vulgare	41.10	0.00
TRINITY_KXZ4568	hypothet	Gonium_pectorale	41.10	0.00
TRINITY_GAX7446	hypothet	Chlamydomonas_eusti	41.10	0.00
TRINITY_GAX8574	hypothet	Chlamydomonas_eusti	41.10	0.00
TRINITY_GAX7637	hypothet	Chlamydomonas_eusti	41.10	0.00
TRINITY_GAX7766	hypothet	Chlamydomonas_eusti	41.10	0.00
TRINITY_KXZ4300	hypothet	Gonium_pectorale	41.10	0.00
TRINITY_PNW7951	hypothet	Chlamydomonas_reinh	41.10	0.00
TRINITY_XP_0238	probable	Quercus_suber	41.10	0.00
TRINITY_GAX8498	hypothet	Chlamydomonas_eusti	41.10	0.00
TRINITY_OAE2128	hypothet	Marchantia_polymorp	41.10	0.00
TRINITY_XP_0093	PREDICTE	Pyrus_x_bretschneid	41.10	0.00
TRINITY_BAK0219	predicte	Hordeum_vulgare	41.10	0.00
TRINITY_XP_0029	hypothet	Volvox_carteri	41.10	0.00
TRINITY_GAX7408	hypothet	Chlamydomonas_eusti	41.10	0.00
TRINITY_GAX8445	hypothet	Chlamydomonas_eusti	41.10	0.00
TRINITY_XP_0109	PREDICTE	Elaeis_guineensis	41.10	0.00
TRINITY_ABK4147	catalase	Haematococcus_lacus	41.10	0.00
TRINITY_KXZ5011	hypothet	Gonium_pectorale	41.10	0.00
TRINITY_GAQ8317	UDP-Gly	Klebsormidium_niten	41.10	0.00
TRINITY_KXZ5199	hypothet	Gonium_pectorale	41.10	0.00
TRINITY_XP_0161	G2/mito	Arachis_ipaensis	41.10	0.00
TRINITY_XP_0109	PREDICTE	Elaeis_guineensis	41.10	0.00
TRINITY_GAX8434	hypothet	Chlamydomonas_eusti	41.10	0.00
TRINITY_PNH0528	ATP-depe	Tetrabaena_socialis	41.10	0.00
TRINITY_XP_0104	PREDICTE	Camelina_sativa	41.10	0.00
TRINITY_XP_0109	PREDICTE	Elaeis_guineensis	41.00	0.00
TRINITY_XP_0042	PREDICTE	Fragaria_vesca	41.00	0.00
TRINITY_XP_0229	calcium-	Cucurbita_moschata	41.00	0.00
TRINITY_KNA0344	hypothet	Spinacia_oleracea	41.00	0.00
TRINITY_XP_0030	Adenyly	Ostreococcus_tauri	41.00	0.00
TRINITY_EFJ1230	hypothet	Selaginella_moellen	41.00	0.00
TRINITY_XP_0025	predicte	Micromonas_commoda	41.00	0.00
TRINITY_PIA5563	hypothet	Aquilegia_coerulea	41.00	0.00
TRINITY_GAX7531	hypothet	Chlamydomonas_eusti	41.00	0.00
TRINITY_XP_0024	protein	Sorghum_bicolor	41.00	0.00
TRINITY_GAQ7965	Signal t	Klebsormidium_niten	41.00	0.00
TRINITY_XP_0228	TatD fa	Ostreococcus_tauri	41.00	0.00
TRINITY_XP_0105	PREDICTE	Tarenaya_hasslerian	41.00	0.00
TRINITY_RWW0460	hypothet	Ensete_ventricosum	41.00	0.00
TRINITY_XP_0243	xanthine	Physcomitrella_pate	41.00	0.00
TRINITY_GAQ7861	Amidase	Klebsormidium_niten	41.00	0.00
TRINITY_XP_0104	PREDICTE	Camelina_sativa	41.00	0.00
TRINITY_KXZ4817	hypothet	Gonium_pectorale	41.00	0.00
TRINITY_XP_0229	low-tem	Cucurbita_moschata	41.00	0.00
TRINITY_XP_0271	triacyl	Cicer_arietinum	41.00	0.00

TRINITY_XP_01389	zinc-met	Monoraphidium_negle	41.00	0.00
TRINITY_KDP21262	hypothet	Jatropha_curcas	41.00	0.00
TRINITY_XP_00169	predicte	Chlamydomonas_reinh	41.00	0.00
TRINITY_GAX80565	hypothet	Chlamydomonas_eusti	41.00	0.00
TRINITY_GAX80348	hypothet	Chlamydomonas_eusti	41.00	0.00
TRINITY_PSC69251	methylt	Micractinium_conduc	41.00	0.00
TRINITY_EFJ30367	hypothet	Selaginella_moellen	41.00	0.00
TRINITY_RLM92426	phosphol	Panicum_miliaceum	41.00	0.00
TRINITY_XP_00713	hypothet	Phaseolus_vulgaris	41.00	0.00
TRINITY_GAX75940	hypothet	Chlamydomonas_eusti	41.00	0.00
TRINITY_EFJ13093	hypothet	Selaginella_moellen	41.00	0.00
TRINITY_KZV17081	putative	Dorcoceras_hygromet	41.00	0.00
TRINITY_GAV91327	Pkinase	Cephalotus_follicul	41.00	0.00
TRINITY_GAX80359	hypothet	Chlamydomonas_eusti	41.00	0.00
TRINITY_XP_02419	dual spe	Rosa_chinensis	41.00	0.00
TRINITY_XP_02665	unchara	Phoenix_dactylifera	41.00	0.00
TRINITY_PNW78446	hypothet	Chlamydomonas_reinh	41.00	0.00
TRINITY_GAX86243	hypothet	Chlamydomonas_eusti	41.00	0.00
TRINITY_P0C8M8.1	RecName:	Zea_mays	41.00	0.00
TRINITY_XP_01024	unchara	Brachypodium_distac	41.00	0.00
TRINITY_KXZ41591	hypothet	Gonium_pectorale	41.00	0.00
TRINITY_GAQ93207	protein	Klebsormidium_niten	41.00	0.00
TRINITY_XP_02010	acyl-CoA	Ananas_comosus	41.00	0.00
TRINITY_KZM86035	hypothet	Daucus_carota	41.00	0.00
TRINITY_XP_00295	strictos	Volvox_carteri	41.00	0.00
TRINITY_GAX72982	hypothet	Chlamydomonas_eusti	41.00	0.00
TRINITY_PSS11853	ELMO dor	Actinidia_chinensis	41.00	0.00
TRINITY_XP_01066	PREDICTE	Vitis_vinifera	41.00	0.00
TRINITY_PNW69910	hypothet	Chlamydomonas_reinh	41.00	0.00
TRINITY_XP_00751	unnamed	Bathycoccus_prasino	41.00	0.00
TRINITY_GBF94558	hypothet	Raphidocelis_subcap	41.00	0.00
TRINITY_KXZ43998	hypothet	Gonium_pectorale	41.00	0.00
TRINITY_GBG00627	hypothet	Raphidocelis_subcap	41.00	0.00
TRINITY_XP_00294	hypothet	Volvox_carteri	41.00	0.00
TRINITY_KMZ71014	Calcium-	Zostera_marina	41.00	0.00
TRINITY_XP_02176	zinc fin	Chenopodium_quinoa	41.00	0.00
TRINITY_GAX83985	hypothet	Chlamydomonas_eusti	41.00	0.00
TRINITY_XP_00564	cytochro	Coccomyxa_subellips	41.00	0.00
TRINITY_PNW80448	hypothet	Chlamydomonas_reinh	41.00	0.00
TRINITY_PRW60421	RAP doma	Chlorella_sorokinia	41.00	0.00
TRINITY_XP_00294	hypothet	Volvox_carteri	41.00	0.00
TRINITY_XP_00294	omega-6-	Volvox_carteri	41.00	0.00
TRINITY_XP_00564	JmjC-dor	Coccomyxa_subellips	41.00	0.00
TRINITY_GAX77889	hypothet	Chlamydomonas_eusti	41.00	0.00
TRINITY_GAX77008	hypothet	Chlamydomonas_eusti	41.00	0.00
TRINITY_PNW88477	hypothet	Chlamydomonas_reinh	41.00	0.00
TRINITY_GBF94196	calcium	Raphidocelis_subcap	41.00	0.00
TRINITY_PRW55974	Ca-trans	Chlorella_sorokinia	41.00	0.00
TRINITY_GAX76574	hypothet	Chlamydomonas_eusti	41.00	0.00
TRINITY_KXZ49031	hypothet	Gonium_pectorale	41.00	0.00
TRINITY_XP_00294	hypothet	Volvox_carteri	41.00	0.00
TRINITY_XP_00299	ubiquiti	Selaginella_moellen	41.00	0.00
TRINITY_GAX75601	hypothet	Chlamydomonas_eusti	41.00	0.00
TRINITY_GAX77772	hypothet	Chlamydomonas_eusti	41.00	0.00
TRINITY_GBF99801	hypothet	Raphidocelis_subcap	41.00	0.00
TRINITY_GAX75027	hypothet	Chlamydomonas_eusti	41.00	0.00
TRINITY_GAX84689	hypothet	Chlamydomonas_eusti	41.00	0.00
TRINITY_XP_01109	chaperon	Sesamum_indicum	41.00	0.00
TRINITY_XP_01067	PREDICTE	Beta_vulgaris	40.90	0.00
TRINITY_XP_02128	peroxire	Herrania_umbratica	40.90	0.00
TRINITY_PTQ38538	hypothet	Marchantia_polymorp	40.90	0.00
TRINITY_XP_02708	UMP-CMP	Coffea_arabica	40.90	0.00
TRINITY_XP_02172	chaperon	Chenopodium_quinoa	40.90	0.00
TRINITY_GAX82389	hypothet	Chlamydomonas_eusti	40.90	0.00
TRINITY_XP_00844	PREDICTE	Cucumis_melo	40.90	0.00
TRINITY_XP_02387	unchara	Quercus_suber	40.90	0.00

TRINITY_XP_0219(	11-beta-	Carica_papaya	40.90	0.00
TRINITY_XP_0244!	serine/t	Populus_trichocarpa	40.90	0.00
TRINITY_GAQ8278'	phosphoị	Klebsormidium_niten	40.90	0.00
TRINITY_XP_0110:	PREDICTI	Populus_euphratica	40.90	0.00
TRINITY_PRW3353;	E3 ubiqu	Chlorella_sorokinia	40.90	0.00
TRINITY_XP_0105	PREDICTI	Tarenaya_hasslerian	40.90	0.00
TRINITY_KRG9833	hypothet	Glycine_max	40.90	0.00
TRINITY_XP_0202;	calcium-	Cajanus_cajan	40.90	0.00
TRINITY_EFJ3734'	hypothet	Selaginella_moellen	40.90	0.00
TRINITY_XP_0264!	ras-rela	Papaver_somniferum	40.90	0.00
TRINITY_XP_0241(	probable	Rosa_chinensis	40.90	0.00
TRINITY_XP_0106{	PREDICTI	Beta_vulgaris	40.90	0.00
TRINITY_XP_0227	nucleoli	Durio_zibethinus	40.90	0.00
TRINITY_GAX8512:	hypothet	Chlamydomonas_eusti	40.90	0.00
TRINITY_XP_0243{	sterol :	Physcomitrella_pate	40.90	0.00
TRINITY_XP_0238	cystath:	Quercus_suber	40.90	0.00
TRINITY_XP_0243	carboxyp	Physcomitrella_pate	40.90	0.00
TRINITY_RWR7659(	serine/t	Cinnamomum_micranth	40.90	0.00
TRINITY_PRW3265;	Transcri	Chlorella_sorokinia	40.90	0.00
TRINITY_AEU0470(	fatty ac	Lobosphaera_incisa	40.90	0.00
TRINITY_XP_0029	hypothet	Volvox_carteri	40.90	0.00
TRINITY_GAX8259!	hypothet	Chlamydomonas_eusti	40.90	0.00
TRINITY_AIY6275{	caroteno	Lycium_chinense	40.90	0.00
TRINITY_GBF9026:	hypothet	Raphidocelis_subcap	40.90	0.00
TRINITY_PSC7276;	ATP bind	Micractinium_conduc	40.90	0.00
TRINITY_GAX8151;	hypothet	Chlamydomonas_eusti	40.90	0.00
TRINITY_XP_0042	18.2 kDa	Solanum_lycopersicu	40.90	0.00
TRINITY_OAE2441!	hypothet	Marchantia_polymorp	40.90	0.00
TRINITY_GAQ9062(	hypothet	Klebsormidium_niten	40.90	0.00
TRINITY_PKA6229	ABC tran	Apostasia_shenzheni	40.90	0.00
TRINITY_RDX8666(	Serine/t	Mucuna_pruriens	40.90	0.00
TRINITY_GAQ8918:	hypothet	Klebsormidium_niten	40.90	0.00
TRINITY_XP_0150'	serine (	Solanum_pennellii	40.90	0.00
TRINITY_KDD7307(	hypothet	Helicosporidium_sp.	40.90	0.00
TRINITY_XP_0218:	annexin	Spinacia_oleracea	40.90	0.00
TRINITY_GAX8069;	hypothet	Chlamydomonas_eusti	40.90	0.00
TRINITY_XP_0102:	zinc tra	Brachypodium_distac	40.90	0.00
TRINITY_PNH0146{	hypothet	Tetrabaena_socialis	40.90	0.00
TRINITY_PNW7810'	hypothet	Chlamydomonas_reinh	40.90	0.00
TRINITY_KZM9479{	hypothet	Daucus_carota	40.90	0.00
TRINITY_XP_0243'	GTPase I	Physcomitrella_pate	40.90	0.00
TRINITY_XP_0083!	PREDICTI	Malus_domestica	40.90	0.00
TRINITY_PNW7497(	hypothet	Chlamydomonas_reinh	40.90	0.00
TRINITY_XP_0138	hypothet	Monoraphidium_negle	40.90	0.00
TRINITY_GAX8366{	hypothet	Chlamydomonas_eusti	40.90	0.00
TRINITY_PNH1114{	Chaperor	Tetrabaena_socialis	40.90	0.00
TRINITY_XP_0228	ras-rela	Olea_europaea	40.90	0.00
TRINITY_XP_0204;	4-alpha-	Prunus_persica	40.90	0.00
TRINITY_PIA5503:	hypothet	Aquilegia_coerulea	40.90	0.00
TRINITY_XP_0138	hypothet	Monoraphidium_negle	40.90	0.00
TRINITY_GAX7906(	hypothet	Chlamydomonas_eusti	40.90	0.00
TRINITY_KXZ5446;	hypothet	Gonium_pectorale	40.90	0.00
TRINITY_GAX8070'	hypothet	Chlamydomonas_eusti	40.90	0.00
TRINITY_GAX8589	hypothet	Chlamydomonas_eusti	40.90	0.00
TRINITY_KXZ4204(	hypothet	Gonium_pectorale	40.90	0.00
TRINITY_KXZ5406;	hypothet	Gonium_pectorale	40.90	0.00
TRINITY_XP_0139(	hypothet	Monoraphidium_negle	40.90	0.00
TRINITY_ABK2501:	unknown	Picea_sitchensis	40.90	0.00
TRINITY_XP_0016	protein	Chlamydomonas_reinh	40.90	0.00
TRINITY_XP_0249;	ADP-ribo	Ziziphus_jujuba	40.90	0.00
TRINITY_GAX8539(	hypothet	Chlamydomonas_eusti	40.90	0.00
TRINITY_XP_0029	hypothet	Volvox_carteri	40.90	0.00
TRINITY_XP_0277'	omega-ar	Solanum_pennellii	40.90	0.00
TRINITY_GAQ9037{	phosphat	Klebsormidium_niten	40.80	0.00
TRINITY_GBF9417!	hypothet	Raphidocelis_subcap	40.80	0.00
TRINITY_GAV8031{	SRR1 dor	Cephalotus_follicul	40.80	0.00

TRINITY_XP_0106(	PREDICTI	Beta_vulgaris	40.80	0.00
TRINITY_KZV2376	hypothet	Dorcoceras_hygromet	40.80	0.00
TRINITY_XP_0217(	WD repea	Chenopodium_quinoa	40.80	0.00
TRINITY_GAX8299	hypothet	Chlamydomonas_eusti	40.80	0.00
TRINITY_XP_0095	PREDICTI	Nicotiana_tomentosi	40.80	0.00
TRINITY_XP_0114(	PREDICTI	Fragaria_vesca	40.80	0.00
TRINITY_XP_0025(	predicte	Micromonas_commoda	40.80	0.00
TRINITY_KXZ4787	hypothet	Gonium_pectorale	40.80	0.00
TRINITY_BAJ8516	predicte	Hordeum_vulgare	40.80	0.00
TRINITY_XP_0124	PREDICTI	Gossypium_raimondii	40.80	0.00
TRINITY_XP_0237	protein	Lactuca_sativa	40.80	0.00
TRINITY_XP_0029	Ca2+/H+	Volvox_carteri	40.80	0.00
TRINITY_GAX7314	hypothet	Chlamydomonas_eusti	40.80	0.00
TRINITY_XP_0058	hypothet	Chlorella_variabili	40.80	0.00
TRINITY_XP_0029	hypothet	Volvox_carteri	40.80	0.00
TRINITY_XP_0029	RWP-RK (	Volvox_carteri	40.80	0.00
TRINITY_XP_0219	uncharac	Helianthus_annuus	40.80	0.00
TRINITY_GBG7539	hypothet	Chara_braunii	40.80	0.00
TRINITY_XP_0239(	uncharac	Quercus_suber	40.80	0.00
TRINITY_XP_0025(	predicte	Micromonas_commoda	40.80	0.00
TRINITY_XP_0271	uncharac	Cicer_arietinum	40.80	0.00
TRINITY_NP_0011	uncharac	Zea_mays	40.80	0.00
TRINITY_PRW5654	Endoglu	Chlorella_sorokinia	40.80	0.00
TRINITY_XP_0029	hypothet	Volvox_carteri	40.80	0.00
TRINITY_GAQ8723(	Dyneins	Klebsormidium_niten	40.80	0.00
TRINITY_XP_0243	transmem	Physcomitrella_pate	40.80	0.00
TRINITY_GAX7638	hypothet	Chlamydomonas_eusti	40.80	0.00
TRINITY_XP_0246	probable	Medicago_truncatula	40.80	0.00
TRINITY_GAQ8234	hypothet	Klebsormidium_niten	40.80	0.00
TRINITY_XP_0138	hypothet	Monoraphidium_negle	40.80	0.00
TRINITY_XP_0058	hypothet	Chlorella_variabili	40.80	0.00
TRINITY_XP_0238	serine (	Quercus_suber	40.80	0.00
TRINITY_XP_0030	Glycosi	Ostreococcus_tauri	40.80	0.00
TRINITY_GBG7070(	hypothet	Chara_braunii	40.80	0.00
TRINITY_XP_0105	PREDICTI	Tarenaya_hasslerian	40.80	0.00
TRINITY_GAX7334	hypothet	Chlamydomonas_eusti	40.80	0.00
TRINITY_ABK2701	unknown	Picea_sitchensis	40.80	0.00
TRINITY_EMS5587(	RNA-bin	Triticum_urartu	40.80	0.00
TRINITY_GBF9484(	hypothet	Raphidocelis_subcap	40.80	0.00
TRINITY_GBF9914	potassi	Raphidocelis_subcap	40.80	0.00
TRINITY_OAE2685	hypothet	Marchantia_polymorp	40.80	0.00
TRINITY_XP_0243	protein-	Physcomitrella_pate	40.80	0.00
TRINITY_GAX8285	hypothet	Chlamydomonas_eusti	40.80	0.00
TRINITY_GAX7282	hypothet	Chlamydomonas_eusti	40.80	0.00
TRINITY_XP_0238	alpha-a	Quercus_suber	40.80	0.00
TRINITY_KXZ4752	hypothet	Gonium_pectorale	40.80	0.00
TRINITY_XP_0100	PREDICTI	Eucalyptus_grandis	40.80	0.00
TRINITY_KXZ5415	hypothet	Gonium_pectorale	40.80	0.00
TRINITY_GAX8230	hypothet	Chlamydomonas_eusti	40.80	0.00
TRINITY_OAE3173	hypothet	Marchantia_polymorp	40.80	0.00
TRINITY_KXZ5450	hypothet	Gonium_pectorale	40.80	0.00
TRINITY_KXZ5303	hypothet	Gonium_pectorale	40.80	0.00
TRINITY_PIN0574	Xanthine	Handroanthus_impeti	40.80	0.00
TRINITY_XP_0068	coatome	Amborella_trichopod	40.80	0.00
TRINITY_GAX8576(	hypothet	Chlamydomonas_eusti	40.80	0.00
TRINITY_XP_0176	PREDICTI	Gossypium_arboreum	40.80	0.00
TRINITY_PNW7395	hypothet	Chlamydomonas_reinh	40.80	0.00
TRINITY_XP_0110(	PREDICTI	Populus_euphratica	40.80	0.00
TRINITY_GAX7389	hypothet	Chlamydomonas_eusti	40.80	0.00
TRINITY_GAX8075	hypothet	Chlamydomonas_eusti	40.80	0.00
TRINITY_PNW8313	hypothet	Chlamydomonas_reinh	40.80	0.00
TRINITY_PNH1207	hypothet	Tetrabaena_socialis	40.80	0.00
TRINITY_RZB6013	Fimbrin-	Glycine_soja	40.80	0.00
TRINITY_XP_0029	hypothet	Volvox_carteri	40.80	0.00
TRINITY_XP_0016	predicte	Chlamydomonas_reinh	40.80	0.00
TRINITY_PNH0830	NFX1-typ	Tetrabaena_socialis	40.80	0.00

TRINITY_PLY6668(	hypothet	Lactuca_sativa	40.80	0.00
TRINITY_GBF9416;	hypothet	Raphidocelis_subcap	40.80	0.00
TRINITY_EFJ0560	hypothet	Selaginella_moellen	40.70	0.00
TRINITY_PRW5753	Ras-rela	Chlorella_sorokinia	40.70	0.00
TRINITY_RXH8570	hypothet	Malus_domestica	40.70	0.00
TRINITY_XP_0186	PREDICTE	Musa_acuminata	40.70	0.00
TRINITY_XP_0192	PREDICTE	Nicotiana_attenuata	40.70	0.00
TRINITY_XP_0113	Pre-mRNA	Auxenochlorella_pro	40.70	0.00
TRINITY_AIU4987	FMN-link	Lilium_brownii	40.70	0.00
TRINITY_PNR4792	hypothet	Physcomitrella_pate	40.70	0.00
TRINITY_XP_0058	hypothet	Chlorella_variabili	40.70	0.00
TRINITY_XP_0049	helicase	Setaria_italica	40.70	0.00
TRINITY_RMZ5429	hypothet	Auxenochlorella_pro	40.70	0.00
TRINITY_XP_0100	PREDICTE	Eucalyptus_grandis	40.70	0.00
TRINITY_ALJ5330	polyubic	Luffa_aegyptiaca	40.70	0.00
TRINITY_XP_0016	transmem	Chlamydomonas_reinh	40.70	0.00
TRINITY_XP_0156	crossove	Oryza_sativa	40.70	0.00
TRINITY_Q9FVI1.1	RecName:	Petunia_x_hybrida	40.70	0.00
TRINITY_XP_0063	mannose-	Populus_trichocarpa	40.70	0.00
TRINITY_VDD4074	unnamed	Brassica_oleracea	40.70	0.00
TRINITY_XP_0201	serine/t	Aegilops_tauschii	40.70	0.00
TRINITY_XP_0217	E3 ubiqu	Chenopodium_quinoa	40.70	0.00
TRINITY_OEL3114	hypothet	Dichanthelium_oligo	40.70	0.00
TRINITY_PNR3027	hypothet	Physcomitrella_pate	40.70	0.00
TRINITY_EFJ2787	hypothet	Selaginella_moellen	40.70	0.00
TRINITY_KXZ5043	hypothet	Gonium_pectorale	40.70	0.00
TRINITY_XP_0056	hypothet	Coccomyxa_subellips	40.70	0.00
TRINITY_GAQ7817	malate s	Klebsormidium_niten	40.70	0.00
TRINITY_AHV8360	ROP5 [Lo	Lotus_japonicus	40.70	0.00
TRINITY_XP_0138	TBC1 dom	Monoraphidium_negle	40.70	0.00
TRINITY_EPS6315	hypothet	Genlisea_aurea	40.70	0.00
TRINITY_PNX7557	flavonol	Trifolium_pratense	40.70	0.00
TRINITY_PSC7551	nuclear	Micractinium_conduc	40.70	0.00
TRINITY_XP_0184	PREDICTE	Raphanus_sativus	40.70	0.00
TRINITY_EPS7422	hypothet	Genlisea_aurea	40.70	0.00
TRINITY_GAX8212	hypothet	Chlamydomonas_eusti	40.70	0.00
TRINITY_GBG6148	hypothet	Chara_braunii	40.70	0.00
TRINITY_GAX8424	hypothet	Chlamydomonas_eusti	40.70	0.00
TRINITY_KXZ4264	hypothet	Gonium_pectorale	40.70	0.00
TRINITY_PSC7203	plancito	Micractinium_conduc	40.70	0.00
TRINITY_PNW8044	hypothet	Chlamydomonas_reinh	40.70	0.00
TRINITY_XP_0239	ras-like	Quercus_suber	40.70	0.00
TRINITY_XP_0062	uncharac	Capsella_rubella	40.70	0.00
TRINITY_XP_0064	beta-ada	Citrus_clementina	40.70	0.00
TRINITY_RLN4351	uncharac	Panicum_miliaceum	40.70	0.00
TRINITY_XP_0220	L-2-hydr	Helianthus_annuus	40.70	0.00
TRINITY_GAX8473	hypothet	Chlamydomonas_eusti	40.70	0.00
TRINITY_XP_0239	ras-rela	Quercus_suber	40.70	0.00
TRINITY_GAQ7902	Ataxin-3	Klebsormidium_niten	40.70	0.00
TRINITY_GAQ8604	Cyclic n	Klebsormidium_niten	40.70	0.00
TRINITY_XP_0030	predicte	Micromonas_pusilla	40.70	0.00
TRINITY_XP_0191	PREDICTE	Ipomoea_nil	40.70	0.00
TRINITY_GAX8175	hypothet	Chlamydomonas_eusti	40.70	0.00
TRINITY_XP_0239	dynein h	Quercus_suber	40.70	0.00
TRINITY_RLM6049	cyclin-c	Panicum_miliaceum	40.70	0.00
TRINITY_XP_0058	hypothet	Chlorella_variabili	40.70	0.00
TRINITY_XP_0030	G-protei	Ostreococcus_tauri	40.70	0.00
TRINITY_XP_0238	calcineu	Quercus_suber	40.70	0.00
TRINITY_GAQ8477	Sensory	Klebsormidium_niten	40.70	0.00
TRINITY_XP_0245	dual spe	Selaginella_moellen	40.70	0.00
TRINITY_KXZ4949	hypothet	Gonium_pectorale	40.70	0.00
TRINITY_BAK0191	predicte	Hordeum_vulgare	40.70	0.00
TRINITY_PNW7198	hypothet	Chlamydomonas_reinh	40.70	0.00
TRINITY_PRW2068	Ubiquiti	Chlorella_sorokinia	40.70	0.00
TRINITY_XP_0159	uncharac	Arachis_duranensis	40.70	0.00
TRINITY_PRW6026	Serine t	Chlorella_sorokinia	40.70	0.00

TRINITY_XP_0139	Photosy	Monoraphidium_negle	40.70	0.00
TRINITY_PNW8719	hypothet	Chlamydomonas_reinh	40.70	0.00
TRINITY_KXZ5550	hypothet	Gonium_pectorale	40.70	0.00
TRINITY_KXZ4337	hypothet	Gonium_pectorale	40.70	0.00
TRINITY_GAX7701	hypothet	Chlamydomonas_eusti	40.70	0.00
TRINITY_RMZ5251	hypothet	Auxenochlorella_pro	40.70	0.00
TRINITY_GAX8399	hypothet	Chlamydomonas_eusti	40.70	0.00
TRINITY_GAX8645	hypothet	Chlamydomonas_eusti	40.70	0.00
TRINITY_PSC7028	Found i	Micractinium_conduc	40.70	0.00
TRINITY_GAX7284	hypothet	Chlamydomonas_eusti	40.70	0.00
TRINITY_GAX7356	hypothet	Chlamydomonas_eusti	40.70	0.00
TRINITY_PNH0281	Vam6/Vp	Tetrabaena_socialis	40.70	0.00
TRINITY_XP_0084	PREDICT	Cucumis_melo	40.70	0.00
TRINITY_XP_0066	PREDICT	Oryza_brachyantha	40.70	0.00
TRINITY_GBF8889	fatty a	Raphidocelis_subca	40.70	0.00
TRINITY_XP_0016	FAB-lik	Chlamydomonas_reinh	40.70	0.00
TRINITY_OVA1450	Bromodor	Macleaya_cordata	40.70	0.00
TRINITY_GAX7596	hypothet	Chlamydomonas_eusti	40.70	0.00
TRINITY_KXZ4467	hypothet	Gonium_pectorale	40.70	0.00
TRINITY_OAE2105	hypothet	Marchantia_polymor	40.70	0.00
TRINITY_XP_0110	PREDICT	Populus_euphratica	40.70	0.00
TRINITY_EPS7208	hypothet	Genlisea_aurea	40.70	0.00
TRINITY_KXZ4844	hypothet	Gonium_pectorale	40.70	0.00
TRINITY_BAK0109	predict	Hordeum_vulgare	40.70	0.00
TRINITY_XP_0016	gametol	Chlamydomonas_reinh	40.70	0.00
TRINITY_XP_0104	PREDICT	Camelina_sativa	40.70	0.00
TRINITY_GAX7897	hypothet	Chlamydomonas_eusti	40.70	0.00
TRINITY_GAX7641	hypothet	Chlamydomonas_eusti	40.70	0.00
TRINITY_GAX7859	hypothet	Chlamydomonas_eusti	40.70	0.00
TRINITY_XP_0058	hypothet	Chlorella_variabili	40.60	0.00
TRINITY_XP_0273	DNA pol	Abrus_precatorius	40.60	0.00
TRINITY_GBF8789	hypothet	Raphidocelis_subca	40.60	0.00
TRINITY_XP_0088	serine/	Phoenix_dactylifera	40.60	0.00
TRINITY_XP_0109	PREDICT	Elaeis_guineensis	40.60	0.00
TRINITY_XP_0238	kinesin	Quercus_suber	40.60	0.00
TRINITY_GBF9460	hypothet	Raphidocelis_subca	40.60	0.00
TRINITY_PRW2077	hypothet	Chlorella_sorokinia	40.60	0.00
TRINITY_RWW8424	hypothet	Ensete_ventricosum	40.60	0.00
TRINITY_RWR9723	MaoC-li	Cinnamomum_micranth	40.60	0.00
TRINITY_XP_0167	PREDICT	Gossypium_hirsutum	40.60	0.00
TRINITY_RLM9133	dual sp	Panicum_miliaceum	40.60	0.00
TRINITY_XP_0172	PREDICT	Daucus_carota	40.60	0.00
TRINITY_XP_0239	carrier	Quercus_suber	40.60	0.00
TRINITY_OMO6959	hypothet	Corchorus_capsulari	40.60	0.00
TRINITY_XP_0162	probabl	Arachis_ipaensis	40.60	0.00
TRINITY_XP_0213	gamma-g	Sorghum_bicolor	40.60	0.00
TRINITY_OAE1994	hypothet	Marchantia_polymor	40.60	0.00
TRINITY_RZC5964	hypothet	Papaver_somniferum	40.60	0.00
TRINITY_XP_0264	ABC tra	Papaver_somniferum	40.60	0.00
TRINITY_XP_0239	serine/	Quercus_suber	40.60	0.00
TRINITY_XP_0249	DNA rep	Cynara_cardunculus	40.60	0.00
TRINITY_AAB3955	nitrate	Agrostemma_githago	40.60	0.00
TRINITY_GAX8254	hypothet	Chlamydomonas_eusti	40.60	0.00
TRINITY_RMZ5775	hypothet	Auxenochlorella_pro	40.60	0.00
TRINITY_KZM8747	hypothet	Daucus_carota	40.60	0.00
TRINITY_EFJ2714	hypothet	Selaginella_moellen	40.60	0.00
TRINITY_EEF2339	conserv	Ricinus_communis	40.60	0.00
TRINITY_XP_0109	PREDICT	Elaeis_guineensis	40.60	0.00
TRINITY_XP_0138	hypothet	Monoraphidium_negle	40.60	0.00
TRINITY_XP_0158	peptidy	Ziziphus_jujuba	40.60	0.00
TRINITY_EFJ3160	hypothet	Selaginella_moellen	40.60	0.00
TRINITY_GAX8274	hypothet	Chlamydomonas_eusti	40.60	0.00
TRINITY_KXZ5697	hypothet	Gonium_pectorale	40.60	0.00
TRINITY_PSC6990	SNF2 fa	Micractinium_conduc	40.60	0.00
TRINITY_GAX7668	hypothet	Chlamydomonas_eusti	40.60	0.00
TRINITY_GAQ8432	UDP-gal	Klebsormidium_niten	40.60	0.00

TRINITY_GAQ8439	hypothet	Klebsormidium_niten	40.60	0.00
TRINITY_KXZ4276	hypothet	Gonium_pectorale	40.60	0.00
TRINITY_PNW8103	hypothet	Chlamydomonas_reinh	40.60	0.00
TRINITY_RXI0409	hypothet	Malus_domestica	40.60	0.00
TRINITY_XP_0136	PREDICTE	Brassica_oleracea	40.60	0.00
TRINITY_PNW8229	hypothet	Chlamydomonas_reinh	40.60	0.00
TRINITY_XP_0258	E3 ubiqu	Oryza_sativa	40.60	0.00
TRINITY_KXZ5483	hypothet	Gonium_pectorale	40.60	0.00
TRINITY_EFJ2472	hypothet	Selaginella_moellen	40.60	0.00
TRINITY_KZV1541	phenylal	Dorcoceras_hygromet	40.60	0.00
TRINITY_ONM3496	CDC5 pro	Zea_mays	40.60	0.00
TRINITY_XP_0245	dihydrol	Selaginella_moellen	40.60	0.00
TRINITY_KYP4658	Lysine-s	Cajanus_cajan	40.60	0.00
TRINITY_GBF9064	p-type a	Raphidocelis_subcap	40.60	0.00
TRINITY_PNH1228	WW domai	Tetrabaena_socialis	40.60	0.00
TRINITY_PNW8805	hypothet	Chlamydomonas_reinh	40.60	0.00
TRINITY_AHA9309	PLC prot	Dunaliella_salina	40.60	0.00
TRINITY_OMO9825	hypothet	Corchorus_capsulari	40.60	0.00
TRINITY_XP_0201	RNA exon	Aegilops_tauschii	40.60	0.00
TRINITY_XP_0029	hypothet	Volvox_carteri	40.60	0.00
TRINITY_GAX8321	hypothet	Chlamydomonas_eusti	40.60	0.00
TRINITY_GBG7190	hypothet	Chara_braunii	40.60	0.00
TRINITY_XP_0029	hypothet	Volvox_carteri	40.60	0.00
TRINITY_XP_0017	R-SNARE	Chlamydomonas_reinh	40.60	0.00
TRINITY_GAX7321	hypothet	Chlamydomonas_eusti	40.60	0.00
TRINITY_GBG8806	hypothet	Chara_braunii	40.60	0.00
TRINITY_PNW8545	hypothet	Chlamydomonas_reinh	40.60	0.00
TRINITY_PNH0637	Protein	Tetrabaena_socialis	40.60	0.00
TRINITY_KXZ5018	hypothet	Gonium_pectorale	40.60	0.00
TRINITY_KXZ4681	hypothet	Gonium_pectorale	40.60	0.00
TRINITY_XP_0058	hypothet	Chlorella_variabili	40.60	0.00
TRINITY_KXZ4535	hypothet	Gonium_pectorale	40.60	0.00
TRINITY_XP_0029	hypothet	Volvox_carteri	40.60	0.00
TRINITY_GBF9841	peptidyl	Raphidocelis_subcap	40.60	0.00
TRINITY_PRW5858	Beta-man	Chlorella_sorokinia	40.60	0.00
TRINITY_KYP6956	Snurport	Cajanus_cajan	40.60	0.00
TRINITY_KXZ4756	hypothet	Gonium_pectorale	40.60	0.00
TRINITY_AEC1371	acetoace	Catharanthus_roseus	40.60	0.00
TRINITY_XP_0191	PREDICTE	Ipomoea_nil	40.50	0.00
TRINITY_PTQ2726	hypothet	Marchantia_polymorp	40.50	0.00
TRINITY_GBG7447	hypothet	Chara_braunii	40.50	0.00
TRINITY_BAJ9457	predicte	Hordeum_vulgare	40.50	0.00
TRINITY_XP_0030	predicte	Micromonas_pusilla	40.50	0.00
TRINITY_XP_0102	PREDICTE	Nelumbo_nucifera	40.50	0.00
TRINITY_GAQ9157	protein	Klebsormidium_niten	40.50	0.00
TRINITY_GAQ8798	Phosphat	Klebsormidium_niten	40.50	0.00
TRINITY_PNH0433	MOXD1 1	Tetrabaena_socialis	40.50	0.00
TRINITY_XP_0110	PREDICTE	Populus_euphratica	40.50	0.00
TRINITY_GAQ8464	protein	Klebsormidium_niten	40.50	0.00
TRINITY_OUS4463	NADH:fla	Ostreococcus_tauri	40.50	0.00
TRINITY_GAQ9054	Ubiquiti	Klebsormidium_niten	40.50	0.00
TRINITY_CAN8063	hypothet	Vitis_vinifera	40.50	0.00
TRINITY_GAQ7793	NMD prot	Klebsormidium_niten	40.50	0.00
TRINITY_EEF2416	two-comp	Ricinus_communis	40.50	0.00
TRINITY_XP_0029	hypothet	Volvox_carteri	40.50	0.00
TRINITY_KXZ5525	hypothet	Gonium_pectorale	40.50	0.00
TRINITY_PRQ1633	putative	Rosa_chinensis	40.50	0.00
TRINITY_GBG6433	hypothet	Chara_braunii	40.50	0.00
TRINITY_XP_0016	ARF-like	Chlamydomonas_reinh	40.50	0.00
TRINITY_ACN8761	kinase-l	Corylus_avellana	40.50	0.00
TRINITY_GAQ7781	branched	Klebsormidium_niten	40.50	0.00
TRINITY_GAX7446	hypothet	Chlamydomonas_eusti	40.50	0.00
TRINITY_RCV2891	hypothet	Setaria_italica	40.50	0.00
TRINITY_GAX7945	hypothet	Chlamydomonas_eusti	40.50	0.00
TRINITY_GAX7604	hypothet	Chlamydomonas_eusti	40.50	0.00
TRINITY_XP_0184	PREDICTE	Raphanus_sativus	40.50	0.00

TRINITY_CBI3147	unnamed	Vitis_vinifera	40.50	0.00
TRINITY_PRW6075	Zn2+-bi	Chlorella_sorokinia	40.50	0.00
TRINITY_OUS4518	tRNA ps	Ostreococcus_tauri	40.50	0.00
TRINITY_PNH0023	hypothet	Tetrabaena_socialis	40.50	0.00
TRINITY_RMZ5649	hypothet	Auxenochlorella_pro	40.50	0.00
TRINITY_XP_0239	ATP-bin	Quercus_suber	40.50	0.00
TRINITY_ONK7286	uncharac	Asparagus_officinal	40.50	0.00
TRINITY_PTQ3906	hypothet	Marchantia_polymorp	40.50	0.00
TRINITY_GAX7697	hypothet	Chlamydomonas_eusti	40.50	0.00
TRINITY_GAX7840	hypothet	Chlamydomonas_eusti	40.50	0.00
TRINITY_EYU3713	hypothet	Erythranthe_guttata	40.50	0.00
TRINITY_XP_0025	thiored	Micromonas_commoda	40.50	0.00
TRINITY_GAX8252	hypothet	Chlamydomonas_eusti	40.50	0.00
TRINITY_XP_0029	hypothet	Volvox_carteri	40.50	0.00
TRINITY_XP_0120	bifuncti	Jatropha_curcas	40.50	0.00
TRINITY_PRW6159	F1F0 AT	Chlorella_sorokinia	40.50	0.00
TRINITY_GAX7396	hypothet	Chlamydomonas_eusti	40.50	0.00
TRINITY_PNW7897	hypothet	Chlamydomonas_reinh	40.50	0.00
TRINITY_KRH2887	hypothet	Glycine_max	40.50	0.00
TRINITY_XP_0238	uncharac	Quercus_suber	40.50	0.00
TRINITY_XP_0241	zinc fi	Rosa_chinensis	40.50	0.00
TRINITY_GBF9753	hypothet	Raphidocelis_subcap	40.50	0.00
TRINITY_XP_0056	hypothet	Coccomyxa_subellips	40.50	0.00
TRINITY_GAV6371	dCMP_cy	Cephalotus_follicul	40.50	0.00
TRINITY_GAX7981	hypothet	Chlamydomonas_eusti	40.50	0.00
TRINITY_XP_0056	mitochor	Coccomyxa_subellips	40.50	0.00
TRINITY_GBF9013	hypothet	Raphidocelis_subcap	40.50	0.00
TRINITY_GAX7859	hypothet	Chlamydomonas_eusti	40.50	0.00
TRINITY_GAX7368	hypothet	Chlamydomonas_eusti	40.50	0.00
TRINITY_XP_0186	PREDICT	Musa_acuminata	40.50	0.00
TRINITY_GBF9435	hypothet	Raphidocelis_subcap	40.40	0.00
TRINITY_KZM9324	hypothet	Daucus_carota	40.40	0.00
TRINITY_XP_0263	CBL-int	Papaver_somniferum	40.40	0.00
TRINITY_XP_0200	UDP-N-a	Ananas_comosus	40.40	0.00
TRINITY_XP_0241	uncharac	Rosa_chinensis	40.40	0.00
TRINITY_XP_0243	uncharac	Physcomitrella_pate	40.40	0.00
TRINITY_XP_0258	EEF1A l	Panicum_hallii	40.40	0.00
TRINITY_XP_0030	flagell	Micromonas_pusilla	40.40	0.00
TRINITY_XP_0243	ABC tra	Physcomitrella_pate	40.40	0.00
TRINITY_XP_0120	fimbrin-	Jatropha_curcas	40.40	0.00
TRINITY_XP_0056	UVB-res	Coccomyxa_subellips	40.40	0.00
TRINITY_XP_0192	PREDICT	Nicotiana_attenuata	40.40	0.00
TRINITY_XP_0029	uncharac	Selaginella_moellen	40.40	0.00
TRINITY_XP_0030	predict	Micromonas_pusilla	40.40	0.00
TRINITY_XP_0029	hypothet	Volvox_carteri	40.40	0.00
TRINITY_PRW5603	Repress	Chlorella_sorokinia	40.40	0.00
TRINITY_KFK2489	hypothet	Arabis_alpina	40.40	0.00
TRINITY_RWR7597	Beta-Ca	Cinnamomum_micranth	40.40	0.00
TRINITY_GAQ8633	lysine-	Klebsormidium_niten	40.40	0.00
TRINITY_GAX8650	hypothet	Chlamydomonas_eusti	40.40	0.00
TRINITY_XP_0243	transcr	Physcomitrella_pate	40.40	0.00
TRINITY_PNW7228	hypothet	Chlamydomonas_reinh	40.40	0.00
TRINITY_PNW7199	hypothet	Chlamydomonas_reinh	40.40	0.00
TRINITY_XP_0029	IAA-ala	Selaginella_moellen	40.40	0.00
TRINITY_XP_0109	PREDICT	Elaeis_guineensis	40.40	0.00
TRINITY_XP_0238	GTP-bin	Quercus_suber	40.40	0.00
TRINITY_EFJ3400	hypothet	Selaginella_moellen	40.40	0.00
TRINITY_XP_0202	GPCR-ty	Asparagus_officinal	40.40	0.00
TRINITY_GAX8611	hypothet	Chlamydomonas_eusti	40.40	0.00
TRINITY_PNW7685	hypothet	Chlamydomonas_reinh	40.40	0.00
TRINITY_BAK0043	predict	Hordeum_vulgare	40.40	0.00
TRINITY_PON3145	Methylt	Parasponia_anderson	40.40	0.00
TRINITY_XP_0245	ran gua	Selaginella_moellen	40.40	0.00
TRINITY_XP_0228	uncharac	Olea_europaea	40.40	0.00
TRINITY_KXZ5328	hypothet	Gonium_pectorale	40.40	0.00
TRINITY_RZR7752	hypothet	Ensete_ventricosum	40.40	0.00

TRINITY_BAJ9884	predicte	Hordeum_vulgare	40.40	0.00
TRINITY_XP_0029	hypothet	Volvox_carteri	40.40	0.00
TRINITY_GAQ7857	elongati	Klebsormidium_niten	40.40	0.00
TRINITY_PRW4436	Poly(A)	Chlorella_sorokinia	40.40	0.00
TRINITY_KXZ4844	hypothet	Gonium_pectorale	40.40	0.00
TRINITY_GAX7378	hypothet	Chlamydomonas_eusti	40.40	0.00
TRINITY_KXZ4645	hypothet	Gonium_pectorale	40.40	0.00
TRINITY_GAX8566	hypothet	Chlamydomonas_eusti	40.40	0.00
TRINITY_RMZ5570	hypothet	Auxenochlorella_prc	40.40	0.00
TRINITY_XP_0138	hypothet	Monoraphidium_negle	40.40	0.00
TRINITY_XP_0014	predicte	Ostreococcus_sp._'l	40.40	0.00
TRINITY_PNH1165	Calcium/	Tetrabaena_socialis	40.40	0.00
TRINITY_XP_0238	pre-mRNA	Quercus_suber	40.40	0.00
TRINITY_PNW8165	hypothet	Chlamydomonas_reinh	40.40	0.00
TRINITY_GAX7532	hypothet	Chlamydomonas_eusti	40.40	0.00
TRINITY_PNH0937	Cytochro	Tetrabaena_socialis	40.40	0.00
TRINITY_GAX7345	hypothet	Chlamydomonas_eusti	40.40	0.00
TRINITY_OAE3416	hypothet	Marchantia_polymorp	40.40	0.00
TRINITY_XP_0016	RWP-RK t	Chlamydomonas_reinh	40.40	0.00
TRINITY_GAX7987	hypothet	Chlamydomonas_eusti	40.40	0.00
TRINITY_GAX7633	hypothet	Chlamydomonas_eusti	40.40	0.00
TRINITY_XP_0264	ELMO dom	Papaver_somniferum	40.40	0.00
TRINITY_PNW7147	hypothet	Chlamydomonas_reinh	40.40	0.00
TRINITY_XP_0161	uncharac	Arachis_ipaensis	40.40	0.00
TRINITY_PNW7797	hypothet	Chlamydomonas_reinh	40.40	0.00
TRINITY_XP_0228	NLI inte	Ostreococcus_tauri	40.40	0.00
TRINITY_XP_0114	PREDICTE	Fragaria_vesca	40.40	0.00
TRINITY_GAX7364	hypothet	Chlamydomonas_eusti	40.40	0.00
TRINITY_XP_0084	PREDICTE	Cucumis_melo	40.40	0.00
TRINITY_GAX8298	hypothet	Chlamydomonas_eusti	40.40	0.00
TRINITY_XP_0238	eukaryot	Quercus_suber	40.40	0.00
TRINITY_XP_0029	hypothet	Volvox_carteri	40.40	0.00
TRINITY_GAX7893	hypothet	Chlamydomonas_eusti	40.40	0.00
TRINITY_XP_0273	putative	Abrus_precatorius	40.40	0.00
TRINITY_XP_0029	hypothet	Volvox_carteri	40.40	0.00
TRINITY_GAX7946	hypothet	Chlamydomonas_eusti	40.40	0.00
TRINITY_XP_0058	hypothet	Chlorella_variabili	40.40	0.00
TRINITY_XP_0239	ras-like	Quercus_suber	40.40	0.00
TRINITY_XP_0258	pre-rRNA	Panicum_hallii	40.40	0.00
TRINITY_XP_0025	predicte	Micromonas_commoda	40.40	0.00
TRINITY_GBF9451	hypothet	Raphidocelis_subcap	40.40	0.00
TRINITY_XP_0243	uncharac	Physcomitrella_pate	40.40	0.00
TRINITY_XP_0243	ATP-depe	Physcomitrella_pate	40.40	0.00
TRINITY_XP_0029	hypothet	Volvox_carteri	40.40	0.00
TRINITY_XP_0244	serine/a	Physcomitrella_pate	40.40	0.00
TRINITY_PNH0734	Bifuncti	Tetrabaena_socialis	40.40	0.00
TRINITY_PNH1169	hypothet	Tetrabaena_socialis	40.40	0.00
TRINITY_GBG7027	hypothet	Chara_braunii	40.40	0.00
TRINITY_GAX7875	hypothet	Chlamydomonas_eusti	40.40	0.00
TRINITY_GAX7612	hypothet	Chlamydomonas_eusti	40.40	0.00
TRINITY_PRW5979	ubiquiti	Chlorella_sorokinia	40.40	0.00
TRINITY_PRW4552	dnaJ-lik	Chlorella_sorokinia	40.40	0.00
TRINITY_PSC7564	exocyst	Micractinium_conduc	40.40	0.00
TRINITY_PNW8443	hypothet	Chlamydomonas_reinh	40.40	0.00
TRINITY_GBG6905	hypothet	Chara_braunii	40.40	0.00
TRINITY_GBF9111	hypothet	Raphidocelis_subcap	40.40	0.00
TRINITY_XP_0029	hypothet	Volvox_carteri	40.40	0.00
TRINITY_XP_0138	hypothet	Monoraphidium_negle	40.40	0.00
TRINITY_XP_0167	PREDICTE	Gossypium_hirsutum	40.40	0.00
TRINITY_XP_0201	uncharac	Aegilops_tauschii	40.40	0.00
TRINITY_GAU4503	hypothet	Trifolium_subterran	40.40	0.00
TRINITY_XP_0238	N-acyl-p	Quercus_suber	40.30	0.00
TRINITY_ESR4742	hypothet	Citrus_clementina	40.30	0.00
TRINITY_PSC7275	glutamyl	Micractinium_conduc	40.30	0.00
TRINITY_RLN4160	hypothet	Panicum_miliaceum	40.30	0.00
TRINITY_XP_0279	integrin	Vigna_unguiculata	40.30	0.00

TRINITY_XP_0161(	LOW QUAI	Arachis_ipaensis	40.30	0.00
TRINITY_GAQ7941(	Na+/H+ a	Klebsormidium_niten	40.30	0.00
TRINITY_XP_0071	hypothet	Phaseolus_vulgaris	40.30	0.00
TRINITY_EFJ2208(	dolichyl	Selaginella_moellen	40.30	0.00
TRINITY_XP_0029	hypothet	Volvox_carteri	40.30	0.00
TRINITY_GAX7580	hypothet	Chlamydomonas_eusti	40.30	0.00
TRINITY_OWM8199	hypothet	Punica_granatum	40.30	0.00
TRINITY_XP_0202	TPR repe	Cajanus_cajan	40.30	0.00
TRINITY_GAX7739	hypothet	Chlamydomonas_eusti	40.30	0.00
TRINITY_PIN0705(	Molecula	Handroanthus_impeti	40.30	0.00
TRINITY_PIN0705(	Molecula	Handroanthus_impeti	40.30	0.00
TRINITY_GAQ8372	vesicle-	Klebsormidium_niten	40.30	0.00
TRINITY_RYR7299	hypothet	Arachis_hypogaea	40.30	0.00
TRINITY_XP_0025(	thioredo	Micromonas_commoda	40.30	0.00
TRINITY_PNW7750	hypothet	Chlamydomonas_reinh	40.30	0.00
TRINITY_PTQ4341	hypothet	Marchantia_polymorp	40.30	0.00
TRINITY_XP_0016	predicte	Chlamydomonas_reinh	40.30	0.00
TRINITY_AMN1485	serine (	Nepenthes_mirabilis	40.30	0.00
TRINITY_XP_0219	actin-re	Helianthus_annuus	40.30	0.00
TRINITY_XP_0271(	protein	Coffea_arabica	40.30	0.00
TRINITY_GAX7348	hypothet	Chlamydomonas_eusti	40.30	0.00
TRINITY_BAK0043	predicte	Hordeum_vulgare	40.30	0.00
TRINITY_XP_0063	40S ribo	Populus_trichocarpa	40.30	0.00
TRINITY_PRW5800	serine (	Chlorella_sorokinia	40.30	0.00
TRINITY_GAX8199	hypothet	Chlamydomonas_eusti	40.30	0.00
TRINITY_XP_0030	predicte	Micromonas_pusilla	40.30	0.00
TRINITY_GAX7499(	hypothet	Chlamydomonas_eusti	40.30	0.00
TRINITY_XP_0201(	short-ch	Ananas_comosus	40.30	0.00
TRINITY_XP_0155	uncharac	Ricinus_communis	40.30	0.00
TRINITY_XP_0239	vacuolar	Quercus_suber	40.30	0.00
TRINITY_GBF9300	hypothet	Raphidocelis_subcap	40.30	0.00
TRINITY_XP_0165	PREDICTE	Capsicum_annuum	40.30	0.00
TRINITY_ERN1694	hypothet	Amborella_trichopod	40.30	0.00
TRINITY_XP_0138	ADP-ribo	Monoraphidium_negle	40.30	0.00
TRINITY_GAX7872	hypothet	Chlamydomonas_eusti	40.30	0.00
TRINITY_GBF9059(	hypothet	Raphidocelis_subcap	40.30	0.00
TRINITY_XP_0056	ubiquiti	Coccomyxa_subellips	40.30	0.00
TRINITY_GAX8284	hypothet	Chlamydomonas_eusti	40.30	0.00
TRINITY_KXZ4574(	hypothet	Gonium_pectorale	40.30	0.00
TRINITY_PRW6160	endoplas	Chlorella_sorokinia	40.30	0.00
TRINITY_PTQ2890(	hypothet	Marchantia_polymorp	40.30	0.00
TRINITY_BAJ9372	predicte	Hordeum_vulgare	40.30	0.00
TRINITY_KHG1546(	1-phosph	Gossypium_arboreum	40.30	0.00
TRINITY_GAX7986	hypothet	Chlamydomonas_eusti	40.30	0.00
TRINITY_KXZ4504	hypothet	Gonium_pectorale	40.30	0.00
TRINITY_XP_0071	hypothet	Phaseolus_vulgaris	40.30	0.00
TRINITY_PNW8565	hypothet	Chlamydomonas_reinh	40.30	0.00
TRINITY_PSC6754(	ribosoma	Micractinium_conduc	40.30	0.00
TRINITY_XP_0249	probable	Ziziphus_jujuba	40.30	0.00
TRINITY_XP_0156	ankyrin	Oryza_sativa	40.30	0.00
TRINITY_GAX7875	hypothet	Chlamydomonas_eusti	40.30	0.00
TRINITY_PNW8013	hypothet	Chlamydomonas_reinh	40.30	0.00
TRINITY_PWA5803(	hypothet	Artemisia_annua	40.30	0.00
TRINITY_GAX8658	hypothet	Chlamydomonas_eusti	40.30	0.00
TRINITY_XP_0014	predicte	Ostreococcus_sp._'l	40.30	0.00
TRINITY_GAQ8098	Anaphase	Klebsormidium_niten	40.30	0.00
TRINITY_XP_0239(	ras-like	Quercus_suber	40.20	0.00
TRINITY_NP_0012	putative	Glycine_max	40.20	0.00
TRINITY_XP_0176	PREDICTE	Gossypium_arboreum	40.20	0.00
TRINITY_XP_0056	RabGAP/T	Coccomyxa_subellips	40.20	0.00
TRINITY_BAK0286	predicte	Hordeum_vulgare	40.20	0.00
TRINITY_GAX7674(	hypothet	Chlamydomonas_eusti	40.20	0.00
TRINITY_GAQ8144(	Hypothet	Klebsormidium_niten	40.20	0.00
TRINITY_XP_0271	serine/t	Coffea_eugenioides	40.20	0.00
TRINITY_PSC6943	Acyl-bir	Micractinium_conduc	40.20	0.00
TRINITY_KZV2910	sufE-lik	Dorcoceras_hygromet	40.20	0.00

TRINITY_XP_0086(	unchara(	Zea_mays	40.20	0.00
TRINITY_XP_0014	predicte	Ostreococcus_sp._'l	40.20	0.00
TRINITY_XP_0243	histidir	Physcomitrella_pate	40.20	0.00
TRINITY_XP_0041	PREDICTI	Cucumis_sativus	40.20	0.00
TRINITY_XP_0025	E3 ubiq	Ricinus_communis	40.20	0.00
TRINITY_GAQ7885	peptide	Klebsormidium_niten	40.20	0.00
TRINITY_XP_0056	hypothet	Coccomyxa_subellips	40.20	0.00
TRINITY_XP_0056	sacchar	Coccomyxa_subellips	40.20	0.00
TRINITY_XP_0014	predicte	Ostreococcus_sp._'l	40.20	0.00
TRINITY_XP_0238	alpha-a	Quercus_suber	40.20	0.00
TRINITY_XP_0066	PREDICTI	Oryza_brachyantha	40.20	0.00
TRINITY_XP_0029	hypothet	Volvox_carteri	40.20	0.00
TRINITY_GAX8535	hypothet	Chlamydomonas_eusti	40.20	0.00
TRINITY_PRW2065	SWI SNF-	Chlorella_sorokinia	40.20	0.00
TRINITY_GAQ7884	alpha-1,	Klebsormidium_niten	40.20	0.00
TRINITY_GBF9670	sulfur s	Raphidocelis_subcap	40.20	0.00
TRINITY_GAX7922	hypothet	Chlamydomonas_eusti	40.20	0.00
TRINITY_XP_0029	hypothet	Volvox_carteri	40.20	0.00
TRINITY_GAX8199	hypothet	Chlamydomonas_eusti	40.20	0.00
TRINITY_XP_0239	probable	Quercus_suber	40.20	0.00
TRINITY_GAX8210	hypothet	Chlamydomonas_eusti	40.20	0.00
TRINITY_XP_0138	hypothet	Monoraphidium_negle	40.20	0.00
TRINITY_KDD7569	hypothet	Helicosporidium_sp.	40.20	0.00
TRINITY_XP_0138	Unchara	Monoraphidium_negle	40.20	0.00
TRINITY_OAE3589	hypothet	Marchantia_polymorp	40.20	0.00
TRINITY_XP_0158	large p	Ziziphus_jujuba	40.20	0.00
TRINITY_XP_0139	WD repea	Monoraphidium_negle	40.20	0.00
TRINITY_OAE2897	hypothet	Marchantia_polymorp	40.20	0.00
TRINITY_XP_0145	ubiquit	Vigna_radiata	40.20	0.00
TRINITY_XP_0029	molecula	Volvox_carteri	40.20	0.00
TRINITY_GAX7465	hypothet	Chlamydomonas_eusti	40.20	0.00
TRINITY_GAX8058	hypothet	Chlamydomonas_eusti	40.20	0.00
TRINITY_XP_0029	hypothet	Volvox_carteri	40.20	0.00
TRINITY_PIN0910	putative	Handroanthus_impeti	40.20	0.00
TRINITY_BAJ9372	predicte	Hordeum_vulgare	40.20	0.00
TRINITY_GAQ8511	hypothet	Klebsormidium_niten	40.20	0.00
TRINITY_PNH1169	hypothet	Tetrabaena_socialis	40.20	0.00
TRINITY_XP_0106	PREDICTI	Beta_vulgaris	40.20	0.00
TRINITY_XP_0029	hypothet	Volvox_carteri	40.20	0.00
TRINITY_GAX7617	hypothet	Chlamydomonas_eusti	40.20	0.00
TRINITY_GBF9254	hypothet	Raphidocelis_subcap	40.20	0.00
TRINITY_GBG8032	hypothet	Chara_braunii	40.20	0.00
TRINITY_BAK0302	predicte	Hordeum_vulgare	40.20	0.00
TRINITY_PNH1226	Tiny mac	Tetrabaena_socialis	40.20	0.00
TRINITY_XP_0016	predicte	Chlamydomonas_reinh	40.20	0.00
TRINITY_XP_0238	alpha-a	Quercus_suber	40.20	0.00
TRINITY_XP_0217	ras-rela	Chenopodium_quinoa	40.20	0.00
TRINITY_EFJ3272	hypothet	Selaginella_moellen	40.20	0.00
TRINITY_PSC6940	symplek	Micractinium_conduc	40.20	0.00
TRINITY_XP_0029	hypothet	Volvox_carteri	40.20	0.00
TRINITY_XP_0045	heat st	Cicer_arietinum	40.20	0.00
TRINITY_GBF8923	DNA repa	Raphidocelis_subcap	40.20	0.00
TRINITY_GAX7821	hypothet	Chlamydomonas_eusti	40.20	0.00
TRINITY_KXZ5444	hypothet	Gonium_pectorale	40.20	0.00
TRINITY_GAQ8723	Dyneins	Klebsormidium_niten	40.20	0.00
TRINITY_OAE2443	hypothet	Marchantia_polymorp	40.20	0.00
TRINITY_PRW5705	guanylyl	Chlorella_sorokinia	40.20	0.00
TRINITY_XP_0279	unchara	Vigna_unguiculata	40.20	0.00
TRINITY_PNW7973	hypothet	Chlamydomonas_reinh	40.20	0.00
TRINITY_XP_0029	hypothet	Volvox_carteri	40.20	0.00
TRINITY_GAQ9315	hypothet	Klebsormidium_niten	40.20	0.00
TRINITY_KXZ4803	hypothet	Gonium_pectorale	40.20	0.00
TRINITY_GAQ8931	Dyneins	Klebsormidium_niten	40.20	0
TRINITY_GAX8431	hypothet	Chlamydomonas_eusti	40.20	0.00
TRINITY_XP_0025	predicte	Micromonas_commoda	40.20	0.00
TRINITY_GAQ9040	multico	Klebsormidium_niten	40.10	0.00

TRINITY_RRT72845	hypothet	Ensete_ventricosum	40.10	0.00
TRINITY_XP_00305	predicte	Micromonas_pusilla	40.10	0.00
TRINITY_KXZ51149	hypothet	Gonium_pectorale	40.10	0.00
TRINITY_OWM78516	hypothet	Punica_granatum	40.10	0.00
TRINITY_GAX79832	hypothet	Chlamydomonas_eusti	40.10	0.00
TRINITY_ADE76687	unknown	Picea_sitchensis	40.10	0.00
TRINITY_XP_00879	pathogen	Phoenix_dactylifera	40.10	0.00
TRINITY_XP_02015	phosphog	Aegilops_tauschii	40.10	0.00
TRINITY_XP_00169	predicte	Chlamydomonas_reinh	40.10	0.00
TRINITY_XP_00295	hypothet	Volvox_carteri	40.10	0.00
TRINITY_BAJ99217	predicte	Hordeum_vulgare	40.10	0.00
TRINITY_XP_00584	hypothet	Chlorella_variabili	40.10	0.00
TRINITY_XP_02389	GTP-binc	Quercus_suber	40.10	0.00
TRINITY_GAX74714	hypothet	Chlamydomonas_eusti	40.10	0.00
TRINITY_XP_02437	glutathi	Physcomitrella_pate	40.10	0.00
TRINITY_GBF92296	hypothet	Raphidocelis_subcap	40.10	0.00
TRINITY_PTQ42579	hypothet	Marchantia_polymorp	40.10	0.00
TRINITY_GAX77149	hypothet	Chlamydomonas_eusti	40.10	0.00
TRINITY_XP_00295	hypothet	Volvox_carteri	40.10	0.00
TRINITY_KXZ51682	hypothet	Gonium_pectorale	40.10	0.00
TRINITY_GAX77550	hypothet	Chlamydomonas_eusti	40.10	0.00
TRINITY_XP_00844	PREDICTE	Cucumis_melo	40.10	0.00
TRINITY_GAX80336	hypothet	Chlamydomonas_eusti	40.10	0.00
TRINITY_GAX80218	hypothet	Chlamydomonas_eusti	40.10	0.00
TRINITY_GAX81593	hypothet	Chlamydomonas_eusti	40.10	0.00
TRINITY_XP_02439	eukaryot	Physcomitrella_pate	40.10	0.00
TRINITY_KXZ47337	hypothet	Gonium_pectorale	40.10	0.00
TRINITY_XP_02436	ribonucl	Physcomitrella_pate	40.10	0.00
TRINITY_PNW87397	hypothet	Chlamydomonas_reinh	40.10	0.00
TRINITY_PRW44471	Peptide-	Chlorella_sorokinia	40.10	0.00
TRINITY_XP_01390	hypothet	Monoraphidium_negle	40.10	0.00
TRINITY_GBF99292	ABC trar	Raphidocelis_subcap	40.10	0.00
TRINITY_XP_00170	high int	Chlamydomonas_reinh	40.10	0.00
TRINITY_PNW85082	hypothet	Chlamydomonas_reinh	40.10	0.00
TRINITY_XP_00564	Nucleoti	Coccomyxa_subellips	40.10	0.00
TRINITY_XP_00169	phosphat	Chlamydomonas_reinh	40.00	0.00
TRINITY_PKA59756	Zinc fir	Apostasia_shenzheni	40.00	0.00
TRINITY_XP_02376	ras-rela	Lactuca_sativa	40.00	0.00
TRINITY_EEF27535	two-comp	Ricinus_communis	40.00	0.00
TRINITY_EFH70792	ubiquiti	Arabidopsis_lyrata	40.00	0.00
TRINITY_PSR91479	Mitogen-	Actinidia_chinensis	40.00	0.00
TRINITY_XP_01090	PREDICTE	Elaeis_guineensis	40.00	0.00
TRINITY_PON91640	Cytochro	Trema_orientale	40.00	0.00
TRINITY_XP_02017	BTB/POZ	Aegilops_tauschii	40.00	0.00
TRINITY_XP_00250	target c	Micromonas_commoda	40.00	0.00
TRINITY_XP_00169	predicte	Chlamydomonas_reinh	40.00	0.00
TRINITY_PWA88159	RING/U-k	Artemisia_annua	40.00	0.00
TRINITY_EPS74144	hypothet	Genlisea_aurea	40.00	0.00
TRINITY_XP_00824	PREDICTE	Prunus_mume	40.00	0.00
TRINITY_GAX77208	hypothet	Chlamydomonas_eusti	40.00	0.00
TRINITY_KZV16597	hypothet	Dorcoceras_hygromet	40.00	0.00
TRINITY_XP_02435	uncharac	Physcomitrella_pate	40.00	0.00
TRINITY_GBG67291	hypothet	Chara_braunii	40.00	0.00
TRINITY_XP_00564	hypothet	Coccomyxa_subellips	40.00	0.00
TRINITY_XP_01206	alpha-N-	Jatropha_curcas	40.00	0.00
TRINITY_XP_00721	calcium-	Prunus_persica	40.00	0.00
TRINITY_GAX75954	hypothet	Chlamydomonas_eusti	40.00	0.00
TRINITY_RAL43982	hypothet	Cuscuta_australis	40.00	0.00
TRINITY_GAX79227	hypothet	Chlamydomonas_eusti	40.00	0.00
TRINITY_XP_00866	beta-ada	Zea_mays	40.00	0.00
TRINITY_GAQ78735	alpha-ar	Klebsormidium_niten	40.00	0.00
TRINITY_RVX01223	Pol poly	Vitis_vinifera	40.00	0.00
TRINITY_GBG59841	hypothet	Chara_braunii	40.00	0.00
TRINITY_XP_00306	predicte	Micromonas_pusilla	40.00	0.00
TRINITY_XP_00979	PREDICTE	Nicotiana_sylvestri	40.00	0.00
TRINITY_PNH12263	hypothet	Tetrabaena_socialis	40.00	0.00

TRINITY_XP_0029	intrafla	Volvox_carteri	40.00	0.00
TRINITY_XP_0082	PREDICTI	Prunus_mume	40.00	0.00
TRINITY_XP_0088	bZIP tra	Phoenix_dactylifera	40.00	0.00
TRINITY_XP_0029	hypothet	Volvox_carteri	40.00	0.00
TRINITY_XP_0145	ras-rela	Vigna_radiata	40.00	0.00
TRINITY_XP_0014	predicte	Ostreococcus_sp._'l	40.00	0.00
TRINITY_XP_0243	regulato	Physcomitrella_pate	40.00	0.00
TRINITY_GBG7998	hypothet	Chara_braunii	40.00	0.00
TRINITY_GAQ7765	ATMRK se	Klebsormidium_niten	40.00	0.00
TRINITY_XP_0158	probable	Ziziphus_jujuba	40.00	0.00

TRINITY_GAX75994	hypothet	Chlamydomonas_eusti	40.00	0.00
TRINITY_AAY31019	low phot	Chlamydomonas_reinh	40.00	0.00
TRINITY_XP_01243	PREDICTE	Gossypium_raimondii	40.00	0.00
TRINITY_XP_00564	Metallo-	Coccomyxa_subellips	40.00	0.00
TRINITY_XP_02438	probable	Physcomitrella_pate	40.00	0.00
TRINITY_OWM73350	hypothet	Punica_granatum	40.00	0.00
TRINITY_GAX74187	hypothet	Chlamydomonas_eusti	40.00	0.00
TRINITY_XP_02498	CTD smal	Cynara_cardunculus	40.00	0.00
TRINITY_GAX83638	hypothet	Chlamydomonas_eusti	40.00	0.00
TRINITY_GBF95162	hypothet	Raphidocelis_subcap	40.00	0.00
TRINITY_KXZ55276	hypothet	Gonium_pectorale	40.00	0.00
TRINITY_XP_01053	PREDICTE	Tarenaya_hasslerian	40.00	0.00
TRINITY_XP_02287	histidin	Olea_europaea	40.00	0.00
TRINITY_XP_01139	L-aminoa	Auxenochlorella_pro	40.00	0.00
TRINITY_XP_00295	hypothet	Volvox_carteri	40.00	0.00
TRINITY_XP_02172	probable	Chenopodium_quinoa	40.00	0.00
TRINITY_RWW01281	hypothet	Ensete_ventricosum	40.00	0.00
TRINITY_EPS65926	hypothet	Genlisea_aurea	40.00	0.00
TRINITY_XP_00250	ankyrin	Micromonas_commoda	40.00	0.00
TRINITY_XP_02059	tubulin-	Phalaenopsis_equest	40.00	0.00
TRINITY_GBG63540	hypothet	Chara_braunii	40.00	0.00
TRINITY_GAQ83607	hypothet	Klebsormidium_niten	40.00	0.00
TRINITY_VDD34005	unnamed	Brassica_oleracea	40.00	0.00
TRINITY_OAE33678	hypothet	Marchantia_polymorp	40.00	0.00
TRINITY_PNW82725	hypothet	Chlamydomonas_reinh	40.00	0.00
TRINITY_KXZ49014	hypothet	Gonium_pectorale	40.00	0.00
TRINITY_GBG75290	hypothet	Chara_braunii	40.00	0.00
TRINITY_PNH05703	hypothet	Tetrabaena_socialis	40.00	0.00
TRINITY_GAX78477	hypothet	Chlamydomonas_eusti	40.00	0.00
TRINITY_XP_00564	glycosid	Coccomyxa_subellips	40.00	0.00
TRINITY_PRW56940	meiotic	Chlorella_sorokinia	40.00	0.00
TRINITY_GAX74230	hypothet	Chlamydomonas_eusti	40.00	0.00
TRINITY_XP_00295	hypothet	Volvox_carteri	40.00	0.00
TRINITY_GAX85199	hypothet	Chlamydomonas_eusti	40.00	0.00
TRINITY_PNW70230	hypothet	Chlamydomonas_reinh	40.00	0.00
TRINITY_XP_00294	hypothet	Volvox_carteri	40.00	0.00
TRINITY_XP_01941	PREDICTE	Lupinus_angustifoli	40.00	0.00
TRINITY_XP_00294	mitochon	Volvox_carteri	40.00	0.00
TRINITY_GAX81125	hypothet	Chlamydomonas_eusti	40.00	0.00
TRINITY_XP_02389	probable	Quercus_suber	40.00	0.00
TRINITY_EYU34376	hypothet	Erythranthe_guttata	40.00	0.00
TRINITY_XP_02027	transcri	Asparagus_officinal	40.00	0.00
TRINITY_PHT37442	Transcri	Capsicum_baccatum	40.00	0.00
TRINITY_XP_02391	dual spe	Quercus_suber	40.00	0.00
TRINITY_XP_00495	cytochro	Setaria_italica	40.00	0.00
TRINITY_XP_01918	PREDICTE	Ipomoea_nil	40.00	0.00
TRINITY_PNW89088	hypothet	Chlamydomonas_reinh	40.00	0.00
TRINITY_GAQ79497	Serine-t	Klebsormidium_niten	40.00	0.00
TRINITY_XP_02167	uncharac	Hevea_brasiliensis	40.00	0.00
TRINITY_XP_00564	MT-A70-c	Coccomyxa_subellips	40.00	0.00
TRINITY_XP_00584	hypothet	Chlorella_variabili	40.00	0.00
TRINITY_XP_01762	PREDICTE	Gossypium_arboreum	40.00	0.00
TRINITY_XP_00294	nitrogen	Volvox_carteri	40.00	0.00
TRINITY_GAX76561	hypothet	Chlamydomonas_eusti	40.00	0.00
TRINITY_ABH09321	leucine	Arachis_hypogaea	40.00	0.00
TRINITY_PKA53662	Peroxiso	Apostasia_shenzheni	40.00	0.00
TRINITY_XP_00584	hypothet	Chlorella_variabili	40.00	0.00
TRINITY_OAE31621	hypothet	Marchantia_polymorp	40.00	0.00
TRINITY_KMT16705	hypothet	Beta_vulgaris	39.90	0.00
TRINITY_RZB89330	Elongato	Glycine_soja	39.90	0.00
TRINITY_KXZ50265	hypothet	Gonium_pectorale	39.90	0.00
TRINITY_XP_02026	cyclin-(	Asparagus_officinal	39.90	0.00
TRINITY_PKA59298	hypothet	Apostasia_shenzheni	39.90	0.00
TRINITY_PTQ44635	hypothet	Marchantia_polymorp	39.90	0.00
TRINITY_PNR50021	hypothet	Physcomitrella_pate	39.90	0.00
TRINITY_XP_00643	serine/t	Citrus_clementina	39.90	0.00

TRINITY_XP_0029(	hybrid s	Selaginella_moellen	39.90	0.00
TRINITY_XP_0042(	PREDICTE	Fragaria_vesca	39.90	0.00
TRINITY_XP_0200(	serine/t	Ananas_comosus	39.90	0.00
TRINITY_EFJ2220:	hypothet	Selaginella_moellen	39.90	0.00
TRINITY_GBG6612(	hypothet	Chara_braunii	39.90	0.00
TRINITY_PPE0288(	hypothet	Gossypium_barbadens	39.90	0.00
TRINITY_XP_0238(	sacchar(	Quercus_suber	39.90	0.00
TRINITY_ERN0084'	hypothet	Amborella_trichopod	39.90	0.00
TRINITY_XP_0025(	predicte	Micromonas_commoda	39.90	0.00
TRINITY_GAX7836(	hypothet	Chlamydomonas_eusti	39.90	0.00
TRINITY_ACU1779(	unknown	Glycine_max	39.90	0.00
TRINITY_GAX8025:	hypothet	Chlamydomonas_eusti	39.90	0.00
TRINITY_GAQ8411(	spla rya	Klebsormidium_niten	39.90	0.00
TRINITY_XP_0029(	ubiquiti	Selaginella_moellen	39.90	0.00
TRINITY_PRW5738(	F-box or	Chlorella_sorokinia	39.90	0.00
TRINITY_GAX8276(	hypothet	Chlamydomonas_eusti	39.90	0.00
TRINITY_PNW8413(	hypothet	Chlamydomonas_reinh	39.90	0.00
TRINITY_PNH0939'	Purple a	Tetrabaena_socialis	39.90	0.00
TRINITY_EFJ3090:	hypothet	Selaginella_moellen	39.90	0.00
TRINITY_XP_0238'	porphobi	Quercus_suber	39.90	0.00
TRINITY_XP_0029(	hypothet	Volvox_carteri	39.90	0.00
TRINITY_KXZ4503(	hypothet	Gonium_pectorale	39.90	0.00
TRINITY_RMZ5446(	hypothet	Auxenochlorella_prc	39.90	0.00
TRINITY_XP_0245:	protein	Selaginella_moellen	39.90	0.00
TRINITY_GAX7569(	hypothet	Chlamydomonas_eusti	39.90	0.00
TRINITY_GAX8612(	hypothet	Chlamydomonas_eusti	39.90	0.00
TRINITY_XP_0138(	hypothet	Monoraphidium_negle	39.90	0.00
TRINITY_XP_0138(	hypothet	Monoraphidium_negle	39.90	0.00
TRINITY_XP_0238'	ras-rela	Quercus_suber	39.90	0.00
TRINITY_PNW7252(	hypothet	Chlamydomonas_reinh	39.90	0.00
TRINITY_KXZ5088(	hypothet	Gonium_pectorale	39.90	0.00
TRINITY_KXZ4954(	hypothet	Gonium_pectorale	39.90	0.00
TRINITY_GAX8151:	hypothet	Chlamydomonas_eusti	39.90	0.00
TRINITY_XP_0202(	LOW QUAI	Asparagus_officinal	39.90	0.00
TRINITY_XP_0225(	unchara(	Brassica_napus	39.90	0.00
TRINITY_XP_0056(	hypothet	Coccomyxa_subellips	39.90	0.00
TRINITY_GAX7290'	hypothet	Chlamydomonas_eusti	39.90	0.00
TRINITY_XP_0158'	PH, RCC:	Ziziphus_jujuba	39.80	0.00
TRINITY_PTQ3548(	hypothet	Marchantia_polymorp	39.80	0.00
TRINITY_XP_0239(	peroxide	Quercus_suber	39.80	0.00
TRINITY_XP_0266(	EVI5-lil	Phoenix_dactylifera	39.80	0.00
TRINITY_XP_0217(	transmem	Chenopodium_quinoa	39.80	0.00
TRINITY_XP_0087(	dnaJ pro	Phoenix_dactylifera	39.80	0.00
TRINITY_XP_0058(	hypothet	Chlorella_variabili	39.80	0.00
TRINITY_GAQ9000(	Serine (	Klebsormidium_niten	39.80	0.00
TRINITY_KXZ4922:	hypothet	Gonium_pectorale	39.80	0.00
TRINITY_XP_0137(	probable	Brassica_napus	39.80	0.00
TRINITY_XP_0056(	hypothet	Coccomyxa_subellips	39.80	0.00
TRINITY_XP_0030(	ROK [Ost	Ostreococcus_tauri	39.80	0.00
TRINITY_PON5488(	Zinc fir	Trema_orientale	39.80	0.00
TRINITY_KZV1389(	histidir	Dorcoceras_hygromet	39.80	0.00
TRINITY_PRQ3407'	putative	Rosa_chinensis	39.80	0.00
TRINITY_OMO8818:	Ribosoma	Corchorus_olitorius	39.80	0.00
TRINITY_EEF2200:	two-comp	Ricinus_communis	39.80	0.00
TRINITY_GAX7763'	hypothet	Chlamydomonas_eusti	39.80	0.00
TRINITY_XP_0238(	protein	Quercus_suber	39.80	0.00
TRINITY_BAK0167:	predicte	Hordeum_vulgare	39.80	0.00
TRINITY_KXZ5694(	hypothet	Gonium_pectorale	39.80	0.00
TRINITY_KDD7538:	hypothet	Helicosporidium_sp.	39.80	0.00
TRINITY_XP_0244(	AP-2 cor	Physcomitrella_pate	39.80	0.00
TRINITY_KXZ5575(	hypothet	Gonium_pectorale	39.80	0.00
TRINITY_XP_0068(	C2 and (	Amborella_trichopod	39.80	0.00
TRINITY_XP_0029(	hypothet	Volvox_carteri	39.80	0.00
TRINITY_XP_0058(	hypothet	Chlorella_variabili	39.80	0.00
TRINITY_XP_0165(	PREDICTE	Capsicum_annuum	39.80	0.00
TRINITY_GAX7466(	hypothet	Chlamydomonas_eusti	39.80	0.00

TRINITY_XP_00878	pre-mRNA	Phoenix_dactylifera	39.80	0.00
TRINITY_XP_00170	predicte	Chlamydomonas_reinh	39.80	0.00
TRINITY_AIA22172	10HGO [(	Gentiana_rigescens	39.80	0.00
TRINITY_XP_00250	predicte	Micromonas_commoda	39.80	0.00
TRINITY_XP_02390	allantoi	Quercus_suber	39.80	0.00
TRINITY_GBF99634	hypothet	Raphidocelis_subcap	39.80	0.00
TRINITY_GAX80339	hypothet	Chlamydomonas_eusti	39.80	0.00
TRINITY_XP_00250	predicte	Micromonas_commoda	39.80	0.00
TRINITY_XP_01026	PREDICTE	Nelumbo_nucifera	39.80	0.00
TRINITY_XP_00938	PREDICTE	Musa_acuminata	39.80	0.00
TRINITY_XP_02436	probable	Physcomitrella_pate	39.80	0.00
TRINITY_XP_01943	PREDICTE	Lupinus_angustifoli	39.80	0.00
TRINITY_XP_02436	protein	Physcomitrella_pate	39.80	0.00
TRINITY_KZV29126	heparan-	Dorcoceras_hygromet	39.80	0.00
TRINITY_XP_00169	phosphat	Chlamydomonas_reinh	39.80	0.00
TRINITY_BAK00754	predicte	Hordeum_vulgare	39.80	0.00
TRINITY_GAX82545	hypothet	Chlamydomonas_eusti	39.80	0.00
TRINITY_KDD75191	hypothet	Helicosporidium_sp.	39.80	0.00
TRINITY_GAX74444	hypothet	Chlamydomonas_eusti	39.80	0.00
TRINITY_BAK01125	predicte	Hordeum_vulgare	39.80	0.00
TRINITY_KXZ53411	hypothet	Gonium_pectorale	39.80	0.00
TRINITY_GAX78267	hypothet	Chlamydomonas_eusti	39.80	0.00
TRINITY_KXZ55409	hypothet	Gonium_pectorale	39.80	0.00
TRINITY_BAG12979	RHYTHM (	Chlamydomonas_reinh	39.80	0.00
TRINITY_XP_00169	predicte	Chlamydomonas_reinh	39.80	0.00
TRINITY_GAX76147	hypothet	Chlamydomonas_eusti	39.80	0.00
TRINITY_XP_02027	ras-rela	Asparagus_officinal	39.80	0.00
TRINITY_KXZ49100	hypothet	Gonium_pectorale	39.80	0.00
TRINITY_GAX85626	hypothet	Chlamydomonas_eusti	39.80	0.00
TRINITY_XP_01053	PREDICTE	Tarenaya_hasslerian	39.80	0.00
TRINITY_XP_01923	PREDICTE	Nicotiana_attenuata	39.80	0.00
TRINITY_GAX74659	hypothet	Chlamydomonas_eusti	39.80	0.00
TRINITY_XP_00294	hypothet	Volvox_carteri	39.80	0.00
TRINITY_GAQ83620	HEAT rep	Klebsormidium_niten	39.80	0.00
TRINITY_BAK00754	predicte	Hordeum_vulgare	39.80	0.00
TRINITY_GAQ83716	NADPH ox	Klebsormidium_niten	39.80	0.00
TRINITY_GAQ78614	Mitochon	Klebsormidium_niten	39.80	0.00
TRINITY_KYP71984	CBL-inte	Cajanus_cajan	39.80	0.00
TRINITY_PNH08303	NFX1-typ	Tetrabaena_socialis	39.80	0.00
TRINITY_XP_02173	tyrosine	Chenopodium_quinoa	39.80	0.00
TRINITY_EEC67154	hypothet	Oryza_sativa	39.80	0.00
TRINITY_GAX74598	hypothet	Chlamydomonas_eusti	39.80	0.00
TRINITY_PNW72017	hypothet	Chlamydomonas_reinh	39.80	0.00
TRINITY_ACG38552	hypothet	Zea_mays	39.80	0.00
TRINITY_PNW77233	hypothet	Chlamydomonas_reinh	39.80	0.00
TRINITY_GAX75575	hypothet	Chlamydomonas_eusti	39.80	0.00
TRINITY_PWA57329	Micro-fi	Artemisia_annua	39.80	0.00
TRINITY_PNW88127	hypothet	Chlamydomonas_reinh	39.80	0.00
TRINITY_XP_02024	probable	Asparagus_officinal	39.80	0.00
TRINITY_OAE34540	hypothet	Marchantia_polymorp	39.80	0.00
TRINITY_XP_02389	probable	Quercus_suber	39.80	0.00
TRINITY_GBF95313	hypothet	Raphidocelis_subcap	39.80	0.00
TRINITY_XP_00168	predicte	Chlamydomonas_reinh	39.80	0.00
TRINITY_GAX73140	hypothet	Chlamydomonas_eusti	39.80	0.00
TRINITY_XP_02167	putative	Hevea_brasiliensis	39.80	0.00
TRINITY_KHG23927	Elongati	Gossypium_arboreum	39.80	0.00
TRINITY_XP_02283	Small GT	Ostreococcus_tauri	39.80	0.00
TRINITY_RYQ89432	hypothet	Arachis_hypogaea	39.80	0.00
TRINITY_GAX77423	hypothet	Chlamydomonas_eusti	39.80	0.00
TRINITY_XP_01390	hypothet	Monoraphidium_negle	39.80	0.00
TRINITY_XP_00295	hypothet	Volvox_carteri	39.80	0.00
TRINITY_XP_02419	kinesin-	Rosa_chinensis	39.80	0.00
TRINITY_OWM72645	hypothet	Punica_granatum	39.80	0.00
TRINITY_CAA49283	gag,prot	Volvox_carteri	39.80	0.00
TRINITY_GBF95577	hypothet	Raphidocelis_subcap	39.80	0.00
TRINITY_GAX82138	hypothet	Chlamydomonas_eusti	39.80	0.00

TRINITY_XP_0058	hypothet	Chlorella_variabili	39.80	0.00
TRINITY_GBF8898	brefeld	Raphidocelis_subcap	39.80	0.00
TRINITY_PNW7977	hypothet	Chlamydomonas_reinh	39.80	0.00
TRINITY_PNW7636	hypothet	Chlamydomonas_reinh	39.80	0.00
TRINITY_XP_0194	PREDICTI	Lupinus_angustifoli	39.80	0.00
TRINITY_CDY1206	BnaC03g	Brassica_napus	39.80	0.00
TRINITY_XP_0166	PREDICTI	Gossypium_hirsutum	39.80	0.00
TRINITY_PRW5887	UVR3 3-	Chlorella_sorokinia	39.70	0.00
TRINITY_XP_0025	predicte	Micromonas_commoda	39.70	0.00
TRINITY_XP_0084	PREDICTI	Cucumis_melo	39.70	0.00
TRINITY_XP_0025	predicte	Micromonas_commoda	39.70	0.00
TRINITY_XP_0088	anaphase	Phoenix_dactylifera	39.70	0.00
TRINITY_OAY7358	Methylt	Ananas_comosus	39.70	0.00
TRINITY_XP_0238	protein-	Quercus_suber	39.70	0.00
TRINITY_PRW5953	MFS gene	Chlorella_sorokinia	39.70	0.00
TRINITY_XP_0147	phospho	Brachypodium_distac	39.70	0.00
TRINITY_XP_0072	ribosoma	Prunus_persica	39.70	0.00
TRINITY_XP_0245	probable	Selaginella_moellen	39.70	0.00
TRINITY_GAX8099	hypothet	Chlamydomonas_eusti	39.70	0.00
TRINITY_XP_0241	translin	Rosa_chinensis	39.70	0.00
TRINITY_XP_0068	probable	Amborella_trichopod	39.70	0.00
TRINITY_OTF8484	putative	Helianthus_annuus	39.70	0.00
TRINITY_RWW8424	hypothet	Ensete_ventricosum	39.70	0.00
TRINITY_EFJ1146	hypothet	Selaginella_moellen	39.70	0.00
TRINITY_XP_0238	probable	Quercus_suber	39.70	0.00
TRINITY_XP_0029	E3 ubiqu	Selaginella_moellen	39.70	0.00
TRINITY_GAQ8161	ABC tran	Klebsormidium_niten	39.70	0.00
TRINITY_GAQ8669	hypothet	Klebsormidium_niten	39.70	0.00
TRINITY_GAX8083	hypothet	Chlamydomonas_eusti	39.70	0.00
TRINITY_RDY0135	putative	Mucuna_pruriens	39.70	0.00
TRINITY_XP_0150	translat	Solanum_pennellii	39.70	0.00
TRINITY_XP_0264	serine/t	Papaver_somniferum	39.70	0.00
TRINITY_XP_0049	kinesin-	Setaria_italica	39.70	0.00
TRINITY_XP_0150	ras-rela	Solanum_pennellii	39.70	0.00
TRINITY_PTQ3609	hypothet	Marchantia_polymorp	39.70	0.00
TRINITY_PNH0537	Hydroger	Tetrabaena_socialis	39.70	0.00
TRINITY_PNW7666	hypothet	Chlamydomonas_reinh	39.70	0.00
TRINITY_PNW7793	hypothet	Chlamydomonas_reinh	39.70	0.00
TRINITY_XP_0243	DNA dama	Physcomitrella_pate	39.70	0.00
TRINITY_PRW4442	Peptidy	Chlorella_sorokinia	39.70	0.00
TRINITY_XP_0044	pirin-l	Cicer_arietinum	39.70	0.00
TRINITY_GAU4840	hypothet	Trifolium_subterran	39.70	0.00
TRINITY_EOY2513	RNA-bind	Theobroma_cacao	39.70	0.00
TRINITY_GAX7964	hypothet	Chlamydomonas_eusti	39.70	0.00
TRINITY_GAQ8408	ABC Acy	Klebsormidium_niten	39.70	0.00
TRINITY_PSC6959	Sucrose-	Micractinium_conduc	39.70	0.00
TRINITY_XP_0238	branchpo	Quercus_suber	39.70	0.00
TRINITY_KXZ5130	hypothet	Gonium_pectorale	39.70	0.00
TRINITY_GAQ8976	mitochon	Klebsormidium_niten	39.70	0.00
TRINITY_GBG7103	hypothet	Chara_braunii	39.70	0.00
TRINITY_PNW8049	hypothet	Chlamydomonas_reinh	39.70	0.00
TRINITY_KXZ4490	hypothet	Gonium_pectorale	39.70	0.00
TRINITY_GAX8523	hypothet	Chlamydomonas_eusti	39.70	0.00
TRINITY_XP_0104	PREDICTI	Camelina_sativa	39.70	0.00
TRINITY_GAX7918	hypothet	Chlamydomonas_eusti	39.70	0.00
TRINITY_PLY9979	hypothet	Lactuca_sativa	39.70	0.00
TRINITY_XP_0264	intermed	Papaver_somniferum	39.70	0.00
TRINITY_XP_0216	afadin-	Hevea_brasiliensis	39.70	0.00
TRINITY_CAA0476	95 kD ba	Spermatozopsis_simi	39.70	0.00
TRINITY_XP_0139	Dynein	Monoraphidium_negle	39.70	0.00
TRINITY_XP_0158	ABC tran	Ziziphus_jujuba	39.70	0.00
TRINITY_KXZ5199	hypothet	Gonium_pectorale	39.70	0.00
TRINITY_KXZ4921	hypothet	Gonium_pectorale	39.70	0.00
TRINITY_XP_0056	hypothet	Coccomyxa_subellips	39.70	0.00
TRINITY_GBG8477	hypothet	Chara_braunii	39.70	0.00
TRINITY_PSC7371	caroten	Micractinium_conduc	39.70	0.00

TRINITY_XP_0016	predicte	Chlamydomonas_reinh	39.70	0.00
TRINITY_KXZ4694	hypothet	Gonium_pectorale	39.70	0.00
TRINITY_ONM5110	Protein	Zea_mays	39.70	0.00
TRINITY_XP_0216	multiple	Manihot_esculenta	39.70	0.00
TRINITY_XP_0029	chromati	Selaginella_moellen	39.60	0.00
TRINITY_RID5033	hypothet	Brassica_rapa	39.60	0.00
TRINITY_EPS6977	hypothet	Genlisea_aurea	39.60	0.00
TRINITY_ACG4585	wee1-lik	Zea_mays	39.60	0.00
TRINITY_XP_0220	DNA poly	Helianthus_annuus	39.60	0.00
TRINITY_GAX7423	hypothet	Chlamydomonas_eusti	39.60	0.00
TRINITY_YP_0094	NADH-pla	Asplenium_prolongat	39.60	0.00
TRINITY_KMZ6040	Abhydrol	Zostera_marina	39.60	0.00
TRINITY_XP_0091	PREDICTE	Brassica_rapa	39.60	0.00
TRINITY_XP_0165	PREDICTE	Nicotiana_tabacum	39.60	0.00
TRINITY_XP_0056	alpha/be	Coccomyxa_subellips	39.60	0.00
TRINITY_XP_0056	Metallo-	Coccomyxa_subellips	39.60	0.00
TRINITY_XP_0229	respirat	Cucurbita_maxima	39.60	0.00
TRINITY_XP_0056	hypothet	Coccomyxa_subellips	39.60	0.00
TRINITY_ABK2149	unknown	Picea_sitchensis	39.60	0.00
TRINITY_XP_0064	eukaryot	Citrus_clementina	39.60	0.00
TRINITY_XP_0056	hypothet	Coccomyxa_subellips	39.60	0.00
TRINITY_YP_6657	NADH deh	Mesostigma_viride	39.60	0.00
TRINITY_XP_0191	PREDICTE	Ipomoea_nil	39.60	0.00
TRINITY_XP_0024	predicte	Micromonas_commoda	39.60	0.00
TRINITY_XP_0093	PREDICTE	Pyrus_x_bretschneid	39.60	0.00
TRINITY_XP_0068	mannose-	Amborella_trichopod	39.60	0.00
TRINITY_KZV3010	hypothet	Dorcoceras_hygromet	39.60	0.00
TRINITY_KXZ5422	hypothet	Gonium_pectorale	39.60	0.00
TRINITY_XP_0058	hypothet	Chlorella_variabili	39.60	0.00
TRINITY_GAX8078	hypothet	Chlamydomonas_eusti	39.60	0.00
TRINITY_GAQ8232	Acyl-CoA	Klebsormidium_niten	39.60	0.00
TRINITY_PNH0830	Leucine-	Tetrabaena_socialis	39.60	0.00
TRINITY_GAQ8209	hypothet	Klebsormidium_niten	39.60	0.00
TRINITY_PNW8820	hypothet	Chlamydomonas_reinh	39.60	0.00
TRINITY_BAJ9701	predicte	Hordeum_vulgare	39.60	0.00
TRINITY_XP_0029	hypothet	Volvox_carteri	39.60	0.00
TRINITY_PSC7535	putative	Micractinium_conduc	39.60	0.00
TRINITY_PNH0193	hypothet	Tetrabaena_socialis	39.60	0.00
TRINITY_EEE6198	hypothet	Oryza_sativa	39.60	0.00
TRINITY_XP_0029	protein	Selaginella_moellen	39.60	0.00
TRINITY_KXZ5052	hypothet	Gonium_pectorale	39.60	0.00
TRINITY_XP_0062	transcri	Capsella_rubella	39.60	0.00
TRINITY_XP_0104	PREDICTE	Camelina_sativa	39.60	0.00
TRINITY_XP_0202	serine/t	Asparagus_officinal	39.60	0.00
TRINITY_GAX8498	hypothet	Chlamydomonas_eusti	39.60	0.00
TRINITY_XP_0138	hypothet	Monoraphidium_negle	39.60	0.00
TRINITY_KXZ5451	hypothet	Gonium_pectorale	39.60	0.00
TRINITY_XP_0227	calcium-	Durio_zibethinus	39.60	0.00
TRINITY_XP_0056	CDP-Etha	Coccomyxa_subellips	39.60	0.00
TRINITY_RXH9768	hypothet	Malus_domestica	39.60	0.00
TRINITY_XP_0237	integrin	Lactuca_sativa	39.60	0.00
TRINITY_GBF9230	hypothet	Raphidocelis_subcap	39.60	0.00
TRINITY_GBF8771	hypothet	Raphidocelis_subcap	39.60	0.00
TRINITY_KXZ4183	hypothet	Gonium_pectorale	39.60	0.00
TRINITY_XP_0017	predicte	Chlamydomonas_reinh	39.60	0.00
TRINITY_XP_0124	PREDICTE	Gossypium_raimondii	39.60	0.00
TRINITY_XP_0105	PREDICTE	Tarenaya_hasslerian	39.60	0.00
TRINITY_XP_0029	uncharac	Selaginella_moellen	39.60	0.00
TRINITY_PON9401	Sirtuin	Trema_orientale	39.60	0.00
TRINITY_GAX7820	hypothet	Chlamydomonas_eusti	39.60	0.00
TRINITY_XP_0016	predicte	Chlamydomonas_reinh	39.60	0.00
TRINITY_XP_0244	DEAD-box	Physcomitrella_pate	39.60	0.00
TRINITY_KMZ5842	SWI/SNF-	Zostera_marina	39.60	0.00
TRINITY_PNW7547	hypothet	Chlamydomonas_reinh	39.60	0.00
TRINITY_XP_0025	predicte	Micromonas_commoda	39.60	0.00
TRINITY_PNW7858	hypothet	Chlamydomonas_reinh	39.60	0.00

TRINITY_PNW8185	hypothet	Chlamydomonas_reinh	39.60	0.00
TRINITY_XP_0145	protein	Vigna_radiata	39.60	0.00
TRINITY_GAQ8511	hypothet	Klebsormidium_niten	39.60	0.00
TRINITY_XP_0029	amino a	Volvox_carteri	39.60	0.00
TRINITY_PNH1172	hypothet	Tetrabaena_socialis	39.60	0.00
TRINITY_GAX7429	hypothet	Chlamydomonas_eusti	39.60	0.00
TRINITY_GAX7365	hypothet	Chlamydomonas_eusti	39.60	0.00
TRINITY_PSC7662	UPF0553	Micractinium_conduc	39.60	0.00
TRINITY_EFJ2869	hypothet	Selaginella_moellen	39.60	0.00
TRINITY_XP_0016	predicte	Chlamydomonas_reinh	39.60	0.00
TRINITY_PIA6302	hypothet	Aquilegia_coerulea	39.60	0.00
TRINITY_XP_0106	PREDICTE	Beta_vulgaris	39.60	0.00
TRINITY_XP_0063	peptidy	Capsella_rubella	39.60	0.00
TRINITY_GAQ7874	Putative	Klebsormidium_niten	39.60	0.00
TRINITY_GAQ7965	hypothet	Klebsormidium_niten	39.60	0.00
TRINITY_GAX7636	hypothet	Chlamydomonas_eusti	39.60	0.00
TRINITY_EFH6539	hypothet	Arabidopsis_lyrata	39.60	0.00
TRINITY_XP_0120	importin	Jatropha_curcas	39.60	0.00
TRINITY_KXZ4957	hypothet	Gonium_pectorale	39.60	0.00
TRINITY_EFJ0955	hypothet	Selaginella_moellen	39.60	0.00
TRINITY_KXZ4470	hypothet	Gonium_pectorale	39.60	0.00
TRINITY_GAX7758	hypothet	Chlamydomonas_eusti	39.60	0.00
TRINITY_KXZ5521	hypothet	Gonium_pectorale	39.60	0.00
TRINITY_XP_0239	carbamoy	Quercus_suber	39.60	0.00
TRINITY_KXZ4158	hypothet	Gonium_pectorale	39.60	0.00
TRINITY_OMO8754	hypothet	Corchorus_capsulari	39.60	0.00
TRINITY_GBF9064	p-type a	Raphidocelis_subcap	39.60	0.00
TRINITY_PNH0287	hypothet	Tetrabaena_socialis	39.60	0.00
TRINITY_XP_0176	PREDICTE	Gossypium_arboreum	39.60	0.00
TRINITY_GAX8534	hypothet	Chlamydomonas_eusti	39.60	0.00
TRINITY_XP_0084	PREDICTE	Cucumis_melo	39.60	0.00
TRINITY_PNH0992	putative	Tetrabaena_socialis	39.60	0.00
TRINITY_PNH0940	hypothet	Tetrabaena_socialis	39.60	0.00
TRINITY_XP_0104	PREDICTE	Camelina_sativa	39.60	0.00
TRINITY_BAK0277	predicte	Hordeum_vulgare	39.60	0.00
TRINITY_GAX8439	hypothet	Chlamydomonas_eusti	39.60	0.00
TRINITY_XP_0159	vesicle	Arachis_duranensis	39.60	0.00
TRINITY_XP_0243	uncharac	Physcomitrella_pate	39.60	0.00
TRINITY_RVW3215	Protein	Vitis_vinifera	39.50	0.00
TRINITY_XP_0100	PREDICTE	Eucalyptus_grandis	39.50	0.00
TRINITY_XP_0191	PREDICTE	Ipomoea_nil	39.50	0.00
TRINITY_GAV8118	LacAB_rp	Cephalotus_follicul	39.50	0.00
TRINITY_GBF9577	hypothet	Raphidocelis_subcap	39.50	0.00
TRINITY_GAQ9040	multicop	Klebsormidium_niten	39.50	0.00
TRINITY_XP_0249	calcium-	Ziziphus_jujuba	39.50	0.00
TRINITY_RID4657	hypothet	Brassica_rapa	39.50	0.00
TRINITY_XP_0218	protein	Carica_papaya	39.50	0.00
TRINITY_PNX9888	putative	Trifolium_pratense	39.50	0.00
TRINITY_XP_0273	ATP-depe	Abrus_precatorius	39.50	0.00
TRINITY_OAE3475	hypothet	Marchantia_polymorp	39.50	0.00
TRINITY_XP_0029	hypothet	Volvox_carteri	39.50	0.00
TRINITY_XP_0220	ARM REPE	Helianthus_annuus	39.50	0.00
TRINITY_CAI8489	putative	Medicago_sativa	39.50	0.00
TRINITY_XP_0219	HEAT rep	Helianthus_annuus	39.50	0.00
TRINITY_NP_1734	DNA-binc	Arabidopsis_thalian	39.50	0.00
TRINITY_PNW7338	hypothet	Chlamydomonas_reinh	39.50	0.00
TRINITY_XP_0243	mitochor	Physcomitrella_pate	39.50	0.00
TRINITY_GAQ9165	RUN and	Klebsormidium_niten	39.50	0.00
TRINITY_XP_0025	predicte	Micromonas_commoda	39.50	0.00
TRINITY_XP_0228	cactin	Olea_europaea	39.50	0.00
TRINITY_PNH1142	hypothet	Tetrabaena_socialis	39.50	0.00
TRINITY_GAX7594	hypothet	Chlamydomonas_eusti	39.50	0.00
TRINITY_GAX8185	hypothet	Chlamydomonas_eusti	39.50	0.00
TRINITY_EPS7175	alpha-g	Genlisea_aurea	39.50	0.00
TRINITY_PRW5862	chlorop	Chlorella_sorokinia	39.50	0.00
TRINITY_PNW8447	hypothet	Chlamydomonas_reinh	39.50	0.00

TRINITY_XP_0220	pre-mRN	Helianthus_annuus	39.50	0.00
TRINITY_GAX8212	hypothet	Chlamydomonas_eusti	39.50	0.00
TRINITY_KXZ4380	hypothet	Gonium_pectorale	39.50	0.00
TRINITY_GAX7616	hypothet	Chlamydomonas_eusti	39.50	0.00
TRINITY_RLN1236	LRR rece	Panicum_miliaceum	39.50	0.00
TRINITY_XP_0227	calcium-	Durio_zibethinus	39.50	0.00
TRINITY_KXZ4678	hypothet	Gonium_pectorale	39.50	0.00
TRINITY_GBF9674	hypothet	Raphidocelis_subcap	39.50	0.00
TRINITY_GAQ8014	GTPase a	Klebsormidium_niten	39.50	0.00
TRINITY_XP_0238	22.0 kDa	Quercus_suber	39.50	0.00
TRINITY_GAV5996	SNF2_N c	Cephalotus_follicul	39.50	0.00
TRINITY_KXZ4181	hypothet	Gonium_pectorale	39.50	0.00
TRINITY_KXZ4659	hypothet	Gonium_pectorale	39.50	0.00
TRINITY_KCW7333	hypothet	Eucalyptus_grandis	39.50	0.00
TRINITY_P0C8M8.1	RecName:	Zea_mays	39.50	0.00
TRINITY_RCV3883	hypothet	Setaria_italica	39.50	0.00
TRINITY_PNW7730	hypothet	Chlamydomonas_reinh	39.50	0.00
TRINITY_GAX7576	hypothet	Chlamydomonas_eusti	39.50	0.00
TRINITY_GAX8300	hypothet	Chlamydomonas_eusti	39.50	0.00
TRINITY_XP_0016	predicte	Chlamydomonas_reinh	39.50	0.00
TRINITY_XP_0029	hypothet	Volvox_carteri	39.50	0.00
TRINITY_XP_0165	PREDICTE	Capsicum_annuum	39.50	0.00
TRINITY_XP_0025	predicte	Micromonas_commoda	39.50	0.00
TRINITY_XP_0029	microtub	Selaginella_moellen	39.50	0.00
TRINITY_GAX8498	hypothet	Chlamydomonas_eusti	39.50	0.00
TRINITY_PNH1021	Glucosid	Tetrabaena_socialis	39.50	0.00
TRINITY_XP_0045	mitochon	Cicer_arietinum	39.50	0.00
TRINITY_XP_0105	PREDICTE	Tarenaya_hasslerian	39.50	0.00
TRINITY_GAX8441	hypothet	Chlamydomonas_eusti	39.50	0.00
TRINITY_PNW7884	hypothet	Chlamydomonas_reinh	39.50	0.00
TRINITY_XP_0237	tRNA pse	Lactuca_sativa	39.50	0.00
TRINITY_GBF9064	p-type a	Raphidocelis_subcap	39.50	0.00
TRINITY_GAX7464	hypothet	Chlamydomonas_eusti	39.50	0.00
TRINITY_XP_0017	predicte	Chlamydomonas_reinh	39.50	0.00
TRINITY_XP_0273	eukaryot	Abrus_precatorius	39.50	0.00
TRINITY_KXZ5328	hypothet	Gonium_pectorale	39.50	0.00
TRINITY_NP_0013	uncharac	Zea_mays	39.40	0.00
TRINITY_RWR7608	ADP-ribo	Cinnamomum_micranth	39.40	0.00
TRINITY_XP_0072	ABC tran	Prunus_persica	39.40	0.00
TRINITY_XP_0030	Plectin/	Ostreococcus_tauri	39.40	0.00
TRINITY_PSC7639	Jumonji	Micractinium_conduc	39.40	0.00
TRINITY_XP_0244	serine/t	Physcomitrella_pate	39.40	0.00
TRINITY_XP_0217	transcri	Chenopodium_quinoa	39.40	0.00
TRINITY_XP_0045	CTD smal	Cicer_arietinum	39.40	0.00
TRINITY_XP_0271	NADH deh	Coffea_arabica	39.40	0.00
TRINITY_XP_0025	predicte	Micromonas_commoda	39.40	0.00
TRINITY_GAQ8968	hypothet	Klebsormidium_niten	39.40	0.00
TRINITY_OVA1939	zinc fin	Macleaya_cordata	39.40	0.00
TRINITY_XP_0205	DCN1-lik	Phalaenopsis_equest	39.40	0.00
TRINITY_XP_0124	PREDICTE	Gossypium_raimondii	39.40	0.00
TRINITY_XP_0243	dual spe	Physcomitrella_pate	39.40	0.00
TRINITY_XP_0172	PREDICTE	Daucus_carota	39.40	0.00
TRINITY_GAQ8534	Ca2+-bin	Klebsormidium_niten	39.40	0.00
TRINITY_AFN4283	intrafla	Marsilea_vestita	39.40	0.00
TRINITY_GAX8563	hypothet	Chlamydomonas_eusti	39.40	0.00
TRINITY_PNW7852	hypothet	Chlamydomonas_reinh	39.40	0.00
TRINITY_CDY1681	BnaA09g0	Brassica_napus	39.40	0.00
TRINITY_XP_0139	hypothet	Monoraphidium_negle	39.40	0.00
TRINITY_GAX7576	hypothet	Chlamydomonas_eusti	39.40	0.00
TRINITY_PSC7520	thiamine	Micractinium_conduc	39.40	0.00
TRINITY_ABK2332	unknown	Picea_sitchensis	39.40	0.00
TRINITY_KXZ4951	hypothet	Gonium_pectorale	39.40	0.00
TRINITY_XP_0064	importin	Citrus_sinensis	39.40	0.00
TRINITY_XP_0016	predicte	Chlamydomonas_reinh	39.40	0.00
TRINITY_KDP3059	hypothet	Jatropha_curcas	39.40	0.00
TRINITY_PNW8129	hypothet	Chlamydomonas_reinh	39.40	0.00

TRINITY_XP_0063(	heat sh(	Capsella_rubella	39.40	0.00
TRINITY_XP_0029!	protein	Selaginella_moellen	39.40	0.00
TRINITY_GBG7026(	hypothet	Chara_braunii	39.40	0.00
TRINITY_GAX7542	hypothet	Chlamydomonas_eusti	39.40	0.00
TRINITY_KXZ4590!	DHC7 pro	Gonium_pectorale	39.40	0.00
TRINITY_PIA3564	hypothet	Aquilegia_coerulea	39.40	0.00
TRINITY_EEF2298(	glucosy	Ricinus_communis	39.40	0.00
TRINITY_XP_0056	ubiquit	Coccomyxa_subellips	39.40	0.00
TRINITY_GAX8319	hypothet	Chlamydomonas_eusti	39.40	0.00
TRINITY_XP_0056	laminin	Coccomyxa_subellips	39.40	0.00
TRINITY_GAQ8246!	aldehyde	Klebsormidium_niten	39.40	0.00
TRINITY_KMZ5717!	Acyl-Co	Zostera_marina	39.40	0.00
TRINITY_PNW8623(	hypothet	Chlamydomonas_reinh	39.40	0.00
TRINITY_PSC7707(	putative	Micractinium_conduc	39.40	0.00
TRINITY_GAX8022	hypothet	Chlamydomonas_eusti	39.40	0.00
TRINITY_XP_0226	protein	Vigna_radiata	39.40	0.00
TRINITY_XP_0100	PREDICTI	Eucalyptus_grandis	39.40	0.00
TRINITY_ATB1973!	putative	Calocedrus_decurren	39.40	0.00
TRINITY_XP_0025(	predicte	Micromonas_commoda	39.40	0.00
TRINITY_XP_0239	guanine	Quercus_suber	39.40	0.00
TRINITY_GBF9172	hypothet	Raphidocelis_subcap	39.40	0.00
TRINITY_XP_0062!	uncharac	Capsella_rubella	39.40	0.00
TRINITY_GAX7302	hypothet	Chlamydomonas_eusti	39.40	0.00
TRINITY_XP_0043(	PREDICTI	Fragaria_vesca	39.40	0.00
TRINITY_GAX7394	hypothet	Chlamydomonas_eusti	39.40	0.00
TRINITY_XP_0029!	hypothet	Volvox_carteri	39.40	0.00
TRINITY_PNW6988(	hypothet	Chlamydomonas_reinh	39.40	0.00
TRINITY_PNW8058!	hypothet	Chlamydomonas_reinh	39.40	0.00
TRINITY_XP_0167!	PREDICTI	Gossypium_hirsutum	39.40	0.00
TRINITY_PNH1161	hypothet	Tetrabaena_socialis	39.40	0.00
TRINITY_PTQ3094	hypothet	Marchantia_polymorp	39.40	0.00
TRINITY_KXZ4844(	hypothet	Gonium_pectorale	39.40	0.00
TRINITY_XP_0239(	ras-like	Quercus_suber	39.40	0.00
TRINITY_PSS2679(	Mannitol	Actinidia_chinensis	39.40	0.00
TRINITY_CBI3794	unnamed	Vitis_vinifera	39.40	0.00
TRINITY_GBF8765	ABC tran	Raphidocelis_subcap	39.40	0.00
TRINITY_EPS7086	hypothet	Genlisea_aurea	39.40	0.00
TRINITY_XP_0029!	hypothet	Volvox_carteri	39.40	0.00
TRINITY_P0C8M8.	RecName:	Zea_mays	39.40	0.00
TRINITY_PKA6280	Protein	Apostasia_shenzheni	39.40	0.00
TRINITY_PSC7683(	U3 small	Micractinium_conduc	39.40	0.00
TRINITY_GAX8167(	hypothet	Chlamydomonas_eusti	39.40	0.00
TRINITY_XP_0238	peroxide	Quercus_suber	39.40	0.00
TRINITY_XP_0056	serine/t	Coccomyxa_subellips	39.40	0.00
TRINITY_PSC7125!	transpor	Micractinium_conduc	39.40	0.00
TRINITY_XP_0102(	PREDICTI	Nelumbo_nucifera	39.40	0.00
TRINITY_XP_0124	PREDICTI	Gossypium_raimondii	39.40	0.00
TRINITY_XP_0088(	vacuolar	Phoenix_dactylifera	39.40	0.00
TRINITY_GAX7517!	hypothet	Chlamydomonas_eusti	39.40	0.00
TRINITY_CDM8581	unnamed	Triticum_aestivum	39.30	0.00
TRINITY_KXZ4854(	hypothet	Gonium_pectorale	39.30	0.00
TRINITY_EFJ1033	hypothet	Selaginella_moellen	39.30	0.00
TRINITY_GBG6841	hypothet	Chara_braunii	39.30	0.00
TRINITY_PSC7632	inorgani	Micractinium_conduc	39.30	0.00
TRINITY_XP_0058!	hypothet	Chlorella_variabili	39.30	0.00
TRINITY_PIM9699	hypothet	Handroanthus_impeti	39.30	0.00
TRINITY_ABK2507(	unknown	Picea_sitchensis	39.30	0.00
TRINITY_XP_0093(	PREDICTI	Pyrus_x_bretschneid	39.30	0.00
TRINITY_XP_0206	PITH dor	Dendrobium_catenatu	39.30	0.00
TRINITY_XP_0124(	PREDICTI	Gossypium_raimondii	39.30	0.00
TRINITY_PRW2086	cinnamyl	Chlorella_sorokinia	39.30	0.00
TRINITY_XP_0184	PREDICTI	Raphanus_sativus	39.30	0.00
TRINITY_RLN0467(	putative	Panicum_miliaceum	39.30	0.00
TRINITY_XP_0064	cysteine	Citrus_sinensis	39.30	0.00
TRINITY_XP_0029	uncharac	Selaginella_moellen	39.30	0.00
TRINITY_BAK0230!	predicte	Hordeum_vulgare	39.30	0.00

TRINITY_XP_0056	AP endor	Coccomyxa_subellips	39.30	0.00
TRINITY_XP_0244	von Wil	Physcomitrella_pate	39.30	0.00
TRINITY_XP_0029	hypothet	Volvox_carteri	39.30	0.00
TRINITY_GBG7574	hypothet	Chara_braunii	39.30	0.00
TRINITY_XP_0228	G-prote	Ostreococcus_tauri	39.30	0.00
TRINITY_GAU2328	hypothet	Trifolium_subterran	39.30	0.00
TRINITY_XP_0165	PREDICTE	Capsicum_annuum	39.30	0.00
TRINITY_XP_0243	COP9 si	Physcomitrella_pate	39.30	0.00
TRINITY_GAX8392	hypothet	Chlamydomonas_eusti	39.30	0.00
TRINITY_KXZ4566	hypothet	Gonium_pectorale	39.30	0.00
TRINITY_EFJ2430	hypothet	Selaginella_moellen	39.30	0.00
TRINITY_PNW8260	hypothet	Chlamydomonas_reinh	39.30	0.00
TRINITY_XP_0158	probable	Ziziphus_jujuba	39.30	0.00
TRINITY_KXZ5496	hypothet	Gonium_pectorale	39.30	0.00
TRINITY_XP_0029	bola-li	Volvox_carteri	39.30	0.00
TRINITY_XP_0239	guanine	Quercus_suber	39.30	0.00
TRINITY_XP_0029	hypothet	Volvox_carteri	39.30	0.00
TRINITY_XP_0241	probable	Rosa_chinensis	39.30	0.00
TRINITY_GAX8080	hypothet	Chlamydomonas_eusti	39.30	0.00
TRINITY_XP_0128	PREDICTE	Erythranthe_guttata	39.30	0.00
TRINITY_KMZ5665	putative	Zostera_marina	39.30	0.00
TRINITY_XP_0191	PREDICTE	Ipomoea_nil	39.30	0.00
TRINITY_GAX8410	hypothet	Chlamydomonas_eusti	39.30	0.00
TRINITY_PSC6826	p21-carb	Micractinium_conduc	39.30	0.00
TRINITY_PRW5840	Rab8 -fa	Chlorella_sorokinia	39.30	0.00
TRINITY_KYP5499	Sec14 cy	Cajanus_cajan	39.30	0.00
TRINITY_GBG8642	hypothet	Chara_braunii	39.30	0.00
TRINITY_XP_0098	PREDICTE	Nicotiana_sylvestri	39.30	0.00
TRINITY_XP_0024	probable	Sorghum_bicolor	39.30	0.00
TRINITY_XP_0264	serine/t	Papaver_somniferum	39.30	0.00
TRINITY_XP_0239	probable	Quercus_suber	39.30	0.00
TRINITY_XP_0190	PREDICTE	Camelina_sativa	39.30	0.00
TRINITY_GAX8048	hypothet	Chlamydomonas_eusti	39.30	0.00
TRINITY_PRW5856	cereblon	Chlorella_sorokinia	39.30	0.00
TRINITY_BAJ9967	predicte	Hordeum_vulgare	39.30	0.00
TRINITY_GAX8394	hypothet	Chlamydomonas_eusti	39.30	0.00
TRINITY_GAX8035	hypothet	Chlamydomonas_eusti	39.30	0.00
TRINITY_RWW9070	hypothet	Ensete_ventricosum	39.30	0.00
TRINITY_GAX8309	hypothet	Chlamydomonas_eusti	39.30	0.00
TRINITY_KXZ5550	hypothet	Gonium_pectorale	39.30	0.00
TRINITY_XP_0056	sarcosin	Coccomyxa_subellips	39.30	0.00
TRINITY_PRW5658	eukaryot	Chlorella_sorokinia	39.30	0.00
TRINITY_XP_0042	PREDICTE	Fragaria_vesca	39.30	0.00
TRINITY_PNW8474	hypothet	Chlamydomonas_reinh	39.30	0.00
TRINITY_GAX8027	hypothet	Chlamydomonas_eusti	39.30	0.00
TRINITY_PNW8805	hypothet	Chlamydomonas_reinh	39.30	0.00
TRINITY_XP_0167	PREDICTE	Gossypium_hirsutum	39.30	0.00
TRINITY_PNH0563	Carboxyp	Tetrabaena_socialis	39.30	0.00
TRINITY_KXZ4621	hypothet	Gonium_pectorale	39.30	0.00
TRINITY_XP_0238	probable	Quercus_suber	39.30	0.00
TRINITY_GAX8501	hypothet	Chlamydomonas_eusti	39.30	0.00
TRINITY_XP_0016	MAP kina	Chlamydomonas_reinh	39.30	0.00
TRINITY_GAQ8687	Cysteine	Klebsormidium_niten	39.30	0.00
TRINITY_PSC7257	acyl-act	Micractinium_conduc	39.30	0.00
TRINITY_GAX8368	hypothet	Chlamydomonas_eusti	39.30	0.00
TRINITY_PNH1226	Tiny mac	Tetrabaena_socialis	39.30	0.00
TRINITY_EFJ3351	hypothet	Selaginella_moellen	39.30	0.00
TRINITY_RWW9104	hypothet	Ensete_ventricosum	39.30	0.00
TRINITY_XP_0075	predicte	Bathycoccus_prasino	39.20	0.00
TRINITY_XP_0030	predicte	Micromonas_pusilla	39.20	0.00
TRINITY_KXZ5556	hypothet	Gonium_pectorale	39.20	0.00
TRINITY_PIA5147	hypothet	Aquilegia_coerulea	39.20	0.00
TRINITY_BAJ9457	predicte	Hordeum_vulgare	39.20	0.00
TRINITY_XP_0030	flagella	Micromonas_pusilla	39.20	0.00
TRINITY_GAX7293	hypothet	Chlamydomonas_eusti	39.20	0.00
TRINITY_XP_0113	Protein	Auxenochlorella_pro	39.20	0.00

TRINITY_EOA1305	hypothet	Capsella_rubella	39.20	0.00
TRINITY_GBG7765	hypothet	Chara_braunii	39.20	0.00
TRINITY_PTQ2712	hypothet	Marchantia_polymorp	39.20	0.00
TRINITY_AFK4638	unknown	Lotus_japonicus	39.20	0.00
TRINITY_GAX8292	hypothet	Chlamydomonas_eusti	39.20	0.00
TRINITY_XP_0135	PREDICTI	Brassica_oleracea	39.20	0.00
TRINITY_GAQ8986	putative	Klebsormidium_niten	39.20	0.00
TRINITY_XP_0041	PREDICTI	Cucumis_sativus	39.20	0.00
TRINITY_XP_0102	probable	Brachypodium_distac	39.20	0.00
TRINITY_GBG6449	hypothet	Chara_braunii	39.20	0.00
TRINITY_KXZ5524	hypothet	Gonium_pectorale	39.20	0.00
TRINITY_XP_0138	triacylg	Monoraphidium_negle	39.20	0.00
TRINITY_XP_0058	hypothet	Chlorella_variabili	39.20	0.00
TRINITY_XP_0239	altered	Quercus_suber	39.20	0.00
TRINITY_XP_0228	serine/t	Olea_europaea	39.20	0.00
TRINITY_XP_0217	CBL-inte	Chenopodium_quinoa	39.20	0.00
TRINITY_PNH0466	putative	Tetrabaena_socialis	39.20	0.00
TRINITY_XP_0075	predicte	Bathycoccus_prasino	39.20	0.00
TRINITY_GAX7949	hypothet	Chlamydomonas_eusti	39.20	0.00
TRINITY_EFJ1820	hypothet	Selaginella_moellen	39.20	0.00
TRINITY_PKA6070	RNA-dire	Apostasia_shenzheni	39.20	0.00
TRINITY_GAQ8867	20S prot	Klebsormidium_niten	39.20	0.00
TRINITY_PTQ4458	hypothet	Marchantia_polymorp	39.20	0.00
TRINITY_PTQ3538	hypothet	Marchantia_polymorp	39.20	0.00
TRINITY_XP_0030	serine/t	Micromonas_pusilla	39.20	0.00
TRINITY_XP_0041	PREDICTI	Cucumis_sativus	39.20	0.00
TRINITY_XP_0029	sphingos	Selaginella_moellen	39.20	0.00
TRINITY_GAX7267	hypothet	Chlamydomonas_eusti	39.20	0.00
TRINITY_KXZ4539	hypothet	Gonium_pectorale	39.20	0.00
TRINITY_XP_0093	PREDICTI	Pyrus_x_bretschneid	39.20	0.00
TRINITY_XP_0093	PREDICTI	Pyrus_x_bretschneid	39.20	0.00
TRINITY_KMZ6455	Chaperon	Zostera_marina	39.20	0.00
TRINITY_GAX8494	hypothet	Chlamydomonas_eusti	39.20	0.00
TRINITY_XP_0239	coronin-	Quercus_suber	39.20	0.00
TRINITY_XP_0017	COP-II c	Chlamydomonas_reinh	39.20	0.00
TRINITY_XP_0138	WD repea	Monoraphidium_negle	39.20	0.00
TRINITY_PNH1055	Sulfhydr	Tetrabaena_socialis	39.20	0.00
TRINITY_XP_0109	PREDICTI	Elaeis_guineensis	39.20	0.00
TRINITY_OWM8231	hypothet	Punica_granatum	39.20	0.00
TRINITY_PRW5604	multidru	Chlorella_sorokinia	39.20	0.00
TRINITY_XP_0139	hypothet	Monoraphidium_negle	39.20	0.00
TRINITY_XP_0270	serine/t	Coffea_arabica	39.20	0.00
TRINITY_XP_0017	predicte	Chlamydomonas_reinh	39.20	0.00
TRINITY_BAK0219	predicte	Hordeum_vulgare	39.20	0.00
TRINITY_GAQ8288	ubiquiti	Klebsormidium_niten	39.20	0.00
TRINITY_XP_0104	PREDICTI	Camelina_sativa	39.20	0.00
TRINITY_PRW6034	(1-3)-be	Chlorella_sorokinia	39.20	0.00
TRINITY_XP_0093	PREDICTI	Pyrus_x_bretschneid	39.20	0.00
TRINITY_GAX8133	hypothet	Chlamydomonas_eusti	39.20	0.00
TRINITY_BAK0289	predicte	Hordeum_vulgare	39.20	0.00
TRINITY_XP_0029	SC5D, C-	Volvox_carteri	39.20	0.00
TRINITY_GAX7384	hypothet	Chlamydomonas_eusti	39.20	0.00
TRINITY_XP_0138	hypothet	Monoraphidium_negle	39.20	0.00
TRINITY_GAX7389	hypothet	Chlamydomonas_eusti	39.20	0.00
TRINITY_GAX7889	hypothet	Chlamydomonas_eusti	39.20	0.00
TRINITY_PUZ6020	hypothet	Panicum_hallii	39.20	0.00
TRINITY_PIA3098	hypothet	Aquilegia_coerulea	39.20	0.00
TRINITY_XP_0227	cytidine	Durio_zibethinus	39.20	0.00
TRINITY_XP_0243	ATP-depe	Physcomitrella_pate	39.20	0.00
TRINITY_PNW7214	hypothet	Chlamydomonas_reinh	39.20	0.00
TRINITY_XP_0016	ABC tran	Chlamydomonas_reinh	39.20	0.00
TRINITY_GAX7690	hypothet	Chlamydomonas_eusti	39.20	0.00
TRINITY_GBF9783	serine p	Raphidocelis_subcap	39.20	0.00
TRINITY_XP_0044	mitochon	Cicer_arietinum	39.20	0.00
TRINITY_PSC7321	ubiquiti	Micractinium_conduc	39.10	0.00
TRINITY_GAX8020	hypothet	Chlamydomonas_eusti	39.10	0.00

TRINITY_XP_0043(	PREDICTI	Fragaria_vesca	39.10	0.00
TRINITY_XP_0120'	peroxis(	Jatropha_curcas	39.10	0.00
TRINITY_XP_0106(	PREDICTI	Beta_vulgaris	39.10	0.00
TRINITY_XP_0241'	elicito:	Rosa_chinensis	39.10	0.00
TRINITY_XP_0221(	nitrate	Momordica_charantia	39.10	0.00
TRINITY_XP_0277'	serine/t	Solanum_pennellii	39.10	0.00
TRINITY_GBF9284:	hypothet	Raphidocelis_subcap	39.10	0.00
TRINITY_XP_0083'	PREDICTI	Malus_domestica	39.10	0.00
TRINITY_PNW7640(	hypothet	Chlamydomonas_reinh	39.10	0.00
TRINITY_XP_0068!	zerumbor	Amborella_trichopod	39.10	0.00
TRINITY_XP_0243!	Down syr	Physcomitrella_pate	39.10	0.00
TRINITY_XP_0125'	protein	Cicer_arietinum	39.10	0.00
TRINITY_KXZ5576:	hypothet	Gonium_pectorale	39.10	0.00
TRINITY_EFJ0955:	hypothet	Selaginella_moellen	39.10	0.00
TRINITY_XP_0239:	carrier	Quercus_suber	39.10	0.00
TRINITY_XP_0066(	PREDICTI	Oryza_brachyantha	39.10	0.00
TRINITY_XP_0044!	kinesin-	Cicer_arietinum	39.10	0.00
TRINITY_XP_0105(	PREDICTI	Tarenaya_hasslerian	39.10	0.00
TRINITY_XP_0167(	PREDICTI	Gossypium_hirsutum	39.10	0.00
TRINITY_PKA5695:	putative	Apostasia_shenzheni	39.10	0.00
TRINITY_AQK9473!	AGC (cAI	Zea_mays	39.10	0.00
TRINITY_PRW5616(	DNA pol;	Chlorella_sorokinia	39.10	0.00
TRINITY_XP_0030(	Alpha-k(	Ostreococcus_tauri	39.10	0.00
TRINITY_GAX7303:	hypothet	Chlamydomonas_eusti	39.10	0.00
TRINITY_GBG7265:	hypothet	Chara_braunii	39.10	0.00
TRINITY_KMZ6529(	putative	Zostera_marina	39.10	0.00
TRINITY_KXZ4580:	hypothet	Gonium_pectorale	39.10	0.00
TRINITY_GAQ8089!	hypothet	Klebsormidium_niten	39.10	0.00
TRINITY_XP_0227(	calcium-	Durio_zibethinus	39.10	0.00
TRINITY_XP_0158'	estradi(	Ziziphus_jujuba	39.10	0.00
TRINITY_XP_0165'	PREDICTI	Capsicum_annuum	39.10	0.00
TRINITY_XP_0138!	hypothet	Monoraphidium_negle	39.10	0.00
TRINITY_OAE3008!	hypothet	Marchantia_polymorp	39.10	0.00
TRINITY_XP_0029(	molecula	Volvox_carteri	39.10	0.00
TRINITY_KXZ5476(	hypothet	Gonium_pectorale	39.10	0.00
TRINITY_GAX7305!	hypothet	Chlamydomonas_eusti	39.10	0.00
TRINITY_OAE3461!	hypothet	Marchantia_polymorp	39.10	0.00
TRINITY_XP_0064:	endochit	Citrus_clementina	39.10	0.00
TRINITY_XP_0243(	serine/t	Physcomitrella_pate	39.10	0.00
TRINITY_GAX7992!	hypothet	Chlamydomonas_eusti	39.10	0.00
TRINITY_RAL4555(	hypothet	Cuscuta_australis	39.10	0.00
TRINITY_GAX8494(	hypothet	Chlamydomonas_eusti	39.10	0.00
TRINITY_PNH0476(	hypothet	Tetrabaena_socialis	39.10	0.00
TRINITY_GAX8645!	hypothet	Chlamydomonas_eusti	39.10	0.00
TRINITY_XP_0139(	hypothet	Monoraphidium_negle	39.10	0.00
TRINITY_PNX9623:	inactive	Trifolium_pratense	39.10	0.00
TRINITY_XP_0017(	predicte	Chlamydomonas_reinh	39.10	0.00
TRINITY_PNH1245:	hypothet	Tetrabaena_socialis	39.10	0.00
TRINITY_KXZ4887!	hypothet	Gonium_pectorale	39.10	0.00
TRINITY_XP_0029!	hypothet	Volvox_carteri	39.10	0.00
TRINITY_XP_0239:	kinesin-	Quercus_suber	39.10	0.00
TRINITY_XP_0106!	PREDICTI	Beta_vulgaris	39.10	0.00
TRINITY_XP_0056(	Rhodane:	Coccomyxa_subellips	39.10	0.00
TRINITY_EPS6017:	hypothet	Genlisea_aurea	39.10	0.00
TRINITY_XP_0029!	hypothet	Volvox_carteri	39.10	0.00
TRINITY_XP_0239:	coronin-	Quercus_suber	39.10	0.00
TRINITY_XP_0016!	predicte	Chlamydomonas_reinh	39.10	0.00
TRINITY_GAX7591!	hypothet	Chlamydomonas_eusti	39.10	0.00
TRINITY_EAY8804(	hypothet	Oryza_sativa	39.10	0.00
TRINITY_XP_0113!	FACT cor	Auxenochlorella_prc	39.10	0.00
TRINITY_XP_0188(	PREDICTI	Juglans_regia	39.10	0.00
TRINITY_XP_0243(	katanin	Physcomitrella_pate	39.10	0.00
TRINITY_XP_0106!	PREDICTI	Vitis_vinifera	39.10	0.00
TRINITY_RAL5374(	hypothet	Cuscuta_australis	39.10	0.00
TRINITY_GAX8069'	hypothet	Chlamydomonas_eusti	39.10	0.00
TRINITY_OTG1944(	putative	Helianthus_annuus	39.10	0.00

TRINITY_XP_01025	PREDICTE	Nelumbo_nucifera	39.10	0.00
TRINITY_PRW44237	WD40 rep	Chlorella_sorokinia	39.10	0.00
TRINITY_XP_00564	hypothet	Coccomyxa_subellips	39.10	0.00
TRINITY_PWZ17754	Phosphat	Zea_mays	39.10	0.00
TRINITY_XP_00169	predicte	Chlamydomonas_reinh	39.10	0.00
TRINITY_GAX76831	hypothet	Chlamydomonas_eusti	39.10	0.00
TRINITY_XP_02392	ubiquiti	Quercus_suber	39.10	0.00
TRINITY_PNW75103	hypothet	Chlamydomonas_reinh	39.10	0.00
TRINITY_PTQ38960	hypothet	Marchantia_polymorp	39.10	0.00
TRINITY_GAX81632	hypothet	Chlamydomonas_eusti	39.10	0.00
TRINITY_XP_00169	predicte	Chlamydomonas_reinh	39.10	0.00
TRINITY_GBF92842	hypothet	Raphidocelis_subcap	39.10	0.00
TRINITY_XP_00584	hypothet	Chlorella_variabili	39.00	0.00
TRINITY_RDX67316	putative	Mucuna_pruriens	39.00	0.00
TRINITY_PNR39758	hypothet	Physcomitrella_pate	39.00	0.00
TRINITY_PNY16260	protein	Trifolium_pratense	39.00	0.00
TRINITY_XP_00867	uncharac	Zea_mays	39.00	0.00
TRINITY_XP_00249	radical	Micromonas_commoda	39.00	0.00
TRINITY_EPS71418	hypothet	Genlisea_aurea	39.00	0.00
TRINITY_XP_00636	PREDICTE	Solanum_tuberosum	39.00	0.00
TRINITY_GAQ82857	RNA poly	Klebsormidium_niten	39.00	0.00
TRINITY_XP_00938	PREDICTE	Musa_acuminata	39.00	0.00
TRINITY_XP_02390	uncharac	Quercus_suber	39.00	0.00
TRINITY_XP_01644	PREDICTE	Nicotiana_tabacum	39.00	0.00
TRINITY_GBF87640	hypothet	Raphidocelis_subcap	39.00	0.00
TRINITY_XP_00414	PREDICTE	Cucumis_sativus	39.00	0.00
TRINITY_XP_00141	predicte	Ostreococcus_sp._'l	39.00	0.00
TRINITY_XP_00564	hypothet	Coccomyxa_subellips	39.00	0.00
TRINITY_XP_01724	PREDICTE	Daucus_carota	39.00	0.00
TRINITY_GAX75954	hypothet	Chlamydomonas_eusti	39.00	0.00
TRINITY_XP_02376	ubiquiti	Lactuca_sativa	39.00	0.00
TRINITY_KZM82365	hypothet	Daucus_carota	39.00	0.00
TRINITY_GAX75954	hypothet	Chlamydomonas_eusti	39.00	0.00
TRINITY_XP_02733	CBL-inte	Abrus_precatorius	39.00	0.00
TRINITY_PRW58541	disulfid	Chlorella_sorokinia	39.00	0.00
TRINITY_XP_02390	uricase-	Quercus_suber	39.00	0.00
TRINITY_XP_00169	predicte	Chlamydomonas_reinh	39.00	0.00
TRINITY_XP_00564	DUF500-c	Coccomyxa_subellips	39.00	0.00
TRINITY_AAC79104	putative	Arabidopsis_thalian	39.00	0.00
TRINITY_GBF98898	methylma	Raphidocelis_subcap	39.00	0.00
TRINITY_KXZ49240	hypothet	Gonium_pectorale	39.00	0.00
TRINITY_XP_02296	uncharac	Cucurbita_maxima	39.00	0.00
TRINITY_XP_00451	uncharac	Cicer_arietinum	39.00	0.00
TRINITY_OAE24723	hypothet	Marchantia_polymorp	39.00	0.00
TRINITY_GAX78023	hypothet	Chlamydomonas_eusti	39.00	0.00
TRINITY_PNH12420	Protein	Tetrabaena_socialis	39.00	0.00
TRINITY_GAX84107	hypothet	Chlamydomonas_eusti	39.00	0.00
TRINITY_KZV17117	putative	Dorcoceras_hygromet	39.00	0.00
TRINITY_XP_02019	serine/t	Aegilops_tauschii	39.00	0.00
TRINITY_XP_01597	long cha	Arachis_duranensis	39.00	0.00
TRINITY_PNW75650	hypothet	Chlamydomonas_reinh	39.00	0.00
TRINITY_XP_00295	hypothet	Volvox_carteri	39.00	0.00
TRINITY_XP_00430	PREDICTE	Fragaria_vesca	39.00	0.00
TRINITY_XP_01368	long cha	Brassica_napus	39.00	0.00
TRINITY_XP_00296	3-phosph	Selaginella_moellen	39.00	0.00
TRINITY_PNH11652	Calcium/	Tetrabaena_socialis	39.00	0.00
TRINITY_XP_00141	predicte	Ostreococcus_sp._'l	39.00	0.00
TRINITY_GAX78644	hypothet	Chlamydomonas_eusti	39.00	0.00
TRINITY_XP_01051	PREDICTE	Tarenaya_hasslerian	39.00	0.00
TRINITY_ACF87913	unknown	Zea_mays	39.00	0.00
TRINITY_XP_00306	predicte	Micromonas_pusilla	39.00	0.00
TRINITY_XP_01006	PREDICTE	Eucalyptus_grandis	39.00	0.00
TRINITY_PNH09397	Purple a	Tetrabaena_socialis	39.00	0.00
TRINITY_XP_01067	PREDICTE	Beta_vulgaris	39.00	0.00
TRINITY_XP_01908	PREDICTE	Camelina_sativa	39.00	0.00
TRINITY_GAX78232	hypothet	Chlamydomonas_eusti	39.00	0.00

TRINITY_GAX8070	hypothet	Chlamydomonas_eusti	39.00	0.00
TRINITY_PRW6034	(1-3)-be	Chlorella_sorokinia	39.00	0.00
TRINITY_XP_0245	probable	Selaginella_moellen	39.00	0.00
TRINITY_GAX8130	hypothet	Chlamydomonas_eusti	39.00	0.00
TRINITY_XP_0179	PREDICTE	Theobroma_cacao	39.00	0.00
TRINITY_XP_0156	protein-	Oryza_sativa	39.00	0.00
TRINITY_GAX8634	hypothet	Chlamydomonas_eusti	39.00	0.00
TRINITY_XP_0218	dual spe	Spinacia_oleracea	39.00	0.00
TRINITY_GAX7818	hypothet	Chlamydomonas_eusti	39.00	0.00
TRINITY_PNW8202	hypothet	Chlamydomonas_reinh	39.00	0.00
TRINITY_GAX7818	hypothet	Chlamydomonas_eusti	39.00	0.00
TRINITY_PNW7334	hypothet	Chlamydomonas_reinh	39.00	0.00
TRINITY_GAX7353	hypothet	Chlamydomonas_eusti	39.00	0.00
TRINITY_GAQ8280	Chromodo	Klebsormidium_niten	39.00	0.00
TRINITY_ATG2999	CYP866A1	Taxus_chinensis	39.00	0.00
TRINITY_OMP0739	hypothet	Corchorus_olitorius	39.00	0.00
TRINITY_CDP1769	unnamed	Coffea_canephora	38.90	0.00
TRINITY_GAX7770	hypothet	Chlamydomonas_eusti	38.90	0.00
TRINITY_RAL4284	hypothet	Cuscuta_australis	38.90	0.00
TRINITY_KYP6920	Serine/t	Cajanus_cajan	38.90	0.00
TRINITY_EEF2536	3-hydrox	Ricinus_communis	38.90	0.00
TRINITY_XP_0167	PREDICTE	Gossypium_hirsutum	38.90	0.00
TRINITY_BAJ9497	predicte	Hordeum_vulgare	38.90	0.00
TRINITY_PUZ7155	hypothet	Panicum_hallii	38.90	0.00
TRINITY_XP_0156	protein	Oryza_sativa	38.90	0.00
TRINITY_GAX8607	hypothet	Chlamydomonas_eusti	38.90	0.00
TRINITY_EEC8259	hypothet	Oryza_sativa	38.90	0.00
TRINITY_KXZ5286	hypothet	Gonium_pectorale	38.90	0.00
TRINITY_XP_0056	hypothet	Coccomyxa_subellips	38.90	0.00
TRINITY_KZV1488	Thermost	Dorcoceras_hygromet	38.90	0.00
TRINITY_XP_0075	alcohol	Bathycoccus_prasino	38.90	0.00
TRINITY_XP_0110	PREDICTE	Populus_euphratica	38.90	0.00
TRINITY_EEF2753	two-comp	Ricinus_communis	38.90	0.00
TRINITY_XP_0064	probable	Eutrema_salsugineum	38.90	0.00
TRINITY_ACN5017	dispropo	Annona_cherimola	38.90	0.00
TRINITY_OAE2507	hypothet	Marchantia_polymorp	38.90	0.00
TRINITY_GAX7645	hypothet	Chlamydomonas_eusti	38.90	0.00
TRINITY_PSC7421	20S prot	Micractinium_conduc	38.90	0.00
TRINITY_RRT3799	hypothet	Ensete_ventricosum	38.90	0.00
TRINITY_GBG6964	hypothet	Chara_braunii	38.90	0.00
TRINITY_KXZ4640	hypothet	Gonium_pectorale	38.90	0.00
TRINITY_XP_0058	hypothet	Chlorella_variabili	38.90	0.00
TRINITY_KXZ5512	hypothet	Gonium_pectorale	38.90	0.00
TRINITY_PNH1024	Chaperon	Tetrabaena_socialis	38.90	0.00
TRINITY_XP_0124	PREDICTE	Gossypium_raimondii	38.90	0.00
TRINITY_XP_0029	signal p	Selaginella_moellen	38.90	0.00
TRINITY_XP_0238	tubulin	Quercus_suber	38.90	0.00
TRINITY_GAX8318	hypothet	Chlamydomonas_eusti	38.90	0.00
TRINITY_GAX8283	hypothet	Chlamydomonas_eusti	38.90	0.00
TRINITY_EFJ1003	hypothet	Selaginella_moellen	38.90	0.00
TRINITY_XP_0273	EEF1A ly	Abrus_precatorius	38.90	0.00
TRINITY_XP_0200	serine/t	Ananas_comosus	38.90	0.00
TRINITY_GAX8038	hypothet	Chlamydomonas_eusti	38.90	0.00
TRINITY_PNW7795	hypothet	Chlamydomonas_reinh	38.90	0.00
TRINITY_GAX7456	hypothet	Chlamydomonas_eusti	38.90	0.00
TRINITY_OAE2015	hypothet	Marchantia_polymorp	38.90	0.00
TRINITY_GAX7943	hypothet	Chlamydomonas_eusti	38.90	0.00
TRINITY_XP_0206	uncharac	Dendrobium_catenatu	38.90	0.00
TRINITY_XP_0238	uncharac	Quercus_suber	38.90	0.00
TRINITY_XP_0056	ArfGap-d	Coccomyxa_subellips	38.90	0.00
TRINITY_PSC6706	Gag-Pol	Micractinium_conduc	38.90	0.00
TRINITY_KXZ4249	hypothet	Gonium_pectorale	38.90	0.00
TRINITY_XP_0243	myb-like	Physcomitrella_pate	38.90	0.00
TRINITY_XP_0071	hypothet	Phaseolus_vulgaris	38.90	0.00
TRINITY_KXZ4912	hypothet	Gonium_pectorale	38.90	0.00
TRINITY_GAX7500	hypothet	Chlamydomonas_eusti	38.90	0.00

TRINITY_XP_00169	protein	Chlamydomonas_reinh	38.90	0.00
TRINITY_XP_00294	hypothet	Volvox_carteri	38.90	0.00
TRINITY_XP_02439	ABC tran	Physcomitrella_pate	38.90	0.00
TRINITY_XP_02087	E3 ubiqu	Arabidopsis_lyrata	38.90	0.00
TRINITY_GBG84821	hypothet	Chara_braunii	38.90	0.00
TRINITY_XP_01916	PREDICTE	Ipomoea_nil	38.90	0.00
TRINITY_GAQ89011	Putative	Klebsormidium_niten	38.80	0.00
TRINITY_BAF04650	Os01g027	Oryza_sativa	38.80	0.00
TRINITY_XP_02068	protein	Dendrobium_catenatu	38.80	0.00
TRINITY_XP_00565	translat	Coccomyxa_subellips	38.80	0.00
TRINITY_XP_02255	dual spe	Brassica_napus	38.80	0.00
TRINITY_XP_01108	8-hydrox	Sesamum_indicum	38.80	0.00
TRINITY_XP_02213	actin-de	Momordica_charantia	38.80	0.00
TRINITY_XP_02213	actin-de	Momordica_charantia	38.80	0.00
TRINITY_KXZ49519	hypothet	Gonium_pectorale	38.80	0.00
TRINITY_KXZ49240	hypothet	Gonium_pectorale	38.80	0.00
TRINITY_XP_00751	AMP dear	Bathycoccus_prasino	38.80	0.00
TRINITY_EPS62777	hypothet	Genlisea_aurea	38.80	0.00
TRINITY_VDC97471	unnamed	Brassica_rapa	38.80	0.00
TRINITY_XP_02388	uncharac	Quercus_suber	38.80	0.00
TRINITY_RRT79998	hypothet	Ensete_ventricosum	38.80	0.00
TRINITY_XP_00295	hypothet	Volvox_carteri	38.80	0.00
TRINITY_GAX79100	hypothet	Chlamydomonas_eusti	38.80	0.00
TRINITY_GAX83833	hypothet	Chlamydomonas_eusti	38.80	0.00
TRINITY_XP_00751	predicte	Bathycoccus_prasino	38.80	0.00
TRINITY_ACJ38195	chitinas	Malus_hupehensis	38.80	0.00
TRINITY_KMZ63072	Acyl-CoA	Zostera_marina	38.80	0.00
TRINITY_XP_00630	phosphol	Capsella_rubella	38.80	0.00
TRINITY_KMZ58900	Protein	Zostera_marina	38.80	0.00
TRINITY_ACO87660	protein	Brachypodium_sylvat	38.80	0.00
TRINITY_XP_00141	predicte	Ostreococcus_sp._'l	38.80	0.00
TRINITY_XP_00360	selT-lik	Medicago_truncatula	38.80	0.00
TRINITY_GAX75461	hypothet	Chlamydomonas_eusti	38.80	0.00
TRINITY_XP_02099	ubiquiti	Arachis_duranensis	38.80	0.00
TRINITY_OAE34501	hypothet	Marchantia_polymorp	38.80	0.00
TRINITY_XP_02203	60S acid	Helianthus_annuus	38.80	0.00
TRINITY_KXZ49814	hypothet	Gonium_pectorale	38.80	0.00
TRINITY_XP_00169	histidin	Chlamydomonas_reinh	38.80	0.00
TRINITY_PWA57359	3'-5'-ex	Artemisia_annua	38.80	0.00
TRINITY_PNW75853	hypothet	Chlamydomonas_reinh	38.80	0.00
TRINITY_XP_00168	componen	Chlamydomonas_reinh	38.80	0.00
TRINITY_KXZ56449	hypothet	Gonium_pectorale	38.80	0.00
TRINITY_GAX83778	hypothet	Chlamydomonas_eusti	38.80	0.00
TRINITY_GAX81186	hypothet	Chlamydomonas_eusti	38.80	0.00
TRINITY_XP_00636	PREDICTE	Solanum_tuberosum	38.80	0.00
TRINITY_PNW75861	hypothet	Chlamydomonas_reinh	38.80	0.00
TRINITY_PKA55206	DNA cros	Apostasia_shenzheni	38.80	0.00
TRINITY_KXZ42694	hypothet	Gonium_pectorale	38.80	0.00
TRINITY_GAX78191	hypothet	Chlamydomonas_eusti	38.80	0.00
TRINITY_XP_00295	hypothet	Volvox_carteri	38.80	0.00
TRINITY_XP_02283	unnamed	Ostreococcus_tauri	38.80	0.00
TRINITY_XP_02189	U3 small	Carica_papaya	38.80	0.00
TRINITY_OUS44692	hypothet	Ostreococcus_tauri	38.80	0.00
TRINITY_GAQ82320	Acyl-CoA	Klebsormidium_niten	38.80	0.00
TRINITY_GAX81922	hypothet	Chlamydomonas_eusti	38.80	0.00
TRINITY_KXZ47460	hypothet	Gonium_pectorale	38.80	0.00
TRINITY_BAK00269	predicte	Hordeum_vulgare	38.80	0.00
TRINITY_XP_02706	choline	Coffea_arabica	38.80	0.00
TRINITY_XP_01390	hypothet	Monoraphidium_negle	38.80	0.00
TRINITY_PNW83574	hypothet	Chlamydomonas_reinh	38.80	0.00
TRINITY_GAX79608	hypothet	Chlamydomonas_eusti	38.80	0.00
TRINITY_KZV28802	Rab5-int	Dorcoceras_hygromet	38.80	0.00
TRINITY_GAX72593	hypothet	Chlamydomonas_eusti	38.80	0.00
TRINITY_XP_01389	Cyclin-A	Monoraphidium_negle	38.80	0.00
TRINITY_GAX79892	hypothet	Chlamydomonas_eusti	38.80	0.00
TRINITY_PSC72510	multidru	Micractinium_conduc	38.80	0.00

TRINITY_PTQ3202	hypothet	Marchantia_polymorp	38.80	0.00
TRINITY_GAX8086	hypothet	Chlamydomonas_eusti	38.80	0.00
TRINITY_GAX8244	hypothet	Chlamydomonas_eusti	38.80	0.00
TRINITY_GAX7836	hypothet	Chlamydomonas_eusti	38.80	0.00
TRINITY_EFJ1424	hypothet	Selaginella_moellen	38.80	0.00
TRINITY_GBF8754	serine	Raphidocelis_subcap	38.80	0.00
TRINITY_XP_0219	tRNA:m(	Carica_papaya	38.80	0.00
TRINITY_XP_0014	predict	Ostreococcus_sp._'l	38.70	0.00
TRINITY_XP_0094	PREDICT	Musa_acuminata	38.70	0.00
TRINITY_RWR9037	protein	Cinnamomum_micranth	38.70	0.00
TRINITY_XP_0136	PREDICT	Brassica_oleracea	38.70	0.00
TRINITY_XP_0228	Homoser	Ostreococcus_tauri	38.70	0.00
TRINITY_XP_0229	WD repe	Cucurbita_moschata	38.70	0.00
TRINITY_XP_0158	ATP-dep	Ziziphus_jujuba	38.70	0.00
TRINITY_KZN0666	hypothet	Daucus_carota	38.70	0.00
TRINITY_XP_0110	PREDICT	Populus_euphratica	38.70	0.00
TRINITY_GAY3471	hypothet	Citrus_unshiu	38.70	0.00
TRINITY_XP_0093	PREDICT	Pyrus_x_bretschneid	38.70	0.00
TRINITY_GAU2300	hypothet	Trifolium_subterran	38.70	0.00
TRINITY_RID6164	hypothet	Brassica_rapa	38.70	0.00
TRINITY_EPS6688	hypothet	Genlisea_aurea	38.70	0.00
TRINITY_XP_0109	PREDICT	Elaeis_guineensis	38.70	0.00
TRINITY_XP_0217	CBL-int	Chenopodium_quinoa	38.70	0.00
TRINITY_XP_0239	mitocho	Quercus_suber	38.70	0.00
TRINITY_XP_0176	PREDICT	Gossypium_arboreum	38.70	0.00
TRINITY_XP_0029	hypothet	Volvox_carteri	38.70	0.00
TRINITY_KXZ5244	hypothet	Gonium_pectorale	38.70	0.00
TRINITY_GBF9064	p-type	Raphidocelis_subcap	38.70	0.00
TRINITY_GBG7673	hypothet	Chara_braunii	38.70	0.00
TRINITY_KDD7717	hypothet	Helicosporidium_sp.	38.70	0.00
TRINITY_XP_0056	hypothet	Coccomyxa_subellips	38.70	0.00
TRINITY_PWA8669	serine/	Artemisia_annua	38.70	0.00
TRINITY_PRW3371	DNA lig	Chlorella_sorokinia	38.70	0.00
TRINITY_GBF9793	hypothet	Raphidocelis_subcap	38.70	0.00
TRINITY_XP_0165	PREDICT	Capsicum_annuum	38.70	0.00
TRINITY_GAX7569	hypothet	Chlamydomonas_eusti	38.70	0.00
TRINITY_PRW5845	guanylat	Chlorella_sorokinia	38.70	0.00
TRINITY_PNW7309	hypothet	Chlamydomonas_reinh	38.70	0.00
TRINITY_XP_0239	GPI eth	Quercus_suber	38.70	0.00
TRINITY_XP_0216	polyade	Hevea_brasiliensis	38.70	0.00
TRINITY_GAX8132	hypothet	Chlamydomonas_eusti	38.70	0.00
TRINITY_PNW8369	hypothet	Chlamydomonas_reinh	38.70	0.00
TRINITY_OAE2522	hypothet	Marchantia_polymorp	38.70	0.00
TRINITY_XP_0184	PREDICT	Raphanus_sativus	38.70	0.00
TRINITY_PNW8597	hypothet	Chlamydomonas_reinh	38.70	0.00
TRINITY_KXZ5093	hypothet	Gonium_pectorale	38.70	0.00
TRINITY_PTQ3895	hypothet	Marchantia_polymorp	38.70	0.00
TRINITY_PNH0871	Protein	Tetrabaena_socialis	38.70	0.00
TRINITY_PRW6042	RAP dom	Chlorella_sorokinia	38.70	0.00
TRINITY_PWZ5389	hypothet	Zea_mays	38.70	0.00
TRINITY_XP_0205	LOW QUA	Phalaenopsis_equest	38.70	0.00
TRINITY_PNH1023	hypothet	Tetrabaena_socialis	38.70	0.00
TRINITY_PNW7070	hypothet	Chlamydomonas_reinh	38.70	0.00
TRINITY_GBF8986	eukaryot	Raphidocelis_subcap	38.70	0.00
TRINITY_GAX7593	hypothet	Chlamydomonas_eusti	38.70	0.00
TRINITY_GAX7638	hypothet	Chlamydomonas_eusti	38.70	0.00
TRINITY_EFJ0747	hypothet	Selaginella_moellen	38.70	0.00
TRINITY_OAY6401	TBC1 do	Ananas_comosus	38.70	0.00
TRINITY_GAX8025	hypothet	Chlamydomonas_eusti	38.70	0.00
TRINITY_PNW8465	hypothet	Chlamydomonas_reinh	38.70	0.00
TRINITY_GAX8188	hypothet	Chlamydomonas_eusti	38.70	0.00
TRINITY_PNW8082	hypothet	Chlamydomonas_reinh	38.70	0.00
TRINITY_XP_0028	short-c	Arabidopsis_lyrata	38.70	0.00
TRINITY_XP_0128	PREDICT	Erythranthe_guttata	38.60	0.00
TRINITY_XP_0273	aldehyd	Abrus_precatorius	38.60	0.00
TRINITY_XP_0113	hypothet	Auxenochlorella_pro	38.60	0.00

TRINITY_XP_0044!	RNA pse	Cicer_arietinum	38.60	0.00
TRINITY_XP_0086	BTB/POZ	Zea_mays	38.60	0.00
TRINITY_XP_0111(	ER memb	Sesamum_indicum	38.60	0.00
TRINITY_GAX8049	hypothet	Chlamydomonas_eusti	38.60	0.00
TRINITY_ATB1984	metallo	Callitropsis_nootka	38.60	0.00
TRINITY_XP_0172	PREDICTI	Daucus_carota	38.60	0.00
TRINITY_BAK0043!	predicte	Hordeum_vulgare	38.60	0.00
TRINITY_XP_0093!	PREDICTI	Pyrus_x_bretschneid	38.60	0.00
TRINITY_PNR3656(	hypothet	Physcomitrella_pate	38.60	0.00
TRINITY_XP_0116!	PREDICTI	Cucumis_sativus	38.60	0.00

TRINITY_XP_0066	PREDICTI	Oryza_brachyantha	38.60	0.00
TRINITY_XP_0220	probable	Helianthus_annuus	38.60	0.00
TRINITY_XP_0200	phosphor	Ananas_comosus	38.60	0.00
TRINITY_XP_0016	predicte	Chlamydomonas_reinh	38.60	0.00
TRINITY_GBG8855	hypothet	Chara_braunii	38.60	0.00
TRINITY_XP_0213	rac-like	Sorghum_bicolor	38.60	0.00
TRINITY_XP_0243	serine (	Physcomitrella_pate	38.60	0.00
TRINITY_XP_0216	RING-H2	Hevea_brasiliensis	38.60	0.00
TRINITY_XP_0191	PREDICTI	Beta_vulgaris	38.60	0.00
TRINITY_PWA4422	alanine-	Artemisia_annua	38.60	0.00
TRINITY_PNH0829	Katanin	Tetrabaena_socialis	38.60	0.00
TRINITY_XP_0238	geranylg	Quercus_suber	38.60	0.00
TRINITY_XP_0206	AP-4 cor	Dendrobium_catenatu	38.60	0.00
TRINITY_PSS3000	Serine/t	Actinidia_chinensis	38.60	0.00
TRINITY_XP_0017	predicte	Chlamydomonas_reinh	38.60	0.00
TRINITY_XP_0243	PAN2-PAN	Physcomitrella_pate	38.60	0.00
TRINITY_XP_0056	hypothet	Coccomyxa_subellips	38.60	0.00
TRINITY_KXZ5406	hypothet	Gonium_pectorale	38.60	0.00
TRINITY_OAE3319	hypothet	Marchantia_polymorp	38.60	0.00
TRINITY_GAX7829	hypothet	Chlamydomonas_eusti	38.60	0.00
TRINITY_KZV1521	petal de	Dorcoceras_hygromet	38.60	0.00
TRINITY_BAJ9884	predicte	Hordeum_vulgare	38.60	0.00
TRINITY_CDP0682	unnamed	Coffea_canephora	38.60	0.00
TRINITY_XP_0029	hypothet	Volvox_carteri	38.60	0.00
TRINITY_GAX8404	hypothet	Chlamydomonas_eusti	38.60	0.00
TRINITY_GAX7553	hypothet	Chlamydomonas_eusti	38.60	0.00
TRINITY_XP_0244	probable	Physcomitrella_pate	38.60	0.00
TRINITY_XP_0030	predicte	Micromonas_pusilla	38.60	0.00
TRINITY_XP_0172	PREDICTI	Daucus_carota	38.60	0.00
TRINITY_XP_0017	predicte	Chlamydomonas_reinh	38.60	0.00
TRINITY_XP_0025	ammonium	Micromonas_commoda	38.60	0.00
TRINITY_GBF9843	hypothet	Raphidocelis_subcap	38.60	0.00
TRINITY_XP_0188	PREDICTI	Juglans_regia	38.60	0.00
TRINITY_PNW7694	hypothet	Chlamydomonas_reinh	38.60	0.00
TRINITY_XP_0184	PREDICTI	Raphanus_sativus	38.60	0.00
TRINITY_KXZ4639	hypothet	Gonium_pectorale	38.60	0.00
TRINITY_GAX7441	hypothet	Chlamydomonas_eusti	38.60	0.00
TRINITY_KXZ4933	hypothet	Gonium_pectorale	38.60	0.00
TRINITY_XP_0094	PREDICTI	Musa_acuminata	38.60	0.00
TRINITY_NP_1895	high mol	Arabidopsis_thalian	38.60	0.00
TRINITY_KXZ4474	hypothet	Gonium_pectorale	38.60	0.00
TRINITY_XP_0016	componen	Chlamydomonas_reinh	38.60	0.00
TRINITY_XP_0134	FACT com	Medicago_truncatula	38.60	0.00
TRINITY_XP_0245	uncharac	Selaginella_moellen	38.60	0.00
TRINITY_XP_0139	WD-40 re	Monoraphidium_negle	38.60	0.00
TRINITY_XP_0208	vesicle-	Arabidopsis_lyrata	38.60	0.00
TRINITY_XP_0025	predicte	Micromonas_commoda	38.60	0.00
TRINITY_OVA1817	Mini-chr	Macleaya_cordata	38.60	0.00
TRINITY_PNW7076	hypothet	Chlamydomonas_reinh	38.60	0.00
TRINITY_XP_0205	protein	Amborella_trichopod	38.60	0.00
TRINITY_OUS4953	adenylat	Ostreococcus_tauri	38.60	0.00
TRINITY_XP_0243	poly [AI	Physcomitrella_pate	38.60	0.00
TRINITY_XP_0029	hypothet	Volvox_carteri	38.60	0.00
TRINITY_KNA1683	hypothet	Spinacia_oleracea	38.60	0.00
TRINITY_XP_0239	putative	Quercus_suber	38.60	0.00
TRINITY_GAX8275	hypothet	Chlamydomonas_eusti	38.60	0.00
TRINITY_XP_0238	uncharac	Quercus_suber	38.60	0.00
TRINITY_GAX8540	hypothet	Chlamydomonas_eusti	38.60	0.00
TRINITY_XP_0279	probable	Vigna_unguiculata	38.60	0.00
TRINITY_PNH1128	Amine ox	Tetrabaena_socialis	38.60	0.00
TRINITY_XP_0238	protein	Quercus_suber	38.60	0.00
TRINITY_XP_0243	ALA-inte	Physcomitrella_pate	38.60	0.00
TRINITY_PNW7955	hypothet	Chlamydomonas_reinh	38.60	0.00
TRINITY_PNW7578	hypothet	Chlamydomonas_reinh	38.60	0.00
TRINITY_KFK4014	hypothet	Arabis_alpina	38.60	0.00
TRINITY_PNH0765	Spermato	Tetrabaena_socialis	38.60	0.00

TRINITY_ADE7668	unknown	Picea_sitchensis	38.60	0.00
TRINITY_KXZ5149	hypothet	Gonium_pectorale	38.60	0.00
TRINITY_XP_0239	cytoplas	Quercus_suber	38.60	0.00
TRINITY_GAX8187	hypothet	Chlamydomonas_eusti	38.60	0.00
TRINITY_XP_0201	calcineu	Ananas_comosus	38.50	0.00
TRINITY_OIW1844	hypothet	Lupinus_angustifoli	38.50	0.00
TRINITY_XP_0167	PREDICTE	Gossypium_hirsutum	38.50	0.00
TRINITY_BAK0286	predicte	Hordeum_vulgare	38.50	0.00
TRINITY_XP_0029	hypothet	Volvox_carteri	38.50	0.00
TRINITY_PRW3353	E3 ubiqu	Chlorella_sorokinia	38.50	0.00
TRINITY_RLM8669	Serine/t	Panicum_miliaceum	38.50	0.00
TRINITY_OAE2258	hypothet	Marchantia_polymorp	38.50	0.00
TRINITY_GBG6698	hypothet	Chara_braunii	38.50	0.00
TRINITY_XP_0110	uncharac	Sesamum_indicum	38.50	0.00
TRINITY_RQO9871	hypothet	Populus_trichocarpa	38.50	0.00
TRINITY_PRW6160	lysozyme	Chlorella_sorokinia	38.50	0.00
TRINITY_ESQ2998	hypothet	Eutrema_salsugineum	38.50	0.00
TRINITY_XP_0243	glutathi	Physcomitrella_pate	38.50	0.00
TRINITY_PNH0433	MOXD1 1	Tetrabaena_socialis	38.50	0.00
TRINITY_GAQ8238	delta(3)	Klebsormidium_niten	38.50	0.00
TRINITY_XP_0029	E3 ubiqu	Selaginella_moellen	38.50	0.00
TRINITY_GAV6491	YTH doma	Cephalotus_follicul	38.50	0.00
TRINITY_BAK0043	predicte	Hordeum_vulgare	38.50	0.00
TRINITY_AAS9246	Fe-super	Lotus_japonicus	38.50	0.00
TRINITY_XP_0244	protein	Physcomitrella_pate	38.50	0.00
TRINITY_XP_0137	pectin a	Brassica_napus	38.50	0.00
TRINITY_XP_0029	hypothet	Volvox_carteri	38.50	0.00
TRINITY_XP_0239	protein	Quercus_suber	38.50	0.00
TRINITY_CDP1798	unnamed	Coffea_canephora	38.50	0.00
TRINITY_AFK4786	unknown	Medicago_truncatula	38.50	0.00
TRINITY_RDY0275	hypothet	Mucuna_pruriens	38.50	0.00
TRINITY_BAJ9457	predicte	Hordeum_vulgare	38.50	0.00
TRINITY_KXZ4437	hypothet	Gonium_pectorale	38.50	0.00
TRINITY_XP_0042	PREDICTE	Fragaria_vesca	38.50	0.00
TRINITY_XP_0029	hypothet	Volvox_carteri	38.50	0.00
TRINITY_BAK0323	predicte	Hordeum_vulgare	38.50	0.00
TRINITY_KXZ4594	hypothet	Gonium_pectorale	38.50	0.00
TRINITY_PNH0808	BTB/POZ	Tetrabaena_socialis	38.50	0.00
TRINITY_XP_0104	PREDICTE	Camelina_sativa	38.50	0.00
TRINITY_GBG8139	hypothet	Chara_braunii	38.50	0.00
TRINITY_EEF2753	two-comp	Ricinus_communis	38.50	0.00
TRINITY_KXZ4799	hypothet	Gonium_pectorale	38.50	0.00
TRINITY_XP_0238	leucine	Quercus_suber	38.50	0.00
TRINITY_PNW8645	hypothet	Chlamydomonas_reinh	38.50	0.00
TRINITY_XP_0086	diacylgl	Zea_mays	38.50	0.00
TRINITY_XP_0194	PREDICTE	Lupinus_angustifoli	38.50	0.00
TRINITY_GAQ7842	hypothet	Klebsormidium_niten	38.50	0.00
TRINITY_OUS4179	hypothet	Ostreococcus_tauri	38.50	0.00
TRINITY_XP_0218	pre-rRNA	Prunus_avium	38.50	0.00
TRINITY_XP_0239	externa	Quercus_suber	38.50	0.00
TRINITY_XP_0243	ras-rela	Physcomitrella_pate	38.50	0.00
TRINITY_PNW7849	hypothet	Chlamydomonas_reinh	38.50	0.00
TRINITY_XP_0056	mitochor	Coccomyxa_subellips	38.50	0.00
TRINITY_GBF8936	diacylgl	Raphidocelis_subcap	38.50	0.00
TRINITY_GAX7767	hypothet	Chlamydomonas_eusti	38.50	0.00
TRINITY_BAK0455	predicte	Hordeum_vulgare	38.50	0.00
TRINITY_XP_0056	Xaa-Pro	Coccomyxa_subellips	38.50	0.00
TRINITY_XP_0239	LOW QUAI	Quercus_suber	38.50	0.00
TRINITY_GAX8172	hypothet	Chlamydomonas_eusti	38.50	0.00
TRINITY_XP_0239	N-acyl-p	Quercus_suber	38.50	0.00
TRINITY_XP_0104	PREDICTE	Camelina_sativa	38.50	0.00
TRINITY_KXZ5505	hypothet	Gonium_pectorale	38.50	0.00
TRINITY_XP_0016	componer	Chlamydomonas_reinh	38.50	0.00
TRINITY_PSS2676	Receptor	Actinidia_chinensis	38.50	0.00
TRINITY_KXZ5031	hypothet	Gonium_pectorale	38.50	0.00
TRINITY_KXZ4447	hypothet	Gonium_pectorale	38.50	0.00

TRINITY_KXZ4755	hypothet	Gonium_pectorale	38.50	0.00
TRINITY_XP_0245	probable	Selaginella_moellen	38.50	0.00
TRINITY_XP_0176	PREDICTI	Gossypium_arboreum	38.50	0.00
TRINITY_GAX8345	hypothet	Chlamydomonas_eusti	38.50	0.00
TRINITY_XP_0016	predicte	Chlamydomonas_reinh	38.50	0.00
TRINITY_XP_0266	CTD smal	Phoenix_dactylifera	38.50	0.00
TRINITY_GAV8014	Kelch_3	Cephalotus_follicul	38.50	0.00
TRINITY_XP_0058	hypothet	Chlorella_variabili	38.50	0.00
TRINITY_PNW7983	hypothet	Chlamydomonas_reinh	38.50	0.00
TRINITY_XP_0029	hypothet	Volvox_carteri	38.50	0.00
TRINITY_GBG7673	hypothet	Chara_braunii	38.50	0.00
TRINITY_GAX8129	hypothet	Chlamydomonas_eusti	38.50	0.00
TRINITY_KXZ4939	hypothet	Gonium_pectorale	38.50	0.00
TRINITY_GAX7448	hypothet	Chlamydomonas_eusti	38.50	0.00
TRINITY_XP_0220	CBL-inte	Helianthus_annuus	38.50	0.00
TRINITY_GAX8545	hypothet	Chlamydomonas_eusti	38.50	0.00
TRINITY_GAX7507	hypothet	Chlamydomonas_eusti	38.50	0.00
TRINITY_XP_0202	uncharac	Asparagus_officinal	38.50	0.00
TRINITY_GAX8055	hypothet	Chlamydomonas_eusti	38.50	0.00
TRINITY_OIW0149	hypothet	Lupinus_angustifoli	38.50	0.00
TRINITY_XP_0229	CTD smal	Cucurbita_moschata	38.50	0.00
TRINITY_GAX8103	hypothet	Chlamydomonas_eusti	38.50	0.00
TRINITY_PNH1181	Serine/t	Tetrabaena_socialis	38.50	0.00
TRINITY_GAX7850	hypothet	Chlamydomonas_eusti	38.50	0.00
TRINITY_GAX8251	hypothet	Chlamydomonas_eusti	38.50	0.00
TRINITY_XP_0110	7-methyl	Sesamum_indicum	38.40	0.00
TRINITY_GBF8892	translat	Raphidocelis_subcap	38.40	0.00
TRINITY_EFJ2815	hypothet	Selaginella_moellen	38.40	0.00
TRINITY_PTQ5055	hypothet	Marchantia_polymorp	38.40	0.00
TRINITY_GAX8530	hypothet	Chlamydomonas_eusti	38.40	0.00
TRINITY_PSS0620	DnaJ pro	Actinidia_chinensis	38.40	0.00
TRINITY_XP_0114	VAMP-lik	Auxenochlorella_pro	38.40	0.00
TRINITY_KMZ6550	Dedicate	Zostera_marina	38.40	0.00
TRINITY_XP_0113	hypothet	Auxenochlorella_pro	38.40	0.00
TRINITY_RXH7691	hypothet	Malus_domestica	38.40	0.00
TRINITY_OAE3117	hypothet	Marchantia_polymorp	38.40	0.00
TRINITY_XP_0206	tyrosine	Dendrobium_catenatu	38.40	0.00
TRINITY_GAX8194	hypothet	Chlamydomonas_eusti	38.40	0.00
TRINITY_PIA4605	hypothet	Aquilegia_coerulea	38.40	0.00
TRINITY_RDX8325	Sorting	Mucuna_pruriens	38.40	0.00
TRINITY_XP_0225	endonucl	Brassica_napus	38.40	0.00
TRINITY_GAX8289	hypothet	Chlamydomonas_eusti	38.40	0.00
TRINITY_GAX7714	hypothet	Chlamydomonas_eusti	38.40	0.00
TRINITY_XP_0058	hypothet	Chlorella_variabili	38.40	0.00
TRINITY_PRW5837	hypothet	Chlorella_sorokinia	38.40	0.00
TRINITY_XP_0030	predicte	Micromonas_pusilla	38.40	0.00
TRINITY_PNW7874	hypothet	Chlamydomonas_reinh	38.40	0.00
TRINITY_GAX8564	hypothet	Chlamydomonas_eusti	38.40	0.00
TRINITY_OAY8236	ABC tran	Ananas_comosus	38.40	0.00
TRINITY_XP_0243	putative	Physcomitrella_pate	38.40	0.00
TRINITY_XP_0238	pre-mRNA	Quercus_suber	38.40	0.00
TRINITY_XP_0238	serine/t	Quercus_suber	38.40	0.00
TRINITY_XP_0191	PREDICTE	Ipomoea_nil	38.40	0.00
TRINITY_PWA4037	GTP-bind	Artemisia_annua	38.40	0.00
TRINITY_XP_0016	predicte	Chlamydomonas_reinh	38.40	0.00
TRINITY_XP_0017	predicte	Chlamydomonas_reinh	38.40	0.00
TRINITY_XP_0240	SNF1-rel	Morus_notabilis	38.40	0.00
TRINITY_OTF9515	putative	Helianthus_annuus	38.40	0.00
TRINITY_PKU8351	hypothet	Dendrobium_catenatu	38.40	0.00
TRINITY_XP_0227	coiled-c	Durio_zibethinus	38.40	0.00
TRINITY_PNW8474	hypothet	Chlamydomonas_reinh	38.40	0.00
TRINITY_KMZ5751	Alkylate	Zostera_marina	38.40	0.00
TRINITY_CAA0411	cytochro	Helianthus_tuberosu	38.40	0.00
TRINITY_XP_0058	hypothet	Chlorella_variabili	38.40	0.00
TRINITY_KCW5779	hypothet	Eucalyptus_grandis	38.40	0.00
TRINITY_OAE1874	hypothet	Marchantia_polymorp	38.40	0.00

TRINITY_GAX73738	hypothet	Chlamydomonas_eusti	38.40	0.00
TRINITY_KXZ44268	hypothet	Gonium_pectorale	38.40	0.00
TRINITY_XP_00169	predicte	Chlamydomonas_reinh	38.40	0.00
TRINITY_XP_00881	molybden	Phoenix_dactylifera	38.40	0.00
TRINITY_GBF95068	hypothet	Raphidocelis_subcap	38.40	0.00
TRINITY_KCW75830	hypothet	Eucalyptus_grandis	38.40	0.00
TRINITY_XP_01884	PREDICTE	Juglans_regia	38.40	0.00
TRINITY_OWM74209	hypothet	Punica_granatum	38.40	0.00
TRINITY_PRW60379	Aldo ket	Chlorella_sorokinia	38.40	0.00
TRINITY_PIN06792	Transcri	Handroanthus_impeti	38.40	0.00
TRINITY_PNH02583	hypothet	Tetrabaena_socialis	38.40	0.00
TRINITY_PNW86830	hypothet	Chlamydomonas_reinh	38.40	0.00
TRINITY_XP_00496	phosphol	Setaria_italica	38.40	0.00
TRINITY_GAX77871	hypothet	Chlamydomonas_eusti	38.40	0.00
TRINITY_PNW82916	hypothet	Chlamydomonas_reinh	38.40	0.00
TRINITY_GAX79554	hypothet	Chlamydomonas_eusti	38.40	0.00
TRINITY_GAX76899	hypothet	Chlamydomonas_eusti	38.40	0.00
TRINITY_XP_02579	NEDD8-ac	Panicum_hallii	38.40	0.00
TRINITY_GAX78457	hypothet	Chlamydomonas_eusti	38.40	0.00
TRINITY_GBF98480	sucrose	Raphidocelis_subcap	38.40	0.00
TRINITY_XP_00938	PREDICTE	Musa_acuminata	38.40	0.00
TRINITY_XP_00142	predicte	Ostreococcus_sp._'l	38.40	0.00
TRINITY_XP_02665	purine-u	Phoenix_dactylifera	38.30	0.00
TRINITY_ACJ38195	chitinas	Malus_hupehensis	38.30	0.00
TRINITY_EPS72842	hypothet	Genlisea_aurea	38.30	0.00
TRINITY_XP_02388	probable	Quercus_suber	38.30	0.00
TRINITY_ESR35771	hypothet	Citrus_clementina	38.30	0.00
TRINITY_PSC70398	elongati	Micractinium_conduc	38.30	0.00
TRINITY_GAX78279	hypothet	Chlamydomonas_eusti	38.30	0.00
TRINITY_EFJ07915	hypothet	Selaginella_moellen	38.30	0.00
TRINITY_PTQ45672	hypothet	Marchantia_polymorp	38.30	0.00
TRINITY_BAJ92145	predicte	Hordeum_vulgare	38.30	0.00
TRINITY_BAK05971	predicte	Hordeum_vulgare	38.30	0.00
TRINITY_XP_02390	uncharac	Quercus_suber	38.30	0.00
TRINITY_OAE34310	hypothet	Marchantia_polymorp	38.30	0.00
TRINITY_OAE24296	hypothet	Marchantia_polymorp	38.30	0.00
TRINITY_XP_02709	phosphol	Coffea_arabica	38.30	0.00
TRINITY_XP_02389	guanine	Quercus_suber	38.30	0.00
TRINITY_XP_00584	hypothet	Chlorella_variabili	38.30	0.00
TRINITY_GBF96262	cysteine	Raphidocelis_subcap	38.30	0.00
TRINITY_XP_01916	PREDICTE	Ipomoea_nil	38.30	0.00
TRINITY_YP_00943	orf187	Bupleurum_falcatum	38.30	0.00
TRINITY_KXZ43261	hypothet	Gonium_pectorale	38.30	0.00
TRINITY_XP_00306	voltage-	Micromonas_pusilla	38.30	0.00
TRINITY_GAX77780	hypothet	Chlamydomonas_eusti	38.30	0.00
TRINITY_GAX85109	hypothet	Chlamydomonas_eusti	38.30	0.00
TRINITY_BAJ96315	predicte	Hordeum_vulgare	38.30	0.00
TRINITY_XP_00639	probable	Eutrema_salsugineum	38.30	0.00
TRINITY_XP_02022	probable	Cajanus_cajan	38.30	0.00
TRINITY_PNW85660	hypothet	Chlamydomonas_reinh	38.30	0.00
TRINITY_GBG91635	hypothet	Chara_braunii	38.30	0.00
TRINITY_ESR39267	hypothet	Citrus_clementina	38.30	0.00
TRINITY_XP_01389	hypothet	Monoraphidium_negle	38.30	0.00
TRINITY_PNW80026	hypothet	Chlamydomonas_reinh	38.30	0.00
TRINITY_GAX80990	hypothet	Chlamydomonas_eusti	38.30	0.00
TRINITY_GAX78147	hypothet	Chlamydomonas_eusti	38.30	0.00
TRINITY_GAX81343	hypothet	Chlamydomonas_eusti	38.30	0.00
TRINITY_XP_02388	putative	Quercus_suber	38.30	0.00
TRINITY_GAQ83315	acetyltr	Klebsormidium_niten	38.30	0.00
TRINITY_GBF94196	calcium	Raphidocelis_subcap	38.30	0.00
TRINITY_XP_00169	predicte	Chlamydomonas_reinh	38.30	0.00
TRINITY_XP_02387	beta-gal	Quercus_suber	38.30	0.00
TRINITY_PSC71848	Glycosyl	Micractinium_conduc	38.30	0.00
TRINITY_XP_02436	uncharac	Physcomitrella_pate	38.30	0.00
TRINITY_KXZ46787	hypothet	Gonium_pectorale	38.30	0.00
TRINITY_KXZ47425	hypothet	Gonium_pectorale	38.30	0.00

TRINITY_PNW7619	hypothet	Chlamydomonas_reinh	38.30	0.00
TRINITY_XP_0279	protein	Vigna_unguiculata	38.30	0.00
TRINITY_GAX7659	hypothet	Chlamydomonas_eusti	38.30	0.00
TRINITY_GAX7975	hypothet	Chlamydomonas_eusti	38.30	0.00
TRINITY_PNW8565	hypothet	Chlamydomonas_reinh	38.30	0.00
TRINITY_GBF9761	hypothet	Raphidocelis_subcap	38.30	0.00
TRINITY_XP_0062	elongat	Capsella_rubella	38.30	0.00
TRINITY_XP_0016	predicte	Chlamydomonas_reinh	38.30	0.00
TRINITY_KXZ4902	hypothet	Gonium_pectorale	38.30	0.00
TRINITY_PTQ3082	hypothet	Marchantia_polymorp	38.30	0.00
TRINITY_RLM9167	CTD sma	Panicum_miliaceum	38.30	0.00
TRINITY_GAU2515	hypothet	Trifolium_subterran	38.30	0.00
TRINITY_GBF9416	hypothet	Raphidocelis_subcap	38.30	0.00
TRINITY_PNW7741	hypothet	Chlamydomonas_reinh	38.30	0.00
TRINITY_XP_0068	E3 ubiq	Amborella_trichopod	38.30	0.00
TRINITY_XP_0029	serine/t	Selaginella_moellen	38.30	0.00
TRINITY_XP_0243	ABC tra	Physcomitrella_pate	38.30	0.00
TRINITY_GAX7648	hypothet	Chlamydomonas_eusti	38.30	0.00
TRINITY_XP_0097	PREDICT	Nicotiana_sylvestri	38.30	0.00
TRINITY_XP_0229	autopha	Cucurbita_moschata	38.30	0.00
TRINITY_XP_0030	predicte	Micromonas_pusilla	38.30	0.00
TRINITY_GAX7455	hypothet	Chlamydomonas_eusti	38.30	0.00
TRINITY_BAJ9457	predicte	Hordeum_vulgare	38.30	0.00
TRINITY_XP_0083	PREDICT	Malus_domestica	38.30	0.00
TRINITY_XP_0238	LOW QUA	Quercus_suber	38.30	0.00
TRINITY_XP_0017	predicte	Chlamydomonas_reinh	38.30	0.00
TRINITY_AAT9397	unknown	Oryza_sativa	38.30	0.00
TRINITY_PRW5604	multidr	Chlorella_sorokinia	38.30	0.00
TRINITY_GAQ8687	Cystein	Klebsormidium_niten	38.30	0.00
TRINITY_RWW9025	hypothet	Ensete_ventricosum	38.30	0.00
TRINITY_GAX7369	hypothet	Chlamydomonas_eusti	38.30	0.00
TRINITY_GAX7334	hypothet	Chlamydomonas_eusti	38.30	0.00
TRINITY_YP_0028	NADH de	Micromonas_commoda	38.30	0.00
TRINITY_PWZ2217	putative	Zea_mays	38.30	0.00
TRINITY_XP_0230	alpha c	Cucurbita_maxima	38.20	0.00
TRINITY_XP_0202	histone	Asparagus_officinal	38.20	0.00
TRINITY_XP_0104	PREDICT	Camelina_sativa	38.20	0.00
TRINITY_OVA0548	Glycosy	Macleaya_cordata	38.20	0.00
TRINITY_XP_0066	PREDICT	Oryza_brachyantha	38.20	0.00
TRINITY_XP_0237	plant U	Lactuca_sativa	38.20	0.00
TRINITY_GAQ7965	Signal t	Klebsormidium_niten	38.20	0.00
TRINITY_XP_0058	hypothet	Chlorella_variabili	38.20	0.00
TRINITY_AAT5873	putative	Oryza_sativa	38.20	0.00
TRINITY_PRW6125	kinase	Chlorella_sorokinia	38.20	0.00
TRINITY_RWR7918	cytochr	Cinnamomum_micranth	38.20	0.00
TRINITY_XP_0206	protein	Dendrobium_catenatu	38.20	0.00
TRINITY_KCW7937	hypothet	Eucalyptus_grandis	38.20	0.00
TRINITY_OWM7052	hypothet	Punica_granatum	38.20	0.00
TRINITY_PIA3054	hypothet	Aquilegia_coerulea	38.20	0.00
TRINITY_PNW8369	hypothet	Chlamydomonas_reinh	38.20	0.00
TRINITY_XP_0243	ribosom	Physcomitrella_pate	38.20	0.00
TRINITY_BAK0230	predicte	Hordeum_vulgare	38.20	0.00
TRINITY_GBF9994	hypothet	Raphidocelis_subcap	38.20	0.00
TRINITY_OAE2748	hypothet	Marchantia_polymorp	38.20	0.00
TRINITY_PIA4255	hypothet	Aquilegia_coerulea	38.20	0.00
TRINITY_XP_0097	PREDICT	Nicotiana_sylvestri	38.20	0.00
TRINITY_XP_0202	probabl	Asparagus_officinal	38.20	0.00
TRINITY_GBG7211	hypothet	Chara_braunii	38.20	0.00
TRINITY_XP_0058	express	Chlorella_variabili	38.20	0.00
TRINITY_PNW6966	hypothet	Chlamydomonas_reinh	38.20	0.00
TRINITY_XP_0237	probabl	Lactuca_sativa	38.20	0.00
TRINITY_XP_0239	coronin-	Quercus_suber	38.20	0.00
TRINITY_GBG6449	hypothet	Chara_braunii	38.20	0.00
TRINITY_GAQ8825	hypothet	Klebsormidium_niten	38.20	0.00
TRINITY_KXZ5052	hypothet	Gonium_pectorale	38.20	0.00
TRINITY_KXZ5404	hypothet	Gonium_pectorale	38.20	0.00

TRINITY_XP_0056!	hypothet	Coccomyxa_subellips	38.20	0.00
TRINITY_XP_0144!	SCAN dor	Vigna_radiata	38.20	0.00
TRINITY_GAQ9133	DEAD-box	Klebsormidium_niten	38.20	0.00
TRINITY_PSC6761!	glycosi	Micractinium_conduc	38.20	0.00
TRINITY_GAQ8824	kelch re	Klebsormidium_niten	38.20	0.00
TRINITY_XP_0106	PREDICTI	Beta_vulgaris	38.20	0.00
TRINITY_GAQ8271	Nucleic	Klebsormidium_niten	38.20	0.00
TRINITY_BAJ9908	predicte	Hordeum_vulgare	38.20	0.00
TRINITY_KCW8343	hypothet	Eucalyptus_grandis	38.20	0.00
TRINITY_XP_0056	alpha/be	Coccomyxa_subellips	38.20	0.00
TRINITY_GAX7499	hypothet	Chlamydomonas_eusti	38.20	0.00
TRINITY_XP_0088	protein	Phoenix_dactylifera	38.20	0.00
TRINITY_GAX7955	hypothet	Chlamydomonas_eusti	38.20	0.00
TRINITY_XP_0229	oxyster	Cucurbita_maxima	38.20	0.00
TRINITY_KDD7310	2-enoyl-	Helicosporidium_sp.	38.20	0.00
TRINITY_VDD6479	unnamed	Brassica_oleracea	38.20	0.00
TRINITY_XP_0201	protein	Aegilops_tauschii	38.20	0.00
TRINITY_KXZ5282	hypothet	Gonium_pectorale	38.20	0.00
TRINITY_GBG8505	hypothet	Chara_braunii	38.20	0.00
TRINITY_XP_0238	probable	Quercus_suber	38.20	0.00
TRINITY_GAX7537	hypothet	Chlamydomonas_eusti	38.20	0.00
TRINITY_XP_0279	putative	Vigna_unguiculata	38.20	0.00
TRINITY_GAX7907	hypothet	Chlamydomonas_eusti	38.20	0.00
TRINITY_GBF8972	hypothet	Raphidocelis_subcap	38.20	0.00
TRINITY_GAX7508	hypothet	Chlamydomonas_eusti	38.20	0.00
TRINITY_KZV1486	Inosine-	Dorcoceras_hygromet	38.20	0.00
TRINITY_KXZ4844	hypothet	Gonium_pectorale	38.20	0.00
TRINITY_EOY1413	Uncharac	Theobroma_cacao	38.20	0.00
TRINITY_XP_0016	potassiu	Chlamydomonas_reinh	38.20	0.00
TRINITY_XP_0239	ras-rela	Quercus_suber	38.20	0.00
TRINITY_GBF9777	hypothet	Raphidocelis_subcap	38.20	0.00
TRINITY_PNW7775	hypothet	Chlamydomonas_reinh	38.20	0.00
TRINITY_XP_0016	DEAD/DEA	Chlamydomonas_reinh	38.20	0.00
TRINITY_PTQ4299	hypothet	Marchantia_polymorp	38.20	0.00
TRINITY_KXZ5543	hypothet	Gonium_pectorale	38.20	0.00
TRINITY_XP_0188	PREDICTI	Juglans_regia	38.20	0.00
TRINITY_PTQ2799	hypothet	Marchantia_polymorp	38.20	0.00
TRINITY_PNW8462	hypothet	Chlamydomonas_reinh	38.20	0.00
TRINITY_GAX7430	hypothet	Chlamydomonas_eusti	38.20	0.00
TRINITY_XP_0016	voltage-	Chlamydomonas_reinh	38.20	0.00
TRINITY_GAX8062	hypothet	Chlamydomonas_eusti	38.20	0.00
TRINITY_KXZ5238	hypothet	Gonium_pectorale	38.20	0.00
TRINITY_GAX7651	hypothet	Chlamydomonas_eusti	38.20	0.00
TRINITY_XP_0029	hypothet	Volvox_carteri	38.20	0.00
TRINITY_PNW8357	hypothet	Chlamydomonas_reinh	38.20	0.00
TRINITY_GAX7923	hypothet	Chlamydomonas_eusti	38.20	0.00
TRINITY_XP_0029	probable	Selaginella_moellen	38.20	0.00
TRINITY_GAQ8687	Cysteine	Klebsormidium_niten	38.20	0.00
TRINITY_XP_0243	glutathi	Physcomitrella_pate	38.20	0.00
TRINITY_PNW7971	hypothet	Chlamydomonas_reinh	38.20	0.00
TRINITY_XP_0239	zinc tra	Quercus_suber	38.20	0.00
TRINITY_GAQ9280	hypothet	Klebsormidium_niten	38.20	0.00
TRINITY_PNW7890	hypothet	Chlamydomonas_reinh	38.20	0.00
TRINITY_PWA6770	ubiquiti	Artemisia_annua	38.20	0.00
TRINITY_XP_0158	uncharac	Ziziphus_jujuba	38.10	0.00
TRINITY_OMO5717	hypothet	Corchorus_capsulari	38.10	0.00
TRINITY_EEF2753	two-comp	Ricinus_communis	38.10	0.00
TRINITY_RDX6492	Gamma-gl	Mucuna_pruriens	38.10	0.00
TRINITY_BBC2842	cytoplas	Yamagishiella_unicc	38.10	0.00
TRINITY_XP_0212	uncharac	Herrania_umbratica	38.10	0.00
TRINITY_OAY6440	Phosphat	Ananas_comosus	38.10	0.00
TRINITY_XP_0058	hypothet	Chlorella_variabili	38.10	0.00
TRINITY_XP_0245	phosphat	Selaginella_moellen	38.10	0.00
TRINITY_EMS4874	hypothet	Triticum_urartu	38.10	0.00
TRINITY_XP_0263	endochit	Papaver_somniferum	38.10	0.00
TRINITY_XP_0017	sulfite	Chlamydomonas_reinh	38.10	0.00

TRINITY_PSC7595	putative	Micractinium_conduc	38.10	0.00
TRINITY_XP_0058	hypothet	Chlorella_variabili	38.10	0.00
TRINITY_XP_0264	short-ch	Papaver_somniferum	38.10	0.00
TRINITY_XP_0244	pre-mRNA	Physcomitrella_pate	38.10	0.00
TRINITY_XP_0030	Alpha-ke	Ostreococcus_tauri	38.10	0.00
TRINITY_XP_0056	EF-hand	Coccomyxa_subellips	38.10	0.00
TRINITY_XP_0219	solute c	Carica_papaya	38.10	0.00
TRINITY_XP_0238	dipeptic	Quercus_suber	38.10	0.00
TRINITY_GAX7594	hypothet	Chlamydomonas_eusti	38.10	0.00
TRINITY_PNH0180	hypothet	Tetrabaena_socialis	38.10	0.00
TRINITY_XP_0228	phosphat	Olea_europaea	38.10	0.00
TRINITY_YP_0094	hypothet	Ulva_pertusa	38.10	0.00
TRINITY_BAJ9725	predicte	Hordeum_vulgare	38.10	0.00
TRINITY_XP_0016	predicte	Chlamydomonas_reinh	38.10	0.00
TRINITY_RLM5424	hypothet	Panicum_miliaceum	38.10	0.00
TRINITY_PNH0518	Protein	Tetrabaena_socialis	38.10	0.00
TRINITY_GAX8002	hypothet	Chlamydomonas_eusti	38.10	0.00
TRINITY_GAX7633	hypothet	Chlamydomonas_eusti	38.10	0.00
TRINITY_XP_0109	PREDICTE	Elaeis_guineensis	38.10	0.00
TRINITY_XP_0177	beta-hex	Phoenix_dactylifera	38.10	0.00
TRINITY_PNH1019	hypothet	Tetrabaena_socialis	38.10	0.00
TRINITY_GAQ7798	RNA heli	Klebsormidium_niten	38.10	0.00
TRINITY_XP_0088	SEC14 cy	Phoenix_dactylifera	38.10	0.00
TRINITY_XP_0228	VAMP-lik	Olea_europaea	38.10	0.00
TRINITY_KXZ4168	hypothet	Gonium_pectorale	38.10	0.00
TRINITY_XP_0016	predicte	Chlamydomonas_reinh	38.10	0.00
TRINITY_GAX7704	hypothet	Chlamydomonas_eusti	38.10	0.00
TRINITY_XP_0201	phyteps	Aegilops_tauschii	38.10	0.00
TRINITY_XP_0238	queuosir	Quercus_suber	38.10	0.00
TRINITY_XP_0016	predicte	Chlamydomonas_reinh	38.10	0.00
TRINITY_XP_0016	predicte	Chlamydomonas_reinh	38.10	0.00
TRINITY_PRW5620	DNA repa	Chlorella_sorokinia	38.10	0.00
TRINITY_XP_0068	serine/t	Amborella_trichopod	38.10	0.00
TRINITY_XP_0227	phosphat	Durio_zibethinus	38.10	0.00
TRINITY_PRW6027	snRNA-ac	Chlorella_sorokinia	38.10	0.00
TRINITY_GAX7366	hypothet	Chlamydomonas_eusti	38.10	0.00
TRINITY_GAQ9010	E3 ubiqu	Klebsormidium_niten	38.10	0.00
TRINITY_RMZ5708	hypothet	Auxenochlorella_prc	38.10	0.00
TRINITY_XP_0029	protein	Selaginella_moellen	38.10	0.00
TRINITY_XP_0029	hypothet	Volvox_carteri	38.10	0.00
TRINITY_GAX7810	hypothet	Chlamydomonas_eusti	38.10	0.00
TRINITY_XP_0016	predicte	Chlamydomonas_reinh	38.10	0.00
TRINITY_GAX8603	hypothet	Chlamydomonas_eusti	38.10	0.00
TRINITY_GAX7368	hypothet	Chlamydomonas_eusti	38.10	0.00
TRINITY_GAX7703	hypothet	Chlamydomonas_eusti	38.10	0.00
TRINITY_XP_0062	SNF1-rel	Capsella_rubella	38.10	0.00
TRINITY_GAX7784	hypothet	Chlamydomonas_eusti	38.10	0.00
TRINITY_RLN0104	dual spe	Panicum_miliaceum	38.10	0.00
TRINITY_XP_0220	serine/t	Helianthus_annuus	38.10	0.00
TRINITY_PNW7320	hypothet	Chlamydomonas_reinh	38.10	0.00
TRINITY_PRW2065	SWI SNF-	Chlorella_sorokinia	38.10	0.00
TRINITY_PSS2172	Protein	Actinidia_chinensis	38.10	0.00
TRINITY_GAX7318	hypothet	Chlamydomonas_eusti	38.10	0.00
TRINITY_PNH1178	Heat sho	Tetrabaena_socialis	38.10	0.00
TRINITY_PRW6015	bifuncti	Chlorella_sorokinia	38.10	0.00
TRINITY_GAX7870	hypothet	Chlamydomonas_eusti	38.10	0.00
TRINITY_XP_0238	pentafur	Quercus_suber	38.10	0.00
TRINITY_XP_0017	centriol	Chlamydomonas_reinh	38.10	0.00
TRINITY_GAX7898	hypothet	Chlamydomonas_eusti	38.10	0
TRINITY_GAX8014	hypothet	Chlamydomonas_eusti	38.10	0.00
TRINITY_XP_0218	uncharac	Prunus_avium	38.10	0.00
TRINITY_XP_0194	PREDICTE	Lupinus_angustifoli	38.00	0.00
TRINITY_XP_0102	PREDICTE	Nelumbo_nucifera	38.00	0.00
TRINITY_XP_0194	PREDICTE	Lupinus_angustifoli	38.00	0.00
TRINITY_XP_0127	serine/t	Setaria_italica	38.00	0.00
TRINITY_XP_0202	phosphol	Asparagus_officinal	38.00	0.00

TRINITY_XP_0101	probable	Morus_notabilis	38.00	0.00
TRINITY_KXZ4345	hypothet	Gonium_pectorale	38.00	0.00
TRINITY_XP_0258	serine	Panicum_hallii	38.00	0.00
TRINITY_OTF8484	putative	Helianthus_annuus	38.00	0.00
TRINITY_XP_0191	PREDICTE	Ipomoea_nil	38.00	0.00
TRINITY_OAE3001	hypothet	Marchantia_polymorp	38.00	0.00
TRINITY_EOA3410	hypothet	Capsella_rubella	38.00	0.00
TRINITY_GAQ8632	major fa	Klebsormidium_niten	38.00	0.00
TRINITY_KEH2376	ARM repe	Medicago_truncatula	38.00	0.00
TRINITY_XP_0082	PREDICTE	Prunus_mume	38.00	0.00
TRINITY_RXH9621	hypothet	Malus_domestica	38.00	0.00
TRINITY_DAA7995	TPA_inf	Chlamydomonas_reinh	38.00	0.00
TRINITY_GAX8523	hypothet	Chlamydomonas_eusti	38.00	0.00
TRINITY_XP_0030	predicte	Micromonas_pusilla	38.00	0.00
TRINITY_XP_0030	Cytochro	Ostreococcus_tauri	38.00	0.00
TRINITY_XP_0014	predicte	Ostreococcus_sp._'l	38.00	0.00
TRINITY_OAE2003	hypothet	Marchantia_polymorp	38.00	0.00
TRINITY_PNW8679	hypothet	Chlamydomonas_reinh	38.00	0.00
TRINITY_PNW8385	hypothet	Chlamydomonas_reinh	38.00	0.00
TRINITY_BAJ9079	predicte	Hordeum_vulgare	38.00	0.00
TRINITY_RMZ5349	hypothet	Auxenochlorella_pro	38.00	0.00
TRINITY_XP_0029	hypothet	Volvox_carteri	38.00	0.00
TRINITY_XP_0243	putative	Physcomitrella_pate	38.00	0.00
TRINITY_AIU4942	translat	Aristolochia_tagala	38.00	0.00
TRINITY_KXZ5624	hypothet	Gonium_pectorale	38.00	0.00
TRINITY_GBF9145	hypothet	Raphidocelis_subcap	38.00	0.00
TRINITY_KGN6463	hypothet	Cucumis_sativus	38.00	0.00
TRINITY_RXH9440	hypothet	Malus_domestica	38.00	0.00
TRINITY_GBF9734	flagella	Raphidocelis_subcap	38.00	0.00
TRINITY_XP_0029	hypothet	Volvox_carteri	38.00	0.00
TRINITY_KXZ5618	hypothet	Gonium_pectorale	38.00	0.00
TRINITY_XP_0063	PREDICTE	Solanum_tuberosum	38.00	0.00
TRINITY_GAX7817	hypothet	Chlamydomonas_eusti	38.00	0.00
TRINITY_XP_0264	protein	Papaver_somniferum	38.00	0.00
TRINITY_XP_0227	transpo	Durio_zibethinus	38.00	0.00
TRINITY_XP_0239	uncharac	Quercus_suber	38.00	0.00
TRINITY_GAX8451	hypothet	Chlamydomonas_eusti	38.00	0.00
TRINITY_GAX8329	hypothet	Chlamydomonas_eusti	38.00	0.00
TRINITY_XP_0016	flagella	Chlamydomonas_reinh	38.00	0.00
TRINITY_CBI3558	unnamed	Vitis_vinifera	38.00	0.00
TRINITY_PSC7220	ultravio	Micractinium_conduc	38.00	0.00
TRINITY_XP_0158	probable	Ziziphus_jujuba	38.00	0.00
TRINITY_XP_0174	PREDICTE	Vigna_angularis	38.00	0.00
TRINITY_XP_0017	predicte	Chlamydomonas_reinh	38.00	0.00
TRINITY_PRW5781	B Chain	Chlorella_sorokinia	38.00	0.00
TRINITY_OAE1944	hypothet	Marchantia_polymorp	38.00	0.00
TRINITY_XP_0029	hypothet	Volvox_carteri	38.00	0.00
TRINITY_PNW7984	hypothet	Chlamydomonas_reinh	38.00	0.00
TRINITY_XP_0239	carrier	Quercus_suber	38.00	0.00
TRINITY_PTQ4358	hypothet	Marchantia_polymorp	38.00	0.00
TRINITY_EOY3167	Calpain-	Theobroma_cacao	38.00	0.00
TRINITY_XP_0042	acidic	Solanum_lycopersicu	38.00	0.00
TRINITY_GAX7806	hypothet	Chlamydomonas_eusti	38.00	0.00
TRINITY_PRW6135	AP2 doma	Chlorella_sorokinia	38.00	0.00
TRINITY_RZB4932	Importin	Glycine_soja	38.00	0.00
TRINITY_PNH0192	Glycosyl	Tetrabaena_socialis	38.00	0.00
TRINITY_XP_0110	PREDICTE	Populus_euphratica	38.00	0.00
TRINITY_GAX8631	hypothet	Chlamydomonas_eusti	38.00	0.00
TRINITY_XP_0058	hypothet	Chlorella_variabili	38.00	0.00
TRINITY_GAX7668	hypothet	Chlamydomonas_eusti	38.00	0.00
TRINITY_XP_0174	PREDICTE	Vigna_angularis	38.00	0.00
TRINITY_XP_0128	PREDICTE	Erythranthe_guttata	38.00	0.00
TRINITY_XP_0249	AFG1-lik	Cynara_cardunculus	38.00	0.00
TRINITY_KXZ4935	hypothet	Gonium_pectorale	38.00	0.00
TRINITY_PNH0519	Phosphat	Tetrabaena_socialis	38.00	0.00
TRINITY_GAX7790	hypothet	Chlamydomonas_eusti	38.00	0.00

TRINITY_XP_0177(	cyclin-1	Phoenix_dactylifera	38.00	0.00
TRINITY_XP_0110(	non-lys(	Sesamum_indicum	38.00	0.00
TRINITY_XP_0245(	uncharac	Selaginella_moellen	38.00	0.00
TRINITY_GAQ8955(	BTB/POZ	Klebsormidium_niten	37.90	0.00
TRINITY_PTQ2785(	hypothet	Marchantia_polymorp	37.90	0.00
TRINITY_XP_0071(	hypothet	Phaseolus_vulgaris	37.90	0.00
TRINITY_XP_0056(	ClpP/crc	Coccomyxa_subellips	37.90	0.00
TRINITY_XP_0243(	uncharac	Physcomitrella_pate	37.90	0.00
TRINITY_XP_0243(	protein	Physcomitrella_pate	37.90	0.00
TRINITY_XP_0239(	phosphat	Quercus_suber	37.90	0.00
TRINITY_XP_0056(	SM/Sec1-	Coccomyxa_subellips	37.90	0.00
TRINITY_XP_0216(	syntaxin	Manihot_esculenta	37.90	0.00
TRINITY_XP_0239(	uncharac	Quercus_suber	37.90	0.00
TRINITY_XP_0239(	protein	Quercus_suber	37.90	0.00
TRINITY_KXZ4282(	hypothet	Gonium_pectorale	37.90	0.00
TRINITY_OAE2795(	hypothet	Marchantia_polymorp	37.90	0.00
TRINITY_XP_0220(	serine/t	Helianthus_annuus	37.90	0.00
TRINITY_XP_0229(	ubiquit:	Cucurbita_moschata	37.90	0.00
TRINITY_GAQ8660(	peptidas	Klebsormidium_niten	37.90	0.00
TRINITY_XP_0239(	peroxide	Quercus_suber	37.90	0.00
TRINITY_XP_0102(	PREDICTE	Nelumbo_nucifera	37.90	0.00
TRINITY_XP_0212(	serine/t	Herrania_umbratica	37.90	0.00
TRINITY_XP_0068(	mannose-	Amborella_trichopod	37.90	0.00
TRINITY_GAQ7902(	Regulat(	Klebsormidium_niten	37.90	0.00
TRINITY_XP_0042(	PREDICTE	Fragaria_vesca	37.90	0.00
TRINITY_XP_0205(	uncharac	Sesamum_indicum	37.90	0.00
TRINITY_XP_0056(	DnaJ-dor	Coccomyxa_subellips	37.90	0.00
TRINITY_XP_0124(	PREDICTE	Gossypium_raimondii	37.90	0.00
TRINITY_GBG6433(	hypothet	Chara_braunii	37.90	0.00
TRINITY_GBF9453(	ABC trar	Raphidocelis_subcap	37.90	0.00
TRINITY_GAX7676(	hypothet	Chlamydomonas_eusti	37.90	0.00
TRINITY_GAQ8912(	hypothet	Klebsormidium_niten	37.90	0.00
TRINITY_XP_0025(	ChlRe ra	Micromonas_commoda	37.90	0.00
TRINITY_XP_0238(	eukaryot	Quercus_suber	37.90	0.00
TRINITY_XP_0229(	protein	Cucurbita_maxima	37.90	0.00
TRINITY_XP_0083(	PREDICTE	Malus_domestica	37.90	0.00
TRINITY_GAX7455(	hypothet	Chlamydomonas_eusti	37.90	0.00
TRINITY_NP_0012(	phospho(	Glycine_max	37.90	0.00
TRINITY_XP_0025(	predicte	Micromonas_commoda	37.90	0.00
TRINITY_KXZ5635(	hypothet	Gonium_pectorale	37.90	0.00
TRINITY_XP_0029(	hypothet	Volvox_carteri	37.90	0.00
TRINITY_BAJ9408(	predicte	Hordeum_vulgare	37.90	0.00
TRINITY_PNW8662(	hypothet	Chlamydomonas_reinh	37.90	0.00
TRINITY_GAX8151(	hypothet	Chlamydomonas_eusti	37.90	0.00
TRINITY_PNH0884(	Fibrocys	Tetrabaena_socialis	37.90	0.00
TRINITY_XP_0191(	PREDICTE	Ipomoea_nil	37.90	0.00
TRINITY_XP_0201(	probable	Aegilops_tauschii	37.90	0.00
TRINITY_GAX7408(	hypothet	Chlamydomonas_eusti	37.90	0.00
TRINITY_GAQ8522(	ornithir	Klebsormidium_niten	37.90	0.00
TRINITY_XP_0097(	PREDICTE	Nicotiana_sylvestri	37.90	0.00
TRINITY_PNW8775(	hypothet	Chlamydomonas_reinh	37.90	0.00
TRINITY_KXZ5000(	hypothet	Gonium_pectorale	37.90	0.00
TRINITY_KXZ4454(	hypothet	Gonium_pectorale	37.90	0.00
TRINITY_KXZ4322(	hypothet	Gonium_pectorale	37.90	0.00
TRINITY_GBG6548(	hypothet	Chara_braunii	37.90	0.00
TRINITY_GAX7918(	hypothet	Chlamydomonas_eusti	37.90	0.00
TRINITY_GBF8820(	hypothet	Raphidocelis_subcap	37.90	0.00
TRINITY_CDY1402(	BnaC02g(	Brassica_napus	37.90	0.00
TRINITY_GAX8024(	hypothet	Chlamydomonas_eusti	37.90	0.00
TRINITY_XP_0144(	(+)-neor	Vigna_radiata	37.90	0.00
TRINITY_PNW7852(	hypothet	Chlamydomonas_reinh	37.90	0.00
TRINITY_GAX8386(	hypothet	Chlamydomonas_eusti	37.90	0.00
TRINITY_XP_0110(	fimbrin-	Sesamum_indicum	37.90	0.00
TRINITY_GAX7570(	hypothet	Chlamydomonas_eusti	37.90	0.00
TRINITY_PTQ4978(	hypothet	Marchantia_polymorp	37.90	0.00
TRINITY_GAX7494(	hypothet	Chlamydomonas_eusti	37.90	0.00

TRINITY_XP_0025	aminope	Ricinus_communis	37.90	0.00
TRINITY_XP_0138	type I	Monoraphidium_negle	37.90	0.00
TRINITY_KXZ4379	hypothet	Gonium_pectorale	37.90	0.00
TRINITY_ACZ3725	Mn supe	Chlamydomonas_reinh	37.90	0.00
TRINITY_XP_0029	hypothet	Volvox_carteri	37.90	0.00
TRINITY_ABK2593	unknown	Picea_sitchensis	37.90	0.00
TRINITY_XP_0245	5'-AMP-a	Selaginella_moellen	37.90	0.00
TRINITY_GAX8547	hypothet	Chlamydomonas_eusti	37.90	0.00
TRINITY_XP_0056	flag-ta	Coccomyxa_subellips	37.90	0.00
TRINITY_GAX8016	hypothet	Chlamydomonas_eusti	37.90	0.00
TRINITY_KXZ4398	hypothet	Gonium_pectorale	37.90	0.00
TRINITY_PSC6737	54S rib	Micractinium_conduc	37.80	0.00
TRINITY_XP_0172	PREDICTE	Daucus_carota	37.80	0.00
TRINITY_XP_0150	probable	Solanum_pennellii	37.80	0.00
TRINITY_PNR4725	hypothet	Physcomitrella_pate	37.80	0.00
TRINITY_XP_0219	uncharac	Helianthus_annuus	37.80	0.00
TRINITY_XP_0042	pathoger	Solanum_lycopersicu	37.80	0.00
TRINITY_VDD0662	unnamed	Brassica_rapa	37.80	0.00
TRINITY_GAQ7758	Cyclic r	Klebsormidium_niten	37.80	0.00
TRINITY_PRW6025	ser thr	Chlorella_sorokinia	37.80	0.00
TRINITY_XP_0083	PREDICTE	Malus_domestica	37.80	0.00
TRINITY_XP_0191	PREDICTE	Ipomoea_nil	37.80	0.00
TRINITY_KEH1543	pyrrolir	Medicago_truncatula	37.80	0.00
TRINITY_XP_0082	PREDICTE	Prunus_mume	37.80	0.00
TRINITY_KXZ5669	hypothet	Gonium_pectorale	37.80	0.00
TRINITY_XP_0239	tRNA-dil	Quercus_suber	37.80	0.00
TRINITY_RHN4201	putative	Medicago_truncatula	37.80	0.00
TRINITY_XP_0029	serine/t	Selaginella_moellen	37.80	0.00
TRINITY_OAE2310	hypothet	Marchantia_polymorp	37.80	0.00
TRINITY_GAQ7783	Protein	Klebsormidium_niten	37.80	0.00
TRINITY_XP_0218	mitogen-	Carica_papaya	37.80	0.00
TRINITY_PNW7850	hypothet	Chlamydomonas_reinh	37.80	0.00
TRINITY_GAX8518	hypothet	Chlamydomonas_eusti	37.80	0.00
TRINITY_XP_0029	hypothet	Volvox_carteri	37.80	0.00
TRINITY_PNW8519	hypothet	Chlamydomonas_reinh	37.80	0.00
TRINITY_PNH0843	Nucleop	Tetrabaena_socialis	37.80	0.00
TRINITY_GAX7277	hypothet	Chlamydomonas_eusti	37.80	0.00
TRINITY_GAX7289	hypothet	Chlamydomonas_eusti	37.80	0.00
TRINITY_PKA5227	putative	Apostasia_shenzheni	37.80	0.00
TRINITY_PSS3156	Serine/t	Actinidia_chinensis	37.80	0.00
TRINITY_GBG7950	hypothet	Chara_braunii	37.80	0.00
TRINITY_OIW0457	hypothet	Lupinus_angustifoli	37.80	0.00
TRINITY_XP_0156	stomatir	Oryza_sativa	37.80	0.00
TRINITY_XP_0016	Rh prote	Chlamydomonas_reinh	37.80	0.00
TRINITY_PSC7035	long cha	Micractinium_conduc	37.80	0.00
TRINITY_XP_0165	PREDICTE	Capsicum_annuum	37.80	0.00
TRINITY_PNW7039	hypothet	Chlamydomonas_reinh	37.80	0.00
TRINITY_PRQ1591	putative	Rosa_chinensis	37.80	0.00
TRINITY_XP_0208	cyclin-c	Arabidopsis_lyrata	37.80	0.00
TRINITY_KMZ6376	ATPase,	Zostera_marina	37.80	0.00
TRINITY_GAX8376	hypothet	Chlamydomonas_eusti	37.80	0.00
TRINITY_PNW7872	hypothet	Chlamydomonas_reinh	37.80	0.00
TRINITY_GBG7104	hypothet	Chara_braunii	37.80	0.00
TRINITY_PNW8775	hypothet	Chlamydomonas_reinh	37.80	0.00
TRINITY_GAX8517	hypothet	Chlamydomonas_eusti	37.80	0.00
TRINITY_XP_0243	phosphat	Physcomitrella_pate	37.80	0.00
TRINITY_XP_0167	PREDICTE	Gossypium_hirsutum	37.80	0.00
TRINITY_XP_0139	hypothet	Monoraphidium_negle	37.80	0.00
TRINITY_XP_0016	voltage-	Chlamydomonas_reinh	37.80	0.00
TRINITY_XP_0218	dual spe	Prunus_avium	37.80	0.00
TRINITY_XP_0036	probable	Medicago_truncatula	37.80	0.00
TRINITY_XP_0056	glutath	Coccomyxa_subellips	37.80	0.00
TRINITY_PNH0752	Leucine-	Tetrabaena_socialis	37.80	0.00
TRINITY_PNW8844	hypothet	Chlamydomonas_reinh	37.80	0.00
TRINITY_GBF9150	sulfur s	Raphidocelis_subcap	37.80	0.00
TRINITY_XP_0270	proteas	Coffea_arabica	37.80	0.00

TRINITY_GAX7573	hypothet	Chlamydomonas_eusti	37.80	0.00
TRINITY_GAX7635	hypothet	Chlamydomonas_eusti	37.80	0.00
TRINITY_XP_0243	unchara	Physcomitrella_pate	37.80	0.00
TRINITY_GAX8387	hypothet	Chlamydomonas_eusti	37.80	0.00
TRINITY_PIA3416	hypothet	Aquilegia_coerulea	37.80	0.00
TRINITY_KMZ5753	putative	Zostera_marina	37.80	0.00
TRINITY_XP_0042	transmem	Solanum_lycopersicu	37.80	0.00
TRINITY_GBF9761	hypothet	Raphidocelis_subcap	37.80	0.00
TRINITY_GAX8574	hypothet	Chlamydomonas_eusti	37.80	0.00
TRINITY_XP_0029	vesicle-	Selaginella_moellen	37.80	0.00
TRINITY_XP_0025	predicte	Micromonas_commoda	37.80	0.00
TRINITY_KHG1409	LON pept	Gossypium_arboreum	37.70	0.00
TRINITY_XP_0136	PREDICTE	Brassica_oleracea	37.70	0.00
TRINITY_GAQ8032	hypothet	Klebsormidium_niten	37.70	0.00
TRINITY_XP_0238	vacuola	Quercus_suber	37.70	0.00
TRINITY_XP_0237	elongati	Lactuca_sativa	37.70	0.00
TRINITY_XP_0194	PREDICTE	Lupinus_angustifoli	37.70	0.00
TRINITY_XP_0228	ABC tran	Ostreococcus_tauri	37.70	0.00
TRINITY_XP_0110	PREDICTE	Populus_euphratica	37.70	0.00
TRINITY_XP_0239	unchara	Quercus_suber	37.70	0.00
TRINITY_PNW8088	hypothet	Chlamydomonas_reinh	37.70	0.00
TRINITY_XP_0237	ubiquiti	Lactuca_sativa	37.70	0.00
TRINITY_XP_0188	PREDICTE	Juglans_regia	37.70	0.00
TRINITY_XP_0110	V-type	Sesamum_indicum	37.70	0.00
TRINITY_PRQ2836	putative	Rosa_chinensis	37.70	0.00
TRINITY_XP_0219	3-phosph	Helianthus_annuus	37.70	0.00
TRINITY_PNH0094	Protein	Tetrabaena_socialis	37.70	0.00
TRINITY_KXZ4409	hypothet	Gonium_pectorale	37.70	0.00
TRINITY_XP_0105	PREDICTE	Tarenaya_hasslerian	37.70	0.00
TRINITY_XP_0238	ubiquiti	Quercus_suber	37.70	0.00
TRINITY_GAX7667	hypothet	Chlamydomonas_eusti	37.70	0.00
TRINITY_XP_0194	PREDICTE	Lupinus_angustifoli	37.70	0.00
TRINITY_GAX7457	hypothet	Chlamydomonas_eusti	37.70	0.00
TRINITY_XP_0016	predicte	Chlamydomonas_reinh	37.70	0.00
TRINITY_XP_0238	serine/t	Quercus_suber	37.70	0.00
TRINITY_GBF9198	hypothet	Raphidocelis_subcap	37.70	0.00
TRINITY_XP_0056	hypothet	Coccomyxa_subellips	37.70	0.00
TRINITY_PRW5962	origin	Chlorella_sorokinia	37.70	0.00
TRINITY_GAQ8595	GTP-bind	Klebsormidium_niten	37.70	0.00
TRINITY_PNW7750	hypothet	Chlamydomonas_reinh	37.70	0.00
TRINITY_PPR9314	hypothet	Gossypium_barbadens	37.70	0.00
TRINITY_XP_0188	PREDICTE	Juglans_regia	37.70	0.00
TRINITY_XP_0024	unchara	Sorghum_bicolor	37.70	0.00
TRINITY_XP_0075	predicte	Bathycoccus_prasino	37.70	0.00
TRINITY_RAL4069	hypothet	Cuscuta_australis	37.70	0.00
TRINITY_KXZ5392	hypothet	Gonium_pectorale	37.70	0.00
TRINITY_KXZ4282	hypothet	Gonium_pectorale	37.70	0.00
TRINITY_XP_0056	DnaJ-dom	Coccomyxa_subellips	37.70	0.00
TRINITY_GAX7866	hypothet	Chlamydomonas_eusti	37.70	0.00
TRINITY_PNW8794	hypothet	Chlamydomonas_reinh	37.70	0.00
TRINITY_GAX8057	hypothet	Chlamydomonas_eusti	37.70	0.00
TRINITY_GAX8439	hypothet	Chlamydomonas_eusti	37.70	0.00
TRINITY_GAQ7755	hypothet	Klebsormidium_niten	37.70	0.00
TRINITY_PNH1015	Aladin,	Tetrabaena_socialis	37.70	0.00
TRINITY_GAX8317	hypothet	Chlamydomonas_eusti	37.70	0.00
TRINITY_ACN2726	unknown	Zea_mays	37.70	0.00
TRINITY_RWR8971	cyclin-	Cinnamomum_micranth	37.70	0.00
TRINITY_XP_0172	PREDICTE	Daucus_carota	37.70	0.00
TRINITY_PNW8523	hypothet	Chlamydomonas_reinh	37.70	0.00
TRINITY_XP_0094	PREDICTE	Musa_acuminata	37.70	0.00
TRINITY_XP_0217	SNF1-rel	Chenopodium_quinoa	37.70	0.00
TRINITY_XP_0205	small G	Phalaenopsis_equest	37.70	0.00
TRINITY_GAX8435	hypothet	Chlamydomonas_eusti	37.70	0.00
TRINITY_XP_0102	PREDICTE	Nelumbo_nucifera	37.70	0.00
TRINITY_GAQ8293	Calmodul	Klebsormidium_niten	37.70	0.00
TRINITY_PSC7398	CAMK CA	Micractinium_conduc	37.70	0.00

TRINITY_KHG0551(	Putative	Gossypium_arboreum	37.70	0.00
TRINITY_KXZ4732	hypothet	Gonium_pectorale	37.70	0.00
TRINITY_PRW5896(	calcium-	Chlorella_sorokinia	37.70	0.00
TRINITY_KMZ5753	putative	Zostera_marina	37.70	0.00
TRINITY_GAX8017(	hypothet	Chlamydomonas_eusti	37.70	0.00
TRINITY_GAQ7758(	Cyclic r	Klebsormidium_niten	37.70	0.00
TRINITY_XP_0138	hypothet	Monoraphidium_negle	37.70	0.00
TRINITY_KFK3661	hypothet	Arabis_alpina	37.70	0.00
TRINITY_XP_0263	protein-	Papaver_somniferum	37.70	0.00
TRINITY_GAX7362(	hypothet	Chlamydomonas_eusti	37.70	0.00
TRINITY_KXZ5396	hypothet	Gonium_pectorale	37.70	0.00
TRINITY_XP_0029	guanylyl	Volvox_carteri	37.70	0.00
TRINITY_KXZ5700	hypothet	Gonium_pectorale	37.70	0.00
TRINITY_XP_0185(	PREDICTE	Pyrus_x_bretschneid	37.70	0.00
TRINITY_XP_0043	PREDICTE	Fragaria_vesca	37.60	0.00
TRINITY_XP_0030	RmlC-lik	Ostreococcus_tauri	37.60	0.00
TRINITY_GAX7458	hypothet	Chlamydomonas_eusti	37.60	0.00
TRINITY_KMZ5707	SEC13-re	Zostera_marina	37.60	0.00
TRINITY_PNW8237(	hypothet	Chlamydomonas_reinh	37.60	0.00
TRINITY_XP_0174	PREDICTE	Vigna_angularis	37.60	0.00
TRINITY_GBF8742	5-formyl	Raphidocelis_subcap	37.60	0.00
TRINITY_XP_0068	phosphoe	Amborella_trichopod	37.60	0.00
TRINITY_PIN0056	Cytochro	Handroanthus_impeti	37.60	0.00
TRINITY_XP_0238	protein	Quercus_suber	37.60	0.00
TRINITY_GAX8091	hypothet	Chlamydomonas_eusti	37.60	0.00
TRINITY_XP_0204	NEDD8-sp	Prunus_persica	37.60	0.00
TRINITY_XP_0172	PREDICTE	Daucus_carota	37.60	0.00
TRINITY_OWM6656(	hypothet	Punica_granatum	37.60	0.00
TRINITY_RWR9135	histidin	Cinnamomum_micranth	37.60	0.00
TRINITY_XP_0025(	predicte	Micromonas_commoda	37.60	0.00
TRINITY_XP_0016	pre-apo	Chlamydomonas_reinh	37.60	0.00
TRINITY_XP_0241	ubiquiti	Rosa_chinensis	37.60	0.00
TRINITY_GAX7315	hypothet	Chlamydomonas_eusti	37.60	0.00
TRINITY_GAQ9156	hypothet	Klebsormidium_niten	37.60	0.00
TRINITY_XP_0200	polyader	Ananas_comosus	37.60	0.00
TRINITY_BAK0219	predicte	Hordeum_vulgare	37.60	0.00
TRINITY_OAY7654	COP9 sig	Ananas_comosus	37.60	0.00
TRINITY_PNW7037	hypothet	Chlamydomonas_reinh	37.60	0.00
TRINITY_XP_0228	DEAD-box	Olea_europaea	37.60	0.00
TRINITY_KZN0701	hypothet	Daucus_carota	37.60	0.00
TRINITY_XP_0056	hypothet	Coccomyxa_subellips	37.60	0.00
TRINITY_PNR3537	hypothet	Physcomitrella_pate	37.60	0.00
TRINITY_XP_0093	PREDICTE	Musa_acuminata	37.60	0.00
TRINITY_XP_0228	Serine-t	Ostreococcus_tauri	37.60	0.00
TRINITY_XP_0024	ribosome	Micromonas_commoda	37.60	0.00
TRINITY_GAX7470	hypothet	Chlamydomonas_eusti	37.60	0.00
TRINITY_PSS2860	Sphingos	Actinidia_chinensis	37.60	0.00
TRINITY_XP_0239	deoxyrib	Quercus_suber	37.60	0.00
TRINITY_GAQ8163	cullin 3	Klebsormidium_niten	37.60	0.00
TRINITY_XP_0205	NADPH-de	Jatropha_curcas	37.60	0.00
TRINITY_PON7268(	Endoplas	Parasponia_anderson	37.60	0.00
TRINITY_XP_0239	60 kDa I	Quercus_suber	37.60	0.00
TRINITY_GAX7306	hypothet	Chlamydomonas_eusti	37.60	0.00
TRINITY_XP_0075	alpha-1,	Bathycoccus_prasino	37.60	0.00
TRINITY_GAX7515	hypothet	Chlamydomonas_eusti	37.60	0.00
TRINITY_XP_0243	GDT1-lik	Physcomitrella_pate	37.60	0.00
TRINITY_EFJ3152	hypothet	Selaginella_moellen	37.60	0.00
TRINITY_XP_0271	LOW QUAL	Coffea_eugenioides	37.60	0.00
TRINITY_GBF8885	hypothet	Raphidocelis_subcap	37.60	0.00
TRINITY_EOA2547(	hypothet	Capsella_rubella	37.60	0.00
TRINITY_XP_0238	protein	Quercus_suber	37.60	0.00
TRINITY_PNH0125(	Calcium-	Tetrabaena_socialis	37.60	0.00
TRINITY_XP_0194	PREDICTE	Lupinus_angustifoli	37.60	0.00
TRINITY_GAX8280	hypothet	Chlamydomonas_eusti	37.60	0.00
TRINITY_GAX7334	hypothet	Chlamydomonas_eusti	37.60	0.00
TRINITY_PNH0706(	Fibrone	Tetrabaena_socialis	37.60	0.00

TRINITY_XP_00664	PREDICTE	Oryza_brachyantha	37.60	0.00
TRINITY_XP_01257	serine/t	Cicer_arietinum	37.60	0.00
TRINITY_GBF89792	hypothet	Raphidocelis_subcap	37.60	0.00
TRINITY_GAX72689	hypothet	Chlamydomonas_eusti	37.60	0.00
TRINITY_XP_00295	hypothet	Volvox_carteri	37.60	0.00
TRINITY_GAX82518	hypothet	Chlamydomonas_eusti	37.60	0.00
TRINITY_GBG70502	hypothet	Chara_braunii	37.60	0.00
TRINITY_XP_01390	hypothet	Monoraphidium_negle	37.60	0.00
TRINITY_PNW82735	hypothet	Chlamydomonas_reinh	37.60	0.00
TRINITY_PVH47630	hypothet	Panicum_hallii	37.60	0.00
TRINITY_XP_02300	serine/t	Cucurbita_maxima	37.60	0.00
TRINITY_XP_00295	hypothet	Volvox_carteri	37.60	0.00
TRINITY_GAX74704	hypothet	Chlamydomonas_eusti	37.60	0.00
TRINITY_KXZ55758	hypothet	Gonium_pectorale	37.60	0.00
TRINITY_GBG78109	hypothet	Chara_braunii	37.60	0.00
TRINITY_XP_01047	PREDICTE	Camelina_sativa	37.60	0.00
TRINITY_XP_00355	probable	Brachypodium_distac	37.60	0.00
TRINITY_XP_02452	uncharac	Selaginella_moellen	37.60	0.00
TRINITY_GBF90935	voltage-	Raphidocelis_subcap	37.60	0.00
TRINITY_PNH04143	30S ribo	Tetrabaena_socialis	37.60	0.00
TRINITY_KXZ45430	hypothet	Gonium_pectorale	37.60	0.00
TRINITY_GBF92514	hypothet	Raphidocelis_subcap	37.60	0.00
TRINITY_ADJ19186	lysine k	Triticum_turgidum	37.60	0.00
TRINITY_XP_00170	flagella	Chlamydomonas_reinh	37.60	0.00
TRINITY_PNH00238	hypothet	Tetrabaena_socialis	37.60	0.00
TRINITY_XP_01562	CBL-inte	Oryza_sativa	37.60	0.00
TRINITY_XP_00249	predicte	Micromonas_commoda	37.60	0.00
TRINITY_XP_02200	UDP-suga	Helianthus_annuus	37.60	0.00
TRINITY_XP_00294	hypothet	Volvox_carteri	37.60	0.00
TRINITY_XP_01885	PREDICTE	Juglans_regia	37.60	0.00
TRINITY_PNW86401	hypothet	Chlamydomonas_reinh	37.60	0.00
TRINITY_XP_02733	lipoamid	Abrus_precatorius	37.60	0.00
TRINITY_GAX84471	hypothet	Chlamydomonas_eusti	37.60	0.00
TRINITY_XP_02440	elongati	Physcomitrella_pate	37.60	0.00
TRINITY_PNH03960	ATP-bind	Tetrabaena_socialis	37.60	0.00
TRINITY_XP_02014	GTP-bind	Aegilops_tauschii	37.60	0.00
TRINITY_GAX81814	hypothet	Chlamydomonas_eusti	37.60	0.00
TRINITY_PTQ36409	hypothet	Marchantia_polymorp	37.60	0.00
TRINITY_GAX83352	hypothet	Chlamydomonas_eusti	37.60	0.00
TRINITY_GAX78457	hypothet	Chlamydomonas_eusti	37.60	0.00
TRINITY_OAE18089	hypothet	Marchantia_polymorp	37.60	0.00
TRINITY_AAD22158	polyprot	Sorghum_bicolor	37.60	0.00
TRINITY_XP_01390	cellulos	Monoraphidium_negle	37.60	0.00
TRINITY_XP_00243	calcium	Sorghum_bicolor	37.60	0.00
TRINITY_XP_02389	glutared	Quercus_suber	37.60	0.00
TRINITY_GAQ80653	Lysophos	Klebsormidium_niten	37.50	0.00
TRINITY_GAQ87383	hypothet	Klebsormidium_niten	37.50	0.00
TRINITY_XP_02392	ras-rela	Quercus_suber	37.50	0.00
TRINITY_GAQ80065	hypothet	Klebsormidium_niten	37.50	0.00
TRINITY_PRW60250	ser thr	Chlorella_sorokinia	37.50	0.00
TRINITY_XP_00142	predicte	Ostreococcus_sp._'l	37.50	0.00
TRINITY_GAX84953	hypothet	Chlamydomonas_eusti	37.50	0.00
TRINITY_XP_00564	SFT2-dom	Coccomyxa_subellips	37.50	0.00
TRINITY_XP_01102	PREDICTE	Populus_euphratica	37.50	0.00
TRINITY_XP_01669	PREDICTE	Gossypium_hirsutum	37.50	0.00
TRINITY_KZV15325	hypothet	Dorcoceras_hygromet	37.50	0.00
TRINITY_EPS72909	pseudour	Genlisea_aurea	37.50	0.00
TRINITY_XP_02735	synaptot	Abrus_precatorius	37.50	0.00
TRINITY_BAJ99754	predicte	Hordeum_vulgare	37.50	0.00
TRINITY_PON64947	Phytochr	Parasponia_anderson	37.50	0.00
TRINITY_PSR85928	SWR1-com	Actinidia_chinensis	37.50	0.00
TRINITY_XP_02588	ras-rela	Solanum_lycopersicu	37.50	0.00
TRINITY_OAE18507	hypothet	Marchantia_polymorp	37.50	0.00
TRINITY_PWA32070	Micro-fi	Artemisia_annua	37.50	0.00
TRINITY_XP_02388	glutamin	Quercus_suber	37.50	0.00
TRINITY_XP_01025	PREDICTE	Nelumbo_nucifera	37.50	0.00

TRINITY_GAX7341	hypothet	Chlamydomonas_eusti	37.50	0.00
TRINITY_PNW7355	hypothet	Chlamydomonas_reinh	37.50	0.00
TRINITY_GAQ9068	ubiquin	Klebsormidium_niten	37.50	0.00
TRINITY_OMO8514	hypothet	Corchorus_capsulari	37.50	0.00
TRINITY_PIA2797	hypothet	Aquilegia_coerulea	37.50	0.00
TRINITY_XP_0106	PREDICT	Beta_vulgaris	37.50	0.00
TRINITY_PRW4448	chitotr	Chlorella_sorokinia	37.50	0.00
TRINITY_XP_0066	PREDICT	Oryza_brachyantha	37.50	0.00
TRINITY_XP_0241	serpin-2	Rosa_chinensis	37.50	0.00
TRINITY_BAJ9694	predicte	Hordeum_vulgare	37.50	0.00
TRINITY_GAX7942	hypothet	Chlamydomonas_eusti	37.50	0.00
TRINITY_GAX7953	hypothet	Chlamydomonas_eusti	37.50	0.00
TRINITY_XP_0200	CBL-inte	Ananas_comosus	37.50	0.00
TRINITY_XP_0238	uncharac	Quercus_suber	37.50	0.00
TRINITY_XP_0243	serine/t	Physcomitrella_pate	37.50	0.00
TRINITY_GAQ7839	HSP70/HS	Klebsormidium_niten	37.50	0.00
TRINITY_XP_0014	predicte	Ostreococcus_sp._'l	37.50	0.00
TRINITY_PNW7736	hypothet	Chlamydomonas_reinh	37.50	0.00
TRINITY_GBG6179	hypothet	Chara_braunii	37.50	0.00
TRINITY_XP_0056	putative	Coccomyxa_subellips	37.50	0.00
TRINITY_BAK0036	predicte	Hordeum_vulgare	37.50	0.00
TRINITY_XP_0056	hypothet	Coccomyxa_subellips	37.50	0.00
TRINITY_XP_0165	PREDICT	Capsicum_annuum	37.50	0.00
TRINITY_XP_0025	predicte	Micromonas_commoda	37.50	0.00
TRINITY_PRW4512	tRNA (u	Chlorella_sorokinia	37.50	0.00
TRINITY_XP_0042	acidic	Solanum_lycopersicu	37.50	0.00
TRINITY_PNW7018	hypothet	Chlamydomonas_reinh	37.50	0.00
TRINITY_XP_0202	xaa-Pro	Asparagus_officinal	37.50	0.00
TRINITY_XP_0042	PREDICT	Fragaria_vesca	37.50	0.00
TRINITY_ABK2437	unknown	Picea_sitchensis	37.50	0.00
TRINITY_XP_0139	hypothet	Monoraphidium_negle	37.50	0.00
TRINITY_KXZ4879	hypothet	Gonium_pectorale	37.50	0.00
TRINITY_XP_0243	uncharac	Physcomitrella_pate	37.50	0.00
TRINITY_PTQ4978	hypothet	Marchantia_polymorp	37.50	0.00
TRINITY_XP_0030	predicte	Micromonas_pusilla	37.50	0.00
TRINITY_KXZ4276	hypothet	Gonium_pectorale	37.50	0.00
TRINITY_GAX8541	hypothet	Chlamydomonas_eusti	37.50	0.00
TRINITY_XP_0238	NAD/NAD	Quercus_suber	37.50	0.00
TRINITY_BAJ9694	predicte	Hordeum_vulgare	37.50	0.00
TRINITY_PRW2090	Oxidored	Chlorella_sorokinia	37.50	0.00
TRINITY_GAQ8739	Serine	Klebsormidium_niten	37.50	0.00
TRINITY_KXZ5249	hypothet	Gonium_pectorale	37.50	0.00
TRINITY_PNW7532	hypothet	Chlamydomonas_reinh	37.50	0.00
TRINITY_PNW7747	hypothet	Chlamydomonas_reinh	37.50	0.00
TRINITY_BBC2842	hypothet	Yamagishiella_unicc	37.50	0.00
TRINITY_XP_0016	predicte	Chlamydomonas_reinh	37.50	0.00
TRINITY_PRW6156	Retrovi	Chlorella_sorokinia	37.50	0.00
TRINITY_XP_0151	PREDICT	Solanum_tuberosum	37.50	0.00
TRINITY_XP_0106	PREDICT	Beta_vulgaris	37.40	0.00
TRINITY_XP_0239	uncharac	Quercus_suber	37.40	0.00
TRINITY_ADR5168	phytoche	Pteris_vittata	37.40	0.00
TRINITY_XP_0105	PREDICT	Tarenaya_hasslerian	37.40	0.00
TRINITY_PSC7623	cyclin-A	Micractinium_conduc	37.40	0.00
TRINITY_XP_0029	Golgi ap	Selaginella_moellen	37.40	0.00
TRINITY_XP_0238	homoser	Quercus_suber	37.40	0.00
TRINITY_EPS6544	hypothet	Genlisea_aurea	37.40	0.00
TRINITY_XP_0023	splicin	Populus_trichocarpa	37.40	0.00
TRINITY_XP_0202	uncharac	Asparagus_officinal	37.40	0.00
TRINITY_RLM8600	hypothet	Panicum_miliaceum	37.40	0.00
TRINITY_XP_0194	PREDICT	Lupinus_angustifoli	37.40	0.00
TRINITY_XP_0273	CBL-inte	Abrus_precatorius	37.40	0.00
TRINITY_XP_0056	P-loop	Coccomyxa_subellips	37.40	0.00
TRINITY_ONI3229	hypothet	Prunus_persica	37.40	0.00
TRINITY_KMZ6219	hypothet	Zostera_marina	37.40	0.00
TRINITY_GBG9044	hypothet	Chara_braunii	37.40	0.00
TRINITY_XP_0093	PREDICT	Pyrus_x_bretschneid	37.40	0.00

TRINITY_XP_0016	predicte	Chlamydomonas_reinh	37.40	0.00
TRINITY_KDO6922	hypothet	Citrus_sinensis	37.40	0.00
TRINITY_XP_0228	TatD far	Ostreococcus_tauri	37.40	0.00
TRINITY_GAX7922	hypothet	Chlamydomonas_eusti	37.40	0.00
TRINITY_KXZ4662	hypothet	Gonium_pectorale	37.40	0.00
TRINITY_GAU1625	hypothet	Trifolium_subterran	37.40	0.00
TRINITY_PNW7414	hypothet	Chlamydomonas_reinh	37.40	0.00
TRINITY_XP_0216	ras-rela	Manihot_esculenta	37.40	0.00
TRINITY_GAQ8722	CCR4-NOT	Klebsormidium_niten	37.40	0.00
TRINITY_EFJ2770	hypothet	Selaginella_moellen	37.40	0.00
TRINITY_XP_0029	hypothet	Volvox_carteri	37.40	0.00
TRINITY_XP_0110	PREDICTE	Populus_euphratica	37.40	0.00
TRINITY_XP_0238	alpha-ac	Quercus_suber	37.40	0.00
TRINITY_XP_0241	mitogen-	Rosa_chinensis	37.40	0.00
TRINITY_XP_0071	hypothet	Phaseolus_vulgaris	37.40	0.00
TRINITY_GAX7929	hypothet	Chlamydomonas_eusti	37.40	0.00
TRINITY_XP_0025	predicte	Micromonas_commoda	37.40	0.00
TRINITY_KMS9362	hypothet	Beta_vulgaris	37.40	0.00
TRINITY_GBF9686	glucosid	Raphidocelis_subcap	37.40	0.00
TRINITY_PNH1169	hypothet	Tetrabaena_socialis	37.40	0.00
TRINITY_XP_0237	poly [AD	Lactuca_sativa	37.40	0.00
TRINITY_XP_0215	actin-re	Manihot_esculenta	37.40	0.00
TRINITY_PIA5972	hypothet	Aquilegia_coerulea	37.40	0.00
TRINITY_XP_0029	hypothet	Volvox_carteri	37.40	0.00
TRINITY_GAX7498	hypothet	Chlamydomonas_eusti	37.40	0.00
TRINITY_XP_0029	hypothet	Volvox_carteri	37.40	0.00
TRINITY_XP_0056	hypothet	Coccomyxa_subellips	37.40	0.00
TRINITY_XP_0110	E3 ubiqu	Sesamum_indicum	37.40	0.00
TRINITY_PNH1190	Phosphog	Tetrabaena_socialis	37.40	0.00
TRINITY_PNW8283	hypothet	Chlamydomonas_reinh	37.40	0.00
TRINITY_XP_0058	hypothet	Chlorella_variabili	37.40	0.00
TRINITY_KZV1541	phenylal	Dorcoceras_hygromet	37.40	0.00
TRINITY_BAK0191	predicte	Hordeum_vulgare	37.40	0.00
TRINITY_XP_0056	putative	Coccomyxa_subellips	37.40	0.00
TRINITY_XP_0029	hypothet	Volvox_carteri	37.40	0.00
TRINITY_XP_0104	PREDICTE	Camelina_sativa	37.40	0.00
TRINITY_PRW3265	Transcri	Chlorella_sorokinia	37.40	0.00
TRINITY_XP_0029	RWP-RK d	Volvox_carteri	37.40	0.00
TRINITY_XP_0014	predicte	Ostreococcus_sp._'l	37.40	0.00
TRINITY_KXZ5177	hypothet	Gonium_pectorale	37.40	0.00
TRINITY_GAX7402	hypothet	Chlamydomonas_eusti	37.40	0.00
TRINITY_XP_0192	PREDICTE	Nicotiana_attenuata	37.40	0.00
TRINITY_XP_0238	inositol	Quercus_suber	37.40	0.00
TRINITY_OAE1957	hypothet	Marchantia_polymorp	37.40	0.00
TRINITY_XP_0025	predicte	Micromonas_commoda	37.40	0.00
TRINITY_GAX7304	hypothet	Chlamydomonas_eusti	37.40	0.00
TRINITY_GAQ8216	hypothet	Klebsormidium_niten	37.40	0
TRINITY_BAJ9457	predicte	Hordeum_vulgare	37.40	0.00
TRINITY_XP_0144	ABC tran	Vigna_radiata	37.40	0.00
TRINITY_GAX7324	hypothet	Chlamydomonas_eusti	37.40	0.00
TRINITY_GAQ9127	etoposid	Klebsormidium_niten	37.40	0.00
TRINITY_ABU5296	hypothet	Dunaliella_viridis	37.40	0.00
TRINITY_GAU3053	hypothet	Trifolium_subterran	37.40	0.00
TRINITY_ACY0631	class II	Pseudotsuga_menzies	37.40	0.00
TRINITY_XP_0075	predicte	Bathycoccus_prasino	37.30	0.00
TRINITY_KMS9340	hypothet	Beta_vulgaris	37.30	0.00
TRINITY_XP_0084	PREDICTE	Cucumis_melo	37.30	0.00
TRINITY_KEH4374	elongati	Medicago_truncatula	37.30	0.00
TRINITY_PON8492	Dolichyl	Trema_orientale	37.30	0.00
TRINITY_RMZ5763	hypothet	Auxenochlorella_pro	37.30	0.00
TRINITY_EFJ2046	hypothet	Selaginella_moellen	37.30	0.00
TRINITY_XP_0219	GTP-bind	Helianthus_annuus	37.30	0.00
TRINITY_OIW0847	hypothet	Lupinus_angustifoli	37.30	0.00
TRINITY_XP_0029	gamma ca	Selaginella_moellen	37.30	0.00
TRINITY_GAQ8700	RNA poly	Klebsormidium_niten	37.30	0.00
TRINITY_GAX7813	hypothet	Chlamydomonas_eusti	37.30	0.00

TRINITY_KQK2146[illegible]	hypothet	Brachypodium_distac	37.30	0.00
TRINITY_XP_0041[illegible]	PREDICTI	Cucumis_sativus	37.30	0.00
TRINITY_XP_0109[illegible]	PREDICTI	Elaeis_guineensis	37.30	0.00
TRINITY_KHN3954[illegible]	Trans-2,	Glycine_soja	37.30	0.00
TRINITY_GBF8753[illegible]	hypothet	Raphidocelis_subcap	37.30	0.00
TRINITY_XP_0093[illegible]	PREDICTI	Pyrus_x_bretschneid	37.30	0.00
TRINITY_GAX8558[illegible]	hypothet	Chlamydomonas_eusti	37.30	0.00
TRINITY_NP_0011[illegible]	peptide	Zea_mays	37.30	0.00
TRINITY_XP_0014[illegible]	predict[illegible]	Ostreococcus_sp._'l	37.30	0.00
TRINITY_XP_0041[illegible]	PREDICTI	Cucumis_sativus	37.30	0.00

TRINITY_XP_0025(predicte	Micromonas_commoda	37.30	0.00
TRINITY_XP_0056⁴MFS gene	Coccomyxa_subellips	37.30	0.00
TRINITY_OVA2030⁹zinc fir	Macleaya_cordata	37.30	0.00
TRINITY_XP_0216⁸fatty ac	Hevea_brasiliensis	37.30	0.00
TRINITY_XP_0218⁵random s	Spinacia_oleracea	37.30	0.00
TRINITY_KXZ4844⁶hypothet	Gonium_pectorale	37.30	0.00
TRINITY_XP_0200⁸probable	Ananas_comosus	37.30	0.00
TRINITY_PSR8627⁸LEC14B p	Actinidia_chinensis	37.30	0.00
TRINITY_XP_0139(leukotri	Monoraphidium_negle	37.30	0.00
TRINITY_GAX8028⁶hypothet	Chlamydomonas_eusti	37.30	0.00
TRINITY_XP_0239(uncharac	Quercus_suber	37.30	0.00
TRINITY_XP_0056⁴MFS gene	Coccomyxa_subellips	37.30	0.00
TRINITY_XP_0218⁴protein	Spinacia_oleracea	37.30	0.00
TRINITY_XP_0017(DP1 tran	Chlamydomonas_reinh	37.30	0.00
TRINITY_RWW8424³hypothet	Ensete_ventricosum	37.30	0.00
TRINITY_PNH0808⁷BTB/POZ	Tetrabaena_socialis	37.30	0.00
TRINITY_PRW5736⁷glycosic	Chlorella_sorokinia	37.30	0.00
TRINITY_PIA5154⁸hypothet	Aquilegia_coerulea	37.30	0.00
TRINITY_BAJ9095(predicte	Hordeum_vulgare	37.30	0.00
TRINITY_GAX7511³hypothet	Chlamydomonas_eusti	37.30	0.00
TRINITY_EPS6438⁴hypothet	Genlisea_aurea	37.30	0.00
TRINITY_GAX8350⁹hypothet	Chlamydomonas_eusti	37.30	0.00
TRINITY_GBF9057(nitrite	Raphidocelis_subcap	37.30	0.00
TRINITY_GAX7891⁶hypothet	Chlamydomonas_eusti	37.30	0.00
TRINITY_KXZ5191³hypothet	Gonium_pectorale	37.30	0.00
TRINITY_EEF2753⁵two-comp	Ricinus_communis	37.30	0.00
TRINITY_OUS4796(hypothet	Ostreococcus_tauri	37.30	0.00
TRINITY_XP_0237⁵alpha-gl	Lactuca_sativa	37.30	0.00
TRINITY_XP_0124⁸PREDICTE	Gossypium_raimondii	37.30	0.00
TRINITY_KXZ4902⁹hypothet	Gonium_pectorale	37.30	0.00
TRINITY_XP_0024⁹predicte	Micromonas_commoda	37.30	0.00
TRINITY_BAK0036⁵predicte	Hordeum_vulgare	37.30	0.00
TRINITY_GBG8470⁴hypothet	Chara_braunii	37.30	0.00
TRINITY_GAX7976⁵hypothet	Chlamydomonas_eusti	37.30	0.00
TRINITY_XP_0228⁶ras-rela	Olea_europaea	37.30	0.00
TRINITY_OAE1897⁵hypothet	Marchantia_polymorp	37.30	0.00
TRINITY_GAX8465⁵hypothet	Chlamydomonas_eusti	37.30	0.00
TRINITY_GAX8222⁹hypothet	Chlamydomonas_eusti	37.30	0.00
TRINITY_GAX8233⁸hypothet	Chlamydomonas_eusti	37.30	0.00
TRINITY_GAQ8664³Zinc-fir	Klebsormidium_niten	37.30	0.00
TRINITY_OIW2075⁷hypothet	Lupinus_angustifoli	37.30	0.00
TRINITY_XP_0042⁹PREDICTE	Fragaria_vesca	37.30	0.00
TRINITY_EFH4948⁹hypothet	Arabidopsis_lyrata	37.30	0.00
TRINITY_XP_0016⁹amidase	Chlamydomonas_reinh	37.30	0.00
TRINITY_GAX8225⁴hypothet	Chlamydomonas_eusti	37.30	0.00
TRINITY_XP_0191⁷PREDICTE	Ipomoea_nil	37.30	0.00
TRINITY_GAQ8024(Dihydrop	Klebsormidium_niten	37.30	0.00
TRINITY_ADE7668⁷unknown	Picea_sitchensis	37.30	0.00
TRINITY_XP_0138⁹hypothet	Monoraphidium_negle	37.30	0.00
TRINITY_XP_0243⁸uncharac	Physcomitrella_pate	37.30	0.00
TRINITY_KXZ4511⁴hypothet	Gonium_pectorale	37.30	0.00
TRINITY_XP_0029⁴hypothet	Volvox_carteri	37.30	0.00
TRINITY_XP_0110⁸8-hydrox	Sesamum_indicum	37.30	0.00
TRINITY_OAY7998⁶Serine/t	Ananas_comosus	37.30	0.00
TRINITY_GAX8591⁵hypothet	Chlamydomonas_eusti	37.30	0.00
TRINITY_XP_0042⁴phosphog	Solanum_lycopersicu	37.30	0.00
TRINITY_GAX7827⁸hypothet	Chlamydomonas_eusti	37.30	0.00
TRINITY_GAX7959⁹hypothet	Chlamydomonas_eusti	37.30	0.00
TRINITY_KXZ5210⁵hypothet	Gonium_pectorale	37.30	0.00
TRINITY_GAX8430(hypothet	Chlamydomonas_eusti	37.30	0.00
TRINITY_AOV8590⁴ABCC1, p	Triticum_polonicum	37.30	0.00
TRINITY_GAX7827⁹hypothet	Chlamydomonas_eusti	37.30	0.00
TRINITY_EFJ3405⁸hypothet	Selaginella_moellen	37.20	0.00
TRINITY_XP_0058⁵hypothet	Chlorella_variabili	37.20	0.00
TRINITY_XP_0139(nuclear	Monoraphidium_negle	37.20	0.00
TRINITY_EPS7163¹hypothet	Genlisea_aurea	37.20	0.00

TRINITY_KMZ7512	dolichy	Zostera_marina	37.20	0.00
TRINITY_XP_0102	serine/	Brachypodium_distac	37.20	0.00
TRINITY_OMO8441	Nicotin	Corchorus_olitorius	37.20	0.00
TRINITY_OAO8910	hypothe	Arabidopsis_thalian	37.20	0.00
TRINITY_XP_0201	SWR1 co	Ananas_comosus	37.20	0.00
TRINITY_XP_0191	PREDICT	Ipomoea_nil	37.20	0.00
TRINITY_XP_0028	calcium-	Arabidopsis_lyrata	37.20	0.00
TRINITY_YP_0088	NADH de	Roya_obtusa	37.20	0.00
TRINITY_XP_0093	PREDICT	Pyrus_x_bretschneid	37.20	0.00
TRINITY_XP_0105	PREDICT	Tarenaya_hasslerian	37.20	0.00
TRINITY_XP_0101	uridine	Morus_notabilis	37.20	0.00
TRINITY_XP_0075	predict	Bathycoccus_prasinc	37.20	0.00
TRINITY_RMZ5414	hypothe	Auxenochlorella_prc	37.20	0.00
TRINITY_GBG8052	hypothe	Chara_braunii	37.20	0.00
TRINITY_PRW4429	ABC tra	Chlorella_sorokinia	37.20	0.00
TRINITY_XP_0110	PREDICT	Populus_euphratica	37.20	0.00
TRINITY_BAK0219	predict	Hordeum_vulgare	37.20	0.00
TRINITY_PRQ5311	putative	Rosa_chinensis	37.20	0.00
TRINITY_GAQ8204	hypothe	Klebsormidium_niten	37.20	0.00
TRINITY_XP_0238	probable	Quercus_suber	37.20	0.00
TRINITY_XP_0029	alpha-1,	Volvox_carteri	37.20	0.00
TRINITY_GAX7591	hypothe	Chlamydomonas_eusti	37.20	0.00
TRINITY_OAE2772	hypothe	Marchantia_polymorp	37.20	0.00
TRINITY_GAX7559	hypothe	Chlamydomonas_eusti	37.20	0.00
TRINITY_XP_0025	predict	Micromonas_commoda	37.20	0.00
TRINITY_EFJ3395	hypothe	Selaginella_moellen	37.20	0.00
TRINITY_XP_0217	ABC tra	Chenopodium_quinoa	37.20	0.00
TRINITY_NP_1988	catalyt	Arabidopsis_thalian	37.20	0.00
TRINITY_GAU1786	hypothe	Trifolium_subterran	37.20	0.00
TRINITY_PNW7311	hypothe	Chlamydomonas_reinh	37.20	0.00
TRINITY_EMS4734	Ubiquit	Triticum_urartu	37.20	0.00
TRINITY_XP_0194	PREDICT	Lupinus_angustifoli	37.20	0.00
TRINITY_XP_0238	lipase	Quercus_suber	37.20	0.00
TRINITY_GBG7313	hypothe	Chara_braunii	37.20	0.00
TRINITY_GBG6775	hypothe	Chara_braunii	37.20	0.00
TRINITY_PTQ4705	hypothe	Marchantia_polymorp	37.20	0.00
TRINITY_GAX7803	hypothe	Chlamydomonas_eusti	37.20	0.00
TRINITY_GAX7276	hypothe	Chlamydomonas_eusti	37.20	0.00
TRINITY_XP_0041	PREDICT	Cucumis_sativus	37.20	0.00
TRINITY_GAX7976	hypothe	Chlamydomonas_eusti	37.20	0.00
TRINITY_XP_0029	hypothe	Volvox_carteri	37.20	0.00
TRINITY_XP_0016	predict	Chlamydomonas_reinh	37.20	0.00
TRINITY_GAX8348	hypothe	Chlamydomonas_eusti	37.20	0.00
TRINITY_XP_0056	hypothe	Coccomyxa_subellips	37.20	0.00
TRINITY_XP_0138	E3 ubiq	Monoraphidium_negle	37.20	0.00
TRINITY_RWR9049	Small G	Cinnamomum_micranth	37.20	0.00
TRINITY_PNW8826	hypothe	Chlamydomonas_reinh	37.20	0.00
TRINITY_XP_0169	PREDICT	Cucumis_melo	37.20	0.00
TRINITY_PHU2780	Serine/	Capsicum_chinense	37.20	0.00
TRINITY_XP_0172	PREDICT	Daucus_carota	37.20	0.00
TRINITY_GBF9238	hypothe	Raphidocelis_subcap	37.20	0.00
TRINITY_PKA5023	Peroxis	Apostasia_shenzheni	37.20	0.00
TRINITY_GAX7380	hypothe	Chlamydomonas_eusti	37.20	0.00
TRINITY_XP_0238	cystath	Quercus_suber	37.20	0.00
TRINITY_XP_0239	stress	Quercus_suber	37.20	0.00
TRINITY_KXZ5111	hypothe	Gonium_pectorale	37.20	0.00
TRINITY_GAX8503	hypothe	Chlamydomonas_eusti	37.20	0.00
TRINITY_GBF8917	hypothe	Raphidocelis_subcap	37.20	0.00
TRINITY_XP_0238	dynein	Quercus_suber	37.20	0.00
TRINITY_XP_0219	probable	Helianthus_annuus	37.20	0.00
TRINITY_GAX8435	hypothe	Chlamydomonas_eusti	37.20	0.00
TRINITY_GAX8524	hypothe	Chlamydomonas_eusti	37.20	0.00
TRINITY_XP_0056	hypothe	Coccomyxa_subellips	37.20	0.00
TRINITY_XP_0243	cluster	Physcomitrella_pate	37.20	0.00
TRINITY_GAQ8542	Putative	Klebsormidium_niten	37.20	0.00
TRINITY_GAX7561	hypothe	Chlamydomonas_eusti	37.20	0.00

TRINITY_XP_0029	hypothet	Volvox_carteri	37.20	0.00
TRINITY_BAK0125	predicte	Hordeum_vulgare	37.20	0.00
TRINITY_BAJ9570	predicte	Hordeum_vulgare	37.20	0.00
TRINITY_GBG7683	hypothet	Chara_braunii	37.10	0.00
TRINITY_XP_0221	host ce	Momordica_charantia	37.10	0.00
TRINITY_PWA5808	protein	Artemisia_annua	37.10	0.00
TRINITY_EFJ3535	hypothet	Selaginella_moellen	37.10	0.00
TRINITY_XP_0212	probable	Herrania_umbratica	37.10	0.00
TRINITY_BAJ9372	predicte	Hordeum_vulgare	37.10	0.00
TRINITY_XP_0058	hypothet	Chlorella_variabili	37.10	0.00
TRINITY_XP_0120	uncharac	Jatropha_curcas	37.10	0.00
TRINITY_XP_0138	DNA-bin	Monoraphidium_negle	37.10	0.00
TRINITY_XP_0093	PREDICT	Pyrus_x_bretschneid	37.10	0.00
TRINITY_XP_0239	endosom	Quercus_suber	37.10	0.00
TRINITY_NP_0013	ras-rel	Nicotiana_tabacum	37.10	0.00
TRINITY_GAQ8852	hypothet	Klebsormidium_niten	37.10	0.00
TRINITY_GAQ9057	Uncharac	Klebsormidium_niten	37.10	0.00
TRINITY_XP_0063	PREDICT	Solanum_tuberosum	37.10	0.00
TRINITY_XP_0238	translat	Quercus_suber	37.10	0.00
TRINITY_XP_0205	UTP:RNA	Phalaenopsis_equest	37.10	0.00
TRINITY_NP_0011	ARP prot	Arabidopsis_thalian	37.10	0.00
TRINITY_XP_0218	pyruvate	Spinacia_oleracea	37.10	0.00
TRINITY_XP_0030	predicte	Micromonas_pusilla	37.10	0.00
TRINITY_BAK0043	predicte	Hordeum_vulgare	37.10	0.00
TRINITY_XP_0188	PREDICT	Juglans_regia	37.10	0.00
TRINITY_RWR8955	vacuola	Cinnamomum_micranth	37.10	0.00
TRINITY_GAX7792	hypothet	Chlamydomonas_eusti	37.10	0.00
TRINITY_GAX8187	hypothet	Chlamydomonas_eusti	37.10	0.00
TRINITY_GBG8621	hypothet	Chara_braunii	37.10	0.00
TRINITY_XP_0091	PREDICT	Brassica_rapa	37.10	0.00
TRINITY_XP_0243	uncharac	Physcomitrella_pate	37.10	0.00
TRINITY_ABD6314	Retrotr	Asparagus_officinal	37.10	0.00
TRINITY_XP_0016	predicte	Chlamydomonas_reinh	37.10	0.00
TRINITY_GAX7279	hypothet	Chlamydomonas_eusti	37.10	0.00
TRINITY_PSC6933	CTD sma	Micractinium_conduc	37.10	0.00
TRINITY_GBF9579	hypothet	Raphidocelis_subcap	37.10	0.00
TRINITY_XP_0030	predicte	Micromonas_pusilla	37.10	0.00
TRINITY_KCW7715	hypothet	Eucalyptus_grandis	37.10	0.00
TRINITY_XP_0025	predicte	Micromonas_commoda	37.10	0.00
TRINITY_XP_0030	glycosy	Micromonas_pusilla	37.10	0.00
TRINITY_PSC7565	tyrosin	Micractinium_conduc	37.10	0.00
TRINITY_GAQ8499	NAD(P)-	Klebsormidium_niten	37.10	0.00
TRINITY_XP_0058	hypothet	Chlorella_variabili	37.10	0.00
TRINITY_KXZ5438	hypothet	Gonium_pectorale	37.10	0.00
TRINITY_PTQ3701	hypothet	Marchantia_polymorp	37.10	0.00
TRINITY_GAX8434	hypothet	Chlamydomonas_eusti	37.10	0.00
TRINITY_XP_0030	predicte	Micromonas_pusilla	37.10	0.00
TRINITY_GAX8307	hypothet	Chlamydomonas_eusti	37.10	0.00
TRINITY_XP_0029	hypothet	Volvox_carteri	37.10	0.00
TRINITY_XP_0114	PREDICT	Fragaria_vesca	37.10	0.00
TRINITY_XP_0016	glycosy	Chlamydomonas_reinh	37.10	0.00
TRINITY_XP_0237	ubiquit	Lactuca_sativa	37.10	0.00
TRINITY_OAE3304	hypothet	Marchantia_polymorp	37.10	0.00
TRINITY_PNH0595	Photosy	Tetrabaena_socialis	37.10	0.00
TRINITY_PNW8098	hypothet	Chlamydomonas_reinh	37.10	0.00
TRINITY_GAX8003	hypothet	Chlamydomonas_eusti	37.10	0.00
TRINITY_XP_0104	PREDICT	Camelina_sativa	37.10	0.00
TRINITY_XP_0237	peptidy	Lactuca_sativa	37.10	0.00
TRINITY_XP_0213	folylpo	Sorghum_bicolor	37.10	0.00
TRINITY_PSC7098	Sho1p i	Micractinium_conduc	37.10	0.00
TRINITY_GAX7976	hypothet	Chlamydomonas_eusti	37.10	0.00
TRINITY_XP_0017	carboxy	Chlamydomonas_reinh	37.10	0.00
TRINITY_GAX7438	hypothet	Chlamydomonas_eusti	37.10	0.00
TRINITY_XP_0201	syntaxi	Aegilops_tauschii	37.10	0.00
TRINITY_GBF9117	hypothet	Raphidocelis_subcap	37.10	0.00
TRINITY_PNH0798	hypothet	Tetrabaena_socialis	37.10	0.00

TRINITY_XP_0029	hypothet	Volvox_carteri	37.10	0.00
TRINITY_XP_0056	hypothet	Coccomyxa_subellips	37.10	0.00
TRINITY_XP_0194	PREDICTI	Lupinus_angustifoli	37.10	0.00
TRINITY_KXZ4922	hypothet	Gonium_pectorale	37.10	0.00
TRINITY_KXZ5329	hypothet	Gonium_pectorale	37.10	0.00
TRINITY_XP_0273	ABC tra	Abrus_precatorius	37.10	0.00
TRINITY_GAX7650	hypothet	Chlamydomonas_eusti	37.10	0.00
TRINITY_GAX7442	hypothet	Chlamydomonas_eusti	37.10	0.00
TRINITY_XP_0176	PREDICTI	Gossypium_arboreum	37.10	0.00
TRINITY_XP_0023	NF-X1-t	Populus_trichocarpa	37.10	0.00
TRINITY_GAX8552	hypothet	Chlamydomonas_eusti	37.10	0.00
TRINITY_XP_0238	putative	Quercus_suber	37.10	0.00
TRINITY_BAJ9457	predicte	Hordeum_vulgare	37.10	0.00
TRINITY_GAX8180	hypothet	Chlamydomonas_eusti	37.10	0.00
TRINITY_GAX7397	hypothet	Chlamydomonas_eusti	37.10	0.00
TRINITY_GAX7738	hypothet	Chlamydomonas_eusti	37.10	0.00
TRINITY_GAX7827	hypothet	Chlamydomonas_eusti	37.10	0.00
TRINITY_AFK3980	unknown	Medicago_truncatula	37.00	0.00
TRINITY_XP_0194	PREDICTI	Lupinus_angustifoli	37.00	0.00
TRINITY_PSC6911	sorboso	Micractinium_conduc	37.00	0.00
TRINITY_PWA9714	cytochr	Artemisia_annua	37.00	0.00
TRINITY_KXZ4706	hypothet	Gonium_pectorale	37.00	0.00
TRINITY_BAJ9887	predicte	Hordeum_vulgare	37.00	0.00
TRINITY_VDC6929	unnamed	Brassica_rapa	37.00	0.00
TRINITY_PKA5716	hypothet	Apostasia_shenzheni	37.00	0.00
TRINITY_XP_0094	PREDICTI	Musa_acuminata	37.00	0.00
TRINITY_PPD8182	hypothet	Gossypium_barbadens	37.00	0.00
TRINITY_XP_0245	choline/	Selaginella_moellen	37.00	0.00
TRINITY_XP_0017	fructose	Chlamydomonas_reinh	37.00	0.00
TRINITY_OAE2799	hypothet	Marchantia_polymorp	37.00	0.00
TRINITY_AAT7666	5-alpha-	Pisum_sativum	37.00	0.00
TRINITY_XP_0237	extracel	Lactuca_sativa	37.00	0.00
TRINITY_EYU2374	hypothet	Erythranthe_guttata	37.00	0.00
TRINITY_GAQ7782	Zinc-bi	Klebsormidium_niten	37.00	0.00
TRINITY_PWA8269	sec7 do	Artemisia_annua	37.00	0.00
TRINITY_XP_0029	hypothet	Volvox_carteri	37.00	0.00
TRINITY_ABK2491	unknown	Picea_sitchensis	37.00	0.00
TRINITY_BAK0244	predicte	Hordeum_vulgare	37.00	0.00
TRINITY_PNW8081	hypothet	Chlamydomonas_reinh	37.00	0.00
TRINITY_GAX7601	hypothet	Chlamydomonas_eusti	37.00	0.00
TRINITY_XP_0258	short-c	Panicum_hallii	37.00	0.00
TRINITY_XP_0172	PREDICTI	Daucus_carota	37.00	0.00
TRINITY_GAX8459	hypothet	Chlamydomonas_eusti	37.00	0.00
TRINITY_EPS6939	hypothet	Genlisea_aurea	37.00	0.00
TRINITY_EPS7200	hypothet	Genlisea_aurea	37.00	0.00
TRINITY_XP_0016	asparty	Chlamydomonas_reinh	37.00	0.00
TRINITY_XP_0105	PREDICTI	Tarenaya_hasslerian	37.00	0.00
TRINITY_GAX7785	hypothet	Chlamydomonas_eusti	37.00	0.00
TRINITY_XP_0238	GTP-bin	Quercus_suber	37.00	0.00
TRINITY_KYP4767	Transcr	Cajanus_cajan	37.00	0.00
TRINITY_XP_0243	serine/	Physcomitrella_pate	37.00	0.00
TRINITY_KXZ4255	hypothet	Gonium_pectorale	37.00	0.00
TRINITY_EPS5921	hypothet	Genlisea_aurea	37.00	0.00
TRINITY_GAX8367	hypothet	Chlamydomonas_eusti	37.00	0.00
TRINITY_BAJ9782	predicte	Hordeum_vulgare	37.00	0.00
TRINITY_GAX7692	hypothet	Chlamydomonas_eusti	37.00	0.00
TRINITY_PSC6878	water d	Micractinium_conduc	37.00	0.00
TRINITY_XP_0104	PREDICTI	Camelina_sativa	37.00	0.00
TRINITY_XP_0029	hypothet	Volvox_carteri	37.00	0.00
TRINITY_GBG8116	hypothet	Chara_braunii	37.00	0.00
TRINITY_GAQ8002	delta-a	Klebsormidium_niten	37.00	0.00
TRINITY_PIA5184	hypothet	Aquilegia_coerulea	37.00	0.00
TRINITY_XP_0238	sodium/	Quercus_suber	37.00	0.00
TRINITY_ESR5390	hypothet	Citrus_clementina	37.00	0.00
TRINITY_XP_0029	hypothet	Volvox_carteri	37.00	0.00
TRINITY_XP_0083	PREDICTI	Malus_domestica	37.00	0.00

TRINITY_XP_0238	vacuola	Quercus_suber	37.00	0.00
TRINITY_GAQ8257	hypothet	Klebsormidium_niten	37.00	0.00
TRINITY_KXZ5525	hypothet	Gonium_pectorale	37.00	0.00
TRINITY_KXZ5612	hypothet	Gonium_pectorale	37.00	0.00
TRINITY_XP_0229	kelch-l	Cucurbita_moschata	37.00	0.00
TRINITY_XP_0243	ultravi	Physcomitrella_pate	37.00	0.00
TRINITY_XP_0030	predict	Micromonas_pusilla	37.00	0.00
TRINITY_PSC6833	sugar k	Micractinium_conduc	37.00	0.00
TRINITY_PTQ2759	hypothet	Marchantia_polymorp	37.00	0.00
TRINITY_GAX8316	hypothet	Chlamydomonas_eusti	37.00	0.00
TRINITY_XP_0102	PREDICT	Nelumbo_nucifera	37.00	0.00
TRINITY_GAX7926	hypothet	Chlamydomonas_eusti	37.00	0.00
TRINITY_XP_0056	hypothet	Coccomyxa_subellips	37.00	0.00
TRINITY_KXZ4981	hypothet	Gonium_pectorale	37.00	0.00
TRINITY_XP_0218	long ch	Spinacia_oleracea	37.00	0.00
TRINITY_CAI8489	putative	Medicago_sativa	37.00	0.00
TRINITY_ACY0631	class I	Pseudotsuga_menzies	37.00	0.00
TRINITY_OWM6628	hypothet	Punica_granatum	37.00	0.00
TRINITY_GBG8341	hypothet	Chara_braunii	37.00	0.00
TRINITY_GAX7745	hypothet	Chlamydomonas_eusti	37.00	0.00
TRINITY_PTQ4116	hypothet	Marchantia_polymorp	37.00	0.00
TRINITY_GAX7497	hypothet	Chlamydomonas_eusti	37.00	0.00
TRINITY_GAX8214	hypothet	Chlamydomonas_eusti	37.00	0.00
TRINITY_XP_0279	cytochr	Vigna_unguiculata	37.00	0.00
TRINITY_GAX7450	hypothet	Chlamydomonas_eusti	37.00	0.00
TRINITY_XP_0243	glutath	Physcomitrella_pate	37.00	0.00
TRINITY_PNW7613	hypothet	Chlamydomonas_reinh	37.00	0.00
TRINITY_XP_0087	protein	Phoenix_dactylifera	37.00	0.00
TRINITY_GAX7875	hypothet	Chlamydomonas_eusti	37.00	0.00
TRINITY_XP_0045	WD repe	Cicer_arietinum	37.00	0.00
TRINITY_GAX7363	hypothet	Chlamydomonas_eusti	37.00	0
TRINITY_ADE7736	unknown	Picea_sitchensis	37.00	0.00
TRINITY_XP_0030	predict	Micromonas_pusilla	37.00	0.00
TRINITY_XP_0216	autopha	Hevea_brasiliensis	37.00	0.00
TRINITY_XP_0017	predict	Chlamydomonas_reinh	36.90	0.00
TRINITY_XP_0213	transcr	Sorghum_bicolor	36.90	0.00
TRINITY_BAJ9782	predict	Hordeum_vulgare	36.90	0.00
TRINITY_XP_0056	Metallo-	Coccomyxa_subellips	36.90	0.00
TRINITY_XP_0238	eIF-2-a	Quercus_suber	36.90	0.00
TRINITY_KDD7648	sugar t	Helicosporidium_sp.	36.90	0.00
TRINITY_XP_0029	hypothet	Volvox_carteri	36.90	0.00
TRINITY_XP_0120	serine/t	Jatropha_curcas	36.90	0.00
TRINITY_KHN4337	G patch	Glycine_soja	36.90	0.00
TRINITY_OAE3257	hypothet	Marchantia_polymorp	36.90	0.00
TRINITY_GAQ9160	Beta-tu	Klebsormidium_niten	36.90	0.00
TRINITY_XP_0116	autopha	Amborella_trichopod	36.90	0.00
TRINITY_PRW5819	coatome	Chlorella_sorokinia	36.90	0.00
TRINITY_RCU6142	hypothet	Setaria_italica	36.90	0.00
TRINITY_ERN1786	hypothet	Amborella_trichopod	36.90	0.00
TRINITY_XP_0138	tRNA ur	Monoraphidium_negle	36.90	0.00
TRINITY_XP_0243	ABC tra	Physcomitrella_pate	36.90	0.00
TRINITY_XP_0172	PREDICT	Daucus_carota	36.90	0.00
TRINITY_PNW7452	hypothet	Chlamydomonas_reinh	36.90	0.00
TRINITY_OAE1880	hypothet	Marchantia_polymorp	36.90	0.00
TRINITY_RMZ5593	hypothet	Auxenochlorella_pro	36.90	0.00
TRINITY_XP_0243	uncharac	Physcomitrella_pate	36.90	0.00
TRINITY_XP_0105	PREDICT	Tarenaya_hasslerian	36.90	0.00
TRINITY_GAX7653	hypothet	Chlamydomonas_eusti	36.90	0.00
TRINITY_OMO5717	hypothet	Corchorus_capsulari	36.90	0.00
TRINITY_XP_0071	hypothet	Phaseolus_vulgaris	36.90	0.00
TRINITY_XP_0188	PREDICT	Juglans_regia	36.90	0.00
TRINITY_XP_0016	predict	Chlamydomonas_reinh	36.90	0.00
TRINITY_XP_0058	hypothet	Chlorella_variabili	36.90	0.00
TRINITY_XP_0105	PREDICT	Tarenaya_hasslerian	36.90	0.00
TRINITY_GAQ8054	hypothet	Klebsormidium_niten	36.90	0.00
TRINITY_PRW5969	ankyrin	Chlorella_sorokinia	36.90	0.00

TRINITY_PIA5043:	hypothet	Aquilegia_coerulea	36.90	0.00
TRINITY_XP_0023:	phospho(	Populus_trichocarpa	36.90	0.00
TRINITY_GAQ8293(	ABC tra	Klebsormidium_niten	36.90	0.00
TRINITY_GAX8391!	hypothet	Chlamydomonas_eusti	36.90	0.00
TRINITY_PTQ3485!	hypothet	Marchantia_polymorp	36.90	0.00
TRINITY_PNW7445!	hypothet	Chlamydomonas_reinh	36.90	0.00
TRINITY_GAX7688'	hypothet	Chlamydomonas_eusti	36.90	0.00
TRINITY_PNW7383(	hypothet	Chlamydomonas_reinh	36.90	0.00
TRINITY_GBG7843<	hypothet	Chara_braunii	36.90	0.00
TRINITY_PSS0845:	Asparti(	Actinidia_chinensis	36.90	0.00
TRINITY_XP_0058!	hypothet	Chlorella_variabili	36.90	0.00
TRINITY_XP_0029!	hypothet	Volvox_carteri	36.90	0.00
TRINITY_RWR7659(	serine/t	Cinnamomum_micranth	36.90	0.00
TRINITY_GAX8529(	hypothet	Chlamydomonas_eusti	36.90	0.00
TRINITY_XP_0237(	mitochor	Lactuca_sativa	36.90	0.00
TRINITY_XP_0098(	PREDICTE	Nicotiana_sylvestri	36.90	0.00
TRINITY_GAQ8432(	UDP-gala	Klebsormidium_niten	36.90	0.00
TRINITY_GAX7643'	hypothet	Chlamydomonas_eusti	36.90	0.00
TRINITY_XP_0030!	predicte	Micromonas_pusilla	36.90	0.00
TRINITY_XP_0025(	predicte	Micromonas_commoda	36.90	0.00
TRINITY_XP_0155(	unchara(	Ricinus_communis	36.90	0.00
TRINITY_GAX8597(	hypothet	Chlamydomonas_eusti	36.90	0.00
TRINITY_GAX8130!	hypothet	Chlamydomonas_eusti	36.90	0.00
TRINITY_GAX7400<	hypothet	Chlamydomonas_eusti	36.90	0.00
TRINITY_XP_0172:	PREDICTE	Daucus_carota	36.90	0.00
TRINITY_XP_0066!	PREDICTE	Oryza_brachyantha	36.90	0.00
TRINITY_XP_0244(	cleavage	Physcomitrella_pate	36.90	0.00
TRINITY_GAX7335(	hypothet	Chlamydomonas_eusti	36.90	0.00
TRINITY_OAE3037(	hypothet	Marchantia_polymorp	36.90	0.00
TRINITY_PNW7260(	hypothet	Chlamydomonas_reinh	36.90	0.00
TRINITY_KXZ4714(	hypothet	Gonium_pectorale	36.90	0.00
TRINITY_XP_0087(	luc7-lik	Phoenix_dactylifera	36.90	0.00
TRINITY_GAX7682<	hypothet	Chlamydomonas_eusti	36.90	0.00
TRINITY_PNW7545(	hypothet	Chlamydomonas_reinh	36.90	0.00
TRINITY_XP_0029!	hypothet	Volvox_carteri	36.90	0.00
TRINITY_GAX7970!	hypothet	Chlamydomonas_eusti	36.90	0.00
TRINITY_GAQ8755(	Leucyl-t	Klebsormidium_niten	36.90	0.00
TRINITY_GAX7878:	hypothet	Chlamydomonas_eusti	36.90	0.00
TRINITY_PNW7140!	hypothet	Chlamydomonas_reinh	36.90	0.00
TRINITY_GAX8569(	hypothet	Chlamydomonas_eusti	36.90	0.00
TRINITY_GAQ8253!	hypothet	Klebsormidium_niten	36.90	0.00
TRINITY_BAJ9457!	predicte	Hordeum_vulgare	36.90	0.00
TRINITY_PNH1141(	Mitogen-	Tetrabaena_socialis	36.90	0.00
TRINITY_XP_0029<	hypothet	Volvox_carteri	36.90	0.00
TRINITY_KXZ5048!	hypothet	Gonium_pectorale	36.90	0.00
TRINITY_GAX8028'	hypothet	Chlamydomonas_eusti	36.90	0.00
TRINITY_ONI1792:	hypothet	Prunus_persica	36.90	0.00
TRINITY_XP_0024<	ubiquit:	Sorghum_bicolor	36.90	0.00
TRINITY_XP_0035!	purine-u	Medicago_truncatula	36.80	0.00
TRINITY_AFG5609(	hypothet	Pinus_taeda	36.80	0.00
TRINITY_XP_0216<	ras-rela	Hevea_brasiliensis	36.80	0.00
TRINITY_XP_0016!	predicte	Chlamydomonas_reinh	36.80	0.00
TRINITY_GBF9349(	hypothet	Raphidocelis_subcap	36.80	0.00
TRINITY_XP_0097(	PREDICTE	Nicotiana_sylvestri	36.80	0.00
TRINITY_XP_0137:	importir	Brassica_napus	36.80	0.00
TRINITY_PON8174(	Phospho:	Trema_orientale	36.80	0.00
TRINITY_XP_0213:	rho GTPa	Sorghum_bicolor	36.80	0.00
TRINITY_XP_0137:	phosphat	Brassica_napus	36.80	0.00
TRINITY_GAQ8020:	protein	Klebsormidium_niten	36.80	0.00
TRINITY_XP_0017(	Snf1-lik	Chlamydomonas_reinh	36.80	0.00
TRINITY_GAQ7988:	hypothet	Klebsormidium_niten	36.80	0.00
TRINITY_XP_0165<	PREDICTE	Capsicum_annuum	36.80	0.00
TRINITY_XP_0164!	PREDICTE	Nicotiana_tabacum	36.80	0.00
TRINITY_PSS0299!	Recepto:	Actinidia_chinensis	36.80	0.00
TRINITY_OAE3474'	hypothet	Marchantia_polymorp	36.80	0.00
TRINITY_EPS5758(	hypothet	Genlisea_aurea	36.80	0.00

TRINITY_XP_0114(	PREDICTE	Fragaria_vesca	36.80	0.00
TRINITY_GAX7575	hypothet	Chlamydomonas_eusti	36.80	0.00
TRINITY_XP_0191(	PREDICTE	Ipomoea_nil	36.80	0.00
TRINITY_XP_0024	neo-calr	Sorghum_bicolor	36.80	0.00
TRINITY_GBG6166	hypothet	Chara_braunii	36.80	0.00
TRINITY_GAX8056	hypothet	Chlamydomonas_eusti	36.80	0.00
TRINITY_ABK2228	unknown	Picea_sitchensis	36.80	0.00
TRINITY_XP_0239	60S ribo	Quercus_suber	36.80	0.00
TRINITY_PSC6870	Transcri	Micractinium_conduc	36.80	0.00
TRINITY_XP_0029	hypothet	Volvox_carteri	36.80	0.00
TRINITY_GAX7770	hypothet	Chlamydomonas_eusti	36.80	0.00
TRINITY_RLN2903	transcri	Panicum_miliaceum	36.80	0.00
TRINITY_NP_0013	uncharac	Zea_mays	36.80	0.00
TRINITY_XP_0100	PREDICTE	Eucalyptus_grandis	36.80	0.00
TRINITY_XP_0030	protofil	Micromonas_pusilla	36.80	0.00
TRINITY_XP_0016	transcri	Chlamydomonas_reinh	36.80	0.00
TRINITY_RVW4277	Pumilio-	Vitis_vinifera	36.80	0.00
TRINITY_XP_0245	kinesin-	Selaginella_moellen	36.80	0.00
TRINITY_XP_0029	hypothet	Volvox_carteri	36.80	0.00
TRINITY_KXZ5665	hypothet	Gonium_pectorale	36.80	0.00
TRINITY_GAQ8691	Beta-tub	Klebsormidium_niten	36.80	0.00
TRINITY_GAX7659	hypothet	Chlamydomonas_eusti	36.80	0.00
TRINITY_GAX8018	hypothet	Chlamydomonas_eusti	36.80	0.00
TRINITY_KXZ4441	hypothet	Gonium_pectorale	36.80	0.00
TRINITY_EOY0131	CHASE do	Theobroma_cacao	36.80	0.00
TRINITY_PSC6774	E3 ubiqu	Micractinium_conduc	36.80	0.00
TRINITY_GAQ8348	phosphol	Klebsormidium_niten	36.80	0.00
TRINITY_GBG0030	sulfur s	Raphidocelis_subcap	36.80	0.00
TRINITY_GAX8258	hypothet	Chlamydomonas_eusti	36.80	0.00
TRINITY_XP_0219	cyclin-U	Carica_papaya	36.80	0.00
TRINITY_XP_0016	potentia	Chlamydomonas_reinh	36.80	0.00
TRINITY_PON7841	Zinc fin	Parasponia_anderson	36.80	0.00
TRINITY_QAB4729	CIPK [Ca	Camellia_sinensis	36.80	0.00
TRINITY_GBF9599	hypothet	Raphidocelis_subcap	36.80	0.00
TRINITY_PNW7464	hypothet	Chlamydomonas_reinh	36.80	0.00
TRINITY_XP_0056	guanine	Coccomyxa_subellips	36.80	0.00
TRINITY_XP_0029	hypothet	Volvox_carteri	36.80	0.00
TRINITY_AES6409	ABC tran	Medicago_truncatula	36.80	0.00
TRINITY_XP_0190	PREDICTE	Camelina_sativa	36.80	0.00
TRINITY_EEF2234	enteroch	Ricinus_communis	36.80	0.00
TRINITY_P0C8M8.1	RecName:	Zea_mays	36.80	0.00
TRINITY_XP_0029	hypothet	Volvox_carteri	36.80	0.00
TRINITY_XP_0094	PREDICTE	Musa_acuminata	36.80	0.00
TRINITY_XP_0096	PREDICTE	Nicotiana_tomentosi	36.80	0.00
TRINITY_GAQ8059	15-cis-z	Klebsormidium_niten	36.80	0.00
TRINITY_KXZ5603	hypothet	Gonium_pectorale	36.80	0.00
TRINITY_OAE2909	hypothet	Marchantia_polymorp	36.80	0.00
TRINITY_GAX8401	hypothet	Chlamydomonas_eusti	36.80	0.00
TRINITY_GAX7907	hypothet	Chlamydomonas_eusti	36.80	0.00
TRINITY_OTF8705	putative	Helianthus_annuus	36.80	0.00
TRINITY_PUZ4082	hypothet	Panicum_hallii	36.80	0.00
TRINITY_PNW8279	hypothet	Chlamydomonas_reinh	36.80	0.00
TRINITY_PNW8370	hypothet	Chlamydomonas_reinh	36.80	0.00
TRINITY_GAX7918	hypothet	Chlamydomonas_eusti	36.80	0.00
TRINITY_XP_0029	hypothet	Volvox_carteri	36.80	0.00
TRINITY_XP_0017	predicte	Chlamydomonas_reinh	36.80	0.00
TRINITY_GAX8583	hypothet	Chlamydomonas_eusti	36.80	0.00
TRINITY_XP_0091	PREDICTE	Brassica_rapa	36.80	0.00
TRINITY_GBG8650	hypothet	Chara_braunii	36.80	0.00
TRINITY_ACA2413	eukaryot	Lolium_temulentum	36.80	0.00
TRINITY_XP_0071	hypothet	Phaseolus_vulgaris	36.80	0.00
TRINITY_XP_0030	predicte	Micromonas_pusilla	36.80	0.00
TRINITY_XP_0243	serine/t	Physcomitrella_pate	36.80	0.00
TRINITY_GAQ8632	major fa	Klebsormidium_niten	36.70	0.00
TRINITY_GAV8615	Syntaxin	Cephalotus_follicul	36.70	0.00
TRINITY_XP_0249	phosphat	Ziziphus_jujuba	36.70	0.00

TRINITY_PNW8042	hypothet	Chlamydomonas_reinh	36.70	0.00
TRINITY_GAX8088	hypothet	Chlamydomonas_eusti	36.70	0.00
TRINITY_RQL7547	hypothet	Brassica_cretica	36.70	0.00
TRINITY_PLY9118	hypothet	Lactuca_sativa	36.70	0.00
TRINITY_XP_0094	PREDICT	Musa_acuminata	36.70	0.00
TRINITY_XP_0063	protein	Eutrema_salsugineum	36.70	0.00
TRINITY_XP_0239	ras-like	Quercus_suber	36.70	0.00
TRINITY_XP_0235	ran-bin	Cucurbita_pepo	36.70	0.00
TRINITY_XP_0101	mitochon	Morus_notabilis	36.70	0.00
TRINITY_XP_0200	26S prot	Ananas_comosus	36.70	0.00
TRINITY_GAQ7952	hypothet	Klebsormidium_niten	36.70	0.00
TRINITY_GAX8669	hypothet	Chlamydomonas_eusti	36.70	0.00
TRINITY_GAX8265	hypothet	Chlamydomonas_eusti	36.70	0.00
TRINITY_XP_0172	PREDICT	Daucus_carota	36.70	0.00
TRINITY_XP_0205	probable	Phalaenopsis_equest	36.70	0.00
TRINITY_PNW7668	hypothet	Chlamydomonas_reinh	36.70	0.00
TRINITY_XP_0217	FACT cor	Chenopodium_quinoa	36.70	0.00
TRINITY_XP_0217	uncharac	Chenopodium_quinoa	36.70	0.00
TRINITY_XP_0030	predicte	Micromonas_pusilla	36.70	0.00
TRINITY_XP_0159	serine/t	Arachis_duranensis	36.70	0.00
TRINITY_XP_0029	hypothet	Volvox_carteri	36.70	0.00
TRINITY_XP_0238	alpha-a	Quercus_suber	36.70	0.00
TRINITY_XP_0238	alpha-a	Quercus_suber	36.70	0.00
TRINITY_KDD7286	hypothet	Helicosporidium_sp.	36.70	0.00
TRINITY_XP_0056	hypothet	Coccomyxa_subellips	36.70	0.00
TRINITY_OUS4793	Amidinot	Ostreococcus_tauri	36.70	0.00
TRINITY_XP_0093	PREDICT	Musa_acuminata	36.70	0.00
TRINITY_PNY0848	U3 smal	Trifolium_pratense	36.70	0.00
TRINITY_GAX8156	hypothet	Chlamydomonas_eusti	36.70	0.00
TRINITY_XP_0245	serine/t	Selaginella_moellen	36.70	0.00
TRINITY_GAQ9350	hypothet	Klebsormidium_niten	36.70	0.00
TRINITY_XP_0202	probable	Asparagus_officinal	36.70	0.00
TRINITY_XP_0238	DNA mis	Quercus_suber	36.70	0.00
TRINITY_OAE3519	hypothet	Marchantia_polymorp	36.70	0.00
TRINITY_GAX7644	hypothet	Chlamydomonas_eusti	36.70	0.00
TRINITY_KCW8314	hypothet	Eucalyptus_grandis	36.70	0.00
TRINITY_GAX8125	hypothet	Chlamydomonas_eusti	36.70	0.00
TRINITY_XP_0075	predicte	Bathycoccus_prasinc	36.70	0.00
TRINITY_EFJ3625	hypothet	Selaginella_moellen	36.70	0.00
TRINITY_XP_0165	PREDICT	Capsicum_annuum	36.70	0.00
TRINITY_XP_0058	hypothet	Chlorella_variabili	36.70	0.00
TRINITY_XP_0243	guanylat	Physcomitrella_pate	36.70	0.00
TRINITY_XP_0138	dynein	Monoraphidium_negle	36.70	0.00
TRINITY_GAX8462	hypothet	Chlamydomonas_eusti	36.70	0.00
TRINITY_GAX7714	hypothet	Chlamydomonas_eusti	36.70	0.00
TRINITY_XP_0229	SWI/SNF	Cucurbita_moschata	36.70	0.00
TRINITY_XP_0058	hypothet	Chlorella_variabili	36.70	0.00
TRINITY_GAX7525	hypothet	Chlamydomonas_eusti	36.70	0.00
TRINITY_XP_0218	uncharac	Spinacia_oleracea	36.70	0.00
TRINITY_XP_0245	NFX1-ty	Selaginella_moellen	36.70	0.00
TRINITY_XP_0144	(+)-neor	Vigna_radiata	36.70	0.00
TRINITY_PNW7636	hypothet	Chlamydomonas_reinh	36.70	0.00
TRINITY_GAX7554	hypothet	Chlamydomonas_eusti	36.70	0.00
TRINITY_PNW7054	hypothet	Chlamydomonas_reinh	36.70	0.00
TRINITY_GBF9701	hypothet	Raphidocelis_subcap	36.70	0.00
TRINITY_XP_0239	protein	Quercus_suber	36.70	0.00
TRINITY_GAX7965	hypothet	Chlamydomonas_eusti	36.70	0.00
TRINITY_XP_0172	PREDICT	Daucus_carota	36.70	0.00
TRINITY_XP_0016	predicte	Chlamydomonas_reinh	36.70	0.00
TRINITY_GAX7924	hypothet	Chlamydomonas_eusti	36.70	0.00
TRINITY_XP_0016	predicte	Chlamydomonas_reinh	36.70	0.00
TRINITY_XP_0016	protein	Chlamydomonas_reinh	36.70	0.00
TRINITY_GAX7775	hypothet	Chlamydomonas_eusti	36.70	0.00
TRINITY_PSR8627	LEC14B	Actinidia_chinensis	36.70	0.00
TRINITY_GAX8019	hypothet	Chlamydomonas_eusti	36.70	0.00
TRINITY_XP_0016	predicte	Chlamydomonas_reinh	36.70	0.00

TRINITY_GAQ81304	hypothet	Klebsormidium_niten	36.60	0.00
TRINITY_XP_0241(	probable	Rosa_chinensis	36.60	0.00
TRINITY_XP_0238	probable	Quercus_suber	36.60	0.00
TRINITY_EEC7424	hypothet	Oryza_sativa	36.60	0.00
TRINITY_XP_0161	zinc fi	Arachis_ipaensis	36.60	0.00
TRINITY_PWA9360	histidi	Artemisia_annua	36.60	0.00
TRINITY_XP_0212	seven t	Herrania_umbratica	36.60	0.00
TRINITY_RWW9074	hypothet	Ensete_ventricosum	36.60	0.00
TRINITY_ABK2175(	unknown	Picea_sitchensis	36.60	0.00
TRINITY_XP_0191	PREDICT	Ipomoea_nil	36.60	0.00
TRINITY_XP_0219	ARM REP	Helianthus_annuus	36.60	0.00
TRINITY_XP_0239(	tRNA-spe	Quercus_suber	36.60	0.00
TRINITY_XP_0084	PREDICT	Cucumis_melo	36.60	0.00
TRINITY_RWR9065	Protein	Cinnamomum_micranth	36.60	0.00
TRINITY_GAX7609	hypothet	Chlamydomonas_eusti	36.60	0.00
TRINITY_XP_0095	PREDICT	Nicotiana_tomentosi	36.60	0.00
TRINITY_XP_0097	PREDICT	Nicotiana_sylvestri	36.60	0.00
TRINITY_OAE2666	hypothet	Marchantia_polymorp	36.60	0.00
TRINITY_GAQ9029	hypothet	Klebsormidium_niten	36.60	0.00
TRINITY_XP_0207(	V-type	Dendrobium_catenatu	36.60	0.00
TRINITY_GAQ8442	hypothet	Klebsormidium_niten	36.60	0.00
TRINITY_PRW5887(	kynureni	Chlorella_sorokinia	36.60	0.00
TRINITY_XP_0237	protein-	Lactuca_sativa	36.60	0.00
TRINITY_XP_0029	alkylate	Selaginella_moellen	36.60	0.00
TRINITY_GAX8451	hypothet	Chlamydomonas_eusti	36.60	0.00
TRINITY_RLN0008	E3 ubiq	Panicum_miliaceum	36.60	0.00
TRINITY_KMZ5849	Polyubi	Zostera_marina	36.60	0.00
TRINITY_PRW5955	anaphase	Chlorella_sorokinia	36.60	0.00
TRINITY_GAQ9325	E3 ubiq	Klebsormidium_niten	36.60	0.00
TRINITY_KXZ5655	hypothet	Gonium_pectorale	36.60	0.00
TRINITY_GAQ7773	Leishma	Klebsormidium_niten	36.60	0.00
TRINITY_XP_0237	uncharac	Lactuca_sativa	36.60	0.00
TRINITY_GBF9978	16S rRN	Raphidocelis_subcap	36.60	0.00
TRINITY_GAX8625	hypothet	Chlamydomonas_eusti	36.60	0.00
TRINITY_VDD6353	unnamed	Brassica_oleracea	36.60	0.00
TRINITY_RMZ5496	hypothet	Auxenochlorella_prc	36.60	0.00
TRINITY_XP_0106	PREDICT	Beta_vulgaris	36.60	0.00
TRINITY_XP_0202	(+)-neor	Cajanus_cajan	36.60	0.00
TRINITY_GAX7806(	hypothet	Chlamydomonas_eusti	36.60	0.00
TRINITY_GAX7557(	hypothet	Chlamydomonas_eusti	36.60	0.00
TRINITY_XP_0244(	transcr	Physcomitrella_pate	36.60	0.00
TRINITY_XP_0017(	predicte	Chlamydomonas_reinh	36.60	0.00
TRINITY_KXZ5130	hypothet	Gonium_pectorale	36.60	0.00
TRINITY_RLN3541	NADPH--	Panicum_miliaceum	36.60	0.00
TRINITY_GAX8484	hypothet	Chlamydomonas_eusti	36.60	0.00
TRINITY_PSS0170	Regulat	Actinidia_chinensis	36.60	0.00
TRINITY_NP_0013	eukaryot	Nicotiana_tabacum	36.60	0.00
TRINITY_XP_0191	PREDICT	Ipomoea_nil	36.60	0.00
TRINITY_GBF9101(	hypothet	Raphidocelis_subcap	36.60	0.00
TRINITY_KYP5151	Sugar p	Cajanus_cajan	36.60	0.00
TRINITY_GBF8909	hypothet	Raphidocelis_subcap	36.60	0.00
TRINITY_XP_0158	dnaJ ho	Ziziphus_jujuba	36.60	0.00
TRINITY_BAK0218	predicte	Hordeum_vulgare	36.60	0.00
TRINITY_RDX9877	Ethylene	Mucuna_pruriens	36.60	0.00
TRINITY_PTQ3871	hypothet	Marchantia_polymorp	36.60	0.00
TRINITY_GAQ7873	alpha-a	Klebsormidium_niten	36.60	0.00
TRINITY_EFJ3330	hypothet	Selaginella_moellen	36.60	0.00
TRINITY_XP_0029	hypothet	Volvox_carteri	36.60	0.00
TRINITY_XP_0114	PREDICT	Fragaria_vesca	36.60	0.00
TRINITY_GAU5086	hypothet	Trifolium_subterran	36.60	0.00
TRINITY_GAX8410	hypothet	Chlamydomonas_eusti	36.60	0.00
TRINITY_KXZ4609	hypothet	Gonium_pectorale	36.60	0.00
TRINITY_GAX7376	hypothet	Chlamydomonas_eusti	36.60	0.00
TRINITY_GAX8325	hypothet	Chlamydomonas_eusti	36.60	0.00
TRINITY_GAX8474(	hypothet	Chlamydomonas_eusti	36.60	0.00
TRINITY_KXZ5578	hypothet	Gonium_pectorale	36.60	0.00

TRINITY_PIA2542	hypothet	Aquilegia_coerulea	36.60	0.00
TRINITY_XP_0016	predicte	Chlamydomonas_reinh	36.60	0.00
TRINITY_XP_0016	predicte	Chlamydomonas_reinh	36.60	0.00
TRINITY_PNH1169	hypothet	Tetrabaena_socialis	36.60	0.00
TRINITY_XP_0110	cytochr	Sesamum_indicum	36.60	0.00
TRINITY_XP_0243	retinol	Physcomitrella_pate	36.60	0.00
TRINITY_XP_0063	enoyl-C	Capsella_rubella	36.50	0.00
TRINITY_XP_0216	alpha-g	Manihot_esculenta	36.50	0.00
TRINITY_XP_0075	predicte	Bathycoccus_prasinc	36.50	0.00
TRINITY_OMP1004	hypothet	Corchorus_olitorius	36.50	0.00
TRINITY_AFN8985	tyrosin	Rhodiola_crenulata	36.50	0.00
TRINITY_XP_0156	DNA-dam	Oryza_sativa	36.50	0.00
TRINITY_GAX8245	hypothet	Chlamydomonas_eusti	36.50	0.00
TRINITY_OMO9884	hypothet	Corchorus_capsulari	36.50	0.00
TRINITY_XP_0205	pre-mRN	Phalaenopsis_equest	36.50	0.00
TRINITY_XP_0243	uncharac	Physcomitrella_pate	36.50	0.00
TRINITY_RAL4622	hypothet	Cuscuta_australis	36.50	0.00
TRINITY_XP_0270	adenine	Coffea_arabica	36.50	0.00
TRINITY_PSC7090	pre-mRN	Micractinium_conduc	36.50	0.00
TRINITY_PNX9421	putative	Trifolium_pratense	36.50	0.00
TRINITY_PNW7717	hypothet	Chlamydomonas_reinh	36.50	0.00
TRINITY_XP_0191	PREDICT	Ipomoea_nil	36.50	0.00
TRINITY_GBG7470	hypothet	Chara_braunii	36.50	0.00
TRINITY_EMS5024	Nuclear	Triticum_urartu	36.50	0.00
TRINITY_GBG6155	hypothet	Chara_braunii	36.50	0.00
TRINITY_ADC4537	actin [	Cleistogenes_songor	36.50	0.00
TRINITY_XP_0110	PREDICT	Populus_euphratica	36.50	0.00
TRINITY_GBF9858	hypothet	Raphidocelis_subcap	36.50	0.00
TRINITY_XP_0243	syntaxi	Physcomitrella_pate	36.50	0.00
TRINITY_PTQ2901	hypothet	Marchantia_polymorp	36.50	0.00
TRINITY_XP_0220	mitogen-	Helianthus_annuus	36.50	0.00
TRINITY_RXI0625	hypothet	Malus_domestica	36.50	0.00
TRINITY_RVW2326	E3 ubiq	Vitis_vinifera	36.50	0.00
TRINITY_GAQ7994	Putative	Klebsormidium_niten	36.50	0.00
TRINITY_GAX7915	hypothet	Chlamydomonas_eusti	36.50	0.00
TRINITY_GAX8018	hypothet	Chlamydomonas_eusti	36.50	0.00
TRINITY_XP_0109	PREDICT	Elaeis_guineensis	36.50	0.00
TRINITY_KXZ5423	hypothet	Gonium_pectorale	36.50	0.00
TRINITY_XP_0062	2-(3-am	Capsella_rubella	36.50	0.00
TRINITY_KXZ4624	hypothet	Gonium_pectorale	36.50	0.00
TRINITY_XP_0056	Pkinase-	Coccomyxa_subellips	36.50	0.00
TRINITY_RWW9047	hypothet	Ensete_ventricosum	36.50	0.00
TRINITY_XP_0042	PREDICT	Fragaria_vesca	36.50	0.00
TRINITY_EFJ2274	hypothet	Selaginella_moellen	36.50	0.00
TRINITY_KMZ7634	putative	Zostera_marina	36.50	0.00
TRINITY_XP_0239	bifunct	Quercus_suber	36.50	0.00
TRINITY_EFJ1759	hypothet	Selaginella_moellen	36.50	0.00
TRINITY_PNW7441	hypothet	Chlamydomonas_reinh	36.50	0.00
TRINITY_XP_0042	pathoge	Solanum_lycopersicu	36.50	0.00
TRINITY_XP_0058	hypothet	Chlorella_variabili	36.50	0.00
TRINITY_PNW8754	hypothet	Chlamydomonas_reinh	36.50	0.00
TRINITY_XP_0029	hypothet	Volvox_carteri	36.50	0.00
TRINITY_PNH0455	hypothet	Tetrabaena_socialis	36.50	0.00
TRINITY_XP_0238	sodium/	Quercus_suber	36.50	0.00
TRINITY_PHT8407	hypothet	Capsicum_annuum	36.50	0.00
TRINITY_CDY5875	BnaCnng	Brassica_napus	36.50	0.00
TRINITY_EPS5790	mitogen-	Genlisea_aurea	36.50	0.00
TRINITY_XP_0240	serine/	Morus_notabilis	36.50	0.00
TRINITY_XP_0139	Unchara	Monoraphidium_negle	36.50	0.00
TRINITY_XP_0016	RWP-RK	Chlamydomonas_reinh	36.50	0.00
TRINITY_GAX7302	hypothet	Chlamydomonas_eusti	36.50	0.00
TRINITY_XP_0029	hypothet	Volvox_carteri	36.50	0.00
TRINITY_XP_0218	uncharac	Spinacia_oleracea	36.50	0.00
TRINITY_KXZ4132	hypothet	Gonium_pectorale	36.50	0.00
TRINITY_XP_0113	Protein	Auxenochlorella_prc	36.50	0.00
TRINITY_XP_0056	hypothet	Coccomyxa_subellips	36.50	0.00

TRINITY_GAX84137	hypothet	Chlamydomonas_eusti	36.50	0.00
TRINITY_PNW77527	hypothet	Chlamydomonas_reinh	36.50	0.00
TRINITY_VDD45001	unnamed	Brassica_oleracea	36.50	0.00
TRINITY_GAX78754	hypothet	Chlamydomonas_eusti	36.50	0.00
TRINITY_XP_00298	E3 ubiqu	Selaginella_moellen	36.50	0.00
TRINITY_GBF94337	hypothet	Raphidocelis_subcap	36.50	0.00
TRINITY_PWA87491	acyl-CoA	Artemisia_annua	36.50	0.00
TRINITY_GAQ87785	ER-Golgi	Klebsormidium_niten	36.50	0.00
TRINITY_KXZ55647	hypothet	Gonium_pectorale	36.50	0.00
TRINITY_XP_01003	PREDICTE	Eucalyptus_grandis	36.40	0.00
TRINITY_XP_02641	elicitor	Papaver_somniferum	36.40	0.00
TRINITY_XP_00251	purine-u	Ricinus_communis	36.40	0.00
TRINITY_XP_00565	hypothet	Coccomyxa_subellips	36.40	0.00
TRINITY_OAE31917	hypothet	Marchantia_polymorp	36.40	0.00
TRINITY_XP_01140	PREDICTE	Fragaria_vesca	36.40	0.00
TRINITY_PHT35692	hypothet	Capsicum_baccatum	36.40	0.00
TRINITY_XP_02445	E3 ubiqu	Populus_trichocarpa	36.40	0.00
TRINITY_ERM98344	hypothet	Amborella_trichopod	36.40	0.00
TRINITY_XP_00305	predicte	Micromonas_pusilla	36.40	0.00
TRINITY_OIW15361	hypothet	Lupinus_angustifoli	36.40	0.00
TRINITY_XP_02452	phosphat	Selaginella_moellen	36.40	0.00
TRINITY_XP_00244	nuclear	Sorghum_bicolor	36.40	0.00
TRINITY_GAQ80764	hypothet	Klebsormidium_niten	36.40	0.00
TRINITY_XP_02392	anthrani	Quercus_suber	36.40	0.00
TRINITY_PSC73006	diaminop	Micractinium_conduc	36.40	0.00
TRINITY_XP_00295	hypothet	Volvox_carteri	36.40	0.00
TRINITY_XP_02792	putative	Vigna_unguiculata	36.40	0.00
TRINITY_XP_00297	ESCRT-re	Selaginella_moellen	36.40	0.00
TRINITY_XP_00142	predicte	Ostreococcus_sp._'l	36.40	0.00
TRINITY_GAX85947	hypothet	Chlamydomonas_eusti	36.40	0.00
TRINITY_AXF41554	CCP430 p	Chlorella_sp._ArM00	36.40	0.00
TRINITY_XP_02067	ras-rela	Dendrobium_catenatu	36.40	0.00
TRINITY_GAQ82363	Telomere	Klebsormidium_niten	36.40	0.00
TRINITY_RWW91059	hypothet	Ensete_ventricosum	36.40	0.00
TRINITY_GAQ84765	hypothet	Klebsormidium_niten	36.40	0.00
TRINITY_XP_00721	low-temp	Prunus_persica	36.40	0.00
TRINITY_PWA41598	BRCT dom	Artemisia_annua	36.40	0.00
TRINITY_XP_01654	PREDICTE	Capsicum_annuum	36.40	0.00
TRINITY_XP_02390	probable	Quercus_suber	36.40	0.00
TRINITY_GAQ78212	exopolyp	Klebsormidium_niten	36.40	0.00
TRINITY_XP_00168	predicte	Chlamydomonas_reinh	36.40	0.00
TRINITY_GAX81320	hypothet	Chlamydomonas_eusti	36.40	0.00
TRINITY_XP_02296	diphosph	Cucurbita_maxima	36.40	0.00
TRINITY_XP_02284	UBA-like	Ostreococcus_tauri	36.40	0.00
TRINITY_GBF93240	hypothet	Raphidocelis_subcap	36.40	0.00
TRINITY_RLN24754	hypothet	Panicum_miliaceum	36.40	0.00
TRINITY_GAX76911	hypothet	Chlamydomonas_eusti	36.40	0.00
TRINITY_XP_02392	mitochon	Quercus_suber	36.40	0.00
TRINITY_XP_00564	MFS gene	Coccomyxa_subellips	36.40	0.00
TRINITY_XP_02058	vacuolar	Phalaenopsis_equest	36.40	0.00
TRINITY_KXZ54883	hypothet	Gonium_pectorale	36.40	0.00
TRINITY_XP_02390	pumilio	Quercus_suber	36.40	0.00
TRINITY_AML78291	putative	Pleurastrum_insigne	36.40	0.00
TRINITY_PIA50207	hypothet	Aquilegia_coerulea	36.40	0.00
TRINITY_GAX77500	hypothet	Chlamydomonas_eusti	36.40	0.00
TRINITY_XP_02707	KIN17-li	Coffea_arabica	36.40	0.00
TRINITY_BAJ94121	predicte	Hordeum_vulgare	36.40	0.00
TRINITY_KXZ50009	hypothet	Gonium_pectorale	36.40	0.00
TRINITY_XP_01047	PREDICTE	Camelina_sativa	36.40	0.00
TRINITY_XP_02388	serine/t	Quercus_suber	36.40	0.00
TRINITY_P0C8M8.1	RecName:	Zea_mays	36.40	0.00
TRINITY_EFJ27865	hypothet	Selaginella_moellen	36.40	0.00
TRINITY_XP_01054	PREDICTE	Tarenaya_hasslerian	36.40	0.00
TRINITY_XP_01674	PREDICTE	Gossypium_hirsutum	36.40	0.00
TRINITY_XP_00169	hydroxyp	Chlamydomonas_reinh	36.40	0.00
TRINITY_PNH04613	Inositol	Tetrabaena_socialis	36.40	0.00

TRINITY_XP_0238	protein	Quercus_suber	36.40	0.00
TRINITY_GBG7039	hypothet	Chara_braunii	36.40	0.00
TRINITY_GAX7958	hypothet	Chlamydomonas_eusti	36.40	0.00
TRINITY_XP_0238	probable	Quercus_suber	36.40	0.00
TRINITY_KXZ4729	hypothet	Gonium_pectorale	36.40	0.00
TRINITY_PIN0277	hypothet	Handroanthus_impeti	36.40	0.00
TRINITY_XP_0217	DNA mism	Chenopodium_quinoa	36.40	0.00
TRINITY_XP_0028	uncharac	Arabidopsis_lyrata	36.40	0.00
TRINITY_XP_0025	calcineu	Micromonas_commoda	36.40	0.00
TRINITY_XP_0093	PREDICTE	Pyrus_x_bretschneid	36.40	0.00
TRINITY_GAX7968	hypothet	Chlamydomonas_eusti	36.40	0.00
TRINITY_KXZ4879	hypothet	Gonium_pectorale	36.40	0.00
TRINITY_KXZ5109	hypothet	Gonium_pectorale	36.40	0.00
TRINITY_PSR9879	Charged	Actinidia_chinensis	36.40	0.00
TRINITY_XP_0244	ESF1 hom	Physcomitrella_pate	36.40	0.00
TRINITY_XP_0238	respirat	Quercus_suber	36.40	0.00
TRINITY_KXZ4437	hypothet	Gonium_pectorale	36.40	0.00
TRINITY_GAQ8682	Leishman	Klebsormidium_niten	36.40	0.00
TRINITY_AJN0522	serine t	Piper_colubrinum	36.40	0.00
TRINITY_GAQ7999	Mediator	Klebsormidium_niten	36.40	0.00
TRINITY_GAX8144	hypothet	Chlamydomonas_eusti	36.40	0.00
TRINITY_GAX7960	hypothet	Chlamydomonas_eusti	36.40	0.00
TRINITY_OMO6141	hypothet	Corchorus_capsulari	36.40	0.00
TRINITY_PNW7501	hypothet	Chlamydomonas_reinh	36.40	0.00
TRINITY_GAX7747	hypothet	Chlamydomonas_eusti	36.40	0.00
TRINITY_GAX8345	hypothet	Chlamydomonas_eusti	36.40	0.00
TRINITY_PNW7136	hypothet	Chlamydomonas_reinh	36.40	0.00
TRINITY_XP_0017	predicte	Chlamydomonas_reinh	36.40	0.00
TRINITY_XP_0016	predicte	Chlamydomonas_reinh	36.40	0.00
TRINITY_XP_0029	hypothet	Volvox_carteri	36.40	0.00
TRINITY_GAX7367	hypothet	Chlamydomonas_eusti	36.40	0.00
TRINITY_XP_0241	zinc fin	Rosa_chinensis	36.40	0.00
TRINITY_PNH1024	Chaperon	Tetrabaena_socialis	36.40	0.00
TRINITY_PNW8649	hypothet	Chlamydomonas_reinh	36.40	0.00
TRINITY_XP_0056	hypothet	Coccomyxa_subellips	36.40	0.00
TRINITY_XP_0091	PREDICTE	Brassica_rapa	36.40	0.00
TRINITY_GAX8254	hypothet	Chlamydomonas_eusti	36.40	0.00
TRINITY_EFH5576	multidru	Arabidopsis_lyrata	36.40	0.00
TRINITY_XP_0029	hypothet	Volvox_carteri	36.40	0.00
TRINITY_XP_0139	cytochro	Monoraphidium_negle	36.40	0.00
TRINITY_PNW7938	hypothet	Chlamydomonas_reinh	36.40	0.00
TRINITY_XP_0172	PREDICTE	Daucus_carota	36.40	0.00
TRINITY_KFK4213	hypothet	Arabis_alpina	36.40	0.00
TRINITY_GAX7735	hypothet	Chlamydomonas_eusti	36.40	0.00
TRINITY_GAX8505	hypothet	Chlamydomonas_eusti	36.40	0.00
TRINITY_XP_0029	hypothet	Volvox_carteri	36.40	0.00
TRINITY_XP_0029	guanylyl	Volvox_carteri	36.40	0.00
TRINITY_XP_0105	PREDICTE	Camelina_sativa	36.40	0.00
TRINITY_EXB7493	Histidin	Morus_notabilis	36.40	0.00
TRINITY_RXH9446	hypothet	Malus_domestica	36.40	0.00
TRINITY_XP_0239	carrier	Quercus_suber	36.30	0.00
TRINITY_PTQ2712	hypothet	Marchantia_polymorp	36.30	0.00
TRINITY_XP_0017	protein	Chlamydomonas_reinh	36.30	0.00
TRINITY_XP_0056	mitochon	Coccomyxa_subellips	36.30	0.00
TRINITY_CAA9007	small G	Pisum_sativum	36.30	0.00
TRINITY_KXZ4873	hypothet	Gonium_pectorale	36.30	0.00
TRINITY_XP_0030	predicte	Micromonas_pusilla	36.30	0.00
TRINITY_GAX7617	hypothet	Chlamydomonas_eusti	36.30	0.00
TRINITY_XP_0147	mitochon	Brachypodium_distac	36.30	0.00
TRINITY_XP_0228	probable	Olea_europaea	36.30	0.00
TRINITY_ADE7742	unknown	Picea_sitchensis	36.30	0.00
TRINITY_XP_0202	LON pept	Asparagus_officinal	36.30	0.00
TRINITY_XP_0239	protein	Quercus_suber	36.30	0.00
TRINITY_BAK0138	predicte	Hordeum_vulgare	36.30	0.00
TRINITY_XP_0025	flagella	Micromonas_commoda	36.30	0.00
TRINITY_GAX8070	hypothet	Chlamydomonas_eusti	36.30	0.00

TRINITY_RAL5435(	hypothet	Cuscuta_australis	36.30	0.00
TRINITY_XP_0014:	predicte	Ostreococcus_sp._'l	36.30	0.00
TRINITY_RYR7147!	hypothet	Arachis_hypogaea	36.30	0.00
TRINITY_GAQ7771:	HECT dor	Klebsormidium_niten	36.30	0.00
TRINITY_RID7762!	hypothet	Brassica_rapa	36.30	0.00
TRINITY_XP_0025(	formate-	Micromonas_commoda	36.30	0.00
TRINITY_PLY8512:	hypothet	Lactuca_sativa	36.30	0.00
TRINITY_XP_0243!	putative	Physcomitrella_pate	36.30	0.00
TRINITY_RYQ9573·	hypothet	Arachis_hypogaea	36.30	0.00
TRINITY_XP_0239(	probable	Quercus_suber	36.30	0.00
TRINITY_GAX7335	hypothet	Chlamydomonas_eusti	36.30	0.00
TRINITY_XP_0025(	predicte	Micromonas_commoda	36.30	0.00
TRINITY_XP_0205!	periodic	Phalaenopsis_equest	36.30	0.00
TRINITY_XP_0025(	hypothet	Micromonas_commoda	36.30	0.00
TRINITY_XP_0264!	poly [AI	Papaver_somniferum	36.30	0.00
TRINITY_XP_0239:	ran-binc	Quercus_suber	36.30	0.00
TRINITY_XP_0245:	WD repea	Selaginella_moellen	36.30	0.00
TRINITY_RWR8613:	methylst	Cinnamomum_micranth	36.30	0.00
TRINITY_XP_0016!	fumarate	Chlamydomonas_reinh	36.30	0.00
TRINITY_PNH1169!	hypothet	Tetrabaena_socialis	36.30	0.00
TRINITY_GAQ7949(	long-acy	Klebsormidium_niten	36.30	0.00
TRINITY_XP_0202:	unchara(	Cajanus_cajan	36.30	0.00
TRINITY_XP_0128:	PREDICTI	Erythranthe_guttata	36.30	0.00
TRINITY_PNH1233!	hypothet	Tetrabaena_socialis	36.30	0.00
TRINITY_XP_0025(	predicte	Micromonas_commoda	36.30	0.00
TRINITY_XP_0029·	hypothet	Volvox_carteri	36.30	0.00
TRINITY_PTQ3206	hypothet	Marchantia_polymorp	36.30	0.00
TRINITY_OVA0100·	WD40 rep	Macleaya_cordata	36.30	0.00
TRINITY_KXZ5594:	hypothet	Gonium_pectorale	36.30	0.00
TRINITY_XP_0030!	glutamir	Micromonas_pusilla	36.30	0.00
TRINITY_XP_0139(	hypothet	Monoraphidium_negle	36.30	0.00
TRINITY_KXZ5153	hypothet	Gonium_pectorale	36.30	0.00
TRINITY_GAX7787:	hypothet	Chlamydomonas_eusti	36.30	0.00
TRINITY_GAX7868:	hypothet	Chlamydomonas_eusti	36.30	0.00
TRINITY_GAX7272:	hypothet	Chlamydomonas_eusti	36.30	0.00
TRINITY_GAX7561(	hypothet	Chlamydomonas_eusti	36.30	0.00
TRINITY_GBF9083:	apyrase-	Raphidocelis_subcap	36.30	0.00
TRINITY_XP_0220:	ubiquit:	Helianthus_annuus	36.30	0.00
TRINITY_XP_0110	mediato:	Sesamum_indicum	36.30	0.00
TRINITY_PRW3908:	putative	Chlorella_sorokinia	36.30	0.00
TRINITY_GAX8431(	hypothet	Chlamydomonas_eusti	36.30	0.00
TRINITY_PNW8057:	hypothet	Chlamydomonas_reinh	36.30	0.00
TRINITY_XP_0239:	coronin-	Quercus_suber	36.30	0.00
TRINITY_GAX7666!	hypothet	Chlamydomonas_eusti	36.30	0.00
TRINITY_XP_0058·	hypothet	Chlorella_variabili	36.30	0.00
TRINITY_KXZ4705!	hypothet	Gonium_pectorale	36.30	0.00
TRINITY_XP_0138!	GPI ino:	Monoraphidium_negle	36.30	0.00
TRINITY_XP_0138!	hypothet	Monoraphidium_negle	36.30	0.00
TRINITY_PRW5599	heat shc	Chlorella_sorokinia	36.30	0.00
TRINITY_XP_0144!	ABC trar	Vigna_radiata	36.30	0.00
TRINITY_XP_0058·	hypothet	Chlorella_variabili	36.30	0.00
TRINITY_XP_0273:	C2 doma:	Abrus_precatorius	36.20	0.00
TRINITY_ABK2164!	unknown	Picea_sitchensis	36.20	0.00
TRINITY_KXZ4981·	hypothet	Gonium_pectorale	36.20	0.00
TRINITY_XP_0042!	PREDICTI	Fragaria_vesca	36.20	0.00
TRINITY_XP_0043(	PREDICTI	Fragaria_vesca	36.20	0.00
TRINITY_PNW8078!	hypothet	Chlamydomonas_reinh	36.20	0.00
TRINITY_OWM8558(	hypothet	Punica_granatum	36.20	0.00
TRINITY_XP_0120	pre-rRNA	Jatropha_curcas	36.20	0.00
TRINITY_XP_0271:	mitochor	Coffea_arabica	36.20	0.00
TRINITY_XP_0075:	predicte	Bathycoccus_prasinc	36.20	0.00
TRINITY_XP_0156!	PREDICTI	Oryza_brachyantha	36.20	0.00
TRINITY_GAQ8731!	F-box ar	Klebsormidium_niten	36.20	0.00
TRINITY_XP_0030!	predicte	Micromonas_pusilla	36.20	0.00
TRINITY_PNR3642:	hypothet	Physcomitrella_pate	36.20	0.00
TRINITY_GAQ8841:	Phosphat	Klebsormidium_niten	36.20	0.00

TRINITY_XP_02162	uncharac	Manihot_esculenta	36.20	0.00
TRINITY_XP_00229	ribosoma	Populus_trichocarpa	36.20	0.00
TRINITY_GAQ87481	putative	Klebsormidium_niten	36.20	0.00
TRINITY_GAQ85231	hypothet	Klebsormidium_niten	36.20	0.00
TRINITY_EEC83485	hypothet	Oryza_sativa	36.20	0.00
TRINITY_XP_01139	Gluconat	Auxenochlorella_pro	36.20	0.00
TRINITY_PNW83851	hypothet	Chlamydomonas_reinh	36.20	0.00
TRINITY_RAL50799	hypothet	Cuscuta_australis	36.20	0.00
TRINITY_KXZ40924	hypothet	Gonium_pectorale	36.20	0.00
TRINITY_XP_00250	predicte	Micromonas_commoda	36.20	0.00
TRINITY_PNH04420	Heme-bin	Tetrabaena_socialis	36.20	0.00
TRINITY_XP_01068	PREDICTE	Beta_vulgaris	36.20	0.00
TRINITY_XP_00228	PREDICTE	Vitis_vinifera	36.20	0.00
TRINITY_XP_00294	hypothet	Volvox_carteri	36.20	0.00
TRINITY_XP_00414	PREDICTE	Cucumis_sativus	36.20	0.00
TRINITY_PSC71593	tubulin-	Micractinium_conduc	36.20	0.00
TRINITY_XP_00451	serine c	Cicer_arietinum	36.20	0.00
TRINITY_XP_02437	acyl-coe	Physcomitrella_pate	36.20	0.00
TRINITY_GBF99212	hypothet	Raphidocelis_subcap	36.20	0.00
TRINITY_XP_02388	probable	Quercus_suber	36.20	0.00
TRINITY_PTQ27799	hypothet	Marchantia_polymorp	36.20	0.00
TRINITY_PRW58839	L-cystei	Chlorella_sorokinia	36.20	0.00
TRINITY_XP_01044	PREDICTE	Camelina_sativa	36.20	0.00
TRINITY_XP_00230	probable	Populus_trichocarpa	36.20	0.00
TRINITY_GBF95018	hypothet	Raphidocelis_subcap	36.20	0.00
TRINITY_XP_00295	hypothet	Volvox_carteri	36.20	0.00
TRINITY_GAQ91376	L-aminoa	Klebsormidium_niten	36.20	0.00
TRINITY_PSR99715	COP9 sig	Actinidia_chinensis	36.20	0.00
TRINITY_XP_02437	DNA repa	Physcomitrella_pate	36.20	0.00
TRINITY_XP_00142	predicte	Ostreococcus_sp._'l	36.20	0.00
TRINITY_XP_00430	PREDICTE	Fragaria_vesca	36.20	0.00
TRINITY_XP_00295	hypothet	Volvox_carteri	36.20	0.00
TRINITY_EOY06934	ATP bind	Theobroma_cacao	36.20	0.00
TRINITY_ACH87585	hypothet	Dunaliella_viridis	36.20	0.00
TRINITY_GAX79090	hypothet	Chlamydomonas_eusti	36.20	0.00
TRINITY_GAQ89752	hypothet	Klebsormidium_niten	36.20	0.00
TRINITY_XP_00141	predicte	Ostreococcus_sp._'l	36.20	0.00
TRINITY_PNW84401	hypothet	Chlamydomonas_reinh	36.20	0.00
TRINITY_XP_00295	hypothet	Volvox_carteri	36.20	0.00
TRINITY_BAJ98875	predicte	Hordeum_vulgare	36.20	0.00
TRINITY_KXZ46180	hypothet	Gonium_pectorale	36.20	0.00
TRINITY_XP_02451	tubulin	Selaginella_moellen	36.20	0.00
TRINITY_GAX84841	hypothet	Chlamydomonas_eusti	36.20	0.00
TRINITY_KXZ53106	hypothet	Gonium_pectorale	36.20	0.00
TRINITY_BAJ94575	predicte	Hordeum_vulgare	36.20	0.00
TRINITY_GBF92152	peptidyl	Raphidocelis_subcap	36.20	0.00
TRINITY_XP_01847	PREDICTE	Raphanus_sativus	36.20	0.00
TRINITY_GAX75360	hypothet	Chlamydomonas_eusti	36.20	0.00
TRINITY_XP_00294	hypothet	Volvox_carteri	36.20	0.00
TRINITY_XP_02437	nucleola	Physcomitrella_pate	36.20	0.00
TRINITY_GAX77971	hypothet	Chlamydomonas_eusti	36.20	0.00
TRINITY_GBG71494	hypothet	Chara_braunii	36.20	0.00
TRINITY_GBF95931	hypothet	Raphidocelis_subcap	36.20	0.00
TRINITY_KXZ54601	hypothet	Gonium_pectorale	36.20	0.00
TRINITY_XP_00170	predicte	Chlamydomonas_reinh	36.20	0.00
TRINITY_GAX78301	hypothet	Chlamydomonas_eusti	36.20	0.00
TRINITY_XP_01941	PREDICTE	Lupinus_angustifoli	36.20	0.00
TRINITY_GAQ81618	ABC tran	Klebsormidium_niten	36.20	0.00
TRINITY_GAX85402	hypothet	Chlamydomonas_eusti	36.20	0.00
TRINITY_PIA31617	hypothet	Aquilegia_coerulea	36.20	0.00
TRINITY_GAX79901	hypothet	Chlamydomonas_eusti	36.10	0.00
TRINITY_XP_01284	PREDICTE	Erythranthe_guttata	36.10	0.00
TRINITY_XP_01365	E3 ubiqu	Brassica_napus	36.10	0.00
TRINITY_EFJ10024	hypothet	Selaginella_moellen	36.10	0.00
TRINITY_XP_01166	PREDICTE	Cucumis_sativus	36.10	0.00
TRINITY_XP_00250	predicte	Micromonas_commoda	36.10	0.00

TRINITY_XP_0042	protein	Solanum_lycopersicu	36.10	0.00
TRINITY_CDY5875	BnaCnng	Brassica_napus	36.10	0.00
TRINITY_GBG6140	hypothet	Chara_braunii	36.10	0.00
TRINITY_XP_0229	phosphat	Cucurbita_maxima	36.10	0.00
TRINITY_RYQ9844	hypothet	Arachis_hypogaea	36.10	0.00
TRINITY_XP_0030	predicte	Micromonas_pusilla	36.10	0.00
TRINITY_XP_0025	hypothet	Micromonas_commoda	36.10	0.00
TRINITY_NP_0013	uncharac	Zea_mays	36.10	0.00
TRINITY_XP_0030	mitochor	Micromonas_pusilla	36.10	0.00
TRINITY_KDD7327	hypothet	Helicosporidium_sp.	36.10	0.00
TRINITY_RAL4722	hypothet	Cuscuta_australis	36.10	0.00
TRINITY_XP_0239	probable	Quercus_suber	36.10	0.00
TRINITY_XP_0017	predicte	Chlamydomonas_reinh	36.10	0.00
TRINITY_PSC7166	Pre-mRN	Micractinium_conduc	36.10	0.00
TRINITY_GAX8096	hypothet	Chlamydomonas_eusti	36.10	0.00
TRINITY_XP_0042	phospho	Solanum_lycopersicu	36.10	0.00
TRINITY_XP_0071	hypothet	Phaseolus_vulgaris	36.10	0.00
TRINITY_XP_0238	repress	Quercus_suber	36.10	0.00
TRINITY_XP_0202	heptahe	Aegilops_tauschii	36.10	0.00
TRINITY_PTQ4114	hypothet	Marchantia_polymorp	36.10	0.00
TRINITY_OUS4793	Amidinot	Ostreococcus_tauri	36.10	0.00
TRINITY_XP_0056	hypothet	Coccomyxa_subellips	36.10	0.00
TRINITY_KXZ4321	hypothet	Gonium_pectorale	36.10	0.00
TRINITY_PRW2090	Oxidore	Chlorella_sorokinia	36.10	0.00
TRINITY_KZV1512	hypothet	Dorcoceras_hygromet	36.10	0.00
TRINITY_PNW7536	hypothet	Chlamydomonas_reinh	36.10	0.00
TRINITY_XP_0245	serine/t	Selaginella_moellen	36.10	0.00
TRINITY_XP_0025	dynein	Micromonas_commoda	36.10	0.00
TRINITY_XP_0238	uncharac	Quercus_suber	36.10	0.00
TRINITY_XP_0243	dihydro	Physcomitrella_pate	36.10	0.00
TRINITY_XP_0017	predicte	Chlamydomonas_reinh	36.10	0.00
TRINITY_XP_0016	predicte	Chlamydomonas_reinh	36.10	0.00
TRINITY_XP_0228	pectin a	Olea_europaea	36.10	0.00
TRINITY_BAK0240	predicte	Hordeum_vulgare	36.10	0.00
TRINITY_XP_0238	phosphat	Quercus_suber	36.10	0.00
TRINITY_XP_0056	AAA-ATPa	Coccomyxa_subellips	36.10	0.00
TRINITY_AQK6524	hAT trar	Zea_mays	36.10	0.00
TRINITY_XP_0063	PREDICTE	Solanum_tuberosum	36.10	0.00
TRINITY_RDY1141	Wee1-lik	Mucuna_pruriens	36.10	0.00
TRINITY_XP_0105	PREDICTE	Tarenaya_hasslerian	36.10	0.00
TRINITY_KXZ4544	hypothet	Gonium_pectorale	36.10	0.00
TRINITY_PSC7145	CHY zinc	Micractinium_conduc	36.10	0.00
TRINITY_XP_0215	serine/t	Manihot_esculenta	36.10	0.00
TRINITY_KXZ5638	hypothet	Gonium_pectorale	36.10	0.00
TRINITY_XP_0058	hypothet	Chlorella_variabili	36.10	0.00
TRINITY_PON7458	Knotted	Parasponia_anderson	36.10	0.00
TRINITY_XP_0029	hypothet	Volvox_carteri	36.10	0.00
TRINITY_XP_0029	hypothet	Volvox_carteri	36.10	0.00
TRINITY_KXZ5410	hypothet	Gonium_pectorale	36.10	0.00
TRINITY_XP_0064	uncharac	Citrus_sinensis	36.10	0.00
TRINITY_GAQ8826	mercapto	Klebsormidium_niten	36.10	0.00
TRINITY_GAX7924	hypothet	Chlamydomonas_eusti	36.10	0.00
TRINITY_PRW5923	nucleol	Chlorella_sorokinia	36.10	0.00
TRINITY_PNW7758	hypothet	Chlamydomonas_reinh	36.10	0.00
TRINITY_XP_0063	ubiquit	Eutrema_salsugineum	36.10	0.00
TRINITY_GAQ9054	Ubiquit	Klebsormidium_niten	36.10	0.00
TRINITY_PNX7611	calcium-	Trifolium_pratense	36.10	0.00
TRINITY_XP_0029	hypothet	Volvox_carteri	36.10	0.00
TRINITY_KXZ4546	hypothet	Gonium_pectorale	36.10	0.00
TRINITY_GAX7714	hypothet	Chlamydomonas_eusti	36.10	0.00
TRINITY_XP_0041	PREDICTE	Cucumis_sativus	36.10	0.00
TRINITY_EFJ1301	hypothet	Selaginella_moellen	36.10	0.00
TRINITY_XP_0109	PREDICTE	Elaeis_guineensis	36.00	0.00
TRINITY_XP_0239	vacuolar	Quercus_suber	36.00	0.00
TRINITY_XP_0244	calpain-	Physcomitrella_pate	36.00	0.00
TRINITY_XP_0025	predicte	Micromonas_commoda	36.00	0.00

TRINITY_PRW6018	DNA repa	Chlorella_sorokinia	36.00	0.00
TRINITY_XP_0176	PREDICTI	Gossypium_arboreum	36.00	0.00
TRINITY_OAE3353	hypothet	Marchantia_polymorp	36.00	0.00
TRINITY_KMZ7395	putative	Zostera_marina	36.00	0.00
TRINITY_RWR7314	ubiquit	Cinnamomum_micranth	36.00	0.00
TRINITY_XP_0070	PREDICTI	Theobroma_cacao	36.00	0.00
TRINITY_XP_0124	PREDICTI	Gossypium_raimondii	36.00	0.00
TRINITY_OAE2007	hypothet	Marchantia_polymorp	36.00	0.00
TRINITY_XP_0016	predicte	Chlamydomonas_reinh	36.00	0.00
TRINITY_XP_0239	peroxide	Quercus_suber	36.00	0.00

TRINITY_XP_0239(	LOW QUAI	Quercus_suber	36.00	0.00
TRINITY_XP_0243(	solute (	Physcomitrella_pate	36.00	0.00
TRINITY_XP_0239]	protein	Quercus_suber	36.00	0.00
TRINITY_XP_0102'	PREDICTI	Nelumbo_nucifera	36.00	0.00
TRINITY_XP_0102!	PREDICTI	Nelumbo_nucifera	36.00	0.00
TRINITY_OWM8465(	hypothet	Punica_granatum	36.00	0.00
TRINITY_XP_0264!	CST comp	Papaver_somniferum	36.00	0.00
TRINITY_GAQ8734!	delta14-	Klebsormidium_niten	36.00	0.00
TRINITY_XP_0024!	unchara(	Sorghum_bicolor	36.00	0.00
TRINITY_EFJ0677]	hypothet	Selaginella_moellen	36.00	0.00
TRINITY_ONK6653(	unchara(	Asparagus_officinal	36.00	0.00
TRINITY_GAX7771(	hypothet	Chlamydomonas_eusti	36.00	0.00
TRINITY_XP_0114(	PREDICTI	Fragaria_vesca	36.00	0.00
TRINITY_XP_0016!	predicte	Chlamydomonas_reinh	36.00	0.00
TRINITY_PIA4735(	hypothet	Aquilegia_coerulea	36.00	0.00
TRINITY_PHT3327!	Amino-a(	Capsicum_baccatum	36.00	0.00
TRINITY_GAX7916(	hypothet	Chlamydomonas_eusti	36.00	0.00
TRINITY_RHN6755!	putative	Medicago_truncatula	36.00	0.00
TRINITY_KXZ4475!	hypothet	Gonium_pectorale	36.00	0.00
TRINITY_P0C8M8.]	RecName:	Zea_mays	36.00	0.00
TRINITY_KHG0315]	ADP-rib(	Gossypium_arboreum	36.00	0.00
TRINITY_XP_0105!	PREDICTI	Tarenaya_hasslerian	36.00	0.00
TRINITY_GAX7994!	hypothet	Chlamydomonas_eusti	36.00	0.00
TRINITY_XP_0239(	ras-like	Quercus_suber	36.00	0.00
TRINITY_GAX8353]	hypothet	Chlamydomonas_eusti	36.00	0.00
TRINITY_XP_0075]	heat sh(	Bathycoccus_prasinc	36.00	0.00
TRINITY_XP_0072]	transcr]	Prunus_persica	36.00	0.00
TRINITY_XP_0271(	histidir	Coffea_arabica	36.00	0.00
TRINITY_PNW7076!	hypothet	Chlamydomonas_reinh	36.00	0.00
TRINITY_XP_0128(	PREDICTI	Erythranthe_guttata	36.00	0.00
TRINITY_EFJ1927'	hypothet	Selaginella_moellen	36.00	0.00
TRINITY_XP_0058(	hypothet	Chlorella_variabili	36.00	0.00
TRINITY_PSC7129!	ATP-binc	Micractinium_conduc	36.00	0.00
TRINITY_GAX7415!	hypothet	Chlamydomonas_eusti	36.00	0.00
TRINITY_EEC7236]	hypothet	Oryza_sativa	36.00	0.00
TRINITY_GAX8182(	hypothet	Chlamydomonas_eusti	36.00	0.00
TRINITY_XP_0045]	unchara(	Cicer_arietinum	36.00	0.00
TRINITY_GAX8325]	hypothet	Chlamydomonas_eusti	36.00	0.00
TRINITY_OMO5004(	hypothet	Corchorus_capsulari	36.00	0.00
TRINITY_XP_0271(	peptide-	Cicer_arietinum	36.00	0.00
TRINITY_PRW5686!	NADPH:q	Chlorella_sorokinia	36.00	0.00
TRINITY_RYR2416'	hypothet	Arachis_hypogaea	36.00	0.00
TRINITY_XP_0243(	unchara(	Physcomitrella_pate	36.00	0.00
TRINITY_XP_0016!	predicte	Chlamydomonas_reinh	36.00	0.00
TRINITY_XP_0215!	LON pept	Manihot_esculenta	36.00	0.00
TRINITY_XP_0218(	serine/t	Spinacia_oleracea	36.00	0.00
TRINITY_GAX8074!	hypothet	Chlamydomonas_eusti	36.00	0.00
TRINITY_GAX7328!	hypothet	Chlamydomonas_eusti	36.00	0.00
TRINITY_GAX7750!	hypothet	Chlamydomonas_eusti	36.00	0.00
TRINITY_XP_0264(	activat(	Papaver_somniferum	36.00	0.00
TRINITY_GAX7470]	hypothet	Chlamydomonas_eusti	36.00	0.00
TRINITY_KZM8319]	hypothet	Daucus_carota	36.00	0.00
TRINITY_GAX8480(	hypothet	Chlamydomonas_eusti	36.00	0.00
TRINITY_PNW8291(	hypothet	Chlamydomonas_reinh	36.00	0.00
TRINITY_PSC6980!	phospho]	Micractinium_conduc	36.00	0.00
TRINITY_ABQ4435!	polyprot	Zea_mays	36.00	0.00
TRINITY_XP_0093!	PREDICTI	Musa_acuminata	36.00	0.00
TRINITY_XP_0016!	selenop:	Chlamydomonas_reinh	36.00	0.00
TRINITY_OAE2006]	hypothet	Marchantia_polymorp	36.00	0.00
TRINITY_XP_0104!	PREDICTI	Camelina_sativa	35.90	0.00
TRINITY_XP_0238'	eukaryot	Quercus_suber	35.90	0.00
TRINITY_PWA7854(	ADP-rib(	Artemisia_annua	35.90	0.00
TRINITY_XP_0237]	peroxis(	Lactuca_sativa	35.90	0.00
TRINITY_XP_0201(	putative	Aegilops_tauschii	35.90	0.00
TRINITY_CAC4838(	ethylene	Fragaria_x_ananassa	35.90	0.00
TRINITY_XP_0113!	MON2-li}	Auxenochlorella_prc	35.90	0.00

TRINITY_PTQ48912	hypothet	Marchantia_polymorp	35.90	0.00
TRINITY_XP_0238	peroxide	Quercus_suber	35.90	0.00
TRINITY_XP_0086	probable	Zea_mays	35.90	0.00
TRINITY_PKA5793	Golgin (	Apostasia_shenzheni	35.90	0.00
TRINITY_XP_0156	pathoger	Oryza_sativa	35.90	0.00
TRINITY_XP_0243	choline,	Physcomitrella_pate	35.90	0.00
TRINITY_XP_0191	PREDICTE	Ipomoea_nil	35.90	0.00
TRINITY_XP_0114	PREDICTE	Fragaria_vesca	35.90	0.00
TRINITY_GAQ8798	Phosphat	Klebsormidium_niten	35.90	0.00
TRINITY_XP_0106	PREDICTE	Beta_vulgaris	35.90	0.00
TRINITY_XP_0245	poly(ADI	Selaginella_moellen	35.90	0.00
TRINITY_BAJ9694	predicte	Hordeum_vulgare	35.90	0.00
TRINITY_XP_0209	uncharac	Arachis_ipaensis	35.90	0.00
TRINITY_XP_0227	CBL-inte	Durio_zibethinus	35.90	0.00
TRINITY_XP_0235	ran-bind	Cucurbita_pepo	35.90	0.00
TRINITY_XP_0058	hypothet	Chlorella_variabili	35.90	0.00
TRINITY_ABR1829	unknown	Picea_sitchensis	35.90	0.00
TRINITY_XP_0243	splicing	Physcomitrella_pate	35.90	0.00
TRINITY_XP_0243	quinone	Physcomitrella_pate	35.90	0.00
TRINITY_KXZ4153	hypothet	Gonium_pectorale	35.90	0.00
TRINITY_XP_0029	flagella	Volvox_carteri	35.90	0.00
TRINITY_GAX7374	hypothet	Chlamydomonas_eusti	35.90	0.00
TRINITY_XP_0029	probable	Selaginella_moellen	35.90	0.00
TRINITY_XP_0043	PREDICTE	Fragaria_vesca	35.90	0.00
TRINITY_ESR5882	hypothet	Citrus_clementina	35.90	0.00
TRINITY_PSC6972	U4 U6 sr	Micractinium_conduc	35.90	0.00
TRINITY_PNW7723	hypothet	Chlamydomonas_reinh	35.90	0.00
TRINITY_EFH5887	hypothet	Arabidopsis_lyrata	35.90	0.00
TRINITY_PNW7038	hypothet	Chlamydomonas_reinh	35.90	0.00
TRINITY_GAX7255	hypothet	Chlamydomonas_eusti	35.90	0.00
TRINITY_XP_0218	EVI5-lik	Spinacia_oleracea	35.90	0.00
TRINITY_XP_0016	glutared	Chlamydomonas_reinh	35.90	0.00
TRINITY_XP_0016	predicte	Chlamydomonas_reinh	35.90	0.00
TRINITY_XP_0194	PREDICTE	Lupinus_angustifoli	35.90	0.00
TRINITY_GAQ8096	M13 fami	Klebsormidium_niten	35.90	0.00
TRINITY_GAX7862	hypothet	Chlamydomonas_eusti	35.90	0.00
TRINITY_GAQ8310	e3 ubiqu	Klebsormidium_niten	35.90	0.00
TRINITY_GAQ8856	hypothet	Klebsormidium_niten	35.90	0.00
TRINITY_VDC6340	unnamed	Brassica_rapa	35.90	0.00
TRINITY_GAQ8772	cystinos	Klebsormidium_niten	35.90	0.00
TRINITY_XP_0229	putative	Cucurbita_moschata	35.90	0.00
TRINITY_PNH0666	Bax inhi	Tetrabaena_socialis	35.90	0.00
TRINITY_XP_0120	pumilio	Jatropha_curcas	35.90	0.00
TRINITY_GBF9522	hypothet	Raphidocelis_subcap	35.90	0.00
TRINITY_GAQ8520	Pumilio	Klebsormidium_niten	35.90	0.00
TRINITY_XP_0184	PREDICTE	Raphanus_sativus	35.90	0.00
TRINITY_KXZ5088	hypothet	Gonium_pectorale	35.90	0.00
TRINITY_PNH1169	hypothet	Tetrabaena_socialis	35.90	0.00
TRINITY_XP_0128	PREDICTE	Erythranthe_guttata	35.90	0.00
TRINITY_XP_0044	phosphol	Cicer_arietinum	35.90	0.00
TRINITY_KXZ4432	hypothet	Gonium_pectorale	35.90	0.00
TRINITY_RID8024	hypothet	Brassica_rapa	35.90	0.00
TRINITY_KXZ4498	hypothet	Gonium_pectorale	35.90	0.00
TRINITY_PNW8886	hypothet	Chlamydomonas_reinh	35.90	0.00
TRINITY_EMS6261	ABC trar	Triticum_urartu	35.90	0.00
TRINITY_GAX7291	hypothet	Chlamydomonas_eusti	35.90	0.00
TRINITY_GAX8569	hypothet	Chlamydomonas_eusti	35.90	0.00
TRINITY_XP_0201	BTB/POZ	Aegilops_tauschii	35.80	0.00
TRINITY_XP_0172	PREDICTE	Daucus_carota	35.80	0.00
TRINITY_XP_0229	sulfite	Cucurbita_moschata	35.80	0.00
TRINITY_XP_0238	oxyster	Quercus_suber	35.80	0.00
TRINITY_PIA6310	hypothet	Aquilegia_coerulea	35.80	0.00
TRINITY_XP_0266	zinc fir	Phoenix_dactylifera	35.80	0.00
TRINITY_XP_0075	peptide	Bathycoccus_prasinc	35.80	0.00
TRINITY_GAX7385	hypothet	Chlamydomonas_eusti	35.80	0.00
TRINITY_XP_0058	hypothet	Chlorella_variabili	35.80	0.00

TRINITY_GBG8341!	hypothet	Chara_braunii	35.80	0.00
TRINITY_BAJ9782!	predicte	Hordeum_vulgare	35.80	0.00
TRINITY_XP_0167!	PREDICTI	Gossypium_hirsutum	35.80	0.00
TRINITY_XP_0156!	DEAD-box	Oryza_sativa	35.80	0.00
TRINITY_PON9549!	Mitogen-	Trema_orientale	35.80	0.00
TRINITY_XP_0264!	protein-	Papaver_somniferum	35.80	0.00
TRINITY_PNX9187!	serine (	Trifolium_pratense	35.80	0.00
TRINITY_XP_0202!	uncharac	Cajanus_cajan	35.80	0.00
TRINITY_EFJ1424!	hypothet	Selaginella_moellen	35.80	0.00
TRINITY_ABU5296!	hypothet	Dunaliella_viridis	35.80	0.00
TRINITY_PNW8871!	hypothet	Chlamydomonas_reinh	35.80	0.00
TRINITY_OAE2772!	hypothet	Marchantia_polymorp	35.80	0.00
TRINITY_XP_0238!	uncharac	Quercus_suber	35.80	0.00
TRINITY_XP_0243!	plant UI	Physcomitrella_pate	35.80	0.00
TRINITY_RMZ5614!	hypothet	Auxenochlorella_pro	35.80	0.00
TRINITY_PSC7691!	kinase !	Micractinium_conduc	35.80	0.00
TRINITY_XP_0139!	translat	Monoraphidium_negle	35.80	0.00
TRINITY_XP_0072!	enhance(	Prunus_persica	35.80	0.00
TRINITY_GAQ8097!	GTPase-a	Klebsormidium_niten	35.80	0.00
TRINITY_XP_0036!	serine/t	Medicago_truncatula	35.80	0.00
TRINITY_PQP9495!	phospho!	Prunus_yedoensis	35.80	0.00
TRINITY_KXZ5530!	hypothet	Gonium_pectorale	35.80	0.00
TRINITY_XP_0016!	predicte	Chlamydomonas_reinh	35.80	0.00
TRINITY_XP_0106!	PREDICTI	Beta_vulgaris	35.80	0.00
TRINITY_RDX7269!	hypothet	Mucuna_pruriens	35.80	0.00
TRINITY_KXZ4297!	hypothet	Gonium_pectorale	35.80	0.00
TRINITY_PRW6123!	cytochro	Chlorella_sorokinia	35.80	0.00
TRINITY_KZV1512!	hypothet	Dorcoceras_hygromet	35.80	0.00
TRINITY_KXZ5656!	hypothet	Gonium_pectorale	35.80	0.00
TRINITY_XP_0258!	pleckst!	Panicum_hallii	35.80	0.00
TRINITY_XP_0025!	predicte	Micromonas_commoda	35.80	0.00
TRINITY_XP_0218!	EVI5-lil	Carica_papaya	35.80	0.00
TRINITY_PRW5906!	secernir	Chlorella_sorokinia	35.80	0.00
TRINITY_KYP3719!	hypothet	Cajanus_cajan	35.80	0.00
TRINITY_XP_0025!	phosphat	Ricinus_communis	35.80	0.00
TRINITY_XP_0243!	programr	Physcomitrella_pate	35.80	0.00
TRINITY_XP_0072!	aldehyde	Prunus_persica	35.80	0.00
TRINITY_GAX8092!	hypothet	Chlamydomonas_eusti	35.80	0.00
TRINITY_PSC6956!	glutath!	Micractinium_conduc	35.80	0.00
TRINITY_GAX7413!	hypothet	Chlamydomonas_eusti	35.80	0.00
TRINITY_XP_0058!	hypothet	Chlorella_variabili	35.80	0.00
TRINITY_XP_0158!	uncharac	Ziziphus_jujuba	35.80	0.00
TRINITY_GAX7515!	hypothet	Chlamydomonas_eusti	35.80	0.00
TRINITY_GAX8016!	hypothet	Chlamydomonas_eusti	35.80	0.00
TRINITY_GAX8150!	hypothet	Chlamydomonas_eusti	35.80	0.00
TRINITY_XP_0029!	hypothet	Volvox_carteri	35.80	0.00
TRINITY_RXH8247!	hypothet	Malus_domestica	35.80	0.00
TRINITY_GAX8345!	hypothet	Chlamydomonas_eusti	35.80	0.00
TRINITY_EFJ1204!	hypothet	Selaginella_moellen	35.80	0.00
TRINITY_GAX7539!	hypothet	Chlamydomonas_eusti	35.80	0.00
TRINITY_GBG8242!	hypothet	Chara_braunii	35.80	0.00
TRINITY_PNH1169!	hypothet	Tetrabaena_socialis	35.80	0.00
TRINITY_GAQ8531!	purple a	Klebsormidium_niten	35.80	0.00
TRINITY_GAX7292!	hypothet	Chlamydomonas_eusti	35.80	0.00
TRINITY_PRW5816!	PAB-depe	Chlorella_sorokinia	35.80	0.00
TRINITY_XP_0216!	uncharac	Hevea_brasiliensis	35.80	0.00
TRINITY_BAJ9585!	predicte	Hordeum_vulgare	35.80	0.00
TRINITY_GAX7492!	hypothet	Chlamydomonas_eusti	35.80	0.00
TRINITY_PNW7534!	hypothet	Chlamydomonas_reinh	35.80	0.00
TRINITY_GAQ8047!	Chromat!	Klebsormidium_niten	35.80	0.00
TRINITY_XP_0087!	cyclin-!	Phoenix_dactylifera	35.80	0.00
TRINITY_XP_0191!	PREDICTI	Beta_vulgaris	35.80	0.00
TRINITY_GAX7701!	hypothet	Chlamydomonas_eusti	35.80	0.00
TRINITY_BAJ9457!	predicte	Hordeum_vulgare	35.80	0.00
TRINITY_PNW7452!	hypothet	Chlamydomonas_reinh	35.80	0.00
TRINITY_PPD9625!	hypothet	Gossypium_barbadens	35.80	0.00

TRINITY_RWR7844	protein	Cinnamomum_micranth	35.70	0.00
TRINITY_KZV3804	F-box/F	Dorcoceras_hygromet	35.70	0.00
TRINITY_BAK0043	predicte	Hordeum_vulgare	35.70	0.00
TRINITY_ABB1352	target (	Chlamydomonas_reinh	35.70	0.00
TRINITY_OAY6599	Abscisi	Ananas_comosus	35.70	0.00
TRINITY_PRW6118	U3 smal	Chlorella_sorokinia	35.70	0.00
TRINITY_XP_0064	actin-re	Citrus_clementina	35.70	0.00
TRINITY_XP_0104	PREDICT	Camelina_sativa	35.70	0.00
TRINITY_OTG3244	putative	Helianthus_annuus	35.70	0.00
TRINITY_XP_0049	serine/t	Setaria_italica	35.70	0.00
TRINITY_KMS9419	hypothet	Beta_vulgaris	35.70	0.00
TRINITY_XP_0172	PREDICT	Daucus_carota	35.70	0.00
TRINITY_XP_0243	uncharac	Physcomitrella_pate	35.70	0.00
TRINITY_GAQ8835	alpha-sc	Klebsormidium_niten	35.70	0.00
TRINITY_PLY6511	hypothet	Lactuca_sativa	35.70	0.00
TRINITY_XP_0212	conserve	Herrania_umbratica	35.70	0.00
TRINITY_XP_0245	serine/t	Selaginella_moellen	35.70	0.00
TRINITY_XP_0245	probable	Selaginella_moellen	35.70	0.00
TRINITY_GAX7965	hypothet	Chlamydomonas_eusti	35.70	0.00
TRINITY_OAE2772	hypothet	Marchantia_polymorp	35.70	0.00
TRINITY_XP_0135	PREDICT	Brassica_oleracea	35.70	0.00
TRINITY_XP_0056	Rab8/Rab	Coccomyxa_subellips	35.70	0.00
TRINITY_OAY7795	ABC tra	Ananas_comosus	35.70	0.00
TRINITY_XP_0220	cytochr	Helianthus_annuus	35.70	0.00
TRINITY_XP_0124	PREDICT	Gossypium_raimondii	35.70	0.00
TRINITY_OAE1812	hypothet	Marchantia_polymorp	35.70	0.00
TRINITY_XP_0035	probable	Brachypodium_distac	35.70	0.00
TRINITY_XP_0258	ras-rela	Panicum_hallii	35.70	0.00
TRINITY_XP_0106	PREDICT	Beta_vulgaris	35.70	0.00
TRINITY_XP_0100	PREDICT	Eucalyptus_grandis	35.70	0.00
TRINITY_KRG9416	hypothet	Glycine_max	35.70	0.00
TRINITY_XP_0110	transmer	Sesamum_indicum	35.70	0.00
TRINITY_XP_0104	PREDICT	Camelina_sativa	35.70	0.00
TRINITY_BAJ9984	predicte	Hordeum_vulgare	35.70	0.00
TRINITY_PWA9232	BRCT do	Artemisia_annua	35.70	0.00
TRINITY_XP_0139	hypothet	Monoraphidium_negle	35.70	0.00
TRINITY_GAX7762	hypothet	Chlamydomonas_eusti	35.70	0.00
TRINITY_GAX8473	hypothet	Chlamydomonas_eusti	35.70	0.00
TRINITY_XP_0030	DNA mis	Micromonas_pusilla	35.70	0.00
TRINITY_PNW7584	hypothet	Chlamydomonas_reinh	35.70	0.00
TRINITY_PNW7724	hypothet	Chlamydomonas_reinh	35.70	0.00
TRINITY_XP_0017	Qb-SNAR	Chlamydomonas_reinh	35.70	0.00
TRINITY_XP_0056	Pkinase-	Coccomyxa_subellips	35.70	0.00
TRINITY_XP_0263	E3 ubiq	Papaver_somniferum	35.70	0.00
TRINITY_GAX7345	hypothet	Chlamydomonas_eusti	35.70	0.00
TRINITY_GBG7277	hypothet	Chara_braunii	35.70	0.00
TRINITY_KXZ5011	hypothet	Gonium_pectorale	35.70	0.00
TRINITY_XP_0205	tubulin-	Phalaenopsis_equest	35.70	0.00
TRINITY_KXZ4165	hypothet	Gonium_pectorale	35.70	0.00
TRINITY_GAQ8919	Putative	Klebsormidium_niten	35.70	0.00
TRINITY_GAX7542	hypothet	Chlamydomonas_eusti	35.70	0.00
TRINITY_PIA4834	hypothet	Aquilegia_coerulea	35.70	0.00
TRINITY_KXZ5460	hypothet	Gonium_pectorale	35.70	0.00
TRINITY_KXZ5199	hypothet	Gonium_pectorale	35.70	0.00
TRINITY_XP_0025	brefeld	Ricinus_communis	35.70	0.00
TRINITY_GAX8304	hypothet	Chlamydomonas_eusti	35.70	0.00
TRINITY_XP_0174	PREDICT	Vigna_angularis	35.70	0.00
TRINITY_GAX8261	hypothet	Chlamydomonas_eusti	35.70	0.00
TRINITY_GBF9588	hypothet	Raphidocelis_subcap	35.70	0.00
TRINITY_GAX7754	hypothet	Chlamydomonas_eusti	35.70	0.00
TRINITY_XP_0243	exonucl	Physcomitrella_pate	35.70	0.00
TRINITY_XP_0066	PREDICT	Oryza_brachyantha	35.70	0.00
TRINITY_XP_0102	PREDICT	Nelumbo_nucifera	35.70	0.00
TRINITY_XP_0029	hypothet	Volvox_carteri	35.70	0.00
TRINITY_PNW6984	hypothet	Chlamydomonas_reinh	35.70	0.00
TRINITY_GAX7336	hypothet	Chlamydomonas_eusti	35.70	0.00

TRINITY_XP_0258(	alkylate	Oryza_sativa	35.70	0.00
TRINITY_XP_0227(	calcium-	Durio_zibethinus	35.60	0.00
TRINITY_XP_0075(	predicte	Bathycoccus_prasinc	35.60	0.00
TRINITY_XP_0030(	Equilib:	Ostreococcus_tauri	35.60	0.00
TRINITY_EEF2755(	conserve	Ricinus_communis	35.60	0.00
TRINITY_OWM9037(	hypothet	Punica_granatum	35.60	0.00
TRINITY_XP_0221(	plant-sp	Momordica_charantia	35.60	0.00
TRINITY_XP_0206(	28 kDa	Dendrobium_catenatu	35.60	0.00
TRINITY_PRW5951(	Rab fam:	Chlorella_sorokinia	35.60	0.00
TRINITY_EFJ1130(	hypothet	Selaginella_moellen	35.60	0.00
TRINITY_GAQ8741(	hypothet	Klebsormidium_niten	35.60	0.00
TRINITY_OAE2041(	hypothet	Marchantia_polymorp	35.60	0.00
TRINITY_GAX7487(	hypothet	Chlamydomonas_eusti	35.60	0.00
TRINITY_XP_0239(	uncharac	Quercus_suber	35.60	0.00
TRINITY_ESQ2959(	hypothet	Eutrema_salsugineum	35.60	0.00
TRINITY_GAX8535(	hypothet	Chlamydomonas_eusti	35.60	0.00
TRINITY_PRW5608(	TPR and	Chlorella_sorokinia	35.60	0.00
TRINITY_XP_0029(	hypothet	Volvox_carteri	35.60	0.00
TRINITY_XP_0029(	hypothet	Volvox_carteri	35.60	0.00
TRINITY_RZC2025(	CCR4-NO'	Glycine_soja	35.60	0.00
TRINITY_PNW7244(	hypothet	Chlamydomonas_reinh	35.60	0.00
TRINITY_AHF5859(	proline	Chrysanthemum_lavan	35.60	0.00
TRINITY_XP_0024(	catalyt:	Micromonas_commoda	35.60	0.00
TRINITY_XP_0238(	membrane	Quercus_suber	35.60	0.00
TRINITY_XP_0016(	predicte	Chlamydomonas_reinh	35.60	0.00
TRINITY_XP_0029(	hypothet	Volvox_carteri	35.60	0.00
TRINITY_GAQ8741(	hypothet	Klebsormidium_niten	35.60	0.00
TRINITY_EMS5886(	hypothet	Triticum_urartu	35.60	0.00
TRINITY_OAE2496(	hypothet	Marchantia_polymorp	35.60	0.00
TRINITY_GBG6335(	hypothet	Chara_braunii	35.60	0.00
TRINITY_GAX7306(	hypothet	Chlamydomonas_eusti	35.60	0.00
TRINITY_GBF9683(	hypothet	Raphidocelis_subcap	35.60	0.00
TRINITY_GBF9829(	hypothet	Raphidocelis_subcap	35.60	0.00
TRINITY_XP_0243(	uncharac	Physcomitrella_pate	35.60	0.00
TRINITY_XP_0113(	Protein	Auxenochlorella_prc	35.60	0.00
TRINITY_PNW8631(	hypothet	Chlamydomonas_reinh	35.60	0.00
TRINITY_KJB2912(	hypothet	Gossypium_raimondii	35.60	0.00
TRINITY_XP_0017(	predicte	Chlamydomonas_reinh	35.60	0.00
TRINITY_KXZ4348(	hypothet	Gonium_pectorale	35.60	0.00
TRINITY_PNH1169(	hypothet	Tetrabaena_socialis	35.60	0.00
TRINITY_KCW5391(	hypothet	Eucalyptus_grandis	35.60	0.00
TRINITY_XP_0075(	predicte	Bathycoccus_prasinc	35.60	0.00
TRINITY_XP_0029(	hypothet	Volvox_carteri	35.60	0.00
TRINITY_XP_0016(	RWP-RK t	Chlamydomonas_reinh	35.60	0.00
TRINITY_XP_0016(	predicte	Chlamydomonas_reinh	35.60	0.00
TRINITY_GBF9561(	guanylat	Raphidocelis_subcap	35.60	0.00
TRINITY_GAX7480(	hypothet	Chlamydomonas_eusti	35.60	0.00
TRINITY_KXZ5237(	hypothet	Gonium_pectorale	35.60	0.00
TRINITY_XP_0014(	predicte	Ostreococcus_sp._'l	35.60	0.00
TRINITY_GAX7642(	hypothet	Chlamydomonas_eusti	35.60	0.00
TRINITY_XP_0243(	diphthir	Physcomitrella_pate	35.60	0.00
TRINITY_PNW7332(	hypothet	Chlamydomonas_reinh	35.60	0.00
TRINITY_GAX8179(	hypothet	Chlamydomonas_eusti	35.60	0.00
TRINITY_XP_0134(	zinc fir	Medicago_truncatula	35.60	0.00
TRINITY_GAX7546(	hypothet	Chlamydomonas_eusti	35.60	0.00
TRINITY_GAX7942(	hypothet	Chlamydomonas_eusti	35.60	0.00
TRINITY_PSC6746(	hypothet	Micractinium_conduc	35.60	0.00
TRINITY_GAX7627(	hypothet	Chlamydomonas_eusti	35.60	0.00
TRINITY_GAQ8108(	hypothet	Klebsormidium_niten	35.60	0.00
TRINITY_GAX7670(	hypothet	Chlamydomonas_eusti	35.60	0.00
TRINITY_GAX7983(	hypothet	Chlamydomonas_eusti	35.60	0.00
TRINITY_XP_0025(	predicte	Micromonas_commoda	35.50	0.00
TRINITY_XP_0110(	PREDICTI	Populus_euphratica	35.50	0.00
TRINITY_XP_0075(	predicte	Bathycoccus_prasinc	35.50	0.00
TRINITY_AJR2238(	CBL-inte	Triticum_aestivum	35.50	0.00
TRINITY_CBI3001(	unnamed	Vitis_vinifera	35.50	0.00

TRINITY_KMZ7409(	Calmodul	Zostera_marina	35.50	0.00
TRINITY_EFJ1972]	hypothet	Selaginella_moellen	35.50	0.00
TRINITY_XP_0209(	CBL-inte	Arachis_duranensis	35.50	0.00
TRINITY_XP_0279(	uncharac	Vigna_unguiculata	35.50	0.00
TRINITY_XP_0088]	uncharac	Phoenix_dactylifera	35.50	0.00
TRINITY_OAE2597]	hypothet	Marchantia_polymorp	35.50	0.00
TRINITY_PRW3402]	alpha be	Chlorella_sorokinia	35.50	0.00
TRINITY_ESQ5007]	hypothet	Eutrema_salsugineum	35.50	0.00
TRINITY_XP_0190'	PREDICTE	Vitis_vinifera	35.50	0.00
TRINITY_AIU4993'	carbohyc	Magnolia_denudata	35.50	0.00
TRINITY_XP_0109]	PREDICTE	Elaeis_guineensis	35.50	0.00
TRINITY_XP_0217'	7-methyl	Chenopodium_quinoa	35.50	0.00
TRINITY_XP_0023(	putative	Populus_trichocarpa	35.50	0.00
TRINITY_XP_0238(	uncharac	Quercus_suber	35.50	0.00
TRINITY_XP_0066(	PREDICTE	Oryza_brachyantha	35.50	0.00
TRINITY_XP_0243(	ubiquit:	Physcomitrella_pate	35.50	0.00
TRINITY_GAQ8789(	calpain-	Klebsormidium_niten	35.50	0.00
TRINITY_GAU4699(	hypothet	Trifolium_subterran	35.50	0.00
TRINITY_PNW8524]	hypothet	Chlamydomonas_reinh	35.50	0.00
TRINITY_PNH0873]	Adenylat	Tetrabaena_socialis	35.50	0.00
TRINITY_XP_0029(	phosphat	Selaginella_moellen	35.50	0.00
TRINITY_GAX8177(	hypothet	Chlamydomonas_eusti	35.50	0.00
TRINITY_GBF9892(	appr-1-p	Raphidocelis_subcap	35.50	0.00
TRINITY_EPS7191(	calcium	Genlisea_aurea	35.50	0.00
TRINITY_XP_0217(	CTD smal	Chenopodium_quinoa	35.50	0.00
TRINITY_XP_0241(	E3 ubiqu	Rosa_chinensis	35.50	0.00
TRINITY_KXZ4684'	TTL3 pro	Gonium_pectorale	35.50	0.00
TRINITY_XP_0206'	uncharac	Dendrobium_catenatu	35.50	0.00
TRINITY_PTQ3924(	hypothet	Marchantia_polymorp	35.50	0.00
TRINITY_PNW8633(	hypothet	Chlamydomonas_reinh	35.50	0.00
TRINITY_XP_0044(	ABC tran	Cicer_arietinum	35.50	0.00
TRINITY_GAX7416]	hypothet	Chlamydomonas_eusti	35.50	0.00
TRINITY_ERN1588]	hypothet	Amborella_trichopod	35.50	0.00
TRINITY_XP_0216'	cinnamoy	Hevea_brasiliensis	35.50	0.00
TRINITY_GAX8289(	hypothet	Chlamydomonas_eusti	35.50	0.00
TRINITY_PNH1190(	Phosphoç	Tetrabaena_socialis	35.50	0.00
TRINITY_GAX8066(	hypothet	Chlamydomonas_eusti	35.50	0.00
TRINITY_KZV1544(	programm	Dorcoceras_hygromet	35.50	0.00
TRINITY_GAX7261]	hypothet	Chlamydomonas_eusti	35.50	0.00
TRINITY_XP_0238(	uncharac	Quercus_suber	35.50	0.00
TRINITY_PNW8754]	hypothet	Chlamydomonas_reinh	35.50	0.00
TRINITY_XP_0187]	PREDICTE	Eucalyptus_grandis	35.50	0.00
TRINITY_OAE2023'	hypothet	Marchantia_polymorp	35.50	0.00
TRINITY_XP_0016(	predicte	Chlamydomonas_reinh	35.50	0.00
TRINITY_XP_0016(	glycerol	Chlamydomonas_reinh	35.50	0.00
TRINITY_GAX7767(	hypothet	Chlamydomonas_eusti	35.50	0.00
TRINITY_XP_0104]	PREDICTE	Camelina_sativa	35.50	0.00
TRINITY_XP_0218(	serine/t	Spinacia_oleracea	35.50	0.00
TRINITY_XP_0206(	serine/t	Dendrobium_catenatu	35.50	0.00
TRINITY_P53385.]	RecName:	Trifolium_repens	35.50	0.00
TRINITY_XP_0029(	sodium t	Selaginella_moellen	35.50	0.00
TRINITY_RWW8282(	hypothet	Ensete_ventricosum	35.50	0.00
TRINITY_OAY6870(	Chromosc	Ananas_comosus	35.50	0.00
TRINITY_XP_0228]	Small G]	Ostreococcus_tauri	35.50	0.00
TRINITY_KXZ4752]	hypothet	Gonium_pectorale	35.50	0.00
TRINITY_GAQ9002(	hypothet	Klebsormidium_niten	35.50	0.00
TRINITY_GBG8296(	hypothet	Chara_braunii	35.50	0.00
TRINITY_GAX7919(	hypothet	Chlamydomonas_eusti	35.50	0.00
TRINITY_XP_0150(	probable	Solanum_pennellii	35.50	0.00
TRINITY_GAX7977(	hypothet	Chlamydomonas_eusti	35.50	0.00
TRINITY_KXZ5107'	hypothet	Gonium_pectorale	35.50	0.00
TRINITY_PNW7371(	hypothet	Chlamydomonas_reinh	35.50	0.00
TRINITY_XP_0138(	hypothet	Monoraphidium_negle	35.50	0.00
TRINITY_OWM6336(	hypothet	Punica_granatum	35.50	0.00
TRINITY_PNH1057(	Retrovi:	Tetrabaena_socialis	35.50	0.00
TRINITY_RLN1196(	elongat:	Panicum_miliaceum	35.50	0.00

TRINITY_GAX76741	hypothet	Chlamydomonas_eusti	35.40	0.00
TRINITY_OAE22293	hypothet	Marchantia_polymorp	35.40	0.00
TRINITY_PSS31546	Transcri	Actinidia_chinensis	35.40	0.00
TRINITY_XP_01055	PREDICTE	Tarenaya_hassleriar	35.40	0.00
TRINITY_KDD72733	GTP-binc	Helicosporidium_sp.	35.40	0.00
TRINITY_RLN30080	calcium-	Panicum_miliaceum	35.40	0.00
TRINITY_KXZ42812	hypothet	Gonium_pectorale	35.40	0.00
TRINITY_GAQ80201	protein	Klebsormidium_niten	35.40	0.00
TRINITY_XP_00750	predicte	Bathycoccus_prasinc	35.40	0.00
TRINITY_XP_02392	probable	Quercus_suber	35.40	0.00
TRINITY_XP_00141	predicte	Ostreococcus_sp._'l	35.40	0.00
TRINITY_XP_02454	MAPK/MAE	Selaginella_moellen	35.40	0.00
TRINITY_XP_01284	PREDICTE	Erythranthe_guttata	35.40	0.00
TRINITY_GBG46512	hypothet	Chara_braunii	35.40	0.00
TRINITY_XP_00961	PREDICTE	Nicotiana_tomentosi	35.40	0.00
TRINITY_EFJ20989	hypothet	Selaginella_moellen	35.40	0.00
TRINITY_GAQ79529	hypothet	Klebsormidium_niten	35.40	0.00
TRINITY_PNH07767	hypothet	Tetrabaena_socialis	35.40	0.00
TRINITY_XP_02174	probable	Chenopodium_quinoa	35.40	0.00
TRINITY_GBF97724	hypothet	Raphidocelis_subcap	35.40	0.00
TRINITY_KXZ44435	hypothet	Gonium_pectorale	35.40	0.00
TRINITY_XP_02014	transcri	Aegilops_tauschii	35.40	0.00
TRINITY_XP_02438	pheromor	Physcomitrella_pate	35.40	0.00
TRINITY_XP_01920	PREDICTE	Ipomoea_nil	35.40	0.00
TRINITY_PNW74657	hypothet	Chlamydomonas_reinh	35.40	0.00
TRINITY_XP_00250	predicte	Micromonas_commoda	35.40	0.00
TRINITY_XP_00170	predicte	Chlamydomonas_reinh	35.40	0.00
TRINITY_XP_00657	tyrosyl-	Glycine_max	35.40	0.00
TRINITY_PSC73989	CAMK CAM	Micractinium_conduc	35.40	0.00
TRINITY_XP_02388	alpha-ac	Quercus_suber	35.40	0.00
TRINITY_BAK02869	predicte	Hordeum_vulgare	35.40	0.00
TRINITY_XP_00636	PREDICTE	Solanum_tuberosum	35.40	0.00
TRINITY_KXZ50905	hypothet	Gonium_pectorale	35.40	0.00
TRINITY_PSC74291	NAD(P)-k	Micractinium_conduc	35.40	0.00
TRINITY_PNH03126	hypothet	Tetrabaena_socialis	35.40	0.00
TRINITY_PNW71157	hypothet	Chlamydomonas_reinh	35.40	0.00
TRINITY_PNW77124	hypothet	Chlamydomonas_reinh	35.40	0.00
TRINITY_XP_00564	hypothet	Coccomyxa_subellips	35.40	0.00
TRINITY_XP_02059	plant UE	Phalaenopsis_equest	35.40	0.00
TRINITY_KCW65146	hypothet	Eucalyptus_grandis	35.40	0.00
TRINITY_GBG66666	hypothet	Chara_braunii	35.40	0.00
TRINITY_GAX82263	hypothet	Chlamydomonas_eusti	35.40	0.00
TRINITY_AEJ07934	Xilon1 g	Zea_mays	35.40	0.00
TRINITY_XP_02189	RNA poly	Carica_papaya	35.40	0.00
TRINITY_GBG64059	hypothet	Chara_braunii	35.40	0.00
TRINITY_EFJ32831	hypothet	Selaginella_moellen	35.40	0.00
TRINITY_ACN40292	unknown	Picea_sitchensis	35.40	0.00
TRINITY_XP_02452	pre-mRNA	Selaginella_moellen	35.40	0.00
TRINITY_EFJ19555	glycosyl	Selaginella_moellen	35.40	0.00
TRINITY_PNH00238	hypothet	Tetrabaena_socialis	35.40	0.00
TRINITY_XP_02068	aldehyde	Dendrobium_catenatu	35.40	0.00
TRINITY_GAX82984	hypothet	Chlamydomonas_eusti	35.40	0.00
TRINITY_GAX80114	hypothet	Chlamydomonas_eusti	35.40	0.00
TRINITY_XP_02069	phosphol	Dendrobium_catenatu	35.40	0.00
TRINITY_GAQ83756	Cyclin f	Klebsormidium_niten	35.40	0.00
TRINITY_XP_00939	PREDICTE	Musa_acuminata	35.40	0.00
TRINITY_XP_02287	ABC trar	Olea_europaea	35.40	0.00
TRINITY_XP_00169	predicte	Chlamydomonas_reinh	35.40	0.00
TRINITY_XP_02438	Fanconi	Physcomitrella_pate	35.40	0.00
TRINITY_KXZ40924	hypothet	Gonium_pectorale	35.40	0.00
TRINITY_XP_02199	3-phosph	Helianthus_annuus	35.40	0.00
TRINITY_PNW74794	hypothet	Chlamydomonas_reinh	35.40	0.00
TRINITY_XP_00169	predicte	Chlamydomonas_reinh	35.40	0.00
TRINITY_KXZ56109	hypothet	Gonium_pectorale	35.40	0.00
TRINITY_GBG65685	hypothet	Chara_braunii	35.40	0.00
TRINITY_PSC69984	ubiquiti	Micractinium_conduc	35.30	0.00

TRINITY_PRW34039	carotene	Chlorella_sorokinia	35.30	0.00
TRINITY_XP_0045	kinesin-	Cicer_arietinum	35.30	0.00
TRINITY_XP_0239	uncharac	Quercus_suber	35.30	0.00
TRINITY_GAQ81304	hypothet	Klebsormidium_niten	35.30	0.00
TRINITY_XP_0066	PREDICTE	Oryza_brachyantha	35.30	0.00
TRINITY_XP_0273	probable	Abrus_precatorius	35.30	0.00
TRINITY_OAE3073	hypothet	Marchantia_polymorp	35.30	0.00
TRINITY_GAQ7878	hypothet	Klebsormidium_niten	35.30	0.00
TRINITY_XP_0228	uncharac	Olea_europaea	35.30	0.00
TRINITY_XP_0109	PREDICTE	Elaeis_guineensis	35.30	0.00
TRINITY_XP_0064	protein	Citrus_clementina	35.30	0.00
TRINITY_XP_0017	CTR type	Chlamydomonas_reinh	35.30	0.00
TRINITY_XP_0241	GTP-binc	Rosa_chinensis	35.30	0.00
TRINITY_PKI3995	hypothet	Punica_granatum	35.30	0.00
TRINITY_KZV4357	phospho	Dorcoceras_hygromet	35.30	0.00
TRINITY_XP_0194	PREDICTE	Lupinus_angustifoli	35.30	0.00
TRINITY_PIA5719	hypothet	Aquilegia_coerulea	35.30	0.00
TRINITY_XP_0245	protein	Selaginella_moellen	35.30	0.00
TRINITY_XP_0025	dynein	Micromonas_commoda	35.30	0.00
TRINITY_EEF2641	lipid A	Ricinus_communis	35.30	0.00
TRINITY_XP_0165	PREDICTE	Capsicum_annuum	35.30	0.00
TRINITY_GAQ8420	Transmem	Klebsormidium_niten	35.30	0.00
TRINITY_XP_0017	ABC tran	Chlamydomonas_reinh	35.30	0.00
TRINITY_KNA0929	hypothet	Spinacia_oleracea	35.30	0.00
TRINITY_XP_0208	calcium-	Arabidopsis_lyrata	35.30	0.00
TRINITY_XP_0239	ras-like	Quercus_suber	35.30	0.00
TRINITY_GAX8152	hypothet	Chlamydomonas_eusti	35.30	0.00
TRINITY_GAX7608	hypothet	Chlamydomonas_eusti	35.30	0.00
TRINITY_XP_0273	uncharac	Abrus_precatorius	35.30	0.00
TRINITY_XP_0104	PREDICTE	Camelina_sativa	35.30	0.00
TRINITY_GBF9155	hypothet	Raphidocelis_subcap	35.30	0.00
TRINITY_XP_0030	predicte	Micromonas_pusilla	35.30	0.00
TRINITY_GBF9439	lipase r	Raphidocelis_subcap	35.30	0.00
TRINITY_XP_0036	cytosoli	Medicago_truncatula	35.30	0.00
TRINITY_KXZ4931	PI3kinas	Gonium_pectorale	35.30	0.00
TRINITY_GAX7714	hypothet	Chlamydomonas_eusti	35.30	0.00
TRINITY_GAQ8204	hypothet	Klebsormidium_niten	35.30	0.00
TRINITY_XP_0201	protein	Aegilops_tauschii	35.30	0.00
TRINITY_PNW7466	hypothet	Chlamydomonas_reinh	35.30	0.00
TRINITY_XP_0025	predicte	Micromonas_commoda	35.30	0.00
TRINITY_GAX8563	hypothet	Chlamydomonas_eusti	35.30	0.00
TRINITY_KXZ4910	hypothet	Gonium_pectorale	35.30	0.00
TRINITY_GAX7787	hypothet	Chlamydomonas_eusti	35.30	0.00
TRINITY_GBF8771	hypothet	Raphidocelis_subcap	35.30	0.00
TRINITY_GAX7401	hypothet	Chlamydomonas_eusti	35.30	0.00
TRINITY_XP_0136	uncharac	Brassica_napus	35.30	0.00
TRINITY_EOY1806	3-oxo-5-	Theobroma_cacao	35.30	0.00
TRINITY_XP_0238	alpha-ac	Quercus_suber	35.30	0.00
TRINITY_XP_0271	protein	Coffea_arabica	35.30	0.00
TRINITY_XP_0016	predicte	Chlamydomonas_reinh	35.30	0.00
TRINITY_XP_0017	predicte	Chlamydomonas_reinh	35.30	0.00
TRINITY_XP_0029	hypothet	Volvox_carteri	35.30	0.00
TRINITY_GAY3426	hypothet	Citrus_unshiu	35.30	0.00
TRINITY_GAQ8004	Predicte	Klebsormidium_niten	35.30	0.00
TRINITY_KXZ5291	hypothet	Gonium_pectorale	35.30	0.00
TRINITY_PNW7327	hypothet	Chlamydomonas_reinh	35.30	0.00
TRINITY_GBF9217	hypothet	Raphidocelis_subcap	35.30	0.00
TRINITY_GAQ8067	TOUSLED-	Klebsormidium_niten	35.30	0.00
TRINITY_KMS6523	hypothet	Beta_vulgaris	35.30	0.00
TRINITY_XP_0029	hypothet	Volvox_carteri	35.30	0.00
TRINITY_GAX8350	hypothet	Chlamydomonas_eusti	35.30	0.00
TRINITY_GAQ7785	protein	Klebsormidium_niten	35.30	0.00
TRINITY_XP_0220	transcri	Helianthus_annuus	35.30	0.00
TRINITY_XP_0205	ubiquiti	Amborella_trichopod	35.30	0.00
TRINITY_GAX7950	hypothet	Chlamydomonas_eusti	35.30	0.00
TRINITY_XP_0100	PREDICTE	Eucalyptus_grandis	35.30	0.00

TRINITY_KXZ5481	hypothet	Gonium_pectorale	35.30	0.00
TRINITY_PNW8764	hypothet	Chlamydomonas_reinh	35.30	0.00
TRINITY_XP_0105	PREDICTE	Tarenaya_hasslerian	35.30	0.00
TRINITY_PNW8479	hypothet	Chlamydomonas_reinh	35.30	0.00
TRINITY_XP_0017	haloper	Chlamydomonas_reinh	35.30	0.00
TRINITY_PNH0869	Cytoplas	Tetrabaena_socialis	35.30	0.00
TRINITY_GAX8207	hypothet	Chlamydomonas_eusti	35.30	0.00
TRINITY_GAX7758	hypothet	Chlamydomonas_eusti	35.30	0.00
TRINITY_GAX7939	hypothet	Chlamydomonas_eusti	35.30	0.00
TRINITY_AOT8215	defender	Botryococcus_brauni	35.20	0.00
TRINITY_XP_0239	ras-rela	Quercus_suber	35.20	0.00
TRINITY_KMZ5734	Casein	Zostera_marina	35.20	0.00
TRINITY_EAY9103	hypothet	Oryza_sativa	35.20	0.00
TRINITY_PNX9503	hypothet	Trifolium_pratense	35.20	0.00
TRINITY_PSC7503	MORN rep	Micractinium_conduc	35.20	0.00
TRINITY_OAY7417	Transfor	Ananas_comosus	35.20	0.00
TRINITY_XP_0237	ras-rela	Lactuca_sativa	35.20	0.00
TRINITY_XP_0042	probable	Solanum_lycopersicu	35.20	0.00
TRINITY_XP_0066	PREDICTE	Oryza_brachyantha	35.20	0.00
TRINITY_KDD7334	hypothet	Helicosporidium_sp.	35.20	0.00
TRINITY_PKI6301	hypothet	Punica_granatum	35.20	0.00
TRINITY_XP_0106	PREDICTE	Beta_vulgaris	35.20	0.00
TRINITY_PIA2643	hypothet	Aquilegia_coerulea	35.20	0.00
TRINITY_PTQ3303	hypothet	Marchantia_polymorp	35.20	0.00
TRINITY_XP_0279	serine/t	Vigna_unguiculata	35.20	0.00
TRINITY_XP_0264	pre-mRNA	Papaver_somniferum	35.20	0.00
TRINITY_XP_0228	hsp70 n	Olea_europaea	35.20	0.00
TRINITY_XP_0017	predicte	Chlamydomonas_reinh	35.20	0.00
TRINITY_XP_0113	putative	Auxenochlorella_pro	35.20	0.00
TRINITY_PNW7033	hypothet	Chlamydomonas_reinh	35.20	0.00
TRINITY_GAX8459	hypothet	Chlamydomonas_eusti	35.20	0.00
TRINITY_GAQ9029	hypothet	Klebsormidium_niten	35.20	0.00
TRINITY_PNH1268	Chloroph	Tetrabaena_socialis	35.20	0.00
TRINITY_XP_0279	serine/t	Vigna_unguiculata	35.20	0.00
TRINITY_PIN0879	hypothet	Handroanthus_impeti	35.20	0.00
TRINITY_GAX7701	hypothet	Chlamydomonas_eusti	35.20	0.00
TRINITY_XP_0249	triacylg	Ziziphus_jujuba	35.20	0.00
TRINITY_GAX7376	hypothet	Chlamydomonas_eusti	35.20	0.00
TRINITY_XP_0263	pathogen	Papaver_somniferum	35.20	0.00
TRINITY_XP_0029	hypothet	Volvox_carteri	35.20	0.00
TRINITY_OAE2618	hypothet	Marchantia_polymorp	35.20	0.00
TRINITY_OAE2772	hypothet	Marchantia_polymorp	35.20	0.00
TRINITY_PTQ2712	hypothet	Marchantia_polymorp	35.20	0.00
TRINITY_OAE3199	hypothet	Marchantia_polymorp	35.20	0.00
TRINITY_GAX7599	hypothet	Chlamydomonas_eusti	35.20	0.00
TRINITY_XP_0238	dihydrox	Quercus_suber	35.20	0.00
TRINITY_GAQ8593	hypothet	Klebsormidium_niten	35.20	0.00
TRINITY_KXZ4733	hypothet	Gonium_pectorale	35.20	0.00
TRINITY_XP_0238	pheromon	Quercus_suber	35.20	0.00
TRINITY_XP_0159	uncharac	Arachis_duranensis	35.20	0.00
TRINITY_XP_0025	predicte	Micromonas_commoda	35.20	0.00
TRINITY_KXZ4398	hypothet	Gonium_pectorale	35.20	0.00
TRINITY_XP_0016	predicte	Chlamydomonas_reinh	35.20	0.00
TRINITY_XP_0136	uncharac	Brassica_napus	35.20	0.00
TRINITY_XP_0113	Beta-hex	Auxenochlorella_pro	35.20	0.00
TRINITY_XP_0239	solute c	Quercus_suber	35.20	0.00
TRINITY_GBF9399	1-phosph	Raphidocelis_subcap	35.20	0.00
TRINITY_GBG7005	hypothet	Chara_braunii	35.20	0.00
TRINITY_GAX7504	hypothet	Chlamydomonas_eusti	35.20	0.00
TRINITY_XP_0029	hypothet	Volvox_carteri	35.20	0.00
TRINITY_XP_0239	ATP-bind	Quercus_suber	35.20	0.00
TRINITY_XP_0238	serine/t	Quercus_suber	35.20	0.00
TRINITY_PNW8663	hypothet	Chlamydomonas_reinh	35.20	0.00
TRINITY_PNH0800	Traffick	Tetrabaena_socialis	35.20	0.00
TRINITY_XP_0093	PREDICTE	Pyrus_x_bretschneid	35.20	0.00
TRINITY_XP_0102	PREDICTE	Nelumbo_nucifera	35.20	0.00

TRINITY_ATA62862	putative	Juniperus_phoenicea	35.20	0.00
TRINITY_XP_0139(	cytochro	Monoraphidium_negle	35.20	0.00
TRINITY_XP_0114!	PREDICTE	Fragaria_vesca	35.20	0.00
TRINITY_RLN0414!	putative	Panicum_miliaceum	35.20	0.00
TRINITY_PNW7686(	hypothet	Chlamydomonas_reinh	35.20	0.00
TRINITY_XP_01882	PREDICTE	Juglans_regia	35.20	0.00
TRINITY_AAT8566	polyunsa	Marchantia_polymorp	35.20	0.00
TRINITY_GAQ8067(	hypothet	Klebsormidium_niten	35.20	0.00
TRINITY_GAQ9104(	calcium-	Klebsormidium_niten	35.20	0.00
TRINITY_XP_0218(	uncharac	Spinacia_oleracea	35.20	0.00
TRINITY_PWA7946!	trimeric	Artemisia_annua	35.20	0.00
TRINITY_XP_0273	uncharac	Abrus_precatorius	35.20	0.00
TRINITY_XP_0029	DNA repl	Selaginella_moellen	35.10	0.00
TRINITY_EPS6635(	hypothet	Genlisea_aurea	35.10	0.00
TRINITY_BAJ9663(	predicte	Hordeum_vulgare	35.10	0.00
TRINITY_XP_0016!	NimA-rel	Chlamydomonas_reinh	35.10	0.00
TRINITY_GAQ8170!	hypothet	Klebsormidium_niten	35.10	0.00
TRINITY_XP_0102	PREDICTE	Nelumbo_nucifera	35.10	0.00
TRINITY_GAX7677(	hypothet	Chlamydomonas_eusti	35.10	0.00
TRINITY_ABR1725(	unknown	Picea_sitchensis	35.10	0.00
TRINITY_RWR7184	biogenes	Cinnamomum_micranth	35.10	0.00
TRINITY_RWR9478(	ras-rela	Cinnamomum_micranth	35.10	0.00
TRINITY_XP_0239	rho-type	Quercus_suber	35.10	0.00
TRINITY_KZV1754(	multidru	Dorcoceras_hygromet	35.10	0.00
TRINITY_PLY7556!	hypothet	Lactuca_sativa	35.10	0.00
TRINITY_EFJ3104!	hypothet	Selaginella_moellen	35.10	0.00
TRINITY_KFK3233	hypothet	Arabis_alpina	35.10	0.00
TRINITY_XP_0016!	flagella	Chlamydomonas_reinh	35.10	0.00
TRINITY_PTQ3094(	hypothet	Marchantia_polymorp	35.10	0.00
TRINITY_GAQ7873!	alpha-am	Klebsormidium_niten	35.10	0.00
TRINITY_XP_0024!	predicte	Micromonas_commoda	35.10	0.00
TRINITY_GAQ82062	hypothet	Klebsormidium_niten	35.10	0.00
TRINITY_XP_0056(	hypothet	Coccomyxa_subellips	35.10	0.00
TRINITY_XP_0029(	monothic	Selaginella_moellen	35.10	0.00
TRINITY_XP_0093(	PREDICTE	Musa_acuminata	35.10	0.00
TRINITY_PRW5589!	kinase	Chlorella_sorokinia	35.10	0.00
TRINITY_PWA5808	protein	Artemisia_annua	35.10	0.00
TRINITY_XP_0100(	PREDICTE	Eucalyptus_grandis	35.10	0.00
TRINITY_PNH0400(	Proteaso	Tetrabaena_socialis	35.10	0.00
TRINITY_XP_0238!	uncharac	Quercus_suber	35.10	0.00
TRINITY_XP_0029(	hypothet	Volvox_carteri	35.10	0.00
TRINITY_XP_0201(	BTB/POZ	Aegilops_tauschii	35.10	0.00
TRINITY_EEF2753!	two-comp	Ricinus_communis	35.10	0.00
TRINITY_KMZ6090	CBL-inte	Zostera_marina	35.10	0.00
TRINITY_GAQ8293(	ABC tran	Klebsormidium_niten	35.10	0.00
TRINITY_GAQ9250	hypothet	Klebsormidium_niten	35.10	0.00
TRINITY_XP_0029(	SCY1-lik	Selaginella_moellen	35.10	0.00
TRINITY_GAX8578	hypothet	Chlamydomonas_eusti	35.10	0.00
TRINITY_XP_0174	PREDICTE	Vigna_angularis	35.10	0.00
TRINITY_XP_0029(	serine/t	Selaginella_moellen	35.10	0.00
TRINITY_PSC7370	NAD-depe	Micractinium_conduc	35.10	0.00
TRINITY_XP_0071!	hypothet	Phaseolus_vulgaris	35.10	0.00
TRINITY_XP_0016!	hypothet	Chlamydomonas_reinh	35.10	0.00
TRINITY_XP_0244(	transcri	Physcomitrella_pate	35.10	0.00
TRINITY_GBG8268	hypothet	Chara_braunii	35.10	0.00
TRINITY_XP_0239	coronin-	Quercus_suber	35.10	0.00
TRINITY_XP_0258	serine/t	Panicum_hallii	35.10	0.00
TRINITY_GAX7466(	hypothet	Chlamydomonas_eusti	35.10	0.00
TRINITY_GAQ8123(	Aminotra	Klebsormidium_niten	35.10	0.00
TRINITY_XP_0205	alkylate	Phalaenopsis_equest	35.10	0.00
TRINITY_GAX7746	hypothet	Chlamydomonas_eusti	35.10	0.00
TRINITY_GAX8107(	hypothet	Chlamydomonas_eusti	35.10	0.00
TRINITY_XP_0029	protein	Selaginella_moellen	35.10	0.00
TRINITY_BAK06922	predicte	Hordeum_vulgare	35.10	0.00
TRINITY_XP_0138!	hypothet	Monoraphidium_negle	35.10	0.00
TRINITY_XP_0238!	pre-mRNA	Quercus_suber	35.10	0.00

TRINITY_GBF96504	hypothet	Raphidocelis_subcap	35.10	0.00
TRINITY_XP_00628	transcri	Capsella_rubella	35.10	0.00
TRINITY_XP_02718	LOW QUAI	Coffea_eugenioides	35.10	0.00
TRINITY_PTQ36584	hypothet	Marchantia_polymorp	35.10	0.00
TRINITY_KZV15279	hypothet	Dorcoceras_hygromet	35.10	0.00
TRINITY_PNW76789	hypothet	Chlamydomonas_reinh	35.10	0.00
TRINITY_PNH09773	Sulfatec	Tetrabaena_socialis	35.10	0.00
TRINITY_PNH06788	Chloroph	Tetrabaena_socialis	35.10	0.00
TRINITY_XP_01848	PREDICTI	Raphanus_sativus	35.10	0.00
TRINITY_XP_01918	PREDICTI	Ipomoea_nil	35.10	0.00
TRINITY_XP_00880	LOW QUAI	Phoenix_dactylifera	35.10	0.00
TRINITY_XP_00295	hypothet	Volvox_carteri	35.10	0.00
TRINITY_CBI29214	unnamed	Vitis_vinifera	35.10	0.00
TRINITY_PTQ39778	hypothet	Marchantia_polymorp	35.10	0.00
TRINITY_GAX84018	hypothet	Chlamydomonas_eusti	35.10	0.00
TRINITY_XP_00294	hypothet	Volvox_carteri	35.10	0.00
TRINITY_PON78660	Metallo-	Parasponia_anderson	35.10	0.00
TRINITY_KXZ56031	hypothet	Gonium_pectorale	35.10	0.00
TRINITY_XP_00294	hypothet	Volvox_carteri	35.10	0.00
TRINITY_XP_01283	PREDICTI	Erythranthe_guttata	35.00	0.00
TRINITY_PTQ36683	hypothet	Marchantia_polymorp	35.00	0.00
TRINITY_XP_01724	PREDICTI	Daucus_carota	35.00	0.00
TRINITY_XP_00565	putative	Coccomyxa_subellips	35.00	0.00
TRINITY_GAX80280	hypothet	Chlamydomonas_eusti	35.00	0.00
TRINITY_KZV25292	hypothet	Dorcoceras_hygromet	35.00	0.00
TRINITY_XP_02390	GTP-binc	Quercus_suber	35.00	0.00
TRINITY_NP_00131	E3 ubiqu	Capsicum_annuum	35.00	0.00
TRINITY_XP_00430	PREDICTI	Fragaria_vesca	35.00	0.00
TRINITY_PNW84678	hypothet	Chlamydomonas_reinh	35.00	0.00
TRINITY_XP_00305	predicte	Micromonas_pusilla	35.00	0.00
TRINITY_PRW50772	Fanconi	Chlorella_sorokinia	35.00	0.00
TRINITY_GBG60729	hypothet	Chara_braunii	35.00	0.00
TRINITY_PNW70067	hypothet	Chlamydomonas_reinh	35.00	0.00
TRINITY_XP_02734	probable	Abrus_precatorius	35.00	0.00
TRINITY_XP_01389	Histone	Monoraphidium_negle	35.00	0.00
TRINITY_GAX74044	hypothet	Chlamydomonas_eusti	35.00	0.00
TRINITY_RVW52073	Stomatin	Vitis_vinifera	35.00	0.00
TRINITY_XP_00297	mitochon	Selaginella_moellen	35.00	0.00
TRINITY_GAQ85806	phosphat	Klebsormidium_niten	35.00	0.00
TRINITY_AAO73552	putative	Chlamydomonas_reinh	35.00	0.00
TRINITY_GAX86120	hypothet	Chlamydomonas_eusti	35.00	0.00
TRINITY_OAE31294	hypothet	Marchantia_polymorp	35.00	0.00
TRINITY_PNW87945	hypothet	Chlamydomonas_reinh	35.00	0.00
TRINITY_PRW58094	GPN-loop	Chlorella_sorokinia	35.00	0.00
TRINITY_XP_00294	hypothet	Volvox_carteri	35.00	0.00
TRINITY_OAE32490	hypothet	Marchantia_polymorp	35.00	0.00
TRINITY_XP_02436	rRNA-pro	Physcomitrella_pate	35.00	0.00
TRINITY_PKA46458	hypothet	Apostasia_shenzheni	35.00	0.00
TRINITY_GBF96838	hypothet	Raphidocelis_subcap	35.00	0.00
TRINITY_XP_00295	hypothet	Volvox_carteri	35.00	0.00
TRINITY_PNW82298	hypothet	Chlamydomonas_reinh	35.00	0.00
TRINITY_GAX83109	hypothet	Chlamydomonas_eusti	35.00	0.00
TRINITY_GAX79974	hypothet	Chlamydomonas_eusti	35.00	0.00
TRINITY_ACF87335	unknown	Zea_mays	35.00	0.00
TRINITY_KMZ72055	THUMP dc	Zostera_marina	35.00	0.00
TRINITY_XP_01067	PREDICTI	Beta_vulgaris	35.00	0.00
TRINITY_XP_01724	PREDICTI	Daucus_carota	35.00	0.00
TRINITY_XP_02638	probable	Papaver_somniferum	35.00	0.00
TRINITY_GAX81772	hypothet	Chlamydomonas_eusti	35.00	0.00
TRINITY_PTQ28716	hypothet	Marchantia_polymorp	35.00	0.00
TRINITY_GAX78082	hypothet	Chlamydomonas_eusti	35.00	0.00
TRINITY_XP_01048	PREDICTI	Camelina_sativa	35.00	0.00
TRINITY_XP_00295	hypothet	Volvox_carteri	35.00	0.00
TRINITY_EFJ27745	hypothet	Selaginella_moellen	35.00	0.00
TRINITY_XP_00294	hypothet	Volvox_carteri	35.00	0.00
TRINITY_AGT17129	embryoge	Saccharum_hybrid_cu	35.00	0.00

TRINITY_GAX8207	hypothet	Chlamydomonas_eusti	35.00	0.00
TRINITY_GAX8195	hypothet	Chlamydomonas_eusti	35.00	0.00
TRINITY_XP_0106	PREDICTE	Beta_vulgaris	35.00	0.00
TRINITY_RMZ5701	hypothet	Auxenochlorella_prc	35.00	0.00
TRINITY_GAX7956	hypothet	Chlamydomonas_eusti	35.00	0.00
TRINITY_GAX7547	hypothet	Chlamydomonas_eusti	35.00	0.00
TRINITY_XP_0239	peroxide	Quercus_suber	35.00	0.00
TRINITY_XP_0202	VAMP-lik	Asparagus_officinal	35.00	0.00
TRINITY_XP_0104	PREDICTE	Camelina_sativa	35.00	0.00
TRINITY_XP_0237	dynamin-	Lactuca_sativa	35.00	0.00
TRINITY_GAX8614	hypothet	Chlamydomonas_eusti	35.00	0.00
TRINITY_GAX7472	hypothet	Chlamydomonas_eusti	35.00	0.00
TRINITY_XP_0029	hypothet	Volvox_carteri	35.00	0.00
TRINITY_XP_0206	importin	Dendrobium_catenatu	35.00	0.00
TRINITY_KDD7501	LEM3/CDC	Helicosporidium_sp.	35.00	0.00
TRINITY_KXZ5161	hypothet	Gonium_pectorale	35.00	0.00
TRINITY_XP_0249	ADP-ribo	Ziziphus_jujuba	35.00	0.00
TRINITY_PPR9275	hypothet	Gossypium_barbadens	35.00	0.00
TRINITY_GAX7715	hypothet	Chlamydomonas_eusti	35.00	0.00
TRINITY_GAQ9244	hypothet	Klebsormidium_niten	35.00	0.00
TRINITY_GAX8445	hypothet	Chlamydomonas_eusti	35.00	0.00
TRINITY_PSC7356	Hydroxyl	Micractinium_conduc	34.90	0.00
TRINITY_GAQ8602	Serine T	Klebsormidium_niten	34.90	0.00
TRINITY_XP_0063	PREDICTE	Solanum_tuberosum	34.90	0.00
TRINITY_XP_0264	high mol	Papaver_somniferum	34.90	0.00
TRINITY_PRW5643	transpor	Chlorella_sorokinia	34.90	0.00
TRINITY_KNA0322	hypothet	Spinacia_oleracea	34.90	0.00
TRINITY_PNW7109	hypothet	Chlamydomonas_reinh	34.90	0.00
TRINITY_PRW3257	Cycloeuc	Chlorella_sorokinia	34.90	0.00
TRINITY_XP_0029	2-oxoglu	Selaginella_moellen	34.90	0.00
TRINITY_XP_0093	PREDICTE	Pyrus_x_bretschneid	34.90	0.00
TRINITY_PRW5985	5 -nucle	Chlorella_sorokinia	34.90	0.00
TRINITY_PTQ3181	hypothet	Marchantia_polymorp	34.90	0.00
TRINITY_XP_0243	glutathi	Physcomitrella_pate	34.90	0.00
TRINITY_GAQ8020	hypothet	Klebsormidium_niten	34.90	0.00
TRINITY_XP_0202	U-box do	Cajanus_cajan	34.90	0.00
TRINITY_XP_0243	glutamat	Physcomitrella_pate	34.90	0.00
TRINITY_XP_0238	probable	Quercus_suber	34.90	0.00
TRINITY_XP_0025	hypothet	Micromonas_commoda	34.90	0.00
TRINITY_GBF9725	hypothet	Raphidocelis_subcap	34.90	0.00
TRINITY_XP_0243	tetratri	Physcomitrella_pate	34.90	0.00
TRINITY_XP_0035	embryoge	Medicago_truncatula	34.90	0.00
TRINITY_PSC6843	phosphor	Micractinium_conduc	34.90	0.00
TRINITY_GAX7328	hypothet	Chlamydomonas_eusti	34.90	0.00
TRINITY_GAX7296	hypothet	Chlamydomonas_eusti	34.90	0.00
TRINITY_PNW8416	hypothet	Chlamydomonas_reinh	34.90	0.00
TRINITY_BAJ9317	predicte	Hordeum_vulgare	34.90	0.00
TRINITY_GAQ8172	Protein	Klebsormidium_niten	34.90	0.00
TRINITY_XP_0239	serine/t	Quercus_suber	34.90	0.00
TRINITY_XP_0241	protein	Rosa_chinensis	34.90	0.00
TRINITY_PNW7273	hypothet	Chlamydomonas_reinh	34.90	0.00
TRINITY_XP_0029	hypothet	Volvox_carteri	34.90	0.00
TRINITY_XP_0239	carrier	Quercus_suber	34.90	0.00
TRINITY_GAX8018	hypothet	Chlamydomonas_eusti	34.90	0.00
TRINITY_RMZ5412	hypothet	Auxenochlorella_prc	34.90	0.00
TRINITY_XP_0041	PREDICTE	Cucumis_sativus	34.90	0.00
TRINITY_KXZ5513	hypothet	Gonium_pectorale	34.90	0.00
TRINITY_KZV1544	programm	Dorcoceras_hygromet	34.90	0.00
TRINITY_KGN6006	hypothet	Cucumis_sativus	34.90	0.00
TRINITY_GBG9306	hypothet	Chara_braunii	34.90	0.00
TRINITY_XP_0243	uncharac	Physcomitrella_pate	34.90	0.00
TRINITY_KXZ4519	hypothet	Gonium_pectorale	34.90	0.00
TRINITY_KXZ5571	DHC8 pro	Gonium_pectorale	34.90	0.00
TRINITY_PNW7849	hypothet	Chlamydomonas_reinh	34.90	0.00
TRINITY_XP_0093	PREDICTE	Musa_acuminata	34.90	0.00
TRINITY_XP_0029	hypothet	Volvox_carteri	34.90	0.00

TRINITY_PNH10445	putative	Tetrabaena_socialis	34.90	0.00
TRINITY_XP_01763	PREDICTE	Gossypium_arboreum	34.90	0.00
TRINITY_XP_02392	cullin-1	Quercus_suber	34.90	0.00
TRINITY_BAJ96688	predicte	Hordeum_vulgare	34.90	0.00
TRINITY_PTQ27624	hypothet	Marchantia_polymorp	34.90	0.00
TRINITY_XP_01390	hypothet	Monoraphidium_negle	34.90	0.00
TRINITY_XP_01368	ras-rela	Brassica_napus	34.90	0.00
TRINITY_XP_01675	PREDICTE	Gossypium_hirsutum	34.90	0.00
TRINITY_GAX76742	hypothet	Chlamydomonas_eusti	34.90	0.00
TRINITY_XP_00169	solanesy	Chlamydomonas_reinh	34.90	0.00
TRINITY_GAX81325	hypothet	Chlamydomonas_eusti	34.90	0.00
TRINITY_RVW70653	Retrovir	Vitis_vinifera	34.90	0.00
TRINITY_GAX75393	hypothet	Chlamydomonas_eusti	34.90	0.00
TRINITY_PNW86099	hypothet	Chlamydomonas_reinh	34.90	0.00
TRINITY_AIU49038	LOS4, pa	Illicium_henryi	34.90	0.00
TRINITY_XP_02017	U3 small	Aegilops_tauschii	34.90	0.00
TRINITY_GBG00107	hypothet	Raphidocelis_subcap	34.90	0.00
TRINITY_GAX77497	hypothet	Chlamydomonas_eusti	34.90	0.00
TRINITY_PRW56431	Peptide-	Chlorella_sorokinia	34.90	0.00
TRINITY_PNH03819	hypothet	Tetrabaena_socialis	34.90	0.00
TRINITY_XP_01390	hypothet	Monoraphidium_negle	34.90	0.00
TRINITY_GAX76125	hypothet	Chlamydomonas_eusti	34.90	0.00
TRINITY_BAJ95402	predicte	Hordeum_vulgare	34.80	0.00
TRINITY_XP_02130	uncharac	Sorghum_bicolor	34.80	0.00
TRINITY_OAE25115	hypothet	Marchantia_polymorp	34.80	0.00
TRINITY_XP_02440	protein	Physcomitrella_pate	34.80	0.00
TRINITY_XP_01595	LOW QUAL	Arachis_duranensis	34.80	0.00
TRINITY_PRW60250	ser thr	Chlorella_sorokinia	34.80	0.00
TRINITY_XP_02059	LOW QUAL	Phalaenopsis_equest	34.80	0.00
TRINITY_XP_01041	PREDICTE	Camelina_sativa	34.80	0.00
TRINITY_GBG92058	hypothet	Chara_braunii	34.80	0.00
TRINITY_PHT83098	hypothet	Capsicum_annuum	34.80	0.00
TRINITY_KXZ44781	hypothet	Gonium_pectorale	34.80	0.00
TRINITY_GAQ87417	hypothet	Klebsormidium_niten	34.80	0.00
TRINITY_GAQ88885	NAD-depe	Klebsormidium_niten	34.80	0.00
TRINITY_PRW61158	GTPase I	Chlorella_sorokinia	34.80	0.00
TRINITY_ACO50733	NADH deh	Micromonas_pusilla	34.80	0.00
TRINITY_KXZ53246	hypothet	Gonium_pectorale	34.80	0.00
TRINITY_GAQ80689	Serine T	Klebsormidium_niten	34.80	0.00
TRINITY_XP_02068	putative	Dendrobium_catenatu	34.80	0.00
TRINITY_XP_01620	synaptot	Arachis_ipaensis	34.80	0.00
TRINITY_XP_00294	hypothet	Volvox_carteri	34.80	0.00
TRINITY_PRW59174	calcium	Chlorella_sorokinia	34.80	0.00
TRINITY_GAQ77713	HECT dom	Klebsormidium_niten	34.80	0.00
TRINITY_PNW79650	hypothet	Chlamydomonas_reinh	34.80	0.00
TRINITY_PNH11697	Bifuncti	Tetrabaena_socialis	34.80	0.00
TRINITY_PRW60243	charged	Chlorella_sorokinia	34.80	0.00
TRINITY_XP_00169	Qc-SNARE	Chlamydomonas_reinh	34.80	0.00
TRINITY_OAE18657	hypothet	Marchantia_polymorp	34.80	0.00
TRINITY_GAQ86100	cation e	Klebsormidium_niten	34.80	0.00
TRINITY_XP_00295	hypothet	Volvox_carteri	34.80	0.00
TRINITY_XP_02173	phosphol	Chenopodium_quinoa	34.80	0.00
TRINITY_BAJ93724	predicte	Hordeum_vulgare	34.80	0.00
TRINITY_KXZ50586	hypothet	Gonium_pectorale	34.80	0.00
TRINITY_XP_00141	predicte	Ostreococcus_sp._'l	34.80	0.00
TRINITY_PIA36270	hypothet	Aquilegia_coerulea	34.80	0.00
TRINITY_PRW56585	Vacuolar	Chlorella_sorokinia	34.80	0.00
TRINITY_XP_02295	RNA-binc	Cucurbita_moschata	34.80	0.00
TRINITY_XP_01389	F-box pr	Monoraphidium_negle	34.80	0.00
TRINITY_GAX77840	hypothet	Chlamydomonas_eusti	34.80	0.00
TRINITY_XP_00295	hypothet	Volvox_carteri	34.80	0.00
TRINITY_XP_00635	PREDICTE	Solanum_tuberosum	34.80	0.00
TRINITY_BAK02517	predicte	Hordeum_vulgare	34.80	0.00
TRINITY_XP_02295	probable	Cucurbita_moschata	34.80	0.00
TRINITY_GAX77686	hypothet	Chlamydomonas_eusti	34.80	0.00
TRINITY_GAX75480	hypothet	Chlamydomonas_eusti	34.80	0.00

TRINITY_PIA5562(	hypothet	Aquilegia_coerulea	34.80	0.00
TRINITY_PNH1146(	hypothet	Tetrabaena_socialis	34.80	0.00
TRINITY_AFK4767	unknown	Medicago_truncatula	34.80	0.00
TRINITY_GAX8439	hypothet	Chlamydomonas_eusti	34.80	0.00
TRINITY_OAE1928	hypothet	Marchantia_polymorp	34.80	0.00
TRINITY_XP_0029	hypothet	Volvox_carteri	34.80	0.00
TRINITY_XP_0016	predicte	Chlamydomonas_reinh	34.80	0.00
TRINITY_XP_0124	PREDICTE	Gossypium_raimondii	34.80	0.00
TRINITY_RWR8770	ABC tran	Cinnamomum_micranth	34.80	0.00
TRINITY_XP_0029	ATM/ATR-	Volvox_carteri	34.80	0.00
TRINITY_RWR9474	putative	Cinnamomum_micranth	34.80	0.00
TRINITY_XP_0058	hypothet	Chlorella_variabili	34.80	0.00
TRINITY_XP_0238	uncharac	Quercus_suber	34.80	0.00
TRINITY_GAX8285	hypothet	Chlamydomonas_eusti	34.80	0.00
TRINITY_RYR1017	hypothet	Arachis_hypogaea	34.80	0.00
TRINITY_PSC6944	arginine	Micractinium_conduc	34.80	0.00
TRINITY_RID5033	hypothet	Brassica_rapa	34.80	0.00
TRINITY_XP_0056	DnaJ-dom	Coccomyxa_subellips	34.80	0.00
TRINITY_XP_0244	calpain-	Physcomitrella_pate	34.80	0.00
TRINITY_RZC0066	Calcium-	Glycine_soja	34.80	0.00
TRINITY_XP_0028	pathogen	Arabidopsis_lyrata	34.80	0.00
TRINITY_GAQ8096	M13 fami	Klebsormidium_niten	34.80	0.00
TRINITY_PRW5900	5 -3 exo	Chlorella_sorokinia	34.80	0.00
TRINITY_RMZ5517	hypothet	Auxenochlorella_pro	34.80	0.00
TRINITY_AIU4162	ABC tran	Hevea_brasiliensis	34.80	0.00
TRINITY_OWM6383	hypothet	Punica_granatum	34.70	0.00
TRINITY_OAE2597	hypothet	Marchantia_polymorp	34.70	0.00
TRINITY_XP_0257	serine/t	Panicum_hallii	34.70	0.00
TRINITY_OAE2957	hypothet	Marchantia_polymorp	34.70	0.00
TRINITY_XP_0216	tetraket	Hevea_brasiliensis	34.70	0.00
TRINITY_XP_0120	probable	Jatropha_curcas	34.70	0.00
TRINITY_KXZ4302	hypothet	Gonium_pectorale	34.70	0.00
TRINITY_XP_0239	DNA mism	Quercus_suber	34.70	0.00
TRINITY_AML7930	putative	Prasinococcus_capsu	34.70	0.00
TRINITY_XP_0116	BTB/POZ	Amborella_trichopod	34.70	0.00
TRINITY_GAQ9165	hypothet	Klebsormidium_niten	34.70	0.00
TRINITY_XP_0156	mitochon	Oryza_sativa	34.70	0.00
TRINITY_PSC7680	alpha be	Micractinium_conduc	34.70	0.00
TRINITY_PPD8108	hypothet	Gossypium_barbadens	34.70	0.00
TRINITY_EMS5136	WD and t	Triticum_urartu	34.70	0.00
TRINITY_GBG8825	hypothet	Chara_braunii	34.70	0.00
TRINITY_XP_0024	histone-	Sorghum_bicolor	34.70	0.00
TRINITY_XP_0172	PREDICTE	Daucus_carota	34.70	0.00
TRINITY_XP_0238	protein	Quercus_suber	34.70	0.00
TRINITY_XP_0202	rac-like	Asparagus_officinal	34.70	0.00
TRINITY_KJB4190	hypothet	Gossypium_raimondii	34.70	0.00
TRINITY_XP_0238	ubiquiti	Quercus_suber	34.70	0.00
TRINITY_XP_0228	calcineu	Olea_europaea	34.70	0.00
TRINITY_EFJ2052	hypothet	Selaginella_moellen	34.70	0.00
TRINITY_GAQ8786	sphingom	Klebsormidium_niten	34.70	0.00
TRINITY_RAL3909	hypothet	Cuscuta_australis	34.70	0.00
TRINITY_PNH1266	Acyl-pro	Tetrabaena_socialis	34.70	0.00
TRINITY_XP_0030	Aspartat	Micromonas_pusilla	34.70	0.00
TRINITY_PIN1367	Zuotin	Handroanthus_impeti	34.70	0.00
TRINITY_OAE2441	hypothet	Marchantia_polymorp	34.70	0.00
TRINITY_XP_0243	serine/t	Physcomitrella_pate	34.70	0.00
TRINITY_XP_0014	predicte	Ostreococcus_sp._'l	34.70	0.00
TRINITY_XP_0238	alpha-ac	Quercus_suber	34.70	0.00
TRINITY_EFJ3720	hypothet	Selaginella_moellen	34.70	0.00
TRINITY_XP_0029	hypothet	Volvox_carteri	34.70	0.00
TRINITY_GAX8092	hypothet	Chlamydomonas_eusti	34.70	0.00
TRINITY_GAX7592	hypothet	Chlamydomonas_eusti	34.70	0.00
TRINITY_XP_0056	hypothet	Coccomyxa_subellips	34.70	0.00
TRINITY_GAQ8635	exoribon	Klebsormidium_niten	34.70	0.00
TRINITY_XP_0202	uncharac	Asparagus_officinal	34.70	0.00
TRINITY_GBG5905	hypothet	Chara_braunii	34.70	0.00

TRINITY_GAX7986	hypothet	Chlamydomonas_eusti	34.70	0.00
TRINITY_OAE2762	hypothet	Marchantia_polymorp	34.70	0.00
TRINITY_XP_0023	phospho	Populus_trichocarpa	34.70	0.00
TRINITY_XP_0029	hypothet	Volvox_carteri	34.70	0.00
TRINITY_OMO5889	hypothet	Corchorus_capsulari	34.70	0.00
TRINITY_GAX7267	hypothet	Chlamydomonas_eusti	34.70	0.00
TRINITY_OAE2555	hypothet	Marchantia_polymorp	34.70	0.00
TRINITY_XP_0270	mRNA-dec	Coffea_arabica	34.70	0.00
TRINITY_XP_0068	anaphase	Amborella_trichopod	34.70	0.00
TRINITY_XP_0014	predicte	Ostreococcus_sp._'l	34.70	0.00
TRINITY_XP_0217	ankyrin-	Chenopodium_quinoa	34.70	0.00
TRINITY_XP_0238	DNA repa	Quercus_suber	34.70	0.00
TRINITY_XP_0243	ATP-depe	Physcomitrella_pate	34.70	0.00
TRINITY_XP_0201	protein	Aegilops_tauschii	34.70	0.00
TRINITY_PNW7758	hypothet	Chlamydomonas_reinh	34.70	0.00
TRINITY_XP_0217	CBL-inte	Chenopodium_quinoa	34.70	0.00
TRINITY_VDD5784	unnamed	Brassica_oleracea	34.70	0.00
TRINITY_PKA6656	Serine/t	Apostasia_shenzheni	34.70	0.00
TRINITY_GAX7714	hypothet	Chlamydomonas_eusti	34.70	0.00
TRINITY_GBF9459	hypothet	Raphidocelis_subcap	34.70	0.00
TRINITY_XP_0029	hypothet	Volvox_carteri	34.70	0.00
TRINITY_XP_0138	hypothet	Monoraphidium_negle	34.70	0.00
TRINITY_BAK0177	predicte	Hordeum_vulgare	34.70	0.00
TRINITY_XP_0238	uncharac	Quercus_suber	34.70	0.00
TRINITY_PRW6126	DNAJ hea	Chlorella_sorokinia	34.70	0.00
TRINITY_GAX7450	hypothet	Chlamydomonas_eusti	34.70	0.00
TRINITY_GBF9660	hypothet	Raphidocelis_subcap	34.70	0.00
TRINITY_GAX7422	hypothet	Chlamydomonas_eusti	34.70	0.00
TRINITY_PWA9317	Protein	Artemisia_annua	34.70	0.00
TRINITY_KMZ5949	E3 ubiqu	Zostera_marina	34.60	0.00
TRINITY_XP_0243	uncharac	Physcomitrella_pate	34.60	0.00
TRINITY_XP_0128	PREDICTE	Erythranthe_guttata	34.60	0.00
TRINITY_GBF9293	cytochro	Raphidocelis_subcap	34.60	0.00
TRINITY_OAE3248	hypothet	Marchantia_polymorp	34.60	0.00
TRINITY_PSC6993	Copper b	Micractinium_conduc	34.60	0.00
TRINITY_GAX7865	hypothet	Chlamydomonas_eusti	34.60	0.00
TRINITY_GBG9114	hypothet	Chara_braunii	34.60	0.00
TRINITY_XP_0104	PREDICTE	Camelina_sativa	34.60	0.00
TRINITY_GAX8389	hypothet	Chlamydomonas_eusti	34.60	0.00
TRINITY_PLY9770	hypothet	Lactuca_sativa	34.60	0.00
TRINITY_XP_0217	actin-7-	Chenopodium_quinoa	34.60	0.00
TRINITY_XP_0075	PHD tran	Bathycoccus_prasino	34.60	0.00
TRINITY_XP_0172	PREDICTE	Daucus_carota	34.60	0.00
TRINITY_XP_0075	predicte	Bathycoccus_prasino	34.60	0.00
TRINITY_XP_0159	peroxiso	Arachis_duranensis	34.60	0.00
TRINITY_XP_0217	probable	Chenopodium_quinoa	34.60	0.00
TRINITY_XP_0058	hypothet	Chlorella_variabili	34.60	0.00
TRINITY_PRW6126	DNAJ hea	Chlorella_sorokinia	34.60	0.00
TRINITY_XP_0273	very-lon	Abrus_precatorius	34.60	0.00
TRINITY_OMO7319	hypothet	Corchorus_capsulari	34.60	0.00
TRINITY_GAX8038	hypothet	Chlamydomonas_eusti	34.60	0.00
TRINITY_PNW8427	hypothet	Chlamydomonas_reinh	34.60	0.00
TRINITY_KXZ5407	hypothet	Gonium_pectorale	34.60	0.00
TRINITY_XP_0205	LETM1 an	Sesamum_indicum	34.60	0.00
TRINITY_GBF9269	hypothet	Raphidocelis_subcap	34.60	0.00
TRINITY_GAQ9104	calcium-	Klebsormidium_niten	34.60	0.00
TRINITY_OWM6913	hypothet	Punica_granatum	34.60	0.00
TRINITY_BAK0219	predicte	Hordeum_vulgare	34.60	0.00
TRINITY_XP_0239	cell div	Quercus_suber	34.60	0.00
TRINITY_GAX8457	hypothet	Chlamydomonas_eusti	34.60	0.00
TRINITY_EFJ0963	hypothet	Selaginella_moellen	34.60	0.00
TRINITY_GBF8810	hypothet	Raphidocelis_subcap	34.60	0.00
TRINITY_XP_0029	SCY1-lik	Selaginella_moellen	34.60	0.00
TRINITY_KXZ4840	hypothet	Gonium_pectorale	34.60	0.00
TRINITY_XP_0058	hypothet	Chlorella_variabili	34.60	0.00
TRINITY_GAQ8989	hypothet	Klebsormidium_niten	34.60	0.00

TRINITY_XP_0066	PREDICTI	Oryza_brachyantha	34.60	0.00
TRINITY_PNW7307	hypothet	Chlamydomonas_reinh	34.60	0.00
TRINITY_PNW6989	hypothet	Chlamydomonas_reinh	34.60	0.00
TRINITY_XP_0138	hypothet	Monoraphidium_negle	34.60	0.00
TRINITY_PTQ3629	hypothet	Marchantia_polymorp	34.60	0.00
TRINITY_PNR6013	hypothet	Physcomitrella_pate	34.60	0.00
TRINITY_KXZ5006	hypothet	Gonium_pectorale	34.60	0.00
TRINITY_PNH1158	Serine/t	Tetrabaena_socialis	34.60	0.00
TRINITY_XP_0202	uncharac	Asparagus_officinal	34.60	0.00
TRINITY_GAX7327	hypothet	Chlamydomonas_eusti	34.60	0.00

TRINITY_XP_0056	alpha/b	Coccomyxa_subellips	34.60	0.00
TRINITY_XP_0139	hypothet	Monoraphidium_negle	34.60	0.00
TRINITY_XP_0029	hypothet	Volvox_carteri	34.60	0.00
TRINITY_XP_0245	gem-ass	Selaginella_moellen	34.60	0.00
TRINITY_GAX8281	hypothet	Chlamydomonas_eusti	34.60	0.00
TRINITY_GAX7463	hypothet	Chlamydomonas_eusti	34.60	0.00
TRINITY_GAX8373	hypothet	Chlamydomonas_eusti	34.60	0.00
TRINITY_GAX7595	hypothet	Chlamydomonas_eusti	34.60	0.00
TRINITY_BAJ9694	predict	Hordeum_vulgare	34.60	0.00
TRINITY_GAX7842	hypothet	Chlamydomonas_eusti	34.60	0.00
TRINITY_PWA8786	multipl	Artemisia_annua	34.60	0.00
TRINITY_GBF9650	hypothet	Raphidocelis_subcap	34.60	0.00
TRINITY_GBG6428	hypothet	Chara_braunii	34.60	0.00
TRINITY_GAX7670	hypothet	Chlamydomonas_eusti	34.60	0.00
TRINITY_GAX8191	hypothet	Chlamydomonas_eusti	34.60	0.00
TRINITY_XP_0029	hypothet	Volvox_carteri	34.60	0.00
TRINITY_XP_0139	putativ	Monoraphidium_negle	34.60	0.00
TRINITY_PNW7702	hypothet	Chlamydomonas_reinh	34.60	0.00
TRINITY_XP_0030	predict	Micromonas_pusilla	34.60	0.00
TRINITY_OAE2597	hypothet	Marchantia_polymorp	34.50	0.00
TRINITY_GAQ8237	GTPase	Klebsormidium_niten	34.50	0.00
TRINITY_RHN3997	putativ	Medicago_truncatula	34.50	0.00
TRINITY_KXZ4330	hypothet	Gonium_pectorale	34.50	0.00
TRINITY_XP_0217	leucine-	Chenopodium_quinoa	34.50	0.00
TRINITY_KDO6609	hypothet	Citrus_sinensis	34.50	0.00
TRINITY_GAQ8194	hypothet	Klebsormidium_niten	34.50	0.00
TRINITY_PHT8550	26S prot	Capsicum_annuum	34.50	0.00
TRINITY_GAX7413	hypothet	Chlamydomonas_eusti	34.50	0.00
TRINITY_XP_0025	predict	Micromonas_commoda	34.50	0.00
TRINITY_EFJ3104	hypothet	Selaginella_moellen	34.50	0.00
TRINITY_XP_0104	PREDICT	Camelina_sativa	34.50	0.00
TRINITY_GAV6461	PB1 dom	Cephalotus_follicul	34.50	0.00
TRINITY_XP_0239	uncharac	Quercus_suber	34.50	0.00
TRINITY_XP_0216	lysosom	Manihot_esculenta	34.50	0.00
TRINITY_ADE7668	unknown	Picea_sitchensis	34.50	0.00
TRINITY_XP_0109	PREDICT	Elaeis_guineensis	34.50	0.00
TRINITY_GBG8282	hypothet	Chara_braunii	34.50	0.00
TRINITY_KXZ4138	hypothet	Gonium_pectorale	34.50	0.00
TRINITY_KXZ4748	hypothet	Gonium_pectorale	34.50	0.00
TRINITY_XP_0238	probabl	Quercus_suber	34.50	0.00
TRINITY_XP_0239	protein	Quercus_suber	34.50	0.00
TRINITY_GAX8539	hypothet	Chlamydomonas_eusti	34.50	0.00
TRINITY_XP_0058	hypothet	Chlorella_variabili	34.50	0.00
TRINITY_XP_0091	PREDICT	Brassica_rapa	34.50	0.00
TRINITY_PNR5000	hypothet	Physcomitrella_pate	34.50	0.00
TRINITY_XP_0029	cyclin-	Selaginella_moellen	34.50	0.00
TRINITY_XP_0016	compone	Chlamydomonas_reinh	34.50	0.00
TRINITY_GAX7930	hypothet	Chlamydomonas_eusti	34.50	0.00
TRINITY_XP_0096	PREDICT	Nicotiana_tomentosi	34.50	0.00
TRINITY_XP_0166	PREDICT	Gossypium_hirsutum	34.50	0.00
TRINITY_GAX8351	hypothet	Chlamydomonas_eusti	34.50	0.00
TRINITY_XP_0244	11-beta-	Populus_trichocarpa	34.50	0.00
TRINITY_XP_0063	uncharac	Eutrema_salsugineum	34.50	0.00
TRINITY_XP_0014	predict	Ostreococcus_sp._'l	34.50	0.00
TRINITY_OEL3448	hypothet	Dichanthelium_oligo	34.50	0.00
TRINITY_XP_0042	PREDICT	Fragaria_vesca	34.50	0.00
TRINITY_KZV4685	hypothet	Dorcoceras_hygromet	34.50	0.00
TRINITY_GAX7757	hypothet	Chlamydomonas_eusti	34.50	0.00
TRINITY_GAX8373	hypothet	Chlamydomonas_eusti	34.50	0.00
TRINITY_XP_0030	voltage-	Micromonas_pusilla	34.50	0.00
TRINITY_XP_0125	protein	Cicer_arietinum	34.50	0.00
TRINITY_XP_0158	TLD dom	Ziziphus_jujuba	34.50	0.00
TRINITY_XP_0025	predict	Micromonas_commoda	34.50	0.00
TRINITY_OMO7495	Zinc fi	Corchorus_olitorius	34.50	0.00
TRINITY_KXZ4775	hypothet	Gonium_pectorale	34.50	0.00
TRINITY_XP_0025	predict	Micromonas_commoda	34.50	0.00

TRINITY_NP_0012:	phospho€	Glycine_max	34.50	0.00
TRINITY_PNH1285:	hypothet	Tetrabaena_socialis	34.50	0.00
TRINITY_GAX8019:	hypothet	Chlamydomonas_eusti	34.50	0.00
TRINITY_XP_0258:	probabl€	Panicum_hallii	34.50	0.00
TRINITY_XP_0017(	predict€	Chlamydomonas_reinh	34.50	0.00
TRINITY_GAQ9090:	hypothet	Klebsormidium_niten	34.50	0.00
TRINITY_GAX7714:	hypothet	Chlamydomonas_eusti	34.50	0.00
TRINITY_XP_0056	DUF1740-	Coccomyxa_subellips	34.50	0.00
TRINITY_OWM6644	hypothet	Punica_granatum	34.50	0.00
TRINITY_KXZ5024:	hypothet	Gonium_pectorale	34.50	0.00
TRINITY_CAA7464	putativ€	Sorghum_bicolor	34.50	0.00
TRINITY_NP_1727:	RHOMBOII	Arabidopsis_thalian	34.50	0.00
TRINITY_OAE3087	hypothet	Marchantia_polymorp	34.50	0.00
TRINITY_GAX7694	hypothet	Chlamydomonas_eusti	34.50	0.00
TRINITY_XP_0056	HD-doma:	Coccomyxa_subellips	34.50	0.00
TRINITY_GAX7902:	hypothet	Chlamydomonas_eusti	34.50	0.00
TRINITY_XP_0016:	predict€	Chlamydomonas_reinh	34.50	0.00
TRINITY_GAX7561	hypothet	Chlamydomonas_eusti	34.50	0.00
TRINITY_KXZ5134:	hypothet	Gonium_pectorale	34.50	0.00
TRINITY_XP_0017(	cytochr(	Chlamydomonas_reinh	34.50	0.00
TRINITY_XP_0191	PREDICTI	Ipomoea_nil	34.40	0.00
TRINITY_XP_0056:	vesicle-	Coccomyxa_subellips	34.40	0.00
TRINITY_XP_0022	PREDICTI	Vitis_vinifera	34.40	0.00
TRINITY_KMZ5853(	Ras-rel:	Zostera_marina	34.40	0.00
TRINITY_XP_0239(	unchara(	Quercus_suber	34.40	0.00
TRINITY_GAQ8194	hypothet	Klebsormidium_niten	34.40	0.00
TRINITY_XP_0150	bidirect	Solanum_pennellii	34.40	0.00
TRINITY_GAQ8786	sphingor	Klebsormidium_niten	34.40	0.00
TRINITY_XP_0014:	predict€	Ostreococcus_sp._'l	34.40	0.00
TRINITY_XP_0194	PREDICTI	Lupinus_angustifoli	34.40	0.00
TRINITY_XP_0103:	mRNA-ca|	Solanum_lycopersicu	34.40	0.00
TRINITY_PTQ3007:	hypothet	Marchantia_polymorp	34.40	0.00
TRINITY_PSC6872:	ubiquit:	Micractinium_conduc	34.40	0.00
TRINITY_PNR4682	hypothet	Physcomitrella_pate	34.40	0.00
TRINITY_NP_1799	Class I	Arabidopsis_thalian	34.40	0.00
TRINITY_XP_0029:	hypothet	Volvox_carteri	34.40	0.00
TRINITY_XP_0109	PREDICTI	Elaeis_guineensis	34.40	0.00
TRINITY_XP_0191(	PREDICTI	Ipomoea_nil	34.40	0.00
TRINITY_XP_0014:	predict€	Ostreococcus_sp._'l	34.40	0.00
TRINITY_GBF9744	bifunct:	Raphidocelis_subcap	34.40	0.00
TRINITY_PRW0592:	melanot:	Chlorella_sorokinia	34.40	0.00
TRINITY_XP_0029:	hypothet	Volvox_carteri	34.40	0.00
TRINITY_PPS1007(	hypothet	Gossypium_barbadens	34.40	0.00
TRINITY_PNH0419	putativ€	Tetrabaena_socialis	34.40	0.00
TRINITY_CBI2075:	unnamed	Vitis_vinifera	34.40	0.00
TRINITY_OMO7004(	hypothet	Corchorus_capsulari	34.40	0.00
TRINITY_XP_0202	beta-ad:	Asparagus_officinal	34.40	0.00
TRINITY_GAQ8172	Protein	Klebsormidium_niten	34.40	0.00
TRINITY_PNW8504:	hypothet	Chlamydomonas_reinh	34.40	0.00
TRINITY_GAX8408:	hypothet	Chlamydomonas_eusti	34.40	0.00
TRINITY_GAX7847	hypothet	Chlamydomonas_eusti	34.40	0.00
TRINITY_GBF9918(	eukaryot	Raphidocelis_subcap	34.40	0.00
TRINITY_XP_0049	ubiquit:	Setaria_italica	34.40	0.00
TRINITY_RMZ5207	hypothet	Auxenochlorella_prc	34.40	0.00
TRINITY_XP_0217:	ADP-rib(	Chenopodium_quinoa	34.40	0.00
TRINITY_GBF9463:	peptidy:	Raphidocelis_subcap	34.40	0.00
TRINITY_VDD5732:	unnamed	Brassica_oleracea	34.40	0.00
TRINITY_OMP0778:	hypothet	Corchorus_olitorius	34.40	0.00
TRINITY_OAE3570:	hypothet	Marchantia_polymorp	34.40	0.00
TRINITY_PNW8135:	hypothet	Chlamydomonas_reinh	34.40	0.00
TRINITY_PWA7854:	Alkalin€	Artemisia_annua	34.40	0.00
TRINITY_KXZ4322(	hypothet	Gonium_pectorale	34.40	0.00
TRINITY_XP_0113:	E3 ubiq(	Auxenochlorella_prc	34.40	0.00
TRINITY_GAX7732	hypothet	Chlamydomonas_eusti	34.40	0.00
TRINITY_XP_0258:	pleckst:	Panicum_hallii	34.40	0.00
TRINITY_XP_0208	RNA-bin(	Arabidopsis_lyrata	34.40	0.00

TRINITY_XP_0106(	PREDICTI	Vitis_vinifera	34.40	0.00
TRINITY_GBF9197(	methylt:	Raphidocelis_subcap	34.40	0.00
TRINITY_PNW7758(	hypothet	Chlamydomonas_reinh	34.40	0.00
TRINITY_GAX7823(	hypothet	Chlamydomonas_eusti	34.40	0.00
TRINITY_GAQ9104(	calcium-	Klebsormidium_niten	34.40	0.00
TRINITY_XP_0029(	hypothet	Volvox_carteri	34.40	0.00
TRINITY_XP_0056(	hypothet	Coccomyxa_subellips	34.40	0.00
TRINITY_GAQ7940(	Prolyl (	Klebsormidium_niten	34.40	0.00
TRINITY_KXZ4138(	hypothet	Gonium_pectorale	34.40	0.00
TRINITY_XP_0093(	PREDICTI	Musa_acuminata	34.40	0.00
TRINITY_GAX7879(	hypothet	Chlamydomonas_eusti	34.40	0.00
TRINITY_PSC7140(	zinc ph(	Micractinium_conduc	34.40	0.00
TRINITY_XP_0029(	hypothet	Volvox_carteri	34.40	0.00
TRINITY_KXZ4386(	hypothet	Gonium_pectorale	34.40	0.00
TRINITY_XP_0029(	hypothet	Volvox_carteri	34.40	0.00
TRINITY_GBF9005(	hypothet	Raphidocelis_subcap	34.40	0.00
TRINITY_XP_0228(	TLDc [O(	Ostreococcus_tauri	34.40	0.00
TRINITY_XP_0264(	uncharac	Papaver_somniferum	34.40	0.00
TRINITY_GAX8540(	hypothet	Chlamydomonas_eusti	34.40	0.00
TRINITY_XP_0016(	predicte	Chlamydomonas_reinh	34.40	0.00
TRINITY_GAX8353(	hypothet	Chlamydomonas_eusti	34.40	0.00
TRINITY_XP_0216(	ras-rela	Manihot_esculenta	34.30	0.00
TRINITY_OEL3435(	Nucleos(	Dichanthelium_oligo	34.30	0.00
TRINITY_AJR2238(	CBL-inte	Triticum_aestivum	34.30	0.00
TRINITY_XP_0110(	PREDICTI	Populus_euphratica	34.30	0.00
TRINITY_BAT7633(	hypothet	Vigna_angularis	34.30	0.00
TRINITY_XP_0256(	mitochor	Arachis_hypogaea	34.30	0.00
TRINITY_XP_0159(	eukaryot	Arachis_duranensis	34.30	0.00
TRINITY_XP_0058(	hypothet	Chlorella_variabili	34.30	0.00
TRINITY_XP_0229(	uncharac	Cucurbita_moschata	34.30	0.00
TRINITY_XP_0238(	nitroger	Quercus_suber	34.30	0.00
TRINITY_PIA6009(	hypothet	Aquilegia_coerulea	34.30	0.00
TRINITY_RXH9144(	hypothet	Malus_domestica	34.30	0.00
TRINITY_XP_0279(	polyader	Vigna_unguiculata	34.30	0.00
TRINITY_RID6607(	hypothet	Brassica_rapa	34.30	0.00
TRINITY_GAQ8886(	hypothet	Klebsormidium_niten	34.30	0.00
TRINITY_XP_0110(	serine (	Sesamum_indicum	34.30	0.00
TRINITY_XP_0158(	C2 and (	Ziziphus_jujuba	34.30	0.00
TRINITY_GAX7401(	hypothet	Chlamydomonas_eusti	34.30	0.00
TRINITY_OAE2856(	hypothet	Marchantia_polymorp	34.30	0.00
TRINITY_GAX8292(	hypothet	Chlamydomonas_eusti	34.30	0.00
TRINITY_XP_0191(	PREDICTI	Ipomoea_nil	34.30	0.00
TRINITY_PNH0323(	putative	Tetrabaena_socialis	34.30	0.00
TRINITY_GAX7503(	hypothet	Chlamydomonas_eusti	34.30	0.00
TRINITY_XP_0216(	uncharac	Hevea_brasiliensis	34.30	0.00
TRINITY_GAX7344(	hypothet	Chlamydomonas_eusti	34.30	0.00
TRINITY_XP_0058(	hypothet	Chlorella_variabili	34.30	0.00
TRINITY_XP_0025(	predicte	Micromonas_commoda	34.30	0.00
TRINITY_XP_0238(	uncharac	Quercus_suber	34.30	0.00
TRINITY_XP_0100(	PREDICTI	Eucalyptus_grandis	34.30	0.00
TRINITY_XP_0075(	predicte	Bathycoccus_prasino	34.30	0.00
TRINITY_XP_0025(	predicte	Micromonas_commoda	34.30	0.00
TRINITY_KXZ4682(	hypothet	Gonium_pectorale	34.30	0.00
TRINITY_XP_0029(	hypothet	Volvox_carteri	34.30	0.00
TRINITY_PNW8129(	hypothet	Chlamydomonas_reinh	34.30	0.00
TRINITY_XP_0139(	molybder	Monoraphidium_negle	34.30	0.00
TRINITY_CDY4333(	BnaC04g(	Brassica_napus	34.30	0.00
TRINITY_ADU0461(	NADH del	Mesostigma_viride	34.30	0.00
TRINITY_XP_0158(	uncharac	Ziziphus_jujuba	34.30	0.00
TRINITY_KXZ5509(	hypothet	Gonium_pectorale	34.30	0.00
TRINITY_EFJ3376(	hypothet	Selaginella_moellen	34.30	0.00
TRINITY_GAX8010(	hypothet	Chlamydomonas_eusti	34.30	0.00
TRINITY_XP_0238(	H(+)/Cl	Quercus_suber	34.30	0.00
TRINITY_KDD7518(	hypothet	Helicosporidium_sp.	34.30	0.00
TRINITY_KCW8763(	hypothet	Eucalyptus_grandis	34.30	0.00
TRINITY_PTQ4778(	hypothet	Marchantia_polymorp	34.30	0.00

TRINITY_GBF9188	hypothet	Raphidocelis_subcap	34.30	0.00
TRINITY_OMO6250	hypothet	Corchorus_capsulari	34.30	0.00
TRINITY_XP_0238	uncharac	Quercus_suber	34.30	0.00
TRINITY_XP_0042	COP9 sig	Solanum_lycopersicu	34.30	0.00
TRINITY_GAX7629	hypothet	Chlamydomonas_eusti	34.30	0.00
TRINITY_GAX8552	hypothet	Chlamydomonas_eusti	34.30	0.00
TRINITY_BAK0175	predicte	Hordeum_vulgare	34.30	0.00
TRINITY_XP_0030	predicte	Micromonas_pusilla	34.30	0.00
TRINITY_AAV4420	unknow p	Oryza_sativa	34.30	0.00
TRINITY_EFJ3336	hypothet	Selaginella_moellen	34.30	0.00
TRINITY_RZC2511	RNA-bind	Glycine_soja	34.30	0.00
TRINITY_KXZ5527	hypothet	Gonium_pectorale	34.30	0.00
TRINITY_XP_0243	serine/t	Physcomitrella_pate	34.30	0.00
TRINITY_XP_0056	hypothet	Coccomyxa_subellips	34.30	0.00
TRINITY_AVY9163	cytosine	Saccharum_hybrid_cu	34.30	0.00
TRINITY_GAQ7765	ATMRK se	Klebsormidium_niten	34.30	0.00
TRINITY_OEL1411	BTB/POZ	Dichanthelium_oligo	34.30	0.00
TRINITY_GAQ8975	hypothet	Klebsormidium_niten	34.30	0.00
TRINITY_XP_0230	nuclear	Cucurbita_maxima	34.30	0.00
TRINITY_EFJ1371	hypothet	Selaginella_moellen	34.30	0.00
TRINITY_XP_0062	translat	Capsella_rubella	34.30	0.00
TRINITY_XP_0240	serine/t	Morus_notabilis	34.30	0.00
TRINITY_XP_0212	transcri	Herrania_umbratica	34.30	0.00
TRINITY_KXZ5676	hypothet	Gonium_pectorale	34.30	0.00
TRINITY_XP_0139	E3 ubiqu	Monoraphidium_negle	34.30	0.00
TRINITY_PNW7099	hypothet	Chlamydomonas_reinh	34.30	0.00
TRINITY_GAX7770	hypothet	Chlamydomonas_eusti	34.30	0.00
TRINITY_GAX7863	hypothet	Chlamydomonas_eusti	34.30	0.00
TRINITY_VDC9952	unnamed	Brassica_rapa	34.30	0.00
TRINITY_GAX7923	hypothet	Chlamydomonas_eusti	34.30	0.00
TRINITY_GAX8337	hypothet	Chlamydomonas_eusti	34.30	0.00
TRINITY_XP_0239	probable	Quercus_suber	34.30	0.00
TRINITY_XP_0174	PREDICTE	Vigna_angularis	34.30	0.00
TRINITY_GAX8212	hypothet	Chlamydomonas_eusti	34.30	0.00
TRINITY_XP_0238	probable	Quercus_suber	34.30	0.00
TRINITY_KXZ4946	hypothet	Gonium_pectorale	34.30	0.00
TRINITY_GAX7284	hypothet	Chlamydomonas_eusti	34.30	0.00
TRINITY_PKA6366	Nudix hy	Apostasia_shenzheni	34.20	0.00
TRINITY_PQM3942	sodium-c	Prunus_yedoensis	34.20	0.00
TRINITY_XP_0238	autophag	Quercus_suber	34.20	0.00
TRINITY_PWA7407	alcohol	Artemisia_annua	34.20	0.00
TRINITY_XP_0208	SEC1 far	Arabidopsis_lyrata	34.20	0.00
TRINITY_XP_0094	PREDICTE	Musa_acuminata	34.20	0.00
TRINITY_XP_0063	14-3-3-l	Capsella_rubella	34.20	0.00
TRINITY_ONK7344	uncharac	Asparagus_officinal	34.20	0.00
TRINITY_VDC7278	unnamed	Brassica_rapa	34.20	0.00
TRINITY_XP_0093	PREDICTE	Musa_acuminata	34.20	0.00
TRINITY_GAQ9158	6-phosph	Klebsormidium_niten	34.20	0.00
TRINITY_XP_0014	predicte	Ostreococcus_sp._'l	34.20	0.00
TRINITY_XP_0194	PREDICTE	Lupinus_angustifoli	34.20	0.00
TRINITY_XP_0025	flagella	Micromonas_commoda	34.20	0.00
TRINITY_OAE2778	hypothet	Marchantia_polymorp	34.20	0.00
TRINITY_XP_0062	short-ch	Capsella_rubella	34.20	0.00
TRINITY_XP_0036	ABC tran	Medicago_truncatula	34.20	0.00
TRINITY_EEC7134	hypothet	Oryza_sativa	34.20	0.00
TRINITY_XP_0244	uncharac	Physcomitrella_pate	34.20	0.00
TRINITY_CDY6748	BnaCnng5	Brassica_napus	34.20	0.00
TRINITY_XP_0030	basal bo	Micromonas_pusilla	34.20	0.00
TRINITY_GAQ7824	cytochro	Klebsormidium_niten	34.20	0.00
TRINITY_XP_0056	DUF833-d	Coccomyxa_subellips	34.20	0.00
TRINITY_XP_0025	outer dy	Micromonas_commoda	34.20	0.00
TRINITY_PNR4508	hypothet	Physcomitrella_pate	34.20	0.00
TRINITY_GAX7735	hypothet	Chlamydomonas_eusti	34.20	0.00
TRINITY_XP_0202	trans-re	Asparagus_officinal	34.20	0.00
TRINITY_XP_0184	PREDICTE	Raphanus_sativus	34.20	0.00
TRINITY_XP_0271	serine/t	Cicer_arietinum	34.20	0.00

TRINITY_XP_01764	PREDICTE	Gossypium_arboreum	34.20	0.00
TRINITY_XP_00297	sphingol	Selaginella_moellen	34.20	0.00
TRINITY_GAX80288	hypothet	Chlamydomonas_eusti	34.20	0.00
TRINITY_GAX73983	hypothet	Chlamydomonas_eusti	34.20	0.00
TRINITY_GAX82524	hypothet	Chlamydomonas_eusti	34.20	0.00
TRINITY_KXZ55369	hypothet	Gonium_pectorale	34.20	0.00
TRINITY_PNH00402	hypothet	Tetrabaena_socialis	34.20	0.00
TRINITY_PKA60957	alcohol	Apostasia_shenzheni	34.20	0.00
TRINITY_GAX85012	hypothet	Chlamydomonas_eusti	34.20	0.00
TRINITY_KJB70285	hypothet	Gossypium_raimondii	34.20	0.00
TRINITY_PRW59105	iodothyr	Chlorella_sorokinia	34.20	0.00
TRINITY_GAX83386	hypothet	Chlamydomonas_eusti	34.20	0.00
TRINITY_XP_02437	probable	Physcomitrella_pate	34.20	0.00
TRINITY_XP_02186	SNF1-rel	Spinacia_oleracea	34.20	0.00
TRINITY_GAQ77713	HECT dom	Klebsormidium_niten	34.20	0.00
TRINITY_GAX78374	hypothet	Chlamydomonas_eusti	34.20	0.00
TRINITY_XP_00169	flagella	Chlamydomonas_reinh	34.20	0.00
TRINITY_GAX75692	hypothet	Chlamydomonas_eusti	34.20	0.00
TRINITY_PNX79740	putative	Trifolium_pratense	34.20	0.00
TRINITY_XP_01054	PREDICTE	Tarenaya_hasslerian	34.20	0.00
TRINITY_GBF98601	hypothet	Raphidocelis_subcap	34.20	0.00
TRINITY_CBI38863	unnamed	Vitis_vinifera	34.20	0.00
TRINITY_XP_01390	guanylat	Monoraphidium_negle	34.20	0.00
TRINITY_XP_00170	predicte	Chlamydomonas_reinh	34.20	0.00
TRINITY_XP_01024	PREDICTE	Nelumbo_nucifera	34.20	0.00
TRINITY_XP_01595	uncharac	Arachis_duranensis	34.20	0.00
TRINITY_XP_00294	hypothet	Volvox_carteri	34.20	0.00
TRINITY_XP_00297	protein	Selaginella_moellen	34.20	0.00
TRINITY_PNW77306	hypothet	Chlamydomonas_reinh	34.20	0.00
TRINITY_XP_00683	uncharac	Amborella_trichopod	34.20	0.00
TRINITY_GAX83657	hypothet	Chlamydomonas_eusti	34.20	0.00
TRINITY_GAX84849	hypothet	Chlamydomonas_eusti	34.20	0.00
TRINITY_XP_00845	PREDICTE	Cucumis_melo	34.20	0.00
TRINITY_XP_00294	hypothet	Volvox_carteri	34.20	0.00
TRINITY_GAX77232	hypothet	Chlamydomonas_eusti	34.20	0.00
TRINITY_KXZ43615	hypothet	Gonium_pectorale	34.20	0.00
TRINITY_GAV82588	zf-DHHC	Cephalotus_follicul	34.20	0.00
TRINITY_XP_02709	V-type p	Coffea_arabica	34.20	0.00
TRINITY_PNW78527	hypothet	Chlamydomonas_reinh	34.20	0.00
TRINITY_XP_02009	protein	Ananas_comosus	34.20	0.00
TRINITY_GAX80735	hypothet	Chlamydomonas_eusti	34.20	0.00
TRINITY_PNG99787	hypothet	Tetrabaena_socialis	34.20	0.00
TRINITY_AEP39605	heat sho	Haematococcus_lacus	34.20	0.00
TRINITY_GAX82662	hypothet	Chlamydomonas_eusti	34.20	0.00
TRINITY_XP_00169	predicte	Chlamydomonas_reinh	34.20	0.00
TRINITY_ONM39222	hypothet	Zea_mays	34.20	0.00
TRINITY_KYP64267	Neurobea	Cajanus_cajan	34.10	0.00
TRINITY_BAA95359	S-like P	Volvox_carteri	34.10	0.00
TRINITY_EFJ25810	hypothet	Selaginella_moellen	34.10	0.00
TRINITY_AML77083	putative	Interfilum_paradoxu	34.10	0.00
TRINITY_XP_00296	3-phosph	Selaginella_moellen	34.10	0.00
TRINITY_KZV18018	hypothet	Dorcoceras_hygromet	34.10	0.00
TRINITY_XP_02057	G patch	Phalaenopsis_equest	34.10	0.00
TRINITY_XP_00169	predicte	Chlamydomonas_reinh	34.10	0.00
TRINITY_KFK44002	hypothet	Arabis_alpina	34.10	0.00
TRINITY_XP_00584	hypothet	Chlorella_variabili	34.10	0.00
TRINITY_XP_02440	uncharac	Physcomitrella_pate	34.10	0.00
TRINITY_XP_02276	lecithin	Durio_zibethinus	34.10	0.00
TRINITY_XP_02283	ABC tran	Ostreococcus_tauri	34.10	0.00
TRINITY_GAX75393	hypothet	Chlamydomonas_eusti	34.10	0.00
TRINITY_XP_00169	predicte	Chlamydomonas_reinh	34.10	0.00
TRINITY_ESQ46946	hypothet	Eutrema_salsugineum	34.10	0.00
TRINITY_KXZ54829	hypothet	Gonium_pectorale	34.10	0.00
TRINITY_PNR60001	hypothet	Physcomitrella_pate	34.10	0.00
TRINITY_GAQ91212	Calcium	Klebsormidium_niten	34.10	0.00
TRINITY_XP_02392	ras-rela	Quercus_suber	34.10	0.00

TRINITY_GAX7749	hypothet	Chlamydomonas_eusti	34.10	0.00
TRINITY_OAE2790	hypothet	Marchantia_polymorp	34.10	0.00
TRINITY_GAX7555	hypothet	Chlamydomonas_eusti	34.10	0.00
TRINITY_XP_0176	PREDICTI	Gossypium_arboreum	34.10	0.00
TRINITY_PTQ3120	hypothet	Marchantia_polymorp	34.10	0.00
TRINITY_GAX7480	hypothet	Chlamydomonas_eusti	34.10	0.00
TRINITY_XP_0194	PREDICTI	Lupinus_angustifoli	34.10	0.00
TRINITY_XP_0238	wiskott-	Quercus_suber	34.10	0.00
TRINITY_OAE2146	hypothet	Marchantia_polymorp	34.10	0.00
TRINITY_PNH0292	hypothet	Tetrabaena_socialis	34.10	0.00
TRINITY_XP_0029	hypothet	Volvox_carteri	34.10	0.00
TRINITY_XP_0202	uncharac	Cajanus_cajan	34.10	0.00
TRINITY_XP_0235	coiled-c	Cucurbita_pepo	34.10	0.00
TRINITY_XP_0029	hypothet	Volvox_carteri	34.10	0.00
TRINITY_XP_0029	hypothet	Volvox_carteri	34.10	0.00
TRINITY_XP_0228	RNA (C5-	Ostreococcus_tauri	34.10	0.00
TRINITY_KXZ5652	hypothet	Gonium_pectorale	34.10	0.00
TRINITY_GAQ8325	GTP-binc	Klebsormidium_niten	34.10	0.00
TRINITY_XP_0264	probable	Papaver_somniferum	34.10	0.00
TRINITY_XP_0243	nicalin-	Physcomitrella_pate	34.10	0.00
TRINITY_XP_0058	hypothet	Chlorella_variabili	34.10	0.00
TRINITY_GAX7393	hypothet	Chlamydomonas_eusti	34.10	0.00
TRINITY_XP_0212	uncharac	Herrania_umbratica	34.10	0.00
TRINITY_EFJ3299	hypothet	Selaginella_moellen	34.10	0.00
TRINITY_GAX7385	hypothet	Chlamydomonas_eusti	34.10	0.00
TRINITY_XP_0245	spindle	Selaginella_moellen	34.10	0.00
TRINITY_OWM7893	hypothet	Punica_granatum	34.10	0.00
TRINITY_XP_0075	predicte	Bathycoccus_prasino	34.10	0.00
TRINITY_XP_0029	chaperor	Volvox_carteri	34.10	0.00
TRINITY_GAX8369	hypothet	Chlamydomonas_eusti	34.10	0.00
TRINITY_GAX8033	hypothet	Chlamydomonas_eusti	34.10	0.00
TRINITY_KXZ4987	hypothet	Gonium_pectorale	34.10	0.00
TRINITY_PSC6949	Magnesiu	Micractinium_conduc	34.10	0.00
TRINITY_XP_0245	nucleola	Selaginella_moellen	34.10	0.00
TRINITY_XP_0194	PREDICTI	Lupinus_angustifoli	34.10	0.00
TRINITY_XP_0105	PREDICTI	Camelina_sativa	34.10	0.00
TRINITY_ACY0631	class I	Pseudotsuga_menzies	34.10	0.00
TRINITY_XP_0200	peflin-l	Ananas_comosus	34.10	0.00
TRINITY_XP_0243	uncharac	Physcomitrella_pate	34.10	0.00
TRINITY_PNW7228	hypothet	Chlamydomonas_reinh	34.10	0.00
TRINITY_GAX8032	hypothet	Chlamydomonas_eusti	34.10	0.00
TRINITY_PNH0145	hypothet	Tetrabaena_socialis	34.10	0.00
TRINITY_XP_0216	uncharac	Hevea_brasiliensis	34.10	0.00
TRINITY_XP_0243	CCR4-NOT	Physcomitrella_pate	34.10	0.00
TRINITY_GBG8073	hypothet	Chara_braunii	34.10	0.00
TRINITY_GAX7879	hypothet	Chlamydomonas_eusti	34.10	0.00
TRINITY_GAX7774	hypothet	Chlamydomonas_eusti	34.10	0.00
TRINITY_GAX8463	hypothet	Chlamydomonas_eusti	34.10	0.00
TRINITY_CDO9867	unnamed	Coffea_canephora	34.00	0.00
TRINITY_XP_0184	PREDICTI	Raphanus_sativus	34.00	0.00
TRINITY_XP_0025	predicte	Micromonas_commoda	34.00	0.00
TRINITY_RXH9465	hypothet	Malus_domestica	34.00	0.00
TRINITY_XP_0243	embryoge	Physcomitrella_pate	34.00	0.00
TRINITY_XP_0109	PREDICTI	Elaeis_guineensis	34.00	0.00
TRINITY_XP_0217	ribonucl	Chenopodium_quinoa	34.00	0.00
TRINITY_OVA1226	Telomere	Macleaya_cordata	34.00	0.00
TRINITY_AIY6075	rhomboic	Alisma_plantago-aqu	34.00	0.00
TRINITY_XP_0239	WD repea	Quercus_suber	34.00	0.00
TRINITY_XP_0243	E3 ubiqu	Physcomitrella_pate	34.00	0.00
TRINITY_XP_0058	hypothet	Chlorella_variabili	34.00	0.00
TRINITY_KDD7228	NLI inte	Helicosporidium_sp.	34.00	0.00
TRINITY_XP_0029	eukaryot	Volvox_carteri	34.00	0.00
TRINITY_GAX7539	hypothet	Chlamydomonas_eusti	34.00	0.00
TRINITY_XP_0191	PREDICTI	Ipomoea_nil	34.00	0.00
TRINITY_XP_0207	nodal mc	Dendrobium_catenatu	34.00	0.00
TRINITY_GAX8337	hypothet	Chlamydomonas_eusti	34.00	0.00

TRINITY_XP_00169	sodium/p	Chlamydomonas_reinh	34.00	0.00
TRINITY_KZV47203	serine (	Dorcoceras_hygromet	34.00	0.00
TRINITY_XP_02163	ABC tran	Manihot_esculenta	34.00	0.00
TRINITY_AAQ07255	putative	Oryza_sativa	34.00	0.00
TRINITY_PSC74621	Phospho]	Micractinium_conduc	34.00	0.00
TRINITY_KDD76370	pyridoxa	Helicosporidium_sp.	34.00	0.00
TRINITY_XP_02438	methylt	Physcomitrella_pate	34.00	0.00
TRINITY_KXZ51014	hypothet	Gonium_pectorale	34.00	0.00
TRINITY_XP_00585	hypothet	Chlorella_variabili	34.00	0.00
TRINITY_XP_02388	unchara(	Quercus_suber	34.00	0.00
TRINITY_XP_02009	polyubi(	Ananas_comosus	34.00	0.00
TRINITY_GBG79985	hypothet	Chara_braunii	34.00	0.00
TRINITY_GAQ77732	Leishmar	Klebsormidium_niten	34.00	0.00
TRINITY_KDP39615	hypothet	Jatropha_curcas	34.00	0.00
TRINITY_XP_01562	guanylat	Oryza_sativa	34.00	0.00
TRINITY_PON33097	Riboph o	Parasponia_anderson	34.00	0.00
TRINITY_XP_02214	RNA poly	Momordica_charantia	34.00	0.00
TRINITY_GAU35392	hypothet	Trifolium_subterran	34.00	0.00
TRINITY_XP_00584	hypothet	Chlorella_variabili	34.00	0.00
TRINITY_PNY16455	sphingos	Trifolium_pratense	34.00	0.00
TRINITY_XP_02203	metal t	Helianthus_annuus	34.00	0.00
TRINITY_XP_00169	predicte	Chlamydomonas_reinh	34.00	0.00
TRINITY_GAX77641	hypothet	Chlamydomonas_eusti	34.00	0.00
TRINITY_XP_02287	casein k	Olea_europaea	34.00	0.00
TRINITY_XP_01093	PREDICTE	Elaeis_guineensis	34.00	0.00
TRINITY_XP_01050	PREDICTE	Camelina_sativa	34.00	0.00
TRINITY_XP_00295	hypothet	Volvox_carteri	34.00	0.00
TRINITY_KXZ53253	hypothet	Gonium_pectorale	34.00	0.00
TRINITY_XP_02351	phospho]	Cucurbita_pepo	34.00	0.00
TRINITY_GAQ80294	calcium-	Klebsormidium_niten	34.00	0.00
TRINITY_XP_02172	GPI tran	Chenopodium_quinoa	34.00	0.00
TRINITY_BAK03025	predicte	Hordeum_vulgare	34.00	0.00
TRINITY_GAX78575	hypothet	Chlamydomonas_eusti	34.00	0.00
TRINITY_GBG68706	hypothet	Chara_braunii	34.00	0.00
TRINITY_GAQ81443	Spc97 /	Klebsormidium_niten	34.00	0.00
TRINITY_PNH05534	Sulfate(	Tetrabaena_socialis	34.00	0.00
TRINITY_PON99467	Phosphat	Trema_orientale	34.00	0.00
TRINITY_GAX76558	hypothet	Chlamydomonas_eusti	34.00	0.00
TRINITY_PSS33168	Serine/t	Actinidia_chinensis	34.00	0.00
TRINITY_OAE31917	hypothet	Marchantia_polymorp	34.00	0.00
TRINITY_GAX75056	hypothet	Chlamydomonas_eusti	34.00	0.00
TRINITY_KVH90510	MORN mot	Cynara_cardunculus	34.00	0.00
TRINITY_XP_00565	AP2-doma	Coccomyxa_subellips	34.00	0.00
TRINITY_KXZ57015	hypothet	Gonium_pectorale	34.00	0.00
TRINITY_XP_00169	predicte	Chlamydomonas_reinh	34.00	0.00
TRINITY_XP_00564	Rft-1-do	Coccomyxa_subellips	34.00	0.00
TRINITY_XP_00294	hypothet	Volvox_carteri	34.00	0.00
TRINITY_GAQ88528	hypothet	Klebsormidium_niten	34.00	0.00
TRINITY_XP_02793	beta-hex	Vigna_unguiculata	34.00	0.00
TRINITY_OAE30546	hypothet	Marchantia_polymorp	34.00	0.00
TRINITY_XP_00584	hypothet	Chlorella_variabili	34.00	0.00
TRINITY_XP_00169	predicte	Chlamydomonas_reinh	34.00	0.00
TRINITY_XP_00360	unchara(	Medicago_truncatula	33.90	0.00
TRINITY_XP_02008	serine/t	Ananas_comosus	33.90	0.00
TRINITY_GAX77354	hypothet	Chlamydomonas_eusti	33.90	0.00
TRINITY_XP_02401	tRNA:m(4	Morus_notabilis	33.90	0.00
TRINITY_OAE34901	hypothet	Marchantia_polymorp	33.90	0.00
TRINITY_XP_01721	PREDICTE	Daucus_carota	33.90	0.00
TRINITY_XP_02643	unchara(	Papaver_somniferum	33.90	0.00
TRINITY_XP_01944	PREDICTE	Lupinus_angustifoli	33.90	0.00
TRINITY_EFJ20958	hypothet	Selaginella_moellen	33.90	0.00
TRINITY_XP_00684	unchara(	Amborella_trichopod	33.90	0.00
TRINITY_PIA61902	hypothet	Aquilegia_coerulea	33.90	0.00
TRINITY_XP_01843	PREDICTE	Raphanus_sativus	33.90	0.00
TRINITY_GAQ89611	hypothet	Klebsormidium_niten	33.90	0.00
TRINITY_GBF97600	acidstal	Raphidocelis_subcap	33.90	0.00

TRINITY_XP_0106	PREDICTE	Beta_vulgaris	33.90	0.00
TRINITY_XP_0192	PREDICTE	Ipomoea_nil	33.90	0.00
TRINITY_KZV1512	protein	Dorcoceras_hygromet	33.90	0.00
TRINITY_PSC7676	phosphol	Micractinium_conduc	33.90	0.00
TRINITY_GAQ7952	hypothet	Klebsormidium_niten	33.90	0.00
TRINITY_XP_0270	cytochro	Coffea_arabica	33.90	0.00
TRINITY_XP_0097	PREDICTE	Nicotiana_sylvestri	33.90	0.00
TRINITY_GAQ9014	hypothet	Klebsormidium_niten	33.90	0.00
TRINITY_GAX7635	hypothet	Chlamydomonas_eusti	33.90	0.00
TRINITY_GAQ8783	RNA-binc	Klebsormidium_niten	33.90	0.00
TRINITY_XP_0029	hypothet	Volvox_carteri	33.90	0.00
TRINITY_GAQ8852	hypothet	Klebsormidium_niten	33.90	0.00
TRINITY_GAV8293	RdRP dor	Cephalotus_follicul	33.90	0.00
TRINITY_OAE2130	hypothet	Marchantia_polymorp	33.90	0.00
TRINITY_XP_0024	tRNA (ac	Sorghum_bicolor	33.90	0.00
TRINITY_XP_0279	(+)-neor	Vigna_unguiculata	33.90	0.00
TRINITY_GAX8116	hypothet	Chlamydomonas_eusti	33.90	0.00
TRINITY_GBG7298	hypothet	Chara_braunii	33.90	0.00
TRINITY_KXZ4748	hypothet	Gonium_pectorale	33.90	0.00
TRINITY_KXZ5509	hypothet	Gonium_pectorale	33.90	0.00
TRINITY_XP_0237	phospho	Lactuca_sativa	33.90	0.00
TRINITY_GAX8180	hypothet	Chlamydomonas_eusti	33.90	0.00
TRINITY_XP_0056	hypothet	Coccomyxa_subellips	33.90	0.00
TRINITY_P0C8M8.	RecName:	Zea_mays	33.90	0.00
TRINITY_XP_0229	purple a	Cucurbita_moschata	33.90	0.00
TRINITY_PNW8887	hypothet	Chlamydomonas_reinh	33.90	0.00
TRINITY_GBG6823	hypothet	Chara_braunii	33.90	0.00
TRINITY_GAQ9291	hypothet	Klebsormidium_niten	33.90	0.00
TRINITY_KXZ4287	hypothet	Gonium_pectorale	33.90	0.00
TRINITY_XP_0167	PREDICTE	Gossypium_hirsutum	33.90	0.00
TRINITY_GAX8266	hypothet	Chlamydomonas_eusti	33.90	0.00
TRINITY_PNW7298	hypothet	Chlamydomonas_reinh	33.90	0.00
TRINITY_GAX7301	hypothet	Chlamydomonas_eusti	33.90	0.00
TRINITY_XP_0042	PREDICTE	Fragaria_vesca	33.90	0.00
TRINITY_XP_0025	predicte	Micromonas_commoda	33.90	0.00
TRINITY_XP_0024	calcium-	Sorghum_bicolor	33.90	0.00
TRINITY_XP_0029	hypothet	Volvox_carteri	33.90	0.00
TRINITY_GAV5777	Cyclin c	Cephalotus_follicul	33.90	0.00
TRINITY_PTQ3202	hypothet	Marchantia_polymorp	33.90	0.00
TRINITY_XP_0029	hypothet	Volvox_carteri	33.90	0.00
TRINITY_PNW8260	hypothet	Chlamydomonas_reinh	33.90	0.00
TRINITY_GBF9763	hypothet	Raphidocelis_subcap	33.90	0.00
TRINITY_PNW7058	hypothet	Chlamydomonas_reinh	33.90	0.00
TRINITY_GAX7981	hypothet	Chlamydomonas_eusti	33.90	0.00
TRINITY_GAX7751	hypothet	Chlamydomonas_eusti	33.90	0.00
TRINITY_XP_0146	pre-mRNA	Glycine_max	33.90	0.00
TRINITY_XP_0016	predicte	Chlamydomonas_reinh	33.90	0.00
TRINITY_NP_0011	glossy 8	Zea_mays	33.90	0.00
TRINITY_GAX8199	hypothet	Chlamydomonas_eusti	33.90	0.00
TRINITY_XP_0237	dehydrog	Lactuca_sativa	33.90	0.00
TRINITY_XP_0017	flagella	Chlamydomonas_reinh	33.90	0.00
TRINITY_XP_0208	succinat	Arabidopsis_lyrata	33.90	0.00
TRINITY_XP_0016	predicte	Chlamydomonas_reinh	33.90	0.00
TRINITY_GAQ8606	hypothet	Klebsormidium_niten	33.90	0.00
TRINITY_XP_0156	elicitor	Oryza_sativa	33.90	0.00
TRINITY_KXZ4557	hypothet	Gonium_pectorale	33.90	0.00
TRINITY_NP_0013	E2F-asso	Zea_mays	33.90	0.00
TRINITY_XP_0220	serine/t	Helianthus_annuus	33.90	0.00
TRINITY_KXZ5158	hypothet	Gonium_pectorale	33.90	0.00
TRINITY_GAX8451	hypothet	Chlamydomonas_eusti	33.90	0.00
TRINITY_XP_0110	PREDICTE	Populus_euphratica	33.90	0.00
TRINITY_XP_0109	PREDICTE	Elaeis_guineensis	33.80	0.00
TRINITY_PIN2131	Dipeptic	Handroanthus_impeti	33.80	0.00
TRINITY_XP_0238	protein	Quercus_suber	33.80	0.00
TRINITY_AAQ0725	putative	Oryza_sativa	33.80	0.00
TRINITY_XP_0014	DMT fami	Ostreococcus_sp._'l	33.80	0.00

TRINITY_GBG7395	hypothet	Chara_braunii	33.80	0.00
TRINITY_KXZ4973	hypothet	Gonium_pectorale	33.80	0.00
TRINITY_EFJ3104	hypothet	Selaginella_moellen	33.80	0.00
TRINITY_XP_0238	leucine	Quercus_suber	33.80	0.00
TRINITY_OMO8650	Peptida	Corchorus_olitorius	33.80	0.00
TRINITY_XP_0109	PREDICT	Elaeis_guineensis	33.80	0.00
TRINITY_XP_0029	hypothet	Volvox_carteri	33.80	0.00
TRINITY_XP_0025	predict	Micromonas_commoda	33.80	0.00
TRINITY_PWA4920	thiol p	Artemisia_annua	33.80	0.00
TRINITY_XP_0017	protofi	Chlamydomonas_reinh	33.80	0.00
TRINITY_KXZ4348	hypothet	Gonium_pectorale	33.80	0.00
TRINITY_GBF9965	hypothet	Raphidocelis_subcap	33.80	0.00
TRINITY_XP_0239	kynureni	Quercus_suber	33.80	0.00
TRINITY_GAX7762	hypothet	Chlamydomonas_eusti	33.80	0.00
TRINITY_GAQ8731	F-box a	Klebsormidium_niten	33.80	0.00
TRINITY_GAU2924	hypothet	Trifolium_subterran	33.80	0.00
TRINITY_PWA4829	protein	Artemisia_annua	33.80	0.00
TRINITY_OAE2772	hypothet	Marchantia_polymorp	33.80	0.00
TRINITY_GAX7875	hypothet	Chlamydomonas_eusti	33.80	0.00
TRINITY_XP_0238	E3 ubiq	Quercus_suber	33.80	0.00
TRINITY_EOY2629	Long-ch	Theobroma_cacao	33.80	0.00
TRINITY_GAV8890	Abhydro	Cephalotus_follicul	33.80	0.00
TRINITY_XP_0150	PH, RCC	Solanum_pennellii	33.80	0.00
TRINITY_GBF9581	hypothet	Raphidocelis_subcap	33.80	0.00
TRINITY_EFJ1806	hypothet	Selaginella_moellen	33.80	0.00
TRINITY_XP_0016	flagell	Chlamydomonas_reinh	33.80	0.00
TRINITY_PNW8453	hypothet	Chlamydomonas_reinh	33.80	0.00
TRINITY_XP_0025	predict	Micromonas_commoda	33.80	0.00
TRINITY_XP_0029	hypothet	Volvox_carteri	33.80	0.00
TRINITY_XP_0029	hypothet	Volvox_carteri	33.80	0.00
TRINITY_PKI6080	hypothet	Punica_granatum	33.80	0.00
TRINITY_EFJ3502	WD repe	Selaginella_moellen	33.80	0.00
TRINITY_KMS6540	hypothet	Beta_vulgaris	33.80	0.00
TRINITY_XP_0025	Drug/Met	Micromonas_commoda	33.80	0.00
TRINITY_XP_0202	60S rib	Cajanus_cajan	33.80	0.00
TRINITY_EPS6064	hypothet	Genlisea_aurea	33.80	0.00
TRINITY_XP_0105	PREDICT	Tarenaya_hasslerian	33.80	0.00
TRINITY_GAX8465	hypothet	Chlamydomonas_eusti	33.80	0.00
TRINITY_GAQ8553	Nuclear	Klebsormidium_niten	33.80	0.00
TRINITY_PNW8448	hypothet	Chlamydomonas_reinh	33.80	0.00
TRINITY_GBF9436	hypothet	Raphidocelis_subcap	33.80	0.00
TRINITY_XP_0106	PREDICT	Vitis_vinifera	33.80	0.00
TRINITY_XP_0245	phosphat	Selaginella_moellen	33.80	0.00
TRINITY_PRW5874	vacuola	Chlorella_sorokinia	33.80	0.00
TRINITY_XP_0194	PREDICT	Lupinus_angustifoli	33.80	0.00
TRINITY_GAX7608	hypothet	Chlamydomonas_eusti	33.80	0.00
TRINITY_XP_0139	putative	Monoraphidium_negle	33.80	0.00
TRINITY_GAX7603	hypothet	Chlamydomonas_eusti	33.80	0.00
TRINITY_KXZ4429	hypothet	Gonium_pectorale	33.80	0.00
TRINITY_GAQ9019	DEAD/DE	Klebsormidium_niten	33.80	0.00
TRINITY_XP_0239	L-2-ami	Quercus_suber	33.80	0.00
TRINITY_XP_0229	ribonuc	Cucurbita_moschata	33.70	0.00
TRINITY_RWR7865	squamou	Cinnamomum_micranth	33.70	0.00
TRINITY_GAX7531	hypothet	Chlamydomonas_eusti	33.70	0.00
TRINITY_OMO8352	Condens	Corchorus_capsulari	33.70	0.00
TRINITY_XP_0238	guanine	Quercus_suber	33.70	0.00
TRINITY_XP_0114	Protein	Auxenochlorella_pro	33.70	0.00
TRINITY_XP_0102	lysopho	Brachypodium_distac	33.70	0.00
TRINITY_OAY8416	putative	Ananas_comosus	33.70	0.00
TRINITY_XP_0016	predict	Chlamydomonas_reinh	33.70	0.00
TRINITY_XP_0056	hypothet	Coccomyxa_subellips	33.70	0.00
TRINITY_XP_0243	unchara	Physcomitrella_pate	33.70	0.00
TRINITY_OUS4188	ankyrin	Ostreococcus_tauri	33.70	0.00
TRINITY_XP_0217	sugar t	Chenopodium_quinoa	33.70	0.00
TRINITY_OUS4833	peptida	Ostreococcus_tauri	33.70	0.00
TRINITY_XP_0238	leucine	Quercus_suber	33.70	0.00

TRINITY_XP_00228	PREDICTE	Vitis_vinifera	33.70	0.00
TRINITY_BAJ94575	predicte	Hordeum_vulgare	33.70	0.00
TRINITY_AIT94139	probable	Marsupiomonas_sp._N	33.70	0.00
TRINITY_XP_01055	PREDICTE	Tarenaya_hasslerian	33.70	0.00
TRINITY_XP_02440	uncharac	Physcomitrella_pate	33.70	0.00
TRINITY_GAX82126	hypothet	Chlamydomonas_eusti	33.70	0.00
TRINITY_XP_00169	predicte	Chlamydomonas_reinh	33.70	0.00
TRINITY_GAX72829	hypothet	Chlamydomonas_eusti	33.70	0.00
TRINITY_GAX81059	hypothet	Chlamydomonas_eusti	33.70	0.00
TRINITY_XP_02161	uncharac	Manihot_esculenta	33.70	0.00
TRINITY_KXZ52061	hypothet	Gonium_pectorale	33.70	0.00
TRINITY_XP_00295	hypothet	Volvox_carteri	33.70	0.00
TRINITY_XP_01054	PREDICTE	Tarenaya_hasslerian	33.70	0.00
TRINITY_GAX80360	hypothet	Chlamydomonas_eusti	33.70	0.00
TRINITY_GBF98142	hypothet	Raphidocelis_subcap	33.70	0.00
TRINITY_XP_02388	ras-rela	Quercus_suber	33.70	0.00
TRINITY_GAX82253	hypothet	Chlamydomonas_eusti	33.70	0.00
TRINITY_GBG72173	hypothet	Chara_braunii	33.70	0.00
TRINITY_GAX80129	hypothet	Chlamydomonas_eusti	33.70	0.00
TRINITY_XP_02199	actin-de	Helianthus_annuus	33.70	0.00
TRINITY_XP_00169	radial s	Chlamydomonas_reinh	33.70	0.00
TRINITY_GAV66326	Pyr_redo	Cephalotus_follicul	33.70	0.00
TRINITY_XP_01139	E3 ISG15	Auxenochlorella_pro	33.70	0.00
TRINITY_GBF87987	hypothet	Raphidocelis_subcap	33.70	0.00
TRINITY_GAQ80294	calcium-	Klebsormidium_niten	33.70	0.00
TRINITY_PWA74912	asparti	Artemisia_annua	33.70	0.00
TRINITY_XP_00252	serine/t	Ricinus_communis	33.70	0.00
TRINITY_XP_01867	PREDICTE	Musa_acuminata	33.70	0.00
TRINITY_GAQ82936	ABC tran	Klebsormidium_niten	33.70	0.00
TRINITY_PWA94916	C2 calci	Artemisia_annua	33.70	0.00
TRINITY_GAU20147	hypothet	Trifolium_subterran	33.70	0.00
TRINITY_PNW72745	hypothet	Chlamydomonas_reinh	33.70	0.00
TRINITY_GAX84352	hypothet	Chlamydomonas_eusti	33.70	0.00
TRINITY_GAQ81526	Zinc fin	Klebsormidium_niten	33.70	0.00
TRINITY_PNW88549	hypothet	Chlamydomonas_reinh	33.70	0.00
TRINITY_XP_00295	hypothet	Volvox_carteri	33.70	0.00
TRINITY_GAX84888	hypothet	Chlamydomonas_eusti	33.70	0.00
TRINITY_KMZ57392	Exosome	Zostera_marina	33.70	0.00
TRINITY_GAX75417	hypothet	Chlamydomonas_eusti	33.70	0.00
TRINITY_XP_02639	ABC tran	Papaver_somniferum	33.70	0.00
TRINITY_XP_01283	PREDICTE	Erythranthe_guttata	33.60	0.00
TRINITY_XP_02389	PITH dom	Quercus_suber	33.60	0.00
TRINITY_XP_02171	gamma ca	Chenopodium_quinoa	33.60	0.00
TRINITY_PSC73951	hypothet	Micractinium_conduc	33.60	0.00
TRINITY_PNH09427	FGFR1 on	Tetrabaena_socialis	33.60	0.00
TRINITY_XP_01006	PREDICTE	Eucalyptus_grandis	33.60	0.00
TRINITY_XP_02017	nitrile-	Aegilops_tauschii	33.60	0.00
TRINITY_RAL39962	hypothet	Cuscuta_australis	33.60	0.00
TRINITY_PRW56764	Golgi ap	Chlorella_sorokinia	33.60	0.00
TRINITY_XP_01284	PREDICTE	Erythranthe_guttata	33.60	0.00
TRINITY_XP_00305	predicte	Micromonas_pusilla	33.60	0.00
TRINITY_PPS07213	hypothet	Gossypium_barbadens	33.60	0.00
TRINITY_RZB55578	CBL-inte	Glycine_soja	33.60	0.00
TRINITY_XP_02127	E3 ubiqu	Herrania_umbratica	33.60	0.00
TRINITY_RLN09150	CBL-inte	Panicum_miliaceum	33.60	0.00
TRINITY_ONM41602	CBL-inte	Zea_mays	33.60	0.00
TRINITY_OMP00818	Transcri	Corchorus_olitorius	33.60	0.00
TRINITY_XP_00497	ATP-depe	Setaria_italica	33.60	0.00
TRINITY_BAJ96920	predicte	Hordeum_vulgare	33.60	0.00
TRINITY_XP_02392	threonyl	Quercus_suber	33.60	0.00
TRINITY_XP_02175	lysosoma	Chenopodium_quinoa	33.60	0.00
TRINITY_XP_01915	PREDICTE	Ipomoea_nil	33.60	0.00
TRINITY_XP_00431	PREDICTE	Fragaria_vesca	33.60	0.00
TRINITY_GBG91863	hypothet	Chara_braunii	33.60	0.00
TRINITY_GAQ90139	hypothet	Klebsormidium_niten	33.60	0.00
TRINITY_XP_02130	putative	Sorghum_bicolor	33.60	0.00

TRINITY_XP_0145(	probable	Vigna_radiata	33.60	0.00
TRINITY_XP_0238!	uncharac	Quercus_suber	33.60	0.00
TRINITY_PSC7360	D-aspart	Micractinium_conduc	33.60	0.00
TRINITY_XP_0243{	uncharac	Physcomitrella_pate	33.60	0.00
TRINITY_XP_0036]	nucleola	Medicago_truncatula	33.60	0.00
TRINITY_GAV6231]	Abhydro_	Cephalotus_follicul	33.60	0.00
TRINITY_XP_0243	DNA repa	Physcomitrella_pate	33.60	0.00
TRINITY_GAQ7977:	nitric c	Klebsormidium_niten	33.60	0.00
TRINITY_XP_0239(	nuclear	Quercus_suber	33.60	0.00
TRINITY_XP_0176]	PREDICTE	Gossypium_arboreum	33.60	0.00
TRINITY_XP_0238{	serine/t	Quercus_suber	33.60	0.00
TRINITY_XP_0159(	probable	Ziziphus_jujuba	33.60	0.00
TRINITY_GAQ8091{	Calcium-	Klebsormidium_niten	33.60	0.00
TRINITY_GAQ8789{	calpain-	Klebsormidium_niten	33.60	0.00
TRINITY_XP_0238{	dnaJ-re]	Quercus_suber	33.60	0.00
TRINITY_XP_0093{	PREDICTE	Musa_acuminata	33.60	0.00
TRINITY_GBG8928	hypothet	Chara_braunii	33.60	0.00
TRINITY_XP_0120{	pollen-s	Jatropha_curcas	33.60	0.00
TRINITY_PNW7251{	hypothet	Chlamydomonas_reinh	33.60	0.00
TRINITY_GAV8014]	Kelch_3	Cephalotus_follicul	33.60	0.00
TRINITY_GAQ9163{	DnaJ hea	Klebsormidium_niten	33.60	0.00
TRINITY_GAX8356{	hypothet	Chlamydomonas_eusti	33.60	0.00
TRINITY_XP_0056	hypothet	Coccomyxa_subellips	33.60	0.00
TRINITY_GBG6519:	hypothet	Chara_braunii	33.60	0.00
TRINITY_PNW7181(	hypothet	Chlamydomonas_reinh	33.60	0.00
TRINITY_PLY9704{	hypothet	Lactuca_sativa	33.60	0.00
TRINITY_GAX8157!	hypothet	Chlamydomonas_eusti	33.60	0.00
TRINITY_KXZ5393!	hypothet	Gonium_pectorale	33.60	0.00
TRINITY_XP_0249{	histidir	Cynara_cardunculus	33.60	0.00
TRINITY_XP_0172	PREDICTE	Daucus_carota	33.60	0.00
TRINITY_PRW6063	SCF ubic	Chlorella_sorokinia	33.60	0.00
TRINITY_XP_0271(	protein	Coffea_arabica	33.60	0.00
TRINITY_XP_0188	PREDICTE	Juglans_regia	33.60	0.00
TRINITY_XP_0238	uncharac	Quercus_suber	33.60	0.00
TRINITY_KZN0873	hypothet	Daucus_carota	33.60	0.00
TRINITY_XP_0029{	ultravic	Selaginella_moellen	33.60	0.00
TRINITY_XP_0216	helicase	Hevea_brasiliensis	33.60	0.00
TRINITY_EFJ2331	hypothet	Selaginella_moellen	33.60	0.00
TRINITY_GAX7764	hypothet	Chlamydomonas_eusti	33.60	0.00
TRINITY_XP_0088(	plant cy	Phoenix_dactylifera	33.60	0.00
TRINITY_PNR2701:	hypothet	Physcomitrella_pate	33.60	0.00
TRINITY_AAT0251{	unknown	Chlamydomonas_reinh	33.60	0.00
TRINITY_PSC7000]	DIS3-lik	Micractinium_conduc	33.60	0.00
TRINITY_RLN0555:	hypothet	Panicum_miliaceum	33.60	0.00
TRINITY_GAX8282]	hypothet	Chlamydomonas_eusti	33.60	0.00
TRINITY_RQO9048	hypothet	Populus_trichocarpa	33.60	0.00
TRINITY_GAX8297	hypothet	Chlamydomonas_eusti	33.60	0.00
TRINITY_XP_0056	S1-domai	Coccomyxa_subellips	33.60	0.00
TRINITY_GBF9358!	hypothet	Raphidocelis_subcap	33.60	0.00
TRINITY_XP_0029	hypothet	Volvox_carteri	33.60	0.00
TRINITY_XP_0243	uncharac	Physcomitrella_pate	33.60	0.00
TRINITY_XP_0063!	uncharac	Eutrema_salsugineum	33.60	0.00
TRINITY_XP_0167:	PREDICTE	Gossypium_hirsutum	33.60	0.00
TRINITY_GAX7555{	hypothet	Chlamydomonas_eusti	33.60	0.00
TRINITY_XP_0237!	DNA repa	Lactuca_sativa	33.60	0.00
TRINITY_XP_0238	E3 ubiqu	Quercus_suber	33.60	0.00
TRINITY_XP_0239(	uncharac	Quercus_suber	33.60	0.00
TRINITY_XP_0243!	phosphat	Physcomitrella_pate	33.60	0.00
TRINITY_GAX7970:	hypothet	Chlamydomonas_eusti	33.60	0.00
TRINITY_GBF9182	hypothet	Raphidocelis_subcap	33.60	0.00
TRINITY_GBF9624!	adenylat	Raphidocelis_subcap	33.60	0.00
TRINITY_PPD6701	hypothet	Gossypium_barbadens	33.50	0.00
TRINITY_XP_0243{	haloacic	Physcomitrella_pate	33.50	0.00
TRINITY_XP_0239(	uncharac	Quercus_suber	33.50	0.00
TRINITY_XP_0220:	heat st	Helianthus_annuus	33.50	0.00
TRINITY_XP_0249{	ras-rela	Cynara_cardunculus	33.50	0.00

TRINITY_OTG3433(	putative	Helianthus_annuus	33.50	0.00
TRINITY_XP_0238(	tripepti	Quercus_suber	33.50	0.00
TRINITY_RXH6734(	hypothet	Malus_domestica	33.50	0.00
TRINITY_XP_0025(	flagella	Micromonas_commoda	33.50	0.00
TRINITY_EAZ0599(	hypothet	Oryza_sativa	33.50	0.00
TRINITY_PRQ4933(	putative	Rosa_chinensis	33.50	0.00
TRINITY_PIA4469(	hypothet	Aquilegia_coerulea	33.50	0.00
TRINITY_XP_0235(	nuclear	Cucurbita_pepo	33.50	0.00
TRINITY_OVA0734(	Protein	Macleaya_cordata	33.50	0.00
TRINITY_XP_0238(	probable	Quercus_suber	33.50	0.00
TRINITY_GAX7431(	hypothet	Chlamydomonas_eusti	33.50	0.00
TRINITY_OAE3098(	hypothet	Marchantia_polymorp	33.50	0.00
TRINITY_PNW8863(	hypothet	Chlamydomonas_reinh	33.50	0.00
TRINITY_XP_0243(	uncharac	Physcomitrella_pate	33.50	0.00
TRINITY_XP_0041(	PREDICTI	Cucumis_sativus	33.50	0.00
TRINITY_XP_0209(	CBL-inte	Arachis_duranensis	33.50	0.00
TRINITY_XP_0029(	hypothet	Volvox_carteri	33.50	0.00
TRINITY_GAQ8650(	eukaryot	Klebsormidium_niten	33.50	0.00
TRINITY_XP_0139(	hypothet	Monoraphidium_negle	33.50	0.00
TRINITY_GBF8922(	hypothet	Raphidocelis_subcap	33.50	0.00
TRINITY_XP_0113(	putative	Auxenochlorella_prc	33.50	0.00
TRINITY_XP_0063(	transmem	Capsella_rubella	33.50	0.00
TRINITY_PKA4989(	hypothet	Apostasia_shenzheni	33.50	0.00
TRINITY_NP_0013(	eukaryot	Nicotiana_tabacum	33.50	0.00
TRINITY_GAX8533(	hypothet	Chlamydomonas_eusti	33.50	0.00
TRINITY_GBG7574(	hypothet	Chara_braunii	33.50	0.00
TRINITY_XP_0035(	dual spe	Glycine_max	33.50	0.00
TRINITY_XP_0110(	apoptosi	Sesamum_indicum	33.50	0.00
TRINITY_XP_0017(	predicte	Chlamydomonas_reinh	33.50	0.00
TRINITY_BAJ9497(	predicte	Hordeum_vulgare	33.50	0.00
TRINITY_XP_0243(	beta-1,3	Physcomitrella_pate	33.50	0.00
TRINITY_GAX7945(	hypothet	Chlamydomonas_eusti	33.50	0.00
TRINITY_XP_0056(	V0/A0 co	Coccomyxa_subellips	33.50	0.00
TRINITY_PSC6758(	type A v	Micractinium_conduc	33.50	0.00
TRINITY_GAQ8511(	hypothet	Klebsormidium_niten	33.50	0.00
TRINITY_RWR7886(	importin	Cinnamomum_micranth	33.50	0.00
TRINITY_GAQ8068(	hypothet	Klebsormidium_niten	33.50	0.00
TRINITY_GAX7729(	hypothet	Chlamydomonas_eusti	33.50	0.00
TRINITY_XP_0014(	predicte	Ostreococcus_sp._'l	33.50	0.00
TRINITY_PRW0575(	phosphol	Chlorella_sorokinia	33.50	0.00
TRINITY_PRW3297(	hypothet	Chlorella_sorokinia	33.50	0.00
TRINITY_GAX7815(	hypothet	Chlamydomonas_eusti	33.50	0.00
TRINITY_OAE2928(	hypothet	Marchantia_polymorp	33.50	0.00
TRINITY_XP_0083(	PREDICTI	Malus_domestica	33.50	0.00
TRINITY_XP_0239(	tuberous	Quercus_suber	33.50	0.00
TRINITY_EYU2599(	hypothet	Erythranthe_guttata	33.50	0.00
TRINITY_KXZ4470(	hypothet	Gonium_pectorale	33.50	0.00
TRINITY_GAX7317(	hypothet	Chlamydomonas_eusti	33.50	0.00
TRINITY_GBG6927(	hypothet	Chara_braunii	33.50	0.00
TRINITY_PPD8214(	hypothet	Gossypium_barbadens	33.50	0.00
TRINITY_GAX7818(	hypothet	Chlamydomonas_eusti	33.50	0.00
TRINITY_XP_0016(	predicte	Chlamydomonas_reinh	33.50	0.00
TRINITY_XP_0056(	hypothet	Coccomyxa_subellips	33.50	0.00
TRINITY_PRW4519(	carboxyl	Chlorella_sorokinia	33.40	0.00
TRINITY_PIN1750(	Protein	Handroanthus_impeti	33.40	0.00
TRINITY_XP_0082(	PREDICTI	Prunus_mume	33.40	0.00
TRINITY_AAV3108(	1,2-diac	Euonymus_alatus	33.40	0.00
TRINITY_XP_0029(	choline/	Selaginella_moellen	33.40	0.00
TRINITY_EFJ2940(	hypothet	Selaginella_moellen	33.40	0.00
TRINITY_BAK0119(	predicte	Hordeum_vulgare	33.40	0.00
TRINITY_GAQ7773(	Leishman	Klebsormidium_niten	33.40	0.00
TRINITY_GAX7270(	hypothet	Chlamydomonas_eusti	33.40	0.00
TRINITY_PNW8504(	hypothet	Chlamydomonas_reinh	33.40	0.00
TRINITY_GAX8467(	hypothet	Chlamydomonas_eusti	33.40	0.00
TRINITY_XP_0029(	hypothet	Volvox_carteri	33.40	0.00
TRINITY_GAX7282(	hypothet	Chlamydomonas_eusti	33.40	0.00

TRINITY_XP_0063!	PREDICTI	Solanum_tuberosum	33.40	0.00
TRINITY_KDO8172(	hypothet	Citrus_sinensis	33.40	0.00
TRINITY_XP_0016!	predicte	Chlamydomonas_reinh	33.40	0.00
TRINITY_XP_0068!	uncharac	Amborella_trichopod	33.40	0.00
TRINITY_GAX7969	hypothet	Chlamydomonas_eusti	33.40	0.00
TRINITY_XP_0088(	transcri	Phoenix_dactylifera	33.40	0.00
TRINITY_XP_0228!	uncharac	Olea_europaea	33.40	0.00
TRINITY_XP_0218(	putative	Spinacia_oleracea	33.40	0.00
TRINITY_KXZ4569	hypothet	Gonium_pectorale	33.40	0.00
TRINITY_XP_0245	DEAD-box	Selaginella_moellen	33.40	0.00
TRINITY_GAY4438	hypothet	Citrus_unshiu	33.40	0.00
TRINITY_GAX7266	hypothet	Chlamydomonas_eusti	33.40	0.00
TRINITY_GBF9400!	MFS tran	Raphidocelis_subcap	33.40	0.00
TRINITY_PNR4203	hypothet	Physcomitrella_pate	33.30	0.00
TRINITY_XP_0212!	macropha	Herrania_umbratica	33.30	0.00
TRINITY_XP_0158	protein	Ziziphus_jujuba	33.30	0.00
TRINITY_BAJ9694	predicte	Hordeum_vulgare	33.30	0.00
TRINITY_XP_0205!	LOW QUAI	Phalaenopsis_equest	33.30	0.00
TRINITY_RLM9138	hypothet	Panicum_miliaceum	33.30	0.00
TRINITY_XP_0239(	elongatc	Quercus_suber	33.30	0.00
TRINITY_XP_0063	PREDICTI	Solanum_tuberosum	33.30	0.00
TRINITY_XP_0025(	flagella	Micromonas_commoda	33.30	0.00
TRINITY_PIA3915!	hypothet	Aquilegia_coerulea	33.30	0.00
TRINITY_XP_0014	predicte	Ostreococcus_sp._'l	33.30	0.00
TRINITY_GAX7668(	hypothet	Chlamydomonas_eusti	33.30	0.00
TRINITY_XP_0239	uncharac	Quercus_suber	33.30	0.00
TRINITY_XP_0056	hypothet	Coccomyxa_subellips	33.30	0.00
TRINITY_XP_0264!	CBL-inte	Papaver_somniferum	33.30	0.00
TRINITY_XP_0097(	PREDICTI	Nicotiana_sylvestri	33.30	0.00
TRINITY_XP_0030(	predicte	Micromonas_pusilla	33.30	0.00
TRINITY_GBG7422	hypothet	Chara_braunii	33.30	0.00
TRINITY_XP_0056	alpha/be	Coccomyxa_subellips	33.30	0.00
TRINITY_XP_0064	endonucl	Eutrema_salsugineum	33.30	0.00
TRINITY_XP_0239(	sphingor	Quercus_suber	33.30	0.00
TRINITY_XP_0216	ARF guar	Hevea_brasiliensis	33.30	0.00
TRINITY_EOA1450!	hypothet	Capsella_rubella	33.30	0.00
TRINITY_XP_0014	predicte	Ostreococcus_sp._'l	33.30	0.00
TRINITY_GAX7753	hypothet	Chlamydomonas_eusti	33.30	0.00
TRINITY_PPR9125(	hypothet	Gossypium_barbadens	33.30	0.00
TRINITY_XP_0156	DNA repa	Oryza_sativa	33.30	0.00
TRINITY_EFJ2948!	hypothet	Selaginella_moellen	33.30	0.00
TRINITY_BAJ8801!	predicte	Hordeum_vulgare	33.30	0.00
TRINITY_RRT6583(	hypothet	Ensete_ventricosum	33.30	0.00
TRINITY_OAE2205	hypothet	Marchantia_polymorp	33.30	0.00
TRINITY_XP_0086	protein	Zea_mays	33.30	0.00
TRINITY_XP_0258(	myosin-2	Panicum_hallii	33.30	0.00
TRINITY_OAE3207	hypothet	Marchantia_polymorp	33.30	0.00
TRINITY_RID6171!	hypothet	Brassica_rapa	33.30	0.00
TRINITY_GAX7976(	hypothet	Chlamydomonas_eusti	33.30	0.00
TRINITY_XP_0016!	predicte	Chlamydomonas_reinh	33.30	0.00
TRINITY_GAX7306	hypothet	Chlamydomonas_eusti	33.30	0.00
TRINITY_GBG6905	hypothet	Chara_braunii	33.30	0.00
TRINITY_XP_0139(	hypothet	Monoraphidium_negle	33.30	0.00
TRINITY_XP_0273!	cystinos	Abrus_precatorius	33.30	0.00
TRINITY_GBG5916(	hypothet	Chara_braunii	33.30	0.00
TRINITY_XP_0227	peroxisc	Durio_zibethinus	33.30	0.00
TRINITY_XP_0243!	uncharac	Physcomitrella_pate	33.30	0.00
TRINITY_XP_0113!	Kelch-li	Auxenochlorella_prc	33.30	0.00
TRINITY_XP_0257!	probable	Panicum_hallii	33.30	0.00
TRINITY_GBF9941(	hypothet	Raphidocelis_subcap	33.30	0.00
TRINITY_GBG6294(	hypothet	Chara_braunii	33.30	0.00
TRINITY_AGT1621(	CIPK-lik	Saccharum_hybrid_cu	33.30	0.00
TRINITY_XP_0138!	hypothet	Monoraphidium_negle	33.30	0.00
TRINITY_XP_0200!	serine/t	Ananas_comosus	33.30	0.00
TRINITY_XP_0220	serine (	Helianthus_annuus	33.30	0.00
TRINITY_PWA9683	Peptidas	Artemisia_annua	33.30	0.00

TRINITY_GAQ9276(	hypothet	Klebsormidium_niten	33.30	0.00
TRINITY_XP_0264!	mitotic	Papaver_somniferum	33.30	0.00
TRINITY_XP_0243(	putative	Physcomitrella_pate	33.30	0.00
TRINITY_GBG9136(	hypothet	Chara_braunii	33.30	0.00
TRINITY_GAX8507(	hypothet	Chlamydomonas_eusti	33.30	0.00
TRINITY_GAX8414(	hypothet	Chlamydomonas_eusti	33.30	0.00
TRINITY_KHF9901	Shaggy-1	Gossypium_arboreum	33.30	0.00
TRINITY_KZV1487	hypothet	Dorcoceras_hygromet	33.30	0.00
TRINITY_XP_0153(	protein	Citrus_sinensis	33.30	0.00
TRINITY_XP_0105	PREDICTI	Tarenaya_hasslerian	33.30	0.00
TRINITY_PTQ4164	hypothet	Marchantia_polymorp	33.30	0.00
TRINITY_XP_0068	BTB/POZ	Amborella_trichopod	33.30	0.00
TRINITY_PRW3286(	retinol	Chlorella_sorokinia	33.30	0.00
TRINITY_XP_0145(	elicitor	Vigna_radiata	33.30	0.00
TRINITY_XP_0205	CBL-inte	Sesamum_indicum	33.30	0.00
TRINITY_XP_0188	PREDICTI	Juglans_regia	33.30	0.00
TRINITY_GBF9405	hypothet	Raphidocelis_subcap	33.30	0.00
TRINITY_GAX8224	hypothet	Chlamydomonas_eusti	33.30	0.00
TRINITY_XP_0155(	ethylene	Ricinus_communis	33.30	0.00
TRINITY_PSC7608	serine p	Micractinium_conduc	33.30	0.00
TRINITY_KXZ4771	hypothet	Gonium_pectorale	33.30	0.00
TRINITY_XP_0238	tyrosina	Quercus_suber	33.30	0.00
TRINITY_OAE2873(	hypothet	Marchantia_polymorp	33.30	0.00
TRINITY_GAX7510(	hypothet	Chlamydomonas_eusti	33.30	0.00
TRINITY_GAX8214	hypothet	Chlamydomonas_eusti	33.30	0.00
TRINITY_KXZ4918	hypothet	Gonium_pectorale	33.30	0.00
TRINITY_OAE1874!	hypothet	Marchantia_polymorp	33.30	0.00
TRINITY_XP_0070!	PREDICTI	Theobroma_cacao	33.30	0.00
TRINITY_GAX7745	hypothet	Chlamydomonas_eusti	33.30	0.00
TRINITY_GAX7894	hypothet	Chlamydomonas_eusti	33.30	0.00
TRINITY_PIA3915!	hypothet	Aquilegia_coerulea	33.30	0.00
TRINITY_XP_0144	uncharac	Vigna_radiata	33.30	0.00
TRINITY_PNT7108	hypothet	Brachypodium_distac	33.30	0.00
TRINITY_XP_0155	V-type p	Ricinus_communis	33.30	0.00
TRINITY_PSC7112	serine t	Micractinium_conduc	33.30	0.00
TRINITY_XP_0243	thyroid	Physcomitrella_pate	33.30	0.00
TRINITY_XP_0243!	long cha	Physcomitrella_pate	33.30	0.00
TRINITY_XP_0139(	hypothet	Monoraphidium_negle	33.30	0.00
TRINITY_XP_0239(	nuclear	Quercus_suber	33.30	0.00
TRINITY_BAJ9109(	predicte	Hordeum_vulgare	33.30	0.00
TRINITY_PNH0930!	Glycolip	Tetrabaena_socialis	33.30	0.00
TRINITY_GAX7290!	hypothet	Chlamydomonas_eusti	33.30	0.00
TRINITY_KXZ5015(	hypothet	Gonium_pectorale	33.30	0.00
TRINITY_XP_0229	zinc fin	Cucurbita_moschata	33.30	0.00
TRINITY_GBG6870(	hypothet	Chara_braunii	33.30	0.00
TRINITY_XP_0016	predicte	Chlamydomonas_reinh	33.30	0.00
TRINITY_GAQ8593	hypothet	Klebsormidium_niten	33.30	0.00
TRINITY_KQK0377	hypothet	Brachypodium_distac	33.30	0.00
TRINITY_GAX7980	hypothet	Chlamydomonas_eusti	33.30	0.00
TRINITY_XP_0056!	WD40 rep	Coccomyxa_subellips	33.30	0.00
TRINITY_XP_0091(	PREDICTI	Brassica_rapa	33.30	0.00
TRINITY_PNH1018(	Myelin e	Tetrabaena_socialis	33.30	0.00
TRINITY_PNH0052	hypothet	Tetrabaena_socialis	33.30	0.00
TRINITY_GAX7276	hypothet	Chlamydomonas_eusti	33.30	0.00
TRINITY_XP_0025(	glutathi	Ricinus_communis	33.30	0.00
TRINITY_GAX7791(	hypothet	Chlamydomonas_eusti	33.30	0.00
TRINITY_GAX7441(	hypothet	Chlamydomonas_eusti	33.30	0.00
TRINITY_XP_0029!	hypothet	Volvox_carteri	33.30	0.00
TRINITY_XP_0025(	predicte	Micromonas_commoda	33.20	0.00
TRINITY_GAX7610	hypothet	Chlamydomonas_eusti	33.20	0.00
TRINITY_PSC7162!	fungal	Micractinium_conduc	33.20	0.00
TRINITY_XP_0071	hypothet	Phaseolus_vulgaris	33.20	0.00
TRINITY_XP_0216(	sulfite	Manihot_esculenta	33.20	0.00
TRINITY_OVA1464(	Tetratri	Macleaya_cordata	33.20	0.00
TRINITY_XP_0245	flagella	Selaginella_moellen	33.20	0.00
TRINITY_XP_0227	CBL-inte	Durio_zibethinus	33.20	0.00

TRINITY_GAX75815	hypothet	Chlamydomonas_eusti	33.20	0.00
TRINITY_XP_00141	predicte	Ostreococcus_sp._'l	33.20	0.00
TRINITY_ADE77590	unknown	Picea_sitchensis	33.20	0.00
TRINITY_XP_00844	PREDICTE	Cucumis_melo	33.20	0.00
TRINITY_XP_02436	uncharac	Physcomitrella_pate	33.20	0.00
TRINITY_XP_02127	serine/t	Herrania_umbratica	33.20	0.00
TRINITY_XP_02389	methyltr	Quercus_suber	33.20	0.00
TRINITY_PNW79243	hypothet	Chlamydomonas_reinh	33.20	0.00
TRINITY_EFJ34954	hypothet	Selaginella_moellen	33.20	0.00
TRINITY_BAJ94976	predicte	Hordeum_vulgare	33.20	0.00
TRINITY_XP_02436	serine/t	Physcomitrella_pate	33.20	0.00
TRINITY_XP_00169	componen	Chlamydomonas_reinh	33.20	0.00
TRINITY_PSC73189	barwin-l	Micractinium_conduc	33.20	0.00
TRINITY_GAX80514	hypothet	Chlamydomonas_eusti	33.20	0.00
TRINITY_XP_00295	hypothet	Volvox_carteri	33.20	0.00
TRINITY_XP_01390	hypothet	Monoraphidium_negle	33.20	0.00
TRINITY_GAX81284	hypothet	Chlamydomonas_eusti	33.20	0.00
TRINITY_XP_01390	Serine/a	Monoraphidium_negle	33.20	0.00
TRINITY_XP_00246	26S prot	Sorghum_bicolor	33.20	0.00
TRINITY_GAX78964	hypothet	Chlamydomonas_eusti	33.20	0.00
TRINITY_GAX75513	hypothet	Chlamydomonas_eusti	33.20	0.00
TRINITY_XP_02452	phosphat	Selaginella_moellen	33.20	0.00
TRINITY_GAX79865	hypothet	Chlamydomonas_eusti	33.20	0.00
TRINITY_PRW58725	putative	Chlorella_sorokinia	33.20	0.00
TRINITY_XP_02451	E3 ubiqu	Selaginella_moellen	33.20	0.00
TRINITY_XP_02387	peroxiso	Quercus_suber	33.20	0.00
TRINITY_XP_00295	hypothet	Volvox_carteri	33.20	0.00
TRINITY_XP_01868	PREDICTE	Musa_acuminata	33.20	0.00
TRINITY_PKA59391	putative	Apostasia_shenzheni	33.20	0.00
TRINITY_XP_01049	PREDICTE	Camelina_sativa	33.20	0.00
TRINITY_GAX79856	hypothet	Chlamydomonas_eusti	33.20	0.00
TRINITY_KXZ47672	hypothet	Gonium_pectorale	33.20	0.00
TRINITY_ABR16149	unknown	Picea_sitchensis	33.20	0.00
TRINITY_XP_00585	hypothet	Chlorella_variabili	33.20	0.00
TRINITY_XP_00940	PREDICTE	Musa_acuminata	33.10	0.00
TRINITY_XP_01066	PREDICTE	Beta_vulgaris	33.10	0.00
TRINITY_XP_00306	dihydrop	Micromonas_pusilla	33.10	0.00
TRINITY_XP_02436	carbonyl	Physcomitrella_pate	33.10	0.00
TRINITY_OAE24892	hypothet	Marchantia_polymorp	33.10	0.00
TRINITY_VDD20602	unnamed	Brassica_oleracea	33.10	0.00
TRINITY_PIN11902	putative	Handroanthus_impeti	33.10	0.00
TRINITY_PRW56764	Golgi ap	Chlorella_sorokinia	33.10	0.00
TRINITY_XP_00169	flagella	Chlamydomonas_reinh	33.10	0.00
TRINITY_OAE29281	hypothet	Marchantia_polymorp	33.10	0.00
TRINITY_GAX77716	hypothet	Chlamydomonas_eusti	33.10	0.00
TRINITY_KYP77380	UPF0341-	Cajanus_cajan	33.10	0.00
TRINITY_GBF96988	nucleoli	Raphidocelis_subcap	33.10	0.00
TRINITY_XP_00250	predicte	Micromonas_commoda	33.10	0.00
TRINITY_XP_00843	PREDICTE	Cucumis_melo	33.10	0.00
TRINITY_EFJ12042	hypothet	Selaginella_moellen	33.10	0.00
TRINITY_XP_02463	SNF1-rel	Medicago_truncatula	33.10	0.00
TRINITY_PRW55936	baculovi	Chlorella_sorokinia	33.10	0.00
TRINITY_XP_00250	predicte	Micromonas_commoda	33.10	0.00
TRINITY_RQP02555	hypothet	Populus_trichocarpa	33.10	0.00
TRINITY_XP_00141	predicte	Ostreococcus_sp._'l	33.10	0.00
TRINITY_XP_02438	snurport	Physcomitrella_pate	33.10	0.00
TRINITY_GAX82950	hypothet	Chlamydomonas_eusti	33.10	0.00
TRINITY_XP_00356	flavin-c	Brachypodium_distac	33.10	0.00
TRINITY_XP_02198	CBL-inte	Helianthus_annuus	33.10	0.00
TRINITY_XP_02008	chromati	Ananas_comosus	33.10	0.00
TRINITY_PRW59743	non-canc	Chlorella_sorokinia	33.10	0.00
TRINITY_KXZ45198	hypothet	Gonium_pectorale	33.10	0.00
TRINITY_CAI99406	P-type A	Flabellia_petiolata	33.10	0.00
TRINITY_XP_00640	syntaxin	Eutrema_salsugineum	33.10	0.00
TRINITY_XP_02389	uncharac	Quercus_suber	33.10	0.00
TRINITY_KMZ61122	Cytokini	Zostera_marina	33.10	0.00

TRINITY_OAO8912(	hypothet	Arabidopsis_thalian	33.10	0.00
TRINITY_XP_0058!	hypothet	Chlorella_variabili	33.10	0.00
TRINITY_XP_0093(	PREDICTI	Pyrus_x_bretschneid	33.10	0.00
TRINITY_XP_0029!	hypothet	Volvox_carteri	33.10	0.00
TRINITY_XP_0070(	PREDICTI	Theobroma_cacao	33.10	0.00
TRINITY_GAX7459(	hypothet	Chlamydomonas_eusti	33.10	0.00
TRINITY_XP_0243!	calcium	Physcomitrella_pate	33.10	0.00
TRINITY_KXZ4279(	hypothet	Gonium_pectorale	33.10	0.00
TRINITY_KXZ5655(	hypothet	Gonium_pectorale	33.10	0.00
TRINITY_XP_0139(	hypothet	Monoraphidium_negle	33.10	0.00

TRINITY_GAX8279(	hypothet	Chlamydomonas_eusti	33.10	0.00
TRINITY_BAJ9372‹	predicte	Hordeum_vulgare	33.10	0.00
TRINITY_XP_0029‹	hypothet	Volvox_carteri	33.10	0.00
TRINITY_XP_0206ˇ	calmodul	Dendrobium_catenatu	33.10	0.00
TRINITY_GAX8376(	hypothet	Chlamydomonas_eusti	33.10	0.00
TRINITY_XP_0029‹	hypothet	Volvox_carteri	33.10	0.00
TRINITY_XP_0245ℑ	probable	Selaginella_moellen	33.10	0.00
TRINITY_XP_0217(	uncharac	Chenopodium_quinoa	33.10	0.00
TRINITY_XP_0220ℑ	nodal mo	Helianthus_annuus	33.10	0.00
TRINITY_XP_0017(	flagella	Chlamydomonas_reinh	33.10	0.00
TRINITY_GAX7828(	hypothet	Chlamydomonas_eusti	33.10	0.00
TRINITY_RXI0250⁵	hypothet	Malus_domestica	33.10	0.00
TRINITY_GAX7452(	hypothet	Chlamydomonas_eusti	33.10	0.00
TRINITY_GAQ9313(	hypothet	Klebsormidium_niten	33.10	0.00
TRINITY_XP_0238⁹	cytochro	Quercus_suber	33.10	0.00
TRINITY_XP_0030⁵	predicte	Micromonas_pusilla	33.10	0.00
TRINITY_XP_0172²	PREDICTE	Daucus_carota	33.00	0.00
TRINITY_GAX8352ℑ	hypothet	Chlamydomonas_eusti	33.00	0.00
TRINITY_XP_0243⁹	guanylat	Physcomitrella_pate	33.00	0.00
TRINITY_XP_0238⁸	UBP9-bir	Quercus_suber	33.00	0.00
TRINITY_XP_0238⁹	succinat	Quercus_suber	33.00	0.00
TRINITY_XP_0263⁸	BTB/POZ	Papaver_somniferum	33.00	0.00
TRINITY_XP_0024‹	survival	Sorghum_bicolor	33.00	0.00
TRINITY_RCV0490⁹	hypothet	Setaria_italica	33.00	0.00
TRINITY_XP_0167ℑ	PREDICTE	Gossypium_hirsutum	33.00	0.00
TRINITY_XP_0138⁹	hypothet	Monoraphidium_negle	33.00	0.00
TRINITY_XP_0030⁵	zeaxanth	Micromonas_pusilla	33.00	0.00
TRINITY_PTQ4186‹	hypothet	Marchantia_polymorp	33.00	0.00
TRINITY_GAX7597⁸	hypothet	Chlamydomonas_eusti	33.00	0.00
TRINITY_XP_0220‹	RNA-binc	Helianthus_annuus	33.00	0.00
TRINITY_GBF9821(	hypothet	Raphidocelis_subcap	33.00	0.00
TRINITY_XP_0237ℑ	probable	Lactuca_sativa	33.00	0.00
TRINITY_XP_0030(	zinc per	Micromonas_pusilla	33.00	0.00
TRINITY_XP_0244(	serine/t	Physcomitrella_pate	33.00	0.00
TRINITY_PLY6726²	hypothet	Lactuca_sativa	33.00	0.00
TRINITY_XP_0215⁹	endonucl	Manihot_esculenta	33.00	0.00
TRINITY_GAV6646²	Sterile	Cephalotus_follicul	33.00	0.00
TRINITY_GAX8134ℑ	hypothet	Chlamydomonas_eusti	33.00	0.00
TRINITY_KMZ7346ℑ	Two pore	Zostera_marina	33.00	0.00
TRINITY_EEC6766⁵	hypothet	Oryza_sativa	33.00	0.00
TRINITY_XP_0139(	hypothet	Monoraphidium_negle	33.00	0.00
TRINITY_PPD6649ℑ	hypothet	Gossypium_barbadens	33.00	0.00
TRINITY_XP_0165‹	PREDICTE	Capsicum_annuum	33.00	0.00
TRINITY_RWR8100ℑ	NADH del	Cinnamomum_micranth	33.00	0.00
TRINITY_XP_0029⁵	hypothet	Volvox_carteri	33.00	0.00
TRINITY_OWM8826⁸	hypothet	Punica_granatum	33.00	0.00
TRINITY_KXZ5641(	hypothet	Gonium_pectorale	33.00	0.00
TRINITY_XP_0075ℑ	ubiquiti	Bathycoccus_prasinc	33.00	0.00
TRINITY_GAX7618²	hypothet	Chlamydomonas_eusti	33.00	0.00
TRINITY_XP_0205⁹	glutathi	Phalaenopsis_equest	33.00	0.00
TRINITY_EEF2753⁵	two-comp	Ricinus_communis	33.00	0.00
TRINITY_XP_0062⁸	SNF1-rel	Capsella_rubella	33.00	0.00
TRINITY_EYU2921⁹	hypothet	Erythranthe_guttata	33.00	0.00
TRINITY_ADK8955⁸	spermidi	Marsilea_vestita	33.00	0.00
TRINITY_PIA5751ℑ	hypothet	Aquilegia_coerulea	33.00	0.00
TRINITY_GBG6619⁹	hypothet	Chara_braunii	33.00	0.00
TRINITY_XP_0029‹	hypothet	Volvox_carteri	33.00	0.00
TRINITY_PHT4061ˇ	Glyoxyso	Capsicum_baccatum	33.00	0.00
TRINITY_BAK0302⁵	predicte	Hordeum_vulgare	33.00	0.00
TRINITY_KXZ4643(	hypothet	Gonium_pectorale	33.00	0.00
TRINITY_PTQ3519(	hypothet	Marchantia_polymorp	33.00	0.00
TRINITY_XP_0104‹	PREDICTE	Camelina_sativa	33.00	0.00
TRINITY_KXZ5329ℑ	hypothet	Gonium_pectorale	33.00	0.00
TRINITY_GAX7452ℑ	hypothet	Chlamydomonas_eusti	33.00	0.00
TRINITY_XP_0227ℑ	plant cy	Durio_zibethinus	33.00	0.00
TRINITY_GAX7602ℑ	hypothet	Chlamydomonas_eusti	33.00	0.00

TRINITY_KXZ4746	hypothet	Gonium_pectorale	33.00	0.00
TRINITY_OVA1278	Ubiquiti	Macleaya_cordata	33.00	0.00
TRINITY_KXZ5147	hypothet	Gonium_pectorale	33.00	0.00
TRINITY_PNH1093	Protein	Tetrabaena_socialis	33.00	0.00
TRINITY_XP_0025	dynein	Micromonas_commoda	33.00	0.00
TRINITY_XP_0066	PREDICTE	Oryza_brachyantha	33.00	0.00
TRINITY_KCW5499	hypothet	Eucalyptus_grandis	32.90	0.00
TRINITY_XP_0217	probable	Chenopodium_quinoa	32.90	0.00
TRINITY_XP_0264	putative	Papaver_somniferum	32.90	0.00
TRINITY_XP_0066	PREDICTE	Oryza_brachyantha	32.90	0.00
TRINITY_OAE2059	hypothet	Marchantia_polymorp	32.90	0.00
TRINITY_XP_0056	hypothet	Coccomyxa_subellips	32.90	0.00
TRINITY_XP_0024	alpha-2	Micromonas_commoda	32.90	0.00
TRINITY_XP_0217	protein	Chenopodium_quinoa	32.90	0.00
TRINITY_GBF9524	TKL kina	Raphidocelis_subcap	32.90	0.00
TRINITY_GBG6360	hypothet	Chara_braunii	32.90	0.00
TRINITY_XP_0192	PREDICTE	Ipomoea_nil	32.90	0.00
TRINITY_KMZ7538	putative	Zostera_marina	32.90	0.00
TRINITY_GAQ8753	hypothet	Klebsormidium_niten	32.90	0.00
TRINITY_PRW5628	T-comple	Chlorella_sorokinia	32.90	0.00
TRINITY_XP_0063	PREDICTE	Solanum_tuberosum	32.90	0.00
TRINITY_GBF9011	hypothet	Raphidocelis_subcap	32.90	0.00
TRINITY_XP_0217	DNA-dire	Chenopodium_quinoa	32.90	0.00
TRINITY_XP_0241	probable	Rosa_chinensis	32.90	0.00
TRINITY_RAL5465	hypothet	Cuscuta_australis	32.90	0.00
TRINITY_XP_0243	gamma-gl	Physcomitrella_pate	32.90	0.00
TRINITY_XP_0029	hypothet	Volvox_carteri	32.90	0.00
TRINITY_OAO9886	SMO1-2	Arabidopsis_thalian	32.90	0.00
TRINITY_PNW8536	hypothet	Chlamydomonas_reinh	32.90	0.00
TRINITY_XP_0245	transcri	Selaginella_moellen	32.90	0.00
TRINITY_RYR3698	hypothet	Arachis_hypogaea	32.90	0.00
TRINITY_PNH0023	hypothet	Tetrabaena_socialis	32.90	0.00
TRINITY_GAX7761	hypothet	Chlamydomonas_eusti	32.90	0.00
TRINITY_RWR7791	pathogen	Cinnamomum_micranth	32.90	0.00
TRINITY_GAX8483	hypothet	Chlamydomonas_eusti	32.90	0.00
TRINITY_XP_0029	gamma ca	Selaginella_moellen	32.90	0.00
TRINITY_XP_0087	uncharac	Phoenix_dactylifera	32.90	0.00
TRINITY_GAX7623	hypothet	Chlamydomonas_eusti	32.90	0.00
TRINITY_KZV1487	hypothet	Dorcoceras_hygromet	32.90	0.00
TRINITY_XP_0166	PREDICTE	Gossypium_hirsutum	32.90	0.00
TRINITY_OAE3191	hypothet	Marchantia_polymorp	32.90	0.00
TRINITY_GAX7324	hypothet	Chlamydomonas_eusti	32.90	0.00
TRINITY_XP_0245	uncharac	Selaginella_moellen	32.90	0.00
TRINITY_XP_0083	PREDICTE	Malus_domestica	32.90	0.00
TRINITY_GAX7422	hypothet	Chlamydomonas_eusti	32.90	0.00
TRINITY_GAQ7992	Putative	Klebsormidium_niten	32.90	0.00
TRINITY_GBG7407	hypothet	Chara_braunii	32.90	0.00
TRINITY_PNH0981	Peroxiso	Tetrabaena_socialis	32.90	0.00
TRINITY_PRW5843	anaphase	Chlorella_sorokinia	32.90	0.00
TRINITY_XP_0030	predicte	Micromonas_pusilla	32.90	0.00
TRINITY_XP_0279	guanylat	Vigna_unguiculata	32.90	0.00
TRINITY_GAX8369	hypothet	Chlamydomonas_eusti	32.90	0.00
TRINITY_GAX8428	hypothet	Chlamydomonas_eusti	32.90	0.00
TRINITY_XP_0105	PREDICTE	Tarenaya_hasslerian	32.90	0.00
TRINITY_OWM7723	hypothet	Punica_granatum	32.90	0.00
TRINITY_GAX7887	hypothet	Chlamydomonas_eusti	32.90	0.00
TRINITY_XP_0106	PREDICTE	Beta_vulgaris	32.90	0.00
TRINITY_XP_0139	Putative	Monoraphidium_negle	32.90	0.00
TRINITY_XP_0191	PREDICTE	Ipomoea_nil	32.80	0.00
TRINITY_XP_0188	PREDICTE	Juglans_regia	32.80	0.00
TRINITY_XP_0243	E3 ubiqu	Physcomitrella_pate	32.80	0.00
TRINITY_BAK0050	predicte	Hordeum_vulgare	32.80	0.00
TRINITY_XP_0243	zinc fir	Physcomitrella_pate	32.80	0.00
TRINITY_XP_0100	PREDICTE	Eucalyptus_grandis	32.80	0.00
TRINITY_PRW5816	TBC1 dor	Chlorella_sorokinia	32.80	0.00
TRINITY_XP_0237	uncharac	Lactuca_sativa	32.80	0.00

TRINITY_XP_02283	ABC trar	Ostreococcus_tauri	32.80	0.00
TRINITY_XP_01843	PREDICTI	Raphanus_sativus	32.80	0.00
TRINITY_XP_0239(	probable	Quercus_suber	32.80	0.00
TRINITY_PNW7131(	hypothet	Chlamydomonas_reinh	32.80	0.00
TRINITY_GAQ8931(	hypothet	Klebsormidium_niten	32.80	0.00
TRINITY_XP_0205(	protein	Phalaenopsis_equest	32.80	0.00
TRINITY_XP_0245(	choline,	Selaginella_moellen	32.80	0.00
TRINITY_XP_0227	11-beta-	Durio_zibethinus	32.80	0.00
TRINITY_RDY0562	hypothet	Mucuna_pruriens	32.80	0.00
TRINITY_XP_0102(	PREDICTI	Nelumbo_nucifera	32.80	0.00
TRINITY_GBG8323	hypothet	Chara_braunii	32.80	0.00
TRINITY_XP_0056	hypothet	Coccomyxa_subellips	32.80	0.00
TRINITY_XP_0056	GBP-doma	Coccomyxa_subellips	32.80	0.00
TRINITY_GAX8413	hypothet	Chlamydomonas_eusti	32.80	0.00
TRINITY_PNR5511	hypothet	Physcomitrella_pate	32.80	0.00
TRINITY_ADE7731	unknown	Picea_sitchensis	32.80	0.00
TRINITY_XP_0264	ER membr	Papaver_somniferum	32.80	0.00
TRINITY_XP_0066(	PREDICTI	Oryza_brachyantha	32.80	0.00
TRINITY_PNH1169	hypothet	Tetrabaena_socialis	32.80	0.00
TRINITY_XP_0200(	uncharac	Ananas_comosus	32.80	0.00
TRINITY_OAY3338	hypothet	Manihot_esculenta	32.80	0.00
TRINITY_EFJ3343(	hypothet	Selaginella_moellen	32.80	0.00
TRINITY_GAX7476	hypothet	Chlamydomonas_eusti	32.80	0.00
TRINITY_RWW4448	hypothet	Ensete_ventricosum	32.80	0.00
TRINITY_KDD7543	3-beta h	Helicosporidium_sp.	32.80	0.00
TRINITY_XP_0202	SNF1-rel	Asparagus_officinal	32.80	0.00
TRINITY_XP_0188	PREDICTI	Juglans_regia	32.80	0.00
TRINITY_GAQ8170	hypothet	Klebsormidium_niten	32.80	0.00
TRINITY_GAQ8875	hypothet	Klebsormidium_niten	32.80	0.00
TRINITY_PLY7099(	hypothet	Lactuca_sativa	32.80	0.00
TRINITY_XP_0194	PREDICTI	Lupinus_angustifoli	32.80	0.00
TRINITY_XP_0200	uncharac	Ananas_comosus	32.80	0.00
TRINITY_XP_0243(	actin-de	Physcomitrella_pate	32.80	0.00
TRINITY_AFK4221(	unknown	Medicago_truncatula	32.80	0.00
TRINITY_EFJ1806(	hypothet	Selaginella_moellen	32.80	0.00
TRINITY_XP_0166(	PREDICTI	Gossypium_hirsutum	32.80	0.00
TRINITY_XP_0075	thiored	Bathycoccus_prasinc	32.80	0.00
TRINITY_GAQ7771	HECT dor	Klebsormidium_niten	32.80	0.00
TRINITY_XP_0264(	protein	Papaver_somniferum	32.80	0.00
TRINITY_XP_0025(	predicte	Micromonas_commoda	32.80	0.00
TRINITY_XP_0238	PQ-loop	Quercus_suber	32.80	0.00
TRINITY_XP_0058	hypothet	Chlorella_variabili	32.80	0.00
TRINITY_GAX8280(	hypothet	Chlamydomonas_eusti	32.80	0.00
TRINITY_XP_0245	uncharac	Selaginella_moellen	32.80	0.00
TRINITY_RRT5311(	hypothet	Ensete_ventricosum	32.80	0.00
TRINITY_GAX8211	hypothet	Chlamydomonas_eusti	32.80	0.00
TRINITY_GAQ7878	hypothet	Klebsormidium_niten	32.80	0.00
TRINITY_VDD1622	unnamed	Brassica_rapa	32.80	0.00
TRINITY_GAX7625	hypothet	Chlamydomonas_eusti	32.80	0.00
TRINITY_GAX8230	hypothet	Chlamydomonas_eusti	32.80	0.00
TRINITY_RWR8908(	Peptidas	Cinnamomum_micranth	32.80	0.00
TRINITY_PNH1165	Calcium/	Tetrabaena_socialis	32.80	0.00
TRINITY_PNW8868	hypothet	Chlamydomonas_reinh	32.80	0.00
TRINITY_XP_0239(	oxyster	Quercus_suber	32.80	0.00
TRINITY_XP_0243(	purple a	Physcomitrella_pate	32.80	0.00
TRINITY_XP_0243(	probable	Physcomitrella_pate	32.80	0.00
TRINITY_XP_0273	putative	Abrus_precatorius	32.80	0.00
TRINITY_GAX7650(	hypothet	Chlamydomonas_eusti	32.80	0.00
TRINITY_GAQ8454(	3'-5'-e	Klebsormidium_niten	32.70	0.00
TRINITY_XP_0056	hypothet	Coccomyxa_subellips	32.70	0.00
TRINITY_XP_0091	PREDICTI	Brassica_rapa	32.70	0.00
TRINITY_PPD8915	hypothet	Gossypium_barbadens	32.70	0.00
TRINITY_XP_0201(	BTB/POZ	Aegilops_tauschii	32.70	0.00
TRINITY_XP_0172	PREDICTI	Daucus_carota	32.70	0.00
TRINITY_BAF9574	ethylene	Physcomitrella_pate	32.70	0.00
TRINITY_XP_0109	PREDICTI	Elaeis_guineensis	32.70	0.00

TRINITY_RAL4233	hypothet	Cuscuta_australis	32.70	0.00
TRINITY_GAQ7773	Leishmar	Klebsormidium_niten	32.70	0.00
TRINITY_GBG9067	hypothet	Chara_braunii	32.70	0.00
TRINITY_XP_0239	ATP-bin	Quercus_suber	32.70	0.00
TRINITY_GAQ8388	CCR4-NO	Klebsormidium_niten	32.70	0.00
TRINITY_KVI0527	Nucleot	Cynara_cardunculus	32.70	0.00
TRINITY_XP_0071	hypothet	Phaseolus_vulgaris	32.70	0.00
TRINITY_XP_0216	activat	Manihot_esculenta	32.70	0.00
TRINITY_KXZ5105	hypothet	Gonium_pectorale	32.70	0.00
TRINITY_XP_0029	hypothet	Volvox_carteri	32.70	0.00
TRINITY_XP_0075	predicte	Bathycoccus_prasinc	32.70	0.00
TRINITY_CDM8520	unnamed	Triticum_aestivum	32.70	0.00
TRINITY_XP_0202	UTP:RNA	Asparagus_officinal	32.70	0.00
TRINITY_XP_0165	PREDICTE	Capsicum_annuum	32.70	0.00
TRINITY_GAX7370	hypothet	Chlamydomonas_eusti	32.70	0.00
TRINITY_XP_0202	major fa	Asparagus_officinal	32.70	0.00
TRINITY_GAQ7764	hypothet	Klebsormidium_niten	32.70	0.00
TRINITY_XP_0264	importir	Papaver_somniferum	32.70	0.00
TRINITY_RMZ5380	hypothet	Auxenochlorella_prc	32.70	0.00
TRINITY_ONM0780	Cox19-li	Zea_mays	32.70	0.00
TRINITY_XP_0190	PREDICTE	Camelina_sativa	32.70	0.00
TRINITY_XP_0243	heat sho	Physcomitrella_pate	32.70	0.00
TRINITY_GAX8069	hypothet	Chlamydomonas_eusti	32.70	0.00
TRINITY_GAX8090	hypothet	Chlamydomonas_eusti	32.70	0.00
TRINITY_XP_0218	BRCA1-as	Carica_papaya	32.70	0.00
TRINITY_XP_0056	hypothet	Coccomyxa_subellips	32.70	0.00
TRINITY_ABJ9122	CBL-inte	Populus_trichocarpa	32.70	0.00
TRINITY_GAQ7872	Notchles	Klebsormidium_niten	32.70	0.00
TRINITY_PIA6174	hypothet	Aquilegia_coerulea	32.70	0.00
TRINITY_GAX7418	hypothet	Chlamydomonas_eusti	32.70	0.00
TRINITY_XP_0049	BTB/POZ	Setaria_italica	32.70	0.00
TRINITY_XP_0191	PREDICTE	Ipomoea_nil	32.70	0.00
TRINITY_GAQ8975	hypothet	Klebsormidium_niten	32.70	0.00
TRINITY_KXZ5619	hypothet	Gonium_pectorale	32.70	0.00
TRINITY_XP_0217	aromatic	Chenopodium_quinoa	32.70	0.00
TRINITY_RLN2791	membrane	Panicum_miliaceum	32.70	0.00
TRINITY_OMO8137	hypothet	Corchorus_olitorius	32.70	0.00
TRINITY_GAQ8975	hypothet	Klebsormidium_niten	32.70	0.00
TRINITY_PNW8511	hypothet	Chlamydomonas_reinh	32.70	0.00
TRINITY_GAQ7941	carbonic	Klebsormidium_niten	32.70	0.00
TRINITY_XP_0029	hypothet	Volvox_carteri	32.70	0.00
TRINITY_GAX7829	hypothet	Chlamydomonas_eusti	32.70	0.00
TRINITY_GAX8338	hypothet	Chlamydomonas_eusti	32.70	0.00
TRINITY_OMO6279	Kelch re	Corchorus_olitorius	32.70	0.00
TRINITY_PNH0317	hypothet	Tetrabaena_socialis	32.70	0.00
TRINITY_XP_0238	inositol	Quercus_suber	32.70	0.00
TRINITY_XP_0056	GTP-bin	Coccomyxa_subellips	32.70	0.00
TRINITY_KZV3170	hypothet	Dorcoceras_hygromet	32.70	0.00
TRINITY_XP_0029	hypothet	Volvox_carteri	32.70	0.00
TRINITY_XP_0192	PREDICTE	Nicotiana_attenuata	32.70	0.00
TRINITY_PNW8454	hypothet	Chlamydomonas_reinh	32.70	0.00
TRINITY_KZV1488	penicil	Dorcoceras_hygromet	32.70	0.00
TRINITY_XP_0239	unchara	Quercus_suber	32.70	0.00
TRINITY_EYU3860	hypothet	Erythranthe_guttata	32.70	0.00
TRINITY_XP_0190	ADP-ribo	Solanum_lycopersicu	32.70	0.00
TRINITY_XP_0025	predicte	Micromonas_commoda	32.70	0.00
TRINITY_GAX8399	hypothet	Chlamydomonas_eusti	32.70	0.00
TRINITY_XP_0240	ras-rela	Morus_notabilis	32.60	0.00
TRINITY_RRT7214	hypothet	Ensete_ventricosum	32.60	0.00
TRINITY_XP_0227	sphingos	Durio_zibethinus	32.60	0.00
TRINITY_XP_0243	unchara	Physcomitrella_pate	32.60	0.00
TRINITY_XP_0172	PREDICTE	Daucus_carota	32.60	0.00
TRINITY_XP_0161	ultravio	Arachis_ipaensis	32.60	0.00
TRINITY_XP_0086	ABC trar	Zea_mays	32.60	0.00
TRINITY_XP_0191	PREDICTE	Ipomoea_nil	32.60	0.00
TRINITY_PWZ5394	Cation/	Zea_mays	32.60	0.00

TRINITY_XP_00294	hypothet	Volvox_carteri	32.60	0.00
TRINITY_OUS43849	leucine-	Ostreococcus_tauri	32.60	0.00
TRINITY_OVA07140	Pyridine	Macleaya_cordata	32.60	0.00
TRINITY_XP_01562	CBL-inte	Oryza_sativa	32.60	0.00
TRINITY_XP_00564	NAD(P)-b	Coccomyxa_subellips	32.60	0.00
TRINITY_XP_01140	Epoxide	Auxenochlorella_prc	32.60	0.00
TRINITY_GBG88259	hypothet	Chara_braunii	32.60	0.00
TRINITY_OAE26797	hypothet	Marchantia_polymorp	32.60	0.00
TRINITY_GAX74997	hypothet	Chlamydomonas_eusti	32.60	0.00
TRINITY_XP_02389	ran-bind	Quercus_suber	32.60	0.00
TRINITY_XP_00250	voltage-	Micromonas_commoda	32.60	0.00
TRINITY_XP_02289	uncharac	Olea_europaea	32.60	0.00
TRINITY_XP_02430	gamma-gl	Physcomitrella_pate	32.60	0.00
TRINITY_EPS57898	hypothet	Genlisea_aurea	32.60	0.00
TRINITY_GAX78374	hypothet	Chlamydomonas_eusti	32.60	0.00
TRINITY_XP_02288	ATP-depe	Olea_europaea	32.60	0.00
TRINITY_EPS64880	hypothet	Genlisea_aurea	32.60	0.00
TRINITY_XP_01054	PREDICTE	Tarenaya_hasslerian	32.60	0.00
TRINITY_XP_01140	hypothet	Auxenochlorella_prc	32.60	0.00
TRINITY_XP_01052	PREDICTE	Tarenaya_hasslerian	32.60	0.00
TRINITY_PNH08303	NFX1-typ	Tetrabaena_socialis	32.60	0.00
TRINITY_XP_01093	PREDICTE	Elaeis_guineensis	32.60	0.00
TRINITY_XP_02388	saccharo	Quercus_suber	32.60	0.00
TRINITY_PRW20805	glutamat	Chlorella_sorokinia	32.60	0.00
TRINITY_XP_02452	uncharac	Selaginella_moellen	32.60	0.00
TRINITY_XP_02392	D-xylose	Quercus_suber	32.60	0.00
TRINITY_XP_00584	hypothet	Chlorella_variabili	32.60	0.00
TRINITY_XP_02287	C2 and (	Olea_europaea	32.60	0.00
TRINITY_XP_01363	PREDICTE	Brassica_oleracea	32.60	0.00
TRINITY_PWZ20812	CBL-inte	Zea_mays	32.60	0.00
TRINITY_XP_01139	Importin	Auxenochlorella_prc	32.60	0.00
TRINITY_XP_02391	uncharac	Quercus_suber	32.60	0.00
TRINITY_GAX77845	hypothet	Chlamydomonas_eusti	32.60	0.00
TRINITY_XP_00290	uncharac	Selaginella_moellen	32.60	0.00
TRINITY_XP_00250	predicte	Micromonas_commoda	32.60	0.00
TRINITY_PSC70167	beta-asp	Micractinium_conduc	32.60	0.00
TRINITY_GAX74960	hypothet	Chlamydomonas_eusti	32.60	0.00
TRINITY_XP_00295	hypothet	Volvox_carteri	32.60	0.00
TRINITY_KXZ47290	hypothet	Gonium_pectorale	32.60	0.00
TRINITY_PNY13150	sterol 1	Trifolium_pratense	32.60	0.00
TRINITY_RLM73830	BTB/POZ	Panicum_miliaceum	32.60	0.00
TRINITY_PTQ32550	hypothet	Marchantia_polymorp	32.60	0.00
TRINITY_GAX74920	hypothet	Chlamydomonas_eusti	32.60	0.00
TRINITY_XP_00294	hypothet	Volvox_carteri	32.60	0.00
TRINITY_PNH11454	Microtub	Tetrabaena_socialis	32.60	0.00
TRINITY_XP_01109	gamma-gl	Sesamum_indicum	32.60	0.00
TRINITY_XP_00295	hypothet	Volvox_carteri	32.60	0.00
TRINITY_XP_00564	hypothet	Coccomyxa_subellips	32.60	0.00
TRINITY_PPD85795	hypothet	Gossypium_barbadens	32.60	0.00
TRINITY_PSC71341	putative	Micractinium_conduc	32.60	0.00
TRINITY_XP_02453	cAMP-dep	Selaginella_moellen	32.60	0.00
TRINITY_XP_00170	flagella	Chlamydomonas_reinh	32.60	0.00
TRINITY_XP_00142	ZIP fami	Ostreococcus_sp._'l	32.60	0.00
TRINITY_XP_02402	serine/t	Morus_notabilis	32.60	0.00
TRINITY_XP_00170	predicte	Chlamydomonas_reinh	32.60	0.00
TRINITY_GAQ91585	RNA poly	Klebsormidium_niten	32.60	0.00
TRINITY_GAX75074	hypothet	Chlamydomonas_eusti	32.60	0.00
TRINITY_PAN13885	hypothet	Panicum_hallii	32.60	0.00
TRINITY_OAE18128	hypothet	Marchantia_polymorp	32.60	0.00
TRINITY_XP_00940	PREDICTE	Musa_acuminata	32.60	0.00
TRINITY_XP_02454	WASH com	Selaginella_moellen	32.60	0.00
TRINITY_XP_00564	hypothet	Coccomyxa_subellips	32.60	0.00
TRINITY_XP_01102	PREDICTE	Populus_euphratica	32.60	0.00
TRINITY_ACR38892	ubiquiti	Apopellia_endiviifc	32.50	0.00
TRINITY_XP_01283	PREDICTE	Erythranthe_guttata	32.50	0.00
TRINITY_NP_18722	importin	Arabidopsis_thalian	32.50	0.00

TRINITY_GAQ8197	oxidore	Klebsormidium_niten	32.50	0.00
TRINITY_AKM7674	RNA-bin	Pelargonium_x_hortc	32.50	0.00
TRINITY_XP_0190	PREDICTI	Camelina_sativa	32.50	0.00
TRINITY_KXZ4567	hypothet	Gonium_pectorale	32.50	0.00
TRINITY_PNW7987	hypothet	Chlamydomonas_reinh	32.50	0.00
TRINITY_XP_0256	uncharac	Arachis_hypogaea	32.50	0.00
TRINITY_RLM5576	hypothet	Panicum_miliaceum	32.50	0.00
TRINITY_PLY7589	hypothet	Lactuca_sativa	32.50	0.00
TRINITY_PRW5843	anaphase	Chlorella_sorokinia	32.50	0.00
TRINITY_BAB5608	putative	Oryza_sativa	32.50	0.00
TRINITY_GAQ8710	cellulos	Klebsormidium_niten	32.50	0.00
TRINITY_EEF2262	carboni	Ricinus_communis	32.50	0.00
TRINITY_OVA1265	Protein	Macleaya_cordata	32.50	0.00
TRINITY_XP_0016	predicte	Chlamydomonas_reinh	32.50	0.00
TRINITY_KHG1428	putative	Gossypium_arboreum	32.50	0.00
TRINITY_XP_0083	PREDICTI	Malus_domestica	32.50	0.00
TRINITY_GAX7969	hypothet	Chlamydomonas_eusti	32.50	0.00
TRINITY_GAQ8152	Vesicle	Klebsormidium_niten	32.50	0.00
TRINITY_GBG6905	hypothet	Chara_braunii	32.50	0.00
TRINITY_XP_0271	uncharac	Cicer_arietinum	32.50	0.00
TRINITY_GAQ8673	hypothet	Klebsormidium_niten	32.50	0.00
TRINITY_GAX7819	hypothet	Chlamydomonas_eusti	32.50	0.00
TRINITY_XP_0100	PREDICTI	Eucalyptus_grandis	32.50	0.00
TRINITY_XP_0128	PREDICTI	Erythranthe_guttata	32.50	0.00
TRINITY_PNW8254	hypothet	Chlamydomonas_reinh	32.50	0.00
TRINITY_PIN1745	WD40 re	Handroanthus_impeti	32.50	0.00
TRINITY_XP_0186	PREDICTI	Nicotiana_tomentosi	32.50	0.00
TRINITY_GBF8875	hypothet	Raphidocelis_subcap	32.50	0.00
TRINITY_GBF9085	DEAD-bo	Raphidocelis_subcap	32.50	0.00
TRINITY_XP_0165	PREDICTI	Capsicum_annuum	32.50	0.00
TRINITY_PTQ3905	hypothet	Marchantia_polymorp	32.50	0.00
TRINITY_PTQ2804	hypothet	Marchantia_polymorp	32.50	0.00
TRINITY_OAE2948	hypothet	Marchantia_polymorp	32.50	0.00
TRINITY_XP_0029	hypothet	Volvox_carteri	32.50	0.00
TRINITY_GAX8311	hypothet	Chlamydomonas_eusti	32.50	0.00
TRINITY_XP_0023	ultravi	Populus_trichocarpa	32.50	0.00
TRINITY_AML7829	putative	Pleurastrum_insigne	32.50	0.00
TRINITY_GAX7635	hypothet	Chlamydomonas_eusti	32.50	0.00
TRINITY_PNH0534	hypothet	Tetrabaena_socialis	32.50	0.00
TRINITY_RWR8862	HSP-inte	Cinnamomum_micranth	32.50	0.00
TRINITY_KYP4918	Cytochr	Cajanus_cajan	32.50	0.00
TRINITY_XP_0235	lysine-	Cucurbita_pepo	32.50	0.00
TRINITY_XP_0058	hypothet	Chlorella_variabili	32.50	0.00
TRINITY_RXH8695	hypothet	Malus_domestica	32.50	0.00
TRINITY_XP_0064	protein	Citrus_clementina	32.50	0.00
TRINITY_XP_0191	PREDICTI	Ipomoea_nil	32.50	0.00
TRINITY_XP_0029	hypothet	Volvox_carteri	32.50	0.00
TRINITY_XP_0245	probable	Selaginella_moellen	32.50	0.00
TRINITY_BAK0043	predicte	Hordeum_vulgare	32.50	0.00
TRINITY_KMZ7365	hypothet	Zostera_marina	32.50	0.00
TRINITY_GAQ8320	vacuola	Klebsormidium_niten	32.50	0.00
TRINITY_XP_0029	hypothet	Volvox_carteri	32.50	0.00
TRINITY_XP_0030	Peptida	Ostreococcus_tauri	32.50	0.00
TRINITY_XP_0192	PREDICTI	Ipomoea_nil	32.50	0.00
TRINITY_PTQ4067	hypothet	Marchantia_polymorp	32.50	0.00
TRINITY_XP_0029	hypothet	Volvox_carteri	32.50	0.00
TRINITY_XP_0192	PREDICTI	Nicotiana_attenuata	32.50	0.00
TRINITY_XP_0249	vesicle-	Cynara_cardunculus	32.50	0.00
TRINITY_XP_0036	serine	Medicago_truncatula	32.40	0.00
TRINITY_XP_0105	PREDICTI	Tarenaya_hasslerian	32.40	0.00
TRINITY_XP_0017	predicte	Chlamydomonas_reinh	32.40	0.00
TRINITY_PWA3241	PDI-like	Artemisia_annua	32.40	0.00
TRINITY_XP_0184	PREDICTI	Raphanus_sativus	32.40	0.00
TRINITY_XP_0094	PREDICTI	Musa_acuminata	32.40	0.00
TRINITY_XP_0241	mitochor	Rosa_chinensis	32.40	0.00
TRINITY_XP_0016	componen	Chlamydomonas_reinh	32.40	0.00

TRINITY_XP_0212	protein	Herrania_umbratica	32.40	0.00
TRINITY_XP_0172	PREDICTI	Daucus_carota	32.40	0.00
TRINITY_GAX8545	hypothet	Chlamydomonas_eusti	32.40	0.00
TRINITY_XP_0110	PREDICTI	Populus_euphratica	32.40	0.00
TRINITY_XP_0109	PREDICTI	Elaeis_guineensis	32.40	0.00
TRINITY_PLY8691	hypothet	Lactuca_sativa	32.40	0.00
TRINITY_XP_0239	uncharac	Quercus_suber	32.40	0.00
TRINITY_EEF2753	two-comp	Ricinus_communis	32.40	0.00
TRINITY_XP_0064	CBL-inte	Citrus_clementina	32.40	0.00
TRINITY_PTQ2779	hypothet	Marchantia_polymorp	32.40	0.00
TRINITY_XP_0264	BTB/POZ	Papaver_somniferum	32.40	0.00
TRINITY_KXZ5265	hypothet	Gonium_pectorale	32.40	0.00
TRINITY_XP_0239	60 kDa	Quercus_suber	32.40	0.00
TRINITY_PSC6862	acidstal	Micractinium_conduc	32.40	0.00
TRINITY_BAK0112	predicte	Hordeum_vulgare	32.40	0.00
TRINITY_XP_0239	guanine	Quercus_suber	32.40	0.00
TRINITY_PNW7576	hypothet	Chlamydomonas_reinh	32.40	0.00
TRINITY_XP_0025	predicte	Micromonas_commoda	32.40	0.00
TRINITY_GAQ7970	hypothet	Klebsormidium_niten	32.40	0.00
TRINITY_XP_0238	bifuncti	Quercus_suber	32.40	0.00
TRINITY_XP_0025	DEK_C do	Micromonas_commoda	32.40	0.00
TRINITY_XP_0120	calcium-	Jatropha_curcas	32.40	0.00
TRINITY_GBF9530	mitochon	Raphidocelis_subcap	32.40	0.00
TRINITY_XP_0201	DENN dom	Ananas_comosus	32.40	0.00
TRINITY_XP_0056	hypothet	Coccomyxa_subellips	32.40	0.00
TRINITY_PRW4510	inactive	Chlorella_sorokinia	32.40	0.00
TRINITY_XP_0025	predicte	Micromonas_commoda	32.40	0.00
TRINITY_GAX7986	hypothet	Chlamydomonas_eusti	32.40	0.00
TRINITY_XP_0056	hypothet	Coccomyxa_subellips	32.40	0.00
TRINITY_GAX8138	hypothet	Chlamydomonas_eusti	32.40	0.00
TRINITY_RWR7878	arginyl-	Cinnamomum_micranth	32.40	0.00
TRINITY_XP_0058	hypothet	Chlorella_variabili	32.40	0.00
TRINITY_KXZ4377	hypothet	Gonium_pectorale	32.40	0.00
TRINITY_BAJ9457	predicte	Hordeum_vulgare	32.40	0.00
TRINITY_XP_0266	probable	Phoenix_dactylifera	32.40	0.00
TRINITY_PNW8414	hypothet	Chlamydomonas_reinh	32.40	0.00
TRINITY_XP_0216	calcineu	Hevea_brasiliensis	32.40	0.00
TRINITY_XP_0221	WD repea	Momordica_charantia	32.40	0.00
TRINITY_OUS4311	5'-nucle	Ostreococcus_tauri	32.40	0.00
TRINITY_XP_0205	tubulin-	Phalaenopsis_equest	32.40	0.00
TRINITY_XP_0243	ribonucl	Physcomitrella_pate	32.40	0.00
TRINITY_XP_0217	probable	Chenopodium_quinoa	32.40	0.00
TRINITY_XP_0217	probable	Chenopodium_quinoa	32.40	0.00
TRINITY_XP_0243	THO comp	Physcomitrella_pate	32.40	0.00
TRINITY_XP_0221	probable	Momordica_charantia	32.40	0.00
TRINITY_XP_0056	hypothet	Coccomyxa_subellips	32.40	0.00
TRINITY_XP_0194	PREDICTI	Lupinus_angustifoli	32.40	0.00
TRINITY_XP_0202	leishman	Cajanus_cajan	32.40	0.00
TRINITY_XP_0151	PREDICTI	Solanum_tuberosum	32.40	0.00
TRINITY_XP_0191	PREDICTI	Ipomoea_nil	32.40	0.00
TRINITY_KZV1512	protein	Dorcoceras_hygromet	32.40	0.00
TRINITY_GAX8183	hypothet	Chlamydomonas_eusti	32.40	0.00
TRINITY_KXZ5292	hypothet	Gonium_pectorale	32.40	0.00
TRINITY_KXZ5559	hypothet	Gonium_pectorale	32.40	0.00
TRINITY_XP_0056	hypothet	Coccomyxa_subellips	32.40	0.00
TRINITY_XP_0165	PREDICTI	Capsicum_annuum	32.30	0.00
TRINITY_RQM0400	hypothet	Brassica_cretica	32.30	0.00
TRINITY_XP_0106	PREDICTI	Beta_vulgaris	32.30	0.00
TRINITY_XP_0192	PREDICTI	Ipomoea_nil	32.30	0.00
TRINITY_XP_0238	uncharac	Quercus_suber	32.30	0.00
TRINITY_XP_0056	AMPKBI-c	Coccomyxa_subellips	32.30	0.00
TRINITY_XP_0029	COP9 sig	Selaginella_moellen	32.30	0.00
TRINITY_EFH5772	binding	Arabidopsis_lyrata	32.30	0.00
TRINITY_GAQ9014	hypothet	Klebsormidium_niten	32.30	0.00
TRINITY_VDD3317	unnamed	Brassica_oleracea	32.30	0.00
TRINITY_XP_0238	delta(3,	Quercus_suber	32.30	0.00

TRINITY_XP_02439	acyl-CoA	Physcomitrella_pate	32.30	0.00
TRINITY_GAX78863	hypothet	Chlamydomonas_eusti	32.30	0.00
TRINITY_GAX79903	hypothet	Chlamydomonas_eusti	32.30	0.00
TRINITY_GAX86324	hypothet	Chlamydomonas_eusti	32.30	0.00
TRINITY_XP_02711	phosphoe	Coffea_arabica	32.30	0.00
TRINITY_RLN34130	synaptot	Panicum_miliaceum	32.30	0.00
TRINITY_XP_01665	PREDICTE	Prunus_mume	32.30	0.00
TRINITY_KMZ57039	Varicose	Zostera_marina	32.30	0.00
TRINITY_PNW78294	hypothet	Chlamydomonas_reinh	32.30	0.00
TRINITY_GAQ89579	Putative	Klebsormidium_niten	32.30	0.00
TRINITY_AJT59571	GABAT2	Veratrum_californic	32.30	0.00
TRINITY_KZV15308	alpha-ar	Dorcoceras_hygromet	32.30	0.00
TRINITY_GBF91111	water di	Raphidocelis_subcap	32.30	0.00
TRINITY_OMO61411	Endoplas	Corchorus_capsulari	32.30	0.00
TRINITY_GAX72893	hypothet	Chlamydomonas_eusti	32.30	0.00
TRINITY_XP_02271	embryoge	Durio_zibethinus	32.30	0.00
TRINITY_GAX78245	hypothet	Chlamydomonas_eusti	32.30	0.00
TRINITY_PRW59996	serine t	Chlorella_sorokinia	32.30	0.00
TRINITY_ADE76140	unknown	Picea_sitchensis	32.30	0.00
TRINITY_XP_01917	PREDICTE	Ipomoea_nil	32.30	0.00
TRINITY_GAX84678	hypothet	Chlamydomonas_eusti	32.30	0.00
TRINITY_XP_02391	cytoplas	Quercus_suber	32.30	0.00
TRINITY_XP_01027	PREDICTE	Nelumbo_nucifera	32.30	0.00
TRINITY_PTQ33009	hypothet	Marchantia_polymorp	32.30	0.00
TRINITY_XP_00424	transcri	Solanum_lycopersicu	32.30	0.00
TRINITY_OTG12379	putative	Helianthus_annuus	32.30	0.00
TRINITY_PIA37221	hypothet	Aquilegia_coerulea	32.30	0.00
TRINITY_XP_02452	enoyl-[a	Selaginella_moellen	32.30	0.00
TRINITY_XP_02163	probable	Hevea_brasiliensis	32.30	0.00
TRINITY_XP_02497	protein	Cynara_cardunculus	32.30	0.00
TRINITY_XP_01867	PREDICTE	Musa_acuminata	32.30	0.00
TRINITY_GAX83980	hypothet	Chlamydomonas_eusti	32.30	0.00
TRINITY_XP_00430	PREDICTE	Fragaria_vesca	32.30	0.00
TRINITY_XP_00297	intrafla	Selaginella_moellen	32.30	0.00
TRINITY_XP_00169	predicte	Chlamydomonas_reinh	32.30	0.00
TRINITY_XP_02640	kelch do	Papaver_somniferum	32.30	0.00
TRINITY_KCW64455	hypothet	Eucalyptus_grandis	32.30	0.00
TRINITY_KMZ65276	Deoxyhyp	Zostera_marina	32.30	0.00
TRINITY_XP_02387	calcium-	Quercus_suber	32.30	0.00
TRINITY_XP_02375	nudix hy	Lactuca_sativa	32.30	0.00
TRINITY_GAX80971	hypothet	Chlamydomonas_eusti	32.30	0.00
TRINITY_XP_02389	uncharac	Quercus_suber	32.30	0.00
TRINITY_XP_02435	probable	Physcomitrella_pate	32.30	0.00
TRINITY_XP_00497	BTB/POZ	Setaria_italica	32.30	0.00
TRINITY_PNW78545	hypothet	Chlamydomonas_reinh	32.30	0.00
TRINITY_XP_01047	PREDICTE	Camelina_sativa	32.30	0.00
TRINITY_XP_00168	tRNA (ur	Chlamydomonas_reinh	32.30	0.00
TRINITY_GAX78814	hypothet	Chlamydomonas_eusti	32.30	0.00
TRINITY_GAX75631	hypothet	Chlamydomonas_eusti	32.30	0.00
TRINITY_GAX76527	hypothet	Chlamydomonas_eusti	32.30	0.00
TRINITY_GAX84396	hypothet	Chlamydomonas_eusti	32.30	0.00
TRINITY_PNW87622	hypothet	Chlamydomonas_reinh	32.30	0.00
TRINITY_AAD51632	KNOX1 ho	Acetabularia_acetab	32.30	0.00
TRINITY_XP_02463	tobamovi	Medicago_truncatula	32.30	0.00
TRINITY_XP_01915	PREDICTE	Ipomoea_nil	32.30	0.00
TRINITY_PNH11552	30S ribo	Tetrabaena_socialis	32.30	0.00
TRINITY_GAX75916	hypothet	Chlamydomonas_eusti	32.30	0.00
TRINITY_GAX81130	hypothet	Chlamydomonas_eusti	32.30	0.00
TRINITY_PTQ37557	hypothet	Marchantia_polymorp	32.30	0.00
TRINITY_XP_01649	PREDICTE	Nicotiana_tabacum	32.30	0.00
TRINITY_XP_02435	ATP synt	Physcomitrella_pate	32.20	0.00
TRINITY_XP_01846	PREDICTE	Raphanus_sativus	32.20	0.00
TRINITY_PNX93661	ABC tran	Trifolium_pratense	32.20	0.00
TRINITY_EFJ33436	hypothet	Selaginella_moellen	32.20	0.00
TRINITY_XP_02445	ABC tran	Populus_trichocarpa	32.20	0.00
TRINITY_XP_02289	tubulin-	Olea_europaea	32.20	0.00

TRINITY_PWA4330(	protein	Artemisia_annua	32.20	0.00
TRINITY_EFJ0925!	hypothet	Selaginella_moellen	32.20	0.00
TRINITY_XP_0235	ran-binc	Cucurbita_pepo	32.20	0.00
TRINITY_GAQ7952!	hypothet	Klebsormidium_niten	32.20	0.00
TRINITY_XP_0220	serine (	Helianthus_annuus	32.20	0.00
TRINITY_XP_0042	ran-binc	Solanum_lycopersicu	32.20	0.00
TRINITY_GAQ8786(	sphingor	Klebsormidium_niten	32.20	0.00
TRINITY_XP_0029!	hypothet	Volvox_carteri	32.20	0.00
TRINITY_PWA8028	SKP1/BTI	Artemisia_annua	32.20	0.00
TRINITY_PSC7251(	multidrı	Micractinium_conduc	32.20	0.00
TRINITY_XP_0243!	ATP synt	Physcomitrella_pate	32.20	0.00
TRINITY_XP_0124	PREDICTI	Gossypium_raimondii	32.20	0.00
TRINITY_XP_0138!	hypothet	Monoraphidium_negle	32.20	0.00
TRINITY_XP_0168!	PREDICTI	Cucumis_melo	32.20	0.00
TRINITY_GAX7735(	hypothet	Chlamydomonas_eusti	32.20	0.00
TRINITY_GAX7417(	hypothet	Chlamydomonas_eusti	32.20	0.00
TRINITY_PRW3381	transcri	Chlorella_sorokinia	32.20	0.00
TRINITY_GAX8112	hypothet	Chlamydomonas_eusti	32.20	0.00
TRINITY_GAX7377(	hypothet	Chlamydomonas_eusti	32.20	0.00
TRINITY_XP_0184!	PREDICTI	Raphanus_sativus	32.20	0.00
TRINITY_OIW2165	hypothet	Lupinus_angustifoli	32.20	0.00
TRINITY_KXZ4627	hypothet	Gonium_pectorale	32.20	0.00
TRINITY_PNW8536(	hypothet	Chlamydomonas_reinh	32.20	0.00
TRINITY_XP_0238	transcri	Quercus_suber	32.20	0.00
TRINITY_OVA0836(	Cytochr(	Macleaya_cordata	32.20	0.00
TRINITY_XP_0239	protein	Quercus_suber	32.20	0.00
TRINITY_KEH4009	metallo	Medicago_truncatula	32.20	0.00
TRINITY_GAX7608	hypothet	Chlamydomonas_eusti	32.20	0.00
TRINITY_XP_0200!	unchara(	Ananas_comosus	32.20	0.00
TRINITY_PNW8077	hypothet	Chlamydomonas_reinh	32.20	0.00
TRINITY_GAQ8842(	Putative	Klebsormidium_niten	32.20	0.00
TRINITY_XP_0239(	myosin-1	Quercus_suber	32.20	0.00
TRINITY_BAJ9694(	predicte	Hordeum_vulgare	32.20	0.00
TRINITY_GAX8351	hypothet	Chlamydomonas_eusti	32.20	0.00
TRINITY_XP_0184!	PREDICTI	Raphanus_sativus	32.20	0.00
TRINITY_XP_0097!	PREDICTI	Nicotiana_sylvestri	32.20	0.00
TRINITY_GAX8031(	hypothet	Chlamydomonas_eusti	32.20	0.00
TRINITY_GBF9892	serine p	Raphidocelis_subcap	32.20	0.00
TRINITY_XP_0156	unchara(	Oryza_sativa	32.20	0.00
TRINITY_GAX8061	hypothet	Chlamydomonas_eusti	32.20	0.00
TRINITY_GAX8439!	hypothet	Chlamydomonas_eusti	32.20	0.00
TRINITY_AJR2238(	CBL-inte	Triticum_aestivum	32.20	0.00
TRINITY_XP_0056	hypothet	Coccomyxa_subellips	32.20	0.00
TRINITY_XP_0029	hypothet	Volvox_carteri	32.20	0.00
TRINITY_KXZ5566	hypothet	Gonium_pectorale	32.20	0.00
TRINITY_GAX7778(	hypothet	Chlamydomonas_eusti	32.20	0.00
TRINITY_GBG8341!	hypothet	Chara_braunii	32.20	0.00
TRINITY_GAQ9153	hypothet	Klebsormidium_niten	32.10	0.00
TRINITY_GBF9737	hypothet	Raphidocelis_subcap	32.10	0.00
TRINITY_XP_0075	predicte	Bathycoccus_prasinc	32.10	0.00
TRINITY_ADE7648!	unknown	Picea_sitchensis	32.10	0.00
TRINITY_XP_0238	calcium-	Quercus_suber	32.10	0.00
TRINITY_GAQ8161	ABC trar	Klebsormidium_niten	32.10	0.00
TRINITY_RWW9088	hypothet	Ensete_ventricosum	32.10	0.00
TRINITY_XP_0243	alkaline	Physcomitrella_pate	32.10	0.00
TRINITY_XP_0128	PREDICTI	Erythranthe_guttata	32.10	0.00
TRINITY_EFJ0991	hypothet	Selaginella_moellen	32.10	0.00
TRINITY_PPR8930!	hypothet	Gossypium_barbadens	32.10	0.00
TRINITY_GAX7287!	hypothet	Chlamydomonas_eusti	32.10	0.00
TRINITY_XP_0264!	BTB/POZ	Papaver_somniferum	32.10	0.00
TRINITY_XP_0164	PREDICTI	Nicotiana_tabacum	32.10	0.00
TRINITY_RWW9078	hypothet	Ensete_ventricosum	32.10	0.00
TRINITY_GBF8754(	serine p	Raphidocelis_subcap	32.10	0.00
TRINITY_PNW7658	hypothet	Chlamydomonas_reinh	32.10	0.00
TRINITY_PUZ4524	hypothet	Panicum_hallii	32.10	0.00
TRINITY_XP_0138!	hypothet	Monoraphidium_negle	32.10	0.00

TRINITY_XP_01725	PREDICTE	Daucus_carota	32.10	0.00
TRINITY_XP_02439	serine/t	Physcomitrella_pate	32.10	0.00
TRINITY_XP_00305	predicte	Micromonas_pusilla	32.10	0.00
TRINITY_XP_01595	C2 and (	Arachis_duranensis	32.10	0.00
TRINITY_XP_01146	PREDICTE	Fragaria_vesca	32.10	0.00
TRINITY_XP_02719	triacylg	Cicer_arietinum	32.10	0.00
TRINITY_OWM65262	hypothet	Punica_granatum	32.10	0.00
TRINITY_BAK01536	predicte	Hordeum_vulgare	32.10	0.00
TRINITY_XP_01941	PREDICTE	Lupinus_angustifoli	32.10	0.00
TRINITY_XP_02057	ubiquiti	Phalaenopsis_equest	32.10	0.00
TRINITY_KXZ56254	hypothet	Gonium_pectorale	32.10	0.00
TRINITY_XP_02666	pectin a	Phoenix_dactylifera	32.10	0.00
TRINITY_XP_01883	PREDICTE	Juglans_regia	32.10	0.00
TRINITY_PTQ37558	hypothet	Marchantia_polymorp	32.10	0.00
TRINITY_XP_02289	ubiquiti	Olea_europaea	32.10	0.00
TRINITY_KJB24464	hypothet	Gossypium_raimondii	32.10	0.00
TRINITY_KXZ48828	hypothet	Gonium_pectorale	32.10	0.00
TRINITY_XP_00295	hypothet	Volvox_carteri	32.10	0.00
TRINITY_GAX83502	hypothet	Chlamydomonas_eusti	32.10	0.00
TRINITY_OUS44004	hypothet	Ostreococcus_tauri	32.10	0.00
TRINITY_GAX76015	hypothet	Chlamydomonas_eusti	32.10	0.00
TRINITY_PNH07438	hypothet	Tetrabaena_socialis	32.10	0.00
TRINITY_XP_00169	predicte	Chlamydomonas_reinh	32.10	0.00
TRINITY_AIF73520	WD40 pro	Volvox_ferrisii	32.10	0.00
TRINITY_XP_02014	GTPase-a	Aegilops_tauschii	32.10	0.00
TRINITY_XP_02199	nudix hy	Helianthus_annuus	32.10	0.00
TRINITY_GAQ77855	S1 RNA b	Klebsormidium_niten	32.10	0.00
TRINITY_PNT39989	hypothet	Populus_trichocarpa	32.10	0.00
TRINITY_XP_01247	PREDICTE	Gossypium_raimondii	32.10	0.00
TRINITY_GAX83758	hypothet	Chlamydomonas_eusti	32.10	0.00
TRINITY_XP_01562	CBL-inte	Oryza_sativa	32.10	0.00
TRINITY_RWR78527	EPS15 EH	Cinnamomum_micranth	32.10	0.00
TRINITY_GAX78297	hypothet	Chlamydomonas_eusti	32.10	0.00
TRINITY_XP_00934	PREDICTE	Pyrus_x_bretschneid	32.10	0.00
TRINITY_XP_01451	SNF1-rel	Vigna_radiata	32.10	0.00
TRINITY_ACR09632	chitinas	Capsicum_chinense	32.10	0.00
TRINITY_GAX77017	hypothet	Chlamydomonas_eusti	32.10	0.00
TRINITY_RMZ55400	hypothet	Auxenochlorella_pro	32.10	0.00
TRINITY_PSC68179	Acyl-coe	Micractinium_conduc	32.10	0.00
TRINITY_BAK00769	predicte	Hordeum_vulgare	32.10	0.00
TRINITY_XP_00565	cysteine	Coccomyxa_subellips	32.10	0.00
TRINITY_XP_02390	ras-like	Quercus_suber	32.10	0.00
TRINITY_GAX84105	hypothet	Chlamydomonas_eusti	32.10	0.00
TRINITY_GBG91646	hypothet	Chara_braunii	32.10	0.00
TRINITY_XP_02454	E3 ubiqu	Selaginella_moellen	32.10	0.00
TRINITY_XP_01507	beta-hex	Solanum_pennellii	32.10	0.00
TRINITY_XP_00170	predicte	Chlamydomonas_reinh	32.10	0.00
TRINITY_GBF87540	serine p	Raphidocelis_subcap	32.10	0.00
TRINITY_XP_00169	predicte	Chlamydomonas_reinh	32.10	0.00
TRINITY_PNH04175	Tiny mac	Tetrabaena_socialis	32.10	0.00
TRINITY_RWR92909	hypothet	Cinnamomum_micranth	32.00	0.00
TRINITY_XP_00939	PREDICTE	Musa_acuminata	32.00	0.00
TRINITY_XP_01100	PREDICTE	Populus_euphratica	32.00	0.00
TRINITY_NP_18160	RING-fin	Arabidopsis_thalian	32.00	0.00
TRINITY_XP_00357	probable	Brachypodium_distac	32.00	0.00
TRINITY_ABK25616	unknown	Picea_sitchensis	32.00	0.00
TRINITY_XP_00750	predicte	Bathycoccus_prasino	32.00	0.00
TRINITY_XP_02637	DExH-box	Papaver_somniferum	32.00	0.00
TRINITY_XP_00713	hypothet	Phaseolus_vulgaris	32.00	0.00
TRINITY_XP_02387	uncharac	Quercus_suber	32.00	0.00
TRINITY_XP_00960	PREDICTE	Nicotiana_tomentosi	32.00	0.00
TRINITY_XP_00169	glycogen	Chlamydomonas_reinh	32.00	0.00
TRINITY_XP_00565	F1F0 ATP	Coccomyxa_subellips	32.00	0.00
TRINITY_XP_02437	phosphol	Physcomitrella_pate	32.00	0.00
TRINITY_XP_02058	BTB/POZ	Phalaenopsis_equest	32.00	0.00
TRINITY_XP_00880	methylst	Phoenix_dactylifera	32.00	0.00

TRINITY_XP_00564	eIF2A-do	Coccomyxa_subellips	32.00	0.00
TRINITY_XP_02288	uncharac	Olea_europaea	32.00	0.00
TRINITY_GAQ84563	hypothet	Klebsormidium_niten	32.00	0.00
TRINITY_EFJ29529	hypothet	Selaginella_moellen	32.00	0.00
TRINITY_PNW73085	hypothet	Chlamydomonas_reinh	32.00	0.00
TRINITY_PNW84649	hypothet	Chlamydomonas_reinh	32.00	0.00
TRINITY_XP_02437	autocrin	Physcomitrella_pate	32.00	0.00
TRINITY_XP_00306	predicte	Micromonas_pusilla	32.00	0.00
TRINITY_GAX84542	hypothet	Chlamydomonas_eusti	32.00	0.00
TRINITY_XP_02387	GTP-bind	Quercus_suber	32.00	0.00
TRINITY_XP_00565	NAD(P)-b	Coccomyxa_subellips	32.00	0.00
TRINITY_XP_01053	PREDICTE	Tarenaya_hasslerian	32.00	0.00
TRINITY_XP_01358	PREDICTE	Brassica_oleracea	32.00	0.00
TRINITY_NP_00117	putative	Zea_mays	32.00	0.00
TRINITY_GAX72787	hypothet	Chlamydomonas_eusti	32.00	0.00
TRINITY_GAX79837	hypothet	Chlamydomonas_eusti	32.00	0.00
TRINITY_GAX77336	hypothet	Chlamydomonas_eusti	32.00	0.00
TRINITY_XP_01023	lysophos	Brachypodium_distac	32.00	0.00
TRINITY_GBF94787	hypothet	Raphidocelis_subcap	32.00	0.00
TRINITY_GAX74786	hypothet	Chlamydomonas_eusti	32.00	0.00
TRINITY_XP_01557	LOW QUAL	Ricinus_communis	32.00	0.00
TRINITY_GAX82840	hypothet	Chlamydomonas_eusti	32.00	0.00
TRINITY_XP_02388	vacuolar	Quercus_suber	32.00	0.00
TRINITY_GAQ88152	Pumilio	Klebsormidium_niten	32.00	0.00
TRINITY_KNA03261	hypothet	Spinacia_oleracea	32.00	0.00
TRINITY_XP_00430	PREDICTE	Fragaria_vesca	32.00	0.00
TRINITY_XP_02202	uncharac	Helianthus_annuus	32.00	0.00
TRINITY_GAX79942	hypothet	Chlamydomonas_eusti	32.00	0.00
TRINITY_XP_01916	PREDICTE	Ipomoea_nil	32.00	0.00
TRINITY_XP_01450	ribonucl	Vigna_radiata	32.00	0.00
TRINITY_XP_02640	C2 and (	Papaver_somniferum	32.00	0.00
TRINITY_XP_01048	PREDICTE	Camelina_sativa	32.00	0.00
TRINITY_XP_02444	E3 ubiqu	Populus_trichocarpa	32.00	0.00
TRINITY_PNH12703	HEAT rep	Tetrabaena_socialis	32.00	0.00
TRINITY_CDY42101	BnaA09g2	Brassica_napus	32.00	0.00
TRINITY_XP_02390	protease	Quercus_suber	32.00	0.00
TRINITY_KXZ52318	hypothet	Gonium_pectorale	32.00	0.00
TRINITY_GAX85978	hypothet	Chlamydomonas_eusti	32.00	0.00
TRINITY_GAX82496	hypothet	Chlamydomonas_eusti	32.00	0.00
TRINITY_XP_00429	PREDICTE	Fragaria_vesca	32.00	0.00
TRINITY_XP_02058	serine/t	Phalaenopsis_equest	31.90	0.00
TRINITY_XP_02715	serine (	Coffea_eugenioides	31.90	0.00
TRINITY_XP_02439	ras-rela	Physcomitrella_pate	31.90	0.00
TRINITY_XP_00287	calmodul	Arabidopsis_lyrata	31.90	0.00
TRINITY_OWM81532	hypothet	Punica_granatum	31.90	0.00
TRINITY_GAQ86466	putative	Klebsormidium_niten	31.90	0.00
TRINITY_XP_00296	vacuolar	Selaginella_moellen	31.90	0.00
TRINITY_XP_01569	PREDICTE	Oryza_brachyantha	31.90	0.00
TRINITY_XP_02391	queuine	Quercus_suber	31.90	0.00
TRINITY_XP_02008	tRNA (ac	Ananas_comosus	31.90	0.00
TRINITY_XP_00564	Emopamil	Coccomyxa_subellips	31.90	0.00
TRINITY_PNY10868	NAD kina	Trifolium_pratense	31.90	0.00
TRINITY_XP_01918	PREDICTE	Ipomoea_nil	31.90	0.00
TRINITY_PRW45201	WD repea	Chlorella_sorokinia	31.90	0.00
TRINITY_KZV16058	hypothet	Dorcoceras_hygromet	31.90	0.00
TRINITY_KXZ50592	hypothet	Gonium_pectorale	31.90	0.00
TRINITY_XP_00142	predicte	Ostreococcus_sp._'l	31.90	0.00
TRINITY_GAQ89697	hypothet	Klebsormidium_niten	31.90	0.00
TRINITY_GAX84124	hypothet	Chlamydomonas_eusti	31.90	0.00
TRINITY_PWA74502	Chaperon	Artemisia_annua	31.90	0.00
TRINITY_XP_00305	predicte	Micromonas_pusilla	31.90	0.00
TRINITY_XP_02791	calcineu	Vigna_unguiculata	31.90	0.00
TRINITY_NP_00115	uncharac	Zea_mays	31.90	0.00
TRINITY_GAQ80087	hypothet	Klebsormidium_niten	31.90	0.00
TRINITY_BAJ89288	predicte	Hordeum_vulgare	31.90	0.00
TRINITY_XP_01593	F-box pr	Arachis_duranensis	31.90	0.00

TRINITY_XP_0201(	cinnamoy	Ananas_comosus	31.90	0.00
TRINITY_NP_0012(	probable	Vitis_vinifera	31.90	0.00
TRINITY_XP_0174:	PREDICTE	Vigna_angularis	31.90	0.00
TRINITY_XP_0194!	PREDICTE	Lupinus_angustifoli	31.90	0.00
TRINITY_PRW5633(	nucleola	Chlorella_sorokinia	31.90	0.00
TRINITY_KXZ4336(	hypothet	Gonium_pectorale	31.90	0.00
TRINITY_XP_0056(	RNB-doma	Coccomyxa_subellips	31.90	0.00
TRINITY_VDD4724:	unnamed	Brassica_oleracea	31.90	0.00
TRINITY_GAQ8968!	Eukaryot	Klebsormidium_niten	31.90	0.00
TRINITY_XP_0239(	endosoma	Quercus_suber	31.90	0.00
TRINITY_PIA5673:	hypothet	Aquilegia_coerulea	31.90	0.00
TRINITY_XP_0030!	predicte	Micromonas_pusilla	31.90	0.00
TRINITY_KNA0701:	hypothet	Spinacia_oleracea	31.90	0.00
TRINITY_EPS6771:	hypothet	Genlisea_aurea	31.90	0.00
TRINITY_XP_0238!	nuclear	Quercus_suber	31.90	0.00
TRINITY_KZV4221(	Para-nit	Dorcoceras_hygromet	31.90	0.00
TRINITY_XP_0058(	hypothet	Chlorella_variabili	31.90	0.00
TRINITY_XP_0056(	TLD-doma	Coccomyxa_subellips	31.90	0.00
TRINITY_XP_0216(	probable	Hevea_brasiliensis	31.90	0.00
TRINITY_XP_0029!	hypothet	Volvox_carteri	31.90	0.00
TRINITY_GAX8118:	hypothet	Chlamydomonas_eusti	31.90	0.00
TRINITY_XP_0156(	E3 ubiqu	Oryza_sativa	31.90	0.00
TRINITY_XP_0058(	hypothet	Chlorella_variabili	31.90	0.00
TRINITY_PWA5047!	plant UE	Artemisia_annua	31.90	0.00
TRINITY_GAU4455:	hypothet	Trifolium_subterran	31.90	0.00
TRINITY_KXZ4582(	hypothet	Gonium_pectorale	31.90	0.00
TRINITY_KXZ4856!	hypothet	Gonium_pectorale	31.90	0.00
TRINITY_XP_0238!	H(+)/Cl	Quercus_suber	31.90	0.00
TRINITY_XP_0239(	vacuola	Quercus_suber	31.90	0.00
TRINITY_XP_0238!	hydrolas	Quercus_suber	31.90	0.00
TRINITY_GAX8372:	hypothet	Chlamydomonas_eusti	31.90	0.00
TRINITY_PLY6708!	hypothet	Lactuca_sativa	31.90	0.00
TRINITY_PTQ3599'	hypothet	Marchantia_polymorp	31.90	0.00
TRINITY_KXZ4357'	hypothet	Gonium_pectorale	31.90	0.00
TRINITY_XP_0217!	condens:	Chenopodium_quinoa	31.90	0.00
TRINITY_GBG7777!	hypothet	Chara_braunii	31.90	0.00
TRINITY_XP_0063!	chaperor	Eutrema_salsugineum	31.90	0.00
TRINITY_XP_0238{	SWI/SNF	Quercus_suber	31.90	0.00
TRINITY_GAX7875!	hypothet	Chlamydomonas_eusti	31.90	0.00
TRINITY_PNT4881(	hypothet	Populus_trichocarpa	31.90	0.00
TRINITY_XP_0245:	ABC trar	Selaginella_moellen	31.90	0.00
TRINITY_XP_0093!	PREDICTE	Musa_acuminata	31.90	0.00
TRINITY_KXZ4971(	hypothet	Gonium_pectorale	31.90	0.00
TRINITY_GAX8313(	hypothet	Chlamydomonas_eusti	31.90	0.00
TRINITY_KMZ5872:	putative	Zostera_marina	31.90	0.00
TRINITY_XP_0093!	PREDICTE	Pyrus_x_bretschneid	31.90	0.00
TRINITY_XP_0014:	predicte	Ostreococcus_sp._'l	31.80	0.00
TRINITY_XP_0029(	hypothet	Volvox_carteri	31.80	0.00
TRINITY_XP_0025(	predicte	Micromonas_commoda	31.80	0.00
TRINITY_XP_0056(	small ra	Coccomyxa_subellips	31.80	0.00
TRINITY_GAQ8852{	hypothet	Klebsormidium_niten	31.80	0.00
TRINITY_XP_0207(	5'-methy	Dendrobium_catenatu	31.80	0.00
TRINITY_XP_0243(	uncharac	Physcomitrella_pate	31.80	0.00
TRINITY_XP_0100{	binding	Morus_notabilis	31.80	0.00
TRINITY_XP_0045:	carbon (	Cicer_arietinum	31.80	0.00
TRINITY_KMZ6669!	C-4 meth	Zostera_marina	31.80	0.00
TRINITY_XP_0238'	geranyl(	Quercus_suber	31.80	0.00
TRINITY_KXZ5579!	hypothet	Gonium_pectorale	31.80	0.00
TRINITY_XP_0030'	Glutath:	Ostreococcus_tauri	31.80	0.00
TRINITY_PTQ2779!	hypothet	Marchantia_polymorp	31.80	0.00
TRINITY_GAQ8682(	Leishmar	Klebsormidium_niten	31.80	0.00
TRINITY_GBF8976(	hypothet	Raphidocelis_subcap	31.80	0.00
TRINITY_KDD7675(	hypothet	Helicosporidium_sp.	31.80	0.00
TRINITY_XP_0029!	hypothet	Volvox_carteri	31.80	0.00
TRINITY_XP_0025(	predicte	Micromonas_commoda	31.80	0.00
TRINITY_XP_0243'	autocrir	Physcomitrella_pate	31.80	0.00

TRINITY_XP_0016	predicte	Chlamydomonas_reinh	31.80	0.00
TRINITY_PWA9599	taxilin	Artemisia_annua	31.80	0.00
TRINITY_XP_0138	leishmar	Monoraphidium_negle	31.80	0.00
TRINITY_GAQ8632	major fa	Klebsormidium_niten	31.80	0.00
TRINITY_GBF8769	hypothet	Raphidocelis_subcap	31.80	0.00
TRINITY_XP_0206	CBL-inte	Dendrobium_catenatu	31.80	0.00
TRINITY_XP_0029	PR-1 lik	Volvox_carteri	31.80	0.00
TRINITY_XP_0029	hypothet	Volvox_carteri	31.80	0.00
TRINITY_KMZ6320	putative	Zostera_marina	31.80	0.00
TRINITY_XP_0035	CBL-inte	Medicago_truncatula	31.80	0.00
TRINITY_XP_0202	inositol	Cajanus_cajan	31.80	0.00
TRINITY_CDY4333	BnaC04g	Brassica_napus	31.80	0.00
TRINITY_GAQ8806	Iron tra	Klebsormidium_niten	31.80	0.00
TRINITY_XP_0016	BAH prot	Chlamydomonas_reinh	31.80	0.00
TRINITY_XP_0216	uncharac	Manihot_esculenta	31.80	0.00
TRINITY_GAX7431	hypothet	Chlamydomonas_eusti	31.80	0.00
TRINITY_KXZ5165	hypothet	Gonium_pectorale	31.80	0.00
TRINITY_KXZ5462	hypothet	Gonium_pectorale	31.80	0.00
TRINITY_XP_0024	calcium	Sorghum_bicolor	31.80	0.00
TRINITY_GAQ8593	hypothet	Klebsormidium_niten	31.80	0.00
TRINITY_GAX7486	hypothet	Chlamydomonas_eusti	31.80	0.00
TRINITY_VDD0508	unnamed	Brassica_rapa	31.80	0.00
TRINITY_PNW7900	hypothet	Chlamydomonas_reinh	31.80	0.00
TRINITY_PTQ4082	hypothet	Marchantia_polymorp	31.80	0.00
TRINITY_XP_0238	protein	Quercus_suber	31.80	0.00
TRINITY_XP_0030	Methylt	Ostreococcus_tauri	31.80	0.00
TRINITY_RAL4695	hypothet	Cuscuta_australis	31.80	0.00
TRINITY_XP_0243	apolipo	Physcomitrella_pate	31.80	0.00
TRINITY_XP_0205	serine/t	Phalaenopsis_equest	31.80	0.00
TRINITY_XP_0159	ras-rela	Arachis_duranensis	31.80	0.00
TRINITY_PNW7926	hypothet	Chlamydomonas_reinh	31.80	0.00
TRINITY_XP_0138	hypothet	Monoraphidium_negle	31.80	0.00
TRINITY_GAQ7773	Leishmar	Klebsormidium_niten	31.80	0.00
TRINITY_GAX7481	hypothet	Chlamydomonas_eusti	31.80	0.00
TRINITY_XP_0104	PREDICTE	Camelina_sativa	31.80	0.00
TRINITY_XP_0105	PREDICTE	Tarenaya_hasslerian	31.80	0.00
TRINITY_PRW5774	nuclear	Chlorella_sorokinia	31.80	0.00
TRINITY_PRW5993	Oxyster	Chlorella_sorokinia	31.80	0.00
TRINITY_PNW8197	hypothet	Chlamydomonas_reinh	31.80	0.00
TRINITY_GAQ8607	hypothet	Klebsormidium_niten	31.80	0.00
TRINITY_CAH6715	H0717B1	Oryza_sativa	31.80	0.00
TRINITY_XP_0243	ABC tran	Physcomitrella_pate	31.80	0.00
TRINITY_GAQ8533	hypothet	Klebsormidium_niten	31.80	0.00
TRINITY_XP_0029	hypothet	Volvox_carteri	31.80	0.00
TRINITY_XP_0238	ras-rela	Quercus_suber	31.70	0.00
TRINITY_XP_0228	iron-sul	Olea_europaea	31.70	0.00
TRINITY_PSC7230	phospho	Micractinium_conduc	31.70	0.00
TRINITY_OAE3458	hypothet	Marchantia_polymorp	31.70	0.00
TRINITY_XP_0243	putative	Physcomitrella_pate	31.70	0.00
TRINITY_XP_0190	PREDICTE	Vitis_vinifera	31.70	0.00
TRINITY_PRW5998	putative	Chlorella_sorokinia	31.70	0.00
TRINITY_CAA6705	calmodul	Capsicum_annuum	31.70	0.00
TRINITY_GAX7798	hypothet	Chlamydomonas_eusti	31.70	0.00
TRINITY_XP_0105	PREDICTE	Tarenaya_hasslerian	31.70	0.00
TRINITY_EFJ1186	hypothet	Selaginella_moellen	31.70	0.00
TRINITY_EEF2254	conserve	Ricinus_communis	31.70	0.00
TRINITY_PRW1836	Kelch do	Chlorella_sorokinia	31.70	0.00
TRINITY_PNH0781	putative	Tetrabaena_socialis	31.70	0.00
TRINITY_XP_0188	PREDICTE	Juglans_regia	31.70	0.00
TRINITY_BAK0153	predicte	Hordeum_vulgare	31.70	0.00
TRINITY_GAQ7771	HECT dom	Klebsormidium_niten	31.70	0.00
TRINITY_GAQ8682	Leishmar	Klebsormidium_niten	31.70	0.00
TRINITY_ADB7780	actin, p	Boergesenia_forbesi	31.70	0.00
TRINITY_GAV8197	Asp doma	Cephalotus_follicul	31.70	0.00
TRINITY_PUZ6861	hypothet	Panicum_hallii	31.70	0.00
TRINITY_XP_0200	HVA22-li	Ananas_comosus	31.70	0.00

TRINITY_PTQ40795	hypothet	Marchantia_polymorp	31.70	0.00
TRINITY_GAQ82851	hypothet	Klebsormidium_niten	31.70	0.00
TRINITY_GBG87220	hypothet	Chara_braunii	31.70	0.00
TRINITY_XP_02791	probable	Vigna_unguiculata	31.70	0.00
TRINITY_GAQ84649	protein	Klebsormidium_niten	31.70	0.00
TRINITY_GBF89222	hypothet	Raphidocelis_subcap	31.70	0.00
TRINITY_XP_02026	LOW QUAL	Asparagus_officinal	31.70	0.00
TRINITY_ABK23827	unknown	Picea_sitchensis	31.70	0.00
TRINITY_XP_00170	predicte	Chlamydomonas_reinh	31.70	0.00
TRINITY_XP_01023	ubiquiti	Brachypodium_distac	31.70	0.00
TRINITY_XP_00496	poly [AI	Setaria_italica	31.70	0.00
TRINITY_KXZ47521	hypothet	Gonium_pectorale	31.70	0.00
TRINITY_XP_02202	embryoge	Helianthus_annuus	31.70	0.00
TRINITY_PNT71083	hypothet	Brachypodium_distac	31.70	0.00
TRINITY_XP_00584	hypothet	Chlorella_variabili	31.70	0.00
TRINITY_XP_01915	PREDICTE	Ipomoea_nil	31.70	0.00
TRINITY_GAX83293	hypothet	Chlamydomonas_eusti	31.70	0.00
TRINITY_XP_00250	resistar	Micromonas_commoda	31.70	0.00
TRINITY_XP_02391	endocyto	Quercus_suber	31.70	0.00
TRINITY_GAQ81982	Mitotic	Klebsormidium_niten	31.70	0.00
TRINITY_XP_02637	HVA22-li	Papaver_somniferum	31.70	0.00
TRINITY_XP_02173	phosphat	Chenopodium_quinoa	31.70	0.00
TRINITY_GBG87333	hypothet	Chara_braunii	31.70	0.00
TRINITY_KXZ56559	hypothet	Gonium_pectorale	31.70	0.00
TRINITY_XP_01103	PREDICTE	Populus_euphratica	31.70	0.00
TRINITY_XP_02452	uncharac	Selaginella_moellen	31.70	0.00
TRINITY_GAX81388	hypothet	Chlamydomonas_eusti	31.70	0.00
TRINITY_XP_00564	hypothet	Coccomyxa_subellips	31.70	0.00
TRINITY_GAX85978	hypothet	Chlamydomonas_eusti	31.70	0.00
TRINITY_XP_02389	uncharac	Quercus_suber	31.70	0.00
TRINITY_XP_01390	VAMP-lik	Monoraphidium_negle	31.70	0.00
TRINITY_BAJ94575	predicte	Hordeum_vulgare	31.70	0.00
TRINITY_GAX74187	hypothet	Chlamydomonas_eusti	31.70	0.00
TRINITY_GAQ88727	exopolyp	Klebsormidium_niten	31.70	0.00
TRINITY_XP_02440	von Will	Physcomitrella_pate	31.70	0.00
TRINITY_KXZ44356	hypothet	Gonium_pectorale	31.70	0.00
TRINITY_PSC71750	DNase I	Micractinium_conduc	31.70	0.00
TRINITY_PWA91220	protein	Artemisia_annua	31.60	0.00
TRINITY_GAQ79315	cytochro	Klebsormidium_niten	31.60	0.00
TRINITY_ADE75631	unknown	Picea_sitchensis	31.60	0.00
TRINITY_KFK30221	hypothet	Arabis_alpina	31.60	0.00
TRINITY_XP_01048	PREDICTE	Camelina_sativa	31.60	0.00
TRINITY_GAQ78497	dual spe	Klebsormidium_niten	31.60	0.00
TRINITY_XP_02389	uncharac	Quercus_suber	31.60	0.00
TRINITY_KCW71454	hypothet	Eucalyptus_grandis	31.60	0.00
TRINITY_XP_01090	PREDICTE	Elaeis_guineensis	31.60	0.00
TRINITY_OAE34065	hypothet	Marchantia_polymorp	31.60	0.00
TRINITY_AAF74566	hexose t	Nicotiana_tabacum	31.60	0.00
TRINITY_XP_02389	probable	Quercus_suber	31.60	0.00
TRINITY_XP_00646	aminopep	Citrus_sinensis	31.60	0.00
TRINITY_XP_00295	hypothet	Volvox_carteri	31.60	0.00
TRINITY_PRW33795	Zn-deper	Chlorella_sorokinia	31.60	0.00
TRINITY_GAQ92386	hypothet	Klebsormidium_niten	31.60	0.00
TRINITY_NP_00131	eukaryot	Nicotiana_tabacum	31.60	0.00
TRINITY_XP_02439	Fanconi	Physcomitrella_pate	31.60	0.00
TRINITY_GAQ87165	hypothet	Klebsormidium_niten	31.60	0.00
TRINITY_XP_01052	PREDICTE	Tarenaya_hasslerian	31.60	0.00
TRINITY_XP_00168	predicte	Chlamydomonas_reinh	31.60	0.00
TRINITY_NP_56527	CHASE do	Arabidopsis_thalian	31.60	0.00
TRINITY_RAL45585	hypothet	Cuscuta_australis	31.60	0.00
TRINITY_XP_02068	CBL-inte	Dendrobium_catenatu	31.60	0.00
TRINITY_GAX83834	hypothet	Chlamydomonas_eusti	31.60	0.00
TRINITY_AAF86529	F21B7.23	Arabidopsis_thalian	31.60	0.00
TRINITY_XP_02277	uncharac	Durio_zibethinus	31.60	0.00
TRINITY_PRW32633	Peptidyl	Chlorella_sorokinia	31.60	0.00
TRINITY_PTQ49864	hypothet	Marchantia_polymorp	31.60	0.00

TRINITY_XP_0213	bromodor	Sorghum_bicolor	31.60	0.00
TRINITY_XP_0075	alpha-1,	Bathycoccus_prasino	31.60	0.00
TRINITY_CDY1474	BnaC04g	Brassica_napus	31.60	0.00
TRINITY_PTQ3895	hypothet	Marchantia_polymorp	31.60	0.00
TRINITY_GBF9859	hypothet	Raphidocelis_subcap	31.60	0.00
TRINITY_GBG0019	hypothet	Raphidocelis_subcap	31.60	0.00
TRINITY_GAQ8017	hypothet	Klebsormidium_niten	31.60	0.00
TRINITY_GAX7835	hypothet	Chlamydomonas_eusti	31.60	0.00
TRINITY_EFJ3431	hypothet	Selaginella_moellen	31.60	0.00
TRINITY_XP_0161	uncharac	Arachis_ipaensis	31.60	0.00
TRINITY_XP_0216	oxyster	Manihot_esculenta	31.60	0.00
TRINITY_XP_0110	nudix hy	Sesamum_indicum	31.60	0.00
TRINITY_PNW8733	hypothet	Chlamydomonas_reinh	31.60	0.00
TRINITY_KXZ4200	hypothet	Gonium_pectorale	31.60	0.00
TRINITY_GAX7872	hypothet	Chlamydomonas_eusti	31.60	0.00
TRINITY_XP_0263	nucleol	Papaver_somniferum	31.60	0.00
TRINITY_XP_0093	PREDICT	Pyrus_x_bretschneid	31.60	0.00
TRINITY_GAX7560	hypothet	Chlamydomonas_eusti	31.60	0.00
TRINITY_KXZ4838	hypothet	Gonium_pectorale	31.60	0.00
TRINITY_OAE2957	hypothet	Marchantia_polymorp	31.60	0.00
TRINITY_XP_0191	PREDICT	Ipomoea_nil	31.60	0.00
TRINITY_GAX8465	hypothet	Chlamydomonas_eusti	31.60	0.00
TRINITY_XP_0023	probable	Populus_trichocarpa	31.60	0.00
TRINITY_XP_0017	alpha-1,	Chlamydomonas_reinh	31.60	0.00
TRINITY_KXZ5358	hypothet	Gonium_pectorale	31.60	0.00
TRINITY_XP_0029	hypothet	Volvox_carteri	31.60	0.00
TRINITY_XP_0238	uncharac	Quercus_suber	31.50	0.00
TRINITY_XP_0100	PREDICT	Eucalyptus_grandis	31.50	0.00
TRINITY_XP_0159	vacuola	Arachis_duranensis	31.50	0.00
TRINITY_PTQ3902	hypothet	Marchantia_polymorp	31.50	0.00
TRINITY_XP_0220	zinc fi	Helianthus_annuus	31.50	0.00
TRINITY_GBG8317	hypothet	Chara_braunii	31.50	0.00
TRINITY_XP_0106	PREDICT	Beta_vulgaris	31.50	0.00
TRINITY_XP_0218	binding	Prunus_avium	31.50	0.00
TRINITY_XP_0243	serpin-	Physcomitrella_pate	31.50	0.00
TRINITY_CAH6823	H0306F0	Oryza_sativa	31.50	0.00
TRINITY_GAQ8383	hypothet	Klebsormidium_niten	31.50	0.00
TRINITY_RWR8577	Rhodanes	Cinnamomum_micranth	31.50	0.00
TRINITY_XP_0200	indole-	Ananas_comosus	31.50	0.00
TRINITY_XP_0105	PREDICT	Tarenaya_hasslerian	31.50	0.00
TRINITY_XP_0068	UV-stim	Amborella_trichopod	31.50	0.00
TRINITY_XP_0064	protein	Eutrema_salsugineum	31.50	0.00
TRINITY_XP_0158	uncharac	Ziziphus_jujuba	31.50	0.00
TRINITY_GAX7604	hypothet	Chlamydomonas_eusti	31.50	0.00
TRINITY_XP_0237	ubiquit	Lactuca_sativa	31.50	0.00
TRINITY_XP_0238	uncharac	Quercus_suber	31.50	0.00
TRINITY_XP_0172	PREDICT	Daucus_carota	31.50	0.00
TRINITY_XP_0194	PREDICT	Lupinus_angustifoli	31.50	0.00
TRINITY_XP_0014	[Ostreo	Ostreococcus_sp._'l	31.50	0.00
TRINITY_GAX7595	hypothet	Chlamydomonas_eusti	31.50	0.00
TRINITY_XP_0243	uncharac	Physcomitrella_pate	31.50	0.00
TRINITY_ADE7643	unknown	Picea_sitchensis	31.50	0.00
TRINITY_CDP1798	unnamed	Coffea_canephora	31.50	0.00
TRINITY_BAK0153	predicte	Hordeum_vulgare	31.50	0.00
TRINITY_GBF9705	hypothet	Raphidocelis_subcap	31.50	0.00
TRINITY_XP_0235	LIMR fa	Cucurbita_pepo	31.50	0.00
TRINITY_GAQ7855	hypothet	Klebsormidium_niten	31.50	0.00
TRINITY_XP_0025	predicte	Micromonas_commoda	31.50	0.00
TRINITY_XP_0212	serine/t	Herrania_umbratica	31.50	0.00
TRINITY_KXZ5031	hypothet	Gonium_pectorale	31.50	0.00
TRINITY_GAQ8257	hypothet	Klebsormidium_niten	31.50	0.00
TRINITY_XP_0147	TIP41-l	Brachypodium_distac	31.50	0.00
TRINITY_KYP4513	hypothet	Cajanus_cajan	31.50	0.00
TRINITY_GAX8451	hypothet	Chlamydomonas_eusti	31.50	0.00
TRINITY_XP_0058	hypothet	Chlorella_variabili	31.50	0.00
TRINITY_XP_0016	predicte	Chlamydomonas_reinh	31.50	0.00

TRINITY_XP_0105⁴	PREDICTI	Tarenaya_hasslerian	31.50	0.00
TRINITY_GAX8163(	hypothet	Chlamydomonas_eusti	31.50	0.00
TRINITY_XP_0030(	SNF2 sup	Micromonas_pusilla	31.50	0.00
TRINITY_BAJ9694(	predicte	Hordeum_vulgare	31.50	0.00
TRINITY_PNW8850(	hypothet	Chlamydomonas_reinh	31.50	0.00
TRINITY_GAX7650!	hypothet	Chlamydomonas_eusti	31.50	0.00
TRINITY_XP_0239(	protein	Quercus_suber	31.50	0.00
TRINITY_GAX8120(	hypothet	Chlamydomonas_eusti	31.50	0.00
TRINITY_XP_0084⁴	PREDICTI	Cucumis_melo	31.50	0.00
TRINITY_PNH0225:	Vegetati	Tetrabaena_socialis	31.50	0.00

TRINITY_XP_00714	hypothet	Phaseolus_vulgaris	31.50	0.00
TRINITY_PNW86482	hypothet	Chlamydomonas_reinh	31.50	0.00
TRINITY_GBF97507	hypothet	Raphidocelis_subcap	31.50	0.00
TRINITY_GAX84454	hypothet	Chlamydomonas_eusti	31.50	0.00
TRINITY_XP_00565	hypothet	Coccomyxa_subellips	31.50	0.00
TRINITY_PNW78753	hypothet	Chlamydomonas_reinh	31.50	0.00
TRINITY_PTQ41656	hypothet	Marchantia_polymorp	31.50	0.00
TRINITY_PRW61284	Casein k	Chlorella_sorokinia	31.50	0.00
TRINITY_KZV56815	hypothet	Dorcoceras_hygromet	31.50	0.00
TRINITY_XP_00306	glycosyl	Micromonas_pusilla	31.50	0.00
TRINITY_XP_02182	GTP-binc	Prunus_avium	31.50	0.00
TRINITY_GAX81744	hypothet	Chlamydomonas_eusti	31.50	0.00
TRINITY_KXZ44759	hypothet	Gonium_pectorale	31.50	0.00
TRINITY_XP_01010	very-lor	Morus_notabilis	31.50	0.00
TRINITY_GAQ82532	translat	Klebsormidium_niten	31.50	0.00
TRINITY_XP_02438	alpha-tu	Physcomitrella_pate	31.50	0.00
TRINITY_GAX74990	hypothet	Chlamydomonas_eusti	31.50	0.00
TRINITY_XP_01009	uncharac	Morus_notabilis	31.50	0.00
TRINITY_XP_00978	PREDICTE	Nicotiana_sylvestri	31.40	0.00
TRINITY_GAQ83828	M-phase	Klebsormidium_niten	31.40	0.00
TRINITY_RRT72140	hypothet	Ensete_ventricosum	31.40	0.00
TRINITY_XP_01101	PREDICTE	Populus_euphratica	31.40	0.00
TRINITY_GBF96504	hypothet	Raphidocelis_subcap	31.40	0.00
TRINITY_RWR79768	protein	Cinnamomum_micranth	31.40	0.00
TRINITY_XP_01586	uncharac	Ziziphus_jujuba	31.40	0.00
TRINITY_GAQ79007	hypothet	Klebsormidium_niten	31.40	0.00
TRINITY_XP_02388	vacuolar	Quercus_suber	31.40	0.00
TRINITY_XP_02017	chaperon	Aegilops_tauschii	31.40	0.00
TRINITY_XP_00701	PREDICTE	Theobroma_cacao	31.40	0.00
TRINITY_XP_02008	E3 ubiqu	Ananas_comosus	31.40	0.00
TRINITY_XP_00308	Protein-	Ostreococcus_tauri	31.40	0.00
TRINITY_XP_02392	vacuolar	Quercus_suber	31.40	0.00
TRINITY_KNA07012	hypothet	Spinacia_oleracea	31.40	0.00
TRINITY_XP_02389	wiskott-	Quercus_suber	31.40	0.00
TRINITY_XP_02177	ABC tran	Chenopodium_quinoa	31.40	0.00
TRINITY_XP_00564	CHR fami	Coccomyxa_subellips	31.40	0.00
TRINITY_GAQ90902	hypothet	Klebsormidium_niten	31.40	0.00
TRINITY_XP_02389	probable	Quercus_suber	31.40	0.00
TRINITY_XP_02439	lysine-s	Physcomitrella_pate	31.40	0.00
TRINITY_AGY48886	MIP1.4a	Nicotiana_benthamia	31.40	0.00
TRINITY_XP_02023	probable	Cajanus_cajan	31.40	0.00
TRINITY_GAX75913	hypothet	Chlamydomonas_eusti	31.40	0.00
TRINITY_XP_00295	hypothet	Volvox_carteri	31.40	0.00
TRINITY_XP_02436	probable	Physcomitrella_pate	31.40	0.00
TRINITY_GAX82756	hypothet	Chlamydomonas_eusti	31.40	0.00
TRINITY_GAX83819	hypothet	Chlamydomonas_eusti	31.40	0.00
TRINITY_NP_20140	Phosphol	Arabidopsis_thalian	31.40	0.00
TRINITY_OWM65158	hypothet	Punica_granatum	31.40	0.00
TRINITY_XP_02437	probable	Physcomitrella_pate	31.40	0.00
TRINITY_GAQ80862	SANT/Myb	Klebsormidium_niten	31.40	0.00
TRINITY_GAX75036	hypothet	Chlamydomonas_eusti	31.40	0.00
TRINITY_PRW58183	ubiquiti	Chlorella_sorokinia	31.40	0.00
TRINITY_OAE20521	hypothet	Marchantia_polymorp	31.40	0.00
TRINITY_XP_00940	PREDICTE	Musa_acuminata	31.40	0.00
TRINITY_XP_00564	hypothet	Coccomyxa_subellips	31.40	0.00
TRINITY_GAX75907	hypothet	Chlamydomonas_eusti	31.40	0.00
TRINITY_GAX82511	hypothet	Chlamydomonas_eusti	31.40	0.00
TRINITY_XP_00295	hypothet	Volvox_carteri	31.40	0.00
TRINITY_OWM89049	hypothet	Punica_granatum	31.40	0.00
TRINITY_XP_02453	cullin-1	Selaginella_moellen	31.40	0.00
TRINITY_XP_00305	major fa	Micromonas_pusilla	31.40	0.00
TRINITY_XP_00170	predicte	Chlamydomonas_reinh	31.40	0.00
TRINITY_PNW76310	hypothet	Chlamydomonas_reinh	31.40	0.00
TRINITY_GAQ85930	hypothet	Klebsormidium_niten	31.40	0.00
TRINITY_XP_00865	uncharac	Zea_mays	31.40	0.00
TRINITY_XP_00295	hypothet	Volvox_carteri	31.40	0.00

TRINITY_XP_0049(	unchara(	Setaria_italica	31.40	0.00
TRINITY_BAJ9497(	predicte	Hordeum_vulgare	31.40	0.00
TRINITY_OAP0318:	AT-ACS1	Arabidopsis_thalian	31.40	0.00
TRINITY_PNH0529'	Type I :	Tetrabaena_socialis	31.40	0.00
TRINITY_OAE3081:	hypothet	Marchantia_polymorp	31.40	0.00
TRINITY_XP_0139(	putative	Monoraphidium_negle	31.40	0.00
TRINITY_GAX7347:	hypothet	Chlamydomonas_eusti	31.40	0.00
TRINITY_POO0216‹	SNU66/S/	Trema_orientale	31.40	0.00
TRINITY_PQP9337(	tRNA (gu	Prunus_yedoensis	31.40	0.00
TRINITY_XP_0035(	lysophos	Brachypodium_distac	31.40	0.00
TRINITY_XP_0113!	hypothet	Auxenochlorella_pro	31.40	0.00
TRINITY_XP_0029!	hypothet	Volvox_carteri	31.40	0.00
TRINITY_XP_0024!	predicte	Micromonas_commoda	31.40	0.00
TRINITY_PNH0856(	hypothet	Tetrabaena_socialis	31.40	0.00
TRINITY_XP_0056‹	hypothet	Coccomyxa_subellips	31.40	0.00
TRINITY_XP_0041‹	PREDICTI	Cucumis_sativus	31.30	0.00
TRINITY_XP_0218(	N-carbar	Carica_papaya	31.30	0.00
TRINITY_BAJ9694(	predicte	Hordeum_vulgare	31.30	0.00
TRINITY_XP_0217(	cytokin:	Chenopodium_quinoa	31.30	0.00
TRINITY_XP_0105!	PREDICTI	Tarenaya_hasslerian	31.30	0.00
TRINITY_PIN1219(	putative	Handroanthus_impeti	31.30	0.00
TRINITY_PNR5511:	hypothet	Physcomitrella_pate	31.30	0.00
TRINITY_XP_0239(	unchara(	Quercus_suber	31.30	0.00
TRINITY_GAX7757(	hypothet	Chlamydomonas_eusti	31.30	0.00
TRINITY_XP_0263!	endoplas	Papaver_somniferum	31.30	0.00
TRINITY_GAX8625!	hypothet	Chlamydomonas_eusti	31.30	0.00
TRINITY_XP_0029‹	hypothet	Volvox_carteri	31.30	0.00
TRINITY_XP_0029!	hypothet	Volvox_carteri	31.30	0.00
TRINITY_XP_0016!	actin [(	Chlamydomonas_reinh	31.30	0.00
TRINITY_KXZ4690‹	hypothet	Gonium_pectorale	31.30	0.00
TRINITY_PNW8279(	hypothet	Chlamydomonas_reinh	31.30	0.00
TRINITY_BAF1598!	Os04g06‹	Oryza_sativa	31.30	0.00
TRINITY_KJB4442'	hypothet	Gossypium_raimondii	31.30	0.00
TRINITY_PIA2546:	hypothet	Aquilegia_coerulea	31.30	0.00
TRINITY_XP_0014:	predicte	Ostreococcus_sp._'l	31.30	0.00
TRINITY_KXZ5384:	hypothet	Gonium_pectorale	31.30	0.00
TRINITY_AEB7155!	CBL-inte	Solanum_chacoense	31.30	0.00
TRINITY_RWR8260:	NADPH--(	Cinnamomum_micranth	31.30	0.00
TRINITY_XP_0216!	fatty a(	Hevea_brasiliensis	31.30	0.00
TRINITY_PNW8248(	hypothet	Chlamydomonas_reinh	31.30	0.00
TRINITY_XP_0030(	Tetratr:	Ostreococcus_tauri	31.30	0.00
TRINITY_PTQ4672(	hypothet	Marchantia_polymorp	31.30	0.00
TRINITY_BAJ9408!	predicte	Hordeum_vulgare	31.30	0.00
TRINITY_XP_0243(	programr	Physcomitrella_pate	31.30	0.00
TRINITY_PKI5182(	hypothet	Punica_granatum	31.30	0.00
TRINITY_XP_0138!	hypothet	Monoraphidium_negle	31.30	0.00
TRINITY_ABY6073:	zygote-(	Chlamydomonas_reinh	31.30	0.00
TRINITY_XP_0245:	transmer	Selaginella_moellen	31.30	0.00
TRINITY_XP_0029!	hypothet	Volvox_carteri	31.30	0.00
TRINITY_KXZ4394:	hypothet	Gonium_pectorale	31.30	0.00
TRINITY_GAX8259(	hypothet	Chlamydomonas_eusti	31.30	0.00
TRINITY_XP_0025(	predicte	Micromonas_commoda	31.30	0.00
TRINITY_XP_0104(	PREDICTI	Camelina_sativa	31.30	0.00
TRINITY_EEE5242(	hypothet	Oryza_sativa	31.30	0.00
TRINITY_GAX7367:	hypothet	Chlamydomonas_eusti	31.30	0.00
TRINITY_PNH0987(	Dopamine	Tetrabaena_socialis	31.30	0.00
TRINITY_OAE1838‹	hypothet	Marchantia_polymorp	31.30	0.00
TRINITY_PNW8203'	hypothet	Chlamydomonas_reinh	31.30	0.00
TRINITY_XP_0029‹	hypothet	Volvox_carteri	31.30	0.00
TRINITY_PRW5863!	hypothet	Chlorella_sorokinia	31.30	0.00
TRINITY_PWA7508(	ARM repe	Artemisia_annua	31.30	0.00
TRINITY_GAX7869'	hypothet	Chlamydomonas_eusti	31.30	0.00
TRINITY_ATB1975‹	putative	Callitropsis_vietna	31.30	0.00
TRINITY_EPS7208(	hypothet	Genlisea_aurea	31.30	0.00
TRINITY_XP_0237!	peptidy:	Lactuca_sativa	31.30	0.00
TRINITY_GAQ8434!	cytoplas	Klebsormidium_niten	31.30	0.00

TRINITY_PWA9714	cytochr	Artemisia_annua	31.20	0.00
TRINITY_XP_0228	histidi	Olea_europaea	31.20	0.00
TRINITY_YP_0091	H-trans	Lobosphaera_incisa	31.20	0.00
TRINITY_XP_0111	transcr	Sesamum_indicum	31.20	0.00
TRINITY_GAQ8852	hypothet	Klebsormidium_niten	31.20	0.00
TRINITY_XP_0044	E3 ubiq	Cicer_arietinum	31.20	0.00
TRINITY_OAE3454	hypothet	Marchantia_polymorp	31.20	0.00
TRINITY_GAX7779	hypothet	Chlamydomonas_eusti	31.20	0.00
TRINITY_XP_0239	ras-like	Quercus_suber	31.20	0.00
TRINITY_GAX7326	hypothet	Chlamydomonas_eusti	31.20	0.00
TRINITY_PTQ3266	hypothet	Marchantia_polymorp	31.20	0.00
TRINITY_XP_0228	NADH de	Ostreococcus_tauri	31.20	0.00
TRINITY_XP_0098	PREDICT	Nicotiana_sylvestri	31.20	0.00
TRINITY_XP_0145	traffic	Vigna_radiata	31.20	0.00
TRINITY_XP_0094	PREDICT	Musa_acuminata	31.20	0.00
TRINITY_GAX8084	hypothet	Chlamydomonas_eusti	31.20	0.00
TRINITY_XP_0217	tRNA wy	Chenopodium_quinoa	31.20	0.00
TRINITY_KDD7228	NLI int	Helicosporidium_sp.	31.20	0.00
TRINITY_XP_0239	CBP3-li	Quercus_suber	31.20	0.00
TRINITY_XP_0104	PREDICT	Camelina_sativa	31.20	0.00
TRINITY_XP_0014	predict	Ostreococcus_sp._'l	31.20	0.00
TRINITY_OUS4889	MET-10+	Ostreococcus_tauri	31.20	0.00
TRINITY_XP_0024	vacuola	Micromonas_commoda	31.20	0.00
TRINITY_PRW6105	2-dehyd	Chlorella_sorokinia	31.20	0.00
TRINITY_XP_0184	PREDICT	Raphanus_sativus	31.20	0.00
TRINITY_XP_0271	serine/t	Coffea_arabica	31.20	0.00
TRINITY_XP_0264	cytochr	Papaver_somniferum	31.20	0.00
TRINITY_GAQ7773	Leishma	Klebsormidium_niten	31.20	0.00
TRINITY_XP_0029	hypothet	Volvox_carteri	31.20	0.00
TRINITY_PNH0747	hypothet	Tetrabaena_socialis	31.20	0.00
TRINITY_XP_0228	RING-H2	Olea_europaea	31.20	0.00
TRINITY_XP_0025	predict	Micromonas_commoda	31.20	0.00
TRINITY_XP_0238	non-red	Quercus_suber	31.20	0.00
TRINITY_XP_0139	hypothet	Monoraphidium_negle	31.20	0.00
TRINITY_XP_0218	ran-bin	Spinacia_oleracea	31.20	0.00
TRINITY_GAQ8376	cytochr	Klebsormidium_niten	31.20	0.00
TRINITY_KXZ4798	hypothet	Gonium_pectorale	31.20	0.00
TRINITY_XP_0101	DNA rep	Morus_notabilis	31.20	0.00
TRINITY_XP_0042	PREDICT	Fragaria_vesca	31.20	0.00
TRINITY_XP_0184	PREDICT	Raphanus_sativus	31.20	0.00
TRINITY_XP_0128	PREDICT	Erythranthe_guttata	31.20	0.00
TRINITY_XP_0257	vacuola	Panicum_hallii	31.20	0.00
TRINITY_GBF9063	hypothet	Raphidocelis_subcap	31.20	0.00
TRINITY_XP_0139	hypothet	Monoraphidium_negle	31.20	0.00
TRINITY_GAV8014	Kelch_3	Cephalotus_follicul	31.20	0.00
TRINITY_GBF9405	hypothet	Raphidocelis_subcap	31.20	0.00
TRINITY_XP_0041	PREDICT	Cucumis_sativus	31.20	0.00
TRINITY_GAX8002	hypothet	Chlamydomonas_eusti	31.20	0.00
TRINITY_BAJ8727	predict	Hordeum_vulgare	31.20	0.00
TRINITY_BAJ9694	predict	Hordeum_vulgare	31.20	0.00
TRINITY_XP_0017	predict	Chlamydomonas_reinh	31.20	0.00
TRINITY_GAX8467	hypothet	Chlamydomonas_eusti	31.20	0.00
TRINITY_XP_0058	hypothet	Chlorella_variabili	31.20	0.00
TRINITY_GAX8138	hypothet	Chlamydomonas_eusti	31.20	0.00
TRINITY_XP_0239	uncharac	Quercus_suber	31.20	0.00
TRINITY_XP_0094	PREDICT	Musa_acuminata	31.20	0.00
TRINITY_RWW6408	hypothet	Ensete_ventricosum	31.20	0.00
TRINITY_PSC6788	prolyl	Micractinium_conduc	31.20	0.00
TRINITY_XP_0025	predict	Micromonas_commoda	31.20	0.00
TRINITY_KXZ5562	hypothet	Gonium_pectorale	31.20	0.00
TRINITY_GAQ8454	hypothet	Klebsormidium_niten	31.20	0.00
TRINITY_XP_0279	uncharac	Vigna_unguiculata	31.20	0.00
TRINITY_XP_0029	hypothet	Volvox_carteri	31.20	0.00
TRINITY_GAX7666	hypothet	Chlamydomonas_eusti	31.20	0.00
TRINITY_RWR7220	serine/t	Cinnamomum_micranth	31.10	0.00
TRINITY_KYP7308	Ankyrin-	Cajanus_cajan	31.10	0.00

TRINITY_XP_0063{	uncharac	Populus_trichocarpa	31.10	0.00
TRINITY_XP_0075(	ABC tran	Bathycoccus_prasinc	31.10	0.00
TRINITY_XP_0116:	LOW QUAI	Amborella_trichopod	31.10	0.00
TRINITY_XP_0030(	predicte	Micromonas_pusilla	31.10	0.00
TRINITY_BAJ9550:	predicte	Hordeum_vulgare	31.10	0.00
TRINITY_PNW7472(	hypothet	Chlamydomonas_reinh	31.10	0.00
TRINITY_GBF9687(	hypothet	Raphidocelis_subcap	31.10	0.00
TRINITY_XP_0110:	PREDICTE	Populus_euphratica	31.10	0.00
TRINITY_EOY3132{	Adenylat	Theobroma_cacao	31.10	0.00
TRINITY_PRW4519{	carboxyl	Chlorella_sorokinia	31.10	0.00
TRINITY_XP_0237	serine/t	Lactuca_sativa	31.10	0.00
TRINITY_PRW0592:	melanotr	Chlorella_sorokinia	31.10	0.00
TRINITY_EYU4160{	hypothet	Erythranthe_guttata	31.10	0.00
TRINITY_PNR4419{	hypothet	Physcomitrella_pate	31.10	0.00
TRINITY_GAQ7773:	Leishmar	Klebsormidium_niten	31.10	0.00
TRINITY_XP_0220:	serine/t	Helianthus_annuus	31.10	0.00
TRINITY_XP_0016{	predicte	Chlamydomonas_reinh	31.10	0.00
TRINITY_XP_0150	ribonucl	Solanum_pennellii	31.10	0.00
TRINITY_XP_0245:	probable	Selaginella_moellen	31.10	0.00
TRINITY_XP_0228	UBA-like	Ostreococcus_tauri	31.10	0.00
TRINITY_XP_0029	hypothet	Volvox_carteri	31.10	0.00
TRINITY_GBF9960	hypothet	Raphidocelis_subcap	31.10	0.00
TRINITY_EOY0891:	Vps51/Vp	Theobroma_cacao	31.10	0.00
TRINITY_XP_0097{	PREDICTE	Nicotiana_sylvestri	31.10	0.00
TRINITY_GAX7665:	hypothet	Chlamydomonas_eusti	31.10	0.00
TRINITY_XP_0202(	uncharac	Aegilops_tauschii	31.10	0.00
TRINITY_XP_0219{	phosphat	Helianthus_annuus	31.10	0.00
TRINITY_GAX8301	hypothet	Chlamydomonas_eusti	31.10	0.00
TRINITY_PNW7678(	hypothet	Chlamydomonas_reinh	31.10	0.00
TRINITY_BAA9700	unnamed	Arabidopsis_thalian	31.10	0.00
TRINITY_PRW5746:	serine t	Chlorella_sorokinia	31.10	0.00
TRINITY_OWM6913(	hypothet	Punica_granatum	31.10	0.00
TRINITY_RXI0312(	hypothet	Malus_domestica	31.10	0.00
TRINITY_XP_0056	pectin l	Coccomyxa_subellips	31.10	0.00
TRINITY_PNH1006(	Gamma-se	Tetrabaena_socialis	31.10	0.00
TRINITY_GAX8147!	hypothet	Chlamydomonas_eusti	31.10	0.00
TRINITY_GAX8229{	hypothet	Chlamydomonas_eusti	31.10	0.00
TRINITY_KXZ4949(	hypothet	Gonium_pectorale	31.10	0.00
TRINITY_GAX8539(	hypothet	Chlamydomonas_eusti	31.10	0.00
TRINITY_XP_0014:	predicte	Ostreococcus_sp._'l	31.10	0.00
TRINITY_GAX8297(	hypothet	Chlamydomonas_eusti	31.10	0.00
TRINITY_XP_0264:	actin-2-	Papaver_somniferum	31.00	0.00
TRINITY_XP_0216	uncharac	Hevea_brasiliensis	31.00	0.00
TRINITY_GAX8336(	hypothet	Chlamydomonas_eusti	31.00	0.00
TRINITY_XP_0264(	afadin-	Papaver_somniferum	31.00	0.00
TRINITY_GAQ7960{	WD repea	Klebsormidium_niten	31.00	0.00
TRINITY_KDD7271	hypothet	Helicosporidium_sp.	31.00	0.00
TRINITY_XP_0220:	asparti	Helianthus_annuus	31.00	0.00
TRINITY_XP_0109:	PREDICTE	Elaeis_guineensis	31.00	0.00
TRINITY_XP_0030!	predicte	Micromonas_pusilla	31.00	0.00
TRINITY_PHT3669:	1-acylgl	Capsicum_baccatum	31.00	0.00
TRINITY_XP_0238{	Niemann-	Quercus_suber	31.00	0.00
TRINITY_ANE1053:	carbonic	Dunaliella_salina	31.00	0.00
TRINITY_XP_0239	erythroc	Quercus_suber	31.00	0.00
TRINITY_OMP0869(	hypothet	Corchorus_olitorius	31.00	0.00
TRINITY_XP_0241{	mediator	Rosa_chinensis	31.00	0.00
TRINITY_XP_0016{	aminotra	Chlamydomonas_reinh	31.00	0.00
TRINITY_XP_0159	cytokini	Arachis_duranensis	31.00	0.00
TRINITY_EFJ0568(	hypothet	Selaginella_moellen	31.00	0.00
TRINITY_GAQ7773:	Leishmar	Klebsormidium_niten	31.00	0.00
TRINITY_OAE2090	hypothet	Marchantia_polymorp	31.00	0.00
TRINITY_XP_0238{	nuclear	Quercus_suber	31.00	0.00
TRINITY_GAX7974:	hypothet	Chlamydomonas_eusti	31.00	0.00
TRINITY_XP_0190!	PREDICTE	Tarenaya_hasslerian	31.00	0.00
TRINITY_XP_0058	hypothet	Chlorella_variabili	31.00	0.00
TRINITY_XP_0084!	PREDICTE	Cucumis_melo	31.00	0.00

TRINITY_GAQ80269	vacuolar	Klebsormidium_niten	31.00	0.00
TRINITY_GAX75179	hypothet	Chlamydomonas_eusti	31.00	0.00
TRINITY_GAX78777	hypothet	Chlamydomonas_eusti	31.00	0.00
TRINITY_XP_02390	uncharac	Quercus_suber	31.00	0.00
TRINITY_PNG99137	hypothet	Tetrabaena_socialis	31.00	0.00
TRINITY_PWZ37686	Fatty ac	Zea_mays	31.00	0.00
TRINITY_GAQ86232	hypothet	Klebsormidium_niten	31.00	0.00
TRINITY_GAQ77713	HECT dom	Klebsormidium_niten	31.00	0.00
TRINITY_XP_00295	hypothet	Volvox_carteri	31.00	0.00
TRINITY_XP_00287	clustere	Arabidopsis_lyrata	31.00	0.00
TRINITY_XP_01390	hypothet	Monoraphidium_negle	31.00	0.00
TRINITY_XP_02181	putative	Prunus_avium	31.00	0.00
TRINITY_PNH12309	hypothet	Tetrabaena_socialis	31.00	0.00
TRINITY_XP_01004	PREDICTE	Eucalyptus_grandis	31.00	0.00
TRINITY_EFJ16813	hypothet	Selaginella_moellen	31.00	0.00
TRINITY_XP_00496	beta-hex	Setaria_italica	31.00	0.00
TRINITY_EFJ20463	hypothet	Selaginella_moellen	31.00	0.00
TRINITY_BAJ93724	predicte	Hordeum_vulgare	31.00	0.00
TRINITY_XP_02390	uncharac	Quercus_suber	31.00	0.00
TRINITY_GAX75650	hypothet	Chlamydomonas_eusti	31.00	0.00
TRINITY_RXH73843	hypothet	Malus_domestica	31.00	0.00
TRINITY_OMO59531	hypothet	Corchorus_olitorius	31.00	0.00
TRINITY_GAX77566	hypothet	Chlamydomonas_eusti	31.00	0.00
TRINITY_RDX65566	Sphingos	Mucuna_pruriens	31.00	0.00
TRINITY_XP_02388	tripepti	Quercus_suber	31.00	0.00
TRINITY_GAX75531	hypothet	Chlamydomonas_eusti	31.00	0.00
TRINITY_GAX75796	hypothet	Chlamydomonas_eusti	31.00	0.00
TRINITY_XP_02284	Methyltr	Ostreococcus_tauri	31.00	0.00
TRINITY_XP_02439	choline/	Physcomitrella_pate	31.00	0.00
TRINITY_XP_02188	EVI5-lik	Carica_papaya	31.00	0.00
TRINITY_KXZ52652	hypothet	Gonium_pectorale	31.00	0.00
TRINITY_XP_02390	probable	Quercus_suber	31.00	0.00
TRINITY_GAX78316	hypothet	Chlamydomonas_eusti	31.00	0.00
TRINITY_XP_02024	inositol	Asparagus_officinal	31.00	0.00
TRINITY_XP_00169	predicte	Chlamydomonas_reinh	31.00	0.00
TRINITY_XP_02390	cell div	Quercus_suber	31.00	0.00
TRINITY_EFJ11300	hypothet	Selaginella_moellen	31.00	0.00
TRINITY_XP_00414	PREDICTE	Cucumis_sativus	31.00	0.00
TRINITY_PNW75800	hypothet	Chlamydomonas_reinh	31.00	0.00
TRINITY_PNH10401	putative	Tetrabaena_socialis	31.00	0.00
TRINITY_GAX81253	hypothet	Chlamydomonas_eusti	31.00	0
TRINITY_XP_02438	gamma-gl	Physcomitrella_pate	31.00	0.00
TRINITY_GAX79641	hypothet	Chlamydomonas_eusti	31.00	0.00
TRINITY_RZC06069	Sister c	Glycine_soja	30.90	0.00
TRINITY_XP_00251	protein	Ricinus_communis	30.90	0.00
TRINITY_XP_02389	ribonucl	Quercus_suber	30.90	0.00
TRINITY_PNH12097	putative	Tetrabaena_socialis	30.90	0.00
TRINITY_XP_02021	putative	Cajanus_cajan	30.90	0.00
TRINITY_XP_01653	PREDICTE	Capsicum_annuum	30.90	0.00
TRINITY_XP_00430	PREDICTE	Fragaria_vesca	30.90	0.00
TRINITY_RWR95994	exosome	Cinnamomum_micranth	30.90	0.00
TRINITY_XP_02438	phosphat	Physcomitrella_pate	30.90	0.00
TRINITY_XP_02390	probable	Quercus_suber	30.90	0.00
TRINITY_GAQ77732	Leishman	Klebsormidium_niten	30.90	0.00
TRINITY_XP_01042	PREDICTE	Camelina_sativa	30.90	0.00
TRINITY_XP_02284	S15/NS1,	Ostreococcus_tauri	30.90	0.00
TRINITY_XP_02285	guanine	Olea_europaea	30.90	0.00
TRINITY_KQK18356	hypothet	Brachypodium_distac	30.90	0.00
TRINITY_RVW39526	ABC tran	Vitis_vinifera	30.90	0.00
TRINITY_XP_00423	ATP-depe	Solanum_lycopersicu	30.90	0.00
TRINITY_XP_02388	ergothio	Quercus_suber	30.90	0.00
TRINITY_XP_02436	protein	Physcomitrella_pate	30.90	0.00
TRINITY_GAX76945	hypothet	Chlamydomonas_eusti	30.90	0.00
TRINITY_PNW76773	hypothet	Chlamydomonas_reinh	30.90	0.00
TRINITY_XP_02440	WASH com	Physcomitrella_pate	30.90	0.00
TRINITY_EEF23764	conserve	Ricinus_communis	30.90	0.00

TRINITY_XP_0239	cAMP-dep	Quercus_suber	30.90	0.00
TRINITY_GBG6140	hypothet	Chara_braunii	30.90	0.00
TRINITY_GAX8215	hypothet	Chlamydomonas_eusti	30.90	0.00
TRINITY_GAX7701	hypothet	Chlamydomonas_eusti	30.90	0.00
TRINITY_XP_0244	von Wil	Physcomitrella_pate	30.90	0.00
TRINITY_XP_0016	predicte	Chlamydomonas_reinh	30.90	0.00
TRINITY_XP_0066	PREDICTE	Oryza_brachyantha	30.90	0.00
TRINITY_PRW3927	hypothet	Chlorella_sorokinia	30.90	0.00
TRINITY_KDO3908	hypothet	Citrus_sinensis	30.90	0.00
TRINITY_GAU2797	hypothet	Trifolium_subterran	30.90	0.00
TRINITY_XP_0056	Emopami	Coccomyxa_subellips	30.90	0.00
TRINITY_XP_0110	thioredo	Sesamum_indicum	30.90	0.00
TRINITY_XP_0062	sulfate	Capsella_rubella	30.90	0.00
TRINITY_GAX8297	hypothet	Chlamydomonas_eusti	30.90	0.00
TRINITY_XP_0030	predicte	Micromonas_pusilla	30.90	0.00
TRINITY_XP_0056	hypothet	Coccomyxa_subellips	30.90	0.00
TRINITY_EOY0820	Purple a	Theobroma_cacao	30.90	0.00
TRINITY_OAE2524	hypothet	Marchantia_polymorp	30.90	0.00
TRINITY_GAQ7842	Hypothet	Klebsormidium_niten	30.90	0.00
TRINITY_GAX7500	hypothet	Chlamydomonas_eusti	30.90	0.00
TRINITY_RXH9867	hypothet	Malus_domestica	30.90	0.00
TRINITY_XP_0238	vacuola	Quercus_suber	30.90	0.00
TRINITY_GAX7545	hypothet	Chlamydomonas_eusti	30.90	0.00
TRINITY_XP_0056	phosphat	Coccomyxa_subellips	30.90	0.00
TRINITY_PWA5677	alpha/be	Artemisia_annua	30.90	0.00
TRINITY_XP_0239	ATP-depe	Quercus_suber	30.90	0.00
TRINITY_OAE1929	hypothet	Marchantia_polymorp	30.90	0.00
TRINITY_XP_0029	hypothet	Volvox_carteri	30.90	0.00
TRINITY_GAX8545	hypothet	Chlamydomonas_eusti	30.90	0.00
TRINITY_PNH0198	Ankyrin-	Tetrabaena_socialis	30.90	0.00
TRINITY_KXZ4402	hypothet	Gonium_pectorale	30.90	0.00
TRINITY_PNW8070	hypothet	Chlamydomonas_reinh	30.90	0.00
TRINITY_GAX8068	hypothet	Chlamydomonas_eusti	30.80	0.00
TRINITY_XP_0243	quinone	Physcomitrella_pate	30.80	0.00
TRINITY_XP_0030	predicte	Micromonas_pusilla	30.80	0.00
TRINITY_OEL2227	hypothet	Dichanthelium_oligo	30.80	0.00
TRINITY_KMZ6794	hypothet	Zostera_marina	30.80	0.00
TRINITY_XP_0264	ultravio	Papaver_somniferum	30.80	0.00
TRINITY_EPS5984	hypothet	Genlisea_aurea	30.80	0.00
TRINITY_XP_0030	predicte	Micromonas_pusilla	30.80	0.00
TRINITY_XP_0192	PREDICTE	Nicotiana_attenuata	30.80	0.00
TRINITY_EPS6671	hypothet	Genlisea_aurea	30.80	0.00
TRINITY_XP_0228	Ubiquiti	Ostreococcus_tauri	30.80	0.00
TRINITY_OMP0244	hypothet	Corchorus_olitorius	30.80	0.00
TRINITY_XP_0104	PREDICTE	Camelina_sativa	30.80	0.00
TRINITY_GAX7606	hypothet	Chlamydomonas_eusti	30.80	0.00
TRINITY_XP_0049	EKC/KEOP	Setaria_italica	30.80	0.00
TRINITY_XP_0096	PREDICTE	Nicotiana_tomentosi	30.80	0.00
TRINITY_XP_0239	uncharac	Quercus_suber	30.80	0.00
TRINITY_RWR8397	lysine-s	Cinnamomum_micranth	30.80	0.00
TRINITY_XP_0155	uncharac	Ricinus_communis	30.80	0.00
TRINITY_PHT8468	Serpin-Z	Capsicum_annuum	30.80	0.00
TRINITY_GAX8523	hypothet	Chlamydomonas_eusti	30.80	0.00
TRINITY_GAX7375	hypothet	Chlamydomonas_eusti	30.80	0.00
TRINITY_PRW5855	DUF21 do	Chlorella_sorokinia	30.80	0.00
TRINITY_XP_0056	DnaJ-dom	Coccomyxa_subellips	30.80	0.00
TRINITY_GAX7818	hypothet	Chlamydomonas_eusti	30.80	0.00
TRINITY_XP_0083	PREDICTE	Malus_domestica	30.80	0.00
TRINITY_XP_0205	immune-a	Phalaenopsis_equest	30.80	0.00
TRINITY_GBF9210	hypothet	Raphidocelis_subcap	30.80	0.00
TRINITY_PTQ4072	hypothet	Marchantia_polymorp	30.80	0.00
TRINITY_GAQ8290	hypothet	Klebsormidium_niten	30.80	0.00
TRINITY_XP_0184	PREDICTE	Raphanus_sativus	30.80	0.00
TRINITY_KXZ5395	hypothet	Gonium_pectorale	30.80	0.00
TRINITY_GAX7516	hypothet	Chlamydomonas_eusti	30.80	0.00
TRINITY_KXZ4302	hypothet	Gonium_pectorale	30.80	0.00

TRINITY_GAQ7921	putativ	Klebsormidium_niten	30.80	0.00
TRINITY_PIM9994	Protein	Handroanthus_impeti	30.80	0.00
TRINITY_AAV9062	Rab7 [C	Cenchrus_americanus	30.80	0.00
TRINITY_RWR8509	phospho	Cinnamomum_micranth	30.80	0.00
TRINITY_XP_0024	structu	Sorghum_bicolor	30.80	0.00
TRINITY_XP_0029	hypothet	Volvox_carteri	30.80	0.00
TRINITY_KDD7696	Proline	Helicosporidium_sp.	30.80	0.00
TRINITY_PKA5738	hypothet	Apostasia_shenzheni	30.80	0.00
TRINITY_PNW7797	hypothet	Chlamydomonas_reinh	30.80	0.00
TRINITY_RAL4467	hypothet	Cuscuta_australis	30.80	0.00
TRINITY_NP_0012	uncharac	Glycine_max	30.80	0.00
TRINITY_XP_0139	glycosy	Monoraphidium_negle	30.80	0.00
TRINITY_PKA4927	hypothet	Apostasia_shenzheni	30.80	0.00
TRINITY_GAQ9280	hypothet	Klebsormidium_niten	30.80	0.00
TRINITY_XP_0025	predict	Micromonas_commoda	30.80	0.00
TRINITY_XP_0245	enoyl-[	Selaginella_moellen	30.70	0.00
TRINITY_XP_0219	CBL-int	Helianthus_annuus	30.70	0.00
TRINITY_OAE2589	hypothet	Marchantia_polymorp	30.70	0.00
TRINITY_XP_0229	protein	Cucurbita_moschata	30.70	0.00
TRINITY_XP_0205	serine/	Phalaenopsis_equest	30.70	0.00
TRINITY_PWA4200	Sodium/	Artemisia_annua	30.70	0.00
TRINITY_EFJ1395	hypothet	Selaginella_moellen	30.70	0.00
TRINITY_XP_0136	probabl	Brassica_napus	30.70	0.00
TRINITY_RRT7405	hypothet	Ensete_ventricosum	30.70	0.00
TRINITY_KDD7471	subtila	Helicosporidium_sp.	30.70	0.00
TRINITY_PTQ3352	hypothet	Marchantia_polymorp	30.70	0.00
TRINITY_GAX8073	hypothet	Chlamydomonas_eusti	30.70	0.00
TRINITY_KXZ4509	hypothet	Gonium_pectorale	30.70	0.00
TRINITY_XP_0024	coatome	Sorghum_bicolor	30.70	0.00
TRINITY_XP_0238	uncharac	Quercus_suber	30.70	0.00
TRINITY_KZV4938	hypothet	Dorcoceras_hygromet	30.70	0.00
TRINITY_GAX7418	hypothet	Chlamydomonas_eusti	30.70	0.00
TRINITY_GAQ8190	SMT3/SU	Klebsormidium_niten	30.70	0.00
TRINITY_GAX8225	hypothet	Chlamydomonas_eusti	30.70	0.00
TRINITY_GBG5987	hypothet	Chara_braunii	30.70	0.00
TRINITY_PNW8639	hypothet	Chlamydomonas_reinh	30.70	0.00
TRINITY_GAQ8645	hypothet	Klebsormidium_niten	30.70	0.00
TRINITY_XP_0238	uncharac	Quercus_suber	30.70	0.00
TRINITY_XP_0016	predict	Chlamydomonas_reinh	30.70	0.00
TRINITY_XP_0201	patatin-	Aegilops_tauschii	30.70	0.00
TRINITY_XP_0058	hypothet	Chlorella_variabili	30.70	0.00
TRINITY_OAE3533	hypothet	Marchantia_polymorp	30.70	0.00
TRINITY_XP_0176	PREDICT	Gossypium_arboreum	30.70	0.00
TRINITY_PSC6921	FAD-lin	Micractinium_conduc	30.70	0.00
TRINITY_XP_0212	serine/	Herrania_umbratica	30.70	0.00
TRINITY_KXZ4353	hypothet	Gonium_pectorale	30.70	0.00
TRINITY_XP_0100	PREDICT	Eucalyptus_grandis	30.70	0.00
TRINITY_XP_0049	aminope	Setaria_italica	30.70	0.00
TRINITY_BAK0524	predict	Hordeum_vulgare	30.70	0.00
TRINITY_XP_0045	protein	Cicer_arietinum	30.70	0.00
TRINITY_XP_0218	branchp	Spinacia_oleracea	30.70	0.00
TRINITY_XP_0228	ABC tra	Ostreococcus_tauri	30.70	0.00
TRINITY_XP_0030	predict	Micromonas_pusilla	30.70	0.00
TRINITY_PRW3322	coatome	Chlorella_sorokinia	30.70	0.00
TRINITY_PWA9830	Helicas	Artemisia_annua	30.70	0.00
TRINITY_GBF9045	peptida	Raphidocelis_subcap	30.70	0.00
TRINITY_XP_0029	hypothet	Volvox_carteri	30.70	0.00
TRINITY_GAX7392	hypothet	Chlamydomonas_eusti	30.70	0.00
TRINITY_XP_0016	predict	Chlamydomonas_reinh	30.70	0.00
TRINITY_OMO7303	Serine	Corchorus_olitorius	30.70	0.00
TRINITY_XP_0167	PREDICT	Gossypium_hirsutum	30.70	0.00
TRINITY_GAQ8456	type I	Klebsormidium_niten	30.70	0.00
TRINITY_XP_0239	vacuola	Quercus_suber	30.70	0.00
TRINITY_XP_0239	vacuola	Quercus_suber	30.70	0.00
TRINITY_XP_0016	predict	Chlamydomonas_reinh	30.70	0.00
TRINITY_GAX7535	hypothet	Chlamydomonas_eusti	30.70	0.00

TRINITY_PLY7174	hypothet	Lactuca_sativa	30.70	0.00
TRINITY_KXZ4873	hypothet	Gonium_pectorale	30.70	0.00
TRINITY_XP_0244	BTB/POZ	Physcomitrella_pate	30.70	0.00
TRINITY_GAX7636	hypothet	Chlamydomonas_eusti	30.70	0.00
TRINITY_KXZ4169	hypothet	Gonium_pectorale	30.70	0.00
TRINITY_GAX8155	hypothet	Chlamydomonas_eusti	30.70	0.00
TRINITY_XP_0279	peroxis	Vigna_unguiculata	30.70	0.00
TRINITY_GAX7484	hypothet	Chlamydomonas_eusti	30.70	0.00
TRINITY_GAX7726	hypothet	Chlamydomonas_eusti	30.70	0.00
TRINITY_XP_0239	unchara	Quercus_suber	30.70	0.00
TRINITY_XP_0029	hypothet	Volvox_carteri	30.70	0.00
TRINITY_XP_0106	PREDICT	Beta_vulgaris	30.70	0.00
TRINITY_XP_0139	hypothet	Monoraphidium_negle	30.70	0.00
TRINITY_PNH0781	putative	Tetrabaena_socialis	30.70	0.00
TRINITY_XP_0094	PREDICT	Musa_acuminata	30.60	0.00
TRINITY_XP_0056	laminin	Coccomyxa_subellips	30.60	0.00
TRINITY_XP_0025	predict	Micromonas_commoda	30.60	0.00
TRINITY_BAA9535	S-like	Volvox_carteri	30.60	0.00
TRINITY_GAX7948	hypothet	Chlamydomonas_eusti	30.60	0.00
TRINITY_OTF9704	putative	Helianthus_annuus	30.60	0.00
TRINITY_XP_0238	cell di	Quercus_suber	30.60	0.00
TRINITY_XP_0100	PREDICT	Eucalyptus_grandis	30.60	0.00
TRINITY_GAQ8111	Zincin-	Klebsormidium_niten	30.60	0.00
TRINITY_GAQ8751	hypothet	Klebsormidium_niten	30.60	0.00
TRINITY_XP_0227	coatome	Durio_zibethinus	30.60	0.00
TRINITY_XP_0056	TBC-dom	Coccomyxa_subellips	30.60	0.00
TRINITY_XP_0167	PREDICT	Gossypium_hirsutum	30.60	0.00
TRINITY_RAL5156	hypothet	Cuscuta_australis	30.60	0.00
TRINITY_XP_0237	cytochr	Lactuca_sativa	30.60	0.00
TRINITY_NP_0011	putative	Zea_mays	30.60	0.00
TRINITY_OAE2817	hypothet	Marchantia_polymorp	30.60	0.00
TRINITY_XP_0139	hypothet	Monoraphidium_negle	30.60	0.00
TRINITY_XP_0202	WD repe	Cajanus_cajan	30.60	0.00
TRINITY_XP_0042	PREDICT	Fragaria_vesca	30.60	0.00
TRINITY_PRW5883	L-cyste	Chlorella_sorokinia	30.60	0.00
TRINITY_GAX7631	hypothet	Chlamydomonas_eusti	30.60	0.00
TRINITY_KXZ4638	hypothet	Gonium_pectorale	30.60	0.00
TRINITY_AJR1677	defecti	Petunia_integrifoli	30.60	0.00
TRINITY_GAY3472	hypothet	Citrus_unshiu	30.60	0.00
TRINITY_XP_0263	ribonuc	Papaver_somniferum	30.60	0.00
TRINITY_XP_0087	probabl	Phoenix_dactylifera	30.60	0.00
TRINITY_GAX7416	hypothet	Chlamydomonas_eusti	30.60	0.00
TRINITY_PNW7065	hypothet	Chlamydomonas_reinh	30.60	0.00
TRINITY_OAE1974	hypothet	Marchantia_polymorp	30.60	0.00
TRINITY_XP_0216	pumilio	Hevea_brasiliensis	30.60	0.00
TRINITY_XP_0216	unchara	Hevea_brasiliensis	30.60	0.00
TRINITY_GAX7644	hypothet	Chlamydomonas_eusti	30.60	0.00
TRINITY_XP_0084	PREDICT	Cucumis_melo	30.60	0.00
TRINITY_XP_0244	von Wil	Physcomitrella_pate	30.60	0.00
TRINITY_BAK0109	predict	Hordeum_vulgare	30.60	0.00
TRINITY_GAX7634	hypothet	Chlamydomonas_eusti	30.60	0.00
TRINITY_GAX7968	hypothet	Chlamydomonas_eusti	30.60	0.00
TRINITY_XP_0030	Aminoac	Ostreococcus_tauri	30.60	0.00
TRINITY_XP_0219	serpin-	Helianthus_annuus	30.60	0.00
TRINITY_XP_0238	unchara	Quercus_suber	30.60	0.00
TRINITY_XP_0042	probabl	Solanum_lycopersicu	30.60	0.00
TRINITY_XP_0238	unchara	Quercus_suber	30.60	0.00
TRINITY_PNW7631	hypothet	Chlamydomonas_reinh	30.60	0.00
TRINITY_GAX7950	hypothet	Chlamydomonas_eusti	30.60	0.00
TRINITY_RYR5998	hypothet	Arachis_hypogaea	30.60	0.00
TRINITY_XP_0024	probabl	Sorghum_bicolor	30.60	0.00
TRINITY_GAX8046	hypothet	Chlamydomonas_eusti	30.60	0.00
TRINITY_XP_0120	actin [	Jatropha_curcas	30.60	0.00
TRINITY_XP_0244	E3 ubiq	Physcomitrella_pate	30.60	0.00
TRINITY_XP_0016	seryl-t	Chlamydomonas_reinh	30.60	0.00
TRINITY_XP_0156	PREDICT	Oryza_brachyantha	30.60	0.00

TRINITY_PSC7672	Extende	Micractinium_conduc	30.60	0.00
TRINITY_PIA2615	hypothet	Aquilegia_coerulea	30.60	0.00
TRINITY_XP_0016	predicte	Chlamydomonas_reinh	30.60	0.00
TRINITY_GAX8301	hypothet	Chlamydomonas_eusti	30.60	0.00
TRINITY_XP_0238	vacuola	Quercus_suber	30.60	0.00
TRINITY_GAX8096	hypothet	Chlamydomonas_eusti	30.60	0.00
TRINITY_XP_0264	uncharac	Papaver_somniferum	30.50	0.00
TRINITY_XP_0184	PREDICTE	Raphanus_sativus	30.50	0.00
TRINITY_GAQ8432	UDP-gal	Klebsormidium_niten	30.50	0.00
TRINITY_XP_0110	WD repea	Sesamum_indicum	30.50	0.00
TRINITY_XP_0218	protein	Carica_papaya	30.50	0.00
TRINITY_PIN0466	Protein	Handroanthus_impeti	30.50	0.00
TRINITY_XP_0049	dual spe	Setaria_italica	30.50	0.00
TRINITY_XP_0063	serine/t	Eutrema_salsugineum	30.50	0.00
TRINITY_GBG8372	hypothet	Chara_braunii	30.50	0.00
TRINITY_BAB7181	hypothet	Marchantia_polymorp	30.50	0.00
TRINITY_XP_0249	integrat	Ziziphus_jujuba	30.50	0.00
TRINITY_XP_0106	PREDICTE	Beta_vulgaris	30.50	0.00
TRINITY_XP_0191	PREDICTE	Ipomoea_nil	30.50	0.00
TRINITY_BAK0112	predicte	Hordeum_vulgare	30.50	0.00
TRINITY_GAX8097	hypothet	Chlamydomonas_eusti	30.50	0.00
TRINITY_BAU7112	solute (	Parachlorella_kessl	30.50	0.00
TRINITY_XP_0239	coronin-	Quercus_suber	30.50	0.00
TRINITY_BAK0200	predicte	Hordeum_vulgare	30.50	0.00
TRINITY_XP_0025	predicte	Micromonas_commoda	30.50	0.00
TRINITY_XP_0025	gamma-g	Ricinus_communis	30.50	0.00
TRINITY_XP_0165	PREDICTE	Nicotiana_tabacum	30.50	0.00
TRINITY_KXZ4340	hypothet	Gonium_pectorale	30.50	0.00
TRINITY_PPD9035	hypothet	Gossypium_barbadens	30.50	0.00
TRINITY_XP_0024	polygal	Sorghum_bicolor	30.50	0.00
TRINITY_XP_0100	PREDICTE	Eucalyptus_grandis	30.50	0.00
TRINITY_XP_0243	uncharac	Physcomitrella_pate	30.50	0.00
TRINITY_KZV4221	Para-nit	Dorcoceras_hygromet	30.50	0.00
TRINITY_PSC7366	glucose-	Micractinium_conduc	30.50	0.00
TRINITY_GAX7378	hypothet	Chlamydomonas_eusti	30.50	0.00
TRINITY_XP_0029	hypothet	Volvox_carteri	30.50	0.00
TRINITY_XP_0218	putative	Spinacia_oleracea	30.50	0.00
TRINITY_XP_0058	hypothet	Chlorella_variabili	30.50	0.00
TRINITY_XP_0109	PREDICTE	Elaeis_guineensis	30.50	0.00
TRINITY_XP_0201	E3 ubiq	Ananas_comosus	30.50	0.00
TRINITY_XP_0156	ER memb	Oryza_sativa	30.50	0.00
TRINITY_XP_0110	tubulin-	Sesamum_indicum	30.50	0.00
TRINITY_PTQ3160	hypothet	Marchantia_polymorp	30.50	0.00
TRINITY_XP_0049	transcr	Setaria_italica	30.50	0.00
TRINITY_XP_0172	PREDICTE	Daucus_carota	30.50	0.00
TRINITY_XP_0071	hypothet	Phaseolus_vulgaris	30.50	0.00
TRINITY_XP_0239	uncharac	Quercus_suber	30.50	0.00
TRINITY_RQL7622	hypothet	Brassica_cretica	30.50	0.00
TRINITY_AAK6320	C2H2 zi	Zea_mays	30.50	0.00
TRINITY_XP_0016	predicte	Chlamydomonas_reinh	30.50	0.00
TRINITY_OAE3191	hypothet	Marchantia_polymorp	30.50	0.00
TRINITY_GBF9688	hypothet	Raphidocelis_subcap	30.50	0.00
TRINITY_XP_0025	predicte	Micromonas_commoda	30.50	0.00
TRINITY_GBG6025	hypothet	Chara_braunii	30.40	0.00
TRINITY_XP_0243	uncharac	Physcomitrella_pate	30.40	0.00
TRINITY_XP_0030	predicte	Micromonas_pusilla	30.40	0.00
TRINITY_XP_0228	serine/t	Olea_europaea	30.40	0.00
TRINITY_GAX8445	hypothet	Chlamydomonas_eusti	30.40	0.00
TRINITY_XP_0028	probable	Arabidopsis_lyrata	30.40	0.00
TRINITY_GAQ8047	hypothet	Klebsormidium_niten	30.40	0.00
TRINITY_GAV8617	Metallo	Cephalotus_follicul	30.40	0.00
TRINITY_XP_0218	peroxis	Spinacia_oleracea	30.40	0.00
TRINITY_XP_0016	predicte	Chlamydomonas_reinh	30.40	0.00
TRINITY_PWA9837	PDI-like	Artemisia_annua	30.40	0.00
TRINITY_OAO8982	RMV1 [A	Arabidopsis_thalian	30.40	0.00
TRINITY_GAX7987	hypothet	Chlamydomonas_eusti	30.40	0.00

TRINITY_GAX7878(	hypothet	Chlamydomonas_eusti	30.40	0.00
TRINITY_XP_0238{	vacuola1	Quercus_suber	30.40	0.00
TRINITY_XP_0029{	ultravi(	Selaginella_moellen	30.40	0.00
TRINITY_RID5249]	hypothet	Brassica_rapa	30.40	0.00
TRINITY_XP_0094(	PREDICTI	Musa_acuminata	30.40	0.00
TRINITY_XP_0239(	LOW QUAI	Quercus_suber	30.40	0.00
TRINITY_XP_0238'	uncharac	Quercus_suber	30.40	0.00
TRINITY_RAL4405]	hypothet	Cuscuta_australis	30.40	0.00
TRINITY_XP_0120(	5'-nucle	Jatropha_curcas	30.40	0.00
TRINITY_GAQ8632(	major fa	Klebsormidium_niten	30.40	0.00
TRINITY_KXZ5329]	hypothet	Gonium_pectorale	30.40	0.00
TRINITY_KEH4009:	metallo(	Medicago_truncatula	30.40	0.00
TRINITY_XP_0109(	PREDICTI	Elaeis_guineensis	30.40	0.00
TRINITY_PWA9587]	Galacto:	Artemisia_annua	30.40	0.00
TRINITY_OEL2357'	Kinesin-	Dichanthelium_oligo	30.40	0.00
TRINITY_XP_0043(	PREDICTI	Fragaria_vesca	30.40	0.00
TRINITY_XP_0245:	uncharac	Selaginella_moellen	30.40	0.00
TRINITY_XP_0239(	beclin-1	Quercus_suber	30.40	0.00
TRINITY_XP_0238(	5'-nucle	Quercus_suber	30.40	0.00
TRINITY_GAX7839(	hypothet	Chlamydomonas_eusti	30.40	0.00
TRINITY_PLY6867'	hypothet	Lactuca_sativa	30.40	0.00
TRINITY_NP_0392'	NADH del	Marchantia_paleacea	30.40	0.00
TRINITY_ERN1625{	hypothet	Amborella_trichopod	30.40	0.00
TRINITY_GAX8429{	hypothet	Chlamydomonas_eusti	30.40	0.00
TRINITY_PRW2110:	Phospho]	Chlorella_sorokinia	30.40	0.00
TRINITY_EFJ1978:	hypothet	Selaginella_moellen	30.40	0.00
TRINITY_BAJ8945]	predicte	Hordeum_vulgare	30.40	0.00
TRINITY_GAX8292]	hypothet	Chlamydomonas_eusti	30.40	0.00
TRINITY_GAX7418{	hypothet	Chlamydomonas_eusti	30.40	0.00
TRINITY_KYP7030]	Signal t	Cajanus_cajan	30.40	0.00
TRINITY_PNW8195(	hypothet	Chlamydomonas_reinh	30.40	0.00
TRINITY_XP_0029{	hypothet	Volvox_carteri	30.40	0.00
TRINITY_GBG7364(	hypothet	Chara_braunii	30.40	0.00
TRINITY_BAK0112{	predicte	Hordeum_vulgare	30.40	0.00
TRINITY_XP_0124(	PREDICTI	Gossypium_raimondii	30.40	0.00
TRINITY_PNW8309'	hypothet	Chlamydomonas_reinh	30.40	0.00
TRINITY_XP_0239]	uncharac	Quercus_suber	30.40	0.00
TRINITY_GAX8362{	hypothet	Chlamydomonas_eusti	30.40	0.00
TRINITY_KMZ7153(	putative	Zostera_marina	30.40	0.00
TRINITY_XP_0124(	PREDICTI	Gossypium_raimondii	30.40	0.00
TRINITY_GAX8277'	hypothet	Chlamydomonas_eusti	30.40	0.00
TRINITY_XP_0024{	vacuola1	Sorghum_bicolor	30.40	0.00
TRINITY_PWA5516:	cytochr(	Artemisia_annua	30.40	0.00
TRINITY_PSC6949]	Magnesi1	Micractinium_conduc	30.40	0.00
TRINITY_GAX7480{	hypothet	Chlamydomonas_eusti	30.40	0.00
TRINITY_PIN2036(	Acyl-CoA	Handroanthus_impeti	30.30	0.00
TRINITY_RZC3030(	Alpha-ma	Glycine_soja	30.30	0.00
TRINITY_AXR8532:	UFD2 [Sa	Sanionia_uncinata	30.30	0.00
TRINITY_XP_0238'	ceramide	Quercus_suber	30.30	0.00
TRINITY_ERM9799{	hypothet	Amborella_trichopod	30.30	0.00
TRINITY_XP_0016(	peptidy]	Chlamydomonas_reinh	30.30	0.00
TRINITY_GBF8822(	cystath]	Raphidocelis_subcap	30.30	0.00
TRINITY_XP_0093(	PREDICTI	Pyrus_x_bretschneid	30.30	0.00
TRINITY_XP_0064{	uncharac	Citrus_sinensis	30.30	0.00
TRINITY_XP_0237]	alkylate	Lactuca_sativa	30.30	0.00
TRINITY_XP_0239:	rRNA met	Quercus_suber	30.30	0.00
TRINITY_XP_0166{	PREDICTI	Prunus_mume	30.30	0.00
TRINITY_GAY5734{	hypothet	Citrus_unshiu	30.30	0.00
TRINITY_XP_0106(	PREDICTI	Beta_vulgaris	30.30	0.00
TRINITY_GAQ8511(	hypothet	Klebsormidium_niten	30.30	0.00
TRINITY_XP_0093{	PREDICTI	Pyrus_x_bretschneid	30.30	0.00
TRINITY_XP_0029(	hypothet	Volvox_carteri	30.30	0.00
TRINITY_PRW4511(	RNA psei	Chlorella_sorokinia	30.30	0.00
TRINITY_GAX8276(	hypothet	Chlamydomonas_eusti	30.30	0.00
TRINITY_XP_0042(	phospho(	Solanum_lycopersicu	30.30	0.00
TRINITY_KXZ5120'	hypothet	Gonium_pectorale	30.30	0.00

TRINITY_XP_0049	protein	Setaria_italica	30.30	0.00
TRINITY_GAX7977	hypothet	Chlamydomonas_eusti	30.30	0.00
TRINITY_XP_0029	hypothet	Volvox_carteri	30.30	0.00
TRINITY_RMZ5257	hypothet	Auxenochlorella_prc	30.30	0.00
TRINITY_XP_0029	hypothet	Volvox_carteri	30.30	0.00
TRINITY_XP_0056	hypothet	Coccomyxa_subellips	30.30	0.00
TRINITY_KFK4344	hypothet	Arabis_alpina	30.30	0.00
TRINITY_XP_0239	indolear	Quercus_suber	30.30	0.00
TRINITY_GAQ8852	hypothet	Klebsormidium_niten	30.30	0.00
TRINITY_XP_0042	PREDICTE	Fragaria_vesca	30.30	0.00
TRINITY_XP_0243	elongat	Physcomitrella_pate	30.30	0.00
TRINITY_XP_0188	PREDICTE	Juglans_regia	30.30	0.00
TRINITY_XP_0202	pathoger	Asparagus_officinal	30.30	0.00
TRINITY_KEH2760	ACT-like	Medicago_truncatula	30.30	0.00
TRINITY_XP_0113	putative	Auxenochlorella_prc	30.30	0.00
TRINITY_XP_0056	hypothet	Coccomyxa_subellips	30.30	0.00
TRINITY_PNW7523	hypothet	Chlamydomonas_reinh	30.30	0.00
TRINITY_PNY0885	ribofla	Trifolium_pratense	30.30	0.00
TRINITY_KMZ7201	Glyoxys	Zostera_marina	30.30	0.00
TRINITY_XP_0025	resistar	Micromonas_commoda	30.30	0.00
TRINITY_XP_0239	probable	Quercus_suber	30.30	0.00
TRINITY_XP_0194	PREDICTE	Lupinus_angustifoli	30.30	0.00
TRINITY_XP_0029	hypothet	Volvox_carteri	30.30	0.00
TRINITY_KXZ4622	hypothet	Gonium_pectorale	30.30	0.00
TRINITY_GAX7806	hypothet	Chlamydomonas_eusti	30.30	0.00
TRINITY_PNH0263	hypothet	Tetrabaena_socialis	30.30	0.00
TRINITY_PTQ4197	hypothet	Marchantia_polymorp	30.30	0.00
TRINITY_XP_0022	PREDICTE	Vitis_vinifera	30.30	0.00
TRINITY_XP_0056	RabGAP/T	Coccomyxa_subellips	30.30	0.00
TRINITY_XP_0016	guanylat	Chlamydomonas_reinh	30.30	0.00
TRINITY_EEF2641	lipid A	Ricinus_communis	30.30	0.00
TRINITY_XP_0064	protein	Citrus_clementina	30.30	0.00
TRINITY_XP_0238	oxyster	Quercus_suber	30.30	0.00
TRINITY_OAE2861	hypothet	Marchantia_polymorp	30.30	0.00
TRINITY_GAX7266	hypothet	Chlamydomonas_eusti	30.30	0.00
TRINITY_XP_0029	hypothet	Volvox_carteri	30.30	0.00
TRINITY_PRW1840	vacuola	Chlorella_sorokinia	30.30	0.00
TRINITY_KXZ4796	hypothet	Gonium_pectorale	30.30	0.00
TRINITY_GAX7905	hypothet	Chlamydomonas_eusti	30.30	0.00
TRINITY_XP_0239	dipeptic	Quercus_suber	30.20	0.00
TRINITY_SAI7601	ATP synt	Botryococcus_brauni	30.20	0.00
TRINITY_XP_0029	hypothet	Volvox_carteri	30.20	0.00
TRINITY_XP_0075	mitochor	Bathycoccus_prasinc	30.20	0.00
TRINITY_XP_0104	PREDICTE	Camelina_sativa	30.20	0.00
TRINITY_PRW2090	Oxidorec	Chlorella_sorokinia	30.20	0.00
TRINITY_XP_0205	nuclear	Phalaenopsis_equest	30.20	0.00
TRINITY_XP_0243	unchara	Physcomitrella_pate	30.20	0.00
TRINITY_GBF9297	hypothet	Raphidocelis_subcap	30.20	0.00
TRINITY_XP_0063	PREDICTE	Solanum_tuberosum	30.20	0.00
TRINITY_XP_0273	unchara	Abrus_precatorius	30.20	0.00
TRINITY_XP_0227	CBL-inte	Durio_zibethinus	30.20	0.00
TRINITY_XP_0049	E3 ubiq	Setaria_italica	30.20	0.00
TRINITY_PON5157	Glucosi	Parasponia_anderson	30.20	0.00
TRINITY_PRW4519	carboxy	Chlorella_sorokinia	30.20	0.00
TRINITY_PNW8555	hypothet	Chlamydomonas_reinh	30.20	0.00
TRINITY_XP_0205	ASC1-li	Phalaenopsis_equest	30.20	0.00
TRINITY_GBG8386	hypothet	Chara_braunii	30.20	0.00
TRINITY_PON4598	Cytopla	Parasponia_anderson	30.20	0.00
TRINITY_XP_0271	protein	Coffea_arabica	30.20	0.00
TRINITY_EFJ0959	hypothet	Selaginella_moellen	30.20	0.00
TRINITY_XP_0017	predicte	Chlamydomonas_reinh	30.20	0.00
TRINITY_KCW8113	hypothet	Eucalyptus_grandis	30.20	0.00
TRINITY_XP_0016	predicte	Chlamydomonas_reinh	30.20	0.00
TRINITY_OMO7505	hypothet	Corchorus_capsulari	30.20	0.00
TRINITY_PNW8810	hypothet	Chlamydomonas_reinh	30.20	0.00
TRINITY_OAE2955	hypothet	Marchantia_polymorp	30.20	0.00

TRINITY_GAX7485	hypothet	Chlamydomonas_eusti	30.20	0.00
TRINITY_GAX8420	hypothet	Chlamydomonas_eusti	30.20	0.00
TRINITY_PRW5855	amidohy	Chlorella_sorokinia	30.20	0.00
TRINITY_XP_0100	PREDICTE	Eucalyptus_grandis	30.20	0.00
TRINITY_KXZ4214	hypothet	Gonium_pectorale	30.20	0.00
TRINITY_PNH1228	WW doma	Tetrabaena_socialis	30.20	0.00
TRINITY_XP_0097	PREDICTE	Nicotiana_sylvestri	30.20	0.00
TRINITY_PNH0846	hypothet	Tetrabaena_socialis	30.20	0.00
TRINITY_GBG7609	hypothet	Chara_braunii	30.20	0.00
TRINITY_XP_0029	rRNA bi	Selaginella_moellen	30.20	0.00
TRINITY_KMZ7257	Eukaryot	Zostera_marina	30.20	0.00
TRINITY_XP_0068	F-box p	Amborella_trichopod	30.20	0.00
TRINITY_GAX7446	hypothet	Chlamydomonas_eusti	30.20	0.00
TRINITY_OAE2275	hypothet	Marchantia_polymorp	30.20	0.00
TRINITY_PNW6984	hypothet	Chlamydomonas_reinh	30.20	0.00
TRINITY_XP_0192	PREDICTE	Nicotiana_attenuata	30.20	0.00
TRINITY_GAQ8029	calcium-	Klebsormidium_niten	30.20	0.00
TRINITY_XP_0279	heat st	Vigna_unguiculata	30.20	0.00
TRINITY_GAX7987	hypothet	Chlamydomonas_eusti	30.20	0.00
TRINITY_GAX8495	hypothet	Chlamydomonas_eusti	30.20	0.00
TRINITY_XP_0201	ALG-2 i	Aegilops_tauschii	30.20	0.00
TRINITY_XP_0172	PREDICTE	Daucus_carota	30.20	0.00
TRINITY_XP_0029	hypothet	Volvox_carteri	30.20	0.00
TRINITY_KXZ4577	hypothet	Gonium_pectorale	30.20	0.00
TRINITY_XP_0016	predicte	Chlamydomonas_reinh	30.20	0.00
TRINITY_KZM9824	hypothet	Daucus_carota	30.20	0.00
TRINITY_XP_0106	PREDICTE	Beta_vulgaris	30.20	0.00
TRINITY_PNW8060	hypothet	Chlamydomonas_reinh	30.20	0.00
TRINITY_GAX7768	hypothet	Chlamydomonas_eusti	30.20	0.00
TRINITY_GAQ8542	Putative	Klebsormidium_niten	30.20	0.00
TRINITY_GAX8259	hypothet	Chlamydomonas_eusti	30.20	0.00
TRINITY_XP_0238	ankyrin	Quercus_suber	30.20	0.00
TRINITY_GAX7334	hypothet	Chlamydomonas_eusti	30.20	0.00
TRINITY_GAQ9115	hypothet	Klebsormidium_niten	30.20	0.00
TRINITY_GAQ8825	hypothet	Klebsormidium_niten	30.20	0.00
TRINITY_XP_0238	DNA dama	Quercus_suber	30.20	0.00
TRINITY_EFJ1198	hypothet	Selaginella_moellen	30.10	0.00
TRINITY_PRW5686	NADPH:q	Chlorella_sorokinia	30.10	0.00
TRINITY_XP_0238	cluster	Quercus_suber	30.10	0.00
TRINITY_XP_0205	LOW QUA	Phalaenopsis_equest	30.10	0.00
TRINITY_XP_0029	hypothet	Volvox_carteri	30.10	0.00
TRINITY_XP_0279	LOW QUA	Vigna_unguiculata	30.10	0.00
TRINITY_XP_0205	uncharac	Amborella_trichopod	30.10	0.00
TRINITY_OUS4211	hypothet	Ostreococcus_tauri	30.10	0.00
TRINITY_XP_0194	PREDICTE	Lupinus_angustifoli	30.10	0.00
TRINITY_XP_0120	3-hydro	Jatropha_curcas	30.10	0.00
TRINITY_OTF8549	putative	Helianthus_annuus	30.10	0.00
TRINITY_EFJ2066	hypothet	Selaginella_moellen	30.10	0.00
TRINITY_OVA1762	WD40 re	Macleaya_cordata	30.10	0.00
TRINITY_GAX7300	hypothet	Chlamydomonas_eusti	30.10	0.00
TRINITY_XP_0097	PREDICTE	Nicotiana_sylvestri	30.10	0.00
TRINITY_KYP4756	DNA mis	Cajanus_cajan	30.10	0.00
TRINITY_XP_0029	hypothet	Volvox_carteri	30.10	0.00
TRINITY_XP_0056	mitochor	Coccomyxa_subellips	30.10	0.00
TRINITY_KXZ4384	hypothet	Gonium_pectorale	30.10	0.00
TRINITY_XP_0237	importi	Lactuca_sativa	30.10	0.00
TRINITY_EFJ1820	hypothet	Selaginella_moellen	30.10	0.00
TRINITY_XP_0191	PREDICTE	Ipomoea_nil	30.10	0.00
TRINITY_PSC7421	ubiquit	Micractinium_conduc	30.10	0.00
TRINITY_GAX7381	hypothet	Chlamydomonas_eusti	30.10	0.00
TRINITY_GAX8414	hypothet	Chlamydomonas_eusti	30.10	0.00
TRINITY_XP_0082	PREDICTE	Prunus_mume	30.10	0.00
TRINITY_BAK0043	predicte	Hordeum_vulgare	30.10	0.00
TRINITY_GAX7446	hypothet	Chlamydomonas_eusti	30.10	0.00
TRINITY_RID7514	hypothet	Brassica_rapa	30.10	0.00
TRINITY_XP_0068	eukaryot	Amborella_trichopod	30.10	0.00

TRINITY_XP_0237	xanthohu	Lactuca_sativa	30.10	0.00
TRINITY_XP_0239	uncharac	Quercus_suber	30.10	0.00
TRINITY_RAL4247	hypothet	Cuscuta_australis	30.10	0.00
TRINITY_XP_0065	tyrosine	Glycine_max	30.10	0.00
TRINITY_XP_0239	dynein h	Quercus_suber	30.10	0.00
TRINITY_GAX7875	hypothet	Chlamydomonas_eusti	30.10	0.00
TRINITY_PNW8352	hypothet	Chlamydomonas_reinh	30.10	0.00
TRINITY_XP_0156	mitochon	Oryza_sativa	30.10	0.00
TRINITY_XP_0036	uncharac	Medicago_truncatula	30.10	0.00
TRINITY_XP_0056	galactos	Coccomyxa_subellips	30.10	0.00
TRINITY_GAX7930	hypothet	Chlamydomonas_eusti	30.10	0.00
TRINITY_PNH0421	Peroxiso	Tetrabaena_socialis	30.10	0.00
TRINITY_GAX7756	hypothet	Chlamydomonas_eusti	30.10	0.00
TRINITY_GAX7347	hypothet	Chlamydomonas_eusti	30.10	0.00
TRINITY_XP_0056	2-amino-	Coccomyxa_subellips	30.10	0.00
TRINITY_XP_0093	PREDICTE	Musa_acuminata	30.10	0.00
TRINITY_PSC6825	putative	Micractinium_conduc	30.10	0.00
TRINITY_XP_0124	PREDICTE	Gossypium_raimondii	30.10	0.00
TRINITY_XP_0097	PREDICTE	Nicotiana_sylvestri	30.10	0.00
TRINITY_GAX8142	hypothet	Chlamydomonas_eusti	30.10	0.00
TRINITY_GAX7996	hypothet	Chlamydomonas_eusti	30.10	0.00
TRINITY_GAX7266	hypothet	Chlamydomonas_eusti	30.10	0.00
TRINITY_GBF9005	hypothet	Raphidocelis_subcap	30.10	0.00
TRINITY_PNH0268	hypothet	Tetrabaena_socialis	30.10	0.00
TRINITY_PRW6086	elongati	Chlorella_sorokinia	30.00	0.00
TRINITY_KXZ5395	hypothet	Gonium_pectorale	30.00	0.00
TRINITY_EFH5187	BTB-POZ	Arabidopsis_lyrata	30.00	0.00
TRINITY_GBG6428	hypothet	Chara_braunii	30.00	0.00
TRINITY_XP_0202	ultravio	Asparagus_officinal	30.00	0.00
TRINITY_PNR5083	hypothet	Physcomitrella_pate	30.00	0.00
TRINITY_XP_0201	ultravio	Ananas_comosus	30.00	0.00
TRINITY_XP_0087	uncharac	Phoenix_dactylifera	30.00	0.00
TRINITY_XP_0202	arginyl-	Asparagus_officinal	30.00	0.00
TRINITY_PTQ3556	hypothet	Marchantia_polymorp	30.00	0.00
TRINITY_XP_0017	zinc-met	Chlamydomonas_reinh	30.00	0.00
TRINITY_EEC8123	hypothet	Oryza_sativa	30.00	0.00
TRINITY_GAQ8951	hypothet	Klebsormidium_niten	30.00	0.00
TRINITY_XP_0030	profilin	Micromonas_pusilla	30.00	0.00
TRINITY_XP_0083	PREDICTE	Malus_domestica	30.00	0.00
TRINITY_GBF9026	hypothet	Raphidocelis_subcap	30.00	0.00
TRINITY_GAQ7773	Leishman	Klebsormidium_niten	30.00	0.00
TRINITY_EPS7353	calcium-	Genlisea_aurea	30.00	0.00
TRINITY_XP_0138	hypothet	Monoraphidium_negle	30.00	0.00
TRINITY_XP_0106	PREDICTE	Beta_vulgaris	30.00	0.00
TRINITY_GAQ8144	hypothet	Klebsormidium_niten	30.00	0.00
TRINITY_XP_0243	excitato	Physcomitrella_pate	30.00	0.00
TRINITY_XP_0056	hypothet	Coccomyxa_subellips	30.00	0.00
TRINITY_PSC7514	general	Micractinium_conduc	30.00	0.00
TRINITY_XP_0191	PREDICTE	Ipomoea_nil	30.00	0.00
TRINITY_XP_0017	hypothet	Chlamydomonas_reinh	30.00	0.00
TRINITY_VDC8966	unnamed	Brassica_oleracea	30.00	0.00
TRINITY_XP_0029	hypothet	Volvox_carteri	30.00	0.00
TRINITY_XP_0029	replicat	Selaginella_moellen	30.00	0.00
TRINITY_XP_0144	probable	Vigna_radiata	30.00	0.00
TRINITY_PNH0830	NFX1-typ	Tetrabaena_socialis	30.00	0.00
TRINITY_XP_0030	C2 calci	Ostreococcus_tauri	30.00	0.00
TRINITY_ATB1973	putative	Calocedrus_decurren	30.00	0.00
TRINITY_XP_0109	PREDICTE	Elaeis_guineensis	30.00	0.00
TRINITY_XP_0167	PREDICTE	Gossypium_hirsutum	30.00	0.00
TRINITY_XP_0106	PREDICTE	Beta_vulgaris	30.00	0.00
TRINITY_XP_0064	probable	Citrus_clementina	30.00	0.00
TRINITY_NP_0013	uncharac	Zea_mays	30.00	0.00
TRINITY_KXZ4576	hypothet	Gonium_pectorale	30.00	0.00
TRINITY_GAX7404	hypothet	Chlamydomonas_eusti	30.00	0.00
TRINITY_XP_0016	predicte	Chlamydomonas_reinh	30.00	0.00
TRINITY_KXZ4682	hypothet	Gonium_pectorale	30.00	0.00

TRINITY_XP_0106	PREDICTE	Beta_vulgaris	30.00	0.00
TRINITY_NP_0013	vesicle-	Solanum_lycopersicu	29.90	0.00
TRINITY_PRW0585	copine-3	Chlorella_sorokinia	29.90	0.00
TRINITY_GAQ8101	hypothet	Klebsormidium_niten	29.90	0.00
TRINITY_PNH0657	Succinat	Tetrabaena_socialis	29.90	0.00
TRINITY_XP_0201	probable	Aegilops_tauschii	29.90	0.00
TRINITY_XP_0094	PREDICTE	Musa_acuminata	29.90	0.00
TRINITY_XP_0243	NEDD8 u	Physcomitrella_pate	29.90	0.00
TRINITY_XP_0238	acetami	Quercus_suber	29.90	0.00
TRINITY_XP_0101	RHOMBOI	Morus_notabilis	29.90	0.00
TRINITY_GAU3857	hypothet	Trifolium_subterran	29.90	0.00
TRINITY_KMZ6238	putative	Zostera_marina	29.90	0.00
TRINITY_XP_0245	uncharac	Selaginella_moellen	29.90	0.00
TRINITY_XP_0229	probable	Cucurbita_maxima	29.90	0.00
TRINITY_GAQ8295	tRNA int	Klebsormidium_niten	29.90	0.00
TRINITY_OMO6391	hypothet	Corchorus_capsulari	29.90	0.00
TRINITY_XP_0029	hypothet	Volvox_carteri	29.90	0.00
TRINITY_EYU3814	hypothet	Erythranthe_guttata	29.90	0.00
TRINITY_PTQ3249	hypothet	Marchantia_polymorp	29.90	0.00
TRINITY_XP_0192	PREDICTE	Nicotiana_attenuata	29.90	0.00
TRINITY_XP_0218	peroxiso	Spinacia_oleracea	29.90	0.00
TRINITY_AOS4962	hypothet	Davallia_tyermannii	29.90	0.00
TRINITY_XP_0017	zinc-met	Chlamydomonas_reinh	29.90	0.00
TRINITY_GBF9649	hypothet	Raphidocelis_subcap	29.90	0.00
TRINITY_PSC6780	transcri	Micractinium_conduc	29.90	0.00
TRINITY_GBG7535	hypothet	Chara_braunii	29.90	0.00
TRINITY_GBF9421	hypothet	Raphidocelis_subcap	29.90	0.00
TRINITY_BAK0735	predicte	Hordeum_vulgare	29.90	0.00
TRINITY_EYU3810	hypothet	Erythranthe_guttata	29.90	0.00
TRINITY_ABK2513	unknown	Picea_sitchensis	29.90	0.00
TRINITY_CDY1867	BnaA09g	Brassica_napus	29.90	0.00
TRINITY_GBG8987	hypothet	Chara_braunii	29.90	0.00
TRINITY_XP_0145	uncharac	Vigna_radiata	29.90	0.00
TRINITY_XP_0043	PREDICTE	Fragaria_vesca	29.90	0.00
TRINITY_KXZ5652	hypothet	Gonium_pectorale	29.90	0.00
TRINITY_GAX7488	hypothet	Chlamydomonas_eusti	29.90	0.00
TRINITY_PSC7350	vacuolar	Micractinium_conduc	29.90	0.00
TRINITY_XP_0239	tripepti	Quercus_suber	29.90	0.00
TRINITY_XP_0110	ubiquiti	Sesamum_indicum	29.90	0.00
TRINITY_GAQ9296	hypothet	Klebsormidium_niten	29.90	0.00
TRINITY_GAX7661	hypothet	Chlamydomonas_eusti	29.90	0.00
TRINITY_GAX7361	hypothet	Chlamydomonas_eusti	29.90	0.00
TRINITY_GAX7377	hypothet	Chlamydomonas_eusti	29.90	0.00
TRINITY_XP_0110	PREDICTE	Populus_euphratica	29.90	0.00
TRINITY_XP_0218	polyader	Carica_papaya	29.90	0.00
TRINITY_XP_0029	hypothet	Volvox_carteri	29.90	0.00
TRINITY_XP_0187	PREDICTE	Eucalyptus_grandis	29.90	0.00
TRINITY_KDP2581	hypothet	Jatropha_curcas	29.90	0.00
TRINITY_PIN1942	Speckle-	Handroanthus_impeti	29.90	0.00
TRINITY_XP_0106	PREDICTE	Beta_vulgaris	29.90	0.00
TRINITY_XP_0029	hypothet	Volvox_carteri	29.90	0.00
TRINITY_XP_0075	predicte	Bathycoccus_prasino	29.90	0.00
TRINITY_XP_0139	hypothet	Monoraphidium_negle	29.90	0.00
TRINITY_XP_0030	predicte	Micromonas_pusilla	29.90	0.00
TRINITY_XP_0075	alpha-1,	Bathycoccus_prasino	29.90	0.00
TRINITY_XP_0016	UNC-50 f	Chlamydomonas_reinh	29.90	0.00
TRINITY_PQM4255	high mol	Prunus_yedoensis	29.90	0.00
TRINITY_XP_0058	hypothet	Chlorella_variabili	29.90	0.00
TRINITY_PRW5593	baculovi	Chlorella_sorokinia	29.90	0.00
TRINITY_KXZ5461	hypothet	Gonium_pectorale	29.90	0.00
TRINITY_GAX8219	hypothet	Chlamydomonas_eusti	29.90	0.00
TRINITY_PTQ3330	hypothet	Marchantia_polymorp	29.90	0.00
TRINITY_KXZ5375	hypothet	Gonium_pectorale	29.90	0.00
TRINITY_PTQ4387	hypothet	Marchantia_polymorp	29.90	0.00
TRINITY_XP_0084	PREDICTE	Cucumis_melo	29.90	0.00
TRINITY_GAX8075	hypothet	Chlamydomonas_eusti	29.90	0.00

TRINITY_OAE2772	hypothet	Marchantia_polymorp	29.90	0.00
TRINITY_GAX8218	hypothet	Chlamydomonas_eusti	29.90	0.00
TRINITY_PTQ4621	hypothet	Marchantia_polymorp	29.80	0.00
TRINITY_XP_0058	hypothet	Chlorella_variabili	29.80	0.00
TRINITY_PNR5511	hypothet	Physcomitrella_pate	29.80	0.00
TRINITY_XP_0128	PREDICTE	Erythranthe_guttata	29.80	0.00
TRINITY_EFJ1681	hypothet	Selaginella_moellen	29.80	0.00
TRINITY_XP_0025	predicte	Micromonas_commoda	29.80	0.00
TRINITY_XP_0083	PREDICTE	Malus_domestica	29.80	0.00
TRINITY_GAX8100	hypothet	Chlamydomonas_eusti	29.80	0.00
TRINITY_KZV2223	putative	Dorcoceras_hygromet	29.80	0.00
TRINITY_GBG9067	hypothet	Chara_braunii	29.80	0.00
TRINITY_GAQ8937	Phosphat	Klebsormidium_niten	29.80	0.00
TRINITY_KXZ5329	hypothet	Gonium_pectorale	29.80	0.00
TRINITY_GAQ7773	Leishmar	Klebsormidium_niten	29.80	0.00
TRINITY_GAX7658	hypothet	Chlamydomonas_eusti	29.80	0.00
TRINITY_GAX7848	hypothet	Chlamydomonas_eusti	29.80	0.00
TRINITY_GAQ8580	phosphat	Klebsormidium_niten	29.80	0.00
TRINITY_PNW8293	hypothet	Chlamydomonas_reinh	29.80	0.00
TRINITY_BAJ9585	predicte	Hordeum_vulgare	29.80	0.00
TRINITY_RLM6049	putative	Panicum_miliaceum	29.80	0.00
TRINITY_XP_0029	kelch re	Volvox_carteri	29.80	0.00
TRINITY_XP_0138	hypothet	Monoraphidium_negle	29.80	0.00
TRINITY_GAX7734	hypothet	Chlamydomonas_eusti	29.80	0.00
TRINITY_RVW1929	LRR rece	Vitis_vinifera	29.80	0.00
TRINITY_GAQ7952	hypothet	Klebsormidium_niten	29.80	0.00
TRINITY_GAQ7762	glycerol	Klebsormidium_niten	29.80	0.00
TRINITY_PNX9407	hypothet	Trifolium_pratense	29.80	0.00
TRINITY_XP_0025	predicte	Micromonas_commoda	29.80	0.00
TRINITY_GBG6828	hypothet	Chara_braunii	29.80	0.00
TRINITY_XP_0028	serine	Arabidopsis_lyrata	29.80	0.00
TRINITY_XP_0102	PREDICTE	Nelumbo_nucifera	29.80	0.00
TRINITY_GBG8048	hypothet	Chara_braunii	29.80	0.00
TRINITY_XP_0243	uncharac	Physcomitrella_pate	29.80	0.00
TRINITY_OAE3179	hypothet	Marchantia_polymorp	29.80	0.00
TRINITY_GAX8298	hypothet	Chlamydomonas_eusti	29.80	0.00
TRINITY_XP_0191	PREDICTE	Ipomoea_nil	29.80	0.00
TRINITY_PNW7207	hypothet	Chlamydomonas_reinh	29.80	0.00
TRINITY_XP_0042	PREDICTE	Fragaria_vesca	29.80	0.00
TRINITY_XP_0197	PREDICTE	Elaeis_guineensis	29.80	0.00
TRINITY_XP_0139	hypothet	Monoraphidium_negle	29.80	0.00
TRINITY_XP_0029	hypothet	Volvox_carteri	29.80	0.00
TRINITY_GAQ8912	putative	Klebsormidium_niten	29.80	0.00
TRINITY_GAX8401	hypothet	Chlamydomonas_eusti	29.80	0.00
TRINITY_PNW7307	hypothet	Chlamydomonas_reinh	29.80	0.00
TRINITY_PNH0921	Tiny mac	Tetrabaena_socialis	29.80	0.00
TRINITY_OTF8705	putative	Helianthus_annuus	29.70	0.00
TRINITY_XP_0102	PREDICTE	Nelumbo_nucifera	29.70	0.00
TRINITY_XP_0025	exocyst	Ricinus_communis	29.70	0.00
TRINITY_GBF9556	hypoxant	Raphidocelis_subcap	29.70	0.00
TRINITY_XP_0029	tRNA-dih	Selaginella_moellen	29.70	0.00
TRINITY_GAQ8146	Putative	Klebsormidium_niten	29.70	0.00
TRINITY_XP_0239	uncharac	Quercus_suber	29.70	0.00
TRINITY_XP_0208	probable	Arabidopsis_lyrata	29.70	0.00
TRINITY_XP_0202	histone	Asparagus_officinal	29.70	0.00
TRINITY_PIA4562	hypothet	Aquilegia_coerulea	29.70	0.00
TRINITY_XP_0030	predicte	Micromonas_pusilla	29.70	0.00
TRINITY_GAQ8569	Targetir	Klebsormidium_niten	29.70	0.00
TRINITY_OAE3042	hypothet	Marchantia_polymorp	29.70	0.00
TRINITY_VDD4156	unnamed	Brassica_oleracea	29.70	0.00
TRINITY_XP_0029	hypothet	Volvox_carteri	29.70	0.00
TRINITY_XP_0016	flagella	Chlamydomonas_reinh	29.70	0.00
TRINITY_XP_0058	hypothet	Chlorella_variabili	29.70	0.00
TRINITY_OMP0480	hypothet	Corchorus_olitorius	29.70	0.00
TRINITY_XP_0014	predicte	Ostreococcus_sp._'l	29.70	0.00
TRINITY_XP_0238	E3 ubiqu	Quercus_suber	29.70	0.00

TRINITY_XP_0207(	ras-rela	Dendrobium_catenatu	29.70	0.00
TRINITY_XP_0243(	ABC trar	Physcomitrella_pate	29.70	0.00
TRINITY_PQM4255(	high mol	Prunus_yedoensis	29.70	0.00
TRINITY_CAN8104(	hypothet	Vitis_vinifera	29.70	0.00
TRINITY_XP_0176(	PREDICTE	Gossypium_arboreum	29.70	0.00
TRINITY_RQL8713(	hypothet	Brassica_cretica	29.70	0.00
TRINITY_XP_0029(	hypothet	Volvox_carteri	29.70	0.00
TRINITY_XP_0205(	ultravic	Amborella_trichopod	29.70	0.00
TRINITY_RAL4492(	hypothet	Cuscuta_australis	29.70	0.00
TRINITY_XP_0263(	LOW QUAI	Papaver_somniferum	29.70	0.00

TRINITY_PRW5601(	kinesin	Chlorella_sorokinia	29.70	0.00
TRINITY_RRT4336(	hypothet	Ensete_ventricosum	29.70	0.00
TRINITY_KFK3098	hypothet	Arabis_alpina	29.70	0.00
TRINITY_OAE3582	hypothet	Marchantia_polymorp	29.70	0.00
TRINITY_XP_0016	predicte	Chlamydomonas_reinh	29.70	0.00
TRINITY_PNW7911	hypothet	Chlamydomonas_reinh	29.70	0.00
TRINITY_XP_0058	hypothet	Chlorella_variabili	29.70	0.00
TRINITY_XP_0249	ras-rela	Cynara_cardunculus	29.70	0.00
TRINITY_XP_0029	monodehy	Selaginella_moellen	29.70	0.00
TRINITY_GAX7557	hypothet	Chlamydomonas_eusti	29.70	0.00
TRINITY_BAJ9457	predicte	Hordeum_vulgare	29.70	0.00
TRINITY_XP_0029	hypothet	Volvox_carteri	29.70	0.00
TRINITY_GAX8199	hypothet	Chlamydomonas_eusti	29.70	0.00
TRINITY_PTQ4804(	hypothet	Marchantia_polymorp	29.70	0.00
TRINITY_XP_0201	V-type p	Aegilops_tauschii	29.60	0.00
TRINITY_XP_0063	actin-re	Populus_trichocarpa	29.60	0.00
TRINITY_XP_0104	PREDICTE	Camelina_sativa	29.60	0.00
TRINITY_XP_0202	cytokini	Asparagus_officinal	29.60	0.00
TRINITY_XP_0238	threonin	Quercus_suber	29.60	0.00
TRINITY_PTQ3088	hypothet	Marchantia_polymorp	29.60	0.00
TRINITY_XP_0227	uncharac	Durio_zibethinus	29.60	0.00
TRINITY_XP_0043(	PREDICTE	Fragaria_vesca	29.60	0.00
TRINITY_XP_0161	ultravio	Arachis_ipaensis	29.60	0.00
TRINITY_PNR5750	hypothet	Physcomitrella_pate	29.60	0.00
TRINITY_RAL3995	hypothet	Cuscuta_australis	29.60	0.00
TRINITY_AMO0273	ATP synt	Chlorella_sp._KQ-20	29.60	0.00
TRINITY_XP_0191	PREDICTE	Ipomoea_nil	29.60	0.00
TRINITY_XP_0201(	uncharac	Ananas_comosus	29.60	0.00
TRINITY_XP_0238	putative	Quercus_suber	29.60	0.00
TRINITY_PWA9448	emp24/gp	Artemisia_annua	29.60	0.00
TRINITY_XP_0176	PREDICTE	Gossypium_arboreum	29.60	0.00
TRINITY_XP_0093	PREDICTE	Musa_acuminata	29.60	0.00
TRINITY_OMP0270	UDP-gluc	Corchorus_capsulari	29.60	0.00
TRINITY_EFJ2336	hypothet	Selaginella_moellen	29.60	0.00
TRINITY_XP_0025(	predicte	Micromonas_commoda	29.60	0.00
TRINITY_RZC8835	hypothet	Papaver_somniferum	29.60	0.00
TRINITY_XP_0245	WD and t	Selaginella_moellen	29.60	0.00
TRINITY_GAX7935	hypothet	Chlamydomonas_eusti	29.60	0.00
TRINITY_PRW5869(	hypothet	Chlorella_sorokinia	29.60	0.00
TRINITY_KXZ4570	hypothet	Gonium_pectorale	29.60	0.00
TRINITY_XP_0068	coiled-c	Amborella_trichopod	29.60	0.00
TRINITY_PNW7042	hypothet	Chlamydomonas_reinh	29.60	0.00
TRINITY_CDP0033	unnamed	Coffea_canephora	29.60	0.00
TRINITY_PSC7292(	hypothet	Micractinium_conduc	29.60	0.00
TRINITY_PNS9888	hypothet	Populus_trichocarpa	29.60	0.00
TRINITY_GAQ8520	Pumilio	Klebsormidium_niten	29.60	0.00
TRINITY_PTQ3752	hypothet	Marchantia_polymorp	29.60	0.00
TRINITY_GAX8356	hypothet	Chlamydomonas_eusti	29.60	0.00
TRINITY_XP_0029	hypothet	Volvox_carteri	29.60	0.00
TRINITY_PIA4118	hypothet	Aquilegia_coerulea	29.60	0.00
TRINITY_XP_0239(	uncharac	Quercus_suber	29.60	0.00
TRINITY_PSC7272	2-oxoglu	Micractinium_conduc	29.60	0.00
TRINITY_KHN4809	Serine/t	Glycine_soja	29.60	0.00
TRINITY_PKI7262	hypothet	Punica_granatum	29.60	0.00
TRINITY_PNW7924	hypothet	Chlamydomonas_reinh	29.60	0.00
TRINITY_PNW8794	hypothet	Chlamydomonas_reinh	29.60	0.00
TRINITY_GAX8413	hypothet	Chlamydomonas_eusti	29.60	0.00
TRINITY_XP_0016	predicte	Chlamydomonas_reinh	29.60	0.00
TRINITY_PNH0069	hypothet	Tetrabaena_socialis	29.60	0.00
TRINITY_GAX7875	hypothet	Chlamydomonas_eusti	29.60	0.00
TRINITY_CDY1105	BnaA03g	Brassica_napus	29.60	0.00
TRINITY_RAL3975	hypothet	Cuscuta_australis	29.60	0.00
TRINITY_GBG9164	hypothet	Chara_braunii	29.60	0.00
TRINITY_KXZ4407	hypothet	Gonium_pectorale	29.60	0.00
TRINITY_KXZ4377	hypothet	Gonium_pectorale	29.60	0.00
TRINITY_GAU1563	hypothet	Trifolium_subterran	29.60	0.00

TRINITY_XP_0071	hypothet	Phaseolus_vulgaris	29.50	0.00
TRINITY_PSC7491	Cystein	Micractinium_conduc	29.50	0.00
TRINITY_XP_0243	pre-mRN	Physcomitrella_pate	29.50	0.00
TRINITY_XP_0279	probabl	Vigna_unguiculata	29.50	0.00
TRINITY_GAQ8065	hypothet	Klebsormidium_niten	29.50	0.00
TRINITY_VDD4876	unnamed	Brassica_oleracea	29.50	0.00
TRINITY_PSC7209	outer d	Micractinium_conduc	29.50	0.00
TRINITY_XP_0194	PREDICT	Lupinus_angustifoli	29.50	0.00
TRINITY_GAQ8383	hypothet	Klebsormidium_niten	29.50	0.00
TRINITY_XP_0273	amino a	Abrus_precatorius	29.50	0.00
TRINITY_XP_0071	hypothet	Phaseolus_vulgaris	29.50	0.00
TRINITY_PTQ3789	hypothet	Marchantia_polymorp	29.50	0.00
TRINITY_PTQ4082	hypothet	Marchantia_polymorp	29.50	0.00
TRINITY_XP_0206	BRCA1-A	Dendrobium_catenatu	29.50	0.00
TRINITY_OAE2630	hypothet	Marchantia_polymorp	29.50	0.00
TRINITY_XP_0172	PREDICT	Daucus_carota	29.50	0.00
TRINITY_XP_0016	predict	Chlamydomonas_reinh	29.50	0.00
TRINITY_XP_0205	AP-2 co	Phalaenopsis_equest	29.50	0.00
TRINITY_XP_0243	protein	Physcomitrella_pate	29.50	0.00
TRINITY_GAQ7853	hypothet	Klebsormidium_niten	29.50	0.00
TRINITY_XP_0239	kexin-l	Quercus_suber	29.50	0.00
TRINITY_XP_0245	uncharac	Selaginella_moellen	29.50	0.00
TRINITY_GBF9836	sacchar	Raphidocelis_subcap	29.50	0.00
TRINITY_XP_0244	triacyl	Populus_trichocarpa	29.50	0.00
TRINITY_GAQ9146	NAD(P)-	Klebsormidium_niten	29.50	0.00
TRINITY_PNW8140	hypothet	Chlamydomonas_reinh	29.50	0.00
TRINITY_XP_0145	nucleol	Vigna_radiata	29.50	0.00
TRINITY_XP_0174	PREDICT	Vigna_angularis	29.50	0.00
TRINITY_GAQ8753	hypothet	Klebsormidium_niten	29.50	0.00
TRINITY_XP_0202	vacuola	Cajanus_cajan	29.50	0.00
TRINITY_XP_0238	uncharac	Quercus_suber	29.50	0.00
TRINITY_XP_0243	uncharac	Physcomitrella_pate	29.50	0.00
TRINITY_GAX7866	hypothet	Chlamydomonas_eusti	29.50	0.00
TRINITY_GBG6501	hypothet	Chara_braunii	29.50	0.00
TRINITY_XP_0025	voltage-	Micromonas_commoda	29.50	0.00
TRINITY_GAX7334	hypothet	Chlamydomonas_eusti	29.50	0.00
TRINITY_XP_0217	uncharac	Chenopodium_quinoa	29.50	0.00
TRINITY_XP_0025	predict	Micromonas_commoda	29.50	0.00
TRINITY_PNW7222	hypothet	Chlamydomonas_reinh	29.50	0.00
TRINITY_GAX7775	hypothet	Chlamydomonas_eusti	29.50	0.00
TRINITY_XP_0097	PREDICT	Nicotiana_sylvestri	29.50	0.00
TRINITY_PRW4499	elongat	Chlorella_sorokinia	29.50	0.00
TRINITY_XP_0056	WD40 re	Coccomyxa_subellips	29.50	0.00
TRINITY_BAJ9694	predict	Hordeum_vulgare	29.50	0.00
TRINITY_KXZ5018	hypothet	Gonium_pectorale	29.50	0.00
TRINITY_BAK0302	predict	Hordeum_vulgare	29.50	0.00
TRINITY_GAX7349	hypothet	Chlamydomonas_eusti	29.50	0.00
TRINITY_XP_0243	uncharac	Physcomitrella_pate	29.50	0.00
TRINITY_KXZ4808	hypothet	Gonium_pectorale	29.50	0.00
TRINITY_GBG7407	hypothet	Chara_braunii	29.50	0.00
TRINITY_XP_0205	uncharac	Phalaenopsis_equest	29.50	0.00
TRINITY_PHU0000	hypothet	Capsicum_chinense	29.50	0.00
TRINITY_XP_0029	hypothet	Volvox_carteri	29.50	0.00
TRINITY_OAE3042	hypothet	Marchantia_polymorp	29.40	0.00
TRINITY_KXZ4483	hypothet	Gonium_pectorale	29.40	0.00
TRINITY_XP_0110	elicito	Sesamum_indicum	29.40	0.00
TRINITY_ABK2205	unknown	Picea_sitchensis	29.40	0.00
TRINITY_XP_0239	cell di	Quercus_suber	29.40	0.00
TRINITY_XP_0062	mitocho	Capsella_rubella	29.40	0.00
TRINITY_XP_0213	random	Sorghum_bicolor	29.40	0.00
TRINITY_PNH0781	putativ	Tetrabaena_socialis	29.40	0.00
TRINITY_XP_0109	PREDICT	Elaeis_guineensis	29.40	0.00
TRINITY_ERN1753	hypothet	Amborella_trichopod	29.40	0.00
TRINITY_XP_0110	PREDICT	Populus_euphratica	29.40	0.00
TRINITY_XP_0240	protein	Morus_notabilis	29.40	0.00
TRINITY_RCW1931	hypothet	Glycine_max	29.40	0.00

TRINITY_XP_02388	dihydrox	Quercus_suber	29.40	0.00
TRINITY_OAE22427	hypothet	Marchantia_polymorp	29.40	0.00
TRINITY_XP_00415	PREDICTE	Cucumis_sativus	29.40	0.00
TRINITY_KZV52696	hypothet	Dorcoceras_hygromet	29.40	0.00
TRINITY_GAX84113	hypothet	Chlamydomonas_eusti	29.40	0.00
TRINITY_GAQ88855	purple a	Klebsormidium_niten	29.40	0.00
TRINITY_BAA22845	HCR2 [A]	Alvikia_littoralis	29.40	0.00
TRINITY_XP_00250	flagella	Micromonas_commoda	29.40	0.00
TRINITY_XP_02452	enoyl-[a	Selaginella_moellen	29.40	0.00
TRINITY_XP_02498	lipid di	Cynara_cardunculus	29.40	0.00
TRINITY_NP_00132	Protein	Arabidopsis_thalian	29.40	0.00
TRINITY_KXZ43942	hypothet	Gonium_pectorale	29.40	0.00
TRINITY_GAX83988	hypothet	Chlamydomonas_eusti	29.40	0.00
TRINITY_XP_02351	uncharac	Cucurbita_pepo	29.40	0.00
TRINITY_XP_00298	uncharac	Selaginella_moellen	29.40	0.00
TRINITY_KXZ56546	hypothet	Gonium_pectorale	29.40	0.00
TRINITY_XP_02390	protein	Quercus_suber	29.40	0.00
TRINITY_ONK63328	uncharac	Asparagus_officinal	29.40	0.00
TRINITY_AML79213	putative	Volvox_aureus	29.40	0.00
TRINITY_NP_00115	transpar	Zea_mays	29.40	0.00
TRINITY_GAX74494	hypothet	Chlamydomonas_eusti	29.40	0.00
TRINITY_KYP48465	hypothet	Cajanus_cajan	29.40	0.00
TRINITY_GAX81333	hypothet	Chlamydomonas_eusti	29.40	0.00
TRINITY_KMZ62392	Major fa	Zostera_marina	29.40	0.00
TRINITY_XP_00250	predicte	Micromonas_commoda	29.40	0.00
TRINITY_XP_02172	alanine-	Chenopodium_quinoa	29.40	0.00
TRINITY_XP_02391	uncharac	Quercus_suber	29.40	0.00
TRINITY_XP_02390	uncharac	Quercus_suber	29.40	0.00
TRINITY_PRW56206	DNA repa	Chlorella_sorokinia	29.40	0.00
TRINITY_XP_00169	predicte	Chlamydomonas_reinh	29.40	0.00
TRINITY_KXG32452	hypothet	Sorghum_bicolor	29.40	0.00
TRINITY_PKA61888	Elongato	Apostasia_shenzheni	29.40	0.00
TRINITY_PTQ30235	hypothet	Marchantia_polymorp	29.40	0.00
TRINITY_XP_01022	heptahel	Brachypodium_distac	29.40	0.00
TRINITY_PNW76910	hypothet	Chlamydomonas_reinh	29.40	0.00
TRINITY_PNW73105	hypothet	Chlamydomonas_reinh	29.40	0.00
TRINITY_XP_01884	PREDICTE	Juglans_regia	29.40	0.00
TRINITY_KXZ54727	hypothet	Gonium_pectorale	29.40	0.00
TRINITY_GAX73342	hypothet	Chlamydomonas_eusti	29.40	0.00
TRINITY_PRW20874	U3 small	Chlorella_sorokinia	29.40	0.00
TRINITY_XP_02373	formin-l	Lactuca_sativa	29.40	0.00
TRINITY_NP_00114	uncharac	Zea_mays	29.40	0.00
TRINITY_PSR86716	Rab5-int	Actinidia_chinensis	29.40	0.00
TRINITY_XP_01390	Dynein l	Monoraphidium_negle	29.30	0.00
TRINITY_XP_02040	uncharac	Zea_mays	29.30	0.00
TRINITY_XP_00498	lipase	Setaria_italica	29.30	0.00
TRINITY_XP_00960	PREDICTE	Nicotiana_tomentosi	29.30	0.00
TRINITY_BAJ96946	predicte	Hordeum_vulgare	29.30	0.00
TRINITY_PIA30414	hypothet	Aquilegia_coerulea	29.30	0.00
TRINITY_RMZ55606	hypothet	Auxenochlorella_pro	29.30	0.00
TRINITY_XP_01538	DNA oxid	Citrus_sinensis	29.30	0.00
TRINITY_EEF25649	NAD(P)H	Ricinus_communis	29.30	0.00
TRINITY_GAX75700	hypothet	Chlamydomonas_eusti	29.30	0.00
TRINITY_XP_01365	phosphog	Brassica_napus	29.30	0.00
TRINITY_XP_01919	PREDICTE	Ipomoea_nil	29.30	0.00
TRINITY_XP_00169	predicte	Chlamydomonas_reinh	29.30	0.00
TRINITY_GAQ80294	calcium-	Klebsormidium_niten	29.30	0.00
TRINITY_GAQ84320	UDP-gala	Klebsormidium_niten	29.30	0.00
TRINITY_YP_66655	NADH deh	Nephroselmis_olivac	29.30	0.00
TRINITY_XP_02022	probable	Cajanus_cajan	29.30	0.00
TRINITY_KXZ53870	hypothet	Gonium_pectorale	29.30	0.00
TRINITY_XP_01248	PREDICTE	Gossypium_raimondii	29.30	0.00
TRINITY_GAX77327	hypothet	Chlamydomonas_eusti	29.30	0.00
TRINITY_XP_00142	predicte	Ostreococcus_sp._'l	29.30	0.00
TRINITY_RYQ87183	hypothet	Arachis_hypogaea	29.30	0.00
TRINITY_BAJ95858	predicte	Hordeum_vulgare	29.30	0.00

TRINITY_XP_0239(	calcium/	Quercus_suber	29.30	0.00
TRINITY_Q43492.2	RecName:	Hordeum_vulgare	29.30	0.00
TRINITY_XP_0023]	sorbitol	Populus_trichocarpa	29.30	0.00
TRINITY_GBF8969]	PKHD-typ	Raphidocelis_subcap	29.30	0.00
TRINITY_XP_0029!	hypothet	Volvox_carteri	29.30	0.00
TRINITY_XP_0110:	PREDICTE	Populus_euphratica	29.30	0.00
TRINITY_GAX8082(	hypothet	Chlamydomonas_eusti	29.30	0.00
TRINITY_XP_0137:	pumilio	Brassica_napus	29.30	0.00
TRINITY_XP_0030!	predicte	Micromonas_pusilla	29.30	0.00
TRINITY_GAX8052{	hypothet	Chlamydomonas_eusti	29.30	0.00
TRINITY_GAX8486]	hypothet	Chlamydomonas_eusti	29.30	0.00
TRINITY_XP_0243'	ankyrin	Physcomitrella_pate	29.30	0.00
TRINITY_RWR7736{	subtilis	Cinnamomum_micranth	29.30	0.00
TRINITY_XP_0056/	alpha/be	Coccomyxa_subellips	29.30	0.00
TRINITY_GAX7406]	hypothet	Chlamydomonas_eusti	29.30	0.00
TRINITY_XP_0238{	vacuolar	Quercus_suber	29.30	0.00
TRINITY_EFJ0959!	hypothet	Selaginella_moellen	29.30	0.00
TRINITY_KXZ4211(	hypothet	Gonium_pectorale	29.30	0.00
TRINITY_PNW8618/	hypothet	Chlamydomonas_reinh	29.30	0.00
TRINITY_XP_0072]	elicitor	Prunus_persica	29.30	0.00
TRINITY_XP_0088(	derlin-1	Phoenix_dactylifera	29.30	0.00
TRINITY_GAX7309!	hypothet	Chlamydomonas_eusti	29.30	0.00
TRINITY_XP_0105!	PREDICTE	Tarenaya_hasslerian	29.30	0.00
TRINITY_EFJ3104(	hypothet	Selaginella_moellen	29.30	0.00
TRINITY_XP_0058/	hypothet	Chlorella_variabili	29.30	0.00
TRINITY_PNW7414!	hypothet	Chlamydomonas_reinh	29.30	0.00
TRINITY_PRW5679]	Pentatri	Chlorella_sorokinia	29.30	0.00
TRINITY_XP_0104'	PREDICTE	Camelina_sativa	29.30	0.00
TRINITY_GAX8411:	hypothet	Chlamydomonas_eusti	29.30	0.00
TRINITY_XP_0056/	hypothet	Coccomyxa_subellips	29.30	0.00
TRINITY_PNW8854!	hypothet	Chlamydomonas_reinh	29.30	0.00
TRINITY_GAX7894]	hypothet	Chlamydomonas_eusti	29.30	0.00
TRINITY_XP_0145]	cyprosin	Vigna_radiata	29.20	0.00
TRINITY_XP_0202/	major fa	Asparagus_officinal	29.20	0.00
TRINITY_XP_0029!	hypothet	Volvox_carteri	29.20	0.00
TRINITY_GAQ9235(	mitogen-	Klebsormidium_niten	29.20	0.00
TRINITY_RXH7298:	hypothet	Malus_domestica	29.20	0.00
TRINITY_XP_0155'	asparti(	Ricinus_communis	29.20	0.00
TRINITY_RWR8047:	nardilys	Cinnamomum_micranth	29.20	0.00
TRINITY_GBG8463'	hypothet	Chara_braunii	29.20	0.00
TRINITY_AAM3365:	mastigon	Chlamydomonas_reinh	29.20	0.00
TRINITY_KZV1647!	hypothet	Dorcoceras_hygromet	29.20	0.00
TRINITY_RDY1206]	hypothet	Mucuna_pruriens	29.20	0.00
TRINITY_GAX8645]	hypothet	Chlamydomonas_eusti	29.20	0.00
TRINITY_XP_0229:	transcri	Cucurbita_moschata	29.20	0.00
TRINITY_XP_0029!	hypothet	Volvox_carteri	29.20	0.00
TRINITY_XP_0128]	PREDICTE	Erythranthe_guttata	29.20	0.00
TRINITY_XP_0243{	methylt	Physcomitrella_pate	29.20	0.00
TRINITY_GBF9705/	hypothet	Raphidocelis_subcap	29.20	0.00
TRINITY_XP_0201!	probable	Aegilops_tauschii	29.20	0.00
TRINITY_XP_0273(	SNF1-rel	Abrus_precatorius	29.20	0.00
TRINITY_GBG8498(	hypothet	Chara_braunii	29.20	0.00
TRINITY_XP_0128]	PREDICTE	Erythranthe_guttata	29.20	0.00
TRINITY_XP_0035]	suppress	Glycine_max	29.20	0.00
TRINITY_XP_0042]	ras-rela	Solanum_lycopersicu	29.20	0.00
TRINITY_XP_0161{	vacuolar	Arachis_ipaensis	29.20	0.00
TRINITY_XP_0238{	protein	Quercus_suber	29.20	0.00
TRINITY_GAX8169(	hypothet	Chlamydomonas_eusti	29.20	0.00
TRINITY_GAX7472/	hypothet	Chlamydomonas_eusti	29.20	0.00
TRINITY_PNW8247'	hypothet	Chlamydomonas_reinh	29.20	0.00
TRINITY_XP_0025(	predicte	Micromonas_commoda	29.20	0.00
TRINITY_GAX7620'	hypothet	Chlamydomonas_eusti	29.20	0.00
TRINITY_GAX7884]	hypothet	Chlamydomonas_eusti	29.20	0.00
TRINITY_KXZ4316]	hypothet	Gonium_pectorale	29.20	0.00
TRINITY_RWR7684]	putative	Cinnamomum_micranth	29.20	0.00
TRINITY_XP_0056/	putative	Coccomyxa_subellips	29.20	0.00

TRINITY_OUS4267(	hypothet	Ostreococcus_tauri	29.20	0.00
TRINITY_XP_0264	probable	Papaver_somniferum	29.20	0.00
TRINITY_XP_0202(	BTB/POZ	Aegilops_tauschii	29.20	0.00
TRINITY_KZV4221(	Para-nit	Dorcoceras_hygromet	29.20	0.00
TRINITY_XP_0095(	PREDICTI	Nicotiana_tomentosi	29.20	0.00
TRINITY_XP_0025(	predicte	Micromonas_commoda	29.20	0.00
TRINITY_KXZ5015(	hypothet	Gonium_pectorale	29.20	0.00
TRINITY_XP_0229(	geraniol	Cucurbita_moschata	29.20	0.00
TRINITY_XP_0243(	von Wil	Physcomitrella_pate	29.20	0.00
TRINITY_RLN0109	eukaryot	Panicum_miliaceum	29.20	0.00
TRINITY_XP_0094(	PREDICTI	Musa_acuminata	29.20	0.00
TRINITY_XP_0056	WD40 re	Coccomyxa_subellips	29.10	0.00
TRINITY_XP_0271	probable	Coffea_eugenioides	29.10	0.00
TRINITY_XP_0216	CBL-inte	Manihot_esculenta	29.10	0.00
TRINITY_EPS6524(	hypothet	Genlisea_aurea	29.10	0.00
TRINITY_P0C8M8.	RecName:	Zea_mays	29.10	0.00
TRINITY_XP_0239(	acyl-pro	Quercus_suber	29.10	0.00
TRINITY_XP_0105	PREDICTI	Tarenaya_hasslerian	29.10	0.00
TRINITY_PNY0946	putative	Trifolium_pratense	29.10	0.00
TRINITY_XP_0238	cell di	Quercus_suber	29.10	0.00
TRINITY_GAX7666(	hypothet	Chlamydomonas_eusti	29.10	0.00
TRINITY_XP_0100(	PREDICTI	Eucalyptus_grandis	29.10	0.00
TRINITY_XP_0014	predicte	Ostreococcus_sp._'l	29.10	0.00
TRINITY_XP_0029	hypothet	Volvox_carteri	29.10	0.00
TRINITY_XP_0266	ATP-depe	Phoenix_dactylifera	29.10	0.00
TRINITY_PSC7130	mitochon	Micractinium_conduc	29.10	0.00
TRINITY_GAU1451	hypothet	Trifolium_subterran	29.10	0.00
TRINITY_GAX7666	hypothet	Chlamydomonas_eusti	29.10	0.00
TRINITY_XP_0109	PREDICTI	Elaeis_guineensis	29.10	0.00
TRINITY_AES9460	eukaryot	Medicago_truncatula	29.10	0.00
TRINITY_EFJ3778	hypothet	Selaginella_moellen	29.10	0.00
TRINITY_XP_0237	probable	Lactuca_sativa	29.10	0.00
TRINITY_GAX7467	hypothet	Chlamydomonas_eusti	29.10	0.00
TRINITY_PNW8845(	hypothet	Chlamydomonas_reinh	29.10	0.00
TRINITY_XP_0239	thiol-sp	Quercus_suber	29.10	0.00
TRINITY_PHT5215	hypothet	Capsicum_baccatum	29.10	0.00
TRINITY_XP_0139(	hypothet	Monoraphidium_negle	29.10	0.00
TRINITY_XP_0237	uncharac	Lactuca_sativa	29.10	0.00
TRINITY_XP_0245	retinobl	Selaginella_moellen	29.10	0.00
TRINITY_XP_0239(	tuberous	Quercus_suber	29.10	0.00
TRINITY_XP_0244(	uncharac	Physcomitrella_pate	29.10	0.00
TRINITY_XP_0227(	heparan-	Durio_zibethinus	29.10	0.00
TRINITY_PNH1150(	Glycosyl	Tetrabaena_socialis	29.10	0.00
TRINITY_PNH0738(	Histone-	Tetrabaena_socialis	29.10	0.00
TRINITY_XP_0064	ABC tran	Citrus_clementina	29.10	0.00
TRINITY_XP_0206	mediator	Dendrobium_catenatu	29.10	0.00
TRINITY_XP_0206	uncharac	Dendrobium_catenatu	29.10	0.00
TRINITY_XP_0045(	oxystero	Cicer_arietinum	29.10	0.00
TRINITY_GAX8391	hypothet	Chlamydomonas_eusti	29.10	0.00
TRINITY_GAX7618(	hypothet	Chlamydomonas_eusti	29.10	0.00
TRINITY_PRW5657	peptidas	Chlorella_sorokinia	29.10	0.00
TRINITY_PNH1279	putative	Tetrabaena_socialis	29.10	0.00
TRINITY_GAX7401	hypothet	Chlamydomonas_eusti	29.10	0.00
TRINITY_XP_0174	PREDICTI	Vigna_angularis	29.10	0.00
TRINITY_VDD5198(	unnamed	Brassica_oleracea	29.10	0.00
TRINITY_PSC7053	pumilio-	Micractinium_conduc	29.10	0.00
TRINITY_XP_0138	zinc fi	Monoraphidium_negle	29.10	0.00
TRINITY_GAX7691	hypothet	Chlamydomonas_eusti	29.10	0.00
TRINITY_GAX7517	hypothet	Chlamydomonas_eusti	29.10	0.00
TRINITY_PNW7463	hypothet	Chlamydomonas_reinh	29.10	0.00
TRINITY_XP_0239	uncharac	Quercus_suber	29.10	0.00
TRINITY_XP_0237	probable	Lactuca_sativa	29.10	0.00
TRINITY_XP_0110	PREDICTI	Populus_euphratica	29.10	0.00
TRINITY_XP_0102	PREDICTI	Nelumbo_nucifera	29.10	0.00
TRINITY_GAX7381	hypothet	Chlamydomonas_eusti	29.10	0.00
TRINITY_GAX7757	hypothet	Chlamydomonas_eusti	29.10	0.00

TRINITY_XP_00295	hypothet	Volvox_carteri	29.10	0.00
TRINITY_PNH12265	Tiny mac	Tetrabaena_socialis	29.10	0.00
TRINITY_GAQ85117	hypothet	Klebsormidium_niten	29.00	0.00
TRINITY_XP_00751	predicte	Bathycoccus_prasino	29.00	0.00
TRINITY_XP_01139	Oxystero	Auxenochlorella_pro	29.00	0.00
TRINITY_RLM60461	70 kDa p	Panicum_miliaceum	29.00	0.00
TRINITY_XP_00585	hypothet	Chlorella_variabili	29.00	0.00
TRINITY_GBG70448	hypothet	Chara_braunii	29.00	0.00
TRINITY_XP_02297	uncharac	Cucurbita_maxima	29.00	0.00
TRINITY_XP_02437	uncharac	Physcomitrella_pate	29.00	0.00
TRINITY_PTQ27799	hypothet	Marchantia_polymorp	29.00	0.00
TRINITY_XP_02451	uncharac	Selaginella_moellen	29.00	0.00
TRINITY_GAQ83394	inositol	Klebsormidium_niten	29.00	0.00
TRINITY_RAL39532	hypothet	Cuscuta_australis	29.00	0.00
TRINITY_XP_02374	probable	Lactuca_sativa	29.00	0.00
TRINITY_XP_00141	VIC fami	Ostreococcus_sp._'l	29.00	0.00
TRINITY_PRW61037	SUMO-act	Chlorella_sorokinia	29.00	0.00
TRINITY_XP_00430	PREDICTE	Fragaria_vesca	29.00	0.00
TRINITY_GAQ84320	UDP-gala	Klebsormidium_niten	29.00	0.00
TRINITY_XP_01346	putative	Medicago_truncatula	29.00	0.00
TRINITY_PNW72631	hypothet	Chlamydomonas_reinh	29.00	0.00
TRINITY_OAE23249	hypothet	Marchantia_polymorp	29.00	0.00
TRINITY_XP_00305	predicte	Micromonas_pusilla	29.00	0.00
TRINITY_XP_00360	F-box pr	Medicago_truncatula	29.00	0.00
TRINITY_PNR28420	hypothet	Physcomitrella_pate	29.00	0.00
TRINITY_KFK33156	hypothet	Arabis_alpina	29.00	0.00
TRINITY_BAK00797	predicte	Hordeum_vulgare	29.00	0.00
TRINITY_XP_01909	PREDICTE	Camelina_sativa	29.00	0.00
TRINITY_XP_02419	probable	Rosa_chinensis	29.00	0.00
TRINITY_XP_01742	PREDICTE	Vigna_angularis	29.00	0.00
TRINITY_RWR86483	14-3-3-l	Cinnamomum_micranth	29.00	0.00
TRINITY_GAQ84224	hypothet	Klebsormidium_niten	29.00	0.00
TRINITY_CDY67315	BnaCnng5	Brassica_napus	29.00	0.00
TRINITY_XP_02387	histidin	Quercus_suber	29.00	0.00
TRINITY_OAE19212	hypothet	Marchantia_polymorp	29.00	0.00
TRINITY_XP_00584	hypothet	Chlorella_variabili	29.00	0.00
TRINITY_XP_02452	DNA poly	Selaginella_moellen	29.00	0.00
TRINITY_BAJ90795	predicte	Hordeum_vulgare	29.00	0.00
TRINITY_GAX72899	hypothet	Chlamydomonas_eusti	29.00	0.00
TRINITY_KZM88819	hypothet	Daucus_carota	29.00	0.00
TRINITY_KMZ76438	Beta-fru	Zostera_marina	29.00	0.00
TRINITY_XP_00250	predicte	Micromonas_commoda	29.00	0.00
TRINITY_XP_02067	protein	Dendrobium_catenatu	29.00	0.00
TRINITY_XP_02790	serine/t	Vigna_unguiculata	29.00	0.00
TRINITY_XP_01452	vacuolar	Vigna_radiata	29.00	0.00
TRINITY_PNH13009	Peroxyur	Tetrabaena_socialis	28.90	0.00
TRINITY_XP_00298	ribosoma	Selaginella_moellen	28.90	0.00
TRINITY_XP_01067	PREDICTE	Beta_vulgaris	28.90	0.00
TRINITY_OAY66327	Calcium-	Ananas_comosus	28.90	0.00
TRINITY_XP_00226	PREDICTE	Vitis_vinifera	28.90	0.00
TRINITY_NP_00132	Transduc	Arabidopsis_thalian	28.90	0.00
TRINITY_XP_01941	PREDICTE	Lupinus_angustifoli	28.90	0.00
TRINITY_PNR34217	hypothet	Physcomitrella_pate	28.90	0.00
TRINITY_GAQ83723	vesicle-	Klebsormidium_niten	28.90	0.00
TRINITY_XP_00565	TPR-like	Coccomyxa_subellips	28.90	0.00
TRINITY_XP_00250	predicte	Micromonas_commoda	28.90	0.00
TRINITY_OAE32122	hypothet	Marchantia_polymorp	28.90	0.00
TRINITY_XP_02436	protein	Physcomitrella_pate	28.90	0.00
TRINITY_XP_00844	PREDICTE	Cucumis_melo	28.90	0.00
TRINITY_XP_02392	tip elon	Quercus_suber	28.90	0.00
TRINITY_XP_00295	hypothet	Volvox_carteri	28.90	0.00
TRINITY_XP_00751	predicte	Bathycoccus_prasino	28.90	0.00
TRINITY_GBG59286	hypothet	Chara_braunii	28.90	0.00
TRINITY_XP_02022	cell div	Cajanus_cajan	28.90	0.00
TRINITY_XP_01872	PREDICTE	Eucalyptus_grandis	28.90	0.00
TRINITY_PNW80411	hypothet	Chlamydomonas_reinh	28.90	0.00

TRINITY_PNH0786(	hypothet	Tetrabaena_socialis	28.90	0.00
TRINITY_XP_0093(	PREDICTI	Musa_acuminata	28.90	0.00
TRINITY_EMS5845(	hypothet	Triticum_urartu	28.90	0.00
TRINITY_XP_0249(	elicito	Cynara_cardunculus	28.90	0.00
TRINITY_XP_0243(	oxidatic	Physcomitrella_pate	28.90	0.00
TRINITY_XP_0238(	putative	Quercus_suber	28.90	0.00
TRINITY_XP_0271(	peroxis	Coffea_arabica	28.90	0.00
TRINITY_XP_0174]	PREDICTI	Vigna_angularis	28.90	0.00
TRINITY_GAX8228(	hypothet	Chlamydomonas_eusti	28.90	0.00
TRINITY_XP_0029(	hypothet	Volvox_carteri	28.90	0.00
TRINITY_GBG9183(	hypothet	Chara_braunii	28.90	0.00
TRINITY_GAX7477]	hypothet	Chlamydomonas_eusti	28.90	0.00
TRINITY_XP_0238(	eIF-2-a]	Quercus_suber	28.90	0.00
TRINITY_GAX8104(	hypothet	Chlamydomonas_eusti	28.90	0.00
TRINITY_PNW7980]	hypothet	Chlamydomonas_reinh	28.90	0.00
TRINITY_XP_0016(	aromatic	Chlamydomonas_reinh	28.90	0.00
TRINITY_ONK6616(	uncharac	Asparagus_officinal	28.90	0.00
TRINITY_XP_0228(	prostam	Olea_europaea	28.90	0.00
TRINITY_EFJ1324(	hypothet	Selaginella_moellen	28.90	0.00
TRINITY_XP_0029(	hypothet	Volvox_carteri	28.90	0.00
TRINITY_PTQ3755(	hypothet	Marchantia_polymorp	28.90	0.00
TRINITY_XP_0056(	CBS-doma	Coccomyxa_subellips	28.90	0.00
TRINITY_OMO6644(	hypothet	Corchorus_capsulari	28.90	0.00
TRINITY_KXZ4543(	hypothet	Gonium_pectorale	28.90	0.00
TRINITY_KXZ4168(	hypothet	Gonium_pectorale	28.90	0.00
TRINITY_XP_0245]	cysteine	Selaginella_moellen	28.80	0.00
TRINITY_KFK3518(	hypothet	Arabis_alpina	28.80	0.00
TRINITY_EFJ1381(	hypothet	Selaginella_moellen	28.80	0.00
TRINITY_XP_0172]	PREDICTI	Daucus_carota	28.80	0.00
TRINITY_XP_0238(	uncharac	Quercus_suber	28.80	0.00
TRINITY_OAE2065(	hypothet	Marchantia_polymorp	28.80	0.00
TRINITY_XP_0264(	ras-rela	Papaver_somniferum	28.80	0.00
TRINITY_XP_0238(	aurofusa	Quercus_suber	28.80	0.00
TRINITY_XP_0056(	IBR-doma	Coccomyxa_subellips	28.80	0.00
TRINITY_XP_0016(	predicte	Chlamydomonas_reinh	28.80	0.00
TRINITY_XP_0106(	PREDICTI	Beta_vulgaris	28.80	0.00
TRINITY_XP_0218]	translat	Prunus_avium	28.80	0.00
TRINITY_CDP0389(	unnamed	Coffea_canephora	28.80	0.00
TRINITY_XP_0042]	glycosyl	Solanum_lycopersicu	28.80	0.00
TRINITY_XP_0239(	uncharac	Quercus_suber	28.80	0.00
TRINITY_XP_0016(	predicte	Chlamydomonas_reinh	28.80	0.00
TRINITY_XP_0064(	cytoplas	Eutrema_salsugineum	28.80	0.00
TRINITY_GAX7458(	hypothet	Chlamydomonas_eusti	28.80	0.00
TRINITY_OAY3962]	hypothet	Manihot_esculenta	28.80	0.00
TRINITY_XP_0030(	mRNA cap	Ostreococcus_tauri	28.80	0.00
TRINITY_XP_0164(	PREDICTI	Nicotiana_tabacum	28.80	0.00
TRINITY_GAX7329(	hypothet	Chlamydomonas_eusti	28.80	0.00
TRINITY_XP_0239(	probable	Quercus_suber	28.80	0.00
TRINITY_XP_0017(	flagella	Chlamydomonas_reinh	28.80	0.00
TRINITY_XP_0041(	PREDICTI	Cucumis_sativus	28.80	0.00
TRINITY_GAX7600(	hypothet	Chlamydomonas_eusti	28.80	0.00
TRINITY_XP_0056(	NAD(P)-b	Coccomyxa_subellips	28.80	0.00
TRINITY_GAX7701(	hypothet	Chlamydomonas_eusti	28.80	0.00
TRINITY_KXZ4728]	hypothet	Gonium_pectorale	28.80	0.00
TRINITY_XP_0238(	E3 ubiqu	Quercus_suber	28.80	0.00
TRINITY_GAQ8368(	hypothet	Klebsormidium_niten	28.80	0.00
TRINITY_PNW7012(	hypothet	Chlamydomonas_reinh	28.80	0.00
TRINITY_PHU0149(	hypothet	Capsicum_chinense	28.80	0.00
TRINITY_PRQ3184]	putative	Rosa_chinensis	28.80	0.00
TRINITY_PNW8193(	hypothet	Chlamydomonas_reinh	28.80	0.00
TRINITY_XP_0071(	hypothet	Phaseolus_vulgaris	28.80	0.00
TRINITY_BAB1810(	cyclic r	Chlamydomonas_reinh	28.80	0.00
TRINITY_GAX7787(	hypothet	Chlamydomonas_eusti	28.80	0.00
TRINITY_GAX7979(	hypothet	Chlamydomonas_eusti	28.80	0.00
TRINITY_XP_0056(	hypothet	Coccomyxa_subellips	28.80	0.00
TRINITY_XP_0239(	paxillir	Quercus_suber	28.80	0.00

TRINITY_GAX80532	hypothet	Chlamydomonas_eusti	28.80	0.00
TRINITY_GAX83680	hypothet	Chlamydomonas_eusti	28.80	0.00
TRINITY_XP_01069	PREDICTE	Beta_vulgaris	28.80	0.00
TRINITY_PIN05760	hypothet	Handroanthus_impeti	28.80	0.00
TRINITY_PUZ43418	hypothet	Panicum_hallii	28.80	0.00
TRINITY_XP_00294	hypothet	Volvox_carteri	28.80	0.00
TRINITY_XP_01027	PREDICTE	Nelumbo_nucifera	28.80	0.00
TRINITY_GAX83361	hypothet	Chlamydomonas_eusti	28.80	0.00
TRINITY_KXZ55666	hypothet	Gonium_pectorale	28.80	0.00
TRINITY_PON40532	Fumarate	Parasponia_anderson	28.70	0.00
TRINITY_KMZ67992	LMBR1-li	Zostera_marina	28.70	0.00
TRINITY_XP_01390	putative	Monoraphidium_negle	28.70	0.00
TRINITY_XP_01594	probable	Arachis_duranensis	28.70	0.00
TRINITY_XP_01743	PREDICTE	Vigna_angularis	28.70	0.00
TRINITY_XP_02353	vacuolar	Cucurbita_pepo	28.70	0.00
TRINITY_GBF92574	hypothet	Raphidocelis_subcap	28.70	0.00
TRINITY_XP_02387	geranylg	Quercus_suber	28.70	0.00
TRINITY_XP_00864	thiamin	Zea_mays	28.70	0.00
TRINITY_XP_02581	UPF0587	Panicum_hallii	28.70	0.00
TRINITY_ABR18287	unknown	Picea_sitchensis	28.70	0.00
TRINITY_XP_02198	calcium	Helianthus_annuus	28.70	0.00
TRINITY_XP_02452	protein	Selaginella_moellen	28.70	0.00
TRINITY_GAX73151	hypothet	Chlamydomonas_eusti	28.70	0.00
TRINITY_ABD52325	mitochon	Amaranthus_tubercul	28.70	0.00
TRINITY_XP_00170	predicte	Chlamydomonas_reinh	28.70	0.00
TRINITY_KMZ70968	hypothet	Zostera_marina	28.70	0.00
TRINITY_GBF88192	hypothet	Raphidocelis_subcap	28.70	0.00
TRINITY_XP_01041	PREDICTE	Camelina_sativa	28.70	0.00
TRINITY_XP_00713	hypothet	Phaseolus_vulgaris	28.70	0.00
TRINITY_GAQ87539	hypothet	Klebsormidium_niten	28.70	0.00
TRINITY_XP_02436	protein	Physcomitrella_pate	28.70	0.00
TRINITY_PNW85671	hypothet	Chlamydomonas_reinh	28.70	0.00
TRINITY_KMS93578	hypothet	Beta_vulgaris	28.70	0.00
TRINITY_GAQ83334	translat	Klebsormidium_niten	28.70	0.00
TRINITY_BAK00002	predicte	Hordeum_vulgare	28.70	0.00
TRINITY_XP_00564	phosphat	Coccomyxa_subellips	28.70	0.00
TRINITY_RWW90240	hypothet	Ensete_ventricosum	28.70	0.00
TRINITY_GBF91567	hypothet	Raphidocelis_subcap	28.70	0.00
TRINITY_XP_02375	serine (	Lactuca_sativa	28.70	0.00
TRINITY_GAX79654	hypothet	Chlamydomonas_eusti	28.70	0.00
TRINITY_RLM59005	patatin-	Panicum_miliaceum	28.70	0.00
TRINITY_OEL37612	CBL-inte	Dichanthelium_oligo	28.70	0.00
TRINITY_ABU52967	hypothet	Dunaliella_viridis	28.70	0.00
TRINITY_XP_00170	hypothet	Chlamydomonas_reinh	28.70	0.00
TRINITY_GAX75196	hypothet	Chlamydomonas_eusti	28.70	0.00
TRINITY_XP_02052	RWD doma	Amborella_trichopod	28.70	0.00
TRINITY_GAX80310	hypothet	Chlamydomonas_eusti	28.70	0.00
TRINITY_XP_00250	predicte	Micromonas_commoda	28.70	0.00
TRINITY_XP_01389	ERD4-rel	Monoraphidium_negle	28.70	0.00
TRINITY_PNH12261	Tiny mac	Tetrabaena_socialis	28.70	0.00
TRINITY_GAX73388	hypothet	Chlamydomonas_eusti	28.70	0.00
TRINITY_XP_00250	predicte	Micromonas_commoda	28.70	0.00
TRINITY_XP_00295	hypothet	Volvox_carteri	28.70	0.00
TRINITY_XP_00294	structur	Volvox_carteri	28.70	0.00
TRINITY_GAX79620	hypothet	Chlamydomonas_eusti	28.70	0.00
TRINITY_KXZ55911	hypothet	Gonium_pectorale	28.70	0.00
TRINITY_GAQ89181	hypothet	Klebsormidium_niten	28.70	0.00
TRINITY_XP_01024	PREDICTE	Nelumbo_nucifera	28.60	0.00
TRINITY_OEL24868	Acyl-CoA	Dichanthelium_oligo	28.60	0.00
TRINITY_OMP03068	hypothet	Corchorus_capsulari	28.60	0.00
TRINITY_XP_02645	BTB/POZ	Papaver_somniferum	28.60	0.00
TRINITY_XP_01725	PREDICTE	Daucus_carota	28.60	0.00
TRINITY_XP_00169	predicte	Chlamydomonas_reinh	28.60	0.00
TRINITY_XP_00356	uncharac	Brachypodium_distac	28.60	0.00
TRINITY_AAM77646	hypothet	Arabidopsis_thalian	28.60	0.00
TRINITY_XP_02175	actin-re	Chenopodium_quinoa	28.60	0.00

TRINITY_EEC7691{	hypothet	Oryza_sativa	28.60	0.00
TRINITY_XP_0058	hypothet	Chlorella_variabili	28.60	0.00
TRINITY_GAQ8006	DNA repa	Klebsormidium_niten	28.60	0.00
TRINITY_XP_0256	cytokin	Arachis_hypogaea	28.60	0.00
TRINITY_XP_0138	hypothet	Monoraphidium_negle	28.60	0.00
TRINITY_GAQ9128	hypothet	Klebsormidium_niten	28.60	0.00
TRINITY_XP_0113	Protein	Auxenochlorella_prc	28.60	0.00
TRINITY_PNH0201	ARM REPI	Tetrabaena_socialis	28.60	0.00
TRINITY_XP_0066	PREDICTI	Oryza_brachyantha	28.60	0.00
TRINITY_XP_0245	DNA repa	Selaginella_moellen	28.60	0.00
TRINITY_XP_0056	RNA 3'-t	Coccomyxa_subellips	28.60	0.00
TRINITY_GAQ9134	Glycine	Klebsormidium_niten	28.60	0.00
TRINITY_XP_0191	PREDICTI	Ipomoea_nil	28.60	0.00
TRINITY_XP_0075	predicte	Bathycoccus_prasinc	28.60	0.00
TRINITY_XP_0029	hypothet	Volvox_carteri	28.60	0.00
TRINITY_OAE1844	hypothet	Marchantia_polymorp	28.60	0.00
TRINITY_XP_0238	vacuola	Quercus_suber	28.60	0.00
TRINITY_AAV5930	putative	Oryza_sativa	28.60	0.00
TRINITY_XP_0064	probable	Citrus_clementina	28.60	0.00
TRINITY_GAQ8449	cytochro	Klebsormidium_niten	28.60	0.00
TRINITY_XP_0102	PREDICTI	Nelumbo_nucifera	28.60	0.00
TRINITY_XP_0243	uncharac	Physcomitrella_pate	28.60	0.00
TRINITY_XP_0075	predicte	Bathycoccus_prasinc	28.60	0.00
TRINITY_KCW8417	hypothet	Eucalyptus_grandis	28.60	0.00
TRINITY_GAX7674	hypothet	Chlamydomonas_eusti	28.60	0.00
TRINITY_PNH0172	hypothet	Tetrabaena_socialis	28.60	0.00
TRINITY_GAX7699	hypothet	Chlamydomonas_eusti	28.60	0.00
TRINITY_GAQ8879	RNA poly	Klebsormidium_niten	28.60	0.00
TRINITY_OEL1605	BTB/POZ	Dichanthelium_oligc	28.60	0.00
TRINITY_XP_0016	predicte	Chlamydomonas_reinh	28.60	0.00
TRINITY_GAX7353	hypothet	Chlamydomonas_eusti	28.60	0.00
TRINITY_XP_0218	ran-binc	Spinacia_oleracea	28.60	0.00
TRINITY_KXZ4397	hypothet	Gonium_pectorale	28.60	0.00
TRINITY_GAX7431	hypothet	Chlamydomonas_eusti	28.60	0.00
TRINITY_XP_0156	LMBR1 dc	Oryza_sativa	28.60	0.00
TRINITY_XP_0264	E3 ubiqu	Papaver_somniferum	28.60	0.00
TRINITY_XP_0016	predicte	Chlamydomonas_reinh	28.50	0.00
TRINITY_XP_0235	polyader	Cucurbita_pepo	28.50	0.00
TRINITY_RWR8097	calmodul	Cinnamomum_micranth	28.50	0.00
TRINITY_OIV9972	hypothet	Lupinus_angustifoli	28.50	0.00
TRINITY_XP_0093	PREDICTI	Pyrus_x_bretschneid	28.50	0.00
TRINITY_PTQ4118	hypothet	Marchantia_polymorp	28.50	0.00
TRINITY_XP_0025	predicte	Micromonas_commoda	28.50	0.00
TRINITY_XP_0191	PREDICTI	Ipomoea_nil	28.50	0.00
TRINITY_XP_0218	transcri	Spinacia_oleracea	28.50	0.00
TRINITY_XP_0075	alpha-1,	Bathycoccus_prasinc	28.50	0.00
TRINITY_XP_0075	unnamed	Bathycoccus_prasinc	28.50	0.00
TRINITY_GAQ7816	hypothet	Klebsormidium_niten	28.50	0.00
TRINITY_XP_0216	cytochro	Hevea_brasiliensis	28.50	0.00
TRINITY_GAQ8975	hypothet	Klebsormidium_niten	28.50	0.00
TRINITY_XP_0239	aorsin-1	Quercus_suber	28.50	0.00
TRINITY_KDD7696	Proline	Helicosporidium_sp.	28.50	0.00
TRINITY_KXZ5058	hypothet	Gonium_pectorale	28.50	0.00
TRINITY_XP_0227	1-phosph	Durio_zibethinus	28.50	0.00
TRINITY_PTQ4457	hypothet	Marchantia_polymorp	28.50	0.00
TRINITY_XP_0029	hypothet	Volvox_carteri	28.50	0.00
TRINITY_GAX7846	hypothet	Chlamydomonas_eusti	28.50	0.00
TRINITY_XP_0058	hypothet	Chlorella_variabili	28.50	0.00
TRINITY_XP_0240	PH, RCC1	Morus_notabilis	28.50	0.00
TRINITY_GAX7441	hypothet	Chlamydomonas_eusti	28.50	0.00
TRINITY_GAQ7797	hypothet	Klebsormidium_niten	28.50	0.00
TRINITY_XP_0025	predicte	Micromonas_commoda	28.50	0.00
TRINITY_XP_0016	guanylat	Chlamydomonas_reinh	28.50	0.00
TRINITY_KHG3038	EVI5-lik	Gossypium_arboreum	28.50	0.00
TRINITY_OWM6293	hypothet	Punica_granatum	28.50	0.00
TRINITY_PNW7980	hypothet	Chlamydomonas_reinh	28.50	0.00

TRINITY_XP_02453	platelet	Selaginella_moellen	28.50	0.00
TRINITY_XP_02353	BTB/POZ	Cucurbita_pepo	28.50	0.00
TRINITY_GAX77363	hypothet	Chlamydomonas_eusti	28.50	0.00
TRINITY_GAX85087	hypothet	Chlamydomonas_eusti	28.50	0.00
TRINITY_KXZ44382	hypothet	Gonium_pectorale	28.50	0.00
TRINITY_XP_00585	hypothet	Chlorella_variabili	28.50	0.00
TRINITY_PSC67599	Gag-Pol	Micractinium_conduc	28.50	0.00
TRINITY_XP_02643	myosin-6	Papaver_somniferum	28.50	0.00
TRINITY_PNW77234	hypothet	Chlamydomonas_reinh	28.50	0.00
TRINITY_PNW78788	hypothet	Chlamydomonas_reinh	28.50	0.00
TRINITY_PNW74393	hypothet	Chlamydomonas_reinh	28.50	0.00
TRINITY_KDP23404	hypothet	Jatropha_curcas	28.40	0.00
TRINITY_XP_00142	predicte	Ostreococcus_sp._'l	28.40	0.00
TRINITY_XP_01923	PREDICTE	Nicotiana_attenuata	28.40	0.00
TRINITY_XP_02289	polyol t	Olea_europaea	28.40	0.00
TRINITY_EPS73375	hypothet	Genlisea_aurea	28.40	0.00
TRINITY_XP_00585	hypothet	Chlorella_variabili	28.40	0.00
TRINITY_XP_02446	sorting	Populus_trichocarpa	28.40	0.00
TRINITY_XP_01910	PREDICTE	Beta_vulgaris	28.40	0.00
TRINITY_GAQ83939	glycerop	Klebsormidium_niten	28.40	0.00
TRINITY_XP_02009	uncharac	Ananas_comosus	28.40	0.00
TRINITY_XP_00448	gamma-so	Cicer_arietinum	28.40	0.00
TRINITY_XP_02454	probable	Selaginella_moellen	28.40	0.00
TRINITY_GAQ91271	etoposic	Klebsormidium_niten	28.40	0.00
TRINITY_KMZ70092	Ethanola	Zostera_marina	28.40	0.00
TRINITY_KYP36096	Ankyrin	Cajanus_cajan	28.40	0.00
TRINITY_XP_02453	uncharac	Selaginella_moellen	28.40	0.00
TRINITY_XP_00413	PREDICTE	Cucumis_sativus	28.40	0.00
TRINITY_PRW59105	iodothyr	Chlorella_sorokinia	28.40	0.00
TRINITY_OIW18785	hypothet	Lupinus_angustifoli	28.40	0.00
TRINITY_PNW88185	hypothet	Chlamydomonas_reinh	28.40	0.00
TRINITY_XP_00878	histone-	Phoenix_dactylifera	28.40	0.00
TRINITY_XP_02709	paired a	Coffea_arabica	28.40	0.00
TRINITY_XP_02499	zeaxanth	Cynara_cardunculus	28.40	0.00
TRINITY_XP_02019	ASC1-lik	Aegilops_tauschii	28.40	0.00
TRINITY_XP_00423	F-box pr	Solanum_lycopersicu	28.40	0.00
TRINITY_PSR86819	Calcium-	Actinidia_chinensis	28.40	0.00
TRINITY_XP_02390	cell div	Quercus_suber	28.40	0.00
TRINITY_XP_00295	hypothet	Volvox_carteri	28.40	0.00
TRINITY_XP_02790	DNA cros	Vigna_unguiculata	28.40	0.00
TRINITY_XP_00845	PREDICTE	Cucumis_melo	28.40	0.00
TRINITY_PRW58827	Pol [Chl	Chlorella_sorokinia	28.40	0.00
TRINITY_GBF88338	hypothet	Raphidocelis_subcap	28.40	0.00
TRINITY_KZM98893	hypothet	Daucus_carota	28.40	0.00
TRINITY_KDD75499	hypothet	Helicosporidium_sp.	28.40	0.00
TRINITY_GAQ83084	hypothet	Klebsormidium_niten	28.40	0.00
TRINITY_XP_00838	PREDICTE	Malus_domestica	28.40	0.00
TRINITY_XP_00308	DENN dom	Ostreococcus_tauri	28.40	0.00
TRINITY_XP_00306	predicte	Micromonas_pusilla	28.40	0.00
TRINITY_XP_02402	putative	Morus_notabilis	28.40	0.00
TRINITY_XP_00305	predicte	Micromonas_pusilla	28.40	0.00
TRINITY_XP_02292	transcri	Cucurbita_moschata	28.40	0.00
TRINITY_EMS63337	Tubulin-	Triticum_urartu	28.40	0.00
TRINITY_XP_01923	PREDICTE	Nicotiana_attenuata	28.40	0.00
TRINITY_BAJ96946	predicte	Hordeum_vulgare	28.40	0.00
TRINITY_XP_02200	glutathi	Helianthus_annuus	28.40	0.00
TRINITY_XP_00938	PREDICTE	Musa_acuminata	28.40	0.00
TRINITY_KXZ55667	hypothet	Gonium_pectorale	28.40	0.00
TRINITY_KXZ44745	hypothet	Gonium_pectorale	28.40	0.00
TRINITY_GAX82115	hypothet	Chlamydomonas_eusti	28.40	0.00
TRINITY_XP_02567	flavin-c	Arachis_hypogaea	28.30	0.00
TRINITY_GBG86742	hypothet	Chara_braunii	28.30	0.00
TRINITY_PSC68023	serine t	Micractinium_conduc	28.30	0.00
TRINITY_GAX77541	hypothet	Chlamydomonas_eusti	28.30	0.00
TRINITY_KXZ52652	hypothet	Gonium_pectorale	28.30	0.00
TRINITY_ASU47403	ethylene	Paeonia_suffruticos	28.30	0.00

TRINITY_EFJ0560(	hypothet	Selaginella_moellen	28.30	0.00
TRINITY_XP_0238(	non-redu	Quercus_suber	28.30	0.00
TRINITY_GAX8118(	hypothet	Chlamydomonas_eusti	28.30	0.00
TRINITY_PIA3041(	hypothet	Aquilegia_coerulea	28.30	0.00
TRINITY_XP_0206(	polypyri	Dendrobium_catenatu	28.30	0.00
TRINITY_XP_0244(	von Will	Physcomitrella_pate	28.30	0.00
TRINITY_XP_0243(	lysine-s	Physcomitrella_pate	28.30	0.00
TRINITY_XP_0025(	calcium-	Micromonas_commoda	28.30	0.00
TRINITY_XP_0238(	dihydrox	Quercus_suber	28.30	0.00
TRINITY_PRW4450(	major fa	Chlorella_sorokinia	28.30	0.00
TRINITY_KXZ4625(	hypothet	Gonium_pectorale	28.30	0.00
TRINITY_RQM0337(	hypothet	Brassica_cretica	28.30	0.00
TRINITY_XP_0072(	ras-rela	Prunus_persica	28.30	0.00
TRINITY_GAX7389(	hypothet	Chlamydomonas_eusti	28.30	0.00
TRINITY_PNW7319(	hypothet	Chlamydomonas_reinh	28.30	0.00
TRINITY_XP_0238(	repressi	Quercus_suber	28.30	0.00
TRINITY_XP_0239(	U5 small	Quercus_suber	28.30	0.00
TRINITY_XP_0184(	PREDICTE	Raphanus_sativus	28.30	0.00
TRINITY_XP_0096(	PREDICTE	Nicotiana_tomentosi	28.30	0.00
TRINITY_PAN4968(	hypothet	Panicum_hallii	28.30	0.00
TRINITY_XP_0238(	indolear	Quercus_suber	28.30	0.00
TRINITY_GAX8569(	hypothet	Chlamydomonas_eusti	28.30	0.00
TRINITY_XP_0104(	PREDICTE	Camelina_sativa	28.30	0.00
TRINITY_XP_0103(	serine i	Solanum_lycopersicu	28.30	0.00
TRINITY_GAX7517(	hypothet	Chlamydomonas_eusti	28.30	0.00
TRINITY_PNW7532(	hypothet	Chlamydomonas_reinh	28.30	0.00
TRINITY_XP_0029(	coatomer	Selaginella_moellen	28.30	0.00
TRINITY_XP_0238(	DSC E3 u	Quercus_suber	28.30	0.00
TRINITY_XP_0025(	predicte	Micromonas_commoda	28.30	0.00
TRINITY_PNW6984(	hypothet	Chlamydomonas_reinh	28.30	0.00
TRINITY_GAX8337(	hypothet	Chlamydomonas_eusti	28.30	0.00
TRINITY_XP_0238(	E3 ubiqu	Quercus_suber	28.20	0.00
TRINITY_XP_0279(	calcium-	Vigna_unguiculata	28.20	0.00
TRINITY_XP_0100(	PREDICTE	Eucalyptus_grandis	28.20	0.00
TRINITY_XP_0172(	PREDICTE	Daucus_carota	28.20	0.00
TRINITY_XP_0200(	putative	Ananas_comosus	28.20	0.00
TRINITY_OTG1563(	putative	Helianthus_annuus	28.20	0.00
TRINITY_XP_0030(	predicte	Micromonas_pusilla	28.20	0.00
TRINITY_XP_0072(	reticulo	Prunus_persica	28.20	0.00
TRINITY_PNW8311(	hypothet	Chlamydomonas_reinh	28.20	0.00
TRINITY_KXZ4620(	hypothet	Gonium_pectorale	28.20	0.00
TRINITY_PNW8427(	hypothet	Chlamydomonas_reinh	28.20	0.00
TRINITY_OMO9715(	hypothet	Corchorus_olitorius	28.20	0.00
TRINITY_XP_0263(	cation/H	Papaver_somniferum	28.20	0.00
TRINITY_KYP6027(	putative	Cajanus_cajan	28.20	0.00
TRINITY_KMZ7291(	vacuolar	Zostera_marina	28.20	0.00
TRINITY_GAX8411(	hypothet	Chlamydomonas_eusti	28.20	0.00
TRINITY_XP_0036(	eukaryot	Medicago_truncatula	28.20	0.00
TRINITY_KXZ4844(	hypothet	Gonium_pectorale	28.20	0.00
TRINITY_GAQ8215(	hypothet	Klebsormidium_niten	28.20	0.00
TRINITY_XP_0201(	uncharac	Aegilops_tauschii	28.20	0.00
TRINITY_XP_0025(	predicte	Micromonas_commoda	28.20	0.00
TRINITY_XP_0216(	mitochon	Hevea_brasiliensis	28.20	0.00
TRINITY_RLN4056(	hypothet	Panicum_miliaceum	28.20	0.00
TRINITY_GBF9832(	hypothet	Raphidocelis_subcap	28.20	0.00
TRINITY_GAQ8879(	RNA poly	Klebsormidium_niten	28.20	0.00
TRINITY_GAX8497(	hypothet	Chlamydomonas_eusti	28.20	0.00
TRINITY_XP_0062(	phosphat	Capsella_rubella	28.20	0.00
TRINITY_XP_0190(	PREDICTE	Camelina_sativa	28.20	0.00
TRINITY_RCV2951(	hypothet	Setaria_italica	28.20	0.00
TRINITY_XP_0216(	vacuolar	Hevea_brasiliensis	28.20	0.00
TRINITY_XP_0029(	hypothet	Volvox_carteri	28.20	0.00
TRINITY_PNH0942(	FGFR1 or	Tetrabaena_socialis	28.20	0.00
TRINITY_XP_0236(	ecotropi	Capsella_rubella	28.20	0.00
TRINITY_XP_0016(	oligoenc	Chlamydomonas_reinh	28.20	0.00
TRINITY_ABU5296(	hypothet	Dunaliella_viridis	28.20	0.00

TRINITY_GAX8204[illegible]	hypothet	Chlamydomonas_eusti	28.20	0.00
TRINITY_OAE2258[illegible]	hypothet	Marchantia_polymorp	28.20	0.00
TRINITY_GAX8535[illegible]	hypothet	Chlamydomonas_eusti	28.20	0.00
TRINITY_XP_0029[illegible]	hypothet	Volvox_carteri	28.20	0.00
TRINITY_GBG8916[illegible]	hypothet	Chara_braunii	28.20	0.00
TRINITY_XP_0029[illegible]	hypothet	Volvox_carteri	28.20	0.00
TRINITY_EPS6606[illegible]	hypothet	Genlisea_aurea	28.10	0.00
TRINITY_PRW5787[illegible]	zinc fir	Chlorella_sorokinia	28.10	0.00
TRINITY_XP_0237[illegible]	probable	Lactuca_sativa	28.10	0.00
TRINITY_PKA6513[illegible]	Putative	Apostasia_shenzheni	28.10	0.00
TRINITY_XP_0239[illegible]	26S prot	Quercus_suber	28.10	0.00
TRINITY_XP_0041[illegible]	PREDICTE	Cucumis_sativus	28.10	0.00
TRINITY_GAX8005[illegible]	hypothet	Chlamydomonas_eusti	28.10	0.00
TRINITY_RMZ5250[illegible]	hypothet	Auxenochlorella_pro	28.10	0.00
TRINITY_XP_0156[illegible]	acylamir	Oryza_sativa	28.10	0.00
TRINITY_XP_0075[illegible]	predicte	Bathycoccus_prasino	28.10	0.00
TRINITY_GAX8212[illegible]	hypothet	Chlamydomonas_eusti	28.10	0.00
TRINITY_PTQ3755[illegible]	hypothet	Marchantia_polymorp	28.10	0.00
TRINITY_XP_0241[illegible]	villin-[illegible]	Rosa_chinensis	28.10	0.00
TRINITY_OAE2631[illegible]	hypothet	Marchantia_polymorp	28.10	0.00
TRINITY_XP_0093[illegible]	PREDICTE	Musa_acuminata	28.10	0.00
TRINITY_XP_0064[illegible]	cation/[illegible]	Citrus_clementina	28.10	0.00
TRINITY_PNT1552[illegible]	hypothet	Populus_trichocarpa	28.10	0.00
TRINITY_EYU3495[illegible]	hypothet	Erythranthe_guttata	28.10	0.00
TRINITY_XP_0068[illegible]	protein	Amborella_trichopod	28.10	0.00
TRINITY_ONK6616[illegible]	uncharac	Asparagus_officinal	28.10	0.00
TRINITY_PRW2022[illegible]	AGC PKA	Chlorella_sorokinia	28.10	0.00
TRINITY_XP_0042[illegible]	PREDICTE	Fragaria_vesca	28.10	0.00
TRINITY_EEF2428[illegible]	Extracel	Ricinus_communis	28.10	0.00
TRINITY_XP_0237[illegible]	elicitor	Lactuca_sativa	28.10	0.00
TRINITY_XP_0238[illegible]	non-redu	Quercus_suber	28.10	0.00
TRINITY_PNW7299[illegible]	hypothet	Chlamydomonas_reinh	28.10	0.00
TRINITY_GAX7787[illegible]	hypothet	Chlamydomonas_eusti	28.10	0.00
TRINITY_XP_0058[illegible]	hypothet	Chlorella_variabili	28.10	0.00
TRINITY_XP_0017[illegible]	predicte	Chlamydomonas_reinh	28.10	0.00
TRINITY_XP_0063[illegible]	PREDICTE	Solanum_tuberosum	28.00	0.00
TRINITY_GAX7666[illegible]	hypothet	Chlamydomonas_eusti	28.00	0.00
TRINITY_XP_0075[illegible]	predicte	Bathycoccus_prasino	28.00	0.00
TRINITY_XP_0188[illegible]	PREDICTE	Juglans_regia	28.00	0.00
TRINITY_ASR7378[illegible]	acyl-CoA	Camellia_sinensis	28.00	0.00
TRINITY_XP_0239[illegible]	aorsin-[illegible]	Quercus_suber	28.00	0.00
TRINITY_GAQ7902[illegible]	Polypyri	Klebsormidium_niten	28.00	0.00
TRINITY_XP_0239[illegible]	rho1 gua	Quercus_suber	28.00	0.00
TRINITY_XP_0239[illegible]	TPR repe	Quercus_suber	28.00	0.00
TRINITY_XP_0093[illegible]	PREDICTE	Pyrus_x_bretschneid	28.00	0.00
TRINITY_XP_0022[illegible]	PREDICTE	Vitis_vinifera	28.00	0.00
TRINITY_GAQ8449[illegible]	cytochro	Klebsormidium_niten	28.00	0.00
TRINITY_OAY7703[illegible]	Beta-glu	Ananas_comosus	28.00	0.00
TRINITY_XP_0227[illegible]	equilibr	Durio_zibethinus	28.00	0.00
TRINITY_OAE2988[illegible]	hypothet	Marchantia_polymorp	28.00	0.00
TRINITY_RLN2459[illegible]	ras-rela	Panicum_miliaceum	28.00	0.00
TRINITY_BAJ9099[illegible]	predicte	Hordeum_vulgare	28.00	0.00
TRINITY_GAX8356[illegible]	hypothet	Chlamydomonas_eusti	28.00	0.00
TRINITY_XP_0238[illegible]	receptor	Quercus_suber	28.00	0.00
TRINITY_XP_0172[illegible]	PREDICTE	Daucus_carota	28.00	0.00
TRINITY_XP_0264[illegible]	serine/t	Papaver_somniferum	28.00	0.00
TRINITY_XP_0238[illegible]	uncharac	Quercus_suber	28.00	0.00
TRINITY_XP_0029[illegible]	basal bo	Volvox_carteri	28.00	0.00
TRINITY_KMZ6146[illegible]	putative	Zostera_marina	28.00	0.00
TRINITY_GAX8028[illegible]	hypothet	Chlamydomonas_eusti	28.00	0.00
TRINITY_PNW8684[illegible]	hypothet	Chlamydomonas_reinh	28.00	0.00
TRINITY_BAK0142[illegible]	predicte	Hordeum_vulgare	28.00	0.00
TRINITY_BAJ8758[illegible]	predicte	Hordeum_vulgare	28.00	0.00
TRINITY_GAX7601[illegible]	hypothet	Chlamydomonas_eusti	28.00	0.00
TRINITY_XP_0238[illegible]	tripepti	Quercus_suber	28.00	0.00
TRINITY_XP_0014[illegible]	predicte	Ostreococcus_sp._'l	28.00	0.00

TRINITY_XP_0042	synaptot	Solanum_lycopersicu	28.00	0.00
TRINITY_GAX7351	hypothet	Chlamydomonas_eusti	28.00	0.00
TRINITY_GAX7586	hypothet	Chlamydomonas_eusti	28.00	0.00
TRINITY_XP_0139	hypothet	Monoraphidium_negle	28.00	0.00
TRINITY_XP_0243	probable	Physcomitrella_pate	28.00	0.00
TRINITY_XP_0075	predicte	Bathycoccus_prasino	28.00	0.00
TRINITY_GAY3817	hypothet	Citrus_unshiu	28.00	0.00
TRINITY_XP_0244	B3 doma	Physcomitrella_pate	28.00	0.00
TRINITY_XP_0029	hypothet	Volvox_carteri	28.00	0.00
TRINITY_GAQ9115	hypothet	Klebsormidium_niten	28.00	0.00
TRINITY_GAQ8144	hypothet	Klebsormidium_niten	28.00	0.00
TRINITY_XP_0166	PREDICTE	Gossypium_hirsutum	28.00	0.00
TRINITY_XP_0075	predicte	Bathycoccus_prasino	27.90	0.00
TRINITY_XP_0102	PREDICTE	Nelumbo_nucifera	27.90	0.00
TRINITY_CAA7070	Kap alph	Arabidopsis_thalian	27.90	0.00
TRINITY_XP_0205	lysine-s	Phalaenopsis_equest	27.90	0.00
TRINITY_GBF9435	alpha be	Raphidocelis_subcap	27.90	0.00
TRINITY_PNW8058	hypothet	Chlamydomonas_reinh	27.90	0.00
TRINITY_XP_0100	PREDICTE	Eucalyptus_grandis	27.90	0.00
TRINITY_EPS7322	hypothet	Genlisea_aurea	27.90	0.00
TRINITY_KXZ4684	TTL3 pro	Gonium_pectorale	27.90	0.00
TRINITY_PNH0606	hypothet	Tetrabaena_socialis	27.90	0.00
TRINITY_XP_0238	fascicli	Quercus_suber	27.90	0.00
TRINITY_GAQ8261	RNA poly	Klebsormidium_niten	27.90	0.00
TRINITY_NP_0011	uncharac	Zea_mays	27.90	0.00
TRINITY_GAQ8957	Putative	Klebsormidium_niten	27.90	0.00
TRINITY_GBG7281	hypothet	Chara_braunii	27.90	0.00
TRINITY_NP_0011	uncharac	Zea_mays	27.90	0.00
TRINITY_PKA5855	hypothet	Apostasia_shenzheni	27.90	0.00
TRINITY_XP_0258	7-dehydr	Panicum_hallii	27.90	0.00
TRINITY_XP_0239	uncharac	Quercus_suber	27.90	0.00
TRINITY_GAX8411	hypothet	Chlamydomonas_eusti	27.90	0.00
TRINITY_XP_0105	PREDICTE	Tarenaya_hasslerian	27.90	0.00
TRINITY_XP_0030	predicte	Micromonas_pusilla	27.90	0.00
TRINITY_XP_0194	PREDICTE	Lupinus_angustifoli	27.90	0.00
TRINITY_PNW8841	hypothet	Chlamydomonas_reinh	27.90	0.00
TRINITY_BAJ9694	predicte	Hordeum_vulgare	27.90	0.00
TRINITY_CBI3339	unnamed	Vitis_vinifera	27.90	0.00
TRINITY_GAX7817	hypothet	Chlamydomonas_eusti	27.90	0.00
TRINITY_GAX8545	hypothet	Chlamydomonas_eusti	27.90	0.00
TRINITY_PSC7251	multidru	Micractinium_conduc	27.90	0.00
TRINITY_KXZ5105	hypothet	Gonium_pectorale	27.90	0.00
TRINITY_GBF9890	hypothet	Raphidocelis_subcap	27.90	0.00
TRINITY_XP_0025	predicte	Micromonas_commoda	27.90	0.00
TRINITY_GAQ8185	caroteno	Klebsormidium_niten	27.90	0.00
TRINITY_GBG6916	hypothet	Chara_braunii	27.90	0.00
TRINITY_XP_0029	hypothet	Volvox_carteri	27.90	0.00
TRINITY_XP_0158	uncharac	Ziziphus_jujuba	27.80	0.00
TRINITY_KXZ5512	hypothet	Gonium_pectorale	27.80	0.00
TRINITY_XP_0025	predicte	Micromonas_commoda	27.80	0.00
TRINITY_GAQ9003	U3 small	Klebsormidium_niten	27.80	0.00
TRINITY_GAQ8151	hypothet	Klebsormidium_niten	27.80	0.00
TRINITY_PRW3289	SDA1 [Ch	Chlorella_sorokinia	27.80	0.00
TRINITY_XP_0035	aspartyl	Brachypodium_distac	27.80	0.00
TRINITY_GBG7069	hypothet	Chara_braunii	27.80	0.00
TRINITY_XP_0228	Saposin-	Ostreococcus_tauri	27.80	0.00
TRINITY_GAQ7773	Leishman	Klebsormidium_niten	27.80	0.00
TRINITY_GAX7861	hypothet	Chlamydomonas_eusti	27.80	0.00
TRINITY_GAX7925	hypothet	Chlamydomonas_eusti	27.80	0.00
TRINITY_BAX0357	galactos	Hyoscyamus_albus	27.80	0.00
TRINITY_GAX7299	hypothet	Chlamydomonas_eusti	27.80	0.00
TRINITY_XP_0184	PREDICTE	Raphanus_sativus	27.80	0.00
TRINITY_RLN1785	aspartat	Panicum_miliaceum	27.80	0.00
TRINITY_XP_0217	uncharac	Chenopodium_quinoa	27.80	0.00
TRINITY_XP_0217	probable	Chenopodium_quinoa	27.80	0.00
TRINITY_XP_0228	Erv1/Alr	Ostreococcus_tauri	27.80	0.00

TRINITY_XP_0200(	asparti(	Ananas_comosus	27.80	0.00
TRINITY_OUS4872!	ribonuc]	Ostreococcus_tauri	27.80	0.00
TRINITY_OAE1911(	hypothet	Marchantia_polymorp	27.80	0.00
TRINITY_KGN4705	Ubiquit:	Cucumis_sativus	27.80	0.00
TRINITY_RDX9032	Protein	Mucuna_pruriens	27.80	0.00
TRINITY_XP_0056!	galactos	Coccomyxa_subellips	27.80	0.00
TRINITY_NP_0013(	uncharac	Zea_mays	27.80	0.00
TRINITY_XP_0056	alpha/be	Coccomyxa_subellips	27.80	0.00
TRINITY_EFJ1537(	hypothet	Selaginella_moellen	27.80	0.00
TRINITY_XP_0202	11-oxo-k	Cajanus_cajan	27.80	0.00
TRINITY_GAX7494	hypothet	Chlamydomonas_eusti	27.80	0.00
TRINITY_PNW7182(	hypothet	Chlamydomonas_reinh	27.80	0.00
TRINITY_XP_0138	hypothet	Monoraphidium_negle	27.80	0.00
TRINITY_PNX9255(	eukaryot	Trifolium_pratense	27.80	0.00
TRINITY_PNH1226!	Tiny mac	Tetrabaena_socialis	27.80	0.00
TRINITY_XP_0245	RING fi	Selaginella_moellen	27.70	0.00
TRINITY_XP_0202	putative	Cajanus_cajan	27.70	0.00
TRINITY_XP_0110	PREDICTE	Populus_euphratica	27.70	0.00
TRINITY_RID4471	hypothet	Brassica_rapa	27.70	0.00
TRINITY_XP_0238(	eIF-2-al	Quercus_suber	27.70	0.00
TRINITY_OAY6684	TOM1-lil	Ananas_comosus	27.70	0.00
TRINITY_XP_0029	extracel	Volvox_carteri	27.70	0.00
TRINITY_XP_0138	hypothet	Monoraphidium_negle	27.70	0.00
TRINITY_GAX7391(	hypothet	Chlamydomonas_eusti	27.70	0.00
TRINITY_GAX8061	hypothet	Chlamydomonas_eusti	27.70	0.00
TRINITY_KXZ4648(	hypothet	Gonium_pectorale	27.70	0.00
TRINITY_XP_0100	mitochor	Morus_notabilis	27.70	0.00
TRINITY_GAQ8773(	hypothet	Klebsormidium_niten	27.70	0.00
TRINITY_GAX7331(	hypothet	Chlamydomonas_eusti	27.70	0.00
TRINITY_XP_0188	PREDICTE	Juglans_regia	27.70	0.00
TRINITY_KXZ5566	hypothet	Gonium_pectorale	27.70	0.00
TRINITY_EPS6414	hypothet	Genlisea_aurea	27.70	0.00
TRINITY_KXZ5031	hypothet	Gonium_pectorale	27.70	0.00
TRINITY_XP_0049(	RPA-inte	Setaria_italica	27.70	0.00
TRINITY_XP_0243	histone-	Physcomitrella_pate	27.70	0.00
TRINITY_XP_0243(	cation/(	Physcomitrella_pate	27.70	0.00
TRINITY_XP_0245	uncharac	Selaginella_moellen	27.70	0.00
TRINITY_AAM3365	mastigor	Chlamydomonas_reinh	27.70	0.00
TRINITY_GAX7271(	hypothet	Chlamydomonas_eusti	27.70	0.00
TRINITY_PNW7076!	hypothet	Chlamydomonas_reinh	27.70	0.00
TRINITY_XP_0022(	PREDICTE	Vitis_vinifera	27.60	0.00
TRINITY_XP_0084	PREDICTE	Cucumis_melo	27.60	0.00
TRINITY_GAQ8525	Mitotic	Klebsormidium_niten	27.60	0.00
TRINITY_BAJ9878	predicte	Hordeum_vulgare	27.60	0.00
TRINITY_KVH9317	CHASE-li	Cynara_cardunculus	27.60	0.00
TRINITY_XP_0236	crossove	Capsella_rubella	27.60	0.00
TRINITY_XP_0105	PREDICTE	Tarenaya_hasslerian	27.60	0.00
TRINITY_PNH0774	Calcium-	Tetrabaena_socialis	27.60	0.00
TRINITY_GAQ8477	Sensory	Klebsormidium_niten	27.60	0.00
TRINITY_XP_0243(	histidir	Physcomitrella_pate	27.60	0.00
TRINITY_PTQ3267(	hypothet	Marchantia_polymorp	27.60	0.00
TRINITY_GAQ8319	Cysteine	Klebsormidium_niten	27.60	0.00
TRINITY_GAX7928!	hypothet	Chlamydomonas_eusti	27.60	0.00
TRINITY_XP_0238	tRNA (u	Quercus_suber	27.60	0.00
TRINITY_XP_0243	syntaxir	Physcomitrella_pate	27.60	0.00
TRINITY_XP_0238(	tryprost	Quercus_suber	27.60	0.00
TRINITY_XP_0202!	cullin-a	Asparagus_officinal	27.60	0.00
TRINITY_GAQ7773	Leishmar	Klebsormidium_niten	27.60	0.00
TRINITY_XP_0245	protein	Selaginella_moellen	27.60	0.00
TRINITY_KXZ4772	hypothet	Gonium_pectorale	27.60	0.00
TRINITY_PIN1964(	Exocyst	Handroanthus_impeti	27.60	0.00
TRINITY_GAX8255(	hypothet	Chlamydomonas_eusti	27.60	0.00
TRINITY_VDD0982	unnamed	Brassica_oleracea	27.60	0.00
TRINITY_XP_0025(	predicte	Micromonas_commoda	27.60	0.00
TRINITY_KXZ5180!	hypothet	Gonium_pectorale	27.60	0.00
TRINITY_XP_0097(	PREDICTE	Nicotiana_sylvestri	27.60	0.00

TRINITY_XP_0104	PREDICTE	Camelina_sativa	27.60	0.00
TRINITY_XP_0239	aorsin-	Quercus_suber	27.60	0.00
TRINITY_XP_0191	PREDICTE	Ipomoea_nil	27.60	0.00
TRINITY_PRW5865	myb fam	Chlorella_sorokinia	27.60	0.00
TRINITY_GAQ7798	proteas	Klebsormidium_niten	27.60	0.00
TRINITY_XP_0238	uncharac	Quercus_suber	27.60	0.00
TRINITY_GAX8635	hypothet	Chlamydomonas_eusti	27.60	0.00
TRINITY_XP_0025	high-mol	Micromonas_commoda	27.60	0.00
TRINITY_XP_0243	uncharac	Physcomitrella_pate	27.60	0.00
TRINITY_XP_0125	calcine	Cicer_arietinum	27.60	0.00
TRINITY_GAX8282	hypothet	Chlamydomonas_eusti	27.60	0.00
TRINITY_BAJ9694	predicte	Hordeum_vulgare	27.60	0.00
TRINITY_EFJ0648	hypothet	Selaginella_moellen	27.60	0.00
TRINITY_XP_0202	CDK5RAP	Cajanus_cajan	27.60	0.00
TRINITY_XP_0110	PREDICTE	Populus_euphratica	27.50	0.00
TRINITY_GAQ8456	hypothet	Klebsormidium_niten	27.50	0.00
TRINITY_XP_0105	PREDICTE	Tarenaya_hasslerian	27.50	0.00
TRINITY_KZM9872	hypothet	Daucus_carota	27.50	0.00
TRINITY_RMZ5340	hypothet	Auxenochlorella_prc	27.50	0.00
TRINITY_XP_0029	hypothet	Volvox_carteri	27.50	0.00
TRINITY_XP_0134	quinone	Medicago_truncatula	27.50	0.00
TRINITY_XP_0029	hypothet	Volvox_carteri	27.50	0.00
TRINITY_PSC7362	FAM98A	Micractinium_conduc	27.50	0.00
TRINITY_PIA5251	hypothet	Aquilegia_coerulea	27.50	0.00
TRINITY_XP_0017	carbonic	Chlamydomonas_reinh	27.50	0.00
TRINITY_OAE2722	hypothet	Marchantia_polymorp	27.50	0.00
TRINITY_BAS0126	syntaxir	Marchantia_polymorp	27.50	0.00
TRINITY_GAX8230	hypothet	Chlamydomonas_eusti	27.50	0.00
TRINITY_XP_0049	tropinor	Setaria_italica	27.50	0.00
TRINITY_XP_0279	uncharac	Vigna_unguiculata	27.50	0.00
TRINITY_XP_0016	predicte	Chlamydomonas_reinh	27.50	0.00
TRINITY_XP_0093	PREDICTE	Musa_acuminata	27.50	0.00
TRINITY_GAX8058	hypothet	Chlamydomonas_eusti	27.50	0.00
TRINITY_XP_0105	PREDICTE	Camelina_sativa	27.50	0.00
TRINITY_NP_0011	uncharac	Zea_mays	27.50	0.00
TRINITY_GAQ8449	cytochr	Klebsormidium_niten	27.50	0.00
TRINITY_XP_0239	vacuola	Quercus_suber	27.50	0.00
TRINITY_XP_0243	26S prot	Physcomitrella_pate	27.50	0.00
TRINITY_XP_0102	PREDICTE	Nelumbo_nucifera	27.50	0.00
TRINITY_GAX8219	hypothet	Chlamydomonas_eusti	27.50	0.00
TRINITY_XP_0029	hypothet	Volvox_carteri	27.50	0.00
TRINITY_XP_0025	predicte	Micromonas_commoda	27.50	0.00
TRINITY_XP_0022	PREDICTE	Vitis_vinifera	27.50	0.00
TRINITY_OAE2538	hypothet	Marchantia_polymorp	27.40	0.00
TRINITY_XP_0094	PREDICTE	Musa_acuminata	27.40	0.00
TRINITY_XP_0094	PREDICTE	Musa_acuminata	27.40	0.00
TRINITY_GAQ8300	WD40 re	Klebsormidium_niten	27.40	0.00
TRINITY_GBG7023	hypothet	Chara_braunii	27.40	0.00
TRINITY_XP_0116	PREDICTE	Cucumis_sativus	27.40	0.00
TRINITY_XP_0245	histone-	Selaginella_moellen	27.40	0.00
TRINITY_XP_0029	hypothet	Volvox_carteri	27.40	0.00
TRINITY_RDX8986	Random	Mucuna_pruriens	27.40	0.00
TRINITY_GAX7760	hypothet	Chlamydomonas_eusti	27.40	0.00
TRINITY_GAQ8568	putative	Klebsormidium_niten	27.40	0.00
TRINITY_XP_0025	actin [	Ricinus_communis	27.40	0.00
TRINITY_GAX7432	hypothet	Chlamydomonas_eusti	27.40	0.00
TRINITY_PTQ3784	hypothet	Marchantia_polymorp	27.40	0.00
TRINITY_XP_0205	uncharac	Phalaenopsis_equest	27.40	0.00
TRINITY_EEC7364	hypothet	Oryza_sativa	27.40	0.00
TRINITY_XP_0128	PREDICTE	Erythranthe_guttata	27.40	0.00
TRINITY_PON8281	Transmem	Trema_orientale	27.40	0.00
TRINITY_XP_0279	seed lin	Vigna_unguiculata	27.40	0.00
TRINITY_GAX7629	hypothet	Chlamydomonas_eusti	27.40	0.00
TRINITY_XP_0245	platelet	Selaginella_moellen	27.40	0.00
TRINITY_XP_0171	PREDICTE	Malus_domestica	27.40	0.00
TRINITY_KVH9493	Ankyrin	Cynara_cardunculus	27.40	0.00

TRINITY_XP_0159:	PP2A reç	Arachis_duranensis	27.40	0.00
TRINITY_XP_0238(	tyrosina	Quercus_suber	27.40	0.00
TRINITY_XP_0190!	PREDICTI	Tarenaya_hasslerian	27.40	0.00
TRINITY_XP_0209'	uncharac	Arachis_ipaensis	27.40	0.00
TRINITY_XP_0139(	hypothet	Monoraphidium_negle	27.40	0.00
TRINITY_XP_0058	hypothet	Chlorella_variabili	27.40	0.00
TRINITY_XP_0045(	uncharac	Cicer_arietinum	27.40	0.00
TRINITY_XP_0243(	probable	Physcomitrella_pate	27.40	0.00
TRINITY_XP_0158'	interfe:	Ziziphus_jujuba	27.40	0.00
TRINITY_XP_0202:	poly(A)-	Cajanus_cajan	27.40	0.00

TRINITY_GAX8016(	hypothet	Chlamydomonas_eusti	27.40	0.00
TRINITY_KXZ4886	hypothet	Gonium_pectorale	27.40	0.00
TRINITY_XP_0205	probable	Phalaenopsis_equest	27.40	0.00
TRINITY_RWR9529	F-actin-	Cinnamomum_micranth	27.40	0.00
TRINITY_GAX8016	hypothet	Chlamydomonas_eusti	27.40	0.00
TRINITY_XP_0029	hypothet	Volvox_carteri	27.40	0.00
TRINITY_GAX8028(	hypothet	Chlamydomonas_eusti	27.40	0.00
TRINITY_PNW8601(	hypothet	Chlamydomonas_reinh	27.40	0.00
TRINITY_GBG9340	hypothet	Chara_braunii	27.40	0.00
TRINITY_XP_0239	cytopla	Quercus_suber	27.40	0.00
TRINITY_XP_0243	alpha-x	Physcomitrella_pate	27.30	0.00
TRINITY_XP_0239(	vacuola	Quercus_suber	27.30	0.00
TRINITY_XP_0249	unchara	Cynara_cardunculus	27.30	0.00
TRINITY_XP_0249	probable	Cynara_cardunculus	27.30	0.00
TRINITY_XP_0042	Fanconi	Solanum_lycopersicu	27.30	0.00
TRINITY_XP_0264(	coiled-c	Papaver_somniferum	27.30	0.00
TRINITY_KMS9355	hypothet	Beta_vulgaris	27.30	0.00
TRINITY_XP_0238	mitochon	Quercus_suber	27.30	0.00
TRINITY_XP_0030	Translat	Ostreococcus_tauri	27.30	0.00
TRINITY_BAJ8945	predicte	Hordeum_vulgare	27.30	0.00
TRINITY_GAQ8860	hypothet	Klebsormidium_niten	27.30	0.00
TRINITY_XP_0167	PREDICTE	Gossypium_hirsutum	27.30	0.00
TRINITY_XP_0184	PREDICTE	Raphanus_sativus	27.30	0.00
TRINITY_RWR7859	putative	Cinnamomum_micranth	27.30	0.00
TRINITY_EFJ2488	hypothet	Selaginella_moellen	27.30	0.00
TRINITY_RYQ8223	hypothet	Arachis_hypogaea	27.30	0.00
TRINITY_XP_0150	hydroxy	Solanum_pennellii	27.30	0.00
TRINITY_XP_0056	SM/Sec1-	Coccomyxa_subellips	27.30	0.00
TRINITY_BAT9835	hypothet	Vigna_angularis	27.30	0.00
TRINITY_PSC7145	spindle	Micractinium_conduc	27.30	0.00
TRINITY_XP_0025(	predicte	Micromonas_commoda	27.30	0.00
TRINITY_PTQ3267	hypothet	Marchantia_polymorp	27.30	0.00
TRINITY_XP_0184	PREDICTE	Raphanus_sativus	27.30	0.00
TRINITY_GBG6920	hypothet	Chara_braunii	27.30	0.00
TRINITY_XP_0270	extrace	Coffea_arabica	27.30	0.00
TRINITY_XP_0022	PREDICTE	Vitis_vinifera	27.30	0.00
TRINITY_PIN0102(	Karyophe	Handroanthus_impeti	27.30	0.00
TRINITY_XP_0239	probable	Quercus_suber	27.30	0.00
TRINITY_GAX8132	hypothet	Chlamydomonas_eusti	27.20	0.00
TRINITY_XP_0106	PREDICTE	Beta_vulgaris	27.20	0.00
TRINITY_XP_0128	PREDICTE	Erythranthe_guttata	27.20	0.00
TRINITY_GAQ8083	deoxyrib	Klebsormidium_niten	27.20	0.00
TRINITY_XP_0075	predicte	Bathycoccus_prasino	27.20	0.00
TRINITY_XP_0014	IISP fa	Ostreococcus_sp._'l	27.20	0.00
TRINITY_XP_0270	probable	Coffea_arabica	27.20	0.00
TRINITY_PNR4686	hypothet	Physcomitrella_pate	27.20	0.00
TRINITY_PTQ5024	hypothet	Marchantia_polymorp	27.20	0.00
TRINITY_XP_0025(	Ca2+:Cat	Micromonas_commoda	27.20	0.00
TRINITY_XP_0229	asparti	Cucurbita_maxima	27.20	0.00
TRINITY_XP_0172	PREDICTE	Daucus_carota	27.20	0.00
TRINITY_OAY5709	hypothet	Manihot_esculenta	27.20	0.00
TRINITY_GAX8411	hypothet	Chlamydomonas_eusti	27.20	0.00
TRINITY_XP_0025(	flagella	Micromonas_commoda	27.20	0.00
TRINITY_XP_0229	amino a	Cucurbita_maxima	27.20	0.00
TRINITY_XP_0030	choline	Micromonas_pusilla	27.20	0.00
TRINITY_XP_0245	unchara	Selaginella_moellen	27.20	0.00
TRINITY_XP_0245	probable	Selaginella_moellen	27.20	0.00
TRINITY_GAX8545	hypothet	Chlamydomonas_eusti	27.20	0.00
TRINITY_XP_0226	unchara	Vigna_radiata	27.20	0.00
TRINITY_GAX8312	hypothet	Chlamydomonas_eusti	27.20	0.00
TRINITY_RWR7623	THO com	Cinnamomum_micranth	27.20	0.00
TRINITY_GAX8635	hypothet	Chlamydomonas_eusti	27.20	0.00
TRINITY_XP_0029	hypothet	Volvox_carteri	27.20	0.00
TRINITY_KXZ4986	hypothet	Gonium_pectorale	27.20	0.00
TRINITY_PNH0069	hypothet	Tetrabaena_socialis	27.20	0.00
TRINITY_KMZ6181	hypothet	Zostera_marina	27.20	0.00

TRINITY_XP_0194	PREDICTI	Lupinus_angustifoli	27.20	0.00
TRINITY_GAX7344	hypothet	Chlamydomonas_eusti	27.20	0.00
TRINITY_XP_0194	PREDICTI	Lupinus_angustifoli	27.20	0.00
TRINITY_GAQ9061	pre-mRNA	Klebsormidium_niten	27.10	0.00
TRINITY_XP_0029	hypothet	Volvox_carteri	27.10	0.00
TRINITY_GBG8987	hypothet	Chara_braunii	27.10	0.00
TRINITY_GBG7671	hypothet	Chara_braunii	27.10	0.00
TRINITY_XP_0150	elongat	Solanum_pennellii	27.10	0.00
TRINITY_GAQ9128	hypothet	Klebsormidium_niten	27.10	0.00
TRINITY_PSC7272	2-oxogl	Micractinium_conduc	27.10	0.00
TRINITY_RAL4350	hypothet	Cuscuta_australis	27.10	0.00
TRINITY_XP_0271	putative	Coffea_arabica	27.10	0.00
TRINITY_EFJ3404	hypothet	Selaginella_moellen	27.10	0.00
TRINITY_PNH0498	Shaggy-	Tetrabaena_socialis	27.10	0.00
TRINITY_XP_0217	phospho	Chenopodium_quinoa	27.10	0.00
TRINITY_XP_0023	PP2A re	Populus_trichocarpa	27.10	0.00
TRINITY_GAQ8804	Zinc fi	Klebsormidium_niten	27.10	0.00
TRINITY_XP_0243	uncharac	Physcomitrella_pate	27.10	0.00
TRINITY_EEF2629	alpha-x	Ricinus_communis	27.10	0.00
TRINITY_BAN5814	putative	Hyoscyamus_albus	27.10	0.00
TRINITY_XP_0243	synapto	Physcomitrella_pate	27.10	0.00
TRINITY_XP_0204	uncharac	Prunus_persica	27.10	0.00
TRINITY_PNR3731	hypothet	Physcomitrella_pate	27.10	0.00
TRINITY_XP_0029	G-prote	Selaginella_moellen	27.10	0.00
TRINITY_PTQ4773	hypothet	Marchantia_polymorp	27.10	0.00
TRINITY_GAQ8753	hypothet	Klebsormidium_niten	27.10	0.00
TRINITY_XP_0218	lysopho	Spinacia_oleracea	27.10	0.00
TRINITY_XP_0113	Syntaxi	Auxenochlorella_pro	27.10	0.00
TRINITY_XP_0063	PREDICTI	Solanum_tuberosum	27.10	0.00
TRINITY_XP_0229	lipase-	Cucurbita_maxima	27.10	0.00
TRINITY_OAE2115	hypothet	Marchantia_polymorp	27.10	0.00
TRINITY_GAX8058	hypothet	Chlamydomonas_eusti	27.10	0.00
TRINITY_AHG2954	ats1, p	Pyramimonas_parkeae	27.10	0.00
TRINITY_EAY7938	hypothet	Oryza_sativa	27.00	0.00
TRINITY_PRW5107	Platelet	Chlorella_sorokinia	27.00	0.00
TRINITY_XP_0035	probable	Glycine_max	27.00	0.00
TRINITY_PTQ4567	hypothet	Marchantia_polymorp	27.00	0.00
TRINITY_GAX7562	hypothet	Chlamydomonas_eusti	27.00	0.00
TRINITY_GAX7776	hypothet	Chlamydomonas_eusti	27.00	0.00
TRINITY_XP_0238	protein	Quercus_suber	27.00	0.00
TRINITY_PTQ4669	hypothet	Marchantia_polymorp	27.00	0.00
TRINITY_KFK2909	hypothet	Arabis_alpina	27.00	0.00
TRINITY_PNW7337	hypothet	Chlamydomonas_reinh	27.00	0.00
TRINITY_XP_0239	rho-type	Quercus_suber	27.00	0.00
TRINITY_EXB8225	Casein	Morus_notabilis	27.00	0.00
TRINITY_GAU2303	hypothet	Trifolium_subterran	27.00	0.00
TRINITY_OAE3436	hypothet	Marchantia_polymorp	27.00	0.00
TRINITY_GAQ8842	mRNA ca	Klebsormidium_niten	27.00	0.00
TRINITY_XP_0205	WD repe	Phalaenopsis_equest	27.00	0.00
TRINITY_XP_0029	hypothet	Volvox_carteri	27.00	0.00
TRINITY_PNW8588	hypothet	Chlamydomonas_reinh	27.00	0.00
TRINITY_GAX8335	hypothet	Chlamydomonas_eusti	27.00	0.00
TRINITY_PNY0554	putative	Trifolium_pratense	27.00	0.00
TRINITY_XP_0243	amino a	Physcomitrella_pate	27.00	0.00
TRINITY_XP_0273	asparti	Abrus_precatorius	27.00	0.00
TRINITY_XP_0202	Down sy	Asparagus_officinal	27.00	0.00
TRINITY_XP_0245	poly [A	Selaginella_moellen	27.00	0.00
TRINITY_PRW4506	HD-doma	Chlorella_sorokinia	26.90	0.00
TRINITY_XP_0029	hypothet	Volvox_carteri	26.90	0.00
TRINITY_KXZ4261	hypothet	Gonium_pectorale	26.90	0.00
TRINITY_XP_0138	sulfide	Monoraphidium_negle	26.90	0.00
TRINITY_XP_0029	acyl-Co	Selaginella_moellen	26.90	0.00
TRINITY_XP_0239	L-pipec	Quercus_suber	26.90	0.00
TRINITY_XP_0056	CBS-doma	Coccomyxa_subellips	26.90	0.00
TRINITY_XP_0238	fascicl	Quercus_suber	26.90	0.00
TRINITY_OAE2990	hypothet	Marchantia_polymorp	26.90	0.00

TRINITY_RVX2142	Ultravi	Vitis_vinifera	26.90	0.00
TRINITY_PSC7399	heme ox	Micractinium_conduc	26.90	0.00
TRINITY_XP_0202	putative	Asparagus_officinal	26.90	0.00
TRINITY_GAX7342	hypothet	Chlamydomonas_eusti	26.90	0.00
TRINITY_XP_0184	PREDICTE	Raphanus_sativus	26.90	0.00
TRINITY_PTQ4862	hypothet	Marchantia_polymorp	26.90	0.00
TRINITY_XP_0238	protein	Quercus_suber	26.90	0.00
TRINITY_XP_0229	centrome	Cucurbita_moschata	26.90	0.00
TRINITY_GAQ8376	cytochr	Klebsormidium_niten	26.90	0.00
TRINITY_GAX8447	hypothet	Chlamydomonas_eusti	26.90	0.00
TRINITY_XP_0216	vacuola	Hevea_brasiliensis	26.90	0.00
TRINITY_KMZ5971	hypothet	Zostera_marina	26.90	0.00
TRINITY_XP_0075	predicte	Bathycoccus_prasinc	26.90	0.00
TRINITY_XP_0110	protein	Sesamum_indicum	26.90	0.00
TRINITY_GAQ8144	hypothet	Klebsormidium_niten	26.90	0.00
TRINITY_PNW8041	hypothet	Chlamydomonas_reinh	26.90	0.00
TRINITY_PRW5831	deoxyri	Chlorella_sorokinia	26.90	0.00
TRINITY_XP_0113	hypothet	Auxenochlorella_prc	26.90	0.00
TRINITY_XP_0030	predicte	Micromonas_pusilla	26.90	0.00
TRINITY_XP_0243	importi	Physcomitrella_pate	26.90	0.00
TRINITY_GAX7649	hypothet	Chlamydomonas_eusti	26.90	0.00
TRINITY_GAX7273	hypothet	Chlamydomonas_eusti	26.90	0.00
TRINITY_XP_0228	Patatin/	Ostreococcus_tauri	26.90	0.00
TRINITY_GAQ8819	alkalin	Klebsormidium_niten	26.90	0.00
TRINITY_GAX7618	hypothet	Chlamydomonas_eusti	26.80	0.00
TRINITY_XP_0238	uncharac	Quercus_suber	26.80	0.00
TRINITY_PSR9804	Phosphat	Actinidia_chinensis	26.80	0.00
TRINITY_RAL3692	hypothet	Cuscuta_australis	26.80	0.00
TRINITY_XP_0072	ARF guar	Prunus_persica	26.80	0.00
TRINITY_KXZ4534	hypothet	Gonium_pectorale	26.80	0.00
TRINITY_XP_0243	DNA repa	Physcomitrella_pate	26.80	0.00
TRINITY_XP_0228	Putative	Ostreococcus_tauri	26.80	0.00
TRINITY_XP_0205	sucrose	Phalaenopsis_equest	26.80	0.00
TRINITY_GBF9172	shaggy-r	Raphidocelis_subcap	26.80	0.00
TRINITY_XP_0205	uncharac	Amborella_trichopod	26.80	0.00
TRINITY_XP_0105	PREDICTE	Tarenaya_hasslerian	26.80	0.00
TRINITY_BAJ9585	predicte	Hordeum_vulgare	26.80	0.00
TRINITY_AAC4937	GmCK3p,	Glycine_max	26.80	0.00
TRINITY_XP_0104	PREDICTE	Camelina_sativa	26.80	0.00
TRINITY_RWR9162	transket	Cinnamomum_micranth	26.80	0.00
TRINITY_XP_0016	predicte	Chlamydomonas_reinh	26.80	0.00
TRINITY_GAQ7888	hypothet	Klebsormidium_niten	26.80	0.00
TRINITY_EEE5433	hypothet	Oryza_sativa	26.80	0.00
TRINITY_XP_0056	CBS-doma	Coccomyxa_subellips	26.80	0.00
TRINITY_GAQ8918	hypothet	Klebsormidium_niten	26.80	0.00
TRINITY_GAX7896	hypothet	Chlamydomonas_eusti	26.80	0.00
TRINITY_XP_0056	hypothet	Coccomyxa_subellips	26.80	0.00
TRINITY_GBF9327	hypothet	Raphidocelis_subcap	26.80	0.00
TRINITY_GAX7875	hypothet	Chlamydomonas_eusti	26.80	0.00
TRINITY_KXZ5277	hypothet	Gonium_pectorale	26.80	0.00
TRINITY_XP_0263	ubiquit	Papaver_somniferum	26.80	0.00
TRINITY_XP_0239	vacuola	Quercus_suber	26.80	0.00
TRINITY_KGN4656	hypothet	Cucumis_sativus	26.80	0.00
TRINITY_XP_0072	protein	Prunus_persica	26.80	0.00
TRINITY_GAX8046	hypothet	Chlamydomonas_eusti	26.80	0.00
TRINITY_EEC7909	hypothet	Oryza_sativa	26.80	0.00
TRINITY_PWA9113	regulat	Artemisia_annua	26.80	0.00
TRINITY_GAX7338	hypothet	Chlamydomonas_eusti	26.80	0.00
TRINITY_XP_0239	reducin	Quercus_suber	26.80	0.00
TRINITY_PRW5700	vacuola	Chlorella_sorokinia	26.70	0.00
TRINITY_XP_0197	PREDICTE	Elaeis_guineensis	26.70	0.00
TRINITY_XP_0016	subunit	Chlamydomonas_reinh	26.70	0.00
TRINITY_XP_0093	PREDICTE	Pyrus_x_bretschneid	26.70	0.00
TRINITY_XP_0245	protein	Selaginella_moellen	26.70	0.00
TRINITY_XP_0016	flagell	Chlamydomonas_reinh	26.70	0.00
TRINITY_XP_0202	probable	Cajanus_cajan	26.70	0.00

TRINITY_XP_0245	unchara	Selaginella_moellen	26.70	0.00
TRINITY_XP_0172	PREDICTI	Daucus_carota	26.70	0.00
TRINITY_XP_0249	elicito	Cynara_cardunculus	26.70	0.00
TRINITY_ESQ4334	hypothet	Eutrema_salsugineum	26.70	0.00
TRINITY_XP_0194	PREDICTI	Lupinus_angustifoli	26.70	0.00
TRINITY_GAQ8528	Inositol	Klebsormidium_niten	26.70	0.00
TRINITY_XP_0243	ADP-rib	Physcomitrella_pate	26.70	0.00
TRINITY_BAS0126	syntaxi	Marchantia_polymorp	26.70	0.00
TRINITY_XP_0029	hypothet	Volvox_carteri	26.70	0.00
TRINITY_XP_0212	topless-	Herrania_umbratica	26.70	0.00
TRINITY_GBF9379	hypothet	Raphidocelis_subcap	26.70	0.00
TRINITY_PNW7304	hypothet	Chlamydomonas_reinh	26.70	0.00
TRINITY_RZC0356	Sulfite	Glycine_soja	26.70	0.00
TRINITY_XP_0206	immune-a	Dendrobium_catenatu	26.70	0.00
TRINITY_GAX8548	hypothet	Chlamydomonas_eusti	26.70	0.00
TRINITY_KZV2810	calmodul	Dorcoceras_hygromet	26.70	0.00
TRINITY_PAN3587	hypothet	Panicum_hallii	26.60	0.00
TRINITY_XP_0270	E3 ubiq	Coffea_arabica	26.60	0.00
TRINITY_XP_0228	Armadil	Ostreococcus_tauri	26.60	0.00
TRINITY_OTG3118	putative	Helianthus_annuus	26.60	0.00
TRINITY_GAQ8376	cytochr	Klebsormidium_niten	26.60	0.00
TRINITY_KJB1944	hypothet	Gossypium_raimondii	26.60	0.00
TRINITY_XP_0056	hypothet	Coccomyxa_subellips	26.60	0.00
TRINITY_GAX7313	hypothet	Chlamydomonas_eusti	26.60	0.00
TRINITY_XP_0113	Eukaryot	Auxenochlorella_pro	26.60	0.00
TRINITY_XP_0243	SNARE-i	Physcomitrella_pate	26.60	0.00
TRINITY_XP_0243	amino a	Physcomitrella_pate	26.60	0.00
TRINITY_XP_0029	unchara	Selaginella_moellen	26.60	0.00
TRINITY_BAJ9694	predicte	Hordeum_vulgare	26.60	0.00
TRINITY_XP_0184	PREDICTI	Raphanus_sativus	26.60	0.00
TRINITY_XP_0243	cyclin-A	Physcomitrella_pate	26.60	0.00
TRINITY_XP_0217	syntaxi	Chenopodium_quinoa	26.60	0.00
TRINITY_XP_0109	PREDICTI	Elaeis_guineensis	26.60	0.00
TRINITY_GAQ8912	putative	Klebsormidium_niten	26.60	0.00
TRINITY_KYP3878	Retrovi	Cajanus_cajan	26.60	0.00
TRINITY_XP_0102	PREDICTI	Nelumbo_nucifera	26.60	0.00
TRINITY_GAX7891	hypothet	Chlamydomonas_eusti	26.50	0.00
TRINITY_XP_0024	predicte	Micromonas_commoda	26.50	0.00
TRINITY_XP_0058	hypothet	Chlorella_variabili	26.50	0.00
TRINITY_XP_0030	flagella	Micromonas_pusilla	26.50	0.00
TRINITY_XP_0025	zinc pe	Micromonas_commoda	26.50	0.00
TRINITY_XP_0134	exporti	Medicago_truncatula	26.50	0.00
TRINITY_GAQ8682	Leishma	Klebsormidium_niten	26.50	0.00
TRINITY_XP_0239	unchara	Quercus_suber	26.50	0.00
TRINITY_PIA4107	hypothet	Aquilegia_coerulea	26.50	0.00
TRINITY_PTQ3064	hypothet	Marchantia_polymorp	26.50	0.00
TRINITY_XP_0239	peroxis	Quercus_suber	26.50	0.00
TRINITY_GAQ8006	interfe	Klebsormidium_niten	26.50	0.00
TRINITY_XP_0030	predicte	Micromonas_pusilla	26.50	0.00
TRINITY_XP_0172	PREDICTI	Daucus_carota	26.50	0.00
TRINITY_PNH0023	hypothet	Tetrabaena_socialis	26.50	0.00
TRINITY_GAX7587	hypothet	Chlamydomonas_eusti	26.50	0.00
TRINITY_XP_0150	alpha-d	Solanum_pennellii	26.50	0.00
TRINITY_XP_0219	piezo-t	Carica_papaya	26.50	0.00
TRINITY_XP_0161	DNA top	Arachis_ipaensis	26.50	0.00
TRINITY_XP_0239	1-phosp	Quercus_suber	26.50	0.00
TRINITY_XP_0166	PREDICTI	Gossypium_hirsutum	26.50	0.00
TRINITY_XP_0025	predicte	Micromonas_commoda	26.50	0.00
TRINITY_GAQ8632	major f	Klebsormidium_niten	26.40	0.00
TRINITY_OAE2451	hypothet	Marchantia_polymorp	26.40	0.00
TRINITY_XP_0216	endopla	Hevea_brasiliensis	26.40	0.00
TRINITY_XP_0238	beta-1,	Quercus_suber	26.40	0.00
TRINITY_PSC7661	hypothet	Micractinium_conduc	26.40	0.00
TRINITY_XP_0188	PREDICTI	Juglans_regia	26.40	0.00
TRINITY_XP_0202	calcium-	Asparagus_officinal	26.40	0.00
TRINITY_GAX8598	hypothet	Chlamydomonas_eusti	26.40	0.00

TRINITY_PIA4425(	hypothet	Aquilegia_coerulea	26.40	0.00
TRINITY_GBF8792!	importir	Raphidocelis_subcap	26.40	0.00
TRINITY_XP_0243(	nicotiar	Physcomitrella_pate	26.40	0.00
TRINITY_GBG6236(	hypothet	Chara_braunii	26.40	0.00
TRINITY_PTQ2834	hypothet	Marchantia_polymorp	26.40	0.00
TRINITY_XP_0258(	importir	Panicum_hallii	26.40	0.00
TRINITY_GAX7335(	hypothet	Chlamydomonas_eusti	26.40	0.00
TRINITY_XP_0104	PREDICTI	Camelina_sativa	26.40	0.00
TRINITY_XP_0246	cyclin-T	Medicago_truncatula	26.40	0.00
TRINITY_PNW8453(	hypothet	Chlamydomonas_reinh	26.40	0.00
TRINITY_EYU2626	hypothet	Erythranthe_guttata	26.40	0.00
TRINITY_XP_0164	PREDICTI	Nicotiana_tabacum	26.40	0.00
TRINITY_XP_0066	PREDICTI	Oryza_brachyantha	26.40	0.00
TRINITY_ONI3318	hypothet	Prunus_persica	26.40	0.00
TRINITY_RYR2447(	hypothet	Arachis_hypogaea	26.40	0.00
TRINITY_PNW7259(	hypothet	Chlamydomonas_reinh	26.40	0.00
TRINITY_XP_0017(	E1-E2 AT	Chlamydomonas_reinh	26.40	0.00
TRINITY_XP_0058	hypothet	Chlorella_variabili	26.40	0.00
TRINITY_PNW7311	hypothet	Chlamydomonas_reinh	26.40	0.00
TRINITY_XP_0147	transmem	Brachypodium_distac	26.30	0.00
TRINITY_XP_0025(	predicte	Micromonas_commoda	26.30	0.00
TRINITY_KXZ4917	hypothet	Gonium_pectorale	26.30	0.00
TRINITY_XP_0243(	SET doma	Physcomitrella_pate	26.30	0.00
TRINITY_XP_0202(	cyclase-	Asparagus_officinal	26.30	0.00
TRINITY_GAQ8669	hypothet	Klebsormidium_niten	26.30	0.00
TRINITY_XP_0166	PREDICTI	Prunus_mume	26.30	0.00
TRINITY_GBG6781(	hypothet	Chara_braunii	26.30	0.00
TRINITY_XP_0228(	SCY1-lik	Olea_europaea	26.30	0.00
TRINITY_GAQ8608	Zinc fir	Klebsormidium_niten	26.30	0.00
TRINITY_PON8451	Ubiquiti	Trema_orientale	26.30	0.00
TRINITY_GAX7872(	hypothet	Chlamydomonas_eusti	26.30	0.00
TRINITY_KMZ6580(	hypothet	Zostera_marina	26.30	0.00
TRINITY_RYR2251	hypothet	Arachis_hypogaea	26.30	0.00
TRINITY_OAE2726(	hypothet	Marchantia_polymorp	26.30	0.00
TRINITY_AAC4937(	GmCK3p,	Glycine_max	26.30	0.00
TRINITY_XP_0134	uncharac	Medicago_truncatula	26.30	0.00
TRINITY_XP_0273	clustere	Abrus_precatorius	26.30	0.00
TRINITY_XP_0124(	PREDICTI	Gossypium_raimondii	26.30	0.00
TRINITY_BAK0073	predicte	Hordeum_vulgare	26.30	0.00
TRINITY_XP_0030(	predicte	Micromonas_pusilla	26.30	0.00
TRINITY_GAQ9234	Sas10/Ut	Klebsormidium_niten	26.30	0.00
TRINITY_PHT4849(	Structur	Capsicum_baccatum	26.30	0.00
TRINITY_XP_0029	hypothet	Volvox_carteri	26.30	0.00
TRINITY_GAX7441(	hypothet	Chlamydomonas_eusti	26.30	0.00
TRINITY_GAQ7964(	hypothet	Klebsormidium_niten	26.30	0.00
TRINITY_XP_0188	PREDICTI	Juglans_regia	26.30	0.00
TRINITY_XP_0056	hypothet	Coccomyxa_subellips	26.30	0.00
TRINITY_XP_0025(	zinc per	Micromonas_commoda	26.20	0.00
TRINITY_XP_0145	protein	Vigna_radiata	26.20	0.00
TRINITY_XP_0150(	cationic	Solanum_pennellii	26.20	0.00
TRINITY_PRW5860	alkaline	Chlorella_sorokinia	26.20	0.00
TRINITY_GAX8375	hypothet	Chlamydomonas_eusti	26.20	0.00
TRINITY_XP_0068	phosphol	Amborella_trichopod	26.20	0.00
TRINITY_XP_0238	ras-rela	Quercus_suber	26.20	0.00
TRINITY_XP_0239(	ribosome	Quercus_suber	26.20	0.00
TRINITY_EPS7152	hypothet	Genlisea_aurea	26.20	0.00
TRINITY_KZV1541	penicill	Dorcoceras_hygromet	26.20	0.00
TRINITY_XP_0202	cytochrc	Cajanus_cajan	26.20	0.00
TRINITY_XP_0134	sulfite	Medicago_truncatula	26.20	0.00
TRINITY_XP_0064(	TOM1-lik	Eutrema_salsugineum	26.20	0.00
TRINITY_PNH0433	MOXD1 1	Tetrabaena_socialis	26.20	0.00
TRINITY_PTQ3267(	hypothet	Marchantia_polymorp	26.20	0.00
TRINITY_ABR1638(	unknown	Picea_sitchensis	26.20	0.00
TRINITY_XP_0239	RHO GTPa	Quercus_suber	26.20	0.00
TRINITY_XP_0110(	PREDICTI	Populus_euphratica	26.20	0.00
TRINITY_PON9045(	Voltage	Trema_orientale	26.20	0.00

TRINITY_XP_0279	pyruvat	Vigna_unguiculata	26.20	0.00
TRINITY_XP_0049	cytochr	Setaria_italica	26.20	0.00
TRINITY_BAD3449	Gag-Pol	Ipomoea_batatas	26.20	0.00
TRINITY_XP_0125	protein	Cicer_arietinum	26.10	0.00
TRINITY_GAX7624	hypothet	Chlamydomonas_eusti	26.10	0.00
TRINITY_XP_0243	choline,	Physcomitrella_pate	26.10	0.00
TRINITY_GBG8236	hypothet	Chara_braunii	26.10	0.00
TRINITY_GAQ8416	Nodal m	Klebsormidium_niten	26.10	0.00
TRINITY_XP_0093	PREDICT	Musa_acuminata	26.10	0.00
TRINITY_XP_0238	probabl	Quercus_suber	26.10	0.00
TRINITY_GBG7699	hypothet	Chara_braunii	26.10	0.00
TRINITY_KJB5982	hypothet	Gossypium_raimondii	26.10	0.00
TRINITY_XP_0227	SCY1-li	Durio_zibethinus	26.10	0.00
TRINITY_EFJ1056	hypothet	Selaginella_moellen	26.10	0.00
TRINITY_XP_0083	PREDICT	Malus_domestica	26.10	0.00
TRINITY_XP_0106	PREDICT	Beta_vulgaris	26.10	0.00
TRINITY_XP_0238	putativ	Quercus_suber	26.10	0.00
TRINITY_XP_0249	acyl-Co	Cynara_cardunculus	26.10	0.00
TRINITY_RWR7545	serine/t	Cinnamomum_micranth	26.10	0.00
TRINITY_CAB7247	putativ	Arabidopsis_thalian	26.10	0.00
TRINITY_GAQ8159	Cation	Klebsormidium_niten	26.10	0.00
TRINITY_XP_0239	phosphat	Quercus_suber	26.10	0.00
TRINITY_GAX8374	hypothet	Chlamydomonas_eusti	26.10	0.00
TRINITY_GBG6890	hypothet	Chara_braunii	26.10	0.00
TRINITY_XP_0241	TOM1-li	Rosa_chinensis	26.10	0.00
TRINITY_PTQ4426	hypothet	Marchantia_polymorp	26.10	0.00
TRINITY_PSC7220	ultravi	Micractinium_conduc	26.10	0.00
TRINITY_EFJ2072	hypothet	Selaginella_moellen	26.10	0.00
TRINITY_PRW5776	DEAD DE	Chlorella_sorokinia	26.10	0.00
TRINITY_GAQ8372	vesicle-	Klebsormidium_niten	26.10	0.00
TRINITY_XP_0249	11-beta-	Ziziphus_jujuba	26.00	0.00
TRINITY_XP_0029	mitochor	Selaginella_moellen	26.00	0.00
TRINITY_XP_0205	LOW QUA	Phalaenopsis_equest	26.00	0.00
TRINITY_XP_0192	PREDICT	Nicotiana_attenuata	26.00	0.00
TRINITY_XP_0167	PREDICT	Gossypium_hirsutum	26.00	0.00
TRINITY_GBG8205	hypothet	Chara_braunii	26.00	0.00
TRINITY_XP_0156	PREDICT	Oryza_brachyantha	26.00	0.00
TRINITY_XP_0071	hypothet	Phaseolus_vulgaris	26.00	0.00
TRINITY_XP_0172	PREDICT	Daucus_carota	26.00	0.00
TRINITY_XP_0025	mechanos	Ricinus_communis	26.00	0.00
TRINITY_XP_0094	PREDICT	Musa_acuminata	26.00	0.00
TRINITY_XP_0263	calcium-	Papaver_somniferum	26.00	0.00
TRINITY_XP_0022	PREDICT	Vitis_vinifera	26.00	0.00
TRINITY_GAX8118	hypothet	Chlamydomonas_eusti	26.00	0.00
TRINITY_OAY7554	Calmodu	Ananas_comosus	26.00	0.00
TRINITY_XP_0056	hypothet	Coccomyxa_subellips	26.00	0.00
TRINITY_XP_0239	unchara	Quercus_suber	26.00	0.00
TRINITY_PIM9994	Cytochr	Handroanthus_impeti	26.00	0.00
TRINITY_PNH0268	AP2-lik	Tetrabaena_socialis	26.00	0.00
TRINITY_BAI6794	tubulin	Chlamydomonas_reinh	26.00	0.00
TRINITY_XP_0056	concana	Coccomyxa_subellips	26.00	0.00
TRINITY_XP_0245	probabl	Selaginella_moellen	26.00	0.00
TRINITY_XP_0094	PREDICT	Musa_acuminata	26.00	0.00
TRINITY_XP_0056	hypothet	Coccomyxa_subellips	26.00	0.00
TRINITY_XP_0025	predict	Micromonas_commoda	26.00	0.00
TRINITY_PNW7498	hypothet	Chlamydomonas_reinh	26.00	0.00
TRINITY_PNW7844	hypothet	Chlamydomonas_reinh	26.00	0.00
TRINITY_XP_0075	predict	Bathycoccus_prasino	25.90	0.00
TRINITY_BAJ9908	predict	Hordeum_vulgare	25.90	0.00
TRINITY_KMZ6579	Translat	Zostera_marina	25.90	0.00
TRINITY_XP_0093	PREDICT	Pyrus_x_bretschneid	25.90	0.00
TRINITY_PSC6969	Transdu	Micractinium_conduc	25.90	0.00
TRINITY_OAE3262	hypothet	Marchantia_polymorp	25.90	0.00
TRINITY_CBI2319	unnamed	Vitis_vinifera	25.90	0.00
TRINITY_XP_0044	exonucl	Cicer_arietinum	25.90	0.00
TRINITY_GAQ8661	Mitochor	Klebsormidium_niten	25.90	0.00

TRINITY_KXZ5601	hypothet	Gonium_pectorale	25.90	0.00
TRINITY_XP_0228	heparan-	Olea_europaea	25.90	0.00
TRINITY_OMO8904	hypothet	Corchorus_capsulari	25.90	0.00
TRINITY_XP_0024	SPX doma	Sorghum_bicolor	25.90	0.00
TRINITY_XP_0030	unnamed	Ostreococcus_tauri	25.90	0.00
TRINITY_XP_0172	PREDICTE	Daucus_carota	25.90	0.00
TRINITY_GAQ9251	hypothet	Klebsormidium_niten	25.90	0.00
TRINITY_GAX7310	hypothet	Chlamydomonas_eusti	25.90	0.00
TRINITY_XP_0016	predicte	Chlamydomonas_reinh	25.90	0.00
TRINITY_XP_0030	predicte	Micromonas_pusilla	25.90	0.00
TRINITY_GAX7413	hypothet	Chlamydomonas_eusti	25.90	0.00
TRINITY_PIA5430	hypothet	Aquilegia_coerulea	25.90	0.00
TRINITY_GAQ8957	Putative	Klebsormidium_niten	25.90	0.00
TRINITY_KXZ4320	hypothet	Gonium_pectorale	25.80	0.00
TRINITY_XP_0229	sugar tı	Cucurbita_maxima	25.80	0.00
TRINITY_OWM8025	hypothet	Punica_granatum	25.80	0.00
TRINITY_PSC7662	wd g-bet	Micractinium_conduc	25.80	0.00
TRINITY_XP_0106	PREDICTE	Beta_vulgaris	25.80	0.00
TRINITY_PTQ5063	hypothet	Marchantia_polymorp	25.80	0.00
TRINITY_XP_0016	flagella	Chlamydomonas_reinh	25.80	0.00
TRINITY_GAQ9220	Ribonucl	Klebsormidium_niten	25.80	0.00
TRINITY_GBF9157	leishmar	Raphidocelis_subcap	25.80	0.00
TRINITY_XP_0094	PREDICTE	Musa_acuminata	25.80	0.00
TRINITY_AES9460	eukaryot	Medicago_truncatula	25.80	0.00
TRINITY_PIA5527	hypothet	Aquilegia_coerulea	25.80	0.00
TRINITY_KXZ4519	hypothet	Gonium_pectorale	25.80	0.00
TRINITY_XP_0071	hypothet	Phaseolus_vulgaris	25.80	0.00
TRINITY_XP_0238	adenosir	Quercus_suber	25.80	0.00
TRINITY_XP_0271	protein	Coffea_eugenioides	25.80	0.00
TRINITY_GAQ9205	hypothet	Klebsormidium_niten	25.80	0.00
TRINITY_XP_0125	phosphat	Cicer_arietinum	25.80	0.00
TRINITY_GBG8052	hypothet	Chara_braunii	25.80	0.00
TRINITY_XP_0139	hypothet	Monoraphidium_negle	25.80	0.00
TRINITY_XP_0202	cytochrc	Asparagus_officinal	25.80	0.00
TRINITY_GAX7405	hypothet	Chlamydomonas_eusti	25.80	0.00
TRINITY_XP_0096	PREDICTE	Nicotiana_tomentosi	25.80	0.00
TRINITY_PNR3728	hypothet	Physcomitrella_pate	25.80	0.00
TRINITY_XP_0238	putative	Quercus_suber	25.80	0.00
TRINITY_XP_0103	transcri	Solanum_lycopersicu	25.80	0.00
TRINITY_XP_0245	2-hydrox	Selaginella_moellen	25.80	0.00
TRINITY_XP_0106	PREDICTE	Vitis_vinifera	25.80	0.00
TRINITY_XP_0239	GTPase-a	Quercus_suber	25.80	0.00
TRINITY_KXZ4284	hypothet	Gonium_pectorale	25.80	0.00
TRINITY_GAQ8542	Putative	Klebsormidium_niten	25.70	0.00
TRINITY_XP_0110	3-oxo-5-	Sesamum_indicum	25.70	0.00
TRINITY_XP_0188	PREDICTE	Juglans_regia	25.70	0.00
TRINITY_BAJ9319	predicte	Hordeum_vulgare	25.70	0.00
TRINITY_GAQ8449	cytochrc	Klebsormidium_niten	25.70	0.00
TRINITY_EFJ1510	hypothet	Selaginella_moellen	25.70	0.00
TRINITY_XP_0025	selenide	Micromonas_commoda	25.70	0.00
TRINITY_XP_0025	8-amino-	Micromonas_commoda	25.70	0.00
TRINITY_XP_0110	PREDICTE	Populus_euphratica	25.70	0.00
TRINITY_ERN0600	hypothet	Amborella_trichopod	25.70	0.00
TRINITY_OWM7028	hypothet	Punica_granatum	25.70	0.00
TRINITY_XP_0241	protein	Rosa_chinensis	25.70	0.00
TRINITY_XP_0030	tRNA (gu	Ostreococcus_tauri	25.70	0.00
TRINITY_XP_0238	1-phosph	Quercus_suber	25.70	0.00
TRINITY_GAQ8302	hypothet	Klebsormidium_niten	25.70	0.00
TRINITY_GAX8022	hypothet	Chlamydomonas_eusti	25.70	0.00
TRINITY_XP_0205	uncharac	Phalaenopsis_equest	25.70	0.00
TRINITY_BAJ9585	predicte	Hordeum_vulgare	25.70	0.00
TRINITY_OAY7181	Ankyrin-	Ananas_comosus	25.70	0.00
TRINITY_XP_0244	protein	Populus_trichocarpa	25.70	0.00
TRINITY_PTQ4936	hypothet	Marchantia_polymorp	25.70	0.00
TRINITY_XP_0156	PREDICTE	Oryza_brachyantha	25.70	0.00
TRINITY_XP_0279	phosphoc	Vigna_unguiculata	25.70	0.00

TRINITY_XP_0238	uncharac	Quercus_suber	25.70	0.00
TRINITY_PIA5074	hypothet	Aquilegia_coerulea	25.70	0.00
TRINITY_OAE3319	hypothet	Marchantia_polymorp	25.70	0.00
TRINITY_XP_0063	PREDICTE	Solanum_tuberosum	25.70	0.00
TRINITY_XP_0016	predicte	Chlamydomonas_reinh	25.70	0.00
TRINITY_XP_0191	PREDICTE	Ipomoea_nil	25.70	0.00
TRINITY_GAX7334	hypothet	Chlamydomonas_eusti	25.70	0.00
TRINITY_OUS4375	hypothet	Ostreococcus_tauri	25.70	0.00
TRINITY_BAJ9457	predicte	Hordeum_vulgare	25.60	0.00
TRINITY_NP_0013	uncharac	Zea_mays	25.60	0.00
TRINITY_XP_0245	probable	Selaginella_moellen	25.60	0.00
TRINITY_GAQ7853	ARM repe	Klebsormidium_niten	25.60	0.00
TRINITY_GAQ9014	Putative	Klebsormidium_niten	25.60	0.00
TRINITY_XP_0035	cytoplas	Glycine_max	25.60	0.00
TRINITY_GAX8118	hypothet	Chlamydomonas_eusti	25.60	0.00
TRINITY_GAQ8044	hypothet	Klebsormidium_niten	25.60	0.00
TRINITY_RZR8506	hypothet	Ensete_ventricosum	25.60	0.00
TRINITY_AFO8408	sucrose	Actinidia_deliciosa	25.60	0.00
TRINITY_KFK2549	hypothet	Arabis_alpina	25.60	0.00
TRINITY_RXH9191	hypothet	Malus_domestica	25.60	0.00
TRINITY_BAI6794	tubulin	Chlamydomonas_reinh	25.60	0.00
TRINITY_XP_0102	PREDICTE	Nelumbo_nucifera	25.60	0.00
TRINITY_XP_0239	low affi	Quercus_suber	25.60	0.00
TRINITY_GAQ8124	hypothet	Klebsormidium_niten	25.60	0.00
TRINITY_XP_0120	tRNAse Z	Jatropha_curcas	25.60	0.00
TRINITY_XP_0096	PREDICTE	Nicotiana_tomentosi	25.60	0.00
TRINITY_NP_0013	two pore	Nicotiana_tabacum	25.60	0.00
TRINITY_BAJ9694	predicte	Hordeum_vulgare	25.60	0.00
TRINITY_GAX8496	hypothet	Chlamydomonas_eusti	25.60	0.00
TRINITY_OTG2536	putative	Helianthus_annuus	25.60	0.00
TRINITY_XP_0227	uncharac	Durio_zibethinus	25.60	0.00
TRINITY_KXZ4822	hypothet	Gonium_pectorale	25.60	0.00
TRINITY_GAV5921	Sec6 dom	Cephalotus_follicul	25.60	0.00
TRINITY_XP_0029	hypothet	Volvox_carteri	25.60	0.00
TRINITY_KZV4209	nicotina	Dorcoceras_hygromet	25.50	0.00
TRINITY_GAX8461	hypothet	Chlamydomonas_eusti	25.50	0.00
TRINITY_XP_0264	cytochro	Papaver_somniferum	25.50	0.00
TRINITY_CDM8690	unnamed	Triticum_aestivum	25.50	0.00
TRINITY_RDX6560	Acid bet	Mucuna_pruriens	25.50	0.00
TRINITY_XP_0271	tip elon	Cicer_arietinum	25.50	0.00
TRINITY_KDP4090	hypothet	Jatropha_curcas	25.50	0.00
TRINITY_NP_0011	SH2 doma	Arabidopsis_thalian	25.50	0.00
TRINITY_XP_0016	predicte	Chlamydomonas_reinh	25.50	0.00
TRINITY_RVX1717	Retrovir	Vitis_vinifera	25.50	0.00
TRINITY_XP_0030	predicte	Micromonas_pusilla	25.50	0.00
TRINITY_GAX8052	hypothet	Chlamydomonas_eusti	25.50	0.00
TRINITY_GAX8016	hypothet	Chlamydomonas_eusti	25.50	0.00
TRINITY_XP_0024	cullin-3	Sorghum_bicolor	25.50	0.00
TRINITY_XP_0042	PREDICTE	Fragaria_vesca	25.40	0.00
TRINITY_GAQ8293	prefoldi	Klebsormidium_niten	25.40	0.00
TRINITY_XP_0237	ELMO dom	Lactuca_sativa	25.40	0.00
TRINITY_GBF9764	hypothet	Raphidocelis_subcap	25.40	0.00
TRINITY_XP_0058	hypothet	Chlorella_variabili	25.40	0.00
TRINITY_PIA3220	hypothet	Aquilegia_coerulea	25.40	0.00
TRINITY_RAL5341	hypothet	Cuscuta_australis	25.40	0.00
TRINITY_XP_0058	hypothet	Chlorella_variabili	25.40	0.00
TRINITY_KXZ5596	hypothet	Gonium_pectorale	25.40	0.00
TRINITY_GAY5680	hypothet	Citrus_unshiu	25.40	0.00
TRINITY_NP_0011	uncharac	Zea_mays	25.40	0.00
TRINITY_XP_0225	ATP-depe	Brassica_napus	25.40	0.00
TRINITY_XP_0049	putative	Setaria_italica	25.40	0.00
TRINITY_XP_0218	poly(A)-	Spinacia_oleracea	25.40	0.00
TRINITY_XP_0239	GTPase-a	Quercus_suber	25.40	0.00
TRINITY_BBE4939	squalene	Polystichum_makinoi	25.40	0.00
TRINITY_GAX8016	hypothet	Chlamydomonas_eusti	25.40	0.00
TRINITY_XP_0025	choline	Micromonas_commoda	25.40	0.00

TRINITY_KXZ5229	hypothet	Gonium_pectorale	25.40	0.00
TRINITY_XP_0243	protein	Physcomitrella_pate	25.40	0.00
TRINITY_PTQ3670	hypothet	Marchantia_polymorp	25.40	0.00
TRINITY_AIU4929	AT2G1841	Sarcandra_glabra	25.40	0.00
TRINITY_PNH1295	A disint	Tetrabaena_socialis	25.40	0.00
TRINITY_XP_0025	5'-nucle	Ricinus_communis	25.30	0.00
TRINITY_GBG9158	hypothet	Chara_braunii	25.30	0.00
TRINITY_XP_0245	nucleola	Selaginella_moellen	25.30	0.00
TRINITY_XP_0200	syntaxir	Ananas_comosus	25.30	0.00
TRINITY_NP_5682	calmodul	Arabidopsis_thalian	25.30	0.00
TRINITY_XP_0042	unchara	Solanum_lycopersicu	25.30	0.00
TRINITY_XP_0237	calcium-	Lactuca_sativa	25.30	0.00
TRINITY_KXZ4330	hypothet	Gonium_pectorale	25.30	0.00
TRINITY_XP_0241	transcri	Rosa_chinensis	25.30	0.00
TRINITY_XP_0237	protein	Lactuca_sativa	25.30	0.00
TRINITY_XP_0056	hypothet	Coccomyxa_subellips	25.30	0.00
TRINITY_XP_0208	putative	Arabidopsis_lyrata	25.30	0.00
TRINITY_GAX7881	hypothet	Chlamydomonas_eusti	25.30	0.00
TRINITY_RYR2251	hypothet	Arachis_hypogaea	25.30	0.00
TRINITY_PTQ4483	hypothet	Marchantia_polymorp	25.30	0.00
TRINITY_XP_0159	ras-rela	Arachis_duranensis	25.30	0.00
TRINITY_XP_0068	protein	Amborella_trichopod	25.30	0.00
TRINITY_RAL4839	hypothet	Cuscuta_australis	25.30	0.00
TRINITY_GBF9955	hypothet	Raphidocelis_subcap	25.30	0.00
TRINITY_XP_0066	PREDICTI	Oryza_brachyantha	25.30	0.00
TRINITY_XP_0238	protein	Quercus_suber	25.30	0.00
TRINITY_GAX7967	hypothet	Chlamydomonas_eusti	25.30	0.00
TRINITY_ONI3142	hypothet	Prunus_persica	25.20	0.00
TRINITY_XP_0184	PREDICTI	Raphanus_sativus	25.20	0.00
TRINITY_BAK0692	predicte	Hordeum_vulgare	25.20	0.00
TRINITY_XP_0228	1,3-beta	Ostreococcus_tauri	25.20	0.00
TRINITY_PNR3636	hypothet	Physcomitrella_pate	25.20	0.00
TRINITY_KDP4068	hypothet	Jatropha_curcas	25.20	0.00
TRINITY_OAE2910	hypothet	Marchantia_polymorp	25.20	0.00
TRINITY_XP_0205	protein	Amborella_trichopod	25.20	0.00
TRINITY_XP_0245	dynamin-	Selaginella_moellen	25.20	0.00
TRINITY_GAQ7801	hypothet	Klebsormidium_niten	25.20	0.00
TRINITY_PNH0921	Tiny mac	Tetrabaena_socialis	25.20	0.00
TRINITY_PKA6398	Protein	Apostasia_shenzheni	25.20	0.00
TRINITY_XP_0202	ubiquit	Asparagus_officinal	25.20	0.00
TRINITY_OTG1794	putative	Helianthus_annuus	25.10	0.00
TRINITY_XP_0110	SEC12-li	Sesamum_indicum	25.10	0.00
TRINITY_XP_0094	PREDICTI	Musa_acuminata	25.10	0.00
TRINITY_XP_0239	vacuolar	Quercus_suber	25.10	0.00
TRINITY_AFK3511	unknown	Medicago_truncatula	25.10	0.00
TRINITY_XP_0205	WD repea	Phalaenopsis_equest	25.10	0.00
TRINITY_XP_0166	PREDICTI	Gossypium_hirsutum	25.10	0.00
TRINITY_XP_0273	ultravio	Abrus_precatorius	25.10	0.00
TRINITY_OAE2772	hypothet	Marchantia_polymorp	25.10	0.00
TRINITY_XP_0174	PREDICTI	Vigna_angularis	25.10	0.00
TRINITY_XP_0229	protein	Cucurbita_moschata	25.10	0.00
TRINITY_XP_0110	PREDICTI	Populus_euphratica	25.10	0.00
TRINITY_GBG8204	hypothet	Chara_braunii	25.10	0.00
TRINITY_PRW5993	sterol 1	Chlorella_sorokinia	25.10	0.00
TRINITY_EFJ2945	hypothet	Selaginella_moellen	25.10	0.00
TRINITY_GAX7285	hypothet	Chlamydomonas_eusti	25.10	0.00
TRINITY_GBF9841	hypothet	Raphidocelis_subcap	25.10	0.00
TRINITY_XP_0213	alpha-L-	Sorghum_bicolor	25.10	0.00
TRINITY_XP_0279	cytochro	Vigna_unguiculata	25.10	0.00
TRINITY_XP_0066	PREDICTI	Oryza_brachyantha	25.10	0.00
TRINITY_PNW7691	hypothet	Chlamydomonas_reinh	25.10	0.00
TRINITY_XP_0066	PREDICTI	Oryza_brachyantha	25.00	0.00
TRINITY_PKU7185	Calmodul	Dendrobium_catenatu	25.00	0.00
TRINITY_KMS9427	hypothet	Beta_vulgaris	25.00	0.00
TRINITY_XP_0101	probable	Morus_notabilis	25.00	0.00
TRINITY_GAX8147	hypothet	Chlamydomonas_eusti	25.00	0.00

TRINITY_GAX7970	hypothet	Chlamydomonas_eusti	25.00	0.00
TRINITY_EYU3799	hypothet	Erythranthe_guttata	25.00	0.00
TRINITY_GAQ8144	hypothet	Klebsormidium_niten	25.00	0.00
TRINITY_XP_0201	transmem	Aegilops_tauschii	25.00	0.00
TRINITY_KFK3556	hypothet	Arabis_alpina	25.00	0.00
TRINITY_PTQ3989	hypothet	Marchantia_polymorp	25.00	0.00
TRINITY_GBF9764	hypothet	Raphidocelis_subcap	25.00	0.00
TRINITY_XP_0030	predicte	Micromonas_pusilla	25.00	0.00
TRINITY_RXH7911	hypothet	Malus_domestica	25.00	0.00
TRINITY_GAQ9025	hypothet	Klebsormidium_niten	25.00	0.00
TRINITY_XP_0016	predicte	Chlamydomonas_reinh	25.00	0.00
TRINITY_GAX7657	hypothet	Chlamydomonas_eusti	25.00	0.00
TRINITY_XP_0238	von Wil	Quercus_suber	25.00	0.00
TRINITY_PNW7090	hypothet	Chlamydomonas_reinh	25.00	0.00
TRINITY_XP_0202	homocyst	Cajanus_cajan	25.00	0.00
TRINITY_XP_0237	importir	Lactuca_sativa	25.00	0.00
TRINITY_XP_0110	protein	Sesamum_indicum	24.90	0.00
TRINITY_RWR9253	preseque	Cinnamomum_micranth	24.90	0.00
TRINITY_XP_0245	neutral	Selaginella_moellen	24.90	0.00
TRINITY_XP_0228	G-patch	Ostreococcus_tauri	24.90	0.00
TRINITY_XP_0161	DNA repa	Arachis_ipaensis	24.90	0.00
TRINITY_XP_0203	leucine	Zea_mays	24.90	0.00
TRINITY_BAK0175	predicte	Hordeum_vulgare	24.90	0.00
TRINITY_OAE2578	hypothet	Marchantia_polymorp	24.90	0.00
TRINITY_ABB4538	diacylgl	Oryza_sativa	24.90	0.00
TRINITY_XP_0239	tuberous	Quercus_suber	24.90	0.00
TRINITY_XP_0270	SNF1-rel	Coffea_arabica	24.90	0.00
TRINITY_PHT7034	Dynamin-	Capsicum_annuum	24.90	0.00
TRINITY_XP_0025	predicte	Micromonas_commoda	24.90	0.00
TRINITY_A2YFR6.	RecName:	Oryza_sativa	24.90	0.00
TRINITY_XP_0258	UBX doma	Panicum_hallii	24.80	0.00
TRINITY_XP_0243	ethylene	Physcomitrella_pate	24.80	0.00
TRINITY_GAX8522	hypothet	Chlamydomonas_eusti	24.80	0.00
TRINITY_KEH1899	shrunker	Medicago_truncatula	24.80	0.00
TRINITY_OAE2967	hypothet	Marchantia_polymorp	24.80	0.00
TRINITY_XP_0208	importir	Arabidopsis_lyrata	24.80	0.00
TRINITY_XP_0075	predicte	Bathycoccus_prasinc	24.80	0.00
TRINITY_GAQ8753	hypothet	Klebsormidium_niten	24.80	0.00
TRINITY_XP_0058	hypothet	Chlorella_variabili	24.80	0.00
TRINITY_XP_0239	LOW QUAI	Quercus_suber	24.80	0.00
TRINITY_XP_0202	EVI5-lik	Asparagus_officinal	24.80	0.00
TRINITY_XP_0139	hypothet	Monoraphidium_negle	24.80	0.00
TRINITY_GAX7736	hypothet	Chlamydomonas_eusti	24.80	0.00
TRINITY_PNW7538	hypothet	Chlamydomonas_reinh	24.80	0.00
TRINITY_KXZ5566	hypothet	Gonium_pectorale	24.80	0.00
TRINITY_XP_0016	predicte	Chlamydomonas_reinh	24.70	0.00
TRINITY_XP_0194	PREDICTI	Lupinus_angustifoli	24.70	0.00
TRINITY_XP_0014	predicte	Ostreococcus_sp._'l	24.70	0.00
TRINITY_XP_0105	PREDICTI	Tarenaya_hasslerian	24.70	0.00
TRINITY_KXZ4772	hypothet	Gonium_pectorale	24.70	0.00
TRINITY_RMZ5536	hypothet	Auxenochlorella_prc	24.70	0.00
TRINITY_RMZ5514	hypothet	Auxenochlorella_prc	24.70	0.00
TRINITY_XP_0030	predicte	Micromonas_pusilla	24.60	0.00
TRINITY_PNW7907	hypothet	Chlamydomonas_reinh	24.60	0.00
TRINITY_XP_0240	phosphol	Eutrema_salsugineum	24.60	0.00
TRINITY_PNH0047	hypothet	Tetrabaena_socialis	24.60	0.00
TRINITY_XP_0243	von Wil	Physcomitrella_pate	24.60	0.00
TRINITY_XP_0016	predicte	Chlamydomonas_reinh	24.60	0.00
TRINITY_XP_0065	uncharac	Glycine_max	24.60	0.00
TRINITY_GAQ8434	transcri	Klebsormidium_niten	24.60	0.00
TRINITY_EFJ1844	hypothet	Selaginella_moellen	24.60	0.00
TRINITY_PWA4376	Armadil	Artemisia_annua	24.60	0.00
TRINITY_XP_0025	predicte	Micromonas_commoda	24.60	0.00
TRINITY_NP_0013	elongati	Gossypium_hirsutum	24.60	0.00
TRINITY_XP_0025	predicte	Micromonas_commoda	24.50	0.00
TRINITY_KRH7392	hypothet	Glycine_max	24.50	0.00

TRINITY_XP_0072(	L-2-hydı	Prunus_persica	24.50	0.00
TRINITY_EEE6370	hypothet	Oryza_sativa	24.50	0.00
TRINITY_XP_0156	pre-mRN/	Oryza_sativa	24.50	0.00
TRINITY_XP_0218	ABC trai	Prunus_avium	24.50	0.00
TRINITY_RLN4070	hypothet	Panicum_miliaceum	24.50	0.00
TRINITY_XP_0106(	PREDICTI	Beta_vulgaris	24.50	0.00
TRINITY_PNW7167	hypothet	Chlamydomonas_reinh	24.50	0.00
TRINITY_XP_0161(	protein	Arachis_ipaensis	24.50	0.00
TRINITY_XP_0238	RNA poly	Quercus_suber	24.50	0.00
TRINITY_XP_0094(	PREDICTI	Musa_acuminata	24.50	0.00
TRINITY_GAX7636	hypothet	Chlamydomonas_eusti	24.50	0.00
TRINITY_XP_0036	mitochor	Medicago_truncatula	24.40	0.00
TRINITY_PNR5773	hypothet	Physcomitrella_pate	24.40	0.00
TRINITY_XP_0029	hypothet	Volvox_carteri	24.40	0.00
TRINITY_XP_0075	predicte	Bathycoccus_prasinc	24.40	0.00
TRINITY_XP_0025(	predicte	Micromonas_commoda	24.40	0.00
TRINITY_PWA7447	organic	Artemisia_annua	24.40	0.00
TRINITY_EPS7053	hypothet	Genlisea_aurea	24.40	0.00
TRINITY_GAX8125(	hypothet	Chlamydomonas_eusti	24.40	0.00
TRINITY_CDP1355	unnamed	Coffea_canephora	24.40	0.00
TRINITY_XP_0216	uncharac	Hevea_brasiliensis	24.40	0.00
TRINITY_KXZ5030(	hypothet	Gonium_pectorale	24.40	0.00
TRINITY_XP_0201(	obtusifc	Aegilops_tauschii	24.40	0.00
TRINITY_XP_0036(	calcium	Medicago_truncatula	24.40	0.00
TRINITY_XP_0029	hypothet	Volvox_carteri	24.40	0.00
TRINITY_GBG7461	hypothet	Chara_braunii	24.30	0.00
TRINITY_KXZ4275	hypothet	Gonium_pectorale	24.30	0.00
TRINITY_KXZ4856	hypothet	Gonium_pectorale	24.30	0.00
TRINITY_PRW6044(	pathoger	Chlorella_sorokinia	24.30	0.00
TRINITY_RAL5397	hypothet	Cuscuta_australis	24.30	0.00
TRINITY_GAQ8019	serine/t	Klebsormidium_niten	24.30	0.00
TRINITY_XP_0246	uncharac	Medicago_truncatula	24.30	0.00
TRINITY_XP_0238	tripept	Quercus_suber	24.30	0.00
TRINITY_XP_0066	PREDICTI	Oryza_brachyantha	24.30	0.00
TRINITY_XP_0258	patatin-	Panicum_hallii	24.30	0.00
TRINITY_KXZ4994	hypothet	Gonium_pectorale	24.30	0.00
TRINITY_KRH4875	hypothet	Glycine_max	24.30	0.00
TRINITY_BAS3104	hypothet	Klebsormidium_flacc	24.30	0.00
TRINITY_XP_0102	PREDICTI	Nelumbo_nucifera	24.30	0.00
TRINITY_XP_0279	2-amino-	Vigna_unguiculata	24.30	0.00
TRINITY_KVI0637	Major fa	Cynara_cardunculus	24.30	0.00
TRINITY_XP_0110	uncharac	Sesamum_indicum	24.30	0.00
TRINITY_XP_0139(	hypothet	Monoraphidium_negle	24.30	0.00
TRINITY_XP_0043(	PREDICTI	Fragaria_vesca	24.30	0.00
TRINITY_XP_0237(	protein	Lactuca_sativa	24.30	0.00
TRINITY_GAX7285	hypothet	Chlamydomonas_eusti	24.30	0.00
TRINITY_PSC7039	Leukotri	Micractinium_conduc	24.30	0.00
TRINITY_XP_0023(	importir	Populus_trichocarpa	24.30	0.00
TRINITY_GAQ8473	Cysteine	Klebsormidium_niten	24.20	0.00
TRINITY_XP_0243	platelet	Physcomitrella_pate	24.20	0.00
TRINITY_XP_0029	hypothet	Volvox_carteri	24.20	0.00
TRINITY_XP_0194	PREDICTI	Lupinus_angustifoli	24.20	0.00
TRINITY_XP_0058	hypothet	Chlorella_variabili	24.20	0.00
TRINITY_XP_0245	retinob	Selaginella_moellen	24.20	0.00
TRINITY_XP_0014	MFS fam	Ostreococcus_sp._'l	24.20	0.00
TRINITY_OAE2688	hypothet	Marchantia_polymorp	24.20	0.00
TRINITY_XP_0201	organic	Aegilops_tauschii	24.20	0.00
TRINITY_GAQ9169	HCP-like	Klebsormidium_niten	24.20	0.00
TRINITY_XP_0025	polyader	Ricinus_communis	24.20	0.00
TRINITY_XP_0016	predicte	Chlamydomonas_reinh	24.20	0.00
TRINITY_EFJ2153	hypothet	Selaginella_moellen	24.10	0.00
TRINITY_EFJ3848	hypothet	Selaginella_moellen	24.10	0.00
TRINITY_PHT5402	hypothet	Capsicum_baccatum	24.10	0.00
TRINITY_XP_0128	PREDICTI	Erythranthe_guttata	24.10	0.00
TRINITY_XP_0238	uncharac	Quercus_suber	24.10	0.00
TRINITY_RYR2251	hypothet	Arachis_hypogaea	24.10	0.00

TRINITY_ONK5620	uncharac	Asparagus_officinal	24.10	0.00
TRINITY_GAQ8574	hypothet	Klebsormidium_niten	24.10	0.00
TRINITY_XP_0172	PREDICTI	Daucus_carota	24.10	0.00
TRINITY_XP_0030	predicte	Micromonas_pusilla	24.10	0.00
TRINITY_XP_0030	Amino ac	Ostreococcus_tauri	24.10	0.00
TRINITY_XP_0106	PREDICTI	Beta_vulgaris	24.10	0.00
TRINITY_XP_0091	PREDICTI	Brassica_rapa	24.10	0.00
TRINITY_BAJ9585	predicte	Hordeum_vulgare	24.10	0.00
TRINITY_KXZ4621	hypothet	Gonium_pectorale	24.10	0.00
TRINITY_XP_0212	alanine-	Herrania_umbratica	24.10	0.00
TRINITY_GBG8599	hypothet	Chara_braunii	24.10	0.00
TRINITY_KMZ6328	Alkaline	Zostera_marina	24.00	0.00
TRINITY_XP_0202	pentatri	Asparagus_officinal	24.00	0.00
TRINITY_XP_0029	hypothet	Volvox_carteri	24.00	0.00
TRINITY_PWA4953	heavy me	Artemisia_annua	24.00	0.00
TRINITY_XP_0104	PREDICTI	Camelina_sativa	24.00	0.00
TRINITY_GAX7884	hypothet	Chlamydomonas_eusti	24.00	0.00
TRINITY_XP_0030	predicte	Micromonas_pusilla	24.00	0.00
TRINITY_OAE2254	hypothet	Marchantia_polymorp	24.00	0.00
TRINITY_XP_0167	PREDICTI	Gossypium_hirsutum	24.00	0.00
TRINITY_XP_0228	Amino ac	Ostreococcus_tauri	23.90	0.00
TRINITY_XP_0014	predicte	Ostreococcus_sp._'l	23.90	0.00
TRINITY_XP_0063	RNA poly	Eutrema_salsugineum	23.90	0.00
TRINITY_KYP5950	Histone-	Cajanus_cajan	23.90	0.00
TRINITY_CDY4010	BnaC02g	Brassica_napus	23.90	0.00
TRINITY_XP_0068	DNA dama	Amborella_trichopod	23.90	0.00
TRINITY_XP_0030	predicte	Micromonas_pusilla	23.90	0.00
TRINITY_XP_0029	RINT1-li	Selaginella_moellen	23.90	0.00
TRINITY_XP_0218	traffick	Spinacia_oleracea	23.90	0.00
TRINITY_XP_0134	ent-kaur	Medicago_truncatula	23.90	0.00
TRINITY_KFK4343	hypothet	Arabis_alpina	23.90	0.00
TRINITY_GAX8310	hypothet	Chlamydomonas_eusti	23.90	0.00
TRINITY_GBF9540	helicase	Raphidocelis_subcap	23.90	0.00
TRINITY_XP_0091	PREDICTI	Brassica_rapa	23.90	0.00
TRINITY_XP_0029	Qb-SNARE	Volvox_carteri	23.90	0.00
TRINITY_PWA4326	protein	Artemisia_annua	23.80	0.00
TRINITY_EFH6841	Serine/t	Arabidopsis_lyrata	23.80	0.00
TRINITY_XP_0246	uncharac	Medicago_truncatula	23.80	0.00
TRINITY_PWA4009	Concanav	Artemisia_annua	23.80	0.00
TRINITY_XP_0264	SNF1-rel	Papaver_somniferum	23.80	0.00
TRINITY_XP_0218	protein	Spinacia_oleracea	23.80	0.00
TRINITY_AYM3274	DNA poly	Silene_vulgaris	23.80	0.00
TRINITY_KEH2791	LAG1 lon	Medicago_truncatula	23.80	0.00
TRINITY_XP_0029	hypothet	Volvox_carteri	23.80	0.00
TRINITY_XP_0075	polycyst	Bathycoccus_prasino	23.80	0.00
TRINITY_PRW6156	Retrovir	Chlorella_sorokinia	23.80	0.00
TRINITY_XP_0044	ribosoma	Cicer_arietinum	23.80	0.00
TRINITY_XP_0243	EVI5-lik	Physcomitrella_pate	23.80	0.00
TRINITY_XP_0245	eukaryot	Selaginella_moellen	23.80	0.00
TRINITY_XP_0238	rho1 gua	Quercus_suber	23.80	0.00
TRINITY_KZV4713	cation/c	Dorcoceras_hygromet	23.80	0.00
TRINITY_PNW8306	hypothet	Chlamydomonas_reinh	23.80	0.00
TRINITY_XP_0025	adenylat	Micromonas_commoda	23.70	0.00
TRINITY_ONL9257	transcri	Zea_mays	23.70	0.00
TRINITY_XP_0064	protein	Citrus_sinensis	23.70	0.00
TRINITY_PIA5351	hypothet	Aquilegia_coerulea	23.70	0.00
TRINITY_ALM5498	DEAD box	Chlamydomonas_sp._I	23.70	0.00
TRINITY_EMS6811	Obtusifo	Triticum_urartu	23.70	0.00
TRINITY_XP_0228	RNA poly	Ostreococcus_tauri	23.70	0.00
TRINITY_XP_0043	PREDICTI	Fragaria_vesca	23.70	0.00
TRINITY_XP_0158	uncharac	Ziziphus_jujuba	23.70	0.00
TRINITY_XP_0014	predicte	Ostreococcus_sp._'l	23.70	0.00
TRINITY_XP_0030	Twin-arg	Ostreococcus_tauri	23.70	0.00
TRINITY_XP_0243	cilia- a	Physcomitrella_pate	23.70	0.00
TRINITY_XP_0172	PREDICTI	Daucus_carota	23.60	0.00
TRINITY_XP_0188	PREDICTI	Juglans_regia	23.60	0.00

TRINITY_XP_0029	uncharac	Selaginella_moellen	23.60	0.00
TRINITY_XP_0042	signal	Solanum_lycopersicu	23.60	0.00
TRINITY_XP_0191	PREDICTI	Ipomoea_nil	23.60	0.00
TRINITY_XP_0188	PREDICTI	Juglans_regia	23.60	0.00
TRINITY_XP_0083	PREDICTI	Malus_domestica	23.60	0.00
TRINITY_XP_0250	LOW QUAI	Ricinus_communis	23.60	0.00
TRINITY_XP_0239	uncharac	Quercus_suber	23.60	0.00
TRINITY_NP_1816	Calcium-	Arabidopsis_thalian	23.60	0.00
TRINITY_XP_0114	PREDICTI	Fragaria_vesca	23.50	0.00
TRINITY_XP_0030	Insulin-	Ostreococcus_tauri	23.50	0.00
TRINITY_XP_0273	fatty a	Abrus_precatorius	23.50	0.00
TRINITY_XP_0239	vacuola	Quercus_suber	23.50	0.00
TRINITY_KXZ5294	hypothet	Gonium_pectorale	23.50	0.00
TRINITY_GAQ8383	RAN GTPa	Klebsormidium_niten	23.50	0.00
TRINITY_XP_0238	probable	Quercus_suber	23.50	0.00
TRINITY_OAE3505	hypothet	Marchantia_polymorp	23.50	0.00
TRINITY_GAX7270	hypothet	Chlamydomonas_eusti	23.50	0.00
TRINITY_XP_0049	UNC93-l	Setaria_italica	23.50	0.00
TRINITY_PRW6030	Mitochon	Chlorella_sorokinia	23.50	0.00
TRINITY_XP_0058	hypothet	Chlorella_variabili	23.40	0.00
TRINITY_XP_0188	PREDICTI	Juglans_regia	23.40	0.00
TRINITY_XP_0172	PREDICTI	Daucus_carota	23.40	0.00
TRINITY_PKI7856	hypothet	Punica_granatum	23.40	0.00
TRINITY_XP_0035	phosphat	Glycine_max	23.40	0.00
TRINITY_PKA5568	hypothet	Apostasia_shenzheni	23.40	0.00
TRINITY_XP_0243	RNA poly	Physcomitrella_pate	23.40	0.00
TRINITY_GAQ7914	major fa	Klebsormidium_niten	23.40	0.00
TRINITY_GAQ8476	hypothet	Klebsormidium_niten	23.40	0.00
TRINITY_PNW7907	hypothet	Chlamydomonas_reinh	23.40	0.00
TRINITY_GAX8583	hypothet	Chlamydomonas_eusti	23.40	0.00
TRINITY_XP_0029	hypothet	Volvox_carteri	23.40	0.00
TRINITY_XP_0091	PREDICTI	Brassica_rapa	23.30	0.00
TRINITY_PTQ4308	hypothet	Marchantia_polymorp	23.30	0.00
TRINITY_CDO9704	unnamed	Coffea_canephora	23.30	0.00
TRINITY_XP_0068	protein	Amborella_trichopod	23.30	0.00
TRINITY_XP_0029	hypothet	Volvox_carteri	23.30	0.00
TRINITY_GAX7986	hypothet	Chlamydomonas_eusti	23.30	0.00
TRINITY_XP_0030	predicte	Micromonas_pusilla	23.30	0.00
TRINITY_EFJ1377	hypothet	Selaginella_moellen	23.30	0.00
TRINITY_PNH0238	hypothet	Tetrabaena_socialis	23.30	0.00
TRINITY_XP_0245	probable	Selaginella_moellen	23.30	0.00
TRINITY_XP_0016	Qa-SNARI	Chlamydomonas_reinh	23.30	0.00
TRINITY_KXZ5058	hypothet	Gonium_pectorale	23.30	0.00
TRINITY_GAQ7992	Putative	Klebsormidium_niten	23.20	0.00
TRINITY_XP_0084	PREDICTI	Cucumis_melo	23.20	0.00
TRINITY_XP_0091	PREDICTI	Brassica_rapa	23.20	0.00
TRINITY_XP_0155	heparan-	Ricinus_communis	23.20	0.00
TRINITY_GAX8053	hypothet	Chlamydomonas_eusti	23.20	0.00
TRINITY_OAE2524	hypothet	Marchantia_polymorp	23.20	0.00
TRINITY_XP_0229	gamma-sc	Cucurbita_moschata	23.20	0.00
TRINITY_XP_0120	uncharac	Jatropha_curcas	23.20	0.00
TRINITY_RVW1784	F-box pı	Vitis_vinifera	23.20	0.00
TRINITY_EEE6639	hypothet	Oryza_sativa	23.20	0.00
TRINITY_PNW8751	hypothet	Chlamydomonas_reinh	23.20	0.00
TRINITY_XP_0158	cytochrc	Ziziphus_jujuba	23.10	0.00
TRINITY_GAX8557	hypothet	Chlamydomonas_eusti	23.10	0.00
TRINITY_GAQ9169	HCP-like	Klebsormidium_niten	23.10	0.00
TRINITY_XP_0216	uncharac	Manihot_esculenta	23.10	0.00
TRINITY_GAX7821	hypothet	Chlamydomonas_eusti	23.10	0.00
TRINITY_GAX7691	hypothet	Chlamydomonas_eusti	23.10	0.00
TRINITY_XP_0239	importir	Quercus_suber	23.00	0.00
TRINITY_XP_0216	transpo	Hevea_brasiliensis	23.00	0.00
TRINITY_XP_0245	uncharac	Selaginella_moellen	23.00	0.00
TRINITY_XP_0158	activat	Ziziphus_jujuba	23.00	0.00
TRINITY_AJE2937	putative	Coffea_canephora	23.00	0.00
TRINITY_XP_0110	PREDICTI	Populus_euphratica	23.00	0.00

TRINITY_GBG60121	hypothet	Chara_braunii	23.00	0.00
TRINITY_XP_02392	ras GTPa	Quercus_suber	23.00	0.00
TRINITY_KXZ50306	hypothet	Gonium_pectorale	23.00	0.00
TRINITY_XP_01596	DNA-dire	Arachis_duranensis	22.90	0.00
TRINITY_ABK23995	unknown	Picea_sitchensis	22.90	0.00
TRINITY_XP_02439	uncharac	Physcomitrella_pate	22.90	0.00
TRINITY_GAX80492	hypothet	Chlamydomonas_eusti	22.90	0.00
TRINITY_KXZ56458	hypothet	Gonium_pectorale	22.90	0.00
TRINITY_XP_02643	putative	Papaver_somniferum	22.90	0.00
TRINITY_BAJ96946	predicte	Hordeum_vulgare	22.90	0.00
TRINITY_XP_02561	pyruvate	Arachis_hypogaea	22.90	0.00
TRINITY_XP_00449	importir	Cicer_arietinum	22.90	0.00
TRINITY_PON37203	Endonucl	Parasponia_anderson	22.80	0.00
TRINITY_XP_00305	predicte	Micromonas_pusilla	22.80	0.00
TRINITY_GAV80154	Ribophor	Cephalotus_follicul	22.80	0.00
TRINITY_XP_00751	peptidas	Bathycoccus_prasinc	22.80	0.00
TRINITY_EEF27535	two-comp	Ricinus_communis	22.70	0.00
TRINITY_BAK01751	predicte	Hordeum_vulgare	22.70	0.00
TRINITY_ABR17135	unknown	Picea_sitchensis	22.70	0.00
TRINITY_XP_01620	sodium-c	Arachis_ipaensis	22.70	0.00
TRINITY_RZB45948	Alpha-d	Glycine_soja	22.70	0.00
TRINITY_XP_00640	EIN3-bir	Eutrema_salsugineum	22.70	0.00
TRINITY_XP_01146	PREDICTE	Fragaria_vesca	22.70	0.00
TRINITY_XP_02438	carbon c	Physcomitrella_pate	22.60	0.00
TRINITY_BAJ96807	predicte	Hordeum_vulgare	22.60	0.00
TRINITY_XP_00938	PREDICTE	Musa_acuminata	22.60	0.00
TRINITY_XP_01006	PREDICTE	Eucalyptus_grandis	22.60	0.00
TRINITY_XP_02057	BTB/POZ	Phalaenopsis_equest	22.60	0.00
TRINITY_RLN41400	hypothet	Panicum_miliaceum	22.60	0.00
TRINITY_PRW39140	kinase c	Chlorella_sorokinia	22.60	0.00
TRINITY_GBG67866	hypothet	Chara_braunii	22.60	0.00
TRINITY_PNH09214	Tiny mac	Tetrabaena_socialis	22.60	0.00
TRINITY_GAQ79003	hypothet	Klebsormidium_niten	22.50	0.00
TRINITY_GBF98776	hypothet	Raphidocelis_subcap	22.50	0.00
TRINITY_XP_00305	predicte	Micromonas_pusilla	22.50	0.00
TRINITY_XP_01924	PREDICTE	Nicotiana_attenuata	22.50	0.00
TRINITY_OAY63206	COP9 sig	Ananas_comosus	22.50	0.00
TRINITY_GAX74416	hypothet	Chlamydomonas_eusti	22.50	0.00
TRINITY_XP_00249	alpha-2	Micromonas_commoda	22.40	0.00
TRINITY_XP_02389	DNA repa	Quercus_suber	22.40	0.00
TRINITY_PSC70656	actin-re	Micractinium_conduc	22.40	0.00
TRINITY_PRW39140	kinase c	Chlorella_sorokinia	22.40	0.00
TRINITY_KYP60255	putative	Cajanus_cajan	22.40	0.00
TRINITY_XP_02377	brefeldi	Lactuca_sativa	22.40	0.00
TRINITY_RQL93479	hypothet	Brassica_cretica	22.30	0.00
TRINITY_XP_02598	serine/t	Glycine_max	22.30	0.00
TRINITY_PSC76429	WD40 rep	Micractinium_conduc	22.20	0.00
TRINITY_CAN61039	hypothet	Vitis_vinifera	22.20	0.00
TRINITY_XP_01047	PREDICTE	Camelina_sativa	22.20	0.00
TRINITY_XP_00294	hypothet	Volvox_carteri	22.10	0.00
TRINITY_XP_00305	predicte	Micromonas_pusilla	22.10	0.00
TRINITY_GAQ79300	hypothet	Klebsormidium_niten	22.10	0.00
TRINITY_ABR17822	unknown	Picea_sitchensis	22.10	0.00
TRINITY_XP_01003	PREDICTE	Eucalyptus_grandis	22.10	0.00
TRINITY_XP_00231	importir	Populus_trichocarpa	22.10	0.00
TRINITY_XP_00682	probable	Amborella_trichopod	22.10	0.00
TRINITY_XP_01006	PREDICTE	Eucalyptus_grandis	22.10	0.00
TRINITY_KZV47137	cation/c	Dorcoceras_hygromet	22.10	0.00
TRINITY_XP_02391	PX domai	Quercus_suber	22.10	0.00
TRINITY_GBG90296	hypothet	Chara_braunii	22.00	0.00
TRINITY_GAU25964	hypothet	Trifolium_subterran	22.00	0.00
TRINITY_GAX73762	hypothet	Chlamydomonas_eusti	22.00	0.00
TRINITY_XP_01918	PREDICTE	Ipomoea_nil	21.90	0.00
TRINITY_XP_00751	predicte	Bathycoccus_prasinc	21.90	0.00
TRINITY_XP_02284	beta-glu	Olea_europaea	21.80	0.00
TRINITY_PNW84206	hypothet	Chlamydomonas_reinh	21.80	0.00

TRINITY_OAE27533	hypothet	Marchantia_polymorp	21.80	0.00
TRINITY_RWR79461	hypothet	Cinnamomum_micranth	21.80	0.00
TRINITY_KDD75286	hypothet	Helicosporidium_sp.	21.80	0.00
TRINITY_KHG27277	Equilibı	Gossypium_arboreum	21.80	0.00
TRINITY_RMZ53223	hypothet	Auxenochlorella_prc	21.80	0.00
TRINITY_GBG76716	hypothet	Chara_braunii	21.70	0.00
TRINITY_XP_01053	PREDICTI	Tarenaya_hasslerian	21.70	0.00
TRINITY_PNY01822	WD repea	Trifolium_pratense	21.60	0.00
TRINITY_EAZ26064	hypothet	Oryza_sativa	21.60	0.00
TRINITY_GAX74708	hypothet	Chlamydomonas_eusti	21.50	0.00
TRINITY_XP_00169	intrafla	Chlamydomonas_reinh	21.40	0.00
TRINITY_KZM87265	hypothet	Daucus_carota	21.30	0.00
TRINITY_XP_02169	SNF1-rel	Hevea_brasiliensis	21.30	0.00
TRINITY_GAQ91638	DnaJ hea	Klebsormidium_niten	21.30	0.00
TRINITY_XP_02168	E3 ubiqu	Hevea_brasiliensis	21.30	0.00
TRINITY_XP_02390	5'-AMP-a	Quercus_suber	21.20	0.00
TRINITY_EOX94415	Ribophoı	Theobroma_cacao	21.20	0.00
TRINITY_GAX73349	hypothet	Chlamydomonas_eusti	21.20	0.00
TRINITY_XP_02375	syndetir	Lactuca_sativa	21.20	0.00
TRINITY_XP_00307	Amino ac	Ostreococcus_tauri	21.10	0.00
TRINITY_PRW58586	glucuror	Chlorella_sorokinia	21.10	0.00
TRINITY_RQL92630	hypothet	Brassica_cretica	21.00	0.00
TRINITY_GAQ81405	hypothet	Klebsormidium_niten	21.00	0.00
TRINITY_XP_01107	beta-glı	Sesamum_indicum	20.90	0.00
TRINITY_XP_01147	PREDICTI	Fragaria_vesca	20.90	0.00
TRINITY_XP_00584	hypothet	Chlorella_variabili	20.90	0.00
TRINITY_XP_02390	uncharac	Quercus_suber	20.90	0.00
TRINITY_KXZ50583	hypothet	Gonium_pectorale	20.90	0.00
TRINITY_GBF87488	sorting	Raphidocelis_subcap	20.90	0.00
TRINITY_XP_02736	polypyri	Abrus_precatorius	20.80	0.00
TRINITY_RQL92630	hypothet	Brassica_cretica	20.80	0.00
TRINITY_XP_02277	pentatri	Durio_zibethinus	20.80	0.00
TRINITY_GBG67933	hypothet	Chara_braunii	20.80	0.00
TRINITY_GAV78730	RRM_5 dc	Cephalotus_follicul	20.80	0.00
TRINITY_XP_00429	PREDICTI	Fragaria_vesca	20.70	0.00
TRINITY_XP_00934	PREDICTI	Pyrus_x_bretschneid	20.60	0.00
TRINITY_XP_02392	ras GTPa	Quercus_suber	20.60	0.00
TRINITY_XP_01725	PREDICTI	Daucus_carota	20.50	0.00
TRINITY_XP_02452	importir	Selaginella_moellen	20.50	0.00
TRINITY_RAL44982	hypothet	Cuscuta_australis	20.50	0.00
TRINITY_XP_02190	SNF1-rel	Carica_papaya	20.30	0.00
TRINITY_ERN05728	hypothet	Amborella_trichopod	20.20	0.00
