# Supplementary material for: Transcriptome Analysis of the Accumulation of Astaxanthin in Haematococcus pluvialis Treated with White and Blue Lights as well as Salicylic Acid
Source: Biomed Res Int. 2022 Jul 14;2022:4827595. doi: 10.1155/2022/4827595 (PMC9315456; doi:10.1155/2022/4827595)
Supplement: Supplementary 5 — Table S3: enrichment analysis of differentially expressed genes (DEGs) identified in Haematococcus pluvialis treated with blue light, white light, and blue light with salicylic acid based on the Pfam database. [file 4827595.f5.pdf]

Query	Pfam_hit	Pfam_description	Pfam_identity	Pfam_evalue
TRINITY	G3RL79_GORGO/46-117	G3RL79.1 PF00538.17	100.00	0.00
TRINITY	F5GY11_HUMAN/7-180	F5GY11.1 PF01852.17	100.00	0.00
TRINITY	B5APV0_PIG/10-151	B5APV0.1 PF04699.12	100.00	0.00
TRINITY	G3V4X6_HUMAN/21-91	G3V4X6.1 PF00076.20	100.00	0.00
TRINITY	K7ES79_HUMAN/7-59	K7ES79.1 PF10251.7;	100.00	0.00
TRINITY	F7A064_MONDO/5-148	F7A064.2 PF04756.11	100.00	0.00
TRINITY	SAMP_MOUSE/27-220	P12246.2 PF00354.15	100.00	0.00
TRINITY	S9XXD8_9CETA/75-183	S9XXD8.1 PF03143.15	100.00	0.00
TRINITY	H0XMX2_OTOGA/1-98	H0XMX2.1 PF01133.15	100.00	0.00
TRINITY	M3WEJ8_FELCA/453-607	M3WEJ8.1 PF09316.8;	100.00	0.00
TRINITY	G3NEV3_GASAC/89-756	G3NEV3.1 PF00063.19	100.00	0.00
TRINITY	CX6B1_MOUSE/19-83	P56391.2 PF02297.15	100.00	0.00
TRINITY	CX6B1_HUMAN/19-83	P14854.2 PF02297.15	100.00	0.00
TRINITY	I3L505_HUMAN/9-68	I3L505.1 PF00550.23	100.00	0.00
TRINITY	G3TKW3_LOXAF/10-66	G3TKW3.1 PF00412.20	100.00	0.00
TRINITY	F7IN08_CALJA/38-180	F7IN08.1 PF13589.4;	100.00	0.00
TRINITY	L5LTN0_MYODS/23-100	L5LTN0.1 PF00935.17	100.00	0.00
TRINITY	PZP_MOUSE/1395-1482	Q61838.3 PF07677.12	100.00	0.00
TRINITY	F7HPI2_MACMU/91-154	F7HPI2.1 PF17136.2;	100.00	0.00
TRINITY	H9H5K1_MACMU/1-60	H9H5K1.1 PF08069.10	100.00	0.00
TRINITY	BCAM_MOUSE/357-438	Q9R069.1 PF13895.4;	100.00	0.00
TRINITY	RNT2_MOUSE/41-217	Q9CQ01.1 PF00445.16	100.00	0.00
TRINITY	A2ZJY0_ORYSI/12-165	A2ZJY0.1 PF02605.13	100.00	0.00
TRINITY	G3ILI3_CRIGR/248-444	G3ILI3.1 PF00587.23	100.00	0.00
TRINITY	Q549D0_MOUSE/10-230	Q549D0.1 PF00335.18	100.00	0.00
TRINITY	F7HVS5_CALJA/27-103	F7HVS5.1 PF15477.4;	100.00	0.00
TRINITY	ECHP_MOUSE/297-471	Q9DBM2.4 PF02737.16	100.00	0.00
TRINITY	F1PM36_CANLF/72-349	F1PM36.2 PF00009.25	100.00	0.00
TRINITY	A0A0A0MSK8_HUMAN/216-291	A0A0A0MSK8.1 PF0527	100.00	0.00
TRINITY	F7BSJ5_CALJA/19-378	F7BSJ5.1 PF01384.18	100.00	0.00
TRINITY	F6S9T4_CALJA/1-194	F6S9T4.1 PF05805.10	100.00	0.00
TRINITY	H0XHG1_OTOGA/55-154	H0XHG1.1 PF03311.12	100.00	0.00
TRINITY	M7ZE25_TRIUA/85-215	M7ZE25.1 PF05479.9;	100.00	0.00
TRINITY	AMBP_MOUSE/229-281	Q07456.2 PF00014.21	100.00	0.00
TRINITY	AMBP_MOUSE/40-185	Q07456.2 PF00061.21	100.00	0.00
TRINITY	E9QAD6_MOUSE/1-92	E9QAD6.7 PF05511.9;	100.00	0.00
TRINITY	M3Z3C9_MUSPF/131-264	M3Z3C9.1 PF04042.14	100.00	0.00
TRINITY	H3BUY0_HUMAN/10-105	H3BUY0.1 PF00829.19	100.00	0.00
TRINITY	I3MWD9_ICTTR/201-270	I3MWD9.1 PF00076.20	100.00	0.00
TRINITY	H2NEP2_PONAB/105-188	H2NEP2.1 PF00189.18	100.00	0.00
TRINITY	H2PV47_PONAB/14-145	H2PV47.1 PF00583.23	100.00	0.00
TRINITY	G3HKP3_CRIGR/95-280	G3HKP3.1 PF05193.19	100.00	0.00
TRINITY	G3UYY2_MOUSE/57-410	G3UYY2.1 PF05694.9;	100.00	0.00
TRINITY	A0A0R4J0X5_MOUSE/46-410	A0A0R4J0X5.1 PF0007	100.00	0.00
TRINITY	H2RDP4_PANTR/47-456	H2RDP4.1 PF03345.12	100.00	0.00
TRINITY	Q5EEY3_RAT/13-172	Q5EEY3.1 PF00503.18	100.00	0.00
TRINITY	HCDH_MOUSE/29-214	Q61425.2 PF02737.16	100.00	0.00
TRINITY	E9PM14_HUMAN/7-105	E9PM14.1 PF06374.9;	100.00	0.00
TRINITY	J9NYN2_CANLF/10-66	J9NYN2.1 PF00412.20	100.00	0.00
TRINITY	Q5SZE1_HUMAN/132-304	Q5SZE1.2 PF03798.14	100.00	0.00
TRINITY	L5JQI7_PTEAL/54-212	L5JQI7.1 PF06703.9;	100.00	0.00
TRINITY	Q548X8_MOUSE/86-130	Q548X8.1 PF00095.19	100.00	0.00
TRINITY	H0Y5V3_HUMAN/43-231	H0Y5V3.2 PF02492.17	100.00	0.00
TRINITY	H2PDB8_PONAB/56-161	H2PDB8.2 PF07679.14	100.00	0.00
TRINITY	U3JNI8_FICAL/14-98	U3JNI8.1 PF00030.17	100.00	0.00
TRINITY	G3UWP5_MOUSE/66-192	G3UWP5.1 PF07731.12	100.00	0.00
TRINITY	R4GLP9_CHICK/2-146	R4GLP9.1 PF05808.9;	100.00	0.00
TRINITY	H0Y8L3_HUMAN/104-218	H0Y8L3.1 PF02469.20	100.00	0.00
TRINITY	H0W268_CAVPO/15-120	H0W268.1 PF02991.14	100.00	0.00
TRINITY	G3TA84_LOXAF/2-182	G3TA84.1 PF00227.24	100.00	0.00
TRINITY	I3L3X7_HUMAN/28-100	I3L3X7.1 PF00227.24	100.00	0.00
TRINITY	G1PPD8_MYOLU/37-149	G1PPD8.1 PF01398.19	100.00	0.00
TRINITY	A0A091E506_FUKDA/6-83	A0A091E506.1 PF0172	100.00	0.00
TRINITY	H2NWY7_PONAB/6-105	H2NWY7.1 PF03920.13	100.00	0.00
TRINITY	H2NWY7_PONAB/6-105	H2NWY7.1 PF03920.13	100.00	0.00
TRINITY	H2NXT2_PONAB/616-875	H2NXT2.1 PF00069.23	100.00	0.00
TRINITY	E9PKV2_HUMAN/58-102	E9PKV2.1 PF01196.17	100.00	0.00
TRINITY	L5M7Y0_MYODS/228-371	L5M7Y0.1 PF00022.17	100.00	0.00

TRINITY_R4GFH1_CHICK/21-262	R4GFH1.1 PF15237.4;	100.00	0.00
TRINITY_G3WTB6_SARHA/7-144	G3WTB6.1 PF00179.24	100.00	0.00
TRINITY_F1NJT3_CHICK/616-697	F1NJT3.1 PF00041.19	100.00	0.00
TRINITY_F7B5X8_MACMU/75-289	F7B5X8.1 PF01564.15	100.00	0.00
TRINITY_A0A096N120_PAPAN/1-167	A0A096N120.1 PF0585	100.00	0.00
TRINITY_F162A_MOUSE/13-154	Q9D6U8.1 PF06388.9;	100.00	0.00
TRINITY_F8W7Q4_HUMAN/3-143	F8W7Q4.1 PF06388.9;	100.00	0.00
TRINITY_H2QFH9_PANTR/4-66	H2QFH9.1 PF00240.21	100.00	0.00
TRINITY_F5GWF6_HUMAN/35-525	F5GWF6.2 PF00118.22	100.00	0.00
TRINITY_D3Z2F8_MOUSE/3-158	D3Z2F8.1 PF04062.12	100.00	0.00
TRINITY_COX7C_GORGO/1-62	P61638.1 PF02935.14	100.00	0.00
TRINITY_M3ZLY8_XIPMA/2389-2535	M3ZLY8.1 PF01608.15	100.00	0.00
TRINITY_H2PHY9_PONAB/172-234	H2PHY9.1 PF08157.9;	100.00	0.00
TRINITY_A0A0G2JSP8_RAT/120-367	A0A0G2JSP8.1 PF0021	100.00	0.00
TRINITY_A0A0G2JSP8_RAT/120-367	A0A0G2JSP8.1 PF0021	100.00	0.00
TRINITY_FETUA_MOUSE/149-239	P29699.1 PF00031.19	100.00	0.00
TRINITY_G1S4R1_NOMLE/186-327	G1S4R1.1 PF10075.7;	100.00	0.00
TRINITY_F1NJT4_CHICK/1575-1655	F1NJT4.2 PF00041.19	100.00	0.00
TRINITY_G3S6Y7_GORGO/199-323	G3S6Y7.1 PF03770.14	100.00	0.00
TRINITY_A0A0A0KW25_CUCSA/102-181	A0A0A0KW25.1 PF0034	100.00	0.00
TRINITY_F7DGD8_MACMU/19-97	F7DGD8.1 PF01423.20	100.00	0.00
TRINITY_THIL_MOUSE/39-296	Q8QZT1.1 PF00108.21	100.00	0.00
TRINITY_L5LKN3_MYODS/5-103	L5LKN3.1 PF03669.11	100.00	0.00
TRINITY_USMG5_MOUSE/1-51	Q78IK2.1 PF14960.4;	100.00	0.00
TRINITY_M3ZAZ1_NOMLE/17-114	M3ZAZ1.1 PF00428.17	100.00	0.00
TRINITY_G3RZ96_GORGO/653-744	G3RZ96.1 PF03129.18	100.00	0.00
TRINITY_D6RIL6_MOUSE/45-249	D6RIL6.1 PF00180.18	100.00	0.00
TRINITY_B1AHL2_HUMAN/394-435	B1AHL2.1 PF07645.13	100.00	0.00
TRINITY_G3RS10_GORGO/133-470	G3RS10.1 PF07992.12	100.00	0.00
TRINITY_F6TEH0_CALJA/109-163	F6TEH0.1 PF00010.24	100.00	0.00
TRINITY_G5BXT5_HETGA/8-114	G5BXT5.1 PF00179.24	100.00	0.00
TRINITY_Q5SYQ7_HUMAN/29-203	Q5SYQ7.1 PF00171.20	100.00	0.00
TRINITY_W0L7Y8_9ENTR/10-203	W0L7Y8.1 PF00009.25	100.00	0.00
TRINITY_H0Z6P7_TAEGU/9-406	H0Z6P7.1 PF00162.17	100.00	0.00
TRINITY_L5LY13_MYODS/82-282	L5LY13.1 PF03388.11	100.00	0.00
TRINITY_H0YAH8_HUMAN/1-43	H0YAH8.1 PF02469.20	100.00	0.00
TRINITY_W5NHJ2_LEPOC/22-154	W5NHJ2.1 PF00380.17	100.00	0.00
TRINITY_D2I822_AILME/2430-2519	D2I822.1 PF00630.17	100.00	0.00
TRINITY_H0YZT3_TAEGU/189-305	H0YZT3.1 PF01873.15	100.00	0.00
TRINITY_H0YZT3_TAEGU/189-305	H0YZT3.1 PF01873.15	100.00	0.00
TRINITY_A0A0D9QYQ0_CHLSB/23-547	A0A0D9QYQ0.1 PF0013	100.00	0.00
TRINITY_PGS2_HUMAN/150-218	P07585.1 PF13855.4;	100.00	0.00
TRINITY_G1NQC5_MELGA/25-137	G1NQC5.1 PF05348.9;	100.00	0.00
TRINITY_G1RJR7_NOMLE/3-183	G1RJR7.1 PF00227.24	100.00	0.00
TRINITY_Q804W0_DANRE/41-108	Q804W0.1 PF13499.4;	100.00	0.00
TRINITY_CP4AE_MOUSE/51-503	O35728.1 PF00067.20	100.00	0.00
TRINITY_H0WAW0_CAVPO/10-74	H0WAW0.1 PF00808.21	100.00	0.00
TRINITY_F7HDP5_CALJA/34-173	F7HDP5.1 PF00179.24	100.00	0.00
TRINITY_F1NJT4_CHICK/1847-1927	F1NJT4.2 PF00041.19	100.00	0.00
TRINITY_A0A0D9RJC0_CHLSB/28-535	A0A0D9RJC0.1 PF0011	100.00	0.00
TRINITY_U3JNI1_FICAL/171-263	U3JNI1.1 PF00153.25	100.00	0.00
TRINITY_F6RMI8_HORSE/171-325	F6RMI8.1 PF08648.10	100.00	0.00
TRINITY_Q3UEN8_MOUSE/38-494	Q3UEN8.1 PF00067.20	100.00	0.00
TRINITY_Q3UEN8_MOUSE/38-494	Q3UEN8.1 PF00067.20	100.00	0.00
TRINITY_HEMO_MOUSE/187-232	Q91X72.2 PF00045.17	100.00	0.00
TRINITY_HEMO_RAT/97-140	P20059.3 PF00045.17	100.00	0.00
TRINITY_A4_HUMAN/31-131	P05067.3 PF02177.14	100.00	0.00
TRINITY_M3W1X5_FELCA/11-113	M3W1X5.1 PF14974.4;	100.00	0.00
TRINITY_M3W1X5_FELCA/11-113	M3W1X5.1 PF14974.4;	100.00	0.00
TRINITY_G3NYQ9_GASAC/8-191	G3NYQ9.1 PF01214.16	100.00	0.00
TRINITY_G3H853_CRIGR/273-360	G3H853.1 PF00027.27	100.00	0.00
TRINITY_PP4C_HUMAN/47-242	P60510.1 PF00149.26	100.00	0.00
TRINITY_M0R1T5_HUMAN/1-164	M0R1T5.1 PF03357.19	100.00	0.00
TRINITY_G1P4C9_MYOLU/5-44	G1P4C9.1 PF01023.17	100.00	0.00
TRINITY_SMD3_HUMAN/8-73	P62318.1 PF01423.20	100.00	0.00
TRINITY_H0WRZ4_OTOGA/107-300	H0WRZ4.1 PF01556.16	100.00	0.00
TRINITY_G1TVQ3_RABIT/3-93	G1TVQ3.1 PF03259.15	100.00	0.00
TRINITY_I3L3K5_HUMAN/27-196	I3L3K5.1 PF03031.16	100.00	0.00
TRINITY_HPPD_MOUSE/180-335	P49429.3 PF00903.23	100.00	0.00

TRINITY_F7G9F5_MONDO/95-128	F7G9F5.2 PF16211.3;	100.00	0.00
TRINITY_B7Z4C8_HUMAN/19-100	B7Z4C8.1 PF01198.17	100.00	0.00
TRINITY_B7Z4C8_HUMAN/19-100	B7Z4C8.1 PF01198.17	100.00	0.00
TRINITY_H3BJQ7_MOUSE/56-210	H3BJQ7.1 PF08534.8;	100.00	0.00
TRINITY_RBM39_HUMAN/341-442	Q14498.2 PF15519.4;	100.00	0.00
TRINITY_A0A0U1RQH7_HUMAN/99-169	A0A0U1RQH7.1 PF0007	100.00	0.00
TRINITY_RL18A_BOVIN/7-130	Q3T003.1 PF01775.15	100.00	0.00
TRINITY_RL18A_BOVIN/7-130	Q3T003.1 PF01775.15	100.00	0.00
TRINITY_A2A848_MOUSE/443-623	A2A848.1 PF01756.17	100.00	0.00
TRINITY_CP2CT_MOUSE/30-487	Q64458.2 PF00067.20	100.00	0.00
TRINITY_A0A0D9RIQ2_CHLSB/227-299	A0A0D9RIQ2.1 PF0456	100.00	0.00
TRINITY_H2R2G1_PANTR/35-615	H2R2G1.1 PF03999.10	100.00	0.00
TRINITY_A0A091E6T2_FUKDA/85-122	A0A091E6T2.1 PF1027	100.00	0.00
TRINITY_M3XM19_MUSPF/109-368	M3XM19.1 PF01564.15	100.00	0.00
TRINITY_E2RFC7_CANLF/2-244	E2RFC7.1 PF10036.7;	100.00	0.00
TRINITY_E6WFD0_PANSA/4-69	E6WFD0.1 PF00313.20	100.00	0.00
TRINITY_Q91VB8_MOUSE/7-107	Q91VB8.1 PF00042.20	100.00	0.00
TRINITY_G1U007_RABIT/16-143	G1U007.1 PF00134.21	100.00	0.00
TRINITY_A0A0N4SVE0_MOUSE/26-209	A0A0N4SVE0.1 PF0101	100.00	0.00
TRINITY_A0A067LBG5_JATCU/18-243	A0A067LBG5.1 PF0024	100.00	0.00
TRINITY_H2NCG8_PONAB/153-239	H2NCG8.1 PF00335.18	100.00	0.00
TRINITY_A0A0A0L4W3_CUCSA/62-154	A0A0A0L4W3.1 PF0124	100.00	0.00
TRINITY_A0A0P7THG7_9TELE/300-367	A0A0P7THG7.1 PF0000	100.00	0.00
TRINITY_H0VI16_CAVPO/1-74	H0VI16.1 PF00240.21	100.00	0.00
TRINITY_A0A0P7WJQ7_9TELE/172-305	A0A0P7WJQ7.1 PF0000	100.00	0.00
TRINITY_RL39_HUMAN/9-50	P62891.2 PF00832.18	100.00	0.00
TRINITY_H3BJT2_MOUSE/19-113	H3BJT2.1 PF00086.16	100.00	0.00
TRINITY_A0A0G2JGM6_MOUSE/54-118	A0A0G2JGM6.1 PF0916	100.00	0.00
TRINITY_H2N6A0_PONAB/757-788	H2N6A0.2 PF12901.5;	100.00	0.00
TRINITY_I3N616_ICTTR/10-94	I3N616.1 PF00855.15	100.00	0.00
TRINITY_G1LLX4_AILME/564-609	G1LLX4.1 PF00628.27	100.00	0.00
TRINITY_H2QR05_PANTR/300-418	H2QR05.1 PF02984.17	100.00	0.00
TRINITY_G1RH69_NOMLE/10-52	G1RH69.1 PF01023.17	100.00	0.00
TRINITY_C5YBI0_SORBI/14-343	C5YBI0.1 PF01536.14	100.00	0.00
TRINITY_H2QV39_PANTR/45-83	H2QV39.1 PF00400.30	100.00	0.00
TRINITY_K7EQR7_HUMAN/8-78	K7EQR7.1 PF00076.20	100.00	0.00
TRINITY_ITB1_HUMAN/640-728	P05556.2 PF07965.10	100.00	0.00
TRINITY_F7GR58_CALJA/5-143	F7GR58.1 PF00025.19	100.00	0.00
TRINITY_F8WD96_HUMAN/78-276	F8WD96.1 PF00026.21	100.00	0.00
TRINITY_G1RG90_NOMLE/368-452	G1RG90.1 PF09173.9;	100.00	0.00
TRINITY_E1BIB4_BOVIN/355-479	E1BIB4.2 PF13893.4;	100.00	0.00
TRINITY_F7G013_ORNAN/1-53	F7G013.1 PF01907.17	100.00	0.00
TRINITY_A0A0E0A8W0_9ORYZ/123-306	A0A0E0A8W0.1 PF0050	100.00	0.00
TRINITY_A0A093DM08_CHAPE/20-114	A0A093DM08.1 PF0033	100.00	0.00
TRINITY_G1PSJ7_MYOLU/1-157	G1PSJ7.1 PF12850.5;	100.00	0.00
TRINITY_H2QSV7_PANTR/18-212	H2QSV7.1 PF00687.19	100.00	0.00
TRINITY_F6QC10_CALJA/247-359	F6QC10.1 PF13012.4;	100.00	0.00
TRINITY_COX8A_MOUSE/26-68	Q64445.1 PF02285.13	100.00	0.00
TRINITY_L5M755_MYODS/142-226	L5M755.1 PF00736.17	100.00	0.00
TRINITY_NDUS5_PANTR/1-96	Q0MQH4.3 PF10200.7;	100.00	0.00
TRINITY_G3SIR0_GORGO/26-148	G3SIR0.1 PF00787.22	100.00	0.00
TRINITY_A0A0D9RKH7_CHLSB/54-148	A0A0D9RKH7.1 PF0222	100.00	0.00
TRINITY_G3I5Q4_CRIGR/499-567	G3I5Q4.1 PF00076.20	100.00	0.00
TRINITY_H3BJZ9_MOUSE/217-328	H3BJZ9.1 PF02803.16	100.00	0.00
TRINITY_H2M7W2_ORYLA/32-84	H2M7W2.1 PF13833.4;	100.00	0.00
TRINITY_A0A0H2UI42_RAT/86-136	A0A0H2UI42.1 PF0032	100.00	0.00
TRINITY_E1BS_ADE02/1-135	P03247.2 PF01691.14	100.00	0.00
TRINITY_F2Z4Z8_PIG/143-181	F2Z4Z8.2 PF00400.30	100.00	0.00
TRINITY_H2PGJ8_PONAB/3-185	H2PGJ8.1 PF01459.20	100.00	0.00
TRINITY_G1NTC8_MYOLU/1-96	G1NTC8.1 PF01199.16	100.00	0.00
TRINITY_HG2A_MOUSE/1-112	P04441.3 PF09307.8;	100.00	0.00
TRINITY_H2P9V3_PONAB/17-82	H2P9V3.1 PF01246.18	100.00	0.00
TRINITY_H2P9V3_PONAB/17-82	H2P9V3.1 PF01246.18	100.00	0.00
TRINITY_H2P9V3_PONAB/17-82	H2P9V3.1 PF01246.18	100.00	0.00
TRINITY_AL1A1_MOUSE/29-492	P24549.5 PF00171.20	100.00	0.00
TRINITY_AL1A1_MOUSE/29-492	P24549.5 PF00171.20	100.00	0.00
TRINITY_H0YED6_HUMAN/1-249	H0YED6.1 PF06409.9;	100.00	0.00
TRINITY_ACADL_MOUSE/54-165	P51174.2 PF02771.14	100.00	0.00
TRINITY_G3RQY0_GORGO/25-107	G3RQY0.1 PF07654.13	100.00	0.00

TRINITY_M7AQ77_CHEMY/82-163	M7AQ77.1 PF07679.14	100.00	0.00
TRINITY_RL27_BOVIN/52-136	P61356.2 PF01777.16	100.00	0.00
TRINITY_RL27_BOVIN/52-136	P61356.2 PF01777.16	100.00	0.00
TRINITY_RL27_BOVIN/52-136	P61356.2 PF01777.16	100.00	0.00
TRINITY_F7HMJ4_CALJA/5-178	F7HMJ4.1 PF00071.20	100.00	0.00
TRINITY_L5MEY5_MYODS/26-121	L5MEY5.1 PF03501.13	100.00	0.00
TRINITY_F6YIK2_HORSE/12-87	F6YIK2.1 PF00347.21	100.00	0.00
TRINITY_G3THP4_LOXAF/2-67	G3THP4.1 PF03931.13	100.00	0.00
TRINITY_A0DTL7_PARTE/135-189	A0DTL7.1 PF12838.5;	100.00	0.00
TRINITY_D2I822_AILME/2430-2519	D2I822.1 PF00630.17	100.00	0.00
TRINITY_H2R2G1_PANTR/35-615	H2R2G1.1 PF03999.10	100.00	0.00
TRINITY_A0A087WVA0_HUMAN/1287-1352	A0A087WVA0.1 PF0675	100.00	0.00
TRINITY_G3RMT1_GORGO/8-122	G3RMT1.1 PF16906.3;	100.00	0.00
TRINITY_G3RMT1_GORGO/8-122	G3RMT1.1 PF16906.3;	100.00	0.00
TRINITY_G3RMT1_GORGO/8-122	G3RMT1.1 PF16906.3;	100.00	0.00
TRINITY_F2Z3T9_RAT/399-460	F2Z3T9.1 PF00076.20	100.00	0.00
TRINITY_A0A078D2I6_BRANA/5-177	A0A078D2I6.1 PF0002	100.00	0.00
TRINITY_A0A0P7TIS5_9TELE/50-201	A0A0P7TIS5.1 PF0017	100.00	0.00
TRINITY_GAG_XMRV6/224-434	Q27ID9.1 PF02093.14	100.00	0.00
TRINITY_POL_XMRV6/758-930	A1Z651.1 PF00078.25	100.00	0.00
TRINITY_L5LTN0_MYODS/23-100	L5LTN0.1 PF00935.17	100.00	0.00
TRINITY_L5LTN0_MYODS/23-100	L5LTN0.1 PF00935.17	100.00	0.00
TRINITY_R4G9A5_ANOCA/12-65	R4G9A5.1 PF00584.18	100.00	0.00
TRINITY_F7EK10_MACMU/8-102	F7EK10.1 PF01248.24	100.00	0.00
TRINITY_LEG3_MOUSE/131-261	P16110.3 PF00337.20	100.00	0.00
TRINITY_G3X1G4_SARHA/19-112	G3X1G4.1 PF00300.20	100.00	0.00
TRINITY_W5MUG1_LEPOC/12-106	W5MUG1.1 PF01248.24	100.00	0.00
TRINITY_W5MUG1_LEPOC/12-106	W5MUG1.1 PF01248.24	100.00	0.00
TRINITY_C3YW48_BRAFL/174-284	C3YW48.1 PF03953.15	100.00	0.00
TRINITY_ATP5L_MOUSE/10-102	Q9CPQ8.1 PF04718.13	100.00	0.00
TRINITY_G1L912_AILME/106-333	G1L912.1 PF04045.12	100.00	0.00
TRINITY_L8YGF1_TUPCH/18-81	L8YGF1.1 PF00076.20	100.00	0.00
TRINITY_Q3UKV9_MOUSE/24-90	Q3UKV9.1 PF01099.15	100.00	0.00
TRINITY_Q6P8P8_MOUSE/96-192	Q6P8P8.1 PF04089.12	100.00	0.00
TRINITY_Q3UBS3_MOUSE/103-340	Q3UBS3.1 PF00089.24	100.00	0.00
TRINITY_FIBA_MOUSE/392-457	E9PV24.1 PF12160.6;	100.00	0.00
TRINITY_Q546G4_MOUSE/30-202	Q546G4.1 PF00273.18	100.00	0.00
TRINITY_A0A096NPT5_PAPAN/107-178	A0A096NPT5.1 PF0007	100.00	0.00
TRINITY_F7G013_ORNAN/1-53	F7G013.1 PF01907.17	100.00	0.00
TRINITY_F7G013_ORNAN/1-53	F7G013.1 PF01907.17	100.00	0.00
TRINITY_SAA3_MOUSE/22-122	P04918.1 PF00277.16	100.00	0.00
TRINITY_G1SHK7_RABIT/1-88	G1SHK7.1 PF16672.3;	100.00	0.00
TRINITY_U3KGV4_FICAL/17-124	U3KGV4.1 PF01655.16	100.00	0.00
TRINITY_F6YS48_MACMU/6-93	F6YS48.1 PF00630.17	100.00	0.00
TRINITY_G3RMM9_GORGO/11-151	G3RMM9.1 PF00241.18	100.00	0.00
TRINITY_OSTP_RAT/251-317	P08721.2 PF00865.16	100.00	0.00
TRINITY_APOH_MOUSE/261-345	Q01339.1 PF09014.8;	100.00	0.00
TRINITY_U3K6L2_FICAL/41-97	U3K6L2.1 PF01849.16	100.00	0.00
TRINITY_F6V795_HORSE/5-118	F6V795.1 PF06320.11	100.00	0.00
TRINITY_H2NEP2_PONAB/105-188	H2NEP2.1 PF00189.18	100.00	0.00
TRINITY_G1LSC2_AILME/16-112	G1LSC2.1 PF03645.11	100.00	0.00
TRINITY_H2NEP2_PONAB/105-188	H2NEP2.1 PF00189.18	100.00	0.00
TRINITY_H2NEP2_PONAB/105-188	H2NEP2.1 PF00189.18	100.00	0.00
TRINITY_A0A0D9RFF7_CHLSB/95-169	A0A0D9RFF7.1 PF0090	100.00	0.00
TRINITY_A0A0D9RFF7_CHLSB/95-169	A0A0D9RFF7.1 PF0090	100.00	0.00
TRINITY_G7PFI0_MACFA/5-305	G7PFI0.1 PF00009.25	100.00	0.00
TRINITY_A0A067K9W4_JATCU/5-205	A0A067K9W4.1 PF0000	100.00	0.00
TRINITY_M4AKG1_XIPMA/171-242	M4AKG1.1 PF03719.13	100.00	0.00
TRINITY_M4AKG1_XIPMA/171-242	M4AKG1.1 PF03719.13	100.00	0.00
TRINITY_M4AKG1_XIPMA/171-242	M4AKG1.1 PF03719.13	100.00	0.00
TRINITY_IGEB_MOUSE/236-426	P03975.1 PF00607.18	100.00	0.00
TRINITY_M4AKG1_XIPMA/171-242	M4AKG1.1 PF03719.13	100.00	0.00
TRINITY_F9W6J4_TRYCI/477-606	F9W6J4.1 PF00075.22	100.00	0.00
TRINITY_F6U187_HORSE/186-405	F6U187.1 PF00006.23	100.00	0.00
TRINITY_H2QR48_PANTR/9-95	H2QR48.1 PF02970.14	100.00	0.00
TRINITY_F6PUM4_MACMU/541-672	F6PUM4.1 PF09733.7;	100.00	0.00
TRINITY_G2HGS6_PANTR/257-780	G2HGS6.1 PF00183.16	100.00	0.00
TRINITY_F6UYZ4_MONDO/3-146	F6UYZ4.2 PF01280.18	100.00	0.00
TRINITY_F6UYZ4_MONDO/3-146	F6UYZ4.2 PF01280.18	100.00	0.00

TRINITY_RL5_BOVIN/14-176	Q58DW5.3 PF17144.2;	100.00	0.00
TRINITY_G1SFR8_RABIT/16-111	G1SFR8.1 PF01248.24	100.00	0.00
TRINITY_A0A0A6YW77_MOUSE/134-271	A0A0A6YW77.1 PF0006	100.00	0.00
TRINITY_A0A093DM08_CHAPE/20-114	A0A093DM08.1 PF0033	100.00	0.00
TRINITY_A9V2B9_MONBE/227-497	A9V2B9.1 PF13598.4;	100.00	0.00
TRINITY_A9V2B9_MONBE/227-497	A9V2B9.1 PF13598.4;	100.00	0.00
TRINITY_HMCS2_MOUSE/224-506	P54869.2 PF08540.8;	100.00	0.00
TRINITY_A0A0G4IGV5_PLABS/15-86	A0A0G4IGV5.1 PF0024	100.00	0.00
TRINITY_G7PCE3_MACFA/80-329	G7PCE3.1 PF00112.21	100.00	0.00
TRINITY_H2R8D1_PANTR/200-266	H2R8D1.1 PF00191.18	100.00	0.00
TRINITY_C6TD73_SOYBN/65-232	C6TD73.1 PF00504.19	100.00	0.00
TRINITY_V7C1G4_PHAVU/2-60	V7C1G4.1 PF00504.19	100.00	0.00
TRINITY_A0A0L6ULP7_9BASI/85-157	A0A0L6ULP7.1 PF0024	100.00	0.00
TRINITY_A0A0G4IGV5_PLABS/15-86	A0A0G4IGV5.1 PF0024	100.00	0.00
TRINITY_G3RX79_GORGO/70-324	G3RX79.1 PF04921.12	100.00	0.00
TRINITY_HMGB1_HORSE/6-78	Q08IE6.3 PF09011.8;	100.00	0.00
TRINITY_INMT_MOUSE/1-260	P40936.1 PF01234.15	100.00	0.00
TRINITY_PDIA3_MOUSE/164-355	P27773.2 PF13848.4;	100.00	0.00
TRINITY_F7ALA4_CALJA/16-106	F7ALA4.1 PF00085.18	100.00	0.00
TRINITY_F7AUW2_HORSE/7-613	F7AUW2.1 PF00012.18	100.00	0.00
TRINITY_GRP78_ICTTR/30-636	Q3S4T7.1 PF00012.18	100.00	0.00
TRINITY_K4A861_SETIT/222-535	K4A861.1 PF01293.18	100.00	0.00
TRINITY_A0A096MQE4_PAPAN/39-155	A0A096MQE4.1 PF0536	100.00	0.00
TRINITY_F7AUW2_HORSE/7-613	F7AUW2.1 PF00012.18	100.00	0.00
TRINITY_G1RY49_NOMLE/118-289	G1RY49.1 PF00270.27	100.00	0.00
TRINITY_G7PQQ4_MACFA/151-381	G7PQQ4.1 PF02854.17	100.00	0.00
TRINITY_G1M7U8_AILME/876-954	G1M7U8.1 PF02020.16	100.00	0.00
TRINITY_G7PQQ4_MACFA/151-381	G7PQQ4.1 PF02854.17	100.00	0.00
TRINITY_A0A0G4IGV5_PLABS/15-86	A0A0G4IGV5.1 PF0024	100.00	0.00
TRINITY_A0A0G4IGV5_PLABS/15-86	A0A0G4IGV5.1 PF0024	100.00	0.00
TRINITY_G1S9L1_NOMLE/1-38	G1S9L1.1 PF04419.12	100.00	0.00
TRINITY_H2P181_PONAB/37-132	H2P181.1 PF00031.19	100.00	0.00
TRINITY_FABPL_MOUSE/4-127	P12710.2 PF00061.21	100.00	0.00
TRINITY_F2Z3Q5_HUMAN/45-104	F2Z3Q5.1 PF07970.10	100.00	0.00
TRINITY_G3QNU5_GORGO/309-377	G3QNU5.1 PF00076.20	100.00	0.00
TRINITY_G1PQS7_MYOLU/16-221	G1PQS7.1 PF01015.16	100.00	0.00
TRINITY_G1PQS7_MYOLU/16-221	G1PQS7.1 PF01015.16	100.00	0.00
TRINITY_B7NZC3_RABIT/5-238	B7NZC3.1 PF00009.25	100.00	0.00
TRINITY_B7NZC3_RABIT/5-238	B7NZC3.1 PF00009.25	100.00	0.00
TRINITY_F1Q1K6_CANLF/114-183	F1Q1K6.2 PF00076.20	100.00	0.00
TRINITY_CKS2_HUMAN/6-73	P33552.1 PF01111.17	100.00	0.00
TRINITY_F1NJT3_CHICK/2201-2277	F1NJT3.1 PF00041.19	100.00	0.00
TRINITY_E9Q035_MOUSE/346-668	E9Q035.1 PF00405.15	100.00	0.00
TRINITY_W5PSW9_SHEEP/97-234	W5PSW9.1 PF03947.16	100.00	0.00
TRINITY_W5PSW9_SHEEP/97-234	W5PSW9.1 PF03947.16	100.00	0.00
TRINITY_V9FDA4_PHYPR/18-99	V9FDA4.1 PF15511.4;	100.00	0.00
TRINITY_V9FDA4_PHYPR/18-99	V9FDA4.1 PF15511.4;	100.00	0.00
TRINITY_G1TRM4_RABIT/75-144	G1TRM4.1 PF00366.18	100.00	0.00
TRINITY_G1TRM4_RABIT/75-144	G1TRM4.1 PF00366.18	100.00	0.00
TRINITY_PETD_CHLRE/65-158	P23230.1 PF00032.15	100.00	0.00
TRINITY_S9YV02_9CETA/358-560	S9YV02.1 PF00009.25	100.00	0.00
TRINITY_ALDR_HUMAN/15-294	P15121.3 PF00248.19	100.00	0.00
TRINITY_A0A075B5P2_MOUSE/6-94	A0A075B5P2.1 PF0765	100.00	0.00
TRINITY_L9KY99_TUPCH/5-130	L9KY99.1 PF00410.17	100.00	0.00
TRINITY_L9KY99_TUPCH/5-130	L9KY99.1 PF00410.17	100.00	0.00
TRINITY_F6YP53_CALJA/5-106	F6YP53.1 PF00466.18	100.00	0.00
TRINITY_F6YP53_CALJA/5-106	F6YP53.1 PF00466.18	100.00	0.00
TRINITY_H2P488_PONAB/431-515	H2P488.1 PF05091.10	100.00	0.00
TRINITY_L7N0B2_CANLF/263-392	L7N0B2.1 PF03953.15	100.00	0.00
TRINITY_C3YW48_BRAFL/174-284	C3YW48.1 PF03953.15	100.00	0.00
TRINITY_F7ID26_CALJA/33-108	F7ID26.1 PF01423.20	100.00	0.00
TRINITY_W5PHW0_SHEEP/191-713	W5PHW0.1 PF00183.16	100.00	0.00
TRINITY_F1PM36_CANLF/72-349	F1PM36.2 PF00009.25	100.00	0.00
TRINITY_G3R288_GORGO/157-314	G3R288.1 PF02800.18	100.00	0.00
TRINITY_NDUB1_MOUSE/1-57	P0DN34.1 PF08040.9;	100.00	0.00
TRINITY_F1NH87_CHICK/1-95	F1NH87.1 PF00044.22	100.00	0.00
TRINITY_D8TT41_VOLCA/30-634	D8TT41.1 PF00012.18	100.00	0.00
TRINITY_A0A087WP98_MOUSE/2-51	A0A087WP98.1 PF0324	100.00	0.00
TRINITY_M3WAB9_FELCA/110-175	M3WAB9.1 PF00076.20	100.00	0.00

TRINITY_G5B0K5_HETGA/23-177	G5B0K5.1 PF00071.20	100.00	0.00
TRINITY_G3RDP2_GORGO/75-371	G3RDP2.1 PF00648.19	100.00	0.00
TRINITY_CD9_MOUSE/10-218	P40240.2 PF00335.18	100.00	0.00
TRINITY_H0V674_CAVPO/5-283	H0V674.1 PF01213.17	100.00	0.00
TRINITY_S9XHQ6_9CETA/9-194	S9XHQ6.1 PF01105.22	100.00	0.00
TRINITY_L5LRD5_MYODS/242-351	L5LRD5.1 PF00271.29	100.00	0.00
TRINITY_RL6_HUMAN/181-288	Q02878.3 PF01159.17	100.00	0.00
TRINITY_Q3UCH0_MOUSE/189-296	Q3UCH0.1 PF01159.17	100.00	0.00
TRINITY_A0A0A0KAJ9_CUCSA/1-370	A0A0A0KAJ9.1 PF0029	100.00	0.00
TRINITY_B9SHX2_RICCO/28-293	B9SHX2.1 PF00124.17	100.00	0.00
TRINITY_K3Z2Q6_SETIT/28-329	K3Z2Q6.1 PF00124.17	100.00	0.00
TRINITY_H0XAT6_OTOGA/83-150	H0XAT6.1 PF01287.18	100.00	0.00
TRINITY_H2PXL0_PONAB/5-70	H2PXL0.1 PF01423.20	100.00	0.00
TRINITY_A0A0G4IGV5_PLABS/15-86	A0A0G4IGV5.1 PF0024	100.00	0.00
TRINITY_G3RCC0_GORGO/1-436	G3RCC0.1 PF00996.16	100.00	0.00
TRINITY_H9H7J0_MONDO/585-641	H9H7J0.2 PF01849.16	100.00	0.00
TRINITY_G3NI20_GASAC/296-364	G3NI20.1 PF00076.20	100.00	0.00
TRINITY_LEG1_MOUSE/5-134	P16045.3 PF00337.20	100.00	0.00
TRINITY_H2QBR4_PANTR/8-187	H2QBR4.1 PF01294.16	100.00	0.00
TRINITY_H2PUE2_PONAB/191-233	H2PUE2.2 PF00400.30	100.00	0.00
TRINITY_E1A_ADE02/1-289	P03254.1 PF02703.12	100.00	0.00
TRINITY_F6RQK7_HORSE/5-62	F6RQK7.1 PF05160.11	100.00	0.00
TRINITY_A0A091CUE5_FUKDA/106-143	A0A091CUE5.1 PF0622	100.00	0.00
TRINITY_H0ZBH5_TAEGU/1-124	H0ZBH5.1 PF01201.20	100.00	0.00
TRINITY_NDUAA_MOUSE/60-287	Q99LC3.1 PF01712.17	100.00	0.00
TRINITY_NDUAA_PANTR/60-287	Q0MQB7.1 PF01712.17	100.00	0.00
TRINITY_Q3TX21_MOUSE/281-374	Q3TX21.1 PF00007.20	100.00	0.00
TRINITY_G1TF14_RABIT/3-41	G1TF14.1 PF01290.18	100.00	0.00
TRINITY_G3W6H2_SARHA/91-390	G3W6H2.1 PF00557.22	100.00	0.00
TRINITY_HMGB1_PAPAN/95-163	A9RA84.1 PF00505.17	100.00	0.00
TRINITY_RL39_HUMAN/9-50	P62891.2 PF00832.18	100.00	0.00
TRINITY_RL39_HUMAN/9-50	P62891.2 PF00832.18	100.00	0.00
TRINITY_H2PDR8_PONAB/150-219	H2PDR8.1 PF00076.20	100.00	0.00
TRINITY_F7FMB4_MACMU/7-96	F7FMB4.1 PF12926.5;	100.00	0.00
TRINITY_M3W902_FELCA/7-72	M3W902.1 PF01423.20	100.00	0.00
TRINITY_A8JFE2_CHLRE/94-156	A8JFE2.1 PF00137.19	100.00	0.00
TRINITY_A8JFE2_CHLRE/94-156	A8JFE2.1 PF00137.19	100.00	0.00
TRINITY_E9Q5L2_MOUSE/762-925	E9Q5L2.1 PF06668.10	100.00	0.00
TRINITY_ECHP_MOUSE/614-710	Q9DBM2.4 PF00725.20	100.00	0.00
TRINITY_D8UHM6_VOLCA/586-795	D8UHM6.1 PF00149.26	100.00	0.00
TRINITY_Q8T8P2_DICDI/364-491	Q8T8P2.1 PF00005.25	100.00	0.00
TRINITY_CP2F2_MOUSE/31-488	P33267.1 PF00067.20	100.00	0.00
TRINITY_CAPP1_MAIZE/167-970	P04711.2 PF00311.15	100.00	0.00
TRINITY_CXL15_MOUSE/28-86	Q9WVL7.1 PF00048.18	100.00	0.00
TRINITY_G1LL23_AILME/28-81	G1LL23.1 PF01667.15	100.00	0.00
TRINITY_A0DTL7_PARTE/135-189	A0DTL7.1 PF12838.5;	100.00	0.00
TRINITY_H0WK33_OTOGA/530-965	H0WK33.1 PF03378.13	100.00	0.00
TRINITY_NDUB3_MOUSE/48-104	Q9CQZ6.1 PF08122.10	100.00	0.00
TRINITY_A9V2B9_MONBE/227-497	A9V2B9.1 PF13598.4;	100.00	0.00
TRINITY_ACTG_EMENI/2-375	P20359.2 PF00022.17	100.00	0.00
TRINITY_ACTG_EMENI/2-375	P20359.2 PF00022.17	100.00	0.00
TRINITY_A0A096T531_MAIZE/104-344	A0A096T531.1 PF0132	100.00	0.00
TRINITY_IGKC_HUMAN/5-93	P01834.1 PF07654.13	100.00	0.00
TRINITY_F7HJT3_CALJA/1-210	F7HJT3.1 PF00244.18	100.00	0.00
TRINITY_TYB4_PIG/3-41	Q95274.3 PF01290.18	100.00	0.00
TRINITY_W5P0L8_SHEEP/639-742	W5P0L8.1 PF00435.19	100.00	0.00
TRINITY_D8U1V2_VOLCA/50-218	D8U1V2.1 PF00504.19	100.00	0.00
TRINITY_D8UHM6_VOLCA/586-795	D8UHM6.1 PF00149.26	100.00	0.00
TRINITY_A9U2Q2_PHYPA/17-739	A9U2Q2.1 PF03030.14	100.00	0.00
TRINITY_S4R239_MOUSE/66-103	S4R239.1 PF05184.13	100.00	0.00
TRINITY_S4R2L6_MOUSE/306-339	S4R2L6.1 PF03489.15	100.00	0.00
TRINITY_BPIA1_MOUSE/83-256	P97361.3 PF01273.23	100.00	0.00
TRINITY_MLP3A_HUMAN/15-120	Q9H492.2 PF02991.14	100.00	0.00
TRINITY_CRBB2_CHICK/18-100	Q05714.2 PF00030.17	100.00	0.00
TRINITY_A0A093DM08_CHAPE/20-114	A0A093DM08.1 PF0033	100.00	0.00
TRINITY_VIME_MOUSE/6-101	P20152.3 PF04732.12	100.00	0.00
TRINITY_D3YUE2_MOUSE/378-482	D3YUE2.1 PF01759.19	100.00	0.00
TRINITY_S9XHD7_9CETA/1601-1813	S9XHD7.1 PF04054.13	100.00	0.00
TRINITY_E1BW78_CHICK/5-74	E1BW78.2 PF02937.13	100.00	0.00

TRINITY_NDUB2_HUMAN/37-104	O95178.1 PF14813.4;	100.00	0.00
TRINITY_ATP5I_MOUSE/2-69	Q06185.2 PF05680.10	100.00	0.00
TRINITY_CD36_MOUSE/14-463	Q08857.2 PF01130.19	100.00	0.00
TRINITY_M0R547_RAT/27-87	M0R547.1 PF04691.10	100.00	0.00
TRINITY_R4GH93_CHICK/10-52	R4GH93.1 PF01023.17	100.00	0.00
TRINITY_E1C2Y5_CHICK/30-87	E1C2Y5.1 PF00048.18	100.00	0.00
TRINITY_E5RIM2_HUMAN/34-197	E5RIM2.1 PF03096.12	100.00	0.00
TRINITY_Q5ZL96_CHICK/5-160	Q5ZL96.1 PF13238.4;	100.00	0.00
TRINITY_A0A061GBH7_THECC/240-474	A0A061GBH7.1 PF0145	100.00	0.00
TRINITY_L5K196_PTEAL/449-501	L5K196.1 PF13833.4;	100.00	0.00
TRINITY_CX6A2_MOUSE/10-90	P43023.2 PF02046.13	100.00	0.00
TRINITY_G3SH69_GORGO/24-117	G3SH69.1 PF00236.16	100.00	0.00
TRINITY_I3MGJ2_ICTTR/24-139	I3MGJ2.1 PF00307.29	100.00	0.00
TRINITY_F6UPR5_CALJA/74-352	F6UPR5.1 PF00464.17	100.00	0.00
TRINITY_ATX10_PONAB/371-468	Q5RE06.1 PF09759.7;	100.00	0.00
TRINITY_SG3A2_MOUSE/30-98	Q920H1.1 PF01099.15	100.00	0.00
TRINITY_H2NVE2_PONAB/39-168	H2NVE2.2 PF00735.16	100.00	0.00
TRINITY_A0A0A6YWV4_MOUSE/9-99	A0A0A6YWV4.1 PF0765	100.00	0.00
TRINITY_Q792A4_MOUSE/24-76	Q792A4.1 PF02238.13	100.00	0.00
TRINITY_Q5M9K1_MOUSE/32-139	Q5M9K1.1 PF00576.19	100.00	0.00
TRINITY_H2QBN5_PANTR/30-168	H2QBN5.1 PF02936.12	100.00	0.00
TRINITY_NDUA1_HUMAN/2-56	O15239.1 PF15879.3;	100.00	0.00
TRINITY_Q3UP42_MOUSE/9-51	Q3UP42.1 PF01023.17	100.00	0.00
TRINITY_THIM_MOUSE/7-266	Q8BWT1.3 PF00108.21	100.00	0.00
TRINITY_HG2A_HUMAN/17-129	P04233.3 PF09307.8;	100.00	0.00
TRINITY_H2PLJ1_PONAB/12-90	H2PLJ1.1 PF10206.7;	100.00	0.00
TRINITY_B4F8P6_MAIZE/352-605	B4F8P6.1 PF03949.13	100.00	0.00
TRINITY_G1RJV7_NOMLE/26-333	G1RJV7.1 PF00365.18	100.00	0.00
TRINITY_H0WVZ0_OTOGA/333-390	H0WVZ0.1 PF00412.20	100.00	0.00
TRINITY_F1SL58_PIG/19-338	F1SL58.2 PF04912.12	100.00	0.00
TRINITY_B6SUC4_MAIZE/56-238	B6SUC4.1 PF00504.19	100.00	0.00
TRINITY_APOA2_MOUSE/24-98	P09813.2 PF04711.11	100.00	0.00
TRINITY_F6WVD1_HORSE/7-68	F6WVD1.1 PF01200.16	100.00	0.00
TRINITY_F6WVD1_HORSE/7-68	F6WVD1.1 PF01200.16	100.00	0.00
TRINITY_F6TZ17_MACMU/13-123	F6TZ17.1 PF02466.17	100.00	0.00
TRINITY_G5B2Q8_HETGA/465-594	G5B2Q8.1 PF14721.4;	100.00	0.00
TRINITY_CAPP1_MAIZE/167-970	P04711.2 PF00311.15	100.00	0.00
TRINITY_VTDB_MOUSE/218-385	P21614.2 PF00273.18	100.00	0.00
TRINITY_PCKA_MAIZE/145-612	Q9SLZ0.1 PF01293.18	100.00	0.00
TRINITY_H0YC35_HUMAN/1-284	H0YC35.1 PF01093.15	100.00	0.00
TRINITY_ATPK_MOUSE/6-84	P56135.3 PF10206.7;	100.00	0.00
TRINITY_K3Z2Q6_SETIT/28-329	K3Z2Q6.1 PF00124.17	100.00	0.00
TRINITY_G1QGN9_NOMLE/633-827	G1QGN9.2 PF05010.12	100.00	0.00
TRINITY_OLA1_BOVIN/305-388	Q2HJ33.1 PF06071.11	100.00	0.00
TRINITY_B6SQV5_MAIZE/32-127	B6SQV5.1 PF04725.10	100.00	0.00
TRINITY_FGL1_MOUSE/81-307	Q71KU9.2 PF00147.16	100.00	0.00
TRINITY_E9PV44_MOUSE/1-69	E9PV44.1 PF04568.10	100.00	0.00
TRINITY_CP2CT_MOUSE/30-487	Q64458.2 PF00067.20	100.00	0.00
TRINITY_E9Q4M7_MOUSE/32-98	E9Q4M7.2 PF07546.11	100.00	0.00
TRINITY_G1MZP6_MELGA/1-55	G1MZP6.1 PF00525.16	100.00	0.00
TRINITY_NLTP_MOUSE/437-539	P32020.3 PF02036.15	100.00	0.00
TRINITY_PPDPF_HUMAN/1-110	Q9H3Y8.1 PF15060.4;	100.00	0.00
TRINITY_A0A0R4IJT8_DANRE/39-529	A0A0R4IJT8.1 PF0011	100.00	0.00
TRINITY_A0A093DIW8_CHAPE/21-702	A0A093DIW8.1 PF0006	100.00	0.00
TRINITY_M3WDW3_FELCA/115-184	M3WDW3.1 PF00076.20	100.00	0.00
TRINITY_G3RV56_GORGO/258-343	G3RV56.1 PF08542.9;	100.00	0.00
TRINITY_G3TGB1_LOXAF/57-276	G3TGB1.1 PF00149.26	100.00	0.00
TRINITY_PGS1_RABIT/48-107	O46377.1 PF13855.4;	100.00	0.00
TRINITY_ANRE_MOUSE/19-121	P61110.1 PF15222.4;	100.00	0.00
TRINITY_E1C440_CHICK/681-731	E1C440.2 PF10515.7;	100.00	0.00
TRINITY_ASSY_HUMAN/8-403	P00966.2 PF00764.17	100.00	0.00
TRINITY_T4S1_MOUSE/1-194	Q64302.1 PF05805.10	100.00	0.00
TRINITY_F7AUW2_HORSE/7-613	F7AUW2.1 PF00012.18	99.70	0.00
TRINITY_TPIS_PONAB/7-245	Q5R928.1 PF00121.16	99.60	0.00
TRINITY_A0A0D9R9Y5_CHLSB/39-529	A0A0D9R9Y5.1 PF0011	99.50	0.00
TRINITY_E1B55_ADE02/110-492	P03244.2 PF01696.15	99.50	0.00
TRINITY_F2Q9A3_PIG/7-163	F2Q9A3.1 PF00160.19	99.40	0.00
TRINITY_GRP78_ICTTR/30-636	Q3S4T7.1 PF00012.18	99.40	0.00
TRINITY_F6TD92_MACMU/1-168	F6TD92.1 PF00838.15	99.40	0.00

TRINITY_G3X9L6_MOUSE/3-154	G3X9L6.1 PF05873.10	99.30	0.00
TRINITY_A1AG1_MOUSE/41-181	Q60590.1 PF00061.21	99.30	0.00
TRINITY_M3YA62_MUSPF/154-401	M3YA62.1 PF00217.17	99.30	0.00
TRINITY_L5KVY8_PTEAL/1-137	L5KVY8.1 PF00179.24	99.30	0.00
TRINITY_U6M5D0_EIMMA/34-255	U6M5D0.1 PF01353.20	99.30	0.00
TRINITY_FRIL1_MOUSE/14-155	P29391.2 PF00210.22	99.30	0.00
TRINITY_RET4_RAT/39-185	P04916.1 PF00061.21	99.30	0.00
TRINITY_A0A096NG97_PAPAN/18-159	A0A096NG97.1 PF0021	99.30	0.00
TRINITY_H0ZQ12_TAEGU/1-160	H0ZQ12.1 PF00838.15	99.30	0.00
TRINITY_G1NIT7_MELGA/6-134	G1NIT7.1 PF00337.20	99.20	0.00
TRINITY_M0R5K9_RAT/13-141	M0R5K9.1 PF00416.20	99.20	0.00
TRINITY_M0R5K9_RAT/13-141	M0R5K9.1 PF00416.20	99.20	0.00
TRINITY_M3XQI1_MUSPF/22-154	M3XQI1.1 PF00237.17	99.20	0.00
TRINITY_F6XWI2_MACMU/41-255	F6XWI2.1 PF03731.13	99.20	0.00
TRINITY_W5PSW9_SHEEP/97-234	W5PSW9.1 PF03947.16	99.20	0.00
TRINITY_L9KY99_TUPCH/5-130	L9KY99.1 PF00410.17	99.20	0.00
TRINITY_H2QBR4_PANTR/8-187	H2QBR4.1 PF01294.16	99.20	0.00
TRINITY_G1PWJ3_MYOLU/28-150	G1PWJ3.1 PF02996.15	99.20	0.00
TRINITY_EFTU_RAT/55-249	P85834.1 PF00009.25	99.10	0.00
TRINITY_U3IHY7_ANAPL/25-161	U3IHY7.1 PF00210.22	99.10	0.00
TRINITY_H2P3T5_PONAB/2-115	H2P3T5.1 PF01187.16	99.10	0.00
TRINITY_H2R1B6_PANTR/5-118	H2R1B6.1 PF00572.16	99.10	0.00
TRINITY_M3ZZZ0_XIPMA/8-121	M3ZZZ0.1 PF00572.16	99.00	0.00
TRINITY_G1NTC8_MYOLU/1-96	G1NTC8.1 PF01199.16	99.00	0.00
TRINITY_L5MEY5_MYODS/26-121	L5MEY5.1 PF03501.13	99.00	0.00
TRINITY_H2ZUX4_LATCH/581-734	H2ZUX4.1 PF00022.17	99.00	0.00
TRINITY_H0Z1N0_TAEGU/1-124	H0Z1N0.1 PF01092.17	99.00	0.00
TRINITY_M4AL71_XIPMA/2-376	M4AL71.1 PF00022.17	99.00	0.00
TRINITY_EIF3L_XENTR/150-548	Q6P878.1 PF10255.7;	99.00	0.00
TRINITY_F7DD44_MONDO/67-165	F7DD44.2 PF00673.19	99.00	0.00
TRINITY_D4A771_RAT/5-104	D4A771.1 PF01247.16	99.00	0.00
TRINITY_H2PNG6_PONAB/8-109	H2PNG6.1 PF01990.15	98.90	0.00
TRINITY_A0A0D9RE68_CHLSB/24-326	A0A0D9RE68.1 PF0092	98.90	0.00
TRINITY_G1M6P5_AILME/22-113	G1M6P5.1 PF00428.17	98.90	0.00
TRINITY_F7EK10_MACMU/8-102	F7EK10.1 PF01248.24	98.90	0.00
TRINITY_M3XXR0_MUSPF/2-188	M3XXR0.1 PF17135.2;	98.90	0.00
TRINITY_G3I2I9_CRIGR/1-375	G3I2I9.1 PF00297.20	98.90	0.00
TRINITY_G7NTL1_MACFA/283-375	G7NTL1.1 PF00007.20	98.90	0.00
TRINITY_A0A0A0KN28_CUCSA/47-151	A0A0A0KN28.1 PF0041	98.80	0.00
TRINITY_M4AHJ8_XIPMA/43-128	M4AHJ8.1 PF00203.19	98.80	0.00
TRINITY_PSBQ1_MAIZE/17-217	Q41048.1 PF05757.9;	98.80	0.00
TRINITY_M0YJF4_HORVD/1-185	M0YJF4.1 PF00091.23	98.80	0.00
TRINITY_F7HUZ7_CALJA/70-149	F7HUZ7.1 PF00312.20	98.80	0.00
TRINITY_F7HUZ7_CALJA/70-149	F7HUZ7.1 PF00312.20	98.80	0.00
TRINITY_G3IMP5_CRIGR/132-269	G3IMP5.1 PF03096.12	98.80	0.00
TRINITY_L5M755_MYODS/142-226	L5M755.1 PF00736.17	98.80	0.00
TRINITY_B2MG_MOUSE/25-107	P01887.2 PF07654.13	98.80	0.00
TRINITY_COX3_DANRE/6-261	Q9MIY4.1 PF00510.16	98.80	0.00
TRINITY_J9PA46_CANLF/65-309	J9PA46.1 PF03372.21	98.80	0.00
TRINITY_G3UPT8_MELGA/1-557	G3UPT8.1 PF05817.12	98.80	0.00
TRINITY_F7FZB5_MONDO/57-243	F7FZB5.1 PF00149.26	98.80	0.00
TRINITY_G3SJE0_GORGO/59-488	G3SJE0.1 PF13520.4;	98.80	0.00
TRINITY_A0A0D2T6A5_GOSRA/46-177	A0A0D2T6A5.1 PF0012	98.80	0.00
TRINITY_A0A0P7UH33_9TELE/3-126	A0A0P7UH33.1 PF0177	98.80	0.00
TRINITY_G3H5D5_CRIGR/35-115	G3H5D5.1 PF00887.17	98.80	0.00
TRINITY_D8UC08_VOLCA/219-349	D8UC08.1 PF00004.27	98.80	0.00
TRINITY_A0A0A0LII5_CUCSA/157-331	A0A0A0LII5.1 PF0286	98.80	0.00
TRINITY_M3XNU4_MUSPF/47-141	M3XNU4.1 PF01248.24	98.70	0.00
TRINITY_G3SUA8_LOXAF/1-231	G3SUA8.1 PF04889.10	98.70	0.00
TRINITY_G7P3C6_MACFA/87-182	G7P3C6.1 PF00153.25	98.70	0.00
TRINITY_F7BCF7_HORSE/368-446	F7BCF7.1 PF00076.20	98.70	0.00
TRINITY_F6YIK2_HORSE/12-87	F6YIK2.1 PF00347.21	98.70	0.00
TRINITY_G3RMT1_GORGO/8-122	G3RMT1.1 PF16906.3;	98.70	0.00
TRINITY_LY6A_MOUSE/29-105	P05533.1 PF00021.19	98.70	0.00
TRINITY_A0A0P7TIS5_9TELE/50-201	A0A0P7TIS5.1 PF0017	98.70	0.00
TRINITY_A0A0P7TIS5_9TELE/50-201	A0A0P7TIS5.1 PF0017	98.70	0.00
TRINITY_L5LTN0_MYODS/23-100	L5LTN0.1 PF00935.17	98.70	0.00
TRINITY_L7MZL4_ANOCA/27-103	L7MZL4.1 PF01253.20	98.70	0.00
TRINITY_Q7R952_PLAYO/98-199	Q7R952.1 PF00665.24	98.70	0.00

TRINITY_G7Q217_MACFA/12-166	G7Q217.1 PF00252.16	98.70	0.00
TRINITY_G7Q217_MACFA/12-166	G7Q217.1 PF00252.16	98.70	0.00
TRINITY_G3IA94_CRIGR/92-168	G3IA94.1 PF08976.9;	98.70	0.00
TRINITY_A0A096QE22_MAIZE/258-391	A0A096QE22.1 PF0000	98.70	0.00
TRINITY_G3RMI7_GORGO/58-144	G3RMI7.1 PF12643.5;	98.70	0.00
TRINITY_A2A8T1_MOUSE/51-504	A2A8T1.1 PF00067.20	98.70	0.00
TRINITY_F6WA72_MACMU/10-156	F6WA72.1 PF03073.13	98.70	0.00
TRINITY_H2QYU1_PANTR/1-79	H2QYU1.1 PF05392.9;	98.70	0.00
TRINITY_G3RM14_GORGO/251-598	G3RM14.1 PF00155.19	98.60	0.00
TRINITY_V8PGM8_OPHHA/114-283	V8PGM8.1 PF00270.27	98.60	0.00
TRINITY_A0A067EX57_CITSI/59-220	A0A067EX57.1 PF0050	98.60	0.00
TRINITY_K4CL74_SOLLC/605-715	K4CL74.1 PF03764.16	98.60	0.00
TRINITY_F6YZP7_HORSE/813-944	F6YZP7.1 PF14806.4;	98.60	0.00
TRINITY_M0SVE5_MUSAM/148-297	M0SVE5.1 PF00485.16	98.60	0.00
TRINITY_A0A0P7V782_9TELE/147-328	A0A0P7V782.1 PF0139	98.60	0.00
TRINITY_G3RMM9_GORGO/11-151	G3RMM9.1 PF00241.18	98.60	0.00
TRINITY_U5DGY2_AMBTC/84-465	U5DGY2.1 PF00162.17	98.60	0.00
TRINITY_A0A0A0MXP7_PONAB/14-155	A0A0A0MXP7.1 PF0021	98.60	0.00
TRINITY_F7AEF4_MACMU/42-111	F7AEF4.1 PF00298.17	98.60	0.00
TRINITY_F7AEF4_MACMU/42-111	F7AEF4.1 PF00298.17	98.60	0.00
TRINITY_RA1L2_HUMAN/107-178	Q32P51.2 PF00076.20	98.60	0.00
TRINITY_G3TK94_LOXAF/138-382	G3TK94.1 PF00362.16	98.60	0.00
TRINITY_ATPA_CHLRE/150-365	P26526.3 PF00006.23	98.60	0.00
TRINITY_A0A091DQJ6_FUKDA/193-262	A0A091DQJ6.1 PF0007	98.60	0.00
TRINITY_MUP6_MOUSE/34-173	P02762.2 PF00061.21	98.60	0.00
TRINITY_A0A0B2VWK9_TOXCA/3-379	A0A0B2VWK9.1 PF0002	98.60	0.00
TRINITY_H0VEY6_CAVPO/29-111	H0VEY6.1 PF04683.11	98.60	0.00
TRINITY_H2NXP8_PONAB/31-242	H2NXP8.1 PF01351.16	98.60	0.00
TRINITY_OCAD1_PONAB/23-109	Q5RD48.1 PF07051.9;	98.60	0.00
TRINITY_A0A078EE19_BRANA/13-361	A0A078EE19.1 PF0107	98.50	0.00
TRINITY_F7IEY6_CALJA/62-241	F7IEY6.1 PF09439.8;	98.50	0.00
TRINITY_M3ZZZ0_XIPMA/8-121	M3ZZZ0.1 PF00572.16	98.50	0.00
TRINITY_A0A087X0J4_HUMAN/1-131	A0A087X0J4.1 PF0527	98.50	0.00
TRINITY_E7ERK6_HUMAN/30-204	E7ERK6.1 PF01093.15	98.50	0.00
TRINITY_F6SKX4_HORSE/4-77	F6SKX4.1 PF09032.9;	98.50	0.00
TRINITY_F6VCK9_MACMU/151-261	F6VCK9.1 PF00355.24	98.50	0.00
TRINITY_F7HUZ7_CALJA/70-149	F7HUZ7.1 PF00312.20	98.50	0.00
TRINITY_G3P017_GASAC/29-225	G3P017.1 PF00687.19	98.50	0.00
TRINITY_A0A0P7UES3_9TELE/23-149	A0A0P7UES3.1 PF0007	98.50	0.00
TRINITY_I3MLN0_ICTTR/12-183	I3MLN0.1 PF07074.10	98.50	0.00
TRINITY_L5MI42_MYODS/11-201	L5MI42.1 PF02115.15	98.50	0.00
TRINITY_F6UZP9_MACMU/774-938	F6UZP9.1 PF02453.15	98.50	0.00
TRINITY_G5AMB0_HETGA/863-929	G5AMB0.1 PF08726.8;	98.50	0.00
TRINITY_G1RWQ5_NOMLE/8-142	G1RWQ5.1 PF00578.19	98.50	0.00
TRINITY_M3XQI1_MUSPF/22-154	M3XQI1.1 PF00237.17	98.50	0.00
TRINITY_W5NHJ2_LEPOC/22-154	W5NHJ2.1 PF00380.17	98.50	0.00
TRINITY_A0A067F1H7_CITSI/37-101	A0A067F1H7.1 PF1349	98.50	0.00
TRINITY_I1R765_ORYGL/23-136	I1R765.1 PF02788.14	98.50	0.00
TRINITY_L5M568_MYODS/476-543	L5M568.1 PF00658.16	98.50	0.00
TRINITY_H0ZBH5_TAEGU/1-124	H0ZBH5.1 PF01201.20	98.50	0.00
TRINITY_W5NHJ2_LEPOC/22-154	W5NHJ2.1 PF00380.17	98.50	0.00
TRINITY_A0A0D9S2M2_CHLSB/17-83	A0A0D9S2M2.1 PF1026	98.40	0.00
TRINITY_F7BGS8_HORSE/1542-1633	F7BGS8.1 PF00630.17	98.40	0.00
TRINITY_RPB1C_HUMAN/29-103	Q9H1A7.1 PF13656.4;	98.40	0.00
TRINITY_M3WS54_FELCA/477-588	M3WS54.1 PF00435.19	98.40	0.00
TRINITY_H0Z1N0_TAEGU/1-124	H0Z1N0.1 PF01092.17	98.40	0.00
TRINITY_H0Z1N0_TAEGU/1-124	H0Z1N0.1 PF01092.17	98.40	0.00
TRINITY_G3PZS2_GASAC/74-143	G3PZS2.1 PF00298.17	98.40	0.00
TRINITY_G7NV07_MACFA/28-91	G7NV07.1 PF02320.14	98.40	0.00
TRINITY_D3ZE63_RAT/2-115	D3ZE63.1 PF01187.16	98.40	0.00
TRINITY_D8UE88_VOLCA/81-358	D8UE88.1 PF13292.4;	98.40	0.00
TRINITY_A0A0A0LH25_CUCSA/269-577	A0A0A0LH25.1 PF0088	98.40	0.00
TRINITY_V7C1G4_PHAVU/2-60	V7C1G4.1 PF00504.19	98.30	0.00
TRINITY_G3RMT1_GORGO/8-122	G3RMT1.1 PF16906.3;	98.30	0.00
TRINITY_H9H3Z5_MACMU/352-419	H9H3Z5.1 PF00076.20	98.30	0.00
TRINITY_A0A091D8G1_FUKDA/75-132	A0A091D8G1.1 PF0475	98.30	0.00
TRINITY_T1G5M3_HELRO/18-99	T1G5M3.1 PF15511.4;	98.30	0.00
TRINITY_C6SY38_SOYBN/27-103	C6SY38.1 PF01253.20	98.30	0.00
TRINITY_E1ZPD3_CHLVA/47-106	E1ZPD3.1 PF12678.5;	98.30	0.00

TRINITY_J3QL01_HUMAN/2-67	J3QL01.1 PF01781.16	98.30	0.00
TRINITY_W5QC41_SHEEP/76-431	W5QC41.1 PF00224.19	98.30	0.00
TRINITY_D8U1P4_VOLCA/539-653	D8U1P4.1 PF00072.22	98.30	0.00
TRINITY_F1ML34_BOVIN/120-332	F1ML34.2 PF09325.8;	98.30	0.00
TRINITY_G1RGJ9_NOMLE/20-85	G1RGJ9.2 PF00191.18	98.20	0.00
TRINITY_G3HWI4_CRIGR/37-255	G3HWI4.1 PF02167.13	98.20	0.00
TRINITY_A4HU11_LEIIN/209-280	A4HU11.2 PF00240.21	98.20	0.00
TRINITY_G3QF51_GORGO/160-365	G3QF51.1 PF00022.17	98.20	0.00
TRINITY_G3H5C9_CRIGR/28-82	G3H5C9.1 PF01667.15	98.20	0.00
TRINITY_L9KY99_TUPCH/5-130	L9KY99.1 PF00410.17	98.20	0.00
TRINITY_D8TRN2_VOLCA/650-880	D8TRN2.1 PF12774.5;	98.20	0.00
TRINITY_ALDR_RAT/13-294	P07943.3 PF00248.19	98.20	0.00
TRINITY_G3H5C9_CRIGR/28-82	G3H5C9.1 PF01667.15	98.20	0.00
TRINITY_A0A0D9QVZ4_CHLSB/36-211	A0A0D9QVZ4.1 PF0302	98.20	0.00
TRINITY_H9GP74_ANOCA/21-88	H9GP74.1 PF13417.4;	98.10	0.00
TRINITY_M3ZAZ1_NOMLE/17-114	M3ZAZ1.1 PF00428.17	98.10	0.00
TRINITY_L9JWV9_TUPCH/67-127	L9JWV9.1 PF05871.10	98.10	0.00
TRINITY_F1PBH2_CANLF/1-558	F1PBH2.2 PF05817.12	98.10	0.00
TRINITY_A0A0B0NIH5_GOSAR/59-258	A0A0B0NIH5.1 PF0083	98.10	0.00
TRINITY_F7BQY8_CALJA/102-410	F7BQY8.1 PF00038.19	98.10	0.00
TRINITY_A0A0D2N2G9_GOSRA/4-73	A0A0D2N2G9.1 PF0024	98.10	0.00
TRINITY_M7AY47_CHEMY/29-139	M7AY47.1 PF00411.17	98.10	0.00
TRINITY_G1TVL1_RABIT/275-381	G1TVL1.1 PF00647.17	98.10	0.00
TRINITY_G3HYE0_CRIGR/503-663	G3HYE0.1 PF00850.17	98.10	0.00
TRINITY_G3NUM7_GASAC/5-110	G3NUM7.1 PF03297.13	98.00	0.00
TRINITY_R4GHB8_CHICK/261-313	R4GHB8.1 PF10531.7;	98.00	0.00
TRINITY_A0A0A0KH05_CUCSA/438-554	A0A0A0KH05.1 PF0376	98.00	0.00
TRINITY_G5C2A0_HETGA/3-52	G5C2A0.1 PF04135.10	98.00	0.00
TRINITY_D4A771_RAT/5-104	D4A771.1 PF01247.16	98.00	0.00
TRINITY_M3Z9M8_NOMLE/8-106	M3Z9M8.1 PF02271.14	98.00	0.00
TRINITY_H2ZUX4_LATCH/581-734	H2ZUX4.1 PF00022.17	98.00	0.00
TRINITY_HA12_MOUSE/25-203	P01900.1 PF00129.16	98.00	0.00
TRINITY_F7I6G6_CALJA/17-116	F7I6G6.1 PF03066.13	98.00	0.00
TRINITY_F7I6G6_CALJA/17-116	F7I6G6.1 PF03066.13	98.00	0.00
TRINITY_A0A0A6YY34_MOUSE/1-71	A0A0A6YY34.1 PF0025	98.00	0.00
TRINITY_G3VTA9_SARHA/6-55	G3VTA9.1 PF00253.19	98.00	0.00
TRINITY_G3VTA9_SARHA/6-55	G3VTA9.1 PF00253.19	98.00	0.00
TRINITY_M3XDW2_FELCA/6-204	M3XDW2.1 PF01088.19	97.90	0.00
TRINITY_E0CQ75_VITVI/216-332	E0CQ75.1 PF00175.19	97.90	0.00
TRINITY_A4XS71_PSEMY/104-200	A4XS71.1 PF00691.18	97.90	0.00
TRINITY_CO1A1_BOVIN/1226-1462	P02453.3 PF01410.16	97.90	0.00
TRINITY_H3BW39_TETNG/360-490	H3BW39.1 PF00012.18	97.90	0.00
TRINITY_D8TM26_VOLCA/141-457	D8TM26.1 PF00291.23	97.90	0.00
TRINITY_U3IV89_ANAPL/366-728	U3IV89.1 PF00702.24	97.90	0.00
TRINITY_B9TH38_RICCO/1-189	B9TH38.1 PF00702.24	97.90	0.00
TRINITY_A0A0G4IGV5_PLABS/15-86	A0A0G4IGV5.1 PF0024	97.90	0.00
TRINITY_W5MUG1_LEPOC/12-106	W5MUG1.1 PF01248.24	97.90	0.00
TRINITY_Q28EQ0_XENTR/6-122	Q28EQ0.1 PF06495.9;	97.80	0.00
TRINITY_G1M6P5_AILME/22-113	G1M6P5.1 PF00428.17	97.80	0.00
TRINITY_A0A0A0LHZ4_CUCSA/124-191	A0A0A0LHZ4.1 PF0054	97.80	0.00
TRINITY_H0W4B9_CAVPO/5-142	H0W4B9.1 PF00179.24	97.80	0.00
TRINITY_R0I9P3_9BRAS/205-338	R0I9P3.1 PF00004.27	97.80	0.00
TRINITY_H2QBR4_PANTR/8-187	H2QBR4.1 PF01294.16	97.80	0.00
TRINITY_A9PHA0_POPTR/48-292	A9PHA0.1 PF00141.21	97.80	0.00
TRINITY_H0YSG2_TAEGU/99-167	H0YSG2.1 PF00076.20	97.80	0.00
TRINITY_B4F8L7_MAIZE/241-398	B4F8L7.1 PF02800.18	97.70	0.00
TRINITY_G5ASK3_HETGA/1066-1145	G5ASK3.1 PF16294.3;	97.70	0.00
TRINITY_A0A0A0K7Z3_CUCSA/57-231	A0A0A0K7Z3.1 PF0021	97.70	0.00
TRINITY_F6PJT9_CALJA/48-116	F6PJT9.1 PF00047.23	97.70	0.00
TRINITY_F1PBH2_CANLF/1-558	F1PBH2.2 PF05817.12	97.70	0.00
TRINITY_A0A0P7UM42_9TELE/16-192	A0A0P7UM42.1 PF0014	97.70	0.00
TRINITY_H2QF02_PANTR/17-361	H2QF02.1 PF00009.25	97.70	0.00
TRINITY_G1PHQ0_MYOLU/70-419	G1PHQ0.1 PF00274.17	97.70	0.00
TRINITY_A0A0B2VWK9_TOXCA/3-379	A0A0B2VWK9.1 PF0002	97.70	0.00
TRINITY_B9GFK3_POPTR/114-208	B9GFK3.1 PF00338.20	97.70	0.00
TRINITY_A0A0D2T6A5_GOSRA/46-177	A0A0D2T6A5.1 PF0012	97.70	0.00
TRINITY_M3W4Z9_FELCA/478-521	M3W4Z9.1 PF00045.17	97.70	0.00
TRINITY_L5KE65_PTEAL/473-1091	L5KE65.1 PF01268.17	97.60	0.00
TRINITY_G1KRM6_ANOCA/120-176	G1KRM6.1 PF00412.20	97.60	0.00

TRINITY_HEMO_RAT/306-347	P20059.3 PF00045.17	97.60	0.00
TRINITY_G3HCL6_CRIGR/1025-1249	G3HCL6.1 PF07678.12	97.60	0.00
TRINITY_M0RF02_MUSAM/1-152	M0RF02.1 PF00009.25	97.60	0.00
TRINITY_G7PIU2_MACFA/24-107	G7PIU2.1 PF02046.13	97.60	0.00
TRINITY_A9TYS8_PHYPA/8-96	A9TYS8.1 PF00125.22	97.60	0.00
TRINITY_F6Y6B1_CALJA/7-215	F6Y6B1.1 PF10500.7;	97.60	0.00
TRINITY_V9FDA4_PHYPR/18-99	V9FDA4.1 PF15511.4;	97.60	0.00
TRINITY_V9FDA4_PHYPR/18-99	V9FDA4.1 PF15511.4;	97.60	0.00
TRINITY_V9FDA4_PHYPR/18-99	V9FDA4.1 PF15511.4;	97.60	0.00
TRINITY_A7YY10_DANRE/4-88	A7YY10.1 PF01780.17	97.60	0.00
TRINITY_M3YML1_MUSPF/345-387	M3YML1.1 PF16300.3;	97.60	0.00
TRINITY_A2A8T1_MOUSE/51-504	A2A8T1.1 PF00067.20	97.50	0.00
TRINITY_K1Q9R2_CRAGI/1-107	K1Q9R2.1 PF00004.27	97.50	0.00
TRINITY_A0A0A0LHJ8_CUCSA/59-212	A0A0A0LHJ8.1 PF0260	97.50	0.00
TRINITY_G3P1S2_GASAC/24-102	G3P1S2.1 PF01282.17	97.50	0.00
TRINITY_D8UDD4_VOLCA/254-660	D8UDD4.1 PF08083.9;	97.50	0.00
TRINITY_A0A0D9RJC0_CHLSB/28-535	A0A0D9RJC0.1 PF0011	97.50	0.00
TRINITY_F6VPL0_MACMU/3-42	F6VPL0.1 PF01779.15	97.50	0.00
TRINITY_G7PUZ7_MACFA/251-352	G7PUZ7.1 PF00153.25	97.50	0.00
TRINITY_D3ZSE0_RAT/1-80	D3ZSE0.1 PF01249.16	97.50	0.00
TRINITY_F1P975_CANLF/510-569	F1P975.2 PF13833.4;	97.40	0.00
TRINITY_G5BZ40_HETGA/57-353	G5BZ40.1 PF00676.18	97.40	0.00
TRINITY_A0A067F640_CITSI/78-358	A0A067F640.1 PF0016	97.40	0.00
TRINITY_D8UHM6_VOLCA/586-795	D8UHM6.1 PF00149.26	97.40	0.00
TRINITY_F6Z5G8_MONDO/2136-2173	F6Z5G8.2 PF00039.16	97.40	0.00
TRINITY_A0A0B2VWK9_TOXCA/3-379	A0A0B2VWK9.1 PF0002	97.40	0.00
TRINITY_A0A087SRQ1_AUXPR/82-562	A0A087SRQ1.1 PF0321	97.40	0.00
TRINITY_D8TPX8_VOLCA/54-249	D8TPX8.1 PF00149.26	97.40	0.00
TRINITY_A8J1K0_CHLRE/37-850	A8J1K0.1 PF01496.17	97.40	0.00
TRINITY_F1A084_DICPU/5-178	F1A084.1 PF00071.20	97.40	0.00
TRINITY_H2R1B6_PANTR/5-118	H2R1B6.1 PF00572.16	97.40	0.00
TRINITY_C1E7K7_MICSR/4-377	C1E7K7.1 PF00022.17	97.40	0.00
TRINITY_A0A096NZS4_PAPAN/59-171	A0A096NZS4.1 PF0086	97.30	0.00
TRINITY_H9GBF8_ANOCA/8-116	H9GBF8.2 PF08662.9;	97.30	0.00
TRINITY_SPTB2_HUMAN/2198-2307	Q01082.2 PF00169.27	97.30	0.00
TRINITY_L5MI42_MYODS/11-201	L5MI42.1 PF02115.15	97.30	0.00
TRINITY_A0A061E6G2_THECC/61-461	A0A061E6G2.1 PF0015	97.30	0.00
TRINITY_A0A0D3E2U4_BRAOL/10-357	A0A0D3E2U4.1 PF0026	97.30	0.00
TRINITY_M3XXR0_MUSPF/2-188	M3XXR0.1 PF17135.2;	97.30	0.00
TRINITY_H2PQL6_PONAB/1-52	H2PQL6.1 PF04272.12	97.30	0.00
TRINITY_E3M3Z3_CAERE/46-173	E3M3Z3.1 PF00125.22	97.30	0.00
TRINITY_E3M3Z3_CAERE/46-173	E3M3Z3.1 PF00125.22	97.30	0.00
TRINITY_Q8UWG7_CHICK/45-105	Q8UWG7.1 PF03868.13	97.30	0.00
TRINITY_A0A0R4J0X5_MOUSE/46-410	A0A0R4J0X5.1 PF0007	97.30	0.00
TRINITY_G1NI74_MELGA/12-87	G1NI74.1 PF00347.21	97.30	0.00
TRINITY_A8GCL8_SERP5/232-328	A8GCL8.1 PF00691.18	97.20	0.00
TRINITY_H2MYQ5_ORYLA/19-127	H2MYQ5.1 PF01776.15	97.20	0.00
TRINITY_M4A568_XIPMA/12-87	M4A568.1 PF00347.21	97.20	0.00
TRINITY_A4HU11_LEIIN/209-280	A4HU11.2 PF00240.21	97.20	0.00
TRINITY_M7C0E7_CHEMY/15-364	M7C0E7.1 PF00274.17	97.20	0.00
TRINITY_A4HU11_LEIIN/209-280	A4HU11.2 PF00240.21	97.20	0.00
TRINITY_A4HU11_LEIIN/209-280	A4HU11.2 PF00240.21	97.20	0.00
TRINITY_D8UJB9_VOLCA/2-55	D8UJB9.1 PF00504.19	97.20	0.00
TRINITY_H2LDR4_ORYLA/18-89	H2LDR4.1 PF11976.6;	97.20	0.00
TRINITY_F6YAF9_ORNAN/151-288	F6YAF9.1 PF10591.7;	97.20	0.00
TRINITY_A4HU11_LEIIN/209-280	A4HU11.2 PF00240.21	97.20	0.00
TRINITY_D8UDE0_VOLCA/442-555	D8UDE0.1 PF02852.20	97.20	0.00
TRINITY_W5MIL4_LEPOC/2-191	W5MIL4.1 PF00827.15	97.10	0.00
TRINITY_L5MAB4_MYODS/4-73	L5MAB4.1 PF00240.21	97.10	0.00
TRINITY_L5MAB4_MYODS/4-73	L5MAB4.1 PF00240.21	97.10	0.00
TRINITY_Q0IIA3_BOVIN/89-122	Q0IIA3.1 PF13405.4;	97.10	0.00
TRINITY_V8P9W0_OPHHA/423-500	V8P9W0.1 PF00630.17	97.10	0.00
TRINITY_S9WF89_9CETA/747-1243	S9WF89.1 PF00343.18	97.10	0.00
TRINITY_A0A0A0K7N4_CUCSA/48-220	A0A0A0K7N4.1 PF0050	97.10	0.00
TRINITY_A0A0A0KTG0_CUCSA/59-127	A0A0A0KTG0.1 PF0267	97.10	0.00
TRINITY_H2MYQ5_ORYLA/19-127	H2MYQ5.1 PF01776.15	97.10	0.00
TRINITY_F7GDB5_CALJA/110-421	F7GDB5.1 PF00038.19	97.10	0.00
TRINITY_V8NNL1_OPHHA/28-271	V8NNL1.1 PF00573.20	97.10	0.00
TRINITY_F6X099_ORNAN/196-725	F6X099.1 PF00183.16	97.10	0.00

TRINITY_H2RQJ9_TAKRU/278-345	H2RQJ9.1 PF00076.20	97.10	0.00
TRINITY_G7PPJ2_MACFA/3-72	G7PPJ2.1 PF00240.21	97.10	0.00
TRINITY_G7PPJ2_MACFA/3-72	G7PPJ2.1 PF00240.21	97.10	0.00
TRINITY_G1PQS7_MYOLU/16-221	G1PQS7.1 PF01015.16	97.10	0.00
TRINITY_B5SNM5_OTOGA/1-34	B5SNM5.1 PF10215.7;	97.10	0.00
TRINITY_RL11_SERP5/72-140	A8G8E3.1 PF00298.17	97.10	0.00
TRINITY_A7LGV1_CHLRE/13-336	A7LGV1.1 PF00225.21	97.10	0.00
TRINITY_A0A0R4J0X5_MOUSE/46-410	A0A0R4J0X5.1 PF0007	97.10	0.00
TRINITY_A8HN02_CHLRE/89-380	A8HN02.1 PF03151.14	97.10	0.00
TRINITY_A8J9W4_CHLRE/107-254	A8J9W4.1 PF00069.23	97.10	0.00
TRINITY_D7UAI8_VITVI/2-38	D7UAI8.1 PF05627.9;	97.10	0.00
TRINITY_A0A0A0KZ66_CUCSA/60-278	A0A0A0KZ66.1 PF0137	97.00	0.00
TRINITY_G1P4R8_MYOLU/332-406	G1P4R8.1 PF13620.4;	97.00	0.00
TRINITY_C5XVB4_SORBI/4-88	C5XVB4.1 PF01780.17	97.00	0.00
TRINITY_H2RBF9_PANTR/73-291	H2RBF9.1 PF06413.9;	97.00	0.00
TRINITY_A0A096NJ44_PAPAN/57-425	A0A096NJ44.1 PF0015	97.00	0.00
TRINITY_A0A0D2N2G9_GOSRA/4-73	A0A0D2N2G9.1 PF0024	97.00	0.00
TRINITY_A0A0P7XD74_9TELE/8-73	A0A0P7XD74.1 PF0142	97.00	0.00
TRINITY_A0A0R4J0I1_MOUSE/50-417	A0A0R4J0I1.1 PF0007	97.00	0.00
TRINITY_A0A096M3R4_POEFO/19-124	A0A096M3R4.1 PF0030	97.00	0.00
TRINITY_G7PL40_MACFA/47-552	G7PL40.1 PF00118.22	97.00	0.00
TRINITY_F7ANH3_CALJA/45-388	F7ANH3.1 PF00079.18	97.00	0.00
TRINITY_G3RF20_GORGO/38-156	G3RF20.1 PF06487.10	97.00	0.00
TRINITY_U3JH28_FICAL/63-162	U3JH28.1 PF00011.19	97.00	0.00
TRINITY_M4DCR8_BRARP/4-92	M4DCR8.1 PF00125.22	97.00	0.00
TRINITY_K4C9L4_SOLLC/518-651	K4C9L4.1 PF00004.27	97.00	0.00
TRINITY_A0A0D9RGN3_CHLSB/84-165	A0A0D9RGN3.1 PF0018	97.00	0.00
TRINITY_G3S6B4_GORGO/11-78	G3S6B4.1 PF06522.9;	97.00	0.00
TRINITY_A0A096N8T4_PAPAN/56-462	A0A096N8T4.1 PF0160	97.00	0.00
TRINITY_A0A0D9R7L0_CHLSB/1-69	A0A0D9R7L0.1 PF0980	97.00	0.00
TRINITY_G3UCE6_LOXAF/569-663	G3UCE6.1 PF03129.18	96.90	0.00
TRINITY_A0A0A0MSS8_HUMAN/18-301	A0A0A0MSS8.1 PF0024	96.90	0.00
TRINITY_A0A0E0ALT2_9ORYZ/67-234	A0A0E0ALT2.1 PF0050	96.90	0.00
TRINITY_H2QSV7_PANTR/18-212	H2QSV7.1 PF00687.19	96.90	0.00
TRINITY_F6VK43_ORNAN/165-196	F6VK43.1 PF00641.16	96.90	0.00
TRINITY_H0VS47_CAVPO/1-226	H0VS47.1 PF00285.19	96.90	0.00
TRINITY_COX5A_PAPAN/45-147	B0VYX8.1 PF02284.14	96.90	0.00
TRINITY_M1CHF5_SOLTU/116-222	M1CHF5.1 PF01245.18	96.90	0.00
TRINITY_G1MR67_MELGA/12-251	G1MR67.2 PF00180.18	96.90	0.00
TRINITY_CB22_MAIZE/65-232	P06671.1 PF00504.19	96.90	0.00
TRINITY_C5MHS9_CANTT/231-297	C5MHS9.1 PF00240.21	96.90	0.00
TRINITY_G1TLE3_RABIT/9-403	G1TLE3.2 PF00162.17	96.90	0.00
TRINITY_A0A0A0K812_CUCSA/22-267	A0A0A0K812.1 PF0150	96.90	0.00
TRINITY_A0A075ARL7_9FUNG/3-136	A0A075ARL7.1 PF0009	96.90	0.00
TRINITY_R4GNK3_RAT/4-103	R4GNK3.1 PF00085.18	96.90	0.00
TRINITY_G1QW63_NOMLE/45-179	G1QW63.2 PF01124.16	96.90	0.00
TRINITY_F7DRD0_XENTR/11-84	F7DRD0.1 PF01423.20	96.80	0.00
TRINITY_A0A022RAK2_ERYGU/82-222	A0A022RAK2.1 PF0008	96.80	0.00
TRINITY_A0A0B0NIH5_GOSAR/59-258	A0A0B0NIH5.1 PF0083	96.80	0.00
TRINITY_G3VWC6_SARHA/595-698	G3VWC6.1 PF00435.19	96.80	0.00
TRINITY_E1ZE03_CHLVA/44-639	E1ZE03.1 PF00012.18	96.80	0.00
TRINITY_B3RIQ7_TRIAD/50-112	B3RIQ7.1 PF00137.19	96.80	0.00
TRINITY_A0A0K9PG33_ZOSMR/608-680	A0A0K9PG33.1 PF0000	96.80	0.00
TRINITY_F6PNM7_HORSE/36-255	F6PNM7.1 PF03731.13	96.80	0.00
TRINITY_B3RIQ7_TRIAD/50-112	B3RIQ7.1 PF00137.19	96.80	0.00
TRINITY_B3RIQ7_TRIAD/50-112	B3RIQ7.1 PF00137.19	96.80	0.00
TRINITY_W5M5B4_LEPOC/1-188	W5M5B4.1 PF01201.20	96.80	0.00
TRINITY_W5M5B4_LEPOC/1-188	W5M5B4.1 PF01201.20	96.80	0.00
TRINITY_H2QLK6_PANTR/1691-1866	H2QLK6.1 PF01576.17	96.80	0.00
TRINITY_C1FFH7_MICSR/896-1009	C1FFH7.1 PF00271.29	96.80	0.00
TRINITY_F6WVD1_HORSE/7-68	F6WVD1.1 PF01200.16	96.80	0.00
TRINITY_W5PUB0_SHEEP/107-140	W5PUB0.1 PF06747.11	96.80	0.00
TRINITY_F7FIK4_CALJA/290-331	F7FIK4.1 PF00396.16	96.80	0.00
TRINITY_F6QBE9_CALJA/1-100	F6QBE9.1 PF15679.3;	96.80	0.00
TRINITY_A0A078IWH5_BRANA/48-178	A0A078IWH5.1 PF0017	96.70	0.00
TRINITY_A0A0B0NIH5_GOSAR/59-258	A0A0B0NIH5.1 PF0083	96.70	0.00
TRINITY_K7J528_NASVI/11-101	K7J528.1 PF00125.22	96.70	0.00
TRINITY_A0A0R4J0I1_MOUSE/50-417	A0A0R4J0I1.1 PF0007	96.70	0.00
TRINITY_H2MYQ5_ORYLA/19-127	H2MYQ5.1 PF01776.15	96.70	0.00

TRINITY_S1N2X0_9ENTE/57-222	S1N2X0.1 PF13481.4;	96.70	0.00
TRINITY_R1F0V7_EMIHU/59-255	R1F0V7.1 PF00091.23	96.70	0.00
TRINITY_A0A0D2T6A5_GOSRA/46-177	A0A0D2T6A5.1 PF0012	96.70	0.00
TRINITY_D8UI03_VOLCA/42-637	D8UI03.1 PF00012.18	96.70	0.00
TRINITY_Q7GTU4_CHICK/15-161	Q7GTU4.1 PF00499.18	96.70	0.00
TRINITY_G3HDE3_CRIGR/11-71	G3HDE3.1 PF02251.16	96.70	0.00
TRINITY_G1SP54_RABIT/465-608	G1SP54.1 PF09127.9;	96.70	0.00
TRINITY_G7PSM9_MACFA/114-332	G7PSM9.1 PF00112.21	96.70	0.00
TRINITY_H2PKA0_PONAB/1-88	H2PKA0.1 PF15510.4;	96.70	0.00
TRINITY_A0A0A0KTF7_CUCSA/5-63	A0A0A0KTF7.1 PF0040	96.60	0.00
TRINITY_F7EK10_MACMU/8-102	F7EK10.1 PF01248.24	96.60	0.00
TRINITY_M7BIM0_CHEMY/246-347	M7BIM0.1 PF01399.25	96.60	0.00
TRINITY_V4U239_9ROSI/24-484	V4U239.1 PF00171.20	96.60	0.00
TRINITY_A0A0D2T6A5_GOSRA/46-177	A0A0D2T6A5.1 PF0012	96.60	0.00
TRINITY_A0A0A0KF04_CUCSA/11-355	A0A0A0KF04.1 PF0026	96.60	0.00
TRINITY_H0Z1N0_TAEGU/1-124	H0Z1N0.1 PF01092.17	96.60	0.00
TRINITY_A0A067F1H7_CITSI/37-101	A0A067F1H7.1 PF1349	96.60	0.00
TRINITY_D8TW30_VOLCA/1-104	D8TW30.1 PF03660.12	96.60	0.00
TRINITY_A8IVW9_CHLRE/30-148	A8IVW9.1 PF01873.15	96.60	0.00
TRINITY_K3ZXD9_SETIT/116-174	K3ZXD9.1 PF02427.15	96.60	0.00
TRINITY_H0Y009_OTOGA/4-61	H0Y009.1 PF00412.20	96.60	0.00
TRINITY_A7YY10_DANRE/4-88	A7YY10.1 PF01780.17	96.50	0.00
TRINITY_A7YY10_DANRE/4-88	A7YY10.1 PF01780.17	96.50	0.00
TRINITY_SODC_RAT/9-150	P07632.2 PF00080.18	96.50	0.00
TRINITY_T1NAL0_TRIUA/39-132	T1NAL0.1 PF15511.4;	96.50	0.00
TRINITY_A0A022RV28_ERYGU/89-313	A0A022RV28.1 PF0012	96.50	0.00
TRINITY_D8TUD2_VOLCA/222-448	D8TUD2.1 PF00006.23	96.50	0.00
TRINITY_G4VCP4_SCHMA/79-149	G4VCP4.1 PF00240.21	96.50	0.00
TRINITY_L9L693_TUPCH/352-607	L9L693.1 PF00118.22	96.50	0.00
TRINITY_Q6S9I0_MOUSE/265-359	Q6S9I0.1 PF00031.19	96.50	0.00
TRINITY_A8GCJ4_SERP5/28-369	A8GCJ4.1 PF00267.19	96.50	0.00
TRINITY_A0A0A0KZ66_CUCSA/60-278	A0A0A0KZ66.1 PF0137	96.40	0.00
TRINITY_G1MGJ0_AILME/27-134	G1MGJ0.1 PF00085.18	96.40	0.00
TRINITY_T0NMM6_9CETA/12-70	T0NMM6.1 PF01847.14	96.40	0.00
TRINITY_Q96399_CUCSA/11-226	Q96399.1 PF00141.21	96.40	0.00
TRINITY_A0A0A0LAW4_CUCSA/7-144	A0A0A0LAW4.1 PF0324	96.40	0.00
TRINITY_A0A0A0LUC2_CUCSA/32-231	A0A0A0LUC2.1 PF0575	96.40	0.00
TRINITY_Q4SRW2_TETNG/43-136	Q4SRW2.1 PF01158.16	96.40	0.00
TRINITY_A7RIF0_NEMVE/31-523	A7RIF0.1 PF00118.22	96.40	0.00
TRINITY_IF4G2_RABIT/544-657	P79398.1 PF02847.15	96.40	0.00
TRINITY_A0A067EMC5_CITSI/162-298	A0A067EMC5.1 PF0000	96.40	0.00
TRINITY_D8U7W6_VOLCA/1473-1703	D8U7W6.1 PF12774.5;	96.40	0.00
TRINITY_G1M1K7_AILME/535-899	G1M1K7.1 PF00503.18	96.40	0.00
TRINITY_W5P5T8_SHEEP/1-171	W5P5T8.1 PF01251.16	96.40	0.00
TRINITY_F7FFN3_MACMU/106-250	F7FFN3.1 PF02252.16	96.40	0.00
TRINITY_D8TSK6_VOLCA/99-334	D8TSK6.1 PF04884.12	96.40	0.00
TRINITY_D8TL86_VOLCA/577-686	D8TL86.1 PF00153.25	96.40	0.00
TRINITY_UCP2_BOVIN/112-208	Q3SZI5.1 PF00153.25	96.40	0.00
TRINITY_A8I2W2_CHLRE/20-271	A8I2W2.1 PF00378.18	96.40	0.00
TRINITY_A8J0A5_CHLRE/308-363	A8J0A5.1 PF13513.4;	96.40	0.00
TRINITY_A0A0A0L215_CUCSA/81-499	A0A0A0L215.1 PF0137	96.40	0.00
TRINITY_A0A0A0LT87_CUCSA/120-203	A0A0A0LT87.1 PF1454	96.40	0.00
TRINITY_E1B8G4_BOVIN/8-142	E1B8G4.2 PF00164.23	96.30	0.00
TRINITY_E1B8G4_BOVIN/8-142	E1B8G4.2 PF00164.23	96.30	0.00
TRINITY_I3MP66_ICTTR/445-498	I3MP66.1 PF00050.19	96.30	0.00
TRINITY_M5WHJ3_PRUPE/57-228	M5WHJ3.1 PF02507.13	96.30	0.00
TRINITY_A0A078IWH5_BRANA/48-178	A0A078IWH5.1 PF0017	96.30	0.00
TRINITY_F6STS3_HORSE/21-76	F6STS3.1 PF12678.5;	96.30	0.00
TRINITY_H2NIV0_PONAB/5-139	H2NIV0.1 PF00334.17	96.30	0.00
TRINITY_V7CDA9_PHAVU/215-331	V7CDA9.1 PF00175.19	96.30	0.00
TRINITY_G3NDY7_GASAC/17-124	G3NDY7.1 PF01655.16	96.30	0.00
TRINITY_A4HU11_LEIIN/209-280	A4HU11.2 PF00240.21	96.30	0.00
TRINITY_C1E7K7_MICSR/4-377	C1E7K7.1 PF00022.17	96.30	0.00
TRINITY_PETN_CHLRE/8-35	P0C1D4.2 PF03742.12	96.30	0.00
TRINITY_F7G013_ORNAN/1-53	F7G013.1 PF01907.17	96.20	0.00
TRINITY_H0XY96_OTOGA/56-175	H0XY96.1 PF12353.6;	96.20	0.00
TRINITY_A0A078I4J7_BRANA/809-889	A0A078I4J7.1 PF1043	96.20	0.00
TRINITY_H2R2N7_PANTR/87-768	H2R2N7.1 PF00063.19	96.20	0.00
TRINITY_K4CHE1_SOLLC/26-102	K4CHE1.1 PF01253.20	96.20	0.00

TRINITY_E2RSI5_CANLF/58-205	E2RSI5.2 PF00675.18	96.20	0.00
TRINITY_W5KPP6_ASTMX/1-375	W5KPP6.1 PF00297.20	96.20	0.00
TRINITY_A8JDN1_CHLRE/47-242	A8JDN1.1 PF00149.26	96.20	0.00
TRINITY_U3KDZ5_FICAL/31-121	U3KDZ5.1 PF13499.4;	96.20	0.00
TRINITY_A0A0C4BF04_WHEAT/1-57	A0A0C4BF04.1 PF0042	96.20	0.00
TRINITY_G7PFD7_MACFA/2-131	G7PFD7.1 PF00337.20	96.20	0.00
TRINITY_G0R1A4_ICHMG/23-114	G0R1A4.1 PF15511.4;	96.20	0.00
TRINITY_D8UIY5_VOLCA/224-471	D8UIY5.1 PF02074.13	96.20	0.00
TRINITY_G3H5P2_CRIGR/101-158	G3H5P2.1 PF00412.20	96.10	0.00
TRINITY_G1R7H0_NOMLE/3-157	G1R7H0.1 PF03079.12	96.10	0.00
TRINITY_U3K9V4_FICAL/115-310	U3K9V4.1 PF13848.4;	96.10	0.00
TRINITY_RBL_CHLRE/154-462	P00877.1 PF00016.18	96.10	0.00
TRINITY_M0SPM5_MUSAM/73-205	M0SPM5.1 PF02531.14	96.10	0.00
TRINITY_A0A087SGZ4_AUXPR/391-442	A0A087SGZ4.1 PF0084	96.10	0.00
TRINITY_B3RKR4_TRIAD/3-376	B3RKR4.1 PF00022.17	96.10	0.00
TRINITY_H0X010_OTOGA/2-104	H0X010.1 PF00085.18	96.10	0.00
TRINITY_A0A0A0LNS8_CUCSA/321-391	A0A0A0LNS8.1 PF0007	96.00	0.00
TRINITY_F6VFT5_XENTR/28-216	F6VFT5.1 PF00227.24	96.00	0.00
TRINITY_LT_SV40/265-699	P03070.2 PF06431.9;	96.00	0.00
TRINITY_VATG1_HUMAN/3-107	O75348.3 PF03179.13	96.00	0.00
TRINITY_E2QZV2_CANLF/191-379	E2QZV2.2 PF09756.7;	96.00	0.00
TRINITY_R8BT89_TOGMI/373-570	R8BT89.1 PF00587.23	96.00	0.00
TRINITY_H2S7F0_TAKRU/5-104	H2S7F0.1 PF01247.16	96.00	0.00
TRINITY_L9L3I8_TUPCH/26-170	L9L3I8.1 PF10208.7;	95.90	0.00
TRINITY_A0A0A0LHJ8_CUCSA/59-212	A0A0A0LHJ8.1 PF0260	95.90	0.00
TRINITY_A0A0A0KXH3_CUCSA/40-137	A0A0A0KXH3.1 PF0472	95.90	0.00
TRINITY_PDCD5_MOUSE/9-114	P56812.3 PF01984.18	95.90	0.00
TRINITY_V4BUJ1_LOTGI/38-643	V4BUJ1.1 PF00012.18	95.90	0.00
TRINITY_E9PKZ0_HUMAN/11-90	E9PKZ0.1 PF00181.21	95.90	0.00
TRINITY_RPAC2_BOVIN/39-113	Q1RMG8.1 PF13656.4;	95.90	0.00
TRINITY_D8TQT3_VOLCA/16-138	D8TQT3.1 PF03463.13	95.90	0.00
TRINITY_W4Z8Z2_STRPU/152-277	W4Z8Z2.1 PF00091.23	95.90	0.00
TRINITY_G1R5J2_NOMLE/42-98	G1R5J2.1 PF08122.10	95.90	0.00
TRINITY_B4FPT8_MAIZE/12-166	B4FPT8.1 PF00252.16	95.80	0.00
TRINITY_G1Q4U0_MYOLU/4-99	G1Q4U0.1 PF01158.16	95.80	0.00
TRINITY_G1Q4U0_MYOLU/4-99	G1Q4U0.1 PF01158.16	95.80	0.00
TRINITY_W5MIL4_LEPOC/2-191	W5MIL4.1 PF00827.15	95.80	0.00
TRINITY_L5LZ73_MYODS/202-297	L5LZ73.1 PF00153.25	95.80	0.00
TRINITY_T1NAL0_TRIUA/39-132	T1NAL0.1 PF15511.4;	95.80	0.00
TRINITY_T1NAL0_TRIUA/39-132	T1NAL0.1 PF15511.4;	95.80	0.00
TRINITY_A0A0P7UBW1_9TELE/13-153	A0A0P7UBW1.1 PF0007	95.80	0.00
TRINITY_A8JGV6_CHLRE/11-235	A8JGV6.1 PF00244.18	95.80	0.00
TRINITY_A0A096NG97_PAPAN/18-159	A0A096NG97.1 PF0021	95.80	0.00
TRINITY_D4A771_RAT/5-104	D4A771.1 PF01247.16	95.80	0.00
TRINITY_A8JGC8_CHLRE/114-229	A8JGC8.1 PF02770.17	95.80	0.00
TRINITY_W5MIL4_LEPOC/2-191	W5MIL4.1 PF00827.15	95.70	0.00
TRINITY_G3UYY2_MOUSE/57-410	G3UYY2.1 PF05694.9;	95.70	0.00
TRINITY_A0A068Z9R6_9ENTR/48-231	A0A068Z9R6.1 PF0138	95.70	0.00
TRINITY_E1C2S1_CHICK/2383-2529	E1C2S1.2 PF01608.15	95.70	0.00
TRINITY_A0A0K9P3Y3_ZOSMR/71-507	A0A0K9P3Y3.1 PF0147	95.70	0.00
TRINITY_H9H0U7_MELGA/1-103	H9H0U7.1 PF00503.18	95.70	0.00
TRINITY_CO3_RAT/1051-1284	P01026.3 PF07678.12	95.70	0.00
TRINITY_G1PLV4_MYOLU/98-167	G1PLV4.1 PF00076.20	95.70	0.00
TRINITY_F6TEX7_HORSE/185-258	F6TEX7.1 PF13534.4;	95.70	0.00
TRINITY_L1IEU9_GUITH/3-212	L1IEU9.1 PF00091.23	95.70	0.00
TRINITY_A0A067DN09_CITSI/76-235	A0A067DN09.1 PF0012	95.70	0.00
TRINITY_A0A077ZVL1_STYLE/1856-2091	A0A077ZVL1.1 PF1277	95.70	0.00
TRINITY_I7LXR5_TETTS/41-162	I7LXR5.1 PF00004.27	95.70	0.00
TRINITY_K4BFU1_SOLLC/124-171	K4BFU1.1 PF04839.11	95.70	0.00
TRINITY_D8QT27_SELML/18-399	D8QT27.1 PF00199.17	95.70	0.00
TRINITY_L5MEY5_MYODS/26-121	L5MEY5.1 PF03501.13	95.70	0.00
TRINITY_S9WBV3_9CETA/6-245	S9WBV3.1 PF00899.19	95.70	0.00
TRINITY_B6DYQ4_RAT/16-150	B6DYQ4.1 PF01124.16	95.60	0.00
TRINITY_A0A0D2S4P7_GOSRA/19-152	A0A0D2S4P7.1 PF0023	95.60	0.00
TRINITY_A0A0A0KV36_CUCSA/105-177	A0A0A0KV36.1 PF0294	95.60	0.00
TRINITY_G3SE29_GORGO/4-163	G3SE29.1 PF00261.18	95.60	0.00
TRINITY_A0A0B2VWK9_TOXCA/3-379	A0A0B2VWK9.1 PF0002	95.60	0.00
TRINITY_A0A067DVK1_CITSI/45-478	A0A067DVK1.1 PF0799	95.60	0.00
TRINITY_G3R288_GORGO/157-314	G3R288.1 PF02800.18	95.60	0.00

TRINITY_F1PCH3_CANLF/142-427	F1PCH3.2 PF00113.20	95.60	0.00
TRINITY_A0BNX9_PARTE/3-187	A0BNX9.1 PF00091.23	95.60	0.00
TRINITY_D8TZ23_VOLCA/810-916	D8TZ23.1 PF06421.10	95.60	0.00
TRINITY_F6UHG8_HORSE/39-219	F6UHG8.1 PF03997.10	95.50	0.00
TRINITY_A0A096MP41_PAPAN/24-209	A0A096MP41.1 PF0062	95.50	0.00
TRINITY_G3IA94_CRIGR/92-168	G3IA94.1 PF08976.9;	95.50	0.00
TRINITY_F1NYJ1_CHICK/132-352	F1NYJ1.2 PF00112.21	95.50	0.00
TRINITY_J9P098_CANLF/23-89	J9P098.1 PF01423.20	95.50	0.00
TRINITY_EIF3L_XENTR/150-548	Q6P878.1 PF10255.7;	95.50	0.00
TRINITY_A0A0A0LG06_CUCSA/156-541	A0A0A0LG06.1 PF0105	95.50	0.00
TRINITY_V8NNL1_OPHHA/28-271	V8NNL1.1 PF00573.20	95.50	0.00
TRINITY_F2Q9A3_PIG/7-163	F2Q9A3.1 PF00160.19	95.50	0.00
TRINITY_M3YFZ4_MUSPF/399-551	M3YFZ4.1 PF02910.18	95.50	0.00
TRINITY_H2QHY6_PANTR/46-120	H2QHY6.1 PF01929.15	95.50	0.00
TRINITY_H0X6J1_OTOGA/2-68	H0X6J1.1 PF05680.10	95.50	0.00
TRINITY_G3GXQ3_CRIGR/363-542	G3GXQ3.1 PF02737.16	95.40	0.00
TRINITY_S9WTM8_9CETA/46-170	S9WTM8.1 PF02747.13	95.40	0.00
TRINITY_A0A022PSH7_ERYGU/244-1363	A0A022PSH7.1 PF0251	95.40	0.00
TRINITY_B9HA54_POPTR/51-129	B9HA54.1 PF01241.16	95.40	0.00
TRINITY_QCR6_RAT/26-89	Q5M9I5.1 PF02320.14	95.30	0.00
TRINITY_M4AHJ8_XIPMA/43-128	M4AHJ8.1 PF00203.19	95.30	0.00
TRINITY_M4AHJ8_XIPMA/43-128	M4AHJ8.1 PF00203.19	95.30	0.00
TRINITY_RL27_BOVIN/52-136	P61356.2 PF01777.16	95.30	0.00
TRINITY_G1TMV1_RABIT/24-151	G1TMV1.1 PF00241.18	95.30	0.00
TRINITY_A0A067FSK7_CITSI/152-299	A0A067FSK7.1 PF0000	95.30	0.00
TRINITY_Q6PBZ1_DANRE/122-216	Q6PBZ1.1 PF01248.24	95.30	0.00
TRINITY_A0A078AUW2_STYLE/5-93	A0A078AUW2.1 PF0012	95.30	0.00
TRINITY_U3IV89_ANAPL/366-728	U3IV89.1 PF00702.24	95.30	0.00
TRINITY_A0A0A0L4M8_CUCSA/48-109	A0A0A0L4M8.1 PF0907	95.20	0.00
TRINITY_M4AWY7_XIPMA/258-469	M4AWY7.1 PF00112.21	95.20	0.00
TRINITY_G5ASL9_HETGA/87-646	G5ASL9.1 PF00063.19	95.20	0.00
TRINITY_A0A0D2S420_GOSRA/148-443	A0A0D2S420.1 PF0011	95.20	0.00
TRINITY_A0A022RGL6_ERYGU/67-245	A0A022RGL6.1 PF0050	95.20	0.00
TRINITY_B9RD45_RICCO/6-154	B9RD45.1 PF00056.21	95.20	0.00

TRINITY_A0A088AN14_APIME/50-115	A0A088AN14.1 PF0393	95.20	0.00
TRINITY_POL_MLVMS/1340-1441	P03355.4 PF16721.3;	95.20	0.00
TRINITY_F6TD92_MACMU/1-168	F6TD92.1 PF00838.15	95.20	0.00
TRINITY_A8JHQ7_CHLRE/10-171	A8JHQ7.1 PF00071.20	95.20	0.00
TRINITY_R0JLZ8_ANAPL/2-115	R0JLZ8.1 PF01187.16	95.20	0.00
TRINITY_D8U1K6_VOLCA/789-919	D8U1K6.1 PF00004.27	95.20	0.00
TRINITY_B4ID84_DROSE/5-88	B4ID84.1 PF01221.16	95.20	0.00
TRINITY_E1ZSB3_CHLVA/11-74	E1ZSB3.1 PF13499.4;	95.20	0.00
TRINITY_A0A0A0L6C0_CUCSA/11-226	A0A0A0L6C0.1 PF0564	95.20	0.00
TRINITY_Q84LQ5_SORBI/161-342	Q84LQ5.1 PF00390.17	95.20	0.00
TRINITY_G3V8V1_RAT/306-347	G3V8V1.1 PF00396.16	95.20	0.00
TRINITY_ATP6_CHICK/18-224	P14092.1 PF00119.18	95.10	0.00
TRINITY_G3UYY2_MOUSE/57-410	G3UYY2.1 PF05694.9;	95.10	0.00
TRINITY_H3BI72_LATCH/4-133	H3BI72.1 PF01912.16	95.10	0.00
TRINITY_A0A096NQK6_PAPAN/193-463	A0A096NQK6.1 PF0034	95.10	0.00
TRINITY_ACTN2_CHICK/284-394	P20111.1 PF00435.19	95.10	0.00
TRINITY_D8TSR8_VOLCA/68-251	D8TSR8.1 PF00504.19	95.10	0.00
TRINITY_D8U1R3_VOLCA/806-886	D8U1R3.1 PF10431.7;	95.10	0.00
TRINITY_V9FDA4_PHYPR/18-99	V9FDA4.1 PF15511.4;	95.10	0.00
TRINITY_L1IEU9_GUITH/3-212	L1IEU9.1 PF00091.23	95.10	0.00
TRINITY_D8U7C0_VOLCA/84-372	D8U7C0.1 PF01370.19	95.10	0.00
TRINITY_A8I175_CHLRE/238-379	A8I175.1 PF00004.27	95.10	0.00
TRINITY_A0A067EH05_CITSI/1-394	A0A067EH05.1 PF0011	95.10	0.00
TRINITY_H2LCX2_ORYLA/101-168	H2LCX2.1 PF00076.20	95.10	0.00
TRINITY_F7HUG3_MACMU/11-111	F7HUG3.1 PF03297.13	95.00	0.00
TRINITY_COX5B_RAT/7-128	P12075.2 PF01215.17	95.00	0.00
TRINITY_A7YY10_DANRE/4-88	A7YY10.1 PF01780.17	95.00	0.00
TRINITY_M5Y333_PRUPE/3-42	M5Y333.1 PF01779.15	95.00	0.00
TRINITY_K3Z2Q6_SETIT/28-329	K3Z2Q6.1 PF00124.17	95.00	0.00
TRINITY_A0A0B2VWK9_TOXCA/3-379	A0A0B2VWK9.1 PF0002	95.00	0.00
TRINITY_A0A0D3F687_9ORYZ/19-78	A0A0D3F687.1 PF0013	95.00	0.00
TRINITY_J9FW75_9SPIT/261-382	J9FW75.1 PF03953.15	95.00	0.00
TRINITY_J3N2K8_ORYBR/25-86	J3N2K8.1 PF01200.16	95.00	0.00
TRINITY_A0A0E0PRJ8_ORYRU/753-911	A0A0E0PRJ8.1 PF0007	95.00	0.00
TRINITY_E1ZBK2_CHLVA/5-250	E1ZBK2.1 PF00009.25	95.00	0.00
TRINITY_A0CPD4_PARTE/151-369	A0CPD4.1 PF00006.23	95.00	0.00
TRINITY_D8TP98_VOLCA/80-363	D8TP98.1 PF00069.23	95.00	0.00
TRINITY_F6VPL0_MACMU/3-42	F6VPL0.1 PF01779.15	95.00	0.00
TRINITY_D3FF62_CONWI/28-99	D3FF62.1 PF00240.21	95.00	0.00
TRINITY_D8UII1_VOLCA/1129-1247	D8UII1.1 PF13863.4;	95.00	0.00
TRINITY_D8U1R3_VOLCA/621-800	D8U1R3.1 PF07724.12	95.00	0.00
TRINITY_A0A093BLW0_CHAPE/1-95	A0A093BLW0.1 PF0008	95.00	0.00
TRINITY_F7EX57_CALJA/231-380	F7EX57.1 PF00441.22	95.00	0.00
TRINITY_RBM42_DANRE/305-375	Q6DRG1.2 PF00076.20	94.90	0.00
TRINITY_A0A0D2ZPE9_BRAOL/222-378	A0A0D2ZPE9.1 PF0280	94.90	0.00
TRINITY_G3JHC2_CORMM/44-102	G3JHC2.1 PF12678.5;	94.90	0.00
TRINITY_A0A087SS30_AUXPR/289-426	A0A087SS30.1 PF0000	94.90	0.00
TRINITY_A4HU11_LEIIN/209-280	A4HU11.2 PF00240.21	94.90	0.00
TRINITY_G1PYK6_MYOLU/29-147	G1PYK6.1 PF00411.17	94.90	0.00
TRINITY_G1PYK6_MYOLU/29-147	G1PYK6.1 PF00411.17	94.90	0.00
TRINITY_RL18A_BOVIN/7-130	Q3T003.1 PF01775.15	94.90	0.00
TRINITY_G0R1A4_ICHMG/23-114	G0R1A4.1 PF15511.4;	94.90	0.00
TRINITY_V8N9Z2_OPHHA/1-80	V8N9Z2.1 PF01249.16	94.90	0.00
TRINITY_M5VMG6_PRUPE/455-581	M5VMG6.1 PF01077.20	94.90	0.00
TRINITY_G3TN32_LOXAF/277-342	G3TN32.1 PF00191.18	94.90	0.00
TRINITY_MYPC_RAT/1067-1151	P56741.2 PF00041.19	94.90	0.00
TRINITY_G1Q784_MYOLU/41-148	G1Q784.1 PF13085.4;	94.80	0.00
TRINITY_A0A024WRW2_PLAFA/3-212	A0A024WRW2.1 PF0009	94.80	0.00
TRINITY_D8U5B3_VOLCA/587-644	D8U5B3.1 PF00839.15	94.80	0.00
TRINITY_E1B8G4_BOVIN/8-142	E1B8G4.2 PF00164.23	94.80	0.00
TRINITY_D3ZN79_RAT/7-63	D3ZN79.1 PF00831.21	94.70	0.00

TRINITY_D3ZN79_RAT/7-63	D3ZN79.1 PF00831.21	94.70	0.00
TRINITY_W5K8M5_ASTMX/444-522	W5K8M5.1 PF13893.4;	94.70	0.00
TRINITY_A0A0D3DHL1_BRAOL/45-257	A0A0D3DHL1.1 PF0159	94.70	0.00
TRINITY_W9SP37_9ROSA/96-288	W9SP37.1 PF13419.4;	94.70	0.00
TRINITY_G1N3Z1_MELGA/6-179	G1N3Z1.1 PF00025.19	94.70	0.00
TRINITY_A0A0D2T6A5_GOSRA/46-177	A0A0D2T6A5.1 PF0012	94.70	0.00
TRINITY_L8H9R9_ACACA/411-532	L8H9R9.1 PF00004.27	94.70	0.00
TRINITY_A8IM74_CHLRE/17-92	A8IM74.1 PF00935.17	94.70	0.00
TRINITY_Q96550_CHLRE/212-435	Q96550.1 PF00006.23	94.70	0.00
TRINITY_A8I339_CHLRE/243-280	A8I339.1 PF00400.30	94.70	0.00
TRINITY_A8I339_CHLRE/243-280	A8I339.1 PF00400.30	94.70	0.00
TRINITY_M3WCB9_FELCA/3-167	M3WCB9.1 PF09470.8;	94.60	0.00
TRINITY_A0A0A0L4W3_CUCSA/62-154	A0A0A0L4W3.1 PF0124	94.60	0.00
TRINITY_A0A022RGL6_ERYGU/67-245	A0A022RGL6.1 PF0050	94.60	0.00
TRINITY_CB22_MAIZE/65-232	P06671.1 PF00504.19	94.60	0.00
TRINITY_Q231U8_TETTS/387-428	Q231U8.1 PF13414.4;	94.60	0.00
TRINITY_I0Z842_9CHLO/17-489	I0Z842.1 PF05761.12	94.60	0.00
TRINITY_B9GL51_POPTR/6-330	B9GL51.2 PF16363.3;	94.60	0.00
TRINITY_W5LGK8_ASTMX/193-262	W5LGK8.1 PF00076.20	94.60	0.00
TRINITY_D8TT53_VOLCA/164-293	D8TT53.1 PF00004.27	94.60	0.00
TRINITY_NENF_MOUSE/47-143	Q9CQ45.1 PF00173.26	94.50	0.00
TRINITY_Q5XFV4_RAT/6-132	Q5XFV4.1 PF00061.21	94.50	0.00
TRINITY_I3MAW3_ICTTR/6-395	I3MAW3.1 PF03036.14	94.40	0.00
TRINITY_RT21_BOVIN/11-64	P82920.3 PF01165.18	94.40	0.00
TRINITY_K4BVV2_SOLLC/483-697	K4BVV2.1 PF01434.16	94.40	0.00
TRINITY_I3KGW5_ORENI/24-166	I3KGW5.1 PF01090.17	94.40	0.00
TRINITY_F1PXY4_CANLF/62-151	F1PXY4.2 PF00153.25	94.40	0.00
TRINITY_U3KGV4_FICAL/17-124	U3KGV4.1 PF01655.16	94.40	0.00
TRINITY_A4HU11_LEIIN/209-280	A4HU11.2 PF00240.21	94.40	0.00
TRINITY_A4HU11_LEIIN/209-280	A4HU11.2 PF00240.21	94.40	0.00
TRINITY_A4HU11_LEIIN/209-280	A4HU11.2 PF00240.21	94.40	0.00
TRINITY_A4HU11_LEIIN/209-280	A4HU11.2 PF00240.21	94.40	0.00
TRINITY_A4HU11_LEIIN/209-280	A4HU11.2 PF00240.21	94.40	0.00
TRINITY_A0A067F7U2_CITSI/267-400	A0A067F7U2.1 PF0000	94.40	0.00
TRINITY_D8UCX2_VOLCA/54-221	D8UCX2.1 PF00504.19	94.40	0.00
TRINITY_D8U4Q2_VOLCA/144-214	D8U4Q2.1 PF02325.15	94.40	0.00
TRINITY_A4HU11_LEIIN/209-280	A4HU11.2 PF00240.21	94.40	0.00
TRINITY_I0YZ00_9CHLO/56-109	I0YZ00.1 PF01192.20	94.40	0.00
TRINITY_G1QDM7_MYOLU/51-104	G1QDM7.1 PF01192.20	94.40	0.00
TRINITY_A4HU11_LEIIN/209-280	A4HU11.2 PF00240.21	94.40	0.00
TRINITY_A4HU11_LEIIN/209-280	A4HU11.2 PF00240.21	94.40	0.00
TRINITY_A4HU11_LEIIN/209-280	A4HU11.2 PF00240.21	94.40	0.00
TRINITY_I0Z930_9CHLO/134-373	I0Z930.1 PF01063.17	94.40	0.00
TRINITY_I1I8C1_BRADI/184-698	I1I8C1.1 PF00183.16	94.40	0.00
TRINITY_A8J4U2_CHLRE/5-255	A8J4U2.1 PF00069.23	94.40	0.00
TRINITY_A0A0A0L7F8_CUCSA/7-334	A0A0A0L7F8.1 PF0153	94.40	0.00
TRINITY_G3S832_GORGO/1-107	G3S832.1 PF01283.17	94.40	0.00
TRINITY_G3PM62_GASAC/6-77	G3PM62.1 PF16205.3;	94.40	0.00
TRINITY_K4B1Z2_SOLLC/287-348	K4B1Z2.1 PF12796.5;	94.30	0.00
TRINITY_A0A0P7UBW1_9TELE/13-153	A0A0P7UBW1.1 PF0007	94.30	0.00
TRINITY_A0A059CKD9_EUCGR/85-154	A0A059CKD9.1 PF0128	94.30	0.00
TRINITY_F7FAM8_ORNAN/92-189	F7FAM8.1 PF02770.17	94.30	0.00
TRINITY_A0A0A0M1E9_CUCSA/541-641	A0A0A0M1E9.1 PF0815	94.30	0.00
TRINITY_J9JUK1_ACYPI/24-182	J9JUK1.1 PF00071.20	94.30	0.00
TRINITY_D8TUE4_VOLCA/239-343	D8TUE4.1 PF14748.4;	94.30	0.00
TRINITY_A8JB67_CHLRE/42-130	A8JB67.1 PF01248.24	94.30	0.00
TRINITY_I3KA95_ORENI/133-501	I3KA95.1 PF00079.18	94.30	0.00
TRINITY_A8JHV0_CHLRE/5-92	A8JHV0.1 PF16561.3;	94.30	0.00
TRINITY_EFTU_CHLRE/10-212	P17746.1 PF00009.25	94.30	0.00
TRINITY_X6M4L5_RETFI/6-65	X6M4L5.1 PF04193.12	94.30	0.00
TRINITY_D8UDD4_VOLCA/254-660	D8UDD4.1 PF08083.9;	94.30	0.00

TRINITY_A0A0E0PCG8_ORYRU/10-121	A0A0E0PCG8.1 PF0025	94.30	0.00
TRINITY_H0YJT9_HUMAN/49-234	H0YJT9.1 PF03096.12	94.20	0.00
TRINITY_M3XXR0_MUSPF/2-188	M3XXR0.1 PF17135.2;	94.20	0.00
TRINITY_M3YPC3_MUSPF/58-404	M3YPC3.1 PF01208.15	94.20	0.00
TRINITY_I0Z680_9CHLO/82-215	I0Z680.1 PF02915.15	94.20	0.00
TRINITY_M4DCR8_BRARP/4-92	M4DCR8.1 PF00125.22	94.20	0.00
TRINITY_A0A0A0LI67_CUCSA/41-161	A0A0A0LI67.1 PF0159	94.20	0.00
TRINITY_G3S832_GORGO/1-107	G3S832.1 PF01283.17	94.20	0.00
TRINITY_V4SL90_9ROSI/35-270	V4SL90.1 PF00230.18	94.10	0.00
TRINITY_PSBC_CHLRE/19-460	P10898.1 PF00421.17	94.10	0.00
TRINITY_G3U9M4_LOXAF/290-364	G3U9M4.1 PF08564.8;	94.10	0.00
TRINITY_S8C602_9LAMI/1-50	S8C602.1 PF08132.9;	94.10	0.00
TRINITY_C1E7K7_MICSR/4-377	C1E7K7.1 PF00022.17	94.10	0.00
TRINITY_A8IYM0_CHLRE/156-461	A8IYM0.1 PF00365.18	94.10	0.00
TRINITY_D8TRN2_VOLCA/650-880	D8TRN2.1 PF12774.5;	94.10	0.00
TRINITY_G3NG58_GASAC/2-69	G3NG58.1 PF01781.16	94.10	0.00
TRINITY_U3JKK9_FICAL/8-187	U3JKK9.1 PF01294.16	94.10	0.00
TRINITY_F7D978_ORNAN/13-75	F7D978.1 PF00035.24	94.10	0.00
TRINITY_I3MZP8_ICTTR/13-253	I3MZP8.1 PF03998.11	94.10	0.00
TRINITY_G3TEH2_LOXAF/278-427	G3TEH2.1 PF00441.22	94.00	0.00
TRINITY_A0A0D2WJE0_CAPO3/24-1290	A0A0D2WJE0.1 PF0246	94.00	0.00
TRINITY_H2LK88_ORYLA/5-120	H2LK88.1 PF01778.15	94.00	0.00
TRINITY_A0A078IS30_BRANA/9-618	A0A078IS30.1 PF0001	94.00	0.00
TRINITY_M3XQI1_MUSPF/22-154	M3XQI1.1 PF00237.17	94.00	0.00
TRINITY_A0A077ZMR8_STYLE/232-365	A0A077ZMR8.1 PF0000	94.00	0.00
TRINITY_B4ID84_DROSE/5-88	B4ID84.1 PF01221.16	94.00	0.00
TRINITY_Q6DF03_XENTR/47-542	Q6DF03.1 PF00118.22	94.00	0.00
TRINITY_M7BM18_CHEMY/38-279	M7BM18.1 PF00224.19	94.00	0.00
TRINITY_D8TSY5_VOLCA/481-711	D8TSY5.1 PF12774.5;	94.00	0.00
TRINITY_A0A0A0LGH3_CUCSA/70-226	A0A0A0LGH3.1 PF0853	94.00	0.00
TRINITY_F6S9T4_CALJA/1-194	F6S9T4.1 PF05805.10	93.90	0.00
TRINITY_A0A067JHW3_JATCU/4-139	A0A067JHW3.1 PF0395	93.90	0.00
TRINITY_M4CX64_BRARP/472-560	M4CX64.1 PF00679.22	93.90	0.00
TRINITY_H2USG6_TAKRU/3-189	H2USG6.1 PF17135.2;	93.90	0.00
TRINITY_M0R983_RAT/14-242	M0R983.1 PF13561.4;	93.90	0.00
TRINITY_A0A067FSK7_CITSI/152-299	A0A067FSK7.1 PF0000	93.90	0.00
TRINITY_S9X3R9_9CETA/7-191	S9X3R9.1 PF01251.16	93.90	0.00
TRINITY_S9X3R9_9CETA/7-191	S9X3R9.1 PF01251.16	93.90	0.00
TRINITY_A8J6T1_CHLRE/139-200	A8J6T1.1 PF00957.19	93.90	0.00
TRINITY_D8TKM7_VOLCA/539-587	D8TKM7.1 PF02042.13	93.90	0.00
TRINITY_A8I2Z6_CHLRE/31-214	A8I2Z6.1 PF10274.7;	93.90	0.00
TRINITY_D8TZU3_VOLCA/134-281	D8TZU3.1 PF00004.27	93.90	0.00
TRINITY_E1ZGU1_CHLVA/5-70	E1ZGU1.1 PF00808.21	93.90	0.00
TRINITY_A0D6B2_PARTE/1-101	A0D6B2.1 PF00125.22	93.90	0.00
TRINITY_A0BQ95_PARTE/28-93	A0BQ95.1 PF00808.21	93.90	0.00
TRINITY_A0BQ95_PARTE/28-93	A0BQ95.1 PF00808.21	93.90	0.00
TRINITY_H2ZAU4_CIOSA/5-242	H2ZAU4.1 PF00022.17	93.90	0.00
TRINITY_A8JDH8_CHLRE/1129-1278	A8JDH8.1 PF12774.5;	93.90	0.00
TRINITY_H0V077_CAVPO/420-467	H0V077.1 PF00045.17	93.80	0.00
TRINITY_A0A0D9VP32_9ORYZ/78-181	A0A0D9VP32.1 PF0004	93.80	0.00
TRINITY_B0BMH7_XENTR/142-432	B0BMH7.1 PF00113.20	93.80	0.00
TRINITY_Q76L11_CHICK/4-114	Q76L11.1 PF00507.17	93.80	0.00
TRINITY_A0A059CCR0_EUCGR/12-121	A0A059CCR0.1 PF0041	93.80	0.00
TRINITY_M0R601_RAT/3-50	M0R601.2 PF04627.11	93.80	0.00
TRINITY_H0WWF4_OTOGA/105-201	H0WWF4.1 PF14497.4;	93.80	0.00
TRINITY_H0XHY4_OTOGA/3-99	H0XHY4.1 PF01157.16	93.80	0.00
TRINITY_A0A0A0LUC2_CUCSA/32-231	A0A0A0LUC2.1 PF0575	93.80	0.00
TRINITY_G1SEL8_RABIT/3-50	G1SEL8.1 PF04627.11	93.80	0.00
TRINITY_L8GW23_ACACA/109-156	L8GW23.1 PF01466.17	93.80	0.00
TRINITY_G3PUN8_GASAC/99-178	G3PUN8.1 PF00347.21	93.80	0.00
TRINITY_Q94SG9_GASAC/258-359	Q94SG9.1 PF00032.15	93.80	0.00

TRINITY_QCR8_RAT/4-79	Q7TQ16.1 PF02939.14	93.80	0.00
TRINITY_W5F886_WHEAT/120-167	W5F886.1 PF01466.17	93.80	0.00
TRINITY_D8TKQ0_VOLCA/40-167	D8TKQ0.1 PF00004.27	93.80	0.00
TRINITY_G3W7E3_SARHA/117-211	G3W7E3.1 PF04800.10	93.80	0.00
TRINITY_F7FZ45_MONDO/7-163	F7FZ45.1 PF00160.19	93.80	0.00
TRINITY_A0A087SI34_AUXPR/663-868	A0A087SI34.1 PF0058	93.80	0.00
TRINITY_A0A0R4IZM7_DANRE/23-263	A0A0R4IZM7.1 PF0026	93.80	0.00
TRINITY_E6WHM1_PANSA/3-101	E6WHM1.1 PF00466.18	93.80	0.00
TRINITY_G3HNJ3_CRIGR/29-445	G3HNJ3.1 PF01093.15	93.80	0.00
TRINITY_L9JB67_TUPCH/58-136	L9JB67.1 PF01282.17	93.70	0.00
TRINITY_L9JB67_TUPCH/58-136	L9JB67.1 PF01282.17	93.70	0.00
TRINITY_F7I4U3_CALJA/222-574	F7I4U3.1 PF00152.18	93.70	0.00
TRINITY_L9JB67_TUPCH/58-136	L9JB67.1 PF01282.17	93.70	0.00
TRINITY_M3YFZ4_MUSPF/399-551	M3YFZ4.1 PF02910.18	93.70	0.00
TRINITY_A8I4H4_CHLRE/32-94	A8I4H4.1 PF01176.17	93.70	0.00
TRINITY_G3H8L7_CRIGR/22-183	G3H8L7.1 PF00071.20	93.70	0.00
TRINITY_FABPH_RAT/6-132	P07483.2 PF00061.21	93.70	0.00
TRINITY_A8J8M9_CHLRE/23-117	A8J8M9.1 PF00338.20	93.70	0.00
TRINITY_Q6V9B0_CHLRE/38-148	Q6V9B0.1 PF01058.20	93.70	0.00
TRINITY_POL_MLVMS/1445-1560	P03355.4 PF00665.24	93.60	0.00
TRINITY_M5Y9Z3_PRUPE/87-375	M5Y9Z3.1 PF00291.23	93.60	0.00
TRINITY_GPX1_RABIT/13-127	P11909.2 PF00255.17	93.60	0.00
TRINITY_F6ZUW6_CALJA/8-100	F6ZUW6.1 PF00166.19	93.50	0.00
TRINITY_A0A078BXA8_BRANA/111-207	A0A078BXA8.1 PF0119	93.50	0.00
TRINITY_J9J1V2_9SPIT/5-143	J9J1V2.1 PF00179.24	93.50	0.00
TRINITY_COX41_RAT/30-168	P10888.1 PF02936.12	93.50	0.00
TRINITY_Q23JY6_TETTS/610-893	Q23JY6.2 PF00481.19	93.50	0.00
TRINITY_G1TAJ3_RABIT/163-331	G1TAJ3.1 PF02866.16	93.50	0.00
TRINITY_H0VN18_CAVPO/2-140	H0VN18.1 PF00235.17	93.50	0.00
TRINITY_L9LE99_TUPCH/40-207	L9LE99.1 PF00270.27	93.50	0.00
TRINITY_D8U1V2_VOLCA/50-218	D8U1V2.1 PF00504.19	93.50	0.00
TRINITY_D8U7W6_VOLCA/1473-1703	D8U7W6.1 PF12774.5;	93.50	0.00
TRINITY_A0A094CTC8_9PEZI/28-146	A0A094CTC8.1 PF0041	93.50	0.00
TRINITY_L5LRC3_MYODS/155-445	L5LRC3.1 PF00113.20	93.50	0.00
TRINITY_A8I4F6_CHLRE/84-227	A8I4F6.1 PF00475.16	93.50	0.00
TRINITY_D8U1V2_VOLCA/50-218	D8U1V2.1 PF00504.19	93.50	0.00
TRINITY_K5YKK7_9PSED/11-75	K5YKK7.1 PF01668.16	93.50	0.00
TRINITY_A0A061E5Y0_THECC/92-252	A0A061E5Y0.1 PF0016	93.40	0.00
TRINITY_TM100_RAT/1-133	Q569C0.1 PF16311.3;	93.40	0.00
TRINITY_V7BFF7_PHAVU/72-305	V7BFF7.1 PF01946.15	93.40	0.00
TRINITY_V4TB21_9ROSI/54-161	V4TB21.1 PF01655.16	93.40	0.00
TRINITY_M4AK76_XIPMA/95-169	M4AK76.1 PF00900.18	93.40	0.00
TRINITY_L8H4F8_ACACA/1-130	L8H4F8.1 PF00125.22	93.40	0.00
TRINITY_W5JTF2_ANODA/105-287	W5JTF2.1 PF00022.17	93.40	0.00
TRINITY_A0A087SCD3_AUXPR/7-162	A0A087SCD3.1 PF0007	93.40	0.00
TRINITY_A1SZ00_PSYIN/273-347	A1SZ00.1 PF00575.21	93.30	0.00
TRINITY_H2UAE0_TAKRU/46-120	H2UAE0.1 PF01929.15	93.30	0.00
TRINITY_POL_XMRV6/1173-1318	A1Z651.1 PF00075.22	93.30	0.00
TRINITY_H2L7Q5_ORYLA/1-119	H2L7Q5.1 PF00833.16	93.30	0.00
TRINITY_H2L7Q5_ORYLA/1-119	H2L7Q5.1 PF00833.16	93.30	0.00
TRINITY_A4ZYQ6_SOLLC/154-253	A4ZYQ6.1 PF03171.18	93.30	0.00
TRINITY_A0A0D2TQ56_GOSRA/8-171	A0A0D2TQ56.1 PF0016	93.30	0.00
TRINITY_Q7JCY9_MOUSE/14-223	Q7JCY9.1 PF00119.18	93.30	0.00
TRINITY_A0A0D9X9G6_9ORYZ/11-357	A0A0D9X9G6.1 PF0026	93.30	0.00
TRINITY_B9RCE0_RICCO/440-822	B9RCE0.1 PF11940.6;	93.30	0.00
TRINITY_D8RBB7_SELML/307-489	D8RBB7.1 PF04055.19	93.30	0.00
TRINITY_I0Z4M5_9CHLO/3-60	I0Z4M5.1 PF04758.12	93.30	0.00
TRINITY_A8J6V1_CHLRE/43-381	A8J6V1.1 PF00180.18	93.30	0.00
TRINITY_V4TDA4_9ROSI/7-102	V4TDA4.1 PF01158.16	93.20	0.00
TRINITY_M3WL90_FELCA/1230-1465	M3WL90.1 PF01410.16	93.20	0.00
TRINITY_CYB5_BOVIN/13-85	P00171.3 PF00173.26	93.20	0.00

TRINITY_A8I3K8_CHLRE/124-290	A8I3K8.1 PF04055.19	93.20	0.00
TRINITY_PSBB_CHLRE/1-500	P37255.1 PF00421.17	93.20	0.00
TRINITY_Q4T4T6_TETNG/14-155	Q4T4T6.1 PF00210.22	93.20	0.00
TRINITY_K4B7P1_SOLLC/96-331	K4B7P1.1 PF00112.21	93.10	0.00
TRINITY_D7SN77_VITVI/673-993	D7SN77.1 PF02171.15	93.10	0.00
TRINITY_A0A0A0KFM1_CUCSA/42-171	A0A0A0KFM1.1 PF0547	93.10	0.00
TRINITY_W9QHC5_9ROSA/44-274	W9QHC5.1 PF00230.18	93.10	0.00
TRINITY_I1I505_BRADI/3-74	I1I505.1 PF00240.21	93.10	0.00
TRINITY_I1I505_BRADI/3-74	I1I505.1 PF00240.21	93.10	0.00
TRINITY_POL_XMRV6/1338-1439	A1Z651.1 PF16721.3;	93.10	0.00
TRINITY_I1I505_BRADI/3-74	I1I505.1 PF00240.21	93.10	0.00
TRINITY_I1I505_BRADI/3-74	I1I505.1 PF00240.21	93.10	0.00
TRINITY_I1I505_BRADI/3-74	I1I505.1 PF00240.21	93.10	0.00
TRINITY_A0A0A0LD02_CUCSA/156-315	A0A0A0LD02.1 PF0048	93.10	0.00
TRINITY_Q4T4T6_TETNG/14-155	Q4T4T6.1 PF00210.22	93.10	0.00
TRINITY_J9G0L1_9SPIT/1308-1538	J9G0L1.1 PF12774.5;	93.10	0.00
TRINITY_M7BGH9_CHEMY/41-136	M7BGH9.1 PF00428.17	93.10	0.00
TRINITY_Q23QN9_TETTS/1894-2035	Q23QN9.3 PF07728.12	93.00	0.00
TRINITY_A0A0A0LJ63_CUCSA/53-397	A0A0A0LJ63.1 PF0027	93.00	0.00
TRINITY_G3HC29_CRIGR/5-47	G3HC29.1 PF01023.17	93.00	0.00
TRINITY_S10AB_RAT/5-47	Q6B345.1 PF01023.17	93.00	0.00
TRINITY_D3ZN79_RAT/7-63	D3ZN79.1 PF00831.21	93.00	0.00
TRINITY_Q88M03_PSEPK/35-105	Q88M03.1 PF00575.21	93.00	0.00
TRINITY_M0T2W8_MUSAM/84-464	M0T2W8.1 PF00162.17	93.00	0.00
TRINITY_Q22BJ7_TETTS/366-422	Q22BJ7.2 PF01485.19	93.00	0.00
TRINITY_H0YWU1_TAEGU/27-83	H0YWU1.1 PF03946.12	93.00	0.00
TRINITY_A0A0P7Y4Y7_9TELE/114-175	A0A0P7Y4Y7.1 PF0103	93.00	0.00
TRINITY_A8JDN3_CHLRE/50-236	A8JDN3.1 PF10211.7;	93.00	0.00
TRINITY_D8UCX2_VOLCA/54-221	D8UCX2.1 PF00504.19	93.00	0.00
TRINITY_A0A087SJA2_AUXPR/5-146	A0A087SJA2.1 PF0016	93.00	0.00
TRINITY_F0IGB2_9FLAO/54-124	F0IGB2.1 PF04542.12	93.00	0.00
TRINITY_A0A0G2KAQ5_RAT/1-264	A0A0G2KAQ5.1 PF0555	93.00	0.00
TRINITY_D8UGK6_VOLCA/377-504	D8UGK6.1 PF02887.14	93.00	0.00
TRINITY_D8TYR4_VOLCA/520-670	D8TYR4.1 PF00160.19	93.00	0.00
TRINITY_M3WEJ8_FELCA/453-607	M3WEJ8.1 PF09316.8;	92.90	0.00
TRINITY_A0A078HSG2_BRANA/78-138	A0A078HSG2.1 PF0163	92.90	0.00
TRINITY_K4D2K3_SOLLC/87-277	K4D2K3.1 PF13419.4;	92.90	0.00
TRINITY_K4CY74_SOLLC/111-399	K4CY74.1 PF03151.14	92.90	0.00
TRINITY_E1ZBK2_CHLVA/352-461	E1ZBK2.1 PF03143.15	92.90	0.00
TRINITY_H2AV_TETTS/17-104	P08992.2 PF00125.22	92.90	0.00
TRINITY_PPX3_PARTE/46-241	A0C1E4.1 PF00149.26	92.90	0.00
TRINITY_B4ID84_DROSE/5-88	B4ID84.1 PF01221.16	92.90	0.00
TRINITY_D8UIE7_VOLCA/49-105	D8UIE7.1 PF01849.16	92.90	0.00
TRINITY_D8TZD4_VOLCA/92-119	D8TZD4.1 PF01239.20	92.90	0.00
TRINITY_W6UTT4_ECHGR/105-314	W6UTT4.1 PF00091.23	92.90	0.00
TRINITY_K3Z3V7_SETIT/480-828	K3Z3V7.1 PF02896.16	92.90	0.00
TRINITY_C1E9Z7_MICSR/4-73	C1E9Z7.1 PF00240.21	92.90	0.00
TRINITY_CYB6_CHLRE/21-210	Q00471.1 PF00033.17	92.90	0.00
TRINITY_L9L2M7_TUPCH/1-213	L9L2M7.1 PF01201.20	92.80	0.00
TRINITY_K4BPD2_SOLLC/121-342	K4BPD2.1 PF01556.16	92.80	0.00
TRINITY_M0SBM9_MUSAM/63-245	M0SBM9.1 PF00504.19	92.80	0.00
TRINITY_D8TNJ7_VOLCA/63-244	D8TNJ7.1 PF00574.21	92.80	0.00
TRINITY_A8IED9_CHLRE/276-349	A8IED9.1 PF01472.18	92.80	0.00
TRINITY_D8TSX4_VOLCA/66-247	D8TSX4.1 PF00574.21	92.80	0.00
TRINITY_F6ZUK7_MACMU/15-305	F6ZUK7.1 PF15302.4;	92.80	0.00
TRINITY_G3SAL4_GORGO/12-187	G3SAL4.1 PF03357.19	92.70	0.00
TRINITY_A0A0D2QID2_GOSRA/148-348	A0A0D2QID2.1 PF0011	92.70	0.00
TRINITY_A0A0B0NFV8_GOSAR/97-330	A0A0B0NFV8.1 PF0171	92.70	0.00
TRINITY_A0A078FRB1_BRANA/142-225	A0A078FRB1.1 PF0018	92.70	0.00
TRINITY_D8TLE3_VOLCA/7-618	D8TLE3.1 PF00012.18	92.70	0.00
TRINITY_A8IEF7_CHLRE/166-398	A8IEF7.1 PF01786.15	92.70	0.00

TRINITY_A8J576_CHLRE/30-84	A8J576.1 PF01667.15	92.70	0.00
TRINITY_A0A0B2VWK9_TOXCA/3-379	A0A0B2VWK9.1 PF0002	92.70	0.00
TRINITY_A0A059BPK8_EUCGR/13-332	A0A059BPK8.1 PF0107	92.60	0.00
TRINITY_A0A0D9RER4_CHLSB/5-61	A0A0D9RER4.1 PF0040	92.60	0.00
TRINITY_F7G4E6_MONDO/1812-2123	F7G4E6.1 PF02889.14	92.60	0.00
TRINITY_K4AXN3_SOLLC/2-226	K4AXN3.1 PF12481.6;	92.60	0.00
TRINITY_A0A0D9SD52_CHLSB/54-121	A0A0D9SD52.1 PF0450	92.60	0.00
TRINITY_I7MHZ8_TETTS/193-219	I7MHZ8.2 PF00641.16	92.60	0.00
TRINITY_H0Y009_OTOGA/4-61	H0Y009.1 PF00412.20	92.60	0.00
TRINITY_E1Z7R6_CHLVA/686-715	E1Z7R6.1 PF00641.16	92.60	0.00
TRINITY_A0A0A0KHT4_CUCSA/362-461	A0A0A0KHT4.1 PF0008	92.60	0.00
TRINITY_A0E561_PARTE/79-379	A0E561.1 PF03133.13	92.60	0.00
TRINITY_D8TP13_VOLCA/1-259	D8TP13.1 PF01201.20	92.60	0.00
TRINITY_D8TTD9_VOLCA/56-452	D8TTD9.1 PF00890.22	92.60	0.00
TRINITY_A8II71_CHLRE/20-411	A8II71.1 PF06068.11	92.60	0.00
TRINITY_D8TME4_VOLCA/86-178	D8TME4.1 PF00355.24	92.60	0.00
TRINITY_D8SB57_SELML/11-37	D8SB57.1 PF00642.22	92.60	0.00
TRINITY_G5C229_HETGA/7-130	G5C229.1 PF05615.11	92.60	0.00
TRINITY_G3NG58_GASAC/2-69	G3NG58.1 PF01781.16	92.60	0.00
TRINITY_D8UBV4_VOLCA/12-65	D8UBV4.1 PF00584.18	92.60	0.00
TRINITY_NLPD_PSEAE/195-290	P45682.1 PF01551.20	92.60	0.00
TRINITY_L5KK36_PTEAL/499-829	L5KK36.1 PF12036.6;	92.50	0.00
TRINITY_E2RKW9_CANLF/192-258	E2RKW9.2 PF00191.18	92.50	0.00
TRINITY_A0DYC6_PARTE/574-750	A0DYC6.1 PF13870.4;	92.50	0.00
TRINITY_CYB_HUMAN/18-204	P00156.2 PF00033.17	92.50	0.00
TRINITY_V8NDK0_OPHHA/202-296	V8NDK0.1 PF16122.3;	92.50	0.00
TRINITY_D8TVB8_VOLCA/3-42	D8TVB8.1 PF01779.15	92.50	0.00
TRINITY_T0NQQ8_9CETA/120-410	T0NQQ8.1 PF00113.20	92.50	0.00
TRINITY_R0I9P3_9BRAS/205-338	R0I9P3.1 PF00004.27	92.50	0.00
TRINITY_B0WJZ3_CULQU/85-125	B0WJZ3.1 PF00400.30	92.50	0.00
TRINITY_A0A0K0D2L7_ANGCA/7-170	A0A0K0D2L7.1 PF0016	92.50	0.00
TRINITY_A0A0F7TIL7_9EURO/203-336	A0A0F7TIL7.1 PF0000	92.50	0.00
TRINITY_A0A096QE22_MAIZE/258-391	A0A096QE22.1 PF0000	92.50	0.00
TRINITY_L1IB53_GUITH/507-640	L1IB53.1 PF00004.27	92.50	0.00
TRINITY_A0DM44_PARTE/173-306	A0DM44.1 PF00004.27	92.50	0.00
TRINITY_E1ZP98_CHLVA/97-149	E1ZP98.1 PF02861.18	92.50	0.00
TRINITY_Q6PBJ9_DANRE/17-114	Q6PBJ9.1 PF00428.17	92.50	0.00
TRINITY_A0A0G2JY64_RAT/1-107	A0A0G2JY64.1 PF0128	92.50	0.00
TRINITY_A0A0A0KM69_CUCSA/96-241	A0A0A0KM69.1 PF0021	92.40	0.00
TRINITY_IF4G2_RABIT/544-657	P79398.1 PF02847.15	92.40	0.00
TRINITY_F6WXR1_MONDO/47-126	F6WXR1.1 PF00313.20	92.40	0.00
TRINITY_M7ZS43_TRIUA/18-164	M7ZS43.1 PF00025.19	92.40	0.00
TRINITY_Q96550_CHLRE/212-435	Q96550.1 PF00006.23	92.40	0.00
TRINITY_A8I5J8_CHLRE/112-334	A8I5J8.1 PF01063.17	92.40	0.00
TRINITY_A5D984_BOVIN/410-528	A5D984.1 PF02887.14	92.40	0.00
TRINITY_CP2E1_RAT/33-489	P05182.4 PF00067.20	92.40	0.00
TRINITY_A0A0A0MU43_PAPAN/1-55	A0A0A0MU43.1 PF0899	92.30	0.00
TRINITY_A0A0D3FDE6_9ORYZ/18-399	A0A0D3FDE6.1 PF0019	92.30	0.00
TRINITY_L8HK22_ACACA/54-249	L8HK22.1 PF00149.26	92.30	0.00
TRINITY_S10A6_RABIT/5-43	P30801.2 PF01023.17	92.30	0.00
TRINITY_M1BV08_SOLTU/45-274	M1BV08.1 PF00230.18	92.30	0.00
TRINITY_SODC_CALJA/9-150	Q8HXP8.3 PF00080.18	92.30	0.00
TRINITY_G3ICC6_CRIGR/7-183	G3ICC6.1 PF00244.18	92.30	0.00
TRINITY_A0A087SQT0_AUXPR/413-546	A0A087SQT0.1 PF0291	92.30	0.00
TRINITY_A8JG07_CHLRE/219-371	A8JG07.1 PF00733.19	92.30	0.00
TRINITY_A8IZK3_CHLRE/12-166	A8IZK3.1 PF00252.16	92.30	0.00
TRINITY_A8JDL8_CHLRE/10-207	A8JDL8.1 PF00106.23	92.30	0.00
TRINITY_A0A078CAC2_BRANA/90-211	A0A078CAC2.1 PF0038	92.30	0.00
TRINITY_NU2M_CHICK/23-289	P18937.1 PF00361.18	92.20	0.00
TRINITY_D8TM24_VOLCA/202-332	D8TM24.1 PF00004.27	92.20	0.00
TRINITY_A0A087SHS8_AUXPR/40-206	A0A087SHS8.1 PF0027	92.20	0.00

TRINITY_V4B5Y6_LOTGI/4-136	V4B5Y6.1 PF02966.14	92.20	0.00
TRINITY_A0A0D2PY75_GOSRA/6-56	A0A0D2PY75.1 PF0025	92.20	0.00
TRINITY_A8JF93_CHLRE/567-861	A8JF93.1 PF00069.23	92.20	0.00
TRINITY_A0A0B0MYY3_GOSAR/1-191	A0A0B0MYY3.1 PF0000	92.20	0.00
TRINITY_M4BI31_HYAAE/430-836	M4BI31.1 PF08083.9;	92.10	0.00
TRINITY_A0A0A0LIL7_CUCSA/85-122	A0A0A0LIL7.1 PF0659	92.10	0.00
TRINITY_A0A0K9PW16_ZOSMR/64-231	A0A0K9PW16.1 PF0050	92.10	0.00
TRINITY_GRP78_ICTTR/30-636	Q3S4T7.1 PF00012.18	92.10	0.00
TRINITY_A8J2L7_CHLRE/18-83	A8J2L7.1 PF01423.20	92.10	0.00
TRINITY_H0X981_OTOGA/3-41	H0X981.1 PF01290.18	92.10	0.00
TRINITY_Q9FPR7_CHLRE/85-235	Q9FPR7.1 PF05201.13	92.10	0.00
TRINITY_MT1_MAIZE/1-76	P30571.1 PF01439.16	92.10	0.00
TRINITY_G3HQV4_CRIGR/1-239	G3HQV4.1 PF00108.21	92.00	0.00
TRINITY_TNNT2_RABIT/107-243	P09741.3 PF00992.18	92.00	0.00
TRINITY_A0A087XF96_POEFO/12-187	A0A087XF96.1 PF0707	92.00	0.00
TRINITY_A0A067LAI0_JATCU/54-452	A0A067LAI0.1 PF0046	92.00	0.00
TRINITY_J9J1V2_9SPIT/5-143	J9J1V2.1 PF00179.24	92.00	0.00
TRINITY_M3ZLS7_XIPMA/80-191	M3ZLS7.1 PF00059.19	92.00	0.00
TRINITY_U3KDZ5_FICAL/31-121	U3KDZ5.1 PF13499.4;	92.00	0.00
TRINITY_I0Z6P1_9CHLO/3-77	I0Z6P1.1 PF03671.12	92.00	0.00
TRINITY_T0QGI5_9STRA/83-121	T0QGI5.1 PF01096.16	92.00	0.00
TRINITY_A8IE95_CHLRE/299-348	A8IE95.1 PF13920.4;	92.00	0.00
TRINITY_D8U524_VOLCA/56-156	D8U524.1 PF14772.4;	92.00	0.00
TRINITY_NU3M_MOUSE/13-113	P03899.3 PF00507.17	92.00	0.00
TRINITY_F7EPH9_ORNAN/96-158	F7EPH9.2 PF02935.14	91.90	0.00
TRINITY_G3NMQ4_GASAC/274-360	G3NMQ4.1 PF14374.4;	91.90	0.00
TRINITY_A0A078AUW2_STYLE/5-93	A0A078AUW2.1 PF0012	91.90	0.00
TRINITY_D8TGX0_VOLCA/2751-2972	D8TGX0.1 PF12781.5;	91.90	0.00
TRINITY_A8HPW8_CHLRE/149-319	A8HPW8.1 PF00270.27	91.90	0.00
TRINITY_D8UHM6_VOLCA/586-795	D8UHM6.1 PF00149.26	91.90	0.00
TRINITY_A8J336_CHLRE/1680-1932	A8J336.1 PF12774.5;	91.90	0.00
TRINITY_M5XDP6_PRUPE/116-200	M5XDP6.1 PF14159.4;	91.80	0.00
TRINITY_A0A0G2QC06_RAT/25-333	A0A0G2QC06.1 PF0040	91.80	0.00
TRINITY_A0A075B1U4_9FUNG/51-246	A0A075B1U4.1 PF0014	91.80	0.00
TRINITY_A0A087SA88_AUXPR/261-382	A0A087SA88.1 PF0395	91.80	0.00
TRINITY_A0A0D2PQ59_GOSRA/198-375	A0A0D2PQ59.1 PF0073	91.80	0.00
TRINITY_A0A061FNF4_THECC/4-359	A0A061FNF4.1 PF0002	91.80	0.00
TRINITY_D8TLE3_VOLCA/7-618	D8TLE3.1 PF00012.18	91.80	0.00
TRINITY_A0A0B2VWK9_TOXCA/3-379	A0A0B2VWK9.1 PF0002	91.80	0.00
TRINITY_D8TKM7_VOLCA/539-587	D8TKM7.1 PF02042.13	91.80	0.00
TRINITY_A0A0D9YNU9_9ORYZ/177-310	A0A0D9YNU9.1 PF0000	91.80	0.00
TRINITY_D8TRN2_VOLCA/650-880	D8TRN2.1 PF12774.5;	91.80	0.00
TRINITY_G3RDU2_GORGO/237-489	G3RDU2.1 PF00147.16	91.70	0.00
TRINITY_A0A096NJ44_PAPAN/57-425	A0A096NJ44.1 PF0015	91.70	0.00
TRINITY_I2GDG2_9BACT/157-192	I2GDG2.1 PF00132.22	91.70	0.00
TRINITY_I1I505_BRADI/3-74	I1I505.1 PF00240.21	91.70	0.00
TRINITY_I0Z900_9CHLO/1-105	I0Z900.1 PF01283.17	91.70	0.00
TRINITY_F4PA54_BATDJ/8-55	F4PA54.1 PF00319.16	91.70	0.00
TRINITY_D8UI03_VOLCA/42-637	D8UI03.1 PF00012.18	91.70	0.00
TRINITY_G0R1A3_ICHMG/15-146	G0R1A3.1 PF00125.22	91.70	0.00
TRINITY_E1Z3L7_CHLVA/246-388	E1Z3L7.1 PF00004.27	91.70	0.00
TRINITY_A0DN76_PARTE/2168-2307	A0DN76.1 PF07728.12	91.70	0.00
TRINITY_A0A072V649_MEDTR/88-243	A0A072V649.1 PF0178	91.70	0.00
TRINITY_MYG_RAT/7-113	Q9QZ76.3 PF00042.20	91.60	0.00
TRINITY_Q7TQ70_RAT/50-192	Q7TQ70.1 PF08702.8;	91.60	0.00
TRINITY_Q9MD82_MOUSE/134-419	Q9MD82.1 PF00361.18	91.60	0.00
TRINITY_FABP5_RAT/8-134	P55053.3 PF00061.21	91.60	0.00
TRINITY_A0BZX6_PARTE/224-448	A0BZX6.1 PF00006.23	91.60	0.00
TRINITY_D8TWM9_VOLCA/678-883	D8TWM9.1 PF07724.12	91.60	0.00
TRINITY_F6TKI4_ORNAN/46-120	F6TKI4.1 PF01929.15	91.50	0.00
TRINITY_B9HEA7_POPTR/2-78	B9HEA7.1 PF14705.4;	91.50	0.00

TRINITY_A0A0A0KRY8_CUCSA/191-283	A0A0A0KRY8.1 PF1420	91.50	0.00
TRINITY_Q9XF12_SOLTU/7-170	Q9XF12.1 PF00160.19	91.50	0.00
TRINITY_A0A0G2JY66_RAT/22-544	A0A0G2JY66.1 PF0013	91.50	0.00
TRINITY_QCR8_RAT/4-79	Q7TQ16.1 PF02939.14	91.50	0.00
TRINITY_X0KQC1_FUSOX/12-121	X0KQC1.1 PF00411.17	91.50	0.00
TRINITY_D8THK6_VOLCA/55-335	D8THK6.1 PF00682.17	91.50	0.00
TRINITY_A8ICV4_CHLRE/37-96	A8ICV4.1 PF02427.15	91.50	0.00
TRINITY_A0DQI4_PARTE/1027-1259	A0DQI4.1 PF12774.5;	91.50	0.00
TRINITY_A5ASF9_VITVI/1-131	A5ASF9.1 PF00235.17	91.50	0.00
TRINITY_I3LWM8_ICTTR/419-453	I3LWM8.1 PF15880.3;	91.40	0.00
TRINITY_A0A061DHH4_THECC/3-37	A0A061DHH4.1 PF0441	91.40	0.00
TRINITY_H9H5V6_MONDO/5-120	H9H5V6.1 PF01778.15	91.40	0.00
TRINITY_H9H5V6_MONDO/5-120	H9H5V6.1 PF01778.15	91.40	0.00
TRINITY_W1PPQ1_AMBTC/65-262	W1PPQ1.1 PF00504.19	91.40	0.00
TRINITY_B7FM94_MEDTR/85-154	B7FM94.1 PF01287.18	91.40	0.00
TRINITY_A0A0G2JSW3_RAT/8-112	A0A0G2JSW3.1 PF0004	91.40	0.00
TRINITY_NU4M_HUMAN/112-403	P03905.1 PF00361.18	91.40	0.00
TRINITY_LYZ1_MOUSE/19-146	P17897.1 PF00062.18	91.40	0.00
TRINITY_A0A087Y8Q1_POEFO/134-502	A0A087Y8Q1.2 PF0007	91.40	0.00
TRINITY_D8U1V2_VOLCA/50-218	D8U1V2.1 PF00504.19	91.40	0.00
TRINITY_I0Z7H9_9CHLO/17-285	I0Z7H9.1 PF00069.23	91.40	0.00
TRINITY_D8TKP3_VOLCA/332-655	D8TKP3.1 PF00493.21	91.40	0.00
TRINITY_A0A0A0LRJ5_CUCSA/304-396	A0A0A0LRJ5.1 PF0866	91.40	0.00
TRINITY_K4B0V2_SOLLC/532-861	K4B0V2.1 PF00305.17	91.30	0.00
TRINITY_M0SPM5_MUSAM/73-205	M0SPM5.1 PF02531.14	91.30	0.00
TRINITY_A0A0M0JFA6_9EUKA/1-104	A0A0M0JFA6.1 PF0366	91.30	0.00
TRINITY_G3H0L9_CRIGR/80-329	G3H0L9.1 PF00112.21	91.30	0.00
TRINITY_F1PP57_CANLF/11-79	F1PP57.2 PF06522.9;	91.30	0.00
TRINITY_G5CAD0_HETGA/29-214	G5CAD0.1 PF00850.17	91.20	0.00
TRINITY_M1A4X3_SOLTU/45-274	M1A4X3.1 PF00230.18	91.20	0.00
TRINITY_F4PT70_DICFS/108-466	F4PT70.1 PF00128.22	91.20	0.00
TRINITY_M1CSI8_SOLTU/8-617	M1CSI8.1 PF00012.18	91.20	0.00
TRINITY_A0A0A0L7F8_CUCSA/7-334	A0A0A0L7F8.1 PF0153	91.20	0.00
TRINITY_O22578_VOLCA/97-266	O22578.1 PF02776.16	91.20	0.00
TRINITY_I0Z6L9_9CHLO/1788-2022	I0Z6L9.1 PF12774.5;	91.20	0.00
TRINITY_D8TQE1_VOLCA/778-879	D8TQE1.1 PF13676.4;	91.20	0.00
TRINITY_A8IAC7_CHLRE/1074-1288	A8IAC7.1 PF00009.25	91.20	0.00
TRINITY_G0QLT6_ICHMG/132-333	G0QLT6.1 PF00112.21	91.20	0.00
TRINITY_H2RKP1_TAKRU/1-80	H2RKP1.1 PF01249.16	91.20	0.00
TRINITY_A0A061FNG9_THECC/55-211	A0A061FNG9.1 PF0050	91.10	0.00
TRINITY_SAA2_MOUSE/22-122	P05367.1 PF00277.16	91.10	0.00
TRINITY_G1NZD3_MYOLU/16-125	G1NZD3.1 PF01248.24	91.10	0.00
TRINITY_G1NZD3_MYOLU/16-125	G1NZD3.1 PF01248.24	91.10	0.00
TRINITY_A8J8T7_CHLRE/374-658	A8J8T7.1 PF00069.23	91.10	0.00
TRINITY_A8IZI2_CHLRE/11-100	A8IZI2.1 PF00447.15	91.10	0.00
TRINITY_D8U2D8_VOLCA/26-126	D8U2D8.1 PF02678.14	91.10	0.00
TRINITY_A0A0J8BN64_BETVU/71-310	A0A0J8BN64.1 PF0012	91.00	0.00
TRINITY_COX3_TETNG/6-261	Q4JQI1.1 PF00510.16	91.00	0.00
TRINITY_A0A067NJE7_PLEOS/48-158	A0A067NJE7.1 PF0105	91.00	0.00
TRINITY_A0A0D9YNU9_9ORYZ/177-310	A0A0D9YNU9.1 PF0000	91.00	0.00
TRINITY_A0A077ZMR8_STYLE/232-365	A0A077ZMR8.1 PF0000	91.00	0.00
TRINITY_I0YZL1_9CHLO/41-196	I0YZL1.1 PF00177.19	91.00	0.00
TRINITY_A0A0E0PU53_ORYRU/199-332	A0A0E0PU53.1 PF0000	91.00	0.00
TRINITY_G0QVF4_ICHMG/209-342	G0QVF4.1 PF00004.27	91.00	0.00
TRINITY_F0ZNF3_DICPU/193-326	F0ZNF3.1 PF00004.27	91.00	0.00
TRINITY_G6D595_DANPL/10-142	G6D595.1 PF00163.17	91.00	0.00
TRINITY_I0YZT8_9CHLO/82-214	I0YZT8.1 PF00004.27	91.00	0.00
TRINITY_A0CUJ6_PARTE/172-305	A0CUJ6.1 PF00004.27	91.00	0.00
TRINITY_D8TK99_VOLCA/5-264	D8TK99.1 PF00069.23	91.00	0.00
TRINITY_H2M1Q0_ORYLA/264-492	H2M1Q0.1 PF01823.17	91.00	0.00
TRINITY_I3KPH0_ORENI/20-237	I3KPH0.1 PF00147.16	90.90	0.00

TRINITY_SEPP1_RAT/23-248	P25236.2 PF04592.12	90.90	0.00
TRINITY_A8J1Q8_CHLRE/9-74	A8J1Q8.1 PF01106.15	90.90	0.00
TRINITY_Q4S0J2_TETNG/65-141	Q4S0J2.1 PF00076.20	90.90	0.00
TRINITY_D8TSX4_VOLCA/66-247	D8TSX4.1 PF00574.21	90.90	0.00
TRINITY_V4SU74_9ROSI/165-197	V4SU74.1 PF06298.9;	90.90	0.00
TRINITY_A8IWK2_CHLRE/38-139	A8IWK2.1 PF02943.13	90.90	0.00
TRINITY_A8HYG8_CHLRE/42-152	A8HYG8.1 PF00694.17	90.90	0.00
TRINITY_A0A067KAE2_JATCU/632-743	A0A067KAE2.1 PF0278	90.90	0.00
TRINITY_A0A0A0LAK4_CUCSA/1-76	A0A0A0LAK4.1 PF0143	90.80	0.00
TRINITY_Q23QN9_TETTS/1577-1811	Q23QN9.3 PF12774.5;	90.80	0.00
TRINITY_A0A067KRB0_JATCU/18-272	A0A067KRB0.1 PF0006	90.80	0.00
TRINITY_F1NI02_CHICK/10-304	F1NI02.1 PF00206.18	90.80	0.00
TRINITY_I3JB49_ORENI/739-828	I3JB49.1 PF00679.22	90.80	0.00
TRINITY_B4MUC5_DROWI/1-185	B4MUC5.1 PF05018.11	90.80	0.00
TRINITY_A0DN20_PARTE/59-254	A0DN20.1 PF00149.26	90.80	0.00
TRINITY_A8JGT9_CHLRE/34-185	A8JGT9.1 PF00270.27	90.80	0.00
TRINITY_H3C991_TETNG/1-108	H3C991.1 PF15060.4;	90.70	0.00
TRINITY_H9GT67_ANOCA/1-121	H9GT67.1 PF00183.16	90.70	0.00
TRINITY_A0A059CJ54_EUCGR/634-745	A0A059CJ54.1 PF0278	90.70	0.00
TRINITY_K4BY59_SOLLC/61-461	K4BY59.1 PF00155.19	90.70	0.00
TRINITY_A8IV40_CHLRE/38-112	A8IV40.1 PF00111.25	90.70	0.00
TRINITY_I0Z5P9_9CHLO/186-228	I0Z5P9.1 PF13639.4;	90.70	0.00
TRINITY_D8TRQ6_VOLCA/46-142	D8TRQ6.1 PF00004.27	90.70	0.00
TRINITY_D8UDZ4_VOLCA/41-219	D8UDZ4.1 PF00406.20	90.70	0.00
TRINITY_W5JTF2_ANODA/105-287	W5JTF2.1 PF00022.17	90.70	0.00
TRINITY_A0A059CRL9_EUCGR/25-483	A0A059CRL9.1 PF0017	90.60	0.00
TRINITY_A0A0D9Y7Q4_9ORYZ/41-103	A0A0D9Y7Q4.1 PF1283	90.60	0.00
TRINITY_L9KQV0_TUPCH/347-411	L9KQV0.1 PF00327.18	90.60	0.00
TRINITY_L9KQV0_TUPCH/347-411	L9KQV0.1 PF00327.18	90.60	0.00
TRINITY_L5JWI9_PTEAL/176-305	L5JWI9.1 PF00271.29	90.60	0.00
TRINITY_A0A0K9PFT4_ZOSMR/197-265	A0A0K9PFT4.1 PF0110	90.60	0.00
TRINITY_K5WBW0_PHACS/168-441	K5WBW0.1 PF01189.15	90.60	0.00
TRINITY_M1D0L4_SOLTU/3-214	M1D0L4.1 PF00091.23	90.60	0.00
TRINITY_D8UE88_VOLCA/81-358	D8UE88.1 PF13292.4;	90.60	0.00
TRINITY_D8TV61_VOLCA/7-133	D8TV61.1 PF07977.11	90.60	0.00
TRINITY_C1E7K7_MICSR/4-377	C1E7K7.1 PF00022.17	90.60	0.00
TRINITY_D8TXK9_VOLCA/940-1129	D8TXK9.1 PF07059.10	90.60	0.00
TRINITY_H2T4S6_TAKRU/22-148	H2T4S6.1 PF14651.4;	90.50	0.00
TRINITY_A0A067KHP2_JATCU/204-278	A0A067KHP2.1 PF1324	90.50	0.00
TRINITY_E2RSI5_CANLF/58-205	E2RSI5.2 PF00675.18	90.50	0.00
TRINITY_A0A078AUW2_STYLE/5-93	A0A078AUW2.1 PF0012	90.50	0.00
TRINITY_A8JG03_CHLRE/50-478	A8JG03.1 PF00330.18	90.50	0.00
TRINITY_A0A067G8J6_CITSI/1-130	A0A067G8J6.1 PF0083	90.40	0.00
TRINITY_B3RPL6_TRIAD/15-135	B3RPL6.1 PF00238.17	90.40	0.00
TRINITY_A0BGV0_PARTE/13-330	A0BGV0.1 PF00225.21	90.40	0.00
TRINITY_G7PXQ3_MACFA/787-996	G7PXQ3.1 PF00689.19	90.40	0.00
TRINITY_D8TJ87_VOLCA/1503-1555	D8TJ87.1 PF00847.18	90.40	0.00
TRINITY_Q9S807_CHLRE/189-240	Q9S807.1 PF00249.29	90.40	0.00
TRINITY_A8IDM5_CHLRE/58-130	A8IDM5.1 PF04564.13	90.40	0.00
TRINITY_D8TTF2_VOLCA/544-720	D8TTF2.1 PF13307.4;	90.40	0.00
TRINITY_A8IVW3_CHLRE/152-558	A8IVW3.1 PF00962.20	90.40	0.00
TRINITY_V4KUH4_EUTSA/48-219	V4KUH4.1 PF02507.13	90.30	0.00
TRINITY_A0A0A0KZG4_CUCSA/303-466	A0A0A0KZG4.1 PF0357	90.30	0.00
TRINITY_A0A0G2KAQ5_RAT/1-264	A0A0G2KAQ5.1 PF0555	90.30	0.00
TRINITY_B4MUC5_DROWI/1-185	B4MUC5.1 PF05018.11	90.30	0.00
TRINITY_E1Z8J7_CHLVA/1-96	E1Z8J7.1 PF00125.22	90.30	0.00
TRINITY_D8TTR5_VOLCA/8-69	D8TTR5.1 PF02320.14	90.30	0.00
TRINITY_I0Z1F8_9CHLO/53-248	I0Z1F8.1 PF00149.26	90.30	0.00
TRINITY_A8IRQ5_CHLRE/1584-1704	A8IRQ5.1 PF00271.29	90.30	0.00
TRINITY_D8TPK3_VOLCA/16-177	D8TPK3.1 PF01734.20	90.30	0.00
TRINITY_E1Z342_CHLVA/8-214	E1Z342.1 PF01680.15	90.30	0.00

TRINITY_I7MF81_TETTS/221-354	I7MF81.1 PF00004.27	90.30	0.00
TRINITY_A8J646_CHLRE/72-215	A8J646.1 PF03255.12	90.30	0.00
TRINITY_I0YQM7_9CHLO/2-69	I0YQM7.1 PF01781.16	90.30	0.00
TRINITY_D8TZA1_VOLCA/137-270	D8TZA1.1 PF02915.15	90.30	0.00
TRINITY_G3PRZ3_GASAC/46-86	G3PRZ3.1 PF00594.18	90.20	0.00
TRINITY_A0A0A0LE99_CUCSA/68-166	A0A0A0LE99.1 PF0012	90.20	0.00
TRINITY_M5VP59_PRUPE/88-148	M5VP59.1 PF02427.15	90.20	0.00
TRINITY_F1RWV4_PIG/22-141	F1RWV4.2 PF10183.7;	90.20	0.00
TRINITY_I7ML74_TETTS/4924-5082	I7ML74.2 PF00179.24	90.20	0.00
TRINITY_G1TNA5_RABIT/24-484	G1TNA5.2 PF00171.20	90.20	0.00
TRINITY_F1P1D5_CHICK/838-1919	F1P1D5.2 PF01576.17	90.20	0.00
TRINITY_G3PA1_ARATH/62-165	P25856.3 PF00044.22	90.20	0.00
TRINITY_M3YFZ4_MUSPF/399-551	M3YFZ4.1 PF02910.18	90.20	0.00
TRINITY_Q22NW7_TETTS/437-569	Q22NW7.1 PF00004.27	90.20	0.00
TRINITY_D8TS50_VOLCA/105-300	D8TS50.1 PF00448.20	90.20	0.00
TRINITY_F0ZT81_DICPU/1933-2183	F0ZT81.1 PF00454.25	90.20	0.00
TRINITY_A8JD99_CHLRE/45-85	A8JD99.1 PF00400.30	90.20	0.00
TRINITY_F0VGJ4_NEOCL/156-288	F0VGJ4.1 PF00004.27	90.20	0.00
TRINITY_A4RZA4_OSTLU/27-279	A4RZA4.1 PF00069.23	90.20	0.00
TRINITY_D8TXK9_VOLCA/940-1129	D8TXK9.1 PF07059.10	90.20	0.00
TRINITY_A0A0A0KVC0_CUCSA/90-262	A0A0A0KVC0.1 PF0178	90.10	0.00
TRINITY_Q7JCY6_MOUSE/112-403	Q7JCY6.1 PF00361.18	90.10	0.00
TRINITY_A8JEW4_CHLRE/29-213	A8JEW4.1 PF00227.24	90.10	0.00
TRINITY_A8J5R1_CHLRE/19-89	A8J5R1.1 PF12265.6;	90.10	0.00
TRINITY_A0A0J7KRI5_LASNI/1798-2027	A0A0J7KRI5.1 PF1213	90.10	0.00
TRINITY_D8UDW8_VOLCA/449-599	D8UDW8.1 PF00270.27	90.10	0.00
TRINITY_G3N9H8_GASAC/5-85	G3N9H8.1 PF00887.17	90.10	0.00
TRINITY_A8IHF4_CHLRE/27-285	A8IHF4.1 PF00069.23	90.10	0.00
TRINITY_B9HFI4_POPTR/52-396	B9HFI4.1 PF00274.17	90.00	0.00
TRINITY_A0BQZ8_PARTE/123-182	A0BQZ8.1 PF00137.19	90.00	0.00
TRINITY_A0A078CCC6_BRANA/617-728	A0A078CCC6.1 PF0278	90.00	0.00
TRINITY_B3RPL6_TRIAD/15-135	B3RPL6.1 PF00238.17	90.00	0.00
TRINITY_B3RPL6_TRIAD/15-135	B3RPL6.1 PF00238.17	90.00	0.00
TRINITY_B3RPL6_TRIAD/15-135	B3RPL6.1 PF00238.17	90.00	0.00
TRINITY_A0A067R0U6_ZOONE/121-338	A0A067R0U6.1 PF0011	90.00	0.00
TRINITY_A0A0D2RCT0_GOSRA/461-583	A0A0D2RCT0.1 PF0001	90.00	0.00
TRINITY_A0A087ST59_AUXPR/1-177	A0A087ST59.1 PF0372	90.00	0.00
TRINITY_D8U7J1_VOLCA/93-463	D8U7J1.1 PF00285.19	90.00	0.00
TRINITY_I0Z6X0_9CHLO/70-149	I0Z6X0.1 PF00312.20	90.00	0.00
TRINITY_D8TUF6_VOLCA/1-78	D8TUF6.1 PF13086.4;	90.00	0.00
TRINITY_L8GUL2_ACACA/162-264	L8GUL2.1 PF13271.4;	90.00	0.00
TRINITY_Q8WBA9_CRIGR/95-214	Q8WBA9.1 PF00116.18	90.00	0.00
TRINITY_D8TXK9_VOLCA/940-1129	D8TXK9.1 PF07059.10	90.00	0.00
TRINITY_C1MQU0_MICPC/100-429	C1MQU0.1 PF00291.23	90.00	0.00
TRINITY_D8TZ51_VOLCA/86-293	D8TZ51.1 PF04055.19	90.00	0.00
TRINITY_G3NRQ4_GASAC/18-307	G3NRQ4.1 PF02574.14	90.00	0.00
TRINITY_D8TPH8_VOLCA/443-483	D8TPH8.1 PF00400.30	90.00	0.00
TRINITY_A8IK98_CHLRE/902-1013	A8IK98.1 PF09111.8;	90.00	0.00
TRINITY_D8U455_VOLCA/119-328	D8U455.1 PF01039.20	90.00	0.00
TRINITY_H2UZK3_TAKRU/43-171	H2UZK3.1 PF00386.19	89.90	0.00
TRINITY_D8TR40_VOLCA/7-265	D8TR40.1 PF00069.23	89.90	0.00
TRINITY_I0YV56_9CHLO/97-234	I0YV56.1 PF03947.16	89.90	0.00
TRINITY_S9Y661_9CETA/4-112	S9Y661.1 PF03297.13	89.90	0.00
TRINITY_S9Y661_9CETA/4-112	S9Y661.1 PF03297.13	89.90	0.00
TRINITY_D8UI03_VOLCA/42-637	D8UI03.1 PF00012.18	89.90	0.00
TRINITY_D8UE17_VOLCA/9-77	D8UE17.1 PF00076.20	89.90	0.00
TRINITY_A0A087XAX0_POEFO/563-785	A0A087XAX0.2 PF0008	89.80	0.00
TRINITY_Q7H115_RAT/6-261	Q7H115.1 PF00510.16	89.80	0.00
TRINITY_D8TN93_VOLCA/54-268	D8TN93.1 PF01656.21	89.80	0.00
TRINITY_D2VQU5_NAEGR/47-242	D2VQU5.1 PF00149.26	89.80	0.00
TRINITY_D8U7P9_VOLCA/335-461	D8U7P9.1 PF00004.27	89.80	0.00

TRINITY_A8IMN5_CHLRE/273-450	A8IMN5.1 PF00117.26	89.80	0.00
TRINITY_Q65Z16_CHLRE/138-186	Q65Z16.1 PF00010.24	89.80	0.00
TRINITY_A8IU73_CHLRE/235-452	A8IU73.1 PF01786.15	89.80	0.00
TRINITY_F0ZFE1_DICPU/808-922	F0ZFE1.1 PF00271.29	89.80	0.00
TRINITY_M1D577_SOLTU/10-557	M1D577.1 PF01268.17	89.70	0.00
TRINITY_D8U2T6_VOLCA/81-168	D8U2T6.1 PF12159.6;	89.70	0.00
TRINITY_A8IDD8_CHLRE/6-73	A8IDD8.1 PF01111.17	89.70	0.00
TRINITY_D8TQ61_VOLCA/95-253	D8TQ61.1 PF00730.23	89.70	0.00
TRINITY_B4MUC5_DROWI/1-185	B4MUC5.1 PF05018.11	89.70	0.00
TRINITY_A9XBD9_MELGA/11-312	A9XBD9.1 PF00146.19	89.70	0.00
TRINITY_A0A078ATG3_STYLE/388-562	A0A078ATG3.1 PF0151	89.70	0.00
TRINITY_D8UIN9_VOLCA/2094-2451	D8UIN9.1 PF00224.19	89.70	0.00
TRINITY_A0A0C4BF04_WHEAT/1-57	A0A0C4BF04.1 PF0042	89.70	0.00
TRINITY_D8U2B6_VOLCA/139-206	D8U2B6.1 PF00013.27	89.70	0.00
TRINITY_A8J236_CHLRE/31-178	A8J236.1 PF00994.22	89.70	0.00
TRINITY_D8U261_VOLCA/199-237	D8U261.1 PF00400.30	89.70	0.00
TRINITY_D8TXE1_VOLCA/535-669	D8TXE1.1 PF00004.27	89.70	0.00
TRINITY_G0QLK7_ICHMG/233-271	G0QLK7.1 PF00400.30	89.70	0.00
TRINITY_A8JEQ4_CHLRE/455-493	A8JEQ4.1 PF00400.30	89.70	0.00
TRINITY_A8JCU1_CHLRE/33-71	A8JCU1.1 PF00400.30	89.70	0.00
TRINITY_D8U9G8_VOLCA/63-351	D8U9G8.1 PF00069.23	89.70	0.00
TRINITY_M0YRV8_HORVD/7-107	M0YRV8.1 PF00044.22	89.60	0.00
TRINITY_A0A0A0KVC0_CUCSA/90-262	A0A0A0KVC0.1 PF0178	89.60	0.00
TRINITY_G3RK79_GORGO/19-133	G3RK79.1 PF00318.18	89.60	0.00
TRINITY_G3RK79_GORGO/19-133	G3RK79.1 PF00318.18	89.60	0.00
TRINITY_F6HBE6_VITVI/138-329	F6HBE6.1 PF00687.19	89.60	0.00
TRINITY_C1FIS7_MICSR/1373-1603	C1FIS7.1 PF12774.5;	89.60	0.00
TRINITY_D8TR37_VOLCA/4-286	D8TR37.1 PF00069.23	89.60	0.00
TRINITY_K4D1X7_SOLLC/2-115	K4D1X7.1 PF01187.16	89.60	0.00
TRINITY_F6U187_HORSE/186-405	F6U187.1 PF00006.23	89.60	0.00
TRINITY_A8JDN6_CHLRE/158-224	A8JDN6.1 PF00076.20	89.60	0.00
TRINITY_D8U8H5_VOLCA/359-460	D8U8H5.1 PF13646.4;	89.60	0.00
TRINITY_D8UDK7_VOLCA/14-415	D8UDK7.1 PF06068.11	89.60	0.00
TRINITY_A0A0A0LNS8_CUCSA/227-297	A0A0A0LNS8.1 PF0007	89.50	0.00
TRINITY_G0QUJ4_ICHMG/9-139	G0QUJ4.1 PF00004.27	89.50	0.00
TRINITY_D8UD37_VOLCA/4-41	D8UD37.1 PF00400.30	89.50	0.00
TRINITY_I0Z8B0_9CHLO/1-146	I0Z8B0.1 PF01125.15	89.50	0.00
TRINITY_CONS_ARATH/306-349	Q39057.1 PF06203.12	89.50	0.00
TRINITY_A8JFB1_CHLRE/43-255	A8JFB1.1 PF01379.18	89.50	0.00
TRINITY_PSAA_OSTTA/35-745	Q0P3K1.1 PF00223.17	89.50	0
TRINITY_M5WHJ0_PRUPE/43-529	M5WHJ0.1 PF00939.17	89.40	0.00
TRINITY_H0YK49_HUMAN/3-94	H0YK49.1 PF01012.19	89.40	0.00
TRINITY_U6M5D0_EIMMA/34-255	U6M5D0.1 PF01353.20	89.40	0.00
TRINITY_D8UH40_VOLCA/4-88	D8UH40.1 PF01780.17	89.40	0.00
TRINITY_A0E1Y7_PARTE/546-611	A0E1Y7.1 PF00659.16	89.40	0.00
TRINITY_D8UF54_VOLCA/157-365	D8UF54.1 PF02786.15	89.40	0.00
TRINITY_D8TWJ5_VOLCA/51-238	D8TWJ5.1 PF00574.21	89.40	0.00
TRINITY_A8JCS9_CHLRE/4-69	A8JCS9.1 PF03931.13	89.40	0.00
TRINITY_A8IYU6_CHLRE/15-276	A8IYU6.1 PF04078.11	89.40	0.00
TRINITY_D8TP73_VOLCA/50-337	D8TP73.1 PF00069.23	89.40	0.00
TRINITY_A0A0A0L3C6_CUCSA/421-486	A0A0A0L3C6.1 PF0662	89.40	0.00
TRINITY_C0HIK4_MAIZE/38-197	C0HIK4.1 PF00484.17	89.40	0.00
TRINITY_G0QJ15_ICHMG/1-104	G0QJ15.1 PF03660.12	89.30	0.00
TRINITY_A8HMC0_CHLRE/261-336	A8HMC0.1 PF00262.16	89.30	0.00
TRINITY_L8GWF5_ACACA/44-239	L8GWF5.1 PF00149.26	89.30	0.00
TRINITY_B3RJ13_TRIAD/149-287	B3RJ13.1 PF00004.27	89.30	0.00
TRINITY_A8J8P5_CHLRE/4-283	A8J8P5.1 PF00069.23	89.30	0.00
TRINITY_A8J463_CHLRE/170-432	A8J463.1 PF01761.18	89.30	0.00
TRINITY_D8U050_VOLCA/128-492	D8U050.1 PF07690.14	89.30	0.00
TRINITY_D8U4D0_VOLCA/72-192	D8U4D0.1 PF00004.27	89.30	0.00
TRINITY_G3PLD2_GASAC/44-236	G3PLD2.1 PF00106.23	89.20	0.00

TRINITY_I0Z1F8_9CHLO/53-248	I0Z1F8.1 PF00149.26	89.20	0.00
TRINITY_E1ZEE3_CHLVA/49-214	E1ZEE3.1 PF04055.19	89.20	0.00
TRINITY_A8J834_CHLRE/11-75	A8J834.1 PF01423.20	89.20	0.00
TRINITY_D8U0F1_VOLCA/204-242	D8U0F1.1 PF00400.30	89.20	0.00
TRINITY_G0QK34_ICHMG/408-472	G0QK34.1 PF00808.21	89.20	0.00
TRINITY_Q5BKM2_XENTR/24-139	Q5BKM2.1 PF00307.29	89.20	0.00
TRINITY_D8TK99_VOLCA/5-264	D8TK99.1 PF00069.23	89.20	0.00
TRINITY_A8J063_CHLRE/819-1225	A8J063.1 PF08393.11	89.20	0.00
TRINITY_I0ZAR7_9CHLO/6-129	I0ZAR7.1 PF01775.15	89.20	0.00
TRINITY_A8J1M5_CHLRE/2842-3109	A8J1M5.1 PF12780.5;	89.20	0.00
TRINITY_A9XBE2_MELGA/94-213	A9XBE2.1 PF00116.18	89.20	0.00
TRINITY_G1TBU9_RABIT/273-395	G1TBU9.1 PF02803.16	89.20	0.00
TRINITY_M7BKA0_CHEMY/333-438	M7BKA0.1 PF00085.18	89.10	0.00
TRINITY_A0A0A0KDR1_CUCSA/2-226	A0A0A0KDR1.1 PF1248	89.10	0.00
TRINITY_C3UVK6_PAPAN/95-214	C3UVK6.1 PF00116.18	89.10	0.00
TRINITY_K4B3H9_SOLLC/8-78	K4B3H9.1 PF00076.20	89.10	0.00
TRINITY_D3AWF9_POLPA/277-448	D3AWF9.1 PF00270.27	89.10	0.00
TRINITY_H0XX22_OTOGA/11-231	H0XX22.1 PF00244.18	89.10	0.00
TRINITY_D8UC16_VOLCA/69-480	D8UC16.1 PF01603.18	89.10	0.00
TRINITY_D8U1W9_VOLCA/67-112	D8U1W9.1 PF00249.29	89.10	0.00
TRINITY_A8JJA7_CHLRE/56-157	A8JJA7.1 PF05670.11	89.10	0.00
TRINITY_D8UHL8_VOLCA/145-256	D8UHL8.1 PF00355.24	89.10	0.00
TRINITY_E1Z9H5_CHLVA/152-279	E1Z9H5.1 PF07728.12	89.10	0.00
TRINITY_D8UFS7_VOLCA/20-140	D8UFS7.1 PF00238.17	89.10	0.00
TRINITY_E1Z7U1_CHLVA/98-225	E1Z7U1.1 PF07728.12	89.10	0.00
TRINITY_A8HUE0_CHLRE/4-204	A8HUE0.1 PF01912.16	89.10	0.00
TRINITY_CTL2B_MOUSE/16-75	P12400.2 PF08246.10	89.10	0.00
TRINITY_A8HMQ7_CHLRE/38-278	A8HMQ7.1 PF00398.18	89.00	0.00
TRINITY_D8TZX7_VOLCA/742-874	D8TZX7.1 PF04153.16	89.00	0.00
TRINITY_V4MN77_EUTSA/334-504	V4MN77.1 PF00004.27	89.00	0.00
TRINITY_F6TTJ6_XENTR/319-345	F6TTJ6.1 PF13912.4;	88.90	0.00
TRINITY_D8UHE6_VOLCA/1187-1213	D8UHE6.1 PF00642.22	88.90	0.00
TRINITY_A8HME4_CHLRE/171-242	A8HME4.1 PF03719.13	88.90	0.00
TRINITY_I7MHZ8_TETTS/193-219	I7MHZ8.2 PF00641.16	88.90	0.00
TRINITY_G0R1A4_ICHMG/23-114	G0R1A4.1 PF15511.4;	88.90	0.00
TRINITY_A8HY93_CHLRE/120-191	A8HY93.1 PF00076.20	88.90	0.00
TRINITY_A8HS14_CHLRE/72-351	A8HS14.1 PF00483.21	88.90	0.00
TRINITY_D8UBQ8_VOLCA/81-359	D8UBQ8.1 PF00483.21	88.90	0.00
TRINITY_RS3A_CHLRE/15-221	A8HS48.1 PF01015.16	88.90	0.00
TRINITY_A8HQ63_CHLRE/1148-1210	A8HQ63.1 PF13812.4;	88.90	0.00
TRINITY_I7MA05_TETTS/930-1324	I7MA05.1 PF02463.17	88.90	0.00
TRINITY_D8U3G1_VOLCA/9-261	D8U3G1.1 PF00069.23	88.90	0.00
TRINITY_D8TZJ7_VOLCA/599-781	D8TZJ7.1 PF07724.12	88.90	0.00
TRINITY_A0A0A0L4Q3_CUCSA/352-462	A0A0A0L4Q3.1 PF0285	88.90	0.00
TRINITY_D8U1V2_VOLCA/50-218	D8U1V2.1 PF00504.19	88.90	0.00
TRINITY_A0A0D2SDW8_GOSRA/41-138	A0A0D2SDW8.1 PF0472	88.80	0.00
TRINITY_G3PXC5_GASAC/167-408	G3PXC5.1 PF00147.16	88.80	0.00
TRINITY_G1QC71_MYOLU/1-157	G1QC71.1 PF01410.16	88.80	0.00
TRINITY_A8II51_CHLRE/79-194	A8II51.1 PF13640.4;	88.80	0.00
TRINITY_D8TPJ2_VOLCA/104-278	D8TPJ2.1 PF01512.15	88.80	0.00
TRINITY_V7ADT7_PHAVU/141-332	V7ADT7.1 PF00687.19	88.70	0.00
TRINITY_D8UBW6_VOLCA/43-243	D8UBW6.1 PF01078.19	88.70	0.00
TRINITY_D8TT41_VOLCA/30-634	D8TT41.1 PF00012.18	88.70	0.00
TRINITY_G1RXI8_NOMLE/5-239	G1RXI8.1 PF03957.11	88.70	0.00
TRINITY_D8TPS0_VOLCA/28-217	D8TPS0.1 PF00227.24	88.70	0.00
TRINITY_I0YNP8_9CHLO/54-249	I0YNP8.1 PF00149.26	88.70	0.00
TRINITY_D8TV86_VOLCA/43-433	D8TV86.1 PF00764.17	88.70	0.00
TRINITY_D8TLV0_VOLCA/71-352	D8TLV0.1 PF00696.26	88.70	0.00
TRINITY_D8TVM9_VOLCA/542-619	D8TVM9.1 PF00041.19	88.70	0.00
TRINITY_D8U9H4_VOLCA/17-113	D8U9H4.1 PF03645.11	88.70	0.00
TRINITY_L9JAS9_TUPCH/5-153	L9JAS9.1 PF05255.9;	88.70	0.00

TRINITY_J9IKG6_9SPIT/91-160	J9IKG6.1 PF13499.4;	88.60	0.00
TRINITY_K4C8Q1_SOLLC/79-345	K4C8Q1.1 PF00009.25	88.60	0.00
TRINITY_A0A0D3B7Z7_BRAOL/1-206	A0A0D3B7Z7.1 PF0023	88.60	0.00
TRINITY_HOP_PIG/6-60	Q8MJD6.1 PF00046.27	88.60	0.00
TRINITY_I3JNN4_ORENI/944-1066	I3JNN4.1 PF07731.12	88.60	0.00
TRINITY_D8U5G1_VOLCA/71-166	D8U5G1.1 PF01588.18	88.60	0.00
TRINITY_F4PJV4_DICFS/105-265	F4PJV4.1 PF00071.20	88.60	0.00
TRINITY_K9ZI44_ANACC/27-325	K9ZI44.1 PF00124.17	88.60	0.00
TRINITY_A0A0A0LZ00_CUCSA/480-628	A0A0A0LZ00.1 PF0018	88.60	0.00
TRINITY_D8U4T8_VOLCA/39-168	D8U4T8.1 PF08240.10	88.60	0.00
TRINITY_A8J3G2_CHLRE/137-307	A8J3G2.1 PF10520.7;	88.60	0.00
TRINITY_D8TZH6_VOLCA/22-283	D8TZH6.1 PF00233.17	88.60	0.00
TRINITY_G0SUD9_RHOG2/12-336	G0SUD9.1 PF00225.21	88.60	0.00
TRINITY_E1ZAQ7_CHLVA/229-423	E1ZAQ7.1 PF01747.15	88.60	0.00
TRINITY_A8II54_CHLRE/359-510	A8II54.1 PF00005.25	88.60	0.00
TRINITY_A8J6C7_CHLRE/445-655	A8J6C7.1 PF01434.16	88.60	0.00
TRINITY_A8JEP8_CHLRE/290-333	A8JEP8.1 PF13639.4;	88.60	0.00
TRINITY_M0U469_MUSAM/40-141	M0U469.1 PF02943.13	88.60	0.00
TRINITY_A0A0A0K738_CUCSA/57-403	A0A0A0K738.1 PF0027	88.50	0.00
TRINITY_A0A067K4A7_JATCU/4-107	A0A067K4A7.1 PF0004	88.50	0.00
TRINITY_D8U3K8_VOLCA/43-175	D8U3K8.1 PF02531.14	88.50	0.00
TRINITY_A8IGK7_CHLRE/465-500	A8IGK7.1 PF00320.25	88.50	0.00
TRINITY_J9JB18_9SPIT/620-757	J9JB18.1 PF00271.29	88.50	0.00
TRINITY_L0AWE3_THEEQ/51-246	L0AWE3.1 PF00149.26	88.50	0.00
TRINITY_A8HMH7_CHLRE/403-577	A8HMH7.1 PF00488.19	88.50	0.00
TRINITY_Q9FE86_CHLRE/46-178	Q9FE86.1 PF00578.19	88.50	0.00
TRINITY_A8HP84_CHLRE/195-351	A8HP84.1 PF02800.18	88.50	0.00
TRINITY_D8TSK6_VOLCA/99-334	D8TSK6.1 PF04884.12	88.50	0.00
TRINITY_I7ML74_TETTS/4924-5082	I7ML74.2 PF00179.24	88.50	0.00
TRINITY_A0A0P7UM42_9TELE/16-192	A0A0P7UM42.1 PF0014	88.50	0.00
TRINITY_D8TMB5_VOLCA/111-375	D8TMB5.1 PF00069.23	88.50	0.00
TRINITY_A8JGB0_CHLRE/102-444	A8JGB0.1 PF02374.13	88.50	0.00
TRINITY_D8U0B6_VOLCA/1-312	D8U0B6.1 PF01916.15	88.50	0.00
TRINITY_D8TGX0_VOLCA/2112-2380	D8TGX0.1 PF12780.5;	88.50	0.00
TRINITY_A0A059LQJ1_9CHLO/20-72	A0A059LQJ1.1 PF0084	88.50	0.00
TRINITY_D8UHW6_VOLCA/966-1338	D8UHW6.1 PF01645.15	88.50	0.00
TRINITY_A0A078EX89_BRANA/18-356	A0A078EX89.1 PF0038	88.40	0.00
TRINITY_ESTD_HUMAN/23-275	P10768.2 PF00756.18	88.40	0.00
TRINITY_COX1_TETNG/14-460	Q4JQI5.1 PF00115.18	88.40	0.00
TRINITY_A0A078AUP9_STYLE/47-242	A0A078AUP9.1 PF0014	88.40	0.00
TRINITY_D8UA83_VOLCA/10-199	D8UA83.1 PF04511.13	88.40	0.00
TRINITY_A8I8Z4_CHLRE/90-280	A8I8Z4.1 PF00687.19	88.40	0.00
TRINITY_D8UD94_VOLCA/31-142	D8UD94.1 PF01398.19	88.40	0.00
TRINITY_D8TNL4_VOLCA/39-305	D8TNL4.1 PF00218.19	88.40	0.00
TRINITY_G0QVY0_ICHMG/15-74	G0QVY0.1 PF00137.19	88.30	0.00
TRINITY_A0A0D3FG20_9ORYZ/20-114	A0A0D3FG20.1 PF0124	88.30	0.00
TRINITY_PPX3_PARTE/46-241	A0C1E4.1 PF00149.26	88.30	0.00
TRINITY_A0DN20_PARTE/59-254	A0DN20.1 PF00149.26	88.30	0.00
TRINITY_D8TI03_VOLCA/117-294	D8TI03.1 PF00800.16	88.30	0.00
TRINITY_D8UAD0_VOLCA/191-338	D8UAD0.1 PF00004.27	88.30	0.00
TRINITY_D8TIT2_VOLCA/124-335	D8TIT2.1 PF02405.14	88.30	0.00
TRINITY_Q8HLX2_ORYLA/95-214	Q8HLX2.1 PF00116.18	88.30	0.00
TRINITY_D8U1H5_VOLCA/12-199	D8U1H5.1 PF01145.23	88.30	0.00
TRINITY_A0A078EX89_BRANA/18-356	A0A078EX89.1 PF0038	88.30	0.00
TRINITY_F6X8Y4_MONDO/7-131	F6X8Y4.1 PF06212.10	88.20	0.00
TRINITY_Q3SEC5_PARTE/16-270	Q3SEC5.1 PF00069.23	88.20	0.00
TRINITY_F1P5V2_CHICK/1-375	F1P5V2.2 PF00297.20	88.20	0.00
TRINITY_A0A0D2ND66_GOSRA/3-237	A0A0D2ND66.1 PF0000	88.20	0.00
TRINITY_G2HGS6_PANTR/257-780	G2HGS6.1 PF00183.16	88.20	0.00
TRINITY_D8TSD9_VOLCA/1-76	D8TSD9.1 PF01926.21	88.20	0.00
TRINITY_A0A0M3K0T4_ANISI/271-353	A0A0M3K0T4.1 PF0046	88.20	0.00

TRINITY_D8TLR9_VOLCA/72-156	D8TLR9.1 PF16561.3;	88.20	0.00
TRINITY_D8ULS6_VOLCA/67-321	D8ULS6.1 PF01636.21	88.20	0.00
TRINITY_A8J1K0_CHLRE/37-850	A8J1K0.1 PF01496.17	88.20	0.00
TRINITY_A8INY3_CHLRE/4-215	A8INY3.1 PF00091.23	88.20	0.00
TRINITY_Q695H0_CHLRE/703-962	Q695H0.1 PF00069.23	88.20	0.00
TRINITY_J9IAN9_9SPIT/1140-1273	J9IAN9.1 PF10597.7;	88.20	0.00
TRINITY_A0A0P7VDE8_9TELE/7-132	A0A0P7VDE8.1 PF0006	88.10	0.00
TRINITY_D3BKC8_POLPA/353-519	D3BKC8.1 PF00623.18	88.10	0.00
TRINITY_R1E4U8_EMIHU/1-59	R1E4U8.1 PF01194.15	88.10	0.00
TRINITY_C5XW23_SORBI/48-277	C5XW23.1 PF00230.18	88.10	0.00
TRINITY_D8TIA7_VOLCA/10-137	D8TIA7.1 PF00125.22	88.10	0.00
TRINITY_R1FJW2_EMIHU/3-75	R1FJW2.1 PF13499.4;	88.10	0.00
TRINITY_D8TUF3_VOLCA/1145-1203	D8TUF3.1 PF14599.4;	88.10	0.00
TRINITY_L5L525_PTEAL/240-323	L5L525.1 PF00766.17	88.10	0.00
TRINITY_PRS8B_ARATH/198-332	Q94BQ2.1 PF00004.27	88.10	0.00
TRINITY_D8UDZ0_VOLCA/36-283	D8UDZ0.1 PF00977.19	88.10	0.00
TRINITY_A0E1Y7_PARTE/546-611	A0E1Y7.1 PF00659.16	88.10	0.00
TRINITY_D8U867_VOLCA/249-377	D8U867.1 PF01080.15	88.10	0.00
TRINITY_A0A0A0K996_CUCSA/1-100	A0A0A0K996.1 PF0324	88.00	0.00
TRINITY_K7ISA7_NASVI/4-78	K7ISA7.1 PF03671.12	88.00	0.00
TRINITY_I3NEG2_ICTTR/7-98	I3NEG2.1 PF13499.4;	88.00	0.00
TRINITY_A0DN29_PARTE/563-813	A0DN29.1 PF00454.25	88.00	0.00
TRINITY_Q22NW7_TETTS/437-569	Q22NW7.1 PF00004.27	88.00	0.00
TRINITY_A0A0P7USS3_9TELE/192-283	A0A0P7USS3.1 PF1420	88.00	0.00
TRINITY_A9TWG4_PHYPA/100-199	A9TWG4.1 PF00153.25	88.00	0.00
TRINITY_D8TK62_VOLCA/157-248	D8TK62.1 PF00153.25	88.00	0.00
TRINITY_D8UIA3_VOLCA/333-466	D8UIA3.1 PF00004.27	88.00	0.00
TRINITY_I7LXT6_TETTS/73-325	I7LXT6.2 PF00069.23	87.90	0.00
TRINITY_A0A0C4BF04_WHEAT/1-57	A0A0C4BF04.1 PF0042	87.90	0.00
TRINITY_A0DHL1_PARTE/171-388	A0DHL1.1 PF00006.23	87.90	0.00
TRINITY_A8JF84_CHLRE/1-66	A8JF84.1 PF01423.20	87.90	0.00
TRINITY_D8TYY1_VOLCA/9-66	D8TYY1.1 PF03946.12	87.90	0.00
TRINITY_Q9FNS5_CHLRE/71-219	Q9FNS5.2 PF00056.21	87.90	0.00
TRINITY_I0YR35_9CHLO/282-398	I0YR35.1 PF02373.20	87.90	0.00
TRINITY_M0SVG7_MUSAM/12-129	M0SVG7.1 PF00583.23	87.90	0.00
TRINITY_NU3M_PANTR/14-113	Q9T9V8.1 PF00507.17	87.90	0.00
TRINITY_H0WIL4_OTOGA/1-191	H0WIL4.1 PF05805.10	87.90	0.00
TRINITY_A0A0D3FDE6_9ORYZ/18-399	A0A0D3FDE6.1 PF0019	87.80	0.00
TRINITY_G3H239_CRIGR/250-578	G3H239.1 PF00702.24	87.80	0.00
TRINITY_I0YWP9_9CHLO/9-50	I0YWP9.1 PF00832.18	87.80	0.00
TRINITY_G0R640_ICHMG/538-830	G0R640.1 PF00176.21	87.80	0.00
TRINITY_A8HX04_CHLRE/200-273	A8HX04.1 PF13534.4;	87.80	0.00
TRINITY_D8TQC6_VOLCA/13-173	D8TQC6.1 PF00071.20	87.80	0.00
TRINITY_D8TR15_VOLCA/33-180	D8TR15.1 PF00005.25	87.80	0.00
TRINITY_A8J8S4_CHLRE/235-305	A8J8S4.1 PF00076.20	87.80	0.00
TRINITY_A8IKB7_CHLRE/113-377	A8IKB7.1 PF02548.13	87.80	0.00
TRINITY_D8TV21_VOLCA/63-250	D8TV21.1 PF00574.21	87.80	0.00
TRINITY_K4CH79_SOLLC/101-165	K4CH79.1 PF17136.2;	87.70	0.00
TRINITY_I1CFD8_RHIO9/1968-2218	I1CFD8.1 PF00454.25	87.70	0.00
TRINITY_D8UA83_VOLCA/10-199	D8UA83.1 PF04511.13	87.70	0.00
TRINITY_D8TY74_VOLCA/1-228	D8TY74.1 PF00696.26	87.70	0.00
TRINITY_A0A022QM18_ERYGU/141-356	A0A022QM18.1 PF0011	87.60	0.00
TRINITY_COX3_TETNG/6-261	Q4JQI1.1 PF00510.16	87.60	0.00
TRINITY_G0R1A3_ICHMG/15-146	G0R1A3.1 PF00125.22	87.60	0.00
TRINITY_I3K9D6_ORENI/259-508	I3K9D6.1 PF00147.16	87.60	0.00
TRINITY_A8J3T9_CHLRE/82-178	A8J3T9.1 PF04900.10	87.60	0.00
TRINITY_A0A087SHA0_AUXPR/2088-2338	A0A087SHA0.1 PF0045	87.60	0.00
TRINITY_L8GPL0_ACACA/13-116	L8GPL0.1 PF02991.14	87.60	0.00
TRINITY_G0R4Q5_ICHMG/82-212	G0R4Q5.1 PF00004.27	87.50	0.00
TRINITY_G3PUE8_GASAC/3-98	G3PUE8.1 PF00031.19	87.50	0.00
TRINITY_COX3_PONAB/6-261	P92696.1 PF00510.16	87.50	0.00

TRINITY_Q24FD5_TETTS/644-902	Q24FD5.2 PF00069.23	87.50	0.00
TRINITY_A0A078A3T6_STYLE/165-275	A0A078A3T6.1 PF0277	87.50	0.00
TRINITY_E1ZTK8_CHLVA/117-212	E1ZTK8.1 PF01248.24	87.50	0.00
TRINITY_F4JRR2_ARATH/858-989	F4JRR2.1 PF00004.27	87.50	0.00
TRINITY_A8J6C7_CHLRE/445-655	A8J6C7.1 PF01434.16	87.50	0.00
TRINITY_B9GGM5_POPTR/108-155	B9GGM5.1 PF00249.29	87.50	0.00
TRINITY_A8IRR6_CHLRE/8-178	A8IRR6.1 PF03357.19	87.50	0.00
TRINITY_A0BGV0_PARTE/13-330	A0BGV0.1 PF00225.21	87.50	0.00
TRINITY_D8TV46_VOLCA/84-259	D8TV46.1 PF06026.12	87.50	0.00
TRINITY_A7SY88_NEMVE/13-94	A7SY88.1 PF00254.26	87.50	0.00
TRINITY_A8J282_CHLRE/56-219	A8J282.1 PF00160.19	87.50	0.00
TRINITY_A8JHR9_CHLRE/189-346	A8JHR9.1 PF02800.18	87.50	0.00
TRINITY_A0A087G6J3_ARAAL/21-88	A0A087G6J3.1 PF0007	87.50	0.00
TRINITY_A8IXE0_CHLRE/12-482	A8IXE0.1 PF05221.15	87.50	0.00
TRINITY_A0A0D2ND66_GOSRA/3-237	A0A0D2ND66.1 PF0000	87.40	0.00
TRINITY_A0A0G2JSG4_PONAB/132-417	A0A0G2JSG4.1 PF0036	87.40	0.00
TRINITY_A8I1A7_CHLRE/57-238	A8I1A7.1 PF00227.24	87.40	0.00
TRINITY_I0YQE6_9CHLO/136-317	I0YQE6.1 PF04055.19	87.40	0.00
TRINITY_A8J8R7_CHLRE/15-93	A8J8R7.1 PF01423.20	87.30	0.00
TRINITY_S9XEV4_9CETA/20-102	S9XEV4.1 PF15511.4;	87.30	0.00
TRINITY_A8IED9_CHLRE/86-203	A8IED9.1 PF01509.16	87.30	0.00
TRINITY_A4RY33_OSTLU/22-139	A4RY33.1 PF00917.24	87.30	0.00
TRINITY_A8J6H1_CHLRE/25-182	A8J6H1.1 PF00071.20	87.30	0.00
TRINITY_D8TKY6_VOLCA/5-130	D8TKY6.1 PF00410.17	87.30	0.00
TRINITY_D8TII3_VOLCA/37-178	D8TII3.1 PF02878.14	87.30	0.00
TRINITY_Q4VSM4_ENTHI/149-376	Q4VSM4.1 PF00006.23	87.30	0.00
TRINITY_B6AA62_CRYMR/283-321	B6AA62.1 PF01096.16	87.20	0.00
TRINITY_D3BPS9_POLPA/6-54	D3BPS9.1 PF02042.13	87.20	0.00
TRINITY_I0Z5M2_9CHLO/94-172	I0Z5M2.1 PF01472.18	87.20	0.00
TRINITY_SULP2_CHLRE/167-362	Q6QJE2.1 PF00528.20	87.20	0.00
TRINITY_Q9SWQ6_CHLRE/6-99	Q9SWQ6.1 PF03259.15	87.20	0.00
TRINITY_E1ZNN0_CHLVA/86-426	E1ZNN0.1 PF01494.17	87.20	0.00
TRINITY_W5QHB4_SHEEP/377-415	W5QHB4.1 PF00400.30	87.20	0.00
TRINITY_D8TWJ5_VOLCA/51-238	D8TWJ5.1 PF00574.21	87.20	0.00
TRINITY_D8UIJ8_VOLCA/100-207	D8UIJ8.1 PF02777.16	87.20	0.00
TRINITY_F6GXM7_VITVI/49-134	F6GXM7.1 PF00203.19	87.20	0.00
TRINITY_D8UDU8_VOLCA/4-90	D8UDU8.1 PF00125.22	87.20	0.00
TRINITY_D8TNB7_VOLCA/1-175	D8TNB7.1 PF08235.11	87.20	0.00
TRINITY_KAD1_HUMAN/13-169	P00568.3 PF00406.20	87.10	0.00
TRINITY_G5BBC6_HETGA/71-178	G5BBC6.1 PF02100.15	87.10	0.00
TRINITY_F4P7D3_BATDJ/1985-2235	F4P7D3.1 PF00454.25	87.10	0.00
TRINITY_I3JN34_ORENI/202-307	I3JN34.1 PF16122.3;	87.10	0.00
TRINITY_Q8HID1_RAT/4-305	Q8HID1.1 PF00146.19	87.10	0.00
TRINITY_A8J7J2_CHLRE/40-534	A8J7J2.1 PF00118.22	87.10	0.00
TRINITY_C1MPP7_MICPC/194-1278	C1MPP7.1 PF02514.14	87.10	0.00
TRINITY_I7LTY1_TETTS/166-289	I7LTY1.1 PF00549.17	87.10	0.00
TRINITY_D8U3Z9_VOLCA/23-116	D8U3Z9.1 PF00572.16	87.10	0.00
TRINITY_D8U772_VOLCA/53-247	D8U772.1 PF01171.18	87.10	0.00
TRINITY_H34_TETTS/1-132	Q22RG7.1 PF00125.22	87.10	0.00
TRINITY_D8U7Q0_VOLCA/2395-2426	D8U7Q0.1 PF02260.18	87.10	0.00
TRINITY_D8TTG5_VOLCA/143-328	D8TTG5.1 PF04802.13	87.10	0.00
TRINITY_I3J359_ORENI/61-188	I3J359.1 PF00061.21	87.00	0.00
TRINITY_Q94SI1_GASAC/10-311	Q94SI1.1 PF00146.19	87.00	0.00
TRINITY_D8U458_VOLCA/46-299	D8U458.1 PF00348.15	87.00	0.00
TRINITY_I7LX18_TETTS/11-362	I7LX18.1 PF00285.19	87.00	0.00
TRINITY_A9XBE8_MELGA/112-403	A9XBE8.1 PF00361.18	87.00	0.00
TRINITY_F6X6V7_XENTR/651-838	F6X6V7.1 PF00488.19	87.00	0.00
TRINITY_I1JHS3_SOYBN/60-105	I1JHS3.1 PF00249.29	87.00	0.00
TRINITY_A8IJV1_CHLRE/241-351	A8IJV1.1 PF00271.29	87.00	0.00
TRINITY_D8TTQ7_VOLCA/53-350	D8TTQ7.1 PF00698.19	87.00	0.00
TRINITY_A8J795_CHLRE/1-217	A8J795.1 PF14713.4;	87.00	0.00

TRINITY_A0A068SAQ1_9FUNG/6-143	A0A068SAQ1.1 PF0017	87.00	0.00
TRINITY_Q6V9A8_CHLRE/197-467	Q6V9A8.1 PF00346.17	87.00	0.00
TRINITY_M4AKT4_XIPMA/10-146	M4AKT4.1 PF01090.17	86.90	0.00
TRINITY_A0A096SBF4_MAIZE/68-128	A0A096SBF4.1 PF0907	86.90	0.00
TRINITY_V4N6Q8_EUTSA/185-283	V4N6Q8.1 PF00153.25	86.90	0.00
TRINITY_L8GW25_ACACA/462-582	L8GW25.1 PF00004.27	86.90	0.00
TRINITY_D8U0E2_VOLCA/388-547	D8U0E2.1 PF09239.9;	86.90	0.00
TRINITY_D8TSY5_VOLCA/2163-2385	D8TSY5.1 PF12781.5;	86.90	0.00
TRINITY_G3N599_GASAC/369-596	G3N599.1 PF00089.24	86.80	0.00
TRINITY_A0A0J9YUB0_MOUSE/1-87	A0A0J9YUB0.1 PF0048	86.80	0.00
TRINITY_A0A087SQA3_AUXPR/18-83	A0A087SQA3.1 PF0124	86.80	0.00
TRINITY_I0YNB1_9CHLO/84-235	I0YNB1.1 PF00270.27	86.80	0.00
TRINITY_A8J924_CHLRE/124-212	A8J924.1 PF00957.19	86.80	0.00
TRINITY_A8HMQ1_CHLRE/72-518	A8HMQ1.1 PF00330.18	86.80	0.00
TRINITY_D8U1F3_VOLCA/56-419	D8U1F3.1 PF00155.19	86.80	0.00
TRINITY_A0A067JX33_JATCU/90-265	A0A067JX33.1 PF1330	86.70	0.00
TRINITY_COX1_TETNG/14-460	Q4JQI5.1 PF00115.18	86.70	0.00
TRINITY_G3H5I7_CRIGR/174-428	G3H5I7.1 PF00147.16	86.70	0.00
TRINITY_D8UC17_VOLCA/28-143	D8UC17.1 PF04410.12	86.70	0.00
TRINITY_A8J1G8_CHLRE/1-128	A8J1G8.1 PF01092.17	86.70	0.00
TRINITY_A8IZU0_CHLRE/1-580	A8IZU0.1 PF00012.18	86.70	0.00
TRINITY_A8JDP6_CHLRE/44-148	A8JDP6.1 PF00416.20	86.70	0.00
TRINITY_A0A059A8J9_EUCGR/8-103	A0A059A8J9.1 PF0016	86.70	0.00
TRINITY_D8TUY8_VOLCA/54-331	D8TUY8.1 PF02091.13	86.70	0.00
TRINITY_D8U7G0_VOLCA/150-194	D8U7G0.1 PF14237.4;	86.70	0.00
TRINITY_I3K3J8_ORENI/921-1100	I3K3J8.1 PF07678.12	86.60	0.00
TRINITY_A0A087SMB5_AUXPR/365-655	A0A087SMB5.1 PF0017	86.60	0.00
TRINITY_A8J282_CHLRE/56-219	A8J282.1 PF00160.19	86.60	0.00
TRINITY_A8IPI7_CHLRE/50-384	A8IPI7.1 PF00389.28	86.60	0.00
TRINITY_D8U6J2_VOLCA/7-178	D8U6J2.1 PF00270.27	86.60	0.00
TRINITY_W5MUS3_LEPOC/577-614	W5MUS3.1 PF00400.30	86.50	0.00
TRINITY_A0A077ZNX5_STYLE/95-246	A0A077ZNX5.1 PF0808	86.50	0.00
TRINITY_A0A0E9NKU1_9ASCO/134-175	A0A0E9NKU1.1 PF0166	86.50	0.00
TRINITY_A8JAP8_CHLRE/26-124	A8JAP8.1 PF00169.27	86.50	0.00
TRINITY_A0C195_PARTE/19-56	A0C195.1 PF02197.15	86.50	0.00
TRINITY_Q9MD59_MOUSE/23-289	Q9MD59.1 PF00361.18	86.50	0.00
TRINITY_M0U837_MUSAM/336-468	M0U837.1 PF00005.25	86.50	0.00
TRINITY_A8IVQ7_CHLRE/22-186	A8IVQ7.1 PF00160.19	86.50	0.00
TRINITY_I7MDR8_TETTS/76-329	I7MDR8.1 PF08423.9;	86.50	0.00
TRINITY_A8IX54_CHLRE/271-486	A8IX54.1 PF05762.12	86.50	0.00
TRINITY_A8IT48_CHLRE/123-211	A8IT48.1 PF00957.19	86.50	0.00
TRINITY_Q944M9_CHLRE/32-158	Q944M9.1 PF01592.14	86.50	0.00
TRINITY_D8UIF0_VOLCA/2-231	D8UIF0.1 PF04981.11	86.50	0.00
TRINITY_A0A061D9F1_BABBI/1-38	A0A061D9F1.1 PF0622	86.50	0.00
TRINITY_Q8W4V3_CHLRE/61-460	Q8W4V3.1 PF00464.17	86.50	0.00
TRINITY_A0A087SEI7_AUXPR/596-725	A0A087SEI7.1 PF0000	86.50	0.00
TRINITY_S9WFR9_9CETA/1-120	S9WFR9.1 PF00992.18	86.50	0.00
TRINITY_V7D0Z7_PHAVU/61-131	V7D0Z7.1 PF02672.13	86.40	0.00
TRINITY_C1E7K7_MICSR/4-377	C1E7K7.1 PF00022.17	86.40	0.00
TRINITY_L1J975_GUITH/2190-2311	L1J975.1 PF08084.9;	86.40	0.00
TRINITY_W4XGW2_STRPU/51-248	W4XGW2.1 PF01171.18	86.40	0.00
TRINITY_D8TZE6_VOLCA/59-216	D8TZE6.1 PF00005.25	86.40	0.00
TRINITY_A0A0D2T6A5_GOSRA/46-177	A0A0D2T6A5.1 PF0012	86.40	0.00
TRINITY_A8HPS2_CHLRE/32-274	A8HPS2.1 PF01370.19	86.40	0.00
TRINITY_J9IET5_9SPIT/50-247	J9IET5.1 PF01171.18	86.40	0.00
TRINITY_D8U8V9_VOLCA/44-395	D8U8V9.1 PF00180.18	86.40	0.00
TRINITY_A0E1Y7_PARTE/546-611	A0E1Y7.1 PF00659.16	86.40	0.00
TRINITY_D8UI40_VOLCA/118-183	D8UI40.1 PF00288.24	86.40	0.00
TRINITY_D8U8F2_VOLCA/1654-1922	D8U8F2.1 PF12780.5;	86.40	0.00
TRINITY_C1EFJ8_MICSR/128-280	C1EFJ8.1 PF13365.4;	86.40	0.00
TRINITY_A8JA79_CHLRE/25-184	A8JA79.1 PF00069.23	86.40	0.00

TRINITY_H3C6N1_TETNG/30-217	H3C6N1.1 PF06668.10	86.30	0.00
TRINITY_U3IP28_ANAPL/102-154	U3IP28.1 PF00013.27	86.30	0.00
TRINITY_W7HS64_9PEZI/130-229	W7HS64.1 PF00307.29	86.30	0.00
TRINITY_D8U148_VOLCA/297-348	D8U148.1 PF00847.18	86.30	0.00
TRINITY_F4PKY3_DICFS/231-456	F4PKY3.1 PF00006.23	86.30	0.00
TRINITY_I0ZAR7_9CHLO/6-129	I0ZAR7.1 PF01775.15	86.30	0.00
TRINITY_A4VDG5_TETTS/317-447	A4VDG5.2 PF00004.27	86.30	0.00
TRINITY_D8TLB0_VOLCA/190-695	D8TLB0.1 PF00183.16	86.30	0.00
TRINITY_I3JKE7_ORENI/148-288	I3JKE7.1 PF01014.16	86.20	0.00
TRINITY_A0A0A0K7Z3_CUCSA/57-231	A0A0A0K7Z3.1 PF0021	86.20	0.00
TRINITY_L9KQV0_TUPCH/347-411	L9KQV0.1 PF00327.18	86.20	0.00
TRINITY_L1LBY9_THEEQ/5-142	L1LBY9.1 PF00179.24	86.20	0.00
TRINITY_Q22MC3_TETTS/176-305	Q22MC3.2 PF00004.27	86.20	0.00
TRINITY_G0QVV1_ICHMG/11-394	G0QVV1.1 PF00199.17	86.20	0.00
TRINITY_D8U3K8_VOLCA/43-175	D8U3K8.1 PF02531.14	86.20	0.00
TRINITY_Q22YY0_TETTS/3-176	Q22YY0.2 PF00025.19	86.20	0.00
TRINITY_E1Z606_CHLVA/55-218	E1Z606.1 PF00504.19	86.20	0.00
TRINITY_G0QLJ0_ICHMG/34-150	G0QLJ0.1 PF00271.29	86.20	0.00
TRINITY_D8TLD6_VOLCA/1883-2121	D8TLD6.1 PF00809.20	86.20	0.00
TRINITY_D8THR5_VOLCA/234-422	D8THR5.1 PF01163.20	86.20	0.00
TRINITY_G0R1I5_ICHMG/26-90	G0R1I5.1 PF13499.4;	86.20	0.00
TRINITY_G0R1I5_ICHMG/26-90	G0R1I5.1 PF13499.4;	86.20	0.00
TRINITY_A8J955_CHLRE/778-1101	A8J955.1 PF00225.21	86.20	0.00
TRINITY_A8J2Z6_CHLRE/38-393	A8J2Z6.1 PF01264.19	86.20	0.00

TRINITY_1A03_GORGO/25-203	P30377.1 PF00129.16	86.20	0.00
TRINITY_E1ZBK2_CHLVA/352-461	E1ZBK2.1 PF03143.15	86.20	0.00
TRINITY_B2VY78_PYRTR/382-413	B2VY78.1 PF16503.3;	86.20	0.00
TRINITY_COX1_TETNG/14-460	Q4JQI5.1 PF00115.18	86.10	0.00
TRINITY_A0C1B9_PARTE/591-787	A0C1B9.1 PF13087.4;	86.10	0.00
TRINITY_I3MMA5_ICTTR/25-347	I3MMA5.1 PF00405.15	86.10	0.00
TRINITY_L8HIH2_ACACA/30-238	L8HIH2.1 PF04055.19	86.10	0.00
TRINITY_A0A0M3HES0_PANTR/23-289	A0A0M3HES0.1 PF0036	86.10	0.00
TRINITY_I0YRI9_9CHLO/53-188	I0YRI9.1 PF00004.27	86.10	0.00
TRINITY_D4AMJ9_ARTBC/81-137	D4AMJ9.1 PF00352.19	86.10	0.00
TRINITY_R1FJW2_EMIHU/3-75	R1FJW2.1 PF13499.4;	86.10	0.00
TRINITY_A8JFK6_CHLRE/147-182	A8JFK6.1 PF00132.22	86.10	0.00
TRINITY_A8JFP1_CHLRE/2267-2971	A8JFP1.1 PF03028.13	86.10	0
TRINITY_D8U5P9_VOLCA/16-143	D8U5P9.1 PF00270.27	86.10	0.00
TRINITY_A0A0C4BF04_WHEAT/1-57	A0A0C4BF04.1 PF0042	86.10	0.00
TRINITY_A0A078B9W6_STYLE/1811-2043	A0A078B9W6.1 PF1277	86.10	0.00
TRINITY_RR4_CHLRE/3-109	P48270.1 PF00163.17	86.00	0.00
TRINITY_D8TWU0_VOLCA/4-176	D8TWU0.1 PF00025.19	86.00	0.00
TRINITY_CFA52_CHLRE/47-89	A8ILK1.1 PF00400.30	86.00	0.00
TRINITY_D8U6Y1_VOLCA/19-310	D8U6Y1.1 PF00069.23	86.00	0.00
TRINITY_A8HUP5_CHLRE/61-235	A8HUP5.1 PF01853.16	86.00	0.00
TRINITY_I0YLK3_9CHLO/155-290	I0YLK3.1 PF00549.17	86.00	0.00
TRINITY_A8J1U1_CHLRE/30-185	A8J1U1.1 PF02518.24	85.90	0.00
TRINITY_METK_CHLRE/243-385	A8HYU5.1 PF02773.14	85.90	0.00
TRINITY_A8ILJ9_CHLRE/59-200	A8ILJ9.1 PF02729.19	85.90	0.00
TRINITY_D8U5K5_VOLCA/67-406	D8U5K5.1 PF00282.17	85.90	0.00
TRINITY_Q4XXN0_PLACH/10-74	Q4XXN0.1 PF13499.4;	85.90	0.00
TRINITY_I0Z723_9CHLO/8-238	I0Z723.1 PF01269.15	85.90	0.00
TRINITY_A8IJK3_CHLRE/10-175	A8IJK3.1 PF03371.13	85.90	0.00
TRINITY_D8TKB5_VOLCA/35-538	D8TKB5.1 PF01602.18	85.90	0.00
TRINITY_D8TH65_VOLCA/254-559	D8TH65.1 PF00749.19	85.90	0.00
TRINITY_A8IRT6_CHLRE/227-416	A8IRT6.1 PF00587.23	85.90	0.00
TRINITY_I7M6E0_TETTS/153-378	I7M6E0.2 PF00006.23	85.80	0.00
TRINITY_W9S1N3_9ROSA/135-360	W9S1N3.1 PF00112.21	85.80	0.00
TRINITY_G3RRH4_GORGO/4-305	G3RRH4.1 PF00146.19	85.80	0.00
TRINITY_A8J1M5_CHLRE/2495-2760	A8J1M5.1 PF12775.5;	85.80	0.00
TRINITY_I0Z3D7_9CHLO/8-145	I0Z3D7.1 PF00179.24	85.80	0.00
TRINITY_A8IZ29_CHLRE/9-270	A8IZ29.1 PF00069.23	85.80	0.00
TRINITY_D8U9R8_VOLCA/28-210	D8U9R8.1 PF00227.24	85.80	0.00
TRINITY_A8HP25_CHLRE/47-243	A8HP25.1 PF01435.16	85.80	0.00
TRINITY_A0A087Y5N8_POEFO/731-917	A0A087Y5N8.2 PF0666	85.70	0.00
TRINITY_A0A087SL21_AUXPR/78-127	A0A087SL21.1 PF0102	85.70	0.00
TRINITY_D8TR78_VOLCA/80-154	D8TR78.1 PF02617.15	85.70	0.00
TRINITY_A0A0C4BF04_WHEAT/1-57	A0A0C4BF04.1 PF0042	85.70	0.00
TRINITY_Q229R5_TETTS/53-249	Q229R5.1 PF00149.26	85.70	0.00
TRINITY_A8J8S4_CHLRE/235-305	A8J8S4.1 PF00076.20	85.70	0.00
TRINITY_L8GP31_ACACA/22-272	L8GP31.1 PF00069.23	85.70	0.00
TRINITY_V8NKP1_OPHHA/13-58	V8NKP1.1 PF00253.19	85.70	0.00
TRINITY_I7M4K4_TETTS/553-594	I7M4K4.1 PF06203.12	85.70	0.00
TRINITY_A8J1X7_CHLRE/48-89	A8J1X7.1 PF05186.11	85.70	0.00
TRINITY_D8TN53_VOLCA/184-476	D8TN53.1 PF00113.20	85.70	0.00
TRINITY_D8TIC0_VOLCA/100-143	D8TIC0.1 PF01599.17	85.70	0.00
TRINITY_A0A087SNW4_AUXPR/143-177	A0A087SNW4.1 PF0080	85.70	0.00
TRINITY_L8GWH4_ACACA/172-255	L8GWH4.1 PF00352.19	85.70	0.00
TRINITY_A8IAT4_CHLRE/78-259	A8IAT4.1 PF07991.10	85.70	0.00
TRINITY_D8UEE8_VOLCA/616-650	D8UEE8.1 PF00320.25	85.70	0.00
TRINITY_A8I6Z0_CHLRE/171-226	A8I6Z0.1 PF13370.4;	85.70	0.00
TRINITY_A8IWN3_CHLRE/1-184	A8IWN3.1 PF00248.19	85.70	0.00
TRINITY_A0D7P1_PARTE/26-628	A0D7P1.1 PF00012.18	85.70	0.00
TRINITY_D8THY1_VOLCA/170-472	D8THY1.1 PF00365.18	85.70	0.00
TRINITY_Q22C26_TETTS/11-618	Q22C26.1 PF00012.18	85.70	0.00

TRINITY_D8TNW2_VOLCA/70-451	D8TNW2.1 PF07517.12	85.70	0.00
TRINITY_D8UAE3_VOLCA/85-246	D8UAE3.1 PF00091.23	85.70	0.00
TRINITY_A0DQI4_PARTE/1027-1259	A0DQI4.1 PF12774.5;	85.70	0.00
TRINITY_Q1AL73_CHLSB/14-460	Q1AL73.1 PF00115.18	85.60	0.00
TRINITY_K3XYF0_SETIT/47-242	K3XYF0.1 PF00149.26	85.60	0.00
TRINITY_D8UG79_VOLCA/1109-1205	D8UG79.1 PF04059.10	85.60	0.00
TRINITY_D8TXI3_VOLCA/14-304	D8TXI3.1 PF00069.23	85.60	0.00
TRINITY_Q2VIY5_CHLRE/20-116	Q2VIY5.1 PF03645.11	85.60	0.00
TRINITY_D8U4Q8_VOLCA/37-227	D8U4Q8.1 PF01145.23	85.60	0.00
TRINITY_I7MJ63_TETTS/11-618	I7MJ63.1 PF00012.18	85.60	0.00
TRINITY_A0A0E0LPA6_ORYPU/816-1223	A0A0E0LPA6.1 PF0164	85.60	0.00
TRINITY_B3MU37_DROAN/4-136	B3MU37.1 PF02966.14	85.60	0.00
TRINITY_F0NXV5_WEEVC/279-362	F0NXV5.1 PF06071.11	85.50	0.00
TRINITY_Q1AL73_CHLSB/14-460	Q1AL73.1 PF00115.18	85.50	0.00
TRINITY_L8H9R9_ACACA/411-532	L8H9R9.1 PF00004.27	85.50	0.00
TRINITY_H2S6P8_TAKRU/137-591	H2S6P8.1 PF00654.18	85.50	0.00
TRINITY_A0A022RWC8_ERYGU/7-335	A0A022RWC8.1 PF0153	85.50	0.00
TRINITY_A0A022RUE4_ERYGU/32-494	A0A022RUE4.1 PF0017	85.50	0.00
TRINITY_D8U6J5_VOLCA/46-261	D8U6J5.1 PF07722.11	85.50	0.00
TRINITY_A8HXM5_CHLRE/38-220	A8HXM5.1 PF02854.17	85.50	0.00
TRINITY_D8TKY9_VOLCA/30-526	D8TKY9.1 PF00118.22	85.50	0.00
TRINITY_E1ZHQ0_CHLVA/130-191	E1ZHQ0.1 PF04406.12	85.50	0.00
TRINITY_G0QVU1_ICHMG/165-205	G0QVU1.1 PF00400.30	85.40	0.00
TRINITY_D8TNS7_VOLCA/60-317	D8TNS7.1 PF00069.23	85.40	0.00
TRINITY_I7MDR8_TETTS/76-329	I7MDR8.1 PF08423.9;	85.40	0.00
TRINITY_A8II51_CHLRE/79-194	A8II51.1 PF13640.4;	85.40	0.00
TRINITY_J9J844_9SPIT/63-315	J9J844.1 PF00069.23	85.40	0.00
TRINITY_D8TH71_VOLCA/349-485	D8TH71.1 PF01926.21	85.40	0.00
TRINITY_Q6VPQ2_CHLRE/114-209	Q6VPQ2.1 PF08241.10	85.40	0.00
TRINITY_D8TKA7_VOLCA/50-555	D8TKA7.1 PF00118.22	85.40	0.00
TRINITY_A0A0A0KKI5_CUCSA/1042-1193	A0A0A0KKI5.1 PF0000	85.40	0.00
TRINITY_A8JIR0_CHLRE/663-867	A8JIR0.1 PF02786.15	85.40	0.00
TRINITY_Q23DJ9_TETTS/19-409	Q23DJ9.1 PF06068.11	85.40	0.00
TRINITY_K7KEX4_SOYBN/167-222	K7KEX4.1 PF03763.11	85.30	0.00
TRINITY_A0A0G4EDU2_9ALVE/16-83	A0A0G4EDU2.1 PF0007	85.30	0.00
TRINITY_M7SQA8_EUTLA/156-313	M7SQA8.1 PF02800.18	85.30	0.00
TRINITY_A8J8S4_CHLRE/93-160	A8J8S4.1 PF00076.20	85.30	0.00
TRINITY_CDC37_RAT/1-164	Q63692.2 PF03234.12	85.30	0.00
TRINITY_Q84U22_CHLRE/46-233	Q84U22.1 PF00573.20	85.30	0.00
TRINITY_A8JGT9_CHLRE/34-185	A8JGT9.1 PF00270.27	85.30	0.00
TRINITY_G0R0N9_ICHMG/20-230	G0R0N9.1 PF04055.19	85.30	0.00
TRINITY_A8ICF1_CHLRE/16-301	A8ICF1.1 PF01212.19	85.30	0.00
TRINITY_D8UFM9_VOLCA/59-361	D8UFM9.1 PF00069.23	85.30	0.00
TRINITY_D8UEB9_VOLCA/1-192	D8UEB9.1 PF03721.12	85.30	0.00
TRINITY_A8IVF2_CHLRE/123-156	A8IVF2.1 PF00515.26	85.30	0.00
TRINITY_F6HK77_VITVI/53-127	F6HK77.1 PF00111.25	85.30	0.00
TRINITY_A0A059C9R8_EUCGR/40-85	A0A059C9R8.1 PF0040	85.20	0.00
TRINITY_M1ADH3_SOLTU/299-359	M1ADH3.1 PF03822.12	85.20	0.00
TRINITY_U5FMG5_POPTR/187-342	U5FMG5.1 PF02800.18	85.20	0.00
TRINITY_I3MYE1_ICTTR/21-309	I3MYE1.1 PF00865.16	85.20	0.00
TRINITY_A9PD27_POPTR/4-84	A9PD27.1 PF00887.17	85.20	0.00
TRINITY_E1ZDK7_CHLVA/305-332	E1ZDK7.1 PF01239.20	85.20	0.00
TRINITY_E0VF66_PEDHC/55-108	E0VF66.1 PF01192.20	85.20	0.00
TRINITY_A0DDZ2_PARTE/53-248	A0DDZ2.1 PF00149.26	85.20	0.00
TRINITY_D8TWR6_VOLCA/213-543	D8TWR6.1 PF01808.16	85.20	0.00
TRINITY_D8TIB5_VOLCA/323-533	D8TIB5.1 PF01189.15	85.20	0.00
TRINITY_A0DN20_PARTE/59-254	A0DN20.1 PF00149.26	85.20	0.00
TRINITY_DRC3_CHLRE/68-218	A8IVX2.1 PF14580.4;	85.20	0.00
TRINITY_L8H9R9_ACACA/411-532	L8H9R9.1 PF00004.27	85.20	0.00
TRINITY_M3Z1A3_MUSPF/155-208	M3Z1A3.1 PF00013.27	85.20	0.00
TRINITY_A0A0N5DF24_TRIMR/12-173	A0A0N5DF24.1 PF0007	85.20	0.00

TRINITY_A0A0G2K8Q1_RAT/22-90	A0A0G2K8Q1.1 PF0577	85.10	0.00
TRINITY_A0A078B606_STYLE/511-644	A0A078B606.1 PF0000	85.10	0.00
TRINITY_D8TWX0_VOLCA/13-81	D8TWX0.1 PF01423.20	85.10	0.00
TRINITY_D8TUG4_VOLCA/65-460	D8TUG4.1 PF00202.19	85.10	0.00
TRINITY_A8IMV2_CHLRE/138-586	A8IMV2.1 PF01373.15	85.10	0.00
TRINITY_D8U0G5_VOLCA/9-55	D8U0G5.1 PF00471.18	85.10	0.00
TRINITY_I0YR29_9CHLO/95-516	I0YR29.1 PF01964.16	85.10	0.00
TRINITY_L8GW25_ACACA/462-582	L8GW25.1 PF00004.27	85.10	0.00
TRINITY_D8TL59_VOLCA/3854-4560	D8TL59.1 PF03028.13	85.10	0
TRINITY_K0SL03_THAOC/17-64	K0SL03.1 PF01466.17	85.10	0.00
TRINITY_RSSA_CHLRE/13-113	A8IB25.1 PF00318.18	85.10	0.00
TRINITY_A0A087SEJ0_AUXPR/164-389	A0A087SEJ0.1 PF0000	85.10	0.00
TRINITY_D8UCM0_VOLCA/35-314	D8UCM0.1 PF05681.12	85.10	0.00
TRINITY_A8I7P5_CHLRE/260-1381	A8I7P5.1 PF02514.14	85.10	0
TRINITY_Q233N6_TETTS/33-289	Q233N6.2 PF00069.23	85.00	0.00
TRINITY_NUOI_LEGPH/62-117	Q5ZRU6.1 PF13237.4;	85.00	0.00
TRINITY_COX1_TETNG/14-460	Q4JQI5.1 PF00115.18	85.00	0.00
TRINITY_J9F4M2_9SPIT/6-613	J9F4M2.1 PF00012.18	85.00	0.00
TRINITY_I0YVG4_9CHLO/65-231	I0YVG4.1 PF00270.27	85.00	0.00
TRINITY_D8TSH4_VOLCA/33-219	D8TSH4.1 PF00406.20	85.00	0.00
TRINITY_Q2HZ21_CHLRE/191-266	Q2HZ21.1 PF00111.25	85.00	0.00
TRINITY_A8IA39_CHLRE/24-297	A8IA39.1 PF00009.25	85.00	0.00
TRINITY_D8U105_VOLCA/3812-4513	D8U105.1 PF03028.13	85.00	0.00
TRINITY_D8U8K4_VOLCA/2780-3052	D8U8K4.1 PF12780.5;	85.00	0.00
TRINITY_F0ZD36_DICPU/3594-3814	F0ZD36.1 PF12781.5;	84.90	0.00
TRINITY_G0R293_ICHMG/5-142	G0R293.1 PF00179.24	84.90	0.00
TRINITY_G0R293_ICHMG/5-142	G0R293.1 PF00179.24	84.90	0.00
TRINITY_Q3SDI8_PARTE/14-172	Q3SDI8.1 PF00071.20	84.90	0.00
TRINITY_J9J3W2_9SPIT/62-136	J9J3W2.1 PF13921.4;	84.90	0.00
TRINITY_B5G4T9_CUCSA/25-766	B5G4T9.1 PF05691.10	84.90	0.00
TRINITY_D8UJ34_VOLCA/5-275	D8UJ34.1 PF06418.12	84.90	0.00
TRINITY_W5FF67_WHEAT/1-334	W5FF67.1 PF00012.18	84.80	0.00
TRINITY_A0A074SBD1_9HOMO/2-375	A0A074SBD1.1 PF0002	84.80	0.00
TRINITY_D8TTA1_VOLCA/184-262	D8TTA1.1 PF02148.17	84.80	0.00
TRINITY_D8UCI9_VOLCA/73-289	D8UCI9.1 PF00230.18	84.80	0.00
TRINITY_H3BH26_LATCH/745-813	H3BH26.1 PF13499.4;	84.80	0.00
TRINITY_A8J239_CHLRE/9-58	A8J239.1 PF03939.11	84.80	0.00
TRINITY_I0YT55_9CHLO/2-191	I0YT55.1 PF00827.15	84.80	0.00
TRINITY_A8IXF1_CHLRE/150-366	A8IXF1.1 PF01747.15	84.80	0.00
TRINITY_I7M9J2_TETTS/1863-2094	I7M9J2.1 PF12774.5;	84.80	0.00
TRINITY_E4WQH8_OIKDI/91-124	E4WQH8.1 PF16211.3;	84.80	0.00
TRINITY_D8U602_VOLCA/225-402	D8U602.1 PF00587.23	84.80	0.00
TRINITY_H3CR06_TETNG/259-485	H3CR06.1 PF01823.17	84.80	0.00
TRINITY_A0A022RNE1_ERYGU/178-211	A0A022RNE1.1 PF0348	84.80	0.00
TRINITY_I7MAH0_TETTS/182-498	I7MAH0.2 PF00225.21	84.80	0.00
TRINITY_A0A022R9J9_ERYGU/2-169	A0A022R9J9.1 PF1487	84.70	0.00
TRINITY_D8U4D9_VOLCA/38-135	D8U4D9.1 PF03645.11	84.70	0.00
TRINITY_A0A0N4ZJP5_PARTI/3-212	A0A0N4ZJP5.1 PF0009	84.70	0.00
TRINITY_D8TZN4_VOLCA/3-146	D8TZN4.1 PF01280.18	84.70	0.00
TRINITY_A0A0P7VZ44_9TELE/155-312	A0A0P7VZ44.1 PF0280	84.70	0.00
TRINITY_A0DN20_PARTE/59-254	A0DN20.1 PF00149.26	84.70	0.00
TRINITY_E1ZQX5_CHLVA/216-755	E1ZQX5.1 PF02867.13	84.70	0.00
TRINITY_D8TT68_VOLCA/237-400	D8TT68.1 PF01554.16	84.70	0.00
TRINITY_A8HST2_CHLRE/1-460	A8HST2.1 PF05761.12	84.70	0.00
TRINITY_D8STQ6_SELML/100-164	D8STQ6.1 PF13499.4;	84.60	0.00
TRINITY_Q23DE7_TETTS/476-808	Q23DE7.1 PF00773.17	84.60	0.00
TRINITY_Q6V502_CHLRE/181-277	Q6V502.1 PF00507.17	84.60	0.00
TRINITY_G0R4L1_ICHMG/394-433	G0R4L1.1 PF00400.30	84.60	0.00
TRINITY_D8TS73_VOLCA/76-176	D8TS73.1 PF00829.19	84.60	0.00
TRINITY_T0QGI5_9STRA/83-121	T0QGI5.1 PF01096.16	84.60	0.00
TRINITY_D8UKG8_VOLCA/2-27	D8UKG8.1 PF00642.22	84.60	0.00

TRINITY_I0YUS7_9CHLO/717-842	I0YUS7.1 PF00271.29	84.60	0.00
TRINITY_I0YS26_9CHLO/99-190	I0YS26.1 PF00686.17	84.60	0.00
TRINITY_G3PKN9_GASAC/78-171	G3PKN9.1 PF00217.17	84.60	0.00
TRINITY_R0MHK8_NOSB1/42-237	R0MHK8.1 PF00149.26	84.60	0.00
TRINITY_F4QBR0_DICFS/571-1176	F4QBR0.1 PF00012.18	84.60	0.00
TRINITY_D8U5J7_VOLCA/107-371	D8U5J7.1 PF00487.22	84.60	0.00
TRINITY_D8TW10_VOLCA/76-386	D8TW10.1 PF00676.18	84.60	0.00
TRINITY_D8U4A4_VOLCA/176-409	D8U4A4.1 PF01798.16	84.60	0.00
TRINITY_A8IV17_CHLRE/6-83	A8IV17.1 PF07189.9;	84.60	0.00
TRINITY_A0A078AD66_STYLE/99-125	A0A078AD66.1 PF0064	84.60	0.00
TRINITY_D8U148_VOLCA/297-348	D8U148.1 PF00847.18	84.60	0.00
TRINITY_A0A0G4FBC1_9ALVE/1551-1781	A0A0G4FBC1.1 PF1277	84.60	0.00
TRINITY_D8TNM7_VOLCA/1-119	D8TNM7.1 PF00833.16	84.50	0.00
TRINITY_A8I980_CHLRE/57-385	A8I980.1 PF00490.19	84.50	0.00
TRINITY_D8U105_VOLCA/2459-2729	D8U105.1 PF12775.5;	84.50	0.00
TRINITY_C1MTS7_MICPC/1754-1900	C1MTS7.1 PF07728.12	84.50	0.00
TRINITY_A0A0D2TSB0_GOSRA/10-80	A0A0D2TSB0.1 PF0007	84.50	0.00
TRINITY_A8IQB8_CHLRE/65-395	A8IQB8.1 PF03405.12	84.50	0.00
TRINITY_L1J975_GUITH/373-779	L1J975.1 PF08083.9;	84.50	0.00
TRINITY_NU4M_TETNG/114-405	Q4JQH8.1 PF00361.18	84.40	0.00
TRINITY_M1BV08_SOLTU/45-274	M1BV08.1 PF00230.18	84.40	0.00
TRINITY_A1YIP5_XIPMA/18-204	A1YIP5.1 PF00033.17	84.40	0.00
TRINITY_NBEA_CAEEL/1830-2106	Q19317.3 PF02138.16	84.40	0.00
TRINITY_D8THQ0_VOLCA/4-99	D8THQ0.1 PF01157.16	84.40	0.00
TRINITY_D8TS41_VOLCA/87-348	D8TS41.1 PF01040.16	84.40	0.00
TRINITY_L8H9R9_ACACA/411-532	L8H9R9.1 PF00004.27	84.40	0.00
TRINITY_D8TJ87_VOLCA/1589-1639	D8TJ87.1 PF00847.18	84.40	0.00
TRINITY_D8UAM0_VOLCA/836-924	D8UAM0.1 PF08512.10	84.40	0.00
TRINITY_A0DRI8_PARTE/1-143	A0DRI8.1 PF01217.18	84.40	0.00
TRINITY_A8J933_CHLRE/64-454	A8J933.1 PF00202.19	84.40	0.00
TRINITY_D8UIW1_VOLCA/41-264	D8UIW1.1 PF13460.4;	84.40	0.00
TRINITY_I1BLY1_RHIO9/326-360	I1BLY1.1 PF00806.17	84.40	0.00
TRINITY_A0A087SP99_AUXPR/16-273	A0A087SP99.1 PF0337	84.40	0.00
TRINITY_D8U3K7_VOLCA/43-340	D8U3K7.1 PF00579.23	84.40	0.00
TRINITY_D8U394_VOLCA/10-73	D8U394.1 PF00226.29	84.40	0.00
TRINITY_A8JG07_CHLRE/48-170	A8JG07.1 PF13537.4;	84.40	0.00
TRINITY_Q22P63_TETTS/283-414	Q22P63.2 PF00004.27	84.30	0.00
TRINITY_L8H8Q1_ACACA/224-401	L8H8Q1.1 PF01853.16	84.30	0.00
TRINITY_D8UGV3_VOLCA/80-368	D8UGV3.1 PF01062.19	84.30	0.00
TRINITY_D8UCI9_VOLCA/73-289	D8UCI9.1 PF00230.18	84.30	0.00
TRINITY_A8I5X7_CHLRE/198-285	A8I5X7.1 PF00153.25	84.30	0.00
TRINITY_K1PWD4_CRAGI/494-626	K1PWD4.1 PF00004.27	84.30	0.00
TRINITY_D8U346_VOLCA/654-723	D8U346.1 PF00076.20	84.30	0.00
TRINITY_D8U5N9_VOLCA/490-623	D8U5N9.1 PF00005.25	84.30	0.00
TRINITY_A4VDG5_TETTS/317-447	A4VDG5.2 PF00004.27	84.30	0.00
TRINITY_D8UJ31_VOLCA/60-237	D8UJ31.1 PF00587.23	84.30	0.00
TRINITY_D8UI03_VOLCA/42-637	D8UI03.1 PF00012.18	84.30	0.00
TRINITY_H2T398_TAKRU/20-237	H2T398.1 PF00147.16	84.20	0.00
TRINITY_I7LXE3_TETTS/981-1239	I7LXE3.2 PF00069.23	84.20	0.00
TRINITY_A8ICK6_CHLRE/46-171	A8ICK6.1 PF00578.19	84.20	0.00
TRINITY_Q240K0_TETTS/222-351	Q240K0.1 PF00004.27	84.20	0.00
TRINITY_A0A0C4BF04_WHEAT/1-57	A0A0C4BF04.1 PF0042	84.20	0.00
TRINITY_A8JFP4_CHLRE/222-560	A8JFP4.1 PF03372.21	84.20	0.00
TRINITY_D8TJN9_VOLCA/7-63	D8TJN9.1 PF00831.21	84.20	0.00
TRINITY_A0A0N4VLJ8_ENTVE/2-54	A0A0N4VLJ8.1 PF0190	84.20	0.00
TRINITY_A0DN20_PARTE/59-254	A0DN20.1 PF00149.26	84.20	0.00
TRINITY_G0QK69_ICHMG/550-609	G0QK69.1 PF14775.4;	84.20	0.00
TRINITY_D8UD98_VOLCA/79-316	D8UD98.1 PF00696.26	84.20	0.00
TRINITY_Q22GC3_TETTS/35-213	Q22GC3.2 PF02779.22	84.20	0.00
TRINITY_A8I7J8_CHLRE/1-142	A8I7J8.1 PF01217.18	84.20	0.00
TRINITY_L8GTU3_ACACA/71-222	L8GTU3.1 PF00270.27	84.20	0.00

TRINITY_B7KET3_CYAP7/17-206	B7KET3.1 PF01078.19	84.20	0.00
TRINITY_A0DN20_PARTE/59-254	A0DN20.1 PF00149.26	84.20	0.00
TRINITY_A0EA59_PARTE/58-385	A0EA59.1 PF00225.21	84.20	0.00
TRINITY_A0A087SMB5_AUXPR/919-1032	A0A087SMB5.1 PF0027	84.20	0.00
TRINITY_A8IBR6_CHLRE/5-397	A8IBR6.1 PF01134.20	84.20	0.00
TRINITY_D8TWP9_VOLCA/1-107	D8TWP9.1 PF01491.14	84.20	0.00
TRINITY_D8U1I4_VOLCA/34-292	D8U1I4.1 PF00069.23	84.20	0.00
TRINITY_U9U289_RHIID/1145-1338	U9U289.1 PF13087.4;	84.10	0.00
TRINITY_A8J8T9_CHLRE/254-460	A8J8T9.1 PF07724.12	84.10	0.00
TRINITY_A8J856_CHLRE/7-148	A8J856.1 PF00179.24	84.10	0.00
TRINITY_A0A087SQB6_AUXPR/6-118	A0A087SQB6.1 PF0177	84.10	0.00
TRINITY_U7PX74_SPOS1/97-178	U7PX74.1 PF00352.19	84.10	0.00
TRINITY_D8TSR8_VOLCA/68-251	D8TSR8.1 PF00504.19	84.10	0.00
TRINITY_W5KUA0_ASTMX/113-181	W5KUA0.1 PF00050.19	84.10	0.00
TRINITY_C1FDC1_MICSR/161-407	C1FDC1.1 PF01136.17	84.10	0.00
TRINITY_D8TQA9_VOLCA/68-111	D8TQA9.1 PF00432.19	84.10	0.00
TRINITY_Q6J213_CHLRE/83-523	Q6J213.1 PF01593.22	84.10	0.00
TRINITY_D8U303_VOLCA/63-392	D8U303.1 PF00069.23	84.10	0.00
TRINITY_I7M962_TETTS/71-323	I7M962.2 PF00069.23	84.10	0.00
TRINITY_D8U5Q2_VOLCA/457-644	D8U5Q2.1 PF00488.19	84.10	0.00
TRINITY_D8TR19_VOLCA/121-252	D8TR19.1 PF00004.27	84.10	0.00
TRINITY_Q248H8_TETTS/1944-2174	Q248H8.1 PF12774.5;	84.10	0.00
TRINITY_F6VZW7_CALJA/62-322	F6VZW7.1 PF00378.18	84.00	0.00
TRINITY_A0A0D1C1G0_USTMA/616-775	A0A0D1C1G0.1 PF0027	84.00	0.00
TRINITY_E1Z972_CHLVA/57-265	E1Z972.1 PF00902.16	84.00	0.00
TRINITY_D8TW13_VOLCA/585-635	D8TW13.1 PF00847.18	84.00	0.00
TRINITY_Q9ZSM9_CHLRE/23-285	Q9ZSM9.1 PF00069.23	84.00	0.00
TRINITY_A8JFF7_CHLRE/174-248	A8JFF7.1 PF01169.17	84.00	0.00
TRINITY_A8J0E4_CHLRE/58-290	A8J0E4.1 PF01716.16	84.00	0.00
TRINITY_A8IBS4_CHLRE/250-480	A8IBS4.1 PF00069.23	84.00	0.00
TRINITY_A8HP28_CHLRE/42-229	A8HP28.1 PF00117.26	84.00	0.00
TRINITY_A8JH35_CHLRE/53-199	A8JH35.1 PF13365.4;	84.00	0.00
TRINITY_D8TMC4_VOLCA/861-948	D8TMC4.1 PF00679.22	84.00	0.00
TRINITY_D8M985_BLAHO/78-127	D8M985.1 PF01020.15	84.00	0.00
TRINITY_D8M985_BLAHO/78-127	D8M985.1 PF01020.15	84.00	0.00
TRINITY_M4AL71_XIPMA/2-376	M4AL71.1 PF00022.17	84.00	0.00
TRINITY_A0A0D9WZB4_9ORYZ/74-294	A0A0D9WZB4.1 PF0150	84.00	0.00
TRINITY_DCOR_CHICK/34-267	P27118.1 PF02784.14	83.90	0.00
TRINITY_A0A0A1NEL1_9FUNG/19-112	A0A0A1NEL1.1 PF0124	83.90	0.00
TRINITY_A0A087XB41_POEFO/15-364	A0A087XB41.1 PF0027	83.90	0.00
TRINITY_Q3SDY2_PARTE/16-270	Q3SDY2.1 PF00069.23	83.90	0.00
TRINITY_D8TZD3_VOLCA/82-391	D8TZD3.1 PF03320.11	83.90	0.00
TRINITY_A0A061F6M9_THECC/11-124	A0A061F6M9.1 PF0233	83.90	0.00
TRINITY_A8J8B3_CHLRE/34-155	A8J8B3.1 PF01678.17	83.90	0.00
TRINITY_H2SPL5_TAKRU/365-613	H2SPL5.1 PF00089.24	83.90	0.00
TRINITY_A8I972_CHLRE/729-901	A8I972.1 PF07724.12	83.90	0.00
TRINITY_I0YMI8_9CHLO/391-532	I0YMI8.1 PF00637.18	83.90	0.00
TRINITY_E1Z4F7_CHLVA/133-410	E1Z4F7.1 PF01494.17	83.90	0.00
TRINITY_I3JNZ6_ORENI/13-146	I3JNZ6.1 PF01124.16	83.80	0.00
TRINITY_C6T9R8_SOYBN/244-401	C6T9R8.1 PF02800.18	83.80	0.00
TRINITY_A0A078IUM5_BRANA/135-325	A0A078IUM5.1 PF0068	83.80	0.00
TRINITY_J9ETJ6_9SPIT/34-321	J9ETJ6.1 PF00069.23	83.80	0.00
TRINITY_A8HMC0_CHLRE/22-264	A8HMC0.1 PF00262.16	83.80	0.00
TRINITY_I0Z2E4_9CHLO/16-194	I0Z2E4.1 PF00270.27	83.80	0.00
TRINITY_A8I9D7_CHLRE/561-758	A8I9D7.1 PF13087.4;	83.80	0.00
TRINITY_D8U239_VOLCA/807-905	D8U239.1 PF12796.5;	83.80	0.00
TRINITY_D8UD58_VOLCA/3-178	D8UD58.1 PF01105.22	83.80	0.00
TRINITY_A8HQG8_CHLRE/150-407	A8HQG8.1 PF00481.19	83.80	0.00
TRINITY_E1ZBA8_CHLVA/153-189	E1ZBA8.1 PF03638.13	83.80	0.00
TRINITY_A0A0D3DUG5_BRAOL/129-428	A0A0D3DUG5.1 PF0055	83.80	0.00
TRINITY_A0E9G8_PARTE/8-75	A0E9G8.1 PF01111.17	83.80	0.00

TRINITY_D8UH33_VOLCA/82-118	D8UH33.1 PF00400.30	83.80	0.00
TRINITY_A8J236_CHLRE/31-178	A8J236.1 PF00994.22	83.80	0.00
TRINITY_D8TLJ6_VOLCA/4-294	D8TLJ6.1 PF00069.23	83.80	0.00
TRINITY_D8U1L9_VOLCA/159-275	D8U1L9.1 PF01926.21	83.80	0.00
TRINITY_Q2VZC8_MAGSA/26-510	Q2VZC8.1 PF01039.20	83.80	0.00
TRINITY_A8HW48_CHLRE/10-108	A8HW48.1 PF00153.25	83.80	0.00
TRINITY_B9H5A1_POPTR/60-257	B9H5A1.2 PF01171.18	83.80	0.00
TRINITY_A8IYM0_CHLRE/156-461	A8IYM0.1 PF00365.18	83.80	0.00
TRINITY_D8U6F9_VOLCA/1848-1955	D8U6F9.1 PF01326.17	83.80	0.00
TRINITY_D8UFR9_VOLCA/717-924	D8UFR9.1 PF00481.19	83.80	0.00
TRINITY_G5BQ42_HETGA/19-112	G5BQ42.1 PF12265.6;	83.70	0.00
TRINITY_D8TWE9_VOLCA/37-86	D8TWE9.1 PF00847.18	83.70	0.00
TRINITY_P93106_CHLRE/182-347	P93106.1 PF02866.16	83.70	0.00
TRINITY_D8UB15_VOLCA/264-324	D8UB15.1 PF03105.17	83.70	0.00
TRINITY_D8U684_VOLCA/90-267	D8U684.1 PF00719.17	83.70	0.00
TRINITY_D8U556_VOLCA/57-406	D8U556.1 PF00266.17	83.70	0.00
TRINITY_A8IX80_CHLRE/79-600	A8IX80.1 PF00920.19	83.70	0.00
TRINITY_A8IYR7_CHLRE/1-99	A8IYR7.1 PF00383.21	83.70	0.00
TRINITY_D8TQ33_VOLCA/623-831	D8TQ33.1 PF00924.16	83.70	0.00
TRINITY_D8UIN5_VOLCA/335-512	D8UIN5.1 PF01853.16	83.70	0.00
TRINITY_Q3SDI8_PARTE/14-172	Q3SDI8.1 PF00071.20	83.60	0.00
TRINITY_I7M1Z0_TETTS/100-295	I7M1Z0.1 PF00448.20	83.60	0.00
TRINITY_Q22F12_TETTS/2057-2327	Q22F12.2 PF12775.5;	83.60	0.00
TRINITY_D8UDW8_VOLCA/692-831	D8UDW8.1 PF00271.29	83.60	0.00
TRINITY_E9GBJ8_DAPPU/35-163	E9GBJ8.1 PF08240.10	83.60	0.00
TRINITY_A8JDA5_CHLRE/11-126	A8JDA5.1 PF01412.16	83.60	0.00
TRINITY_A0A0D2S1I5_GOSRA/100-245	A0A0D2S1I5.1 PF0000	83.60	0.00
TRINITY_Q8LRU3_CHLRE/4-223	Q8LRU3.1 PF00091.23	83.60	0.00
TRINITY_A8J4Q7_CHLRE/74-189	A8J4Q7.1 PF13806.4;	83.60	0.00
TRINITY_DC2L1_CHLRE/43-159	Q7XA07.1 PF08477.11	83.60	0.00
TRINITY_L8HGI6_ACACA/131-203	L8HGI6.1 PF01191.17	83.60	0.00
TRINITY_L5K2L6_PTEAL/7-264	L5K2L6.1 PF01180.19	83.60	0.00
TRINITY_A8JCY4_CHLRE/40-377	A8JCY4.1 PF00274.17	83.60	0.00
TRINITY_A8IRV0_CHLRE/377-444	A8IRV0.1 PF13432.4;	83.60	0.00
TRINITY_Q75VY8_CHLRE/41-205	Q75VY8.1 PF00504.19	83.60	0.00
TRINITY_A8J0X7_CHLRE/68-433	A8J0X7.1 PF00266.17	83.60	0.00
TRINITY_A8ICS4_CHLRE/173-343	A8ICS4.1 PF00270.27	83.60	0.00
TRINITY_Q3SDY2_PARTE/16-270	Q3SDY2.1 PF00069.23	83.50	0.00
TRINITY_V4U7H7_9ROSI/1-89	V4U7H7.1 PF01434.16	83.50	0.00
TRINITY_G4ZB41_PHYSP/21-141	G4ZB41.1 PF00238.17	83.50	0.00
TRINITY_D8TJ80_VOLCA/167-396	D8TJ80.1 PF01798.16	83.50	0.00
TRINITY_A8IBY2_CHLRE/168-349	A8IBY2.1 PF00270.27	83.50	0.00
TRINITY_D8U2B5_VOLCA/102-199	D8U2B5.1 PF00153.25	83.50	0.00
TRINITY_Q41641_VOLCA/200-315	Q41641.1 PF00175.19	83.50	0.00
TRINITY_Q6QJE1_CHLRE/131-273	Q6QJE1.1 PF00005.25	83.50	0.00
TRINITY_A0A0A0KR46_CUCSA/71-179	A0A0A0KR46.1 PF0010	83.50	0.00
TRINITY_A8IBY2_CHLRE/168-349	A8IBY2.1 PF00270.27	83.50	0.00
TRINITY_A8HVQ1_CHLRE/1-188	A8HVQ1.1 PF01201.20	83.50	0.00
TRINITY_I0YMX9_9CHLO/58-221	I0YMX9.1 PF04055.19	83.50	0.00
TRINITY_D8TNN2_VOLCA/238-334	D8TNN2.1 PF07109.9;	83.50	0.00
TRINITY_D8UC50_VOLCA/133-380	D8UC50.1 PF00120.22	83.40	0.00
TRINITY_D8TJK2_VOLCA/852-1116	D8TJK2.1 PF02913.17	83.40	0.00
TRINITY_D8TH19_VOLCA/4-256	D8TH19.1 PF00069.23	83.40	0.00
TRINITY_G0QJ15_ICHMG/1-104	G0QJ15.1 PF03660.12	83.30	0.00
TRINITY_B9H869_POPTR/5-70	B9H869.1 PF01423.20	83.30	0.00
TRINITY_L8HJG6_ACACA/1-66	L8HJG6.1 PF01246.18	83.30	0.00
TRINITY_G0QVY0_ICHMG/92-151	G0QVY0.1 PF00137.19	83.30	0.00
TRINITY_G2IVL3_PSEUL/3-119	G2IVL3.1 PF02678.14	83.30	0.00
TRINITY_A0A0C4BF04_WHEAT/1-57	A0A0C4BF04.1 PF0042	83.30	0.00
TRINITY_D8TXT2_VOLCA/30-122	D8TXT2.1 PF01329.17	83.30	0.00
TRINITY_I0YYT0_9CHLO/28-209	I0YYT0.1 PF00227.24	83.30	0.00

TRINITY_H2MFG8_ORYLA/207-262	H2MFG8.1 PF00084.18	83.30	0.00
TRINITY_L8H946_ACACA/11-172	L8H946.1 PF00071.20	83.30	0.00
TRINITY_A8J8L3_CHLRE/10-81	A8J8L3.1 PF00173.26	83.30	0.00
TRINITY_D8U6J6_VOLCA/71-117	D8U6J6.1 PF00301.18	83.30	0.00
TRINITY_A8JID6_CHLRE/76-553	A8JID6.1 PF03219.12	83.30	0.00
TRINITY_D8TWW0_VOLCA/346-387	D8TWW0.1 PF00400.30	83.30	0.00
TRINITY_A8J8I4_CHLRE/47-327	A8J8I4.1 PF00069.23	83.30	0.00
TRINITY_I0YRK9_9CHLO/3-162	I0YRK9.1 PF01734.20	83.30	0.00
TRINITY_A0A087SJD8_AUXPR/7-268	A0A087SJD8.1 PF0014	83.30	0.00
TRINITY_I0YJ54_9CHLO/13-175	I0YJ54.1 PF00071.20	83.30	0.00
TRINITY_D8TSW4_VOLCA/28-79	D8TSW4.1 PF05047.14	83.30	0.00
TRINITY_K9UHQ5_9CHRO/8-726	K9UHQ5.1 PF00223.17	83.30	0
TRINITY_I0Z4C2_9CHLO/18-311	I0Z4C2.1 PF07992.12	83.30	0.00
TRINITY_D8TY18_VOLCA/1-283	D8TY18.1 PF00128.22	83.30	0.00
TRINITY_D8UFY8_VOLCA/167-203	D8UFY8.1 PF00400.30	83.30	0.00
TRINITY_A8J5T5_CHLRE/1176-1312	A8J5T5.1 PF07304.9;	83.30	0.00
TRINITY_D8U425_VOLCA/27-321	D8U425.1 PF00579.23	83.30	0.00
TRINITY_G0QQR7_ICHMG/565-788	G0QQR7.1 PF00149.26	83.30	0.00
TRINITY_A8JD38_CHLRE/263-591	A8JD38.1 PF01179.18	83.30	0.00
TRINITY_A0A059LSQ0_9CHLO/402-533	A0A059LSQ0.1 PF0000	83.30	0.00
TRINITY_A8JEV0_CHLRE/9-145	A8JEV0.1 PF00179.24	83.30	0.00
TRINITY_D8TGX0_VOLCA/2112-2380	D8TGX0.1 PF12780.5;	83.30	0.00
TRINITY_A8IV22_CHLRE/1629-1900	A8IV22.1 PF12775.5;	83.30	0.00
TRINITY_U5GTV8_POPTR/134-247	U5GTV8.1 PF02770.17	83.30	0.00
TRINITY_I3KSH1_ORENI/53-153	I3KSH1.1 PF01551.20	83.20	0.00
TRINITY_I7MG82_TETTS/90-413	I7MG82.2 PF00225.21	83.20	0.00
TRINITY_A8I1Q9_CHLRE/4-259	A8I1Q9.1 PF00069.23	83.20	0.00
TRINITY_D8U848_VOLCA/22-165	D8U848.1 PF00056.21	83.20	0.00
TRINITY_D8TRJ8_VOLCA/7-191	D8TRJ8.1 PF00025.19	83.20	0.00
TRINITY_A8I3T3_CHLRE/33-204	A8I3T3.1 PF03637.15	83.20	0.00
TRINITY_E2B3F6_HARSA/38-132	E2B3F6.1 PF00338.20	83.20	0.00
TRINITY_A8HVM3_CHLRE/412-558	A8HVM3.1 PF00587.23	83.20	0.00
TRINITY_Q6VTH1_CHLRE/660-810	Q6VTH1.1 PF00005.25	83.20	0.00
TRINITY_D8UEF1_VOLCA/38-210	D8UEF1.1 PF00213.16	83.20	0.00
TRINITY_D8TRV6_VOLCA/279-993	D8TRV6.1 PF00343.18	83.20	0
TRINITY_A0A022RWV0_ERYGU/192-356	A0A022RWV0.1 PF0286	83.10	0.00
TRINITY_I7M317_TETTS/63-246	I7M317.1 PF10274.7;	83.10	0.00
TRINITY_A0A074SSQ8_HAMHA/28-92	A0A074SSQ8.1 PF1349	83.10	0.00
TRINITY_A0A074SSQ8_HAMHA/28-92	A0A074SSQ8.1 PF1349	83.10	0.00
TRINITY_F4PTT9_DICFS/32-203	F4PTT9.1 PF03637.15	83.10	0.00
TRINITY_Q22MC3_TETTS/176-305	Q22MC3.2 PF00004.27	83.10	0.00
TRINITY_D8R512_SELML/19-85	D8R512.1 PF01423.20	83.10	0.00
TRINITY_D8TPA3_VOLCA/158-317	D8TPA3.1 PF01210.21	83.10	0.00
TRINITY_W5Q6E7_SHEEP/39-245	W5Q6E7.1 PF00009.25	83.10	0.00
TRINITY_D8TPM9_VOLCA/13-107	D8TPM9.1 PF01248.24	83.10	0.00
TRINITY_D8TQG6_VOLCA/33-138	D8TQG6.1 PF00116.18	83.10	0.00
TRINITY_A8JDW2_CHLRE/95-212	A8JDW2.1 PF04536.12	83.10	0.00
TRINITY_I7M491_TETTS/56-185	I7M491.1 PF00134.21	83.10	0.00
TRINITY_H0WZK7_OTOGA/180-497	H0WZK7.1 PF00728.20	83.10	0.00
TRINITY_A8IZV8_CHLRE/1-142	A8IZV8.1 PF01217.18	83.10	0.00
TRINITY_A8IZE7_CHLRE/354-483	A8IZE7.1 PF01380.20	83.10	0.00
TRINITY_A0D281_PARTE/96-237	A0D281.1 PF07717.14	83.00	0.00
TRINITY_A8JH12_CHLRE/1-129	A8JH12.1 PF00334.17	83.00	0.00
TRINITY_D8UHK4_VOLCA/936-1344	D8UHK4.1 PF02901.13	83.00	0.00
TRINITY_ALFC_CHLRE/33-377	Q42690.2 PF00274.17	83.00	0.00
TRINITY_A8J0I0_CHLRE/22-263	A8J0I0.1 PF00573.20	83.00	0.00
TRINITY_A8JE85_CHLRE/1-165	A8JE85.1 PF04502.11	83.00	0.00
TRINITY_A8JDH8_CHLRE/1697-1852	A8JDH8.1 PF12775.5;	83.00	0.00
TRINITY_F0ZQ39_DICPU/536-700	F0ZQ39.1 PF07724.12	83.00	0.00
TRINITY_C1FIS7_MICSR/1373-1603	C1FIS7.1 PF12774.5;	83.00	0.00
TRINITY_C1E0R4_MICSR/3145-3366	C1E0R4.1 PF12781.5;	83.00	0.00

TRINITY_C1E9Z7_MICSR/4-73	C1E9Z7.1 PF00240.21	83.00	0.00
TRINITY_A0A077ZWC4_STYLE/46-188	A0A077ZWC4.1 PF0058	82.90	0.00
TRINITY_E8RSH0_ASTEC/64-174	E8RSH0.1 PF01058.20	82.90	0.00
TRINITY_A9SLZ5_PHYPA/42-82	A9SLZ5.1 PF00400.30	82.90	0.00
TRINITY_L1IT40_GUITH/528-609	L1IT40.1 PF00005.25	82.90	0.00
TRINITY_D8TIC8_VOLCA/15-143	D8TIC8.1 PF00416.20	82.90	0.00
TRINITY_A8HQ42_CHLRE/53-87	A8HQ42.1 PF00320.25	82.90	0.00
TRINITY_Q24HK6_TETTS/40-325	Q24HK6.2 PF00069.23	82.90	0.00
TRINITY_D8TPZ8_VOLCA/37-142	D8TPZ8.1 PF00085.18	82.90	0.00
TRINITY_A8HWM1_CHLRE/550-584	A8HWM1.1 PF00320.25	82.90	0.00
TRINITY_A8JG07_CHLRE/219-371	A8JG07.1 PF00733.19	82.90	0.00
TRINITY_D8U3Y9_VOLCA/216-326	D8U3Y9.1 PF01926.21	82.90	0.00
TRINITY_A8JDY6_CHLRE/296-407	A8JDY6.1 PF00004.27	82.90	0.00
TRINITY_D8TYN2_VOLCA/14-312	D8TYN2.1 PF01593.22	82.90	0.00
TRINITY_D8TTY1_VOLCA/522-604	D8TTY1.1 PF04232.10	82.90	0.00
TRINITY_A8J6A7_CHLRE/70-251	A8J6A7.1 PF01507.17	82.90	0.00
TRINITY_D8TM85_VOLCA/1-134	D8TM85.1 PF00125.22	82.90	0.00
TRINITY_I0YXS6_9CHLO/40-191	I0YXS6.1 PF14538.4;	82.90	0.00
TRINITY_A0A0A0LUC2_CUCSA/32-231	A0A0A0LUC2.1 PF0575	82.80	0.00
TRINITY_D8TUP1_VOLCA/244-467	D8TUP1.1 PF00198.21	82.80	0.00
TRINITY_L8HBF0_ACACA/20-205	L8HBF0.1 PF16661.3;	82.80	0.00
TRINITY_M2Y563_GALSU/5-612	M2Y563.1 PF00012.18	82.80	0.00
TRINITY_L5K105_PTEAL/5-361	L5K105.1 PF00009.25	82.80	0.00
TRINITY_A0A087SET3_AUXPR/66-164	A0A087SET3.1 PF0067	82.80	0.00
TRINITY_D8TY17_VOLCA/76-176	D8TY17.1 PF14772.4;	82.80	0.00
TRINITY_A8J3Q6_CHLRE/61-217	A8J3Q6.1 PF01583.18	82.80	0.00
TRINITY_CCR1_MAIZE/36-291	P0C8M8.1 PF00069.23	82.80	0.00
TRINITY_E1Z1Z9_CHLVA/129-187	E1Z1Z9.1 PF00288.24	82.80	0.00
TRINITY_D8TS23_VOLCA/1180-1488	D8TS23.1 PF13086.4;	82.80	0.00
TRINITY_I7M2W8_TETTS/134-424	I7M2W8.2 PF00069.23	82.80	0.00
TRINITY_A4SAL7_OSTLU/51-446	A4SAL7.1 PF00890.22	82.80	0.00
TRINITY_A8J6B1_CHLRE/63-155	A8J6B1.1 PF16531.3;	82.80	0.00
TRINITY_D8UAY5_VOLCA/11-407	D8UAY5.1 PF01053.18	82.80	0.00
TRINITY_A8JFR3_CHLRE/124-216	A8JFR3.1 PF12894.5;	82.80	0.00
TRINITY_I3MAK6_ICTTR/1-58	I3MAK6.1 PF08039.9;	82.80	0.00
TRINITY_Q23KE9_TETTS/6-80	Q23KE9.1 PF03671.12	82.70	0.00
TRINITY_G0QTM5_ICHMG/183-312	G0QTM5.1 PF00004.27	82.70	0.00
TRINITY_A8JF35_CHLRE/233-544	A8JF35.1 PF00749.19	82.70	0.00
TRINITY_D8U6Z7_VOLCA/40-340	D8U6Z7.1 PF00069.23	82.70	0.00
TRINITY_D8TRT7_VOLCA/572-667	D8TRT7.1 PF12796.5;	82.70	0.00
TRINITY_E1Z8B3_CHLVA/25-324	E1Z8B3.1 PF00557.22	82.70	0.00
TRINITY_E1Z1V8_CHLVA/12-192	E1Z1V8.1 PF04321.15	82.70	0.00
TRINITY_A8I629_CHLRE/36-353	A8I629.1 PF00749.19	82.70	0.00
TRINITY_A8J5U1_CHLRE/87-377	A8J5U1.1 PF00579.23	82.70	0.00
TRINITY_I0YV56_9CHLO/97-234	I0YV56.1 PF03947.16	82.70	0.00
TRINITY_A8HQ21_CHLRE/63-562	A8HQ21.1 PF02990.14	82.70	0.00
TRINITY_D8U5Y2_VOLCA/348-477	D8U5Y2.1 PF07728.12	82.70	0.00
TRINITY_H3AVQ3_LATCH/6-99	H3AVQ3.1 PF01329.17	82.60	0.00
TRINITY_E1Z9Y1_CHLVA/87-258	E1Z9Y1.1 PF00270.27	82.60	0.00
TRINITY_I0Z4A3_9CHLO/67-238	I0Z4A3.1 PF00270.27	82.60	0.00
TRINITY_W1NKM0_AMBTC/30-75	W1NKM0.1 PF00249.29	82.60	0.00
TRINITY_V4B2P9_LOTGI/634-783	V4B2P9.1 PF07717.14	82.60	0.00
TRINITY_A0A061E963_THECC/10-335	A0A061E963.1 PF0153	82.60	0.00
TRINITY_D8TSR8_VOLCA/68-251	D8TSR8.1 PF00504.19	82.60	0.00
TRINITY_A0CZ26_PARTE/38-173	A0CZ26.1 PF07728.12	82.60	0.00
TRINITY_A9RUJ1_PHYPA/231-300	A9RUJ1.1 PF07883.9;	82.60	0.00
TRINITY_A8I982_CHLRE/2-191	A8I982.1 PF00827.15	82.60	0.00
TRINITY_D8TKY7_VOLCA/129-300	D8TKY7.1 PF13522.4;	82.60	0.00
TRINITY_A8HN92_CHLRE/243-462	A8HN92.1 PF00215.22	82.60	0.00
TRINITY_D8THW4_VOLCA/5-250	D8THW4.1 PF00009.25	82.60	0.00
TRINITY_M4EX21_BRARP/977-1108	M4EX21.1 PF00004.27	82.60	0.00

TRINITY_I0Z891_9CHLO/1-317	I0Z891.1 PF07992.12	82.60	0.00
TRINITY_G1NHD0_MELGA/86-213	G1NHD0.2 PF04774.13	82.50	0.00
TRINITY_A0A0B0NLB6_GOSAR/4-84	A0A0B0NLB6.1 PF0088	82.50	0.00
TRINITY_F0XVD2_AURAN/21-77	F0XVD2.1 PF08207.10	82.50	0.00
TRINITY_D8SVX9_SELML/23-119	D8SVX9.1 PF02678.14	82.50	0.00
TRINITY_D8TK99_VOLCA/5-264	D8TK99.1 PF00069.23	82.50	0.00
TRINITY_E1Z8R1_CHLVA/55-111	E1Z8R1.1 PF00886.17	82.50	0.00
TRINITY_G3H8L7_CRIGR/22-183	G3H8L7.1 PF00071.20	82.50	0.00
TRINITY_A0A0B2VM41_TOXCA/6-611	A0A0B2VM41.1 PF0001	82.50	0.00
TRINITY_D8TJ34_VOLCA/10-443	D8TJ34.1 PF04209.11	82.50	0.00
TRINITY_E1ZSY5_CHLVA/84-144	E1ZSY5.1 PF01381.20	82.50	0.00
TRINITY_A8ISP7_CHLRE/3-65	A8ISP7.1 PF01176.17	82.50	0.00
TRINITY_H2TQQ7_TAKRU/1040-1270	H2TQQ7.1 PF07678.12	82.50	0.00
TRINITY_T1IWV5_STRMM/90-186	T1IWV5.1 PF04900.10	82.50	0.00
TRINITY_D8U8D1_VOLCA/1822-1862	D8U8D1.1 PF01465.18	82.50	0.00
TRINITY_L8GSE6_ACACA/5-142	L8GSE6.1 PF00179.24	82.50	0.00
TRINITY_V4N6Q8_EUTSA/185-283	V4N6Q8.1 PF00153.25	82.50	0.00
TRINITY_A8JCY4_CHLRE/40-377	A8JCY4.1 PF00274.17	82.50	0.00
TRINITY_A8IA77_CHLRE/87-390	A8IA77.1 PF04107.11	82.50	0.00
TRINITY_A0A0B2VWK9_TOXCA/3-379	A0A0B2VWK9.1 PF0002	82.50	0.00
TRINITY_A8HXR2_CHLRE/224-293	A8HXR2.1 PF03144.23	82.50	0.00
TRINITY_D8UHI3_VOLCA/791-830	D8UHI3.1 PF00400.30	82.50	0.00
TRINITY_A0A078ARV8_STYLE/1404-1634	A0A078ARV8.1 PF1277	82.50	0.00
TRINITY_G3NTN6_GASAC/1-127	G3NTN6.1 PF14651.4;	82.40	0.00
TRINITY_D8U526_VOLCA/675-725	D8U526.1 PF00249.29	82.40	0.00
TRINITY_B3RK37_TRIAD/426-460	B3RK37.1 PF00066.15	82.40	0.00
TRINITY_Q7CG93_YERPE/8-147	Q7CG93.1 PF05638.10	82.40	0.00
TRINITY_A0A058ZDS7_9EUKA/266-414	A0A058ZDS7.1 PF0044	82.40	0.00
TRINITY_D8TYV7_VOLCA/69-451	D8TYV7.1 PF00162.17	82.40	0.00
TRINITY_D8U419_VOLCA/199-357	D8U419.1 PF02774.16	82.40	0.00
TRINITY_D8THU8_VOLCA/20-213	D8THU8.1 PF09366.8;	82.40	0.00
TRINITY_D8UIX5_VOLCA/108-320	D8UIX5.1 PF02190.14	82.40	0.00
TRINITY_D8UHM3_VOLCA/170-329	D8UHM3.1 PF02731.13	82.40	0.00
TRINITY_D8TJB2_VOLCA/33-542	D8TJB2.1 PF00118.22	82.40	0.00
TRINITY_D8U7E0_VOLCA/85-160	D8U7E0.1 PF07444.9;	82.40	0.00
TRINITY_A9U381_PHYPA/155-222	A9U381.1 PF00462.22	82.40	0.00
TRINITY_C1N2E9_MICPC/1-67	C1N2E9.1 PF02325.15	82.40	0.00
TRINITY_I0YRZ2_9CHLO/5-72	I0YRZ2.1 PF01423.20	82.40	0.00
TRINITY_I0J182_9ORYZ/7-170	I0J182.1 PF00160.19	82.40	0.00
TRINITY_E1Z3X9_CHLVA/250-317	E1Z3X9.1 PF01485.19	82.40	0.00
TRINITY_L8HCL2_ACACA/4-88	L8HCL2.1 PF01780.17	82.40	0.00
TRINITY_A8HSJ3_CHLRE/66-324	A8HSJ3.1 PF00069.23	82.40	0.00
TRINITY_A8I2V5_CHLRE/11-180	A8I2V5.1 PF03031.16	82.40	0.00
TRINITY_J9HRP7_9SPIT/567-697	J9HRP7.1 PF00004.27	82.40	0.00
TRINITY_M1BFN4_SOLTU/162-322	M1BFN4.1 PF00078.25	82.40	0.00
TRINITY_A0A0G4F730_9ALVE/203-338	A0A0G4F730.1 PF0000	82.40	0.00
TRINITY_K4B790_SOLLC/10-78	K4B790.1 PF06522.9;	82.30	0.00
TRINITY_D8U7R9_VOLCA/21-206	D8U7R9.1 PF01728.17	82.30	0.00
TRINITY_L8GQF1_ACACA/612-741	L8GQF1.1 PF13086.4;	82.30	0.00
TRINITY_E1ZKW0_CHLVA/235-347	E1ZKW0.1 PF00271.29	82.30	0.00
TRINITY_D8TKH8_VOLCA/19-197	D8TKH8.1 PF02779.22	82.30	0.00
TRINITY_A8J507_CHLRE/124-185	A8J507.1 PF00226.29	82.30	0.00
TRINITY_A8IZ79_CHLRE/73-247	A8IZ79.1 PF16166.3;	82.30	0.00
TRINITY_D7L8A8_ARALL/361-535	D7L8A8.1 PF00270.27	82.30	0.00
TRINITY_I0Z7A5_9CHLO/134-364	I0Z7A5.1 PF00198.21	82.30	0.00
TRINITY_I7LXE3_TETTS/981-1239	I7LXE3.2 PF00069.23	82.20	0.00
TRINITY_W8F233_9BACT/10-269	W8F233.1 PF00795.20	82.20	0.00
TRINITY_A8IKZ2_CHLRE/2-187	A8IKZ2.1 PF17135.2;	82.20	0.00
TRINITY_D3BR60_POLPA/271-389	D3BR60.1 PF10996.6;	82.20	0.00
TRINITY_I7MIQ7_TETTS/80-231	I7MIQ7.1 PF00005.25	82.20	0.00
TRINITY_A8IZW6_CHLRE/85-202	A8IZW6.1 PF13793.4;	82.20	0.00

TRINITY_I7MA96_TETTS/697-955	I7MA96.1 PF00069.23	82.20	0.00
TRINITY_DYHC2_CHLRE/269-725	Q9SMH5.2 PF08385.10	82.20	0.00
TRINITY_F0ZT81_DICPU/1933-2183	F0ZT81.1 PF00454.25	82.20	0.00
TRINITY_D8TTF3_VOLCA/31-178	D8TTF3.1 PF00156.25	82.20	0.00
TRINITY_D8TSK6_VOLCA/99-334	D8TSK6.1 PF04884.12	82.20	0.00
TRINITY_A8JID6_CHLRE/76-553	A8JID6.1 PF03219.12	82.20	0.00
TRINITY_A8IS14_CHLRE/97-427	A8IS14.1 PF00344.18	82.20	0.00
TRINITY_W7XBY2_TETTS/200-272	W7XBY2.1 PF00317.19	82.20	0.00
TRINITY_D6X1Y7_TRICA/282-433	D6X1Y7.1 PF00160.19	82.20	0.00
TRINITY_A8IC95_CHLRE/519-881	A8IC95.1 PF02896.16	82.20	0.00
TRINITY_D8TUL0_VOLCA/18-146	D8TUL0.1 PF04857.18	82.20	0.00
TRINITY_A8HX70_CHLRE/175-478	A8HX70.1 PF00365.18	82.20	0.00
TRINITY_D8TWX6_VOLCA/34-164	D8TWX6.1 PF01467.24	82.20	0.00
TRINITY_Q9AU05_CHLRE/145-232	Q9AU05.1 PF02790.13	82.20	0.00
TRINITY_A0EGX1_PARTE/32-77	A0EGX1.1 PF00249.29	82.20	0.00
TRINITY_D8U8F5_VOLCA/8-541	D8U8F5.1 PF01602.18	82.20	0.00
TRINITY_A8J015_CHLRE/55-285	A8J015.1 PF00487.22	82.20	0.00
TRINITY_V8NPK8_OPHHA/4-98	V8NPK8.1 PF00255.17	82.10	0.00
TRINITY_I7MMG8_TETTS/8-169	I7MMG8.1 PF00071.20	82.10	0.00
TRINITY_R1FJW2_EMIHU/3-75	R1FJW2.1 PF13499.4;	82.10	0.00
TRINITY_D8U2Q4_VOLCA/55-171	D8U2Q4.1 PF13793.4;	82.10	0.00
TRINITY_I7MMG8_TETTS/8-169	I7MMG8.1 PF00071.20	82.10	0.00
TRINITY_Q22ZH2_TETTS/394-433	Q22ZH2.1 PF00400.30	82.10	0.00
TRINITY_A0A0D2W168_CAPO3/23-192	A0A0D2W168.1 PF0007	82.10	0.00
TRINITY_J9EM83_9SPIT/2083-2229	J9EM83.1 PF07728.12	82.10	0.00
TRINITY_G0QXJ5_ICHMG/145-200	G0QXJ5.1 PF13418.4;	82.10	0.00
TRINITY_A8IQ53_CHLRE/2-175	A8IQ53.1 PF01184.17	82.10	0.00
TRINITY_A8HXD8_CHLRE/76-155	A8HXD8.1 PF13417.4;	82.10	0.00
TRINITY_D8UID5_VOLCA/1-40	D8UID5.1 PF06203.12	82.10	0.00
TRINITY_A8IJP9_CHLRE/151-256	A8IJP9.1 PF13640.4;	82.10	0.00
TRINITY_A0A022QEC8_ERYGU/82-317	A0A022QEC8.1 PF1356	82.10	0.00
TRINITY_I4Y8U9_WALMC/106-438	I4Y8U9.1 PF00069.23	82.10	0.00
TRINITY_Q234I5_TETTS/11-618	Q234I5.1 PF00012.18	82.10	0.00
TRINITY_D8TRZ2_VOLCA/509-630	D8TRZ2.1 PF03109.14	82.10	0.00
TRINITY_L8H0I9_ACACA/149-226	L8H0I9.1 PF01883.17	82.10	0.00
TRINITY_G3AEY0_SPAPN/798-1287	G3AEY0.1 PF00940.17	82.10	0.00
TRINITY_I7LV38_TETTS/14-414	I7LV38.1 PF06068.11	82.10	0.00
TRINITY_A8JDY0_CHLRE/19-363	A8JDY0.1 PF00224.19	82.10	0.00
TRINITY_D8U9Q4_VOLCA/207-245	D8U9Q4.1 PF00400.30	82.10	0.00
TRINITY_A8JHU7_CHLRE/2550-2822	A8JHU7.1 PF12775.5;	82.10	0.00
TRINITY_G3PS60_GASAC/17-104	G3PS60.1 PF00428.17	82.00	0.00
TRINITY_E8RSH0_ASTEC/64-174	E8RSH0.1 PF01058.20	82.00	0.00
TRINITY_W1Q9H4_OGAPD/48-245	W1Q9H4.1 PF00149.26	82.00	0.00
TRINITY_G0R338_ICHMG/5-176	G0R338.1 PF00025.19	82.00	0.00
TRINITY_G0R338_ICHMG/5-176	G0R338.1 PF00025.19	82.00	0.00
TRINITY_G0R338_ICHMG/5-176	G0R338.1 PF00025.19	82.00	0.00
TRINITY_D8TSB4_VOLCA/10-142	D8TSB4.1 PF00380.17	82.00	0.00
TRINITY_D8U1I4_VOLCA/34-292	D8U1I4.1 PF00069.23	82.00	0.00
TRINITY_D8UJB1_VOLCA/3-238	D8UJB1.1 PF00463.19	82.00	0.00
TRINITY_A8IKK2_CHLRE/89-210	A8IKK2.1 PF05542.9;	82.00	0.00
TRINITY_G0QMQ9_ICHMG/30-285	G0QMQ9.1 PF00069.23	82.00	0.00
TRINITY_E1ZQ07_CHLVA/11-271	E1ZQ07.1 PF00899.19	82.00	0.00
TRINITY_D8TQN3_VOLCA/192-374	D8TQN3.1 PF01077.20	82.00	0.00
TRINITY_D8UA51_VOLCA/129-220	D8UA51.1 PF00153.25	82.00	0.00
TRINITY_A8J063_CHLRE/3352-4065	A8J063.1 PF03028.13	82.00	0.00
TRINITY_A0A087SEC8_AUXPR/36-432	A0A087SEC8.1 PF0089	82.00	0.00
TRINITY_H0V7Z8_CAVPO/523-660	H0V7Z8.1 PF00748.17	82.00	0.00
TRINITY_J9IEB5_9SPIT/3-74	J9IEB5.1 PF00240.21	81.90	0.00
TRINITY_Q22B43_TETTS/7-144	Q22B43.2 PF00179.24	81.90	0.00
TRINITY_H3CZ55_TETNG/1-146	H3CZ55.1 PF01429.17	81.90	0.00
TRINITY_A0CRG1_PARTE/30-317	A0CRG1.1 PF00069.23	81.90	0.00

TRINITY_A8HVE6_CHLRE/210-429	A8HVE6.1 PF02138.16	81.90	0.00
TRINITY_A8JBC9_CHLRE/56-551	A8JBC9.1 PF02990.14	81.90	0.00
TRINITY_A8I9B0_CHLRE/60-311	A8I9B0.1 PF00378.18	81.90	0.00
TRINITY_D8TGZ9_VOLCA/33-210	D8TGZ9.1 PF02779.22	81.90	0.00
TRINITY_A8IME8_CHLRE/59-180	A8IME8.1 PF02657.13	81.90	0.00
TRINITY_D8U6R9_VOLCA/23-342	D8U6R9.1 PF13393.4;	81.90	0.00
TRINITY_D8U105_VOLCA/3812-4513	D8U105.1 PF03028.13	81.90	0.00
TRINITY_D8U1V6_VOLCA/36-163	D8U1V6.1 PF01553.19	81.90	0.00
TRINITY_I1I505_BRADI/3-74	I1I505.1 PF00240.21	81.90	0.00
TRINITY_D8TT62_VOLCA/15-98	D8TT62.1 PF02195.16	81.90	0.00
TRINITY_R4XHB2_TAPDE/18-493	R4XHB2.1 PF03155.13	81.80	0.00
TRINITY_M0ZQ44_SOLTU/158-223	M0ZQ44.1 PF01529.18	81.80	0.00
TRINITY_A0A067MUJ0_9HOMO/48-517	A0A067MUJ0.1 PF0129	81.80	0.00
TRINITY_D8R071_SELML/400-560	D8R071.1 PF00270.27	81.80	0.00
TRINITY_Q24HK6_TETTS/40-325	Q24HK6.2 PF00069.23	81.80	0.00
TRINITY_A8JHU6_CHLRE/79-333	A8JHU6.1 PF00348.15	81.80	0.00
TRINITY_A0A0B0PX49_GOSAR/4-58	A0A0B0PX49.1 PF0520	81.80	0.00
TRINITY_D8UCV1_VOLCA/7-83	D8UCV1.1 PF01423.20	81.80	0.00
TRINITY_D8TUY4_VOLCA/175-207	D8TUY4.1 PF00023.28	81.80	0.00
TRINITY_A0A078BA60_STYLE/56-451	A0A078BA60.1 PF0089	81.80	0.00
TRINITY_I0Z282_9CHLO/43-174	I0Z282.1 PF00004.27	81.80	0.00
TRINITY_Q23TW8_TETTS/466-626	Q23TW8.2 PF05063.12	81.80	0.00
TRINITY_G7E302_MIXOS/5-177	G7E302.1 PF00163.17	81.80	0.00
TRINITY_E4WQH8_OIKDI/91-124	E4WQH8.1 PF16211.3;	81.80	0.00
TRINITY_A8J605_CHLRE/116-269	A8J605.1 PF13883.4;	81.80	0.00
TRINITY_I0Z7A6_9CHLO/48-210	I0Z7A6.1 PF00504.19	81.80	0.00
TRINITY_A8JIK2_CHLRE/145-177	A8JIK2.1 PF08209.9;	81.80	0.00
TRINITY_A8J082_CHLRE/38-315	A8J082.1 PF07651.14	81.80	0.00
TRINITY_D8TQJ1_VOLCA/374-494	D8TQJ1.1 PF02852.20	81.80	0.00
TRINITY_A8HN02_CHLRE/89-380	A8HN02.1 PF03151.14	81.80	0.00
TRINITY_W4XSP0_STRPU/530-678	W4XSP0.1 PF00005.25	81.80	0.00
TRINITY_H2ZTY4_LATCH/445-478	H2ZTY4.1 PF01422.15	81.80	0.00
TRINITY_A9RMZ2_PHYPA/19-84	A9RMZ2.1 PF01918.19	81.80	0.00
TRINITY_D7FRY4_ECTSI/164-585	D7FRY4.1 PF01964.16	81.80	0.00
TRINITY_A8IUF0_CHLRE/3102-3438	A8IUF0.1 PF12777.5;	81.80	0.00
TRINITY_D8U7P6_VOLCA/2512-2779	D8U7P6.1 PF12780.5;	81.80	0.00
TRINITY_A8ISE0_CHLRE/683-777	A8ISE0.1 PF00004.27	81.70	0.00
TRINITY_A0BZZ4_PARTE/21-91	A0BZZ4.1 PF00076.20	81.70	0.00
TRINITY_G0R0B5_ICHMG/45-346	G0R0B5.1 PF03133.13	81.70	0.00
TRINITY_E3T5C1_CROVB/3-74	E3T5C1.1 PF00240.21	81.70	0.00
TRINITY_F2E9J3_HORVD/38-108	F2E9J3.1 PF00076.20	81.70	0.00
TRINITY_A8JGX0_CHLRE/17-261	A8JGX0.1 PF01564.15	81.70	0.00
TRINITY_A8IY81_CHLRE/272-383	A8IY81.1 PF00012.18	81.70	0.00
TRINITY_D8U7Y0_VOLCA/201-343	D8U7Y0.1 PF07479.12	81.70	0.00
TRINITY_I0Z604_9CHLO/123-286	I0Z604.1 PF03483.15	81.70	0.00
TRINITY_A8IEE5_CHLRE/59-347	A8IEE5.1 PF00291.23	81.70	0.00
TRINITY_A0A068S968_9FUNG/271-412	A0A068S968.1 PF0000	81.70	0.00
TRINITY_A8HRV5_CHLRE/9-80	A8HRV5.1 PF00076.20	81.70	0.00
TRINITY_D8U459_VOLCA/3934-4647	D8U459.1 PF03028.13	81.70	0
TRINITY_D3BML8_POLPA/780-958	D3BML8.1 PF02779.22	81.60	0.00
TRINITY_A8HMX2_CHLRE/97-689	A8HMX2.1 PF02901.13	81.60	0.00
TRINITY_I7MHK8_TETTS/244-385	I7MHK8.1 PF02773.14	81.60	0.00
TRINITY_D8U553_VOLCA/3-176	D8U553.1 PF00025.19	81.60	0.00
TRINITY_L8GIX6_ACACA/63-236	L8GIX6.1 PF01512.15	81.60	0.00
TRINITY_I7M1K4_TETTS/30-285	I7M1K4.1 PF00069.23	81.60	0.00
TRINITY_D8U4L6_VOLCA/1038-1075	D8U4L6.1 PF06596.9;	81.60	0.00
TRINITY_A8J2T5_CHLRE/13-90	A8J2T5.1 PF03061.20	81.60	0.00
TRINITY_M0S879_MUSAM/54-249	M0S879.1 PF00149.26	81.60	0.00
TRINITY_A0A087SIS7_AUXPR/115-152	A0A087SIS7.1 PF0363	81.60	0.00
TRINITY_M0TRI3_MUSAM/8-121	M0TRI3.1 PF16906.3;	81.60	0.00
TRINITY_W5JLT4_ANODA/1951-2064	W5JLT4.1 PF00271.29	81.60	0.00

TRINITY_A0A096P9N6_OSTTA/415-452	A0A096P9N6.1 PF0062	81.60	0.00
TRINITY_A8HMC4_CHLRE/105-371	A8HMC4.1 PF00487.22	81.60	0.00
TRINITY_A0D3A2_PARTE/89-647	A0D3A2.1 PF01175.16	81.60	0.00
TRINITY_D8U5R4_VOLCA/23-96	D8U5R4.1 PF00076.20	81.60	0.00
TRINITY_G0QQT8_ICHMG/33-151	G0QQT8.1 PF00411.17	81.50	0.00
TRINITY_I0Z847_9CHLO/542-718	I0Z847.1 PF13307.4;	81.50	0.00
TRINITY_A0A078AUP9_STYLE/47-242	A0A078AUP9.1 PF0014	81.50	0.00
TRINITY_J9J3Q5_9SPIT/1074-1171	J9J3Q5.1 PF13646.4;	81.50	0.00
TRINITY_D8TNM0_VOLCA/732-959	D8TNM0.1 PF00889.17	81.50	0.00
TRINITY_D3BG64_POLPA/3687-3964	D3BG64.1 PF00454.25	81.50	0.00
TRINITY_D8RPX7_SELML/10-74	D8RPX7.1 PF01423.20	81.50	0.00
TRINITY_A0A059AI06_EUCGR/56-358	A0A059AI06.1 PF0799	81.50	0.00
TRINITY_D8TL84_VOLCA/15-209	D8TL84.1 PF01813.15	81.50	0.00
TRINITY_D8TGV2_VOLCA/203-294	D8TGV2.1 PF00153.25	81.50	0.00
TRINITY_A8I972_CHLRE/729-901	A8I972.1 PF07724.12	81.50	0.00
TRINITY_D8UFN6_VOLCA/191-315	D8UFN6.1 PF00241.18	81.50	0.00
TRINITY_D8THN9_VOLCA/231-404	D8THN9.1 PF04055.19	81.50	0.00
TRINITY_A8J9S7_CHLRE/76-471	A8J9S7.1 PF00180.18	81.50	0.00
TRINITY_D8UJ87_VOLCA/13-309	D8UJ87.1 PF00009.25	81.50	0.00
TRINITY_D3BL86_POLPA/20-181	D3BL86.1 PF00071.20	81.50	0.00
TRINITY_D8TZ99_VOLCA/294-358	D8TZ99.1 PF13499.4;	81.50	0.00
TRINITY_A8J758_CHLRE/166-360	A8J758.1 PF00448.20	81.50	0.00
TRINITY_A0A0A0LNL8_CUCSA/1-153	A0A0A0LNL8.1 PF0040	81.40	0.00
TRINITY_Q23DZ8_TETTS/171-267	Q23DZ8.2 PF04900.10	81.40	0.00
TRINITY_Q9FXT7_CHLRE/58-346	Q9FXT7.2 PF00069.23	81.40	0.00
TRINITY_I0YV74_9CHLO/205-486	I0YV74.1 PF01409.18	81.40	0.00
TRINITY_A8JBE4_CHLRE/65-161	A8JBE4.1 PF12755.5;	81.40	0.00
TRINITY_G3P5J3_GASAC/87-458	G3P5J3.1 PF00079.18	81.40	0.00
TRINITY_B2U8M9_RALPJ/281-391	B2U8M9.1 PF00271.29	81.40	0.00
TRINITY_I0YQE6_9CHLO/136-317	I0YQE6.1 PF04055.19	81.40	0.00
TRINITY_D8TQK5_VOLCA/45-211	D8TQK5.1 PF00504.19	81.40	0.00
TRINITY_T0S5Y6_9STRA/243-565	T0S5Y6.1 PF00152.18	81.40	0.00
TRINITY_D8UAL1_VOLCA/25-320	D8UAL1.1 PF00850.17	81.40	0.00
TRINITY_D8TYS1_VOLCA/90-261	D8TYS1.1 PF04427.16	81.40	0.00
TRINITY_A8IT25_CHLRE/55-221	A8IT25.1 PF00270.27	81.40	0.00
TRINITY_A8J5T0_CHLRE/12-111	A8J5T0.1 PF00034.19	81.40	0.00
TRINITY_D8TGX0_VOLCA/3111-3458	D8TGX0.1 PF03028.13	81.40	0.00
TRINITY_Q237I3_TETTS/916-1130	Q237I3.3 PF00454.25	81.30	0.00
TRINITY_A0A059BWI6_EUCGR/53-161	A0A059BWI6.1 PF0050	81.30	0.00
TRINITY_I7MFA4_TETTS/258-407	I7MFA4.1 PF00441.22	81.30	0.00
TRINITY_L1IQG0_GUITH/248-387	L1IQG0.1 PF02773.14	81.30	0.00
TRINITY_D8U6W2_VOLCA/98-313	D8U6W2.1 PF01866.15	81.30	0.00
TRINITY_E1ZQW9_CHLVA/398-553	E1ZQW9.1 PF00106.23	81.30	0.00
TRINITY_A8IHF4_CHLRE/27-285	A8IHF4.1 PF00069.23	81.30	0.00
TRINITY_I3K9D2_ORENI/40-182	I3K9D2.1 PF08702.8;	81.30	0.00
TRINITY_A8IBN0_CHLRE/112-364	A8IBN0.1 PF00591.19	81.30	0.00
TRINITY_L8GGD8_ACACA/60-491	L8GGD8.1 PF00330.18	81.30	0.00
TRINITY_Q2VA40_CHLRE/141-865	Q2VA40.1 PF00343.18	81.30	0
TRINITY_I0YW71_9CHLO/295-460	I0YW71.1 PF00441.22	81.30	0.00
TRINITY_D8U6E0_VOLCA/780-945	D8U6E0.1 PF00326.19	81.30	0.00
TRINITY_Q23RV4_TETTS/6-262	Q23RV4.2 PF00069.23	81.30	0.00
TRINITY_A8J7F6_CHLRE/189-415	A8J7F6.1 PF00198.21	81.30	0.00
TRINITY_D3BGP2_POLPA/71-119	D3BGP2.1 PF02751.12	81.20	0.00
TRINITY_V4TUF1_9ROSI/17-82	V4TUF1.1 PF01423.20	81.20	0.00
TRINITY_L8HHU3_ACACA/70-149	L8HHU3.1 PF00312.20	81.20	0.00
TRINITY_A0A078BB17_STYLE/30-281	A0A078BB17.1 PF0006	81.20	0.00
TRINITY_H2KQ45_CLOSI/93-730	H2KQ45.1 PF00133.20	81.20	0.00
TRINITY_L5K105_PTEAL/5-361	L5K105.1 PF00009.25	81.20	0.00
TRINITY_I0YXJ4_9CHLO/6-427	I0YXJ4.1 PF00275.18	81.20	0.00
TRINITY_D8U105_VOLCA/3451-3673	D8U105.1 PF12781.5;	81.20	0.00
TRINITY_D8TR42_VOLCA/24-332	D8TR42.1 PF01180.19	81.20	0.00

TRINITY_A8HMH4_CHLRE/20-281	A8HMH4.1 PF00069.23	81.20	0.00
TRINITY_I0Z4F7_9CHLO/47-479	I0Z4F7.1 PF00083.22	81.20	0.00
TRINITY_E1ZJ29_CHLVA/40-443	E1ZJ29.1 PF01704.16	81.20	0.00
TRINITY_G7I3M2_MEDTR/57-271	G7I3M2.1 PF10609.7;	81.20	0.00
TRINITY_D8TM81_VOLCA/17-635	D8TM81.1 PF01268.17	81.20	0.00
TRINITY_A8J1X8_CHLRE/220-540	A8J1X8.1 PF00152.18	81.20	0.00
TRINITY_A8J7D3_CHLRE/129-245	A8J7D3.1 PF13640.4;	81.20	0.00
TRINITY_D8UBA1_VOLCA/246-322	D8UBA1.1 PF13242.4;	81.20	0.00
TRINITY_A8JET6_CHLRE/2-50	A8JET6.1 PF12678.5;	81.20	0.00
TRINITY_A8HYM8_CHLRE/69-313	A8HYM8.1 PF01564.15	81.20	0.00
TRINITY_D8TRN2_VOLCA/1684-1952	D8TRN2.1 PF12780.5;	81.20	0.00
TRINITY_K3XZA2_SETIT/49-212	K3XZA2.1 PF00160.19	81.10	0.00
TRINITY_E1ZBA8_CHLVA/229-266	E1ZBA8.1 PF03638.13	81.10	0.00
TRINITY_L1JC58_GUITH/10-104	L1JC58.1 PF01248.24	81.10	0.00
TRINITY_A0A078L4N2_9GAMM/92-465	A0A078L4N2.1 PF0154	81.10	0.00
TRINITY_A0D8A1_PARTE/9-283	A0D8A1.1 PF00069.23	81.10	0.00
TRINITY_I0YQV6_9CHLO/231-344	I0YQV6.1 PF00271.29	81.10	0.00
TRINITY_I0YZ44_9CHLO/240-402	I0YZ44.1 PF03572.16	81.10	0.00
TRINITY_D8U9J6_VOLCA/163-268	D8U9J6.1 PF01159.17	81.10	0.00
TRINITY_D8TZ17_VOLCA/10-83	D8TZ17.1 PF01423.20	81.10	0.00
TRINITY_I0YN31_9CHLO/2-190	I0YN31.1 PF02492.17	81.10	0.00
TRINITY_D8UA83_VOLCA/10-199	D8UA83.1 PF04511.13	81.10	0.00
TRINITY_D8U2G8_VOLCA/79-404	D8U2G8.1 PF00155.19	81.10	0.00
TRINITY_D8TP05_VOLCA/1-283	D8TP05.1 PF03151.14	81.10	0.00
TRINITY_J9J3W2_9SPIT/62-136	J9J3W2.1 PF13921.4;	81.10	0.00
TRINITY_D8UDE1_VOLCA/6-201	D8UDE1.1 PF00596.19	81.10	0.00
TRINITY_A0A087SNR2_AUXPR/933-1107	A0A087SNR2.1 PF0771	81.10	0.00
TRINITY_A0A058ZCL3_9EUKA/111-205	A0A058ZCL3.1 PF0835	81.10	0.00
TRINITY_A8HTA8_CHLRE/94-167	A8HTA8.1 PF01502.16	81.10	0.00
TRINITY_E1ZT56_CHLVA/160-286	E1ZT56.1 PF04037.11	81.10	0.00
TRINITY_A8HU66_CHLRE/628-764	A8HU66.1 PF13692.4;	81.10	0.00
TRINITY_D8U6I5_VOLCA/93-564	D8U6I5.1 PF01593.22	81.10	0.00
TRINITY_I7MA96_TETTS/697-955	I7MA96.1 PF00069.23	81.10	0.00
TRINITY_A0D3Z3_PARTE/470-617	A0D3Z3.1 PF00004.27	81.10	0.00
TRINITY_Q24BY8_TETTS/76-334	Q24BY8.2 PF00069.23	81.10	0.00
TRINITY_Q230V5_TETTS/10-142	Q230V5.2 PF00164.23	81.10	0.00
TRINITY_D8U7P6_VOLCA/3595-4325	D8U7P6.1 PF03028.13	81.10	0
TRINITY_D8UA93_VOLCA/87-305	D8UA93.1 PF02167.13	81.10	0.00
TRINITY_I3N4U7_ICTTR/1-231	I3N4U7.1 PF01086.15	81.10	0.00
TRINITY_A0DM47_PARTE/17-70	A0DM47.1 PF00584.18	81.00	0.00
TRINITY_G0R5N9_ICHMG/540-773	G0R5N9.1 PF00211.18	81.00	0.00
TRINITY_A4ZYQ6_SOLLC/4-116	A4ZYQ6.1 PF14226.4;	81.00	0.00
TRINITY_C1E8P1_MICSR/77-432	C1E8P1.1 PF00224.19	81.00	0.00
TRINITY_E1Z8S9_CHLVA/74-231	E1Z8S9.1 PF01257.17	81.00	0.00
TRINITY_D8TSZ4_VOLCA/47-423	D8TSZ4.1 PF07690.14	81.00	0.00
TRINITY_A8J2J7_CHLRE/38-313	A8J2J7.1 PF06098.9;	81.00	0.00
TRINITY_F0ZS91_DICPU/16-283	F0ZS91.1 PF00268.19	81.00	0.00
TRINITY_A8HP55_CHLRE/14-176	A8HP55.1 PF17144.2;	81.00	0.00
TRINITY_A8IGM2_CHLRE/69-532	A8IGM2.1 PF02127.13	81.00	0.00
TRINITY_A7SZE7_NEMVE/170-316	A7SZE7.1 PF00004.27	81.00	0.00
TRINITY_A0A078B4I2_STYLE/807-896	A0A078B4I2.1 PF0440	81.00	0.00
TRINITY_D2VN06_NAEGR/1846-2096	D2VN06.1 PF00454.25	81.00	0.00
TRINITY_A0E3I8_PARTE/46-110	A0E3I8.1 PF02319.18	81.00	0.00
TRINITY_A8I9L6_CHLRE/481-524	A8I9L6.1 PF06203.12	81.00	0.00
TRINITY_C1MLT3_MICPC/8-189	C1MLT3.1 PF01251.16	81.00	0.00
TRINITY_A8J1W1_CHLRE/101-346	A8J1W1.1 PF02153.15	81.00	0.00
TRINITY_E1Z2D1_CHLVA/116-358	E1Z2D1.1 PF10609.7;	81.00	0.00
TRINITY_A8JJ58_CHLRE/3-65	A8JJ58.1 PF01246.18	81.00	0.00
TRINITY_D8TWH4_VOLCA/198-328	D8TWH4.1 PF04116.11	81.00	0.00
TRINITY_A8II27_CHLRE/82-353	A8II27.1 PF00481.19	81.00	0.00
TRINITY_I0YM36_9CHLO/389-499	I0YM36.1 PF00271.29	81.00	0.00

TRINITY_D8UHD0_VOLCA/68-343	D8UHD0.1 PF00009.25	81.00	0.00
TRINITY_A0CSV7_PARTE/8-75	A0CSV7.1 PF01111.17	80.90	0.00
TRINITY_A0A0P7UUX0_9TELE/93-232	A0A0P7UUX0.1 PF0057	80.90	0.00
TRINITY_I7M317_TETTS/63-246	I7M317.1 PF10274.7;	80.90	0.00
TRINITY_D8TZQ9_VOLCA/142-279	D8TZQ9.1 PF13365.4;	80.90	0.00
TRINITY_I0Z0S3_9CHLO/1-196	I0Z0S3.1 PF01201.20	80.90	0.00
TRINITY_I7MHK8_TETTS/244-385	I7MHK8.1 PF02773.14	80.90	0.00
TRINITY_Q230X9_TETTS/859-1272	Q230X9.2 PF08393.11	80.90	0.00
TRINITY_K3WWU1_PYTUL/688-1128	K3WWU1.1 PF05577.10	80.90	0.00
TRINITY_D8U1J1_VOLCA/76-126	D8U1J1.1 PF00847.18	80.90	0.00
TRINITY_I7LZN5_TETTS/2-176	I7LZN5.2 PF01154.15	80.90	0.00
TRINITY_D8UCC9_VOLCA/42-157	D8UCC9.1 PF01118.22	80.90	0.00
TRINITY_A8ILS2_CHLRE/1-120	A8ILS2.1 PF04133.12	80.90	0.00
TRINITY_F0ZAV4_DICPU/115-266	F0ZAV4.1 PF14538.4;	80.90	0.00
TRINITY_M5VSA1_PRUPE/108-155	M5VSA1.1 PF01466.17	80.90	0.00
TRINITY_D8TMI8_VOLCA/5-161	D8TMI8.1 PF00106.23	80.90	0.00
TRINITY_I7M490_TETTS/13-195	I7M490.2 PF01214.16	80.90	0.00
TRINITY_G0R0V1_ICHMG/15-209	G0R0V1.1 PF00687.19	80.80	0.00
TRINITY_A4S2L7_OSTLU/50-230	A4S2L7.1 PF02146.15	80.80	0.00
TRINITY_L8HE96_ACACA/156-285	L8HE96.1 PF00004.27	80.80	0.00
TRINITY_D8TNT2_VOLCA/1-125	D8TNT2.1 PF00705.16	80.80	0.00
TRINITY_I0YIQ0_9CHLO/7-69	I0YIQ0.1 PF01423.20	80.80	0.00
TRINITY_A8JAG1_CHLRE/62-407	A8JAG1.1 PF01494.17	80.80	0.00
TRINITY_A8J7E4_CHLRE/9-227	A8J7E4.1 PF00009.25	80.80	0.00
TRINITY_D8THH6_VOLCA/51-306	D8THH6.1 PF00108.21	80.80	0.00
TRINITY_A8JG07_CHLRE/48-170	A8JG07.1 PF13537.4;	80.80	0.00
TRINITY_J9ITR7_9SPIT/200-226	J9ITR7.1 PF00642.22	80.80	0.00
TRINITY_J9ITR7_9SPIT/200-226	J9ITR7.1 PF00642.22	80.80	0.00
TRINITY_D8TWM5_VOLCA/25-651	D8TWM5.1 PF00133.20	80.80	0.00
TRINITY_L8GW25_ACACA/462-582	L8GW25.1 PF00004.27	80.80	0.00
TRINITY_A8JDQ7_CHLRE/375-660	A8JDQ7.1 PF00069.23	80.80	0.00
TRINITY_A0A068S496_9FUNG/66-248	A0A068S496.1 PF0022	80.70	0.00
TRINITY_H2MPL0_ORYLA/24-329	H2MPL0.1 PF00405.15	80.70	0.00
TRINITY_A0A0D2T6A5_GOSRA/46-177	A0A0D2T6A5.1 PF0012	80.70	0.00
TRINITY_A8II42_CHLRE/31-524	A8II42.1 PF00118.22	80.70	0.00
TRINITY_A8I2Z6_CHLRE/31-214	A8I2Z6.1 PF10274.7;	80.70	0.00
TRINITY_A8IG95_CHLRE/188-279	A8IG95.1 PF00153.25	80.70	0.00
TRINITY_D8TYR4_VOLCA/520-670	D8TYR4.1 PF00160.19	80.70	0.00
TRINITY_A8IG39_CHLRE/29-85	A8IG39.1 PF13920.4;	80.70	0.00
TRINITY_B9GQE7_POPTR/27-285	B9GQE7.1 PF00704.26	80.60	0.00
TRINITY_G7P1S1_MACFA/437-567	G7P1S1.1 PF00004.27	80.60	0.00
TRINITY_A0A087GB61_ARAAL/187-373	A0A087GB61.1 PF1626	80.60	0.00
TRINITY_A4HU11_LEIIN/209-280	A4HU11.2 PF00240.21	80.60	0.00
TRINITY_Q3SE81_PARTE/5-59	Q3SE81.1 PF05207.11	80.60	0.00
TRINITY_A8I3K3_CHLRE/264-300	A8I3K3.1 PF00400.30	80.60	0.00
TRINITY_A8IUW5_CHLRE/92-128	A8IUW5.1 PF06803.10	80.60	0.00
TRINITY_CCR1_MAIZE/36-291	P0C8M8.1 PF00069.23	80.60	0.00
TRINITY_D8TQ02_VOLCA/410-543	D8TQ02.1 PF00271.29	80.60	0.00
TRINITY_A8IFM3_CHLRE/1-128	A8IFM3.1 PF00005.25	80.60	0.00
TRINITY_D8TW46_VOLCA/2-156	D8TW46.1 PF00160.19	80.60	0.00
TRINITY_A8ISU1_CHLRE/27-279	A8ISU1.1 PF00069.23	80.60	0.00
TRINITY_A8I9R1_CHLRE/126-419	A8I9R1.1 PF00291.23	80.60	0.00
TRINITY_A8HR79_CHLRE/44-247	A8HR79.1 PF11264.6;	80.60	0.00
TRINITY_I0YK21_9CHLO/195-355	I0YK21.1 PF00350.21	80.60	0.00
TRINITY_T0Q7Y3_9STRA/23-90	T0Q7Y3.1 PF00076.20	80.60	0.00
TRINITY_D8UCI1_VOLCA/284-350	D8UCI1.1 PF13432.4;	80.60	0.00
TRINITY_G0R532_ICHMG/920-1260	G0R532.1 PF00443.27	80.60	0.00
TRINITY_CLH2_ORYSJ/1288-1433	Q2QYW2.1 PF00637.18	80.60	0.00
TRINITY_A8J760_CHLRE/2-161	A8J760.1 PF00160.19	80.60	0.00
TRINITY_F4PA68_BATDJ/23-64	F4PA68.1 PF00832.18	80.50	0.00
TRINITY_F4PA68_BATDJ/23-64	F4PA68.1 PF00832.18	80.50	0.00

TRINITY_A0A075B209_9FUNG/263-414	A0A075B209.1 PF0027	80.50	0.00
TRINITY_E1Z4X7_CHLVA/42-439	E1Z4X7.1 PF00155.19	80.50	0.00
TRINITY_D8UJ10_VOLCA/159-332	D8UJ10.1 PF00270.27	80.50	0.00
TRINITY_I7M416_TETTS/67-333	I7M416.1 PF00268.19	80.50	0.00
TRINITY_K3XYF0_SETIT/47-242	K3XYF0.1 PF00149.26	80.50	0.00
TRINITY_E1ZME9_CHLVA/252-386	E1ZME9.1 PF02518.24	80.50	0.00
TRINITY_G0QY93_ICHMG/11-206	G0QY93.1 PF00149.26	80.50	0.00
TRINITY_A0C7Z2_PARTE/19-59	A0C7Z2.1 PF00097.23	80.50	0.00
TRINITY_D8U454_VOLCA/174-337	D8U454.1 PF02866.16	80.50	0.00
TRINITY_A8JIN8_CHLRE/110-448	A8JIN8.1 PF01546.26	80.50	0.00
TRINITY_A8IN76_CHLRE/1169-1379	A8IN76.1 PF00454.25	80.50	0.00
TRINITY_D8UGN1_VOLCA/338-770	D8UGN1.1 PF04053.12	80.50	0.00
TRINITY_A8J5Z8_CHLRE/55-516	A8J5Z8.1 PF00171.20	80.50	0.00
TRINITY_D8UB79_VOLCA/85-551	D8UB79.1 PF00939.17	80.50	0.00
TRINITY_I0YYL9_9CHLO/377-418	I0YYL9.1 PF00400.30	80.50	0.00
TRINITY_D8TJG5_VOLCA/52-487	D8TJG5.1 PF01474.14	80.50	0.00
TRINITY_I0Z2A7_9CHLO/114-589	I0Z2A7.1 PF01293.18	80.50	0.00
TRINITY_D8TQJ5_VOLCA/85-663	D8TQJ5.1 PF01411.17	80.50	0.00
TRINITY_D8U961_VOLCA/31-279	D8U961.1 PF00487.22	80.50	0.00
TRINITY_A0C963_PARTE/227-297	A0C963.1 PF00076.20	80.40	0.00
TRINITY_A0CJS0_PARTE/128-311	A0CJS0.1 PF10274.7;	80.40	0.00
TRINITY_A0A0D2WYE3_CAPO3/196-251	A0A0D2WYE3.1 PF0204	80.40	0.00
TRINITY_D8U0B1_VOLCA/355-464	D8U0B1.1 PF00271.29	80.40	0.00
TRINITY_A0BLS3_PARTE/98-348	A0BLS3.1 PF00012.18	80.40	0.00
TRINITY_A8J9S9_CHLRE/9-334	A8J9S9.1 PF16363.3;	80.40	0.00
TRINITY_D8TJT9_VOLCA/206-373	D8TJT9.1 PF02882.17	80.40	0.00
TRINITY_D8TVA7_VOLCA/191-460	D8TVA7.1 PF04095.14	80.40	0.00
TRINITY_CCR1_MAIZE/36-291	P0C8M8.1 PF00069.23	80.40	0.00
TRINITY_A8HSI6_CHLRE/10-133	A8HSI6.1 PF04628.11	80.40	0.00
TRINITY_D8U105_VOLCA/201-765	D8U105.1 PF08385.10	80.40	0.00
TRINITY_D0MVU0_PHYIT/128-254	D0MVU0.1 PF13401.4;	80.40	0.00
TRINITY_D8TXL7_VOLCA/1-353	D8TXL7.1 PF00999.19	80.40	0.00
TRINITY_A8HXG5_CHLRE/9-120	A8HXG5.1 PF04725.10	80.40	0.00
TRINITY_A8ISN7_CHLRE/59-260	A8ISN7.1 PF06862.10	80.40	0.00
TRINITY_A4RWN5_OSTLU/13-58	A4RWN5.1 PF00249.29	80.40	0.00
TRINITY_A0D2P6_PARTE/10-80	A0D2P6.1 PF11976.6;	80.30	0.00
TRINITY_A0A0D2SQL4_GOSRA/11-138	A0A0D2SQL4.1 PF0165	80.30	0.00
TRINITY_D8TVN2_VOLCA/11-127	D8TVN2.1 PF00583.23	80.30	0.00
TRINITY_A8I0E2_CHLRE/13-78	A8I0E2.1 PF01423.20	80.30	0.00
TRINITY_D8TJU9_VOLCA/121-523	D8TJU9.1 PF00202.19	80.30	0.00
TRINITY_D8TV85_VOLCA/114-189	D8TV85.1 PF00347.21	80.30	0.00
TRINITY_A8HS40_CHLRE/377-576	A8HS40.1 PF01434.16	80.30	0.00
TRINITY_A8JD57_CHLRE/4-264	A8JD57.1 PF00069.23	80.30	0.00
TRINITY_D8TIY1_VOLCA/66-126	D8TIY1.1 PF00226.29	80.30	0.00
TRINITY_A0C160_PARTE/124-184	A0C160.1 PF13921.4;	80.30	0.00
TRINITY_A8IC93_CHLRE/350-587	A8IC93.1 PF00122.18	80.30	0.00
TRINITY_D8UJ71_VOLCA/128-336	D8UJ71.1 PF00696.26	80.30	0.00
TRINITY_D8UJI7_VOLCA/128-574	D8UJI7.1 PF00067.20	80.30	0.00
TRINITY_A0A068S2Z4_9FUNG/67-138	A0A068S2Z4.1 PF0036	80.30	0.00
TRINITY_Q5Z816_ORYSJ/21-147	Q5Z816.1 PF01398.19	80.30	0.00
TRINITY_D8U0U0_VOLCA/56-133	D8U0U0.1 PF02470.18	80.30	0.00
TRINITY_A8IJK9_CHLRE/110-170	A8IJK9.1 PF00839.15	80.30	0.00
TRINITY_I0YLH8_9CHLO/371-639	I0YLH8.1 PF00128.22	80.30	0.00
TRINITY_J7KJQ3_CUCSA/91-250	J7KJQ3.1 PF01699.22	80.20	0.00
TRINITY_G3HPC9_CRIGR/75-286	G3HPC9.1 PF01442.16	80.20	0.00
TRINITY_D8U9J6_VOLCA/163-268	D8U9J6.1 PF01159.17	80.20	0.00
TRINITY_I7MMG7_TETTS/4-265	I7MMG7.1 PF00069.23	80.20	0.00
TRINITY_Q3SDL5_PARTE/12-173	Q3SDL5.1 PF00071.20	80.20	0.00
TRINITY_D8TY97_VOLCA/1017-1137	D8TY97.1 PF00271.29	80.20	0.00
TRINITY_D8UGK6_VOLCA/18-362	D8UGK6.1 PF00224.19	80.20	0.00
TRINITY_D8TJY2_VOLCA/214-457	D8TJY2.1 PF08323.9;	80.20	0.00

TRINITY_I0YTF8_9CHLO/16-117	I0YTF8.1 PF06047.9;	80.20	0.00
TRINITY_A8JJ39_CHLRE/244-948	A8JJ39.1 PF03028.13	80.20	0.00
TRINITY_D8UBX0_VOLCA/59-219	D8UBX0.1 PF10436.7;	80.20	0.00
TRINITY_I0YWV0_9CHLO/22-317	I0YWV0.1 PF00206.18	80.20	0.00
TRINITY_D8U2A7_VOLCA/24-316	D8U2A7.1 PF00850.17	80.20	0.00
TRINITY_D8TJ97_VOLCA/1128-1233	D8TJ97.1 PF03912.12	80.20	0.00
TRINITY_A8IWA1_CHLRE/6-256	A8IWA1.1 PF00069.23	80.20	0.00
TRINITY_D8TM85_VOLCA/1-134	D8TM85.1 PF00125.22	80.20	0.00
TRINITY_A8JFI9_CHLRE/69-288	A8JFI9.1 PF01940.14	80.20	0.00
TRINITY_Q22Z01_TETTS/63-210	Q22Z01.2 PF00005.25	80.10	0.00
TRINITY_Q8HC78_ORYLA/88-253	Q8HC78.1 PF13631.4;	80.10	0.00
TRINITY_L8GSP6_ACACA/24-185	L8GSP6.1 PF00071.20	80.10	0.00
TRINITY_L8GI40_ACACA/53-338	L8GI40.1 PF00069.23	80.10	0.00
TRINITY_I0YZL1_9CHLO/41-196	I0YZL1.1 PF00177.19	80.10	0.00
TRINITY_A8J5V8_CHLRE/42-223	A8J5V8.1 PF00574.21	80.10	0.00
TRINITY_G7E302_MIXOS/5-177	G7E302.1 PF00163.17	80.10	0.00
TRINITY_G7E302_MIXOS/5-177	G7E302.1 PF00163.17	80.10	0.00
TRINITY_E2BY81_HARSA/5-166	E2BY81.1 PF00071.20	80.10	0.00
TRINITY_A8J233_CHLRE/533-721	A8J233.1 PF08323.9;	80.10	0.00
TRINITY_B0XCU4_CULQU/79-178	B0XCU4.1 PF13012.4;	80.00	0.00
TRINITY_W7XI20_TETTS/141-304	W7XI20.1 PF05118.13	80.00	0.00
TRINITY_A0A078AY45_STYLE/121-266	A0A078AY45.1 PF0078	80.00	0.00
TRINITY_G5C260_HETGA/95-194	G5C260.1 PF01092.17	80.00	0.00
TRINITY_J9JCA5_9SPIT/125-339	J9JCA5.1 PF00112.21	80.00	0.00
TRINITY_V4T5X4_9ROSI/18-400	V4T5X4.1 PF00199.17	80.00	0.00
TRINITY_D8TQ63_VOLCA/33-344	D8TQ63.1 PF01406.17	80.00	0.00
TRINITY_B6JZU2_SCHJY/70-149	B6JZU2.1 PF00312.20	80.00	0.00
TRINITY_Q8LLV9_CHLRE/7-125	Q8LLV9.1 PF14931.4;	80.00	0.00
TRINITY_Q22FZ7_TETTS/267-312	Q22FZ7.2 PF00249.29	80.00	0.00
TRINITY_D8TY17_VOLCA/592-651	D8TY17.1 PF14775.4;	80.00	0.00
TRINITY_A8JDZ5_CHLRE/12-188	A8JDZ5.1 PF02492.17	80.00	0.00
TRINITY_A8JHA6_CHLRE/288-368	A8JHA6.1 PF05002.13	80.00	0.00
TRINITY_A8HNE3_CHLRE/3-84	A8HNE3.1 PF13418.4;	80.00	0.00
TRINITY_D8TJ43_VOLCA/761-1018	D8TJ43.1 PF00860.18	80.00	0.00
TRINITY_A8ISR9_CHLRE/1482-1562	A8ISR9.1 PF00355.24	80.00	0.00
TRINITY_RTCB_MICSR/76-513	C1E9Y5.1 PF01139.15	80.00	0.00
TRINITY_Q22W82_TETTS/189-703	Q22W82.1 PF00183.16	80.00	0.00
TRINITY_A8HZ94_CHLRE/348-474	A8HZ94.1 PF01529.18	80.00	0.00
TRINITY_A8I923_CHLRE/9-53	A8I923.1 PF04851.13	80.00	0.00
TRINITY_D8U3U0_VOLCA/167-326	D8U3U0.1 PF02800.18	80.00	0.00
TRINITY_A9STB1_PHYPA/391-767	A9STB1.1 PF00632.23	80.00	0.00
TRINITY_A8J6L8_CHLRE/50-117	A8J6L8.1 PF12265.6;	80.00	0.00
TRINITY_M5G5C9_DACSP/14-101	M5G5C9.1 PF01172.16	80.00	0.00
TRINITY_A8I2Y6_CHLRE/11-265	A8I2Y6.1 PF00069.23	80.00	0.00
TRINITY_A8J2I0_CHLRE/322-387	A8J2I0.1 PF00076.20	80.00	0.00
TRINITY_E1ZM02_CHLVA/424-457	E1ZM02.1 PF00320.25	80.00	0.00
TRINITY_D8TTR8_VOLCA/511-575	D8TTR8.1 PF00333.18	80.00	0.00
TRINITY_L1K0C3_GUITH/211-380	L1K0C3.1 PF00270.27	80.00	0.00
TRINITY_A0A0D2WMI0_CAPO3/1822-2191	A0A0D2WMI0.1 PF0044	80.00	0.00
TRINITY_A8HP34_CHLRE/80-131	A8HP34.1 PF00847.18	80.00	0.00
TRINITY_A8JFJ4_CHLRE/5-302	A8JFJ4.1 PF00248.19	80.00	0.00
TRINITY_C1MW35_MICPC/52-81	C1MW35.1 PF00397.24	80.00	0.00
TRINITY_W4FG10_9STRA/195-296	W4FG10.1 PF00106.23	80.00	0.00
TRINITY_D8UDB9_VOLCA/93-250	D8UDB9.1 PF14580.4;	80.00	0.00
TRINITY_W5E4P6_WHEAT/293-485	W5E4P6.1 PF13087.4;	80.00	0.00
TRINITY_A8HX70_CHLRE/175-478	A8HX70.1 PF00365.18	80.00	0.00
TRINITY_R1C1A7_EMIHU/23-281	R1C1A7.1 PF04078.11	80.00	0.00
TRINITY_A8HNC0_CHLRE/92-335	A8HNC0.1 PF02784.14	80.00	0.00
TRINITY_A8HYE1_CHLRE/47-92	A8HYE1.1 PF00319.16	80.00	0.00
TRINITY_D8TKN4_VOLCA/2144-2253	D8TKN4.1 PF01477.21	80.00	0.00
TRINITY_D8TNB7_VOLCA/1-175	D8TNB7.1 PF08235.11	80.00	0.00

TRINITY_D8U5N1_VOLCA/45-382	D8U5N1.1 PF00155.19	80.00	0.00
TRINITY_D8U796_VOLCA/81-170	D8U796.1 PF00153.25	80.00	0.00
TRINITY_Q23YD4_TETTS/543-688	Q23YD4.2 PF00005.25	80.00	0.00
TRINITY_A0A0E9NEH2_9ASCO/48-379	A0A0E9NEH2.1 PF0020	79.90	0.00
TRINITY_A8J5P7_CHLRE/222-410	A8J5P7.1 PF05193.19	79.90	0.00
TRINITY_D8TZ96_VOLCA/167-360	D8TZ96.1 PF00528.20	79.90	0.00
TRINITY_A8JIJ6_CHLRE/8-182	A8JIJ6.1 PF01595.18	79.90	0.00
TRINITY_D8U4Q9_VOLCA/42-434	D8U4Q9.1 PF09334.9;	79.90	0.00
TRINITY_A8JEU8_CHLRE/26-190	A8JEU8.1 PF00005.25	79.90	0.00
TRINITY_A8JDK4_CHLRE/36-199	A8JDK4.1 PF00091.23	79.90	0.00
TRINITY_D8TLJ7_VOLCA/75-601	D8TLJ7.1 PF10433.7;	79.90	0.00
TRINITY_D8U5M8_VOLCA/537-670	D8U5M8.1 PF00454.25	79.90	0.00
TRINITY_A8HMX2_CHLRE/97-689	A8HMX2.1 PF02901.13	79.90	0.00
TRINITY_D8TIC8_VOLCA/15-143	D8TIC8.1 PF00416.20	79.80	0.00
TRINITY_D8TTE0_VOLCA/103-388	D8TTE0.1 PF00069.23	79.80	0.00
TRINITY_E1ZSL7_CHLVA/153-376	E1ZSL7.1 PF07298.9;	79.80	0.00
TRINITY_C4Y0F4_CLAL4/385-494	C4Y0F4.1 PF02852.20	79.80	0.00
TRINITY_D8U4Q1_VOLCA/54-389	D8U4Q1.1 PF00456.19	79.80	0.00
TRINITY_E4WYV9_OIKDI/12-104	E4WYV9.1 PF00254.26	79.80	0.00
TRINITY_D8UF47_VOLCA/128-336	D8UF47.1 PF02786.15	79.80	0.00
TRINITY_D8U5K1_VOLCA/20-262	D8U5K1.1 PF00262.16	79.80	0.00
TRINITY_RBS0_SOLTU/70-178	P10647.1 PF00101.18	79.80	0.00
TRINITY_D8TJS9_VOLCA/99-464	D8TJS9.1 PF00155.19	79.80	0.00
TRINITY_T1L205_TETUR/246-439	T1L205.1 PF13087.4;	79.80	0.00

TRINITY_D8UEF2_VOLCA/54-237	D8UEF2.1 PF00504.19	79.80	0.00
TRINITY_A0D0A0_PARTE/1-59	A0D0A0.1 PF01194.15	79.70	0.00
TRINITY_J9HR66_9SPIT/678-1055	J9HR66.1 PF00562.26	79.70	0.00
TRINITY_U9U1U0_RHIID/17-315	U9U1U0.1 PF00248.19	79.70	0.00
TRINITY_D8UD42_VOLCA/5-141	D8UD42.1 PF00578.19	79.70	0.00
TRINITY_A0A0M0JU91_9EUKA/180-253	A0A0M0JU91.1 PF0038	79.70	0.00
TRINITY_D8TXC5_VOLCA/44-118	D8TXC5.1 PF00111.25	79.70	0.00
TRINITY_Q4VKB4_CHLRE/119-229	Q4VKB4.1 PF00487.22	79.70	0.00
TRINITY_D8THF5_VOLCA/15-73	D8THF5.1 PF03946.12	79.70	0.00
TRINITY_G0QVV1_ICHMG/11-394	G0QVV1.1 PF00199.17	79.70	0.00
TRINITY_A0A015JQR0_9GLOM/33-111	A0A015JQR0.1 PF0142	79.70	0.00
TRINITY_A8JGJ4_CHLRE/324-589	A8JGJ4.1 PF00069.23	79.70	0.00
TRINITY_R1BXE3_EMIHU/7-213	R1BXE3.1 PF01680.15	79.70	0.00
TRINITY_A8IHL3_CHLRE/124-246	A8IHL3.1 PF00329.17	79.70	0.00
TRINITY_D8TPH7_VOLCA/96-190	D8TPH7.1 PF00887.17	79.70	0.00
TRINITY_D8U949_VOLCA/297-418	D8U949.1 PF01926.21	79.70	0.00
TRINITY_D8UBI5_VOLCA/7-202	D8UBI5.1 PF01174.17	79.70	0.00
TRINITY_I4MUM8_9BURK/78-146	I4MUM8.1 PF02560.12	79.70	0.00
TRINITY_A0EGB9_PARTE/32-287	A0EGB9.1 PF00069.23	79.70	0.00
TRINITY_D8TJA7_VOLCA/116-184	D8TJA7.1 PF00076.20	79.70	0.00
TRINITY_D8U638_VOLCA/106-284	D8U638.1 PF01163.20	79.70	0.00
TRINITY_Q22T05_TETTS/33-102	Q22T05.2 PF13499.4;	79.70	0.00
TRINITY_Q22GC0_TETTS/259-427	Q22GC0.3 PF05063.12	79.60	0.00
TRINITY_A8ILP2_CHLRE/73-166	A8ILP2.1 PF10674.7;	79.60	0.00
TRINITY_G0QRK4_ICHMG/34-87	G0QRK4.1 PF00013.27	79.60	0.00
TRINITY_A8J8U1_CHLRE/4-117	A8J8U1.1 PF13519.4;	79.60	0.00
TRINITY_I3IVP4_ORENI/91-461	I3IVP4.1 PF00079.18	79.60	0.00
TRINITY_Q7X7A7_CHLRE/13-236	Q7X7A7.1 PF00244.18	79.60	0.00
TRINITY_D8TWE9_VOLCA/37-86	D8TWE9.1 PF00847.18	79.60	0.00
TRINITY_A0A078AIG8_STYLE/34-284	A0A078AIG8.1 PF0006	79.60	0.00
TRINITY_B9R7I0_RICCO/93-145	B9R7I0.1 PF13921.4;	79.60	0.00
TRINITY_D3B847_POLPA/531-837	D3B847.1 PF01180.19	79.60	0.00
TRINITY_D8THH0_VOLCA/35-423	D8THH0.1 PF04137.13	79.60	0.00
TRINITY_A8I0V1_CHLRE/57-346	A8I0V1.1 PF00696.26	79.60	0.00
TRINITY_Q3SDL5_PARTE/12-173	Q3SDL5.1 PF00071.20	79.60	0.00
TRINITY_T1IN01_STRMM/160-213	T1IN01.1 PF00013.27	79.60	0.00
TRINITY_D8TI21_VOLCA/104-201	D8TI21.1 PF00153.25	79.60	0.00
TRINITY_A8JFG3_CHLRE/16-353	A8JFG3.1 PF00225.21	79.60	0.00
TRINITY_A8JBB8_CHLRE/327-420	A8JBB8.1 PF01926.21	79.60	0.00
TRINITY_D8TP57_VOLCA/83-523	D8TP57.1 PF01593.22	79.60	0.00
TRINITY_A0D389_PARTE/1494-1652	A0D389.1 PF00179.24	79.50	0.00
TRINITY_G0QMD1_ICHMG/4-286	G0QMD1.1 PF00069.23	79.50	0.00
TRINITY_M7XBH8_RHOT1/80-182	M7XBH8.1 PF08576.8;	79.50	0.00
TRINITY_L9L401_TUPCH/11-101	L9L401.1 PF16122.3;	79.50	0.00
TRINITY_Q24F10_TETTS/11-394	Q24F10.2 PF00199.17	79.50	0.00
TRINITY_L8HHV0_ACACA/268-340	L8HHV0.1 PF01485.19	79.50	0.00
TRINITY_E9HFT7_DAPPU/108-146	E9HFT7.1 PF00400.30	79.50	0.00
TRINITY_K1QG22_CRAGI/7-612	K1QG22.1 PF00012.18	79.50	0.00
TRINITY_D8TUC0_VOLCA/231-593	D8TUC0.1 PF09359.8;	79.50	0.00
TRINITY_I0HYJ0_CALAS/264-416	I0HYJ0.1 PF13336.4;	79.50	0.00
TRINITY_F0ZA78_DICPU/90-206	F0ZA78.1 PF00692.17	79.50	0.00
TRINITY_A0BMZ0_PARTE/208-296	A0BMZ0.1 PF00153.25	79.50	0.00
TRINITY_A0A0G4IJU7_PLABS/245-283	A0A0G4IJU7.1 PF0040	79.50	0.00
TRINITY_A8JDK0_CHLRE/6-78	A8JDK0.1 PF15906.3;	79.50	0.00
TRINITY_Q54WP9_DICDI/197-235	Q54WP9.1 PF00400.30	79.50	0.00
TRINITY_D8TSR3_VOLCA/221-391	D8TSR3.1 PF10520.7;	79.50	0.00
TRINITY_Q22XT4_TETTS/475-635	Q22XT4.1 PF08662.9;	79.50	0.00
TRINITY_A8J7C8_CHLRE/230-580	A8J7C8.1 PF00152.18	79.50	0.00
TRINITY_A8HNQ7_CHLRE/83-374	A8HNQ7.1 PF07992.12	79.50	0.00
TRINITY_D8UGM2_VOLCA/23-242	D8UGM2.1 PF14234.4;	79.50	0.00
TRINITY_A8J876_CHLRE/179-295	A8J876.1 PF00270.27	79.50	0.00

TRINITY_I7M9M6_TETTS/1-66	I7M9M6.1 PF01246.18	79.40	0.00
TRINITY_L8GRV5_ACACA/10-232	L8GRV5.1 PF00244.18	79.40	0.00
TRINITY_Q6UKY4_CHLRE/54-121	Q6UKY4.1 PF00550.23	79.40	0.00
TRINITY_E1Z857_CHLVA/95-169	E1Z857.1 PF00900.18	79.40	0.00
TRINITY_A0A0B2VWK9_TOXCA/3-379	A0A0B2VWK9.1 PF0002	79.40	0.00
TRINITY_A0CSV7_PARTE/8-75	A0CSV7.1 PF01111.17	79.40	0.00
TRINITY_D8TVL1_VOLCA/2020-2517	D8TVL1.1 PF00443.27	79.40	0.00
TRINITY_A8I3W3_CHLRE/45-321	A8I3W3.1 PF00701.20	79.40	0.00
TRINITY_D8TJU4_VOLCA/48-144	D8TJU4.1 PF00127.18	79.40	0.00
TRINITY_L1JBT9_GUITH/1-408	L1JBT9.1 PF00474.15	79.40	0.00
TRINITY_I7M280_TETTS/444-558	I7M280.2 PF00271.29	79.40	0.00
TRINITY_Q23DR0_TETTS/3463-3683	Q23DR0.2 PF12781.5;	79.40	0.00
TRINITY_D8U2R0_VOLCA/13-111	D8U2R0.1 PF03226.12	79.40	0.00
TRINITY_A0A075AMU6_9FUNG/696-926	A0A075AMU6.1 PF0000	79.40	0.00
TRINITY_A8ISK5_CHLRE/26-155	A8ISK5.1 PF00692.17	79.30	0.00
TRINITY_H9G7K1_ANOCA/1-175	H9G7K1.1 PF03657.11	79.30	0.00
TRINITY_A8IKV5_CHLRE/91-356	A8IKV5.1 PF00348.15	79.30	0.00
TRINITY_F4PL27_DICFS/55-204	F4PL27.1 PF00005.25	79.30	0.00
TRINITY_A8JBA2_CHLRE/102-475	A8JBA2.1 PF07690.14	79.30	0.00
TRINITY_A8JF47_CHLRE/300-389	A8JF47.1 PF00153.25	79.30	0.00
TRINITY_A8J073_CHLRE/5-146	A8J073.1 PF02792.12	79.30	0.00
TRINITY_A8I1R6_CHLRE/74-190	A8I1R6.1 PF00903.23	79.30	0.00
TRINITY_A8J8V5_CHLRE/3-116	A8J8V5.1 PF01398.19	79.30	0.00
TRINITY_J0XGN0_LOALO/211-239	J0XGN0.1 PF01436.19	79.30	0.00
TRINITY_A0A0G4FW13_9ALVE/156-200	A0A0G4FW13.1 PF1363	79.30	0.00
TRINITY_A8J0P9_CHLRE/8-152	A8J0P9.1 PF00179.24	79.30	0.00
TRINITY_A8IV22_CHLRE/2986-3540	A8IV22.1 PF03028.13	79.30	0.00
TRINITY_A8JD57_CHLRE/4-264	A8JD57.1 PF00069.23	79.30	0.00
TRINITY_A0A0K9PRK9_ZOSMR/90-295	A0A0K9PRK9.1 PF1308	79.30	0.00
TRINITY_A0EEF5_PARTE/29-120	A0EEF5.1 PF00447.15	79.30	0.00
TRINITY_H3EDZ8_PRIPA/7-170	H3EDZ8.1 PF00160.19	79.30	0.00
TRINITY_I1MZC1_SOYBN/304-468	I1MZC1.2 PF08662.9;	79.30	0.00
TRINITY_D8TYB6_VOLCA/31-225	D8TYB6.1 PF00926.17	79.30	0.00
TRINITY_C0HHR1_MAIZE/337-528	C0HHR1.1 PF00270.27	79.30	0.00
TRINITY_I7MLX5_TETTS/12-131	I7MLX5.1 PF00583.23	79.30	0.00
TRINITY_I0YWB4_9CHLO/16-484	I0YWB4.1 PF01602.18	79.30	0.00
TRINITY_A8HWS8_CHLRE/35-94	A8HWS8.1 PF00830.17	79.30	0.00
TRINITY_M2XL41_GALSU/5-130	M2XL41.1 PF00410.17	79.20	0.00
TRINITY_D8UFG8_VOLCA/50-168	D8UFG8.1 PF01042.19	79.20	0.00
TRINITY_A8J2G4_CHLRE/15-122	A8J2G4.1 PF01655.16	79.20	0.00
TRINITY_L8GYZ5_ACACA/65-112	L8GYZ5.1 PF00319.16	79.20	0.00
TRINITY_A0E3B2_PARTE/312-359	A0E3B2.1 PF00400.30	79.20	0.00
TRINITY_H2KQ92_CLOSI/161-232	H2KQ92.1 PF03719.13	79.20	0.00
TRINITY_A8IJJ8_CHLRE/185-357	A8IJJ8.1 PF02222.20	79.20	0.00
TRINITY_A8J032_CHLRE/151-204	A8J032.1 PF13415.4;	79.20	0.00
TRINITY_D8TW13_VOLCA/585-635	D8TW13.1 PF00847.18	79.20	0.00
TRINITY_D8U5K1_VOLCA/20-262	D8U5K1.1 PF00262.16	79.20	0.00
TRINITY_D8TJI5_VOLCA/29-431	D8TJI5.1 PF00464.17	79.20	0.00
TRINITY_L1INW0_GUITH/3-74	L1INW0.1 PF00240.21	79.20	0.00
TRINITY_A0A087X9F3_POEFO/403-631	A0A087X9F3.2 PF0008	79.10	0.00
TRINITY_A0DSX8_PARTE/29-200	A0DSX8.1 PF03637.15	79.10	0.00
TRINITY_C6SYH7_SOYBN/19-85	C6SYH7.1 PF01423.20	79.10	0.00
TRINITY_Q235C4_TETTS/81-281	Q235C4.2 PF00149.26	79.10	0.00
TRINITY_G0QZY1_ICHMG/82-282	G0QZY1.1 PF00149.26	79.10	0.00
TRINITY_L8H109_ACACA/6-140	L8H109.1 PF00164.23	79.10	0.00
TRINITY_R1F613_EMIHU/4-105	R1F613.1 PF00009.25	79.10	0.00
TRINITY_D8UK47_VOLCA/1-286	D8UK47.1 PF03151.14	79.10	0.00
TRINITY_A0A087SU54_AUXPR/193-346	A0A087SU54.1 PF0218	79.10	0.00
TRINITY_Q4SCH8_TETNG/32-122	Q4SCH8.1 PF13233.4;	79.10	0.00
TRINITY_A0A078AVM5_STYLE/320-363	A0A078AVM5.1 PF0056	79.10	0.00
TRINITY_Q27GW2_TAEGU/6-261	Q27GW2.1 PF00510.16	79.10	0.00

TRINITY_Q23DN8_TETTS/448-708	Q23DN8.2 PF00069.23	79.10	0.00
TRINITY_A8HTG4_CHLRE/160-250	A8HTG4.1 PF00254.26	79.10	0.00
TRINITY_D8TMW6_VOLCA/26-233	D8TMW6.1 PF00566.16	79.10	0.00
TRINITY_D8TTJ7_VOLCA/82-358	D8TTJ7.1 PF06485.9;	79.10	0.00
TRINITY_D8UDC0_VOLCA/413-856	D8UDC0.1 PF00136.19	79.10	0.00
TRINITY_A8IVV9_CHLRE/238-371	A8IVV9.1 PF00258.23	79.10	0.00
TRINITY_D8U144_VOLCA/1279-1546	D8U144.1 PF12780.5;	79.10	0.00
TRINITY_E1Z257_CHLVA/49-201	E1Z257.1 PF00293.26	79.10	0.00
TRINITY_S8DQK5_9LAMI/94-308	S8DQK5.1 PF00112.21	79.00	0.00
TRINITY_A0A067M4U7_9HOMO/12-132	A0A067M4U7.1 PF0164	79.00	0.00
TRINITY_A0A075AZ01_9FUNG/2-191	A0A075AZ01.1 PF0082	79.00	0.00
TRINITY_D8U965_VOLCA/235-359	D8U965.1 PF00293.26	79.00	0.00
TRINITY_L8H9J2_ACACA/952-1127	L8H9J2.1 PF07717.14	79.00	0.00
TRINITY_H2MRT2_ORYLA/669-855	H2MRT2.1 PF06668.10	78.90	0.00
TRINITY_A0A077ZNX5_STYLE/437-843	A0A077ZNX5.1 PF0808	78.90	0.00
TRINITY_A0DJM8_PARTE/67-104	A0DJM8.1 PF09360.8;	78.90	0.00
TRINITY_V4BQF1_LOTGI/175-215	V4BQF1.1 PF00400.30	78.90	0.00
TRINITY_D8TTE7_VOLCA/32-219	D8TTE7.1 PF00227.24	78.90	0.00
TRINITY_I7MHK8_TETTS/244-385	I7MHK8.1 PF02773.14	78.90	0.00
TRINITY_T1G269_HELRO/148-280	T1G269.1 PF03464.13	78.90	0.00
TRINITY_C5LBM1_PERM5/36-106	C5LBM1.1 PF00076.20	78.90	0.00
TRINITY_ABCH2_DICDI/58-209	Q8T664.1 PF00005.25	78.90	0.00
TRINITY_E3T5C1_CROVB/3-74	E3T5C1.1 PF00240.21	78.90	0.00
TRINITY_A0A059LMI3_9CHLO/7-112	A0A059LMI3.1 PF0038	78.90	0.00
TRINITY_A8IRK4_CHLRE/68-247	A8IRK4.1 PF00316.18	78.90	0.00
TRINITY_D3LUB1_9FIRM/225-389	D3LUB1.1 PF00278.20	78.90	0.00
TRINITY_A0A077ZY01_STYLE/5-283	A0A077ZY01.1 PF0006	78.90	0.00
TRINITY_A0C6S9_PARTE/9-150	A0C6S9.1 PF02792.12	78.90	0.00
TRINITY_Q247W8_TETTS/1117-1244	Q247W8.2 PF00069.23	78.90	0.00
TRINITY_B3MU37_DROAN/4-136	B3MU37.1 PF02966.14	78.90	0.00
TRINITY_I7M0D5_TETTS/4-286	I7M0D5.2 PF00069.23	78.90	0.00
TRINITY_Q22XT4_TETTS/475-635	Q22XT4.1 PF08662.9;	78.90	0.00
TRINITY_D8UF46_VOLCA/295-494	D8UF46.1 PF03441.12	78.90	0.00
TRINITY_L8GKJ8_ACACA/14-84	L8GKJ8.1 PF00076.20	78.90	0.00
TRINITY_D8U5N8_VOLCA/270-308	D8U5N8.1 PF00400.30	78.90	0.00
TRINITY_A8HVU9_CHLRE/18-546	A8HVU9.1 PF01676.16	78.90	0.00
TRINITY_I7M1K4_TETTS/30-285	I7M1K4.1 PF00069.23	78.90	0.00
TRINITY_Q240X8_TETTS/533-757	Q240X8.2 PF00149.26	78.90	0.00
TRINITY_D8U105_VOLCA/1269-1674	D8U105.1 PF08393.11	78.90	0.00
TRINITY_I0YU70_9CHLO/4-458	I0YU70.1 PF00982.19	78.90	0.00
TRINITY_J9IVJ5_9SPIT/452-505	J9IVJ5.1 PF13821.4;	78.90	0.00
TRINITY_D8UKB1_VOLCA/988-1273	D8UKB1.1 PF03133.13	78.90	0.00
TRINITY_D8U8K4_VOLCA/199-750	D8U8K4.1 PF08385.10	78.90	0.00
TRINITY_D8UIG2_VOLCA/332-549	D8UIG2.1 PF00557.22	78.90	0.00
TRINITY_D8U1K8_VOLCA/108-374	D8U1K8.1 PF00664.21	78.90	0.00
TRINITY_A8IZE7_CHLRE/525-659	A8IZE7.1 PF01380.20	78.90	0.00
TRINITY_D8U4L6_VOLCA/1038-1075	D8U4L6.1 PF06596.9;	78.90	0.00
TRINITY_Q22UL2_TETTS/160-319	Q22UL2.1 PF02800.18	78.80	0.00
TRINITY_G0QXI7_ICHMG/211-314	G0QXI7.1 PF03133.13	78.80	0.00
TRINITY_L8HAC7_ACACA/15-184	L8HAC7.1 PF00071.20	78.80	0.00
TRINITY_D8TMT4_VOLCA/58-161	D8TMT4.1 PF08241.10	78.80	0.00
TRINITY_A0A087SF84_AUXPR/359-554	A0A087SF84.1 PF0022	78.80	0.00
TRINITY_M3ZWP6_XIPMA/261-348	M3ZWP6.1 PF09014.8;	78.80	0.00
TRINITY_A8IGD6_CHLRE/40-206	A8IGD6.1 PF03193.14	78.80	0.00
TRINITY_A8IGD6_CHLRE/40-206	A8IGD6.1 PF03193.14	78.80	0.00
TRINITY_A9T9B7_PHYPA/138-500	A9T9B7.1 PF07690.14	78.80	0.00
TRINITY_A0DEE1_PARTE/205-289	A0DEE1.1 PF00766.17	78.80	0.00
TRINITY_A8IC88_CHLRE/107-224	A8IC88.1 PF13640.4;	78.80	0.00
TRINITY_A8ITV3_CHLRE/37-177	A8ITV3.1 PF00504.19	78.80	0.00
TRINITY_A8ITV3_CHLRE/37-177	A8ITV3.1 PF00504.19	78.80	0.00
TRINITY_A8J6P9_CHLRE/60-111	A8J6P9.1 PF00253.19	78.80	0.00

TRINITY_C1E5A6_MICSR/30-340	C1E5A6.1 PF00814.23	78.80	0.00
TRINITY_Q9FNS5_CHLRE/71-219	Q9FNS5.2 PF00056.21	78.80	0.00
TRINITY_D8TZ80_VOLCA/552-683	D8TZ80.1 PF00004.27	78.80	0.00
TRINITY_D8TVR1_VOLCA/102-271	D8TVR1.1 PF02866.16	78.80	0.00
TRINITY_A0A068S232_9FUNG/48-162	A0A068S232.1 PF0139	78.80	0.00
TRINITY_A8JCQ8_CHLRE/128-575	A8JCQ8.1 PF00501.26	78.80	0.00
TRINITY_A8JHB4_CHLRE/27-450	A8JHB4.1 PF00310.19	78.80	0.00
TRINITY_A8HWI6_CHLRE/5-189	A8HWI6.1 PF00227.24	78.80	0.00
TRINITY_D8UC43_VOLCA/47-387	D8UC43.1 PF01208.15	78.80	0.00
TRINITY_D8TK54_VOLCA/171-394	D8TK54.1 PF01786.15	78.80	0.00
TRINITY_A0A067LGR7_JATCU/96-526	A0A067LGR7.1 PF0085	78.70	0.00
TRINITY_G0R2E2_ICHMG/1-217	G0R2E2.1 PF00069.23	78.70	0.00
TRINITY_G3P285_GASAC/259-398	G3P285.1 PF01794.17	78.70	0.00
TRINITY_G3NMY8_GASAC/1113-1201	G3NMY8.1 PF07677.12	78.70	0.00
TRINITY_F4Q6S2_DICFS/285-332	F4Q6S2.1 PF02042.13	78.70	0.00
TRINITY_D8UEU8_VOLCA/4-164	D8UEU8.1 PF07162.9;	78.70	0.00
TRINITY_D8U432_VOLCA/29-347	D8U432.1 PF00899.19	78.70	0.00
TRINITY_L8H3I3_ACACA/86-340	L8H3I3.1 PF08423.9;	78.70	0.00
TRINITY_L1JTT7_GUITH/13-303	L1JTT7.1 PF00069.23	78.70	0.00
TRINITY_Q22GZ5_TETTS/22-280	Q22GZ5.3 PF00069.23	78.70	0.00
TRINITY_A0A078AUP9_STYLE/47-242	A0A078AUP9.1 PF0014	78.70	0.00
TRINITY_A0CVD7_PARTE/716-762	A0CVD7.1 PF00400.30	78.70	0.00
TRINITY_A0BWV5_PARTE/13-191	A0BWV5.1 PF02779.22	78.70	0.00
TRINITY_Q24CB9_TETTS/192-468	Q24CB9.1 PF08634.8;	78.70	0.00
TRINITY_A8HRZ4_CHLRE/83-157	A8HRZ4.1 PF01215.17	78.70	0.00
TRINITY_E1ZEE7_CHLVA/19-66	E1ZEE7.1 PF13637.4;	78.70	0.00
TRINITY_A0A0A0L764_CUCSA/57-131	A0A0A0L764.1 PF0011	78.70	0.00
TRINITY_D8TKE7_VOLCA/124-297	D8TKE7.1 PF00270.27	78.70	0.00
TRINITY_M0RS96_MUSAM/58-188	M0RS96.1 PF01467.24	78.70	0.00
TRINITY_H2R700_PANTR/122-413	H2R700.1 PF00850.17	78.70	0.00
TRINITY_Q6Q242_CHLRE/87-341	Q6Q242.1 PF08423.9;	78.70	0.00
TRINITY_D8TRD4_VOLCA/130-335	D8TRD4.1 PF00753.25	78.70	0.00
TRINITY_D8UB22_VOLCA/201-483	D8UB22.1 PF00303.17	78.70	0.00
TRINITY_D8UDT3_VOLCA/121-255	D8UDT3.1 PF00254.26	78.70	0.00
TRINITY_D8U4X7_VOLCA/4-295	D8U4X7.1 PF05653.12	78.70	0.00
TRINITY_A0CP41_PARTE/49-236	A0CP41.1 PF10211.7;	78.70	0.00
TRINITY_Q22XN2_TETTS/43-220	Q22XN2.1 PF02146.15	78.60	0.00
TRINITY_S8CD37_9LAMI/458-616	S8CD37.1 PF02769.20	78.60	0.00
TRINITY_A0A087SF33_AUXPR/5-71	A0A087SF33.1 PF0142	78.60	0.00
TRINITY_G0QZY1_ICHMG/82-282	G0QZY1.1 PF00149.26	78.60	0.00
TRINITY_D8U9S6_VOLCA/313-473	D8U9S6.1 PF00270.27	78.60	0.00
TRINITY_D8U4F1_VOLCA/2-252	D8U4F1.1 PF01265.15	78.60	0.00
TRINITY_ARL3_CHLRE/3-176	A8ISN6.2 PF00025.19	78.60	0.00
TRINITY_A8J2Q0_CHLRE/7-70	A8J2Q0.1 PF13499.4;	78.60	0.00
TRINITY_D8U2G0_VOLCA/149-253	D8U2G0.1 PF00639.19	78.60	0.00
TRINITY_A8INI6_CHLRE/46-246	A8INI6.1 PF01728.17	78.60	0.00
TRINITY_W9QWG9_9ROSA/873-975	W9QWG9.1 PF05187.11	78.60	0.00
TRINITY_D8U5I0_VOLCA/499-857	D8U5I0.1 PF00654.18	78.60	0.00
TRINITY_I3K9D2_ORENI/434-667	I3K9D2.1 PF00147.16	78.60	0.00
TRINITY_D8UAK0_VOLCA/1-437	D8UAK0.1 PF00996.16	78.60	0.00
TRINITY_A0A0D2U5B7_CAPO3/9-50	A0A0D2U5B7.1 PF0083	78.60	0.00
TRINITY_A0A059LFD6_9CHLO/1-99	A0A059LFD6.1 PF0075	78.60	0.00
TRINITY_E1Z6L6_CHLVA/245-426	E1Z6L6.1 PF00270.27	78.60	0.00
TRINITY_A8I3C6_CHLRE/191-368	A8I3C6.1 PF00504.19	78.60	0.00
TRINITY_A8HPZ8_CHLRE/149-557	A8HPZ8.1 PF01432.18	78.60	0.00
TRINITY_A8J7I6_CHLRE/28-195	A8J7I6.1 PF03420.11	78.60	0.00
TRINITY_D8U9G8_VOLCA/63-351	D8U9G8.1 PF00069.23	78.60	0.00
TRINITY_RSP2_CHLRE/8-49	Q6UBQ3.1 PF05186.11	78.60	0.00
TRINITY_D8TR40_VOLCA/7-265	D8TR40.1 PF00069.23	78.60	0.00
TRINITY_J9IIV2_9SPIT/39-80	J9IIV2.1 PF05186.11	78.60	0.00
TRINITY_J9EW47_9SPIT/841-1143	J9EW47.1 PF00176.21	78.50	0.00

TRINITY_D8U4F8_VOLCA/24-200	D8U4F8.1 PF01728.17	78.50	0.00
TRINITY_D8THR4_VOLCA/73-174	D8THR4.1 PF00237.17	78.50	0.00
TRINITY_A0E956_PARTE/98-269	A0E956.1 PF00270.27	78.50	0.00
TRINITY_G5C1N0_HETGA/39-137	G5C1N0.1 PF00276.18	78.50	0.00
TRINITY_G5C1N0_HETGA/39-137	G5C1N0.1 PF00276.18	78.50	0.00
TRINITY_G0QMQ9_ICHMG/30-285	G0QMQ9.1 PF00069.23	78.50	0.00
TRINITY_L8GG56_ACACA/6-615	L8GG56.1 PF00012.18	78.50	0.00
TRINITY_A8JIA7_CHLRE/141-205	A8JIA7.1 PF00462.22	78.50	0.00
TRINITY_D8TJI4_VOLCA/127-194	D8TJI4.1 PF00542.17	78.50	0.00
TRINITY_D8U506_VOLCA/14-279	D8U506.1 PF10234.7;	78.50	0.00
TRINITY_A0A087SQR6_AUXPR/26-169	A0A087SQR6.1 PF0272	78.50	0.00
TRINITY_M5WZQ0_PRUPE/537-711	M5WZQ0.1 PF00270.27	78.50	0.00
TRINITY_A0A0D2U3Q1_CAPO3/577-723	A0A0D2U3Q1.1 PF0063	78.50	0.00
TRINITY_D8U402_VOLCA/85-470	D8U402.1 PF01053.18	78.50	0.00
TRINITY_A8J1X8_CHLRE/220-540	A8J1X8.1 PF00152.18	78.50	0.00
TRINITY_A8HNG4_CHLRE/29-172	A8HNG4.1 PF00810.16	78.50	0.00
TRINITY_E1Z5U7_CHLVA/169-492	E1Z5U7.1 PF02445.14	78.50	0.00
TRINITY_D8S4V0_SELML/2-136	D8S4V0.1 PF00334.17	78.50	0.00
TRINITY_I0YUV4_9CHLO/265-351	I0YUV4.1 PF08542.9;	78.50	0.00
TRINITY_A8J336_CHLRE/1141-1550	A8J336.1 PF08393.11	78.50	0.00
TRINITY_G5C1N0_HETGA/39-137	G5C1N0.1 PF00276.18	78.50	0.00
TRINITY_M0ZZP3_SOLTU/47-121	M0ZZP3.1 PF00111.25	78.40	0.00
TRINITY_A8HU20_CHLRE/6-257	A8HU20.1 PF00069.23	78.40	0.00
TRINITY_D0LH24_HALO1/11-135	D0LH24.1 PF02915.15	78.40	0.00
TRINITY_D8TT10_VOLCA/33-270	D8TT10.1 PF13561.4;	78.40	0.00
TRINITY_Q3SD32_PARTE/10-176	Q3SD32.1 PF00071.20	78.40	0.00
TRINITY_I0YYF9_9CHLO/476-527	I0YYF9.1 PF12801.5;	78.40	0.00
TRINITY_F0Y3G2_AURAN/97-212	F0Y3G2.1 PF01509.16	78.40	0.00
TRINITY_V7BE91_PHAVU/88-552	V7BE91.1 PF00501.26	78.40	0.00
TRINITY_Q3SEA7_PARTE/155-448	Q3SEA7.1 PF00113.20	78.40	0.00
TRINITY_A4VDB5_TETTS/67-400	A4VDB5.2 PF00225.21	78.40	0.00
TRINITY_D8TIK7_VOLCA/420-560	D8TIK7.1 PF04784.12	78.40	0.00
TRINITY_A0A078FZF9_BRANA/30-122	A0A078FZF9.1 PF0024	78.40	0.00
TRINITY_D8TUW1_VOLCA/298-409	D8TUW1.1 PF00271.29	78.40	0.00
TRINITY_D8TRN2_VOLCA/120-524	D8TRN2.1 PF08393.11	78.40	0.00
TRINITY_A8IDU2_CHLRE/877-1024	A8IDU2.1 PF03330.16	78.40	0.00
TRINITY_A8JI25_CHLRE/1010-1316	A8JI25.1 PF02889.14	78.40	0.00
TRINITY_A0A078B908_STYLE/61-227	A0A078B908.1 PF0027	78.40	0.00
TRINITY_I0Z4H9_9CHLO/20-93	I0Z4H9.1 PF05066.11	78.40	0.00
TRINITY_I0YI54_9CHLO/262-456	I0YI54.1 PF02786.15	78.40	0.00
TRINITY_D8TW33_VOLCA/256-307	D8TW33.1 PF05739.17	78.40	0.00
TRINITY_V4AVB2_LOTGI/9-91	V4AVB2.1 PF02319.18	78.40	0.00
TRINITY_A8I702_CHLRE/1-254	A8I702.1 PF08392.10	78.40	0.00
TRINITY_D8TV96_VOLCA/405-705	D8TV96.1 PF00069.23	78.40	0.00
TRINITY_D8TL75_VOLCA/715-913	D8TL75.1 PF00742.17	78.40	0.00
TRINITY_D8U8F2_VOLCA/2665-3369	D8U8F2.1 PF03028.13	78.40	0.00
TRINITY_A0DN76_PARTE/190-761	A0DN76.1 PF08385.10	78.30	0.00
TRINITY_D8UCQ9_VOLCA/86-148	D8UCQ9.1 PF00226.29	78.30	0.00
TRINITY_A0CJS0_PARTE/128-311	A0CJS0.1 PF10274.7;	78.30	0.00
TRINITY_A8JID6_CHLRE/76-553	A8JID6.1 PF03219.12	78.30	0.00
TRINITY_Q22B78_TETTS/72-140	Q22B78.1 PF00366.18	78.30	0.00
TRINITY_Q23YD7_TETTS/956-1005	Q23YD7.2 PF13920.4;	78.30	0.00
TRINITY_A8IK98_CHLRE/74-458	A8IK98.1 PF00176.21	78.30	0.00
TRINITY_A8I3H2_CHLRE/1028-1073	A8I3H2.1 PF13920.4;	78.30	0.00
TRINITY_G0QPJ7_ICHMG/85-154	G0QPJ7.1 PF13499.4;	78.30	0.00
TRINITY_A8HYD5_CHLRE/39-159	A8HYD5.1 PF01597.17	78.30	0.00
TRINITY_D8TMT3_VOLCA/73-165	D8TMT3.1 PF00254.26	78.30	0.00
TRINITY_W8ET04_9BACT/216-743	W8ET04.1 PF02867.13	78.30	0.00
TRINITY_C1MHN6_MICPC/12-80	C1MHN6.1 PF01423.20	78.30	0.00
TRINITY_A8JF79_CHLRE/207-326	A8JF79.1 PF01926.21	78.30	0.00
TRINITY_A8J063_CHLRE/2338-2608	A8J063.1 PF12780.5;	78.30	0.00

TRINITY_D8TIE7_VOLCA/109-200	D8TIE7.1 PF00153.25	78.30	0.00
TRINITY_I7MJG2_TETTS/1-142	I7MJG2.1 PF01217.18	78.20	0.00
TRINITY_D8TRK3_VOLCA/46-386	D8TRK3.1 PF01208.15	78.20	0.00
TRINITY_D8TGZ0_VOLCA/1-268	D8TGZ0.1 PF06732.9;	78.20	0.00
TRINITY_Q22BR5_TETTS/56-213	Q22BR5.3 PF00071.20	78.20	0.00
TRINITY_A8IRU0_CHLRE/5-311	A8IRU0.1 PF02535.20	78.20	0.00
TRINITY_D8U584_VOLCA/45-218	D8U584.1 PF03092.14	78.20	0.00
TRINITY_Q23MC3_TETTS/274-362	Q23MC3.2 PF13499.4;	78.20	0.00
TRINITY_A8J6V5_CHLRE/201-406	A8J6V5.1 PF01872.15	78.20	0.00
TRINITY_D8U298_VOLCA/104-201	D8U298.1 PF00254.26	78.20	0.00
TRINITY_E1ZK76_CHLVA/225-502	E1ZK76.1 PF00425.16	78.20	0.00
TRINITY_M1CWT7_SOLTU/7-120	M1CWT7.1 PF05564.10	78.10	0.00
TRINITY_Q22BJ8_TETTS/71-135	Q22BJ8.1 PF02319.18	78.10	0.00
TRINITY_Q23DE7_TETTS/476-808	Q23DE7.1 PF00773.17	78.10	0.00
TRINITY_A0A0D2UQH2_CAPO3/305-337	A0A0D2UQH2.1 PF0040	78.10	0.00
TRINITY_ERK2_DICDI/14-304	Q54QB1.1 PF00069.23	78.10	0.00
TRINITY_A8IA03_CHLRE/16-271	A8IA03.1 PF00069.23	78.10	0.00
TRINITY_C1N1E2_MICPC/2-69	C1N1E2.1 PF01781.16	78.10	0.00
TRINITY_D8TK59_VOLCA/100-237	D8TK59.1 PF11371.6;	78.10	0.00
TRINITY_D8U8N7_VOLCA/113-236	D8U8N7.1 PF01398.19	78.10	0.00
TRINITY_A8ITN4_CHLRE/17-48	A8ITN4.1 PF03604.11	78.10	0.00
TRINITY_A9S6B6_PHYPA/102-247	A9S6B6.1 PF00210.22	78.10	0.00
TRINITY_D8UJB1_VOLCA/236-413	D8UJB1.1 PF00463.19	78.10	0.00
TRINITY_D8TLJ9_VOLCA/109-448	D8TLJ9.1 PF02401.16	78.10	0.00
TRINITY_IF6_TETTS/4-204	Q245F2.1 PF01912.16	78.10	0.00
TRINITY_A0A087SRR6_AUXPR/74-458	A0A087SRR6.1 PF0046	78.10	0.00
TRINITY_G0QZY1_ICHMG/82-282	G0QZY1.1 PF00149.26	78.10	0.00
TRINITY_A0A022R695_ERYGU/119-408	A0A022R695.1 PF0006	78.10	0.00
TRINITY_A0A060SKG4_PYCCI/750-782	A0A060SKG4.1 PF0185	78.10	0.00
TRINITY_D8U552_VOLCA/128-397	D8U552.1 PF00009.25	78.10	0.00
TRINITY_A8IVM9_CHLRE/82-510	A8IVM9.1 PF02347.14	78.10	0.00
TRINITY_I7M490_TETTS/13-195	I7M490.2 PF01214.16	78.10	0.00
TRINITY_A8IRU2_CHLRE/1046-1118	A8IRU2.1 PF02135.14	78.10	0.00
TRINITY_I0YKR7_9CHLO/86-150	I0YKR7.1 PF00462.22	78.10	0.00
TRINITY_D8UJD9_VOLCA/37-534	D8UJD9.1 PF00118.22	78.10	0.00
TRINITY_K4CP64_SOLLC/29-227	K4CP64.1 PF00314.15	78.00	0.00
TRINITY_G0QYX8_ICHMG/137-263	G0QYX8.1 PF04037.11	78.00	0.00
TRINITY_C1M2T9_SCHMA/1-81	C1M2T9.2 PF00077.18	78.00	0.00
TRINITY_E1ZP27_CHLVA/687-1184	E1ZP27.1 PF00136.19	78.00	0.00
TRINITY_J9I560_9SPIT/4-261	J9I560.1 PF00069.23	78.00	0.00
TRINITY_A8JBP5_CHLRE/14-117	A8JBP5.1 PF00153.25	78.00	0.00
TRINITY_I0YMS5_9CHLO/72-517	I0YMS5.1 PF08801.9;	78.00	0.00
TRINITY_F1A2J8_DICPU/141-248	F1A2J8.1 PF11380.6;	78.00	0.00
TRINITY_A0DGG3_PARTE/16-124	A0DGG3.1 PF01655.16	78.00	0.00
TRINITY_A8IZM3_CHLRE/15-118	A8IZM3.1 PF04178.10	78.00	0.00
TRINITY_I7MA96_TETTS/697-955	I7MA96.1 PF00069.23	78.00	0.00
TRINITY_L8HB18_ACACA/915-1031	L8HB18.1 PF02518.24	78.00	0.00
TRINITY_D8U9R9_VOLCA/298-402	D8U9R9.1 PF01399.25	78.00	0.00
TRINITY_A8J0E4_CHLRE/58-290	A8J0E4.1 PF01716.16	78.00	0.00
TRINITY_A0A072W136_MEDTR/465-514	A0A072W136.1 PF0184	78.00	0.00
TRINITY_Q6BGF1_PARTE/393-606	Q6BGF1.1 PF07859.11	78.00	0.00
TRINITY_D8UKQ0_VOLCA/3-52	D8UKQ0.1 PF04135.10	78.00	0.00
TRINITY_E1Z7M6_CHLVA/19-145	E1Z7M6.1 PF00134.21	78.00	0.00
TRINITY_L8GNZ7_ACACA/132-341	L8GNZ7.1 PF02786.15	78.00	0.00
TRINITY_D3BED6_POLPA/317-420	D3BED6.1 PF04191.11	78.00	0.00
TRINITY_D8TL96_VOLCA/131-458	D8TL96.1 PF00225.21	78.00	0.00
TRINITY_D8TGX0_VOLCA/3455-3807	D8TGX0.1 PF03028.13	78.00	0.00
TRINITY_D8UEQ7_VOLCA/74-205	D8UEQ7.1 PF00430.16	78.00	0.00
TRINITY_L8GKP4_ACACA/9-182	L8GKP4.1 PF00025.19	78.00	0.00
TRINITY_A8JCK1_CHLRE/44-293	A8JCK1.1 PF00109.24	78.00	0.00
TRINITY_I1G401_AMPQE/41-90	I1G401.1 PF04135.10	78.00	0.00

TRINITY_I7M8W6_TETTS/24-336	I7M8W6.1 PF00850.17	77.90	0.00
TRINITY_A0BLS3_PARTE/98-348	A0BLS3.1 PF00012.18	77.90	0.00
TRINITY_D2VBJ0_NAEGR/31-212	D2VBJ0.1 PF00227.24	77.90	0.00
TRINITY_C3XQ45_BRAFL/27-200	C3XQ45.1 PF04055.19	77.90	0.00
TRINITY_E1ZRE9_CHLVA/65-132	E1ZRE9.1 PF08606.9;	77.90	0.00
TRINITY_A8J3B5_CHLRE/12-175	A8J3B5.1 PF07162.9;	77.90	0.00
TRINITY_A9RG89_PHYPA/2-191	A9RG89.1 PF00827.15	77.90	0.00
TRINITY_D8U582_VOLCA/34-431	D8U582.1 PF01960.16	77.90	0.00
TRINITY_A8JD41_CHLRE/76-172	A8JD41.1 PF11460.6;	77.90	0.00
TRINITY_D8TJA9_VOLCA/41-757	D8TJA9.1 PF05879.10	77.90	0.00
TRINITY_Q22UG4_TETTS/45-302	Q22UG4.1 PF00899.19	77.90	0.00
TRINITY_A0A078AG04_STYLE/8-75	A0A078AG04.1 PF0111	77.90	0.00
TRINITY_A8J0N1_CHLRE/179-359	A8J0N1.1 PF11891.6;	77.90	0.00
TRINITY_D8TJA5_VOLCA/53-597	D8TJA5.1 PF02990.14	77.90	0.00
TRINITY_D8U702_VOLCA/48-293	D8U702.1 PF07714.15	77.90	0.00
TRINITY_D8TJK0_VOLCA/116-341	D8TJK0.1 PF03407.14	77.90	0.00
TRINITY_D8TXN0_VOLCA/8-251	D8TXN0.1 PF03029.15	77.90	0.00
TRINITY_D8TWW9_VOLCA/964-1103	D8TWW9.1 PF07766.11	77.90	0.00
TRINITY_D8UHG2_VOLCA/134-264	D8UHG2.1 PF00648.19	77.90	0.00
TRINITY_Q9SNV7_CHLRE/301-445	Q9SNV7.1 PF00004.27	77.90	0.00
TRINITY_D8UA15_VOLCA/77-144	D8UA15.1 PF02416.14	77.90	0.00
TRINITY_A8JF47_CHLRE/300-389	A8JF47.1 PF00153.25	77.90	0.00
TRINITY_J9GB80_9SPIT/69-225	J9GB80.1 PF12146.6;	77.80	0.00
TRINITY_W7XI20_TETTS/141-304	W7XI20.1 PF05118.13	77.80	0.00
TRINITY_G1K265_CANLF/19-146	G1K265.1 PF00062.18	77.80	0.00
TRINITY_F0VG80_NEOCL/32-94	F0VG80.1 PF01176.17	77.80	0.00
TRINITY_R1FJW2_EMIHU/3-75	R1FJW2.1 PF13499.4;	77.80	0.00
TRINITY_D3BI40_POLPA/29-280	D3BI40.1 PF00398.18	77.80	0.00
TRINITY_A0A067CLG0_SAPPC/220-264	A0A067CLG0.1 PF0040	77.80	0.00
TRINITY_D8TVR5_VOLCA/136-250	D8TVR5.1 PF00164.23	77.80	0.00
TRINITY_W5KCF3_ASTMX/94-120	W5KCF3.1 PF07974.11	77.80	0.00
TRINITY_A8HS59_CHLRE/19-153	A8HS59.1 PF00237.17	77.80	0.00
TRINITY_A8HMS2_CHLRE/493-591	A8HMS2.1 PF08417.10	77.80	0.00
TRINITY_A8J2A5_CHLRE/185-273	A8J2A5.1 PF00153.25	77.80	0.00
TRINITY_A8IIJ6_CHLRE/68-1078	A8IIJ6.1 PF02463.17	77.80	0.00
TRINITY_A0A0G4EHW8_9ALVE/46-108	A0A0G4EHW8.1 PF0184	77.80	0.00
TRINITY_D8U352_VOLCA/483-540	D8U352.1 PF07683.12	77.80	0.00
TRINITY_A0A0G4J440_PLABS/4-216	A0A0G4J440.1 PF0009	77.80	0.00
TRINITY_F7DC37_XENTR/13-192	F7DC37.1 PF00009.25	77.80	0.00
TRINITY_A8J4P9_CHLRE/106-285	A8J4P9.1 PF00174.17	77.80	0.00
TRINITY_D8TQ33_VOLCA/623-831	D8TQ33.1 PF00924.16	77.80	0.00
TRINITY_I7MHZ8_TETTS/193-219	I7MHZ8.2 PF00641.16	77.80	0.00
TRINITY_D8TH31_VOLCA/617-688	D8TH31.1 PF04564.13	77.80	0.00
TRINITY_D8UIJ8_VOLCA/100-207	D8UIJ8.1 PF02777.16	77.80	0.00
TRINITY_A8J0U5_CHLRE/110-307	A8J0U5.1 PF00696.26	77.80	0.00
TRINITY_D8U5H7_VOLCA/6-339	D8U5H7.1 PF00225.21	77.80	0.00
TRINITY_A8IQT7_CHLRE/9-103	A8IQT7.1 PF01230.21	77.80	0.00
TRINITY_A0A0N0JZT4_9SPHN/9-246	A0A0N0JZT4.1 PF0631	77.80	0.00
TRINITY_A8IZW8_CHLRE/9-89	A8IZW8.1 PF01722.16	77.80	0.00
TRINITY_R1DHZ4_EMIHU/98-166	R1DHZ4.1 PF00076.20	77.80	0.00
TRINITY_D8UGM1_VOLCA/330-419	D8UGM1.1 PF11715.6;	77.80	0.00
TRINITY_A8J6C7_CHLRE/445-655	A8J6C7.1 PF01434.16	77.80	0.00
TRINITY_A8I8D2_CHLRE/105-225	A8I8D2.1 PF13640.4;	77.80	0.00
TRINITY_D8UBP7_VOLCA/46-407	D8UBP7.1 PF00225.21	77.80	0.00
TRINITY_L8GRI5_ACACA/69-274	L8GRI5.1 PF14681.4;	77.70	0.00
TRINITY_A8JHW4_CHLRE/9-147	A8JHW4.1 PF00179.24	77.70	0.00
TRINITY_C3YQF4_BRAFL/20-294	C3YQF4.1 PF00682.17	77.70	0.00
TRINITY_R4X8G3_TAPDE/22-366	R4X8G3.1 PF00884.21	77.70	0.00
TRINITY_M5WAG6_PRUPE/2-191	M5WAG6.1 PF00827.15	77.70	0.00
TRINITY_D8UGN1_VOLCA/816-1223	D8UGN1.1 PF06957.9;	77.70	0.00
TRINITY_A0A078AUP9_STYLE/47-242	A0A078AUP9.1 PF0014	77.70	0.00

TRINITY_A8I311_CHLRE/4-113	A8I311.1 PF03650.11	77.70	0.00
TRINITY_E1Z3T4_CHLVA/233-526	E1Z3T4.1 PF00977.19	77.70	0.00
TRINITY_D8TSY5_VOLCA/2523-3232	D8TSY5.1 PF03028.13	77.70	0.00
TRINITY_H2SQV4_TAKRU/30-137	H2SQV4.1 PF00576.19	77.60	0.00
TRINITY_A0A078AB47_STYLE/144-465	A0A078AB47.1 PF0064	77.60	0.00
TRINITY_G5C1N0_HETGA/39-137	G5C1N0.1 PF00276.18	77.60	0.00
TRINITY_I7MFR0_TETTS/5-80	I7MFR0.1 PF01423.20	77.60	0.00
TRINITY_W5LAM7_ASTMX/226-330	W5LAM7.1 PF00042.20	77.60	0.00
TRINITY_G3I8F7_CRIGR/82-393	G3I8F7.1 PF00038.19	77.60	0.00
TRINITY_IF6_TETTS/4-204	Q245F2.1 PF01912.16	77.60	0.00
TRINITY_I7M274_TETTS/211-384	I7M274.2 PF04055.19	77.60	0.00
TRINITY_D8QU58_SELML/11-233	D8QU58.1 PF00244.18	77.60	0.00
TRINITY_F0ZPW4_DICPU/4-230	F0ZPW4.1 PF04670.10	77.60	0.00
TRINITY_L8HHR2_ACACA/1-660	L8HHR2.1 PF00063.19	77.60	0.00
TRINITY_A8IE29_CHLRE/43-319	A8IE29.1 PF00494.17	77.60	0.00
TRINITY_A0A0F4JLE5_9ACTN/315-372	A0A0F4JLE5.1 PF0067	77.60	0.00
TRINITY_D8U3H0_VOLCA/426-550	D8U3H0.1 PF01624.18	77.60	0.00
TRINITY_E1Z2Y2_CHLVA/150-352	E1Z2Y2.1 PF13561.4;	77.60	0.00
TRINITY_D8THH0_VOLCA/35-423	D8THH0.1 PF04137.13	77.60	0.00
TRINITY_A8J3M3_CHLRE/633-784	A8J3M3.1 PF00441.22	77.60	0.00
TRINITY_F4QA18_DICFS/63-236	F4QA18.1 PF00071.20	77.60	0.00
TRINITY_D3BG38_POLPA/598-767	D3BG38.1 PF07724.12	77.60	0.00
TRINITY_NVL_DICDI/603-736	Q54SY2.1 PF00004.27	77.60	0.00
TRINITY_D8U895_VOLCA/50-293	D8U895.1 PF01409.18	77.60	0.00
TRINITY_D8THC9_VOLCA/332-462	D8THC9.1 PF00004.27	77.60	0.00
TRINITY_D8TNS4_VOLCA/81-459	D8TNS4.1 PF00285.19	77.60	0.00
TRINITY_A0A0M8YG60_9ACTN/194-252	A0A0M8YG60.1 PF0067	77.60	0.00
TRINITY_A0A0M8YG60_9ACTN/194-252	A0A0M8YG60.1 PF0067	77.60	0.00
TRINITY_A8I0M0_CHLRE/350-501	A8I0M0.1 PF00160.19	77.60	0.00
TRINITY_D8TWQ0_VOLCA/4-293	D8TWQ0.1 PF00069.23	77.60	0.00
TRINITY_A8JB97_CHLRE/128-243	A8JB97.1 PF02770.17	77.60	0.00
TRINITY_Q9FNS5_CHLRE/222-394	Q9FNS5.2 PF02866.16	77.60	0.00
TRINITY_L8H3Q7_ACACA/13-83	L8H3Q7.1 PF00076.20	77.50	0.00
TRINITY_E1Z6A7_CHLVA/6-94	E1Z6A7.1 PF10693.7;	77.50	0.00
TRINITY_L8HAX2_ACACA/110-468	L8HAX2.1 PF00128.22	77.50	0.00
TRINITY_A8IW09_CHLRE/104-199	A8IW09.1 PF00254.26	77.50	0.00
TRINITY_L1JHZ5_GUITH/4-287	L1JHZ5.1 PF00069.23	77.50	0.00
TRINITY_Q84X75_CHLRE/86-321	Q84X75.1 PF13561.4;	77.50	0.00
TRINITY_L8GLD6_ACACA/908-1049	L8GLD6.1 PF00004.27	77.50	0.00
TRINITY_A0CW82_PARTE/237-374	A0CW82.1 PF00334.17	77.50	0.00
TRINITY_Q3SD87_PARTE/6-613	Q3SD87.1 PF00012.18	77.50	0.00
TRINITY_A8HSB7_CHLRE/6-257	A8HSB7.1 PF00069.23	77.50	0.00
TRINITY_G0QK16_ICHMG/92-301	G0QK16.1 PF02786.15	77.50	0.00
TRINITY_I0Z5H1_9CHLO/149-188	I0Z5H1.1 PF00400.30	77.50	0.00
TRINITY_A0A099NYT5_PICKU/5-177	A0A099NYT5.1 PF0002	77.50	0.00
TRINITY_A0CJF4_PARTE/1-142	A0CJF4.1 PF01217.18	77.50	0.00
TRINITY_A8I3X6_CHLRE/2565-3295	A8I3X6.1 PF03028.13	77.50	0.00
TRINITY_I7MIS2_TETTS/15-160	I7MIS2.2 PF00179.24	77.40	0.00
TRINITY_A8JHU7_CHLRE/3186-3525	A8JHU7.1 PF12777.5;	77.40	0.00
TRINITY_F7DLT4_XENTR/97-236	F7DLT4.1 PF01565.21	77.40	0.00
TRINITY_A7SWF7_NEMVE/319-432	A7SWF7.1 PF00271.29	77.40	0.00
TRINITY_L8HJG0_ACACA/21-206	L8HJG0.1 PF01728.17	77.40	0.00
TRINITY_I0YKX7_9CHLO/155-269	I0YKX7.1 PF00504.19	77.40	0.00
TRINITY_ENV_XMRV6/21-615	Q27ID8.1 PF00429.17	77.40	0.00
TRINITY_A0EE13_PARTE/19-311	A0EE13.1 PF00069.23	77.40	0.00
TRINITY_C3YQF4_BRAFL/20-294	C3YQF4.1 PF00682.17	77.40	0.00
TRINITY_D8TRR8_VOLCA/35-118	D8TRR8.1 PF13774.4;	77.40	0.00
TRINITY_D8TWJ2_VOLCA/29-194	D8TWJ2.1 PF03190.13	77.40	0.00
TRINITY_A8HX77_CHLRE/10-172	A8HX77.1 PF00071.20	77.40	0.00
TRINITY_A8J8P6_CHLRE/148-236	A8J8P6.1 PF13225.4;	77.40	0.00
TRINITY_D8TSD5_VOLCA/46-204	D8TSD5.1 PF03737.13	77.40	0.00

TRINITY_Q4VKB4_CHLRE/119-229	Q4VKB4.1 PF00487.22	77.40	0.00
TRINITY_A0D389_PARTE/1494-1652	A0D389.1 PF00179.24	77.40	0.00
TRINITY_E1ZGV8_CHLVA/118-161	E1ZGV8.1 PF14634.4;	77.40	0.00
TRINITY_B9SMB4_RICCO/1034-1192	B9SMB4.1 PF07717.14	77.40	0.00
TRINITY_M5EDE1_MALS4/87-196	M5EDE1.1 PF08242.10	77.40	0.00
TRINITY_A0A087SCP9_AUXPR/199-389	A0A087SCP9.1 PF0163	77.40	0.00
TRINITY_D8TSY5_VOLCA/1513-1777	D8TSY5.1 PF12780.5;	77.40	0.00
TRINITY_A8JCQ8_CHLRE/128-575	A8JCQ8.1 PF00501.26	77.40	0.00
TRINITY_D8U8Q0_VOLCA/72-213	D8U8Q0.1 PF05981.10	77.40	0.00
TRINITY_D2R9J4_PIRSD/426-749	D2R9J4.1 PF01717.16	77.40	0.00
TRINITY_DRC7_CHLRE/349-401	A8JAM0.1 PF13418.4;	77.40	0.00
TRINITY_I0YUW3_9CHLO/17-344	I0YUW3.1 PF00009.25	77.40	0.00
TRINITY_A0BKT3_PARTE/6-401	A0BKT3.1 PF00180.18	77.30	0.00
TRINITY_K3XYF0_SETIT/47-242	K3XYF0.1 PF00149.26	77.30	0.00
TRINITY_D8TPT6_VOLCA/374-448	D8TPT6.1 PF03110.12	77.30	0.00
TRINITY_A0A022QS20_ERYGU/21-601	A0A022QS20.1 PF0251	77.30	0.00
TRINITY_D8UBE7_VOLCA/20-150	D8UBE7.1 PF01590.24	77.30	0.00
TRINITY_D8UB59_VOLCA/11-326	D8UB59.1 PF03151.14	77.30	0.00
TRINITY_D8UF24_VOLCA/49-303	D8UF24.1 PF01571.19	77.30	0.00
TRINITY_D8U9G9_VOLCA/27-224	D8U9G9.1 PF03587.12	77.30	0.00
TRINITY_E1FZR6_LOALO/5-178	E1FZR6.2 PF00071.20	77.30	0.00
TRINITY_A8IS88_CHLRE/409-505	A8IS88.1 PF07683.12	77.30	0.00
TRINITY_A8JE84_CHLRE/107-213	A8JE84.1 PF04367.11	77.30	0.00
TRINITY_Q22T92_TETTS/412-618	Q22T92.1 PF00448.20	77.30	0.00
TRINITY_D8TZ80_VOLCA/552-683	D8TZ80.1 PF00004.27	77.30	0.00
TRINITY_I0YRG7_9CHLO/240-386	I0YRG7.1 PF00441.22	77.30	0.00
TRINITY_D8UBV1_VOLCA/61-192	D8UBV1.1 PF00782.18	77.30	0.00
TRINITY_D8TWV0_VOLCA/249-578	D8TWV0.1 PF00676.18	77.30	0.00
TRINITY_D8UH93_VOLCA/209-296	D8UH93.1 PF00153.25	77.30	0.00
TRINITY_L8GSG1_ACACA/9-65	L8GSG1.1 PF00412.20	77.20	0.00
TRINITY_Q229R5_TETTS/53-249	Q229R5.1 PF00149.26	77.20	0.00
TRINITY_T1IG80_RHOPR/112-327	T1IG80.1 PF00112.21	77.20	0.00
TRINITY_A0C4G5_PARTE/13-301	A0C4G5.1 PF00069.23	77.20	0.00
TRINITY_A8HR70_CHLRE/53-293	A8HR70.1 PF07992.12	77.20	0.00
TRINITY_D8UIE7_VOLCA/49-105	D8UIE7.1 PF01849.16	77.20	0.00
TRINITY_D8TKH5_VOLCA/385-739	D8TKH5.1 PF03016.13	77.20	0.00
TRINITY_E1ZQD5_CHLVA/445-541	E1ZQD5.1 PF00271.29	77.20	0.00
TRINITY_A8I4C2_CHLRE/272-455	A8I4C2.1 PF07970.10	77.20	0.00
TRINITY_H2WL12_CAEJA/163-320	H2WL12.2 PF02800.18	77.20	0.00
TRINITY_I0YSN7_9CHLO/8-121	I0YSN7.1 PF16906.3;	77.20	0.00
TRINITY_D8U1W1_VOLCA/105-319	D8U1W1.1 PF00590.18	77.20	0.00
TRINITY_A8IID7_CHLRE/10-66	A8IID7.1 PF05383.15	77.20	0.00
TRINITY_A8HQS1_CHLRE/2-471	A8HQS1.1 PF00171.20	77.20	0.00
TRINITY_D8TWM5_VOLCA/25-651	D8TWM5.1 PF00133.20	77.20	0.00
TRINITY_A0C460_PARTE/18-360	A0C460.1 PF07992.12	77.10	0.00
TRINITY_M1BIS3_SOLTU/3-146	M1BIS3.1 PF01280.18	77.10	0.00
TRINITY_L1JLA4_GUITH/13-82	L1JLA4.1 PF01423.20	77.10	0.00
TRINITY_D8U423_VOLCA/1-108	D8U423.1 PF00752.15	77.10	0.00
TRINITY_V3ZNN7_LOTGI/92-552	V3ZNN7.1 PF00501.26	77.10	0.00
TRINITY_A0A0D9VUL1_9ORYZ/7-183	A0A0D9VUL1.1 PF0016	77.10	0.00
TRINITY_D8TIC8_VOLCA/15-143	D8TIC8.1 PF00416.20	77.10	0.00
TRINITY_D8U5F4_VOLCA/15-132	D8U5F4.1 PF01894.15	77.10	0.00
TRINITY_D8U9Z9_VOLCA/12-172	D8U9Z9.1 PF03248.11	77.10	0.00
TRINITY_G0QZY5_ICHMG/317-510	G0QZY5.1 PF00122.18	77.10	0.00
TRINITY_D8U8Z5_VOLCA/2-189	D8U8Z5.1 PF05023.12	77.10	0.00
TRINITY_A9SYE1_PHYPA/28-246	A9SYE1.1 PF03332.11	77.10	0.00
TRINITY_A8ITC4_CHLRE/117-174	A8ITC4.1 PF03106.13	77.10	0.00
TRINITY_C7PC87_CHIPD/216-737	C7PC87.1 PF02867.13	77.10	0.00
TRINITY_Q23YQ6_TETTS/11-294	Q23YQ6.2 PF00069.23	77.10	0.00
TRINITY_D8UJ04_VOLCA/181-416	D8UJ04.1 PF02784.14	77.10	0.00
TRINITY_D8TJQ8_VOLCA/18-148	D8TJQ8.1 PF01138.19	77.10	0.00

TRINITY_D8UFM7_VOLCA/27-322	D8UFM7.1 PF00676.18	77.10	0.00
TRINITY_F0ZKW9_DICPU/428-462	F0ZKW9.1 PF00320.25	77.10	0.00
TRINITY_F2UG01_SALR5/39-88	F2UG01.1 PF10509.7;	77.10	0.00
TRINITY_Q22GZ5_TETTS/22-280	Q22GZ5.3 PF00069.23	77.10	0.00
TRINITY_Q22GZ5_TETTS/22-280	Q22GZ5.3 PF00069.23	77.10	0.00
TRINITY_L8GEU6_ACACA/8-121	L8GEU6.1 PF01398.19	77.10	0.00
TRINITY_A0A087SC77_AUXPR/518-566	A0A087SC77.1 PF1392	77.10	0.00
TRINITY_G0R442_ICHMG/8-260	G0R442.1 PF00069.23	77.10	0.00
TRINITY_D8U242_VOLCA/4-260	D8U242.1 PF00069.23	77.10	0.00
TRINITY_A8JGJ4_CHLRE/324-589	A8JGJ4.1 PF00069.23	77.10	0.00
TRINITY_A8JCF0_CHLRE/439-785	A8JCF0.1 PF00493.21	77.10	0.00
TRINITY_A8HYE1_CHLRE/47-92	A8HYE1.1 PF00319.16	77.10	0.00
TRINITY_A8ISA6_CHLRE/1125-1393	A8ISA6.1 PF12775.5;	77.10	0.00
TRINITY_A8IZ65_CHLRE/1-328	A8IZ65.1 PF00069.23	77.10	0.00
TRINITY_D8TLE6_VOLCA/14-651	D8TLE6.1 PF00888.20	77.10	0.00
TRINITY_Q24IM5_TETTS/56-282	Q24IM5.2 PF02167.13	77.10	0.00
TRINITY_M0TMY2_MUSAM/144-235	M0TMY2.1 PF13499.4;	77.00	0.00
TRINITY_A0BRW1_PARTE/570-819	A0BRW1.1 PF07714.15	77.00	0.00
TRINITY_X6LPL6_RETFI/63-180	X6LPL6.1 PF00271.29	77.00	0.00
TRINITY_G0R088_ICHMG/41-328	G0R088.1 PF00069.23	77.00	0.00
TRINITY_G4TRT7_PIRID/13-103	G4TRT7.1 PF00125.22	77.00	0.00
TRINITY_L8GMP5_ACACA/121-288	L8GMP5.1 PF05063.12	77.00	0.00
TRINITY_A8HP58_CHLRE/36-138	A8HP58.1 PF00085.18	77.00	0.00
TRINITY_F4PTS1_DICFS/797-935	F4PTS1.1 PF00271.29	77.00	0.00
TRINITY_I0Z2P7_9CHLO/20-284	I0Z2P7.1 PF00268.19	77.00	0.00
TRINITY_A8JCJ9_CHLRE/432-566	A8JCJ9.1 PF00005.25	77.00	0.00
TRINITY_A8J286_CHLRE/100-222	A8J286.1 PF00293.26	77.00	0.00
TRINITY_A8IWQ7_CHLRE/13-138	A8IWQ7.1 PF01641.16	77.00	0.00
TRINITY_D8TNU1_VOLCA/13-146	D8TNU1.1 PF04061.12	77.00	0.00
TRINITY_I0YLK3_9CHLO/155-290	I0YLK3.1 PF00549.17	77.00	0.00
TRINITY_D8U6M8_VOLCA/25-206	D8U6M8.1 PF01105.22	77.00	0.00
TRINITY_A8HWK6_CHLRE/1-239	A8HWK6.1 PF03029.15	77.00	0.00
TRINITY_A0A077ZY60_STYLE/6352-6887	A0A077ZY60.1 PF0089	77.00	0.00
TRINITY_D8UIG4_VOLCA/308-609	D8UIG4.1 PF10637.7;	77.00	0.00
TRINITY_I0Z5Q6_9CHLO/192-252	I0Z5Q6.1 PF03987.13	77.00	0.00
TRINITY_A8J1B8_CHLRE/7-119	A8J1B8.1 PF01412.16	77.00	0.00
TRINITY_D8U5C4_VOLCA/126-200	D8U5C4.1 PF00400.30	77.00	0.00
TRINITY_E1ZGX8_CHLVA/47-144	E1ZGX8.1 PF13649.4;	77.00	0.00
TRINITY_I7ML77_TETTS/37-532	I7ML77.2 PF00118.22	77.00	0.00
TRINITY_B6AFJ7_CRYMR/406-632	B6AFJ7.1 PF00587.23	76.90	0.00
TRINITY_A0A078AVA8_STYLE/23-100	A0A078AVA8.1 PF0142	76.90	0.00
TRINITY_A0A0N0VDQ6_9TRYP/66-95	A0A0N0VDQ6.1 PF0039	76.90	0.00
TRINITY_Q22UW8_TETTS/5-177	Q22UW8.2 PF00025.19	76.90	0.00
TRINITY_G0QX06_ICHMG/22-142	G0QX06.1 PF00238.17	76.90	0.00
TRINITY_G0QIW1_ICHMG/26-90	G0QIW1.1 PF13499.4;	76.90	0.00
TRINITY_G0R0B5_ICHMG/45-346	G0R0B5.1 PF03133.13	76.90	0.00
TRINITY_Q240K0_TETTS/222-351	Q240K0.1 PF00004.27	76.90	0.00
TRINITY_K1R1B5_CRAGI/204-242	K1R1B5.1 PF00400.30	76.90	0.00
TRINITY_A0BZY6_PARTE/125-176	A0BZY6.1 PF01805.18	76.90	0.00
TRINITY_D8U4H3_VOLCA/13-347	D8U4H3.1 PF00225.21	76.90	0.00
TRINITY_A0A0Q9WFX2_DROVI/329-367	A0A0Q9WFX2.1 PF0040	76.90	0.00
TRINITY_Q6DN05_CHLRE/283-659	Q6DN05.1 PF11899.6;	76.90	0.00
TRINITY_A8IZW5_CHLRE/26-133	A8IZW5.1 PF00638.16	76.90	0.00
TRINITY_D8TS39_VOLCA/55-405	D8TS39.1 PF00266.17	76.90	0.00
TRINITY_D8TPF5_VOLCA/5-177	D8TPF5.1 PF00025.19	76.90	0.00
TRINITY_I0Z2K1_9CHLO/72-136	I0Z2K1.1 PF00462.22	76.90	0.00
TRINITY_G0QQR7_ICHMG/565-788	G0QQR7.1 PF00149.26	76.90	0.00
TRINITY_A8IV98_CHLRE/12-185	A8IV98.1 PF00270.27	76.90	0.00
TRINITY_I1E6H0_AMPQE/52-122	I1E6H0.1 PF00076.20	76.90	0.00
TRINITY_A0A058ZDS7_9EUKA/266-414	A0A058ZDS7.1 PF0044	76.90	0.00
TRINITY_A8HNX7_CHLRE/110-174	A8HNX7.1 PF13499.4;	76.90	0.00

TRINITY_Q84UB2_CHLRE/13-201	Q84UB2.1 PF00117.26	76.90	0.00
TRINITY_D8U9X3_VOLCA/116-609	D8U9X3.1 PF00067.20	76.90	0.00
TRINITY_D8UF52_VOLCA/60-419	D8UF52.1 PF02274.15	76.90	0.00
TRINITY_I0YR35_9CHLO/282-398	I0YR35.1 PF02373.20	76.90	0.00
TRINITY_D8TQ16_VOLCA/99-254	D8TQ16.1 PF14566.4;	76.90	0.00
TRINITY_E1ZQQ3_CHLVA/47-298	E1ZQQ3.1 PF00109.24	76.90	0.00
TRINITY_A0A078AVA8_STYLE/23-100	A0A078AVA8.1 PF0142	76.90	0.00
TRINITY_Q230X9_TETTS/859-1272	Q230X9.2 PF08393.11	76.80	0.00
TRINITY_L8GNF0_ACACA/4-155	L8GNF0.1 PF00160.19	76.80	0.00
TRINITY_D8T1I5_SELML/13-265	D8T1I5.1 PF07714.15	76.80	0.00
TRINITY_Q9FNS5_CHLRE/222-394	Q9FNS5.2 PF02866.16	76.80	0.00
TRINITY_A0A0D2S7C8_GOSRA/11-92	A0A0D2S7C8.1 PF0119	76.80	0.00
TRINITY_A8HWY9_CHLRE/4-85	A8HWY9.1 PF00076.20	76.80	0.00
TRINITY_V9FDA4_PHYPR/18-99	V9FDA4.1 PF15511.4;	76.80	0.00
TRINITY_A8IJE8_CHLRE/219-330	A8IJE8.1 PF00271.29	76.80	0.00
TRINITY_A0C4G5_PARTE/13-301	A0C4G5.1 PF00069.23	76.80	0.00
TRINITY_A8IX41_CHLRE/50-316	A8IX41.1 PF00348.15	76.80	0.00
TRINITY_A0A095ET61_CRYGA/8-69	A0A095ET61.1 PF0120	76.80	0.00
TRINITY_D8TZS1_VOLCA/168-450	D8TZS1.1 PF00928.19	76.80	0.00
TRINITY_D8UI61_VOLCA/124-252	D8UI61.1 PF01926.21	76.80	0.00
TRINITY_Q8GV23_CHLRE/172-240	Q8GV23.2 PF00076.20	76.80	0.00
TRINITY_D8TIC7_VOLCA/60-159	D8TIC7.1 PF01230.21	76.80	0.00
TRINITY_T1FMH1_HELRO/101-156	T1FMH1.1 PF00288.24	76.80	0.00
TRINITY_L8H292_ACACA/97-234	L8H292.1 PF03947.16	76.80	0.00
TRINITY_G0R1B4_ICHMG/8-259	G0R1B4.1 PF00069.23	76.80	0.00
TRINITY_G0QUB8_ICHMG/510-575	G0QUB8.1 PF00659.16	76.80	0.00
TRINITY_D8UKJ5_VOLCA/169-433	D8UKJ5.1 PF00069.23	76.80	0.00
TRINITY_D8TRG5_VOLCA/311-495	D8TRG5.1 PF02737.16	76.80	0.00
TRINITY_A9TV53_PHYPA/249-398	A9TV53.1 PF00005.25	76.80	0.00
TRINITY_A0A078ATE7_STYLE/90-132	A0A078ATE7.1 PF0184	76.70	0.00
TRINITY_Q8HQM8_LEPOC/14-224	Q8HQM8.1 PF00119.18	76.70	0.00
TRINITY_G3NMP4_GASAC/39-401	G3NMP4.1 PF00079.18	76.70	0.00
TRINITY_A0A0D3F687_9ORYZ/19-78	A0A0D3F687.1 PF0013	76.70	0.00
TRINITY_I7MMG7_TETTS/4-265	I7MMG7.1 PF00069.23	76.70	0.00
TRINITY_D8U5Y2_VOLCA/2458-2558	D8U5Y2.1 PF07728.12	76.70	0.00
TRINITY_A8IQE3_CHLRE/44-120	A8IQE3.1 PF01929.15	76.70	0.00
TRINITY_G1R190_NOMLE/177-520	G1R190.1 PF12631.5;	76.70	0.00
TRINITY_T0R7J0_9STRA/183-466	T0R7J0.1 PF00303.17	76.70	0.00
TRINITY_D8UIB6_VOLCA/245-417	D8UIB6.1 PF00160.19	76.70	0.00
TRINITY_A8J805_CHLRE/1-86	A8J805.1 PF03871.12	76.70	0.00
TRINITY_A8J9T6_CHLRE/99-204	A8J9T6.1 PF01903.15	76.70	0.00
TRINITY_L8GTU7_ACACA/11-186	L8GTU7.1 PF01725.14	76.70	0.00
TRINITY_I0Z594_9CHLO/787-1217	I0Z594.1 PF02514.14	76.70	0.00
TRINITY_D8U4H1_VOLCA/23-228	D8U4H1.1 PF01926.21	76.70	0.00
TRINITY_U9T7M3_RHIID/51-209	U9T7M3.1 PF00441.22	76.70	0.00
TRINITY_A8IRP4_CHLRE/21-110	A8IRP4.1 PF01221.16	76.70	0.00
TRINITY_A0A0G4FEP8_9ALVE/497-648	A0A0G4FEP8.1 PF0016	76.70	0.00
TRINITY_D8TR38_VOLCA/281-452	D8TR38.1 PF13535.4;	76.70	0.00
TRINITY_D8TUT6_VOLCA/541-613	D8TUT6.1 PF04818.11	76.70	0.00
TRINITY_D8TY83_VOLCA/1-61	D8TY83.1 PF01809.16	76.70	0.00
TRINITY_E1ZDA6_CHLVA/103-407	E1ZDA6.1 PF13249.4;	76.70	0.00
TRINITY_D8U3I9_VOLCA/67-225	D8U3I9.1 PF00005.25	76.70	0.00
TRINITY_E1ZG99_CHLVA/288-324	E1ZG99.1 PF00400.30	76.70	0.00
TRINITY_D8U8H2_VOLCA/795-1749	D8U8H2.1 PF02514.14	76.70	0.00
TRINITY_A8JES3_CHLRE/59-192	A8JES3.1 PF01565.21	76.70	0.00
TRINITY_A8HYY7_CHLRE/43-300	A8HYY7.1 PF00069.23	76.70	0.00
TRINITY_D8U4V3_VOLCA/737-1265	D8U4V3.1 PF02738.16	76.70	0.00
TRINITY_A0BII3_PARTE/8-68	A0BII3.1 PF01200.16	76.70	0.00
TRINITY_G0QZY1_ICHMG/82-282	G0QZY1.1 PF00149.26	76.60	0.00
TRINITY_A8HUE0_CHLRE/4-204	A8HUE0.1 PF01912.16	76.60	0.00
TRINITY_I3J4H8_ORENI/18-261	I3J4H8.1 PF00089.24	76.60	0.00

TRINITY_A8J599_CHLRE/1-258	A8J599.1 PF00290.18	76.60	0.00
TRINITY_E5SFT9_TRISP/12-88	E5SFT9.1 PF00753.25	76.60	0.00
TRINITY_A8ILX2_CHLRE/10-178	A8ILX2.1 PF00071.20	76.60	0.00
TRINITY_A8IWK9_CHLRE/41-219	A8IWK9.1 PF02779.22	76.60	0.00
TRINITY_D8TLU9_VOLCA/60-150	D8TLU9.1 PF02575.14	76.60	0.00
TRINITY_D8TWC4_VOLCA/5-141	D8TWC4.1 PF00334.17	76.60	0.00
TRINITY_Q22BJ8_TETTS/71-135	Q22BJ8.1 PF02319.18	76.60	0.00
TRINITY_D8TIX5_VOLCA/233-373	D8TIX5.1 PF00334.17	76.60	0.00
TRINITY_D8TIG9_VOLCA/366-536	D8TIG9.1 PF00069.23	76.60	0.00
TRINITY_Q6UPR4_CHLRE/11-266	Q6UPR4.1 PF00069.23	76.60	0.00
TRINITY_L8GGD8_ACACA/60-491	L8GGD8.1 PF00330.18	76.60	0.00
TRINITY_I7MD55_TETTS/531-788	I7MD55.2 PF00069.23	76.60	0.00
TRINITY_A8IQ05_CHLRE/30-238	A8IQ05.1 PF08442.8;	76.60	0.00
TRINITY_K8Z0E9_9STRA/3-376	K8Z0E9.1 PF00022.17	76.60	0.00
TRINITY_A8I9T2_CHLRE/11-335	A8I9T2.1 PF00225.21	76.60	0.00
TRINITY_A0CNJ8_PARTE/131-199	A0CNJ8.1 PF01106.15	76.50	0.00
TRINITY_A0CNJ8_PARTE/131-199	A0CNJ8.1 PF01106.15	76.50	0.00
TRINITY_G0QSG1_ICHMG/5-55	G0QSG1.1 PF00253.19	76.50	0.00
TRINITY_A0A0N1J4Z6_9TRYP/9-151	A0A0N1J4Z6.1 PF0005	76.50	0.00
TRINITY_A4RUI2_OSTLU/224-344	A4RUI2.1 PF08443.9;	76.50	0.00
TRINITY_D8U6J5_VOLCA/46-261	D8U6J5.1 PF07722.11	76.50	0.00
TRINITY_A8J4P1_CHLRE/462-577	A8J4P1.1 PF01077.20	76.50	0.00
TRINITY_D8U306_VOLCA/17-305	D8U306.1 PF03151.14	76.50	0.00
TRINITY_D8TP48_VOLCA/38-177	D8TP48.1 PF00504.19	76.50	0.00
TRINITY_D8UEE2_VOLCA/1087-1274	D8UEE2.1 PF00211.18	76.50	0.00
TRINITY_D8U166_VOLCA/32-148	D8U166.1 PF00583.23	76.50	0.00
TRINITY_I7M985_TETTS/75-236	I7M985.2 PF00071.20	76.50	0.00
TRINITY_D8UAR0_VOLCA/58-357	D8UAR0.1 PF00676.18	76.50	0.00
TRINITY_I4VNC1_9GAMM/20-121	I4VNC1.1 PF02678.14	76.50	0.00
TRINITY_I0Z7U0_9CHLO/75-417	I0Z7U0.1 PF00155.19	76.50	0.00
TRINITY_A0A059LFB7_9CHLO/326-378	A0A059LFB7.1 PF0084	76.50	0.00
TRINITY_D8TRA0_VOLCA/161-328	D8TRA0.1 PF02882.17	76.50	0.00
TRINITY_D8TYL0_VOLCA/24-429	D8TYL0.1 PF00815.18	76.50	0.00
TRINITY_M2XNT1_GALSU/32-204	M2XNT1.1 PF00227.24	76.50	0.00
TRINITY_D8U638_VOLCA/106-284	D8U638.1 PF01163.20	76.50	0.00
TRINITY_D8U877_VOLCA/429-566	D8U877.1 PF03133.13	76.50	0.00
TRINITY_E1ZGF1_CHLVA/201-497	E1ZGF1.1 PF02781.14	76.50	0.00
TRINITY_D8TPX3_VOLCA/433-615	D8TPX3.1 PF01369.18	76.50	0.00
TRINITY_A0A0D2R821_GOSRA/25-263	A0A0D2R821.1 PF0150	76.50	0.00
TRINITY_A0D4P4_PARTE/26-312	A0D4P4.1 PF01256.15	76.50	0.00
TRINITY_I3K6P5_ORENI/355-431	I3K6P5.1 PF14478.4;	76.40	0.00
TRINITY_F7IT74_CALJA/2-372	F7IT74.1 PF00022.17	76.40	0.00
TRINITY_R1FLF2_EMIHU/43-137	R1FLF2.1 PF01248.24	76.40	0.00
TRINITY_I7M0D5_TETTS/4-286	I7M0D5.2 PF00069.23	76.40	0.00
TRINITY_D8THD0_VOLCA/31-170	D8THD0.1 PF01743.18	76.40	0.00
TRINITY_A9TJ83_PHYPA/70-219	A9TJ83.1 PF00675.18	76.40	0.00
TRINITY_A8HN23_CHLRE/7-146	A8HN23.1 PF00179.24	76.40	0.00
TRINITY_L8GYL0_ACACA/10-208	L8GYL0.1 PF00106.23	76.40	0.00
TRINITY_F4PJ23_DICFS/87-365	F4PJ23.1 PF00795.20	76.40	0.00
TRINITY_D8U986_VOLCA/307-378	D8U986.1 PF00575.21	76.40	0.00
TRINITY_D8TTM8_VOLCA/232-379	D8TTM8.1 PF04280.13	76.40	0.00
TRINITY_D8TI16_VOLCA/38-358	D8TI16.1 PF00231.17	76.40	0.00
TRINITY_A8IVN2_CHLRE/121-243	A8IVN2.1 PF00254.26	76.40	0.00
TRINITY_A8IW49_CHLRE/70-357	A8IW49.1 PF01326.17	76.40	0.00
TRINITY_D8UBC2_VOLCA/92-232	D8UBC2.1 PF00588.17	76.40	0.00
TRINITY_D8TV35_VOLCA/36-170	D8TV35.1 PF00293.26	76.40	0.00
TRINITY_A8JEP1_CHLRE/46-106	A8JEP1.1 PF01632.17	76.40	0.00
TRINITY_A0A0N5DF24_TRIMR/12-173	A0A0N5DF24.1 PF0007	76.40	0.00
TRINITY_D8TVU0_VOLCA/499-857	D8TVU0.1 PF02896.16	76.40	0.00
TRINITY_A8ISA6_CHLRE/1125-1393	A8ISA6.1 PF12775.5;	76.40	0.00
TRINITY_S9XF47_9CETA/18-125	S9XF47.1 PF02466.17	76.40	0.00

TRINITY_E1ZGU4_CHLVA/29-104	E1ZGU4.1 PF00935.17	76.30	0.00
TRINITY_G5AVB9_HETGA/45-142	G5AVB9.1 PF05300.9;	76.30	0.00
TRINITY_L8HI18_ACACA/1-38	L8HI18.1 PF06220.10	76.30	0.00
TRINITY_A8J2R7_CHLRE/2-241	A8J2R7.1 PF13714.4;	76.30	0.00
TRINITY_D8U3E0_VOLCA/2-238	D8U3E0.1 PF00590.18	76.30	0.00
TRINITY_L8GVB5_ACACA/24-212	L8GVB5.1 PF13869.4;	76.30	0.00
TRINITY_C1E342_MICSR/2-60	C1E342.1 PF13921.4;	76.30	0.00
TRINITY_I1FRI7_AMPQE/5-178	I1FRI7.1 PF00071.20	76.30	0.00
TRINITY_J9EWA0_9SPIT/4-255	J9EWA0.1 PF00069.23	76.30	0.00
TRINITY_W8EXB1_9BACT/43-393	W8EXB1.1 PF00155.19	76.30	0.00
TRINITY_A8I4Q7_CHLRE/4-177	A8I4Q7.1 PF00025.19	76.30	0.00
TRINITY_D8U7W0_VOLCA/188-291	D8U7W0.1 PF08241.10	76.30	0.00
TRINITY_A8HPT4_CHLRE/518-732	A8HPT4.1 PF01434.16	76.30	0.00
TRINITY_A8IS66_CHLRE/157-216	A8IS66.1 PF04117.10	76.30	0.00
TRINITY_E1ZPD8_CHLVA/677-1060	E1ZPD8.1 PF00562.26	76.30	0.00
TRINITY_F0ZMF1_DICPU/9-531	F0ZMF1.1 PF01602.18	76.30	0.00
TRINITY_A8ITU7_CHLRE/41-352	A8ITU7.1 PF00389.28	76.30	0.00
TRINITY_A0CWH0_PARTE/374-647	A0CWH0.1 PF12780.5;	76.30	0.00
TRINITY_D8TRK3_VOLCA/46-386	D8TRK3.1 PF01208.15	76.30	0.00
TRINITY_D8U934_VOLCA/3-96	D8U934.1 PF03501.13	76.30	0.00
TRINITY_D7TZR0_VITVI/54-112	D7TZR0.1 PF12678.5;	76.30	0.00
TRINITY_D7TZR0_VITVI/54-112	D7TZR0.1 PF12678.5;	76.30	0.00
TRINITY_A8JFT4_CHLRE/24-412	A8JFT4.1 PF00162.17	76.30	0.00
TRINITY_D8TIT4_VOLCA/17-171	D8TIT4.1 PF14580.4;	76.30	0.00
TRINITY_A0A0G4EX02_9ALVE/207-341	A0A0G4EX02.1 PF0033	76.30	0.00
TRINITY_B4GHX2_DROPE/12-232	B4GHX2.1 PF00244.18	76.30	0.00
TRINITY_D8UKL0_VOLCA/70-414	D8UKL0.1 PF02374.13	76.30	0.00
TRINITY_A8IND8_CHLRE/519-556	A8IND8.1 PF00400.30	76.30	0.00
TRINITY_W0SBT4_9RHOO/9-264	W0SBT4.1 PF00378.18	76.30	0.00
TRINITY_E1ZPG6_CHLVA/203-291	E1ZPG6.1 PF05706.10	76.30	0.00
TRINITY_M0ZX54_SOLTU/141-203	M0ZX54.1 PF00403.24	76.20	0.00
TRINITY_D8TXY7_VOLCA/1011-1403	D8TXY7.1 PF00176.21	76.20	0.00
TRINITY_M2XL41_GALSU/5-130	M2XL41.1 PF00410.17	76.20	0.00
TRINITY_C3Y8A7_BRAFL/261-303	C3Y8A7.1 PF00400.30	76.20	0.00
TRINITY_Q234J0_TETTS/316-420	Q234J0.2 PF00271.29	76.20	0.00
TRINITY_L8GV49_ACACA/58-474	L8GV49.1 PF13520.4;	76.20	0.00
TRINITY_B2WIM4_PYRTR/6-216	B2WIM4.1 PF00091.23	76.20	0.00
TRINITY_D8UCZ9_VOLCA/61-318	D8UCZ9.1 PF00795.20	76.20	0.00
TRINITY_A8JJ39_CHLRE/244-948	A8JJ39.1 PF03028.13	76.20	0.00
TRINITY_A8JBN7_CHLRE/43-209	A8JBN7.1 PF00270.27	76.20	0.00
TRINITY_D8UEF3_VOLCA/334-375	D8UEF3.1 PF13445.4;	76.20	0.00
TRINITY_L8GRZ9_ACACA/142-435	L8GRZ9.1 PF00113.20	76.20	0.00
TRINITY_F1A2W8_DICPU/72-134	F1A2W8.1 PF01485.19	76.20	0.00
TRINITY_A8I2G3_CHLRE/18-202	A8I2G3.1 PF04427.16	76.20	0.00
TRINITY_A8IA89_CHLRE/65-459	A8IA89.1 PF00815.18	76.20	0.00
TRINITY_A8JCV0_CHLRE/49-298	A8JCV0.1 PF01406.17	76.20	0.00
TRINITY_A8JGD1_CHLRE/214-448	A8JGD1.1 PF00208.19	76.20	0.00
TRINITY_A0A078AD32_STYLE/224-475	A0A078AD32.1 PF0006	76.20	0.00
TRINITY_A8HQ52_CHLRE/508-594	A8HQ52.1 PF14874.4;	76.20	0.00
TRINITY_I7MK75_TETTS/365-471	I7MK75.1 PF01926.21	76.20	0.00
TRINITY_D8U4M5_VOLCA/265-345	D8U4M5.1 PF08544.11	76.20	0.00
TRINITY_E1AXT4_SOLTU/503-586	E1AXT4.1 PF08244.10	76.20	0.00
TRINITY_Q7G6A5_ORYSJ/539-699	Q7G6A5.1 PF00078.25	76.20	0.00
TRINITY_D8UEQ7_VOLCA/74-205	D8UEQ7.1 PF00430.16	76.20	0.00
TRINITY_D8U7H4_VOLCA/6-301	D8U7H4.1 PF02219.15	76.20	0.00
TRINITY_A8I263_CHLRE/54-421	A8I263.1 PF00155.19	76.20	0.00
TRINITY_D8TGV1_VOLCA/625-668	D8TGV1.1 PF06203.12	76.20	0.00
TRINITY_D8U4B1_VOLCA/1-175	D8U4B1.1 PF03657.11	76.20	0.00
TRINITY_D8TU13_VOLCA/649-861	D8TU13.1 PF04192.10	76.20	0.00
TRINITY_A0DSX8_PARTE/29-200	A0DSX8.1 PF03637.15	76.20	0.00
TRINITY_B5DNJ2_DROPS/2-103	B5DNJ2.1 PF00125.22	76.10	0.00

TRINITY_K5VRE2_PHACS/69-290	K5VRE2.1 PF12708.5;	76.10	0.00
TRINITY_A8JFI9_CHLRE/69-288	A8JFI9.1 PF01940.14	76.10	0.00
TRINITY_M4ANS3_XIPMA/340-670	M4ANS3.1 PF00405.15	76.10	0.00
TRINITY_D3BP99_POLPA/174-329	D3BP99.1 PF00903.23	76.10	0.00
TRINITY_A0A015LRD9_9GLOM/3-430	A0A015LRD9.1 PF0522	76.10	0.00
TRINITY_Q23YD7_TETTS/956-1005	Q23YD7.2 PF13920.4;	76.10	0.00
TRINITY_A8I818_CHLRE/8-120	A8I818.1 PF04502.11	76.10	0.00
TRINITY_J9J908_9SPIT/40-295	J9J908.1 PF00069.23	76.10	0.00
TRINITY_A0A0M0J786_9EUKA/14-143	A0A0M0J786.1 PF0069	76.10	0.00
TRINITY_D8TZS1_VOLCA/168-450	D8TZS1.1 PF00928.19	76.10	0.00
TRINITY_D8U6R7_VOLCA/9-163	D8U6R7.1 PF08694.9;	76.10	0.00
TRINITY_A8ING4_CHLRE/129-210	A8ING4.1 PF00173.26	76.10	0.00
TRINITY_E1Z4U0_CHLVA/313-482	E1Z4U0.1 PF00587.23	76.10	0.00
TRINITY_D8M6A4_BLAHO/3-74	D8M6A4.1 PF00240.21	76.10	0.00
TRINITY_A0C4G5_PARTE/13-301	A0C4G5.1 PF00069.23	76.10	0.00
TRINITY_E1Z8Y9_CHLVA/43-348	E1Z8Y9.1 PF00206.18	76.10	0.00
TRINITY_D8TSZ1_VOLCA/11-476	D8TSZ1.1 PF00171.20	76.10	0.00
TRINITY_M1AQS2_SOLTU/173-845	M1AQS2.1 PF00305.17	76.10	0.00
TRINITY_E1ZIK2_CHLVA/502-660	E1ZIK2.1 PF00005.25	76.10	0.00
TRINITY_D8U219_VOLCA/225-500	D8U219.1 PF01031.18	76.10	0.00
TRINITY_Q24IL9_TETTS/1333-1477	Q24IL9.2 PF00005.25	76.10	0.00
TRINITY_A9UP30_MONBE/304-428	A9UP30.1 PF10155.7;	76.00	0.00
TRINITY_D8UGT4_VOLCA/12-87	D8UGT4.1 PF02656.13	76.00	0.00
TRINITY_D8TSY5_VOLCA/2523-3232	D8TSY5.1 PF03028.13	76.00	0.00
TRINITY_Q6UP32_CHLRE/19-136	Q6UP32.1 PF06212.10	76.00	0.00
TRINITY_G0QS48_ICHMG/679-1134	G0QS48.1 PF00521.18	76.00	0.00
TRINITY_A8HQF3_CHLRE/474-628	A8HQF3.1 PF00406.20	76.00	0.00
TRINITY_M4B726_HYAAE/14-415	M4B726.1 PF06068.11	76.00	0.00
TRINITY_F4QCM9_DICFS/35-305	F4QCM9.1 PF06418.12	76.00	0.00
TRINITY_D8TX08_VOLCA/83-228	D8TX08.1 PF00210.22	76.00	0.00
TRINITY_D8TW41_VOLCA/100-374	D8TW41.1 PF08491.8;	76.00	0.00
TRINITY_G1U3X6_RABIT/1-186	G1U3X6.1 PF00149.26	76.00	0.00
TRINITY_A0D8A1_PARTE/9-283	A0D8A1.1 PF00069.23	76.00	0.00
TRINITY_A0A078A7I6_STYLE/615-789	A0A078A7I6.1 PF0027	76.00	0.00
TRINITY_D8TV94_VOLCA/34-289	D8TV94.1 PF00069.23	76.00	0.00
TRINITY_D8UFG9_VOLCA/1-363	D8UFG9.1 PF01053.18	76.00	0.00
TRINITY_D8TKP3_VOLCA/332-655	D8TKP3.1 PF00493.21	76.00	0.00
TRINITY_CLH2_ORYSJ/1288-1433	Q2QYW2.1 PF00637.18	76.00	0.00
TRINITY_A0A0M0JVC1_9EUKA/11-89	A0A0M0JVC1.1 PF0142	75.90	0.00
TRINITY_A0A078BCR6_STYLE/356-465	A0A078BCR6.1 PF0027	75.90	0.00
TRINITY_C0ZND5_RHOE4/8-137	C0ZND5.1 PF07080.9;	75.90	0.00
TRINITY_A0DJ53_PARTE/197-334	A0DJ53.1 PF00004.27	75.90	0.00
TRINITY_A0EGT3_PARTE/436-493	A0EGT3.1 PF12678.5;	75.90	0.00
TRINITY_D8TSZ5_VOLCA/22-132	D8TSZ5.1 PF03099.17	75.90	0.00
TRINITY_D8TRG6_VOLCA/25-136	D8TRG6.1 PF02771.14	75.90	0.00
TRINITY_A8IYS1_CHLRE/103-187	A8IYS1.1 PF03948.12	75.90	0.00
TRINITY_I0YZ68_9CHLO/57-510	I0YZ68.1 PF00067.20	75.90	0.00
TRINITY_D8U8F2_VOLCA/90-504	D8U8F2.1 PF08393.11	75.90	0.00
TRINITY_J9IGQ2_9SPIT/61-567	J9IGQ2.1 PF00330.18	75.90	0.00
TRINITY_D8U1Y7_VOLCA/234-391	D8U1Y7.1 PF04432.11	75.90	0.00
TRINITY_A8ITV3_CHLRE/37-177	A8ITV3.1 PF00504.19	75.90	0.00
TRINITY_A8I2Y6_CHLRE/11-265	A8I2Y6.1 PF00069.23	75.90	0.00
TRINITY_D8U3R3_VOLCA/60-256	D8U3R3.1 PF00149.26	75.90	0.00
TRINITY_Q2HZ24_CHLRE/64-143	Q2HZ24.1 PF00111.25	75.90	0.00
TRINITY_A0A075ASD4_9FUNG/87-203	A0A075ASD4.1 PF0150	75.90	0.00
TRINITY_A8HXL0_CHLRE/18-320	A8HXL0.1 PF08449.9;	75.90	0.00
TRINITY_D8TRA1_VOLCA/2-140	D8TRA1.1 PF05903.12	75.90	0.00
TRINITY_D8U988_VOLCA/273-500	D8U988.1 PF04172.14	75.90	0.00
TRINITY_A0A0D2VXG2_CAPO3/659-863	A0A0D2VXG2.1 PF0278	75.90	0.00
TRINITY_G0R4M5_ICHMG/51-307	G0R4M5.1 PF00481.19	75.90	0.00
TRINITY_D8RNT4_SELML/251-418	D8RNT4.1 PF00009.25	75.90	0.00

TRINITY_H2RPI2_TAKRU/18-127	H2RPI2.1 PF00059.19	75.90	0.00
TRINITY_Q229D4_TETTS/255-368	Q229D4.2 PF00128.22	75.80	0.00
TRINITY_A7SI02_NEMVE/42-268	A7SI02.1 PF04670.10	75.80	0.00
TRINITY_D3AXY2_POLPA/180-413	D3AXY2.1 PF01702.16	75.80	0.00
TRINITY_I7MGP0_TETTS/37-294	I7MGP0.2 PF00069.23	75.80	0.00
TRINITY_F1A0B5_DICPU/3-610	F1A0B5.1 PF00012.18	75.80	0.00
TRINITY_F4NU28_BATDJ/37-541	F4NU28.1 PF12070.6;	75.80	0.00
TRINITY_A0BIC4_PARTE/12-166	A0BIC4.1 PF00252.16	75.80	0.00
TRINITY_I0Z468_9CHLO/23-220	I0Z468.1 PF13460.4;	75.80	0.00
TRINITY_F4QED8_DICFS/130-220	F4QED8.1 PF13640.4;	75.80	0.00
TRINITY_H2T8S7_TAKRU/1125-1273	H2T8S7.1 PF00005.25	75.80	0.00
TRINITY_I7M4M7_TETTS/184-245	I7M4M7.1 PF00333.18	75.80	0.00
TRINITY_A8J3Y2_CHLRE/3-98	A8J3Y2.1 PF01588.18	75.80	0.00
TRINITY_C1MT80_MICPC/15-105	C1MT80.1 PF00254.26	75.80	0.00
TRINITY_D8TWF1_VOLCA/136-314	D8TWF1.1 PF00953.19	75.80	0.00
TRINITY_A0CVB3_PARTE/54-148	A0CVB3.1 PF01248.24	75.80	0.00
TRINITY_D8U5S6_VOLCA/155-429	D8U5S6.1 PF00487.22	75.80	0.00
TRINITY_G0QR65_ICHMG/31-179	G0QR65.1 PF00056.21	75.80	0.00
TRINITY_A8IN92_CHLRE/84-145	A8IN92.1 PF02297.15	75.80	0.00
TRINITY_A9TAW8_PHYPA/108-286	A9TAW8.1 PF02390.15	75.80	0.00
TRINITY_G0QMQ9_ICHMG/30-285	G0QMQ9.1 PF00069.23	75.80	0.00
TRINITY_I7M1K4_TETTS/571-636	I7M1K4.1 PF00659.16	75.80	0.00
TRINITY_A8JGJ4_CHLRE/324-589	A8JGJ4.1 PF00069.23	75.80	0.00
TRINITY_A8IYM0_CHLRE/156-461	A8IYM0.1 PF00365.18	75.80	0.00
TRINITY_D8TUH9_VOLCA/43-166	D8TUH9.1 PF00782.18	75.80	0.00
TRINITY_D8TKZ3_VOLCA/45-224	D8TKZ3.1 PF01300.16	75.80	0.00
TRINITY_E1ZCY8_CHLVA/152-300	E1ZCY8.1 PF00004.27	75.80	0.00
TRINITY_G3P746_GASAC/463-714	G3P746.1 PF00089.24	75.70	0.00
TRINITY_I7LZU3_TETTS/215-299	I7LZU3.1 PF04433.15	75.70	0.00
TRINITY_A0A077ZRC7_STYLE/176-249	A0A077ZRC7.1 PF0038	75.70	0.00
TRINITY_L8HE18_ACACA/7-180	L8HE18.1 PF00071.20	75.70	0.00
TRINITY_B7P363_IXOSC/6-160	B7P363.1 PF08694.9;	75.70	0.00
TRINITY_K3XF99_SETIT/554-612	K3XF99.1 PF08492.10	75.70	0.00
TRINITY_A8IUV7_CHLRE/7-197	A8IUV7.1 PF01294.16	75.70	0.00
TRINITY_A4XEZ9_NOVAD/8-304	A4XEZ9.1 PF00732.17	75.70	0.00
TRINITY_A8HUW4_CHLRE/288-362	A8HUW4.1 PF00454.25	75.70	0.00
TRINITY_G0QW25_ICHMG/9-148	G0QW25.1 PF00179.24	75.70	0.00
TRINITY_V4SC53_9ROSI/29-634	V4SC53.1 PF00012.18	75.70	0.00
TRINITY_I1FRI7_AMPQE/5-178	I1FRI7.1 PF00071.20	75.70	0.00
TRINITY_D8TX39_VOLCA/119-281	D8TX39.1 PF04055.19	75.70	0.00
TRINITY_D8UJC6_VOLCA/308-381	D8UJC6.1 PF14360.4;	75.70	0.00
TRINITY_E1ZFZ0_CHLVA/25-169	E1ZFZ0.1 PF00903.23	75.70	0.00
TRINITY_HELCL_DICDI/553-735	Q55CI8.1 PF00270.27	75.70	0.00
TRINITY_D8TXJ3_VOLCA/100-289	D8TXJ3.1 PF04427.16	75.70	0.00
TRINITY_A8J3F9_CHLRE/2-235	A8J3F9.1 PF00483.21	75.70	0.00
TRINITY_A0A0E0M8B7_ORYPU/856-992	A0A0E0M8B7.1 PF0772	75.70	0.00
TRINITY_I0YM50_9CHLO/67-252	I0YM50.1 PF00350.21	75.70	0.00
TRINITY_A8J921_CHLRE/1156-1265	A8J921.1 PF04679.13	75.70	0.00
TRINITY_E1ZSN6_CHLVA/219-424	E1ZSN6.1 PF01189.15	75.70	0.00
TRINITY_T1J8J3_STRMM/220-397	T1J8J3.1 PF16114.3;	75.70	0.00
TRINITY_A8JCR6_CHLRE/402-475	A8JCR6.1 PF10369.7;	75.70	0.00
TRINITY_A0A0A0K487_CUCSA/26-486	A0A0A0K487.1 PF0008	75.70	0.00
TRINITY_H1YIQ7_9SPHI/5-125	H1YIQ7.1 PF01678.17	75.70	0.00
TRINITY_G6FQG6_9CYAN/32-122	G6FQG6.1 PF04828.12	75.60	0.00
TRINITY_A0A059ACX2_EUCGR/217-885	A0A059ACX2.1 PF0030	75.60	0.00
TRINITY_H9G6B0_ANOCA/135-193	H9G6B0.2 PF13921.4;	75.60	0.00
TRINITY_I1KVB3_SOYBN/319-519	I1KVB3.2 PF03441.12	75.60	0.00
TRINITY_Q23U84_TETTS/23-120	Q23U84.1 PF02678.14	75.60	0.00
TRINITY_I1G7J1_AMPQE/19-190	I1G7J1.1 PF00535.24	75.60	0.00
TRINITY_A0EGX1_PARTE/32-77	A0EGX1.1 PF00249.29	75.60	0.00
TRINITY_F4PX79_DICFS/6-241	F4PX79.1 PF01115.15	75.60	0.00

TRINITY_W2Q1K5_PHYPN/251-447	W2Q1K5.1 PF00149.26	75.60	0.00
TRINITY_I7LVE7_TETTS/507-549	I7LVE7.1 PF06203.12	75.60	0.00
TRINITY_D8UIX5_VOLCA/682-891	D8UIX5.1 PF05362.11	75.60	0.00
TRINITY_D8U1A7_VOLCA/64-292	D8U1A7.1 PF01063.17	75.60	0.00
TRINITY_A9UV86_MONBE/39-341	A9UV86.1 PF01916.15	75.60	0.00
TRINITY_D8TKX0_VOLCA/10-138	D8TKX0.1 PF00179.24	75.60	0.00
TRINITY_D8U0I3_VOLCA/1-208	D8U0I3.1 PF01591.16	75.60	0.00
TRINITY_A8HPW5_CHLRE/282-345	A8HPW5.1 PF13432.4;	75.60	0.00
TRINITY_A8J5W8_CHLRE/385-462	A8J5W8.1 PF00533.24	75.60	0.00
TRINITY_E1ZKB0_CHLVA/3-85	E1ZKB0.1 PF12861.5;	75.60	0.00
TRINITY_D8U697_VOLCA/106-335	D8U697.1 PF01798.16	75.60	0.00
TRINITY_D8U4I5_VOLCA/433-551	D8U4I5.1 PF02880.14	75.60	0.00
TRINITY_A0C997_PARTE/746-831	A0C997.1 PF05002.13	75.60	0.00
TRINITY_P93664_CHLRE/45-210	P93664.1 PF00504.19	75.60	0.00
TRINITY_D8UA87_VOLCA/31-116	D8UA87.1 PF14560.4;	75.60	0.00
TRINITY_A9V2B9_MONBE/227-497	A9V2B9.1 PF13598.4;	75.60	0.00
TRINITY_A8HTQ6_CHLRE/420-597	A8HTQ6.1 PF00534.18	75.60	0.00
TRINITY_A8HPZ8_CHLRE/149-557	A8HPZ8.1 PF01432.18	75.60	0.00
TRINITY_A8J9S8_CHLRE/93-225	A8J9S8.1 PF01138.19	75.60	0.00
TRINITY_C1E5T3_MICSR/260-304	C1E5T3.1 PF00432.19	75.60	0.00
TRINITY_A8HVE0_CHLRE/77-289	A8HVE0.1 PF07722.11	75.60	0.00
TRINITY_Q24IL9_TETTS/1333-1477	Q24IL9.2 PF00005.25	75.60	0.00
TRINITY_G0QWM3_ICHMG/44-239	G0QWM3.1 PF00149.26	75.60	0.00
TRINITY_D8U4H3_VOLCA/13-347	D8U4H3.1 PF00225.21	75.60	0.00
TRINITY_Q24HK9_TETTS/22-280	Q24HK9.3 PF00069.23	75.60	0.00
TRINITY_A0A0H4VV80_9BACT/19-171	A0A0H4VV80.1 PF0071	75.50	0.00
TRINITY_D7TIM9_VITVI/17-69	D7TIM9.1 PF09713.8;	75.50	0.00
TRINITY_A0C3G9_PARTE/1-172	A0C3G9.1 PF05063.12	75.50	0.00
TRINITY_A0A0M0JC73_9EUKA/85-238	A0A0M0JC73.1 PF0000	75.50	0.00
TRINITY_A0BX73_PARTE/218-318	A0BX73.1 PF00153.25	75.50	0.00
TRINITY_A8JEL2_CHLRE/7-186	A8JEL2.1 PF01725.14	75.50	0.00
TRINITY_L8HH61_ACACA/28-215	L8HH61.1 PF00227.24	75.50	0.00
TRINITY_F1RYD2_PIG/14-120	F1RYD2.2 PF04538.10	75.50	0.00
TRINITY_D8UAM0_VOLCA/1269-1323	D8UAM0.1 PF04083.14	75.50	0.00
TRINITY_D3B6Z8_POLPA/513-595	D3B6Z8.1 PF13965.4;	75.50	0.00
TRINITY_W1NRE8_AMBTC/73-524	W1NRE8.1 PF01425.19	75.50	0.00
TRINITY_O24451_CHLRE/244-338	O24451.1 PF00153.25	75.50	0.00
TRINITY_A8JH01_CHLRE/228-534	A8JH01.1 PF01968.16	75.50	0.00
TRINITY_A0A087XHI0_POEFO/37-89	A0A087XHI0.1 PF0644	75.50	0.00
TRINITY_A8J875_CHLRE/1-139	A8J875.1 PF00588.17	75.50	0.00
TRINITY_A8ISN1_CHLRE/11-205	A8ISN1.1 PF11539.6;	75.50	0.00
TRINITY_A8J2U2_CHLRE/141-287	A8J2U2.1 PF04884.12	75.50	0.00
TRINITY_C1EBY8_MICSR/14-108	C1EBY8.1 PF00125.22	75.50	0.00
TRINITY_D8TRB6_VOLCA/114-319	D8TRB6.1 PF03741.14	75.50	0.00
TRINITY_E1Z3E2_CHLVA/59-108	E1Z3E2.1 PF13920.4;	75.50	0.00
TRINITY_J9HIS0_9SPIT/571-794	J9HIS0.1 PF00246.22	75.50	0.00
TRINITY_A0A087SEE1_AUXPR/1-254	A0A087SEE1.1 PF0315	75.50	0.00
TRINITY_D8TSV4_VOLCA/211-455	D8TSV4.1 PF04577.12	75.50	0.00
TRINITY_D8TXR9_VOLCA/16-116	D8TXR9.1 PF00153.25	75.50	0.00
TRINITY_A0A0D2VS83_CAPO3/3068-3348	A0A0D2VS83.1 PF0213	75.50	0.00
TRINITY_A8HZ73_CHLRE/59-189	A8HZ73.1 PF00004.27	75.50	0.00
TRINITY_M4BMA1_HYAAE/108-159	M4BMA1.1 PF00400.30	75.50	0.00
TRINITY_J9HVB4_9SPIT/36-297	J9HVB4.1 PF02263.17	75.40	0.00
TRINITY_Q4RZF0_TETNG/46-171	Q4RZF0.1 PF00062.18	75.40	0.00
TRINITY_L8HKH2_ACACA/7-148	L8HKH2.1 PF02792.12	75.40	0.00
TRINITY_F1A4Z4_DICPU/29-207	F1A4Z4.1 PF02779.22	75.40	0.00
TRINITY_G0R0Y0_ICHMG/1-155	G0R0Y0.1 PF04729.11	75.40	0.00
TRINITY_A0A087SNS0_AUXPR/84-161	A0A087SNS0.1 PF0125	75.40	0.00
TRINITY_D8U4U2_VOLCA/17-73	D8U4U2.1 PF00076.20	75.40	0.00
TRINITY_I7MHN4_TETTS/205-383	I7MHN4.2 PF00069.23	75.40	0.00
TRINITY_I0Z3Y1_9CHLO/595-662	I0Z3Y1.1 PF00658.16	75.40	0.00

TRINITY_Q23YJ0_TETTS/28-372	Q23YJ0.1 PF16499.3;	75.40	0.00
TRINITY_A0A0A1NY27_9FUNG/482-615	A0A0A1NY27.1 PF0000	75.40	0.00
TRINITY_Q245E7_TETTS/76-189	Q245E7.2 PF00572.16	75.40	0.00
TRINITY_D8U1L8_VOLCA/197-355	D8U1L8.1 PF02582.12	75.40	0.00
TRINITY_K3WS96_PYTUL/7-71	K3WS96.1 PF01423.20	75.40	0.00
TRINITY_A8J0V0_CHLRE/67-183	A8J0V0.1 PF02033.16	75.40	0.00
TRINITY_A8J6V1_CHLRE/43-381	A8J6V1.1 PF00180.18	75.40	0.00
TRINITY_A8J2A7_CHLRE/355-468	A8J2A7.1 PF08241.10	75.40	0.00
TRINITY_A8HZG4_CHLRE/44-193	A8HZG4.1 PF00650.18	75.40	0.00
TRINITY_G0R207_ICHMG/11-210	G0R207.1 PF00106.23	75.40	0.00
TRINITY_D8U4N3_VOLCA/87-204	D8U4N3.1 PF13640.4;	75.40	0.00
TRINITY_L8H2N3_ACACA/58-547	L8H2N3.1 PF02990.14	75.40	0.00
TRINITY_G0QNL1_ICHMG/140-204	G0QNL1.1 PF02319.18	75.40	0.00
TRINITY_J9IKD3_9SPIT/98-195	J9IKD3.1 PF02678.14	75.40	0.00
TRINITY_I0Z203_9CHLO/625-693	I0Z203.1 PF05066.11	75.40	0.00
TRINITY_D8UI14_VOLCA/133-315	D8UI14.1 PF00390.17	75.40	0.00
TRINITY_A8JI60_CHLRE/78-408	A8JI60.1 PF07992.12	75.40	0.00
TRINITY_A8ID17_CHLRE/21-404	A8ID17.1 PF00225.21	75.40	0.00
TRINITY_A8J1C1_CHLRE/443-962	A8J1C1.1 PF00899.19	75.40	0.00
TRINITY_A0A0D2VU79_GOSRA/16-132	A0A0D2VU79.1 PF0534	75.40	0.00
TRINITY_Q3SD60_PARTE/9-170	Q3SD60.1 PF00071.20	75.30	0.00
TRINITY_A0A078AXW8_STYLE/449-814	A0A078AXW8.1 PF0049	75.30	0.00
TRINITY_A0A0B0NZY7_GOSAR/4-219	A0A0B0NZY7.1 PF0002	75.30	0.00
TRINITY_G7IKP7_MEDTR/296-491	G7IKP7.2 PF10596.7;	75.30	0.00

TRINITY_A7STE6_NEMVE/3-176	A7STE6.1 PF00025.19	75.30	0.00
TRINITY_Q22AX6_TETTS/547-627	Q22AX6.2 PF12895.5;	75.30	0.00
TRINITY_A4S6E6_OSTLU/13-171	A4S6E6.1 PF00071.20	75.30	0.00
TRINITY_A0D8A1_PARTE/9-283	A0D8A1.1 PF00069.23	75.30	0.00
TRINITY_A8IV98_CHLRE/12-185	A8IV98.1 PF00270.27	75.30	0.00
TRINITY_E1ZLA8_CHLVA/543-623	E1ZLA8.1 PF13193.4;	75.30	0.00
TRINITY_D8UEM2_VOLCA/42-134	D8UEM2.1 PF00254.26	75.30	0.00
TRINITY_A8HYL9_CHLRE/331-803	A8HYL9.1 PF00939.17	75.30	0.00
TRINITY_E1ZQN2_CHLVA/1-80	E1ZQN2.1 PF00153.25	75.30	0.00
TRINITY_D8U9S9_VOLCA/599-793	D8U9S9.1 PF00211.18	75.30	0.00
TRINITY_A8HUS6_CHLRE/825-1086	A8HUS6.1 PF00756.18	75.30	0.00
TRINITY_A0A078ALR3_STYLE/13-94	A0A078ALR3.1 PF0119	75.30	0.00
TRINITY_A8JDL5_CHLRE/42-199	A8JDL5.1 PF00160.19	75.30	0.00
TRINITY_I0YYI2_9CHLO/35-113	I0YYI2.1 PF00013.27	75.30	0.00
TRINITY_A8HYM2_CHLRE/1-109	A8HYM2.1 PF01873.15	75.30	0.00
TRINITY_E1ZRS1_CHLVA/4-340	E1ZRS1.1 PF03214.11	75.30	0.00
TRINITY_I0Z5Q1_9CHLO/68-237	I0Z5Q1.1 PF00270.27	75.30	0.00
TRINITY_A8JDX8_CHLRE/570-937	A8JDX8.1 PF00224.19	75.30	0.00
TRINITY_A8ISQ5_CHLRE/2275-2409	A8ISQ5.1 PF00004.27	75.30	0.00
TRINITY_I0Z7K3_9CHLO/694-887	I0Z7K3.1 PF13844.4;	75.30	0.00
TRINITY_D8TV66_VOLCA/112-305	D8TV66.1 PF07910.11	75.30	0.00
TRINITY_D8TJV1_VOLCA/64-213	D8TJV1.1 PF13401.4;	75.30	0.00
TRINITY_A8J063_CHLRE/3352-4065	A8J063.1 PF03028.13	75.30	0.00
TRINITY_D8UEF2_VOLCA/54-237	D8UEF2.1 PF00504.19	75.30	0.00
TRINITY_E9NPS9_9CHLO/22-449	E9NPS9.1 PF00148.17	75.30	0.00
TRINITY_V3ZIU2_LOTGI/248-550	V3ZIU2.1 PF01968.16	75.20	0.00
TRINITY_F7AX64_CALJA/58-219	F7AX64.1 PF00071.20	75.20	0.00
TRINITY_G0QVK1_ICHMG/8-402	G0QVK1.1 PF00180.18	75.20	0.00
TRINITY_H2YYX9_CIOSA/52-184	H2YYX9.1 PF04116.11	75.20	0.00
TRINITY_I7M7Z9_TETTS/616-760	I7M7Z9.1 PF00005.25	75.20	0.00
TRINITY_G0QJ62_ICHMG/5-158	G0QJ62.1 PF08694.9;	75.20	0.00
TRINITY_I7LXC5_TETTS/178-436	I7LXC5.2 PF00069.23	75.20	0.00
TRINITY_C1MJ87_MICPC/531-707	C1MJ87.1 PF13307.4;	75.20	0.00
TRINITY_Q241B6_TETTS/1199-1363	Q241B6.2 PF03031.16	75.20	0.00
TRINITY_A0D7P1_PARTE/26-628	A0D7P1.1 PF00012.18	75.20	0.00
TRINITY_A9RII8_PHYPA/238-353	A9RII8.1 PF00004.27	75.20	0.00
TRINITY_D3B730_POLPA/591-823	D3B730.1 PF01145.23	75.20	0.00
TRINITY_F4PMV2_DICFS/89-458	F4PMV2.1 PF00285.19	75.20	0.00
TRINITY_D8TIW6_VOLCA/23-485	D8TIW6.1 PF00171.20	75.20	0.00
TRINITY_I1NBE6_SOYBN/60-224	I1NBE6.1 PF00270.27	75.20	0.00
TRINITY_A8J3N8_CHLRE/21-579	A8J3N8.1 PF01602.18	75.20	0.00
TRINITY_A8IQM3_CHLRE/52-382	A8IQM3.1 PF03133.13	75.20	0.00
TRINITY_D8TLN4_VOLCA/325-501	D8TLN4.1 PF11931.6;	75.20	0.00
TRINITY_A0A087STB9_AUXPR/309-471	A0A087STB9.1 PF0044	75.20	0.00
TRINITY_A8ISQ8_CHLRE/1-122	A8ISQ8.1 PF00004.27	75.20	0.00
TRINITY_L8H6M4_ACACA/340-668	L8H6M4.1 PF00291.23	75.10	0.00
TRINITY_E1Z5T2_CHLVA/130-322	E1Z5T2.1 PF01071.17	75.10	0.00
TRINITY_A8I469_CHLRE/47-260	A8I469.1 PF03619.14	75.10	0.00
TRINITY_D8TVU1_VOLCA/240-780	D8TVU1.1 PF01411.17	75.10	0.00
TRINITY_A0DIN0_PARTE/9-195	A0DIN0.1 PF03256.14	75.00	0.00
TRINITY_G4NB93_MAGO7/2-131	G4NB93.1 PF00179.24	75.00	0.00
TRINITY_Q22DN2_TETTS/565-703	Q22DN2.1 PF00004.27	75.00	0.00
TRINITY_A0A077Z0G9_TRITR/23-130	A0A077Z0G9.1 PF0058	75.00	0.00
TRINITY_L8HHX5_ACACA/8-250	L8HHX5.1 PF00454.25	75.00	0.00
TRINITY_K1QIK0_CRAGI/470-596	K1QIK0.1 PF00271.29	75.00	0.00
TRINITY_A0A0J8BJB0_BETVU/262-392	A0A0J8BJB0.1 PF0000	75.00	0.00
TRINITY_A0E7M3_PARTE/77-115	A0E7M3.1 PF01096.16	75.00	0.00
TRINITY_I7MCG1_TETTS/113-177	I7MCG1.1 PF13499.4;	75.00	0.00
TRINITY_A9UFY1_VITVI/1-78	A9UFY1.1 PF01439.16	75.00	0.00
TRINITY_F4PSK6_DICFS/2895-3184	F4PSK6.1 PF12780.5;	75.00	0.00
TRINITY_D5GEP6_TUBMM/30-69	D5GEP6.1 PF05920.9;	75.00	0.00

TRINITY_A0CJS0_PARTE/128-311	A0CJS0.1 PF10274.7;	75.00	0.00
TRINITY_D7FRF0_ECTSI/508-545	D7FRF0.1 PF03638.13	75.00	0.00
TRINITY_F0ZEC6_DICPU/169-248	F0ZEC6.1 PF05093.11	75.00	0.00
TRINITY_A0A087SJR8_AUXPR/1-94	A0A087SJR8.1 PF0119	75.00	0.00
TRINITY_A9SBZ9_PHYPA/193-324	A9SBZ9.1 PF00004.27	75.00	0.00
TRINITY_A5DZ68_LODEL/36-147	A5DZ68.1 PF00355.24	75.00	0.00
TRINITY_I0YMB4_9CHLO/135-172	I0YMB4.1 PF01428.14	75.00	0.00
TRINITY_A0A087YN06_POEFO/122-182	A0A087YN06.2 PF1385	75.00	0.00
TRINITY_G7LGQ6_MEDTR/160-317	G7LGQ6.2 PF13481.4;	75.00	0.00
TRINITY_D8UIB3_VOLCA/764-970	D8UIB3.1 PF08214.9;	75.00	0.00
TRINITY_Q241P5_TETTS/77-120	Q241P5.1 PF00432.19	75.00	0.00
TRINITY_A0A059LHG8_9CHLO/1-220	A0A059LHG8.1 PF1356	75.00	0.00
TRINITY_A8J8C0_CHLRE/51-194	A8J8C0.1 PF06799.9;	75.00	0.00
TRINITY_D8UIB7_VOLCA/15-77	D8UIB7.1 PF02953.13	75.00	0.00
TRINITY_Q22C41_TETTS/46-301	Q22C41.2 PF00069.23	75.00	0.00
TRINITY_D8TVP4_VOLCA/24-342	D8TVP4.1 PF00294.22	75.00	0.00
TRINITY_D8TW00_VOLCA/426-561	D8TW00.1 PF00005.25	75.00	0.00
TRINITY_A8HXG5_CHLRE/9-120	A8HXG5.1 PF04725.10	75.00	0.00
TRINITY_A0DM52_PARTE/64-171	A0DM52.1 PF13085.4;	75.00	0.00
TRINITY_Q22AV9_TETTS/108-191	Q22AV9.3 PF00189.18	75.00	0.00
TRINITY_A8I8Z1_CHLRE/75-214	A8I8Z1.1 PF00885.17	75.00	0.00
TRINITY_E1Z3R4_CHLVA/40-76	E1Z3R4.1 PF00301.18	75.00	0.00
TRINITY_D8UDF4_VOLCA/15-102	D8UDF4.1 PF01172.16	75.00	0.00
TRINITY_T1EEY3_HELRO/12-76	T1EEY3.1 PF13499.4;	75.00	0.00
TRINITY_A8JI10_CHLRE/2-116	A8JI10.1 PF01981.14	75.00	0.00
TRINITY_D8TR30_VOLCA/194-314	D8TR30.1 PF03109.14	75.00	0.00
TRINITY_A0CLB3_PARTE/139-190	A0CLB3.1 PF13415.4;	75.00	0.00
TRINITY_D8U1I4_VOLCA/34-292	D8U1I4.1 PF00069.23	75.00	0.00
TRINITY_D8TJ66_VOLCA/196-251	D8TJ66.1 PF00415.16	75.00	0.00
TRINITY_W1NXB9_AMBTC/258-407	W1NXB9.1 PF00441.22	75.00	0.00
TRINITY_A8JCQ6_CHLRE/38-110	A8JCQ6.1 PF05022.10	75.00	0.00
TRINITY_K9T4C4_9CYAN/507-830	K9T4C4.1 PF02896.16	75.00	0.00
TRINITY_A8I1P7_CHLRE/40-217	A8I1P7.1 PF08648.10	75.00	0.00
TRINITY_D8THB0_VOLCA/261-500	D8THB0.1 PF00814.23	75.00	0.00
TRINITY_W5F886_WHEAT/120-167	W5F886.1 PF01466.17	75.00	0.00
TRINITY_D8TY64_VOLCA/74-307	D8TY64.1 PF00270.27	75.00	0.00
TRINITY_D8TZ40_VOLCA/1-297	D8TZ40.1 PF04997.10	75.00	0.00
TRINITY_D8U3B6_VOLCA/1-119	D8U3B6.1 PF06991.9;	75.00	0.00
TRINITY_D8UFR3_VOLCA/25-120	D8UFR3.1 PF01248.24	75.00	0.00
TRINITY_L5KKQ1_PTEAL/2440-3138	L5KKQ1.1 PF03028.13	75.00	0.00
TRINITY_E1Z299_CHLVA/24-373	E1Z299.1 PF04371.13	75.00	0.00
TRINITY_E1ZI46_CHLVA/12-125	E1ZI46.1 PF02338.17	75.00	0.00
TRINITY_A8IHF2_CHLRE/77-270	A8IHF2.1 PF12146.6;	75.00	0.00
TRINITY_A8HNN6_CHLRE/41-220	A8HNN6.1 PF12710.5;	75.00	0.00
TRINITY_D8TZE9_VOLCA/31-143	D8TZE9.1 PF09430.8;	75.00	0.00
TRINITY_D8UJV3_VOLCA/399-442	D8UJV3.1 PF13639.4;	75.00	0.00
TRINITY_A0A0K0D2L7_ANGCA/7-170	A0A0K0D2L7.1 PF0016	75.00	0.00
TRINITY_M1VGQ3_CYAME/65-161	M1VGQ3.1 PF02678.14	75.00	0.00
TRINITY_A0A0E9NLA7_9ASCO/465-503	A0A0E9NLA7.1 PF0040	75.00	0.00
TRINITY_A9TA03_PHYPA/319-420	A9TA03.1 PF13499.4;	75.00	0.00
TRINITY_D8TS74_VOLCA/68-345	D8TS74.1 PF03982.11	75.00	0.00
TRINITY_D8U8W1_VOLCA/276-479	D8U8W1.1 PF01068.19	75.00	0.00
TRINITY_E1ZH40_CHLVA/421-558	E1ZH40.1 PF02910.18	75.00	0.00
TRINITY_A8HYQ1_CHLRE/25-184	A8HYQ1.1 PF01233.17	75.00	0.00
TRINITY_A0BH68_PARTE/9-286	A0BH68.1 PF00069.23	75.00	0.00
TRINITY_Q22DR0_TETTS/17-338	Q22DR0.2 PF00009.25	75.00	0.00
TRINITY_D8U7P6_VOLCA/2512-2779	D8U7P6.1 PF12780.5;	75.00	0.00
TRINITY_D8TM29_VOLCA/67-234	D8TM29.1 PF00504.19	75.00	0.00
TRINITY_D8UF95_VOLCA/53-225	D8UF95.1 PF04055.19	75.00	0.00
TRINITY_D8TIK5_VOLCA/329-452	D8TIK5.1 PF03720.13	75.00	0.00
TRINITY_A0D9S1_PARTE/381-707	A0D9S1.1 PF00176.21	75.00	0.00

TRINITY_J9JC92_9SPIT/14-110	J9JC92.1 PF06294.9;	75.00	0.00
TRINITY_C1KRG0_MICSR/128-417	C1KRG0.1 PF00361.18	75.00	0.00
TRINITY_D2UXF6_NAEGR/45-227	D2UXF6.1 PF00227.24	74.90	0.00
TRINITY_A8IWQ2_CHLRE/123-328	A8IWQ2.1 PF00566.16	74.90	0.00
TRINITY_D7FR97_ECTSI/1-259	D7FR97.1 PF01201.20	74.90	0.00
TRINITY_L8GWF5_ACACA/44-239	L8GWF5.1 PF00149.26	74.90	0.00
TRINITY_Q22T35_TETTS/4-288	Q22T35.2 PF00069.23	74.90	0.00
TRINITY_Q22A40_TETTS/44-239	Q22A40.1 PF00149.26	74.90	0.00
TRINITY_Q22C41_TETTS/46-301	Q22C41.2 PF00069.23	74.90	0.00
TRINITY_D8U8C2_VOLCA/29-358	D8U8C2.1 PF07992.12	74.90	0.00
TRINITY_A8JGX1_CHLRE/153-390	A8JGX1.1 PF01702.16	74.90	0.00
TRINITY_D8U5N9_VOLCA/157-326	D8U5N9.1 PF00005.25	74.90	0.00
TRINITY_A8HZZ4_CHLRE/109-509	A8HZZ4.1 PF00155.19	74.90	0.00
TRINITY_D8TT90_VOLCA/105-315	D8TT90.1 PF02096.18	74.90	0.00
TRINITY_U4U808_DENPD/10-213	U4U808.1 PF00106.23	74.80	0.00
TRINITY_Q229U9_TETTS/47-305	Q229U9.2 PF00069.23	74.80	0.00
TRINITY_D8TNY1_VOLCA/72-191	D8TNY1.1 PF00622.26	74.80	0.00
TRINITY_D8UB59_VOLCA/11-326	D8UB59.1 PF03151.14	74.80	0.00
TRINITY_I3KFH3_ORENI/512-716	I3KFH3.1 PF02786.15	74.80	0.00
TRINITY_D8TXZ1_VOLCA/127-249	D8TXZ1.1 PF07541.10	74.80	0.00
TRINITY_D8TQS7_VOLCA/109-309	D8TQS7.1 PF01812.18	74.80	0.00
TRINITY_D8U428_VOLCA/222-371	D8U428.1 PF00005.25	74.80	0.00
TRINITY_D2VEP3_NAEGR/22-152	D2VEP3.1 PF01138.19	74.80	0.00
TRINITY_Q3SEA9_PARTE/47-550	Q3SEA9.1 PF00118.22	74.80	0.00
TRINITY_G0QTY4_ICHMG/126-725	G0QTY4.1 PF00012.18	74.80	0.00
TRINITY_D8U437_VOLCA/2317-2440	D8U437.1 PF01769.14	74.80	0.00
TRINITY_A8JGP9_CHLRE/57-171	A8JGP9.1 PF00101.18	74.80	0.00
TRINITY_A0A0K9NW20_ZOSMR/209-321	A0A0K9NW20.1 PF0844	74.80	0.00
TRINITY_A8J1T4_CHLRE/38-363	A8J1T4.1 PF07992.12	74.80	0.00
TRINITY_D8U4E6_VOLCA/258-549	D8U4E6.1 PF00393.17	74.80	0.00
TRINITY_J9HW86_9SPIT/36-291	J9HW86.1 PF00069.23	74.80	0.00
TRINITY_D8UJ68_VOLCA/5-122	D8UJ68.1 PF00072.22	74.80	0.00
TRINITY_A8J5D8_CHLRE/5-419	A8J5D8.1 PF01676.16	74.80	0.00
TRINITY_E9GFP0_DAPPU/8-159	E9GFP0.1 PF00179.24	74.70	0.00
TRINITY_I7ML63_TETTS/2127-2377	I7ML63.1 PF00454.25	74.70	0.00
TRINITY_R9PA35_PSEHS/301-626	R9PA35.1 PF00176.21	74.70	0.00
TRINITY_A0C4G5_PARTE/13-301	A0C4G5.1 PF00069.23	74.70	0.00
TRINITY_A0CFW1_PARTE/22-104	A0CFW1.1 PF01016.17	74.70	0.00
TRINITY_D8TYH5_VOLCA/16-426	D8TYH5.1 PF00860.18	74.70	0.00
TRINITY_A8IZ83_CHLRE/1-162	A8IZ83.1 PF07714.15	74.70	0.00
TRINITY_A8I1B6_CHLRE/82-217	A8I1B6.1 PF02146.15	74.70	0.00
TRINITY_A0CVB3_PARTE/54-148	A0CVB3.1 PF01248.24	74.70	0.00
TRINITY_Q84K56_CHLRE/133-225	Q84K56.1 PF00420.22	74.70	0.00
TRINITY_W4GN10_9STRA/1046-1365	W4GN10.1 PF00225.21	74.70	0.00
TRINITY_D8TZS0_VOLCA/209-505	D8TZS0.1 PF00069.23	74.70	0.00
TRINITY_D8TVB0_VOLCA/25-224	D8TVB0.1 PF13419.4;	74.70	0.00
TRINITY_D8UI27_VOLCA/1-188	D8UI27.1 PF13898.4;	74.70	0.00
TRINITY_K9ZZN9_DEIPD/36-214	K9ZZN9.1 PF01227.20	74.70	0.00
TRINITY_D8TJI0_VOLCA/186-764	D8TJI0.1 PF00133.20	74.70	0.00
TRINITY_A8IYW8_CHLRE/488-717	A8IYW8.1 PF00326.19	74.70	0.00
TRINITY_A4VCZ3_TETTS/109-258	A4VCZ3.1 PF00817.18	74.60	0.00
TRINITY_A0BP58_PARTE/344-595	A0BP58.1 PF12775.5;	74.60	0.00
TRINITY_Q3SE39_PARTE/146-316	Q3SE39.1 PF05063.12	74.60	0.00
TRINITY_I7MA75_TETTS/15-152	I7MA75.2 PF00179.24	74.60	0.00
TRINITY_I3KGU8_ORENI/68-273	I3KGU8.1 PF01442.16	74.60	0.00
TRINITY_I7M2Z5_TETTS/227-524	I7M2Z5.2 PF03133.13	74.60	0.00
TRINITY_W4YD10_STRPU/46-116	W4YD10.1 PF00076.20	74.60	0.00
TRINITY_G0R1C5_ICHMG/3-376	G0R1C5.1 PF00022.17	74.60	0.00
TRINITY_A0A0P7UBI8_9TELE/235-418	A0A0P7UBI8.1 PF0144	74.60	0.00
TRINITY_D8TGW6_VOLCA/193-334	D8TGW6.1 PF05173.12	74.60	0.00
TRINITY_I0Z2P4_9CHLO/20-90	I0Z2P4.1 PF00076.20	74.60	0.00

TRINITY_A2PZC3_CHLRE/329-452	A2PZC3.1	PF03720.13	74.60	0.00
TRINITY_E4XDI4_OIKDI/17-84	E4XDI4.1	PF12265.6;	74.60	0.00
TRINITY_A0DGN9_PARTE/20-152	A0DGN9.1	PF00237.17	74.60	0.00
TRINITY_L8GKM5_ACACA/5-229	L8GKM5.1	PF00108.21	74.60	0.00
TRINITY_D8UCR2_VOLCA/7-144	D8UCR2.1	PF00258.23	74.60	0.00
TRINITY_D8UBN7_VOLCA/1293-1410	D8UBN7.1	PF00271.29	74.60	0.00
TRINITY_D8TQK0_VOLCA/137-253	D8TQK0.1	PF13640.4;	74.60	0.00
TRINITY_A0E893_PARTE/36-101	A0E893.1	PF00550.23	74.60	0.00
TRINITY_A0E1E7_PARTE/8-354	A0E1E7.1	PF00225.21	74.60	0.00
TRINITY_H3ECV4_PRIPA/376-525	H3ECV4.1	PF00005.25	74.60	0.00
TRINITY_Q24F69_TETTS/174-303	Q24F69.2	PF00004.27	74.60	0.00
TRINITY_I0Z1U6_9CHLO/11-413	I0Z1U6.1	PF03069.13	74.60	0.00
TRINITY_L8H393_ACACA/1839-1910	L8H393.1	PF00240.21	74.60	0.00
TRINITY_H2MTU0_ORYLA/52-111	H2MTU0.1	PF00137.19	74.60	0.00
TRINITY_D8TIZ0_VOLCA/253-366	D8TIZ0.1	PF06565.10	74.60	0.00
TRINITY_D3BKQ8_POLPA/426-602	D3BKQ8.1	PF13307.4;	74.60	0.00
TRINITY_I0Z7W7_9CHLO/156-336	I0Z7W7.1	PF00270.27	74.60	0.00
TRINITY_D8U581_VOLCA/133-272	D8U581.1	PF01529.18	74.60	0.00
TRINITY_D8U964_VOLCA/41-174	D8U964.1	PF01894.15	74.60	0.00
TRINITY_Q22XT4_TETTS/475-635	Q22XT4.1	PF08662.9;	74.50	0.00
TRINITY_A0A066WFV8_9BASI/183-237	A0A066WFV8.1	PF0001	74.50	0.00
TRINITY_F4PRU3_DICFS/168-564	F4PRU3.1	PF00180.18	74.50	0.00
TRINITY_I7LXE3_TETTS/981-1239	I7LXE3.2	PF00069.23	74.50	0.00
TRINITY_C1EFV0_MICSR/16-345	C1EFV0.1	PF00009.25	74.50	0.00
TRINITY_A8IY85_CHLRE/275-397	A8IY85.1	PF13288.4;	74.50	0.00
TRINITY_D8U8G7_VOLCA/220-419	D8U8G7.1	PF13851.4;	74.50	0.00
TRINITY_A8JCS0_CHLRE/101-261	A8JCS0.1	PF04442.12	74.50	0.00
TRINITY_T1G1B1_HELRO/13-404	T1G1B1.1	PF06068.11	74.50	0.00
TRINITY_D8TNC2_VOLCA/225-271	D8TNC2.1	PF01479.23	74.50	0.00
TRINITY_A8IDN1_CHLRE/5-97	A8IDN1.1	PF00166.19	74.50	0.00
TRINITY_E1Z766_CHLVA/250-301	E1Z766.1	PF00415.16	74.50	0.00
TRINITY_D8U7J1_VOLCA/93-463	D8U7J1.1	PF00285.19	74.50	0.00
TRINITY_F0ZD57_DICPU/18-72	F0ZD57.1	PF06961.11	74.50	0.00
TRINITY_A8I6F3_CHLRE/385-587	A8I6F3.1	PF07714.15	74.50	0.00
TRINITY_A8J8V7_CHLRE/28-176	A8J8V7.1	PF00069.23	74.50	0.00
TRINITY_I7ML74_TETTS/5678-6228	I7ML74.2	PF00899.19	74.50	0.00
TRINITY_L8GUU1_ACACA/68-321	L8GUU1.1	PF00069.23	74.50	0.00
TRINITY_C1GTN3_PARBA/179-359	C1GTN3.2	PF13945.4;	74.50	0.00
TRINITY_M2XTV4_GALSU/218-264	M2XTV4.1	PF00249.29	74.50	0.00
TRINITY_L8HKU1_ACACA/362-412	L8HKU1.1	PF00249.29	74.50	0.00
TRINITY_A8J4T9_CHLRE/27-180	A8J4T9.1	PF00211.18	74.50	0.00
TRINITY_A8J4N6_CHLRE/18-68	A8J4N6.1	PF00249.29	74.50	0.00
TRINITY_D8TSQ7_VOLCA/51-360	D8TSQ7.1	PF01008.15	74.50	0.00
TRINITY_A8HPU0_CHLRE/161-472	A8HPU0.1	PF04111.10	74.50	0.00
TRINITY_A8JA79_CHLRE/25-184	A8JA79.1	PF00069.23	74.50	0.00
TRINITY_D8TJR2_VOLCA/372-757	D8TJR2.1	PF00155.19	74.50	0.00
TRINITY_A8JFG0_CHLRE/36-148	A8JFG0.1	PF10184.7;	74.50	0.00
TRINITY_G0R248_ICHMG/14-502	G0R248.1	PF00443.27	74.40	0.00
TRINITY_L8H2M8_ACACA/18-150	L8H2M8.1	PF00380.17	74.40	0.00
TRINITY_A0CXK7_PARTE/16-143	A0CXK7.1	PF01592.14	74.40	0.00
TRINITY_A7HJC7_FERNB/27-141	A7HJC7.1	PF01661.19	74.40	0.00
TRINITY_M3ZY45_XIPMA/53-299	M3ZY45.1	PF00795.20	74.40	0.00
TRINITY_F0YCW2_AURAN/9-90	F0YCW2.1	PF01198.17	74.40	0.00
TRINITY_I7M0D5_TETTS/4-286	I7M0D5.2	PF00069.23	74.40	0.00
TRINITY_A8J0Z1_CHLRE/44-418	A8J0Z1.1	PF00155.19	74.40	0.00
TRINITY_A8JC68_CHLRE/6-45	A8JC68.1	PF10013.7;	74.40	0.00
TRINITY_F4Q1T9_DICFS/260-472	F4Q1T9.1	PF00300.20	74.40	0.00
TRINITY_A0BSN3_PARTE/14-266	A0BSN3.1	PF00069.23	74.40	0.00
TRINITY_C3Y522_BRAFL/967-1080	C3Y522.1	PF00271.29	74.40	0.00
TRINITY_G4TRT7_PIRID/13-103	G4TRT7.1	PF00125.22	74.40	0.00
TRINITY_V2X5W0_MONRO/112-261	V2X5W0.1	PF00849.20	74.40	0.00

TRINITY_L8HD92_ACACA/1328-1501	L8HD92.1 PF00270.27	74.40	0.00
TRINITY_L8FWR5_PSED2/349-387	L8FWR5.1 PF00400.30	74.40	0.00
TRINITY_B3RRL1_TRIAD/533-571	B3RRL1.1 PF00400.30	74.40	0.00
TRINITY_D3B5B2_POLPA/50-303	D3B5B2.1 PF00069.23	74.40	0.00
TRINITY_L1J966_GUITH/6-44	L1J966.1 PF00400.30	74.40	0.00
TRINITY_Q22T35_TETTS/4-288	Q22T35.2 PF00069.23	74.40	0.00
TRINITY_D8TKS9_VOLCA/633-722	D8TKS9.1 PF00626.20	74.40	0.00
TRINITY_B8B024_ORYSI/165-472	B8B024.1 PF00365.18	74.40	0.00
TRINITY_D8U2W7_VOLCA/234-432	D8U2W7.1 PF08662.9;	74.40	0.00
TRINITY_D8SX17_SELML/2-91	D8SX17.1 PF00447.15	74.40	0.00
TRINITY_D8UBE0_VOLCA/3-108	D8UBE0.1 PF00163.17	74.40	0.00
TRINITY_E1ZRS1_CHLVA/4-340	E1ZRS1.1 PF03214.11	74.40	0.00
TRINITY_E1Z6G3_CHLVA/49-392	E1Z6G3.1 PF01433.18	74.40	0.00
TRINITY_D8U2V0_VOLCA/461-542	D8U2V0.1 PF00069.23	74.40	0.00
TRINITY_A8J044_CHLRE/35-198	A8J044.1 PF03031.16	74.40	0.00
TRINITY_A8HXJ4_CHLRE/89-561	A8HXJ4.1 PF00939.17	74.40	0.00
TRINITY_A8JHC0_CHLRE/32-77	A8JHC0.1 PF00249.29	74.40	0.00
TRINITY_A8JH37_CHLRE/445-772	A8JH37.1 PF01717.16	74.40	0.00
TRINITY_A8JCD7_CHLRE/88-172	A8JCD7.1 PF04676.12	74.40	0.00
TRINITY_D8TUT1_VOLCA/28-147	D8TUT1.1 PF03420.11	74.40	0.00
TRINITY_U5D9W5_AMBTC/184-696	U5D9W5.1 PF00183.16	74.40	0.00
TRINITY_E1ZGY2_CHLVA/153-316	E1ZGY2.1 PF03031.16	74.40	0.00
TRINITY_Q01GS9_OSTTA/26-245	Q01GS9.1 PF03332.11	74.40	0.00
TRINITY_J3MIZ1_ORYBR/565-602	J3MIZ1.1 PF03638.13	74.30	0.00
TRINITY_L1J8G2_GUITH/21-282	L1J8G2.1 PF00069.23	74.30	0.00
TRINITY_I7M4Q4_TETTS/47-304	I7M4Q4.1 PF00069.23	74.30	0.00
TRINITY_A0A0A0LJA2_CUCSA/2-134	A0A0A0LJA2.1 PF0712	74.30	0.00
TRINITY_D7FLX8_ECTSI/53-219	D7FLX8.1 PF00270.27	74.30	0.00
TRINITY_Q22NA0_TETTS/1407-1551	Q22NA0.2 PF00005.25	74.30	0.00
TRINITY_Q24BW0_TETTS/593-630	Q24BW0.2 PF03638.13	74.30	0.00
TRINITY_K4BS77_SOLLC/165-309	K4BS77.1 PF03033.18	74.30	0.00
TRINITY_A0A0F4ZLK4_9PEZI/293-327	A0A0F4ZLK4.1 PF0032	74.30	0.00
TRINITY_I7MFA4_TETTS/258-407	I7MFA4.1 PF00441.22	74.30	0.00
TRINITY_D8TSX7_VOLCA/266-378	D8TSX7.1 PF00271.29	74.30	0.00
TRINITY_K1QRR1_CRAGI/6-78	K1QRR1.1 PF13499.4;	74.30	0.00
TRINITY_A0BZN0_PARTE/60-429	A0BZN0.1 PF00285.19	74.30	0.00
TRINITY_F4PRV3_DICFS/4-411	F4PRV3.1 PF00022.17	74.30	0.00
TRINITY_D8TP32_VOLCA/174-390	D8TP32.1 PF01556.16	74.30	0.00
TRINITY_A8HYH9_CHLRE/34-242	A8HYH9.1 PF01027.18	74.30	0.00
TRINITY_A8JEN1_CHLRE/1-122	A8JEN1.1 PF00293.26	74.30	0.00
TRINITY_A8JEA1_CHLRE/40-292	A8JEA1.1 PF14779.4;	74.30	0.00
TRINITY_D8TLA7_VOLCA/379-474	D8TLA7.1 PF01926.21	74.30	0.00
TRINITY_D8TL59_VOLCA/198-793	D8TL59.1 PF08385.10	74.30	0.00
TRINITY_Q546J6_CHLRE/30-276	Q546J6.1 PF06705.9;	74.30	0.00
TRINITY_A8IZI8_CHLRE/163-626	A8IZI8.1 PF00136.19	74.30	0.00
TRINITY_A8HQT1_CHLRE/33-146	A8HQT1.1 PF00085.18	74.30	0.00
TRINITY_A8J7D6_CHLRE/247-423	A8J7D6.1 PF13870.4;	74.30	0.00
TRINITY_E1ZQ58_CHLVA/40-113	E1ZQ58.1 PF00076.20	74.30	0.00
TRINITY_A8IAH1_CHLRE/287-469	A8IAH1.1 PF03441.12	74.30	0.00
TRINITY_A0A0D2X4N9_CAPO3/412-1052	A0A0D2X4N9.1 PF0032	74.20	0.00
TRINITY_G0QV49_ICHMG/2561-2592	G0QV49.1 PF02260.18	74.20	0.00
TRINITY_D8UEC0_VOLCA/1-102	D8UEC0.1 PF00543.20	74.20	0.00
TRINITY_A0D8A1_PARTE/9-283	A0D8A1.1 PF00069.23	74.20	0.00
TRINITY_L8HDV5_ACACA/6-380	L8HDV5.1 PF00022.17	74.20	0.00
TRINITY_A0D8A1_PARTE/9-283	A0D8A1.1 PF00069.23	74.20	0.00
TRINITY_C3Z6V7_BRAFL/18-180	C3Z6V7.1 PF03190.13	74.20	0.00
TRINITY_A0D8A1_PARTE/9-283	A0D8A1.1 PF00069.23	74.20	0.00
TRINITY_D8UCL7_VOLCA/162-371	D8UCL7.1 PF00297.20	74.20	0.00
TRINITY_T1FMA9_HELRO/17-344	T1FMA9.1 PF00009.25	74.20	0.00
TRINITY_S2J4P9_MUCC1/361-519	S2J4P9.1 PF00441.22	74.20	0.00
TRINITY_A0CS93_PARTE/11-301	A0CS93.1 PF00069.23	74.20	0.00

TRINITY_Q231W2_TETTS/18-753	Q231W2.1 PF03030.14	74.20	0.00
TRINITY_W7TIE3_9STRA/305-432	W7TIE3.1 PF00004.27	74.20	0.00
TRINITY_CCR1_MAIZE/36-291	P0C8M8.1 PF00069.23	74.20	0.00
TRINITY_G0R1B4_ICHMG/8-259	G0R1B4.1 PF00069.23	74.20	0.00
TRINITY_A8HVJ9_CHLRE/1-298	A8HVJ9.1 PF14870.4;	74.20	0.00
TRINITY_A8J0R2_CHLRE/160-318	A8J0R2.1 PF01553.19	74.20	0.00
TRINITY_A8IEU2_CHLRE/29-295	A8IEU2.1 PF01026.19	74.20	0.00
TRINITY_Q3SD87_PARTE/6-613	Q3SD87.1 PF00012.18	74.20	0.00
TRINITY_I0Z664_9CHLO/510-765	I0Z664.1 PF16203.3;	74.20	0.00
TRINITY_A8IU68_CHLRE/98-653	A8IU68.1 PF00133.20	74.20	0.00
TRINITY_Q3SD10_PARTE/7-191	Q3SD10.1 PF00025.19	74.10	0.00
TRINITY_X6M4L5_RETFI/6-65	X6M4L5.1 PF04193.12	74.10	0.00
TRINITY_A8JI35_CHLRE/4-300	A8JI35.1 PF00069.23	74.10	0.00
TRINITY_A8IFG0_CHLRE/344-605	A8IFG0.1 PF00899.19	74.10	0.00
TRINITY_D8UEB9_VOLCA/1-192	D8UEB9.1 PF03721.12	74.10	0.00
TRINITY_A0A077YX33_TRITR/1118-1518	A0A077YX33.1 PF0390	74.10	0.00
TRINITY_I0YWF3_9CHLO/229-309	I0YWF3.1 PF04000.13	74.10	0.00
TRINITY_F4PMV2_DICFS/89-458	F4PMV2.1 PF00285.19	74.10	0.00
TRINITY_Q23D84_TETTS/68-437	Q23D84.1 PF00285.19	74.10	0.00
TRINITY_F4QE85_DICFS/5-97	F4QE85.1 PF05670.11	74.10	0.00
TRINITY_D8TKZ3_VOLCA/45-224	D8TKZ3.1 PF01300.16	74.10	0.00
TRINITY_A8I087_CHLRE/147-226	A8I087.1 PF00595.22	74.10	0.00
TRINITY_A8JG58_CHLRE/250-555	A8JG58.1 PF00587.23	74.10	0.00
TRINITY_A8HYW8_CHLRE/207-276	A8HYW8.1 PF14360.4;	74.10	0.00
TRINITY_I0Z473_9CHLO/7-181	I0Z473.1 PF00025.19	74.10	0.00
TRINITY_N4VI40_COLOR/14-178	N4VI40.1 PF00160.19	74.10	0.00
TRINITY_A8HPZ4_CHLRE/321-635	A8HPZ4.1 PF00493.21	74.10	0.00
TRINITY_D8UG85_VOLCA/40-517	D8UG85.1 PF01532.18	74.10	0.00
TRINITY_D8TQZ7_VOLCA/265-459	D8TQZ7.1 PF08323.9;	74.10	0.00
TRINITY_D8TRJ9_VOLCA/14-234	D8TRJ9.1 PF04012.10	74.10	0.00
TRINITY_D8U694_VOLCA/30-303	D8U694.1 PF01459.20	74.10	0.00
TRINITY_D8U013_VOLCA/37-329	D8U013.1 PF02934.13	74.10	0.00
TRINITY_A0A0D2U007_CAPO3/379-490	A0A0D2U007.1 PF0030	74.10	0.00
TRINITY_D2KP25_ORENI/23-288	D2KP25.1 PF00361.18	74.10	0.00
TRINITY_A8IUI9_CHLRE/197-379	A8IUI9.1 PF04055.19	74.10	0.00
TRINITY_E1Z6D1_CHLVA/2002-2055	E1Z6D1.1 PF11793.6;	74.10	0.00
TRINITY_E1Z6D1_CHLVA/2002-2055	E1Z6D1.1 PF11793.6;	74.10	0.00
TRINITY_D8U7B0_VOLCA/64-740	D8U7B0.1 PF00063.19	74.10	0.00
TRINITY_A0A0A1NSM9_9FUNG/1-126	A0A0A1NSM9.1 PF0109	74.00	0.00
TRINITY_A8JA79_CHLRE/25-184	A8JA79.1 PF00069.23	74.00	0.00
TRINITY_D3BNV2_POLPA/438-587	D3BNV2.1 PF00441.22	74.00	0.00
TRINITY_G0QVV1_ICHMG/11-394	G0QVV1.1 PF00199.17	74.00	0.00
TRINITY_L8H3G5_ACACA/1-175	L8H3G5.1 PF00025.19	74.00	0.00
TRINITY_Q22Z33_TETTS/46-240	Q22Z33.1 PF00149.26	74.00	0.00
TRINITY_D8UC36_VOLCA/80-302	D8UC36.1 PF03407.14	74.00	0.00
TRINITY_D8TLB0_VOLCA/190-695	D8TLB0.1 PF00183.16	74.00	0.00
TRINITY_D8TRN2_VOLCA/1964-2313	D8TRN2.1 PF12777.5;	74.00	0.00
TRINITY_G8JRJ8_ERECY/6-56	G8JRJ8.1 PF00253.19	74.00	0.00
TRINITY_A0A087SRH3_AUXPR/169-242	A0A087SRH3.1 PF0007	74.00	0.00
TRINITY_I0Z3A0_9CHLO/204-277	I0Z3A0.1 PF00173.26	74.00	0.00
TRINITY_D8U288_VOLCA/1-154	D8U288.1 PF00211.18	74.00	0.00
TRINITY_A8JH47_CHLRE/50-264	A8JH47.1 PF13460.4;	74.00	0.00
TRINITY_D8U0B6_VOLCA/1-312	D8U0B6.1 PF01916.15	74.00	0.00
TRINITY_A0A067R1A6_ZOONE/18-144	A0A067R1A6.1 PF0146	74.00	0.00
TRINITY_D8TVP7_VOLCA/20-322	D8TVP7.1 PF02374.13	74.00	0.00
TRINITY_D8U5I0_VOLCA/499-857	D8U5I0.1 PF00654.18	74.00	0.00
TRINITY_D8UGV0_VOLCA/186-450	D8UGV0.1 PF00122.18	74.00	0.00
TRINITY_D8TSA8_VOLCA/761-1236	D8TSA8.1 PF01432.18	74.00	0.00
TRINITY_Q22S16_TETTS/15-271	Q22S16.3 PF00069.23	73.90	0.00
TRINITY_A0EEF5_PARTE/29-120	A0EEF5.1 PF00447.15	73.90	0.00
TRINITY_Q22NA0_TETTS/1407-1551	Q22NA0.2 PF00005.25	73.90	0.00

TRINITY_F4QD81_DICFS/62-323	F4QD81.1 PF00295.15	73.90	0.00
TRINITY_F4PKB5_DICFS/4-285	F4PKB5.1 PF00069.23	73.90	0.00
TRINITY_A0A068S232_9FUNG/48-162	A0A068S232.1 PF0139	73.90	0.00
TRINITY_A0D3T1_PARTE/120-234	A0D3T1.1 PF00355.24	73.90	0.00
TRINITY_Q23QT9_TETTS/74-119	Q23QT9.2 PF00249.29	73.90	0.00
TRINITY_E1Z207_CHLVA/8-77	E1Z207.1 PF01423.20	73.90	0.00
TRINITY_I7LVI5_TETTS/8-141	I7LVI5.2 PF00179.24	73.90	0.00
TRINITY_D8TJ16_VOLCA/47-488	D8TJ16.1 PF00067.20	73.90	0.00
TRINITY_D8TS39_VOLCA/55-405	D8TS39.1 PF00266.17	73.90	0.00
TRINITY_D8TUW4_VOLCA/64-325	D8TUW4.1 PF02127.13	73.90	0.00
TRINITY_D8U2M3_VOLCA/84-222	D8U2M3.1 PF02577.12	73.90	0.00
TRINITY_A8IS13_CHLRE/197-399	A8IS13.1 PF14681.4;	73.90	0.00
TRINITY_A0A015ISW7_9GLOM/163-273	A0A015ISW7.1 PF0277	73.90	0.00
TRINITY_Q6Y682_CHLRE/61-280	Q6Y682.1 PF01370.19	73.90	0.00
TRINITY_E1ZQE8_CHLVA/39-192	E1ZQE8.1 PF02605.13	73.90	0.00
TRINITY_A8IC95_CHLRE/519-881	A8IC95.1 PF02896.16	73.90	0.00
TRINITY_I7MA75_TETTS/15-152	I7MA75.2 PF00179.24	73.90	0.00
TRINITY_A8IY43_CHLRE/85-241	A8IY43.1 PF00160.19	73.90	0.00
TRINITY_V4C6I3_LOTGI/521-639	V4C6I3.1 PF11978.6;	73.90	0.00
TRINITY_A0CR98_PARTE/229-416	A0CR98.1 PF00587.23	73.90	0.00
TRINITY_A0A087SHG3_AUXPR/29-204	A0A087SHG3.1 PF0027	73.90	0.00
TRINITY_A2EC21_TRIVA/8-171	A2EC21.1 PF00160.19	73.90	0.00
TRINITY_D8U6G0_VOLCA/102-178	D8U6G0.1 PF13417.4;	73.90	0.00
TRINITY_A8ITP6_CHLRE/676-966	A8ITP6.1 PF00069.23	73.90	0.00
TRINITY_D8UJ42_VOLCA/430-567	D8UJ42.1 PF07683.12	73.90	0.00
TRINITY_I3TW47_TISMK/50-437	I3TW47.1 PF01979.18	73.80	0.00
TRINITY_A0A067JRP4_JATCU/28-285	A0A067JRP4.1 PF0070	73.80	0.00
TRINITY_A0D8A1_PARTE/9-283	A0D8A1.1 PF00069.23	73.80	0.00
TRINITY_A0DD78_PARTE/6-291	A0DD78.1 PF00069.23	73.80	0.00
TRINITY_L8GIQ2_ACACA/74-303	L8GIQ2.1 PF01269.15	73.80	0.00
TRINITY_T1H7D4_MEGSC/107-186	T1H7D4.1 PF00318.18	73.80	0.00
TRINITY_L8HHR2_ACACA/1-660	L8HHR2.1 PF00063.19	73.80	0.00
TRINITY_D8U9J9_VOLCA/8-234	D8U9J9.1 PF01168.18	73.80	0.00
TRINITY_A8J7A7_CHLRE/10-173	A8J7A7.1 PF00071.20	73.80	0.00
TRINITY_W7TGG0_9STRA/84-381	W7TGG0.1 PF00291.23	73.80	0.00
TRINITY_D8U6J2_VOLCA/7-178	D8U6J2.1 PF00270.27	73.80	0.00
TRINITY_I0Z5Y6_9CHLO/110-236	I0Z5Y6.1 PF01553.19	73.80	0.00
TRINITY_D8TIF3_VOLCA/121-253	D8TIF3.1 PF00252.16	73.80	0.00
TRINITY_A0E600_PARTE/46-194	A0E600.1 PF01257.17	73.80	0.00
TRINITY_A8JGB0_CHLRE/102-444	A8JGB0.1 PF02374.13	73.80	0.00
TRINITY_I0YTU8_9CHLO/23-358	I0YTU8.1 PF16363.3;	73.80	0.00
TRINITY_A8JGP9_CHLRE/57-171	A8JGP9.1 PF00101.18	73.80	0.00
TRINITY_D8TSX7_VOLCA/62-231	D8TSX7.1 PF00270.27	73.80	0.00
TRINITY_A8ITH8_CHLRE/54-560	A8ITH8.1 PF00118.22	73.80	0.00
TRINITY_I0YT93_9CHLO/120-251	I0YT93.1 PF12923.5;	73.80	0.00
TRINITY_A8II74_CHLRE/185-405	A8II74.1 PF13086.4;	73.80	0.00
TRINITY_Q23QY9_TETTS/11-178	Q23QY9.2 PF00160.19	73.80	0.00
TRINITY_D8U7B6_VOLCA/44-255	D8U7B6.1 PF13419.4;	73.80	0.00
TRINITY_A0A0F0KX75_9MICO/11-148	A0A0F0KX75.1 PF0561	73.70	0.00
TRINITY_D8TPG3_VOLCA/100-302	D8TPG3.1 PF00004.27	73.70	0.00
TRINITY_A0A0D3FXD7_9ORYZ/682-788	A0A0D3FXD7.1 PF0500	73.70	0.00
TRINITY_A9SRD0_PHYPA/81-141	A9SRD0.1 PF05030.10	73.70	0.00
TRINITY_D8U4I7_VOLCA/96-133	D8U4I7.1 PF02861.18	73.70	0.00
TRINITY_K4CG47_SOLLC/29-213	K4CG47.1 PF00227.24	73.70	0.00
TRINITY_T1K154_TETUR/223-477	T1K154.1 PF00122.18	73.70	0.00
TRINITY_E1Z2U6_CHLVA/123-591	E1Z2U6.1 PF01293.18	73.70	0.00
TRINITY_R1DSV0_EMIHU/23-79	R1DSV0.1 PF08207.10	73.70	0.00
TRINITY_A8IQH3_CHLRE/1-180	A8IQH3.1 PF01184.17	73.70	0.00
TRINITY_T1FYE9_HELRO/23-315	T1FYE9.1 PF00850.17	73.70	0.00
TRINITY_D3BFF5_POLPA/368-556	D3BFF5.1 PF01163.20	73.70	0.00
TRINITY_G0QXX8_ICHMG/49-106	G0QXX8.1 PF01849.16	73.70	0.00

TRINITY_A8IXP8_CHLRE/25-340	A8IXP8.1 PF00225.21	73.70	0.00
TRINITY_F1A3H0_DICPU/186-253	F1A3H0.1 PF01485.19	73.70	0.00
TRINITY_D8UDW8_VOLCA/1298-1470	D8UDW8.1 PF08148.10	73.70	0.00
TRINITY_A8HPY3_CHLRE/45-165	A8HPY3.1 PF03567.12	73.70	0.00
TRINITY_D8U0P6_VOLCA/36-243	D8U0P6.1 PF01189.15	73.70	0.00
TRINITY_A8HT53_CHLRE/18-325	A8HT53.1 PF00069.23	73.70	0.00
TRINITY_I7MGJ7_TETTS/43-393	I7MGJ7.1 PF00225.21	73.70	0.00
TRINITY_E4U4P6_OCEP5/129-184	E4U4P6.1 PF09285.9;	73.70	0.00
TRINITY_W1PYU1_AMBTC/723-855	W1PYU1.1 PF00004.27	73.70	0.00
TRINITY_A8IQW5_CHLRE/92-259	A8IQW5.1 PF13911.4;	73.70	0.00
TRINITY_D8UCT7_VOLCA/816-914	D8UCT7.1 PF13640.4;	73.70	0.00
TRINITY_E1ZR84_CHLVA/4-63	E1ZR84.1 PF00076.20	73.70	0.00
TRINITY_A8I6T5_CHLRE/1202-1411	A8I6T5.1 PF01326.17	73.70	0.00
TRINITY_A8JAC5_CHLRE/85-308	A8JAC5.1 PF01128.17	73.70	0.00
TRINITY_Q22T35_TETTS/4-288	Q22T35.2 PF00069.23	73.70	0.00
TRINITY_D8TYY0_VOLCA/15-360	D8TYY0.1 PF03630.12	73.70	0.00
TRINITY_A4VCZ6_TETTS/85-391	A4VCZ6.2 PF01866.15	73.70	0.00
TRINITY_A0A0N4XWF5_NIPBR/74-172	A0A0N4XWF5.1 PF0067	73.70	0.00
TRINITY_A8I501_CHLRE/9-221	A8I501.1 PF01988.17	73.70	0.00
TRINITY_D8TS97_VOLCA/30-272	D8TS97.1 PF00069.23	73.70	0.00
TRINITY_D8UAJ8_VOLCA/135-267	D8UAJ8.1 PF03464.13	73.70	0.00
TRINITY_A8IZZ9_CHLRE/452-770	A8IZZ9.1 PF01515.17	73.70	0.00
TRINITY_A8JFV3_CHLRE/95-388	A8JFV3.1 PF00069.23	73.70	0.00
TRINITY_A8J481_CHLRE/187-343	A8J481.1 PF00005.25	73.70	0.00
TRINITY_A0BE74_PARTE/27-121	A0BE74.1 PF01247.16	73.70	0.00
TRINITY_A8NCG5_COPC7/2-119	A8NCG5.1 PF00125.22	73.60	0.00
TRINITY_Q236T8_TETTS/38-401	Q236T8.2 PF00266.17	73.60	0.00
TRINITY_M0U8S7_MUSAM/347-404	M0U8S7.1 PF03106.13	73.60	0.00
TRINITY_A8I960_CHLRE/24-194	A8I960.1 PF00270.27	73.60	0.00
TRINITY_D8TM23_VOLCA/34-204	D8TM23.1 PF00925.18	73.60	0.00
TRINITY_D8TI63_VOLCA/177-320	D8TI63.1 PF01764.23	73.60	0.00
TRINITY_D8TWK7_VOLCA/1-154	D8TWK7.1 PF00211.18	73.60	0.00
TRINITY_F0ZN98_DICPU/6-241	F0ZN98.1 PF00149.26	73.60	0.00
TRINITY_D8UDZ8_VOLCA/216-360	D8UDZ8.1 PF08920.8;	73.60	0.00
TRINITY_D8UAL7_VOLCA/12-470	D8UAL7.1 PF02516.12	73.60	0.00
TRINITY_E1Z2X5_CHLVA/270-543	E1Z2X5.1 PF00128.22	73.60	0.00
TRINITY_A8IRU2_CHLRE/81-151	A8IRU2.1 PF02135.14	73.60	0.00
TRINITY_D8U8A9_VOLCA/17-742	D8U8A9.1 PF03030.14	73.60	0.00
TRINITY_D8U263_VOLCA/31-215	D8U263.1 PF00227.24	73.60	0.00
TRINITY_K1QWS3_CRAGI/14-101	K1QWS3.1 PF01172.16	73.60	0.00
TRINITY_D8TKM1_VOLCA/60-237	D8TKM1.1 PF11891.6;	73.60	0.00
TRINITY_D8U945_VOLCA/3-117	D8U945.1 PF11527.6;	73.60	0.00
TRINITY_E1ZF83_CHLVA/18-386	E1ZF83.1 PF00199.17	73.60	0.00
TRINITY_A8I6R1_CHLRE/72-158	A8I6R1.1 PF04158.12	73.60	0.00
TRINITY_L8HBG2_ACACA/50-121	L8HBG2.1 PF00717.21	73.60	0.00
TRINITY_Q9ZTA7_CHLRE/91-554	Q9ZTA7.1 PF01593.22	73.60	0.00
TRINITY_G0QRP9_ICHMG/62-152	G0QRP9.1 PF01215.17	73.60	0.00
TRINITY_Q22A67_TETTS/3863-4617	Q22A67.1 PF03028.13	73.60	0.00
TRINITY_D8U477_VOLCA/18-657	D8U477.1 PF04551.12	73.60	0.00
TRINITY_F4QEL1_DICFS/1211-1425	F4QEL1.1 PF00454.25	73.60	0.00
TRINITY_D8TTG0_VOLCA/101-205	D8TTG0.1 PF00085.18	73.60	0.00
TRINITY_A0A0N4ZT82_PARTI/13-66	A0A0N4ZT82.1 PF0058	73.60	0.00
TRINITY_N1PJZ1_DOTSN/106-334	N1PJZ1.1 PF12706.5;	73.50	0.00
TRINITY_K7IYF0_NASVI/3312-4022	K7IYF0.1 PF03028.13	73.50	0.00
TRINITY_J9FK96_9SPIT/795-1052	J9FK96.1 PF00069.23	73.50	0.00
TRINITY_A0E3Q5_PARTE/29-357	A0E3Q5.1 PF00850.17	73.50	0.00
TRINITY_G0QPV5_ICHMG/91-228	G0QPV5.1 PF14671.4;	73.50	0.00
TRINITY_I7MN19_TETTS/35-532	I7MN19.1 PF00118.22	73.50	0.00
TRINITY_U5H7A1_USTV1/26-191	U5H7A1.1 PF00071.20	73.50	0.00
TRINITY_A8J9P9_CHLRE/14-167	A8J9P9.1 PF00160.19	73.50	0.00
TRINITY_A8J836_CHLRE/61-158	A8J836.1 PF10084.7;	73.50	0.00

TRINITY_D8U3S6_VOLCA/175-279	D8U3S6.1 PF00085.18	73.50	0.00
TRINITY_A0A0D2X2D5_CAPO3/103-590	A0A0D2X2D5.1 PF0103	73.50	0.00
TRINITY_D8TLR7_VOLCA/44-93	D8TLR7.1 PF00584.18	73.50	0.00
TRINITY_H2TQQ9_TAKRU/1524-1641	H2TQQ9.1 PF01759.19	73.50	0.00
TRINITY_H3FJT1_PRIPA/252-319	H3FJT1.1 PF01423.20	73.50	0.00
TRINITY_L8HC03_ACACA/63-447	L8HC03.1 PF00344.18	73.50	0.00
TRINITY_L8H2K8_ACACA/208-276	L8H2K8.1 PF00382.17	73.50	0.00
TRINITY_A8IFM3_CHLRE/1-128	A8IFM3.1 PF00005.25	73.50	0.00
TRINITY_D8TKA0_VOLCA/255-358	D8TKA0.1 PF03109.14	73.50	0.00
TRINITY_A0A067FJA4_CITSI/608-722	A0A067FJA4.1 PF0027	73.50	0.00
TRINITY_D8TKQ2_VOLCA/556-871	D8TKQ2.1 PF00176.21	73.50	0.00
TRINITY_D8UCL3_VOLCA/40-194	D8UCL3.1 PF08534.8;	73.50	0.00
TRINITY_A0A0D2WX19_CAPO3/13-116	A0A0D2WX19.1 PF0299	73.50	0.00
TRINITY_A8J905_CHLRE/234-329	A8J905.1 PF01575.17	73.50	0.00
TRINITY_D8TPV9_VOLCA/41-385	D8TPV9.1 PF01208.15	73.50	0.00
TRINITY_A8J9F3_CHLRE/20-663	A8J9F3.1 PF00133.20	73.50	0.00
TRINITY_D8UJP2_VOLCA/71-428	D8UJP2.1 PF01237.16	73.50	0.00
TRINITY_R4X6B7_TAPDE/246-279	R4X6B7.1 PF00806.17	73.50	0.00
TRINITY_A8IYS5_CHLRE/107-400	A8IYS5.1 PF00735.16	73.50	0.00
TRINITY_M2SVV7_COCH5/53-264	M2SVV7.1 PF13945.4;	73.50	0.00
TRINITY_G0R1F8_ICHMG/1-722	G0R1F8.1 PF03030.14	73.50	0.00
TRINITY_A0A059LRV1_9CHLO/348-513	A0A059LRV1.1 PF0010	73.50	0.00
TRINITY_D0MZ70_PHYIT/6-73	D0MZ70.1 PF00076.20	73.50	0.00
TRINITY_I0Z0C9_9CHLO/187-220	I0Z0C9.1 PF08238.10	73.50	0.00
TRINITY_G0QNU9_ICHMG/5-258	G0QNU9.1 PF01201.20	73.50	0.00
TRINITY_I0Z952_9CHLO/60-323	I0Z952.1 PF00082.20	73.50	0.00
TRINITY_D3BE51_POLPA/540-684	D3BE51.1 PF00005.25	73.50	0.00
TRINITY_D8U8F7_VOLCA/192-323	D8U8F7.1 PF00005.25	73.50	0.00
TRINITY_A4VDV3_TETTS/129-225	A4VDV3.1 PF00153.25	73.40	0.00
TRINITY_J9IFT1_9SPIT/16-145	J9IFT1.1 PF00828.17	73.40	0.00
TRINITY_A0BKT3_PARTE/6-401	A0BKT3.1 PF00180.18	73.40	0.00
TRINITY_H2XVN3_CIOIN/20-314	H2XVN3.1 PF00291.23	73.40	0.00
TRINITY_W1NFB7_AMBTC/7-616	W1NFB7.1 PF00012.18	73.40	0.00
TRINITY_A0A0N1E2S5_9FLAO/26-441	A0A0N1E2S5.1 PF0020	73.40	0.00
TRINITY_A0C4G5_PARTE/13-301	A0C4G5.1 PF00069.23	73.40	0.00
TRINITY_D8TRL9_VOLCA/216-387	D8TRL9.1 PF08547.10	73.40	0.00
TRINITY_D7FNK5_ECTSI/311-454	D7FNK5.1 PF00475.16	73.40	0.00
TRINITY_A8J4S1_CHLRE/44-219	A8J4S1.1 PF02507.13	73.40	0.00
TRINITY_D8TZI0_VOLCA/70-329	D8TZI0.1 PF04921.12	73.40	0.00
TRINITY_A8JEM3_CHLRE/48-224	A8JEM3.1 PF00702.24	73.40	0.00
TRINITY_D8U637_VOLCA/15-321	D8U637.1 PF00176.21	73.40	0.00
TRINITY_E5SX20_TRISP/61-244	E5SX20.1 PF01926.21	73.40	0.00
TRINITY_A8J063_CHLRE/819-1225	A8J063.1 PF08393.11	73.40	0.00
TRINITY_D8U9A5_VOLCA/30-553	D8U9A5.1 PF00118.22	73.40	0.00
TRINITY_A8IS11_CHLRE/406-740	A8IS11.1 PF00702.24	73.40	0.00
TRINITY_A8HYR2_CHLRE/947-1224	A8HYR2.1 PF02965.15	73.40	0.00
TRINITY_L8HJA5_ACACA/2-388	L8HJA5.1 PF00022.17	73.40	0.00
TRINITY_D8U3G5_VOLCA/95-425	D8U3G5.1 PF07992.12	73.40	0.00
TRINITY_E1ZTL2_CHLVA/961-1039	E1ZTL2.1 PF12812.5;	73.40	0.00
TRINITY_F2UBE5_SALR5/5-166	F2UBE5.1 PF00071.20	73.30	0.00
TRINITY_A0CR44_PARTE/304-440	A0CR44.1 PF08443.9;	73.30	0.00
TRINITY_C5L4E2_PERM5/63-92	C5L4E2.1 PF00397.24	73.30	0.00
TRINITY_A0A058ZDU1_9EUKA/786-1000	A0A058ZDU1.1 PF0045	73.30	0.00
TRINITY_D8U7Q8_VOLCA/24-125	D8U7Q8.1 PF01521.18	73.30	0.00
TRINITY_MOB1A_DICDI/31-202	Q54XJ0.1 PF03637.15	73.30	0.00
TRINITY_Q241B6_TETTS/1199-1363	Q241B6.2 PF03031.16	73.30	0.00
TRINITY_A0A078ALM0_STYLE/473-767	A0A078ALM0.1 PF0006	73.30	0.00
TRINITY_D3B6T5_POLPA/268-312	D3B6T5.1 PF00569.15	73.30	0.00
TRINITY_D8U5N0_VOLCA/679-740	D8U5N0.1 PF08240.10	73.30	0.00
TRINITY_L1ICM6_GUITH/49-87	L1ICM6.1 PF00400.30	73.30	0.00
TRINITY_D8UIG4_VOLCA/163-267	D8UIG4.1 PF13661.4;	73.30	0.00

TRINITY_D8TYL1_VOLCA/3-133	D8TYL1.1 PF04099.10	73.30	0.00
TRINITY_Q6QJE0_CHLRE/143-398	Q6QJE0.1 PF13531.4;	73.30	0.00
TRINITY_A8I8F2_CHLRE/3-187	A8I8F2.1 PF00625.19	73.30	0.00
TRINITY_D8UFN4_VOLCA/1689-1806	D8UFN4.1 PF01661.19	73.30	0.00
TRINITY_A8IPJ0_CHLRE/119-217	A8IPJ0.1 PF00153.25	73.30	0.00
TRINITY_D8UA41_VOLCA/65-182	D8UA41.1 PF09745.7;	73.30	0.00
TRINITY_G0R088_ICHMG/41-328	G0R088.1 PF00069.23	73.30	0.00
TRINITY_D8U2T7_VOLCA/62-151	D8U2T7.1 PF06831.12	73.30	0.00
TRINITY_D8TGS3_VOLCA/766-928	D8TGS3.1 PF03572.16	73.30	0.00
TRINITY_D8TM31_VOLCA/342-683	D8TM31.1 PF00493.21	73.30	0.00
TRINITY_A8IJT8_CHLRE/9-135	A8IJT8.1 PF01014.16	73.30	0.00
TRINITY_A0A061DMP8_THECC/350-439	A0A061DMP8.1 PF0007	73.30	0.00
TRINITY_D8R9B3_SELML/325-369	D8R9B3.1 PF00400.30	73.30	0.00
TRINITY_D8TN03_VOLCA/51-349	D8TN03.1 PF03133.13	73.30	0.00
TRINITY_D8TKI2_VOLCA/1226-1356	D8TKI2.1 PF02732.13	73.30	0.00
TRINITY_E1ZE87_CHLVA/19-257	E1ZE87.1 PF01946.15	73.30	0.00
TRINITY_R1FM73_EMIHU/100-139	R1FM73.1 PF01753.16	73.30	0.00
TRINITY_Q23D15_TETTS/14-179	Q23D15.1 PF00160.19	73.20	0.00
TRINITY_A0C3X5_PARTE/32-114	A0C3X5.1 PF00111.25	73.20	0.00
TRINITY_KLP3_SCHPO/9-325	Q9US60.1 PF00225.21	73.20	0.00
TRINITY_V7CY33_PHAVU/13-85	V7CY33.1 PF13304.4;	73.20	0.00
TRINITY_S9X060_9CETA/78-191	S9X060.1 PF00828.17	73.20	0.00
TRINITY_A0A0D2WI65_CAPO3/65-186	A0A0D2WI65.1 PF0000	73.20	0.00
TRINITY_M4DRK5_BRARP/69-297	M4DRK5.1 PF01269.15	73.20	0.00
TRINITY_D8UIY4_VOLCA/1-274	D8UIY4.1 PF00225.21	73.20	0.00
TRINITY_A0A068YEU6_ECHMU/52-247	A0A068YEU6.1 PF0014	73.20	0.00
TRINITY_I7ML30_TETTS/420-576	I7ML30.2 PF00271.29	73.20	0.00
TRINITY_A8JFM7_CHLRE/12-149	A8JFM7.1 PF00179.24	73.20	0.00
TRINITY_I0Z9R9_9CHLO/51-217	I0Z9R9.1 PF00009.25	73.20	0.00
TRINITY_D8UDS8_VOLCA/42-152	D8UDS8.1 PF10184.7;	73.20	0.00
TRINITY_A8I4N7_CHLRE/69-107	A8I4N7.1 PF01096.16	73.20	0.00
TRINITY_E1ZMJ7_CHLVA/38-377	E1ZMJ7.1 PF01979.18	73.20	0.00
TRINITY_G3PD63_GASAC/48-411	G3PD63.1 PF00079.18	73.20	0.00
TRINITY_A0A087SPZ3_AUXPR/56-212	A0A087SPZ3.1 PF0000	73.20	0.00
TRINITY_D7MGC5_ARALL/4-104	D7MGC5.1 PF03297.13	73.20	0.00
TRINITY_A8JAY5_CHLRE/85-391	A8JAY5.1 PF00579.23	73.20	0.00
TRINITY_D8TQE6_VOLCA/1663-1897	D8TQE6.1 PF13087.4;	73.20	0.00
TRINITY_I0YQT6_9CHLO/40-326	I0YQT6.1 PF02535.20	73.20	0.00
TRINITY_A8ISA5_CHLRE/9-212	A8ISA5.1 PF00106.23	73.20	0.00
TRINITY_D8TYG6_VOLCA/7-70	D8TYG6.1 PF08154.10	73.20	0.00
TRINITY_I7LXG7_TETTS/35-529	I7LXG7.1 PF00118.22	73.20	0.00
TRINITY_D8UCZ9_VOLCA/61-318	D8UCZ9.1 PF00795.20	73.20	0.00
TRINITY_D8U367_VOLCA/1-178	D8U367.1 PF02630.12	73.20	0.00
TRINITY_A0A0A1PIE4_9FUNG/6-143	A0A0A1PIE4.1 PF0017	73.20	0.00
TRINITY_A8IDM1_CHLRE/29-241	A8IDM1.1 PF02919.13	73.20	0.00
TRINITY_D8TRR9_VOLCA/403-468	D8TRR9.1 PF04825.11	73.20	0.00
TRINITY_A8IFQ9_CHLRE/596-707	A8IFQ9.1 PF12014.6;	73.20	0.00
TRINITY_A8IVZ2_CHLRE/70-222	A8IVZ2.1 PF00005.25	73.20	0.00
TRINITY_B4L337_DROMO/117-166	B4L337.1 PF06221.11	73.20	0.00
TRINITY_D8UCJ4_VOLCA/672-743	D8UCJ4.1 PF12253.6;	73.10	0.00
TRINITY_Q23R84_TETTS/138-345	Q23R84.2 PF00009.25	73.10	0.00
TRINITY_I7M0C0_TETTS/39-287	I7M0C0.1 PF00378.18	73.10	0.00
TRINITY_I7MN19_TETTS/35-532	I7MN19.1 PF00118.22	73.10	0.00
TRINITY_Q22RL3_TETTS/148-199	Q22RL3.2 PF01805.18	73.10	0.00
TRINITY_A8I6J1_CHLRE/10-264	A8I6J1.1 PF00069.23	73.10	0.00
TRINITY_Q22MB4_TETTS/42-542	Q22MB4.3 PF00118.22	73.10	0.00
TRINITY_A8JGG4_CHLRE/505-786	A8JGG4.1 PF02626.13	73.10	0.00
TRINITY_D8U0Y4_VOLCA/61-183	D8U0Y4.1 PF02678.14	73.10	0.00
TRINITY_G7KRK6_MEDTR/200-274	G7KRK6.1 PF00364.20	73.10	0.00
TRINITY_D8UJ05_VOLCA/69-344	D8UJ05.1 PF00899.19	73.10	0.00
TRINITY_K1RE71_CRAGI/11-77	K1RE71.1 PF01423.20	73.10	0.00

TRINITY_I0Z6A9_9CHLO/124-387	I0Z6A9.1 PF00069.23	73.10	0.00
TRINITY_E1Z4R7_CHLVA/63-124	E1Z4R7.1 PF00013.27	73.10	0.00
TRINITY_D8UCJ5_VOLCA/37-155	D8UCJ5.1 PF04178.10	73.10	0.00
TRINITY_A0A0A2VTW1_BEABA/102-706	A0A0A2VTW1.1 PF1403	73.10	0.00
TRINITY_A8IH85_CHLRE/30-562	A8IH85.1 PF02516.12	73.10	0.00
TRINITY_D8UIN9_VOLCA/2094-2451	D8UIN9.1 PF00224.19	73.10	0.00
TRINITY_D8UIP0_VOLCA/9-539	D8UIP0.1 PF01274.20	73.10	0.00
TRINITY_A0A068RG97_9FUNG/2-54	A0A068RG97.1 PF0190	73.10	0.00
TRINITY_A0A068RG97_9FUNG/2-54	A0A068RG97.1 PF0190	73.10	0.00
TRINITY_A8IFC8_CHLRE/9-477	A8IFC8.1 PF05694.9;	73.10	0.00
TRINITY_D8TVF8_VOLCA/3-77	D8TVF8.1 PF03110.12	73.10	0.00
TRINITY_I1KVB3_SOYBN/319-519	I1KVB3.2 PF03441.12	73.00	0.00
TRINITY_B9GKY3_POPTR/440-477	B9GKY3.2 PF03638.13	73.00	0.00
TRINITY_A0A0F7KCK0_9PROT/27-164	A0A0F7KCK0.1 PF1286	73.00	0.00
TRINITY_D3BAK5_POLPA/88-483	D3BAK5.1 PF01134.20	73.00	0.00
TRINITY_I3K0S0_ORENI/15-104	I3K0S0.1 PF05825.9;	73.00	0.00
TRINITY_Q234P9_TETTS/544-689	Q234P9.1 PF00004.27	73.00	0.00
TRINITY_F0Z9M2_DICPU/415-584	F0Z9M2.1 PF00270.27	73.00	0.00
TRINITY_D8U2H8_VOLCA/2-319	D8U2H8.1 PF07992.12	73.00	0.00
TRINITY_A0A067JZZ9_JATCU/119-156	A0A067JZZ9.1 PF0142	73.00	0.00
TRINITY_A0A078AS87_STYLE/137-297	A0A078AS87.1 PF0027	73.00	0.00
TRINITY_G0QUI7_ICHMG/220-415	G0QUI7.1 PF00149.26	73.00	0.00
TRINITY_A0A0D2WIE4_CAPO3/71-135	A0A0D2WIE4.1 PF0231	73.00	0.00
TRINITY_A0A0F5YAJ2_9CYAN/93-184	A0A0F5YAJ2.1 PF0025	73.00	0.00
TRINITY_L8HFG1_ACACA/38-503	L8HFG1.1 PF02516.12	73.00	0.00
TRINITY_A8JHA9_CHLRE/39-103	A8JHA9.1 PF00462.22	73.00	0.00
TRINITY_A8IH03_CHLRE/49-392	A8IH03.1 PF00266.17	73.00	0.00
TRINITY_W9RSB6_9ROSA/51-120	W9RSB6.1 PF00076.20	73.00	0.00
TRINITY_K4BVU7_SOLLC/2-153	K4BVU7.1 PF00257.17	73.00	0.00
TRINITY_A8ITU1_CHLRE/159-280	A8ITU1.1 PF10502.7;	73.00	0.00
TRINITY_P93664_CHLRE/45-210	P93664.1 PF00504.19	73.00	0.00
TRINITY_M0ZYF5_SOLTU/142-469	M0ZYF5.1 PF00733.19	73.00	0.00
TRINITY_A8I3M7_CHLRE/1181-1220	A8I3M7.1 PF00226.29	73.00	0.00
TRINITY_A8IV05_CHLRE/51-224	A8IV05.1 PF00227.24	73.00	0.00
TRINITY_D8U7K4_VOLCA/714-1036	D8U7K4.1 PF08767.9;	73.00	0.00
TRINITY_M2WSH9_GALSU/397-434	M2WSH9.1 PF03638.13	73.00	0.00
TRINITY_A0DIN0_PARTE/9-195	A0DIN0.1 PF03256.14	73.00	0.00
TRINITY_I7M129_TETTS/32-216	I7M129.1 PF01145.23	72.90	0.00
TRINITY_G4MSD5_MAGO7/19-339	G4MSD5.1 PF00303.17	72.90	0.00
TRINITY_A9V782_MONBE/6-53	A9V782.1 PF16891.3;	72.90	0.00
TRINITY_Q22G04_TETTS/58-197	Q22G04.2 PF00578.19	72.90	0.00
TRINITY_I7M3W6_TETTS/237-558	I7M3W6.2 PF00152.18	72.90	0.00
TRINITY_A0A096LTZ0_POEFO/22-80	A0A096LTZ0.1 PF0005	72.90	0.00
TRINITY_A0A074XWJ0_AURPU/12-166	A0A074XWJ0.1 PF0025	72.90	0.00
TRINITY_F0ZTH4_DICPU/47-356	F0ZTH4.1 PF01406.17	72.90	0.00
TRINITY_A0A078AK98_STYLE/24-200	A0A078AK98.1 PF0172	72.90	0.00
TRINITY_L8HBV7_ACACA/83-337	L8HBV7.1 PF00069.23	72.90	0.00
TRINITY_A0DZP1_PARTE/30-624	A0DZP1.1 PF00012.18	72.90	0.00
TRINITY_A8J625_CHLRE/26-151	A8J625.1 PF01926.21	72.90	0.00
TRINITY_A8J654_CHLRE/51-361	A8J654.1 PF00248.19	72.90	0.00
TRINITY_A0A078A9T0_STYLE/80-292	A0A078A9T0.1 PF0011	72.90	0.00
TRINITY_A8IBJ5_CHLRE/140-260	A8IBJ5.1 PF03109.14	72.90	0.00
TRINITY_D8U7F8_VOLCA/1707-1755	D8U7F8.1 PF02042.13	72.90	0.00
TRINITY_D8UFL9_VOLCA/279-388	D8UFL9.1 PF00271.29	72.90	0.00
TRINITY_D8TJB9_VOLCA/44-317	D8TJB9.1 PF06472.13	72.90	0.00
TRINITY_G0QSP9_ICHMG/8-66	G0QSP9.1 PF03946.12	72.90	0.00
TRINITY_A8IMT5_CHLRE/325-394	A8IMT5.1 PF00240.21	72.90	0.00
TRINITY_A7SH82_NEMVE/14-142	A7SH82.1 PF00416.20	72.90	0.00
TRINITY_G3NQ97_GASAC/152-245	G3NQ97.1 PF00031.19	72.90	0.00
TRINITY_C5YXL4_SORBI/281-330	C5YXL4.1 PF13920.4;	72.90	0.00
TRINITY_D8U2K3_VOLCA/185-439	D8U2K3.1 PF00928.19	72.90	0.00

TRINITY_Q23FU4_TETTS/44-246	Q23FU4.2 PF00106.23	72.90	0.00
TRINITY_D8U1Y8_VOLCA/89-217	D8U1Y8.1 PF07386.9;	72.90	0.00
TRINITY_A0A061EEG7_THECC/589-749	A0A061EEG7.1 PF0007	72.90	0.00
TRINITY_A0A090M9L0_OSTTA/651-839	A0A090M9L0.1 PF0048	72.90	0.00
TRINITY_E1ZIE5_CHLVA/8-245	E1ZIE5.1 PF00294.22	72.90	0.00
TRINITY_A0A078DMU8_BRANA/411-458	A0A078DMU8.1 PF0040	72.90	0.00
TRINITY_A0CXB5_PARTE/27-357	A0CXB5.1 PF00206.18	72.90	0.00
TRINITY_E1Z768_CHLVA/311-358	E1Z768.1 PF13920.4;	72.90	0.00
TRINITY_F2UC19_SALR5/15-73	F2UC19.1 PF08207.10	72.90	0.00
TRINITY_I7LZU3_TETTS/215-299	I7LZU3.1 PF04433.15	72.90	0.00
TRINITY_A0A077ZVC4_STYLE/74-162	A0A077ZVC4.1 PF0313	72.90	0.00
TRINITY_I0YVW2_9CHLO/10-94	I0YVW2.1 PF00867.16	72.90	0.00
TRINITY_A8HY12_CHLRE/2-258	A8HY12.1 PF08323.9;	72.90	0.00
TRINITY_D8TWM9_VOLCA/889-969	D8TWM9.1 PF10431.7;	72.80	0.00
TRINITY_D8U4R5_VOLCA/799-913	D8U4R5.1 PF00271.29	72.80	0.00
TRINITY_D8UCK6_VOLCA/1279-1453	D8UCK6.1 PF01789.14	72.80	0.00
TRINITY_Q238Z8_TETTS/20-275	Q238Z8.2 PF00069.23	72.80	0.00
TRINITY_J9IGQ2_9SPIT/61-567	J9IGQ2.1 PF00330.18	72.80	0.00
TRINITY_W2RAX8_PHYPN/14-175	W2RAX8.1 PF00071.20	72.80	0.00
TRINITY_K5VRE6_PHACS/17-308	K5VRE6.1 PF00248.19	72.80	0.00
TRINITY_D8TLB0_VOLCA/190-695	D8TLB0.1 PF00183.16	72.80	0.00
TRINITY_E1ZFX9_CHLVA/15-322	E1ZFX9.1 PF00248.19	72.80	0.00
TRINITY_I0YTS3_9CHLO/36-300	I0YTS3.1 PF02263.17	72.80	0.00
TRINITY_I7MD69_TETTS/14-307	I7MD69.1 PF00069.23	72.80	0.00
TRINITY_A0A0G4IHD2_PLABS/115-310	A0A0G4IHD2.1 PF0044	72.80	0.00
TRINITY_Q7XJ66_CHLRE/90-384	Q7XJ66.1 PF03151.14	72.80	0.00
TRINITY_T1IGS0_STRMM/121-585	T1IGS0.1 PF00221.17	72.80	0.00
TRINITY_L8GRD1_ACACA/668-1044	L8GRD1.1 PF00562.26	72.80	0.00
TRINITY_C1D3B0_DEIDV/63-298	C1D3B0.1 PF13561.4;	72.80	0.00
TRINITY_A8JHP9_CHLRE/44-235	A8JHP9.1 PF00551.17	72.80	0.00
TRINITY_D8U2E0_VOLCA/10-182	D8U2E0.1 PF01145.23	72.80	0.00
TRINITY_A8IB12_CHLRE/4-161	A8IB12.1 PF01018.20	72.80	0.00
TRINITY_D8UIF2_VOLCA/72-354	D8UIF2.1 PF06472.13	72.80	0.00
TRINITY_A8IZE7_CHLRE/74-209	A8IZE7.1 PF13522.4;	72.80	0.00
TRINITY_I7MMN9_TETTS/8-128	I7MMN9.1 PF00583.23	72.70	0.00
TRINITY_I7MD88_TETTS/227-422	I7MD88.2 PF04055.19	72.70	0.00
TRINITY_A0A087SPT2_AUXPR/14-167	A0A087SPT2.1 PF0016	72.70	0.00
TRINITY_A0A0D2WLP0_CAPO3/348-479	A0A0D2WLP0.1 PF0000	72.70	0.00
TRINITY_A0A0D2WXT2_CAPO3/716-1103	A0A0D2WXT2.1 PF0238	72.70	0.00
TRINITY_E1Z3B9_CHLVA/3-112	E1Z3B9.1 PF10497.7;	72.70	0.00
TRINITY_K2RSE9_MACPH/140-207	K2RSE9.1 PF08606.9;	72.70	0.00
TRINITY_D8U7J5_VOLCA/6-105	D8U7J5.1 PF03650.11	72.70	0.00
TRINITY_J4CD27_THEOR/10-74	J4CD27.1 PF13499.4;	72.70	0.00
TRINITY_F0ZCH2_DICPU/18-531	F0ZCH2.1 PF01642.20	72.70	0.00
TRINITY_D8TWR1_VOLCA/23-311	D8TWR1.1 PF00069.23	72.70	0.00
TRINITY_L5K105_PTEAL/5-361	L5K105.1 PF00009.25	72.70	0.00
TRINITY_D8TJ19_VOLCA/158-319	D8TJ19.1 PF09416.8;	72.70	0.00
TRINITY_A8J345_CHLRE/96-451	A8J345.1 PF00224.19	72.70	0.00
TRINITY_Q8GV23_CHLRE/7-73	Q8GV23.2 PF00313.20	72.70	0.00
TRINITY_D8U4C5_VOLCA/313-528	D8U4C5.1 PF00246.22	72.70	0.00
TRINITY_E1ZHW8_CHLVA/50-209	E1ZHW8.1 PF00270.27	72.70	0.00
TRINITY_D8UEK9_VOLCA/130-230	D8UEK9.1 PF01205.17	72.70	0.00
TRINITY_A0A059LM82_9CHLO/191-257	A0A059LM82.1 PF0148	72.70	0.00
TRINITY_A0A078B7E7_STYLE/11-282	A0A078B7E7.1 PF0046	72.70	0.00
TRINITY_D8TJI5_VOLCA/29-431	D8TJI5.1 PF00464.17	72.70	0.00
TRINITY_C5D5D3_GEOSW/5-336	C5D5D3.1 PF00180.18	72.70	0.00
TRINITY_C1FG00_MICSR/21-97	C1FG00.1 PF07650.15	72.70	0.00
TRINITY_I7MD55_TETTS/531-788	I7MD55.2 PF00069.23	72.70	0.00
TRINITY_D3BF00_POLPA/218-261	D3BF00.1 PF01505.16	72.70	0.00
TRINITY_D8TUR7_VOLCA/529-606	D8TUR7.1 PF03061.20	72.70	0.00
TRINITY_Q75JR6_DICDI/887-1109	Q75JR6.1 PF13236.4;	72.70	0.00

TRINITY_A0D3A2_PARTE/89-647	A0D3A2.1 PF01175.16	72.70	0.00
TRINITY_A8HN56_CHLRE/225-326	A8HN56.1 PF13499.4;	72.70	0.00
TRINITY_A8INJ9_CHLRE/159-309	A8INJ9.1 PF14252.4;	72.70	0.00
TRINITY_D8TW92_VOLCA/91-227	D8TW92.1 PF00472.18	72.70	0.00
TRINITY_A0CEN8_PARTE/455-521	A0CEN8.1 PF14559.4;	72.70	0.00
TRINITY_A8IE23_CHLRE/119-618	A8IE23.1 PF00342.17	72.70	0.00
TRINITY_D8U4W9_VOLCA/246-364	D8U4W9.1 PF00012.18	72.70	0.00
TRINITY_Q241B6_TETTS/1199-1363	Q241B6.2 PF03031.16	72.70	0.00
TRINITY_I0YY61_9CHLO/79-297	I0YY61.1 PF01940.14	72.70	0.00
TRINITY_A0A024WRW2_PLAFA/3-212	A0A024WRW2.1 PF0009	72.60	0.00
TRINITY_A0BVN2_PARTE/135-194	A0BVN2.1 PF04968.10	72.60	0.00
TRINITY_G0QRF2_ICHMG/154-323	G0QRF2.1 PF05063.12	72.60	0.00
TRINITY_I3K2Q4_ORENI/65-126	I3K2Q4.1 PF07448.9;	72.60	0.00
TRINITY_G0R463_ICHMG/40-325	G0R463.1 PF00069.23	72.60	0.00
TRINITY_B8C7U0_THAPS/3-129	B8C7U0.1 PF01592.14	72.60	0.00
TRINITY_Q22UW8_TETTS/5-177	Q22UW8.2 PF00025.19	72.60	0.00
TRINITY_C1FFF0_MICSR/114-280	C1FFF0.1 PF00270.27	72.60	0.00
TRINITY_Q235X6_TETTS/109-170	Q235X6.2 PF00226.29	72.60	0.00
TRINITY_A0A075AY65_9FUNG/29-112	A0A075AY65.1 PF0011	72.60	0.00
TRINITY_B6TKH0_MAIZE/220-416	B6TKH0.1 PF00149.26	72.60	0.00
TRINITY_A8HMH4_CHLRE/20-281	A8HMH4.1 PF00069.23	72.60	0.00
TRINITY_Q695H0_CHLRE/184-287	Q695H0.1 PF00027.27	72.60	0.00
TRINITY_A0A087SUC5_AUXPR/436-614	A0A087SUC5.1 PF0027	72.60	0.00
TRINITY_G0QMT4_ICHMG/1-243	G0QMT4.1 PF13714.4;	72.60	0.00
TRINITY_A0BK54_PARTE/463-606	A0BK54.1 PF00005.25	72.60	0.00
TRINITY_A8IR24_CHLRE/83-353	A8IR24.1 PF00487.22	72.60	0.00
TRINITY_A8I5C3_CHLRE/1467-1736	A8I5C3.1 PF12775.5;	72.60	0.00
TRINITY_A0CBT9_PARTE/24-142	A0CBT9.1 PF13863.4;	72.60	0.00
TRINITY_A8HSF6_CHLRE/65-181	A8HSF6.1 PF00072.22	72.60	0.00
TRINITY_D8TVI0_VOLCA/15-318	D8TVI0.1 PF05890.10	72.60	0.00
TRINITY_A9SDI8_PHYPA/535-607	A9SDI8.1 PF14492.4;	72.60	0.00
TRINITY_I1M2V2_SOYBN/6-137	I1M2V2.1 PF06314.9;	72.60	0.00
TRINITY_D8UGY5_VOLCA/1551-1798	D8UGY5.1 PF07059.10	72.60	0.00
TRINITY_I7MH10_TETTS/124-450	I7MH10.2 PF00225.21	72.60	0.00
TRINITY_F0Y6L3_AURAN/5-178	F0Y6L3.1 PF00071.20	72.60	0.00
TRINITY_W0IXY9_9BACT/20-136	W0IXY9.1 PF01894.15	72.60	0.00
TRINITY_H2KS42_CLOSI/133-205	H2KS42.1 PF01191.17	72.60	0.00
TRINITY_A8JIN8_CHLRE/110-448	A8JIN8.1 PF01546.26	72.60	0.00
TRINITY_A0A090M7N4_OSTTA/22-313	A0A090M7N4.1 PF0085	72.60	0.00
TRINITY_D8UJG7_VOLCA/41-266	D8UJG7.1 PF05368.11	72.60	0.00
TRINITY_A8JD62_CHLRE/10-122	A8JD62.1 PF13673.5;	72.60	0.00
TRINITY_D8TNS1_VOLCA/9-82	D8TNS1.1 PF02953.13	72.60	0.00
TRINITY_A8J5M7_CHLRE/219-336	A8J5M7.1 PF09273.9;	72.60	0.00
TRINITY_A8IVJ7_CHLRE/320-643	A8IVJ7.1 PF00384.20	72.60	0.00
TRINITY_D8TP15_VOLCA/86-557	D8TP15.1 PF00501.26	72.60	0.00
TRINITY_A8I8D2_CHLRE/105-225	A8I8D2.1 PF13640.4;	72.60	0.00
TRINITY_A8JFL7_CHLRE/275-425	A8JFL7.1 PF00069.23	72.60	0.00
TRINITY_E2AUA4_CAMFO/24-104	E2AUA4.1 PF00887.17	72.50	0.00
TRINITY_A0A0M3IK75_ASCLU/22-197	A0A0M3IK75.1 PF0172	72.50	0.00
TRINITY_W4H832_9STRA/799-914	W4H832.1 PF01398.19	72.50	0.00
TRINITY_A0BJM5_PARTE/1-113	A0BJM5.1 PF01283.17	72.50	0.00
TRINITY_L9L3D3_TUPCH/116-174	L9L3D3.1 PF13921.4;	72.50	0.00
TRINITY_J9IZT8_9SPIT/23-368	J9IZT8.1 PF00224.19	72.50	0.00
TRINITY_S9X060_9CETA/78-191	S9X060.1 PF00828.17	72.50	0.00
TRINITY_D8UDZ9_VOLCA/100-179	D8UDZ9.1 PF00347.21	72.50	0.00
TRINITY_A0C9U4_PARTE/24-299	A0C9U4.1 PF00009.25	72.50	0.00
TRINITY_D8TYV3_VOLCA/51-266	D8TYV3.1 PF10243.7;	72.50	0.00
TRINITY_A8J5F6_CHLRE/25-200	A8J5F6.1 PF00270.27	72.50	0.00
TRINITY_D8TP13_VOLCA/1-259	D8TP13.1 PF01201.20	72.50	0.00
TRINITY_A8J9M4_CHLRE/411-506	A8J9M4.1 PF01619.16	72.50	0.00
TRINITY_A8I3J2_CHLRE/862-901	A8I3J2.1 PF05920.9;	72.50	0.00

TRINITY_D2VBG3_NAEGR/61-468	D2VBG3.1 PF00962.20	72.50	0.00
TRINITY_E3M8I4_CAERE/11-77	E3M8I4.1 PF00076.20	72.50	0.00
TRINITY_I7LVT5_TETTS/1-175	I7LVT5.1 PF03657.11	72.50	0.00
TRINITY_A0A0D9Y8J9_9ORYZ/216-512	A0A0D9Y8J9.1 PF0017	72.50	0.00
TRINITY_A8INX1_CHLRE/68-250	A8INX1.1 PF00574.21	72.50	0.00
TRINITY_E5SX20_TRISP/61-244	E5SX20.1 PF01926.21	72.50	0.00
TRINITY_D8U3K0_VOLCA/855-1058	D8U3K0.1 PF00689.19	72.50	0.00
TRINITY_A8ISS7_CHLRE/217-307	A8ISS7.1 PF11360.6;	72.50	0.00
TRINITY_A8IGG5_CHLRE/567-774	A8IGG5.1 PF07714.15	72.50	0.00
TRINITY_H2ZAB9_CIOSA/257-398	H2ZAB9.1 PF00637.18	72.50	0.00
TRINITY_I0Z267_9CHLO/112-345	I0Z267.1 PF01544.16	72.50	0.00
TRINITY_D3B6V8_POLPA/15-181	D3B6V8.1 PF00071.20	72.50	0.00
TRINITY_A8J2V7_CHLRE/26-121	A8J2V7.1 PF12796.5;	72.50	0.00
TRINITY_A0A0N5CDG6_STREA/630-765	A0A0N5CDG6.1 PF0000	72.50	0.00
TRINITY_V5I3U6_BYSSN/3-58	V5I3U6.1 PF04758.12	72.50	0.00
TRINITY_D8UJ31_VOLCA/253-352	D8UJ31.1 PF03129.18	72.50	0.00
TRINITY_V3ZIM1_LOTGI/157-566	V3ZIM1.1 PF00654.18	72.50	0.00
TRINITY_A0A0D2QXC4_GOSRA/53-198	A0A0D2QXC4.1 PF1335	72.50	0.00
TRINITY_M1C2L8_SOLTU/13-92	M1C2L8.1 PF16845.3;	72.50	0.00
TRINITY_G5B4D5_HETGA/1-90	G5B4D5.1 PF03227.14	72.50	0.00
TRINITY_W7XI32_TETTS/8-116	W7XI32.1 PF01412.16	72.50	0.00
TRINITY_C3K7J0_PSEFS/2-81	C3K7J0.1 PF02798.18	72.50	0.00
TRINITY_A0DJ53_PARTE/197-334	A0DJ53.1 PF00004.27	72.40	0.00
TRINITY_D8LFS8_ECTSI/11-287	D8LFS8.1 PF00009.25	72.40	0.00
TRINITY_I7LUR8_TETTS/853-986	I7LUR8.2 PF00637.18	72.40	0.00
TRINITY_I7MGN0_TETTS/178-378	I7MGN0.2 PF00856.26	72.40	0.00
TRINITY_A0A067R7B6_ZOONE/53-150	A0A067R7B6.1 PF0123	72.40	0.00
TRINITY_L8GQE5_ACACA/87-221	L8GQE5.1 PF00334.17	72.40	0.00
TRINITY_I7M7Z9_TETTS/616-760	I7M7Z9.1 PF00005.25	72.40	0.00
TRINITY_Q7G6A5_ORYSJ/539-699	Q7G6A5.1 PF00078.25	72.40	0.00
TRINITY_D8U0L4_VOLCA/423-486	D8U0L4.1 PF03457.12	72.40	0.00
TRINITY_A8JA68_CHLRE/1-273	A8JA68.1 PF01008.15	72.40	0.00
TRINITY_G0QSX6_ICHMG/4-221	G0QSX6.1 PF00112.21	72.40	0.00
TRINITY_I7M998_TETTS/451-480	I7M998.2 PF01422.15	72.40	0.00
TRINITY_B3RNF8_TRIAD/14-176	B3RNF8.1 PF17144.2;	72.40	0.00
TRINITY_A0C124_PARTE/465-540	A0C124.1 PF03144.23	72.40	0.00
TRINITY_A8J3C4_CHLRE/30-226	A8J3C4.1 PF03462.16	72.40	0.00
TRINITY_L1J321_GUITH/32-638	L1J321.1 PF00012.18	72.40	0.00
TRINITY_D8U2M6_VOLCA/20-246	D8U2M6.1 PF00069.23	72.40	0.00
TRINITY_Q8W4V3_CHLRE/61-460	Q8W4V3.1 PF00464.17	72.40	0.00
TRINITY_A8I6G3_CHLRE/189-516	A8I6G3.1 PF00493.21	72.40	0.00
TRINITY_A8JH69_CHLRE/34-92	A8JH69.1 PF08642.8;	72.40	0.00
TRINITY_A8HN88_CHLRE/81-140	A8HN88.1 PF13812.4;	72.40	0.00
TRINITY_Q23GA8_TETTS/212-398	Q23GA8.3 PF01529.18	72.40	0.00
TRINITY_A8ITP1_CHLRE/141-245	A8ITP1.1 PF11987.6;	72.40	0.00
TRINITY_D8TMX0_VOLCA/175-376	D8TMX0.1 PF00448.20	72.40	0.00
TRINITY_L8HI43_ACACA/81-359	L8HI43.1 PF01761.18	72.40	0.00
TRINITY_A8J3Y6_CHLRE/1133-1398	A8J3Y6.1 PF13507.4;	72.40	0.00
TRINITY_A8HXX4_CHLRE/114-363	A8HXX4.1 PF07228.10	72.40	0.00
TRINITY_A8IYR9_CHLRE/1221-1493	A8IYR9.1 PF12775.5;	72.40	0.00
TRINITY_W7X5J4_TETTS/21-67	W7X5J4.1 PF12554.6;	72.30	0.00
TRINITY_Q24IM0_TETTS/4374-5086	Q24IM0.2 PF03028.13	72.30	0.00
TRINITY_A0C046_PARTE/90-390	A0C046.1 PF03133.13	72.30	0.00
TRINITY_Q6BFM8_PARTE/531-718	Q6BFM8.1 PF00488.19	72.30	0.00
TRINITY_F4PL91_DICFS/32-143	F4PL91.1 PF13669.4;	72.30	0.00
TRINITY_A0DVW7_PARTE/13-297	A0DVW7.1 PF00069.23	72.30	0.00
TRINITY_D2UXS3_NAEGR/418-464	D2UXS3.1 PF00249.29	72.30	0.00
TRINITY_Q234I5_TETTS/11-618	Q234I5.1 PF00012.18	72.30	0.00
TRINITY_G0QW64_ICHMG/273-469	G0QW64.1 PF13086.4;	72.30	0.00
TRINITY_F4Q6S2_DICFS/285-332	F4Q6S2.1 PF02042.13	72.30	0.00
TRINITY_D8U9E1_VOLCA/862-912	D8U9E1.1 PF13920.4;	72.30	0.00

TRINITY_A8JAR0_CHLRE/16-136	A8JAR0.1 PF01641.16	72.30	0.00
TRINITY_I0YRK9_9CHLO/3-162	I0YRK9.1 PF01734.20	72.30	0.00
TRINITY_I7MF70_TETTS/40-534	I7MF70.1 PF00118.22	72.30	0.00
TRINITY_D8UAR4_VOLCA/160-278	D8UAR4.1 PF00271.29	72.30	0.00
TRINITY_D8TIQ5_VOLCA/106-517	D8TIQ5.1 PF05834.10	72.30	0.00
TRINITY_G0QL09_ICHMG/65-248	G0QL09.1 PF01926.21	72.30	0.00
TRINITY_D8TQ59_VOLCA/165-475	D8TQ59.1 PF05291.9;	72.30	0.00
TRINITY_I7MGM4_TETTS/21-207	I7MGM4.2 PF01728.17	72.30	0.00
TRINITY_A4S316_OSTLU/9-74	A4S316.1 PF01423.20	72.30	0.00
TRINITY_A8IYL4_CHLRE/471-625	A8IYL4.1 PF13844.4;	72.30	0.00
TRINITY_F4PMI7_DICFS/27-634	F4PMI7.1 PF00012.18	72.30	0.00
TRINITY_A0CZ26_PARTE/1343-1557	A0CZ26.1 PF12781.5;	72.30	0.00
TRINITY_N1RCT6_FUSC4/14-178	N1RCT6.1 PF00160.19	72.30	0.00
TRINITY_A0BVS2_PARTE/98-234	A0BVS2.1 PF03947.16	72.30	0.00
TRINITY_D2VLQ1_NAEGR/5-192	D2VLQ1.1 PF02492.17	72.30	0.00
TRINITY_D8U252_VOLCA/5-52	D8U252.1 PF12906.5;	72.30	0.00
TRINITY_Q247Z0_TETTS/1359-1503	Q247Z0.3 PF00005.25	72.30	0.00
TRINITY_D8U751_VOLCA/1183-1591	D8U751.1 PF08393.11	72.30	0.00
TRINITY_A0DGD7_PARTE/230-572	A0DGD7.1 PF03133.13	72.30	0.00
TRINITY_G0R0I9_ICHMG/1-266	G0R0I9.1 PF00009.25	72.20	0.00
TRINITY_Q22C84_TETTS/307-580	Q22C84.2 PF02138.16	72.20	0.00
TRINITY_H2V759_TAKRU/249-485	H2V759.1 PF00089.24	72.20	0.00
TRINITY_L8H3W5_ACACA/971-1093	L8H3W5.1 PF00271.29	72.20	0.00
TRINITY_F4QB04_DICFS/230-484	F4QB04.1 PF00069.23	72.20	0.00
TRINITY_A0A095CHZ4_CRYGA/228-554	A0A095CHZ4.1 PF0015	72.20	0.00
TRINITY_L5K105_PTEAL/5-361	L5K105.1 PF00009.25	72.20	0.00
TRINITY_F0Y200_AURAN/464-581	F0Y200.1 PF01751.20	72.20	0.00
TRINITY_Q241B6_TETTS/1199-1363	Q241B6.2 PF03031.16	72.20	0.00
TRINITY_D8TUF5_VOLCA/117-328	D8TUF5.1 PF12317.6;	72.20	0.00
TRINITY_Q9ATK5_CHLRE/2095-2192	Q9ATK5.1 PF14874.4;	72.20	0.00
TRINITY_I0Z8B5_9CHLO/174-487	I0Z8B5.1 PF00282.17	72.20	0.00
TRINITY_Q22HF4_TETTS/267-471	Q22HF4.2 PF01189.15	72.20	0.00
TRINITY_Q0V5H8_PHANO/386-544	Q0V5H8.1 PF00441.22	72.20	0.00
TRINITY_A8JDH3_CHLRE/10-84	A8JDH3.1 PF04969.14	72.20	0.00
TRINITY_A0A087SMM2_AUXPR/532-621	A0A087SMM2.1 PF0854	72.20	0.00
TRINITY_D8TN75_VOLCA/173-409	D8TN75.1 PF00233.17	72.20	0.00
TRINITY_A8IMJ7_CHLRE/9-44	A8IMJ7.1 PF06220.10	72.20	0.00
TRINITY_L8GGT2_ACACA/354-435	L8GGT2.1 PF00017.22	72.20	0.00
TRINITY_D8TUM3_VOLCA/223-475	D8TUM3.1 PF00266.17	72.20	0.00
TRINITY_A0A077ZY60_STYLE/6352-6887	A0A077ZY60.1 PF0089	72.20	0.00
TRINITY_D8TV39_VOLCA/305-753	D8TV39.1 PF01055.24	72.20	0.00
TRINITY_E1ZL40_CHLVA/67-105	E1ZL40.1 PF00400.30	72.20	0.00
TRINITY_J9FVJ9_9SPIT/25-280	J9FVJ9.1 PF00069.23	72.20	0.00
TRINITY_F0ZR55_DICPU/66-326	F0ZR55.1 PF01650.16	72.20	0.00
TRINITY_Q23TU2_TETTS/1994-2229	Q23TU2.2 PF12774.5;	72.20	0.00
TRINITY_A9S9W1_PHYPA/526-565	A9S9W1.1 PF00400.30	72.20	0.00
TRINITY_A8HTI5_CHLRE/224-447	A8HTI5.1 PF00149.26	72.20	0.00
TRINITY_A0A059LHQ1_9CHLO/78-113	A0A059LHQ1.1 PF0109	72.20	0.00
TRINITY_C1E5F4_MICSR/209-312	C1E5F4.1 PF12796.5;	72.20	0.00
TRINITY_D8TJG1_VOLCA/7-287	D8TJG1.1 PF01940.14	72.20	0.00
TRINITY_D8TTD9_VOLCA/507-641	D8TTD9.1 PF02910.18	72.20	0.00
TRINITY_A0A024URK1_9STRA/85-138	A0A024URK1.1 PF0138	72.20	0.00
TRINITY_J7U8K5_MORMO/156-262	J7U8K5.1 PF13583.4;	72.10	0.00
TRINITY_A0BP79_PARTE/515-640	A0BP79.1 PF00069.23	72.10	0.00
TRINITY_A0C160_PARTE/124-184	A0C160.1 PF13921.4;	72.10	0.00
TRINITY_G0QQH8_ICHMG/137-204	G0QQH8.1 PF00542.17	72.10	0.00
TRINITY_D8QMW4_SELML/2-69	D8QMW4.1 PF01111.17	72.10	0.00
TRINITY_I0YM48_9CHLO/123-208	I0YM48.1 PF00203.19	72.10	0.00
TRINITY_Q7PXP8_ANOGA/255-297	Q7PXP8.5 PF05182.11	72.10	0.00
TRINITY_F0Z8J7_DICPU/441-569	F0Z8J7.1 PF13091.4;	72.10	0.00
TRINITY_D8TQL7_VOLCA/34-100	D8TQL7.1 PF01197.16	72.10	0.00

TRINITY_D8TXZ2_VOLCA/49-343	D8TXZ2.1 PF03151.14	72.10	0.00
TRINITY_L8GYQ9_ACACA/8-73	L8GYQ9.1 PF03931.13	72.10	0.00
TRINITY_D8TY52_VOLCA/729-877	D8TY52.1 PF00733.19	72.10	0.00
TRINITY_A8JFR4_CHLRE/54-441	A8JFR4.1 PF00202.19	72.10	0.00
TRINITY_A9S2N9_PHYPA/6-369	A9S2N9.1 PF00266.17	72.10	0.00
TRINITY_A8HQC2_CHLRE/23-259	A8HQC2.1 PF13561.4;	72.10	0.00
TRINITY_A0A015M072_9GLOM/14-82	A0A015M072.1 PF0142	72.10	0.00
TRINITY_HSTC_CHLRE/91-359	A1JHN0.1 PF01040.16	72.10	0.00
TRINITY_A8I6R4_CHLRE/209-373	A8I6R4.1 PF02769.20	72.10	0.00
TRINITY_Q0F4X0_9RHOB/25-475	Q0F4X0.1 PF01425.19	72.10	0.00
TRINITY_D8TNT6_VOLCA/43-690	D8TNT6.1 PF00995.21	72.10	0.00
TRINITY_D8U4F3_VOLCA/38-173	D8U4F3.1 PF02681.12	72.10	0.00
TRINITY_I0YZ53_9CHLO/51-202	I0YZ53.1 PF11016.6;	72.10	0.00
TRINITY_D8UF47_VOLCA/826-1058	D8UF47.1 PF02682.14	72.10	0.00
TRINITY_D8TIL1_VOLCA/2-62	D8TIL1.1 PF04193.12	72.10	0.00
TRINITY_A8JF38_CHLRE/108-269	A8JF38.1 PF00293.26	72.10	0.00
TRINITY_A8J6D4_CHLRE/100-167	A8J6D4.1 PF03776.12	72.10	0.00
TRINITY_A0A0F6W2V7_9DELT/32-163	A0A0F6W2V7.1 PF1318	72.10	0.00
TRINITY_I1G913_AMPQE/207-506	I1G913.1 PF00557.22	72.10	0.00
TRINITY_D8TX39_VOLCA/111-220	D8TX39.1 PF13353.4;	72.10	0.00
TRINITY_H2R1J3_PANTR/113-216	H2R1J3.1 PF02777.16	72.10	0.00
TRINITY_M3ZEG1_XIPMA/149-244	M3ZEG1.1 PF00031.19	72.10	0.00
TRINITY_D8U435_VOLCA/275-493	D8U435.1 PF00069.23	72.10	0.00
TRINITY_A0A078A0I1_STYLE/51-321	A0A078A0I1.1 PF0015	72.10	0.00
TRINITY_F4P5I7_BATDJ/163-273	F4P5I7.1 PF02770.17	72.10	0.00
TRINITY_A0A0G4FWU4_9ALVE/233-404	A0A0G4FWU4.1 PF0027	72.10	0.00
TRINITY_A8ITF3_CHLRE/22-440	A8ITF3.1 PF01704.16	72.10	0.00
TRINITY_A8ISQ5_CHLRE/527-928	A8ISQ5.1 PF13086.4;	72.10	0.00
TRINITY_A8IE47_CHLRE/154-333	A8IE47.1 PF01852.17	72.10	0.00
TRINITY_A0A0K9NTT1_ZOSMR/20-330	A0A0K9NTT1.1 PF0006	72.10	0.00
TRINITY_A9TN58_PHYPA/199-274	A9TN58.1 PF00076.20	72.10	0.00
TRINITY_G0QN37_ICHMG/30-529	G0QN37.1 PF00118.22	72.10	0.00
TRINITY_A8I996_CHLRE/133-271	A8I996.1 PF00004.27	72.10	0.00
TRINITY_Q247Z0_TETTS/1359-1503	Q247Z0.3 PF00005.25	72.10	0.00
TRINITY_I7M713_TETTS/140-385	I7M713.1 PF10609.7;	72.10	0.00
TRINITY_B7KET3_CYAP7/17-206	B7KET3.1 PF01078.19	72.10	0.00
TRINITY_A0BH68_PARTE/9-286	A0BH68.1 PF00069.23	72.00	0.00
TRINITY_W9RJG5_9ROSA/175-865	W9RJG5.1 PF00305.17	72.00	0.00
TRINITY_L8HHX5_ACACA/8-250	L8HHX5.1 PF00454.25	72.00	0.00
TRINITY_W2PQP4_PHYPN/64-184	W2PQP4.1 PF00004.27	72.00	0.00
TRINITY_I7MMJ3_TETTS/101-375	I7MMJ3.2 PF00682.17	72.00	0.00
TRINITY_A0D3K4_PARTE/152-201	A0D3K4.1 PF13920.4;	72.00	0.00
TRINITY_L8HGT5_ACACA/61-145	L8HGT5.1 PF03061.20	72.00	0.00
TRINITY_J9HY32_9SPIT/83-139	J9HY32.1 PF13418.4;	72.00	0.00
TRINITY_D3AXW6_POLPA/88-287	D3AXW6.1 PF07002.14	72.00	0.00
TRINITY_A8HVQ1_CHLRE/1-188	A8HVQ1.1 PF01201.20	72.00	0.00
TRINITY_A0A015LHU2_9GLOM/54-365	A0A015LHU2.1 PF0191	72.00	0.00
TRINITY_D8UDE6_VOLCA/151-243	D8UDE6.1 PF00153.25	72.00	0.00
TRINITY_Q64FN7_CHLRE/295-369	Q64FN7.1 PF13424.4;	72.00	0.00
TRINITY_A8IDE6_CHLRE/59-491	A8IDE6.1 PF07994.10	72.00	0.00
TRINITY_A0A0D3END7_9ORYZ/154-260	A0A0D3END7.1 PF0008	72.00	0.00
TRINITY_E2BY81_HARSA/5-166	E2BY81.1 PF00071.20	72.00	0.00
TRINITY_A8JHT1_CHLRE/5710-5825	A8JHT1.1 PF06101.9;	72.00	0.00
TRINITY_I0YUC4_9CHLO/62-396	I0YUC4.1 PF01207.15	72.00	0.00
TRINITY_G0QQ59_ICHMG/1-119	G0QQ59.1 PF00833.16	72.00	0.00
TRINITY_M4DND9_BRARP/82-228	M4DND9.1 PF02383.16	72.00	0.00
TRINITY_D8TMZ9_VOLCA/1-149	D8TMZ9.1 PF09752.7;	72.00	0.00
TRINITY_E1ZGN4_CHLVA/213-289	E1ZGN4.1 PF13242.4;	72.00	0.00
TRINITY_A8J9F6_CHLRE/116-201	A8J9F6.1 PF02536.12	72.00	0.00
TRINITY_D8TTC2_VOLCA/10-183	D8TTC2.1 PF07714.15	72.00	0.00
TRINITY_A8JGY0_CHLRE/5-117	A8JGY0.1 PF01984.18	72.00	0.00

TRINITY_D8UGT4_VOLCA/323-605	D8UGT4.1 PF09359.8;	72.00	0.00
TRINITY_D8U2V0_VOLCA/461-542	D8U2V0.1 PF00069.23	72.00	0.00
TRINITY_A0A0A0LAX1_CUCSA/2-152	A0A0A0LAX1.1 PF0040	72.00	0.00
TRINITY_A0EB42_PARTE/37-322	A0EB42.1 PF00069.23	71.90	0.00
TRINITY_G0R1B7_ICHMG/7-66	G0R1B7.1 PF04193.12	71.90	0.00
TRINITY_L8GMA0_ACACA/2496-2774	L8GMA0.1 PF02138.16	71.90	0.00
TRINITY_D2VGW7_NAEGR/11-149	D2VGW7.1 PF00179.24	71.90	0.00
TRINITY_G0R3Y7_ICHMG/212-270	G0R3Y7.1 PF14050.4;	71.90	0.00
TRINITY_T1FNM2_HELRO/28-123	T1FNM2.1 PF01248.24	71.90	0.00
TRINITY_F1A122_DICPU/10-288	F1A122.1 PF00069.23	71.90	0.00
TRINITY_D3BDY0_POLPA/106-283	D3BDY0.1 PF01163.20	71.90	0.00
TRINITY_F1A075_DICPU/24-207	F1A075.1 PF01012.19	71.90	0.00
TRINITY_M4BGE8_HYAAE/184-223	M4BGE8.1 PF00400.30	71.90	0.00
TRINITY_A8IN95_CHLRE/29-214	A8IN95.1 PF00227.24	71.90	0.00
TRINITY_D8UCG3_VOLCA/61-471	D8UCG3.1 PF05889.11	71.90	0.00
TRINITY_A8JEV1_CHLRE/5-198	A8JEV1.1 PF05757.9;	71.90	0.00
TRINITY_D3B072_POLPA/93-386	D3B072.1 PF00579.23	71.90	0.00
TRINITY_A8IA75_CHLRE/44-181	A8IA75.1 PF03330.16	71.90	0.00
TRINITY_D8TP03_VOLCA/143-238	D8TP03.1 PF00166.19	71.90	0.00
TRINITY_D8TIL2_VOLCA/2158-2243	D8TIL2.1 PF01068.19	71.90	0.00
TRINITY_A8ICA8_CHLRE/27-323	A8ICA8.1 PF00294.22	71.90	0.00
TRINITY_L8H556_ACACA/732-1068	L8H556.1 PF00476.18	71.90	0.00
TRINITY_E1Z4M5_CHLVA/81-379	E1Z4M5.1 PF04106.10	71.90	0.00
TRINITY_I0YMY0_9CHLO/259-447	I0YMY0.1 PF00300.20	71.90	0.00

TRINITY_M1D4E4_SOLTU/4-67	M1D4E4.1 PF00226.29	71.90	0.00
TRINITY_I0Z462_9CHLO/34-319	I0Z462.1 PF00069.23	71.90	0.00
TRINITY_D8TSR3_VOLCA/221-391	D8TSR3.1 PF10520.7;	71.90	0.00
TRINITY_A0A0G4IUP9_PLABS/33-64	A0A0G4IUP9.1 PF0360	71.90	0.00
TRINITY_A8HTL0_CHLRE/8-65	A8HTL0.1 PF05347.13	71.90	0.00
TRINITY_G0QU91_ICHMG/11-177	G0QU91.1 PF00071.20	71.90	0.00
TRINITY_L8GW32_ACACA/63-498	L8GW32.1 PF07994.10	71.90	0.00
TRINITY_G1RUC5_NOMLE/44-205	G1RUC5.1 PF00071.20	71.90	0.00
TRINITY_Q4VKB5_CHLRE/128-265	Q4VKB5.1 PF04116.11	71.90	0.00
TRINITY_A8JA32_CHLRE/65-238	A8JA32.1 PF01425.19	71.90	0.00
TRINITY_D8U4A4_VOLCA/176-409	D8U4A4.1 PF01798.16	71.90	0.00
TRINITY_I0YMS5_9CHLO/72-517	I0YMS5.1 PF08801.9;	71.90	0.00
TRINITY_S8CCD2_9LAMI/30-65	S8CCD2.1 PF00320.25	71.90	0.00
TRINITY_D8TQS8_VOLCA/150-597	D8TQS8.1 PF01593.22	71.90	0.00
TRINITY_A0A059LLW8_9CHLO/225-320	A0A059LLW8.1 PF0158	71.90	0.00
TRINITY_I0YPT8_9CHLO/2-115	I0YPT8.1 PF03834.12	71.90	0.00
TRINITY_D8TIE9_VOLCA/450-754	D8TIE9.1 PF00176.21	71.90	0.00
TRINITY_I0YT34_9CHLO/31-160	I0YT34.1 PF08240.10	71.90	0.00
TRINITY_H3EXF1_PRIPA/197-267	H3EXF1.1 PF00076.20	71.90	0.00
TRINITY_G0R280_ICHMG/52-338	G0R280.1 PF01704.16	71.80	0.00
TRINITY_A4VEZ5_TETTS/23-342	A4VEZ5.1 PF00814.23	71.80	0.00
TRINITY_R1DYD4_EMIHU/16-142	R1DYD4.1 PF00134.21	71.80	0.00
TRINITY_A0BID5_PARTE/6-91	A0BID5.1 PF01388.19	71.80	0.00
TRINITY_C3XPH3_BRAFL/317-387	C3XPH3.1 PF00076.20	71.80	0.00
TRINITY_I7MEJ2_TETTS/122-336	I7MEJ2.1 PF00112.21	71.80	0.00
TRINITY_W7XB73_TETTS/470-511	W7XB73.1 PF05186.11	71.80	0.00
TRINITY_I0ZAV6_9CHLO/30-82	I0ZAV6.1 PF07491.9;	71.80	0.00
TRINITY_D8U802_VOLCA/6-45	D8U802.1 PF01428.14	71.80	0.00
TRINITY_U6MZR3_9EIME/1-71	U6MZR3.1 PF00076.20	71.80	0.00
TRINITY_A0A0C4BF04_WHEAT/1-57	A0A0C4BF04.1 PF0042	71.80	0.00
TRINITY_D8TTL6_VOLCA/6-263	D8TTL6.1 PF00069.23	71.80	0.00
TRINITY_A8HQB8_CHLRE/129-294	A8HQB8.1 PF04055.19	71.80	0.00
TRINITY_D7SYC1_VITVI/39-112	D7SYC1.1 PF00364.20	71.80	0.00
TRINITY_D3BD64_POLPA/685-724	D3BD64.1 PF00400.30	71.80	0.00
TRINITY_K1PDE3_CRAGI/12-173	K1PDE3.1 PF00071.20	71.80	0.00
TRINITY_W4FBG8_9STRA/93-138	W4FBG8.1 PF10502.7;	71.80	0.00
TRINITY_A8I087_CHLRE/296-471	A8I087.1 PF03572.16	71.80	0.00
TRINITY_A8I696_CHLRE/1-144	A8I696.1 PF00566.16	71.80	0.00
TRINITY_E1ZL16_CHLVA/23-140	E1ZL16.1 PF06487.10	71.80	0.00
TRINITY_L8GT97_ACACA/137-369	L8GT97.1 PF00198.21	71.80	0.00
TRINITY_G0QNJ3_ICHMG/13-115	G0QNJ3.1 PF01990.15	71.80	0.00
TRINITY_A0BPU1_PARTE/40-457	A0BPU1.1 PF01603.18	71.80	0.00
TRINITY_D8UHF7_VOLCA/11-188	D8UHF7.1 PF00270.27	71.80	0.00
TRINITY_E1Z6F1_CHLVA/152-254	E1Z6F1.1 PF07978.11	71.80	0.00
TRINITY_D8TLQ5_VOLCA/580-703	D8TLQ5.1 PF13640.4;	71.80	0.00
TRINITY_D8UCK6_VOLCA/1279-1453	D8UCK6.1 PF01789.14	71.80	0.00
TRINITY_D8U3H0_VOLCA/1215-1429	D8U3H0.1 PF00488.19	71.80	0.00
TRINITY_MVPA_DICDI/504-621	P34118.1 PF11978.6;	71.80	0.00
TRINITY_A8HMR0_CHLRE/112-281	A8HMR0.1 PF04055.19	71.80	0.00
TRINITY_A8INM9_CHLRE/26-403	A8INM9.1 PF07690.14	71.80	0.00
TRINITY_A8J3U8_CHLRE/18-191	A8J3U8.1 PF13177.4;	71.80	0.00
TRINITY_D8U2W0_VOLCA/10-80	D8U2W0.1 PF00076.20	71.80	0.00
TRINITY_D8TSY5_VOLCA/1-349	D8TSY5.1 PF08393.11	71.80	0.00
TRINITY_G0QK68_ICHMG/86-259	G0QK68.1 PF00025.19	71.80	0.00
TRINITY_I7M8Y1_TETTS/109-271	I7M8Y1.2 PF03483.15	71.80	0.00
TRINITY_C1E9S6_MICSR/1-354	C1E9S6.1 PF16363.3;	71.80	0.00
TRINITY_B9SQE3_RICCO/5-254	B9SQE3.1 PF00335.18	71.80	0.00
TRINITY_H3BB87_LATCH/126-221	H3BB87.2 PF00153.25	71.70	0.00
TRINITY_D3B0E6_POLPA/9-108	D3B0E6.1 PF00034.19	71.70	0.00
TRINITY_U9UN07_RHIID/1-145	U9UN07.1 PF01125.15	71.70	0.00
TRINITY_A0A061FSZ2_THECC/367-414	A0A061FSZ2.1 PF1392	71.70	0.00

TRINITY_G0R0B5_ICHMG/45-346	G0R0B5.1 PF03133.13	71.70	0.00
TRINITY_I7M0D5_TETTS/4-286	I7M0D5.2 PF00069.23	71.70	0.00
TRINITY_A8JA07_CHLRE/449-848	A8JA07.1 PF03016.13	71.70	0.00
TRINITY_A0A067QDD7_9HOMO/1-367	A0A067QDD7.1 PF0029	71.70	0.00
TRINITY_D8TTM4_VOLCA/754-812	D8TTM4.1 PF03959.11	71.70	0.00
TRINITY_D8TTV8_VOLCA/81-324	D8TTV8.1 PF00233.17	71.70	0.00
TRINITY_A8I2G1_CHLRE/183-305	A8I2G1.1 PF00254.26	71.70	0.00
TRINITY_E1ZG76_CHLVA/1-138	E1ZG76.1 PF02580.14	71.70	0.00
TRINITY_A8HTE8_CHLRE/130-195	A8HTE8.1 PF12352.6;	71.70	0.00
TRINITY_D8TXR9_VOLCA/225-319	D8TXR9.1 PF00153.25	71.70	0.00
TRINITY_Q22DR0_TETTS/17-338	Q22DR0.2 PF00009.25	71.70	0.00
TRINITY_D8U2C8_VOLCA/51-288	D8U2C8.1 PF05721.11	71.70	0.00
TRINITY_D3BI11_POLPA/90-647	D3BI11.1 PF01175.16	71.70	0.00
TRINITY_A0A0M9UC29_9CHLR/9-254	A0A0M9UC29.1 PF0046	71.70	0.00
TRINITY_D8U240_VOLCA/226-324	D8U240.1 PF13180.4;	71.70	0.00
TRINITY_A0CGU9_PARTE/3-228	A0CGU9.1 PF00108.21	71.70	0.00
TRINITY_E1ZB53_CHLVA/77-203	E1ZB53.1 PF00828.17	71.70	0.00
TRINITY_R4XA01_TAPDE/306-366	R4XA01.1 PF12706.5;	71.70	0.00
TRINITY_D8TQL1_VOLCA/71-359	D8TQL1.1 PF00291.23	71.70	0.00
TRINITY_A0A066WM51_9BASI/47-207	A0A066WM51.1 PF0303	71.70	0.00
TRINITY_A8JBV3_CHLRE/4-269	A8JBV3.1 PF00069.23	71.70	0.00
TRINITY_D8U2E3_VOLCA/120-479	D8U2E3.1 PF01733.16	71.70	0.00
TRINITY_D8TQR2_VOLCA/38-197	D8TQR2.1 PF00160.19	71.70	0.00
TRINITY_V4ACB1_LOTGI/2-54	V4ACB1.1 PF01907.17	71.70	0.00
TRINITY_D8U6D1_VOLCA/94-140	D8U6D1.1 PF10447.7;	71.70	0.00
TRINITY_L8H1C8_ACACA/343-532	L8H1C8.1 PF00350.21	71.70	0.00
TRINITY_D8U5W7_VOLCA/1-134	D8U5W7.1 PF00005.25	71.70	0.00
TRINITY_A0EEF5_PARTE/29-120	A0EEF5.1 PF00447.15	71.70	0.00
TRINITY_A8JEN2_CHLRE/214-443	A8JEN2.1 PF00487.22	71.70	0.00
TRINITY_A0A078AEX3_STYLE/8-351	A0A078AEX3.1 PF0022	71.60	0.00
TRINITY_G4UHU0_NEUT9/78-207	G4UHU0.1 PF00043.23	71.60	0.00
TRINITY_Q2JQ46_SYNJB/2-89	Q2JQ46.1 PF04248.10	71.60	0.00
TRINITY_H3EZQ6_PRIPA/42-207	H3EZQ6.1 PF04055.19	71.60	0.00
TRINITY_D3AYM5_POLPA/12-208	D3AYM5.1 PF00596.19	71.60	0.00
TRINITY_A8ISQ5_CHLRE/2007-2137	A8ISQ5.1 PF00004.27	71.60	0.00
TRINITY_I0Z615_9CHLO/3-475	I0Z615.1 PF05694.9;	71.60	0.00
TRINITY_A0A077ZZ83_STYLE/13-301	A0A077ZZ83.1 PF0006	71.60	0.00
TRINITY_A0A061DMP8_THECC/350-439	A0A061DMP8.1 PF0007	71.60	0.00
TRINITY_D8UEZ1_VOLCA/81-175	D8UEZ1.1 PF00338.20	71.60	0.00
TRINITY_PI3K4_DICDI/557-762	P54676.2 PF00454.25	71.60	0.00
TRINITY_A0A0N4UII4_DRAME/113-211	A0A0N4UII4.1 PF0015	71.60	0.00
TRINITY_F0YCA9_AURAN/13-174	F0YCA9.1 PF00071.20	71.60	0.00
TRINITY_D8U154_VOLCA/1-138	D8U154.1 PF05419.10	71.60	0.00
TRINITY_Q24IB1_TETTS/24-305	Q24IB1.1 PF00069.23	71.60	0.00
TRINITY_A8IIE9_CHLRE/4-130	A8IIE9.1 PF12165.6;	71.60	0.00
TRINITY_I7MGZ7_TETTS/104-197	I7MGZ7.2 PF01588.18	71.60	0.00
TRINITY_D8TRG3_VOLCA/135-229	D8TRG3.1 PF07683.12	71.60	0.00
TRINITY_G0QY52_ICHMG/59-125	G0QY52.1 PF04716.12	71.60	0.00
TRINITY_A8IUY7_CHLRE/197-421	A8IUY7.1 PF03407.14	71.60	0.00
TRINITY_G0R0V1_ICHMG/15-209	G0R0V1.1 PF00687.19	71.60	0.00
TRINITY_A8INT0_CHLRE/1165-1307	A8INT0.1 PF00211.18	71.60	0.00
TRINITY_D3BEH4_POLPA/456-651	D3BEH4.1 PF00270.27	71.60	0.00
TRINITY_I0YRB1_9CHLO/194-267	I0YRB1.1 PF00382.17	71.60	0.00
TRINITY_D8TLL8_VOLCA/22-269	D8TLL8.1 PF00557.22	71.60	0.00
TRINITY_A8IZA8_CHLRE/4-79	A8IZA8.1 PF12701.5;	71.60	0.00
TRINITY_A8JCM5_CHLRE/592-791	A8JCM5.1 PF01369.18	71.60	0.00
TRINITY_D8UBI0_VOLCA/14-440	D8UBI0.1 PF00067.20	71.60	0.00
TRINITY_A0A078AHP9_STYLE/355-421	A0A078AHP9.1 PF0022	71.60	0.00
TRINITY_A0CCX5_PARTE/60-126	A0CCX5.1 PF00550.23	71.60	0.00
TRINITY_A0CCX5_PARTE/60-126	A0CCX5.1 PF00550.23	71.60	0.00
TRINITY_A8IS11_CHLRE/406-740	A8IS11.1 PF00702.24	71.60	0.00

TRINITY_Q241B6_TETTS/1199-1363	Q241B6.2 PF03031.16	71.50	0.00
TRINITY_D8UKJ5_VOLCA/169-433	D8UKJ5.1 PF00069.23	71.50	0.00
TRINITY_F4QBH6_DICFS/16-280	F4QBH6.1 PF00069.23	71.50	0.00
TRINITY_D8TIH5_VOLCA/37-194	D8TIH5.1 PF00504.19	71.50	0.00
TRINITY_RL5_TETTS/14-176	Q231U7.2 PF17144.2;	71.50	0.00
TRINITY_I7LSV4_TETTS/3173-3398	I7LSV4.2 PF12781.5;	71.50	0.00
TRINITY_I7MJT9_TETTS/19-342	I7MJT9.1 PF00225.21	71.50	0.00
TRINITY_DEGP2_ARATH/121-308	O82261.2 PF00089.24	71.50	0.00
TRINITY_B7G964_PHATC/54-201	B7G964.1 PF01257.17	71.50	0.00
TRINITY_PGM1_DICDI/18-161	Q23919.1 PF02878.14	71.50	0.00
TRINITY_B4FBC2_MAIZE/32-271	B4FBC2.1 PF01370.19	71.50	0.00
TRINITY_A8IBF9_CHLRE/92-269	A8IBF9.1 PF00551.17	71.50	0.00
TRINITY_A0A0E3ZJV1_9BACT/33-379	A0A0E3ZJV1.1 PF1028	71.40	0.00
TRINITY_G8YBA3_PICSO/217-342	G8YBA3.1 PF00383.21	71.40	0.00
TRINITY_A0A096T653_MAIZE/226-297	A0A096T653.1 PF1343	71.40	0.00
TRINITY_I7M8Q5_TETTS/67-185	I7M8Q5.2 PF01641.16	71.40	0.00
TRINITY_K7MTZ1_SOYBN/120-251	K7MTZ1.1 PF01529.18	71.40	0.00
TRINITY_D8TZC1_VOLCA/821-877	D8TZC1.1 PF00004.27	71.40	0.00
TRINITY_D8TSH1_VOLCA/17-104	D8TSH1.1 PF00153.25	71.40	0.00
TRINITY_D3BK65_POLPA/49-450	D3BK65.1 PF05889.11	71.40	0.00
TRINITY_I7MGT3_TETTS/477-511	I7MGT3.2 PF13831.4;	71.40	0.00
TRINITY_J9HXH4_9SPIT/314-525	J9HXH4.1 PF00211.18	71.40	0.00
TRINITY_R1FJW2_EMIHU/3-75	R1FJW2.1 PF13499.4;	71.40	0.00
TRINITY_Q6UKY7_CHLRE/11-73	Q6UKY7.1 PF05676.11	71.40	0.00
TRINITY_U3J4Z9_ANAPL/87-548	U3J4Z9.1 PF00501.26	71.40	0.00
TRINITY_DNAJH_SYNY3/8-70	P50027.1 PF00226.29	71.40	0.00
TRINITY_A0DZP1_PARTE/30-624	A0DZP1.1 PF00012.18	71.40	0.00
TRINITY_D8TGW1_VOLCA/127-365	D8TGW1.1 PF00557.22	71.40	0.00
TRINITY_G0R0D8_ICHMG/37-295	G0R0D8.1 PF04078.11	71.40	0.00
TRINITY_D8TYS8_VOLCA/1-137	D8TYS8.1 PF02527.13	71.40	0.00
TRINITY_A0A0A1MD11_9BACI/7-124	A0A0A1MD11.1 PF0058	71.40	0.00
TRINITY_D8UC57_VOLCA/315-398	D8UC57.1 PF01494.17	71.40	0.00
TRINITY_E1ZN42_CHLVA/148-241	E1ZN42.1 PF00166.19	71.40	0.00
TRINITY_A0DAK7_PARTE/9-358	A0DAK7.1 PF00285.19	71.40	0.00
TRINITY_W5MEL6_LEPOC/11-266	W5MEL6.1 PF03437.13	71.40	0.00
TRINITY_D8U1V2_VOLCA/50-218	D8U1V2.1 PF00504.19	71.40	0.00
TRINITY_E9HEH0_DAPPU/125-181	E9HEH0.1 PF00013.27	71.40	0.00
TRINITY_I7MA65_TETTS/30-100	I7MA65.1 PF00076.20	71.40	0.00
TRINITY_A8IWF9_CHLRE/284-500	A8IWF9.1 PF05127.12	71.40	0.00
TRINITY_E1ZKU2_CHLVA/285-636	E1ZKU2.1 PF00493.21	71.40	0.00
TRINITY_A0A059CC78_EUCGR/285-348	A0A059CC78.1 PF0007	71.40	0.00
TRINITY_Q235I9_TETTS/1375-1547	Q235I9.2 PF00270.27	71.40	0.00
TRINITY_A8J3Q2_CHLRE/82-165	A8J3Q2.1 PF03061.20	71.40	0.00
TRINITY_A0A0B2VWK9_TOXCA/3-379	A0A0B2VWK9.1 PF0002	71.40	0.00
TRINITY_PDC2_ORYSJ/44-218	Q10MW3.1 PF02776.16	71.40	0.00
TRINITY_L8H006_ACACA/393-494	L8H006.1 PF06047.9;	71.40	0.00
TRINITY_I7MFC1_TETTS/454-574	I7MFC1.2 PF02852.20	71.40	0.00
TRINITY_I0YMS5_9CHLO/72-517	I0YMS5.1 PF08801.9;	71.40	0.00
TRINITY_A8I3M7_CHLRE/1181-1220	A8I3M7.1 PF00226.29	71.40	0.00
TRINITY_A0A0D2VWK4_CAPO3/60-99	A0A0D2VWK4.1 PF0142	71.40	0.00
TRINITY_A0A0D2VWK4_CAPO3/60-99	A0A0D2VWK4.1 PF0142	71.40	0.00
TRINITY_A8I3X6_CHLRE/1-372	A8I3X6.1 PF08393.11	71.40	0.00
TRINITY_D8TF39_SELML/12-144	D8TF39.1 PF00380.17	71.40	0.00
TRINITY_A8IDT8_CHLRE/297-359	A8IDT8.1 PF00076.20	71.40	0.00
TRINITY_A8ISP5_CHLRE/116-218	A8ISP5.1 PF00153.25	71.40	0.00
TRINITY_A8JAI8_CHLRE/4-182	A8JAI8.1 PF00227.24	71.40	0.00
TRINITY_O24426_CHLRE/6-380	O24426.1 PF00022.17	71.40	0.00
TRINITY_I0Z955_9CHLO/143-197	I0Z955.1 PF10309.7;	71.40	0.00
TRINITY_D8UK85_VOLCA/265-463	D8UK85.1 PF00587.23	71.40	0.00
TRINITY_D8TTJ4_VOLCA/17-94	D8TTJ4.1 PF12894.5;	71.40	0.00
TRINITY_D8TH65_VOLCA/561-753	D8TH65.1 PF03950.16	71.40	0.00

TRINITY_D8UDL2_VOLCA/636-712	D8UDL2.1 PF00069.23	71.40	0.00
TRINITY_A0A0G4GWI5_9ALVE/604-813	A0A0G4GWI5.1 PF0021	71.40	0.00
TRINITY_V4B5R1_LOTGI/1-115	V4B5R1.1 PF00583.23	71.30	0.00
TRINITY_D8LJW0_ECTSI/3-176	D8LJW0.1 PF00025.19	71.30	0.00
TRINITY_Q23BX7_TETTS/77-169	Q23BX7.2 PF00254.26	71.30	0.00
TRINITY_D8QXE2_SELML/8-171	D8QXE2.1 PF00160.19	71.30	0.00
TRINITY_F4PLV5_DICFS/13-332	F4PLV5.1 PF00180.18	71.30	0.00
TRINITY_C1MQM4_MICPC/64-230	C1MQM4.1 PF00270.27	71.30	0.00
TRINITY_Q23W06_TETTS/135-298	Q23W06.3 PF03167.17	71.30	0.00
TRINITY_U9T4N2_RHIID/27-275	U9T4N2.1 PF00753.25	71.30	0.00
TRINITY_A8JB54_CHLRE/70-297	A8JB54.1 PF01237.16	71.30	0.00
TRINITY_I3ZC34_TERRK/3-275	I3ZC34.1 PF00749.19	71.30	0.00
TRINITY_L8H6J5_ACACA/267-374	L8H6J5.1 PF01661.19	71.30	0.00
TRINITY_D8TRN4_VOLCA/77-333	D8TRN4.1 PF08704.8;	71.30	0.00
TRINITY_A8I9T2_CHLRE/11-335	A8I9T2.1 PF00225.21	71.30	0.00
TRINITY_B6AB19_CRYMR/57-223	B6AB19.1 PF00270.27	71.30	0.00
TRINITY_A0A061E0L6_THECC/1443-1653	A0A061E0L6.1 PF0045	71.30	0.00
TRINITY_G1X9L3_ARTOA/2-143	G1X9L3.1 PF00125.22	71.30	0.00
TRINITY_W7TKS0_9STRA/126-321	W7TKS0.1 PF03171.18	71.30	0.00
TRINITY_A8HY76_CHLRE/17-265	A8HY76.1 PF02110.13	71.30	0.00
TRINITY_A8JEV1_CHLRE/5-198	A8JEV1.1 PF05757.9;	71.30	0.00
TRINITY_A8NC61_COPC7/99-247	A8NC61.2 PF00005.25	71.30	0.00
TRINITY_E1ZTT0_CHLVA/507-717	E1ZTT0.1 PF00326.19	71.30	0.00
TRINITY_A8I1M5_CHLRE/630-818	A8I1M5.1 PF07724.12	71.30	0.00
TRINITY_D8UE51_VOLCA/16-238	D8UE51.1 PF09084.9;	71.30	0.00
TRINITY_D8U6Y4_VOLCA/85-387	D8U6Y4.1 PF00128.22	71.30	0.00
TRINITY_A0D6H4_PARTE/25-310	A0D6H4.1 PF00069.23	71.30	0.00
TRINITY_A8J3J7_CHLRE/7-187	A8J3J7.1 PF00857.18	71.30	0.00
TRINITY_J9JUQ1_ACYPI/18-466	J9JUQ1.1 PF01425.19	71.20	0.00
TRINITY_A0A0G4IP29_PLABS/13-302	A0A0G4IP29.1 PF0006	71.20	0.00
TRINITY_U1MDL2_ASCSU/20-114	U1MDL2.1 PF01248.24	71.20	0.00
TRINITY_H2LRL9_ORYLA/1096-1185	H2LRL9.1 PF07677.12	71.20	0.00
TRINITY_L8HFM0_ACACA/458-593	L8HFM0.1 PF02867.13	71.20	0.00
TRINITY_M4B270_HYAAE/28-128	M4B270.1 PF13302.5;	71.20	0.00
TRINITY_F0VJ54_NEOCL/73-222	F0VJ54.1 PF12146.6;	71.20	0.00
TRINITY_F4Q137_DICFS/5-143	F4Q137.1 PF00179.24	71.20	0.00
TRINITY_Q8LNT1_ORYSJ/143-278	Q8LNT1.1 PF00892.18	71.20	0.00
TRINITY_I3J7Q1_ORENI/42-187	I3J7Q1.1 PF00061.21	71.20	0.00
TRINITY_I7MHK8_TETTS/244-385	I7MHK8.1 PF02773.14	71.20	0.00
TRINITY_F0ZP19_DICPU/70-509	F0ZP19.1 PF13520.4;	71.20	0.00
TRINITY_G0R2W8_ICHMG/56-116	G0R2W8.1 PF00288.24	71.20	0.00
TRINITY_A0BKI0_PARTE/327-399	A0BKI0.1 PF00313.20	71.20	0.00
TRINITY_E1ZGT7_CHLVA/875-1022	E1ZGT7.1 PF00004.27	71.20	0.00
TRINITY_D8U0I3_VOLCA/1-208	D8U0I3.1 PF01591.16	71.20	0.00
TRINITY_I0YSG4_9CHLO/1-79	I0YSG4.1 PF01249.16	71.20	0.00
TRINITY_C3XQV0_BRAFL/8-73	C3XQV0.1 PF01423.20	71.20	0.00
TRINITY_A0A078ACN6_STYLE/494-552	A0A078ACN6.1 PF1392	71.20	0.00
TRINITY_D8TTC3_VOLCA/645-758	D8TTC3.1 PF01061.22	71.20	0.00
TRINITY_D8TSK9_VOLCA/4-203	D8TSK9.1 PF01513.19	71.20	0.00
TRINITY_A1D7V8_NEOFI/6-162	A1D7V8.1 PF00160.19	71.20	0.00
TRINITY_I0YIS4_9CHLO/494-644	I0YIS4.1 PF00160.19	71.20	0.00
TRINITY_I0YN57_9CHLO/106-225	I0YN57.1 PF09335.9;	71.20	0.00
TRINITY_J9JCA5_9SPIT/125-339	J9JCA5.1 PF00112.21	71.20	0.00
TRINITY_D8TRH3_VOLCA/858-1070	D8TRH3.1 PF01061.22	71.20	0.00
TRINITY_E3T5C1_CROVB/3-74	E3T5C1.1 PF00240.21	71.20	0.00
TRINITY_Q4RHD1_TETNG/8-109	Q4RHD1.1 PF00042.20	71.20	0.00
TRINITY_A0A0G4ET17_9ALVE/1-249	A0A0G4ET17.1 PF0096	71.20	0.00
TRINITY_A0BFU1_PARTE/28-283	A0BFU1.1 PF00069.23	71.20	0.00
TRINITY_S7XFB7_SPRLO/42-237	S7XFB7.1 PF00149.26	71.20	0.00
TRINITY_A8J9I1_CHLRE/67-139	A8J9I1.1 PF04640.12	71.20	0.00
TRINITY_A0A058ZGC8_9EUKA/17-258	A0A058ZGC8.1 PF0057	71.20	0.00

TRINITY_A0DS30_PARTE/165-395	A0DS30.1 PF00198.21	71.20	0.00
TRINITY_D8TVM2_VOLCA/291-443	D8TVM2.1 PF00441.22	71.20	0.00
TRINITY_D8TQE1_VOLCA/181-346	D8TQE1.1 PF04127.13	71.20	0.00
TRINITY_A8IWM0_CHLRE/93-193	A8IWM0.1 PF00561.18	71.20	0.00
TRINITY_A8INC7_CHLRE/152-211	A8INC7.1 PF12656.5;	71.20	0.00
TRINITY_E1Z8K6_CHLVA/428-581	E1Z8K6.1 PF01343.16	71.20	0.00
TRINITY_D8UA96_VOLCA/198-448	D8UA96.1 PF07766.11	71.20	0.00
TRINITY_Q3SDM6_PARTE/17-177	Q3SDM6.1 PF00071.20	71.20	0.00
TRINITY_D8U2N0_VOLCA/133-249	D8U2N0.1 PF00128.22	71.20	0.00
TRINITY_G3SDF1_GORGO/734-1359	G3SDF1.1 PF01576.17	71.20	0.00
TRINITY_F7II54_CALJA/13-214	F7II54.1 PF00330.18	71.20	0.00
TRINITY_A0BSI3_PARTE/7-123	A0BSI3.1 PF00235.17	71.10	0.00
TRINITY_I7MMF7_TETTS/570-607	I7MMF7.2 PF03638.13	71.10	0.00
TRINITY_F4PRW5_DICFS/17-294	F4PRW5.1 PF00398.18	71.10	0.00
TRINITY_A0A068SDF3_9FUNG/19-94	A0A068SDF3.1 PF0093	71.10	0.00
TRINITY_A8JC77_CHLRE/1-279	A8JC77.1 PF00225.21	71.10	0.00
TRINITY_E1ZHV9_CHLVA/66-201	E1ZHV9.1 PF04116.11	71.10	0.00
TRINITY_M4ASI0_XIPMA/32-183	M4ASI0.1 PF11032.6;	71.10	0.00
TRINITY_A0A067HHJ2_CITSI/382-426	A0A067HHJ2.1 PF0040	71.10	0.00
TRINITY_I7MLB6_TETTS/13-264	I7MLB6.1 PF00069.23	71.10	0.00
TRINITY_J9HVL7_9SPIT/73-307	J9HVL7.1 PF01674.16	71.10	0.00
TRINITY_I7M3G5_TETTS/1235-1331	I7M3G5.1 PF04059.10	71.10	0.00
TRINITY_A8I872_CHLRE/401-536	A8I872.1 PF00005.25	71.10	0.00
TRINITY_E1ZR27_CHLVA/519-691	E1ZR27.1 PF07992.12	71.10	0.00
TRINITY_A0A059LLR6_9CHLO/7-71	A0A059LLR6.1 PF1343	71.10	0.00
TRINITY_D8TZC2_VOLCA/19-63	D8TZC2.1 PF01585.21	71.10	0.00
TRINITY_I0Z7E8_9CHLO/53-147	I0Z7E8.1 PF13409.4;	71.10	0.00
TRINITY_A8J212_CHLRE/65-251	A8J212.1 PF00227.24	71.10	0.00
TRINITY_G0R4K3_ICHMG/302-340	G0R4K3.1 PF00400.30	71.10	0.00
TRINITY_A0BFU1_PARTE/28-283	A0BFU1.1 PF00069.23	71.10	0.00
TRINITY_M1UVS2_CYAME/1080-1282	M1UVS2.1 PF02786.15	71.10	0.00
TRINITY_A0A0J8D431_BETVU/1134-1171	A0A0J8D431.1 PF0062	71.10	0.00
TRINITY_D8UJ23_VOLCA/23-144	D8UJ23.1 PF00572.16	71.10	0.00
TRINITY_D8U5K4_VOLCA/61-210	D8U5K4.1 PF08231.10	71.10	0.00
TRINITY_D8TQ42_VOLCA/214-445	D8TQ42.1 PF01262.19	71.10	0.00
TRINITY_I7MD69_TETTS/14-307	I7MD69.1 PF00069.23	71.10	0.00
TRINITY_Q22XN2_TETTS/43-220	Q22XN2.1 PF02146.15	71.10	0.00
TRINITY_Q236J1_TETTS/86-573	Q236J1.1 PF01039.20	71.10	0.00
TRINITY_A8INK2_CHLRE/17-54	A8INK2.1 PF01753.16	71.10	0.00
TRINITY_U6M3F7_EIMMA/104-182	U6M3F7.1 PF01529.18	71.10	0.00
TRINITY_G0QRB2_ICHMG/101-199	G0QRB2.1 PF00153.25	71.10	0.00
TRINITY_Q24DR4_TETTS/102-148	Q24DR4.2 PF00249.29	71.10	0.00
TRINITY_Q24DR4_TETTS/102-148	Q24DR4.2 PF00249.29	71.10	0.00
TRINITY_I0YM50_9CHLO/67-252	I0YM50.1 PF00350.21	71.10	0.00
TRINITY_D8TWL3_VOLCA/5-253	D8TWL3.1 PF02361.14	71.10	0.00
TRINITY_A8HX99_CHLRE/214-381	A8HX99.1 PF00160.19	71.10	0.00
TRINITY_A0A0D3B882_BRAOL/178-215	A0A0D3B882.1 PF0142	71.10	0.00
TRINITY_D3BHK7_POLPA/1514-1634	D3BHK7.1 PF02687.19	71.10	0.00
TRINITY_I0YNX0_9CHLO/469-606	I0YNX0.1 PF02910.18	71.10	0.00
TRINITY_A0A0D3F9S3_9ORYZ/694-790	A0A0D3F9S3.1 PF0405	71.10	0.00
TRINITY_H1SFD6_9BURK/7-160	H1SFD6.1 PF01625.19	71.10	0.00
TRINITY_W2RAX8_PHYPN/14-175	W2RAX8.1 PF00071.20	71.00	0.00
TRINITY_D8UEM2_VOLCA/42-134	D8UEM2.1 PF00254.26	71.00	0.00
TRINITY_J9HWN9_9SPIT/176-304	J9HWN9.1 PF01467.24	71.00	0.00
TRINITY_S7UK71_TOXGO/169-306	S7UK71.1 PF00179.24	71.00	0.00
TRINITY_A0A067QKL9_9HOMO/6-183	A0A067QKL9.1 PF0172	71.00	0.00
TRINITY_J9HWY1_9SPIT/263-327	J9HWY1.1 PF13499.4;	71.00	0.00
TRINITY_D8TS54_VOLCA/44-151	D8TS54.1 PF01521.18	71.00	0.00
TRINITY_J9IU65_9SPIT/608-801	J9IU65.1 PF05057.12	71.00	0.00
TRINITY_G0QX71_ICHMG/303-372	G0QX71.1 PF00076.20	71.00	0.00
TRINITY_A0A0D9VXY0_9ORYZ/17-139	A0A0D9VXY0.1 PF0346	71.00	0.00

TRINITY_A0A0D3DJ59_BRAOL/54-157	A0A0D3DJ59.1 PF0648	71.00	0.00
TRINITY_I0ANS2_IGNAJ/4-227	I0ANS2.1 PF00300.20	71.00	0.00
TRINITY_S8CMQ1_9LAMI/383-615	S8CMQ1.1 PF00149.26	71.00	0.00
TRINITY_A0A087SC98_AUXPR/3-72	A0A087SC98.1 PF0548	71.00	0.00
TRINITY_D8UJC9_VOLCA/697-758	D8UJC9.1 PF00226.29	71.00	0.00
TRINITY_G5EDD4_CAEEL/3-212	G5EDD4.1 PF00091.23	71.00	0.00
TRINITY_J9IF18_9SPIT/297-439	J9IF18.1 PF02840.13	71.00	0.00
TRINITY_A0D319_PARTE/71-135	A0D319.1 PF13499.4;	71.00	0.00
TRINITY_M1UVD9_CYAME/1-104	M1UVD9.1 PF03656.11	71.00	0.00
TRINITY_F7G4M4_MONDO/5-252	F7G4M4.2 PF01086.15	71.00	0.00
TRINITY_T1G8U8_HELRO/132-239	T1G8U8.1 PF00307.29	71.00	0.00
TRINITY_A8IXI7_CHLRE/236-341	A8IXI7.1 PF00153.25	71.00	0.00
TRINITY_A0A078A3F5_STYLE/487-630	A0A078A3F5.1 PF0000	71.00	0.00
TRINITY_A8J7T7_CHLRE/102-321	A8J7T7.1 PF00112.21	71.00	0.00
TRINITY_A0A068RH38_9FUNG/6-60	A0A068RH38.1 PF0520	70.90	0.00
TRINITY_G0QXR4_ICHMG/82-136	G0QXR4.1 PF01192.20	70.90	0.00
TRINITY_Q23QF2_TETTS/248-571	Q23QF2.2 PF03133.13	70.90	0.00
TRINITY_U4LU72_PYROM/47-258	U4LU72.1 PF01184.17	70.90	0.00
TRINITY_E1Z343_CHLVA/26-211	E1Z343.1 PF01012.19	70.90	0.00
TRINITY_M2R1T0_CERS8/1-126	M2R1T0.1 PF01092.17	70.90	0.00
TRINITY_B3PDE1_CELJU/181-470	B3PDE1.1 PF00393.17	70.90	0.00
TRINITY_A8IGM5_CHLRE/7-296	A8IGM5.1 PF00795.20	70.90	0.00
TRINITY_A8J911_CHLRE/146-200	A8J911.1 PF13639.4;	70.90	0.00
TRINITY_A0A078ATQ4_STYLE/166-308	A0A078ATQ4.1 PF0551	70.90	0.00
TRINITY_G0QX41_ICHMG/40-335	G0QX41.1 PF00069.23	70.90	0.00
TRINITY_TCPB_DICDI/34-524	Q54ES9.1 PF00118.22	70.90	0.00
TRINITY_U6GK02_EIMAC/106-385	U6GK02.1 PF08648.10	70.90	0.00
TRINITY_FEN1_NEMVE/1-107	A7RRJ0.1 PF00752.15	70.90	0.00
TRINITY_A8JIS8_CHLRE/44-194	A8JIS8.1 PF00005.25	70.90	0.00
TRINITY_A8J7C8_CHLRE/230-580	A8J7C8.1 PF00152.18	70.90	0.00
TRINITY_J9HXX1_9SPIT/130-420	J9HXX1.1 PF00069.23	70.90	0.00
TRINITY_S8BVD4_9LAMI/137-311	S8BVD4.1 PF07717.14	70.90	0.00
TRINITY_E1ZEY4_CHLVA/1-175	E1ZEY4.1 PF14580.4;	70.90	0.00
TRINITY_A8IE17_CHLRE/15-164	A8IE17.1 PF03073.13	70.90	0.00
TRINITY_A0A090MCW7_OSTTA/6-240	A0A090MCW7.1 PF0137	70.90	0.00
TRINITY_UGGG_DICDI/1372-1605	Q8T191.2 PF01501.18	70.90	0.00
TRINITY_W1P3N8_AMBTC/981-1177	W1P3N8.1 PF12717.5;	70.90	0.00
TRINITY_A8IJF1_CHLRE/7-265	A8IJF1.1 PF03619.14	70.90	0.00
TRINITY_A8HN56_CHLRE/225-326	A8HN56.1 PF13499.4;	70.90	0.00
TRINITY_A0A023B7Q3_GRENI/27-81	A0A023B7Q3.1 PF0166	70.90	0.00
TRINITY_A0A023B7Q3_GRENI/27-81	A0A023B7Q3.1 PF0166	70.90	0.00
TRINITY_G7IDZ4_MEDTR/189-687	G7IDZ4.1 PF00183.16	70.90	0.00
TRINITY_D8TJG4_VOLCA/48-173	D8TJG4.1 PF10155.7;	70.90	0.00
TRINITY_A8J2W0_CHLRE/9-227	A8J2W0.1 PF00171.20	70.90	0.00
TRINITY_Q84X76_CHLRE/163-272	Q84X76.1 PF10507.7;	70.90	0.00
TRINITY_I7M700_TETTS/181-443	I7M700.2 PF00069.23	70.90	0.00
TRINITY_K9T3X2_9CYAN/32-477	K9T3X2.1 PF00521.18	70.90	0.00
TRINITY_A8HZR1_CHLRE/19353-19622	A8HZR1.1 PF00975.18	70.90	0.00
TRINITY_Q24IL9_TETTS/1333-1477	Q24IL9.2 PF00005.25	70.90	0.00
TRINITY_D8U7E7_VOLCA/13-187	D8U7E7.1 PF00270.27	70.90	0.00
TRINITY_I7M967_TETTS/407-548	I7M967.1 PF04377.13	70.90	0.00
TRINITY_D8TZN5_VOLCA/93-195	D8TZN5.1 PF16131.3;	70.90	0.00
TRINITY_A0E5G8_PARTE/143-683	A0E5G8.1 PF03571.13	70.90	0.00
TRINITY_G0QJ46_ICHMG/1-145	G0QJ46.1 PF01125.15	70.90	0.00
TRINITY_A0DXT9_PARTE/396-460	A0DXT9.1 PF13432.4;	70.80	0.00
TRINITY_D3BF71_POLPA/219-289	D3BF71.1 PF15906.3;	70.80	0.00
TRINITY_F7AX64_CALJA/58-219	F7AX64.1 PF00071.20	70.80	0.00
TRINITY_A0A0K9Q6D6_ZOSMR/82-266	A0A0K9Q6D6.1 PF0475	70.80	0.00
TRINITY_B7Q170_IXOSC/51-317	B7Q170.1 PF00899.19	70.80	0.00
TRINITY_L8H7L6_ACACA/2-162	L8H7L6.1 PF00160.19	70.80	0.00
TRINITY_D8TWN7_VOLCA/6-278	D8TWN7.1 PF00795.20	70.80	0.00

TRINITY_Q3SD81_PARTE/16-176	Q3SD81.1	PF00071.20	70.80	0.00
TRINITY_Q23KG1_TETTS/160-231	Q23KG1.3	PF03719.13	70.80	0.00
TRINITY_C1E0B3_MICSR/14-152	C1E0B3.1	PF00179.24	70.80	0.00
TRINITY_W2QSL5_PHYPN/1-107	W2QSL5.1	PF04825.11	70.80	0.00
TRINITY_D8TVT1_VOLCA/62-167	D8TVT1.1	PF00085.18	70.80	0.00
TRINITY_D8U345_VOLCA/113-255	D8U345.1	PF00650.18	70.80	0.00
TRINITY_A0E153_PARTE/125-437	A0E153.1	PF00012.18	70.80	0.00
TRINITY_A8JHP0_CHLRE/60-465	A8JHP0.1	PF01593.22	70.80	0.00
TRINITY_D8TM87_VOLCA/30-104	D8TM87.1	PF13656.4;	70.80	0.00
TRINITY_A8JI35_CHLRE/4-300	A8JI35.1	PF00069.23	70.80	0.00
TRINITY_D8UFI6_VOLCA/112-190	D8UFI6.1	PF00622.26	70.80	0.00
TRINITY_Q5TNJ4_ANOGA/104-168	Q5TNJ4.3	PF00327.18	70.80	0.00
TRINITY_W7XBY2_TETTS/275-798	W7XBY2.1	PF02867.13	70.80	0.00
TRINITY_O64927_CHLRE/149-401	O64927.2	PF08323.9;	70.80	0.00
TRINITY_D8U7F8_VOLCA/1707-1755	D8U7F8.1	PF02042.13	70.80	0.00
TRINITY_I0YNU2_9CHLO/91-261	I0YNU2.1	PF13439.4;	70.80	0.00
TRINITY_D8U986_VOLCA/307-378	D8U986.1	PF00575.21	70.80	0.00
TRINITY_D8TSH3_VOLCA/615-663	D8TSH3.1	PF10403.7;	70.80	0.00
TRINITY_A0A077ZPL9_STYLE/99-171	A0A077ZPL9.1	PF1349	70.80	0.00
TRINITY_D8UGC4_VOLCA/18-338	D8UGC4.1	PF00225.21	70.80	0.00
TRINITY_V7C1H6_PHAVU/153-219	V7C1H6.1	PF14559.4;	70.70	0.00
TRINITY_A0A0D2WL90_CAPO3/1704-1922	A0A0D2WL90.1	PF0214	70.70	0.00
TRINITY_G0QZ21_ICHMG/64-162	G0QZ21.1	PF00673.19	70.70	0.00
TRINITY_A0A067CH25_SAPPC/30-278	A0A067CH25.1	PF0037	70.70	0.00
TRINITY_G3IVK3_9GAMM/389-530	G3IVK3.1	PF02775.19	70.70	0.00
TRINITY_M4C049_HYAAE/417-566	M4C049.1	PF00005.25	70.70	0.00
TRINITY_T1IA37_RHOPR/3-169	T1IA37.1	PF05856.10	70.70	0.00
TRINITY_A0E741_PARTE/638-836	A0E741.1	PF01434.16	70.70	0.00
TRINITY_A9TGY2_PHYPA/315-454	A9TGY2.1	PF00004.27	70.70	0.00
TRINITY_E1ZMC1_CHLVA/1-86	E1ZMC1.1	PF00111.25	70.70	0.00
TRINITY_A0A058ZR90_EUCGR/392-535	A0A058ZR90.1	PF0277	70.70	0.00
TRINITY_A8I815_CHLRE/125-257	A8I815.1	PF00730.23	70.70	0.00
TRINITY_Q234I5_TETTS/11-618	Q234I5.1	PF00012.18	70.70	0.00
TRINITY_F4Q6H9_DICFS/339-402	F4Q6H9.1	PF13921.4;	70.70	0.00
TRINITY_A8J921_CHLRE/279-389	A8J921.1	PF07522.12	70.70	0.00
TRINITY_G0R210_ICHMG/224-577	G0R210.1	PF00152.18	70.70	0.00
TRINITY_D8TZV2_VOLCA/32-154	D8TZV2.1	PF13589.4;	70.70	0.00
TRINITY_E1ZEH1_CHLVA/412-551	E1ZEH1.1	PF00004.27	70.70	0.00
TRINITY_D8U3B8_VOLCA/16-330	D8U3B8.1	PF00225.21	70.70	0.00
TRINITY_D8TPK4_VOLCA/13-76	D8TPK4.1	PF01066.19	70.70	0.00
TRINITY_L8GZ33_ACACA/1-430	L8GZ33.1	PF01532.18	70.70	0.00
TRINITY_T1KVR0_TETUR/210-496	T1KVR0.1	PF01409.18	70.70	0.00
TRINITY_B6BIT4_SULGG/4-65	B6BIT4.1	PF00226.29	70.70	0.00
TRINITY_D8UHW6_VOLCA/104-529	D8UHW6.1	PF00310.19	70.70	0.00
TRINITY_B8ALV9_ORYSI/25-308	B8ALV9.1	PF00069.23	70.70	0.00
TRINITY_A0A015KWK9_9GLOM/362-402	A0A015KWK9.1	PF1363	70.70	0.00
TRINITY_I7M0F7_TETTS/1-239	I7M0F7.2	PF00149.26	70.70	0.00
TRINITY_D8UHR3_VOLCA/53-145	D8UHR3.1	PF02225.20	70.70	0.00
TRINITY_D8TT81_VOLCA/280-477	D8TT81.1	PF01734.20	70.70	0.00
TRINITY_I0YZT6_9CHLO/13-422	I0YZT6.1	PF05834.10	70.70	0.00
TRINITY_D8TZK2_VOLCA/16946-17196	D8TZK2.1	PF00109.24	70.70	0.00
TRINITY_A8HQT5_CHLRE/54-428	A8HQT5.1	PF01933.16	70.70	0.00
TRINITY_A8JGY0_CHLRE/5-117	A8JGY0.1	PF01984.18	70.70	0.00
TRINITY_D8TW13_VOLCA/228-326	D8TW13.1	PF13640.4;	70.70	0.00
TRINITY_I0YWU5_9CHLO/707-938	I0YWU5.1	PF00005.25	70.70	0.00
TRINITY_A8HTX4_CHLRE/6-135	A8HTX4.1	PF04446.10	70.70	0.00
TRINITY_D8UGG9_VOLCA/958-1056	D8UGG9.1	PF04676.12	70.70	0.00
TRINITY_B1Y7Z0_LEPCP/762-878	B1Y7Z0.1	PF02518.24	70.70	0.00
TRINITY_G0R4S1_ICHMG/27-120	G0R4S1.1	PF01248.24	70.60	0.00
TRINITY_A0EH23_PARTE/630-898	A0EH23.1	PF00365.18	70.60	0.00
TRINITY_I7MEY9_TETTS/1352-1465	I7MEY9.2	PF00271.29	70.60	0.00

TRINITY_F6WMP0_MACMU/1-57	F6WMP0.1 PF16273.3;	70.60	0.00
TRINITY_A0DJ02_PARTE/58-309	A0DJ02.1 PF10609.7;	70.60	0.00
TRINITY_A0A0M0JX63_9EUKA/192-467	A0A0M0JX63.1 PF0035	70.60	0.00
TRINITY_A0DN29_PARTE/563-813	A0DN29.1 PF00454.25	70.60	0.00
TRINITY_J9HT64_9SPIT/322-441	J9HT64.1 PF00622.26	70.60	0.00
TRINITY_G0QZX4_ICHMG/13-301	G0QZX4.1 PF00069.23	70.60	0.00
TRINITY_L8HDT1_ACACA/11-405	L8HDT1.1 PF00464.17	70.60	0.00
TRINITY_D8TS86_VOLCA/309-393	D8TS86.1 PF00677.15	70.60	0.00
TRINITY_I7MLN0_TETTS/228-363	I7MLN0.2 PF04116.11	70.60	0.00
TRINITY_J9FXA2_9SPIT/25-291	J9FXA2.1 PF02263.17	70.60	0.00
TRINITY_A8I901_CHLRE/262-511	A8I901.1 PF00928.19	70.60	0.00
TRINITY_D8TI05_VOLCA/66-119	D8TI05.1 PF04193.12	70.60	0.00
TRINITY_D8SD29_SELML/9-310	D8SD29.1 PF00069.23	70.60	0.00
TRINITY_D8U8W9_VOLCA/2-69	D8U8W9.1 PF01781.16	70.60	0.00
TRINITY_A9T0A6_PHYPA/52-137	A9T0A6.1 PF00717.21	70.60	0.00
TRINITY_E1ZQ41_CHLVA/90-248	E1ZQ41.1 PF00005.25	70.60	0.00
TRINITY_A8IAU3_CHLRE/769-853	A8IAU3.1 PF00069.23	70.60	0.00
TRINITY_I0YW34_9CHLO/120-188	I0YW34.1 PF13424.4;	70.60	0.00
TRINITY_A8J092_CHLRE/159-261	A8J092.1 PF00781.22	70.60	0.00
TRINITY_WDR12_CHLRE/266-301	A8IR43.2 PF00400.30	70.60	0.00
TRINITY_I0YP88_9CHLO/51-213	I0YP88.1 PF00270.27	70.60	0.00
TRINITY_I0YJB2_9CHLO/12-175	I0YJB2.1 PF00994.22	70.60	0.00
TRINITY_D8TL09_VOLCA/12-62	D8TL09.1 PF00327.18	70.60	0.00
TRINITY_D8UG84_VOLCA/141-461	D8UG84.1 PF00443.27	70.60	0.00
TRINITY_D8R568_SELML/322-423	D8R568.1 PF13499.4;	70.60	0.00
TRINITY_D8TNY1_VOLCA/72-191	D8TNY1.1 PF00622.26	70.60	0.00
TRINITY_A8HPH6_CHLRE/474-677	A8HPH6.1 PF00488.19	70.60	0.00
TRINITY_A8IVJ6_CHLRE/868-1120	A8IVJ6.1 PF16212.3;	70.60	0.00
TRINITY_A8HYQ4_CHLRE/1-259	A8HYQ4.1 PF00069.23	70.60	0.00
TRINITY_A8IRU2_CHLRE/549-764	A8IRU2.1 PF08214.9;	70.60	0.00
TRINITY_A8IIK4_CHLRE/40-249	A8IIK4.1 PF01370.19	70.60	0.00
TRINITY_A0BUE7_PARTE/26-60	A0BUE7.1 PF06747.11	70.60	0.00
TRINITY_Q22XN5_TETTS/43-220	Q22XN5.1 PF02146.15	70.60	0.00
TRINITY_Q22W27_TETTS/68-135	Q22W27.2 PF08606.9;	70.50	0.00
TRINITY_D8UD96_VOLCA/47-207	D8UD96.1 PF00270.27	70.50	0.00
TRINITY_Q23FJ3_TETTS/38-349	Q23FJ3.2 PF00206.18	70.50	0.00
TRINITY_Q247Z0_TETTS/1359-1503	Q247Z0.3 PF00005.25	70.50	0.00
TRINITY_A0A022RGC4_ERYGU/34-77	A0A022RGC4.1 PF0147	70.50	0.00
TRINITY_E1Z7Y2_CHLVA/358-468	E1Z7Y2.1 PF00271.29	70.50	0.00
TRINITY_A0A059LKY6_9CHLO/227-377	A0A059LKY6.1 PF0044	70.50	0.00
TRINITY_G0R5N5_ICHMG/142-439	G0R5N5.1 PF00176.21	70.50	0.00
TRINITY_L8HBZ4_ACACA/484-607	L8HBZ4.1 PF09732.7;	70.50	0.00
TRINITY_I0ZA76_9CHLO/1744-1888	I0ZA76.1 PF13901.4;	70.50	0.00
TRINITY_A0A0G4EPF7_9ALVE/3-474	A0A0G4EPF7.1 PF0522	70.50	0.00
TRINITY_H2R1B6_PANTR/5-118	H2R1B6.1 PF00572.16	70.50	0.00
TRINITY_D8TH42_VOLCA/90-214	D8TH42.1 PF13911.4;	70.50	0.00
TRINITY_D8THK9_VOLCA/34-111	D8THK9.1 PF04232.10	70.50	0.00
TRINITY_D8UFJ8_VOLCA/9-406	D8UFJ8.1 PF00999.19	70.50	0.00
TRINITY_A8J5W8_CHLRE/599-740	A8J5W8.1 PF00004.27	70.50	0.00
TRINITY_D8THC5_VOLCA/241-456	D8THC5.1 PF01435.16	70.50	0.00
TRINITY_AP1S2_DICDI/2-143	B0G185.1 PF01217.18	70.50	0.00
TRINITY_A8I3X6_CHLRE/2565-3295	A8I3X6.1 PF03028.13	70.50	0.00
TRINITY_D8UHE9_VOLCA/1-77	D8UHE9.1 PF10163.7;	70.50	0.00
TRINITY_A0D1R8_PARTE/167-469	A0D1R8.1 PF00365.18	70.50	0.00
TRINITY_E1ZFK1_CHLVA/59-444	E1ZFK1.1 PF01979.18	70.50	0.00
TRINITY_D8TVC0_VOLCA/1983-2096	D8TVC0.1 PF00665.24	70.50	0.00
TRINITY_T1NNB4_TRIUA/316-490	T1NNB4.1 PF00270.27	70.50	0.00
TRINITY_I9BWL2_9RALS/2-80	I9BWL2.1 PF02798.18	70.50	0.00
TRINITY_A0A0G4EJ89_9ALVE/444-1017	A0A0G4EJ89.1 PF0518	70.50	0.00
TRINITY_A8HTK2_CHLRE/419-543	A8HTK2.1 PF13460.4;	70.50	0.00
TRINITY_D8U4B9_VOLCA/79-456	D8U4B9.1 PF00890.22	70.50	0.00

TRINITY_D8TQE1_VOLCA/778-879	D8TQE1.1 PF13676.4;	70.50	0.00
TRINITY_A8IJK9_CHLRE/110-170	A8IJK9.1 PF00839.15	70.50	0.00
TRINITY_D8U1W0_VOLCA/944-987	D8U1W0.1 PF13920.4;	70.50	0.00
TRINITY_A8JCJ2_CHLRE/191-376	A8JCJ2.1 PF10300.7;	70.50	0.00
TRINITY_D8TQ46_VOLCA/1-152	D8TQ46.1 PF01965.22	70.50	0.00
TRINITY_W1QA96_OGAPD/109-203	W1QA96.1 PF08351.9;	70.50	0.00
TRINITY_A8I5C3_CHLRE/2838-3549	A8I5C3.1 PF03028.13	70.50	0.00
TRINITY_A8IWH9_CHLRE/172-322	A8IWH9.1 PF01764.23	70.50	0.00
TRINITY_E6X0L4_NITSE/5-67	E6X0L4.1 PF00226.29	70.50	0.00
TRINITY_A0A0C4DX99_MAGP6/8-389	A0A0C4DX99.1 PF0126	70.40	0.00
TRINITY_E9HRY0_DAPPU/700-829	E9HRY0.1 PF00694.17	70.40	0.00
TRINITY_W4Y2A5_STRPU/36-106	W4Y2A5.1 PF00076.20	70.40	0.00
TRINITY_F4PJR8_DICFS/7-158	F4PJR8.1 PF00179.24	70.40	0.00
TRINITY_A8ICK6_CHLRE/46-171	A8ICK6.1 PF00578.19	70.40	0.00
TRINITY_A8IWT7_CHLRE/8-115	A8IWT7.1 PF00635.24	70.40	0.00
TRINITY_G0R010_ICHMG/505-961	G0R010.1 PF00136.19	70.40	0.00
TRINITY_D8U162_VOLCA/140-368	D8U162.1 PF02475.14	70.40	0.00
TRINITY_A0A0M0K079_9EUKA/14-84	A0A0M0K079.1 PF1226	70.40	0.00
TRINITY_A0A068YEU6_ECHMU/52-247	A0A068YEU6.1 PF0014	70.40	0.00
TRINITY_D8UG57_VOLCA/22-359	D8UG57.1 PF00443.27	70.40	0.00
TRINITY_A8IGZ3_CHLRE/12-113	A8IGZ3.1 PF12680.5;	70.40	0.00
TRINITY_D8UAD2_VOLCA/233-313	D8UAD2.1 PF08240.10	70.40	0.00
TRINITY_J9ISJ3_9SPIT/90-258	J9ISJ3.1 PF04427.16	70.40	0.00
TRINITY_D8TZ23_VOLCA/49-119	D8TZ23.1 PF00076.20	70.40	0.00
TRINITY_L8GW25_ACACA/462-582	L8GW25.1 PF00004.27	70.40	0.00
TRINITY_D8TPR9_VOLCA/5-90	D8TPR9.1 PF06294.9;	70.40	0.00
TRINITY_A4VDB5_TETTS/67-400	A4VDB5.2 PF00225.21	70.40	0.00
TRINITY_A0C095_PARTE/256-397	A0C095.1 PF00004.27	70.40	0.00
TRINITY_E9FVW5_DAPPU/14-220	E9FVW5.1 PF00149.26	70.40	0.00
TRINITY_A8J0S3_CHLRE/81-215	A8J0S3.1 PF13301.4;	70.40	0.00
TRINITY_D8U986_VOLCA/307-378	D8U986.1 PF00575.21	70.40	0.00
TRINITY_A8I6V0_CHLRE/188-301	A8I6V0.1 PF02797.13	70.40	0.00
TRINITY_I7LSW1_TETTS/10-170	I7LSW1.1 PF00071.20	70.40	0.00
TRINITY_A8I282_CHLRE/52-291	A8I282.1 PF01709.18	70.40	0.00
TRINITY_D8TL67_VOLCA/78-499	D8TL67.1 PF00654.18	70.40	0.00
TRINITY_A8JH52_CHLRE/89-557	A8JH52.1 PF02446.15	70.40	0.00
TRINITY_A0A0G4GMJ0_9ALVE/266-561	A0A0G4GMJ0.1 PF0006	70.40	0.00
TRINITY_D8UIN4_VOLCA/109-467	D8UIN4.1 PF00750.17	70.40	0.00
TRINITY_A8J9X1_CHLRE/71-151	A8J9X1.1 PF02823.14	70.40	0.00
TRINITY_A8IQ53_CHLRE/2-175	A8IQ53.1 PF01184.17	70.40	0.00
TRINITY_E1Z2U6_CHLVA/123-591	E1Z2U6.1 PF01293.18	70.40	0.00
TRINITY_A8JBN5_CHLRE/42-346	A8JBN5.1 PF01180.19	70.40	0.00
TRINITY_K1QM08_CRAGI/25-134	K1QM08.1 PF11969.6;	70.30	0.00
TRINITY_W7X4E2_TETTS/398-434	W7X4E2.1 PF01753.16	70.30	0.00
TRINITY_A0CVD0_PARTE/83-383	A0CVD0.1 PF03133.13	70.30	0.00
TRINITY_F0VMA6_NEOCL/118-188	F0VMA6.1 PF00076.20	70.30	0.00
TRINITY_A0E0K1_PARTE/14-270	A0E0K1.1 PF00069.23	70.30	0.00
TRINITY_A0CW82_PARTE/237-374	A0CW82.1 PF00334.17	70.30	0.00
TRINITY_F4B373_DOKS4/28-145	F4B373.1 PF08240.10	70.30	0.00
TRINITY_F4PL90_DICFS/95-474	F4PL90.1 PF00285.19	70.30	0.00
TRINITY_V2XHJ7_MONRO/11-110	V2XHJ7.1 PF00153.25	70.30	0.00
TRINITY_CLPP_ANAMF/29-210	B9KHZ4.1 PF00574.21	70.30	0.00
TRINITY_W7XDP3_TETTS/1821-2090	W7XDP3.1 PF12775.5;	70.30	0.00
TRINITY_B2VT60_PYRTR/488-668	B2VT60.1 PF00623.18	70.30	0.00
TRINITY_Q244Y3_TETTS/6-149	Q244Y3.1 PF05517.10	70.30	0.00
TRINITY_H3S9A0_9BACL/16-466	H3S9A0.1 PF03972.12	70.30	0.00
TRINITY_R4X8G3_TAPDE/22-366	R4X8G3.1 PF00884.21	70.30	0.00
TRINITY_D8U2F3_VOLCA/264-593	D8U2F3.1 PF12631.5;	70.30	0.00
TRINITY_D8TSF9_VOLCA/9-302	D8TSF9.1 PF02569.13	70.30	0.00
TRINITY_D8TM65_VOLCA/36-136	D8TM65.1 PF02362.19	70.30	0.00
TRINITY_D8UIV9_VOLCA/632-668	D8UIV9.1 PF00400.30	70.30	0.00

TRINITY_A0A0L6VHX5_9BASI/108-172	A0A0L6VHX5.1 PF1349	70.30	0.00
TRINITY_B4N953_DROWI/43-80	B4N953.2 PF00400.30	70.30	0.00
TRINITY_A8J0S3_CHLRE/81-215	A8J0S3.1 PF13301.4;	70.30	0.00
TRINITY_J9I207_9SPIT/218-497	J9I207.1 PF01409.18	70.30	0.00
TRINITY_E9FQX1_DAPPU/3-76	E9FQX1.1 PF08783.9;	70.30	0.00
TRINITY_I4YDX4_WALMC/34-201	I4YDX4.1 PF00270.27	70.30	0.00
TRINITY_A8HUN7_CHLRE/420-490	A8HUN7.1 PF08164.10	70.30	0.00
TRINITY_D8UE34_VOLCA/786-893	D8UE34.1 PF07683.12	70.30	0.00
TRINITY_A0A0A1NV14_9FUNG/72-508	A0A0A1NV14.1 PF0033	70.30	0.00
TRINITY_D8TLS6_VOLCA/130-240	D8TLS6.1 PF00581.18	70.30	0.00
TRINITY_B8PGK3_POSPM/1159-1305	B8PGK3.1 PF00005.25	70.30	0.00
TRINITY_D8TL05_VOLCA/10-212	D8TL05.1 PF02146.15	70.30	0.00
TRINITY_D8SBS3_SELML/9-153	D8SBS3.1 PF00179.24	70.30	0.00
TRINITY_D8TJA7_VOLCA/116-184	D8TJA7.1 PF00076.20	70.30	0.00
TRINITY_A8IU30_CHLRE/6-70	A8IU30.1 PF00226.29	70.30	0.00
TRINITY_D8U9R6_VOLCA/505-633	D8U9R6.1 PF03109.14	70.30	0.00
TRINITY_A0A0K9NXN2_ZOSMR/88-219	A0A0K9NXN2.1 PF0078	70.30	0.00
TRINITY_A0A068RG59_9FUNG/1-366	A0A068RG59.1 PF0029	70.30	0.00
TRINITY_D8TW88_VOLCA/1436-1578	D8TW88.1 PF00637.18	70.30	0.00
TRINITY_B3LBC1_PLAKH/255-779	B3LBC1.1 PF02867.13	70.20	0.00
TRINITY_A0A0D2UB67_CAPO3/112-282	A0A0D2UB67.1 PF0172	70.20	0.00
TRINITY_L8H934_ACACA/59-311	L8H934.1 PF10609.7;	70.20	0.00
TRINITY_Q231N6_TETTS/18-179	Q231N6.3 PF00071.20	70.20	0.00
TRINITY_Q00ZX4_OSTTA/54-141	Q00ZX4.1 PF05175.12	70.20	0.00
TRINITY_D8TNH8_VOLCA/9-55	D8TNH8.1 PF13639.4;	70.20	0.00
TRINITY_K4BTQ3_SOLLC/13-107	K4BTQ3.1 PF01248.24	70.20	0.00
TRINITY_Q3SD81_PARTE/16-176	Q3SD81.1 PF00071.20	70.20	0.00
TRINITY_L8GWV4_ACACA/132-255	L8GWV4.1 PF03153.11	70.20	0.00
TRINITY_A8HWF0_CHLRE/35-291	A8HWF0.1 PF00069.23	70.20	0.00
TRINITY_A0A059LRS6_9CHLO/159-279	A0A059LRS6.1 PF0192	70.20	0.00
TRINITY_F4Q6S2_DICFS/285-332	F4Q6S2.1 PF02042.13	70.20	0.00
TRINITY_D8TR11_VOLCA/20-187	D8TR11.1 PF07933.12	70.20	0.00
TRINITY_Q00TP1_OSTTA/98-216	Q00TP1.1 PF00383.21	70.20	0.00
TRINITY_D8U2L0_VOLCA/73-217	D8U2L0.1 PF01699.22	70.20	0.00
TRINITY_B9IH29_POPTR/44-89	B9IH29.1 PF00569.15	70.20	0.00
TRINITY_A8J594_CHLRE/10-113	A8J594.1 PF00085.18	70.20	0.00
TRINITY_L8GP72_ACACA/123-377	L8GP72.1 PF00069.23	70.20	0.00
TRINITY_J9FRH7_9SPIT/20-284	J9FRH7.1 PF01650.16	70.20	0.00
TRINITY_A8IE32_CHLRE/452-798	A8IE32.1 PF01103.21	70.20	0.00
TRINITY_D8UJ42_VOLCA/36-250	D8UJ42.1 PF02492.17	70.20	0.00
TRINITY_A0A0F2R845_9PROT/30-434	A0A0F2R845.1 PF0050	70.20	0.00
TRINITY_L1IH70_GUITH/122-177	L1IH70.1 PF13513.4;	70.20	0.00
TRINITY_L8GJY4_ACACA/379-528	L8GJY4.1 PF00005.25	70.20	0.00
TRINITY_D8TX75_VOLCA/101-214	D8TX75.1 PF13640.4;	70.20	0.00
TRINITY_A0A077ZY60_STYLE/5897-6330	A0A077ZY60.1 PF0089	70.20	0.00
TRINITY_D8TUH4_VOLCA/83-248	D8TUH4.1 PF01734.20	70.20	0.00
TRINITY_J9IEB5_9SPIT/3-74	J9IEB5.1 PF00240.21	70.20	0.00
TRINITY_D8UFW2_VOLCA/35-207	D8UFW2.1 PF00270.27	70.20	0.00
TRINITY_D8S3D3_SELML/139-309	D8S3D3.1 PF00270.27	70.20	0.00
TRINITY_A8IU49_CHLRE/6-199	A8IU49.1 PF13460.4;	70.20	0.00
TRINITY_D8U874_VOLCA/184-307	D8U874.1 PF09335.9;	70.20	0.00
TRINITY_E1ZCG2_CHLVA/71-434	E1ZCG2.1 PF01704.16	70.20	0.00
TRINITY_D8UIZ7_VOLCA/369-849	D8UIZ7.1 PF00759.17	70.20	0.00
TRINITY_D8TGX0_VOLCA/2112-2380	D8TGX0.1 PF12780.5;	70.20	0.00
TRINITY_W0E7C0_MARPU/48-414	W0E7C0.1 PF00285.19	70.10	0.00
TRINITY_L8GF06_ACACA/751-1260	L8GF06.1 PF02538.12	70.10	0.00
TRINITY_I7MFU9_TETTS/14-114	I7MFU9.1 PF01230.21	70.10	0.00
TRINITY_G0QY62_ICHMG/14-153	G0QY62.1 PF01565.21	70.10	0.00
TRINITY_I7M316_TETTS/3-177	I7M316.1 PF00025.19	70.10	0.00
TRINITY_J9HXH4_9SPIT/314-525	J9HXH4.1 PF00211.18	70.10	0.00
TRINITY_D8U2P2_VOLCA/607-721	D8U2P2.1 PF00271.29	70.10	0.00

TRINITY_A0A068RQB0_9FUNG/13-231	A0A068RQB0.1 PF0255	70.10	0.00
TRINITY_S8EWC3_FOMPI/732-811	S8EWC3.1 PF08282.10	70.10	0.00
TRINITY_A0BN60_PARTE/55-373	A0BN60.1 PF00225.21	70.10	0.00
TRINITY_L8H6F1_ACACA/387-1227	L8H6F1.1 PF05994.9;	70.10	0.00
TRINITY_E1ZKP9_CHLVA/344-410	E1ZKP9.1 PF01106.15	70.10	0.00
TRINITY_I0Z960_9CHLO/524-668	I0Z960.1 PF02775.19	70.10	0.00
TRINITY_U1HHE9_ENDPU/1158-1366	U1HHE9.1 PF02786.15	70.10	0.00
TRINITY_D8UFN5_VOLCA/1-213	D8UFN5.1 PF00069.23	70.10	0.00
TRINITY_E1ZPH9_CHLVA/94-719	E1ZPH9.1 PF00133.20	70.10	0.00
TRINITY_A8HP10_CHLRE/27-189	A8HP10.1 PF00160.19	70.10	0.00
TRINITY_A8HMJ8_CHLRE/48-474	A8HMJ8.1 PF13520.4;	70.10	0.00
TRINITY_A8JI17_CHLRE/255-345	A8JI17.1 PF00153.25	70.10	0.00
TRINITY_Q3SDT3_PARTE/63-246	Q3SDT3.1 PF01926.21	70.10	0.00
TRINITY_D8TYF3_VOLCA/286-402	D8TYF3.1 PF02373.20	70.10	0.00
TRINITY_A8J940_CHLRE/199-654	A8J940.1 PF09418.8;	70.10	0.00
TRINITY_D8TJ29_VOLCA/745-825	D8TJ29.1 PF10431.7;	70.10	0.00
TRINITY_D8U9Q6_VOLCA/12-318	D8U9Q6.1 PF08449.9;	70.10	0.00
TRINITY_A8JFB8_CHLRE/12-388	A8JFB8.1 PF07690.14	70.10	0.00
TRINITY_D8TLP0_VOLCA/19-126	D8TLP0.1 PF00255.17	70.10	0.00
TRINITY_A0A0G4G804_9ALVE/77-459	A0A0G4G804.1 PF0034	70.10	0.00
TRINITY_A8J9E9_CHLRE/74-564	A8J9E9.1 PF01593.22	70.10	0.00
TRINITY_I0YVT0_9CHLO/110-216	I0YVT0.1 PF00675.18	70.10	0.00
TRINITY_C1MIV7_MICPC/34-170	C1MIV7.1 PF00179.24	70.00	0.00
TRINITY_A0DY12_PARTE/14-183	A0DY12.1 PF00071.20	70.00	0.00
TRINITY_A0BR59_PARTE/14-240	A0BR59.1 PF14713.4;	70.00	0.00
TRINITY_Q22A43_TETTS/570-685	Q22A43.2 PF01412.16	70.00	0.00
TRINITY_J3N297_ORYBR/120-184	J3N297.1 PF13499.4;	70.00	0.00
TRINITY_A0A0M0JPE1_9EUKA/348-379	A0A0M0JPE1.1 PF1318	70.00	0.00
TRINITY_A8PUV0_MALGO/95-164	A8PUV0.1 PF00076.20	70.00	0.00
TRINITY_W4YTR2_STRPU/82-118	W4YTR2.1 PF10276.7;	70.00	0.00
TRINITY_I7M3D8_TETTS/206-439	I7M3D8.2 PF01702.16	70.00	0.00
TRINITY_D8TZ40_VOLCA/777-1279	D8TZ40.1 PF04998.15	70.00	0.00
TRINITY_D8RBM7_SELML/270-310	D8RBM7.1 PF00400.30	70.00	0.00
TRINITY_A0A075B1B2_9FUNG/246-507	A0A075B1B2.1 PF0107	70.00	0.00
TRINITY_D8UG12_VOLCA/197-259	D8UG12.1 PF00010.24	70.00	0.00
TRINITY_D8UCL7_VOLCA/162-371	D8UCL7.1 PF00297.20	70.00	0.00
TRINITY_A0A068RHT9_9FUNG/63-102	A0A068RHT9.1 PF0017	70.00	0.00
TRINITY_A0A067DKF7_CITSI/10-139	A0A067DKF7.1 PF0012	70.00	0.00
TRINITY_L8GTX7_ACACA/29-258	L8GTX7.1 PF01144.21	70.00	0.00
TRINITY_C1E009_MICSR/281-415	C1E009.1 PF13679.4;	70.00	0.00
TRINITY_Q23DM3_TETTS/193-382	Q23DM3.1 PF01163.20	70.00	0.00
TRINITY_A8JE89_CHLRE/422-617	A8JE89.1 PF04055.19	70.00	0.00
TRINITY_S0ES67_CHTCT/49-539	S0ES67.1 PF00342.17	70.00	0.00
TRINITY_I0Z062_9CHLO/726-884	I0Z062.1 PF00179.24	70.00	0.00
TRINITY_D8UJC6_VOLCA/308-381	D8UJC6.1 PF14360.4;	70.00	0.00
TRINITY_D8TJK7_VOLCA/147-198	D8TJK7.1 PF03909.15	70.00	0.00
TRINITY_A8J6J4_CHLRE/41-303	A8J6J4.1 PF00459.23	70.00	0.00
TRINITY_A8JJY7_CHLRE/9-161	A8JJY7.1 PF00004.27	70.00	0.00
TRINITY_A0A090M7B0_OSTTA/51-202	A0A090M7B0.1 PF0260	70.00	0.00
TRINITY_A0A023BBI7_GRENI/52-309	A0A023BBI7.1 PF0006	70.00	0.00
TRINITY_D8TN51_VOLCA/181-224	D8TN51.1 PF15985.3;	70.00	0.00
TRINITY_D8TN51_VOLCA/181-224	D8TN51.1 PF15985.3;	70.00	0.00
TRINITY_D8TKS9_VOLCA/120-393	D8TKS9.1 PF04811.13	70.00	0.00
TRINITY_I7MH10_TETTS/124-450	I7MH10.2 PF00225.21	70.00	0.00
TRINITY_Q22YU1_TETTS/85-594	Q22YU1.3 PF00118.22	70.00	0.00
TRINITY_D8TZ28_VOLCA/2137-2238	D8TZ28.1 PF01248.24	70.00	0.00
TRINITY_H3H405_PHYRM/37-151	H3H405.1 PF00665.24	70.00	0.00
TRINITY_F4PYF0_DICFS/1573-1749	F4PYF0.1 PF00689.19	70.00	0.00
TRINITY_D8U3B9_VOLCA/286-484	D8U3B9.1 PF03016.13	70.00	0.00
TRINITY_D8UBS2_VOLCA/5-73	D8UBS2.1 PF13921.4;	70.00	0.00
TRINITY_A0A067KCA8_JATCU/387-527	A0A067KCA8.1 PF0053	70.00	0.00

TRINITY_D8TJP7_VOLCA/78-344	D8TJP7.1 PF08323.9;	70.00	0.00
TRINITY_C1E9S6_MICSR/1-354	C1E9S6.1 PF16363.3;	70.00	0.00
TRINITY_G0R4M5_ICHMG/51-307	G0R4M5.1 PF00481.19	70.00	0.00
TRINITY_A8IZR1_CHLRE/67-638	A8IZR1.1 PF03063.18	70.00	0.00
TRINITY_A0A059LEQ3_9CHLO/1-225	A0A059LEQ3.1 PF0315	70.00	0.00
TRINITY_D8U6E1_VOLCA/443-651	D8U6E1.1 PF01061.22	70.00	0.00
TRINITY_W5E6X5_WHEAT/258-297	W5E6X5.1 PF05920.9;	70.00	0.00
TRINITY_A8J1F4_CHLRE/136-240	A8J1F4.1 PF12894.5;	70.00	0.00
TRINITY_A0D4J6_PARTE/38-180	A0D4J6.1 PF00080.18	69.90	0.00
TRINITY_L8GGG2_ACACA/628-752	L8GGG2.1 PF00754.23	69.90	0.00
TRINITY_A0BXM4_PARTE/6-118	A0BXM4.1 PF01778.15	69.90	0.00
TRINITY_G6DM41_DANPL/13-105	G6DM41.1 PF00254.26	69.90	0.00
TRINITY_J9IXJ6_9SPIT/12-173	J9IXJ6.1 PF00071.20	69.90	0.00
TRINITY_A0A098C1Y0_9PORP/5-398	A0A098C1Y0.1 PF0018	69.90	0.00
TRINITY_I7M0Y2_TETTS/35-440	I7M0Y2.1 PF01134.20	69.90	0.00
TRINITY_D8TWR1_VOLCA/23-311	D8TWR1.1 PF00069.23	69.90	0.00
TRINITY_L8GYI0_ACACA/117-249	L8GYI0.1 PF04116.11	69.90	0.00
TRINITY_E1ZK31_CHLVA/55-147	E1ZK31.1 PF00254.26	69.90	0.00
TRINITY_G0QUB8_ICHMG/9-264	G0QUB8.1 PF00069.23	69.90	0.00
TRINITY_J9HQW6_9SPIT/1043-1343	J9HQW6.1 PF03133.13	69.90	0.00
TRINITY_D2VBK4_NAEGR/81-263	D2VBK4.1 PF00227.24	69.90	0.00
TRINITY_A0A077ZT83_STYLE/26-281	A0A077ZT83.1 PF0006	69.90	0.00
TRINITY_A8JIG3_CHLRE/178-353	A8JIG3.1 PF13870.4;	69.90	0.00
TRINITY_A8ID84_CHLRE/1-367	A8ID84.1 PF00297.20	69.90	0.00
TRINITY_A8JFE1_CHLRE/108-243	A8JFE1.1 PF00730.23	69.90	0.00
TRINITY_I0YQQ2_9CHLO/61-427	I0YQQ2.1 PF00155.19	69.90	0.00
TRINITY_I7M251_TETTS/85-252	I7M251.1 PF00650.18	69.90	0.00
TRINITY_D8TN16_VOLCA/470-582	D8TN16.1 PF00168.28	69.90	0.00
TRINITY_Q22RW2_TETTS/60-133	Q22RW2.1 PF13656.4;	69.90	0.00
TRINITY_C5D5D3_GEOSW/5-336	C5D5D3.1 PF00180.18	69.90	0.00
TRINITY_A0A087S9Z1_AUXPR/19-207	A0A087S9Z1.1 PF0003	69.90	0.00
TRINITY_D8UAU0_VOLCA/6-256	D8UAU0.1 PF00069.23	69.90	0.00
TRINITY_F4PGI6_DICFS/51-216	F4PGI6.1 PF02776.16	69.90	0.00
TRINITY_D8TQW5_VOLCA/248-390	D8TQW5.1 PF08005.10	69.90	0.00
TRINITY_A8JD76_CHLRE/119-191	A8JD76.1 PF00226.29	69.90	0.00
TRINITY_R1FIW4_EMIHU/154-427	R1FIW4.1 PF00928.19	69.90	0.00
TRINITY_D8U9E2_VOLCA/144-305	D8U9E2.1 PF06472.13	69.90	0.00
TRINITY_Q23DD6_TETTS/62-499	Q23DD6.2 PF04997.10	69.90	0.00
TRINITY_E1ZN95_CHLVA/455-604	E1ZN95.1 PF00005.25	69.90	0.00
TRINITY_J9HWA9_9SPIT/8-351	J9HWA9.1 PF00225.21	69.80	0.00
TRINITY_B9L955_NAUPA/2-64	B9L955.1 PF00226.29	69.80	0.00
TRINITY_A0A0G4EHW8_9ALVE/46-108	A0A0G4EHW8.1 PF0184	69.80	0.00
TRINITY_B3S9B3_TRIAD/85-147	B3S9B3.1 PF13499.4;	69.80	0.00
TRINITY_V4A936_LOTGI/166-580	V4A936.1 PF01131.18	69.80	0.00
TRINITY_G8P069_GRAMM/78-242	G8P069.1 PF13472.4;	69.80	0.00
TRINITY_F4PJU0_DICFS/308-467	F4PJU0.1 PF00441.22	69.80	0.00
TRINITY_F6HVS8_VITVI/215-258	F6HVS8.1 PF13639.4;	69.80	0.00
TRINITY_F6HVS8_VITVI/215-258	F6HVS8.1 PF13639.4;	69.80	0.00
TRINITY_D3PBZ6_DEFDS/311-436	D3PBZ6.1 PF16921.3;	69.80	0.00
TRINITY_A0A0P7ZDF3_9TELE/26-88	A0A0P7ZDF3.1 PF0046	69.80	0.00
TRINITY_G0QR69_ICHMG/1-229	G0QR69.1 PF00069.23	69.80	0.00
TRINITY_A8JGV6_CHLRE/11-235	A8JGV6.1 PF00244.18	69.80	0.00
TRINITY_A0A061E403_THECC/222-265	A0A061E403.1 PF1363	69.80	0.00
TRINITY_G0QP27_ICHMG/30-508	G0QP27.1 PF01293.18	69.80	0.00
TRINITY_D8U5C9_VOLCA/33-191	D8U5C9.1 PF00160.19	69.80	0.00
TRINITY_A8JFG8_CHLRE/29-537	A8JFG8.1 PF00759.17	69.80	0.00
TRINITY_D8TZK0_VOLCA/36-151	D8TZK0.1 PF01981.14	69.80	0.00
TRINITY_D8THU5_VOLCA/1538-1582	D8THU5.1 PF00249.29	69.80	0.00
TRINITY_A0A078AVM5_STYLE/320-363	A0A078AVM5.1 PF0056	69.80	0.00
TRINITY_I0YNI1_9CHLO/1161-1216	I0YNI1.1 PF01844.21	69.80	0.00
TRINITY_L8HIM0_ACACA/5-177	L8HIM0.1 PF00025.19	69.80	0.00

TRINITY_I7M8Z5_TETTS/107-277	I7M8Z5.2 PF00270.27	69.80	0.00
TRINITY_E1ZH39_CHLVA/14-56	E1ZH39.1 PF08271.10	69.80	0.00
TRINITY_A8IF21_CHLRE/105-379	A8IF21.1 PF00069.23	69.80	0.00
TRINITY_TTL3C_TETTS/705-1030	Q23FE2.1 PF03133.13	69.80	0.00
TRINITY_A0A087SIH8_AUXPR/181-244	A0A087SIH8.1 PF0041	69.80	0.00
TRINITY_A8I8C6_CHLRE/135-217	A8I8C6.1 PF01169.17	69.80	0.00
TRINITY_I7M2W8_TETTS/134-424	I7M2W8.2 PF00069.23	69.80	0.00
TRINITY_W1P857_AMBTC/33-199	W1P857.1 PF05019.11	69.80	0.00
TRINITY_D8U0H5_VOLCA/63-256	D8U0H5.1 PF00535.24	69.80	0.00
TRINITY_E1ZRG2_CHLVA/108-338	E1ZRG2.1 PF04493.12	69.80	0.00
TRINITY_D8UHK6_VOLCA/116-180	D8UHK6.1 PF13499.4;	69.80	0.00
TRINITY_F4PYU5_DICFS/82-558	F4PYU5.1 PF01039.20	69.80	0.00
TRINITY_A8IRR7_CHLRE/290-417	A8IRR7.1 PF00271.29	69.80	0.00
TRINITY_D8U4P2_VOLCA/8-128	D8U4P2.1 PF13238.4;	69.80	0.00
TRINITY_UNG_DICDI/172-334	P53766.2 PF03167.17	69.80	0.00
TRINITY_A8HWW0_CHLRE/6-166	A8HWW0.1 PF00005.25	69.80	0.00
TRINITY_D8TVI5_VOLCA/233-449	D8TVI5.1 PF01786.15	69.80	0.00
TRINITY_A8I1A4_CHLRE/52-365	A8I1A4.1 PF00248.19	69.80	0.00
TRINITY_L1LGC8_THEEQ/39-193	L1LGC8.1 PF00177.19	69.70	0.00
TRINITY_A0EA65_PARTE/11-205	A0EA65.1 PF01813.15	69.70	0.00
TRINITY_L8HDP2_ACACA/6-81	L8HDP2.1 PF01722.16	69.70	0.00
TRINITY_B4FN08_MAIZE/21-336	B4FN08.1 PF02463.17	69.70	0.00
TRINITY_A0A0A0LF44_CUCSA/15-117	A0A0A0LF44.1 PF0023	69.70	0.00
TRINITY_E3KZV8_PUCGT/77-528	E3KZV8.2 PF01406.17	69.70	0.00
TRINITY_S0ETZ9_CHTCT/482-606	S0ETZ9.1 PF02780.18	69.70	0.00
TRINITY_A0A0D2WLA8_CAPO3/9-128	A0A0D2WLA8.1 PF0058	69.70	0.00
TRINITY_L8GWD8_ACACA/49-315	L8GWD8.1 PF00561.18	69.70	0.00
TRINITY_F0ZME5_DICPU/115-445	F0ZME5.1 PF00351.19	69.70	0.00
TRINITY_C4ZM53_THASP/1-77	C4ZM53.1 PF02798.18	69.70	0.00
TRINITY_J9JC12_9SPIT/231-416	J9JC12.1 PF00587.23	69.70	0.00
TRINITY_A8IMK5_CHLRE/545-666	A8IMK5.1 PF04153.16	69.70	0.00
TRINITY_A0A099NQ59_PICKU/19-56	A0A099NQ59.1 PF0040	69.70	0.00
TRINITY_B3RW99_TRIAD/199-299	B3RW99.1 PF00153.25	69.70	0.00
TRINITY_A0A0G4G3B3_9ALVE/2-145	A0A0G4G3B3.1 PF0128	69.70	0.00
TRINITY_D3B3W9_POLPA/31-182	D3B3W9.1 PF13177.4;	69.70	0.00
TRINITY_A8ISQ5_CHLRE/1729-1856	A8ISQ5.1 PF00004.27	69.70	0.00
TRINITY_C5KE35_PERM5/655-693	C5KE35.1 PF00400.30	69.70	0.00
TRINITY_A0A0A1NLD6_9FUNG/567-720	A0A0A1NLD6.1 PF0027	69.70	0.00
TRINITY_D8U1M4_VOLCA/81-232	D8U1M4.1 PF00297.20	69.70	0.00
TRINITY_A0A068S9F6_9FUNG/6-450	A0A068S9F6.1 PF0089	69.70	0.00
TRINITY_A8ISZ7_CHLRE/11-105	A8ISZ7.1 PF05477.9;	69.70	0.00
TRINITY_GTAA_DICDI/294-329	Q550D5.1 PF00320.25	69.70	0.00
TRINITY_A8IYK0_CHLRE/1-155	A8IYK0.1 PF07714.15	69.70	0.00
TRINITY_A8I2Y4_CHLRE/16-324	A8I2Y4.1 PF13393.4;	69.70	0.00
TRINITY_A0A078AZN1_STYLE/5-70	A0A078AZN1.1 PF0142	69.70	0.00
TRINITY_Q22DI1_TETTS/70-229	Q22DI1.1 PF02731.13	69.70	0.00
TRINITY_D8TRS5_VOLCA/107-172	D8TRS5.1 PF12352.6;	69.70	0.00
TRINITY_I0YKJ9_9CHLO/163-335	I0YKJ9.1 PF17177.2;	69.70	0.00
TRINITY_J9HQ37_9SPIT/2176-2315	J9HQ37.1 PF07728.12	69.70	0.00
TRINITY_D8U9E2_VOLCA/144-305	D8U9E2.1 PF06472.13	69.70	0.00
TRINITY_L8H251_ACACA/215-485	L8H251.1 PF00082.20	69.70	0.00
TRINITY_A8JGG6_CHLRE/51-459	A8JGG6.1 PF00474.15	69.70	0.00
TRINITY_D8U3R6_VOLCA/859-896	D8U3R6.1 PF03638.13	69.70	0.00
TRINITY_D8TSA4_VOLCA/2733-3552	D8TSA4.1 PF02364.13	69.70	0.00
TRINITY_D8UIV0_VOLCA/25-394	D8UIV0.1 PF00224.19	69.70	0.00
TRINITY_E1X1Z7_HALMS/34-321	E1X1Z7.1 PF00294.22	69.60	0.00
TRINITY_K4M9S9_9EURY/28-275	K4M9S9.1 PF02784.14	69.60	0.00
TRINITY_X6MSS0_RETFI/8-145	X6MSS0.1 PF00179.24	69.60	0.00
TRINITY_A0EEF5_PARTE/29-120	A0EEF5.1 PF00447.15	69.60	0.00
TRINITY_D8U5N5_VOLCA/206-284	D8U5N5.1 PF00789.18	69.60	0.00
TRINITY_A8I2Y6_CHLRE/11-265	A8I2Y6.1 PF00069.23	69.60	0.00

TRINITY_A8IDE3_CHLRE/126-260	A8IDE3.1 PF00258.23	69.60	0.00
TRINITY_A0A087SCB0_AUXPR/70-204	A0A087SCB0.1 PF0033	69.60	0.00
TRINITY_L8GEL6_ACACA/329-543	L8GEL6.1 PF13236.4;	69.60	0.00
TRINITY_L8H3V4_ACACA/180-304	L8H3V4.1 PF09668.8;	69.60	0.00
TRINITY_L8HM80_ACACA/121-301	L8HM80.1 PF00390.17	69.60	0.00
TRINITY_D8TS98_VOLCA/47-118	D8TS98.1 PF00173.26	69.60	0.00
TRINITY_A0CS57_PARTE/161-431	A0CS57.1 PF00928.19	69.60	0.00
TRINITY_A8I8C3_CHLRE/53-289	A8I8C3.1 PF01209.16	69.60	0.00
TRINITY_A8IY03_CHLRE/43-427	A8IY03.1 PF04563.13	69.60	0.00
TRINITY_A8J131_CHLRE/55-173	A8J131.1 PF00622.26	69.60	0.00
TRINITY_A8J9A9_CHLRE/9-118	A8J9A9.1 PF01398.19	69.60	0.00
TRINITY_A8IWG9_CHLRE/37-167	A8IWG9.1 PF06658.10	69.60	0.00
TRINITY_A8JC72_CHLRE/4-278	A8JC72.1 PF00069.23	69.60	0.00
TRINITY_D8TT01_VOLCA/22-159	D8TT01.1 PF05832.10	69.60	0.00
TRINITY_Q6BGF1_PARTE/393-606	Q6BGF1.1 PF07859.11	69.60	0.00
TRINITY_A8JAK4_CHLRE/54-555	A8JAK4.1 PF02990.14	69.60	0.00
TRINITY_Q9FV97_CHLRE/132-382	Q9FV97.1 PF00510.16	69.60	0.00
TRINITY_A5UM58_METS3/427-631	A5UM58.1 PF13087.4;	69.60	0.00
TRINITY_A8HPQ4_CHLRE/133-377	A8HPQ4.1 PF05770.9;	69.60	0.00
TRINITY_A8I481_CHLRE/44-106	A8I481.1 PF01276.18	69.60	0.00
TRINITY_E1ZR28_CHLVA/100-466	E1ZR28.1 PF02906.12	69.60	0.00
TRINITY_B8HKC8_CYAP4/160-335	B8HKC8.1 PF14518.4;	69.60	0.00
TRINITY_A0A077ZY60_STYLE/6352-6887	A0A077ZY60.1 PF0089	69.60	0.00
TRINITY_A0A0J8BYD9_BETVU/76-190	A0A0J8BYD9.1 PF0000	69.60	0.00
TRINITY_A8ISQ5_CHLRE/1729-1856	A8ISQ5.1 PF00004.27	69.60	0.00
TRINITY_A0A0D2WTS7_CAPO3/32-443	A0A0D2WTS7.1 PF0020	69.50	0.00
TRINITY_Q23A33_TETTS/2-240	Q23A33.1 PF00590.18	69.50	0.00
TRINITY_F4QD63_DICFS/2-233	F4QD63.1 PF00483.21	69.50	0.00
TRINITY_A0A0E3UWF6_9BACT/52-529	A0A0E3UWF6.1 PF0120	69.50	0.00
TRINITY_A8IY85_CHLRE/58-186	A8IY85.1 PF02670.14	69.50	0.00
TRINITY_I0Z0I1_9CHLO/15-145	I0Z0I1.1 PF00828.17	69.50	0.00
TRINITY_A8I795_CHLRE/8-120	A8I795.1 PF02391.15	69.50	0.00
TRINITY_D8U027_VOLCA/4-158	D8U027.1 PF01265.15	69.50	0.00
TRINITY_E2C7C6_HARSA/378-471	E2C7C6.1 PF01926.21	69.50	0.00
TRINITY_D8TJ71_VOLCA/102-211	D8TJ71.1 PF15739.3;	69.50	0.00
TRINITY_A0A075ARR7_9FUNG/247-456	A0A075ARR7.1 PF0118	69.50	0.00
TRINITY_L8GE89_ACACA/64-237	L8GE89.1 PF01154.15	69.50	0.00
TRINITY_A8HQ52_CHLRE/1511-1569	A8HQ52.1 PF14874.4;	69.50	0.00
TRINITY_Q24GH8_TETTS/10-259	Q24GH8.2 PF00398.18	69.50	0.00
TRINITY_U5H8V4_USTV1/11-407	U5H8V4.1 PF00162.17	69.50	0.00
TRINITY_A8IWD2_CHLRE/25-261	A8IWD2.1 PF13561.4;	69.50	0.00
TRINITY_A9S0R9_PHYPA/98-345	A9S0R9.1 PF00120.22	69.50	0.00
TRINITY_A8J1K6_CHLRE/27-159	A8J1K6.1 PF06148.9;	69.50	0.00
TRINITY_Q4KX33_CHLRE/41-243	Q4KX33.1 PF00702.24	69.50	0.00
TRINITY_A0EEU3_PARTE/113-206	A0EEU3.1 PF01588.18	69.50	0.00
TRINITY_D8U260_VOLCA/79-183	D8U260.1 PF06426.12	69.50	0.00
TRINITY_D8U9R0_VOLCA/29-115	D8U9R0.1 PF17146.2;	69.50	0.00
TRINITY_D8U459_VOLCA/305-868	D8U459.1 PF08385.10	69.50	0.00
TRINITY_D8TQE8_VOLCA/51-168	D8TQE8.1 PF13815.4;	69.50	0.00
TRINITY_D8TIT3_VOLCA/179-414	D8TIT3.1 PF13641.4;	69.50	0.00
TRINITY_D8TZ32_VOLCA/66-240	D8TZ32.1 PF02776.16	69.50	0.00
TRINITY_C1MVG4_MICPC/1087-1191	C1MVG4.1 PF00856.26	69.50	0.00
TRINITY_E1X1Z7_HALMS/34-321	E1X1Z7.1 PF00294.22	69.40	0.00
TRINITY_Q3SDM6_PARTE/17-177	Q3SDM6.1 PF00071.20	69.40	0.00
TRINITY_U5HFI2_USTV1/242-348	U5HFI2.1 PF12894.5;	69.40	0.00
TRINITY_E3RIZ4_PYRTT/432-675	E3RIZ4.1 PF12708.5;	69.40	0.00
TRINITY_ENV_XMRV6/21-615	Q27ID8.1 PF00429.17	69.40	0.00
TRINITY_M2Y135_GALSU/20-178	M2Y135.1 PF00071.20	69.40	0.00
TRINITY_I0Z0T5_9CHLO/103-228	I0Z0T5.1 PF10497.7;	69.40	0.00
TRINITY_R9AAJ6_WALI9/2-179	R9AAJ6.1 PF13869.4;	69.40	0.00
TRINITY_A8IAF7_CHLRE/62-257	A8IAF7.1 PF00849.20	69.40	0.00

TRINITY_Q235I3_TETTS/2-101	Q235I3.1 PF00453.16	69.40	0.00
TRINITY_D8UAY8_VOLCA/20-183	D8UAY8.1 PF00929.22	69.40	0.00
TRINITY_D8U841_VOLCA/324-612	D8U841.1 PF00122.18	69.40	0.00
TRINITY_I0Z393_9CHLO/25-477	I0Z393.1 PF01384.18	69.40	0.00
TRINITY_D8UAY5_VOLCA/11-407	D8UAY5.1 PF01053.18	69.40	0.00
TRINITY_A8J1H5_CHLRE/26-174	A8J1H5.1 PF07690.14	69.40	0.00
TRINITY_Q65Z16_CHLRE/138-186	Q65Z16.1 PF00010.24	69.40	0.00
TRINITY_I7M435_TETTS/17-177	I7M435.2 PF00071.20	69.40	0.00
TRINITY_M4E1Q7_BRARP/63-99	M4E1Q7.1 PF00504.19	69.40	0.00
TRINITY_A0EGB9_PARTE/32-287	A0EGB9.1 PF00069.23	69.40	0.00
TRINITY_A8I2K8_CHLRE/37-330	A8I2K8.1 PF00850.17	69.40	0.00
TRINITY_D8UDF3_VOLCA/894-1029	D8UDF3.1 PF13472.4;	69.40	0.00
TRINITY_A8HS81_CHLRE/45-155	A8HS81.1 PF11255.6;	69.40	0.00
TRINITY_A9U198_PHYPA/427-463	A9U198.1 PF01851.20	69.40	0.00
TRINITY_D8U656_VOLCA/537-572	D8U656.1 PF00320.25	69.40	0.00
TRINITY_B3RRA4_TRIAD/270-311	B3RRA4.1 PF00569.15	69.40	0.00
TRINITY_E1Z5P6_CHLVA/24-206	E1Z5P6.1 PF13439.4;	69.40	0.00
TRINITY_A0A087SKJ3_AUXPR/234-390	A0A087SKJ3.1 PF0280	69.40	0.00
TRINITY_D8UC15_VOLCA/233-379	D8UC15.1 PF02840.13	69.40	0.00
TRINITY_D8UD29_VOLCA/129-190	D8UD29.1 PF04117.10	69.40	0.00
TRINITY_D8TVX6_VOLCA/1-304	D8TVX6.1 PF00225.21	69.40	0.00
TRINITY_A8JBT8_CHLRE/1-130	A8JBT8.1 PF12146.6;	69.40	0.00
TRINITY_A8IWB5_CHLRE/1-288	A8IWB5.1 PF04733.12	69.40	0.00
TRINITY_A8IT69_CHLRE/27-375	A8IT69.1 PF06437.9;	69.40	0.00
TRINITY_A8JDL8_CHLRE/10-207	A8JDL8.1 PF00106.23	69.40	0.00
TRINITY_I0YXZ8_9CHLO/18-247	I0YXZ8.1 PF00149.26	69.40	0.00
TRINITY_W7XDP3_TETTS/3204-3907	W7XDP3.1 PF03028.13	69.40	0.00
TRINITY_B9GSN5_POPTR/530-637	B9GSN5.2 PF00651.29	69.40	0.00
TRINITY_D8TZG7_VOLCA/127-396	D8TZG7.1 PF00557.22	69.40	0.00
TRINITY_Q22BJ7_TETTS/366-422	Q22BJ7.2 PF01485.19	69.40	0.00
TRINITY_L8GGG2_ACACA/628-752	L8GGG2.1 PF00754.23	69.30	0.00
TRINITY_A0A074ST19_HAMHA/95-169	A0A074ST19.1 PF0090	69.30	0.00
TRINITY_G3NGT4_GASAC/24-99	G3NGT4.1 PF05355.9;	69.30	0.00
TRINITY_A0A0G4GIW3_9ALVE/170-245	A0A0G4GIW3.1 PF0509	69.30	0.00
TRINITY_M1PDW1_DESSD/226-515	M1PDW1.1 PF00977.19	69.30	0.00
TRINITY_A0D6Q8_PARTE/170-399	A0D6Q8.1 PF01798.16	69.30	0.00
TRINITY_D3BJU4_POLPA/5-155	D3BJU4.1 PF00179.24	69.30	0.00
TRINITY_S8EWC3_FOMPI/732-811	S8EWC3.1 PF08282.10	69.30	0.00
TRINITY_C1EF17_MICSR/72-185	C1EF17.1 PF08241.10	69.30	0.00
TRINITY_V4BFL7_LOTGI/22-203	V4BFL7.1 PF16661.3;	69.30	0.00
TRINITY_A8J1Z7_CHLRE/52-192	A8J1Z7.1 PF03188.14	69.30	0.00
TRINITY_A8HPM0_CHLRE/19-202	A8HPM0.1 PF01214.16	69.30	0.00
TRINITY_V4NAF4_EUTSA/460-556	V4NAF4.1 PF13432.4;	69.30	0.00
TRINITY_D8U977_VOLCA/128-254	D8U977.1 PF01529.18	69.30	0.00
TRINITY_D8UB96_VOLCA/670-833	D8UB96.1 PF01544.16	69.30	0.00
TRINITY_D8UJT5_VOLCA/505-722	D8UJT5.1 PF00112.21	69.30	0.00
TRINITY_I0YMU2_9CHLO/78-152	I0YMU2.1 PF03110.12	69.30	0.00
TRINITY_A8HRK2_CHLRE/43-301	A8HRK2.1 PF00664.21	69.30	0.00
TRINITY_A8IGH4_CHLRE/1709-1823	A8IGH4.1 PF01477.21	69.30	0.00
TRINITY_G0QW84_ICHMG/19-190	G0QW84.1 PF13177.4;	69.20	0.00
TRINITY_M1CAV2_SOLTU/42-171	M1CAV2.1 PF00692.17	69.20	0.00
TRINITY_MAAI_DICDI/8-87	Q54YN2.1 PF13417.4;	69.20	0.00
TRINITY_Q22GZ6_TETTS/169-289	Q22GZ6.1 PF14833.4;	69.20	0.00
TRINITY_A0A0R4IDH6_DANRE/19-507	A0A0R4IDH6.1 PF0315	69.20	0.00
TRINITY_A0A0G4F3P2_9ALVE/864-1138	A0A0G4F3P2.1 PF0313	69.20	0.00
TRINITY_A0E388_PARTE/154-420	A0E388.1 PF08014.9;	69.20	0.00
TRINITY_A9T7V0_PHYPA/35-73	A9T7V0.1 PF13923.4;	69.20	0.00
TRINITY_A8IVC2_CHLRE/79-199	A8IVC2.1 PF08292.10	69.20	0.00
TRINITY_A8IVC2_CHLRE/79-199	A8IVC2.1 PF08292.10	69.20	0.00
TRINITY_G0QK71_ICHMG/189-227	G0QK71.1 PF00400.30	69.20	0.00
TRINITY_A8JHW9_CHLRE/42-387	A8JHW9.1 PF07690.14	69.20	0.00

TRINITY_A0A078AAD9_STYLE/328-454	A0A078AAD9.1 PF0013	69.20	0.00
TRINITY_A0A022PWX0_ERYGU/13-52	A0A022PWX0.1 PF0142	69.20	0.00
TRINITY_G0QXX3_ICHMG/245-365	G0QXX3.1 PF00622.26	69.20	0.00
TRINITY_D8UCK7_VOLCA/66-172	D8UCK7.1 PF16589.3;	69.20	0.00
TRINITY_A8HV10_CHLRE/101-275	A8HV10.1 PF01612.18	69.20	0.00
TRINITY_A0A077ZY10_STYLE/590-848	A0A077ZY10.1 PF0006	69.20	0.00
TRINITY_L8HIH5_ACACA/40-527	L8HIH5.1 PF00118.22	69.20	0.00
TRINITY_B8MET7_TALSN/73-243	B8MET7.1 PF00270.27	69.20	0.00
TRINITY_Q55AW0_DICDI/246-414	Q55AW0.2 PF01612.18	69.20	0.00
TRINITY_D8UDE2_VOLCA/11-283	D8UDE2.1 PF00069.23	69.20	0.00
TRINITY_A8HSY2_CHLRE/37-412	A8HSY2.1 PF02535.20	69.20	0.00
TRINITY_A8IFJ6_CHLRE/65-283	A8IFJ6.1 PF00856.26	69.20	0.00
TRINITY_A8HVC8_CHLRE/11-102	A8HVC8.1 PF05670.11	69.20	0.00
TRINITY_GLRX3_DICDI/156-220	Q86H62.1 PF00462.22	69.20	0.00
TRINITY_A8JCU9_CHLRE/1-62	A8JCU9.1 PF10203.7;	69.20	0.00
TRINITY_D8TH61_VOLCA/13-486	D8TH61.1 PF00478.23	69.20	0.00
TRINITY_A8I4A8_CHLRE/416-481	A8I4A8.1 PF00076.20	69.20	0.00
TRINITY_A9U358_PHYPA/745-1059	A9U358.1 PF03178.13	69.20	0.00
TRINITY_D8TJI1_VOLCA/348-401	D8TJI1.1 PF13432.4;	69.20	0.00
TRINITY_D8THC3_VOLCA/74-255	D8THC3.1 PF01758.14	69.20	0.00
TRINITY_A0E493_PARTE/11-338	A0E493.1 PF00225.21	69.20	0.00
TRINITY_A8J3Z7_CHLRE/1-51	A8J3Z7.1 PF03055.13	69.20	0.00
TRINITY_U6N203_9EIME/5-88	U6N203.1 PF01221.16	69.20	0.00
TRINITY_D8TX81_VOLCA/1-135	D8TX81.1 PF03656.11	69.20	0.00
TRINITY_A8I8K9_CHLRE/173-286	A8I8K9.1 PF01416.18	69.20	0.00
TRINITY_D8UCL0_VOLCA/1-120	D8UCL0.1 PF00238.17	69.20	0.00
TRINITY_G0R213_ICHMG/16-192	G0R213.1 PF01433.18	69.20	0.00
TRINITY_I0Z554_9CHLO/96-515	I0Z554.1 PF01373.15	69.20	0.00
TRINITY_D8U295_VOLCA/98-278	D8U295.1 PF11891.6;	69.20	0.00
TRINITY_C3Z927_BRAFL/251-427	C3Z927.1 PF01979.18	69.20	0.00
TRINITY_D8U133_VOLCA/3-322	D8U133.1 PF03405.12	69.20	0.00
TRINITY_D8TMW4_VOLCA/135-459	D8TMW4.1 PF00009.25	69.20	0.00
TRINITY_L5LTN0_MYODS/23-100	L5LTN0.1 PF00935.17	69.20	0.00
TRINITY_D8UG85_VOLCA/594-710	D8UG85.1 PF09725.7;	69.20	0.00
TRINITY_I1HS90_BRADI/568-920	I1HS90.1 PF00176.21	69.20	0.00
TRINITY_G0R081_ICHMG/66-109	G0R081.1 PF00643.22	69.20	0.00
TRINITY_A8HVJ4_CHLRE/171-515	A8HVJ4.1 PF00009.25	69.20	0.00
TRINITY_I7LSV4_TETTS/3534-4237	I7LSV4.2 PF03028.13	69.20	0.00
TRINITY_I7LUR8_TETTS/995-1140	I7LUR8.2 PF00637.18	69.20	0.00
TRINITY_F0ZPW7_DICPU/88-747	F0ZPW7.1 PF00063.19	69.20	0.00
TRINITY_J9JAX5_9SPIT/137-263	J9JAX5.1 PF00134.21	69.20	0.00
TRINITY_A0A059LDR7_9CHLO/55-122	A0A059LDR7.1 PF0055	69.10	0.00
TRINITY_G8Q5U0_PSEFL/3-184	G8Q5U0.1 PF01965.22	69.10	0.00
TRINITY_Q23DW5_TETTS/611-969	Q23DW5.2 PF00476.18	69.10	0.00
TRINITY_A0A078AEL1_STYLE/19-303	A0A078AEL1.1 PF0006	69.10	0.00
TRINITY_I4C388_DESTA/23-372	I4C388.1 PF00266.17	69.10	0.00
TRINITY_A0A0E9NPS3_9ASCO/349-566	A0A0E9NPS3.1 PF1270	69.10	0.00
TRINITY_A0EGU8_PARTE/540-749	A0EGU8.1 PF00211.18	69.10	0.00
TRINITY_Q7XLE8_ORYSJ/857-1017	Q7XLE8.2 PF00078.25	69.10	0.00
TRINITY_Q54BQ2_DICDI/95-233	Q54BQ2.1 PF01743.18	69.10	0.00
TRINITY_D8TIX1_VOLCA/41-296	D8TIX1.1 PF12697.5;	69.10	0.00
TRINITY_I7MH21_TETTS/71-139	I7MH21.2 PF00076.20	69.10	0.00
TRINITY_I7M3G5_TETTS/1235-1331	I7M3G5.1 PF04059.10	69.10	0.00
TRINITY_A8JEJ8_CHLRE/56-123	A8JEJ8.1 PF01066.19	69.10	0.00
TRINITY_L8GNJ4_ACACA/100-377	L8GNJ4.1 PF00069.23	69.10	0.00
TRINITY_D8TZS1_VOLCA/168-450	D8TZS1.1 PF00928.19	69.10	0.00
TRINITY_D8U8M8_VOLCA/2-115	D8U8M8.1 PF07123.10	69.10	0.00
TRINITY_Q23AP4_TETTS/312-462	Q23AP4.1 PF00160.19	69.10	0.00
TRINITY_F1A1I2_DICPU/3-99	F1A1I2.1 PF01157.16	69.10	0.00
TRINITY_D8U2V4_VOLCA/3-96	D8U2V4.1 PF00203.19	69.10	0.00
TRINITY_A9VAA2_MONBE/9-170	A9VAA2.1 PF00071.20	69.10	0.00

TRINITY_A8JC02_CHLRE/1-216	A8JC02.1 PF13621.4;	69.10	0.00
TRINITY_D8TVS8_VOLCA/777-1067	D8TVS8.1 PF04227.10	69.10	0.00
TRINITY_A8II54_CHLRE/359-510	A8II54.1 PF00005.25	69.10	0.00
TRINITY_A8I1U5_CHLRE/107-229	A8I1U5.1 PF00254.26	69.10	0.00
TRINITY_D8UD45_VOLCA/445-567	D8UD45.1 PF00306.25	69.10	0.00
TRINITY_I7MHS8_TETTS/328-395	I7MHS8.2 PF00498.24	69.10	0.00
TRINITY_D8U8T8_VOLCA/50-657	D8U8T8.1 PF00888.20	69.10	0.00
TRINITY_Q23H40_TETTS/63-592	Q23H40.1 PF02516.12	69.10	0.00
TRINITY_A8JBB9_CHLRE/9-104	A8JBB9.1 PF06294.9;	69.10	0.00
TRINITY_A0A060QCK4_9PROT/41-485	A0A060QCK4.1 PF0008	69.10	0.00
TRINITY_A8IFS5_CHLRE/189-410	A8IFS5.1 PF07944.10	69.10	0.00
TRINITY_D8UEC8_VOLCA/43-247	D8UEC8.1 PF02683.13	69.10	0.00
TRINITY_D3BDQ8_POLPA/12-329	D3BDQ8.1 PF00225.21	69.10	0.00
TRINITY_G0QQF6_ICHMG/8-146	G0QQF6.1 PF00179.24	69.10	0.00
TRINITY_Q23KA2_TETTS/98-168	Q23KA2.1 PF13499.4;	69.00	0.00
TRINITY_Q01V52_SOLUE/29-174	Q01V52.1 PF13673.5;	69.00	0.00
TRINITY_Q24HY7_TETTS/120-163	Q24HY7.3 PF13639.4;	69.00	0.00
TRINITY_A8HSS0_CHLRE/293-723	A8HSS0.1 PF01432.18	69.00	0.00
TRINITY_J9J9Q5_9SPIT/339-382	J9J9Q5.1 PF00569.15	69.00	0.00
TRINITY_RL34_NAUPA/1-44	B9L9D4.1 PF00468.15	69.00	0.00
TRINITY_A0BX73_PARTE/422-514	A0BX73.1 PF00153.25	69.00	0.00
TRINITY_Q22KA9_TETTS/18-102	Q22KA9.1 PF12895.5;	69.00	0.00
TRINITY_I7M9X1_TETTS/613-683	I7M9X1.1 PF00313.20	69.00	0.00
TRINITY_A8J5L3_CHLRE/85-203	A8J5L3.1 PF01926.21	69.00	0.00

TRINITY_D8UFH7_VOLCA/121-308	D8UFH7.1 PF10294.7;	69.00	0.00
TRINITY_G3UE99_LOXAF/2-91	G3UE99.1 PF00125.22	69.00	0.00
TRINITY_D8UJN9_VOLCA/195-305	D8UJN9.1 PF00271.29	69.00	0.00
TRINITY_D8TUY1_VOLCA/58-157	D8TUY1.1 PF00970.22	69.00	0.00
TRINITY_D8T0N5_SELML/36-106	D8T0N5.1 PF00076.20	69.00	0.00
TRINITY_L8GWE7_ACACA/35-528	L8GWE7.1 PF00118.22	69.00	0.00
TRINITY_A0A0E9NBB2_9ASCO/551-738	A0A0E9NBB2.1 PF0027	69.00	0.00
TRINITY_D8TI60_VOLCA/24-107	D8TI60.1 PF03083.14	69.00	0.00
TRINITY_A8I9I0_CHLRE/1727-1832	A8I9I0.1 PF00211.18	69.00	0.00
TRINITY_KCY_DICDI/12-169	P20425.2 PF00406.20	69.00	0.00
TRINITY_D8U2F3_VOLCA/264-593	D8U2F3.1 PF12631.5;	69.00	0.00
TRINITY_D8U9M9_VOLCA/28-86	D8U9M9.1 PF14444.4;	69.00	0.00
TRINITY_A8IWN3_CHLRE/1-184	A8IWN3.1 PF00248.19	69.00	0.00
TRINITY_A8IJT8_CHLRE/9-135	A8IJT8.1 PF01014.16	69.00	0.00
TRINITY_Q24FH6_TETTS/26-513	Q24FH6.2 PF03972.12	69.00	0.00
TRINITY_E1Z9E9_CHLVA/207-314	E1Z9E9.1 PF05834.10	69.00	0.00
TRINITY_A8I338_CHLRE/75-399	A8I338.1 PF00648.19	69.00	0.00
TRINITY_D8TNP8_VOLCA/116-223	D8TNP8.1 PF00091.23	69.00	0.00
TRINITY_A0A0H4KYG8_9RHOB/7-354	A0A0H4KYG8.1 PF0045	69.00	0.00
TRINITY_A8J9B2_CHLRE/52-177	A8J9B2.1 PF10357.7;	69.00	0.00
TRINITY_A8HM35_CHLRE/39-83	A8HM35.1 PF13445.4;	69.00	0.00
TRINITY_D8U7V5_VOLCA/43-236	D8U7V5.1 PF00106.23	69.00	0.00
TRINITY_D8TLG6_VOLCA/250-413	D8TLG6.1 PF05179.12	69.00	0.00
TRINITY_J9IV20_9SPIT/3490-4193	J9IV20.1 PF03028.13	69.00	0.00
TRINITY_SYTC_ENCCU/314-524	Q8SRH2.1 PF00587.23	68.90	0.00
TRINITY_I7M3P1_TETTS/107-196	I7M3P1.1 PF00027.27	68.90	0.00
TRINITY_Q240X5_TETTS/179-268	Q240X5.2 PF00027.27	68.90	0.00
TRINITY_I7MDC9_TETTS/35-225	I7MDC9.2 PF00316.18	68.90	0.00
TRINITY_A8IZ65_CHLRE/1-328	A8IZ65.1 PF00069.23	68.90	0.00
TRINITY_A0CVD0_PARTE/83-383	A0CVD0.1 PF03133.13	68.90	0.00
TRINITY_G4NJ54_MAGO7/479-523	G4NJ54.1 PF00400.30	68.90	0.00
TRINITY_J9IU65_9SPIT/608-801	J9IU65.1 PF05057.12	68.90	0.00
TRINITY_A0DFK5_PARTE/51-306	A0DFK5.1 PF00069.23	68.90	0.00
TRINITY_A8IVB9_CHLRE/354-458	A8IVB9.1 PF04003.10	68.90	0.00
TRINITY_D8U7F8_VOLCA/1707-1755	D8U7F8.1 PF02042.13	68.90	0.00
TRINITY_D8TLT5_VOLCA/18-126	D8TLT5.1 PF01412.16	68.90	0.00
TRINITY_NDK_CYAP4/2-136	B8HUM7.1 PF00334.17	68.90	0.00
TRINITY_D8TZE2_VOLCA/21-65	D8TZE2.1 PF07496.13	68.90	0.00
TRINITY_F4PHW4_DICFS/249-555	F4PHW4.1 PF00749.19	68.90	0.00
TRINITY_D8TSJ5_VOLCA/2597-3305	D8TSJ5.1 PF03028.13	68.90	0.00
TRINITY_D8U1P8_VOLCA/49-480	D8U1P8.1 PF00909.19	68.90	0.00
TRINITY_I0Z4M3_9CHLO/127-427	I0Z4M3.1 PF00176.21	68.90	0.00
TRINITY_A0A068RKL8_9FUNG/26-202	A0A068RKL8.1 PF0172	68.90	0.00
TRINITY_D8TLV9_VOLCA/362-640	D8TLV9.1 PF00702.24	68.90	0.00
TRINITY_D8UIB3_VOLCA/764-970	D8UIB3.1 PF08214.9;	68.90	0.00
TRINITY_A0BQ47_PARTE/3-95	A0BQ47.1 PF03259.15	68.80	0.00
TRINITY_M4AU00_XIPMA/1-323	M4AU00.1 PF01122.17	68.80	0.00
TRINITY_U9TLQ1_RHIID/331-370	U9TLQ1.1 PF00400.30	68.80	0.00
TRINITY_H3G9K1_PHYRM/83-147	H3G9K1.1 PF13499.4;	68.80	0.00
TRINITY_A0A0E0PRJ8_ORYRU/753-911	A0A0E0PRJ8.1 PF0007	68.80	0.00
TRINITY_Q3SDM6_PARTE/17-177	Q3SDM6.1 PF00071.20	68.80	0.00
TRINITY_A0A094F0M8_9PEZI/64-193	A0A094F0M8.1 PF0069	68.80	0.00
TRINITY_W4YVI5_STRPU/36-147	W4YVI5.1 PF08240.10	68.80	0.00
TRINITY_J9I6B6_9SPIT/8-264	J9I6B6.1 PF00069.23	68.80	0.00
TRINITY_D8U6W6_VOLCA/80-177	D8U6W6.1 PF01196.17	68.80	0.00
TRINITY_B3S900_TRIAD/188-252	B3S900.1 PF00462.22	68.80	0.00
TRINITY_A8J4P1_CHLRE/202-362	A8J4P1.1 PF01077.20	68.80	0.00
TRINITY_Q23AM3_TETTS/375-524	Q23AM3.2 PF00005.25	68.80	0.00
TRINITY_A8ISQ5_CHLRE/1729-1856	A8ISQ5.1 PF00004.27	68.80	0.00
TRINITY_I0RRG9_MYCPH/20-169	I0RRG9.1 PF00005.25	68.80	0.00
TRINITY_D8U3D7_VOLCA/774-888	D8U3D7.1 PF00072.22	68.80	0.00

TRINITY_D8TT93_VOLCA/60-222	D8TT93.1 PF01195.17	68.80	0.00
TRINITY_A8HWS1_CHLRE/2584-2904	A8HWS1.1 PF12777.5;	68.80	0.00
TRINITY_D8U7H9_VOLCA/70-449	D8U7H9.1 PF00632.23	68.80	0.00
TRINITY_D8TWN6_VOLCA/49-376	D8TWN6.1 PF00012.18	68.80	0.00
TRINITY_D3BC33_POLPA/5-166	D3BC33.1 PF00071.20	68.80	0.00
TRINITY_A0A068RLI7_9FUNG/34-530	A0A068RLI7.1 PF0011	68.80	0.00
TRINITY_D8UKL7_VOLCA/84-133	D8UKL7.1 PF00415.16	68.80	0.00
TRINITY_I7LTC5_TETTS/314-458	I7LTC5.2 PF00005.25	68.80	0.00
TRINITY_D3BDM9_POLPA/5-36	D3BDM9.1 PF03604.11	68.80	0.00
TRINITY_L2FVU6_COLGN/36-309	L2FVU6.1 PF00264.18	68.80	0.00
TRINITY_D8TSI9_VOLCA/91-251	D8TSI9.1 PF00753.25	68.80	0.00
TRINITY_A8I4W2_CHLRE/1-181	A8I4W2.1 PF00211.18	68.80	0.00
TRINITY_D8TMK9_VOLCA/311-362	D8TMK9.1 PF01846.17	68.80	0.00
TRINITY_A7RU37_NEMVE/31-537	A7RU37.1 PF00330.18	68.80	0.00
TRINITY_D8ULS6_VOLCA/67-321	D8ULS6.1 PF01636.21	68.80	0.00
TRINITY_D8U5R7_VOLCA/7-126	D8U5R7.1 PF08477.11	68.80	0.00
TRINITY_Q247W5_TETTS/30-198	Q247W5.1 PF00227.24	68.80	0.00
TRINITY_D8TMI8_VOLCA/5-161	D8TMI8.1 PF00106.23	68.80	0.00
TRINITY_Q22DC6_TETTS/70-165	Q22DC6.2 PF00338.20	68.80	0.00
TRINITY_D8U7F8_VOLCA/1707-1755	D8U7F8.1 PF02042.13	68.80	0.00
TRINITY_A8I985_CHLRE/223-459	A8I985.1 PF04811.13	68.80	0.00
TRINITY_G3WLT7_SARHA/41-278	G3WLT7.1 PF04884.12	68.80	0.00
TRINITY_J0KPW8_9BURK/278-481	J0KPW8.1 PF03441.12	68.80	0.00
TRINITY_A0BWL4_PARTE/26-288	A0BWL4.1 PF00069.23	68.80	0.00
TRINITY_E1ZFN2_CHLVA/22-277	E1ZFN2.1 PF01040.16	68.80	0.00
TRINITY_D8TQB0_VOLCA/1630-1755	D8TQB0.1 PF00211.18	68.80	0.00
TRINITY_A0A0I9NA30_BRUMA/16-81	A0A0I9NA30.1 PF0142	68.80	0.00
TRINITY_L8HC09_ACACA/233-325	L8HC09.1 PF05761.12	68.80	0.00
TRINITY_Q22W11_TETTS/50-150	Q22W11.1 PF00111.25	68.70	0.00
TRINITY_F0Z713_DICPU/3-213	F0Z713.1 PF00091.23	68.70	0.00
TRINITY_D3BPE3_POLPA/1-135	D3BPE3.1 PF01217.18	68.70	0.00
TRINITY_D0MQG3_PHYIT/261-376	D0MQG3.1 PF02801.20	68.70	0.00
TRINITY_A0A078AHP9_STYLE/355-421	A0A078AHP9.1 PF0022	68.70	0.00
TRINITY_A0A078AR09_STYLE/8-169	A0A078AR09.1 PF0007	68.70	0.00
TRINITY_I0YV20_9CHLO/60-294	I0YV20.1 PF01370.19	68.70	0.00
TRINITY_A8IX31_CHLRE/146-272	A8IX31.1 PF00134.21	68.70	0.00
TRINITY_W4GSZ0_9STRA/1585-1861	W4GSZ0.1 PF02138.16	68.70	0.00
TRINITY_A8JFN4_CHLRE/30-188	A8JFN4.1 PF00160.19	68.70	0.00
TRINITY_D3BSA4_POLPA/117-365	D3BSA4.1 PF01063.17	68.70	0.00
TRINITY_D8UAD8_VOLCA/126-306	D8UAD8.1 PF00270.27	68.70	0.00
TRINITY_A0A078AQC9_STYLE/701-959	A0A078AQC9.1 PF0006	68.70	0.00
TRINITY_L8H1T2_ACACA/189-371	L8H1T2.1 PF00117.26	68.70	0.00
TRINITY_G5EDF9_CAEEL/417-566	G5EDF9.1 PF00005.25	68.70	0.00
TRINITY_C1MZF2_MICPC/167-287	C1MZF2.1 PF00271.29	68.70	0.00
TRINITY_I0Z246_9CHLO/667-749	I0Z246.1 PF09369.8;	68.70	0.00
TRINITY_Q6BG73_PARTE/7-73	Q6BG73.1 PF13921.4;	68.70	0.00
TRINITY_G3RKE6_GORGO/13-813	G3RKE6.1 PF04563.13	68.70	0.00
TRINITY_D8U2W9_VOLCA/23-287	D8U2W9.1 PF00459.23	68.70	0.00
TRINITY_A8HYK7_CHLRE/141-358	A8HYK7.1 PF02096.18	68.70	0.00
TRINITY_D8TJ80_VOLCA/167-396	D8TJ80.1 PF01798.16	68.70	0.00
TRINITY_A8IXP1_CHLRE/1840-2022	A8IXP1.1 PF00211.18	68.70	0.00
TRINITY_A0A015JFC3_9GLOM/15-346	A0A015JFC3.1 PF0020	68.70	0.00
TRINITY_E1Z466_CHLVA/57-351	E1Z466.1 PF00248.19	68.70	0.00
TRINITY_F2IAW3_FLUTR/160-316	F2IAW3.1 PF02800.18	68.70	0.00
TRINITY_A8JAJ2_CHLRE/92-306	A8JAJ2.1 PF01036.16	68.70	0.00
TRINITY_L8GQL0_ACACA/48-299	L8GQL0.1 PF00069.23	68.70	0.00
TRINITY_Q9A8K3_CAUCR/227-377	Q9A8K3.1 PF00441.22	68.70	0.00
TRINITY_D3BDQ8_POLPA/12-329	D3BDQ8.1 PF00225.21	68.70	0.00
TRINITY_A8J6U8_CHLRE/3-261	A8J6U8.1 PF00069.23	68.70	0.00
TRINITY_G0QJ07_ICHMG/10-191	G0QJ07.1 PF00227.24	68.70	0.00
TRINITY_W7X227_TETTS/314-349	W7X227.1 PF08238.10	68.60	0.00

TRINITY_F4PRE1_DICFS/194-228	F4PRE1.1 PF00320.25	68.60	0.00
TRINITY_S2KFF9_MUCC1/19-136	S2KFF9.1 PF01894.15	68.60	0.00
TRINITY_S2KFF9_MUCC1/19-136	S2KFF9.1 PF01894.15	68.60	0.00
TRINITY_A0A0G4LJ84_9PEZI/114-328	A0A0G4LJ84.1 PF0009	68.60	0.00
TRINITY_A0A0P7VAB1_9TELE/21-192	A0A0P7VAB1.1 PF0026	68.60	0.00
TRINITY_I7MJD5_TETTS/89-391	I7MJD5.1 PF00676.18	68.60	0.00
TRINITY_A0D712_PARTE/195-452	A0D712.1 PF00069.23	68.60	0.00
TRINITY_A0A0G4J5Y5_PLABS/8-361	A0A0G4J5Y5.1 PF0126	68.60	0.00
TRINITY_I7M7Z9_TETTS/616-760	I7M7Z9.1 PF00005.25	68.60	0.00
TRINITY_D8THB0_VOLCA/261-500	D8THB0.1 PF00814.23	68.60	0.00
TRINITY_K7U0V6_MAIZE/58-353	K7U0V6.1 PF00579.23	68.60	0.00
TRINITY_A8J673_CHLRE/1-121	A8J673.1 PF08315.10	68.60	0.00
TRINITY_L8GVQ4_ACACA/573-690	L8GVQ4.1 PF02310.17	68.60	0.00
TRINITY_Q22UZ7_TETTS/14-118	Q22UZ7.2 PF00307.29	68.60	0.00
TRINITY_A0BED7_PARTE/44-252	A0BED7.1 PF08442.8;	68.60	0.00
TRINITY_D8UI45_VOLCA/1-169	D8UI45.1 PF01545.19	68.60	0.00
TRINITY_A0A087SQB4_AUXPR/47-116	A0A087SQB4.1 PF0267	68.60	0.00
TRINITY_D8TUF3_VOLCA/32-168	D8TUF3.1 PF01814.21	68.60	0.00
TRINITY_D8U5R4_VOLCA/306-375	D8U5R4.1 PF00076.20	68.60	0.00
TRINITY_A0DHP1_PARTE/551-761	A0DHP1.1 PF00211.18	68.60	0.00
TRINITY_Q233N6_TETTS/33-289	Q233N6.2 PF00069.23	68.60	0.00
TRINITY_M4BMA1_HYAAE/108-159	M4BMA1.1 PF00400.30	68.60	0.00
TRINITY_A0A078A864_STYLE/29-333	A0A078A864.1 PF0313	68.60	0.00
TRINITY_L8GUT5_ACACA/47-135	L8GUT5.1 PF00062.18	68.60	0.00
TRINITY_A0A0D2TAC2_GOSRA/51-457	A0A0D2TAC2.1 PF0518	68.60	0.00
TRINITY_A0A0G4J868_PLABS/11-48	A0A0G4J868.1 PF0040	68.60	0.00
TRINITY_A8HVF8_CHLRE/9-127	A8HVF8.1 PF02338.17	68.60	0.00
TRINITY_A7SGD9_NEMVE/51-86	A7SGD9.1 PF00400.30	68.60	0.00
TRINITY_I7MD55_TETTS/531-788	I7MD55.2 PF00069.23	68.60	0.00
TRINITY_G0QLS3_ICHMG/25-209	G0QLS3.1 PF00227.24	68.60	0.00
TRINITY_U6PHM9_HAECO/501-829	U6PHM9.1 PF00176.21	68.60	0.00
TRINITY_I0YVN5_9CHLO/16-242	I0YVN5.1 PF00850.17	68.60	0.00
TRINITY_A0E493_PARTE/11-338	A0E493.1 PF00225.21	68.60	0.00
TRINITY_D8TJP6_VOLCA/149-473	D8TJP6.1 PF00443.27	68.60	0.00
TRINITY_K1QHT8_CRAGI/74-144	K1QHT8.1 PF00076.20	68.60	0.00
TRINITY_M2Y471_GALSU/29-219	M2Y471.1 PF00227.24	68.60	0.00
TRINITY_A8JFW5_CHLRE/24-180	A8JFW5.1 PF05351.9;	68.60	0.00
TRINITY_A0A0M2HVV0_9MICO/28-144	A0A0M2HVV0.1 PF0824	68.60	0.00
TRINITY_D8TII1_VOLCA/7-490	D8TII1.1 PF00982.19	68.60	0.00
TRINITY_I1K7D4_SOYBN/18-391	I1K7D4.1 PF00199.17	68.60	0.00
TRINITY_M1VCC4_CYAME/278-464	M1VCC4.1 PF07724.12	68.60	0.00
TRINITY_A8I0B3_CHLRE/1-207	A8I0B3.1 PF00481.19	68.60	0.00
TRINITY_A9U311_PHYPA/469-571	A9U311.1 PF00085.18	68.60	0.00
TRINITY_D8TUH3_VOLCA/26-235	D8TUH3.1 PF12706.5;	68.60	0.00
TRINITY_I7MDE4_TETTS/57-274	I7MDE4.2 PF03332.11	68.60	0.00
TRINITY_A8IPH2_CHLRE/150-269	A8IPH2.1 PF04982.11	68.60	0.00
TRINITY_G7ZEL6_AZOL4/114-433	G7ZEL6.1 PF01546.26	68.50	0.00
TRINITY_E0VEF9_PEDHC/79-132	E0VEF9.1 PF01381.20	68.50	0.00
TRINITY_A0CNM0_PARTE/7-99	A0CNM0.1 PF00153.25	68.50	0.00
TRINITY_A8HME1_CHLRE/44-213	A8HME1.1 PF16166.3;	68.50	0.00
TRINITY_A0BXQ9_PARTE/10-344	A0BXQ9.1 PF00225.21	68.50	0.00
TRINITY_M2Y2Z1_GALSU/166-292	M2Y2Z1.1 PF00134.21	68.50	0.00
TRINITY_A0DZP1_PARTE/30-624	A0DZP1.1 PF00012.18	68.50	0.00
TRINITY_I7MG82_TETTS/90-413	I7MG82.2 PF00225.21	68.50	0.00
TRINITY_E1ZKP2_CHLVA/286-366	E1ZKP2.1 PF00498.24	68.50	0.00
TRINITY_D8TIW2_VOLCA/287-378	D8TIW2.1 PF02517.14	68.50	0.00
TRINITY_A8IRP0_CHLRE/1-360	A8IRP0.1 PF07690.14	68.50	0.00
TRINITY_A0DQV4_PARTE/2-236	A0DQV4.1 PF00483.21	68.50	0.00
TRINITY_D8TQV2_VOLCA/457-709	D8TQV2.1 PF12697.5;	68.50	0.00
TRINITY_A8JGJ4_CHLRE/324-589	A8JGJ4.1 PF00069.23	68.50	0.00
TRINITY_D3B882_POLPA/43-231	D3B882.1 PF00069.23	68.50	0.00

TRINITY_A8I8K4_CHLRE/36-301	A8I8K4.1 PF00459.23	68.50	0.00
TRINITY_B0THU3_HELMI/5-266	B0THU3.1 PF00795.20	68.50	0.00
TRINITY_E1ZFM0_CHLVA/58-200	E1ZFM0.1 PF13365.4;	68.50	0.00
TRINITY_I0Z2W4_9CHLO/48-225	I0Z2W4.1 PF01634.16	68.50	0.00
TRINITY_I0Z2Z0_9CHLO/345-422	I0Z2Z0.1 PF02020.16	68.50	0.00
TRINITY_D8TQ08_VOLCA/100-172	D8TQ08.1 PF02325.15	68.50	0.00
TRINITY_I7LUG9_TETTS/17-335	I7LUG9.2 PF00225.21	68.50	0.00
TRINITY_A8IPI3_CHLRE/31-103	A8IPI3.1 PF07883.9;	68.50	0.00
TRINITY_D8U3X9_VOLCA/1-254	D8U3X9.1 PF00069.23	68.50	0.00
TRINITY_L8HFC7_ACACA/637-816	L8HFC7.1 PF05063.12	68.50	0.00
TRINITY_A8HX99_CHLRE/214-381	A8HX99.1 PF00160.19	68.50	0.00
TRINITY_A8IAK1_CHLRE/13-231	A8IAK1.1 PF01031.18	68.50	0.00
TRINITY_A8J6W3_CHLRE/6-208	A8J6W3.1 PF02854.17	68.50	0.00
TRINITY_W4Z930_STRPU/42-190	W4Z930.1 PF00005.25	68.50	0.00
TRINITY_I7LSV4_TETTS/3534-4237	I7LSV4.2 PF03028.13	68.50	0.00
TRINITY_A8JGN8_CHLRE/797-969	A8JGN8.1 PF02460.16	68.50	0.00
TRINITY_U4U9B7_DENPD/47-258	U4U9B7.1 PF01351.16	68.40	0.00
TRINITY_G0R5C0_ICHMG/323-360	G0R5C0.1 PF00400.30	68.40	0.00
TRINITY_V8NPF4_OPHHA/193-277	V8NPF4.1 PF01974.15	68.40	0.00
TRINITY_G0QQX6_ICHMG/619-733	G0QQX6.1 PF00271.29	68.40	0.00
TRINITY_I7M0P6_TETTS/42-98	I7M0P6.1 PF01849.16	68.40	0.00
TRINITY_F0Z6W5_DICPU/32-323	F0Z6W5.1 PF00850.17	68.40	0.00
TRINITY_I7M4Q4_TETTS/47-304	I7M4Q4.1 PF00069.23	68.40	0.00
TRINITY_B9XKX6_PEDPL/39-443	B9XKX6.1 PF00202.19	68.40	0.00
TRINITY_A8IDE3_CHLRE/567-680	A8IDE3.1 PF00175.19	68.40	0.00
TRINITY_Q23JY6_TETTS/610-893	Q23JY6.2 PF00481.19	68.40	0.00
TRINITY_A8IYH5_CHLRE/1-189	A8IYH5.1 PF00551.17	68.40	0.00
TRINITY_A0A067RA84_ZOONE/106-735	A0A067RA84.1 PF0013	68.40	0.00
TRINITY_A8IV51_CHLRE/123-238	A8IV51.1 PF13640.4;	68.40	0.00
TRINITY_A0A059LHG8_9CHLO/1-220	A0A059LHG8.1 PF1356	68.40	0.00
TRINITY_I0YXK4_9CHLO/3-146	I0YXK4.1 PF06799.9;	68.40	0.00
TRINITY_G3PBA6_GASAC/5-62	G3PBA6.1 PF00412.20	68.40	0.00
TRINITY_A0A094GNB2_9PEZI/42-212	A0A094GNB2.1 PF0214	68.40	0.00
TRINITY_Q24GE4_TETTS/399-611	Q24GE4.3 PF00644.18	68.40	0.00
TRINITY_A0A094GNB2_9PEZI/42-212	A0A094GNB2.1 PF0214	68.40	0.00
TRINITY_RL4_TETTS/23-269	P0DJ55.1 PF00573.20	68.40	0.00
TRINITY_A0A061DMP8_THECC/350-439	A0A061DMP8.1 PF0007	68.40	0.00
TRINITY_Q22UI8_TETTS/2041-2254	Q22UI8.2 PF00454.25	68.40	0.00
TRINITY_D8UJL1_VOLCA/640-868	D8UJL1.1 PF00326.19	68.40	0.00
TRINITY_I0YLA3_9CHLO/43-441	I0YLA3.1 PF00464.17	68.40	0.00
TRINITY_A8HME9_CHLRE/130-227	A8HME9.1 PF00561.18	68.40	0.00
TRINITY_A9T8K5_PHYPA/288-484	A9T8K5.1 PF04389.15	68.40	0.00
TRINITY_F4Q5T3_DICFS/12-408	F4Q5T3.1 PF00162.17	68.40	0.00
TRINITY_A7EX43_SCLS1/411-558	A7EX43.1 PF00731.18	68.40	0.00
TRINITY_A8J6C7_CHLRE/445-655	A8J6C7.1 PF01434.16	68.40	0.00
TRINITY_A7SC77_NEMVE/56-189	A7SC77.1 PF00578.19	68.40	0.00
TRINITY_D8TI22_VOLCA/166-394	D8TI22.1 PF03040.12	68.40	0.00
TRINITY_A8IL08_CHLRE/489-703	A8IL08.1 PF01434.16	68.40	0.00
TRINITY_D8U5Z4_VOLCA/87-202	D8U5Z4.1 PF13640.4;	68.40	0.00
TRINITY_D8TLC8_VOLCA/60-639	D8TLC8.1 PF05470.10	68.40	0.00
TRINITY_D8U4P6_VOLCA/58-162	D8U4P6.1 PF00588.17	68.40	0.00
TRINITY_L8GQL5_ACACA/626-1215	L8GQL5.1 PF00821.16	68.40	0.00
TRINITY_L8GQL5_ACACA/626-1215	L8GQL5.1 PF00821.16	68.40	0.00
TRINITY_A8I318_CHLRE/2-286	A8I318.1 PF03151.14	68.40	0.00
TRINITY_A0A0B2VWK9_TOXCA/3-379	A0A0B2VWK9.1 PF0002	68.40	0.00
TRINITY_A0A0G1MNI9_9BACT/9-281	A0A0G1MNI9.1 PF0000	68.30	0.00
TRINITY_Q245G5_TETTS/3-166	Q245G5.2 PF08613.9;	68.30	0.00
TRINITY_Q245G5_TETTS/3-166	Q245G5.2 PF08613.9;	68.30	0.00
TRINITY_I0YWU4_9CHLO/8-153	I0YWU4.1 PF01751.20	68.30	0.00
TRINITY_I7MJ35_TETTS/25-211	I7MJ35.1 PF01012.19	68.30	0.00
TRINITY_I0XNV4_9LEPT/1-77	I0XNV4.1 PF02798.18	68.30	0.00

TRINITY_G0QQF6_ICHMG/8-146	G0QQF6.1 PF00179.24	68.30	0.00
TRINITY_A0EGS7_PARTE/54-95	A0EGS7.1 PF13923.4;	68.30	0.00
TRINITY_Q24DJ0_TETTS/30-131	Q24DJ0.1 PF14772.4;	68.30	0.00
TRINITY_D7FTN9_ECTSI/26-139	D7FTN9.1 PF01398.19	68.30	0.00
TRINITY_G0QPV5_ICHMG/284-405	G0QPV5.1 PF00782.18	68.30	0.00
TRINITY_D3AWC6_POLPA/24-347	D3AWC6.1 PF00899.19	68.30	0.00
TRINITY_A8IFE0_CHLRE/525-1063	A8IFE0.1 PF04998.15	68.30	0.00
TRINITY_D8TNA1_VOLCA/128-418	D8TNA1.1 PF06814.11	68.30	0.00
TRINITY_A0BK26_PARTE/10-356	A0BK26.1 PF00225.21	68.30	0.00
TRINITY_D8UD65_VOLCA/308-348	D8UD65.1 PF00400.30	68.30	0.00
TRINITY_W5AA68_WHEAT/3-184	W5AA68.1 PF00012.18	68.30	0.00
TRINITY_F0ZR56_DICPU/72-332	F0ZR56.1 PF00122.18	68.30	0.00
TRINITY_D8UEP9_VOLCA/1198-1364	D8UEP9.1 PF00270.27	68.30	0.00
TRINITY_A8I1W3_CHLRE/2979-3226	A8I1W3.1 PF00454.25	68.30	0.00
TRINITY_Q84TR5_CHLRE/17-117	Q84TR5.1 PF00307.29	68.30	0.00
TRINITY_Q22AZ2_TETTS/11-309	Q22AZ2.2 PF00463.19	68.30	0.00
TRINITY_D8TUE1_VOLCA/55-325	D8TUE1.1 PF01513.19	68.30	0.00
TRINITY_A8J629_CHLRE/229-374	A8J629.1 PF01699.22	68.30	0.00
TRINITY_A0A087SAG6_AUXPR/336-376	A0A087SAG6.1 PF0040	68.30	0.00
TRINITY_L8HKC2_ACACA/226-268	L8HKC2.1 PF00569.15	68.30	0.00
TRINITY_A8J7U7_CHLRE/2-344	A8J7U7.1 PF01137.19	68.30	0.00
TRINITY_A0EDS7_PARTE/465-664	A0EDS7.1 PF01434.16	68.30	0.00
TRINITY_D8UGS8_VOLCA/11-249	D8UGS8.1 PF00899.19	68.30	0.00
TRINITY_A0A0D2X094_CAPO3/2-82	A0A0D2X094.1 PF0088	68.30	0.00
TRINITY_A4VDD6_TETTS/15-172	A4VDD6.1 PF02099.15	68.20	0.00
TRINITY_D2VUH3_NAEGR/405-524	D2VUH3.1 PF00917.24	68.20	0.00
TRINITY_W7TKH0_9STRA/1-223	W7TKH0.1 PF06418.12	68.20	0.00
TRINITY_D3AX77_POLPA/30-357	D3AX77.1 PF04909.12	68.20	0.00
TRINITY_G0R585_ICHMG/543-822	G0R585.1 PF12775.5;	68.20	0.00
TRINITY_Q24IJ5_TETTS/374-418	Q24IJ5.1 PF06203.12	68.20	0.00
TRINITY_MYOD_DICDI/9-674	P34109.2 PF00063.19	68.20	0.00
TRINITY_J9JCA5_9SPIT/125-339	J9JCA5.1 PF00112.21	68.20	0.00
TRINITY_I1CHR9_RHIO9/197-291	I1CHR9.1 PF00153.25	68.20	0.00
TRINITY_I0Z1Z3_9CHLO/21-112	I0Z1Z3.1 PF00428.17	68.20	0.00
TRINITY_I0YZR3_9CHLO/5-159	I0YZR3.1 PF00929.22	68.20	0.00
TRINITY_F4K151_ARATH/29-74	F4K151.1 PF13639.4;	68.20	0.00
TRINITY_A8HQW1_CHLRE/5-232	A8HQW1.1 PF01182.18	68.20	0.00
TRINITY_E1Z3R6_CHLVA/113-623	E1Z3R6.1 PF07969.9;	68.20	0.00
TRINITY_G0QN21_ICHMG/543-674	G0QN21.1 PF00005.25	68.20	0.00
TRINITY_A0CMX3_PARTE/57-210	A0CMX3.1 PF00005.25	68.20	0.00
TRINITY_D8UAS6_VOLCA/335-465	D8UAS6.1 PF00566.16	68.20	0.00
TRINITY_I0YML0_9CHLO/1-132	I0YML0.1 PF00504.19	68.20	0.00
TRINITY_L8GZG8_ACACA/214-302	L8GZG8.1 PF07571.11	68.20	0.00
TRINITY_I7LTN8_TETTS/14-165	I7LTN8.1 PF00179.24	68.20	0.00
TRINITY_A0A087SME0_AUXPR/3-322	A0A087SME0.1 PF0369	68.20	0.00
TRINITY_D8U3F5_VOLCA/48-269	D8U3F5.1 PF01370.19	68.20	0.00
TRINITY_A8J201_CHLRE/78-123	A8J201.1 PF00400.30	68.20	0.00
TRINITY_A8IIZ5_CHLRE/19-278	A8IIZ5.1 PF00069.23	68.20	0.00
TRINITY_A9TN94_PHYPA/43-165	A9TN94.1 PF00903.23	68.20	0.00
TRINITY_A0A0D9VUL1_9ORYZ/7-183	A0A0D9VUL1.1 PF0016	68.20	0.00
TRINITY_A0A0F5L2E0_9RHIZ/426-749	A0A0F5L2E0.1 PF0171	68.20	0.00
TRINITY_A8IHQ2_CHLRE/12-181	A8IHQ2.1 PF04893.15	68.20	0.00
TRINITY_L8H6I3_ACACA/416-480	L8H6I3.1 PF01485.19	68.20	0.00
TRINITY_D8TY38_VOLCA/1581-1689	D8TY38.1 PF00856.26	68.20	0.00
TRINITY_D8UE48_VOLCA/38-167	D8UE48.1 PF00271.29	68.20	0.00
TRINITY_Q23K21_TETTS/369-636	Q23K21.2 PF00487.22	68.20	0.00
TRINITY_D7G1J2_ECTSI/274-437	D7G1J2.1 PF00270.27	68.20	0.00
TRINITY_A0A0M9UDC4_9CHLR/12-275	A0A0M9UDC4.1 PF1356	68.20	0.00
TRINITY_L8GF06_ACACA/12-222	L8GF06.1 PF05378.11	68.10	0.00
TRINITY_A0A068XFX1_HYMMI/1-132	A0A068XFX1.1 PF0012	68.10	0.00
TRINITY_A8JBC8_CHLRE/11-125	A8JBC8.1 PF01398.19	68.10	0.00

TRINITY_I0YWD0_9CHLO/8-264	I0YWD0.1 PF00069.23	68.10	0.00
TRINITY_A0A087SA88_AUXPR/261-382	A0A087SA88.1 PF0395	68.10	0.00
TRINITY_L8GVQ7_ACACA/9-125	L8GVQ7.1 PF02678.14	68.10	0.00
TRINITY_A8IYL8_CHLRE/50-137	A8IYL8.1 PF05172.11	68.10	0.00
TRINITY_D8TKT9_VOLCA/549-804	D8TKT9.1 PF00425.16	68.10	0.00
TRINITY_D8TZ47_VOLCA/242-358	D8TZ47.1 PF00932.17	68.10	0.00
TRINITY_L8GW58_ACACA/230-524	L8GW58.1 PF02781.14	68.10	0.00
TRINITY_D8U5Y2_VOLCA/952-1077	D8U5Y2.1 PF07728.12	68.10	0.00
TRINITY_I7MLL9_TETTS/291-547	I7MLL9.2 PF00069.23	68.10	0.00
TRINITY_K1QCY0_CRAGI/35-433	K1QCY0.1 PF00464.17	68.10	0.00
TRINITY_C4JNN2_UNCRE/73-160	C4JNN2.1 PF04488.13	68.10	0.00
TRINITY_Q4QCN8_LEIMA/134-211	Q4QCN8.1 PF05002.13	68.10	0.00
TRINITY_Q239M6_TETTS/768-1156	Q239M6.2 PF00940.17	68.10	0.00
TRINITY_K4AE64_SETIT/14-140	K4AE64.1 PF12165.6;	68.10	0.00
TRINITY_F4Q353_DICFS/600-717	F4Q353.1 PF02466.17	68.10	0.00
TRINITY_Q239R2_TETTS/10-57	Q239R2.2 PF00319.16	68.10	0.00
TRINITY_I0Z920_9CHLO/37-305	I0Z920.1 PF00494.17	68.10	0.00
TRINITY_C5XJE3_SORBI/299-460	C5XJE3.1 PF08662.9;	68.10	0.00
TRINITY_FEN11_PARTE/1-111	A0CXT3.1 PF00752.15	68.10	0.00
TRINITY_L8GT20_ACACA/17-342	L8GT20.1 PF00009.25	68.10	0.00
TRINITY_I7MDB1_TETTS/296-654	I7MDB1.2 PF03133.13	68.10	0.00
TRINITY_D6WWC9_TRICA/1052-1123	D6WWC9.1 PF04564.13	68.10	0.00
TRINITY_A8J3T4_CHLRE/72-440	A8J3T4.1 PF01979.18	68.10	0.00
TRINITY_A0BWW1_PARTE/272-340	A0BWW1.1 PF00498.24	68.10	0.00
TRINITY_D3B0D6_POLPA/8-284	D3B0D6.1 PF03643.13	68.10	0.00
TRINITY_A0A087YGB1_POEFO/20-271	A0A087YGB1.2 PF0006	68.10	0.00
TRINITY_A8J326_CHLRE/65-436	A8J326.1 PF01979.18	68.10	0.00
TRINITY_D8U0A7_VOLCA/524-587	D8U0A7.1 PF13432.4;	68.10	0.00
TRINITY_B3S5T2_TRIAD/107-153	B3S5T2.1 PF13921.4;	68.10	0.00
TRINITY_D8U9P4_VOLCA/143-212	D8U9P4.1 PF00076.20	68.10	0.00
TRINITY_D3BR06_POLPA/461-695	D3BR06.1 PF00198.21	68.10	0.00
TRINITY_PR38A_DANRE/10-175	Q6DHU4.1 PF03371.13	68.10	0.00
TRINITY_I0YP89_9CHLO/15-422	I0YP89.1 PF06838.9;	68.10	0.00
TRINITY_D3BTL7_POLPA/1505-1792	D3BTL7.1 PF01031.18	68.10	0.00
TRINITY_Q24C33_TETTS/16-62	Q24C33.1 PF01281.17	68.10	0.00
TRINITY_D8UK57_VOLCA/430-594	D8UK57.1 PF00534.18	68.10	0.00
TRINITY_I7M700_TETTS/181-443	I7M700.2 PF00069.23	68.10	0.00
TRINITY_D8U2Q8_VOLCA/673-763	D8U2Q8.1 PF03982.11	68.10	0.00
TRINITY_A0A0E9NBZ0_9ASCO/15-61	A0A0E9NBZ0.1 PF1290	68.10	0.00
TRINITY_A8J537_CHLRE/18-399	A8J537.1 PF00199.17	68.10	0.00
TRINITY_I0Z8F4_9CHLO/651-787	I0Z8F4.1 PF00005.25	68.10	0.00
TRINITY_D8TI09_VOLCA/114-277	D8TI09.1 PF04116.11	68.10	0.00
TRINITY_D8UCZ7_VOLCA/23-229	D8UCZ7.1 PF00106.23	68.10	0.00
TRINITY_D8TZ40_VOLCA/777-1279	D8TZ40.1 PF04998.15	68.10	0.00
TRINITY_A8J046_CHLRE/643-936	A8J046.1 PF03715.11	68.10	0.00
TRINITY_I7LV68_TETTS/19-199	I7LV68.1 PF13439.4;	68.10	0.00
TRINITY_X6MRY1_RETFI/247-315	X6MRY1.1 PF04564.13	68.00	0.00
TRINITY_B8HSG8_CYAP4/8-137	B8HSG8.1 PF07080.9;	68.00	0.00
TRINITY_ITPA_MICSR/9-188	C1FI13.1 PF01725.14	68.00	0.00
TRINITY_G0QL25_ICHMG/300-478	G0QL25.1 PF11931.6;	68.00	0.00
TRINITY_A0A072PK09_9EURO/17-112	A0A072PK09.1 PF0042	68.00	0.00
TRINITY_A0DAW7_PARTE/169-243	A0DAW7.1 PF01169.17	68.00	0.00
TRINITY_D8TKV7_VOLCA/614-688	D8TKV7.1 PF11732.6;	68.00	0.00
TRINITY_A8IV40_CHLRE/38-112	A8IV40.1 PF00111.25	68.00	0.00
TRINITY_Q238V7_TETTS/58-226	Q238V7.2 PF00270.27	68.00	0.00
TRINITY_E1ZKT8_CHLVA/93-167	E1ZKT8.1 PF03110.12	68.00	0.00
TRINITY_I0ZAA1_9CHLO/56-167	I0ZAA1.1 PF00072.22	68.00	0.00
TRINITY_I7MLG1_TETTS/1-175	I7MLG1.1 PF14580.4;	68.00	0.00
TRINITY_A8IVD4_CHLRE/52-173	A8IVD4.1 PF13409.4;	68.00	0.00
TRINITY_Q7XLE8_ORYSJ/857-1017	Q7XLE8.2 PF00078.25	68.00	0.00
TRINITY_D8TQK9_VOLCA/19-93	D8TQK9.1 PF04969.14	68.00	0.00

TRINITY_I0YMS0_9CHLO/96-197	I0YMS0.1 PF00153.25	68.00	0.00
TRINITY_Q24HK9_TETTS/22-280	Q24HK9.3 PF00069.23	68.00	0.00
TRINITY_A8IHL8_CHLRE/15-117	A8IHL8.1 PF13815.4;	68.00	0.00
TRINITY_D8TU26_VOLCA/70-514	D8TU26.1 PF00899.19	68.00	0.00
TRINITY_E1Z439_CHLVA/15-334	E1Z439.1 PF03747.12	68.00	0.00
TRINITY_A8IMP0_CHLRE/47-242	A8IMP0.1 PF00149.26	68.00	0.00
TRINITY_I7M3W6_TETTS/237-558	I7M3W6.2 PF00152.18	68.00	0.00
TRINITY_D8UIV0_VOLCA/25-394	D8UIV0.1 PF00224.19	68.00	0.00
TRINITY_A8JAT5_CHLRE/39-89	A8JAT5.1 PF00847.18	68.00	0.00
TRINITY_F0ZZF5_DICPU/9-255	F0ZZF5.1 PF13714.4;	68.00	0.00
TRINITY_D8TP06_VOLCA/164-311	D8TP06.1 PF03031.16	68.00	0.00
TRINITY_A8IIN4_CHLRE/53-510	A8IIN4.1 PF01425.19	68.00	0.00
TRINITY_D8TH36_VOLCA/208-334	D8TH36.1 PF01980.14	68.00	0.00
TRINITY_D8UFG0_VOLCA/350-786	D8UFG0.1 PF00654.18	68.00	0.00
TRINITY_A0A0N4TT30_BRUPA/1-101	A0A0N4TT30.1 PF0012	68.00	0.00
TRINITY_G0QXH1_ICHMG/83-135	G0QXH1.1 PF01381.20	67.90	0.00
TRINITY_B3S8A3_TRIAD/15-255	B3S8A3.1 PF13621.4;	67.90	0.00
TRINITY_B2ZPK7_SOLLC/151-363	B2ZPK7.1 PF03181.13	67.90	0.00
TRINITY_Q4S823_TETNG/14-176	Q4S823.1 PF00160.19	67.90	0.00
TRINITY_A0A078A5I6_STYLE/47-225	A0A078A5I6.1 PF0035	67.90	0.00
TRINITY_D8M736_BLAHO/107-315	D8M736.1 PF00112.21	67.90	0.00
TRINITY_Q24GG1_TETTS/54-209	Q24GG1.2 PF09416.8;	67.90	0.00
TRINITY_I7LZX7_TETTS/8-264	I7LZX7.2 PF00069.23	67.90	0.00
TRINITY_X6NYM3_RETFI/392-506	X6NYM3.1 PF00188.24	67.90	0.00
TRINITY_A8J2D1_CHLRE/1-56	A8J2D1.1 PF12796.5;	67.90	0.00
TRINITY_A8ISQ5_CHLRE/1729-1856	A8ISQ5.1 PF00004.27	67.90	0.00
TRINITY_B4J6T2_DROGR/47-154	B4J6T2.1 PF13085.4;	67.90	0.00
TRINITY_A0BWW1_PARTE/272-340	A0BWW1.1 PF00498.24	67.90	0.00
TRINITY_I2EPL2_EMTOG/3-295	I2EPL2.1 PF00732.17	67.90	0.00
TRINITY_A8HXG2_CHLRE/256-343	A8HXG2.1 PF16561.3;	67.90	0.00
TRINITY_J9JCA5_9SPIT/125-339	J9JCA5.1 PF00112.21	67.90	0.00
TRINITY_E1ZE86_CHLVA/64-141	E1ZE86.1 PF00076.20	67.90	0.00
TRINITY_A0E7B4_PARTE/247-358	A0E7B4.1 PF06565.10	67.90	0.00
TRINITY_E1Z4H4_CHLVA/267-425	E1Z4H4.1 PF00204.23	67.90	0.00
TRINITY_G0QTD0_ICHMG/10-221	G0QTD0.1 PF01145.23	67.90	0.00
TRINITY_D8TQW2_VOLCA/49-126	D8TQW2.1 PF00173.26	67.90	0.00
TRINITY_T1KP71_TETUR/34-290	T1KP71.1 PF01650.16	67.90	0.00
TRINITY_A8IKY9_CHLRE/2-121	A8IKY9.1 PF03966.14	67.90	0.00
TRINITY_D8UDL2_VOLCA/636-712	D8UDL2.1 PF00069.23	67.90	0.00
TRINITY_A0A087SBP1_AUXPR/23-164	A0A087SBP1.1 PF0014	67.90	0.00
TRINITY_D8U1K7_VOLCA/238-345	D8U1K7.1 PF00085.18	67.90	0.00
TRINITY_A0A0J7KVW5_LASNI/257-464	A0A0J7KVW5.1 PF0113	67.90	0.00
TRINITY_G0QPY8_ICHMG/8-406	G0QPY8.1 PF01264.19	67.90	0.00
TRINITY_D8TW87_VOLCA/17-209	D8TW87.1 PF00687.19	67.90	0.00
TRINITY_A8IZP3_CHLRE/4-466	A8IZP3.1 PF07779.10	67.90	0.00
TRINITY_A8HPY4_CHLRE/80-358	A8HPY4.1 PF00795.20	67.90	0.00
TRINITY_L5LTN0_MYODS/23-100	L5LTN0.1 PF00935.17	67.90	0.00
TRINITY_A8IQD6_CHLRE/150-339	A8IQD6.1 PF04802.13	67.90	0.00
TRINITY_A8J4P3_CHLRE/37-399	A8J4P3.1 PF07690.14	67.90	0.00
TRINITY_A8JGG5_CHLRE/37-505	A8JGG5.1 PF01425.19	67.90	0.00
TRINITY_D8UJC8_VOLCA/1-224	D8UJC8.1 PF01902.15	67.90	0.00
TRINITY_G0R5X3_ICHMG/62-122	G0R5X3.1 PF10203.7;	67.80	0.00
TRINITY_K1PGI9_CRAGI/524-659	K1PGI9.1 PF00005.25	67.80	0.00
TRINITY_D8TRX1_VOLCA/123-216	D8TRX1.1 PF00166.19	67.80	0.00
TRINITY_A8IQW5_CHLRE/92-259	A8IQW5.1 PF13911.4;	67.80	0.00
TRINITY_G0QVM2_ICHMG/72-361	G0QVM2.1 PF00579.23	67.80	0.00
TRINITY_K3WQ17_PYTUL/63-407	K3WQ17.1 PF00155.19	67.80	0.00
TRINITY_D8U1E9_VOLCA/47-105	D8U1E9.1 PF05383.15	67.80	0.00
TRINITY_D8U0M7_VOLCA/159-303	D8U0M7.1 PF01699.22	67.80	0.00
TRINITY_A8IU88_CHLRE/35-156	A8IU88.1 PF08311.10	67.80	0.00
TRINITY_D8TNQ4_VOLCA/48-346	D8TNQ4.1 PF00732.17	67.80	0.00

TRINITY_I3BVD7_9GAMM/3-177	I3BVD7.1 PF00227.24	67.80	0.00
TRINITY_A0BEL1_PARTE/237-357	A0BEL1.1 PF00622.26	67.80	0.00
TRINITY_L5K105_PTEAL/5-361	L5K105.1 PF00009.25	67.80	0.00
TRINITY_A0CC74_PARTE/73-372	A0CC74.1 PF03133.13	67.80	0.00
TRINITY_F4XNB4_9CYAN/28-490	F4XNB4.1 PF00171.20	67.80	0.00
TRINITY_C1N1C6_MICPC/1681-1949	C1N1C6.1 PF12780.5;	67.80	0.00
TRINITY_D8TX36_VOLCA/31-175	D8TX36.1 PF00005.25	67.80	0.00
TRINITY_U5D0K8_AMBTC/138-226	U5D0K8.1 PF00867.16	67.80	0.00
TRINITY_C5KAN2_PERM5/114-259	C5KAN2.1 PF00005.25	67.80	0.00
TRINITY_C6Y1V7_PEDHD/4-261	C6Y1V7.1 PF00108.21	67.80	0.00
TRINITY_A8IFT8_CHLRE/600-698	A8IFT8.1 PF00211.18	67.80	0.00
TRINITY_I7M6Y9_TETTS/9-179	I7M6Y9.1 PF00535.24	67.80	0.00
TRINITY_Q240X5_TETTS/179-268	Q240X5.2 PF00027.27	67.80	0.00
TRINITY_Q22XN5_TETTS/43-220	Q22XN5.1 PF02146.15	67.80	0.00
TRINITY_R4K6C0_CLOPA/9-298	R4K6C0.1 PF00251.18	67.80	0.00
TRINITY_I0YJB7_9CHLO/10-248	I0YJB7.1 PF13621.4;	67.80	0.00
TRINITY_D8U097_VOLCA/530-621	D8U097.1 PF12796.5;	67.80	0.00
TRINITY_A0A0E0QA79_ORYRU/263-392	A0A0E0QA79.1 PF0395	67.80	0.00
TRINITY_D8TII4_VOLCA/175-245	D8TII4.1 PF04845.11	67.80	0.00
TRINITY_D8U9Y0_VOLCA/1-143	D8U9Y0.1 PF00588.17	67.80	0.00
TRINITY_F4B1G5_DOKS4/2-110	F4B1G5.1 PF00903.23	67.70	0.00
TRINITY_L8GNF3_ACACA/465-529	L8GNF3.1 PF06628.10	67.70	0.00
TRINITY_A0BSN3_PARTE/14-266	A0BSN3.1 PF00069.23	67.70	0.00
TRINITY_D8SX26_SELML/44-116	D8SX26.1 PF00173.26	67.70	0.00
TRINITY_I7MH42_TETTS/165-435	I7MH42.1 PF00928.19	67.70	0.00
TRINITY_Q54FV6_DICDI/11-71	Q54FV6.1 PF00412.20	67.70	0.00
TRINITY_M2Y2Z1_GALSU/166-292	M2Y2Z1.1 PF00134.21	67.70	0.00
TRINITY_D8TS03_VOLCA/34-195	D8TS03.1 PF02815.17	67.70	0.00
TRINITY_G0QZL5_ICHMG/10-170	G0QZL5.1 PF00071.20	67.70	0.00
TRINITY_F1A1K2_DICPU/35-199	F1A1K2.1 PF00160.19	67.70	0.00
TRINITY_Q23JY6_TETTS/610-893	Q23JY6.2 PF00481.19	67.70	0.00
TRINITY_A0CDX9_PARTE/544-846	A0CDX9.1 PF01180.19	67.70	0.00
TRINITY_I7M6T2_TETTS/301-464	I7M6T2.2 PF00160.19	67.70	0.00
TRINITY_A0BN60_PARTE/55-373	A0BN60.1 PF00225.21	67.70	0.00
TRINITY_L8GVX9_ACACA/3-80	L8GVX9.1 PF03650.11	67.70	0.00
TRINITY_D8UD76_VOLCA/695-824	D8UD76.1 PF06398.9;	67.70	0.00
TRINITY_I0Z8B4_9CHLO/5-176	I0Z8B4.1 PF00929.22	67.70	0.00
TRINITY_A8J5W6_CHLRE/31-285	A8J5W6.1 PF12697.5;	67.70	0.00
TRINITY_D8U0G0_VOLCA/59-397	D8U0G0.1 PF00206.18	67.70	0.00
TRINITY_A0A0E0LJL9_ORYPU/262-459	A0A0E0LJL9.1 PF0278	67.70	0.00
TRINITY_I0Z6M3_9CHLO/18-112	I0Z6M3.1 PF08698.9;	67.70	0.00
TRINITY_A8HQN4_CHLRE/363-427	A8HQN4.1 PF00226.29	67.70	0.00
TRINITY_D8U0E7_VOLCA/59-182	D8U0E7.1 PF02466.17	67.70	0.00
TRINITY_D8TKS8_VOLCA/334-429	D8TKS8.1 PF08295.10	67.70	0.00
TRINITY_IFT27_CHLRE/18-188	A8HN58.1 PF00071.20	67.70	0.00
TRINITY_D8UIP0_VOLCA/9-539	D8UIP0.1 PF01274.20	67.70	0.00
TRINITY_D8UE50_VOLCA/48-270	D8UE50.1 PF03407.14	67.70	0.00
TRINITY_I0YPL0_9CHLO/97-349	I0YPL0.1 PF08323.9;	67.70	0.00
TRINITY_E1ZCV9_CHLVA/573-1684	E1ZCV9.1 PF02463.17	67.70	0.00
TRINITY_Q245X8_TETTS/386-454	Q245X8.1 PF00076.20	67.70	0.00
TRINITY_U6JDZ7_ECHGR/13-174	U6JDZ7.1 PF00071.20	67.60	0.00
TRINITY_A0A0D1VZF4_ANEMI/9-178	A0A0D1VZF4.1 PF0088	67.60	0.00
TRINITY_E1ZQY0_CHLVA/78-247	E1ZQY0.1 PF14543.4;	67.60	0.00
TRINITY_F2U9H6_SALR5/181-233	F2U9H6.1 PF00071.20	67.60	0.00
TRINITY_F4PIQ2_DICFS/4-144	F4PIQ2.1 PF00179.24	67.60	0.00
TRINITY_T1IXR9_STRMM/18-91	T1IXR9.1 PF01423.20	67.60	0.00
TRINITY_V5EMR8_PSEBG/280-319	V5EMR8.1 PF05920.9;	67.60	0.00
TRINITY_X1W3S7_TETTS/4-262	X1W3S7.1 PF00069.23	67.60	0.00
TRINITY_I0YKX7_9CHLO/155-269	I0YKX7.1 PF00504.19	67.60	0.00
TRINITY_A0A077ZRC7_STYLE/176-249	A0A077ZRC7.1 PF0038	67.60	0.00
TRINITY_A0A0B2VWK9_TOXCA/3-379	A0A0B2VWK9.1 PF0002	67.60	0.00

TRINITY_G0QW93_ICHMG/62-317	G0QW93.1 PF00069.23	67.60	0.00
TRINITY_A0A0N4Y0F3_NIPBR/36-320	A0A0N4Y0F3.1 PF0006	67.60	0.00
TRINITY_A8JDI7_CHLRE/86-341	A8JDI7.1 PF08423.9;	67.60	0.00
TRINITY_Q238R8_TETTS/18-286	Q238R8.1 PF02263.17	67.60	0.00
TRINITY_E1ZKT8_CHLVA/93-167	E1ZKT8.1 PF03110.12	67.60	0.00
TRINITY_D8U8W6_VOLCA/27-494	D8U8W6.1 PF01384.18	67.60	0.00
TRINITY_A0A088A820_APIME/55-202	A0A088A820.1 PF0067	67.60	0.00
TRINITY_A9S530_PHYPA/43-80	A9S530.1 PF00400.30	67.60	0.00
TRINITY_A8IBQ0_CHLRE/60-244	A8IBQ0.1 PF01728.17	67.60	0.00
TRINITY_Q944P3_CHLRE/38-319	Q944P3.1 PF01430.17	67.60	0.00
TRINITY_A0A061FIQ2_THECC/17-196	A0A061FIQ2.1 PF0050	67.60	0.00
TRINITY_I7LTH0_TETTS/41-222	I7LTH0.1 PF00350.21	67.60	0.00
TRINITY_F2U3A2_SALR5/20-125	F2U3A2.1 PF00085.18	67.60	0.00
TRINITY_K1QNG9_CRAGI/93-166	K1QNG9.1 PF00276.18	67.60	0.00
TRINITY_G0QX41_ICHMG/40-335	G0QX41.1 PF00069.23	67.60	0.00
TRINITY_D8U9K9_VOLCA/5-174	D8U9K9.1 PF07106.11	67.60	0.00
TRINITY_A0A0N4Z578_PARTI/31-148	A0A0N4Z578.1 PF0018	67.60	0.00
TRINITY_Q110A4_TRIEI/111-328	Q110A4.1 PF13604.4;	67.60	0.00
TRINITY_Q3M0X3_PARTE/2-375	Q3M0X3.1 PF00022.17	67.60	0.00
TRINITY_D8UAX7_VOLCA/1116-1281	D8UAX7.1 PF00246.22	67.60	0.00
TRINITY_J9HZY6_9SPIT/1-105	J9HZY6.1 PF00125.22	67.60	0.00
TRINITY_W7XKA0_TETTS/123-161	W7XKA0.1 PF00400.30	67.60	0.00
TRINITY_A8HQB8_CHLRE/129-294	A8HQB8.1 PF04055.19	67.60	0.00
TRINITY_A8I637_CHLRE/938-1005	A8I637.1 PF13499.4;	67.60	0.00
TRINITY_A0A0L0P6V1_9ASCO/1164-1325	A0A0L0P6V1.1 PF0000	67.60	0.00
TRINITY_D3BUM8_POLPA/833-1106	D3BUM8.1 PF00176.21	67.60	0.00
TRINITY_Q23JK8_TETTS/655-953	Q23JK8.2 PF03133.13	67.50	0.00
TRINITY_F0YGR3_AURAN/343-780	F0YGR3.1 PF04053.12	67.50	0.00
TRINITY_B9N2M6_POPTR/20-67	B9N2M6.1 PF06221.11	67.50	0.00
TRINITY_A0A0G4FWU4_9ALVE/233-404	A0A0G4FWU4.1 PF0027	67.50	0.00
TRINITY_A0A0A0LUG4_CUCSA/256-295	A0A0A0LUG4.1 PF0040	67.50	0.00
TRINITY_L8GJF7_ACACA/99-177	L8GJF7.1 PF00347.21	67.50	0.00
TRINITY_V4BDG5_LOTGI/47-164	V4BDG5.1 PF09725.7;	67.50	0.00
TRINITY_L0PE19_PNEJ8/2-181	L0PE19.1 PF13177.4;	67.50	0.00
TRINITY_W1PU38_AMBTC/88-250	W1PU38.1 PF04055.19	67.50	0.00
TRINITY_K7M393_SOYBN/193-235	K7M393.1 PF00400.30	67.50	0.00
TRINITY_D8THZ0_VOLCA/58-178	D8THZ0.1 PF09335.9;	67.50	0.00
TRINITY_A0A067R639_ZOONE/156-480	A0A067R639.1 PF0107	67.50	0.00
TRINITY_D8TQN1_VOLCA/5-273	D8TQN1.1 PF00481.19	67.50	0.00
TRINITY_H3DL28_TETNG/1337-1431	H3DL28.1 PF07677.12	67.50	0.00
TRINITY_L8H7N6_ACACA/177-303	L8H7N6.1 PF04037.11	67.50	0.00
TRINITY_I7LUR8_TETTS/1292-1437	I7LUR8.2 PF00637.18	67.50	0.00
TRINITY_D8TKU4_VOLCA/79-204	D8TKU4.1 PF04536.12	67.50	0.00
TRINITY_F0ZB76_DICPU/5-166	F0ZB76.1 PF00071.20	67.50	0.00
TRINITY_L8HDT0_ACACA/49-350	L8HDT0.1 PF00579.23	67.50	0.00
TRINITY_A0CRV2_PARTE/124-203	A0CRV2.1 PF00013.27	67.50	0.00
TRINITY_A0A0E0KYK6_ORYPU/693-820	A0A0E0KYK6.1 PF0500	67.50	0.00
TRINITY_Q233B2_TETTS/10-51	Q233B2.1 PF05186.11	67.50	0.00
TRINITY_D8TPE8_VOLCA/146-261	D8TPE8.1 PF03556.13	67.50	0.00
TRINITY_A0A084W1R2_ANOSI/121-283	A0A084W1R2.1 PF0348	67.50	0.00
TRINITY_D8U3E4_VOLCA/58-142	D8U3E4.1 PF14159.4;	67.50	0.00
TRINITY_A0A0F2NWY3_9FLAO/24-465	A0A0F2NWY3.1 PF0142	67.50	0.00
TRINITY_A8IW37_CHLRE/116-195	A8IW37.1 PF04176.11	67.50	0.00
TRINITY_A0CHQ4_PARTE/1987-2260	A0CHQ4.1 PF12775.5;	67.50	0.00
TRINITY_J5P7M9_SACK1/210-249	J5P7M9.1 PF05920.9;	67.50	0.00
TRINITY_A8JCC7_CHLRE/57-180	A8JCC7.1 PF01553.19	67.50	0.00
TRINITY_A8IZM7_CHLRE/65-202	A8IZM7.1 PF14580.4;	67.50	0.00
TRINITY_A8JGW6_CHLRE/119-413	A8JGW6.1 PF01008.15	67.50	0.00
TRINITY_D8TNC3_VOLCA/359-437	D8TNC3.1 PF08149.9;	67.50	0.00
TRINITY_Q22A67_TETTS/2454-2736	Q22A67.1 PF12775.5;	67.50	0.00
TRINITY_H2XYB1_CIOIN/109-154	H2XYB1.1 PF00249.29	67.40	0.00

TRINITY_R1DB01_EMIHU/11-56	R1DB01.1 PF05051.11	67.40	0.00
TRINITY_A9TSQ4_PHYPA/264-675	A9TSQ4.1 PF00962.20	67.40	0.00
TRINITY_Q245S5_TETTS/6-262	Q245S5.1 PF00069.23	67.40	0.00
TRINITY_I0YML2_9CHLO/47-91	I0YML2.1 PF01585.21	67.40	0.00
TRINITY_A0EEF5_PARTE/29-120	A0EEF5.1 PF00447.15	67.40	0.00
TRINITY_Q24FC7_TETTS/25-161	Q24FC7.2 PF00782.18	67.40	0.00
TRINITY_S8BVD4_9LAMI/137-311	S8BVD4.1 PF07717.14	67.40	0.00
TRINITY_A8J990_CHLRE/3-117	A8J990.1 PF01042.19	67.40	0.00
TRINITY_K3WH50_PYTUL/48-360	K3WH50.1 PF01916.15	67.40	0.00
TRINITY_A0A078AVM5_STYLE/320-363	A0A078AVM5.1 PF0056	67.40	0.00
TRINITY_A8I2G1_CHLRE/183-305	A8I2G1.1 PF00254.26	67.40	0.00
TRINITY_I1GC88_AMPQE/167-380	I1GC88.1 PF00112.21	67.40	0.00
TRINITY_D8UD64_VOLCA/3-606	D8UD64.1 PF00443.27	67.40	0.00
TRINITY_F0ZXI3_DICPU/2-96	F0ZXI3.1 PF09138.9;	67.40	0.00
TRINITY_HUTU_DICDI/87-647	Q86AX3.2 PF01175.16	67.40	0.00
TRINITY_W4YTW3_STRPU/244-336	W4YTW3.1 PF00153.25	67.40	0.00
TRINITY_D8UBT5_VOLCA/37-279	D8UBT5.1 PF00956.16	67.40	0.00
TRINITY_D3BKC2_POLPA/110-529	D3BKC2.1 PF00368.16	67.40	0.00
TRINITY_A0A061DMP8_THECC/350-439	A0A061DMP8.1 PF0007	67.40	0.00
TRINITY_W7XAD3_TETTS/339-665	W7XAD3.1 PF00493.21	67.40	0.00
TRINITY_I1CF79_RHIO9/7-240	I1CF79.1 PF00149.26	67.40	0.00
TRINITY_A0A0J8CE11_BETVU/16-293	A0A0J8CE11.1 PF1362	67.40	0.00
TRINITY_A8IQH3_CHLRE/1-180	A8IQH3.1 PF01184.17	67.40	0.00
TRINITY_Q235M5_TETTS/736-779	Q235M5.2 PF06203.12	67.40	0.00
TRINITY_PURA_PARTE/13-428	A0BD92.1 PF00709.19	67.40	0.00
TRINITY_A0A0E9NMV7_9ASCO/188-280	A0A0E9NMV7.1 PF0015	67.40	0.00
TRINITY_A8I6B9_CHLRE/19-260	A8I6B9.1 PF13561.4;	67.40	0.00
TRINITY_B8AEH2_ORYSI/853-895	B8AEH2.1 PF00400.30	67.40	0.00
TRINITY_D8UBG9_VOLCA/219-399	D8UBG9.1 PF04055.19	67.40	0.00
TRINITY_A8ISV4_CHLRE/26-122	A8ISV4.1 PF11378.6;	67.40	0.00
TRINITY_A0A077ZWA5_STYLE/272-315	A0A077ZWA5.1 PF0056	67.40	0.00
TRINITY_A0A078AVM5_STYLE/320-363	A0A078AVM5.1 PF0056	67.40	0.00
TRINITY_A0A078AVM5_STYLE/320-363	A0A078AVM5.1 PF0056	67.40	0.00
TRINITY_D8TU46_VOLCA/1-365	D8TU46.1 PF10498.7;	67.40	0.00
TRINITY_L8GXZ4_ACACA/90-225	L8GXZ4.1 PF00179.24	67.40	0.00
TRINITY_F4NTH8_BATDJ/623-677	F4NTH8.1 PF00628.27	67.40	0.00
TRINITY_D8UFJ1_VOLCA/103-391	D8UFJ1.1 PF00664.21	67.40	0.00
TRINITY_F4PBP5_BATDJ/207-387	F4PBP5.1 PF00270.27	67.40	0.00
TRINITY_D8TT36_VOLCA/1047-1091	D8TT36.1 PF01585.21	67.40	0.00
TRINITY_D8TN57_VOLCA/1-144	D8TN57.1 PF01762.19	67.40	0.00
TRINITY_A9UWF4_MONBE/13-244	A9UWF4.1 PF00141.21	67.40	0.00
TRINITY_A0A017SEC6_9EURO/5-68	A0A017SEC6.1 PF0907	67.30	0.00
TRINITY_B2B5E3_PODAN/1665-1801	B2B5E3.1 PF00271.29	67.30	0.00
TRINITY_A0DZ19_PARTE/27-125	A0DZ19.1 PF00438.18	67.30	0.00
TRINITY_A0DQI4_PARTE/1345-1481	A0DQI4.1 PF07728.12	67.30	0.00
TRINITY_F4QE82_DICFS/231-284	F4QE82.1 PF07653.15	67.30	0.00
TRINITY_F1A3Q8_DICPU/206-262	F1A3Q8.1 PF00046.27	67.30	0.00
TRINITY_Q54F04_DICDI/45-467	Q54F04.1 PF13520.4;	67.30	0.00
TRINITY_Q24FR9_TETTS/28-281	Q24FR9.2 PF00069.23	67.30	0.00
TRINITY_K1Q6X5_CRAGI/23-127	K1Q6X5.1 PF00085.18	67.30	0.00
TRINITY_A0A087YQG5_POEFO/33-182	A0A087YQG5.2 PF1103	67.30	0.00
TRINITY_E1ZDD6_CHLVA/131-250	E1ZDD6.1 PF00006.23	67.30	0.00
TRINITY_D8U4C5_VOLCA/313-528	D8U4C5.1 PF00246.22	67.30	0.00
TRINITY_G0QXV0_ICHMG/52-760	G0QXV0.1 PF03030.14	67.30	0.00
TRINITY_L8GP92_ACACA/40-250	L8GP92.1 PF02786.15	67.30	0.00
TRINITY_A0A087SC76_AUXPR/80-254	A0A087SC76.1 PF0178	67.30	0.00
TRINITY_D8UG79_VOLCA/1109-1205	D8UG79.1 PF04059.10	67.30	0.00
TRINITY_A8ISQ5_CHLRE/1729-1856	A8ISQ5.1 PF00004.27	67.30	0.00
TRINITY_G0R663_ICHMG/137-188	G0R663.1 PF13415.4;	67.30	0.00
TRINITY_D8U0P6_VOLCA/36-243	D8U0P6.1 PF01189.15	67.30	0.00
TRINITY_A0A078ANA5_STYLE/18-270	A0A078ANA5.1 PF0006	67.30	0.00

TRINITY_F6GVT0_VITVI/52-378	F6GVT0.1 PF01536.14	67.30	0.00
TRINITY_A8IN76_CHLRE/1169-1379	A8IN76.1 PF00454.25	67.30	0.00
TRINITY_C1ECL1_MICSR/22-70	C1ECL1.1 PF12906.5;	67.30	0.00
TRINITY_A8J1E5_CHLRE/97-206	A8J1E5.1 PF00254.26	67.30	0.00
TRINITY_A8IEW6_CHLRE/22-317	A8IEW6.1 PF16363.3;	67.30	0.00
TRINITY_D8U6V0_VOLCA/134-351	D8U6V0.1 PF00112.21	67.30	0.00
TRINITY_A5GJW6_SYNPW/37-145	A5GJW6.1 PF08241.10	67.30	0.00
TRINITY_E1Z4H4_CHLVA/693-1146	E1Z4H4.1 PF00521.18	67.30	0.00
TRINITY_D8U9Z2_VOLCA/59-107	D8U9Z2.1 PF00415.16	67.30	0.00
TRINITY_A8JIJ1_CHLRE/64-233	A8JIJ1.1 PF00270.27	67.30	0.00
TRINITY_D8U3L8_VOLCA/25-270	D8U3L8.1 PF00248.19	67.30	0.00
TRINITY_D8U8A6_VOLCA/8-169	D8U8A6.1 PF03446.13	67.30	0.00
TRINITY_D8U532_VOLCA/3-106	D8U532.1 PF01408.20	67.30	0.00
TRINITY_A0A087SI38_AUXPR/89-535	A0A087SI38.1 PF0050	67.30	0.00
TRINITY_A8HME7_CHLRE/455-567	A8HME7.1 PF00175.19	67.30	0.00
TRINITY_A0A0N0RRX5_9BASI/35-89	A0A0N0RRX5.1 PF0166	67.30	0.00
TRINITY_A8IWW5_CHLRE/1531-1644	A8IWW5.1 PF00856.26	67.30	0.00
TRINITY_I0Z062_9CHLO/726-884	I0Z062.1 PF00179.24	67.30	0.00
TRINITY_A0A078AJ02_STYLE/72-396	A0A078AJ02.1 PF0022	67.30	0.00
TRINITY_Q8LLV9_CHLRE/7-125	Q8LLV9.1 PF14931.4;	67.30	0.00
TRINITY_D8UF49_VOLCA/74-482	D8UF49.1 PF00474.15	67.30	0.00
TRINITY_I0YNB1_9CHLO/845-1015	I0YNB1.1 PF08148.10	67.30	0.00
TRINITY_D8UCT8_VOLCA/75-127	D8UCT8.1 PF01805.18	67.30	0.00
TRINITY_W0SBT4_9RHOO/9-264	W0SBT4.1 PF00378.18	67.30	0.00
TRINITY_A8IYC3_CHLRE/398-620	A8IYC3.1 PF01061.22	67.30	0.00
TRINITY_A0E9C2_PARTE/3-52	A0E9C2.1 PF04135.10	67.30	0.00
TRINITY_J9IZV4_9SPIT/54-108	J9IZV4.1 PF09597.8;	67.30	0.00
TRINITY_G0QLR8_ICHMG/22-143	G0QLR8.1 PF13238.4;	67.20	0.00
TRINITY_G0R1F7_ICHMG/29-105	G0R1F7.1 PF12895.5;	67.20	0.00
TRINITY_A0A0P7UBI8_9TELE/235-418	A0A0P7UBI8.1 PF0144	67.20	0.00
TRINITY_Q23FV5_TETTS/249-318	Q23FV5.1 PF01485.19	67.20	0.00
TRINITY_Q22LN3_TETTS/139-201	Q22LN3.1 PF00957.19	67.20	0.00
TRINITY_F6X9E5_CIOIN/19-486	F6X9E5.2 PF05694.9;	67.20	0.00
TRINITY_I7M250_TETTS/101-222	I7M250.1 PF09335.9;	67.20	0.00
TRINITY_A0DIT9_PARTE/34-353	A0DIT9.1 PF00180.18	67.20	0.00
TRINITY_A0E0K1_PARTE/14-270	A0E0K1.1 PF00069.23	67.20	0.00
TRINITY_A0BFU1_PARTE/28-283	A0BFU1.1 PF00069.23	67.20	0.00
TRINITY_F4PZT8_DICFS/26-206	F4PZT8.1 PF01183.18	67.20	0.00
TRINITY_Q6BG73_PARTE/7-73	Q6BG73.1 PF13921.4;	67.20	0.00
TRINITY_A8I3J5_CHLRE/51-318	A8I3J5.1 PF00459.23	67.20	0.00
TRINITY_A0BIB7_PARTE/16-98	A0BIB7.1 PF10163.7;	67.20	0.00
TRINITY_A0A078BB17_STYLE/30-281	A0A078BB17.1 PF0006	67.20	0.00
TRINITY_D8TJB0_VOLCA/34-172	D8TJB0.1 PF07534.14	67.20	0.00
TRINITY_G0QZ58_ICHMG/8-69	G0QZ58.1 PF00226.29	67.20	0.00
TRINITY_C1E976_MICSR/1-199	C1E976.1 PF01135.17	67.20	0.00
TRINITY_A8J163_CHLRE/127-188	A8J163.1 PF04117.10	67.20	0.00
TRINITY_I0YQ45_9CHLO/43-196	I0YQ45.1 PF00504.19	67.20	0.00
TRINITY_Q23KJ6_TETTS/622-886	Q23KJ6.2 PF00069.23	67.20	0.00
TRINITY_A8JEV0_CHLRE/9-145	A8JEV0.1 PF00179.24	67.20	0.00
TRINITY_Q23RV4_TETTS/6-262	Q23RV4.2 PF00069.23	67.20	0.00
TRINITY_R4FL09_RHOPR/3-66	R4FL09.1 PF00226.29	67.20	0.00
TRINITY_A8ITR2_CHLRE/55-186	A8ITR2.1 PF03641.12	67.20	0.00
TRINITY_T1FNL1_HELRO/3-168	T1FNL1.1 PF12038.6;	67.20	0.00
TRINITY_A0A072V987_MEDTR/257-421	A0A072V987.1 PF0196	67.20	0.00
TRINITY_D8UD36_VOLCA/42-510	D8UD36.1 PF01490.16	67.20	0.00
TRINITY_M1VG33_CYAME/32-94	M1VG33.1 PF01176.17	67.20	0.00
TRINITY_A0A067M391_9HOMO/1310-1466	A0A067M391.1 PF0000	67.20	0.00
TRINITY_D8UBS1_VOLCA/47-242	D8UBS1.1 PF04427.16	67.20	0.00
TRINITY_F4PLW9_DICFS/425-485	F4PLW9.1 PF04193.12	67.20	0.00
TRINITY_I0Z8F4_9CHLO/1266-1414	I0Z8F4.1 PF00005.25	67.20	0.00
TRINITY_A8JEB7_CHLRE/516-889	A8JEB7.1 PF00856.26	67.20	0.00

TRINITY_G0QTV5_ICHMG/76-136	G0QTV5.1 PF00226.29	67.20	0.00
TRINITY_A8JFA2_CHLRE/28-287	A8JFA2.1 PF12697.5;	67.20	0.00
TRINITY_D8TK69_VOLCA/36-388	D8TK69.1 PF01070.16	67.20	0.00
TRINITY_A0DFK5_PARTE/51-306	A0DFK5.1 PF00069.23	67.20	0.00
TRINITY_A8J1A7_CHLRE/15-272	A8J1A7.1 PF00069.23	67.20	0.00
TRINITY_A0A060SK24_PYCCI/128-195	A0A060SK24.1 PF1343	67.20	0.00
TRINITY_D8TPM1_VOLCA/127-271	D8TPM1.1 PF07823.9;	67.20	0.00
TRINITY_A8J5C4_CHLRE/18-103	A8J5C4.1 PF01221.16	67.20	0.00
TRINITY_D8TI23_VOLCA/231-520	D8TI23.1 PF14308.4;	67.20	0.00
TRINITY_A0A087ST01_AUXPR/256-320	A0A087ST01.1 PF0084	67.20	0.00
TRINITY_D8TMY5_VOLCA/48-525	D8TMY5.1 PF00501.26	67.20	0.00
TRINITY_I0YQM2_9CHLO/15-121	I0YQM2.1 PF00255.17	67.20	0.00
TRINITY_U5H3W5_USTV1/1325-1473	U5H3W5.1 PF00005.25	67.20	0.00
TRINITY_I7M423_TETTS/244-392	I7M423.2 PF01751.20	67.10	0.00
TRINITY_A0D9E0_PARTE/137-400	A0D9E0.1 PF00481.19	67.10	0.00
TRINITY_D2VMQ6_NAEGR/60-212	D2VMQ6.1 PF00182.17	67.10	0.00
TRINITY_V2XH51_MONRO/1-79	V2XH51.1 PF01249.16	67.10	0.00
TRINITY_D8TLR6_VOLCA/28-288	D8TLR6.1 PF00069.23	67.10	0.00
TRINITY_G0R640_ICHMG/538-830	G0R640.1 PF00176.21	67.10	0.00
TRINITY_Q22W55_TETTS/12-233	Q22W55.2 PF00244.18	67.10	0.00
TRINITY_Q22W55_TETTS/12-233	Q22W55.2 PF00244.18	67.10	0.00
TRINITY_I0Z225_9CHLO/1827-2140	I0Z225.1 PF02889.14	67.10	0.00
TRINITY_A8IRT1_CHLRE/1-144	A8IRT1.1 PF10229.7;	67.10	0.00
TRINITY_A8HVT9_CHLRE/12-303	A8HVT9.1 PF17184.2;	67.10	0.00
TRINITY_A0A0D2WKM4_CAPO3/125-381	A0A0D2WKM4.1 PF0102	67.10	0.00
TRINITY_A8I5C3_CHLRE/2838-3549	A8I5C3.1 PF03028.13	67.10	0.00
TRINITY_D8UAG0_VOLCA/22-183	D8UAG0.1 PF00071.20	67.10	0.00
TRINITY_D8U140_VOLCA/48-120	D8U140.1 PF00226.29	67.10	0.00
TRINITY_Q84XW3_CHLRE/83-381	Q84XW3.1 PF03151.14	67.10	0.00
TRINITY_G0R646_ICHMG/414-563	G0R646.1 PF00005.25	67.10	0.00
TRINITY_Q23R92_TETTS/152-318	Q23R92.1 PF00071.20	67.10	0.00
TRINITY_L8GUA1_ACACA/32-127	L8GUA1.1 PF03650.11	67.10	0.00
TRINITY_Q7G6A5_ORYSJ/539-699	Q7G6A5.1 PF00078.25	67.10	0.00
TRINITY_D8TTV8_VOLCA/81-324	D8TTV8.1 PF00233.17	67.10	0.00
TRINITY_D3BNL1_POLPA/140-395	D3BNL1.1 PF07714.15	67.10	0.00
TRINITY_A8I843_CHLRE/4-339	A8I843.1 PF02628.13	67.10	0.00
TRINITY_D8U8S0_VOLCA/51-352	D8U8S0.1 PF01513.19	67.10	0.00
TRINITY_D8TUM3_VOLCA/511-627	D8TUM3.1 PF03476.14	67.10	0.00
TRINITY_D8U9J7_VOLCA/303-375	D8U9J7.1 PF03451.12	67.10	0.00
TRINITY_A8J629_CHLRE/49-208	A8J629.1 PF01699.22	67.10	0.00
TRINITY_A0A0N1E411_9FLAO/19-231	A0A0N1E411.1 PF0357	67.10	0.00
TRINITY_L8GVZ3_ACACA/2654-2901	L8GVZ3.1 PF00454.25	67.10	0.00
TRINITY_A0A077ZYU8_STYLE/71-382	A0A077ZYU8.1 PF0313	67.10	0.00
TRINITY_D8U1S9_VOLCA/42-131	D8U1S9.1 PF13671.4;	67.10	0.00
TRINITY_D8TI91_VOLCA/241-409	D8TI91.1 PF08547.10	67.10	0.00
TRINITY_D8U303_VOLCA/63-392	D8U303.1 PF00069.23	67.10	0.00
TRINITY_A0A0D2X0N5_CAPO3/122-252	A0A0D2X0N5.1 PF0411	67.00	0.00
TRINITY_Q23D14_TETTS/13-112	Q23D14.2 PF03226.12	67.00	0.00
TRINITY_COQ5_DICDI/49-313	Q54VN2.1 PF01209.16	67.00	0.00
TRINITY_D8UK42_VOLCA/1182-1274	D8UK42.1 PF00271.29	67.00	0.00
TRINITY_L8WZS6_THACA/41-156	L8WZS6.1 PF02678.14	67.00	0.00
TRINITY_I7MCU1_TETTS/89-194	I7MCU1.1 PF06565.10	67.00	0.00
TRINITY_Q29DY2_DROPS/156-498	Q29DY2.2 PF00155.19	67.00	0.00
TRINITY_W5KX88_ASTMX/131-240	W5KX88.1 PF12894.5;	67.00	0.00
TRINITY_D8UDA8_VOLCA/127-234	D8UDA8.1 PF13640.4;	67.00	0.00
TRINITY_A0BYK2_PARTE/116-313	A0BYK2.1 PF00406.20	67.00	0.00
TRINITY_L8HGY3_ACACA/42-251	L8HGY3.1 PF05023.12	67.00	0.00
TRINITY_T1JNG4_STRMM/9-146	T1JNG4.1 PF00258.23	67.00	0.00
TRINITY_D8TY75_VOLCA/150-249	D8TY75.1 PF07707.13	67.00	0.00
TRINITY_D8TQ02_VOLCA/606-696	D8TQ02.1 PF04408.21	67.00	0.00
TRINITY_Q23QN9_TETTS/2588-2848	Q23QN9.3 PF12780.5;	67.00	0.00

TRINITY_J9IBL8_9SPIT/60-394	J9IBL8.1 PF00225.21	67.00	0.00
TRINITY_D8UDW1_VOLCA/207-377	D8UDW1.1 PF00534.18	67.00	0.00
TRINITY_L8GI45_ACACA/286-561	L8GI45.1 PF00069.23	67.00	0.00
TRINITY_A8I8X5_CHLRE/91-196	A8I8X5.1 PF12680.5;	67.00	0.00
TRINITY_D8TR32_VOLCA/127-263	D8TR32.1 PF00293.26	67.00	0.00
TRINITY_D8TSE8_VOLCA/554-647	D8TSE8.1 PF13640.4;	67.00	0.00
TRINITY_D8THF4_VOLCA/99-388	D8THF4.1 PF08392.10	67.00	0.00
TRINITY_A0DN76_PARTE/3850-4562	A0DN76.1 PF03028.13	67.00	0.00
TRINITY_L1JNZ4_GUITH/284-466	L1JNZ4.1 PF04055.19	67.00	0.00
TRINITY_A8J129_CHLRE/50-423	A8J129.1 PF00155.19	67.00	0.00
TRINITY_A0A0D3B3Z8_BRAOL/218-303	A0A0D3B3Z8.1 PF0001	67.00	0.00
TRINITY_A0A078AP91_STYLE/413-592	A0A078AP91.1 PF0660	67.00	0.00
TRINITY_D8TR18_VOLCA/4-367	D8TR18.1 PF05631.12	67.00	0.00
TRINITY_A8JHX8_CHLRE/134-251	A8JHX8.1 PF13673.5;	67.00	0.00
TRINITY_A0EA85_PARTE/16-242	A0EA85.1 PF04981.11	67.00	0.00
TRINITY_D8TYV3_VOLCA/154-527	D8TYV3.1 PF10243.7;	67.00	0.00
TRINITY_A0A087SRM2_AUXPR/556-752	A0A087SRM2.1 PF1308	67.00	0.00
TRINITY_A8J2H1_CHLRE/591-689	A8J2H1.1 PF02806.16	67.00	0.00
TRINITY_A0A0D9VUL1_9ORYZ/7-183	A0A0D9VUL1.1 PF0016	66.90	0.00
TRINITY_A0A015MDK1_9GLOM/369-543	A0A015MDK1.1 PF0286	66.90	0.00
TRINITY_A7RH52_NEMVE/525-960	A7RH52.1 PF00136.19	66.90	0.00
TRINITY_A0A059AUW9_EUCGR/17-411	A0A059AUW9.1 PF0933	66.90	0.00
TRINITY_A0DVW7_PARTE/13-297	A0DVW7.1 PF00069.23	66.90	0.00
TRINITY_A0A024TW57_9STRA/26-146	A0A024TW57.1 PF0082	66.90	0.00
TRINITY_D2W2Z5_NAEGR/163-328	D2W2Z5.1 PF09414.8;	66.90	0.00
TRINITY_J3PCN6_GAGT3/124-422	J3PCN6.1 PF00128.22	66.90	0.00
TRINITY_D8UC91_VOLCA/67-241	D8UC91.1 PF07798.9;	66.90	0.00
TRINITY_D8UCJ1_VOLCA/46-240	D8UCJ1.1 PF01557.16	66.90	0.00
TRINITY_E1Z565_CHLVA/12-286	E1Z565.1 PF00682.17	66.90	0.00
TRINITY_A8I6U1_CHLRE/29-336	A8I6U1.1 PF00291.23	66.90	0.00
TRINITY_G0QPV4_ICHMG/536-846	G0QPV4.1 PF04950.10	66.90	0.00
TRINITY_Q4J8D7_SULAC/8-161	Q4J8D7.1 PF04008.12	66.90	0.00
TRINITY_F4PA58_BATDJ/14-174	F4PA58.1 PF00071.20	66.90	0.00
TRINITY_D8TXV2_VOLCA/2-122	D8TXV2.1 PF12624.5;	66.90	0.00
TRINITY_D8TH53_VOLCA/54-179	D8TH53.1 PF13638.4;	66.90	0.00
TRINITY_A0A0L6V9S5_9BASI/44-217	A0A0L6V9S5.1 PF0002	66.90	0.00
TRINITY_A8JH58_CHLRE/39-518	A8JH58.1 PF00501.26	66.90	0.00
TRINITY_B8HGH0_ARTCA/28-144	B8HGH0.1 PF08240.10	66.90	0.00
TRINITY_A8HQ44_CHLRE/7-158	A8HQ44.1 PF13419.4;	66.90	0.00
TRINITY_K4C8Q1_SOLLC/79-345	K4C8Q1.1 PF00009.25	66.90	0.00
TRINITY_D8TGZ7_VOLCA/449-690	D8TGZ7.1 PF00122.18	66.90	0.00
TRINITY_E1ZRK0_CHLVA/806-926	E1ZRK0.1 PF04607.15	66.90	0.00
TRINITY_F2CS06_HORVD/31-248	F2CS06.1 PF03332.11	66.80	0.00
TRINITY_Q247W0_TETTS/149-864	Q247W0.1 PF00343.18	66.80	0.00
TRINITY_D8TTQ5_VOLCA/98-351	D8TTQ5.1 PF04305.12	66.80	0.00
TRINITY_D8U1I3_VOLCA/56-475	D8U1I3.1 PF00709.19	66.80	0.00
TRINITY_PSA1_DICDI/29-216	Q27562.1 PF00227.24	66.80	0.00
TRINITY_E1ZL76_CHLVA/91-362	E1ZL76.1 PF00860.18	66.80	0.00
TRINITY_A8J6H8_CHLRE/243-427	A8J6H8.1 PF05193.19	66.80	0.00
TRINITY_D8TPE1_VOLCA/50-346	D8TPE1.1 PF01207.15	66.80	0.00
TRINITY_D8UIN9_VOLCA/1371-1729	D8UIN9.1 PF00224.19	66.80	0.00
TRINITY_G0R1C4_ICHMG/49-235	G0R1C4.1 PF10211.7;	66.80	0.00
TRINITY_Q22XN2_TETTS/43-220	Q22XN2.1 PF02146.15	66.70	0.00
TRINITY_E9G2X2_DAPPU/421-573	E9G2X2.1 PF00005.25	66.70	0.00
TRINITY_A0DHQ8_PARTE/13-161	A0DHQ8.1 PF02099.15	66.70	0.00
TRINITY_Q0IIY3_XENTR/9-72	Q0IIY3.1 PF01066.19	66.70	0.00
TRINITY_J9HZC4_9SPIT/10-65	J9HZC4.1 PF14777.4;	66.70	0.00
TRINITY_J9I9P2_9SPIT/356-394	J9I9P2.1 PF00400.30	66.70	0.00
TRINITY_S6AIB6_9PROT/3-43	S6AIB6.1 PF09360.8;	66.70	0.00
TRINITY_W7XD08_TETTS/123-343	W7XD08.1 PF02475.14	66.70	0.00
TRINITY_ACYP2_MELGA/14-101	P00821.2 PF00708.16	66.70	0.00

TRINITY_I7M304_TETTS/2743-3020	I7M304.2 PF12780.5;	66.70	0.00
TRINITY_I3IW28_ORENI/1426-1537	I3IW28.1 PF01759.19	66.70	0.00
TRINITY_J4CDA2_THEOR/29-74	J4CDA2.1 PF05051.11	66.70	0.00
TRINITY_Q241N4_TETTS/523-658	Q241N4.2 PF00005.25	66.70	0.00
TRINITY_A0CLD6_PARTE/82-333	A0CLD6.1 PF00069.23	66.70	0.00
TRINITY_A0A0A7LLI1_9BACT/4-177	A0A0A7LLI1.1 PF0053	66.70	0.00
TRINITY_A0CVG6_PARTE/11-268	A0CVG6.1 PF00069.23	66.70	0.00
TRINITY_I0YPN5_9CHLO/742-942	I0YPN5.1 PF11865.6;	66.70	0.00
TRINITY_A0DBM3_PARTE/273-356	A0DBM3.1 PF00439.23	66.70	0.00
TRINITY_F0YPS5_AURAN/7-63	F0YPS5.1 PF00831.21	66.70	0.00
TRINITY_D8M3T0_BLAHO/27-92	D8M3T0.1 PF00808.21	66.70	0.00
TRINITY_G0QU80_ICHMG/220-439	G0QU80.1 PF07002.14	66.70	0.00
TRINITY_L8H083_ACACA/9-144	L8H083.1 PF00578.19	66.70	0.00
TRINITY_A0A0D2UG50_CAPO3/388-425	A0A0D2UG50.1 PF0040	66.70	0.00
TRINITY_C3YUB2_BRAFL/188-372	C3YUB2.1 PF02145.13	66.70	0.00
TRINITY_A0A067Q3X5_9HOMO/17-111	A0A067Q3X5.1 PF0042	66.70	0.00
TRINITY_B7P6Q1_IXOSC/390-431	B7P6Q1.1 PF00396.16	66.70	0.00
TRINITY_Q5KI84_CRYNJ/11-366	Q5KI84.1 PF00180.18	66.70	0.00
TRINITY_L8H3W5_ACACA/971-1093	L8H3W5.1 PF00271.29	66.70	0.00
TRINITY_K3WM84_PYTUL/304-379	K3WM84.1 PF02138.16	66.70	0.00
TRINITY_SYAM_DICDI/32-589	Q86H43.1 PF01411.17	66.70	0.00
TRINITY_G0R5F1_ICHMG/174-302	G0R5F1.1 PF00454.25	66.70	0.00
TRINITY_A0A078AC84_STYLE/371-406	A0A078AC84.1 PF0040	66.70	0.00
TRINITY_F6PXA9_CIOIN/291-438	F6PXA9.2 PF00271.29	66.70	0.00
TRINITY_PI3K3_DICDI/1331-1545	P54675.2 PF00454.25	66.70	0.00
TRINITY_Q22NA0_TETTS/1407-1551	Q22NA0.2 PF00005.25	66.70	0.00
TRINITY_A0A0K9PDE5_ZOSMR/104-150	A0A0K9PDE5.1 PF1363	66.70	0.00
TRINITY_R4XLQ6_TAPDE/115-205	R4XLQ6.1 PF00043.23	66.70	0.00
TRINITY_A0A078AVM5_STYLE/320-363	A0A078AVM5.1 PF0056	66.70	0.00
TRINITY_A8IQI1_CHLRE/1-179	A8IQI1.1 PF01184.17	66.70	0.00
TRINITY_L8GZS8_ACACA/644-714	L8GZS8.1 PF04564.13	66.70	0.00
TRINITY_D3B725_POLPA/72-139	D3B725.1 PF08606.9;	66.70	0.00
TRINITY_F6SC57_CIOIN/2-375	F6SC57.2 PF00022.17	66.70	0.00
TRINITY_A0BTN4_PARTE/489-626	A0BTN4.1 PF00005.25	66.70	0.00
TRINITY_B2A9X4_PODAN/55-130	B2A9X4.1 PF01066.19	66.70	0.00
TRINITY_A8I7G7_CHLRE/107-226	A8I7G7.1 PF13640.4;	66.70	0.00
TRINITY_A8J4N6_CHLRE/18-68	A8J4N6.1 PF00249.29	66.70	0.00
TRINITY_M4BL21_HYAAE/239-277	M4BL21.1 PF13923.4;	66.70	0.00
TRINITY_F0ZAQ3_DICPU/6-54	F0ZAQ3.1 PF01679.15	66.70	0.00
TRINITY_D8UJW1_VOLCA/2648-2740	D8UJW1.1 PF04777.11	66.70	0.00
TRINITY_E1Z986_CHLVA/102-243	E1Z986.1 PF10075.7;	66.70	0.00
TRINITY_H9IZ84_BOMMO/144-251	H9IZ84.1 PF00085.18	66.70	0.00
TRINITY_A8IEC7_CHLRE/155-247	A8IEC7.1 PF04777.11	66.70	0.00
TRINITY_K7IPJ4_NASVI/12-388	K7IPJ4.1 PF01433.18	66.70	0.00
TRINITY_L8H164_ACACA/6-54	L8H164.1 PF02042.13	66.70	0.00
TRINITY_D8RRS9_SELML/20-117	D8RRS9.1 PF03226.12	66.70	0.00
TRINITY_I0YQM8_9CHLO/239-347	I0YQM8.1 PF01399.25	66.70	0.00
TRINITY_A8HNX9_CHLRE/48-218	A8HNX9.1 PF04755.10	66.70	0.00
TRINITY_L8GRQ4_ACACA/222-320	L8GRQ4.1 PF13661.4;	66.70	0.00
TRINITY_U9TF24_RHIID/25-265	U9TF24.1 PF03029.15	66.70	0.00
TRINITY_A0A061DMP8_THECC/350-439	A0A061DMP8.1 PF0007	66.70	0.00
TRINITY_D8TJ35_VOLCA/507-704	D8TJ35.1 PF04916.11	66.70	0.00
TRINITY_H9GHR5_ANOCA/124-192	H9GHR5.2 PF01302.23	66.70	0.00
TRINITY_A0DAW7_PARTE/169-243	A0DAW7.1 PF01169.17	66.70	0.00
TRINITY_I1JJ06_SOYBN/511-549	I1JJ06.1 PF00400.30	66.70	0.00
TRINITY_D8U6D5_VOLCA/376-492	D8U6D5.1 PF00271.29	66.70	0.00
TRINITY_C3Z3Z3_BRAFL/43-143	C3Z3Z3.1 PF13589.4;	66.70	0.00
TRINITY_H9H038_HORSE/164-207	H9H038.1 PF13639.4;	66.70	0.00
TRINITY_I0ZAW8_9CHLO/360-815	I0ZAW8.1 PF14648.4;	66.70	0.00
TRINITY_A0A0J8D1N5_BETVU/217-475	A0A0J8D1N5.1 PF1047	66.70	0.00
TRINITY_A0A0J8CX97_BETVU/25-97	A0A0J8CX97.1 PF0437	66.70	0.00

TRINITY_A8I268_CHLRE/191-326	A8I268.1 PF00005.25	66.70	0.00
TRINITY_A0BYR7_PARTE/34-293	A0BYR7.1 PF00069.23	66.70	0.00
TRINITY_D8TIU4_VOLCA/101-307	D8TIU4.1 PF13460.4;	66.70	0.00
TRINITY_D8U288_VOLCA/1-154	D8U288.1 PF00211.18	66.70	0.00
TRINITY_A8IXA1_CHLRE/102-140	A8IXA1.1 PF00400.30	66.70	0.00
TRINITY_D8UDB0_VOLCA/268-499	D8UDB0.1 PF04791.14	66.70	0.00
TRINITY_A8J9I5_CHLRE/244-339	A8J9I5.1 PF00153.25	66.70	0.00
TRINITY_J9FDL8_9SPIT/54-95	J9FDL8.1 PF00400.30	66.70	0.00
TRINITY_A8J3V0_CHLRE/8-70	A8J3V0.1 PF00808.21	66.70	0.00
TRINITY_I0YYL9_9CHLO/15-52	I0YYL9.1 PF00400.30	66.70	0.00
TRINITY_A9SCH2_PHYPA/219-298	A9SCH2.1 PF00107.24	66.70	0.00
TRINITY_E1Z774_CHLVA/14-243	E1Z774.1 PF00149.26	66.70	0.00
TRINITY_A0A072U9H1_MEDTR/209-254	A0A072U9H1.1 PF0024	66.70	0.00
TRINITY_Q23RG9_TETTS/116-209	Q23RG9.1 PF16531.3;	66.70	0.00
TRINITY_I0YX02_9CHLO/106-280	I0YX02.1 PF03572.16	66.70	0.00
TRINITY_A8J2B6_CHLRE/53-121	A8J2B6.1 PF15247.4;	66.70	0.00
TRINITY_V2XVC4_MONRO/112-449	V2XVC4.1 PF03104.17	66.70	0.00
TRINITY_U9V1C7_RHIID/19-122	U9V1C7.1 PF08241.10	66.70	0.00
TRINITY_A0E1G8_PARTE/540-795	A0E1G8.1 PF00069.23	66.70	0.00
TRINITY_E1ZGI7_CHLVA/158-356	E1ZGI7.1 PF04989.10	66.70	0.00
TRINITY_D8TL86_VOLCA/716-874	D8TL86.1 PF00153.25	66.70	0.00
TRINITY_A8HPU2_CHLRE/70-200	A8HPU2.1 PF01127.20	66.70	0.00
TRINITY_D3BK04_POLPA/1050-1098	D3BK04.1 PF14604.4;	66.70	0.00
TRINITY_A8JH92_CHLRE/203-385	A8JH92.1 PF02417.13	66.70	0.00
TRINITY_D8THW1_VOLCA/32-257	D8THW1.1 PF01255.17	66.70	0.00
TRINITY_A8JAW8_CHLRE/513-575	A8JAW8.1 PF07714.15	66.70	0.00
TRINITY_W4XZG3_STRPU/29-79	W4XZG3.1 PF13920.4;	66.70	0.00
TRINITY_Q22DX0_TETTS/130-389	Q22DX0.1 PF00494.17	66.70	0.00
TRINITY_I7MAL0_TETTS/12-201	I7MAL0.1 PF00596.19	66.70	0.00
TRINITY_D8UG37_VOLCA/206-269	D8UG37.1 PF00170.19	66.70	0.00
TRINITY_I0YR66_9CHLO/426-473	I0YR66.1 PF02891.18	66.70	0.00
TRINITY_A0A087SB57_AUXPR/89-364	A0A087SB57.1 PF0032	66.70	0.00
TRINITY_A0BWW1_PARTE/272-340	A0BWW1.1 PF00498.24	66.70	0.00
TRINITY_A8I6V8_CHLRE/39-74	A8I6V8.1 PF00320.25	66.70	0.00
TRINITY_Q4U0V8_CHLRE/49-349	Q4U0V8.1 PF01218.16	66.70	0.00
TRINITY_D8TPP1_VOLCA/104-246	D8TPP1.1 PF02469.20	66.70	0.00
TRINITY_Q8S3U7_CHLRE/18-278	Q8S3U7.1 PF00069.23	66.70	0.00
TRINITY_I7M280_TETTS/857-971	I7M280.2 PF09111.8;	66.70	0.00
TRINITY_K1QTP1_CRAGI/434-645	K1QTP1.1 PF00326.19	66.70	0.00
TRINITY_A0BWW1_PARTE/272-340	A0BWW1.1 PF00498.24	66.70	0.00
TRINITY_A0A075AS88_9FUNG/581-798	A0A075AS88.1 PF0032	66.70	0.00
TRINITY_RL26_TETTS/8-121	Q23F79.2 PF16906.3;	66.70	0.00
TRINITY_RL26_TETTS/8-121	Q23F79.2 PF16906.3;	66.70	0.00
TRINITY_A0DZS9_PARTE/39-80	A0DZS9.1 PF13445.4;	66.70	0.00
TRINITY_R1C902_EMIHU/442-490	R1C902.1 PF00628.27	66.70	0.00
TRINITY_M0TZ95_MUSAM/623-690	M0TZ95.1 PF00658.16	66.70	0.00
TRINITY_A0A059LFT7_9CHLO/113-267	A0A059LFT7.1 PF0050	66.70	0.00
TRINITY_M5VM98_PRUPE/561-708	M5VM98.1 PF00078.25	66.70	0.00
TRINITY_C4Y7D3_CLAL4/242-402	C4Y7D3.1 PF00078.25	66.70	0.00
TRINITY_I0YP34_9CHLO/86-231	I0YP34.1 PF00210.22	66.70	0.00
TRINITY_A8J8S4_CHLRE/3-71	A8J8S4.1 PF00076.20	66.70	0.00
TRINITY_A8HN56_CHLRE/225-326	A8HN56.1 PF13499.4;	66.70	0.00
TRINITY_A8HN56_CHLRE/225-326	A8HN56.1 PF13499.4;	66.70	0.00
TRINITY_Q22UJ1_TETTS/89-234	Q22UJ1.1 PF14580.4;	66.70	0.00
TRINITY_A8IS63_CHLRE/7-142	A8IS63.1 PF00258.23	66.70	0.00
TRINITY_I0Z2C4_9CHLO/1-304	I0Z2C4.1 PF00248.19	66.70	0.00
TRINITY_D8TNN8_VOLCA/63-306	D8TNN8.1 PF00112.21	66.70	0.00
TRINITY_A0A087SBJ9_AUXPR/105-178	A0A087SBJ9.1 PF0128	66.70	0.00
TRINITY_B9J6Z0_AGRRK/36-313	B9J6Z0.1 PF00150.16	66.70	0.00
TRINITY_E1Z9K0_CHLVA/64-322	E1Z9K0.1 PF08145.10	66.70	0.00
TRINITY_D8TYX9_VOLCA/666-861	D8TYX9.1 PF00211.18	66.70	0.00

TRINITY_D2V0W5_NAEGR/206-247	D2V0W5.1 PF00400.30	66.70	0.00
TRINITY_A8J9D9_CHLRE/105-169	A8J9D9.1 PF17136.2;	66.70	0.00
TRINITY_A8I6F3_CHLRE/385-587	A8I6F3.1 PF07714.15	66.70	0.00
TRINITY_I0Z7K6_9CHLO/17-331	I0Z7K6.1 PF00225.21	66.70	0.00
TRINITY_D8TTV8_VOLCA/81-324	D8TTV8.1 PF00233.17	66.70	0.00
TRINITY_W7X0K6_TETTS/22-140	W7X0K6.1 PF01894.15	66.70	0.00
TRINITY_F4PWL2_DICFS/91-392	F4PWL2.1 PF00676.18	66.60	0.00
TRINITY_A4RRK6_OSTLU/9-300	A4RRK6.1 PF00069.23	66.60	0.00
TRINITY_D8TMH6_VOLCA/62-346	D8TMH6.1 PF00632.23	66.60	0.00
TRINITY_A8J6K6_CHLRE/229-518	A8J6K6.1 PF01031.18	66.60	0.00
TRINITY_A8HYZ5_CHLRE/53-384	A8HYZ5.1 PF00069.23	66.60	0.00
TRINITY_M1CRH4_SOLTU/15-176	M1CRH4.1 PF00071.20	66.50	0.00
TRINITY_A0A096P9X9_OSTTA/129-296	A0A096P9X9.1 PF0288	66.50	0.00
TRINITY_A8JHW6_CHLRE/43-718	A8JHW6.1 PF00995.21	66.50	0.00
TRINITY_E1ZTF6_CHLVA/45-198	E1ZTF6.1 PF00406.20	66.50	0.00
TRINITY_D8UI94_VOLCA/26-188	D8UI94.1 PF01652.16	66.50	0.00
TRINITY_A9TB22_PHYPA/75-590	A9TB22.1 PF10433.7;	66.50	0.00
TRINITY_A8HMX2_CHLRE/97-689	A8HMX2.1 PF02901.13	66.50	0.00
TRINITY_D3B3X6_POLPA/215-540	D3B3X6.1 PF00733.19	66.50	0.00
TRINITY_D8TSD0_VOLCA/116-318	D8TSD0.1 PF00590.18	66.50	0.00
TRINITY_A0A087SKD7_AUXPR/233-504	A0A087SKD7.1 PF0066	66.50	0.00
TRINITY_D8UHE6_VOLCA/620-857	D8UHE6.1 PF00069.23	66.50	0.00
TRINITY_D3B2W9_POLPA/20-667	D3B2W9.1 PF00888.20	66.50	0.00
TRINITY_Q7XX43_ORYSJ/585-745	Q7XX43.2 PF00078.25	66.50	0.00
TRINITY_Q7XX43_ORYSJ/585-745	Q7XX43.2 PF00078.25	66.50	0.00
TRINITY_A0A0A1NYQ7_9FUNG/96-537	A0A0A1NYQ7.1 PF0050	66.40	0.00
TRINITY_Q22EZ7_TETTS/48-509	Q22EZ7.1 PF00171.20	66.40	0.00
TRINITY_S8CW06_9LAMI/33-141	S8CW06.1 PF00255.17	66.40	0.00
TRINITY_A0EID1_PARTE/75-324	A0EID1.1 PF00069.23	66.40	0.00
TRINITY_C1E1A5_MICSR/1-125	C1E1A5.1 PF00705.16	66.40	0.00
TRINITY_D8TVF4_VOLCA/25-437	D8TVF4.1 PF01704.16	66.40	0.00
TRINITY_E1G463_LOALO/14-142	E1G463.1 PF00416.20	66.40	0.00
TRINITY_A0EG30_PARTE/10-359	A0EG30.1 PF00274.17	66.40	0.00
TRINITY_A8J0U4_CHLRE/27-488	A8J0U4.1 PF01384.18	66.40	0.00
TRINITY_A0A0D2WUX5_CAPO3/529-755	A0A0D2WUX5.1 PF1371	66.40	0.00
TRINITY_A0BL44_PARTE/620-730	A0BL44.1 PF13426.5;	66.40	0.00
TRINITY_I7ME83_TETTS/11-124	I7ME83.1 PF01398.19	66.40	0.00
TRINITY_A0A078ABM6_STYLE/46-281	A0A078ABM6.1 PF0011	66.40	0.00
TRINITY_A7SK20_NEMVE/1-113	A7SK20.1 PF01778.15	66.40	0.00
TRINITY_E1ZA18_CHLVA/75-196	E1ZA18.1 PF00581.18	66.40	0.00
TRINITY_F8PG43_SERL3/67-171	F8PG43.1 PF00639.19	66.30	0.00
TRINITY_A9S740_PHYPA/3098-3376	A9S740.1 PF02138.16	66.30	0.00
TRINITY_L8HD25_ACACA/7-190	L8HD25.1 PF00025.19	66.30	0.00
TRINITY_I1C5V8_RHIO9/33-220	I1C5V8.1 PF00227.24	66.30	0.00
TRINITY_A8I1Y7_CHLRE/6-281	A8I1Y7.1 PF14938.4;	66.30	0.00
TRINITY_G0R3T4_ICHMG/1-117	G0R3T4.1 PF12775.5;	66.30	0.00
TRINITY_Q23GD1_TETTS/1838-1959	Q23GD1.2 PF00069.23	66.30	0.00
TRINITY_Q23VX2_TETTS/36-127	Q23VX2.2 PF00447.15	66.30	0.00
TRINITY_ABCB4_DICDI/542-691	Q54W24.1 PF00005.25	66.30	0.00
TRINITY_Q55CR6_DICDI/72-241	Q55CR6.1 PF00270.27	66.30	0.00
TRINITY_D8TII8_VOLCA/13-280	D8TII8.1 PF00459.23	66.30	0.00
TRINITY_D8TKB0_VOLCA/20-165	D8TKB0.1 PF00005.25	66.30	0.00
TRINITY_A0CSP0_PARTE/193-289	A0CSP0.1 PF01248.24	66.30	0.00
TRINITY_A0DZ73_PARTE/168-416	A0DZ73.1 PF00069.23	66.30	0.00
TRINITY_F4PMI7_DICFS/27-634	F4PMI7.1 PF00012.18	66.30	0.00
TRINITY_W5LQY3_ASTMX/1-167	W5LQY3.1 PF00838.15	66.30	0.00
TRINITY_A8J8B2_CHLRE/72-172	A8J8B2.1 PF02482.17	66.30	0.00
TRINITY_I7MAY1_TETTS/3674-4379	I7MAY1.1 PF03028.13	66.30	0.00
TRINITY_D8U5C6_VOLCA/1-392	D8U5C6.1 PF13520.4;	66.30	0.00
TRINITY_A0A077ZW97_STYLE/111-205	A0A077ZW97.1 PF1683	66.30	0.00
TRINITY_D8TJ79_VOLCA/552-647	D8TJ79.1 PF12936.5;	66.30	0.00

TRINITY_A8J924_CHLRE/124-212	A8J924.1 PF00957.19	66.30	0.00
TRINITY_K1QUF8_CRAGI/40-110	K1QUF8.1 PF00076.20	66.20	0.00
TRINITY_I7MIS4_TETTS/1-83	I7MIS4.2 PF12861.5;	66.20	0.00
TRINITY_M4AYU5_XIPMA/24-136	M4AYU5.1 PF00074.18	66.20	0.00
TRINITY_L8H823_ACACA/184-369	L8H823.1 PF07065.12	66.20	0.00
TRINITY_Q2RWI6_RHORT/16-80	Q2RWI6.1 PF00462.22	66.20	0.00
TRINITY_F0ZR69_DICPU/692-883	F0ZR69.1 PF08282.10	66.20	0.00
TRINITY_A0DC90_PARTE/163-363	A0DC90.1 PF00856.26	66.20	0.00
TRINITY_SUMO1_ARATH/18-91	P55852.2 PF00240.21	66.20	0.00
TRINITY_Q236N2_TETTS/32-570	Q236N2.1 PF00995.21	66.20	0.00
TRINITY_Q22V76_TETTS/529-664	Q22V76.2 PF00005.25	66.20	0.00
TRINITY_A0A0M2SZE4_9BACI/4-217	A0A0M2SZE4.1 PF0179	66.20	0.00
TRINITY_A0CFQ7_PARTE/349-467	A0CFQ7.1 PF00622.26	66.20	0.00
TRINITY_L8GPS3_ACACA/400-478	L8GPS3.1 PF08149.9;	66.20	0.00
TRINITY_B1XJD9_SYNP2/730-795	B1XJD9.1 PF00512.23	66.20	0.00
TRINITY_H2TE27_TAKRU/12-130	H2TE27.1 PF07686.15	66.20	0.00
TRINITY_A0E741_PARTE/638-836	A0E741.1 PF01434.16	66.20	0.00
TRINITY_D8U5Y4_VOLCA/1-248	D8U5Y4.1 PF00225.21	66.20	0.00
TRINITY_D8U650_VOLCA/3-70	D8U650.1 PF01066.19	66.20	0.00
TRINITY_L8HGI8_ACACA/89-695	L8HGI8.1 PF00133.20	66.20	0.00
TRINITY_A0A087SBU5_AUXPR/287-356	A0A087SBU5.1 PF0877	66.20	0.00
TRINITY_A8JGT2_CHLRE/86-154	A8JGT2.1 PF02201.16	66.20	0.00
TRINITY_A0CWR1_PARTE/401-628	A0CWR1.1 PF00198.21	66.20	0.00
TRINITY_D8U9G4_VOLCA/52-168	D8U9G4.1 PF03099.17	66.20	0.00

TRINITY_A0A015J5F3_9GLOM/2-79	A0A015J5F3.1 PF0718	66.20	0.00
TRINITY_A8HQD2_CHLRE/65-209	A8HQD2.1 PF10607.7;	66.20	0.00
TRINITY_D3BRS6_POLPA/217-623	D3BRS6.1 PF00962.20	66.20	0.00
TRINITY_D8UK61_VOLCA/227-395	D8UK61.1 PF04832.10	66.20	0.00
TRINITY_D8U6D4_VOLCA/109-491	D8U6D4.1 PF00632.23	66.20	0.00
TRINITY_L7WAY7_NONDD/31-309	L7WAY7.1 PF00248.19	66.20	0.00
TRINITY_D8TXM0_VOLCA/44-111	D8TXM0.1 PF00076.20	66.20	0.00
TRINITY_RS3A_CHLRE/15-221	A8HS48.1 PF01015.16	66.20	0.00
TRINITY_G0R6B9_ICHMG/35-104	G0R6B9.1 PF10407.7;	66.20	0.00
TRINITY_F4NW08_BATDJ/1-138	F4NW08.1 PF00179.24	66.20	0.00
TRINITY_Q3SDM6_PARTE/17-177	Q3SDM6.1 PF00071.20	66.20	0.00
TRINITY_A8IIS8_CHLRE/45-114	A8IIS8.1 PF02416.14	66.20	0.00
TRINITY_D8U4B9_VOLCA/79-456	D8U4B9.1 PF00890.22	66.20	0.00
TRINITY_A0A078AJC6_STYLE/38-250	A0A078AJC6.1 PF0072	66.20	0.00
TRINITY_D8U0H6_VOLCA/123-187	D8U0H6.1 PF00076.20	66.20	0.00
TRINITY_I7M2D2_TETTS/40-113	I7M2D2.2 PF02798.18	66.20	0.00
TRINITY_A8I459_CHLRE/89-433	A8I459.1 PF01546.26	66.20	0.00
TRINITY_A0A0A1MQX3_9FUNG/93-171	A0A0A1MQX3.1 PF0147	66.20	0.00
TRINITY_E1ZLP7_CHLVA/204-432	E1ZLP7.1 PF00557.22	66.20	0.00
TRINITY_K1PGH0_CRAGI/9-165	K1PGH0.1 PF12850.5;	66.20	0.00
TRINITY_I0YN96_9CHLO/60-564	I0YN96.1 PF00118.22	66.20	0.00
TRINITY_I0YKC2_9CHLO/1-154	I0YKC2.1 PF04729.11	66.20	0.00
TRINITY_G0QMF6_ICHMG/19-283	G0QMF6.1 PF02263.17	66.20	0.00
TRINITY_X5D8J8_9BACT/110-281	X5D8J8.1 PF00589.20	66.20	0.00
TRINITY_U3J5I5_ANAPL/285-901	U3J5I5.1 PF00443.27	66.10	0.00
TRINITY_A0A075AZL7_9FUNG/132-459	A0A075AZL7.1 PF0049	66.10	0.00
TRINITY_I7MMR4_TETTS/183-264	I7MMR4.1 PF12895.5;	66.10	0.00
TRINITY_D3B5F9_POLPA/16-194	D3B5F9.1 PF02301.16	66.10	0.00
TRINITY_S7UK71_TOXGO/169-306	S7UK71.1 PF00179.24	66.10	0.00
TRINITY_H1YCL7_9SPHI/51-213	H1YCL7.1 PF03167.17	66.10	0.00
TRINITY_A0A0D2VTJ8_CAPO3/132-305	A0A0D2VTJ8.1 PF0602	66.10	0.00
TRINITY_Q22W39_TETTS/56-311	Q22W39.2 PF00069.23	66.10	0.00
TRINITY_I7M1U7_TETTS/515-650	I7M1U7.1 PF00005.25	66.10	0.00
TRINITY_F0Z8T4_DICPU/179-349	F0Z8T4.1 PF02866.16	66.10	0.00
TRINITY_C8X1V8_DESRD/5-119	C8X1V8.1 PF09996.7;	66.10	0.00
TRINITY_A8IWK6_CHLRE/17-269	A8IWK6.1 PF07714.15	66.10	0.00
TRINITY_A0BTM6_PARTE/247-554	A0BTM6.1 PF00749.19	66.10	0.00
TRINITY_U6NK01_HAECO/73-145	U6NK01.1 PF02594.14	66.10	0.00
TRINITY_E1ZKP1_CHLVA/20-212	E1ZKP1.1 PF00857.18	66.10	0.00
TRINITY_A8IYH3_CHLRE/127-188	A8IYH3.1 PF04117.10	66.10	0.00
TRINITY_U3K0G6_FICAL/1325-1473	U3K0G6.1 PF00005.25	66.10	0.00
TRINITY_CCR1_MAIZE/36-291	P0C8M8.1 PF00069.23	66.10	0.00
TRINITY_A8J1X1_CHLRE/197-257	A8J1X1.1 PF04117.10	66.10	0.00
TRINITY_A8JHQ1_CHLRE/328-388	A8JHQ1.1 PF00226.29	66.10	0.00
TRINITY_V4TVT0_9ROSI/84-377	V4TVT0.1 PF05185.14	66.10	0.00
TRINITY_A8IUC0_CHLRE/192-310	A8IUC0.1 PF01544.16	66.10	0.00
TRINITY_D8TMQ9_VOLCA/165-600	D8TMQ9.1 PF01131.18	66.10	0.00
TRINITY_A0BD97_PARTE/9-70	A0BD97.1 PF00886.17	66.10	0.00
TRINITY_I7LZU3_TETTS/215-299	I7LZU3.1 PF04433.15	66.10	0.00
TRINITY_A8J0A8_CHLRE/143-217	A8J0A8.1 PF09368.8;	66.10	0.00
TRINITY_R8BQX2_TOGMI/2-183	R8BQX2.1 PF17135.2;	66.10	0.00
TRINITY_I7LU51_TETTS/84-400	I7LU51.1 PF01406.17	66.10	0.00
TRINITY_A0BDP7_PARTE/12-138	A0BDP7.1 PF00179.24	66.10	0.00
TRINITY_M0S5G6_MUSAM/335-651	M0S5G6.1 PF02540.15	66.10	0.00
TRINITY_D8U751_VOLCA/2314-2574	D8U751.1 PF12775.5;	66.10	0.00
TRINITY_I1QGW5_ORYGL/64-128	I1QGW5.1 PF00076.20	66.10	0.00
TRINITY_M0Z181_HORVD/16-118	M0Z181.1 PF13304.4;	66.10	0.00
TRINITY_I0YK34_9CHLO/173-228	I0YK34.1 PF13370.4;	66.10	0.00
TRINITY_A8HNX9_CHLRE/48-218	A8HNX9.1 PF04755.10	66.10	0.00
TRINITY_F0ZAE5_DICPU/489-544	F0ZAE5.1 PF00412.20	66.10	0.00
TRINITY_D8UGC1_VOLCA/164-275	D8UGC1.1 PF01205.17	66.10	0.00

TRINITY_D8U821_VOLCA/5-290	D8U821.1 PF00069.23	66.10	0.00
TRINITY_A8IBU9_CHLRE/138-261	A8IBU9.1 PF03109.14	66.10	0.00
TRINITY_I0Z2E4_9CHLO/522-831	I0Z2E4.1 PF02889.14	66.10	0.00
TRINITY_I0YL17_9CHLO/29-208	I0YL17.1 PF00270.27	66.10	0.00
TRINITY_C1FII7_MICSR/12-58	C1FII7.1 PF12554.6;	66.00	0.00
TRINITY_A0BMT1_PARTE/17-71	A0BMT1.1 PF00226.29	66.00	0.00
TRINITY_I3KEJ4_ORENI/13-139	I3KEJ4.1 PF00828.17	66.00	0.00
TRINITY_Q22YP6_TETTS/3235-3331	Q22YP6.2 PF09333.9;	66.00	0.00
TRINITY_I7LY30_TETTS/325-418	I7LY30.1 PF03147.12	66.00	0.00
TRINITY_I1GBR9_AMPQE/103-201	I1GBR9.1 PF14497.4;	66.00	0.00
TRINITY_A0A022PSY2_ERYGU/17-69	A0A022PSY2.1 PF0971	66.00	0.00
TRINITY_A0BX73_PARTE/218-318	A0BX73.1 PF00153.25	66.00	0.00
TRINITY_I3JLH7_ORENI/145-239	I3JLH7.1 PF00031.19	66.00	0.00
TRINITY_D8M2T5_BLAHO/134-181	D8M2T5.1 PF13920.4;	66.00	0.00
TRINITY_A0A0M8K7X2_9CHLR/384-533	A0A0M8K7X2.1 PF0000	66.00	0.00
TRINITY_A0A068RSS3_9FUNG/161-210	A0A068RSS3.1 PF0354	66.00	0.00
TRINITY_A0A067DPW2_CITSI/95-292	A0A067DPW2.1 PF1308	66.00	0.00
TRINITY_A0E1G8_PARTE/540-795	A0E1G8.1 PF00069.23	66.00	0.00
TRINITY_A0D3K4_PARTE/152-201	A0D3K4.1 PF13920.4;	66.00	0.00
TRINITY_Q22W39_TETTS/56-311	Q22W39.2 PF00069.23	66.00	0.00
TRINITY_A0A058ZA67_9EUKA/9-170	A0A058ZA67.1 PF0007	66.00	0.00
TRINITY_L1IJY6_GUITH/93-250	L1IJY6.1 PF02518.24	66.00	0.00
TRINITY_A0A078AYS6_STYLE/9-55	A0A078AYS6.1 PF0047	66.00	0.00
TRINITY_C1E1U5_MICSR/94-250	C1E1U5.1 PF02535.20	66.00	0.00
TRINITY_A8IQH3_CHLRE/1-180	A8IQH3.1 PF01184.17	66.00	0.00
TRINITY_U6N408_9EIME/83-147	U6N408.1 PF13499.4;	66.00	0.00
TRINITY_A0DFK5_PARTE/51-306	A0DFK5.1 PF00069.23	66.00	0.00
TRINITY_E1ZHU6_CHLVA/51-276	E1ZHU6.1 PF00082.20	66.00	0.00
TRINITY_D8UJW1_VOLCA/1579-1721	D8UJW1.1 PF12842.5;	66.00	0.00
TRINITY_W7XFH3_TETTS/470-624	W7XFH3.1 PF00005.25	66.00	0.00
TRINITY_G1N1B4_MELGA/1-241	G1N1B4.2 PF03437.13	66.00	0.00
TRINITY_Q23MM8_TETTS/343-397	Q23MM8.2 PF00076.20	66.00	0.00
TRINITY_C1MZY5_MICPC/483-532	C1MZY5.1 PF00847.18	66.00	0.00
TRINITY_A8IRP8_CHLRE/2-206	A8IRP8.1 PF00091.23	66.00	0.00
TRINITY_E1Z4J6_CHLVA/166-333	E1Z4J6.1 PF13640.4;	66.00	0.00
TRINITY_A8IA47_CHLRE/9-142	A8IA47.1 PF05871.10	66.00	0.00
TRINITY_A8JGF9_CHLRE/112-208	A8JGF9.1 PF13864.4;	66.00	0.00
TRINITY_D0P397_PHYIT/201-459	D0P397.1 PF00069.23	66.00	0.00
TRINITY_D3B6E7_POLPA/21-441	D3B6E7.1 PF04209.11	66.00	0.00
TRINITY_D8TNU6_VOLCA/93-344	D8TNU6.1 PF01040.16	66.00	0.00
TRINITY_A8I6Y0_CHLRE/141-334	A8I6Y0.1 PF01556.16	66.00	0.00
TRINITY_A0A075ATC3_9FUNG/49-177	A0A075ATC3.1 PF0152	66.00	0.00
TRINITY_Q239R2_TETTS/10-57	Q239R2.2 PF00319.16	66.00	0.00
TRINITY_D8TPJ7_VOLCA/169-322	D8TPJ7.1 PF01556.16	66.00	0.00
TRINITY_I0Z4D4_9CHLO/20-126	I0Z4D4.1 PF11267.6;	66.00	0.00
TRINITY_A8ID30_CHLRE/13-183	A8ID30.1 PF10237.7;	66.00	0.00
TRINITY_A8JC80_CHLRE/16-196	A8JC80.1 PF03357.19	66.00	0.00
TRINITY_R6T066_9CLOT/673-721	R6T066.1 PF13538.4;	66.00	0.00
TRINITY_Q23CW4_TETTS/196-365	Q23CW4.2 PF02866.16	66.00	0.00
TRINITY_A8IRJ6_CHLRE/159-209	A8IRJ6.1 PF13639.4;	66.00	0.00
TRINITY_D8TKW7_VOLCA/131-373	D8TKW7.1 PF00112.21	66.00	0.00
TRINITY_Q23YB5_TETTS/199-456	Q23YB5.2 PF00069.23	66.00	0.00
TRINITY_D8TLB9_VOLCA/79-355	D8TLB9.1 PF02353.18	66.00	0.00
TRINITY_PGM2_DICDI/44-187	Q54UQ2.1 PF02878.14	66.00	0.00
TRINITY_F4PRM5_DICFS/283-491	F4PRM5.1 PF04055.19	66.00	0.00
TRINITY_I0YSE8_9CHLO/45-385	I0YSE8.1 PF00274.17	66.00	0.00
TRINITY_A8HWC2_CHLRE/332-590	A8HWC2.1 PF03949.13	66.00	0.00
TRINITY_A0A0G2ZWH9_9DELT/1116-1168	A0A0G2ZWH9.1 PF0067	66.00	0.00
TRINITY_A8J1E8_CHLRE/66-271	A8J1E8.1 PF07002.14	66.00	0.00
TRINITY_A8INZ7_CHLRE/69-346	A8INZ7.1 PF03982.11	66.00	0.00
TRINITY_L8HMU1_ACACA/91-775	L8HMU1.1 PF00063.19	66.00	0.00

TRINITY_I7ML74_TETTS/5678-6228	I7ML74.2 PF00899.19	66.00	0.00
TRINITY_A9RQZ4_PHYPA/20-489	A9RQZ4.1 PF00171.20	66.00	0.00
TRINITY_A0A0D2VUK7_CAPO3/553-737	A0A0D2VUK7.1 PF0027	66.00	0.00
TRINITY_A9U1T0_PHYPA/12-337	A9U1T0.1 PF00069.23	66.00	0.00
TRINITY_W5JBT3_ANODA/175-435	W5JBT3.1 PF01189.15	66.00	0.00
TRINITY_A9UUZ7_MONBE/1923-2066	A9UUZ7.1 PF07728.12	66.00	0.00
TRINITY_A8I3J5_CHLRE/51-318	A8I3J5.1 PF00459.23	66.00	0.00
TRINITY_Q8YV84_NOSS1/37-134	Q8YV84.1 PF00355.24	66.00	0.00
TRINITY_A8JGS8_CHLRE/319-763	A8JGS8.1 PF04053.12	66.00	0.00
TRINITY_M5VM98_PRUPE/561-708	M5VM98.1 PF00078.25	66.00	0.00
TRINITY_J9HJ22_9SPIT/3-99	J9HJ22.1 PF01157.16	66.00	0.00
TRINITY_MANA_DICDI/43-353	P34098.2 PF01074.20	65.90	0.00
TRINITY_U5DAJ6_AMBTC/68-108	U5DAJ6.1 PF01096.16	65.90	0.00
TRINITY_L8HHK4_ACACA/13-94	L8HHK4.1 PF04241.13	65.90	0.00
TRINITY_A0A077ZVL1_STYLE/189-765	A0A077ZVL1.1 PF0838	65.90	0.00
TRINITY_G0QPU0_ICHMG/54-487	G0QPU0.1 PF07994.10	65.90	0.00
TRINITY_Q23AF8_TETTS/106-149	Q23AF8.3 PF13639.4;	65.90	0.00
TRINITY_W4XZV9_STRPU/157-237	W4XZV9.1 PF01336.23	65.90	0.00
TRINITY_J9EN44_9SPIT/738-778	J9EN44.1 PF05186.11	65.90	0.00
TRINITY_H3CEP8_TETNG/9-186	H3CEP8.1 PF11032.6;	65.90	0.00
TRINITY_G0QX41_ICHMG/40-335	G0QX41.1 PF00069.23	65.90	0.00
TRINITY_A7SMN7_NEMVE/5-178	A7SMN7.1 PF00071.20	65.90	0.00
TRINITY_A9UP20_MONBE/90-256	A9UP20.1 PF04427.16	65.90	0.00
TRINITY_L8HD99_ACACA/100-180	L8HD99.1 PF03366.14	65.90	0.00
TRINITY_I0YKX7_9CHLO/155-269	I0YKX7.1 PF00504.19	65.90	0.00
TRINITY_I0YKX7_9CHLO/155-269	I0YKX7.1 PF00504.19	65.90	0.00
TRINITY_A0A0N4TTI4_BRUPA/409-452	A0A0N4TTI4.1 PF1363	65.90	0.00
TRINITY_A0A0F3IMW9_9GAMM/81-520	A0A0F3IMW9.1 PF0050	65.90	0.00
TRINITY_K9TKX8_9CYAN/243-286	K9TKX8.1 PF01505.16	65.90	0.00
TRINITY_A8INH4_CHLRE/30-266	A8INH4.1 PF00069.23	65.90	0.00
TRINITY_A8IG04_CHLRE/28-151	A8IG04.1 PF00067.20	65.90	0.00
TRINITY_D8UFH7_VOLCA/121-308	D8UFH7.1 PF10294.7;	65.90	0.00
TRINITY_A0A078AVM5_STYLE/320-363	A0A078AVM5.1 PF0056	65.90	0.00
TRINITY_F1NKR8_CHICK/217-260	F1NKR8.2 PF01505.16	65.90	0.00
TRINITY_G0R673_ICHMG/55-221	G0R673.1 PF00270.27	65.90	0.00
TRINITY_A0B6T0_METTP/484-615	A0B6T0.1 PF00004.27	65.90	0.00
TRINITY_Q23JR2_TETTS/338-482	Q23JR2.2 PF00005.25	65.90	0.00
TRINITY_A0BL44_PARTE/620-730	A0BL44.1 PF13426.5;	65.90	0.00
TRINITY_A0A067FM35_CITSI/738-950	A0A067FM35.1 PF0064	65.90	0.00
TRINITY_A8I7V2_CHLRE/44-214	A8I7V2.1 PF08534.8;	65.90	0.00
TRINITY_A0A0G4IWK8_PLABS/55-128	A0A0G4IWK8.1 PF0071	65.90	0.00
TRINITY_F4PTV7_DICFS/4405-4494	F4PTV7.1 PF09333.9;	65.90	0.00
TRINITY_A8I8G1_CHLRE/21-65	A8I8G1.1 PF01585.21	65.90	0.00
TRINITY_Q23CU3_TETTS/69-513	Q23CU3.1 PF00501.26	65.90	0.00
TRINITY_A8I032_CHLRE/24-135	A8I032.1 PF02466.17	65.90	0.00
TRINITY_A0A087SJT0_AUXPR/71-167	A0A087SJT0.1 PF1289	65.90	0.00
TRINITY_A8HTA5_CHLRE/677-924	A8HTA5.1 PF07714.15	65.90	0.00
TRINITY_W7AJD3_9APIC/6-229	W7AJD3.1 PF00300.20	65.90	0.00
TRINITY_A0A058Z5I0_9EUKA/950-1099	A0A058Z5I0.1 PF0000	65.90	0.00
TRINITY_A8ICB6_CHLRE/231-427	A8ICB6.1 PF12315.6;	65.90	0.00
TRINITY_D8U6J8_VOLCA/489-599	D8U6J8.1 PF01926.21	65.90	0.00
TRINITY_E1ZEN1_CHLVA/289-465	E1ZEN1.1 PF00270.27	65.90	0.00
TRINITY_A8JFD5_CHLRE/394-482	A8JFD5.1 PF08544.11	65.90	0.00
TRINITY_A8J6D5_CHLRE/102-360	A8J6D5.1 PF00122.18	65.90	0.00
TRINITY_A8ISI4_CHLRE/11-98	A8ISI4.1 PF02970.14	65.90	0.00
TRINITY_A8IQI1_CHLRE/1-179	A8IQI1.1 PF01184.17	65.90	0.00
TRINITY_L8HBF6_ACACA/1-126	L8HBF6.1 PF00235.17	65.90	0.00
TRINITY_A8HPA3_CHLRE/760-803	A8HPA3.1 PF13920.4;	65.90	0.00
TRINITY_Q23ZH6_TETTS/1205-1354	Q23ZH6.2 PF00005.25	65.90	0.00
TRINITY_A8IC88_CHLRE/107-224	A8IC88.1 PF13640.4;	65.90	0.00
TRINITY_Q6UKY8_CHLRE/13-72	Q6UKY8.1 PF05347.13	65.90	0.00

TRINITY_I1EYR0_AMPQE/51-129	I1EYR0.1 PF00717.21	65.80	0.00
TRINITY_I7LZR6_TETTS/63-261	I7LZR6.1 PF00106.23	65.80	0.00
TRINITY_A0A078B7E7_STYLE/269-506	A0A078B7E7.1 PF0046	65.80	0.00
TRINITY_A0A058ZFB3_9EUKA/1-108	A0A058ZFB3.1 PF0128	65.80	0.00
TRINITY_C4R522_PICPG/162-202	C4R522.1 PF00514.21	65.80	0.00
TRINITY_L1JX35_GUITH/119-245	L1JX35.1 PF04116.11	65.80	0.00
TRINITY_B4GNZ7_DROPE/46-86	B4GNZ7.1 PF00400.30	65.80	0.00
TRINITY_D8SL27_SELML/138-242	D8SL27.1 PF12894.5;	65.80	0.00
TRINITY_A0D3E5_PARTE/45-322	A0D3E5.1 PF00069.23	65.80	0.00
TRINITY_A0A0D2VJK4_CAPO3/673-820	A0A0D2VJK4.1 PF0029	65.80	0.00
TRINITY_B8D016_HALOH/8-124	B8D016.1 PF13793.4;	65.80	0.00
TRINITY_L8GJP7_ACACA/9-182	L8GJP7.1 PF02776.16	65.80	0.00
TRINITY_MOC2A_CHLRE/5-83	A8JJB2.1 PF02597.18	65.80	0.00
TRINITY_I1G9X5_AMPQE/63-217	I1G9X5.1 PF00675.18	65.80	0.00
TRINITY_Q230X5_TETTS/14-75	Q230X5.3 PF13499.4;	65.80	0.00
TRINITY_A0A059CW53_EUCGR/96-133	A0A059CW53.1 PF0142	65.80	0.00
TRINITY_A0BNB7_PARTE/164-432	A0BNB7.1 PF00928.19	65.80	0.00
TRINITY_Q09B48_STIAD/174-291	Q09B48.1 PF03171.18	65.80	0.00
TRINITY_L8HFI6_ACACA/84-198	L8HFI6.1 PF02338.17	65.80	0.00
TRINITY_Q3SD81_PARTE/16-176	Q3SD81.1 PF00071.20	65.80	0.00
TRINITY_Q23FP2_TETTS/142-252	Q23FP2.2 PF02770.17	65.80	0.00
TRINITY_F1A0D6_DICPU/2-259	F1A0D6.1 PF00069.23	65.80	0.00
TRINITY_A8IN84_CHLRE/1-86	A8IN84.1 PF12796.5;	65.80	0.00
TRINITY_A0E5C3_PARTE/963-1060	A0E5C3.1 PF00581.18	65.80	0.00
TRINITY_G0QJA0_ICHMG/157-432	G0QJA0.1 PF08634.8;	65.80	0.00
TRINITY_Q21MT3_SACD2/4-326	Q21MT3.1 PF16363.3;	65.80	0.00
TRINITY_W4K881_9HOMO/629-923	W4K881.1 PF04950.10	65.80	0.00
TRINITY_A0A059LMP9_9CHLO/127-248	A0A059LMP9.1 PF1340	65.80	0.00
TRINITY_D8TMZ3_VOLCA/1-193	D8TMZ3.1 PF00069.23	65.80	0.00
TRINITY_A8JES1_CHLRE/78-260	A8JES1.1 PF01025.17	65.80	0.00
TRINITY_I1G913_AMPQE/207-506	I1G913.1 PF00557.22	65.80	0.00
TRINITY_T1KZR4_TETUR/12-476	T1KZR4.1 PF00221.17	65.80	0.00
TRINITY_A7SJM7_NEMVE/332-369	A7SJM7.1 PF00400.30	65.80	0.00
TRINITY_A8HNJ4_CHLRE/1-185	A8HNJ4.1 PF02492.17	65.80	0.00
TRINITY_I7MIX1_TETTS/178-297	I7MIX1.2 PF02373.20	65.80	0.00
TRINITY_A8J448_CHLRE/16-348	A8J448.2 PF07992.12	65.80	0.00
TRINITY_F1A3F4_DICPU/109-243	F1A3F4.1 PF01926.21	65.80	0.00
TRINITY_A8HSI6_CHLRE/10-133	A8HSI6.1 PF04628.11	65.80	0.00
TRINITY_B3RKR4_TRIAD/3-376	B3RKR4.1 PF00022.17	65.80	0.00
TRINITY_I7M3D3_TETTS/42-666	I7M3D3.2 PF00133.20	65.80	0.00
TRINITY_A8I478_CHLRE/307-344	A8I478.1 PF05184.13	65.80	0.00
TRINITY_A8JH04_CHLRE/12-203	A8JH04.1 PF13385.4;	65.80	0.00
TRINITY_D3BFW2_POLPA/33-76	D3BFW2.1 PF01585.21	65.80	0.00
TRINITY_I0Z4V2_9CHLO/13-278	I0Z4V2.1 PF01263.18	65.80	0.00
TRINITY_A0A075B3Z7_9FUNG/21-207	A0A075B3Z7.1 PF0040	65.80	0.00
TRINITY_A0A0D1XZS4_ANEMI/11-85	A0A0D1XZS4.1 PF0399	65.80	0.00
TRINITY_A8IAZ1_CHLRE/51-88	A8IAZ1.1 PF00400.30	65.80	0.00
TRINITY_A0A074S0Z7_9HOMO/1-227	A0A074S0Z7.1 PF0315	65.80	0.00
TRINITY_D8U1P8_VOLCA/49-480	D8U1P8.1 PF00909.19	65.80	0.00
TRINITY_A0A061DMP8_THECC/350-439	A0A061DMP8.1 PF0007	65.80	0.00
TRINITY_A0BKI0_PARTE/327-399	A0BKI0.1 PF00313.20	65.80	0.00
TRINITY_A8I3X6_CHLRE/2565-3295	A8I3X6.1 PF03028.13	65.80	0.00
TRINITY_J9I8E2_9SPIT/28-186	J9I8E2.1 PF00071.20	65.80	0.00
TRINITY_I7MFR5_TETTS/26-354	I7MFR5.2 PF00180.18	65.70	0.00
TRINITY_A0A0D2VGN8_CAPO3/875-1090	A0A0D2VGN8.1 PF1308	65.70	0.00
TRINITY_A0A096M5E4_POEFO/325-433	A0A096M5E4.1 PF0062	65.70	0.00
TRINITY_M1CRH4_SOLTU/15-176	M1CRH4.1 PF00071.20	65.70	0.00
TRINITY_I3KUZ0_ORENI/24-92	I3KUZ0.1 PF00021.19	65.70	0.00
TRINITY_Q22UZ7_TETTS/14-118	Q22UZ7.2 PF00307.29	65.70	0.00
TRINITY_A0E1G8_PARTE/540-795	A0E1G8.1 PF00069.23	65.70	0.00
TRINITY_A0A022PWX0_ERYGU/13-52	A0A022PWX0.1 PF0142	65.70	0.00

TRINITY_T1FMX8_HELRO/221-290	T1FMX8.1 PF00076.20	65.70	0.00
TRINITY_D5BHW7_ZUNPS/132-464	D5BHW7.1 PF05089.10	65.70	0.00
TRINITY_A0BK25_PARTE/80-121	A0BK25.1 PF00643.22	65.70	0.00
TRINITY_G0QPV5_ICHMG/91-228	G0QPV5.1 PF14671.4;	65.70	0.00
TRINITY_A9TV44_PHYPA/95-219	A9TV44.1 PF00583.23	65.70	0.00
TRINITY_A0A078ANA5_STYLE/18-270	A0A078ANA5.1 PF0006	65.70	0.00
TRINITY_Q22UZ7_TETTS/14-118	Q22UZ7.2 PF00307.29	65.70	0.00
TRINITY_A8IFW1_CHLRE/4-233	A8IFW1.1 PF01048.18	65.70	0.00
TRINITY_A0A0F7HNP9_9STAP/9-44	A0A0F7HNP9.1 PF0013	65.70	0.00
TRINITY_R4G4X7_RHOPR/10-44	R4G4X7.1 PF06842.10	65.70	0.00
TRINITY_L8GTY7_ACACA/7-254	L8GTY7.1 PF03029.15	65.70	0.00
TRINITY_D8THU3_VOLCA/43-122	D8THU3.1 PF00312.20	65.70	0.00
TRINITY_J9HU22_9SPIT/4-283	J9HU22.1 PF00069.23	65.70	0.00
TRINITY_D8UJZ6_VOLCA/269-443	D8UJZ6.1 PF12430.6;	65.70	0.00
TRINITY_A0A061DMP8_THECC/350-439	A0A061DMP8.1 PF0007	65.70	0.00
TRINITY_F4PHG1_DICFS/31-284	F4PHG1.1 PF00069.23	65.70	0.00
TRINITY_D8TLU3_VOLCA/9-79	D8TLU3.1 PF00076.20	65.70	0.00
TRINITY_D8UD36_VOLCA/42-510	D8UD36.1 PF01490.16	65.70	0.00
TRINITY_A8HP70_CHLRE/54-170	A8HP70.1 PF03099.17	65.70	0.00
TRINITY_D8QP39_SELML/16-117	D8QP39.1 PF01990.15	65.70	0.00
TRINITY_A8HVA6_CHLRE/314-418	A8HVA6.1 PF01399.25	65.70	0.00
TRINITY_A0CH51_PARTE/341-417	A0CH51.1 PF08544.11	65.70	0.00
TRINITY_B9N0P5_POPTR/8-88	B9N0P5.1 PF14438.4;	65.70	0.00
TRINITY_A0DKF0_PARTE/2-336	A0DKF0.1 PF00225.21	65.70	0.00
TRINITY_A8IA67_CHLRE/15-425	A8IA67.1 PF01222.15	65.70	0.00
TRINITY_A8HYH4_CHLRE/412-639	A8HYH4.1 PF00198.21	65.70	0.00
TRINITY_A9RTR4_PHYPA/354-424	A9RTR4.1 PF04542.12	65.70	0.00
TRINITY_D8TJY1_VOLCA/90-344	D8TJY1.1 PF00149.26	65.70	0.00
TRINITY_D8TVC0_VOLCA/1983-2096	D8TVC0.1 PF00665.24	65.70	0.00
TRINITY_A8JHT2_CHLRE/90-361	A8JHT2.1 PF01650.16	65.70	0.00
TRINITY_A0BHN3_PARTE/4-73	A0BHN3.1 PF00076.20	65.70	0.00
TRINITY_D8UBS2_VOLCA/5-73	D8UBS2.1 PF13921.4;	65.70	0.00
TRINITY_Q22A43_TETTS/570-685	Q22A43.2 PF01412.16	65.70	0.00
TRINITY_A0A067RUW4_ZOONE/3-74	A0A067RUW4.1 PF0024	65.70	0.00
TRINITY_D2VS08_NAEGR/119-258	D2VS08.1 PF00182.17	65.70	0.00
TRINITY_J9J2V1_9SPIT/660-878	J9J2V1.1 PF07859.11	65.60	0.00
TRINITY_K2K6U2_9PROT/14-98	K2K6U2.1 PF06155.10	65.60	0.00
TRINITY_F0ZZ65_DICPU/7-70	F0ZZ65.1 PF13499.4;	65.60	0.00
TRINITY_G0R6G4_ICHMG/17-176	G0R6G4.1 PF00071.20	65.60	0.00
TRINITY_G0QVD2_ICHMG/39-208	G0QVD2.1 PF00009.25	65.60	0.00
TRINITY_Q3SDY9_PARTE/141-230	Q3SDY9.1 PF00027.27	65.60	0.00
TRINITY_L8GZ36_ACACA/502-627	L8GZ36.1 PF00271.29	65.60	0.00
TRINITY_L8HKV9_ACACA/23-322	L8HKV9.1 PF02374.13	65.60	0.00
TRINITY_Q24FR9_TETTS/28-281	Q24FR9.2 PF00069.23	65.60	0.00
TRINITY_F0VH16_NEOCL/49-92	F0VH16.1 PF00400.30	65.60	0.00
TRINITY_H3A085_LATCH/794-1004	H3A085.1 PF00644.18	65.60	0.00
TRINITY_A0A0G4ILL0_PLABS/1-93	A0A0G4ILL0.1 PF0119	65.60	0.00
TRINITY_A0CY99_PARTE/217-391	A0CY99.1 PF14572.4;	65.60	0.00
TRINITY_G4ZMR6_PHYSP/3-95	G4ZMR6.1 PF03501.13	65.60	0.00
TRINITY_J9J1K8_9SPIT/166-255	J9J1K8.1 PF00027.27	65.60	0.00
TRINITY_Q5TNJ4_ANOGA/104-168	Q5TNJ4.3 PF00327.18	65.60	0.00
TRINITY_A0A061EEG7_THECC/589-749	A0A061EEG7.1 PF0007	65.60	0.00
TRINITY_C1FIS7_MICSR/3449-4152	C1FIS7.1 PF03028.13	65.60	0.00
TRINITY_A8IGF0_CHLRE/107-282	A8IGF0.1 PF01202.20	65.60	0.00
TRINITY_I1CGX7_RHIO9/526-586	I1CGX7.1 PF13432.4;	65.60	0.00
TRINITY_A0A087SRH6_AUXPR/15-285	A0A087SRH6.1 PF0089	65.60	0.00
TRINITY_A4CPY6_ROBBH/31-262	A4CPY6.1 PF01182.18	65.60	0.00
TRINITY_I7MMC0_TETTS/13-345	I7MMC0.2 PF00225.21	65.60	0.00
TRINITY_A0A087SSS1_AUXPR/190-294	A0A087SSS1.1 PF1275	65.60	0.00
TRINITY_D8UI18_VOLCA/50-224	D8UI18.1 PF05042.11	65.60	0.00
TRINITY_D8UHJ0_VOLCA/23-604	D8UHJ0.1 PF00995.21	65.60	0.00

TRINITY_Q3SDY9_PARTE/141-230	Q3SDY9.1 PF00027.27	65.60	0.00
TRINITY_A0ZLZ1_NODSP/3-97	A0ZLZ1.1 PF12766.5;	65.60	0.00
TRINITY_A8JJ74_CHLRE/617-738	A8JJ74.1 PF06419.9;	65.60	0.00
TRINITY_D8TS24_VOLCA/54-242	D8TS24.1 PF13460.4;	65.60	0.00
TRINITY_A0A0D3CVX9_BRAOL/315-377	A0A0D3CVX9.1 PF1343	65.60	0.00
TRINITY_I0Z645_9CHLO/68-454	I0Z645.1 PF01266.22	65.60	0.00
TRINITY_A8IX31_CHLRE/146-272	A8IX31.1 PF00134.21	65.60	0.00
TRINITY_D2V9F0_NAEGR/46-106	D2V9F0.1 PF13921.4;	65.60	0.00
TRINITY_D8TS22_VOLCA/30-108	D8TS22.1 PF01016.17	65.60	0.00
TRINITY_S2JSH4_MUCC1/92-190	S2JSH4.1 PF01841.17	65.60	0.00
TRINITY_E1Z570_CHLVA/75-366	E1Z570.1 PF03095.13	65.60	0.00
TRINITY_D8TWL1_VOLCA/694-951	D8TWL1.1 PF00520.29	65.60	0.00
TRINITY_A8IE86_CHLRE/144-413	A8IE86.1 PF00664.21	65.60	0.00
TRINITY_A0CW61_PARTE/9-264	A0CW61.1 PF00069.23	65.60	0.00
TRINITY_I3JY17_ORENI/66-120	I3JY17.1 PF00084.18	65.50	0.00
TRINITY_L0PGP6_PNEJ8/12-172	L0PGP6.1 PF00071.20	65.50	0.00
TRINITY_W1QIU2_OGAPD/64-189	W1QIU2.1 PF02585.15	65.50	0.00
TRINITY_W4YHL3_STRPU/16-61	W4YHL3.1 PF13445.4;	65.50	0.00
TRINITY_Q246D1_TETTS/522-749	Q246D1.2 PF00326.19	65.50	0.00
TRINITY_F0V7R9_NEOCL/93-188	F0V7R9.1 PF01217.18	65.50	0.00
TRINITY_Q22A67_TETTS/206-780	Q22A67.1 PF08385.10	65.50	0.00
TRINITY_G0R500_ICHMG/297-509	G0R500.1 PF00454.25	65.50	0.00
TRINITY_A0A015LBY6_9GLOM/353-631	A0A015LBY6.1 PF0213	65.50	0.00
TRINITY_L8GQX7_ACACA/1030-1086	L8GQX7.1 PF00412.20	65.50	0.00
TRINITY_Q23K61_TETTS/4970-5279	Q23K61.2 PF00632.23	65.50	0.00
TRINITY_A0BTN4_PARTE/489-626	A0BTN4.1 PF00005.25	65.50	0.00
TRINITY_A0BX57_PARTE/51-189	A0BX57.1 PF01743.18	65.50	0.00
TRINITY_I7MH21_TETTS/71-139	I7MH21.2 PF00076.20	65.50	0.00
TRINITY_D8THB0_VOLCA/261-500	D8THB0.1 PF00814.23	65.50	0.00
TRINITY_W3WQH0_9PEZI/17-317	W3WQH0.1 PF00248.19	65.50	0.00
TRINITY_D8TZ47_VOLCA/242-358	D8TZ47.1 PF00932.17	65.50	0.00
TRINITY_I0Z7R4_9CHLO/183-296	I0Z7R4.1 PF01624.18	65.50	0.00
TRINITY_D8TZX3_VOLCA/5-184	D8TZX3.1 PF00025.19	65.50	0.00
TRINITY_D8UJ11_VOLCA/25-281	D8UJ11.1 PF00756.18	65.50	0.00
TRINITY_L8GVQ7_ACACA/9-125	L8GVQ7.1 PF02678.14	65.50	0.00
TRINITY_Q24CJ1_TETTS/143-542	Q24CJ1.1 PF14740.4;	65.50	0.00
TRINITY_A8I3J7_CHLRE/208-407	A8I3J7.1 PF00742.17	65.50	0.00
TRINITY_W5FCD2_WHEAT/10-175	W5FCD2.1 PF03371.13	65.50	0.00
TRINITY_I7LVW0_TETTS/6-146	I7LVW0.1 PF05903.12	65.50	0.00
TRINITY_B3RU20_TRIAD/28-82	B3RU20.1 PF01667.15	65.50	0.00
TRINITY_Q23U85_TETTS/23-120	Q23U85.2 PF02678.14	65.50	0.00
TRINITY_A8I1M1_CHLRE/91-288	A8I1M1.1 PF13469.4;	65.50	0.00
TRINITY_A0A077ZUD0_STYLE/232-495	A0A077ZUD0.1 PF0006	65.50	0.00
TRINITY_I0YSG3_9CHLO/50-231	I0YSG3.1 PF01625.19	65.50	0.00
TRINITY_A8IL88_CHLRE/8-153	A8IL88.1 PF14186.4;	65.50	0.00
TRINITY_A0A0F5PDJ0_9SPHN/29-120	A0A0F5PDJ0.1 PF0267	65.50	0.00
TRINITY_A8I5K4_CHLRE/78-192	A8I5K4.1 PF02338.17	65.50	0.00
TRINITY_D8TX35_VOLCA/4-148	D8TX35.1 PF00005.25	65.50	0.00
TRINITY_L8GNB3_ACACA/14-388	L8GNB3.1 PF01053.18	65.50	0.00
TRINITY_D8TUH7_VOLCA/1-350	D8TUH7.1 PF02005.14	65.50	0.00
TRINITY_A8J0L0_CHLRE/98-184	A8J0L0.1 PF00153.25	65.50	0.00
TRINITY_A0A087SB36_AUXPR/128-225	A0A087SB36.1 PF0610	65.50	0.00
TRINITY_A8J9K7_CHLRE/163-468	A8J9K7.1 PF00069.23	65.50	0.00
TRINITY_A8JFV7_CHLRE/13-425	A8JFV7.1 PF00909.19	65.50	0.00
TRINITY_D8TSC2_VOLCA/521-578	D8TSC2.1 PF15911.3;	65.50	0.00
TRINITY_A8IB95_CHLRE/25-150	A8IB95.1 PF08240.10	65.50	0.00
TRINITY_G0R3C8_ICHMG/145-194	G0R3C8.1 PF01344.23	65.40	0.00
TRINITY_A3ZKW9_9PLAN/33-196	A3ZKW9.1 PF00160.19	65.40	0.00
TRINITY_W7XKA4_TETTS/3-80	W7XKA4.1 PF07189.9;	65.40	0.00
TRINITY_A0EB42_PARTE/37-322	A0EB42.1 PF00069.23	65.40	0.00
TRINITY_D8U4E0_VOLCA/45-212	D8U4E0.1 PF00650.18	65.40	0.00

TRINITY_A0D476_PARTE/34-353	A0D476.1 PF00180.18	65.40	0.00
TRINITY_A0BDP7_PARTE/12-138	A0BDP7.1 PF00179.24	65.40	0.00
TRINITY_F6RA07_XENTR/86-142	F6RA07.1 PF01494.17	65.40	0.00
TRINITY_A8HS11_CHLRE/7-114	A8HS11.1 PF03694.11	65.40	0.00
TRINITY_A0A0A1NWK7_9FUNG/31-221	A0A0A1NWK7.1 PF0022	65.40	0.00
TRINITY_A0DS37_PARTE/1-284	A0DS37.1 PF00579.23	65.40	0.00
TRINITY_I7MLL9_TETTS/291-547	I7MLL9.2 PF00069.23	65.40	0.00
TRINITY_D8TJF1_VOLCA/39-225	D8TJF1.1 PF03193.14	65.40	0.00
TRINITY_T1EGA9_HELRO/31-538	T1EGA9.1 PF00118.22	65.40	0.00
TRINITY_D8U2X0_VOLCA/43-149	D8U2X0.1 PF09799.7;	65.40	0.00
TRINITY_A8IIG3_CHLRE/28-135	A8IIG3.1 PF00255.17	65.40	0.00
TRINITY_I0Z4G6_9CHLO/5-111	I0Z4G6.1 PF00466.18	65.40	0.00
TRINITY_D3AYJ1_POLPA/449-567	D3AYJ1.1 PF01926.21	65.40	0.00
TRINITY_E1ZGX1_CHLVA/16-177	E1ZGX1.1 PF04755.10	65.40	0.00
TRINITY_E1Z4P6_CHLVA/409-639	E1Z4P6.1 PF00198.21	65.40	0.00
TRINITY_E4X7T3_OIKDI/17-108	E4X7T3.1 PF00428.17	65.40	0.00
TRINITY_D8UED0_VOLCA/39-399	D8UED0.1 PF00262.16	65.40	0.00
TRINITY_D8UJR1_VOLCA/47-221	D8UJR1.1 PF16166.3;	65.40	0.00
TRINITY_D8TS46_VOLCA/39-121	D8TS46.1 PF07713.11	65.40	0.00
TRINITY_D8TLB2_VOLCA/1-167	D8TLB2.1 PF01956.14	65.40	0.00
TRINITY_Q5SKY7_THET8/6-520	Q5SKY7.1 PF01274.20	65.40	0.00
TRINITY_I1P1S4_ORYGL/57-108	I1P1S4.1 PF00847.18	65.40	0.00
TRINITY_I0Z6L3_9CHLO/345-550	I0Z6L3.1 PF02475.14	65.40	0.00
TRINITY_F4PUZ7_DICFS/7-168	F4PUZ7.1 PF00071.20	65.40	0.00
TRINITY_I7M3D3_TETTS/42-666	I7M3D3.2 PF00133.20	65.40	0.00
TRINITY_F4QBL1_DICFS/1326-1509	F4QBL1.1 PF00211.18	65.40	0.00
TRINITY_M0TY87_MUSAM/442-714	M0TY87.1 PF13476.4;	65.40	0.00
TRINITY_A8J6A5_CHLRE/74-203	A8J6A5.1 PF07738.11	65.40	0.00
TRINITY_A8ISI1_CHLRE/223-484	A8ISI1.1 PF01167.16	65.40	0.00
TRINITY_A9RL89_PHYPA/3-207	A9RL89.1 PF01088.19	65.40	0.00
TRINITY_A8I2P1_CHLRE/73-218	A8I2P1.1 PF09424.8;	65.40	0.00
TRINITY_D8U635_VOLCA/284-414	D8U635.1 PF03109.14	65.40	0.00
TRINITY_A8IW47_CHLRE/16-224	A8IW47.1 PF01991.16	65.40	0.00
TRINITY_D8U8L3_VOLCA/556-682	D8U8L3.1 PF00565.15	65.40	0.00
TRINITY_I0YVX4_9CHLO/3-155	I0YVX4.1 PF00005.25	65.40	0.00
TRINITY_A8NKQ4_COPC7/200-349	A8NKQ4.2 PF02518.24	65.30	0.00
TRINITY_I7LT01_TETTS/50-170	I7LT01.1 PF00293.26	65.30	0.00
TRINITY_L8GTD8_ACACA/607-806	L8GTD8.1 PF00326.19	65.30	0.00
TRINITY_A0CYG1_PARTE/160-260	A0CYG1.1 PF13864.4;	65.30	0.00
TRINITY_A0A0E0LCQ0_ORYPU/799-847	A0A0E0LCQ0.1 PF0040	65.30	0.00
TRINITY_A0DAW7_PARTE/169-243	A0DAW7.1 PF01169.17	65.30	0.00
TRINITY_G6CNF7_DANPL/112-206	G6CNF7.1 PF16835.3;	65.30	0.00
TRINITY_I7M7P8_TETTS/17-342	I7M7P8.2 PF00225.21	65.30	0.00
TRINITY_D8UAZ8_VOLCA/185-234	D8UAZ8.1 PF07496.13	65.30	0.00
TRINITY_E1ZJY5_CHLVA/229-278	E1ZJY5.1 PF13639.4;	65.30	0.00
TRINITY_A0A090M4H2_OSTTA/116-285	A0A090M4H2.1 PF0000	65.30	0.00
TRINITY_D2VA17_NAEGR/157-329	D2VA17.1 PF02866.16	65.30	0.00
TRINITY_I0YHZ8_9CHLO/11-411	I0YHZ8.1 PF03155.13	65.30	0.00
TRINITY_I7M6H6_TETTS/54-166	I7M6H6.1 PF10908.6;	65.30	0.00
TRINITY_D8THC5_VOLCA/241-456	D8THC5.1 PF01435.16	65.30	0.00
TRINITY_A8ITN7_CHLRE/25-107	A8ITN7.1 PF12895.5;	65.30	0.00
TRINITY_L8HBG2_ACACA/50-121	L8HBG2.1 PF00717.21	65.30	0.00
TRINITY_A8HXX1_CHLRE/172-245	A8HXX1.1 PF07915.11	65.30	0.00
TRINITY_D8TSR6_VOLCA/479-964	D8TSR6.1 PF00311.15	65.30	0.00
TRINITY_L8GZY2_ACACA/223-485	L8GZY2.1 PF00082.20	65.30	0.00
TRINITY_E1ZC58_CHLVA/255-304	E1ZC58.1 PF01805.18	65.30	0.00
TRINITY_D8TJX1_VOLCA/79-335	D8TJX1.1 PF09353.8;	65.30	0.00
TRINITY_D8TNV0_VOLCA/25-197	D8TNV0.1 PF13579.4;	65.30	0.00
TRINITY_I1IPJ0_BRADI/59-130	I1IPJ0.1 PF00240.21	65.30	0.00
TRINITY_A7RKB9_NEMVE/20-70	A7RKB9.1 PF01805.18	65.30	0.00
TRINITY_A8J822_CHLRE/27-277	A8J822.1 PF07714.15	65.30	0.00

TRINITY_D8TV48_VOLCA/298-451	D8TV48.1 PF14688.4;	65.30	0.00
TRINITY_E1ZCL0_CHLVA/62-357	E1ZCL0.1 PF00698.19	65.30	0.00
TRINITY_A8HWI3_CHLRE/14-351	A8HWI3.1 PF07289.9;	65.30	0.00
TRINITY_I7M6H4_TETTS/3452-4165	I7M6H4.2 PF03028.13	65.30	0.00
TRINITY_H2L7Q5_ORYLA/1-119	H2L7Q5.1 PF00833.16	65.30	0.00
TRINITY_D8U0H6_VOLCA/6-77	D8U0H6.1 PF00076.20	65.30	0.00
TRINITY_D8UDF2_VOLCA/95-323	D8UDF2.1 PF00561.18	65.30	0.00
TRINITY_L8H475_ACACA/6-151	L8H475.1 PF01699.22	65.30	0.00
TRINITY_T1KPR8_TETUR/102-147	T1KPR8.1 PF01599.17	65.30	0.00
TRINITY_I7M700_TETTS/181-443	I7M700.2 PF00069.23	65.30	0.00
TRINITY_H2SY51_TAKRU/39-177	H2SY51.1 PF00988.20	65.30	0.00
TRINITY_I0Z456_9CHLO/86-232	I0Z456.1 PF00929.22	65.30	0.00
TRINITY_A8IZH9_CHLRE/236-411	A8IZH9.1 PF11891.6;	65.30	0.00
TRINITY_D8UE71_VOLCA/1-261	D8UE71.1 PF00069.23	65.30	0.00
TRINITY_D8UAX2_VOLCA/22-298	D8UAX2.1 PF01326.17	65.30	0.00
TRINITY_D8THA5_VOLCA/511-744	D8THA5.1 PF00005.25	65.30	0.00
TRINITY_G0QRB2_ICHMG/101-199	G0QRB2.1 PF00153.25	65.30	0.00
TRINITY_E7RMK9_9BACT/29-164	E7RMK9.1 PF13580.4;	65.20	0.00
TRINITY_A0A0G4LJ84_9PEZI/114-328	A0A0G4LJ84.1 PF0009	65.20	0.00
TRINITY_F0ZLS3_DICPU/481-616	F0ZLS3.1 PF00005.25	65.20	0.00
TRINITY_D3BAH5_POLPA/202-293	D3BAH5.1 PF00153.25	65.20	0.00
TRINITY_E1VQD3_9GAMM/331-445	E1VQD3.1 PF08328.9;	65.20	0.00
TRINITY_A9SBP8_PHYPA/3-68	A9SBP8.1 PF01246.18	65.20	0.00
TRINITY_A0A096STN8_MAIZE/6-117	A0A096STN8.1 PF1351	65.20	0.00
TRINITY_A0BTC1_PARTE/366-624	A0BTC1.1 PF00069.23	65.20	0.00
TRINITY_A0A0D2QTE0_GOSRA/31-120	A0A0D2QTE0.1 PF0029	65.20	0.00
TRINITY_A0D788_PARTE/126-474	A0D788.1 PF13868.4;	65.20	0.00
TRINITY_A0BWW1_PARTE/272-340	A0BWW1.1 PF00498.24	65.20	0.00
TRINITY_L8GQD6_ACACA/30-104	L8GQD6.1 PF13656.4;	65.20	0.00
TRINITY_D8TJU1_VOLCA/648-709	D8TJU1.1 PF08492.10	65.20	0.00
TRINITY_A0A0D2WGZ6_CAPO3/307-538	A0A0D2WGZ6.1 PF0019	65.20	0.00
TRINITY_Q6CC81_YARLI/184-256	Q6CC81.1 PF00173.26	65.20	0.00
TRINITY_D8UG12_VOLCA/197-259	D8UG12.1 PF00010.24	65.20	0.00
TRINITY_D8U0B6_VOLCA/1-312	D8U0B6.1 PF01916.15	65.20	0.00
TRINITY_D8U4L8_VOLCA/947-1031	D8U4L8.1 PF01733.16	65.20	0.00
TRINITY_A8IRU0_CHLRE/5-311	A8IRU0.1 PF02535.20	65.20	0.00
TRINITY_A8I3Q3_CHLRE/2-108	A8I3Q3.1 PF02109.14	65.20	0.00
TRINITY_A0E6B4_PARTE/180-334	A0E6B4.1 PF00903.23	65.20	0.00
TRINITY_A8HQI0_CHLRE/478-646	A8HQI0.1 PF03441.12	65.20	0.00
TRINITY_A8IA75_CHLRE/44-181	A8IA75.1 PF03330.16	65.20	0.00
TRINITY_J9ITL9_9SPIT/13-268	J9ITL9.1 PF00069.23	65.20	0.00
TRINITY_U4KWQ2_PYROM/4781-4944	U4KWQ2.1 PF00092.26	65.20	0.00
TRINITY_A8J3R6_CHLRE/36-222	A8J3R6.1 PF03997.10	65.20	0.00
TRINITY_D8UII8_VOLCA/1-89	D8UII8.1 PF08216.9;	65.20	0.00
TRINITY_I0Z9Z0_9CHLO/140-959	I0Z9Z0.1 PF00311.15	65.20	0.00
TRINITY_D8ULA0_VOLCA/109-171	D8ULA0.1 PF00415.16	65.20	0.00
TRINITY_A8I1T0_CHLRE/166-281	A8I1T0.1 PF03171.18	65.20	0.00
TRINITY_I7MFT0_TETTS/25-577	I7MFT0.2 PF01602.18	65.20	0.00
TRINITY_A8JBW3_CHLRE/80-235	A8JBW3.1 PF00650.18	65.20	0.00
TRINITY_A8I406_CHLRE/61-235	A8I406.1 PF06003.10	65.20	0.00
TRINITY_J9IWR3_9SPIT/8-73	J9IWR3.1 PF01423.20	65.20	0.00
TRINITY_L1I441_GUITH/17-320	L1I441.1 PF00069.23	65.20	0.00
TRINITY_A0A015KUS6_9GLOM/82-193	A0A015KUS6.1 PF0754	65.20	0.00
TRINITY_D8TUL8_VOLCA/9-255	D8TUL8.1 PF04427.16	65.20	0.00
TRINITY_H2Y0X5_CIOIN/22-223	H2Y0X5.1 PF01557.16	65.20	0.00
TRINITY_G0QPH9_ICHMG/237-360	G0QPH9.1 PF00069.23	65.20	0.00
TRINITY_I1L1L2_SOYBN/717-1087	I1L1L2.1 PF00562.26	65.20	0.00
TRINITY_I0ZAQ2_9CHLO/12-272	I0ZAQ2.1 PF03803.13	65.20	0.00
TRINITY_I7MAB4_TETTS/36-500	I7MAB4.1 PF00171.20	65.20	0.00
TRINITY_A0A059D3Y5_EUCGR/395-511	A0A059D3Y5.1 PF0237	65.20	0.00
TRINITY_A8HQB6_CHLRE/32-408	A8HQB6.1 PF00282.17	65.20	0.00

TRINITY_D8TPF9_VOLCA/18-597	D8TPF9.1 PF01602.18	65.20	0.00
TRINITY_K9VEN6_9CYAN/467-898	K9VEN6.1 PF02347.14	65.20	0.00
TRINITY_A0A087SHE2_AUXPR/23-163	A0A087SHE2.1 PF1468	65.20	0.00
TRINITY_F4PZ54_DICFS/50-234	F4PZ54.1 PF00485.16	65.20	0.00
TRINITY_D8TKQ5_VOLCA/87-279	D8TKQ5.1 PF04056.12	65.20	0.00
TRINITY_A0A078AR09_STYLE/8-169	A0A078AR09.1 PF0007	65.20	0.00
TRINITY_A7T106_NEMVE/7-254	A7T106.1 PF04641.10	65.10	0.00
TRINITY_L8GUT5_ACACA/47-135	L8GUT5.1 PF00062.18	65.10	0.00
TRINITY_Q22RW5_TETTS/292-401	Q22RW5.2 PF00472.18	65.10	0.00
TRINITY_J9HYH4_9SPIT/23-307	J9HYH4.1 PF00069.23	65.10	0.00
TRINITY_H1Y7W2_9SPHI/49-435	H1Y7W2.1 PF01979.18	65.10	0.00
TRINITY_D7FUD3_ECTSI/69-384	D7FUD3.1 PF00225.21	65.10	0.00
TRINITY_W7XI32_TETTS/8-116	W7XI32.1 PF01412.16	65.10	0.00
TRINITY_T1J7U2_STRMM/83-145	T1J7U2.1 PF13499.4;	65.10	0.00
TRINITY_A0A0P7UC39_9TELE/461-503	A0A0P7UC39.1 PF0009	65.10	0.00
TRINITY_DNAK_OLICO/4-599	B6JCI3.1 PF00012.18	65.10	0.00
TRINITY_S2KI81_MUCC1/12-84	S2KI81.1 PF00173.26	65.10	0.00
TRINITY_A0A0G4EQP5_9ALVE/78-141	A0A0G4EQP5.1 PF0007	65.10	0.00
TRINITY_L8HES9_ACACA/21-69	L8HES9.1 PF13920.4;	65.10	0.00
TRINITY_D8TL86_VOLCA/577-686	D8TL86.1 PF00153.25	65.10	0.00
TRINITY_E1ZI90_CHLVA/2-165	E1ZI90.1 PF11965.6;	65.10	0.00
TRINITY_Q235M5_TETTS/736-779	Q235M5.2 PF06203.12	65.10	0.00
TRINITY_K7IWT7_NASVI/282-433	K7IWT7.1 PF00160.19	65.10	0.00
TRINITY_L8HET6_ACACA/43-369	L8HET6.1 PF07992.12	65.10	0.00
TRINITY_D8U3H2_VOLCA/1-587	D8U3H2.1 PF04734.11	65.10	0.00
TRINITY_Q22VV2_TETTS/3889-4192	Q22VV2.2 PF00632.23	65.10	0.00
TRINITY_I7M1V6_TETTS/314-533	I7M1V6.1 PF07002.14	65.10	0.00
TRINITY_K3YA69_SETIT/111-155	K3YA69.1 PF13639.4;	65.10	0.00
TRINITY_A0A061F7D5_THECC/54-176	A0A061F7D5.1 PF1270	65.10	0.00
TRINITY_A8JIJ6_CHLRE/8-182	A8JIJ6.1 PF01595.18	65.10	0.00
TRINITY_A8JHC6_CHLRE/3-255	A8JHC6.1 PF03618.12	65.10	0.00
TRINITY_A8HMF3_CHLRE/52-279	A8HMF3.1 PF04755.10	65.10	0.00
TRINITY_D8UD74_VOLCA/17-256	D8UD74.1 PF13561.4;	65.10	0.00
TRINITY_D2V2M8_NAEGR/181-417	D2V2M8.1 PF00009.25	65.10	0.00
TRINITY_I0YS81_9CHLO/13-161	I0YS81.1 PF00179.24	65.10	0.00
TRINITY_A8JD42_CHLRE/86-357	A8JD42.1 PF07082.9;	65.10	0.00
TRINITY_A0A087SPC4_AUXPR/77-448	A0A087SPC4.1 PF0769	65.10	0.00
TRINITY_E1ZI46_CHLVA/12-125	E1ZI46.1 PF02338.17	65.10	0.00
TRINITY_L8GX47_ACACA/1-273	L8GX47.1 PF04005.10	65.10	0.00
TRINITY_A0A078AVM5_STYLE/320-363	A0A078AVM5.1 PF0056	65.10	0.00
TRINITY_A0CMB4_PARTE/14-269	A0CMB4.1 PF00069.23	65.10	0.00
TRINITY_D8UAB6_VOLCA/19-231	D8UAB6.1 PF00834.17	65.10	0.00
TRINITY_T1L5E2_TETUR/34-246	T1L5E2.1 PF00063.19	65.10	0.00
TRINITY_A8IW20_CHLRE/203-431	A8IW20.1 PF02854.17	65.10	0.00
TRINITY_A0CMB4_PARTE/14-269	A0CMB4.1 PF00069.23	65.10	0.00
TRINITY_A0CVP4_PARTE/188-358	A0CVP4.1 PF02317.15	65.10	0.00
TRINITY_Q22BB4_TETTS/128-254	Q22BB4.1 PF02747.13	65.10	0.00
TRINITY_A0E1L0_PARTE/62-435	A0E1L0.1 PF00285.19	65.00	0.00
TRINITY_A0A077ZY01_STYLE/5-283	A0A077ZY01.1 PF0006	65.00	0.00
TRINITY_H2MN76_ORYLA/1-231	H2MN76.1 PF00069.23	65.00	0.00
TRINITY_V4S761_9ROSI/13-52	V4S761.1 PF01428.14	65.00	0.00
TRINITY_C6XKL0_HIRBI/7-61	C6XKL0.1 PF16177.3;	65.00	0.00
TRINITY_X1X5I6_ACYPI/1-138	X1X5I6.1 PF00005.25	65.00	0.00
TRINITY_G0R6G4_ICHMG/17-176	G0R6G4.1 PF00071.20	65.00	0.00
TRINITY_A5CF32_ORITB/23-470	A5CF32.1 PF00115.18	65.00	0.00
TRINITY_A0DA51_PARTE/476-579	A0DA51.1 PF02770.17	65.00	0.00
TRINITY_A0A0G4GY06_9ALVE/17-104	A0A0G4GY06.1 PF0015	65.00	0.00
TRINITY_L8GYL0_ACACA/427-549	L8GYL0.1 PF01575.17	65.00	0.00
TRINITY_W4Z2W7_STRPU/52-200	W4Z2W7.1 PF00005.25	65.00	0.00
TRINITY_D8TZR1_VOLCA/88-258	D8TZR1.1 PF01126.18	65.00	0.00
TRINITY_C1MVV9_MICPC/172-288	C1MVV9.1 PF02373.20	65.00	0.00

TRINITY_I7MHC4_TETTS/1048-1120	I7MHC4.1 PF14418.4;	65.00	0.00
TRINITY_Q22W19_TETTS/115-323	Q22W19.3 PF00112.21	65.00	0.00
TRINITY_D8U8R3_VOLCA/137-279	D8U8R3.1 PF00849.20	65.00	0.00
TRINITY_B6AAR2_CRYMR/38-343	B6AAR2.1 PF00749.19	65.00	0.00
TRINITY_D8TN79_VOLCA/33-213	D8TN79.1 PF00069.23	65.00	0.00
TRINITY_D8TT40_VOLCA/253-805	D8TT40.1 PF00183.16	65.00	0.00
TRINITY_A8JGC0_CHLRE/1-221	A8JGC0.1 PF04889.10	65.00	0.00
TRINITY_I0YL34_9CHLO/34-94	I0YL34.1 PF03810.17	65.00	0.00
TRINITY_A0A059A3L1_EUCGR/62-101	A0A059A3L1.1 PF0040	65.00	0.00
TRINITY_F5SG62_9BACL/6-145	F5SG62.1 PF08719.9;	65.00	0.00
TRINITY_F0ZHZ2_DICPU/221-566	F0ZHZ2.1 PF00155.19	65.00	0.00
TRINITY_A5BGC5_VITVI/9-145	A5BGC5.1 PF01090.17	65.00	0.00
TRINITY_I0Z7R1_9CHLO/62-270	I0Z7R1.1 PF05577.10	65.00	0.00
TRINITY_A0A077ZUV7_STYLE/56-208	A0A077ZUV7.1 PF1473	65.00	0.00
TRINITY_D8UHA1_VOLCA/14-431	D8UHA1.1 PF00067.20	65.00	0.00
TRINITY_I0Z3M4_9CHLO/311-447	I0Z3M4.1 PF00271.29	65.00	0.00
TRINITY_A8IBM5_CHLRE/42-236	A8IBM5.1 PF05721.11	65.00	0.00
TRINITY_D8UBE1_VOLCA/26-435	D8UBE1.1 PF00232.16	65.00	0.00
TRINITY_D3BG33_POLPA/4-85	D3BG33.1 PF00887.17	65.00	0.00
TRINITY_A0A0M3JTP0_ANISI/349-388	A0A0M3JTP0.1 PF0592	65.00	0.00
TRINITY_A0A0M3JTP0_ANISI/349-388	A0A0M3JTP0.1 PF0592	65.00	0.00
TRINITY_V4T5T4_9ROSI/257-296	V4T5T4.1 PF05920.9;	65.00	0.00
TRINITY_A8J3L0_CHLRE/13-395	A8J3L0.1 PF07690.14	65.00	0.00
TRINITY_B0TB11_HELMI/616-802	B0TB11.1 PF00488.19	65.00	0.00
TRINITY_A9SIZ2_PHYPA/68-108	A9SIZ2.1 PF01096.16	65.00	0.00
TRINITY_A0BJU5_PARTE/1-81	A0BJU5.1 PF12861.5;	64.90	0.00
TRINITY_K5XTP2_AGABU/3-50	K5XTP2.1 PF09791.7;	64.90	0.00
TRINITY_A0A078B4S2_STYLE/39-101	A0A078B4S2.1 PF0232	64.90	0.00
TRINITY_G0QX38_ICHMG/832-928	G0QX38.1 PF08454.9;	64.90	0.00
TRINITY_I1BX44_RHIO9/11-67	I1BX44.1 PF00412.20	64.90	0.00
TRINITY_G0QK53_ICHMG/367-497	G0QK53.1 PF00004.27	64.90	0.00
TRINITY_L8GRH2_ACACA/50-526	L8GRH2.1 PF01039.20	64.90	0.00
TRINITY_J9FK96_9SPIT/795-1052	J9FK96.1 PF00069.23	64.90	0.00
TRINITY_B6KHB5_TOXGO/12-50	B6KHB5.1 PF13917.4;	64.90	0.00
TRINITY_W7TVR0_9STRA/422-494	W7TVR0.1 PF04564.13	64.90	0.00
TRINITY_G3Q173_GASAC/268-353	G3Q173.1 PF12894.5;	64.90	0.00
TRINITY_L8GWB2_ACACA/300-470	L8GWB2.1 PF12430.6;	64.90	0.00
TRINITY_C3XQS7_BRAFL/192-491	C3XQS7.1 PF00557.22	64.90	0.00
TRINITY_Q23D68_TETTS/517-710	Q23D68.2 PF05057.12	64.90	0.00
TRINITY_Q23MP4_TETTS/227-406	Q23MP4.2 PF00534.18	64.90	0.00
TRINITY_D8U7B6_VOLCA/44-255	D8U7B6.1 PF13419.4;	64.90	0.00
TRINITY_F4PH56_DICFS/34-638	F4PH56.1 PF00888.20	64.90	0.00
TRINITY_A0A0D2UHJ2_CAPO3/1461-1609	A0A0D2UHJ2.1 PF0000	64.90	0.00
TRINITY_G0QPK0_ICHMG/7-249	G0QPK0.1 PF00121.16	64.90	0.00
TRINITY_A8IB95_CHLRE/25-150	A8IB95.1 PF08240.10	64.90	0.00
TRINITY_A0A0D2V4C6_GOSRA/379-806	A0A0D2V4C6.1 PF0096	64.90	0.00
TRINITY_U3J7M9_ANAPL/142-256	U3J7M9.1 PF11969.6;	64.90	0.00
TRINITY_A0A078AAI9_STYLE/21-285	A0A078AAI9.1 PF0165	64.90	0.00
TRINITY_C4LUE1_ENTHI/13-268	C4LUE1.1 PF00069.23	64.90	0.00
TRINITY_D3AXV1_POLPA/114-372	D3AXV1.1 PF02548.13	64.90	0.00
TRINITY_E1ZIK2_CHLVA/502-660	E1ZIK2.1 PF00005.25	64.90	0.00
TRINITY_A8I4K1_CHLRE/197-369	A8I4K1.1 PF05050.10	64.90	0.00
TRINITY_A0A078BYM5_BRANA/260-333	A0A078BYM5.1 PF0007	64.90	0.00
TRINITY_V4UIP7_9ROSI/302-358	V4UIP7.1 PF03106.13	64.90	0.00
TRINITY_A0A078B2G5_STYLE/98-245	A0A078B2G5.1 PF0303	64.90	0.00
TRINITY_A0A078ARC7_STYLE/92-264	A0A078ARC7.1 PF0092	64.90	0.00
TRINITY_C0W8U7_9ACTO/1-271	C0W8U7.1 PF00365.18	64.90	0.00
TRINITY_F4PPP9_DICFS/27-217	F4PPP9.1 PF01145.23	64.90	0.00
TRINITY_G0QYA3_ICHMG/572-716	G0QYA3.1 PF04053.12	64.90	0.00
TRINITY_J9HNV3_9SPIT/116-153	J9HNV3.1 PF01428.14	64.90	0.00
TRINITY_A0A087SKT9_AUXPR/14-192	A0A087SKT9.1 PF0475	64.90	0.00

TRINITY_A8J9F3_CHLRE/20-663	A8J9F3.1 PF00133.20	64.90	0.00
TRINITY_J9J1F3_9SPIT/102-627	J9J1F3.1 PF01411.17	64.90	0.00
TRINITY_F4Q586_DICFS/676-789	F4Q586.1 PF13271.4;	64.90	0.00
TRINITY_D8U6S6_VOLCA/68-306	D8U6S6.1 PF00233.17	64.90	0.00
TRINITY_A8J637_CHLRE/761-993	A8J637.1 PF00889.17	64.90	0.00
TRINITY_A8J3P1_CHLRE/954-1161	A8J3P1.1 PF00211.18	64.90	0.00
TRINITY_Q9LLJ1_CHLRE/37-302	Q9LLJ1.1 PF00069.23	64.90	0.00
TRINITY_A0A0N5DQK4_TRIMR/409-552	A0A0N5DQK4.1 PF0000	64.90	0.00
TRINITY_Q23KJ6_TETTS/622-886	Q23KJ6.2 PF00069.23	64.80	0.00
TRINITY_A0A0N1J0J1_9BACT/4-124	A0A0N1J0J1.1 PF0090	64.80	0.00
TRINITY_Q23A33_TETTS/2-240	Q23A33.1 PF00590.18	64.80	0.00
TRINITY_G0QWD0_ICHMG/622-801	G0QWD0.1 PF08282.10	64.80	0.00
TRINITY_L8HD92_ACACA/1814-2187	L8HD92.1 PF02889.14	64.80	0.00
TRINITY_G0QKV0_ICHMG/1158-1563	G0QKV0.1 PF08393.11	64.80	0.00
TRINITY_A0A087SSH8_AUXPR/237-346	A0A087SSH8.1 PF0027	64.80	0.00
TRINITY_A8J2F0_CHLRE/19-89	A8J2F0.1 PF00076.20	64.80	0.00
TRINITY_A8J3A8_CHLRE/15-85	A8J3A8.1 PF11976.6;	64.80	0.00
TRINITY_I0HZS9_CALAS/5-265	I0HZS9.1 PF00682.17	64.80	0.00
TRINITY_L1IFX3_GUITH/4-62	L1IFX3.1 PF06624.10	64.80	0.00
TRINITY_A0CWR1_PARTE/401-628	A0CWR1.1 PF00198.21	64.80	0.00
TRINITY_CCR1_MAIZE/36-291	P0C8M8.1 PF00069.23	64.80	0.00
TRINITY_D8UAM1_VOLCA/99-303	D8UAM1.1 PF00856.26	64.80	0.00
TRINITY_A0A0J8FQX1_BETVU/72-133	A0A0J8FQX1.1 PF1392	64.80	0.00
TRINITY_D8TN79_VOLCA/305-478	D8TN79.1 PF00069.23	64.80	0.00
TRINITY_D8TYZ4_VOLCA/83-263	D8TYZ4.1 PF01789.14	64.80	0.00
TRINITY_W7TZE3_9STRA/267-428	W7TZE3.1 PF03031.16	64.80	0.00
TRINITY_I7M9A5_TETTS/443-496	I7M9A5.1 PF13639.4;	64.80	0.00
TRINITY_F0ZQL5_DICPU/271-341	F0ZQL5.1 PF00076.20	64.80	0.00
TRINITY_D8TJ36_VOLCA/107-195	D8TJ36.1 PF12333.6;	64.80	0.00
TRINITY_D8U0Y8_VOLCA/115-261	D8U0Y8.1 PF00069.23	64.80	0.00
TRINITY_A8IHL6_CHLRE/3-133	A8IHL6.1 PF08332.8;	64.80	0.00
TRINITY_A8JDN4_CHLRE/48-138	A8JDN4.1 PF01649.16	64.80	0.00
TRINITY_A9SKW3_PHYPA/217-440	A9SKW3.1 PF00667.18	64.80	0.00
TRINITY_W4Z475_STRPU/2-390	W4Z475.1 PF02463.17	64.80	0.00
TRINITY_D3B3G3_POLPA/31-240	D3B3G3.1 PF08442.8;	64.80	0.00
TRINITY_D8TP55_VOLCA/67-324	D8TP55.1 PF00233.17	64.80	0.00
TRINITY_L8GDT9_ACACA/329-399	L8GDT9.1 PF00076.20	64.80	0.00
TRINITY_L8GVK4_ACACA/31-192	L8GVK4.1 PF00071.20	64.80	0.00
TRINITY_D4Z5U1_SPHJU/54-215	D4Z5U1.1 PF03167.17	64.80	0.00
TRINITY_A8ICZ0_CHLRE/1-181	A8ICZ0.1 PF07714.15	64.80	0.00
TRINITY_B9RF67_RICCO/181-854	B9RF67.1 PF00305.17	64.80	0.00
TRINITY_A8JEP4_CHLRE/267-445	A8JEP4.1 PF00174.17	64.80	0.00
TRINITY_D8UG75_VOLCA/130-306	D8UG75.1 PF00005.25	64.80	0.00
TRINITY_I7M3Y4_TETTS/103-224	I7M3Y4.1 PF09335.9;	64.80	0.00
TRINITY_I7MLH2_TETTS/45-262	I7MLH2.2 PF01370.19	64.70	0.00
TRINITY_K1QRR1_CRAGI/6-78	K1QRR1.1 PF13499.4;	64.70	0.00
TRINITY_Q22UZ5_TETTS/25-175	Q22UZ5.1 PF02878.14	64.70	0.00
TRINITY_L8H577_ACACA/11-86	L8H577.1 PF01280.18	64.70	0.00
TRINITY_D8U4C0_VOLCA/255-304	D8U4C0.1 PF00415.16	64.70	0.00
TRINITY_Q23PV4_TETTS/135-245	Q23PV4.2 PF02770.17	64.70	0.00
TRINITY_D8UFN8_VOLCA/399-518	D8UFN8.1 PF04677.13	64.70	0.00
TRINITY_I1FHQ1_AMPQE/555-690	I1FHQ1.1 PF00005.25	64.70	0.00
TRINITY_Q22TC3_TETTS/11-164	Q22TC3.1 PF05833.9;	64.70	0.00
TRINITY_D8TL16_VOLCA/14-549	D8TL16.1 PF01602.18	64.70	0.00
TRINITY_D8TVA7_VOLCA/191-460	D8TVA7.1 PF04095.14	64.70	0.00
TRINITY_D8TPF9_VOLCA/18-597	D8TPF9.1 PF01602.18	64.70	0.00
TRINITY_A8JG53_CHLRE/62-529	A8JG53.1 PF00067.20	64.70	0.00
TRINITY_A8JE75_CHLRE/448-533	A8JE75.1 PF00083.22	64.70	0.00
TRINITY_S2KFH5_MUCC1/273-308	S2KFH5.1 PF00320.25	64.70	0.00
TRINITY_I7M1P1_TETTS/6-124	I7M1P1.1 PF14931.4;	64.70	0.00
TRINITY_A8I5X7_CHLRE/77-195	A8I5X7.1 PF00153.25	64.70	0.00

TRINITY_A2FFU6_TRIVA/124-180	A2FFU6.1 PF04193.12	64.70	0.00
TRINITY_A8J7C2_CHLRE/241-448	A8J7C2.1 PF00962.20	64.70	0.00
TRINITY_A8IDY0_CHLRE/16-346	A8IDY0.1 PF01992.14	64.70	0.00
TRINITY_I0YJA4_9CHLO/37-324	I0YJA4.1 PF04142.13	64.70	0.00
TRINITY_I0YVN9_9CHLO/162-510	I0YVN9.1 PF07992.12	64.70	0.00
TRINITY_CORO_SCHPO/345-387	O13923.1 PF16300.3;	64.70	0.00
TRINITY_G0QY17_ICHMG/18-250	G0QY17.1 PF01144.21	64.70	0.00
TRINITY_A0A087SDA1_AUXPR/398-670	A0A087SDA1.1 PF0006	64.70	0.00
TRINITY_A0A061EEG7_THECC/589-749	A0A061EEG7.1 PF0007	64.70	0.00
TRINITY_D8TSY5_VOLCA/2523-3232	D8TSY5.1 PF03028.13	64.70	0.00
TRINITY_A0EB84_PARTE/306-460	A0EB84.1 PF00609.17	64.70	0.00
TRINITY_I7MMI9_TETTS/391-540	I7MMI9.2 PF00005.25	64.70	0.00
TRINITY_D8SNX9_SELML/224-529	D8SNX9.1 PF00749.19	64.70	0.00
TRINITY_A8J3K3_CHLRE/55-544	A8J3K3.1 PF01593.22	64.70	0.00
TRINITY_S3DNG1_GLAL2/173-275	S3DNG1.1 PF01363.19	64.70	0.00
TRINITY_C3Z2P3_BRAFL/305-514	C3Z2P3.1 PF00644.18	64.70	0.00
TRINITY_A7SDH9_NEMVE/209-352	A7SDH9.1 PF00388.17	64.70	0.00
TRINITY_D5BV10_NITHN/89-190	D5BV10.1 PF02777.16	64.70	0.00
TRINITY_A8IGT6_CHLRE/67-356	A8IGT6.1 PF04258.11	64.70	0.00
TRINITY_F0Y476_AURAN/8-251	F0Y476.1 PF00348.15	64.70	0.00
TRINITY_D8U2L0_VOLCA/468-621	D8U2L0.1 PF01699.22	64.70	0.00
TRINITY_A8HPK7_CHLRE/79-270	A8HPK7.1 PF01300.16	64.70	0.00
TRINITY_A0A061EEG7_THECC/589-749	A0A061EEG7.1 PF0007	64.70	0.00
TRINITY_D8TYC0_VOLCA/26-210	D8TYC0.1 PF01184.17	64.70	0.00
TRINITY_T1JZP5_TETUR/104-171	T1JZP5.1 PF03847.11	64.70	0.00
TRINITY_D8UFL5_VOLCA/67-164	D8UFL5.1 PF01521.18	64.70	0.00
TRINITY_A8I0B3_CHLRE/1-207	A8I0B3.1 PF00481.19	64.70	0.00
TRINITY_U9UC80_RHIID/1-89	U9UC80.1 PF00125.22	64.70	0.00
TRINITY_M5VQ69_PRUPE/1-98	M5VQ69.1 PF03242.11	64.60	0.00
TRINITY_D8RUT0_SELML/117-215	D8RUT0.1 PF00153.25	64.60	0.00
TRINITY_Q22FZ3_TETTS/111-209	Q22FZ3.1 PF00153.25	64.60	0.00
TRINITY_Q22DP0_TETTS/27-164	Q22DP0.2 PF00675.18	64.60	0.00
TRINITY_D8TW91_VOLCA/13-143	D8TW91.1 PF13673.5;	64.60	0.00
TRINITY_M2XTQ8_GALSU/187-325	M2XTQ8.1 PF13365.4;	64.60	0.00
TRINITY_A8JHA9_CHLRE/39-103	A8JHA9.1 PF00462.22	64.60	0.00
TRINITY_A0A067QAD5_9HOMO/41-256	A0A067QAD5.1 PF0035	64.60	0.00
TRINITY_I7MAF1_TETTS/67-145	I7MAF1.1 PF05046.12	64.60	0.00
TRINITY_D8THX6_VOLCA/7-109	D8THX6.1 PF02271.14	64.60	0.00
TRINITY_A0A0F5YF43_9CYAN/223-601	A0A0F5YF43.1 PF0072	64.60	0.00
TRINITY_D2W338_NAEGR/12-250	D2W338.1 PF08713.9;	64.60	0.00
TRINITY_ASNA2_PARTE/14-318	A0E7A5.1 PF02374.13	64.60	0.00
TRINITY_A0A0G4ER15_9ALVE/428-778	A0A0G4ER15.1 PF0015	64.60	0.00
TRINITY_Q23QZ5_TETTS/6-263	Q23QZ5.2 PF00069.23	64.60	0.00
TRINITY_W4XFX6_STRPU/58-308	W4XFX6.1 PF10609.7;	64.60	0.00
TRINITY_A3VAW8_9RHOB/509-588	A3VAW8.1 PF13193.4;	64.60	0.00
TRINITY_D8UHV8_VOLCA/129-323	D8UHV8.1 PF07970.10	64.60	0.00
TRINITY_D8RM97_SELML/70-667	D8RM97.1 PF00888.20	64.60	0.00
TRINITY_L8GIJ1_ACACA/425-561	L8GIJ1.1 PF00005.25	64.60	0.00
TRINITY_J9JBC1_9SPIT/37-248	J9JBC1.1 PF00722.19	64.60	0.00
TRINITY_L8GIA8_ACACA/26-458	L8GIA8.1 PF00450.20	64.60	0.00
TRINITY_D8G576_9CYAN/39-86	D8G576.1 PF04839.11	64.60	0.00
TRINITY_I0YRT6_9CHLO/78-159	I0YRT6.1 PF00575.21	64.60	0.00
TRINITY_D8U625_VOLCA/163-241	D8U625.1 PF11998.6;	64.60	0.00
TRINITY_D8U746_VOLCA/113-207	D8U746.1 PF16835.3;	64.60	0.00
TRINITY_A0DNX8_PARTE/1-308	A0DNX8.1 PF00443.27	64.60	0.00
TRINITY_D8U4G0_VOLCA/558-765	D8U4G0.1 PF00623.18	64.60	0.00
TRINITY_A0A0D9VCE0_9ORYZ/14-321	A0A0D9VCE0.1 PF0000	64.60	0.00
TRINITY_I4C074_DESTA/30-442	I4C074.1 PF00501.26	64.60	0.00
TRINITY_A0A0G4F7G8_9ALVE/22-121	A0A0G4F7G8.1 PF1289	64.60	0.00
TRINITY_J9G5M8_9SPIT/464-784	J9G5M8.1 PF13393.4;	64.60	0.00
TRINITY_D8TVC0_VOLCA/1983-2096	D8TVC0.1 PF00665.24	64.60	0.00

TRINITY_R1BWN0_EMIHU/31-209	R1BWN0.1 PF00955.19	64.60	0.00
TRINITY_A0A078C9S1_BRANA/4-98	A0A078C9S1.1 PF1420	64.50	0.00
TRINITY_J9ENV1_9SPIT/415-758	J9ENV1.1 PF00493.21	64.50	0.00
TRINITY_Q240Q4_TETTS/341-560	Q240Q4.3 PF00557.22	64.50	0.00
TRINITY_F4PX31_DICFS/215-337	F4PX31.1 PF02984.17	64.50	0.00
TRINITY_Q23YB5_TETTS/199-456	Q23YB5.2 PF00069.23	64.50	0.00
TRINITY_I7MHS8_TETTS/213-274	I7MHS8.2 PF12906.5;	64.50	0.00
TRINITY_M2XX77_GALSU/665-775	M2XX77.1 PF00271.29	64.50	0.00
TRINITY_D8TL55_VOLCA/18-128	D8TL55.1 PF00173.26	64.50	0.00
TRINITY_I7LWN1_TETTS/4-96	I7LWN1.1 PF05670.11	64.50	0.00
TRINITY_S8E0Y0_9LAMI/146-269	S8E0Y0.1 PF01553.19	64.50	0.00
TRINITY_L8H5N4_ACACA/10-130	L8H5N4.1 PF13238.4;	64.50	0.00
TRINITY_D8T9A7_SELML/78-232	D8T9A7.1 PF00504.19	64.50	0.00
TRINITY_D8TS14_VOLCA/10-152	D8TS14.1 PF01625.19	64.50	0.00
TRINITY_D8UJQ9_VOLCA/63-496	D8UJQ9.1 PF00854.19	64.50	0.00
TRINITY_D8UD76_VOLCA/695-824	D8UD76.1 PF06398.9;	64.50	0.00
TRINITY_A0E1Y7_PARTE/446-512	A0E1Y7.1 PF00659.16	64.50	0.00
TRINITY_A0CS16_PARTE/344-450	A0CS16.1 PF02847.15	64.50	0.00
TRINITY_D8TL36_VOLCA/61-378	D8TL36.1 PF16363.3;	64.50	0.00
TRINITY_A4VF59_TETTS/56-131	A4VF59.1 PF01066.19	64.50	0.00
TRINITY_C1EIK2_MICSR/1-193	C1EIK2.1 PF00069.23	64.50	0.00
TRINITY_J4CD27_THEOR/10-74	J4CD27.1 PF13499.4;	64.50	0.00
TRINITY_A8JGC9_CHLRE/17-78	A8JGC9.1 PF00226.29	64.50	0.00
TRINITY_A8I5X1_CHLRE/126-374	A8I5X1.1 PF01896.17	64.50	0.00
TRINITY_E1ZND9_CHLVA/929-1188	E1ZND9.1 PF00069.23	64.50	0.00
TRINITY_A0A0G1W8L6_9BACT/210-391	A0A0G1W8L6.1 PF0053	64.50	0.00
TRINITY_T0PW96_9STRA/35-391	T0PW96.1 PF09334.9;	64.50	0.00
TRINITY_A8IKM0_CHLRE/45-119	A8IKM0.1 PF02656.13	64.50	0.00
TRINITY_L8GYB8_ACACA/255-364	L8GYB8.1 PF08022.10	64.50	0.00
TRINITY_A8IZ48_CHLRE/45-159	A8IZ48.1 PF00293.26	64.50	0.00
TRINITY_C7PLA0_CHIPD/8-216	C7PLA0.1 PF00753.25	64.50	0.00
TRINITY_Q66YD1_CHLRE/72-133	Q66YD1.1 PF00226.29	64.50	0.00
TRINITY_A8I3P4_CHLRE/1-384	A8I3P4.1 PF13520.4;	64.50	0.00
TRINITY_D8U719_VOLCA/28-288	D8U719.1 PF12697.5;	64.50	0.00
TRINITY_A8HYR6_CHLRE/286-348	A8HYR6.1 PF00013.27	64.50	0.00
TRINITY_I0Z8B5_9CHLO/174-487	I0Z8B5.1 PF00282.17	64.50	0.00
TRINITY_E1Z5Q9_CHLVA/247-324	E1Z5Q9.1 PF05684.10	64.50	0.00
TRINITY_D8TIT7_VOLCA/772-837	D8TIT7.1 PF13432.4;	64.50	0.00
TRINITY_A8HMG4_CHLRE/48-108	A8HMG4.1 PF13691.4;	64.50	0.00
TRINITY_J2IF11_9BACL/46-290	J2IF11.1 PF02274.15	64.50	0.00
TRINITY_A0A087H1X4_ARAAL/15-137	A0A087H1X4.1 PF0017	64.40	0.00
TRINITY_J9HU22_9SPIT/4-283	J9HU22.1 PF00069.23	64.40	0.00
TRINITY_A0A0G4H4N9_9ALVE/11-150	A0A0G4H4N9.1 PF1458	64.40	0.00
TRINITY_A0A0G4GQ90_9ALVE/50-668	A0A0G4GQ90.1 PF0044	64.40	0.00
TRINITY_L8GUS5_ACACA/34-120	L8GUS5.1 PF04110.11	64.40	0.00
TRINITY_W7XEI3_TETTS/144-320	W7XEI3.1 PF04427.16	64.40	0.00
TRINITY_A0A0C4EL35_PUCT1/283-363	A0A0C4EL35.1 PF0049	64.40	0.00
TRINITY_Q22XT4_TETTS/475-635	Q22XT4.1 PF08662.9;	64.40	0.00
TRINITY_A0CYG1_PARTE/160-260	A0CYG1.1 PF13864.4;	64.40	0.00
TRINITY_A0A067CLG0_SAPPC/220-264	A0A067CLG0.1 PF0040	64.40	0.00
TRINITY_Q247Z0_TETTS/1359-1503	Q247Z0.3 PF00005.25	64.40	0.00
TRINITY_A0DI20_PARTE/19-543	A0DI20.1 PF01602.18	64.40	0.00
TRINITY_J9J7C7_9SPIT/46-148	J9J7C7.1 PF08241.10	64.40	0.00
TRINITY_E8QYX7_ISOPI/16-175	E8QYX7.1 PF03446.13	64.40	0.00
TRINITY_G0R1V5_ICHMG/624-842	G0R1V5.1 PF12781.5;	64.40	0.00
TRINITY_E9HWN6_DAPPU/1040-1188	E9HWN6.1 PF00005.25	64.40	0.00
TRINITY_A0A059LH28_9CHLO/1-116	A0A059LH28.1 PF0073	64.40	0.00
TRINITY_D8TT44_VOLCA/311-511	D8TT44.1 PF01425.19	64.40	0.00
TRINITY_I1FVG2_AMPQE/134-296	I1FVG2.1 PF00317.19	64.40	0.00
TRINITY_A8IZU0_CHLRE/1-580	A8IZU0.1 PF00012.18	64.40	0.00
TRINITY_D7SIR1_VITVI/13-371	D7SIR1.1 PF04371.13	64.40	0.00

TRINITY_D2VPF0_NAEGR/10-183	D2VPF0.1 PF00071.20	64.40	0.00
TRINITY_A0A087SK44_AUXPR/609-723	A0A087SK44.1 PF0027	64.40	0.00
TRINITY_I0YPX2_9CHLO/757-1117	I0YPX2.1 PF00328.20	64.40	0.00
TRINITY_A0A0M2LPS2_9SPHN/343-477	A0A0M2LPS2.1 PF0000	64.40	0.00
TRINITY_L8H7U8_ACACA/492-702	L8H7U8.1 PF00326.19	64.40	0.00
TRINITY_D8TV14_VOLCA/61-218	D8TV14.1 PF10436.7;	64.40	0.00
TRINITY_E9FRU0_DAPPU/24-130	E9FRU0.1 PF13616.4;	64.40	0.00
TRINITY_A8HMH6_CHLRE/121-284	A8HMH6.1 PF01596.15	64.40	0.00
TRINITY_E1Z9L4_CHLVA/22-155	E1Z9L4.1 PF07231.10	64.40	0.00
TRINITY_W7XHC6_TETTS/183-324	W7XHC6.1 PF07479.12	64.40	0.00
TRINITY_H3AVK4_LATCH/596-1043	H3AVK4.1 PF13086.4;	64.40	0.00
TRINITY_W4Z2W7_STRPU/52-200	W4Z2W7.1 PF00005.25	64.40	0.00
TRINITY_J9I4R1_9SPIT/251-324	J9I4R1.1 PF03451.12	64.40	0.00
TRINITY_I0YXD9_9CHLO/216-510	I0YXD9.1 PF02781.14	64.40	0.00
TRINITY_D8U7B5_VOLCA/451-777	D8U7B5.1 PF00225.21	64.40	0.00
TRINITY_A8IU68_CHLRE/707-855	A8IU68.1 PF08264.11	64.40	0.00
TRINITY_A8J838_CHLRE/2-97	A8J838.1 PF10084.7;	64.40	0.00
TRINITY_L1JER9_GUITH/328-856	L1JER9.1 PF00443.27	64.40	0.00
TRINITY_A0A093XHF8_9PEZI/60-146	A0A093XHF8.1 PF0062	64.40	0.00
TRINITY_D8UJD9_VOLCA/37-534	D8UJD9.1 PF00118.22	64.40	0.00
TRINITY_I7MD55_TETTS/531-788	I7MD55.2 PF00069.23	64.40	0.00
TRINITY_A0A0G4EVB2_9ALVE/139-300	A0A0G4EVB2.1 PF0476	64.40	0.00
TRINITY_A0A0D1DZ70_USTMA/33-109	A0A0D1DZ70.1 PF0125	64.40	0.00
TRINITY_L0G510_ECHVK/25-388	L0G510.1 PF14587.4;	64.40	0.00
TRINITY_A0BNQ0_PARTE/32-276	A0BNQ0.1 PF00069.23	64.30	0.00
TRINITY_L8GXT7_ACACA/1726-1939	L8GXT7.1 PF00454.25	64.30	0.00
TRINITY_A0A087SPW5_AUXPR/32-101	A0A087SPW5.1 PF1524	64.30	0.00
TRINITY_A8J5Y5_CHLRE/211-445	A8J5Y5.1 PF01167.16	64.30	0.00
TRINITY_E2AZT8_CAMFO/5-107	E2AZT8.1 PF00466.18	64.30	0.00
TRINITY_A0C2G3_PARTE/131-256	A0C2G3.1 PF09335.9;	64.30	0.00
TRINITY_F4PTP3_DICFS/137-548	F4PTP3.1 PF00465.17	64.30	0.00
TRINITY_A4VCZ9_TETTS/28-319	A4VCZ9.2 PF01026.19	64.30	0.00
TRINITY_A8IZS5_CHLRE/5-74	A8IZS5.1 PF00076.20	64.30	0.00
TRINITY_A8JDF8_CHLRE/1-213	A8JDF8.1 PF02517.14	64.30	0.00
TRINITY_A0EE63_PARTE/3584-4294	A0EE63.1 PF03028.13	64.30	0.00
TRINITY_W3WQH0_9PEZI/17-317	W3WQH0.1 PF00248.19	64.30	0.00
TRINITY_Q22XT4_TETTS/475-635	Q22XT4.1 PF08662.9;	64.30	0.00
TRINITY_A0CMB4_PARTE/14-269	A0CMB4.1 PF00069.23	64.30	0.00
TRINITY_A8IR22_CHLRE/1-180	A8IR22.1 PF00230.18	64.30	0.00
TRINITY_B4D029_9BACT/5-275	B4D029.1 PF00749.19	64.30	0.00
TRINITY_I7LUG9_TETTS/17-335	I7LUG9.2 PF00225.21	64.30	0.00
TRINITY_K0KSV0_WICCF/346-415	K0KSV0.1 PF00076.20	64.30	0.00
TRINITY_A8HNH0_CHLRE/596-829	A8HNH0.1 PF07717.14	64.30	0.00
TRINITY_D8UJ01_VOLCA/7-76	D8UJ01.1 PF11605.6;	64.30	0.00
TRINITY_F4Q9S8_DICFS/3-60	F4Q9S8.1 PF04758.12	64.30	0.00
TRINITY_D8TU63_VOLCA/154-209	D8TU63.1 PF12646.5;	64.30	0.00
TRINITY_Q238R8_TETTS/848-963	Q238R8.1 PF00787.22	64.30	0.00
TRINITY_T1G6V1_HELRO/297-341	T1G6V1.1 PF01585.21	64.30	0.00
TRINITY_D8ULV7_VOLCA/1-185	D8ULV7.1 PF06966.10	64.30	0.00
TRINITY_C3YCG6_BRAFL/2-105	C3YCG6.1 PF00085.18	64.30	0.00
TRINITY_A0A0D9ZS63_9ORYZ/74-185	A0A0D9ZS63.1 PF0413	64.30	0.00
TRINITY_A8IZN0_CHLRE/131-439	A8IZN0.1 PF03486.12	64.30	0.00
TRINITY_E1ZFF3_CHLVA/46-344	E1ZFF3.1 PF01866.15	64.30	0.00
TRINITY_V4UDH9_9ROSI/86-370	V4UDH9.1 PF00009.25	64.30	0.00
TRINITY_A0A075AR84_9FUNG/64-192	A0A075AR84.1 PF0017	64.30	0.00
TRINITY_A0A077ZUV7_STYLE/56-208	A0A077ZUV7.1 PF1473	64.30	0.00
TRINITY_A0CCB3_PARTE/50-120	A0CCB3.1 PF00076.20	64.30	0.00
TRINITY_A0A0E9NI79_9ASCO/1503-1793	A0A0E9NI79.1 PF0006	64.30	0.00
TRINITY_Q24GF5_TETTS/192-235	Q24GF5.1 PF13639.4;	64.30	0.00
TRINITY_A0A022RCY7_ERYGU/331-611	A0A022RCY7.1 PF0120	64.30	0.00
TRINITY_A8J3T4_CHLRE/72-440	A8J3T4.1 PF01979.18	64.30	0.00

TRINITY_L8H8M4_ACACA/72-337	L8H8M4.1 PF01193.22	64.30	0.00
TRINITY_C4Y7D3_CLAL4/242-402	C4Y7D3.1 PF00078.25	64.30	0.00
TRINITY_A8I268_CHLRE/466-743	A8I268.1 PF00664.21	64.30	0.00
TRINITY_A0A087SFG2_AUXPR/609-698	A0A087SFG2.1 PF0015	64.30	0.00
TRINITY_D8U968_VOLCA/279-518	D8U968.1 PF04884.12	64.30	0.00
TRINITY_I0YJJ0_9CHLO/27-182	I0YJJ0.1 PF02518.24	64.30	0.00
TRINITY_A0BIT3_PARTE/166-286	A0BIT3.1 PF01575.17	64.20	0.00
TRINITY_A0D270_PARTE/367-467	A0D270.1 PF12796.5;	64.20	0.00
TRINITY_D3B1V2_POLPA/5-372	D3B1V2.1 PF00022.17	64.20	0.00
TRINITY_J9FBS9_9SPIT/259-507	J9FBS9.1 PF00009.25	64.20	0.00
TRINITY_C3Z578_BRAFL/7-211	C3Z578.1 PF03575.15	64.20	0.00
TRINITY_L8GX50_ACACA/28-221	L8GX50.1 PF00227.24	64.20	0.00
TRINITY_F4QBM2_DICFS/48-337	F4QBM2.1 PF01008.15	64.20	0.00
TRINITY_I7MG57_TETTS/680-735	I7MG57.2 PF17120.3;	64.20	0.00
TRINITY_C3Z9A3_BRAFL/297-569	C3Z9A3.1 PF02138.16	64.20	0.00
TRINITY_L8GDZ9_ACACA/65-381	L8GDZ9.1 PF00749.19	64.20	0.00
TRINITY_A0A078ABS3_STYLE/96-356	A0A078ABS3.1 PF0006	64.20	0.00
TRINITY_L8H6C4_ACACA/201-373	L8H6C4.1 PF00174.17	64.20	0.00
TRINITY_D8TVI1_VOLCA/43-160	D8TVI1.1 PF06417.10	64.20	0.00
TRINITY_A8I3V3_CHLRE/47-202	A8I3V3.1 PF14306.4;	64.20	0.00
TRINITY_D8TUZ2_VOLCA/101-232	D8TUZ2.1 PF04366.10	64.20	0.00
TRINITY_G0QLJ9_ICHMG/51-234	G0QLJ9.1 PF05063.12	64.20	0.00
TRINITY_D8TK70_VOLCA/76-461	D8TK70.1 PF00344.18	64.20	0.00
TRINITY_D3PD64_DEFDS/53-528	D3PD64.1 PF00171.20	64.20	0.00
TRINITY_T0RQL2_9STRA/132-184	T0RQL2.1 PF13964.4;	64.20	0.00
TRINITY_A0A096SHJ9_MAIZE/112-192	A0A096SHJ9.1 PF0035	64.20	0.00
TRINITY_D8TIB1_VOLCA/100-431	D8TIB1.1 PF07887.9;	64.20	0.00
TRINITY_F4PVZ6_DICFS/12-183	F4PVZ6.1 PF00071.20	64.20	0.00
TRINITY_Q24FC7_TETTS/25-161	Q24FC7.2 PF00782.18	64.20	0.00
TRINITY_PAP_DICDI/54-396	Q54J73.1 PF04928.15	64.20	0.00
TRINITY_I7LSV4_TETTS/3534-4237	I7LSV4.2 PF03028.13	64.20	0.00
TRINITY_A0A087SAS6_AUXPR/214-289	A0A087SAS6.1 PF0620	64.20	0.00
TRINITY_D8U5J5_VOLCA/53-475	D8U5J5.1 PF00654.18	64.20	0.00
TRINITY_A8IZ05_CHLRE/23-214	A8IZ05.1 PF08856.9;	64.20	0.00
TRINITY_A1ZSD1_9BACT/38-190	A1ZSD1.1 PF00484.17	64.20	0.00
TRINITY_A8JA31_CHLRE/67-347	A8JA31.1 PF00246.22	64.20	0.00
TRINITY_E1ZM44_CHLVA/121-407	E1ZM44.1 PF05007.11	64.20	0.00
TRINITY_D8UIV1_VOLCA/157-391	D8UIV1.1 PF09325.8;	64.20	0.00
TRINITY_D8UB93_VOLCA/33-525	D8UB93.1 PF00118.22	64.20	0.00
TRINITY_I0REP9_MYCPH/59-501	I0REP9.1 PF00501.26	64.20	0.00
TRINITY_A8J304_CHLRE/23-170	A8J304.1 PF04051.14	64.20	0.00
TRINITY_A8HZX4_CHLRE/9-174	A8HZX4.1 PF03453.15	64.20	0.00
TRINITY_W4XGG4_STRPU/640-750	W4XGG4.1 PF00271.29	64.20	0.00
TRINITY_Q8LPN9_CHLRE/133-237	Q8LPN9.1 PF06426.12	64.20	0.00
TRINITY_A0A078DNT7_BRANA/370-586	A0A078DNT7.1 PF0500	64.20	0.00
TRINITY_A8HM32_CHLRE/167-302	A8HM32.1 PF00107.24	64.20	0.00
TRINITY_Q23KL1_TETTS/74-139	Q23KL1.1 PF15393.4;	64.10	0.00
TRINITY_A0E2W5_PARTE/196-452	A0E2W5.1 PF00069.23	64.10	0.00
TRINITY_G0R500_ICHMG/297-509	G0R500.1 PF00454.25	64.10	0.00
TRINITY_I0YWV4_9CHLO/88-151	I0YWV4.1 PF00076.20	64.10	0.00
TRINITY_J9J9Q5_9SPIT/339-382	J9J9Q5.1 PF00569.15	64.10	0.00
TRINITY_Q23JY6_TETTS/610-893	Q23JY6.2 PF00481.19	64.10	0.00
TRINITY_A8ITW3_CHLRE/1-127	A8ITW3.1 PF03364.18	64.10	0.00
TRINITY_D8TNP4_VOLCA/20-98	D8TNP4.1 PF16131.3;	64.10	0.00
TRINITY_L8HFB8_ACACA/127-166	L8HFB8.1 PF13923.4;	64.10	0.00
TRINITY_H9JBI6_BOMMO/283-544	H9JBI6.1 PF00069.23	64.10	0.00
TRINITY_A8J635_CHLRE/81-251	A8J635.1 PF13301.4;	64.10	0.00
TRINITY_A8J9P0_CHLRE/74-378	A8J9P0.1 PF00248.19	64.10	0.00
TRINITY_G7JHY7_MEDTR/29-69	G7JHY7.1 PF00514.21	64.10	0.00
TRINITY_G7JHY7_MEDTR/29-69	G7JHY7.1 PF00514.21	64.10	0.00
TRINITY_J9JBX5_9SPIT/149-408	J9JBX5.1 PF00069.23	64.10	0.00

TRINITY_A8IYY3_CHLRE/2-287	A8IYY3.1 PF00069.23	64.10	0.00
TRINITY_D3B161_POLPA/45-433	D3B161.1 PF00202.19	64.10	0.00
TRINITY_A8I7J0_CHLRE/17-187	A8I7J0.1 PF03357.19	64.10	0.00
TRINITY_I1CSZ3_RHIO9/260-602	I1CSZ3.1 PF00750.17	64.10	0.00
TRINITY_K1VK41_TRIAC/10-74	K1VK41.1 PF13499.4;	64.10	0.00
TRINITY_D8S3L2_SELML/19-84	D8S3L2.1 PF01918.19	64.10	0.00
TRINITY_Q23R92_TETTS/152-318	Q23R92.1 PF00071.20	64.10	0.00
TRINITY_D8U6W4_VOLCA/9-110	D8U6W4.1 PF12624.5;	64.10	0.00
TRINITY_A9RZL7_PHYPA/11-637	A9RZL7.1 PF03169.13	64.10	0.00
TRINITY_A0A024TID3_9STRA/5-178	A0A024TID3.1 PF0007	64.10	0.00
TRINITY_D8TQN2_VOLCA/1042-1224	D8TQN2.1 PF02581.15	64.10	0.00
TRINITY_A8I960_CHLRE/24-194	A8I960.1 PF00270.27	64.10	0.00
TRINITY_A8HXA6_CHLRE/5-349	A8HXA6.1 PF07992.12	64.10	0.00
TRINITY_A0A075B4E8_9FUNG/35-330	A0A075B4E8.1 PF0029	64.10	0.00
TRINITY_A8J7F2_CHLRE/937-1258	A8J7F2.1 PF00632.23	64.10	0.00
TRINITY_D8UIV0_VOLCA/408-525	D8UIV0.1 PF02887.14	64.10	0.00
TRINITY_A0A0C2XP57_BACBA/17-186	A0A0C2XP57.1 PF0088	64.10	0.00
TRINITY_D8TN49_VOLCA/90-232	D8TN49.1 PF04116.11	64.10	0.00
TRINITY_A8JGJ4_CHLRE/324-589	A8JGJ4.1 PF00069.23	64.10	0.00
TRINITY_A0DZ75_PARTE/8-105	A0DZ75.1 PF00153.25	64.10	0.00
TRINITY_B3MHS2_DROAN/42-244	B3MHS2.2 PF00106.23	64.10	0.00
TRINITY_A0CNX9_PARTE/179-246	A0CNX9.1 PF13833.4;	64.00	0.00
TRINITY_Q23BV8_TETTS/160-349	Q23BV8.1 PF13489.4;	64.00	0.00
TRINITY_W2RHI5_PHYPN/46-548	W2RHI5.1 PF00342.17	64.00	0.00
TRINITY_A0A074XB46_AURPU/51-136	A0A074XB46.1 PF0020	64.00	0.00
TRINITY_A0A024TNL1_9STRA/29-108	A0A024TNL1.1 PF1377	64.00	0.00
TRINITY_Q22W55_TETTS/12-233	Q22W55.2 PF00244.18	64.00	0.00
TRINITY_A0CVD0_PARTE/83-383	A0CVD0.1 PF03133.13	64.00	0.00
TRINITY_J9HU22_9SPIT/4-283	J9HU22.1 PF00069.23	64.00	0.00
TRINITY_D8UE16_VOLCA/2-127	D8UE16.1 PF03244.12	64.00	0.00
TRINITY_A0A087YCQ2_POEFO/12-263	A0A087YCQ2.2 PF0079	64.00	0.00
TRINITY_A2AU08_MOUSE/14-76	A2AU08.1 PF04851.13	64.00	0.00
TRINITY_A9RGF0_PHYPA/71-122	A9RGF0.1 PF00847.18	64.00	0.00
TRINITY_I7LX18_TETTS/11-362	I7LX18.1 PF00285.19	64.00	0.00
TRINITY_D8U1I5_VOLCA/8-145	D8U1I5.1 PF01090.17	64.00	0.00
TRINITY_A0DPK2_PARTE/224-334	A0DPK2.1 PF16131.3;	64.00	0.00
TRINITY_A8I7F2_CHLRE/54-181	A8I7F2.1 PF11317.6;	64.00	0.00
TRINITY_G0J100_CYCMS/29-323	G0J100.1 PF00294.22	64.00	0.00
TRINITY_D8UEP5_VOLCA/160-219	D8UEP5.1 PF04900.10	64.00	0.00
TRINITY_A8HYJ1_CHLRE/93-253	A8HYJ1.1 PF04548.14	64.00	0.00
TRINITY_D8TMW1_VOLCA/578-754	D8TMW1.1 PF04072.12	64.00	0.00
TRINITY_D8UCG1_VOLCA/148-249	D8UCG1.1 PF02204.16	64.00	0.00
TRINITY_D8UD64_VOLCA/3-606	D8UD64.1 PF00443.27	64.00	0.00
TRINITY_A0A059D108_EUCGR/1001-1077	A0A059D108.1 PF0549	64.00	0.00
TRINITY_W4Y3K6_STRPU/371-574	W4Y3K6.1 PF02190.14	64.00	0.00
TRINITY_D8UHR4_VOLCA/55-338	D8UHR4.1 PF02089.13	64.00	0.00
TRINITY_A8HQJ4_CHLRE/170-255	A8HQJ4.1 PF03439.11	64.00	0.00
TRINITY_L8H2W6_ACACA/144-363	L8H2W6.1 PF07970.10	64.00	0.00
TRINITY_A8JG42_CHLRE/1-100	A8JG42.1 PF04134.10	64.00	0.00
TRINITY_D8TPT6_VOLCA/374-448	D8TPT6.1 PF03110.12	64.00	0.00
TRINITY_D8TPT6_VOLCA/374-448	D8TPT6.1 PF03110.12	64.00	0.00
TRINITY_D8TQW6_VOLCA/21-192	D8TQW6.1 PF06925.9;	64.00	0.00
TRINITY_A0A067F6F8_CITSI/271-345	A0A067F6F8.1 PF0040	64.00	0.00
TRINITY_I7M858_TETTS/25-110	I7M858.1 PF00439.23	64.00	0.00
TRINITY_G0QX38_ICHMG/832-928	G0QX38.1 PF08454.9;	64.00	0.00
TRINITY_A8JFS8_CHLRE/138-226	A8JFS8.1 PF00867.16	64.00	0.00
TRINITY_I0YNH7_9CHLO/13-109	I0YNH7.1 PF00153.25	64.00	0.00
TRINITY_A8JAX0_CHLRE/230-448	A8JAX0.1 PF00233.17	64.00	0.00
TRINITY_A0A067QQ98_ZOONE/85-133	A0A067QQ98.1 PF0040	64.00	0.00
TRINITY_W7X4T7_TETTS/1-455	W7X4T7.1 PF00171.20	63.90	0.00
TRINITY_A0A068RPI6_9FUNG/147-230	A0A068RPI6.1 PF0122	63.90	0.00

TRINITY_A0A0M0K115_9EUKA/22-129	A0A0M0K115.1 PF0177	63.90	0.00
TRINITY_E3FDX6_STIAD/55-174	E3FDX6.1 PF01641.16	63.90	0.00
TRINITY_A8HWD0_CHLRE/106-251	A8HWD0.1 PF00535.24	63.90	0.00
TRINITY_Q22LN3_TETTS/139-201	Q22LN3.1 PF00957.19	63.90	0.00
TRINITY_Q2WAP0_MAGSA/27-227	Q2WAP0.1 PF00596.19	63.90	0.00
TRINITY_A7S548_NEMVE/26-81	A7S548.1 PF00412.20	63.90	0.00
TRINITY_G0QU69_ICHMG/12-144	G0QU69.1 PF05222.13	63.90	0.00
TRINITY_A0D657_PARTE/575-719	A0D657.1 PF00005.25	63.90	0.00
TRINITY_A0A098GFW5_TATMI/92-465	A0A098GFW5.1 PF0154	63.90	0.00
TRINITY_A8HWB1_CHLRE/6-66	A8HWB1.1 PF09493.8;	63.90	0.00
TRINITY_I7LX13_TETTS/4-205	I7LX13.1 PF01088.19	63.90	0.00
TRINITY_D7TBY5_VITVI/191-298	D7TBY5.1 PF00175.19	63.90	0.00
TRINITY_C1FIQ0_MICSR/78-400	C1FIQ0.1 PF00152.18	63.90	0.00
TRINITY_D3B236_POLPA/93-355	D3B236.1 PF00154.19	63.90	0.00
TRINITY_Q244Y1_TETTS/6-149	Q244Y1.1 PF05517.10	63.90	0.00
TRINITY_A8JBQ5_CHLRE/72-323	A8JBQ5.1 PF01259.16	63.90	0.00
TRINITY_Q24C80_TETTS/314-509	Q24C80.2 PF00122.18	63.90	0.00
TRINITY_D8TH82_VOLCA/1-111	D8TH82.1 PF01477.21	63.90	0.00
TRINITY_M4BFE1_HYAAE/23-104	M4BFE1.1 PF00081.20	63.90	0.00
TRINITY_A8J2P9_CHLRE/180-424	A8J2P9.1 PF13087.4;	63.90	0.00
TRINITY_G0QX42_ICHMG/1-451	G0QX42.1 PF00996.16	63.90	0.00
TRINITY_A0CWY4_PARTE/48-300	A0CWY4.1 PF00069.23	63.90	0.00
TRINITY_D8TJL4_VOLCA/30-90	D8TJL4.1 PF13499.4;	63.90	0.00
TRINITY_A8IF29_CHLRE/93-176	A8IF29.1 PF00439.23	63.90	0.00

TRINITY_E1ZJ01_CHLVA/214-327	E1ZJ01.1 PF00271.29	63.90	0.00
TRINITY_A0A0G0NFV6_9BACT/197-257	A0A0G0NFV6.1 PF1319	63.90	0.00
TRINITY_A8IDN7_CHLRE/1-167	A8IDN7.1 PF03371.13	63.90	0.00
TRINITY_D8U986_VOLCA/307-378	D8U986.1 PF00575.21	63.90	0.00
TRINITY_A8HW46_CHLRE/34-172	A8HW46.1 PF00388.17	63.90	0.00
TRINITY_X6N4P2_RETFI/201-618	X6N4P2.1 PF00962.20	63.90	0.00
TRINITY_A8IRI7_CHLRE/381-479	A8IRI7.1 PF08544.11	63.90	0.00
TRINITY_A8JGJ7_CHLRE/158-219	A8JGJ7.1 PF00415.16	63.90	0.00
TRINITY_D8TX00_VOLCA/7-257	D8TX00.1 PF00483.21	63.90	0.00
TRINITY_F4Q412_DICFS/123-377	F4Q412.1 PF00069.23	63.90	0.00
TRINITY_F4PYF0_DICFS/912-1172	F4PYF0.1 PF00122.18	63.90	0.00
TRINITY_G0QN11_ICHMG/255-400	G0QN11.1 PF00441.22	63.90	0.00
TRINITY_E1ZH11_CHLVA/9-91	E1ZH11.1 PF08640.9;	63.90	0.00
TRINITY_A9STA8_PHYPA/204-241	A9STA8.1 PF00400.30	63.90	0.00
TRINITY_I1GHB5_AMPQE/60-719	I1GHB5.1 PF00063.19	63.80	0.00
TRINITY_I7MLR3_TETTS/9-205	I7MLR3.2 PF00834.17	63.80	0.00
TRINITY_G0R1J7_ICHMG/20-328	G0R1J7.1 PF00557.22	63.80	0.00
TRINITY_L8GVZ4_ACACA/237-292	L8GVZ4.1 PF08635.8;	63.80	0.00
TRINITY_W4H922_9STRA/11-79	W4H922.1 PF01423.20	63.80	0.00
TRINITY_G2PSK8_MURRD/55-200	G2PSK8.1 PF10067.7;	63.80	0.00
TRINITY_Q22MG9_TETTS/410-664	Q22MG9.2 PF00069.23	63.80	0.00
TRINITY_Q54EU1_DICDI/7-167	Q54EU1.1 PF09419.8;	63.80	0.00
TRINITY_C5Z7K5_SORBI/93-212	C5Z7K5.1 PF01641.16	63.80	0.00
TRINITY_Q23MB9_TETTS/114-293	Q23MB9.2 PF02390.15	63.80	0.00
TRINITY_F0ZR16_DICPU/2-153	F0ZR16.1 PF00160.19	63.80	0.00
TRINITY_L8GCF9_ACACA/109-300	L8GCF9.1 PF02750.12	63.80	0.00
TRINITY_K1PZI7_CRAGI/291-611	K1PZI7.1 PF00493.21	63.80	0.00
TRINITY_Q239R2_TETTS/10-57	Q239R2.2 PF00319.16	63.80	0.00
TRINITY_Q23YB5_TETTS/199-456	Q23YB5.2 PF00069.23	63.80	0.00
TRINITY_C3Y0X8_BRAFL/1013-1161	C3Y0X8.1 PF00005.25	63.80	0.00
TRINITY_L1J3Y8_GUITH/9-322	L1J3Y8.1 PF13393.4;	63.80	0.00
TRINITY_Q229U9_TETTS/47-305	Q229U9.2 PF00069.23	63.80	0.00
TRINITY_D8U5A6_VOLCA/371-559	D8U5A6.1 PF01764.23	63.80	0.00
TRINITY_A8HT69_CHLRE/689-903	A8HT69.1 PF07569.9;	63.80	0.00
TRINITY_I0Z539_9CHLO/162-258	I0Z539.1 PF00173.26	63.80	0.00
TRINITY_A0A0M2U5X9_9FIRM/45-442	A0A0M2U5X9.1 PF0020	63.80	0.00
TRINITY_K9ZX98_DEIPD/466-536	K9ZX98.1 PF03725.13	63.80	0.00
TRINITY_E1Z860_CHLVA/90-389	E1Z860.1 PF00632.23	63.80	0.00
TRINITY_D8TZH6_VOLCA/22-283	D8TZH6.1 PF00233.17	63.80	0.00
TRINITY_D8TZH6_VOLCA/22-283	D8TZH6.1 PF00233.17	63.80	0.00
TRINITY_GABT_DICDI/61-489	Q55FI1.1 PF00202.19	63.80	0.00
TRINITY_A8JFF9_CHLRE/17-202	A8JFF9.1 PF01105.22	63.80	0.00
TRINITY_F2SQ31_TRIRC/53-111	F2SQ31.1 PF12678.5;	63.80	0.00
TRINITY_D8UF31_VOLCA/562-668	D8UF31.1 PF01426.16	63.80	0.00
TRINITY_A8HZ94_CHLRE/10-103	A8HZ94.1 PF12796.5;	63.80	0.00
TRINITY_D3BV36_POLPA/91-309	D3BV36.1 PF01591.16	63.80	0.00
TRINITY_I0K5M5_9BACT/33-364	I0K5M5.1 PF00251.18	63.80	0.00
TRINITY_A0A058Z1P5_9EUKA/8-261	A0A058Z1P5.1 PF0006	63.80	0.00
TRINITY_D8TKM3_VOLCA/1072-1235	D8TKM3.1 PF08623.8;	63.80	0.00
TRINITY_D8TMS2_VOLCA/103-407	D8TMS2.1 PF00850.17	63.80	0.00
TRINITY_A8JEM7_CHLRE/230-394	A8JEM7.1 PF03407.14	63.80	0.00
TRINITY_E1ZFR6_CHLVA/491-840	E1ZFR6.1 PF00773.17	63.80	0.00
TRINITY_D8TZZ9_VOLCA/109-201	D8TZZ9.1 PF12796.5;	63.80	0.00
TRINITY_D8U9I3_VOLCA/6-105	D8U9I3.1 PF00168.28	63.80	0.00
TRINITY_E1ZP23_CHLVA/396-454	E1ZP23.1 PF01485.19	63.80	0.00
TRINITY_D8TPC7_VOLCA/39-285	D8TPC7.1 PF00566.16	63.80	0.00
TRINITY_A8IW93_CHLRE/555-749	A8IW93.1 PF00211.18	63.80	0.00
TRINITY_L8HMU1_ACACA/812-1162	L8HMU1.1 PF01576.17	63.80	0.00
TRINITY_A8JFA1_CHLRE/3-102	A8JFA1.1 PF00235.17	63.80	0.00
TRINITY_A8IX73_CHLRE/114-562	A8IX73.1 PF00083.22	63.80	0.00
TRINITY_F4P307_BATDJ/345-413	F4P307.1 PF15247.4;	63.80	0.00

TRINITY_D8TUI3_VOLCA/202-270	D8TUI3.1 PF00498.24	63.80	0.00
TRINITY_A0A067QPP7_ZOONE/268-558	A0A067QPP7.1 PF0066	63.80	0.00
TRINITY_Q24BB2_TETTS/349-579	Q24BB2.2 PF01028.18	63.80	0.00
TRINITY_L1JJ97_GUITH/358-487	L1JJ97.1 PF00005.25	63.80	0.00
TRINITY_A8HMQ9_CHLRE/1-129	A8HMQ9.1 PF05255.9;	63.80	0.00
TRINITY_D8TMU9_VOLCA/6-157	D8TMU9.1 PF09349.8;	63.80	0.00
TRINITY_E1ZDY4_CHLVA/478-638	E1ZDY4.1 PF02518.24	63.80	0.00
TRINITY_E1ZEH7_CHLVA/121-367	E1ZEH7.1 PF00561.18	63.80	0.00
TRINITY_F0ZR48_DICPU/670-1006	F0ZR48.1 PF00176.21	63.80	0.00
TRINITY_G0R4A1_ICHMG/1-230	G0R4A1.1 PF00225.21	63.80	0.00
TRINITY_W7T228_9STRA/208-331	W7T228.1 PF09668.8;	63.70	0.00
TRINITY_Q42475_SOLLC/1-90	Q42475.1 PF03242.11	63.70	0.00
TRINITY_A0A0M9F2X8_9HYPO/223-518	A0A0M9F2X8.1 PF0012	63.70	0.00
TRINITY_U3K139_FICAL/18-188	U3K139.1 PF13177.4;	63.70	0.00
TRINITY_D8U1P8_VOLCA/49-480	D8U1P8.1 PF00909.19	63.70	0.00
TRINITY_I7LTP7_TETTS/3726-4428	I7LTP7.2 PF03028.13	63.70	0.00
TRINITY_A0A078A9T0_STYLE/80-292	A0A078A9T0.1 PF0011	63.70	0.00
TRINITY_I7MCU1_TETTS/405-506	I7MCU1.1 PF06565.10	63.70	0.00
TRINITY_M2UE92_COCH5/492-660	M2UE92.1 PF00009.25	63.70	0.00
TRINITY_A8JHD4_CHLRE/855-967	A8JHD4.1 PF13832.4;	63.70	0.00
TRINITY_D8UIJ0_VOLCA/8-357	D8UIJ0.1 PF00274.17	63.70	0.00
TRINITY_A0A087SH64_AUXPR/7-119	A0A087SH64.1 PF0213	63.70	0.00
TRINITY_A0A0D2X2H1_CAPO3/607-950	A0A0D2X2H1.1 PF1474	63.70	0.00
TRINITY_D8THE7_VOLCA/190-448	D8THE7.1 PF04258.11	63.70	0.00
TRINITY_E1ZBC0_CHLVA/20-351	E1ZBC0.1 PF00225.21	63.70	0.00
TRINITY_A0A068S2T4_9FUNG/35-224	A0A068S2T4.1 PF0442	63.70	0.00
TRINITY_A8IUN1_CHLRE/1083-1429	A8IUN1.1 PF04054.13	63.70	0.00
TRINITY_A0A087ST14_AUXPR/88-233	A0A087ST14.1 PF0081	63.70	0.00
TRINITY_I7M6H6_TETTS/54-166	I7M6H6.1 PF10908.6;	63.70	0.00
TRINITY_A0ECC8_PARTE/8-135	A0ECC8.1 PF00782.18	63.60	0.00
TRINITY_D3AVJ6_POLPA/582-722	D3AVJ6.1 PF00005.25	63.60	0.00
TRINITY_Q22T35_TETTS/4-288	Q22T35.2 PF00069.23	63.60	0.00
TRINITY_I7MMM1_TETTS/27-188	I7MMM1.1 PF05351.9;	63.60	0.00
TRINITY_F0VNN1_NEOCL/211-337	F0VNN1.1 PF00782.18	63.60	0.00
TRINITY_D3BVL0_POLPA/330-389	D3BVL0.1 PF00076.20	63.60	0.00
TRINITY_K0KIU4_WICCF/2-270	K0KIU4.1 PF06418.12	63.60	0.00
TRINITY_S3CAE7_OPHP1/691-1013	S3CAE7.1 PF00176.21	63.60	0.00
TRINITY_F4PQE4_DICFS/63-138	F4PQE4.1 PF01066.19	63.60	0.00
TRINITY_A0A084WUY1_ANOSI/27-70	A0A084WUY1.1 PF0040	63.60	0.00
TRINITY_G7I2B1_MEDTR/142-202	G7I2B1.1 PF04193.12	63.60	0.00
TRINITY_E1Z692_CHLVA/4-137	E1Z692.1 PF00271.29	63.60	0.00
TRINITY_Q54GD2_DICDI/145-273	Q54GD2.1 PF01467.24	63.60	0.00
TRINITY_URM1_CHLRE/3-99	A8IC48.1 PF09138.9;	63.60	0.00
TRINITY_I7MMT5_TETTS/48-124	I7MMT5.2 PF01066.19	63.60	0.00
TRINITY_F4PQK3_DICFS/22-352	F4PQK3.1 PF01992.14	63.60	0.00
TRINITY_Q23FG2_TETTS/1702-2050	Q23FG2.2 PF00443.27	63.60	0.00
TRINITY_W9SPR0_9ROSA/192-235	W9SPR0.1 PF00432.19	63.60	0.00
TRINITY_G0QXE3_ICHMG/23-411	G0QXE3.1 PF00202.19	63.60	0.00
TRINITY_Q240Q4_TETTS/341-560	Q240Q4.3 PF00557.22	63.60	0.00
TRINITY_A8ICV4_CHLRE/37-96	A8ICV4.1 PF02427.15	63.60	0.00
TRINITY_Q23DV6_TETTS/25-350	Q23DV6.2 PF07992.12	63.60	0.00
TRINITY_A9SFG7_PHYPA/138-291	A9SFG7.1 PF10602.7;	63.60	0.00
TRINITY_A8HVT9_CHLRE/12-303	A8HVT9.1 PF17184.2;	63.60	0.00
TRINITY_W5JTG6_ANODA/381-471	W5JTG6.1 PF12894.5;	63.60	0.00
TRINITY_A0A087SAE1_AUXPR/105-205	A0A087SAE1.1 PF0147	63.60	0.00
TRINITY_A8IAF7_CHLRE/62-257	A8IAF7.1 PF00849.20	63.60	0.00
TRINITY_A0CMX3_PARTE/57-210	A0CMX3.1 PF00005.25	63.60	0.00
TRINITY_A0A087STB2_AUXPR/21-106	A0A087STB2.1 PF0103	63.60	0.00
TRINITY_L8GSN9_ACACA/40-123	L8GSN9.1 PF04683.11	63.60	0.00
TRINITY_A8HYJ4_CHLRE/45-302	A8HYJ4.1 PF08282.10	63.60	0.00
TRINITY_A0A078B0Q9_STYLE/402-508	A0A078B0Q9.1 PF1289	63.60	0.00

TRINITY_J9HU22_9SPIT/4-283	J9HU22.1 PF00069.23	63.60	0.00
TRINITY_R6FU95_9CLOT/5-67	R6FU95.1 PF00226.29	63.60	0.00
TRINITY_NFYB_DICDI/51-116	Q54WV0.1 PF00808.21	63.60	0.00
TRINITY_D8THP0_VOLCA/118-279	D8THP0.1 PF01789.14	63.60	0.00
TRINITY_U4KTV3_PYROM/215-258	U4KTV3.1 PF10590.7;	63.60	0.00
TRINITY_J9ICB1_9SPIT/267-310	J9ICB1.1 PF00569.15	63.60	0.00
TRINITY_I4A2S0_ORNRL/43-393	I4A2S0.1 PF00155.19	63.60	0.00
TRINITY_A0E826_PARTE/289-506	A0E826.1 PF07002.14	63.60	0.00
TRINITY_D8TX79_VOLCA/1-108	D8TX79.1 PF04825.11	63.60	0.00
TRINITY_D8U1X0_VOLCA/12-381	D8U1X0.1 PF07690.14	63.60	0.00
TRINITY_A9RIN7_PHYPA/44-288	A9RIN7.1 PF00743.17	63.60	0.00
TRINITY_L8HGD4_ACACA/42-536	L8HGD4.1 PF00478.23	63.60	0.00
TRINITY_D8TLP7_VOLCA/868-976	D8TLP7.1 PF00069.23	63.60	0.00
TRINITY_D8TJV3_VOLCA/38-154	D8TJV3.1 PF02466.17	63.60	0.00
TRINITY_I7M308_TETTS/37-322	I7M308.1 PF06098.9;	63.60	0.00
TRINITY_D3B5K0_POLPA/337-650	D3B5K0.1 PF02540.15	63.60	0.00
TRINITY_A8IF06_CHLRE/80-459	A8IF06.1 PF01490.16	63.60	0.00
TRINITY_F0ZLJ3_DICPU/142-259	F0ZLJ3.1 PF02770.17	63.60	0.00
TRINITY_A0C0W6_PARTE/7-154	A0C0W6.1 PF00056.21	63.60	0.00
TRINITY_I1E641_AMPQE/154-314	I1E641.1 PF00078.25	63.60	0.00
TRINITY_G0R468_ICHMG/39-145	G0R468.1 PF01521.18	63.60	0.00
TRINITY_D8UAB4_VOLCA/54-366	D8UAB4.1 PF03133.13	63.60	0.00
TRINITY_C1E8T4_MICSR/55-208	C1E8T4.1 PF13365.4;	63.60	0.00
TRINITY_D8TPF9_VOLCA/18-597	D8TPF9.1 PF01602.18	63.60	0.00
TRINITY_A8JHU5_CHLRE/130-356	A8JHU5.1 PF04499.13	63.60	0.00
TRINITY_D8TX83_VOLCA/28-241	D8TX83.1 PF13532.4;	63.60	0.00
TRINITY_A8IA75_CHLRE/44-181	A8IA75.1 PF03330.16	63.60	0.00
TRINITY_A8IF64_CHLRE/192-351	A8IF64.1 PF05673.11	63.60	0.00
TRINITY_I1KBT9_SOYBN/27-466	I1KBT9.1 PF05602.10	63.60	0.00
TRINITY_D8U5G0_VOLCA/3-122	D8U5G0.1 PF00188.24	63.60	0.00
TRINITY_J9IT19_9SPIT/9-63	J9IT19.1 PF13637.4;	63.60	0.00
TRINITY_L8GWB5_ACACA/1009-1313	L8GWB5.1 PF02889.14	63.60	0.00
TRINITY_A8HZ69_CHLRE/55-159	A8HZ69.1 PF08241.10	63.60	0.00
TRINITY_G0QZT8_ICHMG/236-838	G0QZT8.1 PF08385.10	63.50	0.00
TRINITY_A0BTN4_PARTE/489-626	A0BTN4.1 PF00005.25	63.50	0.00
TRINITY_Q22L38_TETTS/79-141	Q22L38.2 PF00226.29	63.50	0.00
TRINITY_L8GF06_ACACA/751-1260	L8GF06.1 PF02538.12	63.50	0.00
TRINITY_G4ZC07_PHYSP/4-55	G4ZC07.1 PF00471.18	63.50	0.00
TRINITY_A0E6G5_PARTE/4-110	A0E6G5.1 PF00307.29	63.50	0.00
TRINITY_I7MJ19_TETTS/50-149	I7MJ19.2 PF01230.21	63.50	0.00
TRINITY_A0A072UGZ0_MEDTR/7-71	A0A072UGZ0.1 PF0080	63.50	0.00
TRINITY_D8TXD2_VOLCA/290-341	D8TXD2.1 PF14279.4;	63.50	0.00
TRINITY_A0A0G4IGU5_PLABS/58-161	A0A0G4IGU5.1 PF0824	63.50	0.00
TRINITY_I0YUP2_9CHLO/269-343	I0YUP2.1 PF07572.10	63.50	0.00
TRINITY_M0VBU1_HORVD/11-359	M0VBU1.1 PF16499.3;	63.50	0.00
TRINITY_A8JBZ1_CHLRE/43-328	A8JBZ1.1 PF03151.14	63.50	0.00
TRINITY_A0BH07_PARTE/232-358	A0BH07.1 PF00169.27	63.50	0.00
TRINITY_I7MIF3_TETTS/425-676	I7MIF3.1 PF00756.18	63.50	0.00
TRINITY_L0PE19_PNEJ8/2-181	L0PE19.1 PF13177.4;	63.50	0.00
TRINITY_A8JGM8_CHLRE/17-401	A8JGM8.1 PF01546.26	63.50	0.00
TRINITY_A8J567_CHLRE/116-213	A8J567.1 PF01248.24	63.50	0.00
TRINITY_A5H8I1_CHLRE/64-268	A5H8I1.1 PF11152.6;	63.50	0.00
TRINITY_A8JBM0_CHLRE/4-366	A8JBM0.1 PF05631.12	63.50	0.00
TRINITY_G0QU54_ICHMG/58-355	G0QU54.1 PF00676.18	63.50	0.00
TRINITY_L8GUM8_ACACA/94-179	L8GUM8.1 PF04719.12	63.50	0.00
TRINITY_A8HYU2_CHLRE/3-363	A8HYU2.1 PF03223.13	63.50	0.00
TRINITY_E1ZGK8_CHLVA/274-460	E1ZGK8.1 PF02782.14	63.50	0.00
TRINITY_I0ZAC1_9CHLO/31-369	I0ZAC1.1 PF00155.19	63.50	0.00
TRINITY_D8TZ18_VOLCA/229-407	D8TZ18.1 PF00225.21	63.50	0.00
TRINITY_L8GV46_ACACA/115-166	L8GV46.1 PF01805.18	63.50	0.00
TRINITY_E1ZN61_CHLVA/1018-1299	E1ZN61.1 PF00664.21	63.50	0.00

TRINITY_I7ME40_TETTS/13-116	I7ME40.1 PF00318.18	63.50	0.00
TRINITY_B3RQS2_TRIAD/688-825	B3RQS2.1 PF03764.16	63.50	0.00
TRINITY_D8U9D7_VOLCA/311-620	D8U9D7.1 PF05192.16	63.50	0.00
TRINITY_D8TKH5_VOLCA/385-739	D8TKH5.1 PF03016.13	63.50	0.00
TRINITY_L8H2P4_ACACA/120-1087	L8H2P4.1 PF02463.17	63.50	0.00
TRINITY_C1MIT9_MICPC/12-181	C1MIT9.1 PF13439.4;	63.50	0.00
TRINITY_A8IYH2_CHLRE/1-172	A8IYH2.1 PF00211.18	63.50	0.00
TRINITY_I7MHY7_TETTS/368-473	I7MHY7.1 PF00085.18	63.50	0.00
TRINITY_Q22AX2_TETTS/19-81	Q22AX2.3 PF02320.14	63.50	0.00
TRINITY_G0QLU3_ICHMG/4-88	G0QLU3.1 PF01780.17	63.50	0.00
TRINITY_G0QLU3_ICHMG/4-88	G0QLU3.1 PF01780.17	63.50	0.00
TRINITY_D8TWX1_VOLCA/342-415	D8TWX1.1 PF03451.12	63.50	0.00
TRINITY_A8JFG8_CHLRE/29-537	A8JFG8.1 PF00759.17	63.50	0.00
TRINITY_D8UB72_VOLCA/8-263	D8UB72.1 PF08433.8;	63.50	0.00
TRINITY_A8IXP1_CHLRE/1840-2022	A8IXP1.1 PF00211.18	63.50	0.00
TRINITY_D8TTV8_VOLCA/81-324	D8TTV8.1 PF00233.17	63.50	0.00
TRINITY_M5VM98_PRUPE/561-708	M5VM98.1 PF00078.25	63.50	0.00
TRINITY_G0QP92_ICHMG/6-205	G0QP92.1 PF00227.24	63.50	0.00
TRINITY_A0C010_PARTE/19-116	A0C010.1 PF00034.19	63.40	0.00
TRINITY_W6V622_ECHGR/367-406	W6V622.1 PF00400.30	63.40	0.00
TRINITY_D8U578_VOLCA/555-682	D8U578.1 PF16898.3;	63.40	0.00
TRINITY_L8HDU4_ACACA/23-77	L8HDU4.1 PF10152.7;	63.40	0.00
TRINITY_A0A087SCI6_AUXPR/115-156	A0A087SCI6.1 PF0051	63.40	0.00
TRINITY_A0A078AQ35_STYLE/5-179	A0A078AQ35.1 PF0002	63.40	0.00
TRINITY_G0QSX0_ICHMG/7-148	G0QSX0.1 PF05517.10	63.40	0.00
TRINITY_A9V2B9_MONBE/227-497	A9V2B9.1 PF13598.4;	63.40	0.00
TRINITY_U5H3G6_USTV1/2-375	U5H3G6.1 PF00022.17	63.40	0.00
TRINITY_G7Y3S9_CLOSI/3-391	G7Y3S9.1 PF00022.17	63.40	0.00
TRINITY_Q22NA0_TETTS/1407-1551	Q22NA0.2 PF00005.25	63.40	0.00
TRINITY_D8UGV1_VOLCA/161-275	D8UGV1.1 PF00583.23	63.40	0.00
TRINITY_E5SFZ6_TRISP/46-134	E5SFZ6.1 PF15663.3;	63.40	0.00
TRINITY_A0A068SB59_9FUNG/249-388	A0A068SB59.1 PF0027	63.40	0.00
TRINITY_M4A3Z5_XIPMA/37-87	M4A3Z5.1 PF06446.10	63.40	0.00
TRINITY_PAN2_SCHPO/860-1033	Q09798.2 PF00929.22	63.40	0.00
TRINITY_M3JB19_CANMX/6-167	M3JB19.1 PF00071.20	63.40	0.00
TRINITY_D8TUM3_VOLCA/223-475	D8TUM3.1 PF00266.17	63.40	0.00
TRINITY_PIRA_DICDI/393-1224	Q6UK63.1 PF05994.9;	63.40	0.00
TRINITY_A8J2Y1_CHLRE/341-490	A8J2Y1.1 PF13532.4;	63.40	0.00
TRINITY_B6IMU6_RHOCS/29-494	B6IMU6.1 PF00171.20	63.40	0.00
TRINITY_J9J9Q5_9SPIT/339-382	J9J9Q5.1 PF00569.15	63.40	0.00
TRINITY_T0RJF7_9STRA/23-99	T0RJF7.1 PF01253.20	63.40	0.00
TRINITY_D8TP18_VOLCA/1-249	D8TP18.1 PF00795.20	63.40	0.00
TRINITY_L8HFT5_ACACA/271-321	L8HFT5.1 PF02272.17	63.40	0.00
TRINITY_B3SAC1_TRIAD/191-351	B3SAC1.1 PF02731.13	63.40	0.00
TRINITY_A0A0G4EI57_9ALVE/1306-1711	A0A0G4EI57.1 PF0839	63.40	0.00
TRINITY_A0A024RE44_VAVCU/259-521	A0A024RE44.1 PF0058	63.40	0.00
TRINITY_J9IG73_9SPIT/121-352	J9IG73.1 PF00009.25	63.40	0.00
TRINITY_D8UF21_VOLCA/109-298	D8UF21.1 PF16575.3;	63.40	0.00
TRINITY_Q7MGD2_VIBVY/76-156	Q7MGD2.1 PF01035.18	63.40	0.00
TRINITY_Q22CT9_TETTS/7-296	Q22CT9.3 PF00069.23	63.40	0.00
TRINITY_A8PC17_COPC7/8-56	A8PC17.1 PF01679.15	63.40	0.00
TRINITY_A8JGN0_CHLRE/32-131	A8JGN0.1 PF04430.12	63.40	0.00
TRINITY_D3B8N7_POLPA/197-466	D3B8N7.1 PF00069.23	63.40	0.00
TRINITY_A8IRC0_CHLRE/5-225	A8IRC0.1 PF04157.14	63.40	0.00
TRINITY_FEN11_PARTE/1-111	A0CXT3.1 PF00752.15	63.40	0.00
TRINITY_I0YPW0_9CHLO/282-626	I0YPW0.1 PF00493.21	63.40	0.00
TRINITY_F4P3W7_BATDJ/274-383	F4P3W7.1 PF01398.19	63.40	0.00
TRINITY_A8HSD3_CHLRE/90-188	A8HSD3.1 PF00383.21	63.40	0.00
TRINITY_A8JGL3_CHLRE/53-302	A8JGL3.1 PF01370.19	63.40	0.00
TRINITY_D8UHI9_VOLCA/117-368	D8UHI9.1 PF12697.5;	63.40	0.00
TRINITY_E1ZFR6_CHLVA/491-840	E1ZFR6.1 PF00773.17	63.40	0.00

TRINITY_L8GIF1_ACACA/60-448	L8GIF1.1 PF01979.18	63.40	0.00
TRINITY_B3SC84_TRIAD/226-266	B3SC84.1 PF13639.4;	63.40	0.00
TRINITY_A0A0D3FK07_9ORYZ/574-631	A0A0D3FK07.1 PF0837	63.40	0.00
TRINITY_A0A0G4J8S9_PLABS/29-325	A0A0G4J8S9.1 PF0799	63.40	0.00
TRINITY_A8J979_CHLRE/177-389	A8J979.1 PF02786.15	63.40	0.00
TRINITY_D8TKR0_VOLCA/126-399	D8TKR0.1 PF01189.15	63.40	0.00
TRINITY_E1Z854_CHLVA/803-925	E1Z854.1 PF03109.14	63.40	0.00
TRINITY_F0ZN13_DICPU/38-111	F0ZN13.1 PF01722.16	63.40	0.00
TRINITY_A0EDI7_PARTE/1191-1469	A0EDI7.1 PF00069.23	63.40	0.00
TRINITY_Q23K21_TETTS/369-636	Q23K21.2 PF00487.22	63.40	0.00
TRINITY_RDRP_ASPVP/1752-2169	Q64962.1 PF00978.19	63.40	0.00
TRINITY_M1A3V7_SOLTU/2-114	M1A3V7.1 PF00183.16	63.30	0.00
TRINITY_A7GU23_BACCN/2-118	A7GU23.1 PF00903.23	63.30	0.00
TRINITY_A0DJ75_PARTE/483-760	A0DJ75.1 PF00702.24	63.30	0.00
TRINITY_A0A0M2R937_9PROT/8-99	A0A0M2R937.1 PF0132	63.30	0.00
TRINITY_M2Y724_GALSU/9-281	M2Y724.1 PF00069.23	63.30	0.00
TRINITY_Q234R4_TETTS/265-337	Q234R4.3 PF00173.26	63.30	0.00
TRINITY_A0A0D2X5C2_CAPO3/59-309	A0A0D2X5C2.1 PF0037	63.30	0.00
TRINITY_H2TE24_TAKRU/18-131	H2TE24.1 PF07686.15	63.30	0.00
TRINITY_D3BAR7_POLPA/506-673	D3BAR7.1 PF00270.27	63.30	0.00
TRINITY_A0E5F0_PARTE/57-215	A0E5F0.1 PF03034.13	63.30	0.00
TRINITY_A0EFQ7_PARTE/75-330	A0EFQ7.1 PF00069.23	63.30	0.00
TRINITY_L1K3Q8_GUITH/147-198	L1K3Q8.1 PF00249.29	63.30	0.00
TRINITY_A0A0R4IU56_DANRE/1-241	A0A0R4IU56.1 PF0059	63.30	0.00
TRINITY_D3B058_POLPA/665-713	D3B058.1 PF17102.3;	63.30	0.00
TRINITY_Q5AWT4_EMENI/461-784	Q5AWT4.1 PF13086.4;	63.30	0.00
TRINITY_H3HAJ2_PHYRM/359-478	H3HAJ2.1 PF00665.24	63.30	0.00
TRINITY_Q23DI7_TETTS/1868-2219	Q23DI7.2 PF00443.27	63.30	0.00
TRINITY_D3BJ98_POLPA/447-728	D3BJ98.1 PF00176.21	63.30	0.00
TRINITY_E1Z419_CHLVA/40-506	E1Z419.1 PF00171.20	63.30	0.00
TRINITY_A8JII1_CHLRE/35-212	A8JII1.1 PF09439.8;	63.30	0.00
TRINITY_C3XZD0_BRAFL/41-209	C3XZD0.1 PF10263.7;	63.30	0.00
TRINITY_D8UF13_VOLCA/742-963	D8UF13.1 PF13725.4;	63.30	0.00
TRINITY_Q22RC8_TETTS/135-363	Q22RC8.1 PF00557.22	63.30	0.00
TRINITY_Q52WZ8_CHLRE/11-107	Q52WZ8.1 PF06294.9;	63.30	0.00
TRINITY_E9G068_DAPPU/1-436	E9G068.1 PF00996.16	63.30	0.00
TRINITY_A8IZ85_CHLRE/350-640	A8IZ85.1 PF02836.15	63.30	0.00
TRINITY_G0QLE7_ICHMG/123-414	G0QLE7.1 PF00009.25	63.30	0.00
TRINITY_G0QKJ0_ICHMG/93-465	G0QKJ0.1 PF01546.26	63.30	0.00
TRINITY_Q228S4_TETTS/411-674	Q228S4.2 PF00069.23	63.30	0.00
TRINITY_L8H3G3_ACACA/12-254	L8H3G3.1 PF00348.15	63.30	0.00
TRINITY_A8J5R5_CHLRE/1031-1196	A8J5R5.1 PF01504.16	63.30	0.00
TRINITY_A8JIJ9_CHLRE/11-261	A8JIJ9.1 PF00069.23	63.30	0.00
TRINITY_D8TWI2_VOLCA/58-251	D8TWI2.1 PF01928.19	63.30	0.00
TRINITY_F4QEF1_DICFS/291-617	F4QEF1.1 PF00676.18	63.30	0.00
TRINITY_A8ICT4_CHLRE/7-55	A8ICT4.1 PF04627.11	63.30	0.00
TRINITY_C1N2K6_MICPC/50-260	C1N2K6.1 PF00590.18	63.30	0.00
TRINITY_A0A0K9P8S9_ZOSMR/111-254	A0A0K9P8S9.1 PF0303	63.30	0.00
TRINITY_E1Z9L2_CHLVA/59-244	E1Z9L2.1 PF00350.21	63.30	0.00
TRINITY_D8TZN2_VOLCA/94-268	D8TZN2.1 PF01595.18	63.30	0.00
TRINITY_Q234E6_TETTS/453-713	Q234E6.2 PF00069.23	63.30	0.00
TRINITY_I7MIX1_TETTS/178-297	I7MIX1.2 PF02373.20	63.30	0.00
TRINITY_D8UF85_VOLCA/179-263	D8UF85.1 PF05699.12	63.30	0.00
TRINITY_Q23DW6_TETTS/27-380	Q23DW6.1 PF16499.3;	63.20	0.00
TRINITY_Q23K54_TETTS/999-1188	Q23K54.1 PF00488.19	63.20	0.00
TRINITY_D8M0V1_BLAHO/40-78	D8M0V1.1 PF00400.30	63.20	0.00
TRINITY_A0A078B9J1_STYLE/6-262	A0A078B9J1.1 PF0006	63.20	0.00
TRINITY_F8EPR6_RUNSL/17-162	F8EPR6.1 PF00106.23	63.20	0.00
TRINITY_L1I7I0_GUITH/5-178	L1I7I0.1 PF00071.20	63.20	0.00
TRINITY_A0A0G4H1C2_9ALVE/10-381	A0A0G4H1C2.1 PF0002	63.20	0.00
TRINITY_A3DCA3_CLOTH/160-383	A3DCA3.1 PF03949.13	63.20	0.00

TRINITY_H0VVA8_CAVPO/123-413	H0VVA8.1 PF01557.16	63.20	0.00
TRINITY_A7S107_NEMVE/113-152	A7S107.1 PF00400.30	63.20	0.00
TRINITY_I0YNI5_9CHLO/35-101	I0YNI5.1 PF00498.24	63.20	0.00
TRINITY_A8ISQ5_CHLRE/2275-2409	A8ISQ5.1 PF00004.27	63.20	0.00
TRINITY_O24426_CHLRE/6-380	O24426.1 PF00022.17	63.20	0.00
TRINITY_A8JDF6_CHLRE/27-233	A8JDF6.1 PF03741.14	63.20	0.00
TRINITY_D3BL62_POLPA/284-465	D3BL62.1 PF02146.15	63.20	0.00
TRINITY_A0A087SS47_AUXPR/18-112	A0A087SS47.1 PF0612	63.20	0.00
TRINITY_Q229Y3_TETTS/2010-2423	Q229Y3.2 PF00856.26	63.20	0.00
TRINITY_A8MK44_ALKOO/8-124	A8MK44.1 PF13793.4;	63.20	0.00
TRINITY_TTL3E_TETTS/968-1246	Q23AS2.1 PF03133.13	63.20	0.00
TRINITY_I0YVI1_9CHLO/44-119	I0YVI1.1 PF01066.19	63.20	0.00
TRINITY_D7TJ66_VITVI/10-48	D7TJ66.1 PF00400.30	63.20	0.00
TRINITY_J9FM16_9SPIT/6-163	J9FM16.1 PF00790.17	63.20	0.00
TRINITY_A0C636_PARTE/191-324	A0C636.1 PF14580.4;	63.20	0.00
TRINITY_D2VE41_NAEGR/1-342	D2VE41.1 PF00022.17	63.20	0.00
TRINITY_A9TYS8_PHYPA/8-96	A9TYS8.1 PF00125.22	63.20	0.00
TRINITY_A0A087SGF1_AUXPR/12-87	A0A087SGF1.1 PF1015	63.20	0.00
TRINITY_D8TU89_VOLCA/319-430	D8TU89.1 PF00787.22	63.20	0.00
TRINITY_G0QYS8_ICHMG/16-216	G0QYS8.1 PF00069.23	63.20	0.00
TRINITY_L8HG59_ACACA/44-379	L8HG59.1 PF07992.12	63.20	0.00
TRINITY_D8TKC8_VOLCA/6-187	D8TKC8.1 PF12263.6;	63.20	0.00
TRINITY_A8J688_CHLRE/146-349	A8J688.1 PF00106.23	63.20	0.00
TRINITY_D8UBS2_VOLCA/5-73	D8UBS2.1 PF13921.4;	63.20	0.00
TRINITY_E1ZAL6_CHLVA/652-690	E1ZAL6.1 PF00400.30	63.20	0.00
TRINITY_A9TT36_PHYPA/88-262	A9TT36.1 PF10602.7;	63.20	0.00
TRINITY_K8GGY5_9CYAN/3-257	K8GGY5.1 PF01926.21	63.20	0.00
TRINITY_D8UJA4_VOLCA/186-243	D8UJA4.1 PF03909.15	63.20	0.00
TRINITY_D8UJ40_VOLCA/721-962	D8UJ40.1 PF00122.18	63.20	0.00
TRINITY_Q22TJ1_TETTS/178-486	Q22TJ1.1 PF00365.18	63.20	0.00
TRINITY_A8IYU9_CHLRE/9-204	A8IYU9.1 PF05648.12	63.20	0.00
TRINITY_A9PD24_POPTR/207-295	A9PD24.1 PF13813.4;	63.20	0.00
TRINITY_D8U9Z0_VOLCA/954-1167	D8U9Z0.1 PF05153.13	63.20	0.00
TRINITY_A8JES7_CHLRE/33-169	A8JES7.1 PF00179.24	63.20	0.00
TRINITY_M2WZS1_GALSU/23-243	M2WZS1.1 PF00817.18	63.20	0.00
TRINITY_A8J3R3_CHLRE/414-557	A8J3R3.1 PF13602.4;	63.20	0.00
TRINITY_A0A087SL28_AUXPR/20-209	A0A087SL28.1 PF0249	63.20	0.00
TRINITY_C1MY91_MICPC/103-141	C1MY91.1 PF00400.30	63.20	0.00
TRINITY_D8UFN6_VOLCA/191-315	D8UFN6.1 PF00241.18	63.20	0.00
TRINITY_A0BMI9_PARTE/267-334	A0BMI9.1 PF00498.24	63.20	0.00
TRINITY_A0CKF8_PARTE/1118-1269	A0CKF8.1 PF00005.25	63.20	0.00
TRINITY_A8I6F0_CHLRE/131-262	A8I6F0.1 PF00782.18	63.20	0.00
TRINITY_D8RCY3_SELML/4-188	D8RCY3.1 PF01351.16	63.20	0.00
TRINITY_D8TQU3_VOLCA/613-764	D8TQU3.1 PF09127.9;	63.20	0.00
TRINITY_D8S9R5_SELML/151-188	D8S9R5.1 PF00400.30	63.20	0.00
TRINITY_D8UBB8_VOLCA/40-453	D8UBB8.1 PF01406.17	63.20	0.00
TRINITY_A8JGX6_CHLRE/3-96	A8JGX6.1 PF01250.15	63.20	0.00
TRINITY_D8TY45_VOLCA/121-196	D8TY45.1 PF03703.12	63.20	0.00
TRINITY_E1ZNE5_CHLVA/1-246	E1ZNE5.1 PF00225.21	63.20	0.00
TRINITY_A0A0D2M173_GOSRA/148-215	A0A0D2M173.1 PF0049	63.20	0.00
TRINITY_G0QKD4_ICHMG/49-354	G0QKD4.1 PF00899.19	63.20	0.00
TRINITY_D8UEF4_VOLCA/478-657	D8UEF4.1 PF07724.12	63.20	0.00
TRINITY_H9GJL0_ANOCA/451-488	H9GJL0.2 PF00400.30	63.20	0.00
TRINITY_A0A0F7JRH6_9DEIO/27-525	A0A0F7JRH6.1 PF0013	63.20	0.00
TRINITY_A0A0J8CJE8_BETVU/66-103	A0A0J8CJE8.1 PF0142	63.20	0.00
TRINITY_A0C0W6_PARTE/7-154	A0C0W6.1 PF00056.21	63.10	0.00
TRINITY_G0R3Z3_ICHMG/38-122	G0R3Z3.1 PF16561.3;	63.10	0.00
TRINITY_F4Q976_DICFS/77-141	F4Q976.1 PF00808.21	63.10	0.00
TRINITY_A7RQX5_NEMVE/83-227	A7RQX5.1 PF02252.16	63.10	0.00
TRINITY_Q21EK4_SACD2/60-238	Q21EK4.1 PF13847.4;	63.10	0.00
TRINITY_L8H813_ACACA/26-277	L8H813.1 PF00069.23	63.10	0.00

TRINITY_A8J629_CHLRE/49-208	A8J629.1 PF01699.22	63.10	0.00
TRINITY_I0YQJ9_9CHLO/124-485	I0YQJ9.1 PF01594.14	63.10	0.00
TRINITY_A8J6F4_CHLRE/46-300	A8J6F4.1 PF00474.15	63.10	0.00
TRINITY_L8H1T2_ACACA/1503-1836	L8H1T2.1 PF01979.18	63.10	0.00
TRINITY_D8UGZ5_VOLCA/112-246	D8UGZ5.1 PF15305.4;	63.10	0.00
TRINITY_A8JH46_CHLRE/26-513	A8JH46.1 PF01039.20	63.10	0.00
TRINITY_I0YYU3_9CHLO/67-131	I0YYU3.1 PF01066.19	63.10	0.00
TRINITY_A8IW16_CHLRE/695-1085	A8IW16.1 PF03372.21	63.10	0.00
TRINITY_H3G886_PHYRM/24-302	H3G886.1 PF00814.23	63.10	0.00
TRINITY_E1Z786_CHLVA/443-591	E1Z786.1 PF02775.19	63.10	0.00
TRINITY_K9QZ12_NOSS7/12-223	K9QZ12.1 PF01379.18	63.10	0.00
TRINITY_A8JCE7_CHLRE/177-241	A8JCE7.1 PF00076.20	63.10	0.00
TRINITY_E1ZGM8_CHLVA/298-409	E1ZGM8.1 PF00004.27	63.10	0.00
TRINITY_D8TSL7_VOLCA/63-224	D8TSL7.1 PF00160.19	63.10	0.00
TRINITY_L8GNA6_ACACA/12-76	L8GNA6.1 PF13499.4;	63.10	0.00
TRINITY_A0A075ASD8_9FUNG/454-785	A0A075ASD8.1 PF0077	63.10	0.00
TRINITY_D8UE04_VOLCA/56-615	D8UE04.1 PF02990.14	63.10	0.00
TRINITY_I0YZG5_9CHLO/15-98	I0YZG5.1 PF00887.17	63.10	0.00
TRINITY_DNAJ_CLOCE/6-68	B8I304.1 PF00226.29	63.00	0.00
TRINITY_A0A0G4KUT2_9PEZI/4-132	A0A0G4KUT2.1 PF1587	63.00	0.00
TRINITY_D7FM58_ECTSI/182-316	D7FM58.1 PF00730.23	63.00	0.00
TRINITY_G0QU93_ICHMG/188-379	G0QU93.1 PF00227.24	63.00	0.00
TRINITY_B3QYP7_CHLT3/38-191	B3QYP7.1 PF01625.19	63.00	0.00
TRINITY_A0A0D2X5I7_CAPO3/54-299	A0A0D2X5I7.1 PF1060	63.00	0.00
TRINITY_J9IXT9_9SPIT/144-217	J9IXT9.1 PF13499.4;	63.00	0.00
TRINITY_A0A0E0BMZ8_9ORYZ/15-114	A0A0E0BMZ8.1 PF0023	63.00	0.00
TRINITY_A0A0G4IZR2_PLABS/16-150	A0A0G4IZR2.1 PF0017	63.00	0.00
TRINITY_A8IL90_CHLRE/74-226	A8IL90.1 PF12638.5;	63.00	0.00
TRINITY_Q22BV7_TETTS/174-334	Q22BV7.2 PF00120.22	63.00	0.00
TRINITY_A0A0G4GQ90_9ALVE/50-668	A0A0G4GQ90.1 PF0044	63.00	0.00
TRINITY_D8U6Q6_VOLCA/22-225	D8U6Q6.1 PF01105.22	63.00	0.00
TRINITY_I7M9W8_TETTS/2-179	I7M9W8.2 PF17135.2;	63.00	0.00
TRINITY_A0A0G4KUT2_9PEZI/4-132	A0A0G4KUT2.1 PF1587	63.00	0.00
TRINITY_A8HX98_CHLRE/44-144	A8HX98.1 PF00027.27	63.00	0.00
TRINITY_A0A067NBV4_PLEOS/4-111	A0A067NBV4.1 PF0025	63.00	0.00
TRINITY_G0QLX6_ICHMG/31-195	G0QLX6.1 PF03031.16	63.00	0.00
TRINITY_A8I4G8_CHLRE/5-77	A8I4G8.1 PF05251.10	63.00	0.00
TRINITY_D8UIY0_VOLCA/88-344	D8UIY0.1 PF01207.15	63.00	0.00
TRINITY_J9J4F0_9SPIT/9-265	J9J4F0.1 PF00069.23	63.00	0.00
TRINITY_G0R323_ICHMG/28-216	G0R323.1 PF00227.24	63.00	0.00
TRINITY_A0CEN2_PARTE/1195-1399	A0CEN2.1 PF00069.23	63.00	0.00
TRINITY_D8U258_VOLCA/26-155	D8U258.1 PF01184.17	63.00	0.00
TRINITY_J9I4F7_9SPIT/364-418	J9I4F7.1 PF00046.27	63.00	0.00
TRINITY_D8U380_VOLCA/93-229	D8U380.1 PF01569.19	63.00	0.00
TRINITY_F4QBH8_DICFS/1949-2123	F4QBH8.1 PF01504.16	63.00	0.00
TRINITY_RL23A_TETTS/69-142	P0DJ57.1 PF00276.18	63.00	0.00
TRINITY_A8IMV6_CHLRE/56-113	A8IMV6.1 PF03109.14	63.00	0.00
TRINITY_Q23QY4_TETTS/796-1116	Q23QY4.2 PF03133.13	63.00	0.00
TRINITY_U9UZZ9_RHIID/23-241	U9UZZ9.1 PF00557.22	63.00	0.00
TRINITY_L1JMU5_GUITH/70-166	L1JMU5.1 PF12755.5;	63.00	0.00
TRINITY_D8U338_VOLCA/274-367	D8U338.1 PF05705.12	63.00	0.00
TRINITY_D8TLH6_VOLCA/190-248	D8TLH6.1 PF07529.11	63.00	0.00
TRINITY_L1ISC9_GUITH/1329-1522	L1ISC9.1 PF13087.4;	63.00	0.00
TRINITY_I0YLB0_9CHLO/54-356	I0YLB0.1 PF01866.15	63.00	0.00
TRINITY_I0Z0B7_9CHLO/68-241	I0Z0B7.1 PF10602.7;	63.00	0.00
TRINITY_F0ZQQ8_DICPU/161-436	F0ZQQ8.1 PF00928.19	63.00	0.00
TRINITY_D8TGS7_VOLCA/23-187	D8TGS7.1 PF13023.4;	63.00	0.00
TRINITY_L8GYS4_ACACA/31-525	L8GYS4.1 PF00118.22	63.00	0.00
TRINITY_A8I975_CHLRE/1-86	A8I975.1 PF00153.25	63.00	0.00
TRINITY_S2KHY2_MUCC1/130-422	S2KHY2.1 PF00012.18	63.00	0.00
TRINITY_D8SD29_SELML/9-310	D8SD29.1 PF00069.23	63.00	0.00

TRINITY_Q5CPQ8_CRYPI/271-319	Q5CPQ8.1 PF00628.27	63.00	0.00
TRINITY_A8HWS1_CHLRE/874-1281	A8HWS1.1 PF08393.11	63.00	0.00
TRINITY_A8J510_CHLRE/41-713	A8J510.1 PF00063.19	63.00	0.00
TRINITY_A4VE98_TETTS/337-580	A4VE98.1 PF00112.21	63.00	0.00
TRINITY_A8I3V9_CHLRE/1060-1326	A8I3V9.1 PF12775.5;	63.00	0.00
TRINITY_Q248E9_TETTS/284-336	Q248E9.1 PF13418.4;	63.00	0.00
TRINITY_A0A0A1NFX3_9FUNG/158-239	A0A0A1NFX3.1 PF0268	63.00	0.00
TRINITY_Q5CPQ8_CRYPI/271-319	Q5CPQ8.1 PF00628.27	63.00	0.00
TRINITY_S3ECM3_GLAL2/20-228	S3ECM3.1 PF01557.16	63.00	0.00
TRINITY_C6XYG7_PEDHD/132-254	C6XYG7.1 PF01678.17	63.00	0.00
TRINITY_G0R3C8_ICHMG/804-1213	G0R3C8.1 PF08393.11	62.90	0.00
TRINITY_S8CJ07_9LAMI/313-454	S8CJ07.1 PF01926.21	62.90	0.00
TRINITY_I7M372_TETTS/24-109	I7M372.1 PF00439.23	62.90	0.00
TRINITY_A0E666_PARTE/19-269	A0E666.1 PF00248.19	62.90	0.00
TRINITY_F8KYZ3_PARAV/9-533	F8KYZ3.1 PF01274.20	62.90	0.00
TRINITY_H6SIN7_RHOPH/14-298	H6SIN7.1 PF02934.13	62.90	0.00
TRINITY_G0R128_ICHMG/15-444	G0R128.1 PF04209.11	62.90	0.00
TRINITY_A0A0D2WHX7_CAPO3/174-264	A0A0D2WHX7.1 PF0195	62.90	0.00
TRINITY_L8HFB9_ACACA/8-70	L8HFB9.1 PF00076.20	62.90	0.00
TRINITY_B6AHC4_CRYMR/589-680	B6AHC4.1 PF02212.16	62.90	0.00
TRINITY_W4XHX1_STRPU/26-367	W4XHX1.1 PF00270.27	62.90	0.00
TRINITY_D3AXW6_POLPA/88-287	D3AXW6.1 PF07002.14	62.90	0.00
TRINITY_G4CNT7_9NEIS/3-185	G4CNT7.1 PF01965.22	62.90	0.00
TRINITY_A8JBH7_CHLRE/27-131	A8JBH7.1 PF00085.18	62.90	0.00
TRINITY_A8J098_CHLRE/134-233	A8J098.1 PF13864.4;	62.90	0.00
TRINITY_Q3SD32_PARTE/10-176	Q3SD32.1 PF00071.20	62.90	0.00
TRINITY_D8U9U4_VOLCA/6-78	D8U9U4.1 PF05915.10	62.90	0.00
TRINITY_E1Z4R7_CHLVA/63-124	E1Z4R7.1 PF00013.27	62.90	0.00
TRINITY_I7MB24_TETTS/12-253	I7MB24.2 PF01370.19	62.90	0.00
TRINITY_I7ML74_TETTS/5678-6228	I7ML74.2 PF00899.19	62.90	0.00
TRINITY_G0R3B1_ICHMG/35-559	G0R3B1.1 PF00022.17	62.90	0.00
TRINITY_D8TPI3_VOLCA/152-267	D8TPI3.1 PF00753.25	62.90	0.00
TRINITY_A0BKF7_PARTE/129-420	A0BKF7.1 PF01557.16	62.90	0.00
TRINITY_D8TSA2_VOLCA/92-565	D8TSA2.1 PF05140.12	62.90	0.00
TRINITY_I1FI58_AMPQE/22-257	I1FI58.1 PF00262.16	62.90	0.00
TRINITY_B3RKH2_TRIAD/61-247	B3RKH2.1 PF00149.26	62.90	0.00
TRINITY_I7MHS8_TETTS/213-274	I7MHS8.2 PF12906.5;	62.90	0.00
TRINITY_I7M2Q0_TETTS/35-469	I7M2Q0.2 PF00999.19	62.90	0.00
TRINITY_L8HA37_ACACA/375-633	L8HA37.1 PF00069.23	62.90	0.00
TRINITY_A8J3W4_CHLRE/78-147	A8J3W4.1 PF13499.4;	62.90	0.00
TRINITY_I0YQS8_9CHLO/608-694	I0YQS8.1 PF02077.13	62.90	0.00
TRINITY_A8I7W6_CHLRE/7-257	A8I7W6.1 PF03029.15	62.90	0.00
TRINITY_A8J6R9_CHLRE/56-154	A8J6R9.1 PF00276.18	62.90	0.00
TRINITY_A0A0M9G5U1_9TRYP/169-257	A0A0M9G5U1.1 PF1289	62.90	0.00
TRINITY_D8TU20_VOLCA/455-524	D8TU20.1 PF00076.20	62.90	0.00
TRINITY_J3B5Q2_9BACL/17-217	J3B5Q2.1 PF01596.15	62.90	0.00
TRINITY_D8TN85_VOLCA/552-651	D8TN85.1 PF05517.10	62.90	0.00
TRINITY_I7MDL9_TETTS/3-99	I7MDL9.2 PF03650.11	62.90	0.00
TRINITY_D3BK16_POLPA/30-526	D3BK16.1 PF00118.22	62.90	0.00
TRINITY_I1QGW5_ORYGL/64-128	I1QGW5.1 PF00076.20	62.90	0.00
TRINITY_I7M9J2_TETTS/3860-4581	I7M9J2.1 PF03028.13	62.90	0.00
TRINITY_D8TTL6_VOLCA/6-263	D8TTL6.1 PF00069.23	62.90	0.00
TRINITY_I2GJL0_9BACT/4-261	I2GJL0.1 PF00108.21	62.90	0.00
TRINITY_K4AZ06_SOLLC/54-343	K4AZ06.1 PF00176.21	62.90	0.00
TRINITY_A0CR10_PARTE/25-70	A0CR10.1 PF02953.13	62.80	0.00
TRINITY_J9IP56_9SPIT/145-317	J9IP56.1 PF00953.19	62.80	0.00
TRINITY_A0A0G0GHG9_9BACT/10-349	A0A0G0GHG9.1 PF0769	62.80	0.00
TRINITY_L8GSJ5_ACACA/191-458	L8GSJ5.1 PF08811.9;	62.80	0.00
TRINITY_G0R5D6_ICHMG/106-164	G0R5D6.1 PF13921.4;	62.80	0.00
TRINITY_D8LQ28_ECTSI/3-89	D8LQ28.1 PF00125.22	62.80	0.00
TRINITY_I7M7Z9_TETTS/616-760	I7M7Z9.1 PF00005.25	62.80	0.00

TRINITY_E1ZTK5_CHLVA/222-684	E1ZTK5.1 PF05292.9;	62.80	0.00
TRINITY_D6RK63_COPC7/12-263	D6RK63.1 PF00069.23	62.80	0.00
TRINITY_I7MLG9_TETTS/376-605	I7MLG9.2 PF00112.21	62.80	0.00
TRINITY_L8H9B8_ACACA/1085-1287	L8H9B8.1 PF02786.15	62.80	0.00
TRINITY_L8HAB6_ACACA/85-471	L8HAB6.1 PF02463.17	62.80	0.00
TRINITY_A8IU23_CHLRE/7-84	A8IU23.1 PF09429.8;	62.80	0.00
TRINITY_G4T6T5_PIRID/31-151	G4T6T5.1 PF01408.20	62.80	0.00
TRINITY_E1X1Z7_HALMS/34-321	E1X1Z7.1 PF00294.22	62.80	0.00
TRINITY_I7M6F2_TETTS/272-314	I7M6F2.1 PF00400.30	62.80	0.00
TRINITY_L8GUU1_ACACA/68-321	L8GUU1.1 PF00069.23	62.80	0.00
TRINITY_D8UEG7_VOLCA/1411-1882	D8UEG7.1 PF02738.16	62.80	0.00
TRINITY_A8JHW6_CHLRE/43-718	A8JHW6.1 PF00995.21	62.80	0.00
TRINITY_A8HWL3_CHLRE/1-192	A8HWL3.1 PF07837.10	62.80	0.00
TRINITY_A0D2Y8_PARTE/22-645	A0D2Y8.1 PF00133.20	62.80	0.00
TRINITY_C1MW67_MICPC/420-627	C1MW67.1 PF00448.20	62.80	0.00
TRINITY_E1Z9J1_CHLVA/157-454	E1Z9J1.1 PF00632.23	62.80	0.00
TRINITY_D8UC10_VOLCA/180-416	D8UC10.1 PF03407.14	62.80	0.00
TRINITY_G0R514_ICHMG/672-757	G0R514.1 PF00867.16	62.80	0.00
TRINITY_F4PZD1_DICFS/58-409	F4PZD1.1 PF00285.19	62.80	0.00
TRINITY_K1PMZ3_CRAGI/4622-4780	K1PMZ3.1 PF00179.24	62.80	0.00
TRINITY_E1Z2A8_CHLVA/13-426	E1Z2A8.1 PF01593.22	62.80	0.00
TRINITY_D8TVP0_VOLCA/34-178	D8TVP0.1 PF08190.10	62.80	0.00
TRINITY_D8UBI0_VOLCA/14-440	D8UBI0.1 PF00067.20	62.80	0.00
TRINITY_E1ZBC0_CHLVA/20-351	E1ZBC0.1 PF00225.21	62.80	0.00
TRINITY_I0YPG1_9CHLO/7-423	I0YPG1.1 PF00501.26	62.80	0.00
TRINITY_G0R4A1_ICHMG/1-230	G0R4A1.1 PF00225.21	62.80	0.00
TRINITY_A0A078AB47_STYLE/144-465	A0A078AB47.1 PF0064	62.80	0.00
TRINITY_F4PJU2_DICFS/59-319	F4PJU2.1 PF00069.23	62.80	0.00
TRINITY_F2UBG1_SALR5/68-126	F2UBG1.1 PF05608.10	62.70	0.00
TRINITY_G7Q415_9DELT/111-229	G7Q415.1 PF00877.17	62.70	0.00
TRINITY_A0D4V9_PARTE/19-283	A0D4V9.1 PF02263.17	62.70	0.00
TRINITY_C5LAN5_PERM5/8-168	C5LAN5.1 PF00406.20	62.70	0.00
TRINITY_D3BFW6_POLPA/46-147	D3BFW6.1 PF00011.19	62.70	0.00
TRINITY_H2XN33_CIOIN/273-341	H2XN33.1 PF00076.20	62.70	0.00
TRINITY_M0QSG0_ACACA/39-129	M0QSG0.1 PF02214.20	62.70	0.00
TRINITY_F1A589_DICPU/12-113	F1A589.1 PF01990.15	62.70	0.00
TRINITY_S8CF35_9LAMI/516-629	S8CF35.1 PF00271.29	62.70	0.00
TRINITY_TTL3C_TETTS/705-1030	Q23FE2.1 PF03133.13	62.70	0.00
TRINITY_Q5ZUW7_LEGPH/33-284	Q5ZUW7.1 PF01259.16	62.70	0.00
TRINITY_A9RI14_PHYPA/120-458	A9RI14.1 PF01074.20	62.70	0.00
TRINITY_E7KVV6_YEASL/41-142	E7KVV6.1 PF13649.4;	62.70	0.00
TRINITY_F4S555_MELLP/761-969	F4S555.1 PF00702.24	62.70	0.00
TRINITY_Q22V76_TETTS/529-664	Q22V76.2 PF00005.25	62.70	0.00
TRINITY_L8HKM6_ACACA/8-83	L8HKM6.1 PF12701.5;	62.70	0.00
TRINITY_F0ZGG2_DICPU/3-254	F0ZGG2.1 PF10609.7;	62.70	0.00
TRINITY_A8J6H2_CHLRE/100-254	A8J6H2.1 PF14996.4;	62.70	0.00
TRINITY_Q23DJ0_TETTS/477-557	Q23DJ0.1 PF02319.18	62.70	0.00
TRINITY_D8TZE1_VOLCA/133-328	D8TZE1.1 PF09991.7;	62.70	0.00
TRINITY_MYOD_DICDI/9-674	P34109.2 PF00063.19	62.70	0.00
TRINITY_E1ZTC6_CHLVA/34-107	E1ZTC6.1 PF03703.12	62.70	0.00
TRINITY_A0E719_PARTE/9-357	A0E719.1 PF00225.21	62.70	0.00
TRINITY_A8IC88_CHLRE/107-224	A8IC88.1 PF13640.4;	62.70	0.00
TRINITY_G3W6X4_SARHA/274-326	G3W6X4.1 PF00628.27	62.70	0.00
TRINITY_A0A0K0E3Y2_STRER/6-65	A0A0K0E3Y2.1 PF0022	62.70	0.00
TRINITY_A8JH94_CHLRE/12-74	A8JH94.1 PF01423.20	62.70	0.00
TRINITY_F2K4M5_MARM1/466-865	F2K4M5.1 PF02347.14	62.70	0.00
TRINITY_A8JFK5_CHLRE/351-433	A8JFK5.1 PF00789.18	62.70	0.00
TRINITY_A9STP2_PHYPA/980-1079	A9STP2.1 PF11923.6;	62.70	0.00
TRINITY_A8JHW5_CHLRE/324-436	A8JHW5.1 PF04780.10	62.70	0.00
TRINITY_A0DWB8_PARTE/118-215	A0DWB8.1 PF00581.18	62.70	0.00
TRINITY_Q23GD1_TETTS/1838-1959	Q23GD1.2 PF00069.23	62.70	0.00

TRINITY_D8U9E7_VOLCA/173-708	D8U9E7.1 PF14740.4;	62.70	0.00
TRINITY_L8GP31_ACACA/22-272	L8GP31.1 PF00069.23	62.70	0.00
TRINITY_D8THK9_VOLCA/34-111	D8THK9.1 PF04232.10	62.70	0.00
TRINITY_D7FVB1_ECTSI/366-501	D7FVB1.1 PF00005.25	62.70	0.00
TRINITY_B8C9T3_THAPS/326-385	B8C9T3.1 PF01485.19	62.70	0.00
TRINITY_A0BL44_PARTE/620-730	A0BL44.1 PF13426.5;	62.70	0.00
TRINITY_A8IUJ0_CHLRE/149-489	A8IUJ0.1 PF00883.19	62.70	0.00
TRINITY_D8U239_VOLCA/1-248	D8U239.1 PF00294.22	62.70	0.00
TRINITY_F0ZP53_DICPU/58-468	F0ZP53.1 PF01704.16	62.70	0.00
TRINITY_G0QQZ2_ICHMG/14-154	G0QQZ2.1 PF00080.18	62.70	0.00
TRINITY_D2VEZ6_NAEGR/6-109	D2VEZ6.1 PF00307.29	62.70	0.00
TRINITY_A0A087SI65_AUXPR/343-497	A0A087SI65.1 PF1308	62.70	0.00
TRINITY_E1Z7M6_CHLVA/19-145	E1Z7M6.1 PF00134.21	62.70	0.00
TRINITY_Q8LSS7_ORYSJ/928-1073	Q8LSS7.1 PF00078.25	62.70	0.00
TRINITY_E1ZEY2_CHLVA/58-141	E1ZEY2.1 PF04359.12	62.70	0.00
TRINITY_E1ZDD9_CHLVA/24-466	E1ZDD9.1 PF05577.10	62.70	0.00
TRINITY_A0DL63_PARTE/5-202	A0DL63.1 PF02114.14	62.70	0.00
TRINITY_D8UGY5_VOLCA/697-931	D8UGY5.1 PF07059.10	62.70	0.00
TRINITY_U6PTD8_HAECO/13-83	U6PTD8.1 PF13499.4;	62.70	0.00
TRINITY_G2PN37_MURRD/2-184	G2PN37.1 PF01965.22	62.60	0.00
TRINITY_K3X9M8_PYTUL/42-511	K3X9M8.1 PF00171.20	62.60	0.00
TRINITY_D8TL53_VOLCA/30-163	D8TL53.1 PF00782.18	62.60	0.00
TRINITY_PHB2_DICDI/42-223	Q54Q31.1 PF01145.23	62.60	0.00
TRINITY_D8TP55_VOLCA/67-324	D8TP55.1 PF00233.17	62.60	0.00
TRINITY_G0MGC2_CAEBE/1-132	G0MGC2.1 PF00125.22	62.60	0.00
TRINITY_C7M9I1_CAPOD/34-265	C7M9I1.1 PF01182.18	62.60	0.00
TRINITY_A8HPM8_CHLRE/71-161	A8HPM8.1 PF14347.4;	62.60	0.00
TRINITY_F6HY26_VITVI/73-235	F6HY26.1 PF03446.13	62.60	0.00
TRINITY_E1ZKG3_CHLVA/126-281	E1ZKG3.1 PF13847.4;	62.60	0.00
TRINITY_J9I6X9_9SPIT/47-180	J9I6X9.1 PF00578.19	62.60	0.00
TRINITY_A0EEH1_PARTE/11-437	A0EEH1.1 PF00171.20	62.60	0.00
TRINITY_H1YF66_9SPHI/5-196	H1YF66.1 PF00316.18	62.60	0.00
TRINITY_F0ZMB0_DICPU/159-249	F0ZMB0.1 PF02214.20	62.60	0.00
TRINITY_A0A0N4Z2A3_PARTI/161-322	A0A0N4Z2A3.1 PF0007	62.60	0.00
TRINITY_PI3K4_DICDI/557-762	P54676.2 PF00454.25	62.60	0.00
TRINITY_A7RU37_NEMVE/31-537	A7RU37.1 PF00330.18	62.60	0.00
TRINITY_M2XX62_GALSU/4-177	M2XX62.1 PF00163.17	62.60	0.00
TRINITY_D8TL22_VOLCA/37-856	D8TL22.1 PF01496.17	62.60	0.00
TRINITY_A8ISU4_CHLRE/484-681	A8ISU4.1 PF01061.22	62.60	0.00
TRINITY_D8TNC6_VOLCA/84-266	D8TNC6.1 PF04755.10	62.60	0.00
TRINITY_A8I832_CHLRE/131-229	A8I832.1 PF03091.13	62.60	0.00
TRINITY_A8JGE8_CHLRE/187-322	A8JGE8.1 PF01170.16	62.60	0.00
TRINITY_D8TIC3_VOLCA/148-427	D8TIC3.1 PF00009.25	62.60	0.00
TRINITY_J3MXD5_ORYBR/40-148	J3MXD5.1 PF13616.4;	62.60	0.00
TRINITY_A0A068XBT7_HYMMI/1021-1170	A0A068XBT7.2 PF0000	62.60	0.00
TRINITY_W5KLE2_ASTMX/189-285	W5KLE2.1 PF11069.6;	62.60	0.00
TRINITY_A0A0M3JBN3_ANISI/3-59	A0A0M3JBN3.1 PF0456	62.50	0.00
TRINITY_W5M8K1_LEPOC/13-52	W5M8K1.1 PF03966.14	62.50	0.00
TRINITY_E4T484_PALPW/44-249	E4T484.1 PF04402.12	62.50	0.00
TRINITY_B3RL36_TRIAD/117-506	B3RL36.1 PF01979.18	62.50	0.00
TRINITY_B4QP44_DROSI/37-109	B4QP44.1 PF02594.14	62.50	0.00
TRINITY_A0A0A0KW25_CUCSA/102-181	A0A0A0KW25.1 PF0034	62.50	0.00
TRINITY_Q231M6_TETTS/6-197	Q231M6.1 PF11539.6;	62.50	0.00
TRINITY_D8TIL0_VOLCA/2037-2086	D8TIL0.1 PF00847.18	62.50	0.00
TRINITY_B3S565_TRIAD/8-71	B3S565.1 PF01066.19	62.50	0.00
TRINITY_I7M0D5_TETTS/4-286	I7M0D5.2 PF00069.23	62.50	0.00
TRINITY_A0CCP6_PARTE/592-655	A0CCP6.1 PF13432.4;	62.50	0.00
TRINITY_D8TH10_VOLCA/161-276	D8TH10.1 PF01565.21	62.50	0.00
TRINITY_A0D7X4_PARTE/11-52	A0D7X4.1 PF05186.11	62.50	0.00
TRINITY_Q0CVY7_ASPTN/153-264	Q0CVY7.1 PF00970.22	62.50	0.00
TRINITY_A0A078AAI9_STYLE/21-285	A0A078AAI9.1 PF0165	62.50	0.00

TRINITY_M3ZYP5_XIPMA/1-230	M3ZYP5.1 PF00149.26	62.50	0.00
TRINITY_D8U203_VOLCA/5-111	D8U203.1 PF03179.13	62.50	0.00
TRINITY_I7MMG7_TETTS/4-265	I7MMG7.1 PF00069.23	62.50	0.00
TRINITY_D8U467_VOLCA/4-95	D8U467.1 PF12796.5;	62.50	0.00
TRINITY_Q23BQ5_TETTS/133-203	Q23BQ5.1 PF01287.18	62.50	0.00
TRINITY_A8J2V3_CHLRE/15-193	A8J2V3.1 PF01871.15	62.50	0.00
TRINITY_Q234R3_TETTS/8-301	Q234R3.1 PF04502.11	62.50	0.00
TRINITY_D8TSJ5_VOLCA/2597-3305	D8TSJ5.1 PF03028.13	62.50	0.00
TRINITY_U9UGD1_RHIID/9-71	U9UGD1.1 PF08154.10	62.50	0.00
TRINITY_D8TPH2_VOLCA/7-288	D8TPH2.1 PF01148.18	62.50	0.00
TRINITY_A8JB70_CHLRE/362-427	A8JB70.1 PF00226.29	62.50	0.00
TRINITY_RUB2_ARATH/79-150	Q8RUC6.3 PF00240.21	62.50	0.00
TRINITY_A0CYP7_PARTE/16-152	A0CYP7.1 PF00782.18	62.50	0.00
TRINITY_A0A078B2X8_STYLE/192-239	A0A078B2X8.1 PF1341	62.50	0.00
TRINITY_A8IYC3_CHLRE/90-243	A8IYC3.1 PF00005.25	62.50	0.00
TRINITY_S8C461_9LAMI/592-752	S8C461.1 PF00078.25	62.50	0.00
TRINITY_K1RXS2_CRAGI/10-121	K1RXS2.1 PF03874.14	62.50	0.00
TRINITY_F4PER5_BATDJ/27-305	F4PER5.1 PF00814.23	62.50	0.00
TRINITY_W5KQJ9_ASTMX/424-719	W5KQJ9.1 PF00176.21	62.50	0.00
TRINITY_A0A0D2X315_CAPO3/79-542	A0A0D2X315.1 PF0022	62.50	0.00
TRINITY_A8HPD4_CHLRE/13-273	A8HPD4.1 PF00520.29	62.50	0.00
TRINITY_A8JBE4_CHLRE/465-642	A8JBE4.1 PF11916.6;	62.50	0.00
TRINITY_A8HYL9_CHLRE/331-803	A8HYL9.1 PF00939.17	62.50	0.00
TRINITY_A0BV71_PARTE/2-85	A0BV71.1 PF08648.10	62.50	0.00
TRINITY_D8TX09_VOLCA/154-320	D8TX09.1 PF04851.13	62.50	0.00
TRINITY_A8J0A5_CHLRE/308-363	A8J0A5.1 PF13513.4;	62.50	0.00
TRINITY_A8IQI1_CHLRE/1-179	A8IQI1.1 PF01184.17	62.50	0.00
TRINITY_I0Z6C9_9CHLO/39-314	I0Z6C9.1 PF00850.17	62.50	0.00
TRINITY_Q1WWC9_DROME/1031-1079	Q1WWC9.1 PF14604.4;	62.50	0.00
TRINITY_L8H2N6_ACACA/244-611	L8H2N6.1 PF00916.18	62.50	0.00
TRINITY_LONM_OSTLU/725-932	A4S6Y4.1 PF05362.11	62.50	0.00
TRINITY_A8HSY0_CHLRE/44-461	A8HSY0.1 PF00474.15	62.50	0.00
TRINITY_D3BLR6_POLPA/274-329	D3BLR6.1 PF00412.20	62.50	0.00
TRINITY_A8HVT9_CHLRE/12-303	A8HVT9.1 PF17184.2;	62.50	0.00
TRINITY_D5GEP6_TUBMM/30-69	D5GEP6.1 PF05920.9;	62.50	0.00
TRINITY_D8U978_VOLCA/2-203	D8U978.1 PF07714.15	62.50	0.00
TRINITY_A0BIX8_PARTE/187-226	A0BIX8.1 PF05920.9;	62.50	0.00
TRINITY_A0A058ZZ18_EUCGR/78-186	A0A058ZZ18.1 PF0149	62.50	0.00
TRINITY_A0A078AEL1_STYLE/19-303	A0A078AEL1.1 PF0006	62.50	0.00
TRINITY_C5Z583_SORBI/934-1179	C5Z583.1 PF07727.12	62.50	0.00
TRINITY_G0IY52_CYCMS/2-184	G0IY52.1 PF01965.22	62.40	0.00
TRINITY_L8GXR0_ACACA/7-117	L8GXR0.1 PF02136.18	62.40	0.00
TRINITY_F0ZGK2_DICPU/47-224	F0ZGK2.1 PF00719.17	62.40	0.00
TRINITY_F0ZTG5_DICPU/6-231	F0ZTG5.1 PF03731.13	62.40	0.00
TRINITY_A0A088AP06_APIME/31-216	A0A088AP06.1 PF0022	62.40	0.00
TRINITY_F0Z9J5_DICPU/99-202	F0Z9J5.1 PF04191.11	62.40	0.00
TRINITY_F0Z6C1_DICPU/120-348	F0Z6C1.1 PF00557.22	62.40	0.00
TRINITY_A0E226_PARTE/313-438	A0E226.1 PF00134.21	62.40	0.00
TRINITY_A0A0A1NXR9_9FUNG/178-611	A0A0A1NXR9.1 PF0015	62.40	0.00
TRINITY_A0A078ATQ4_STYLE/166-308	A0A078ATQ4.1 PF0551	62.40	0.00
TRINITY_A8HVE3_CHLRE/27-112	A8HVE3.1 PF03083.14	62.40	0.00
TRINITY_L8HGG8_ACACA/19-143	L8HGG8.1 PF14226.4;	62.40	0.00
TRINITY_L8GYR2_ACACA/26-254	L8GYR2.1 PF04981.11	62.40	0.00
TRINITY_D8UDG7_VOLCA/130-319	D8UDG7.1 PF00119.18	62.40	0.00
TRINITY_A0A078BAI8_STYLE/6-263	A0A078BAI8.1 PF0006	62.40	0.00
TRINITY_A8IAG6_CHLRE/48-294	A8IAG6.1 PF01080.15	62.40	0.00
TRINITY_D8SLV4_SELML/2-167	D8SLV4.1 PF01217.18	62.40	0.00
TRINITY_A0A0N4UQK4_DRAME/19-197	A0A0N4UQK4.1 PF0122	62.40	0.00
TRINITY_M2N7K9_BAUCO/19-318	M2N7K9.1 PF00248.19	62.40	0.00
TRINITY_G0QPK0_ICHMG/7-249	G0QPK0.1 PF00121.16	62.40	0.00
TRINITY_Q22A08_TETTS/126-234	Q22A08.2 PF04430.12	62.40	0.00

TRINITY_A0A0N4UWM2_ENTVE/608-775	A0A0N4UWM2.1 PF1330	62.40	0.00
TRINITY_A8HMI1_CHLRE/46-131	A8HMI1.1 PF01627.21	62.40	0.00
TRINITY_A8HY41_CHLRE/145-368	A8HY41.1 PF07228.10	62.40	0.00
TRINITY_A0A068S0Q9_9FUNG/99-436	A0A068S0Q9.1 PF0310	62.40	0.00
TRINITY_A8JFP4_CHLRE/222-560	A8JFP4.1 PF03372.21	62.40	0.00
TRINITY_I0YML0_9CHLO/1-132	I0YML0.1 PF00504.19	62.40	0.00
TRINITY_D3B782_POLPA/7-248	D3B782.1 PF00121.16	62.40	0.00
TRINITY_E1ZKH7_CHLVA/16-179	E1ZKH7.1 PF12706.5;	62.40	0.00
TRINITY_D3AXW6_POLPA/88-287	D3AXW6.1 PF07002.14	62.40	0.00
TRINITY_D8TUX7_VOLCA/78-160	D8TUX7.1 PF16282.3;	62.40	0.00
TRINITY_A1ZPA1_9BACT/45-169	A1ZPA1.1 PF01661.19	62.40	0.00
TRINITY_D8LEI9_ECTSI/185-413	D8LEI9.1 PF13621.4;	62.40	0.00
TRINITY_A0A078B8U4_STYLE/32-494	A0A078B8U4.1 PF0017	62.40	0.00
TRINITY_D8U584_VOLCA/216-459	D8U584.1 PF03092.14	62.40	0.00
TRINITY_D8UBC2_VOLCA/92-232	D8UBC2.1 PF00588.17	62.40	0.00
TRINITY_Q23YB5_TETTS/199-456	Q23YB5.2 PF00069.23	62.30	0.00
TRINITY_I7MGI4_TETTS/15-68	I7MGI4.1 PF05347.13	62.30	0.00
TRINITY_Q54PB7_DICDI/1358-1684	Q54PB7.1 PF06920.11	62.30	0.00
TRINITY_I7MDU0_TETTS/12-274	I7MDU0.1 PF00069.23	62.30	0.00
TRINITY_L8GRH2_ACACA/50-526	L8GRH2.1 PF01039.20	62.30	0.00
TRINITY_H8Z269_9GAMM/13-315	H8Z269.1 PF00923.17	62.30	0.00
TRINITY_TTL6A_TETTS/399-697	Q23MT7.1 PF03133.13	62.30	0.00
TRINITY_F0ZGW6_DICPU/11-678	F0ZGW6.1 PF00063.19	62.30	0.00
TRINITY_I7MLH4_TETTS/52-165	I7MLH4.1 PF11527.6;	62.30	0.00
TRINITY_A9RB21_PHYPA/45-204	A9RB21.1 PF01233.17	62.30	0.00
TRINITY_F4Q3F3_DICFS/139-199	F4Q3F3.1 PF00957.19	62.30	0.00
TRINITY_K9VUP7_9CYAN/15-318	K9VUP7.1 PF00248.19	62.30	0.00
TRINITY_A8HQ67_CHLRE/8-327	A8HQ67.1 PF16499.3;	62.30	0.00
TRINITY_A8II54_CHLRE/13-296	A8II54.1 PF00664.21	62.30	0.00
TRINITY_D8TID0_VOLCA/38-138	D8TID0.1 PF00466.18	62.30	0.00
TRINITY_D8U7J2_VOLCA/97-150	D8U7J2.1 PF01344.23	62.30	0.00
TRINITY_A0EG50_PARTE/304-525	A0EG50.1 PF07002.14	62.30	0.00
TRINITY_I0YS07_9CHLO/29-89	I0YS07.1 PF04193.12	62.30	0.00
TRINITY_C2M6V4_CAPGI/2-222	C2M6V4.1 PF01026.19	62.30	0.00
TRINITY_A8I5W8_CHLRE/36-112	A8I5W8.1 PF03992.14	62.30	0.00
TRINITY_D8TYP4_VOLCA/93-412	D8TYP4.1 PF01546.26	62.30	0.00
TRINITY_I7M700_TETTS/181-443	I7M700.2 PF00069.23	62.30	0.00
TRINITY_D8U5B9_VOLCA/23-226	D8U5B9.1 PF01965.22	62.30	0.00
TRINITY_A0EFU5_PARTE/59-386	A0EFU5.1 PF01148.18	62.30	0.00
TRINITY_A8IHD9_CHLRE/504-670	A8IHD9.1 PF07717.14	62.30	0.00
TRINITY_B1H117_XENTR/33-212	B1H117.1 PF00270.27	62.30	0.00
TRINITY_D8UJ84_VOLCA/1008-1082	D8UJ84.1 PF04640.12	62.30	0.00
TRINITY_Q22T18_TETTS/2-176	Q22T18.1 PF00025.19	62.30	0.00
TRINITY_A0A015IDK6_9GLOM/201-443	A0A015IDK6.1 PF1362	62.30	0.00
TRINITY_Q23Q10_TETTS/220-310	Q23Q10.2 PF15963.3;	62.30	0.00
TRINITY_Q54G77_DICDI/24-165	Q54G77.1 PF09286.9;	62.30	0.00
TRINITY_G0QQT1_ICHMG/68-119	G0QQT1.1 PF00415.16	62.30	0.00
TRINITY_A0A077ZVL1_STYLE/3835-4552	A0A077ZVL1.1 PF0302	62.30	0.00
TRINITY_I1C2M6_RHIO9/30-175	I1C2M6.1 PF00293.26	62.30	0.00
TRINITY_F0ZMG6_DICPU/616-703	F0ZMG6.1 PF00679.22	62.30	0.00
TRINITY_D6WW15_TRICA/43-94	D6WW15.1 PF02229.14	62.30	0.00
TRINITY_L8GES2_ACACA/67-143	L8GES2.1 PF13499.4;	62.30	0.00
TRINITY_D8UHF7_VOLCA/536-669	D8UHF7.1 PF07717.14	62.30	0.00
TRINITY_E1ZK06_CHLVA/262-317	E1ZK06.1 PF00226.29	62.30	0.00
TRINITY_G0QL61_ICHMG/18-265	G0QL61.1 PF06705.9;	62.30	0.00
TRINITY_D8TJS4_VOLCA/266-380	D8TJS4.1 PF02576.15	62.30	0.00
TRINITY_A8J4K4_CHLRE/57-279	A8J4K4.1 PF02602.13	62.30	0.00
TRINITY_A8IR64_CHLRE/183-235	A8IR64.1 PF05739.17	62.30	0.00
TRINITY_A0A0A1PIP5_9FUNG/166-244	A0A0A1PIP5.1 PF1364	62.30	0.00
TRINITY_D8TVC0_VOLCA/1983-2096	D8TVC0.1 PF00665.24	62.30	0.00
TRINITY_F8JKS3_STREN/564-731	F8JKS3.1 PF08760.9;	62.20	0.00

TRINITY_G2G1L5_9FIRM/28-276	G2G1L5.1 PF02784.14	62.20	0.00
TRINITY_A0A0G4F0S1_9ALVE/11-50	A0A0G4F0S1.1 PF0142	62.20	0.00
TRINITY_A0D3Y9_PARTE/54-129	A0D3Y9.1 PF08241.10	62.20	0.00
TRINITY_A0EBG4_PARTE/41-332	A0EBG4.1 PF05185.14	62.20	0.00
TRINITY_L1IB99_GUITH/5-101	L1IB99.1 PF01133.15	62.20	0.00
TRINITY_A0A0J7K787_LASNI/141-178	A0A0J7K787.1 PF0933	62.20	0.00
TRINITY_C1EFY8_MICSR/1-98	C1EFY8.1 PF00453.16	62.20	0.00
TRINITY_A0D476_PARTE/34-353	A0D476.1 PF00180.18	62.20	0.00
TRINITY_A0A0D2UGV5_CAPO3/32-187	A0A0D2UGV5.1 PF0163	62.20	0.00
TRINITY_CLCE_DICDI/235-609	Q54LQ4.1 PF00654.18	62.20	0.00
TRINITY_Q7V4P6_PROMM/458-658	Q7V4P6.1 PF00106.23	62.20	0.00
TRINITY_D2VMQ6_NAEGR/60-212	D2VMQ6.1 PF00182.17	62.20	0.00
TRINITY_F0ZJT3_DICPU/35-109	F0ZJT3.1 PF13656.4;	62.20	0.00
TRINITY_C1E4S7_MICSR/834-960	C1E4S7.1 PF14806.4;	62.20	0.00
TRINITY_Q23QY3_TETTS/416-917	Q23QY3.2 PF00899.19	62.20	0.00
TRINITY_A8IHC4_CHLRE/607-855	A8IHC4.1 PF00082.20	62.20	0.00
TRINITY_G1KBH5_ANOCA/186-475	G1KBH5.2 PF05291.9;	62.20	0.00
TRINITY_I7MGA5_TETTS/126-350	I7MGA5.1 PF00687.19	62.20	0.00
TRINITY_D8TTK6_VOLCA/730-956	D8TTK6.1 PF12576.6;	62.20	0.00
TRINITY_L8HCN5_ACACA/51-374	L8HCN5.1 PF08627.8;	62.20	0.00
TRINITY_D8TJC9_VOLCA/6-275	D8TJC9.1 PF00109.24	62.20	0.00
TRINITY_E1Z921_CHLVA/46-198	E1Z921.1 PF00929.22	62.20	0.00
TRINITY_K3Y5M4_SETIT/355-646	K3Y5M4.1 PF00069.23	62.20	0.00
TRINITY_W5NAE3_LEPOC/86-270	W5NAE3.1 PF02146.15	62.20	0.00
TRINITY_D8U8B9_VOLCA/387-523	D8U8B9.1 PF00069.23	62.20	0.00
TRINITY_A0E876_PARTE/28-215	A0E876.1 PF00227.24	62.20	0.00
TRINITY_I7LUR8_TETTS/1292-1437	I7LUR8.2 PF00637.18	62.20	0.00
TRINITY_M1VII6_CYAME/556-744	M1VII6.1 PF13481.4;	62.20	0.00
TRINITY_D3B3I9_POLPA/42-544	D3B3I9.1 PF00118.22	62.20	0.00
TRINITY_D8UBW1_VOLCA/31-522	D8UBW1.1 PF00118.22	62.20	0.00
TRINITY_A8IV13_CHLRE/26-108	A8IV13.1 PF08700.9;	62.20	0.00
TRINITY_E3LME5_CAERE/1-148	E3LME5.1 PF01217.18	62.20	0.00
TRINITY_L8GEZ6_ACACA/756-1044	L8GEZ6.1 PF00176.21	62.20	0.00
TRINITY_D8U9B6_VOLCA/193-239	D8U9B6.1 PF13415.4;	62.20	0.00
TRINITY_F4QCT6_DICFS/606-750	F4QCT6.1 PF00005.25	62.20	0.00
TRINITY_D3AYP3_POLPA/1472-1615	D3AYP3.1 PF00588.17	62.20	0.00
TRINITY_I1LXZ7_SOYBN/198-371	I1LXZ7.1 PF02866.16	62.20	0.00
TRINITY_D8U962_VOLCA/204-366	D8U962.1 PF06733.13	62.20	0.00
TRINITY_D8TQZ7_VOLCA/265-459	D8TQZ7.1 PF08323.9;	62.20	0.00
TRINITY_H3ERC5_PRIPA/29-149	H3ERC5.1 PF01873.15	62.20	0.00
TRINITY_X1W3S0_TETTS/318-363	X1W3S0.1 PF13639.4;	62.20	0.00
TRINITY_D8TNU9_VOLCA/13-614	D8TNU9.1 PF00888.20	62.20	0.00
TRINITY_B8CFV7_THAPS/409-453	B8CFV7.1 PF13639.4;	62.20	0.00
TRINITY_A8JHU5_CHLRE/130-356	A8JHU5.1 PF04499.13	62.20	0.00
TRINITY_A0BML3_PARTE/159-391	A0BML3.1 PF00122.18	62.20	0.00
TRINITY_E1Z4Y7_CHLVA/643-686	E1Z4Y7.1 PF00400.30	62.20	0.00
TRINITY_D8UB19_VOLCA/380-475	D8UB19.1 PF13650.4;	62.20	0.00
TRINITY_J3NDH4_ORYBR/147-405	J3NDH4.1 PF00069.23	62.20	0.00
TRINITY_D8U1P0_VOLCA/190-448	D8U1P0.1 PF00704.26	62.20	0.00
TRINITY_Q8LP67_CHLRE/27-494	Q8LP67.2 PF01384.18	62.20	0.00
TRINITY_D8U2S1_VOLCA/86-258	D8U2S1.1 PF10294.7;	62.20	0.00
TRINITY_I1NBN5_SOYBN/673-757	I1NBN5.1 PF08033.10	62.20	0.00
TRINITY_H0HLR9_9RHIZ/181-299	H0HLR9.1 PF13673.5;	62.20	0.00
TRINITY_G0S1D0_CHATD/503-547	G0S1D0.1 PF00400.30	62.20	0.00
TRINITY_I7M465_TETTS/267-321	I7M465.1 PF01485.19	62.20	0.00
TRINITY_L8HG70_ACACA/1131-1300	L8HG70.1 PF13091.4;	62.20	0.00
TRINITY_A0DRN1_PARTE/90-336	A0DRN1.1 PF03372.21	62.10	0.00
TRINITY_A0A087SA88_AUXPR/3-212	A0A087SA88.1 PF0009	62.10	0.00
TRINITY_A0C046_PARTE/90-390	A0C046.1 PF03133.13	62.10	0.00
TRINITY_A0A0J8BP99_BETVU/27-294	A0A0J8BP99.1 PF0006	62.10	0.00
TRINITY_I7MKU0_TETTS/11-96	I7MKU0.1 PF10235.7;	62.10	0.00

TRINITY_E1ZC14_CHLVA/145-340	E1ZC14.1 PF07859.11	62.10	0.00
TRINITY_V5BPK4_TRYCR/251-504	V5BPK4.1 PF00069.23	62.10	0.00
TRINITY_G8BY52_TETPH/1063-1540	G8BY52.1 PF06202.12	62.10	0.00
TRINITY_F1A4R1_DICPU/94-192	F1A4R1.1 PF00153.25	62.10	0.00
TRINITY_L8H350_ACACA/3-284	L8H350.1 PF03643.13	62.10	0.00
TRINITY_A0A0G4ETT9_9ALVE/1700-1885	A0A0G4ETT9.1 PF0053	62.10	0.00
TRINITY_L8HGA2_ACACA/177-385	L8HGA2.1 PF02475.14	62.10	0.00
TRINITY_W7TKS0_9STRA/126-321	W7TKS0.1 PF03171.18	62.10	0.00
TRINITY_A8JH37_CHLRE/6-324	A8JH37.1 PF08267.10	62.10	0.00
TRINITY_B2RZH9_VOLCA/39-279	B2RZH9.1 PF00121.16	62.10	0.00
TRINITY_A8J9T2_CHLRE/21-86	A8J9T2.1 PF12254.6;	62.10	0.00
TRINITY_A0A068RW80_9FUNG/216-433	A0A068RW80.1 PF0514	62.10	0.00
TRINITY_SPTC2_DICDI/110-470	Q54EX5.1 PF00155.19	62.10	0.00
TRINITY_A8JBV9_CHLRE/72-227	A8JBV9.1 PF10294.7;	62.10	0.00
TRINITY_I7LTE1_TETTS/844-1165	I7LTE1.1 PF03178.13	62.10	0.00
TRINITY_D8TRM7_VOLCA/133-258	D8TRM7.1 PF13640.4;	62.10	0.00
TRINITY_D8UJ39_VOLCA/18-298	D8UJ39.1 PF06027.10	62.10	0.00
TRINITY_H0ZFW0_TAEGU/1642-1785	H0ZFW0.1 PF07728.12	62.10	0.00
TRINITY_M3XQ36_MUSPF/73-326	M3XQ36.1 PF03159.16	62.10	0.00
TRINITY_D3B6M3_POLPA/229-533	D3B6M3.1 PF00082.20	62.10	0.00
TRINITY_G0QUB2_ICHMG/81-382	G0QUB2.1 PF00999.19	62.10	0.00
TRINITY_A0A087SJE4_AUXPR/92-310	A0A087SJE4.1 PF0216	62.10	0.00
TRINITY_A0E4T2_PARTE/382-534	A0E4T2.1 PF00005.25	62.10	0.00
TRINITY_A8IZE1_CHLRE/504-623	A8IZE1.1 PF13474.4;	62.10	0.00
TRINITY_G4QHL6_GLANF/22-153	G4QHL6.1 PF13185.4;	62.10	0.00
TRINITY_A8JHB2_CHLRE/220-447	A8JHB2.1 PF01458.15	62.10	0.00
TRINITY_A8IAC7_CHLRE/163-265	A8IAC7.1 PF01588.18	62.10	0.00
TRINITY_E1ZTC4_CHLVA/4-307	E1ZTC4.1 PF00923.17	62.10	0.00
TRINITY_Q22N18_TETTS/6443-6855	Q22N18.2 PF00899.19	62.10	0.00
TRINITY_D8TQZ7_VOLCA/498-682	D8TQZ7.1 PF00534.18	62.10	0.00
TRINITY_R1CA39_EMIHU/22-90	R1CA39.1 PF04564.13	62.10	0.00
TRINITY_A8IBR0_CHLRE/1139-1279	A8IBR0.1 PF05843.12	62.10	0.00
TRINITY_A0A059LLR0_9CHLO/29-278	A0A059LLR0.1 PF0075	62.10	0.00
TRINITY_A8JHJ5_CHLRE/128-220	A8JHJ5.1 PF00153.25	62.10	0.00
TRINITY_Q22TY7_TETTS/3-233	Q22TY7.1 PF04670.10	62.10	0.00
TRINITY_SULT2_CHLRE/98-492	A8J6J0.1 PF00916.18	62.10	0.00
TRINITY_E4X5J3_OIKDI/140-434	E4X5J3.1 PF00082.20	62.10	0.00
TRINITY_D8UI03_VOLCA/42-637	D8UI03.1 PF00012.18	62.10	0.00
TRINITY_R6EU84_9FIRM/363-512	R6EU84.1 PF00005.25	62.10	0.00
TRINITY_C3YGH6_BRAFL/5-211	C3YGH6.1 PF00149.26	62.10	0.00
TRINITY_Q22RG0_TETTS/26-78	Q22RG0.2 PF05047.14	62.00	0.00
TRINITY_R4XG17_TAPDE/5-54	R4XG17.1 PF01679.15	62.00	0.00
TRINITY_A7GM30_BACCN/8-529	A7GM30.1 PF01274.20	62.00	0.00
TRINITY_I7MD37_TETTS/11-258	I7MD37.1 PF03029.15	62.00	0.00
TRINITY_T1FNC7_HELRO/9-149	T1FNC7.1 PF00080.18	62.00	0.00
TRINITY_L8HJC1_ACACA/5-75	L8HJC1.1 PF00076.20	62.00	0.00
TRINITY_E2AJR5_CAMFO/1620-1767	E2AJR5.1 PF00005.25	62.00	0.00
TRINITY_W2RL36_9EURO/311-410	W2RL36.1 PF01545.19	62.00	0.00
TRINITY_E1ZQC0_CHLVA/1-314	E1ZQC0.1 PF16420.3;	62.00	0.00
TRINITY_G0R3K3_ICHMG/10-172	G0R3K3.1 PF00071.20	62.00	0.00
TRINITY_U6M9K5_EIMMA/2543-2722	U6M9K5.1 PF00534.18	62.00	0.00
TRINITY_M2N7K9_BAUCO/19-318	M2N7K9.1 PF00248.19	62.00	0.00
TRINITY_D8U0F4_VOLCA/142-300	D8U0F4.1 PF01556.16	62.00	0.00
TRINITY_A9USC1_MONBE/6-76	A9USC1.1 PF00076.20	62.00	0.00
TRINITY_F4PLK7_DICFS/36-501	F4PLK7.1 PF00171.20	62.00	0.00
TRINITY_A0A0G4EB95_9ALVE/641-835	A0A0G4EB95.1 PF0505	62.00	0.00
TRINITY_C1MLP4_MICPC/766-989	C1MLP4.1 PF01136.17	62.00	0.00
TRINITY_D8TUL4_VOLCA/2-165	D8TUL4.1 PF03453.15	62.00	0.00
TRINITY_F0VN86_NEOCL/225-295	F0VN86.1 PF00076.20	62.00	0.00
TRINITY_F0ZTE2_DICPU/105-183	F0ZTE2.1 PF00076.20	62.00	0.00
TRINITY_A0A078L386_9CHLA/610-815	A0A078L386.1 PF0536	62.00	0.00

TRINITY_E2C0I9_HARSA/25-117	E2C0I9.1 PF01243.18	62.00	0.00
TRINITY_Q237J4_TETTS/105-303	Q237J4.1 PF03587.12	62.00	0.00
TRINITY_D8UJ72_VOLCA/109-179	D8UJ72.1 PF00076.20	62.00	0.00
TRINITY_D8U4C4_VOLCA/2-132	D8U4C4.1 PF04756.11	62.00	0.00
TRINITY_A8IMU0_CHLRE/150-241	A8IMU0.1 PF00153.25	62.00	0.00
TRINITY_D8TX10_VOLCA/3-160	D8TX10.1 PF13328.4;	62.00	0.00
TRINITY_G0QL09_ICHMG/65-248	G0QL09.1 PF01926.21	62.00	0.00
TRINITY_I0YJB4_9CHLO/65-154	I0YJB4.1 PF13881.4;	62.00	0.00
TRINITY_Q23CU3_TETTS/521-600	Q23CU3.1 PF13193.4;	62.00	0.00
TRINITY_D8UFK2_VOLCA/39-209	D8UFK2.1 PF00504.19	62.00	0.00
TRINITY_D8TY55_VOLCA/277-416	D8TY55.1 PF02797.13	62.00	0.00
TRINITY_S2K6K5_MUCC1/522-772	S2K6K5.1 PF16203.3;	62.00	0.00
TRINITY_I1IJT0_BRADI/17-352	I1IJT0.1 PF00248.19	62.00	0.00
TRINITY_M2Y7F6_GALSU/41-353	M2Y7F6.1 PF00389.28	62.00	0.00
TRINITY_D3B5K0_POLPA/337-650	D3B5K0.1 PF02540.15	62.00	0.00
TRINITY_A8JKB8_CHLRE/3-82	A8JKB8.1 PF08386.8;	62.00	0.00
TRINITY_I0YI54_9CHLO/1666-2217	I0YI54.1 PF01039.20	62.00	0.00
TRINITY_D8TN96_VOLCA/20-315	D8TN96.1 PF05653.12	62.00	0.00
TRINITY_I7LXP9_TETTS/257-308	I7LXP9.2 PF13920.4;	62.00	0.00
TRINITY_T1KAI4_TETUR/159-262	T1KAI4.1 PF04857.18	62.00	0.00
TRINITY_Q6J214_CHLRE/95-363	Q6J214.1 PF00494.17	62.00	0.00
TRINITY_A8IVU1_CHLRE/157-347	A8IVU1.1 PF01758.14	62.00	0.00
TRINITY_D8TTW5_VOLCA/2-253	D8TTW5.1 PF00999.19	62.00	0.00
TRINITY_A0A074SRS4_HAMHA/863-1002	A0A074SRS4.1 PF0063	62.00	0.00
TRINITY_G0R4U1_ICHMG/30-190	G0R4U1.1 PF00160.19	61.90	0.00
TRINITY_Q22ZI1_TETTS/49-356	Q22ZI1.1 PF00443.27	61.90	0.00
TRINITY_I4B1X5_TURPD/4-137	I4B1X5.1 PF00578.19	61.90	0.00
TRINITY_A0A067K6N7_JATCU/94-157	A0A067K6N7.1 PF1349	61.90	0.00
TRINITY_F2TZG9_SALR5/133-174	F2TZG9.1 PF13639.4;	61.90	0.00
TRINITY_A0CMB2_PARTE/43-298	A0CMB2.1 PF00069.23	61.90	0.00
TRINITY_I7MGY2_TETTS/301-415	I7MGY2.1 PF02338.17	61.90	0.00
TRINITY_H3CEP1_TETNG/170-286	H3CEP1.1 PF02373.20	61.90	0.00
TRINITY_M0QSG3_ACACA/116-206	M0QSG3.1 PF02214.20	61.90	0.00
TRINITY_A0A0G4GU86_9ALVE/492-612	A0A0G4GU86.1 PF0268	61.90	0.00
TRINITY_J9HMM9_9SPIT/852-1080	J9HMM9.1 PF16212.3;	61.90	0.00
TRINITY_A0A096LR37_POEFO/605-648	A0A096LR37.1 PF1363	61.90	0.00
TRINITY_D8TKR5_VOLCA/1743-2169	D8TKR5.1 PF08490.10	61.90	0.00
TRINITY_D8TIU9_VOLCA/1-243	D8TIU9.1 PF00149.26	61.90	0.00
TRINITY_D8U7H9_VOLCA/70-449	D8U7H9.1 PF00632.23	61.90	0.00
TRINITY_C1EGQ2_MICSR/3-282	C1EGQ2.1 PF00069.23	61.90	0.00
TRINITY_A8JIP7_CHLRE/12-146	A8JIP7.1 PF03435.16	61.90	0.00
TRINITY_A8HVV8_CHLRE/131-217	A8HVV8.1 PF03083.14	61.90	0.00
TRINITY_A0DTL9_PARTE/204-384	A0DTL9.1 PF01853.16	61.90	0.00
TRINITY_T1JDX2_STRMM/461-504	T1JDX2.1 PF13639.4;	61.90	0.00
TRINITY_M0QSG3_ACACA/116-206	M0QSG3.1 PF02214.20	61.90	0.00
TRINITY_I7LWI9_TETTS/119-217	I7LWI9.2 PF00581.18	61.90	0.00
TRINITY_A8IHH9_CHLRE/174-286	A8IHH9.1 PF01789.14	61.90	0.00
TRINITY_A8HX98_CHLRE/512-668	A8HX98.1 PF01734.20	61.90	0.00
TRINITY_Q23RV6_TETTS/6-277	Q23RV6.1 PF06732.9;	61.90	0.00
TRINITY_A8I678_CHLRE/1-190	A8I678.1 PF05158.10	61.90	0.00
TRINITY_I0Z859_9CHLO/371-414	I0Z859.1 PF13639.4;	61.90	0.00
TRINITY_A0A096UDI9_MAIZE/100-144	A0A096UDI9.1 PF1363	61.90	0.00
TRINITY_A8J4Z9_CHLRE/223-598	A8J4Z9.1 PF07690.14	61.90	0.00
TRINITY_F4Q1X8_DICFS/1246-1394	F4Q1X8.1 PF00005.25	61.90	0.00
TRINITY_I0Z601_9CHLO/442-816	I0Z601.1 PF03016.13	61.90	0.00
TRINITY_A2DG97_TRIVA/91-155	A2DG97.1 PF13499.4;	61.90	0.00
TRINITY_D8TZS8_VOLCA/96-370	D8TZS8.1 PF00685.25	61.90	0.00
TRINITY_D8THI7_VOLCA/30-155	D8THI7.1 PF10392.7;	61.90	0.00
TRINITY_A8J3N0_CHLRE/60-401	A8J3N0.1 PF07690.14	61.90	0.00
TRINITY_D8UAV1_VOLCA/6-306	D8UAV1.1 PF04189.11	61.90	0.00
TRINITY_D8U8Q8_VOLCA/981-1095	D8U8Q8.1 PF04560.18	61.90	0.00

TRINITY_I7LXC9_TETTS/121-511	I7LXC9.1 PF00155.19	61.80	0.00
TRINITY_L8GWU5_ACACA/543-592	L8GWU5.1 PF13920.4;	61.80	0.00
TRINITY_I7MLG5_TETTS/24-278	I7MLG5.2 PF02263.17	61.80	0.00
TRINITY_D3BLH3_POLPA/671-830	D3BLH3.1 PF11865.6;	61.80	0.00
TRINITY_A0A078G387_BRANA/225-511	A0A078G387.1 PF0794	61.80	0.00
TRINITY_K3WV80_PYTUL/3-175	K3WV80.1 PF01923.16	61.80	0.00
TRINITY_A0A0B5AJA9_9BACL/336-460	A0A0B5AJA9.1 PF1692	61.80	0.00
TRINITY_F0Z7I3_DICPU/9-801	F0Z7I3.1 PF00063.19	61.80	0.00
TRINITY_I7ML76_TETTS/9-131	I7ML76.1 PF01873.15	61.80	0.00
TRINITY_D2V9R9_NAEGR/5-190	D2V9R9.1 PF00227.24	61.80	0.00
TRINITY_J9JA03_9SPIT/172-345	J9JA03.1 PF08662.9;	61.80	0.00
TRINITY_A0A0L0NS74_9ASCO/167-414	A0A0L0NS74.1 PF0118	61.80	0.00
TRINITY_A0A067CC13_SAPPC/5-298	A0A067CC13.1 PF0000	61.80	0.00
TRINITY_A8J3E7_CHLRE/65-166	A8J3E7.1 PF00630.17	61.80	0.00
TRINITY_E1ZHJ7_CHLVA/1-134	E1ZHJ7.1 PF13344.4;	61.80	0.00
TRINITY_F1A552_DICPU/32-163	F1A552.1 PF01966.20	61.80	0.00
TRINITY_A0A068SCF9_9FUNG/35-101	A0A068SCF9.1 PF0069	61.80	0.00
TRINITY_A0A087SC48_AUXPR/130-229	A0A087SC48.1 PF1364	61.80	0.00
TRINITY_D8TSV2_VOLCA/52-209	D8TSV2.1 PF01789.14	61.80	0.00
TRINITY_G0R0J2_ICHMG/131-378	G0R0J2.1 PF00122.18	61.80	0.00
TRINITY_I7M3G1_TETTS/208-776	I7M3G1.1 PF00183.16	61.80	0.00
TRINITY_D8M1W7_BLAHO/168-334	D8M1W7.1 PF02421.16	61.80	0.00
TRINITY_E1ZH58_CHLVA/1-248	E1ZH58.1 PF00454.25	61.80	0.00
TRINITY_A0A067QLN0_ZOONE/42-257	A0A067QLN0.1 PF0014	61.80	0.00
TRINITY_D8UBW9_VOLCA/5-216	D8UBW9.1 PF08442.8;	61.80	0.00
TRINITY_I0Z3G2_9CHLO/789-892	I0Z3G2.1 PF02984.17	61.80	0.00
TRINITY_A0CBE5_PARTE/106-283	A0CBE5.1 PF01163.20	61.80	0.00
TRINITY_I7MD12_TETTS/59-126	I7MD12.1 PF00550.23	61.80	0.00
TRINITY_A8JEK6_CHLRE/155-260	A8JEK6.1 PF00254.26	61.80	0.00
TRINITY_D8UBS2_VOLCA/5-73	D8UBS2.1 PF13921.4;	61.80	0.00
TRINITY_D8TJJ9_VOLCA/4-113	D8TJJ9.1 PF00411.17	61.80	0.00
TRINITY_A0A078B4I1_STYLE/16-344	A0A078B4I1.1 PF0089	61.80	0.00
TRINITY_F2KY65_PREDF/3-203	F2KY65.1 PF00590.18	61.80	0.00
TRINITY_Q235L2_TETTS/74-150	Q235L2.1 PF03134.17	61.80	0.00
TRINITY_D8TJ25_VOLCA/8-123	D8TJ25.1 PF14580.4;	61.80	0.00
TRINITY_L8H3W5_ACACA/971-1093	L8H3W5.1 PF00271.29	61.80	0.00
TRINITY_Q551W4_DICDI/546-624	Q551W4.1 PF13193.4;	61.80	0.00
TRINITY_Q5W9T0_CHLRE/210-282	Q5W9T0.1 PF00504.19	61.80	0.00
TRINITY_D8UCI5_VOLCA/121-196	D8UCI5.1 PF00076.20	61.80	0.00
TRINITY_Q5AWT4_EMENI/461-784	Q5AWT4.1 PF13086.4;	61.80	0.00
TRINITY_I9BU49_9RALS/186-452	I9BU49.1 PF03313.13	61.80	0.00
TRINITY_A0A0E9NMI7_9ASCO/175-287	A0A0E9NMI7.1 PF0295	61.80	0.00
TRINITY_I7MMG8_TETTS/8-169	I7MMG8.1 PF00071.20	61.70	0.00
TRINITY_L8GUK1_ACACA/237-488	L8GUK1.1 PF07714.15	61.70	0.00
TRINITY_R9AGT5_WALI9/349-415	R9AGT5.1 PF01423.20	61.70	0.00
TRINITY_A9U389_PHYPA/2-143	A9U389.1 PF01217.18	61.70	0.00
TRINITY_F4XSE4_9CYAN/117-379	F4XSE4.1 PF07995.9;	61.70	0.00
TRINITY_G0QMA7_ICHMG/113-358	G0QMA7.1 PF02906.12	61.70	0.00
TRINITY_Q1IJR4_KORVE/50-364	Q1IJR4.1 PF00251.18	61.70	0.00
TRINITY_Q22T22_TETTS/520-735	Q22T22.3 PF07859.11	61.70	0.00
TRINITY_A7S5U3_NEMVE/23-275	A7S5U3.1 PF00756.18	61.70	0.00
TRINITY_M2Y664_GALSU/13-174	M2Y664.1 PF00071.20	61.70	0.00
TRINITY_A0A022QSG7_ERYGU/141-295	A0A022QSG7.1 PF0444	61.70	0.00
TRINITY_A0A0D2UGE9_CAPO3/18-165	A0A0D2UGE9.1 PF0405	61.70	0.00
TRINITY_K1RU04_CRAGI/3-376	K1RU04.1 PF00022.17	61.70	0.00
TRINITY_D8TX33_VOLCA/7-126	D8TX33.1 PF00637.18	61.70	0.00
TRINITY_A0A0N1HZ48_9EURO/127-265	A0A0N1HZ48.1 PF0000	61.70	0.00
TRINITY_A0A078AF24_STYLE/251-493	A0A078AF24.1 PF0012	61.70	0.00
TRINITY_W5MEL6_LEPOC/11-266	W5MEL6.1 PF03437.13	61.70	0.00
TRINITY_A0CC48_PARTE/14-196	A0CC48.1 PF00227.24	61.70	0.00
TRINITY_A0CC48_PARTE/14-196	A0CC48.1 PF00227.24	61.70	0.00

TRINITY_I4YCC2_WALMC/1210-1553	I4YCC2.1 PF00176.21	61.70	0.00
TRINITY_A0BN60_PARTE/55-373	A0BN60.1 PF00225.21	61.70	0.00
TRINITY_D8TTV8_VOLCA/81-324	D8TTV8.1 PF00233.17	61.70	0.00
TRINITY_A4VCZ5_TETTS/564-728	A4VCZ5.1 PF12931.5;	61.70	0.00
TRINITY_A0EAW6_PARTE/188-236	A0EAW6.1 PF13833.4;	61.70	0.00
TRINITY_L8H537_ACACA/119-311	L8H537.1 PF01071.17	61.70	0.00
TRINITY_E1Z882_CHLVA/22-272	E1Z882.1 PF12740.5;	61.70	0.00
TRINITY_A8I243_CHLRE/5-153	A8I243.1 PF03801.11	61.70	0.00
TRINITY_Q23QI5_TETTS/56-189	Q23QI5.1 PF13350.4;	61.70	0.00
TRINITY_A0D3K4_PARTE/152-201	A0D3K4.1 PF13920.4;	61.70	0.00
TRINITY_D8UEU2_VOLCA/503-670	D8UEU2.1 PF01885.14	61.70	0.00
TRINITY_A0A078D7A8_BRANA/47-111	A0A078D7A8.1 PF1349	61.70	0.00
TRINITY_E7F643_DANRE/401-761	E7F643.1 PF00493.21	61.70	0.00
TRINITY_M1AI07_SOLTU/29-88	M1AI07.1 PF00400.30	61.70	0.00
TRINITY_D8UAY6_VOLCA/22-101	D8UAY6.1 PF10273.7;	61.70	0.00
TRINITY_A0A061FPA9_THECC/161-335	A0A061FPA9.1 PF0073	61.70	0.00
TRINITY_F6I015_VITVI/6-146	F6I015.1 PF05903.12	61.70	0.00
TRINITY_C6VWI0_DYAFD/52-521	C6VWI0.1 PF00171.20	61.70	0.00
TRINITY_I7M3R0_TETTS/275-389	I7M3R0.1 PF01926.21	61.70	0.00
TRINITY_D8TZH6_VOLCA/22-283	D8TZH6.1 PF00233.17	61.70	0.00
TRINITY_I0YUL0_9CHLO/44-186	I0YUL0.1 PF13365.4;	61.70	0.00
TRINITY_W4GI63_9STRA/1-186	W4GI63.1 PF01201.20	61.70	0.00
TRINITY_L8GSW4_ACACA/36-82	L8GSW4.1 PF01281.17	61.70	0.00
TRINITY_L8GL86_ACACA/16-585	L8GL86.1 PF01411.17	61.70	0.00

TRINITY_G0QU69_ICHMG/12-144	G0QU69.1 PF05222.13	61.70	0.00
TRINITY_A8HN56_CHLRE/225-326	A8HN56.1 PF13499.4;	61.70	0.00
TRINITY_A7REQ2_NEMVE/4-157	A7REQ2.1 PF00005.25	61.70	0.00
TRINITY_D8TQB4_VOLCA/266-516	D8TQB4.1 PF00069.23	61.70	0.00
TRINITY_F1A0B3_DICPU/165-273	F1A0B3.1 PF08616.8;	61.70	0.00
TRINITY_A8IBL5_CHLRE/76-217	A8IBL5.1 PF01529.18	61.70	0.00
TRINITY_A8J5V2_CHLRE/8-268	A8J5V2.1 PF08323.9;	61.70	0.00
TRINITY_A0A059B6L1_EUCGR/13-172	A0A059B6L1.1 PF0410	61.70	0.00
TRINITY_L8GDC4_ACACA/12-153	L8GDC4.1 PF00080.18	61.70	0.00
TRINITY_C1E8C1_MICSR/212-300	C1E8C1.1 PF03908.11	61.70	0.00
TRINITY_D8UF73_VOLCA/251-332	D8UF73.1 PF00211.18	61.70	0.00
TRINITY_C1E160_MICSR/172-281	C1E160.1 PF13640.4;	61.70	0.00
TRINITY_E1Z915_CHLVA/484-803	E1Z915.1 PF01937.17	61.70	0.00
TRINITY_D8UEF2_VOLCA/54-237	D8UEF2.1 PF00504.19	61.70	0.00
TRINITY_W7X957_TETTS/108-419	W7X957.1 PF02636.15	61.70	0.00
TRINITY_Q23AM3_TETTS/1054-1231	Q23AM3.2 PF00005.25	61.60	0.00
TRINITY_G6CIB4_DANPL/49-293	G6CIB4.1 PF01209.16	61.60	0.00
TRINITY_A0A068RTD5_9FUNG/12-173	A0A068RTD5.1 PF0007	61.60	0.00
TRINITY_G0R4W3_ICHMG/13-112	G0R4W3.1 PF03226.12	61.60	0.00
TRINITY_A0A078A3V7_STYLE/126-338	A0A078A3V7.1 PF0011	61.60	0.00
TRINITY_I4AK14_FLELS/6-265	I4AK14.1 PF00108.21	61.60	0.00
TRINITY_I7M9L6_TETTS/1063-1226	I7M9L6.1 PF00005.25	61.60	0.00
TRINITY_L8GNP9_ACACA/1-74	L8GNP9.1 PF05251.10	61.60	0.00
TRINITY_M1MTR0_9CLOT/74-285	M1MTR0.1 PF07859.11	61.60	0.00
TRINITY_A0A059LP53_9CHLO/51-227	A0A059LP53.1 PF0438	61.60	0.00
TRINITY_A8HTI5_CHLRE/224-447	A8HTI5.1 PF00149.26	61.60	0.00
TRINITY_F0Z9X7_DICPU/19-269	F0Z9X7.1 PF00069.23	61.60	0.00
TRINITY_A8JI17_CHLRE/39-132	A8JI17.1 PF00153.25	61.60	0.00
TRINITY_U3IHR3_ANAPL/140-303	U3IHR3.1 PF00005.25	61.60	0.00
TRINITY_W5BIS0_WHEAT/1-160	W5BIS0.1 PF00899.19	61.60	0.00
TRINITY_J2ZWG3_9CAUL/2-181	J2ZWG3.1 PF01121.18	61.60	0.00
TRINITY_E1ZGE3_CHLVA/24-296	E1ZGE3.1 PF00069.23	61.60	0.00
TRINITY_S8CD85_9LAMI/5-102	S8CD85.1 PF00125.22	61.60	0.00
TRINITY_A0A096Q8F0_MAIZE/61-420	A0A096Q8F0.1 PF0933	61.60	0.00
TRINITY_A8JFQ2_CHLRE/9-158	A8JFQ2.1 PF02099.15	61.60	0.00
TRINITY_A0A0J8D1N5_BETVU/217-475	A0A0J8D1N5.1 PF1047	61.60	0.00
TRINITY_D8TZF0_VOLCA/126-211	D8TZF0.1 PF03083.14	61.60	0.00
TRINITY_L8HBC4_ACACA/1006-1169	L8HBC4.1 PF08623.8;	61.60	0.00
TRINITY_D8UFR6_VOLCA/71-385	D8UFR6.1 PF00069.23	61.60	0.00
TRINITY_A8JC77_CHLRE/1-279	A8JC77.1 PF00225.21	61.60	0.00
TRINITY_DST2_DICDI/20-276	Q55GC2.1 PF00069.23	61.60	0.00
TRINITY_I0YS28_9CHLO/336-547	I0YS28.1 PF01434.16	61.60	0.00
TRINITY_A0A078B3U9_STYLE/406-642	A0A078B3U9.1 PF0006	61.60	0.00
TRINITY_E0UPF7_SULAO/21-158	E0UPF7.1 PF12867.5;	61.50	0.00
TRINITY_I7MCW0_TETTS/37-114	I7MCW0.1 PF09398.8;	61.50	0.00
TRINITY_A0A059CK21_EUCGR/37-128	A0A059CK21.1 PF0188	61.50	0.00
TRINITY_F4NYW3_BATDJ/653-1037	F4NYW3.1 PF05192.16	61.50	0.00
TRINITY_B6JVL7_SCHJY/88-450	B6JVL7.1 PF00291.23	61.50	0.00
TRINITY_K9R9X6_9CYAN/33-125	K9R9X6.1 PF04248.10	61.50	0.00
TRINITY_X1W3S8_TETTS/285-401	X1W3S8.1 PF02373.20	61.50	0.00
TRINITY_Q22EY8_TETTS/112-209	Q22EY8.1 PF00153.25	61.50	0.00
TRINITY_M4BMA1_HYAAE/108-159	M4BMA1.1 PF00400.30	61.50	0.00
TRINITY_Q239R9_TETTS/1112-1262	Q239R9.2 PF00005.25	61.50	0.00
TRINITY_T1J7S0_STRMM/622-667	T1J7S0.1 PF13920.4;	61.50	0.00
TRINITY_L8HAK4_ACACA/43-223	L8HAK4.1 PF02737.16	61.50	0.00
TRINITY_GPX7_ARATH/76-184	Q9SZ54.2 PF00255.17	61.50	0.00
TRINITY_L8GLU7_ACACA/23-201	L8GLU7.1 PF01300.16	61.50	0.00
TRINITY_L8H4R8_ACACA/22-218	L8H4R8.1 PF04664.11	61.50	0.00
TRINITY_H2YP11_CIOSA/230-438	H2YP11.1 PF00005.25	61.50	0.00
TRINITY_L8H7T0_ACACA/33-396	L8H7T0.1 PF00155.19	61.50	0.00
TRINITY_A0A096MH05_POEFO/1172-1320	A0A096MH05.1 PF0000	61.50	0.00

TRINITY_A9TQ97_PHYPA/269-325	A9TQ97.1 PF09280.9;	61.50	0.00
TRINITY_J9HPY2_9SPIT/10-48	J9HPY2.1 PF13917.4;	61.50	0.00
TRINITY_A0A087SBZ8_AUXPR/221-410	A0A087SBZ8.1 PF0058	61.50	0.00
TRINITY_A0DIP7_PARTE/2051-2134	A0DIP7.1 PF05406.13	61.50	0.00
TRINITY_A8HMM6_CHLRE/139-249	A8HMM6.1 PF02517.14	61.50	0.00
TRINITY_A8JEB7_CHLRE/516-889	A8JEB7.1 PF00856.26	61.50	0.00
TRINITY_A8J8D4_CHLRE/40-220	A8J8D4.1 PF13419.4;	61.50	0.00
TRINITY_C6W4K0_DYAFD/4-370	C6W4K0.1 PF01663.20	61.50	0.00
TRINITY_M5GC36_DACSP/667-845	M5GC36.1 PF11916.6;	61.50	0.00
TRINITY_Y3393_SYNFM/110-252	A0LNR4.1 PF07286.10	61.50	0.00
TRINITY_K9VY32_9CYAN/312-350	K9VY32.1 PF00400.30	61.50	0.00
TRINITY_Q5TNJ4_ANOGA/104-168	Q5TNJ4.3 PF00327.18	61.50	0.00
TRINITY_K1QEC4_CRAGI/17-106	K1QEC4.1 PF02214.20	61.50	0.00
TRINITY_D8TX29_VOLCA/96-436	D8TX29.1 PF00724.18	61.50	0.00
TRINITY_D8UIZ7_VOLCA/369-849	D8UIZ7.1 PF00759.17	61.50	0.00
TRINITY_L8HLI2_ACACA/228-282	L8HLI2.1 PF08063.10	61.50	0.00
TRINITY_V4L9F1_EUTSA/130-411	V4L9F1.1 PF00176.21	61.50	0.00
TRINITY_D8TUG8_VOLCA/629-854	D8TUG8.1 PF00149.26	61.50	0.00
TRINITY_A0A068S411_9FUNG/317-355	A0A068S411.1 PF0481	61.50	0.00
TRINITY_A8JCE5_CHLRE/121-255	A8JCE5.1 PF04073.13	61.50	0.00
TRINITY_Q24JG1_TETTS/16-326	Q24JG1.1 PF07992.12	61.50	0.00
TRINITY_AP1G_DICDI/21-578	Q8I8U2.1 PF01602.18	61.50	0.00
TRINITY_I7M6H6_TETTS/54-166	I7M6H6.1 PF10908.6;	61.50	0.00
TRINITY_A0A0D1VZF4_ANEMI/9-178	A0A0D1VZF4.1 PF0088	61.50	0.00
TRINITY_F4PYU2_DICFS/181-889	F4PYU2.1 PF00343.18	61.50	0.00
TRINITY_A0EBG4_PARTE/41-332	A0EBG4.1 PF05185.14	61.50	0.00
TRINITY_D8UBE1_VOLCA/26-435	D8UBE1.1 PF00232.16	61.50	0.00
TRINITY_D8U9S9_VOLCA/599-793	D8U9S9.1 PF00211.18	61.50	0.00
TRINITY_D8UCM4_VOLCA/1-110	D8UCM4.1 PF07001.9;	61.50	0.00
TRINITY_C1E5J4_MICSR/37-128	C1E5J4.1 PF01248.24	61.50	0.00
TRINITY_D8TTX4_VOLCA/18-408	D8TTX4.1 PF07690.14	61.50	0.00
TRINITY_A0A0A0KIY4_CUCSA/103-167	A0A0A0KIY4.1 PF1349	61.50	0.00
TRINITY_F0SSI4_RUBBR/11-136	F0SSI4.1 PF00578.19	61.50	0.00
TRINITY_A0E189_PARTE/446-495	A0E189.1 PF13920.4;	61.40	0.00
TRINITY_Q22RZ8_TETTS/183-242	Q22RZ8.2 PF13921.4;	61.40	0.00
TRINITY_J9ELK2_9SPIT/58-353	J9ELK2.1 PF00069.23	61.40	0.00
TRINITY_M2PPP9_CERS8/675-986	M2PPP9.1 PF05843.12	61.40	0.00
TRINITY_A0A0J8B2Q8_BETVU/356-401	A0A0J8B2Q8.1 PF1363	61.40	0.00
TRINITY_A0EGG4_PARTE/7-89	A0EGG4.1 PF01221.16	61.40	0.00
TRINITY_A0A0A7LLI1_9BACT/4-177	A0A0A7LLI1.1 PF0053	61.40	0.00
TRINITY_G0R374_ICHMG/25-316	G0R374.1 PF03095.13	61.40	0.00
TRINITY_B9PQT4_TOXGO/12-383	B9PQT4.1 PF00022.17	61.40	0.00
TRINITY_D8U0Q2_VOLCA/308-569	D8U0Q2.1 PF10672.7;	61.40	0.00
TRINITY_L8GUR2_ACACA/284-351	L8GUR2.1 PF02207.18	61.40	0.00
TRINITY_J9FRH7_9SPIT/20-284	J9FRH7.1 PF01650.16	61.40	0.00
TRINITY_B8BVC5_THAPS/7-64	B8BVC5.1 PF13921.4;	61.40	0.00
TRINITY_I0YKX7_9CHLO/155-269	I0YKX7.1 PF00504.19	61.40	0.00
TRINITY_L1JEC9_GUITH/16-205	L1JEC9.1 PF00270.27	61.40	0.00
TRINITY_D3BGU2_POLPA/134-214	D3BGU2.1 PF00071.20	61.40	0.00
TRINITY_TILS_THEEB/24-201	Q8DIH2.1 PF01171.18	61.40	0.00
TRINITY_C3ZMR5_BRAFL/4-377	C3ZMR5.1 PF00022.17	61.40	0.00
TRINITY_C1DZQ0_MICSR/2034-2102	C1DZQ0.1 PF02207.18	61.40	0.00
TRINITY_F1A2B8_DICPU/35-339	F1A2B8.1 PF00069.23	61.40	0.00
TRINITY_I7M864_TETTS/33-294	I7M864.1 PF00069.23	61.40	0.00
TRINITY_A0A0J6R8B7_9RHIZ/33-83	A0A0J6R8B7.1 PF1139	61.40	0.00
TRINITY_A0BFS0_PARTE/154-197	A0BFS0.1 PF00569.15	61.40	0.00
TRINITY_A0D197_PARTE/10-69	A0D197.1 PF06624.10	61.40	0.00
TRINITY_D8UHB1_VOLCA/404-473	D8UHB1.1 PF00571.26	61.40	0.00
TRINITY_D8UGG6_VOLCA/17-191	D8UGG6.1 PF10188.7;	61.40	0.00
TRINITY_Q248H8_TETTS/3600-3822	Q248H8.1 PF12781.5;	61.40	0.00
TRINITY_A8HP31_CHLRE/4-166	A8HP31.1 PF04573.10	61.40	0.00

TRINITY_L8HMW5_ACACA/38-177	L8HMW5.1 PF08719.9;	61.40	0.00
TRINITY_W3WQH0_9PEZI/17-317	W3WQH0.1 PF00248.19	61.40	0.00
TRINITY_U1MTI7_ASCSU/188-330	U1MTI7.1 PF00156.25	61.40	0.00
TRINITY_A0A0D2WMT8_CAPO3/13-354	A0A0D2WMT8.1 PF0112	61.40	0.00
TRINITY_D8TVC0_VOLCA/1983-2096	D8TVC0.1 PF00665.24	61.40	0.00
TRINITY_D8RGS1_SELML/17-144	D8RGS1.1 PF13625.4;	61.40	0.00
TRINITY_L8HJK9_ACACA/14-635	L8HJK9.1 PF00888.20	61.40	0.00
TRINITY_L8H7E1_ACACA/163-365	L8H7E1.1 PF02750.12	61.40	0.00
TRINITY_G0R1B7_ICHMG/7-66	G0R1B7.1 PF04193.12	61.40	0.00
TRINITY_L8GIR2_ACACA/45-227	L8GIR2.1 PF13532.4;	61.40	0.00
TRINITY_B7XKC3_ENTBH/234-373	B7XKC3.1 PF00004.27	61.40	0.00
TRINITY_D8UB15_VOLCA/264-324	D8UB15.1 PF03105.17	61.40	0.00
TRINITY_A0A0A1NH53_9FUNG/12-62	A0A0A1NH53.1 PF0038	61.40	0.00
TRINITY_D8TT41_VOLCA/30-634	D8TT41.1 PF00012.18	61.40	0.00
TRINITY_E1Z5J0_CHLVA/71-358	E1Z5J0.1 PF01207.15	61.40	0.00
TRINITY_K4CET7_SOLLC/200-244	K4CET7.1 PF13396.4;	61.40	0.00
TRINITY_A0A077AV43_9RICK/4-107	A0A077AV43.1 PF0152	61.40	0.00
TRINITY_D8U1I6_VOLCA/153-493	D8U1I6.1 PF00152.18	61.40	0.00
TRINITY_F0ZKN4_DICPU/472-543	F0ZKN4.1 PF02825.18	61.40	0.00
TRINITY_I0YXA9_9CHLO/19-419	I0YXA9.1 PF00202.19	61.40	0.00
TRINITY_W5K6K0_ASTMX/59-146	W5K6K0.1 PF08712.9;	61.40	0.00
TRINITY_V4TQM7_9ROSI/538-738	V4TQM7.1 PF12931.5;	61.40	0.00
TRINITY_A0A0D2WMY3_CAPO3/287-433	A0A0D2WMY3.1 PF0062	61.40	0.00
TRINITY_J9F683_9SPIT/731-774	J9F683.1 PF06203.12	61.40	0.00
TRINITY_C1MV11_MICPC/439-838	C1MV11.1 PF00940.17	61.40	0.00
TRINITY_D8TMB1_VOLCA/383-522	D8TMB1.1 PF01399.25	61.40	0.00
TRINITY_G0R454_ICHMG/13-82	G0R454.1 PF01423.20	61.40	0.00
TRINITY_B0W062_CULQU/317-754	B0W062.1 PF00501.26	61.40	0.00
TRINITY_Q16I11_AEDAE/993-1140	Q16I11.1 PF00005.25	61.40	0.00
TRINITY_E4T5G0_PALPW/31-384	E4T5G0.1 PF16499.3;	61.30	0.00
TRINITY_Q3SDL5_PARTE/12-173	Q3SDL5.1 PF00071.20	61.30	0.00
TRINITY_W9YR47_9EURO/37-188	W9YR47.1 PF01743.18	61.30	0.00
TRINITY_A0CW36_PARTE/237-420	A0CW36.1 PF02799.13	61.30	0.00
TRINITY_E1ZQ79_CHLVA/143-400	E1ZQ79.1 PF10672.7;	61.30	0.00
TRINITY_L8GIJ7_ACACA/461-567	L8GIJ7.1 PF00175.19	61.30	0.00
TRINITY_A0A067CFX7_SAPPC/106-352	A0A067CFX7.1 PF0012	61.30	0.00
TRINITY_I7MHS8_TETTS/213-274	I7MHS8.2 PF12906.5;	61.30	0.00
TRINITY_A0A059LS92_9CHLO/560-705	A0A059LS92.1 PF0251	61.30	0.00
TRINITY_I0Z6H7_9CHLO/21-95	I0Z6H7.1 PF00364.20	61.30	0.00
TRINITY_A8J5Y7_CHLRE/75-168	A8J5Y7.1 PF01250.15	61.30	0.00
TRINITY_C6RNY4_ACIRA/4-65	C6RNY4.1 PF00226.29	61.30	0.00
TRINITY_G0GEA9_SPITZ/216-397	G0GEA9.1 PF00587.23	61.30	0.00
TRINITY_V9G1A7_PHYPR/152-221	V9G1A7.1 PF13424.4;	61.30	0.00
TRINITY_I7MDU0_TETTS/12-274	I7MDU0.1 PF00069.23	61.30	0.00
TRINITY_R4XC98_TAPDE/138-274	R4XC98.1 PF04116.11	61.30	0.00
TRINITY_D8TJL5_VOLCA/90-167	D8TJL5.1 PF09420.8;	61.30	0.00
TRINITY_A0A024RE44_VAVCU/259-521	A0A024RE44.1 PF0058	61.30	0.00
TRINITY_Q66YD1_CHLRE/72-133	Q66YD1.1 PF00226.29	61.30	0.00
TRINITY_W3WQH0_9PEZI/17-317	W3WQH0.1 PF00248.19	61.30	0.00
TRINITY_D8TL16_VOLCA/14-549	D8TL16.1 PF01602.18	61.30	0.00
TRINITY_D8UI50_VOLCA/3-153	D8UI50.1 PF04099.10	61.30	0.00
TRINITY_A0A0N4UMD3_DRAME/5-177	A0A0N4UMD3.1 PF0002	61.30	0.00
TRINITY_A0A078A6A0_STYLE/8-70	A0A078A6A0.1 PF0007	61.30	0.00
TRINITY_A0EA10_PARTE/59-340	A0EA10.1 PF06472.13	61.30	0.00
TRINITY_D0MXE0_PHYIT/188-370	D0MXE0.1 PF00270.27	61.30	0.00
TRINITY_Q23R00_TETTS/6-298	Q23R00.2 PF00579.23	61.30	0.00
TRINITY_F0ZYM8_DICPU/393-643	F0ZYM8.1 PF07714.15	61.30	0.00
TRINITY_Q8MML7_DICDI/240-382	Q8MML7.1 PF02840.13	61.30	0.00
TRINITY_A0A0G4EZU4_9ALVE/291-621	A0A0G4EZU4.1 PF0081	61.30	0.00
TRINITY_FYV1_DICDI/935-1227	B0G126.1 PF00118.22	61.30	0.00
TRINITY_D8UF04_VOLCA/7-220	D8UF04.1 PF01088.19	61.30	0.00

TRINITY_Q23VX7_TETTS/222-459	Q23VX7.1 PF00198.21	61.30	0.00
TRINITY_K9VES7_9CYAN/141-246	K9VES7.1 PF08241.10	61.30	0.00
TRINITY_D8UJ00_VOLCA/198-351	D8UJ00.1 PF13328.4;	61.30	0.00
TRINITY_D8UHT9_VOLCA/5-258	D8UHT9.1 PF00069.23	61.30	0.00
TRINITY_A0A078AA42_STYLE/1-191	A0A078AA42.1 PF0120	61.30	0.00
TRINITY_D1VX60_9BACT/15-351	D1VX60.1 PF13393.4;	61.20	0.00
TRINITY_Q234S6_TETTS/45-667	Q234S6.3 PF00443.27	61.20	0.00
TRINITY_Q3SDT4_PARTE/8-173	Q3SDT4.1 PF00071.20	61.20	0.00
TRINITY_G0R2Q6_ICHMG/2-287	G0R2Q6.1 PF00248.19	61.20	0.00
TRINITY_L8GF85_ACACA/2138-2306	L8GF85.1 PF00520.29	61.20	0.00
TRINITY_J9HXH4_9SPIT/314-525	J9HXH4.1 PF00211.18	61.20	0.00
TRINITY_L0G6R7_ECHVK/36-378	L0G6R7.1 PF07221.9;	61.20	0.00
TRINITY_E7KVV6_YEASL/41-142	E7KVV6.1 PF13649.4;	61.20	0.00
TRINITY_C6SYH7_SOYBN/19-85	C6SYH7.1 PF01423.20	61.20	0.00
TRINITY_C6SYH7_SOYBN/19-85	C6SYH7.1 PF01423.20	61.20	0.00
TRINITY_Q22BY0_TETTS/1870-1918	Q22BY0.2 PF00415.16	61.20	0.00
TRINITY_D5DVZ6_BACMQ/25-194	D5DVZ6.1 PF00881.22	61.20	0.00
TRINITY_C5LIW9_PERM5/8-85	C5LIW9.1 PF01423.20	61.20	0.00
TRINITY_A7REQ2_NEMVE/4-157	A7REQ2.1 PF00005.25	61.20	0.00
TRINITY_I0YYW7_9CHLO/18-115	I0YYW7.1 PF16455.3;	61.20	0.00
TRINITY_T1K519_TETUR/34-291	T1K519.1 PF01650.16	61.20	0.00
TRINITY_L8HHJ0_ACACA/66-247	L8HHJ0.1 PF00535.24	61.20	0.00
TRINITY_D8U2P4_VOLCA/55-250	D8U2P4.1 PF00406.20	61.20	0.00
TRINITY_A0A0D2WP71_CAPO3/367-660	A0A0D2WP71.1 PF0089	61.20	0.00
TRINITY_D8TVL1_VOLCA/2020-2517	D8TVL1.1 PF00443.27	61.20	0.00
TRINITY_E7NPP2_TREPH/23-227	E7NPP2.1 PF01145.23	61.20	0.00
TRINITY_I7MIU6_TETTS/36-84	I7MIU6.2 PF00415.16	61.20	0.00
TRINITY_A0A077JEV5_9CYAN/11-217	A0A077JEV5.1 PF0049	61.20	0.00
TRINITY_F6TKY7_HORSE/75-371	F6TKY7.1 PF00291.23	61.20	0.00
TRINITY_I0Z554_9CHLO/96-515	I0Z554.1 PF01373.15	61.20	0.00
TRINITY_D8UE55_VOLCA/49-359	D8UE55.1 PF00294.22	61.20	0.00
TRINITY_A0D712_PARTE/195-452	A0D712.1 PF00069.23	61.20	0.00
TRINITY_M0YB45_HORVD/257-445	M0YB45.1 PF03062.17	61.20	0.00
TRINITY_A0BF49_PARTE/4-177	A0BF49.1 PF00265.16	61.20	0.00
TRINITY_I7LUR8_TETTS/1292-1437	I7LUR8.2 PF00637.18	61.20	0.00
TRINITY_Q24FS1_TETTS/14-305	Q24FS1.1 PF04227.10	61.20	0.00
TRINITY_A8J5A3_CHLRE/88-237	A8J5A3.1 PF02544.14	61.20	0.00
TRINITY_D8U3J0_VOLCA/3-143	D8U3J0.1 PF08610.8;	61.20	0.00
TRINITY_I0YYY9_9CHLO/17-98	I0YYY9.1 PF12895.5;	61.20	0.00
TRINITY_A0DD95_PARTE/1-247	A0DD95.1 PF01902.15	61.20	0.00
TRINITY_I0Z389_9CHLO/124-275	I0Z389.1 PF00817.18	61.20	0.00
TRINITY_D8THF9_VOLCA/214-341	D8THF9.1 PF12014.6;	61.20	0.00
TRINITY_D8TTV8_VOLCA/81-324	D8TTV8.1 PF00233.17	61.20	0.00
TRINITY_D8TK71_VOLCA/3-185	D8TK71.1 PF04055.19	61.20	0.00
TRINITY_I0Z9X6_9CHLO/59-388	I0Z9X6.1 PF00176.21	61.20	0.00
TRINITY_A0A0E9NR88_9ASCO/216-330	A0A0E9NR88.1 PF0141	61.20	0.00
TRINITY_W2R8A8_PHYPN/163-447	W2R8A8.1 PF04095.14	61.20	0.00
TRINITY_A8JGU7_CHLRE/38-240	A8JGU7.1 PF00349.19	61.20	0.00
TRINITY_Q22GW0_TETTS/1651-1813	Q22GW0.2 PF00069.23	61.20	0.00
TRINITY_A8HVM9_CHLRE/80-432	A8HVM9.1 PF07690.14	61.20	0.00
TRINITY_TTL3C_TETTS/705-1030	Q23FE2.1 PF03133.13	61.20	0.00
TRINITY_I0YRN9_9CHLO/94-384	I0YRN9.1 PF00487.22	61.20	0.00
TRINITY_A8IWA3_CHLRE/107-492	A8IWA3.1 PF01979.18	61.20	0.00
TRINITY_D8TL05_VOLCA/10-212	D8TL05.1 PF02146.15	61.20	0.00
TRINITY_A0A059A4T3_EUCGR/566-712	A0A059A4T3.1 PF0000	61.20	0.00
TRINITY_A8HZR1_CHLRE/17086-17364	A8HZR1.1 PF00109.24	61.20	0.00
TRINITY_I7MAW2_TETTS/928-1107	I7MAW2.2 PF13091.4;	61.20	0.00
TRINITY_W5JZ43_ASTMX/344-429	W5JZ43.1 PF08544.11	61.20	0.00
TRINITY_J9HT96_9SPIT/260-331	J9HT96.1 PF02187.15	61.20	0.00
TRINITY_I0YQL2_9CHLO/174-274	I0YQL2.1 PF06552.10	61.20	0.00
TRINITY_C1E0R4_MICSR/940-1343	C1E0R4.1 PF08393.11	61.20	0.00

TRINITY_A8IE97_CHLRE/193-399	A8IE97.1 PF08543.10	61.20	0.00
TRINITY_A0BJW5_PARTE/673-824	A0BJW5.1 PF00005.25	61.20	0.00
TRINITY_A8IYR9_CHLRE/24-434	A8IYR9.1 PF08393.11	61.20	0.00
TRINITY_D8UC96_VOLCA/594-693	D8UC96.1 PF00211.18	61.20	0.00
TRINITY_L0G510_ECHVK/25-388	L0G510.1 PF14587.4;	61.20	0.00
TRINITY_A0CYK6_PARTE/20-129	A0CYK6.1 PF00255.17	61.20	0.00
TRINITY_A0A078B887_STYLE/93-437	A0A078B887.1 PF0310	61.10	0.00
TRINITY_T0QVA0_9STRA/14-121	T0QVA0.1 PF01776.15	61.10	0.00
TRINITY_E1Z6L6_CHLVA/1126-1433	E1Z6L6.1 PF02889.14	61.10	0.00
TRINITY_G0R0T4_ICHMG/1-228	G0R0T4.1 PF00069.23	61.10	0.00
TRINITY_K1Q151_CRAGI/43-150	K1Q151.1 PF01655.16	61.10	0.00
TRINITY_A8MJ89_ALKOO/5-288	A8MJ89.1 PF01212.19	61.10	0.00
TRINITY_A0DPJ4_PARTE/293-338	A0DPJ4.1 PF13639.4;	61.10	0.00
TRINITY_S2JF03_MUCC1/11-106	S2JF03.1 PF01230.21	61.10	0.00
TRINITY_A0A078B0I2_STYLE/23-113	A0A078B0I2.1 PF0496	61.10	0.00
TRINITY_F9XQ77_ZYMTI/189-369	F9XQ77.1 PF02774.16	61.10	0.00
TRINITY_D0NKN9_PHYIT/305-429	D0NKN9.1 PF10155.7;	61.10	0.00
TRINITY_G3TRR7_LOXAF/493-813	G3TRR7.1 PF03372.21	61.10	0.00
TRINITY_G0QJK3_ICHMG/16-196	G0QJK3.1 PF02737.16	61.10	0.00
TRINITY_A0DTP8_PARTE/77-166	A0DTP8.1 PF16531.3;	61.10	0.00
TRINITY_I7MCP6_TETTS/244-601	I7MCP6.2 PF00152.18	61.10	0.00
TRINITY_V4UHG4_9ROSI/55-448	V4UHG4.1 PF05577.10	61.10	0.00
TRINITY_A7RL90_NEMVE/6-148	A7RL90.1 PF00179.24	61.10	0.00
TRINITY_F4PJZ2_DICFS/426-527	F4PJZ2.1 PF00616.17	61.10	0.00
TRINITY_I0HZT5_CALAS/6-348	I0HZT5.1 PF00180.18	61.10	0.00
TRINITY_T0R6L1_9STRA/24-351	T0R6L1.1 PF01137.19	61.10	0.00
TRINITY_W2SCP1_9EURO/803-1296	W2SCP1.1 PF02463.17	61.10	0.00
TRINITY_A0A0G4ERS4_9ALVE/381-452	A0A0G4ERS4.1 PF0007	61.10	0.00
TRINITY_E1ZBF1_CHLVA/23-168	E1ZBF1.1 PF00254.26	61.10	0.00
TRINITY_A0A0D2WME3_CAPO3/65-361	A0A0D2WME3.1 PF0067	61.10	0.00
TRINITY_D8TUZ0_VOLCA/326-458	D8TUZ0.1 PF13679.4;	61.10	0.00
TRINITY_G0QRJ3_ICHMG/395-534	G0QRJ3.1 PF01529.18	61.10	0.00
TRINITY_A0A0G4IR21_PLABS/101-470	A0A0G4IR21.1 PF0156	61.10	0.00
TRINITY_A8HZW8_CHLRE/157-347	A8HZW8.1 PF01612.18	61.10	0.00
TRINITY_D8TRW7_VOLCA/284-377	D8TRW7.1 PF12894.5;	61.10	0.00
TRINITY_DAAF1_CHLRE/86-224	Q09JZ4.1 PF14580.4;	61.10	0.00
TRINITY_A8J807_CHLRE/44-241	A8J807.1 PF01812.18	61.10	0.00
TRINITY_A8IKA6_CHLRE/72-154	A8IKA6.1 PF04359.12	61.10	0.00
TRINITY_B9XKL5_PEDPL/646-757	B9XKL5.1 PF00072.22	61.10	0.00
TRINITY_D8U610_VOLCA/52-269	D8U610.1 PF00856.26	61.10	0.00
TRINITY_F4PLR0_DICFS/9-266	F4PLR0.1 PF00069.23	61.10	0.00
TRINITY_D8U6E5_VOLCA/457-510	D8U6E5.1 PF13637.4;	61.10	0.00
TRINITY_E1ZC14_CHLVA/145-340	E1ZC14.1 PF07859.11	61.10	0.00
TRINITY_F7EQM6_XENTR/5-178	F7EQM6.1 PF00071.20	61.10	0.00
TRINITY_Q23YB7_TETTS/26-283	Q23YB7.2 PF00108.21	61.10	0.00
TRINITY_H9JDW8_BOMMO/655-700	H9JDW8.1 PF13920.4;	61.10	0.00
TRINITY_A0A022QK02_ERYGU/391-605	A0A022QK02.1 PF1342	61.10	0.00
TRINITY_A8ISR6_CHLRE/237-390	A8ISR6.1 PF05057.12	61.10	0.00
TRINITY_I7M6I7_TETTS/33-261	I7M6I7.1 PF04981.11	61.10	0.00
TRINITY_C1DYN4_MICSR/252-390	C1DYN4.1 PF07690.14	61.10	0.00
TRINITY_I1FN95_AMPQE/387-832	I1FN95.1 PF01055.24	61.10	0.00
TRINITY_E1ZLM6_CHLVA/510-646	E1ZLM6.1 PF00754.23	61.10	0.00
TRINITY_D8TWG5_VOLCA/75-164	D8TWG5.1 PF00027.27	61.10	0.00
TRINITY_D8TY97_VOLCA/648-998	D8TY97.1 PF00176.21	61.10	0.00
TRINITY_W8QWE2_PSEST/13-147	W8QWE2.1 PF13302.5;	61.10	0.00
TRINITY_D8THN5_VOLCA/82-424	D8THN5.1 PF00520.29	61.10	0.00
TRINITY_Q234R4_TETTS/265-337	Q234R4.3 PF00173.26	61.10	0.00
TRINITY_J6LG81_9RHOB/17-264	J6LG81.1 PF13561.4;	61.10	0.00
TRINITY_Q6LRN4_PHOPR/16-142	Q6LRN4.1 PF01553.19	61.10	0.00
TRINITY_I0Z1R1_9CHLO/33-297	I0Z1R1.1 PF00069.23	61.10	0.00
TRINITY_A8IZ72_CHLRE/38-253	A8IZ72.1 PF01487.13	61.10	0.00

TRINITY_W2PXB2_PHYPN/493-609	W2PXB2.1 PF00665.24	61.10	0.00
TRINITY_A4VCU9_TETTS/541-621	A4VCU9.1 PF14714.4;	61.10	0.00
TRINITY_M5BMA8_THACB/1-217	M5BMA8.1 PF00795.20	61.00	0.00
TRINITY_A0A023BDG7_GRENI/225-368	A0A023BDG7.1 PF0006	61.00	0.00
TRINITY_A0A0N5BS40_STREA/255-384	A0A0N5BS40.1 PF0000	61.00	0.00
TRINITY_K9QKU9_9NOSO/8-132	K9QKU9.1 PF14226.4;	61.00	0.00
TRINITY_A0E0J0_PARTE/110-343	A0E0J0.1 PF01702.16	61.00	0.00
TRINITY_I7MB50_TETTS/88-420	I7MB50.1 PF02628.13	61.00	0.00
TRINITY_J9JA50_9SPIT/52-203	J9JA50.1 PF00675.18	61.00	0.00
TRINITY_A0A0G4EV97_9ALVE/284-324	A0A0G4EV97.1 PF1363	61.00	0.00
TRINITY_L1JU42_GUITH/47-90	L1JU42.1 PF00432.19	61.00	0.00
TRINITY_F4PZT8_DICFS/26-206	F4PZT8.1 PF01183.18	61.00	0.00
TRINITY_L8HAC7_ACACA/15-184	L8HAC7.1 PF00071.20	61.00	0.00
TRINITY_G0R0F8_ICHMG/378-537	G0R0F8.1 PF00406.20	61.00	0.00
TRINITY_MYOC_DICDI/17-685	P42522.2 PF00063.19	61.00	0.00
TRINITY_F4R630_MELLP/9-83	F4R630.1 PF00575.21	61.00	0.00
TRINITY_D8TIL1_VOLCA/194-254	D8TIL1.1 PF04193.12	61.00	0.00
TRINITY_A0A087SB97_AUXPR/41-161	A0A087SB97.1 PF1477	61.00	0.00
TRINITY_Q23JV0_TETTS/48-415	Q23JV0.2 PF00155.19	61.00	0.00
TRINITY_A8HUC0_CHLRE/205-556	A8HUC0.1 PF02636.15	61.00	0.00
TRINITY_M2WUI5_GALSU/24-368	M2WUI5.1 PF00224.19	61.00	0.00
TRINITY_A0DG60_PARTE/75-304	A0DG60.1 PF01798.16	61.00	0.00
TRINITY_F0ZDG1_DICPU/32-320	F0ZDG1.1 PF00069.23	61.00	0.00
TRINITY_F6VHV0_HORSE/118-447	F6VHV0.1 PF01074.20	61.00	0.00
TRINITY_D3BIP5_POLPA/728-993	D3BIP5.1 PF00069.23	61.00	0.00
TRINITY_M0RRI2_MUSAM/5-212	M0RRI2.1 PF04722.11	61.00	0.00
TRINITY_M5X2N3_PRUPE/33-178	M5X2N3.1 PF02580.14	61.00	0.00
TRINITY_W6QM76_PENRO/104-144	W6QM76.1 PF00187.17	61.00	0.00
TRINITY_I7MB53_TETTS/73-236	I7MB53.2 PF00071.20	61.00	0.00
TRINITY_W1NHW5_AMBTC/75-225	W1NHW5.1 PF00005.25	61.00	0.00
TRINITY_M2XK48_GALSU/162-472	M2XK48.1 PF00365.18	61.00	0.00
TRINITY_F1A642_DICPU/62-295	F1A642.1 PF00112.21	61.00	0.00
TRINITY_D8U3D7_VOLCA/992-1109	D8U3D7.1 PF00072.22	61.00	0.00
TRINITY_L8HBT1_ACACA/46-185	L8HBT1.1 PF08719.9;	61.00	0.00
TRINITY_D8UES1_VOLCA/58-162	D8UES1.1 PF00970.22	61.00	0.00
TRINITY_G0QUX8_ICHMG/208-464	G0QUX8.1 PF00069.23	61.00	0.00
TRINITY_I0YTA5_9CHLO/317-481	I0YTA5.1 PF00994.22	61.00	0.00
TRINITY_A8IGD3_CHLRE/51-240	A8IGD3.1 PF00270.27	61.00	0.00
TRINITY_A0A0G4IYM2_PLABS/106-147	A0A0G4IYM2.1 PF0051	61.00	0.00
TRINITY_D2VS08_NAEGR/119-258	D2VS08.1 PF00182.17	61.00	0.00
TRINITY_B8AIK5_ORYSI/9-129	B8AIK5.1 PF00581.18	61.00	0.00
TRINITY_D8U6E5_VOLCA/949-1048	D8U6E5.1 PF12796.5;	61.00	0.00
TRINITY_A8IVP3_CHLRE/1-172	A8IVP3.1 PF02737.16	61.00	0.00
TRINITY_U9TU81_RHIID/109-418	U9TU81.1 PF02574.14	61.00	0.00
TRINITY_FTSH_THET1/233-367	D1CDT8.1 PF00004.27	61.00	0.00
TRINITY_I0YNZ6_9CHLO/32-378	I0YNZ6.1 PF00773.17	61.00	0.00
TRINITY_A8JFV5_CHLRE/17-152	A8JFV5.1 PF00892.18	61.00	0.00
TRINITY_A8J5F2_CHLRE/110-374	A8J5F2.1 PF00069.23	61.00	0.00
TRINITY_L8GPA8_ACACA/1396-1737	L8GPA8.1 PF00443.27	61.00	0.00
TRINITY_A2DCC3_TRIVA/3-376	A2DCC3.1 PF00022.17	60.90	0.00
TRINITY_A0A074RR78_9HOMO/382-674	A0A074RR78.1 PF0012	60.90	0.00
TRINITY_A0DSW5_PARTE/265-406	A0DSW5.1 PF05728.10	60.90	0.00
TRINITY_D6WWM4_TRICA/124-287	D6WWM4.1 PF08662.9;	60.90	0.00
TRINITY_F4QEP8_DICFS/752-1151	F4QEP8.1 PF00176.21	60.90	0.00
TRINITY_A4CAA3_9GAMM/193-448	A4CAA3.1 PF03781.14	60.90	0.00
TRINITY_C5LVG8_PERM5/4-72	C5LVG8.1 PF13304.4;	60.90	0.00
TRINITY_L8HKS3_ACACA/4-70	L8HKS3.1 PF01423.20	60.90	0.00
TRINITY_A0E309_PARTE/27-98	A0E309.1 PF16209.3;	60.90	0.00
TRINITY_G0R1C5_ICHMG/3-376	G0R1C5.1 PF00022.17	60.90	0.00
TRINITY_I7M601_TETTS/1212-1358	I7M601.2 PF03031.16	60.90	0.00
TRINITY_I7M8T5_TETTS/163-273	I7M8T5.2 PF02770.17	60.90	0.00

TRINITY_V4AVQ0_LOTGI/421-889	V4AVQ0.1 PF01055.24	60.90	0.00
TRINITY_A0A0J8D5U2_BETVU/39-155	A0A0J8D5U2.1 PF0390	60.90	0.00
TRINITY_F0ZWT6_DICPU/16-309	F0ZWT6.1 PF07159.10	60.90	0.00
TRINITY_D3B8P7_POLPA/35-160	D3B8P7.1 PF00102.25	60.90	0.00
TRINITY_E1ZJU5_CHLVA/389-681	E1ZJU5.1 PF00850.17	60.90	0.00
TRINITY_M2XYK1_GALSU/109-358	M2XYK1.1 PF00120.22	60.90	0.00
TRINITY_E3HN58_ACHXA/11-258	E3HN58.1 PF13561.4;	60.90	0.00
TRINITY_A0DTL9_PARTE/31-95	A0DTL9.1 PF11717.6;	60.90	0.00
TRINITY_E1Z6G9_CHLVA/128-298	E1Z6G9.1 PF00892.18	60.90	0.00
TRINITY_D8TJ36_VOLCA/107-195	D8TJ36.1 PF12333.6;	60.90	0.00
TRINITY_Q24F10_TETTS/11-394	Q24F10.2 PF00199.17	60.90	0.00
TRINITY_W2RAX8_PHYPN/14-175	W2RAX8.1 PF00071.20	60.90	0.00
TRINITY_Q54J76_DICDI/150-326	Q54J76.1 PF04055.19	60.90	0.00
TRINITY_A0DG06_PARTE/625-771	A0DG06.1 PF00454.25	60.90	0.00
TRINITY_A0BRT5_PARTE/364-413	A0BRT5.1 PF13920.4;	60.90	0.00
TRINITY_A0BNF3_PARTE/145-507	A0BNF3.1 PF03133.13	60.90	0.00
TRINITY_D8TWQ7_VOLCA/44-273	D8TWQ7.1 PF00566.16	60.90	0.00
TRINITY_D3PQC1_MEIRD/29-378	D3PQC1.1 PF00155.19	60.90	0.00
TRINITY_A8I7G0_CHLRE/1-160	A8I7G0.1 PF03031.16	60.90	0.00
TRINITY_D8TST4_VOLCA/59-324	D8TST4.1 PF01263.18	60.90	0.00
TRINITY_J9I4N9_9SPIT/622-711	J9I4N9.1 PF00626.20	60.90	0.00
TRINITY_F4PQQ2_DICFS/9-262	F4PQQ2.1 PF00069.23	60.90	0.00
TRINITY_M2XX62_GALSU/4-177	M2XX62.1 PF00163.17	60.90	0.00
TRINITY_A8HY85_CHLRE/42-92	A8HY85.1 PF13920.4;	60.90	0.00
TRINITY_L1JZL8_GUITH/135-326	L1JZL8.1 PF01734.20	60.90	0.00
TRINITY_A0A078ARV0_STYLE/502-593	A0A078ARV0.1 PF1289	60.90	0.00
TRINITY_D8U2F3_VOLCA/264-593	D8U2F3.1 PF12631.5;	60.90	0.00
TRINITY_D8THH3_VOLCA/345-424	D8THH3.1 PF01494.17	60.90	0.00
TRINITY_C1MK75_MICPC/173-283	C1MK75.1 PF02770.17	60.90	0.00
TRINITY_C1EGA3_MICSR/16-192	C1EGA3.1 PF02776.16	60.90	0.00
TRINITY_V4N6Q8_EUTSA/185-283	V4N6Q8.1 PF00153.25	60.90	0.00
TRINITY_A8IAC0_CHLRE/12-77	A8IAC0.1 PF13499.4;	60.90	0.00
TRINITY_D8UB42_VOLCA/33-193	D8UB42.1 PF00156.25	60.90	0.00
TRINITY_D8U9R1_VOLCA/444-517	D8U9R1.1 PF08409.9;	60.90	0.00
TRINITY_A0C1Q8_PARTE/313-381	A0C1Q8.1 PF00076.20	60.90	0.00
TRINITY_A0A0M2ZCZ3_9MYCO/13-413	A0A0M2ZCZ3.1 PF0050	60.90	0.00
TRINITY_I0YWV4_9CHLO/88-151	I0YWV4.1 PF00076.20	60.90	0.00
TRINITY_C1FH03_MICSR/16-120	C1FH03.1 PF04695.11	60.90	0.00
TRINITY_Q8LP67_CHLRE/27-494	Q8LP67.2 PF01384.18	60.90	0.00
TRINITY_Q23FV5_TETTS/249-318	Q23FV5.1 PF01485.19	60.90	0.00
TRINITY_C1FG26_MICSR/197-263	C1FG26.1 PF01302.23	60.90	0.00
TRINITY_F7DDC1_XENTR/62-218	F7DDC1.1 PF00179.24	60.90	0.00
TRINITY_G0R3Z2_ICHMG/42-178	G0R3Z2.1 PF00768.18	60.90	0.00
TRINITY_I0YXL0_9CHLO/17-195	I0YXL0.1 PF02441.17	60.90	0.00
TRINITY_A0A0J8C200_BETVU/11-357	A0A0J8C200.1 PF0027	60.90	0.00
TRINITY_D8TW17_VOLCA/26-284	D8TW17.1 PF00454.25	60.90	0.00
TRINITY_A0D8S8_PARTE/638-896	A0D8S8.1 PF07714.15	60.90	0.00
TRINITY_B2J555_NOSP7/29-290	B2J555.1 PF00657.20	60.90	0.00
TRINITY_A8IZN8_CHLRE/348-602	A8IZN8.1 PF07690.14	60.90	0.00
TRINITY_G4YV14_PHYSP/6-75	G4YV14.1 PF02798.18	60.90	0.00
TRINITY_I0YLU4_9CHLO/93-211	I0YLU4.1 PF02906.12	60.90	0.00
TRINITY_D8U9S9_VOLCA/599-793	D8U9S9.1 PF00211.18	60.90	0.00
TRINITY_I7M4Q4_TETTS/47-304	I7M4Q4.1 PF00069.23	60.90	0.00
TRINITY_C3YMX3_BRAFL/10-217	C3YMX3.1 PF01545.19	60.80	0.00
TRINITY_L8GHY8_ACACA/6-92	L8GHY8.1 PF00708.16	60.80	0.00
TRINITY_J9HMM9_9SPIT/852-1080	J9HMM9.1 PF16212.3;	60.80	0.00
TRINITY_G0QUG8_ICHMG/87-410	G0QUG8.1 PF00069.23	60.80	0.00
TRINITY_A8IQL7_CHLRE/1-150	A8IQL7.1 PF00316.18	60.80	0.00
TRINITY_G0QSE6_ICHMG/15-273	G0QSE6.1 PF00248.19	60.80	0.00
TRINITY_Q22AH3_TETTS/20-208	Q22AH3.2 PF00485.16	60.80	0.00
TRINITY_D0MSW7_PHYIT/76-243	D0MSW7.1 PF00270.27	60.80	0.00

TRINITY_A0A0A1TYI7_ENTIV/482-603	A0A0A1TYI7.1 PF0000	60.80	0.00
TRINITY_A0A068RPQ1_9FUNG/48-417	A0A068RPQ1.1 PF0015	60.80	0.00
TRINITY_G0QVP9_ICHMG/2-103	G0QVP9.1 PF01158.16	60.80	0.00
TRINITY_C8X4A4_DESRD/265-456	C8X4A4.1 PF02782.14	60.80	0.00
TRINITY_A8J7F6_CHLRE/35-108	A8J7F6.1 PF00364.20	60.80	0.00
TRINITY_D8U511_VOLCA/389-562	D8U511.1 PF09736.7;	60.80	0.00
TRINITY_M7B3B1_CHEMY/73-178	M7B3B1.1 PF01661.19	60.80	0.00
TRINITY_A8HNU5_CHLRE/78-128	A8HNU5.1 PF00415.16	60.80	0.00
TRINITY_W4ZKD8_STRPU/281-385	W4ZKD8.1 PF01399.25	60.80	0.00
TRINITY_Q23AM3_TETTS/1054-1231	Q23AM3.2 PF00005.25	60.80	0.00
TRINITY_D8TYH1_VOLCA/97-221	D8TYH1.1 PF04178.10	60.80	0.00
TRINITY_I0Z3D4_9CHLO/59-178	I0Z3D4.1 PF00622.26	60.80	0.00
TRINITY_G3IFJ2_CRIGR/33-252	G3IFJ2.1 PF00149.26	60.80	0.00
TRINITY_M2XRY9_GALSU/223-392	M2XRY9.1 PF12710.5;	60.80	0.00
TRINITY_A5GB05_GEOUR/54-510	A5GB05.1 PF00171.20	60.80	0.00
TRINITY_D3BLA2_POLPA/49-377	D3BLA2.1 PF00128.22	60.80	0.00
TRINITY_E1ZBM9_CHLVA/55-150	E1ZBM9.1 PF10187.7;	60.80	0.00
TRINITY_L8HHI9_ACACA/243-535	L8HHI9.1 PF01504.16	60.80	0.00
TRINITY_A8HX34_CHLRE/56-171	A8HX34.1 PF13911.4;	60.80	0.00
TRINITY_A0EFQ7_PARTE/75-330	A0EFQ7.1 PF00069.23	60.80	0.00
TRINITY_D8UIC0_VOLCA/170-480	D8UIC0.1 PF00176.21	60.80	0.00
TRINITY_A8J925_CHLRE/343-430	A8J925.1 PF04720.10	60.80	0.00
TRINITY_A8IQV5_CHLRE/92-496	A8IQV5.1 PF10255.7;	60.80	0.00
TRINITY_A0A023X102_9ACTN/521-633	A0A023X102.1 PF0251	60.80	0.00
TRINITY_A8IQK4_CHLRE/15-342	A8IQK4.1 PF00248.19	60.80	0.00
TRINITY_A8IPE0_CHLRE/268-418	A8IPE0.1 PF00441.22	60.80	0.00
TRINITY_A0A024HC74_PSEKB/11-476	A0A024HC74.1 PF0017	60.80	0.00
TRINITY_A8JAW8_CHLRE/513-575	A8JAW8.1 PF07714.15	60.80	0.00
TRINITY_D8UCY0_VOLCA/560-694	D8UCY0.1 PF03188.14	60.80	0.00
TRINITY_A0A087SB57_AUXPR/89-364	A0A087SB57.1 PF0032	60.80	0.00
TRINITY_G0R4H4_ICHMG/196-460	G0R4H4.1 PF00557.22	60.80	0.00
TRINITY_I7M643_TETTS/75-442	I7M643.1 PF01207.15	60.80	0.00
TRINITY_A8HVG5_CHLRE/56-124	A8HVG5.1 PF00076.20	60.80	0.00
TRINITY_I0ZA66_9CHLO/57-248	I0ZA66.1 PF01545.19	60.80	0.00
TRINITY_I1HGN0_BRADI/83-162	I1HGN0.1 PF00111.25	60.80	0.00
TRINITY_Q22RI9_TETTS/289-441	Q22RI9.1 PF01467.24	60.80	0.00
TRINITY_E1ZCS4_CHLVA/37-115	E1ZCS4.1 PF03810.17	60.80	0.00
TRINITY_F4PSM6_DICFS/3-127	F4PSM6.1 PF00583.23	60.80	0.00
TRINITY_A0A087SSY7_AUXPR/12268-12330	A0A087SSY7.1 PF0055	60.80	0.00
TRINITY_A8JJX1_CHLRE/1-168	A8JJX1.1 PF00211.18	60.80	0.00
TRINITY_M0T8F5_MUSAM/4-79	M0T8F5.1 PF01722.16	60.80	0.00
TRINITY_D8RT90_SELML/13-293	D8RT90.1 PF00069.23	60.80	0.00
TRINITY_A0DUD1_PARTE/60-138	A0DUD1.1 PF03061.20	60.80	0.00
TRINITY_I7MA19_TETTS/70-345	I7MA19.2 PF00487.22	60.70	0.00
TRINITY_A0BUS1_PARTE/18-141	A0BUS1.1 PF01775.15	60.70	0.00
TRINITY_F0ZZ51_DICPU/1-174	F0ZZ51.1 PF04062.12	60.70	0.00
TRINITY_V4AWN0_LOTGI/221-327	V4AWN0.1 PF02770.17	60.70	0.00
TRINITY_I7MIK7_TETTS/172-349	I7MIK7.1 PF02866.16	60.70	0.00
TRINITY_L1IH70_GUITH/122-177	L1IH70.1 PF13513.4;	60.70	0.00
TRINITY_Q240X8_TETTS/533-757	Q240X8.2 PF00149.26	60.70	0.00
TRINITY_A8IJ00_CHLRE/2-163	A8IJ00.1 PF12850.5;	60.70	0.00
TRINITY_I7M700_TETTS/181-443	I7M700.2 PF00069.23	60.70	0.00
TRINITY_D8UBI3_VOLCA/2-142	D8UBI3.1 PF00179.24	60.70	0.00
TRINITY_GELA_DICDI/12-118	P13466.1 PF00307.29	60.70	0.00
TRINITY_D8TIM4_VOLCA/675-744	D8TIM4.1 PF16326.3;	60.70	0.00
TRINITY_U3JZB3_FICAL/200-466	U3JZB3.1 PF00557.22	60.70	0.00
TRINITY_A8ISQ5_CHLRE/1729-1856	A8ISQ5.1 PF00004.27	60.70	0.00
TRINITY_J9HY32_9SPIT/83-139	J9HY32.1 PF13418.4;	60.70	0.00
TRINITY_L8GUI1_ACACA/1063-1317	L8GUI1.1 PF01504.16	60.70	0.00
TRINITY_A8J7D5_CHLRE/111-230	A8J7D5.1 PF12146.6;	60.70	0.00
TRINITY_M2VXJ2_GALSU/118-200	M2VXJ2.1 PF15963.3;	60.70	0.00

TRINITY_F4PRM6_DICFS/415-693	F4PRM6.1 PF02089.13	60.70	0.00
TRINITY_A8J3T8_CHLRE/773-845	A8J3T8.1 PF02214.20	60.70	0.00
TRINITY_D8U0X3_VOLCA/27-233	D8U0X3.1 PF00664.21	60.70	0.00
TRINITY_Q5LUM3_RUEPO/28-222	Q5LUM3.1 PF00753.25	60.70	0.00
TRINITY_I7MCM8_TETTS/22-110	I7MCM8.1 PF01221.16	60.70	0.00
TRINITY_D8UAR2_VOLCA/40-165	D8UAR2.1 PF06979.10	60.70	0.00
TRINITY_I0YY21_9CHLO/23-435	I0YY21.1 PF03486.12	60.70	0.00
TRINITY_H0XNX4_OTOGA/140-573	H0XNX4.1 PF00654.18	60.70	0.00
TRINITY_A8IZN8_CHLRE/149-294	A8IZN8.1 PF07690.14	60.70	0.00
TRINITY_A0A087SDT6_AUXPR/1023-1248	A0A087SDT6.1 PF0246	60.70	0.00
TRINITY_D8TH51_VOLCA/203-334	D8TH51.1 PF00012.18	60.70	0.00
TRINITY_D8TZ46_VOLCA/4-152	D8TZ46.1 PF02469.20	60.70	0.00
TRINITY_L8HJ83_ACACA/415-598	L8HJ83.1 PF02145.13	60.70	0.00
TRINITY_D8U2I5_VOLCA/121-481	D8U2I5.1 PF07690.14	60.70	0.00
TRINITY_A8HQN2_CHLRE/716-773	A8HQN2.1 PF00839.15	60.70	0.00
TRINITY_E1ZLM9_CHLVA/205-324	E1ZLM9.1 PF13410.4;	60.70	0.00
TRINITY_D8TS44_VOLCA/61-200	D8TS44.1 PF00328.20	60.70	0.00
TRINITY_Q4KX33_CHLRE/41-243	Q4KX33.1 PF00702.24	60.70	0.00
TRINITY_E1ZE44_CHLVA/390-768	E1ZE44.1 PF00702.24	60.70	0.00
TRINITY_E1ZNG0_CHLVA/135-423	E1ZNG0.1 PF00664.21	60.70	0.00
TRINITY_A8IM09_CHLRE/15-199	A8IM09.1 PF13419.4;	60.70	0.00
TRINITY_I7MMC6_TETTS/8-267	I7MMC6.1 PF00795.20	60.70	0.00
TRINITY_A0A078ATQ4_STYLE/10-147	A0A078ATQ4.1 PF0551	60.70	0.00
TRINITY_A0BWW1_PARTE/272-340	A0BWW1.1 PF00498.24	60.60	0.00
TRINITY_I7MLH5_TETTS/110-313	I7MLH5.1 PF02630.12	60.60	0.00
TRINITY_G0R559_ICHMG/162-325	G0R559.1 PF03031.16	60.60	0.00
TRINITY_A0A022QPS6_ERYGU/47-118	A0A022QPS6.1 PF0017	60.60	0.00
TRINITY_O96212_PLAF7/4-69	O96212.1 PF00226.29	60.60	0.00
TRINITY_I7MKM3_TETTS/42-327	I7MKM3.2 PF01875.15	60.60	0.00
TRINITY_Q3SDM6_PARTE/17-177	Q3SDM6.1 PF00071.20	60.60	0.00
TRINITY_A6CKQ5_9BACI/6-206	A6CKQ5.1 PF00106.23	60.60	0.00
TRINITY_B7G8G3_PHATC/71-206	B7G8G3.1 PF00179.24	60.60	0.00
TRINITY_A0A077ZRM4_STYLE/9-353	A0A077ZRM4.1 PF0072	60.60	0.00
TRINITY_G1XAK0_ARTOA/259-586	G1XAK0.1 PF00152.18	60.60	0.00
TRINITY_D2V2H2_NAEGR/29-165	D2V2H2.1 PF01138.19	60.60	0.00
TRINITY_F4PWL5_DICFS/65-135	F4PWL5.1 PF07258.12	60.60	0.00
TRINITY_L0PEQ1_PNEJ8/1982-2232	L0PEQ1.1 PF00454.25	60.60	0.00
TRINITY_A8IV68_CHLRE/10-170	A8IV68.1 PF01554.16	60.60	0.00
TRINITY_W5L3X6_ASTMX/15-361	W5L3X6.1 PF01208.15	60.60	0.00
TRINITY_D8UCM9_VOLCA/16-368	D8UCM9.1 PF14249.4;	60.60	0.00
TRINITY_I0Z8B5_9CHLO/174-487	I0Z8B5.1 PF00282.17	60.60	0.00
TRINITY_D8UB55_VOLCA/53-527	D8UB55.1 PF03917.15	60.60	0.00
TRINITY_E1ZCZ4_CHLVA/177-413	E1ZCZ4.1 PF13621.4;	60.60	0.00
TRINITY_A8IZX0_CHLRE/194-290	A8IZX0.1 PF00153.25	60.60	0.00
TRINITY_A0A078AR09_STYLE/8-169	A0A078AR09.1 PF0007	60.60	0.00
TRINITY_I3SWQ0_MEDTR/4-177	I3SWQ0.1 PF00025.19	60.60	0.00
TRINITY_Q22W33_TETTS/214-501	Q22W33.2 PF01031.18	60.60	0.00
TRINITY_L8GTX7_ACACA/29-258	L8GTX7.1 PF01144.21	60.60	0.00
TRINITY_A0A067CN16_SAPPC/176-246	A0A067CN16.1 PF0007	60.60	0.00
TRINITY_D8TQR8_VOLCA/39-141	D8TQR8.1 PF06984.11	60.60	0.00
TRINITY_D8TTV8_VOLCA/81-324	D8TTV8.1 PF00233.17	60.60	0.00
TRINITY_A8J5G1_CHLRE/62-132	A8J5G1.1 PF00076.20	60.60	0.00
TRINITY_D8TIW7_VOLCA/25-159	D8TIW7.1 PF10358.7;	60.60	0.00
TRINITY_A8IIK8_CHLRE/22-646	A8IIK8.1 PF00012.18	60.60	0.00
TRINITY_W7XI32_TETTS/8-116	W7XI32.1 PF01412.16	60.60	0.00
TRINITY_NC2B_DICDI/11-76	Q55DJ5.1 PF00808.21	60.60	0.00
TRINITY_A8IP41_CHLRE/256-440	A8IP41.1 PF17177.2;	60.60	0.00
TRINITY_D8UHG2_VOLCA/303-495	D8UHG2.1 PF00625.19	60.60	0.00
TRINITY_A8JGR9_CHLRE/78-301	A8JGR9.1 PF07859.11	60.60	0.00
TRINITY_D8UBU5_VOLCA/35-166	D8UBU5.1 PF00582.24	60.60	0.00
TRINITY_G0QP90_ICHMG/68-301	G0QP90.1 PF08709.9;	60.60	0.00

TRINITY_I7LXC1_TETTS/15-327	I7LXC1.1 PF00248.19	60.60	0.00
TRINITY_A0A087SN26_AUXPR/253-511	A0A087SN26.1 PF0042	60.60	0.00
TRINITY_A8I2W2_CHLRE/20-271	A8I2W2.1 PF00378.18	60.60	0.00
TRINITY_D8TVC0_VOLCA/1983-2096	D8TVC0.1 PF00665.24	60.60	0.00
TRINITY_I7M910_TETTS/11-285	I7M910.1 PF00069.23	60.60	0.00
TRINITY_I1FJ82_AMPQE/74-164	I1FJ82.1 PF02214.20	60.60	0.00
TRINITY_I0YIK0_9CHLO/2-1118	I0YIK0.1 PF02463.17	60.60	0.00
TRINITY_G0QNX2_ICHMG/458-558	G0QNX2.1 PF13246.4;	60.60	0.00
TRINITY_A8JH01_CHLRE/760-1287	A8JH01.1 PF02538.12	60.60	0.00
TRINITY_D8THC3_VOLCA/345-463	D8THC3.1 PF03398.12	60.60	0.00
TRINITY_C6X4G5_FLAB3/3-109	C6X4G5.1 PF00453.16	60.60	0.00
TRINITY_A0E1Y7_PARTE/546-611	A0E1Y7.1 PF00659.16	60.60	0.00
TRINITY_A0D0Z9_PARTE/173-301	A0D0Z9.1 PF17207.1;	60.60	0.00
TRINITY_B9HAK1_POPTR/1-38	B9HAK1.1 PF00444.16	60.50	0.00
TRINITY_W4GIQ7_9STRA/55-461	W4GIQ7.1 PF03901.15	60.50	0.00
TRINITY_Q24GF5_TETTS/192-235	Q24GF5.1 PF13639.4;	60.50	0.00
TRINITY_F4PQP3_DICFS/61-212	F4PQP3.1 PF12146.6;	60.50	0.00
TRINITY_Q22BR3_TETTS/13-180	Q22BR3.2 PF00071.20	60.50	0.00
TRINITY_D3BKU3_POLPA/99-247	D3BKU3.1 PF04051.14	60.50	0.00
TRINITY_L1IBT4_GUITH/87-131	L1IBT4.1 PF00400.30	60.50	0.00
TRINITY_G4VDS7_SCHMA/18-67	G4VDS7.1 PF00385.22	60.50	0.00
TRINITY_A0A015JS03_9GLOM/219-473	A0A015JS03.1 PF0042	60.50	0.00
TRINITY_W7TBG8_9STRA/23-109	W7TBG8.1 PF10217.7;	60.50	0.00
TRINITY_PAKF_DICDI/394-646	Q869T7.1 PF00069.23	60.50	0.00
TRINITY_A4RWA9_OSTLU/932-974	A4RWA9.1 PF00569.15	60.50	0.00
TRINITY_A0A078BAY1_STYLE/2-421	A0A078BAY1.1 PF0002	60.50	0.00
TRINITY_F8KZ09_PARAV/42-385	F8KZ09.1 PF00155.19	60.50	0.00
TRINITY_S7RV90_GLOTA/2-92	S7RV90.1 PF00153.25	60.50	0.00
TRINITY_D8RSP9_SELML/19-278	D8RSP9.1 PF00108.21	60.50	0.00
TRINITY_E4Y0P8_OIKDI/192-320	E4Y0P8.1 PF14833.4;	60.50	0.00
TRINITY_D3BAY6_POLPA/1281-1386	D3BAY6.1 PF00307.29	60.50	0.00
TRINITY_F4PVW8_DICFS/49-173	F4PVW8.1 PF00782.18	60.50	0.00
TRINITY_L8GHG3_ACACA/31-111	L8GHG3.1 PF13774.4;	60.50	0.00
TRINITY_D8UFD4_VOLCA/1148-1364	D8UFD4.1 PF02301.16	60.50	0.00
TRINITY_D8TN22_VOLCA/1491-1533	D8TN22.1 PF00400.30	60.50	0.00
TRINITY_A8J576_CHLRE/30-84	A8J576.1 PF01667.15	60.50	0.00
TRINITY_V4AHX0_LOTGI/87-328	V4AHX0.1 PF08627.8;	60.50	0.00
TRINITY_A0A075AW01_9FUNG/299-343	A0A075AW01.1 PF1363	60.50	0.00
TRINITY_Q6U9W9_CHLRE/45-120	Q6U9W9.1 PF01066.19	60.50	0.00
TRINITY_D8U396_VOLCA/10-273	D8U396.1 PF04641.10	60.50	0.00
TRINITY_A8IL82_CHLRE/88-164	A8IL82.1 PF14933.4;	60.50	0.00
TRINITY_F2UFQ3_SALR5/2479-2561	F2UFQ3.1 PF05406.13	60.50	0.00
TRINITY_H3HAJ2_PHYRM/359-478	H3HAJ2.1 PF00665.24	60.50	0.00
TRINITY_A0A0F4JJD4_9ACTN/29-473	A0A0F4JJD4.1 PF1352	60.50	0.00
TRINITY_A0A0F8BLB8_CERFI/630-920	A0A0F8BLB8.1 PF0519	60.50	0.00
TRINITY_A0A0D2WVY6_CAPO3/6-314	A0A0D2WVY6.1 PF0337	60.50	0.00
TRINITY_L8H169_ACACA/11-527	L8H169.1 PF01019.19	60.50	0.00
TRINITY_L8HA47_ACACA/343-485	L8HA47.1 PF13768.4;	60.50	0.00
TRINITY_A8IFT8_CHLRE/600-698	A8IFT8.1 PF00211.18	60.50	0.00
TRINITY_A8HQ85_CHLRE/29-108	A8HQ85.1 PF00717.21	60.50	0.00
TRINITY_A8HPM7_CHLRE/39-76	A8HPM7.1 PF12907.5;	60.50	0.00
TRINITY_A8J940_CHLRE/199-654	A8J940.1 PF09418.8;	60.50	0.00
TRINITY_A0BI43_PARTE/13-346	A0BI43.1 PF00225.21	60.50	0.00
TRINITY_Q22T22_TETTS/520-735	Q22T22.3 PF07859.11	60.50	0.00
TRINITY_K5W5T9_PHACS/160-202	K5W5T9.1 PF15985.3;	60.50	0.00
TRINITY_D8UJA5_VOLCA/42-123	D8UJA5.1 PF03366.14	60.50	0.00
TRINITY_D8TZ34_VOLCA/1-77	D8TZ34.1 PF00300.20	60.50	0.00
TRINITY_Q5CXU5_CRYPI/210-254	Q5CXU5.1 PF13639.4;	60.50	0.00
TRINITY_D8U7H0_VOLCA/304-342	D8U7H0.1 PF00400.30	60.50	0.00
TRINITY_E1ZGK4_CHLVA/25-99	E1ZGK4.1 PF12895.5;	60.50	0.00
TRINITY_R7JIC3_9PORP/14-318	R7JIC3.1 PF00206.18	60.50	0.00

TRINITY_A4S165_OSTLU/201-315	A4S165.1 PF00387.17	60.50	0.00
TRINITY_K2K6U2_9PROT/14-98	K2K6U2.1 PF06155.10	60.50	0.00
TRINITY_I0YWB9_9CHLO/268-310	I0YWB9.1 PF05182.11	60.50	0.00
TRINITY_D8UJ17_VOLCA/237-357	D8UJ17.1 PF08294.9;	60.50	0.00
TRINITY_D8TNV3_VOLCA/57-246	D8TNV3.1 PF02431.13	60.50	0.00
TRINITY_D8THM5_VOLCA/20-316	D8THM5.1 PF00291.23	60.50	0.00
TRINITY_I0YYP7_9CHLO/26-829	I0YYP7.1 PF01496.17	60.50	0.00
TRINITY_F0ZPV3_DICPU/326-403	F0ZPV3.1 PF00626.20	60.50	0.00
TRINITY_I1KAV1_SOYBN/23-103	I1KAV1.1 PF03810.17	60.50	0.00
TRINITY_D8UER9_VOLCA/17-281	D8UER9.1 PF00069.23	60.50	0.00
TRINITY_D8TJA9_VOLCA/41-757	D8TJA9.1 PF05879.10	60.50	0.00
TRINITY_A0A0D2RPE2_GOSRA/79-123	A0A0D2RPE2.1 PF1363	60.50	0.00
TRINITY_D8TM14_VOLCA/44-264	D8TM14.1 PF01036.16	60.50	0.00
TRINITY_A0DEC4_PARTE/157-332	A0DEC4.1 PF00297.20	60.50	0.00
TRINITY_J9FGP4_9SPIT/3326-3635	J9FGP4.1 PF00520.29	60.50	0.00
TRINITY_W5B1M5_WHEAT/1031-1109	W5B1M5.1 PF16770.3;	60.50	0.00
TRINITY_A0BJW5_PARTE/673-824	A0BJW5.1 PF00005.25	60.50	0.00
TRINITY_A8HMX4_CHLRE/2800-2846	A8HMX4.1 PF13920.4;	60.50	0.00
TRINITY_I0YVX4_9CHLO/299-509	I0YVX4.1 PF01061.22	60.50	0.00
TRINITY_A8J0U4_CHLRE/27-488	A8J0U4.1 PF01384.18	60.50	0.00
TRINITY_I7LZH4_TETTS/16-180	I7LZH4.1 PF10237.7;	60.50	0.00
TRINITY_A0CM91_PARTE/146-237	A0CM91.1 PF02770.17	60.40	0.00
TRINITY_L8GN11_ACACA/11-328	L8GN11.1 PF16363.3;	60.40	0.00
TRINITY_A0A090N2X0_OSTTA/7-430	A0A090N2X0.1 PF0390	60.40	0.00
TRINITY_D8Q147_SCHCM/127-219	D8Q147.1 PF13640.4;	60.40	0.00
TRINITY_Q24FD5_TETTS/395-493	Q24FD5.2 PF12796.5;	60.40	0.00
TRINITY_G3ASL6_SPAPN/310-357	G3ASL6.1 PF14604.4;	60.40	0.00
TRINITY_C7PLA0_CHIPD/8-216	C7PLA0.1 PF00753.25	60.40	0.00
TRINITY_V7BFG5_PHAVU/653-788	V7BFG5.1 PF00005.25	60.40	0.00
TRINITY_D3B1V2_POLPA/5-372	D3B1V2.1 PF00022.17	60.40	0.00
TRINITY_Q236N6_TETTS/11-335	Q236N6.2 PF00225.21	60.40	0.00
TRINITY_D2UXL3_NAEGR/231-284	D2UXL3.1 PF13639.4;	60.40	0.00
TRINITY_A8I3E9_CHLRE/6-101	A8I3E9.1 PF00168.28	60.40	0.00
TRINITY_A0DXZ0_PARTE/196-452	A0DXZ0.1 PF00069.23	60.40	0.00
TRINITY_A0CMJ9_PARTE/173-229	A0CMJ9.1 PF08063.10	60.40	0.00
TRINITY_F4Q879_DICFS/343-583	F4Q879.1 PF00122.18	60.40	0.00
TRINITY_D8TH82_VOLCA/2119-2230	D8TH82.1 PF01477.21	60.40	0.00
TRINITY_K1RLC3_CRAGI/178-234	K1RLC3.1 PF03909.15	60.40	0.00
TRINITY_E1ZSH0_CHLVA/182-287	E1ZSH0.1 PF13813.4;	60.40	0.00
TRINITY_A8HTI5_CHLRE/224-447	A8HTI5.1 PF00149.26	60.40	0.00
TRINITY_J9F675_9SPIT/89-719	J9F675.1 PF00133.20	60.40	0.00
TRINITY_C1FHE0_MICSR/238-341	C1FHE0.1 PF13266.4;	60.40	0.00
TRINITY_E1Z4Q4_CHLVA/32-185	E1Z4Q4.1 PF02590.15	60.40	0.00
TRINITY_A0A067QXN5_ZOONE/105-425	A0A067QXN5.1 PF0017	60.40	0.00
TRINITY_B4N4L3_DROWI/10-97	B4N4L3.1 PF00708.16	60.40	0.00
TRINITY_D8TWB2_VOLCA/396-443	D8TWB2.1 PF13639.4;	60.40	0.00
TRINITY_K2JWG6_9GAMM/175-508	K2JWG6.1 PF00883.19	60.40	0.00
TRINITY_G3PJM8_GASAC/67-736	G3PJM8.1 PF00063.19	60.40	0.00
TRINITY_Q22TJ1_TETTS/178-486	Q22TJ1.1 PF00365.18	60.40	0.00
TRINITY_D8SAX2_SELML/206-432	D8SAX2.1 PF13621.4;	60.40	0.00
TRINITY_A0BD09_PARTE/2285-2969	A0BD09.1 PF03028.13	60.40	0.00
TRINITY_D8TN99_VOLCA/23-445	D8TN99.1 PF04597.12	60.40	0.00
TRINITY_I1N9L1_SOYBN/10-174	I1N9L1.1 PF00160.19	60.40	0.00
TRINITY_D8TVU2_VOLCA/33-362	D8TVU2.1 PF00180.18	60.40	0.00
TRINITY_A8HXN9_CHLRE/61-524	A8HXN9.1 PF04100.10	60.40	0.00
TRINITY_D8TIK2_VOLCA/20-442	D8TIK2.1 PF00999.19	60.40	0.00
TRINITY_B3ESW8_AMOA5/41-239	B3ESW8.1 PF01734.20	60.40	0.00
TRINITY_D8U1G9_VOLCA/1333-1663	D8U1G9.1 PF00225.21	60.40	0.00
TRINITY_H3B4W6_LATCH/552-647	H3B4W6.1 PF05495.10	60.40	0.00
TRINITY_I1G3Y8_AMPQE/366-838	I1G3Y8.1 PF03200.14	60.40	0.00
TRINITY_I7MHC4_TETTS/1048-1120	I7MHC4.1 PF14418.4;	60.40	0.00

TRINITY_Q5AH29_CANAL/74-136	Q5AH29.1 PF00462.22	60.40	0.00
TRINITY_A8IVY2_CHLRE/426-558	A8IVY2.1 PF07732.13	60.40	0.00
TRINITY_S8E968_9LAMI/38-286	S8E968.1 PF03372.21	60.40	0.00
TRINITY_A0A068RY16_9FUNG/1113-1219	A0A068RY16.1 PF0085	60.40	0.00
TRINITY_I0Z9K0_9CHLO/143-285	I0Z9K0.1 PF10607.7;	60.40	0.00
TRINITY_CFA74_CHLRE/607-707	D4P3R7.1 PF14874.4;	60.40	0.00
TRINITY_T1JNQ3_STRMM/6-283	T1JNQ3.1 PF00795.20	60.30	0.00
TRINITY_A0A0D3HDK8_9ORYZ/63-128	A0A0D3HDK8.1 PF1620	60.30	0.00
TRINITY_A1U9N9_MYCSK/11-219	A1U9N9.1 PF00743.17	60.30	0.00
TRINITY_A0A058Z427_9EUKA/28-247	A0A058Z427.1 PF0332	60.30	0.00
TRINITY_K4B118_SOLLC/7-112	K4B118.1 PF05564.10	60.30	0.00
TRINITY_A0A078B9W6_STYLE/3791-4496	A0A078B9W6.1 PF0302	60.30	0.00
TRINITY_L8HMW5_ACACA/38-177	L8HMW5.1 PF08719.9;	60.30	0.00
TRINITY_W4GV92_9STRA/64-139	W4GV92.1 PF01066.19	60.30	0.00
TRINITY_A0DS94_PARTE/230-586	A0DS94.1 PF08613.9;	60.30	0.00
TRINITY_A9TLF9_PHYPA/34-95	A9TLF9.1 PF00226.29	60.30	0.00
TRINITY_G0R0U0_ICHMG/14-286	G0R0U0.1 PF10234.7;	60.30	0.00
TRINITY_C1MKD3_MICPC/15-79	C1MKD3.1 PF13499.4;	60.30	0.00
TRINITY_I7LV14_TETTS/122-336	I7LV14.1 PF00112.21	60.30	0.00
TRINITY_A0A0G4H4N9_9ALVE/11-150	A0A0G4H4N9.1 PF1458	60.30	0.00
TRINITY_A0A0G1QZP5_9BACT/13-346	A0A0G1QZP5.1 PF0120	60.30	0.00
TRINITY_G4YIK5_PHYSP/252-375	G4YIK5.1 PF01926.21	60.30	0.00
TRINITY_L8GEQ6_ACACA/44-387	L8GEQ6.1 PF00724.18	60.30	0.00
TRINITY_D8TUM3_VOLCA/223-475	D8TUM3.1 PF00266.17	60.30	0.00
TRINITY_I7M198_TETTS/52-316	I7M198.2 PF00069.23	60.30	0.00
TRINITY_D8TNS7_VOLCA/490-809	D8TNS7.1 PF10498.7;	60.30	0.00
TRINITY_YR260_MIMIV/7-69	Q5UP23.1 PF00226.29	60.30	0.00
TRINITY_A0DBR4_PARTE/173-230	A0DBR4.1 PF00415.16	60.30	0.00
TRINITY_C1MN06_MICPC/482-595	C1MN06.1 PF00271.29	60.30	0.00
TRINITY_A8IGG5_CHLRE/30-160	A8IGG5.1 PF01590.24	60.30	0.00
TRINITY_D3BTH5_POLPA/151-413	D3BTH5.1 PF00566.16	60.30	0.00
TRINITY_L8GZC6_ACACA/162-341	L8GZC6.1 PF01612.18	60.30	0.00
TRINITY_F1A249_DICPU/27-235	F1A249.1 PF08442.8;	60.30	0.00
TRINITY_D3B5K6_POLPA/1245-1393	D3B5K6.1 PF00005.25	60.30	0.00
TRINITY_K9T8B5_9CYAN/155-580	K9T8B5.1 PF00501.26	60.30	0.00
TRINITY_A0A0D2WIU1_CAPO3/627-784	A0A0D2WIU1.1 PF0703	60.30	0.00
TRINITY_D3B5L4_POLPA/76-144	D3B5L4.1 PF12265.6;	60.30	0.00
TRINITY_C1MVV9_MICPC/172-288	C1MVV9.1 PF02373.20	60.30	0.00
TRINITY_I0YP20_9CHLO/96-251	I0YP20.1 PF01625.19	60.30	0.00
TRINITY_A0E1B7_PARTE/222-546	A0E1B7.1 PF00676.18	60.30	0.00
TRINITY_A0ZJS6_NODSP/13-341	A0ZJS6.1 PF07690.14	60.30	0.00
TRINITY_D8U9J7_VOLCA/756-823	D8U9J7.1 PF12894.5;	60.30	0.00
TRINITY_A0A024WRW2_PLAFA/3-212	A0A024WRW2.1 PF0009	60.30	0.00
TRINITY_Q22AH3_TETTS/20-208	Q22AH3.2 PF00485.16	60.30	0.00
TRINITY_Q3SEQ6_PARTE/138-200	Q3SEQ6.1 PF00957.19	60.30	0.00
TRINITY_A8J0I9_CHLRE/27-230	A8J0I9.1 PF00106.23	60.30	0.00
TRINITY_A0A0D2U0A2_CAPO3/664-793	A0A0D2U0A2.1 PF0063	60.30	0.00
TRINITY_D8TNS2_VOLCA/256-775	D8TNS2.1 PF00183.16	60.30	0.00
TRINITY_D8UDU0_VOLCA/334-939	D8UDU0.1 PF02446.15	60.30	0.00
TRINITY_A0A068XM71_HYMMI/64-359	A0A068XM71.1 PF0154	60.30	0.00
TRINITY_A8HP50_CHLRE/3-278	A8HP50.1 PF03194.13	60.30	0.00
TRINITY_I0YX80_9CHLO/2-489	I0YX80.1 PF02074.13	60.30	0.00
TRINITY_A8ITR3_CHLRE/395-621	A8ITR3.1 PF00067.20	60.30	0.00
TRINITY_G7KCZ8_MEDTR/8-70	G7KCZ8.1 PF00280.16	60.30	0.00
TRINITY_I1BT58_RHIO9/516-691	I1BT58.1 PF01725.14	60.20	0.00
TRINITY_I7M706_TETTS/16-370	I7M706.2 PF00225.21	60.20	0.00
TRINITY_D3B006_POLPA/20-124	D3B006.1 PF00307.29	60.20	0.00
TRINITY_A0A0G4ESR1_9ALVE/93-308	A0A0G4ESR1.1 PF0214	60.20	0.00
TRINITY_D8TUF1_VOLCA/74-355	D8TUF1.1 PF00850.17	60.20	0.00
TRINITY_D8UBW7_VOLCA/3-270	D8UBW7.1 PF01459.20	60.20	0.00
TRINITY_A8J6V4_CHLRE/40-273	A8J6V4.1 PF00923.17	60.20	0.00

TRINITY_W2RUV3_9EURO/292-396	W2RUV3.1 PF00307.29	60.20	0.00
TRINITY_D8U7L1_VOLCA/68-150	D8U7L1.1 PF12796.5;	60.20	0.00
TRINITY_A0A091E6Y3_FUKDA/802-907	A0A091E6Y3.1 PF0828	60.20	0.00
TRINITY_A8HQ95_CHLRE/22-183	A8HQ95.1 PF01554.16	60.20	0.00
TRINITY_A5FUJ8_ACICJ/536-886	A5FUJ8.1 PF01212.19	60.20	0.00
TRINITY_E1Z7H0_CHLVA/96-228	E1Z7H0.1 PF00293.26	60.20	0.00
TRINITY_V7BY08_PHAVU/6-93	V7BY08.1 PF14368.4;	60.20	0.00
TRINITY_A6C7R0_9PLAN/13-219	A6C7R0.1 PF02492.17	60.20	0.00
TRINITY_L8GQT8_ACACA/655-748	L8GQT8.1 PF13661.4;	60.20	0.00
TRINITY_K9T216_9CYAN/49-161	K9T216.1 PF03641.12	60.20	0.00
TRINITY_D3B9Q6_POLPA/99-347	D3B9Q6.1 PF07714.15	60.20	0.00
TRINITY_C4WTZ1_ACYPI/29-118	C4WTZ1.1 PF02269.14	60.20	0.00
TRINITY_G0QZK0_ICHMG/203-416	G0QZK0.1 PF00208.19	60.20	0.00
TRINITY_A0A087XSU4_POEFO/226-404	A0A087XSU4.1 PF0017	60.20	0.00
TRINITY_I7ML66_TETTS/12-99	I7ML66.1 PF01172.16	60.20	0.00
TRINITY_A0A0E3UWF6_9BACT/52-529	A0A0E3UWF6.1 PF0120	60.20	0.00
TRINITY_D8TY39_VOLCA/74-187	D8TY39.1 PF01245.18	60.20	0.00
TRINITY_I7M1V6_TETTS/314-533	I7M1V6.1 PF07002.14	60.20	0.00
TRINITY_A0DCA2_PARTE/43-238	A0DCA2.1 PF00149.26	60.20	0.00
TRINITY_D8TQ52_VOLCA/268-455	D8TQ52.1 PF00534.18	60.20	0.00
TRINITY_V4TM29_9ROSI/102-251	V4TM29.1 PF06574.10	60.20	0.00
TRINITY_A8J9U6_CHLRE/66-326	A8J9U6.1 PF12697.5;	60.20	0.00
TRINITY_A0A068S3R7_9FUNG/169-382	A0A068S3R7.1 PF0291	60.20	0.00
TRINITY_D8TRM7_VOLCA/133-258	D8TRM7.1 PF13640.4;	60.20	0.00
TRINITY_A0A0F3K961_9GAMM/71-236	A0A0F3K961.1 PF1347	60.20	0.00
TRINITY_D8THQ9_VOLCA/562-677	D8THQ9.1 PF10366.7;	60.20	0.00
TRINITY_A0CWH1_PARTE/397-1150	A0CWH1.1 PF03028.13	60.20	0.00
TRINITY_D8TKW0_VOLCA/57-415	D8TKW0.1 PF01566.16	60.20	0.00
TRINITY_A8IBB6_CHLRE/141-344	A8IBB6.1 PF00211.18	60.20	0.00
TRINITY_A8WKQ6_CAEBR/1-344	A8WKQ6.2 PF00022.17	60.20	0.00
TRINITY_K8YSS9_9STRA/22-327	K8YSS9.1 PF16363.3;	60.10	0.00
TRINITY_A0BP85_PARTE/12-233	A0BP85.1 PF00244.18	60.10	0.00
TRINITY_Q22HI3_TETTS/105-331	Q22HI3.2 PF13347.4;	60.10	0.00
TRINITY_A0BWL4_PARTE/26-288	A0BWL4.1 PF00069.23	60.10	0.00
TRINITY_D8UGD2_VOLCA/9-340	D8UGD2.1 PF01536.14	60.10	0.00
TRINITY_Q7X7A7_CHLRE/13-236	Q7X7A7.1 PF00244.18	60.10	0.00
TRINITY_V9FL50_PHYPR/153-293	V9FL50.1 PF00107.24	60.10	0.00
TRINITY_B3RPW9_TRIAD/5-178	B3RPW9.1 PF00071.20	60.10	0.00
TRINITY_D3BKT1_POLPA/724-1258	D3BKT1.1 PF02738.16	60.10	0.00
TRINITY_J9J3B4_9SPIT/124-338	J9J3B4.1 PF00112.21	60.10	0.00
TRINITY_GPA1_DICDI/15-344	P16894.1 PF00503.18	60.10	0.00
TRINITY_A0A087SHY5_AUXPR/4-168	A0A087SHY5.1 PF0196	60.10	0.00
TRINITY_L1JN59_GUITH/23-218	L1JN59.1 PF03587.12	60.10	0.00
TRINITY_F0ZJQ6_DICPU/27-492	F0ZJQ6.1 PF00171.20	60.10	0.00
TRINITY_A8HY17_CHLRE/88-376	A8HY17.1 PF01643.15	60.10	0.00
TRINITY_E1Z5A8_CHLVA/8-148	E1Z5A8.1 PF03870.13	60.10	0.00
TRINITY_E1Z551_CHLVA/1297-1614	E1Z551.1 PF13871.4;	60.10	0.00
TRINITY_Q9ATG8_CHLRE/87-411	Q9ATG8.1 PF00762.17	60.10	0.00
TRINITY_D8TSA1_VOLCA/1283-1531	D8TSA1.1 PF02714.13	60.10	0.00
TRINITY_D8U342_VOLCA/534-910	D8U342.1 PF03378.13	60.10	0.00
TRINITY_C1E430_MICSR/135-271	C1E430.1 PF13673.5;	60.10	0.00
TRINITY_A8J110_CHLRE/37-324	A8J110.1 PF03982.11	60.10	0.00
TRINITY_A8HV32_CHLRE/124-295	A8HV32.1 PF02582.12	60.10	0.00
TRINITY_I7MK02_TETTS/82-122	I7MK02.1 PF10276.7;	60.00	0.00
TRINITY_U3KCU9_FICAL/38-238	U3KCU9.1 PF01965.22	60.00	0.00
TRINITY_B8C4W0_THAPS/3589-3983	B8C4W0.1 PF07394.10	60.00	0.00
TRINITY_A0EDX8_PARTE/605-720	A0EDX8.1 PF00472.18	60.00	0.00
TRINITY_A0A072PFG7_9EURO/171-283	A0A072PFG7.1 PF0017	60.00	0.00
TRINITY_I7MKM3_TETTS/42-327	I7MKM3.2 PF01875.15	60.00	0.00
TRINITY_K7J0G1_NASVI/47-96	K7J0G1.1 PF07896.10	60.00	0.00
TRINITY_Q54TE8_DICDI/628-748	Q54TE8.1 PF00271.29	60.00	0.00

TRINITY_W5LA12_ASTMX/72-254	W5LA12.1 PF00535.24	60.00	0.00
TRINITY_F4PNZ2_DICFS/423-671	F4PNZ2.1 PF07714.15	60.00	0.00
TRINITY_A0A060S5Q7_PYCCI/11-142	A0A060S5Q7.1 PF0864	60.00	0.00
TRINITY_A0BZD1_PARTE/27-212	A0BZD1.1 PF01145.23	60.00	0.00
TRINITY_GNA1_DICDI/17-140	Q54WR8.1 PF00583.23	60.00	0.00
TRINITY_G0QWD0_ICHMG/622-801	G0QWD0.1 PF08282.10	60.00	0.00
TRINITY_D3B339_POLPA/183-305	D3B339.1 PF00241.18	60.00	0.00
TRINITY_A0A096M629_POEFO/648-698	A0A096M629.1 PF1460	60.00	0.00
TRINITY_I7M0Y2_TETTS/442-655	I7M0Y2.1 PF13932.4;	60.00	0.00
TRINITY_W4XXC1_STRPU/103-275	W4XXC1.1 PF00953.19	60.00	0.00
TRINITY_A0A0M2CU82_9MICC/240-462	A0A0M2CU82.1 PF0012	60.00	0.00
TRINITY_A0C7D7_PARTE/9-285	A0C7D7.1 PF00069.23	60.00	0.00
TRINITY_D8TZ58_VOLCA/138-226	D8TZ58.1 PF00867.16	60.00	0.00
TRINITY_D3BJT3_POLPA/369-583	D3BJT3.1 PF00300.20	60.00	0.00
TRINITY_A8J165_CHLRE/133-193	A8J165.1 PF04117.10	60.00	0.00
TRINITY_A0A0D2WUV3_CAPO3/23-228	A0A0D2WUV3.1 PF0254	60.00	0.00
TRINITY_D8U5Z6_VOLCA/23-152	D8U5Z6.1 PF01288.18	60.00	0.00
TRINITY_A0A074RY28_9HOMO/35-123	A0A074RY28.1 PF1289	60.00	0.00
TRINITY_G0R4A3_ICHMG/52-199	G0R4A3.1 PF13847.4;	60.00	0.00
TRINITY_D8TU06_VOLCA/34-230	D8TU06.1 PF03798.14	60.00	0.00
TRINITY_Q238R8_TETTS/848-963	Q238R8.1 PF00787.22	60.00	0.00
TRINITY_V7D020_PHAVU/173-249	V7D020.1 PF13646.4;	60.00	0.00
TRINITY_F6HZ81_VITVI/16-90	F6HZ81.1 PF00538.17	60.00	0.00
TRINITY_I7MKT7_TETTS/1324-1364	I7MKT7.2 PF00400.30	60.00	0.00
TRINITY_A0A0A1MD11_9BACI/7-124	A0A0A1MD11.1 PF0058	60.00	0.00
TRINITY_Q54EU5_DICDI/201-291	Q54EU5.1 PF02214.20	60.00	0.00
TRINITY_A0CCE1_PARTE/35-105	A0CCE1.1 PF00076.20	60.00	0.00
TRINITY_I0Z0H4_9CHLO/506-817	I0Z0H4.1 PF00069.23	60.00	0.00
TRINITY_B4S9S9_PELPB/85-400	B4S9S9.1 PF01546.26	60.00	0.00
TRINITY_E1ZEQ5_CHLVA/621-660	E1ZEQ5.1 PF00400.30	60.00	0.00
TRINITY_D8U575_VOLCA/212-449	D8U575.1 PF03399.14	60.00	0.00
TRINITY_D7T402_VITVI/254-312	D7T402.1 PF01485.19	60.00	0.00
TRINITY_B3MNH4_DROAN/1482-1833	B3MNH4.1 PF02259.21	60.00	0.00
TRINITY_E1Z4Y8_CHLVA/180-244	E1Z4Y8.1 PF00076.20	60.00	0.00
TRINITY_E1ZP48_CHLVA/105-230	E1ZP48.1 PF00782.18	60.00	0.00
TRINITY_U4LGE2_PYROM/440-494	U4LGE2.1 PF13513.4;	60.00	0.00
TRINITY_D8TZB4_VOLCA/16-197	D8TZB4.1 PF10294.7;	60.00	0.00
TRINITY_F2UMI5_SALR5/10-176	F2UMI5.1 PF00071.20	60.00	0.00
TRINITY_A9RDN7_PHYPA/1-342	A9RDN7.1 PF00443.27	60.00	0.00
TRINITY_R0FNM0_9BRAS/503-587	R0FNM0.1 PF00439.23	60.00	0.00
TRINITY_H3F2B5_PRIPA/28-132	H3F2B5.1 PF00085.18	60.00	0.00
TRINITY_Q23QT6_TETTS/242-612	Q23QT6.2 PF03074.14	60.00	0.00
TRINITY_D8UF65_VOLCA/7-108	D8UF65.1 PF01416.18	60.00	0.00
TRINITY_J3LD11_ORYBR/535-693	J3LD11.1 PF00730.23	60.00	0.00
TRINITY_I0YPQ0_9CHLO/7-122	I0YPQ0.1 PF00072.22	60.00	0.00
TRINITY_A0A078B7W0_STYLE/1247-1559	A0A078B7W0.1 PF0313	60.00	0.00
TRINITY_K3WGZ0_PYTUL/6-414	K3WGZ0.1 PF00202.19	60.00	0.00
TRINITY_A8IU24_CHLRE/114-221	A8IU24.1 PF02517.14	60.00	0.00
TRINITY_Q8LPN9_CHLRE/133-237	Q8LPN9.1 PF06426.12	60.00	0.00
TRINITY_D8U4M4_VOLCA/4-64	D8U4M4.1 PF15459.4;	60.00	0.00
TRINITY_E1ZBV4_CHLVA/379-589	E1ZBV4.1 PF04959.11	60.00	0.00
TRINITY_L8GR31_ACACA/27-95	L8GR31.1 PF00076.20	60.00	0.00
TRINITY_Q22RE7_TETTS/435-519	Q22RE7.2 PF02319.18	60.00	0.00
TRINITY_D8TMS6_VOLCA/1-185	D8TMS6.1 PF00488.19	60.00	0.00
TRINITY_M1V6V4_CYAME/377-456	M1V6V4.1 PF15963.3;	60.00	0.00
TRINITY_C6D5M5_PAESJ/50-181	C6D5M5.1 PF01565.21	60.00	0.00
TRINITY_D2W495_NAEGR/37-143	D2W495.1 PF00307.29	60.00	0.00
TRINITY_J9JBL9_9SPIT/441-611	J9JBL9.1 PF00271.29	60.00	0.00
TRINITY_G0QYI6_ICHMG/319-574	G0QYI6.1 PF01636.21	60.00	0.00
TRINITY_A8JF94_CHLRE/124-282	A8JF94.1 PF01694.20	60.00	0.00
TRINITY_W7A303_9APIC/239-649	W7A303.1 PF00962.20	60.00	0.00

TRINITY_I7M450_TETTS/2253-2375	I7M450.2 PF00069.23	60.00	0.00
TRINITY_A0A096MBY0_POEFO/238-281	A0A096MBY0.1 PF1363	60.00	0.00
TRINITY_A8HQM3_CHLRE/111-155	A8HQM3.1 PF00400.30	60.00	0.00
TRINITY_I7MKS8_TETTS/312-371	I7MKS8.1 PF13297.4;	60.00	0.00
TRINITY_D8UGA8_VOLCA/34-275	D8UGA8.1 PF15669.3;	60.00	0.00
TRINITY_D8U274_VOLCA/495-541	D8U274.1 PF00249.29	60.00	0.00
TRINITY_E1ZE56_CHLVA/4456-4520	E1ZE56.1 PF00550.23	60.00	0.00
TRINITY_Q7XW39_ORYSJ/722-882	Q7XW39.2 PF00078.25	60.00	0.00
TRINITY_C7RG14_ANAPD/573-776	C7RG14.1 PF05362.11	60.00	0.00
TRINITY_D8TVK0_VOLCA/1-60	D8TVK0.1 PF13616.4;	60.00	0.00
TRINITY_I7LUE3_TETTS/406-451	I7LUE3.2 PF13639.4;	60.00	0.00
TRINITY_D8TZ90_VOLCA/77-184	D8TZ90.1 PF00583.23	60.00	0.00
TRINITY_X6MAS1_RETFI/199-243	X6MAS1.1 PF00569.15	60.00	0.00
TRINITY_X6MAS1_RETFI/199-243	X6MAS1.1 PF00569.15	60.00	0.00
TRINITY_X6MAS1_RETFI/199-243	X6MAS1.1 PF00569.15	60.00	0.00
TRINITY_C5YN68_SORBI/639-832	C5YN68.1 PF13844.4;	60.00	0.00
TRINITY_A8IRU1_CHLRE/249-321	A8IRU1.1 PF14559.4;	60.00	0.00
TRINITY_I7LX26_TETTS/136-483	I7LX26.2 PF03969.14	60.00	0.00
TRINITY_G0QN11_ICHMG/255-400	G0QN11.1 PF00441.22	60.00	0.00
TRINITY_A8HY85_CHLRE/42-92	A8HY85.1 PF13920.4;	60.00	0.00
TRINITY_A8J9K5_CHLRE/137-186	A8J9K5.1 PF13920.4;	60.00	0.00
TRINITY_D8UF60_VOLCA/4-191	D8UF60.1 PF01007.18	60.00	0.00
TRINITY_I7M3R1_TETTS/59-238	I7M3R1.2 PF01728.17	60.00	0.00
TRINITY_A8JF41_CHLRE/171-378	A8JF41.1 PF07250.9;	60.00	0.00
TRINITY_A0C1X6_PARTE/38-292	A0C1X6.1 PF00069.23	60.00	0.00
TRINITY_E1ZHH1_CHLVA/57-281	E1ZHH1.1 PF04884.12	60.00	0.00
TRINITY_A0A067H4J3_CITSI/1156-1204	A0A067H4J3.1 PF0062	60.00	0.00
TRINITY_D8RLJ6_SELML/4-105	D8RLJ6.1 PF03297.13	60.00	0.00
TRINITY_D8TJV4_VOLCA/5-99	D8TJV4.1 PF06294.9;	60.00	0.00
TRINITY_A0A0D2NSD1_GOSRA/24-141	A0A0D2NSD1.1 PF0025	60.00	0.00
TRINITY_L1JQL2_GUITH/8-233	L1JQL2.1 PF00817.18	60.00	0.00
TRINITY_T0Q1R5_9STRA/36-222	T0Q1R5.1 PF00406.20	60.00	0.00
TRINITY_I7LU65_TETTS/183-282	I7LU65.1 PF00168.28	60.00	0.00
TRINITY_I7LTY0_TETTS/1086-1488	I7LTY0.2 PF03133.13	59.90	0.00
TRINITY_Q3SDL0_PARTE/12-173	Q3SDL0.1 PF00071.20	59.90	0.00
TRINITY_I7M185_TETTS/116-375	I7M185.1 PF00348.15	59.90	0.00
TRINITY_L8GYQ1_ACACA/45-195	L8GYQ1.1 PF00179.24	59.90	0.00
TRINITY_I1G1K5_AMPQE/1-295	I1G1K5.1 PF06524.10	59.90	0.00
TRINITY_A0A0G4G1J3_9ALVE/251-434	A0A0G4G1J3.1 PF0027	59.90	0.00
TRINITY_I1LMF9_SOYBN/316-479	I1LMF9.2 PF08662.9;	59.90	0.00
TRINITY_A0A098VUP0_9MICR/2-375	A0A098VUP0.1 PF0002	59.90	0.00
TRINITY_A8I5A0_CHLRE/41-174	A8I5A0.1 PF13326.4;	59.90	0.00
TRINITY_D8U414_VOLCA/35-318	D8U414.1 PF01545.19	59.90	0.00
TRINITY_A7S629_NEMVE/52-373	A7S629.1 PF00762.17	59.90	0.00
TRINITY_D8SD29_SELML/9-310	D8SD29.1 PF00069.23	59.90	0.00
TRINITY_V7CQ85_PHAVU/113-314	V7CQ85.1 PF01786.15	59.90	0.00
TRINITY_G2LEJ3_CHLTF/49-201	G2LEJ3.1 PF00484.17	59.90	0.00
TRINITY_L8H813_ACACA/26-277	L8H813.1 PF00069.23	59.90	0.00
TRINITY_A8J2S4_CHLRE/1-395	A8J2S4.1 PF00743.17	59.90	0.00
TRINITY_A8IWG3_CHLRE/30-177	A8IWG3.1 PF13419.4;	59.90	0.00
TRINITY_UNC50_DICDI/33-257	Q54DD7.1 PF05216.11	59.90	0.00
TRINITY_E1Z3M6_CHLVA/53-252	E1Z3M6.1 PF13489.4;	59.90	0.00
TRINITY_A9TIH6_PHYPA/37-298	A9TIH6.1 PF01650.16	59.90	0.00
TRINITY_A8JF21_CHLRE/228-378	A8JF21.1 PF00650.18	59.90	0.00
TRINITY_D8TWK2_VOLCA/969-1135	D8TWK2.1 PF14738.4;	59.90	0.00
TRINITY_A8IIZ1_CHLRE/10-171	A8IIZ1.1 PF01554.16	59.90	0.00
TRINITY_T2GA88_DESGI/300-467	T2GA88.1 PF03441.12	59.90	0.00
TRINITY_A8I3S3_CHLRE/106-302	A8I3S3.1 PF04389.15	59.90	0.00
TRINITY_A8JDA6_CHLRE/844-1310	A8JDA6.1 PF08016.10	59.90	0.00
TRINITY_E1ZJ92_CHLVA/81-283	E1ZJ92.1 PF12706.5;	59.90	0.00
TRINITY_A0A0J8BB47_BETVU/26-282	A0A0J8BB47.1 PF0006	59.90	0.00

TRINITY_A0DWM6_PARTE/516-794	A0DWM6.1 PF03133.13	59.80	0.00
TRINITY_I2K3U4_DEKBR/161-284	I2K3U4.1 PF10996.6;	59.80	0.00
TRINITY_Q54P30_DICDI/1396-1570	Q54P30.1 PF02145.13	59.80	0.00
TRINITY_F0ZDF3_DICPU/52-230	F0ZDF3.1 PF02390.15	59.80	0.00
TRINITY_A0BQ47_PARTE/3-95	A0BQ47.1 PF03259.15	59.80	0.00
TRINITY_A0A0G0YDQ8_9BACT/6-244	A0A0G0YDQ8.1 PF0137	59.80	0.00
TRINITY_I3K5J1_ORENI/93-208	I3K5J1.1 PF00059.19	59.80	0.00
TRINITY_L8GXI1_ACACA/313-483	L8GXI1.1 PF13091.4;	59.80	0.00
TRINITY_CCA31_ARATH/90-217	Q9FMH5.1 PF00134.21	59.80	0.00
TRINITY_I7M3Q7_TETTS/390-506	I7M3Q7.2 PF02373.20	59.80	0.00
TRINITY_Q24GE4_TETTS/399-611	Q24GE4.3 PF00644.18	59.80	0.00
TRINITY_A8J255_CHLRE/171-445	A8J255.1 PF05116.11	59.80	0.00
TRINITY_D8SJV7_SELML/197-318	D8SJV7.1 PF09335.9;	59.80	0.00
TRINITY_A8IXD5_CHLRE/72-207	A8IXD5.1 PF01694.20	59.80	0.00
TRINITY_I7M9T0_TETTS/42-205	I7M9T0.2 PF14908.4;	59.80	0.00
TRINITY_D8TXV6_VOLCA/1-121	D8TXV6.1 PF14822.4;	59.80	0.00
TRINITY_Q23AM3_TETTS/1054-1231	Q23AM3.2 PF00005.25	59.80	0.00
TRINITY_E1ZLB2_CHLVA/322-442	E1ZLB2.1 PF04379.12	59.80	0.00
TRINITY_D8U6U2_VOLCA/208-422	D8U6U2.1 PF10672.7;	59.80	0.00
TRINITY_D8TZ82_VOLCA/36-233	D8TZ82.1 PF00106.23	59.80	0.00
TRINITY_M0U8F6_MUSAM/1022-1128	M0U8F6.1 PF00856.26	59.80	0.00
TRINITY_I0Z2E6_9CHLO/384-490	I0Z2E6.1 PF13426.5;	59.80	0.00
TRINITY_A8HXK0_CHLRE/277-519	A8HXK0.1 PF14308.4;	59.80	0.00
TRINITY_M0QSG0_ACACA/39-129	M0QSG0.1 PF02214.20	59.80	0.00
TRINITY_A0A024TBZ2_9STRA/126-336	A0A024TBZ2.1 PF0247	59.80	0.00
TRINITY_D8TIL2_VOLCA/453-563	D8TIL2.1 PF07522.12	59.80	0.00
TRINITY_T1EDA0_HELRO/8-286	T1EDA0.1 PF00009.25	59.80	0.00
TRINITY_H3H405_PHYRM/37-151	H3H405.1 PF00665.24	59.80	0.00
TRINITY_E3EIC2_PAEPS/11-245	E3EIC2.1 PF06314.9;	59.80	0.00
TRINITY_I0YLG4_9CHLO/199-335	I0YLG4.1 PF01764.23	59.80	0.00
TRINITY_A8IR25_CHLRE/1-289	A8IR25.1 PF08433.8;	59.80	0.00
TRINITY_A8I874_CHLRE/64-181	A8I874.1 PF07000.9;	59.80	0.00
TRINITY_D8TRY1_VOLCA/329-426	D8TRY1.1 PF00153.25	59.80	0.00
TRINITY_B3RTR0_TRIAD/6-257	B3RTR0.1 PF00795.20	59.80	0.00
TRINITY_L8H3S2_ACACA/849-997	L8H3S2.1 PF08676.9;	59.70	0.00
TRINITY_I7ME32_TETTS/19-92	I7ME32.1 PF07491.9;	59.70	0.00
TRINITY_D7G4D0_ECTSI/79-145	D7G4D0.1 PF01193.22	59.70	0.00
TRINITY_W7XI32_TETTS/8-116	W7XI32.1 PF01412.16	59.70	0.00
TRINITY_F4Q960_DICFS/60-352	F4Q960.1 PF00128.22	59.70	0.00
TRINITY_A0A0F6W723_9DELT/34-204	A0A0F6W723.1 PF0085	59.70	0.00
TRINITY_A8IVY4_CHLRE/15-196	A8IVY4.1 PF00227.24	59.70	0.00
TRINITY_I1G7D7_AMPQE/8-475	I1G7D7.1 PF05694.9;	59.70	0.00
TRINITY_A0A075AZB6_9FUNG/55-199	A0A075AZB6.1 PF0073	59.70	0.00
TRINITY_R1D4T2_EMIHU/10-171	R1D4T2.1 PF00071.20	59.70	0.00
TRINITY_F2U4H2_SALR5/23-112	F2U4H2.1 PF11175.6;	59.70	0.00
TRINITY_A0A0M0KA22_9EUKA/51-144	A0A0M0KA22.1 PF1029	59.70	0.00
TRINITY_D8U0Z9_VOLCA/65-259	D8U0Z9.1 PF02676.12	59.70	0.00
TRINITY_D2VP14_NAEGR/98-301	D2VP14.1 PF00149.26	59.70	0.00
TRINITY_Q00XW1_OSTTA/2-187	Q00XW1.1 PF00625.19	59.70	0.00
TRINITY_Q22MP1_TETTS/37-190	Q22MP1.1 PF00005.25	59.70	0.00
TRINITY_W5D8K4_WHEAT/654-1020	W5D8K4.1 PF02463.17	59.70	0.00
TRINITY_E1ZCH3_CHLVA/293-451	E1ZCH3.1 PF00534.18	59.70	0.00
TRINITY_I7ME23_TETTS/19-90	I7ME23.1 PF13499.4;	59.70	0.00
TRINITY_G0QVT5_ICHMG/238-337	G0QVT5.1 PF08454.9;	59.70	0.00
TRINITY_A8JHX5_CHLRE/2-78	A8JHX5.1 PF00498.24	59.70	0.00
TRINITY_T1G2P3_HELRO/1-333	T1G2P3.1 PF05185.14	59.70	0.00
TRINITY_L8H0B4_ACACA/93-357	L8H0B4.1 PF04811.13	59.70	0.00
TRINITY_E3JVN0_PUCGT/1-80	E3JVN0.1 PF01249.16	59.70	0.00
TRINITY_D8TR89_VOLCA/21-149	D8TR89.1 PF01138.19	59.70	0.00
TRINITY_D2VAE1_NAEGR/114-496	D2VAE1.1 PF00199.17	59.70	0.00
TRINITY_G0QXL5_ICHMG/691-767	G0QXL5.1 PF14559.4;	59.70	0.00

TRINITY_D8TUM3_VOLCA/655-775	D8TUM3.1	PF03473.15	59.70	0.00
TRINITY_D8THW2_VOLCA/9-219	D8THW2.1	PF05648.12	59.70	0.00
TRINITY_A8ID15_CHLRE/20-207	A8ID15.1	PF10248.7;	59.70	0.00
TRINITY_F0ZGT3_DICPU/243-315	F0ZGT3.1	PF02825.18	59.70	0.00
TRINITY_Q23A17_TETTS/301-520	Q23A17.1	PF07002.14	59.70	0.00
TRINITY_Q7NC33_MYCGA/9-70	Q7NC33.1	PF00226.29	59.70	0.00
TRINITY_F4PGE7_DICFS/977-1179	F4PGE7.1	PF01434.16	59.70	0.00
TRINITY_D8TJI7_VOLCA/7-220	D8TJI7.1	PF01738.16	59.70	0.00
TRINITY_A0E4A1_PARTE/27-303	A0E4A1.1	PF00231.17	59.70	0.00
TRINITY_L8H0T8_ACACA/98-471	L8H0T8.1	PF01546.26	59.70	0.00
TRINITY_A8ICB6_CHLRE/231-427	A8ICB6.1	PF12315.6;	59.70	0.00
TRINITY_A8I754_CHLRE/177-244	A8I754.1	PF00170.19	59.70	0.00
TRINITY_L8GUS4_ACACA/70-276	L8GUS4.1	PF09511.8;	59.70	0.00
TRINITY_A8IZS5_CHLRE/5-74	A8IZS5.1	PF00076.20	59.70	0.00
TRINITY_D8UCY0_VOLCA/560-694	D8UCY0.1	PF03188.14	59.70	0.00
TRINITY_A8JAY4_CHLRE/1-198	A8JAY4.1	PF00009.25	59.70	0.00
TRINITY_W4YUT1_STRPU/82-421	W4YUT1.1	PF01979.18	59.70	0.00
TRINITY_D2VQ41_NAEGR/1223-1371	D2VQ41.1	PF00005.25	59.70	0.00
TRINITY_R5PLD7_9CLOT/4-290	R5PLD7.1	PF00009.25	59.70	0.00
TRINITY_A8J7D7_CHLRE/1-212	A8J7D7.1	PF00850.17	59.70	0.00
TRINITY_A4VE98_TETTS/337-580	A4VE98.1	PF00112.21	59.70	0.00
TRINITY_A8J3P5_CHLRE/6-664	A8J3P5.1	PF00012.18	59.70	0.00
TRINITY_I7MB77_TETTS/156-227	I7MB77.2	PF01529.18	59.70	0.00
TRINITY_D2VMY7_NAEGR/253-729	D2VMY7.1	PF01055.24	59.70	0.00

TRINITY_D8U6R8_VOLCA/6-435	D8U6R8.1 PF00266.17	59.70	0.00
TRINITY_H3G570_PHYRM/11-232	H3G570.1 PF00009.25	59.70	0.00
TRINITY_A0C4X3_PARTE/1548-1824	A0C4X3.1 PF02138.16	59.60	0.00
TRINITY_R4GF85_CHICK/1-160	R4GF85.1 PF00071.20	59.60	0.00
TRINITY_A0A0A6D1I8_9SPHN/14-256	A0A0A6D1I8.1 PF1356	59.60	0.00
TRINITY_J9J9I1_9SPIT/470-590	J9J9I1.1 PF02518.24	59.60	0.00
TRINITY_K1RSZ6_CRAGI/7-189	K1RSZ6.1 PF01251.16	59.60	0.00
TRINITY_T1JTS6_TETUR/100-385	T1JTS6.1 PF12257.6;	59.60	0.00
TRINITY_W5MYE7_LEPOC/651-786	W5MYE7.1 PF00005.25	59.60	0.00
TRINITY_J9FIJ3_9SPIT/747-905	J9FIJ3.1 PF03031.16	59.60	0.00
TRINITY_F4Q7L2_DICFS/13-96	F4Q7L2.1 PF01453.22	59.60	0.00
TRINITY_A0A0G4IP82_PLABS/175-385	A0A0G4IP82.1 PF0154	59.60	0.00
TRINITY_D8U4L8_VOLCA/670-733	D8U4L8.1 PF01733.16	59.60	0.00
TRINITY_D8TUT5_VOLCA/57-145	D8TUT5.1 PF13649.4;	59.60	0.00
TRINITY_L8GLW0_ACACA/708-815	L8GLW0.1 PF00651.29	59.60	0.00
TRINITY_A0A099TFJ7_9RHOB/40-177	A0A099TFJ7.1 PF0156	59.60	0.00
TRINITY_A0A084VF30_ANOSI/103-309	A0A084VF30.1 PF0014	59.60	0.00
TRINITY_M4BMA1_HYAAE/108-159	M4BMA1.1 PF00400.30	59.60	0.00
TRINITY_M4BMA1_HYAAE/108-159	M4BMA1.1 PF00400.30	59.60	0.00
TRINITY_A0E741_PARTE/638-836	A0E741.1 PF01434.16	59.60	0.00
TRINITY_D5DX48_BACMQ/160-384	D5DX48.1 PF03949.13	59.60	0.00
TRINITY_T1FYY7_HELRO/62-264	T1FYY7.1 PF00149.26	59.60	0.00
TRINITY_I7MIE8_TETTS/22-184	I7MIE8.1 PF01926.21	59.60	0.00
TRINITY_I7M9A1_TETTS/31-209	I7M9A1.2 PF14753.4;	59.60	0.00
TRINITY_A0A078ANK0_STYLE/101-417	A0A078ANK0.1 PF0154	59.60	0.00
TRINITY_A0A087STU0_AUXPR/427-677	A0A087STU0.1 PF0063	59.60	0.00
TRINITY_W1QF11_OGAPD/365-424	W1QF11.1 PF01485.19	59.60	0.00
TRINITY_G0QSM7_ICHMG/602-816	G0QSM7.1 PF05362.11	59.60	0.00
TRINITY_I7ME97_TETTS/54-318	I7ME97.1 PF00069.23	59.60	0.00
TRINITY_J9G2U8_9SPIT/24-114	J9G2U8.1 PF00153.25	59.60	0.00
TRINITY_A0A0G4FJZ0_9ALVE/12-173	A0A0G4FJZ0.1 PF0007	59.60	0.00
TRINITY_A8I382_CHLRE/52-192	A8I382.1 PF00581.18	59.60	0.00
TRINITY_S2J9M9_MUCC1/73-173	S2J9M9.1 PF13646.4;	59.60	0.00
TRINITY_L1IH70_GUITH/122-177	L1IH70.1 PF13513.4;	59.60	0.00
TRINITY_M7ZZU9_TRIUA/103-332	M7ZZU9.1 PF12706.5;	59.60	0.00
TRINITY_Q6WEE4_CHLRE/187-285	Q6WEE4.1 PF08241.10	59.60	0.00
TRINITY_A0E308_PARTE/95-202	A0E308.1 PF10262.7;	59.60	0.00
TRINITY_W7XFG4_TETTS/26-279	W7XFG4.1 PF01571.19	59.60	0.00
TRINITY_A8IFZ9_CHLRE/100-433	A8IFZ9.1 PF00291.23	59.60	0.00
TRINITY_A8JCU7_CHLRE/396-559	A8JCU7.1 PF03914.15	59.60	0.00
TRINITY_A8HYT2_CHLRE/1-283	A8HYT2.1 PF13593.4;	59.60	0.00
TRINITY_D8U819_VOLCA/63-369	D8U819.1 PF02383.16	59.60	0.00
TRINITY_C5DFZ2_LACTC/2-122	C5DFZ2.1 PF00125.22	59.60	0.00
TRINITY_D8TSE6_VOLCA/605-849	D8TSE6.1 PF02535.20	59.60	0.00
TRINITY_D8THV1_VOLCA/93-361	D8THV1.1 PF00069.23	59.60	0.00
TRINITY_A8J0N3_CHLRE/123-316	A8J0N3.1 PF01729.17	59.60	0.00
TRINITY_I0YJD2_9CHLO/402-453	I0YJD2.1 PF01805.18	59.60	0.00
TRINITY_D8UEF4_VOLCA/45-208	D8UEF4.1 PF00004.27	59.60	0.00
TRINITY_J9I105_9SPIT/748-1060	J9I105.1 PF03133.13	59.60	0.00
TRINITY_E1Z917_CHLVA/42-215	E1Z917.1 PF00504.19	59.60	0.00
TRINITY_D8TTV8_VOLCA/81-324	D8TTV8.1 PF00233.17	59.60	0.00
TRINITY_A8JBS8_CHLRE/56-197	A8JBS8.1 PF01612.18	59.60	0.00
TRINITY_A8HP62_CHLRE/179-430	A8HP62.1 PF05548.9;	59.60	0.00
TRINITY_A0A059LDC2_9CHLO/19-223	A0A059LDC2.1 PF0430	59.60	0.00
TRINITY_D8RK80_SELML/210-263	D8RK80.1 PF12417.6;	59.60	0.00
TRINITY_A8IDF6_CHLRE/121-317	A8IDF6.1 PF03167.17	59.60	0.00
TRINITY_D8TTV8_VOLCA/81-324	D8TTV8.1 PF00233.17	59.60	0.00
TRINITY_A0A078B4E1_STYLE/16-67	A0A078B4E1.1 PF1344	59.60	0.00
TRINITY_A0DUV3_PARTE/152-197	A0DUV3.1 PF13920.4;	59.50	0.00
TRINITY_G6CHT9_DANPL/16-65	G6CHT9.1 PF00385.22	59.50	0.00
TRINITY_J9J1V2_9SPIT/5-143	J9J1V2.1 PF00179.24	59.50	0.00

TRINITY_D3SGJ3_THISK/5-83	D3SGJ3.1 PF14216.4;	59.50	0.00
TRINITY_R6I2T3_9FIRM/263-453	R6I2T3.1 PF02782.14	59.50	0.00
TRINITY_S8BXP8_9LAMI/37-157	S8BXP8.1 PF01597.17	59.50	0.00
TRINITY_G0QZI5_ICHMG/1-443	G0QZI5.1 PF00342.17	59.50	0.00
TRINITY_L8GGX4_ACACA/142-246	L8GGX4.1 PF00085.18	59.50	0.00
TRINITY_A0A015M2R0_9GLOM/42-194	A0A015M2R0.1 PF0405	59.50	0.00
TRINITY_A0A0D2X2K3_CAPO3/532-638	A0A0D2X2K3.1 PF0073	59.50	0.00
TRINITY_E2BY81_HARSA/5-166	E2BY81.1 PF00071.20	59.50	0.00
TRINITY_A0A0F4ZAI8_9PEZI/94-131	A0A0F4ZAI8.1 PF0017	59.50	0.00
TRINITY_A0BEH9_PARTE/36-77	A0BEH9.1 PF13639.4;	59.50	0.00
TRINITY_X6M8Y4_RETFI/359-412	X6M8Y4.1 PF00385.22	59.50	0.00
TRINITY_D8TMA0_VOLCA/15-196	D8TMA0.1 PF00227.24	59.50	0.00
TRINITY_B9SDZ9_RICCO/29-158	B9SDZ9.1 PF00917.24	59.50	0.00
TRINITY_A0A015II56_9GLOM/376-548	A0A015II56.1 PF1421	59.50	0.00
TRINITY_A8IY12_CHLRE/26-105	A8IY12.1 PF04241.13	59.50	0.00
TRINITY_D2VQU5_NAEGR/47-242	D2VQU5.1 PF00149.26	59.50	0.00
TRINITY_W5P0R0_SHEEP/22-244	W5P0R0.1 PF01602.18	59.50	0.00
TRINITY_I1BKV5_RHIO9/319-714	I1BKV5.1 PF06862.10	59.50	0.00
TRINITY_D8TJW5_VOLCA/14-164	D8TJW5.1 PF13302.5;	59.50	0.00
TRINITY_J0LGA5_9BACT/1-296	J0LGA5.1 PF01204.16	59.50	0.00
TRINITY_M0QSG3_ACACA/116-206	M0QSG3.1 PF02214.20	59.50	0.00
TRINITY_A8JHY4_CHLRE/94-135	A8JHY4.1 PF00132.22	59.50	0.00
TRINITY_Q23QF2_TETTS/248-571	Q23QF2.2 PF03133.13	59.50	0.00
TRINITY_I0YJY8_9CHLO/292-439	I0YJY8.1 PF02880.14	59.50	0.00
TRINITY_A8IKU4_CHLRE/95-317	A8IKU4.1 PF02141.19	59.50	0.00
TRINITY_Q23JY6_TETTS/610-893	Q23JY6.2 PF00481.19	59.50	0.00
TRINITY_I7LZN5_TETTS/536-926	I7LZN5.2 PF00368.16	59.50	0.00
TRINITY_C1N665_MICPC/20-107	C1N665.1 PF00428.17	59.50	0.00
TRINITY_Q24DE2_TETTS/709-750	Q24DE2.2 PF00400.30	59.50	0.00
TRINITY_M9LX83_PSEA3/276-328	M9LX83.1 PF00385.22	59.50	0.00
TRINITY_G0QKI2_ICHMG/2-112	G0QKI2.1 PF03966.14	59.50	0.00
TRINITY_A8IWB7_CHLRE/38-199	A8IWB7.1 PF01507.17	59.50	0.00
TRINITY_A8IS72_CHLRE/29-323	A8IS72.1 PF04086.11	59.50	0.00
TRINITY_D8TZH6_VOLCA/22-283	D8TZH6.1 PF00233.17	59.50	0.00
TRINITY_D2V0F2_NAEGR/56-208	D2V0F2.1 PF00005.25	59.50	0.00
TRINITY_A8HNI5_CHLRE/39-425	A8HNI5.1 PF00155.19	59.50	0.00
TRINITY_A0A088A591_APIME/114-257	A0A088A591.1 PF1214	59.50	0.00
TRINITY_A0A078G3N8_BRANA/161-203	A0A078G3N8.1 PF0040	59.50	0.00
TRINITY_A0A087SL58_AUXPR/133-369	A0A087SL58.1 PF0024	59.50	0.00
TRINITY_A0CWH1_PARTE/397-1150	A0CWH1.1 PF03028.13	59.50	0.00
TRINITY_A0CYU6_PARTE/98-357	A0CYU6.1 PF00069.23	59.40	0.00
TRINITY_A0A0C4DW14_MAGP6/5-147	A0A0C4DW14.1 PF0017	59.40	0.00
TRINITY_Q238Z1_TETTS/96-250	Q238Z1.1 PF00650.18	59.40	0.00
TRINITY_A7RPZ7_NEMVE/42-111	A7RPZ7.1 PF00076.20	59.40	0.00
TRINITY_U5QIH0_9CYAN/30-331	U5QIH0.1 PF00248.19	59.40	0.00
TRINITY_G0QSW4_ICHMG/29-97	G0QSW4.1 PF00366.18	59.40	0.00
TRINITY_Q23FX2_TETTS/36-451	Q23FX2.3 PF00999.19	59.40	0.00
TRINITY_A0A0N0D2H4_9DELT/189-254	A0A0N0D2H4.1 PF0051	59.40	0.00
TRINITY_B0WZR8_CULQU/14-182	B0WZR8.1 PF00071.20	59.40	0.00
TRINITY_L8HLP2_ACACA/9-202	L8HLP2.1 PF00834.17	59.40	0.00
TRINITY_B9ICR1_POPTR/84-179	B9ICR1.2 PF00173.26	59.40	0.00
TRINITY_F0ZN65_DICPU/59-351	F0ZN65.1 PF00128.22	59.40	0.00
TRINITY_B3RN54_TRIAD/23-224	B3RN54.1 PF07992.12	59.40	0.00
TRINITY_F0ZXI3_DICPU/2-96	F0ZXI3.1 PF09138.9;	59.40	0.00
TRINITY_C1FIF0_MICSR/52-437	C1FIF0.1 PF04563.13	59.40	0.00
TRINITY_D7FTM7_ECTSI/5-252	D7FTM7.1 PF00370.19	59.40	0.00
TRINITY_A8J650_CHLRE/49-258	A8J650.1 PF00697.20	59.40	0.00
TRINITY_Q3SE66_PARTE/97-259	Q3SE66.1 PF03167.17	59.40	0.00
TRINITY_Q24HI2_TETTS/127-344	Q24HI2.1 PF00112.21	59.40	0.00
TRINITY_D8TJD1_VOLCA/814-918	D8TJD1.1 PF04003.10	59.40	0.00
TRINITY_L8H9Z0_ACACA/196-375	L8H9Z0.1 PF00270.27	59.40	0.00

TRINITY_A8I8N0_CHLRE/95-286	A8I8N0.1 PF00300.20	59.40	0.00
TRINITY_A0DTL9_PARTE/31-95	A0DTL9.1 PF11717.6;	59.40	0.00
TRINITY_D8TWM8_VOLCA/153-220	D8TWM8.1 PF10260.7;	59.40	0.00
TRINITY_A8IR47_CHLRE/9-342	A8IR47.1 PF08569.9;	59.40	0.00
TRINITY_A8IZH0_CHLRE/174-387	A8IZH0.1 PF04042.14	59.40	0.00
TRINITY_D8U8M1_VOLCA/7-310	D8U8M1.1 PF03372.21	59.40	0.00
TRINITY_D8U9S9_VOLCA/599-793	D8U9S9.1 PF00211.18	59.40	0.00
TRINITY_D8U3H2_VOLCA/1-587	D8U3H2.1 PF04734.11	59.40	0.00
TRINITY_A0A0M2R709_9PROT/218-408	A0A0M2R709.1 PF0769	59.40	0.00
TRINITY_D8U273_VOLCA/4-107	D8U273.1 PF00085.18	59.40	0.00
TRINITY_D8TJ78_VOLCA/60-314	D8TJ78.1 PF10275.7;	59.40	0.00
TRINITY_G0QU48_ICHMG/662-757	G0QU48.1 PF09333.9;	59.40	0.00
TRINITY_A0A075AVH6_9FUNG/98-266	A0A075AVH6.1 PF0442	59.40	0.00
TRINITY_D8U6Q5_VOLCA/76-242	D8U6Q5.1 PF00650.18	59.40	0.00
TRINITY_D8UEW3_VOLCA/53-435	D8UEW3.1 PF13621.4;	59.40	0.00
TRINITY_A8J7T0_CHLRE/100-165	A8J7T0.1 PF03719.13	59.40	0.00
TRINITY_D8R7I0_SELML/13-121	D8R7I0.1 PF01920.18	59.40	0.00
TRINITY_D8U168_VOLCA/87-282	D8U168.1 PF13369.4;	59.40	0.00
TRINITY_C3YHE8_BRAFL/385-428	C3YHE8.1 PF13639.4;	59.40	0.00
TRINITY_G0QZ46_ICHMG/4-87	G0QZ46.1 PF01221.16	59.30	0.00
TRINITY_W7XGD4_TETTS/87-322	W7XGD4.1 PF12146.6;	59.30	0.00
TRINITY_A0CZ01_PARTE/4-96	A0CZ01.1 PF03501.13	59.30	0.00
TRINITY_X6M4L5_RETFI/6-65	X6M4L5.1 PF04193.12	59.30	0.00
TRINITY_Q09B48_STIAD/174-291	Q09B48.1 PF03171.18	59.30	0.00
TRINITY_F6USE7_XENTR/29-200	F6USE7.1 PF00270.27	59.30	0.00
TRINITY_J9IMT2_9SPIT/146-246	J9IMT2.1 PF00153.25	59.30	0.00
TRINITY_B3RKM6_TRIAD/5-291	B3RKM6.1 PF01875.15	59.30	0.00
TRINITY_A0A0H2UIW8_WHEAT/1-223	A0A0H2UIW8.1 PF0450	59.30	0.00
TRINITY_A0D7P8_PARTE/1491-1674	A0D7P8.1 PF00534.18	59.30	0.00
TRINITY_A0DI20_PARTE/19-543	A0DI20.1 PF01602.18	59.30	0.00
TRINITY_N4W6M1_COLOR/267-416	N4W6M1.1 PF04410.12	59.30	0.00
TRINITY_I0I634_CALAS/15-339	I0I634.1 PF00266.17	59.30	0.00
TRINITY_D6WV22_TRICA/26-271	D6WV22.1 PF01145.23	59.30	0.00
TRINITY_INT9_NEMVE/90-246	A7SBF0.1 PF16661.3;	59.30	0.00
TRINITY_J9EFC8_9SPIT/704-837	J9EFC8.1 PF02518.24	59.30	0.00
TRINITY_A0E7K0_PARTE/175-280	A0E7K0.1 PF01416.18	59.30	0.00
TRINITY_F4Q1R8_DICFS/16-344	F4Q1R8.1 PF00503.18	59.30	0.00
TRINITY_D8TTC1_VOLCA/228-356	D8TTC1.1 PF04097.12	59.30	0.00
TRINITY_E1ZGH5_CHLVA/16-177	E1ZGH5.1 PF13419.4;	59.30	0.00
TRINITY_Q23FG2_TETTS/1702-2050	Q23FG2.2 PF00443.27	59.30	0.00
TRINITY_A0BSP4_PARTE/28-177	A0BSP4.1 PF00179.24	59.30	0.00
TRINITY_D8U8H6_VOLCA/1-97	D8U8H6.1 PF13664.4;	59.30	0.00
TRINITY_A0A0M0K094_9EUKA/27-120	A0A0M0K094.1 PF1390	59.30	0.00
TRINITY_J9HZM9_9SPIT/78-296	J9HZM9.1 PF00112.21	59.30	0.00
TRINITY_D8U6I4_VOLCA/26-255	D8U6I4.1 PF00687.19	59.30	0.00
TRINITY_Q22EH7_TETTS/100-154	Q22EH7.2 PF03909.15	59.30	0.00
TRINITY_A0BKD0_PARTE/99-237	A0BKD0.1 PF14580.4;	59.30	0.00
TRINITY_G0QL35_ICHMG/210-567	G0QL35.1 PF00246.22	59.30	0.00
TRINITY_A8J3M3_CHLRE/46-296	A8J3M3.1 PF01636.21	59.30	0.00
TRINITY_D8THE4_VOLCA/708-799	D8THE4.1 PF02212.16	59.30	0.00
TRINITY_D8TTU4_VOLCA/420-563	D8TTU4.1 PF01612.18	59.30	0.00
TRINITY_L8H244_ACACA/118-237	L8H244.1 PF00622.26	59.30	0.00
TRINITY_A0CN21_PARTE/290-662	A0CN21.1 PF03074.14	59.30	0.00
TRINITY_A8HSR5_CHLRE/161-392	A8HSR5.1 PF00246.22	59.30	0.00
TRINITY_D8U9F4_VOLCA/173-306	D8U9F4.1 PF13012.4;	59.30	0.00
TRINITY_A0A0F2RJP9_9PROT/13-396	A0A0F2RJP9.1 PF0019	59.30	0.00
TRINITY_B4FYT5_MAIZE/176-324	B4FYT5.1 PF00485.16	59.30	0.00
TRINITY_A0BL44_PARTE/620-730	A0BL44.1 PF13426.5;	59.30	0.00
TRINITY_A7RSI0_NEMVE/8-120	A7RSI0.1 PF02391.15	59.30	0.00
TRINITY_I1E296_9GAMM/80-160	I1E296.1 PF01035.18	59.30	0.00
TRINITY_Q23YT4_TETTS/253-339	Q23YT4.2 PF00736.17	59.30	0.00

TRINITY_A0A0N4Z2H2_PARTI/535-738	A0A0N4Z2H2.1 PF0143	59.30	0.00
TRINITY_C1MTS7_MICPC/2072-2343	C1MTS7.1 PF12775.5;	59.30	0.00
TRINITY_D3BIJ9_POLPA/690-861	D3BIJ9.1 PF00270.27	59.30	0.00
TRINITY_E1ZSF7_CHLVA/18-169	E1ZSF7.1 PF00857.18	59.30	0.00
TRINITY_D8TKK5_VOLCA/94-546	D8TKK5.1 PF05185.14	59.30	0.00
TRINITY_A8HSU4_CHLRE/1-143	A8HSU4.1 PF14437.4;	59.30	0.00
TRINITY_A8JCR3_CHLRE/12-76	A8JCR3.1 PF13499.4;	59.30	0.00
TRINITY_A0A087SNS7_AUXPR/51-362	A0A087SNS7.1 PF0315	59.30	0.00
TRINITY_A8HU48_CHLRE/1149-1287	A8HU48.1 PF11816.6;	59.30	0.00
TRINITY_A0A024TZW5_9STRA/77-491	A0A024TZW5.1 PF0070	59.30	0.00
TRINITY_A0BJJ0_PARTE/87-192	A0BJJ0.1 PF06565.10	59.30	0.00
TRINITY_L8GL09_ACACA/247-304	L8GL09.1 PF00571.26	59.30	0.00
TRINITY_A8HZD1_CHLRE/1-350	A8HZD1.1 PF00225.21	59.30	0.00
TRINITY_W2PXB2_PHYPN/493-609	W2PXB2.1 PF00665.24	59.30	0.00
TRINITY_K9SQ14_9CYAN/405-542	K9SQ14.1 PF02518.24	59.20	0.00
TRINITY_I7M2D2_TETTS/40-113	I7M2D2.2 PF02798.18	59.20	0.00
TRINITY_A0A024UAY1_9STRA/8-329	A0A024UAY1.1 PF0293	59.20	0.00
TRINITY_D5GJQ0_TUBMM/674-726	D5GJQ0.1 PF13513.4;	59.20	0.00
TRINITY_Q238R8_TETTS/18-286	Q238R8.1 PF02263.17	59.20	0.00
TRINITY_A4VCS2_TETTS/102-221	A4VCS2.1 PF01981.14	59.20	0.00
TRINITY_A9TAJ1_PHYPA/1-185	A9TAJ1.1 PF05018.11	59.20	0.00
TRINITY_G4QFB8_GLANF/33-277	G4QFB8.1 PF02784.14	59.20	0.00
TRINITY_M0XYK6_HORVD/40-120	M0XYK6.1 PF01016.17	59.20	0.00
TRINITY_U9SSL5_RHIID/3-1230	U9SSL5.1 PF02463.17	59.20	0.00
TRINITY_D8UIB4_VOLCA/392-592	D8UIB4.1 PF08662.9;	59.20	0.00
TRINITY_I0YKW1_9CHLO/8-84	I0YKW1.1 PF13499.4;	59.20	0.00
TRINITY_L8H1J5_ACACA/387-531	L8H1J5.1 PF00300.20	59.20	0.00
TRINITY_E1ZE86_CHLVA/64-141	E1ZE86.1 PF00076.20	59.20	0.00
TRINITY_F2U697_SALR5/3015-3236	F2U697.1 PF12781.5;	59.20	0.00
TRINITY_G0QMS4_ICHMG/137-465	G0QMS4.1 PF00069.23	59.20	0.00
TRINITY_J9J5P5_9SPIT/28-172	J9J5P5.1 PF00810.16	59.20	0.00
TRINITY_Q23KJ6_TETTS/622-886	Q23KJ6.2 PF00069.23	59.20	0.00
TRINITY_G0R119_ICHMG/152-200	G0R119.1 PF00400.30	59.20	0.00
TRINITY_I0Z997_9CHLO/17-136	I0Z997.1 PF13671.4;	59.20	0.00
TRINITY_A0BGA7_PARTE/140-295	A0BGA7.1 PF13847.4;	59.20	0.00
TRINITY_A0A0D2VQT8_CAPO3/19-143	A0A0D2VQT8.1 PF0029	59.20	0.00
TRINITY_A0A0C4DL18_MAGP6/21-224	A0A0C4DL18.1 PF0642	59.20	0.00
TRINITY_D8TSH3_VOLCA/788-863	D8TSH3.1 PF10405.7;	59.20	0.00
TRINITY_A8JBY6_CHLRE/203-662	A8JBY6.1 PF04130.11	59.20	0.00
TRINITY_AMT1_DICDI/41-440	Q9BLG4.1 PF00909.19	59.20	0.00
TRINITY_D8TRH3_VOLCA/1836-2055	D8TRH3.1 PF01061.22	59.20	0.00
TRINITY_I0YR51_9CHLO/128-422	I0YR51.1 PF01557.16	59.20	0.00
TRINITY_U9SW31_RHIID/110-183	U9SW31.1 PF05495.10	59.20	0.00
TRINITY_I2FW02_USTH4/107-155	I2FW02.1 PF00628.27	59.20	0.00
TRINITY_L8GLJ2_ACACA/616-664	L8GLJ2.1 PF14604.4;	59.20	0.00
TRINITY_F4PHT1_DICFS/491-542	F4PHT1.1 PF13833.4;	59.20	0.00
TRINITY_L8H670_ACACA/88-235	L8H670.1 PF10609.7;	59.20	0.00
TRINITY_E1ZD03_CHLVA/322-419	E1ZD03.1 PF13180.4;	59.20	0.00
TRINITY_G0QWP0_ICHMG/124-181	G0QWP0.1 PF01485.19	59.20	0.00
TRINITY_D8U9N9_VOLCA/98-267	D8U9N9.1 PF00270.27	59.20	0.00
TRINITY_PCDP1_CHLRE/112-209	A8J6X7.1 PF14874.4;	59.20	0.00
TRINITY_D3B6M3_POLPA/229-533	D3B6M3.1 PF00082.20	59.20	0.00
TRINITY_Q8YWS5_NOSS1/35-264	Q8YWS5.1 PF02522.12	59.20	0.00
TRINITY_A8JDY7_CHLRE/10-58	A8JDY7.1 PF12906.5;	59.20	0.00
TRINITY_A0A087SBU1_AUXPR/606-800	A0A087SBU1.1 PF1341	59.20	0.00
TRINITY_L8GJZ1_ACACA/243-460	L8GJZ1.1 PF01189.15	59.20	0.00
TRINITY_I0Z9A9_9CHLO/745-1340	I0Z9A9.1 PF04998.15	59.20	0.00
TRINITY_D8UI70_VOLCA/213-633	D8UI70.1 PF00171.20	59.20	0.00
TRINITY_Q9ZSJ4_CHLRE/68-236	Q9ZSJ4.2 PF00504.19	59.20	0.00
TRINITY_A8J6I8_CHLRE/888-1113	A8J6I8.1 PF05193.19	59.20	0.00
TRINITY_Q8S653_ORYSJ/452-609	Q8S653.1 PF00078.25	59.20	0.00

TRINITY_I7MA77_TETTS/29-80	I7MA77.2 PF05047.14	59.20	0.00
TRINITY_M1CZP3_SOLTU/72-309	M1CZP3.1 PF01255.17	59.10	0.00
TRINITY_A0A067R4S1_ZOONE/93-369	A0A067R4S1.1 PF0364	59.10	0.00
TRINITY_L8HJK0_ACACA/186-408	L8HJK0.1 PF13236.4;	59.10	0.00
TRINITY_L1IRT5_GUITH/383-589	L1IRT5.1 PF01061.22	59.10	0.00
TRINITY_I1CH46_RHIO9/26-333	I1CH46.1 PF01266.22	59.10	0.00
TRINITY_A0A0E3UY03_9BACT/4-308	A0A0E3UY03.1 PF0111	59.10	0.00
TRINITY_A0A0E4GD53_9FIRM/6-68	A0A0E4GD53.1 PF0022	59.10	0.00
TRINITY_M2Y8C7_GALSU/442-709	M2Y8C7.1 PF01513.19	59.10	0.00
TRINITY_A0A078ERX2_BRANA/26-250	A0A078ERX2.1 PF0192	59.10	0.00
TRINITY_A0A078ARM2_STYLE/135-202	A0A078ARM2.1 PF0022	59.10	0.00
TRINITY_H2LXA0_ORYLA/83-257	H2LXA0.1 PF02146.15	59.10	0.00
TRINITY_L8GLB6_ACACA/1279-1454	L8GLB6.1 PF00092.26	59.10	0.00
TRINITY_N1PE77_DOTSN/19-318	N1PE77.1 PF00248.19	59.10	0.00
TRINITY_F1A219_DICPU/111-281	F1A219.1 PF02769.20	59.10	0.00
TRINITY_G0R1F0_ICHMG/20-315	G0R1F0.1 PF00069.23	59.10	0.00
TRINITY_B9S9S4_RICCO/112-239	B9S9S4.1 PF00293.26	59.10	0.00
TRINITY_D2UZX2_NAEGR/143-328	D2UZX2.1 PF13521.4;	59.10	0.00
TRINITY_H6L1T3_SAPGL/18-402	H6L1T3.1 PF00368.16	59.10	0.00
TRINITY_J9J3B4_9SPIT/124-338	J9J3B4.1 PF00112.21	59.10	0.00
TRINITY_A0DIQ1_PARTE/466-597	A0DIQ1.1 PF00782.18	59.10	0.00
TRINITY_K0IJ19_NITGG/6-472	K0IJ19.1 PF00478.23	59.10	0.00
TRINITY_A0A0G4IUN0_PLABS/94-139	A0A0G4IUN0.1 PF0041	59.10	0.00
TRINITY_I0YKJ3_9CHLO/305-395	I0YKJ3.1 PF00027.27	59.10	0.00
TRINITY_D8TNV9_VOLCA/175-458	D8TNV9.1 PF00656.20	59.10	0.00
TRINITY_A0A0D2X3T7_CAPO3/36-185	A0A0D2X3T7.1 PF0853	59.10	0.00
TRINITY_A0A059CL54_EUCGR/64-347	A0A059CL54.1 PF0085	59.10	0.00
TRINITY_D8U9T2_VOLCA/248-480	D8U9T2.1 PF00069.23	59.10	0.00
TRINITY_H3G684_PHYRM/1-81	H3G684.1 PF00665.24	59.10	0.00
TRINITY_A0A0L7KPY5_9NEOP/11-106	A0A0L7KPY5.1 PF0014	59.10	0.00
TRINITY_D8TRJ9_VOLCA/14-234	D8TRJ9.1 PF04012.10	59.10	0.00
TRINITY_E1ZGW5_CHLVA/631-675	E1ZGW5.1 PF00249.29	59.10	0.00
TRINITY_A8IPD5_CHLRE/146-189	A8IPD5.1 PF01753.16	59.10	0.00
TRINITY_B0W537_CULQU/638-773	B0W537.1 PF00005.25	59.10	0.00
TRINITY_D8UCA1_VOLCA/10-375	D8UCA1.1 PF07851.11	59.10	0.00
TRINITY_A0A0M9G5U1_9TRYP/169-257	A0A0M9G5U1.1 PF1289	59.10	0.00
TRINITY_M1BYH8_SOLTU/55-116	M1BYH8.1 PF06325.11	59.10	0.00
TRINITY_A0A087SQ99_AUXPR/131-532	A0A087SQ99.1 PF0091	59.10	0.00
TRINITY_I0YXN6_9CHLO/225-641	I0YXN6.1 PF01179.18	59.10	0.00
TRINITY_C1FFN1_MICSR/350-393	C1FFN1.1 PF13639.4;	59.10	0.00
TRINITY_D8TL19_VOLCA/110-460	D8TL19.1 PF00654.18	59.10	0.00
TRINITY_A8IXY4_CHLRE/14-432	A8IXY4.1 PF02897.13	59.10	0.00
TRINITY_R4GCI3_ANOCA/1-108	R4GCI3.1 PF00665.24	59.10	0.00
TRINITY_G0R3W3_ICHMG/1142-1282	G0R3W3.1 PF00637.18	59.10	0.00
TRINITY_Q23AS6_TETTS/7-98	Q23AS6.1 PF00166.19	59.10	0.00
TRINITY_A0DNI8_PARTE/201-510	A0DNI8.1 PF00632.23	59.00	0.00
TRINITY_K9V0E6_9CYAN/31-123	K9V0E6.1 PF04248.10	59.00	0.00
TRINITY_L8GVJ0_ACACA/36-118	L8GVJ0.1 PF12861.5;	59.00	0.00
TRINITY_C5LTF4_PERM5/86-148	C5LTF4.1 PF13499.4;	59.00	0.00
TRINITY_Q54C20_DICDI/221-281	Q54C20.1 PF04193.12	59.00	0.00
TRINITY_A0A0A2TVU3_9BACL/316-444	A0A0A2TVU3.1 PF0283	59.00	0.00
TRINITY_F2UQY4_SALR5/39-238	F2UQY4.1 PF00106.23	59.00	0.00
TRINITY_M7XBN2_RHOT1/1-127	M7XBN2.1 PF00125.22	59.00	0.00
TRINITY_I0YSC3_9CHLO/14-127	I0YSC3.1 PF07934.10	59.00	0.00
TRINITY_I2H725_TETBL/29-519	I2H725.1 PF00118.22	59.00	0.00
TRINITY_M4BRB5_HYAAE/27-197	M4BRB5.1 PF00586.22	59.00	0.00
TRINITY_D8TRF2_VOLCA/63-520	D8TRF2.1 PF13520.4;	59.00	0.00
TRINITY_J9J7F2_9SPIT/261-301	J9J7F2.1 PF00400.30	59.00	0.00
TRINITY_H2LMM4_ORYLA/473-721	H2LMM4.1 PF00089.24	59.00	0.00
TRINITY_D8TZA2_VOLCA/167-347	D8TZA2.1 PF00849.20	59.00	0.00
TRINITY_E1ZEL2_CHLVA/1-142	E1ZEL2.1 PF01217.18	59.00	0.00

TRINITY_D8U4X1_VOLCA/43-120	D8U4X1.1 PF08561.8;	59.00	0.00
TRINITY_T1EDY3_HELRO/130-445	T1EDY3.1 PF00009.25	59.00	0.00
TRINITY_A8IA80_CHLRE/1-365	A8IA80.1 PF05684.10	59.00	0.00
TRINITY_B3RRA4_TRIAD/270-311	B3RRA4.1 PF00569.15	59.00	0.00
TRINITY_Q8V3N1_SWPV1/8-179	Q8V3N1.1 PF00265.16	59.00	0.00
TRINITY_A0D5K9_PARTE/135-221	A0D5K9.1 PF12796.5;	59.00	0.00
TRINITY_A8JAQ3_CHLRE/38-173	A8JAQ3.1 PF00583.23	59.00	0.00
TRINITY_D8TNL8_VOLCA/36-226	D8TNL8.1 PF01145.23	59.00	0.00
TRINITY_I0Z9Z8_9CHLO/47-196	I0Z9Z8.1 PF01553.19	59.00	0.00
TRINITY_D8UB59_VOLCA/11-326	D8UB59.1 PF03151.14	59.00	0.00
TRINITY_D8TRB5_VOLCA/1-106	D8TRB5.1 PF08585.10	59.00	0.00
TRINITY_I0YVN9_9CHLO/162-510	I0YVN9.1 PF07992.12	59.00	0.00
TRINITY_L8GUU1_ACACA/68-321	L8GUU1.1 PF00069.23	59.00	0.00
TRINITY_A0A090N2N5_OSTTA/40-101	A0A090N2N5.1 PF0022	59.00	0.00
TRINITY_A0A0N4V4F1_ENTVE/4-73	A0A0N4V4F1.1 PF0279	59.00	0.00
TRINITY_I0Z2M9_9CHLO/3-327	I0Z2M9.1 PF00294.22	59.00	0.00
TRINITY_D8TZF9_VOLCA/151-233	D8TZF9.1 PF01833.22	59.00	0.00
TRINITY_G0QQJ8_ICHMG/9-131	G0QQJ8.1 PF08477.11	59.00	0.00
TRINITY_D8UEL6_VOLCA/79-252	D8UEL6.1 PF02330.14	59.00	0.00
TRINITY_A8IHE7_CHLRE/70-516	A8IHE7.1 PF13520.4;	59.00	0.00
TRINITY_V3Z559_LOTGI/256-294	V3Z559.1 PF04810.13	59.00	0.00
TRINITY_D8UIC2_VOLCA/42-312	D8UIC2.1 PF07942.10	59.00	0.00
TRINITY_I0YPH9_9CHLO/124-475	I0YPH9.1 PF04109.14	59.00	0.00
TRINITY_E1ZAY8_CHLVA/25-236	E1ZAY8.1 PF02492.17	59.00	0.00
TRINITY_D8UCG7_VOLCA/266-518	D8UCG7.1 PF03133.13	59.00	0.00
TRINITY_D8TKS7_VOLCA/1-197	D8TKS7.1 PF16879.3;	59.00	0.00
TRINITY_K9SEK3_9CYAN/14-209	K9SEK3.1 PF00106.23	59.00	0.00
TRINITY_G0QJK2_ICHMG/26-210	G0QJK2.1 PF00227.24	59.00	0.00
TRINITY_I0Z8J3_9CHLO/4-317	I0Z8J3.1 PF08267.10	59.00	0.00
TRINITY_D8TXI8_VOLCA/4-119	D8TXI8.1 PF09822.7;	59.00	0.00
TRINITY_D8TLS2_VOLCA/270-308	D8TLS2.1 PF00400.30	59.00	0.00
TRINITY_I7ME62_TETTS/523-742	I7ME62.2 PF13718.4;	59.00	0.00
TRINITY_A8HQG9_CHLRE/32-344	A8HQG9.1 PF00459.23	59.00	0.00
TRINITY_V7B9E6_PHAVU/90-153	V7B9E6.1 PF13499.4;	59.00	0.00
TRINITY_W7XD08_TETTS/123-343	W7XD08.1 PF02475.14	58.90	0.00
TRINITY_A2FR27_TRIVA/28-204	A2FR27.1 PF00350.21	58.90	0.00
TRINITY_I7ML70_TETTS/56-343	I7ML70.2 PF01875.15	58.90	0.00
TRINITY_Q22W36_TETTS/25-114	Q22W36.2 PF06984.11	58.90	0.00
TRINITY_A0A087X566_POEFO/714-938	A0A087X566.2 PF1308	58.90	0.00
TRINITY_S2J0U7_MUCC1/7-79	S2J0U7.1 PF00173.26	58.90	0.00
TRINITY_I7M0N5_TETTS/125-295	I7M0N5.1 PF00092.26	58.90	0.00
TRINITY_A0A023AY35_GRENI/3-118	A0A023AY35.1 PF0141	58.90	0.00
TRINITY_W4HC62_9STRA/62-464	W4HC62.1 PF05889.11	58.90	0.00
TRINITY_A8J3K1_CHLRE/1842-2127	A8J3K1.1 PF07714.15	58.90	0.00
TRINITY_B9PZT4_TOXGO/9-211	B9PZT4.1 PF00687.19	58.90	0.00
TRINITY_A0A067CAF5_SAPPC/81-424	A0A067CAF5.1 PF0197	58.90	0.00
TRINITY_Q118R4_TRIEI/27-139	Q118R4.1 PF08240.10	58.90	0.00
TRINITY_Q54Z02_DICDI/259-353	Q54Z02.1 PF00153.25	58.90	0.00
TRINITY_E0VRM3_PEDHC/699-848	E0VRM3.1 PF08282.10	58.90	0.00
TRINITY_A8ILL9_CHLRE/66-359	A8ILL9.1 PF03151.14	58.90	0.00
TRINITY_A8IIZ1_CHLRE/237-400	A8IIZ1.1 PF01554.16	58.90	0.00
TRINITY_A0LMI0_SYNFM/73-377	A0LMI0.1 PF00365.18	58.90	0.00
TRINITY_E3GGZ4_EUBLK/17-113	E3GGZ4.1 PF01196.17	58.90	0.00
TRINITY_Q22XT4_TETTS/475-635	Q22XT4.1 PF08662.9;	58.90	0.00
TRINITY_I0YVT0_9CHLO/20-89	I0YVT0.1 PF00675.18	58.90	0.00
TRINITY_D8U5D4_VOLCA/100-363	D8U5D4.1 PF04104.12	58.90	0.00
TRINITY_A8ITF1_CHLRE/137-265	A8ITF1.1 PF00107.24	58.90	0.00
TRINITY_D3BAA7_POLPA/15-484	D3BAA7.1 PF00171.20	58.90	0.00
TRINITY_I7M1V6_TETTS/314-533	I7M1V6.1 PF07002.14	58.90	0.00
TRINITY_L1IH70_GUITH/122-177	L1IH70.1 PF13513.4;	58.90	0.00
TRINITY_D8TIQ3_VOLCA/163-237	D8TIQ3.1 PF08511.9;	58.90	0.00

TRINITY_F0ZA00_DICPU/59-694	F0ZA00.1 PF05693.11	58.90	0.00
TRINITY_L8GTG9_ACACA/36-446	L8GTG9.1 PF01055.24	58.90	0.00
TRINITY_A8J8V3_CHLRE/161-386	A8J8V3.1 PF10253.7;	58.90	0.00
TRINITY_K4D4D0_SOLLC/8-307	K4D4D0.1 PF10243.7;	58.90	0.00
TRINITY_D8U5V9_VOLCA/54-144	D8U5V9.1 PF00153.25	58.90	0.00
TRINITY_A0A024URQ3_9STRA/30-121	A0A024URQ3.1 PF0482	58.90	0.00
TRINITY_A8J979_CHLRE/401-517	A8J979.1 PF02785.17	58.90	0.00
TRINITY_I0YKY3_9CHLO/718-905	I0YKY3.1 PF01369.18	58.90	0.00
TRINITY_A0A0D2WKM9_CAPO3/329-594	A0A0D2WKM9.1 PF0048	58.90	0.00
TRINITY_D8UJW1_VOLCA/1103-1292	D8UJW1.1 PF16415.3;	58.90	0.00
TRINITY_I7LUL6_TETTS/1643-1715	I7LUL6.2 PF03451.12	58.90	0.00
TRINITY_Q23RK6_TETTS/259-331	Q23RK6.2 PF11523.6;	58.90	0.00
TRINITY_L8GT26_ACACA/11-396	L8GT26.1 PF01433.18	58.90	0.00
TRINITY_A9SF14_PHYPA/48-159	A9SF14.1 PF02847.15	58.90	0.00
TRINITY_C3XYC3_BRAFL/5-378	C3XYC3.1 PF00022.17	58.90	0.00
TRINITY_A8IZI4_CHLRE/95-364	A8IZI4.1 PF00685.25	58.90	0.00
TRINITY_A8JGI3_CHLRE/270-557	A8JGI3.1 PF00233.17	58.90	0.00
TRINITY_D2VS08_NAEGR/119-258	D2VS08.1 PF00182.17	58.90	0.00
TRINITY_D8TNR8_VOLCA/2040-2146	D8TNR8.1 PF00856.26	58.90	0.00
TRINITY_A0A077KKX4_9FLAO/38-144	A0A077KKX4.1 PF0025	58.90	0.00
TRINITY_A8IK88_CHLRE/163-299	A8IK88.1 PF00892.18	58.90	0.00
TRINITY_A8JAY8_CHLRE/277-401	A8JAY8.1 PF01189.15	58.90	0.00
TRINITY_I1GAF7_AMPQE/406-501	I1GAF7.1 PF08295.10	58.90	0.00
TRINITY_L8GY02_ACACA/175-566	L8GY02.1 PF00962.20	58.90	0.00
TRINITY_I0YWY4_9CHLO/7-508	I0YWY4.1 PF05833.9;	58.90	0.00
TRINITY_D8U6I8_VOLCA/1149-1343	D8U6I8.1 PF00211.18	58.90	0.00
TRINITY_DEGP3_ARATH/100-287	Q9SHZ1.1 PF00089.24	58.90	0.00
TRINITY_D8UEZ9_VOLCA/123-392	D8UEZ9.1 PF11708.6;	58.90	0.00
TRINITY_D8TKA2_VOLCA/1-90	D8TKA2.1 PF00153.25	58.90	0.00
TRINITY_A0BRK1_PARTE/6-155	A0BRK1.1 PF00588.17	58.80	0.00
TRINITY_L8H6N0_ACACA/39-378	L8H6N0.1 PF04563.13	58.80	0.00
TRINITY_Q23QF5_TETTS/161-229	Q23QF5.2 PF00505.17	58.80	0.00
TRINITY_A0BJW5_PARTE/673-824	A0BJW5.1 PF00005.25	58.80	0.00
TRINITY_C1FIZ8_MICSR/58-109	C1FIZ8.1 PF13920.4;	58.80	0.00
TRINITY_A0A0D2U5L7_CAPO3/45-198	A0A0D2U5L7.1 PF0472	58.80	0.00
TRINITY_J9HVL1_9SPIT/989-1056	J9HVL1.1 PF00498.24	58.80	0.00
TRINITY_I7LZU8_TETTS/276-465	I7LZU8.1 PF00743.17	58.80	0.00
TRINITY_F1A3E2_DICPU/47-206	F1A3E2.1 PF04893.15	58.80	0.00
TRINITY_G7Y3S9_CLOSI/3-391	G7Y3S9.1 PF00022.17	58.80	0.00
TRINITY_Q22UZ4_TETTS/669-923	Q22UZ4.2 PF00069.23	58.80	0.00
TRINITY_L8GM48_ACACA/6-105	L8GM48.1 PF02036.15	58.80	0.00
TRINITY_Q8I0X9_OIKDI/2-90	Q8I0X9.1 PF00125.22	58.80	0.00
TRINITY_I7M6M9_TETTS/644-940	I7M6M9.2 PF00069.23	58.80	0.00
TRINITY_I7LX53_TETTS/117-484	I7LX53.1 PF00750.17	58.80	0.00
TRINITY_J9HM31_9SPIT/21-117	J9HM31.1 PF00169.27	58.80	0.00
TRINITY_RPAB3_DICDI/7-141	Q54YW8.1 PF03870.13	58.80	0.00
TRINITY_K1PRQ6_CRAGI/12-109	K1PRQ6.1 PF02214.20	58.80	0.00
TRINITY_F9W9Y1_TRYCI/1-98	F9W9Y1.1 PF00665.24	58.80	0.00
TRINITY_D3BIA3_POLPA/39-260	D3BIA3.1 PF00112.21	58.80	0.00
TRINITY_D8THD1_VOLCA/120-495	D8THD1.1 PF00443.27	58.80	0.00
TRINITY_D8TML8_VOLCA/431-482	D8TML8.1 PF13639.4;	58.80	0.00
TRINITY_I1FU93_AMPQE/114-413	I1FU93.1 PF13532.4;	58.80	0.00
TRINITY_C0NA16_TETTS/219-378	C0NA16.1 PF00005.25	58.80	0.00
TRINITY_D8UC51_VOLCA/360-524	D8UC51.1 PF08757.9;	58.80	0.00
TRINITY_Q23AM3_TETTS/1054-1231	Q23AM3.2 PF00005.25	58.80	0.00
TRINITY_F2UJ11_SALR5/237-437	F2UJ11.1 PF00149.26	58.80	0.00
TRINITY_A7SAJ2_NEMVE/1-231	A7SAJ2.1 PF04884.12	58.80	0.00
TRINITY_A0A096TLD8_MAIZE/76-538	A0A096TLD8.1 PF1043	58.80	0.00
TRINITY_A0EE63_PARTE/3584-4294	A0EE63.1 PF03028.13	58.80	0.00
TRINITY_K6VRX0_9MICO/27-143	K6VRX0.1 PF08240.10	58.80	0.00
TRINITY_A0DTA6_PARTE/314-534	A0DTA6.1 PF07002.14	58.80	0.00

TRINITY_A8HPP9_CHLRE/43-139	A8HPP9.1 PF04140.12	58.80	0.00
TRINITY_A0A015JQY8_9GLOM/125-534	A0A015JQY8.1 PF0160	58.80	0.00
TRINITY_A8IA75_CHLRE/44-181	A8IA75.1 PF03330.16	58.80	0.00
TRINITY_A8J0B6_CHLRE/58-327	A8J0B6.1 PF00248.19	58.80	0.00
TRINITY_D8TQ06_VOLCA/22-86	D8TQ06.1 PF13499.4;	58.80	0.00
TRINITY_D8UDE3_VOLCA/306-378	D8UDE3.1 PF04564.13	58.80	0.00
TRINITY_Q45QX4_CHLRE/57-154	Q45QX4.1 PF14769.4;	58.80	0.00
TRINITY_I7MMI9_TETTS/391-540	I7MMI9.2 PF00005.25	58.80	0.00
TRINITY_I0I3H4_CALAS/35-165	I0I3H4.1 PF03641.12	58.80	0.00
TRINITY_C3ZLG7_BRAFL/44-214	C3ZLG7.1 PF01595.18	58.80	0.00
TRINITY_A6G8G3_9DELT/10-78	A6G8G3.1 PF13450.4;	58.80	0.00
TRINITY_D8TKI2_VOLCA/1226-1356	D8TKI2.1 PF02732.13	58.80	0.00
TRINITY_I0YK64_9CHLO/2-459	I0YK64.1 PF00232.16	58.80	0.00
TRINITY_I0YPH7_9CHLO/3-233	I0YPH7.1 PF04065.13	58.80	0.00
TRINITY_A0A024UJ54_9STRA/4-73	A0A024UJ54.1 PF0279	58.80	0.00
TRINITY_D8TT27_VOLCA/101-313	D8TT27.1 PF12481.6;	58.80	0.00
TRINITY_I0Z7G6_9CHLO/812-1273	I0Z7G6.1 PF00136.19	58.80	0.00
TRINITY_D9S262_THEOJ/493-710	D9S262.1 PF00702.24	58.80	0.00
TRINITY_D8UBQ5_VOLCA/27-120	D8UBQ5.1 PF00686.17	58.80	0.00
TRINITY_A8HSE8_CHLRE/44-130	A8HSE8.1 PF01388.19	58.80	0.00
TRINITY_A8JHU2_CHLRE/3-98	A8JHU2.1 PF01158.16	58.80	0.00
TRINITY_E1Z8X2_CHLVA/426-620	E1Z8X2.1 PF03602.13	58.80	0.00
TRINITY_I0YM63_9CHLO/9-169	I0YM63.1 PF03031.16	58.80	0.00
TRINITY_A0A067KM87_JATCU/330-380	A0A067KM87.1 PF0062	58.80	0.00
TRINITY_G7L177_MEDTR/186-400	G7L177.1 PF00270.27	58.80	0.00
TRINITY_C1MZV2_MICPC/102-152	C1MZV2.1 PF01753.16	58.80	0.00
TRINITY_D8U2N0_VOLCA/476-575	D8U2N0.1 PF02806.16	58.80	0.00
TRINITY_L8GWW2_ACACA/318-616	L8GWW2.1 PF00069.23	58.80	0.00
TRINITY_A0A0M0JE94_9EUKA/117-208	A0A0M0JE94.1 PF0027	58.70	0.00
TRINITY_J9HYF8_9SPIT/799-1284	J9HYF8.1 PF00136.19	58.70	0.00
TRINITY_D2VK18_NAEGR/919-1071	D2VK18.1 PF00005.25	58.70	0.00
TRINITY_G7JZ43_MEDTR/9-81	G7JZ43.1 PF00173.26	58.70	0.00
TRINITY_B3S2H1_TRIAD/213-338	B3S2H1.1 PF00782.18	58.70	0.00
TRINITY_L8HH73_ACACA/11-114	L8HH73.1 PF04178.10	58.70	0.00
TRINITY_H3CEP8_TETNG/9-186	H3CEP8.1 PF11032.6;	58.70	0.00
TRINITY_D3B844_POLPA/641-686	D3B844.1 PF13920.4;	58.70	0.00
TRINITY_R9AGL6_WALI9/521-692	R9AGL6.1 PF07717.14	58.70	0.00
TRINITY_I7M700_TETTS/181-443	I7M700.2 PF00069.23	58.70	0.00
TRINITY_A0A0N8JXW8_9TELE/798-962	A0A0N8JXW8.1 PF0519	58.70	0.00
TRINITY_U3KN62_RABIT/56-225	U3KN62.1 PF00078.25	58.70	0.00
TRINITY_J9I2R8_9SPIT/282-345	J9I2R8.1 PF00498.24	58.70	0.00
TRINITY_F4PTY5_DICFS/4-286	F4PTY5.1 PF00069.23	58.70	0.00
TRINITY_A0CMD2_PARTE/512-586	A0CMD2.1 PF08059.11	58.70	0.00
TRINITY_A0A0G2ZF02_9DELT/207-312	A0A0G2ZF02.1 PF0255	58.70	0.00
TRINITY_S2J4F2_MUCC1/516-759	S2J4F2.1 PF00122.18	58.70	0.00
TRINITY_L8GRY6_ACACA/17-481	L8GRY6.1 PF01602.18	58.70	0.00
TRINITY_Q23JX7_TETTS/316-378	Q23JX7.2 PF00170.19	58.70	0.00
TRINITY_I7M286_TETTS/153-256	I7M286.2 PF12756.5;	58.70	0.00
TRINITY_L8GH56_ACACA/26-138	L8GH56.1 PF08487.8;	58.70	0.00
TRINITY_G0QXL5_ICHMG/691-767	G0QXL5.1 PF14559.4;	58.70	0.00
TRINITY_L8H6M2_ACACA/5-273	L8H6M2.1 PF00218.19	58.70	0.00
TRINITY_G7EA73_MIXOS/494-542	G7EA73.1 PF00628.27	58.70	0.00
TRINITY_A0A0A1TYJ5_ENTIV/244-291	A0A0A1TYJ5.1 PF1460	58.70	0.00
TRINITY_Q3SDT3_PARTE/63-246	Q3SDT3.1 PF01926.21	58.70	0.00
TRINITY_A4VDB2_TETTS/31-294	A4VDB2.2 PF00069.23	58.70	0.00
TRINITY_D3BE54_POLPA/200-587	D3BE54.1 PF00155.19	58.70	0.00
TRINITY_Q5NYT6_AROAE/134-284	Q5NYT6.1 PF05175.12	58.70	0.00
TRINITY_J9J3B4_9SPIT/124-338	J9J3B4.1 PF00112.21	58.70	0.00
TRINITY_H2XK01_CIOIN/56-103	H2XK01.1 PF00628.27	58.70	0.00
TRINITY_W7X4G9_TETTS/95-250	W7X4G9.1 PF00650.18	58.70	0.00
TRINITY_Q24DL3_TETTS/233-295	Q24DL3.2 PF07647.15	58.70	0.00

TRINITY_D8TIL0_VOLCA/40-231	D8TIL0.1 PF03942.13	58.70	0.00
TRINITY_I7M7B9_TETTS/137-405	I7M7B9.2 PF00561.18	58.70	0.00
TRINITY_A8ILB4_CHLRE/605-717	A8ILB4.1 PF00251.18	58.70	0.00
TRINITY_A0A078B325_STYLE/32-117	A0A078B325.1 PF0042	58.70	0.00
TRINITY_D3B4H6_POLPA/4-75	D3B4H6.1 PF00240.21	58.70	0.00
TRINITY_D8TMW1_VOLCA/578-754	D8TMW1.1 PF04072.12	58.70	0.00
TRINITY_E1Z7K9_CHLVA/14-439	E1Z7K9.1 PF04209.11	58.70	0.00
TRINITY_D8U7A0_VOLCA/662-914	D8U7A0.1 PF03914.15	58.70	0.00
TRINITY_H2Z6A0_CIOSA/284-404	H2Z6A0.1 PF00293.26	58.70	0.00
TRINITY_I7M9C4_TETTS/167-241	I7M9C4.2 PF00900.18	58.70	0.00
TRINITY_M2N7K9_BAUCO/19-318	M2N7K9.1 PF00248.19	58.70	0.00
TRINITY_D0MTJ2_PHYIT/91-154	D0MTJ2.1 PF05180.10	58.70	0.00
TRINITY_D8UF71_VOLCA/1141-1369	D8UF71.1 PF09353.8;	58.70	0.00
TRINITY_I1YUQ4_PREI7/5-67	I1YUQ4.1 PF00226.29	58.70	0.00
TRINITY_D8TK63_VOLCA/1-121	D8TK63.1 PF01926.21	58.70	0.00
TRINITY_E1Z2V1_CHLVA/15-376	E1Z2V1.1 PF00248.19	58.70	0.00
TRINITY_I0Z2E6_9CHLO/627-761	I0Z2E6.1 PF02518.24	58.70	0.00
TRINITY_A8IT01_CHLRE/39-374	A8IT01.1 PF00194.19	58.70	0.00
TRINITY_A8IVZ4_CHLRE/1-150	A8IVZ4.1 PF11911.6;	58.70	0.00
TRINITY_I0Z6A9_9CHLO/124-387	I0Z6A9.1 PF00069.23	58.70	0.00
TRINITY_L8H2X2_ACACA/22-576	L8H2X2.1 PF01602.18	58.70	0.00
TRINITY_D8UIZ7_VOLCA/369-849	D8UIZ7.1 PF00759.17	58.70	0.00
TRINITY_D8UHA9_VOLCA/879-1087	D8UHA9.1 PF07250.9;	58.70	0.00
TRINITY_H0EQ64_GLAL7/39-118	H0EQ64.1 PF02878.14	58.70	0.00
TRINITY_Q248E9_TETTS/284-336	Q248E9.1 PF13418.4;	58.70	0.00
TRINITY_A0A0A0KIY4_CUCSA/103-167	A0A0A0KIY4.1 PF1349	58.70	0.00
TRINITY_L8HG26_ACACA/287-428	L8HG26.1 PF01189.15	58.60	0.00
TRINITY_M2Y4X3_GALSU/55-262	M2Y4X3.1 PF00106.23	58.60	0.00
TRINITY_A0D2I3_PARTE/30-100	A0D2I3.1 PF05347.13	58.60	0.00
TRINITY_I4YPI0_9RHIZ/293-408	I4YPI0.1 PF02518.24	58.60	0.00
TRINITY_A0A078AKP0_STYLE/68-336	A0A078AKP0.1 PF0226	58.60	0.00
TRINITY_J9FGN9_9SPIT/840-1097	J9FGN9.1 PF00481.19	58.60	0.00
TRINITY_Q54NK9_DICDI/51-227	Q54NK9.1 PF10294.7;	58.60	0.00
TRINITY_W4G762_9STRA/203-335	W4G762.1 PF00583.23	58.60	0.00
TRINITY_G0QKY7_ICHMG/168-327	G0QKY7.1 PF00406.20	58.60	0.00
TRINITY_F4QCM6_DICFS/1249-1547	F4QCM6.1 PF00069.23	58.60	0.00
TRINITY_L8HJG4_ACACA/72-245	L8HJG4.1 PF10602.7;	58.60	0.00
TRINITY_F4PIH5_DICFS/22-331	F4PIH5.1 PF01008.15	58.60	0.00
TRINITY_F4PVI5_DICFS/14-70	F4PVI5.1 PF00412.20	58.60	0.00
TRINITY_F0ZDJ6_DICPU/473-685	F0ZDJ6.1 PF13236.4;	58.60	0.00
TRINITY_L8HDC4_ACACA/76-192	L8HDC4.1 PF01661.19	58.60	0.00
TRINITY_A7SPF1_NEMVE/30-122	A7SPF1.1 PF00153.25	58.60	0.00
TRINITY_I7MJV9_TETTS/69-545	I7MJV9.1 PF00501.26	58.60	0.00
TRINITY_L8GPW4_ACACA/35-152	L8GPW4.1 PF08240.10	58.60	0.00
TRINITY_I7M700_TETTS/181-443	I7M700.2 PF00069.23	58.60	0.00
TRINITY_J9HVL7_9SPIT/73-307	J9HVL7.1 PF01674.16	58.60	0.00
TRINITY_B0X3U8_CULQU/65-234	B0X3U8.1 PF00270.27	58.60	0.00
TRINITY_Q22Y91_TETTS/368-602	Q22Y91.1 PF01167.16	58.60	0.00
TRINITY_G0QQC9_ICHMG/712-856	G0QQC9.1 PF00005.25	58.60	0.00
TRINITY_A0A0F6W8X8_9DELT/415-575	A0A0F6W8X8.1 PF0044	58.60	0.00
TRINITY_A0A067CPB0_SAPPC/160-415	A0A067CPB0.1 PF0409	58.60	0.00
TRINITY_B5G2K2_TAEGU/24-121	B5G2K2.1 PF01230.21	58.60	0.00
TRINITY_A0A078AVZ2_STYLE/100-248	A0A078AVZ2.1 PF0000	58.60	0.00
TRINITY_A8I3K8_CHLRE/124-290	A8I3K8.1 PF04055.19	58.60	0.00
TRINITY_D8TZ27_VOLCA/330-623	D8TZ27.1 PF00899.19	58.60	0.00
TRINITY_A8J6D5_CHLRE/484-760	A8J6D5.1 PF00702.24	58.60	0.00
TRINITY_A8JB50_CHLRE/10-265	A8JB50.1 PF00069.23	58.60	0.00
TRINITY_I0YPE8_9CHLO/863-1042	I0YPE8.1 PF00689.19	58.60	0.00
TRINITY_F4R632_MELLP/44-195	F4R632.1 PF04098.13	58.60	0.00
TRINITY_Q22W25_TETTS/269-397	Q22W25.2 PF04153.16	58.60	0.00
TRINITY_M5XT00_PRUPE/24-151	M5XT00.1 PF04073.13	58.60	0.00

TRINITY_D8U496_VOLCA/89-239	D8U496.1 PF12146.6;	58.60	0.00
TRINITY_A8J7G2_CHLRE/615-923	A8J7G2.1 PF00176.21	58.60	0.00
TRINITY_E1ZTF1_CHLVA/187-273	E1ZTF1.1 PF01909.21	58.60	0.00
TRINITY_C0Z502_BREBN/4-157	C0Z502.1 PF13420.5;	58.60	0.00
TRINITY_A0DTM2_PARTE/98-446	A0DTM2.1 PF13868.4;	58.60	0.00
TRINITY_A0DGY7_PARTE/114-212	A0DGY7.1 PF00153.25	58.60	0.00
TRINITY_A8J9X5_CHLRE/165-251	A8J9X5.1 PF00189.18	58.60	0.00
TRINITY_A0A078A0I1_STYLE/51-321	A0A078A0I1.1 PF0015	58.60	0.00
TRINITY_E1ZLK8_CHLVA/56-427	E1ZLK8.1 PF01137.19	58.60	0.00
TRINITY_L8GDU5_ACACA/21-186	L8GDU5.1 PF03357.19	58.60	0.00
TRINITY_L8HJK9_ACACA/14-635	L8HJK9.1 PF00888.20	58.60	0.00
TRINITY_A0CXN8_PARTE/37-405	A0CXN8.1 PF00155.19	58.60	0.00
TRINITY_D8THI6_VOLCA/146-208	D8THI6.1 PF01485.19	58.60	0.00
TRINITY_E1ZB62_CHLVA/52-175	E1ZB62.1 PF01575.17	58.60	0.00
TRINITY_I0YP30_9CHLO/11-241	I0YP30.1 PF02602.13	58.60	0.00
TRINITY_D8TU75_VOLCA/97-213	D8TU75.1 PF10184.7;	58.60	0.00
TRINITY_I0Z7P1_9CHLO/7-77	I0Z7P1.1 PF00076.20	58.60	0.00
TRINITY_L8HHG5_ACACA/403-695	L8HHG5.1 PF00632.23	58.60	0.00
TRINITY_A0A087STE3_AUXPR/40-137	A0A087STE3.1 PF0812	58.60	0.00
TRINITY_A0A087SC18_AUXPR/51-204	A0A087SC18.1 PF0975	58.60	0.00
TRINITY_I1FC34_AMPQE/179-503	I1FC34.1 PF00176.21	58.60	0.00
TRINITY_D3B9B5_POLPA/307-358	D3B9B5.1 PF00415.16	58.50	0.00
TRINITY_A0A078A9A6_STYLE/490-629	A0A078A9A6.1 PF1467	58.50	0.00
TRINITY_G0R4M5_ICHMG/51-307	G0R4M5.1 PF00481.19	58.50	0.00
TRINITY_W4XEW9_STRPU/5-113	W4XEW9.1 PF02109.14	58.50	0.00
TRINITY_A4BFQ8_9GAMM/22-387	A4BFQ8.1 PF00916.18	58.50	0.00
TRINITY_I7LV57_TETTS/19-212	I7LV57.1 PF00106.23	58.50	0.00
TRINITY_D8M9G4_BLAHO/200-250	D8M9G4.1 PF02820.16	58.50	0.00
TRINITY_A0EAY1_PARTE/296-618	A0EAY1.1 PF00384.20	58.50	0.00
TRINITY_A0DYN1_PARTE/250-413	A0DYN1.1 PF03031.16	58.50	0.00
TRINITY_A0A0G4IXE7_PLABS/3-137	A0A0G4IXE7.1 PF0121	58.50	0.00
TRINITY_A0A0D2WU03_CAPO3/526-643	A0A0D2WU03.1 PF1197	58.50	0.00
TRINITY_L8H1U1_ACACA/20-480	L8H1U1.1 PF00171.20	58.50	0.00
TRINITY_SLT2_CHLRE/16-750	D2K6F1.1 PF03600.14	58.50	0.00
TRINITY_A0CDQ2_PARTE/31-219	A0CDQ2.1 PF00227.24	58.50	0.00
TRINITY_A0A0D2WU03_CAPO3/526-643	A0A0D2WU03.1 PF1197	58.50	0.00
TRINITY_H2L694_ORYLA/710-1243	H2L694.1 PF02738.16	58.50	0.00
TRINITY_A8J0M8_CHLRE/597-713	A8J0M8.1 PF00271.29	58.50	0.00
TRINITY_F0YD11_AURAN/754-825	F0YD11.1 PF04564.13	58.50	0.00
TRINITY_A8IJ64_CHLRE/11-302	A8IJ64.1 PF03151.14	58.50	0.00
TRINITY_B0WMS8_CULQU/14-158	B0WMS8.1 PF14580.4;	58.50	0.00
TRINITY_B3RT61_TRIAD/41-306	B3RT61.1 PF00561.18	58.50	0.00
TRINITY_I7LTG4_TETTS/318-462	I7LTG4.2 PF00388.17	58.50	0.00
TRINITY_F4P3I1_BATDJ/9-283	F4P3I1.1 PF02900.16	58.50	0.00
TRINITY_A4VDB2_TETTS/31-294	A4VDB2.2 PF00069.23	58.50	0.00
TRINITY_A8J3C8_CHLRE/1-296	A8J3C8.1 PF03942.13	58.50	0.00
TRINITY_R9AMY5_WALI9/88-713	R9AMY5.1 PF00133.20	58.50	0.00
TRINITY_A0D5K9_PARTE/135-221	A0D5K9.1 PF12796.5;	58.50	0.00
TRINITY_A0E576_PARTE/650-695	A0E576.1 PF13920.4;	58.50	0.00
TRINITY_A0A0N4T425_BRUPA/42-84	A0A0N4T425.1 PF1363	58.50	0.00
TRINITY_A0A0N4T425_BRUPA/42-84	A0A0N4T425.1 PF1363	58.50	0.00
TRINITY_L8GZN5_ACACA/388-506	L8GZN5.1 PF00106.23	58.50	0.00
TRINITY_F4PR82_DICFS/446-698	F4PR82.1 PF00069.23	58.50	0.00
TRINITY_W7X4G9_TETTS/95-250	W7X4G9.1 PF00650.18	58.50	0.00
TRINITY_A9SWK3_PHYPA/96-205	A9SWK3.1 PF00583.23	58.50	0.00
TRINITY_Q22D35_TETTS/926-1042	Q22D35.2 PF02518.24	58.50	0.00
TRINITY_F8ELC3_RUNSL/306-442	F8ELC3.1 PF04030.12	58.50	0.00
TRINITY_L8GYA7_ACACA/28-169	L8GYA7.1 PF00810.16	58.50	0.00
TRINITY_D8TSW2_VOLCA/15-187	D8TSW2.1 PF14580.4;	58.50	0.00
TRINITY_L8H399_ACACA/66-353	L8H399.1 PF05185.14	58.50	0.00
TRINITY_D8TUH4_VOLCA/83-248	D8TUH4.1 PF01734.20	58.50	0.00

TRINITY_I1AXL1_9RHOB/5-268	I1AXL1.1 PF02900.16	58.50	0.00
TRINITY_D8UHD7_VOLCA/269-321	D8UHD7.1 PF13920.4;	58.50	0.00
TRINITY_A8JFH1_CHLRE/93-580	A8JFH1.1 PF00759.17	58.50	0.00
TRINITY_D8TV82_VOLCA/6-218	D8TV82.1 PF13460.4;	58.50	0.00
TRINITY_A8IGE2_CHLRE/6-210	A8IGE2.1 PF01975.15	58.50	0.00
TRINITY_M1VCC4_CYAME/278-464	M1VCC4.1 PF07724.12	58.50	0.00
TRINITY_I0YVX4_9CHLO/3-155	I0YVX4.1 PF00005.25	58.50	0.00
TRINITY_D8UCK7_VOLCA/66-172	D8UCK7.1 PF16589.3;	58.50	0.00
TRINITY_F4Q2H1_DICFS/1503-1853	F4Q2H1.1 PF00476.18	58.50	0.00
TRINITY_A0C2J6_PARTE/65-132	A0C2J6.1 PF00550.23	58.50	0.00
TRINITY_E7KA58_YEASA/419-459	E7KA58.1 PF00400.30	58.50	0.00
TRINITY_Q3SD57_PARTE/13-173	Q3SD57.1 PF00071.20	58.50	0.00
TRINITY_I7M7V0_TETTS/141-205	I7M7V0.2 PF07258.12	58.50	0.00
TRINITY_I0Z3Z5_9CHLO/270-624	I0Z3Z5.1 PF03016.13	58.50	0.00
TRINITY_A0A0A0KIY4_CUCSA/103-167	A0A0A0KIY4.1 PF1349	58.50	0.00
TRINITY_A0A0A0KIY4_CUCSA/103-167	A0A0A0KIY4.1 PF1349	58.50	0.00
TRINITY_A0CYQ1_PARTE/321-362	A0CYQ1.1 PF00400.30	58.50	0.00
TRINITY_J9IT22_9SPIT/499-822	J9IT22.1 PF00702.24	58.40	0.00
TRINITY_MPCP_CAEEL/40-130	P40614.1 PF00153.25	58.40	0.00
TRINITY_J9IZ27_9SPIT/31-320	J9IZ27.1 PF04616.12	58.40	0.00
TRINITY_F3ZVQ2_MAHA5/29-163	F3ZVQ2.1 PF14871.4;	58.40	0.00
TRINITY_J9IQR8_9SPIT/20-190	J9IQR8.1 PF01871.15	58.40	0.00
TRINITY_D3BTW3_POLPA/48-402	D3BTW3.1 PF01207.15	58.40	0.00
TRINITY_F5SKU2_9BACL/11-163	F5SKU2.1 PF01625.19	58.40	0.00
TRINITY_L8GGJ1_ACACA/139-335	L8GGJ1.1 PF02750.12	58.40	0.00
TRINITY_G0R2T0_ICHMG/33-283	G0R2T0.1 PF00069.23	58.40	0.00
TRINITY_A0A077ZUP3_STYLE/22-211	A0A077ZUP3.1 PF0130	58.40	0.00
TRINITY_F0ZPW4_DICPU/4-230	F0ZPW4.1 PF04670.10	58.40	0.00
TRINITY_Q225Q9_TETTS/99-223	Q225Q9.1 PF13091.4;	58.40	0.00
TRINITY_A1DIM3_NEOFI/533-969	A1DIM3.1 PF00136.19	58.40	0.00
TRINITY_D5X5V5_THIK1/71-152	D5X5V5.1 PF01035.18	58.40	0.00
TRINITY_V4BJX4_LOTGI/7-249	V4BJX4.1 PF01564.15	58.40	0.00
TRINITY_Q9XUW5_CAEEL/153-324	Q9XUW5.1 PF00270.27	58.40	0.00
TRINITY_A8IAU3_CHLRE/93-264	A8IAU3.1 PF00069.23	58.40	0.00
TRINITY_L8H9J9_ACACA/133-458	L8H9J9.1 PF01808.16	58.40	0.00
TRINITY_A7SFF2_NEMVE/4-136	A7SFF2.1 PF02966.14	58.40	0.00
TRINITY_I0YLR2_9CHLO/8-326	I0YLR2.1 PF13872.4;	58.40	0.00
TRINITY_U6GK02_EIMAC/106-385	U6GK02.1 PF08648.10	58.40	0.00
TRINITY_A8JHD4_CHLRE/503-591	A8JHD4.1 PF01342.19	58.40	0.00
TRINITY_I1KQH1_SOYBN/78-445	I1KQH1.1 PF00009.25	58.40	0.00
TRINITY_D8TJA8_VOLCA/81-422	D8TJA8.1 PF04124.10	58.40	0.00
TRINITY_D8U9S2_VOLCA/5-143	D8U9S2.1 PF00179.24	58.40	0.00
TRINITY_A0A0G4ENF6_9ALVE/238-543	A0A0G4ENF6.1 PF0058	58.40	0.00
TRINITY_A8HUP3_CHLRE/217-445	A8HUP3.1 PF05996.10	58.40	0.00
TRINITY_D3B897_POLPA/546-622	D3B897.1 PF00017.22	58.40	0.00
TRINITY_Q23DR0_TETTS/3824-4544	Q23DR0.2 PF03028.13	58.40	0.00
TRINITY_A8HQP1_CHLRE/46-376	A8HQP1.1 PF02685.14	58.40	0.00
TRINITY_D8U980_VOLCA/175-378	D8U980.1 PF08801.9;	58.40	0.00
TRINITY_I7MMT5_TETTS/48-124	I7MMT5.2 PF01066.19	58.40	0.00
TRINITY_L8H0E9_ACACA/65-222	L8H0E9.1 PF01652.16	58.40	0.00
TRINITY_B0WPI5_CULQU/202-326	B0WPI5.1 PF00781.22	58.40	0.00
TRINITY_L8H505_ACACA/79-640	L8H505.1 PF03063.18	58.40	0.00
TRINITY_I0KFX4_9BACT/26-138	I0KFX4.1 PF01661.19	58.40	0.00
TRINITY_R4X8K9_TAPDE/13-174	R4X8K9.1 PF00071.20	58.40	0.00
TRINITY_I7MMT5_TETTS/48-124	I7MMT5.2 PF01066.19	58.40	0.00
TRINITY_A8IUI1_CHLRE/36-146	A8IUI1.1 PF08240.10	58.40	0.00
TRINITY_W2PXB2_PHYPN/493-609	W2PXB2.1 PF00665.24	58.40	0.00
TRINITY_A8IDR1_CHLRE/59-159	A8IDR1.1 PF03330.16	58.40	0.00
TRINITY_D8TJ30_VOLCA/555-818	D8TJ30.1 PF05346.9;	58.40	0.00
TRINITY_L8GX59_ACACA/136-414	L8GX59.1 PF00494.17	58.40	0.00
TRINITY_L8GRS0_ACACA/1563-1914	L8GRS0.1 PF00443.27	58.40	0.00

TRINITY_E1ZFM0_CHLVA/58-200	E1ZFM0.1 PF13365.4;	58.40	0.00
TRINITY_A8IQU4_CHLRE/3-468	A8IQU4.1 PF03219.12	58.40	0.00
TRINITY_A0A0D2WTL5_CAPO3/513-630	A0A0D2WTL5.1 PF0141	58.30	0.00
TRINITY_D8QNB6_SELML/77-462	D8QNB6.1 PF05292.9;	58.30	0.00
TRINITY_F0ZLB7_DICPU/2-271	F0ZLB7.1 PF06432.9;	58.30	0.00
TRINITY_D2UXT8_NAEGR/264-327	D2UXT8.1 PF13432.4;	58.30	0.00
TRINITY_Q22UZ4_TETTS/669-923	Q22UZ4.2 PF00069.23	58.30	0.00
TRINITY_V4ARF4_LOTGI/299-354	V4ARF4.1 PF00412.20	58.30	0.00
TRINITY_G0QX12_ICHMG/518-692	G0QX12.1 PF04983.16	58.30	0.00
TRINITY_A0DYY1_PARTE/126-253	A0DYY1.1 PF00134.21	58.30	0.00
TRINITY_A8N9L1_COPC7/1112-1588	A8N9L1.1 PF06202.12	58.30	0.00
TRINITY_D2VT76_NAEGR/265-345	D2VT76.1 PF04000.13	58.30	0.00
TRINITY_M5EBX6_MALS4/8-169	M5EBX6.1 PF00071.20	58.30	0.00
TRINITY_D8TY67_VOLCA/261-527	D8TY67.1 PF00664.21	58.30	0.00
TRINITY_A0EID1_PARTE/75-324	A0EID1.1 PF00069.23	58.30	0.00
TRINITY_M5XT60_PRUPE/38-168	M5XT60.1 PF00782.18	58.30	0.00
TRINITY_L8GZB8_ACACA/129-440	L8GZB8.1 PF16653.3;	58.30	0.00
TRINITY_SRFC_DICDI/10-57	Q55F37.2 PF00319.16	58.30	0.00
TRINITY_A0A0E9N7P2_9ASCO/94-222	A0A0E9N7P2.1 PF0029	58.30	0.00
TRINITY_I1EQY4_AMPQE/281-440	I1EQY4.1 PF00441.22	58.30	0.00
TRINITY_A8IZ85_CHLRE/54-229	A8IZ85.1 PF02837.16	58.30	0.00
TRINITY_A0CQU3_PARTE/310-530	A0CQU3.1 PF07002.14	58.30	0.00
TRINITY_F4QBZ3_DICFS/1-155	F4QBZ3.1 PF06052.10	58.30	0.00
TRINITY_G0PC99_CAEBE/259-308	G0PC99.1 PF02891.18	58.30	0.00
TRINITY_T1HHY4_RHOPR/39-363	T1HHY4.1 PF01074.20	58.30	0.00
TRINITY_I7M483_TETTS/113-361	I7M483.1 PF01063.17	58.30	0.00
TRINITY_I0YID6_9CHLO/101-197	I0YID6.1 PF00153.25	58.30	0.00
TRINITY_E1Z4D4_CHLVA/17-278	E1Z4D4.1 PF01073.17	58.30	0.00
TRINITY_D8U4P4_VOLCA/210-295	D8U4P4.1 PF14693.4;	58.30	0.00
TRINITY_C1DY55_MICSR/32-253	C1DY55.1 PF05721.11	58.30	0.00
TRINITY_I4MN02_9BURK/9-328	I4MN02.1 PF00962.20	58.30	0.00
TRINITY_D3AW49_POLPA/91-217	D3AW49.1 PF04034.11	58.30	0.00
TRINITY_L8GWB9_ACACA/441-524	L8GWB9.1 PF00017.22	58.30	0.00
TRINITY_D8U0T9_VOLCA/66-570	D8U0T9.1 PF02696.12	58.30	0.00
TRINITY_E1ZNN0_CHLVA/86-426	E1ZNN0.1 PF01494.17	58.30	0.00
TRINITY_D1C2X0_SPHTD/3-118	D1C2X0.1 PF06983.11	58.30	0.00
TRINITY_Q22T03_TETTS/214-383	Q22T03.1 PF00270.27	58.30	0.00
TRINITY_A8JCF5_CHLRE/31-360	A8JCF5.1 PF00150.16	58.30	0.00
TRINITY_W4G9W3_9STRA/32-202	W4G9W3.1 PF04893.15	58.30	0.00
TRINITY_I1CDY0_RHIO9/132-234	I1CDY0.1 PF01159.17	58.30	0.00
TRINITY_A8NTI7_COPC7/176-350	A8NTI7.2 PF00150.16	58.30	0.00
TRINITY_L8HAN6_ACACA/270-325	L8HAN6.1 PF00571.26	58.30	0.00
TRINITY_A0A059LHS0_9CHLO/2-51	A0A059LHS0.1 PF0040	58.30	0.00
TRINITY_L8GZ17_ACACA/7-220	L8GZ17.1 PF00300.20	58.30	0.00
TRINITY_D8U315_VOLCA/132-268	D8U315.1 PF03330.16	58.30	0.00
TRINITY_Q8T6B8_DICDI/197-358	Q8T6B8.1 PF00005.25	58.30	0.00
TRINITY_D8TL13_VOLCA/212-337	D8TL13.1 PF14580.4;	58.30	0.00
TRINITY_A0A087SDQ3_AUXPR/1-246	A0A087SDQ3.1 PF0014	58.30	0.00
TRINITY_B3S5N5_TRIAD/577-704	B3S5N5.1 PF13646.4;	58.30	0.00
TRINITY_A0D515_PARTE/43-239	A0D515.1 PF00149.26	58.30	0.00
TRINITY_A0A0D2VGU2_CAPO3/80-369	A0A0D2VGU2.1 PF0647	58.30	0.00
TRINITY_J3NKC5_GAGT3/341-390	J3NKC5.1 PF02891.18	58.30	0.00
TRINITY_D8TZL1_VOLCA/29-94	D8TZL1.1 PF00536.28	58.30	0.00
TRINITY_Q22BL2_TETTS/10-119	Q22BL2.2 PF00255.17	58.30	0.00
TRINITY_A0A078AE02_STYLE/23-329	A0A078AE02.1 PF0024	58.30	0.00
TRINITY_D8U9E6_VOLCA/223-308	D8U9E6.1 PF00707.20	58.30	0.00
TRINITY_L8GWW6_ACACA/39-76	L8GWW6.1 PF12907.5;	58.30	0.00
TRINITY_A8JHW9_CHLRE/42-387	A8JHW9.1 PF07690.14	58.30	0.00
TRINITY_L8H8K0_ACACA/18-112	L8H8K0.1 PF06127.9;	58.30	0.00
TRINITY_A0A0L6VS53_9BASI/21-185	A0A0L6VS53.1 PF0010	58.30	0.00
TRINITY_D8UA75_VOLCA/248-321	D8UA75.1 PF04564.13	58.30	0.00

TRINITY_A8HZR1_CHLRE/14486-14621	A8HZR1.1 PF13602.4;	58.30	0.00
TRINITY_J9J5P5_9SPIT/28-172	J9J5P5.1 PF00810.16	58.30	0.00
TRINITY_B3S5N5_TRIAD/577-704	B3S5N5.1 PF13646.4;	58.30	0.00
TRINITY_I0Z0D9_9CHLO/180-344	I0Z0D9.1 PF00160.19	58.30	0.00
TRINITY_I7M1U7_TETTS/1176-1331	I7M1U7.1 PF00005.25	58.30	0.00
TRINITY_C5LPP9_PERM5/1-205	C5LPP9.1 PF00022.17	58.30	0.00
TRINITY_E9HTT4_DAPPU/1052-1100	E9HTT4.1 PF00628.27	58.30	0.00
TRINITY_K3XBN6_PYTUL/60-178	K3XBN6.1 PF07986.10	58.30	0.00
TRINITY_Q235W0_TETTS/37-189	Q235W0.2 PF05071.14	58.30	0.00
TRINITY_A0A095C721_CRYGA/74-315	A0A095C721.2 PF0000	58.20	0.00
TRINITY_J9HMM9_9SPIT/101-388	J9HMM9.1 PF00122.18	58.20	0.00
TRINITY_L8H2L6_ACACA/21-154	L8H2L6.1 PF00237.17	58.20	0.00
TRINITY_Q22SK7_TETTS/32-174	Q22SK7.1 PF00485.16	58.20	0.00
TRINITY_E3HZV5_RHOVT/102-346	E3HZV5.1 PF10609.7;	58.20	0.00
TRINITY_F4PJM4_DICFS/12-398	F4PJM4.1 PF01053.18	58.20	0.00
TRINITY_A0A0D2WNJ4_CAPO3/773-827	A0A0D2WNJ4.1 PF0041	58.20	0.00
TRINITY_G0QVH9_ICHMG/93-465	G0QVH9.1 PF01546.26	58.20	0.00
TRINITY_Q54G51_DICDI/238-559	Q54G51.1 PF03630.12	58.20	0.00
TRINITY_E2BIM3_HARSA/11-266	E2BIM3.1 PF00370.19	58.20	0.00
TRINITY_I0APD5_IGNAJ/4-186	I0APD5.1 PF01965.22	58.20	0.00
TRINITY_A8IJV9_CHLRE/373-692	A8IJV9.1 PF00702.24	58.20	0.00
TRINITY_B9SNT8_RICCO/1030-1139	B9SNT8.1 PF00005.25	58.20	0.00
TRINITY_D8TMX3_VOLCA/1-97	D8TMX3.1 PF03073.13	58.20	0.00
TRINITY_L8GJ62_ACACA/8-255	L8GJ62.1 PF03029.15	58.20	0.00
TRINITY_A0A074WMF9_9PEZI/42-261	A0A074WMF9.1 PF0035	58.20	0.00
TRINITY_A0A067SYC7_9AGAR/3-126	A0A067SYC7.1 PF0177	58.20	0.00
TRINITY_H2LJS9_ORYLA/16-322	H2LJS9.1 PF01556.16	58.20	0.00
TRINITY_A0A067NIU5_PLEOS/110-373	A0A067NIU5.1 PF0006	58.20	0.00
TRINITY_J9IGI9_9SPIT/49-304	J9IGI9.1 PF00069.23	58.20	0.00
TRINITY_D8U0B5_VOLCA/34-237	D8U0B5.1 PF05997.10	58.20	0.00
TRINITY_D8U2V5_VOLCA/32-176	D8U2V5.1 PF00071.20	58.20	0.00
TRINITY_L8GS12_ACACA/6-211	L8GS12.1 PF00108.21	58.20	0.00
TRINITY_C4M4R8_ENTHI/24-138	C4M4R8.1 PF01412.16	58.20	0.00
TRINITY_D8UBJ0_VOLCA/157-235	D8UBJ0.1 PF11282.6;	58.20	0.00
TRINITY_A0A0D2T6A5_GOSRA/46-177	A0A0D2T6A5.1 PF0012	58.20	0.00
TRINITY_NOXA_DICDI/237-347	Q9XYS3.1 PF08022.10	58.20	0.00
TRINITY_A0A087SLA0_AUXPR/25-104	A0A087SLA0.1 PF0128	58.20	0.00
TRINITY_E1Z9P0_CHLVA/12-226	E1Z9P0.1 PF04757.12	58.20	0.00
TRINITY_L8GYB8_ACACA/255-364	L8GYB8.1 PF08022.10	58.20	0.00
TRINITY_D8UG67_VOLCA/120-260	D8UG67.1 PF13365.4;	58.20	0.00
TRINITY_D8U0B7_VOLCA/5-118	D8U0B7.1 PF06094.10	58.20	0.00
TRINITY_F4PWV9_DICFS/586-847	F4PWV9.1 PF07714.15	58.20	0.00
TRINITY_D8TMZ1_VOLCA/7-443	D8TMZ1.1 PF05577.10	58.20	0.00
TRINITY_MLH1_MOUSE/23-248	Q9JK91.2 PF02518.24	58.20	0.00
TRINITY_Q6UKY3_CHLRE/53-119	Q6UKY3.1 PF04716.12	58.20	0.00
TRINITY_L8HLC2_ACACA/17-345	L8HLC2.1 PF00503.18	58.20	0.00
TRINITY_E1ZXV4_CAMFO/514-1046	E1ZXV4.1 PF13423.4;	58.20	0.00
TRINITY_L8HJG5_ACACA/67-313	L8HJG5.1 PF03381.13	58.20	0.00
TRINITY_A8HW34_CHLRE/1-390	A8HW34.1 PF01373.15	58.20	0.00
TRINITY_C1MTS7_MICPC/2072-2343	C1MTS7.1 PF12775.5;	58.20	0.00
TRINITY_A0A078GK44_BRANA/667-868	A0A078GK44.1 PF0285	58.20	0.00
TRINITY_A0A078AYX4_STYLE/85-816	A0A078AYX4.1 PF0303	58.20	0.00
TRINITY_A0CF26_PARTE/22-275	A0CF26.1 PF03803.13	58.20	0.00
TRINITY_D8TZI8_VOLCA/219-453	D8TZI8.1 PF03407.14	58.20	0.00
TRINITY_D8M1W7_BLAHO/168-334	D8M1W7.1 PF02421.16	58.20	0.00
TRINITY_H2AZ07_KAZAF/7-297	H2AZ07.1 PF00069.23	58.20	0.00
TRINITY_E1Z4L0_CHLVA/312-586	E1Z4L0.1 PF00082.20	58.20	0.00
TRINITY_A9SNT8_PHYPA/19-274	A9SNT8.1 PF00069.23	58.20	0.00
TRINITY_H3HAJ2_PHYRM/359-478	H3HAJ2.1 PF00665.24	58.20	0.00
TRINITY_R4GCI3_ANOCA/1-108	R4GCI3.1 PF00665.24	58.20	0.00
TRINITY_A4VCZ6_TETTS/85-391	A4VCZ6.2 PF01866.15	58.20	0.00

TRINITY_D8TCQ6_SELML/752-1283	D8TCQ6.1 PF02538.12	58.10	0.00
TRINITY_J9IZ27_9SPIT/31-320	J9IZ27.1 PF04616.12	58.10	0.00
TRINITY_A0A078AMJ2_STYLE/15-197	A0A078AMJ2.1 PF0502	58.10	0.00
TRINITY_B4RDU7_PHEZH/59-204	B4RDU7.1 PF00903.23	58.10	0.00
TRINITY_E0W122_PEDHC/14-87	E0W122.1 PF01722.16	58.10	0.00
TRINITY_I1G153_AMPQE/19-61	I1G153.1 PF02150.14	58.10	0.00
TRINITY_I7LVW0_TETTS/6-146	I7LVW0.1 PF05903.12	58.10	0.00
TRINITY_M0V1V7_HORVD/41-207	M0V1V7.1 PF08662.9;	58.10	0.00
TRINITY_L8GJG4_ACACA/20-138	L8GJG4.1 PF00307.29	58.10	0.00
TRINITY_C7PPF7_CHIPD/460-723	C7PPF7.1 PF12972.5;	58.10	0.00
TRINITY_D3B612_POLPA/43-240	D3B612.1 PF00106.23	58.10	0.00
TRINITY_A0A068RSW5_9FUNG/641-776	A0A068RSW5.1 PF0000	58.10	0.00
TRINITY_D8UES2_VOLCA/60-121	D8UES2.1 PF04818.11	58.10	0.00
TRINITY_G0R0U0_ICHMG/14-286	G0R0U0.1 PF10234.7;	58.10	0.00
TRINITY_A0A078A7J6_STYLE/762-1058	A0A078A7J6.1 PF0006	58.10	0.00
TRINITY_Q23YX7_TETTS/1-171	Q23YX7.2 PF05856.10	58.10	0.00
TRINITY_W4XI36_STRPU/662-777	W4XI36.1 PF00665.24	58.10	0.00
TRINITY_G4TH90_PIRID/51-445	G4TH90.1 PF00999.19	58.10	0.00
TRINITY_D8THX1_VOLCA/227-448	D8THX1.1 PF13672.4;	58.10	0.00
TRINITY_A0A0G4H2P4_9ALVE/364-449	A0A0G4H2P4.1 PF1349	58.10	0.00
TRINITY_A8J288_CHLRE/392-604	A8J288.1 PF07690.14	58.10	0.00
TRINITY_A8HNT1_CHLRE/52-191	A8HNT1.2 PF10262.7;	58.10	0.00
TRINITY_D8U5R3_VOLCA/259-485	D8U5R3.1 PF00667.18	58.10	0.00
TRINITY_Q23Q10_TETTS/220-310	Q23Q10.2 PF15963.3;	58.10	0.00
TRINITY_K4C2L5_SOLLC/85-129	K4C2L5.1 PF13639.4;	58.10	0.00
TRINITY_A5D7H4_BOVIN/614-657	A5D7H4.1 PF13639.4;	58.10	0.00
TRINITY_D8TUC5_VOLCA/7-195	D8TUC5.1 PF13869.4;	58.10	0.00
TRINITY_G0QUA3_ICHMG/83-385	G0QUA3.1 PF00648.19	58.10	0.00
TRINITY_L8HFB9_ACACA/8-70	L8HFB9.1 PF00076.20	58.10	0.00
TRINITY_E1VQB4_9GAMM/598-665	E1VQB4.1 PF00364.20	58.10	0.00
TRINITY_A8JEK5_CHLRE/22-141	A8JEK5.1 PF02221.13	58.10	0.00
TRINITY_B7RZD3_9GAMM/4-382	B7RZD3.1 PF02515.15	58.10	0.00
TRINITY_E1ZPN0_CHLVA/23-186	E1ZPN0.1 PF09419.8;	58.10	0.00
TRINITY_A4VCU9_TETTS/362-480	A4VCU9.1 PF01926.21	58.10	0.00
TRINITY_I7MCL6_TETTS/651-743	I7MCL6.2 PF13424.4;	58.10	0.00
TRINITY_D8TRZ5_VOLCA/14-244	D8TRZ5.1 PF12740.5;	58.10	0.00
TRINITY_A0A0G4IX38_PLABS/3-60	A0A0G4IX38.1 PF0475	58.10	0.00
TRINITY_A8IRK5_CHLRE/40-113	A8IRK5.1 PF00364.20	58.10	0.00
TRINITY_D8TRG8_VOLCA/542-742	D8TRG8.1 PF00488.19	58.10	0.00
TRINITY_I0YJE2_9CHLO/24-288	I0YJE2.1 PF01193.22	58.10	0.00
TRINITY_J9J7E3_9SPIT/2162-2244	J9J7E3.1 PF05406.13	58.10	0.00
TRINITY_D8TI56_VOLCA/411-534	D8TI56.1 PF05285.10	58.10	0.00
TRINITY_F4PV19_DICFS/1034-1076	F4PV19.1 PF01585.21	58.10	0.00
TRINITY_L8H3C4_ACACA/231-487	L8H3C4.1 PF00069.23	58.10	0.00
TRINITY_U9T9G2_RHIID/228-351	U9T9G2.1 PF00271.29	58.10	0.00
TRINITY_A0A0M8SM37_9ACTN/11-450	A0A0M8SM37.1 PF1352	58.10	0.00
TRINITY_D8TJ74_VOLCA/20-139	D8TJ74.1 PF13537.4;	58.10	0.00
TRINITY_D8UI89_VOLCA/116-212	D8UI89.1 PF13649.4;	58.10	0.00
TRINITY_D8TS95_VOLCA/19-308	D8TS95.1 PF00069.23	58.10	0.00
TRINITY_A8J1Y2_CHLRE/46-453	A8J1Y2.1 PF00450.20	58.10	0.00
TRINITY_I7M910_TETTS/11-285	I7M910.1 PF00069.23	58.10	0.00
TRINITY_I0Z955_9CHLO/143-197	I0Z955.1 PF10309.7;	58.10	0.00
TRINITY_Q2H7U0_CHAGB/401-534	Q2H7U0.1 PF00005.25	58.10	0.00
TRINITY_L8GEL6_ACACA/329-543	L8GEL6.1 PF13236.4;	58.10	0.00
TRINITY_A0A0G4EL00_9ALVE/28-158	A0A0G4EL00.1 PF0411	58.10	0.00
TRINITY_S9UGY6_9TRYP/384-515	S9UGY6.1 PF09976.7;	58.10	0.00
TRINITY_D8U2P2_VOLCA/281-578	D8U2P2.1 PF00176.21	58.10	0.00
TRINITY_D8U3D7_VOLCA/31-246	D8U3D7.1 PF01036.16	58.10	0.00
TRINITY_L8H393_ACACA/160-347	L8H393.1 PF00644.18	58.10	0.00
TRINITY_A8I807_CHLRE/1018-1101	A8I807.1 PF00211.18	58.10	0.00
TRINITY_Q232M9_TETTS/5-90	Q232M9.3 PF00887.17	58.10	0.00

TRINITY_G0QWU3_ICHMG/12-121	G0QWU3.1 PF00787.22	58.10	0.00
TRINITY_A0CNX9_PARTE/179-246	A0CNX9.1 PF13833.4;	58.00	0.00
TRINITY_F0XXR9_AURAN/4-179	F0XXR9.1 PF00025.19	58.00	0.00
TRINITY_F0ZDJ6_DICPU/473-685	F0ZDJ6.1 PF13236.4;	58.00	0.00
TRINITY_R1E6D9_BOTPV/7-94	R1E6D9.1 PF01035.18	58.00	0.00
TRINITY_G0QRM1_ICHMG/13-169	G0QRM1.1 PF12850.5;	58.00	0.00
TRINITY_L8GGG2_ACACA/628-752	L8GGG2.1 PF00754.23	58.00	0.00
TRINITY_J9INC1_9SPIT/455-710	J9INC1.1 PF00069.23	58.00	0.00
TRINITY_A0A085N172_9BILA/8-121	A0A085N172.1 PF1690	58.00	0.00
TRINITY_G0QW14_ICHMG/165-434	G0QW14.1 PF00928.19	58.00	0.00
TRINITY_I0YIA4_9CHLO/3-165	I0YIA4.1 PF00071.20	58.00	0.00
TRINITY_FTSZ2_RHIME/15-176	P45484.2 PF00091.23	58.00	0.00
TRINITY_S8ED18_9LAMI/13-126	S8ED18.1 PF01981.14	58.00	0.00
TRINITY_F0ZA90_DICPU/22-375	F0ZA90.1 PF04757.12	58.00	0.00
TRINITY_L8HG30_ACACA/14-111	L8HG30.1 PF00153.25	58.00	0.00
TRINITY_A0DTP8_PARTE/77-166	A0DTP8.1 PF16531.3;	58.00	0.00
TRINITY_E1ZLK8_CHLVA/56-427	E1ZLK8.1 PF01137.19	58.00	0.00
TRINITY_I1G7R9_AMPQE/53-103	I1G7R9.1 PF00415.16	58.00	0.00
TRINITY_I7MDY6_TETTS/636-751	I7MDY6.2 PF00188.24	58.00	0.00
TRINITY_A0E9I5_PARTE/35-123	A0E9I5.1 PF01221.16	58.00	0.00
TRINITY_D8UBS2_VOLCA/5-73	D8UBS2.1 PF13921.4;	58.00	0.00
TRINITY_A0A0F4ZED4_9PEZI/281-347	A0A0F4ZED4.1 PF0148	58.00	0.00
TRINITY_T2GA88_DESGI/300-467	T2GA88.1 PF03441.12	58.00	0.00
TRINITY_A0EBQ7_PARTE/465-697	A0EBQ7.1 PF00233.17	58.00	0.00
TRINITY_A0D8S8_PARTE/638-896	A0D8S8.1 PF07714.15	58.00	0.00
TRINITY_A4AGI9_9ACTN/14-329	A4AGI9.1 PF07993.10	58.00	0.00
TRINITY_D8TTJ2_VOLCA/574-753	D8TTJ2.1 PF13532.4;	58.00	0.00
TRINITY_F0Z8I9_DICPU/17-186	F0Z8I9.1 PF00071.20	58.00	0.00
TRINITY_A0A0B2UU04_TOXCA/25-95	A0A0B2UU04.1 PF0007	58.00	0.00
TRINITY_A0A0G4ERG2_9ALVE/5-294	A0A0G4ERG2.1 PF0121	58.00	0.00
TRINITY_F4QFF3_DICFS/61-180	F4QFF3.1 PF02338.17	58.00	0.00
TRINITY_A0DYA9_PARTE/9-80	A0DYA9.1 PF00240.21	58.00	0.00
TRINITY_A0A078AMN3_STYLE/203-269	A0A078AMN3.1 PF1392	58.00	0.00
TRINITY_H2V6H5_TAKRU/370-643	H2V6H5.1 PF02913.17	58.00	0.00
TRINITY_A8IS78_CHLRE/5-74	A8IS78.1 PF02325.15	58.00	0.00
TRINITY_D8TUV8_VOLCA/6-258	D8TUV8.1 PF03343.11	58.00	0.00
TRINITY_G0QKW1_ICHMG/111-281	G0QKW1.1 PF00092.26	58.00	0.00
TRINITY_M4BXD5_HYAAE/4-455	M4BXD5.1 PF00171.20	58.00	0.00
TRINITY_A0A067MEM0_9HOMO/6-117	A0A067MEM0.1 PF1351	58.00	0.00
TRINITY_H0X010_OTOGA/2-104	H0X010.1 PF00085.18	58.00	0.00
TRINITY_D8MBA7_BLAHO/1-174	D8MBA7.1 PF00025.19	58.00	0.00
TRINITY_E1ZSC6_CHLVA/5-192	E1ZSC6.1 PF13460.4;	58.00	0.00
TRINITY_D8UBS2_VOLCA/5-73	D8UBS2.1 PF13921.4;	58.00	0.00
TRINITY_A8ICB8_CHLRE/5-295	A8ICB8.1 PF00225.21	58.00	0.00
TRINITY_A8JGI3_CHLRE/270-557	A8JGI3.1 PF00233.17	58.00	0.00
TRINITY_CMT1_DICDI/4-361	Q54JH6.1 PF00145.15	58.00	0.00
TRINITY_C1ECL1_MICSR/22-70	C1ECL1.1 PF12906.5;	58.00	0.00
TRINITY_A0A0A0LPU3_CUCSA/26-143	A0A0A0LPU3.1 PF0115	58.00	0.00
TRINITY_G1PQS7_MYOLU/16-221	G1PQS7.1 PF01015.16	58.00	0.00
TRINITY_E0VFJ1_PEDHC/31-114	E0VFJ1.1 PF00027.27	58.00	0.00
TRINITY_E1ZJ62_CHLVA/25-310	E1ZJ62.1 PF00862.17	58.00	0.00
TRINITY_L8HEV2_ACACA/41-347	L8HEV2.1 PF01074.20	58.00	0.00
TRINITY_E1Z5N4_CHLVA/1185-1342	E1Z5N4.1 PF00179.24	58.00	0.00
TRINITY_D8TZJ0_VOLCA/13-112	D8TZJ0.1 PF00651.29	58.00	0.00
TRINITY_Q23FR5_TETTS/51-305	Q23FR5.2 PF00069.23	58.00	0.00
TRINITY_I7LWK7_TETTS/31-345	I7LWK7.1 PF00850.17	58.00	0.00
TRINITY_A0CEE8_PARTE/74-215	A0CEE8.1 PF01966.20	58.00	0.00
TRINITY_A8IYR9_CHLRE/2598-3304	A8IYR9.1 PF03028.13	58.00	0.00
TRINITY_I3ITW0_ORENI/22-89	I3ITW0.1 PF00021.19	58.00	0.00
TRINITY_J9JBC0_9SPIT/113-238	J9JBC0.1 PF00293.26	57.90	0.00
TRINITY_A0A0F8DL43_CERFI/735-872	A0A0F8DL43.1 PF0027	57.90	0.00

TRINITY_A0A078B349_STYLE/444-804	A0A078B349.1 PF0070	57.90	0.00
TRINITY_G0QW14_ICHMG/165-434	G0QW14.1 PF00928.19	57.90	0.00
TRINITY_A8HQ22_CHLRE/163-586	A8HQ22.1 PF12739.5;	57.90	0.00
TRINITY_A0BHV8_PARTE/64-106	A0BHV8.1 PF00643.22	57.90	0.00
TRINITY_A0A091DU58_FUKDA/11-162	A0A091DU58.1 PF1332	57.90	0.00
TRINITY_F4Q487_DICFS/425-550	F4Q487.1 PF04628.11	57.90	0.00
TRINITY_E9PR47_HUMAN/6-165	E9PR47.1 PF04430.12	57.90	0.00
TRINITY_D8UEB8_VOLCA/42-168	D8UEB8.1 PF00067.20	57.90	0.00
TRINITY_G0R409_ICHMG/421-565	G0R409.1 PF00005.25	57.90	0.00
TRINITY_F4PSP2_DICFS/256-385	F4PSP2.1 PF04494.13	57.90	0.00
TRINITY_A7SBR8_NEMVE/13-144	A7SBR8.1 PF13589.4;	57.90	0.00
TRINITY_RPA1_DICDI/622-799	Q86H36.1 PF04983.16	57.90	0.00
TRINITY_L8GUP7_ACACA/25-206	L8GUP7.1 PF04664.11	57.90	0.00
TRINITY_I7MJV9_TETTS/69-545	I7MJV9.1 PF00501.26	57.90	0.00
TRINITY_A0A0G4FB00_9ALVE/8-65	A0A0G4FB00.1 PF0083	57.90	0.00
TRINITY_L8HCD5_ACACA/44-209	L8HCD5.1 PF09414.8;	57.90	0.00
TRINITY_D8TLS0_VOLCA/13-212	D8TLS0.1 PF09778.7;	57.90	0.00
TRINITY_E1ZH81_CHLVA/26-178	E1ZH81.1 PF01509.16	57.90	0.00
TRINITY_I7MI70_TETTS/14-137	I7MI70.1 PF13893.4;	57.90	0.00
TRINITY_A0A077ZZ86_STYLE/16-83	A0A077ZZ86.1 PF1586	57.90	0.00
TRINITY_L8GQX7_ACACA/1030-1086	L8GQX7.1 PF00412.20	57.90	0.00
TRINITY_L8GGE2_ACACA/32-145	L8GGE2.1 PF07876.10	57.90	0.00
TRINITY_D3BSL6_POLPA/4-431	D3BSL6.1 PF00171.20	57.90	0.00
TRINITY_A8J6Q3_CHLRE/107-166	A8J6Q3.1 PF05421.9;	57.90	0.00
TRINITY_A8IJQ1_CHLRE/86-172	A8IJQ1.1 PF08712.9;	57.90	0.00
TRINITY_A8J5M0_CHLRE/19-212	A8J5M0.1 PF13419.4;	57.90	0.00
TRINITY_L8GHB5_ACACA/140-315	L8GHB5.1 PF00270.27	57.90	0.00
TRINITY_D8U5V1_VOLCA/37-396	D8U5V1.1 PF07944.10	57.90	0.00
TRINITY_A8J8U9_CHLRE/45-302	A8J8U9.1 PF07082.9;	57.90	0.00
TRINITY_L8GRF8_ACACA/491-786	L8GRF8.1 PF03635.15	57.90	0.00
TRINITY_A8JIV5_CHLRE/68-245	A8JIV5.1 PF01612.18	57.90	0.00
TRINITY_B2IX05_NOSP7/90-197	B2IX05.1 PF13578.4;	57.90	0.00
TRINITY_A0A0D2PZV3_GOSRA/11-126	A0A0D2PZV3.1 PF0141	57.90	0.00
TRINITY_D8U3Y1_VOLCA/28-189	D8U3Y1.1 PF11947.6;	57.90	0.00
TRINITY_A8J3R1_CHLRE/1-130	A8J3R1.1 PF09366.8;	57.90	0.00
TRINITY_F0ZRZ2_DICPU/4-79	F0ZRZ2.1 PF07297.10	57.90	0.00
TRINITY_D8TKB8_VOLCA/41-663	D8TKB8.1 PF01602.18	57.90	0.00
TRINITY_D8UF05_VOLCA/50-301	D8UF05.1 PF00494.17	57.90	0.00
TRINITY_Q111M9_TRIEI/24-486	Q111M9.1 PF00171.20	57.90	0.00
TRINITY_A0E493_PARTE/11-338	A0E493.1 PF00225.21	57.90	0.00
TRINITY_W7TAA3_9STRA/119-293	W7TAA3.1 PF00270.27	57.90	0.00
TRINITY_A8IBK6_CHLRE/1-131	A8IBK6.1 PF14811.4;	57.90	0.00
TRINITY_A8J1A0_CHLRE/250-682	A8J1A0.1 PF00152.18	57.90	0.00
TRINITY_D8ULY9_VOLCA/128-258	D8ULY9.1 PF01156.17	57.90	0.00
TRINITY_D2VS08_NAEGR/119-258	D2VS08.1 PF00182.17	57.90	0.00
TRINITY_U9SW55_RHIID/233-302	U9SW55.1 PF00013.27	57.90	0.00
TRINITY_D8TKH0_VOLCA/161-515	D8TKH0.1 PF01103.21	57.90	0.00
TRINITY_F7AW79_CALJA/105-327	F7AW79.1 PF00009.25	57.90	0.00
TRINITY_D8TMY3_VOLCA/29-292	D8TMY3.1 PF00069.23	57.90	0.00
TRINITY_A8J4W1_CHLRE/1-418	A8J4W1.1 PF00202.19	57.90	0.00
TRINITY_G0R409_ICHMG/421-565	G0R409.1 PF00005.25	57.90	0.00
TRINITY_A8INF6_CHLRE/2101-2396	A8INF6.1 PF00069.23	57.90	0.00
TRINITY_T0PYF1_9STRA/70-184	T0PYF1.1 PF01981.14	57.90	0.00
TRINITY_D8TH37_VOLCA/318-449	D8TH37.1 PF07683.12	57.90	0.00
TRINITY_G1N8F2_MELGA/80-381	G1N8F2.2 PF00561.18	57.80	0.00
TRINITY_A0C6C2_PARTE/5-172	A0C6C2.1 PF05301.9;	57.80	0.00
TRINITY_B5EG20_GEOBB/14-188	B5EG20.1 PF00857.18	57.80	0.00
TRINITY_A0A0D9WUG4_9ORYZ/254-302	A0A0D9WUG4.1 PF0062	57.80	0.00
TRINITY_A0A0M0JS54_9EUKA/57-649	A0A0M0JS54.1 PF0363	57.80	0.00
TRINITY_Q23FD8_TETTS/19-112	Q23FD8.4 PF01230.21	57.80	0.00
TRINITY_A0A078AU69_STYLE/106-169	A0A078AU69.1 PF0518	57.80	0.00

TRINITY_F0ZUP2_DICPU/5-195	F0ZUP2.1 PF02545.12	57.80	0.00
TRINITY_GCST_DICDI/29-290	Q54DD3.1 PF01571.19	57.80	0.00
TRINITY_D3BJV1_POLPA/136-322	D3BJV1.1 PF00485.16	57.80	0.00
TRINITY_Q86HU8_DICDI/6-243	Q86HU8.1 PF01370.19	57.80	0.00
TRINITY_A8J3K1_CHLRE/1842-2127	A8J3K1.1 PF07714.15	57.80	0.00
TRINITY_J9IA35_9SPIT/13-289	J9IA35.1 PF07993.10	57.80	0.00
TRINITY_A0A0F9ZEW7_9MICR/186-229	A0A0F9ZEW7.1 PF1363	57.80	0.00
TRINITY_F4NSD2_BATDJ/224-374	F4NSD2.1 PF00005.25	57.80	0.00
TRINITY_F4PRR1_DICFS/28-120	F4PRR1.1 PF02214.20	57.80	0.00
TRINITY_W7X388_TETTS/56-115	W7X388.1 PF01363.19	57.80	0.00
TRINITY_D8U8Q9_VOLCA/3-158	D8U8Q9.1 PF13019.4;	57.80	0.00
TRINITY_L8HFJ6_ACACA/43-464	L8HFJ6.1 PF05577.10	57.80	0.00
TRINITY_L8GKG8_ACACA/21-183	L8GKG8.1 PF00071.20	57.80	0.00
TRINITY_A0A0A1PCH1_9FUNG/7-327	A0A0A1PCH1.1 PF0049	57.80	0.00
TRINITY_W1NUB4_AMBTC/284-525	W1NUB4.1 PF02913.17	57.80	0.00
TRINITY_D8UHG2_VOLCA/1220-1413	D8UHG2.1 PF00406.20	57.80	0.00
TRINITY_A0A078A9T0_STYLE/80-292	A0A078A9T0.1 PF0011	57.80	0.00
TRINITY_Q23Q10_TETTS/220-310	Q23Q10.2 PF15963.3;	57.80	0.00
TRINITY_I0YX02_9CHLO/106-280	I0YX02.1 PF03572.16	57.80	0.00
TRINITY_D2VRR8_NAEGR/260-481	D2VRR8.1 PF00215.22	57.80	0.00
TRINITY_W7X994_TETTS/109-415	W7X994.1 PF00648.19	57.80	0.00
TRINITY_Q1DEU4_MYXXD/197-302	Q1DEU4.1 PF02557.15	57.80	0.00
TRINITY_A0A0E9NBT1_9ASCO/21-211	A0A0E9NBT1.1 PF0092	57.80	0.00
TRINITY_A0A0N4XS36_NIPBR/5-53	A0A0N4XS36.1 PF0062	57.80	0.00

TRINITY_A0A087SPB6_AUXPR/165-516	A0A087SPB6.1 PF0410	57.80	0.00
TRINITY_A8HP45_CHLRE/1-215	A8HP45.1 PF04305.12	57.80	0.00
TRINITY_E1ZCK6_CHLVA/58-340	E1ZCK6.1 PF00009.25	57.80	0.00
TRINITY_L8HCT5_ACACA/1-304	L8HCT5.1 PF00009.25	57.80	0.00
TRINITY_ABCD2_DICDI/93-376	Q8T8P3.1 PF06472.13	57.80	0.00
TRINITY_I0Z472_9CHLO/55-358	I0Z472.1 PF00225.21	57.80	0.00
TRINITY_PR46B_CHLRE/99-205	Q94EY1.1 PF13877.4;	57.80	0.00
TRINITY_A8I2T6_CHLRE/51-181	A8I2T6.1 PF13398.4;	57.80	0.00
TRINITY_W5NEL6_LEPOC/257-501	W5NEL6.1 PF00481.19	57.80	0.00
TRINITY_A0A078B349_STYLE/444-804	A0A078B349.1 PF0070	57.80	0.00
TRINITY_A0BF61_PARTE/17-269	A0BF61.1 PF00069.23	57.80	0.00
TRINITY_A8IQ02_CHLRE/1471-1681	A8IQ02.1 PF14228.4;	57.80	0.00
TRINITY_A0BJ29_PARTE/7-340	A0BJ29.1 PF07992.12	57.80	0.00
TRINITY_A8JEL8_CHLRE/56-300	A8JEL8.1 PF09295.8;	57.80	0.00
TRINITY_A8J3S4_CHLRE/110-273	A8J3S4.1 PF00211.18	57.80	0.00
TRINITY_L8HI43_ACACA/398-832	L8HI43.1 PF00275.18	57.80	0.00
TRINITY_I0I8C5_CALAS/40-411	I0I8C5.1 PF00155.19	57.80	0.00
TRINITY_R1DE31_EMIHU/14-210	R1DE31.1 PF00106.23	57.80	0.00
TRINITY_A8HPW9_CHLRE/1369-1561	A8HPW9.1 PF00211.18	57.80	0.00
TRINITY_A8HXY9_CHLRE/27-123	A8HXY9.1 PF00153.25	57.80	0.00
TRINITY_CALL3_RAT/10-74	Q5U206.1 PF13499.4;	57.80	0.00
TRINITY_A8HZR1_CHLRE/6512-6779	A8HZR1.1 PF00109.24	57.70	0.00
TRINITY_Q22GW0_TETTS/1651-1813	Q22GW0.2 PF00069.23	57.70	0.00
TRINITY_G0QXC5_ICHMG/9-406	G0QXC5.1 PF00162.17	57.70	0.00
TRINITY_G0QQR1_ICHMG/26-120	G0QQR1.1 PF00995.21	57.70	0.00
TRINITY_C1EF89_MICSR/177-433	C1EF89.1 PF00176.21	57.70	0.00
TRINITY_A0A0A0KTZ4_CUCSA/17-227	A0A0A0KTZ4.1 PF0135	57.70	0.00
TRINITY_A0A0N4WHM5_HAEPC/23-118	A0A0N4WHM5.1 PF0124	57.70	0.00
TRINITY_T2L481_9GAMM/25-120	T2L481.1 PF04828.12	57.70	0.00
TRINITY_D2VS08_NAEGR/119-258	D2VS08.1 PF00182.17	57.70	0.00
TRINITY_Q239A0_TETTS/806-868	Q239A0.1 PF02187.15	57.70	0.00
TRINITY_J9IM53_9SPIT/388-673	J9IM53.1 PF00481.19	57.70	0.00
TRINITY_TTL3C_TETTS/705-1030	Q23FE2.1 PF03133.13	57.70	0.00
TRINITY_U6GK02_EIMAC/106-385	U6GK02.1 PF08648.10	57.70	0.00
TRINITY_K1QS40_CRAGI/89-337	K1QS40.1 PF00112.21	57.70	0.00
TRINITY_Q60DB4_ORYSJ/141-303	Q60DB4.1 PF00078.25	57.70	0.00
TRINITY_I7M3Q0_TETTS/233-414	I7M3Q0.1 PF00270.27	57.70	0.00
TRINITY_J9HZ29_9SPIT/335-483	J9HZ29.1 PF00817.18	57.70	0.00
TRINITY_A8J818_CHLRE/57-109	A8J818.1 PF05739.17	57.70	0.00
TRINITY_S2J644_MUCC1/47-117	S2J644.1 PF01480.15	57.70	0.00
TRINITY_E0VFT2_PEDHC/270-407	E0VFT2.1 PF04146.13	57.70	0.00
TRINITY_A8IZL0_CHLRE/600-696	A8IZL0.1 PF07801.9;	57.70	0.00
TRINITY_I1LMF9_SOYBN/316-479	I1LMF9.2 PF08662.9;	57.70	0.00
TRINITY_A0A087SDW0_AUXPR/327-464	A0A087SDW0.1 PF0010	57.70	0.00
TRINITY_A0A0F6YJJ3_9DELT/331-518	A0A0F6YJJ3.1 PF1360	57.70	0.00
TRINITY_A8IZS3_CHLRE/136-240	A8IZS3.1 PF02338.17	57.70	0.00
TRINITY_A8JD03_CHLRE/54-312	A8JD03.1 PF00069.23	57.70	0.00
TRINITY_D8TYJ0_VOLCA/80-323	D8TYJ0.1 PF01226.15	57.70	0.00
TRINITY_D8TIN2_VOLCA/1-163	D8TIN2.1 PF10436.7;	57.70	0.00
TRINITY_Q01D64_OSTTA/169-411	Q01D64.1 PF01136.17	57.70	0.00
TRINITY_D3BKA7_POLPA/113-448	D3BKA7.1 PF07992.12	57.70	0.00
TRINITY_E1Z2I0_CHLVA/60-159	E1Z2I0.1 PF03330.16	57.70	0.00
TRINITY_J9HT96_9SPIT/260-331	J9HT96.1 PF02187.15	57.70	0.00
TRINITY_J9I2Q5_9SPIT/540-610	J9I2Q5.1 PF14791.4;	57.70	0.00
TRINITY_D8U6I8_VOLCA/629-717	D8U6I8.1 PF00211.18	57.70	0.00
TRINITY_A8JDX0_CHLRE/164-273	A8JDX0.1 PF06859.10	57.70	0.00
TRINITY_A8JB50_CHLRE/10-265	A8JB50.1 PF00069.23	57.70	0.00
TRINITY_A8J1Z2_CHLRE/36-257	A8J1Z2.1 PF13460.4;	57.70	0.00
TRINITY_I1HKP9_BRADI/1-52	I1HKP9.1 PF13920.4;	57.70	0.00
TRINITY_B8C531_THAPS/4-166	B8C531.1 PF00071.20	57.70	0.00
TRINITY_A8IRK2_CHLRE/356-957	A8IRK2.1 PF00328.20	57.70	0.00

TRINITY_D8U2N8_VOLCA/151-247	D8U2N8.1 PF00211.18	57.70	0.00
TRINITY_D3BVE1_POLPA/180-887	D3BVE1.1 PF00343.18	57.70	0.00
TRINITY_DYHC2_CHLRE/3624-4332	Q9SMH5.2 PF03028.13	57.70	0.00
TRINITY_D8TZ09_VOLCA/273-394	D8TZ09.1 PF01412.16	57.70	0.00
TRINITY_A8HYE3_CHLRE/960-1164	A8HYE3.1 PF07714.15	57.70	0.00
TRINITY_I0ZAL4_9CHLO/164-311	I0ZAL4.1 PF12422.6;	57.70	0.00
TRINITY_M0ZZV1_SOLTU/323-401	M0ZZV1.1 PF09409.8;	57.70	0.00
TRINITY_Q22SL3_TETTS/348-483	Q22SL3.3 PF01590.24	57.70	0.00
TRINITY_G0R2T3_ICHMG/22-99	G0R2T3.1 PF01282.17	57.70	0.00
TRINITY_A0C9R8_PARTE/6-92	A0C9R8.1 PF08700.9;	57.70	0.00
TRINITY_E1ZSQ4_CHLVA/98-176	E1ZSQ4.1 PF01472.18	57.70	0.00
TRINITY_G0QP08_ICHMG/102-340	G0QP08.1 PF00069.23	57.60	0.00
TRINITY_L8GIS7_ACACA/19-169	L8GIS7.1 PF13768.4;	57.60	0.00
TRINITY_Q23K28_TETTS/1-286	Q23K28.1 PF02586.12	57.60	0.00
TRINITY_A0BVN4_PARTE/6-78	A0BVN4.1 PF02798.18	57.60	0.00
TRINITY_I7MFS0_TETTS/260-418	I7MFS0.1 PF03367.11	57.60	0.00
TRINITY_MNMA_ALKMQ/8-359	A6TUB2.1 PF03054.14	57.60	0.00
TRINITY_G0R1C1_ICHMG/28-126	G0R1C1.1 PF03645.11	57.60	0.00
TRINITY_A0CNM0_PARTE/7-99	A0CNM0.1 PF00153.25	57.60	0.00
TRINITY_G0QK50_ICHMG/177-323	G0QK50.1 PF05843.12	57.60	0.00
TRINITY_V9FEZ6_PHYPR/29-201	V9FEZ6.1 PF03853.13	57.60	0.00
TRINITY_G0R2V0_ICHMG/53-221	G0R2V0.1 PF04893.15	57.60	0.00
TRINITY_E9FY13_DAPPU/3-101	E9FY13.1 PF03091.13	57.60	0.00
TRINITY_A0A015JV36_9GLOM/16-189	A0A015JV36.1 PF0335	57.60	0.00
TRINITY_L8GFE5_ACACA/8-113	L8GFE5.1 PF01920.18	57.60	0.00
TRINITY_L8HCT8_ACACA/124-189	L8HCT8.1 PF00076.20	57.60	0.00
TRINITY_L8GRN2_ACACA/498-555	L8GRN2.1 PF13884.4;	57.60	0.00
TRINITY_A0A0J8BHR0_BETVU/21-308	A0A0J8BHR0.1 PF0024	57.60	0.00
TRINITY_A0A0M8K8G3_9CHLR/9-154	A0A0M8K8G3.1 PF0099	57.60	0.00
TRINITY_I7MLG9_TETTS/376-605	I7MLG9.2 PF00112.21	57.60	0.00
TRINITY_M2XPD2_GALSU/207-497	M2XPD2.1 PF00291.23	57.60	0.00
TRINITY_F4PK47_DICFS/824-1029	F4PK47.1 PF00454.25	57.60	0.00
TRINITY_I7LV96_TETTS/890-1181	I7LV96.1 PF00069.23	57.60	0.00
TRINITY_C1EDW5_MICSR/3-188	C1EDW5.1 PF00625.19	57.60	0.00
TRINITY_A0A0G4GZH6_9ALVE/419-599	A0A0G4GZH6.1 PF0281	57.60	0.00
TRINITY_G0R0N7_ICHMG/32-549	G0R0N7.1 PF01602.18	57.60	0.00
TRINITY_T1IG80_RHOPR/112-327	T1IG80.1 PF00112.21	57.60	0.00
TRINITY_L8GE59_ACACA/389-656	L8GE59.1 PF00702.24	57.60	0.00
TRINITY_A0A061GVN5_THECC/418-662	A0A061GVN5.1 PF0006	57.60	0.00
TRINITY_F0ZHE3_DICPU/392-643	F0ZHE3.1 PF00069.23	57.60	0.00
TRINITY_A0A0G4GAG3_9ALVE/133-198	A0A0G4GAG3.1 PF1392	57.60	0.00
TRINITY_I7MLL9_TETTS/291-547	I7MLL9.2 PF00069.23	57.60	0.00
TRINITY_G0QQ97_ICHMG/55-152	G0QQ97.1 PF12894.5;	57.60	0.00
TRINITY_E1ZCZ4_CHLVA/177-413	E1ZCZ4.1 PF13621.4;	57.60	0.00
TRINITY_A0DRB0_PARTE/269-386	A0DRB0.1 PF03764.16	57.60	0.00
TRINITY_A0A0D2WKM9_CAPO3/329-594	A0A0D2WKM9.1 PF0048	57.60	0.00
TRINITY_A8JF65_CHLRE/335-864	A8JF65.1 PF05691.10	57.60	0.00
TRINITY_D8U9U1_VOLCA/4-89	D8U9U1.1 PF02290.13	57.60	0.00
TRINITY_A8HV44_CHLRE/53-401	A8HV44.1 PF00294.22	57.60	0.00
TRINITY_D8UFI3_VOLCA/48-224	D8UFI3.1 PF08660.9;	57.60	0.00
TRINITY_C1MGE7_MICPC/335-709	C1MGE7.1 PF01433.18	57.60	0.00
TRINITY_W1PKI2_AMBTC/46-184	W1PKI2.1 PF01743.18	57.60	0.00
TRINITY_A8IVN6_CHLRE/8-192	A8IVN6.1 PF06552.10	57.60	0.00
TRINITY_L8GQY9_ACACA/530-672	L8GQY9.1 PF00620.25	57.60	0.00
TRINITY_I1NGX0_SOYBN/765-943	I1NGX0.2 PF05063.12	57.60	0.00
TRINITY_D8TJ88_VOLCA/347-405	D8TJ88.1 PF08711.9;	57.60	0.00
TRINITY_A8HZ59_CHLRE/31-292	A8HZ59.1 PF01697.25	57.60	0.00
TRINITY_Q23YR9_TETTS/7-439	Q23YR9.1 PF00171.20	57.60	0.00
TRINITY_A8HYK1_CHLRE/19-314	A8HYK1.1 PF15294.4;	57.60	0.00
TRINITY_D8TH36_VOLCA/208-334	D8TH36.1 PF01980.14	57.60	0.00
TRINITY_K1PUG4_CRAGI/1381-1529	K1PUG4.1 PF00005.25	57.60	0.00

TRINITY_D3BDQ8_POLPA/12-329	D3BDQ8.1 PF00225.21	57.60	0.00
TRINITY_H3B651_LATCH/318-461	H3B651.1 PF00388.17	57.60	0.00
TRINITY_A8IZM6_CHLRE/14-437	A8IZM6.1 PF01593.22	57.60	0.00
TRINITY_I0YYK1_9CHLO/49-324	I0YYK1.1 PF00664.21	57.60	0.00
TRINITY_F0VD63_NEOCL/195-288	F0VD63.1 PF01209.16	57.60	0.00
TRINITY_B7PEW3_IXOSC/119-180	B7PEW3.1 PF00226.29	57.60	0.00
TRINITY_D8UK61_VOLCA/227-395	D8UK61.1 PF04832.10	57.60	0.00
TRINITY_T1ELD1_HELRO/10-231	T1ELD1.1 PF00244.18	57.50	0.00
TRINITY_D2W2M3_NAEGR/33-71	D2W2M3.1 PF00172.16	57.50	0.00
TRINITY_Q241V8_TETTS/718-905	Q241V8.2 PF00613.18	57.50	0.00
TRINITY_A0A0G4EYE2_9ALVE/19-94	A0A0G4EYE2.1 PF0093	57.50	0.00
TRINITY_L8HFE9_ACACA/3-181	L8HFE9.1 PF13236.4;	57.50	0.00
TRINITY_J9I4X4_9SPIT/1061-1316	J9I4X4.1 PF00069.23	57.50	0.00
TRINITY_A0A0N4XCC7_NIPBR/3-253	A0A0N4XCC7.1 PF0037	57.50	0.00
TRINITY_F2U7V2_SALR5/92-306	F2U7V2.1 PF01786.15	57.50	0.00
TRINITY_M0RJC8_MUSAM/108-180	M0RJC8.1 PF00076.20	57.50	0.00
TRINITY_C6SWC1_SOYBN/9-121	C6SWC1.1 PF01984.18	57.50	0.00
TRINITY_D3BLJ5_POLPA/1315-1446	D3BLJ5.1 PF08477.11	57.50	0.00
TRINITY_H8N2F2_CORCM/104-196	H8N2F2.1 PF13640.4;	57.50	0.00
TRINITY_MYLKB_DICDI/20-278	Q86AD7.1 PF00069.23	57.50	0.00
TRINITY_A0A0G3A017_9DELT/39-267	A0A0G3A017.1 PF0048	57.50	0.00
TRINITY_L8H2S3_ACACA/18-72	L8H2S3.1 PF14813.4;	57.50	0.00
TRINITY_F6HT97_VITVI/91-430	F6HT97.1 PF01546.26	57.50	0.00
TRINITY_A9UR38_MONBE/5-142	A9UR38.1 PF00179.24	57.50	0.00
TRINITY_H2A2_PICGU/4-90	A5DJJ2.1 PF00125.22	57.50	0.00
TRINITY_J9G2U8_9SPIT/24-114	J9G2U8.1 PF00153.25	57.50	0.00
TRINITY_D3B4M5_POLPA/129-174	D3B4M5.1 PF09360.8;	57.50	0.00
TRINITY_I7M1V6_TETTS/314-533	I7M1V6.1 PF07002.14	57.50	0.00
TRINITY_D8TWK4_VOLCA/491-577	D8TWK4.1 PF04232.10	57.50	0.00
TRINITY_A0A072NXK1_9EURO/5-192	A0A072NXK1.1 PF0010	57.50	0.00
TRINITY_I0YW85_9CHLO/2-252	I0YW85.1 PF00218.19	57.50	0.00
TRINITY_A8JGM1_CHLRE/218-338	A8JGM1.1 PF00581.18	57.50	0.00
TRINITY_D8TMB4_VOLCA/171-394	D8TMB4.1 PF16953.3;	57.50	0.00
TRINITY_A0A087SPY8_AUXPR/251-525	A0A087SPY8.1 PF0291	57.50	0.00
TRINITY_I0Z9F9_9CHLO/439-555	I0Z9F9.1 PF05064.11	57.50	0.00
TRINITY_B4H991_DROPE/139-212	B4H991.1 PF12861.5;	57.50	0.00
TRINITY_F1A4V5_DICPU/233-486	F1A4V5.1 PF07714.15	57.50	0.00
TRINITY_G0R1C4_ICHMG/49-235	G0R1C4.1 PF10211.7;	57.50	0.00
TRINITY_H2CAX0_9LEPT/178-396	H2CAX0.1 PF00120.22	57.50	0.00
TRINITY_A0A0D9MX36_ASPFL/286-390	A0A0D9MX36.1 PF0030	57.50	0.00
TRINITY_D8TIB7_VOLCA/36-439	D8TIB7.1 PF01150.15	57.50	0.00
TRINITY_A8JGZ8_CHLRE/33-118	A8JGZ8.1 PF03795.12	57.50	0.00
TRINITY_A0DSS8_PARTE/4-75	A0DSS8.1 PF15906.3;	57.50	0.00
TRINITY_D8TKU7_VOLCA/132-339	D8TKU7.1 PF00300.20	57.50	0.00
TRINITY_D8U4A8_VOLCA/140-199	D8U4A8.1 PF00137.19	57.50	0.00
TRINITY_MYLKB_DICDI/20-278	Q86AD7.1 PF00069.23	57.50	0.00
TRINITY_D8TMG1_VOLCA/789-869	D8TMG1.1 PF10431.7;	57.50	0.00
TRINITY_E4UN52_ARTGP/42-227	E4UN52.1 PF04055.19	57.50	0.00
TRINITY_Q23JD9_TETTS/681-821	Q23JD9.3 PF07718.10	57.50	0.00
TRINITY_A0A087ST35_AUXPR/103-356	A0A087ST35.1 PF1346	57.50	0.00
TRINITY_H3H405_PHYRM/37-151	H3H405.1 PF00665.24	57.50	0.00
TRINITY_I7MHV9_TETTS/142-248	I7MHV9.2 PF04857.18	57.50	0.00
TRINITY_D8TUT9_VOLCA/1-195	D8TUT9.1 PF03159.16	57.50	0.00
TRINITY_E1ZG54_CHLVA/38-210	E1ZG54.1 PF00856.26	57.50	0.00
TRINITY_V4TVT0_9ROSI/84-377	V4TVT0.1 PF05185.14	57.50	0.00
TRINITY_W2SCP1_9EURO/803-1296	W2SCP1.1 PF02463.17	57.50	0.00
TRINITY_D8U9I9_VOLCA/104-190	D8U9I9.1 PF04719.12	57.50	0.00
TRINITY_I7LXR5_TETTS/227-312	I7LXR5.1 PF08542.9;	57.50	0.00
TRINITY_F2ACK3_RHIET/646-719	F2ACK3.1 PF00575.21	57.50	0.00
TRINITY_A0D750_PARTE/381-527	A0D750.1 PF03031.16	57.50	0.00
TRINITY_G3Q7G3_GASAC/183-328	G3Q7G3.1 PF00856.26	57.40	0.00

TRINITY_C5LTF4_PERM5/86-148	C5LTF4.1 PF13499.4;	57.40	0.00
TRINITY_D3AWY3_POLPA/164-210	D3AWY3.1 PF01585.21	57.40	0.00
TRINITY_A0A0G4G959_9ALVE/6-133	A0A0G4G959.1 PF0111	57.40	0.00
TRINITY_M1LXF0_9PROT/150-600	M1LXF0.1 PF01131.18	57.40	0.00
TRINITY_Q7QIH6_ANOGA/17-126	Q7QIH6.3 PF00651.29	57.40	0.00
TRINITY_K1P9S7_CRAGI/54-229	K1P9S7.1 PF04427.16	57.40	0.00
TRINITY_I7M0N6_TETTS/161-343	I7M0N6.1 PF04678.11	57.40	0.00
TRINITY_A0A0C2XP57_BACBA/17-186	A0A0C2XP57.1 PF0088	57.40	0.00
TRINITY_A0A0M9DYW8_9DELT/250-366	A0A0M9DYW8.1 PF0251	57.40	0.00
TRINITY_B9SNT8_RICCO/1030-1139	B9SNT8.1 PF00005.25	57.40	0.00
TRINITY_A0A0A0LKP7_CUCSA/67-317	A0A0A0LKP7.1 PF0515	57.40	0.00
TRINITY_A0A068RJJ4_9FUNG/154-202	A0A068RJJ4.1 PF1598	57.40	0.00
TRINITY_A0A0D2WWS7_CAPO3/58-459	A0A0D2WWS7.1 PF0557	57.40	0.00
TRINITY_A0A067CCZ7_SAPPC/230-458	A0A067CCZ7.1 PF0170	57.40	0.00
TRINITY_Q233Z3_TETTS/947-1211	Q233Z3.2 PF00069.23	57.40	0.00
TRINITY_I1FL82_AMPQE/474-536	I1FL82.1 PF13432.4;	57.40	0.00
TRINITY_G7DWQ1_MIXOS/278-403	G7DWQ1.1 PF00004.27	57.40	0.00
TRINITY_G0QUI0_ICHMG/278-349	G0QUI0.1 PF00498.24	57.40	0.00
TRINITY_A8JFJ3_CHLRE/162-254	A8JFJ3.1 PF05684.10	57.40	0.00
TRINITY_A9TK03_PHYPA/460-553	A9TK03.1 PF14578.4;	57.40	0.00
TRINITY_R4GEK9_DANRE/100-220	R4GEK9.1 PF01980.14	57.40	0.00
TRINITY_Q239R2_TETTS/10-57	Q239R2.2 PF00319.16	57.40	0.00
TRINITY_A8IDR1_CHLRE/59-159	A8IDR1.1 PF03330.16	57.40	0.00
TRINITY_E1Z307_CHLVA/23-382	E1Z307.1 PF01231.16	57.40	0.00
TRINITY_A8HRZ0_CHLRE/47-116	A8HRZ0.1 PF00538.17	57.40	0.00
TRINITY_C5XSH9_SORBI/8-261	C5XSH9.1 PF05116.11	57.40	0.00
TRINITY_D8TYS8_VOLCA/1-137	D8TYS8.1 PF02527.13	57.40	0.00
TRINITY_L8HGY3_ACACA/42-251	L8HGY3.1 PF05023.12	57.40	0.00
TRINITY_Q22MB3_TETTS/37-521	Q22MB3.3 PF00118.22	57.40	0.00
TRINITY_D8TRJ9_VOLCA/14-234	D8TRJ9.1 PF04012.10	57.40	0.00
TRINITY_A0A0J8D2W5_BETVU/290-546	A0A0J8D2W5.1 PF0171	57.40	0.00
TRINITY_E0W224_PEDHC/4-85	E0W224.1 PF12796.5;	57.40	0.00
TRINITY_G0QPV5_ICHMG/91-228	G0QPV5.1 PF14671.4;	57.40	0.00
TRINITY_D3BT40_POLPA/479-585	D3BT40.1 PF00307.29	57.40	0.00
TRINITY_EPN_DICDI/19-141	Q54EH1.1 PF01417.18	57.40	0.00
TRINITY_E4V3H0_ARTGP/597-670	E4V3H0.1 PF10405.7;	57.40	0.00
TRINITY_D8UFI1_VOLCA/32-328	D8UFI1.1 PF00246.22	57.40	0.00
TRINITY_D8UDF3_VOLCA/894-1029	D8UDF3.1 PF13472.4;	57.40	0.00
TRINITY_Q8YRK3_NOSS1/427-682	Q8YRK3.1 PF00425.16	57.40	0.00
TRINITY_D8UH56_VOLCA/268-335	D8UH56.1 PF00347.21	57.40	0.00
TRINITY_G7Y450_CLOSI/319-372	G7Y450.1 PF00400.30	57.40	0.00
TRINITY_A8I3I6_CHLRE/67-485	A8I3I6.1 PF01266.22	57.40	0.00
TRINITY_A8J8G2_CHLRE/1-322	A8J8G2.1 PF07690.14	57.40	0.00
TRINITY_A8J3P3_CHLRE/1-295	A8J3P3.1 PF08449.9;	57.40	0.00
TRINITY_D8TNW8_VOLCA/29-302	D8TNW8.1 PF01073.17	57.40	0.00
TRINITY_F2UBE5_SALR5/5-166	F2UBE5.1 PF00071.20	57.40	0.00
TRINITY_I7LV68_TETTS/201-381	I7LV68.1 PF00534.18	57.40	0.00
TRINITY_Q23PY2_TETTS/45-118	Q23PY2.2 PF02798.18	57.40	0.00
TRINITY_I0ZAK7_9CHLO/38-179	I0ZAK7.1 PF00561.18	57.40	0.00
TRINITY_Q23FM4_TETTS/126-440	Q23FM4.1 PF00009.25	57.40	0.00
TRINITY_D8U7I1_VOLCA/1020-1138	D8U7I1.1 PF02466.17	57.40	0.00
TRINITY_A0A067JC81_JATCU/108-271	A0A067JC81.1 PF0176	57.40	0.00
TRINITY_Q23RK4_TETTS/1793-2067	Q23RK4.2 PF00069.23	57.40	0.00
TRINITY_A8JFK2_CHLRE/13-113	A8JFK2.1 PF01738.16	57.40	0.00
TRINITY_I7M1M3_TETTS/515-650	I7M1M3.2 PF00005.25	57.40	0.00
TRINITY_D8U5A8_VOLCA/79-306	D8U5A8.1 PF00069.23	57.40	0.00
TRINITY_F4PQQ2_DICFS/9-262	F4PQQ2.1 PF00069.23	57.40	0.00
TRINITY_M0TYW1_MUSAM/165-261	M0TYW1.1 PF08241.10	57.40	0.00
TRINITY_DNAJ_LACRD/5-66	A5VJE8.1 PF00226.29	57.40	0.00
TRINITY_E1Z6X5_CHLVA/472-776	E1Z6X5.1 PF07690.14	57.40	0.00
TRINITY_D7G7W1_ECTSI/280-355	D7G7W1.1 PF14437.4;	57.40	0.00

TRINITY_F7AX64_CALJA/58-219	F7AX64.1 PF00071.20	57.40	0.00
TRINITY_D8TJV1_VOLCA/246-390	D8TJV1.1 PF00271.29	57.40	0.00
TRINITY_D8TTV8_VOLCA/81-324	D8TTV8.1 PF00233.17	57.40	0.00
TRINITY_G8Y0G9_PICSO/1-94	G8Y0G9.1 PF01199.16	57.40	0.00
TRINITY_I7LXC9_TETTS/121-511	I7LXC9.1 PF00155.19	57.30	0.00
TRINITY_A0A0F6YLI4_9DELT/74-167	A0A0F6YLI4.1 PF0255	57.30	0.00
TRINITY_M5W4R7_PRUPE/93-315	M5W4R7.1 PF01255.17	57.30	0.00
TRINITY_A0A0G4FZ83_9ALVE/13-108	A0A0G4FZ83.1 PF0016	57.30	0.00
TRINITY_G0QVG9_ICHMG/32-198	G0QVG9.1 PF00071.20	57.30	0.00
TRINITY_L8HET6_ACACA/43-369	L8HET6.1 PF07992.12	57.30	0.00
TRINITY_J9HZN8_9SPIT/420-538	J9HZN8.1 PF00581.18	57.30	0.00
TRINITY_H2BU57_9FLAO/15-223	H2BU57.1 PF00106.23	57.30	0.00
TRINITY_A0A067QDS9_9HOMO/615-750	A0A067QDS9.1 PF0000	57.30	0.00
TRINITY_F0ZD36_DICPU/3232-3570	F0ZD36.1 PF12777.5;	57.30	0.00
TRINITY_A6C3U5_9PLAN/115-458	A6C3U5.1 PF00152.18	57.30	0.00
TRINITY_M7XC99_RHOT1/988-1131	M7XC99.1 PF12842.5;	57.30	0.00
TRINITY_G0R213_ICHMG/186-430	G0R213.1 PF01433.18	57.30	0.00
TRINITY_I7M0N5_TETTS/125-295	I7M0N5.1 PF00092.26	57.30	0.00
TRINITY_A0DJ78_PARTE/1961-2231	A0DJ78.1 PF12775.5;	57.30	0.00
TRINITY_A7RV33_NEMVE/13-133	A7RV33.1 PF01124.16	57.30	0.00
TRINITY_F4PVW8_DICFS/49-173	F4PVW8.1 PF00782.18	57.30	0.00
TRINITY_A8J6L3_CHLRE/82-185	A8J6L3.1 PF00355.24	57.30	0.00
TRINITY_A0A015JAT3_9GLOM/292-422	A0A015JAT3.1 PF0000	57.30	0.00
TRINITY_D8TI00_VOLCA/18-121	D8TI00.1 PF00466.18	57.30	0.00
TRINITY_A0A0G0IMV6_9BACT/8-139	A0A0G0IMV6.1 PF0146	57.30	0.00
TRINITY_E1ZR28_CHLVA/100-466	E1ZR28.1 PF02906.12	57.30	0.00
TRINITY_F2UFQ3_SALR5/2479-2561	F2UFQ3.1 PF05406.13	57.30	0.00
TRINITY_F2UGU8_SALR5/3442-4146	F2UGU8.1 PF03028.13	57.30	0.00
TRINITY_I7LT49_TETTS/89-213	I7LT49.2 PF01926.21	57.30	0.00
TRINITY_D3BR25_POLPA/200-289	D3BR25.1 PF00153.25	57.30	0.00
TRINITY_A0EFQ7_PARTE/75-330	A0EFQ7.1 PF00069.23	57.30	0.00
TRINITY_E1ZC52_CHLVA/15-403	E1ZC52.1 PF01433.18	57.30	0.00
TRINITY_L8GTQ9_ACACA/202-506	L8GTQ9.1 PF00443.27	57.30	0.00
TRINITY_D8U6T6_VOLCA/1-119	D8U6T6.1 PF12796.5;	57.30	0.00
TRINITY_D2VMS7_NAEGR/158-405	D2VMS7.1 PF00756.18	57.30	0.00
TRINITY_E1ZN16_CHLVA/259-441	E1ZN16.1 PF05193.19	57.30	0.00
TRINITY_D8UIH0_VOLCA/82-265	D8UIH0.1 PF13506.4;	57.30	0.00
TRINITY_L8H3W5_ACACA/971-1093	L8H3W5.1 PF00271.29	57.30	0.00
TRINITY_A8IDU2_CHLRE/877-1024	A8IDU2.1 PF03330.16	57.30	0.00
TRINITY_D8TJR4_VOLCA/503-639	D8TJR4.1 PF00892.18	57.30	0.00
TRINITY_D8U2G1_VOLCA/130-249	D8U2G1.1 PF01553.19	57.30	0.00
TRINITY_A0CYK6_PARTE/20-129	A0CYK6.1 PF00255.17	57.30	0.00
TRINITY_A9TT62_PHYPA/30-132	A9TT62.1 PF00355.24	57.30	0.00
TRINITY_D8U0R7_VOLCA/48-409	D8U0R7.1 PF07690.14	57.30	0.00
TRINITY_W4YY43_STRPU/1-194	W4YY43.1 PF13087.4;	57.30	0.00
TRINITY_A0A087SLT3_AUXPR/8-175	A0A087SLT3.1 PF0398	57.30	0.00
TRINITY_Q235L2_TETTS/74-150	Q235L2.1 PF03134.17	57.30	0.00
TRINITY_A0DN21_PARTE/1115-1266	A0DN21.1 PF00005.25	57.30	0.00
TRINITY_A8I6Q2_CHLRE/10-242	A8I6Q2.1 PF08392.10	57.30	0.00
TRINITY_V4B0M8_LOTGI/419-833	V4B0M8.1 PF00702.24	57.30	0.00
TRINITY_M1MTR0_9CLOT/74-285	M1MTR0.1 PF07859.11	57.30	0.00
TRINITY_I0Z0T0_9CHLO/131-282	I0Z0T0.1 PF00588.17	57.30	0.00
TRINITY_J9HVL7_9SPIT/73-307	J9HVL7.1 PF01674.16	57.30	0.00
TRINITY_I0YK42_9CHLO/4-285	I0YK42.1 PF00069.23	57.30	0.00
TRINITY_I7M699_TETTS/80-309	I7M699.1 PF00557.22	57.20	0.00
TRINITY_L8H2Q9_ACACA/7-182	L8H2Q9.1 PF00071.20	57.20	0.00
TRINITY_A5KCF1_PLAVS/5-177	A5KCF1.1 PF00025.19	57.20	0.00
TRINITY_D5EN24_CORAD/1-160	D5EN24.1 PF01408.20	57.20	0.00
TRINITY_D8TSN5_VOLCA/38-217	D8TSN5.1 PF03798.14	57.20	0.00
TRINITY_D8UJW0_VOLCA/85-273	D8UJW0.1 PF07714.15	57.20	0.00
TRINITY_G0R6H5_ICHMG/16-510	G0R6H5.1 PF00478.23	57.20	0.00

TRINITY_D3BB15_POLPA/229-481	D3BB15.1 PF00348.15	57.20	0.00
TRINITY_L8H2S4_ACACA/76-413	L8H2S4.1 PF08627.8;	57.20	0.00
TRINITY_G0R409_ICHMG/421-565	G0R409.1 PF00005.25	57.20	0.00
TRINITY_B8HSK0_CYAP4/26-176	B8HSK0.1 PF02481.13	57.20	0.00
TRINITY_E1ZLF7_CHLVA/28-387	E1ZLF7.1 PF16499.3;	57.20	0.00
TRINITY_I0YIQ3_9CHLO/3-277	I0YIQ3.1 PF00370.19	57.20	0.00
TRINITY_F4NRF8_BATDJ/944-1152	F4NRF8.1 PF05362.11	57.20	0.00
TRINITY_D8TSE2_VOLCA/53-220	D8TSE2.1 PF08819.9;	57.20	0.00
TRINITY_A0A098VY53_9MICR/66-218	A0A098VY53.1 PF0000	57.20	0.00
TRINITY_A0A0K9PUS7_ZOSMR/4-377	A0A0K9PUS7.1 PF0002	57.20	0.00
TRINITY_A0A0G4J1G0_PLABS/106-352	A0A0G4J1G0.1 PF0012	57.20	0.00
TRINITY_D8UAB7_VOLCA/60-464	D8UAB7.1 PF00450.20	57.20	0.00
TRINITY_L8H3Y8_ACACA/315-570	L8H3Y8.1 PF00069.23	57.20	0.00
TRINITY_A8IL75_CHLRE/24-301	A8IL75.1 PF06454.9;	57.20	0.00
TRINITY_J9JJ17_ACYPI/10-171	J9JJ17.1 PF00071.20	57.20	0.00
TRINITY_G0QP95_ICHMG/6-183	G0QP95.1 PF05677.10	57.10	0.00
TRINITY_A0CY63_PARTE/292-365	A0CY63.1 PF00027.27	57.10	0.00
TRINITY_W2PTU3_PHYPN/17-113	W2PTU3.1 PF00708.16	57.10	0.00
TRINITY_A0A0G4GH78_9ALVE/276-543	A0A0G4GH78.1 PF0120	57.10	0.00
TRINITY_S7QMW6_GLOTA/32-147	S7QMW6.1 PF08240.10	57.10	0.00
TRINITY_A0A0E3UWF6_9BACT/52-529	A0A0E3UWF6.1 PF0120	57.10	0.00
TRINITY_A0A078B4M6_STYLE/289-456	A0A078B4M6.1 PF0053	57.10	0.00
TRINITY_Q3SDK9_PARTE/13-174	Q3SDK9.1 PF00071.20	57.10	0.00
TRINITY_E6X099_NITSE/253-371	E6X099.1 PF02518.24	57.10	0.00
TRINITY_J9EG36_9SPIT/111-295	J9EG36.1 PF00270.27	57.10	0.00
TRINITY_A0A068RVW5_9FUNG/1289-1437	A0A068RVW5.1 PF0000	57.10	0.00
TRINITY_L8H823_ACACA/184-369	L8H823.1 PF07065.12	57.10	0.00
TRINITY_B4VZR7_9CYAN/533-655	B4VZR7.1 PF02518.24	57.10	0.00
TRINITY_COAE_BACAN/3-184	Q6HSG2.1 PF01121.18	57.10	0.00
TRINITY_A0EHM3_PARTE/2237-2575	A0EHM3.1 PF12777.5;	57.10	0.00
TRINITY_F0SM98_RUBBR/33-177	F0SM98.1 PF00160.19	57.10	0.00
TRINITY_G0QW14_ICHMG/165-434	G0QW14.1 PF00928.19	57.10	0.00
TRINITY_G0R1C5_ICHMG/3-376	G0R1C5.1 PF00022.17	57.10	0.00
TRINITY_I7ME97_TETTS/54-318	I7ME97.1 PF00069.23	57.10	0.00
TRINITY_A0A077C591_9RICK/4-61	A0A077C591.1 PF0820	57.10	0.00
TRINITY_G0QJL0_ICHMG/3-152	G0QJL0.1 PF06602.12	57.10	0.00
TRINITY_A0A078AVM5_STYLE/320-363	A0A078AVM5.1 PF0056	57.10	0.00
TRINITY_S7RV90_GLOTA/2-92	S7RV90.1 PF00153.25	57.10	0.00
TRINITY_TALA_DICDI/163-275	P0CE94.1 PF00373.16	57.10	0.00
TRINITY_G0R099_ICHMG/58-319	G0R099.1 PF03034.13	57.10	0.00
TRINITY_I7LW20_TETTS/148-308	I7LW20.2 PF03031.16	57.10	0.00
TRINITY_L8H642_ACACA/613-682	L8H642.1 PF01480.15	57.10	0.00
TRINITY_I2F868_9BACT/3-157	I2F868.1 PF01327.19	57.10	0.00
TRINITY_A0DTP8_PARTE/77-166	A0DTP8.1 PF16531.3;	57.10	0.00
TRINITY_A0A094F571_9PEZI/310-422	A0A094F571.1 PF0066	57.10	0.00
TRINITY_L8GUB9_ACACA/284-439	L8GUB9.1 PF13768.4;	57.10	0.00
TRINITY_Q1GRJ8_SPHAL/1-286	Q1GRJ8.1 PF00579.23	57.10	0.00
TRINITY_A0D1K9_PARTE/24-590	A0D1K9.1 PF01602.18	57.10	0.00
TRINITY_A3XP40_LEEBM/28-145	A3XP40.1 PF08240.10	57.10	0.00
TRINITY_I7MMA1_TETTS/1281-1428	I7MMA1.2 PF00005.25	57.10	0.00
TRINITY_A0A0N4UEW4_DRAME/61-102	A0A0N4UEW4.1 PF1363	57.10	0.00
TRINITY_G0QNE2_ICHMG/99-359	G0QNE2.1 PF00069.23	57.10	0.00
TRINITY_A4VD54_TETTS/1086-1248	A4VD54.1 PF00005.25	57.10	0.00
TRINITY_G0QVI6_ICHMG/266-473	G0QVI6.1 PF03133.13	57.10	0.00
TRINITY_A0A098EUG0_9BACI/4-122	A0A098EUG0.1 PF1323	57.10	0.00
TRINITY_I7M198_TETTS/52-316	I7M198.2 PF00069.23	57.10	0.00
TRINITY_I0Z997_9CHLO/17-136	I0Z997.1 PF13671.4;	57.10	0.00
TRINITY_U9UB77_RHIID/104-402	U9UB77.1 PF01866.15	57.10	0.00
TRINITY_Q53QL2_ORYSJ/1085-1197	Q53QL2.1 PF00665.24	57.10	0.00
TRINITY_D8UBS2_VOLCA/5-73	D8UBS2.1 PF13921.4;	57.10	0.00
TRINITY_A0D8S8_PARTE/638-896	A0D8S8.1 PF07714.15	57.10	0.00

TRINITY_F0ZJ03_DICPU/494-612	F0ZJ03.1 PF01412.16	57.10	0.00
TRINITY_F2U3N6_SALR5/48-132	F2U3N6.1 PF08240.10	57.10	0.00
TRINITY_Q22Y34_TETTS/17-463	Q22Y34.1 PF02127.13	57.10	0.00
TRINITY_D8TSL6_VOLCA/36-126	D8TSL6.1 PF01241.16	57.10	0.00
TRINITY_W5MWV1_LEPOC/272-471	W5MWV1.1 PF13901.4;	57.10	0.00
TRINITY_A0A0K9PHI9_ZOSMR/94-282	A0A0K9PHI9.1 PF0975	57.10	0.00
TRINITY_C6XKL0_HIRBI/7-61	C6XKL0.1 PF16177.3;	57.10	0.00
TRINITY_A4RTE2_OSTLU/31-370	A4RTE2.1 PF04997.10	57.10	0.00
TRINITY_A0EEN4_PARTE/1565-1920	A0EEN4.1 PF12777.5;	57.10	0.00
TRINITY_A0BDU6_PARTE/323-596	A0BDU6.1 PF02913.17	57.10	0.00
TRINITY_Q23A17_TETTS/139-248	Q23A17.1 PF00168.28	57.10	0.00
TRINITY_L8GN02_ACACA/559-628	L8GN02.1 PF00017.22	57.10	0.00
TRINITY_Q23GZ7_TETTS/211-419	Q23GZ7.2 PF00112.21	57.10	0.00
TRINITY_D8TUC1_VOLCA/8-180	D8TUC1.1 PF00566.16	57.10	0.00
TRINITY_D8U9Z0_VOLCA/954-1167	D8U9Z0.1 PF05153.13	57.10	0.00
TRINITY_M0WFQ1_HORVD/44-111	M0WFQ1.1 PF16209.3;	57.10	0.00
TRINITY_A8J7H2_CHLRE/1264-1437	A8J7H2.1 PF07228.10	57.10	0.00
TRINITY_Q5KHY4_CRYNJ/528-577	Q5KHY4.1 PF14604.4;	57.10	0.00
TRINITY_D8SVI8_SELML/691-882	D8SVI8.1 PF00488.19	57.10	0.00
TRINITY_A0A024TF60_9STRA/347-446	A0A024TF60.1 PF0016	57.10	0.00
TRINITY_E1ZKX2_CHLVA/8-198	E1ZKX2.1 PF13460.4;	57.10	0.00
TRINITY_A0A0A1T3C9_9HYPO/440-598	A0A0A1T3C9.1 PF0000	57.10	0.00
TRINITY_G0QWH7_ICHMG/52-110	G0QWH7.1 PF08063.10	57.10	0.00
TRINITY_Q7NUF0_CHRVO/47-137	Q7NUF0.1 PF08241.10	57.10	0.00
TRINITY_A0A087SIT8_AUXPR/25-103	A0A087SIT8.1 PF0142	57.10	0.00
TRINITY_B3RKH4_TRIAD/45-507	B3RKH4.1 PF01593.22	57.10	0.00
TRINITY_A8IFT4_CHLRE/30-162	A8IFT4.1 PF14908.4;	57.10	0.00
TRINITY_I0YS12_9CHLO/16-86	I0YS12.1 PF00076.20	57.10	0.00
TRINITY_D8UD04_VOLCA/2-346	D8UD04.1 PF00443.27	57.10	0.00
TRINITY_D8TZD6_VOLCA/11-124	D8TZD6.1 PF06058.11	57.10	0.00
TRINITY_I1G374_AMPQE/612-798	I1G374.1 PF00211.18	57.10	0.00
TRINITY_A8J5U5_CHLRE/85-240	A8J5U5.1 PF00650.18	57.10	0.00
TRINITY_D8UEG1_VOLCA/25-627	D8UEG1.1 PF00995.21	57.10	0.00
TRINITY_A8JB50_CHLRE/10-265	A8JB50.1 PF00069.23	57.10	0.00
TRINITY_E1ZB42_CHLVA/13-89	E1ZB42.1 PF00575.21	57.10	0.00
TRINITY_C1E8Y5_MICSR/20-201	C1E8Y5.1 PF00483.21	57.10	0.00
TRINITY_T1K5B5_TETUR/776-1081	T1K5B5.1 PF00632.23	57.10	0.00
TRINITY_A8ISN7_CHLRE/59-260	A8ISN7.1 PF06862.10	57.10	0.00
TRINITY_I0YKL1_9CHLO/222-315	I0YKL1.1 PF00153.25	57.10	0.00
TRINITY_D8QR38_SELML/15-176	D8QR38.1 PF00270.27	57.10	0.00
TRINITY_F4PYX8_DICFS/631-809	F4PYX8.1 PF00617.17	57.10	0.00
TRINITY_Q23U84_TETTS/23-120	Q23U84.1 PF02678.14	57.10	0.00
TRINITY_I0YJU5_9CHLO/516-725	I0YJU5.1 PF03441.12	57.10	0.00
TRINITY_Q22KG5_TETTS/34-173	Q22KG5.3 PF00782.18	57.10	0.00
TRINITY_D8TR90_VOLCA/25-230	D8TR90.1 PF05700.9;	57.10	0.00
TRINITY_I0Z904_9CHLO/17-303	I0Z904.1 PF03416.17	57.10	0.00
TRINITY_D8U978_VOLCA/2-203	D8U978.1 PF07714.15	57.10	0.00
TRINITY_A8I807_CHLRE/1018-1101	A8I807.1 PF00211.18	57.10	0.00
TRINITY_D8TTV8_VOLCA/81-324	D8TTV8.1 PF00233.17	57.10	0.00
TRINITY_D8TW00_VOLCA/697-980	D8TW00.1 PF00664.21	57.10	0.00
TRINITY_W0EN80_9PORP/5-192	W0EN80.1 PF04011.10	57.10	0.00
TRINITY_A0BQ47_PARTE/3-95	A0BQ47.1 PF03259.15	57.00	0.00
TRINITY_L8HM13_ACACA/10-146	L8HM13.1 PF04756.11	57.00	0.00
TRINITY_A0A078AVJ8_STYLE/16-272	A0A078AVJ8.1 PF0006	57.00	0.00
TRINITY_F1A482_DICPU/1767-1976	F1A482.1 PF16095.3;	57.00	0.00
TRINITY_A0A0A1U6N2_ENTIV/1420-1674	A0A0A1U6N2.1 PF0771	57.00	0.00
TRINITY_C3Z661_BRAFL/3-348	C3Z661.1 PF02515.15	57.00	0.00
TRINITY_Q86K39_DICDI/356-457	Q86K39.1 PF12894.5;	57.00	0.00
TRINITY_D8TK01_VOLCA/12-360	D8TK01.1 PF01148.18	57.00	0.00
TRINITY_I7MHC3_TETTS/22-425	I7MHC3.2 PF01433.18	57.00	0.00
TRINITY_B7GD27_PHATC/164-234	B7GD27.1 PF00382.17	57.00	0.00

TRINITY_L8GUH2_ACACA/267-438	L8GUH2.1 PF01979.18	57.00	0.00
TRINITY_C3XRT7_BRAFL/49-255	C3XRT7.1 PF00106.23	57.00	0.00
TRINITY_Q23TS6_TETTS/41-176	Q23TS6.2 PF00005.25	57.00	0.00
TRINITY_A8HQJ9_CHLRE/14-109	A8HQJ9.1 PF06916.11	57.00	0.00
TRINITY_D8UED2_VOLCA/2081-2182	D8UED2.1 PF01477.21	57.00	0.00
TRINITY_D8TQN2_VOLCA/16-126	D8TQN2.1 PF03070.14	57.00	0.00
TRINITY_D8TNB9_VOLCA/33-337	D8TNB9.1 PF03006.18	57.00	0.00
TRINITY_C4LSY9_ENTHI/11-169	C4LSY9.1 PF00071.20	57.00	0.00
TRINITY_A0A0K6G6D9_9HOMO/306-907	A0A0K6G6D9.1 PF0519	57.00	0.00
TRINITY_A8INS0_CHLRE/4-845	A8INS0.1 PF16399.3;	57.00	0.00
TRINITY_J9IGI9_9SPIT/49-304	J9IGI9.1 PF00069.23	57.00	0.00
TRINITY_D8THQ9_VOLCA/1195-1302	D8THQ9.1 PF10367.7;	57.00	0.00
TRINITY_L1JQA2_GUITH/374-479	L1JQA2.1 PF00085.18	57.00	0.00
TRINITY_Q231W2_TETTS/18-753	Q231W2.1 PF03030.14	57.00	0.00
TRINITY_A8HQC1_CHLRE/216-346	A8HQC1.1 PF02338.17	57.00	0.00
TRINITY_I7M3W2_TETTS/27-163	I7M3W2.2 PF00782.18	57.00	0.00
TRINITY_D3BS03_POLPA/40-147	D3BS03.1 PF00635.24	57.00	0.00
TRINITY_D8TVH0_VOLCA/91-285	D8TVH0.1 PF13875.4;	57.00	0.00
TRINITY_W4FH29_9STRA/206-638	W4FH29.1 PF01532.18	57.00	0.00
TRINITY_D8U030_VOLCA/188-279	D8U030.1 PF11919.6;	57.00	0.00
TRINITY_A6LNE4_THEM4/221-510	A6LNE4.1 PF02906.12	57.00	0.00
TRINITY_D8TM63_VOLCA/78-222	D8TM63.1 PF00810.16	57.00	0.00
TRINITY_D8TU52_VOLCA/1-101	D8TU52.1 PF14226.4;	57.00	0.00
TRINITY_A8IPU0_CHLRE/155-397	A8IPU0.1 PF03878.13	57.00	0.00
TRINITY_L8GSC4_ACACA/45-257	L8GSC4.1 PF08245.10	57.00	0.00
TRINITY_D8UB01_VOLCA/95-192	D8UB01.1 PF01588.18	57.00	0.00
TRINITY_A8IB22_CHLRE/22-195	A8IB22.1 PF14825.4;	57.00	0.00
TRINITY_D8TMD0_VOLCA/54-312	D8TMD0.1 PF00069.23	57.00	0.00
TRINITY_F4PT49_DICFS/46-223	F4PT49.1 PF00719.17	57.00	0.00
TRINITY_A8I6F3_CHLRE/385-587	A8I6F3.1 PF07714.15	57.00	0.00
TRINITY_D8UBW5_VOLCA/500-606	D8UBW5.1 PF13519.4;	57.00	0.00
TRINITY_C1DIX7_AZOVD/10-205	C1DIX7.1 PF00753.25	57.00	0.00
TRINITY_B7JVL0_CYAP8/1-129	B7JVL0.1 PF14437.4;	57.00	0.00
TRINITY_A8JFH1_CHLRE/93-580	A8JFH1.1 PF00759.17	57.00	0.00
TRINITY_B8C1N5_THAPS/11-175	B8C1N5.1 PF00270.27	57.00	0.00
TRINITY_A0A087SCV9_AUXPR/36-318	A0A087SCV9.1 PF0006	57.00	0.00
TRINITY_E0UDP4_CYAP2/129-203	E0UDP4.1 PF01169.17	56.90	0.00
TRINITY_A0A087SR84_AUXPR/814-890	A0A087SR84.1 PF0134	56.90	0.00
TRINITY_R1C984_EMIHU/989-1276	R1C984.1 PF08811.9;	56.90	0.00
TRINITY_F4Q879_DICFS/343-583	F4Q879.1 PF00122.18	56.90	0.00
TRINITY_E1X552_HALMS/50-182	E1X552.1 PF01380.20	56.90	0.00
TRINITY_A0E309_PARTE/27-98	A0E309.1 PF16209.3;	56.90	0.00
TRINITY_C5D3X8_GEOSW/2-205	C5D3X8.1 PF01323.18	56.90	0.00
TRINITY_T1G126_HELRO/39-90	T1G126.1 PF04588.11	56.90	0.00
TRINITY_Q22T22_TETTS/520-735	Q22T22.3 PF07859.11	56.90	0.00
TRINITY_M4EKQ8_BRARP/82-247	M4EKQ8.1 PF13012.4;	56.90	0.00
TRINITY_F4PXA4_DICFS/1-331	F4PXA4.1 PF01207.15	56.90	0.00
TRINITY_F2G9F8_ALTMD/624-740	F2G9F8.1 PF02518.24	56.90	0.00
TRINITY_I7M967_TETTS/407-548	I7M967.1 PF04377.13	56.90	0.00
TRINITY_D3BU33_POLPA/111-235	D3BU33.1 PF04495.12	56.90	0.00
TRINITY_T0SBM8_9STRA/625-911	T0SBM8.1 PF00128.22	56.90	0.00
TRINITY_R4FNG2_RHOPR/51-244	R4FNG2.1 PF00753.25	56.90	0.00
TRINITY_J9IJ53_9SPIT/76-190	J9IJ53.1 PF00622.26	56.90	0.00
TRINITY_A0A078AI59_STYLE/31-95	A0A078AI59.1 PF1349	56.90	0.00
TRINITY_A0A0M0JB56_9EUKA/14-175	A0A0M0JB56.1 PF0007	56.90	0.00
TRINITY_A0A078A1V0_STYLE/258-516	A0A078A1V0.1 PF0006	56.90	0.00
TRINITY_E1ZQ85_CHLVA/21-84	E1ZQ85.1 PF01176.17	56.90	0.00
TRINITY_A8JBG1_CHLRE/74-210	A8JBG1.1 PF13716.4;	56.90	0.00
TRINITY_A0A067Q0T2_9HOMO/33-148	A0A067Q0T2.1 PF0824	56.90	0.00
TRINITY_F4PLJ9_DICFS/696-840	F4PLJ9.1 PF01553.19	56.90	0.00
TRINITY_A8JCT0_CHLRE/35-235	A8JCT0.1 PF01027.18	56.90	0.00

TRINITY_A0EB06_PARTE/1-70	A0EB06.1 PF01302.23	56.90	0.00
TRINITY_A8IT21_CHLRE/54-219	A8IT21.1 PF02622.13	56.90	0.00
TRINITY_W9QMK2_9ROSA/322-692	W9QMK2.1 PF00890.22	56.90	0.00
TRINITY_I0YVF7_9CHLO/505-604	I0YVF7.1 PF00300.20	56.90	0.00
TRINITY_Q22YS0_TETTS/11-186	Q22YS0.1 PF05603.10	56.90	0.00
TRINITY_A0DCS5_PARTE/76-348	A0DCS5.1 PF00248.19	56.90	0.00
TRINITY_M7NNX9_PNEMU/12-172	M7NNX9.1 PF00071.20	56.90	0.00
TRINITY_Q6UP31_CHLRE/49-151	Q6UP31.1 PF05071.14	56.90	0.00
TRINITY_A0A078AAI9_STYLE/21-285	A0A078AAI9.1 PF0165	56.90	0.00
TRINITY_G0QJD2_ICHMG/409-632	G0QJD2.1 PF13236.4;	56.90	0.00
TRINITY_A0BZD8_PARTE/231-430	A0BZD8.1 PF13851.4;	56.90	0.00
TRINITY_A0A075AS09_9FUNG/185-265	A0A075AS09.1 PF0400	56.90	0.00
TRINITY_A0A0K0DKC6_ANGCA/267-394	A0A0K0DKC6.1 PF0023	56.90	0.00
TRINITY_U6LJF0_9EIME/8-73	U6LJF0.1 PF01246.18	56.90	0.00
TRINITY_A8N0R4_COPC7/3-111	A8N0R4.1 PF03966.14	56.90	0.00
TRINITY_I0YJW0_9CHLO/18-124	I0YJW0.1 PF14765.4;	56.90	0.00
TRINITY_Q22T22_TETTS/520-735	Q22T22.3 PF07859.11	56.90	0.00
TRINITY_I2GE79_9BACT/5-172	I2GE79.1 PF00535.24	56.90	0.00
TRINITY_F4PLI7_DICFS/1581-2117	F4PLI7.1 PF03571.13	56.90	0.00
TRINITY_J9I6A1_9SPIT/73-210	J9I6A1.1 PF01966.20	56.90	0.00
TRINITY_E1Z438_CHLVA/277-336	E1Z438.1 PF13855.4;	56.90	0.00
TRINITY_D8U1T7_VOLCA/393-574	D8U1T7.1 PF09273.9;	56.90	0.00
TRINITY_D8TX27_VOLCA/36-313	D8TX27.1 PF00481.19	56.90	0.00
TRINITY_A8IH64_CHLRE/46-108	A8IH64.1 PF17136.2;	56.90	0.00
TRINITY_A0BHS7_PARTE/77-199	A0BHS7.1 PF00638.16	56.90	0.00
TRINITY_D8U2U8_VOLCA/40-107	D8U2U8.1 PF00013.27	56.90	0.00
TRINITY_D8UJY1_VOLCA/524-588	D8UJY1.1 PF00536.28	56.90	0.00
TRINITY_D8U252_VOLCA/5-52	D8U252.1 PF12906.5;	56.90	0.00
TRINITY_A8JF24_CHLRE/5-76	A8JF24.1 PF04969.14	56.90	0.00
TRINITY_A8IZ74_CHLRE/179-435	A8IZ74.1 PF03781.14	56.90	0.00
TRINITY_A0BIL8_PARTE/1261-1405	A0BIL8.1 PF12842.5;	56.90	0.00
TRINITY_CFA45_CHLRE/140-488	A8I9E8.1 PF13868.4;	56.90	0.00
TRINITY_I0YNX6_9CHLO/3-292	I0YNX6.1 PF05890.10	56.90	0.00
TRINITY_D8STS7_SELML/825-1160	D8STS7.1 PF12777.5;	56.90	0.00
TRINITY_A0A087SU36_AUXPR/3-309	A0A087SU36.1 PF0050	56.90	0.00
TRINITY_Q22HG2_TETTS/74-222	Q22HG2.1 PF04442.12	56.90	0.00
TRINITY_C9RD40_AMMDK/6-118	C9RD40.1 PF00072.22	56.80	0.00
TRINITY_A0A0K9NRU0_ZOSMR/130-174	A0A0K9NRU0.1 PF1363	56.80	0.00
TRINITY_A0A077ZN15_STYLE/119-156	A0A077ZN15.1 PF0142	56.80	0.00
TRINITY_I7LTY4_TETTS/276-599	I7LTY4.2 PF04515.10	56.80	0.00
TRINITY_U9UTZ8_RHIID/86-324	U9UTZ8.1 PF00899.19	56.80	0.00
TRINITY_E6ZR54_SPORE/209-444	E6ZR54.1 PF00069.23	56.80	0.00
TRINITY_A0A015LBX0_9GLOM/11-97	A0A015LBX0.1 PF1023	56.80	0.00
TRINITY_D3BHH6_POLPA/16-194	D3BHH6.1 PF03357.19	56.80	0.00
TRINITY_D2VIA4_NAEGR/392-466	D2VIA4.1 PF14492.4;	56.80	0.00
TRINITY_A0A0B0NP41_GOSAR/40-125	A0A0B0NP41.1 PF0698	56.80	0.00
TRINITY_A0E8S4_PARTE/1-104	A0E8S4.1 PF12763.5;	56.80	0.00
TRINITY_V3ZA01_LOTGI/240-411	V3ZA01.1 PF01068.19	56.80	0.00
TRINITY_A0A0G4J1G0_PLABS/106-352	A0A0G4J1G0.1 PF0012	56.80	0.00
TRINITY_A0A084WUY1_ANOSI/27-70	A0A084WUY1.1 PF0040	56.80	0.00
TRINITY_F7BXQ2_ORNAN/101-144	F7BXQ2.1 PF00569.15	56.80	0.00
TRINITY_G0QR70_ICHMG/24-173	G0QR70.1 PF00782.18	56.80	0.00
TRINITY_F0ZJW2_DICPU/563-743	F0ZJW2.1 PF02145.13	56.80	0.00
TRINITY_D8U077_VOLCA/4-112	D8U077.1 PF12998.5;	56.80	0.00
TRINITY_Q24HH9_TETTS/52-312	Q24HH9.2 PF00069.23	56.80	0.00
TRINITY_A0A0D2QGM6_GOSRA/224-355	A0A0D2QGM6.1 PF0004	56.80	0.00
TRINITY_A0A088AMB1_APIME/35-225	A0A088AMB1.1 PF0114	56.80	0.00
TRINITY_L8GTG1_ACACA/168-315	L8GTG1.1 PF00588.17	56.80	0.00
TRINITY_L8GUM9_ACACA/46-140	L8GUM9.1 PF01158.16	56.80	0.00
TRINITY_A0A072V8C4_MEDTR/140-183	A0A072V8C4.1 PF1363	56.80	0.00
TRINITY_E1Z921_CHLVA/46-198	E1Z921.1 PF00929.22	56.80	0.00

TRINITY_I7LTD5_TETTS/235-367	I7LTD5.2 PF14580.4;	56.80	0.00
TRINITY_A8JB02_CHLRE/91-278	A8JB02.1 PF02698.15	56.80	0.00
TRINITY_J9ICA8_9SPIT/818-1100	J9ICA8.1 PF00481.19	56.80	0.00
TRINITY_Q23RI2_TETTS/875-1059	Q23RI2.2 PF00689.19	56.80	0.00
TRINITY_G0QK00_ICHMG/9-339	G0QK00.1 PF01148.18	56.80	0.00
TRINITY_A0A078G0Q8_BRANA/273-317	A0A078G0Q8.1 PF1363	56.80	0.00
TRINITY_D8U0P8_VOLCA/37-131	D8U0P8.1 PF13905.4;	56.80	0.00
TRINITY_I7LXS6_TETTS/57-758	I7LXS6.2 PF00133.20	56.80	0.00
TRINITY_A8INB8_CHLRE/529-609	A8INB8.1 PF01253.20	56.80	0.00
TRINITY_A0A0P7YEZ4_9TELE/146-540	A0A0P7YEZ4.1 PF0113	56.80	0.00
TRINITY_G6D343_DANPL/652-725	G6D343.1 PF10405.7;	56.80	0.00
TRINITY_G2E1S7_9GAMM/532-648	G2E1S7.1 PF02518.24	56.80	0.00
TRINITY_I0YRN9_9CHLO/94-384	I0YRN9.1 PF00487.22	56.80	0.00
TRINITY_K3WRI4_PYTUL/1-142	K3WRI4.1 PF14753.4;	56.80	0.00
TRINITY_V4B2C3_LOTGI/12-106	V4B2C3.1 PF02214.20	56.80	0.00
TRINITY_U6LJD0_9EIME/21-65	U6LJD0.1 PF01585.21	56.80	0.00
TRINITY_A0A067L5Q9_JATCU/65-285	A0A067L5Q9.1 PF0027	56.80	0.00
TRINITY_G8BJW8_CANPC/6-167	G8BJW8.1 PF00071.20	56.80	0.00
TRINITY_D8UCP4_VOLCA/360-468	D8UCP4.1 PF00271.29	56.80	0.00
TRINITY_E1Z1W2_CHLVA/3-141	E1Z1W2.1 PF03800.12	56.80	0.00
TRINITY_I0YP56_9CHLO/10-161	I0YP56.1 PF00293.26	56.80	0.00
TRINITY_D8TUN2_VOLCA/1-188	D8TUN2.1 PF00069.23	56.80	0.00
TRINITY_D3BC33_POLPA/5-166	D3BC33.1 PF00071.20	56.80	0.00
TRINITY_C5Z583_SORBI/934-1179	C5Z583.1 PF07727.12	56.80	0.00
TRINITY_D8SPU1_SELML/284-446	D8SPU1.1 PF00270.27	56.80	0.00
TRINITY_D8TMP7_VOLCA/11-642	D8TMP7.1 PF03600.14	56.80	0.00
TRINITY_A0D1C3_PARTE/64-223	A0D1C3.1 PF01343.16	56.80	0.00
TRINITY_E0UFG5_CYAP2/7-202	E0UFG5.1 PF00106.23	56.80	0.00
TRINITY_Q23QY4_TETTS/796-1116	Q23QY4.2 PF03133.13	56.70	0.00
TRINITY_A0A068SEF3_9FUNG/34-341	A0A068SEF3.1 PF0337	56.70	0.00
TRINITY_J9IZ27_9SPIT/31-320	J9IZ27.1 PF04616.12	56.70	0.00
TRINITY_G0R4J0_ICHMG/295-619	G0R4J0.1 PF00493.21	56.70	0.00
TRINITY_Q22GW0_TETTS/1651-1813	Q22GW0.2 PF00069.23	56.70	0.00
TRINITY_K7MLD4_SOYBN/76-138	K7MLD4.1 PF09415.8;	56.70	0.00
TRINITY_J9J5F8_9SPIT/511-624	J9J5F8.1 PF02518.24	56.70	0.00
TRINITY_B4J7Z6_DROGR/290-495	B4J7Z6.1 PF00106.23	56.70	0.00
TRINITY_Q23QF2_TETTS/248-571	Q23QF2.2 PF03133.13	56.70	0.00
TRINITY_Q22U45_TETTS/225-538	Q22U45.1 PF00883.19	56.70	0.00
TRINITY_A0A078AHC7_STYLE/13-268	A0A078AHC7.1 PF0006	56.70	0.00
TRINITY_D8TKG8_VOLCA/103-423	D8TKG8.1 PF00398.18	56.70	0.00
TRINITY_A0A0D2X0P2_CAPO3/15-243	A0A0D2X0P2.1 PF1356	56.70	0.00
TRINITY_B0C408_ACAM1/40-133	B0C408.1 PF01243.18	56.70	0.00
TRINITY_A0A015J2R3_9GLOM/17-196	A0A015J2R3.1 PF1343	56.70	0.00
TRINITY_A0A0M0JR79_9EUKA/247-386	A0A0M0JR79.1 PF0139	56.70	0.00
TRINITY_D8LJ67_ECTSI/5-155	D8LJ67.1 PF00176.21	56.70	0.00
TRINITY_G8BTR7_TETPH/129-225	G8BTR7.1 PF04140.12	56.70	0.00
TRINITY_A8J4I9_CHLRE/17-270	A8J4I9.1 PF02900.16	56.70	0.00
TRINITY_A8ISQ5_CHLRE/941-1186	A8ISQ5.1 PF13087.4;	56.70	0.00
TRINITY_B2ACQ8_PODAN/66-442	B2ACQ8.1 PF00155.19	56.70	0.00
TRINITY_G0QZF2_ICHMG/23-289	G0QZF2.1 PF02263.17	56.70	0.00
TRINITY_D2VTI9_NAEGR/37-143	D2VTI9.1 PF00307.29	56.70	0.00
TRINITY_G0QKW1_ICHMG/111-281	G0QKW1.1 PF00092.26	56.70	0.00
TRINITY_I7M0N5_TETTS/125-295	I7M0N5.1 PF00092.26	56.70	0.00
TRINITY_R0GVR1_9BRAS/47-473	R0GVR1.1 PF01532.18	56.70	0.00
TRINITY_I0YJ19_9CHLO/201-328	I0YJ19.1 PF04366.10	56.70	0.00
TRINITY_Q22A31_TETTS/163-484	Q22A31.4 PF01207.15	56.70	0.00
TRINITY_A0A0D2WM34_CAPO3/100-531	A0A0D2WM34.1 PF0147	56.70	0.00
TRINITY_A0DC59_PARTE/3-141	A0DC59.1 PF01217.18	56.70	0.00
TRINITY_W9XM97_9EURO/41-128	W9XM97.1 PF07713.11	56.70	0.00
TRINITY_D8T044_SELML/198-454	D8T044.1 PF01704.16	56.70	0.00
TRINITY_L8HF73_ACACA/111-202	L8HF73.1 PF13426.5;	56.70	0.00

TRINITY_D8TZS8_VOLCA/96-370	D8TZS8.1 PF00685.25	56.70	0.00
TRINITY_A0E9L0_PARTE/73-288	A0E9L0.1 PF12317.6;	56.70	0.00
TRINITY_A0BR56_PARTE/569-683	A0BR56.1 PF00387.17	56.70	0.00
TRINITY_F4NRF8_BATDJ/944-1152	F4NRF8.1 PF05362.11	56.70	0.00
TRINITY_H2LJS9_ORYLA/16-322	H2LJS9.1 PF01556.16	56.70	0.00
TRINITY_Q6IYG1_CHLRE/156-401	Q6IYG1.1 PF01226.15	56.70	0.00
TRINITY_M2PRS3_CERS8/7-68	M2PRS3.1 PF00226.29	56.70	0.00
TRINITY_M7XE34_RHOT1/468-848	M7XE34.1 PF04109.14	56.70	0.00
TRINITY_A8J3P1_CHLRE/954-1161	A8J3P1.1 PF00211.18	56.70	0.00
TRINITY_A0C6I2_PARTE/147-212	A0C6I2.1 PF13499.4;	56.70	0.00
TRINITY_I0YWU5_9CHLO/707-938	I0YWU5.1 PF00005.25	56.70	0.00
TRINITY_G0QJS6_ICHMG/1-248	G0QJS6.1 PF03159.16	56.60	0.00
TRINITY_W7XI36_TETTS/344-791	W7XI36.1 PF03200.14	56.60	0.00
TRINITY_A0BUC8_PARTE/5-143	A0BUC8.1 PF00179.24	56.60	0.00
TRINITY_I7LTA1_TETTS/47-227	I7LTA1.1 PF01965.22	56.60	0.00
TRINITY_A0A072UTH7_MEDTR/65-168	A0A072UTH7.1 PF0023	56.60	0.00
TRINITY_Q23DI0_TETTS/65-244	Q23DI0.3 PF00535.24	56.60	0.00
TRINITY_H1VJD0_COLHI/32-109	H1VJD0.1 PF14821.4;	56.60	0.00
TRINITY_H0XZR6_OTOGA/23-105	H0XZR6.1 PF01336.23	56.60	0.00
TRINITY_J9GC40_9SPIT/788-1282	J9GC40.1 PF04998.15	56.60	0.00
TRINITY_D3BLA2_POLPA/49-377	D3BLA2.1 PF00128.22	56.60	0.00
TRINITY_A0A0F5AT32_9GAMM/449-560	A0A0F5AT32.1 PF0251	56.60	0.00
TRINITY_D8U5N3_VOLCA/4-176	D8U5N3.1 PF00929.22	56.60	0.00
TRINITY_F0ZJT2_DICPU/38-322	F0ZJT2.1 PF01207.15	56.60	0.00
TRINITY_A0A0D9V0U0_9ORYZ/1-326	A0A0D9V0U0.1 PF0450	56.60	0.00
TRINITY_N4U6Y1_FUSC1/73-209	N4U6Y1.1 PF14342.4;	56.60	0.00
TRINITY_A0E9Q1_PARTE/19-396	A0E9Q1.1 PF01053.18	56.60	0.00
TRINITY_A0A0F7N102_9ACTN/18-155	A0A0F7N102.1 PF0058	56.60	0.00
TRINITY_D8U023_VOLCA/49-190	D8U023.1 PF09335.9;	56.60	0.00
TRINITY_C3XTE8_BRAFL/65-760	C3XTE8.1 PF00063.19	56.60	0.00
TRINITY_D8TH09_VOLCA/55-197	D8TH09.1 PF01764.23	56.60	0.00
TRINITY_A0A067Q261_9HOMO/198-480	A0A067Q261.1 PF0500	56.60	0.00
TRINITY_W7TSN9_9STRA/70-230	W7TSN9.1 PF01451.19	56.60	0.00
TRINITY_E1Z6A2_CHLVA/331-642	E1Z6A2.1 PF08637.8;	56.60	0.00
TRINITY_E1ZRP5_CHLVA/44-345	E1ZRP5.1 PF04950.10	56.60	0.00
TRINITY_J9HSA4_9SPIT/276-557	J9HSA4.1 PF00481.19	56.60	0.00
TRINITY_A0CNI6_PARTE/314-523	A0CNI6.1 PF07002.14	56.60	0.00
TRINITY_D8UJM0_VOLCA/106-206	D8UJM0.1 PF03330.16	56.60	0.00
TRINITY_J9I3N5_9SPIT/288-363	J9I3N5.1 PF00076.20	56.60	0.00
TRINITY_Q23R92_TETTS/152-318	Q23R92.1 PF00071.20	56.60	0.00
TRINITY_A0A087SIL7_AUXPR/175-500	A0A087SIL7.1 PF0186	56.60	0.00
TRINITY_I1CSK8_RHIO9/13-66	I1CSK8.1 PF00385.22	56.60	0.00
TRINITY_A8J2K2_CHLRE/34-168	A8J2K2.1 PF02114.14	56.60	0.00
TRINITY_I0YLA5_9CHLO/2-167	I0YLA5.1 PF00005.25	56.60	0.00
TRINITY_W7X3C5_TETTS/169-244	W7X3C5.1 PF00789.18	56.60	0.00
TRINITY_D1AFY6_SEBTE/11-254	D1AFY6.1 PF08543.10	56.60	0.00
TRINITY_A8HMP3_CHLRE/281-499	A8HMP3.1 PF07774.11	56.60	0.00
TRINITY_D8TPX3_VOLCA/983-1066	D8TPX3.1 PF09324.8;	56.60	0.00
TRINITY_C1MRF4_MICPC/139-213	C1MRF4.1 PF00076.20	56.60	0.00
TRINITY_E1Z8K9_CHLVA/36-403	E1Z8K9.1 PF01276.18	56.60	0.00
TRINITY_C5LTF4_PERM5/86-148	C5LTF4.1 PF13499.4;	56.60	0.00
TRINITY_Q1IJR4_KORVE/50-364	Q1IJR4.1 PF00251.18	56.60	0.00
TRINITY_A0A0L1KUH4_9EUGL/17-343	A0A0L1KUH4.1 PF0000	56.60	0.00
TRINITY_A8HVZ5_CHLRE/96-382	A8HVZ5.1 PF00069.23	56.60	0.00
TRINITY_A8IHW3_CHLRE/282-550	A8IHW3.1 PF00520.29	56.60	0.00
TRINITY_F4PN45_DICFS/1769-1851	F4PN45.1 PF01833.22	56.60	0.00
TRINITY_E1ZL55_CHLVA/97-362	E1ZL55.1 PF00069.23	56.60	0.00
TRINITY_I7LTH0_TETTS/231-521	I7LTH0.1 PF01031.18	56.60	0.00
TRINITY_Q23WQ1_TETTS/51-171	Q23WQ1.3 PF02996.15	56.60	0.00
TRINITY_L8GJP4_ACACA/126-202	L8GJP4.1 PF06384.9;	56.60	0.00
TRINITY_D8U580_VOLCA/69-435	D8U580.1 PF02383.16	56.60	0.00

TRINITY_D8TTV8_VOLCA/81-324	D8TTV8.1 PF00233.17	56.60	0.00
TRINITY_D8TTV8_VOLCA/81-324	D8TTV8.1 PF00233.17	56.60	0.00
TRINITY_L7UL82_MYXSD/6-194	L7UL82.1 PF00106.23	56.60	0.00
TRINITY_DPOL_HBVCJ/601-843	Q69028.2 PF00336.16	56.60	0.00
TRINITY_Q1ZXK4_DICDI/7-55	Q1ZXK4.1 PF01679.15	56.50	0.00
TRINITY_C5L9M0_PERM5/63-132	C5L9M0.1 PF13499.4;	56.50	0.00
TRINITY_I7MGN3_TETTS/143-312	I7MGN3.1 PF01556.16	56.50	0.00
TRINITY_L9KTK3_TUPCH/396-587	L9KTK3.1 PF00587.23	56.50	0.00
TRINITY_Q23UD8_TETTS/821-870	Q23UD8.2 PF13920.4;	56.50	0.00
TRINITY_D3BU00_POLPA/7-62	D3BU00.1 PF00412.20	56.50	0.00
TRINITY_U1ZQL5_9BURK/517-630	U1ZQL5.2 PF02518.24	56.50	0.00
TRINITY_A0BXR0_PARTE/306-355	A0BXR0.1 PF13920.4;	56.50	0.00
TRINITY_A0A068S287_9FUNG/6-65	A0A068S287.1 PF0022	56.50	0.00
TRINITY_A0EHV9_PARTE/4-278	A0EHV9.1 PF03643.13	56.50	0.00
TRINITY_A0A077ZZ82_STYLE/17-255	A0A077ZZ82.1 PF0089	56.50	0.00
TRINITY_L8GVF1_ACACA/95-436	L8GVF1.1 PF01244.19	56.50	0.00
TRINITY_L5K396_PTEAL/915-1387	L5K396.1 PF06202.12	56.50	0.00
TRINITY_M2Y3H7_GALSU/69-317	M2Y3H7.1 PF00378.18	56.50	0.00
TRINITY_Q8Y8K3_LISMO/12-197	Q8Y8K3.1 PF09371.8;	56.50	0.00
TRINITY_Q54Y48_DICDI/54-183	Q54Y48.1 PF00561.18	56.50	0.00
TRINITY_D8TZL4_VOLCA/118-181	D8TZL4.1 PF13499.4;	56.50	0.00
TRINITY_Q22D97_TETTS/1219-1386	Q22D97.1 PF00005.25	56.50	0.00
TRINITY_L8H107_ACACA/542-664	L8H107.1 PF04153.16	56.50	0.00
TRINITY_TTL3C_TETTS/705-1030	Q23FE2.1 PF03133.13	56.50	0.00
TRINITY_G4VIN8_SCHMA/422-755	G4VIN8.1 PF03372.21	56.50	0.00
TRINITY_A9TR69_PHYPA/255-298	A9TR69.1 PF13639.4;	56.50	0.00
TRINITY_E1ZGD0_CHLVA/314-359	E1ZGD0.1 PF00646.31	56.50	0.00
TRINITY_A0A068RMS3_9FUNG/104-467	A0A068RMS3.1 PF0026	56.50	0.00
TRINITY_B3RLQ8_TRIAD/9-325	B3RLQ8.1 PF07992.12	56.50	0.00
TRINITY_A0A059LG41_9CHLO/232-413	A0A059LG41.1 PF0027	56.50	0.00
TRINITY_A0A0L6V5C6_9BASI/107-175	A0A0L6V5C6.1 PF0022	56.50	0.00
TRINITY_A8I4W2_CHLRE/1-181	A8I4W2.1 PF00211.18	56.50	0.00
TRINITY_C4M9I2_ENTHI/311-357	C4M9I2.1 PF14604.4;	56.50	0.00
TRINITY_A0A0A1MD11_9BACI/7-124	A0A0A1MD11.1 PF0058	56.50	0.00
TRINITY_C1EI16_MICSR/132-379	C1EI16.1 PF02475.14	56.50	0.00
TRINITY_A0A059LJY7_9CHLO/64-125	A0A059LJY7.1 PF0295	56.50	0.00
TRINITY_I0Z657_9CHLO/15-263	I0Z657.1 PF00149.26	56.50	0.00
TRINITY_A0CWK3_PARTE/1832-1933	A0CWK3.1 PF12796.5;	56.50	0.00
TRINITY_F4Q9B2_DICFS/36-104	F4Q9B2.1 PF00076.20	56.50	0.00
TRINITY_B3MPW9_DROAN/206-266	B3MPW9.1 PF13921.4;	56.50	0.00
TRINITY_A0A0G4EJ55_9ALVE/242-541	A0A0G4EJ55.1 PF0942	56.50	0.00
TRINITY_A0A078ARV0_STYLE/502-593	A0A078ARV0.1 PF1289	56.50	0.00
TRINITY_R1CP06_EMIHU/11-154	R1CP06.1 PF00071.20	56.50	0.00
TRINITY_G0QR87_ICHMG/122-270	G0QR87.1 PF00817.18	56.50	0.00
TRINITY_A8ISE1_CHLRE/178-331	A8ISE1.1 PF06963.10	56.50	0.00
TRINITY_A0A088AAZ0_APIME/237-405	A0A088AAZ0.1 PF0492	56.50	0.00
TRINITY_I7M1S5_TETTS/8-201	I7M1S5.1 PF00149.26	56.50	0.00
TRINITY_I7MLI1_TETTS/1260-1444	I7MLI1.2 PF00005.25	56.50	0.00
TRINITY_D8TJ08_VOLCA/9-196	D8TJ08.1 PF00106.23	56.50	0.00
TRINITY_A0BW49_PARTE/79-330	A0BW49.1 PF00069.23	56.50	0.00
TRINITY_F3ZXB2_MAHA5/6-321	F3ZXB2.1 PF00389.28	56.50	0.00
TRINITY_H3GG74_PHYRM/74-440	H3GG74.1 PF01266.22	56.50	0.00
TRINITY_D3B6M3_POLPA/229-533	D3B6M3.1 PF00082.20	56.50	0.00
TRINITY_F0ZSA3_DICPU/25-166	F0ZSA3.1 PF09286.9;	56.50	0.00
TRINITY_E1ZQH6_CHLVA/17-108	E1ZQH6.1 PF06831.12	56.50	0.00
TRINITY_Q22AQ5_TETTS/371-416	Q22AQ5.1 PF13639.4;	56.50	0.00
TRINITY_E1ZC64_CHLVA/294-347	E1ZC64.1 PF13821.4;	56.50	0.00
TRINITY_F2TYB8_SALR5/166-420	F2TYB8.1 PF01358.16	56.50	0.00
TRINITY_G0QYY5_ICHMG/38-207	G0QYY5.1 PF13847.4;	56.50	0.00
TRINITY_A0A015L9B6_9GLOM/3-226	A0A015L9B6.1 PF0187	56.50	0.00
TRINITY_K3WIE3_PYTUL/441-526	K3WIE3.1 PF00439.23	56.50	0.00

TRINITY_D8TYP9_VOLCA/272-328	D8TYP9.1 PF07576.10	56.50	0.00
TRINITY_D8U7A0_VOLCA/276-367	D8U7A0.1 PF07540.9;	56.50	0.00
TRINITY_B0WL15_CULQU/916-1063	B0WL15.1 PF00005.25	56.50	0.00
TRINITY_A0A087SN34_AUXPR/433-529	A0A087SN34.1 PF1400	56.50	0.00
TRINITY_A8IZF1_CHLRE/32-101	A8IZF1.1 PF01480.15	56.50	0.00
TRINITY_K9SV99_9SYNE/229-400	K9SV99.1 PF00204.23	56.50	0.00
TRINITY_D8U9H2_VOLCA/8-391	D8U9H2.1 PF03097.16	56.50	0.00
TRINITY_D8TW13_VOLCA/585-635	D8TW13.1 PF00847.18	56.50	0.00
TRINITY_D8U9V6_VOLCA/76-298	D8U9V6.1 PF03531.12	56.50	0.00
TRINITY_I0Z0M5_9CHLO/2-126	I0Z0M5.1 PF04031.11	56.50	0.00
TRINITY_A8I7L6_CHLRE/263-616	A8I7L6.1 PF03016.13	56.50	0.00
TRINITY_E1Z7K2_CHLVA/29-386	E1Z7K2.1 PF00704.26	56.50	0.00
TRINITY_D8TNL7_VOLCA/339-763	D8TNL7.1 PF03372.21	56.50	0.00
TRINITY_G2PLE0_MURRD/6-68	G2PLE0.1 PF06902.9;	56.50	0.00
TRINITY_B3E2J7_GEOLS/5-304	B3E2J7.1 PF00698.19	56.40	0.00
TRINITY_C3XYC3_BRAFL/5-378	C3XYC3.1 PF00022.17	56.40	0.00
TRINITY_F4QB04_DICFS/230-484	F4QB04.1 PF00069.23	56.40	0.00
TRINITY_A0DVJ8_PARTE/823-943	A0DVJ8.1 PF00069.23	56.40	0.00
TRINITY_A0A0F5I701_9BACI/4-145	A0A0F5I701.1 PF0145	56.40	0.00
TRINITY_A0A015LL06_9GLOM/224-430	A0A015LL06.1 PF0143	56.40	0.00
TRINITY_K1PYU3_CRAGI/13-269	K1PYU3.1 PF00069.23	56.40	0.00
TRINITY_Q22P17_TETTS/146-401	Q22P17.3 PF00069.23	56.40	0.00
TRINITY_F4Q0T3_DICFS/285-699	F4Q0T3.1 PF01373.15	56.40	0.00
TRINITY_F4PJP8_DICFS/360-420	F4PJP8.1 PF00571.26	56.40	0.00
TRINITY_Q23KI3_TETTS/3416-4122	Q23KI3.2 PF03028.13	56.40	0.00
TRINITY_A0A0D2UDJ0_CAPO3/193-604	A0A0D2UDJ0.1 PF0065	56.40	0.00
TRINITY_A0A078AB65_STYLE/310-782	A0A078AB65.1 PF0044	56.40	0.00
TRINITY_A8JET7_CHLRE/10-155	A8JET7.1 PF01217.18	56.40	0.00
TRINITY_A0A077ZUB0_STYLE/515-998	A0A077ZUB0.1 PF0023	56.40	0.00
TRINITY_A8HTT6_CHLRE/6-60	A8HTT6.1 PF05347.13	56.40	0.00
TRINITY_L8HEJ0_ACACA/46-141	L8HEJ0.1 PF10046.7;	56.40	0.00
TRINITY_Q22W19_TETTS/115-323	Q22W19.3 PF00112.21	56.40	0.00
TRINITY_M2XZ12_GALSU/232-284	M2XZ12.1 PF13639.4;	56.40	0.00
TRINITY_D8TQW1_VOLCA/73-212	D8TQW1.1 PF03188.14	56.40	0.00
TRINITY_A8HXJ4_CHLRE/89-561	A8HXJ4.1 PF00939.17	56.40	0.00
TRINITY_A8JB14_CHLRE/440-619	A8JB14.1 PF02734.15	56.40	0.00
TRINITY_E1ZGP5_CHLVA/978-1270	E1ZGP5.1 PF13621.4;	56.40	0.00
TRINITY_I7MD14_TETTS/11-177	I7MD14.1 PF00071.20	56.40	0.00
TRINITY_D2VB32_NAEGR/32-70	D2VB32.1 PF00172.16	56.40	0.00
TRINITY_Q23A17_TETTS/301-520	Q23A17.1 PF07002.14	56.40	0.00
TRINITY_A0EHU5_PARTE/441-644	A0EHU5.1 PF00454.25	56.40	0.00
TRINITY_W5LE58_ASTMX/270-502	W5LE58.1 PF00198.21	56.40	0.00
TRINITY_G0QT51_ICHMG/105-433	G0QT51.1 PF00676.18	56.40	0.00
TRINITY_I0Z1Z9_9CHLO/189-441	I0Z1Z9.1 PF00809.20	56.40	0.00
TRINITY_D3AW13_POLPA/79-419	D3AW13.1 PF01494.17	56.40	0.00
TRINITY_A0CRZ5_PARTE/180-218	A0CRZ5.1 PF04810.13	56.40	0.00
TRINITY_K3ZVR6_SETIT/13-52	K3ZVR6.1 PF01428.14	56.40	0.00
TRINITY_Q8S6Z2_ORYSJ/980-1121	Q8S6Z2.1 PF00078.25	56.40	0.00
TRINITY_L8HK05_ACACA/283-533	L8HK05.1 PF00069.23	56.40	0.00
TRINITY_A8J9D1_CHLRE/1-152	A8J9D1.1 PF00588.17	56.40	0.00
TRINITY_V4UAJ7_9ROSI/161-416	V4UAJ7.1 PF05193.19	56.40	0.00
TRINITY_H0YR09_TAEGU/495-601	H0YR09.1 PF02847.15	56.40	0.00
TRINITY_I7ML74_TETTS/5678-6228	I7ML74.2 PF00899.19	56.40	0.00
TRINITY_A8JF16_CHLRE/28-294	A8JF16.1 PF00069.23	56.40	0.00
TRINITY_L8GWI1_ACACA/131-259	L8GWI1.1 PF08920.8;	56.40	0.00
TRINITY_E1Z4H3_CHLVA/1661-1859	E1Z4H3.1 PF13378.4;	56.40	0.00
TRINITY_A0A0N0NL56_9EURO/60-256	A0A0N0NL56.1 PF1348	56.40	0.00
TRINITY_K9VGI6_9CYAN/8-301	K9VGI6.1 PF00291.23	56.40	0.00
TRINITY_I7MHS0_TETTS/26-176	I7MHS0.1 PF04446.10	56.30	0.00
TRINITY_G0QV33_ICHMG/81-320	G0QV33.1 PF00443.27	56.30	0.00
TRINITY_A0CXP2_PARTE/13-372	A0CXP2.1 PF01992.14	56.30	0.00

TRINITY_G1XR80_ARTOA/12-95	G1XR80.1 PF01221.16	56.30	0.00
TRINITY_L8GGP9_ACACA/18-187	L8GGP9.1 PF01923.16	56.30	0.00
TRINITY_D6U7R5_9CHLR/11-187	D6U7R5.1 PF00857.18	56.30	0.00
TRINITY_K1VQR1_TRIAC/143-213	K1VQR1.1 PF06741.11	56.30	0.00
TRINITY_F0ZRT4_DICPU/137-269	F0ZRT4.1 PF04116.11	56.30	0.00
TRINITY_O93325_CHICK/2-88	O93325.1 PF00125.22	56.30	0.00
TRINITY_L8HHR2_ACACA/1-660	L8HHR2.1 PF00063.19	56.30	0.00
TRINITY_F4NUZ2_BATDJ/3-112	F4NUZ2.1 PF00168.28	56.30	0.00
TRINITY_I7MGE9_TETTS/1162-1351	I7MGE9.2 PF00488.19	56.30	0.00
TRINITY_I7M483_TETTS/113-361	I7M483.1 PF01063.17	56.30	0.00
TRINITY_E1ZM59_CHLVA/232-413	E1ZM59.1 PF00160.19	56.30	0.00
TRINITY_Q01B63_OSTTA/364-434	Q01B63.1 PF00076.20	56.30	0.00
TRINITY_D8TKM2_VOLCA/17-87	D8TKM2.1 PF00076.20	56.30	0.00
TRINITY_B6K341_SCHJY/21-455	B6K341.1 PF02127.13	56.30	0.00
TRINITY_I1EPA9_AMPQE/11-98	I1EPA9.1 PF14216.4;	56.30	0.00
TRINITY_A8JEV7_CHLRE/99-216	A8JEV7.1 PF03367.11	56.30	0.00
TRINITY_M2T2S0_COCH5/23-450	M2T2S0.1 PF01937.17	56.30	0.00
TRINITY_A8J137_CHLRE/416-726	A8J137.1 PF01103.21	56.30	0.00
TRINITY_D8U1U1_VOLCA/14-201	D8U1U1.1 PF01227.20	56.30	0.00
TRINITY_D8TVF4_VOLCA/25-437	D8TVF4.1 PF01704.16	56.30	0.00
TRINITY_A8JCM8_CHLRE/16-589	A8JCM8.1 PF00995.21	56.30	0.00
TRINITY_A0A068RTH5_9FUNG/5-178	A0A068RTH5.1 PF0007	56.30	0.00
TRINITY_I7M6G5_TETTS/59-200	I7M6G5.2 PF08719.9;	56.30	0.00
TRINITY_A8IW64_CHLRE/56-142	A8IW64.1 PF00041.19	56.30	0.00
TRINITY_A8IVV9_CHLRE/238-371	A8IVV9.1 PF00258.23	56.30	0.00
TRINITY_A8IGR6_CHLRE/225-527	A8IGR6.1 PF04258.11	56.30	0.00
TRINITY_D8UG07_VOLCA/87-173	D8UG07.1 PF09793.7;	56.30	0.00
TRINITY_A0A089LV66_9BACL/4-116	A0A089LV66.1 PF0038	56.20	0.00
TRINITY_G0QRN4_ICHMG/6-105	G0QRN4.1 PF00153.25	56.20	0.00
TRINITY_Q3SDM6_PARTE/17-177	Q3SDM6.1 PF00071.20	56.20	0.00
TRINITY_G0R4F8_ICHMG/80-145	G0R4F8.1 PF00462.22	56.20	0.00
TRINITY_A0A0J8BS38_BETVU/8-71	A0A0J8BS38.1 PF0028	56.20	0.00
TRINITY_E1ZEF9_CHLVA/278-352	E1ZEF9.1 PF03110.12	56.20	0.00
TRINITY_I7MMG8_TETTS/8-169	I7MMG8.1 PF00071.20	56.20	0.00
TRINITY_C4LV99_ENTHI/13-76	C4LV99.1 PF00226.29	56.20	0.00
TRINITY_KIF3_DICDI/9-329	Q54UC9.1 PF00225.21	56.20	0.00
TRINITY_Q22BA7_TETTS/59-282	Q22BA7.1 PF07228.10	56.20	0.00
TRINITY_U9SYG8_RHIID/7-102	U9SYG8.1 PF05728.10	56.20	0.00
TRINITY_D3RQW6_ALLVD/164-268	D3RQW6.1 PF14748.4;	56.20	0.00
TRINITY_L8H286_ACACA/20-197	L8H286.1 PF03997.10	56.20	0.00
TRINITY_A0A0G3BNF0_9BURK/610-828	A0A0G3BNF0.1 PF0936	56.20	0.00
TRINITY_A0A0A1P208_9FUNG/115-327	A0A0A1P208.1 PF0155	56.20	0.00
TRINITY_T1KBR7_TETUR/161-209	T1KBR7.1 PF14604.4;	56.20	0.00
TRINITY_B2VWQ3_PYRTR/385-719	B2VWQ3.1 PF00176.21	56.20	0.00
TRINITY_I7M328_TETTS/23-132	I7M328.1 PF00787.22	56.20	0.00
TRINITY_F4PTE6_DICFS/521-632	F4PTE6.1 PF00072.22	56.20	0.00
TRINITY_D8TPR6_VOLCA/558-621	D8TPR6.1 PF08590.8;	56.20	0.00
TRINITY_I7MGY5_TETTS/3550-4238	I7MGY5.2 PF03028.13	56.20	0.00
TRINITY_A0A0A1NH74_9FUNG/78-129	A0A0A1NH74.1 PF0798	56.20	0.00
TRINITY_H8MT64_CORCM/655-766	H8MT64.1 PF00072.22	56.20	0.00
TRINITY_D0P397_PHYIT/201-459	D0P397.1 PF00069.23	56.20	0.00
TRINITY_L8GIH5_ACACA/118-300	L8GIH5.1 PF02146.15	56.20	0.00
TRINITY_L8GJV6_ACACA/286-373	L8GJV6.1 PF00789.18	56.20	0.00
TRINITY_A8HY85_CHLRE/42-92	A8HY85.1 PF13920.4;	56.20	0.00
TRINITY_L1I701_GUITH/69-270	L1I701.1 PF07002.14	56.20	0.00
TRINITY_D8U7K5_VOLCA/154-487	D8U7K5.1 PF00155.19	56.20	0.00
TRINITY_Q3SDK9_PARTE/13-174	Q3SDK9.1 PF00071.20	56.20	0.00
TRINITY_D8U6A9_VOLCA/77-347	D8U6A9.1 PF01193.22	56.20	0.00
TRINITY_D6TNU0_9CHLR/3-300	D6TNU0.1 PF01156.17	56.20	0.00
TRINITY_F1A1R9_DICPU/992-1069	F1A1R9.1 PF00626.20	56.20	0.00
TRINITY_B0WL15_CULQU/916-1063	B0WL15.1 PF00005.25	56.20	0.00

TRINITY_A8IK66_CHLRE/240-312	A8IK66.1 PF07743.11	56.20	0.00
TRINITY_D8TRT9_VOLCA/27-343	D8TRT9.1 PF00856.26	56.20	0.00
TRINITY_L8HHD7_ACACA/399-693	L8HHD7.1 PF01008.15	56.20	0.00
TRINITY_I0Z2I2_9CHLO/14-356	I0Z2I2.1 PF00009.25	56.20	0.00
TRINITY_R5Y7H5_9CLOT/4-325	R5Y7H5.1 PF16363.3;	56.20	0.00
TRINITY_G0R2Y4_ICHMG/103-174	G0R2Y4.1 PF04564.13	56.20	0.00
TRINITY_A0A077AUY9_9RICK/43-114	A0A077AUY9.1 PF0456	56.20	0.00
TRINITY_B9HRF2_POPTR/13-184	B9HRF2.2 PF03152.12	56.20	0.00
TRINITY_Q248A8_TETTS/75-338	Q248A8.2 PF08145.10	56.20	0.00
TRINITY_D8U978_VOLCA/2-203	D8U978.1 PF07714.15	56.20	0.00
TRINITY_F5HH60_NEUCR/868-998	F5HH60.1 PF00271.29	56.20	0.00
TRINITY_E1Z3T0_CHLVA/1-183	E1Z3T0.1 PF04072.12	56.20	0.00
TRINITY_L1INW0_GUITH/3-74	L1INW0.1 PF00240.21	56.20	0.00
TRINITY_A0A0A7LN38_9BACT/16-380	A0A0A7LN38.1 PF0026	56.20	0.00
TRINITY_H3AKG0_LATCH/49-338	H3AKG0.1 PF02219.15	56.20	0.00
TRINITY_D3BH48_POLPA/164-480	D3BH48.1 PF00728.20	56.20	0.00
TRINITY_I0Z502_9CHLO/30-503	I0Z502.1 PF10243.7;	56.20	0.00
TRINITY_A0A0D3AF02_BRAOL/4-67	A0A0D3AF02.1 PF0022	56.20	0.00
TRINITY_T1KGR1_TETUR/6-612	T1KGR1.1 PF00012.18	56.20	0.00
TRINITY_L8H2N3_ACACA/58-547	L8H2N3.1 PF02990.14	56.20	0.00
TRINITY_A8HV32_CHLRE/124-295	A8HV32.1 PF02582.12	56.20	0.00
TRINITY_I1FCH5_AMPQE/13-174	I1FCH5.1 PF00071.20	56.20	0.00
TRINITY_I4C4E4_DESTA/14-154	I4C4E4.1 PF01625.19	56.20	0.00
TRINITY_C1EBY4_MICSR/311-401	C1EBY4.1 PF12894.5;	56.20	0.00
TRINITY_J9J3D6_9SPIT/1-244	J9J3D6.1 PF01902.15	56.20	0.00
TRINITY_A0A087G4R9_ARAAL/635-730	A0A087G4R9.1 PF0158	56.20	0.00
TRINITY_L8GPZ7_ACACA/15-171	L8GPZ7.1 PF13563.4;	56.20	0.00
TRINITY_I1GC88_AMPQE/167-380	I1GC88.1 PF00112.21	56.20	0.00
TRINITY_D8UK64_VOLCA/8-471	D8UK64.1 PF13520.4;	56.20	0.00
TRINITY_A8IHW3_CHLRE/2254-2527	A8IHW3.1 PF00520.29	56.20	0.00
TRINITY_D3B1V2_POLPA/5-372	D3B1V2.1 PF00022.17	56.20	0.00
TRINITY_A0A089LV66_9BACL/4-116	A0A089LV66.1 PF0038	56.20	0.00
TRINITY_G0QXL9_ICHMG/177-426	G0QXL9.1 PF07714.15	56.10	0.00
TRINITY_A0CGP3_PARTE/111-168	A0CGP3.1 PF13921.4;	56.10	0.00
TRINITY_Q230X4_TETTS/30-358	Q230X4.2 PF16113.3;	56.10	0.00
TRINITY_A0D0V0_PARTE/71-112	A0D0V0.1 PF05186.11	56.10	0.00
TRINITY_S6EFY1_ZYGB2/247-337	S6EFY1.1 PF08544.11	56.10	0.00
TRINITY_Q23D11_TETTS/154-431	Q23D11.1 PF00069.23	56.10	0.00
TRINITY_Q5XJM7_DANRE/18-275	Q5XJM7.1 PF00248.19	56.10	0.00
TRINITY_L8H0M8_ACACA/8-74	L8H0M8.1 PF00076.20	56.10	0.00
TRINITY_J9I3N0_9SPIT/1-183	J9I3N0.1 PF00069.23	56.10	0.00
TRINITY_F0ZG66_DICPU/173-232	F0ZG66.1 PF00786.26	56.10	0.00
TRINITY_D8SSH7_SELML/15-372	D8SSH7.1 PF00176.21	56.10	0.00
TRINITY_F1A2J8_DICPU/1032-1088	F1A2J8.1 PF17103.3;	56.10	0.00
TRINITY_D8UFU5_VOLCA/644-695	D8UFU5.1 PF12906.5;	56.10	0.00
TRINITY_A8I807_CHLRE/1018-1101	A8I807.1 PF00211.18	56.10	0.00
TRINITY_L8HER1_ACACA/228-321	L8HER1.1 PF02148.17	56.10	0.00
TRINITY_I7MIQ2_TETTS/1022-1280	I7MIQ2.2 PF07714.15	56.10	0.00
TRINITY_D8U7H1_VOLCA/144-209	D8U7H1.1 PF13432.4;	56.10	0.00
TRINITY_A0CTX8_PARTE/90-309	A0CTX8.1 PF07970.10	56.10	0.00
TRINITY_X6N8Z0_RETFI/66-153	X6N8Z0.1 PF01541.22	56.10	0.00
TRINITY_L8GTU8_ACACA/76-547	L8GTU8.1 PF07969.9;	56.10	0.00
TRINITY_I7M4K8_TETTS/73-244	I7M4K8.2 PF01595.18	56.10	0.00
TRINITY_Q245G5_TETTS/3-166	Q245G5.2 PF08613.9;	56.10	0.00
TRINITY_C3YX76_BRAFL/17-261	C3YX76.1 PF03372.21	56.10	0.00
TRINITY_R1D5U8_EMIHU/83-299	R1D5U8.1 PF05023.12	56.10	0.00
TRINITY_F6RV21_ORNAN/110-245	F6RV21.1 PF04116.11	56.10	0.00
TRINITY_I0Z1R7_9CHLO/5-502	I0Z1R7.1 PF05091.10	56.10	0.00
TRINITY_D8U6H7_VOLCA/9-152	D8U6H7.1 PF07933.12	56.10	0.00
TRINITY_I7MHY1_TETTS/1126-1276	I7MHY1.2 PF00005.25	56.10	0.00
TRINITY_I7MHY1_TETTS/1126-1276	I7MHY1.2 PF00005.25	56.10	0.00

TRINITY_K6XR77_9ALTE/18-317	K6XR77.1 PF01156.17	56.10	0.00
TRINITY_C5GMC3_AJEDR/96-249	C5GMC3.1 PF13673.5;	56.10	0.00
TRINITY_A0A0K0DNX8_ANGCA/156-282	A0A0K0DNX8.1 PF1324	56.10	0.00
TRINITY_D8TGX6_VOLCA/877-1013	D8TGX6.1 PF01925.17	56.10	0.00
TRINITY_A8I2P4_CHLRE/206-295	A8I2P4.1 PF04488.13	56.10	0.00
TRINITY_D2UDI3_XANAP/14-122	D2UDI3.1 PF00856.26	56.10	0.00
TRINITY_I0YQY8_9CHLO/23-161	I0YQY8.1 PF00781.22	56.10	0.00
TRINITY_Q23DB7_TETTS/8-263	Q23DB7.2 PF00069.23	56.10	0.00
TRINITY_L8HAJ3_ACACA/30-143	L8HAJ3.1 PF01398.19	56.10	0.00
TRINITY_A0A087SQ95_AUXPR/6-172	A0A087SQ95.1 PF0778	56.10	0.00
TRINITY_T1HIN0_RHOPR/617-1085	T1HIN0.1 PF13844.4;	56.10	0.00
TRINITY_A0A061FR92_THECC/58-102	A0A061FR92.1 PF1392	56.10	0.00
TRINITY_D8UFU1_VOLCA/1-281	D8UFU1.1 PF00481.19	56.10	0.00
TRINITY_B4FJK8_MAIZE/8-69	B4FJK8.1 PF02320.14	56.10	0.00
TRINITY_K3WHU2_PYTUL/657-699	K3WHU2.1 PF00400.30	56.10	0.00
TRINITY_Q22A96_TETTS/376-441	Q22A96.2 PF00498.24	56.10	0.00
TRINITY_A8HQF7_CHLRE/968-1179	A8HQF7.1 PF01061.22	56.10	0.00
TRINITY_E1Z8Z0_CHLVA/13-192	E1Z8Z0.1 PF02223.15	56.10	0.00
TRINITY_D8SYE6_SELML/15-206	D8SYE6.1 PF07910.11	56.10	0.00
TRINITY_I7MAE9_TETTS/49-204	I7MAE9.1 PF13847.4;	56.10	0.00
TRINITY_A8HN19_CHLRE/83-198	A8HN19.1 PF05195.14	56.10	0.00
TRINITY_A8JIA0_CHLRE/148-349	A8JIA0.1 PF07478.11	56.10	0.00
TRINITY_J9HQD5_9SPIT/229-287	J9HQD5.1 PF00498.24	56.10	0.00
TRINITY_D2V2A3_NAEGR/415-919	D2V2A3.1 PF00899.19	56.10	0.00
TRINITY_A0A0M0L002_9EUKA/1987-2131	A0A0M0L002.1 PF0000	56.10	0.00
TRINITY_G3WMB5_SARHA/62-225	G3WMB5.1 PF08662.9;	56.10	0.00
TRINITY_J9J3B4_9SPIT/124-338	J9J3B4.1 PF00112.21	56.10	0.00
TRINITY_S8CKT9_9LAMI/21-340	S8CKT9.1 PF02733.15	56.10	0.00
TRINITY_I4ECZ1_9CHLR/3-117	I4ECZ1.1 PF06983.11	56.10	0.00
TRINITY_Q22P17_TETTS/11-125	Q22P17.3 PF00072.22	56.00	0.00
TRINITY_BGAL_SOLLC/30-336	P48980.1 PF01301.17	56.00	0.00
TRINITY_V4AWS4_LOTGI/3-64	V4AWS4.1 PF00226.29	56.00	0.00
TRINITY_A0A0D2WMX1_CAPO3/127-230	A0A0D2WMX1.1 PF0061	56.00	0.00
TRINITY_D3F3H7_CONWI/123-182	D3F3H7.1 PF13376.4;	56.00	0.00
TRINITY_L8H880_ACACA/28-209	L8H880.1 PF01183.18	56.00	0.00
TRINITY_D8UDD9_VOLCA/30-246	D8UDD9.1 PF00856.26	56.00	0.00
TRINITY_J9H4G8_9ACTN/395-544	J9H4G8.1 PF00005.25	56.00	0.00
TRINITY_D7FSC0_ECTSI/20-203	D7FSC0.1 PF02223.15	56.00	0.00
TRINITY_C7PPF7_CHIPD/460-723	C7PPF7.1 PF12972.5;	56.00	0.00
TRINITY_J9IMT2_9SPIT/146-246	J9IMT2.1 PF00153.25	56.00	0.00
TRINITY_J9FRH7_9SPIT/20-284	J9FRH7.1 PF01650.16	56.00	0.00
TRINITY_A0A0G2ZF02_9DELT/207-312	A0A0G2ZF02.1 PF0255	56.00	0.00
TRINITY_D1B7C5_THEAS/8-349	D1B7C5.1 PF00266.17	56.00	0.00
TRINITY_A0A087XGU5_POEFO/8-68	A0A087XGU5.1 PF0022	56.00	0.00
TRINITY_A0A0D2WQ34_CAPO3/2-186	A0A0D2WQ34.1 PF0273	56.00	0.00
TRINITY_Q22W19_TETTS/115-323	Q22W19.3 PF00112.21	56.00	0.00
TRINITY_A0CUS1_PARTE/180-292	A0CUS1.1 PF05708.10	56.00	0.00
TRINITY_A0CWV2_PARTE/507-606	A0CWV2.1 PF04815.13	56.00	0.00
TRINITY_K9X806_9CHRO/230-637	K9X806.1 PF01179.18	56.00	0.00
TRINITY_I1FHZ4_AMPQE/488-551	I1FHZ4.1 PF01485.19	56.00	0.00
TRINITY_A0A087XHP4_POEFO/512-659	A0A087XHP4.2 PF0911	56.00	0.00
TRINITY_A0CH03_PARTE/40-316	A0CH03.1 PF00664.21	56.00	0.00
TRINITY_I8UA81_9BACI/56-152	I8UA81.1 PF08241.10	56.00	0.00
TRINITY_D8U3Z3_VOLCA/6-409	D8U3Z3.1 PF03372.21	56.00	0.00
TRINITY_A8JEQ7_CHLRE/115-265	A8JEQ7.1 PF00581.18	56.00	0.00
TRINITY_D8TCM5_SELML/343-475	D8TCM5.1 PF01553.19	56.00	0.00
TRINITY_F0V8T6_NEOCL/5-88	F0V8T6.1 PF01221.16	56.00	0.00
TRINITY_A0A087SQ98_AUXPR/82-179	A0A087SQ98.1 PF0419	56.00	0.00
TRINITY_A8J937_CHLRE/104-222	A8J937.1 PF00188.24	56.00	0.00
TRINITY_W2RL69_9EURO/448-506	W2RL69.1 PF01485.19	56.00	0.00
TRINITY_A2SS06_METLZ/5-66	A2SS06.1 PF00226.29	56.00	0.00

TRINITY_D3B6M3_POLPA/229-533	D3B6M3.1 PF00082.20	56.00	0.00
TRINITY_A0A0D2WKR6_CAPO3/303-496	A0A0D2WKR6.1 PF0438	56.00	0.00
TRINITY_L8GQL5_ACACA/626-1215	L8GQL5.1 PF00821.16	56.00	0.00
TRINITY_I1BK57_RHIO9/844-1034	I1BK57.1 PF13087.4;	56.00	0.00
TRINITY_Q22X13_TETTS/1-295	Q22X13.2 PF08610.8;	56.00	0.00
TRINITY_A0CX64_PARTE/177-410	A0CX64.1 PF01798.16	56.00	0.00
TRINITY_D8UG46_VOLCA/440-574	D8UG46.1 PF00581.18	56.00	0.00
TRINITY_A0CLD6_PARTE/82-333	A0CLD6.1 PF00069.23	56.00	0.00
TRINITY_A0A077FR81_9RICK/8-137	A0A077FR81.1 PF0192	56.00	0.00
TRINITY_A0A0G4IH02_PLABS/188-236	A0A0G4IH02.1 PF1392	56.00	0.00
TRINITY_A8JI29_CHLRE/455-744	A8JI29.1 PF06046.11	56.00	0.00
TRINITY_I0YS50_9CHLO/95-197	I0YS50.1 PF02777.16	56.00	0.00
TRINITY_F4P7G3_BATDJ/1816-1998	F4P7G3.1 PF12348.6;	56.00	0.00
TRINITY_A0A0A0KJ41_CUCSA/48-198	A0A0A0KJ41.1 PF1429	55.90	0.00
TRINITY_B7FQH6_PHATC/4-92	B7FQH6.1 PF00153.25	55.90	0.00
TRINITY_H3CFC6_TETNG/1489-1604	H3CFC6.1 PF00271.29	55.90	0.00
TRINITY_F4QDR0_DICFS/205-312	F4QDR0.1 PF00175.19	55.90	0.00
TRINITY_C1MIT9_MICPC/12-181	C1MIT9.1 PF13439.4;	55.90	0.00
TRINITY_A6L9N3_PARD8/670-787	A6L9N3.1 PF00072.22	55.90	0.00
TRINITY_L8GS45_ACACA/110-198	L8GS45.1 PF00957.19	55.90	0.00
TRINITY_J9F9Y8_9SPIT/10-381	J9F9Y8.1 PF00022.17	55.90	0.00
TRINITY_G7WIC3_DESOD/8-126	G7WIC3.1 PF01575.17	55.90	0.00
TRINITY_DHE3_DICDI/217-453	Q54KB7.1 PF00208.19	55.90	0.00
TRINITY_G0QW95_ICHMG/250-437	G0QW95.1 PF02799.13	55.90	0.00

TRINITY_J9J0J0_9SPIT/1450-1691	J9J0J0.1 PF00520.29	55.90	0.00
TRINITY_F4PSS6_DICFS/422-518	F4PSS6.1 PF00153.25	55.90	0.00
TRINITY_D3AVR4_POLPA/17-262	D3AVR4.1 PF00069.23	55.90	0.00
TRINITY_A0DAI9_PARTE/12-124	A0DAI9.1 PF01412.16	55.90	0.00
TRINITY_A0A0J7KD70_LASNI/25-145	A0A0J7KD70.1 PF0289	55.90	0.00
TRINITY_Q758Y6_ASHGO/4-130	Q758Y6.1 PF00125.22	55.90	0.00
TRINITY_A0A022QYL1_ERYGU/109-178	A0A022QYL1.1 PF0697	55.90	0.00
TRINITY_I7M706_TETTS/16-370	I7M706.2 PF00225.21	55.90	0.00
TRINITY_A0CK56_PARTE/2-375	A0CK56.1 PF01222.15	55.90	0.00
TRINITY_D8UIG4_VOLCA/308-609	D8UIG4.1 PF10637.7;	55.90	0.00
TRINITY_A0E2M3_PARTE/3-103	A0E2M3.1 PF00168.28	55.90	0.00
TRINITY_D8TKG8_VOLCA/103-423	D8TKG8.1 PF00398.18	55.90	0.00
TRINITY_D8TXM1_VOLCA/46-287	D8TXM1.1 PF07714.15	55.90	0.00
TRINITY_D8TR61_VOLCA/1-236	D8TR61.1 PF06105.10	55.90	0.00
TRINITY_G0QVH9_ICHMG/93-465	G0QVH9.1 PF01546.26	55.90	0.00
TRINITY_A8JHW6_CHLRE/43-718	A8JHW6.1 PF00995.21	55.90	0.00
TRINITY_L8GKG4_ACACA/104-164	L8GKG4.1 PF12656.5;	55.90	0.00
TRINITY_D8TRX0_VOLCA/247-317	D8TRX0.1 PF00575.21	55.90	0.00
TRINITY_I0Z981_9CHLO/3-133	I0Z981.1 PF02130.15	55.90	0.00
TRINITY_L8GLR0_ACACA/875-1195	L8GLR0.1 PF03178.13	55.90	0.00
TRINITY_G0R216_ICHMG/467-534	G0R216.1 PF01480.15	55.90	0.00
TRINITY_A0A087SL48_AUXPR/2-291	A0A087SL48.1 PF0315	55.90	0.00
TRINITY_Q24F23_TETTS/322-390	Q24F23.1 PF08059.11	55.90	0.00
TRINITY_G0QNE2_ICHMG/99-359	G0QNE2.1 PF00069.23	55.90	0.00
TRINITY_W4G762_9STRA/203-335	W4G762.1 PF00583.23	55.90	0.00
TRINITY_D8U4A9_VOLCA/393-582	D8U4A9.1 PF07887.9;	55.90	0.00
TRINITY_W5MNV1_LEPOC/4-107	W5MNV1.1 PF14933.4;	55.90	0.00
TRINITY_C3XRT7_BRAFL/49-255	C3XRT7.1 PF00106.23	55.90	0.00
TRINITY_M1BNR5_SOLTU/95-164	M1BNR5.1 PF00076.20	55.90	0.00
TRINITY_I7MLL9_TETTS/291-547	I7MLL9.2 PF00069.23	55.90	0.00
TRINITY_G3XRB0_ASPNA/5-106	G3XRB0.1 PF00085.18	55.90	0.00
TRINITY_Q22T22_TETTS/520-735	Q22T22.3 PF07859.11	55.90	0.00
TRINITY_A0A087SP99_AUXPR/16-273	A0A087SP99.1 PF0337	55.90	0.00
TRINITY_Q00SW9_OSTTA/582-755	Q00SW9.1 PF02769.20	55.90	0.00
TRINITY_I0YV30_9CHLO/201-384	I0YV30.1 PF05193.19	55.90	0.00
TRINITY_GELA_DICDI/12-118	P13466.1 PF00307.29	55.90	0.00
TRINITY_M7C5U4_CHEMY/2-373	M7C5U4.1 PF00022.17	55.90	0.00
TRINITY_A0A096PAY4_OSTTA/138-206	A0A096PAY4.1 PF0001	55.90	0.00
TRINITY_A0A0D2VRM0_CAPO3/28-335	A0A0D2VRM0.1 PF0036	55.90	0.00
TRINITY_J9I2R8_9SPIT/282-345	J9I2R8.1 PF00498.24	55.90	0.00
TRINITY_L8H0X3_ACACA/523-817	L8H0X3.1 PF04950.10	55.90	0.00
TRINITY_L8H4Q3_ACACA/5-358	L8H4Q3.1 PF00443.27	55.90	0.00
TRINITY_D3BDQ8_POLPA/12-329	D3BDQ8.1 PF00225.21	55.90	0.00
TRINITY_K9QCP6_9NOSO/32-144	K9QCP6.1 PF00355.24	55.90	0.00
TRINITY_A0A059A334_EUCGR/77-151	A0A059A334.1 PF0311	55.90	0.00
TRINITY_A0A0L0C6F7_LUCCU/717-852	A0A0L0C6F7.1 PF0000	55.90	0.00
TRINITY_D8UFR9_VOLCA/717-924	D8UFR9.1 PF00481.19	55.90	0.00
TRINITY_C5KZM2_PERM5/29-216	C5KZM2.1 PF00227.24	55.90	0.00
TRINITY_E1ZAZ4_CHLVA/6-98	E1ZAZ4.1 PF00166.19	55.90	0.00
TRINITY_A0C0W6_PARTE/7-154	A0C0W6.1 PF00056.21	55.80	0.00
TRINITY_B9RB46_RICCO/80-124	B9RB46.1 PF13639.4;	55.80	0.00
TRINITY_A0A0K6GHT1_9HOMO/109-457	A0A0K6GHT1.1 PF0310	55.80	0.00
TRINITY_S2J9M4_MUCC1/226-394	S2J9M4.1 PF01612.18	55.80	0.00
TRINITY_J9IXJ6_9SPIT/12-173	J9IXJ6.1 PF00071.20	55.80	0.00
TRINITY_H2N283_ORYLA/37-97	H2N283.1 PF00050.19	55.80	0.00
TRINITY_F0ZCS1_DICPU/21-68	F0ZCS1.1 PF13920.4;	55.80	0.00
TRINITY_F4PMN3_DICFS/350-629	F4PMN3.1 PF12257.6;	55.80	0.00
TRINITY_I7M1S8_TETTS/492-689	I7M1S8.2 PF05958.9;	55.80	0.00
TRINITY_D8FWW1_9CYAN/72-190	D8FWW1.1 PF09335.9;	55.80	0.00
TRINITY_B7ZX64_MAIZE/53-527	B7ZX64.1 PF00501.26	55.80	0.00
TRINITY_A0DD10_PARTE/379-525	A0DD10.1 PF03031.16	55.80	0.00

TRINITY_J9DM28_EDHAE/188-231	J9DM28.1 PF13639.4;	55.80	0.00
TRINITY_B8BES4_ORYSI/707-805	B8BES4.1 PF13304.4;	55.80	0.00
TRINITY_A0A015J9E1_9GLOM/123-174	A0A015J9E1.1 PF1363	55.80	0.00
TRINITY_A0A022RBP7_ERYGU/435-478	A0A022RBP7.1 PF1363	55.80	0.00
TRINITY_J9HVL7_9SPIT/73-307	J9HVL7.1 PF01674.16	55.80	0.00
TRINITY_A0A087SEF3_AUXPR/73-194	A0A087SEF3.1 PF0828	55.80	0.00
TRINITY_K9X7H8_9NOST/344-533	K9X7H8.1 PF13604.4;	55.80	0.00
TRINITY_I0YUK2_9CHLO/10-84	I0YUK2.1 PF13656.4;	55.80	0.00
TRINITY_W9X2X5_9EURO/161-524	W9X2X5.1 PF00266.17	55.80	0.00
TRINITY_A0A087XF22_POEFO/159-287	A0A087XF22.2 PF0010	55.80	0.00
TRINITY_A0A067BDQ4_SAPPC/403-574	A0A067BDQ4.1 PF0814	55.80	0.00
TRINITY_A0A087SQU6_AUXPR/2-78	A0A087SQU6.1 PF0512	55.80	0.00
TRINITY_A3QAA4_SHELP/5-371	A3QAA4.1 PF01266.22	55.80	0.00
TRINITY_F0ZB10_DICPU/389-622	F0ZB10.1 PF01545.19	55.80	0.00
TRINITY_D8U0A7_VOLCA/66-160	D8U0A7.1 PF12542.6;	55.80	0.00
TRINITY_Q24F54_TETTS/168-427	Q24F54.4 PF02263.17	55.80	0.00
TRINITY_U4KVX6_PYROM/277-320	U4KVX6.1 PF13639.4;	55.80	0.00
TRINITY_D8TIZ1_VOLCA/51-145	D8TIZ1.1 PF00686.17	55.80	0.00
TRINITY_A0A087SSY0_AUXPR/20-105	A0A087SSY0.1 PF0796	55.80	0.00
TRINITY_A8J8P1_CHLRE/31-280	A8J8P1.1 PF00225.21	55.80	0.00
TRINITY_D8THZ3_VOLCA/156-424	D8THZ3.1 PF00928.19	55.80	0.00
TRINITY_D8UGL3_VOLCA/426-506	D8UGL3.1 PF00168.28	55.80	0.00
TRINITY_A8J3V3_CHLRE/10-183	A8J3V3.1 PF03547.16	55.80	0.00
TRINITY_A0A087SBA0_AUXPR/25-190	A0A087SBA0.1 PF1366	55.80	0.00
TRINITY_A0A078ADF2_STYLE/166-294	A0A078ADF2.1 PF0076	55.80	0.00
TRINITY_L8H4I8_ACACA/10-1020	L8H4I8.1 PF02463.17	55.80	0.00
TRINITY_A0A077ZVR1_STYLE/127-198	A0A077ZVR1.1 PF0007	55.80	0.00
TRINITY_Q5CQG6_CRYPI/335-477	Q5CQG6.1 PF04893.15	55.80	0.00
TRINITY_W5MNV1_LEPOC/4-107	W5MNV1.1 PF14933.4;	55.80	0.00
TRINITY_A0A0C4ENE9_PUCT1/252-356	A0A0C4ENE9.1 PF0030	55.80	0.00
TRINITY_I3L5Q2_PIG/13-60	I3L5Q2.1 PF13920.4;	55.80	0.00
TRINITY_Q23DI7_TETTS/1868-2219	Q23DI7.2 PF00443.27	55.80	0.00
TRINITY_A8JAR8_CHLRE/1-204	A8JAR8.1 PF05057.12	55.80	0.00
TRINITY_S2K4R7_MUCC1/8-126	S2K4R7.1 PF01042.19	55.80	0.00
TRINITY_A8J8W0_CHLRE/288-488	A8J8W0.1 PF03441.12	55.80	0.00
TRINITY_D2V9F0_NAEGR/1-51	D2V9F0.1 PF13921.4;	55.80	0.00
TRINITY_E1Z8J3_CHLVA/1-97	E1Z8J3.1 PF02410.13	55.80	0.00
TRINITY_Q22KE9_TETTS/144-692	Q22KE9.1 PF03571.13	55.80	0.00
TRINITY_A0C2C1_PARTE/372-421	A0C2C1.1 PF13920.4;	55.80	0.00
TRINITY_Q229B1_TETTS/1-270	Q229B1.1 PF08433.8;	55.70	0.00
TRINITY_I7ML74_TETTS/5240-5676	I7ML74.2 PF00899.19	55.70	0.00
TRINITY_D2VA04_NAEGR/1-326	D2VA04.1 PF13393.4;	55.70	0.00
TRINITY_S8ANP7_PENO1/505-638	S8ANP7.1 PF03404.14	55.70	0.00
TRINITY_A0C2G1_PARTE/125-233	A0C2G1.1 PF02770.17	55.70	0.00
TRINITY_C3Z4S4_BRAFL/15-164	C3Z4S4.1 PF00857.18	55.70	0.00
TRINITY_L7U781_MYXSD/88-193	L7U781.1 PF01553.19	55.70	0.00
TRINITY_L8H7V4_ACACA/94-172	L8H7V4.1 PF00076.20	55.70	0.00
TRINITY_I7M0V3_TETTS/377-638	I7M0V3.2 PF00069.23	55.70	0.00
TRINITY_D3AYR5_POLPA/92-343	D3AYR5.1 PF00069.23	55.70	0.00
TRINITY_A8ILB2_CHLRE/23-298	A8ILB2.1 PF00251.18	55.70	0.00
TRINITY_K5VG38_PHACS/42-103	K5VG38.1 PF00403.24	55.70	0.00
TRINITY_F4PJV7_DICFS/77-370	F4PJV7.1 PF12705.5;	55.70	0.00
TRINITY_A0A015ICW4_9GLOM/276-552	A0A015ICW4.1 PF0066	55.70	0.00
TRINITY_W9XVR6_9EURO/26-98	W9XVR6.1 PF13499.4;	55.70	0.00
TRINITY_I0Z970_9CHLO/3-151	I0Z970.1 PF13365.4;	55.70	0.00
TRINITY_W1PAH0_AMBTC/16-78	W1PAH0.1 PF00226.29	55.70	0.00
TRINITY_A8LQG8_DINSH/4-242	A8LQG8.1 PF01144.21	55.70	0.00
TRINITY_G7J420_MEDTR/11-290	G7J420.1 PF00370.19	55.70	0.00
TRINITY_V6LTL1_9EUKA/3-74	V6LTL1.1 PF00240.21	55.70	0.00
TRINITY_A0A0N4VA03_ENTVE/17-187	A0A0N4VA03.1 PF0335	55.70	0.00
TRINITY_R0IAX6_9BRAS/128-200	R0IAX6.1 PF01472.18	55.70	0.00

TRINITY_I0YPZ4_9CHLO/5-140	I0YPZ4.1 PF00504.19	55.70	0.00
TRINITY_F4PHG1_DICFS/31-284	F4PHG1.1 PF00069.23	55.70	0.00
TRINITY_W7XBL0_TETTS/482-665	W7XBL0.1 PF11916.6;	55.70	0.00
TRINITY_A0A0N1NYS6_9EURO/109-167	A0A0N1NYS6.1 PF0411	55.70	0.00
TRINITY_Q229Q4_TETTS/33-368	Q229Q4.2 PF00225.21	55.70	0.00
TRINITY_L8HBF0_ACACA/20-205	L8HBF0.1 PF16661.3;	55.70	0.00
TRINITY_N1JGE9_BLUG1/99-248	N1JGE9.1 PF00005.25	55.70	0.00
TRINITY_RECA_BURCJ/7-269	B4EBY3.1 PF00154.19	55.70	0.00
TRINITY_A8JG04_CHLRE/72-269	A8JG04.1 PF07466.9;	55.70	0.00
TRINITY_B9ICS6_POPTR/118-434	B9ICS6.2 PF00251.18	55.70	0.00
TRINITY_D8TZS9_VOLCA/306-373	D8TZS9.1 PF00789.18	55.70	0.00
TRINITY_A8IA75_CHLRE/44-181	A8IA75.1 PF03330.16	55.70	0.00
TRINITY_L8HFF6_ACACA/21-280	L8HFF6.1 PF00491.19	55.70	0.00
TRINITY_W9SCN3_9ROSA/200-348	W9SCN3.1 PF00929.22	55.70	0.00
TRINITY_B9GVF4_POPTR/64-407	B9GVF4.2 PF00009.25	55.70	0.00
TRINITY_F0ZY14_DICPU/351-612	F0ZY14.1 PF07714.15	55.70	0.00
TRINITY_GELA_DICDI/12-118	P13466.1 PF00307.29	55.70	0.00
TRINITY_A0A059LJ23_9CHLO/282-790	A0A059LJ23.1 PF0017	55.70	0.00
TRINITY_A0A0R4I9K8_DANRE/12-105	A0A0R4I9K8.1 PF1017	55.70	0.00
TRINITY_A8I7P2_CHLRE/17-260	A8I7P2.1 PF06966.10	55.70	0.00
TRINITY_D8U494_VOLCA/3-139	D8U494.1 PF10294.7;	55.70	0.00
TRINITY_K5VY91_PHACS/357-763	K5VY91.1 PF00962.20	55.70	0.00
TRINITY_G0QRM1_ICHMG/13-169	G0QRM1.1 PF12850.5;	55.70	0.00
TRINITY_A0CG28_PARTE/9-130	A0CG28.1 PF01042.19	55.70	0.00
TRINITY_Q7XLT8_ORYSJ/503-697	Q7XLT8.2 PF00078.25	55.70	0.00
TRINITY_D3B1V2_POLPA/5-372	D3B1V2.1 PF00022.17	55.70	0.00
TRINITY_G0QX82_ICHMG/451-647	G0QX82.1 PF00566.16	55.70	0.00
TRINITY_A0EEX5_PARTE/21-575	A0EEX5.1 PF01602.18	55.60	0.00
TRINITY_F4QED2_DICFS/611-718	F4QED2.1 PF00307.29	55.60	0.00
TRINITY_A4VEZ3_TETTS/248-296	A4VEZ3.1 PF00400.30	55.60	0.00
TRINITY_D8UBJ9_VOLCA/160-331	D8UBJ9.1 PF16575.3;	55.60	0.00
TRINITY_C6J521_9BACL/48-380	C6J521.1 PF02055.14	55.60	0.00
TRINITY_I7M1V6_TETTS/314-533	I7M1V6.1 PF07002.14	55.60	0.00
TRINITY_Q5TNJ4_ANOGA/104-168	Q5TNJ4.3 PF00327.18	55.60	0.00
TRINITY_Q3SDW1_PARTE/11-74	Q3SDW1.1 PF13499.4;	55.60	0.00
TRINITY_G0QUC0_ICHMG/33-271	G0QUC0.1 PF08704.8;	55.60	0.00
TRINITY_B4UXL3_9ACTN/7-233	B4UXL3.1 PF05368.11	55.60	0.00
TRINITY_I0Z580_9CHLO/57-483	I0Z580.1 PF01222.15	55.60	0.00
TRINITY_D8TDQ2_SELML/11-266	D8TDQ2.1 PF00069.23	55.60	0.00
TRINITY_Q22CC8_TETTS/4-483	Q22CC8.2 PF04791.14	55.60	0.00
TRINITY_D3B6M3_POLPA/229-533	D3B6M3.1 PF00082.20	55.60	0.00
TRINITY_D3B581_POLPA/295-339	D3B581.1 PF01084.18	55.60	0.00
TRINITY_F7E8Y2_ORNAN/252-299	F7E8Y2.1 PF13920.4;	55.60	0.00
TRINITY_L1JWW5_GUITH/129-200	L1JWW5.1 PF04564.13	55.60	0.00
TRINITY_L8HGS9_ACACA/1-197	L8HGS9.1 PF02115.15	55.60	0.00
TRINITY_A0A078FHA5_BRANA/210-310	A0A078FHA5.1 PF1289	55.60	0.00
TRINITY_A0A0D2X3R4_CAPO3/24-592	A0A0D2X3R4.1 PF0160	55.60	0.00
TRINITY_D3BNN2_POLPA/173-243	D3BNN2.1 PF15159.4;	55.60	0.00
TRINITY_A7STF6_NEMVE/2-108	A7STF6.1 PF01521.18	55.60	0.00
TRINITY_E1Z750_CHLVA/56-323	E1Z750.1 PF00122.18	55.60	0.00
TRINITY_A0A077FJN5_9RICK/1-283	A0A077FJN5.1 PF0057	55.60	0.00
TRINITY_U5H3G6_USTV1/2-375	U5H3G6.1 PF00022.17	55.60	0.00
TRINITY_A0A0G4ERS4_9ALVE/381-452	A0A0G4ERS4.1 PF0007	55.60	0.00
TRINITY_D3BT91_POLPA/163-335	D3BT91.1 PF10520.7;	55.60	0.00
TRINITY_A0EFQ7_PARTE/75-330	A0EFQ7.1 PF00069.23	55.60	0.00
TRINITY_J9J754_9SPIT/543-600	J9J754.1 PF01485.19	55.60	0.00
TRINITY_L8GG36_ACACA/72-255	L8GG36.1 PF02145.13	55.60	0.00
TRINITY_Q54RS0_DICDI/66-205	Q54RS0.1 PF00849.20	55.60	0.00
TRINITY_G0QT22_ICHMG/18-134	G0QT22.1 PF00583.23	55.60	0.00
TRINITY_S2JKZ2_MUCC1/571-773	S2JKZ2.1 PF00566.16	55.60	0.00
TRINITY_A0A0M3J5J0_ANISI/4-73	A0A0M3J5J0.1 PF0279	55.60	0.00

TRINITY_A8I5Y8_CHLRE/3-173	A8I5Y8.1 PF05078.10	55.60	0.00
TRINITY_A0A078B8T9_STYLE/13-150	A0A078B8T9.1 PF1458	55.60	0.00
TRINITY_V7AXD5_PHAVU/35-93	V7AXD5.1 PF00013.27	55.60	0.00
TRINITY_Q54HY5_DICDI/490-673	Q54HY5.2 PF00644.18	55.60	0.00
TRINITY_M3WQG6_FELCA/66-392	M3WQG6.1 PF00009.25	55.60	0.00
TRINITY_L8H3W5_ACACA/971-1093	L8H3W5.1 PF00271.29	55.60	0.00
TRINITY_L8H3W5_ACACA/971-1093	L8H3W5.1 PF00271.29	55.60	0.00
TRINITY_F0ZXF1_DICPU/1-269	F0ZXF1.1 PF01545.19	55.60	0.00
TRINITY_H3CHL6_TETNG/976-1284	H3CHL6.1 PF02889.14	55.60	0.00
TRINITY_B3RWZ4_TRIAD/73-117	B3RWZ4.1 PF00569.15	55.60	0.00
TRINITY_M7ZW29_TRIUA/635-826	M7ZW29.1 PF13844.4;	55.60	0.00
TRINITY_Q23PW9_TETTS/29-162	Q23PW9.2 PF01138.19	55.60	0.00
TRINITY_D8UFX2_VOLCA/571-829	D8UFX2.1 PF01734.20	55.60	0.00
TRINITY_E1ZK98_CHLVA/2-185	E1ZK98.1 PF06941.10	55.60	0.00
TRINITY_A0A0G4FGY8_9ALVE/32-100	A0A0G4FGY8.1 PF0050	55.60	0.00
TRINITY_B6AEW6_CRYMR/10-73	B6AEW6.1 PF01423.20	55.60	0.00
TRINITY_A0A0G3QF21_KLUIN/97-209	A0A0G3QF21.1 PF0018	55.60	0.00
TRINITY_D0N363_PHYIT/406-475	D0N363.1 PF02798.18	55.60	0.00
TRINITY_C1E094_MICSR/52-268	C1E094.1 PF01988.17	55.60	0.00
TRINITY_Q8GWA5_ARATH/2-329	Q8GWA5.1 PF00022.17	55.60	0.00
TRINITY_A7RR70_NEMVE/7-107	A7RR70.1 PF01247.16	55.60	0.00
TRINITY_I1BK57_RHIO9/844-1034	I1BK57.1 PF13087.4;	55.60	0.00
TRINITY_F2U7V2_SALR5/92-306	F2U7V2.1 PF01786.15	55.60	0.00
TRINITY_R5NRG8_9FIRM/195-265	R5NRG8.1 PF00575.21	55.60	0.00
TRINITY_W4K5L4_9HOMO/103-194	W4K5L4.1 PF00153.25	55.60	0.00
TRINITY_A8IKD4_CHLRE/117-217	A8IKD4.1 PF13410.4;	55.60	0.00
TRINITY_Q23G19_TETTS/51-248	Q23G19.1 PF02330.14	55.60	0.00
TRINITY_G0R646_ICHMG/414-563	G0R646.1 PF00005.25	55.60	0.00
TRINITY_C5VRP7_CLOBO/5-67	C5VRP7.1 PF00226.29	55.60	0.00
TRINITY_A0D2X6_PARTE/2-135	A0D2X6.1 PF05914.10	55.60	0.00
TRINITY_D8TZ80_VOLCA/781-992	D8TZ80.1 PF01434.16	55.60	0.00
TRINITY_E1ZQ10_CHLVA/38-341	E1ZQ10.1 PF00443.27	55.60	0.00
TRINITY_E1ZB02_CHLVA/101-362	E1ZB02.1 PF03009.15	55.60	0.00
TRINITY_D8U3E6_VOLCA/14-218	D8U3E6.1 PF05057.12	55.60	0.00
TRINITY_C7RCK1_KANKD/4-154	C7RCK1.1 PF08534.8;	55.60	0.00
TRINITY_Q247Y9_TETTS/39-137	Q247Y9.2 PF00034.19	55.60	0.00
TRINITY_D3BAQ9_POLPA/142-437	D3BAQ9.1 PF06027.10	55.60	0.00
TRINITY_A0DYC6_PARTE/574-750	A0DYC6.1 PF13870.4;	55.60	0.00
TRINITY_A8IS90_CHLRE/98-398	A8IS90.1 PF00248.19	55.60	0.00
TRINITY_D8UHA9_VOLCA/879-1087	D8UHA9.1 PF07250.9;	55.60	0.00
TRINITY_A0A059BGQ8_EUCGR/38-100	A0A059BGQ8.1 PF0184	55.60	0.00
TRINITY_A8IYP3_CHLRE/14-85	A8IYP3.1 PF00240.21	55.60	0.00
TRINITY_A8HTU8_CHLRE/13-251	A8HTU8.1 PF00856.26	55.60	0.00
TRINITY_A8J851_CHLRE/66-237	A8J851.1 PF00891.16	55.60	0.00
TRINITY_I1C775_RHIO9/8-129	I1C775.1 PF02291.13	55.60	0.00
TRINITY_D8U9S9_VOLCA/599-793	D8U9S9.1 PF00211.18	55.60	0.00
TRINITY_A8JJ05_CHLRE/562-633	A8JJ05.1 PF05699.12	55.60	0.00
TRINITY_Q23M99_TETTS/10-108	Q23M99.1 PF00153.25	55.60	0.00
TRINITY_Q23QF2_TETTS/248-571	Q23QF2.2 PF03133.13	55.60	0.00
TRINITY_W7X719_TETTS/9-66	W7X719.1 PF00076.20	55.60	0.00
TRINITY_A0A068S3W9_9FUNG/253-296	A0A068S3W9.1 PF1363	55.60	0.00
TRINITY_Q22WQ3_TETTS/116-190	Q22WQ3.2 PF12796.5;	55.60	0.00
TRINITY_A0DNM9_PARTE/10-137	A0DNM9.1 PF00293.26	55.50	0.00
TRINITY_L8H455_ACACA/1-182	L8H455.1 PF00227.24	55.50	0.00
TRINITY_L1J820_GUITH/223-386	L1J820.1 PF01399.25	55.50	0.00
TRINITY_F0Y6L3_AURAN/5-178	F0Y6L3.1 PF00071.20	55.50	0.00
TRINITY_A0A068RTH5_9FUNG/5-178	A0A068RTH5.1 PF0007	55.50	0.00
TRINITY_A0A0D2WM63_CAPO3/25-134	A0A0D2WM63.1 PF0196	55.50	0.00
TRINITY_F4PH79_DICFS/17-684	F4PH79.1 PF00063.19	55.50	0.00
TRINITY_D8TWR3_VOLCA/83-425	D8TWR3.1 PF02383.16	55.50	0.00
TRINITY_A0DQM7_PARTE/243-406	A0DQM7.1 PF03031.16	55.50	0.00

TRINITY_B6QFY4_TALMQ/58-194	B6QFY4.1 PF00293.26	55.50	0.00
TRINITY_E2R9S3_CANLF/155-266	E2R9S3.2 PF07757.11	55.50	0.00
TRINITY_I0YMP7_9CHLO/538-648	I0YMP7.1 PF02847.15	55.50	0.00
TRINITY_G0R5C3_ICHMG/2-671	G0R5C3.1 PF02463.17	55.50	0.00
TRINITY_Q22U45_TETTS/225-538	Q22U45.1 PF00883.19	55.50	0.00
TRINITY_C1MVB0_MICPC/12-130	C1MVB0.1 PF13863.4;	55.50	0.00
TRINITY_B9S046_RICCO/3-206	B9S046.1 PF01088.19	55.50	0.00
TRINITY_A0C8M5_PARTE/110-338	A0C8M5.1 PF00487.22	55.50	0.00
TRINITY_U5QLE8_9CYAN/15-160	U5QLE8.1 PF05118.13	55.50	0.00
TRINITY_A8IDU2_CHLRE/877-1024	A8IDU2.1 PF03330.16	55.50	0.00
TRINITY_Q22RI1_TETTS/11-256	Q22RI1.1 PF13561.4;	55.50	0.00
TRINITY_A0A087Y149_POEFO/231-464	A0A087Y149.1 PF1362	55.50	0.00
TRINITY_D3AY16_POLPA/236-600	D3AY16.1 PF03074.14	55.50	0.00
TRINITY_U9SUN3_RHIID/5-178	U9SUN3.1 PF00071.20	55.50	0.00
TRINITY_D8UAV7_VOLCA/928-1103	D8UAV7.1 PF13874.4;	55.50	0.00
TRINITY_A0A077ZUB0_STYLE/515-998	A0A077ZUB0.1 PF0023	55.50	0.00
TRINITY_M0S8C6_MUSAM/344-789	M0S8C6.1 PF04053.12	55.50	0.00
TRINITY_D8TH58_VOLCA/24-423	D8TH58.1 PF03345.12	55.50	0.00
TRINITY_A8IGG2_CHLRE/2-183	A8IGG2.1 PF01121.18	55.50	0.00
TRINITY_C1N091_MICPC/229-368	C1N091.1 PF00160.19	55.50	0.00
TRINITY_E1ZP63_CHLVA/17-231	E1ZP63.1 PF00817.18	55.50	0.00
TRINITY_A0A098EUG0_9BACI/4-122	A0A098EUG0.1 PF1323	55.50	0.00
TRINITY_D3BDQ8_POLPA/12-329	D3BDQ8.1 PF00225.21	55.50	0.00
TRINITY_I7M9T0_TETTS/42-205	I7M9T0.2 PF14908.4;	55.50	0.00
TRINITY_A8IG04_CHLRE/236-482	A8IG04.1 PF00067.20	55.50	0.00
TRINITY_I7M328_TETTS/23-132	I7M328.1 PF00787.22	55.40	0.00
TRINITY_M2Y4W0_GALSU/84-230	M2Y4W0.1 PF00588.17	55.40	0.00
TRINITY_A0A078A3V7_STYLE/126-338	A0A078A3V7.1 PF0011	55.40	0.00
TRINITY_W4ZJ67_STRPU/6-295	W4ZJ67.1 PF01026.19	55.40	0.00
TRINITY_D8LST0_ECTSI/14-138	D8LST0.1 PF00572.16	55.40	0.00
TRINITY_F4Q0Q7_DICFS/10-147	F4Q0Q7.1 PF00179.24	55.40	0.00
TRINITY_F4Q0M1_DICFS/50-482	F4Q0M1.1 PF13520.4;	55.40	0.00
TRINITY_D2VAL1_NAEGR/10-175	D2VAL1.1 PF00071.20	55.40	0.00
TRINITY_F4PMG8_DICFS/24-1090	F4PMG8.1 PF10266.7;	55.40	0.00
TRINITY_Q22P73_TETTS/71-322	Q22P73.2 PF00069.23	55.40	0.00
TRINITY_F8I5P3_SULAT/13-80	F8I5P3.1 PF00366.18	55.40	0.00
TRINITY_I0Z8F4_9CHLO/937-1204	I0Z8F4.1 PF00664.21	55.40	0.00
TRINITY_I7M0U2_TETTS/23-1098	I7M0U2.2 PF10266.7;	55.40	0.00
TRINITY_L8GF13_ACACA/142-338	L8GF13.1 PF01068.19	55.40	0.00
TRINITY_I0YU42_9CHLO/7-203	I0YU42.1 PF00270.27	55.40	0.00
TRINITY_L8H8X8_ACACA/120-212	L8H8X8.1 PF00153.25	55.40	0.00
TRINITY_D8TGY3_VOLCA/165-303	D8TGY3.1 PF02926.15	55.40	0.00
TRINITY_D8U3Q5_VOLCA/24-179	D8U3Q5.1 PF00160.19	55.40	0.00
TRINITY_J9GCX4_9SPIT/78-251	J9GCX4.1 PF01068.19	55.40	0.00
TRINITY_D8U172_VOLCA/142-280	D8U172.1 PF00258.23	55.40	0.00
TRINITY_D0MK82_RHOM4/15-320	D0MK82.1 PF00248.19	55.40	0.00
TRINITY_I0YXC4_9CHLO/1-58	I0YXC4.1 PF04564.13	55.40	0.00
TRINITY_Q54T47_DICDI/30-211	Q54T47.1 PF00596.19	55.40	0.00
TRINITY_D4ZZ36_ARTPN/26-390	D4ZZ36.1 PF03738.12	55.40	0.00
TRINITY_L8HBE6_ACACA/360-488	L8HBE6.1 PF04784.12	55.40	0.00
TRINITY_A0BIT4_PARTE/126-209	A0BIT4.1 PF01529.18	55.40	0.00
TRINITY_G7YR19_CLOSI/2-103	G7YR19.1 PF00085.18	55.40	0.00
TRINITY_D8UIV0_VOLCA/25-394	D8UIV0.1 PF00224.19	55.40	0.00
TRINITY_Q240Y1_TETTS/863-931	Q240Y1.3 PF13499.4;	55.40	0.00
TRINITY_A0A077ZYN3_STYLE/16-177	A0A077ZYN3.1 PF0007	55.40	0.00
TRINITY_F1A0J6_DICPU/33-136	F1A0J6.1 PF10205.7;	55.40	0.00
TRINITY_D2V342_NAEGR/575-752	D2V342.1 PF13761.4;	55.40	0.00
TRINITY_Q2JH92_SYNJB/25-89	Q2JH92.1 PF01066.19	55.40	0.00
TRINITY_I7MHC8_TETTS/479-543	I7MHC8.2 PF13499.4;	55.40	0.00
TRINITY_A0A0D2WTS7_CAPO3/32-443	A0A0D2WTS7.1 PF0020	55.40	0.00
TRINITY_A0DZ84_PARTE/87-248	A0DZ84.1 PF01746.19	55.40	0.00

TRINITY_D8TQ27_VOLCA/156-320	D8TQ27.1 PF04072.12	55.40	0.00
TRINITY_A8IWR0_CHLRE/61-406	A8IWR0.1 PF05625.9;	55.40	0.00
TRINITY_A8IVP1_CHLRE/64-130	A8IVP1.1 PF00498.24	55.40	0.00
TRINITY_M7WHB3_RHOT1/20-83	M7WHB3.1 PF01817.19	55.40	0.00
TRINITY_E1ZGP7_CHLVA/103-232	E1ZGP7.1 PF08695.8;	55.40	0.00
TRINITY_Q24FP1_TETTS/133-395	Q24FP1.1 PF00069.23	55.40	0.00
TRINITY_D8TRX0_VOLCA/247-317	D8TRX0.1 PF00575.21	55.40	0.00
TRINITY_I0Z7G6_9CHLO/812-1273	I0Z7G6.1 PF00136.19	55.40	0.00
TRINITY_A0A0G4IJ65_PLABS/11-68	A0A0G4IJ65.1 PF0538	55.40	0.00
TRINITY_E1Z8Z3_CHLVA/100-197	E1Z8Z3.1 PF10294.7;	55.40	0.00
TRINITY_S8EGD3_9LAMI/11-122	S8EGD3.1 PF02338.17	55.40	0.00
TRINITY_B9SQ25_RICCO/695-1026	B9SQ25.1 PF00009.25	55.40	0.00
TRINITY_I7MF85_TETTS/30-143	I7MF85.1 PF00307.29	55.40	0.00
TRINITY_A0A0E0PZ67_ORYRU/562-682	A0A0E0PZ67.1 PF1444	55.40	0.00
TRINITY_A8IAF3_CHLRE/81-429	A8IAF3.1 PF01546.26	55.40	0.00
TRINITY_A0A087SL44_AUXPR/222-322	A0A087SL44.1 PF0035	55.40	0.00
TRINITY_Q22RV0_TETTS/39-90	Q22RV0.1 PF05186.11	55.40	0.00
TRINITY_L1IMU1_GUITH/84-255	L1IMU1.1 PF02816.16	55.40	0.00
TRINITY_Q237J4_TETTS/105-303	Q237J4.1 PF03587.12	55.40	0.00
TRINITY_A0A078ATQ4_STYLE/166-308	A0A078ATQ4.1 PF0551	55.40	0.00
TRINITY_I7MDT7_TETTS/18-115	I7MDT7.2 PF03650.11	55.30	0.00
TRINITY_A0A0F4XC52_HANUV/18-299	A0A0F4XC52.1 PF0024	55.30	0.00
TRINITY_L8GKQ1_ACACA/188-236	L8GKQ1.1 PF13415.4;	55.30	0.00
TRINITY_A0E5L3_PARTE/49-221	A0E5L3.1 PF00270.27	55.30	0.00
TRINITY_Q22AQ2_TETTS/63-169	Q22AQ2.2 PF00027.27	55.30	0.00
TRINITY_C3Y972_BRAFL/27-164	C3Y972.1 PF01951.14	55.30	0.00
TRINITY_G3NLB9_GASAC/22-115	G3NLB9.1 PF00031.19	55.30	0.00
TRINITY_D3BU71_POLPA/11-282	D3BU71.1 PF01267.15	55.30	0.00
TRINITY_J9HR05_9SPIT/1567-1710	J9HR05.1 PF00588.17	55.30	0.00
TRINITY_K3Z3R1_SETIT/635-784	K3Z3R1.1 PF00179.24	55.30	0.00
TRINITY_X6MRN4_RETFI/13-98	X6MRN4.1 PF00439.23	55.30	0.00
TRINITY_F4Q7L2_DICFS/13-96	F4Q7L2.1 PF01453.22	55.30	0.00
TRINITY_A0A078AVG4_STYLE/25-176	A0A078AVG4.1 PF0010	55.30	0.00
TRINITY_F6T7M4_XENTR/323-476	F6T7M4.1 PF13091.4;	55.30	0.00
TRINITY_F4Q468_DICFS/146-420	F4Q468.1 PF02353.18	55.30	0.00
TRINITY_Q22P73_TETTS/71-322	Q22P73.2 PF00069.23	55.30	0.00
TRINITY_Q7XRL9_ORYSJ/564-738	Q7XRL9.2 PF00078.25	55.30	0.00
TRINITY_D8U5A6_VOLCA/371-559	D8U5A6.1 PF01764.23	55.30	0.00
TRINITY_A0A0D2QGM6_GOSRA/224-355	A0A0D2QGM6.1 PF0004	55.30	0.00
TRINITY_F2UBX6_SALR5/366-627	F2UBX6.1 PF00218.19	55.30	0.00
TRINITY_A8HYT0_CHLRE/62-334	A8HYT0.1 PF01256.15	55.30	0.00
TRINITY_A0A067QTF4_ZOONE/4-97	A0A067QTF4.1 PF1279	55.30	0.00
TRINITY_A0A078D6D3_BRANA/113-500	A0A078D6D3.1 PF0583	55.30	0.00
TRINITY_G0R4Y1_ICHMG/49-124	G0R4Y1.1 PF01929.15	55.30	0.00
TRINITY_E1Z768_CHLVA/16-250	E1Z768.1 PF04757.12	55.30	0.00
TRINITY_D8TGY2_VOLCA/2-216	D8TGY2.1 PF06244.10	55.30	0.00
TRINITY_B7J4U6_ACIF2/43-145	B7J4U6.1 PF00011.19	55.30	0.00
TRINITY_A8IDR3_CHLRE/6-149	A8IDR3.1 PF01554.16	55.30	0.00
TRINITY_L8HL85_ACACA/435-627	L8HL85.1 PF02902.17	55.30	0.00
TRINITY_A0D6X3_PARTE/79-164	A0D6X3.1 PF00153.25	55.30	0.00
TRINITY_L8HHG1_ACACA/13-59	L8HHG1.1 PF13920.4;	55.30	0.00
TRINITY_D8UHN3_VOLCA/9-149	D8UHN3.1 PF10914.6;	55.30	0.00
TRINITY_B9RNS0_RICCO/406-607	B9RNS0.1 PF08662.9;	55.30	0.00
TRINITY_A0A0Q9WQD4_DROWI/21-227	A0A0Q9WQD4.1 PF0010	55.30	0.00
TRINITY_D8UJA2_VOLCA/1384-1512	D8UJA2.1 PF13640.4;	55.30	0.00
TRINITY_A0A0M0JSM2_9EUKA/51-589	A0A0M0JSM2.1 PF0299	55.30	0.00
TRINITY_A0A078AVM5_STYLE/320-363	A0A078AVM5.1 PF0056	55.30	0.00
TRINITY_A8IRW3_CHLRE/249-516	A8IRW3.1 PF00566.16	55.30	0.00
TRINITY_A8ISP8_CHLRE/33-146	A8ISP8.1 PF01152.19	55.30	0.00
TRINITY_G0QNS9_ICHMG/23-353	G0QNS9.1 PF00389.28	55.30	0.00
TRINITY_D8TZE8_VOLCA/51-367	D8TZE8.1 PF13000.5;	55.30	0.00

TRINITY_H9JRI2_BOMMO/756-948	H9JRI2.1 PF03200.14	55.30	0.00
TRINITY_D8U7U1_VOLCA/35-300	D8U7U1.1 PF01036.16	55.30	0.00
TRINITY_A8JFC7_CHLRE/3-207	A8JFC7.1 PF03932.12	55.30	0.00
TRINITY_D8U618_VOLCA/659-764	D8U618.1 PF13426.5;	55.30	0.00
TRINITY_A0A0M0LRY4_9EUKA/18-190	A0A0M0LRY4.1 PF1018	55.20	0.00
TRINITY_Q23CX7_TETTS/74-325	Q23CX7.2 PF00378.18	55.20	0.00
TRINITY_E4XG21_OIKDI/6-78	E4XG21.1 PF00173.26	55.20	0.00
TRINITY_A0A0F5PWD3_9RHIZ/5-331	A0A0F5PWD3.1 PF0799	55.20	0.00
TRINITY_G4ZR71_PHYSP/17-181	G4ZR71.1 PF13023.4;	55.20	0.00
TRINITY_L8HHA3_ACACA/7-64	L8HHA3.1 PF00403.24	55.20	0.00
TRINITY_A0DC59_PARTE/3-141	A0DC59.1 PF01217.18	55.20	0.00
TRINITY_A0DRY7_PARTE/472-713	A0DRY7.1 PF01504.16	55.20	0.00
TRINITY_I7M0W4_TETTS/279-641	I7M0W4.2 PF06602.12	55.20	0.00
TRINITY_K3W8N7_PYTUL/8-329	K3W8N7.1 PF01406.17	55.20	0.00
TRINITY_Q9EMN2_AMEPV/3-74	Q9EMN2.1 PF00240.21	55.20	0.00
TRINITY_A8IWS7_CHLRE/8-76	A8IWS7.1 PF03992.14	55.20	0.00
TRINITY_I7M1N7_TETTS/84-192	I7M1N7.1 PF14580.4;	55.20	0.00
TRINITY_D2VTJ0_NAEGR/20-144	D2VTJ0.1 PF13671.4;	55.20	0.00
TRINITY_G0QUU8_ICHMG/120-541	G0QUU8.1 PF00443.27	55.20	0.00
TRINITY_L8GTU6_ACACA/1-159	L8GTU6.1 PF00071.20	55.20	0.00
TRINITY_D8UJ40_VOLCA/967-1176	D8UJ40.1 PF00702.24	55.20	0.00
TRINITY_I7MIT2_TETTS/90-275	I7MIT2.2 PF01121.18	55.20	0.00
TRINITY_F0Z9K8_DICPU/44-444	F0Z9K8.1 PF06728.11	55.20	0.00
TRINITY_B9SNT8_RICCO/1030-1139	B9SNT8.1 PF00005.25	55.20	0.00
TRINITY_D8TP89_VOLCA/472-662	D8TP89.1 PF11833.6;	55.20	0.00
TRINITY_I7MMX0_TETTS/304-523	I7MMX0.1 PF07002.14	55.20	0.00
TRINITY_Q233Z3_TETTS/947-1211	Q233Z3.2 PF00069.23	55.20	0.00
TRINITY_L8H9I7_ACACA/66-504	L8H9I7.1 PF01425.19	55.20	0.00
TRINITY_E5S5P0_TRISP/24-128	E5S5P0.1 PF00085.18	55.20	0.00
TRINITY_Q24DL3_TETTS/233-295	Q24DL3.2 PF07647.15	55.20	0.00
TRINITY_L8HGS4_ACACA/419-928	L8HGS4.1 PF00899.19	55.20	0.00
TRINITY_A0BW08_PARTE/27-287	A0BW08.1 PF02263.17	55.20	0.00
TRINITY_F1A0J6_DICPU/33-136	F1A0J6.1 PF10205.7;	55.20	0.00
TRINITY_I0Z055_9CHLO/36-156	I0Z055.1 PF00856.26	55.20	0.00
TRINITY_D8TJE4_VOLCA/96-272	D8TJE4.1 PF04063.12	55.20	0.00
TRINITY_C3YCJ5_BRAFL/1-175	C3YCJ5.1 PF14580.4;	55.20	0.00
TRINITY_A8J3C6_CHLRE/33-205	A8J3C6.1 PF00270.27	55.20	0.00
TRINITY_A0A0N4VKG2_ENTVE/3-458	A0A0N4VKG2.1 PF0022	55.20	0.00
TRINITY_K4C1X2_SOLLC/67-276	K4C1X2.1 PF08245.10	55.20	0.00
TRINITY_H1XSX2_9BACT/4-365	H1XSX2.1 PF00266.17	55.20	0.00
TRINITY_B3S5N5_TRIAD/577-704	B3S5N5.1 PF13646.4;	55.20	0.00
TRINITY_A0CS29_PARTE/2561-2834	A0CS29.1 PF00520.29	55.20	0.00
TRINITY_L8H3C5_ACACA/115-368	L8H3C5.1 PF07714.15	55.20	0.00
TRINITY_L8GUU7_ACACA/465-928	L8GUU7.1 PF12157.6;	55.20	0.00
TRINITY_M5XN62_PRUPE/53-307	M5XN62.1 PF00108.21	55.20	0.00
TRINITY_A8JJ05_CHLRE/562-633	A8JJ05.1 PF05699.12	55.20	0.00
TRINITY_D8UC61_VOLCA/12-133	D8UC61.1 PF03009.15	55.20	0.00
TRINITY_Q22RZ8_TETTS/183-242	Q22RZ8.2 PF13921.4;	55.20	0.00
TRINITY_A8JB06_CHLRE/182-297	A8JB06.1 PF00581.18	55.20	0.00
TRINITY_K7L0E8_SOYBN/435-562	K7L0E8.1 PF01909.21	55.20	0.00
TRINITY_A8I807_CHLRE/1018-1101	A8I807.1 PF00211.18	55.20	0.00
TRINITY_M2W5E2_GALSU/2-375	M2W5E2.1 PF00022.17	55.20	0.00
TRINITY_Q22DU3_TETTS/53-268	Q22DU3.1 PF01988.17	55.20	0.00
TRINITY_G0R147_ICHMG/150-282	G0R147.1 PF06003.10	55.10	0.00
TRINITY_G0QTU9_ICHMG/187-497	G0QTU9.1 PF00883.19	55.10	0.00
TRINITY_J3QRK3_HUMAN/11-61	J3QRK3.1 PF13920.4;	55.10	0.00
TRINITY_F4PTN1_DICFS/42-388	F4PTN1.1 PF07690.14	55.10	0.00
TRINITY_G0QRM1_ICHMG/13-169	G0QRM1.1 PF12850.5;	55.10	0.00
TRINITY_I1F0T5_AMPQE/143-214	I1F0T5.1 PF07258.12	55.10	0.00
TRINITY_A0A0N5BCJ9_STREA/12-342	A0A0N5BCJ9.1 PF0050	55.10	0.00
TRINITY_Q23FX2_TETTS/36-451	Q23FX2.3 PF00999.19	55.10	0.00

TRINITY_G7YTC4_CLOSI/599-734	G7YTC4.1 PF04146.13	55.10	0.00
TRINITY_G8R4J3_OWEHD/54-189	G8R4J3.1 PF02557.15	55.10	0.00
TRINITY_F0ZHR0_DICPU/110-202	F0ZHR0.1 PF04777.11	55.10	0.00
TRINITY_A9SGR7_PHYPA/200-606	A9SGR7.1 PF00962.20	55.10	0.00
TRINITY_A0A077ZQS0_STYLE/68-262	A0A077ZQS0.1 PF1214	55.10	0.00
TRINITY_I2JY54_DEKBR/1-329	I2JY54.1 PF00022.17	55.10	0.00
TRINITY_H0A378_9PROT/13-362	H0A378.1 PF01266.22	55.10	0.00
TRINITY_S2JIV7_MUCC1/97-174	S2JIV7.1 PF16282.3;	55.10	0.00
TRINITY_Q22D97_TETTS/564-699	Q22D97.1 PF00005.25	55.10	0.00
TRINITY_I1FJ82_AMPQE/74-164	I1FJ82.1 PF02214.20	55.10	0.00
TRINITY_K9Z194_CYAAP/83-151	K9Z194.1 PF00610.19	55.10	0.00
TRINITY_I7MJV9_TETTS/69-545	I7MJV9.1 PF00501.26	55.10	0.00
TRINITY_F6QE47_CIOIN/32-189	F6QE47.2 PF03367.11	55.10	0.00
TRINITY_Q22Z04_TETTS/53-204	Q22Z04.1 PF13847.4;	55.10	0.00
TRINITY_A0E0U7_PARTE/391-585	A0E0U7.1 PF05057.12	55.10	0.00
TRINITY_D8TK81_VOLCA/60-235	D8TK81.1 PF01595.18	55.10	0.00
TRINITY_A0A0G3QF21_KLUIN/97-209	A0A0G3QF21.1 PF0018	55.10	0.00
TRINITY_W4FT02_9STRA/188-499	W4FT02.1 PF00883.19	55.10	0.00
TRINITY_D8TNX4_VOLCA/199-291	D8TNX4.1 PF00085.18	55.10	0.00
TRINITY_G0SZ73_RHOG2/396-454	G0SZ73.1 PF00226.29	55.10	0.00
TRINITY_D8TSB6_VOLCA/10-279	D8TSB6.1 PF03998.11	55.10	0.00
TRINITY_J9IHW3_9SPIT/644-695	J9IHW3.1 PF04968.10	55.10	0.00
TRINITY_D3BT40_POLPA/479-585	D3BT40.1 PF00307.29	55.10	0.00
TRINITY_D3B2T4_POLPA/258-512	D3B2T4.1 PF03949.13	55.10	0.00
TRINITY_D3B2K0_POLPA/101-590	D3B2K0.1 PF00118.22	55.10	0.00
TRINITY_D8TGY6_VOLCA/234-486	D8TGY6.1 PF03949.13	55.10	0.00
TRINITY_F4PJE6_DICFS/386-434	F4PJE6.1 PF14604.4;	55.10	0.00
TRINITY_A0C0F4_PARTE/399-612	A0C0F4.1 PF07859.11	55.10	0.00
TRINITY_D8U111_VOLCA/16-117	D8U111.1 PF12796.5;	55.10	0.00
TRINITY_A0A0D2WU03_CAPO3/526-643	A0A0D2WU03.1 PF1197	55.10	0.00
TRINITY_I7LUL2_TETTS/109-268	I7LUL2.1 PF00849.20	55.10	0.00
TRINITY_L8H596_ACACA/587-655	L8H596.1 PF13884.4;	55.10	0.00
TRINITY_A0A059BFV3_EUCGR/483-532	A0A059BFV3.1 PF0041	55.10	0.00
TRINITY_A0A067N164_9HOMO/74-127	A0A067N164.1 PF0222	55.10	0.00
TRINITY_A8HQM9_CHLRE/18-261	A8HQM9.1 PF07264.9;	55.10	0.00
TRINITY_A0A078AWA6_STYLE/56-620	A0A078AWA6.1 PF0044	55.10	0.00
TRINITY_F8L621_SIMNZ/408-626	F8L621.1 PF00702.24	55.10	0.00
TRINITY_M0U7P0_MUSAM/675-724	M0U7P0.1 PF00415.16	55.10	0.00
TRINITY_D8TGX6_VOLCA/877-1013	D8TGX6.1 PF01925.17	55.10	0.00
TRINITY_T1NWZ3_TRIUA/3-405	T1NWZ3.1 PF02897.13	55.10	0.00
TRINITY_A0A078A5E8_STYLE/235-303	A0A078A5E8.1 PF0007	55.10	0.00
TRINITY_Q7XXT3_CHLRE/210-446	Q7XXT3.1 PF00208.19	55.10	0.00
TRINITY_D8TNV2_VOLCA/856-1080	D8TNV2.1 PF00069.23	55.10	0.00
TRINITY_A6G013_9DELT/29-479	A6G013.1 PF02133.13	55.10	0.00
TRINITY_D8UEG9_VOLCA/181-367	D8UEG9.1 PF13848.4;	55.10	0.00
TRINITY_Q7XRL9_ORYSJ/564-738	Q7XRL9.2 PF00078.25	55.10	0.00
TRINITY_L8GJ61_ACACA/82-379	L8GJ61.1 PF00579.23	55.10	0.00
TRINITY_A0A087HDY2_ARAAL/25-123	A0A087HDY2.1 PF0038	55.10	0.00
TRINITY_G0QYG5_ICHMG/141-688	G0QYG5.1 PF03571.13	55.10	0.00
TRINITY_V2XGQ0_MONRO/218-326	V2XGQ0.1 PF00175.19	55.10	0.00
TRINITY_S9WXX3_SCHCR/161-230	S9WXX3.1 PF00076.20	55.00	0.00
TRINITY_L8H857_ACACA/42-209	L8H857.1 PF06884.9;	55.00	0.00
TRINITY_Q23CX0_TETTS/24-63	Q23CX0.2 PF15227.4;	55.00	0.00
TRINITY_A0A024QH81_9BACI/6-150	A0A024QH81.1 PF0871	55.00	0.00
TRINITY_A0A074Y438_9PEZI/553-739	A0A074Y438.1 PF0498	55.00	0.00
TRINITY_I4C884_DESTA/350-499	I4C884.1 PF00005.25	55.00	0.00
TRINITY_Q24CZ5_TETTS/7-168	Q24CZ5.2 PF00071.20	55.00	0.00
TRINITY_Q234R6_TETTS/130-349	Q234R6.1 PF01556.16	55.00	0.00
TRINITY_K1PZP8_CRAGI/95-259	K1PZP8.1 PF01746.19	55.00	0.00
TRINITY_D2V4S4_NAEGR/16-181	D2V4S4.1 PF08534.8;	55.00	0.00
TRINITY_I1BGP6_RHIO9/12-73	I1BGP6.1 PF00226.29	55.00	0.00

TRINITY_A0DYX2_PARTE/54-188	A0DYX2.1 PF04146.13	55.00	0.00
TRINITY_A0C4I4_PARTE/1-218	A0C4I4.1 PF01591.16	55.00	0.00
TRINITY_I7MF78_TETTS/79-205	I7MF78.1 PF00828.17	55.00	0.00
TRINITY_L8H8X3_ACACA/150-369	L8H8X3.1 PF01556.16	55.00	0.00
TRINITY_M1V5K1_CYAME/772-813	M1V5K1.1 PF00400.30	55.00	0.00
TRINITY_A0BJC2_PARTE/318-756	A0BJC2.1 PF04053.12	55.00	0.00
TRINITY_F4QDK7_DICFS/154-257	F4QDK7.1 PF12756.5;	55.00	0.00
TRINITY_L8GLT8_ACACA/131-535	L8GLT8.1 PF13520.4;	55.00	0.00
TRINITY_G0FN68_AMYMS/122-379	G0FN68.1 PF00487.22	55.00	0.00
TRINITY_A0A015LLT8_9GLOM/312-428	A0A015LLT8.1 PF0066	55.00	0.00
TRINITY_F0ZHG6_DICPU/113-449	F0ZHG6.1 PF02628.13	55.00	0.00
TRINITY_Q22W19_TETTS/115-323	Q22W19.3 PF00112.21	55.00	0.00
TRINITY_A8JCM5_CHLRE/592-791	A8JCM5.1 PF01369.18	55.00	0.00
TRINITY_Q23A17_TETTS/301-520	Q23A17.1 PF07002.14	55.00	0.00
TRINITY_I0Z315_9CHLO/276-512	I0Z315.1 PF13641.4;	55.00	0.00
TRINITY_A0DRP1_PARTE/26-544	A0DRP1.1 PF01602.18	55.00	0.00
TRINITY_A0C4I4_PARTE/1-218	A0C4I4.1 PF01591.16	55.00	0.00
TRINITY_A0E5L3_PARTE/49-221	A0E5L3.1 PF00270.27	55.00	0.00
TRINITY_M5GGS4_DACSP/71-254	M5GGS4.1 PF02630.12	55.00	0.00
TRINITY_S7QDQ3_GLOTA/11-231	S7QDQ3.1 PF00710.18	55.00	0.00
TRINITY_A0CRV1_PARTE/99-317	A0CRV1.1 PF01556.16	55.00	0.00
TRINITY_D8U202_VOLCA/350-568	D8U202.1 PF00149.26	55.00	0.00
TRINITY_V9F1A7_PHYPR/115-362	V9F1A7.1 PF00155.19	55.00	0.00
TRINITY_D8TP49_VOLCA/639-682	D8TP49.1 PF13920.4;	55.00	0.00
TRINITY_L8GTJ0_ACACA/1-167	L8GTJ0.1 PF00069.23	55.00	0.00
TRINITY_D2VAN2_NAEGR/6-526	D2VAN2.1 PF04734.11	55.00	0.00
TRINITY_A9U358_PHYPA/745-1059	A9U358.1 PF03178.13	55.00	0.00
TRINITY_Q23MB3_TETTS/79-354	Q23MB3.2 PF02383.16	55.00	0.00
TRINITY_I1FE07_AMPQE/138-289	I1FE07.1 PF00849.20	55.00	0.00
TRINITY_A0A023B855_GRENI/22-64	A0A023B855.1 PF1290	55.00	0.00
TRINITY_D3B601_POLPA/76-445	D3B601.1 PF01979.18	55.00	0.00
TRINITY_K9S7I1_9CYAN/456-567	K9S7I1.1 PF00072.22	55.00	0.00
TRINITY_Q23QY3_TETTS/416-917	Q23QY3.2 PF00899.19	55.00	0.00
TRINITY_A8J018_CHLRE/492-680	A8J018.1 PF13307.4;	55.00	0.00
TRINITY_Q3SDM6_PARTE/17-177	Q3SDM6.1 PF00071.20	55.00	0.00
TRINITY_G0QKM2_ICHMG/159-223	G0QKM2.1 PF13499.4;	55.00	0.00
TRINITY_A8J1V6_CHLRE/56-196	A8J1V6.1 PF11051.6;	55.00	0.00
TRINITY_A0A087SN24_AUXPR/114-276	A0A087SN24.1 PF1384	55.00	0.00
TRINITY_D8TZS8_VOLCA/96-370	D8TZS8.1 PF00685.25	55.00	0.00
TRINITY_Q22RZ8_TETTS/183-242	Q22RZ8.2 PF13921.4;	55.00	0.00
TRINITY_K9VDR2_9CYAN/6-137	K9VDR2.1 PF01124.16	55.00	0.00
TRINITY_D8TMJ4_VOLCA/2-264	D8TMJ4.1 PF00481.19	55.00	0.00
TRINITY_A4XRA0_PSEMY/26-259	A4XRA0.1 PF03575.15	55.00	0.00
TRINITY_D8TNH5_VOLCA/832-942	D8TNH5.1 PF00251.18	55.00	0.00
TRINITY_A7SX93_NEMVE/403-769	A7SX93.1 PF04685.11	55.00	0.00
TRINITY_I7M8Z3_TETTS/14-184	I7M8Z3.2 PF00025.19	55.00	0.00
TRINITY_J9J7M7_9SPIT/238-317	J9J7M7.1 PF10159.7;	55.00	0.00
TRINITY_A0CFP3_PARTE/86-235	A0CFP3.1 PF00650.18	54.90	0.00
TRINITY_A0EG16_PARTE/9-79	A0EG16.1 PF00076.20	54.90	0.00
TRINITY_J9I2N4_9SPIT/16-128	J9I2N4.1 PF02466.17	54.90	0.00
TRINITY_I7MLI6_TETTS/33-173	I7MLI6.1 PF13185.4;	54.90	0.00
TRINITY_D2VK26_NAEGR/13-300	D2VK26.1 PF00069.23	54.90	0.00
TRINITY_W4XPK0_STRPU/4-207	W4XPK0.1 PF00106.23	54.90	0.00
TRINITY_D2VFG0_NAEGR/336-551	D2VFG0.1 PF07002.14	54.90	0.00
TRINITY_A0A078B270_STYLE/2405-2625	A0A078B270.1 PF0064	54.90	0.00
TRINITY_A0A0M0J609_9EUKA/34-265	A0A0M0J609.1 PF0116	54.90	0.00
TRINITY_A0EBN0_PARTE/66-334	A0EBN0.1 PF00069.23	54.90	0.00
TRINITY_Q22BA7_TETTS/59-282	Q22BA7.1 PF07228.10	54.90	0.00
TRINITY_G0QM20_ICHMG/331-399	G0QM20.1 PF00498.24	54.90	0.00
TRINITY_A0A0L0BZ22_LUCCU/8-78	A0A0L0BZ22.1 PF0664	54.90	0.00
TRINITY_D6WFW5_TRICA/366-590	D6WFW5.1 PF00009.25	54.90	0.00

TRINITY_A0A0G4FRX5_9ALVE/6-341	A0A0G4FRX5.1 PF0563	54.90	0.00
TRINITY_A0A0G4F0P3_9ALVE/46-96	A0A0G4F0P3.1 PF1290	54.90	0.00
TRINITY_I7LXB6_TETTS/1143-1299	I7LXB6.2 PF00005.25	54.90	0.00
TRINITY_Q54UP1_DICDI/142-283	Q54UP1.1 PF04851.13	54.90	0.00
TRINITY_D8SIG5_SELML/2-156	D8SIG5.1 PF03079.12	54.90	0.00
TRINITY_G3IVK3_9GAMM/389-530	G3IVK3.1 PF02775.19	54.90	0.00
TRINITY_I7MKW7_TETTS/16-270	I7MKW7.1 PF08450.10	54.90	0.00
TRINITY_W1PZS0_AMBTC/33-443	W1PZS0.1 PF03901.15	54.90	0.00
TRINITY_E3CZV0_9BACT/24-354	E3CZV0.1 PF00155.19	54.90	0.00
TRINITY_G0R6E8_ICHMG/182-459	G0R6E8.1 PF00246.22	54.90	0.00
TRINITY_S3C2H7_OPHP1/1044-1114	S3C2H7.1 PF04564.13	54.90	0.00
TRINITY_L8HEB5_ACACA/599-681	L8HEB5.1 PF03366.14	54.90	0.00
TRINITY_A8J0C9_CHLRE/70-168	A8J0C9.1 PF08576.8;	54.90	0.00
TRINITY_MCFG_DICDI/11-103	Q54BM3.1 PF00153.25	54.90	0.00
TRINITY_A0A0M8MT83_9BASI/126-176	A0A0M8MT83.1 PF0009	54.90	0.00
TRINITY_A0A061E799_THECC/412-703	A0A061E799.1 PF0006	54.90	0.00
TRINITY_K3WDP5_PYTUL/54-135	K3WDP5.1 PF00076.20	54.90	0.00
TRINITY_I0YW03_9CHLO/108-400	I0YW03.1 PF03095.13	54.90	0.00
TRINITY_A4VDQ6_TETTS/51-165	A4VDQ6.1 PF05071.14	54.90	0.00
TRINITY_D8TKX3_VOLCA/242-413	D8TKX3.1 PF13229.4;	54.90	0.00
TRINITY_D8TVJ4_VOLCA/22-281	D8TVJ4.1 PF03372.21	54.90	0.00
TRINITY_I7M323_TETTS/94-382	I7M323.1 PF00579.23	54.90	0.00
TRINITY_L8GTK3_ACACA/495-608	L8GTK3.1 PF00989.23	54.90	0.00
TRINITY_MLH1_MOUSE/23-248	Q9JK91.2 PF02518.24	54.90	0.00
TRINITY_Q22GW0_TETTS/1651-1813	Q22GW0.2 PF00069.23	54.90	0.00
TRINITY_M0TLF4_MUSAM/89-401	M0TLF4.1 PF01074.20	54.90	0.00
TRINITY_L1JHU7_GUITH/1-193	L1JHU7.1 PF08450.10	54.90	0.00
TRINITY_I7M701_TETTS/137-332	I7M701.1 PF00092.26	54.90	0.00
TRINITY_A8IME4_CHLRE/1-185	A8IME4.1 PF05018.11	54.90	0.00
TRINITY_A8I1W3_CHLRE/2374-2753	A8I1W3.1 PF02259.21	54.90	0.00
TRINITY_A8J8G9_CHLRE/153-439	A8J8G9.1 PF00122.18	54.90	0.00
TRINITY_A8JB24_CHLRE/3-73	A8JB24.1 PF00076.20	54.90	0.00
TRINITY_D8UEU3_VOLCA/110-291	D8UEU3.1 PF13419.4;	54.90	0.00
TRINITY_M7NW25_PNEMU/332-655	M7NW25.1 PF00176.21	54.90	0.00
TRINITY_D8TIL7_VOLCA/250-554	D8TIL7.1 PF02274.15	54.90	0.00
TRINITY_L8H601_ACACA/278-728	L8H601.1 PF00183.16	54.90	0.00
TRINITY_A9SFY1_PHYPA/1448-1765	A9SFY1.1 PF00176.21	54.90	0.00
TRINITY_A0A0K9P5E0_ZOSMR/22-116	A0A0K9P5E0.1 PF0566	54.90	0.00
TRINITY_I7LWF2_TETTS/9-141	I7LWF2.1 PF00179.24	54.90	0.00
TRINITY_A0A090L8Y6_STRRB/482-834	A0A090L8Y6.1 PF0000	54.90	0.00
TRINITY_I0YT59_9CHLO/9-172	I0YT59.1 PF01170.16	54.90	0.00
TRINITY_W4FD00_9STRA/147-197	W4FD00.1 PF13432.4;	54.90	0.00
TRINITY_I7LT38_TETTS/33-104	I7LT38.1 PF16209.3;	54.90	0.00
TRINITY_D0MUC9_PHYIT/140-211	D0MUC9.1 PF07572.10	54.90	0.00
TRINITY_D8TUA7_VOLCA/875-1016	D8TUA7.1 PF01764.23	54.90	0.00
TRINITY_A8IU18_CHLRE/645-803	A8IU18.1 PF01764.23	54.90	0.00
TRINITY_A0DVR4_PARTE/17-87	A0DVR4.1 PF13499.4;	54.90	0.00
TRINITY_A0A087SSB9_AUXPR/159-276	A0A087SSB9.1 PF0933	54.90	0.00
TRINITY_A8HMC1_CHLRE/34-479	A8HMC1.1 PF00067.20	54.90	0.00
TRINITY_C1DIX7_AZOVD/10-205	C1DIX7.1 PF00753.25	54.90	0.00
TRINITY_D8UFG3_VOLCA/542-705	D8UFG3.1 PF07534.14	54.90	0.00
TRINITY_D8UJP5_VOLCA/5-122	D8UJP5.1 PF05773.20	54.90	0.00
TRINITY_A0CL90_PARTE/477-726	A0CL90.1 PF07714.15	54.90	0.00
TRINITY_Q5CUZ6_CRYPI/11-183	Q5CUZ6.1 PF00025.19	54.80	0.00
TRINITY_I4EHU7_9CHLR/282-401	I4EHU7.1 PF07731.12	54.80	0.00
TRINITY_G0R1C4_ICHMG/49-235	G0R1C4.1 PF10211.7;	54.80	0.00
TRINITY_A0A078DCT9_BRANA/1-112	A0A078DCT9.1 PF0240	54.80	0.00
TRINITY_F4PGH8_DICFS/309-533	F4PGH8.1 PF13236.4;	54.80	0.00
TRINITY_A0EG50_PARTE/15-118	A0EG50.1 PF00168.28	54.80	0.00
TRINITY_Q5CQI6_CRYPI/19-156	Q5CQI6.1 PF00179.24	54.80	0.00
TRINITY_F0ZFF8_DICPU/18-131	F0ZFF8.1 PF01398.19	54.80	0.00

TRINITY_F2UET0_SALR5/191-280	F2UET0.1 PF09451.8;	54.80	0.00
TRINITY_Q23KI7_TETTS/1600-1806	Q23KI7.2 PF12348.6;	54.80	0.00
TRINITY_L8HIK1_ACACA/126-176	L8HIK1.1 PF13418.4;	54.80	0.00
TRINITY_Q00SL7_OSTTA/43-132	Q00SL7.1 PF00153.25	54.80	0.00
TRINITY_Q22A31_TETTS/163-484	Q22A31.4 PF01207.15	54.80	0.00
TRINITY_B3S0L3_TRIAD/4-66	B3S0L3.1 PF00076.20	54.80	0.00
TRINITY_L8H6V3_ACACA/29-430	L8H6V3.1 PF00450.20	54.80	0.00
TRINITY_A0BEV3_PARTE/80-358	A0BEV3.1 PF02383.16	54.80	0.00
TRINITY_H3G7I0_PHYRM/25-154	H3G7I0.1 PF13589.4;	54.80	0.00
TRINITY_G0R2M8_ICHMG/1378-1595	G0R2M8.1 PF00609.17	54.80	0.00
TRINITY_B7G074_PHATC/413-475	B7G074.1 PF12738.5;	54.80	0.00
TRINITY_I0Z559_9CHLO/145-582	I0Z559.1 PF01131.18	54.80	0.00
TRINITY_K9TMT3_9CYAN/114-396	K9TMT3.1 PF00082.20	54.80	0.00
TRINITY_D0YXL3_PHODD/796-907	D0YXL3.1 PF02518.24	54.80	0.00
TRINITY_A0A061F2E7_THECC/190-438	A0A061F2E7.1 PF0012	54.80	0.00
TRINITY_NDRB_DICDI/130-437	Q54IH8.1 PF00069.23	54.80	0.00
TRINITY_I7MDB1_TETTS/296-654	I7MDB1.2 PF03133.13	54.80	0.00
TRINITY_D8UJN2_VOLCA/21-260	D8UJN2.1 PF00248.19	54.80	0.00
TRINITY_D8TP59_VOLCA/83-228	D8TP59.1 PF00675.18	54.80	0.00
TRINITY_D6CWL4_9BACE/148-449	D6CWL4.1 PF00251.18	54.80	0.00
TRINITY_T1GP12_MEGSC/76-228	T1GP12.1 PF00929.22	54.80	0.00
TRINITY_M0QSM4_ACACA/400-492	M0QSM4.1 PF02214.20	54.80	0.00
TRINITY_L8HIA3_ACACA/6-287	L8HIA3.1 PF04909.12	54.80	0.00
TRINITY_A8J409_CHLRE/3-75	A8J409.1 PF00240.21	54.80	0.00
TRINITY_B0WZR8_CULQU/14-182	B0WZR8.1 PF00071.20	54.80	0.00
TRINITY_D8TUM0_VOLCA/73-555	D8TUM0.1 PF10433.7;	54.80	0.00
TRINITY_E1ZCN5_CHLVA/397-598	E1ZCN5.1 PF12706.5;	54.80	0.00
TRINITY_F0ZF65_DICPU/175-440	F0ZF65.1 PF01189.15	54.80	0.00
TRINITY_I0Z0E5_9CHLO/7-287	I0Z0E5.1 PF01875.15	54.80	0.00
TRINITY_D7KK04_ARALL/107-294	D7KK04.1 PF02698.15	54.80	0.00
TRINITY_A8I4K4_CHLRE/128-297	A8I4K4.1 PF03031.16	54.80	0.00
TRINITY_A8J1H0_CHLRE/65-495	A8J1H0.1 PF00909.19	54.80	0.00
TRINITY_L8GEB3_ACACA/338-766	L8GEB3.1 PF04053.12	54.80	0.00
TRINITY_U3K2G7_FICAL/81-402	U3K2G7.1 PF01546.26	54.80	0.00
TRINITY_G0QNT4_ICHMG/321-534	G0QNT4.1 PF00246.22	54.80	0.00
TRINITY_I0XNB0_9LEPT/41-103	I0XNB0.1 PF03625.12	54.80	0.00
TRINITY_M7WPF2_RHOT1/493-595	M7WPF2.1 PF02204.16	54.80	0.00
TRINITY_A8I9C1_CHLRE/175-267	A8I9C1.1 PF00153.25	54.80	0.00
TRINITY_D8TVL4_VOLCA/254-358	D8TVL4.1 PF01399.25	54.80	0.00
TRINITY_D7TBS7_VITVI/99-223	D7TBS7.1 PF14226.4;	54.80	0.00
TRINITY_E1ZDS3_CHLVA/17-203	E1ZDS3.1 PF10263.7;	54.80	0.00
TRINITY_D7FKH8_ECTSI/433-566	D7FKH8.1 PF01841.17	54.80	0.00
TRINITY_D3B5I3_POLPA/203-296	D3B5I3.1 PF00153.25	54.80	0.00
TRINITY_A8HZG7_CHLRE/8-169	A8HZG7.1 PF03547.16	54.80	0.00
TRINITY_A8IP72_CHLRE/3640-3875	A8IP72.1 PF12781.5;	54.80	0.00
TRINITY_D8U702_VOLCA/48-293	D8U702.1 PF07714.15	54.80	0.00
TRINITY_L8H3W5_ACACA/971-1093	L8H3W5.1 PF00271.29	54.80	0.00
TRINITY_D8UD65_VOLCA/1295-1479	D8UD65.1 PF08148.10	54.80	0.00
TRINITY_A0A080WS87_TRIRC/1-299	A0A080WS87.1 PF0006	54.80	0.00
TRINITY_C3YCQ4_BRAFL/2-75	C3YCQ4.1 PF02798.18	54.80	0.00
TRINITY_A7AS55_BABBO/4-104	A7AS55.1 PF03297.13	54.80	0.00
TRINITY_B4WMW0_9SYNE/1370-1484	B4WMW0.1 PF00072.22	54.80	0.00
TRINITY_D8UEM9_VOLCA/200-272	D8UEM9.1 PF04564.13	54.80	0.00
TRINITY_A8JGH3_CHLRE/759-1028	A8JGH3.1 PF02714.13	54.80	0.00
TRINITY_D8U6I8_VOLCA/629-717	D8U6I8.1 PF00211.18	54.80	0.00
TRINITY_A0A0J7KDP1_LASNI/474-593	A0A0J7KDP1.1 PF0066	54.80	0.00
TRINITY_D8UJ52_VOLCA/83-339	D8UJ52.1 PF01237.16	54.80	0.00
TRINITY_C4M6A4_ENTHI/5-468	C4M6A4.1 PF01039.20	54.80	0.00
TRINITY_I0YVK1_9CHLO/101-520	I0YVK1.1 PF02897.13	54.80	0.00
TRINITY_D2A369_TRICA/29-113	D2A369.1 PF13417.4;	54.80	0.00
TRINITY_T1G2W1_HELRO/10-79	T1G2W1.1 PF02798.18	54.80	0.00

TRINITY_A0A087SNF7_AUXPR/636-724	A0A087SNF7.1 PF0202	54.80	0.00
TRINITY_Q7XRL9_ORYSJ/564-738	Q7XRL9.2 PF00078.25	54.80	0.00
TRINITY_Q23G43_TETTS/140-301	Q23G43.2 PF00326.19	54.70	0.00
TRINITY_R1G8R0_BOTPV/91-297	R1G8R0.1 PF01871.15	54.70	0.00
TRINITY_B3RI21_TRIAD/3-160	B3RI21.1 PF04101.14	54.70	0.00
TRINITY_I7M6P2_TETTS/611-745	I7M6P2.2 PF07717.14	54.70	0.00
TRINITY_L8HHS3_ACACA/744-951	L8HHS3.1 PF00566.16	54.70	0.00
TRINITY_Q23PP8_TETTS/225-367	Q23PP8.1 PF04116.11	54.70	0.00
TRINITY_I3KW27_ORENI/187-334	I3KW27.1 PF00849.20	54.70	0.00
TRINITY_I7MFQ7_TETTS/652-907	I7MFQ7.2 PF00069.23	54.70	0.00
TRINITY_A0A0M0LRP5_9EUKA/1-407	A0A0M0LRP5.1 PF1283	54.70	0.00
TRINITY_A0A0F4ZAA3_9PEZI/198-462	A0A0F4ZAA3.1 PF0006	54.70	0.00
TRINITY_A0A0J8BV14_BETVU/162-227	A0A0J8BV14.1 PF0007	54.70	0.00
TRINITY_Q22T76_TETTS/30-371	Q22T76.2 PF00225.21	54.70	0.00
TRINITY_I8REC5_9FIRM/8-198	I8REC5.1 PF01734.20	54.70	0.00
TRINITY_A8JBZ2_CHLRE/108-313	A8JBZ2.1 PF13460.4;	54.70	0.00
TRINITY_J9IX82_9SPIT/757-1008	J9IX82.1 PF16212.3;	54.70	0.00
TRINITY_A0A059AGJ9_EUCGR/161-422	A0A059AGJ9.1 PF0263	54.70	0.00
TRINITY_L8GZH9_ACACA/38-295	L8GZH9.1 PF04427.16	54.70	0.00
TRINITY_D2VNB4_NAEGR/168-493	D2VNB4.1 PF00176.21	54.70	0.00
TRINITY_M0RXA8_MUSAM/117-180	M0RXA8.1 PF00400.30	54.70	0.00
TRINITY_A0A059LER1_9CHLO/66-239	A0A059LER1.1 PF0599	54.70	0.00
TRINITY_H2MLX9_ORYLA/17-80	H2MLX9.1 PF01442.16	54.70	0.00
TRINITY_F4Q169_DICFS/12-262	F4Q169.1 PF00069.23	54.70	0.00
TRINITY_D8TUL9_VOLCA/147-372	D8TUL9.1 PF09353.8;	54.70	0.00
TRINITY_G0QS55_ICHMG/919-1039	G0QS55.1 PF02687.19	54.70	0.00
TRINITY_Q24GQ7_TETTS/133-301	Q24GQ7.1 PF04664.11	54.70	0.00
TRINITY_Q1IIS4_KORVE/170-433	Q1IIS4.1 PF03571.13	54.70	0.00
TRINITY_E1Z9L5_CHLVA/93-351	E1Z9L5.1 PF13469.4;	54.70	0.00
TRINITY_D3BT40_POLPA/479-585	D3BT40.1 PF00307.29	54.70	0.00
TRINITY_A0E8D8_PARTE/487-711	A0E8D8.1 PF00149.26	54.70	0.00
TRINITY_A0A022QR24_ERYGU/129-199	A0A022QR24.1 PF1026	54.70	0.00
TRINITY_L8H039_ACACA/206-457	L8H039.1 PF00069.23	54.70	0.00
TRINITY_Q5CUZ6_CRYPI/11-183	Q5CUZ6.1 PF00025.19	54.70	0.00
TRINITY_A0A0N4YGY5_NIPBR/309-824	A0A0N4YGY5.1 PF0519	54.70	0.00
TRINITY_B4WRB7_9SYNE/4-82	B4WRB7.1 PF05239.14	54.70	0.00
TRINITY_L8H0X9_ACACA/9-146	L8H0X9.1 PF00179.24	54.70	0.00
TRINITY_L8H0T4_ACACA/417-589	L8H0T4.1 PF00644.18	54.70	0.00
TRINITY_A0CKF8_PARTE/1118-1269	A0CKF8.1 PF00005.25	54.70	0.00
TRINITY_D8TQ93_VOLCA/109-161	D8TQ93.1 PF09834.7;	54.70	0.00
TRINITY_W7X7I9_TETTS/323-408	W7X7I9.1 PF00439.23	54.70	0.00
TRINITY_A0E7H1_PARTE/82-449	A0E7H1.1 PF00155.19	54.70	0.00
TRINITY_I0ZAB0_9CHLO/226-314	I0ZAB0.1 PF00153.25	54.70	0.00
TRINITY_A0A087SSF7_AUXPR/366-672	A0A087SSF7.1 PF0371	54.70	0.00
TRINITY_D8U3D7_VOLCA/31-246	D8U3D7.1 PF01036.16	54.70	0.00
TRINITY_D8UJH8_VOLCA/5-69	D8UJH8.1 PF13499.4;	54.70	0.00
TRINITY_B6IN35_RHOCS/786-841	B6IN35.1 PF13368.4;	54.70	0.00
TRINITY_A7SJK6_NEMVE/4-377	A7SJK6.1 PF00022.17	54.70	0.00
TRINITY_G0R3J0_ICHMG/181-308	G0R3J0.1 PF14833.4;	54.70	0.00
TRINITY_D8U0S3_VOLCA/739-921	D8U0S3.1 PF05193.19	54.70	0.00
TRINITY_R1D9Q6_EMIHU/11-61	R1D9Q6.1 PF00628.27	54.70	0.00
TRINITY_Q3SD15_PARTE/6-168	Q3SD15.1 PF00071.20	54.70	0.00
TRINITY_B9H981_POPTR/146-504	B9H981.2 PF00152.18	54.70	0.00
TRINITY_I1CC05_RHIO9/28-143	I1CC05.1 PF00085.18	54.70	0.00
TRINITY_F4PSU3_DICFS/306-385	F4PSU3.1 PF01453.22	54.70	0.00
TRINITY_L8H446_ACACA/214-362	L8H446.1 PF00179.24	54.70	0.00
TRINITY_J9HMM9_9SPIT/101-388	J9HMM9.1 PF00122.18	54.70	0.00
TRINITY_K2D3R8_9BACT/180-337	K2D3R8.1 PF00128.22	54.70	0.00
TRINITY_Q75JA1_DICDI/1040-1293	Q75JA1.1 PF08235.11	54.70	0.00
TRINITY_A0A075AV78_9FUNG/154-274	A0A075AV78.1 PF0184	54.70	0.00
TRINITY_D8TQI6_VOLCA/239-337	D8TQI6.1 PF00027.27	54.70	0.00

TRINITY_A0A078A9T0_STYLE/80-292	A0A078A9T0.1 PF0011	54.70	0.00
TRINITY_D8TIN2_VOLCA/1-163	D8TIN2.1 PF10436.7;	54.70	0.00
TRINITY_A0DF13_PARTE/219-645	A0DF13.1 PF06862.10	54.70	0.00
TRINITY_S7Q0J7_GLOTA/499-741	S7Q0J7.1 PF16203.3;	54.70	0.00
TRINITY_V6S698_9FLAO/3-238	V6S698.1 PF03721.12	54.70	0.00
TRINITY_E0W0I4_PEDHC/872-1326	E0W0I4.1 PF00145.15	54.60	0.00
TRINITY_DUS1_ARATH/58-188	Q9ZR37.1 PF00782.18	54.60	0.00
TRINITY_H8K3Y5_RICAG/5-395	H8K3Y5.1 PF01134.20	54.60	0.00
TRINITY_G0R101_ICHMG/12-220	G0R101.1 PF00149.26	54.60	0.00
TRINITY_A8IQ53_CHLRE/2-175	A8IQ53.1 PF01184.17	54.60	0.00
TRINITY_E1ZDV7_CHLVA/13-202	E1ZDV7.1 PF02545.12	54.60	0.00
TRINITY_Q22MC1_TETTS/26-195	Q22MC1.2 PF00270.27	54.60	0.00
TRINITY_G0R3E4_ICHMG/224-490	G0R3E4.1 PF05021.13	54.60	0.00
TRINITY_F0ZUT3_DICPU/104-579	F0ZUT3.1 PF05761.12	54.60	0.00
TRINITY_A0A060BGL7_9GAMM/12-358	A0A060BGL7.1 PF0126	54.60	0.00
TRINITY_L8H7B2_ACACA/14-280	L8H7B2.1 PF07714.15	54.60	0.00
TRINITY_I0YLU1_9CHLO/2-217	I0YLU1.1 PF04012.10	54.60	0.00
TRINITY_A0A0G4GZV6_9ALVE/5-178	A0A0G4GZV6.1 PF0002	54.60	0.00
TRINITY_L1JGW3_GUITH/93-242	L1JGW3.1 PF03073.13	54.60	0.00
TRINITY_D8UD75_VOLCA/62-359	D8UD75.1 PF00248.19	54.60	0.00
TRINITY_I0YXH0_9CHLO/4-274	I0YXH0.1 PF14938.4;	54.60	0.00
TRINITY_I1GYV5_BRADI/706-951	I1GYV5.1 PF07727.12	54.60	0.00
TRINITY_I3ZX87_ORNRL/211-533	I3ZX87.1 PF00733.19	54.60	0.00
TRINITY_A3WF44_9SPHN/23-526	A3WF44.1 PF00118.22	54.60	0.00
TRINITY_A0A068RPQ9_9FUNG/64-500	A0A068RPQ9.1 PF0153	54.60	0.00
TRINITY_A2FSG1_TRIVA/297-416	A2FSG1.1 PF00723.19	54.60	0.00
TRINITY_A8HWY6_CHLRE/83-260	A8HWY6.1 PF09366.8;	54.60	0.00
TRINITY_A0A087XPG7_POEFO/49-145	A0A087XPG7.2 PF0219	54.60	0.00
TRINITY_A8J2R2_CHLRE/226-459	A8J2R2.1 PF04194.11	54.60	0.00
TRINITY_N1RA27_FUSC4/247-366	N1RA27.1 PF01399.25	54.60	0.00
TRINITY_A0A068RPY8_9FUNG/55-243	A0A068RPY8.1 PF0022	54.60	0.00
TRINITY_W7XCF2_TETTS/36-200	W7XCF2.1 PF03637.15	54.50	0.00
TRINITY_A0A0E3ZGI9_9BACT/111-270	A0A0E3ZGI9.1 PF0295	54.50	0.00
TRINITY_Q22AH3_TETTS/20-208	Q22AH3.2 PF00485.16	54.50	0.00
TRINITY_F0ZEM8_DICPU/96-468	F0ZEM8.1 PF01546.26	54.50	0.00
TRINITY_A0A0F2T7R1_9ACTN/109-337	A0A0F2T7R1.1 PF0010	54.50	0.00
TRINITY_Q23TC9_TETTS/457-522	Q23TC9.2 PF01485.19	54.50	0.00
TRINITY_L8GVK7_ACACA/26-246	L8GVK7.1 PF01965.22	54.50	0.00
TRINITY_D2A482_TRICA/997-1142	D2A482.1 PF00005.25	54.50	0.00
TRINITY_G0R050_ICHMG/14-167	G0R050.1 PF13302.5;	54.50	0.00
TRINITY_A0DZH7_PARTE/105-216	A0DZH7.1 PF00651.29	54.50	0.00
TRINITY_T1EM35_HELRO/362-1082	T1EM35.1 PF03028.13	54.50	0.00
TRINITY_L8GQ15_ACACA/505-723	L8GQ15.1 PF02450.13	54.50	0.00
TRINITY_A0A015K3X5_9GLOM/1348-1691	A0A015K3X5.1 PF0225	54.50	0.00
TRINITY_Q555X7_DICDI/54-355	Q555X7.1 PF10343.7;	54.50	0.00
TRINITY_I7MGP2_TETTS/91-280	I7MGP2.1 PF00573.20	54.50	0.00
TRINITY_Q22U45_TETTS/225-538	Q22U45.1 PF00883.19	54.50	0.00
TRINITY_K9TLZ5_9CYAN/145-327	K9TLZ5.1 PF00211.18	54.50	0.00
TRINITY_F6B8F6_DESCC/11-109	F6B8F6.1 PF01230.21	54.50	0.00
TRINITY_D8UDD9_VOLCA/30-246	D8UDD9.1 PF00856.26	54.50	0.00
TRINITY_D8M2B1_BLAHO/16-178	D8M2B1.1 PF00071.20	54.50	0.00
TRINITY_L8HGE6_ACACA/7-152	L8HGE6.1 PF00071.20	54.50	0.00
TRINITY_H3DN92_TETNG/14-447	H3DN92.1 PF02005.14	54.50	0.00
TRINITY_A0A087ZSG8_APIME/204-327	A0A087ZSG8.1 PF0966	54.50	0.00
TRINITY_G0QVK5_ICHMG/1-165	G0QVK5.1 PF00838.15	54.50	0.00
TRINITY_G0QNF4_ICHMG/82-412	G0QNF4.1 PF00150.16	54.50	0.00
TRINITY_Q22W19_TETTS/115-323	Q22W19.3 PF00112.21	54.50	0.00
TRINITY_J9J7E3_9SPIT/2429-2649	J9J7E3.1 PF00644.18	54.50	0.00
TRINITY_Q23QF2_TETTS/248-571	Q23QF2.2 PF03133.13	54.50	0.00
TRINITY_I7LW62_TETTS/2-387	I7LW62.1 PF00022.17	54.50	0.00
TRINITY_I7M2I4_TETTS/392-512	I7M2I4.2 PF02687.19	54.50	0.00

TRINITY_T1ELD6_HELRO/5-166	T1ELD6.1 PF00071.20	54.50	0.00
TRINITY_L8H779_ACACA/2-133	L8H779.1 PF13527.5;	54.50	0.00
TRINITY_A8JHR4_CHLRE/13-142	A8JHR4.1 PF01124.16	54.50	0.00
TRINITY_L8HEJ6_ACACA/94-154	L8HEJ6.1 PF00571.26	54.50	0.00
TRINITY_A8ILC7_CHLRE/1-98	A8ILC7.1 PF03556.13	54.50	0.00
TRINITY_A0D0U1_PARTE/12-281	A0D0U1.1 PF00069.23	54.50	0.00
TRINITY_A8IQ59_CHLRE/1465-1608	A8IQ59.1 PF00520.29	54.50	0.00
TRINITY_F4PYE9_DICFS/464-585	F4PYE9.1 PF02338.17	54.50	0.00
TRINITY_K7J987_NASVI/460-578	K7J987.1 PF00665.24	54.50	0.00
TRINITY_I0ZA59_9CHLO/97-564	I0ZA59.1 PF00939.17	54.50	0.00
TRINITY_A8J9U8_CHLRE/258-417	A8J9U8.1 PF01554.16	54.50	0.00
TRINITY_L8H7R1_ACACA/43-388	L8H7R1.1 PF00224.19	54.50	0.00
TRINITY_D7FY41_ECTSI/52-95	D7FY41.1 PF13639.4;	54.50	0.00
TRINITY_D8R2N6_SELML/7-165	D8R2N6.1 PF01699.22	54.50	0.00
TRINITY_C3XRT7_BRAFL/49-255	C3XRT7.1 PF00106.23	54.50	0.00
TRINITY_D8TTV8_VOLCA/81-324	D8TTV8.1 PF00233.17	54.50	0.00
TRINITY_I0YYI3_9CHLO/7-77	I0YYI3.1 PF00076.20	54.50	0.00
TRINITY_D8UGX0_VOLCA/39-423	D8UGX0.1 PF03148.12	54.50	0.00
TRINITY_A8JJ30_CHLRE/1-100	A8JJ30.1 PF01417.18	54.50	0.00
TRINITY_A9TT91_PHYPA/166-357	A9TT91.1 PF00270.27	54.50	0.00
TRINITY_I0YS69_9CHLO/104-193	I0YS69.1 PF00153.25	54.50	0.00
TRINITY_I0ZA39_9CHLO/1-155	I0ZA39.1 PF00535.24	54.50	0.00
TRINITY_F4WL33_ACREC/62-117	F4WL33.1 PF04818.11	54.50	0.00
TRINITY_E1Z7K7_CHLVA/1096-1264	E1Z7K7.1 PF08148.10	54.50	0.00
TRINITY_A0BNU8_PARTE/87-254	A0BNU8.1 PF00650.18	54.50	0.00
TRINITY_M0SHF5_MUSAM/145-250	M0SHF5.1 PF00581.18	54.50	0.00
TRINITY_L8GN02_ACACA/559-628	L8GN02.1 PF00017.22	54.50	0.00
TRINITY_A0A0A1NT77_9FUNG/31-206	A0A0A1NT77.1 PF0027	54.50	0.00
TRINITY_A0BZG0_PARTE/490-812	A0BZG0.1 PF03133.13	54.50	0.00
TRINITY_Q24C42_TETTS/18-248	Q24C42.1 PF03803.13	54.50	0.00
TRINITY_A0A0D9MFL4_9EURO/9-355	A0A0D9MFL4.1 PF0028	54.50	0.00
TRINITY_W5LD68_ASTMX/138-301	W5LD68.1 PF03031.16	54.50	0.00
TRINITY_D8UH89_VOLCA/5-117	D8UH89.1 PF01778.15	54.50	0.00
TRINITY_W7X737_TETTS/315-581	W7X737.1 PF00481.19	54.50	0.00
TRINITY_I0Z500_9CHLO/2-262	I0Z500.1 PF03403.11	54.50	0.00
TRINITY_A0A0D2WR81_CAPO3/170-391	A0A0D2WR81.1 PF0125	54.50	0.00
TRINITY_D9S0F3_THEOJ/6-161	D9S0F3.1 PF00156.25	54.50	0.00
TRINITY_A0A060S8N1_PYCCI/1968-2088	A0A060S8N1.1 PF0066	54.50	0.00
TRINITY_Q55EX4_DICDI/567-736	Q55EX4.1 PF02390.15	54.40	0.00
TRINITY_A0DHI6_PARTE/87-146	A0DHI6.1 PF13921.4;	54.40	0.00
TRINITY_Q245Y0_TETTS/8-169	Q245Y0.1 PF00071.20	54.40	0.00
TRINITY_V4KJ74_EUTSA/268-349	V4KJ74.1 PF00400.30	54.40	0.00
TRINITY_F6QZC4_XENTR/70-368	F6QZC4.1 PF01406.17	54.40	0.00
TRINITY_Q22HF8_TETTS/19-257	Q22HF8.1 PF13561.4;	54.40	0.00
TRINITY_A0A0G4IZI2_PLABS/8-169	A0A0G4IZI2.1 PF0007	54.40	0.00
TRINITY_A0BU06_PARTE/27-216	A0BU06.1 PF01145.23	54.40	0.00
TRINITY_V3ZSB5_LOTGI/10-115	V3ZSB5.1 PF01920.18	54.40	0.00
TRINITY_G0QWP0_ICHMG/124-181	G0QWP0.1 PF01485.19	54.40	0.00
TRINITY_G0QIZ2_ICHMG/16-177	G0QIZ2.1 PF00071.20	54.40	0.00
TRINITY_M2PPD1_CERS8/446-564	M2PPD1.1 PF12708.5;	54.40	0.00
TRINITY_A0A078AR51_STYLE/8-287	A0A078AR51.1 PF0006	54.40	0.00
TRINITY_E8QX25_ISOPI/79-315	E8QX25.1 PF04055.19	54.40	0.00
TRINITY_Q3SDT4_PARTE/8-173	Q3SDT4.1 PF00071.20	54.40	0.00
TRINITY_F1A5Z4_DICPU/161-217	F1A5Z4.1 PF13639.4;	54.40	0.00
TRINITY_G0QVH9_ICHMG/93-465	G0QVH9.1 PF01546.26	54.40	0.00
TRINITY_F4QF57_DICFS/124-262	F4QF57.1 PF03734.12	54.40	0.00
TRINITY_A1WG70_VEREI/650-717	A1WG70.1 PF00364.20	54.40	0.00
TRINITY_L8GGT0_ACACA/221-344	L8GGT0.1 PF00241.18	54.40	0.00
TRINITY_J9J3B4_9SPIT/124-338	J9J3B4.1 PF00112.21	54.40	0.00
TRINITY_A0C099_PARTE/53-181	A0C099.1 PF00877.17	54.40	0.00
TRINITY_E9G222_DAPPU/164-280	E9G222.1 PF01873.15	54.40	0.00

TRINITY_A8JD26_CHLRE/231-393	A8JD26.1 PF01554.16	54.40	0.00
TRINITY_D8UD04_VOLCA/2-346	D8UD04.1 PF00443.27	54.40	0.00
TRINITY_D8TJY8_VOLCA/379-944	D8TJY8.1 PF00328.20	54.40	0.00
TRINITY_L8H309_ACACA/23-135	L8H309.1 PF02338.17	54.40	0.00
TRINITY_A8I807_CHLRE/1018-1101	A8I807.1 PF00211.18	54.40	0.00
TRINITY_A8J8P1_CHLRE/31-280	A8J8P1.1 PF00225.21	54.40	0.00
TRINITY_D8UDM2_VOLCA/126-221	D8UDM2.1 PF00012.18	54.40	0.00
TRINITY_D8G5U1_9CYAN/576-700	D8G5U1.1 PF02518.24	54.40	0.00
TRINITY_A0DIQ1_PARTE/466-597	A0DIQ1.1 PF00782.18	54.40	0.00
TRINITY_E6NUC5_JATCU/13-132	E6NUC5.1 PF00903.23	54.40	0.00
TRINITY_Q23AM3_TETTS/1054-1231	Q23AM3.2 PF00005.25	54.40	0.00
TRINITY_G0QWP0_ICHMG/124-181	G0QWP0.1 PF01485.19	54.40	0.00
TRINITY_I0YL92_9CHLO/41-120	I0YL92.1 PF00504.19	54.40	0.00
TRINITY_A8J998_CHLRE/100-183	A8J998.1 PF04720.10	54.40	0.00
TRINITY_J4C8J7_THEOR/331-460	J4C8J7.1 PF00004.27	54.40	0.00
TRINITY_I1FZF5_AMPQE/424-568	I1FZF5.1 PF00005.25	54.40	0.00
TRINITY_H2YP11_CIOSA/230-438	H2YP11.1 PF00005.25	54.40	0.00
TRINITY_L8HAQ1_ACACA/941-1132	L8HAQ1.1 PF02145.13	54.40	0.00
TRINITY_A0DYC6_PARTE/574-750	A0DYC6.1 PF13870.4;	54.40	0.00
TRINITY_D8TZ80_VOLCA/781-992	D8TZ80.1 PF01434.16	54.40	0.00
TRINITY_D8UBP9_VOLCA/83-318	D8UBP9.1 PF00194.19	54.40	0.00
TRINITY_E1ZLM6_CHLVA/82-177	E1ZLM6.1 PF12248.6;	54.40	0.00
TRINITY_A8JHP2_CHLRE/61-382	A8JHP2.1 PF01546.26	54.40	0.00
TRINITY_A8IZI1_CHLRE/208-298	A8IZI1.1 PF16561.3;	54.40	0.00
TRINITY_E1ZCJ2_CHLVA/69-399	E1ZCJ2.1 PF03405.12	54.40	0.00
TRINITY_D8UGD4_VOLCA/25-305	D8UGD4.1 PF00069.23	54.40	0.00
TRINITY_D8U819_VOLCA/63-369	D8U819.1 PF02383.16	54.40	0.00
TRINITY_A8I9X2_CHLRE/3-151	A8I9X2.1 PF05903.12	54.40	0.00
TRINITY_D8TLD2_VOLCA/16-292	D8TLD2.1 PF06432.9;	54.40	0.00
TRINITY_A0A067CI89_SAPPC/11-172	A0A067CI89.1 PF0007	54.40	0.00
TRINITY_E1ZNA5_CHLVA/106-362	E1ZNA5.1 PF00248.19	54.40	0.00
TRINITY_A0A078KZ82_9CHLA/128-232	A0A078KZ82.1 PF1364	54.40	0.00
TRINITY_F2UJD2_SALR5/129-336	F2UJD2.1 PF00112.21	54.40	0.00
TRINITY_E1ZCH3_CHLVA/293-451	E1ZCH3.1 PF00534.18	54.40	0.00
TRINITY_D8TXF5_VOLCA/622-733	D8TXF5.1 PF00989.23	54.40	0.00
TRINITY_E4XK02_OIKDI/5-72	E4XK02.1 PF01423.20	54.30	0.00
TRINITY_U6JI51_ECHGR/8-80	U6JI51.1 PF00173.26	54.30	0.00
TRINITY_A0DKE3_PARTE/353-402	A0DKE3.1 PF13920.4;	54.30	0.00
TRINITY_A0CDV3_PARTE/213-277	A0CDV3.1 PF01485.19	54.30	0.00
TRINITY_G0QZ29_ICHMG/1143-1601	G0QZ29.1 PF06202.12	54.30	0.00
TRINITY_I7M8J3_TETTS/17-410	I7M8J3.2 PF00450.20	54.30	0.00
TRINITY_A0A059LAX3_9CHLO/2-186	A0A059LAX3.1 PF0070	54.30	0.00
TRINITY_A0C657_PARTE/5-84	A0C657.1 PF09340.8;	54.30	0.00
TRINITY_F0ZD07_DICPU/119-261	F0ZD07.1 PF00620.25	54.30	0.00
TRINITY_A0A0G4FP44_9ALVE/2954-3289	A0A0G4FP44.1 PF1277	54.30	0.00
TRINITY_A0DY68_PARTE/382-462	A0DY68.1 PF13646.4;	54.30	0.00
TRINITY_A0BXR0_PARTE/306-355	A0BXR0.1 PF13920.4;	54.30	0.00
TRINITY_G0QW62_ICHMG/11-232	G0QW62.1 PF00244.18	54.30	0.00
TRINITY_V8NJ50_OPHHA/48-128	V8NJ50.1 PF00887.17	54.30	0.00
TRINITY_S9WXX3_SCHCR/161-230	S9WXX3.1 PF00076.20	54.30	0.00
TRINITY_A0BG07_PARTE/1-82	A0BG07.1 PF14846.4;	54.30	0.00
TRINITY_A0A058ZFN9_9EUKA/412-522	A0A058ZFN9.1 PF0027	54.30	0.00
TRINITY_A0A0A1UCS6_ENTIV/1389-1650	A0A0A1UCS6.1 PF0006	54.30	0.00
TRINITY_A0A067QYX3_ZOONE/5-80	A0A067QYX3.1 PF0279	54.30	0.00
TRINITY_F0ZDN0_DICPU/1-96	F0ZDN0.1 PF12796.5;	54.30	0.00
TRINITY_I7M475_TETTS/2217-2320	I7M475.2 PF08454.9;	54.30	0.00
TRINITY_E1ZLB3_CHLVA/13-82	E1ZLB3.1 PF00076.20	54.30	0.00
TRINITY_I7LZV2_TETTS/151-413	I7LZV2.1 PF00069.23	54.30	0.00
TRINITY_V4RIN0_9ROSI/56-225	V4RIN0.1 PF05042.11	54.30	0.00
TRINITY_M1BGI1_SOLTU/16-105	M1BGI1.1 PF00447.15	54.30	0.00
TRINITY_A0D8S8_PARTE/638-896	A0D8S8.1 PF07714.15	54.30	0.00

TRINITY_G0QPP3_ICHMG/85-222	G0QPP3.1 PF01529.18	54.30	0.00
TRINITY_A0A0P7VT48_9TELE/762-1082	A0A0P7VT48.1 PF0017	54.30	0.00
TRINITY_A0E826_PARTE/128-238	A0E826.1 PF00168.28	54.30	0.00
TRINITY_A0A087HP96_ARAAL/63-131	A0A087HP96.1 PF0007	54.30	0.00
TRINITY_L8GR35_ACACA/37-107	L8GR35.1 PF00076.20	54.30	0.00
TRINITY_A8JAJ9_CHLRE/62-304	A8JAJ9.1 PF04755.10	54.30	0.00
TRINITY_A4VDU7_TETTS/32-438	A4VDU7.2 PF00899.19	54.30	0.00
TRINITY_K3WKL6_PYTUL/299-382	K3WKL6.1 PF00013.27	54.30	0.00
TRINITY_A0EA12_PARTE/35-340	A0EA12.1 PF00456.19	54.30	0.00
TRINITY_D8U7C3_VOLCA/5-569	D8U7C3.1 PF08553.8;	54.30	0.00
TRINITY_W5MNV1_LEPOC/4-107	W5MNV1.1 PF14933.4;	54.30	0.00
TRINITY_E8N328_ANATU/15-318	E8N328.1 PF00248.19	54.30	0.00
TRINITY_D8UCJ2_VOLCA/53-156	D8UCJ2.1 PF06110.9;	54.30	0.00
TRINITY_A8IS69_CHLRE/18-327	A8IS69.1 PF03009.15	54.30	0.00
TRINITY_E1Z240_CHLVA/31-171	E1Z240.1 PF01138.19	54.30	0.00
TRINITY_D8UC96_VOLCA/594-693	D8UC96.1 PF00211.18	54.30	0.00
TRINITY_E1ZE62_CHLVA/196-716	E1ZE62.1 PF12569.6;	54.30	0.00
TRINITY_I7M910_TETTS/11-285	I7M910.1 PF00069.23	54.30	0.00
TRINITY_A0A0A1N3N7_9FUNG/267-511	A0A0A1N3N7.1 PF0118	54.30	0.00
TRINITY_T1G9L5_HELRO/74-372	T1G9L5.1 PF00291.23	54.30	0.00
TRINITY_D8U8R7_VOLCA/29-293	D8U8R7.1 PF00069.23	54.30	0.00
TRINITY_C1N1Y8_MICPC/190-434	C1N1Y8.1 PF08572.8;	54.30	0.00
TRINITY_K1XUG6_MARBU/19-319	K1XUG6.1 PF00248.19	54.30	0.00
TRINITY_D8TNV2_VOLCA/856-1080	D8TNV2.1 PF00069.23	54.30	0.00
TRINITY_A0A0M9E9Y5_9DELT/508-624	A0A0M9E9Y5.1 PF0251	54.30	0.00
TRINITY_D8TKK7_VOLCA/1-188	D8TKK7.1 PF07714.15	54.30	0.00
TRINITY_E1ZE44_CHLVA/390-768	E1ZE44.1 PF00702.24	54.30	0.00
TRINITY_Q22RX1_TETTS/26-322	Q22RX1.2 PF00069.23	54.30	0.00
TRINITY_G0R2T0_ICHMG/33-283	G0R2T0.1 PF00069.23	54.30	0.00
TRINITY_G0QQ97_ICHMG/55-152	G0QQ97.1 PF12894.5;	54.30	0.00
TRINITY_A7TPP5_VANPO/27-100	A7TPP5.1 PF01722.16	54.30	0.00
TRINITY_R4XFE9_TAPDE/681-796	R4XFE9.1 PF00665.24	54.30	0.00
TRINITY_D8TQK7_VOLCA/86-340	D8TQK7.1 PF00856.26	54.30	0.00
TRINITY_Q4UEX5_THEAN/244-287	Q4UEX5.1 PF13639.4;	54.30	0.00
TRINITY_V2XT82_MONRO/20-161	V2XT82.1 PF00125.22	54.30	0.00
TRINITY_M2QZG3_CERS8/31-79	M2QZG3.1 PF01679.15	54.20	0.00
TRINITY_H3GFZ1_PHYRM/18-298	H3GFZ1.1 PF03747.12	54.20	0.00
TRINITY_A0CW58_PARTE/436-485	A0CW58.1 PF13920.4;	54.20	0.00
TRINITY_I7M692_TETTS/132-377	I7M692.2 PF00561.18	54.20	0.00
TRINITY_V3ZQH9_LOTGI/67-180	V3ZQH9.1 PF00581.18	54.20	0.00
TRINITY_G0QY39_ICHMG/147-345	G0QY39.1 PF07992.12	54.20	0.00
TRINITY_L8H314_ACACA/14-297	L8H314.1 PF03151.14	54.20	0.00
TRINITY_L8GYQ1_ACACA/45-195	L8GYQ1.1 PF00179.24	54.20	0.00
TRINITY_Q23DB1_TETTS/874-1050	Q23DB1.2 PF13091.4;	54.20	0.00
TRINITY_C1N7V7_MICPC/1-187	C1N7V7.1 PF01265.15	54.20	0.00
TRINITY_D3BSD2_POLPA/53-310	D3BSD2.1 PF00069.23	54.20	0.00
TRINITY_L8H5Z5_ACACA/38-485	L8H5Z5.1 PF05577.10	54.20	0.00
TRINITY_L8GK67_ACACA/447-561	L8GK67.1 PF00271.29	54.20	0.00
TRINITY_K5UZ20_PHACS/84-444	K5UZ20.1 PF00266.17	54.20	0.00
TRINITY_G0QMH1_ICHMG/62-324	G0QMH1.1 PF00069.23	54.20	0.00
TRINITY_L8GNJ5_ACACA/2442-2524	L8GNJ5.1 PF05406.13	54.20	0.00
TRINITY_Q8S653_ORYSJ/452-609	Q8S653.1 PF00078.25	54.20	0.00
TRINITY_L8HJ51_ACACA/91-488	L8HJ51.1 PF00026.21	54.20	0.00
TRINITY_B3RVA8_TRIAD/484-656	B3RVA8.1 PF07717.14	54.20	0.00
TRINITY_F1NX57_CHICK/16-334	F1NX57.2 PF00389.28	54.20	0.00
TRINITY_C1MMC3_MICPC/125-264	C1MMC3.1 PF13679.4;	54.20	0.00
TRINITY_E1Z307_CHLVA/23-382	E1Z307.1 PF01231.16	54.20	0.00
TRINITY_F4PKS2_DICFS/13-73	F4PKS2.1 PF00076.20	54.20	0.00
TRINITY_A9SSI1_PHYPA/93-303	A9SSI1.1 PF00454.25	54.20	0.00
TRINITY_A0BQA6_PARTE/560-677	A0BQA6.1 PF02518.24	54.20	0.00
TRINITY_A8HYI2_CHLRE/152-298	A8HYI2.1 PF12049.6;	54.20	0.00

TRINITY_I0YWK3_9CHLO/29-205	I0YWK3.1 PF04675.12	54.20	0.00
TRINITY_A0A077ZTQ1_STYLE/305-470	A0A077ZTQ1.1 PF0044	54.20	0.00
TRINITY_A0A087SPK2_AUXPR/569-730	A0A087SPK2.1 PF0007	54.20	0.00
TRINITY_I2GLZ1_9BACT/11-166	I2GLZ1.1 PF14328.4;	54.20	0.00
TRINITY_D8THV5_VOLCA/124-361	D8THV5.1 PF00122.18	54.20	0.00
TRINITY_B0JH77_MICAN/400-538	B0JH77.1 PF00005.25	54.20	0.00
TRINITY_D8U4R6_VOLCA/3-217	D8U4R6.1 PF10191.7;	54.20	0.00
TRINITY_I7MA07_TETTS/32-269	I7MA07.2 PF00481.19	54.20	0.00
TRINITY_E1ZEV3_CHLVA/5-272	E1ZEV3.1 PF01040.16	54.20	0.00
TRINITY_D2VRI8_NAEGR/1028-1193	D2VRI8.1 PF02146.15	54.20	0.00
TRINITY_G4ZCB9_PHYSP/108-179	G4ZCB9.1 PF00240.21	54.20	0.00
TRINITY_B6K6Y0_SCHJY/131-296	B6K6Y0.1 PF00270.27	54.20	0.00
TRINITY_A0A0D2WKC1_CAPO3/200-284	A0A0D2WKC1.1 PF1484	54.20	0.00
TRINITY_A0DIQ1_PARTE/466-597	A0DIQ1.1 PF00782.18	54.20	0.00
TRINITY_S2JN73_MUCC1/38-191	S2JN73.1 PF04051.14	54.20	0.00
TRINITY_A8IWT5_CHLRE/361-504	A8IWT5.1 PF09384.8;	54.20	0.00
TRINITY_I0Z1Z1_9CHLO/36-150	I0Z1Z1.1 PF00254.26	54.20	0.00
TRINITY_F4Q5X2_DICFS/522-639	F4Q5X2.1 PF13671.4;	54.20	0.00
TRINITY_D8TKF3_VOLCA/105-363	D8TKF3.1 PF03235.12	54.20	0.00
TRINITY_F4PTA8_DICFS/9-405	F4PTA8.1 PF01490.16	54.20	0.00
TRINITY_Q22BR3_TETTS/13-180	Q22BR3.2 PF00071.20	54.20	0.00
TRINITY_A0BZ18_PARTE/112-171	A0BZ18.1 PF00076.20	54.20	0.00
TRINITY_A8IXK6_CHLRE/1-265	A8IXK6.1 PF00176.21	54.20	0.00
TRINITY_A0E5D8_PARTE/235-310	A0E5D8.1 PF02798.18	54.20	0.00

TRINITY_A8JI30_CHLRE/12-145	A8JI30.1 PF00588.17	54.20	0.00
TRINITY_D3B3N0_POLPA/42-229	D3B3N0.1 PF03942.13	54.20	0.00
TRINITY_Q22SJ9_TETTS/1450-1600	Q22SJ9.2 PF07534.14	54.20	0.00
TRINITY_A8HUI6_CHLRE/69-145	A8HUI6.1 PF13193.4;	54.20	0.00
TRINITY_A0A0K9P9W3_ZOSMR/623-847	A0A0K9P9W3.1 PF0823	54.20	0.00
TRINITY_Q22MP0_TETTS/38-191	Q22MP0.2 PF00005.25	54.20	0.00
TRINITY_A0A0D2X0S6_CAPO3/711-763	A0A0D2X0S6.1 PF0831	54.20	0.00
TRINITY_Q23FG9_TETTS/2610-2860	Q23FG9.2 PF00520.29	54.20	0.00
TRINITY_Q22AB0_TETTS/141-425	Q22AB0.2 PF00566.16	54.20	0.00
TRINITY_A0A078AY45_STYLE/121-266	A0A078AY45.1 PF0078	54.20	0.00
TRINITY_J9J144_9SPIT/92-349	J9J144.1 PF00069.23	54.20	0.00
TRINITY_A8JFU2_CHLRE/234-292	A8JFU2.1 PF14874.4;	54.20	0.00
TRINITY_A8I1G7_CHLRE/1-184	A8I1G7.1 PF00211.18	54.20	0.00
TRINITY_D8UA57_VOLCA/8-89	D8UA57.1 PF14846.4;	54.20	0.00
TRINITY_A0A0F3K961_9GAMM/71-236	A0A0F3K961.1 PF1347	54.20	0.00
TRINITY_K1PW81_CRAGI/34-495	K1PW81.1 PF00082.20	54.20	0.00
TRINITY_A8JAS9_CHLRE/125-378	A8JAS9.1 PF05007.11	54.20	0.00
TRINITY_A8JIH3_CHLRE/67-169	A8JIH3.1 PF08855.8;	54.20	0.00
TRINITY_D8U6K4_VOLCA/23-594	D8U6K4.1 PF02696.12	54.20	0.00
TRINITY_R4GAB7_ANOCA/908-1154	R4GAB7.1 PF07727.12	54.20	0.00
TRINITY_G0QYX1_ICHMG/10-68	G0QYX1.1 PF13637.4;	54.20	0.00
TRINITY_A0DYF8_PARTE/433-482	A0DYF8.1 PF13920.4;	54.20	0.00
TRINITY_Q22BR3_TETTS/13-180	Q22BR3.2 PF00071.20	54.20	0.00
TRINITY_I7M7D5_TETTS/682-731	I7M7D5.2 PF01344.23	54.20	0.00
TRINITY_B3RZ00_TRIAD/8-190	B3RZ00.1 PF00370.19	54.20	0.00
TRINITY_Q22MH4_TETTS/143-377	Q22MH4.1 PF13532.4;	54.10	0.00
TRINITY_G0QYY5_ICHMG/38-207	G0QYY5.1 PF13847.4;	54.10	0.00
TRINITY_A4I6U0_LEIIN/7-173	A4I6U0.1 PF01931.16	54.10	0.00
TRINITY_Q24IM0_TETTS/4374-5086	Q24IM0.2 PF03028.13	54.10	0.00
TRINITY_H2ZLT1_CIOSA/2-73	H2ZLT1.1 PF02798.18	54.10	0.00
TRINITY_E8R2Z6_ISOPI/209-360	E8R2Z6.1 PF13185.4;	54.10	0.00
TRINITY_W7X2L6_TETTS/41-297	W7X2L6.1 PF01209.16	54.10	0.00
TRINITY_A0A015MTC7_9GLOM/5-187	A0A015MTC7.1 PF0244	54.10	0.00
TRINITY_F0YD47_AURAN/46-120	F0YD47.1 PF01929.15	54.10	0.00
TRINITY_A0A024TP29_9STRA/11-259	A0A024TP29.1 PF0010	54.10	0.00
TRINITY_R0FGT8_9BRAS/11-185	R0FGT8.1 PF00069.23	54.10	0.00
TRINITY_A0A067QIB6_ZOONE/1-91	A0A067QIB6.1 PF0015	54.10	0.00
TRINITY_G6FXD7_9CYAN/19-321	G6FXD7.1 PF00248.19	54.10	0.00
TRINITY_MECR_DICDI/172-303	Q54YT4.1 PF00107.24	54.10	0.00
TRINITY_K7JUI4_NASVI/1045-1110	K7JUI4.1 PF01363.19	54.10	0.00
TRINITY_G1KK58_ANOCA/805-869	G1KK58.2 PF01363.19	54.10	0.00
TRINITY_A4VDD3_TETTS/7-79	A4VDD3.1 PF02798.18	54.10	0.00
TRINITY_L8GUP7_ACACA/25-206	L8GUP7.1 PF04664.11	54.10	0.00
TRINITY_J9FYL2_9SPIT/398-662	J9FYL2.1 PF00069.23	54.10	0.00
TRINITY_L8H4R4_ACACA/43-293	L8H4R4.1 PF01040.16	54.10	0.00
TRINITY_E1ZPZ5_CHLVA/17-232	E1ZPZ5.1 PF00106.23	54.10	0.00
TRINITY_I7M250_TETTS/101-222	I7M250.1 PF09335.9;	54.10	0.00
TRINITY_I7M910_TETTS/11-285	I7M910.1 PF00069.23	54.10	0.00
TRINITY_H3CG24_TETNG/13-278	H3CG24.1 PF07942.10	54.10	0.00
TRINITY_I7MMV5_TETTS/55-352	I7MMV5.2 PF00443.27	54.10	0.00
TRINITY_D0NJV0_PHYIT/56-170	D0NJV0.1 PF00787.22	54.10	0.00
TRINITY_V3ZIZ6_LOTGI/185-317	V3ZIZ6.1 PF00107.24	54.10	0.00
TRINITY_Q8LP67_CHLRE/27-494	Q8LP67.2 PF01384.18	54.10	0.00
TRINITY_D3B388_POLPA/36-371	D3B388.1 PF16113.3;	54.10	0.00
TRINITY_A0A0G4J2X3_PLABS/751-865	A0A0G4J2X3.1 PF0066	54.10	0.00
TRINITY_J9IMY3_9SPIT/15-201	J9IMY3.1 PF13460.4;	54.10	0.00
TRINITY_D3B1I3_POLPA/193-379	D3B1I3.1 PF00852.17	54.10	0.00
TRINITY_A0A060SLW1_PYCCI/25-332	A0A060SLW1.1 PF0024	54.10	0.00
TRINITY_W4FNE7_9STRA/257-695	W4FNE7.1 PF01055.24	54.10	0.00
TRINITY_D8TTM5_VOLCA/12-85	D8TTM5.1 PF00240.21	54.10	0.00
TRINITY_I1G417_AMPQE/105-426	I1G417.1 PF03747.12	54.10	0.00

TRINITY_W4YW78_STRPU/40-245	W4YW78.1 PF00106.23	54.10	0.00
TRINITY_A0A059LKS4_9CHLO/66-176	A0A059LKS4.1 PF1099	54.10	0.00
TRINITY_I0Z8K2_9CHLO/52-140	I0Z8K2.1 PF00504.19	54.10	0.00
TRINITY_A8IDU2_CHLRE/877-1024	A8IDU2.1 PF03330.16	54.10	0.00
TRINITY_D8U5G0_VOLCA/3-122	D8U5G0.1 PF00188.24	54.10	0.00
TRINITY_D8U0W9_VOLCA/157-323	D8U0W9.1 PF05712.11	54.10	0.00
TRINITY_D8TSJ5_VOLCA/1589-1853	D8TSJ5.1 PF12780.5;	54.10	0.00
TRINITY_E1ZED1_CHLVA/12-210	E1ZED1.1 PF13460.4;	54.10	0.00
TRINITY_D8TX44_VOLCA/516-713	D8TX44.1 PF02854.17	54.10	0.00
TRINITY_F0ZV38_DICPU/17-262	F0ZV38.1 PF00069.23	54.10	0.00
TRINITY_H3HAJ2_PHYRM/359-478	H3HAJ2.1 PF00665.24	54.10	0.00
TRINITY_A0A078AKP0_STYLE/68-336	A0A078AKP0.1 PF0226	54.10	0.00
TRINITY_A0BNQ0_PARTE/32-276	A0BNQ0.1 PF00069.23	54.10	0.00
TRINITY_A0A0D3A1E3_BRAOL/880-1124	A0A0D3A1E3.1 PF0772	54.10	0.00
TRINITY_Q8LL16_CHLRE/258-451	Q8LL16.1 PF03239.12	54.10	0.00
TRINITY_I7M055_TETTS/291-404	I7M055.1 PF08616.8;	54.10	0.00
TRINITY_Q17DH5_AEDAE/171-352	Q17DH5.1 PF02145.13	54.10	0.00
TRINITY_B4KVZ2_DROMO/275-344	B4KVZ2.1 PF01485.19	54.10	0.00
TRINITY_D8TWR1_VOLCA/23-311	D8TWR1.1 PF00069.23	54.10	0.00
TRINITY_A0A0D2X543_CAPO3/27-289	A0A0D2X543.1 PF0006	54.10	0.00
TRINITY_A8HNY7_CHLRE/32-329	A8HNY7.1 PF12698.5;	54.10	0.00
TRINITY_R6DR55_9FIRM/122-281	R6DR55.1 PF02882.17	54.10	0.00
TRINITY_W7U2W9_9STRA/85-305	W7U2W9.1 PF05019.11	54.10	0.00
TRINITY_K4BM70_SOLLC/7-93	K4BM70.1 PF14368.4;	54.00	0.00
TRINITY_L8GRF9_ACACA/15-170	L8GRF9.1 PF13563.4;	54.00	0.00
TRINITY_A0CPA4_PARTE/232-506	A0CPA4.1 PF00481.19	54.00	0.00
TRINITY_Q5TNJ4_ANOGA/104-168	Q5TNJ4.3 PF00327.18	54.00	0.00
TRINITY_W7X626_TETTS/95-219	W7X626.1 PF00572.16	54.00	0.00
TRINITY_A0A0G4EH92_9ALVE/233-501	A0A0G4EH92.1 PF0378	54.00	0.00
TRINITY_A0A0N5B4U9_STREA/94-236	A0A0N5B4U9.1 PF0176	54.00	0.00
TRINITY_E9GA05_DAPPU/477-955	E9GA05.1 PF07748.11	54.00	0.00
TRINITY_A0EBQ7_PARTE/465-697	A0EBQ7.1 PF00233.17	54.00	0.00
TRINITY_D2VT99_NAEGR/698-760	D2VT99.1 PF10557.7;	54.00	0.00
TRINITY_A0CWS5_PARTE/255-401	A0CWS5.1 PF05728.10	54.00	0.00
TRINITY_E1ZDP4_CHLVA/818-1116	E1ZDP4.1 PF00664.21	54.00	0.00
TRINITY_V7BFG5_PHAVU/653-788	V7BFG5.1 PF00005.25	54.00	0.00
TRINITY_A0A088AGE7_APIME/51-194	A0A088AGE7.1 PF0057	54.00	0.00
TRINITY_G0QRW1_ICHMG/96-486	G0QRW1.1 PF00155.19	54.00	0.00
TRINITY_A4VEW4_TETTS/134-185	A4VEW4.1 PF13833.4;	54.00	0.00
TRINITY_A0A0D6JB15_9RHIZ/2218-2267	A0A0D6JB15.1 PF1388	54.00	0.00
TRINITY_L8HKJ7_ACACA/259-385	L8HKJ7.1 PF00004.27	54.00	0.00
TRINITY_L8HKJ7_ACACA/259-385	L8HKJ7.1 PF00004.27	54.00	0.00
TRINITY_D8TUF0_VOLCA/155-576	D8TUF0.1 PF03164.12	54.00	0.00
TRINITY_A0E387_PARTE/199-271	A0E387.1 PF00076.20	54.00	0.00
TRINITY_A0A096RCN9_MAIZE/1-168	A0A096RCN9.1 PF0363	54.00	0.00
TRINITY_A0A059LSC9_9CHLO/145-230	A0A059LSC9.1 PF0197	54.00	0.00
TRINITY_D8U0T7_VOLCA/304-642	D8U0T7.1 PF10373.7;	54.00	0.00
TRINITY_C1EE69_MICSR/50-518	C1EE69.1 PF00082.20	54.00	0.00
TRINITY_J9FXX4_9SPIT/3487-3802	J9FXX4.1 PF00520.29	54.00	0.00
TRINITY_A0A078B8E5_STYLE/126-338	A0A078B8E5.1 PF0011	54.00	0.00
TRINITY_K1Q7R8_CRAGI/54-179	K1Q7R8.1 PF10357.7;	54.00	0.00
TRINITY_A0D0E3_PARTE/7-96	A0D0E3.1 PF01118.22	54.00	0.00
TRINITY_A0A077ZT92_STYLE/94-145	A0A077ZT92.1 PF0222	54.00	0.00
TRINITY_A8IZM2_CHLRE/1-136	A8IZM2.1 PF03476.14	54.00	0.00
TRINITY_L8H039_ACACA/206-457	L8H039.1 PF00069.23	54.00	0.00
TRINITY_L8H1J5_ACACA/387-531	L8H1J5.1 PF00300.20	54.00	0.00
TRINITY_A8JJ05_CHLRE/562-633	A8JJ05.1 PF05699.12	54.00	0.00
TRINITY_F7B414_HORSE/11-334	F7B414.1 PF00026.21	54.00	0.00
TRINITY_L8H9D8_ACACA/83-203	L8H9D8.1 PF07992.12	54.00	0.00
TRINITY_A0CBQ5_PARTE/198-342	A0CBQ5.1 PF00929.22	54.00	0.00
TRINITY_A0A058Z6E6_9EUKA/622-686	A0A058Z6E6.1 PF0136	54.00	0.00

TRINITY_S3CIM7_OPHP1/647-699	S3CIM7.1 PF00385.22	54.00	0.00
TRINITY_D8U0I1_VOLCA/250-406	D8U0I1.1 PF01494.17	54.00	0.00
TRINITY_A8IPQ9_CHLRE/171-429	A8IPQ9.1 PF00069.23	54.00	0.00
TRINITY_D8U7A4_VOLCA/874-951	D8U7A4.1 PF00076.20	54.00	0.00
TRINITY_A8IQQ8_CHLRE/597-709	A8IQQ8.1 PF00989.23	54.00	0.00
TRINITY_A0A087SQB7_AUXPR/10-142	A0A087SQB7.1 PF0023	54.00	0.00
TRINITY_A8I9P4_CHLRE/58-131	A8I9P4.1 PF01926.21	54.00	0.00
TRINITY_A8IRB6_CHLRE/37-136	A8IRB6.1 PF08551.8;	54.00	0.00
TRINITY_A0A0K0F1H0_9BILA/906-1108	A0A0K0F1H0.1 PF0061	54.00	0.00
TRINITY_I1CEQ7_RHIO9/64-296	I1CEQ7.1 PF02784.14	54.00	0.00
TRINITY_A0BEV9_PARTE/21-218	A0BEV9.1 PF14753.4;	54.00	0.00
TRINITY_D8U9J1_VOLCA/7-145	D8U9J1.1 PF04658.11	54.00	0.00
TRINITY_A8J200_CHLRE/65-399	A8J200.1 PF00632.23	54.00	0.00
TRINITY_I0Z522_9CHLO/13-186	I0Z522.1 PF01925.17	54.00	0.00
TRINITY_A0A087SL44_AUXPR/222-322	A0A087SL44.1 PF0035	54.00	0.00
TRINITY_I7M6H4_TETTS/2601-2958	I7M6H4.2 PF12777.5;	54.00	0.00
TRINITY_I0YV31_9CHLO/168-401	I0YV31.1 PF00149.26	54.00	0.00
TRINITY_I7LTH0_TETTS/231-521	I7LTH0.1 PF01031.18	54.00	0.00
TRINITY_A8J018_CHLRE/492-680	A8J018.1 PF13307.4;	54.00	0.00
TRINITY_I7M5Z3_TETTS/393-553	I7M5Z3.2 PF00441.22	54.00	0.00
TRINITY_D8U6I8_VOLCA/629-717	D8U6I8.1 PF00211.18	54.00	0.00
TRINITY_D8U3D7_VOLCA/31-246	D8U3D7.1 PF01036.16	54.00	0.00
TRINITY_A8IBW2_CHLRE/139-344	A8IBW2.1 PF00211.18	54.00	0.00
TRINITY_A0A0G4EDW6_9ALVE/4-204	A0A0G4EDW6.1 PF0085	54.00	0.00
TRINITY_I7MFC1_TETTS/56-423	I7MFC1.2 PF07992.12	53.90	0.00
TRINITY_Q23QF2_TETTS/248-571	Q23QF2.2 PF03133.13	53.90	0.00
TRINITY_F0ZAJ2_DICPU/56-156	F0ZAJ2.1 PF00829.19	53.90	0.00
TRINITY_Q1HPR3_BOMMO/39-272	Q1HPR3.1 PF02784.14	53.90	0.00
TRINITY_A0CWG9_PARTE/1279-1697	A0CWG9.1 PF08393.11	53.90	0.00
TRINITY_F6RED4_HORSE/427-619	F6RED4.1 PF00566.16	53.90	0.00
TRINITY_Q23PT3_TETTS/717-870	Q23PT3.3 PF00005.25	53.90	0.00
TRINITY_A0CTJ4_PARTE/53-356	A0CTJ4.1 PF00128.22	53.90	0.00
TRINITY_A8JFB7_CHLRE/26-179	A8JFB7.1 PF04051.14	53.90	0.00
TRINITY_A0A0E9NF63_9ASCO/6-410	A0A0E9NF63.1 PF0050	53.90	0.00
TRINITY_A9ER91_SORC5/76-715	A9ER91.1 PF03639.11	53.90	0.00
TRINITY_A0A078A8V3_STYLE/25-119	A0A078A8V3.1 PF1390	53.90	0.00
TRINITY_I7M6I7_TETTS/33-261	I7M6I7.1 PF04981.11	53.90	0.00
TRINITY_A0A0M8K9R9_9CHLR/21-422	A0A0M8K9R9.1 PF0050	53.90	0.00
TRINITY_A8JI33_CHLRE/71-161	A8JI33.1 PF13832.4;	53.90	0.00
TRINITY_D8UJ06_VOLCA/6-84	D8UJ06.1 PF13417.4;	53.90	0.00
TRINITY_C3XQU6_BRAFL/256-445	C3XQU6.1 PF04389.15	53.90	0.00
TRINITY_D3BFW6_POLPA/46-147	D3BFW6.1 PF00011.19	53.90	0.00
TRINITY_D8TGU6_VOLCA/104-269	D8TGU6.1 PF01789.14	53.90	0.00
TRINITY_A0A0G4IQT5_PLABS/1-127	A0A0G4IQT5.1 PF0000	53.90	0.00
TRINITY_D8M1W7_BLAHO/168-334	D8M1W7.1 PF02421.16	53.90	0.00
TRINITY_D8U5V1_VOLCA/37-396	D8U5V1.1 PF07944.10	53.90	0.00
TRINITY_E1ZF97_CHLVA/15-242	E1ZF97.1 PF00378.18	53.90	0.00
TRINITY_D8U0A8_VOLCA/366-760	D8U0A8.1 PF04515.10	53.90	0.00
TRINITY_H2KSG7_CLOSI/57-218	H2KSG7.1 PF04733.12	53.90	0.00
TRINITY_Q22BR3_TETTS/13-180	Q22BR3.2 PF00071.20	53.90	0.00
TRINITY_A8IR95_CHLRE/401-503	A8IR95.1 PF13426.5;	53.90	0.00
TRINITY_L8HDH4_ACACA/74-421	L8HDH4.1 PF00155.19	53.90	0.00
TRINITY_N8TG76_ACIGI/9-357	N8TG76.1 PF07287.9;	53.90	0.00
TRINITY_A0A096P3C0_PAPAN/1-436	A0A096P3C0.1 PF0099	53.90	0.00
TRINITY_D3BI36_POLPA/1029-1279	D3BI36.1 PF07714.15	53.90	0.00
TRINITY_A8HN65_CHLRE/87-190	A8HN65.1 PF00565.15	53.90	0.00
TRINITY_A9RFT7_PHYPA/99-592	A9RFT7.1 PF01593.22	53.90	0.00
TRINITY_M0T515_MUSAM/223-331	M0T515.1 PF08616.8;	53.90	0.00
TRINITY_D8U5A5_VOLCA/12-547	D8U5A5.1 PF00899.19	53.90	0.00
TRINITY_E1Z904_CHLVA/10-198	E1Z904.1 PF00270.27	53.90	0.00
TRINITY_A0BFP5_PARTE/56-247	A0BFP5.1 PF10243.7;	53.90	0.00

TRINITY_A8J3T6_CHLRE/55-168	A8J3T6.1 PF00504.19	53.90	0.00
TRINITY_F2UBN5_SALR5/152-407	F2UBN5.1 PF04305.12	53.90	0.00
TRINITY_A0A0K9P2B6_ZOSMR/2-865	A0A0K9P2B6.1 PF0246	53.90	0.00
TRINITY_A8J750_CHLRE/1-94	A8J750.1 PF13943.4;	53.90	0.00
TRINITY_D3BJ38_POLPA/101-179	D3BJ38.1 PF01253.20	53.90	0.00
TRINITY_I0YV72_9CHLO/31-534	I0YV72.1 PF05761.12	53.90	0.00
TRINITY_B1ZSD7_OPITP/28-392	B1ZSD7.1 PF00916.18	53.90	0.00
TRINITY_D8U0P0_VOLCA/133-278	D8U0P0.1 PF01694.20	53.90	0.00
TRINITY_A0A078BAP0_STYLE/225-510	A0A078BAP0.1 PF0118	53.90	0.00
TRINITY_D8TIX0_VOLCA/336-697	D8TIX0.1 PF03016.13	53.90	0.00
TRINITY_A0BS93_PARTE/8-191	A0BS93.1 PF14913.4;	53.90	0.00
TRINITY_A0A0J8BSN9_BETVU/38-123	A0A0J8BSN9.1 PF1684	53.90	0.00
TRINITY_I7MIU6_TETTS/87-138	I7MIU6.2 PF00415.16	53.80	0.00
TRINITY_A0A072TZX1_MEDTR/2-1165	A0A072TZX1.1 PF0246	53.80	0.00
TRINITY_M1B5B5_SOLTU/248-328	M1B5B5.1 PF00498.24	53.80	0.00
TRINITY_I1FE35_AMPQE/260-318	I1FE35.1 PF12894.5;	53.80	0.00
TRINITY_B8CA70_THAPS/124-338	B8CA70.1 PF13563.4;	53.80	0.00
TRINITY_J9J7N7_9SPIT/498-715	J9J7N7.1 PF08014.9;	53.80	0.00
TRINITY_F4Q3L0_DICFS/517-661	F4Q3L0.1 PF00005.25	53.80	0.00
TRINITY_I7MCX7_TETTS/268-349	I7MCX7.1 PF13242.4;	53.80	0.00
TRINITY_L8HA43_ACACA/8-290	L8HA43.1 PF00704.26	53.80	0.00
TRINITY_L8GNV1_ACACA/26-201	L8GNV1.1 PF00149.26	53.80	0.00
TRINITY_G0R2V0_ICHMG/53-221	G0R2V0.1 PF04893.15	53.80	0.00
TRINITY_Q22BA7_TETTS/59-282	Q22BA7.1 PF07228.10	53.80	0.00
TRINITY_A0A075AMP7_9FUNG/470-588	A0A075AMP7.1 PF0058	53.80	0.00
TRINITY_A0A063BMC1_9HYPO/7-83	A0A063BMC1.1 PF0040	53.80	0.00
TRINITY_F0ZGL2_DICPU/12-208	F0ZGL2.1 PF04511.13	53.80	0.00
TRINITY_D8U326_VOLCA/8-304	D8U326.1 PF09743.7;	53.80	0.00
TRINITY_L8HEU1_ACACA/217-256	L8HEU1.1 PF00400.30	53.80	0.00
TRINITY_I7M1U7_TETTS/1176-1331	I7M1U7.1 PF00005.25	53.80	0.00
TRINITY_A8JIU8_CHLRE/35-321	A8JIU8.1 PF00520.29	53.80	0.00
TRINITY_A0A086SYF2_ACRCH/60-345	A0A086SYF2.1 PF0012	53.80	0.00
TRINITY_G3VDU7_SARHA/1-103	G3VDU7.1 PF08487.8;	53.80	0.00
TRINITY_D8SE48_SELML/137-562	D8SE48.1 PF01593.22	53.80	0.00
TRINITY_C1FE42_MICSR/328-435	C1FE42.1 PF02002.15	53.80	0.00
TRINITY_F1RVZ1_PIG/396-576	F1RVZ1.2 PF01756.17	53.80	0.00
TRINITY_F1A1K8_DICPU/244-422	F1A1K8.1 PF00621.18	53.80	0.00
TRINITY_I3CDP0_9GAMM/61-411	I3CDP0.1 PF01979.18	53.80	0.00
TRINITY_F0ZQ10_DICPU/33-357	F0ZQ10.1 PF00180.18	53.80	0.00
TRINITY_U5D3H9_AMBTC/48-218	U5D3H9.1 PF07786.10	53.80	0.00
TRINITY_A0BDL2_PARTE/6-167	A0BDL2.1 PF00071.20	53.80	0.00
TRINITY_D8U771_VOLCA/39-168	D8U771.1 PF03435.16	53.80	0.00
TRINITY_W5L8V6_ASTMX/19-139	W5L8V6.1 PF07738.11	53.80	0.00
TRINITY_D8TN24_VOLCA/27-489	D8TN24.1 PF01384.18	53.80	0.00
TRINITY_Q23QF2_TETTS/248-571	Q23QF2.2 PF03133.13	53.80	0.00
TRINITY_D8UIE1_VOLCA/38-142	D8UIE1.1 PF03692.13	53.80	0.00
TRINITY_G3PDN2_GASAC/1400-1649	G3PDN2.1 PF00856.26	53.80	0.00
TRINITY_I7M483_TETTS/113-361	I7M483.1 PF01063.17	53.80	0.00
TRINITY_A8HZ83_CHLRE/125-240	A8HZ83.1 PF07534.14	53.80	0.00
TRINITY_A0A0D2UKD6_GOSRA/51-115	A0A0D2UKD6.1 PF0007	53.80	0.00
TRINITY_D3BSD2_POLPA/53-310	D3BSD2.1 PF00069.23	53.80	0.00
TRINITY_J3AVK9_9CAUL/83-252	J3AVK9.1 PF02837.16	53.80	0.00
TRINITY_A0A087SRZ5_AUXPR/6-131	A0A087SRZ5.1 PF0029	53.80	0.00
TRINITY_D3BL88_POLPA/9-166	D3BL88.1 PF02099.15	53.80	0.00
TRINITY_K1QAJ0_CRAGI/253-292	K1QAJ0.1 PF00400.30	53.80	0.00
TRINITY_I7MDT2_TETTS/149-278	I7MDT2.1 PF00107.24	53.80	0.00
TRINITY_D8TD38_SELML/88-179	D8TD38.1 PF00504.19	53.80	0.00
TRINITY_Q769F7_DICDI/20-209	Q769F7.1 PF01105.22	53.80	0.00
TRINITY_I1CK88_RHIO9/29-463	I1CK88.1 PF02127.13	53.80	0.00
TRINITY_D8TIM3_VOLCA/1-359	D8TIM3.1 PF00443.27	53.80	0.00
TRINITY_L8HG64_ACACA/2211-2633	L8HG64.1 PF12166.6;	53.80	0.00

TRINITY_A0A0K0JY66_BRUMA/478-566	A0A0K0JY66.1 PF0549	53.80	0.00
TRINITY_Q54TP9_DICDI/15-133	Q54TP9.1 PF01412.16	53.80	0.00
TRINITY_D8TY37_VOLCA/354-459	D8TY37.1 PF13307.4;	53.80	0.00
TRINITY_D2VRI8_NAEGR/1028-1193	D2VRI8.1 PF02146.15	53.80	0.00
TRINITY_I7MFT0_TETTS/25-577	I7MFT0.2 PF01602.18	53.80	0.00
TRINITY_I7MLB9_TETTS/842-958	I7MLB9.1 PF00271.29	53.80	0.00
TRINITY_A0A0K6FN84_9HOMO/26-148	A0A0K6FN84.1 PF0063	53.80	0.00
TRINITY_A8JE75_CHLRE/66-190	A8JE75.1 PF00083.22	53.80	0.00
TRINITY_D8U241_VOLCA/101-399	D8U241.1 PF10475.7;	53.80	0.00
TRINITY_M4D8R6_BRARP/19-224	M4D8R6.1 PF02114.14	53.80	0.00
TRINITY_T1IZ26_STRMM/430-565	T1IZ26.1 PF00005.25	53.80	0.00
TRINITY_A0BQP4_PARTE/505-570	A0BQP4.1 PF00659.16	53.80	0.00
TRINITY_I0Z889_9CHLO/3-84	I0Z889.1 PF07011.9;	53.80	0.00
TRINITY_A0BU39_PARTE/129-390	A0BU39.1 PF00069.23	53.80	0.00
TRINITY_D8TZK2_VOLCA/13092-13314	D8TZK2.1 PF00109.24	53.80	0.00
TRINITY_A0A096MFT6_POEFO/324-543	A0A096MFT6.1 PF0700	53.80	0.00
TRINITY_Q23QF2_TETTS/248-571	Q23QF2.2 PF03133.13	53.80	0.00
TRINITY_Q22ML2_TETTS/132-170	Q22ML2.1 PF00400.30	53.80	0.00
TRINITY_S9XQ78_9CETA/2-375	S9XQ78.1 PF00022.17	53.80	0.00
TRINITY_A8J375_CHLRE/133-491	A8J375.1 PF02163.20	53.80	0.00
TRINITY_D3BB49_POLPA/309-549	D3BB49.1 PF00122.18	53.80	0.00
TRINITY_SPKA_DICDI/351-612	Q86AT8.1 PF07714.15	53.80	0.00
TRINITY_D8TKU3_VOLCA/544-633	D8TKU3.1 PF13193.4;	53.80	0.00
TRINITY_A0A087H3I3_ARAAL/153-246	A0A087H3I3.1 PF0015	53.80	0.00
TRINITY_Q22A96_TETTS/376-441	Q22A96.2 PF00498.24	53.80	0.00
TRINITY_A8J9U8_CHLRE/258-417	A8J9U8.1 PF01554.16	53.80	0.00
TRINITY_I0YN04_9CHLO/151-438	I0YN04.1 PF00176.21	53.80	0.00
TRINITY_A8J691_CHLRE/23-294	A8J691.1 PF00069.23	53.80	0.00
TRINITY_A8J3L0_CHLRE/13-395	A8J3L0.1 PF07690.14	53.80	0.00
TRINITY_D8U144_VOLCA/2295-3023	D8U144.1 PF03028.13	53.80	0.00
TRINITY_A0C2G7_PARTE/2-79	A0C2G7.1 PF01781.16	53.80	0.00
TRINITY_Q236Z5_TETTS/302-587	Q236Z5.1 PF05206.12	53.80	0.00
TRINITY_G0QJN1_ICHMG/433-510	G0QJN1.1 PF13424.4;	53.80	0.00
TRINITY_G0GCI4_SPITZ/27-140	G0GCI4.1 PF00072.22	53.70	0.00
TRINITY_A9T0J4_PHYPA/437-479	A9T0J4.1 PF00643.22	53.70	0.00
TRINITY_G0QSQ4_ICHMG/3-73	G0QSQ4.1 PF13802.4;	53.70	0.00
TRINITY_F0ZTE3_DICPU/290-475	F0ZTE3.1 PF00753.25	53.70	0.00
TRINITY_D8UE72_VOLCA/205-506	D8UE72.1 PF00069.23	53.70	0.00
TRINITY_A8J6M3_CHLRE/172-585	A8J6M3.1 PF01131.18	53.70	0.00
TRINITY_A0A0B1P9Z4_UNCNE/3-126	A0A0B1P9Z4.1 PF1389	53.70	0.00
TRINITY_H2LLQ1_ORYLA/60-151	H2LLQ1.1 PF12894.5;	53.70	0.00
TRINITY_T1FTQ4_HELRO/309-373	T1FTQ4.1 PF00076.20	53.70	0.00
TRINITY_F4PWJ3_DICFS/510-572	F4PWJ3.1 PF07647.15	53.70	0.00
TRINITY_I0YKC1_9CHLO/32-104	I0YKC1.1 PF00498.24	53.70	0.00
TRINITY_Q24HI2_TETTS/127-344	Q24HI2.1 PF00112.21	53.70	0.00
TRINITY_L8GCZ7_ACACA/5-159	L8GCZ7.1 PF01965.22	53.70	0.00
TRINITY_S2JMN3_MUCC1/1-135	S2JMN3.1 PF00773.17	53.70	0.00
TRINITY_Q23K82_TETTS/274-327	Q23K82.2 PF01084.18	53.70	0.00
TRINITY_7TMK1_DICDI/363-623	Q54N73.1 PF07714.15	53.70	0.00
TRINITY_Q75JY8_DICDI/192-314	Q75JY8.1 PF00241.18	53.70	0.00
TRINITY_L8HKF4_ACACA/100-146	L8HKF4.1 PF13920.4;	53.70	0.00
TRINITY_D2VQS1_NAEGR/368-482	D2VQS1.1 PF04266.12	53.70	0.00
TRINITY_I7MLV0_TETTS/461-797	I7MLV0.2 PF00443.27	53.70	0.00
TRINITY_A0A0G4J6A5_PLABS/162-268	A0A0G4J6A5.1 PF0120	53.70	0.00
TRINITY_Q24FC3_TETTS/272-351	Q24FC3.3 PF13242.4;	53.70	0.00
TRINITY_V4A1H3_LOTGI/625-760	V4A1H3.1 PF00005.25	53.70	0.00
TRINITY_A0ECD8_PARTE/1092-1212	A0ECD8.1 PF02687.19	53.70	0.00
TRINITY_Q22GW0_TETTS/1651-1813	Q22GW0.2 PF00069.23	53.70	0.00
TRINITY_L8H318_ACACA/10-51	L8H318.1 PF01585.21	53.70	0.00
TRINITY_D2V5A5_NAEGR/214-344	D2V5A5.1 PF00022.17	53.70	0.00
TRINITY_A8IWP9_CHLRE/32-593	A8IWP9.1 PF01204.16	53.70	0.00

TRINITY_I0I796_CALAS/35-409	I0I796.1 PF00282.17	53.70	0.00
TRINITY_M0QSN0_ACACA/73-165	M0QSN0.1 PF02214.20	53.70	0.00
TRINITY_Q24HH9_TETTS/52-312	Q24HH9.2 PF00069.23	53.70	0.00
TRINITY_D8TLE4_VOLCA/425-658	D8TLE4.1 PF08161.10	53.70	0.00
TRINITY_Q4G298_TRIVA/9-169	Q4G298.1 PF00071.20	53.70	0.00
TRINITY_G0QZB3_ICHMG/327-396	G0QZB3.1 PF00498.24	53.70	0.00
TRINITY_C1MW00_MICPC/337-405	C1MW00.1 PF07529.11	53.70	0.00
TRINITY_K0T5T5_THAOC/511-560	K0T5T5.1 PF13920.4;	53.70	0.00
TRINITY_I0YSF3_9CHLO/48-367	I0YSF3.1 PF01937.17	53.70	0.00
TRINITY_A0A078ANA5_STYLE/18-270	A0A078ANA5.1 PF0006	53.70	0.00
TRINITY_A0A0A1P9T2_9FUNG/432-572	A0A0A1P9T2.1 PF0152	53.70	0.00
TRINITY_I3TP22_TISMK/8-195	I3TP22.1 PF07992.12	53.70	0.00
TRINITY_E5SFZ6_TRISP/46-134	E5SFZ6.1 PF15663.3;	53.70	0.00
TRINITY_F1A5J4_DICPU/566-819	F1A5J4.1 PF00069.23	53.70	0.00
TRINITY_A0A075B2H7_9FUNG/53-379	A0A075B2H7.1 PF0799	53.70	0.00
TRINITY_F0ZRD8_DICPU/247-426	F0ZRD8.1 PF00270.27	53.70	0.00
TRINITY_E1ZSA8_CHLVA/103-221	E1ZSA8.1 PF16036.3;	53.70	0.00
TRINITY_A8IXM4_CHLRE/1640-1791	A8IXM4.1 PF03712.13	53.70	0.00
TRINITY_A8ISP2_CHLRE/200-468	A8ISP2.1 PF00248.19	53.70	0.00
TRINITY_D8UFD3_VOLCA/71-660	D8UFD3.1 PF16940.3;	53.70	0.00
TRINITY_D8TP12_VOLCA/546-938	D8TP12.1 PF00773.17	53.70	0.00
TRINITY_F4PMG8_DICFS/24-1090	F4PMG8.1 PF10266.7;	53.70	0.00
TRINITY_Q3SD73_PARTE/8-167	Q3SD73.1 PF00071.20	53.70	0.00
TRINITY_B2J2T9_NOSP7/31-127	B2J2T9.1 PF00355.24	53.70	0.00
TRINITY_D8THR7_VOLCA/300-536	D8THR7.1 PF12697.5;	53.70	0.00
TRINITY_A9FAR4_SORC5/21-203	A9FAR4.1 PF07081.9;	53.70	0.00
TRINITY_D8U819_VOLCA/63-369	D8U819.1 PF02383.16	53.70	0.00
TRINITY_E1ZCK1_CHLVA/40-178	E1ZCK1.1 PF00892.18	53.70	0.00
TRINITY_L8HIS0_ACACA/29-314	L8HIS0.1 PF04258.11	53.70	0.00
TRINITY_A8IHE7_CHLRE/70-516	A8IHE7.1 PF13520.4;	53.70	0.00
TRINITY_F0ZYQ8_DICPU/339-461	F0ZYQ8.1 PF13271.4;	53.70	0.00
TRINITY_B3KYI2_PHAVU/209-536	B3KYI2.1 PF00733.19	53.70	0.00
TRINITY_Q3SDK9_PARTE/13-174	Q3SDK9.1 PF00071.20	53.70	0.00
TRINITY_G0R646_ICHMG/1087-1249	G0R646.1 PF00005.25	53.60	0.00
TRINITY_A0DWJ3_PARTE/548-668	A0DWJ3.1 PF02518.24	53.60	0.00
TRINITY_A0A0G4ETE6_9ALVE/5-177	A0A0G4ETE6.1 PF0002	53.60	0.00
TRINITY_F4PRU3_DICFS/5-70	F4PRU3.1 PF00808.21	53.60	0.00
TRINITY_H3GMZ0_PHYRM/40-372	H3GMZ0.1 PF04922.10	53.60	0.00
TRINITY_E2BNF8_HARSA/50-255	E2BNF8.1 PF00106.23	53.60	0.00
TRINITY_H2XUH7_CIOIN/393-562	H2XUH7.1 PF02182.15	53.60	0.00
TRINITY_L8GMQ5_ACACA/2-258	L8GMQ5.1 PF00398.18	53.60	0.00
TRINITY_E1REZ7_METP4/12-150	E1REZ7.1 PF13302.5;	53.60	0.00
TRINITY_L8GXG2_ACACA/21-262	L8GXG2.1 PF07714.15	53.60	0.00
TRINITY_D3PDK7_DEFDS/861-971	D3PDK7.1 PF00072.22	53.60	0.00
TRINITY_L8GDX3_ACACA/118-294	L8GDX3.1 PF13532.4;	53.60	0.00
TRINITY_D8THX1_VOLCA/227-448	D8THX1.1 PF13672.4;	53.60	0.00
TRINITY_L8GFG9_ACACA/67-280	L8GFG9.1 PF01135.17	53.60	0.00
TRINITY_A0A0F3H0L8_9BACT/58-180	A0A0F3H0L8.1 PF0498	53.60	0.00
TRINITY_G0QS77_ICHMG/9-177	G0QS77.1 PF00188.24	53.60	0.00
TRINITY_A8IAW5_CHLRE/63-149	A8IAW5.1 PF14159.4;	53.60	0.00
TRINITY_A0D9N4_PARTE/343-722	A0D9N4.1 PF00702.24	53.60	0.00
TRINITY_C7LR38_DESBD/5-73	C7LR38.1 PF00076.20	53.60	0.00
TRINITY_D8TQC4_VOLCA/18-543	D8TQC4.1 PF05600.10	53.60	0.00
TRINITY_Q23QF5_TETTS/161-229	Q23QF5.2 PF00505.17	53.60	0.00
TRINITY_C1N8D4_MICPC/206-292	C1N8D4.1 PF04379.12	53.60	0.00
TRINITY_L8GL32_ACACA/1-129	L8GL32.1 PF03463.13	53.60	0.00
TRINITY_K4AT24_SOLLC/39-162	K4AT24.1 PF10238.7;	53.60	0.00
TRINITY_A0A022R7Q1_ERYGU/124-346	A0A022R7Q1.1 PF0155	53.60	0.00
TRINITY_D8TI14_VOLCA/22-1084	D8TI14.1 PF04147.10	53.60	0.00
TRINITY_L8GVQ6_ACACA/315-425	L8GVQ6.1 PF09070.9;	53.60	0.00
TRINITY_A8HVG5_CHLRE/56-124	A8HVG5.1 PF00076.20	53.60	0.00

TRINITY_F4QCC5_DICFS/10-118	F4QCC5.1 PF01398.19	53.60	0.00
TRINITY_A0A067CUV9_SAPPC/9-104	A0A067CUV9.1 PF0864	53.60	0.00
TRINITY_E1Z4P9_CHLVA/76-352	E1Z4P9.1 PF01048.18	53.60	0.00
TRINITY_L1IH70_GUITH/122-177	L1IH70.1 PF13513.4;	53.60	0.00
TRINITY_H3GI85_PHYRM/29-361	H3GI85.1 PF00155.19	53.60	0.00
TRINITY_D8UFK9_VOLCA/270-358	D8UFK9.1 PF07571.11	53.60	0.00
TRINITY_A8JEP4_CHLRE/267-445	A8JEP4.1 PF00174.17	53.60	0.00
TRINITY_A0A078B270_STYLE/2405-2625	A0A078B270.1 PF0064	53.60	0.00
TRINITY_D8TVB7_VOLCA/70-209	D8TVB7.1 PF14108.4;	53.60	0.00
TRINITY_L8HHA4_ACACA/11-122	L8HHA4.1 PF01412.16	53.60	0.00
TRINITY_Q23QF5_TETTS/161-229	Q23QF5.2 PF00505.17	53.60	0.00
TRINITY_D8U5M9_VOLCA/31-238	D8U5M9.1 PF01027.18	53.60	0.00
TRINITY_D8U0W0_VOLCA/18-141	D8U0W0.1 PF09801.7;	53.60	0.00
TRINITY_W7XHC6_TETTS/6-164	W7XHC6.1 PF01210.21	53.60	0.00
TRINITY_F4Q989_DICFS/407-604	F4Q989.1 PF01434.16	53.60	0.00
TRINITY_D8TVJ2_VOLCA/146-262	D8TVJ2.1 PF03129.18	53.60	0.00
TRINITY_L8H1F7_ACACA/71-180	L8H1F7.1 PF02338.17	53.60	0.00
TRINITY_D8TRU7_VOLCA/47-296	D8TRU7.1 PF01571.19	53.60	0.00
TRINITY_E1ZQP7_CHLVA/9-316	E1ZQP7.1 PF07992.12	53.60	0.00
TRINITY_D8M2P3_BLAHO/26-94	D8M2P3.1 PF00505.17	53.60	0.00
TRINITY_F4PJ18_DICFS/1-374	F4PJ18.1 PF00022.17	53.60	0.00
TRINITY_K6VRX0_9MICO/27-143	K6VRX0.1 PF08240.10	53.60	0.00
TRINITY_M1V4Y7_CYAME/154-222	M1V4Y7.1 PF00610.19	53.60	0.00
TRINITY_L8H0B4_ACACA/93-357	L8H0B4.1 PF04811.13	53.60	0.00
TRINITY_D8TNV2_VOLCA/856-1080	D8TNV2.1 PF00069.23	53.60	0.00
TRINITY_F4PL66_DICFS/461-680	F4PL66.1 PF13236.4;	53.60	0.00
TRINITY_G8X9B9_FLACA/1-118	G8X9B9.1 PF01925.17	53.60	0.00
TRINITY_U5GEP5_POPTR/138-331	U5GEP5.1 PF04055.19	53.60	0.00
TRINITY_A8HSZ4_CHLRE/128-229	A8HSZ4.1 PF01909.21	53.60	0.00
TRINITY_J9J3B4_9SPIT/124-338	J9J3B4.1 PF00112.21	53.60	0.00
TRINITY_A8I4J6_CHLRE/57-253	A8I4J6.1 PF00106.23	53.60	0.00
TRINITY_A8J6M5_CHLRE/1-274	A8J6M5.1 PF00664.21	53.60	0.00
TRINITY_A8JF90_CHLRE/1546-1844	A8JF90.1 PF00520.29	53.60	0.00
TRINITY_A8I1G7_CHLRE/1-184	A8I1G7.1 PF00211.18	53.60	0.00
TRINITY_Q2UPL3_ASPOR/566-682	Q2UPL3.1 PF00665.24	53.60	0.00
TRINITY_Q22X69_TETTS/149-192	Q22X69.2 PF13639.4;	53.50	0.00
TRINITY_W9RTZ7_9ROSA/126-170	W9RTZ7.1 PF13639.4;	53.50	0.00
TRINITY_G6DK36_DANPL/398-512	G6DK36.1 PF00856.26	53.50	0.00
TRINITY_Q22LW9_TETTS/480-571	Q22LW9.2 PF07885.14	53.50	0.00
TRINITY_A0CB90_PARTE/31-436	A0CB90.1 PF05577.10	53.50	0.00
TRINITY_A0D2N3_PARTE/21-149	A0D2N3.1 PF02466.17	53.50	0.00
TRINITY_A0A0K9PWV4_ZOSMR/192-235	A0A0K9PWV4.1 PF1363	53.50	0.00
TRINITY_G0QY85_ICHMG/26-111	G0QY85.1 PF01221.16	53.50	0.00
TRINITY_Q245X8_TETTS/495-565	Q245X8.1 PF00076.20	53.50	0.00
TRINITY_A0BEH2_PARTE/1-140	A0BEH2.1 PF00134.21	53.50	0.00
TRINITY_A0A077ZPT0_STYLE/897-969	A0A077ZPT0.1 PF0456	53.50	0.00
TRINITY_F4QED8_DICFS/130-220	F4QED8.1 PF13640.4;	53.50	0.00
TRINITY_A0CQ64_PARTE/35-105	A0CQ64.1 PF13499.4;	53.50	0.00
TRINITY_Q24GQ7_TETTS/133-301	Q24GQ7.1 PF04664.11	53.50	0.00
TRINITY_F0YDD5_AURAN/17-173	F0YDD5.1 PF00156.25	53.50	0.00
TRINITY_A0A0G2ZMH7_9DELT/8-249	A0A0G2ZMH7.1 PF0037	53.50	0.00
TRINITY_A7RPJ4_NEMVE/13-362	A7RPJ4.1 PF05208.11	53.50	0.00
TRINITY_Q23K91_TETTS/486-612	Q23K91.2 PF00134.21	53.50	0.00
TRINITY_A5H8H8_CHLRE/85-256	A5H8H8.1 PF12046.6;	53.50	0.00
TRINITY_D3B3S1_POLPA/40-228	D3B3S1.1 PF10294.7;	53.50	0.00
TRINITY_J9ICB1_9SPIT/267-310	J9ICB1.1 PF00569.15	53.50	0.00
TRINITY_A0E2M3_PARTE/3-103	A0E2M3.1 PF00168.28	53.50	0.00
TRINITY_A0D334_PARTE/1-89	A0D334.1 PF00169.27	53.50	0.00
TRINITY_A0A087SIS3_AUXPR/5-432	A0A087SIS3.1 PF0418	53.50	0.00
TRINITY_V4N0N0_EUTSA/113-157	V4N0N0.1 PF13639.4;	53.50	0.00
TRINITY_F4QED2_DICFS/996-1169	F4QED2.1 PF00621.18	53.50	0.00

TRINITY_D2V036_NAEGR/53-331	D2V036.1 PF05853.10	53.50	0.00
TRINITY_M2VXJ2_GALSU/118-200	M2VXJ2.1 PF15963.3;	53.50	0.00
TRINITY_A8JF91_CHLRE/7-125	A8JF91.1 PF05348.9;	53.50	0.00
TRINITY_L1JI00_GUITH/19-66	L1JI00.1 PF02042.13	53.50	0.00
TRINITY_A0A059CYP0_EUCGR/78-122	A0A059CYP0.1 PF1363	53.50	0.00
TRINITY_A0D6M4_PARTE/91-395	A0D6M4.1 PF02383.16	53.50	0.00
TRINITY_D3BSB4_POLPA/66-254	D3BSB4.1 PF12697.5;	53.50	0.00
TRINITY_A0CEZ8_PARTE/353-396	A0CEZ8.1 PF13639.4;	53.50	0.00
TRINITY_L8GE60_ACACA/531-659	L8GE60.1 PF00241.18	53.50	0.00
TRINITY_W9WAK1_9EURO/306-565	W9WAK1.1 PF13360.4;	53.50	0.00
TRINITY_A0A078AKR1_STYLE/126-337	A0A078AKR1.1 PF0011	53.50	0.00
TRINITY_A8IVU6_CHLRE/3378-3666	A8IVU6.1 PF00520.29	53.50	0.00
TRINITY_A0A087S9P8_AUXPR/95-266	A0A087S9P8.1 PF0161	53.50	0.00
TRINITY_A0A015M8V8_9GLOM/124-353	A0A015M8V8.1 PF0179	53.50	0.00
TRINITY_D8SCH3_SELML/372-473	D8SCH3.1 PF06584.11	53.50	0.00
TRINITY_T1J1G3_STRMM/245-319	T1J1G3.1 PF04564.13	53.50	0.00
TRINITY_A0A0E0PRJ8_ORYRU/753-911	A0A0E0PRJ8.1 PF0007	53.50	0.00
TRINITY_A8IDQ8_CHLRE/8-151	A8IDQ8.1 PF10568.7;	53.50	0.00
TRINITY_G0QWD7_ICHMG/33-211	G0QWD7.1 PF00270.27	53.50	0.00
TRINITY_A0DVR4_PARTE/17-87	A0DVR4.1 PF13499.4;	53.50	0.00
TRINITY_L8GQL5_ACACA/626-1215	L8GQL5.1 PF00821.16	53.50	0.00
TRINITY_D8TNS6_VOLCA/1-284	D8TNS6.1 PF12697.5;	53.50	0.00
TRINITY_H3GEM8_PHYRM/190-315	H3GEM8.1 PF03171.18	53.50	0.00
TRINITY_A8IRU3_CHLRE/135-292	A8IRU3.1 PF02622.13	53.50	0.00
TRINITY_W0RQB7_9BACT/7-153	W0RQB7.1 PF01408.20	53.50	0.00
TRINITY_F0ZXU8_DICPU/314-400	F0ZXU8.1 PF00439.23	53.50	0.00
TRINITY_A0A0M0JR79_9EUKA/247-386	A0A0M0JR79.1 PF0139	53.50	0.00
TRINITY_D8ULK7_VOLCA/8-219	D8ULK7.1 PF00106.23	53.50	0.00
TRINITY_A8IFT8_CHLRE/600-698	A8IFT8.1 PF00211.18	53.50	0.00
TRINITY_I7MJZ2_TETTS/407-470	I7MJZ2.2 PF01485.19	53.50	0.00
TRINITY_A0DA13_PARTE/277-608	A0DA13.1 PF03124.12	53.40	0.00
TRINITY_L8GYY1_ACACA/191-600	L8GYY1.1 PF13520.4;	53.40	0.00
TRINITY_Q22BY0_TETTS/1678-1732	Q22BY0.2 PF00415.16	53.40	0.00
TRINITY_G1PVH4_MYOLU/15-139	G1PVH4.1 PF04857.18	53.40	0.00
TRINITY_H1A007_TAEGU/17-165	H1A007.1 PF00753.25	53.40	0.00
TRINITY_B9RY99_RICCO/88-165	B9RY99.1 PF05347.13	53.40	0.00
TRINITY_A0A078HR88_BRANA/158-310	A0A078HR88.1 PF0254	53.40	0.00
TRINITY_B8C2F6_THAPS/183-287	B8C2F6.1 PF00856.26	53.40	0.00
TRINITY_I7MMC2_TETTS/118-205	I7MMC2.2 PF04969.14	53.40	0.00
TRINITY_D8TIX0_VOLCA/336-697	D8TIX0.1 PF03016.13	53.40	0.00
TRINITY_E1ZMG6_CHLVA/69-319	E1ZMG6.1 PF08704.8;	53.40	0.00
TRINITY_W7U7D9_9STRA/21-262	W7U7D9.1 PF13561.4;	53.40	0.00
TRINITY_A0E8V2_PARTE/129-259	A0E8V2.1 PF00782.18	53.40	0.00
TRINITY_D3BGA1_POLPA/8-299	D3BGA1.1 PF00480.18	53.40	0.00
TRINITY_A0A074RTM7_9HOMO/1276-1423	A0A074RTM7.1 PF0000	53.40	0.00
TRINITY_I7M1N7_TETTS/84-192	I7M1N7.1 PF14580.4;	53.40	0.00
TRINITY_A0A078AAD9_STYLE/456-573	A0A078AAD9.1 PF0298	53.40	0.00
TRINITY_F0ZTN0_DICPU/29-87	F0ZTN0.1 PF00786.26	53.40	0.00
TRINITY_D8UEA8_VOLCA/143-403	D8UEA8.1 PF08007.10	53.40	0.00
TRINITY_Q22GW0_TETTS/1651-1813	Q22GW0.2 PF00069.23	53.40	0.00
TRINITY_D8LJS2_ECTSI/20-262	D8LJS2.1 PF00348.15	53.40	0.00
TRINITY_G0QRW1_ICHMG/96-486	G0QRW1.1 PF00155.19	53.40	0.00
TRINITY_Q22RZ8_TETTS/183-242	Q22RZ8.2 PF13921.4;	53.40	0.00
TRINITY_SUN2_DICDI/534-657	Q54MI3.1 PF07738.11	53.40	0.00
TRINITY_K3W5S1_PYTUL/49-320	K3W5S1.1 PF01545.19	53.40	0.00
TRINITY_A9TT93_PHYPA/1-251	A9TT93.1 PF05742.10	53.40	0.00
TRINITY_B8FAM2_DESAA/4-298	B8FAM2.1 PF00294.22	53.40	0.00
TRINITY_A0E826_PARTE/128-238	A0E826.1 PF00168.28	53.40	0.00
TRINITY_A0A0M8KB64_9CHLR/83-139	A0A0M8KB64.1 PF0057	53.40	0.00
TRINITY_A8IZ02_CHLRE/5-108	A8IZ02.1 PF00504.19	53.40	0.00
TRINITY_I0YML3_9CHLO/74-193	I0YML3.1 PF00622.26	53.40	0.00

TRINITY_Q23DB7_TETTS/8-263	Q23DB7.2 PF00069.23	53.40	0.00
TRINITY_A8IMY7_CHLRE/43-215	A8IMY7.1 PF00445.16	53.40	0.00
TRINITY_D0NV41_PHYIT/12-174	D0NV41.1 PF00071.20	53.40	0.00
TRINITY_A8J233_CHLRE/172-261	A8J233.1 PF16760.3;	53.40	0.00
TRINITY_A0A096TV93_MAIZE/17-168	A0A096TV93.1 PF0006	53.40	0.00
TRINITY_A8IRL5_CHLRE/87-234	A8IRL5.1 PF08670.9;	53.40	0.00
TRINITY_A0C6C2_PARTE/5-172	A0C6C2.1 PF05301.9;	53.40	0.00
TRINITY_D3BNC5_POLPA/1335-1452	D3BNC5.1 PF00072.22	53.40	0.00
TRINITY_A0A022QW50_ERYGU/15-107	A0A022QW50.1 PF1279	53.40	0.00
TRINITY_A8IVU6_CHLRE/482-724	A8IVU6.1 PF00520.29	53.40	0.00
TRINITY_A0CES3_PARTE/634-752	A0CES3.1 PF00072.22	53.40	0.00
TRINITY_F0ZLU7_DICPU/181-297	F0ZLU7.1 PF14792.4;	53.40	0.00
TRINITY_G3BAW3_CANTC/210-276	G3BAW3.1 PF01485.19	53.40	0.00
TRINITY_F4QE14_DICFS/72-540	F4QE14.1 PF00171.20	53.40	0.00
TRINITY_D7FKH8_ECTSI/433-566	D7FKH8.1 PF01841.17	53.40	0.00
TRINITY_A8HZF2_CHLRE/68-224	A8HZF2.1 PF04143.12	53.40	0.00
TRINITY_T0R2U2_9STRA/44-116	T0R2U2.1 PF09398.8;	53.40	0.00
TRINITY_RL27_PARL1/2-81	A7HT69.1 PF01016.17	53.40	0.00
TRINITY_A8J6M6_CHLRE/100-170	A8J6M6.1 PF00076.20	53.40	0.00
TRINITY_A8IJW5_CHLRE/71-276	A8IJW5.1 PF03403.11	53.40	0.00
TRINITY_G0QJ87_ICHMG/6-199	G0QJ87.1 PF00069.23	53.40	0.00
TRINITY_J9FBS9_9SPIT/259-507	J9FBS9.1 PF00009.25	53.40	0.00
TRINITY_G0QKE9_ICHMG/201-285	G0QKE9.1 PF00027.27	53.40	0.00
TRINITY_D8TI57_VOLCA/11-171	D8TI57.1 PF05285.10	53.40	0.00
TRINITY_F4P544_BATDJ/979-1123	F4P544.1 PF00637.18	53.40	0.00
TRINITY_A8IFT8_CHLRE/600-698	A8IFT8.1 PF00211.18	53.40	0.00
TRINITY_Q23KD1_TETTS/1-249	Q23KD1.1 PF04641.10	53.40	0.00
TRINITY_Q235X0_TETTS/59-332	Q235X0.3 PF01193.22	53.30	0.00
TRINITY_I6Z4X1_MELRP/33-329	I6Z4X1.1 PF01263.18	53.30	0.00
TRINITY_A0A0E3ZJV1_9BACT/33-379	A0A0E3ZJV1.1 PF1028	53.30	0.00
TRINITY_A0A067TAS5_9AGAR/33-107	A0A067TAS5.1 PF1289	53.30	0.00
TRINITY_G0QJM6_ICHMG/4-283	G0QJM6.1 PF02383.16	53.30	0.00
TRINITY_A4S6N9_OSTLU/207-255	A4S6N9.1 PF00400.30	53.30	0.00
TRINITY_M0QSN1_ACACA/309-399	M0QSN1.1 PF02214.20	53.30	0.00
TRINITY_G0QW64_ICHMG/554-754	G0QW64.1 PF13087.4;	53.30	0.00
TRINITY_I7LWJ8_TETTS/52-307	I7LWJ8.1 PF00069.23	53.30	0.00
TRINITY_D3BM00_POLPA/28-88	D3BM00.1 PF00830.17	53.30	0.00
TRINITY_Q0YR84_9CHLB/108-286	Q0YR84.1 PF07478.11	53.30	0.00
TRINITY_Q46CR5_METBF/475-543	Q46CR5.1 PF13424.4;	53.30	0.00
TRINITY_L8GYJ5_ACACA/1646-1904	L8GYJ5.1 PF07714.15	53.30	0.00
TRINITY_J9IJY7_9SPIT/252-297	J9IJY7.1 PF13639.4;	53.30	0.00
TRINITY_C7G039_DICDI/1-107	C7G039.1 PF04471.10	53.30	0.00
TRINITY_Q65P25_BACLD/104-279	Q65P25.1 PF02655.12	53.30	0.00
TRINITY_I7MLV0_TETTS/461-797	I7MLV0.2 PF00443.27	53.30	0.00
TRINITY_B7G4K9_PHATC/179-223	B7G4K9.1 PF13639.4;	53.30	0.00
TRINITY_Q29PC4_DROPS/124-168	Q29PC4.1 PF00569.15	53.30	0.00
TRINITY_D8TQ43_VOLCA/10-71	D8TQ43.1 PF05030.10	53.30	0.00
TRINITY_A0A072PHZ5_9EURO/92-153	A0A072PHZ5.1 PF0022	53.30	0.00
TRINITY_L8GMF7_ACACA/729-802	L8GMF7.1 PF00498.24	53.30	0.00
TRINITY_A0A0D2WIY8_CAPO3/885-1002	A0A0D2WIY8.1 PF0141	53.30	0.00
TRINITY_F4Q8K6_DICFS/32-131	F4Q8K6.1 PF05071.14	53.30	0.00
TRINITY_GLU2B_ORYSI/457-614	A2WNF5.1 PF13015.4;	53.30	0.00
TRINITY_K7J1L6_NASVI/543-615	K7J1L6.1 PF05022.10	53.30	0.00
TRINITY_I0ZAS5_9CHLO/1575-1619	I0ZAS5.1 PF00249.29	53.30	0.00
TRINITY_C6J078_9BACL/6-125	C6J078.1 PF01408.20	53.30	0.00
TRINITY_Q24HH9_TETTS/52-312	Q24HH9.2 PF00069.23	53.30	0.00
TRINITY_I1GYV5_BRADI/706-951	I1GYV5.1 PF07727.12	53.30	0.00
TRINITY_I0YRH1_9CHLO/107-438	I0YRH1.1 PF03062.17	53.30	0.00
TRINITY_E1ZGN4_CHLVA/213-289	E1ZGN4.1 PF13242.4;	53.30	0.00
TRINITY_A0CMJ3_PARTE/426-538	A0CMJ3.1 PF14792.4;	53.30	0.00
TRINITY_A1S364_SHEAM/3-328	A1S364.1 PF00389.28	53.30	0.00

TRINITY_C4VA82_NOSCE/28-175	C4VA82.1 PF01926.21	53.30	0.00
TRINITY_SLA2_SCHPO/16-279	Q9P6L5.2 PF07651.14	53.30	0.00
TRINITY_D8U338_VOLCA/274-367	D8U338.1 PF05705.12	53.30	0.00
TRINITY_B6IRA2_RHOCS/37-303	B6IRA2.1 PF01636.21	53.30	0.00
TRINITY_D8U2E9_VOLCA/1-78	D8U2E9.1 PF00153.25	53.30	0.00
TRINITY_L8HA48_ACACA/25-177	L8HA48.1 PF10152.7;	53.30	0.00
TRINITY_J9IA49_9SPIT/254-345	J9IA49.1 PF00447.15	53.30	0.00
TRINITY_A0A087SL84_AUXPR/66-315	A0A087SL84.1 PF0705	53.30	0.00
TRINITY_G0R0B5_ICHMG/45-346	G0R0B5.1 PF03133.13	53.30	0.00
TRINITY_F4PHK8_DICFS/156-470	F4PHK8.1 PF02383.16	53.30	0.00
TRINITY_A0A0N4UL36_DRAME/64-256	A0A0N4UL36.1 PF0113	53.30	0.00
TRINITY_A0A0D3GJH5_9ORYZ/228-328	A0A0D3GJH5.1 PF0104	53.30	0.00
TRINITY_D8TL05_VOLCA/10-212	D8TL05.1 PF02146.15	53.30	0.00
TRINITY_D8TI15_VOLCA/1-167	D8TI15.1 PF05856.10	53.30	0.00
TRINITY_C1E469_MICSR/149-341	C1E469.1 PF08325.8;	53.30	0.00
TRINITY_V9EPL4_PHYPR/52-300	V9EPL4.1 PF06966.10	53.30	0.00
TRINITY_A0A015N5W5_9GLOM/109-260	A0A015N5W5.1 PF0000	53.30	0.00
TRINITY_D8U5Y8_VOLCA/2-366	D8U5Y8.1 PF02005.14	53.30	0.00
TRINITY_E1ZBK3_CHLVA/422-629	E1ZBK3.1 PF00448.20	53.30	0.00
TRINITY_A9SXH5_PHYPA/24-252	A9SXH5.1 PF06966.10	53.30	0.00
TRINITY_A8I3N8_CHLRE/39-150	A8I3N8.1 PF07714.15	53.30	0.00
TRINITY_D8UKY2_VOLCA/1209-1430	D8UKY2.1 PF07714.15	53.30	0.00
TRINITY_A0A066WZL8_COLSU/805-949	A0A066WZL8.1 PF0027	53.30	0.00
TRINITY_I0YYG8_9CHLO/12-184	I0YYG8.1 PF05063.12	53.30	0.00
TRINITY_A0CWK0_PARTE/410-529	A0CWK0.1 PF02518.24	53.20	0.00
TRINITY_I0LED3_9ACTN/92-398	I0LED3.1 PF00082.20	53.20	0.00
TRINITY_S8A3P0_DACHA/824-944	S8A3P0.1 PF01412.16	53.20	0.00
TRINITY_I7M4C2_TETTS/42-276	I7M4C2.1 PF00149.26	53.20	0.00
TRINITY_R1FHC9_EMIHU/26-218	R1FHC9.1 PF04118.12	53.20	0.00
TRINITY_L0PAP9_PNEJ8/2-143	L0PAP9.1 PF01217.18	53.20	0.00
TRINITY_A0A0N0LZU9_9GAMM/969-1089	A0A0N0LZU9.1 PF0007	53.20	0.00
TRINITY_I0YXQ2_9CHLO/111-392	I0YXQ2.1 PF05971.10	53.20	0.00
TRINITY_G0QPH4_ICHMG/437-665	G0QPH4.1 PF13932.4;	53.20	0.00
TRINITY_G0QPY6_ICHMG/30-344	G0QPY6.1 PF01074.20	53.20	0.00
TRINITY_Q54CZ3_DICDI/187-344	Q54CZ3.1 PF00730.23	53.20	0.00
TRINITY_A7RNS4_NEMVE/406-526	A7RNS4.1 PF00083.22	53.20	0.00
TRINITY_F5J0Z1_9PORP/9-132	F5J0Z1.1 PF00583.23	53.20	0.00
TRINITY_D8TZD2_VOLCA/241-481	D8TZD2.1 PF10494.7;	53.20	0.00
TRINITY_T1JIR4_STRMM/41-277	T1JIR4.1 PF01370.19	53.20	0.00
TRINITY_L8GZG2_ACACA/46-186	L8GZG2.1 PF01135.17	53.20	0.00
TRINITY_A0A059LL00_9CHLO/125-202	A0A059LL00.1 PF0275	53.20	0.00
TRINITY_A0A087STY5_AUXPR/124-295	A0A087STY5.1 PF0173	53.20	0.00
TRINITY_D8TT65_VOLCA/80-202	D8TT65.1 PF13401.4;	53.20	0.00
TRINITY_D3BTW1_POLPA/43-94	D3BTW1.1 PF07646.13	53.20	0.00
TRINITY_F4PU36_DICFS/130-410	F4PU36.1 PF03034.13	53.20	0.00
TRINITY_I7LT74_TETTS/2-115	I7LT74.2 PF09822.7;	53.20	0.00
TRINITY_D8TYP6_VOLCA/7-236	D8TYP6.1 PF05764.11	53.20	0.00
TRINITY_D8S292_SELML/247-291	D8S292.1 PF00097.23	53.20	0.00
TRINITY_B8M547_TALSN/177-376	B8M547.1 PF01262.19	53.20	0.00
TRINITY_D8TRM1_VOLCA/9-55	D8TRM1.1 PF08122.10	53.20	0.00
TRINITY_W4FEE0_9STRA/137-198	W4FEE0.1 PF00957.19	53.20	0.00
TRINITY_C1MWU7_MICPC/78-201	C1MWU7.1 PF01769.14	53.20	0.00
TRINITY_A0A068S0X7_9FUNG/67-352	A0A068S0X7.1 PF0006	53.20	0.00
TRINITY_D3BMQ7_POLPA/597-742	D3BMQ7.1 PF01694.20	53.20	0.00
TRINITY_A8INA3_CHLRE/1-158	A8INA3.1 PF08603.9;	53.20	0.00
TRINITY_A0A077ZQW9_STYLE/49-359	A0A077ZQW9.1 PF0024	53.20	0.00
TRINITY_A0CJL2_PARTE/227-274	A0CJL2.1 PF00415.16	53.20	0.00
TRINITY_G0QKW1_ICHMG/111-281	G0QKW1.1 PF00092.26	53.20	0.00
TRINITY_D3BM29_POLPA/88-372	D3BM29.1 PF03308.14	53.20	0.00
TRINITY_A0A099NW38_PICKU/146-302	A0A099NW38.1 PF0007	53.20	0.00
TRINITY_D8TZY9_VOLCA/23-157	D8TZY9.1 PF10063.7;	53.20	0.00

TRINITY_I0Z396_9CHLO/6-329	I0Z396.1 PF07992.12	53.20	0.00
TRINITY_F2JMJ6_CELLD/12-249	F2JMJ6.1 PF13561.4;	53.20	0.00
TRINITY_I7MGV0_TETTS/599-693	I7MGV0.2 PF12796.5;	53.20	0.00
TRINITY_E9GIT2_DAPPU/699-895	E9GIT2.1 PF02259.21	53.20	0.00
TRINITY_F0ZYD6_DICPU/27-173	F0ZYD6.1 PF00483.21	53.20	0.00
TRINITY_D2A1C7_TRICA/865-1023	D2A1C7.1 PF08433.8;	53.20	0.00
TRINITY_E1ZMM7_CHLVA/264-482	E1ZMM7.1 PF07059.10	53.20	0.00
TRINITY_A0A087SAP6_AUXPR/2-67	A0A087SAP6.1 PF0549	53.20	0.00
TRINITY_A8I6K1_CHLRE/3-111	A8I6K1.1 PF03164.12	53.20	0.00
TRINITY_D6WRW1_TRICA/241-291	D6WRW1.1 PF10058.7;	53.20	0.00
TRINITY_D8TR85_VOLCA/7-153	D8TR85.1 PF01925.17	53.20	0.00
TRINITY_A8IKW6_CHLRE/43-242	A8IKW6.1 PF00834.17	53.20	0.00
TRINITY_L8H1U2_ACACA/19-539	L8H1U2.1 PF01602.18	53.20	0.00
TRINITY_A8IYC6_CHLRE/17-176	A8IYC6.1 PF00211.18	53.20	0.00
TRINITY_H2Y4I3_CIOSA/26-621	H2Y4I3.1 PF04563.13	53.20	0.00
TRINITY_A0A0N4Z6H1_PARTI/38-114	A0A0N4Z6H1.1 PF0188	53.20	0.00
TRINITY_D8U0D0_VOLCA/83-393	D8U0D0.1 PF00632.23	53.20	0.00
TRINITY_Q239Q7_TETTS/71-416	Q239Q7.2 PF00225.21	53.20	0.00
TRINITY_A0A0D2X4E2_CAPO3/576-810	A0A0D2X4E2.1 PF0012	53.20	0.00
TRINITY_A0A078BCF8_STYLE/3-64	A0A078BCF8.1 PF0007	53.20	0.00
TRINITY_S3CQ68_OPHP1/12-322	S3CQ68.1 PF00069.23	53.20	0.00
TRINITY_E1Z997_CHLVA/7-108	E1Z997.1 PF00085.18	53.20	0.00
TRINITY_E3MI87_CAERE/184-457	E3MI87.1 PF07714.15	53.10	0.00
TRINITY_V8N9K4_OPHHA/45-170	V8N9K4.1 PF00005.25	53.10	0.00
TRINITY_A0A0C2UC57_BACBA/8-253	A0A0C2UC57.1 PF0227	53.10	0.00
TRINITY_J9K1U4_ACYPI/4-67	J9K1U4.1 PF00226.29	53.10	0.00
TRINITY_Q23QF2_TETTS/248-571	Q23QF2.2 PF03133.13	53.10	0.00
TRINITY_A0DAI9_PARTE/12-124	A0DAI9.1 PF01412.16	53.10	0.00
TRINITY_A0D6M9_PARTE/6-345	A0D6M9.1 PF00724.18	53.10	0.00
TRINITY_D3BE38_POLPA/22-144	D3BE38.1 PF00787.22	53.10	0.00
TRINITY_W7T6I3_9STRA/76-208	W7T6I3.1 PF00472.18	53.10	0.00
TRINITY_F4PHG0_DICFS/285-427	F4PHG0.1 PF00441.22	53.10	0.00
TRINITY_U9UKY8_RHIID/250-425	U9UKY8.1 PF05063.12	53.10	0.00
TRINITY_L8GF10_ACACA/1-543	L8GF10.1 PF04858.11	53.10	0.00
TRINITY_W7XJ81_TETTS/240-289	W7XJ81.1 PF13920.4;	53.10	0.00
TRINITY_T1JR84_TETUR/56-200	T1JR84.1 PF00005.25	53.10	0.00
TRINITY_I7MJV7_TETTS/57-267	I7MJV7.1 PF00710.18	53.10	0.00
TRINITY_Q3JEP1_NITOC/4-348	Q3JEP1.1 PF00266.17	53.10	0.00
TRINITY_I7M2Z5_TETTS/227-524	I7M2Z5.2 PF03133.13	53.10	0.00
TRINITY_K7J0P1_NASVI/185-522	K7J0P1.1 PF05028.12	53.10	0.00
TRINITY_A0A087SJB7_AUXPR/215-475	A0A087SJB7.1 PF0085	53.10	0.00
TRINITY_Q22WB5_TETTS/12-124	Q22WB5.2 PF01412.16	53.10	0.00
TRINITY_G0QWF3_ICHMG/180-406	G0QWF3.1 PF00557.22	53.10	0.00
TRINITY_G0QYC9_ICHMG/6-247	G0QYC9.1 PF01115.15	53.10	0.00
TRINITY_Q22XX1_TETTS/200-263	Q22XX1.2 PF12906.5;	53.10	0.00
TRINITY_G0QQP7_ICHMG/8-263	G0QQP7.1 PF00069.23	53.10	0.00
TRINITY_D8TTG4_VOLCA/36-241	D8TTG4.1 PF01351.16	53.10	0.00
TRINITY_B4FYZ2_MAIZE/186-550	B4FYZ2.1 PF00728.20	53.10	0.00
TRINITY_D8TRM2_VOLCA/1-210	D8TRM2.1 PF01594.14	53.10	0.00
TRINITY_E1Z3Z4_CHLVA/54-215	E1Z3Z4.1 PF04387.12	53.10	0.00
TRINITY_D8U978_VOLCA/2-203	D8U978.1 PF07714.15	53.10	0.00
TRINITY_F1A0T9_DICPU/142-239	F1A0T9.1 PF01833.22	53.10	0.00
TRINITY_I3YYD4_AEQSU/21-199	I3YYD4.1 PF14124.4;	53.10	0.00
TRINITY_F7W7F7_SORMK/277-424	F7W7F7.1 PF13692.4;	53.10	0.00
TRINITY_Q2H7U0_CHAGB/401-534	Q2H7U0.1 PF00005.25	53.10	0.00
TRINITY_F2CVA4_HORVD/116-320	F2CVA4.1 PF00485.16	53.10	0.00
TRINITY_Q23TB4_TETTS/337-449	Q23TB4.1 PF09070.9;	53.10	0.00
TRINITY_A8JA23_CHLRE/167-512	A8JA23.1 PF04910.12	53.10	0.00
TRINITY_D2V826_NAEGR/15-78	D2V826.1 PF01918.19	53.10	0.00
TRINITY_I0Z5Z6_9CHLO/1-400	I0Z5Z6.1 PF00022.17	53.10	0.00
TRINITY_H2Y4I3_CIOSA/26-621	H2Y4I3.1 PF04563.13	53.10	0.00

TRINITY_A0A067EBU9_CITSI/37-101	A0A067EBU9.1 PF1343	53.10	0.00
TRINITY_I7M048_TETTS/1-155	I7M048.1 PF04729.11	53.10	0.00
TRINITY_A0A078AUG7_STYLE/11-91	A0A078AUG7.1 PF1349	53.10	0.00
TRINITY_A8HTS4_CHLRE/73-136	A8HTS4.1 PF13432.4;	53.10	0.00
TRINITY_K1PT87_CRAGI/1655-1734	K1PT87.1 PF13771.4;	53.10	0.00
TRINITY_Q24CZ5_TETTS/7-168	Q24CZ5.2 PF00071.20	53.10	0.00
TRINITY_I0Z8P8_9CHLO/1-153	I0Z8P8.1 PF00566.16	53.10	0.00
TRINITY_C4LVM6_ENTHI/287-578	C4LVM6.1 PF00176.21	53.10	0.00
TRINITY_L8HFN6_ACACA/331-559	L8HFN6.1 PF06314.9;	53.10	0.00
TRINITY_D8UEJ7_VOLCA/8-793	D8UEJ7.1 PF05804.10	53.10	0.00
TRINITY_D8THA7_VOLCA/1-124	D8THA7.1 PF05057.12	53.10	0.00
TRINITY_I1C0E6_RHIO9/491-715	I1C0E6.1 PF16415.3;	53.10	0.00
TRINITY_D8U9S9_VOLCA/599-793	D8U9S9.1 PF00211.18	53.10	0.00
TRINITY_A8IID5_CHLRE/1-302	A8IID5.1 PF10149.7;	53.10	0.00
TRINITY_L8GEL6_ACACA/329-543	L8GEL6.1 PF13236.4;	53.10	0.00
TRINITY_H3D7Q3_TETNG/584-648	H3D7Q3.1 PF01363.19	53.10	0.00
TRINITY_I7MAM9_TETTS/250-530	I7MAM9.2 PF01189.15	53.10	0.00
TRINITY_D8UJZ9_VOLCA/1479-1570	D8UJZ9.1 PF00207.20	53.10	0.00
TRINITY_A8IFT8_CHLRE/600-698	A8IFT8.1 PF00211.18	53.10	0.00
TRINITY_A0A022Q2R9_ERYGU/817-911	A0A022Q2R9.1 PF1270	53.10	0.00
TRINITY_E6TYI3_BACCJ/4-148	E6TYI3.1 PF01451.19	53.10	0.00
TRINITY_Q0UIN5_PHANO/161-364	Q0UIN5.1 PF00742.17	53.00	0.00
TRINITY_A0DZT1_PARTE/4-196	A0DZT1.1 PF01652.16	53.00	0.00
TRINITY_C3YRH6_BRAFL/289-443	C3YRH6.1 PF13768.4;	53.00	0.00
TRINITY_K7ITS3_NASVI/167-504	K7ITS3.1 PF06602.12	53.00	0.00
TRINITY_Q22U45_TETTS/225-538	Q22U45.1 PF00883.19	53.00	0.00
TRINITY_I7LVQ8_TETTS/213-363	I7LVQ8.2 PF07992.12	53.00	0.00
TRINITY_L8HFY7_ACACA/34-232	L8HFY7.1 PF02492.17	53.00	0.00
TRINITY_L8GIV1_ACACA/257-449	L8GIV1.1 PF00270.27	53.00	0.00
TRINITY_A0A0D2WK40_CAPO3/1001-1326	A0A0D2WK40.1 PF0063	53.00	0.00
TRINITY_D3BEH4_POLPA/456-651	D3BEH4.1 PF00270.27	53.00	0.00
TRINITY_G0R410_ICHMG/23-292	G0R410.1 PF00481.19	53.00	0.00
TRINITY_D8TZI3_VOLCA/5-83	D8TZI3.1 PF04969.14	53.00	0.00
TRINITY_A4S9S4_OSTLU/72-247	A4S9S4.1 PF00534.18	53.00	0.00
TRINITY_M7B8U5_CHEMY/293-390	M7B8U5.1 PF08022.10	53.00	0.00
TRINITY_J9FWB3_9SPIT/489-626	J9FWB3.1 PF12146.6;	53.00	0.00
TRINITY_A0A015KH76_9GLOM/26-190	A0A015KH76.1 PF0385	53.00	0.00
TRINITY_A8HT37_CHLRE/1198-1411	A8HT37.1 PF16206.3;	53.00	0.00
TRINITY_L8HIQ5_ACACA/114-277	L8HIQ5.1 PF08645.9;	53.00	0.00
TRINITY_C3Y8X7_BRAFL/7-276	C3Y8X7.1 PF01073.17	53.00	0.00
TRINITY_D8TSG0_VOLCA/222-374	D8TSG0.1 PF01529.18	53.00	0.00
TRINITY_B0RH91_CLAMS/133-220	B0RH91.1 PF16697.3;	53.00	0.00
TRINITY_L8HLI8_ACACA/115-329	L8HLI8.1 PF00112.21	53.00	0.00
TRINITY_A0A0D2T7E2_GOSRA/130-278	A0A0D2T7E2.1 PF0029	53.00	0.00
TRINITY_K8GI83_9CYAN/140-352	K8GI83.1 PF00171.20	53.00	0.00
TRINITY_B9GQT7_POPTR/61-125	B9GQT7.1 PF00226.29	53.00	0.00
TRINITY_I2GZY6_TETBL/58-201	I2GZY6.1 PF08662.9;	53.00	0.00
TRINITY_A9HBS4_GLUDA/13-388	A9HBS4.1 PF04339.10	53.00	0.00
TRINITY_S8CEZ3_9LAMI/164-445	S8CEZ3.1 PF00566.16	53.00	0.00
TRINITY_I0K4V8_9BACT/18-321	I0K4V8.1 PF00294.22	53.00	0.00
TRINITY_F0ZYR6_DICPU/124-420	F0ZYR6.1 PF00069.23	53.00	0.00
TRINITY_D8TU71_VOLCA/262-361	D8TU71.1 PF07707.13	53.00	0.00
TRINITY_A8JJW2_CHLRE/66-256	A8JJW2.1 PF01370.19	53.00	0.00
TRINITY_E1ZLD4_CHLVA/328-548	E1ZLD4.1 PF05362.11	53.00	0.00
TRINITY_D8U9S9_VOLCA/599-793	D8U9S9.1 PF00211.18	53.00	0.00
TRINITY_C1E245_MICSR/28-260	C1E245.1 PF03407.14	53.00	0.00
TRINITY_A0A099NWK8_PICKU/112-302	A0A099NWK8.1 PF0007	53.00	0.00
TRINITY_F4Q691_DICFS/694-1078	F4Q691.1 PF00940.17	53.00	0.00
TRINITY_I7MKZ5_TETTS/850-1202	I7MKZ5.2 PF00773.17	53.00	0.00
TRINITY_L8GWZ4_ACACA/279-461	L8GWZ4.1 PF00270.27	53.00	0.00
TRINITY_A8ISF0_CHLRE/4-391	A8ISF0.1 PF00022.17	53.00	0.00

TRINITY_E1ZE38_CHLVA/277-473	E1ZE38.1 PF00534.18	53.00	0.00
TRINITY_G1N063_MELGA/197-609	G1N063.1 PF01131.18	53.00	0.00
TRINITY_A8ILZ7_CHLRE/84-382	A8ILZ7.1 PF01007.18	53.00	0.00
TRINITY_G7Y6D9_CLOSI/616-802	G7Y6D9.1 PF00702.24	53.00	0.00
TRINITY_A0A091DLJ5_FUKDA/167-876	A0A091DLJ5.1 PF0013	53.00	0.00
TRINITY_R4XFE9_TAPDE/681-796	R4XFE9.1 PF00665.24	53.00	0.00
TRINITY_I0YWD0_9CHLO/8-264	I0YWD0.1 PF00069.23	53.00	0.00
TRINITY_I7MMG8_TETTS/8-169	I7MMG8.1 PF00071.20	53.00	0.00
TRINITY_U9U4H0_RHIID/1-204	U9U4H0.1 PF00122.18	53.00	0.00
TRINITY_J9JBX5_9SPIT/149-408	J9JBX5.1 PF00069.23	53.00	0.00
TRINITY_W7XG98_TETTS/15-84	W7XG98.1 PF13499.4;	52.90	0.00
TRINITY_A0CSI2_PARTE/27-156	A0CSI2.1 PF00782.18	52.90	0.00
TRINITY_K7IV52_NASVI/14-106	K7IV52.1 PF13233.4;	52.90	0.00
TRINITY_A0A0A1P236_9FUNG/284-755	A0A0A1P236.1 PF0620	52.90	0.00
TRINITY_A0A0D9VWR2_9ORYZ/46-175	A0A0D9VWR2.1 PF0851	52.90	0.00
TRINITY_B3QRX7_CHLT3/148-394	B3QRX7.1 PF01564.15	52.90	0.00
TRINITY_C5Y2L6_SORBI/25-334	C5Y2L6.1 PF00899.19	52.90	0.00
TRINITY_A6EQ69_9BACT/162-272	A6EQ69.1 PF00072.22	52.90	0.00
TRINITY_Q24FE9_TETTS/125-249	Q24FE9.2 PF00561.18	52.90	0.00
TRINITY_J9ICA0_9SPIT/1036-1087	J9ICA0.1 PF00415.16	52.90	0.00
TRINITY_D0N3V4_PHYIT/61-209	D0N3V4.1 PF04727.11	52.90	0.00
TRINITY_V3ZKW3_LOTGI/19-108	V3ZKW3.1 PF13646.4;	52.90	0.00
TRINITY_RL24_ACICJ/40-104	A5FZV4.1 PF17136.2;	52.90	0.00
TRINITY_A7S1D0_NEMVE/18-158	A7S1D0.1 PF01217.18	52.90	0.00
TRINITY_DAAF1_CHLRE/86-224	Q09JZ4.1 PF14580.4;	52.90	0.00
TRINITY_D8U9W9_VOLCA/12-348	D8U9W9.1 PF03224.12	52.90	0.00
TRINITY_D8PJF8_9BACT/55-157	D8PJF8.1 PF00011.19	52.90	0.00
TRINITY_H3CZ55_TETNG/1-146	H3CZ55.1 PF01429.17	52.90	0.00
TRINITY_A8JCW7_CHLRE/69-254	A8JCW7.1 PF02431.13	52.90	0.00
TRINITY_I7MH53_TETTS/28-94	I7MH53.1 PF00498.24	52.90	0.00
TRINITY_A8XXM7_CAEBR/23-294	A8XXM7.1 PF01926.21	52.90	0.00
TRINITY_A0A067QYX3_ZOONE/5-80	A0A067QYX3.1 PF0279	52.90	0.00
TRINITY_R1CKZ4_EMIHU/50-141	R1CKZ4.1 PF02214.20	52.90	0.00
TRINITY_Q247R2_TETTS/585-696	Q247R2.2 PF13246.4;	52.90	0.00
TRINITY_I7MLA5_TETTS/7-226	I7MLA5.2 PF01168.18	52.90	0.00
TRINITY_Q22T51_TETTS/44-513	Q22T51.2 PF00067.20	52.90	0.00
TRINITY_K2H6W1_9RHOB/3-233	K2H6W1.1 PF00297.20	52.90	0.00
TRINITY_M0SUF6_MUSAM/11-179	M0SUF6.1 PF06728.11	52.90	0.00
TRINITY_V3ZXS3_LOTGI/97-188	V3ZXS3.1 PF00153.25	52.90	0.00
TRINITY_A0DF57_PARTE/61-385	A0DF57.1 PF00069.23	52.90	0.00
TRINITY_D8MBA7_BLAHO/1-174	D8MBA7.1 PF00025.19	52.90	0.00
TRINITY_Q24FE9_TETTS/125-249	Q24FE9.2 PF00561.18	52.90	0.00
TRINITY_G3VF29_SARHA/15-362	G3VF29.1 PF01070.16	52.90	0.00
TRINITY_D2VYJ1_NAEGR/1343-1528	D2VYJ1.1 PF02145.13	52.90	0.00
TRINITY_F0ZP74_DICPU/221-539	F0ZP74.1 PF00657.20	52.90	0.00
TRINITY_A7BR79_9GAMM/15-298	A7BR79.1 PF03747.12	52.90	0.00
TRINITY_D1CD72_THET1/288-642	D1CD72.1 PF13361.4;	52.90	0.00
TRINITY_D8UJ36_VOLCA/93-448	D8UJ36.1 PF04938.10	52.90	0.00
TRINITY_Q24HK5_TETTS/22-161	Q24HK5.2 PF00501.26	52.90	0.00
TRINITY_X1WIA8_ACYPI/258-307	X1WIA8.1 PF13639.4;	52.90	0.00
TRINITY_D8TKZ7_VOLCA/1579-2019	D8TKZ7.1 PF00443.27	52.90	0.00
TRINITY_I0YTT5_9CHLO/19-259	I0YTT5.1 PF03649.11	52.90	0.00
TRINITY_D3BJZ4_POLPA/1611-1794	D3BJZ4.1 PF02145.13	52.90	0.00
TRINITY_G0R2T0_ICHMG/33-283	G0R2T0.1 PF00069.23	52.90	0.00
TRINITY_L8GPM7_ACACA/156-303	L8GPM7.1 PF01170.16	52.90	0.00
TRINITY_A0A090M4G4_OSTTA/6-76	A0A090M4G4.1 PF0007	52.90	0.00
TRINITY_L8GVV1_ACACA/94-272	L8GVV1.1 PF10602.7;	52.90	0.00
TRINITY_L8GPF5_ACACA/46-355	L8GPF5.1 PF01593.22	52.90	0.00
TRINITY_A0A085NAW1_9BILA/83-135	A0A085NAW1.1 PF0001	52.90	0.00
TRINITY_A0BKH0_PARTE/437-726	A0BKH0.1 PF03133.13	52.90	0.00
TRINITY_L8GD76_ACACA/67-365	L8GD76.1 PF13249.4;	52.90	0.00

TRINITY_I0Z005_9CHLO/54-303	I0Z005.1 PF13343.4;	52.90	0.00
TRINITY_D8TS64_VOLCA/5-517	D8TS64.1 PF00501.26	52.90	0.00
TRINITY_D8TQ64_VOLCA/608-670	D8TQ64.1 PF01424.20	52.90	0.00
TRINITY_M1VG03_CYAME/11-173	M1VG03.1 PF00071.20	52.90	0.00
TRINITY_Q22ZB8_TETTS/896-1015	Q22ZB8.2 PF08578.8;	52.90	0.00
TRINITY_D8TIX0_VOLCA/336-697	D8TIX0.1 PF03016.13	52.90	0.00
TRINITY_A8IP12_CHLRE/72-509	A8IP12.1 PF01490.16	52.90	0.00
TRINITY_D8TYN7_VOLCA/4-93	D8TYN7.1 PF04536.12	52.90	0.00
TRINITY_A0BJC2_PARTE/318-756	A0BJC2.1 PF04053.12	52.90	0.00
TRINITY_E8QX25_ISOPI/79-315	E8QX25.1 PF04055.19	52.90	0.00
TRINITY_W1NZ48_AMBTC/40-177	W1NZ48.1 PF09750.7;	52.90	0.00
TRINITY_C1EBU4_MICSR/64-161	C1EBU4.1 PF12796.5;	52.90	0.00
TRINITY_I0YZS0_9CHLO/5-148	I0YZS0.1 PF03073.13	52.90	0.00
TRINITY_B8CC85_THAPS/233-469	B8CC85.1 PF04811.13	52.90	0.00
TRINITY_A8HRZ0_CHLRE/47-116	A8HRZ0.1 PF00538.17	52.90	0.00
TRINITY_D8TYX9_VOLCA/666-861	D8TYX9.1 PF00211.18	52.90	0.00
TRINITY_L8GAQ0_PSED2/1125-1318	L8GAQ0.1 PF13087.4;	52.90	0.00
TRINITY_A0A060T0U9_PYCCI/1009-1125	A0A060T0U9.1 PF0066	52.90	0.00
TRINITY_D8LMM6_ECTSI/1-175	D8LMM6.1 PF01145.23	52.90	0.00
TRINITY_I7MH25_TETTS/266-611	I7MH25.2 PF03124.12	52.90	0.00
TRINITY_A0A075AXP8_9FUNG/1685-1961	A0A075AXP8.1 PF0006	52.80	0.00
TRINITY_J9I8P6_9SPIT/142-505	J9I8P6.1 PF05028.12	52.80	0.00
TRINITY_Q22E94_TETTS/13-86	Q22E94.1 PF01423.20	52.80	0.00
TRINITY_Q099W1_STIAD/478-664	Q099W1.1 PF01186.15	52.80	0.00
TRINITY_I7LVV2_TETTS/2-75	I7LVV2.1 PF02798.18	52.80	0.00
TRINITY_G0R0U2_ICHMG/19-320	G0R0U2.1 PF00491.19	52.80	0.00
TRINITY_G0QNF3_ICHMG/123-438	G0QNF3.1 PF00351.19	52.80	0.00
TRINITY_A7SWI0_NEMVE/11-95	A7SWI0.1 PF09809.7;	52.80	0.00
TRINITY_I7ME47_TETTS/120-274	I7ME47.1 PF00650.18	52.80	0.00
TRINITY_L0G0W3_ECHVK/170-296	L0G0W3.1 PF00107.24	52.80	0.00
TRINITY_G0QW69_ICHMG/157-289	G0QW69.1 PF00107.24	52.80	0.00
TRINITY_F0VJ54_NEOCL/73-222	F0VJ54.1 PF12146.6;	52.80	0.00
TRINITY_B6JVQ9_SCHJY/193-264	B6JVQ9.1 PF00076.20	52.80	0.00
TRINITY_G0QJQ7_ICHMG/12-286	G0QJQ7.1 PF00069.23	52.80	0.00
TRINITY_C3XWL8_BRAFL/22-271	C3XWL8.1 PF00481.19	52.80	0.00
TRINITY_I7M0X0_TETTS/460-654	I7M0X0.2 PF08662.9;	52.80	0.00
TRINITY_I3YJA3_ALIFI/88-363	I3YJA3.1 PF00078.25	52.80	0.00
TRINITY_E1ZQ10_CHLVA/38-341	E1ZQ10.1 PF00443.27	52.80	0.00
TRINITY_D3B860_POLPA/197-458	D3B860.1 PF07714.15	52.80	0.00
TRINITY_G0QXV0_ICHMG/52-760	G0QXV0.1 PF03030.14	52.80	0.00
TRINITY_Q08MK1_STIAD/195-335	Q08MK1.1 PF03561.13	52.80	0.00
TRINITY_W0J5E9_9BACT/286-592	W0J5E9.1 PF02836.15	52.80	0.00
TRINITY_D8TRD2_VOLCA/436-547	D8TRD2.1 PF01416.18	52.80	0.00
TRINITY_L8H039_ACACA/206-457	L8H039.1 PF00069.23	52.80	0.00
TRINITY_NDH_DICDI/40-362	Q55CD9.2 PF07992.12	52.80	0.00
TRINITY_L8HIJ0_ACACA/40-237	L8HIJ0.1 PF00106.23	52.80	0.00
TRINITY_A0A0M0J5D8_9EUKA/75-587	A0A0M0J5D8.1 PF1043	52.80	0.00
TRINITY_D8U0Z7_VOLCA/39-188	D8U0Z7.1 PF00504.19	52.80	0.00
TRINITY_D3B9P3_POLPA/85-246	D3B9P3.1 PF03031.16	52.80	0.00
TRINITY_I0YJ67_9CHLO/1-387	I0YJ67.1 PF00145.15	52.80	0.00
TRINITY_D8U5L8_VOLCA/41-437	D8U5L8.1 PF00856.26	52.80	0.00
TRINITY_A0C161_PARTE/15-325	A0C161.1 PF00248.19	52.80	0.00
TRINITY_Q6BGF7_PARTE/428-813	Q6BGF7.1 PF00702.24	52.80	0.00
TRINITY_A0DRX0_PARTE/123-371	A0DRX0.1 PF12697.5;	52.80	0.00
TRINITY_A0A0G4IY42_PLABS/1-168	A0A0G4IY42.1 PF0861	52.80	0.00
TRINITY_A8IFT8_CHLRE/600-698	A8IFT8.1 PF00211.18	52.80	0.00
TRINITY_B8P2A8_POSPM/116-288	B8P2A8.1 PF02222.20	52.80	0.00
TRINITY_F4RDE8_MELLP/7-194	F4RDE8.1 PF03357.19	52.80	0.00
TRINITY_D8TR51_VOLCA/679-786	D8TR51.1 PF01909.21	52.80	0.00
TRINITY_A0A0J8BP20_BETVU/10-171	A0A0J8BP20.1 PF0007	52.80	0.00
TRINITY_D8TS58_VOLCA/15-293	D8TS58.1 PF03133.13	52.80	0.00

TRINITY_A8JJ05_CHLRE/562-633	A8JJ05.1 PF05699.12	52.80	0.00
TRINITY_D8THB9_VOLCA/33-139	D8THB9.1 PF09799.7;	52.80	0.00
TRINITY_D0MID8_RHOM4/42-365	D0MID8.1 PF00144.22	52.80	0.00
TRINITY_E1Z4G9_CHLVA/221-385	E1Z4G9.1 PF16575.3;	52.80	0.00
TRINITY_D8TKG0_VOLCA/59-161	D8TKG0.1 PF08241.10	52.80	0.00
TRINITY_Q23YR9_TETTS/7-439	Q23YR9.1 PF00171.20	52.80	0.00
TRINITY_K3XAX2_PYTUL/861-1065	K3XAX2.1 PF00454.25	52.80	0.00
TRINITY_I0YQ38_9CHLO/146-206	I0YQ38.1 PF04117.10	52.80	0.00
TRINITY_H9IV50_BOMMO/11-254	H9IV50.1 PF01545.19	52.80	0.00
TRINITY_G0QJN1_ICHMG/433-510	G0QJN1.1 PF13424.4;	52.80	0.00
TRINITY_B8KSY6_9GAMM/97-212	B8KSY6.1 PF13469.4;	52.80	0.00
TRINITY_C1EIK2_MICSR/1-193	C1EIK2.1 PF00069.23	52.80	0.00
TRINITY_I0Z5Q3_9CHLO/6-741	I0Z5Q3.1 PF03635.15	52.80	0.00
TRINITY_A8J006_CHLRE/165-571	A8J006.1 PF07690.14	52.80	0.00
TRINITY_A8JJ05_CHLRE/562-633	A8JJ05.1 PF05699.12	52.80	0.00
TRINITY_A0BP38_PARTE/6-195	A0BP38.1 PF11539.6;	52.80	0.00
TRINITY_A0A078A8N4_STYLE/65-263	A0A078A8N4.1 PF0010	52.80	0.00
TRINITY_A0A061DVZ4_THECC/15-98	A0A061DVZ4.1 PF1341	52.70	0.00
TRINITY_G0QNT9_ICHMG/146-265	G0QNT9.1 PF04266.12	52.70	0.00
TRINITY_Q24D55_TETTS/15-181	Q24D55.1 PF10294.7;	52.70	0.00
TRINITY_I7LZR1_TETTS/114-210	I7LZR1.1 PF00153.25	52.70	0.00
TRINITY_R5ZF84_9ACTN/12-214	R5ZF84.1 PF14681.4;	52.70	0.00
TRINITY_Q236A7_TETTS/291-667	Q236A7.1 PF00493.21	52.70	0.00
TRINITY_Q3SDP8_PARTE/11-174	Q3SDP8.1 PF00071.20	52.70	0.00
TRINITY_G0QJT1_ICHMG/92-233	G0QJT1.1 PF00005.25	52.70	0.00
TRINITY_L8H8X9_ACACA/269-577	L8H8X9.1 PF06087.10	52.70	0.00
TRINITY_A0A0G4ERH9_9ALVE/48-231	A0A0G4ERH9.1 PF0087	52.70	0.00
TRINITY_I7MHI4_TETTS/19-149	I7MHI4.1 PF14886.4;	52.70	0.00
TRINITY_C4WSL9_ACYPI/25-193	C4WSL9.1 PF03232.11	52.70	0.00
TRINITY_A4VCZ9_TETTS/28-319	A4VCZ9.2 PF01026.19	52.70	0.00
TRINITY_C5LQ52_PERM5/31-288	C5LQ52.1 PF00069.23	52.70	0.00
TRINITY_ACT_ASPOR/2-375	Q2U7A3.1 PF00022.17	52.70	0.00
TRINITY_A0A061DJX7_THECC/104-200	A0A061DJX7.1 PF1289	52.70	0.00
TRINITY_Q241T7_TETTS/122-328	Q241T7.1 PF00112.21	52.70	0.00
TRINITY_L8HFK5_ACACA/20-200	L8HFK5.1 PF13532.4;	52.70	0.00
TRINITY_D8UEW3_VOLCA/53-435	D8UEW3.1 PF13621.4;	52.70	0.00
TRINITY_W7A9C1_9APIC/862-1087	W7A9C1.1 PF08235.11	52.70	0.00
TRINITY_A0A0N1GXE4_9EURO/29-358	A0A0N1GXE4.1 PF0015	52.70	0.00
TRINITY_D3BA29_POLPA/3755-4092	D3BA29.1 PF00632.23	52.70	0.00
TRINITY_Q22CC9_TETTS/27-248	Q22CC9.2 PF00687.19	52.70	0.00
TRINITY_A0A0K8L121_9EURO/998-1115	A0A0K8L121.1 PF0007	52.70	0.00
TRINITY_Q23G35_TETTS/134-438	Q23G35.1 PF03133.13	52.70	0.00
TRINITY_D8UIW9_VOLCA/52-303	D8UIW9.1 PF08538.8;	52.70	0.00
TRINITY_F7IEP9_CALJA/143-253	F7IEP9.1 PF00730.23	52.70	0.00
TRINITY_D3BM92_POLPA/29-281	D3BM92.1 PF00069.23	52.70	0.00
TRINITY_D8UIC1_VOLCA/20-114	D8UIC1.1 PF06127.9;	52.70	0.00
TRINITY_U9SQ30_RHIID/5-408	U9SQ30.1 PF04841.11	52.70	0.00
TRINITY_I0YWU6_9CHLO/805-859	I0YWU6.1 PF00628.27	52.70	0.00
TRINITY_E1ZPX6_CHLVA/248-417	E1ZPX6.1 PF07910.11	52.70	0.00
TRINITY_M2XZ12_GALSU/232-284	M2XZ12.1 PF13639.4;	52.70	0.00
TRINITY_A8HT53_CHLRE/18-325	A8HT53.1 PF00069.23	52.70	0.00
TRINITY_D3F372_CONWI/46-181	D3F372.1 PF01565.21	52.70	0.00
TRINITY_A0D5W6_PARTE/591-1119	A0D5W6.1 PF12157.6;	52.70	0.00
TRINITY_A8I7P7_CHLRE/1-181	A8I7P7.1 PF00609.17	52.70	0.00
TRINITY_D8SYZ7_SELML/836-1427	D8SYZ7.1 PF04998.15	52.70	0.00
TRINITY_A8IDI2_CHLRE/155-305	A8IDI2.1 PF00892.18	52.70	0.00
TRINITY_A8IS85_CHLRE/4-172	A8IS85.1 PF03446.13	52.70	0.00
TRINITY_A8IZH2_CHLRE/548-643	A8IZH2.1 PF00211.18	52.70	0.00
TRINITY_D8LU94_ECTSI/82-277	D8LU94.1 PF11715.6;	52.70	0.00
TRINITY_A8I3U0_CHLRE/22-158	A8I3U0.1 PF03364.18	52.70	0.00
TRINITY_L8HID3_ACACA/81-268	L8HID3.1 PF02141.19	52.70	0.00

TRINITY_A0A0N4XDG5_NIPBR/1-95	A0A0N4XDG5.1 PF1390	52.70	0.00
TRINITY_D1AE16_THECD/254-473	D1AE16.1 PF04389.15	52.70	0.00
TRINITY_A8JBA2_CHLRE/102-475	A8JBA2.1 PF07690.14	52.70	0.00
TRINITY_L8H7Z4_ACACA/62-190	L8H7Z4.1 PF00782.18	52.70	0.00
TRINITY_A8IGG5_CHLRE/30-160	A8IGG5.1 PF01590.24	52.70	0.00
TRINITY_D8UCH3_VOLCA/408-695	D8UCH3.1 PF00632.23	52.70	0.00
TRINITY_E1Z7Y3_CHLVA/54-162	E1Z7Y3.1 PF02893.18	52.70	0.00
TRINITY_G0QJD8_ICHMG/12-266	G0QJD8.1 PF00108.21	52.70	0.00
TRINITY_Q22FY1_TETTS/75-422	Q22FY1.2 PF00443.27	52.70	0.00
TRINITY_S2J338_MUCC1/6-296	S2J338.1 PF03095.13	52.70	0.00
TRINITY_D8TMT3_VOLCA/73-165	D8TMT3.1 PF00254.26	52.70	0.00
TRINITY_H0X7V6_OTOGA/341-578	H0X7V6.1 PF02836.15	52.70	0.00
TRINITY_I1FK56_AMPQE/235-397	I1FK56.1 PF13191.4;	52.70	0.00
TRINITY_A0BYH4_PARTE/37-622	A0BYH4.1 PF00995.21	52.70	0.00
TRINITY_A0A0K0EFA0_STRER/1-526	A0A0K0EFA0.1 PF0074	52.60	0.00
TRINITY_K7J951_NASVI/21-1070	K7J951.1 PF14750.4;	52.60	0.00
TRINITY_A0ECD1_PARTE/120-507	A0ECD1.1 PF00443.27	52.60	0.00
TRINITY_H2ZJF1_CIOSA/98-313	H2ZJF1.1 PF01545.19	52.60	0.00
TRINITY_W1PAY6_AMBTC/20-115	W1PAY6.1 PF00173.26	52.60	0.00
TRINITY_W5LI81_ASTMX/270-330	W5LI81.1 PF04193.12	52.60	0.00
TRINITY_Q22Z31_TETTS/543-620	Q22Z31.4 PF00027.27	52.60	0.00
TRINITY_A0A015K4I0_9GLOM/211-305	A0A015K4I0.1 PF0869	52.60	0.00
TRINITY_F0ZG51_DICPU/29-342	F0ZG51.1 PF03820.15	52.60	0.00
TRINITY_A0A0D2QTE0_GOSRA/31-120	A0A0D2QTE0.1 PF0029	52.60	0.00
TRINITY_A0E5C3_PARTE/963-1060	A0E5C3.1 PF00581.18	52.60	0.00
TRINITY_Q7XCY0_ORYSJ/59-456	Q7XCY0.2 PF05577.10	52.60	0.00
TRINITY_Q6BFT8_PARTE/121-366	Q6BFT8.1 PF11708.6;	52.60	0.00
TRINITY_F4Y204_9CYAN/242-414	F4Y204.1 PF00201.16	52.60	0.00
TRINITY_D3B1Q0_POLPA/79-502	D3B1Q0.1 PF01704.16	52.60	0.00
TRINITY_L8H3W5_ACACA/497-720	L8H3W5.1 PF00176.21	52.60	0.00
TRINITY_I7ML74_TETTS/5678-6228	I7ML74.2 PF00899.19	52.60	0.00
TRINITY_I7MAL7_TETTS/150-223	I7MAL7.1 PF12265.6;	52.60	0.00
TRINITY_A0A0P7UBE8_9TELE/803-1086	A0A0P7UBE8.1 PF0066	52.60	0.00
TRINITY_B4VM58_9CYAN/119-249	B4VM58.1 PF00782.18	52.60	0.00
TRINITY_B7KBM6_CYAP7/9-203	B7KBM6.1 PF00106.23	52.60	0.00
TRINITY_I7M466_TETTS/5-184	I7M466.1 PF06644.9;	52.60	0.00
TRINITY_Q22TJ1_TETTS/178-486	Q22TJ1.1 PF00365.18	52.60	0.00
TRINITY_A0A0J8E2C9_BETVU/94-308	A0A0J8E2C9.1 PF0113	52.60	0.00
TRINITY_I0YKS0_9CHLO/346-500	I0YKS0.1 PF17098.3;	52.60	0.00
TRINITY_W7X994_TETTS/783-919	W7X994.1 PF01067.20	52.60	0.00
TRINITY_I1FE73_AMPQE/40-233	I1FE73.1 PF00149.26	52.60	0.00
TRINITY_I0I7K4_CALAS/1-394	I0I7K4.1 PF03702.12	52.60	0.00
TRINITY_A0A068RPG8_9FUNG/50-335	A0A068RPG8.1 PF1034	52.60	0.00
TRINITY_A0EDK2_PARTE/307-528	A0EDK2.1 PF07002.14	52.60	0.00
TRINITY_A8I8X5_CHLRE/638-797	A8I8X5.1 PF02194.13	52.60	0.00
TRINITY_I7M7L0_TETTS/1-232	I7M7L0.1 PF05648.12	52.60	0.00
TRINITY_I0Z8F8_9CHLO/42-188	I0Z8F8.1 PF00160.19	52.60	0.00
TRINITY_Q024C7_SOLUE/41-331	Q024C7.1 PF00933.19	52.60	0.00
TRINITY_I0Z218_9CHLO/2-265	I0Z218.1 PF00069.23	52.60	0.00
TRINITY_A8IZH2_CHLRE/548-643	A8IZH2.1 PF00211.18	52.60	0.00
TRINITY_M1V4W3_CYAME/268-322	M1V4W3.1 PF13418.4;	52.60	0.00
TRINITY_Q3SDB5_PARTE/105-403	Q3SDB5.1 PF00122.18	52.60	0.00
TRINITY_G3AV41_SPAPN/216-460	G3AV41.1 PF01633.18	52.60	0.00
TRINITY_I0YT05_9CHLO/139-289	I0YT05.1 PF01569.19	52.60	0.00
TRINITY_D3BHH5_POLPA/638-963	D3BHH5.1 PF16653.3;	52.60	0.00
TRINITY_G0QZL8_ICHMG/314-544	G0QZL8.1 PF00233.17	52.60	0.00
TRINITY_D9V6J0_9ACTN/39-381	D9V6J0.1 PF00282.17	52.60	0.00
TRINITY_A0A0E9NJ37_9ASCO/88-221	A0A0E9NJ37.1 PF0029	52.60	0.00
TRINITY_F6GSP4_VITVI/896-1185	F6GSP4.1 PF00069.23	52.60	0.00
TRINITY_Q23CV4_TETTS/238-295	Q23CV4.1 PF13921.4;	52.60	0.00
TRINITY_A8HV78_CHLRE/111-149	A8HV78.1 PF00400.30	52.60	0.00

TRINITY_A0A067KRD3_JATCU/85-435	A0A067KRD3.1 PF0413	52.60	0.00
TRINITY_A8J3Q7_CHLRE/869-1071	A8J3Q7.1 PF00211.18	52.60	0.00
TRINITY_D8TZG3_VOLCA/2436-2604	D8TZG3.1 PF13229.4;	52.60	0.00
TRINITY_A7SAS1_NEMVE/13-174	A7SAS1.1 PF00071.20	52.50	0.00
TRINITY_A7RLD5_NEMVE/3-186	A7RLD5.1 PF02146.15	52.50	0.00
TRINITY_B7PNG4_IXOSC/3-130	B7PNG4.1 PF00091.23	52.50	0.00
TRINITY_A0A0D2UQ09_CAPO3/106-349	A0A0D2UQ09.1 PF0142	52.50	0.00
TRINITY_A0A0D8I9J3_9CLOT/9-150	A0A0D8I9J3.1 PF1330	52.50	0.00
TRINITY_G0QKM2_ICHMG/159-223	G0QKM2.1 PF13499.4;	52.50	0.00
TRINITY_A0A0D5C5Q2_9ARCH/6-67	A0A0D5C5Q2.1 PF0022	52.50	0.00
TRINITY_T1JL22_STRMM/313-418	T1JL22.1 PF00581.18	52.50	0.00
TRINITY_PYRG_DICDI/322-561	Q54V77.1 PF00117.26	52.50	0.00
TRINITY_D3AZJ7_POLPA/170-233	D3AZJ7.1 PF00333.18	52.50	0.00
TRINITY_A0A0K1QN11_PSEFL/5-73	A0A0K1QN11.1 PF0022	52.50	0.00
TRINITY_J9FRH7_9SPIT/20-284	J9FRH7.1 PF01650.16	52.50	0.00
TRINITY_L8GJ67_ACACA/71-172	L8GJ67.1 PF16131.3;	52.50	0.00
TRINITY_L8H8P6_ACACA/5-166	L8H8P6.1 PF00071.20	52.50	0.00
TRINITY_G0QUN0_ICHMG/10-151	G0QUN0.1 PF01090.17	52.50	0.00
TRINITY_K7IQQ6_NASVI/17-131	K7IQQ6.1 PF01412.16	52.50	0.00
TRINITY_D2V297_NAEGR/8-169	D2V297.1 PF00071.20	52.50	0.00
TRINITY_A0A0N0HDI4_THEVU/104-279	A0A0N0HDI4.1 PF0265	52.50	0.00
TRINITY_A5DLF2_PICGU/191-232	A5DLF2.2 PF00400.30	52.50	0.00
TRINITY_I7LV60_TETTS/36-201	I7LV60.1 PF00071.20	52.50	0.00
TRINITY_Q2UPL3_ASPOR/566-682	Q2UPL3.1 PF00665.24	52.50	0.00

TRINITY_J9BIQ0_WUCBA/253-317	J9BIQ0.1 PF01363.19	52.50	0.00
TRINITY_Q24D80_TETTS/178-486	Q24D80.2 PF08014.9;	52.50	0.00
TRINITY_J9ELB2_9SPIT/196-397	J9ELB2.1 PF13532.4;	52.50	0.00
TRINITY_I0YYU8_9CHLO/9-260	I0YYU8.1 PF07714.15	52.50	0.00
TRINITY_A0DE75_PARTE/346-500	A0DE75.1 PF04108.10	52.50	0.00
TRINITY_A0A0D2WQ32_CAPO3/229-428	A0A0D2WQ32.1 PF0832	52.50	0.00
TRINITY_Q23G27_TETTS/573-1166	Q23G27.2 PF00443.27	52.50	0.00
TRINITY_K4C109_SOLLC/15-352	K4C109.1 PF01112.16	52.50	0.00
TRINITY_F0ZPB8_DICPU/23-285	F0ZPB8.1 PF00069.23	52.50	0.00
TRINITY_Q23A17_TETTS/139-248	Q23A17.1 PF00168.28	52.50	0.00
TRINITY_G0QRB8_ICHMG/54-342	G0QRB8.1 PF00069.23	52.50	0.00
TRINITY_M1BNQ0_SOLTU/21-84	M1BNQ0.1 PF02953.13	52.50	0.00
TRINITY_A0A078AMG9_STYLE/130-467	A0A078AMG9.1 PF0024	52.50	0.00
TRINITY_A0A078B8T9_STYLE/13-150	A0A078B8T9.1 PF1458	52.50	0.00
TRINITY_F4Q9D4_DICFS/162-426	F4Q9D4.1 PF00069.23	52.50	0.00
TRINITY_A0A0E9NRK3_9ASCO/115-177	A0A0E9NRK3.1 PF1273	52.50	0.00
TRINITY_A0A0D2WWF4_CAPO3/373-573	A0A0D2WWF4.1 PF0116	52.50	0.00
TRINITY_A0A0M8K9R9_9CHLR/21-422	A0A0M8K9R9.1 PF0050	52.50	0.00
TRINITY_A0A0F0K8V7_9MICO/4-65	A0A0F0K8V7.1 PF0022	52.50	0.00
TRINITY_A8J9Y9_CHLRE/74-289	A8J9Y9.1 PF01036.16	52.50	0.00
TRINITY_C9S5A1_VERA1/326-510	C9S5A1.1 PF00201.16	52.50	0.00
TRINITY_D8U4J9_VOLCA/149-346	D8U4J9.1 PF03839.14	52.50	0.00
TRINITY_W3XP60_9PEZI/112-558	W3XP60.1 PF00083.22	52.50	0.00
TRINITY_L1I579_GUITH/77-199	L1I579.1 PF02996.15	52.50	0.00
TRINITY_L8H6D1_ACACA/645-898	L8H6D1.1 PF07714.15	52.50	0.00
TRINITY_E1ZKG7_CHLVA/267-390	E1ZKG7.1 PF08327.9;	52.50	0.00
TRINITY_A9B7N2_HERA2/4-143	A9B7N2.1 PF08719.9;	52.50	0.00
TRINITY_A8JCQ8_CHLRE/128-575	A8JCQ8.1 PF00501.26	52.50	0.00
TRINITY_D8UFK1_VOLCA/49-416	D8UFK1.1 PF07992.12	52.50	0.00
TRINITY_K1ZUE7_9BACT/6-67	K1ZUE7.1 PF00226.29	52.50	0.00
TRINITY_A4S9Q9_OSTLU/23-87	A4S9Q9.1 PF13499.4;	52.50	0.00
TRINITY_U5D205_AMBTC/686-1008	U5D205.1 PF08767.9;	52.50	0.00
TRINITY_A0EIL2_PARTE/105-216	A0EIL2.1 PF00651.29	52.50	0.00
TRINITY_D8UGT5_VOLCA/205-418	D8UGT5.1 PF01061.22	52.50	0.00
TRINITY_L8H6I7_ACACA/100-313	L8H6I7.1 PF04921.12	52.50	0.00
TRINITY_D8U7U2_VOLCA/1-264	D8U7U2.1 PF04080.11	52.50	0.00
TRINITY_F1R9Z2_DANRE/529-730	F1R9Z2.1 PF03914.15	52.50	0.00
TRINITY_L8HG59_ACACA/44-379	L8HG59.1 PF07992.12	52.50	0.00
TRINITY_K9UDB7_9CHRO/3-129	K9UDB7.1 PF12681.5;	52.50	0.00
TRINITY_A0A087SIS5_AUXPR/23-183	A0A087SIS5.1 PF1270	52.50	0.00
TRINITY_A8J6G7_CHLRE/513-669	A8J6G7.1 PF04727.11	52.50	0.00
TRINITY_A0A090LTL4_STRRB/5-166	A0A090LTL4.1 PF0007	52.50	0.00
TRINITY_A8IZU4_CHLRE/79-157	A8IZU4.1 PF04232.10	52.50	0.00
TRINITY_H3AUA0_LATCH/345-491	H3AUA0.1 PF12781.5;	52.50	0.00
TRINITY_F4QFV0_DICFS/86-241	F4QFV0.1 PF00005.25	52.50	0.00
TRINITY_A8JGR6_CHLRE/140-304	A8JGR6.1 PF01746.19	52.50	0.00
TRINITY_A8IZ91_CHLRE/337-455	A8IZ91.1 PF07714.15	52.50	0.00
TRINITY_E1ZN95_CHLVA/866-1132	E1ZN95.1 PF00664.21	52.50	0.00
TRINITY_D8TPW1_VOLCA/4-124	D8TPW1.1 PF13326.4;	52.50	0.00
TRINITY_A8ICD3_CHLRE/3-145	A8ICD3.1 PF09801.7;	52.50	0.00
TRINITY_A0A078B3M0_STYLE/472-532	A0A078B3M0.1 PF1395	52.50	0.00
TRINITY_H8ZCL5_NEMS1/3-374	H8ZCL5.1 PF00022.17	52.50	0.00
TRINITY_Q3SDP8_PARTE/11-174	Q3SDP8.1 PF00071.20	52.40	0.00
TRINITY_V2XDV5_MONRO/83-147	V2XDV5.1 PF13499.4;	52.40	0.00
TRINITY_I7MGY5_TETTS/2250-2501	I7MGY5.2 PF12775.5;	52.40	0.00
TRINITY_A0D6M9_PARTE/6-345	A0D6M9.1 PF00724.18	52.40	0.00
TRINITY_V7BDZ6_PHAVU/8-70	V7BDZ6.1 PF00280.16	52.40	0.00
TRINITY_L7N3A5_XENTR/13-75	L7N3A5.1 PF00226.29	52.40	0.00
TRINITY_D7FTR8_ECTSI/67-129	D7FTR8.1 PF10181.7;	52.40	0.00
TRINITY_A0A078A3M1_STYLE/248-329	A0A078A3M1.1 PF0496	52.40	0.00
TRINITY_Q8SUM5_ENCCU/189-232	Q8SUM5.1 PF13639.4;	52.40	0.00

TRINITY_J9J5L0_9SPIT/92-259	J9J5L0.1 PF00650.18	52.40	0.00
TRINITY_A0A0J8BQ47_BETVU/55-131	A0A0J8BQ47.1 PF0851	52.40	0.00
TRINITY_L8HEV1_ACACA/56-120	L8HEV1.1 PF13499.4;	52.40	0.00
TRINITY_A0A0D2WX97_CAPO3/45-164	A0A0D2WX97.1 PF0441	52.40	0.00
TRINITY_I7MEA2_TETTS/352-496	I7MEA2.1 PF00005.25	52.40	0.00
TRINITY_F4QFE8_DICFS/272-458	F4QFE8.1 PF13489.4;	52.40	0.00
TRINITY_A0A015JJL8_9GLOM/86-364	A0A015JJL8.1 PF0048	52.40	0.00
TRINITY_B8HP17_CYAP4/998-1116	B8HP17.1 PF02518.24	52.40	0.00
TRINITY_I7M7Z9_TETTS/616-760	I7M7Z9.1 PF00005.25	52.40	0.00
TRINITY_D8TVZ9_VOLCA/7-333	D8TVZ9.1 PF00150.16	52.40	0.00
TRINITY_A0A0N0JQK4_9BRAD/209-298	A0A0N0JQK4.1 PF1456	52.40	0.00
TRINITY_A8I6F3_CHLRE/385-587	A8I6F3.1 PF07714.15	52.40	0.00
TRINITY_D2VCT4_NAEGR/118-362	D2VCT4.1 PF00122.18	52.40	0.00
TRINITY_A0CPL9_PARTE/47-528	A0CPL9.1 PF00501.26	52.40	0.00
TRINITY_A0A0F2QT72_9PSED/790-907	A0A0F2QT72.1 PF0007	52.40	0.00
TRINITY_D8TZA3_VOLCA/30-444	D8TZA3.1 PF01266.22	52.40	0.00
TRINITY_C0ZHF8_BREBN/6-160	C0ZHF8.1 PF00156.25	52.40	0.00
TRINITY_A0A0D2X269_CAPO3/247-328	A0A0D2X269.1 PF0078	52.40	0.00
TRINITY_V4A6R2_LOTGI/19-124	V4A6R2.1 PF00085.18	52.40	0.00
TRINITY_F6SRN2_XENTR/4-66	F6SRN2.1 PF00076.20	52.40	0.00
TRINITY_E1ZII3_CHLVA/133-196	E1ZII3.1 PF04406.12	52.40	0.00
TRINITY_D8TVF5_VOLCA/108-230	D8TVF5.1 PF00293.26	52.40	0.00
TRINITY_D8TQR7_VOLCA/192-273	D8TQR7.1 PF00454.25	52.40	0.00
TRINITY_D8U1I0_VOLCA/272-506	D8U1I0.1 PF01458.15	52.40	0.00
TRINITY_F2U272_SALR5/62-424	F2U272.1 PF00155.19	52.40	0.00
TRINITY_D8UD63_VOLCA/25-106	D8UD63.1 PF09409.8;	52.40	0.00
TRINITY_I0YUX0_9CHLO/2-256	I0YUX0.1 PF02668.14	52.40	0.00
TRINITY_Q172U1_AEDAE/112-338	Q172U1.1 PF00271.29	52.40	0.00
TRINITY_L8GRV1_ACACA/206-432	L8GRV1.1 PF00557.22	52.40	0.00
TRINITY_A0A015K4H0_9GLOM/97-347	A0A015K4H0.1 PF0012	52.40	0.00
TRINITY_A8I2M1_CHLRE/105-395	A8I2M1.1 PF03016.13	52.40	0.00
TRINITY_A8JII7_CHLRE/36-301	A8JII7.1 PF00149.26	52.40	0.00
TRINITY_A0DYM5_PARTE/61-463	A0DYM5.1 PF05889.11	52.40	0.00
TRINITY_D8TX15_VOLCA/501-770	D8TX15.1 PF02714.13	52.40	0.00
TRINITY_B7P150_IXOSC/13-219	B7P150.1 PF00149.26	52.40	0.00
TRINITY_V7BQG8_PHAVU/77-221	V7BQG8.1 PF10607.7;	52.40	0.00
TRINITY_A0A078FAF1_BRANA/608-731	A0A078FAF1.1 PF1383	52.40	0.00
TRINITY_D8TJ49_VOLCA/2822-3161	D8TJ49.1 PF02259.21	52.40	0.00
TRINITY_Q3SDP8_PARTE/11-174	Q3SDP8.1 PF00071.20	52.40	0.00
TRINITY_J9F9C2_9SPIT/12-241	J9F9C2.1 PF13561.4;	52.40	0.00
TRINITY_L8GIU2_ACACA/23-338	L8GIU2.1 PF00248.19	52.40	0.00
TRINITY_A0BMD4_PARTE/522-774	A0BMD4.1 PF01237.16	52.30	0.00
TRINITY_J9FRG5_9SPIT/274-319	J9FRG5.1 PF13639.4;	52.30	0.00
TRINITY_N0BAD0_9EURY/266-337	N0BAD0.1 PF00801.18	52.30	0.00
TRINITY_F0XZ49_AURAN/1-402	F0XZ49.1 PF00171.20	52.30	0.00
TRINITY_A0A0F4XC52_HANUV/18-299	A0A0F4XC52.1 PF0024	52.30	0.00
TRINITY_A9UZN1_MONBE/32-140	A9UZN1.1 PF14113.4;	52.30	0.00
TRINITY_Q229R4_TETTS/829-1227	Q229R4.1 PF06957.9;	52.30	0.00
TRINITY_A0A0M0UMY9_9PEZI/711-849	A0A0M0UMY9.1 PF0273	52.30	0.00
TRINITY_K3XAB2_PYTUL/309-516	K3XAB2.1 PF01925.17	52.30	0.00
TRINITY_L0EIF6_THECK/558-601	L0EIF6.1 PF01476.18	52.30	0.00
TRINITY_K9ZI10_ANACC/19-321	K9ZI10.1 PF00248.19	52.30	0.00
TRINITY_I7MDK2_TETTS/9-142	I7MDK2.1 PF00293.26	52.30	0.00
TRINITY_D3BLJ5_POLPA/1841-2108	D3BLJ5.1 PF07714.15	52.30	0.00
TRINITY_GPX8_ARATH/9-117	Q8LBU2.1 PF00255.17	52.30	0.00
TRINITY_L8HBN0_ACACA/204-390	L8HBN0.1 PF00852.17	52.30	0.00
TRINITY_A0A067R8P5_ZOONE/135-245	A0A067R8P5.1 PF0355	52.30	0.00
TRINITY_G0QJS9_ICHMG/119-291	G0QJS9.1 PF05677.10	52.30	0.00
TRINITY_D2V7B5_NAEGR/69-430	D2V7B5.1 PF05139.12	52.30	0.00
TRINITY_W4XFF3_STRPU/197-480	W4XFF3.1 PF01457.14	52.30	0.00
TRINITY_L8HJR8_ACACA/134-243	L8HJR8.1 PF10998.6;	52.30	0.00

TRINITY_Q247W8_TETTS/1117-1244	Q247W8.2 PF00069.23	52.30	0.00
TRINITY_A4VE41_TETTS/736-919	A4VE41.2 PF01369.18	52.30	0.00
TRINITY_D8LJA3_ECTSI/664-759	D8LJA3.1 PF02852.20	52.30	0.00
TRINITY_R1DYV5_EMIHU/9-182	R1DYV5.1 PF00071.20	52.30	0.00
TRINITY_F5YBL2_TREAZ/367-483	F5YBL2.1 PF00072.22	52.30	0.00
TRINITY_F0ZJ65_DICPU/29-418	F0ZJ65.1 PF00155.19	52.30	0.00
TRINITY_A0A0D2U4K6_CAPO3/73-371	A0A0D2U4K6.1 PF0473	52.30	0.00
TRINITY_A0A0G2JY53_RAT/84-757	A0A0G2JY53.1 PF0006	52.30	0.00
TRINITY_F0Z9F9_DICPU/146-396	F0Z9F9.1 PF00069.23	52.30	0.00
TRINITY_D8U5Y8_VOLCA/2-366	D8U5Y8.1 PF02005.14	52.30	0.00
TRINITY_U6L0Q4_EIMTE/278-348	U6L0Q4.1 PF12906.5;	52.30	0.00
TRINITY_F4Q3G5_DICFS/68-324	F4Q3G5.1 PF05721.11	52.30	0.00
TRINITY_F0ZHG2_DICPU/191-404	F0ZHG2.1 PF01556.16	52.30	0.00
TRINITY_B3RXG2_TRIAD/48-495	B3RXG2.1 PF00067.20	52.30	0.00
TRINITY_S9XAY6_SCHCR/191-411	S9XAY6.1 PF01591.16	52.30	0.00
TRINITY_A8I1G7_CHLRE/1-184	A8I1G7.1 PF00211.18	52.30	0.00
TRINITY_I1FVK3_AMPQE/42-255	I1FVK3.1 PF01135.17	52.30	0.00
TRINITY_F0ZAS8_DICPU/3-229	F0ZAS8.1 PF07786.10	52.30	0.00
TRINITY_R1BIM9_EMIHU/22-255	R1BIM9.1 PF13561.4;	52.30	0.00
TRINITY_U9SKD1_RHIID/65-328	U9SKD1.1 PF00069.23	52.30	0.00
TRINITY_A0A090M731_OSTTA/111-280	A0A090M731.1 PF0027	52.30	0.00
TRINITY_W2PXB2_PHYPN/493-609	W2PXB2.1 PF00665.24	52.30	0.00
TRINITY_G0QP44_ICHMG/256-681	G0QP44.1 PF00183.16	52.30	0.00
TRINITY_D8TPQ3_VOLCA/270-395	D8TPQ3.1 PF00188.24	52.30	0.00
TRINITY_B0VV52_ACIBS/24-262	B0VV52.1 PF13561.4;	52.30	0.00
TRINITY_A0DJI1_PARTE/152-367	A0DJI1.1 PF01556.16	52.30	0.00
TRINITY_W5MLC4_LEPOC/33-284	W5MLC4.1 PF00069.23	52.30	0.00
TRINITY_F0Z8Z6_DICPU/388-581	F0Z8Z6.1 PF01699.22	52.30	0.00
TRINITY_Q8RU52_ORYSJ/606-827	Q8RU52.1 PF07727.12	52.30	0.00
TRINITY_G3HPN4_CRIGR/113-296	G3HPN4.1 PF00517.15	52.30	0.00
TRINITY_Q23VE6_TETTS/45-109	Q23VE6.3 PF01363.19	52.30	0.00
TRINITY_A8HYL5_CHLRE/47-152	A8HYL5.1 PF05916.9;	52.30	0.00
TRINITY_F9W568_TRYCI/42-275	F9W568.1 PF02784.14	52.30	0.00
TRINITY_A0A022PTQ8_ERYGU/28-310	A0A022PTQ8.1 PF0285	52.30	0.00
TRINITY_L8H6U6_ACACA/87-250	L8H6U6.1 PF03371.13	52.30	0.00
TRINITY_A0A077ZQS0_STYLE/68-262	A0A077ZQS0.1 PF1214	52.30	0.00
TRINITY_W4G917_9STRA/20-167	W4G917.1 PF14588.4;	52.30	0.00
TRINITY_N1PWF8_DOTSN/28-220	N1PWF8.1 PF13489.4;	52.30	0.00
TRINITY_A0BE95_PARTE/82-430	A0BE95.1 PF00155.19	52.30	0.00
TRINITY_A8IBR0_CHLRE/860-935	A8IBR0.1 PF00575.21	52.30	0.00
TRINITY_R9PHE7_PSEHS/581-747	R9PHE7.1 PF11875.6;	52.30	0.00
TRINITY_F0ZB76_DICPU/5-166	F0ZB76.1 PF00071.20	52.30	0.00
TRINITY_D8U6T6_VOLCA/1-119	D8U6T6.1 PF12796.5;	52.30	0.00
TRINITY_A0A0G4J2X3_PLABS/751-865	A0A0G4J2X3.1 PF0066	52.30	0.00
TRINITY_A8J5U5_CHLRE/85-240	A8J5U5.1 PF00650.18	52.30	0.00
TRINITY_D3BAA5_POLPA/36-100	D3BAA5.1 PF13499.4;	52.30	0.00
TRINITY_Q22TH1_TETTS/202-459	Q22TH1.2 PF00069.23	52.30	0.00
TRINITY_I0YMW7_9CHLO/2-201	I0YMW7.1 PF00141.21	52.30	0.00
TRINITY_M2XGC5_GALSU/24-440	M2XGC5.1 PF00501.26	52.30	0.00
TRINITY_A8IJG2_CHLRE/26-238	A8IJG2.1 PF15665.3;	52.30	0.00
TRINITY_E1ZH69_CHLVA/262-391	E1ZH69.1 PF15982.3;	52.30	0.00
TRINITY_D8U9S9_VOLCA/599-793	D8U9S9.1 PF00211.18	52.30	0.00
TRINITY_D8UAK9_VOLCA/119-585	D8UAK9.1 PF14643.4;	52.30	0.00
TRINITY_A0A067MKT0_9HOMO/134-519	A0A067MKT0.1 PF0015	52.30	0.00
TRINITY_D8U3J1_VOLCA/85-259	D8U3J1.1 PF12263.6;	52.30	0.00
TRINITY_W7X2I6_TETTS/101-283	W7X2I6.1 PF00069.23	52.30	0.00
TRINITY_I1FX47_AMPQE/13-266	I1FX47.1 PF01370.19	52.30	0.00
TRINITY_A0DJ75_PARTE/483-760	A0DJ75.1 PF00702.24	52.20	0.00
TRINITY_A0CKD6_PARTE/642-939	A0CKD6.1 PF00632.23	52.20	0.00
TRINITY_G7DSU2_MIXOS/241-468	G7DSU2.1 PF01896.17	52.20	0.00
TRINITY_G0QWT9_ICHMG/1-339	G0QWT9.1 PF03345.12	52.20	0.00

TRINITY_Q23UC8_TETTS/103-344	Q23UC8.3 PF08704.8;	52.20	0.00
TRINITY_I7M8J3_TETTS/17-410	I7M8J3.2 PF00450.20	52.20	0.00
TRINITY_A0BMN9_PARTE/5-287	A0BMN9.1 PF00480.18	52.20	0.00
TRINITY_G4MWG4_MAGO7/8-74	G4MWG4.1 PF13432.4;	52.20	0.00
TRINITY_Q6C6K7_YARLI/9-170	Q6C6K7.2 PF00071.20	52.20	0.00
TRINITY_A0CJV4_PARTE/111-373	A0CJV4.1 PF00069.23	52.20	0.00
TRINITY_Q23UD7_TETTS/667-716	Q23UD7.2 PF13920.4;	52.20	0.00
TRINITY_W5JRN5_ANODA/45-254	W5JRN5.1 PF15924.3;	52.20	0.00
TRINITY_G8JNS0_ERECY/265-333	G8JNS0.1 PF00076.20	52.20	0.00
TRINITY_A0A0G4J477_PLABS/25-252	A0A0G4J477.1 PF0475	52.20	0.00
TRINITY_Q9SZ91_ARATH/44-249	Q9SZ91.1 PF00106.23	52.20	0.00
TRINITY_A0EFU1_PARTE/162-228	A0EFU1.1 PF01302.23	52.20	0.00
TRINITY_G0R0Z8_ICHMG/307-442	G0R0Z8.1 PF00005.25	52.20	0.00
TRINITY_D2V443_NAEGR/138-207	D2V443.1 PF00076.20	52.20	0.00
TRINITY_W5NIX2_LEPOC/291-587	W5NIX2.1 PF00069.23	52.20	0.00
TRINITY_D8U0E6_VOLCA/33-194	D8U0E6.1 PF04939.10	52.20	0.00
TRINITY_Q248E9_TETTS/284-336	Q248E9.1 PF13418.4;	52.20	0.00
TRINITY_B3RWZ2_TRIAD/17-264	B3RWZ2.1 PF03372.21	52.20	0.00
TRINITY_I7MGG4_TETTS/247-411	I7MGG4.2 PF07534.14	52.20	0.00
TRINITY_A0C8F2_PARTE/1-86	A0C8F2.1 PF00005.25	52.20	0.00
TRINITY_A4S3C2_OSTLU/53-332	A4S3C2.1 PF02353.18	52.20	0.00
TRINITY_Q22W19_TETTS/115-323	Q22W19.3 PF00112.21	52.20	0.00
TRINITY_D0MQP4_PHYIT/223-288	D0MQP4.1 PF01918.19	52.20	0.00
TRINITY_I0YVX4_9CHLO/299-509	I0YVX4.1 PF01061.22	52.20	0.00
TRINITY_L8GL88_ACACA/966-1225	L8GL88.1 PF00664.21	52.20	0.00
TRINITY_D8UCG0_VOLCA/59-148	D8UCG0.1 PF08241.10	52.20	0.00
TRINITY_I2FS14_USTH4/233-318	I2FS14.1 PF02146.15	52.20	0.00
TRINITY_L8GTU8_ACACA/76-547	L8GTU8.1 PF07969.9;	52.20	0.00
TRINITY_A0A068SIM2_9FUNG/187-303	A0A068SIM2.1 PF0066	52.20	0.00
TRINITY_D8TIM4_VOLCA/675-744	D8TIM4.1 PF16326.3;	52.20	0.00
TRINITY_A0CNX9_PARTE/179-246	A0CNX9.1 PF13833.4;	52.20	0.00
TRINITY_Q24FP1_TETTS/133-395	Q24FP1.1 PF00069.23	52.20	0.00
TRINITY_U9SIS7_RHIID/1-312	U9SIS7.1 PF07992.12	52.20	0.00
TRINITY_Q3SEA4_PARTE/73-258	Q3SEA4.1 PF00625.19	52.20	0.00
TRINITY_I7MDV7_TETTS/219-569	I7MDV7.2 PF00009.25	52.20	0.00
TRINITY_D8TJ10_VOLCA/96-269	D8TJ10.1 PF04727.11	52.20	0.00
TRINITY_M2Y4X3_GALSU/55-262	M2Y4X3.1 PF00106.23	52.20	0.00
TRINITY_I0Z724_9CHLO/211-302	I0Z724.1 PF00153.25	52.20	0.00
TRINITY_A0A0P7V2U8_9TELE/66-330	A0A0P7V2U8.1 PF0842	52.20	0.00
TRINITY_D2VJV1_NAEGR/844-994	D2VJV1.1 PF02275.16	52.20	0.00
TRINITY_V2XS26_MONRO/26-71	V2XS26.1 PF06839.10	52.20	0.00
TRINITY_A7SJL0_NEMVE/206-295	A7SJL0.1 PF01336.23	52.20	0.00
TRINITY_T1ELD6_HELRO/5-166	T1ELD6.1 PF00071.20	52.20	0.00
TRINITY_D8THE3_VOLCA/348-443	D8THE3.1 PF08777.9;	52.20	0.00
TRINITY_E4U659_OCEP5/2-66	E4U659.1 PF00313.20	52.20	0.00
TRINITY_L8H838_ACACA/32-353	L8H838.1 PF01301.17	52.20	0.00
TRINITY_A0A0G4IR31_PLABS/443-537	A0A0G4IR31.1 PF1293	52.20	0.00
TRINITY_A8JIV0_CHLRE/150-309	A8JIV0.1 PF03033.18	52.20	0.00
TRINITY_J9IA49_9SPIT/254-345	J9IA49.1 PF00447.15	52.20	0.00
TRINITY_M2Y4X3_GALSU/55-262	M2Y4X3.1 PF00106.23	52.20	0.00
TRINITY_A8J1D4_CHLRE/32-158	A8J1D4.1 PF09732.7;	52.20	0.00
TRINITY_K9YAW7_HALP7/44-193	K9YAW7.1 PF01947.14	52.20	0.00
TRINITY_D2V0L9_NAEGR/410-476	D2V0L9.1 PF01363.19	52.20	0.00
TRINITY_A0A015LPX9_9GLOM/340-671	A0A015LPX9.1 PF0006	52.20	0.00
TRINITY_A0A015KWK9_9GLOM/362-402	A0A015KWK9.1 PF1363	52.20	0.00
TRINITY_T1FY05_HELRO/14-161	T1FY05.1 PF04098.13	52.20	0.00
TRINITY_D8QZ95_SELML/193-357	D8QZ95.1 PF08007.10	52.20	0.00
TRINITY_D8THU4_VOLCA/76-201	D8THU4.1 PF00572.16	52.20	0.00
TRINITY_A8I1G7_CHLRE/1-184	A8I1G7.1 PF00211.18	52.20	0.00
TRINITY_C1EBE2_MICSR/50-251	C1EBE2.1 PF02545.12	52.20	0.00
TRINITY_E1ZBS9_CHLVA/188-322	E1ZBS9.1 PF04695.11	52.20	0.00

TRINITY_F4QFV0_DICFS/86-241	F4QFV0.1 PF00005.25	52.20	0.00
TRINITY_K9SZG2_9CYAN/110-382	K9SZG2.1 PF01636.21	52.20	0.00
TRINITY_D8U9S9_VOLCA/599-793	D8U9S9.1 PF00211.18	52.20	0.00
TRINITY_J9HZG8_9SPIT/452-565	J9HZG8.1 PF02518.24	52.20	0.00
TRINITY_D0NM42_PHYIT/90-205	D0NM42.1 PF01266.22	52.20	0.00
TRINITY_L8H985_ACACA/67-139	L8H985.1 PF13499.4;	52.10	0.00
TRINITY_Q23CW4_TETTS/196-365	Q23CW4.2 PF02866.16	52.10	0.00
TRINITY_W7X1L8_TETTS/52-266	W7X1L8.1 PF05891.10	52.10	0.00
TRINITY_I1I505_BRADI/3-74	I1I505.1 PF00240.21	52.10	0.00
TRINITY_T1IVB0_STRMM/20-70	T1IVB0.1 PF00385.22	52.10	0.00
TRINITY_GXCDD_DICDI/16-123	Q1ZXH8.3 PF00307.29	52.10	0.00
TRINITY_C5YZE6_SORBI/230-280	C5YZE6.1 PF13920.4;	52.10	0.00
TRINITY_A0BVC3_PARTE/23-117	A0BVC3.1 PF13905.4;	52.10	0.00
TRINITY_G0QUI0_ICHMG/278-349	G0QUI0.1 PF00498.24	52.10	0.00
TRINITY_Q3SDK9_PARTE/13-174	Q3SDK9.1 PF00071.20	52.10	0.00
TRINITY_Q4E658_TRYCC/165-299	Q4E658.1 PF00004.27	52.10	0.00
TRINITY_A8IVT4_CHLRE/510-880	A8IVT4.1 PF00060.24	52.10	0.00
TRINITY_Q0FMV9_PELBH/204-320	Q0FMV9.1 PF07687.12	52.10	0.00
TRINITY_Q22DR4_TETTS/671-764	Q22DR4.2 PF12796.5;	52.10	0.00
TRINITY_M0YGB8_HORVD/122-194	M0YGB8.1 PF04564.13	52.10	0.00
TRINITY_A0A0G4IYR4_PLABS/33-382	A0A0G4IYR4.1 PF0022	52.10	0.00
TRINITY_I7MM56_TETTS/332-571	I7MM56.1 PF00117.26	52.10	0.00
TRINITY_A8JF90_CHLRE/1546-1844	A8JF90.1 PF00520.29	52.10	0.00
TRINITY_ATX10_DICDI/500-598	Q55EI6.1 PF09759.7;	52.10	0.00
TRINITY_A0A0D2VZA5_CAPO3/33-171	A0A0D2VZA5.1 PF0008	52.10	0.00
TRINITY_L8GPY4_ACACA/167-252	L8GPY4.1 PF13640.4;	52.10	0.00
TRINITY_A9TN91_PHYPA/128-246	A9TN91.1 PF01926.21	52.10	0.00
TRINITY_Q54C42_DICDI/342-587	Q54C42.1 PF05958.9;	52.10	0.00
TRINITY_I7MHC3_TETTS/22-425	I7MHC3.2 PF01433.18	52.10	0.00
TRINITY_G0QK71_ICHMG/314-776	G0QK71.1 PF04053.12	52.10	0.00
TRINITY_I1MTB1_SOYBN/15-374	I1MTB1.1 PF04371.13	52.10	0.00
TRINITY_L8HIQ7_ACACA/482-586	L8HIQ7.1 PF00618.18	52.10	0.00
TRINITY_U9U3G2_RHIID/135-534	U9U3G2.1 PF10255.7;	52.10	0.00
TRINITY_L8H874_ACACA/42-307	L8H874.1 PF09423.8;	52.10	0.00
TRINITY_Q6PPG2_CHLRE/1-397	Q6PPG2.1 PF00909.19	52.10	0.00
TRINITY_C1D3B0_DEIDV/63-298	C1D3B0.1 PF13561.4;	52.10	0.00
TRINITY_A0CTM1_PARTE/151-369	A0CTM1.1 PF01556.16	52.10	0.00
TRINITY_A0A087SRN0_AUXPR/74-530	A0A087SRN0.1 PF0052	52.10	0.00
TRINITY_D3B128_POLPA/192-442	D3B128.1 PF00069.23	52.10	0.00
TRINITY_A8HSM1_CHLRE/54-427	A8HSM1.1 PF03222.11	52.10	0.00
TRINITY_Q9EMN2_AMEPV/3-74	Q9EMN2.1 PF00240.21	52.10	0.00
TRINITY_Q9EMN2_AMEPV/3-74	Q9EMN2.1 PF00240.21	52.10	0.00
TRINITY_H2YP11_CIOSA/230-438	H2YP11.1 PF00005.25	52.10	0.00
TRINITY_A0E686_PARTE/138-276	A0E686.1 PF12146.6;	52.10	0.00
TRINITY_A0E0I9_PARTE/102-233	A0E0I9.1 PF00782.18	52.10	0.00
TRINITY_L8H6Z9_ACACA/332-376	L8H6Z9.1 PF06839.10	52.10	0.00
TRINITY_A0A090M6I7_OSTTA/69-139	A0A090M6I7.1 PF0007	52.10	0.00
TRINITY_A8IG60_CHLRE/89-225	A8IG60.1 PF06101.9;	52.10	0.00
TRINITY_A0DHR7_PARTE/216-415	A0DHR7.1 PF13851.4;	52.10	0.00
TRINITY_A8IHE7_CHLRE/70-516	A8IHE7.1 PF13520.4;	52.10	0.00
TRINITY_A0EG50_PARTE/15-118	A0EG50.1 PF00168.28	52.10	0.00
TRINITY_I0YXM8_9CHLO/12-285	I0YXM8.1 PF07714.15	52.10	0.00
TRINITY_G0R3R3_ICHMG/550-644	G0R3R3.1 PF12936.5;	52.10	0.00
TRINITY_CDPK_SOYBN/34-292	P28583.1 PF00069.23	52.10	0.00
TRINITY_J9FBN2_9SPIT/564-662	J9FBN2.1 PF08647.9;	52.10	0.00
TRINITY_A0A0D2X4D0_CAPO3/274-436	A0A0D2X4D0.1 PF0864	52.10	0.00
TRINITY_D8U9M4_VOLCA/314-443	D8U9M4.1 PF12796.5;	52.10	0.00
TRINITY_A8IXU9_CHLRE/109-258	A8IXU9.1 PF01789.14	52.10	0.00
TRINITY_G0R1I2_ICHMG/31-228	G0R1I2.1 PF03462.16	52.10	0.00
TRINITY_M3B6K5_PSEFD/7-258	M3B6K5.1 PF01370.19	52.10	0.00
TRINITY_I1XKK3_METNJ/6-255	I1XKK3.1 PF10127.7;	52.10	0.00

TRINITY_F6UMT9_MACMU/12-284	F6UMT9.1 PF01073.17	52.10	0.00
TRINITY_D3BDG4_POLPA/314-432	D3BDG4.1 PF02518.24	52.10	0.00
TRINITY_A8J691_CHLRE/23-294	A8J691.1 PF00069.23	52.10	0.00
TRINITY_A0A077ZZE8_STYLE/725-771	A0A077ZZE8.1 PF1392	52.10	0.00
TRINITY_G3RZ16_GORGO/55-477	G3RZ16.1 PF05577.10	52.10	0.00
TRINITY_A0A0A0KNH1_CUCSA/285-405	A0A0A0KNH1.1 PF0038	52.10	0.00
TRINITY_YDIU_ECOLI/1-451	P77649.1 PF02696.12	52.10	0.00
TRINITY_D8FWV8_9CYAN/51-244	D8FWV8.1 PF09511.8;	52.10	0.00
TRINITY_D8SD29_SELML/9-310	D8SD29.1 PF00069.23	52.10	0.00
TRINITY_CMTR2_DROME/110-319	Q9UAS6.2 PF01728.17	52.10	0.00
TRINITY_C1EGU9_MICSR/265-402	C1EGU9.1 PF00271.29	52.10	0.00
TRINITY_A0A0G4FAZ2_9ALVE/139-353	A0A0G4FAZ2.1 PF0178	52.10	0.00
TRINITY_A0A0F6SHT8_9DELT/6-95	A0A0F6SHT8.1 PF0058	52.10	0.00
TRINITY_A8IQW3_CHLRE/1244-1324	A8IQW3.1 PF13020.4;	52.10	0.00
TRINITY_D8U7Y5_VOLCA/8-101	D8U7Y5.1 PF03647.11	52.10	0.00
TRINITY_E1ZED9_CHLVA/43-259	E1ZED9.1 PF13500.4;	52.10	0.00
TRINITY_Q86HL7_DICDI/771-841	Q86HL7.1 PF00076.20	52.10	0.00
TRINITY_A8JB60_CHLRE/3-174	A8JB60.1 PF01636.21	52.10	0.00
TRINITY_D2UXN7_NAEGR/381-585	D2UXN7.1 PF14648.4;	52.10	0.00
TRINITY_I0YII0_9CHLO/48-213	I0YII0.1 PF13532.4;	52.10	0.00
TRINITY_A9U271_PHYPA/28-469	A9U271.1 PF07690.14	52.10	0.00
TRINITY_D8TYH3_VOLCA/1126-1230	D8TYH3.1 PF10367.7;	52.10	0.00
TRINITY_B8KT62_9GAMM/38-328	B8KT62.1 PF00850.17	52.10	0.00
TRINITY_W2QLG7_PHYPN/170-298	W2QLG7.1 PF03171.18	52.10	0.00
TRINITY_I0Z5L8_9CHLO/28-198	I0Z5L8.1 PF04979.12	52.10	0.00
TRINITY_K1VH30_TRIAC/1299-1414	K1VH30.1 PF00665.24	52.10	0.00
TRINITY_A0A077ZPB4_STYLE/1-123	A0A077ZPB4.1 PF0141	52.10	0.00
TRINITY_E1Z9C8_CHLVA/364-442	E1Z9C8.1 PF00789.18	52.10	0.00
TRINITY_I0ZAR9_9CHLO/1175-1469	I0ZAR9.1 PF13871.4;	52.10	0.00
TRINITY_I7M1V6_TETTS/314-533	I7M1V6.1 PF07002.14	52.10	0.00
TRINITY_A0A087SGH3_AUXPR/484-710	A0A087SGH3.1 PF0017	52.10	0.00
TRINITY_A0A0A1UBU5_ENTIV/727-986	A0A0A1UBU5.1 PF0771	52.10	0.00
TRINITY_R4XFE9_TAPDE/681-796	R4XFE9.1 PF00665.24	52.10	0.00
TRINITY_D8TXT6_VOLCA/657-764	D8TXT6.1 PF08172.10	52.10	0.00
TRINITY_H3FGM2_PRIPA/3-98	H3FGM2.1 PF13417.4;	52.10	0.00
TRINITY_H3HAJ2_PHYRM/359-478	H3HAJ2.1 PF00665.24	52.10	0.00
TRINITY_D8U2N8_VOLCA/151-247	D8U2N8.1 PF00211.18	52.10	0.00
TRINITY_A0A097QNB7_9VIBR/592-708	A0A097QNB7.1 PF0251	52.10	0.00
TRINITY_W7XG48_TETTS/15-139	W7XG48.1 PF00583.23	52.00	0.00
TRINITY_A0EFY2_PARTE/12-118	A0EFY2.1 PF02109.14	52.00	0.00
TRINITY_E1VPP4_9GAMM/167-285	E1VPP4.1 PF01575.17	52.00	0.00
TRINITY_I7LX02_TETTS/145-266	I7LX02.1 PF13456.4;	52.00	0.00
TRINITY_T1FYL8_HELRO/230-283	T1FYL8.1 PF00400.30	52.00	0.00
TRINITY_A0DK90_PARTE/257-447	A0DK90.1 PF02782.14	52.00	0.00
TRINITY_J9FDB9_9SPIT/10-149	J9FDB9.1 PF01321.16	52.00	0.00
TRINITY_A0BGA7_PARTE/140-295	A0BGA7.1 PF13847.4;	52.00	0.00
TRINITY_L8HHX1_ACACA/206-452	L8HHX1.1 PF13621.4;	52.00	0.00
TRINITY_S2J9M9_MUCC1/73-173	S2J9M9.1 PF13646.4;	52.00	0.00
TRINITY_W4XT28_STRPU/127-252	W4XT28.1 PF04669.11	52.00	0.00
TRINITY_K6Z1A1_9ALTE/225-329	K6Z1A1.1 PF10503.7;	52.00	0.00
TRINITY_D8U4Q3_VOLCA/231-353	D8U4Q3.1 PF02893.18	52.00	0.00
TRINITY_E1ZCM2_CHLVA/47-222	E1ZCM2.1 PF00534.18	52.00	0.00
TRINITY_W7X9P5_TETTS/69-195	W7X9P5.1 PF00782.18	52.00	0.00
TRINITY_A9SG48_PHYPA/246-602	A9SG48.1 PF00443.27	52.00	0.00
TRINITY_A8IW01_CHLRE/83-235	A8IW01.1 PF00781.22	52.00	0.00
TRINITY_F8EN41_RUNSL/15-310	F8EN41.1 PF02274.15	52.00	0.00
TRINITY_A0A0L1JFH3_ASPNO/50-395	A0A0L1JFH3.1 PF0026	52.00	0.00
TRINITY_G0QMQ9_ICHMG/30-285	G0QMQ9.1 PF00069.23	52.00	0.00
TRINITY_Q23U84_TETTS/23-120	Q23U84.1 PF02678.14	52.00	0.00
TRINITY_G0NFV9_CAEBE/23-271	G0NFV9.1 PF00756.18	52.00	0.00
TRINITY_F4PIH2_DICFS/1-99	F4PIH2.1 PF00752.15	52.00	0.00

TRINITY_G0QNE0_ICHMG/208-288	G0QNE0.1 PF05406.13	52.00	0.00
TRINITY_D8TJG2_VOLCA/32-227	D8TJG2.1 PF03798.14	52.00	0.00
TRINITY_B4KUQ9_DROMO/83-359	B4KUQ9.2 PF00248.19	52.00	0.00
TRINITY_F2CVE6_HORVD/260-487	F2CVE6.1 PF00149.26	52.00	0.00
TRINITY_E4WWA2_OIKDI/170-221	E4WWA2.1 PF00385.22	52.00	0.00
TRINITY_NDRA_DICDI/112-406	Q54Y26.1 PF00069.23	52.00	0.00
TRINITY_D8U5Y6_VOLCA/619-729	D8U5Y6.1 PF00989.23	52.00	0.00
TRINITY_I7M6D1_TETTS/15-195	I7M6D1.1 PF00929.22	52.00	0.00
TRINITY_D8U5W2_VOLCA/26-208	D8U5W2.1 PF11209.6;	52.00	0.00
TRINITY_L8GFL6_ACACA/87-162	L8GFL6.1 PF05965.12	52.00	0.00
TRINITY_K9RSW8_SYNP3/378-540	K9RSW8.1 PF00270.27	52.00	0.00
TRINITY_M4FBR7_BRARP/23-349	M4FBR7.1 PF00443.27	52.00	0.00
TRINITY_A0A090LXU0_OSTTA/87-136	A0A090LXU0.1 PF1290	52.00	0.00
TRINITY_J9IVT6_9SPIT/28-125	J9IVT6.1 PF02678.14	52.00	0.00
TRINITY_G0R253_ICHMG/33-82	G0R253.1 PF08583.8;	52.00	0.00
TRINITY_I7MI43_TETTS/43-172	I7MI43.1 PF03100.13	52.00	0.00
TRINITY_D8S477_SELML/399-448	D8S477.1 PF01846.17	52.00	0.00
TRINITY_D8TYX9_VOLCA/666-861	D8TYX9.1 PF00211.18	52.00	0.00
TRINITY_A0A087SGS7_AUXPR/533-739	A0A087SGS7.1 PF1308	52.00	0.00
TRINITY_D8TR57_VOLCA/1-169	D8TR57.1 PF00211.18	52.00	0.00
TRINITY_S3CIM7_OPHP1/647-699	S3CIM7.1 PF00385.22	52.00	0.00
TRINITY_I0YVV6_9CHLO/305-541	I0YVV6.1 PF13641.4;	52.00	0.00
TRINITY_A0D4B0_PARTE/4-259	A0D4B0.1 PF00069.23	52.00	0.00
TRINITY_Q234P9_TETTS/860-1069	Q234P9.1 PF05362.11	51.90	0.00
TRINITY_A0A078B0D1_STYLE/1223-1283	A0A078B0D1.1 PF1385	51.90	0.00
TRINITY_Q23ED0_TETTS/691-811	Q23ED0.2 PF02518.24	51.90	0.00
TRINITY_Q3SDA6_PARTE/83-149	Q3SDA6.1 PF13499.4;	51.90	0.00
TRINITY_J9ICA0_9SPIT/1329-1380	J9ICA0.1 PF00415.16	51.90	0.00
TRINITY_F1A2C1_DICPU/148-386	F1A2C1.1 PF16908.3;	51.90	0.00
TRINITY_D9QQ12_ACEAZ/6-191	D9QQ12.1 PF02245.14	51.90	0.00
TRINITY_D2VZL2_NAEGR/226-473	D2VZL2.1 PF13378.4;	51.90	0.00
TRINITY_D2D956_JATCU/1-77	D2D956.1 PF01439.16	51.90	0.00
TRINITY_Q11Y26_CYTH3/5-471	Q11Y26.1 PF00982.19	51.90	0.00
TRINITY_D2VRY6_NAEGR/12-173	D2VRY6.1 PF00071.20	51.90	0.00
TRINITY_I3KRS9_ORENI/6-194	I3KRS9.1 PF04722.11	51.90	0.00
TRINITY_A0A077ZSC1_STYLE/1-177	A0A077ZSC1.1 PF1458	51.90	0.00
TRINITY_A9UNQ4_MONBE/153-394	A9UNQ4.1 PF11175.6;	51.90	0.00
TRINITY_A0BEL5_PARTE/9-71	A0BEL5.1 PF00280.16	51.90	0.00
TRINITY_A0A096PMV0_MAIZE/266-315	A0A096PMV0.1 PF0041	51.90	0.00
TRINITY_E1VQX9_9GAMM/44-288	E1VQX9.1 PF00561.18	51.90	0.00
TRINITY_F4QCJ4_DICFS/213-689	F4QCJ4.1 PF01055.24	51.90	0.00
TRINITY_M2XFA0_GALSU/177-357	M2XFA0.1 PF00006.23	51.90	0.00
TRINITY_E1ZR18_CHLVA/622-700	E1ZR18.1 PF08170.10	51.90	0.00
TRINITY_D2UZX2_NAEGR/143-328	D2UZX2.1 PF13521.4;	51.90	0.00
TRINITY_A4VD01_TETTS/854-1082	A4VD01.1 PF16212.3;	51.90	0.00
TRINITY_F0ZLB0_DICPU/338-685	F0ZLB0.1 PF12698.5;	51.90	0.00
TRINITY_H3GEM8_PHYRM/190-315	H3GEM8.1 PF03171.18	51.90	0.00
TRINITY_A5K3I1_PLAVS/154-283	A5K3I1.1 PF00004.27	51.90	0.00
TRINITY_L8HEJ6_ACACA/94-154	L8HEJ6.1 PF00571.26	51.90	0.00
TRINITY_A0A0G4J2X3_PLABS/751-865	A0A0G4J2X3.1 PF0066	51.90	0.00
TRINITY_A0DYX2_PARTE/54-188	A0DYX2.1 PF04146.13	51.90	0.00
TRINITY_V9G1A7_PHYPR/152-221	V9G1A7.1 PF13424.4;	51.90	0.00
TRINITY_A0A067LA91_JATCU/277-640	A0A067LA91.1 PF0012	51.90	0.00
TRINITY_A0A0K9NIL4_ZOSMR/222-282	A0A0K9NIL4.1 PF1341	51.90	0.00
TRINITY_A0D6M9_PARTE/6-345	A0D6M9.1 PF00724.18	51.90	0.00
TRINITY_Y0010_DICDI/201-462	Q54WW7.1 PF00069.23	51.90	0.00
TRINITY_A0A0N5DJ00_TRIMR/2-286	A0A0N5DJ00.1 PF0058	51.90	0.00
TRINITY_C3Z2N5_BRAFL/34-323	C3Z2N5.1 PF00850.17	51.90	0.00
TRINITY_M0VP57_HORVD/67-313	M0VP57.1 PF01963.15	51.90	0.00
TRINITY_I0Z256_9CHLO/47-238	I0Z256.1 PF00300.20	51.90	0.00
TRINITY_Q0IXJ3_ORYSJ/567-684	Q0IXJ3.2 PF00665.24	51.90	0.00

TRINITY_A8JGU6_CHLRE/108-203	A8JGU6.1 PF12796.5;	51.90	0.00
TRINITY_U9SIS7_RHIID/1-312	U9SIS7.1 PF07992.12	51.90	0.00
TRINITY_L8HHC6_ACACA/135-311	L8HHC6.1 PF04675.12	51.90	0.00
TRINITY_A0A0G4IR21_PLABS/101-470	A0A0G4IR21.1 PF0156	51.90	0.00
TRINITY_A0A0E0L501_ORYPU/104-218	A0A0E0L501.1 PF0940	51.90	0.00
TRINITY_M2Y4X3_GALSU/55-262	M2Y4X3.1 PF00106.23	51.90	0.00
TRINITY_A0A078A9T0_STYLE/80-292	A0A078A9T0.1 PF0011	51.90	0.00
TRINITY_A0A0P7X2K9_9TELE/123-254	A0A0P7X2K9.1 PF0184	51.90	0.00
TRINITY_L8HJ53_ACACA/762-973	L8HJ53.1 PF00069.23	51.90	0.00
TRINITY_L8H5Y2_ACACA/40-458	L8H5Y2.1 PF02897.13	51.90	0.00
TRINITY_A9TJI8_PHYPA/100-525	A9TJI8.1 PF03092.14	51.90	0.00
TRINITY_B9XKY4_PEDPL/8-164	B9XKY4.1 PF09349.8;	51.90	0.00
TRINITY_A0A068S627_9FUNG/147-231	A0A068S627.1 PF0343	51.90	0.00
TRINITY_D7FUH9_ECTSI/200-254	D7FUH9.1 PF13513.4;	51.90	0.00
TRINITY_C1EHA6_MICSR/457-509	C1EHA6.1 PF02213.14	51.90	0.00
TRINITY_Q9A7U7_CAUCR/13-298	Q9A7U7.1 PF02129.16	51.90	0.00
TRINITY_I0YK20_9CHLO/7-315	I0YK20.1 PF04321.15	51.90	0.00
TRINITY_B9H058_POPTR/125-328	B9H058.2 PF13532.4;	51.90	0.00
TRINITY_G3N717_GASAC/14-224	G3N717.1 PF01130.19	51.90	0.00
TRINITY_W7T2P8_9STRA/149-251	W7T2P8.1 PF13646.4;	51.90	0.00
TRINITY_A8HXA9_CHLRE/38-384	A8HXA9.1 PF01263.18	51.90	0.00
TRINITY_Q5W9T0_CHLRE/210-282	Q5W9T0.1 PF00504.19	51.90	0.00
TRINITY_SPKA_DICDI/351-612	Q86AT8.1 PF07714.15	51.90	0.00
TRINITY_J9KAS1_ACYPI/214-373	J9KAS1.2 PF13359.4;	51.90	0.00
TRINITY_A0YVW9_LYNSP/57-111	A0YVW9.1 PF00856.26	51.90	0.00
TRINITY_I0Z5M5_9CHLO/93-249	I0Z5M5.1 PF00650.18	51.90	0.00
TRINITY_F1P100_CHICK/518-733	F1P100.2 PF12706.5;	51.90	0.00
TRINITY_M0RE52_MUSAM/21-101	M0RE52.1 PF03810.17	51.90	0.00
TRINITY_D8TR57_VOLCA/1-169	D8TR57.1 PF00211.18	51.90	0.00
TRINITY_D8U583_VOLCA/285-594	D8U583.1 PF00883.19	51.90	0.00
TRINITY_S9V190_9TRYP/107-251	S9V190.1 PF00782.18	51.90	0.00
TRINITY_A8J151_CHLRE/549-830	A8J151.1 PF00069.23	51.90	0.00
TRINITY_F2U0X9_SALR5/5-144	F2U0X9.1 PF08719.9;	51.80	0.00
TRINITY_A0A078B3U9_STYLE/406-642	A0A078B3U9.1 PF0006	51.80	0.00
TRINITY_F4NV00_BATDJ/83-227	F4NV00.1 PF10607.7;	51.80	0.00
TRINITY_L8GGE2_ACACA/32-145	L8GGE2.1 PF07876.10	51.80	0.00
TRINITY_I7M6P2_TETTS/41-229	I7M6P2.2 PF00270.27	51.80	0.00
TRINITY_F4PRM8_DICFS/512-641	F4PRM8.1 PF00782.18	51.80	0.00
TRINITY_B3RLP7_TRIAD/67-211	B3RLP7.1 PF13522.4;	51.80	0.00
TRINITY_A0A0D2UJE3_CAPO3/7-91	A0A0D2UJE3.1 PF1341	51.80	0.00
TRINITY_F0ZUX0_DICPU/138-266	F0ZUX0.1 PF09335.9;	51.80	0.00
TRINITY_I7MKW6_TETTS/644-994	I7MKW6.2 PF14744.4;	51.80	0.00
TRINITY_I0Z512_9CHLO/375-519	I0Z512.1 PF01593.22	51.80	0.00
TRINITY_R9S628_SIMAS/59-142	R9S628.1 PF00565.15	51.80	0.00
TRINITY_I1FE51_AMPQE/7-102	I1FE51.1 PF00153.25	51.80	0.00
TRINITY_W4Y616_STRPU/5-95	W4Y616.1 PF02214.20	51.80	0.00
TRINITY_D8UE48_VOLCA/347-514	D8UE48.1 PF07717.14	51.80	0.00
TRINITY_G0R3D4_ICHMG/149-213	G0R3D4.1 PF01485.19	51.80	0.00
TRINITY_A0A099NW38_PICKU/146-302	A0A099NW38.1 PF0007	51.80	0.00
TRINITY_C1N0A8_MICPC/365-557	C1N0A8.1 PF00753.25	51.80	0.00
TRINITY_M0W3Y7_HORVD/194-398	M0W3Y7.1 PF07137.9;	51.80	0.00
TRINITY_I0ZAY0_9CHLO/33-684	I0ZAY0.1 PF06419.9;	51.80	0.00
TRINITY_Q23ZH8_TETTS/79-176	Q23ZH8.1 PF14769.4;	51.80	0.00
TRINITY_PRLA_DICDI/26-330	Q1ZXG4.1 PF00557.22	51.80	0.00
TRINITY_R0GTS5_9BRAS/91-413	R0GTS5.1 PF00291.23	51.80	0.00
TRINITY_W7XAD3_TETTS/166-299	W7XAD3.1 PF17207.1;	51.80	0.00
TRINITY_A8IYH9_CHLRE/107-281	A8IYH9.1 PF01789.14	51.80	0.00
TRINITY_F0ZLY1_DICPU/16-303	F0ZLY1.1 PF03151.14	51.80	0.00
TRINITY_D8UEF8_VOLCA/192-619	D8UEF8.1 PF07517.12	51.80	0.00
TRINITY_A4VEG9_TETTS/264-465	A4VEG9.1 PF02735.14	51.80	0.00
TRINITY_G0R0E4_ICHMG/471-925	G0R0E4.1 PF00521.18	51.80	0.00

TRINITY_F4P040_BATDJ/2-139	F4P040.1 PF05832.10	51.80	0.00
TRINITY_A0A059LM93_9CHLO/128-293	A0A059LM93.1 PF0143	51.80	0.00
TRINITY_A8IGH1_CHLRE/126-228	A8IGH1.1 PF02777.16	51.80	0.00
TRINITY_D8TJZ3_VOLCA/218-459	D8TJZ3.1 PF00294.22	51.80	0.00
TRINITY_A0A0J8CMM6_BETVU/212-323	A0A0J8CMM6.1 PF0027	51.80	0.00
TRINITY_K2A6F5_9BACT/37-285	K2A6F5.1 PF01048.18	51.80	0.00
TRINITY_A0A087SK75_AUXPR/124-221	A0A087SK75.1 PF0335	51.80	0.00
TRINITY_A0A087G746_ARAAL/372-505	A0A087G746.1 PF0113	51.80	0.00
TRINITY_B9I5E6_POPTR/16-160	B9I5E6.1 PF06201.11	51.80	0.00
TRINITY_D8TIB1_VOLCA/100-431	D8TIB1.1 PF07887.9;	51.80	0.00
TRINITY_G7V9B0_THELD/210-320	G7V9B0.1 PF00472.18	51.80	0.00
TRINITY_V4RUS0_9ROSI/993-1308	V4RUS0.1 PF00225.21	51.80	0.00
TRINITY_C5KBW8_PERM5/10-171	C5KBW8.1 PF00071.20	51.80	0.00
TRINITY_A0CS29_PARTE/2561-2834	A0CS29.1 PF00520.29	51.80	0.00
TRINITY_U6GMY8_EIMAC/1849-2009	U6GMY8.1 PF08240.10	51.80	0.00
TRINITY_A8JI07_CHLRE/533-929	A8JI07.1 PF00465.17	51.80	0.00
TRINITY_W4FSJ9_9STRA/1228-1335	W4FSJ9.1 PF00005.25	51.80	0.00
TRINITY_A8I2Z0_CHLRE/359-633	A8I2Z0.1 PF08757.9;	51.80	0.00
TRINITY_D8UAY2_VOLCA/103-259	D8UAY2.1 PF00996.16	51.80	0.00
TRINITY_I0YMI8_9CHLO/391-532	I0YMI8.1 PF00637.18	51.80	0.00
TRINITY_A0BVZ5_PARTE/262-539	A0BVZ5.1 PF00069.23	51.80	0.00
TRINITY_B8NPY1_ASPFN/4-122	B8NPY1.1 PF01814.21	51.70	0.00
TRINITY_Q22RZ8_TETTS/183-242	Q22RZ8.2 PF13921.4;	51.70	0.00
TRINITY_I1CQT4_RHIO9/195-453	I1CQT4.1 PF00069.23	51.70	0.00
TRINITY_G1NSS6_MYOLU/4067-4374	G1NSS6.1 PF00632.23	51.70	0.00
TRINITY_H8MEU3_CORCM/58-379	H8MEU3.1 PF07995.9;	51.70	0.00
TRINITY_Q22RZ8_TETTS/183-242	Q22RZ8.2 PF13921.4;	51.70	0.00
TRINITY_U3IHM8_ANAPL/14-129	U3IHM8.1 PF00583.23	51.70	0.00
TRINITY_F1A021_DICPU/6-65	F1A021.1 PF04667.15	51.70	0.00
TRINITY_A0A0B4HIH8_9HYPO/1107-1229	A0A0B4HIH8.1 PF0007	51.70	0.00
TRINITY_A0A0E0RDB0_ORYRU/61-226	A0A0E0RDB0.1 PF1352	51.70	0.00
TRINITY_Q245N9_TETTS/30-118	Q245N9.1 PF17146.2;	51.70	0.00
TRINITY_A0A087SRA4_AUXPR/51-514	A0A087SRA4.1 PF0008	51.70	0.00
TRINITY_B8C1H9_THAPS/8-265	B8C1H9.1 PF00069.23	51.70	0.00
TRINITY_F1A0L6_DICPU/16-102	F1A0L6.1 PF17146.2;	51.70	0.00
TRINITY_A0DY11_PARTE/22-1088	A0DY11.1 PF10266.7;	51.70	0.00
TRINITY_D8U9I2_VOLCA/41-265	D8U9I2.1 PF00328.20	51.70	0.00
TRINITY_A0A0D2TUG4_GOSRA/1-743	A0A0D2TUG4.1 PF0149	51.70	0.00
TRINITY_D8TQP3_VOLCA/171-273	D8TQP3.1 PF05546.9;	51.70	0.00
TRINITY_D8TZ69_VOLCA/12-134	D8TZ69.1 PF02291.13	51.70	0.00
TRINITY_I7MCV7_TETTS/96-369	I7MCV7.1 PF00248.19	51.70	0.00
TRINITY_D8UCQ9_VOLCA/212-360	D8UCQ9.1 PF01556.16	51.70	0.00
TRINITY_V7B8S5_PHAVU/71-293	V7B8S5.1 PF01255.17	51.70	0.00
TRINITY_A0A087SJ79_AUXPR/628-814	A0A087SJ79.1 PF0048	51.70	0.00
TRINITY_Q22RZ8_TETTS/183-242	Q22RZ8.2 PF13921.4;	51.70	0.00
TRINITY_L8H344_ACACA/275-728	L8H344.1 PF00501.26	51.70	0.00
TRINITY_M2Y425_GALSU/295-353	M2Y425.1 PF00076.20	51.70	0.00
TRINITY_A8JCR3_CHLRE/12-76	A8JCR3.1 PF13499.4;	51.70	0.00
TRINITY_D8TY44_VOLCA/285-608	D8TY44.1 PF09423.8;	51.70	0.00
TRINITY_L8GNW6_ACACA/47-481	L8GNW6.1 PF01406.17	51.70	0.00
TRINITY_A0A0G4J1L3_PLABS/178-263	A0A0G4J1L3.1 PF1596	51.70	0.00
TRINITY_A8J614_CHLRE/570-725	A8J614.1 PF00625.19	51.70	0.00
TRINITY_R4XGA1_TAPDE/6-280	R4XGA1.1 PF06732.9;	51.70	0.00
TRINITY_L8H5V6_ACACA/5-62	L8H5V6.1 PF00412.20	51.70	0.00
TRINITY_I0YMR5_9CHLO/36-344	I0YMR5.1 PF01180.19	51.70	0.00
TRINITY_G0QRB8_ICHMG/54-342	G0QRB8.1 PF00069.23	51.70	0.00
TRINITY_I7M6S3_TETTS/55-312	I7M6S3.1 PF00069.23	51.70	0.00
TRINITY_A0A0K9Q208_ZOSMR/407-497	A0A0K9Q208.1 PF0349	51.70	0.00
TRINITY_Q22RZ8_TETTS/183-242	Q22RZ8.2 PF13921.4;	51.70	0.00
TRINITY_Q22RZ8_TETTS/183-242	Q22RZ8.2 PF13921.4;	51.70	0.00
TRINITY_A8IUJ9_CHLRE/41-331	A8IUJ9.1 PF03151.14	51.70	0.00

TRINITY_A0A059LM16_9CHLO/271-318	A0A059LM16.1 PF0039	51.70	0.00
TRINITY_K1PRU0_CRAGI/549-754	K1PRU0.1 PF01431.19	51.70	0.00
TRINITY_A0A078BB69_STYLE/58-324	A0A078BB69.1 PF0338	51.70	0.00
TRINITY_A8J715_CHLRE/7-153	A8J715.1 PF00211.18	51.70	0.00
TRINITY_DHKD_DICDI/323-440	Q54SP4.1 PF02518.24	51.70	0.00
TRINITY_C1FDK5_MICSR/32-313	C1FDK5.1 PF00483.21	51.70	0.00
TRINITY_A0A060T0U9_PYCCI/1009-1125	A0A060T0U9.1 PF0066	51.70	0.00
TRINITY_Q8LP67_CHLRE/27-494	Q8LP67.2 PF01384.18	51.70	0.00
TRINITY_A0A099NWK8_PICKU/112-302	A0A099NWK8.1 PF0007	51.70	0.00
TRINITY_C6W8M6_ACTMD/11-73	C6W8M6.1 PF00280.16	51.60	0.00
TRINITY_D3AVJ6_POLPA/582-722	D3AVJ6.1 PF00005.25	51.60	0.00
TRINITY_C7LQT5_DESBD/479-544	C7LQT5.1 PF00512.23	51.60	0.00
TRINITY_J9FKD6_9SPIT/7-192	J9FKD6.1 PF01294.16	51.60	0.00
TRINITY_Q22XX1_TETTS/200-263	Q22XX1.2 PF12906.5;	51.60	0.00
TRINITY_A0E1G8_PARTE/540-795	A0E1G8.1 PF00069.23	51.60	0.00
TRINITY_L8GY68_ACACA/20-143	L8GY68.1 PF00241.18	51.60	0.00
TRINITY_G1R190_NOMLE/177-520	G1R190.1 PF12631.5;	51.60	0.00
TRINITY_M0S9Q5_MUSAM/108-171	M0S9Q5.1 PF13432.4;	51.60	0.00
TRINITY_V4AKP0_LOTGI/1-160	V4AKP0.1 PF01956.14	51.60	0.00
TRINITY_A0DPK5_PARTE/1-260	A0DPK5.1 PF04889.10	51.60	0.00
TRINITY_A0C0R9_PARTE/182-243	A0C0R9.1 PF12906.5;	51.60	0.00
TRINITY_V9E928_PHYPR/45-406	V9E928.1 PF00083.22	51.60	0.00
TRINITY_D3B1R6_POLPA/12-311	D3B1R6.1 PF02274.15	51.60	0.00
TRINITY_E1ZR92_CHLVA/447-739	E1ZR92.1 PF00481.19	51.60	0.00
TRINITY_F4PQX3_DICFS/2-227	F4PQX3.1 PF04065.13	51.60	0.00
TRINITY_E4X453_OIKDI/18-179	E4X453.1 PF00071.20	51.60	0.00
TRINITY_Q3SDM6_PARTE/17-177	Q3SDM6.1 PF00071.20	51.60	0.00
TRINITY_A0D1K9_PARTE/24-590	A0D1K9.1 PF01602.18	51.60	0.00
TRINITY_H3AIK0_LATCH/14-212	H3AIK0.1 PF02545.12	51.60	0.00
TRINITY_L8H468_ACACA/39-379	L8H468.1 PF00155.19	51.60	0.00
TRINITY_A0EDK2_PARTE/18-123	A0EDK2.1 PF00168.28	51.60	0.00
TRINITY_Q23H79_TETTS/226-497	Q23H79.1 PF12018.6;	51.60	0.00
TRINITY_Q6LPU2_PHOPR/528-595	Q6LPU2.1 PF00364.20	51.60	0.00
TRINITY_I7M1V6_TETTS/314-533	I7M1V6.1 PF07002.14	51.60	0.00
TRINITY_K3WQA3_PYTUL/86-274	K3WQA3.1 PF00573.20	51.60	0.00
TRINITY_F0ZXH1_DICPU/36-289	F0ZXH1.1 PF00378.18	51.60	0.00
TRINITY_A0A059LQ77_9CHLO/1-219	A0A059LQ77.1 PF0315	51.60	0.00
TRINITY_A0A087SEG7_AUXPR/271-565	A0A087SEG7.1 PF0398	51.60	0.00
TRINITY_D8UCJ0_VOLCA/119-224	D8UCJ0.1 PF09366.8;	51.60	0.00
TRINITY_D8TUF2_VOLCA/178-372	D8TUF2.1 PF00344.18	51.60	0.00
TRINITY_C3YKU6_BRAFL/40-237	C3YKU6.1 PF00106.23	51.60	0.00
TRINITY_H9GH48_ANOCA/17-365	H9GH48.1 PF00009.25	51.60	0.00
TRINITY_GTDC1_DANRE/246-398	Q568B7.2 PF00534.18	51.60	0.00
TRINITY_I0YX01_9CHLO/2-193	I0YX01.1 PF01490.16	51.60	0.00
TRINITY_A0EFU5_PARTE/59-386	A0EFU5.1 PF01148.18	51.60	0.00
TRINITY_G0R1C0_ICHMG/87-270	G0R1C0.1 PF00270.27	51.60	0.00
TRINITY_Q23CN1_TETTS/13-195	Q23CN1.1 PF01885.14	51.60	0.00
TRINITY_A0A0H5C0S1_CYBJA/140-353	A0A0H5C0S1.1 PF0166	51.60	0.00
TRINITY_A0A0D3BCI3_BRAOL/104-536	A0A0D3BCI3.1 PF0153	51.60	0.00
TRINITY_D8U7T5_VOLCA/1-379	D8U7T5.1 PF01238.19	51.60	0.00
TRINITY_D8TJF5_VOLCA/313-455	D8TJF5.1 PF07534.14	51.60	0.00
TRINITY_W1NX64_AMBTC/4-444	W1NX64.1 PF00022.17	51.60	0.00
TRINITY_D3BEB3_POLPA/1642-1881	D3BEB3.1 PF00082.20	51.60	0.00
TRINITY_A8HZR1_CHLRE/15132-15384	A8HZR1.1 PF00109.24	51.60	0.00
TRINITY_C7Q3T8_CATAD/28-144	C7Q3T8.1 PF08240.10	51.60	0.00
TRINITY_G0QWM6_ICHMG/175-428	G0QWM6.1 PF00069.23	51.60	0.00
TRINITY_D8U1A5_VOLCA/27-185	D8U1A5.1 PF06424.10	51.60	0.00
TRINITY_W5MCV8_LEPOC/15-77	W5MCV8.1 PF01066.19	51.60	0.00
TRINITY_A8J3P1_CHLRE/954-1161	A8J3P1.1 PF00211.18	51.60	0.00
TRINITY_A8IUL9_CHLRE/21-122	A8IUL9.1 PF02036.15	51.60	0.00
TRINITY_U1SBP_DANRE/52-122	Q4KMD3.1 PF00076.20	51.60	0.00

TRINITY_A0A0P7XRF3_9TELE/158-268	A0A0P7XRF3.1 PF0355	51.60	0.00
TRINITY_A0A078B196_STYLE/206-298	A0A078B196.1 PF1279	51.60	0.00
TRINITY_A0A0E3ZHW8_9BACT/9-157	A0A0E3ZHW8.1 PF1624	51.60	0.00
TRINITY_E5SVL2_TRISP/135-242	E5SVL2.1 PF00665.24	51.60	0.00
TRINITY_I7LUD3_TETTS/90-229	I7LUD3.1 PF00782.18	51.60	0.00
TRINITY_X5DJF9_9BACT/10-77	X5DJF9.1 PF04542.12	51.50	0.00
TRINITY_Q248F0_TETTS/19-502	Q248F0.2 PF04916.11	51.50	0.00
TRINITY_J9IWV5_9SPIT/47-287	J9IWV5.1 PF00069.23	51.50	0.00
TRINITY_D8QNI9_SELML/8-116	D8QNI9.1 PF00255.17	51.50	0.00
TRINITY_A0A068RWJ7_9FUNG/3-212	A0A068RWJ7.1 PF0009	51.50	0.00
TRINITY_W6FUH4_NODSP/15-183	W6FUH4.1 PF13385.4;	51.50	0.00
TRINITY_J9G9V4_9SPIT/1151-1280	J9G9V4.1 PF04096.12	51.50	0.00
TRINITY_F2UG91_SALR5/227-643	F2UG91.1 PF01432.18	51.50	0.00
TRINITY_W7TKS0_9STRA/126-321	W7TKS0.1 PF03171.18	51.50	0.00
TRINITY_U5NBE3_9BURK/245-311	U5NBE3.1 PF00512.23	51.50	0.00
TRINITY_Q24GQ7_TETTS/133-301	Q24GQ7.1 PF04664.11	51.50	0.00
TRINITY_A8IQQ8_CHLRE/597-709	A8IQQ8.1 PF00989.23	51.50	0.00
TRINITY_F7EQ32_MONDO/78-378	F7EQ32.2 PF00561.18	51.50	0.00
TRINITY_A8JFR7_CHLRE/99-366	A8JFR7.1 PF01062.19	51.50	0.00
TRINITY_H3ATB2_LATCH/105-403	H3ATB2.1 PF00443.27	51.50	0.00
TRINITY_A0A0D2WVS2_CAPO3/1-331	A0A0D2WVS2.1 PF0450	51.50	0.00
TRINITY_W7I460_9PEZI/190-533	W7I460.1 PF00176.21	51.50	0.00
TRINITY_F4QBH5_DICFS/1205-1402	F4QBH5.1 PF00621.18	51.50	0.00
TRINITY_Q22EC6_TETTS/68-359	Q22EC6.2 PF04258.11	51.50	0.00
TRINITY_Q22AR0_TETTS/17-145	Q22AR0.1 PF13529.4;	51.50	0.00
TRINITY_E1Z1Y2_CHLVA/2-106	E1Z1Y2.1 PF00504.19	51.50	0.00
TRINITY_R1D643_EMIHU/94-205	R1D643.1 PF02211.13	51.50	0.00
TRINITY_A0A0G4GZH6_9ALVE/419-599	A0A0G4GZH6.1 PF0281	51.50	0.00
TRINITY_Q24C34_TETTS/105-170	Q24C34.1 PF17136.2;	51.50	0.00
TRINITY_A0CEH0_PARTE/17-332	A0CEH0.1 PF00009.25	51.50	0.00
TRINITY_L8H1H1_ACACA/128-330	L8H1H1.1 PF04389.15	51.50	0.00
TRINITY_D8TCH6_SELML/10-283	D8TCH6.1 PF00696.26	51.50	0.00
TRINITY_B9H0D7_POPTR/559-627	B9H0D7.1 PF00505.17	51.50	0.00
TRINITY_D3B1Q1_POLPA/635-841	D3B1Q1.1 PF00566.16	51.50	0.00
TRINITY_A0A0G0E2J9_9BACT/264-431	A0A0G0E2J9.1 PF0015	51.50	0.00
TRINITY_A8JCT6_CHLRE/204-275	A8JCT6.1 PF06741.11	51.50	0.00
TRINITY_H2SE72_TAKRU/14-1649	H2SE72.1 PF11894.6;	51.50	0.00
TRINITY_I7MA07_TETTS/32-269	I7MA07.2 PF00481.19	51.50	0.00
TRINITY_D8U6N2_VOLCA/596-799	D8U6N2.1 PF07714.15	51.50	0.00
TRINITY_Q2GG22_EHRCR/6-112	Q2GG22.1 PF00436.23	51.50	0.00
TRINITY_K6XR77_9ALTE/18-317	K6XR77.1 PF01156.17	51.50	0.00
TRINITY_Q553C7_DICDI/37-133	Q553C7.1 PF14580.4;	51.50	0.00
TRINITY_Q23YD6_TETTS/29-282	Q23YD6.1 PF00069.23	51.50	0.00
TRINITY_F4Q3D5_DICFS/22-285	F4Q3D5.1 PF01193.22	51.50	0.00
TRINITY_L8HFR9_ACACA/221-419	L8HFR9.1 PF00149.26	51.50	0.00
TRINITY_A7SPU8_NEMVE/95-163	A7SPU8.1 PF00076.20	51.50	0.00
TRINITY_E1Z751_CHLVA/73-748	E1Z751.1 PF00063.19	51.50	0.00
TRINITY_X6NML8_RETFI/12-173	X6NML8.1 PF00071.20	51.50	0.00
TRINITY_A0A078AQU9_STYLE/782-892	A0A078AQU9.1 PF0064	51.50	0.00
TRINITY_F8W2M8_DANRE/214-518	F8W2M8.1 PF00082.20	51.50	0.00
TRINITY_A0A059B9U1_EUCGR/362-547	A0A059B9U1.1 PF0258	51.50	0.00
TRINITY_M2PPP9_CERS8/675-986	M2PPP9.1 PF05843.12	51.50	0.00
TRINITY_Q8YMJ8_NOSS1/554-625	Q8YMJ8.1 PF13448.4;	51.50	0.00
TRINITY_A8I6F3_CHLRE/385-587	A8I6F3.1 PF07714.15	51.50	0.00
TRINITY_J9I7Z3_9SPIT/528-596	J9I7Z3.1 PF02187.15	51.50	0.00
TRINITY_D8TQ79_VOLCA/98-546	D8TQ79.1 PF00501.26	51.50	0.00
TRINITY_A0A0K0F7Z5_9BILA/97-165	A0A0K0F7Z5.1 PF0050	51.50	0.00
TRINITY_L8HAP8_ACACA/12-144	L8HAP8.1 PF01467.24	51.50	0.00
TRINITY_I0YYZ3_9CHLO/38-280	I0YYZ3.1 PF08190.10	51.50	0.00
TRINITY_A8HZ49_CHLRE/38-151	A8HZ49.1 PF01843.17	51.50	0.00
TRINITY_D8TJE3_VOLCA/737-861	D8TJE3.1 PF00134.21	51.50	0.00

TRINITY_D8U7T4_VOLCA/32-384	D8U7T4.1 PF00224.19	51.50	0.00
TRINITY_D8TZG3_VOLCA/2436-2604	D8TZG3.1 PF13229.4;	51.50	0.00
TRINITY_A8J6I6_CHLRE/134-532	A8J6I6.1 PF04137.13	51.50	0.00
TRINITY_A5H8H9_CHLRE/77-306	A5H8H9.1 PF11152.6;	51.50	0.00
TRINITY_A8IBX2_CHLRE/464-568	A8IBX2.1 PF13426.5;	51.50	0.00
TRINITY_A0DMC7_PARTE/1-451	A0DMC7.1 PF00171.20	51.40	0.00
TRINITY_A0A078ACD1_STYLE/54-167	A0A078ACD1.1 PF1152	51.40	0.00
TRINITY_A0A0M2NC82_9FIRM/1438-1555	A0A0M2NC82.1 PF0007	51.40	0.00
TRINITY_J9IJ11_9SPIT/431-592	J9IJ11.1 PF02518.24	51.40	0.00
TRINITY_A0A075MLK6_9RICK/34-371	A0A075MLK6.1 PF0093	51.40	0.00
TRINITY_A0A066VMD9_9BASI/20-182	A0A066VMD9.1 PF0007	51.40	0.00
TRINITY_F0Z7E7_DICPU/1467-1681	F0Z7E7.1 PF00454.25	51.40	0.00
TRINITY_K9TLZ5_9CYAN/145-327	K9TLZ5.1 PF00211.18	51.40	0.00
TRINITY_A0A0D2WGS6_CAPO3/41-325	A0A0D2WGS6.1 PF0065	51.40	0.00
TRINITY_L1IW65_GUITH/6-299	L1IW65.1 PF00443.27	51.40	0.00
TRINITY_Q23JY6_TETTS/610-893	Q23JY6.2 PF00481.19	51.40	0.00
TRINITY_D8UA31_VOLCA/1397-1652	D8UA31.1 PF08326.10	51.40	0.00
TRINITY_A0A067QYX3_ZOONE/5-80	A0A067QYX3.1 PF0279	51.40	0.00
TRINITY_E1R9M4_SPISS/43-153	E1R9M4.1 PF03099.17	51.40	0.00
TRINITY_A0A0G4H6R4_9ALVE/223-367	A0A0G4H6R4.1 PF0073	51.40	0.00
TRINITY_A0BT76_PARTE/42-155	A0BT76.1 PF00970.22	51.40	0.00
TRINITY_A0A0G4GCP6_9ALVE/33-172	A0A0G4GCP6.1 PF0535	51.40	0.00
TRINITY_F4Q197_DICFS/73-310	F4Q197.1 PF00149.26	51.40	0.00
TRINITY_D7MDC3_ARALL/176-362	D7MDC3.1 PF08662.9;	51.40	0.00
TRINITY_G0QNL8_ICHMG/17-231	G0QNL8.1 PF02230.14	51.40	0.00
TRINITY_J9JCA5_9SPIT/125-339	J9JCA5.1 PF00112.21	51.40	0.00
TRINITY_A8IQQ5_CHLRE/39-165	A8IQQ5.1 PF04034.11	51.40	0.00
TRINITY_A6G1S3_9DELT/651-724	A6G1S3.1 PF00575.21	51.40	0.00
TRINITY_A8IXB2_CHLRE/37-327	A8IXB2.1 PF03982.11	51.40	0.00
TRINITY_I0YXW3_9CHLO/68-190	I0YXW3.1 PF13369.4;	51.40	0.00
TRINITY_L8GZX6_ACACA/240-400	L8GZX6.1 PF04152.12	51.40	0.00
TRINITY_I7MFP4_TETTS/185-254	I7MFP4.1 PF00076.20	51.40	0.00
TRINITY_D8TIL5_VOLCA/23-103	D8TIL5.1 PF10785.7;	51.40	0.00
TRINITY_E1ZKT7_CHLVA/421-785	E1ZKT7.1 PF03372.21	51.40	0.00
TRINITY_A0A087SDT2_AUXPR/175-284	A0A087SDT2.1 PF0008	51.40	0.00
TRINITY_V9FIY6_PHYPR/89-393	V9FIY6.1 PF00561.18	51.40	0.00
TRINITY_E1ZGG3_CHLVA/1-323	E1ZGG3.1 PF01207.15	51.40	0.00
TRINITY_R5CVJ7_9FIRM/854-970	R5CVJ7.1 PF00072.22	51.40	0.00
TRINITY_D8UDX8_VOLCA/15-306	D8UDX8.1 PF13872.4;	51.40	0.00
TRINITY_A8J701_CHLRE/150-244	A8J701.1 PF13879.4;	51.40	0.00
TRINITY_L8GL73_ACACA/29-174	L8GL73.1 PF06201.11	51.40	0.00
TRINITY_A0CKH3_PARTE/10-115	A0CKH3.1 PF12796.5;	51.40	0.00
TRINITY_A9V4S2_MONBE/204-426	A9V4S2.1 PF04389.15	51.40	0.00
TRINITY_E1ZIE5_CHLVA/234-347	E1ZIE5.1 PF00294.22	51.40	0.00
TRINITY_B4JDR4_DROGR/30-323	B4JDR4.1 PF04227.10	51.40	0.00
TRINITY_A0A0D2VQ46_CAPO3/350-817	A0A0D2VQ46.1 PF0105	51.40	0.00
TRINITY_E1Z4I8_CHLVA/45-256	E1Z4I8.1 PF02190.14	51.40	0.00
TRINITY_A0A0F2RSV7_9PROT/27-304	A0A0F2RSV7.1 PF0015	51.40	0.00
TRINITY_J9EHC1_9SPIT/1166-1271	J9EHC1.1 PF00168.28	51.40	0.00
TRINITY_I0YY29_9CHLO/32-376	I0YY29.1 PF00155.19	51.40	0.00
TRINITY_A0BDM2_PARTE/435-648	A0BDM2.1 PF07859.11	51.40	0.00
TRINITY_A0A0G4II46_PLABS/21-126	A0A0G4II46.1 PF0030	51.40	0.00
TRINITY_D8UF81_VOLCA/4-73	D8UF81.1 PF02798.18	51.40	0.00
TRINITY_D8U9M0_VOLCA/80-404	D8U9M0.1 PF13896.4;	51.40	0.00
TRINITY_E1ZFK8_CHLVA/344-459	E1ZFK8.1 PF09070.9;	51.40	0.00
TRINITY_D8UKB1_VOLCA/988-1273	D8UKB1.1 PF03133.13	51.40	0.00
TRINITY_A0A078A9T0_STYLE/80-292	A0A078A9T0.1 PF0011	51.40	0.00
TRINITY_A8IRX6_CHLRE/14-154	A8IRX6.1 PF15025.4;	51.40	0.00
TRINITY_W7U6T6_9STRA/37-189	W7U6T6.1 PF00857.18	51.40	0.00
TRINITY_I7M7R1_TETTS/11-180	I7M7R1.1 PF00071.20	51.30	0.00
TRINITY_D8TSD6_VOLCA/425-880	D8TSD6.1 PF11894.6;	51.30	0.00

TRINITY_D3BFK1_POLPA/293-519	D3BFK1.1 PF13236.4;	51.30	0.00
TRINITY_W5WFW8_9PSEU/2-442	W5WFW8.1 PF00743.17	51.30	0.00
TRINITY_W5NJ56_LEPOC/942-1254	W5NJ56.1 PF00664.21	51.30	0.00
TRINITY_A0A0D2USD7_CAPO3/130-207	A0A0D2USD7.1 PF0725	51.30	0.00
TRINITY_I7M2G3_TETTS/68-184	I7M2G3.2 PF00622.26	51.30	0.00
TRINITY_L8GS79_ACACA/32-421	L8GS79.1 PF01053.18	51.30	0.00
TRINITY_L8GTK1_ACACA/146-395	L8GTK1.1 PF07714.15	51.30	0.00
TRINITY_L8GLW3_ACACA/10-209	L8GLW3.1 PF00225.21	51.30	0.00
TRINITY_E8N055_ANATU/196-314	E8N055.1 PF01926.21	51.30	0.00
TRINITY_Q235L2_TETTS/74-150	Q235L2.1 PF03134.17	51.30	0.00
TRINITY_V5SB04_9RHIZ/12-204	V5SB04.1 PF03462.16	51.30	0.00
TRINITY_I7MN18_TETTS/134-383	I7MN18.2 PF00122.18	51.30	0.00
TRINITY_G0QJS9_ICHMG/119-291	G0QJS9.1 PF05677.10	51.30	0.00
TRINITY_A9UT62_MONBE/15-91	A9UT62.1 PF06093.11	51.30	0.00
TRINITY_A0D0R4_PARTE/36-191	A0D0R4.1 PF02815.17	51.30	0.00
TRINITY_A0A0F5FWU6_9RHIZ/3-279	A0A0F5FWU6.1 PF0256	51.30	0.00
TRINITY_S2IV79_MUCC1/38-333	S2IV79.1 PF03151.14	51.30	0.00
TRINITY_A0A060SJ25_PYCCI/64-170	A0A060SJ25.1 PF0058	51.30	0.00
TRINITY_A8JFV1_CHLRE/37-296	A8JFV1.1 PF01062.19	51.30	0.00
TRINITY_D8TIF2_VOLCA/197-723	D8TIF2.1 PF12569.6;	51.30	0.00
TRINITY_L8H293_ACACA/380-718	L8H293.1 PF05028.12	51.30	0.00
TRINITY_W4Y0M1_STRPU/293-645	W4Y0M1.1 PF01457.14	51.30	0.00
TRINITY_Q16K07_AEDAE/76-303	Q16K07.1 PF00557.22	51.30	0.00
TRINITY_A8J3I2_CHLRE/205-360	A8J3I2.1 PF05645.11	51.30	0.00
TRINITY_A0A059LR00_9CHLO/217-347	A0A059LR00.1 PF0023	51.30	0.00
TRINITY_L8HCJ2_ACACA/105-328	L8HCJ2.1 PF01556.16	51.30	0.00
TRINITY_F4Q882_DICFS/91-313	F4Q882.1 PF02811.17	51.30	0.00
TRINITY_A8HN61_CHLRE/2-203	A8HN61.1 PF04127.13	51.30	0.00
TRINITY_Q22X34_TETTS/82-235	Q22X34.2 PF00810.16	51.30	0.00
TRINITY_M0TL63_MUSAM/98-287	M0TL63.1 PF03031.16	51.30	0.00
TRINITY_I0YQQ6_9CHLO/334-489	I0YQQ6.1 PF13671.4;	51.30	0.00
TRINITY_I0YSK5_9CHLO/592-711	I0YSK5.1 PF10193.7;	51.30	0.00
TRINITY_W8ERK8_9BACT/56-809	W8ERK8.1 PF01804.16	51.30	0.00
TRINITY_D8UEF8_VOLCA/1333-1647	D8UEF8.1 PF07516.11	51.30	0.00
TRINITY_F4PGG9_DICFS/359-434	F4PGG9.1 PF00017.22	51.30	0.00
TRINITY_D2VUA2_NAEGR/472-642	D2VUA2.1 PF00025.19	51.30	0.00
TRINITY_Q6BGF7_PARTE/428-813	Q6BGF7.1 PF00702.24	51.30	0.00
TRINITY_H3HAJ2_PHYRM/359-478	H3HAJ2.1 PF00665.24	51.30	0.00
TRINITY_A0A0G4FYC6_9ALVE/127-300	A0A0G4FYC6.1 PF0047	51.30	0.00
TRINITY_D8U5G0_VOLCA/3-122	D8U5G0.1 PF00188.24	51.30	0.00
TRINITY_D8UA76_VOLCA/46-324	D8UA76.1 PF00231.17	51.30	0.00
TRINITY_CDK10_DICDI/7-293	Q55GS4.1 PF00069.23	51.30	0.00
TRINITY_A0A0D3FKV1_9ORYZ/91-246	A0A0D3FKV1.1 PF1456	51.30	0.00
TRINITY_D8R4M1_SELML/2-80	D8R4M1.1 PF05129.11	51.30	0.00
TRINITY_F9GER5_FUSOF/39-215	F9GER5.1 PF00485.16	51.30	0.00
TRINITY_D8TM33_VOLCA/1610-1805	D8TM33.1 PF00211.18	51.30	0.00
TRINITY_D2VI22_NAEGR/414-634	D2VI22.1 PF13236.4;	51.30	0.00
TRINITY_L8HAW3_ACACA/654-873	L8HAW3.1 PF13236.4;	51.30	0.00
TRINITY_C7LRE2_DESBD/427-512	C7LRE2.1 PF00355.24	51.30	0.00
TRINITY_D8TXU8_VOLCA/17-1207	D8TXU8.1 PF02463.17	51.30	0.00
TRINITY_A0A072UZD8_MEDTR/36-152	A0A072UZD8.1 PF0017	51.30	0.00
TRINITY_A0A076K2L8_9RHOB/2-123	A0A076K2L8.1 PF0090	51.30	0.00
TRINITY_W7X7N0_TETTS/5-44	W7X7N0.1 PF06677.10	51.30	0.00
TRINITY_G0QKA0_ICHMG/1960-2256	G0QKA0.1 PF00632.23	51.30	0.00
TRINITY_A0A059LME8_9CHLO/27-307	A0A059LME8.1 PF0363	51.20	0.00
TRINITY_C5KAG4_PERM5/145-190	C5KAG4.1 PF01344.23	51.20	0.00
TRINITY_I7LTA5_TETTS/34-118	I7LTA5.1 PF03795.12	51.20	0.00
TRINITY_I7MCW7_TETTS/50-135	I7MCW7.1 PF12894.5;	51.20	0.00
TRINITY_G0R6D6_ICHMG/98-267	G0R6D6.1 PF10274.7;	51.20	0.00
TRINITY_Q3SD80_PARTE/13-180	Q3SD80.1 PF00071.20	51.20	0.00
TRINITY_A0A078BBM2_STYLE/736-852	A0A078BBM2.1 PF1364	51.20	0.00

TRINITY_M2XR81_GALSU/167-210	M2XR81.1 PF13639.4;	51.20	0.00
TRINITY_F4KT89_HALH1/36-176	F4KT89.1 PF00188.24	51.20	0.00
TRINITY_UXT_DICDI/29-159	Q54ND3.1 PF02996.15	51.20	0.00
TRINITY_M7AP01_CHEMY/434-482	M7AP01.1 PF13920.4;	51.20	0.00
TRINITY_D3BR29_POLPA/314-555	D3BR29.1 PF02913.17	51.20	0.00
TRINITY_I7MID1_TETTS/134-392	I7MID1.2 PF00069.23	51.20	0.00
TRINITY_I7IGD3_BABMI/2-82	I7IGD3.1 PF00081.20	51.20	0.00
TRINITY_F0ZEM4_DICPU/808-1204	F0ZEM4.1 PF06957.9;	51.20	0.00
TRINITY_Q234Q1_TETTS/3-340	Q234Q1.1 PF01137.19	51.20	0.00
TRINITY_D8RRA3_SELML/21-126	D8RRA3.1 PF00085.18	51.20	0.00
TRINITY_G3NA74_GASAC/66-272	G3NA74.1 PF13489.4;	51.20	0.00
TRINITY_A0A0N4ZX25_PARTI/3-213	A0A0N4ZX25.1 PF0009	51.20	0.00
TRINITY_Q22RJ8_TETTS/269-765	Q22RJ8.3 PF01055.24	51.20	0.00
TRINITY_D8TPI1_VOLCA/329-417	D8TPI1.1 PF00686.17	51.20	0.00
TRINITY_Q22Y58_TETTS/31-206	Q22Y58.2 PF03152.12	51.20	0.00
TRINITY_I7MMI7_TETTS/43-214	I7MMI7.1 PF00213.16	51.20	0.00
TRINITY_D3BE39_POLPA/6-244	D3BE39.1 PF01370.19	51.20	0.00
TRINITY_I7MCL9_TETTS/190-488	I7MCL9.2 PF00648.19	51.20	0.00
TRINITY_A0A0F2NX53_9FLAO/37-431	A0A0F2NX53.1 PF0378	51.20	0.00
TRINITY_M0T2D0_MUSAM/307-383	M0T2D0.1 PF09169.8;	51.20	0.00
TRINITY_M2RQ51_CERS8/309-362	M2RQ51.1 PF13821.4;	51.20	0.00
TRINITY_Q22DP0_TETTS/183-364	Q22DP0.2 PF05193.19	51.20	0.00
TRINITY_F0Z6M0_DICPU/1-332	F0Z6M0.1 PF01148.18	51.20	0.00
TRINITY_F1A4S2_DICPU/369-501	F1A4S2.1 PF13716.4;	51.20	0.00
TRINITY_A0A077KMY8_9FLAO/166-484	A0A077KMY8.1 PF0096	51.20	0.00
TRINITY_A0A098GD03_TATMI/503-737	A0A098GD03.1 PF0278	51.20	0.00
TRINITY_A0EC42_PARTE/101-492	A0EC42.1 PF01619.16	51.20	0.00
TRINITY_A0DVX9_PARTE/401-444	A0DVX9.1 PF13639.4;	51.20	0.00
TRINITY_E1ZKA8_CHLVA/18-215	E1ZKA8.1 PF06080.10	51.20	0.00
TRINITY_D7UBL1_VITVI/257-752	D7UBL1.1 PF06862.10	51.20	0.00
TRINITY_A0A024TID3_9STRA/5-178	A0A024TID3.1 PF0007	51.20	0.00
TRINITY_E1ZQM4_CHLVA/233-323	E1ZQM4.1 PF00781.22	51.20	0.00
TRINITY_I1G689_AMPQE/65-366	I1G689.1 PF03747.12	51.20	0.00
TRINITY_D8TMP7_VOLCA/11-642	D8TMP7.1 PF03600.14	51.20	0.00
TRINITY_A8IXQ8_CHLRE/344-468	A8IXQ8.1 PF07986.10	51.20	0.00
TRINITY_B0WVE0_CULQU/175-214	B0WVE0.1 PF07645.13	51.20	0.00
TRINITY_F6I499_VITVI/284-363	F6I499.1 PF00400.30	51.20	0.00
TRINITY_F4Q7L0_DICFS/19-59	F4Q7L0.1 PF13639.4;	51.20	0.00
TRINITY_M3WQG6_FELCA/66-392	M3WQG6.1 PF00009.25	51.20	0.00
TRINITY_L8GT53_ACACA/70-602	L8GT53.1 PF00995.21	51.20	0.00
TRINITY_A8IQV3_CHLRE/555-697	A8IQV3.1 PF05517.10	51.20	0.00
TRINITY_K4CI03_SOLLC/101-144	K4CI03.1 PF13639.4;	51.20	0.00
TRINITY_A8HWI9_CHLRE/348-571	A8HWI9.1 PF07714.15	51.20	0.00
TRINITY_E1ZLH0_CHLVA/22-325	E1ZLH0.1 PF00814.23	51.20	0.00
TRINITY_I7MA05_TETTS/49-723	I7MA05.1 PF02463.17	51.20	0.00
TRINITY_Q0IM09_ORYSJ/93-213	Q0IM09.1 PF00581.18	51.20	0.00
TRINITY_I7LXM8_TETTS/241-404	I7LXM8.1 PF03031.16	51.20	0.00
TRINITY_D8TT51_VOLCA/426-589	D8TT51.1 PF03031.16	51.20	0.00
TRINITY_D8TNM5_VOLCA/493-720	D8TNM5.1 PF11718.6;	51.20	0.00
TRINITY_Q237K5_TETTS/252-442	Q237K5.3 PF13229.4;	51.20	0.00
TRINITY_A8HPG3_CHLRE/275-438	A8HPG3.1 PF01554.16	51.20	0.00
TRINITY_L1IZV4_GUITH/21-244	L1IZV4.1 PF01207.15	51.20	0.00
TRINITY_D2V8A1_NAEGR/1019-1139	D2V8A1.1 PF02687.19	51.20	0.00
TRINITY_D8UJ46_VOLCA/1047-1282	D8UJ46.1 PF07714.15	51.20	0.00
TRINITY_ACT_ASPOR/2-375	Q2U7A3.1 PF00022.17	51.20	0.00
TRINITY_A0A087GQD8_ARAAL/252-615	A0A087GQD8.1 PF0012	51.20	0.00
TRINITY_D8U8D8_VOLCA/174-843	D8U8D8.1 PF04615.11	51.20	0.00
TRINITY_J0LCW4_AURST/382-795	J0LCW4.1 PF05088.10	51.20	0.00
TRINITY_E1ZB58_CHLVA/3-139	E1ZB58.1 PF07047.10	51.20	0.00
TRINITY_S8CJ07_9LAMI/313-454	S8CJ07.1 PF01926.21	51.20	0.00
TRINITY_Q22AR6_TETTS/17-145	Q22AR6.3 PF13529.4;	51.10	0.00

TRINITY_A0A0M0JRU3_9EUKA/3-89	A0A0M0JRU3.1 PF0456	51.10	0.00
TRINITY_A0A078AF27_STYLE/20-227	A0A078AF27.1 PF0155	51.10	0.00
TRINITY_U9UPN8_RHIID/1-404	U9UPN8.1 PF00022.17	51.10	0.00
TRINITY_B0S768_DANRE/21-71	B0S768.1 PF00385.22	51.10	0.00
TRINITY_Q23QF2_TETTS/248-571	Q23QF2.2 PF03133.13	51.10	0.00
TRINITY_W9RKT2_9ROSA/29-165	W9RKT2.1 PF13489.4;	51.10	0.00
TRINITY_A0DBI4_PARTE/123-180	A0DBI4.1 PF13921.4;	51.10	0.00
TRINITY_I0Z4B7_9CHLO/8-125	I0Z4B7.1 PF05348.9;	51.10	0.00
TRINITY_Q231T2_TETTS/85-131	Q231T2.2 PF13445.4;	51.10	0.00
TRINITY_Q22EY1_TETTS/40-131	Q22EY1.1 PF00276.18	51.10	0.00
TRINITY_Q23JD8_TETTS/35-79	Q23JD8.1 PF00097.23	51.10	0.00
TRINITY_G4D1L1_9FIRM/35-287	G4D1L1.1 PF00561.18	51.10	0.00
TRINITY_D3BLA2_POLPA/49-377	D3BLA2.1 PF00128.22	51.10	0.00
TRINITY_L0A9E0_DEIPD/46-181	L0A9E0.1 PF09346.8;	51.10	0.00
TRINITY_I7LXB6_TETTS/1143-1299	I7LXB6.2 PF00005.25	51.10	0.00
TRINITY_G0QW69_ICHMG/157-289	G0QW69.1 PF00107.24	51.10	0.00
TRINITY_I7MFD5_TETTS/394-587	I7MFD5.2 PF13901.4;	51.10	0.00
TRINITY_E1G5M1_LOALO/40-219	E1G5M1.2 PF01294.16	51.10	0.00
TRINITY_Q24DF0_TETTS/748-1037	Q24DF0.2 PF00648.19	51.10	0.00
TRINITY_Q8ESL7_OCEIH/26-192	Q8ESL7.1 PF00270.27	51.10	0.00
TRINITY_M5X747_PRUPE/959-1138	M5X747.1 PF12816.5;	51.10	0.00
TRINITY_Q23QF2_TETTS/248-571	Q23QF2.2 PF03133.13	51.10	0.00
TRINITY_A0A067STQ1_9AGAR/42-221	A0A067STQ1.1 PF0114	51.10	0.00
TRINITY_I7M2G9_TETTS/752-929	I7M2G9.2 PF00069.23	51.10	0.00
TRINITY_A0A059ARR8_EUCGR/219-276	A0A059ARR8.1 PF0148	51.10	0.00
TRINITY_I7M6I7_TETTS/33-261	I7M6I7.1 PF04981.11	51.10	0.00
TRINITY_I0YTS8_9CHLO/855-957	I0YTS8.1 PF00271.29	51.10	0.00
TRINITY_J9IMI2_9SPIT/315-376	J9IMI2.1 PF12906.5;	51.10	0.00
TRINITY_I7LTS7_TETTS/32-217	I7LTS7.2 PF00227.24	51.10	0.00
TRINITY_A8ICH8_CHLRE/72-332	A8ICH8.1 PF12146.6;	51.10	0.00
TRINITY_Q6BGA6_PARTE/227-274	Q6BGA6.1 PF00415.16	51.10	0.00
TRINITY_E1Z4Q7_CHLVA/43-325	E1Z4Q7.1 PF14500.4;	51.10	0.00
TRINITY_I0YTA7_9CHLO/19-233	I0YTA7.1 PF05721.11	51.10	0.00
TRINITY_Q23QF2_TETTS/248-571	Q23QF2.2 PF03133.13	51.10	0.00
TRINITY_B3SD13_TRIAD/235-607	B3SD13.1 PF00587.23	51.10	0.00
TRINITY_A0A0N5C759_STREA/4-334	A0A0N5C759.1 PF0856	51.10	0.00
TRINITY_A0A068SDR8_9FUNG/779-900	A0A068SDR8.1 PF0157	51.10	0.00
TRINITY_G0QPI1_ICHMG/26-576	G0QPI1.1 PF01602.18	51.10	0.00
TRINITY_S2J1Z4_MUCC1/378-695	S2J1Z4.1 PF00069.23	51.10	0.00
TRINITY_F4PGY2_DICFS/41-142	F4PGY2.1 PF00011.19	51.10	0.00
TRINITY_I0YPU3_9CHLO/12-259	I0YPU3.1 PF00561.18	51.10	0.00
TRINITY_A0CM85_PARTE/515-588	A0CM85.1 PF00173.26	51.10	0.00
TRINITY_A4S9E8_OSTLU/168-383	A4S9E8.1 PF12706.5;	51.10	0.00
TRINITY_D8TGV7_VOLCA/12-194	D8TGV7.1 PF00227.24	51.10	0.00
TRINITY_L8HCK5_ACACA/530-801	L8HCK5.1 PF07714.15	51.10	0.00
TRINITY_D8M555_BLAHO/64-112	D8M555.1 PF00628.27	51.10	0.00
TRINITY_W7XFS0_TETTS/147-236	W7XFS0.1 PF02978.17	51.10	0.00
TRINITY_D8TR33_VOLCA/19-284	D8TR33.1 PF03644.11	51.10	0.00
TRINITY_D8TTM9_VOLCA/774-953	D8TTM9.1 PF15625.4;	51.10	0.00
TRINITY_A8J3P1_CHLRE/954-1161	A8J3P1.1 PF00211.18	51.10	0.00
TRINITY_A8JHW6_CHLRE/43-718	A8JHW6.1 PF00995.21	51.10	0.00
TRINITY_I0YZC8_9CHLO/135-227	I0YZC8.1 PF00867.16	51.10	0.00
TRINITY_F0ZJW2_DICPU/563-743	F0ZJW2.1 PF02145.13	51.10	0.00
TRINITY_Q23CZ3_TETTS/246-390	Q23CZ3.2 PF01170.16	51.10	0.00
TRINITY_Q23K98_TETTS/2488-2701	Q23K98.2 PF00520.29	51.10	0.00
TRINITY_C1EB37_MICSR/504-899	C1EB37.1 PF00940.17	51.10	0.00
TRINITY_D8U5A8_VOLCA/79-306	D8U5A8.1 PF00069.23	51.10	0.00
TRINITY_C5L7Q9_PERM5/16-385	C5L7Q9.1 PF00009.25	51.10	0.00
TRINITY_K7V3P2_MAIZE/98-541	K7V3P2.1 PF00067.20	51.10	0.00
TRINITY_D8UG06_VOLCA/2308-2398	D8UG06.1 PF08743.8;	51.10	0.00
TRINITY_L8HLY7_ACACA/2-230	L8HLY7.1 PF00233.17	51.10	0.00

TRINITY_A8JAF1_CHLRE/70-391	A8JAF1.1 PF07992.12	51.10	0.00
TRINITY_V4A8I2_LOTGI/135-269	V4A8I2.1 PF08433.8;	51.10	0.00
TRINITY_A8IZ68_CHLRE/17-192	A8IZ68.1 PF02301.16	51.10	0.00
TRINITY_I7MHV4_TETTS/46-141	I7MHV4.1 PF01329.17	51.00	0.00
TRINITY_Q54JK0_DICDI/144-585	Q54JK0.1 PF00501.26	51.00	0.00
TRINITY_I7MLM4_TETTS/239-295	I7MLM4.2 PF00571.26	51.00	0.00
TRINITY_Q22AW2_TETTS/85-366	Q22AW2.1 PF00664.21	51.00	0.00
TRINITY_G4TRG8_PIRID/542-763	G4TRG8.1 PF00326.19	51.00	0.00
TRINITY_A0A084VZV5_ANOSI/113-165	A0A084VZV5.1 PF0139	51.00	0.00
TRINITY_J9HQC6_9SPIT/15-385	J9HQC6.1 PF01008.15	51.00	0.00
TRINITY_A0BJD5_PARTE/63-126	A0BJD5.1 PF13499.4;	51.00	0.00
TRINITY_W5M5D7_LEPOC/156-505	W5M5D7.1 PF12631.5;	51.00	0.00
TRINITY_J9F1H9_9SPIT/12-116	J9F1H9.1 PF02991.14	51.00	0.00
TRINITY_R8BMD6_TOGMI/112-271	R8BMD6.1 PF00078.25	51.00	0.00
TRINITY_Q22KI6_TETTS/5-326	Q22KI6.2 PF00294.22	51.00	0.00
TRINITY_F0ZG46_DICPU/17-281	F0ZG46.1 PF00248.19	51.00	0.00
TRINITY_A3U3V4_OCEBH/299-401	A3U3V4.1 PF02566.17	51.00	0.00
TRINITY_A0A0G3A7I0_9DELT/1-292	A0A0G3A7I0.1 PF0029	51.00	0.00
TRINITY_A0A087SBN5_AUXPR/15-272	A0A087SBN5.1 PF0079	51.00	0.00
TRINITY_J9J5I9_9SPIT/631-835	J9J5I9.1 PF01852.17	51.00	0.00
TRINITY_A0A0G4FE33_9ALVE/654-908	A0A0G4FE33.1 PF0006	51.00	0.00
TRINITY_Q23CL0_TETTS/804-906	Q23CL0.1 PF04003.10	51.00	0.00
TRINITY_D0LL56_HALO1/26-161	D0LL56.1 PF08240.10	51.00	0.00
TRINITY_I7MI70_TETTS/14-137	I7MI70.1 PF13893.4;	51.00	0.00
TRINITY_A0E768_PARTE/18-136	A0E768.1 PF02466.17	51.00	0.00
TRINITY_A0A0K9P6H7_ZOSMR/143-205	A0A0K9P6H7.1 PF0518	51.00	0.00
TRINITY_D8U183_VOLCA/501-651	D8U183.1 PF01699.22	51.00	0.00
TRINITY_A0E826_PARTE/128-238	A0E826.1 PF00168.28	51.00	0.00
TRINITY_D8UEE4_VOLCA/8-204	D8UEE4.1 PF03959.11	51.00	0.00
TRINITY_E1ZQ47_CHLVA/199-476	E1ZQ47.1 PF00069.23	51.00	0.00
TRINITY_A8JH58_CHLRE/39-518	A8JH58.1 PF00501.26	51.00	0.00
TRINITY_A0A0A7LKC0_9BACT/1-349	A0A0A7LKC0.1 PF0149	51.00	0.00
TRINITY_A0A024WMR2_PLAFA/292-549	A0A024WMR2.1 PF0006	51.00	0.00
TRINITY_A0A0M0K741_9EUKA/109-268	A0A0M0K741.1 PF1384	51.00	0.00
TRINITY_J9IVT6_9SPIT/28-125	J9IVT6.1 PF02678.14	51.00	0.00
TRINITY_W4YLM9_STRPU/197-528	W4YLM9.1 PF00443.27	51.00	0.00
TRINITY_Q6MNK0_BDEBA/4-192	Q6MNK0.1 PF01557.16	51.00	0.00
TRINITY_L8H3B2_ACACA/7-401	L8H3B2.1 PF00266.17	51.00	0.00
TRINITY_K6XR77_9ALTE/18-317	K6XR77.1 PF01156.17	51.00	0.00
TRINITY_A0A0E0NR04_ORYRU/248-372	A0A0E0NR04.1 PF0139	51.00	0.00
TRINITY_I1CAR9_RHIO9/36-149	I1CAR9.1 PF00188.24	51.00	0.00
TRINITY_E1ZFJ6_CHLVA/275-376	E1ZFJ6.1 PF00134.21	51.00	0.00
TRINITY_D8TLU8_VOLCA/1-257	D8TLU8.1 PF01213.17	51.00	0.00
TRINITY_A8J6C9_CHLRE/173-278	A8J6C9.1 PF05548.9;	51.00	0.00
TRINITY_A0A0A1MZZ8_9FUNG/164-296	A0A0A1MZZ8.1 PF0010	51.00	0.00
TRINITY_A0A087SEE8_AUXPR/23-129	A0A087SEE8.1 PF0277	51.00	0.00
TRINITY_I7M104_TETTS/32-282	I7M104.1 PF00069.23	51.00	0.00
TRINITY_A0A094GVQ6_9PEZI/366-473	A0A094GVQ6.1 PF0058	51.00	0.00
TRINITY_K5BAQ8_9MYCO/60-502	K5BAQ8.1 PF00501.26	51.00	0.00
TRINITY_I7LTQ3_TETTS/124-278	I7LTQ3.2 PF00650.18	51.00	0.00
TRINITY_D8UAI2_VOLCA/117-272	D8UAI2.1 PF01746.19	51.00	0.00
TRINITY_L8GQP6_ACACA/131-425	L8GQP6.1 PF07992.12	51.00	0.00
TRINITY_L8HC71_ACACA/163-263	L8HC71.1 PF00085.18	51.00	0.00
TRINITY_D8U6S6_VOLCA/68-306	D8U6S6.1 PF00233.17	51.00	0.00
TRINITY_A8JFF5_CHLRE/13-156	A8JFF5.1 PF00106.23	51.00	0.00
TRINITY_F4Q4A3_DICFS/16-269	F4Q4A3.1 PF00069.23	51.00	0.00
TRINITY_I0Z3R2_9CHLO/102-212	I0Z3R2.1 PF10998.6;	51.00	0.00
TRINITY_I1G6Z0_AMPQE/84-498	I1G6Z0.1 PF00450.20	51.00	0.00
TRINITY_C5XC66_SORBI/97-147	C5XC66.1 PF09465.8;	51.00	0.00
TRINITY_A8ISB0_CHLRE/37-325	A8ISB0.1 PF00291.23	51.00	0.00
TRINITY_A0BML7_PARTE/19-118	A0BML7.1 PF00447.15	51.00	0.00

TRINITY_F0ZY14_DICPU/351-612	F0ZY14.1 PF07714.15	51.00	0.00
TRINITY_A0A067U0W0_9AGAR/21-182	A0A067U0W0.1 PF0007	51.00	0.00
TRINITY_A0A0A1NH53_9FUNG/12-62	A0A0A1NH53.1 PF0038	51.00	0.00
TRINITY_E2A359_CAMFO/103-152	E2A359.1 PF00415.16	51.00	0.00
TRINITY_G0QLT6_ICHMG/132-333	G0QLT6.1 PF00112.21	51.00	0.00
TRINITY_A8IQQ8_CHLRE/597-709	A8IQQ8.1 PF00989.23	51.00	0.00
TRINITY_K7JCF9_NASVI/173-377	K7JCF9.1 PF00078.25	51.00	0.00
TRINITY_V4AE46_LOTGI/10-66	V4AE46.1 PF00412.20	50.90	0.00
TRINITY_A2E3Y5_TRIVA/92-154	A2E3Y5.1 PF01485.19	50.90	0.00
TRINITY_I0YKX7_9CHLO/155-269	I0YKX7.1 PF00504.19	50.90	0.00
TRINITY_A0A0N5DDZ9_TRIMR/71-197	A0A0N5DDZ9.1 PF0403	50.90	0.00
TRINITY_W4HAE7_9STRA/32-262	W4HAE7.1 PF13489.4;	50.90	0.00
TRINITY_B8ANZ6_ORYSI/328-561	B8ANZ6.1 PF08282.10	50.90	0.00
TRINITY_DHX33_MOUSE/219-401	Q80VY9.1 PF00271.29	50.90	0.00
TRINITY_Q17KA5_AEDAE/466-686	Q17KA5.1 PF00888.20	50.90	0.00
TRINITY_Q23RE5_TETTS/230-338	Q23RE5.2 PF01399.25	50.90	0.00
TRINITY_Q2IQ30_ANADE/26-103	Q2IQ30.1 PF03061.20	50.90	0.00
TRINITY_F0XXR9_AURAN/4-179	F0XXR9.1 PF00025.19	50.90	0.00
TRINITY_F4QCM6_DICFS/1657-1787	F4QCM6.1 PF00782.18	50.90	0.00
TRINITY_T1HWY1_RHOPR/29-318	T1HWY1.2 PF00069.23	50.90	0.00
TRINITY_I7M2Q8_TETTS/92-356	I7M2Q8.2 PF00069.23	50.90	0.00
TRINITY_K1W3V0_TRIAC/206-269	K1W3V0.1 PF05970.12	50.90	0.00
TRINITY_G1U9F0_RABIT/4-92	G1U9F0.1 PF00031.19	50.90	0.00
TRINITY_A9SHN8_PHYPA/109-165	A9SHN8.1 PF00412.20	50.90	0.00

TRINITY_D8UJM5_VOLCA/866-973	D8UJM5.1 PF00168.28	50.90	0.00
TRINITY_A0A015LWF2_9GLOM/60-342	A0A015LWF2.1 PF1438	50.90	0.00
TRINITY_B7FVL6_PHATC/17-178	B7FVL6.1 PF00071.20	50.90	0.00
TRINITY_E1ZIJ2_CHLVA/54-219	E1ZIJ2.1 PF00994.22	50.90	0.00
TRINITY_H9GB02_ANOCA/324-379	H9GB02.2 PF00412.20	50.90	0.00
TRINITY_A0A077ZS92_STYLE/199-359	A0A077ZS92.1 PF0303	50.90	0.00
TRINITY_A8ILF4_CHLRE/732-839	A8ILF4.1 PF02883.18	50.90	0.00
TRINITY_M2XFY2_GALSU/50-155	M2XFY2.1 PF00613.18	50.90	0.00
TRINITY_D3AXU6_POLPA/3-143	D3AXU6.1 PF10604.7;	50.90	0.00
TRINITY_A8JCP0_CHLRE/22-190	A8JCP0.1 PF03357.19	50.90	0.00
TRINITY_A8J8D9_CHLRE/22-258	A8J8D9.1 PF07714.15	50.90	0.00
TRINITY_A0A0G4ENY6_9ALVE/191-300	A0A0G4ENY6.1 PF0017	50.90	0.00
TRINITY_A0A099NW38_PICKU/146-302	A0A099NW38.1 PF0007	50.90	0.00
TRINITY_E1ZEX7_CHLVA/18-277	E1ZEX7.1 PF01959.14	50.90	0.00
TRINITY_H2YU38_CIOSA/1-194	H2YU38.1 PF00112.21	50.90	0.00
TRINITY_E5SXV8_TRISP/503-619	E5SXV8.1 PF00665.24	50.90	0.00
TRINITY_G0QWH7_ICHMG/52-110	G0QWH7.1 PF08063.10	50.90	0.00
TRINITY_G4ZRE7_PHYSP/4-181	G4ZRE7.1 PF00025.19	50.90	0.00
TRINITY_D8TSG4_VOLCA/126-300	D8TSG4.1 PF04144.11	50.90	0.00
TRINITY_H0ZQB3_TAEGU/134-313	H0ZQB3.1 PF13429.4;	50.90	0.00
TRINITY_G0QKW1_ICHMG/111-281	G0QKW1.1 PF00092.26	50.90	0.00
TRINITY_A8J114_CHLRE/10-389	A8J114.1 PF00083.22	50.90	0.00
TRINITY_D8U9E7_VOLCA/9-116	D8U9E7.1 PF14737.4;	50.90	0.00
TRINITY_D3BGU0_POLPA/57-295	D3BGU0.1 PF00795.20	50.90	0.00
TRINITY_F5SZQ1_9GAMM/482-668	F5SZQ1.1 PF05272.9;	50.90	0.00
TRINITY_A0A0G3IW95_9MYCO/186-309	A0A0G3IW95.1 PF0010	50.90	0.00
TRINITY_A8IHD2_CHLRE/184-407	A8IHD2.1 PF05686.10	50.90	0.00
TRINITY_D8TTH2_VOLCA/1584-1696	D8TTH2.1 PF00307.29	50.90	0.00
TRINITY_F4Q084_DICFS/88-744	F4Q084.1 PF00063.19	50.90	0.00
TRINITY_A8HW46_CHLRE/352-463	A8HW46.1 PF00168.28	50.90	0.00
TRINITY_D8UFC0_VOLCA/1-516	D8UFC0.1 PF04791.14	50.90	0.00
TRINITY_D8TJ43_VOLCA/551-744	D8TJ43.1 PF00860.18	50.90	0.00
TRINITY_F1N6B1_BOVIN/35-176	F1N6B1.2 PF05175.12	50.90	0.00
TRINITY_A8IGG5_CHLRE/567-774	A8IGG5.1 PF07714.15	50.90	0.00
TRINITY_Q3SDB5_PARTE/105-403	Q3SDB5.1 PF00122.18	50.90	0.00
TRINITY_J9LSZ4_ACYPI/496-633	J9LSZ4.2 PF00004.27	50.90	0.00
TRINITY_G0QW77_ICHMG/43-324	G0QW77.1 PF03619.14	50.80	0.00
TRINITY_A0A0D3ADZ0_BRAOL/16-271	A0A0D3ADZ0.1 PF0006	50.80	0.00
TRINITY_H2KNZ3_CLOSI/10-231	H2KNZ3.1 PF00244.18	50.80	0.00
TRINITY_Q0DG78_ORYSJ/1-179	Q0DG78.2 PF13847.4;	50.80	0.00
TRINITY_U9U2M9_RHIID/923-1112	U9U2M9.1 PF00488.19	50.80	0.00
TRINITY_Q54KJ2_DICDI/332-552	Q54KJ2.1 PF13236.4;	50.80	0.00
TRINITY_C1F1Y1_ACIC5/526-690	C1F1Y1.1 PF08760.9;	50.80	0.00
TRINITY_L8H5H5_ACACA/34-507	L8H5H5.1 PF01229.15	50.80	0.00
TRINITY_Q23FS8_TETTS/33-196	Q23FS8.2 PF14956.4;	50.80	0.00
TRINITY_D9W3Z4_9ACTN/20-100	D9W3Z4.1 PF13304.4;	50.80	0.00
TRINITY_A0A0Q3UWS4_SETIT/75-165	A0A0Q3UWS4.1 PF0015	50.80	0.00
TRINITY_I7M6G4_TETTS/82-228	I7M6G4.2 PF12922.5;	50.80	0.00
TRINITY_D5GLZ2_TUBMM/4-146	D5GLZ2.1 PF00106.23	50.80	0.00
TRINITY_A0A090M6F3_OSTTA/22-82	A0A090M6F3.1 PF0083	50.80	0.00
TRINITY_A0A0N4ZN70_PARTI/54-114	A0A0N4ZN70.1 PF0419	50.80	0.00
TRINITY_C7JBV9_ACEP3/15-302	C7JBV9.1 PF01040.16	50.80	0.00
TRINITY_I7MM31_TETTS/85-222	I7MM31.2 PF01529.18	50.80	0.00
TRINITY_A0A022RE77_ERYGU/162-217	A0A022RE77.1 PF0204	50.80	0.00
TRINITY_K9R703_9CYAN/197-277	K9R703.1 PF08445.8;	50.80	0.00
TRINITY_A0A067QYA9_ZOONE/4-88	A0A067QYA9.1 PF1279	50.80	0.00
TRINITY_B6IVT1_RHOCS/39-312	B6IVT1.1 PF01636.21	50.80	0.00
TRINITY_A0A0D2WXF0_CAPO3/284-676	A0A0D2WXF0.1 PF0149	50.80	0.00
TRINITY_T1EI44_HELRO/49-109	T1EI44.1 PF13921.4;	50.80	0.00
TRINITY_L8HMC9_ACACA/70-852	L8HMC9.1 PF08553.8;	50.80	0.00
TRINITY_F0ZLI6_DICPU/14-141	F0ZLI6.1 PF00134.21	50.80	0.00

TRINITY_I0Z754_9CHLO/80-144	I0Z754.1 PF13432.4;	50.80	0.00
TRINITY_A0D3J7_PARTE/113-382	A0D3J7.1 PF00069.23	50.80	0.00
TRINITY_N6TU53_DENPD/2-117	N6TU53.1 PF03656.11	50.80	0.00
TRINITY_A0DFQ3_PARTE/150-411	A0DFQ3.1 PF00481.19	50.80	0.00
TRINITY_D8U2C8_VOLCA/51-288	D8U2C8.1 PF05721.11	50.80	0.00
TRINITY_C5KE40_PERM5/231-416	C5KE40.1 PF00270.27	50.80	0.00
TRINITY_Q23RD3_TETTS/32-97	Q23RD3.2 PF13499.4;	50.80	0.00
TRINITY_I7M450_TETTS/2253-2375	I7M450.2 PF00069.23	50.80	0.00
TRINITY_RACA_DICDI/5-171	P34147.2 PF00071.20	50.80	0.00
TRINITY_A0A059CIA7_EUCGR/152-219	A0A059CIA7.1 PF1026	50.80	0.00
TRINITY_V9EPL4_PHYPR/52-300	V9EPL4.1 PF06966.10	50.80	0.00
TRINITY_G0QUF2_ICHMG/19-193	G0QUF2.1 PF13439.4;	50.80	0.00
TRINITY_J9IXE9_9SPIT/299-361	J9IXE9.1 PF00170.19	50.80	0.00
TRINITY_A0A0D2WTH3_CAPO3/67-257	A0A0D2WTH3.1 PF0014	50.80	0.00
TRINITY_L8GG94_ACACA/5-162	L8GG94.1 PF07933.12	50.80	0.00
TRINITY_G0QPZ9_ICHMG/16-280	G0QPZ9.1 PF04811.13	50.80	0.00
TRINITY_F2U898_SALR5/16-176	F2U898.1 PF00071.20	50.80	0.00
TRINITY_D8TM37_VOLCA/48-225	D8TM37.1 PF13326.4;	50.80	0.00
TRINITY_A0A0D3B9H3_BRAOL/3-266	A0A0D3B9H3.1 PF0045	50.80	0.00
TRINITY_A0A087SAV1_AUXPR/120-283	A0A087SAV1.1 PF1214	50.80	0.00
TRINITY_A0DC58_PARTE/9-534	A0DC58.1 PF01602.18	50.80	0.00
TRINITY_Q24C26_TETTS/16-259	Q24C26.3 PF00069.23	50.80	0.00
TRINITY_H2ZI06_CIOSA/8-243	H2ZI06.1 PF02784.14	50.80	0.00
TRINITY_G0R442_ICHMG/8-260	G0R442.1 PF00069.23	50.80	0.00
TRINITY_D8ULI0_VOLCA/1-183	D8ULI0.1 PF00160.19	50.80	0.00
TRINITY_A0EC59_PARTE/148-317	A0EC59.1 PF01068.19	50.80	0.00
TRINITY_A8J000_CHLRE/25-420	A8J000.1 PF00871.15	50.80	0.00
TRINITY_E1Z620_CHLVA/19-302	E1Z620.1 PF08538.8;	50.80	0.00
TRINITY_A8J3C2_CHLRE/32-153	A8J3C2.1 PF10218.7;	50.80	0.00
TRINITY_I7MA91_TETTS/672-1130	I7MA91.2 PF00521.18	50.80	0.00
TRINITY_A0A067C5K4_SAPPC/1127-1244	A0A067C5K4.1 PF0007	50.80	0.00
TRINITY_A0A096PAK6_OSTTA/5-143	A0A096PAK6.1 PF0017	50.80	0.00
TRINITY_L8GRR7_ACACA/534-605	L8GRR7.1 PF01485.19	50.80	0.00
TRINITY_D3AWT2_POLPA/361-423	D3AWT2.1 PF13432.4;	50.80	0.00
TRINITY_F7B069_MACMU/20-276	F7B069.1 PF00069.23	50.80	0.00
TRINITY_Q7XRL9_ORYSJ/564-738	Q7XRL9.2 PF00078.25	50.80	0.00
TRINITY_W7XA27_TETTS/236-568	W7XA27.1 PF12309.6;	50.80	0.00
TRINITY_Q23R00_TETTS/6-298	Q23R00.2 PF00579.23	50.80	0.00
TRINITY_Q6CSC0_KLULA/276-338	Q6CSC0.1 PF01485.19	50.80	0.00
TRINITY_Q54F04_DICDI/45-467	Q54F04.1 PF13520.4;	50.80	0.00
TRINITY_G0QWQ0_ICHMG/415-899	G0QWQ0.1 PF00899.19	50.80	0.00
TRINITY_Q8I7P3_DICDI/141-423	Q8I7P3.1 PF03372.21	50.80	0.00
TRINITY_A0A078FU68_BRANA/15-77	A0A078FU68.1 PF0046	50.80	0.00
TRINITY_L8H265_ACACA/43-772	L8H265.1 PF03635.15	50.80	0.00
TRINITY_Q22KI6_TETTS/5-326	Q22KI6.2 PF00294.22	50.80	0.00
TRINITY_L8HJX9_ACACA/36-222	L8HJX9.1 PF02146.15	50.80	0.00
TRINITY_E1ZN61_CHLVA/1401-1550	E1ZN61.1 PF00005.25	50.80	0.00
TRINITY_E9G3A8_DAPPU/52-441	E9G3A8.1 PF00144.22	50.80	0.00
TRINITY_A8IAA5_CHLRE/115-366	A8IAA5.1 PF00566.16	50.80	0.00
TRINITY_I7M9L3_TETTS/26-279	I7M9L3.2 PF02263.17	50.80	0.00
TRINITY_D8TTC1_VOLCA/415-853	D8TTC1.1 PF04097.12	50.80	0.00
TRINITY_D7SRR6_VITVI/26-97	D7SRR6.1 PF00240.21	50.80	0.00
TRINITY_D8TIN0_VOLCA/58-342	D8TIN0.1 PF03381.13	50.80	0.00
TRINITY_L8GPA6_ACACA/21-278	L8GPA6.1 PF00378.18	50.80	0.00
TRINITY_S2J6K5_MUCC1/33-95	S2J6K5.1 PF00076.20	50.80	0.00
TRINITY_K2E0Y4_9BACT/13-145	K2E0Y4.1 PF00390.17	50.80	0.00
TRINITY_A8J8J1_CHLRE/220-658	A8J8J1.1 PF02889.14	50.80	0.00
TRINITY_G0QMZ8_ICHMG/182-250	G0QMZ8.1 PF13424.4;	50.80	0.00
TRINITY_G0QPQ5_ICHMG/48-200	G0QPQ5.1 PF14908.4;	50.80	0.00
TRINITY_E1Z8Z4_CHLVA/291-808	E1Z8Z4.1 PF03200.14	50.80	0.00
TRINITY_A8ISQ5_CHLRE/527-928	A8ISQ5.1 PF13086.4;	50.80	0.00

TRINITY_L8GR08_ACACA/206-388	L8GR08.1 PF02145.13	50.80	0.00
TRINITY_Q23YD4_TETTS/903-1281	Q23YD4.2 PF12698.5;	50.80	0.00
TRINITY_A8J7Q7_CHLRE/89-325	A8J7Q7.1 PF12697.5;	50.80	0.00
TRINITY_A0A0G4EQ84_9ALVE/661-725	A0A0G4EQ84.1 PF1343	50.80	0.00
TRINITY_D8TN37_VOLCA/1910-2160	D8TN37.1 PF00520.29	50.80	0.00
TRINITY_B3RXG0_TRIAD/41-489	B3RXG0.1 PF00067.20	50.80	0.00
TRINITY_L8H072_ACACA/127-268	L8H072.1 PF12014.6;	50.80	0.00
TRINITY_F0ZDP7_DICPU/387-536	F0ZDP7.1 PF00620.25	50.80	0.00
TRINITY_I7MAC8_TETTS/3673-3968	I7MAC8.2 PF00632.23	50.70	0.00
TRINITY_A0CJA5_PARTE/79-275	A0CJA5.1 PF02630.12	50.70	0.00
TRINITY_Q24HP0_TETTS/705-821	Q24HP0.2 PF02518.24	50.70	0.00
TRINITY_A0CN15_PARTE/130-424	A0CN15.1 PF00481.19	50.70	0.00
TRINITY_Q22DR6_TETTS/64-212	Q22DR6.3 PF09758.7;	50.70	0.00
TRINITY_A0DQ58_PARTE/137-400	A0DQ58.1 PF00069.23	50.70	0.00
TRINITY_Q6BG72_PARTE/10-174	Q6BG72.1 PF01370.19	50.70	0.00
TRINITY_I7MMC5_TETTS/472-769	I7MMC5.2 PF00632.23	50.70	0.00
TRINITY_S2J3Z6_MUCC1/14-179	S2J3Z6.1 PF00071.20	50.70	0.00
TRINITY_Q24BS9_TETTS/921-1178	Q24BS9.2 PF00481.19	50.70	0.00
TRINITY_A0A098GEW6_TATMI/6-528	A0A098GEW6.1 PF0127	50.70	0.00
TRINITY_I7M0L2_TETTS/8-119	I7M0L2.1 PF11969.6;	50.70	0.00
TRINITY_A0E1C9_PARTE/22-92	A0E1C9.1 PF13499.4;	50.70	0.00
TRINITY_D3B760_POLPA/17-238	D3B760.1 PF01048.18	50.70	0.00
TRINITY_E3M8I4_CAERE/11-77	E3M8I4.1 PF00076.20	50.70	0.00
TRINITY_A0A078AG39_STYLE/151-365	A0A078AG39.1 PF0155	50.70	0.00
TRINITY_G0QJQ7_ICHMG/12-286	G0QJQ7.1 PF00069.23	50.70	0.00
TRINITY_J4KMF2_BEAB2/252-629	J4KMF2.1 PF00176.21	50.70	0.00
TRINITY_A7RPQ4_NEMVE/135-551	A7RPQ4.1 PF00930.19	50.70	0.00
TRINITY_D8UKL5_VOLCA/150-225	D8UKL5.1 PF00338.20	50.70	0.00
TRINITY_Q54SB7_DICDI/2115-2262	Q54SB7.2 PF00179.24	50.70	0.00
TRINITY_F4QEN5_DICFS/35-320	F4QEN5.1 PF01008.15	50.70	0.00
TRINITY_C4M7N9_ENTHI/808-1067	C4M7N9.1 PF00069.23	50.70	0.00
TRINITY_A0A0A1PID5_9FUNG/30-98	A0A0A1PID5.1 PF0007	50.70	0.00
TRINITY_I7LZQ9_TETTS/100-246	I7LZQ9.2 PF10075.7;	50.70	0.00
TRINITY_Q6BFC5_PARTE/2079-2177	Q6BFC5.1 PF08454.9;	50.70	0.00
TRINITY_A7SPT7_NEMVE/31-241	A7SPT7.1 PF07722.11	50.70	0.00
TRINITY_Q54DZ1_DICDI/232-306	Q54DZ1.1 PF03456.16	50.70	0.00
TRINITY_A0A078B539_STYLE/66-169	A0A078B539.1 PF0007	50.70	0.00
TRINITY_A0A0N4UQT1_DRAME/104-178	A0A0N4UQT1.1 PF1455	50.70	0.00
TRINITY_Q22TZ6_TETTS/170-240	Q22TZ6.1 PF15159.4;	50.70	0.00
TRINITY_D8TLT7_VOLCA/38-166	D8TLT7.1 PF04424.11	50.70	0.00
TRINITY_A0A061G294_THECC/35-107	A0A061G294.1 PF0017	50.70	0.00
TRINITY_Q01DB3_OSTTA/60-134	Q01DB3.1 PF00076.20	50.70	0.00
TRINITY_Q6FXM8_CANGA/10-171	Q6FXM8.1 PF00071.20	50.70	0.00
TRINITY_A8HPQ1_CHLRE/1343-1424	A8HPQ1.1 PF16135.3;	50.70	0.00
TRINITY_A0A0G4EHM0_9ALVE/13-373	A0A0G4EHM0.1 PF0015	50.70	0.00
TRINITY_D8TTJ9_VOLCA/2-172	D8TTJ9.1 PF00106.23	50.70	0.00
TRINITY_I7MMC4_TETTS/63-228	I7MMC4.2 PF08547.10	50.70	0.00
TRINITY_A9TQT2_PHYPA/3-78	A9TQT2.1 PF12894.5;	50.70	0.00
TRINITY_A0A0J8CND6_BETVU/11-209	A0A0J8CND6.1 PF0976	50.70	0.00
TRINITY_Q245Z0_TETTS/9-210	Q245Z0.3 PF01088.19	50.70	0.00
TRINITY_D8R1A8_SELML/10-81	D8R1A8.1 PF12894.5;	50.70	0.00
TRINITY_A8J1Y6_CHLRE/551-620	A8J1Y6.1 PF00226.29	50.70	0.00
TRINITY_A0A0K9P3T5_ZOSMR/29-105	A0A0K9P3T5.1 PF1289	50.70	0.00
TRINITY_D2VSE5_NAEGR/55-399	D2VSE5.1 PF02055.14	50.70	0.00
TRINITY_D8U7P5_VOLCA/18-335	D8U7P5.1 PF00225.21	50.70	0.00
TRINITY_I7LTH0_TETTS/231-521	I7LTH0.1 PF01031.18	50.70	0.00
TRINITY_G0QLN1_ICHMG/579-666	G0QLN1.1 PF12796.5;	50.70	0.00
TRINITY_I0YWL0_9CHLO/10-149	I0YWL0.1 PF00581.18	50.70	0.00
TRINITY_A8JHE7_CHLRE/3-293	A8JHE7.1 PF03881.12	50.70	0.00
TRINITY_W5KP64_ASTMX/256-327	W5KP64.1 PF13424.4;	50.70	0.00
TRINITY_I0Z3G2_9CHLO/789-892	I0Z3G2.1 PF02984.17	50.70	0.00

TRINITY_B0CYV5_LACBS/101-170	B0CYV5.1 PF00076.20	50.70	0.00
TRINITY_D8QXZ8_SELML/9-81	D8QXZ8.1 PF00076.20	50.70	0.00
TRINITY_F4PQV3_DICFS/219-491	F4PQV3.1 PF00082.20	50.70	0.00
TRINITY_A0A078B161_STYLE/1505-1876	A0A078B161.1 PF0044	50.70	0.00
TRINITY_D8TVH3_VOLCA/34-177	D8TVH3.1 PF03208.17	50.70	0.00
TRINITY_L8H596_ACACA/587-655	L8H596.1 PF13884.4;	50.70	0.00
TRINITY_J9HQD5_9SPIT/115-187	J9HQD5.1 PF00498.24	50.70	0.00
TRINITY_D8U2P8_VOLCA/502-654	D8U2P8.1 PF02622.13	50.70	0.00
TRINITY_J9IJC6_9SPIT/230-303	J9IJC6.1 PF08059.11	50.70	0.00
TRINITY_H2V477_TAKRU/309-380	H2V477.1 PF04564.13	50.70	0.00
TRINITY_A0A0A1NSH3_9FUNG/411-613	A0A0A1NSH3.1 PF0438	50.70	0.00
TRINITY_A0CAK3_PARTE/7-76	A0CAK3.1 PF01834.14	50.70	0.00
TRINITY_D7KSF0_ARALL/115-252	D7KSF0.1 PF13365.4;	50.70	0.00
TRINITY_D8U0M2_VOLCA/6-147	D8U0M2.1 PF00582.24	50.70	0.00
TRINITY_W5JHV0_ANODA/13-515	W5JHV0.1 PF01457.14	50.70	0.00
TRINITY_M0RGE8_MUSAM/42-271	M0RGE8.1 PF00566.16	50.70	0.00
TRINITY_E1ZE38_CHLVA/277-473	E1ZE38.1 PF00534.18	50.70	0.00
TRINITY_A9B7L6_HERA2/397-546	A9B7L6.1 PF00005.25	50.70	0.00
TRINITY_C4R3N7_PICPG/16-177	C4R3N7.1 PF00071.20	50.70	0.00
TRINITY_A0A078ADF2_STYLE/8-121	A0A078ADF2.1 PF0076	50.70	0.00
TRINITY_E1ZSR2_CHLVA/168-303	E1ZSR2.1 PF00107.24	50.70	0.00
TRINITY_H8KQX5_SOLCM/9-302	H8KQX5.1 PF02274.15	50.70	0.00
TRINITY_L8GR65_ACACA/345-515	L8GR65.1 PF03770.14	50.70	0.00
TRINITY_A8IXM3_CHLRE/179-474	A8IXM3.1 PF00481.19	50.70	0.00
TRINITY_Q24DF0_TETTS/748-1037	Q24DF0.2 PF00648.19	50.70	0.00
TRINITY_A8JBS8_CHLRE/56-197	A8JBS8.1 PF01612.18	50.70	0.00
TRINITY_L8H596_ACACA/587-655	L8H596.1 PF13884.4;	50.70	0.00
TRINITY_A0A0L6U7J0_9BASI/410-764	A0A0L6U7J0.1 PF0070	50.70	0.00
TRINITY_A8IZN8_CHLRE/149-294	A8IZN8.1 PF07690.14	50.70	0.00
TRINITY_F4S280_MELLP/47-120	F4S280.1 PF01480.15	50.70	0.00
TRINITY_W6QVR6_PENRO/4-74	W6QVR6.1 PF00076.20	50.70	0.00
TRINITY_D8TKZ7_VOLCA/1579-2019	D8TKZ7.1 PF00443.27	50.70	0.00
TRINITY_Q23TU2_TETTS/4051-4801	Q23TU2.2 PF03028.13	50.70	0.00
TRINITY_H3AJZ9_LATCH/39-112	H3AJZ9.2 PF01722.16	50.70	0.00
TRINITY_A0CV13_PARTE/21-225	A0CV13.1 PF05013.10	50.60	0.00
TRINITY_I7MLV3_TETTS/2-96	I7MLV3.2 PF03669.11	50.60	0.00
TRINITY_I0KA28_9BACT/153-420	I0KA28.1 PF07995.9;	50.60	0.00
TRINITY_Q5FUT4_GLUOX/43-224	Q5FUT4.1 PF13489.4;	50.60	0.00
TRINITY_J9J5L0_9SPIT/92-259	J9J5L0.1 PF00650.18	50.60	0.00
TRINITY_G0R049_ICHMG/53-326	G0R049.1 PF00069.23	50.60	0.00
TRINITY_D5XB11_THEPJ/73-253	D5XB11.1 PF13847.4;	50.60	0.00
TRINITY_I7LXU5_TETTS/95-420	I7LXU5.2 PF02535.20	50.60	0.00
TRINITY_A3GFG7_PICST/80-164	A3GFG7.1 PF00575.21	50.60	0.00
TRINITY_C6XSN3_PEDHD/455-717	C6XSN3.1 PF12972.5;	50.60	0.00
TRINITY_A0BE11_PARTE/7-89	A0BE11.1 PF05495.10	50.60	0.00
TRINITY_E0VIL0_PEDHC/279-437	E0VIL0.1 PF02872.16	50.60	0.00
TRINITY_Q23QF2_TETTS/248-571	Q23QF2.2 PF03133.13	50.60	0.00
TRINITY_G5GGH4_9FIRM/7-87	G5GGH4.1 PF00312.20	50.60	0.00
TRINITY_W6FUH4_NODSP/15-183	W6FUH4.1 PF13385.4;	50.60	0.00
TRINITY_Q110A4_TRIEI/111-328	Q110A4.1 PF13604.4;	50.60	0.00
TRINITY_D8TIV9_VOLCA/4-251	D8TIV9.1 PF14926.4;	50.60	0.00
TRINITY_Q23FG9_TETTS/2610-2860	Q23FG9.2 PF00520.29	50.60	0.00
TRINITY_D8UGE5_VOLCA/164-277	D8UGE5.1 PF00644.18	50.60	0.00
TRINITY_L8HF00_ACACA/100-261	L8HF00.1 PF01652.16	50.60	0.00
TRINITY_E3CZV0_9BACT/24-354	E3CZV0.1 PF00155.19	50.60	0.00
TRINITY_A8IH52_CHLRE/113-189	A8IH52.1 PF02536.12	50.60	0.00
TRINITY_K1R4I2_CRAGI/95-237	K1R4I2.1 PF01399.25	50.60	0.00
TRINITY_K1PT59_CRAGI/33-126	K1PT59.1 PF13905.4;	50.60	0.00
TRINITY_G0QV45_ICHMG/138-367	G0QV45.1 PF01798.16	50.60	0.00
TRINITY_R6P4I1_9FIRM/30-106	R6P4I1.1 PF03061.20	50.60	0.00
TRINITY_F4PRX4_DICFS/3-79	F4PRX4.1 PF07524.11	50.60	0.00

TRINITY_C3ZP28_BRAFL/5-178	C3ZP28.1 PF00071.20	50.60	0.00
TRINITY_I7M433_TETTS/1051-1168	I7M433.2 PF02518.24	50.60	0.00
TRINITY_Q4YVP7_PLABA/27-105	Q4YVP7.1 PF05608.10	50.60	0.00
TRINITY_B1W187_STRGG/1-299	B1W187.1 PF00248.19	50.60	0.00
TRINITY_D8U978_VOLCA/2-203	D8U978.1 PF07714.15	50.60	0.00
TRINITY_S8CL60_9LAMI/418-503	S8CL60.1 PF00439.23	50.60	0.00
TRINITY_L8GJE1_ACACA/32-414	L8GJE1.1 PF00144.22	50.60	0.00
TRINITY_I0YPA9_9CHLO/18-167	I0YPA9.1 PF01947.14	50.60	0.00
TRINITY_A4VD11_TETTS/21-261	A4VD11.2 PF00481.19	50.60	0.00
TRINITY_A8J1L6_CHLRE/28-183	A8J1L6.1 PF12796.5;	50.60	0.00
TRINITY_L8H393_ACACA/160-347	L8H393.1 PF00644.18	50.60	0.00
TRINITY_Q23YR9_TETTS/7-439	Q23YR9.1 PF00171.20	50.60	0.00
TRINITY_I7LVZ2_TETTS/119-388	I7LVZ2.2 PF00664.21	50.60	0.00
TRINITY_D7FH31_ECTSI/15-440	D7FH31.1 PF01663.20	50.60	0.00
TRINITY_I0YZY2_9CHLO/181-311	I0YZY2.1 PF00583.23	50.60	0.00
TRINITY_A8JCE4_CHLRE/156-246	A8JCE4.1 PF00041.19	50.60	0.00
TRINITY_A0A0F2RSV7_9PROT/27-304	A0A0F2RSV7.1 PF0015	50.60	0.00
TRINITY_D8U417_VOLCA/198-314	D8U417.1 PF00504.19	50.60	0.00
TRINITY_A0A075AS09_9FUNG/185-265	A0A075AS09.1 PF0400	50.60	0.00
TRINITY_D8TJI7_VOLCA/7-220	D8TJI7.1 PF01738.16	50.60	0.00
TRINITY_D8TU89_VOLCA/319-430	D8TU89.1 PF00787.22	50.60	0.00
TRINITY_A8IP92_CHLRE/3-79	A8IP92.1 PF07524.11	50.60	0.00
TRINITY_A8HM85_CHLRE/59-327	A8HM85.1 PF00069.23	50.60	0.00
TRINITY_I7MCL9_TETTS/190-488	I7MCL9.2 PF00648.19	50.60	0.00
TRINITY_I0YVV3_9CHLO/785-1087	I0YVV3.1 PF07064.11	50.60	0.00
TRINITY_I0YS09_9CHLO/19-96	I0YS09.1 PF03134.17	50.60	0.00
TRINITY_I0Z826_9CHLO/160-291	I0Z826.1 PF00782.18	50.60	0.00
TRINITY_A0DFT1_PARTE/3-85	A0DFT1.1 PF00778.15	50.60	0.00
TRINITY_L8HJ37_ACACA/6-88	L8HJ37.1 PF03259.15	50.60	0.00
TRINITY_I7M104_TETTS/32-282	I7M104.1 PF00069.23	50.60	0.00
TRINITY_F4QF56_DICFS/241-413	F4QF56.1 PF03031.16	50.60	0.00
TRINITY_Q22GW0_TETTS/1651-1813	Q22GW0.2 PF00069.23	50.60	0.00
TRINITY_J9IST0_9SPIT/130-284	J9IST0.1 PF13768.4;	50.60	0.00
TRINITY_H9GIE1_ANOCA/105-454	H9GIE1.2 PF00773.17	50.60	0.00
TRINITY_D8TYV0_VOLCA/326-491	D8TYV0.1 PF10173.7;	50.60	0.00
TRINITY_A8HM51_CHLRE/256-491	A8HM51.1 PF04811.13	50.60	0.00
TRINITY_V4KQR7_EUTSA/287-399	V4KQR7.1 PF03835.13	50.60	0.00
TRINITY_C4WXA7_ACYPI/23-101	C4WXA7.1 PF01423.20	50.60	0.00
TRINITY_L8GCT6_ACACA/23-104	L8GCT6.1 PF14542.4;	50.60	0.00
TRINITY_YTA12_SCHPO/134-230	Q9HGM3.1 PF06480.13	50.60	0.00
TRINITY_A0A078AGR2_STYLE/52-145	A0A078AGR2.1 PF0177	50.50	0.00
TRINITY_I4B3F1_TURPD/61-196	I4B3F1.1 PF00188.24	50.50	0.00
TRINITY_A0A067CMQ3_SAPPC/225-321	A0A067CMQ3.1 PF1654	50.50	0.00
TRINITY_E1ZQW8_CHLVA/294-422	E1ZQW8.1 PF06479.10	50.50	0.00
TRINITY_Q22AV5_TETTS/18-120	Q22AV5.3 PF00466.18	50.50	0.00
TRINITY_I0Z658_9CHLO/14-184	I0Z658.1 PF00270.27	50.50	0.00
TRINITY_L8GXJ8_ACACA/333-582	L8GXJ8.1 PF07065.12	50.50	0.00
TRINITY_M0SCI7_MUSAM/848-955	M0SCI7.1 PF00970.22	50.50	0.00
TRINITY_G8BJW8_CANPC/6-167	G8BJW8.1 PF00071.20	50.50	0.00
TRINITY_Q22DU5_TETTS/61-276	Q22DU5.3 PF01988.17	50.50	0.00
TRINITY_A0DY44_PARTE/5-114	A0DY44.1 PF00787.22	50.50	0.00
TRINITY_A4B9B6_9GAMM/5-197	A4B9B6.1 PF00316.18	50.50	0.00
TRINITY_E1ZQW0_CHLVA/4-287	E1ZQW0.1 PF00069.23	50.50	0.00
TRINITY_I1BWC7_RHIO9/616-710	I1BWC7.1 PF04676.12	50.50	0.00
TRINITY_A9URG4_MONBE/17-320	A9URG4.1 PF00933.19	50.50	0.00
TRINITY_E6TZQ2_BACCJ/5-113	E6TZQ2.1 PF04134.10	50.50	0.00
TRINITY_I7MMY6_TETTS/639-738	I7MMY6.2 PF00989.23	50.50	0.00
TRINITY_A0DMS6_PARTE/43-308	A0DMS6.1 PF03381.13	50.50	0.00
TRINITY_I0YIQ4_9CHLO/34-136	I0YIQ4.1 PF00583.23	50.50	0.00
TRINITY_D8U031_VOLCA/422-597	D8U031.1 PF16507.3;	50.50	0.00
TRINITY_F4PZZ1_DICFS/16-111	F4PZZ1.1 PF00169.27	50.50	0.00

TRINITY_A0BCH6_PARTE/98-199	A0BCH6.1 PF00829.19	50.50	0.00
TRINITY_C3YM49_BRAFL/34-172	C3YM49.1 PF13532.4;	50.50	0.00
TRINITY_D3BVR3_POLPA/201-453	D3BVR3.1 PF07714.15	50.50	0.00
TRINITY_A8IUH2_CHLRE/119-324	A8IUH2.1 PF07714.15	50.50	0.00
TRINITY_D8TQ02_VOLCA/862-1054	D8TQ02.1 PF07717.14	50.50	0.00
TRINITY_I7M279_TETTS/162-262	I7M279.1 PF13864.4;	50.50	0.00
TRINITY_A8HQ93_CHLRE/25-123	A8HQ93.1 PF00085.18	50.50	0.00
TRINITY_A8I341_CHLRE/1-292	A8I341.1 PF04916.11	50.50	0.00
TRINITY_A0A087SS12_AUXPR/84-521	A0A087SS12.1 PF0099	50.50	0.00
TRINITY_A0A0G4FFD9_9ALVE/90-555	A0A0G4FFD9.1 PF0006	50.50	0.00
TRINITY_L1IEU9_GUITH/3-212	L1IEU9.1 PF00091.23	50.50	0.00
TRINITY_D8TKK8_VOLCA/284-440	D8TKK8.1 PF04755.10	50.50	0.00
TRINITY_B0CTS7_LACBS/26-235	B0CTS7.1 PF13489.4;	50.50	0.00
TRINITY_I0YK29_9CHLO/86-684	I0YK29.1 PF03169.13	50.50	0.00
TRINITY_E1Z6K7_CHLVA/53-256	E1Z6K7.1 PF00856.26	50.50	0.00
TRINITY_Q22T22_TETTS/520-735	Q22T22.3 PF07859.11	50.50	0.00
TRINITY_U2FW98_9GAMM/144-399	U2FW98.1 PF00067.20	50.50	0.00
TRINITY_A0CJJ5_PARTE/148-258	A0CJJ5.1 PF00085.18	50.50	0.00
TRINITY_F4PYX7_DICFS/450-662	F4PYX7.1 PF13236.4;	50.50	0.00
TRINITY_D8TV76_VOLCA/263-363	D8TV76.1 PF01399.25	50.50	0.00
TRINITY_D8TXQ4_VOLCA/134-237	D8TXQ4.1 PF02269.14	50.50	0.00
TRINITY_L8HHZ1_ACACA/27-668	L8HHZ1.1 PF01496.17	50.50	0.00
TRINITY_U9T380_RHIID/3-184	U9T380.1 PF01145.23	50.50	0.00
TRINITY_D3BDG4_POLPA/512-623	D3BDG4.1 PF00072.22	50.50	0.00
TRINITY_I0Z9K8_9CHLO/1-199	I0Z9K8.1 PF01545.19	50.50	0.00
TRINITY_D8TQQ3_VOLCA/75-581	D8TQQ3.1 PF04129.10	50.50	0.00
TRINITY_I1CRD3_RHIO9/362-587	I1CRD3.1 PF00133.20	50.50	0.00
TRINITY_V7AZU6_PHAVU/330-774	V7AZU6.1 PF01055.24	50.50	0.00
TRINITY_A8IEX1_CHLRE/352-562	A8IEX1.1 PF01061.22	50.50	0.00
TRINITY_A0A0G4FFD9_9ALVE/90-555	A0A0G4FFD9.1 PF0006	50.50	0.00
TRINITY_A7HR89_PARL1/16-441	A7HR89.1 PF00501.26	50.50	0.00
TRINITY_G0QKR4_ICHMG/620-711	G0QKR4.1 PF03129.18	50.50	0.00
TRINITY_D8U5K2_VOLCA/113-191	D8U5K2.1 PF03061.20	50.50	0.00
TRINITY_A8J3P1_CHLRE/954-1161	A8J3P1.1 PF00211.18	50.50	0.00
TRINITY_Q021V3_SOLUE/2015-2114	Q021V3.1 PF02369.14	50.50	0.00
TRINITY_I7M328_TETTS/23-132	I7M328.1 PF00787.22	50.50	0.00
TRINITY_A0A0P7TRU5_9TELE/547-700	A0A0P7TRU5.1 PF0000	50.50	0.00
TRINITY_A8J6B3_CHLRE/199-368	A8J6B3.1 PF00069.23	50.50	0.00
TRINITY_D8U9S9_VOLCA/599-793	D8U9S9.1 PF00211.18	50.50	0.00
TRINITY_L8HFK7_ACACA/695-881	L8HFK7.1 PF00644.18	50.50	0.00
TRINITY_K3XV70_SETIT/273-738	K3XV70.1 PF01055.24	50.50	0.00
TRINITY_F4Q5I7_DICFS/473-706	F4Q5I7.1 PF00233.17	50.50	0.00
TRINITY_B2IT25_NOSP7/32-490	B2IT25.1 PF03055.13	50.50	0.00
TRINITY_E5SHN1_TRISP/301-751	E5SHN1.1 PF01055.24	50.40	0.00
TRINITY_F1A043_DICPU/1229-1482	F1A043.1 PF07714.15	50.40	0.00
TRINITY_A0A078BB69_STYLE/58-324	A0A078BB69.1 PF0338	50.40	0.00
TRINITY_C4MB22_ENTHI/3-139	C4MB22.1 PF02502.16	50.40	0.00
TRINITY_A0EH94_PARTE/143-486	A0EH94.1 PF03062.17	50.40	0.00
TRINITY_Q234E4_TETTS/260-515	Q234E4.2 PF05677.10	50.40	0.00
TRINITY_Q23YS8_TETTS/179-436	Q23YS8.2 PF02666.13	50.40	0.00
TRINITY_M3ZSR5_XIPMA/122-254	M3ZSR5.1 PF17207.1;	50.40	0.00
TRINITY_A0A0M8K928_9CHLR/19-151	A0A0M8K928.1 PF0258	50.40	0.00
TRINITY_Q54UH9_DICDI/297-423	Q54UH9.1 PF01569.19	50.40	0.00
TRINITY_K3WM25_PYTUL/97-215	K3WM25.1 PF03834.12	50.40	0.00
TRINITY_A0A078AEE4_STYLE/1534-1673	A0A078AEE4.1 PF1027	50.40	0.00
TRINITY_E1ZN56_CHLVA/84-366	E1ZN56.1 PF00664.21	50.40	0.00
TRINITY_G0QSX2_ICHMG/1-334	G0QSX2.1 PF00724.18	50.40	0.00
TRINITY_I0Z307_9CHLO/78-324	I0Z307.1 PF00561.18	50.40	0.00
TRINITY_A0A078AJ77_STYLE/19-206	A0A078AJ77.1 PF0090	50.40	0.00
TRINITY_Q183E8_PEPD6/4-324	Q183E8.1 PF16363.3;	50.40	0.00
TRINITY_A0A022RC66_ERYGU/150-384	A0A022RC66.1 PF1362	50.40	0.00

TRINITY_L8GMG2_ACACA/38-448	L8GMG2.1 PF00450.20	50.40	0.00
TRINITY_D2VII2_NAEGR/70-302	D2VII2.1 PF04185.12	50.40	0.00
TRINITY_I7MMV5_TETTS/55-352	I7MMV5.2 PF00443.27	50.40	0.00
TRINITY_D3B1D9_POLPA/14-486	D3B1D9.1 PF01229.15	50.40	0.00
TRINITY_A0A0H4VMJ0_9BACT/6-246	A0A0H4VMJ0.1 PF0778	50.40	0.00
TRINITY_F2NKK1_MARHT/45-180	F2NKK1.1 PF01565.21	50.40	0.00
TRINITY_A8JEJ5_CHLRE/61-186	A8JEJ5.1 PF02225.20	50.40	0.00
TRINITY_A0A0D3C966_BRAOL/461-593	A0A0D3C966.1 PF0141	50.40	0.00
TRINITY_A0A0A6D1K0_9SPHN/18-142	A0A0A6D1K0.1 PF1358	50.40	0.00
TRINITY_D2VI09_NAEGR/2-296	D2VI09.1 PF00069.23	50.40	0.00
TRINITY_H3CGY9_TETNG/863-1085	H3CGY9.1 PF03178.13	50.40	0.00
TRINITY_I7MA07_TETTS/32-269	I7MA07.2 PF00481.19	50.40	0.00
TRINITY_A0C4Z1_PARTE/13-292	A0C4Z1.1 PF07993.10	50.40	0.00
TRINITY_D8TKH1_VOLCA/3-235	D8TKH1.1 PF08597.8;	50.40	0.00
TRINITY_C5YK16_SORBI/1050-1167	C5YK16.1 PF00665.24	50.40	0.00
TRINITY_A0A067E2Y8_CITSI/13-132	A0A067E2Y8.1 PF0090	50.40	0.00
TRINITY_G8YM70_PICSO/5-126	G8YM70.1 PF03311.12	50.40	0.00
TRINITY_V6LK02_9EUKA/3-212	V6LK02.1 PF00091.23	50.40	0.00
TRINITY_D8UCU9_VOLCA/285-401	D8UCU9.1 PF06565.10	50.40	0.00
TRINITY_D8TXN9_VOLCA/18-203	D8TXN9.1 PF03357.19	50.40	0.00
TRINITY_I0YS22_9CHLO/25-200	I0YS22.1 PF05175.12	50.40	0.00
TRINITY_A8JID7_CHLRE/377-635	A8JID7.1 PF00112.21	50.40	0.00
TRINITY_D8UE69_VOLCA/79-352	D8UE69.1 PF00270.27	50.40	0.00
TRINITY_A8JF64_CHLRE/376-794	A8JF64.1 PF05557.11	50.40	0.00
TRINITY_L8GZ22_ACACA/175-421	L8GZ22.1 PF00149.26	50.40	0.00
TRINITY_Q22SL3_TETTS/348-483	Q22SL3.3 PF01590.24	50.40	0.00
TRINITY_D2UXN1_NAEGR/204-317	D2UXN1.1 PF02373.20	50.40	0.00
TRINITY_I0Z535_9CHLO/41-385	I0Z535.1 PF00266.17	50.40	0.00
TRINITY_A0C0U9_PARTE/95-431	A0C0U9.1 PF03969.14	50.40	0.00
TRINITY_A0A087SKR7_AUXPR/207-323	A0A087SKR7.1 PF0176	50.40	0.00
TRINITY_A8JG06_CHLRE/8-378	A8JG06.1 PF03097.16	50.40	0.00
TRINITY_W5AB19_WHEAT/542-682	W5AB19.1 PF04096.12	50.40	0.00
TRINITY_D8U7H7_VOLCA/11-359	D8U7H7.1 PF04928.15	50.40	0.00
TRINITY_D8TZH6_VOLCA/22-283	D8TZH6.1 PF00233.17	50.40	0.00
TRINITY_A8JHV6_CHLRE/431-702	A8JHV6.1 PF00069.23	50.40	0.00
TRINITY_A0CYT3_PARTE/95-208	A0CYT3.1 PF01159.17	50.40	0.00
TRINITY_A0CJY8_PARTE/436-698	A0CJY8.1 PF00069.23	50.30	0.00
TRINITY_Q245K3_TETTS/327-677	Q245K3.2 PF01237.16	50.30	0.00
TRINITY_L8GHY4_ACACA/84-371	L8GHY4.1 PF07690.14	50.30	0.00
TRINITY_L8GF65_ACACA/32-171	L8GF65.1 PF01138.19	50.30	0.00
TRINITY_T1HIC0_RHOPR/10-171	T1HIC0.1 PF00071.20	50.30	0.00
TRINITY_D3AVJ6_POLPA/226-391	D3AVJ6.1 PF00005.25	50.30	0.00
TRINITY_D3BLJ5_POLPA/1841-2108	D3BLJ5.1 PF07714.15	50.30	0.00
TRINITY_Q54TS5_DICDI/59-351	Q54TS5.1 PF00128.22	50.30	0.00
TRINITY_A8IFG0_CHLRE/3-330	A8IFG0.1 PF16420.3;	50.30	0.00
TRINITY_I7M0N5_TETTS/125-295	I7M0N5.1 PF00092.26	50.30	0.00
TRINITY_I7M6K2_TETTS/65-258	I7M6K2.1 PF01145.23	50.30	0.00
TRINITY_D8TSX5_VOLCA/1-202	D8TSX5.1 PF04139.11	50.30	0.00
TRINITY_D3BMQ2_POLPA/45-229	D3BMQ2.1 PF00406.20	50.30	0.00
TRINITY_Q22W19_TETTS/115-323	Q22W19.3 PF00112.21	50.30	0.00
TRINITY_A0A075AXX5_9FUNG/49-226	A0A075AXX5.1 PF0000	50.30	0.00
TRINITY_D3BP99_POLPA/174-329	D3BP99.1 PF00903.23	50.30	0.00
TRINITY_F4PH07_DICFS/583-780	F4PH07.1 PF00617.17	50.30	0.00
TRINITY_F0ZJW2_DICPU/563-743	F0ZJW2.1 PF02145.13	50.30	0.00
TRINITY_G0QNF3_ICHMG/123-438	G0QNF3.1 PF00351.19	50.30	0.00
TRINITY_A0CKF0_PARTE/1023-1167	A0CKF0.1 PF00005.25	50.30	0.00
TRINITY_L8HEV2_ACACA/41-347	L8HEV2.1 PF01074.20	50.30	0.00
TRINITY_G4V6C8_SCHMA/54-247	G4V6C8.1 PF04733.12	50.30	0.00
TRINITY_Q19VH5_CHLRE/2-151	Q19VH5.1 PF03358.13	50.30	0.00
TRINITY_D8TPF1_VOLCA/22-214	D8TPF1.1 PF16661.3;	50.30	0.00
TRINITY_A7SIF7_NEMVE/134-316	A7SIF7.1 PF02145.13	50.30	0.00

TRINITY_A0A078AN29_STYLE/103-278	A0A078AN29.1 PF0471	50.30	0.00
TRINITY_E1Z3R9_CHLVA/105-275	E1Z3R9.1 PF12068.6;	50.30	0.00
TRINITY_F4PSM9_DICFS/33-212	F4PSM9.1 PF13460.4;	50.30	0.00
TRINITY_A8J2P8_CHLRE/467-630	A8J2P8.1 PF06728.11	50.30	0.00
TRINITY_A0DRY4_PARTE/120-281	A0DRY4.1 PF14580.4;	50.30	0.00
TRINITY_I7MCL9_TETTS/190-488	I7MCL9.2 PF00648.19	50.30	0.00
TRINITY_L8GQN2_ACACA/447-624	L8GQN2.1 PF00617.17	50.30	0.00
TRINITY_W7X4T7_TETTS/1-455	W7X4T7.1 PF00171.20	50.30	0.00
TRINITY_A0A075AWU8_9FUNG/39-183	A0A075AWU8.1 PF0010	50.30	0.00
TRINITY_E1Z6Q9_CHLVA/24-203	E1Z6Q9.1 PF01012.19	50.30	0.00
TRINITY_Q7XLT8_ORYSJ/503-697	Q7XLT8.2 PF00078.25	50.30	0.00
TRINITY_Q23DI7_TETTS/1868-2219	Q23DI7.2 PF00443.27	50.30	0.00
TRINITY_K6XR77_9ALTE/18-317	K6XR77.1 PF01156.17	50.20	0.00
TRINITY_G7Z4V8_AZOL4/33-281	G7Z4V8.1 PF12697.5;	50.20	0.00
TRINITY_D8TSD6_VOLCA/425-880	D8TSD6.1 PF11894.6;	50.20	0.00
TRINITY_I7M7G3_TETTS/21-302	I7M7G3.1 PF00069.23	50.20	0.00
TRINITY_A9U2F6_PHYPA/53-547	A9U2F6.1 PF02990.14	50.20	0.00
TRINITY_C5NXZ0_9BACL/3-212	C5NXZ0.1 PF01791.7;	50.20	0.00
TRINITY_A9V4S2_MONBE/204-426	A9V4S2.1 PF04389.15	50.20	0.00
TRINITY_Q22W19_TETTS/115-323	Q22W19.3 PF00112.21	50.20	0.00
TRINITY_D8TPH6_VOLCA/875-1156	D8TPH6.1 PF10595.7;	50.20	0.00
TRINITY_I0YIS2_9CHLO/89-346	I0YIS2.1 PF00561.18	50.20	0.00
TRINITY_L8HKC9_ACACA/286-508	L8HKC9.1 PF04389.15	50.20	0.00
TRINITY_L8HAW3_ACACA/654-873	L8HAW3.1 PF13236.4;	50.20	0.00
TRINITY_D8TN00_VOLCA/1-276	D8TN00.1 PF01433.18	50.20	0.00
TRINITY_A0A074WT85_9PEZI/321-526	A0A074WT85.1 PF0056	50.20	0.00
TRINITY_D8U819_VOLCA/63-369	D8U819.1 PF02383.16	50.20	0.00
TRINITY_A8J6M5_CHLRE/663-937	A8J6M5.1 PF00664.21	50.20	0.00
TRINITY_A6Q2C0_NITSB/20-221	A6Q2C0.1 PF02146.15	50.20	0.00
TRINITY_D8TTV8_VOLCA/81-324	D8TTV8.1 PF00233.17	50.20	0.00
TRINITY_A8I4W2_CHLRE/1-181	A8I4W2.1 PF00211.18	50.20	0.00
TRINITY_G0QMH5_ICHMG/105-461	G0QMH5.1 PF06602.12	50.10	0.00
TRINITY_Q0DG76_ORYSJ/129-450	Q0DG76.1 PF00069.23	50.10	0.00
TRINITY_D8TKH5_VOLCA/385-739	D8TKH5.1 PF03016.13	50.10	0.00
TRINITY_SLT2_CHLRE/16-750	D2K6F1.1 PF03600.14	50.10	0.00
TRINITY_Q7Z113_PARTE/30-110	Q7Z113.1 PF13774.4;	50.00	0.00
TRINITY_I7MH25_TETTS/266-611	I7MH25.2 PF03124.12	50.00	0.00
TRINITY_L8H823_ACACA/184-369	L8H823.1 PF07065.12	50.00	0.00
TRINITY_D8UBR5_VOLCA/567-935	D8UBR5.1 PF03215.13	50.00	0.00
TRINITY_A0CXJ7_PARTE/74-221	A0CXJ7.1 PF01553.19	50.00	0.00
TRINITY_G0QJ72_ICHMG/112-320	G0QJ72.1 PF00112.21	50.00	0.00
TRINITY_A0A078A070_STYLE/521-739	A0A078A070.1 PF0785	50.00	0.00
TRINITY_Q7RLR7_PLAYO/177-256	Q7RLR7.1 PF03371.13	50.00	0.00
TRINITY_I7M2I2_TETTS/204-299	I7M2I2.1 PF00153.25	50.00	0.00
TRINITY_I7MGE9_TETTS/750-1106	I7MGE9.2 PF05192.16	50.00	0.00
TRINITY_Q1ZXJ3_DICDI/154-439	Q1ZXJ3.1 PF03034.13	50.00	0.00
TRINITY_Q23FD8_TETTS/19-112	Q23FD8.4 PF01230.21	50.00	0.00
TRINITY_J9ICS8_9SPIT/235-354	J9ICS8.1 PF00383.21	50.00	0.00
TRINITY_Q245F6_TETTS/838-909	Q245F6.2 PF14791.4;	50.00	0.00
TRINITY_E1C1F8_CHICK/851-1054	E1C1F8.2 PF00488.19	50.00	0.00
TRINITY_T1KTS2_TETUR/45-205	T1KTS2.1 PF03031.16	50.00	0.00
TRINITY_A0C5H1_PARTE/22-271	A0C5H1.1 PF00378.18	50.00	0.00
TRINITY_J0LG47_9BACT/30-232	J0LG47.1 PF04402.12	50.00	0.00
TRINITY_A0A078LAK8_9CHLA/47-214	A0A078LAK8.1 PF0258	50.00	0.00
TRINITY_V4LMZ4_EUTSA/9-153	V4LMZ4.1 PF00179.24	50.00	0.00
TRINITY_I0Z567_9CHLO/1670-1720	I0Z567.1 PF00847.18	50.00	0.00
TRINITY_A0E9F9_PARTE/141-258	A0E9F9.1 PF12146.6;	50.00	0.00
TRINITY_U9SM67_RHIID/195-278	U9SM67.1 PF12861.5;	50.00	0.00
TRINITY_L8GTT3_ACACA/772-1081	L8GTT3.1 PF00632.23	50.00	0.00
TRINITY_B3E8K8_GEOLS/331-515	B3E8K8.1 PF13489.4;	50.00	0.00
TRINITY_Q232W6_TETTS/660-768	Q232W6.3 PF01248.24	50.00	0.00

TRINITY_L8GW12_ACACA/1-423	L8GW12.1 PF00022.17	50.00	0.00
TRINITY_D2VGX1_NAEGR/83-147	D2VGX1.1 PF13499.4;	50.00	0.00
TRINITY_I1IZ00_BRADI/799-857	I1IZ00.1 PF08672.9;	50.00	0.00
TRINITY_Q246C6_TETTS/4-223	Q246C6.1 PF00300.20	50.00	0.00
TRINITY_A0BPY0_PARTE/414-535	A0BPY0.1 PF02518.24	50.00	0.00
TRINITY_A0CJA5_PARTE/79-275	A0CJA5.1 PF02630.12	50.00	0.00
TRINITY_A0A0D2UQJ9_CAPO3/69-137	A0A0D2UQJ9.1 PF1384	50.00	0.00
TRINITY_H1XQG2_9BACT/3-135	H1XQG2.1 PF02878.14	50.00	0.00
TRINITY_W4K583_9HOMO/427-473	W4K583.1 PF13639.4;	50.00	0.00
TRINITY_K7J0U5_NASVI/298-365	K7J0U5.1 PF00076.20	50.00	0.00
TRINITY_G0R2N3_ICHMG/123-174	G0R2N3.1 PF13833.4;	50.00	0.00
TRINITY_W7X388_TETTS/56-115	W7X388.1 PF01363.19	50.00	0.00
TRINITY_I4ALD6_FLELS/7-286	I4ALD6.1 PF07992.12	50.00	0.00
TRINITY_F6WF51_CIOIN/57-145	F6WF51.2 PF00355.24	50.00	0.00
TRINITY_K1QH83_CRAGI/11-474	K1QH83.1 PF03155.13	50.00	0.00
TRINITY_A0A077ZUK1_STYLE/214-500	A0A077ZUK1.1 PF0103	50.00	0.00
TRINITY_F4Q277_DICFS/68-123	F4Q277.1 PF00412.20	50.00	0.00
TRINITY_A9U107_PHYPA/108-245	A9U107.1 PF12706.5;	50.00	0.00
TRINITY_A4VDX0_TETTS/52-359	A4VDX0.1 PF14664.4;	50.00	0.00
TRINITY_A0A0A1NFD8_9FUNG/129-275	A0A0A1NFD8.1 PF0007	50.00	0.00
TRINITY_A0A0F5Q7C2_9RHIZ/18-222	A0A0F5Q7C2.1 PF1348	50.00	0.00
TRINITY_G3Y0Z8_ASPNA/1-169	G3Y0Z8.1 PF13561.4;	50.00	0.00
TRINITY_C3YID1_BRAFL/211-416	C3YID1.1 PF01189.15	50.00	0.00
TRINITY_U5QRF4_9CYAN/14-91	U5QRF4.1 PF01266.22	50.00	0.00
TRINITY_F1A5F9_DICPU/1-112	F1A5F9.1 PF08510.10	50.00	0.00
TRINITY_I7LY21_TETTS/1717-1892	I7LY21.2 PF00520.29	50.00	0.00
TRINITY_I4BY26_ANAMD/4-474	I4BY26.1 PF00982.19	50.00	0.00
TRINITY_F0Y6L3_AURAN/5-178	F0Y6L3.1 PF00071.20	50.00	0.00
TRINITY_M2XIP2_GALSU/52-123	M2XIP2.1 PF00076.20	50.00	0.00
TRINITY_C1GTA6_PARBA/7-112	C1GTA6.2 PF00383.21	50.00	0.00
TRINITY_A0LZE2_GRAFK/28-145	A0LZE2.1 PF08240.10	50.00	0.00
TRINITY_A0D2X6_PARTE/2-135	A0D2X6.1 PF05914.10	50.00	0.00
TRINITY_A0A094GXC1_9PEZI/767-870	A0A094GXC1.1 PF0015	50.00	0.00
TRINITY_A0A0F9ZX14_TRIHA/65-234	A0A0F9ZX14.1 PF1309	50.00	0.00
TRINITY_V9F301_PHYPR/24-80	V9F301.1 PF08207.10	50.00	0.00
TRINITY_A0A0B1P9Z4_UNCNE/3-126	A0A0B1P9Z4.1 PF1389	50.00	0.00
TRINITY_Q22NP9_TETTS/339-441	Q22NP9.2 PF02204.16	50.00	0.00
TRINITY_APT_UREPA/9-164	Q9PQ02.1 PF00156.25	50.00	0.00
TRINITY_B4VTM8_9CYAN/5-199	B4VTM8.1 PF00300.20	50.00	0.00
TRINITY_J9IMT2_9SPIT/146-246	J9IMT2.1 PF00153.25	50.00	0.00
TRINITY_V8N9K4_OPHHA/45-170	V8N9K4.1 PF00005.25	50.00	0.00
TRINITY_A0BEL5_PARTE/9-71	A0BEL5.1 PF00280.16	50.00	0.00
TRINITY_D8THX1_VOLCA/227-448	D8THX1.1 PF13672.4;	50.00	0.00
TRINITY_G0R4J9_ICHMG/260-436	G0R4J9.1 PF00406.20	50.00	0.00
TRINITY_W9CAR1_9HELO/208-486	W9CAR1.1 PF00122.18	50.00	0.00
TRINITY_I7MDH2_TETTS/627-734	I7MDH2.2 PF13426.5;	50.00	0.00
TRINITY_D2S423_GEOOG/13-252	D2S423.1 PF13561.4;	50.00	0.00
TRINITY_G5AYP1_HETGA/320-591	G5AYP1.1 PF00664.21	50.00	0.00
TRINITY_A0A088ADF8_APIME/634-677	A0A088ADF8.1 PF1363	50.00	0.00
TRINITY_C3XWK4_BRAFL/8-214	C3XWK4.1 PF01088.19	50.00	0.00
TRINITY_B6UHU2_MAIZE/9-57	B6UHU2.1 PF04627.11	50.00	0.00
TRINITY_Q22V58_TETTS/214-314	Q22V58.2 PF00153.25	50.00	0.00
TRINITY_Q231N2_TETTS/513-802	Q231N2.3 PF04950.10	50.00	0.00
TRINITY_A0A078DL47_BRANA/21-630	A0A078DL47.1 PF0088	50.00	0.00
TRINITY_E9QCK7_DANRE/639-797	E9QCK7.1 PF05572.11	50.00	0.00
TRINITY_I0YRC1_9CHLO/125-240	I0YRC1.1 PF13238.4;	50.00	0.00
TRINITY_A0A078A2Y6_STYLE/244-434	A0A078A2Y6.1 PF0506	50.00	0.00
TRINITY_Q24G69_TETTS/13-259	Q24G69.2 PF00069.23	50.00	0.00
TRINITY_I7MID1_TETTS/134-392	I7MID1.2 PF00069.23	50.00	0.00
TRINITY_L8H3W5_ACACA/971-1093	L8H3W5.1 PF00271.29	50.00	0.00
TRINITY_I0YQ01_9CHLO/19-138	I0YQ01.1 PF06212.10	50.00	0.00

TRINITY_W6FUH4_NODSP/15-183	W6FUH4.1 PF13385.4;	50.00	0.00
TRINITY_G0QU69_ICHMG/304-439	G0QU69.1 PF01262.19	50.00	0.00
TRINITY_D3AWB1_POLPA/438-507	D3AWB1.1 PF12796.5;	50.00	0.00
TRINITY_B4JJA0_DROGR/927-982	B4JJA0.1 PF13513.4;	50.00	0.00
TRINITY_C1MH71_MICPC/142-195	C1MH71.1 PF00412.20	50.00	0.00
TRINITY_L8GRJ6_ACACA/5-112	L8GRJ6.1 PF00307.29	50.00	0.00
TRINITY_L8HKM5_ACACA/4-214	L8HKM5.1 PF04139.11	50.00	0.00
TRINITY_G1SQP9_RABIT/65-237	G1SQP9.2 PF01596.15	50.00	0.00
TRINITY_B3S094_TRIAD/15-158	B3S094.1 PF00134.21	50.00	0.00
TRINITY_I7M9Y2_TETTS/44-181	I7M9Y2.1 PF00198.21	50.00	0.00
TRINITY_G0QJ48_ICHMG/856-963	G0QJ48.1 PF10367.7;	50.00	0.00
TRINITY_D8TTV8_VOLCA/81-324	D8TTV8.1 PF00233.17	50.00	0.00
TRINITY_A8HNZ4_CHLRE/178-321	A8HNZ4.1 PF00005.25	50.00	0.00
TRINITY_A0A078A8V3_STYLE/25-119	A0A078A8V3.1 PF1390	50.00	0.00
TRINITY_A0D8R6_PARTE/23-121	A0D8R6.1 PF03645.11	50.00	0.00
TRINITY_B9SD10_RICCO/11-296	B9SD10.1 PF07992.12	50.00	0.00
TRINITY_G0QMR6_ICHMG/167-275	G0QMR6.1 PF00085.18	50.00	0.00
TRINITY_I0Z1I3_9CHLO/25-268	I0Z1I3.1 PF12146.6;	50.00	0.00
TRINITY_G8AWG3_AZOBR/633-755	G8AWG3.1 PF02518.24	50.00	0.00
TRINITY_D8TXQ3_VOLCA/351-421	D8TXQ3.1 PF04818.11	50.00	0.00
TRINITY_D8UA16_VOLCA/457-643	D8UA16.1 PF13469.4;	50.00	0.00
TRINITY_D0MQP4_PHYIT/223-288	D0MQP4.1 PF01918.19	50.00	0.00
TRINITY_Q08MJ9_STIAD/4-113	Q08MJ9.1 PF00576.19	50.00	0.00
TRINITY_G0QJA3_ICHMG/85-432	G0QJA3.1 PF00155.19	50.00	0.00
TRINITY_I0YLH8_9CHLO/764-861	I0YLH8.1 PF02806.16	50.00	0.00
TRINITY_Q23QF2_TETTS/248-571	Q23QF2.2 PF03133.13	50.00	0.00
TRINITY_A9W9Z5_CHLAA/1-117	A9W9Z5.1 PF01408.20	50.00	0.00
TRINITY_Q00YD9_OSTTA/268-339	Q00YD9.1 PF12894.5;	50.00	0.00
TRINITY_D8R7A4_SELML/134-230	D8R7A4.1 PF00153.25	50.00	0.00
TRINITY_Q54R10_DICDI/49-285	Q54R10.1 PF00149.26	50.00	0.00
TRINITY_A7SDR5_NEMVE/83-308	A7SDR5.1 PF00112.21	50.00	0.00
TRINITY_W4H202_9STRA/290-475	W4H202.1 PF00689.19	50.00	0.00
TRINITY_A0A068SAB1_9FUNG/123-211	A0A068SAB1.1 PF0095	50.00	0.00
TRINITY_A0CXJ7_PARTE/74-221	A0CXJ7.1 PF01553.19	50.00	0.00
TRINITY_H2KV20_CLOSI/39-198	H2KV20.1 PF08645.9;	50.00	0.00
TRINITY_A0CIZ1_PARTE/663-715	A0CIZ1.1 PF13833.4;	50.00	0.00
TRINITY_D6WWM4_TRICA/124-287	D6WWM4.1 PF08662.9;	50.00	0.00
TRINITY_A0A024TBB3_9STRA/10-218	A0A024TBB3.1 PF0307	50.00	0.00
TRINITY_A9FAR4_SORC5/21-203	A9FAR4.1 PF07081.9;	50.00	0.00
TRINITY_A6GAQ0_9DELT/22-108	A6GAQ0.1 PF04379.12	50.00	0.00
TRINITY_D8TPQ3_VOLCA/270-395	D8TPQ3.1 PF00188.24	50.00	0.00
TRINITY_G0R0V5_ICHMG/25-299	G0R0V5.1 PF00069.23	50.00	0.00
TRINITY_E1ZTC5_CHLVA/44-473	E1ZTC5.1 PF00856.26	50.00	0.00
TRINITY_A8JBD8_CHLRE/71-321	A8JBD8.1 PF00481.19	50.00	0.00
TRINITY_D8UHG6_VOLCA/270-381	D8UHG6.1 PF08718.9;	50.00	0.00
TRINITY_G0QWF1_ICHMG/47-134	G0QWF1.1 PF05873.10	50.00	0.00
TRINITY_L8GG01_ACACA/221-375	L8GG01.1 PF13768.4;	50.00	0.00
TRINITY_T1KND4_TETUR/244-315	T1KND4.1 PF12894.5;	50.00	0.00
TRINITY_G0QIV3_ICHMG/284-335	G0QIV3.1 PF13418.4;	50.00	0.00
TRINITY_A0A0E9N0F0_9SPHI/271-327	A0A0E9N0F0.1 PF1341	50.00	0.00
TRINITY_D0MJ84_RHOM4/509-745	D0MJ84.1 PF02784.14	50.00	0.00
TRINITY_K7KDK2_SOYBN/252-309	K7KDK2.1 PF13921.4;	50.00	0.00
TRINITY_A0A067C8Q0_SAPPC/815-999	A0A067C8Q0.1 PF0068	50.00	0.00
TRINITY_A0BN18_PARTE/24-154	A0BN18.1 PF00782.18	50.00	0.00
TRINITY_I7M738_TETTS/333-442	I7M738.2 PF00271.29	50.00	0.00
TRINITY_A0E3Z8_PARTE/64-123	A0E3Z8.1 PF13921.4;	50.00	0.00
TRINITY_K3WI24_PYTUL/382-620	K3WI24.1 PF00112.21	50.00	0.00
TRINITY_A8J1W4_CHLRE/95-265	A8J1W4.1 PF01746.19	50.00	0.00
TRINITY_M1APL9_SOLTU/202-287	M1APL9.1 PF00439.23	50.00	0.00
TRINITY_D8RJ89_SELML/135-293	D8RJ89.1 PF01556.16	50.00	0.00
TRINITY_G8BCR4_CANPC/5-179	G8BCR4.1 PF00071.20	50.00	0.00

TRINITY_D8U424_VOLCA/106-437	D8U424.1 PF07093.9;	50.00	0.00
TRINITY_I7MCL9_TETTS/190-488	I7MCL9.2 PF00648.19	50.00	0.00
TRINITY_A0A078AXV4_STYLE/252-312	A0A078AXV4.1 PF1290	50.00	0.00
TRINITY_D8TTG5_VOLCA/143-328	D8TTG5.1 PF04802.13	50.00	0.00
TRINITY_D8TVM4_VOLCA/425-634	D8TVM4.1 PF06963.10	50.00	0.00
TRINITY_V4TJX0_9ROSI/39-395	V4TJX0.1 PF04185.12	50.00	0.00
TRINITY_ATAT_TETTS/4-178	Q22XZ3.2 PF05301.9;	50.00	0.00
TRINITY_I1FWW6_AMPQE/169-305	I1FWW6.1 PF08920.8;	50.00	0.00
TRINITY_W5K2H6_ASTMX/409-514	W5K2H6.1 PF00307.29	50.00	0.00
TRINITY_K3YSJ3_SETIT/132-320	K3YSJ3.1 PF16575.3;	50.00	0.00
TRINITY_B8N7G9_ASPFN/667-720	B8N7G9.1 PF13637.4;	50.00	0.00
TRINITY_A0A096TH44_MAIZE/1-160	A0A096TH44.1 PF1384	50.00	0.00
TRINITY_A9UTS2_MONBE/373-438	A9UTS2.1 PF00226.29	50.00	0.00
TRINITY_A0A077AYK7_9RICK/5-110	A0A077AYK7.1 PF0043	50.00	0.00
TRINITY_M0UB95_MUSAM/328-506	M0UB95.1 PF11931.6;	50.00	0.00
TRINITY_M7NRF1_PNEMU/1365-1713	M7NRF1.1 PF02259.21	50.00	0.00
TRINITY_A9THD7_PHYPA/5-80	A9THD7.1 PF02798.18	50.00	0.00
TRINITY_D8U5L8_VOLCA/41-437	D8U5L8.1 PF00856.26	50.00	0.00
TRINITY_H6N9S4_9BACL/1-342	H6N9S4.1 PF01494.17	50.00	0.00
TRINITY_D8UF31_VOLCA/562-668	D8UF31.1 PF01426.16	50.00	0.00
TRINITY_A0A074RNW8_9HOMO/1398-1514	A0A074RNW8.1 PF0066	50.00	0.00
TRINITY_L8GGC6_ACACA/243-303	L8GGC6.1 PF00571.26	50.00	0.00
TRINITY_T1J3Q6_STRMM/4-138	T1J3Q6.1 PF03874.14	50.00	0.00
TRINITY_G0QUL3_ICHMG/151-280	G0QUL3.1 PF00107.24	50.00	0.00
TRINITY_Q24FR8_TETTS/35-132	Q24FR8.2 PF12796.5;	50.00	0.00
TRINITY_A0CAP2_PARTE/154-305	A0CAP2.1 PF01529.18	50.00	0.00
TRINITY_A7RWL7_NEMVE/44-277	A7RWL7.1 PF02784.14	50.00	0.00
TRINITY_A0A0N4XQ29_NIPBR/39-119	A0A0N4XQ29.1 PF0940	50.00	0.00
TRINITY_I7M433_TETTS/1051-1168	I7M433.2 PF02518.24	50.00	0.00
TRINITY_A0A0J8BFP5_BETVU/98-161	A0A0J8BFP5.1 PF0007	50.00	0.00
TRINITY_U9UA20_RHIID/211-275	U9UA20.1 PF13432.4;	50.00	0.00
TRINITY_G0R363_ICHMG/11-273	G0R363.1 PF00069.23	50.00	0.00
TRINITY_K4DGX4_SOLLC/33-169	K4DGX4.1 PF00179.24	50.00	0.00
TRINITY_A0A066W870_9BASI/701-954	A0A066W870.1 PF0070	50.00	0.00
TRINITY_L8GNP7_ACACA/102-303	L8GNP7.1 PF00149.26	50.00	0.00
TRINITY_D7FKH8_ECTSI/433-566	D7FKH8.1 PF01841.17	50.00	0.00
TRINITY_A0A087SQX9_AUXPR/305-517	A0A087SQX9.1 PF0008	50.00	0.00
TRINITY_F0S119_DESTD/30-140	F0S119.1 PF01661.19	50.00	0.00
TRINITY_W4Z7S6_STRPU/13-77	W4Z7S6.1 PF13499.4;	50.00	0.00
TRINITY_A8I857_CHLRE/5-85	A8I857.1 PF12796.5;	50.00	0.00
TRINITY_L8GSG4_ACACA/468-550	L8GSG4.1 PF01833.22	50.00	0.00
TRINITY_G0QU83_ICHMG/195-287	G0QU83.1 PF00153.25	50.00	0.00
TRINITY_C0BXT1_9FIRM/482-599	C0BXT1.1 PF02518.24	50.00	0.00
TRINITY_C1FJI8_MICSR/203-318	C1FJI8.1 PF13640.4;	50.00	0.00
TRINITY_A8JGE5_CHLRE/63-221	A8JGE5.1 PF00156.25	50.00	0.00
TRINITY_G3PVU7_GASAC/50-255	G3PVU7.1 PF00106.23	50.00	0.00
TRINITY_A0A078AR09_STYLE/8-169	A0A078AR09.1 PF0007	50.00	0.00
TRINITY_D8TPN9_VOLCA/226-466	D8TPN9.1 PF05548.9;	50.00	0.00
TRINITY_L9JBY7_TUPCH/882-984	L9JBY7.1 PF00005.25	50.00	0.00
TRINITY_L8H6D1_ACACA/645-898	L8H6D1.1 PF07714.15	50.00	0.00
TRINITY_J9J3B4_9SPIT/124-338	J9J3B4.1 PF00112.21	50.00	0.00
TRINITY_M5WTV6_PRUPE/36-202	M5WTV6.1 PF04727.11	50.00	0.00
TRINITY_W7XA27_TETTS/236-568	W7XA27.1 PF12309.6;	50.00	0.00
TRINITY_B4MMR6_DROWI/16-333	B4MMR6.1 PF14647.4;	50.00	0.00
TRINITY_D2VGP7_NAEGR/9-261	D2VGP7.1 PF00069.23	50.00	0.00
TRINITY_F1NM51_CHICK/911-1180	F1NM51.2 PF00664.21	50.00	0.00
TRINITY_V7CQS1_PHAVU/168-234	V7CQS1.1 PF00191.18	50.00	0.00
TRINITY_D8TS65_VOLCA/33-205	D8TS65.1 PF04832.10	50.00	0.00
TRINITY_I7LU23_TETTS/37-151	I7LU23.1 PF11527.6;	50.00	0.00
TRINITY_W4HBE9_9STRA/262-331	W4HBE9.1 PF00076.20	50.00	0.00
TRINITY_T1KQA6_TETUR/1588-1665	T1KQA6.1 PF12861.5;	50.00	0.00

TRINITY_L8H6D1_ACACA/645-898	L8H6D1.1 PF07714.15	50.00	0.00
TRINITY_D8TZA0_VOLCA/605-695	D8TZA0.1 PF00211.18	50.00	0.00
TRINITY_I7MAP5_TETTS/532-581	I7MAP5.2 PF13920.4;	50.00	0.00
TRINITY_A8J230_CHLRE/50-257	A8J230.1 PF09353.8;	50.00	0.00
TRINITY_J9B569_WUCBA/23-82	J9B569.1 PF00400.30	50.00	0.00
TRINITY_D8U352_VOLCA/16-208	D8U352.1 PF02492.17	50.00	0.00
TRINITY_F1L5G4_ASCSU/10-99	F1L5G4.1 PF08662.9;	50.00	0.00
TRINITY_A8J3J1_CHLRE/3-109	A8J3J1.1 PF00416.20	50.00	0.00
TRINITY_Q2INW4_ANADE/41-163	Q2INW4.1 PF13456.4;	50.00	0.00
TRINITY_D8U7Q1_VOLCA/108-313	D8U7Q1.1 PF03798.14	50.00	0.00
TRINITY_A0A078A1C7_STYLE/8-93	A0A078A1C7.1 PF1341	50.00	0.00
TRINITY_I0YIM7_9CHLO/330-828	I0YIM7.1 PF00443.27	50.00	0.00
TRINITY_R1CHP5_EMIHU/6-380	R1CHP5.1 PF00266.17	50.00	0.00
TRINITY_L8HAX0_ACACA/6-175	L8HAX0.1 PF00590.18	50.00	0.00
TRINITY_L8HCS9_ACACA/5-130	L8HCS9.1 PF01417.18	50.00	0.00
TRINITY_F4Q375_DICFS/657-1119	F4Q375.1 PF12157.6;	50.00	0.00
TRINITY_M8A0N6_TRIUA/98-345	M8A0N6.1 PF13671.4;	50.00	0.00
TRINITY_I0YT53_9CHLO/313-453	I0YT53.1 PF02182.15	50.00	0.00
TRINITY_A0A0D2WIN4_CAPO3/469-518	A0A0D2WIN4.1 PF0041	50.00	0.00
TRINITY_A8I7E7_CHLRE/1-86	A8I7E7.1 PF08609.8;	50.00	0.00
TRINITY_A0A015JFG3_9GLOM/315-447	A0A015JFG3.1 PF0192	50.00	0.00
TRINITY_D8TJR4_VOLCA/308-429	D8TJR4.1 PF00892.18	50.00	0.00
TRINITY_A8I9T9_CHLRE/72-562	A8I9T9.1 PF03055.13	50.00	0.00
TRINITY_D8QWG6_SELML/2-124	D8QWG6.1 PF10644.7;	50.00	0.00
TRINITY_D7FLF8_ECTSI/553-621	D7FLF8.1 PF00505.17	50.00	0.00
TRINITY_B3ESW8_AMOA5/41-239	B3ESW8.1 PF01734.20	50.00	0.00
TRINITY_I7M280_TETTS/857-971	I7M280.2 PF09111.8;	50.00	0.00
TRINITY_C1MH29_MICPC/526-828	C1MH29.1 PF00069.23	50.00	0.00
TRINITY_A8IZT5_CHLRE/7-125	A8IZT5.1 PF00588.17	50.00	0.00
TRINITY_G0R2T0_ICHMG/33-283	G0R2T0.1 PF00069.23	50.00	0.00
TRINITY_M1VCY5_CYAME/316-387	M1VCY5.1 PF02207.18	50.00	0.00
TRINITY_A0A015IAE9_9GLOM/273-356	A0A015IAE9.1 PF1324	50.00	0.00
TRINITY_L8GPY7_ACACA/4-112	L8GPY7.1 PF03694.11	50.00	0.00
TRINITY_A8XL17_CAEBR/353-625	A8XL17.2 PF07714.15	50.00	0.00
TRINITY_D8UAS3_VOLCA/97-295	D8UAS3.1 PF03942.13	50.00	0.00
TRINITY_T1IZ26_STRMM/430-565	T1IZ26.1 PF00005.25	50.00	0.00
TRINITY_A8IPQ9_CHLRE/171-429	A8IPQ9.1 PF00069.23	50.00	0.00
TRINITY_F0ZW58_DICPU/38-145	F0ZW58.1 PF00307.29	50.00	0.00
TRINITY_A0A0G4J2X3_PLABS/751-865	A0A0G4J2X3.1 PF0066	50.00	0.00
TRINITY_E8RT56_ASTEC/1-240	E8RT56.1 PF03883.12	50.00	0.00
TRINITY_Q22RZ8_TETTS/183-242	Q22RZ8.2 PF13921.4;	50.00	0.00
TRINITY_A8IWD5_CHLRE/101-179	A8IWD5.1 PF05239.14	50.00	0.00
TRINITY_Q23YR9_TETTS/7-439	Q23YR9.1 PF00171.20	50.00	0.00
TRINITY_R1DK67_EMIHU/4-226	R1DK67.1 PF00664.21	50.00	0.00
TRINITY_A0A0D2X385_CAPO3/82-162	A0A0D2X385.1 PF0336	50.00	0.00
TRINITY_G0W4E6_NAUDC/21-89	G0W4E6.1 PF00505.17	50.00	0.00
TRINITY_Q24FJ8_TETTS/86-365	Q24FJ8.2 PF00122.18	50.00	0.00
TRINITY_W6V2S5_ECHGR/32-94	W6V2S5.1 PF01176.17	50.00	0.00
TRINITY_W4FSJ9_9STRA/1228-1335	W4FSJ9.1 PF00005.25	50.00	0.00
TRINITY_K9XSF6_STAC7/476-617	K9XSF6.1 PF02518.24	50.00	0.00
TRINITY_L1JDL6_GUITH/44-269	L1JDL6.1 PF07556.9;	50.00	0.00
TRINITY_A0BIT4_PARTE/126-209	A0BIT4.1 PF01529.18	50.00	0.00
TRINITY_B3S125_TRIAD/544-612	B3S125.1 PF00658.16	50.00	0.00
TRINITY_D6WPU4_TRICA/23-169	D6WPU4.1 PF05608.10	50.00	0.00
TRINITY_C1E5P2_MICSR/25-127	C1E5P2.1 PF08216.9;	50.00	0.00
TRINITY_I0Z7Q3_9CHLO/405-952	I0Z7Q3.1 PF02460.16	50.00	0.00
TRINITY_C1EGU1_MICSR/59-165	C1EGU1.1 PF00504.19	50.00	0.00
TRINITY_K1QIC1_CRAGI/13-50	K1QIC1.1 PF01753.16	50.00	0.00
TRINITY_L8HCA8_ACACA/4208-4473	L8HCA8.1 PF03028.13	50.00	0.00
TRINITY_Q608D7_METCA/190-307	Q608D7.1 PF00581.18	50.00	0.00
TRINITY_C7N732_SLAHD/37-147	C7N732.1 PF08487.8;	50.00	0.00

TRINITY_A9TH59_PHYPA/211-260	A9TH59.1 PF00415.16	50.00	0.00
TRINITY_A0A096P8D0_OSTTA/436-552	A0A096P8D0.1 PF0237	50.00	0.00
TRINITY_A8J1H0_CHLRE/65-495	A8J1H0.1 PF00909.19	50.00	0.00
TRINITY_I3J6Q2_ORENI/316-532	I3J6Q2.1 PF07002.14	50.00	0.00
TRINITY_A0A087XHP4_POEFO/512-659	A0A087XHP4.2 PF0911	50.00	0.00
TRINITY_D8TTP0_VOLCA/1124-1422	D8TTP0.1 PF00069.23	50.00	0.00
TRINITY_A8HZH0_CHLRE/103-256	A8HZH0.1 PF03547.16	50.00	0.00
TRINITY_A0DXZ6_PARTE/485-602	A0DXZ6.1 PF02518.24	50.00	0.00
TRINITY_A0A0B2V3L2_TOXCA/185-251	A0A0B2V3L2.1 PF0007	50.00	0.00
TRINITY_C1EFF0_MICSR/32-123	C1EFF0.1 PF13499.4;	50.00	0.00
TRINITY_D8TN82_VOLCA/692-904	D8TN82.1 PF12931.5;	50.00	0.00
TRINITY_D2A369_TRICA/29-113	D2A369.1 PF13417.4;	50.00	0.00
TRINITY_M4EG11_BRARP/102-377	M4EG11.1 PF09243.8;	50.00	0.00
TRINITY_K1RR22_CRAGI/43-122	K1RR22.1 PF07019.10	50.00	0.00
TRINITY_C4LUZ1_ENTHI/462-722	C4LUZ1.1 PF00069.23	50.00	0.00
TRINITY_E9GBA1_DAPPU/1-214	E9GBA1.1 PF00135.26	50.00	0.00
TRINITY_Q22C39_TETTS/292-540	Q22C39.1 PF02535.20	50.00	0.00
TRINITY_D8TTP0_VOLCA/1124-1422	D8TTP0.1 PF00069.23	50.00	0.00
TRINITY_A0A087SGK8_AUXPR/9-98	A0A087SGK8.1 PF0408	50.00	0.00
TRINITY_D8TZ90_VOLCA/77-184	D8TZ90.1 PF00583.23	50.00	0.00
TRINITY_I7LTQ5_TETTS/169-330	I7LTQ5.1 PF01331.17	50.00	0.00
TRINITY_A8JHV3_CHLRE/30-234	A8JHV3.1 PF00145.15	50.00	0.00
TRINITY_D8UJD9_VOLCA/37-534	D8UJD9.1 PF00118.22	50.00	0.00
TRINITY_D8THW0_VOLCA/1490-1598	D8THW0.1 PF08372.8;	50.00	0.00
TRINITY_A8I1M3_CHLRE/18-152	A8I1M3.1 PF12697.5;	50.00	0.00
TRINITY_A0BKI4_PARTE/27-441	A0BKI4.1 PF07690.14	50.00	0.00
TRINITY_I7M3E0_TETTS/62-213	I7M3E0.2 PF12146.6;	50.00	0.00
TRINITY_A8X9V3_CAEBR/235-307	A8X9V3.2 PF02201.16	50.00	0.00
TRINITY_Q236Z5_TETTS/302-587	Q236Z5.1 PF05206.12	50.00	0.00
TRINITY_W9VIF1_9EURO/36-188	W9VIF1.1 PF14234.4;	50.00	0.00
TRINITY_F2U5U5_SALR5/99-464	F2U5U5.1 PF00295.15	49.90	0.00
TRINITY_A8HP11_CHLRE/1-288	A8HP11.1 PF03969.14	49.90	0.00
TRINITY_E1Z539_CHLVA/9-250	E1Z539.1 PF00149.26	49.80	0.00
TRINITY_J5WTF5_9FIRM/120-356	J5WTF5.1 PF00078.25	49.80	0.00
TRINITY_F4Q4A3_DICFS/16-269	F4Q4A3.1 PF00069.23	49.80	0.00
TRINITY_A8HWI9_CHLRE/348-571	A8HWI9.1 PF07714.15	49.80	0.00
TRINITY_L8H188_ACACA/100-395	L8H188.1 PF06472.13	49.80	0.00
TRINITY_K4D2K3_SOLLC/87-277	K4D2K3.1 PF13419.4;	49.80	0.00
TRINITY_E1ZP80_CHLVA/225-428	E1ZP80.1 PF06733.13	49.80	0.00
TRINITY_D8TTV8_VOLCA/81-324	D8TTV8.1 PF00233.17	49.80	0.00
TRINITY_A8JDM5_CHLRE/862-1055	A8JDM5.1 PF07162.9;	49.80	0.00
TRINITY_A0DKQ5_PARTE/78-362	A0DKQ5.1 PF00069.23	49.80	0.00
TRINITY_A4VD54_TETTS/744-1018	A4VD54.1 PF00664.21	49.80	0.00
TRINITY_Q172U1_AEDAE/112-338	Q172U1.1 PF00271.29	49.80	0.00
TRINITY_L8HCT6_ACACA/45-631	L8HCT6.1 PF00888.20	49.80	0.00
TRINITY_D8TTN7_VOLCA/70-275	D8TTN7.1 PF00856.26	49.80	0.00
TRINITY_M0TWI2_MUSAM/36-556	M0TWI2.1 PF03169.13	49.80	0.00
TRINITY_D8U7B9_VOLCA/953-1207	D8U7B9.1 PF16212.3;	49.80	0.00
TRINITY_C1FHA9_MICSR/36-219	C1FHA9.1 PF02146.15	49.80	0.00
TRINITY_L0DAL9_SINAD/21-273	L0DAL9.1 PF00109.24	49.80	0.00
TRINITY_D8U6M0_VOLCA/747-984	D8U6M0.1 PF16212.3;	49.80	0.00
TRINITY_Q3SDP1_PARTE/8-169	Q3SDP1.1 PF00071.20	49.70	0.00
TRINITY_Q609M8_METCA/632-748	Q609M8.1 PF02518.24	49.70	0.00
TRINITY_G0QLZ6_ICHMG/5-338	G0QLZ6.1 PF03690.11	49.70	0.00
TRINITY_I1G6P3_AMPQE/29-327	I1G6P3.1 PF00850.17	49.70	0.00
TRINITY_A0EG50_PARTE/304-525	A0EG50.1 PF07002.14	49.70	0.00
TRINITY_E9FYH7_DAPPU/1-169	E9FYH7.1 PF02776.16	49.70	0.00
TRINITY_F4PXD0_DICFS/13-162	F4PXD0.1 PF01399.25	49.70	0.00
TRINITY_A0A0D2U7U3_CAPO3/12-174	A0A0D2U7U3.1 PF0007	49.70	0.00
TRINITY_D8TI68_VOLCA/292-458	D8TI68.1 PF00069.23	49.70	0.00
TRINITY_G0QVA6_ICHMG/11-162	G0QVA6.1 PF00179.24	49.70	0.00

TRINITY_A8HSL4_CHLRE/2830-3140	A8HSL4.1 PF00454.25	49.70	0.00
TRINITY_H8KQX5_SOLCM/9-302	H8KQX5.1 PF02274.15	49.70	0.00
TRINITY_A0A087SCV5_AUXPR/231-497	A0A087SCV5.1 PF0935	49.70	0.00
TRINITY_V4AI91_LOTGI/63-217	V4AI91.1 PF01694.20	49.70	0.00
TRINITY_I0Z7U3_9CHLO/233-569	I0Z7U3.1 PF00176.21	49.70	0.00
TRINITY_Q3SDB5_PARTE/105-403	Q3SDB5.1 PF00122.18	49.70	0.00
TRINITY_D8SW38_SELML/1-179	D8SW38.1 PF07466.9;	49.70	0.00
TRINITY_L8H3P8_ACACA/9-435	L8H3P8.1 PF00171.20	49.70	0.00
TRINITY_D3B4H7_POLPA/29-194	D3B4H7.1 PF12385.6;	49.70	0.00
TRINITY_D8UEY3_VOLCA/15-140	D8UEY3.1 PF10513.7;	49.70	0.00
TRINITY_R6GQG5_9CLOT/72-275	R6GQG5.1 PF06745.11	49.70	0.00
TRINITY_A0A0D2X138_CAPO3/11-169	A0A0D2X138.1 PF0007	49.70	0.00
TRINITY_A0A078HL68_BRANA/612-794	A0A078HL68.1 PF0136	49.70	0.00
TRINITY_A8J745_CHLRE/6-189	A8J745.1 PF00211.18	49.70	0.00
TRINITY_A8IZ20_CHLRE/174-481	A8IZ20.1 PF08495.8;	49.70	0.00
TRINITY_I0YTV7_9CHLO/367-731	I0YTV7.1 PF00702.24	49.70	0.00
TRINITY_D8TV10_VOLCA/114-261	D8TV10.1 PF04136.13	49.70	0.00
TRINITY_A9G1A8_SORC5/25-391	A9G1A8.1 PF01433.18	49.70	0.00
TRINITY_A8HMW8_CHLRE/212-403	A8HMW8.1 PF17047.3;	49.70	0.00
TRINITY_D8TJZ3_VOLCA/218-459	D8TJZ3.1 PF00294.22	49.70	0.00
TRINITY_E1ZP54_CHLVA/45-185	E1ZP54.1 PF01966.20	49.70	0.00
TRINITY_I0Z1M1_9CHLO/82-282	I0Z1M1.1 PF00270.27	49.70	0.00
TRINITY_F4PHH2_DICFS/476-791	F4PHH2.1 PF08637.8;	49.60	0.00
TRINITY_I7MFB3_TETTS/236-482	I7MFB3.1 PF02913.17	49.60	0.00
TRINITY_N6TAE8_DENPD/812-987	N6TAE8.1 PF12295.6;	49.60	0.00
TRINITY_G0QKR3_ICHMG/42-350	G0QKR3.1 PF14664.4;	49.60	0.00
TRINITY_Q22AR0_TETTS/17-145	Q22AR0.1 PF13529.4;	49.60	0.00
TRINITY_C4M7N9_ENTHI/808-1067	C4M7N9.1 PF00069.23	49.60	0.00
TRINITY_J0DZ08_LOALO/6-305	J0DZ08.1 PF06682.10	49.60	0.00
TRINITY_TPC2L_DICDI/9-139	Q54CU7.1 PF04628.11	49.60	0.00
TRINITY_Q23QF2_TETTS/248-571	Q23QF2.2 PF03133.13	49.60	0.00
TRINITY_A9V8Y7_MONBE/333-442	A9V8Y7.1 PF00271.29	49.60	0.00
TRINITY_Q22W57_TETTS/127-261	Q22W57.2 PF01641.16	49.60	0.00
TRINITY_G8BJW8_CANPC/6-167	G8BJW8.1 PF00071.20	49.60	0.00
TRINITY_A0A059C0G5_EUCGR/25-162	A0A059C0G5.1 PF0590	49.60	0.00
TRINITY_Q099X4_STIAD/16-298	Q099X4.1 PF00732.17	49.60	0.00
TRINITY_H2URE4_TAKRU/5-121	H2URE4.1 PF00466.18	49.60	0.00
TRINITY_I7LXR4_TETTS/82-199	I7LXR4.1 PF01873.15	49.60	0.00
TRINITY_W7B190_PLAVN/374-631	W7B190.1 PF00069.23	49.60	0.00
TRINITY_I0Z8H2_9CHLO/8-139	I0Z8H2.1 PF03435.16	49.60	0.00
TRINITY_J9IKC1_9SPIT/18-253	J9IKC1.1 PF13561.4;	49.60	0.00
TRINITY_A0A0D2UAY5_CAPO3/55-401	A0A0D2UAY5.1 PF1649	49.60	0.00
TRINITY_Q23K51_TETTS/27-150	Q23K51.2 PF02996.15	49.60	0.00
TRINITY_A0A0M9DW34_9BACT/11-135	A0A0M9DW34.1 PF0058	49.60	0.00
TRINITY_Q23QF2_TETTS/248-571	Q23QF2.2 PF03133.13	49.60	0.00
TRINITY_J9IJQ5_9SPIT/433-601	J9IJQ5.1 PF13561.4;	49.60	0.00
TRINITY_J9IJQ5_9SPIT/433-601	J9IJQ5.1 PF13561.4;	49.60	0.00
TRINITY_A0A015LT99_9GLOM/148-280	A0A015LT99.1 PF0346	49.60	0.00
TRINITY_Q22AQ4_TETTS/550-689	Q22AQ4.1 PF01529.18	49.60	0.00
TRINITY_A0A022RC66_ERYGU/150-384	A0A022RC66.1 PF1362	49.60	0.00
TRINITY_D8UDF3_VOLCA/894-1029	D8UDF3.1 PF13472.4;	49.60	0.00
TRINITY_A0A059LQL1_9CHLO/239-355	A0A059LQL1.1 PF0063	49.60	0.00
TRINITY_I0Z109_9CHLO/256-889	I0Z109.1 PF03343.11	49.60	0.00
TRINITY_Q9RYT7_DEIRA/27-166	Q9RYT7.1 PF09346.8;	49.60	0.00
TRINITY_H2U929_TAKRU/117-405	H2U929.1 PF06079.9;	49.60	0.00
TRINITY_A0A095C3A6_CRYGA/90-202	A0A095C3A6.1 PF1613	49.60	0.00
TRINITY_A0A078A3V7_STYLE/126-338	A0A078A3V7.1 PF0011	49.60	0.00
TRINITY_H3FY79_PRIPA/9-158	H3FY79.1 PF10558.7;	49.60	0.00
TRINITY_A0CQE0_PARTE/146-402	A0CQE0.1 PF00069.23	49.60	0.00
TRINITY_A0A075ANW3_9FUNG/209-660	A0A075ANW3.1 PF0143	49.60	0.00
TRINITY_C3ZL63_BRAFL/350-687	C3ZL63.1 PF06602.12	49.60	0.00

TRINITY_I0ZAP1_9CHLO/34-522	I0ZAP1.1 PF03372.21	49.60	0.00
TRINITY_RIFK_SCHPO/20-147	O74866.1 PF01687.15	49.60	0.00
TRINITY_A0A058Z5C4_9EUKA/106-248	A0A058Z5C4.1 PF0169	49.60	0.00
TRINITY_I7M4R2_TETTS/53-317	I7M4R2.1 PF10343.7;	49.60	0.00
TRINITY_A8HQ35_CHLRE/54-313	A8HQ35.1 PF07714.15	49.60	0.00
TRINITY_A0A0E0AJL1_9ORYZ/253-495	A0A0E0AJL1.1 PF0771	49.60	0.00
TRINITY_G0QVR2_ICHMG/491-907	G0QVR2.1 PF02460.16	49.60	0.00
TRINITY_K3W7P3_PYTUL/115-303	K3W7P3.1 PF09756.7;	49.60	0.00
TRINITY_A8J8I4_CHLRE/47-327	A8J8I4.1 PF00069.23	49.60	0.00
TRINITY_D8TNW0_VOLCA/690-893	D8TNW0.1 PF09787.7;	49.60	0.00
TRINITY_D2A543_TRICA/466-585	D2A543.1 PF00665.24	49.60	0.00
TRINITY_D8TNY3_VOLCA/1-151	D8TNY3.1 PF01878.16	49.60	0.00
TRINITY_A8J4X1_CHLRE/9-288	A8J4X1.1 PF03643.13	49.60	0.00
TRINITY_Q22W19_TETTS/115-323	Q22W19.3 PF00112.21	49.60	0.00
TRINITY_E1ZGK0_CHLVA/160-290	E1ZGK0.1 PF00294.22	49.60	0.00
TRINITY_V4KQ72_EUTSA/102-349	V4KQ72.1 PF12697.5;	49.60	0.00
TRINITY_A0A059LRD4_9CHLO/607-765	A0A059LRD4.1 PF0000	49.50	0.00
TRINITY_A0C3F0_PARTE/8-164	A0C3F0.1 PF07162.9;	49.50	0.00
TRINITY_A0A0A1U1N4_ENTIV/1327-1589	A0A0A1U1N4.1 PF0006	49.50	0.00
TRINITY_F1MYX5_BOVIN/515-625	F1MYX5.1 PF00307.29	49.50	0.00
TRINITY_Q23CY8_TETTS/56-353	Q23CY8.2 PF00480.18	49.50	0.00
TRINITY_D3AW94_POLPA/29-216	D3AW94.1 PF02589.13	49.50	0.00
TRINITY_D8TSZ7_VOLCA/1690-1975	D8TSZ7.1 PF07714.15	49.50	0.00
TRINITY_A0CTS6_PARTE/568-858	A0CTS6.1 PF01237.16	49.50	0.00
TRINITY_A0A0M0JGU0_9EUKA/93-271	A0A0M0JGU0.1 PF0024	49.50	0.00
TRINITY_D3BUX6_POLPA/12-324	D3BUX6.1 PF01218.16	49.50	0.00
TRINITY_Q23DC1_TETTS/461-1153	Q23DC1.2 PF00443.27	49.50	0.00
TRINITY_K4D228_SOLLC/12-104	K4D228.1 PF02214.20	49.50	0.00
TRINITY_L8GTK1_ACACA/146-395	L8GTK1.1 PF07714.15	49.50	0.00
TRINITY_F4Q2J6_DICFS/31-137	F4Q2J6.1 PF00307.29	49.50	0.00
TRINITY_Q3SDI0_PARTE/17-177	Q3SDI0.1 PF00071.20	49.50	0.00
TRINITY_E1Z9Z4_CHLVA/12-204	E1Z9Z4.1 PF03357.19	49.50	0.00
TRINITY_SPKA_DICDI/351-612	Q86AT8.1 PF07714.15	49.50	0.00
TRINITY_A8IRV7_CHLRE/54-272	A8IRV7.1 PF11833.6;	49.50	0.00
TRINITY_A8HWI9_CHLRE/348-571	A8HWI9.1 PF07714.15	49.50	0.00
TRINITY_U6HMV9_ECHMU/75-215	U6HMV9.1 PF00561.18	49.50	0.00
TRINITY_A0CKP0_PARTE/8-263	A0CKP0.1 PF00069.23	49.50	0.00
TRINITY_F0ZH73_DICPU/405-577	F0ZH73.1 PF00621.18	49.50	0.00
TRINITY_A0A0L0BN69_LUCCU/264-405	A0A0L0BN69.1 PF0116	49.50	0.00
TRINITY_D3BJA2_POLPA/20-171	D3BJA2.1 PF14580.4;	49.50	0.00
TRINITY_L8GIW0_ACACA/41-462	L8GIW0.1 PF06824.9;	49.50	0.00
TRINITY_E1ZAE1_CHLVA/18-540	E1ZAE1.1 PF12832.5;	49.50	0.00
TRINITY_I0Z2G1_9CHLO/80-263	I0Z2G1.1 PF01789.14	49.50	0.00
TRINITY_Q22WK1_TETTS/72-180	Q22WK1.2 PF00787.22	49.50	0.00
TRINITY_D7TXF1_VITVI/16-120	D7TXF1.1 PF01920.18	49.50	0.00
TRINITY_C1EEA0_MICSR/148-339	C1EEA0.1 PF13532.4;	49.50	0.00
TRINITY_E5S262_TRISP/8-109	E5S262.1 PF00568.21	49.50	0.00
TRINITY_K1RML1_CRAGI/4-288	K1RML1.1 PF00069.23	49.50	0.00
TRINITY_Q231T5_TETTS/15-118	Q231T5.1 PF03645.11	49.50	0.00
TRINITY_G0VKB1_NAUCC/226-413	G0VKB1.1 PF00300.20	49.50	0.00
TRINITY_M3XGQ6_LATCH/1-113	M3XGQ6.1 PF00568.21	49.50	0.00
TRINITY_ADK_DICDI/23-336	Q54MB5.2 PF00294.22	49.50	0.00
TRINITY_L8GQZ4_ACACA/23-685	L8GQZ4.1 PF07093.9;	49.50	0.00
TRINITY_D8U7C7_VOLCA/52-577	D8U7C7.1 PF03055.13	49.50	0.00
TRINITY_L8GUJ1_ACACA/207-501	L8GUJ1.1 PF04515.10	49.50	0.00
TRINITY_A8IBX2_CHLRE/464-568	A8IBX2.1 PF13426.5;	49.50	0.00
TRINITY_A8IYS4_CHLRE/7-144	A8IYS4.1 PF15011.4;	49.50	0.00
TRINITY_A0A072VKI5_MEDTR/502-835	A0A072VKI5.1 PF0012	49.50	0.00
TRINITY_D8TTG8_VOLCA/39-316	D8TTG8.1 PF00294.22	49.50	0.00
TRINITY_D3B5S9_POLPA/11-170	D3B5S9.1 PF03986.11	49.50	0.00
TRINITY_A8JHT1_CHLRE/167-417	A8JHT1.1 PF16908.3;	49.50	0.00

TRINITY_A1ZQ27_9BACT/9-271	A1ZQ27.1 PF00378.18	49.50	0.00
TRINITY_E1ZEK6_CHLVA/4-111	E1ZEK6.1 PF00300.20	49.50	0.00
TRINITY_C6W3F1_DYAFD/7-229	C6W3F1.1 PF00106.23	49.50	0.00
TRINITY_Q22T22_TETTS/520-735	Q22T22.3 PF07859.11	49.50	0.00
TRINITY_Q23JD9_TETTS/24-552	Q23JD9.3 PF01602.18	49.50	0.00
TRINITY_B8I057_CLOCE/6-188	B8I057.1 PF01195.17	49.50	0.00
TRINITY_Q24GE4_TETTS/399-611	Q24GE4.3 PF00644.18	49.50	0.00
TRINITY_Q22NP9_TETTS/339-441	Q22NP9.2 PF02204.16	49.50	0.00
TRINITY_W7X292_TETTS/58-150	W7X292.1 PF16455.3;	49.50	0.00
TRINITY_I0Z522_9CHLO/13-186	I0Z522.1 PF01925.17	49.50	0.00
TRINITY_B0DSH5_LACBS/234-393	B0DSH5.1 PF01399.25	49.50	0.00
TRINITY_A0A059B6L2_EUCGR/387-543	A0A059B6L2.1 PF0398	49.50	0.00
TRINITY_D2V9V9_NAEGR/18-190	D2V9V9.1 PF00929.22	49.50	0.00
TRINITY_I7M2H5_TETTS/224-317	I7M2H5.1 PF00153.25	49.50	0.00
TRINITY_E1Z994_CHLVA/147-412	E1Z994.1 PF05116.11	49.50	0.00
TRINITY_A8IP12_CHLRE/72-509	A8IP12.1 PF01490.16	49.50	0.00
TRINITY_I7MMY6_TETTS/639-738	I7MMY6.2 PF00989.23	49.50	0.00
TRINITY_A8IR95_CHLRE/401-503	A8IR95.1 PF13426.5;	49.50	0.00
TRINITY_A8IYT2_CHLRE/40-133	A8IYT2.1 PF12894.5;	49.50	0.00
TRINITY_D8TTV8_VOLCA/81-324	D8TTV8.1 PF00233.17	49.50	0.00
TRINITY_Q22S90_TETTS/208-560	Q22S90.1 PF05028.12	49.50	0.00
TRINITY_A0A024T955_9STRA/43-204	A0A024T955.1 PF0007	49.40	0.00
TRINITY_J9FBW6_9SPIT/36-484	J9FBW6.1 PF05577.10	49.40	0.00
TRINITY_Q24E55_TETTS/8-179	Q24E55.1 PF00071.20	49.40	0.00
TRINITY_I7MIE2_TETTS/246-724	I7MIE2.2 PF01055.24	49.40	0.00
TRINITY_A0E388_PARTE/154-420	A0E388.1 PF08014.9;	49.40	0.00
TRINITY_A0A0A1MPB6_9BACI/27-455	A0A0A1MPB6.1 PF0142	49.40	0.00
TRINITY_I7LXL4_TETTS/220-317	I7LXL4.1 PF00153.25	49.40	0.00
TRINITY_T1L3G8_TETUR/55-191	T1L3G8.1 PF07534.14	49.40	0.00
TRINITY_A0A022R0V6_ERYGU/11-123	A0A022R0V6.1 PF0233	49.40	0.00
TRINITY_F6Q9D5_CIOIN/58-324	F6Q9D5.2 PF00149.26	49.40	0.00
TRINITY_L8GVF1_ACACA/95-436	L8GVF1.1 PF01244.19	49.40	0.00
TRINITY_D3BRJ2_POLPA/219-314	D3BRJ2.1 PF00153.25	49.40	0.00
TRINITY_G8BJW8_CANPC/6-167	G8BJW8.1 PF00071.20	49.40	0.00
TRINITY_H3CBK3_TETNG/53-477	H3CBK3.1 PF05577.10	49.40	0.00
TRINITY_J9I759_9SPIT/142-428	J9I759.1 PF00122.18	49.40	0.00
TRINITY_A0A090M864_OSTTA/61-459	A0A090M864.1 PF0588	49.40	0.00
TRINITY_A0D206_PARTE/25-225	A0D206.1 PF00106.23	49.40	0.00
TRINITY_A0A0N5DKI9_TRIMR/456-610	A0A0N5DKI9.1 PF0944	49.40	0.00
TRINITY_H2YP11_CIOSA/230-438	H2YP11.1 PF00005.25	49.40	0.00
TRINITY_W6FUH4_NODSP/15-183	W6FUH4.1 PF13385.4;	49.40	0.00
TRINITY_W6FUH4_NODSP/15-183	W6FUH4.1 PF13385.4;	49.40	0.00
TRINITY_A0A058ZAA1_9EUKA/30-111	A0A058ZAA1.1 PF0282	49.40	0.00
TRINITY_A0A0G4J2X3_PLABS/751-865	A0A0G4J2X3.1 PF0066	49.40	0.00
TRINITY_C6CWU9_PAESJ/29-254	C6CWU9.1 PF06452.9;	49.40	0.00
TRINITY_A0A078ADF6_STYLE/3865-4174	A0A078ADF6.1 PF0063	49.40	0.00
TRINITY_W2PR86_PHYPN/1-93	W2PR86.1 PF08544.11	49.40	0.00
TRINITY_V4CBN3_LOTGI/41-446	V4CBN3.1 PF03164.12	49.40	0.00
TRINITY_E1ZQ29_CHLVA/42-314	E1ZQ29.1 PF00069.23	49.40	0.00
TRINITY_F0ZM09_DICPU/17-351	F0ZM09.1 PF02733.15	49.40	0.00
TRINITY_G4THH4_PIRID/2049-2169	G4THH4.1 PF00665.24	49.40	0.00
TRINITY_DOT1L_DICDI/1230-1434	Q55AX2.2 PF08123.11	49.40	0.00
TRINITY_G0QXG1_ICHMG/263-484	G0QXG1.1 PF07002.14	49.40	0.00
TRINITY_A7SLJ0_NEMVE/4-288	A7SLJ0.1 PF00069.23	49.40	0.00
TRINITY_A8J3E8_CHLRE/4729-4882	A8J3E8.1 PF14309.4;	49.40	0.00
TRINITY_G0QZ92_ICHMG/19-335	G0QZ92.1 PF00069.23	49.40	0.00
TRINITY_V4AGX3_LOTGI/44-222	V4AGX3.1 PF01699.22	49.40	0.00
TRINITY_I7MEA2_TETTS/352-496	I7MEA2.1 PF00005.25	49.40	0.00
TRINITY_D8T7B3_SELML/63-181	D8T7B3.1 PF01926.21	49.40	0.00
TRINITY_NHAAB_MONBE/6-109	A9V2C1.1 PF02211.13	49.40	0.00
TRINITY_A0A078A3V7_STYLE/126-338	A0A078A3V7.1 PF0011	49.40	0.00

TRINITY_A3DPC2_STAMF/1-407	A3DPC2.1 PF00232.16	49.40	0.00
TRINITY_L8HIG4_ACACA/43-454	L8HIG4.1 PF01490.16	49.40	0.00
TRINITY_G0R1Y7_ICHMG/3-188	G0R1Y7.1 PF01251.16	49.40	0.00
TRINITY_V4AJZ4_LOTGI/51-222	V4AJZ4.1 PF14124.4;	49.40	0.00
TRINITY_I7M8M1_TETTS/1021-1109	I7M8M1.1 PF00679.22	49.40	0.00
TRINITY_G0QUA8_ICHMG/3047-3315	G0QUA8.1 PF00454.25	49.40	0.00
TRINITY_A0A087SJ86_AUXPR/163-245	A0A087SJ86.1 PF0306	49.40	0.00
TRINITY_A8J6W0_CHLRE/118-282	A8J6W0.1 PF04678.11	49.40	0.00
TRINITY_D8UDA7_VOLCA/1-149	D8UDA7.1 PF07714.15	49.40	0.00
TRINITY_Q0YR84_9CHLB/108-286	Q0YR84.1 PF07478.11	49.40	0.00
TRINITY_W4YW97_STRPU/1-101	W4YW97.1 PF00125.22	49.40	0.00
TRINITY_B9L4Y8_THERP/1-166	B9L4Y8.1 PF06559.9;	49.40	0.00
TRINITY_L8GFW8_ACACA/286-459	L8GFW8.1 PF00441.22	49.40	0.00
TRINITY_D8TJJ1_VOLCA/1356-1532	D8TJJ1.1 PF02141.19	49.40	0.00
TRINITY_A0A0D2UDJ0_CAPO3/193-604	A0A0D2UDJ0.1 PF0065	49.40	0.00
TRINITY_D8QSN2_SELML/263-400	D8QSN2.1 PF01544.16	49.40	0.00
TRINITY_A4VEL1_TETTS/1008-1157	A4VEL1.2 PF00005.25	49.40	0.00
TRINITY_G3PHL3_GASAC/294-434	G3PHL3.1 PF09112.8;	49.40	0.00
TRINITY_A9SKG6_PHYPA/692-776	A9SKG6.1 PF00637.18	49.40	0.00
TRINITY_D8TLV7_VOLCA/779-974	D8TLV7.1 PF00326.19	49.40	0.00
TRINITY_A8JFP7_CHLRE/157-424	A8JFP7.1 PF04187.11	49.40	0.00
TRINITY_A0E936_PARTE/243-406	A0E936.1 PF03031.16	49.40	0.00
TRINITY_A0A0G4GRG0_9ALVE/2-181	A0A0G4GRG0.1 PF0022	49.40	0.00
TRINITY_D8TP55_VOLCA/67-324	D8TP55.1 PF00233.17	49.40	0.00

TRINITY_I7MJ25_TETTS/230-402	I7MJ25.1 PF05193.19	49.40	0.00
TRINITY_D8TYQ9_VOLCA/13-319	D8TYQ9.1 PF02574.14	49.40	0.00
TRINITY_A0CMN6_PARTE/121-399	A0CMN6.1 PF01457.14	49.40	0.00
TRINITY_I0YRQ7_9CHLO/88-217	I0YRQ7.1 PF00293.26	49.40	0.00
TRINITY_F4PHF0_DICFS/606-854	F4PHF0.1 PF00481.19	49.40	0.00
TRINITY_M7C1Z6_CHEMY/168-264	M7C1Z6.1 PF12894.5;	49.40	0.00
TRINITY_A0A075AXN4_9FUNG/162-414	A0A075AXN4.1 PF0006	49.40	0.00
TRINITY_A0BJW5_PARTE/673-824	A0BJW5.1 PF00005.25	49.40	0.00
TRINITY_L8GSM0_ACACA/1518-1768	L8GSM0.1 PF07714.15	49.40	0.00
TRINITY_I0YUB8_9CHLO/105-257	I0YUB8.1 PF08389.10	49.40	0.00
TRINITY_A8IV18_CHLRE/452-536	A8IV18.1 PF00887.17	49.40	0.00
TRINITY_G0QU25_ICHMG/766-848	G0QU25.1 PF00635.24	49.40	0.00
TRINITY_D8UB11_VOLCA/330-583	D8UB11.1 PF09325.8;	49.40	0.00
TRINITY_D5GFX0_TUBMM/96-251	D5GFX0.1 PF13499.4;	49.40	0.00
TRINITY_Q4BVL1_CROWT/39-452	Q4BVL1.1 PF03092.14	49.40	0.00
TRINITY_G0QKJ2_ICHMG/22-183	G0QKJ2.1 PF00173.26	49.40	0.00
TRINITY_Q23DI7_TETTS/1868-2219	Q23DI7.2 PF00443.27	49.40	0.00
TRINITY_D8UJK8_VOLCA/363-743	D8UJK8.1 PF00702.24	49.40	0.00
TRINITY_L8HM05_ACACA/513-681	L8HM05.1 PF05729.10	49.40	0.00
TRINITY_F8ED73_RUNSL/2-93	F8ED73.1 PF03807.15	49.30	0.00
TRINITY_A0DGX8_PARTE/729-814	A0DGX8.1 PF00027.27	49.30	0.00
TRINITY_Q22BW3_TETTS/18-233	Q22BW3.2 PF02230.14	49.30	0.00
TRINITY_Q22LR2_TETTS/173-344	Q22LR2.3 PF00270.27	49.30	0.00
TRINITY_C3ZY00_BRAFL/336-556	C3ZY00.1 PF01734.20	49.30	0.00
TRINITY_E5WNT4_9BACI/8-148	E5WNT4.1 PF13302.5;	49.30	0.00
TRINITY_A0BG56_PARTE/51-241	A0BG56.1 PF00106.23	49.30	0.00
TRINITY_S2JQ85_MUCC1/13-150	S2JQ85.1 PF00334.17	49.30	0.00
TRINITY_Q22KK3_TETTS/1692-1803	Q22KK3.2 PF00307.29	49.30	0.00
TRINITY_A0A0G4J0S9_PLABS/5-178	A0A0G4J0S9.1 PF0007	49.30	0.00
TRINITY_A5E688_LODEL/12-84	A5E688.1 PF00173.26	49.30	0.00
TRINITY_Q86KA1_DICDI/114-182	Q86KA1.1 PF00542.17	49.30	0.00
TRINITY_I1FE46_AMPQE/127-192	I1FE46.1 PF07258.12	49.30	0.00
TRINITY_Q22EC6_TETTS/68-359	Q22EC6.2 PF04258.11	49.30	0.00
TRINITY_E0VFC0_PEDHC/42-205	E0VFC0.1 PF01652.16	49.30	0.00
TRINITY_B4KIM7_DROMO/984-1256	B4KIM7.2 PF00664.21	49.30	0.00
TRINITY_D7FM24_ECTSI/20-195	D7FM24.1 PF02417.13	49.30	0.00
TRINITY_Q23AY4_TETTS/31-436	Q23AY4.2 PF05577.10	49.30	0.00
TRINITY_G4YQJ5_PHYSP/58-315	G4YQJ5.1 PF02636.15	49.30	0.00
TRINITY_W7XK44_TETTS/3-75	W7XK44.1 PF02798.18	49.30	0.00
TRINITY_A0A072U2E0_MEDTR/103-307	A0A072U2E0.1 PF0048	49.30	0.00
TRINITY_C3ZB99_BRAFL/56-119	C3ZB99.1 PF04818.11	49.30	0.00
TRINITY_L1JMZ5_GUITH/357-427	L1JMZ5.1 PF00076.20	49.30	0.00
TRINITY_A8J1L7_CHLRE/28-111	A8J1L7.1 PF12796.5;	49.30	0.00
TRINITY_A7RRM0_NEMVE/31-173	A7RRM0.1 PF00102.25	49.30	0.00
TRINITY_A7SKD9_NEMVE/156-246	A7SKD9.1 PF12894.5;	49.30	0.00
TRINITY_A8NF86_COPC7/232-300	A8NF86.1 PF00076.20	49.30	0.00
TRINITY_L8GUR2_ACACA/284-351	L8GUR2.1 PF02207.18	49.30	0.00
TRINITY_L8GH74_ACACA/214-289	L8GH74.1 PF00533.24	49.30	0.00
TRINITY_G0QK40_ICHMG/27-393	G0QK40.1 PF07690.14	49.30	0.00
TRINITY_Q24DE0_TETTS/57-384	Q24DE0.2 PF04137.13	49.30	0.00
TRINITY_U9T1H6_RHIID/130-200	U9T1H6.1 PF00076.20	49.30	0.00
TRINITY_G0QNE0_ICHMG/208-288	G0QNE0.1 PF05406.13	49.30	0.00
TRINITY_I7M912_TETTS/429-502	I7M912.1 PF13424.4;	49.30	0.00
TRINITY_A0DUA5_PARTE/170-239	A0DUA5.1 PF14360.4;	49.30	0.00
TRINITY_G5EDF9_CAEEL/417-566	G5EDF9.1 PF00005.25	49.30	0.00
TRINITY_I0Z0B5_9CHLO/6-78	I0Z0B5.1 PF00240.21	49.30	0.00
TRINITY_F4Q1E4_DICFS/225-294	F4Q1E4.1 PF02825.18	49.30	0.00
TRINITY_U5D852_AMBTC/4-79	U5D852.1 PF00808.21	49.30	0.00
TRINITY_A8JCA4_CHLRE/66-273	A8JCA4.1 PF00566.16	49.30	0.00
TRINITY_A0A0E3UVI8_9BACT/37-178	A0A0E3UVI8.1 PF0821	49.30	0.00
TRINITY_L7U9F0_MYXSD/3-287	L7U9F0.1 PF01156.17	49.30	0.00

TRINITY_D8TTV8_VOLCA/81-324	D8TTV8.1 PF00233.17	49.30	0.00
TRINITY_M5CDH7_THACB/489-602	M5CDH7.1 PF00665.24	49.30	0.00
TRINITY_I0YUZ3_9CHLO/2-74	I0YUZ3.1 PF11571.6;	49.30	0.00
TRINITY_E1ZDW0_CHLVA/527-597	E1ZDW0.1 PF00575.21	49.30	0.00
TRINITY_A0A066WFU6_9BASI/3-74	A0A066WFU6.1 PF0024	49.30	0.00
TRINITY_I1FQJ7_AMPQE/21-96	I1FQJ7.1 PF15630.4;	49.30	0.00
TRINITY_D8TTX8_VOLCA/4-119	D8TTX8.1 PF01412.16	49.30	0.00
TRINITY_F4Q8Q1_DICFS/32-805	F4Q8Q1.1 PF01496.17	49.30	0.00
TRINITY_K5VBV7_PHACS/9-130	K5VBV7.1 PF01873.15	49.30	0.00
TRINITY_B3E6I6_GEOLS/27-171	B3E6I6.1 PF08240.10	49.30	0.00
TRINITY_W7X4T7_TETTS/1-455	W7X4T7.1 PF00171.20	49.30	0.00
TRINITY_L8GVS0_ACACA/731-1032	L8GVS0.1 PF03133.13	49.30	0.00
TRINITY_A0A078DNT7_BRANA/370-586	A0A078DNT7.1 PF0500	49.30	0.00
TRINITY_U5HHP4_USTV1/52-122	U5HHP4.1 PF00076.20	49.30	0.00
TRINITY_D8UBB0_VOLCA/187-259	D8UBB0.1 PF00076.20	49.30	0.00
TRINITY_E1Z493_CHLVA/55-440	E1Z493.1 PF00501.26	49.30	0.00
TRINITY_TPPC4_DICDI/3-134	Q54UU1.1 PF04099.10	49.30	0.00
TRINITY_D8TR80_VOLCA/432-709	D8TR80.1 PF02854.17	49.30	0.00
TRINITY_A8HNJ6_CHLRE/92-232	A8HNJ6.1 PF03981.10	49.30	0.00
TRINITY_H2YA27_CIOSA/108-177	H2YA27.1 PF00076.20	49.30	0.00
TRINITY_J9HLW6_9SPIT/39-360	J9HLW6.1 PF00022.17	49.30	0.00
TRINITY_G0R3J1_ICHMG/14-292	G0R3J1.1 PF07993.10	49.30	0.00
TRINITY_A0DG99_PARTE/634-720	A0DG99.1 PF00027.27	49.20	0.00
TRINITY_A0D5U2_PARTE/69-148	A0D5U2.1 PF12796.5;	49.20	0.00
TRINITY_I7MKI3_TETTS/5-69	I7MKI3.1 PF00808.21	49.20	0.00
TRINITY_G0R0W4_ICHMG/156-318	G0R0W4.1 PF01027.18	49.20	0.00
TRINITY_A4ICT1_LEIIN/3-75	A4ICT1.1 PF00240.21	49.20	0.00
TRINITY_I7LXZ0_TETTS/23-169	I7LXZ0.1 PF06201.11	49.20	0.00
TRINITY_J9J0W3_9SPIT/51-328	J9J0W3.1 PF03747.12	49.20	0.00
TRINITY_Q23YB5_TETTS/199-456	Q23YB5.2 PF00069.23	49.20	0.00
TRINITY_D8TJV7_VOLCA/3641-4172	D8TJV7.1 PF02364.13	49.20	0.00
TRINITY_R8BY23_TOGMI/12-74	R8BY23.1 PF05676.11	49.20	0.00
TRINITY_A0DGL4_PARTE/163-424	A0DGL4.1 PF00149.26	49.20	0.00
TRINITY_I1EX18_AMPQE/260-399	I1EX18.1 PF04377.13	49.20	0.00
TRINITY_S8D991_9LAMI/469-530	S8D991.1 PF02297.15	49.20	0.00
TRINITY_C3YIZ2_BRAFL/100-330	C3YIZ2.1 PF14680.4;	49.20	0.00
TRINITY_I7MJ44_TETTS/43-852	I7MJ44.2 PF01804.16	49.20	0.00
TRINITY_W4ZJ46_STRPU/169-293	W4ZJ46.1 PF10996.6;	49.20	0.00
TRINITY_A8IBW2_CHLRE/139-344	A8IBW2.1 PF00211.18	49.20	0.00
TRINITY_A0CT98_PARTE/1951-2193	A0CT98.1 PF00644.18	49.20	0.00
TRINITY_L8HC90_ACACA/101-259	L8HC90.1 PF08231.10	49.20	0.00
TRINITY_Y508_THEMA/445-561	Q9WYX8.1 PF01661.19	49.20	0.00
TRINITY_L8H4H1_ACACA/451-620	L8H4H1.1 PF00069.23	49.20	0.00
TRINITY_A8I4X1_CHLRE/212-340	A8I4X1.1 PF13369.4;	49.20	0.00
TRINITY_D3B009_POLPA/25-161	D3B009.1 PF13529.4;	49.20	0.00
TRINITY_I1Q3F9_ORYGL/20-84	I1Q3F9.1 PF01918.19	49.20	0.00
TRINITY_E4U8M2_OCEP5/55-270	E4U8M2.1 PF08704.8;	49.20	0.00
TRINITY_D3B6M3_POLPA/229-533	D3B6M3.1 PF00082.20	49.20	0.00
TRINITY_A0CS00_PARTE/134-484	A0CS00.1 PF13868.4;	49.20	0.00
TRINITY_I0YZP9_9CHLO/2-210	I0YZP9.1 PF00590.18	49.20	0.00
TRINITY_B9SX98_RICCO/3-71	B9SX98.1 PF00240.21	49.20	0.00
TRINITY_A8HPS6_CHLRE/8-137	A8HPS6.1 PF00179.24	49.20	0.00
TRINITY_E1ZC62_CHLVA/232-333	E1ZC62.1 PF08755.9;	49.20	0.00
TRINITY_I1FPF0_AMPQE/81-258	I1FPF0.1 PF08892.9;	49.20	0.00
TRINITY_A8HQG8_CHLRE/150-407	A8HQG8.1 PF00481.19	49.20	0.00
TRINITY_C3ZMD7_BRAFL/270-567	C3ZMD7.1 PF00632.23	49.20	0.00
TRINITY_A0A0D9YAA1_9ORYZ/29-290	A0A0D9YAA1.1 PF1362	49.20	0.00
TRINITY_G0QPZ9_ICHMG/16-280	G0QPZ9.1 PF04811.13	49.20	0.00
TRINITY_L8GWH7_ACACA/4-194	L8GWH7.1 PF01956.14	49.20	0.00
TRINITY_E1ZID6_CHLVA/915-981	E1ZID6.1 PF00439.23	49.20	0.00
TRINITY_A0A0M9E444_9DELT/340-456	A0A0M9E444.1 PF0251	49.20	0.00

TRINITY_I1HR67_BRADI/14-266	I1HR67.1 PF00069.23	49.20	0.00
TRINITY_Q2H7U0_CHAGB/401-534	Q2H7U0.1 PF00005.25	49.20	0.00
TRINITY_C3YRH6_BRAFL/289-443	C3YRH6.1 PF13768.4;	49.20	0.00
TRINITY_A0BQA0_PARTE/27-116	A0BQA0.1 PF13011.4;	49.20	0.00
TRINITY_A8JJ05_CHLRE/562-633	A8JJ05.1 PF05699.12	49.20	0.00
TRINITY_K3XVA6_SETIT/40-168	K3XVA6.1 PF00266.17	49.20	0.00
TRINITY_D3BKP4_POLPA/83-143	D3BKP4.1 PF00571.26	49.20	0.00
TRINITY_F0Z7L6_DICPU/1-247	F0Z7L6.1 PF02121.16	49.20	0.00
TRINITY_F0ZB76_DICPU/5-166	F0ZB76.1 PF00071.20	49.20	0.00
TRINITY_D8TL05_VOLCA/10-212	D8TL05.1 PF02146.15	49.20	0.00
TRINITY_A8IKB4_CHLRE/123-518	A8IKB4.1 PF01546.26	49.20	0.00
TRINITY_L8HKZ1_ACACA/368-1087	L8HKZ1.1 PF00443.27	49.20	0.00
TRINITY_B8BL22_ORYSI/371-577	B8BL22.1 PF00501.26	49.20	0.00
TRINITY_F1A079_DICPU/82-142	F1A079.1 PF13833.4;	49.20	0.00
TRINITY_D8TIQ8_VOLCA/641-853	D8TIQ8.1 PF07842.10	49.20	0.00
TRINITY_A0A015II85_9GLOM/776-970	A0A015II85.1 PF0068	49.20	0.00
TRINITY_F0ZW32_DICPU/315-390	F0ZW32.1 PF07650.15	49.20	0.00
TRINITY_E1ZNL9_CHLVA/1-372	E1ZNL9.1 PF04916.11	49.20	0.00
TRINITY_A0A0J8BEW9_BETVU/14-203	A0A0J8BEW9.1 PF0407	49.20	0.00
TRINITY_A8IGD3_CHLRE/368-494	A8IGD3.1 PF02469.20	49.20	0.00
TRINITY_D7A267_STAND/16-271	D7A267.1 PF00704.26	49.20	0.00
TRINITY_I0YMU9_9CHLO/133-582	I0YMU9.1 PF00501.26	49.20	0.00
TRINITY_A8IXM4_CHLRE/1640-1791	A8IXM4.1 PF03712.13	49.20	0.00
TRINITY_G4VJB1_SCHMA/183-469	G4VJB1.1 PF00082.20	49.20	0.00
TRINITY_I0YUQ5_9CHLO/33-353	I0YUQ5.1 PF01636.21	49.20	0.00
TRINITY_D8UFU6_VOLCA/12-507	D8UFU6.1 PF07690.14	49.20	0.00
TRINITY_D8TND4_VOLCA/49-240	D8TND4.1 PF00856.26	49.20	0.00
TRINITY_I7M4A5_TETTS/168-343	I7M4A5.2 PF03031.16	49.20	0.00
TRINITY_I7MMP1_TETTS/1561-1691	I7MMP1.2 PF00072.22	49.20	0.00
TRINITY_A0DMK4_PARTE/24-339	A0DMK4.1 PF03372.21	49.10	0.00
TRINITY_Q23BP9_TETTS/506-1002	Q23BP9.2 PF07748.11	49.10	0.00
TRINITY_A5GVV7_SYNR3/127-329	A5GVV7.1 PF13604.4;	49.10	0.00
TRINITY_L8H969_ACACA/5-162	L8H969.1 PF00071.20	49.10	0.00
TRINITY_A0A0G1TKJ1_9BACT/168-323	A0A0G1TKJ1.1 PF0000	49.10	0.00
TRINITY_E7ELN7_MEDTR/632-739	E7ELN7.1 PF00970.22	49.10	0.00
TRINITY_M5A0P1_9ACTN/2-107	M5A0P1.1 PF00085.18	49.10	0.00
TRINITY_A0CJN5_PARTE/521-765	A0CJN5.1 PF01504.16	49.10	0.00
TRINITY_A0A0D2QTE0_GOSRA/31-120	A0A0D2QTE0.1 PF0029	49.10	0.00
TRINITY_A0A0D2WXF0_CAPO3/284-676	A0A0D2WXF0.1 PF0149	49.10	0.00
TRINITY_F0ZYF9_DICPU/41-447	F0ZYF9.1 PF05577.10	49.10	0.00
TRINITY_Q23CV4_TETTS/238-295	Q23CV4.1 PF13921.4;	49.10	0.00
TRINITY_PIRA_DICDI/393-1224	Q6UK63.1 PF05994.9;	49.10	0.00
TRINITY_D2CG46_TRICA/228-284	D2CG46.1 PF13414.4;	49.10	0.00
TRINITY_D2W6C4_NAEGR/55-109	D2W6C4.1 PF09597.8;	49.10	0.00
TRINITY_W9RC43_9ROSA/11-136	W9RC43.1 PF02338.17	49.10	0.00
TRINITY_J3NX84_GAGT3/490-811	J3NX84.1 PF01504.16	49.10	0.00
TRINITY_A0A0U1RQS7_HUMAN/11-163	A0A0U1RQS7.1 PF0078	49.10	0.00
TRINITY_A0A068RIG1_9FUNG/28-137	A0A068RIG1.1 PF0018	49.10	0.00
TRINITY_L8H0Y6_ACACA/58-360	L8H0Y6.1 PF03747.12	49.10	0.00
TRINITY_A0A0D2VRW0_CAPO3/54-160	A0A0D2VRW0.1 PF0190	49.10	0.00
TRINITY_A8JBC4_CHLRE/155-289	A8JBC4.1 PF09753.7;	49.10	0.00
TRINITY_A0ED46_PARTE/12-128	A0ED46.1 PF01412.16	49.10	0.00
TRINITY_A0DEL7_PARTE/173-230	A0DEL7.1 PF13921.4;	49.10	0.00
TRINITY_Q22WK1_TETTS/72-180	Q22WK1.2 PF00787.22	49.10	0.00
TRINITY_F1P4K1_CHICK/113-237	F1P4K1.2 PF04488.13	49.10	0.00
TRINITY_A0A0G0BIW2_9BACT/23-178	A0A0G0BIW2.1 PF0150	49.10	0.00
TRINITY_A8J6H6_CHLRE/198-406	A8J6H6.1 PF04893.15	49.10	0.00
TRINITY_F1A1R9_DICPU/1114-1186	F1A1R9.1 PF00626.20	49.10	0.00
TRINITY_A8IX94_CHLRE/4-104	A8IX94.1 PF05773.20	49.10	0.00
TRINITY_L8GKC3_ACACA/11-128	L8GKC3.1 PF07792.10	49.10	0.00
TRINITY_A0A024U529_9STRA/132-489	A0A024U529.1 PF0006	49.10	0.00

TRINITY_C1MM16_MICPC/148-266	C1MM16.1 PF13679.4;	49.10	0.00
TRINITY_I0YID9_9CHLO/1287-1402	I0YID9.1 PF02373.20	49.10	0.00
TRINITY_G7Q122_MACFA/63-315	G7Q122.1 PF01556.16	49.10	0.00
TRINITY_D8U4G0_VOLCA/1146-1840	D8U4G0.1 PF04998.15	49.10	0.00
TRINITY_K7J987_NASVI/460-578	K7J987.1 PF00665.24	49.10	0.00
TRINITY_I7MJT4_TETTS/15-283	I7MJT4.1 PF01073.17	49.10	0.00
TRINITY_D8U9Y4_VOLCA/2633-2865	D8U9Y4.1 PF01494.17	49.10	0.00
TRINITY_A8ISA5_CHLRE/9-212	A8ISA5.1 PF00106.23	49.10	0.00
TRINITY_Q22BB6_TETTS/30-133	Q22BB6.2 PF01521.18	49.10	0.00
TRINITY_I7LZL5_TETTS/3-180	I7LZL5.1 PF06017.11	49.10	0.00
TRINITY_T5AQP3_OPHSC/13-170	T5AQP3.1 PF06424.10	49.10	0.00
TRINITY_K1R4I2_CRAGI/95-237	K1R4I2.1 PF01399.25	49.10	0.00
TRINITY_B0TDE0_HELMI/812-926	B0TDE0.1 PF00072.22	49.10	0.00
TRINITY_L8GW37_ACACA/108-239	L8GW37.1 PF00828.17	49.10	0.00
TRINITY_W5WET9_9PSEU/46-402	W5WET9.1 PF00561.18	49.10	0.00
TRINITY_I0Z7P4_9CHLO/183-505	I0Z7P4.1 PF00493.21	49.10	0.00
TRINITY_D8TTV8_VOLCA/81-324	D8TTV8.1 PF00233.17	49.10	0.00
TRINITY_D8TPG7_VOLCA/13-263	D8TPG7.1 PF00251.18	49.10	0.00
TRINITY_A0A087SBX9_AUXPR/28-162	A0A087SBX9.1 PF0089	49.10	0.00
TRINITY_A8J6X0_CHLRE/1-220	A8J6X0.1 PF00149.26	49.10	0.00
TRINITY_Q22T22_TETTS/520-735	Q22T22.3 PF07859.11	49.10	0.00
TRINITY_A0A087SCV9_AUXPR/36-318	A0A087SCV9.1 PF0006	49.10	0.00
TRINITY_C5KU79_PERM5/43-101	C5KU79.1 PF05383.15	49.10	0.00
TRINITY_I7LTP2_TETTS/82-283	I7LTP2.1 PF03942.13	49.00	0.00
TRINITY_F0RWF4_SPHGB/73-451	F0RWF4.1 PF00365.18	49.00	0.00
TRINITY_W0SK07_9RHOO/185-250	W0SK07.1 PF14743.4;	49.00	0.00
TRINITY_Q236J0_TETTS/23-252	Q236J0.1 PF05023.12	49.00	0.00
TRINITY_E0V8Z5_PEDHC/129-276	E0V8Z5.1 PF08662.9;	49.00	0.00
TRINITY_A0D0G9_PARTE/14-80	A0D0G9.1 PF02953.13	49.00	0.00
TRINITY_C1E1J6_MICSR/20-116	C1E1J6.1 PF01196.17	49.00	0.00
TRINITY_A0BML7_PARTE/19-118	A0BML7.1 PF00447.15	49.00	0.00
TRINITY_A8HQ25_CHLRE/22-120	A8HQ25.1 PF14726.4;	49.00	0.00
TRINITY_I0YKN3_9CHLO/45-275	I0YKN3.1 PF00149.26	49.00	0.00
TRINITY_S2J9M9_MUCC1/73-173	S2J9M9.1 PF13646.4;	49.00	0.00
TRINITY_L8GRB0_ACACA/166-309	L8GRB0.1 PF00293.26	49.00	0.00
TRINITY_G0QPX7_ICHMG/754-994	G0QPX7.1 PF00520.29	49.00	0.00
TRINITY_Q22HL7_TETTS/92-505	Q22HL7.2 PF02005.14	49.00	0.00
TRINITY_A4VD57_TETTS/84-127	A4VD57.2 PF00097.23	49.00	0.00
TRINITY_Q23JY6_TETTS/610-893	Q23JY6.2 PF00481.19	49.00	0.00
TRINITY_S8BZM3_9LAMI/14-116	S8BZM3.1 PF00307.29	49.00	0.00
TRINITY_L8GHU6_ACACA/376-526	L8GHU6.1 PF00620.25	49.00	0.00
TRINITY_A8HMG5_CHLRE/9-174	A8HMG5.1 PF06813.11	49.00	0.00
TRINITY_L8H5Y2_ACACA/40-458	L8H5Y2.1 PF02897.13	49.00	0.00
TRINITY_I7MJZ2_TETTS/407-470	I7MJZ2.2 PF01485.19	49.00	0.00
TRINITY_K9WA65_9CYAN/3-206	K9WA65.1 PF01126.18	49.00	0.00
TRINITY_A0A077ZUB0_STYLE/515-998	A0A077ZUB0.1 PF0023	49.00	0.00
TRINITY_W9QPY1_9ROSA/325-373	W9QPY1.1 PF02042.13	49.00	0.00
TRINITY_A0A0P7VAT3_9TELE/4-110	A0A0P7VAT3.1 PF0457	49.00	0.00
TRINITY_L8HIE7_ACACA/13-192	L8HIE7.1 PF00929.22	49.00	0.00
TRINITY_D5BNU3_PUNMI/3-294	D5BNU3.1 PF00732.17	49.00	0.00
TRINITY_X6NUE4_RETFI/19-214	X6NUE4.1 PF00929.22	49.00	0.00
TRINITY_L8H9W4_ACACA/92-288	L8H9W4.1 PF13229.4;	49.00	0.00
TRINITY_Q23WL9_TETTS/1175-1377	Q23WL9.2 PF14644.4;	49.00	0.00
TRINITY_A0A0N1LKD1_9SPHN/11-191	A0A0N1LKD1.1 PF0059	49.00	0.00
TRINITY_I7MLV0_TETTS/461-797	I7MLV0.2 PF00443.27	49.00	0.00
TRINITY_A0A095C6U3_CRYGA/156-423	A0A095C6U3.1 PF0092	49.00	0.00
TRINITY_D8U460_VOLCA/264-502	D8U460.1 PF12740.5;	49.00	0.00
TRINITY_D8TQZ7_VOLCA/158-254	D8TQZ7.1 PF16760.3;	49.00	0.00
TRINITY_A0A059LM08_9CHLO/35-237	A0A059LM08.1 PF1348	49.00	0.00
TRINITY_A8IX54_CHLRE/271-486	A8IX54.1 PF05762.12	49.00	0.00
TRINITY_A4ACG2_9GAMM/44-301	A4ACG2.1 PF01040.16	49.00	0.00

TRINITY_L8H4T0_ACACA/615-724	L8H4T0.1 PF00651.29	49.00	0.00
TRINITY_A0A087SMG1_AUXPR/1-155	A0A087SMG1.1 PF1353	49.00	0.00
TRINITY_U4UIV1_DENPD/165-221	U4UIV1.1 PF03909.15	49.00	0.00
TRINITY_D2V5F0_NAEGR/143-242	D2V5F0.1 PF13640.4;	49.00	0.00
TRINITY_B2GUN0_XENTR/32-585	B2GUN0.1 PF01602.18	49.00	0.00
TRINITY_Q65GW7_BACLD/39-349	Q65GW7.1 PF00251.18	49.00	0.00
TRINITY_K1VNU6_TRIAC/870-965	K1VNU6.1 PF00169.27	49.00	0.00
TRINITY_E3KGM4_PUCGT/342-712	E3KGM4.2 PF00702.24	49.00	0.00
TRINITY_A7SCT7_NEMVE/1-157	A7SCT7.1 PF01399.25	49.00	0.00
TRINITY_A0CYB1_PARTE/171-469	A0CYB1.1 PF07992.12	49.00	0.00
TRINITY_I0YLD1_9CHLO/1-298	I0YLD1.1 PF06325.11	49.00	0.00
TRINITY_F4KT89_HALH1/36-176	F4KT89.1 PF00188.24	49.00	0.00
TRINITY_D8UIS1_VOLCA/104-280	D8UIS1.1 PF00753.25	49.00	0.00
TRINITY_K1RMB2_CRAGI/43-137	K1RMB2.1 PF00355.24	49.00	0.00
TRINITY_D8UGR3_VOLCA/153-288	D8UGR3.1 PF01590.24	49.00	0.00
TRINITY_D8UFD3_VOLCA/71-660	D8UFD3.1 PF16940.3;	49.00	0.00
TRINITY_D8UEL0_VOLCA/47-385	D8UEL0.1 PF00648.19	49.00	0.00
TRINITY_A8IR95_CHLRE/401-503	A8IR95.1 PF13426.5;	49.00	0.00
TRINITY_I7MB89_TETTS/43-816	I7MB89.2 PF01804.16	49.00	0.00
TRINITY_F4PCH0_BATDJ/27-278	F4PCH0.1 PF00069.23	49.00	0.00
TRINITY_A0A0J8F0H4_BETVU/49-473	A0A0J8F0H4.1 PF0557	49.00	0.00
TRINITY_G0QXB1_ICHMG/7-102	G0QXB1.1 PF00153.25	48.90	0.00
TRINITY_J9G2B2_9SPIT/13-164	J9G2B2.1 PF01124.16	48.90	0.00
TRINITY_A0EG17_PARTE/4-357	A0EG17.1 PF07690.14	48.90	0.00
TRINITY_A0D0Q8_PARTE/66-238	A0D0Q8.1 PF04727.11	48.90	0.00
TRINITY_Q83CG0_COXBU/22-440	Q83CG0.1 PF01222.15	48.90	0.00
TRINITY_I7M0V8_TETTS/1085-1228	I7M0V8.2 PF00005.25	48.90	0.00
TRINITY_Q22V96_TETTS/83-148	Q22V96.1 PF13424.4;	48.90	0.00
TRINITY_A0E9M6_PARTE/88-133	A0E9M6.1 PF13639.4;	48.90	0.00
TRINITY_F4Q535_DICFS/51-562	F4Q535.1 PF01204.16	48.90	0.00
TRINITY_J9IMT2_9SPIT/146-246	J9IMT2.1 PF00153.25	48.90	0.00
TRINITY_A0A078B161_STYLE/1505-1876	A0A078B161.1 PF0044	48.90	0.00
TRINITY_A0A0J8BQZ2_BETVU/162-500	A0A0J8BQZ2.1 PF0017	48.90	0.00
TRINITY_M2R430_CERS8/103-197	M2R430.1 PF00153.25	48.90	0.00
TRINITY_I7M185_TETTS/116-375	I7M185.1 PF00348.15	48.90	0.00
TRINITY_Q3SD54_PARTE/17-177	Q3SD54.1 PF00071.20	48.90	0.00
TRINITY_E1ZMN4_CHLVA/35-443	E1ZMN4.1 PF03985.11	48.90	0.00
TRINITY_I0Z709_9CHLO/8-263	I0Z709.1 PF12697.5;	48.90	0.00
TRINITY_L8HI67_ACACA/12-196	L8HI67.1 PF04511.13	48.90	0.00
TRINITY_E3MUW2_CAERE/7-97	E3MUW2.1 PF02214.20	48.90	0.00
TRINITY_F0ZP74_DICPU/221-539	F0ZP74.1 PF00657.20	48.90	0.00
TRINITY_I1FUD9_AMPQE/138-228	I1FUD9.1 PF13640.4;	48.90	0.00
TRINITY_A8IUR7_CHLRE/4-95	A8IUR7.1 PF03807.15	48.90	0.00
TRINITY_I0Z981_9CHLO/187-446	I0Z981.1 PF08282.10	48.90	0.00
TRINITY_W7TVN2_9STRA/95-579	W7TVN2.1 PF01425.19	48.90	0.00
TRINITY_Q239M6_TETTS/302-644	Q239M6.2 PF14700.4;	48.90	0.00
TRINITY_A0A0A1NCR0_9FUNG/19-192	A0A0A1NCR0.1 PF0492	48.90	0.00
TRINITY_D3B5Z4_POLPA/62-154	D3B5Z4.1 PF02214.20	48.90	0.00
TRINITY_A0A078AYK1_STYLE/136-395	A0A078AYK1.1 PF0006	48.90	0.00
TRINITY_G0QR68_ICHMG/177-395	G0QR68.1 PF04811.13	48.90	0.00
TRINITY_I1GGB4_AMPQE/46-226	I1GGB4.1 PF00092.26	48.90	0.00
TRINITY_A0C2U9_PARTE/4-97	A0C2U9.1 PF00153.25	48.90	0.00
TRINITY_A7S5F1_NEMVE/15-61	A7S5F1.1 PF13445.4;	48.90	0.00
TRINITY_F4PRL8_DICFS/2-89	F4PRL8.1 PF04908.13	48.90	0.00
TRINITY_D8TZ82_VOLCA/36-233	D8TZ82.1 PF00106.23	48.90	0.00
TRINITY_G0QNV3_ICHMG/510-616	G0QNV3.1 PF00787.22	48.90	0.00
TRINITY_D8UHF5_VOLCA/54-144	D8UHF5.1 PF13772.4;	48.90	0.00
TRINITY_L1IC59_GUITH/17-357	L1IC59.1 PF04506.11	48.90	0.00
TRINITY_Q9M6B0_CHLRE/5-367	Q9M6B0.1 PF05914.10	48.90	0.00
TRINITY_D8U3A8_VOLCA/251-1086	D8U3A8.1 PF00176.21	48.90	0.00
TRINITY_A8IEM4_CHLRE/32-214	A8IEM4.1 PF01150.15	48.90	0.00

TRINITY_F0ZY22_DICPU/659-881	F0ZY22.1 PF02145.13	48.90	0.00
TRINITY_A0A078D6D3_BRANA/113-500	A0A078D6D3.1 PF0583	48.90	0.00
TRINITY_A8J6M5_CHLRE/1-274	A8J6M5.1 PF00664.21	48.90	0.00
TRINITY_A0A059LGR6_9CHLO/125-471	A0A059LGR6.1 PF0306	48.90	0.00
TRINITY_G0QK23_ICHMG/3-104	G0QK23.1 PF00168.28	48.90	0.00
TRINITY_D8UAZ1_VOLCA/74-373	D8UAZ1.1 PF04121.11	48.90	0.00
TRINITY_A0A078AQT8_STYLE/753-1040	A0A078AQT8.1 PF0032	48.90	0.00
TRINITY_F0ZFI0_DICPU/392-647	F0ZFI0.1 PF05990.10	48.90	0.00
TRINITY_A0A067K683_JATCU/55-374	A0A067K683.1 PF0369	48.90	0.00
TRINITY_Q23AC3_TETTS/7-112	Q23AC3.2 PF00787.22	48.90	0.00
TRINITY_A8HZ72_CHLRE/72-253	A8HZ72.1 PF01789.14	48.90	0.00
TRINITY_A8JEW5_CHLRE/404-526	A8JEW5.1 PF07714.15	48.90	0.00
TRINITY_E1ZGI7_CHLVA/158-356	E1ZGI7.1 PF04989.10	48.90	0.00
TRINITY_F0ZYY4_DICPU/16-110	F0ZYY4.1 PF02214.20	48.90	0.00
TRINITY_A0A0G1W2M3_9BACT/3-223	A0A0G1W2M3.1 PF0030	48.90	0.00
TRINITY_A0A0D2PGX0_GOSRA/173-346	A0A0D2PGX0.1 PF0027	48.90	0.00
TRINITY_K4D4D0_SOLLC/8-307	K4D4D0.1 PF10243.7;	48.90	0.00
TRINITY_Q22U39_TETTS/54-238	Q22U39.3 PF13460.4;	48.90	0.00
TRINITY_I1FLG0_AMPQE/819-1036	I1FLG0.1 PF13087.4;	48.90	0.00
TRINITY_A8IUY5_CHLRE/8-128	A8IUY5.1 PF09945.7;	48.90	0.00
TRINITY_Q8S6Z2_ORYSJ/980-1121	Q8S6Z2.1 PF00078.25	48.90	0.00
TRINITY_I7M3H7_TETTS/56-172	I7M3H7.2 PF05348.9;	48.90	0.00
TRINITY_J9F544_9SPIT/10-200	J9F544.1 PF16213.3;	48.80	0.00
TRINITY_A0EAB1_PARTE/28-329	A0EAB1.1 PF01074.20	48.80	0.00
TRINITY_Q22WQ9_TETTS/22-146	Q22WQ9.1 PF07542.9;	48.80	0.00
TRINITY_A7T4E5_NEMVE/13-95	A7T4E5.1 PF04032.14	48.80	0.00
TRINITY_L8GHY4_ACACA/353-545	L8GHY4.1 PF07690.14	48.80	0.00
TRINITY_I7MJM5_TETTS/3-168	I7MJM5.1 PF10602.7;	48.80	0.00
TRINITY_A8JGZ8_CHLRE/33-118	A8JGZ8.1 PF03795.12	48.80	0.00
TRINITY_H2YGU3_CIOSA/59-220	H2YGU3.1 PF04387.12	48.80	0.00
TRINITY_W6FUH4_NODSP/15-183	W6FUH4.1 PF13385.4;	48.80	0.00
TRINITY_I7MJ25_TETTS/230-402	I7MJ25.1 PF05193.19	48.80	0.00
TRINITY_M7SDG0_EUTLA/134-223	M7SDG0.1 PF00153.25	48.80	0.00
TRINITY_A0A0D2UGE6_CAPO3/10-171	A0A0D2UGE6.1 PF0007	48.80	0.00
TRINITY_W5KH01_ASTMX/89-413	W5KH01.1 PF00443.27	48.80	0.00
TRINITY_A0A0D2VFH8_CAPO3/177-259	A0A0D2VFH8.1 PF0791	48.80	0.00
TRINITY_A8HPQ7_CHLRE/1320-1574	A8HPQ7.1 PF00211.18	48.80	0.00
TRINITY_D3BC68_POLPA/41-230	D3BC68.1 PF04427.16	48.80	0.00
TRINITY_G0QVG8_ICHMG/709-842	G0QVG8.1 PF00107.24	48.80	0.00
TRINITY_L8GWQ0_ACACA/940-1189	L8GWQ0.1 PF07714.15	48.80	0.00
TRINITY_G0QUT7_ICHMG/11-305	G0QUT7.1 PF00069.23	48.80	0.00
TRINITY_V4A0Y3_LOTGI/56-179	V4A0Y3.1 PF04424.11	48.80	0.00
TRINITY_D8UJY2_VOLCA/9-141	D8UJY2.1 PF00134.21	48.80	0.00
TRINITY_A0A0D2SG08_GOSRA/184-225	A0A0D2SG08.1 PF0154	48.80	0.00
TRINITY_Q22GY6_TETTS/213-378	Q22GY6.3 PF03031.16	48.80	0.00
TRINITY_L8H9D8_ACACA/203-360	L8H9D8.1 PF07992.12	48.80	0.00
TRINITY_D8UBR3_VOLCA/34-208	D8UBR3.1 PF04755.10	48.80	0.00
TRINITY_D8SDA6_SELML/34-245	D8SDA6.1 PF00106.23	48.80	0.00
TRINITY_Q5CWY7_CRYPI/10-171	Q5CWY7.1 PF00071.20	48.80	0.00
TRINITY_D8U8R9_VOLCA/128-207	D8U8R9.1 PF02319.18	48.80	0.00
TRINITY_A0A0G4IR09_PLABS/500-730	A0A0G4IR09.1 PF1308	48.80	0.00
TRINITY_A0A068S1S4_9FUNG/263-433	A0A068S1S4.1 PF1271	48.80	0.00
TRINITY_A0A060SBA7_PYCCI/770-1215	A0A060SBA7.1 PF0052	48.80	0.00
TRINITY_H9G5X2_ANOCA/44-135	H9G5X2.1 PF10447.7;	48.80	0.00
TRINITY_D8UER0_VOLCA/28-200	D8UER0.1 PF09750.7;	48.80	0.00
TRINITY_I1K7E0_SOYBN/243-326	I1K7E0.1 PF05542.9;	48.80	0.00
TRINITY_L8GTW8_ACACA/410-578	L8GTW8.1 PF00621.18	48.80	0.00
TRINITY_K3WU70_PYTUL/516-749	K3WU70.1 PF00233.17	48.80	0.00
TRINITY_I7MEA1_TETTS/292-382	I7MEA1.1 PF00153.25	48.80	0.00
TRINITY_A7TPK5_VANPO/251-513	A7TPK5.1 PF00122.18	48.80	0.00
TRINITY_A8IAM0_CHLRE/330-429	A8IAM0.1 PF11799.6;	48.80	0.00

TRINITY_I0Z541_9CHLO/270-469	I0Z541.1 PF02190.14	48.80	0.00
TRINITY_A8I668_CHLRE/87-283	A8I668.1 PF06026.12	48.80	0.00
TRINITY_W6FUH4_NODSP/15-183	W6FUH4.1 PF13385.4;	48.80	0.00
TRINITY_G0QQW5_ICHMG/13-98	G0QQW5.1 PF14560.4;	48.80	0.00
TRINITY_A7SU16_NEMVE/160-243	A7SU16.1 PF14768.4;	48.80	0.00
TRINITY_A0A090M5B6_OSTTA/141-247	A0A090M5B6.1 PF1049	48.80	0.00
TRINITY_B7P150_IXOSC/288-446	B7P150.1 PF02872.16	48.80	0.00
TRINITY_L8GL12_ACACA/119-330	L8GL12.1 PF00112.21	48.80	0.00
TRINITY_E1ZGN0_CHLVA/8-431	E1ZGN0.1 PF00890.22	48.80	0.00
TRINITY_A0A0A0KAX5_CUCSA/446-658	A0A0A0KAX5.1 PF0064	48.80	0.00
TRINITY_H5WJN2_9BURK/687-803	H5WJN2.1 PF00072.22	48.80	0.00
TRINITY_H5WJN2_9BURK/687-803	H5WJN2.1 PF00072.22	48.80	0.00
TRINITY_A8J6S3_CHLRE/2038-2196	A8J6S3.1 PF06650.10	48.80	0.00
TRINITY_D8TUB8_VOLCA/469-844	D8TUB8.1 PF00225.21	48.80	0.00
TRINITY_A9T7W1_PHYPA/99-243	A9T7W1.1 PF13365.4;	48.80	0.00
TRINITY_A0A0D2UJE3_CAPO3/7-91	A0A0D2UJE3.1 PF1341	48.80	0.00
TRINITY_A7RRA0_NEMVE/147-272	A7RRA0.1 PF02338.17	48.80	0.00
TRINITY_A8JBX4_CHLRE/56-319	A8JBX4.1 PF13561.4;	48.80	0.00
TRINITY_I0Z1F9_9CHLO/13-192	I0Z1F9.1 PF13489.4;	48.80	0.00
TRINITY_I0YWQ7_9CHLO/58-293	I0YWQ7.1 PF08245.10	48.80	0.00
TRINITY_I7MAC8_TETTS/3673-3968	I7MAC8.2 PF00632.23	48.80	0.00
TRINITY_Q22W19_TETTS/115-323	Q22W19.3 PF00112.21	48.80	0.00
TRINITY_D8TPI1_VOLCA/514-606	D8TPI1.1 PF00686.17	48.80	0.00
TRINITY_I7LXA4_TETTS/158-324	I7LXA4.1 PF10238.7;	48.70	0.00
TRINITY_L1K0L5_GUITH/1758-2072	L1K0L5.1 PF02889.14	48.70	0.00
TRINITY_A0D8H4_PARTE/1416-1749	A0D8H4.1 PF00443.27	48.70	0.00
TRINITY_A0A0E9NGE3_9ASCO/2-116	A0A0E9NGE3.1 PF1262	48.70	0.00
TRINITY_K1QS76_CRAGI/36-177	K1QS76.1 PF09794.7;	48.70	0.00
TRINITY_L8H3Q1_ACACA/13-230	L8H3Q1.1 PF01171.18	48.70	0.00
TRINITY_I1GJ44_AMPQE/19-375	I1GJ44.1 PF00503.18	48.70	0.00
TRINITY_W7XGD4_TETTS/87-322	W7XGD4.1 PF12146.6;	48.70	0.00
TRINITY_G0QPB7_ICHMG/28-214	G0QPB7.1 PF00227.24	48.70	0.00
TRINITY_Q23TU2_TETTS/4051-4801	Q23TU2.2 PF03028.13	48.70	0.00
TRINITY_Q2SAD5_HAHCH/119-542	Q2SAD5.1 PF01425.19	48.70	0.00
TRINITY_I4YMJ8_9RHIZ/5-185	I4YMJ8.1 PF00300.20	48.70	0.00
TRINITY_Y508_THEMA/445-561	Q9WYX8.1 PF01661.19	48.70	0.00
TRINITY_D3B9I0_POLPA/31-228	D3B9I0.1 PF00106.23	48.70	0.00
TRINITY_F0ZZW8_DICPU/16-286	F0ZZW8.1 PF00069.23	48.70	0.00
TRINITY_I0YZV0_9CHLO/1-203	I0YZV0.1 PF00106.23	48.70	0.00
TRINITY_D8SRB3_SELML/118-235	D8SRB3.1 PF00307.29	48.70	0.00
TRINITY_J4D5C0_THEOR/138-214	J4D5C0.1 PF00076.20	48.70	0.00
TRINITY_I0Z4S6_9CHLO/54-216	I0Z4S6.1 PF10294.7;	48.70	0.00
TRINITY_A0A0A1TNZ7_9HYPO/56-138	A0A0A1TNZ7.1 PF0333	48.70	0.00
TRINITY_I7M0X0_TETTS/460-654	I7M0X0.2 PF08662.9;	48.70	0.00
TRINITY_I7LT38_TETTS/868-1116	I7LT38.1 PF16212.3;	48.70	0.00
TRINITY_A4VD11_TETTS/21-261	A4VD11.2 PF00481.19	48.70	0.00
TRINITY_F0Z6W2_DICPU/5-275	F0Z6W2.1 PF03850.12	48.70	0.00
TRINITY_D8U6H1_VOLCA/280-435	D8U6H1.1 PF12576.6;	48.70	0.00
TRINITY_Q24IJ0_TETTS/27-299	Q24IJ0.1 PF00069.23	48.70	0.00
TRINITY_D8THU7_VOLCA/193-274	D8THU7.1 PF00076.20	48.70	0.00
TRINITY_Q24HR9_TETTS/46-305	Q24HR9.2 PF00069.23	48.70	0.00
TRINITY_F0ZYQ5_DICPU/195-300	F0ZYQ5.1 PF12894.5;	48.70	0.00
TRINITY_D8THR7_VOLCA/300-536	D8THR7.1 PF12697.5;	48.70	0.00
TRINITY_D8U951_VOLCA/5-158	D8U951.1 PF05907.11	48.70	0.00
TRINITY_Q23DD6_TETTS/1118-1721	Q23DD6.2 PF04998.15	48.70	0.00
TRINITY_D3B268_POLPA/1-987	D3B268.1 PF04762.10	48.70	0.00
TRINITY_D8U1P4_VOLCA/539-653	D8U1P4.1 PF00072.22	48.70	0.00
TRINITY_A8HX31_CHLRE/21-249	A8HX31.1 PF04757.12	48.70	0.00
TRINITY_J9J0W3_9SPIT/51-328	J9J0W3.1 PF03747.12	48.70	0.00
TRINITY_A0A077ZUV7_STYLE/56-208	A0A077ZUV7.1 PF1473	48.70	0.00
TRINITY_A0A066W1Y4_9HOMO/6-86	A0A066W1Y4.1 PF1341	48.70	0.00

TRINITY_G0QZ92_ICHMG/19-335	G0QZ92.1 PF00069.23	48.70	0.00
TRINITY_D8U7Q0_VOLCA/1416-1774	D8U7Q0.1 PF02259.21	48.70	0.00
TRINITY_D8U4U1_VOLCA/300-526	D8U4U1.1 PF00248.19	48.70	0.00
TRINITY_Q7XLT8_ORYSJ/503-697	Q7XLT8.2 PF00078.25	48.70	0.00
TRINITY_A0A0B5ES46_STRA4/62-371	A0A0B5ES46.1 PF0025	48.70	0.00
TRINITY_A0A085MQK8_9BILA/324-484	A0A085MQK8.1 PF0007	48.70	0.00
TRINITY_F0ZLA6_DICPU/16-296	F0ZLA6.1 PF10222.7;	48.70	0.00
TRINITY_A0A087S9G6_AUXPR/46-310	A0A087S9G6.1 PF0006	48.70	0.00
TRINITY_F0ZCT2_DICPU/601-783	F0ZCT2.1 PF00617.17	48.70	0.00
TRINITY_T1IVC2_STRMM/88-349	T1IVC2.1 PF06246.10	48.70	0.00
TRINITY_D3B1A4_POLPA/306-493	D3B1A4.1 PF14437.4;	48.70	0.00
TRINITY_E1ZN16_CHLVA/784-966	E1ZN16.1 PF05193.19	48.70	0.00
TRINITY_M2Y0I1_GALSU/89-164	M2Y0I1.1 PF03061.20	48.70	0.00
TRINITY_F4QBH6_DICFS/16-280	F4QBH6.1 PF00069.23	48.70	0.00
TRINITY_Q30T31_SULDN/210-589	Q30T31.1 PF00285.19	48.70	0.00
TRINITY_A0A087SJ57_AUXPR/981-1252	A0A087SJ57.1 PF0132	48.70	0.00
TRINITY_D8UFD3_VOLCA/71-660	D8UFD3.1 PF16940.3;	48.70	0.00
TRINITY_A0A0G0MZM8_9BACT/12-671	A0A0G0MZM8.1 PF0013	48.70	0.00
TRINITY_A0A0J7KC47_LASNI/473-592	A0A0J7KC47.1 PF0066	48.70	0.00
TRINITY_A8IZH2_CHLRE/548-643	A8IZH2.1 PF00211.18	48.70	0.00
TRINITY_Q7XLT8_ORYSJ/503-697	Q7XLT8.2 PF00078.25	48.70	0.00
TRINITY_B5JM65_9BACT/782-897	B5JM65.1 PF00072.22	48.60	0.00
TRINITY_I7LVZ5_TETTS/5-189	I7LVZ5.1 PF00106.23	48.60	0.00
TRINITY_M4A9Z6_XIPMA/288-456	M4A9Z6.1 PF01612.18	48.60	0.00
TRINITY_D8QMU7_SELML/18-180	D8QMU7.1 PF00071.20	48.60	0.00
TRINITY_Q6BFJ6_PARTE/12-99	Q6BFJ6.1 PF00153.25	48.60	0.00
TRINITY_BECNB_DICDI/503-810	Q54JI9.1 PF04111.10	48.60	0.00
TRINITY_G0QK40_ICHMG/243-478	G0QK40.1 PF07690.14	48.60	0.00
TRINITY_L8GP39_ACACA/2-191	L8GP39.1 PF01121.18	48.60	0.00
TRINITY_I7M661_TETTS/232-447	I7M661.1 PF04042.14	48.60	0.00
TRINITY_Q22RI2_TETTS/41-476	Q22RI2.2 PF05602.10	48.60	0.00
TRINITY_A0CES7_PARTE/367-473	A0CES7.1 PF13646.4;	48.60	0.00
TRINITY_D6WFE0_TRICA/46-216	D6WFE0.1 PF08660.9;	48.60	0.00
TRINITY_E0W2T7_PEDHC/35-103	E0W2T7.1 PF00076.20	48.60	0.00
TRINITY_H3EEF3_PRIPA/100-216	H3EEF3.1 PF01873.15	48.60	0.00
TRINITY_T0SAN3_9STRA/35-278	T0SAN3.1 PF12697.5;	48.60	0.00
TRINITY_I7MK41_TETTS/120-286	I7MK41.2 PF03062.17	48.60	0.00
TRINITY_C1ECK5_MICSR/1873-2017	C1ECK5.1 PF05843.12	48.60	0.00
TRINITY_Q22B41_TETTS/170-248	Q22B41.1 PF05022.10	48.60	0.00
TRINITY_G0SVM5_RHOG2/116-265	G0SVM5.1 PF13091.4;	48.60	0.00
TRINITY_A0A0G0AZA9_9BACT/22-241	A0A0G0AZA9.1 PF0126	48.60	0.00
TRINITY_A8HQ36_CHLRE/7-119	A8HQ36.1 PF00581.18	48.60	0.00
TRINITY_DNAS1_RABIT/25-273	O18998.1 PF03372.21	48.60	0.00
TRINITY_G2QNY2_MYCTT/196-323	G2QNY2.1 PF00107.24	48.60	0.00
TRINITY_A8JJ05_CHLRE/562-633	A8JJ05.1 PF05699.12	48.60	0.00
TRINITY_E1ZI47_CHLVA/2-294	E1ZI47.1 PF00850.17	48.60	0.00
TRINITY_G0R0N7_ICHMG/32-549	G0R0N7.1 PF01602.18	48.60	0.00
TRINITY_D8U315_VOLCA/132-268	D8U315.1 PF03330.16	48.60	0.00
TRINITY_A0A067QVQ6_ZOONE/155-226	A0A067QVQ6.1 PF0024	48.60	0.00
TRINITY_D8T4Q4_SELML/27-546	D8T4Q4.1 PF01602.18	48.60	0.00
TRINITY_A0A015KSK4_9GLOM/344-503	A0A015KSK4.1 PF0391	48.60	0.00
TRINITY_I1HUJ9_BRADI/39-109	I1HUJ9.1 PF00076.20	48.60	0.00
TRINITY_F4QDB6_DICFS/16-310	F4QDB6.1 PF07159.10	48.60	0.00
TRINITY_A0A087SFY1_AUXPR/677-889	A0A087SFY1.1 PF0006	48.60	0.00
TRINITY_A2EWC0_TRIVA/30-243	A2EWC0.1 PF07002.14	48.60	0.00
TRINITY_D8U4P3_VOLCA/41-332	D8U4P3.1 PF08449.9;	48.60	0.00
TRINITY_A8JGL2_CHLRE/105-341	A8JGL2.1 PF02358.14	48.60	0.00
TRINITY_W1NUJ8_AMBTC/515-760	W1NUJ8.1 PF09258.8;	48.60	0.00
TRINITY_C1FJC7_MICSR/77-321	C1FJC7.1 PF12697.5;	48.60	0.00
TRINITY_Q22T22_TETTS/520-735	Q22T22.3 PF07859.11	48.60	0.00
TRINITY_A8I108_CHLRE/67-274	A8I108.1 PF00566.16	48.60	0.00

TRINITY_A0A0D2X453_CAPO3/177-285	A0A0D2X453.1 PF0572	48.60	0.00
TRINITY_G1PSI8_MYOLU/34-277	G1PSI8.1 PF01593.22	48.60	0.00
TRINITY_D3AY46_POLPA/593-775	D3AY46.1 PF00617.17	48.60	0.00
TRINITY_I1LEZ0_SOYBN/9-79	I1LEZ0.1 PF00076.20	48.60	0.00
TRINITY_B0PH80_9FIRM/979-1096	B0PH80.1 PF00072.22	48.60	0.00
TRINITY_A0A0E9NFD1_9ASCO/67-136	A0A0E9NFD1.1 PF0007	48.60	0.00
TRINITY_D8U0M3_VOLCA/103-328	D8U0M3.1 PF00856.26	48.60	0.00
TRINITY_G7YWE9_CLOSI/1652-1723	G7YWE9.1 PF02207.18	48.60	0.00
TRINITY_Q6C9V2_YARLI/189-300	Q6C9V2.1 PF00188.24	48.60	0.00
TRINITY_A0A0D2UFB5_CAPO3/250-338	A0A0D2UFB5.1 PF0293	48.60	0.00
TRINITY_A0DYX2_PARTE/54-188	A0DYX2.1 PF04146.13	48.60	0.00
TRINITY_A8JH76_CHLRE/10-98	A8JH76.1 PF10270.7;	48.60	0.00
TRINITY_A9S9U0_PHYPA/6-313	A9S9U0.1 PF03372.21	48.60	0.00
TRINITY_W5J229_ANODA/1179-1326	W5J229.1 PF00005.25	48.60	0.00
TRINITY_A0A078CVI2_BRANA/94-239	A0A078CVI2.1 PF0021	48.60	0.00
TRINITY_A0A059LKZ4_9CHLO/1-159	A0A059LKZ4.1 PF0924	48.60	0.00
TRINITY_F4PV04_DICFS/87-528	F4PV04.1 PF00501.26	48.60	0.00
TRINITY_G7KIB0_MEDTR/67-272	G7KIB0.2 PF00106.23	48.60	0.00
TRINITY_D3BQ47_POLPA/174-421	D3BQ47.1 PF04109.14	48.60	0.00
TRINITY_G0R2T0_ICHMG/33-283	G0R2T0.1 PF00069.23	48.60	0.00
TRINITY_D2UY94_NAEGR/292-414	D2UY94.1 PF00271.29	48.60	0.00
TRINITY_A8J5D8_CHLRE/5-419	A8J5D8.1 PF01676.16	48.60	0.00
TRINITY_A0DG32_PARTE/47-189	A0DG32.1 PF03193.14	48.60	0.00
TRINITY_A0A067JIF2_JATCU/9-266	A0A067JIF2.1 PF0006	48.50	0.00
TRINITY_Q09CU7_STIAD/3-227	Q09CU7.1 PF01370.19	48.50	0.00
TRINITY_L8GTK1_ACACA/146-395	L8GTK1.1 PF07714.15	48.50	0.00
TRINITY_W9S2V3_9ROSA/63-340	W9S2V3.1 PF00481.19	48.50	0.00
TRINITY_A0A0D2NWK1_GOSRA/37-167	A0A0D2NWK1.1 PF0078	48.50	0.00
TRINITY_F4Q156_DICFS/452-627	F4Q156.1 PF00621.18	48.50	0.00
TRINITY_A0A0N4X5F8_HAEPC/93-264	A0A0N4X5F8.1 PF0176	48.50	0.00
TRINITY_A0A075B583_9FUNG/12-181	A0A075B583.1 PF0007	48.50	0.00
TRINITY_F0ZSM2_DICPU/404-506	F0ZSM2.1 PF14593.4;	48.50	0.00
TRINITY_Q22X26_TETTS/84-364	Q22X26.2 PF01266.22	48.50	0.00
TRINITY_Q22GH9_TETTS/22-286	Q22GH9.1 PF00481.19	48.50	0.00
TRINITY_F0Y6L3_AURAN/5-178	F0Y6L3.1 PF00071.20	48.50	0.00
TRINITY_A8I788_CHLRE/2-174	A8I788.1 PF13019.4;	48.50	0.00
TRINITY_A0A068S2S3_9FUNG/56-483	A0A068S2S3.1 PF0200	48.50	0.00
TRINITY_I7MFD5_TETTS/394-587	I7MFD5.2 PF13901.4;	48.50	0.00
TRINITY_G0QLX3_ICHMG/12-277	G0QLX3.1 PF07714.15	48.50	0.00
TRINITY_D3BUZ1_POLPA/29-265	D3BUZ1.1 PF01636.21	48.50	0.00
TRINITY_B3RLY5_TRIAD/95-165	B3RLY5.1 PF07258.12	48.50	0.00
TRINITY_I7MLB3_TETTS/170-454	I7MLB3.1 PF01008.15	48.50	0.00
TRINITY_D2VBB5_NAEGR/177-479	D2VBB5.1 PF06079.9;	48.50	0.00
TRINITY_DCAM_DICDI/26-352	Q8T1E3.3 PF01536.14	48.50	0.00
TRINITY_D8UBG5_VOLCA/36-244	D8UBG5.1 PF03896.14	48.50	0.00
TRINITY_F0YD11_AURAN/754-825	F0YD11.1 PF04564.13	48.50	0.00
TRINITY_K6UY88_9APIC/75-189	K6UY88.1 PF16531.3;	48.50	0.00
TRINITY_A8HWH6_CHLRE/477-630	A8HWH6.1 PF01343.16	48.50	0.00
TRINITY_A8J6D2_CHLRE/412-609	A8J6D2.1 PF05057.12	48.50	0.00
TRINITY_A0A0G4IKI4_PLABS/456-623	A0A0G4IKI4.1 PF1309	48.50	0.00
TRINITY_D3AXS4_POLPA/39-263	D3AXS4.1 PF02666.13	48.50	0.00
TRINITY_F1A3H0_DICPU/186-253	F1A3H0.1 PF01485.19	48.50	0.00
TRINITY_M5GEX9_DACSP/52-125	M5GEX9.1 PF13517.4;	48.50	0.00
TRINITY_Q23CN6_TETTS/42-263	Q23CN6.2 PF02230.14	48.50	0.00
TRINITY_D8U4R9_VOLCA/1-136	D8U4R9.1 PF04096.12	48.50	0.00
TRINITY_D3BAG4_POLPA/82-505	D3BAG4.1 PF00450.20	48.50	0.00
TRINITY_E4X3N6_OIKDI/14-792	E4X3N6.1 PF16399.3;	48.50	0.00
TRINITY_A9SNT8_PHYPA/19-274	A9SNT8.1 PF00069.23	48.50	0.00
TRINITY_F0ZLS2_DICPU/66-274	F0ZLS2.1 PF13489.4;	48.50	0.00
TRINITY_C1MNQ1_MICPC/64-209	C1MNQ1.1 PF02367.15	48.50	0.00
TRINITY_J1RVG7_9ACTN/15-188	J1RVG7.1 PF04072.12	48.50	0.00

TRINITY_F6RLE1_BOVIN/37-106	F6RLE1.1 PF00610.19	48.50	0.00
TRINITY_I0Z9R3_9CHLO/262-526	I0Z9R3.1 PF12698.5;	48.50	0.00
TRINITY_Q6BG63_PARTE/135-272	Q6BG63.1 PF01529.18	48.50	0.00
TRINITY_B8HYX6_CYAP4/26-200	B8HYX6.1 PF13532.4;	48.50	0.00
TRINITY_A0CXW7_PARTE/958-1287	A0CXW7.1 PF02889.14	48.50	0.00
TRINITY_E6ZRN3_SPORE/61-227	E6ZRN3.1 PF00270.27	48.50	0.00
TRINITY_F0ZB08_DICPU/112-415	F0ZB08.1 PF02906.12	48.50	0.00
TRINITY_K7V0Y9_MAIZE/158-416	K7V0Y9.1 PF00069.23	48.50	0.00
TRINITY_A0A087SCC4_AUXPR/51-217	A0A087SCC4.1 PF0610	48.50	0.00
TRINITY_G0QYS9_ICHMG/215-317	G0QYS9.1 PF00168.28	48.50	0.00
TRINITY_F6UC79_XENTR/435-628	F6UC79.1 PF00566.16	48.50	0.00
TRINITY_F0ZU77_DICPU/256-473	F0ZU77.1 PF13236.4;	48.50	0.00
TRINITY_D0MZ70_PHYIT/6-73	D0MZ70.1 PF00076.20	48.50	0.00
TRINITY_A0BX75_PARTE/58-413	A0BX75.1 PF01704.16	48.50	0.00
TRINITY_A8IGC6_CHLRE/15-304	A8IGC6.1 PF09139.9;	48.50	0.00
TRINITY_Q22SV8_TETTS/139-277	Q22SV8.1 PF01553.19	48.50	0.00
TRINITY_A8J5P0_CHLRE/384-551	A8J5P0.1 PF09088.9;	48.50	0.00
TRINITY_L8HD35_ACACA/690-920	L8HD35.1 PF00481.19	48.50	0.00
TRINITY_A0A0N5DJ00_TRIMR/2-286	A0A0N5DJ00.1 PF0058	48.50	0.00
TRINITY_K1Q3Y8_CRAGI/69-219	K1Q3Y8.1 PF07534.14	48.50	0.00
TRINITY_D8F589_9DELT/424-537	D8F589.1 PF00072.22	48.50	0.00
TRINITY_D8U5Y6_VOLCA/619-729	D8U5Y6.1 PF00989.23	48.50	0.00
TRINITY_A8J1H0_CHLRE/65-495	A8J1H0.1 PF00909.19	48.50	0.00
TRINITY_A0DMS6_PARTE/43-308	A0DMS6.1 PF03381.13	48.50	0.00
TRINITY_Q16VZ3_AEDAE/83-208	Q16VZ3.1 PF04857.18	48.50	0.00
TRINITY_A8HT24_CHLRE/1413-1659	A8HT24.1 PF02714.13	48.50	0.00
TRINITY_J3KY02_ORYBR/637-893	J3KY02.1 PF12706.5;	48.40	0.00
TRINITY_A0A067LIX5_JATCU/2-151	A0A067LIX5.1 PF0040	48.40	0.00
TRINITY_I7MK61_TETTS/82-178	I7MK61.1 PF04800.10	48.40	0.00
TRINITY_A2EPG3_TRIVA/9-170	A2EPG3.1 PF00071.20	48.40	0.00
TRINITY_I6XSN4_PROPF/19-635	I6XSN4.1 PF00133.20	48.40	0.00
TRINITY_A0A0G1J0E2_9BACT/21-88	A0A0G1J0E2.1 PF0036	48.40	0.00
TRINITY_A0A0M0J556_9EUKA/51-512	A0A0M0J556.1 PF1590	48.40	0.00
TRINITY_A0A077ZY60_STYLE/5897-6330	A0A077ZY60.1 PF0089	48.40	0.00
TRINITY_NDX1_CAEEL/72-197	O45830.1 PF00293.26	48.40	0.00
TRINITY_J9IDU1_9SPIT/332-931	J9IDU1.1 PF08385.10	48.40	0.00
TRINITY_Q22AS9_TETTS/396-460	Q22AS9.1 PF13499.4;	48.40	0.00
TRINITY_E1ZEZ8_CHLVA/496-844	E1ZEZ8.1 PF05192.16	48.40	0.00
TRINITY_Q8T2H6_DICDI/31-218	Q8T2H6.1 PF02589.13	48.40	0.00
TRINITY_H8MEU3_CORCM/58-379	H8MEU3.1 PF07995.9;	48.40	0.00
TRINITY_S8CMD2_9LAMI/36-166	S8CMD2.1 PF00782.18	48.40	0.00
TRINITY_A0A0F4QN62_9GAMM/5-131	A0A0F4QN62.1 PF1443	48.40	0.00
TRINITY_A0A078B8P5_STYLE/295-617	A0A078B8P5.1 PF0123	48.40	0.00
TRINITY_F4R3K5_MELLP/20-115	F4R3K5.1 PF00153.25	48.40	0.00
TRINITY_G0R3P6_ICHMG/8-169	G0R3P6.1 PF01765.17	48.40	0.00
TRINITY_D8R3Q3_SELML/1-64	D8R3Q3.1 PF00280.16	48.40	0.00
TRINITY_L8HDF4_ACACA/1013-1289	L8HDF4.1 PF07714.15	48.40	0.00
TRINITY_D7FM24_ECTSI/244-406	D7FM24.1 PF02417.13	48.40	0.00
TRINITY_J9IE32_9SPIT/16-80	J9IE32.1 PF01918.19	48.40	0.00
TRINITY_G0R592_ICHMG/1-273	G0R592.1 PF00648.19	48.40	0.00
TRINITY_D8UAZ5_VOLCA/71-252	D8UAZ5.1 PF13419.4;	48.40	0.00
TRINITY_A8HVN4_CHLRE/30-193	A8HVN4.1 PF03067.13	48.40	0.00
TRINITY_Q3SD57_PARTE/13-173	Q3SD57.1 PF00071.20	48.40	0.00
TRINITY_I7M4F9_TETTS/51-228	I7M4F9.2 PF01195.17	48.40	0.00
TRINITY_L8H3W5_ACACA/971-1093	L8H3W5.1 PF00271.29	48.40	0.00
TRINITY_E1ZR53_CHLVA/368-431	E1ZR53.1 PF01842.23	48.40	0.00
TRINITY_A4VD54_TETTS/744-1018	A4VD54.1 PF00664.21	48.40	0.00
TRINITY_D8RYU3_SELML/195-363	D8RYU3.1 PF00005.25	48.40	0.00
TRINITY_A0A072V237_MEDTR/45-175	A0A072V237.1 PF0078	48.40	0.00
TRINITY_B6H4J2_PENRW/28-96	B6H4J2.1 PF00505.17	48.40	0.00
TRINITY_A0BDL2_PARTE/6-167	A0BDL2.1 PF00071.20	48.40	0.00

TRINITY_A8JFA9_CHLRE/230-383	A8JFA9.1 PF16016.3;	48.40	0.00
TRINITY_A7RTF3_NEMVE/273-427	A7RTF3.1 PF13768.4;	48.40	0.00
TRINITY_I0YUF8_9CHLO/29-120	I0YUF8.1 PF00581.18	48.40	0.00
TRINITY_D3BAA5_POLPA/36-100	D3BAA5.1 PF13499.4;	48.40	0.00
TRINITY_A0A058ZD03_9EUKA/391-497	A0A058ZD03.1 PF0141	48.40	0.00
TRINITY_E1ZR53_CHLVA/368-431	E1ZR53.1 PF01842.23	48.40	0.00
TRINITY_A0A0G4J3V7_PLABS/3789-4100	A0A0G4J3V7.1 PF0063	48.40	0.00
TRINITY_J9F6J0_9SPIT/6042-6471	J9F6J0.1 PF00899.19	48.40	0.00
TRINITY_A0A044S528_ONCVO/30-143	A0A044S528.1 PF0299	48.40	0.00
TRINITY_D8U5K1_VOLCA/20-262	D8U5K1.1 PF00262.16	48.40	0.00
TRINITY_D8U5M5_VOLCA/197-590	D8U5M5.1 PF01619.16	48.40	0.00
TRINITY_C1E3X7_MICSR/85-301	C1E3X7.1 PF02353.18	48.40	0.00
TRINITY_D8TS04_VOLCA/5-179	D8TS04.1 PF03256.14	48.40	0.00
TRINITY_D3BRC5_POLPA/498-682	D3BRC5.1 PF02145.13	48.40	0.00
TRINITY_I0Z054_9CHLO/1-486	I0Z054.1 PF03055.13	48.40	0.00
TRINITY_A1ZMR1_9BACT/19-139	A1ZMR1.1 PF00583.23	48.40	0.00
TRINITY_I1G2T0_AMPQE/174-530	I1G2T0.1 PF00657.20	48.40	0.00
TRINITY_D8TKF6_VOLCA/24-231	D8TKF6.1 PF07250.9;	48.40	0.00
TRINITY_T1J5U4_STRMM/792-1094	T1J5U4.1 PF13086.4;	48.40	0.00
TRINITY_D8ULQ2_VOLCA/4-208	D8ULQ2.1 PF07714.15	48.40	0.00
TRINITY_A0A060BGL7_9GAMM/12-358	A0A060BGL7.1 PF0126	48.40	0.00
TRINITY_L8H072_ACACA/127-268	L8H072.1 PF12014.6;	48.40	0.00
TRINITY_A0A087SF89_AUXPR/266-435	A0A087SF89.1 PF0146	48.40	0.00
TRINITY_L8GG01_ACACA/221-375	L8GG01.1 PF13768.4;	48.40	0.00
TRINITY_K9VL88_9CYAN/845-958	K9VL88.1 PF00072.22	48.40	0.00
TRINITY_A0E0V7_PARTE/895-1271	A0E0V7.1 PF12698.5;	48.40	0.00
TRINITY_F6VVB7_CIOIN/7-134	F6VVB7.2 PF00134.21	48.40	0.00
TRINITY_Q54VB5_DICDI/255-321	Q54VB5.1 PF01363.19	48.40	0.00
TRINITY_M0RXA8_MUSAM/117-180	M0RXA8.1 PF00400.30	48.40	0.00
TRINITY_B4M409_DROVI/14-110	B4M409.1 PF03645.11	48.40	0.00
TRINITY_I7MDT1_TETTS/158-284	I7MDT1.1 PF00107.24	48.30	0.00
TRINITY_Q240V2_TETTS/2-160	Q240V2.3 PF02580.14	48.30	0.00
TRINITY_I7MMP5_TETTS/4189-4485	I7MMP5.2 PF00632.23	48.30	0.00
TRINITY_Q1NWK7_9DELT/444-563	Q1NWK7.1 PF02518.24	48.30	0.00
TRINITY_D3AYL4_POLPA/358-420	D3AYL4.1 PF13432.4;	48.30	0.00
TRINITY_A0D918_PARTE/28-94	A0D918.1 PF10629.7;	48.30	0.00
TRINITY_L8HMC3_ACACA/10-132	L8HMC3.1 PF00241.18	48.30	0.00
TRINITY_L8GX54_ACACA/364-655	L8GX54.1 PF00176.21	48.30	0.00
TRINITY_U9TPN2_RHIID/70-158	U9TPN2.1 PF01230.21	48.30	0.00
TRINITY_W2QKH8_PHYPN/49-164	W2QKH8.1 PF05916.9;	48.30	0.00
TRINITY_V7AU43_PHAVU/229-326	V7AU43.1 PF00153.25	48.30	0.00
TRINITY_K9T0U6_9CYAN/328-414	K9T0U6.1 PF08447.10	48.30	0.00
TRINITY_MYLKB_DICDI/20-278	Q86AD7.1 PF00069.23	48.30	0.00
TRINITY_A0A0F5W0E3_9ACTN/38-181	A0A0F5W0E3.1 PF0824	48.30	0.00
TRINITY_Q22S98_TETTS/953-1159	Q22S98.1 PF01852.17	48.30	0.00
TRINITY_E9ECE3_METAQ/14-113	E9ECE3.1 PF02271.14	48.30	0.00
TRINITY_F0ZDI2_DICPU/347-532	F0ZDI2.1 PF00617.17	48.30	0.00
TRINITY_G0QKA0_ICHMG/1960-2256	G0QKA0.1 PF00632.23	48.30	0.00
TRINITY_A0A0D2WH60_CAPO3/43-435	A0A0D2WH60.1 PF0045	48.30	0.00
TRINITY_PBX4_MOUSE/216-275	Q99NE9.2 PF00046.27	48.30	0.00
TRINITY_W6FUH4_NODSP/15-183	W6FUH4.1 PF13385.4;	48.30	0.00
TRINITY_F0ZID3_DICPU/66-208	F0ZID3.1 PF01794.17	48.30	0.00
TRINITY_A0CWS5_PARTE/255-401	A0CWS5.1 PF05728.10	48.30	0.00
TRINITY_D3BSH8_POLPA/25-395	D3BSH8.1 PF10142.7;	48.30	0.00
TRINITY_I4YCU4_WALMC/5-65	I4YCU4.1 PF04193.12	48.30	0.00
TRINITY_J9J5M4_9SPIT/47-304	J9J5M4.1 PF00069.23	48.30	0.00
TRINITY_Q236L3_TETTS/620-683	Q236L3.1 PF13499.4;	48.30	0.00
TRINITY_F5Z9A3_ALTSS/30-182	F5Z9A3.1 PF05175.12	48.30	0.00
TRINITY_A8HZL5_CHLRE/1-327	A8HZL5.1 PF03372.21	48.30	0.00
TRINITY_F0ZTN0_DICPU/29-87	F0ZTN0.1 PF00786.26	48.30	0.00
TRINITY_L8GIE1_ACACA/28-436	L8GIE1.1 PF00999.19	48.30	0.00

TRINITY_D8UCH9_VOLCA/147-243	D8UCH9.1 PF00169.27	48.30	0.00
TRINITY_Q22W19_TETTS/115-323	Q22W19.3 PF00112.21	48.30	0.00
TRINITY_D8U6V6_VOLCA/25-262	D8U6V6.1 PF02536.12	48.30	0.00
TRINITY_I0Z3J9_9CHLO/60-210	I0Z3J9.1 PF00849.20	48.30	0.00
TRINITY_C1MHH4_MICPC/147-496	C1MHH4.1 PF13868.4;	48.30	0.00
TRINITY_I1M943_SOYBN/2-287	I1M943.2 PF05653.12	48.30	0.00
TRINITY_Q22RZ8_TETTS/183-242	Q22RZ8.2 PF13921.4;	48.30	0.00
TRINITY_R5PLD7_9CLOT/4-290	R5PLD7.1 PF00009.25	48.30	0.00
TRINITY_E4WR67_OIKDI/552-620	E4WR67.1 PF00505.17	48.30	0.00
TRINITY_A9U0X8_PHYPA/98-557	A9U0X8.1 PF00501.26	48.30	0.00
TRINITY_E1ZGD7_CHLVA/1-261	E1ZGD7.1 PF06246.10	48.30	0.00
TRINITY_A0A067U0K4_9AGAR/63-122	A0A067U0K4.1 PF1392	48.30	0.00
TRINITY_N1PJZ1_DOTSN/106-334	N1PJZ1.1 PF12706.5;	48.30	0.00
TRINITY_D8TNV2_VOLCA/856-1080	D8TNV2.1 PF00069.23	48.30	0.00
TRINITY_D8TL43_VOLCA/13-129	D8TL43.1 PF06487.10	48.30	0.00
TRINITY_E1ZKX7_CHLVA/288-465	E1ZKX7.1 PF07092.10	48.30	0.00
TRINITY_B7G1K5_PHATC/21-93	B7G1K5.1 PF04564.13	48.30	0.00
TRINITY_F4PVF7_DICFS/511-835	F4PVF7.1 PF00176.21	48.30	0.00
TRINITY_D8TNJ5_VOLCA/473-806	D8TNJ5.1 PF00773.17	48.30	0.00
TRINITY_I7MH27_TETTS/188-490	I7MH27.2 PF00069.23	48.30	0.00
TRINITY_Q22RZ8_TETTS/183-242	Q22RZ8.2 PF13921.4;	48.30	0.00
TRINITY_D8THM0_VOLCA/151-388	D8THM0.1 PF00687.19	48.30	0.00
TRINITY_D8UGV7_VOLCA/6-313	D8UGV7.1 PF01008.15	48.30	0.00
TRINITY_D8UJ01_VOLCA/442-703	D8UJ01.1 PF04157.14	48.30	0.00
TRINITY_A0E7K6_PARTE/64-122	A0E7K6.1 PF13921.4;	48.30	0.00
TRINITY_A8IW93_CHLRE/811-1086	A8IW93.1 PF03133.13	48.30	0.00
TRINITY_D8TGV3_VOLCA/197-257	D8TGV3.1 PF04117.10	48.30	0.00
TRINITY_A0A078AT62_STYLE/63-123	A0A078AT62.1 PF0419	48.30	0.00
TRINITY_A8HNP6_CHLRE/11-260	A8HNP6.1 PF07727.12	48.30	0.00
TRINITY_J9ILA3_9SPIT/9-310	J9ILA3.1 PF00069.23	48.30	0.00
TRINITY_D8TY55_VOLCA/277-416	D8TY55.1 PF02797.13	48.30	0.00
TRINITY_D8TYQ8_VOLCA/773-1053	D8TYQ8.1 PF12698.5;	48.30	0.00
TRINITY_W6MMC1_9ASCO/16-368	W6MMC1.1 PF00009.25	48.30	0.00
TRINITY_H2ZGU1_CIOSA/135-218	H2ZGU1.1 PF00017.22	48.30	0.00
TRINITY_L8GGB3_ACACA/1052-1230	L8GGB3.1 PF00617.17	48.30	0.00
TRINITY_A8J6B0_CHLRE/916-1326	A8J6B0.1 PF07714.15	48.30	0.00
TRINITY_I0YX50_9CHLO/460-547	I0YX50.1 PF14791.4;	48.30	0.00
TRINITY_G1NFL7_MELGA/27-141	G1NFL7.2 PF08662.9;	48.30	0.00
TRINITY_G0QQH6_ICHMG/266-462	G0QQH6.1 PF07859.11	48.20	0.00
TRINITY_A3J4K6_9FLAO/31-246	A3J4K6.1 PF00149.26	48.20	0.00
TRINITY_F9VIH1_ARTSS/25-144	F9VIH1.1 PF01926.21	48.20	0.00
TRINITY_A0A077ZW89_STYLE/21-290	A0A077ZW89.1 PF0024	48.20	0.00
TRINITY_I7LVZ5_TETTS/5-189	I7LVZ5.1 PF00106.23	48.20	0.00
TRINITY_I0YJM6_9CHLO/260-497	I0YJM6.1 PF13641.4;	48.20	0.00
TRINITY_Q1N2R9_9GAMM/772-890	Q1N2R9.1 PF00072.22	48.20	0.00
TRINITY_H0EAU9_9ACTN/11-349	H0EAU9.1 PF00724.18	48.20	0.00
TRINITY_D3BPQ9_POLPA/486-595	D3BPQ9.1 PF00271.29	48.20	0.00
TRINITY_A0D6P8_PARTE/189-398	A0D6P8.1 PF00113.20	48.20	0.00
TRINITY_E8RNC8_ASTEC/15-220	E8RNC8.1 PF01738.16	48.20	0.00
TRINITY_Q245E1_TETTS/75-258	Q245E1.1 PF07998.9;	48.20	0.00
TRINITY_A8IDE3_CHLRE/126-260	A8IDE3.1 PF00258.23	48.20	0.00
TRINITY_Q23ML0_TETTS/38-292	Q23ML0.2 PF00069.23	48.20	0.00
TRINITY_A0A0D2UJE3_CAPO3/7-91	A0A0D2UJE3.1 PF1341	48.20	0.00
TRINITY_A0A0H5C5W6_CYBJA/96-182	A0A0H5C5W6.1 PF1289	48.20	0.00
TRINITY_C1EBI2_MICSR/59-135	C1EBI2.1 PF03061.20	48.20	0.00
TRINITY_Q93X47_SOLLC/25-326	Q93X47.1 PF00069.23	48.20	0.00
TRINITY_J3MQ70_ORYBR/4-443	J3MQ70.1 PF00022.17	48.20	0.00
TRINITY_F4QK37_9CAUL/382-492	F4QK37.1 PF00072.22	48.20	0.00
TRINITY_A8JH85_CHLRE/7-146	A8JH85.1 PF02577.12	48.20	0.00
TRINITY_A0A074T522_HAMHA/154-349	A0A074T522.1 PF1214	48.20	0.00
TRINITY_H3GPM7_PHYRM/358-482	H3GPM7.1 PF04677.13	48.20	0.00

TRINITY_A0A077ZQT6_STYLE/504-743	A0A077ZQT6.1 PF0379	48.20	0.00
TRINITY_K7J5V0_NASVI/268-379	K7J5V0.1 PF01399.25	48.20	0.00
TRINITY_A0D747_PARTE/33-224	A0D747.1 PF00227.24	48.20	0.00
TRINITY_A7EJG1_SCLS1/195-540	A7EJG1.1 PF00704.26	48.20	0.00
TRINITY_E6SH94_THEM7/29-344	E6SH94.1 PF12849.5;	48.20	0.00
TRINITY_I7MLH4_TETTS/52-165	I7MLH4.1 PF11527.6;	48.20	0.00
TRINITY_A0A0M3J4C5_ANISI/18-185	A0A0M3J4C5.1 PF0027	48.20	0.00
TRINITY_A0A0G4J2X3_PLABS/751-865	A0A0G4J2X3.1 PF0066	48.20	0.00
TRINITY_A0A072UUB3_MEDTR/313-575	A0A072UUB3.1 PF0771	48.20	0.00
TRINITY_I0Z2L0_9CHLO/1-260	I0Z2L0.1 PF00246.22	48.20	0.00
TRINITY_A0A097QJW1_9VIBR/492-606	A0A097QJW1.1 PF0007	48.20	0.00
TRINITY_A8J1D6_CHLRE/44-170	A8J1D6.1 PF07714.15	48.20	0.00
TRINITY_Q6QAY4_CHLRE/46-164	Q6QAY4.1 PF02466.17	48.20	0.00
TRINITY_K1QUD2_CRAGI/21-580	K1QUD2.1 PF02696.12	48.20	0.00
TRINITY_A0A0M3JA26_ANISI/3-121	A0A0M3JA26.1 PF0007	48.20	0.00
TRINITY_E1ZBK6_CHLVA/5-138	E1ZBK6.1 PF08534.8;	48.20	0.00
TRINITY_A0A078B349_STYLE/444-804	A0A078B349.1 PF0070	48.20	0.00
TRINITY_A8IDJ5_CHLRE/65-423	A8IDJ5.1 PF12832.5;	48.20	0.00
TRINITY_D8Q0R8_SCHCM/345-567	D8Q0R8.1 PF00082.20	48.20	0.00
TRINITY_Q24JG1_TETTS/16-326	Q24JG1.1 PF07992.12	48.20	0.00
TRINITY_D8U9K8_VOLCA/158-468	D8U9K8.1 PF08318.10	48.20	0.00
TRINITY_D8TIY0_VOLCA/27-347	D8TIY0.1 PF05185.14	48.20	0.00
TRINITY_I0Z502_9CHLO/30-503	I0Z502.1 PF10243.7;	48.20	0.00
TRINITY_Q4U9S0_THEAN/10-79	Q4U9S0.1 PF00076.20	48.20	0.00
TRINITY_D8UF48_VOLCA/43-512	D8UF48.1 PF01425.19	48.20	0.00
TRINITY_D8UK64_VOLCA/8-471	D8UK64.1 PF13520.4;	48.20	0.00
TRINITY_D8THA8_VOLCA/22-139	D8THA8.1 PF01152.19	48.20	0.00
TRINITY_Q22RY4_TETTS/17-249	Q22RY4.2 PF13561.4;	48.20	0.00
TRINITY_A0A068RVE3_9FUNG/37-177	A0A068RVE3.1 PF0010	48.20	0.00
TRINITY_G3N717_GASAC/14-224	G3N717.1 PF01130.19	48.20	0.00
TRINITY_A0A099NW38_PICKU/146-302	A0A099NW38.1 PF0007	48.20	0.00
TRINITY_Q24GQ7_TETTS/133-301	Q24GQ7.1 PF04664.11	48.20	0.00
TRINITY_L8HFM8_ACACA/51-472	L8HFM8.1 PF13520.4;	48.10	0.00
TRINITY_F4PIG0_DICFS/3-277	F4PIG0.1 PF09743.7;	48.10	0.00
TRINITY_G0R0S9_ICHMG/8-169	G0R0S9.1 PF00071.20	48.10	0.00
TRINITY_L8HG43_ACACA/2-237	L8HG43.1 PF01026.19	48.10	0.00
TRINITY_D3BLJ5_POLPA/1506-1723	D3BLJ5.1 PF16095.3;	48.10	0.00
TRINITY_G0QLS5_ICHMG/84-228	G0QLS5.1 PF00782.18	48.10	0.00
TRINITY_J9JCA5_9SPIT/125-339	J9JCA5.1 PF00112.21	48.10	0.00
TRINITY_PATS1_DICDI/1724-1858	Q55E58.1 PF08477.11	48.10	0.00
TRINITY_L8GPA8_ACACA/1396-1737	L8GPA8.1 PF00443.27	48.10	0.00
TRINITY_A0CKF8_PARTE/1118-1269	A0CKF8.1 PF00005.25	48.10	0.00
TRINITY_G7UVT4_PSEUP/20-250	G7UVT4.1 PF06966.10	48.10	0.00
TRINITY_W5N4Z1_LEPOC/367-520	W5N4Z1.1 PF13091.4;	48.10	0.00
TRINITY_A0A058ZCU4_9EUKA/174-384	A0A058ZCU4.1 PF0438	48.10	0.00
TRINITY_V4AKP7_LOTGI/34-119	V4AKP7.1 PF05172.11	48.10	0.00
TRINITY_Q232X8_TETTS/36-488	Q232X8.1 PF00067.20	48.10	0.00
TRINITY_I0YRR8_9CHLO/10-222	I0YRR8.1 PF04755.10	48.10	0.00
TRINITY_C7ZFC2_NECH7/12-267	C7ZFC2.1 PF00370.19	48.10	0.00
TRINITY_F4QBI1_DICFS/9-217	F4QBI1.1 PF01135.17	48.10	0.00
TRINITY_A0CXI4_PARTE/131-348	A0CXI4.1 PF00112.21	48.10	0.00
TRINITY_G0QWE9_ICHMG/1-205	G0QWE9.1 PF03531.12	48.10	0.00
TRINITY_X6LEC0_RETFI/128-273	X6LEC0.1 PF07534.14	48.10	0.00
TRINITY_W7U6S8_9STRA/84-164	W7U6S8.1 PF05046.12	48.10	0.00
TRINITY_U9TX15_RHIID/316-561	U9TX15.1 PF00122.18	48.10	0.00
TRINITY_A0BGJ9_PARTE/233-359	A0BGJ9.1 PF00169.27	48.10	0.00
TRINITY_D0MJ84_RHOM4/509-745	D0MJ84.1 PF02784.14	48.10	0.00
TRINITY_D2VCP2_NAEGR/25-231	D2VCP2.1 PF00149.26	48.10	0.00
TRINITY_A0A077JGQ4_9CYAN/5-157	A0A077JGQ4.1 PF0053	48.10	0.00
TRINITY_D8TXT6_VOLCA/540-616	D8TXT6.1 PF08172.10	48.10	0.00
TRINITY_A0A059F104_9MICR/244-374	A0A059F104.1 PF0000	48.10	0.00

TRINITY_L0DQE9_SINAD/83-239	L0DQE9.1 PF12831.5;	48.10	0.00
TRINITY_L8GL12_ACACA/119-330	L8GL12.1 PF00112.21	48.10	0.00
TRINITY_A0A0E0PAX7_ORYRU/784-1270	A0A0E0PAX7.1 PF0499	48.10	0.00
TRINITY_A0A089INQ2_9BACL/3-298	A0A089INQ2.1 PF0115	48.10	0.00
TRINITY_A8JFG1_CHLRE/27-174	A8JFG1.1 PF00005.25	48.10	0.00
TRINITY_D8T0H5_SELML/2010-2704	D8T0H5.1 PF03028.13	48.10	0.00
TRINITY_A0E2J1_PARTE/100-358	A0E2J1.1 PF00069.23	48.10	0.00
TRINITY_S2JXT8_MUCC1/140-216	S2JXT8.1 PF01713.19	48.10	0.00
TRINITY_I7MMU2_TETTS/406-489	I7MMU2.2 PF00173.26	48.10	0.00
TRINITY_D8TMB4_VOLCA/171-394	D8TMB4.1 PF16953.3;	48.10	0.00
TRINITY_D8UB73_VOLCA/14-95	D8UB73.1 PF00439.23	48.10	0.00
TRINITY_D8UKR0_VOLCA/688-843	D8UKR0.1 PF08625.9;	48.10	0.00
TRINITY_D8U810_VOLCA/723-1010	D8U810.1 PF00689.19	48.10	0.00
TRINITY_L8GTS3_ACACA/110-367	L8GTS3.1 PF00082.20	48.10	0.00
TRINITY_E1ZGW7_CHLVA/63-362	E1ZGW7.1 PF00291.23	48.10	0.00
TRINITY_L8GLQ1_ACACA/9-534	L8GLQ1.1 PF00995.21	48.10	0.00
TRINITY_I0ANS2_IGNAJ/4-227	I0ANS2.1 PF00300.20	48.10	0.00
TRINITY_A8ILY4_CHLRE/58-303	A8ILY4.1 PF06966.10	48.10	0.00
TRINITY_D8TJ44_VOLCA/283-744	D8TJ44.1 PF01131.18	48.10	0.00
TRINITY_A0A075B1I0_9FUNG/7-164	A0A075B1I0.1 PF0010	48.10	0.00
TRINITY_A0A058Z850_9EUKA/93-329	A0A058Z850.1 PF0404	48.10	0.00
TRINITY_L8GD76_ACACA/374-704	L8GD76.1 PF13243.4;	48.10	0.00
TRINITY_U6PQX4_HAECO/8-143	U6PQX4.1 PF05871.10	48.10	0.00
TRINITY_G0QU25_ICHMG/766-848	G0QU25.1 PF00635.24	48.10	0.00
TRINITY_A8JCH8_CHLRE/152-639	A8JCH8.1 PF01532.18	48.10	0.00
TRINITY_W2QLZ0_PHYPN/255-412	W2QLZ0.1 PF00903.23	48.10	0.00
TRINITY_I0YUQ5_9CHLO/33-353	I0YUQ5.1 PF01636.21	48.10	0.00
TRINITY_C1KRF4_MICSR/10-318	C1KRF4.1 PF00146.19	48.10	0.00
TRINITY_I7MF85_TETTS/409-515	I7MF85.1 PF00307.29	48.10	0.00
TRINITY_I0Z4A2_9CHLO/348-426	I0Z4A2.1 PF01713.19	48.10	0.00
TRINITY_A0D0G9_PARTE/14-80	A0D0G9.1 PF02953.13	48.10	0.00
TRINITY_G0QZD1_ICHMG/22-199	G0QZD1.1 PF00300.20	48.00	0.00
TRINITY_A9E8E2_9RHOB/379-491	A9E8E2.1 PF02518.24	48.00	0.00
TRINITY_I7MHD1_TETTS/27-277	I7MHD1.1 PF00194.19	48.00	0.00
TRINITY_A0DSD5_PARTE/527-699	A0DSD5.1 PF15901.3;	48.00	0.00
TRINITY_Q6MPK6_BDEBA/12-184	Q6MPK6.1 PF01048.18	48.00	0.00
TRINITY_W9QUK4_9ROSA/10-107	W9QUK4.1 PF04749.15	48.00	0.00
TRINITY_G7I4A8_MEDTR/136-211	G7I4A8.2 PF04969.14	48.00	0.00
TRINITY_Q54G72_DICDI/71-198	Q54G72.1 PF04178.10	48.00	0.00
TRINITY_A0A0K0GA30_9FIRM/79-280	A0A0K0GA30.1 PF0785	48.00	0.00
TRINITY_F4Q2G9_DICFS/1228-1485	F4Q2G9.1 PF00069.23	48.00	0.00
TRINITY_I1CP32_RHIO9/418-584	I1CP32.1 PF08282.10	48.00	0.00
TRINITY_S8ASF3_DACHA/11-257	S8ASF3.1 PF05368.11	48.00	0.00
TRINITY_J9G2T1_9SPIT/184-448	J9G2T1.1 PF00069.23	48.00	0.00
TRINITY_A0A0G2FCS4_9PEZI/613-798	A0A0G2FCS4.1 PF0136	48.00	0.00
TRINITY_A0A0G1TKJ1_9BACT/168-323	A0A0G1TKJ1.1 PF0000	48.00	0.00
TRINITY_D3BKD6_POLPA/841-1356	D3BKD6.1 PF07690.14	48.00	0.00
TRINITY_A9B7L6_HERA2/39-313	A9B7L6.1 PF00664.21	48.00	0.00
TRINITY_F0ZS77_DICPU/34-592	F0ZS77.1 PF01602.18	48.00	0.00
TRINITY_A0A0D2U0H3_CAPO3/48-269	A0A0D2U0H3.1 PF1269	48.00	0.00
TRINITY_L8GUS0_ACACA/40-365	L8GUS0.1 PF05028.12	48.00	0.00
TRINITY_A0A0D2U4X4_CAPO3/129-469	A0A0D2U4X4.1 PF0028	48.00	0.00
TRINITY_I7MJV0_TETTS/846-955	I7MJV0.2 PF00069.23	48.00	0.00
TRINITY_A0BYQ1_PARTE/967-1061	A0BYQ1.1 PF12796.5;	48.00	0.00
TRINITY_G7KDK7_MEDTR/79-1040	G7KDK7.1 PF03813.12	48.00	0.00
TRINITY_A8J5N4_CHLRE/1-277	A8J5N4.1 PF04005.10	48.00	0.00
TRINITY_G3VCG1_SARHA/99-262	G3VCG1.1 PF00160.19	48.00	0.00
TRINITY_CAP_DICDI/7-295	P54654.1 PF01213.17	48.00	0.00
TRINITY_D8TYV9_VOLCA/182-326	D8TYV9.1 PF01547.23	48.00	0.00
TRINITY_I3M983_ICTTR/326-450	I3M983.1 PF00637.18	48.00	0.00
TRINITY_I7M1Q4_TETTS/136-379	I7M1Q4.1 PF02104.13	48.00	0.00

TRINITY_J9J6J8_9SPIT/288-337	J9J6J8.1 PF13920.4;	48.00	0.00
TRINITY_D2V2K6_NAEGR/15-458	D2V2K6.1 PF01532.18	48.00	0.00
TRINITY_B8CEK5_THAPS/115-168	B8CEK5.1 PF13920.4;	48.00	0.00
TRINITY_I0Z4U1_9CHLO/1-239	I0Z4U1.1 PF03029.15	48.00	0.00
TRINITY_F0ZKI2_DICPU/61-525	F0ZKI2.1 PF00501.26	48.00	0.00
TRINITY_E1ZNW4_CHLVA/74-194	E1ZNW4.1 PF03099.17	48.00	0.00
TRINITY_C3Y231_BRAFL/41-301	C3Y231.1 PF13621.4;	48.00	0.00
TRINITY_B9J9K3_AGRRK/3-223	B9J9K3.1 PF01370.19	48.00	0.00
TRINITY_A0A059LER1_9CHLO/66-239	A0A059LER1.1 PF0599	48.00	0.00
TRINITY_D8TQ12_VOLCA/33-150	D8TQ12.1 PF00861.20	48.00	0.00
TRINITY_Q2JN91_SYNJB/3-177	Q2JN91.1 PF00875.16	48.00	0.00
TRINITY_Q23QD2_TETTS/366-575	Q23QD2.2 PF00566.16	48.00	0.00
TRINITY_D8UHP4_VOLCA/36-304	D8UHP4.1 PF00481.19	48.00	0.00
TRINITY_A8IJH7_CHLRE/73-337	A8IJH7.1 PF07690.14	48.00	0.00
TRINITY_D8U114_VOLCA/14-254	D8U114.1 PF04278.10	48.00	0.00
TRINITY_A0A022Q101_ERYGU/250-880	A0A022Q101.1 PF0244	48.00	0.00
TRINITY_D8UGV3_VOLCA/80-368	D8UGV3.1 PF01062.19	48.00	0.00
TRINITY_D8TZA3_VOLCA/30-444	D8TZA3.1 PF01266.22	48.00	0.00
TRINITY_D8TNQ6_VOLCA/75-273	D8TNQ6.1 PF03798.14	48.00	0.00
TRINITY_I0YZT9_9CHLO/111-270	I0YZT9.1 PF10294.7;	48.00	0.00
TRINITY_Q4U0V9_CHLRE/394-516	Q4U0V9.1 PF04145.13	48.00	0.00
TRINITY_I3NI03_HUMAN/25-142	I3NI03.1 PF00085.18	48.00	0.00
TRINITY_I7LZX2_TETTS/778-864	I7LZX2.2 PF15780.3;	47.90	0.00
TRINITY_M0YGB8_HORVD/122-194	M0YGB8.1 PF04564.13	47.90	0.00
TRINITY_K5Y5M0_AGABU/807-904	K5Y5M0.1 PF00439.23	47.90	0.00
TRINITY_U5D445_AMBTC/15-142	U5D445.1 PF04857.18	47.90	0.00
TRINITY_F4KT89_HALH1/36-176	F4KT89.1 PF00188.24	47.90	0.00
TRINITY_A0D8U5_PARTE/465-719	A0D8U5.1 PF01504.16	47.90	0.00
TRINITY_Q23FA1_TETTS/3-173	Q23FA1.2 PF01956.14	47.90	0.00
TRINITY_L8H072_ACACA/127-268	L8H072.1 PF12014.6;	47.90	0.00
TRINITY_F0ZH73_DICPU/405-577	F0ZH73.1 PF00621.18	47.90	0.00
TRINITY_I7LUI2_TETTS/149-309	I7LUI2.1 PF02544.14	47.90	0.00
TRINITY_B7QIS0_IXOSC/91-185	B7QIS0.1 PF04800.10	47.90	0.00
TRINITY_C7PGG8_CHIPD/65-212	C7PGG8.1 PF00174.17	47.90	0.00
TRINITY_L8GHQ5_ACACA/212-274	L8GHQ5.1 PF13840.4;	47.90	0.00
TRINITY_A0C833_PARTE/29-147	A0C833.1 PF07986.10	47.90	0.00
TRINITY_Q24FP1_TETTS/133-395	Q24FP1.1 PF00069.23	47.90	0.00
TRINITY_A2F6E0_TRIVA/259-408	A2F6E0.1 PF00005.25	47.90	0.00
TRINITY_W2Q5C8_PHYPN/67-137	W2Q5C8.1 PF00076.20	47.90	0.00
TRINITY_A0A084G6U9_9PEZI/1792-1911	A0A084G6U9.1 PF0007	47.90	0.00
TRINITY_Q22ZB8_TETTS/896-1015	Q22ZB8.2 PF08578.8;	47.90	0.00
TRINITY_X6PD65_RETFI/417-571	X6PD65.1 PF09445.8;	47.90	0.00
TRINITY_A8IYR6_CHLRE/6-146	A8IYR6.1 PF05903.12	47.90	0.00
TRINITY_A8IA75_CHLRE/44-181	A8IA75.1 PF03330.16	47.90	0.00
TRINITY_A0A085MQK8_9BILA/324-484	A0A085MQK8.1 PF0007	47.90	0.00
TRINITY_A0A0P7WDR4_9TELE/68-471	A0A0P7WDR4.1 PF0045	47.90	0.00
TRINITY_K9RGX6_9CYAN/129-348	K9RGX6.1 PF00891.16	47.90	0.00
TRINITY_Q54Q13_DICDI/172-276	Q54Q13.1 PF00616.17	47.90	0.00
TRINITY_E1ZGY6_CHLVA/11-104	E1ZGY6.1 PF09696.8;	47.90	0.00
TRINITY_Q54QA5_DICDI/3-75	Q54QA5.1 PF00240.21	47.90	0.00
TRINITY_D8TJ90_VOLCA/44-180	D8TJ90.1 PF01138.19	47.90	0.00
TRINITY_D8TQI4_VOLCA/63-382	D8TQI4.1 PF05686.10	47.90	0.00
TRINITY_D8TJ45_VOLCA/16-248	D8TJ45.1 PF13483.4;	47.90	0.00
TRINITY_C7R815_KANKD/38-416	C7R815.1 PF00266.17	47.90	0.00
TRINITY_A0DA23_PARTE/4-77	A0DA23.1 PF00240.21	47.90	0.00
TRINITY_A0A078A398_STYLE/78-203	A0A078A398.1 PF0502	47.90	0.00
TRINITY_L8HHE2_ACACA/28-144	L8HHE2.1 PF00856.26	47.90	0.00
TRINITY_D4ZZ36_ARTPN/26-390	D4ZZ36.1 PF03738.12	47.90	0.00
TRINITY_Q22EG9_TETTS/11-197	Q22EG9.1 PF13460.4;	47.90	0.00
TRINITY_D8U9S9_VOLCA/599-793	D8U9S9.1 PF00211.18	47.90	0.00
TRINITY_A0A0A8JNB0_BACSX/9-297	A0A0A8JNB0.1 PF0070	47.90	0.00

TRINITY_C4M4F2_ENTHI/87-277	C4M4F2.1 PF01545.19	47.90	0.00
TRINITY_I0YPX2_9CHLO/757-1117	I0YPX2.1 PF00328.20	47.90	0.00
TRINITY_A0A0G4EI42_9ALVE/68-343	A0A0G4EI42.1 PF0374	47.90	0.00
TRINITY_M5WT69_PRUPE/136-299	M5WT69.1 PF00156.25	47.90	0.00
TRINITY_A0BSG8_PARTE/16-715	A0BSG8.1 PF03030.14	47.90	0.00
TRINITY_I2G5Z0_USTH4/119-189	I2G5Z0.1 PF00076.20	47.90	0.00
TRINITY_GANAB_DICDI/363-810	Q94502.1 PF01055.24	47.90	0.00
TRINITY_A0A067Q6E0_9HOMO/18-200	A0A067Q6E0.1 PF0524	47.90	0.00
TRINITY_A0D6N7_PARTE/232-422	A0D6N7.1 PF05193.19	47.90	0.00
TRINITY_A4VD54_TETTS/744-1018	A4VD54.1 PF00664.21	47.90	0.00
TRINITY_Q9S7V1_CHLRE/62-365	Q9S7V1.1 PF01218.16	47.90	0.00
TRINITY_A0A087SBU6_AUXPR/25-511	A0A087SBU6.1 PF0305	47.90	0.00
TRINITY_D7KSY9_ARALL/12-364	D7KSY9.1 PF05282.9;	47.90	0.00
TRINITY_I1LWH3_SOYBN/143-260	I1LWH3.1 PF13640.4;	47.90	0.00
TRINITY_E1ZHV4_CHLVA/59-295	E1ZHV4.1 PF01063.17	47.90	0.00
TRINITY_PEX1_DICDI/104-178	Q54GX5.1 PF09262.9;	47.90	0.00
TRINITY_M3XJS3_LATCH/384-728	M3XJS3.1 PF01237.16	47.90	0.00
TRINITY_A8HSS2_CHLRE/75-286	A8HSS2.1 PF00856.26	47.90	0.00
TRINITY_C3ZB32_BRAFL/9-126	C3ZB32.1 PF09748.7;	47.90	0.00
TRINITY_Q4Q2F7_LEIMA/2-211	Q4Q2F7.1 PF00230.18	47.90	0.00
TRINITY_A8IMI9_CHLRE/35-177	A8IMI9.1 PF00810.16	47.90	0.00
TRINITY_K9VG38_9CYAN/46-322	K9VG38.1 PF02016.13	47.90	0.00
TRINITY_A0A0G4F7A0_9ALVE/6-124	A0A0G4F7A0.1 PF1600	47.90	0.00
TRINITY_A0A067RDK5_ZOONE/223-365	A0A067RDK5.1 PF0006	47.90	0.00
TRINITY_J9J3S5_9SPIT/394-881	J9J3S5.1 PF00232.16	47.90	0.00
TRINITY_L8GRD3_ACACA/26-540	L8GRD3.1 PF00135.26	47.90	0.00
TRINITY_D8UJQ1_VOLCA/16-132	D8UJQ1.1 PF00179.24	47.90	0.00
TRINITY_E1ZLR3_CHLVA/1008-1219	E1ZLR3.1 PF01061.22	47.90	0.00
TRINITY_G0R413_ICHMG/18-299	G0R413.1 PF00122.18	47.90	0.00
TRINITY_V4AA96_LOTGI/115-252	V4AA96.1 PF10238.7;	47.90	0.00
TRINITY_A0A0G4GBM8_9ALVE/180-254	A0A0G4GBM8.1 PF0024	47.90	0.00
TRINITY_A0A0G4GVC2_9ALVE/10-176	A0A0G4GVC2.1 PF0007	47.90	0.00
TRINITY_K1PTG4_CRAGI/14-61	K1PTG4.1 PF13920.4;	47.90	0.00
TRINITY_A8IZS4_CHLRE/25-124	A8IZS4.1 PF00583.23	47.90	0.00
TRINITY_L8GWQ5_ACACA/986-1235	L8GWQ5.1 PF07714.15	47.90	0.00
TRINITY_D8U9E8_VOLCA/326-444	D8U9E8.1 PF09273.9;	47.90	0.00
TRINITY_A0A087SQQ5_AUXPR/81-201	A0A087SQQ5.1 PF0933	47.90	0.00
TRINITY_F0NZF5_WEEVC/18-168	F0NZF5.1 PF01368.18	47.80	0.00
TRINITY_F2G2I5_ALTMD/397-513	F2G2I5.1 PF02518.24	47.80	0.00
TRINITY_I7MGN1_TETTS/116-411	I7MGN1.1 PF00648.19	47.80	0.00
TRINITY_G0QL39_ICHMG/187-277	G0QL39.1 PF00153.25	47.80	0.00
TRINITY_Q247V8_TETTS/66-225	Q247V8.2 PF01343.16	47.80	0.00
TRINITY_A9PFT8_POPTR/187-232	A9PFT8.1 PF13920.4;	47.80	0.00
TRINITY_A0A067JGY6_JATCU/15-269	A0A067JGY6.1 PF0006	47.80	0.00
TRINITY_HSCB_RICPR/3-69	Q9ZDW4.2 PF00226.29	47.80	0.00
TRINITY_F4PJK8_DICFS/3330-3423	F4PJK8.1 PF09333.9;	47.80	0.00
TRINITY_J9IZH8_9SPIT/189-265	J9IZH8.1 PF14360.4;	47.80	0.00
TRINITY_C7RHS1_ANAPD/9-172	C7RHS1.1 PF00156.25	47.80	0.00
TRINITY_F2TWN2_SALR5/28-80	F2TWN2.1 PF00130.20	47.80	0.00
TRINITY_E1ZQH4_CHLVA/1-72	E1ZQH4.1 PF00111.25	47.80	0.00
TRINITY_L8H0M8_ACACA/8-74	L8H0M8.1 PF00076.20	47.80	0.00
TRINITY_A0A061EEG7_THECC/589-749	A0A061EEG7.1 PF0007	47.80	0.00
TRINITY_Q232X8_TETTS/36-488	Q232X8.1 PF00067.20	47.80	0.00
TRINITY_M1AV27_SOLTU/1-248	M1AV27.1 PF04614.10	47.80	0.00
TRINITY_D2VUC7_NAEGR/630-746	D2VUC7.1 PF08785.9;	47.80	0.00
TRINITY_C5CFM1_KOSOT/64-152	C5CFM1.1 PF06108.10	47.80	0.00
TRINITY_I7MA61_TETTS/288-628	I7MA61.1 PF02841.12	47.80	0.00
TRINITY_V7AHU9_PHAVU/431-499	V7AHU9.1 PF00076.20	47.80	0.00
TRINITY_E1ZP12_CHLVA/84-124	E1ZP12.1 PF13923.4;	47.80	0.00
TRINITY_H0DKM4_9STAP/5-117	H0DKM4.1 PF02152.16	47.80	0.00
TRINITY_A8BJ08_GIAIC/6-76	A8BJ08.1 PF11976.6;	47.80	0.00

TRINITY_C1MXI4_MICPC/1-152	C1MXI4.1 PF13489.4;	47.80	0.00
TRINITY_L8H0D0_ACACA/265-492	L8H0D0.1 PF13087.4;	47.80	0.00
TRINITY_Q22U39_TETTS/54-238	Q22U39.3 PF13460.4;	47.80	0.00
TRINITY_A8IT81_CHLRE/4-456	A8IT81.1 PF13520.4;	47.80	0.00
TRINITY_F6GXW2_VITVI/197-314	F6GXW2.1 PF07500.12	47.80	0.00
TRINITY_F2Z5T4_PIG/14-105	F2Z5T4.1 PF02214.20	47.80	0.00
TRINITY_A8GEY6_SERP5/149-325	A8GEY6.1 PF02894.15	47.80	0.00
TRINITY_D8UCK8_VOLCA/2-74	D8UCK8.1 PF03937.14	47.80	0.00
TRINITY_K2MXL3_TRYCR/193-267	K2MXL3.1 PF14360.4;	47.80	0.00
TRINITY_B9XNB2_PEDPL/16-309	B9XNB2.1 PF06626.10	47.80	0.00
TRINITY_A8IS78_CHLRE/5-74	A8IS78.1 PF02325.15	47.80	0.00
TRINITY_D8U875_VOLCA/48-175	D8U875.1 PF07967.11	47.80	0.00
TRINITY_B5JCS6_9BACT/448-600	B5JCS6.1 PF13229.4;	47.80	0.00
TRINITY_C1N0B1_MICPC/149-384	C1N0B1.1 PF12850.5;	47.80	0.00
TRINITY_A0DB80_PARTE/91-183	A0DB80.1 PF04777.11	47.80	0.00
TRINITY_A0A0G4EFT4_9ALVE/392-487	A0A0G4EFT4.1 PF1276	47.80	0.00
TRINITY_A0CBK7_PARTE/79-221	A0CBK7.1 PF01694.20	47.80	0.00
TRINITY_V8PBV8_OPHHA/271-441	V8PBV8.1 PF02836.15	47.80	0.00
TRINITY_F4Q2G9_DICFS/1228-1485	F4Q2G9.1 PF00069.23	47.80	0.00
TRINITY_M0S1Q3_MUSAM/282-665	M0S1Q3.1 PF01055.24	47.80	0.00
TRINITY_GBPC_DICDI/2508-2603	Q8MVR1.1 PF00027.27	47.80	0.00
TRINITY_A8HQD3_CHLRE/65-178	A8HQD3.1 PF05916.9;	47.80	0.00
TRINITY_F0RM89_DEIPM/14-368	F0RM89.1 PF07992.12	47.80	0.00
TRINITY_J9IA49_9SPIT/254-345	J9IA49.1 PF00447.15	47.80	0.00

TRINITY_D3BLF1_POLPA/69-500	D3BLF1.1 PF00450.20	47.80	0.00
TRINITY_C1E442_MICSR/168-316	C1E442.1 PF00929.22	47.80	0.00
TRINITY_D8TI14_VOLCA/22-1084	D8TI14.1 PF04147.10	47.80	0.00
TRINITY_A0A0G1VJ21_9BACT/163-331	A0A0G1VJ21.1 PF0053	47.80	0.00
TRINITY_D8UBI0_VOLCA/14-440	D8UBI0.1 PF00067.20	47.80	0.00
TRINITY_G2G577_9ACTN/11-453	G2G577.1 PF13520.4;	47.80	0.00
TRINITY_D8UC02_VOLCA/1132-1256	D8UC02.1 PF07714.15	47.80	0.00
TRINITY_I1R4R8_ORYGL/175-305	I1R4R8.1 PF01419.15	47.80	0.00
TRINITY_W5NJQ1_LEPOC/5-96	W5NJQ1.1 PF03647.11	47.80	0.00
TRINITY_G0R3B2_ICHMG/13-265	G0R3B2.1 PF00069.23	47.80	0.00
TRINITY_G0QK27_ICHMG/116-201	G0QK27.1 PF10785.7;	47.70	0.00
TRINITY_I7MGA5_TETTS/126-350	I7MGA5.1 PF00687.19	47.70	0.00
TRINITY_A0A0G4IIY5_PLABS/444-547	A0A0G4IIY5.1 PF0085	47.70	0.00
TRINITY_Q231W9_TETTS/41-172	Q231W9.2 PF08315.10	47.70	0.00
TRINITY_A4H9T6_LEIBR/28-121	A4H9T6.1 PF01329.17	47.70	0.00
TRINITY_I7M3C5_TETTS/44-281	I7M3C5.1 PF10275.7;	47.70	0.00
TRINITY_A0BLU0_PARTE/56-228	A0BLU0.1 PF07162.9;	47.70	0.00
TRINITY_V7C4E4_PHAVU/12-103	V7C4E4.1 PF02214.20	47.70	0.00
TRINITY_A0C0D4_PARTE/39-227	A0C0D4.1 PF13862.4;	47.70	0.00
TRINITY_W9R525_9ROSA/8-98	W9R525.1 PF02970.14	47.70	0.00
TRINITY_D6W9R7_TRICA/19-113	D6W9R7.1 PF00153.25	47.70	0.00
TRINITY_F0ZA90_DICPU/22-375	F0ZA90.1 PF04757.12	47.70	0.00
TRINITY_A0A078B856_STYLE/13-301	A0A078B856.1 PF0006	47.70	0.00
TRINITY_MYLKB_DICDI/20-278	Q86AD7.1 PF00069.23	47.70	0.00
TRINITY_W4Z0C5_STRPU/373-459	W4Z0C5.1 PF09261.9;	47.70	0.00
TRINITY_B4J7Z6_DROGR/290-495	B4J7Z6.1 PF00106.23	47.70	0.00
TRINITY_Q23QF2_TETTS/248-571	Q23QF2.2 PF03133.13	47.70	0.00
TRINITY_D0MQP4_PHYIT/223-288	D0MQP4.1 PF01918.19	47.70	0.00
TRINITY_V4AY93_LOTGI/391-542	V4AY93.1 PF01699.22	47.70	0.00
TRINITY_U3KB49_FICAL/116-350	U3KB49.1 PF04389.15	47.70	0.00
TRINITY_I0Z8W9_9CHLO/1-165	I0Z8W9.1 PF05653.12	47.70	0.00
TRINITY_A0A0G0EM12_9BACT/22-298	A0A0G0EM12.1 PF0058	47.70	0.00
TRINITY_G0R3B8_ICHMG/412-571	G0R3B8.1 PF00637.18	47.70	0.00
TRINITY_D7MP44_ARALL/704-971	D7MP44.1 PF12931.5;	47.70	0.00
TRINITY_D8UCD6_VOLCA/1046-1104	D8UCD6.1 PF08373.8;	47.70	0.00
TRINITY_A0A068RYC9_9FUNG/57-378	A0A068RYC9.1 PF0186	47.70	0.00
TRINITY_A2FHQ0_TRIVA/5-116	A2FHQ0.1 PF01412.16	47.70	0.00
TRINITY_D8TND4_VOLCA/49-240	D8TND4.1 PF00856.26	47.70	0.00
TRINITY_F1A1W5_DICPU/73-242	F1A1W5.1 PF01553.19	47.70	0.00
TRINITY_L8GFU2_ACACA/577-721	L8GFU2.1 PF00644.18	47.70	0.00
TRINITY_C1MKL4_MICPC/125-232	C1MKL4.1 PF02002.15	47.70	0.00
TRINITY_A8JEZ9_CHLRE/2139-2229	A8JEZ9.1 PF13020.4;	47.70	0.00
TRINITY_A8JJ74_CHLRE/1-248	A8JJ74.1 PF00756.18	47.70	0.00
TRINITY_A0A084W838_ANOSI/22-81	A0A084W838.1 PF1392	47.70	0.00
TRINITY_G0QR68_ICHMG/177-395	G0QR68.1 PF04811.13	47.70	0.00
TRINITY_W9WCQ3_9EURO/72-287	W9WCQ3.1 PF13489.4;	47.70	0.00
TRINITY_E1ZLD4_CHLVA/75-218	E1ZLD4.1 PF00004.27	47.70	0.00
TRINITY_A0CMX0_PARTE/135-272	A0CMX0.1 PF01529.18	47.70	0.00
TRINITY_J9J1V2_9SPIT/5-143	J9J1V2.1 PF00179.24	47.70	0.00
TRINITY_D8U4J4_VOLCA/20-324	D8U4J4.1 PF16363.3;	47.70	0.00
TRINITY_Q22NJ2_TETTS/2-450	Q22NJ2.2 PF00067.20	47.70	0.00
TRINITY_Q6U9W9_CHLRE/45-120	Q6U9W9.1 PF01066.19	47.70	0.00
TRINITY_A0DTA6_PARTE/314-534	A0DTA6.1 PF07002.14	47.70	0.00
TRINITY_A0A0D2VSI5_CAPO3/35-145	A0A0D2VSI5.1 PF0848	47.70	0.00
TRINITY_Q22ZG4_TETTS/603-784	Q22ZG4.1 PF02141.19	47.70	0.00
TRINITY_L8HEV0_ACACA/102-580	L8HEV0.1 PF00890.22	47.70	0.00
TRINITY_I0YXB3_9CHLO/6-211	I0YXB3.1 PF00106.23	47.70	0.00
TRINITY_W7X7I9_TETTS/323-408	W7X7I9.1 PF00439.23	47.70	0.00
TRINITY_A8INQ8_CHLRE/150-374	A8INQ8.1 PF07817.11	47.70	0.00
TRINITY_A8I9K8_CHLRE/78-336	A8I9K8.1 PF01663.20	47.70	0.00
TRINITY_D8TYL3_VOLCA/65-218	D8TYL3.1 PF12499.6;	47.70	0.00

TRINITY_I1GCH0_AMPQE/1-303	I1GCH0.1 PF04641.10	47.70	0.00
TRINITY_B0D263_LACBS/27-567	B0D263.1 PF00135.26	47.70	0.00
TRINITY_D8UA95_VOLCA/22-333	D8UA95.1 PF00814.23	47.70	0.00
TRINITY_D8TMB6_VOLCA/8-677	D8TMB6.1 PF00063.19	47.70	0.00
TRINITY_A0A085MQK8_9BILA/324-484	A0A085MQK8.1 PF0007	47.70	0.00
TRINITY_D8UCG7_VOLCA/266-518	D8UCG7.1 PF03133.13	47.70	0.00
TRINITY_A0A085ND65_9BILA/425-543	A0A085ND65.1 PF0066	47.70	0.00
TRINITY_Q478B9_DECAR/513-628	Q478B9.1 PF00072.22	47.60	0.00
TRINITY_D2VU90_NAEGR/548-662	D2VU90.1 PF00072.22	47.60	0.00
TRINITY_J9ISP5_9SPIT/145-363	J9ISP5.1 PF00566.16	47.60	0.00
TRINITY_A0A0A1U7P7_ENTIV/2307-2608	A0A0A1U7P7.1 PF0063	47.60	0.00
TRINITY_A0A024TZI0_9STRA/11-268	A0A024TZI0.1 PF0006	47.60	0.00
TRINITY_Q3SDP8_PARTE/11-174	Q3SDP8.1 PF00071.20	47.60	0.00
TRINITY_D8TXG7_VOLCA/661-718	D8TXG7.1 PF13517.4;	47.60	0.00
TRINITY_L8HK06_ACACA/25-210	L8HK06.1 PF01467.24	47.60	0.00
TRINITY_J9FR32_9SPIT/300-343	J9FR32.1 PF13639.4;	47.60	0.00
TRINITY_B8GS56_THISH/19-133	B8GS56.1 PF07386.9;	47.60	0.00
TRINITY_A0BZS8_PARTE/33-282	A0BZS8.1 PF00768.18	47.60	0.00
TRINITY_G0QW57_ICHMG/65-210	G0QW57.1 PF00071.20	47.60	0.00
TRINITY_F7EQM6_XENTR/5-178	F7EQM6.1 PF00071.20	47.60	0.00
TRINITY_L8H8S1_ACACA/2-104	L8H8S1.1 PF05456.9;	47.60	0.00
TRINITY_A0A078B349_STYLE/444-804	A0A078B349.1 PF0070	47.60	0.00
TRINITY_G0QQF0_ICHMG/1-230	G0QQF0.1 PF03159.16	47.60	0.00
TRINITY_E1Z6X4_CHLVA/17-92	E1Z6X4.1 PF13450.4;	47.60	0.00
TRINITY_M5WU18_PRUPE/10-171	M5WU18.1 PF00071.20	47.60	0.00
TRINITY_I7M1V6_TETTS/314-533	I7M1V6.1 PF07002.14	47.60	0.00
TRINITY_T1JTJ1_TETUR/61-203	T1JTJ1.1 PF08240.10	47.60	0.00
TRINITY_L1J820_GUITH/223-386	L1J820.1 PF01399.25	47.60	0.00
TRINITY_A0D1Q9_PARTE/17-130	A0D1Q9.1 PF01920.18	47.60	0.00
TRINITY_I0Z997_9CHLO/17-136	I0Z997.1 PF13671.4;	47.60	0.00
TRINITY_W5LHY3_ASTMX/501-587	W5LHY3.1 PF13893.4;	47.60	0.00
TRINITY_A2FHQ0_TRIVA/5-116	A2FHQ0.1 PF01412.16	47.60	0.00
TRINITY_I7MHV7_TETTS/3658-3827	I7MHV7.2 PF16909.3;	47.60	0.00
TRINITY_B9TBU4_RICCO/34-192	B9TBU4.1 PF00043.23	47.60	0.00
TRINITY_A0A087SBP4_AUXPR/75-333	A0A087SBP4.1 PF1214	47.60	0.00
TRINITY_L8GV37_ACACA/70-221	L8GV37.1 PF12694.5;	47.60	0.00
TRINITY_H3FDM9_PRIPA/77-152	H3FDM9.1 PF01066.19	47.60	0.00
TRINITY_L8H219_ACACA/5-289	L8H219.1 PF05833.9;	47.60	0.00
TRINITY_E1ZEB5_CHLVA/404-575	E1ZEB5.1 PF04755.10	47.60	0.00
TRINITY_G0R1F7_ICHMG/29-105	G0R1F7.1 PF12895.5;	47.60	0.00
TRINITY_I7MAB8_TETTS/24-405	I7MAB8.1 PF07690.14	47.60	0.00
TRINITY_G0QR68_ICHMG/177-395	G0QR68.1 PF04811.13	47.60	0.00
TRINITY_A0A067RIS5_ZOONE/12-338	A0A067RIS5.1 PF0113	47.60	0.00
TRINITY_G4V8C5_SCHMA/289-760	G4V8C5.1 PF01055.24	47.60	0.00
TRINITY_G0QTC5_ICHMG/44-199	G0QTC5.1 PF03031.16	47.60	0.00
TRINITY_B7G1K5_PHATC/21-93	B7G1K5.1 PF04564.13	47.60	0.00
TRINITY_D8UBV3_VOLCA/223-296	D8UBV3.1 PF13424.4;	47.60	0.00
TRINITY_I7MA17_TETTS/370-645	I7MA17.1 PF00657.20	47.60	0.00
TRINITY_L8GJH9_ACACA/304-615	L8GJH9.1 PF00632.23	47.60	0.00
TRINITY_I0Z6E5_9CHLO/39-246	I0Z6E5.1 PF07228.10	47.60	0.00
TRINITY_E1ZJI9_CHLVA/117-197	E1ZJI9.1 PF04182.10	47.60	0.00
TRINITY_I0YIF8_9CHLO/21-168	I0YIF8.1 PF06201.11	47.60	0.00
TRINITY_A0A024U5X1_9STRA/115-328	A0A024U5X1.1 PF0011	47.60	0.00
TRINITY_D2QWB2_PIRSD/565-803	D2QWB2.1 PF01593.22	47.60	0.00
TRINITY_AIFM1_DICDI/96-417	Q9GRX6.1 PF07992.12	47.60	0.00
TRINITY_M4DIT4_BRARP/2-121	M4DIT4.1 PF09775.7;	47.60	0.00
TRINITY_M2MER5_BAUCO/1-60	M2MER5.1 PF03105.17	47.60	0.00
TRINITY_L8HE93_ACACA/369-514	L8HE93.1 PF07534.14	47.60	0.00
TRINITY_I0Z4V9_9CHLO/14-136	I0Z4V9.1 PF13806.4;	47.60	0.00
TRINITY_A0DL08_PARTE/182-272	A0DL08.1 PF00153.25	47.60	0.00
TRINITY_W9YHS4_9EURO/205-1398	W9YHS4.1 PF02463.17	47.60	0.00

TRINITY_Q099W1_STIAD/478-664	Q099W1.1 PF01186.15	47.60	0.00
TRINITY_E5SHF7_TRISP/288-377	E5SHF7.1 PF02214.20	47.60	0.00
TRINITY_E5SHF7_TRISP/288-377	E5SHF7.1 PF02214.20	47.60	0.00
TRINITY_D3B4E0_POLPA/113-558	D3B4E0.1 PF14655.4;	47.60	0.00
TRINITY_D3B4U3_POLPA/96-477	D3B4U3.1 PF01080.15	47.60	0.00
TRINITY_D8TR25_VOLCA/152-372	D8TR25.1 PF07970.10	47.60	0.00
TRINITY_J9HX68_9SPIT/57-359	J9HX68.1 PF00648.19	47.60	0.00
TRINITY_L2GCP5_COLGN/682-784	L2GCP5.1 PF12796.5;	47.60	0.00
TRINITY_Q4C263_CROWT/345-440	Q4C263.1 PF02163.20	47.60	0.00
TRINITY_I0Z3K2_9CHLO/34-261	I0Z3K2.1 PF05148.13	47.60	0.00
TRINITY_D8UC02_VOLCA/1132-1256	D8UC02.1 PF07714.15	47.60	0.00
TRINITY_D8TKH0_VOLCA/161-515	D8TKH0.1 PF01103.21	47.60	0.00
TRINITY_D8U9Z7_VOLCA/584-707	D8U9Z7.1 PF10442.7;	47.60	0.00
TRINITY_G8YE80_PICSO/31-93	G8YE80.1 PF00462.22	47.60	0.00
TRINITY_J9J144_9SPIT/92-349	J9J144.1 PF00069.23	47.50	0.00
TRINITY_A0A072P8D0_9EURO/1-106	A0A072P8D0.1 PF0365	47.50	0.00
TRINITY_D8R325_SELML/87-151	D8R325.1 PF13499.4;	47.50	0.00
TRINITY_A0A078BCF8_STYLE/3-64	A0A078BCF8.1 PF0007	47.50	0.00
TRINITY_G0QKX0_ICHMG/51-329	G0QKX0.1 PF03034.13	47.50	0.00
TRINITY_A4VD75_TETTS/10-116	A4VD75.1 PF00085.18	47.50	0.00
TRINITY_W4YRK6_STRPU/145-212	W4YRK6.1 PF07258.12	47.50	0.00
TRINITY_Q229H4_TETTS/1167-1284	Q229H4.2 PF00072.22	47.50	0.00
TRINITY_U4KWA9_PYROM/98-270	U4KWA9.1 PF00775.19	47.50	0.00
TRINITY_J9J078_9SPIT/277-392	J9J078.1 PF02518.24	47.50	0.00
TRINITY_A7REQ2_NEMVE/4-157	A7REQ2.1 PF00005.25	47.50	0.00
TRINITY_A0A0G2ZUL9_9DELT/201-261	A0A0G2ZUL9.1 PF1477	47.50	0.00
TRINITY_K7I8C5_CAEJA/79-206	K7I8C5.1 PF00271.29	47.50	0.00
TRINITY_A0DXS8_PARTE/25-438	A0DXS8.1 PF07690.14	47.50	0.00
TRINITY_E1C6X4_CHICK/60-122	E1C6X4.1 PF13499.4;	47.50	0.00
TRINITY_A0A058Z2L7_9EUKA/41-283	A0A058Z2L7.1 PF0137	47.50	0.00
TRINITY_Q23AY4_TETTS/31-436	Q23AY4.2 PF05577.10	47.50	0.00
TRINITY_A0A0K9NHS8_ZOSMR/73-332	A0A0K9NHS8.1 PF0024	47.50	0.00
TRINITY_L8H2P8_ACACA/253-311	L8H2P8.1 PF00786.26	47.50	0.00
TRINITY_I7M0N5_TETTS/125-295	I7M0N5.1 PF00092.26	47.50	0.00
TRINITY_L1IFE1_GUITH/66-327	L1IFE1.1 PF00591.19	47.50	0.00
TRINITY_W5CIR0_WHEAT/54-184	W5CIR0.1 PF00782.18	47.50	0.00
TRINITY_I1I3M9_BRADI/76-218	I1I3M9.1 PF10607.7;	47.50	0.00
TRINITY_I7M3V2_TETTS/53-319	I7M3V2.1 PF00069.23	47.50	0.00
TRINITY_D8LVM2_BLAHO/81-144	D8LVM2.1 PF00226.29	47.50	0.00
TRINITY_Q84QI2_CHLRE/13-106	Q84QI2.1 PF14966.4;	47.50	0.00
TRINITY_K3WVC7_PYTUL/120-247	K3WVC7.1 PF04116.11	47.50	0.00
TRINITY_A0A015IMC7_9GLOM/126-421	A0A015IMC7.1 PF0056	47.50	0.00
TRINITY_B3RYA0_TRIAD/140-479	B3RYA0.1 PF02163.20	47.50	0.00
TRINITY_I7M3X7_TETTS/14-121	I7M3X7.2 PF14933.4;	47.50	0.00
TRINITY_M5VV76_PRUPE/1-485	M5VV76.1 PF04130.11	47.50	0.00
TRINITY_A0A090BU49_9GAMM/570-686	A0A090BU49.1 PF0251	47.50	0.00
TRINITY_L8H030_ACACA/25-454	L8H030.1 PF05602.10	47.50	0.00
TRINITY_W7AAT9_9APIC/75-218	W7AAT9.1 PF16531.3;	47.50	0.00
TRINITY_A0E7G7_PARTE/121-385	A0E7G7.1 PF00069.23	47.50	0.00
TRINITY_A0A068Y8Y9_ECHMU/356-468	A0A068Y8Y9.1 PF0322	47.50	0.00
TRINITY_E8N3V4_ANATU/2-233	E8N3V4.1 PF03577.13	47.50	0.00
TRINITY_D8TWR1_VOLCA/23-311	D8TWR1.1 PF00069.23	47.50	0.00
TRINITY_M0RXY1_MUSAM/144-265	M0RXY1.1 PF01553.19	47.50	0.00
TRINITY_U9T9S0_RHIID/1-459	U9T9S0.1 PF04006.10	47.50	0.00
TRINITY_A8JG56_CHLRE/85-309	A8JG56.1 PF00141.21	47.50	0.00
TRINITY_A0E2M3_PARTE/3-103	A0E2M3.1 PF00168.28	47.50	0.00
TRINITY_G0QM33_ICHMG/33-214	G0QM33.1 PF00270.27	47.50	0.00
TRINITY_D8UJA5_VOLCA/42-123	D8UJA5.1 PF03366.14	47.50	0.00
TRINITY_D8TKV7_VOLCA/38-199	D8TKV7.1 PF16134.3;	47.50	0.00
TRINITY_I7MGZ3_TETTS/4-164	I7MGZ3.1 PF06127.9;	47.50	0.00
TRINITY_A0A022R067_ERYGU/5-225	A0A022R067.1 PF0415	47.50	0.00

TRINITY_A0A0A1PIM2_9FUNG/7-117	A0A0A1PIM2.1 PF0085	47.50	0.00
TRINITY_L8HEU7_ACACA/28-342	L8HEU7.1 PF14647.4;	47.50	0.00
TRINITY_A0A089JXQ9_9BACL/1-317	A0A089JXQ9.1 PF0242	47.50	0.00
TRINITY_A8IU15_CHLRE/11-266	A8IU15.1 PF00069.23	47.50	0.00
TRINITY_F0XYH0_AURAN/5-252	F0XYH0.1 PF00975.18	47.50	0.00
TRINITY_A0A068XYS6_ECHMU/153-259	A0A068XYS6.1 PF0030	47.50	0.00
TRINITY_C1E082_MICSR/7-89	C1E082.1 PF10785.7;	47.50	0.00
TRINITY_E1ZS88_CHLVA/5-312	E1ZS88.1 PF01156.17	47.50	0.00
TRINITY_D8U792_VOLCA/1973-2038	D8U792.1 PF00415.16	47.50	0.00
TRINITY_A0A087GDX5_ARAAL/331-775	A0A087GDX5.1 PF0105	47.50	0.00
TRINITY_A8JFJ5_CHLRE/243-455	A8JFJ5.1 PF05450.13	47.50	0.00
TRINITY_Q23QF2_TETTS/248-571	Q23QF2.2 PF03133.13	47.50	0.00
TRINITY_A5KBZ1_PLAVS/4-69	A5KBZ1.1 PF00226.29	47.50	0.00
TRINITY_F4Q1F7_DICFS/372-584	F4Q1F7.1 PF07228.10	47.50	0.00
TRINITY_T0R0B8_9STRA/37-164	T0R0B8.1 PF12796.5;	47.50	0.00
TRINITY_Q3SDM9_PARTE/30-193	Q3SDM9.1 PF00071.20	47.50	0.00
TRINITY_A0A078A4D4_STYLE/430-659	A0A078A4D4.1 PF0584	47.50	0.00
TRINITY_E5SHF7_TRISP/288-377	E5SHF7.1 PF02214.20	47.50	0.00
TRINITY_K3YY32_SETIT/31-151	K3YY32.1 PF00188.24	47.50	0.00
TRINITY_Q23KC0_TETTS/243-334	Q23KC0.3 PF07683.12	47.50	0.00
TRINITY_I7MKW8_TETTS/798-1133	I7MKW8.2 PF03133.13	47.50	0.00
TRINITY_A0A075AP28_9FUNG/87-370	A0A075AP28.1 PF0339	47.50	0.00
TRINITY_S2JYD2_MUCC1/27-185	S2JYD2.1 PF01553.19	47.50	0.00
TRINITY_D2UZB4_NAEGR/80-179	D2UZB4.1 PF00173.26	47.50	0.00
TRINITY_A0DMS4_PARTE/255-356	A0DMS4.1 PF06565.10	47.50	0.00
TRINITY_A8I7K9_CHLRE/1-88	A8I7K9.1 PF07714.15	47.50	0.00
TRINITY_PHOP2_XENTR/3-236	Q66KD6.1 PF06888.10	47.50	0.00
TRINITY_H3HAJ2_PHYRM/359-478	H3HAJ2.1 PF00665.24	47.50	0.00
TRINITY_A8J3P1_CHLRE/954-1161	A8J3P1.1 PF00211.18	47.50	0.00
TRINITY_E1Z6G9_CHLVA/128-298	E1Z6G9.1 PF00892.18	47.50	0.00
TRINITY_A0A0J7K0M9_LASNI/335-453	A0A0J7K0M9.1 PF0066	47.50	0.00
TRINITY_A8JDJ8_CHLRE/29-120	A8JDJ8.1 PF05699.12	47.50	0.00
TRINITY_B8HYX6_CYAP4/26-200	B8HYX6.1 PF13532.4;	47.50	0.00
TRINITY_D8U0S2_VOLCA/944-1143	D8U0S2.1 PF07714.15	47.50	0.00
TRINITY_J9J800_9SPIT/592-723	J9J800.1 PF00293.26	47.50	0.00
TRINITY_A0A0A1TNZ7_9HYPO/56-138	A0A0A1TNZ7.1 PF0333	47.50	0.00
TRINITY_Q1N2R9_9GAMM/772-890	Q1N2R9.1 PF00072.22	47.40	0.00
TRINITY_T1FYE9_HELRO/23-315	T1FYE9.1 PF00850.17	47.40	0.00
TRINITY_D3BQP3_POLPA/114-258	D3BQP3.1 PF08737.8;	47.40	0.00
TRINITY_K1Q470_CRAGI/4-98	K1Q470.1 PF14216.4;	47.40	0.00
TRINITY_I7LXT2_TETTS/158-424	I7LXT2.2 PF00069.23	47.40	0.00
TRINITY_B8MPJ7_TALSN/61-536	B8MPJ7.1 PF00067.20	47.40	0.00
TRINITY_A0A078B207_STYLE/111-202	A0A078B207.1 PF0190	47.40	0.00
TRINITY_Q22AI0_TETTS/688-801	Q22AI0.2 PF02887.14	47.40	0.00
TRINITY_D8U320_VOLCA/46-142	D8U320.1 PF00293.26	47.40	0.00
TRINITY_A0A0D9X011_9ORYZ/57-207	A0A0D9X011.1 PF0029	47.40	0.00
TRINITY_D7FNH9_ECTSI/61-176	D7FNH9.1 PF12894.5;	47.40	0.00
TRINITY_A9V494_MONBE/287-459	A9V494.1 PF00534.18	47.40	0.00
TRINITY_A0A0F5ZTC1_9GAMM/1030-1145	A0A0F5ZTC1.1 PF0007	47.40	0.00
TRINITY_I7M1V6_TETTS/314-533	I7M1V6.1 PF07002.14	47.40	0.00
TRINITY_G3XX32_ASPNA/97-234	G3XX32.1 PF13091.4;	47.40	0.00
TRINITY_UBR7_DICDI/239-293	Q54DV0.2 PF00628.27	47.40	0.00
TRINITY_L8H4S7_ACACA/16-207	L8H4S7.1 PF00106.23	47.40	0.00
TRINITY_L8GYG8_ACACA/142-276	L8GYG8.1 PF00782.18	47.40	0.00
TRINITY_E9IRE1_SOLIN/4-173	E9IRE1.1 PF08547.10	47.40	0.00
TRINITY_M0QSM4_ACACA/400-492	M0QSM4.1 PF02214.20	47.40	0.00
TRINITY_L8GDW5_ACACA/38-251	L8GDW5.1 PF09414.8;	47.40	0.00
TRINITY_L8HDA6_ACACA/205-295	L8HDA6.1 PF07885.14	47.40	0.00
TRINITY_I7MJT4_TETTS/15-283	I7MJT4.1 PF01073.17	47.40	0.00
TRINITY_C5GAB2_AJEDR/9-139	C5GAB2.1 PF03435.16	47.40	0.00
TRINITY_A0C4U0_PARTE/156-220	A0C4U0.1 PF00153.25	47.40	0.00

TRINITY_A0CTM1_PARTE/151-369	A0CTM1.1 PF01556.16	47.40	0.00
TRINITY_W7X7D0_TETTS/127-467	W7X7D0.1 PF16653.3;	47.40	0.00
TRINITY_I1JXK9_SOYBN/29-387	I1JXK9.2 PF04185.12	47.40	0.00
TRINITY_A0A0G4B5X6_9BACT/334-445	A0A0G4B5X6.1 PF0251	47.40	0.00
TRINITY_A8J4E2_CHLRE/159-328	A8J4E2.1 PF01223.21	47.40	0.00
TRINITY_I0YX01_9CHLO/213-357	I0YX01.1 PF01490.16	47.40	0.00
TRINITY_D8U8R8_VOLCA/29-111	D8U8R8.1 PF10251.7;	47.40	0.00
TRINITY_G1KFB0_ANOCA/789-999	G1KFB0.2 PF00644.18	47.40	0.00
TRINITY_V4B185_LOTGI/96-380	V4B185.1 PF00443.27	47.40	0.00
TRINITY_K7IQ87_NASVI/181-299	K7IQ87.1 PF00665.24	47.40	0.00
TRINITY_A9S9P1_PHYPA/7-262	A9S9P1.1 PF10160.7;	47.40	0.00
TRINITY_V6LCP3_9EUKA/29-329	V6LCP3.1 PF00009.25	47.40	0.00
TRINITY_D8T3Y6_SELML/60-263	D8T3Y6.1 PF00804.23	47.40	0.00
TRINITY_I7MH91_TETTS/186-488	I7MH91.1 PF00883.19	47.40	0.00
TRINITY_I7MKW7_TETTS/16-270	I7MKW7.1 PF08450.10	47.40	0.00
TRINITY_A0A0D2WTZ0_CAPO3/236-510	A0A0D2WTZ0.1 PF0113	47.40	0.00
TRINITY_D8TP76_VOLCA/24-120	D8TP76.1 PF03704.15	47.40	0.00
TRINITY_K9WN24_9CYAN/827-940	K9WN24.1 PF08448.8;	47.40	0.00
TRINITY_G0R1Y7_ICHMG/3-188	G0R1Y7.1 PF01251.16	47.40	0.00
TRINITY_A0BJW5_PARTE/673-824	A0BJW5.1 PF00005.25	47.40	0.00
TRINITY_F4PQQ1_DICFS/380-541	F4PQQ1.1 PF01343.16	47.40	0.00
TRINITY_A8IR44_CHLRE/424-884	A8IR44.1 PF03547.16	47.40	0.00
TRINITY_D8TYS9_VOLCA/105-194	D8TYS9.1 PF00686.17	47.40	0.00
TRINITY_L1I9Z9_GUITH/167-420	L1I9Z9.1 PF00928.19	47.40	0.00
TRINITY_C1H6W8_PARBA/144-651	C1H6W8.2 PF03098.13	47.40	0.00
TRINITY_D8TKX7_VOLCA/49-142	D8TKX7.1 PF00686.17	47.40	0.00
TRINITY_M2VVT6_GALSU/41-311	M2VVT6.1 PF00664.21	47.40	0.00
TRINITY_I3KHL5_ORENI/590-742	I3KHL5.1 PF00753.25	47.40	0.00
TRINITY_T1IQQ9_STRMM/764-975	T1IQQ9.1 PF00644.18	47.40	0.00
TRINITY_A9TBZ2_PHYPA/187-703	A9TBZ2.1 PF12569.6;	47.40	0.00
TRINITY_A0A0G0FN99_9BACT/7-494	A0A0G0FN99.1 PF0141	47.40	0.00
TRINITY_A0CFL4_PARTE/1647-1747	A0CFL4.1 PF16455.3;	47.40	0.00
TRINITY_A8JBZ0_CHLRE/15-224	A8JBZ0.1 PF01126.18	47.40	0.00
TRINITY_M2Y4X3_GALSU/55-262	M2Y4X3.1 PF00106.23	47.40	0.00
TRINITY_Q22S54_TETTS/173-326	Q22S54.3 PF00929.22	47.40	0.00
TRINITY_C1E862_MICSR/43-180	C1E862.1 PF00293.26	47.40	0.00
TRINITY_A0A0G4FHT3_9ALVE/97-227	A0A0G4FHT3.1 PF0152	47.40	0.00
TRINITY_D8TLC4_VOLCA/28-233	D8TLC4.1 PF13472.4;	47.40	0.00
TRINITY_U7PPQ1_SPOS1/150-654	U7PPQ1.1 PF03098.13	47.40	0.00
TRINITY_F2UC22_SALR5/539-752	F2UC22.1 PF07859.11	47.40	0.00
TRINITY_D2QCA4_SPILD/4-366	D2QCA4.1 PF01266.22	47.40	0.00
TRINITY_L8HI32_ACACA/42-157	L8HI32.1 PF00169.27	47.40	0.00
TRINITY_A8HZZ8_CHLRE/12-186	A8HZZ8.1 PF04752.10	47.40	0.00
TRINITY_A4S6D3_OSTLU/79-383	A4S6D3.1 PF00487.22	47.40	0.00
TRINITY_E1Z3Q9_CHLVA/75-167	E1Z3Q9.1 PF00686.17	47.40	0.00
TRINITY_A8JGS1_CHLRE/156-363	A8JGS1.1 PF04614.10	47.40	0.00
TRINITY_C1MRG7_MICPC/57-273	C1MRG7.1 PF01988.17	47.40	0.00
TRINITY_I7LX71_TETTS/33-164	I7LX71.1 PF00782.18	47.40	0.00
TRINITY_I7MKP3_TETTS/27-347	I7MKP3.2 PF00443.27	47.40	0.00
TRINITY_V7BZ82_PHAVU/8-182	V7BZ82.1 PF01145.23	47.40	0.00
TRINITY_I7M1X2_TETTS/313-443	I7M1X2.2 PF01529.18	47.40	0.00
TRINITY_A8IWM1_CHLRE/227-702	A8IWM1.1 PF01055.24	47.40	0.00
TRINITY_A0A0A0LGR9_CUCSA/173-622	A0A0A0LGR9.1 PF0518	47.40	0.00
TRINITY_W2PE18_PHYPN/147-241	W2PE18.1 PF01922.15	47.40	0.00
TRINITY_I7MD86_TETTS/149-260	I7MD86.1 PF13519.4;	47.40	0.00
TRINITY_A0A090LAM2_STRRB/7-114	A0A090LAM2.1 PF0063	47.40	0.00
TRINITY_G0QVK9_ICHMG/261-559	G0QVK9.1 PF00632.23	47.30	0.00
TRINITY_Q23QF2_TETTS/248-571	Q23QF2.2 PF03133.13	47.30	0.00
TRINITY_I7LWV6_TETTS/492-585	I7LWV6.1 PF12796.5;	47.30	0.00
TRINITY_D3BLH3_POLPA/1312-1636	D3BLH3.1 PF02259.21	47.30	0.00
TRINITY_A0A0N4UDI6_DRAME/543-786	A0A0N4UDI6.1 PF0584	47.30	0.00

TRINITY_A0BJG4_PARTE/43-103	A0BJG4.1 PF10247.7;	47.30	0.00
TRINITY_I7LWC5_TETTS/371-638	I7LWC5.1 PF00481.19	47.30	0.00
TRINITY_K1Q7Z5_CRAGI/2-58	K1Q7Z5.1 PF05347.13	47.30	0.00
TRINITY_W4FUJ0_9STRA/146-253	W4FUJ0.1 PF00071.20	47.30	0.00
TRINITY_A0A024TJX9_9STRA/1-173	A0A024TJX9.1 PF0406	47.30	0.00
TRINITY_B8CBR8_THAPS/90-420	B8CBR8.1 PF00291.23	47.30	0.00
TRINITY_L1IAI5_GUITH/36-218	L1IAI5.1 PF01121.18	47.30	0.00
TRINITY_Q0VSF8_ALCBS/675-792	Q0VSF8.1 PF00072.22	47.30	0.00
TRINITY_D3BA25_POLPA/354-660	D3BA25.1 PF05970.12	47.30	0.00
TRINITY_G0QVI4_ICHMG/212-511	G0QVI4.1 PF03133.13	47.30	0.00
TRINITY_D8LKX8_ECTSI/231-443	D8LKX8.1 PF00149.26	47.30	0.00
TRINITY_L8GW48_ACACA/228-483	L8GW48.1 PF00069.23	47.30	0.00
TRINITY_I7M1B3_TETTS/91-221	I7M1B3.2 PF00782.18	47.30	0.00
TRINITY_J9EMC8_9SPIT/1395-1489	J9EMC8.1 PF01833.22	47.30	0.00
TRINITY_A0A077ZZB5_STYLE/25-119	A0A077ZZB5.1 PF1390	47.30	0.00
TRINITY_L8GNF2_ACACA/21-241	L8GNF2.1 PF00149.26	47.30	0.00
TRINITY_V4TZR8_9ROSI/13-107	V4TZR8.1 PF01922.15	47.30	0.00
TRINITY_I0YW63_9CHLO/16-326	I0YW63.1 PF00225.21	47.30	0.00
TRINITY_D8UL08_VOLCA/9-201	D8UL08.1 PF00300.20	47.30	0.00
TRINITY_D8UJD3_VOLCA/6-53	D8UJD3.1 PF14570.4;	47.30	0.00
TRINITY_Q22WQ4_TETTS/3-444	Q22WQ4.2 PF00022.17	47.30	0.00
TRINITY_I7MHG9_TETTS/819-1072	I7MHG9.2 PF00233.17	47.30	0.00
TRINITY_A8IRK2_CHLRE/356-957	A8IRK2.1 PF00328.20	47.30	0.00
TRINITY_A0BLX9_PARTE/634-738	A0BLX9.1 PF13426.5;	47.30	0.00
TRINITY_A8HT27_CHLRE/2550-3400	A8HT27.1 PF02364.13	47.30	0.00
TRINITY_D8TQS0_VOLCA/3-260	D8TQS0.1 PF00756.18	47.30	0.00
TRINITY_A0E0L6_PARTE/109-206	A0E0L6.1 PF00153.25	47.30	0.00
TRINITY_A8JF52_CHLRE/1721-2039	A8JF52.1 PF00520.29	47.30	0.00
TRINITY_D8TQC8_VOLCA/57-186	D8TQC8.1 PF02338.17	47.30	0.00
TRINITY_A0A0E9NFI9_9ASCO/26-160	A0A0E9NFI9.1 PF1289	47.30	0.00
TRINITY_J9IMI7_9SPIT/716-901	J9IMI7.1 PF04983.16	47.30	0.00
TRINITY_D8TZ68_VOLCA/56-151	D8TZ68.1 PF00153.25	47.30	0.00
TRINITY_M4A3G0_XIPMA/134-562	M4A3G0.1 PF00654.18	47.30	0.00
TRINITY_B9IJH8_POPTR/232-905	B9IJH8.1 PF00443.27	47.30	0.00
TRINITY_A0A077ZQW9_STYLE/49-359	A0A077ZQW9.1 PF0024	47.30	0.00
TRINITY_A8J9V7_CHLRE/33-104	A8J9V7.1 PF00498.24	47.30	0.00
TRINITY_U3KC04_FICAL/85-321	U3KC04.1 PF00704.26	47.30	0.00
TRINITY_E1ZF17_CHLVA/2-516	E1ZF17.1 PF03055.13	47.30	0.00
TRINITY_A0CJY8_PARTE/436-698	A0CJY8.1 PF00069.23	47.30	0.00
TRINITY_M5WP91_PRUPE/57-197	M5WP91.1 PF01597.17	47.30	0.00
TRINITY_W5WA22_9PSEU/53-163	W5WA22.1 PF08448.8;	47.30	0.00
TRINITY_A8HMB1_CHLRE/5-281	A8HMB1.1 PF01062.19	47.30	0.00
TRINITY_A8HNP6_CHLRE/11-260	A8HNP6.1 PF07727.12	47.30	0.00
TRINITY_V6LW21_9EUKA/15-303	V6LW21.1 PF00069.23	47.30	0.00
TRINITY_A0A068RQM9_9FUNG/885-1159	A0A068RQM9.1 PF0006	47.30	0.00
TRINITY_Q24BS9_TETTS/921-1178	Q24BS9.2 PF00481.19	47.30	0.00
TRINITY_A0A0M2PU93_PROHO/94-505	A0A0M2PU93.1 PF0001	47.30	0.00
TRINITY_A0A0D2UV53_GOSRA/10-106	A0A0D2UV53.1 PF1279	47.30	0.00
TRINITY_SODM_DEIRA/100-206	Q9RUV2.3 PF02777.16	47.30	0.00
TRINITY_G3AYK2_CANTC/419-519	G3AYK2.1 PF00153.25	47.30	0.00
TRINITY_A8I5Q6_CHLRE/245-370	A8I5Q6.1 PF00581.18	47.30	0.00
TRINITY_L8GUI1_ACACA/1063-1317	L8GUI1.1 PF01504.16	47.30	0.00
TRINITY_D8U9S9_VOLCA/599-793	D8U9S9.1 PF00211.18	47.30	0.00
TRINITY_J9JBX5_9SPIT/149-408	J9JBX5.1 PF00069.23	47.30	0.00
TRINITY_Q8SB62_ORYSJ/971-1085	Q8SB62.1 PF00665.24	47.30	0.00
TRINITY_J2LGI7_9BURK/6-153	J2LGI7.1 PF03358.13	47.30	0.00
TRINITY_W9SEV9_9ROSA/43-182	W9SEV9.1 PF01743.18	47.20	0.00
TRINITY_K3XBG4_PYTUL/100-168	K3XBG4.1 PF08726.8;	47.20	0.00
TRINITY_I7MM31_TETTS/85-222	I7MM31.2 PF01529.18	47.20	0.00
TRINITY_Q3SDM9_PARTE/30-193	Q3SDM9.1 PF00071.20	47.20	0.00
TRINITY_I1ELT8_AMPQE/210-327	I1ELT8.1 PF03764.16	47.20	0.00

TRINITY_K0KJA7_WICCF/147-254	K0KJA7.1 PF00175.19	47.20	0.00
TRINITY_A0A022RM38_ERYGU/36-166	A0A022RM38.1 PF0078	47.20	0.00
TRINITY_M1UVD9_CYAME/1-104	M1UVD9.1 PF03656.11	47.20	0.00
TRINITY_A0CWT1_PARTE/157-265	A0CWT1.1 PF00787.22	47.20	0.00
TRINITY_F1A2V0_DICPU/25-187	F1A2V0.1 PF13532.4;	47.20	0.00
TRINITY_A0CEP9_PARTE/460-569	A0CEP9.1 PF12796.5;	47.20	0.00
TRINITY_V4B1I8_LOTGI/477-584	V4B1I8.1 PF00085.18	47.20	0.00
TRINITY_F4PKM0_DICFS/1539-1756	F4PKM0.1 PF16095.3;	47.20	0.00
TRINITY_D8G265_9CYAN/13-295	D8G265.1 PF01007.18	47.20	0.00
TRINITY_F4Q916_DICFS/16-247	F4Q916.1 PF00378.18	47.20	0.00
TRINITY_R4LFA6_9ACTN/34-164	R4LFA6.1 PF00293.26	47.20	0.00
TRINITY_A0A0A1UEQ8_ENTIV/1122-1381	A0A0A1UEQ8.1 PF0006	47.20	0.00
TRINITY_Q23AY4_TETTS/31-436	Q23AY4.2 PF05577.10	47.20	0.00
TRINITY_A0A0D2WRU5_CAPO3/136-260	A0A0D2WRU5.1 PF0411	47.20	0.00
TRINITY_A8IIR9_CHLRE/66-192	A8IIR9.1 PF11712.6;	47.20	0.00
TRINITY_I7M1L3_TETTS/318-762	I7M1L3.1 PF04053.12	47.20	0.00
TRINITY_A0C6A0_PARTE/601-713	A0C6A0.1 PF08785.9;	47.20	0.00
TRINITY_W7WZC8_TETTS/6-197	W7WZC8.1 PF04072.12	47.20	0.00
TRINITY_A7RTF3_NEMVE/273-427	A7RTF3.1 PF13768.4;	47.20	0.00
TRINITY_Q22AW2_TETTS/85-366	Q22AW2.1 PF00664.21	47.20	0.00
TRINITY_A0DPD0_PARTE/462-730	A0DPD0.1 PF00149.26	47.20	0.00
TRINITY_A8HUF5_CHLRE/3-152	A8HUF5.1 PF08534.8;	47.20	0.00
TRINITY_D3BKA1_POLPA/2-144	D3BKA1.1 PF09801.7;	47.20	0.00
TRINITY_A0A0J8D496_BETVU/6-286	A0A0J8D496.1 PF0187	47.20	0.00
TRINITY_D2VRY9_NAEGR/382-533	D2VRY9.1 PF00005.25	47.20	0.00
TRINITY_A0DMK4_PARTE/24-339	A0DMK4.1 PF03372.21	47.20	0.00
TRINITY_A8IXA0_CHLRE/596-759	A8IXA0.1 PF01554.16	47.20	0.00
TRINITY_A0DIB1_PARTE/38-559	A0DIB1.1 PF01602.18	47.20	0.00
TRINITY_V4A564_LOTGI/44-223	V4A564.1 PF00092.26	47.20	0.00
TRINITY_E1ZKZ3_CHLVA/56-500	E1ZKZ3.1 PF00083.22	47.20	0.00
TRINITY_U3K101_FICAL/407-570	U3K101.1 PF05572.11	47.20	0.00
TRINITY_B3M057_DROAN/142-468	B3M057.1 PF01074.20	47.20	0.00
TRINITY_Q54VS5_DICDI/550-660	Q54VS5.1 PF02204.16	47.20	0.00
TRINITY_D8UF85_VOLCA/179-263	D8UF85.1 PF05699.12	47.20	0.00
TRINITY_G0R3R5_ICHMG/1-259	G0R3R5.1 PF00069.23	47.20	0.00
TRINITY_F4PKA1_DICFS/40-147	F4PKA1.1 PF00307.29	47.20	0.00
TRINITY_F0ZEI2_DICPU/318-489	F0ZEI2.1 PF04377.13	47.20	0.00
TRINITY_A9RX41_PHYPA/953-1080	A9RX41.1 PF06461.9;	47.20	0.00
TRINITY_L8H896_ACACA/33-195	L8H896.1 PF00071.20	47.20	0.00
TRINITY_H3D6C5_TETNG/114-454	H3D6C5.1 PF00282.17	47.20	0.00
TRINITY_I7M3I9_TETTS/58-293	I7M3I9.1 PF04176.11	47.20	0.00
TRINITY_A8IPD2_CHLRE/123-314	A8IPD2.1 PF00743.17	47.20	0.00
TRINITY_D8TLN5_VOLCA/747-863	D8TLN5.1 PF11969.6;	47.20	0.00
TRINITY_Q6CA40_YARLI/185-295	Q6CA40.1 PF00188.24	47.20	0.00
TRINITY_A0DB80_PARTE/91-183	A0DB80.1 PF04777.11	47.20	0.00
TRINITY_K4CP98_SOLLC/627-679	K4CP98.1 PF02928.14	47.20	0.00
TRINITY_F0ZNF5_DICPU/8-113	F0ZNF5.1 PF12998.5;	47.20	0.00
TRINITY_A8JF96_CHLRE/61-351	A8JF96.1 PF02104.13	47.20	0.00
TRINITY_I7LT94_TETTS/441-529	I7LT94.2 PF08164.10	47.20	0.00
TRINITY_A8TYY1_9PROT/7-196	A8TYY1.1 PF12007.6;	47.20	0.00
TRINITY_D3BM63_POLPA/430-612	D3BM63.1 PF00326.19	47.20	0.00
TRINITY_A0A077ZJP7_TRITR/54-141	A0A077ZJP7.1 PF1364	47.20	0.00
TRINITY_I7MCL9_TETTS/190-488	I7MCL9.2 PF00648.19	47.20	0.00
TRINITY_D8TY82_VOLCA/96-294	D8TY82.1 PF00504.19	47.20	0.00
TRINITY_H0ZIZ5_TAEGU/106-175	H0ZIZ5.1 PF00076.20	47.20	0.00
TRINITY_A8IT81_CHLRE/4-456	A8IT81.1 PF13520.4;	47.20	0.00
TRINITY_B7G8Z0_PHATC/142-411	B7G8Z0.1 PF00389.28	47.20	0.00
TRINITY_D8TIL4_VOLCA/417-993	D8TIL4.1 PF16969.3;	47.20	0.00
TRINITY_V3ZYV1_LOTGI/193-350	V3ZYV1.1 PF00179.24	47.20	0.00
TRINITY_A8HVN4_CHLRE/30-193	A8HVN4.1 PF03067.13	47.20	0.00
TRINITY_D8TPA9_VOLCA/111-230	D8TPA9.1 PF00158.24	47.20	0.00

TRINITY_D8U8W2_VOLCA/347-627	D8U8W2.1 PF07842.10	47.20	0.00
TRINITY_G4LZB0_SCHMA/44-134	G4LZB0.1 PF02214.20	47.20	0.00
TRINITY_E1ZIB9_CHLVA/81-428	E1ZIB9.1 PF00459.23	47.20	0.00
TRINITY_G3WIQ8_SARHA/3-212	G3WIQ8.1 PF00091.23	47.20	0.00
TRINITY_M2Y8Z7_GALSU/16-374	M2Y8Z7.1 PF04928.15	47.20	0.00
TRINITY_K9XCD8_9CHRO/1102-1339	K9XCD8.1 PF07993.10	47.20	0.00
TRINITY_A0BRA8_PARTE/7-108	A0BRA8.1 PF00168.28	47.10	0.00
TRINITY_A0A078ID03_BRANA/153-246	A0A078ID03.1 PF0015	47.10	0.00
TRINITY_A8HZR1_CHLRE/17086-17364	A8HZR1.1 PF00109.24	47.10	0.00
TRINITY_L8H6V3_ACACA/29-430	L8H6V3.1 PF00450.20	47.10	0.00
TRINITY_I7LXH2_TETTS/36-330	I7LXH2.2 PF09423.8;	47.10	0.00
TRINITY_V8PAJ1_OPHHA/50-136	V8PAJ1.1 PF02970.14	47.10	0.00
TRINITY_J9I165_9SPIT/605-907	J9I165.1 PF03372.21	47.10	0.00
TRINITY_A0A068NVT3_9BACT/763-877	A0A068NVT3.1 PF0007	47.10	0.00
TRINITY_I7LXC8_TETTS/30-186	I7LXC8.2 PF01124.16	47.10	0.00
TRINITY_C3Z2P3_BRAFL/166-302	C3Z2P3.1 PF02877.12	47.10	0.00
TRINITY_A0A0N0VH21_9TRYP/4-258	A0A0N0VH21.1 PF0006	47.10	0.00
TRINITY_C4QXV0_PICPG/30-147	C4QXV0.1 PF01042.19	47.10	0.00
TRINITY_S6E3Q8_ZYGB2/57-352	S6E3Q8.1 PF00176.21	47.10	0.00
TRINITY_W6MFQ4_9ASCO/7-198	W6MFQ4.1 PF13462.4;	47.10	0.00
TRINITY_I7LU42_TETTS/219-308	I7LU42.1 PF00153.25	47.10	0.00
TRINITY_R1EJ93_EMIHU/552-944	R1EJ93.1 PF07394.10	47.10	0.00
TRINITY_I7MJZ2_TETTS/407-470	I7MJZ2.2 PF01485.19	47.10	0.00
TRINITY_B4GLJ8_DROPE/29-367	B4GLJ8.1 PF03265.13	47.10	0.00
TRINITY_U6JEY5_ECHGR/5-177	U6JEY5.1 PF00025.19	47.10	0.00
TRINITY_I0Z703_9CHLO/261-382	I0Z703.1 PF03953.15	47.10	0.00
TRINITY_H9GCJ4_ANOCA/1-73	H9GCJ4.1 PF07061.9;	47.10	0.00
TRINITY_M2XSQ2_GALSU/201-287	M2XSQ2.1 PF01388.19	47.10	0.00
TRINITY_V4B961_LOTGI/162-496	V4B961.1 PF00443.27	47.10	0.00
TRINITY_A0A098EUG0_9BACI/4-122	A0A098EUG0.1 PF1323	47.10	0.00
TRINITY_A8JD70_CHLRE/16-151	A8JD70.1 PF04048.12	47.10	0.00
TRINITY_S2JN63_MUCC1/4-378	S2JN63.1 PF01238.19	47.10	0.00
TRINITY_V8PGX3_OPHHA/6-119	V8PGX3.1 PF00696.26	47.10	0.00
TRINITY_I7LUE5_TETTS/10-734	I7LUE5.2 PF03635.15	47.10	0.00
TRINITY_F4PIZ1_DICFS/122-267	F4PIZ1.1 PF13091.4;	47.10	0.00
TRINITY_L8GPA5_ACACA/108-239	L8GPA5.1 PF04116.11	47.10	0.00
TRINITY_G0QK71_ICHMG/834-1225	G0QK71.1 PF06957.9;	47.10	0.00
TRINITY_L8HIU8_ACACA/24-185	L8HIU8.1 PF01327.19	47.10	0.00
TRINITY_Q247R2_TETTS/942-1215	Q247R2.2 PF16212.3;	47.10	0.00
TRINITY_A0BFR4_PARTE/111-265	A0BFR4.1 PF00650.18	47.10	0.00
TRINITY_Q24GJ3_TETTS/1773-1849	Q24GJ3.2 PF16561.3;	47.10	0.00
TRINITY_A0A0P7V782_9TELE/147-328	A0A0P7V782.1 PF0139	47.10	0.00
TRINITY_L9JBY7_TUPCH/882-984	L9JBY7.1 PF00005.25	47.10	0.00
TRINITY_J9JYY0_ACYPI/27-195	J9JYY0.1 PF00043.23	47.10	0.00
TRINITY_A0A0D2WLU9_CAPO3/139-291	A0A0D2WLU9.1 PF1384	47.10	0.00
TRINITY_L1ILZ4_GUITH/20-180	L1ILZ4.1 PF00071.20	47.10	0.00
TRINITY_E5A958_LEPMJ/739-844	E5A958.1 PF04003.10	47.10	0.00
TRINITY_A0D650_PARTE/108-461	A0D650.1 PF00282.17	47.10	0.00
TRINITY_A0A087SU48_AUXPR/329-587	A0A087SU48.1 PF0771	47.10	0.00
TRINITY_A0A067R9Q6_ZOONE/8-135	A0A067R9Q6.1 PF0493	47.10	0.00
TRINITY_A0A077ZV76_STYLE/219-298	A0A077ZV76.1 PF1279	47.10	0.00
TRINITY_F2U0Y4_SALR5/31-184	F2U0Y4.1 PF09295.8;	47.10	0.00
TRINITY_J9IZH8_9SPIT/189-265	J9IZH8.1 PF14360.4;	47.10	0.00
TRINITY_A4RW13_OSTLU/32-429	A4RW13.1 PF01055.24	47.10	0.00
TRINITY_D8TM14_VOLCA/44-264	D8TM14.1 PF01036.16	47.10	0.00
TRINITY_A0BN51_PARTE/187-314	A0BN51.1 PF00107.24	47.10	0.00
TRINITY_I1QCX5_ORYGL/137-224	I1QCX5.1 PF00736.17	47.10	0.00
TRINITY_A8IE07_CHLRE/28-226	A8IE07.1 PF02492.17	47.10	0.00
TRINITY_W6FUH4_NODSP/15-183	W6FUH4.1 PF13385.4;	47.10	0.00
TRINITY_F0ZJL1_DICPU/4-73	F0ZJL1.1 PF07258.12	47.10	0.00
TRINITY_I7LU42_TETTS/123-212	I7LU42.1 PF00153.25	47.10	0.00

TRINITY_G0QS15_ICHMG/223-300	G0QS15.1 PF00789.18	47.10	0.00
TRINITY_A0D2Z5_PARTE/151-399	A0D2Z5.1 PF01457.14	47.10	0.00
TRINITY_L8H1Q6_ACACA/35-109	L8H1Q6.1 PF02148.17	47.10	0.00
TRINITY_A0A015K277_9GLOM/181-420	A0A015K277.1 PF0012	47.10	0.00
TRINITY_A9T5C7_PHYPA/343-459	A9T5C7.1 PF03109.14	47.10	0.00
TRINITY_A0CDK4_PARTE/404-736	A0CDK4.1 PF00702.24	47.10	0.00
TRINITY_I0YR44_9CHLO/12-1196	I0YR44.1 PF02463.17	47.10	0.00
TRINITY_H1VKX7_COLHI/177-458	H1VKX7.1 PF02540.15	47.10	0.00
TRINITY_I0YK30_9CHLO/217-372	I0YK30.1 PF00293.26	47.10	0.00
TRINITY_K9SS25_9SYNE/45-161	K9SS25.1 PF04536.12	47.10	0.00
TRINITY_I7M2R5_TETTS/19-173	I7M2R5.2 PF00406.20	47.10	0.00
TRINITY_D8TND4_VOLCA/49-240	D8TND4.1 PF00856.26	47.10	0.00
TRINITY_A0A0G4F821_9ALVE/39-330	A0A0G4F821.1 PF0006	47.10	0.00
TRINITY_U6PGJ8_HAECO/38-118	U6PGJ8.1 PF01883.17	47.10	0.00
TRINITY_D7FN74_ECTSI/48-147	D7FN74.1 PF00083.22	47.10	0.00
TRINITY_A8IT81_CHLRE/4-456	A8IT81.1 PF13520.4;	47.10	0.00
TRINITY_E1ZGI7_CHLVA/158-356	E1ZGI7.1 PF04989.10	47.10	0.00
TRINITY_H3H0L4_PHYRM/68-590	H3H0L4.1 PF03639.11	47.10	0.00
TRINITY_A8JJ05_CHLRE/562-633	A8JJ05.1 PF05699.12	47.10	0.00
TRINITY_A8I2C0_CHLRE/1-171	A8I2C0.1 PF05620.9;	47.10	0.00
TRINITY_A0A0D8PXB3_9GAMM/581-694	A0A0D8PXB3.1 PF0251	47.00	0.00
TRINITY_A0A0G4EYQ6_9ALVE/5-181	A0A0G4EYQ6.1 PF0087	47.00	0.00
TRINITY_I7MA61_TETTS/288-628	I7MA61.1 PF02841.12	47.00	0.00
TRINITY_G0QV43_ICHMG/249-568	G0QV43.1 PF04515.10	47.00	0.00
TRINITY_A4VDY4_TETTS/34-116	A4VDY4.1 PF08240.10	47.00	0.00
TRINITY_E6SJG6_THEM7/272-355	E6SJG6.1 PF06071.11	47.00	0.00
TRINITY_A0A0N4Y4L7_NIPBR/185-334	A0A0N4Y4L7.1 PF0104	47.00	0.00
TRINITY_A0A078ASQ4_STYLE/1-375	A0A078ASQ4.1 PF0002	47.00	0.00
TRINITY_A0A0G4J6M2_PLABS/10-171	A0A0G4J6M2.1 PF0007	47.00	0.00
TRINITY_A0A0M0K766_9EUKA/14-174	A0A0M0K766.1 PF0324	47.00	0.00
TRINITY_A0A067CDN1_SAPPC/56-432	A0A067CDN1.1 PF1362	47.00	0.00
TRINITY_A0DPQ3_PARTE/89-206	A0DPQ3.1 PF01909.21	47.00	0.00
TRINITY_A0CVZ1_PARTE/75-272	A0CVZ1.1 PF02096.18	47.00	0.00
TRINITY_A0A068RST3_9FUNG/46-164	A0A068RST3.1 PF0413	47.00	0.00
TRINITY_K9WVK1_9NOST/2149-2386	K9WVK1.1 PF07993.10	47.00	0.00
TRINITY_L8HGG6_ACACA/132-402	L8HGG6.1 PF03372.21	47.00	0.00
TRINITY_F4Q1R8_DICFS/16-344	F4Q1R8.1 PF00503.18	47.00	0.00
TRINITY_A0A075ANC9_9FUNG/8-264	A0A075ANC9.1 PF0006	47.00	0.00
TRINITY_A0A0J8CCV8_BETVU/1-132	A0A0J8CCV8.1 PF0169	47.00	0.00
TRINITY_A0A0D2VT98_CAPO3/389-590	A0A0D2VT98.1 PF0273	47.00	0.00
TRINITY_A0A0D2WRW2_CAPO3/74-406	A0A0D2WRW2.1 PF0197	47.00	0.00
TRINITY_A8J3P1_CHLRE/954-1161	A8J3P1.1 PF00211.18	47.00	0.00
TRINITY_A8JFA0_CHLRE/6-129	A8JFA0.1 PF01417.18	47.00	0.00
TRINITY_C5KYR6_PERM5/167-351	C5KYR6.1 PF13868.4;	47.00	0.00
TRINITY_C1MZX9_MICPC/195-329	C1MZX9.1 PF05517.10	47.00	0.00
TRINITY_A0A0J8EDR7_BETVU/401-548	A0A0J8EDR7.1 PF0081	47.00	0.00
TRINITY_A0A067KN01_JATCU/413-773	A0A067KN01.1 PF0070	47.00	0.00
TRINITY_Q246C6_TETTS/4-223	Q246C6.1 PF00300.20	47.00	0.00
TRINITY_I0Z838_9CHLO/271-504	I0Z838.1 PF00149.26	47.00	0.00
TRINITY_Q22NW8_TETTS/199-479	Q22NW8.2 PF06098.9;	47.00	0.00
TRINITY_A8JD26_CHLRE/31-191	A8JD26.1 PF01554.16	47.00	0.00
TRINITY_B6QSQ9_TALMQ/136-379	B6QSQ9.1 PF12706.5;	47.00	0.00
TRINITY_I0YVQ8_9CHLO/75-367	I0YVQ8.1 PF03372.21	47.00	0.00
TRINITY_I0YM78_9CHLO/8-231	I0YM78.1 PF00780.20	47.00	0.00
TRINITY_I0YR65_9CHLO/69-283	I0YR65.1 PF01633.18	47.00	0.00
TRINITY_D3B0Y8_POLPA/74-258	D3B0Y8.1 PF08857.9;	47.00	0.00
TRINITY_F2TZL1_SALR5/109-381	F2TZL1.1 PF04041.11	47.00	0.00
TRINITY_E9EDL0_METAQ/44-330	E9EDL0.1 PF00264.18	47.00	0.00
TRINITY_A0A0B4H7V2_9HYPO/227-516	A0A0B4H7V2.1 PF0012	47.00	0.00
TRINITY_E1ZE61_CHLVA/105-410	E1ZE61.1 PF00443.27	47.00	0.00
TRINITY_L8HLC2_ACACA/17-345	L8HLC2.1 PF00503.18	47.00	0.00

TRINITY_C3YPL1_BRAFL/9-386	C3YPL1.1 PF01433.18	47.00	0.00
TRINITY_H3HA27_PHYRM/82-199	H3HA27.1 PF00665.24	47.00	0.00
TRINITY_DEGP2_ARATH/121-308	O82261.2 PF00089.24	47.00	0.00
TRINITY_G0QN83_ICHMG/542-647	G0QN83.1 PF07717.14	47.00	0.00
TRINITY_J9G2B2_9SPIT/13-164	J9G2B2.1 PF01124.16	47.00	0.00
TRINITY_V4LXM5_EUTSA/193-367	V4LXM5.1 PF00022.17	47.00	0.00
TRINITY_D3B9V6_POLPA/831-1080	D3B9V6.1 PF07714.15	47.00	0.00
TRINITY_Q5TKD7_ORYSJ/846-1091	Q5TKD7.1 PF07727.12	47.00	0.00
TRINITY_D8TVP6_VOLCA/9-279	D8TVP6.1 PF03291.14	47.00	0.00
TRINITY_D0NBG3_PHYIT/336-495	D0NBG3.1 PF16796.3;	47.00	0.00
TRINITY_I7LUW4_TETTS/744-850	I7LUW4.2 PF02847.15	47.00	0.00
TRINITY_A9V2U7_MONBE/303-718	A9V2U7.1 PF01266.22	47.00	0.00
TRINITY_A8HPK0_CHLRE/62-165	A8HPK0.1 PF08241.10	47.00	0.00
TRINITY_D8TPW2_VOLCA/405-522	D8TPW2.1 PF07500.12	47.00	0.00
TRINITY_D8UEK5_VOLCA/70-191	D8UEK5.1 PF00781.22	47.00	0.00
TRINITY_H2XP71_CIOIN/399-482	H2XP71.1 PF00439.23	47.00	0.00
TRINITY_D8TZU6_VOLCA/420-780	D8TZU6.1 PF00155.19	47.00	0.00
TRINITY_D8SEV5_SELML/124-975	D8SEV5.1 PF00311.15	47.00	0.00
TRINITY_LIAS_CHLRE/53-236	A8JGF7.1 PF04055.19	47.00	0.00
TRINITY_A8J7M9_CHLRE/742-1038	A8J7M9.1 PF00069.23	47.00	0.00
TRINITY_Q3SD10_PARTE/7-191	Q3SD10.1 PF00025.19	47.00	0.00
TRINITY_A0CK29_PARTE/31-245	A0CK29.1 PF01027.18	47.00	0.00
TRINITY_A0CG00_PARTE/11-106	A0CG00.1 PF01230.21	46.90	0.00
TRINITY_J9IE32_9SPIT/16-80	J9IE32.1 PF01918.19	46.90	0.00
TRINITY_A0A067QRL2_ZOONE/3-213	A0A067QRL2.1 PF0009	46.90	0.00
TRINITY_L8GTY3_ACACA/3-81	L8GTY3.1 PF16511.3;	46.90	0.00
TRINITY_A0A075AZD4_9FUNG/14-188	A0A075AZD4.1 PF0222	46.90	0.00
TRINITY_R1DMU8_EMIHU/62-422	R1DMU8.1 PF07394.10	46.90	0.00
TRINITY_W4YX96_STRPU/218-379	W4YX96.1 PF05188.15	46.90	0.00
TRINITY_L8WVS7_THACA/438-564	L8WVS7.1 PF00176.21	46.90	0.00
TRINITY_I7LXP5_TETTS/139-427	I7LXP5.2 PF00122.18	46.90	0.00
TRINITY_D2VDY2_NAEGR/834-985	D2VDY2.1 PF01699.22	46.90	0.00
TRINITY_F0ZC70_DICPU/196-325	F0ZC70.1 PF00782.18	46.90	0.00
TRINITY_F0VMP6_NEOCL/39-200	F0VMP6.1 PF13847.4;	46.90	0.00
TRINITY_Q54UM3_DICDI/35-284	Q54UM3.1 PF01048.18	46.90	0.00
TRINITY_R4LFA6_9ACTN/34-164	R4LFA6.1 PF00293.26	46.90	0.00
TRINITY_S8CMD2_9LAMI/36-166	S8CMD2.1 PF00782.18	46.90	0.00
TRINITY_L8GKH0_ACACA/49-150	L8GKH0.1 PF13600.4;	46.90	0.00
TRINITY_D8LNJ0_ECTSI/87-261	D8LNJ0.1 PF04053.12	46.90	0.00
TRINITY_I7MJK6_TETTS/611-875	I7MJK6.1 PF00481.19	46.90	0.00
TRINITY_A0A024UKA9_9STRA/421-527	A0A024UKA9.1 PF0065	46.90	0.00
TRINITY_C1EFA8_MICSR/1-272	C1EFA8.1 PF04997.10	46.90	0.00
TRINITY_G0R646_ICHMG/745-1019	G0R646.1 PF00664.21	46.90	0.00
TRINITY_A0CTS6_PARTE/568-858	A0CTS6.1 PF01237.16	46.90	0.00
TRINITY_D2VIR9_NAEGR/12-171	D2VIR9.1 PF00071.20	46.90	0.00
TRINITY_H3C425_TETNG/9-149	H3C425.1 PF04603.10	46.90	0.00
TRINITY_B0W9I3_CULQU/211-318	B0W9I3.1 PF00651.29	46.90	0.00
TRINITY_F0ZYR6_DICPU/124-420	F0ZYR6.1 PF00069.23	46.90	0.00
TRINITY_L8GQG3_ACACA/1-193	L8GQG3.1 PF04148.11	46.90	0.00
TRINITY_J9IT22_9SPIT/499-822	J9IT22.1 PF00702.24	46.90	0.00
TRINITY_D8TRL5_VOLCA/234-457	D8TRL5.1 PF00520.29	46.90	0.00
TRINITY_A0A0D2WQD1_CAPO3/65-259	A0A0D2WQD1.1 PF0010	46.90	0.00
TRINITY_L9LDS6_TUPCH/20-143	L9LDS6.1 PF04263.14	46.90	0.00
TRINITY_L8H892_ACACA/48-115	L8H892.1 PF00505.17	46.90	0.00
TRINITY_A0E806_PARTE/2-134	A0E806.1 PF07047.10	46.90	0.00
TRINITY_W6FUH4_NODSP/15-183	W6FUH4.1 PF13385.4;	46.90	0.00
TRINITY_Q9FWI2_ORYSJ/795-1030	Q9FWI2.2 PF07727.12	46.90	0.00
TRINITY_I7MDT2_TETTS/149-278	I7MDT2.1 PF00107.24	46.90	0.00
TRINITY_Q22NV2_TETTS/1650-1768	Q22NV2.1 PF08683.9;	46.90	0.00
TRINITY_G0QT52_ICHMG/1139-1415	G0QT52.1 PF00520.29	46.90	0.00
TRINITY_D3B1P0_POLPA/183-439	D3B1P0.1 PF07714.15	46.90	0.00

TRINITY_B6QR46_TALMQ/3-65	B6QR46.1 PF14497.4;	46.90	0.00
TRINITY_A0A087SD81_AUXPR/312-540	A0A087SD81.1 PF0578	46.90	0.00
TRINITY_L8GD19_ACACA/407-451	L8GD19.1 PF13639.4;	46.90	0.00
TRINITY_D8TT91_VOLCA/32-164	D8TT91.1 PF00583.23	46.90	0.00
TRINITY_C1FIS7_MICSR/830-1238	C1FIS7.1 PF08393.11	46.90	0.00
TRINITY_T1FQ89_HELRO/187-525	T1FQ89.1 PF06602.12	46.90	0.00
TRINITY_D2VJC3_NAEGR/5-177	D2VJC3.1 PF00025.19	46.90	0.00
TRINITY_A0D515_PARTE/43-239	A0D515.1 PF00149.26	46.90	0.00
TRINITY_A8IRX8_CHLRE/440-520	A8IRX8.1 PF00789.18	46.90	0.00
TRINITY_C4LZL0_ENTHI/154-328	C4LZL0.1 PF00350.21	46.90	0.00
TRINITY_L8H676_ACACA/180-264	L8H676.1 PF03439.11	46.90	0.00
TRINITY_Q22R76_TETTS/156-952	Q22R76.2 PF04762.10	46.90	0.00
TRINITY_A0A0E0NVU7_ORYRU/96-256	A0A0E0NVU7.1 PF0141	46.90	0.00
TRINITY_I0YQL8_9CHLO/1474-1869	I0YQL8.1 PF00632.23	46.90	0.00
TRINITY_E1VHL4_9GAMM/921-1033	E1VHL4.1 PF00072.22	46.90	0.00
TRINITY_K1ZH36_9BACT/100-185	K1ZH36.1 PF04488.13	46.90	0.00
TRINITY_Q54A44_CYAME/3-377	Q54A44.1 PF00022.17	46.90	0.00
TRINITY_A0A0N5A8P3_9BILA/8-221	A0A0N5A8P3.1 PF0113	46.90	0.00
TRINITY_A8HP62_CHLRE/179-430	A8HP62.1 PF05548.9;	46.90	0.00
TRINITY_D8U9S9_VOLCA/599-793	D8U9S9.1 PF00211.18	46.90	0.00
TRINITY_E1ZSA7_CHLVA/196-504	E1ZSA7.1 PF04030.12	46.90	0.00
TRINITY_A0A067CV48_SAPPC/96-289	A0A067CV48.1 PF0281	46.90	0.00
TRINITY_Q23QD2_TETTS/366-575	Q23QD2.2 PF00566.16	46.90	0.00
TRINITY_R7KSH0_9FIRM/287-491	R7KSH0.1 PF01144.21	46.80	0.00
TRINITY_A0E189_PARTE/446-495	A0E189.1 PF13920.4;	46.80	0.00
TRINITY_A0A059LK99_9CHLO/143-244	A0A059LK99.1 PF1349	46.80	0.00
TRINITY_J9J9I6_9SPIT/56-413	J9J9I6.1 PF15902.3;	46.80	0.00
TRINITY_M1VLE0_CYAME/17-81	M1VLE0.1 PF13432.4;	46.80	0.00
TRINITY_A0A097EGI2_9SPHN/51-177	A0A097EGI2.1 PF0018	46.80	0.00
TRINITY_I7LX25_TETTS/48-128	I7LX25.1 PF09398.8;	46.80	0.00
TRINITY_T1KPW7_TETUR/7-120	T1KPW7.1 PF01412.16	46.80	0.00
TRINITY_K1R1A1_CRAGI/479-586	K1R1A1.1 PF01315.20	46.80	0.00
TRINITY_B3L3A9_PLAKH/641-702	B3L3A9.1 PF12906.5;	46.80	0.00
TRINITY_W5MXN2_LEPOC/10-224	W5MXN2.1 PF00702.24	46.80	0.00
TRINITY_L8GTK1_ACACA/146-395	L8GTK1.1 PF07714.15	46.80	0.00
TRINITY_J9IBV7_9SPIT/751-1030	J9IBV7.1 PF00122.18	46.80	0.00
TRINITY_G5A9X4_PHYSP/228-479	G5A9X4.1 PF07714.15	46.80	0.00
TRINITY_G0R6D5_ICHMG/134-280	G0R6D5.1 PF03031.16	46.80	0.00
TRINITY_A0A024UFZ7_9STRA/209-678	A0A024UFZ7.1 PF0143	46.80	0.00
TRINITY_A0A0A1P476_9FUNG/139-258	A0A0A1P476.1 PF0062	46.80	0.00
TRINITY_Q23Q42_TETTS/887-1010	Q23Q42.2 PF02518.24	46.80	0.00
TRINITY_C3ZVM6_BRAFL/123-491	C3ZVM6.1 PF01546.26	46.80	0.00
TRINITY_Q42420_MAIZE/14-73	Q42420.1 PF00280.16	46.80	0.00
TRINITY_I1XN16_METNJ/199-323	I1XN16.1 PF10017.7;	46.80	0.00
TRINITY_A9SBN3_PHYPA/3-195	A9SBN3.1 PF15251.4;	46.80	0.00
TRINITY_A0A078B349_STYLE/444-804	A0A078B349.1 PF0070	46.80	0.00
TRINITY_A0BDL2_PARTE/6-167	A0BDL2.1 PF00071.20	46.80	0.00
TRINITY_Q965M4_CAEEL/312-493	Q965M4.1 PF02145.13	46.80	0.00
TRINITY_A0A077ZVZ5_STYLE/1412-1476	A0A077ZVZ5.1 PF1349	46.80	0.00
TRINITY_A8I356_CHLRE/64-445	A8I356.1 PF01490.16	46.80	0.00
TRINITY_L8GZ65_ACACA/20-84	L8GZ65.1 PF13499.4;	46.80	0.00
TRINITY_A0A024ULS4_9STRA/32-248	A0A024ULS4.1 PF0223	46.80	0.00
TRINITY_L8GVQ3_ACACA/116-380	L8GVQ3.1 PF07766.11	46.80	0.00
TRINITY_A0DTA6_PARTE/314-534	A0DTA6.1 PF07002.14	46.80	0.00
TRINITY_A0A0D2WRI8_CAPO3/947-1198	A0A0D2WRI8.1 PF0771	46.80	0.00
TRINITY_D8LWE9_BLAHO/35-189	D8LWE9.1 PF01652.16	46.80	0.00
TRINITY_J9I8E2_9SPIT/28-186	J9I8E2.1 PF00071.20	46.80	0.00
TRINITY_I1FZZ4_AMPQE/233-565	I1FZZ4.1 PF05028.12	46.80	0.00
TRINITY_B7PNU9_IXOSC/20-82	B7PNU9.1 PF00226.29	46.80	0.00
TRINITY_A0A078F732_BRANA/8-194	A0A078F732.1 PF0117	46.80	0.00
TRINITY_F0VL60_NEOCL/26-105	F0VL60.1 PF01282.17	46.80	0.00

TRINITY_L7U7N8_MYXSD/32-303	L7U7N8.1 PF00664.21	46.80	0.00
TRINITY_W5JKD9_ANODA/60-214	W5JKD9.1 PF13847.4;	46.80	0.00
TRINITY_F0ZJ03_DICPU/294-386	F0ZJ03.1 PF00169.27	46.80	0.00
TRINITY_F0ZJC1_DICPU/222-612	F0ZJC1.1 PF00916.18	46.80	0.00
TRINITY_S2JH36_MUCC1/189-444	S2JH36.1 PF08145.10	46.80	0.00
TRINITY_K3X2Y4_PYTUL/147-383	K3X2Y4.1 PF00122.18	46.80	0.00
TRINITY_L8GWQ5_ACACA/986-1235	L8GWQ5.1 PF07714.15	46.80	0.00
TRINITY_I1FHP7_AMPQE/40-314	I1FHP7.1 PF00664.21	46.80	0.00
TRINITY_D7G211_ECTSI/397-445	D7G211.1 PF00097.23	46.80	0.00
TRINITY_A0A087SML6_AUXPR/83-264	A0A087SML6.1 PF1346	46.80	0.00
TRINITY_A0A087SQT5_AUXPR/380-517	A0A087SQT5.1 PF0273	46.80	0.00
TRINITY_W7XI32_TETTS/8-116	W7XI32.1 PF01412.16	46.80	0.00
TRINITY_G0R6G3_ICHMG/54-245	G0R6G3.1 PF01145.23	46.80	0.00
TRINITY_A0A062XE64_LEUPS/12-303	A0A062XE64.1 PF0769	46.80	0.00
TRINITY_D8TQT5_VOLCA/180-508	D8TQT5.1 PF00082.20	46.80	0.00
TRINITY_W7X4T7_TETTS/1-455	W7X4T7.1 PF00171.20	46.80	0.00
TRINITY_A8HQN1_CHLRE/395-521	A8HQN1.1 PF05118.13	46.80	0.00
TRINITY_U2E7K8_9BACT/35-264	U2E7K8.1 PF02522.12	46.80	0.00
TRINITY_A0A024WRW2_PLAFA/3-212	A0A024WRW2.1 PF0009	46.80	0.00
TRINITY_W2PUW6_PHYPN/2-375	W2PUW6.1 PF00022.17	46.80	0.00
TRINITY_D8U886_VOLCA/105-335	D8U886.1 PF01728.17	46.80	0.00
TRINITY_H6NE65_9BACL/18-262	H6NE65.1 PF08450.10	46.80	0.00
TRINITY_M7NLM8_PNEMU/837-954	M7NLM8.1 PF04408.21	46.80	0.00
TRINITY_A8JIJ9_CHLRE/11-261	A8JIJ9.1 PF00069.23	46.80	0.00
TRINITY_I0YQM6_9CHLO/1-105	I0YQM6.1 PF00085.18	46.80	0.00
TRINITY_G0QVG7_ICHMG/239-716	G0QVG7.1 PF04791.14	46.80	0.00
TRINITY_A0A096QB17_MAIZE/144-427	A0A096QB17.1 PF0045	46.70	0.00
TRINITY_G0QR55_ICHMG/103-222	G0QR55.1 PF02518.24	46.70	0.00
TRINITY_I7MMW7_TETTS/113-305	I7MMW7.2 PF00270.27	46.70	0.00
TRINITY_I7MHF3_TETTS/13-173	I7MHF3.1 PF00071.20	46.70	0.00
TRINITY_L1LFE5_THEEQ/14-268	L1LFE5.1 PF00069.23	46.70	0.00
TRINITY_A9TXN9_PHYPA/196-318	A9TXN9.1 PF05971.10	46.70	0.00
TRINITY_F8VPK0_MOUSE/10-84	F8VPK0.1 PF13432.4;	46.70	0.00
TRINITY_A0A087SMY6_AUXPR/803-1077	A0A087SMY6.1 PF0066	46.70	0.00
TRINITY_W7X292_TETTS/58-150	W7X292.1 PF16455.3;	46.70	0.00
TRINITY_I4BY13_ANAMD/456-543	I4BY13.1 PF00581.18	46.70	0.00
TRINITY_Q23QF2_TETTS/248-571	Q23QF2.2 PF03133.13	46.70	0.00
TRINITY_F6YGC4_XENTR/241-331	F6YGC4.1 PF08743.8;	46.70	0.00
TRINITY_C3XZI6_BRAFL/131-196	C3XZI6.1 PF12352.6;	46.70	0.00
TRINITY_Q22EI4_TETTS/243-433	Q22EI4.2 PF05193.19	46.70	0.00
TRINITY_G0R080_ICHMG/9-179	G0R080.1 PF00300.20	46.70	0.00
TRINITY_L1I5M4_GUITH/5-195	L1I5M4.1 PF10294.7;	46.70	0.00
TRINITY_D8TXZ2_VOLCA/49-343	D8TXZ2.1 PF03151.14	46.70	0.00
TRINITY_H8KY18_SOLCM/14-377	H8KY18.1 PF01266.22	46.70	0.00
TRINITY_PTP2_DICDI/56-346	P34138.1 PF00102.25	46.70	0.00
TRINITY_F6GD80_LACS5/19-92	F6GD80.1 PF09907.7;	46.70	0.00
TRINITY_M5GGP1_DACSP/1-147	M5GGP1.1 PF00125.22	46.70	0.00
TRINITY_L8H2B5_ACACA/449-511	L8H2B5.1 PF13424.4;	46.70	0.00
TRINITY_Q23QF2_TETTS/248-571	Q23QF2.2 PF03133.13	46.70	0.00
TRINITY_A8JGS5_CHLRE/358-511	A8JGS5.1 PF13692.4;	46.70	0.00
TRINITY_D0NLH6_PHYIT/126-232	D0NLH6.1 PF00307.29	46.70	0.00
TRINITY_F8W2M8_DANRE/214-518	F8W2M8.1 PF00082.20	46.70	0.00
TRINITY_D8TTA0_VOLCA/12-77	D8TTA0.1 PF11470.6;	46.70	0.00
TRINITY_F6QDG2_HORSE/10-84	F6QDG2.1 PF13432.4;	46.70	0.00
TRINITY_L8HGG1_ACACA/468-729	L8HGG1.1 PF00069.23	46.70	0.00
TRINITY_D8UIN7_VOLCA/64-172	D8UIN7.1 PF04800.10	46.70	0.00
TRINITY_E1ZGR2_CHLVA/377-472	E1ZGR2.1 PF03129.18	46.70	0.00
TRINITY_A0DMF5_PARTE/180-251	A0DMF5.1 PF01485.19	46.70	0.00
TRINITY_D8TN27_VOLCA/564-828	D8TN27.1 PF00069.23	46.70	0.00
TRINITY_D8U8K1_VOLCA/1-246	D8U8K1.1 PF05096.10	46.70	0.00
TRINITY_B9H8D7_POPTR/18-295	B9H8D7.2 PF13621.4;	46.70	0.00

TRINITY_D8TNE1_VOLCA/86-242	D8TNE1.1 PF00650.18	46.70	0.00
TRINITY_F0ZBL5_DICPU/1981-2245	F0ZBL5.1 PF07714.15	46.70	0.00
TRINITY_A8J165_CHLRE/133-193	A8J165.1 PF04117.10	46.70	0.00
TRINITY_M5FYW4_DACSP/6-170	M5FYW4.1 PF00071.20	46.70	0.00
TRINITY_M2X230_GALSU/401-613	M2X230.1 PF07859.11	46.70	0.00
TRINITY_E1ZCE5_CHLVA/104-280	E1ZCE5.1 PF00551.17	46.70	0.00
TRINITY_E1Z8Q3_CHLVA/99-250	E1Z8Q3.1 PF08389.10	46.70	0.00
TRINITY_U3JF38_FICAL/133-194	U3JF38.1 PF04117.10	46.70	0.00
TRINITY_D3BKY1_POLPA/404-538	D3BKY1.1 PF07534.14	46.70	0.00
TRINITY_L8H7D9_ACACA/29-322	L8H7D9.1 PF00850.17	46.70	0.00
TRINITY_A8IDW0_CHLRE/76-192	A8IDW0.1 PF10236.7;	46.70	0.00
TRINITY_A0A0G4ENJ3_9ALVE/137-405	A0A0G4ENJ3.1 PF1338	46.70	0.00
TRINITY_I1GSS6_BRADI/9-114	I1GSS6.1 PF05915.10	46.70	0.00
TRINITY_Q23QF2_TETTS/248-571	Q23QF2.2 PF03133.13	46.70	0.00
TRINITY_D8THD3_VOLCA/212-384	D8THD3.1 PF00685.25	46.70	0.00
TRINITY_H3HAM5_PHYRM/167-517	H3HAM5.1 PF00069.23	46.70	0.00
TRINITY_D3B844_POLPA/641-686	D3B844.1 PF13920.4;	46.70	0.00
TRINITY_A8J2X9_CHLRE/125-257	A8J2X9.1 PF00504.19	46.70	0.00
TRINITY_A0A0G4FY22_9ALVE/62-543	A0A0G4FY22.1 PF0050	46.70	0.00
TRINITY_A8J0Z0_CHLRE/6-151	A8J0Z0.1 PF05517.10	46.70	0.00
TRINITY_D3BSK9_POLPA/389-510	D3BSK9.1 PF01569.19	46.70	0.00
TRINITY_Q22TA6_TETTS/252-367	Q22TA6.2 PF01416.18	46.70	0.00
TRINITY_L8HDF4_ACACA/508-622	L8HDF4.1 PF08477.11	46.70	0.00
TRINITY_A8HXD3_CHLRE/54-176	A8HXD3.1 PF02996.15	46.70	0.00
TRINITY_A8J937_CHLRE/104-222	A8J937.1 PF00188.24	46.70	0.00
TRINITY_A0BMS3_PARTE/40-160	A0BMS3.1 PF01597.17	46.70	0.00
TRINITY_Q24I26_TETTS/15-212	Q24I26.2 PF16213.3;	46.70	0.00
TRINITY_L0A9E0_DEIPD/46-181	L0A9E0.1 PF09346.8;	46.70	0.00
TRINITY_A0A078AZ38_STYLE/853-1037	A0A078AZ38.1 PF0068	46.60	0.00
TRINITY_B2AXL9_PODAN/47-390	B2AXL9.1 PF00150.16	46.60	0.00
TRINITY_I7MJV0_TETTS/846-955	I7MJV0.2 PF00069.23	46.60	0.00
TRINITY_F4PQG8_DICFS/394-556	F4PQG8.1 PF03666.11	46.60	0.00
TRINITY_K3W6E1_PYTUL/147-285	K3W6E1.1 PF17207.1;	46.60	0.00
TRINITY_D3HKH9_LEGLN/14-149	D3HKH9.1 PF00583.23	46.60	0.00
TRINITY_E0VTM7_PEDHC/5-96	E0VTM7.1 PF03647.11	46.60	0.00
TRINITY_Q22NP9_TETTS/339-441	Q22NP9.2 PF02204.16	46.60	0.00
TRINITY_E2BCV0_HARSA/63-172	E2BCV0.1 PF07992.12	46.60	0.00
TRINITY_J9FR16_9SPIT/32-356	J9FR16.1 PF04041.11	46.60	0.00
TRINITY_I0YM25_9CHLO/47-337	I0YM25.1 PF03151.14	46.60	0.00
TRINITY_A8I9R7_CHLRE/283-767	A8I9R7.1 PF01432.18	46.60	0.00
TRINITY_L1IVP8_GUITH/472-575	L1IVP8.1 PF14874.4;	46.60	0.00
TRINITY_A8J6P8_CHLRE/44-163	A8J6P8.1 PF07542.9;	46.60	0.00
TRINITY_TM120_DICDI/34-361	Q54IK2.1 PF07851.11	46.60	0.00
TRINITY_J9IMA0_9SPIT/532-645	J9IMA0.1 PF02518.24	46.60	0.00
TRINITY_K1Q3Y8_CRAGI/69-219	K1Q3Y8.1 PF07534.14	46.60	0.00
TRINITY_D8TVT9_VOLCA/521-846	D8TVT9.1 PF03399.14	46.60	0.00
TRINITY_E3FRS4_STIAD/140-391	E3FRS4.1 PF03372.21	46.60	0.00
TRINITY_A8J8Y2_CHLRE/797-855	A8J8Y2.1 PF02358.14	46.60	0.00
TRINITY_I0YMK7_9CHLO/41-221	I0YMK7.1 PF07714.15	46.60	0.00
TRINITY_V8P9E3_OPHHA/130-207	V8P9E3.1 PF03061.20	46.60	0.00
TRINITY_C3ZM89_BRAFL/5-354	C3ZM89.1 PF16317.3;	46.60	0.00
TRINITY_V6S777_9FLAO/37-449	V6S777.1 PF00266.17	46.60	0.00
TRINITY_D8RI41_SELML/492-592	D8RI41.1 PF00168.28	46.60	0.00
TRINITY_A8IMU3_CHLRE/397-552	A8IMU3.1 PF15964.3;	46.60	0.00
TRINITY_A8JD56_CHLRE/362-522	A8JD56.1 PF05698.12	46.60	0.00
TRINITY_D8SM72_SELML/3-368	D8SM72.1 PF01494.17	46.60	0.00
TRINITY_D8UHH6_VOLCA/99-319	D8UHH6.1 PF00573.20	46.60	0.00
TRINITY_A0A024UTX6_9STRA/17-316	A0A024UTX6.1 PF0844	46.60	0.00
TRINITY_I0Z703_9CHLO/261-382	I0Z703.1 PF03953.15	46.60	0.00
TRINITY_K1QA57_CRAGI/136-268	K1QA57.1 PF03464.13	46.60	0.00
TRINITY_E1ZN16_CHLVA/784-966	E1ZN16.1 PF05193.19	46.60	0.00

TRINITY_DUS1_ARATH/58-188	Q9ZR37.1 PF00782.18	46.60	0.00
TRINITY_A8J4N7_CHLRE/147-340	A8J4N7.1 PF00481.19	46.60	0.00
TRINITY_A0A058Z3I3_9EUKA/133-262	A0A058Z3I3.1 PF0000	46.60	0.00
TRINITY_A0A084FUX2_9PEZI/172-302	A0A084FUX2.1 PF0000	46.60	0.00
TRINITY_I0YR17_9CHLO/1-190	I0YR17.1 PF00106.23	46.60	0.00
TRINITY_A0A078A5D1_STYLE/12-162	A0A078A5D1.1 PF0112	46.60	0.00
TRINITY_A0A087SEK8_AUXPR/17-445	A0A087SEK8.1 PF0089	46.60	0.00
TRINITY_SACC_BACSU/39-349	P05656.1 PF00251.18	46.60	0.00
TRINITY_A8HNP6_CHLRE/11-260	A8HNP6.1 PF07727.12	46.60	0.00
TRINITY_Q22D58_TETTS/15-329	Q22D58.1 PF00248.19	46.60	0.00
TRINITY_A6DLQ3_9BACT/17-244	A6DLQ3.1 PF01738.16	46.60	0.00
TRINITY_Q23CV4_TETTS/238-295	Q23CV4.1 PF13921.4;	46.60	0.00
TRINITY_A0A0D2QGM6_GOSRA/224-355	A0A0D2QGM6.1 PF0004	46.60	0.00
TRINITY_I0Z9F5_9CHLO/485-642	I0Z9F5.1 PF07731.12	46.60	0.00
TRINITY_A8HNP6_CHLRE/11-260	A8HNP6.1 PF07727.12	46.60	0.00
TRINITY_E1ZGV4_CHLVA/261-464	E1ZGV4.1 PF01331.17	46.60	0.00
TRINITY_D8TR79_VOLCA/50-236	D8TR79.1 PF05197.11	46.60	0.00
TRINITY_A0A0G2ZK96_9DELT/98-408	A0A0G2ZK96.1 PF0008	46.60	0.00
TRINITY_A0BGA7_PARTE/140-295	A0BGA7.1 PF13847.4;	46.60	0.00
TRINITY_D8UAV2_VOLCA/5-78	D8UAV2.1 PF08783.9;	46.60	0.00
TRINITY_D8TY55_VOLCA/1-316	D8TY55.1 PF08392.10	46.60	0.00
TRINITY_L0PBL5_PNEJ8/22-125	L0PBL5.1 PF13344.4;	46.60	0.00
TRINITY_D8U3Q0_VOLCA/57-316	D8U3Q0.1 PF00069.23	46.60	0.00
TRINITY_A0CPN7_PARTE/24-297	A0CPN7.1 PF00069.23	46.60	0.00
TRINITY_A0CUS6_PARTE/513-811	A0CUS6.1 PF03372.21	46.50	0.00
TRINITY_I7MM85_TETTS/11-175	I7MM85.1 PF13460.4;	46.50	0.00
TRINITY_D2W1D5_NAEGR/81-231	D2W1D5.1 PF00782.18	46.50	0.00
TRINITY_I7LZN5_TETTS/536-926	I7LZN5.2 PF00368.16	46.50	0.00
TRINITY_G1P929_MYOLU/9-432	G1P929.1 PF05577.10	46.50	0.00
TRINITY_G0QX53_ICHMG/28-565	G0QX53.1 PF00995.21	46.50	0.00
TRINITY_M3ZQ64_XIPMA/212-456	M3ZQ64.1 PF00566.16	46.50	0.00
TRINITY_E1ZJM7_CHLVA/29-208	E1ZJM7.1 PF00265.16	46.50	0.00
TRINITY_F4Q9T6_DICFS/1388-1946	F4Q9T6.1 PF06920.11	46.50	0.00
TRINITY_F6TYN3_CIOIN/5-177	F6TYN3.2 PF00025.19	46.50	0.00
TRINITY_R4YTU2_OLEAN/390-509	R4YTU2.1 PF02518.24	46.50	0.00
TRINITY_G4VST9_SCHMA/28-420	G4VST9.1 PF00270.27	46.50	0.00
TRINITY_L8H349_ACACA/353-471	L8H349.1 PF05064.11	46.50	0.00
TRINITY_I7M706_TETTS/16-370	I7M706.2 PF00225.21	46.50	0.00
TRINITY_A0A0G4J5Z2_PLABS/225-268	A0A0G4J5Z2.1 PF1363	46.50	0.00
TRINITY_A0DRJ0_PARTE/9-177	A0DRJ0.1 PF00188.24	46.50	0.00
TRINITY_A0A059LFA5_9CHLO/31-127	A0A059LFA5.1 PF1279	46.50	0.00
TRINITY_A0A0M0JSR8_9EUKA/1-158	A0A0M0JSR8.1 PF0975	46.50	0.00
TRINITY_A0EBT4_PARTE/159-442	A0EBT4.1 PF04712.10	46.50	0.00
TRINITY_A0A067L493_JATCU/87-130	A0A067L493.1 PF1363	46.50	0.00
TRINITY_A0A0C4EPX9_PUCT1/384-738	A0A0C4EPX9.1 PF0070	46.50	0.00
TRINITY_W4XFF3_STRPU/197-480	W4XFF3.1 PF01457.14	46.50	0.00
TRINITY_D8TEF7_SELML/494-813	D8TEF7.1 PF01504.16	46.50	0.00
TRINITY_G4ZY58_PHYSP/182-609	G4ZY58.1 PF05185.14	46.50	0.00
TRINITY_G0QNP0_ICHMG/104-376	G0QNP0.1 PF00233.17	46.50	0.00
TRINITY_JMJCC_DICDI/9-293	Q54LV7.2 PF13621.4;	46.50	0.00
TRINITY_F0Z7K0_DICPU/185-314	F0Z7K0.1 PF00241.18	46.50	0.00
TRINITY_A8JG06_CHLRE/384-671	A8JG06.1 PF13949.4;	46.50	0.00
TRINITY_H2MVB9_ORYLA/351-580	H2MVB9.1 PF02836.15	46.50	0.00
TRINITY_A0A059LFH9_9CHLO/84-174	A0A059LFH9.1 PF0133	46.50	0.00
TRINITY_D8UKC2_VOLCA/33-146	D8UKC2.1 PF01966.20	46.50	0.00
TRINITY_D3BGV1_POLPA/114-495	D3BGV1.1 PF07690.14	46.50	0.00
TRINITY_A0E2J1_PARTE/100-358	A0E2J1.1 PF00069.23	46.50	0.00
TRINITY_D8TZM6_VOLCA/8-296	D8TZM6.1 PF00704.26	46.50	0.00
TRINITY_I0ZAZ9_9CHLO/77-250	I0ZAZ9.1 PF04548.14	46.50	0.00
TRINITY_J9JQJ9_ACYPI/168-488	J9JQJ9.1 PF00069.23	46.50	0.00
TRINITY_A8IVV3_CHLRE/21-122	A8IVV3.1 PF13967.4;	46.50	0.00

TRINITY_D8U9S9_VOLCA/599-793	D8U9S9.1 PF00211.18	46.50	0.00
TRINITY_J9EXT5_9SPIT/263-461	J9EXT5.1 PF00117.26	46.50	0.00
TRINITY_G0QKW1_ICHMG/111-281	G0QKW1.1 PF00092.26	46.50	0.00
TRINITY_D2VTM9_NAEGR/497-639	D2VTM9.1 PF00644.18	46.50	0.00
TRINITY_F4PY90_DICFS/118-394	F4PY90.1 PF00122.18	46.50	0.00
TRINITY_B1WYH5_CYAA5/33-300	B1WYH5.1 PF00756.18	46.50	0.00
TRINITY_A5DMW0_PICGU/24-166	A5DMW0.2 PF10604.7;	46.50	0.00
TRINITY_A0A0D2W2W6_GOSRA/101-248	A0A0D2W2W6.1 PF0081	46.50	0.00
TRINITY_F0ZQX2_DICPU/45-354	F0ZQX2.1 PF00176.21	46.50	0.00
TRINITY_I1IAH5_BRADI/1140-1407	I1IAH5.1 PF00856.26	46.50	0.00
TRINITY_A0E9F9_PARTE/141-258	A0E9F9.1 PF12146.6;	46.50	0.00
TRINITY_D2VGY2_NAEGR/306-540	D2VGY2.1 PF06314.9;	46.50	0.00
TRINITY_W4FKN9_9STRA/2-119	W4FKN9.1 PF13806.4;	46.50	0.00
TRINITY_L8GQB4_ACACA/35-395	L8GQB4.1 PF01433.18	46.50	0.00
TRINITY_L8GYB8_ACACA/73-218	L8GYB8.1 PF01794.17	46.50	0.00
TRINITY_A0A061FSJ2_THECC/107-524	A0A061FSJ2.1 PF0086	46.50	0.00
TRINITY_A9SNE7_PHYPA/3-88	A9SNE7.1 PF13417.4;	46.50	0.00
TRINITY_C1DYL3_MICSR/88-241	C1DYL3.1 PF00730.23	46.50	0.00
TRINITY_A0A0D2URP3_CAPO3/339-607	A0A0D2URP3.1 PF0066	46.50	0.00
TRINITY_A0A077ZR29_STYLE/143-358	A0A077ZR29.1 PF0011	46.50	0.00
TRINITY_I0Z0Z9_9CHLO/830-1158	I0Z0Z9.1 PF01326.17	46.50	0.00
TRINITY_Q22AR0_TETTS/17-145	Q22AR0.1 PF13529.4;	46.50	0.00
TRINITY_A8IBB6_CHLRE/141-344	A8IBB6.1 PF00211.18	46.50	0.00
TRINITY_V5ED09_PSEBG/5-177	V5ED09.1 PF00025.19	46.50	0.00
TRINITY_K3WZX0_PYTUL/2-110	K3WZX0.1 PF10256.7;	46.40	0.00
TRINITY_A4VD00_TETTS/44-155	A4VD00.1 PF09184.9;	46.40	0.00
TRINITY_L0D875_SINAD/48-362	L0D875.1 PF07995.9;	46.40	0.00
TRINITY_C1N3Y2_MICPC/168-228	C1N3Y2.1 PF12656.5;	46.40	0.00
TRINITY_Q54P73_DICDI/98-339	Q54P73.1 PF01636.21	46.40	0.00
TRINITY_A0DZY7_PARTE/71-141	A0DZY7.1 PF10601.7;	46.40	0.00
TRINITY_G0QTH6_ICHMG/49-264	G0QTH6.1 PF01556.16	46.40	0.00
TRINITY_I7LY25_TETTS/21-290	I7LY25.2 PF00069.23	46.40	0.00
TRINITY_Q22WJ4_TETTS/279-392	Q22WJ4.2 PF12796.5;	46.40	0.00
TRINITY_I7M3I8_TETTS/279-794	I7M3I8.1 PF01055.24	46.40	0.00
TRINITY_M4U5L4_9GAMM/2-203	M4U5L4.1 PF03932.12	46.40	0.00
TRINITY_A0A0D2X4A7_CAPO3/26-399	A0A0D2X4A7.1 PF1014	46.40	0.00
TRINITY_Q23QF5_TETTS/161-229	Q23QF5.2 PF00505.17	46.40	0.00
TRINITY_D2V4M3_NAEGR/31-141	D2V4M3.1 PF15891.3;	46.40	0.00
TRINITY_J9HY65_9SPIT/97-190	J9HY65.1 PF12894.5;	46.40	0.00
TRINITY_F4PVG2_DICFS/12-140	F4PVG2.1 PF00782.18	46.40	0.00
TRINITY_L8GLW0_ACACA/708-815	L8GLW0.1 PF00651.29	46.40	0.00
TRINITY_Q54F12_DICDI/45-224	Q54F12.1 PF10294.7;	46.40	0.00
TRINITY_F1A131_DICPU/7-91	F1A131.1 PF04000.13	46.40	0.00
TRINITY_F4Q2P4_DICFS/1-246	F4Q2P4.1 PF02121.16	46.40	0.00
TRINITY_A0DZ98_PARTE/7-67	A0DZ98.1 PF04193.12	46.40	0.00
TRINITY_W6FUH4_NODSP/15-183	W6FUH4.1 PF13385.4;	46.40	0.00
TRINITY_D8TMY6_VOLCA/279-490	D8TMY6.1 PF07714.15	46.40	0.00
TRINITY_Q23ZF6_TETTS/19-311	Q23ZF6.1 PF01008.15	46.40	0.00
TRINITY_R1FX63_EMIHU/5-180	R1FX63.1 PF00929.22	46.40	0.00
TRINITY_D8TJS0_VOLCA/25-143	D8TJS0.1 PF07986.10	46.40	0.00
TRINITY_C1AE84_GEMAT/24-380	C1AE84.1 PF01244.19	46.40	0.00
TRINITY_M0S0V4_MUSAM/34-174	M0S0V4.1 PF11938.6;	46.40	0.00
TRINITY_C3Z710_BRAFL/85-154	C3Z710.1 PF00076.20	46.40	0.00
TRINITY_U3I811_ANAPL/80-1064	U3I811.1 PF03813.12	46.40	0.00
TRINITY_D8UA72_VOLCA/1-143	D8UA72.1 PF00588.17	46.40	0.00
TRINITY_U6LP07_9EIME/637-706	U6LP07.1 PF00076.20	46.40	0.00
TRINITY_M4XKR5_PSEDE/34-276	M4XKR5.1 PF00561.18	46.40	0.00
TRINITY_B1XK59_SYNP2/12-118	B1XK59.1 PF00583.23	46.40	0.00
TRINITY_S2JLV9_MUCC1/30-309	S2JLV9.1 PF01256.15	46.40	0.00
TRINITY_J9J0E5_9SPIT/15-172	J9J0E5.1 PF00071.20	46.40	0.00
TRINITY_L8GN51_ACACA/128-249	L8GN51.1 PF01648.18	46.40	0.00

TRINITY_A0A024U6X7_9STRA/1427-1840	A0A024U6X7.1 PF0170	46.40	0.00
TRINITY_Q22L23_TETTS/258-471	Q22L23.2 PF00667.18	46.40	0.00
TRINITY_I7LW24_TETTS/45-152	I7LW24.1 PF00970.22	46.40	0.00
TRINITY_A0A067NSE5_PLEOS/285-490	A0A067NSE5.1 PF0106	46.40	0.00
TRINITY_A0A061F304_THECC/9-89	A0A061F304.1 PF0870	46.40	0.00
TRINITY_D8UAK9_VOLCA/119-585	D8UAK9.1 PF14643.4;	46.40	0.00
TRINITY_A0A0D2X4U8_CAPO3/256-518	A0A0D2X4U8.1 PF0055	46.40	0.00
TRINITY_D5CQ56_SIDLE/33-306	D5CQ56.1 PF00664.21	46.40	0.00
TRINITY_G0QKB3_ICHMG/70-207	G0QKB3.1 PF05832.10	46.40	0.00
TRINITY_L8HMK7_ACACA/1726-1906	L8HMK7.1 PF00617.17	46.40	0.00
TRINITY_I7MLX1_TETTS/25-110	I7MLX1.1 PF09409.8;	46.40	0.00
TRINITY_A0CJY8_PARTE/436-698	A0CJY8.1 PF00069.23	46.40	0.00
TRINITY_A4VCQ0_TETTS/13-99	A4VCQ0.1 PF10217.7;	46.40	0.00
TRINITY_D8TRM3_VOLCA/14-214	D8TRM3.1 PF05753.12	46.40	0.00
TRINITY_A0A068SAP9_9FUNG/188-562	A0A068SAP9.1 PF0197	46.40	0.00
TRINITY_L8GWR3_ACACA/24-134	L8GWR3.1 PF02893.18	46.40	0.00
TRINITY_M2W0Y8_GALSU/92-216	M2W0Y8.1 PF09377.8;	46.40	0.00
TRINITY_A4VDY4_TETTS/184-313	A4VDY4.1 PF00107.24	46.40	0.00
TRINITY_A0A067MUR2_9HOMO/49-211	A0A067MUR2.1 PF0007	46.40	0.00
TRINITY_E1Z6X0_CHLVA/69-304	E1Z6X0.1 PF05368.11	46.40	0.00
TRINITY_A0A0D2U4E0_CAPO3/653-889	A0A0D2U4E0.1 PF0106	46.40	0.00
TRINITY_A0A0G4J2X3_PLABS/751-865	A0A0G4J2X3.1 PF0066	46.40	0.00
TRINITY_T0RT30_9STRA/311-475	T0RT30.1 PF00004.27	46.40	0.00
TRINITY_I0ZAW8_9CHLO/10-318	I0ZAW8.1 PF14647.4;	46.40	0.00
TRINITY_Q234Z9_TETTS/1982-2340	Q234Z9.2 PF04054.13	46.40	0.00
TRINITY_A9TZT0_PHYPA/35-465	A9TZT0.1 PF00860.18	46.40	0.00
TRINITY_A8J3I8_CHLRE/22-137	A8J3I8.1 PF07386.9;	46.40	0.00
TRINITY_L8GCE7_ACACA/550-749	L8GCE7.1 PF13087.4;	46.40	0.00
TRINITY_F4PDN3_BATDJ/6-85	F4PDN3.1 PF08571.8;	46.40	0.00
TRINITY_L8HLG8_ACACA/502-679	L8HLG8.1 PF00621.18	46.30	0.00
TRINITY_V4L7Y5_EUTSA/13-121	V4L7Y5.1 PF04081.11	46.30	0.00
TRINITY_I7LW70_TETTS/31-393	I7LW70.2 PF07690.14	46.30	0.00
TRINITY_K1QEN4_CRAGI/565-816	K1QEN4.1 PF00664.21	46.30	0.00
TRINITY_A0A0K8NYB5_9BURK/22-285	A0A0K8NYB5.1 PF1373	46.30	0.00
TRINITY_Q23TC9_TETTS/457-522	Q23TC9.2 PF01485.19	46.30	0.00
TRINITY_F8Q1Q8_SERL3/1049-1119	F8Q1Q8.1 PF14668.4;	46.30	0.00
TRINITY_F1A0E5_DICPU/83-317	F1A0E5.1 PF09749.7;	46.30	0.00
TRINITY_A0A0N4UXM7_ENTVE/77-172	A0A0N4UXM7.1 PF1279	46.30	0.00
TRINITY_D8SGK2_SELML/212-377	D8SGK2.1 PF01467.24	46.30	0.00
TRINITY_U9SP46_RHIID/7-70	U9SP46.1 PF00076.20	46.30	0.00
TRINITY_F4Q789_DICFS/9-129	F4Q789.1 PF00241.18	46.30	0.00
TRINITY_K9RX27_SYNP3/7-119	K9RX27.1 PF00583.23	46.30	0.00
TRINITY_D8LCC9_ECTSI/93-216	D8LCC9.1 PF04784.12	46.30	0.00
TRINITY_G0QV97_ICHMG/256-492	G0QV97.1 PF14309.4;	46.30	0.00
TRINITY_G0QTH6_ICHMG/49-264	G0QTH6.1 PF01556.16	46.30	0.00
TRINITY_J9J144_9SPIT/92-349	J9J144.1 PF00069.23	46.30	0.00
TRINITY_Q240Q4_TETTS/176-339	Q240Q4.3 PF16189.3;	46.30	0.00
TRINITY_A0DP15_PARTE/106-362	A0DP15.1 PF00069.23	46.30	0.00
TRINITY_L8H9P5_ACACA/1162-1679	L8H9P5.1 PF06920.11	46.30	0.00
TRINITY_B4J7Z6_DROGR/290-495	B4J7Z6.1 PF00106.23	46.30	0.00
TRINITY_A9RJ33_PHYPA/30-164	A9RJ33.1 PF01694.20	46.30	0.00
TRINITY_A0A087SAY9_AUXPR/242-338	A0A087SAY9.1 PF1049	46.30	0.00
TRINITY_A0A0A2W5J0_BEABA/658-966	A0A0A2W5J0.1 PF0111	46.30	0.00
TRINITY_U9UT29_RHIID/176-382	U9UT29.1 PF04389.15	46.30	0.00
TRINITY_D8M708_BLAHO/4-279	D8M708.1 PF14938.4;	46.30	0.00
TRINITY_D8TRM0_VOLCA/12-148	D8TRM0.1 PF04603.10	46.30	0.00
TRINITY_D8T2B5_SELML/134-422	D8T2B5.1 PF06814.11	46.30	0.00
TRINITY_H2YP11_CIOSA/230-438	H2YP11.1 PF00005.25	46.30	0.00
TRINITY_D3BVD2_POLPA/1706-1973	D3BVD2.1 PF07714.15	46.30	0.00
TRINITY_G0QXS8_ICHMG/51-117	G0QXS8.1 PF08583.8;	46.30	0.00
TRINITY_A7SNI1_NEMVE/16-69	A7SNI1.1 PF05676.11	46.30	0.00

TRINITY_A9RKZ5_PHYPA/61-400	A9RKZ5.1 PF04137.13	46.30	0.00
TRINITY_F4C2F9_SPHS2/61-294	F4C2F9.1 PF00487.22	46.30	0.00
TRINITY_D8UGM7_VOLCA/6-420	D8UGM7.1 PF03547.16	46.30	0.00
TRINITY_A0A061F080_THECC/36-252	A0A061F080.1 PF0113	46.30	0.00
TRINITY_D3AX40_POLPA/311-727	D3AX40.1 PF00083.22	46.30	0.00
TRINITY_A8IRL1_CHLRE/164-299	A8IRL1.1 PF00892.18	46.30	0.00
TRINITY_L8GYS6_ACACA/75-348	L8GYS6.1 PF08423.9;	46.30	0.00
TRINITY_A0A0B1P9Z4_UNCNE/3-126	A0A0B1P9Z4.1 PF1389	46.30	0.00
TRINITY_L8GGG7_ACACA/1114-1258	L8GGG7.1 PF02877.12	46.30	0.00
TRINITY_Q24FR8_TETTS/35-132	Q24FR8.2 PF12796.5;	46.30	0.00
TRINITY_F0ZVY0_DICPU/172-440	F0ZVY0.1 PF08491.8;	46.30	0.00
TRINITY_F0ZLC1_DICPU/453-547	F0ZLC1.1 PF12755.5;	46.30	0.00
TRINITY_E1ZJS1_CHLVA/190-329	E1ZJS1.1 PF04410.12	46.30	0.00
TRINITY_A0A078I2C4_BRANA/104-238	A0A078I2C4.1 PF1720	46.30	0.00
TRINITY_K9SZG2_9CYAN/110-382	K9SZG2.1 PF01636.21	46.30	0.00
TRINITY_D8TJH5_VOLCA/40-247	D8TJH5.1 PF01633.18	46.30	0.00
TRINITY_F4PWQ0_DICFS/222-536	F4PWQ0.1 PF02535.20	46.30	0.00
TRINITY_K1QLY7_CRAGI/16-147	K1QLY7.1 PF00782.18	46.30	0.00
TRINITY_E5WNT4_9BACI/8-148	E5WNT4.1 PF13302.5;	46.30	0.00
TRINITY_A8HQF5_CHLRE/280-399	A8HQF5.1 PF00069.23	46.30	0.00
TRINITY_I7MCL9_TETTS/190-488	I7MCL9.2 PF00648.19	46.30	0.00
TRINITY_A0A059LDS0_9CHLO/34-221	A0A059LDS0.1 PF0176	46.30	0.00
TRINITY_W7TKL6_9STRA/13-174	W7TKL6.1 PF00071.20	46.30	0.00
TRINITY_W7TKL6_9STRA/13-174	W7TKL6.1 PF00071.20	46.30	0.00

TRINITY_D8UBE1_VOLCA/26-435	D8UBE1.1 PF00232.16	46.30	0.00
TRINITY_A0A0L9SYQ2_9HYPO/89-158	A0A0L9SYQ2.1 PF0007	46.30	0.00
TRINITY_F8W2M8_DANRE/214-518	F8W2M8.1 PF00082.20	46.30	0.00
TRINITY_A0A078A3M9_STYLE/52-228	A0A078A3M9.1 PF0009	46.30	0.00
TRINITY_F4Q063_DICFS/153-478	F4Q063.1 PF00676.18	46.30	0.00
TRINITY_H2LIF3_ORYLA/1141-1234	H2LIF3.1 PF12755.5;	46.30	0.00
TRINITY_DGAT2_DICDI/36-330	Q54GC1.1 PF03982.11	46.30	0.00
TRINITY_A0A089IJD8_9BACL/185-308	A0A089IJD8.1 PF0010	46.30	0.00
TRINITY_C4R3S3_PICPG/5-181	C4R3S3.1 PF12999.5;	46.30	0.00
TRINITY_H8MQ77_CORCM/113-222	H8MQ77.1 PF07819.11	46.30	0.00
TRINITY_Q22YP9_TETTS/944-1208	Q22YP9.2 PF16212.3;	46.30	0.00
TRINITY_I7MIP6_TETTS/67-577	I7MIP6.2 PF04791.14	46.30	0.00
TRINITY_A0A0M9E9X1_9DELT/500-583	A0A0M9E9X1.1 PF0148	46.30	0.00
TRINITY_A0BWM8_PARTE/771-894	A0BWM8.1 PF00072.22	46.30	0.00
TRINITY_L8HIS6_ACACA/291-541	L8HIS6.1 PF16203.3;	46.30	0.00
TRINITY_A8HPD6_CHLRE/727-817	A8HPD6.1 PF12796.5;	46.30	0.00
TRINITY_F8F3W2_TRECH/572-683	F8F3W2.1 PF00072.22	46.30	0.00
TRINITY_G0R1G3_ICHMG/80-301	G0R1G3.1 PF00566.16	46.20	0.00
TRINITY_L8HDL6_ACACA/1-64	L8HDL6.1 PF06331.10	46.20	0.00
TRINITY_F7DU67_XENTR/2326-2572	F7DU67.1 PF00454.25	46.20	0.00
TRINITY_H2KSV6_CLOSI/110-1035	H2KSV6.1 PF09742.7;	46.20	0.00
TRINITY_A7AWS0_BABBO/3-252	A7AWS0.1 PF00370.19	46.20	0.00
TRINITY_L8GK68_ACACA/41-236	L8GK68.1 PF05721.11	46.20	0.00
TRINITY_A0E045_PARTE/204-339	A0E045.1 PF01529.18	46.20	0.00
TRINITY_M3XHV6_LATCH/47-162	M3XHV6.1 PF10639.7;	46.20	0.00
TRINITY_A0C4J2_PARTE/42-423	A0C4J2.1 PF00282.17	46.20	0.00
TRINITY_C4LTV2_ENTHI/1486-1746	C4LTV2.1 PF07714.15	46.20	0.00
TRINITY_A0A0D2VQ46_CAPO3/350-817	A0A0D2VQ46.1 PF0105	46.20	0.00
TRINITY_H2UB91_TAKRU/26-129	H2UB91.1 PF00168.28	46.20	0.00
TRINITY_I0YMM8_9CHLO/86-517	I0YMM8.1 PF03069.13	46.20	0.00
TRINITY_G0QNF4_ICHMG/82-412	G0QNF4.1 PF00150.16	46.20	0.00
TRINITY_A0A078A4I9_STYLE/1445-1496	A0A078A4I9.1 PF0041	46.20	0.00
TRINITY_U6PGJ8_HAECO/38-118	U6PGJ8.1 PF01883.17	46.20	0.00
TRINITY_Q23YJ0_TETTS/28-372	Q23YJ0.1 PF16499.3;	46.20	0.00
TRINITY_A0BT05_PARTE/750-873	A0BT05.1 PF00072.22	46.20	0.00
TRINITY_D8UF85_VOLCA/179-263	D8UF85.1 PF05699.12	46.20	0.00
TRINITY_I1JFU9_SOYBN/180-360	I1JFU9.1 PF00270.27	46.20	0.00
TRINITY_E1Z331_CHLVA/1024-1234	E1Z331.1 PF12348.6;	46.20	0.00
TRINITY_A0A078AMG9_STYLE/130-467	A0A078AMG9.1 PF0024	46.20	0.00
TRINITY_I0YYQ8_9CHLO/34-403	I0YYQ8.1 PF00224.19	46.20	0.00
TRINITY_D8UMJ8_VOLCA/352-506	D8UMJ8.1 PF00406.20	46.20	0.00
TRINITY_A0A0P7TRU5_9TELE/547-700	A0A0P7TRU5.1 PF0000	46.20	0.00
TRINITY_L8GYA6_ACACA/206-328	L8GYA6.1 PF10996.6;	46.20	0.00
TRINITY_F4QD49_DICFS/285-430	F4QD49.1 PF01694.20	46.20	0.00
TRINITY_Q23TB4_TETTS/477-736	Q23TB4.1 PF08324.9;	46.20	0.00
TRINITY_D7G144_ECTSI/662-819	D7G144.1 PF04087.12	46.20	0.00
TRINITY_A8JFL7_CHLRE/275-425	A8JFL7.1 PF00069.23	46.20	0.00
TRINITY_D8TXN8_VOLCA/36-217	D8TXN8.1 PF02390.15	46.20	0.00
TRINITY_I0YU58_9CHLO/86-254	I0YU58.1 PF00881.22	46.20	0.00
TRINITY_G0R4M5_ICHMG/51-307	G0R4M5.1 PF00481.19	46.20	0.00
TRINITY_W5HJX8_WHEAT/70-203	W5HJX8.1 PF12894.5;	46.20	0.00
TRINITY_A0A078AV94_STYLE/569-682	A0A078AV94.1 PF0251	46.20	0.00
TRINITY_D8U780_VOLCA/84-209	D8U780.1 PF00583.23	46.20	0.00
TRINITY_Q4G2C6_TRIVA/9-170	Q4G2C6.1 PF00071.20	46.20	0.00
TRINITY_M1V7G0_CYAME/130-294	M1V7G0.1 PF08613.9;	46.20	0.00
TRINITY_C3Y7M2_BRAFL/152-530	C3Y7M2.1 PF00443.27	46.20	0.00
TRINITY_A0A0A0KSA7_CUCSA/61-340	A0A0A0KSA7.1 PF0771	46.20	0.00
TRINITY_Q5W9T0_CHLRE/28-202	Q5W9T0.1 PF00504.19	46.20	0.00
TRINITY_A9SWK6_PHYPA/87-335	A9SWK6.1 PF01963.15	46.20	0.00
TRINITY_D8TST9_VOLCA/621-848	D8TST9.1 PF00211.18	46.20	0.00
TRINITY_C9RQA6_FIBSS/1383-1499	C9RQA6.1 PF00072.22	46.20	0.00

TRINITY_A7RRK8_NEMVE/238-552	A7RRK8.1 PF03372.21	46.20	0.00
TRINITY_A0A0B2UWJ7_TOXCA/194-421	A0A0B2UWJ7.1 PF0010	46.20	0.00
TRINITY_I1KL83_SOYBN/10-108	I1KL83.1 PF04749.15	46.10	0.00
TRINITY_H2MLP4_ORYLA/34-175	H2MLP4.1 PF09286.9;	46.10	0.00
TRINITY_U5QIM0_9CYAN/7-453	U5QIM0.1 PF05694.9;	46.10	0.00
TRINITY_K9UIC8_9CHRO/3-283	K9UIC8.1 PF01430.17	46.10	0.00
TRINITY_J9FPH7_9SPIT/28-192	J9FPH7.1 PF00071.20	46.10	0.00
TRINITY_L1JTR4_GUITH/243-344	L1JTR4.1 PF01248.24	46.10	0.00
TRINITY_Q22CF9_TETTS/26-290	Q22CF9.2 PF06098.9;	46.10	0.00
TRINITY_Q22LY6_TETTS/26-166	Q22LY6.2 PF06201.11	46.10	0.00
TRINITY_A0D0L1_PARTE/195-322	A0D0L1.1 PF00107.24	46.10	0.00
TRINITY_G0R4M8_ICHMG/258-481	G0R4M8.1 PF00454.25	46.10	0.00
TRINITY_B3RX98_TRIAD/8-175	B3RX98.1 PF03167.17	46.10	0.00
TRINITY_Q23D11_TETTS/154-431	Q23D11.1 PF00069.23	46.10	0.00
TRINITY_A0DJ79_PARTE/185-262	A0DJ79.1 PF03127.12	46.10	0.00
TRINITY_L8H5H0_ACACA/20-282	L8H5H0.1 PF00069.23	46.10	0.00
TRINITY_Q22RT0_TETTS/376-631	Q22RT0.2 PF10236.7;	46.10	0.00
TRINITY_A0A059D981_EUCGR/880-1125	A0A059D981.1 PF1621	46.10	0.00
TRINITY_D8TQZ8_VOLCA/95-161	D8TQZ8.1 PF00542.17	46.10	0.00
TRINITY_E1Z7I6_CHLVA/14-139	E1Z7I6.1 PF13673.5;	46.10	0.00
TRINITY_F0ZF52_DICPU/175-363	F0ZF52.1 PF13918.4;	46.10	0.00
TRINITY_A0A0N5A6S5_PARTI/5-166	A0A0N5A6S5.1 PF0007	46.10	0.00
TRINITY_D8U2B3_VOLCA/14-273	D8U2B3.1 PF07160.10	46.10	0.00
TRINITY_L8GR10_ACACA/254-510	L8GR10.1 PF12269.6;	46.10	0.00
TRINITY_K7IQ87_NASVI/181-299	K7IQ87.1 PF00665.24	46.10	0.00
TRINITY_W5JXP9_ASTMX/309-526	W5JXP9.1 PF07002.14	46.10	0.00
TRINITY_D8TW99_VOLCA/1136-1328	D8TW99.1 PF12295.6;	46.10	0.00
TRINITY_A0A0M2LPB6_9SPHN/49-175	A0A0M2LPB6.1 PF0018	46.10	0.00
TRINITY_D8TYC0_VOLCA/26-210	D8TYC0.1 PF01184.17	46.10	0.00
TRINITY_A9V7T3_MONBE/21-266	A9V7T3.1 PF09084.9;	46.10	0.00
TRINITY_A0DXE0_PARTE/145-304	A0DXE0.1 PF03031.16	46.10	0.00
TRINITY_D4ZL63_SHEVD/387-830	D4ZL63.1 PF00305.17	46.10	0.00
TRINITY_D6Y6S1_THEBD/346-498	D6Y6S1.1 PF01343.16	46.10	0.00
TRINITY_L8HIV2_ACACA/313-488	L8HIV2.1 PF00621.18	46.10	0.00
TRINITY_I1KQH1_SOYBN/78-445	I1KQH1.1 PF00009.25	46.10	0.00
TRINITY_F4PX48_DICFS/113-397	F4PX48.1 PF00664.21	46.10	0.00
TRINITY_F0ZT81_DICPU/1933-2183	F0ZT81.1 PF00454.25	46.10	0.00
TRINITY_L8H188_ACACA/100-395	L8H188.1 PF06472.13	46.10	0.00
TRINITY_A0C5M5_PARTE/424-685	A0C5M5.1 PF00069.23	46.10	0.00
TRINITY_E0U6F7_CYAP2/20-261	E0U6F7.1 PF00809.20	46.10	0.00
TRINITY_A0A087SQH9_AUXPR/230-400	A0A087SQH9.1 PF0505	46.10	0.00
TRINITY_A0BR56_PARTE/569-683	A0BR56.1 PF00387.17	46.10	0.00
TRINITY_H9GAZ3_ANOCA/42-445	H9GAZ3.1 PF02487.15	46.10	0.00
TRINITY_A0A077ZWU2_STYLE/13-143	A0A077ZWU2.1 PF0078	46.10	0.00
TRINITY_A9RDU7_PHYPA/693-887	A9RDU7.1 PF02816.16	46.10	0.00
TRINITY_I0YNU5_9CHLO/11-274	I0YNU5.1 PF00248.19	46.10	0.00
TRINITY_A0A0G4EVB2_9ALVE/139-300	A0A0G4EVB2.1 PF0476	46.10	0.00
TRINITY_D3BQ59_POLPA/1691-1830	D3BQ59.1 PF00637.18	46.10	0.00
TRINITY_A0A078ADH0_STYLE/236-312	A0A078ADH0.1 PF1342	46.10	0.00
TRINITY_I7M1V6_TETTS/24-129	I7M1V6.1 PF00168.28	46.10	0.00
TRINITY_ASPC1_HUMAN/386-463	Q9BZE9.1 PF00789.18	46.10	0.00
TRINITY_I0YWI4_9CHLO/461-920	I0YWI4.1 PF07748.11	46.10	0.00
TRINITY_D8UDF3_VOLCA/894-1029	D8UDF3.1 PF13472.4;	46.10	0.00
TRINITY_A8IQQ8_CHLRE/597-709	A8IQQ8.1 PF00989.23	46.10	0.00
TRINITY_A0BQA6_PARTE/560-677	A0BQA6.1 PF02518.24	46.00	0.00
TRINITY_I7MLW3_TETTS/3-82	I7MLW3.1 PF14892.4;	46.00	0.00
TRINITY_Q23TT4_TETTS/537-817	Q23TT4.1 PF03133.13	46.00	0.00
TRINITY_W2Q0J9_PHYPN/239-542	W2Q0J9.1 PF01055.24	46.00	0.00
TRINITY_Q4RUP7_TETNG/12-179	Q4RUP7.1 PF11032.6;	46.00	0.00
TRINITY_D7FVU4_ECTSI/132-361	D7FVU4.1 PF12146.6;	46.00	0.00
TRINITY_J9I3Q1_9SPIT/511-630	J9I3Q1.1 PF01529.18	46.00	0.00

TRINITY_L8GLJ9_ACACA/48-132	L8GLJ9.1 PF01777.16	46.00	0.00
TRINITY_B3S1P2_TRIAD/83-136	B3S1P2.1 PF13920.4;	46.00	0.00
TRINITY_F0ZYB1_DICPU/209-508	F0ZYB1.1 PF03062.17	46.00	0.00
TRINITY_A0A0G4GNN3_9ALVE/161-467	A0A0G4GNN3.1 PF1353	46.00	0.00
TRINITY_L8HH71_ACACA/197-295	L8HH71.1 PF02517.14	46.00	0.00
TRINITY_A0E2M3_PARTE/3-103	A0E2M3.1 PF00168.28	46.00	0.00
TRINITY_L8GCU9_ACACA/1075-1170	L8GCU9.1 PF00169.27	46.00	0.00
TRINITY_D8LW92_BLAHO/1-121	D8LW92.1 PF05477.9;	46.00	0.00
TRINITY_G0R698_ICHMG/1-203	G0R698.1 PF00069.23	46.00	0.00
TRINITY_A0A0J8CE73_BETVU/7-69	A0A0J8CE73.1 PF0028	46.00	0.00
TRINITY_G1KB57_ANOCA/52-264	G1KB57.2 PF01997.14	46.00	0.00
TRINITY_L8HDY5_ACACA/41-161	L8HDY5.1 PF00188.24	46.00	0.00
TRINITY_I0YRC5_9CHLO/366-465	I0YRC5.1 PF09759.7;	46.00	0.00
TRINITY_L8H3W5_ACACA/971-1093	L8H3W5.1 PF00271.29	46.00	0.00
TRINITY_S8E0B0_9LAMI/93-255	S8E0B0.1 PF04893.15	46.00	0.00
TRINITY_J9IRU0_9SPIT/190-239	J9IRU0.1 PF13415.4;	46.00	0.00
TRINITY_I7LWG9_TETTS/79-336	I7LWG9.1 PF00102.25	46.00	0.00
TRINITY_F0ZXX3_DICPU/8-246	F0ZXX3.1 PF01027.18	46.00	0.00
TRINITY_K9XXK3_STAC7/263-498	K9XXK3.1 PF00773.17	46.00	0.00
TRINITY_D8TRD6_VOLCA/204-322	D8TRD6.1 PF13911.4;	46.00	0.00
TRINITY_F4Q9D3_DICFS/167-398	F4Q9D3.1 PF13621.4;	46.00	0.00
TRINITY_L8H5H6_ACACA/18-345	L8H5H6.1 PF00503.18	46.00	0.00
TRINITY_D8THJ3_VOLCA/20-330	D8THJ3.1 PF03747.12	46.00	0.00
TRINITY_I7MGS4_TETTS/171-373	I7MGS4.2 PF00149.26	46.00	0.00
TRINITY_K1PAB4_CRAGI/1904-2205	K1PAB4.1 PF00291.23	46.00	0.00
TRINITY_A0A0F4RGI4_9RHOB/21-269	A0A0F4RGI4.1 PF0075	46.00	0.00
TRINITY_A0CS29_PARTE/2561-2834	A0CS29.1 PF00520.29	46.00	0.00
TRINITY_H3GAE8_PHYRM/7-180	H3GAE8.1 PF00071.20	46.00	0.00
TRINITY_J9G2X7_9SPIT/600-658	J9G2X7.1 PF00498.24	46.00	0.00
TRINITY_L8GXL8_ACACA/109-274	L8GXL8.1 PF00650.18	46.00	0.00
TRINITY_I7LTY4_TETTS/276-599	I7LTY4.2 PF04515.10	46.00	0.00
TRINITY_Q23AC3_TETTS/7-112	Q23AC3.2 PF00787.22	46.00	0.00
TRINITY_A0A078AKC9_STYLE/8-314	A0A078AKC9.1 PF0006	46.00	0.00
TRINITY_A0D8M0_PARTE/6-277	A0D8M0.1 PF05096.10	46.00	0.00
TRINITY_A0A078B3L0_STYLE/265-316	A0A078B3L0.1 PF1392	46.00	0.00
TRINITY_A0D9G0_PARTE/399-612	A0D9G0.1 PF07859.11	46.00	0.00
TRINITY_D8UHD7_VOLCA/269-321	D8UHD7.1 PF13920.4;	46.00	0.00
TRINITY_B8BSZ0_THAPS/4-370	B8BSZ0.1 PF07690.14	46.00	0.00
TRINITY_A0A087SJ81_AUXPR/175-429	A0A087SJ81.1 PF0333	46.00	0.00
TRINITY_D8UJ81_VOLCA/324-420	D8UJ81.1 PF01974.15	46.00	0.00
TRINITY_D8TR08_VOLCA/49-335	D8TR08.1 PF01546.26	46.00	0.00
TRINITY_I7MCL9_TETTS/190-488	I7MCL9.2 PF00648.19	46.00	0.00
TRINITY_I0Z746_9CHLO/49-270	I0Z746.1 PF07808.11	46.00	0.00
TRINITY_K9QM29_NOSS7/573-968	K9QM29.1 PF00476.18	46.00	0.00
TRINITY_D8TYR8_VOLCA/31-351	D8TYR8.1 PF00225.21	46.00	0.00
TRINITY_F2UN84_SALR5/57-134	F2UN84.1 PF03061.20	46.00	0.00
TRINITY_I0YLG8_9CHLO/31-304	I0YLG8.1 PF00069.23	46.00	0.00
TRINITY_A0A0C2MGC1_THEKT/4-177	A0A0C2MGC1.1 PF0002	46.00	0.00
TRINITY_W1NTA4_AMBTC/143-244	W1NTA4.1 PF00254.26	46.00	0.00
TRINITY_D8U7R8_VOLCA/1-335	D8U7R8.1 PF15239.4;	46.00	0.00
TRINITY_K4LIJ4_THEPS/4-116	K4LIJ4.1 PF00072.22	46.00	0.00
TRINITY_Q0AXB8_SYNWW/535-649	Q0AXB8.1 PF00072.22	46.00	0.00
TRINITY_I7M2G7_TETTS/73-181	I7M2G7.1 PF00787.22	45.90	0.00
TRINITY_G0QQY5_ICHMG/243-493	G0QQY5.1 PF13532.4;	45.90	0.00
TRINITY_I7MAC8_TETTS/3673-3968	I7MAC8.2 PF00632.23	45.90	0.00
TRINITY_A0BPY0_PARTE/414-535	A0BPY0.1 PF02518.24	45.90	0.00
TRINITY_Q23QF2_TETTS/248-571	Q23QF2.2 PF03133.13	45.90	0.00
TRINITY_Q39US8_GEOMG/342-457	Q39US8.1 PF00072.22	45.90	0.00
TRINITY_I7M898_TETTS/218-305	I7M898.2 PF00326.19	45.90	0.00
TRINITY_C1EG27_MICSR/13-344	C1EG27.1 PF07289.9;	45.90	0.00
TRINITY_W5I2C6_WHEAT/5-102	W5I2C6.1 PF04749.15	45.90	0.00

TRINITY_A0A0G3WBG6_9CLOT/9-282	A0A0G3WBG6.1 PF1433	45.90	0.00
TRINITY_J9G2B2_9SPIT/13-164	J9G2B2.1 PF01124.16	45.90	0.00
TRINITY_F0ZND0_DICPU/7-67	F0ZND0.1 PF04193.12	45.90	0.00
TRINITY_R5XLM3_9CLOT/612-730	R5XLM3.1 PF00753.25	45.90	0.00
TRINITY_A0A067QRE5_ZOONE/4-182	A0A067QRE5.1 PF0002	45.90	0.00
TRINITY_F9R8T7_VIBSN/691-807	F9R8T7.1 PF00072.22	45.90	0.00
TRINITY_D8TGZ7_VOLCA/449-690	D8TGZ7.1 PF00122.18	45.90	0.00
TRINITY_F0Y1S6_AURAN/100-357	F0Y1S6.1 PF02353.18	45.90	0.00
TRINITY_F4PUI7_DICFS/323-466	F4PUI7.1 PF00892.18	45.90	0.00
TRINITY_F4PYG0_DICFS/69-275	F4PYG0.1 PF09511.8;	45.90	0.00
TRINITY_L8GWW2_ACACA/318-616	L8GWW2.1 PF00069.23	45.90	0.00
TRINITY_A0A0N5CY83_THECL/4-139	A0A0N5CY83.1 PF1613	45.90	0.00
TRINITY_Q86B07_DICDI/215-531	Q86B07.1 PF00657.20	45.90	0.00
TRINITY_A0A077ZZM5_STYLE/548-782	A0A077ZZM5.1 PF0070	45.90	0.00
TRINITY_A0A060QEJ3_9PROT/186-308	A0A060QEJ3.1 PF0010	45.90	0.00
TRINITY_B8HDF0_ARTCA/239-376	B8HDF0.1 PF03641.12	45.90	0.00
TRINITY_L8HHR2_ACACA/700-893	L8HHR2.1 PF06017.11	45.90	0.00
TRINITY_A0A087STE3_AUXPR/40-137	A0A087STE3.1 PF0812	45.90	0.00
TRINITY_TRUA_COPPD/7-102	B5Y955.1 PF01416.18	45.90	0.00
TRINITY_L8GUK1_ACACA/538-796	L8GUK1.1 PF07714.15	45.90	0.00
TRINITY_F4PLH6_DICFS/600-782	F4PLH6.1 PF00617.17	45.90	0.00
TRINITY_Q22KK0_TETTS/166-347	Q22KK0.1 PF13870.4;	45.90	0.00
TRINITY_L8HD01_ACACA/632-698	L8HD01.1 PF00017.22	45.90	0.00
TRINITY_L8GUE5_ACACA/1-157	L8GUE5.1 PF04446.10	45.90	0.00
TRINITY_U3J628_ANAPL/331-776	U3J628.1 PF01055.24	45.90	0.00
TRINITY_A0C6X1_PARTE/171-244	A0C6X1.1 PF13848.4;	45.90	0.00
TRINITY_A0A078ART0_STYLE/361-506	A0A078ART0.1 PF0753	45.90	0.00
TRINITY_E1ZJ28_CHLVA/7-148	E1ZJ28.1 PF01025.17	45.90	0.00
TRINITY_J9FSK1_9SPIT/15-207	J9FSK1.1 PF14753.4;	45.90	0.00
TRINITY_L8GY76_ACACA/11-267	L8GY76.1 PF08217.9;	45.90	0.00
TRINITY_U5RSG0_9CLOT/6-255	U5RSG0.1 PF01073.17	45.90	0.00
TRINITY_A0A0G4IZS4_PLABS/45-153	A0A0G4IZS4.1 PF1613	45.90	0.00
TRINITY_A0A0E9N797_9ASCO/872-1054	A0A0E9N797.1 PF0061	45.90	0.00
TRINITY_D8UHM7_VOLCA/6-148	D8UHM7.1 PF00535.24	45.90	0.00
TRINITY_A8I4W2_CHLRE/1-181	A8I4W2.1 PF00211.18	45.90	0.00
TRINITY_A0A0B0NKV3_GOSAR/34-203	A0A0B0NKV3.1 PF1341	45.90	0.00
TRINITY_L8HH52_ACACA/396-599	L8HH52.1 PF00566.16	45.90	0.00
TRINITY_L8GPZ7_ACACA/15-171	L8GPZ7.1 PF13563.4;	45.90	0.00
TRINITY_W9WAK1_9EURO/306-565	W9WAK1.1 PF13360.4;	45.90	0.00
TRINITY_D8TXA5_VOLCA/213-429	D8TXA5.1 PF00112.21	45.90	0.00
TRINITY_A8HUC0_CHLRE/205-556	A8HUC0.1 PF02636.15	45.90	0.00
TRINITY_D8U408_VOLCA/148-305	D8U408.1 PF01694.20	45.90	0.00
TRINITY_A4HDM4_LEIBR/16-304	A4HDM4.1 PF07993.10	45.90	0.00
TRINITY_A8Q2F7_MALGO/39-328	A8Q2F7.1 PF00069.23	45.90	0.00
TRINITY_A6G2C0_9DELT/175-346	A6G2C0.1 PF12850.5;	45.90	0.00
TRINITY_F0NYY3_WEEVC/121-212	F0NYY3.1 PF00581.18	45.90	0.00
TRINITY_A9V270_MONBE/15-287	A9V270.1 PF01358.16	45.90	0.00
TRINITY_A0A0K9P6B5_ZOSMR/31-105	A0A0K9P6B5.1 PF0609	45.90	0.00
TRINITY_E1ZM81_CHLVA/1-228	E1ZM81.1 PF00089.24	45.90	0.00
TRINITY_G0QV43_ICHMG/249-568	G0QV43.1 PF04515.10	45.90	0.00
TRINITY_F1A0P0_DICPU/1-267	F1A0P0.1 PF03619.14	45.90	0.00
TRINITY_A8ITD7_CHLRE/3-78	A8ITD7.1 PF00240.21	45.90	0.00
TRINITY_A0A078D6D3_BRANA/113-500	A0A078D6D3.1 PF0583	45.90	0.00
TRINITY_A0A087SK32_AUXPR/29-511	A0A087SK32.1 PF0321	45.90	0.00
TRINITY_L1IE17_GUITH/205-315	L1IE17.1 PF00565.15	45.90	0.00
TRINITY_H2TVA2_TAKRU/382-663	H2TVA2.1 PF16187.3;	45.90	0.00
TRINITY_S8EB85_9LAMI/59-422	S8EB85.1 PF03600.14	45.90	0.00
TRINITY_A0A0E0NVU7_ORYRU/96-256	A0A0E0NVU7.1 PF0141	45.90	0.00
TRINITY_A8HT27_CHLRE/2550-3400	A8HT27.1 PF02364.13	45.90	0.00
TRINITY_I0YMZ8_9CHLO/74-403	I0YMZ8.1 PF00155.19	45.90	0.00
TRINITY_V3ZP91_LOTGI/73-422	V3ZP91.1 PF00266.17	45.90	0.00

TRINITY_A8HPX1_CHLRE/2514-2793	A8HPX1.1 PF12777.5;	45.90	0.00
TRINITY_C5Z583_SORBI/934-1179	C5Z583.1 PF07727.12	45.90	0.00
TRINITY_T1IQX5_STRMM/1067-1239	T1IQX5.1 PF08148.10	45.80	0.00
TRINITY_A0BIJ5_PARTE/438-487	A0BIJ5.1 PF13920.4;	45.80	0.00
TRINITY_L8HJG2_ACACA/55-171	L8HJG2.1 PF01661.19	45.80	0.00
TRINITY_Q1N2R9_9GAMM/772-890	Q1N2R9.1 PF00072.22	45.80	0.00
TRINITY_F0ZZ09_DICPU/129-413	F0ZZ09.1 PF01733.16	45.80	0.00
TRINITY_G3H850_CRIGR/668-769	G3H850.1 PF00005.25	45.80	0.00
TRINITY_L8GIS9_ACACA/27-240	L8GIS9.1 PF04055.19	45.80	0.00
TRINITY_C7Q3T8_CATAD/28-144	C7Q3T8.1 PF08240.10	45.80	0.00
TRINITY_B9RBZ2_RICCO/46-164	B9RBZ2.1 PF06487.10	45.80	0.00
TRINITY_I0Z9B3_9CHLO/185-274	I0Z9B3.1 PF01243.18	45.80	0.00
TRINITY_A9SUE0_PHYPA/168-284	A9SUE0.1 PF09745.7;	45.80	0.00
TRINITY_Q23QF2_TETTS/248-571	Q23QF2.2 PF03133.13	45.80	0.00
TRINITY_E1ZN66_CHLVA/1025-1170	E1ZN66.1 PF14566.4;	45.80	0.00
TRINITY_Q6ARD6_DESPS/790-902	Q6ARD6.1 PF00072.22	45.80	0.00
TRINITY_I7MDS7_TETTS/37-166	I7MDS7.2 PF12298.6;	45.80	0.00
TRINITY_D8U1M1_VOLCA/66-180	D8U1M1.1 PF11527.6;	45.80	0.00
TRINITY_A0A068XPV1_HYMMI/83-147	A0A068XPV1.1 PF1349	45.80	0.00
TRINITY_F4P9J4_BATDJ/272-410	F4P9J4.1 PF16418.3;	45.80	0.00
TRINITY_A7RMS4_NEMVE/12-59	A7RMS4.1 PF14604.4;	45.80	0.00
TRINITY_F6XDK0_MACMU/73-203	F6XDK0.1 PF00782.18	45.80	0.00
TRINITY_F4Q7F4_DICFS/111-168	F4Q7F4.1 PF07973.12	45.80	0.00
TRINITY_A4VEE1_TETTS/16-254	A4VEE1.1 PF01370.19	45.80	0.00
TRINITY_A0DVV8_PARTE/1927-2317	A0DVV8.1 PF12166.6;	45.80	0.00
TRINITY_D3BPS2_POLPA/1341-1518	D3BPS2.1 PF00617.17	45.80	0.00
TRINITY_S6AG68_PSERE/47-382	S6AG68.1 PF01244.19	45.80	0.00
TRINITY_B8HT46_CYAP4/58-393	B8HT46.1 PF01244.19	45.80	0.00
TRINITY_I7M4E9_TETTS/11-677	I7M4E9.2 PF00012.18	45.80	0.00
TRINITY_L8GST7_ACACA/99-608	L8GST7.1 PF01019.19	45.80	0.00
TRINITY_F0ZLC1_DICPU/453-547	F0ZLC1.1 PF12755.5;	45.80	0.00
TRINITY_I7LX71_TETTS/33-164	I7LX71.1 PF00782.18	45.80	0.00
TRINITY_D3BJZ6_POLPA/100-462	D3BJZ6.1 PF00155.19	45.80	0.00
TRINITY_W5ANU5_WHEAT/40-246	W5ANU5.1 PF03798.14	45.80	0.00
TRINITY_A7SNT2_NEMVE/8-128	A7SNT2.1 PF01082.18	45.80	0.00
TRINITY_D8U0K1_VOLCA/348-490	D8U0K1.1 PF08646.8;	45.80	0.00
TRINITY_L8GUR2_ACACA/284-351	L8GUR2.1 PF02207.18	45.80	0.00
TRINITY_C3Z7M9_BRAFL/186-281	C3Z7M9.1 PF12894.5;	45.80	0.00
TRINITY_M4B3P6_HYAAE/8-161	M4B3P6.1 PF04031.11	45.80	0.00
TRINITY_A0A087SCX2_AUXPR/9-229	A0A087SCX2.1 PF0149	45.80	0.00
TRINITY_D7FXL4_ECTSI/354-797	D7FXL4.1 PF01432.18	45.80	0.00
TRINITY_I1FBA4_AMPQE/207-465	I1FBA4.1 PF02263.17	45.80	0.00
TRINITY_L8GNJ5_ACACA/2442-2524	L8GNJ5.1 PF05406.13	45.80	0.00
TRINITY_L8HGG6_ACACA/132-402	L8HGG6.1 PF03372.21	45.80	0.00
TRINITY_A0A091D197_FUKDA/478-731	A0A091D197.1 PF0283	45.80	0.00
TRINITY_G3J709_CORMM/29-150	G3J709.1 PF00383.21	45.80	0.00
TRINITY_I0Z872_9CHLO/151-222	I0Z872.1 PF08234.10	45.80	0.00
TRINITY_D3B6Z4_POLPA/15-169	D3B6Z4.1 PF04707.12	45.80	0.00
TRINITY_U9TN42_RHIID/384-782	U9TN42.1 PF00176.21	45.80	0.00
TRINITY_L8HJR7_ACACA/270-398	L8HJR7.1 PF00781.22	45.80	0.00
TRINITY_D8UJ77_VOLCA/630-847	D8UJ77.1 PF00702.24	45.80	0.00
TRINITY_D8TL50_VOLCA/261-380	D8TL50.1 PF01909.21	45.80	0.00
TRINITY_D8UJL0_VOLCA/2-171	D8UJL0.1 PF03009.15	45.80	0.00
TRINITY_A9SQW4_PHYPA/111-371	A9SQW4.1 PF01702.16	45.80	0.00
TRINITY_G1KFK8_ANOCA/161-246	G1KFK8.1 PF12894.5;	45.80	0.00
TRINITY_I7M2F2_TETTS/121-405	I7M2F2.2 PF05291.9;	45.80	0.00
TRINITY_A8IYR0_CHLRE/101-281	A8IYR0.1 PF03770.14	45.80	0.00
TRINITY_W6FUH4_NODSP/15-183	W6FUH4.1 PF13385.4;	45.80	0.00
TRINITY_E1Z6P2_CHLVA/157-430	E1Z6P2.1 PF00557.22	45.80	0.00
TRINITY_D8SKW4_SELML/64-170	D8SKW4.1 PF00134.21	45.80	0.00
TRINITY_D8U5I9_VOLCA/8-186	D8U5I9.1 PF01007.18	45.80	0.00

TRINITY_D8U1I4_VOLCA/34-292	D8U1I4.1 PF00069.23	45.80	0.00
TRINITY_B3S7X0_TRIAD/229-278	B3S7X0.1 PF00415.16	45.80	0.00
TRINITY_D8UF85_VOLCA/179-263	D8UF85.1 PF05699.12	45.80	0.00
TRINITY_D8TJB9_VOLCA/410-574	D8TJB9.1 PF00005.25	45.80	0.00
TRINITY_V8P4L1_OPHHA/51-146	V8P4L1.1 PF02991.14	45.70	0.00
TRINITY_A0CA32_PARTE/9-233	A0CA32.1 PF02230.14	45.70	0.00
TRINITY_D8TQ62_VOLCA/2279-2520	D8TQ62.1 PF02259.21	45.70	0.00
TRINITY_W5MU48_LEPOC/107-374	W5MU48.1 PF07942.10	45.70	0.00
TRINITY_J9ITG1_9SPIT/22-292	J9ITG1.1 PF00248.19	45.70	0.00
TRINITY_TMEDA_DICDI/24-200	Q769F9.1 PF01105.22	45.70	0.00
TRINITY_A0A0J8E325_BETVU/28-210	A0A0J8E325.1 PF1341	45.70	0.00
TRINITY_B9HJ54_POPTR/12-109	B9HJ54.1 PF00153.25	45.70	0.00
TRINITY_H3CEP8_TETNG/9-186	H3CEP8.1 PF11032.6;	45.70	0.00
TRINITY_A0DUA5_PARTE/170-239	A0DUA5.1 PF14360.4;	45.70	0.00
TRINITY_A0BP90_PARTE/47-120	A0BP90.1 PF00076.20	45.70	0.00
TRINITY_E3NLH6_CAERE/106-152	E3NLH6.1 PF13639.4;	45.70	0.00
TRINITY_A8HQ22_CHLRE/163-586	A8HQ22.1 PF12739.5;	45.70	0.00
TRINITY_F1A1E3_DICPU/37-330	F1A1E3.1 PF03982.11	45.70	0.00
TRINITY_S8BLX5_DACHA/1-163	S8BLX5.1 PF00838.15	45.70	0.00
TRINITY_G9A044_TORDC/243-507	G9A044.1 PF00122.18	45.70	0.00
TRINITY_L8H1I6_ACACA/6-1219	L8H1I6.1 PF02463.17	45.70	0.00
TRINITY_D3BN50_POLPA/12-267	D3BN50.1 PF00069.23	45.70	0.00
TRINITY_L8GTS3_ACACA/110-367	L8GTS3.1 PF00082.20	45.70	0.00
TRINITY_A0DER1_PARTE/316-457	A0DER1.1 PF00782.18	45.70	0.00
TRINITY_J9INI5_9SPIT/488-743	J9INI5.1 PF00899.19	45.70	0.00
TRINITY_F0ZC69_DICPU/186-316	F0ZC69.1 PF00782.18	45.70	0.00
TRINITY_F4PM68_DICFS/19-198	F4PM68.1 PF16213.3;	45.70	0.00
TRINITY_D8LBC2_ECTSI/41-175	D8LBC2.1 PF12146.6;	45.70	0.00
TRINITY_A7BR79_9GAMM/15-298	A7BR79.1 PF03747.12	45.70	0.00
TRINITY_W4YIV3_STRPU/135-245	W4YIV3.1 PF03556.13	45.70	0.00
TRINITY_A0BPS9_PARTE/47-528	A0BPS9.1 PF00501.26	45.70	0.00
TRINITY_A0A095DHY2_CRYGA/162-428	A0A095DHY2.1 PF0118	45.70	0.00
TRINITY_F8KXV4_PARAV/23-504	F8KXV4.1 PF02446.15	45.70	0.00
TRINITY_I7M328_TETTS/23-132	I7M328.1 PF00787.22	45.70	0.00
TRINITY_F4PVI6_DICFS/286-442	F4PVI6.1 PF05179.12	45.70	0.00
TRINITY_F2IG28_FLUTR/9-147	F2IG28.1 PF13302.5;	45.70	0.00
TRINITY_G0QNX2_ICHMG/838-1082	G0QNX2.1 PF16212.3;	45.70	0.00
TRINITY_I1BVS2_RHIO9/188-290	I1BVS2.1 PF12894.5;	45.70	0.00
TRINITY_A4VD54_TETTS/76-357	A4VD54.1 PF00664.21	45.70	0.00
TRINITY_A0A0C2N390_THEKT/10-171	A0A0C2N390.1 PF0007	45.70	0.00
TRINITY_D8TMY6_VOLCA/279-490	D8TMY6.1 PF07714.15	45.70	0.00
TRINITY_A0A0G4H0Z9_9ALVE/24-182	A0A0G4H0Z9.1 PF0007	45.70	0.00
TRINITY_A0E0M5_PARTE/573-711	A0E0M5.1 PF00004.27	45.70	0.00
TRINITY_M7BBZ2_CHEMY/8-223	M7BBZ2.1 PF05891.10	45.70	0.00
TRINITY_A0A0D2WMW4_CAPO3/198-359	A0A0D2WMW4.1 PF0673	45.70	0.00
TRINITY_H2U0B8_TAKRU/576-712	H2U0B8.1 PF07534.14	45.70	0.00
TRINITY_D3AY38_POLPA/846-1105	D3AY38.1 PF07714.15	45.70	0.00
TRINITY_F4P893_BATDJ/24-194	F4P893.1 PF01871.15	45.70	0.00
TRINITY_K9VY24_9CYAN/43-487	K9VY24.1 PF03055.13	45.70	0.00
TRINITY_W4FSJ9_9STRA/1228-1335	W4FSJ9.1 PF00005.25	45.70	0.00
TRINITY_D3BL66_POLPA/302-470	D3BL66.1 PF04727.11	45.70	0.00
TRINITY_L8H0C4_ACACA/110-294	L8H0C4.1 PF09757.7;	45.70	0.00
TRINITY_L8HAY2_ACACA/1-176	L8HAY2.1 PF13462.4;	45.70	0.00
TRINITY_A8HPW8_CHLRE/149-319	A8HPW8.1 PF00270.27	45.70	0.00
TRINITY_E0W407_PEDHC/12-180	E0W407.1 PF14580.4;	45.70	0.00
TRINITY_G8TDU0_NIAKG/33-344	G8TDU0.1 PF00251.18	45.70	0.00
TRINITY_Q22UT0_TETTS/175-646	Q22UT0.2 PF00443.27	45.70	0.00
TRINITY_I7LTJ4_TETTS/16-121	I7LTJ4.1 PF01920.18	45.70	0.00
TRINITY_D8THR7_VOLCA/300-536	D8THR7.1 PF12697.5;	45.70	0.00
TRINITY_A0A0G4EWQ1_9ALVE/611-660	A0A0G4EWQ1.1 PF1341	45.70	0.00
TRINITY_F4QPE4_9CAUL/28-149	F4QPE4.1 PF00188.24	45.70	0.00

TRINITY_D8TZH6_VOLCA/22-283	D8TZH6.1 PF00233.17	45.70	0.00
TRINITY_F1LAC2_ASCSU/8-170	F1LAC2.1 PF00071.20	45.70	0.00
TRINITY_C0ZWC4_RHOE4/40-175	C0ZWC4.1 PF01565.21	45.70	0.00
TRINITY_Q6TGW9_DANRE/142-233	Q6TGW9.1 PF06831.12	45.70	0.00
TRINITY_G0QRB2_ICHMG/1-98	G0QRB2.1 PF00153.25	45.70	0.00
TRINITY_D8UJ14_VOLCA/185-336	D8UJ14.1 PF01569.19	45.70	0.00
TRINITY_M1WE29_CLAP2/186-274	M1WE29.1 PF12894.5;	45.70	0.00
TRINITY_D8TSG6_VOLCA/79-180	D8TSG6.1 PF00254.26	45.70	0.00
TRINITY_A0JQX4_ARTS2/5-151	A0JQX4.1 PF16242.3;	45.70	0.00
TRINITY_Q23QD2_TETTS/366-575	Q23QD2.2 PF00566.16	45.70	0.00
TRINITY_I7M6K2_TETTS/65-258	I7M6K2.1 PF01145.23	45.70	0.00
TRINITY_J9J3B4_9SPIT/124-338	J9J3B4.1 PF00112.21	45.70	0.00
TRINITY_D8U469_VOLCA/89-355	D8U469.1 PF00268.19	45.70	0.00
TRINITY_D8TZK2_VOLCA/16462-16640	D8TZK2.1 PF08659.8;	45.70	0.00
TRINITY_D8UDG5_VOLCA/23-294	D8UDG5.1 PF05770.9;	45.70	0.00
TRINITY_G0QNX2_ICHMG/73-307	G0QNX2.1 PF00122.18	45.70	0.00
TRINITY_A8JFG5_CHLRE/4-305	A8JFG5.1 PF00248.19	45.70	0.00
TRINITY_B7GBX5_PHATC/147-215	B7GBX5.1 PF02207.18	45.70	0.00
TRINITY_E9FYK7_DAPPU/332-447	E9FYK7.1 PF00271.29	45.70	0.00
TRINITY_E1ZFY9_CHLVA/64-196	E1ZFY9.1 PF00583.23	45.70	0.00
TRINITY_E4ZXV1_LEPMJ/601-1335	E4ZXV1.1 PF02460.16	45.70	0.00
TRINITY_H3HAJ2_PHYRM/359-478	H3HAJ2.1 PF00665.24	45.70	0.00
TRINITY_I1G2T0_AMPQE/174-530	I1G2T0.1 PF00657.20	45.70	0.00
TRINITY_A8HVG8_CHLRE/54-275	A8HVG8.1 PF00856.26	45.70	0.00
TRINITY_A0A060SIJ8_PYCCI/940-1185	A0A060SIJ8.1 PF0772	45.70	0.00
TRINITY_A0A0M3JRG0_ANISI/33-246	A0A0M3JRG0.1 PF0113	45.70	0.00
TRINITY_I1KBQ5_SOYBN/9-133	I1KBQ5.1 PF04628.11	45.70	0.00
TRINITY_G4ZVE5_PHYSP/23-127	G4ZVE5.1 PF05916.9;	45.60	0.00
TRINITY_G0VGN4_NAUCC/243-508	G0VGN4.1 PF00122.18	45.60	0.00
TRINITY_A0DNI0_PARTE/1-148	A0DNI0.1 PF03656.11	45.60	0.00
TRINITY_A7THQ5_VANPO/285-389	A7THQ5.1 PF00307.29	45.60	0.00
TRINITY_G0QV43_ICHMG/249-568	G0QV43.1 PF04515.10	45.60	0.00
TRINITY_C3KLU7_RHISN/61-162	C3KLU7.1 PF00011.19	45.60	0.00
TRINITY_Q16RK6_AEDAE/467-795	Q16RK6.1 PF07714.15	45.60	0.00
TRINITY_U3IGT3_ANAPL/358-427	U3IGT3.1 PF12894.5;	45.60	0.00
TRINITY_C3XZG9_BRAFL/1-285	C3XZG9.1 PF04189.11	45.60	0.00
TRINITY_A0CSC4_PARTE/899-1093	A0CSC4.1 PF14309.4;	45.60	0.00
TRINITY_B1WQY9_CYAA5/29-196	B1WQY9.1 PF06966.10	45.60	0.00
TRINITY_A0A0M3AW95_9SPHN/64-191	A0A0M3AW95.1 PF0018	45.60	0.00
TRINITY_I1G9N1_AMPQE/18-97	I1G9N1.1 PF09340.8;	45.60	0.00
TRINITY_A0DN99_PARTE/43-299	A0DN99.1 PF00069.23	45.60	0.00
TRINITY_A0CXP9_PARTE/92-386	A0CXP9.1 PF01457.14	45.60	0.00
TRINITY_T1JWJ8_TETUR/18-104	T1JWJ8.1 PF02970.14	45.60	0.00
TRINITY_C1E8K9_MICSR/182-435	C1E8K9.1 PF01189.15	45.60	0.00
TRINITY_K6UY88_9APIC/75-189	K6UY88.1 PF16531.3;	45.60	0.00
TRINITY_D8TXM1_VOLCA/1926-2033	D8TXM1.1 PF07714.15	45.60	0.00
TRINITY_I7LXL4_TETTS/25-124	I7LXL4.1 PF00153.25	45.60	0.00
TRINITY_Q22TN6_TETTS/693-784	Q22TN6.3 PF00027.27	45.60	0.00
TRINITY_D3BM84_POLPA/157-305	D3BM84.1 PF01529.18	45.60	0.00
TRINITY_L8GR65_ACACA/345-515	L8GR65.1 PF03770.14	45.60	0.00
TRINITY_L8GXS7_ACACA/913-1039	L8GXS7.1 PF00241.18	45.60	0.00
TRINITY_A8IHB6_CHLRE/1-172	A8IHB6.1 PF06041.9;	45.60	0.00
TRINITY_Q23CV4_TETTS/238-295	Q23CV4.1 PF13921.4;	45.60	0.00
TRINITY_Q23QV2_TETTS/229-494	Q23QV2.2 PF00122.18	45.60	0.00
TRINITY_A0A0N5AZR8_9BILA/73-158	A0A0N5AZR8.1 PF0073	45.60	0.00
TRINITY_J9IZH8_9SPIT/189-265	J9IZH8.1 PF14360.4;	45.60	0.00
TRINITY_D8U097_VOLCA/432-523	D8U097.1 PF12796.5;	45.60	0.00
TRINITY_A8IDU2_CHLRE/877-1024	A8IDU2.1 PF03330.16	45.60	0.00
TRINITY_W5KDC8_ASTMX/1554-1702	W5KDC8.1 PF05843.12	45.60	0.00
TRINITY_D8UCV0_VOLCA/107-209	D8UCV0.1 PF02536.12	45.60	0.00
TRINITY_Q22NW8_TETTS/199-479	Q22NW8.2 PF06098.9;	45.60	0.00

TRINITY_A0A067QSY9_ZOONE/1-120	A0A067QSY9.1 PF0075	45.60	0.00
TRINITY_D8U5V1_VOLCA/480-704	D8U5V1.1 PF07944.10	45.60	0.00
TRINITY_A0A0G4IX44_PLABS/1187-1362	A0A0G4IX44.1 PF0639	45.60	0.00
TRINITY_A0A087SHX8_AUXPR/61-348	A0A087SHX8.1 PF0029	45.60	0.00
TRINITY_M2W588_GALSU/27-222	M2W588.1 PF00106.23	45.60	0.00
TRINITY_C1MS08_MICPC/71-164	C1MS08.1 PF12796.5;	45.60	0.00
TRINITY_Q232H5_TETTS/29-208	Q232H5.1 PF04939.10	45.60	0.00
TRINITY_A8HNP6_CHLRE/11-260	A8HNP6.1 PF07727.12	45.60	0.00
TRINITY_L8GG55_ACACA/12-237	L8GG55.1 PF05724.9;	45.60	0.00
TRINITY_A0A075AXW7_9FUNG/248-775	A0A075AXW7.1 PF0044	45.60	0.00
TRINITY_S8B4G2_PENO1/609-970	S8B4G2.1 PF00702.24	45.60	0.00
TRINITY_A0A078BAX9_STYLE/384-451	A0A078BAX9.1 PF1349	45.60	0.00
TRINITY_A0A090K3E3_9GAMM/6-288	A0A090K3E3.1 PF0426	45.60	0.00
TRINITY_A0A024TS32_9STRA/50-121	A0A024TS32.1 PF0024	45.60	0.00
TRINITY_A0A0A0L4P3_CUCSA/173-229	A0A0A0L4P3.1 PF0824	45.60	0.00
TRINITY_D8TTV8_VOLCA/81-324	D8TTV8.1 PF00233.17	45.60	0.00
TRINITY_A8J3P1_CHLRE/954-1161	A8J3P1.1 PF00211.18	45.60	0.00
TRINITY_A0A0E9N0F0_9SPHI/271-327	A0A0E9N0F0.1 PF1341	45.60	0.00
TRINITY_Q22T22_TETTS/520-735	Q22T22.3 PF07859.11	45.50	0.00
TRINITY_A0A0D2ULF9_CAPO3/34-349	A0A0D2ULF9.1 PF0190	45.50	0.00
TRINITY_L8H3W1_ACACA/3787-4090	L8H3W1.1 PF00632.23	45.50	0.00
TRINITY_L8GXI9_ACACA/77-276	L8GXI9.1 PF09511.8;	45.50	0.00
TRINITY_A0A061E403_THECC/222-265	A0A061E403.1 PF1363	45.50	0.00
TRINITY_Q23QF2_TETTS/248-571	Q23QF2.2 PF03133.13	45.50	0.00
TRINITY_F0ZLB7_DICPU/2-271	F0ZLB7.1 PF06432.9;	45.50	0.00
TRINITY_I7LY30_TETTS/57-313	I7LY30.1 PF01409.18	45.50	0.00
TRINITY_A0A087XVW6_POEFO/22-145	A0A087XVW6.2 PF0299	45.50	0.00
TRINITY_F0Z7X3_DICPU/435-489	F0Z7X3.1 PF00412.20	45.50	0.00
TRINITY_A0A084GFG0_9PEZI/34-416	A0A084GFG0.1 PF0015	45.50	0.00
TRINITY_Q54XN8_DICDI/4-79	Q54XN8.1 PF12894.5;	45.50	0.00
TRINITY_Q22HI3_TETTS/105-331	Q22HI3.2 PF13347.4;	45.50	0.00
TRINITY_I0Z5F8_9CHLO/188-273	I0Z5F8.1 PF01342.19	45.50	0.00
TRINITY_G0QL84_ICHMG/1-101	G0QL84.1 PF01027.18	45.50	0.00
TRINITY_A0A0D2WPY5_CAPO3/13-453	A0A0D2WPY5.1 PF0307	45.50	0.00
TRINITY_C4R5A8_PICPG/533-633	C4R5A8.1 PF12763.5;	45.50	0.00
TRINITY_F0Z7M0_DICPU/15-209	F0Z7M0.1 PF01813.15	45.50	0.00
TRINITY_W0JY30_9EURY/94-226	W0JY30.1 PF13185.4;	45.50	0.00
TRINITY_L8H3R1_ACACA/85-377	L8H3R1.1 PF00083.22	45.50	0.00
TRINITY_L8GRC5_ACACA/88-246	L8GRC5.1 PF01652.16	45.50	0.00
TRINITY_C0QIE8_DESAH/8-255	C0QIE8.1 PF01226.15	45.50	0.00
TRINITY_Q9ABV9_CAUCR/23-148	Q9ABV9.1 PF04073.13	45.50	0.00
TRINITY_I0Z206_9CHLO/66-412	I0Z206.1 PF12697.5;	45.50	0.00
TRINITY_A0A0J7N2F4_LASNI/2-106	A0A0J7N2F4.1 PF0066	45.50	0.00
TRINITY_V8PI87_OPHHA/3-83	V8PI87.1 PF02798.18	45.50	0.00
TRINITY_A0A0E3S131_9EURY/14-150	A0A0E3S131.1 PF1367	45.50	0.00
TRINITY_D3AX40_POLPA/311-727	D3AX40.1 PF00083.22	45.50	0.00
TRINITY_A8IXV0_CHLRE/95-247	A8IXV0.1 PF01789.14	45.50	0.00
TRINITY_Q22Y90_TETTS/126-273	Q22Y90.2 PF01553.19	45.50	0.00
TRINITY_W4G407_9STRA/22-146	W4G407.1 PF10502.7;	45.50	0.00
TRINITY_K9ZQ45_ANACC/366-572	K9ZQ45.1 PF05729.10	45.50	0.00
TRINITY_F4PN58_DICFS/628-838	F4PN58.1 PF01823.17	45.50	0.00
TRINITY_I7MM74_TETTS/141-434	I7MM74.2 PF00454.25	45.50	0.00
TRINITY_A0A0P7XRF3_9TELE/158-268	A0A0P7XRF3.1 PF0355	45.50	0.00
TRINITY_L8HHX4_ACACA/703-1129	L8HHX4.1 PF00476.18	45.50	0.00
TRINITY_D8UDQ7_VOLCA/593-735	D8UDQ7.1 PF12499.6;	45.50	0.00
TRINITY_A8HTU8_CHLRE/13-251	A8HTU8.1 PF00856.26	45.50	0.00
TRINITY_Q22CW3_TETTS/227-323	Q22CW3.1 PF12796.5;	45.50	0.00
TRINITY_A8IDJ5_CHLRE/422-860	A8IDJ5.1 PF12832.5;	45.50	0.00
TRINITY_C3Z127_BRAFL/108-378	C3Z127.1 PF03629.16	45.50	0.00
TRINITY_I7MMY6_TETTS/639-738	I7MMY6.2 PF00989.23	45.50	0.00
TRINITY_V3ZCW0_LOTGI/106-315	V3ZCW0.1 PF00566.16	45.50	0.00

TRINITY_T1J449_STRMM/360-466	T1J449.1 PF13646.4;	45.50	0.00
TRINITY_D8UK76_VOLCA/37-268	D8UK76.1 PF01156.17	45.50	0.00
TRINITY_A0BIF4_PARTE/119-382	A0BIF4.1 PF00069.23	45.50	0.00
TRINITY_A0A0K0EAQ3_STRER/29-132	A0A0K0EAQ3.1 PF0008	45.50	0.00
TRINITY_A9SM27_PHYPA/231-414	A9SM27.1 PF00941.19	45.50	0.00
TRINITY_D5BYY4_NITHN/24-95	D5BYY4.1 PF11523.6;	45.50	0.00
TRINITY_E1Z9X6_CHLVA/862-1052	E1Z9X6.1 PF07714.15	45.50	0.00
TRINITY_A0A0J8BH52_BETVU/51-165	A0A0J8BH52.1 PF0121	45.50	0.00
TRINITY_A0A089PNS7_PLUGE/1-341	A0A089PNS7.1 PF0149	45.50	0.00
TRINITY_Q1DA84_MYXXD/113-198	Q1DA84.1 PF13640.4;	45.50	0.00
TRINITY_J9IWS4_9SPIT/678-793	J9IWS4.1 PF00168.28	45.50	0.00
TRINITY_I7LZV2_TETTS/151-413	I7LZV2.1 PF00069.23	45.50	0.00
TRINITY_K9U7U1_9CYAN/443-580	K9U7U1.1 PF02518.24	45.50	0.00
TRINITY_I4BB66_TURPD/8-314	I4BB66.1 PF01636.21	45.50	0.00
TRINITY_A8HPJ4_CHLRE/15-97	A8HPJ4.1 PF05542.9;	45.50	0.00
TRINITY_A8I550_CHLRE/6-465	A8I550.1 PF04712.10	45.50	0.00
TRINITY_I1GCR6_AMPQE/41-411	I1GCR6.1 PF04857.18	45.50	0.00
TRINITY_A8J9C1_CHLRE/48-416	A8J9C1.1 PF01237.16	45.50	0.00
TRINITY_A0CV01_PARTE/32-282	A0CV01.1 PF00768.18	45.50	0.00
TRINITY_A0A0J7K1H3_LASNI/67-147	A0A0J7K1H3.1 PF1335	45.50	0.00
TRINITY_A0A0D2VTR1_CAPO3/8-309	A0A0D2VTR1.1 PF0049	45.50	0.00
TRINITY_A9G978_SORC5/236-347	A9G978.1 PF00271.29	45.50	0.00
TRINITY_G0R413_ICHMG/765-1014	G0R413.1 PF16212.3;	45.50	0.00
TRINITY_J9IM35_9SPIT/72-192	J9IM35.1 PF14933.4;	45.50	0.00
TRINITY_A9RJZ5_PHYPA/276-381	A9RJZ5.1 PF12325.6;	45.50	0.00
TRINITY_K3YXY4_SETIT/198-563	K3YXY4.1 PF05970.12	45.50	0.00
TRINITY_R0FJH1_9BRAS/651-744	R0FJH1.1 PF02689.12	45.50	0.00
TRINITY_I7MAC8_TETTS/3673-3968	I7MAC8.2 PF00632.23	45.50	0.00
TRINITY_A8J4Y3_CHLRE/2-209	A8J4Y3.1 PF00069.23	45.50	0.00
TRINITY_D8U1T3_VOLCA/80-649	D8U1T3.1 PF03063.18	45.50	0.00
TRINITY_G0QXM4_ICHMG/12-216	G0QXM4.1 PF01088.19	45.50	0.00
TRINITY_A8JH38_CHLRE/355-450	A8JH38.1 PF08295.10	45.50	0.00
TRINITY_Q1AZH8_RUBXD/36-477	Q1AZH8.1 PF02133.13	45.40	0.00
TRINITY_Q22M73_TETTS/17-124	Q22M73.1 PF02991.14	45.40	0.00
TRINITY_B9TN91_RICCO/190-303	B9TN91.1 PF00072.22	45.40	0.00
TRINITY_J9HSH9_9SPIT/67-426	J9HSH9.1 PF07690.14	45.40	0.00
TRINITY_A0E2J1_PARTE/100-358	A0E2J1.1 PF00069.23	45.40	0.00
TRINITY_D3B9A6_POLPA/115-227	D3B9A6.1 PF00782.18	45.40	0.00
TRINITY_D8TND6_VOLCA/38-192	D8TND6.1 PF02542.14	45.40	0.00
TRINITY_D3BL52_POLPA/124-232	D3BL52.1 PF08240.10	45.40	0.00
TRINITY_G0R0C9_ICHMG/17-291	G0R0C9.1 PF00378.18	45.40	0.00
TRINITY_L8H2P9_ACACA/62-245	L8H2P9.1 PF14308.4;	45.40	0.00
TRINITY_D2A4K7_TRICA/36-208	D2A4K7.1 PF03637.15	45.40	0.00
TRINITY_I7M023_TETTS/534-690	I7M023.1 PF04727.11	45.40	0.00
TRINITY_Q9TVK6_DICDI/465-1321	Q9TVK6.1 PF02460.16	45.40	0.00
TRINITY_D8LU94_ECTSI/82-277	D8LU94.1 PF11715.6;	45.40	0.00
TRINITY_H2KTH9_CLOSI/179-359	H2KTH9.1 PF00270.27	45.40	0.00
TRINITY_A0A015N2M8_9GLOM/217-520	A0A015N2M8.1 PF0597	45.40	0.00
TRINITY_D8TN00_VOLCA/1-276	D8TN00.1 PF01433.18	45.40	0.00
TRINITY_L8H533_ACACA/10-116	L8H533.1 PF00307.29	45.40	0.00
TRINITY_Q22BJ2_TETTS/163-335	Q22BJ2.3 PF07162.9;	45.40	0.00
TRINITY_L8GV43_ACACA/128-238	L8GV43.1 PF00373.16	45.40	0.00
TRINITY_D8UEG8_VOLCA/121-251	D8UEG8.1 PF03364.18	45.40	0.00
TRINITY_W5K4E3_ASTMX/157-414	W5K4E3.1 PF11708.6;	45.40	0.00
TRINITY_A2U192_9FLAO/445-555	A2U192.3 PF00072.22	45.40	0.00
TRINITY_H3GSE5_PHYRM/18-263	H3GSE5.1 PF04305.12	45.40	0.00
TRINITY_D3BL06_POLPA/649-893	D3BL06.1 PF00481.19	45.40	0.00
TRINITY_D8UFJ2_VOLCA/156-276	D8UFJ2.1 PF03407.14	45.40	0.00
TRINITY_J9FBN2_9SPIT/564-662	J9FBN2.1 PF08647.9;	45.40	0.00
TRINITY_A0A087SE41_AUXPR/106-364	A0A087SE41.1 PF0066	45.40	0.00
TRINITY_J9F2X1_9SPIT/418-624	J9F2X1.1 PF04811.13	45.40	0.00

TRINITY_K9X2I1_9NOST/29-219	K9X2I1.1 PF13532.4;	45.40	0.00
TRINITY_W2QGB8_PHYPN/92-396	W2QGB8.1 PF01301.17	45.30	0.00
TRINITY_C8NB28_9GAMM/75-163	C8NB28.1 PF12796.5;	45.30	0.00
TRINITY_I1C5L9_RHIO9/18-625	I1C5L9.1 PF04130.11	45.30	0.00
TRINITY_A0C1H5_PARTE/172-233	A0C1H5.1 PF04117.10	45.30	0.00
TRINITY_A0D0H6_PARTE/190-319	A0D0H6.1 PF00782.18	45.30	0.00
TRINITY_G0QIZ2_ICHMG/16-177	G0QIZ2.1 PF00071.20	45.30	0.00
TRINITY_R1E4R6_EMIHU/5-166	R1E4R6.1 PF00071.20	45.30	0.00
TRINITY_G4ZAG1_PHYSP/270-682	G4ZAG1.1 PF06602.12	45.30	0.00
TRINITY_F2UEK7_SALR5/32-127	F2UEK7.1 PF13879.4;	45.30	0.00
TRINITY_L8GKR3_ACACA/5-118	L8GKR3.1 PF06320.11	45.30	0.00
TRINITY_D2VRY6_NAEGR/12-173	D2VRY6.1 PF00071.20	45.30	0.00
TRINITY_B9SDF9_RICCO/2-119	B9SDF9.1 PF12457.6;	45.30	0.00
TRINITY_A0A0A1U2T7_ENTIV/669-929	A0A0A1U2T7.1 PF0771	45.30	0.00
TRINITY_V4B732_LOTGI/186-369	V4B732.1 PF07714.15	45.30	0.00
TRINITY_J9HIW9_9SPIT/9-83	J9HIW9.1 PF04969.14	45.30	0.00
TRINITY_A7S9M2_NEMVE/16-112	A7S9M2.1 PF03645.11	45.30	0.00
TRINITY_I7LXB1_TETTS/1059-1123	I7LXB1.2 PF13499.4;	45.30	0.00
TRINITY_A0A074W9H8_9PEZI/159-727	A0A074W9H8.1 PF0518	45.30	0.00
TRINITY_D3BAA5_POLPA/36-100	D3BAA5.1 PF13499.4;	45.30	0.00
TRINITY_A0DXK9_PARTE/23-179	A0DXK9.1 PF05477.9;	45.30	0.00
TRINITY_C9JUS5_HUMAN/5-152	C9JUS5.2 PF00780.20	45.30	0.00
TRINITY_E1TJX2_BURSG/177-429	E1TJX2.1 PF03781.14	45.30	0.00
TRINITY_A0DAV4_PARTE/5-111	A0DAV4.1 PF14469.4;	45.30	0.00
TRINITY_G9MNE4_HYPVG/42-440	G9MNE4.1 PF00999.19	45.30	0.00
TRINITY_S0GJQ0_9PORP/664-780	S0GJQ0.1 PF00072.22	45.30	0.00
TRINITY_Q55AW0_DICDI/30-119	Q55AW0.2 PF08066.10	45.30	0.00
TRINITY_A0A078AFI0_STYLE/404-615	A0A078AFI0.1 PF0106	45.30	0.00
TRINITY_D8SAB9_SELML/1092-1203	D8SAB9.1 PF00271.29	45.30	0.00
TRINITY_E1Z6A9_CHLVA/799-867	E1Z6A9.1 PF15613.4;	45.30	0.00
TRINITY_I0YWA1_9CHLO/70-388	I0YWA1.1 PF12698.5;	45.30	0.00
TRINITY_A0DJE9_PARTE/430-486	A0DJE9.1 PF12678.5;	45.30	0.00
TRINITY_D3BAB9_POLPA/27-383	D3BAB9.1 PF16499.3;	45.30	0.00
TRINITY_I7LTE1_TETTS/844-1165	I7LTE1.1 PF03178.13	45.30	0.00
TRINITY_Q23C23_TETTS/1-172	Q23C23.1 PF00025.19	45.30	0.00
TRINITY_A8IC32_CHLRE/122-225	A8IC32.1 PF00085.18	45.30	0.00
TRINITY_B9N5K1_POPTR/44-129	B9N5K1.1 PF01388.19	45.30	0.00
TRINITY_L8H2D7_ACACA/248-333	L8H2D7.1 PF02761.12	45.30	0.00
TRINITY_M2X0R2_GALSU/5-81	M2X0R2.1 PF02798.18	45.30	0.00
TRINITY_A0A0D3A1U9_BRAOL/157-350	A0A0D3A1U9.1 PF1338	45.30	0.00
TRINITY_E1ZM61_CHLVA/236-361	E1ZM61.1 PF06888.10	45.30	0.00
TRINITY_G0QQW5_ICHMG/161-227	G0QQW5.1 PF01302.23	45.30	0.00
TRINITY_D8TJS5_VOLCA/5-98	D8TJS5.1 PF09177.9;	45.30	0.00
TRINITY_W7X474_TETTS/3-234	W7X474.1 PF01876.14	45.30	0.00
TRINITY_D8TJR4_VOLCA/308-429	D8TJR4.1 PF00892.18	45.30	0.00
TRINITY_A0A0G4ILT0_PLABS/5-204	A0A0G4ILT0.1 PF0395	45.30	0.00
TRINITY_I7MJI0_TETTS/1-168	I7MJI0.1 PF14580.4;	45.30	0.00
TRINITY_A0A074RY28_9HOMO/35-123	A0A074RY28.1 PF1289	45.30	0.00
TRINITY_E1Z9P4_CHLVA/171-710	E1Z9P4.1 PF04130.11	45.30	0.00
TRINITY_A0A078A8Z2_STYLE/34-167	A0A078A8Z2.1 PF1021	45.30	0.00
TRINITY_E1ZNR9_CHLVA/1865-2215	E1ZNR9.1 PF02889.14	45.30	0.00
TRINITY_D8U8C2_VOLCA/29-358	D8U8C2.1 PF07992.12	45.30	0.00
TRINITY_A0A0N4UGN0_DRAME/194-461	A0A0N4UGN0.1 PF0055	45.30	0.00
TRINITY_H2XVQ2_CIOIN/227-325	H2XVQ2.1 PF12763.5;	45.30	0.00
TRINITY_Q23QD2_TETTS/366-575	Q23QD2.2 PF00566.16	45.30	0.00
TRINITY_C5X9N6_SORBI/14-139	C5X9N6.1 PF13671.4;	45.30	0.00
TRINITY_G0R0V5_ICHMG/25-299	G0R0V5.1 PF00069.23	45.30	0.00
TRINITY_H2Z7Y9_CIOSA/125-464	H2Z7Y9.1 PF06602.12	45.30	0.00
TRINITY_G0QKW0_ICHMG/98-205	G0QKW0.1 PF00787.22	45.30	0.00
TRINITY_A7BR79_9GAMM/15-298	A7BR79.1 PF03747.12	45.30	0.00
TRINITY_A0A0G2ZDT3_9DELT/10-158	A0A0G2ZDT3.1 PF1624	45.30	0.00

TRINITY_W1NNU8_AMBTC/171-302	W1NNU8.1 PF04696.11	45.30	0.00
TRINITY_A0A066WL70_9BASI/93-431	A0A066WL70.1 PF1277	45.30	0.00
TRINITY_I0YWI5_9CHLO/9-73	I0YWI5.1 PF00226.29	45.30	0.00
TRINITY_A0CIM5_PARTE/204-331	A0CIM5.1 PF01926.21	45.30	0.00
TRINITY_D8TJA9_VOLCA/41-757	D8TJA9.1 PF05879.10	45.30	0.00
TRINITY_T1K5B5_TETUR/776-1081	T1K5B5.1 PF00632.23	45.30	0.00
TRINITY_E1Z8P6_CHLVA/15-119	E1Z8P6.1 PF00581.18	45.30	0.00
TRINITY_S3CQ68_OPHP1/12-322	S3CQ68.1 PF00069.23	45.30	0.00
TRINITY_H2UVR9_TAKRU/12-283	H2UVR9.1 PF01073.17	45.30	0.00
TRINITY_A0A015K817_9GLOM/26-201	A0A015K817.1 PF0114	45.30	0.00
TRINITY_A0A087SE95_AUXPR/53-291	A0A087SE95.1 PF0014	45.30	0.00
TRINITY_S8C461_9LAMI/1422-1487	S8C461.1 PF00385.22	45.30	0.00
TRINITY_G4ZJG1_PHYSP/290-379	G4ZJG1.1 PF05699.12	45.30	0.00
TRINITY_A8IWZ4_CHLRE/275-446	A8IWZ4.1 PF14288.4;	45.30	0.00
TRINITY_I7MHP0_TETTS/475-658	I7MHP0.1 PF01756.17	45.30	0.00
TRINITY_Q3M0Y3_PARTE/32-235	Q3M0Y3.1 PF00804.23	45.20	0.00
TRINITY_D8LF24_ECTSI/946-1351	D8LF24.1 PF08393.11	45.20	0.00
TRINITY_W5MDB7_LEPOC/196-464	W5MDB7.1 PF00664.21	45.20	0.00
TRINITY_A7SRL3_NEMVE/17-270	A7SRL3.1 PF00069.23	45.20	0.00
TRINITY_E4X4L5_OIKDI/27-636	E4X4L5.1 PF00012.18	45.20	0.00
TRINITY_V2WY49_MONRO/2-173	V2WY49.1 PF14437.4;	45.20	0.00
TRINITY_A4VDZ5_TETTS/5-135	A4VDZ5.1 PF05914.10	45.20	0.00
TRINITY_D3BSQ8_POLPA/10-337	D3BSQ8.1 PF05631.12	45.20	0.00
TRINITY_D8S0L6_SELML/388-492	D8S0L6.1 PF03450.15	45.20	0.00
TRINITY_W6KPB0_9TRYP/165-337	W6KPB0.1 PF02475.14	45.20	0.00
TRINITY_J9I759_9SPIT/142-428	J9I759.1 PF00122.18	45.20	0.00
TRINITY_L8GMG2_ACACA/38-448	L8GMG2.1 PF00450.20	45.20	0.00
TRINITY_Q22S98_TETTS/953-1159	Q22S98.1 PF01852.17	45.20	0.00
TRINITY_A0DVX9_PARTE/401-444	A0DVX9.1 PF13639.4;	45.20	0.00
TRINITY_G0R0N7_ICHMG/32-549	G0R0N7.1 PF01602.18	45.20	0.00
TRINITY_A9T2W4_PHYPA/14-175	A9T2W4.1 PF00071.20	45.20	0.00
TRINITY_A4VDX0_TETTS/737-849	A4VDX0.1 PF14663.4;	45.20	0.00
TRINITY_A0A059C1L3_EUCGR/507-588	A0A059C1L3.1 PF0078	45.20	0.00
TRINITY_I1C8R6_RHIO9/64-229	I1C8R6.1 PF10354.7;	45.20	0.00
TRINITY_B9IE05_POPTR/26-171	B9IE05.2 PF09794.7;	45.20	0.00
TRINITY_G0R4V0_ICHMG/469-626	G0R4V0.1 PF00637.18	45.20	0.00
TRINITY_L8GLK0_ACACA/347-813	L8GLK0.1 PF06862.10	45.20	0.00
TRINITY_I7MJI0_TETTS/1-168	I7MJI0.1 PF14580.4;	45.20	0.00
TRINITY_C3ZBN6_BRAFL/12-319	C3ZBN6.1 PF00225.21	45.20	0.00
TRINITY_A8HQK6_CHLRE/313-437	A8HQK6.1 PF07732.13	45.20	0.00
TRINITY_D8UHT4_VOLCA/1070-1142	D8UHT4.1 PF02941.13	45.20	0.00
TRINITY_D8U5V1_VOLCA/480-704	D8U5V1.1 PF07944.10	45.20	0.00
TRINITY_R4X9F3_TAPDE/1782-2011	R4X9F3.1 PF12134.6;	45.20	0.00
TRINITY_Q22RT8_TETTS/1160-1264	Q22RT8.2 PF02204.16	45.20	0.00
TRINITY_D8UCH7_VOLCA/2-93	D8UCH7.1 PF14995.4;	45.20	0.00
TRINITY_RHBG_DANRE/21-432	Q7T070.1 PF00909.19	45.20	0.00
TRINITY_L8HFY8_ACACA/67-479	L8HFY8.1 PF01490.16	45.20	0.00
TRINITY_D5GIM7_TUBMM/269-381	D5GIM7.1 PF12014.6;	45.20	0.00
TRINITY_L8H3W5_ACACA/971-1093	L8H3W5.1 PF00271.29	45.20	0.00
TRINITY_F2TXW1_SALR5/158-287	F2TXW1.1 PF00781.22	45.20	0.00
TRINITY_F4QPE4_9CAUL/28-149	F4QPE4.1 PF00188.24	45.20	0.00
TRINITY_L8H6W3_ACACA/15-134	L8H6W3.1 PF02136.18	45.20	0.00
TRINITY_A0DKS0_PARTE/20-339	A0DKS0.1 PF00328.20	45.20	0.00
TRINITY_F4PH07_DICFS/583-780	F4PH07.1 PF00617.17	45.20	0.00
TRINITY_D8THD3_VOLCA/212-384	D8THD3.1 PF00685.25	45.20	0.00
TRINITY_W5MLC4_LEPOC/33-284	W5MLC4.1 PF00069.23	45.20	0.00
TRINITY_A0A0M0J3K1_9EUKA/23-87	A0A0M0J3K1.1 PF1349	45.20	0.00
TRINITY_G0R2L7_ICHMG/18-285	G0R2L7.1 PF00069.23	45.20	0.00
TRINITY_L8GUY8_ACACA/436-561	L8GUY8.1 PF10162.7;	45.20	0.00
TRINITY_Q23QF2_TETTS/248-571	Q23QF2.2 PF03133.13	45.20	0.00
TRINITY_W2PSX8_PHYPN/461-539	W2PSX8.1 PF04433.15	45.20	0.00

TRINITY_A0A0F5YGZ4_9CYAN/12-376	A0A0F5YGZ4.1 PF0373	45.20	0.00
TRINITY_A8IXG0_CHLRE/21-204	A8IXG0.1 PF00069.23	45.20	0.00
TRINITY_L8H3W5_ACACA/971-1093	L8H3W5.1 PF00271.29	45.20	0.00
TRINITY_A0A015JNE6_9GLOM/190-476	A0A015JNE6.1 PF0154	45.20	0.00
TRINITY_X6NKU2_RETFI/36-170	X6NKU2.1 PF00782.18	45.20	0.00
TRINITY_D0MZ56_PHYIT/6-67	D0MZ56.1 PF00403.24	45.20	0.00
TRINITY_I7MMD4_TETTS/81-328	I7MMD4.2 PF09423.8;	45.20	0.00
TRINITY_A8J2X9_CHLRE/125-257	A8J2X9.1 PF00504.19	45.20	0.00
TRINITY_A0A0M2PJR3_9BACI/381-456	A0A0M2PJR3.1 PF0824	45.20	0.00
TRINITY_A0A0P7Z3L4_9TELE/166-268	A0A0P7Z3L4.1 PF1364	45.20	0.00
TRINITY_AT2B3_RAT/876-1058	Q64568.2 PF00689.19	45.20	0.00
TRINITY_G7KQK6_MEDTR/246-556	G7KQK6.2 PF00201.16	45.20	0.00
TRINITY_M2QL90_CERS8/55-263	M2QL90.1 PF00350.21	45.20	0.00
TRINITY_A0BG56_PARTE/51-241	A0BG56.1 PF00106.23	45.20	0.00
TRINITY_Q23FF9_TETTS/217-398	Q23FF9.3 PF00022.17	45.20	0.00
TRINITY_D8TN37_VOLCA/1910-2160	D8TN37.1 PF00520.29	45.20	0.00
TRINITY_H3C1Y9_TETNG/109-200	H3C1Y9.1 PF12796.5;	45.20	0.00
TRINITY_C3Z6N3_BRAFL/365-470	C3Z6N3.1 PF00085.18	45.10	0.00
TRINITY_A0A0G4ITJ6_PLABS/3944-4038	A0A0G4ITJ6.1 PF0933	45.10	0.00
TRINITY_G0QY02_ICHMG/755-917	G0QY02.1 PF00326.19	45.10	0.00
TRINITY_L8HBE1_ACACA/154-348	L8HBE1.1 PF13472.4;	45.10	0.00
TRINITY_A8JHQ7_CHLRE/10-171	A8JHQ7.1 PF00071.20	45.10	0.00
TRINITY_A0BHW3_PARTE/35-124	A0BHW3.1 PF13011.4;	45.10	0.00
TRINITY_G0QK29_ICHMG/70-205	G0QK29.1 PF02100.15	45.10	0.00
TRINITY_A0EDD8_PARTE/1167-1337	A0EDD8.1 PF01504.16	45.10	0.00
TRINITY_Q23AY4_TETTS/31-436	Q23AY4.2 PF05577.10	45.10	0.00
TRINITY_I0YWR8_9CHLO/31-183	I0YWR8.1 PF03024.12	45.10	0.00
TRINITY_A7RRM0_NEMVE/31-173	A7RRM0.1 PF00102.25	45.10	0.00
TRINITY_D5EJC3_CORAD/442-515	D5EJC3.1 PF00575.21	45.10	0.00
TRINITY_A0A0G0LP85_9BACT/9-100	A0A0G0LP85.1 PF0096	45.10	0.00
TRINITY_B3RU29_TRIAD/10-171	B3RU29.1 PF00071.20	45.10	0.00
TRINITY_I0YWJ1_9CHLO/17-152	I0YWJ1.1 PF04934.12	45.10	0.00
TRINITY_L8GS37_ACACA/378-494	L8GS37.1 PF02518.24	45.10	0.00
TRINITY_G0QJG8_ICHMG/371-552	G0QJG8.1 PF01756.17	45.10	0.00
TRINITY_A0CNU6_PARTE/9-126	A0CNU6.1 PF00855.15	45.10	0.00
TRINITY_A0A067BZ43_SAPPC/32-320	A0A067BZ43.1 PF0022	45.10	0.00
TRINITY_R1EJ93_EMIHU/552-944	R1EJ93.1 PF07394.10	45.10	0.00
TRINITY_A0A0A1PED8_9FUNG/120-190	A0A0A1PED8.1 PF0007	45.10	0.00
TRINITY_Q218F5_RHOPB/27-449	Q218F5.1 PF01425.19	45.10	0.00
TRINITY_A9TR80_PHYPA/1-272	A9TR80.1 PF06454.9;	45.10	0.00
TRINITY_K8GCU9_9CYAN/941-1063	K8GCU9.1 PF02518.24	45.10	0.00
TRINITY_E1Z5J8_CHLVA/185-287	E1Z5J8.1 PF00085.18	45.10	0.00
TRINITY_A8J9C9_CHLRE/114-217	A8J9C9.1 PF01841.17	45.10	0.00
TRINITY_A0A0L0CBW2_LUCCU/28-129	A0A0L0CBW2.1 PF1349	45.10	0.00
TRINITY_L8GE50_ACACA/461-783	L8GE50.1 PF01593.22	45.10	0.00
TRINITY_E4TKK0_MARTH/14-163	E4TKK0.1 PF00849.20	45.10	0.00
TRINITY_MYOJ_DICDI/83-809	P54697.2 PF00063.19	45.10	0.00
TRINITY_A0A078AE25_STYLE/157-303	A0A078AE25.1 PF0753	45.10	0.00
TRINITY_A8I9T9_CHLRE/72-562	A8I9T9.1 PF03055.13	45.10	0.00
TRINITY_R1DRS4_EMIHU/374-577	R1DRS4.1 PF13087.4;	45.10	0.00
TRINITY_A8IYN1_CHLRE/354-606	A8IYN1.1 PF00520.29	45.10	0.00
TRINITY_I7MGR5_TETTS/497-590	I7MGR5.2 PF12796.5;	45.10	0.00
TRINITY_D8TI24_VOLCA/5-120	D8TI24.1 PF13881.4;	45.10	0.00
TRINITY_D8TK82_VOLCA/651-941	D8TK82.1 PF07993.10	45.10	0.00
TRINITY_D8UB98_VOLCA/211-425	D8UB98.1 PF01189.15	45.10	0.00
TRINITY_V3Z5M5_LOTGI/58-380	V3Z5M5.1 PF01866.15	45.10	0.00
TRINITY_D8UK49_VOLCA/4-179	D8UK49.1 PF00025.19	45.10	0.00
TRINITY_E1ZE48_CHLVA/65-128	E1ZE48.1 PF05347.13	45.10	0.00
TRINITY_A0EC62_PARTE/382-547	A0EC62.1 PF01699.22	45.10	0.00
TRINITY_Q54XL4_DICDI/1195-1392	Q54XL4.1 PF00617.17	45.10	0.00
TRINITY_A0E5C3_PARTE/689-806	A0E5C3.1 PF00072.22	45.10	0.00

TRINITY_J9EW10_9SPIT/183-441	J9EW10.1 PF00520.29	45.10	0.00
TRINITY_A0A0N5A7B3_PARTI/375-436	A0A0N5A7B3.1 PF0065	45.00	0.00
TRINITY_T1HH24_RHOPR/1134-1557	T1HH24.1 PF06602.12	45.00	0.00
TRINITY_A0D1W7_PARTE/525-821	A0D1W7.1 PF03372.21	45.00	0.00
TRINITY_I7MB87_TETTS/63-523	I7MB87.2 PF00067.20	45.00	0.00
TRINITY_A0A077ZXH0_STYLE/111-176	A0A077ZXH0.1 PF1349	45.00	0.00
TRINITY_A0A067BHW8_SAPPC/1-131	A0A067BHW8.1 PF0012	45.00	0.00
TRINITY_I7M667_TETTS/167-431	I7M667.1 PF00149.26	45.00	0.00
TRINITY_U6MVI1_9EIME/171-246	U6MVI1.1 PF04969.14	45.00	0.00
TRINITY_I7M790_TETTS/28-207	I7M790.2 PF01734.20	45.00	0.00
TRINITY_A8PX58_MALGO/422-745	A8PX58.1 PF03372.21	45.00	0.00
TRINITY_A0DCZ2_PARTE/117-176	A0DCZ2.1 PF13921.4;	45.00	0.00
TRINITY_G0QYL0_ICHMG/104-390	G0QYL0.1 PF01457.14	45.00	0.00
TRINITY_B9H2M0_POPTR/463-609	B9H2M0.1 PF04408.21	45.00	0.00
TRINITY_G0WAG4_NAUDC/14-392	G0WAG4.1 PF08569.9;	45.00	0.00
TRINITY_S2J574_MUCC1/83-146	S2J574.1 PF13499.4;	45.00	0.00
TRINITY_A7SRS7_NEMVE/41-178	A7SRS7.1 PF00782.18	45.00	0.00
TRINITY_W7XE61_TETTS/598-747	W7XE61.1 PF01927.14	45.00	0.00
TRINITY_I7MG40_TETTS/3-154	I7MG40.1 PF09809.7;	45.00	0.00
TRINITY_A0A078B8P5_STYLE/295-617	A0A078B8P5.1 PF0123	45.00	0.00
TRINITY_A0A061J6Y9_TRYRA/1-229	A0A061J6Y9.1 PF0006	45.00	0.00
TRINITY_PIRA_DICDI/393-1224	Q6UK63.1 PF05994.9;	45.00	0.00
TRINITY_A0A0G4J4J7_PLABS/9-230	A0A0G4J4J7.1 PF0010	45.00	0.00
TRINITY_A8J2E8_CHLRE/68-328	A8J2E8.1 PF00487.22	45.00	0.00
TRINITY_W4XR36_STRPU/437-714	W4XR36.1 PF00069.23	45.00	0.00
TRINITY_A0A0F4G4U9_9PEZI/77-228	A0A0F4G4U9.1 PF0000	45.00	0.00
TRINITY_G0QK40_ICHMG/243-478	G0QK40.1 PF07690.14	45.00	0.00
TRINITY_A9V1G0_MONBE/141-242	A9V1G0.1 PF16656.3;	45.00	0.00
TRINITY_I0Z0T3_9CHLO/10-292	I0Z0T3.1 PF00069.23	45.00	0.00
TRINITY_A8J237_CHLRE/18-201	A8J237.1 PF03357.19	45.00	0.00
TRINITY_A0A0D2W0B3_CAPO3/484-581	A0A0D2W0B3.1 PF0016	45.00	0.00
TRINITY_F0ZP74_DICPU/221-539	F0ZP74.1 PF00657.20	45.00	0.00
TRINITY_Q54EI7_DICDI/72-491	Q54EI7.1 PF01490.16	45.00	0.00
TRINITY_A0A0G4J236_PLABS/53-171	A0A0G4J236.1 PF0309	45.00	0.00
TRINITY_A8HSY5_CHLRE/171-679	A8HSY5.1 PF00654.18	45.00	0.00
TRINITY_A0EFL7_PARTE/156-283	A0EFL7.1 PF00107.24	45.00	0.00
TRINITY_L8HCM1_ACACA/41-284	L8HCM1.1 PF09414.8;	45.00	0.00
TRINITY_I3CDQ7_9GAMM/16-390	I3CDQ7.1 PF01433.18	45.00	0.00
TRINITY_I0YQS5_9CHLO/52-357	I0YQS5.1 PF00291.23	45.00	0.00
TRINITY_A8IL21_CHLRE/65-244	A8IL21.1 PF00574.21	45.00	0.00
TRINITY_A4VD54_TETTS/76-357	A4VD54.1 PF00664.21	45.00	0.00
TRINITY_A0A015K277_9GLOM/181-420	A0A015K277.1 PF0012	45.00	0.00
TRINITY_K6DXZ9_9BACI/45-456	K6DXZ9.1 PF00266.17	45.00	0.00
TRINITY_T0QLF1_9STRA/183-314	T0QLF1.1 PF01553.19	45.00	0.00
TRINITY_Q22D68_TETTS/29-277	Q22D68.3 PF03803.13	45.00	0.00
TRINITY_A9V1U1_MONBE/10-171	A9V1U1.1 PF00071.20	45.00	0.00
TRINITY_A0A0D2X453_CAPO3/177-285	A0A0D2X453.1 PF0572	45.00	0.00
TRINITY_G6DRL2_DANPL/310-440	G6DRL2.1 PF00782.18	45.00	0.00
TRINITY_H5X6P0_9PSEU/244-583	H5X6P0.1 PF00144.22	45.00	0.00
TRINITY_A0A098VPN8_9MICR/44-169	A0A098VPN8.1 PF0405	45.00	0.00
TRINITY_W2QUI9_PHYPN/848-1058	W2QUI9.1 PF00689.19	45.00	0.00
TRINITY_D8UG84_VOLCA/3-124	D8UG84.1 PF00917.24	45.00	0.00
TRINITY_D8TH57_VOLCA/40-476	D8TH57.1 PF12698.5;	45.00	0.00
TRINITY_A8HNP6_CHLRE/11-260	A8HNP6.1 PF07727.12	45.00	0.00
TRINITY_A0DMV3_PARTE/527-841	A0DMV3.1 PF05192.16	44.90	0.00
TRINITY_G0QNM9_ICHMG/8-198	G0QNM9.1 PF03092.14	44.90	0.00
TRINITY_G2YZT3_FLABF/17-401	G2YZT3.1 PF03702.12	44.90	0.00
TRINITY_A0A0J8CZ44_BETVU/15-152	A0A0J8CZ44.1 PF0590	44.90	0.00
TRINITY_G2LEF4_CHLTF/33-507	G2LEF4.1 PF01425.19	44.90	0.00
TRINITY_A0A024UKA9_9STRA/421-527	A0A024UKA9.1 PF0065	44.90	0.00
TRINITY_Q240R7_TETTS/8-280	Q240R7.3 PF05914.10	44.90	0.00

TRINITY_D2VE32_NAEGR/14-151	D2VE32.1 PF00754.23	44.90	0.00
TRINITY_V8NRM8_OPHHA/139-245	V8NRM8.1 PF00307.29	44.90	0.00
TRINITY_Q23A05_TETTS/171-240	Q23A05.1 PF14360.4;	44.90	0.00
TRINITY_Q236N2_TETTS/32-570	Q236N2.1 PF00995.21	44.90	0.00
TRINITY_M0TEA9_MUSAM/325-573	M0TEA9.1 PF07714.15	44.90	0.00
TRINITY_D3AVN9_POLPA/18-506	D3AVN9.1 PF13001.5;	44.90	0.00
TRINITY_Q235I9_TETTS/1861-2196	Q235I9.2 PF02889.14	44.90	0.00
TRINITY_GSTM2_CHICK/3-82	P20136.4 PF02798.18	44.90	0.00
TRINITY_A7RRG5_NEMVE/1069-1295	A7RRG5.1 PF16415.3;	44.90	0.00
TRINITY_D8UC17_VOLCA/28-143	D8UC17.1 PF04410.12	44.90	0.00
TRINITY_A0A078AZH3_STYLE/295-407	A0A078AZH3.1 PF0251	44.90	0.00
TRINITY_I0YQN0_9CHLO/447-610	I0YQN0.1 PF09192.8;	44.90	0.00
TRINITY_J9FYL2_9SPIT/398-662	J9FYL2.1 PF00069.23	44.90	0.00
TRINITY_G3PRG1_GASAC/52-221	G3PRG1.1 PF02146.15	44.90	0.00
TRINITY_A0DAD2_PARTE/11-147	A0DAD2.1 PF00334.17	44.90	0.00
TRINITY_A0A059LS10_9CHLO/247-414	A0A059LS10.1 PF0505	44.90	0.00
TRINITY_K8GCU9_9CYAN/941-1063	K8GCU9.1 PF02518.24	44.90	0.00
TRINITY_D3BUS7_POLPA/1-381	D3BUS7.1 PF04506.11	44.90	0.00
TRINITY_Q54XX2_DICDI/101-415	Q54XX2.1 PF03062.17	44.90	0.00
TRINITY_A0A0B0MCH7_GOSAR/64-178	A0A0B0MCH7.1 PF0190	44.90	0.00
TRINITY_G1KT98_ANOCA/1-292	G1KT98.1 PF05971.10	44.90	0.00
TRINITY_A8IFF5_CHLRE/9-119	A8IFF5.1 PF14138.4;	44.90	0.00
TRINITY_A0BP90_PARTE/47-120	A0BP90.1 PF00076.20	44.90	0.00
TRINITY_L8HGD8_ACACA/51-323	L8HGD8.1 PF07995.9;	44.90	0.00
TRINITY_Q2S2T9_SALRD/5-377	Q2S2T9.1 PF01053.18	44.90	0.00
TRINITY_J7S154_KAZNA/374-462	J7S154.1 PF11715.6;	44.90	0.00
TRINITY_A0CK32_PARTE/4-158	A0CK32.1 PF12796.5;	44.90	0.00
TRINITY_W5JM09_ANODA/76-144	W5JM09.1 PF00505.17	44.90	0.00
TRINITY_K7VF10_MAIZE/107-319	K7VF10.1 PF09747.7;	44.90	0.00
TRINITY_A0C428_PARTE/30-180	A0C428.1 PF09767.7;	44.90	0.00
TRINITY_D8TTK7_VOLCA/78-378	D8TTK7.1 PF13896.4;	44.90	0.00
TRINITY_A9V1F3_MONBE/178-284	A9V1F3.1 PF01416.18	44.90	0.00
TRINITY_V2XSB3_MONRO/27-273	V2XSB3.1 PF11790.6;	44.90	0.00
TRINITY_J9JA41_9SPIT/35-290	J9JA41.1 PF04427.16	44.90	0.00
TRINITY_I0Z7X8_9CHLO/278-395	I0Z7X8.1 PF07986.10	44.90	0.00
TRINITY_I7M003_TETTS/55-364	I7M003.2 PF00962.20	44.90	0.00
TRINITY_Q235I9_TETTS/701-851	Q235I9.2 PF00271.29	44.90	0.00
TRINITY_L8GN46_ACACA/29-281	L8GN46.1 PF00069.23	44.90	0.00
TRINITY_A0CMN7_PARTE/1075-1240	A0CMN7.1 PF00005.25	44.90	0.00
TRINITY_H0ZEN8_TAEGU/280-533	H0ZEN8.1 PF00069.23	44.90	0.00
TRINITY_J9IFZ4_9SPIT/31-129	J9IFZ4.1 PF13499.4;	44.90	0.00
TRINITY_J9HR16_9SPIT/123-375	J9HR16.1 PF00069.23	44.90	0.00
TRINITY_I7M3M7_TETTS/22-131	I7M3M7.1 PF12796.5;	44.90	0.00
TRINITY_D8U5D1_VOLCA/16-278	D8U5D1.1 PF00069.23	44.90	0.00
TRINITY_K4CAU9_SOLLC/154-406	K4CAU9.1 PF05021.13	44.90	0.00
TRINITY_A0A059LRD3_9CHLO/19-199	A0A059LRD3.1 PF0006	44.90	0.00
TRINITY_F4QPE4_9CAUL/28-149	F4QPE4.1 PF00188.24	44.90	0.00
TRINITY_I7M889_TETTS/31-328	I7M889.1 PF09423.8;	44.90	0.00
TRINITY_L1J7H4_GUITH/303-465	L1J7H4.1 PF06699.9;	44.80	0.00
TRINITY_QOR_HUMAN/192-327	Q08257.1 PF13602.4;	44.80	0.00
TRINITY_A0A0K9NKF0_ZOSMR/654-855	A0A0K9NKF0.1 PF0285	44.80	0.00
TRINITY_J9J708_9SPIT/51-217	J9J708.1 PF03009.15	44.80	0.00
TRINITY_A7MCL7_DANRE/6-94	A7MCL7.1 PF00031.19	44.80	0.00
TRINITY_I7LUK8_TETTS/15-119	I7LUK8.1 PF02991.14	44.80	0.00
TRINITY_L8H7C2_ACACA/5-156	L8H7C2.1 PF09468.8;	44.80	0.00
TRINITY_H6NEC5_9BACL/123-181	H6NEC5.1 PF13376.4;	44.80	0.00
TRINITY_K9PGL6_9CYAN/23-254	K9PGL6.1 PF08894.9;	44.80	0.00
TRINITY_F4Q1H1_DICFS/279-467	F4Q1H1.1 PF01331.17	44.80	0.00
TRINITY_I1G2T0_AMPQE/174-530	I1G2T0.1 PF00657.20	44.80	0.00
TRINITY_A0BZT9_PARTE/8-137	A0BZT9.1 PF00782.18	44.80	0.00
TRINITY_A0BUJ1_PARTE/272-330	A0BUJ1.1 PF13432.4;	44.80	0.00

TRINITY_G4UGJ9_NEUT9/259-384	G4UGJ9.1 PF00622.26	44.80	0.00
TRINITY_A0A077ZZ03_STYLE/8-287	A0A077ZZ03.1 PF0006	44.80	0.00
TRINITY_A0A0A0LDE1_CUCSA/322-694	A0A0A0LDE1.1 PF0049	44.80	0.00
TRINITY_D8TA01_SELML/21-165	D8TA01.1 PF06201.11	44.80	0.00
TRINITY_Q9LKH0_CHLRE/34-81	Q9LKH0.1 PF05051.11	44.80	0.00
TRINITY_I1CA35_RHIO9/23-261	I1CA35.1 PF07819.11	44.80	0.00
TRINITY_I0Z3E9_9CHLO/21-155	I0Z3E9.1 PF07884.12	44.80	0.00
TRINITY_C1EIK2_MICSR/1-193	C1EIK2.1 PF00069.23	44.80	0.00
TRINITY_A4RRI5_OSTLU/318-413	A4RRI5.1 PF08295.10	44.80	0.00
TRINITY_I0YSS6_9CHLO/10-67	I0YSS6.1 PF08221.9;	44.80	0.00
TRINITY_B3RKZ3_TRIAD/1-417	B3RKZ3.1 PF04188.11	44.80	0.00
TRINITY_G0QN14_ICHMG/12-182	G0QN14.1 PF00071.20	44.80	0.00
TRINITY_H2YXH4_CIOSA/1120-1184	H2YXH4.1 PF01363.19	44.80	0.00
TRINITY_H8N1Q4_CORCM/22-90	H8N1Q4.1 PF00498.24	44.80	0.00
TRINITY_D8TMY6_VOLCA/279-490	D8TMY6.1 PF07714.15	44.80	0.00
TRINITY_A0CHC8_PARTE/261-354	A0CHC8.1 PF12796.5;	44.80	0.00
TRINITY_A0A067CKA5_SAPPC/69-223	A0A067CKA5.1 PF0864	44.80	0.00
TRINITY_E1Z715_CHLVA/40-133	E1Z715.1 PF02463.17	44.80	0.00
TRINITY_I0ZA89_9CHLO/133-364	I0ZA89.1 PF00198.21	44.80	0.00
TRINITY_A0A085MQK8_9BILA/324-484	A0A085MQK8.1 PF0007	44.80	0.00
TRINITY_A8JEK4_CHLRE/889-1117	A8JEK4.1 PF00069.23	44.80	0.00
TRINITY_L8HB96_ACACA/273-714	L8HB96.1 PF01142.16	44.80	0.00
TRINITY_F2LK13_BURGS/275-390	F2LK13.1 PF07683.12	44.80	0.00
TRINITY_T0QIM3_9STRA/132-379	T0QIM3.1 PF09423.8;	44.80	0.00
TRINITY_A0A068Y2T9_ECHMU/154-303	A0A068Y2T9.1 PF0866	44.80	0.00
TRINITY_F4QFV3_DICFS/1382-1559	F4QFV3.1 PF00617.17	44.80	0.00
TRINITY_Q23J69_TETTS/21-486	Q23J69.2 PF00083.22	44.80	0.00
TRINITY_D3BQH2_POLPA/3-97	D3BQH2.1 PF00168.28	44.80	0.00
TRINITY_M2TMX2_COCH5/60-163	M2TMX2.1 PF00168.28	44.80	0.00
TRINITY_R0GVH4_9BRAS/385-524	R0GVH4.1 PF00534.18	44.80	0.00
TRINITY_A0A077ZQS0_STYLE/68-262	A0A077ZQS0.1 PF1214	44.80	0.00
TRINITY_I0Z5U6_9CHLO/234-483	I0Z5U6.1 PF01068.19	44.80	0.00
TRINITY_G0QYS4_ICHMG/12-127	G0QYS4.1 PF08327.9;	44.80	0.00
TRINITY_G1Q9K0_MYOLU/96-185	G1Q9K0.1 PF00071.20	44.80	0.00
TRINITY_A0EDA4_PARTE/11-172	A0EDA4.1 PF01217.18	44.80	0.00
TRINITY_G0QZC0_ICHMG/11-172	G0QZC0.1 PF00071.20	44.80	0.00
TRINITY_E1ZBW1_CHLVA/41-209	E1ZBW1.1 PF09768.7;	44.80	0.00
TRINITY_I0YIC0_9CHLO/510-978	I0YIC0.1 PF00082.20	44.80	0.00
TRINITY_D8TSZ7_VOLCA/1690-1975	D8TSZ7.1 PF07714.15	44.80	0.00
TRINITY_G0QK71_ICHMG/834-1225	G0QK71.1 PF06957.9;	44.70	0.00
TRINITY_Q6BFM7_PARTE/40-233	Q6BFM7.1 PF00106.23	44.70	0.00
TRINITY_U5QED8_9CYAN/96-245	U5QED8.1 PF00849.20	44.70	0.00
TRINITY_G0R6C8_ICHMG/129-261	G0R6C8.1 PF01926.21	44.70	0.00
TRINITY_D8U1R6_VOLCA/4-150	D8U1R6.1 PF08560.8;	44.70	0.00
TRINITY_C0NPR6_AJECG/38-139	C0NPR6.1 PF00168.28	44.70	0.00
TRINITY_A0A0G4FC78_9ALVE/34-122	A0A0G4FC78.1 PF0221	44.70	0.00
TRINITY_I7MN11_TETTS/232-405	I7MN11.1 PF04925.13	44.70	0.00
TRINITY_F1QTX8_DANRE/374-480	F1QTX8.1 PF00085.18	44.70	0.00
TRINITY_Q57ZK9_TRYB2/21-219	Q57ZK9.1 PF01852.17	44.70	0.00
TRINITY_W7X1C7_TETTS/22-152	W7X1C7.1 PF00782.18	44.70	0.00
TRINITY_A0A0A1UCS6_ENTIV/1389-1650	A0A0A1UCS6.1 PF0006	44.70	0.00
TRINITY_I7MI70_TETTS/14-137	I7MI70.1 PF13893.4;	44.70	0.00
TRINITY_L8GGB3_ACACA/1052-1230	L8GGB3.1 PF00617.17	44.70	0.00
TRINITY_I7MDS7_TETTS/37-166	I7MDS7.2 PF12298.6;	44.70	0.00
TRINITY_D8SUK9_SELML/21-209	D8SUK9.1 PF01612.18	44.70	0.00
TRINITY_F0ZXN7_DICPU/186-461	F0ZXN7.1 PF03151.14	44.70	0.00
TRINITY_V7B491_PHAVU/53-333	V7B491.1 PF01494.17	44.70	0.00
TRINITY_I7MHS7_TETTS/50-527	I7MHS7.1 PF00501.26	44.70	0.00
TRINITY_F4PZV0_DICFS/18-113	F4PZV0.1 PF00169.27	44.70	0.00
TRINITY_F4PN46_DICFS/255-412	F4PN46.1 PF00730.23	44.70	0.00
TRINITY_A0A0N5C217_STREA/160-487	A0A0N5C217.1 PF0107	44.70	0.00

TRINITY_F4Q0L9_DICFS/16-121	F4Q0L9.1 PF00568.21	44.70	0.00
TRINITY_A0A0G4EL58_9ALVE/27-157	A0A0G4EL58.1 PF0407	44.70	0.00
TRINITY_A0A087SHU1_AUXPR/253-525	A0A087SHU1.1 PF0553	44.70	0.00
TRINITY_F0ZLD9_DICPU/606-972	F0ZLD9.1 PF02181.21	44.70	0.00
TRINITY_C3XRT4_BRAFL/8-176	C3XRT4.1 PF00908.15	44.70	0.00
TRINITY_K7LC65_SOYBN/150-507	K7LC65.1 PF03062.17	44.70	0.00
TRINITY_D2VBV7_NAEGR/17-532	D2VBV7.1 PF04916.11	44.70	0.00
TRINITY_D8TUH4_VOLCA/83-248	D8TUH4.1 PF01734.20	44.70	0.00
TRINITY_C5KZH2_PERM5/199-428	C5KZH2.1 PF01728.17	44.70	0.00
TRINITY_D8UK41_VOLCA/70-418	D8UK41.1 PF15902.3;	44.70	0.00
TRINITY_TALA_DICDI/163-275	P0CE94.1 PF00373.16	44.70	0.00
TRINITY_G7J7M5_MEDTR/90-137	G7J7M5.1 PF00249.29	44.70	0.00
TRINITY_A0A0J8B878_BETVU/74-221	A0A0J8B878.1 PF0095	44.70	0.00
TRINITY_A8IS59_CHLRE/1-288	A8IS59.1 PF00483.21	44.70	0.00
TRINITY_E9HK74_DAPPU/229-559	E9HK74.1 PF07992.12	44.70	0.00
TRINITY_I7LVY1_TETTS/23-350	I7LVY1.2 PF00225.21	44.70	0.00
TRINITY_A8IGG5_CHLRE/30-160	A8IGG5.1 PF01590.24	44.70	0.00
TRINITY_D8T5X6_SELML/116-226	D8T5X6.1 PF08389.10	44.70	0.00
TRINITY_F7DM18_ORNAN/143-237	F7DM18.2 PF08698.9;	44.70	0.00
TRINITY_L1JND6_GUITH/150-376	L1JND6.1 PF13621.4;	44.70	0.00
TRINITY_K9TZW6_9CYAN/45-184	K9TZW6.1 PF13847.4;	44.70	0.00
TRINITY_A8IQ02_CHLRE/1240-1463	A8IQ02.1 PF14228.4;	44.70	0.00
TRINITY_Q8HCR1_ORYSJ/11-256	Q8HCR1.1 PF07727.12	44.70	0.00
TRINITY_A0A0A1PIM2_9FUNG/7-117	A0A0A1PIM2.1 PF0085	44.70	0.00
TRINITY_A8HTU8_CHLRE/13-251	A8HTU8.1 PF00856.26	44.70	0.00
TRINITY_A9U123_PHYPA/2-1170	A9U123.1 PF02463.17	44.70	0.00
TRINITY_I8AKI3_9BACI/41-313	I8AKI3.1 PF00082.20	44.70	0.00
TRINITY_A0BRA8_PARTE/7-108	A0BRA8.1 PF00168.28	44.70	0.00
TRINITY_K3XB12_PYTUL/79-318	K3XB12.1 PF04884.12	44.70	0.00
TRINITY_D8THR0_VOLCA/108-289	D8THR0.1 PF01273.23	44.70	0.00
TRINITY_A0A0D6TCS9_9RHOB/2-151	A0A0D6TCS9.1 PF0335	44.70	0.00
TRINITY_RA51C_MOUSE/22-340	Q924H5.1 PF08423.9;	44.70	0.00
TRINITY_K9T4R9_9CYAN/41-162	K9T4R9.1 PF09837.7;	44.70	0.00
TRINITY_A0BYK2_PARTE/116-313	A0BYK2.1 PF00406.20	44.70	0.00
TRINITY_A0A0F0GW99_9ACTN/23-463	A0A0F0GW99.1 PF1403	44.70	0.00
TRINITY_D8U182_VOLCA/674-878	D8U182.1 PF01434.16	44.70	0.00
TRINITY_E3JYQ2_PUCGT/424-778	E3JYQ2.2 PF00702.24	44.70	0.00
TRINITY_A8IL34_CHLRE/21-289	A8IL34.1 PF00069.23	44.70	0.00
TRINITY_D8U3S5_VOLCA/63-138	D8U3S5.1 PF09340.8;	44.70	0.00
TRINITY_D8U978_VOLCA/2-203	D8U978.1 PF07714.15	44.70	0.00
TRINITY_C5Z583_SORBI/934-1179	C5Z583.1 PF07727.12	44.70	0.00
TRINITY_F0VIJ0_NEOCL/1240-1430	F0VIJ0.1 PF00689.19	44.70	0.00
TRINITY_I7LSW7_TETTS/11-177	I7LSW7.1 PF00316.18	44.70	0.00
TRINITY_I7LVZ2_TETTS/119-388	I7LVZ2.2 PF00664.21	44.60	0.00
TRINITY_A0A078A0Q2_STYLE/458-744	A0A078A0Q2.1 PF0337	44.60	0.00
TRINITY_A0BWP2_PARTE/43-433	A0BWP2.1 PF01490.16	44.60	0.00
TRINITY_J9J5F8_9SPIT/511-624	J9J5F8.1 PF02518.24	44.60	0.00
TRINITY_L8HDL6_ACACA/1-64	L8HDL6.1 PF06331.10	44.60	0.00
TRINITY_G0AH21_COLFT/1-90	G0AH21.1 PF00216.19	44.60	0.00
TRINITY_A0A078B7J6_STYLE/170-246	A0A078B7J6.1 PF1342	44.60	0.00
TRINITY_G0QKV4_ICHMG/49-211	G0QKV4.1 PF00067.20	44.60	0.00
TRINITY_K9TP00_9CYAN/15-228	K9TP00.1 PF00106.23	44.60	0.00
TRINITY_F1A2P2_DICPU/37-159	F1A2P2.1 PF03067.13	44.60	0.00
TRINITY_A0A015KUS6_9GLOM/82-193	A0A015KUS6.1 PF0754	44.60	0.00
TRINITY_A0A066VSL3_9HOMO/35-393	A0A066VSL3.1 PF0028	44.60	0.00
TRINITY_W5BB92_WHEAT/25-307	W5BB92.1 PF00487.22	44.60	0.00
TRINITY_K1QG77_CRAGI/54-430	K1QG77.1 PF01979.18	44.60	0.00
TRINITY_L8GDH9_ACACA/994-1253	L8GDH9.1 PF07714.15	44.60	0.00
TRINITY_F2U695_SALR5/75-334	F2U695.1 PF00069.23	44.60	0.00
TRINITY_RAC1_CAEEL/5-178	Q03206.2 PF00071.20	44.60	0.00
TRINITY_L8HL68_ACACA/22-196	L8HL68.1 PF05995.10	44.60	0.00

TRINITY_G4E3U0_9GAMM/20-306	G4E3U0.1 PF00850.17	44.60	0.00
TRINITY_I7MI70_TETTS/14-137	I7MI70.1 PF13893.4;	44.60	0.00
TRINITY_A7RRV3_NEMVE/15-171	A7RRV3.1 PF08662.9;	44.60	0.00
TRINITY_G0QKY6_ICHMG/10-97	G0QKY6.1 PF16561.3;	44.60	0.00
TRINITY_A0A024UKA9_9STRA/421-527	A0A024UKA9.1 PF0065	44.60	0.00
TRINITY_A7RIQ3_NEMVE/3-265	A7RIQ3.1 PF01026.19	44.60	0.00
TRINITY_A0A078BZL6_BRANA/305-450	A0A078BZL6.1 PF0437	44.60	0.00
TRINITY_U3JCM7_FICAL/23-277	U3JCM7.1 PF00069.23	44.60	0.00
TRINITY_F2U297_SALR5/2182-2441	F2U297.1 PF00520.29	44.60	0.00
TRINITY_J0WT80_AURST/119-238	J0WT80.1 PF00168.28	44.60	0.00
TRINITY_A7RGQ3_NEMVE/75-367	A7RGQ3.1 PF00176.21	44.60	0.00
TRINITY_D6WUY8_TRICA/64-189	D6WUY8.1 PF10218.7;	44.60	0.00
TRINITY_A0A0F4NTH2_PSEO7/12-387	A0A0F4NTH2.1 PF0373	44.60	0.00
TRINITY_A0BTW1_PARTE/50-170	A0BTW1.1 PF02338.17	44.60	0.00
TRINITY_D8U2T8_VOLCA/63-195	D8U2T8.1 PF00782.18	44.60	0.00
TRINITY_C1EHB3_MICSR/11-134	C1EHB3.1 PF02678.14	44.60	0.00
TRINITY_L8HLM2_ACACA/705-992	L8HLM2.1 PF01031.18	44.60	0.00
TRINITY_Q0F3F6_9PROT/42-142	Q0F3F6.1 PF00011.19	44.60	0.00
TRINITY_G8M2N6_CLOCD/600-766	G8M2N6.1 PF07724.12	44.60	0.00
TRINITY_I7M2H5_TETTS/224-317	I7M2H5.1 PF00153.25	44.60	0.00
TRINITY_A9T6W9_PHYPA/102-248	A9T6W9.1 PF04116.11	44.60	0.00
TRINITY_K3WTT3_PYTUL/26-297	K3WTT3.1 PF01650.16	44.60	0.00
TRINITY_G6FSF2_9CYAN/1540-1967	G6FSF2.1 PF00501.26	44.60	0.00
TRINITY_F4QE03_DICFS/903-1096	F4QE03.1 PF06017.11	44.60	0.00

TRINITY_A0CK88_PARTE/14-69	A0CK88.1 PF00385.22	44.60	0.00
TRINITY_I0YUQ5_9CHLO/33-353	I0YUQ5.1 PF01636.21	44.60	0.00
TRINITY_V3ZF78_LOTGI/343-420	V3ZF78.1 PF00622.26	44.60	0.00
TRINITY_I7M2X3_TETTS/28-255	I7M2X3.1 PF00149.26	44.60	0.00
TRINITY_U5DFD7_AMBTC/550-607	U5DFD7.1 PF08373.8;	44.60	0.00
TRINITY_A4BKN8_9GAMM/403-468	A4BKN8.1 PF00512.23	44.60	0.00
TRINITY_K1QAL3_CRAGI/448-520	K1QAL3.1 PF00076.20	44.60	0.00
TRINITY_A8I1M3_CHLRE/18-152	A8I1M3.1 PF12697.5;	44.60	0.00
TRINITY_KAD_TOLAT/5-187	C4L8U2.1 PF00406.20	44.60	0.00
TRINITY_F0ZH73_DICPU/405-577	F0ZH73.1 PF00621.18	44.60	0.00
TRINITY_A8JGX3_CHLRE/1139-1204	A8JGX3.1 PF00957.19	44.60	0.00
TRINITY_A9REM2_PHYPA/25-487	A9REM2.1 PF01384.18	44.60	0.00
TRINITY_D8ULH4_VOLCA/183-452	D8ULH4.1 PF00520.29	44.60	0.00
TRINITY_A8J3P1_CHLRE/954-1161	A8J3P1.1 PF00211.18	44.60	0.00
TRINITY_A0A066X8V5_COLSU/39-150	A0A066X8V5.1 PF0038	44.60	0.00
TRINITY_I0ZAW8_9CHLO/360-815	I0ZAW8.1 PF14648.4;	44.60	0.00
TRINITY_A0A015J5P3_9GLOM/26-135	A0A015J5P3.1 PF0888	44.50	0.00
TRINITY_F0Z7U6_DICPU/40-202	F0Z7U6.1 PF10294.7;	44.50	0.00
TRINITY_Q22T22_TETTS/520-735	Q22T22.3 PF07859.11	44.50	0.00
TRINITY_Q7NK64_GLOVI/264-552	Q7NK64.1 PF00773.17	44.50	0.00
TRINITY_G0QQW4_ICHMG/64-237	G0QQW4.1 PF13847.4;	44.50	0.00
TRINITY_A0A0G4INI1_PLABS/60-232	A0A0G4INI1.1 PF0363	44.50	0.00
TRINITY_L8HI53_ACACA/58-628	L8HI53.1 PF01602.18	44.50	0.00
TRINITY_D8UFD3_VOLCA/71-660	D8UFD3.1 PF16940.3;	44.50	0.00
TRINITY_Q23QF2_TETTS/248-571	Q23QF2.2 PF03133.13	44.50	0.00
TRINITY_B9I062_POPTR/27-338	B9I062.1 PF03291.14	44.50	0.00
TRINITY_L8GLB5_ACACA/120-346	L8GLB5.1 PF12146.6;	44.50	0.00
TRINITY_H3HAJ2_PHYRM/359-478	H3HAJ2.1 PF00665.24	44.50	0.00
TRINITY_I7MLC4_TETTS/363-643	I7MLC4.1 PF00657.20	44.50	0.00
TRINITY_A0E0I6_PARTE/13-410	A0E0I6.1 PF00899.19	44.50	0.00
TRINITY_K3XAI6_PYTUL/186-434	K3XAI6.1 PF04042.14	44.50	0.00
TRINITY_A0A078AYT5_STYLE/68-219	A0A078AYT5.1 PF0753	44.50	0.00
TRINITY_G0R646_ICHMG/745-1019	G0R646.1 PF00664.21	44.50	0.00
TRINITY_J9INA7_9SPIT/626-748	J9INA7.1 PF01857.18	44.50	0.00
TRINITY_M0TTX4_MUSAM/42-371	M0TTX4.1 PF00069.23	44.50	0.00
TRINITY_A8IKL2_CHLRE/43-432	A8IKL2.1 PF03416.17	44.50	0.00
TRINITY_D8UCR5_VOLCA/725-974	D8UCR5.1 PF07393.9;	44.50	0.00
TRINITY_D8SJ59_SELML/20-342	D8SJ59.1 PF09139.9;	44.50	0.00
TRINITY_F2TZR2_SALR5/13-207	F2TZR2.1 PF06941.10	44.50	0.00
TRINITY_A8JF89_CHLRE/400-723	A8JF89.1 PF00520.29	44.50	0.00
TRINITY_D3B677_POLPA/110-266	D3B677.1 PF00005.25	44.50	0.00
TRINITY_D8TND4_VOLCA/49-240	D8TND4.1 PF00856.26	44.50	0.00
TRINITY_D8U9M7_VOLCA/253-490	D8U9M7.1 PF11051.6;	44.50	0.00
TRINITY_D6X2B1_TRICA/278-497	D6X2B1.1 PF00667.18	44.50	0.00
TRINITY_M5VSU7_PRUPE/488-813	M5VSU7.1 PF01504.16	44.50	0.00
TRINITY_I0Z502_9CHLO/30-503	I0Z502.1 PF10243.7;	44.50	0.00
TRINITY_I0Z1H2_9CHLO/42-459	I0Z1H2.1 PF00999.19	44.50	0.00
TRINITY_A8HWI9_CHLRE/348-571	A8HWI9.1 PF07714.15	44.50	0.00
TRINITY_A8HXI8_CHLRE/1160-1352	A8HXI8.1 PF00211.18	44.50	0.00
TRINITY_D8TVK8_VOLCA/2148-2411	D8TVK8.1 PF00520.29	44.50	0.00
TRINITY_B3RQW2_TRIAD/45-131	B3RQW2.1 PF14874.4;	44.40	0.00
TRINITY_A0A096QYU0_MAIZE/529-657	A0A096QYU0.1 PF0478	44.40	0.00
TRINITY_C3YKQ0_BRAFL/6-195	C3YKQ0.1 PF01353.20	44.40	0.00
TRINITY_A0A078A2M7_STYLE/3691-4473	A0A078A2M7.1 PF0302	44.40	0.00
TRINITY_Q22A65_TETTS/524-741	Q22A65.2 PF01612.18	44.40	0.00
TRINITY_D2W479_NAEGR/767-891	D2W479.1 PF07714.15	44.40	0.00
TRINITY_Q23TD6_TETTS/82-200	Q23TD6.2 PF01926.21	44.40	0.00
TRINITY_A0A072UR89_MEDTR/58-312	A0A072UR89.1 PF0771	44.40	0.00
TRINITY_I7LT38_TETTS/108-339	I7LT38.1 PF00122.18	44.40	0.00
TRINITY_G4YKZ0_PHYSP/36-341	G4YKZ0.1 PF01237.16	44.40	0.00
TRINITY_U9T7D8_RHIID/265-375	U9T7D8.1 PF01398.19	44.40	0.00

TRINITY_F4PMH8_DICFS/72-202	F4PMH8.1 PF07967.11	44.40	0.00
TRINITY_A0A087SSV0_AUXPR/118-234	A0A087SSV0.1 PF0155	44.40	0.00
TRINITY_I7LXT2_TETTS/158-424	I7LXT2.2 PF00069.23	44.40	0.00
TRINITY_M5EDH3_MALS4/201-396	M5EDH3.1 PF00733.19	44.40	0.00
TRINITY_D8U8N7_VOLCA/113-236	D8U8N7.1 PF01398.19	44.40	0.00
TRINITY_L8HKQ7_ACACA/361-718	L8HKQ7.1 PF00443.27	44.40	0.00
TRINITY_C6W3C2_DYAFD/20-198	C6W3C2.1 PF13532.4;	44.40	0.00
TRINITY_A0A0M8MSS6_9BASI/8-170	A0A0M8MSS6.1 PF0007	44.40	0.00
TRINITY_I0Z2A7_9CHLO/114-589	I0Z2A7.1 PF01293.18	44.40	0.00
TRINITY_F0ZPI4_DICPU/1-102	F0ZPI4.1 PF00168.28	44.40	0.00
TRINITY_I7MAS1_TETTS/209-350	I7MAS1.2 PF00781.22	44.40	0.00
TRINITY_W4GNN6_9STRA/356-454	W4GNN6.1 PF16158.3;	44.40	0.00
TRINITY_D8U1X9_VOLCA/1617-1769	D8U1X9.1 PF07744.11	44.40	0.00
TRINITY_I0YPQ0_9CHLO/7-122	I0YPQ0.1 PF00072.22	44.40	0.00
TRINITY_G0QPQ5_ICHMG/48-200	G0QPQ5.1 PF14908.4;	44.40	0.00
TRINITY_A9VBV3_MONBE/113-301	A9VBV3.1 PF08613.9;	44.40	0.00
TRINITY_D8UDM0_VOLCA/9-413	D8UDM0.1 PF03348.13	44.40	0.00
TRINITY_A0A0G4J3J9_PLABS/33-250	A0A0G4J3J9.1 PF0699	44.40	0.00
TRINITY_L0AVB9_THEEQ/36-143	L0AVB9.1 PF00085.18	44.40	0.00
TRINITY_H0W0T5_CAVPO/402-495	H0W0T5.1 PF12796.5;	44.40	0.00
TRINITY_D8TVK8_VOLCA/245-526	D8TVK8.1 PF00520.29	44.40	0.00
TRINITY_A9S9L2_PHYPA/700-1078	A9S9L2.1 PF13423.4;	44.40	0.00
TRINITY_A0A098VUJ1_9MICR/152-260	A0A098VUJ1.1 PF1487	44.40	0.00
TRINITY_M0RHE9_MUSAM/38-393	M0RHE9.1 PF04185.12	44.40	0.00
TRINITY_L8GLZ0_ACACA/205-444	L8GLZ0.1 PF10486.7;	44.40	0.00
TRINITY_F1RFJ0_PIG/121-202	F1RFJ0.2 PF01822.17	44.40	0.00
TRINITY_E3KL98_PUCGT/81-191	E3KL98.2 PF01491.14	44.40	0.00
TRINITY_Q869V0_DICDI/657-841	Q869V0.1 PF02145.13	44.40	0.00
TRINITY_K0IMK6_NITGG/2-223	K0IMK6.1 PF01269.15	44.40	0.00
TRINITY_C4M0S0_ENTHI/4-74	C4M0S0.1 PF00076.20	44.40	0.00
TRINITY_D2W2V3_NAEGR/71-211	D2W2V3.1 PF00782.18	44.40	0.00
TRINITY_E1Z9C0_CHLVA/522-797	E1Z9C0.1 PF13532.4;	44.40	0.00
TRINITY_D8TIR1_VOLCA/82-185	D8TIR1.1 PF00085.18	44.40	0.00
TRINITY_W5KK44_ASTMX/68-374	W5KK44.1 PF00026.21	44.40	0.00
TRINITY_A0A067JDG7_JATCU/1367-1440	A0A067JDG7.1 PF0057	44.40	0.00
TRINITY_L8HEU4_ACACA/47-335	L8HEU4.1 PF02383.16	44.40	0.00
TRINITY_D8U0J3_VOLCA/3-532	D8U0J3.1 PF00759.17	44.40	0.00
TRINITY_D8TCH9_SELML/127-258	D8TCH9.1 PF01529.18	44.40	0.00
TRINITY_A0A024WH81_PLAFA/121-425	A0A024WH81.1 PF1050	44.40	0.00
TRINITY_E1ZIY9_CHLVA/424-615	E1ZIY9.1 PF14881.4;	44.40	0.00
TRINITY_D8UHE8_VOLCA/111-192	D8UHE8.1 PF09409.8;	44.40	0.00
TRINITY_L8H0K3_ACACA/125-255	L8H0K3.1 PF17207.1;	44.40	0.00
TRINITY_K4MDB8_9EURY/98-153	K4MDB8.1 PF00571.26	44.40	0.00
TRINITY_I7LZV2_TETTS/151-413	I7LZV2.1 PF00069.23	44.40	0.00
TRINITY_E9DJQ9_COCPS/216-269	E9DJQ9.1 PF00249.29	44.40	0.00
TRINITY_T0PTH5_9STRA/27-186	T0PTH5.1 PF04939.10	44.40	0.00
TRINITY_S9VR53_9TRYP/106-249	S9VR53.1 PF00782.18	44.40	0.00
TRINITY_A9TQ85_PHYPA/12-93	A9TQ85.1 PF04683.11	44.40	0.00
TRINITY_D8TKD2_VOLCA/5-408	D8TKD2.1 PF04801.11	44.40	0.00
TRINITY_A0YYD8_LYNSP/2-157	A0YYD8.1 PF05706.10	44.40	0.00
TRINITY_C1E5C1_MICSR/25-305	C1E5C1.1 PF01904.16	44.40	0.00
TRINITY_C0ZCI2_BREBN/109-247	C0ZCI2.1 PF00004.27	44.40	0.00
TRINITY_A8IGD3_CHLRE/368-494	A8IGD3.1 PF02469.20	44.40	0.00
TRINITY_D3BNR7_POLPA/86-176	D3BNR7.1 PF00626.20	44.40	0.00
TRINITY_S8CXI3_9LAMI/320-390	S8CXI3.1 PF13620.4;	44.40	0.00
TRINITY_A0A0M9DTP7_9BACT/3-161	A0A0M9DTP7.1 PF1352	44.40	0.00
TRINITY_B9JG80_AGRRK/156-333	B9JG80.1 PF00271.29	44.40	0.00
TRINITY_D8U4Y7_VOLCA/38-111	D8U4Y7.1 PF00240.21	44.40	0.00
TRINITY_V4RFV4_9ROSI/5-552	V4RFV4.1 PF00862.17	44.40	0.00
TRINITY_A0A072U318_MEDTR/1434-1686	A0A072U318.1 PF0056	44.40	0.00
TRINITY_L1IXT3_GUITH/1-178	L1IXT3.1 PF16909.3;	44.40	0.00

TRINITY_W4FSJ9_9STRA/1228-1335	W4FSJ9.1 PF00005.25	44.40	0.00
TRINITY_I1JF64_SOYBN/83-147	I1JF64.1 PF13499.4;	44.40	0.00
TRINITY_Q245C4_TETTS/76-281	Q245C4.1 PF00566.16	44.40	0.00
TRINITY_W2PF29_PHYPN/5-86	W2PF29.1 PF13417.4;	44.30	0.00
TRINITY_A0A0G4GXE9_9ALVE/227-438	A0A0G4GXE9.1 PF1367	44.30	0.00
TRINITY_M4A6Y6_XIPMA/7-199	M4A6Y6.1 PF11032.6;	44.30	0.00
TRINITY_A0EDD8_PARTE/1167-1337	A0EDD8.1 PF01504.16	44.30	0.00
TRINITY_Q22BW3_TETTS/18-233	Q22BW3.2 PF02230.14	44.30	0.00
TRINITY_H2YT61_CIOSA/436-716	H2YT61.1 PF00702.24	44.30	0.00
TRINITY_A0A078BB17_STYLE/30-281	A0A078BB17.1 PF0006	44.30	0.00
TRINITY_Q245C4_TETTS/76-281	Q245C4.1 PF00566.16	44.30	0.00
TRINITY_M2XTZ6_GALSU/34-173	M2XTZ6.1 PF05832.10	44.30	0.00
TRINITY_K1WDM8_TRIAC/173-476	K1WDM8.1 PF05970.12	44.30	0.00
TRINITY_E1WY84_HALMS/472-586	E1WY84.1 PF00072.22	44.30	0.00
TRINITY_A9S1X0_PHYPA/23-92	A9S1X0.1 PF00076.20	44.30	0.00
TRINITY_M7ARK4_CHEMY/1192-1261	M7ARK4.1 PF00076.20	44.30	0.00
TRINITY_L8HE02_ACACA/133-229	L8HE02.1 PF06916.11	44.30	0.00
TRINITY_Q23DH5_TETTS/185-254	Q23DH5.1 PF13921.4;	44.30	0.00
TRINITY_W5MFJ6_LEPOC/42-401	W5MFJ6.1 PF05208.11	44.30	0.00
TRINITY_K7JH06_NASVI/8-194	K7JH06.1 PF01812.18	44.30	0.00
TRINITY_J9I745_9SPIT/386-468	J9I745.1 PF01833.22	44.30	0.00
TRINITY_A0A0M3K351_ANISI/1167-1240	A0A0M3K351.1 PF1279	44.30	0.00
TRINITY_Q096Q7_STIAD/83-224	Q096Q7.1 PF13847.4;	44.30	0.00
TRINITY_F2UHC5_SALR5/32-203	F2UHC5.1 PF03637.15	44.30	0.00
TRINITY_G6DCU0_DANPL/431-760	G6DCU0.1 PF00702.24	44.30	0.00
TRINITY_I7LU64_TETTS/7-127	I7LU64.2 PF13344.4;	44.30	0.00
TRINITY_Q24DE8_TETTS/129-738	Q24DE8.1 PF00443.27	44.30	0.00
TRINITY_D3BPK6_POLPA/6-168	D3BPK6.1 PF00071.20	44.30	0.00
TRINITY_Q7Z1F7_TOXGO/185-703	Q7Z1F7.1 PF00183.16	44.30	0.00
TRINITY_G1N3M5_MELGA/134-215	G1N3M5.1 PF12796.5;	44.30	0.00
TRINITY_A0DE81_PARTE/17-265	A0DE81.1 PF02263.17	44.30	0.00
TRINITY_I0Z9G3_9CHLO/483-760	I0Z9G3.1 PF08324.9;	44.30	0.00
TRINITY_F4Q2G9_DICFS/1228-1485	F4Q2G9.1 PF00069.23	44.30	0.00
TRINITY_D7FVI2_ECTSI/108-188	D7FVI2.1 PF09262.9;	44.30	0.00
TRINITY_A0CDJ1_PARTE/92-164	A0CDJ1.1 PF13499.4;	44.30	0.00
TRINITY_A0CDJ1_PARTE/92-164	A0CDJ1.1 PF13499.4;	44.30	0.00
TRINITY_F1A0Z3_DICPU/486-804	F1A0Z3.1 PF09243.8;	44.30	0.00
TRINITY_B6Q3N7_TALMQ/30-112	B6Q3N7.1 PF13233.4;	44.30	0.00
TRINITY_G0QZF5_ICHMG/394-482	G0QZF5.1 PF01833.22	44.30	0.00
TRINITY_A0A0G4GZH6_9ALVE/419-599	A0A0G4GZH6.1 PF0281	44.30	0.00
TRINITY_D8U1Y2_VOLCA/14-130	D8U1Y2.1 PF00072.22	44.30	0.00
TRINITY_C7RB04_KANKD/13-210	C7RB04.1 PF01596.15	44.30	0.00
TRINITY_L8GN09_ACACA/119-355	L8GN09.1 PF03878.13	44.30	0.00
TRINITY_E1ZCG5_CHLVA/58-447	E1ZCG5.1 PF05536.9;	44.30	0.00
TRINITY_L8GKH0_ACACA/249-578	L8GKH0.1 PF13598.4;	44.30	0.00
TRINITY_J9IZ86_9SPIT/129-645	J9IZ86.1 PF00443.27	44.30	0.00
TRINITY_D8TPG7_VOLCA/13-263	D8TPG7.1 PF00251.18	44.30	0.00
TRINITY_D8T1I5_SELML/13-265	D8T1I5.1 PF07714.15	44.30	0.00
TRINITY_F8AAY3_THEID/8-88	F8AAY3.1 PF00312.20	44.30	0.00
TRINITY_B9Q6X4_TOXGO/138-221	B9Q6X4.1 PF00867.16	44.30	0.00
TRINITY_D7A4Q9_STAND/67-269	D7A4Q9.1 PF07859.11	44.30	0.00
TRINITY_I0YLQ2_9CHLO/4-298	I0YLQ2.1 PF02598.15	44.30	0.00
TRINITY_Q22P47_TETTS/327-477	Q22P47.2 PF16113.3;	44.30	0.00
TRINITY_L1J437_GUITH/102-337	L1J437.1 PF09423.8;	44.30	0.00
TRINITY_D8TNJ1_VOLCA/217-896	D8TNJ1.1 PF04006.10	44.30	0.00
TRINITY_C3Z710_BRAFL/85-154	C3Z710.1 PF00076.20	44.30	0.00
TRINITY_A0A0D3AIM6_BRAOL/116-259	A0A0D3AIM6.1 PF0246	44.30	0.00
TRINITY_K7E0X2_MONDO/56-126	K7E0X2.1 PF13499.4;	44.30	0.00
TRINITY_D8UKR2_VOLCA/77-344	D8UKR2.1 PF10343.7;	44.30	0.00
TRINITY_I0YPY6_9CHLO/7-342	I0YPY6.1 PF01276.18	44.30	0.00
TRINITY_A0A0D2X453_CAPO3/177-285	A0A0D2X453.1 PF0572	44.30	0.00

TRINITY_L8H4X2_ACACA/32-109	L8H4X2.1 PF09696.8;	44.30	0.00
TRINITY_D8TLD5_VOLCA/44-175	D8TLD5.1 PF00293.26	44.30	0.00
TRINITY_F7EZB8_ORNAN/49-170	F7EZB8.1 PF09335.9;	44.30	0.00
TRINITY_C5YLL1_SORBI/161-406	C5YLL1.1 PF07727.12	44.30	0.00
TRINITY_A8I4F2_CHLRE/117-336	A8I4F2.1 PF07714.15	44.30	0.00
TRINITY_E1ZMP9_CHLVA/411-697	E1ZMP9.1 PF12341.6;	44.30	0.00
TRINITY_I1GG91_AMPQE/394-601	I1GG91.1 PF00644.18	44.30	0.00
TRINITY_A8JBE2_CHLRE/96-202	A8JBE2.1 PF14497.4;	44.30	0.00
TRINITY_A9PEF0_POPTR/65-138	A9PEF0.1 PF02201.16	44.30	0.00
TRINITY_E4XGI0_OIKDI/237-305	E4XGI0.1 PF00076.20	44.30	0.00
TRINITY_F4NSG0_BATDJ/183-312	F4NSG0.1 PF03188.14	44.30	0.00
TRINITY_A0A078ALS8_STYLE/1012-1418	A0A078ALS8.1 PF0839	44.30	0.00
TRINITY_J9F1H9_9SPIT/12-116	J9F1H9.1 PF02991.14	44.20	0.00
TRINITY_I7MJT4_TETTS/15-283	I7MJT4.1 PF01073.17	44.20	0.00
TRINITY_L9KU30_TUPCH/43-263	L9KU30.1 PF09749.7;	44.20	0.00
TRINITY_A0A068RQS6_9FUNG/55-131	A0A068RQS6.1 PF0313	44.20	0.00
TRINITY_A0A087XW94_POEFO/6-94	A0A087XW94.2 PF0003	44.20	0.00
TRINITY_Q23ZD1_TETTS/86-338	Q23ZD1.2 PF00566.16	44.20	0.00
TRINITY_I7LZV2_TETTS/151-413	I7LZV2.1 PF00069.23	44.20	0.00
TRINITY_W5LBQ2_ASTMX/1-271	W5LBQ2.1 PF08433.8;	44.20	0.00
TRINITY_A0CJL8_PARTE/108-301	A0CJL8.1 PF13901.4;	44.20	0.00
TRINITY_Q6LH92_PHOPR/8-198	Q6LH92.1 PF01734.20	44.20	0.00
TRINITY_Q24HR9_TETTS/46-305	Q24HR9.2 PF00069.23	44.20	0.00
TRINITY_G4YL70_PHYSP/103-480	G4YL70.1 PF00443.27	44.20	0.00
TRINITY_B2GPU0_DANRE/4-371	B2GPU0.1 PF03223.13	44.20	0.00
TRINITY_G2E9J5_9FLAO/29-146	G2E9J5.1 PF08240.10	44.20	0.00
TRINITY_A0CDL7_PARTE/665-974	A0CDL7.1 PF00069.23	44.20	0.00
TRINITY_A8ZWM7_DESOH/2-194	A8ZWM7.1 PF00106.23	44.20	0.00
TRINITY_A0DPD0_PARTE/462-730	A0DPD0.1 PF00149.26	44.20	0.00
TRINITY_M0Z034_HORVD/47-150	M0Z034.1 PF00011.19	44.20	0.00
TRINITY_F0YAI9_AURAN/4-240	F0YAI9.1 PF00149.26	44.20	0.00
TRINITY_A0CKQ7_PARTE/20-273	A0CKQ7.1 PF00069.23	44.20	0.00
TRINITY_D7G7A9_ECTSI/222-420	D7G7A9.1 PF01545.19	44.20	0.00
TRINITY_D3BRR7_POLPA/130-385	D3BRR7.1 PF07714.15	44.20	0.00
TRINITY_A8IYP4_CHLRE/38-237	A8IYP4.1 PF00485.16	44.20	0.00
TRINITY_K1S6R2_CRAGI/67-193	K1S6R2.1 PF00583.23	44.20	0.00
TRINITY_D8TZ78_VOLCA/416-537	D8TZ78.1 PF01544.16	44.20	0.00
TRINITY_A0A0D2VSI5_CAPO3/35-145	A0A0D2VSI5.1 PF0848	44.20	0.00
TRINITY_E0CV79_VITVI/40-388	E0CV79.1 PF00856.26	44.20	0.00
TRINITY_A0A0D2UHK5_GOSRA/5-168	A0A0D2UHK5.1 PF0346	44.20	0.00
TRINITY_D4GLL5_PANAM/12-267	D4GLL5.1 PF00899.19	44.20	0.00
TRINITY_C1DZ03_MICSR/595-1008	C1DZ03.1 PF04357.11	44.20	0.00
TRINITY_A8J491_CHLRE/1-108	A8J491.1 PF03969.14	44.20	0.00
TRINITY_I0YPY7_9CHLO/17-104	I0YPY7.1 PF04488.13	44.20	0.00
TRINITY_I7MAX8_TETTS/82-258	I7MAX8.2 PF04727.11	44.20	0.00
TRINITY_I7M4C2_TETTS/42-276	I7M4C2.1 PF00149.26	44.20	0.00
TRINITY_W7X142_TETTS/122-276	W7X142.1 PF00650.18	44.20	0.00
TRINITY_A0E2A8_PARTE/308-600	A0E2A8.1 PF13243.4;	44.20	0.00
TRINITY_F4Q7J1_DICFS/156-472	F4Q7J1.1 PF00728.20	44.20	0.00
TRINITY_J9FZ76_9SPIT/15-176	J9FZ76.1 PF00071.20	44.20	0.00
TRINITY_D8U516_VOLCA/39-285	D8U516.1 PF03407.14	44.20	0.00
TRINITY_D8TTP6_VOLCA/535-692	D8TTP6.1 PF08646.8;	44.20	0.00
TRINITY_A8J0K8_CHLRE/1552-1659	A8J0K8.1 PF00651.29	44.20	0.00
TRINITY_A8ITN3_CHLRE/83-328	A8ITN3.1 PF00233.17	44.20	0.00
TRINITY_D8U645_VOLCA/3-62	D8U645.1 PF06624.10	44.20	0.00
TRINITY_A8HQH0_CHLRE/38-357	A8HQH0.1 PF08627.8;	44.20	0.00
TRINITY_E1Z4T6_CHLVA/749-1168	E1Z4T6.1 PF08326.10	44.20	0.00
TRINITY_D7L4R4_ARALL/68-330	D7L4R4.1 PF00069.23	44.20	0.00
TRINITY_F4Q620_DICFS/392-441	F4Q620.1 PF00415.16	44.20	0.00
TRINITY_D2V5F0_NAEGR/143-242	D2V5F0.1 PF13640.4;	44.20	0.00
TRINITY_A0A087SS49_AUXPR/653-766	A0A087SS49.1 PF0877	44.20	0.00

TRINITY_A8IKA4_CHLRE/2156-2500	A8IKA4.1 PF00443.27	44.20	0.00
TRINITY_L8GS17_ACACA/603-781	L8GS17.1 PF00617.17	44.20	0.00
TRINITY_T0Q7D4_9STRA/138-238	T0Q7D4.1 PF12763.5;	44.20	0.00
TRINITY_A0CL09_PARTE/55-165	A0CL09.1 PF12796.5;	44.20	0.00
TRINITY_D8TKH0_VOLCA/161-515	D8TKH0.1 PF01103.21	44.20	0.00
TRINITY_H3CX28_TETNG/29-156	H3CX28.1 PF00134.21	44.20	0.00
TRINITY_I7M2I7_TETTS/1351-1566	I7M2I7.1 PF00566.16	44.20	0.00
TRINITY_D8TNZ3_VOLCA/555-849	D8TNZ3.1 PF03851.12	44.20	0.00
TRINITY_A0A067FRR0_CITSI/37-121	A0A067FRR0.1 PF1017	44.20	0.00
TRINITY_D8TIE8_VOLCA/640-737	D8TIE8.1 PF02806.16	44.20	0.00
TRINITY_J9JCA5_9SPIT/125-339	J9JCA5.1 PF00112.21	44.20	0.00
TRINITY_A8X9K0_CAEBR/3-166	A8X9K0.2 PF00091.23	44.20	0.00
TRINITY_L0J865_MYCSM/7-158	L0J865.1 PF08768.9;	44.10	0.00
TRINITY_A0BRA8_PARTE/7-108	A0BRA8.1 PF00168.28	44.10	0.00
TRINITY_K9XSY7_STAC7/103-215	K9XSY7.1 PF01841.17	44.10	0.00
TRINITY_Q23G56_TETTS/110-197	Q23G56.2 PF00153.25	44.10	0.00
TRINITY_A0A078EB35_BRANA/617-822	A0A078EB35.1 PF0000	44.10	0.00
TRINITY_M5FWE7_DACSP/103-196	M5FWE7.1 PF07818.11	44.10	0.00
TRINITY_F4Q5E1_DICFS/42-384	F4Q5E1.1 PF00150.16	44.10	0.00
TRINITY_L8GZ24_ACACA/23-205	L8GZ24.1 PF03962.13	44.10	0.00
TRINITY_K1PZ87_CRAGI/39-172	K1PZ87.1 PF00782.18	44.10	0.00
TRINITY_J9HMP7_9SPIT/7-124	J9HMP7.1 PF13815.4;	44.10	0.00
TRINITY_Q6MAQ5_PARUW/27-167	Q6MAQ5.1 PF06962.10	44.10	0.00
TRINITY_A0A0G4IJE1_PLABS/7-102	A0A0G4IJE1.1 PF0141	44.10	0.00
TRINITY_L1J9H4_GUITH/347-522	L1J9H4.1 PF13870.4;	44.10	0.00
TRINITY_D8U3A0_VOLCA/3-209	D8U3A0.1 PF03665.11	44.10	0.00
TRINITY_K1QM54_CRAGI/222-338	K1QM54.1 PF08327.9;	44.10	0.00
TRINITY_A0DSK2_PARTE/44-329	A0DSK2.1 PF01459.20	44.10	0.00
TRINITY_C1MSG6_MICPC/3-207	C1MSG6.1 PF00069.23	44.10	0.00
TRINITY_E1Z5P1_CHLVA/131-317	E1Z5P1.1 PF07714.15	44.10	0.00
TRINITY_E1Z5T3_CHLVA/338-498	E1Z5T3.1 PF01734.20	44.10	0.00
TRINITY_G4M023_SCHMA/13-385	G4M023.1 PF01433.18	44.10	0.00
TRINITY_F0ZPC9_DICPU/681-926	F0ZPC9.1 PF08427.8;	44.10	0.00
TRINITY_M8AXH1_TRIUA/124-183	M8AXH1.1 PF13418.4;	44.10	0.00
TRINITY_K1QD00_CRAGI/691-770	K1QD00.1 PF12796.5;	44.10	0.00
TRINITY_I7LUV4_TETTS/6-94	I7LUV4.2 PF00855.15	44.10	0.00
TRINITY_S5VR79_STRC3/295-393	S5VR79.1 PF01551.20	44.10	0.00
TRINITY_C8VR19_EMENI/36-151	C8VR19.1 PF01571.19	44.10	0.00
TRINITY_D8TKD3_VOLCA/49-222	D8TKD3.1 PF04832.10	44.10	0.00
TRINITY_U9SRI9_RHIID/123-267	U9SRI9.1 PF05188.15	44.10	0.00
TRINITY_A8I8P0_CHLRE/185-745	A8I8P0.1 PF10191.7;	44.10	0.00
TRINITY_G0R643_ICHMG/19-285	G0R643.1 PF02263.17	44.10	0.00
TRINITY_G0R5L7_ICHMG/165-390	G0R5L7.1 PF12146.6;	44.10	0.00
TRINITY_D8SQH8_SELML/67-214	D8SQH8.1 PF04073.13	44.10	0.00
TRINITY_I3JQL1_ORENI/184-358	I3JQL1.1 PF07786.10	44.10	0.00
TRINITY_D8UK64_VOLCA/8-471	D8UK64.1 PF13520.4;	44.10	0.00
TRINITY_R7YJT1_CONA1/48-355	R7YJT1.1 PF00264.18	44.10	0.00
TRINITY_A8J8Y6_CHLRE/19-396	A8J8Y6.1 PF16113.3;	44.10	0.00
TRINITY_H2W2V9_CAEJA/120-371	H2W2V9.1 PF07714.15	44.10	0.00
TRINITY_A0BQW0_PARTE/27-209	A0BQW0.1 PF03357.19	44.10	0.00
TRINITY_G0QJM7_ICHMG/14-137	G0QJM7.1 PF01042.19	44.10	0.00
TRINITY_W2PQT4_PHYPN/122-348	W2PQT4.1 PF00667.18	44.10	0.00
TRINITY_L8GL94_ACACA/389-614	L8GL94.1 PF13672.4;	44.10	0.00
TRINITY_A8HT69_CHLRE/689-903	A8HT69.1 PF07569.9;	44.10	0.00
TRINITY_K3X3N5_PYTUL/83-154	K3X3N5.1 PF03937.14	44.10	0.00
TRINITY_A8HPX0_CHLRE/136-242	A8HPX0.1 PF07818.11	44.10	0.00
TRINITY_D8TNI3_VOLCA/179-415	D8TNI3.1 PF00251.18	44.10	0.00
TRINITY_X1WIT1_ACYPI/8-221	X1WIT1.1 PF01135.17	44.10	0.00
TRINITY_L0A9E0_DEIPD/46-181	L0A9E0.1 PF09346.8;	44.10	0.00
TRINITY_V4AHX0_LOTGI/87-328	V4AHX0.1 PF08627.8;	44.10	0.00
TRINITY_D3B9V6_POLPA/831-1080	D3B9V6.1 PF07714.15	44.10	0.00

TRINITY_F0ZFB5_DICPU/246-376	F0ZFB5.1 PF01529.18	44.10	0.00
TRINITY_I7MG17_TETTS/55-123	I7MG17.2 PF05198.14	44.10	0.00
TRINITY_G8T7W2_NIAKG/266-324	G8T7W2.1 PF13415.4;	44.10	0.00
TRINITY_H9GKH8_ANOCA/45-119	H9GKH8.1 PF01883.17	44.10	0.00
TRINITY_D8TS72_VOLCA/114-369	D8TS72.1 PF10343.7;	44.10	0.00
TRINITY_B4W5E8_9CYAN/32-542	B4W5E8.1 PF03098.13	44.10	0.00
TRINITY_D3BIK5_POLPA/674-1142	D3BIK5.1 PF01055.24	44.10	0.00
TRINITY_A0A067KN01_JATCU/413-773	A0A067KN01.1 PF0070	44.10	0.00
TRINITY_A0C3K9_PARTE/12-186	A0C3K9.1 PF01885.14	44.10	0.00
TRINITY_W5KHN2_ASTMX/504-686	W5KHN2.1 PF02145.13	44.10	0.00
TRINITY_Q22D68_TETTS/29-277	Q22D68.3 PF03803.13	44.10	0.00
TRINITY_F6RBW8_CIOIN/26-130	F6RBW8.2 PF00085.18	44.10	0.00
TRINITY_A0BCB8_PARTE/4-136	A0BCB8.1 PF08613.9;	44.10	0.00
TRINITY_D5GLZ2_TUBMM/4-146	D5GLZ2.1 PF00106.23	44.10	0.00
TRINITY_J9EF05_9SPIT/90-238	J9EF05.1 PF01694.20	44.10	0.00
TRINITY_A8JFH1_CHLRE/93-580	A8JFH1.1 PF00759.17	44.10	0.00
TRINITY_G0QK23_ICHMG/3-104	G0QK23.1 PF00168.28	44.10	0.00
TRINITY_I7MJT4_TETTS/15-283	I7MJT4.1 PF01073.17	44.00	0.00
TRINITY_Q23Q47_TETTS/4-162	Q23Q47.2 PF01652.16	44.00	0.00
TRINITY_A0A0F3L1G8_9GAMM/75-177	A0A0F3L1G8.1 PF0419	44.00	0.00
TRINITY_Q22A65_TETTS/524-741	Q22A65.2 PF01612.18	44.00	0.00
TRINITY_S2J8G5_MUCC1/102-425	S2J8G5.1 PF00176.21	44.00	0.00
TRINITY_F7DT46_MONDO/46-198	F7DT46.2 PF13847.4;	44.00	0.00
TRINITY_A0A023BA03_GRENI/11-172	A0A023BA03.1 PF0007	44.00	0.00
TRINITY_I1FTI4_AMPQE/232-479	I1FTI4.1 PF07714.15	44.00	0.00
TRINITY_A0A077ZQS0_STYLE/68-262	A0A077ZQS0.1 PF1214	44.00	0.00
TRINITY_C3YJZ7_BRAFL/25-191	C3YJZ7.1 PF08740.9;	44.00	0.00
TRINITY_A0A0L7LG53_9NEOP/24-167	A0A0L7LG53.1 PF0146	44.00	0.00
TRINITY_L8H1V7_ACACA/826-948	L8H1V7.1 PF00307.29	44.00	0.00
TRINITY_Q23QF2_TETTS/248-571	Q23QF2.2 PF03133.13	44.00	0.00
TRINITY_A8HQS2_CHLRE/22-83	A8HQS2.1 PF02953.13	44.00	0.00
TRINITY_A9V005_MONBE/102-190	A9V005.1 PF00153.25	44.00	0.00
TRINITY_D3BE01_POLPA/226-333	D3BE01.1 PF04410.12	44.00	0.00
TRINITY_Q23QF2_TETTS/248-571	Q23QF2.2 PF03133.13	44.00	0.00
TRINITY_A8JFV4_CHLRE/12-120	A8JFV4.1 PF03226.12	44.00	0.00
TRINITY_A0A075AVX5_9FUNG/177-435	A0A075AVX5.1 PF1030	44.00	0.00
TRINITY_D2V003_NAEGR/1-93	D2V003.1 PF00169.27	44.00	0.00
TRINITY_F2U0C9_SALR5/32-247	F2U0C9.1 PF04493.12	44.00	0.00
TRINITY_A0BNN4_PARTE/64-327	A0BNN4.1 PF00520.29	44.00	0.00
TRINITY_L8H844_ACACA/109-517	L8H844.1 PF00909.19	44.00	0.00
TRINITY_D3BB70_POLPA/28-221	D3BB70.1 PF02902.17	44.00	0.00
TRINITY_G0R585_ICHMG/1209-1569	G0R585.1 PF12777.5;	44.00	0.00
TRINITY_D8UI53_VOLCA/138-281	D8UI53.1 PF00153.25	44.00	0.00
TRINITY_A0A087SM36_AUXPR/9-296	A0A087SM36.1 PF0315	44.00	0.00
TRINITY_A0A0K3AYF1_CAEEL/1-306	A0A0K3AYF1.1 PF0056	44.00	0.00
TRINITY_A0A078AV48_STYLE/86-356	A0A078AV48.1 PF0012	44.00	0.00
TRINITY_F0ZHQ8_DICPU/126-336	F0ZHQ8.1 PF04757.12	44.00	0.00
TRINITY_A4RU80_OSTLU/392-595	A4RU80.1 PF01612.18	44.00	0.00
TRINITY_PHEA_AQUAE/93-270	O67085.1 PF00800.16	44.00	0.00
TRINITY_L8GCE7_ACACA/328-543	L8GCE7.1 PF13086.4;	44.00	0.00
TRINITY_D8TTZ8_VOLCA/6-184	D8TTZ8.1 PF00025.19	44.00	0.00
TRINITY_B4HHW0_DROSE/31-815	B4HHW0.1 PF16399.3;	44.00	0.00
TRINITY_D8U8X5_VOLCA/76-184	D8U8X5.1 PF14924.4;	44.00	0.00
TRINITY_A8HP02_CHLRE/163-262	A8HP02.1 PF07707.13	44.00	0.00
TRINITY_J9IAJ3_9SPIT/25-140	J9IAJ3.1 PF01966.20	44.00	0.00
TRINITY_L8H2D7_ACACA/248-333	L8H2D7.1 PF02761.12	44.00	0.00
TRINITY_B0C469_ACAM1/438-625	B0C469.1 PF13191.4;	44.00	0.00
TRINITY_Q7YZQ8_BABBO/9-171	Q7YZQ8.1 PF00071.20	44.00	0.00
TRINITY_F1A0P0_DICPU/1-267	F1A0P0.1 PF03619.14	44.00	0.00
TRINITY_A0DTT7_PARTE/327-627	A0DTT7.1 PF00069.23	44.00	0.00
TRINITY_E1Z213_CHLVA/62-322	E1Z213.1 PF09353.8;	44.00	0.00

TRINITY_L8H4T2_ACACA/305-409	L8H4T2.1 PF03792.11	44.00	0.00
TRINITY_D2B907_STRRD/6-123	D2B907.1 PF00581.18	44.00	0.00
TRINITY_F0ZI20_DICPU/3-217	F0ZI20.1 PF00091.23	44.00	0.00
TRINITY_C1EFZ5_MICSR/3-109	C1EFZ5.1 PF00085.18	44.00	0.00
TRINITY_L8H7R1_ACACA/43-388	L8H7R1.1 PF00224.19	44.00	0.00
TRINITY_A0C764_PARTE/28-250	A0C764.1 PF00378.18	44.00	0.00
TRINITY_F4Q5R2_DICFS/72-228	F4Q5R2.1 PF01652.16	44.00	0.00
TRINITY_A8JGZ2_CHLRE/8-91	A8JGZ2.1 PF04110.11	44.00	0.00
TRINITY_A7RXI9_NEMVE/46-278	A7RXI9.1 PF00102.25	44.00	0.00
TRINITY_A8JIU8_CHLRE/35-321	A8JIU8.1 PF00520.29	44.00	0.00
TRINITY_F1A2T0_DICPU/396-578	F1A2T0.1 PF01369.18	44.00	0.00
TRINITY_A0A078AJI8_STYLE/155-505	A0A078AJI8.1 PF1386	44.00	0.00
TRINITY_L8H3C1_ACACA/447-563	L8H3C1.1 PF00651.29	44.00	0.00
TRINITY_B3SBW1_TRIAD/8-182	B3SBW1.1 PF15469.4;	44.00	0.00
TRINITY_NRAM1_DICDI/94-450	Q869V1.1 PF01566.16	44.00	0.00
TRINITY_L8GF84_ACACA/748-1120	L8GF84.1 PF02259.21	44.00	0.00
TRINITY_L8GFU2_ACACA/577-721	L8GFU2.1 PF00644.18	44.00	0.00
TRINITY_A0CV13_PARTE/21-225	A0CV13.1 PF05013.10	44.00	0.00
TRINITY_D8U7M2_VOLCA/12-126	D8U7M2.1 PF00651.29	44.00	0.00
TRINITY_D2V9M8_NAEGR/13-146	D2V9M8.1 PF00782.18	44.00	0.00
TRINITY_D8U5T7_VOLCA/76-294	D8U5T7.1 PF01036.16	44.00	0.00
TRINITY_I0YLN8_9CHLO/172-489	I0YLN8.1 PF11735.6;	44.00	0.00
TRINITY_D8UIN9_VOLCA/1371-1729	D8UIN9.1 PF00224.19	44.00	0.00
TRINITY_A0CB62_PARTE/773-896	A0CB62.1 PF00072.22	44.00	0.00
TRINITY_Q248F0_TETTS/19-502	Q248F0.2 PF04916.11	43.90	0.00
TRINITY_L8H300_ACACA/1-341	L8H300.1 PF12722.5;	43.90	0.00
TRINITY_A0A015JZN3_9GLOM/168-528	A0A015JZN3.1 PF0529	43.90	0.00
TRINITY_Q23PV4_TETTS/274-435	Q23PV4.2 PF00441.22	43.90	0.00
TRINITY_D3BK07_POLPA/84-175	D3BK07.1 PF02214.20	43.90	0.00
TRINITY_TRM6_DICDI/34-336	Q54UB1.1 PF04189.11	43.90	0.00
TRINITY_G0QQ97_ICHMG/55-152	G0QQ97.1 PF12894.5;	43.90	0.00
TRINITY_A0C9T6_PARTE/339-409	A0C9T6.1 PF12872.5;	43.90	0.00
TRINITY_A8P6D5_COPC7/41-435	A8P6D5.1 PF00999.19	43.90	0.00
TRINITY_T1KM47_TETUR/561-617	T1KM47.1 PF00412.20	43.90	0.00
TRINITY_D3BCX7_POLPA/108-185	D3BCX7.1 PF07258.12	43.90	0.00
TRINITY_M0TYP2_MUSAM/266-514	M0TYP2.1 PF07714.15	43.90	0.00
TRINITY_L7UF69_MYXSD/314-383	L7UF69.1 PF00801.18	43.90	0.00
TRINITY_D3AY17_POLPA/19-125	D3AY17.1 PF00568.21	43.90	0.00
TRINITY_I7MJ92_TETTS/146-409	I7MJ92.1 PF00069.23	43.90	0.00
TRINITY_Q22S98_TETTS/953-1159	Q22S98.1 PF01852.17	43.90	0.00
TRINITY_K4C3Y3_SOLLC/332-392	K4C3Y3.1 PF13620.4;	43.90	0.00
TRINITY_L8GNQ8_ACACA/1-226	L8GNQ8.1 PF03009.15	43.90	0.00
TRINITY_A0A0A1NTH4_9FUNG/533-645	A0A0A1NTH4.1 PF0027	43.90	0.00
TRINITY_L8H271_ACACA/165-267	L8H271.1 PF00168.28	43.90	0.00
TRINITY_Q233Z3_TETTS/947-1211	Q233Z3.2 PF00069.23	43.90	0.00
TRINITY_A8ZYB6_DESOH/9-118	A8ZYB6.1 PF13911.4;	43.90	0.00
TRINITY_Q23QF2_TETTS/248-571	Q23QF2.2 PF03133.13	43.90	0.00
TRINITY_I7M4B7_TETTS/191-499	I7M4B7.1 PF00122.18	43.90	0.00
TRINITY_D8U4G0_VOLCA/11-274	D8U4G0.1 PF04997.10	43.90	0.00
TRINITY_L8HI63_ACACA/82-197	L8HI63.1 PF04178.10	43.90	0.00
TRINITY_Q54DL9_DICDI/22-88	Q54DL9.1 PF01176.17	43.90	0.00
TRINITY_A8IYC0_CHLRE/10-303	A8IYC0.1 PF02274.15	43.90	0.00
TRINITY_I0YKL6_9CHLO/50-293	I0YKL6.1 PF13621.4;	43.90	0.00
TRINITY_Q23CN1_TETTS/13-195	Q23CN1.1 PF01885.14	43.90	0.00
TRINITY_I0Z552_9CHLO/14-94	I0Z552.1 PF00076.20	43.90	0.00
TRINITY_I0Z4D9_9CHLO/46-281	I0Z4D9.1 PF00149.26	43.90	0.00
TRINITY_A0A067SDA6_9AGAR/22-283	A0A067SDA6.1 PF0006	43.90	0.00
TRINITY_Q22D68_TETTS/29-277	Q22D68.3 PF03803.13	43.90	0.00
TRINITY_Q24HP0_TETTS/705-821	Q24HP0.2 PF02518.24	43.90	0.00
TRINITY_L8HBF7_ACACA/234-428	L8HBF7.1 PF02958.18	43.90	0.00
TRINITY_R1FX63_EMIHU/5-180	R1FX63.1 PF00929.22	43.90	0.00

TRINITY_A0A0A1NIL7_9FUNG/11-80	A0A0A1NIL7.1 PF0496	43.90	0.00
TRINITY_D2VNG5_NAEGR/186-510	D2VNG5.1 PF00728.20	43.90	0.00
TRINITY_A0BWQ1_PARTE/195-370	A0BWQ1.1 PF13848.4;	43.90	0.00
TRINITY_A8JGL3_CHLRE/53-302	A8JGL3.1 PF01370.19	43.90	0.00
TRINITY_A8J2Q9_CHLRE/69-602	A8J2Q9.1 PF03055.13	43.90	0.00
TRINITY_A0A078EB35_BRANA/617-822	A0A078EB35.1 PF0000	43.90	0.00
TRINITY_L8GPT6_ACACA/118-448	L8GPT6.1 PF00476.18	43.90	0.00
TRINITY_A0A0G4ELM0_9ALVE/32-167	A0A0G4ELM0.1 PF0861	43.90	0.00
TRINITY_D4ZL63_SHEVD/387-830	D4ZL63.1 PF00305.17	43.90	0.00
TRINITY_C3XWS0_BRAFL/2-272	C3XWS0.1 PF00282.17	43.90	0.00
TRINITY_V7BWA6_PHAVU/189-252	V7BWA6.1 PF00097.23	43.90	0.00
TRINITY_A8JCA5_CHLRE/46-396	A8JCA5.1 PF13868.4;	43.90	0.00
TRINITY_A8JFT4_CHLRE/24-412	A8JFT4.1 PF00162.17	43.90	0.00
TRINITY_E1Z9L2_CHLVA/59-244	E1Z9L2.1 PF00350.21	43.90	0.00
TRINITY_B8ALW3_ORYSI/150-251	B8ALW3.1 PF13640.4;	43.90	0.00
TRINITY_B3L869_PLAKH/24-131	B3L869.1 PF00085.18	43.90	0.00
TRINITY_I0YYC7_9CHLO/212-512	I0YYC7.1 PF00176.21	43.90	0.00
TRINITY_A7RHN8_NEMVE/134-237	A7RHN8.1 PF00307.29	43.90	0.00
TRINITY_K2E0Y4_9BACT/13-145	K2E0Y4.1 PF00390.17	43.90	0.00
TRINITY_Q22YA0_TETTS/3-273	Q22YA0.1 PF09743.7;	43.80	0.00
TRINITY_J9J104_9SPIT/9-266	J9J104.1 PF00069.23	43.80	0.00
TRINITY_A0A0D2UG24_GOSRA/94-323	A0A0D2UG24.1 PF0079	43.80	0.00
TRINITY_A0A0A8DUL9_9XANT/87-252	A0A0A8DUL9.1 PF1347	43.80	0.00
TRINITY_A0A0G4FL24_9ALVE/291-470	A0A0G4FL24.1 PF0040	43.80	0.00
TRINITY_X6NSF5_RETFI/88-345	X6NSF5.1 PF07714.15	43.80	0.00
TRINITY_Q23AP5_TETTS/32-425	Q23AP5.1 PF03345.12	43.80	0.00
TRINITY_Q5ALK5_CANAL/697-979	Q5ALK5.1 PF00702.24	43.80	0.00
TRINITY_D2A1W2_TRICA/266-553	D2A1W2.1 PF00702.24	43.80	0.00
TRINITY_Q22UG5_TETTS/31-658	Q22UG5.2 PF05786.12	43.80	0.00
TRINITY_F1A199_DICPU/421-797	F1A199.1 PF00176.21	43.80	0.00
TRINITY_A0A024UAH6_9STRA/9-297	A0A024UAH6.1 PF0048	43.80	0.00
TRINITY_SYHM_DICDI/61-389	Q55D12.1 PF13393.4;	43.80	0.00
TRINITY_B3RNY6_TRIAD/11-248	B3RNY6.1 PF02274.15	43.80	0.00
TRINITY_I7MFM5_TETTS/56-201	I7MFM5.1 PF01699.22	43.80	0.00
TRINITY_Q240L9_TETTS/677-744	Q240L9.2 PF00512.23	43.80	0.00
TRINITY_U3K5A8_FICAL/227-675	U3K5A8.1 PF01432.18	43.80	0.00
TRINITY_Q22V58_TETTS/214-314	Q22V58.2 PF00153.25	43.80	0.00
TRINITY_A6GAH4_9DELT/4-198	A6GAH4.1 PF00106.23	43.80	0.00
TRINITY_L8HFX0_ACACA/26-189	L8HFX0.1 PF09174.8;	43.80	0.00
TRINITY_A0A078AZ95_STYLE/187-469	A0A078AZ95.1 PF0471	43.80	0.00
TRINITY_F4Q2G9_DICFS/1228-1485	F4Q2G9.1 PF00069.23	43.80	0.00
TRINITY_G1N8F2_MELGA/80-381	G1N8F2.2 PF00561.18	43.80	0.00
TRINITY_G3P6H1_GASAC/86-386	G3P6H1.1 PF00561.18	43.80	0.00
TRINITY_D8QYJ4_SELML/155-291	D8QYJ4.1 PF00107.24	43.80	0.00
TRINITY_Q23KB0_TETTS/73-246	Q23KB0.4 PF03357.19	43.80	0.00
TRINITY_A0A022QX58_ERYGU/131-260	A0A022QX58.1 PF0152	43.80	0.00
TRINITY_A0A0L9T1J1_9HYPO/202-254	A0A0L9T1J1.1 PF0038	43.80	0.00
TRINITY_L1JFR0_GUITH/69-276	L1JFR0.1 PF12146.6;	43.80	0.00
TRINITY_I1Q599_ORYGL/52-383	I1Q599.1 PF10186.7;	43.80	0.00
TRINITY_A0A0A1T1D1_9HYPO/1278-1421	A0A0A1T1D1.1 PF0000	43.80	0.00
TRINITY_F1QWW8_DANRE/60-259	F1QWW8.1 PF00106.23	43.80	0.00
TRINITY_L8H8Z5_ACACA/146-294	L8H8Z5.1 PF13358.4;	43.80	0.00
TRINITY_A0A078B3T1_STYLE/585-748	A0A078B3T1.1 PF0570	43.80	0.00
TRINITY_Q233Z6_TETTS/6-165	Q233Z6.1 PF00106.23	43.80	0.00
TRINITY_A0BPW6_PARTE/424-1020	A0BPW6.1 PF00443.27	43.80	0.00
TRINITY_W1QAL9_OGAPD/40-114	W1QAL9.1 PF00364.20	43.80	0.00
TRINITY_A0BGZ6_PARTE/125-266	A0BGZ6.1 PF01467.24	43.80	0.00
TRINITY_D3BT06_POLPA/532-628	D3BT06.1 PF16979.3;	43.80	0.00
TRINITY_D8TZ14_VOLCA/40-253	D8TZ14.1 PF02230.14	43.80	0.00
TRINITY_C3YP19_BRAFL/2-357	C3YP19.1 PF04869.12	43.80	0.00
TRINITY_G0QTZ1_ICHMG/40-256	G0QTZ1.1 PF03731.13	43.80	0.00

TRINITY_A0BIF4_PARTE/119-382	A0BIF4.1 PF00069.23	43.80	0.00
TRINITY_A8IIM2_CHLRE/41-762	A8IIM2.1 PF05879.10	43.80	0.00
TRINITY_K3W600_PYTUL/2-431	K3W600.1 PF04188.11	43.80	0.00
TRINITY_F0ZI24_DICPU/28-214	F0ZI24.1 PF01183.18	43.80	0.00
TRINITY_A0CJY8_PARTE/436-698	A0CJY8.1 PF00069.23	43.80	0.00
TRINITY_I1GJ64_AMPQE/75-338	I1GJ64.1 PF00657.20	43.80	0.00
TRINITY_H3GJI2_PHYRM/6-206	H3GJI2.1 PF05997.10	43.80	0.00
TRINITY_F4QEP8_DICFS/752-1151	F4QEP8.1 PF00176.21	43.80	0.00
TRINITY_Q54L33_DICDI/4881-5216	Q54L33.1 PF00632.23	43.80	0.00
TRINITY_L8H624_ACACA/41-114	L8H624.1 PF09341.8;	43.80	0.00
TRINITY_D8TS96_VOLCA/560-714	D8TS96.1 PF16213.3;	43.80	0.00
TRINITY_I7MDF9_TETTS/292-610	I7MDF9.1 PF04515.10	43.80	0.00
TRINITY_D8U9V5_VOLCA/49-396	D8U9V5.1 PF03124.12	43.80	0.00
TRINITY_I7MHB1_TETTS/6-102	I7MHB1.2 PF03259.15	43.80	0.00
TRINITY_F0ZQK5_DICPU/35-293	F0ZQK5.1 PF12850.5;	43.80	0.00
TRINITY_I0JTM3_HALH3/9-148	I0JTM3.1 PF13302.5;	43.80	0.00
TRINITY_Q6F356_ORYSJ/774-1019	Q6F356.1 PF07727.12	43.80	0.00
TRINITY_C1MIV1_MICPC/248-356	C1MIV1.1 PF06325.11	43.80	0.00
TRINITY_I7LZV2_TETTS/151-413	I7LZV2.1 PF00069.23	43.80	0.00
TRINITY_Q6LH92_PHOPR/8-198	Q6LH92.1 PF01734.20	43.80	0.00
TRINITY_W2R8H4_PHYPN/131-359	W2R8H4.1 PF01633.18	43.80	0.00
TRINITY_L1IW60_GUITH/398-496	L1IW60.1 PF07801.9;	43.80	0.00
TRINITY_A8I728_CHLRE/54-170	A8I728.1 PF13537.4;	43.80	0.00
TRINITY_T0QVQ8_9STRA/216-463	T0QVQ8.1 PF07714.15	43.80	0.00
TRINITY_J9FBN2_9SPIT/564-662	J9FBN2.1 PF08647.9;	43.80	0.00
TRINITY_L8HA43_ACACA/8-290	L8HA43.1 PF00704.26	43.80	0.00
TRINITY_A0A0C2I723_THEKT/9-170	A0A0C2I723.1 PF0007	43.80	0.00
TRINITY_E1ZTC2_CHLVA/158-607	E1ZTC2.1 PF01425.19	43.80	0.00
TRINITY_A0A0G4EYN0_9ALVE/80-431	A0A0G4EYN0.1 PF0862	43.80	0.00
TRINITY_I7M2H5_TETTS/15-113	I7M2H5.1 PF00153.25	43.80	0.00
TRINITY_I0Z4C8_9CHLO/210-521	I0Z4C8.1 PF16858.3;	43.80	0.00
TRINITY_T4ZYL3_OPHSC/1844-1962	T4ZYL3.1 PF00072.22	43.80	0.00
TRINITY_K4IP70_PSYTT/5-127	K4IP70.1 PF14108.4;	43.80	0.00
TRINITY_I7MJI0_TETTS/1-168	I7MJI0.1 PF14580.4;	43.80	0.00
TRINITY_Q23QF2_TETTS/248-571	Q23QF2.2 PF03133.13	43.80	0.00
TRINITY_F0ZG89_DICPU/62-135	F0ZG89.1 PF00498.24	43.70	0.00
TRINITY_Q22KF0_TETTS/10-184	Q22KF0.2 PF00071.20	43.70	0.00
TRINITY_C9JUS5_HUMAN/5-152	C9JUS5.2 PF00780.20	43.70	0.00
TRINITY_H3DIR9_TETNG/11-126	H3DIR9.1 PF10639.7;	43.70	0.00
TRINITY_A0BIV9_PARTE/3-201	A0BIV9.1 PF01956.14	43.70	0.00
TRINITY_W7XGH2_TETTS/51-249	W7XGH2.1 PF00856.26	43.70	0.00
TRINITY_A0A0D2S9F0_GOSRA/348-509	A0A0D2S9F0.1 PF0391	43.70	0.00
TRINITY_A0CV13_PARTE/21-225	A0CV13.1 PF05013.10	43.70	0.00
TRINITY_I7MGN0_TETTS/409-544	I7MGN0.2 PF09273.9;	43.70	0.00
TRINITY_G1KGL2_ANOCA/1108-1607	G1KGL2.2 PF06920.11	43.70	0.00
TRINITY_C3YLA6_BRAFL/5-96	C3YLA6.1 PF02214.20	43.70	0.00
TRINITY_S2K500_MUCC1/52-292	S2K500.1 PF06644.9;	43.70	0.00
TRINITY_C3Z9P2_BRAFL/22-219	C3Z9P2.1 PF01997.14	43.70	0.00
TRINITY_A0A059D5W5_EUCGR/56-151	A0A059D5W5.1 PF0015	43.70	0.00
TRINITY_I7LZV2_TETTS/151-413	I7LZV2.1 PF00069.23	43.70	0.00
TRINITY_F4QEH4_DICFS/16-150	F4QEH4.1 PF04061.12	43.70	0.00
TRINITY_E1ZKX2_CHLVA/8-198	E1ZKX2.1 PF13460.4;	43.70	0.00
TRINITY_Q23GD7_TETTS/81-801	Q23GD7.2 PF00063.19	43.70	0.00
TRINITY_A7T5Z1_NEMVE/16-257	A7T5Z1.1 PF02144.14	43.70	0.00
TRINITY_A0A0A8JIY0_BACSX/1-240	A0A0A8JIY0.1 PF0574	43.70	0.00
TRINITY_A8JFV0_CHLRE/219-502	A8JFV0.1 PF00069.23	43.70	0.00
TRINITY_V9G338_PHYPR/143-625	V9G338.1 PF00152.18	43.70	0.00
TRINITY_A0A068RX79_9FUNG/730-880	A0A068RX79.1 PF0062	43.70	0.00
TRINITY_A0A0G4H0Z9_9ALVE/24-182	A0A0G4H0Z9.1 PF0007	43.70	0.00
TRINITY_A0A087X0J3_HUMAN/238-392	A0A087X0J3.1 PF0860	43.70	0.00
TRINITY_A8I777_CHLRE/173-306	A8I777.1 PF09229.9;	43.70	0.00

TRINITY_D8TRN5_VOLCA/467-662	D8TRN5.1 PF08757.9;	43.70	0.00
TRINITY_E1ZMP2_CHLVA/16-203	E1ZMP2.1 PF08626.9;	43.70	0.00
TRINITY_A0A0N0K611_9PROT/126-271	A0A0N0K611.1 PF0134	43.70	0.00
TRINITY_Q9A4E9_CAUCR/34-259	Q9A4E9.1 PF00266.17	43.70	0.00
TRINITY_F4PI13_DICFS/24-393	F4PI13.1 PF10142.7;	43.70	0.00
TRINITY_F4Q138_DICFS/90-513	F4Q138.1 PF00450.20	43.70	0.00
TRINITY_G2LF82_CHLTF/4-225	G2LF82.1 PF01370.19	43.70	0.00
TRINITY_F3NDJ1_9ACTN/31-272	F3NDJ1.1 PF12697.5;	43.70	0.00
TRINITY_L8HEN7_ACACA/434-510	L8HEN7.1 PF00789.18	43.70	0.00
TRINITY_U9SMA8_RHIID/1-107	U9SMA8.1 PF03254.11	43.70	0.00
TRINITY_A0A096LR09_POEFO/47-485	A0A096LR09.1 PF0170	43.70	0.00
TRINITY_D3BQC1_POLPA/385-635	D3BQC1.1 PF07714.15	43.70	0.00
TRINITY_E1FZR6_LOALO/5-178	E1FZR6.2 PF00071.20	43.70	0.00
TRINITY_K1QKF4_CRAGI/462-667	K1QKF4.1 PF04087.12	43.70	0.00
TRINITY_A0A0M0J9F0_9EUKA/37-326	A0A0M0J9F0.1 PF0058	43.70	0.00
TRINITY_D8TZH4_VOLCA/8-113	D8TZH4.1 PF01920.18	43.70	0.00
TRINITY_E1Z2R8_CHLVA/270-459	E1Z2R8.1 PF05050.10	43.70	0.00
TRINITY_D8TXR4_VOLCA/43-360	D8TXR4.1 PF00194.19	43.70	0.00
TRINITY_I2JNF8_9GAMM/27-136	I2JNF8.1 PF04143.12	43.70	0.00
TRINITY_L8H324_ACACA/42-327	L8H324.1 PF09734.7;	43.70	0.00
TRINITY_I0YW25_9CHLO/7-136	I0YW25.1 PF04049.11	43.70	0.00
TRINITY_C1MUV0_MICPC/10-313	C1MUV0.1 PF07992.12	43.70	0.00
TRINITY_A0A022RVV5_ERYGU/217-302	A0A022RVV5.1 PF0043	43.70	0.00
TRINITY_C1N8L7_MICPC/137-806	C1N8L7.1 PF10408.7;	43.70	0.00
TRINITY_E0VCP7_PEDHC/14-300	E0VCP7.1 PF04118.12	43.70	0.00
TRINITY_A8HPD4_CHLRE/451-711	A8HPD4.1 PF00520.29	43.70	0.00
TRINITY_H2XNP0_CIOIN/2-96	H2XNP0.1 PF16770.3;	43.70	0.00
TRINITY_I4AKQ4_FLELS/53-342	I4AKQ4.1 PF14388.4;	43.70	0.00
TRINITY_I7MIF5_TETTS/84-239	I7MIF5.2 PF04855.10	43.60	0.00
TRINITY_Q0VSF8_ALCBS/675-792	Q0VSF8.1 PF00072.22	43.60	0.00
TRINITY_A0A077ZPS4_STYLE/243-438	A0A077ZPS4.1 PF0011	43.60	0.00
TRINITY_D5G714_TUBMM/57-244	D5G714.1 PF08740.9;	43.60	0.00
TRINITY_A0A0M8K8W8_9CHLR/1-198	A0A0M8K8W8.1 PF0088	43.60	0.00
TRINITY_B9RKG8_RICCO/17-301	B9RKG8.1 PF17184.2;	43.60	0.00
TRINITY_B7JV57_CYAP8/33-307	B7JV57.1 PF01007.18	43.60	0.00
TRINITY_A0A0M0JUD7_9EUKA/134-330	A0A0M0JUD7.1 PF0014	43.60	0.00
TRINITY_W4YMC5_STRPU/24-275	W4YMC5.1 PF13660.4;	43.60	0.00
TRINITY_F0ZET2_DICPU/10-303	F0ZET2.1 PF01213.17	43.60	0.00
TRINITY_A0A0G3BP10_9BURK/5-120	A0A0G3BP10.1 PF0347	43.60	0.00
TRINITY_G2IJ91_9SPHN/64-158	G2IJ91.1 PF08241.10	43.60	0.00
TRINITY_A4VDN1_TETTS/4-430	A4VDN1.1 PF00067.20	43.60	0.00
TRINITY_F0Y6L3_AURAN/5-178	F0Y6L3.1 PF00071.20	43.60	0.00
TRINITY_A0A0M8MZ18_9BASI/12-154	A0A0M8MZ18.1 PF0465	43.60	0.00
TRINITY_I0YRB1_9CHLO/401-489	I0YRB1.1 PF07741.11	43.60	0.00
TRINITY_G0R2W5_ICHMG/613-901	G0R2W5.1 PF00069.23	43.60	0.00
TRINITY_H3H5J1_PHYRM/141-340	H3H5J1.1 PF00078.25	43.60	0.00
TRINITY_W7TRG4_9STRA/152-319	W7TRG4.1 PF01746.19	43.60	0.00
TRINITY_E1ZHL6_CHLVA/325-606	E1ZHL6.1 PF12698.5;	43.60	0.00
TRINITY_V3ZQM8_LOTGI/176-265	V3ZQM8.1 PF12796.5;	43.60	0.00
TRINITY_L8HAY2_ACACA/1-176	L8HAY2.1 PF13462.4;	43.60	0.00
TRINITY_A8I4F2_CHLRE/117-336	A8I4F2.1 PF07714.15	43.60	0.00
TRINITY_A0A087SS85_AUXPR/37-250	A0A087SS85.1 PF0085	43.60	0.00
TRINITY_L8H199_ACACA/70-358	L8H199.1 PF01869.18	43.60	0.00
TRINITY_A8JIC5_CHLRE/17-143	A8JIC5.1 PF09229.9;	43.60	0.00
TRINITY_L8GWP3_ACACA/247-427	L8GWP3.1 PF00621.18	43.60	0.00
TRINITY_A7REZ3_NEMVE/300-780	A7REZ3.1 PF00443.27	43.60	0.00
TRINITY_A8J320_CHLRE/64-185	A8J320.1 PF13578.4;	43.60	0.00
TRINITY_D8REA1_SELML/2457-3170	D8REA1.1 PF03028.13	43.60	0.00
TRINITY_W4XS62_STRPU/124-179	W4XS62.1 PF16558.3;	43.60	0.00
TRINITY_A0E0V7_PARTE/895-1271	A0E0V7.1 PF12698.5;	43.60	0.00
TRINITY_D8U710_VOLCA/574-720	D8U710.1 PF10442.7;	43.60	0.00

TRINITY_A0A0D3CLB3_BRAOL/111-526	A0A0D3CLB3.1 PF0086	43.60	0.00
TRINITY_A0A0G4IJS4_PLABS/64-322	A0A0G4IJS4.1 PF0040	43.60	0.00
TRINITY_I0Z2K8_9CHLO/59-324	I0Z2K8.1 PF11735.6;	43.60	0.00
TRINITY_L1IVP8_GUITH/472-575	L1IVP8.1 PF14874.4;	43.60	0.00
TRINITY_I0Z0I0_9CHLO/150-316	I0Z0I0.1 PF00849.20	43.60	0.00
TRINITY_A8HPD4_CHLRE/451-711	A8HPD4.1 PF00520.29	43.60	0.00
TRINITY_A0A0L7LII1_9NEOP/131-358	A0A0L7LII1.1 PF0245	43.50	0.00
TRINITY_G0R0V4_ICHMG/11-248	G0R0V4.1 PF01991.16	43.50	0.00
TRINITY_A0BLZ4_PARTE/31-180	A0BLZ4.1 PF00810.16	43.50	0.00
TRINITY_H2YAX7_CIOSA/32-94	H2YAX7.1 PF05347.13	43.50	0.00
TRINITY_F0ZT81_DICPU/1337-1660	F0ZT81.1 PF02259.21	43.50	0.00
TRINITY_G9NFL1_HYPAI/175-440	G9NFL1.1 PF13434.4;	43.50	0.00
TRINITY_Q3SDD4_PARTE/8-169	Q3SDD4.1 PF00071.20	43.50	0.00
TRINITY_ABCGC_DICDI/74-229	Q8T685.1 PF00005.25	43.50	0.00
TRINITY_F0ZK99_DICPU/30-201	F0ZK99.1 PF10294.7;	43.50	0.00
TRINITY_G0QR52_ICHMG/10-328	G0QR52.1 PF08449.9;	43.50	0.00
TRINITY_F4PQ87_DICFS/28-89	F4PQ87.1 PF05047.14	43.50	0.00
TRINITY_A0CDK4_PARTE/404-736	A0CDK4.1 PF00702.24	43.50	0.00
TRINITY_I7LTQ3_TETTS/124-278	I7LTQ3.2 PF00650.18	43.50	0.00
TRINITY_I7MMP0_TETTS/53-309	I7MMP0.1 PF03381.13	43.50	0.00
TRINITY_L8GRL4_ACACA/3-152	L8GRL4.1 PF13350.4;	43.50	0.00
TRINITY_C6XHJ4_LIBAP/8-286	C6XHJ4.1 PF00009.25	43.50	0.00
TRINITY_L0J865_MYCSM/7-158	L0J865.1 PF08768.9;	43.50	0.00
TRINITY_S2IWR4_MUCC1/262-511	S2IWR4.1 PF00069.23	43.50	0.00
TRINITY_A0A074X9B9_9PEZI/143-278	A0A074X9B9.1 PF0437	43.50	0.00
TRINITY_H0VK94_CAVPO/73-203	H0VK94.1 PF00782.18	43.50	0.00
TRINITY_L8HDY5_ACACA/41-161	L8HDY5.1 PF00188.24	43.50	0.00
TRINITY_F6CIF3_DESK7/263-470	F6CIF3.1 PF00128.22	43.50	0.00
TRINITY_Q54R70_DICDI/559-832	Q54R70.1 PF08172.10	43.50	0.00
TRINITY_I3KZI4_ORENI/1-183	I3KZI4.1 PF11032.6;	43.50	0.00
TRINITY_K9TYV0_9CYAN/112-235	K9TYV0.1 PF02469.20	43.50	0.00
TRINITY_G1K9V9_ANOCA/32-1199	G1K9V9.2 PF06427.9;	43.50	0.00
TRINITY_T1KE61_TETUR/267-370	T1KE61.1 PF13676.4;	43.50	0.00
TRINITY_Q13R55_BURXL/388-563	Q13R55.1 PF02734.15	43.50	0.00
TRINITY_I3KB29_ORENI/92-239	I3KB29.1 PF14580.4;	43.50	0.00
TRINITY_F6Z156_CIOIN/19-181	F6Z156.2 PF00071.20	43.50	0.00
TRINITY_I7LWR6_TETTS/477-659	I7LWR6.2 PF01756.17	43.50	0.00
TRINITY_D8TIJ3_VOLCA/319-573	D8TIJ3.1 PF02902.17	43.50	0.00
TRINITY_L8GE60_ACACA/531-659	L8GE60.1 PF00241.18	43.50	0.00
TRINITY_F6V9P3_CIOIN/10-137	F6V9P3.2 PF08315.10	43.50	0.00
TRINITY_D8TTP0_VOLCA/1124-1422	D8TTP0.1 PF00069.23	43.50	0.00
TRINITY_I0Z2X7_9CHLO/1496-1903	I0Z2X7.1 PF00443.27	43.50	0.00
TRINITY_H0VK94_CAVPO/73-203	H0VK94.1 PF00782.18	43.50	0.00
TRINITY_Q22P21_TETTS/167-388	Q22P21.2 PF00566.16	43.50	0.00
TRINITY_I7M700_TETTS/181-443	I7M700.2 PF00069.23	43.50	0.00
TRINITY_E1Z8M5_CHLVA/17-221	E1Z8M5.1 PF10232.7;	43.50	0.00
TRINITY_D3BQC1_POLPA/385-635	D3BQC1.1 PF07714.15	43.50	0.00
TRINITY_R0GCG4_9BRAS/595-830	R0GCG4.1 PF02358.14	43.50	0.00
TRINITY_A0A0M2PJ24_9BACI/28-144	A0A0M2PJ24.1 PF0824	43.50	0.00
TRINITY_C3YS02_BRAFL/391-568	C3YS02.1 PF00644.18	43.50	0.00
TRINITY_Q75G45_ORYSJ/950-1193	Q75G45.1 PF07727.12	43.50	0.00
TRINITY_I7ME47_TETTS/120-274	I7ME47.1 PF00650.18	43.50	0.00
TRINITY_I1GFZ8_AMPQE/5-177	I1GFZ8.1 PF00025.19	43.50	0.00
TRINITY_B9GT62_POPTR/123-192	B9GT62.1 PF00076.20	43.50	0.00
TRINITY_J9K7G1_ACYPI/40-226	J9K7G1.1 PF15862.3;	43.50	0.00
TRINITY_D8U3T0_VOLCA/2-136	D8U3T0.1 PF08332.8;	43.50	0.00
TRINITY_A8IW16_CHLRE/695-1085	A8IW16.1 PF03372.21	43.50	0.00
TRINITY_L8GMR3_ACACA/270-526	L8GMR3.1 PF07714.15	43.50	0.00
TRINITY_D8UA01_VOLCA/193-533	D8UA01.1 PF09786.7;	43.50	0.00
TRINITY_A0A061GIX2_THECC/1-133	A0A061GIX2.1 PF0023	43.50	0.00
TRINITY_D3BUV7_POLPA/393-543	D3BUV7.1 PF00620.25	43.50	0.00

TRINITY_D8TU46_VOLCA/1-365	D8TU46.1 PF10498.7;	43.50	0.00
TRINITY_K0S9E4_THAOC/31-347	K0S9E4.1 PF03747.12	43.50	0.00
TRINITY_F2U4V8_SALR5/237-299	F2U4V8.1 PF12738.5;	43.50	0.00
TRINITY_D3AY38_POLPA/846-1105	D3AY38.1 PF07714.15	43.50	0.00
TRINITY_L8GN48_ACACA/67-128	L8GN48.1 PF04117.10	43.50	0.00
TRINITY_J9IHI6_9SPIT/179-269	J9IHI6.1 PF12796.5;	43.50	0.00
TRINITY_A0CDK4_PARTE/404-736	A0CDK4.1 PF00702.24	43.50	0.00
TRINITY_L8H653_ACACA/846-1065	L8H653.1 PF02854.17	43.50	0.00
TRINITY_I0Z7D1_9CHLO/202-409	I0Z7D1.1 PF00924.16	43.50	0.00
TRINITY_A8XGM9_CAEBR/3-73	A8XGM9.1 PF00240.21	43.50	0.00
TRINITY_D8U2Q8_VOLCA/673-763	D8U2Q8.1 PF03982.11	43.50	0.00
TRINITY_D8TSH7_VOLCA/518-608	D8TSH7.1 PF08574.8;	43.50	0.00
TRINITY_I0Z3T1_9CHLO/364-607	I0Z3T1.1 PF03399.14	43.50	0.00
TRINITY_E1ZQK2_CHLVA/53-148	E1ZQK2.1 PF00686.17	43.50	0.00
TRINITY_B9SD55_RICCO/669-811	B9SD55.1 PF02854.17	43.50	0.00
TRINITY_Q23K99_TETTS/9-201	Q23K99.2 PF04511.13	43.50	0.00
TRINITY_A0CWW0_PARTE/35-576	A0CWW0.1 PF00995.21	43.40	0.00
TRINITY_A0A0M0JXL1_9EUKA/39-128	A0A0M0JXL1.1 PF1025	43.40	0.00
TRINITY_U5DG69_AMBTC/13-290	U5DG69.1 PF04321.15	43.40	0.00
TRINITY_F0ZS77_DICPU/34-592	F0ZS77.1 PF01602.18	43.40	0.00
TRINITY_A2DTF0_TRIVA/15-267	A2DTF0.1 PF00069.23	43.40	0.00
TRINITY_A0A0D2WVJ4_CAPO3/267-373	A0A0D2WVJ4.1 PF0030	43.40	0.00
TRINITY_S2K8S0_MUCC1/8-268	S2K8S0.1 PF00483.21	43.40	0.00
TRINITY_F0ZN57_DICPU/39-296	F0ZN57.1 PF02263.17	43.40	0.00
TRINITY_C1MZS9_MICPC/743-954	C1MZS9.1 PF13087.4;	43.40	0.00
TRINITY_J9J7E3_9SPIT/2429-2649	J9J7E3.1 PF00644.18	43.40	0.00
TRINITY_A0A0B0M9H7_GOSAR/41-446	A0A0B0M9H7.1 PF0769	43.40	0.00
TRINITY_I4Y5Q9_WALMC/75-361	I4Y5Q9.1 PF00656.20	43.40	0.00
TRINITY_C7N732_SLAHD/37-147	C7N732.1 PF08487.8;	43.40	0.00
TRINITY_G0QIY3_ICHMG/15-273	G0QIY3.1 PF00069.23	43.40	0.00
TRINITY_G0R4Y1_ICHMG/49-124	G0R4Y1.1 PF01929.15	43.40	0.00
TRINITY_A0A087SN04_AUXPR/283-436	A0A087SN04.1 PF0000	43.40	0.00
TRINITY_A0A078A6D8_STYLE/844-957	A0A078A6D8.1 PF0289	43.40	0.00
TRINITY_A8J7F0_CHLRE/9-153	A8J7F0.1 PF05255.9;	43.40	0.00
TRINITY_A0CQE0_PARTE/146-402	A0CQE0.1 PF00069.23	43.40	0.00
TRINITY_A0A0D2UMU4_CAPO3/32-518	A0A0D2UMU4.1 PF1403	43.40	0.00
TRINITY_A0A067CBT5_SAPPC/7-182	A0A067CBT5.1 PF0014	43.40	0.00
TRINITY_W7MMH3_GIBM7/105-197	W7MMH3.1 PF12796.5;	43.40	0.00
TRINITY_A0A0D2X0U1_CAPO3/1-197	A0A0D2X0U1.1 PF0114	43.40	0.00
TRINITY_A7SLH2_NEMVE/5-134	A7SLH2.1 PF00782.18	43.40	0.00
TRINITY_Q22YV4_TETTS/1100-1325	Q22YV4.3 PF12612.6;	43.40	0.00
TRINITY_I7ML76_TETTS/9-131	I7ML76.1 PF01873.15	43.40	0.00
TRINITY_D5G3Z1_TUBMM/42-168	D5G3Z1.1 PF02466.17	43.40	0.00
TRINITY_D8U1E7_VOLCA/95-427	D8U1E7.1 PF07946.12	43.40	0.00
TRINITY_K1PTD9_CRAGI/42-336	K1PTD9.1 PF00291.23	43.40	0.00
TRINITY_R0I9A7_9BRAS/107-381	R0I9A7.1 PF00122.18	43.40	0.00
TRINITY_CFA53_CHLRE/132-467	A8IRJ7.1 PF13868.4;	43.40	0.00
TRINITY_D8TLP9_VOLCA/889-1060	D8TLP9.1 PF17177.2;	43.40	0.00
TRINITY_A0E7T1_PARTE/778-901	A0E7T1.1 PF00072.22	43.40	0.00
TRINITY_S7VXP5_TOXGO/1598-1905	S7VXP5.1 PF01237.16	43.40	0.00
TRINITY_C4YC96_CLAL4/20-101	C4YC96.1 PF12763.5;	43.40	0.00
TRINITY_A0A067TKA0_9AGAR/99-242	A0A067TKA0.1 PF1007	43.40	0.00
TRINITY_A0CMY4_PARTE/32-295	A0CMY4.1 PF00494.17	43.40	0.00
TRINITY_E0VMP2_PEDHC/38-318	E0VMP2.1 PF13621.4;	43.40	0.00
TRINITY_A0A095CHE4_CRYGA/3-131	A0A095CHE4.1 PF0140	43.40	0.00
TRINITY_Q24HR9_TETTS/46-305	Q24HR9.2 PF00069.23	43.40	0.00
TRINITY_T0QTN3_9STRA/1-216	T0QTN3.1 PF01902.15	43.40	0.00
TRINITY_D8TTC1_VOLCA/415-853	D8TTC1.1 PF04097.12	43.40	0.00
TRINITY_A0A0A7LI04_9BACT/12-194	A0A0A7LI04.1 PF1353	43.40	0.00
TRINITY_A0A0G4GZH6_9ALVE/419-599	A0A0G4GZH6.1 PF0281	43.40	0.00
TRINITY_A0A072TMB4_MEDTR/15-273	A0A072TMB4.1 PF0006	43.40	0.00

TRINITY_A0A015J5I9_9GLOM/468-586	A0A015J5I9.1 PF0066	43.40	0.00
TRINITY_E1Z3S1_CHLVA/369-540	E1Z3S1.1 PF03031.16	43.40	0.00
TRINITY_A4VEM8_TETTS/431-719	A4VEM8.1 PF02995.15	43.40	0.00
TRINITY_D8TQC4_VOLCA/18-543	D8TQC4.1 PF05600.10	43.40	0.00
TRINITY_A8JFG8_CHLRE/29-537	A8JFG8.1 PF00759.17	43.40	0.00
TRINITY_D8UE90_VOLCA/24-180	D8UE90.1 PF05351.9;	43.40	0.00
TRINITY_W5HMB5_WHEAT/6-83	W5HMB5.1 PF00068.17	43.40	0.00
TRINITY_I7MB89_TETTS/43-816	I7MB89.2 PF01804.16	43.30	0.00
TRINITY_A0A0N5C0D5_STREA/59-201	A0A0N5C0D5.1 PF0155	43.30	0.00
TRINITY_Q23R21_TETTS/6-99	Q23R21.2 PF10270.7;	43.30	0.00
TRINITY_A0EGL5_PARTE/997-1236	A0EGL5.1 PF00233.17	43.30	0.00
TRINITY_F2U1H3_SALR5/53-159	F2U1H3.1 PF03226.12	43.30	0.00
TRINITY_E5WNT4_9BACI/8-148	E5WNT4.1 PF13302.5;	43.30	0.00
TRINITY_F2TK29_AJEDA/147-259	F2TK29.1 PF02517.14	43.30	0.00
TRINITY_E4PKM1_MARAH/481-675	E4PKM1.1 PF13847.4;	43.30	0.00
TRINITY_M2Y827_GALSU/146-309	M2Y827.1 PF03031.16	43.30	0.00
TRINITY_Q247T9_TETTS/201-486	Q247T9.2 PF00735.16	43.30	0.00
TRINITY_C4M6G2_ENTHI/341-470	C4M6G2.1 PF00782.18	43.30	0.00
TRINITY_M5ECQ9_MALS4/316-722	M5ECQ9.1 PF00962.20	43.30	0.00
TRINITY_E1ZAR5_CHLVA/7-111	E1ZAR5.1 PF01900.17	43.30	0.00
TRINITY_D8TN37_VOLCA/1910-2160	D8TN37.1 PF00520.29	43.30	0.00
TRINITY_L8H8E7_ACACA/138-352	L8H8E7.1 PF13236.4;	43.30	0.00
TRINITY_D2UYU1_NAEGR/118-218	D2UYU1.1 PF00153.25	43.30	0.00
TRINITY_A0A0P7YAT8_9TELE/393-487	A0A0P7YAT8.1 PF1289	43.30	0.00
TRINITY_F1A0P0_DICPU/1-267	F1A0P0.1 PF03619.14	43.30	0.00
TRINITY_A0A078LBB0_9CHLA/2-141	A0A078LBB0.1 PF0259	43.30	0.00
TRINITY_I7MA30_TETTS/1410-1531	I7MA30.2 PF00072.22	43.30	0.00
TRINITY_F0ZL87_DICPU/588-833	F0ZL87.1 PF07714.15	43.30	0.00
TRINITY_E0VAT2_PEDHC/3-217	E0VAT2.1 PF02230.14	43.30	0.00
TRINITY_A9TJL5_PHYPA/321-494	A9TJL5.1 PF00201.16	43.30	0.00
TRINITY_D2VAE5_NAEGR/8-341	D2VAE5.1 PF00503.18	43.30	0.00
TRINITY_L8HE52_ACACA/224-498	L8HE52.1 PF00928.19	43.30	0.00
TRINITY_A0A087SG51_AUXPR/19-146	A0A087SG51.1 PF1524	43.30	0.00
TRINITY_K3XDM5_PYTUL/113-294	K3XDM5.1 PF00078.25	43.30	0.00
TRINITY_F4PXJ9_DICFS/2-417	F4PXJ9.1 PF13965.4;	43.30	0.00
TRINITY_DES6_PHYPA/230-498	Q9ZNW2.1 PF00487.22	43.30	0.00
TRINITY_A0DZE8_PARTE/290-351	A0DZE8.1 PF01485.19	43.30	0.00
TRINITY_B6SJA3_MAIZE/1-71	B6SJA3.1 PF06645.11	43.30	0.00
TRINITY_J9EPE0_9SPIT/85-154	J9EPE0.1 PF00076.20	43.30	0.00
TRINITY_I1IAH5_BRADI/1140-1407	I1IAH5.1 PF00856.26	43.30	0.00
TRINITY_D8U7B3_VOLCA/6-275	D8U7B3.1 PF02492.17	43.30	0.00
TRINITY_F0XZ84_AURAN/15-150	F0XZ84.1 PF00753.25	43.30	0.00
TRINITY_A7SBN5_NEMVE/204-405	A7SBN5.1 PF05193.19	43.30	0.00
TRINITY_D8U002_VOLCA/1333-1769	D8U002.1 PF02181.21	43.30	0.00
TRINITY_U5HE40_USTV1/555-793	U5HE40.1 PF00270.27	43.30	0.00
TRINITY_F4Q6Y0_DICFS/140-587	F4Q6Y0.1 PF00501.26	43.30	0.00
TRINITY_A0A072V2L0_MEDTR/270-519	A0A072V2L0.1 PF0771	43.30	0.00
TRINITY_A0A0G4EZX3_9ALVE/193-343	A0A0G4EZX3.1 PF1388	43.30	0.00
TRINITY_C5XP01_SORBI/98-317	C5XP01.1 PF01148.18	43.30	0.00
TRINITY_D8RFY2_SELML/510-661	D8RFY2.1 PF08676.9;	43.30	0.00
TRINITY_A0A087SS18_AUXPR/88-276	A0A087SS18.1 PF0057	43.30	0.00
TRINITY_D8TIH2_VOLCA/1283-1461	D8TIH2.1 PF12830.5;	43.30	0.00
TRINITY_A8J0K8_CHLRE/1552-1659	A8J0K8.1 PF00651.29	43.30	0.00
TRINITY_A0A015MLC3_9GLOM/495-577	A0A015MLC3.1 PF0569	43.30	0.00
TRINITY_A0A067NY02_PLEOS/23-184	A0A067NY02.1 PF1029	43.30	0.00
TRINITY_D8TIU1_VOLCA/173-363	D8TIU1.1 PF07103.9;	43.30	0.00
TRINITY_F8W2M8_DANRE/214-518	F8W2M8.1 PF00082.20	43.30	0.00
TRINITY_L8GMG4_ACACA/32-285	L8GMG4.1 PF02265.14	43.30	0.00
TRINITY_Q24JG1_TETTS/16-326	Q24JG1.1 PF07992.12	43.30	0.00
TRINITY_D8UL35_VOLCA/26-349	D8UL35.1 PF12849.5;	43.30	0.00
TRINITY_D8UEG1_VOLCA/25-627	D8UEG1.1 PF00995.21	43.30	0.00

TRINITY_I0YQ81_9CHLO/76-436	I0YQ81.1 PF00251.18	43.30	0.00
TRINITY_A0A087SRL9_AUXPR/40-236	A0A087SRL9.1 PF0290	43.30	0.00
TRINITY_D8U9S7_VOLCA/3-93	D8U9S7.1 PF11527.6;	43.30	0.00
TRINITY_G0QKA0_ICHMG/1960-2256	G0QKA0.1 PF00632.23	43.30	0.00
TRINITY_F2U0M5_SALR5/49-345	F2U0M5.1 PF04616.12	43.30	0.00
TRINITY_A8IT81_CHLRE/4-456	A8IT81.1 PF13520.4;	43.30	0.00
TRINITY_A0A068RWP6_9FUNG/21-124	A0A068RWP6.1 PF1334	43.20	0.00
TRINITY_I7MJV7_TETTS/451-541	I7MJV7.1 PF12796.5;	43.20	0.00
TRINITY_A0A0A0LV37_CUCSA/9-335	A0A0A0LV37.1 PF0126	43.20	0.00
TRINITY_W5BL23_WHEAT/505-672	W5BL23.1 PF00069.23	43.20	0.00
TRINITY_B9Y8M0_9FIRM/44-405	B9Y8M0.1 PF00282.17	43.20	0.00
TRINITY_L8HB13_ACACA/23-289	L8HB13.1 PF00069.23	43.20	0.00
TRINITY_A0A0F8B106_CERFI/176-285	A0A0F8B106.1 PF0572	43.20	0.00
TRINITY_G0R0U2_ICHMG/19-320	G0R0U2.1 PF00491.19	43.20	0.00
TRINITY_X6NP50_RETFI/532-683	X6NP50.1 PF07534.14	43.20	0.00
TRINITY_G5AL54_HETGA/920-1084	G5AL54.1 PF02902.17	43.20	0.00
TRINITY_Q65GW7_BACLD/39-349	Q65GW7.1 PF00251.18	43.20	0.00
TRINITY_A0DKG2_PARTE/35-457	A0DKG2.1 PF00450.20	43.20	0.00
TRINITY_L8H2A4_ACACA/19-179	L8H2A4.1 PF03398.12	43.20	0.00
TRINITY_C7N732_SLAHD/37-147	C7N732.1 PF08487.8;	43.20	0.00
TRINITY_A9VBY4_MONBE/44-295	A9VBY4.1 PF00069.23	43.20	0.00
TRINITY_L8H3W5_ACACA/971-1093	L8H3W5.1 PF00271.29	43.20	0.00
TRINITY_H2U6F6_TAKRU/14-209	H2U6F6.1 PF02545.12	43.20	0.00
TRINITY_D2VK62_NAEGR/37-344	D2VK62.1 PF01207.15	43.20	0.00
TRINITY_A0A0A1P481_9FUNG/102-237	A0A0A1P481.1 PF0056	43.20	0.00
TRINITY_F0ZM27_DICPU/3-173	F0ZM27.1 PF03357.19	43.20	0.00
TRINITY_D3BIF8_POLPA/11-104	D3BIF8.1 PF12796.5;	43.20	0.00
TRINITY_F6ZLJ6_CALJA/11-110	F6ZLJ6.1 PF16661.3;	43.20	0.00
TRINITY_A0A0J8C8A2_BETVU/137-407	A0A0J8C8A2.1 PF0461	43.20	0.00
TRINITY_L8H682_ACACA/25-274	L8H682.1 PF07714.15	43.20	0.00
TRINITY_A0A0A2VUL1_BEABA/1049-1277	A0A0A2VUL1.1 PF1360	43.20	0.00
TRINITY_A0A0D2WGB0_CAPO3/21-228	A0A0D2WGB0.1 PF0681	43.20	0.00
TRINITY_D8UB59_VOLCA/11-326	D8UB59.1 PF03151.14	43.20	0.00
TRINITY_F6ZBY5_CIOIN/51-228	F6ZBY5.2 PF04176.11	43.20	0.00
TRINITY_Q23QU7_TETTS/257-876	Q23QU7.3 PF10408.7;	43.20	0.00
TRINITY_M7XQ60_RHOT1/22-283	M7XQ60.1 PF00069.23	43.20	0.00
TRINITY_A0A059LSB5_9CHLO/2-298	A0A059LSB5.1 PF0015	43.20	0.00
TRINITY_F4PYG0_DICFS/69-275	F4PYG0.1 PF09511.8;	43.20	0.00
TRINITY_A0A0G0JDR4_9BACT/606-722	A0A0G0JDR4.1 PF0251	43.20	0.00
TRINITY_J9IKD2_9SPIT/2-108	J9IKD2.1 PF14469.4;	43.20	0.00
TRINITY_A0A078HKA2_BRANA/474-721	A0A078HKA2.1 PF0006	43.20	0.00
TRINITY_A0A068SAB1_9FUNG/123-211	A0A068SAB1.1 PF0095	43.20	0.00
TRINITY_D3BG64_POLPA/3687-3964	D3BG64.1 PF00454.25	43.20	0.00
TRINITY_I7MIQ2_TETTS/1022-1280	I7MIQ2.2 PF07714.15	43.20	0.00
TRINITY_A0A077ZXU2_STYLE/908-1064	A0A077ZXU2.1 PF1190	43.20	0.00
TRINITY_L8GQB3_ACACA/358-538	L8GQB3.1 PF00621.18	43.20	0.00
TRINITY_B3S3V5_TRIAD/48-274	B3S3V5.1 PF01734.20	43.20	0.00
TRINITY_W4Y1D7_STRPU/18-141	W4Y1D7.1 PF00168.28	43.20	0.00
TRINITY_D2V5F0_NAEGR/143-242	D2V5F0.1 PF13640.4;	43.20	0.00
TRINITY_J9IST0_9SPIT/130-284	J9IST0.1 PF13768.4;	43.20	0.00
TRINITY_M2W2Z2_GALSU/198-337	M2W2Z2.1 PF04116.11	43.20	0.00
TRINITY_A0A0A1PFL8_9FUNG/161-335	A0A0A1PFL8.1 PF1084	43.20	0.00
TRINITY_A9TCM2_PHYPA/150-1138	A9TCM2.1 PF14631.4;	43.20	0.00
TRINITY_W5HJX8_WHEAT/70-203	W5HJX8.1 PF12894.5;	43.20	0.00
TRINITY_Q232X8_TETTS/36-488	Q232X8.1 PF00067.20	43.20	0.00
TRINITY_J0LG47_9BACT/30-232	J0LG47.1 PF04402.12	43.20	0.00
TRINITY_E1ZJ73_CHLVA/493-716	E1ZJ73.1 PF12706.5;	43.20	0.00
TRINITY_E8UXX1_TERSS/201-448	E8UXX1.1 PF04030.12	43.20	0.00
TRINITY_Q1ZXE9_DICDI/3-266	Q1ZXE9.1 PF07651.14	43.20	0.00
TRINITY_A0A087SEE7_AUXPR/2-92	A0A087SEE7.1 PF0008	43.20	0.00
TRINITY_D3BU40_POLPA/287-362	D3BU40.1 PF00626.20	43.20	0.00

TRINITY_A0A024WH81_PLAFA/121-425	A0A024WH81.1 PF1050	43.20	0.00
TRINITY_M7C3J8_CHEMY/100-320	M7C3J8.1 PF00587.23	43.20	0.00
TRINITY_F1A3K1_DICPU/89-240	F1A3K1.1 PF00293.26	43.20	0.00
TRINITY_D2W2P1_NAEGR/1-133	D2W2P1.1 PF10166.7;	43.20	0.00
TRINITY_I7LUZ6_TETTS/119-269	I7LUZ6.1 PF00561.18	43.20	0.00
TRINITY_A8J8J0_CHLRE/1-473	A8J8J0.1 PF03055.13	43.20	0.00
TRINITY_A8HQG8_CHLRE/150-407	A8HQG8.1 PF00481.19	43.20	0.00
TRINITY_L8HJ57_ACACA/262-482	L8HJ57.1 PF13236.4;	43.20	0.00
TRINITY_U4UAC1_DENPD/101-360	U4UAC1.1 PF07714.15	43.20	0.00
TRINITY_H3HAJ2_PHYRM/359-478	H3HAJ2.1 PF00665.24	43.20	0.00
TRINITY_G8MIM5_9BURK/9-199	G8MIM5.1 PF13419.4;	43.20	0.00
TRINITY_D8TN01_VOLCA/432-555	D8TN01.1 PF00069.23	43.20	0.00
TRINITY_Q23RB4_TETTS/70-227	Q23RB4.2 PF01343.16	43.20	0.00
TRINITY_A8IRE4_CHLRE/12-283	A8IRE4.1 PF00069.23	43.20	0.00
TRINITY_K4L188_SIMAS/3-128	K4L188.1 PF01124.16	43.20	0.00
TRINITY_C3YNL3_BRAFL/203-479	C3YNL3.1 PF00067.20	43.20	0.00
TRINITY_I1FCU2_AMPQE/197-343	I1FCU2.1 PF13359.4;	43.20	0.00
TRINITY_Q8YMJ8_NOSS1/554-625	Q8YMJ8.1 PF13448.4;	43.20	0.00
TRINITY_A8HSJ3_CHLRE/66-324	A8HSJ3.1 PF00069.23	43.20	0.00
TRINITY_A0A0M0LS19_9EUKA/162-238	A0A0M0LS19.1 PF0454	43.20	0.00
TRINITY_B9XKX7_PEDPL/29-480	B9XKX7.1 PF02133.13	43.10	0.00
TRINITY_Q23TU2_TETTS/303-899	Q23TU2.2 PF08385.10	43.10	0.00
TRINITY_D0NZW4_PHYIT/6-352	D0NZW4.1 PF07690.14	43.10	0.00
TRINITY_G0QZT8_ICHMG/1370-1783	G0QZT8.1 PF08393.11	43.10	0.00
TRINITY_A0A0L7LVE1_9NEOP/18-286	A0A0L7LVE1.1 PF0014	43.10	0.00
TRINITY_C5LNI1_PERM5/39-107	C5LNI1.1 PF10601.7;	43.10	0.00
TRINITY_F4QFV3_DICFS/1382-1559	F4QFV3.1 PF00617.17	43.10	0.00
TRINITY_A7ST90_NEMVE/96-563	A7ST90.1 PF01457.14	43.10	0.00
TRINITY_E6ZSW6_SPORE/8-72	E6ZSW6.1 PF00808.21	43.10	0.00
TRINITY_M1PCZ5_BARAA/24-345	M1PCZ5.1 PF02628.13	43.10	0.00
TRINITY_L8H5I3_ACACA/1-195	L8H5I3.1 PF05346.9;	43.10	0.00
TRINITY_W7X772_TETTS/112-346	W7X772.1 PF05148.13	43.10	0.00
TRINITY_D3B4Y1_POLPA/156-397	D3B4Y1.1 PF00481.19	43.10	0.00
TRINITY_F4PWT3_DICFS/123-503	F4PWT3.1 PF04801.11	43.10	0.00
TRINITY_L7FPH0_ENTIV/1668-1929	L7FPH0.1 PF00069.23	43.10	0.00
TRINITY_I2FQV0_USTH4/95-298	I2FQV0.1 PF01145.23	43.10	0.00
TRINITY_Q23AY4_TETTS/31-436	Q23AY4.2 PF05577.10	43.10	0.00
TRINITY_L8HI91_ACACA/91-321	L8HI91.1 PF05721.11	43.10	0.00
TRINITY_A0A0D2UJE3_CAPO3/82-201	A0A0D2UJE3.1 PF0004	43.10	0.00
TRINITY_L8H3V3_ACACA/627-901	L8H3V3.1 PF01513.19	43.10	0.00
TRINITY_S2IWW5_MUCC1/154-424	S2IWW5.1 PF08634.8;	43.10	0.00
TRINITY_A0A0A1N5Q2_9FUNG/196-415	A0A0A1N5Q2.1 PF1223	43.10	0.00
TRINITY_A9SVA6_PHYPA/785-918	A9SVA6.1 PF07928.10	43.10	0.00
TRINITY_A0A0F4ZCY2_9PEZI/11-127	A0A0F4ZCY2.1 PF0058	43.10	0.00
TRINITY_D8TNS7_VOLCA/490-809	D8TNS7.1 PF10498.7;	43.10	0.00
TRINITY_I7M4N1_TETTS/619-783	I7M4N1.2 PF13925.4;	43.10	0.00
TRINITY_U9U9M7_RHIID/105-486	U9U9M7.1 PF00443.27	43.10	0.00
TRINITY_R1C984_EMIHU/989-1276	R1C984.1 PF08811.9;	43.10	0.00
TRINITY_Q22MF7_TETTS/48-249	Q22MF7.1 PF04427.16	43.10	0.00
TRINITY_U5VRW5_9ACTN/261-458	U5VRW5.1 PF13489.4;	43.10	0.00
TRINITY_A0A085NEY6_9BILA/25-325	A0A085NEY6.1 PF0844	43.10	0.00
TRINITY_L8HCU8_ACACA/47-175	L8HCU8.1 PF01138.19	43.10	0.00
TRINITY_A8IKE6_CHLRE/67-216	A8IKE6.1 PF01789.14	43.10	0.00
TRINITY_Q22FY9_TETTS/376-440	Q22FY9.3 PF13499.4;	43.10	0.00
TRINITY_A8JFH1_CHLRE/93-580	A8JFH1.1 PF00759.17	43.10	0.00
TRINITY_E4WWA2_OIKDI/170-221	E4WWA2.1 PF00385.22	43.10	0.00
TRINITY_G0QUL3_ICHMG/151-280	G0QUL3.1 PF00107.24	43.10	0.00
TRINITY_Q23QF2_TETTS/248-571	Q23QF2.2 PF03133.13	43.10	0.00
TRINITY_A8JB76_CHLRE/20-573	A8JB76.1 PF03155.13	43.10	0.00
TRINITY_L8H307_ACACA/572-747	L8H307.1 PF00621.18	43.10	0.00
TRINITY_V4LZZ1_EUTSA/32-162	V4LZZ1.1 PF00782.18	43.10	0.00

TRINITY_I0Z586_9CHLO/263-404	I0Z586.1 PF17216.1;	43.10	0.00
TRINITY_Q4QB38_LEIMA/127-278	Q4QB38.1 PF11715.6;	43.10	0.00
TRINITY_A8JFA3_CHLRE/2019-2494	A8JFA3.1 PF00476.18	43.10	0.00
TRINITY_L8HI44_ACACA/353-561	L8HI44.1 PF00616.17	43.10	0.00
TRINITY_F6B7E2_DESCC/215-462	F6B7E2.1 PF07221.9;	43.10	0.00
TRINITY_F1QJH5_DANRE/131-299	F1QJH5.1 PF05175.12	43.10	0.00
TRINITY_C1MLQ6_MICPC/44-247	C1MLQ6.1 PF01018.20	43.10	0.00
TRINITY_I7LWC2_TETTS/50-399	I7LWC2.1 PF00144.22	43.10	0.00
TRINITY_C5XE22_SORBI/580-843	C5XE22.1 PF07714.15	43.10	0.00
TRINITY_L8HJG2_ACACA/55-171	L8HJG2.1 PF01661.19	43.10	0.00
TRINITY_H1XPJ5_9BACT/26-425	H1XPJ5.1 PF02897.13	43.10	0.00
TRINITY_F7DMW1_MACMU/1-281	F7DMW1.1 PF00817.18	43.10	0.00
TRINITY_G4ZJG1_PHYSP/290-379	G4ZJG1.1 PF05699.12	43.10	0.00
TRINITY_A8ISP2_CHLRE/200-468	A8ISP2.1 PF00248.19	43.10	0.00
TRINITY_L8GQB3_ACACA/358-538	L8GQB3.1 PF00621.18	43.10	0.00
TRINITY_U3KBN7_FICAL/1081-1145	U3KBN7.1 PF07647.15	43.10	0.00
TRINITY_A8IGR4_CHLRE/166-1418	A8IGR4.1 PF03813.12	43.10	0.00
TRINITY_D8U024_VOLCA/89-305	D8U024.1 PF02096.18	43.10	0.00
TRINITY_A8JHN4_CHLRE/6-148	A8JHN4.1 PF01217.18	43.10	0.00
TRINITY_A8HZR1_CHLRE/17086-17364	A8HZR1.1 PF00109.24	43.10	0.00
TRINITY_A0A078AZG6_STYLE/1083-1214	A0A078AZG6.1 PF0007	43.10	0.00
TRINITY_Q23BP6_TETTS/28-199	Q23BP6.2 PF01734.20	43.10	0.00
TRINITY_D8TKB1_VOLCA/654-912	D8TKB1.1 PF07714.15	43.10	0.00
TRINITY_A0A059LFP0_9CHLO/13-151	A0A059LFP0.1 PF1035	43.10	0.00
TRINITY_X1WWB9_ACYPI/250-471	X1WWB9.1 PF07727.12	43.10	0.00
TRINITY_E1Z9Q2_CHLVA/1058-1739	E1Z9Q2.1 PF07539.10	43.10	0.00
TRINITY_A8WZJ8_CAEBR/185-244	A8WZJ8.1 PF00046.27	43.10	0.00
TRINITY_Q23YJ0_TETTS/28-372	Q23YJ0.1 PF16499.3;	43.00	0.00
TRINITY_A0CIG8_PARTE/136-677	A0CIG8.1 PF03571.13	43.00	0.00
TRINITY_F7GFD0_MACMU/4-197	F7GFD0.1 PF01501.18	43.00	0.00
TRINITY_R4GBR7_ANOCA/9-144	R4GBR7.1 PF05871.10	43.00	0.00
TRINITY_Q23R88_TETTS/18-272	Q23R88.1 PF00069.23	43.00	0.00
TRINITY_A0A0L7LGD6_9NEOP/3-604	A0A0L7LGD6.1 PF0001	43.00	0.00
TRINITY_A0D0L1_PARTE/195-322	A0D0L1.1 PF00107.24	43.00	0.00
TRINITY_M0SD13_MUSAM/131-217	M0SD13.1 PF03083.14	43.00	0.00
TRINITY_A9T499_PHYPA/25-152	A9T499.1 PF03364.18	43.00	0.00
TRINITY_A0A024UKA9_9STRA/421-527	A0A024UKA9.1 PF0065	43.00	0.00
TRINITY_A0A0D2U9Z3_CAPO3/139-383	A0A0D2U9Z3.1 PF0582	43.00	0.00
TRINITY_F0Y5R3_AURAN/29-109	F0Y5R3.1 PF03366.14	43.00	0.00
TRINITY_L8HDF0_ACACA/65-441	L8HDF0.1 PF07690.14	43.00	0.00
TRINITY_V4B438_LOTGI/23-1106	V4B438.1 PF10266.7;	43.00	0.00
TRINITY_A0D0L1_PARTE/195-322	A0D0L1.1 PF00107.24	43.00	0.00
TRINITY_G0FMT1_AMYMS/32-279	G0FMT1.1 PF12697.5;	43.00	0.00
TRINITY_F0ZQD1_DICPU/175-295	F0ZQD1.1 PF00380.17	43.00	0.00
TRINITY_W4GVX0_9STRA/4-213	W4GVX0.1 PF10229.7;	43.00	0.00
TRINITY_I0YZW7_9CHLO/66-185	I0YZW7.1 PF09335.9;	43.00	0.00
TRINITY_I7MLB9_TETTS/331-784	I7MLB9.1 PF00176.21	43.00	0.00
TRINITY_F0ZHF8_DICPU/558-859	F0ZHF8.1 PF00069.23	43.00	0.00
TRINITY_F6PR32_CIOIN/14-343	F6PR32.2 PF00503.18	43.00	0.00
TRINITY_A0A0D3HPK5_9ORYZ/119-252	A0A0D3HPK5.1 PF1353	43.00	0.00
TRINITY_A0A0D2W094_CAPO3/47-174	A0A0D2W094.1 PF0058	43.00	0.00
TRINITY_I0YYH2_9CHLO/10-274	I0YYH2.1 PF05116.11	43.00	0.00
TRINITY_D2Z5E3_9BACT/601-717	D2Z5E3.1 PF02518.24	43.00	0.00
TRINITY_U3INF1_ANAPL/68-270	U3INF1.1 PF04678.11	43.00	0.00
TRINITY_A0A059LE45_9CHLO/5-92	A0A059LE45.1 PF1656	43.00	0.00
TRINITY_I7MHB3_TETTS/2068-2428	I7MHB3.2 PF00443.27	43.00	0.00
TRINITY_A0A067BK62_SAPPC/136-393	A0A067BK62.1 PF0006	43.00	0.00
TRINITY_K9XJA5_9CHRO/5-202	K9XJA5.1 PF00300.20	43.00	0.00
TRINITY_I7LZV2_TETTS/151-413	I7LZV2.1 PF00069.23	43.00	0.00
TRINITY_L8HBL0_ACACA/285-445	L8HBL0.1 PF02450.13	43.00	0.00
TRINITY_E1ZG57_CHLVA/79-239	E1ZG57.1 PF04832.10	43.00	0.00

TRINITY_D8U0K3_VOLCA/576-800	D8U0K3.1 PF00326.19	43.00	0.00
TRINITY_F2U4X2_SALR5/201-406	F2U4X2.1 PF02979.14	43.00	0.00
TRINITY_F4PI13_DICFS/24-393	F4PI13.1 PF10142.7;	43.00	0.00
TRINITY_A8J1Y1_CHLRE/23-100	A8J1Y1.1 PF12937.5;	43.00	0.00
TRINITY_J9EXT5_9SPIT/263-461	J9EXT5.1 PF00117.26	43.00	0.00
TRINITY_A0A0R4IW43_DANRE/130-277	A0A0R4IW43.1 PF0413	43.00	0.00
TRINITY_Q6MQ84_BDEBA/790-905	Q6MQ84.1 PF00072.22	43.00	0.00
TRINITY_D8TH98_VOLCA/18-105	D8TH98.1 PF13499.4;	43.00	0.00
TRINITY_D3BQC1_POLPA/385-635	D3BQC1.1 PF07714.15	43.00	0.00
TRINITY_U4L329_PYROM/74-160	U4L329.1 PF01336.23	43.00	0.00
TRINITY_A0A073CMD8_PLAAG/265-555	A0A073CMD8.1 PF0077	43.00	0.00
TRINITY_D8TKU7_VOLCA/132-339	D8TKU7.1 PF00300.20	43.00	0.00
TRINITY_A0A0L1J239_ASPNO/736-827	A0A0L1J239.1 PF1279	43.00	0.00
TRINITY_E1Z8V4_CHLVA/5-283	E1Z8V4.1 PF01040.16	43.00	0.00
TRINITY_L1IXT3_GUITH/1-178	L1IXT3.1 PF16909.3;	43.00	0.00
TRINITY_L8GCR5_ACACA/14-522	L8GCR5.1 PF00899.19	43.00	0.00
TRINITY_E1ZNK8_CHLVA/38-143	E1ZNK8.1 PF06179.10	43.00	0.00
TRINITY_L8H8P6_ACACA/5-166	L8H8P6.1 PF00071.20	43.00	0.00
TRINITY_L8GLH3_ACACA/236-467	L8GLH3.1 PF12931.5;	43.00	0.00
TRINITY_A0A061F080_THECC/36-252	A0A061F080.1 PF0113	43.00	0.00
TRINITY_L8GQE8_ACACA/2-240	L8GQE8.1 PF06105.10	43.00	0.00
TRINITY_A8HTQ0_CHLRE/98-251	A8HTQ0.1 PF12146.6;	43.00	0.00
TRINITY_A0A087SFD6_AUXPR/170-379	A0A087SFD6.1 PF0030	43.00	0.00
TRINITY_M4ANV9_XIPMA/26-349	M4ANV9.1 PF01074.20	43.00	0.00

TRINITY_A8ICG2_CHLRE/94-190	A8ICG2.1 PF07052.9;	43.00	0.00
TRINITY_M4AGF1_XIPMA/1107-1209	M4AGF1.1 PF14580.4;	43.00	0.00
TRINITY_K9QPK3_NOSS7/11-206	K9QPK3.1 PF00106.23	43.00	0.00
TRINITY_A8J7M9_CHLRE/742-1038	A8J7M9.1 PF00069.23	43.00	0.00
TRINITY_Q1N2R9_9GAMM/772-890	Q1N2R9.1 PF00072.22	43.00	0.00
TRINITY_Q239Q1_TETTS/221-389	Q239Q1.2 PF07106.11	42.90	0.00
TRINITY_A0A088ADF8_APIME/634-677	A0A088ADF8.1 PF1363	42.90	0.00
TRINITY_F4PNJ7_DICFS/52-298	F4PNJ7.1 PF03381.13	42.90	0.00
TRINITY_B4MX20_DROWI/670-764	B4MX20.2 PF02225.20	42.90	0.00
TRINITY_Q22HC6_TETTS/2408-2508	Q22HC6.2 PF14844.4;	42.90	0.00
TRINITY_B3LD78_PLAKH/163-304	B3LD78.1 PF00179.24	42.90	0.00
TRINITY_F4PQG8_DICFS/89-407	F4PQG8.1 PF03666.11	42.90	0.00
TRINITY_B0CSA0_LACBS/199-340	B0CSA0.1 PF08635.8;	42.90	0.00
TRINITY_A0BG18_PARTE/74-327	A0BG18.1 PF00102.25	42.90	0.00
TRINITY_Q248F0_TETTS/19-502	Q248F0.2 PF04916.11	42.90	0.00
TRINITY_A2F8P2_TRIVA/39-146	A2F8P2.1 PF00168.28	42.90	0.00
TRINITY_F1A0M6_DICPU/22-274	F1A0M6.1 PF07714.15	42.90	0.00
TRINITY_A0A0M8MM38_9BASI/220-393	A0A0M8MM38.1 PF0165	42.90	0.00
TRINITY_L8GQX9_ACACA/44-107	L8GQX9.1 PF04818.11	42.90	0.00
TRINITY_L8HGW6_ACACA/60-130	L8HGW6.1 PF00076.20	42.90	0.00
TRINITY_F4Q1A1_DICFS/125-194	F4Q1A1.1 PF13499.4;	42.90	0.00
TRINITY_PNPP_SCHPO/18-258	Q00472.2 PF00702.24	42.90	0.00
TRINITY_A0A0J8CXM9_BETVU/74-329	A0A0J8CXM9.1 PF0048	42.90	0.00
TRINITY_F8J7A5_HYPSM/5-355	F8J7A5.1 PF01266.22	42.90	0.00
TRINITY_G0R0N7_ICHMG/32-549	G0R0N7.1 PF01602.18	42.90	0.00
TRINITY_Q22M86_TETTS/262-671	Q22M86.1 PF04910.12	42.90	0.00
TRINITY_A0BHW3_PARTE/35-124	A0BHW3.1 PF13011.4;	42.90	0.00
TRINITY_L1JAY0_GUITH/42-332	L1JAY0.1 PF03133.13	42.90	0.00
TRINITY_A9UP34_MONBE/2-79	A9UP34.1 PF14705.4;	42.90	0.00
TRINITY_A6QZS3_AJECN/105-362	A6QZS3.1 PF04857.18	42.90	0.00
TRINITY_Q23QF2_TETTS/248-571	Q23QF2.2 PF03133.13	42.90	0.00
TRINITY_C1E3X7_MICSR/85-301	C1E3X7.1 PF02353.18	42.90	0.00
TRINITY_Q22KS8_TETTS/371-683	Q22KS8.2 PF00443.27	42.90	0.00
TRINITY_I7M7C8_TETTS/9-251	I7M7C8.1 PF05648.12	42.90	0.00
TRINITY_M2TBK4_COCSN/4-242	M2TBK4.1 PF01145.23	42.90	0.00
TRINITY_A8J1F4_CHLRE/136-240	A8J1F4.1 PF12894.5;	42.90	0.00
TRINITY_A9AXR9_HERA2/5-130	A9AXR9.1 PF11528.6;	42.90	0.00
TRINITY_D8TYV9_VOLCA/182-326	D8TYV9.1 PF01547.23	42.90	0.00
TRINITY_A0DEP9_PARTE/295-411	A0DEP9.1 PF16669.3;	42.90	0.00
TRINITY_L8H4Z2_ACACA/119-333	L8H4Z2.1 PF00122.18	42.90	0.00
TRINITY_A1WBB4_ACISJ/43-382	A1WBB4.1 PF03009.15	42.90	0.00
TRINITY_A0A072U329_MEDTR/1630-1731	A0A072U329.1 PF1562	42.90	0.00
TRINITY_E0W3G1_PEDHC/364-462	E0W3G1.1 PF12755.5;	42.90	0.00
TRINITY_Q24DF0_TETTS/748-1037	Q24DF0.2 PF00648.19	42.90	0.00
TRINITY_A4VDR4_TETTS/17-290	A4VDR4.1 PF01193.22	42.90	0.00
TRINITY_E0VDI2_PEDHC/1298-1512	E0VDI2.1 PF14633.4;	42.90	0.00
TRINITY_D8U5E5_VOLCA/603-733	D8U5E5.1 PF00694.17	42.90	0.00
TRINITY_G1MXH8_MELGA/241-296	G1MXH8.1 PF00412.20	42.90	0.00
TRINITY_L1JY95_GUITH/10-156	L1JY95.1 PF06201.11	42.90	0.00
TRINITY_L8GMJ6_ACACA/182-400	L8GMJ6.1 PF00667.18	42.90	0.00
TRINITY_Q3SJU2_THIDA/5-102	Q3SJU2.1 PF00970.22	42.90	0.00
TRINITY_I7LV61_TETTS/61-342	I7LV61.2 PF02383.16	42.90	0.00
TRINITY_D8THR7_VOLCA/300-536	D8THR7.1 PF12697.5;	42.90	0.00
TRINITY_K9TMN2_9CYAN/32-292	K9TMN2.1 PF01636.21	42.90	0.00
TRINITY_Q245G5_TETTS/3-166	Q245G5.2 PF08613.9;	42.90	0.00
TRINITY_G0R421_ICHMG/384-521	G0R421.1 PF07534.14	42.90	0.00
TRINITY_A8JF52_CHLRE/1721-2039	A8JF52.1 PF00520.29	42.90	0.00
TRINITY_A8J3P1_CHLRE/954-1161	A8J3P1.1 PF00211.18	42.90	0.00
TRINITY_F0LTS5_VIBFN/214-279	F0LTS5.1 PF14743.4;	42.90	0.00
TRINITY_A9TQZ8_PHYPA/38-328	A9TQZ8.1 PF00176.21	42.90	0.00
TRINITY_K2E3Q2_9BACT/64-325	K2E3Q2.1 PF12697.5;	42.90	0.00

TRINITY_G0R1C5_ICHMG/3-376	G0R1C5.1 PF00022.17	42.90	0.00
TRINITY_L8GVC8_ACACA/416-697	L8GVC8.1 PF00150.16	42.90	0.00
TRINITY_I7MMP5_TETTS/4189-4485	I7MMP5.2 PF00632.23	42.90	0.00
TRINITY_Q23Q47_TETTS/4-162	Q23Q47.2 PF01652.16	42.90	0.00
TRINITY_A0A0N4ZQ78_PARTI/25-352	A0A0N4ZQ78.1 PF0050	42.90	0.00
TRINITY_G7YND5_CLOSI/13-343	G7YND5.1 PF00503.18	42.90	0.00
TRINITY_B9M6Y2_GEODF/355-673	B9M6Y2.1 PF13243.4;	42.90	0.00
TRINITY_D2A3W1_TRICA/153-237	D2A3W1.1 PF00439.23	42.90	0.00
TRINITY_D3BLW3_POLPA/16-123	D3BLW3.1 PF00307.29	42.90	0.00
TRINITY_A8JAG8_CHLRE/672-990	A8JAG8.1 PF02171.15	42.90	0.00
TRINITY_K4BB11_SOLLC/91-236	K4BB11.1 PF01569.19	42.90	0.00
TRINITY_A0A0B0PG55_GOSAR/152-315	A0A0B0PG55.1 PF0173	42.90	0.00
TRINITY_D3B6P4_POLPA/959-1095	D3B6P4.1 PF07534.14	42.90	0.00
TRINITY_L8GL42_ACACA/354-480	L8GL42.1 PF00644.18	42.90	0.00
TRINITY_E1ZAE1_CHLVA/18-540	E1ZAE1.1 PF12832.5;	42.90	0.00
TRINITY_D8U4A7_VOLCA/26-266	D8U4A7.1 PF00481.19	42.90	0.00
TRINITY_D8U2J0_VOLCA/102-256	D8U2J0.1 PF02453.15	42.90	0.00
TRINITY_F7BFK9_XENTR/9-95	F7BFK9.1 PF02970.14	42.90	0.00
TRINITY_A0A072VFE5_MEDTR/15-426	A0A072VFE5.1 PF0609	42.90	0.00
TRINITY_A8J152_CHLRE/83-238	A8J152.1 PF08645.9;	42.90	0.00
TRINITY_F0Y2A4_AURAN/256-356	F0Y2A4.1 PF13180.4;	42.90	0.00
TRINITY_A5PLC1_DANRE/160-516	A5PLC1.1 PF05686.10	42.90	0.00
TRINITY_R1FS95_EMIHU/3-114	R1FS95.1 PF14770.4;	42.90	0.00
TRINITY_D8UF85_VOLCA/179-263	D8UF85.1 PF05699.12	42.90	0.00
TRINITY_A9RL87_PHYPA/187-534	A9RL87.1 PF01266.22	42.90	0.00
TRINITY_A0A015J5I9_9GLOM/468-586	A0A015J5I9.1 PF0066	42.90	0.00
TRINITY_A0BKA4_PARTE/11-135	A0BKA4.1 PF00241.18	42.90	0.00
TRINITY_W7XE85_TETTS/5-84	W7XE85.1 PF08571.8;	42.90	0.00
TRINITY_I7LXC7_TETTS/17-452	I7LXC7.1 PF00083.22	42.90	0.00
TRINITY_Q6LI57_PHOPR/454-572	Q6LI57.1 PF00072.22	42.90	0.00
TRINITY_A0A015IQG9_9GLOM/18-169	A0A015IQG9.1 PF0482	42.80	0.00
TRINITY_L8HFI2_ACACA/43-231	L8HFI2.1 PF09414.8;	42.80	0.00
TRINITY_G0R0N7_ICHMG/649-793	G0R0N7.1 PF08752.8;	42.80	0.00
TRINITY_I1IG10_BRADI/222-591	I1IG10.1 PF07995.9;	42.80	0.00
TRINITY_D2VZ46_NAEGR/253-405	D2VZ46.1 PF07534.14	42.80	0.00
TRINITY_A0A075B5D3_9FUNG/690-1127	A0A075B5D3.1 PF0105	42.80	0.00
TRINITY_E1ZKY2_CHLVA/1-359	E1ZKY2.1 PF04857.18	42.80	0.00
TRINITY_G1KPC3_ANOCA/381-566	G1KPC3.2 PF05241.10	42.80	0.00
TRINITY_B6AAG7_CRYMR/5264-5570	B6AAG7.1 PF00632.23	42.80	0.00
TRINITY_F9WF79_TRYCI/16-301	F9WF79.1 PF07993.10	42.80	0.00
TRINITY_L8H7W7_ACACA/573-736	L8H7W7.1 PF13692.4;	42.80	0.00
TRINITY_X6MLH9_RETFI/78-383	X6MLH9.1 PF02668.14	42.80	0.00
TRINITY_D8TTP0_VOLCA/1124-1422	D8TTP0.1 PF00069.23	42.80	0.00
TRINITY_A0DJB8_PARTE/29-287	A0DJB8.1 PF00194.19	42.70	0.00
TRINITY_Q22AI5_TETTS/29-127	Q22AI5.3 PF00085.18	42.70	0.00
TRINITY_A0DGY3_PARTE/11-267	A0DGY3.1 PF00069.23	42.70	0.00
TRINITY_F1A489_DICPU/2-138	F1A489.1 PF04699.12	42.70	0.00
TRINITY_A0A0D3VAH0_9BACL/78-279	A0A0D3VAH0.1 PF0785	42.70	0.00
TRINITY_L8H8E7_ACACA/138-352	L8H8E7.1 PF13236.4;	42.70	0.00
TRINITY_L8H6M2_ACACA/339-525	L8H6M2.1 PF00697.20	42.70	0.00
TRINITY_U5FU41_POPTR/28-122	U5FU41.1 PF05669.10	42.70	0.00
TRINITY_L8GIJ5_ACACA/160-302	L8GIJ5.1 PF01467.24	42.70	0.00
TRINITY_A0A078AD16_STYLE/27-478	A0A078AD16.1 PF0045	42.70	0.00
TRINITY_F4Q789_DICFS/9-129	F4Q789.1 PF00241.18	42.70	0.00
TRINITY_A0A0A8K4P5_9RHIZ/34-213	A0A0A8K4P5.1 PF0172	42.70	0.00
TRINITY_U4UR29_DENPD/230-447	U4UR29.1 PF00112.21	42.70	0.00
TRINITY_I7MLV0_TETTS/461-797	I7MLV0.2 PF00443.27	42.70	0.00
TRINITY_I7LXB6_TETTS/1143-1299	I7LXB6.2 PF00005.25	42.70	0.00
TRINITY_I7LZV2_TETTS/151-413	I7LZV2.1 PF00069.23	42.70	0.00
TRINITY_A7RRE4_NEMVE/36-252	A7RRE4.1 PF01370.19	42.70	0.00
TRINITY_G6CJN5_DANPL/396-551	G6CJN5.1 PF12783.5;	42.70	0.00

TRINITY_F4PI13_DICFS/24-393	F4PI13.1 PF10142.7;	42.70	0.00
TRINITY_A0A015LDJ2_9GLOM/73-241	A0A015LDJ2.1 PF0610	42.70	0.00
TRINITY_I1BMW8_RHIO9/7-126	I1BMW8.1 PF10302.7;	42.70	0.00
TRINITY_D8TI14_VOLCA/22-1084	D8TI14.1 PF04147.10	42.70	0.00
TRINITY_I7MDT8_TETTS/9-130	I7MDT8.2 PF03859.14	42.70	0.00
TRINITY_C1E5C7_MICSR/1365-1459	C1E5C7.1 PF03129.18	42.70	0.00
TRINITY_D7FM70_ECTSI/474-608	D7FM70.1 PF08662.9;	42.70	0.00
TRINITY_A0A0M0JCY4_9EUKA/507-827	A0A0M0JCY4.1 PF0063	42.70	0.00
TRINITY_E1Z5Q9_CHLVA/1-227	E1Z5Q9.1 PF05684.10	42.70	0.00
TRINITY_D8TST0_VOLCA/314-634	D8TST0.1 PF14688.4;	42.70	0.00
TRINITY_D3BSH8_POLPA/25-395	D3BSH8.1 PF10142.7;	42.70	0.00
TRINITY_A0CXP9_PARTE/92-386	A0CXP9.1 PF01457.14	42.70	0.00
TRINITY_J9F7V1_9SPIT/154-504	J9F7V1.1 PF13868.4;	42.70	0.00
TRINITY_D7STJ7_VITVI/11-202	D7STJ7.1 PF04511.13	42.70	0.00
TRINITY_A0A0D2VP51_CAPO3/269-513	A0A0D2VP51.1 PF0056	42.70	0.00
TRINITY_Q2IQ35_ANADE/179-509	Q2IQ35.1 PF00171.20	42.70	0.00
TRINITY_D8LD63_ECTSI/224-370	D8LD63.1 PF01694.20	42.70	0.00
TRINITY_G0QT41_ICHMG/24-172	G0QT41.1 PF00782.18	42.70	0.00
TRINITY_A0CPZ2_PARTE/1051-1377	A0CPZ2.1 PF12777.5;	42.70	0.00
TRINITY_F8W2M8_DANRE/214-518	F8W2M8.1 PF00082.20	42.70	0.00
TRINITY_A0A0B2VVD4_TOXCA/13-290	A0A0B2VVD4.1 PF0063	42.70	0.00
TRINITY_K3Z8Q6_SETIT/58-188	K3Z8Q6.1 PF04263.14	42.70	0.00
TRINITY_D8UKB6_VOLCA/44-157	D8UKB6.1 PF07714.15	42.70	0.00
TRINITY_L8GTK1_ACACA/146-395	L8GTK1.1 PF07714.15	42.70	0.00
TRINITY_A2DLY7_TRIVA/10-126	A2DLY7.1 PF01412.16	42.70	0.00
TRINITY_S7XRW0_SPRLO/11-173	S7XRW0.1 PF00071.20	42.70	0.00
TRINITY_D8TMY6_VOLCA/279-490	D8TMY6.1 PF07714.15	42.70	0.00
TRINITY_C3ZE50_BRAFL/11-386	C3ZE50.1 PF00145.15	42.70	0.00
TRINITY_Q22P47_TETTS/327-477	Q22P47.2 PF16113.3;	42.70	0.00
TRINITY_D8U417_VOLCA/198-314	D8U417.1 PF00504.19	42.70	0.00
TRINITY_A2EHA2_TRIVA/171-421	A2EHA2.1 PF07714.15	42.70	0.00
TRINITY_A0A068SEF3_9FUNG/34-341	A0A068SEF3.1 PF0337	42.70	0.00
TRINITY_A0A059LRF5_9CHLO/301-422	A0A059LRF5.1 PF0253	42.70	0.00
TRINITY_Q23GE6_TETTS/664-765	Q23GE6.1 PF04815.13	42.70	0.00
TRINITY_A0A084WBI6_ANOSI/474-580	A0A084WBI6.1 PF0030	42.70	0.00
TRINITY_D8TTD6_VOLCA/12-283	D8TTD6.1 PF01869.18	42.70	0.00
TRINITY_D8TUM1_VOLCA/2-286	D8TUM1.1 PF15239.4;	42.70	0.00
TRINITY_A8JBH7_CHLRE/27-131	A8JBH7.1 PF00085.18	42.70	0.00
TRINITY_A0A0B4GQ28_9HYPO/653-894	A0A0B4GQ28.1 PF0772	42.70	0.00
TRINITY_E3FU34_STIAD/40-322	E3FU34.1 PF01636.21	42.70	0.00
TRINITY_I0Z929_9CHLO/115-362	I0Z929.1 PF01433.18	42.70	0.00
TRINITY_A8JAQ5_CHLRE/488-591	A8JAQ5.1 PF12796.5;	42.70	0.00
TRINITY_T0R0B8_9STRA/37-164	T0R0B8.1 PF12796.5;	42.70	0.00
TRINITY_I0Z190_9CHLO/11-606	I0Z190.1 PF00012.18	42.70	0.00
TRINITY_I7LUV4_TETTS/6-94	I7LUV4.2 PF00855.15	42.70	0.00
TRINITY_A0A077ZZ05_STYLE/13-335	A0A077ZZ05.1 PF0329	42.70	0.00
TRINITY_A0A0G2FVY5_9PEZI/193-453	A0A0G2FVY5.1 PF0006	42.60	0.00
TRINITY_A5V585_SPHWW/37-167	A5V585.1 PF00188.24	42.60	0.00
TRINITY_G0QK23_ICHMG/3-104	G0QK23.1 PF00168.28	42.60	0.00
TRINITY_I7MIJ4_TETTS/15-231	I7MIJ4.2 PF02230.14	42.60	0.00
TRINITY_ACPS_DESVH/4-120	Q72AT2.1 PF01648.18	42.60	0.00
TRINITY_I1KL83_SOYBN/10-108	I1KL83.1 PF04749.15	42.60	0.00
TRINITY_I7MMH9_TETTS/212-523	I7MMH9.2 PF00632.23	42.60	0.00
TRINITY_F1NXC2_CHICK/38-153	F1NXC2.2 PF00615.17	42.60	0.00
TRINITY_D8QV77_SELML/62-307	D8QV77.1 PF08704.8;	42.60	0.00
TRINITY_L8GWQ5_ACACA/986-1235	L8GWQ5.1 PF07714.15	42.60	0.00
TRINITY_S2K3Z9_MUCC1/5-158	S2K3Z9.1 PF05907.11	42.60	0.00
TRINITY_U1MSE1_ASCSU/193-284	U1MSE1.1 PF08321.10	42.60	0.00
TRINITY_A0A078B1Y8_STYLE/319-384	A0A078B1Y8.1 PF0049	42.60	0.00
TRINITY_A0DRK2_PARTE/202-274	A0DRK2.1 PF00173.26	42.60	0.00
TRINITY_G0QVG7_ICHMG/239-716	G0QVG7.1 PF04791.14	42.60	0.00

TRINITY_L8GM38_ACACA/468-720	L8GM38.1 PF07714.15	42.60	0.00
TRINITY_G5APF5_HETGA/1-317	G5APF5.1 PF01201.20	42.60	0.00
TRINITY_K4BAL8_SOLLC/77-206	K4BAL8.1 PF00582.24	42.60	0.00
TRINITY_E1Z947_CHLVA/294-456	E1Z947.1 PF00743.17	42.60	0.00
TRINITY_G0QQC7_ICHMG/138-415	G0QQC7.1 PF00069.23	42.60	0.00
TRINITY_D8TH75_VOLCA/74-173	D8TH75.1 PF13815.4;	42.60	0.00
TRINITY_B3L6C7_PLAKH/14-175	B3L6C7.1 PF00071.20	42.60	0.00
TRINITY_T1K1A2_TETUR/106-536	T1K1A2.1 PF10433.7;	42.60	0.00
TRINITY_W5N955_LEPOC/19-328	W5N955.1 PF16420.3;	42.60	0.00
TRINITY_I0YZV3_9CHLO/123-427	I0YZV3.1 PF00069.23	42.60	0.00
TRINITY_A0A024UX32_9STRA/62-134	A0A024UX32.1 PF0069	42.60	0.00
TRINITY_F4PLA2_DICFS/498-650	F4PLA2.1 PF00620.25	42.60	0.00
TRINITY_M5GEX9_DACSP/52-125	M5GEX9.1 PF13517.4;	42.60	0.00
TRINITY_G6YGX7_9RHIZ/6-251	G6YGX7.1 PF01370.19	42.60	0.00
TRINITY_M0S2P3_MUSAM/119-185	M0S2P3.1 PF00013.27	42.60	0.00
TRINITY_E9IKC8_SOLIN/9-264	E9IKC8.1 PF00069.23	42.60	0.00
TRINITY_A0A0G2ZUL9_9DELT/277-452	A0A0G2ZUL9.1 PF1376	42.60	0.00
TRINITY_A0A078AJL8_STYLE/420-527	A0A078AJL8.1 PF1364	42.60	0.00
TRINITY_I0I884_CALAS/53-389	I0I884.1 PF01547.23	42.60	0.00
TRINITY_A0D8M4_PARTE/180-432	A0D8M4.1 PF00233.17	42.60	0.00
TRINITY_L8HAY2_ACACA/1-176	L8HAY2.1 PF13462.4;	42.60	0.00
TRINITY_A8IRH6_CHLRE/37-466	A8IRH6.1 PF01150.15	42.60	0.00
TRINITY_A7RZS6_NEMVE/21-504	A7RZS6.1 PF03055.13	42.60	0.00
TRINITY_A8HQN7_CHLRE/125-185	A8HQN7.1 PF14295.4;	42.60	0.00
TRINITY_K9UCD6_9CHRO/15-154	K9UCD6.1 PF08719.9;	42.60	0.00
TRINITY_G4VC11_SCHMA/93-420	G4VC11.1 PF00704.26	42.60	0.00
TRINITY_D3BGC6_POLPA/239-445	D3BGC6.1 PF00616.17	42.60	0.00
TRINITY_A0A087SA18_AUXPR/141-251	A0A087SA18.1 PF1364	42.60	0.00
TRINITY_A8HPD4_CHLRE/451-711	A8HPD4.1 PF00520.29	42.60	0.00
TRINITY_F0YCP8_AURAN/53-463	F0YCP8.1 PF00450.20	42.60	0.00
TRINITY_A0A078DXQ9_BRANA/182-281	A0A078DXQ9.1 PF0016	42.60	0.00
TRINITY_J3MBD2_ORYBR/517-715	J3MBD2.1 PF05724.9;	42.60	0.00
TRINITY_D8TTM6_VOLCA/6-138	D8TTM6.1 PF13676.4;	42.60	0.00
TRINITY_G0QV06_ICHMG/23-182	G0QV06.1 PF03357.19	42.60	0.00
TRINITY_A0A024UK03_9STRA/17-187	A0A024UK03.1 PF0335	42.60	0.00
TRINITY_A0A068RN78_9FUNG/262-373	A0A068RN78.1 PF0056	42.60	0.00
TRINITY_F4PV61_DICFS/476-1360	F4PV61.1 PF02460.16	42.60	0.00
TRINITY_I0YVV6_9CHLO/774-1265	I0YVV6.1 PF00759.17	42.60	0.00
TRINITY_A8IEV8_CHLRE/3-288	A8IEV8.1 PF00069.23	42.60	0.00
TRINITY_A0A087SKP2_AUXPR/5-174	A0A087SKP2.1 PF0612	42.60	0.00
TRINITY_F4Q1V6_DICFS/12-291	F4Q1V6.1 PF10127.7;	42.60	0.00
TRINITY_D3B745_POLPA/298-395	D3B745.1 PF16158.3;	42.60	0.00
TRINITY_G0QT60_ICHMG/17-86	G0QT60.1 PF13499.4;	42.60	0.00
TRINITY_Q234R8_TETTS/286-375	Q234R8.2 PF14561.4;	42.50	0.00
TRINITY_L8HET9_ACACA/76-164	L8HET9.1 PF12894.5;	42.50	0.00
TRINITY_Q245E1_TETTS/75-258	Q245E1.1 PF07998.9;	42.50	0.00
TRINITY_L1I7Y3_GUITH/29-102	L1I7Y3.1 PF16093.3;	42.50	0.00
TRINITY_W7X9B2_TETTS/39-342	W7X9B2.1 PF00850.17	42.50	0.00
TRINITY_F1A652_DICPU/115-293	F1A652.1 PF05712.11	42.50	0.00
TRINITY_K4BFL0_SOLLC/116-234	K4BFL0.1 PF00622.26	42.50	0.00
TRINITY_I7LUD1_TETTS/1-227	I7LUD1.1 PF01086.15	42.50	0.00
TRINITY_D8RZS1_SELML/1-142	D8RZS1.1 PF04658.11	42.50	0.00
TRINITY_Q23AY4_TETTS/31-436	Q23AY4.2 PF05577.10	42.50	0.00
TRINITY_Q232X8_TETTS/36-488	Q232X8.1 PF00067.20	42.50	0.00
TRINITY_C3Z5Z8_BRAFL/34-140	C3Z5Z8.1 PF02837.16	42.50	0.00
TRINITY_D8Q0R8_SCHCM/345-567	D8Q0R8.1 PF00082.20	42.50	0.00
TRINITY_M1WE29_CLAP2/186-274	M1WE29.1 PF12894.5;	42.50	0.00
TRINITY_D6WQA3_TRICA/15-387	D6WQA3.1 PF01433.18	42.50	0.00
TRINITY_A8JD96_CHLRE/7-176	A8JD96.1 PF00186.17	42.50	0.00
TRINITY_Q23FR1_TETTS/29-247	Q23FR1.2 PF08442.8;	42.50	0.00
TRINITY_D8TPI1_VOLCA/191-280	D8TPI1.1 PF00686.17	42.50	0.00

TRINITY_D8TMF4_VOLCA/11-131	D8TMF4.1 PF01398.19	42.50	0.00
TRINITY_A7RIR3_NEMVE/12-456	A7RIR3.1 PF03901.15	42.50	0.00
TRINITY_A0A078ANZ7_STYLE/243-431	A0A078ANZ7.1 PF0092	42.50	0.00
TRINITY_Q54PB7_DICDI/1358-1684	Q54PB7.1 PF06920.11	42.50	0.00
TRINITY_D8PFW3_9BACT/753-985	D8PFW3.1 PF00248.19	42.50	0.00
TRINITY_F4PZT7_DICFS/149-338	F4PZT7.1 PF04893.15	42.50	0.00
TRINITY_Q23TZ6_TETTS/592-664	Q23TZ6.1 PF00575.21	42.50	0.00
TRINITY_A2D8V9_TRIVA/448-772	A2D8V9.1 PF00702.24	42.50	0.00
TRINITY_I0Z205_9CHLO/52-262	I0Z205.1 PF01852.17	42.50	0.00
TRINITY_A0A087YKX9_POEFO/31-223	A0A087YKX9.1 PF1356	42.50	0.00
TRINITY_L8GMP4_ACACA/297-555	L8GMP4.1 PF07714.15	42.50	0.00
TRINITY_A8HT24_CHLRE/1-153	A8HT24.1 PF13967.4;	42.50	0.00
TRINITY_A8N128_COPC7/821-1417	A8N128.2 PF04998.15	42.50	0.00
TRINITY_H2AYA1_KAZAF/168-318	H2AYA1.1 PF00650.18	42.50	0.00
TRINITY_A0A0C2MRZ6_THEKT/508-668	A0A0C2MRZ6.1 PF0007	42.50	0.00
TRINITY_GXCDD_DICDI/464-636	Q1ZXH8.3 PF00621.18	42.50	0.00
TRINITY_I1I093_BRADI/214-419	I1I093.1 PF05450.13	42.50	0.00
TRINITY_E1ZG60_CHLVA/124-217	E1ZG60.1 PF00686.17	42.50	0.00
TRINITY_J9EG13_9SPIT/584-905	J9EG13.1 PF05192.16	42.50	0.00
TRINITY_J9I105_9SPIT/748-1060	J9I105.1 PF03133.13	42.50	0.00
TRINITY_A0A087SE27_AUXPR/662-921	A0A087SE27.1 PF0006	42.50	0.00
TRINITY_Q22Y90_TETTS/126-273	Q22Y90.2 PF01553.19	42.50	0.00
TRINITY_D3AXH6_POLPA/88-309	D3AXH6.1 PF03531.12	42.50	0.00
TRINITY_B7PNE9_IXOSC/191-298	B7PNE9.1 PF00651.29	42.50	0.00
TRINITY_L8H6Q9_ACACA/39-219	L8H6Q9.1 PF00621.18	42.50	0.00
TRINITY_A8IBB6_CHLRE/141-344	A8IBB6.1 PF00211.18	42.50	0.00
TRINITY_Q9FXC0_ARATH/9-217	Q9FXC0.1 PF01470.15	42.50	0.00
TRINITY_L8HHV9_ACACA/1409-1536	L8HHV9.1 PF04784.12	42.50	0.00
TRINITY_Q22NX2_TETTS/58-257	Q22NX2.1 PF00106.23	42.50	0.00
TRINITY_A0A0A1TX61_ENTIV/615-880	A0A0A1TX61.1 PF0006	42.50	0.00
TRINITY_D8TPI1_VOLCA/514-606	D8TPI1.1 PF00686.17	42.50	0.00
TRINITY_A0A074S5N7_9HOMO/876-1190	A0A074S5N7.1 PF0020	42.50	0.00
TRINITY_S2J153_MUCC1/962-1236	S2J153.1 PF00664.21	42.50	0.00
TRINITY_Q23QF2_TETTS/248-571	Q23QF2.2 PF03133.13	42.50	0.00
TRINITY_I3MYK3_ICTTR/5-377	I3MYK3.1 PF00022.17	42.50	0.00
TRINITY_R6P451_9FIRM/38-122	R6P451.1 PF13508.5;	42.40	0.00
TRINITY_L8H2W8_ACACA/130-416	L8H2W8.1 PF00955.19	42.40	0.00
TRINITY_Q2KX37_BORA1/12-190	Q2KX37.1 PF00857.18	42.40	0.00
TRINITY_A9RTT7_PHYPA/174-241	A9RTT7.1 PF00076.20	42.40	0.00
TRINITY_F0UUU2_AJEC8/77-464	F0UUU2.1 PF01266.22	42.40	0.00
TRINITY_L8HED3_ACACA/1-118	L8HED3.1 PF13350.4;	42.40	0.00
TRINITY_L8HED3_ACACA/1-118	L8HED3.1 PF13350.4;	42.40	0.00
TRINITY_A0D6N3_PARTE/415-536	A0D6N3.1 PF02518.24	42.40	0.00
TRINITY_J3P2K8_GAGT3/209-270	J3P2K8.1 PF00403.24	42.40	0.00
TRINITY_J9I2H5_9SPIT/998-1122	J9I2H5.1 PF07744.11	42.40	0.00
TRINITY_C1MWE8_MICPC/31-276	C1MWE8.1 PF12706.5;	42.40	0.00
TRINITY_A0A0L0CBX0_LUCCU/65-161	A0A0L0CBX0.1 PF1275	42.40	0.00
TRINITY_A9SK13_PHYPA/14-128	A9SK13.1 PF01416.18	42.40	0.00
TRINITY_K7L0E8_SOYBN/435-562	K7L0E8.1 PF01909.21	42.40	0.00
TRINITY_Q22KA1_TETTS/31-431	Q22KA1.2 PF00155.19	42.40	0.00
TRINITY_F0Z708_DICPU/34-273	F0Z708.1 PF04190.11	42.40	0.00
TRINITY_A0A015MTB8_9GLOM/426-625	A0A015MTB8.1 PF0006	42.40	0.00
TRINITY_G0QP91_ICHMG/89-387	G0QP91.1 PF00648.19	42.40	0.00
TRINITY_A0A0A1U0T6_ENTIV/4-232	A0A0A1U0T6.1 PF0771	42.40	0.00
TRINITY_Q6FQG5_CANGA/368-428	Q6FQG5.1 PF09336.8;	42.40	0.00
TRINITY_A0A088A0P6_APIME/58-404	A0A088A0P6.1 PF0514	42.40	0.00
TRINITY_J9IW09_9SPIT/261-719	J9IW09.1 PF04547.10	42.40	0.00
TRINITY_A0A0A7KLL9_9DEIO/38-159	A0A0A7KLL9.1 PF0246	42.40	0.00
TRINITY_F4PWI6_DICFS/2-205	F4PWI6.1 PF04161.11	42.40	0.00
TRINITY_F2USG6_SALR5/66-240	F2USG6.1 PF00092.26	42.40	0.00
TRINITY_A0Q1G7_CLONN/318-600	A0Q1G7.1 PF00759.17	42.40	0.00

TRINITY_A0E2I7_PARTE/134-394	A0E2I7.1 PF00069.23	42.40	0.00
TRINITY_A0A0D2U6D4_CAPO3/422-639	A0A0D2U6D4.1 PF0315	42.40	0.00
TRINITY_C5XI79_SORBI/15-510	C5XI79.1 PF01593.22	42.40	0.00
TRINITY_G5A3Z6_PHYSP/16-367	G5A3Z6.1 PF00225.21	42.40	0.00
TRINITY_F0XZG0_AURAN/14-151	F0XZG0.1 PF14226.4;	42.40	0.00
TRINITY_Q6MJD8_BDEBA/3-365	Q6MJD8.1 PF04303.11	42.40	0.00
TRINITY_M4BZI1_HYAAE/223-366	M4BZI1.1 PF00892.18	42.40	0.00
TRINITY_W4Z612_STRPU/466-705	W4Z612.1 PF00850.17	42.40	0.00
TRINITY_A0A0D2UI95_GOSRA/75-224	A0A0D2UI95.1 PF0029	42.40	0.00
TRINITY_E0XN34_SOLLC/490-1011	E0XN34.1 PF07748.11	42.40	0.00
TRINITY_E1ZMS8_CHLVA/193-304	E1ZMS8.1 PF00271.29	42.40	0.00
TRINITY_D8TNE3_VOLCA/48-267	D8TNE3.1 PF00856.26	42.40	0.00
TRINITY_I7MGS4_TETTS/171-373	I7MGS4.2 PF00149.26	42.40	0.00
TRINITY_A8IRD9_CHLRE/35-380	A8IRD9.1 PF09766.7;	42.40	0.00
TRINITY_P2XB_DICDI/71-350	Q553Y1.2 PF00864.17	42.40	0.00
TRINITY_I0Z937_9CHLO/634-846	I0Z937.1 PF07714.15	42.40	0.00
TRINITY_A0A0N4Z0N8_PARTI/122-187	A0A0N4Z0N8.1 PF1235	42.40	0.00
TRINITY_Q8S692_ORYSJ/491-673	Q8S692.1 PF00078.25	42.40	0.00
TRINITY_G4T9G5_PIRID/312-596	G4T9G5.1 PF00069.23	42.40	0.00
TRINITY_Q22RP0_TETTS/768-1090	Q22RP0.2 PF08767.9;	42.40	0.00
TRINITY_A8J0L7_CHLRE/8-430	A8J0L7.1 PF04339.10	42.40	0.00
TRINITY_A8J5C3_CHLRE/92-264	A8J5C3.1 PF01997.14	42.40	0.00
TRINITY_A0A067L5Q9_JATCU/65-285	A0A067L5Q9.1 PF0027	42.40	0.00
TRINITY_A9V2B9_MONBE/227-497	A9V2B9.1 PF13598.4;	42.40	0.00
TRINITY_A0DFX3_PARTE/110-382	A0DFX3.1 PF00069.23	42.40	0.00
TRINITY_A0A0M0JT31_9EUKA/18-161	A0A0M0JT31.1 PF1330	42.40	0.00
TRINITY_V2XG74_MONRO/7-157	V2XG74.1 PF02182.15	42.40	0.00
TRINITY_B8BV76_THAPS/393-767	B8BV76.1 PF01266.22	42.40	0.00
TRINITY_B2AQ42_PODAN/293-439	B2AQ42.1 PF13692.4;	42.40	0.00
TRINITY_D8UC19_VOLCA/408-790	D8UC19.1 PF01432.18	42.40	0.00
TRINITY_A0CDK4_PARTE/404-736	A0CDK4.1 PF00702.24	42.40	0.00
TRINITY_M5VZ74_PRUPE/110-250	M5VZ74.1 PF09810.7;	42.40	0.00
TRINITY_D8UJ77_VOLCA/285-526	D8UJ77.1 PF00122.18	42.40	0.00
TRINITY_I7LUV4_TETTS/6-94	I7LUV4.2 PF00855.15	42.40	0.00
TRINITY_A8J3Q7_CHLRE/869-1071	A8J3Q7.1 PF00211.18	42.40	0.00
TRINITY_G6FV49_9CYAN/2-195	G6FV49.1 PF00106.23	42.40	0.00
TRINITY_L1JV11_GUITH/1217-1343	L1JV11.1 PF00580.19	42.40	0.00
TRINITY_A8J7M9_CHLRE/742-1038	A8J7M9.1 PF00069.23	42.40	0.00
TRINITY_A0A0J8CZF3_BETVU/13-293	A0A0J8CZF3.1 PF0107	42.40	0.00
TRINITY_G0R3W2_ICHMG/171-370	G0R3W2.1 PF05019.11	42.40	0.00
TRINITY_D2V0M3_NAEGR/179-278	D2V0M3.1 PF05028.12	42.40	0.00
TRINITY_D8M0W4_BLAHO/11-182	D8M0W4.1 PF00621.18	42.40	0.00
TRINITY_I7MKL6_TETTS/229-436	I7MKL6.2 PF00566.16	42.30	0.00
TRINITY_D7TD95_VITVI/109-584	D7TD95.1 PF00501.26	42.30	0.00
TRINITY_E1Z4B1_CHLVA/527-660	E1Z4B1.1 PF01426.16	42.30	0.00
TRINITY_J9JJ30_ACYPI/268-738	J9JJ30.2 PF01055.24	42.30	0.00
TRINITY_A0A091D0H7_FUKDA/27-157	A0A091D0H7.1 PF0078	42.30	0.00
TRINITY_F4NRB1_BATDJ/133-481	F4NRB1.1 PF01180.19	42.30	0.00
TRINITY_K4D4K1_SOLLC/1-73	K4D4K1.1 PF10200.7;	42.30	0.00
TRINITY_A0A0G2FYM6_9PEZI/56-136	A0A0G2FYM6.1 PF0333	42.30	0.00
TRINITY_F7E2U7_MONDO/5-234	F7E2U7.2 PF13561.4;	42.30	0.00
TRINITY_Q7D467_CLOAB/16-241	Q7D467.1 PF04452.12	42.30	0.00
TRINITY_A6VZR0_MARMS/1161-1276	A6VZR0.1 PF00072.22	42.30	0.00
TRINITY_A0BIL6_PARTE/986-1090	A0BIL6.1 PF02204.16	42.30	0.00
TRINITY_L8GEK5_ACACA/196-340	L8GEK5.1 PF07534.14	42.30	0.00
TRINITY_F0ZYB1_DICPU/209-508	F0ZYB1.1 PF03062.17	42.30	0.00
TRINITY_R6XHY1_9CLOT/12-606	R6XHY1.1 PF02901.13	42.30	0.00
TRINITY_W5MCU5_LEPOC/683-914	W5MCU5.1 PF00102.25	42.30	0.00
TRINITY_M3J9B4_CANMX/7-161	M3J9B4.1 PF01687.15	42.30	0.00
TRINITY_L8GVK1_ACACA/140-464	L8GVK1.1 PF02181.21	42.30	0.00
TRINITY_J9K5B4_ACYPI/832-1061	J9K5B4.2 PF16212.3;	42.30	0.00

TRINITY_Q23QF2_TETTS/248-571	Q23QF2.2 PF03133.13	42.30	0.00
TRINITY_L5KD32_PTEAL/57-213	L5KD32.1 PF10255.7;	42.30	0.00
TRINITY_D8RTW3_SELML/703-814	D8RTW3.1 PF00072.22	42.30	0.00
TRINITY_F7KIS7_9FIRM/862-979	F7KIS7.1 PF00072.22	42.30	0.00
TRINITY_F0ZQR9_DICPU/2019-2148	F0ZQR9.1 PF00782.18	42.30	0.00
TRINITY_C3YXY9_BRAFL/3-377	C3YXY9.1 PF09751.7;	42.30	0.00
TRINITY_I7LZV2_TETTS/151-413	I7LZV2.1 PF00069.23	42.30	0.00
TRINITY_A0CS76_PARTE/16-224	A0CS76.1 PF01991.16	42.30	0.00
TRINITY_F7IR60_CALJA/10-84	F7IR60.1 PF00076.20	42.30	0.00
TRINITY_A0CC42_PARTE/16-287	A0CC42.1 PF00069.23	42.30	0.00
TRINITY_K3X5V1_PYTUL/14-448	K3X5V1.1 PF01474.14	42.30	0.00
TRINITY_W5MXZ8_LEPOC/20-140	W5MXZ8.1 PF05175.12	42.30	0.00
TRINITY_A4VEE1_TETTS/16-254	A4VEE1.1 PF01370.19	42.30	0.00
TRINITY_A0A0G4IT47_PLABS/363-457	A0A0G4IT47.1 PF1289	42.30	0.00
TRINITY_G0R2J9_ICHMG/1-211	G0R2J9.1 PF00069.23	42.30	0.00
TRINITY_I7LWA5_TETTS/1556-1748	I7LWA5.2 PF00406.20	42.30	0.00
TRINITY_A9UQE2_MONBE/13-93	A9UQE2.1 PF12796.5;	42.30	0.00
TRINITY_V9EKI7_PHYPR/73-203	V9EKI7.1 PF00782.18	42.30	0.00
TRINITY_L8HIG4_ACACA/43-454	L8HIG4.1 PF01490.16	42.30	0.00
TRINITY_B5MF71_DANRE/24-281	B5MF71.1 PF00069.23	42.30	0.00
TRINITY_W7X994_TETTS/597-768	W7X994.1 PF01067.20	42.30	0.00
TRINITY_G1RP02_NOMLE/116-185	G1RP02.1 PF00076.20	42.30	0.00
TRINITY_A8IBB6_CHLRE/141-344	A8IBB6.1 PF00211.18	42.30	0.00
TRINITY_I7LUA5_TETTS/33-143	I7LUA5.1 PF08240.10	42.30	0.00
TRINITY_GACH_DICDI/390-541	Q86IG9.1 PF00620.25	42.30	0.00
TRINITY_D9S0T9_THEOJ/4-320	D9S0T9.1 PF00490.19	42.30	0.00
TRINITY_J9ZAU4_LEPFM/4-98	J9ZAU4.1 PF00042.20	42.30	0.00
TRINITY_D3B9E3_POLPA/2-74	D3B9E3.1 PF08520.8;	42.30	0.00
TRINITY_A0A061IX71_TRYRA/308-358	A0A061IX71.1 PF1392	42.30	0.00
TRINITY_A0CUI8_PARTE/50-226	A0CUI8.1 PF03798.14	42.30	0.00
TRINITY_A8HTH4_CHLRE/1-155	A8HTH4.1 PF11894.6;	42.30	0.00
TRINITY_J9EHK3_9SPIT/2-255	J9EHK3.1 PF03194.13	42.30	0.00
TRINITY_I7MAM9_TETTS/250-530	I7MAM9.2 PF01189.15	42.30	0.00
TRINITY_D3BIN2_POLPA/295-392	D3BIN2.1 PF03131.15	42.30	0.00
TRINITY_T1GTY1_MEGSC/38-110	T1GTY1.1 PF02807.13	42.30	0.00
TRINITY_C1N3S6_MICPC/4-201	C1N3S6.1 PF05724.9;	42.30	0.00
TRINITY_B7FYV8_PHATC/342-541	B7FYV8.1 PF03006.18	42.30	0.00
TRINITY_L8GGT2_ACACA/266-351	L8GGT2.1 PF02761.12	42.30	0.00
TRINITY_A0A0M3JWU8_ANISI/1-227	A0A0M3JWU8.1 PF0106	42.30	0.00
TRINITY_I0Z8T8_9CHLO/471-753	I0Z8T8.1 PF07714.15	42.30	0.00
TRINITY_D3BKI9_POLPA/132-385	D3BKI9.1 PF10192.7;	42.30	0.00
TRINITY_F4PIB6_DICFS/31-128	F4PIB6.1 PF01464.18	42.30	0.00
TRINITY_T1IQ34_STRMM/22-421	T1IQ34.1 PF06602.12	42.30	0.00
TRINITY_D8U202_VOLCA/350-568	D8U202.1 PF00149.26	42.30	0.00
TRINITY_D8U7D6_VOLCA/35-449	D8U7D6.1 PF01130.19	42.30	0.00
TRINITY_D8U183_VOLCA/1-169	D8U183.1 PF01699.22	42.30	0.00
TRINITY_E4T0R7_PALPW/19-248	E4T0R7.1 PF06966.10	42.30	0.00
TRINITY_I0YWA1_9CHLO/876-1281	I0YWA1.1 PF12698.5;	42.30	0.00
TRINITY_L8GL94_ACACA/389-614	L8GL94.1 PF13672.4;	42.30	0.00
TRINITY_F6SHW8_XENTR/104-190	F6SHW8.1 PF01248.24	42.30	0.00
TRINITY_I0YVD7_9CHLO/62-468	I0YVD7.1 PF01490.16	42.30	0.00
TRINITY_I1G2V3_AMPQE/8-158	I1G2V3.1 PF10914.6;	42.30	0.00
TRINITY_D3B5Y0_POLPA/16-91	D3B5Y0.1 PF00240.21	42.30	0.00
TRINITY_A0A0M0JU59_9EUKA/376-505	A0A0M0JU59.1 PF1457	42.30	0.00
TRINITY_I7M4Q8_TETTS/30-99	I7M4Q8.2 PF03810.17	42.20	0.00
TRINITY_L8GHP8_ACACA/1394-1625	L8GHP8.1 PF16095.3;	42.20	0.00
TRINITY_H2Z2F3_CIOSA/14-176	H2Z2F3.1 PF00071.20	42.20	0.00
TRINITY_F0Z7W7_DICPU/8-208	F0Z7W7.1 PF01470.15	42.20	0.00
TRINITY_G4HL19_9BACL/13-251	G4HL19.1 PF00149.26	42.20	0.00
TRINITY_F4Q9C3_DICFS/41-125	F4Q9C3.1 PF10241.7;	42.20	0.00
TRINITY_D3AW55_POLPA/307-519	D3AW55.1 PF00149.26	42.20	0.00

TRINITY_L8HGU1_ACACA/324-572	L8HGU1.1 PF07714.15	42.20	0.00
TRINITY_I7MFE1_TETTS/2-85	I7MFE1.2 PF00564.22	42.20	0.00
TRINITY_GXCDD_DICDI/464-636	Q1ZXH8.3 PF00621.18	42.20	0.00
TRINITY_I1FTI4_AMPQE/232-479	I1FTI4.1 PF07714.15	42.20	0.00
TRINITY_D6U0E1_9CHLR/8-365	D6U0E1.1 PF01266.22	42.20	0.00
TRINITY_A0CGH2_PARTE/14-116	A0CGH2.1 PF12796.5;	42.20	0.00
TRINITY_A4VDB7_TETTS/131-351	A4VDB7.2 PF00566.16	42.20	0.00
TRINITY_D8UBZ4_VOLCA/102-215	D8UBZ4.1 PF00072.22	42.20	0.00
TRINITY_R0F5G9_9BRAS/121-269	R0F5G9.1 PF00561.18	42.20	0.00
TRINITY_E1ZEP7_CHLVA/760-891	E1ZEP7.1 PF00782.18	42.20	0.00
TRINITY_F2NQ77_MARHT/11-101	F2NQ77.1 PF04930.13	42.20	0.00
TRINITY_J7S154_KAZNA/36-132	J7S154.1 PF12894.5;	42.20	0.00
TRINITY_B3SD03_TRIAD/19-185	B3SD03.1 PF13439.4;	42.20	0.00
TRINITY_E8R687_ISOPI/422-672	E8R687.1 PF00768.18	42.20	0.00
TRINITY_I7LUY7_TETTS/523-836	I7LUY7.2 PF00443.27	42.20	0.00
TRINITY_G4ZGH2_PHYSP/27-511	G4ZGH2.1 PF03917.15	42.20	0.00
TRINITY_D3BNX3_POLPA/460-588	D3BNX3.1 PF00134.21	42.20	0.00
TRINITY_B6UCH8_MAIZE/25-539	B6UCH8.1 PF12070.6;	42.20	0.00
TRINITY_A0A077ZYF9_STYLE/402-524	A0A077ZYF9.1 PF1389	42.20	0.00
TRINITY_J9INA7_9SPIT/626-748	J9INA7.1 PF01857.18	42.20	0.00
TRINITY_I7MLF7_TETTS/127-357	I7MLF7.2 PF12146.6;	42.20	0.00
TRINITY_C6Y250_PEDHD/31-271	C6Y250.1 PF03372.21	42.20	0.00
TRINITY_E3T5H3_CROVB/671-790	E3T5H3.1 PF00271.29	42.20	0.00
TRINITY_F4PAM0_BATDJ/73-357	F4PAM0.1 PF00069.23	42.20	0.00
TRINITY_A8J7G0_CHLRE/58-189	A8J7G0.1 PF14196.4;	42.20	0.00
TRINITY_A8IQ59_CHLRE/90-279	A8IQ59.1 PF00520.29	42.20	0.00
TRINITY_F2NKM4_MARHT/49-761	F2NKM4.1 PF01804.16	42.20	0.00
TRINITY_B0G157_DICDI/215-603	B0G157.1 PF00916.18	42.20	0.00
TRINITY_I7MER1_TETTS/6-122	I7MER1.1 PF00072.22	42.20	0.00
TRINITY_L8GTK3_ACACA/495-608	L8GTK3.1 PF00989.23	42.20	0.00
TRINITY_I1NIU9_SOYBN/691-943	I1NIU9.2 PF07714.15	42.20	0.00
TRINITY_I7M6A9_TETTS/15-456	I7M6A9.1 PF01593.22	42.20	0.00
TRINITY_A9SW39_PHYPA/275-482	A9SW39.1 PF04042.14	42.20	0.00
TRINITY_D8U4T2_VOLCA/12-139	D8U4T2.1 PF14108.4;	42.20	0.00
TRINITY_A0A0L1KTJ6_9EUGL/1-239	A0A0L1KTJ6.1 PF0190	42.20	0.00
TRINITY_C9SC39_VERA1/285-374	C9SC39.1 PF00400.30	42.20	0.00
TRINITY_D8U5D1_VOLCA/16-278	D8U5D1.1 PF00069.23	42.20	0.00
TRINITY_A0A0J7K9E1_LASNI/612-853	A0A0J7K9E1.1 PF0772	42.20	0.00
TRINITY_B7Q2C5_IXOSC/42-124	B7Q2C5.1 PF10163.7;	42.20	0.00
TRINITY_A0A0G0BZ10_9BACT/74-148	A0A0G0BZ10.1 PF1351	42.20	0.00
TRINITY_G0QUJ8_ICHMG/504-567	G0QUJ8.1 PF13499.4;	42.20	0.00
TRINITY_Q22P47_TETTS/55-293	Q22P47.2 PF16113.3;	42.20	0.00
TRINITY_B3SD12_TRIAD/384-712	B3SD12.1 PF05192.16	42.20	0.00
TRINITY_R1CK05_EMIHU/114-181	R1CK05.1 PF14835.4;	42.10	0.00
TRINITY_COAT_PVMR/117-257	P17529.2 PF00286.18	42.10	0.00
TRINITY_R0HFC0_9BRAS/6-379	R0HFC0.1 PF00022.17	42.10	0.00
TRINITY_A0D650_PARTE/108-461	A0D650.1 PF00282.17	42.10	0.00
TRINITY_A0A0A1RNM4_9FLAO/19-135	A0A0A1RNM4.1 PF0166	42.10	0.00
TRINITY_A0A0F8CZ33_CERFI/22-115	A0A0F8CZ33.1 PF0017	42.10	0.00
TRINITY_F0ZSE8_DICPU/110-250	F0ZSE8.1 PF01764.23	42.10	0.00
TRINITY_D3BF50_POLPA/766-874	D3BF50.1 PF10367.7;	42.10	0.00
TRINITY_M2YK38_PSEFD/995-1117	M2YK38.1 PF00072.22	42.10	0.00
TRINITY_T1K3F8_TETUR/8-287	T1K3F8.1 PF01073.17	42.10	0.00
TRINITY_A7SIC3_NEMVE/419-562	A7SIC3.1 PF00406.20	42.10	0.00
TRINITY_I7LWS5_TETTS/825-940	I7LWS5.1 PF02518.24	42.10	0.00
TRINITY_6PGL_DICDI/11-233	Q54CJ3.1 PF01182.18	42.10	0.00
TRINITY_Q244Z6_TETTS/77-284	Q244Z6.1 PF01027.18	42.10	0.00
TRINITY_I7MKT8_TETTS/187-659	I7MKT8.2 PF12569.6;	42.10	0.00
TRINITY_A0A0D2X4F4_CAPO3/760-1270	A0A0D2X4F4.1 PF0273	42.10	0.00
TRINITY_M2Y0I1_GALSU/89-164	M2Y0I1.1 PF03061.20	42.10	0.00
TRINITY_A2FHQ0_TRIVA/5-116	A2FHQ0.1 PF01412.16	42.10	0.00

TRINITY_S2J5Q1_MUCC1/24-327	S2J5Q1.1 PF03676.12	42.10	0.00
TRINITY_B9IQV9_POPTR/61-193	B9IQV9.1 PF12894.5;	42.10	0.00
TRINITY_A8JFG7_CHLRE/20-187	A8JFG7.1 PF16016.3;	42.10	0.00
TRINITY_D5DUV3_BACMQ/4-208	D5DUV3.1 PF00106.23	42.10	0.00
TRINITY_W1PN16_AMBTC/264-486	W1PN16.1 PF12230.6;	42.10	0.00
TRINITY_A0A0F6W723_9DELT/34-204	A0A0F6W723.1 PF0085	42.10	0.00
TRINITY_F2U7G3_SALR5/6-199	F2U7G3.1 PF03547.16	42.10	0.00
TRINITY_Q7U8B9_SYNPX/585-979	Q7U8B9.1 PF00476.18	42.10	0.00
TRINITY_L8HI91_ACACA/91-321	L8HI91.1 PF05721.11	42.10	0.00
TRINITY_L8H1D6_ACACA/34-1138	L8H1D6.1 PF06427.9;	42.10	0.00
TRINITY_W7TSG7_9STRA/162-344	W7TSG7.1 PF13489.4;	42.10	0.00
TRINITY_B9GG97_POPTR/234-459	B9GG97.2 PF10189.7;	42.10	0.00
TRINITY_K7LGI4_SOYBN/14-124	K7LGI4.1 PF16457.3;	42.10	0.00
TRINITY_D3B127_POLPA/7-702	D3B127.1 PF04563.13	42.10	0.00
TRINITY_L8GMG4_ACACA/32-285	L8GMG4.1 PF02265.14	42.10	0.00
TRINITY_A0DWG3_PARTE/57-420	A0DWG3.1 PF08014.9;	42.10	0.00
TRINITY_D8TQB2_VOLCA/123-576	D8TQB2.1 PF06087.10	42.10	0.00
TRINITY_K6DS30_9BACI/321-446	K6DS30.1 PF16921.3;	42.10	0.00
TRINITY_F0ZPI4_DICPU/1-102	F0ZPI4.1 PF00168.28	42.10	0.00
TRINITY_U5GDM3_POPTR/76-330	U5GDM3.1 PF07714.15	42.10	0.00
TRINITY_D3BLM9_POLPA/5-417	D3BLM9.1 PF03348.13	42.10	0.00
TRINITY_A8IY87_CHLRE/14-419	A8IY87.1 PF00225.21	42.10	0.00
TRINITY_A0A0D2UMU4_CAPO3/32-518	A0A0D2UMU4.1 PF1403	42.10	0.00
TRINITY_A0A072V6I0_MEDTR/24-100	A0A072V6I0.1 PF0313	42.10	0.00
TRINITY_I0Z5H8_9CHLO/124-332	I0Z5H8.1 PF11397.6;	42.10	0.00
TRINITY_L8H174_ACACA/373-591	L8H174.1 PF07690.14	42.10	0.00
TRINITY_A0DS96_PARTE/7-120	A0DS96.1 PF02136.18	42.10	0.00
TRINITY_W6SJQ7_9CLOT/39-179	W6SJQ7.2 PF00388.17	42.10	0.00
TRINITY_F8L2G7_PARAV/112-247	F8L2G7.1 PF14566.4;	42.10	0.00
TRINITY_A0A0E0NR04_ORYRU/248-372	A0A0E0NR04.1 PF0139	42.10	0.00
TRINITY_A8IJV3_CHLRE/389-451	A8IJV3.1 PF13499.4;	42.10	0.00
TRINITY_L1LFM9_THEEQ/430-589	L1LFM9.1 PF00613.18	42.10	0.00
TRINITY_D8TML2_VOLCA/701-1072	D8TML2.1 PF09763.7;	42.10	0.00
TRINITY_W4XUU0_STRPU/19-148	W4XUU0.1 PF00153.25	42.10	0.00
TRINITY_L8H4I8_ACACA/10-1020	L8H4I8.1 PF02463.17	42.10	0.00
TRINITY_A0A0D2X3J6_CAPO3/1040-1286	A0A0D2X3J6.1 PF1621	42.10	0.00
TRINITY_B7KET0_CYAP7/9-251	B7KET0.1 PF09925.7;	42.10	0.00
TRINITY_D8UFJ6_VOLCA/43-258	D8UFJ6.1 PF00005.25	42.10	0.00
TRINITY_Q22GE2_TETTS/34-285	Q22GE2.2 PF00069.23	42.10	0.00
TRINITY_H0Q4S0_9RHOO/6-227	H0Q4S0.1 PF05368.11	42.10	0.00
TRINITY_A7S5W3_NEMVE/30-440	A7S5W3.1 PF03345.12	42.10	0.00
TRINITY_A8IMH9_CHLRE/18-331	A8IMH9.1 PF00246.22	42.10	0.00
TRINITY_I1P6K5_ORYGL/449-713	I1P6K5.1 PF00069.23	42.10	0.00
TRINITY_E1ZTF2_CHLVA/2-283	E1ZTF2.1 PF03399.14	42.10	0.00
TRINITY_A8JGN8_CHLRE/123-615	A8JGN8.1 PF02460.16	42.10	0.00
TRINITY_M5FYF3_DACSP/39-395	M5FYF3.1 PF00067.20	42.10	0.00
TRINITY_A0A0F5YH50_9CYAN/28-136	A0A0F5YH50.1 PF0056	42.10	0.00
TRINITY_A0A0D2WLK3_CAPO3/40-116	A0A0D2WLK3.1 PF0576	42.10	0.00
TRINITY_A0A0N0RFN5_9CHLR/1-451	A0A0N0RFN5.1 PF0017	42.00	0.00
TRINITY_A0E014_PARTE/337-474	A0E014.1 PF07534.14	42.00	0.00
TRINITY_Q233P3_TETTS/56-171	Q233P3.2 PF08487.8;	42.00	0.00
TRINITY_ROCO9_DICDI/795-1404	Q6XHA7.1 PF06602.12	42.00	0.00
TRINITY_G0QIY3_ICHMG/15-273	G0QIY3.1 PF00069.23	42.00	0.00
TRINITY_A0A0G1MVQ3_9BACT/7-179	A0A0G1MVQ3.1 PF0193	42.00	0.00
TRINITY_A0A0D2VT60_CAPO3/8-170	A0A0D2VT60.1 PF0612	42.00	0.00
TRINITY_F8EMT3_RUNSL/38-203	F8EMT3.1 PF01965.22	42.00	0.00
TRINITY_I1BYH5_RHIO9/1238-1353	I1BYH5.1 PF00665.24	42.00	0.00
TRINITY_I1HEB8_BRADI/45-196	I1HEB8.1 PF01694.20	42.00	0.00
TRINITY_I7M4F9_TETTS/51-228	I7M4F9.2 PF01195.17	42.00	0.00
TRINITY_A0A072U3N2_MEDTR/535-642	A0A072U3N2.1 PF0065	42.00	0.00
TRINITY_W4YX03_STRPU/465-606	W4YX03.1 PF01936.16	42.00	0.00

TRINITY_F4QDG7_DICFS/50-329	F4QDG7.1 PF07159.10	42.00	0.00
TRINITY_L8GIZ6_ACACA/66-487	L8GIZ6.1 PF04587.13	42.00	0.00
TRINITY_F4PXK2_DICFS/237-332	F4PXK2.1 PF12796.5;	42.00	0.00
TRINITY_A0DUQ8_PARTE/34-561	A0DUQ8.1 PF16969.3;	42.00	0.00
TRINITY_L8HAQ2_ACACA/138-434	L8HAQ2.1 PF01733.16	42.00	0.00
TRINITY_F4QEK4_DICFS/1-172	F4QEK4.1 PF00149.26	42.00	0.00
TRINITY_M0RWE8_MUSAM/20-395	M0RWE8.1 PF03215.13	42.00	0.00
TRINITY_Q22C26_TETTS/11-618	Q22C26.1 PF00012.18	42.00	0.00
TRINITY_A0A0G4IPY5_PLABS/12-534	A0A0G4IPY5.1 PF0160	42.00	0.00
TRINITY_D8U002_VOLCA/1333-1769	D8U002.1 PF02181.21	42.00	0.00
TRINITY_F1A1Z7_DICPU/11-129	F1A1Z7.1 PF00241.18	42.00	0.00
TRINITY_A8UVI3_9AQUI/7-126	A8UVI3.1 PF03099.17	42.00	0.00
TRINITY_F0ZEH2_DICPU/3-82	F0ZEH2.1 PF00564.22	42.00	0.00
TRINITY_L8GEC7_ACACA/6-324	L8GEC7.1 PF01112.16	42.00	0.00
TRINITY_K3WX01_PYTUL/34-102	K3WX01.1 PF00505.17	42.00	0.00
TRINITY_D3BMY9_POLPA/174-320	D3BMY9.1 PF07534.14	42.00	0.00
TRINITY_L8H1J8_ACACA/532-611	L8H1J8.1 PF13646.4;	42.00	0.00
TRINITY_C5LZF4_PERM5/19-298	C5LZF4.1 PF00664.21	42.00	0.00
TRINITY_A0A0F9Z917_9MICR/131-248	A0A0F9Z917.1 PF0066	42.00	0.00
TRINITY_G0R330_ICHMG/329-430	G0R330.1 PF01399.25	42.00	0.00
TRINITY_L8GH09_ACACA/77-221	L8GH09.1 PF05118.13	42.00	0.00
TRINITY_F0ZVG6_DICPU/9-103	F0ZVG6.1 PF10270.7;	42.00	0.00
TRINITY_A9S6E3_PHYPA/19-193	A9S6E3.1 PF10294.7;	42.00	0.00
TRINITY_A0A087ST44_AUXPR/143-575	A0A087ST44.1 PF0114	42.00	0.00
TRINITY_D8TID5_VOLCA/47-174	D8TID5.1 PF01569.19	42.00	0.00
TRINITY_K4L3H3_9FIRM/7-87	K4L3H3.1 PF00312.20	42.00	0.00
TRINITY_A0A087SE41_AUXPR/106-364	A0A087SE41.1 PF0066	42.00	0.00
TRINITY_A8HMH0_CHLRE/50-171	A8HMH0.1 PF13902.4;	42.00	0.00
TRINITY_D8SX78_SELML/328-890	D8SX78.1 PF05183.10	42.00	0.00
TRINITY_G0R4I1_ICHMG/93-249	G0R4I1.1 PF06705.9;	42.00	0.00
TRINITY_Q23DB0_TETTS/210-405	Q23DB0.2 PF00566.16	42.00	0.00
TRINITY_A0A087SUE7_AUXPR/3-172	A0A087SUE7.1 PF0192	42.00	0.00
TRINITY_A0BGN6_PARTE/41-392	A0BGN6.1 PF13868.4;	42.00	0.00
TRINITY_A0A072TZJ6_MEDTR/418-499	A0A072TZJ6.1 PF0078	42.00	0.00
TRINITY_E1ZBU8_CHLVA/98-365	E1ZBU8.1 PF12697.5;	42.00	0.00
TRINITY_A9TA33_PHYPA/48-150	A9TA33.1 PF13664.4;	42.00	0.00
TRINITY_A8JBE2_CHLRE/96-202	A8JBE2.1 PF14497.4;	42.00	0.00
TRINITY_A8JJ05_CHLRE/562-633	A8JJ05.1 PF05699.12	42.00	0.00
TRINITY_Q24HN9_TETTS/108-178	Q24HN9.1 PF13499.4;	42.00	0.00
TRINITY_A0A0A2VBR5_BEABA/78-552	A0A0A2VBR5.1 PF0142	42.00	0.00
TRINITY_H3SN11_9BACL/78-365	H3SN11.1 PF08450.10	42.00	0.00
TRINITY_W5JTG0_ANODA/6-218	W5JTG0.1 PF13561.4;	41.90	0.00
TRINITY_A9SHY7_PHYPA/7-301	A9SHY7.1 PF01026.19	41.90	0.00
TRINITY_H2XX86_CIOIN/165-293	H2XX86.1 PF04784.12	41.90	0.00
TRINITY_J9I397_9SPIT/144-375	J9I397.1 PF03372.21	41.90	0.00
TRINITY_A0CQ76_PARTE/32-273	A0CQ76.1 PF00481.19	41.90	0.00
TRINITY_A0A078ARK2_STYLE/326-473	A0A078ARK2.1 PF0753	41.90	0.00
TRINITY_W9WL36_9EURO/1-109	W9WL36.1 PF03656.11	41.90	0.00
TRINITY_D3BQD3_POLPA/205-456	D3BQD3.1 PF07714.15	41.90	0.00
TRINITY_A0A059CH48_EUCGR/116-267	A0A059CH48.1 PF0254	41.90	0.00
TRINITY_A0A024TSY5_9STRA/10-156	A0A024TSY5.1 PF0598	41.90	0.00
TRINITY_A0A077ZSF8_STYLE/367-472	A0A077ZSF8.1 PF0008	41.90	0.00
TRINITY_I1IX59_BRADI/330-496	I1IX59.1 PF08585.10	41.90	0.00
TRINITY_F0ZEG5_DICPU/238-412	F0ZEG5.1 PF16575.3;	41.90	0.00
TRINITY_A0A0M2MP12_9ACTN/150-316	A0A0M2MP12.1 PF0089	41.90	0.00
TRINITY_W4GXX6_9STRA/9-527	W4GXX6.1 PF01274.20	41.90	0.00
TRINITY_Q22D68_TETTS/29-277	Q22D68.3 PF03803.13	41.90	0.00
TRINITY_F0ZCT2_DICPU/601-783	F0ZCT2.1 PF00617.17	41.90	0.00
TRINITY_G3WDD0_SARHA/765-949	G3WDD0.1 PF05193.19	41.90	0.00
TRINITY_A0A0A1UBU5_ENTIV/727-986	A0A0A1UBU5.1 PF0771	41.90	0.00
TRINITY_S9X737_SCHCR/3-74	S9X737.1 PF00240.21	41.90	0.00

TRINITY_E0MTL2_9RHOB/76-323	E0MTL2.1 PF05292.9;	41.90	0.00
TRINITY_R6KRQ7_9FIRM/452-569	R6KRQ7.1 PF00072.22	41.90	0.00
TRINITY_ACTG_EMENI/2-375	P20359.2 PF00022.17	41.90	0.00
TRINITY_A0CDL7_PARTE/665-974	A0CDL7.1 PF00069.23	41.90	0.00
TRINITY_Q1QL94_NITHX/18-166	Q1QL94.1 PF00499.18	41.90	0.00
TRINITY_I7M4N1_TETTS/619-783	I7M4N1.2 PF13925.4;	41.90	0.00
TRINITY_A0A059LHX5_9CHLO/154-349	A0A059LHX5.1 PF0048	41.90	0.00
TRINITY_C4LWB1_ENTHI/196-373	C4LWB1.1 PF00644.18	41.90	0.00
TRINITY_A0DVE4_PARTE/154-417	A0DVE4.1 PF00069.23	41.90	0.00
TRINITY_C1MTN3_MICPC/42-154	C1MTN3.1 PF13589.4;	41.90	0.00
TRINITY_H2ZC63_CIOSA/36-102	H2ZC63.1 PF03810.17	41.90	0.00
TRINITY_L8HK60_ACACA/154-481	L8HK60.1 PF07690.14	41.90	0.00
TRINITY_A0A077ZWA2_STYLE/66-158	A0A077ZWA2.1 PF1254	41.90	0.00
TRINITY_L8GSH4_ACACA/37-217	L8GSH4.1 PF00621.18	41.90	0.00
TRINITY_G3NEE7_GASAC/102-172	G3NEE7.1 PF01472.18	41.90	0.00
TRINITY_J0LJG4_9BACT/8-143	J0LJG4.1 PF13673.5;	41.90	0.00
TRINITY_D8TNZ7_VOLCA/521-584	D8TNZ7.1 PF13499.4;	41.90	0.00
TRINITY_B8HKC9_CYAP4/36-145	B8HKC9.1 PF05175.12	41.90	0.00
TRINITY_A0A067LA43_JATCU/589-650	A0A067LA43.1 PF0057	41.90	0.00
TRINITY_K9Y0F8_STAC7/144-341	K9Y0F8.1 PF09414.8;	41.90	0.00
TRINITY_D2VVN4_NAEGR/4-230	D2VVN4.1 PF01370.19	41.90	0.00
TRINITY_W5D0Y2_WHEAT/394-466	W5D0Y2.1 PF00076.20	41.90	0.00
TRINITY_B3QRX7_CHLT3/148-394	B3QRX7.1 PF01564.15	41.90	0.00
TRINITY_D8TH74_VOLCA/222-373	D8TH74.1 PF00582.24	41.90	0.00
TRINITY_H2YJI5_CIOSA/18-123	H2YJI5.1 PF00085.18	41.90	0.00
TRINITY_A0A0D2WXD3_CAPO3/14-111	A0A0D2WXD3.1 PF0016	41.90	0.00
TRINITY_K3X6J3_PYTUL/51-297	K3X6J3.1 PF06966.10	41.90	0.00
TRINITY_A4RUB8_OSTLU/240-304	A4RUB8.1 PF00076.20	41.90	0.00
TRINITY_B3S701_TRIAD/49-334	B3S701.1 PF03133.13	41.90	0.00
TRINITY_A0A0D2X4V9_CAPO3/630-791	A0A0D2X4V9.1 PF0155	41.90	0.00
TRINITY_K3YIM3_SETIT/150-261	K3YIM3.1 PF00651.29	41.90	0.00
TRINITY_G3H9M4_CRIGR/134-352	G3H9M4.1 PF10152.7;	41.90	0.00
TRINITY_A8J7M9_CHLRE/742-1038	A8J7M9.1 PF00069.23	41.90	0.00
TRINITY_A0A015JRC9_9GLOM/187-229	A0A015JRC9.1 PF0874	41.90	0.00
TRINITY_Q22D68_TETTS/29-277	Q22D68.3 PF03803.13	41.90	0.00
TRINITY_Q22CD1_TETTS/91-375	Q22CD1.3 PF15239.4;	41.90	0.00
TRINITY_B4N7D2_DROWI/8-102	B4N7D2.2 PF12763.5;	41.90	0.00
TRINITY_F4PMR5_DICFS/17-275	F4PMR5.1 PF00069.23	41.90	0.00
TRINITY_A8IGG5_CHLRE/30-160	A8IGG5.1 PF01590.24	41.90	0.00
TRINITY_M5DT52_9GAMM/962-1080	M5DT52.1 PF00072.22	41.90	0.00
TRINITY_A0A0B5AK87_9BACL/6-140	A0A0B5AK87.1 PF1367	41.90	0.00
TRINITY_A0C2U9_PARTE/199-297	A0C2U9.1 PF00153.25	41.80	0.00
TRINITY_A0DZU7_PARTE/220-392	A0DZU7.1 PF02141.19	41.80	0.00
TRINITY_F4PX08_DICFS/1189-1360	F4PX08.1 PF00616.17	41.80	0.00
TRINITY_A0CM38_PARTE/187-256	A0CM38.1 PF14360.4;	41.80	0.00
TRINITY_A0BQP2_PARTE/7-162	A0BQP2.1 PF14825.4;	41.80	0.00
TRINITY_UFL1_NEMVE/6-285	A7S2N8.1 PF09743.7;	41.80	0.00
TRINITY_Q236N2_TETTS/32-570	Q236N2.1 PF00995.21	41.80	0.00
TRINITY_A0A0D2X2T3_CAPO3/170-333	A0A0D2X2T3.1 PF0150	41.80	0.00
TRINITY_A7SJ08_NEMVE/1-193	A7SJ08.1 PF05603.10	41.80	0.00
TRINITY_B9Y3S3_9FIRM/34-274	B9Y3S3.1 PF09234.8;	41.80	0.00
TRINITY_G0QR98_ICHMG/111-321	G0QR98.1 PF00481.19	41.80	0.00
TRINITY_E1ZLK8_CHLVA/56-427	E1ZLK8.1 PF01137.19	41.80	0.00
TRINITY_I4B662_TURPD/34-279	I4B662.1 PF00561.18	41.80	0.00
TRINITY_E9FWX6_DAPPU/9-301	E9FWX6.1 PF00459.23	41.80	0.00
TRINITY_A0YRE9_LYNSP/27-168	A0YRE9.1 PF08240.10	41.80	0.00
TRINITY_T0Q0L4_9STRA/2173-2348	T0Q0L4.1 PF00520.29	41.80	0.00
TRINITY_Q238W5_TETTS/26-475	Q238W5.1 PF00067.20	41.80	0.00
TRINITY_L8HFF8_ACACA/783-882	L8HFF8.1 PF04815.13	41.80	0.00
TRINITY_A0A087SM46_AUXPR/22-344	A0A087SM46.1 PF0022	41.80	0.00
TRINITY_I1IXR1_BRADI/35-177	I1IXR1.1 PF10075.7;	41.80	0.00

TRINITY_L8H8K8_ACACA/477-884	L8H8K8.1 PF02450.13	41.80	0.00
TRINITY_D8TQV2_VOLCA/75-216	D8TQV2.1 PF00875.16	41.80	0.00
TRINITY_A8IJP0_CHLRE/1-359	A8IJP0.1 PF03372.21	41.80	0.00
TRINITY_J9ITQ4_9SPIT/400-466	J9ITQ4.1 PF00659.16	41.80	0.00
TRINITY_I0Z1L6_9CHLO/71-262	I0Z1L6.1 PF13489.4;	41.80	0.00
TRINITY_V4MCG7_EUTSA/142-222	V4MCG7.1 PF10609.7;	41.80	0.00
TRINITY_B3RQX0_TRIAD/2-438	B3RQX0.1 PF00067.20	41.80	0.00
TRINITY_J9I4Z2_9SPIT/36-141	J9I4Z2.1 PF00085.18	41.80	0.00
TRINITY_A0A058Z2H3_9EUKA/776-953	A0A058Z2H3.1 PF0061	41.80	0.00
TRINITY_D8TUE9_VOLCA/339-558	D8TUE9.1 PF12348.6;	41.80	0.00
TRINITY_W2QD38_PHYPN/821-913	W2QD38.1 PF12796.5;	41.80	0.00
TRINITY_C1EC92_MICSR/28-237	C1EC92.1 PF00106.23	41.80	0.00
TRINITY_E1Z5Y3_CHLVA/6-105	E1Z5Y3.1 PF05773.20	41.80	0.00
TRINITY_Q23KK9_TETTS/391-552	Q23KK9.1 PF14580.4;	41.80	0.00
TRINITY_A8HPQ8_CHLRE/224-321	A8HPQ8.1 PF01342.19	41.80	0.00
TRINITY_G0QV21_ICHMG/3082-3254	G0QV21.1 PF16909.3;	41.80	0.00
TRINITY_A4RS27_OSTLU/1-507	A4RS27.1 PF01593.22	41.80	0.00
TRINITY_A0A0G4EFT4_9ALVE/392-487	A0A0G4EFT4.1 PF1276	41.80	0.00
TRINITY_A0A067KN01_JATCU/413-773	A0A067KN01.1 PF0070	41.80	0.00
TRINITY_D8TY98_VOLCA/526-656	D8TY98.1 PF00004.27	41.80	0.00
TRINITY_A0A0D2WWJ2_CAPO3/488-794	A0A0D2WWJ2.1 PF0371	41.80	0.00
TRINITY_Q229C1_TETTS/81-547	Q229C1.2 PF00083.22	41.80	0.00
TRINITY_G0R2D7_ICHMG/7-172	G0R2D7.1 PF00071.20	41.80	0.00
TRINITY_L8GQV2_ACACA/6-257	L8GQV2.1 PF03194.13	41.80	0.00
TRINITY_F0ZPA4_DICPU/191-422	F0ZPA4.1 PF00557.22	41.80	0.00
TRINITY_A0A078APK7_STYLE/16-112	A0A078APK7.1 PF0017	41.80	0.00
TRINITY_A0A091CYN1_FUKDA/425-618	A0A091CYN1.1 PF0056	41.80	0.00
TRINITY_D8TUF0_VOLCA/155-576	D8TUF0.1 PF03164.12	41.80	0.00
TRINITY_I0YVY5_9CHLO/13-438	I0YVY5.1 PF01532.18	41.80	0.00
TRINITY_D8UFJ2_VOLCA/156-276	D8UFJ2.1 PF03407.14	41.80	0.00
TRINITY_E1ZIQ9_CHLVA/318-396	E1ZIQ9.1 PF09409.8;	41.70	0.00
TRINITY_G0QJN5_ICHMG/3-324	G0QJN5.1 PF07690.14	41.70	0.00
TRINITY_R9NWW6_PSEHS/42-243	R9NWW6.1 PF00270.27	41.70	0.00
TRINITY_B3S5V4_TRIAD/37-486	B3S5V4.1 PF00067.20	41.70	0.00
TRINITY_A0A0C2N3F1_THEKT/13-174	A0A0C2N3F1.1 PF0007	41.70	0.00
TRINITY_I7M4C2_TETTS/42-276	I7M4C2.1 PF00149.26	41.70	0.00
TRINITY_C5LZF4_PERM5/19-298	C5LZF4.1 PF00664.21	41.70	0.00
TRINITY_L8GMP4_ACACA/297-555	L8GMP4.1 PF07714.15	41.70	0.00
TRINITY_A0A024UP17_9STRA/27-123	A0A024UP17.1 PF1387	41.70	0.00
TRINITY_A0A078AZE2_STYLE/57-170	A0A078AZE2.1 PF0097	41.70	0.00
TRINITY_X6NTL5_RETFI/199-322	X6NTL5.1 PF13271.4;	41.70	0.00
TRINITY_G7YX44_CLOSI/74-306	G7YX44.1 PF00069.23	41.70	0.00
TRINITY_A0A078ART0_STYLE/361-506	A0A078ART0.1 PF0753	41.70	0.00
TRINITY_A0A087Y905_POEFO/1-122	A0A087Y905.1 PF0624	41.70	0.00
TRINITY_L8GMR3_ACACA/270-526	L8GMR3.1 PF07714.15	41.70	0.00
TRINITY_A0DYX2_PARTE/54-188	A0DYX2.1 PF04146.13	41.70	0.00
TRINITY_S2JLP4_MUCC1/13-267	S2JLP4.1 PF00378.18	41.70	0.00
TRINITY_L8HDD8_ACACA/1327-1576	L8HDD8.1 PF07714.15	41.70	0.00
TRINITY_G8NQG5_GRAMM/20-239	G8NQG5.1 PF05368.11	41.70	0.00
TRINITY_C5XSI0_SORBI/565-716	C5XSI0.1 PF08646.8;	41.70	0.00
TRINITY_G0S3W0_CHATD/10-171	G0S3W0.1 PF00071.20	41.70	0.00
TRINITY_X6NSF5_RETFI/88-345	X6NSF5.1 PF07714.15	41.70	0.00
TRINITY_A0DUB6_PARTE/233-698	A0DUB6.1 PF01055.24	41.70	0.00
TRINITY_V3ZNT8_LOTGI/77-368	V3ZNT8.1 PF01008.15	41.70	0.00
TRINITY_J9EMC8_9SPIT/1395-1489	J9EMC8.1 PF01833.22	41.70	0.00
TRINITY_CF50_DICDI/32-218	Q556R7.1 PF01183.18	41.70	0.00
TRINITY_B8MLR9_TALSN/4-463	B8MLR9.1 PF00743.17	41.70	0.00
TRINITY_G0QKT4_ICHMG/33-338	G0QKT4.1 PF04756.11	41.70	0.00
TRINITY_C1N3N9_MICPC/250-346	C1N3N9.1 PF00153.25	41.70	0.00
TRINITY_I0YWK9_9CHLO/100-333	I0YWK9.1 PF00149.26	41.70	0.00
TRINITY_B3RXN7_TRIAD/22-269	B3RXN7.1 PF00481.19	41.70	0.00

TRINITY_A6C4L7_9PLAN/434-549	A6C4L7.1 PF00072.22	41.70	0.00
TRINITY_I4D1P6_DESAJ/150-215	I4D1P6.1 PF00512.23	41.70	0.00
TRINITY_D8U0Z5_VOLCA/66-226	D8U0Z5.1 PF04751.12	41.70	0.00
TRINITY_G0QW56_ICHMG/288-451	G0QW56.1 PF07690.14	41.70	0.00
TRINITY_I0YSB4_9CHLO/407-612	I0YSB4.1 PF09797.7;	41.70	0.00
TRINITY_A0A059LNL8_9CHLO/41-139	A0A059LNL8.1 PF0322	41.70	0.00
TRINITY_A0A0G1R6A7_9BACT/2-122	A0A0G1R6A7.1 PF0871	41.70	0.00
TRINITY_I0YM11_9CHLO/121-499	I0YM11.1 PF09737.7;	41.70	0.00
TRINITY_A0A068X9X2_HYMMI/98-256	A0A068X9X2.1 PF0123	41.70	0.00
TRINITY_X6NZT3_RETFI/649-765	X6NZT3.1 PF00454.25	41.70	0.00
TRINITY_E1ZH45_CHLVA/404-606	E1ZH45.1 PF08719.9;	41.70	0.00
TRINITY_M7NNA1_PNEMU/13-170	M7NNA1.1 PF00071.20	41.70	0.00
TRINITY_A0C3N3_PARTE/5-199	A0C3N3.1 PF03031.16	41.70	0.00
TRINITY_I1IWC9_BRADI/18-209	I1IWC9.1 PF00300.20	41.70	0.00
TRINITY_E1Z7R1_CHLVA/519-776	E1Z7R1.1 PF07714.15	41.70	0.00
TRINITY_A8JIH7_CHLRE/59-225	A8JIH7.1 PF00650.18	41.70	0.00
TRINITY_D8UHI9_VOLCA/117-368	D8UHI9.1 PF12697.5;	41.70	0.00
TRINITY_A0A0G0VBZ0_9BACT/8-133	A0A0G0VBZ0.1 PF0029	41.70	0.00
TRINITY_L8GUF1_ACACA/338-497	L8GUF1.1 PF02136.18	41.70	0.00
TRINITY_A0A060SMB1_PYCCI/3-124	A0A060SMB1.1 PF0024	41.70	0.00
TRINITY_F4QD17_DICFS/238-364	F4QD17.1 PF03188.14	41.70	0.00
TRINITY_A9SXS5_PHYPA/609-839	A9SXS5.1 PF01612.18	41.70	0.00
TRINITY_S9WZ17_SCHCR/171-278	S9WZ17.1 PF00175.19	41.70	0.00
TRINITY_D3BF23_POLPA/294-416	D3BF23.1 PF00917.24	41.70	0.00
TRINITY_I0YPY2_9CHLO/2-154	I0YPY2.1 PF04720.10	41.70	0.00
TRINITY_F4PI07_DICFS/810-1059	F4PI07.1 PF07714.15	41.70	0.00
TRINITY_A0A0L6DIV3_CRYGA/274-399	A0A0L6DIV3.1 PF0007	41.70	0.00
TRINITY_B4N9W5_DROWI/120-193	B4N9W5.2 PF13499.4;	41.70	0.00
TRINITY_B0C469_ACAM1/31-206	B0C469.1 PF00211.18	41.70	0.00
TRINITY_A0A0G4IH87_PLABS/70-292	A0A0G4IH87.1 PF0845	41.70	0.00
TRINITY_A0A0N5AAC6_9BILA/75-207	A0A0N5AAC6.1 PF0078	41.70	0.00
TRINITY_K9TJ96_9CYAN/29-277	K9TJ96.1 PF00657.20	41.70	0.00
TRINITY_A0CHY1_PARTE/11-268	A0CHY1.1 PF00069.23	41.70	0.00
TRINITY_F0ZDD1_DICPU/1-371	F0ZDD1.1 PF03097.16	41.70	0.00
TRINITY_A0EFW2_PARTE/159-373	A0EFW2.1 PF06991.9;	41.70	0.00
TRINITY_D2V295_NAEGR/289-529	D2V295.1 PF00082.20	41.70	0.00
TRINITY_A5V585_SPHWW/37-167	A5V585.1 PF00188.24	41.70	0.00
TRINITY_I7MCJ9_TETTS/89-462	I7MCJ9.1 PF03006.18	41.70	0.00
TRINITY_X6NQF9_RETFI/38-135	X6NQF9.1 PF13640.4;	41.70	0.00
TRINITY_G0R5B0_ICHMG/43-223	G0R5B0.1 PF03357.19	41.70	0.00
TRINITY_D8ULH4_VOLCA/183-452	D8ULH4.1 PF00520.29	41.70	0.00
TRINITY_K3X5W9_PYTUL/770-1032	K3X5W9.1 PF00664.21	41.70	0.00
TRINITY_A8BJ08_GIAIC/6-76	A8BJ08.1 PF11976.6;	41.70	0.00
TRINITY_R0GSU3_9BRAS/9-145	R0GSU3.1 PF00179.24	41.60	0.00
TRINITY_F0VPP9_NEOCL/24-411	F0VPP9.1 PF00022.17	41.60	0.00
TRINITY_Q24GM0_TETTS/267-369	Q24GM0.1 PF16656.3;	41.60	0.00
TRINITY_A0A077ZW35_STYLE/38-421	A0A077ZW35.1 PF0022	41.60	0.00
TRINITY_A0A084WHS3_ANOSI/246-395	A0A084WHS3.1 PF0027	41.60	0.00
TRINITY_I1CC69_RHIO9/32-317	I1CC69.1 PF02383.16	41.60	0.00
TRINITY_F0ZM84_DICPU/129-665	F0ZM84.1 PF04791.14	41.60	0.00
TRINITY_A0DFM4_PARTE/122-222	A0DFM4.1 PF00237.17	41.60	0.00
TRINITY_W4Y4C3_STRPU/3-113	W4Y4C3.1 PF00071.20	41.60	0.00
TRINITY_W7X142_TETTS/122-276	W7X142.1 PF00650.18	41.60	0.00
TRINITY_C1DYI8_MICSR/44-311	C1DYI8.1 PF00248.19	41.60	0.00
TRINITY_G0QVH1_ICHMG/13-130	G0QVH1.1 PF00498.24	41.60	0.00
TRINITY_A9V0E1_MONBE/11-355	A9V0E1.1 PF03283.11	41.60	0.00
TRINITY_A0A0M9UBV5_9CHLR/10-202	A0A0M9UBV5.1 PF0030	41.60	0.00
TRINITY_Q23QF2_TETTS/248-571	Q23QF2.2 PF03133.13	41.60	0.00
TRINITY_J9EXT5_9SPIT/263-461	J9EXT5.1 PF00117.26	41.60	0.00
TRINITY_A8I8T3_CHLRE/1528-1690	A8I8T3.1 PF10475.7;	41.60	0.00
TRINITY_A0A0N5AC28_9BILA/45-151	A0A0N5AC28.1 PF0016	41.60	0.00

TRINITY_D0MI23_RHOM4/25-436	D0MI23.1 PF00245.18	41.60	0.00
TRINITY_D8U611_VOLCA/11-73	D8U611.1 PF00886.17	41.60	0.00
TRINITY_I0Z3X8_9CHLO/19-152	I0Z3X8.1 PF17004.3;	41.60	0.00
TRINITY_A8HWK8_CHLRE/45-673	A8HWK8.1 PF16969.3;	41.60	0.00
TRINITY_D8UIJ4_VOLCA/36-294	D8UIJ4.1 PF02636.15	41.60	0.00
TRINITY_A0A078A9S2_STYLE/86-176	A0A078A9S2.1 PF1279	41.60	0.00
TRINITY_A8I2D8_CHLRE/6-285	A8I2D8.1 PF01875.15	41.60	0.00
TRINITY_C5XNT7_SORBI/39-251	C5XNT7.1 PF12146.6;	41.60	0.00
TRINITY_T1FM94_HELRO/45-137	T1FM94.1 PF00254.26	41.60	0.00
TRINITY_L8H311_ACACA/395-613	L8H311.1 PF08427.8;	41.60	0.00
TRINITY_A0A067TKC9_9AGAR/118-210	A0A067TKC9.1 PF1658	41.60	0.00
TRINITY_A0BRQ6_PARTE/134-489	A0BRQ6.1 PF03133.13	41.60	0.00
TRINITY_F4QHS4_9CAUL/170-343	F4QHS4.1 PF00848.17	41.60	0.00
TRINITY_A0A0A1UAM9_ENTIV/575-835	A0A0A1UAM9.1 PF0771	41.60	0.00
TRINITY_H3FPL3_PRIPA/86-223	H3FPL3.1 PF01764.23	41.60	0.00
TRINITY_D8TKQ1_VOLCA/18-228	D8TKQ1.1 PF03152.12	41.60	0.00
TRINITY_A0A0G4J0Q6_PLABS/20-282	A0A0G4J0Q6.1 PF0048	41.60	0.00
TRINITY_A0A024TZI0_9STRA/11-268	A0A024TZI0.1 PF0006	41.60	0.00
TRINITY_I7M910_TETTS/11-285	I7M910.1 PF00069.23	41.60	0.00
TRINITY_I0Z6W1_9CHLO/46-407	I0Z6W1.1 PF04137.13	41.60	0.00
TRINITY_A0A077ZYF9_STYLE/402-524	A0A077ZYF9.1 PF1389	41.60	0.00
TRINITY_L8GL94_ACACA/389-614	L8GL94.1 PF13672.4;	41.60	0.00
TRINITY_I7M1H9_TETTS/18-275	I7M1H9.1 PF00069.23	41.60	0.00
TRINITY_B9XQP6_PEDPL/33-364	B9XQP6.1 PF00144.22	41.60	0.00
TRINITY_I7LZJ1_TETTS/122-329	I7LZJ1.1 PF01027.18	41.60	0.00
TRINITY_D8TJS9_VOLCA/99-464	D8TJS9.1 PF00155.19	41.60	0.00
TRINITY_E9FWK4_DAPPU/139-266	E9FWK4.1 PF12923.5;	41.60	0.00
TRINITY_A0A0F4XAN2_HANUV/191-322	A0A0F4XAN2.1 PF0013	41.60	0.00
TRINITY_A0C7T4_PARTE/42-157	A0C7T4.1 PF00787.22	41.60	0.00
TRINITY_L8GIS6_ACACA/16-179	L8GIS6.1 PF07106.11	41.60	0.00
TRINITY_Q0C7B1_AEDAE/426-599	Q0C7B1.1 PF00616.17	41.60	0.00
TRINITY_I7ME47_TETTS/120-274	I7ME47.1 PF00650.18	41.60	0.00
TRINITY_L8GTK1_ACACA/146-395	L8GTK1.1 PF07714.15	41.60	0.00
TRINITY_M1D132_SOLTU/15-273	M1D132.1 PF01545.19	41.50	0.00
TRINITY_L8HG15_ACACA/1584-1834	L8HG15.1 PF07714.15	41.50	0.00
TRINITY_A0A078AZG6_STYLE/1083-1214	A0A078AZG6.1 PF0007	41.50	0.00
TRINITY_A0D220_PARTE/555-671	A0D220.1 PF02518.24	41.50	0.00
TRINITY_A0BM91_PARTE/555-881	A0BM91.1 PF01237.16	41.50	0.00
TRINITY_A0A0M0J338_9EUKA/27-132	A0A0M0J338.1 PF0192	41.50	0.00
TRINITY_G0QX56_ICHMG/29-236	G0QX56.1 PF01027.18	41.50	0.00
TRINITY_J9FPR6_9SPIT/21-190	J9FPR6.1 PF01327.19	41.50	0.00
TRINITY_K9U8Y4_9CYAN/3-109	K9U8Y4.1 PF04273.11	41.50	0.00
TRINITY_L8GFV3_ACACA/2-143	L8GFV3.1 PF04099.10	41.50	0.00
TRINITY_E8N2U7_ANATU/5-297	E8N2U7.1 PF01156.17	41.50	0.00
TRINITY_G0QWD0_ICHMG/802-1041	G0QWD0.1 PF16212.3;	41.50	0.00
TRINITY_A0A067CV01_SAPPC/31-409	A0A067CV01.1 PF0799	41.50	0.00
TRINITY_D8T0J5_SELML/168-381	D8T0J5.1 PF04157.14	41.50	0.00
TRINITY_A0A0E0QZI9_ORYRU/480-547	A0A0E0QZI9.1 PF0057	41.50	0.00
TRINITY_K3X1T1_PYTUL/403-594	K3X1T1.1 PF05990.10	41.50	0.00
TRINITY_A4S3R8_OSTLU/32-277	A4S3R8.1 PF04321.15	41.50	0.00
TRINITY_F4PI69_DICFS/362-718	F4PI69.1 PF12698.5;	41.50	0.00
TRINITY_A0A068XMD3_HYMMI/250-356	A0A068XMD3.1 PF0085	41.50	0.00
TRINITY_D6U116_9CHLR/6-340	D6U116.1 PF01494.17	41.50	0.00
TRINITY_Q22YP9_TETTS/108-386	Q22YP9.2 PF00122.18	41.50	0.00
TRINITY_D3B6M3_POLPA/229-533	D3B6M3.1 PF00082.20	41.50	0.00
TRINITY_A0A0A1UBU5_ENTIV/727-986	A0A0A1UBU5.1 PF0771	41.50	0.00
TRINITY_Q23DR5_TETTS/172-553	Q23DR5.2 PF03062.17	41.50	0.00
TRINITY_D8UFJ2_VOLCA/156-276	D8UFJ2.1 PF03407.14	41.50	0.00
TRINITY_I1G2T0_AMPQE/174-530	I1G2T0.1 PF00657.20	41.50	0.00
TRINITY_J9KQP3_ACYPI/152-267	J9KQP3.2 PF00665.24	41.50	0.00
TRINITY_A0A0D2WRJ1_CAPO3/144-365	A0A0D2WRJ1.1 PF0300	41.50	0.00

TRINITY_A0A087STZ8_AUXPR/140-426	A0A087STZ8.1 PF0149	41.50	0.00
TRINITY_D8TTA7_VOLCA/51-181	D8TTA7.1 PF00782.18	41.50	0.00
TRINITY_GEFD_DICDI/436-611	Q8IS19.1 PF00617.17	41.50	0.00
TRINITY_I7M3P8_TETTS/11-168	I7M3P8.1 PF00179.24	41.50	0.00
TRINITY_S8CEZ3_9LAMI/164-445	S8CEZ3.1 PF00566.16	41.50	0.00
TRINITY_D8UDA7_VOLCA/1-149	D8UDA7.1 PF07714.15	41.50	0.00
TRINITY_L8H4J3_ACACA/179-658	L8H4J3.1 PF04636.11	41.50	0.00
TRINITY_A0A0G4J2D0_PLABS/402-510	A0A0G4J2D0.1 PF0861	41.50	0.00
TRINITY_A0BG18_PARTE/74-327	A0BG18.1 PF00102.25	41.50	0.00
TRINITY_A8JAU8_CHLRE/318-547	A8JAU8.1 PF07714.15	41.50	0.00
TRINITY_L8H1Q6_ACACA/140-507	L8H1Q6.1 PF00443.27	41.50	0.00
TRINITY_G8YTL4_PICSO/591-663	G8YTL4.1 PF00173.26	41.50	0.00
TRINITY_V4AI83_LOTGI/253-402	V4AI83.1 PF00620.25	41.50	0.00
TRINITY_A0A088A484_APIME/23-250	A0A088A484.1 PF0984	41.50	0.00
TRINITY_U6JGN1_ECHGR/95-192	U6JGN1.1 PF00043.23	41.50	0.00
TRINITY_E1ZE38_CHLVA/277-473	E1ZE38.1 PF00534.18	41.50	0.00
TRINITY_D8TZ12_VOLCA/52-308	D8TZ12.1 PF03372.21	41.50	0.00
TRINITY_A0CEK2_PARTE/175-290	A0CEK2.1 PF07986.10	41.50	0.00
TRINITY_I1DUT7_9GAMM/34-367	I1DUT7.1 PF00266.17	41.50	0.00
TRINITY_E3LXN4_CAERE/1226-1292	E3LXN4.1 PF02207.18	41.50	0.00
TRINITY_I1ITK1_BRADI/304-435	I1ITK1.1 PF14815.4;	41.50	0.00
TRINITY_D8THX8_VOLCA/233-377	D8THX8.1 PF01569.19	41.50	0.00
TRINITY_A8JDI5_CHLRE/988-1178	A8JDI5.1 PF07714.15	41.50	0.00
TRINITY_D8UF30_VOLCA/42-345	D8UF30.1 PF01223.21	41.50	0.00
TRINITY_A1ZK68_9BACT/4-128	A1ZK68.1 PF14108.4;	41.50	0.00
TRINITY_L8GT04_ACACA/130-378	L8GT04.1 PF02450.13	41.40	0.00
TRINITY_A0A078AD16_STYLE/27-478	A0A078AD16.1 PF0045	41.40	0.00
TRINITY_T1ELF1_HELRO/15-176	T1ELF1.1 PF00071.20	41.40	0.00
TRINITY_W9WAK1_9EURO/306-565	W9WAK1.1 PF13360.4;	41.40	0.00
TRINITY_A0A0H5C2I6_CYBJA/246-390	A0A0H5C2I6.1 PF0677	41.40	0.00
TRINITY_E1FZR6_LOALO/5-178	E1FZR6.2 PF00071.20	41.40	0.00
TRINITY_M7NRF1_PNEMU/1365-1713	M7NRF1.1 PF02259.21	41.40	0.00
TRINITY_A0A0K9PW93_ZOSMR/1-70	A0A0K9PW93.1 PF0858	41.40	0.00
TRINITY_E9HNW2_DAPPU/81-1277	E9HNW2.1 PF02463.17	41.40	0.00
TRINITY_A0A077KKH2_9FLAO/8-245	A0A077KKH2.1 PF0137	41.40	0.00
TRINITY_S2J9M9_MUCC1/73-173	S2J9M9.1 PF13646.4;	41.40	0.00
TRINITY_A0A0F6W7C1_9DELT/4-206	A0A0F6W7C1.1 PF0572	41.40	0.00
TRINITY_FOL1_DICDI/4-113	Q54YD9.1 PF02152.16	41.40	0.00
TRINITY_G0R2J9_ICHMG/1-211	G0R2J9.1 PF00069.23	41.40	0.00
TRINITY_G3TG84_LOXAF/4-92	G3TG84.1 PF00031.19	41.40	0.00
TRINITY_A0A0A8USH3_LEGHA/18-115	A0A0A8USH3.1 PF1717	41.40	0.00
TRINITY_Q24FF7_TETTS/32-192	Q24FF7.1 PF10294.7;	41.40	0.00
TRINITY_I1GJC5_AMPQE/5-178	I1GJC5.1 PF00071.20	41.40	0.00
TRINITY_I1GC88_AMPQE/167-380	I1GC88.1 PF00112.21	41.40	0.00
TRINITY_H1XTY4_9BACT/28-391	H1XTY4.1 PF07690.14	41.40	0.00
TRINITY_C9JUS5_HUMAN/5-152	C9JUS5.2 PF00780.20	41.40	0.00
TRINITY_A0BKL5_PARTE/147-405	A0BKL5.1 PF00069.23	41.40	0.00
TRINITY_F4PW26_DICFS/381-526	F4PW26.1 PF00620.25	41.40	0.00
TRINITY_D3AW03_POLPA/290-359	D3AW03.1 PF04739.13	41.40	0.00
TRINITY_R0GWW4_9BRAS/36-460	R0GWW4.1 PF00450.20	41.40	0.00
TRINITY_M2X4E0_GALSU/1-212	M2X4E0.1 PF01151.16	41.40	0.00
TRINITY_M2Y135_GALSU/20-178	M2Y135.1 PF00071.20	41.40	0.00
TRINITY_W9W9I5_9EURO/31-229	W9W9I5.1 PF13862.4;	41.40	0.00
TRINITY_I7LWM3_TETTS/13-272	I7LWM3.1 PF00069.23	41.40	0.00
TRINITY_I0Z5G3_9CHLO/333-610	I0Z5G3.1 PF16477.3;	41.40	0.00
TRINITY_A0A0F2Q731_9CLOT/5-128	A0A0F2Q731.1 PF1352	41.40	0.00
TRINITY_F4P7Z5_BATDJ/159-301	F4P7Z5.1 PF00107.24	41.40	0.00
TRINITY_Q02718_PODAN/556-712	Q02718.1 PF01348.19	41.40	0.00
TRINITY_H9GR50_ANOCA/3-369	H9GR50.2 PF00022.17	41.40	0.00
TRINITY_W4YEP7_STRPU/160-217	W4YEP7.1 PF03909.15	41.40	0.00
TRINITY_A0A0D2UP69_CAPO3/111-361	A0A0D2UP69.1 PF1214	41.40	0.00

TRINITY_I3KIP7_ORENI/454-610	I3KIP7.1 PF05572.11	41.40	0.00
TRINITY_L8GYM9_ACACA/12-272	L8GYM9.1 PF00149.26	41.40	0.00
TRINITY_A0A074WG56_9PEZI/162-329	A0A074WG56.1 PF0303	41.40	0.00
TRINITY_L8GTJ1_ACACA/5-168	L8GTJ1.1 PF00071.20	41.40	0.00
TRINITY_G3AUB2_SPAPN/40-172	G3AUB2.1 PF12894.5;	41.40	0.00
TRINITY_A4VD54_TETTS/76-357	A4VD54.1 PF00664.21	41.40	0.00
TRINITY_F4Q4B4_DICFS/31-204	F4Q4B4.1 PF02099.15	41.40	0.00
TRINITY_D8UF85_VOLCA/179-263	D8UF85.1 PF05699.12	41.40	0.00
TRINITY_I3CJ36_9GAMM/63-303	I3CJ36.1 PF12697.5;	41.40	0.00
TRINITY_F4PM20_DICFS/434-616	F4PM20.1 PF00621.18	41.40	0.00
TRINITY_A0A087SFP5_AUXPR/37-150	A0A087SFP5.1 PF1015	41.40	0.00
TRINITY_D3HKK6_LEGLN/43-145	D3HKK6.1 PF00011.19	41.40	0.00
TRINITY_I0ZA04_9CHLO/2-117	I0ZA04.1 PF00072.22	41.40	0.00
TRINITY_F4Q481_DICFS/654-807	F4Q481.1 PF03914.15	41.40	0.00
TRINITY_A0A0M0J8Q3_9EUKA/851-1028	A0A0M0J8Q3.1 PF1336	41.40	0.00
TRINITY_A0A0K9PPQ4_ZOSMR/94-333	A0A0K9PPQ4.1 PF0048	41.40	0.00
TRINITY_F0ZUU2_DICPU/1086-1467	F0ZUU2.1 PF00632.23	41.40	0.00
TRINITY_W7XLQ4_TETTS/8-169	W7XLQ4.1 PF00134.21	41.40	0.00
TRINITY_I0YXS8_9CHLO/33-321	I0YXS8.1 PF01062.19	41.40	0.00
TRINITY_G3Z774_9NEIS/14-147	G3Z774.1 PF02182.15	41.40	0.00
TRINITY_C3YIW1_BRAFL/1-95	C3YIW1.1 PF00125.22	41.40	0.00
TRINITY_Q16UY0_AEDAE/1-72	Q16UY0.1 PF05254.10	41.30	0.00
TRINITY_I7MHS2_TETTS/3-96	I7MHS2.1 PF01250.15	41.30	0.00
TRINITY_G0QJQ7_ICHMG/12-286	G0QJQ7.1 PF00069.23	41.30	0.00
TRINITY_V7CMI9_PHAVU/146-307	V7CMI9.1 PF09419.8;	41.30	0.00
TRINITY_A0A0G4GV89_9ALVE/243-432	A0A0G4GV89.1 PF1457	41.30	0.00
TRINITY_K9T4E8_9CYAN/3-112	K9T4E8.1 PF04273.11	41.30	0.00
TRINITY_GLRX2_PONAB/66-128	Q5RC53.1 PF00462.22	41.30	0.00
TRINITY_Q24BX5_TETTS/50-136	Q24BX5.2 PF12796.5;	41.30	0.00
TRINITY_A2G1A1_TRIVA/5-175	A2G1A1.1 PF00071.20	41.30	0.00
TRINITY_L8GZZ5_ACACA/3-97	L8GZZ5.1 PF07876.10	41.30	0.00
TRINITY_A0EIF0_PARTE/631-746	A0EIF0.1 PF00072.22	41.30	0.00
TRINITY_L8HH11_ACACA/217-300	L8HH11.1 PF12371.6;	41.30	0.00
TRINITY_D0MW28_PHYIT/147-399	D0MW28.1 PF01399.25	41.30	0.00
TRINITY_L8GS57_ACACA/211-407	L8GS57.1 PF02190.14	41.30	0.00
TRINITY_K7IP59_NASVI/12-253	K7IP59.1 PF03998.11	41.30	0.00
TRINITY_A0A068RU92_9FUNG/90-540	A0A068RU92.1 PF0006	41.30	0.00
TRINITY_Q22X64_TETTS/2-598	Q22X64.2 PF05804.10	41.30	0.00
TRINITY_A0BZ03_PARTE/2627-2912	A0BZ03.1 PF00520.29	41.30	0.00
TRINITY_SARM1_CAEEL/612-676	Q86DA5.1 PF00536.28	41.30	0.00
TRINITY_I7MES6_TETTS/376-440	I7MES6.1 PF07647.15	41.30	0.00
TRINITY_L8GPR0_ACACA/208-389	L8GPR0.1 PF00621.18	41.30	0.00
TRINITY_I0X8M0_9SPIO/513-588	I0X8M0.1 PF00533.24	41.30	0.00
TRINITY_I7LTL1_TETTS/622-741	I7LTL1.2 PF03351.15	41.30	0.00
TRINITY_W7XCC6_TETTS/200-478	W7XCC6.1 PF03372.21	41.30	0.00
TRINITY_CD123_DICDI/20-363	Q75JF9.1 PF07065.12	41.30	0.00
TRINITY_K3XAQ9_PYTUL/43-155	K3XAQ9.1 PF00583.23	41.30	0.00
TRINITY_A0A078B8J0_STYLE/1532-1877	A0A078B8J0.1 PF0063	41.30	0.00
TRINITY_A0A0D2VJK4_CAPO3/6-268	A0A0D2VJK4.1 PF0037	41.30	0.00
TRINITY_U6PUQ4_HAECO/444-748	U6PUQ4.1 PF00632.23	41.30	0.00
TRINITY_A8IZV4_CHLRE/1072-1423	A8IZV4.1 PF00069.23	41.30	0.00
TRINITY_A0A059D6J6_EUCGR/16-307	A0A059D6J6.1 PF0006	41.30	0.00
TRINITY_A0A087HAW9_ARAAL/186-358	A0A087HAW9.1 PF0084	41.30	0.00
TRINITY_BZPJ_DICDI/516-613	Q554P0.1 PF03131.15	41.30	0.00
TRINITY_L1JXD7_GUITH/351-429	L1JXD7.1 PF00789.18	41.30	0.00
TRINITY_G9P210_HYPAI/74-302	G9P210.1 PF00230.18	41.30	0.00
TRINITY_A8MR40_ARATH/21-274	A8MR40.1 PF01556.16	41.30	0.00
TRINITY_D8TN48_VOLCA/58-162	D8TN48.1 PF00085.18	41.30	0.00
TRINITY_J9JMZ2_ACYPI/277-496	J9JMZ2.1 PF00667.18	41.30	0.00
TRINITY_L1IXT3_GUITH/1-178	L1IXT3.1 PF16909.3;	41.30	0.00
TRINITY_A0A096MUE0_PAPAN/71-222	A0A096MUE0.1 PF0062	41.30	0.00

TRINITY_TPP1_MOUSE/33-176	O89023.2 PF09286.9;	41.30	0.00
TRINITY_A0A0D2WUC6_CAPO3/250-562	A0A0D2WUC6.1 PF0502	41.30	0.00
TRINITY_I0YPN8_9CHLO/5-364	I0YPN8.1 PF03849.12	41.30	0.00
TRINITY_A0A0D9XHN4_9ORYZ/99-189	A0A0D9XHN4.1 PF0179	41.30	0.00
TRINITY_A0A015MLC3_9GLOM/495-577	A0A015MLC3.1 PF0569	41.30	0.00
TRINITY_I7LX25_TETTS/48-128	I7LX25.1 PF09398.8;	41.30	0.00
TRINITY_A0A058Z5X4_9EUKA/12-173	A0A058Z5X4.1 PF0007	41.30	0.00
TRINITY_Q23AE6_TETTS/568-909	Q23AE6.4 PF00069.23	41.30	0.00
TRINITY_A8JFR0_CHLRE/22-142	A8JFR0.1 PF00583.23	41.30	0.00
TRINITY_A8J348_CHLRE/77-181	A8J348.1 PF00085.18	41.30	0.00
TRINITY_H6C292_EXODN/271-406	H6C292.1 PF01399.25	41.30	0.00
TRINITY_J3JUM0_DENPD/17-283	J3JUM0.1 PF02089.13	41.30	0.00
TRINITY_M0TH27_MUSAM/13-150	M0TH27.1 PF05615.11	41.30	0.00
TRINITY_E1Z6H8_CHLVA/853-1217	E1Z6H8.1 PF02929.15	41.30	0.00
TRINITY_F6XRR2_CALJA/672-802	F6XRR2.1 PF04408.21	41.30	0.00
TRINITY_Q8YQC2_NOSS1/14-413	Q8YQC2.1 PF02897.13	41.30	0.00
TRINITY_A0A078B9W1_STYLE/27-329	A0A078B9W1.1 PF0056	41.20	0.00
TRINITY_G0QTM0_ICHMG/757-875	G0QTM0.1 PF13863.4;	41.20	0.00
TRINITY_A0A078ANS2_STYLE/627-760	A0A078ANS2.1 PF0007	41.20	0.00
TRINITY_A0A061DUJ6_THECC/168-405	A0A061DUJ6.1 PF0411	41.20	0.00
TRINITY_A0A084R1W7_9HYPO/206-287	A0A084R1W7.1 PF0243	41.20	0.00
TRINITY_F4PXD0_DICFS/13-162	F4PXD0.1 PF01399.25	41.20	0.00
TRINITY_T1EQ94_HELRO/158-242	T1EQ94.1 PF00027.27	41.20	0.00
TRINITY_F5YP91_TREPZ/683-801	F5YP91.1 PF00072.22	41.20	0.00

TRINITY_R9ACX4_WALI9/10-171	R9ACX4.1 PF00071.20	41.20	0.00
TRINITY_H2LMA6_ORYLA/481-730	H2LMA6.1 PF08367.9;	41.20	0.00
TRINITY_F4QC01_DICFS/33-201	F4QC01.1 PF09348.8;	41.20	0.00
TRINITY_G0QWP1_ICHMG/11-421	G0QWP1.1 PF02005.14	41.20	0.00
TRINITY_D3B882_POLPA/43-231	D3B882.1 PF00069.23	41.20	0.00
TRINITY_A7MPD5_CROS8/10-532	A7MPD5.1 PF01274.20	41.20	0.00
TRINITY_I7LZV2_TETTS/151-413	I7LZV2.1 PF00069.23	41.20	0.00
TRINITY_W4XWB3_STRPU/424-994	W4XWB3.1 PF05994.9;	41.20	0.00
TRINITY_I7M8C4_TETTS/709-1009	I7M8C4.2 PF00069.23	41.20	0.00
TRINITY_I0YMI4_9CHLO/1-105	I0YMI4.1 PF08661.9;	41.20	0.00
TRINITY_R4X8T3_TAPDE/73-125	R4X8T3.1 PF14853.4;	41.20	0.00
TRINITY_U4UBW5_DENPD/321-540	U4UBW5.1 PF12215.6;	41.20	0.00
TRINITY_D8U5W1_VOLCA/1-114	D8U5W1.1 PF08839.9;	41.20	0.00
TRINITY_D3AWK8_POLPA/609-739	D3AWK8.1 PF00782.18	41.20	0.00
TRINITY_L8GVE3_ACACA/437-678	L8GVE3.1 PF06738.10	41.20	0.00
TRINITY_I7M4E9_TETTS/11-677	I7M4E9.2 PF00012.18	41.20	0.00
TRINITY_A0A022QPX2_ERYGU/24-326	A0A022QPX2.1 PF0257	41.20	0.00
TRINITY_M4BKF5_HYAAE/14-94	M4BKF5.1 PF10382.7;	41.20	0.00
TRINITY_A8JDA0_CHLRE/22-318	A8JDA0.1 PF00069.23	41.20	0.00
TRINITY_Q1IPB6_KORVE/51-252	Q1IPB6.1 PF03575.15	41.20	0.00
TRINITY_A0A077B136_9RICK/22-346	A0A077B136.1 PF1611	41.20	0.00
TRINITY_A8IGG5_CHLRE/30-160	A8IGG5.1 PF01590.24	41.20	0.00
TRINITY_D8UEU4_VOLCA/55-178	D8UEU4.1 PF00782.18	41.20	0.00
TRINITY_A0A078A032_STYLE/460-607	A0A078A032.1 PF0753	41.20	0.00
TRINITY_Q0V3A2_PHANO/164-486	Q0V3A2.1 PF01663.20	41.20	0.00
TRINITY_L8HIQ7_ACACA/482-586	L8HIQ7.1 PF00618.18	41.20	0.00
TRINITY_W4XQ31_STRPU/96-216	W4XQ31.1 PF04000.13	41.20	0.00
TRINITY_A0A067HG76_CITSI/48-522	A0A067HG76.1 PF0050	41.20	0.00
TRINITY_J9J4K4_9SPIT/309-521	J9J4K4.1 PF04811.13	41.20	0.00
TRINITY_A0A0N5A6S5_PARTI/5-166	A0A0N5A6S5.1 PF0007	41.20	0.00
TRINITY_I7MKV0_TETTS/148-266	I7MKV0.2 PF13863.4;	41.20	0.00
TRINITY_I7MDX6_TETTS/38-395	I7MDX6.2 PF00225.21	41.20	0.00
TRINITY_A8HPJ5_CHLRE/6-106	A8HPJ5.1 PF00168.28	41.20	0.00
TRINITY_DEGP2_ARATH/121-308	O82261.2 PF00089.24	41.20	0.00
TRINITY_A0DVE4_PARTE/154-417	A0DVE4.1 PF00069.23	41.20	0.00
TRINITY_A7SE78_NEMVE/44-348	A7SE78.1 PF00648.19	41.20	0.00
TRINITY_U3JLY0_FICAL/604-875	U3JLY0.1 PF00118.22	41.20	0.00
TRINITY_A0A078DXQ9_BRANA/182-281	A0A078DXQ9.1 PF0016	41.20	0.00
TRINITY_A0A0A1TUY4_ENTIV/741-999	A0A0A1TUY4.1 PF0771	41.20	0.00
TRINITY_A0A067GH93_CITSI/106-409	A0A067GH93.1 PF0120	41.20	0.00
TRINITY_J9EBV0_WUCBA/232-390	J9EBV0.1 PF09976.7;	41.20	0.00
TRINITY_G0QVP1_ICHMG/862-1120	G0QVP1.1 PF16212.3;	41.20	0.00
TRINITY_A0A087SEM0_AUXPR/418-504	A0A087SEM0.1 PF1677	41.20	0.00
TRINITY_A0A078IIF4_BRANA/385-832	A0A078IIF4.1 PF0020	41.20	0.00
TRINITY_E1ZKI1_CHLVA/80-224	E1ZKI1.1 PF00160.19	41.20	0.00
TRINITY_W4GF38_9STRA/74-355	W4GF38.1 PF00069.23	41.20	0.00
TRINITY_A8JFL7_CHLRE/275-425	A8JFL7.1 PF00069.23	41.20	0.00
TRINITY_A8J2Q9_CHLRE/69-602	A8J2Q9.1 PF03055.13	41.20	0.00
TRINITY_V9E9N6_PHYPR/2756-2844	V9E9N6.1 PF15780.3;	41.20	0.00
TRINITY_D8UF85_VOLCA/179-263	D8UF85.1 PF05699.12	41.20	0.00
TRINITY_A0A077ZVH2_STYLE/355-500	A0A077ZVH2.1 PF0753	41.20	0.00
TRINITY_W7X8R9_TETTS/92-184	W7X8R9.1 PF10167.7;	41.20	0.00
TRINITY_B0WMA2_CULQU/11-178	B0WMA2.1 PF00071.20	41.10	0.00
TRINITY_W7XEN5_TETTS/244-735	W7XEN5.1 PF01055.24	41.10	0.00
TRINITY_A0A078AA00_STYLE/230-356	A0A078AA00.1 PF0085	41.10	0.00
TRINITY_A0C0W0_PARTE/3-412	A0C0W0.1 PF00999.19	41.10	0.00
TRINITY_A0BSD3_PARTE/41-235	A0BSD3.1 PF00106.23	41.10	0.00
TRINITY_A0E014_PARTE/337-474	A0E014.1 PF07534.14	41.10	0.00
TRINITY_M4U5L4_9GAMM/2-203	M4U5L4.1 PF03932.12	41.10	0.00
TRINITY_C1E0I5_MICSR/121-686	C1E0I5.1 PF02364.13	41.10	0.00
TRINITY_A0D0Q8_PARTE/66-238	A0D0Q8.1 PF04727.11	41.10	0.00

TRINITY_A0A067Q6E2_9HOMO/17-139	A0A067Q6E2.1 PF0024	41.10	0.00
TRINITY_A0A0D2R6G2_GOSRA/133-262	A0A0D2R6G2.1 PF0152	41.10	0.00
TRINITY_M2WS09_GALSU/248-425	M2WS09.1 PF03770.14	41.10	0.00
TRINITY_G0R3D4_ICHMG/149-213	G0R3D4.1 PF01485.19	41.10	0.00
TRINITY_L8H1E1_ACACA/5-221	L8H1E1.1 PF01370.19	41.10	0.00
TRINITY_L8GII0_ACACA/41-782	L8GII0.1 PF05786.12	41.10	0.00
TRINITY_A0D9R2_PARTE/374-480	A0D9R2.1 PF00651.29	41.10	0.00
TRINITY_F7CSL0_ORNAN/129-269	F7CSL0.2 PF01014.16	41.10	0.00
TRINITY_Q23QF2_TETTS/248-571	Q23QF2.2 PF03133.13	41.10	0.00
TRINITY_F4PTD3_DICFS/26-214	F4PTD3.1 PF01183.18	41.10	0.00
TRINITY_F4Q2R9_DICFS/75-336	F4Q2R9.1 PF14500.4;	41.10	0.00
TRINITY_K8EP65_CARML/4-162	K8EP65.2 PF13238.4;	41.10	0.00
TRINITY_A8J7I4_CHLRE/1-165	A8J7I4.1 PF07714.15	41.10	0.00
TRINITY_A8IWT9_CHLRE/94-457	A8IWT9.1 PF03747.12	41.10	0.00
TRINITY_A0E5U0_PARTE/139-212	A0E5U0.1 PF01253.20	41.10	0.00
TRINITY_D8U3B5_VOLCA/1008-1129	D8U3B5.1 PF02181.21	41.10	0.00
TRINITY_D2VBV7_NAEGR/17-532	D2VBV7.1 PF04916.11	41.10	0.00
TRINITY_U9SWP3_RHIID/53-221	U9SWP3.1 PF09768.7;	41.10	0.00
TRINITY_E9GZ69_DAPPU/27-448	E9GZ69.1 PF04597.12	41.10	0.00
TRINITY_L8GG64_ACACA/110-167	L8GG64.1 PF00571.26	41.10	0.00
TRINITY_I7MJI0_TETTS/1-168	I7MJI0.1 PF14580.4;	41.10	0.00
TRINITY_W1NUC9_AMBTC/846-918	W1NUC9.1 PF00533.24	41.10	0.00
TRINITY_A0D818_PARTE/140-399	A0D818.1 PF00069.23	41.10	0.00
TRINITY_C4M6R0_ENTHI/343-452	C4M6R0.1 PF00454.25	41.10	0.00
TRINITY_W8F7T7_9BACT/175-491	W8F7T7.1 PF00728.20	41.10	0.00
TRINITY_A0A0H4VLW9_9BACT/5-225	A0A0H4VLW9.1 PF0536	41.10	0.00
TRINITY_B9SFB6_RICCO/56-390	B9SFB6.1 PF07992.12	41.10	0.00
TRINITY_G0R442_ICHMG/8-260	G0R442.1 PF00069.23	41.10	0.00
TRINITY_S2IUY4_MUCC1/31-205	S2IUY4.1 PF09439.8;	41.10	0.00
TRINITY_Q018B9_OSTTA/47-153	Q018B9.1 PF00581.18	41.10	0.00
TRINITY_Q22Z53_TETTS/262-370	Q22Z53.3 PF05517.10	41.10	0.00
TRINITY_A7SV88_NEMVE/34-631	A7SV88.1 PF00995.21	41.10	0.00
TRINITY_A7S6G7_NEMVE/391-615	A7S6G7.1 PF05843.12	41.10	0.00
TRINITY_BBS2_RAT/276-715	Q99MH9.1 PF14782.4;	41.10	0.00
TRINITY_A0A024UBD2_9STRA/265-453	A0A024UBD2.1 PF0223	41.10	0.00
TRINITY_K5WJY7_PHACS/170-321	K5WJY7.1 PF01501.18	41.10	0.00
TRINITY_D8U937_VOLCA/54-200	D8U937.1 PF12499.6;	41.10	0.00
TRINITY_A0A0L1KXQ3_9EUGL/10-206	A0A0L1KXQ3.1 PF0083	41.10	0.00
TRINITY_F7CHL5_XENTR/27-313	F7CHL5.1 PF00443.27	41.10	0.00
TRINITY_D3BT39_POLPA/3-267	D3BT39.1 PF07651.14	41.10	0.00
TRINITY_K9RJU6_9CYAN/1-167	K9RJU6.1 PF05163.10	41.10	0.00
TRINITY_T1F1C4_HELRO/100-183	T1F1C4.1 PF05699.12	41.10	0.00
TRINITY_F6S301_XENTR/259-371	F6S301.1 PF00169.27	41.10	0.00
TRINITY_R0FZ65_9BRAS/178-278	R0FZ65.1 PF02689.12	41.10	0.00
TRINITY_I7MB89_TETTS/43-816	I7MB89.2 PF01804.16	41.10	0.00
TRINITY_A8J8V1_CHLRE/17-362	A8J8V1.1 PF02567.14	41.10	0.00
TRINITY_I0YVN8_9CHLO/1080-1316	I0YVN8.1 PF05623.10	41.10	0.00
TRINITY_I0Z126_9CHLO/17-129	I0Z126.1 PF05916.9;	41.10	0.00
TRINITY_L8GS57_ACACA/211-407	L8GS57.1 PF02190.14	41.10	0.00
TRINITY_I0Z4Q7_9CHLO/119-248	I0Z4Q7.1 PF00134.21	41.10	0.00
TRINITY_I0Z379_9CHLO/120-500	I0Z379.1 PF00176.21	41.10	0.00
TRINITY_E3L492_PUCGT/9-138	E3L492.2 PF01984.18	41.10	0.00
TRINITY_A0A078AK08_STYLE/58-358	A0A078AK08.1 PF0064	41.10	0.00
TRINITY_I0Z893_9CHLO/60-343	I0Z893.1 PF00069.23	41.10	0.00
TRINITY_L8GTK1_ACACA/146-395	L8GTK1.1 PF07714.15	41.10	0.00
TRINITY_D8TQV9_VOLCA/173-366	D8TQV9.1 PF01266.22	41.10	0.00
TRINITY_A8IGB8_CHLRE/18-200	A8IGB8.1 PF06644.9;	41.10	0.00
TRINITY_A8J192_CHLRE/156-505	A8J192.1 PF04515.10	41.10	0.00
TRINITY_D8TZH6_VOLCA/22-283	D8TZH6.1 PF00233.17	41.10	0.00
TRINITY_Q24DA4_TETTS/107-224	Q24DA4.3 PF04153.16	41.00	0.00
TRINITY_L8GTK1_ACACA/146-395	L8GTK1.1 PF07714.15	41.00	0.00

TRINITY_A0A067TTA6_9AGAR/11-133	A0A067TTA6.1 PF0024	41.00	0.00
TRINITY_A0CBG9_PARTE/12-286	A0CBG9.1 PF00069.23	41.00	0.00
TRINITY_A0A078L5Z7_9CHLA/167-375	A0A078L5Z7.1 PF0117	41.00	0.00
TRINITY_L8GK49_ACACA/197-397	L8GK49.1 PF00149.26	41.00	0.00
TRINITY_I1CEB1_RHIO9/6-81	I1CEB1.1 PF00173.26	41.00	0.00
TRINITY_D3BK07_POLPA/84-175	D3BK07.1 PF02214.20	41.00	0.00
TRINITY_A0A0D2VRK4_CAPO3/301-383	A0A0D2VRK4.1 PF0088	41.00	0.00
TRINITY_Q22VU8_TETTS/895-1163	Q22VU8.2 PF00664.21	41.00	0.00
TRINITY_J9IUL5_9SPIT/144-253	J9IUL5.1 PF00787.22	41.00	0.00
TRINITY_F0ZEH9_DICPU/1492-2015	F0ZEH9.1 PF06920.11	41.00	0.00
TRINITY_X6LP84_RETFI/433-580	X6LP84.1 PF07534.14	41.00	0.00
TRINITY_I7M9L6_TETTS/721-995	I7M9L6.1 PF00664.21	41.00	0.00
TRINITY_A0A078APX7_STYLE/277-459	A0A078APX7.1 PF0064	41.00	0.00
TRINITY_W4GC09_9STRA/173-449	W4GC09.1 PF00928.19	41.00	0.00
TRINITY_F0ZS95_DICPU/1020-1248	F0ZS95.1 PF13378.4;	41.00	0.00
TRINITY_B3S4G2_TRIAD/5-337	B3S4G2.1 PF01784.16	41.00	0.00
TRINITY_A0DYB5_PARTE/75-171	A0DYB5.1 PF12796.5;	41.00	0.00
TRINITY_L0KD63_HALHC/5-298	L0KD63.1 PF09820.7;	41.00	0.00
TRINITY_D3BN92_POLPA/515-687	D3BN92.1 PF00637.18	41.00	0.00
TRINITY_X6MX50_RETFI/149-315	X6MX50.1 PF05729.10	41.00	0.00
TRINITY_D3BRW4_POLPA/8-89	D3BRW4.1 PF02825.18	41.00	0.00
TRINITY_B9HER4_POPTR/19-185	B9HER4.2 PF12706.5;	41.00	0.00
TRINITY_U9UI46_RHIID/30-185	U9UI46.1 PF00810.16	41.00	0.00
TRINITY_A7RGR1_NEMVE/4-429	A7RGR1.1 PF16496.3;	41.00	0.00
TRINITY_J9IUG9_9SPIT/1614-1848	J9IUG9.1 PF00069.23	41.00	0.00
TRINITY_T1IM08_STRMM/32-240	T1IM08.1 PF09778.7;	41.00	0.00
TRINITY_A0A085N9K0_9BILA/353-472	A0A085N9K0.1 PF0066	41.00	0.00
TRINITY_M5VUJ3_PRUPE/209-406	M5VUJ3.1 PF10312.7;	41.00	0.00
TRINITY_D8TSC3_VOLCA/26-137	D8TSC3.1 PF00072.22	41.00	0.00
TRINITY_D8U8A1_VOLCA/631-807	D8U8A1.1 PF00995.21	41.00	0.00
TRINITY_D8UCJ9_VOLCA/1-282	D8UCJ9.1 PF00069.23	41.00	0.00
TRINITY_G0QRL3_ICHMG/17-371	G0QRL3.1 PF00899.19	41.00	0.00
TRINITY_D2VPB7_NAEGR/707-816	D2VPB7.1 PF01835.17	40.90	0.00
TRINITY_T0QCJ3_9STRA/346-415	T0QCJ3.1 PF14559.4;	40.90	0.00
TRINITY_D7G343_ECTSI/500-751	D7G343.1 PF00069.23	40.90	0.00
TRINITY_A0A0A8URD3_LEGHA/1119-1355	A0A0A8URD3.1 PF0799	40.90	0.00
TRINITY_K3X250_PYTUL/7-245	K3X250.1 PF01370.19	40.90	0.00
TRINITY_A9G0Q5_SORC5/53-346	A9G0Q5.1 PF14388.4;	40.90	0.00
TRINITY_M3JWD8_CANMX/111-206	M3JWD8.1 PF00153.25	40.90	0.00
TRINITY_Q23CK3_TETTS/552-765	Q23CK3.1 PF00326.19	40.90	0.00
TRINITY_A0A0N0Y9M6_THEVU/137-385	A0A0N0Y9M6.1 PF0008	40.90	0.00
TRINITY_L7FPE7_ENTIV/1-104	L7FPE7.1 PF02078.14	40.90	0.00
TRINITY_Q55BA7_DICDI/1541-1873	Q55BA7.1 PF16206.3;	40.90	0.00
TRINITY_M4B5M7_HYAAE/5-119	M4B5M7.1 PF15490.4;	40.90	0.00
TRINITY_G0QJ48_ICHMG/856-963	G0QJ48.1 PF10367.7;	40.90	0.00
TRINITY_D0MWR3_PHYIT/1-223	D0MWR3.1 PF02586.12	40.90	0.00
TRINITY_A7SSF9_NEMVE/31-101	A7SSF9.1 PF10601.7;	40.90	0.00
TRINITY_D3B9E9_POLPA/16-242	D3B9E9.1 PF00483.21	40.90	0.00
TRINITY_D2QI58_SPILD/10-126	D2QI58.1 PF13704.4;	40.90	0.00
TRINITY_Q24BN5_TETTS/927-1025	Q24BN5.2 PF00169.27	40.90	0.00
TRINITY_D8TKW3_VOLCA/617-803	D8TKW3.1 PF01764.23	40.90	0.00
TRINITY_B0JGD0_MICAN/413-505	B0JGD0.1 PF02517.14	40.90	0.00
TRINITY_W5GW52_WHEAT/103-416	W5GW52.1 PF03062.17	40.90	0.00
TRINITY_Q23Q47_TETTS/4-162	Q23Q47.2 PF01652.16	40.90	0.00
TRINITY_I2K2J3_DEKBR/29-136	I2K2J3.1 PF00307.29	40.90	0.00
TRINITY_A0A077ZWA2_STYLE/66-158	A0A077ZWA2.1 PF1254	40.90	0.00
TRINITY_K9T8N3_9CYAN/5-199	K9T8N3.1 PF00300.20	40.90	0.00
TRINITY_G3X324_SARHA/25-204	G3X324.1 PF09762.7;	40.90	0.00
TRINITY_D8TL16_VOLCA/724-840	D8TL16.1 PF09066.8;	40.90	0.00
TRINITY_I3KIP7_ORENI/454-610	I3KIP7.1 PF05572.11	40.90	0.00
TRINITY_F4PTY6_DICFS/660-885	F4PTY6.1 PF00617.17	40.90	0.00

TRINITY_D8U5S8_VOLCA/69-169	D8U5S8.1 PF00085.18	40.90	0.00
TRINITY_W5MYU2_LEPOC/542-687	W5MYU2.1 PF09113.8;	40.90	0.00
TRINITY_A0A078AKP9_STYLE/375-534	A0A078AKP9.1 PF1458	40.90	0.00
TRINITY_B0WCA4_CULQU/353-814	B0WCA4.1 PF13844.4;	40.90	0.00
TRINITY_I0YTS2_9CHLO/3-284	I0YTS2.1 PF00481.19	40.90	0.00
TRINITY_Q552T7_DICDI/119-554	Q552T7.2 PF11957.6;	40.90	0.00
TRINITY_F4PEL5_BATDJ/37-331	F4PEL5.1 PF03982.11	40.90	0.00
TRINITY_L8HI91_ACACA/91-321	L8HI91.1 PF05721.11	40.90	0.00
TRINITY_A0A093YSK8_9PEZI/134-247	A0A093YSK8.1 PF0322	40.90	0.00
TRINITY_A0A078L9Y9_9CHLA/1-200	A0A078L9Y9.1 PF0023	40.90	0.00
TRINITY_I1FYR5_AMPQE/31-157	I1FYR5.1 PF00134.21	40.90	0.00
TRINITY_A0CXP3_PARTE/26-844	A0CXP3.1 PF01496.17	40.90	0.00
TRINITY_D8M2P3_BLAHO/26-94	D8M2P3.1 PF00505.17	40.90	0.00
TRINITY_Q4C0Q1_CROWT/8-467	Q4C0Q1.1 PF03055.13	40.90	0.00
TRINITY_G0QKY7_ICHMG/168-327	G0QKY7.1 PF00406.20	40.90	0.00
TRINITY_H3BYI0_TETNG/20-174	H3BYI0.1 PF08603.9;	40.90	0.00
TRINITY_Q22DB9_TETTS/354-467	Q22DB9.2 PF08642.8;	40.90	0.00
TRINITY_K3VNH2_FUSPC/87-353	K3VNH2.1 PF00150.16	40.80	0.00
TRINITY_A0A0J7L6J6_LASNI/109-581	A0A0J7L6J6.1 PF0050	40.80	0.00
TRINITY_L8HKG8_ACACA/73-171	L8HKG8.1 PF13649.4;	40.80	0.00
TRINITY_Q234K7_TETTS/37-180	Q234K7.2 PF00179.24	40.80	0.00
TRINITY_G0QNG4_ICHMG/42-584	G0QNG4.1 PF00995.21	40.80	0.00
TRINITY_U3IKK7_ANAPL/406-500	U3IKK7.1 PF12796.5;	40.80	0.00
TRINITY_V3ZY91_LOTGI/30-181	V3ZY91.1 PF10152.7;	40.80	0.00
TRINITY_A0A0G4IY77_PLABS/137-395	A0A0G4IY77.1 PF0006	40.80	0.00
TRINITY_A0DB53_PARTE/121-198	A0DB53.1 PF03061.20	40.80	0.00
TRINITY_A0A0K9PMV7_ZOSMR/131-204	A0A0K9PMV7.1 PF0007	40.80	0.00
TRINITY_A0A067TXX9_9AGAR/165-319	A0A067TXX9.1 PF0317	40.80	0.00
TRINITY_D7CW28_TRURR/18-175	D7CW28.1 PF03073.13	40.80	0.00
TRINITY_A0A015LDZ0_9GLOM/118-260	A0A015LDZ0.1 PF1060	40.80	0.00
TRINITY_Q8INZ8_DROME/49-244	Q8INZ8.2 PF00106.23	40.80	0.00
TRINITY_A0A087YNU4_POEFO/38-433	A0A087YNU4.1 PF0248	40.80	0.00
TRINITY_F4Q3C0_DICFS/169-521	F4Q3C0.1 PF00587.23	40.80	0.00
TRINITY_K2HBN6_9RHOB/157-274	K2HBN6.1 PF00581.18	40.80	0.00
TRINITY_S2JQN6_MUCC1/15-345	S2JQN6.1 PF00503.18	40.80	0.00
TRINITY_E0VFV5_PEDHC/561-878	E0VFV5.1 PF00702.24	40.80	0.00
TRINITY_I7LY38_TETTS/121-191	I7LY38.1 PF13499.4;	40.80	0.00
TRINITY_I0YVA9_9CHLO/319-409	I0YVA9.1 PF02875.19	40.80	0.00
TRINITY_A0A0C4EZ98_PUCT1/115-313	A0A0C4EZ98.1 PF0405	40.80	0.00
TRINITY_I7LSW3_TETTS/19-277	I7LSW3.2 PF00069.23	40.80	0.00
TRINITY_L8GGT0_ACACA/221-344	L8GGT0.1 PF00241.18	40.80	0.00
TRINITY_V4B630_LOTGI/57-315	V4B630.1 PF00266.17	40.80	0.00
TRINITY_A8JCN0_CHLRE/10-224	A8JCN0.1 PF08325.8;	40.80	0.00
TRINITY_A0A0H5BYU1_CYBJA/12-89	A0A0H5BYU1.1 PF0512	40.80	0.00
TRINITY_A0CY07_PARTE/21-127	A0CY07.1 PF09799.7;	40.80	0.00
TRINITY_E1ZBJ1_CHLVA/198-371	E1ZBJ1.1 PF01454.17	40.80	0.00
TRINITY_I0YLU5_9CHLO/46-280	I0YLU5.1 PF00975.18	40.80	0.00
TRINITY_H2SG74_TAKRU/136-465	H2SG74.1 PF00083.22	40.80	0.00
TRINITY_A0BMG4_PARTE/28-194	A0BMG4.1 PF08718.9;	40.80	0.00
TRINITY_H2BU57_9FLAO/15-223	H2BU57.1 PF00106.23	40.80	0.00
TRINITY_C3Y383_BRAFL/1-325	C3Y383.1 PF07857.10	40.80	0.00
TRINITY_F0ZTB8_DICPU/351-556	F0ZTB8.1 PF00566.16	40.80	0.00
TRINITY_D8TMB5_VOLCA/111-375	D8TMB5.1 PF00069.23	40.80	0.00
TRINITY_B9SZ58_RICCO/191-282	B9SZ58.1 PF12796.5;	40.80	0.00
TRINITY_F0Z9A5_DICPU/23-213	F0Z9A5.1 PF01183.18	40.80	0.00
TRINITY_A9USM9_MONBE/264-370	A9USM9.1 PF00307.29	40.80	0.00
TRINITY_A6G384_9DELT/139-255	A6G384.1 PF08546.9;	40.80	0.00
TRINITY_A0CNR5_PARTE/331-380	A0CNR5.1 PF13920.4;	40.80	0.00
TRINITY_W9R965_9ROSA/98-315	W9R965.1 PF04755.10	40.80	0.00
TRINITY_A8J2L9_CHLRE/84-199	A8J2L9.1 PF10294.7;	40.80	0.00
TRINITY_G7I5V8_MEDTR/41-656	G7I5V8.2 PF05470.10	40.80	0.00

TRINITY_G0QR38_ICHMG/43-305	G0QR38.1 PF00069.23	40.80	0.00
TRINITY_L8HAB9_ACACA/836-943	L8HAB9.1 PF00651.29	40.80	0.00
TRINITY_D8U5R0_VOLCA/26-273	D8U5R0.1 PF12146.6;	40.80	0.00
TRINITY_E1ZRG2_CHLVA/108-338	E1ZRG2.1 PF04493.12	40.80	0.00
TRINITY_L8GT01_ACACA/132-437	L8GT01.1 PF08627.8;	40.80	0.00
TRINITY_M0VW02_HORVD/2-176	M0VW02.1 PF07847.10	40.80	0.00
TRINITY_I0Z0F3_9CHLO/402-670	I0Z0F3.1 PF11831.6;	40.80	0.00
TRINITY_M2QW23_CERS8/68-195	M2QW23.1 PF00622.26	40.80	0.00
TRINITY_D8TTP0_VOLCA/1124-1422	D8TTP0.1 PF00069.23	40.80	0.00
TRINITY_A8IU02_CHLRE/22-115	A8IU02.1 PF08123.11	40.80	0.00
TRINITY_D8TV59_VOLCA/8-713	D8TV59.1 PF05327.9;	40.80	0.00
TRINITY_I0S303_MYCPH/715-805	I0S303.1 PF16184.3;	40.80	0.00
TRINITY_T0JXF4_COLGC/1152-1269	T0JXF4.1 PF00072.22	40.80	0.00
TRINITY_I1KVL7_SOYBN/19-273	I1KVL7.1 PF00069.23	40.80	0.00
TRINITY_A0A0N0EC30_9BACI/3-197	A0A0N0EC30.1 PF0598	40.70	0.00
TRINITY_A0DPA1_PARTE/82-191	A0DPA1.1 PF01569.19	40.70	0.00
TRINITY_W7AAT9_9APIC/75-218	W7AAT9.1 PF16531.3;	40.70	0.00
TRINITY_D3BG34_POLPA/95-179	D3BG34.1 PF09793.7;	40.70	0.00
TRINITY_G0QXF6_ICHMG/1-194	G0QXF6.1 PF14309.4;	40.70	0.00
TRINITY_F4Q916_DICFS/16-247	F4Q916.1 PF00378.18	40.70	0.00
TRINITY_D3B803_POLPA/36-206	D3B803.1 PF00445.16	40.70	0.00
TRINITY_A0A0N5ART5_9BILA/657-796	A0A0N5ART5.1 PF0176	40.70	0.00
TRINITY_V4A8R6_LOTGI/8-252	V4A8R6.1 PF01073.17	40.70	0.00
TRINITY_E3MXL2_CAERE/1-286	E3MXL2.1 PF00294.22	40.70	0.00
TRINITY_A0A015M955_9GLOM/179-498	A0A015M955.1 PF0006	40.70	0.00
TRINITY_L8GTK1_ACACA/146-395	L8GTK1.1 PF07714.15	40.70	0.00
TRINITY_D7KB12_ARALL/348-437	D7KB12.1 PF13877.4;	40.70	0.00
TRINITY_A0CMN6_PARTE/121-399	A0CMN6.1 PF01457.14	40.70	0.00
TRINITY_B0CDR8_ACAM1/5-228	B0CDR8.1 PF01148.18	40.70	0.00
TRINITY_Q24E29_TETTS/46-151	Q24E29.1 PF13649.4;	40.70	0.00
TRINITY_U9TNT0_RHIID/22-167	U9TNT0.1 PF13302.5;	40.70	0.00
TRINITY_I7MIN0_TETTS/9-308	I7MIN0.1 PF08449.9;	40.70	0.00
TRINITY_D3BR85_POLPA/18-223	D3BR85.1 PF00117.26	40.70	0.00
TRINITY_F4QBC3_DICFS/131-212	F4QBC3.1 PF07258.12	40.70	0.00
TRINITY_Q22VA7_TETTS/184-474	Q22VA7.2 PF00233.17	40.70	0.00
TRINITY_L8GTS5_ACACA/3-666	L8GTS5.1 PF00012.18	40.70	0.00
TRINITY_G3P0A4_GASAC/214-309	G3P0A4.1 PF00169.27	40.70	0.00
TRINITY_E1ZS39_CHLVA/93-324	E1ZS39.1 PF00889.17	40.70	0.00
TRINITY_A8J4D6_CHLRE/165-312	A8J4D6.1 PF07200.11	40.70	0.00
TRINITY_L8GUA0_ACACA/175-340	L8GUA0.1 PF00609.17	40.70	0.00
TRINITY_A0A067KXP8_JATCU/409-547	A0A067KXP8.1 PF0414	40.70	0.00
TRINITY_A9V2C9_MONBE/525-757	A9V2C9.1 PF00149.26	40.70	0.00
TRINITY_A0A0B0PYI6_GOSAR/18-294	A0A0B0PYI6.1 PF0126	40.70	0.00
TRINITY_F6QWH2_HORSE/2185-2303	F6QWH2.1 PF10162.7;	40.70	0.00
TRINITY_D8U6D8_VOLCA/207-665	D8U6D8.1 PF00450.20	40.70	0.00
TRINITY_I1M679_SOYBN/556-837	I1M679.2 PF12110.6;	40.70	0.00
TRINITY_E1ZU59_CHLVA/469-691	E1ZU59.1 PF05794.11	40.70	0.00
TRINITY_I7LTS5_TETTS/100-452	I7LTS5.2 PF03372.21	40.70	0.00
TRINITY_E1Z5T3_CHLVA/338-498	E1Z5T3.1 PF01734.20	40.70	0.00
TRINITY_I7M4C2_TETTS/42-276	I7M4C2.1 PF00149.26	40.70	0.00
TRINITY_W5LN34_ASTMX/22-386	W5LN34.1 PF09751.7;	40.70	0.00
TRINITY_A0A0G3EAR2_9BACT/17-242	A0A0G3EAR2.1 PF0445	40.70	0.00
TRINITY_A7SVU0_NEMVE/7-118	A7SVU0.1 PF10644.7;	40.70	0.00
TRINITY_D8UK64_VOLCA/8-471	D8UK64.1 PF13520.4;	40.70	0.00
TRINITY_L8HHX4_ACACA/703-1129	L8HHX4.1 PF00476.18	40.70	0.00
TRINITY_A0A087YFA1_POEFO/360-467	A0A087YFA1.2 PF0065	40.70	0.00
TRINITY_D3B6M3_POLPA/229-533	D3B6M3.1 PF00082.20	40.70	0.00
TRINITY_A0A0G1NLR9_9BACT/1-168	A0A0G1NLR9.1 PF0192	40.70	0.00
TRINITY_A8J3M6_CHLRE/1-427	A8J3M6.1 PF00022.17	40.70	0.00
TRINITY_J9HPH3_9SPIT/203-298	J9HPH3.1 PF00787.22	40.70	0.00
TRINITY_E1Z7X4_CHLVA/30-382	E1Z7X4.1 PF01593.22	40.70	0.00

TRINITY_A0A078I297_BRANA/61-293	A0A078I297.1 PF0027	40.70	0.00
TRINITY_F4PZZ1_DICFS/16-111	F4PZZ1.1 PF00169.27	40.70	0.00
TRINITY_B6IP52_RHOCS/51-204	B6IP52.1 PF01738.16	40.70	0.00
TRINITY_D8SKW4_SELML/64-170	D8SKW4.1 PF00134.21	40.60	0.00
TRINITY_A0A015JP33_9GLOM/12-80	A0A015JP33.1 PF0858	40.60	0.00
TRINITY_A0A078B6K5_STYLE/485-620	A0A078B6K5.1 PF0251	40.60	0.00
TRINITY_A0A078A108_STYLE/264-452	A0A078A108.1 PF1717	40.60	0.00
TRINITY_A0A078ASG5_STYLE/209-401	A0A078ASG5.1 PF0009	40.60	0.00
TRINITY_I7MI98_TETTS/575-967	I7MI98.2 PF00176.21	40.60	0.00
TRINITY_D5SX86_PLAL2/131-390	D5SX86.1 PF00069.23	40.60	0.00
TRINITY_R7R886_9FIRM/88-244	R7R886.1 PF00849.20	40.60	0.00
TRINITY_G7YUG8_CLOSI/12-174	G7YUG8.1 PF00071.20	40.60	0.00
TRINITY_F2NDY6_DESAR/54-186	F2NDY6.1 PF13365.4;	40.60	0.00
TRINITY_MUS81_SCHPO/334-469	P87231.2 PF02732.13	40.60	0.00
TRINITY_F6ZX42_XENTR/50-390	F6ZX42.1 PF04124.10	40.60	0.00
TRINITY_U9UZ81_RHIID/6-111	U9UZ81.1 PF01920.18	40.60	0.00
TRINITY_A8J4M1_CHLRE/78-217	A8J4M1.1 PF12499.6;	40.60	0.00
TRINITY_D2VCL5_NAEGR/5-169	D2VCL5.1 PF00071.20	40.60	0.00
TRINITY_L8GEW1_ACACA/53-301	L8GEW1.1 PF03009.15	40.60	0.00
TRINITY_A8JI94_CHLRE/16-125	A8JI94.1 PF01776.15	40.60	0.00
TRINITY_L8GTQ2_ACACA/12-212	L8GTQ2.1 PF00106.23	40.60	0.00
TRINITY_F0Z7L0_DICPU/2-530	F0Z7L0.1 PF04916.11	40.60	0.00
TRINITY_A0A0G1ZDX1_9BACT/68-233	A0A0G1ZDX1.1 PF1338	40.60	0.00
TRINITY_A0A078A1U0_STYLE/3115-3435	A0A078A1U0.1 PF0052	40.60	0.00
TRINITY_Q239B4_TETTS/549-761	Q239B4.2 PF00326.19	40.60	0.00
TRINITY_I7M148_TETTS/147-410	I7M148.1 PF00069.23	40.60	0.00
TRINITY_I0YKK0_9CHLO/8-180	I0YKK0.1 PF01145.23	40.60	0.00
TRINITY_I0ZAX4_9CHLO/60-233	I0ZAX4.1 PF13460.4;	40.60	0.00
TRINITY_A0E2M3_PARTE/3-103	A0E2M3.1 PF00168.28	40.60	0.00
TRINITY_F6QYZ1_XENTR/575-830	F6QYZ1.1 PF00078.25	40.60	0.00
TRINITY_F4PYG0_DICFS/69-275	F4PYG0.1 PF09511.8;	40.60	0.00
TRINITY_D8TPU4_VOLCA/1-251	D8TPU4.1 PF00102.25	40.60	0.00
TRINITY_C3YMS1_BRAFL/32-426	C3YMS1.1 PF07690.14	40.60	0.00
TRINITY_F0ZY85_DICPU/202-311	F0ZY85.1 PF10363.7;	40.60	0.00
TRINITY_I7LUJ4_TETTS/1-176	I7LUJ4.2 PF04062.12	40.60	0.00
TRINITY_D8TWK0_VOLCA/1-143	D8TWK0.1 PF01217.18	40.60	0.00
TRINITY_F1A177_DICPU/18-421	F1A177.1 PF07690.14	40.60	0.00
TRINITY_G1LRV8_AILME/492-789	G1LRV8.1 PF00702.24	40.60	0.00
TRINITY_A7SPM1_NEMVE/19-272	A7SPM1.1 PF00780.20	40.60	0.00
TRINITY_A9VC39_MONBE/7-107	A9VC39.1 PF13646.4;	40.60	0.00
TRINITY_D8TJA5_VOLCA/53-597	D8TJA5.1 PF02990.14	40.60	0.00
TRINITY_W7TE55_9STRA/34-299	W7TE55.1 PF00069.23	40.60	0.00
TRINITY_R1B2R3_EMIHU/13-218	R1B2R3.1 PF05241.10	40.60	0.00
TRINITY_L8HC31_ACACA/19-199	L8HC31.1 PF05241.10	40.60	0.00
TRINITY_V4TVT0_9ROSI/84-377	V4TVT0.1 PF05185.14	40.60	0.00
TRINITY_BTN1_ASHGO/9-400	Q758C3.1 PF02487.15	40.60	0.00
TRINITY_A0CXP9_PARTE/92-386	A0CXP9.1 PF01457.14	40.60	0.00
TRINITY_A0A067G1M8_CITSI/208-309	A0A067G1M8.1 PF0304	40.60	0.00
TRINITY_D7EIK9_TRICA/5-177	D7EIK9.1 PF00025.19	40.60	0.00
TRINITY_A7S4K7_NEMVE/114-484	A7S4K7.1 PF08506.8;	40.60	0.00
TRINITY_D8UEC7_VOLCA/2-394	D8UEC7.1 PF03348.13	40.60	0.00
TRINITY_A8J3P1_CHLRE/954-1161	A8J3P1.1 PF00211.18	40.60	0.00
TRINITY_F8WZF9_9PORP/1-87	F8WZF9.1 PF00686.17	40.60	0.00
TRINITY_A0D5S5_PARTE/19-153	A0D5S5.1 PF04178.10	40.60	0.00
TRINITY_B7G054_PHATC/72-147	B7G054.1 PF03134.17	40.50	0.00
TRINITY_A0A024P1K9_9BACI/18-253	A0A024P1K9.1 PF1356	40.50	0.00
TRINITY_S2K0L0_MUCC1/59-241	S2K0L0.1 PF03467.13	40.50	0.00
TRINITY_C5LE07_PERM5/832-1258	C5LE07.1 PF06957.9;	40.50	0.00
TRINITY_E5SAK8_TRISP/972-1481	E5SAK8.1 PF00118.22	40.50	0.00
TRINITY_Q3SDL5_PARTE/12-173	Q3SDL5.1 PF00071.20	40.50	0.00
TRINITY_K0C3B7_CYCSP/71-273	K0C3B7.1 PF07859.11	40.50	0.00

TRINITY_G0QJQ7_ICHMG/12-286	G0QJQ7.1 PF00069.23	40.50	0.00
TRINITY_F4Q7P1_DICFS/106-270	F4Q7P1.1 PF08389.10	40.50	0.00
TRINITY_Q23K54_TETTS/266-381	Q23K54.1 PF01624.18	40.50	0.00
TRINITY_D3B9Q6_POLPA/99-347	D3B9Q6.1 PF07714.15	40.50	0.00
TRINITY_D8LTQ5_ECTSI/28-199	D8LTQ5.1 PF03152.12	40.50	0.00
TRINITY_L1JFR0_GUITH/69-276	L1JFR0.1 PF12146.6;	40.50	0.00
TRINITY_A0DAB1_PARTE/334-1006	A0DAB1.1 PF00443.27	40.50	0.00
TRINITY_L8GJN4_ACACA/119-306	L8GJN4.1 PF02750.12	40.50	0.00
TRINITY_MKAR_COCIM/71-268	Q1DNC5.2 PF00106.23	40.50	0.00
TRINITY_G0QV75_ICHMG/42-337	G0QV75.1 PF03619.14	40.50	0.00
TRINITY_A7RPW5_NEMVE/6-444	A7RPW5.1 PF00899.19	40.50	0.00
TRINITY_BRD9_XENTR/153-240	Q6NVM8.1 PF00439.23	40.50	0.00
TRINITY_I7M495_TETTS/713-823	I7M495.1 PF02204.16	40.50	0.00
TRINITY_F4NSD5_BATDJ/57-236	F4NSD5.1 PF03467.13	40.50	0.00
TRINITY_I0YXM0_9CHLO/863-1066	I0YXM0.1 PF13087.4;	40.50	0.00
TRINITY_W4FSJ9_9STRA/1228-1335	W4FSJ9.1 PF00005.25	40.50	0.00
TRINITY_T1JPK6_STRMM/21-141	T1JPK6.1 PF05743.11	40.50	0.00
TRINITY_A8J133_CHLRE/27-179	A8J133.1 PF00719.17	40.50	0.00
TRINITY_A8HQC2_CHLRE/23-259	A8HQC2.1 PF13561.4;	40.50	0.00
TRINITY_A0A067C067_SAPPC/20-106	A0A067C067.1 PF0437	40.50	0.00
TRINITY_G0QNV6_ICHMG/23-97	G0QNV6.1 PF03810.17	40.50	0.00
TRINITY_A0A0G4EJ81_9ALVE/34-117	A0A0G4EJ81.1 PF1341	40.50	0.00
TRINITY_M1VCE7_CYAME/19-259	M1VCE7.1 PF01151.16	40.50	0.00
TRINITY_H3GQY3_PHYRM/105-170	H3GQY3.1 PF01424.20	40.50	0.00
TRINITY_K0TJT6_THAOC/257-659	K0TJT6.1 PF01457.14	40.50	0.00
TRINITY_A0A067CKA5_SAPPC/69-223	A0A067CKA5.1 PF0864	40.50	0.00
TRINITY_E1Z593_CHLVA/1-99	E1Z593.1 PF02466.17	40.50	0.00
TRINITY_D5GLZ2_TUBMM/4-146	D5GLZ2.1 PF00106.23	40.50	0.00
TRINITY_F0Z753_DICPU/189-311	F0Z753.1 PF02338.17	40.50	0.00
TRINITY_A0CLK7_PARTE/302-529	A0CLK7.1 PF00149.26	40.50	0.00
TRINITY_M5WBK9_PRUPE/339-411	M5WBK9.1 PF12894.5;	40.50	0.00
TRINITY_D7T1Z1_VITVI/4-834	D7T1Z1.1 PF10191.7;	40.50	0.00
TRINITY_A0D8U5_PARTE/465-719	A0D8U5.1 PF01504.16	40.50	0.00
TRINITY_I7M6F7_TETTS/1178-1445	I7M6F7.2 PF01504.16	40.50	0.00
TRINITY_PNPP_SCHPO/18-258	Q00472.2 PF00702.24	40.50	0.00
TRINITY_D3B954_POLPA/242-587	D3B954.1 PF04910.12	40.50	0.00
TRINITY_A0A074RY28_9HOMO/35-123	A0A074RY28.1 PF1289	40.50	0.00
TRINITY_B8BTM7_THAPS/220-314	B8BTM7.1 PF02984.17	40.50	0.00
TRINITY_H3GGG5_PHYRM/438-525	H3GGG5.1 PF00651.29	40.50	0.00
TRINITY_F2UKM1_SALR5/9-203	F2UKM1.1 PF00009.25	40.50	0.00
TRINITY_I0Z949_9CHLO/14-145	I0Z949.1 PF02996.15	40.50	0.00
TRINITY_A8IDJ5_CHLRE/422-860	A8IDJ5.1 PF12832.5;	40.50	0.00
TRINITY_A0A0N8K258_9TELE/258-340	A0A0N8K258.1 PF1289	40.50	0.00
TRINITY_A0A089KRY0_9BACL/8-416	A0A089KRY0.1 PF0142	40.50	0.00
TRINITY_G0R1V3_ICHMG/699-811	G0R1V3.1 PF00168.28	40.50	0.00
TRINITY_J3NZX9_GAGT3/476-746	J3NZX9.1 PF13476.4;	40.50	0.00
TRINITY_Q23R88_TETTS/18-272	Q23R88.1 PF00069.23	40.50	0.00
TRINITY_D8UJ46_VOLCA/1047-1282	D8UJ46.1 PF07714.15	40.50	0.00
TRINITY_J9J4K4_9SPIT/309-521	J9J4K4.1 PF04811.13	40.50	0.00
TRINITY_A0A078A6L2_STYLE/11-141	A0A078A6L2.1 PF0078	40.50	0.00
TRINITY_A0A0D2UKQ8_CAPO3/188-572	A0A0D2UKQ8.1 PF0065	40.50	0.00
TRINITY_H2PAR9_PONAB/74-692	H2PAR9.1 PF00443.27	40.50	0.00
TRINITY_A8IY68_CHLRE/1-382	A8IY68.1 PF00022.17	40.50	0.00
TRINITY_A0A066VDM6_9BASI/185-469	A0A066VDM6.1 PF0589	40.50	0.00
TRINITY_A8IP72_CHLRE/4180-5004	A8IP72.1 PF03028.13	40.50	0.00
TRINITY_A0A078BBP3_STYLE/94-223	A0A078BBP3.1 PF0922	40.40	0.00
TRINITY_A0A078B2J0_STYLE/41-343	A0A078B2J0.1 PF0064	40.40	0.00
TRINITY_W7X070_TETTS/33-148	W7X070.1 PF06417.10	40.40	0.00
TRINITY_A0DRG1_PARTE/852-1181	A0DRG1.1 PF01237.16	40.40	0.00
TRINITY_K9U4Q0_9CYAN/9-190	K9U4Q0.1 PF06041.9;	40.40	0.00
TRINITY_F0Z756_DICPU/761-1025	F0Z756.1 PF00069.23	40.40	0.00

TRINITY_A3LPJ1_PICST/46-164	A3LPJ1.2 PF06417.10	40.40	0.00
TRINITY_I7M6F7_TETTS/1178-1445	I7M6F7.2 PF01504.16	40.40	0.00
TRINITY_D3B0Q0_POLPA/425-685	D3B0Q0.1 PF00149.26	40.40	0.00
TRINITY_I7MMI6_TETTS/8-379	I7MMI6.1 PF01238.19	40.40	0.00
TRINITY_D8QPW9_SELML/6-146	D8QPW9.1 PF00892.18	40.40	0.00
TRINITY_L8GZD2_ACACA/50-195	L8GZD2.1 PF10021.7;	40.40	0.00
TRINITY_THSA_AERPE/34-529	Q9YDK6.2 PF00118.22	40.40	0.00
TRINITY_Q0AU63_SYNWW/45-196	Q0AU63.1 PF00484.17	40.40	0.00
TRINITY_Q236B5_TETTS/16-110	Q236B5.1 PF12796.5;	40.40	0.00
TRINITY_J9JYL8_ACYPI/216-375	J9JYL8.2 PF01399.25	40.40	0.00
TRINITY_D2VTD5_NAEGR/50-351	D2VTD5.1 PF02685.14	40.40	0.00
TRINITY_W7XH00_TETTS/296-364	W7XH00.1 PF01485.19	40.40	0.00
TRINITY_D2V2D9_NAEGR/1-106	D2V2D9.1 PF00125.22	40.40	0.00
TRINITY_L8GPG1_ACACA/240-559	L8GPG1.1 PF02567.14	40.40	0.00
TRINITY_C9SQ36_VERA1/59-525	C9SQ36.1 PF00443.27	40.40	0.00
TRINITY_A0CJW2_PARTE/47-528	A0CJW2.1 PF00501.26	40.40	0.00
TRINITY_F4Q2R1_DICFS/21-158	F4Q2R1.1 PF02221.13	40.40	0.00
TRINITY_J4KMF2_BEAB2/252-629	J4KMF2.1 PF00176.21	40.40	0.00
TRINITY_I0YPU8_9CHLO/38-335	I0YPU8.1 PF00083.22	40.40	0.00
TRINITY_F0YER8_AURAN/12-109	F0YER8.1 PF01588.18	40.40	0.00
TRINITY_F8WH20_MOUSE/459-803	F8WH20.1 PF01237.16	40.40	0.00
TRINITY_E1ZFJ9_CHLVA/326-429	E1ZFJ9.1 PF11987.6;	40.40	0.00
TRINITY_A0A075B096_9FUNG/18-127	A0A075B096.1 PF0177	40.40	0.00
TRINITY_E1ZIZ3_CHLVA/4-194	E1ZIZ3.1 PF07281.10	40.40	0.00
TRINITY_F0Z871_DICPU/21-257	F0Z871.1 PF00481.19	40.40	0.00
TRINITY_A0A059AJH8_EUCGR/121-227	A0A059AJH8.1 PF0015	40.40	0.00
TRINITY_L8GPR0_ACACA/208-389	L8GPR0.1 PF00621.18	40.40	0.00
TRINITY_A0A0N4TCS9_BRUPA/10-105	A0A0N4TCS9.1 PF0866	40.40	0.00
TRINITY_A0A059LM80_9CHLO/187-411	A0A059LM80.1 PF0403	40.40	0.00
TRINITY_I0Z6Q8_9CHLO/60-252	I0Z6Q8.1 PF03006.18	40.40	0.00
TRINITY_A4VDN1_TETTS/4-430	A4VDN1.1 PF00067.20	40.40	0.00
TRINITY_H3G9F8_PHYRM/463-692	H3G9F8.1 PF05843.12	40.40	0.00
TRINITY_A0A0M9VL61_9MICO/7-194	A0A0M9VL61.1 PF0010	40.40	0.00
TRINITY_Q54EQ6_DICDI/82-222	Q54EQ6.1 PF01764.23	40.40	0.00
TRINITY_F0ZJW2_DICPU/563-743	F0ZJW2.1 PF02145.13	40.40	0.00
TRINITY_D3BLW9_POLPA/1582-1669	D3BLW9.1 PF00027.27	40.40	0.00
TRINITY_D8U0R7_VOLCA/48-409	D8U0R7.1 PF07690.14	40.40	0.00
TRINITY_M0T2K1_MUSAM/14-175	M0T2K1.1 PF00071.20	40.40	0.00
TRINITY_F0ZWR7_DICPU/375-764	F0ZWR7.1 PF11864.6;	40.40	0.00
TRINITY_L8HCV9_ACACA/8-432	L8HCV9.1 PF13347.4;	40.40	0.00
TRINITY_G8T8X8_NIAKG/18-219	G8T8X8.1 PF13568.4;	40.40	0.00
TRINITY_GEFK_DICDI/1319-1498	Q54FF3.1 PF00617.17	40.40	0.00
TRINITY_D8TY73_VOLCA/22-158	D8TY73.1 PF13673.5;	40.40	0.00
TRINITY_A0CA55_PARTE/30-871	A0CA55.1 PF02460.16	40.40	0.00
TRINITY_D8TLJ5_VOLCA/156-794	D8TLJ5.1 PF04762.10	40.40	0.00
TRINITY_A0A022QNZ2_ERYGU/465-709	A0A022QNZ2.1 PF0771	40.40	0.00
TRINITY_I0YSB3_9CHLO/32-246	I0YSB3.1 PF00106.23	40.40	0.00
TRINITY_A0CQG7_PARTE/77-171	A0CQG7.1 PF12796.5;	40.40	0.00
TRINITY_B6HGX9_PENRW/378-750	B6HGX9.1 PF00702.24	40.40	0.00
TRINITY_D8UJW0_VOLCA/85-273	D8UJW0.1 PF07714.15	40.40	0.00
TRINITY_L5KHJ0_PTEAL/55-463	L5KHJ0.1 PF00083.22	40.40	0.00
TRINITY_I4VUZ4_9GAMM/248-396	I4VUZ4.1 PF10282.7;	40.40	0.00
TRINITY_L8GHR5_ACACA/165-276	L8GHR5.1 PF13519.4;	40.40	0.00
TRINITY_D8TST9_VOLCA/621-848	D8TST9.1 PF00211.18	40.40	0.00
TRINITY_A0A088AH46_APIME/4-157	A0A088AH46.1 PF0716	40.40	0.00
TRINITY_S8CX08_9LAMI/494-827	S8CX08.1 PF00120.22	40.40	0.00
TRINITY_D8TNB9_VOLCA/33-337	D8TNB9.1 PF03006.18	40.40	0.00
TRINITY_I7MIX5_TETTS/2-121	I7MIX5.1 PF05915.10	40.40	0.00
TRINITY_A0A078B9W1_STYLE/27-329	A0A078B9W1.1 PF0056	40.30	0.00
TRINITY_T1K4Z2_TETUR/57-124	T1K4Z2.1 PF01722.16	40.30	0.00
TRINITY_L9L202_TUPCH/116-185	L9L202.1 PF00076.20	40.30	0.00

TRINITY_A0EBS1_PARTE/150-392	A0EBS1.1 PF03133.13	40.30	0.00
TRINITY_L8HCK6_ACACA/9-255	L8HCK6.1 PF01370.19	40.30	0.00
TRINITY_I0YKY3_9CHLO/718-905	I0YKY3.1 PF01369.18	40.30	0.00
TRINITY_A0ECR3_PARTE/7-288	A0ECR3.1 PF14938.4;	40.30	0.00
TRINITY_F0Z6A9_DICPU/14-200	F0Z6A9.1 PF00485.16	40.30	0.00
TRINITY_A0BBG7_PARTE/2-367	A0BBG7.1 PF00022.17	40.30	0.00
TRINITY_A0A0A1UBU5_ENTIV/727-986	A0A0A1UBU5.1 PF0771	40.30	0.00
TRINITY_A9RW86_PHYPA/1-409	A9RW86.1 PF03901.15	40.30	0.00
TRINITY_A0A024VJJ5_PLAFA/28-278	A0A024VJJ5.1 PF0192	40.30	0.00
TRINITY_P80_DICDI/393-516	Q7YXD4.1 PF04145.13	40.30	0.00
TRINITY_A8PB07_COPC7/2-375	A8PB07.1 PF00022.17	40.30	0.00
TRINITY_A0A0J8F513_BETVU/156-360	A0A0J8F513.1 PF1438	40.30	0.00
TRINITY_I7MKV0_TETTS/148-266	I7MKV0.2 PF13863.4;	40.30	0.00
TRINITY_J9ISW6_9SPIT/1229-1443	J9ISW6.1 PF14633.4;	40.30	0.00
TRINITY_K9WEX9_9CYAN/10-331	K9WEX9.1 PF13434.4;	40.30	0.00
TRINITY_C3YRI7_BRAFL/24-243	C3YRI7.1 PF00149.26	40.30	0.00
TRINITY_U9TU79_RHIID/30-109	U9TU79.1 PF09409.8;	40.30	0.00
TRINITY_A0DFQ8_PARTE/4-232	A0DFQ8.1 PF04670.10	40.30	0.00
TRINITY_F0ZIS5_DICPU/509-828	F0ZIS5.1 PF04840.10	40.30	0.00
TRINITY_D8UJQ9_VOLCA/63-496	D8UJQ9.1 PF00854.19	40.30	0.00
TRINITY_A0A0P7XD05_9TELE/391-881	A0A0P7XD05.1 PF0320	40.30	0.00
TRINITY_S8DQN0_FOMPI/504-733	S8DQN0.1 PF13604.4;	40.30	0.00
TRINITY_I3LZC5_ICTTR/3-535	I3LZC5.1 PF00743.17	40.30	0.00
TRINITY_Q22GJ3_TETTS/84-228	Q22GJ3.1 PF04658.11	40.30	0.00
TRINITY_A0A0D2UAR8_CAPO3/95-270	A0A0D2UAR8.1 PF0062	40.30	0.00
TRINITY_J9IMZ6_9SPIT/896-1027	J9IMZ6.1 PF00072.22	40.30	0.00
TRINITY_F4PZS0_DICFS/51-299	F4PZS0.1 PF10230.7;	40.30	0.00
TRINITY_G4Z5K0_PHYSP/12-291	G4Z5K0.1 PF07993.10	40.30	0.00
TRINITY_L8H264_ACACA/433-569	L8H264.1 PF06544.10	40.30	0.00
TRINITY_D8U141_VOLCA/23-143	D8U141.1 PF01138.19	40.30	0.00
TRINITY_F4PGZ0_DICFS/296-492	F4PGZ0.1 PF04811.13	40.30	0.00
TRINITY_D8LU94_ECTSI/82-277	D8LU94.1 PF11715.6;	40.30	0.00
TRINITY_E5T190_TRISP/41-158	E5T190.1 PF00581.18	40.30	0.00
TRINITY_F2TYP3_SALR5/9-336	F2TYP3.1 PF00503.18	40.30	0.00
TRINITY_L8GFH7_ACACA/620-829	L8GFH7.1 PF00616.17	40.30	0.00
TRINITY_D8TGX0_VOLCA/1790-1999	D8TGX0.1 PF12775.5;	40.30	0.00
TRINITY_I0Z7Q5_9CHLO/1-366	I0Z7Q5.1 PF00067.20	40.30	0.00
TRINITY_A0A0G4IP25_PLABS/150-297	A0A0G4IP25.1 PF0419	40.30	0.00
TRINITY_G0R0B2_ICHMG/42-369	G0R0B2.1 PF05770.9;	40.30	0.00
TRINITY_F0ZP92_DICPU/298-480	F0ZP92.1 PF00621.18	40.30	0.00
TRINITY_I0YKK8_9CHLO/313-503	I0YKK8.1 PF08737.8;	40.30	0.00
TRINITY_A4RUB8_OSTLU/240-304	A4RUB8.1 PF00076.20	40.30	0.00
TRINITY_C1FFY4_MICSR/1990-2222	C1FFY4.1 PF03568.15	40.30	0.00
TRINITY_A0BK04_PARTE/149-277	A0BK04.1 PF04326.12	40.30	0.00
TRINITY_A0A0D2VIL9_CAPO3/5-77	A0A0D2VIL9.1 PF0525	40.30	0.00
TRINITY_J9GDG0_9SPIT/883-1129	J9GDG0.1 PF16212.3;	40.30	0.00
TRINITY_I0Z6V9_9CHLO/48-349	I0Z6V9.1 PF13896.4;	40.30	0.00
TRINITY_L8GS17_ACACA/603-781	L8GS17.1 PF00617.17	40.30	0.00
TRINITY_A9TCE6_PHYPA/157-467	A9TCE6.1 PF08506.8;	40.30	0.00
TRINITY_L8GR65_ACACA/345-515	L8GR65.1 PF03770.14	40.30	0.00
TRINITY_K4C5W7_SOLLC/32-171	K4C5W7.1 PF01909.21	40.30	0.00
TRINITY_L8H3W5_ACACA/971-1093	L8H3W5.1 PF00271.29	40.30	0.00
TRINITY_A0A059LL35_9CHLO/12-297	A0A059LL35.1 PF0338	40.30	0.00
TRINITY_D8TWQ4_VOLCA/120-450	D8TWQ4.1 PF01593.22	40.30	0.00
TRINITY_H3CA14_TETNG/107-409	H3CA14.1 PF00122.18	40.30	0.00
TRINITY_D3B8P4_POLPA/1391-1620	D3B8P4.1 PF07714.15	40.30	0.00
TRINITY_C3YNI2_BRAFL/38-202	C3YNI2.1 PF00270.27	40.30	0.00
TRINITY_K1PP44_CRAGI/10-83	K1PP44.1 PF00240.21	40.30	0.00
TRINITY_A0A059LS85_9CHLO/131-341	A0A059LS85.1 PF0505	40.30	0.00
TRINITY_C4LXP5_ENTHI/8-168	C4LXP5.1 PF00071.20	40.20	0.00
TRINITY_K0IP37_NITGG/41-257	K0IP37.1 PF12138.6;	40.20	0.00

TRINITY_G0R080_ICHMG/9-179	G0R080.1 PF00300.20	40.20	0.00
TRINITY_H3CMX4_TETNG/13-257	H3CMX4.1 PF03998.11	40.20	0.00
TRINITY_J9EIN9_9SPIT/452-555	J9EIN9.1 PF12796.5;	40.20	0.00
TRINITY_Q16QR4_AEDAE/180-429	Q16QR4.1 PF05206.12	40.20	0.00
TRINITY_A0CJZ7_PARTE/8-163	A0CJZ7.1 PF00795.20	40.20	0.00
TRINITY_B8A5M6_DANRE/403-508	B8A5M6.1 PF00085.18	40.20	0.00
TRINITY_J9ILE1_9SPIT/1169-1300	J9ILE1.1 PF00072.22	40.20	0.00
TRINITY_I7MHJ7_TETTS/1612-1732	I7MHJ7.2 PF00072.22	40.20	0.00
TRINITY_T1JDY6_STRMM/63-161	T1JDY6.1 PF12894.5;	40.20	0.00
TRINITY_A0A058Z459_9EUKA/58-298	A0A058Z459.1 PF0115	40.20	0.00
TRINITY_V4B1M3_LOTGI/23-303	V4B1M3.1 PF00069.23	40.20	0.00
TRINITY_A0BMQ9_PARTE/112-376	A0BMQ9.1 PF00069.23	40.20	0.00
TRINITY_G8BJW8_CANPC/6-167	G8BJW8.1 PF00071.20	40.20	0.00
TRINITY_A0A0P7ZDX1_9TELE/109-398	A0A0P7ZDX1.1 PF0520	40.20	0.00
TRINITY_M7BJX8_CHEMY/357-605	M7BJX8.1 PF09822.7;	40.20	0.00
TRINITY_C5KL65_PERM5/172-239	C5KL65.1 PF00013.27	40.20	0.00
TRINITY_E2BR37_HARSA/2-398	E2BR37.1 PF00450.20	40.20	0.00
TRINITY_Q23QF2_TETTS/248-571	Q23QF2.2 PF03133.13	40.20	0.00
TRINITY_A0A078A0U8_STYLE/283-375	A0A078A0U8.1 PF0183	40.20	0.00
TRINITY_I0YNY9_9CHLO/55-299	I0YNY9.1 PF10494.7;	40.20	0.00
TRINITY_J9K3I7_ACYPI/702-906	J9K3I7.2 PF00069.23	40.20	0.00
TRINITY_A0A024UB11_9STRA/150-422	A0A024UB11.1 PF0066	40.20	0.00
TRINITY_R0GI74_9BRAS/32-488	R0GI74.1 PF00450.20	40.20	0.00
TRINITY_A0D3L9_PARTE/6-126	A0D3L9.1 PF00466.18	40.20	0.00
TRINITY_A0A075API1_9FUNG/162-264	A0A075API1.1 PF1275	40.20	0.00
TRINITY_D3B8L6_POLPA/799-915	D3B8L6.1 PF17034.3;	40.20	0.00
TRINITY_A0A0B0MP59_GOSAR/696-1148	A0A0B0MP59.1 PF0052	40.20	0.00
TRINITY_D8LB42_ECTSI/657-909	D8LB42.1 PF07714.15	40.20	0.00
TRINITY_D3BJM0_POLPA/633-901	D3BJM0.1 PF00069.23	40.20	0.00
TRINITY_A0A0E0N1I3_ORYRU/75-189	A0A0E0N1I3.1 PF1226	40.20	0.00
TRINITY_A0DSX0_PARTE/335-463	A0DSX0.1 PF00565.15	40.20	0.00
TRINITY_A0A078A3P9_STYLE/651-804	A0A078A3P9.1 PF0056	40.20	0.00
TRINITY_A0A078ALJ4_STYLE/354-498	A0A078ALJ4.1 PF0753	40.20	0.00
TRINITY_R1E4R6_EMIHU/5-166	R1E4R6.1 PF00071.20	40.20	0.00
TRINITY_M1VZW3_CLAP2/263-585	M1VZW3.1 PF09423.8;	40.20	0.00
TRINITY_Q23DV4_TETTS/29-454	Q23DV4.1 PF04597.12	40.20	0.00
TRINITY_D2V1E9_NAEGR/25-106	D2V1E9.1 PF03951.17	40.20	0.00
TRINITY_D8LIL3_ECTSI/337-443	D8LIL3.1 PF00651.29	40.20	0.00
TRINITY_E1ZGW5_CHLVA/15-381	E1ZGW5.1 PF16496.3;	40.20	0.00
TRINITY_D3BUV7_POLPA/393-543	D3BUV7.1 PF00620.25	40.20	0.00
TRINITY_I7M682_TETTS/710-925	I7M682.2 PF12807.5;	40.20	0.00
TRINITY_L8GZC0_ACACA/119-285	L8GZC0.1 PF01872.15	40.20	0.00
TRINITY_A0A067QSY9_ZOONE/1-120	A0A067QSY9.1 PF0075	40.20	0.00
TRINITY_D8TZ67_VOLCA/1522-1849	D8TZ67.1 PF03747.12	40.20	0.00
TRINITY_A0A067JUG5_JATCU/42-416	A0A067JUG5.1 PF0028	40.20	0.00
TRINITY_Q22XU8_TETTS/54-237	Q22XU8.3 PF06452.9;	40.20	0.00
TRINITY_D8UEH3_VOLCA/245-468	D8UEH3.1 PF13489.4;	40.20	0.00
TRINITY_H3E6W2_PRIPA/841-961	H3E6W2.1 PF00622.26	40.20	0.00
TRINITY_A9TUH0_PHYPA/288-386	A9TUH0.1 PF13499.4;	40.20	0.00
TRINITY_D8U2Y4_VOLCA/70-217	D8U2Y4.1 PF00106.23	40.20	0.00
TRINITY_Q23R88_TETTS/18-272	Q23R88.1 PF00069.23	40.20	0.00
TRINITY_L8GG36_ACACA/72-255	L8GG36.1 PF02145.13	40.20	0.00
TRINITY_G0SDJ2_CHATD/240-715	G0SDJ2.1 PF06862.10	40.20	0.00
TRINITY_A0EGL5_PARTE/997-1236	A0EGL5.1 PF00233.17	40.10	0.00
TRINITY_A0DTT7_PARTE/327-627	A0DTT7.1 PF00069.23	40.10	0.00
TRINITY_A0A0F3KNK0_9GAMM/14-193	A0A0F3KNK0.1 PF1353	40.10	0.00
TRINITY_F4PJJ7_DICFS/115-484	F4PJJ7.1 PF00854.19	40.10	0.00
TRINITY_A0A0E3ZJB7_9BACT/31-235	A0A0E3ZJB7.1 PF0440	40.10	0.00
TRINITY_F0ZUR8_DICPU/9-209	F0ZUR8.1 PF14938.4;	40.10	0.00
TRINITY_A0A0D2WW75_CAPO3/6-180	A0A0D2WW75.1 PF0080	40.10	0.00
TRINITY_A9RCP9_PHYPA/61-483	A9RCP9.1 PF00450.20	40.10	0.00

TRINITY_A0CUS6_PARTE/513-811	A0CUS6.1 PF03372.21	40.10	0.00
TRINITY_D6W990_TRICA/133-463	D6W990.1 PF05028.12	40.10	0.00
TRINITY_A0CS82_PARTE/14-193	A0CS82.1 PF03248.11	40.10	0.00
TRINITY_I0YMM8_9CHLO/86-517	I0YMM8.1 PF03069.13	40.10	0.00
TRINITY_U4K610_9VIBR/46-287	U4K610.1 PF00561.18	40.10	0.00
TRINITY_G0QK40_ICHMG/27-393	G0QK40.1 PF07690.14	40.10	0.00
TRINITY_L8GMG4_ACACA/32-285	L8GMG4.1 PF02265.14	40.10	0.00
TRINITY_F0ZNA0_DICPU/21-199	F0ZNA0.1 PF01105.22	40.10	0.00
TRINITY_L8GT92_ACACA/55-226	L8GT92.1 PF00092.26	40.10	0.00
TRINITY_D8U3I5_VOLCA/269-427	D8U3I5.1 PF00270.27	40.10	0.00
TRINITY_K7H5G4_CAEJA/381-536	K7H5G4.1 PF03712.13	40.10	0.00
TRINITY_A0A0E3UVI8_9BACT/37-178	A0A0E3UVI8.1 PF0821	40.10	0.00
TRINITY_M3ZMC0_XIPMA/67-318	M3ZMC0.1 PF00069.23	40.10	0.00
TRINITY_A8IXA0_CHLRE/244-369	A8IXA0.1 PF01554.16	40.10	0.00
TRINITY_I0YM35_9CHLO/12-222	I0YM35.1 PF04757.12	40.10	0.00
TRINITY_L8HBF7_ACACA/234-428	L8HBF7.1 PF02958.18	40.10	0.00
TRINITY_K4C6Z0_SOLLC/57-596	K4C6Z0.1 PF02990.14	40.10	0.00
TRINITY_A0BG36_PARTE/5-220	A0BG36.1 PF08713.9;	40.10	0.00
TRINITY_E1ZR23_CHLVA/1-185	E1ZR23.1 PF00582.24	40.10	0.00
TRINITY_T1HYY9_RHOPR/5-259	T1HYY9.1 PF03194.13	40.10	0.00
TRINITY_D8TMZ5_VOLCA/89-222	D8TMZ5.1 PF04832.10	40.10	0.00
TRINITY_A0CXP3_PARTE/26-844	A0CXP3.1 PF01496.17	40.10	0.00
TRINITY_I4B662_TURPD/34-279	I4B662.1 PF00561.18	40.10	0.00
TRINITY_D8TJ68_VOLCA/55-321	D8TJ68.1 PF01073.17	40.10	0.00
TRINITY_D3B9P3_POLPA/85-246	D3B9P3.1 PF03031.16	40.10	0.00
TRINITY_B9S9V5_RICCO/84-363	B9S9V5.1 PF06012.10	40.10	0.00
TRINITY_I1N3M6_SOYBN/7-176	I1N3M6.2 PF00106.23	40.10	0.00
TRINITY_W7UAA9_9STRA/162-337	W7UAA9.1 PF05050.10	40.10	0.00
TRINITY_I0HIU6_ACTM4/505-636	I0HIU6.1 PF00004.27	40.10	0.00
TRINITY_I1G669_AMPQE/284-402	I1G669.1 PF00581.18	40.10	0.00
TRINITY_L8GVV2_ACACA/2-276	L8GVV2.1 PF06454.9;	40.10	0.00
TRINITY_D2VRF8_NAEGR/1-426	D2VRF8.1 PF14727.4;	40.10	0.00
TRINITY_A0A067CDF1_SAPPC/8-67	A0A067CDF1.1 PF0534	40.00	0.00
TRINITY_Q23QF5_TETTS/161-229	Q23QF5.2 PF00505.17	40.00	0.00
TRINITY_H3FEW2_PRIPA/81-220	H3FEW2.1 PF01764.23	40.00	0.00
TRINITY_I7MCK6_TETTS/51-569	I7MCK6.2 PF02990.14	40.00	0.00
TRINITY_I7MHQ9_TETTS/20-306	I7MHQ9.2 PF00069.23	40.00	0.00
TRINITY_G0QXF6_ICHMG/1-194	G0QXF6.1 PF14309.4;	40.00	0.00
TRINITY_I7MMP5_TETTS/4189-4485	I7MMP5.2 PF00632.23	40.00	0.00
TRINITY_D6U2B0_9CHLR/18-113	D6U2B0.1 PF08818.9;	40.00	0.00
TRINITY_A0A0N8K0P3_9TELE/163-383	A0A0N8K0P3.1 PF1308	40.00	0.00
TRINITY_G4VDJ6_SCHMA/5-177	G4VDJ6.1 PF00025.19	40.00	0.00
TRINITY_A0BHK3_PARTE/94-275	A0BHK3.1 PF03357.19	40.00	0.00
TRINITY_W7XFP0_TETTS/100-339	W7XFP0.1 PF00889.17	40.00	0.00
TRINITY_A0A077ZW47_STYLE/354-499	A0A077ZW47.1 PF0753	40.00	0.00
TRINITY_D3BGE8_POLPA/10-440	D3BGE8.1 PF01593.22	40.00	0.00
TRINITY_M0QSN1_ACACA/309-399	M0QSN1.1 PF02214.20	40.00	0.00
TRINITY_L8HBW7_ACACA/952-1197	L8HBW7.1 PF07714.15	40.00	0.00
TRINITY_L8H764_ACACA/17-275	L8H764.1 PF03372.21	40.00	0.00
TRINITY_L8GDV2_ACACA/554-812	L8GDV2.1 PF07714.15	40.00	0.00
TRINITY_M5WNT3_PRUPE/256-392	M5WNT3.1 PF04146.13	40.00	0.00
TRINITY_M4BH02_HYAAE/21-255	M4BH02.1 PF01370.19	40.00	0.00
TRINITY_D8R2B4_SELML/111-306	D8R2B4.1 PF04389.15	40.00	0.00
TRINITY_D8U3Z3_VOLCA/6-409	D8U3Z3.1 PF03372.21	40.00	0.00
TRINITY_A0A0M8K6W4_9CHLR/16-417	A0A0M8K6W4.1 PF0597	40.00	0.00
TRINITY_A0A088APP1_APIME/248-559	A0A088APP1.1 PF0451	40.00	0.00
TRINITY_L8H4P4_ACACA/191-320	L8H4P4.1 PF00293.26	40.00	0.00
TRINITY_A9AVH5_HERA2/104-281	A9AVH5.1 PF02655.12	40.00	0.00
TRINITY_A0A0D2WST2_CAPO3/27-317	A0A0D2WST2.1 PF0842	40.00	0.00
TRINITY_I0KFG2_9BACT/11-118	I0KFG2.1 PF12680.5;	40.00	0.00
TRINITY_A0A0G1X5K3_9BACT/25-151	A0A0G1X5K3.1 PF0164	40.00	0.00

TRINITY_A0A0F5Y7G7_9CYAN/389-507	A0A0F5Y7G7.1 PF0251	40.00	0.00
TRINITY_I7M9T1_TETTS/284-344	I7M9T1.2 PF13499.4;	40.00	0.00
TRINITY_Q9RVJ8_DEIRA/11-370	Q9RVJ8.1 PF00155.19	40.00	0.00
TRINITY_F7BJ78_MACMU/83-176	F7BJ78.1 PF12796.5;	40.00	0.00
TRINITY_F4QD17_DICFS/238-364	F4QD17.1 PF03188.14	40.00	0.00
TRINITY_G2QBT0_MYCTT/406-545	G2QBT0.1 PF00005.25	40.00	0.00
TRINITY_D8TWT3_VOLCA/56-182	D8TWT3.1 PF03517.11	40.00	0.00
TRINITY_F0ZB12_DICPU/1091-1610	F0ZB12.1 PF06920.11	40.00	0.00
TRINITY_A0A075R593_BRELA/182-435	A0A075R593.1 PF0403	40.00	0.00
TRINITY_C3Z2U8_BRAFL/1-91	C3Z2U8.1 PF08285.9;	40.00	0.00
TRINITY_D8SQH8_SELML/67-214	D8SQH8.1 PF04073.13	40.00	0.00
TRINITY_J9IXZ0_9SPIT/597-711	J9IXZ0.1 PF13893.4;	40.00	0.00
TRINITY_Q22CF9_TETTS/26-290	Q22CF9.2 PF06098.9;	40.00	0.00
TRINITY_X6LEC0_RETFI/128-273	X6LEC0.1 PF07534.14	40.00	0.00
TRINITY_I7LV23_TETTS/355-496	I7LV23.2 PF08016.10	40.00	0.00
TRINITY_X1WJ08_ACYPI/105-211	X1WJ08.1 PF00307.29	40.00	0.00
TRINITY_G0QW53_ICHMG/57-236	G0QW53.1 PF03959.11	40.00	0.00
TRINITY_D8TSZ7_VOLCA/1690-1975	D8TSZ7.1 PF07714.15	40.00	0.00
TRINITY_H9UFC8_SPIAZ/7-212	H9UFC8.1 PF00106.23	40.00	0.00
TRINITY_D8REM8_SELML/150-380	D8REM8.1 PF01544.16	40.00	0.00
TRINITY_W7X1I7_TETTS/95-219	W7X1I7.1 PF04178.10	40.00	0.00
TRINITY_Q4DUM3_TRYCC/1-76	Q4DUM3.1 PF02798.18	40.00	0.00
TRINITY_Q0E511_SFAVA/202-508	Q0E511.1 PF05686.10	40.00	0.00
TRINITY_L8GZK0_ACACA/419-595	L8GZK0.1 PF00621.18	40.00	0.00
TRINITY_A0A022R2D1_ERYGU/4-111	A0A022R2D1.1 PF0063	40.00	0.00
TRINITY_W4Y2D5_STRPU/110-495	W4Y2D5.1 PF02696.12	40.00	0.00
TRINITY_A8JGH3_CHLRE/7-182	A8JGH3.1 PF13967.4;	40.00	0.00
TRINITY_A0A0D3FDS0_9ORYZ/161-297	A0A0D3FDS0.1 PF0549	40.00	0.00
TRINITY_L8HDD8_ACACA/713-975	L8HDD8.1 PF07714.15	40.00	0.00
TRINITY_D2V5F8_NAEGR/81-284	D2V5F8.1 PF00795.20	40.00	0.00
TRINITY_K3ZSB1_SETIT/80-514	K3ZSB1.1 PF00067.20	40.00	0.00
TRINITY_A0A074RK62_9HOMO/1-233	A0A074RK62.1 PF0772	40.00	0.00
TRINITY_I1FLM8_AMPQE/195-377	I1FLM8.1 PF00644.18	40.00	0.00
TRINITY_A8J3P1_CHLRE/954-1161	A8J3P1.1 PF00211.18	40.00	0.00
TRINITY_E1ZRM2_CHLVA/1-112	E1ZRM2.1 PF00078.25	40.00	0.00
TRINITY_L8GMP4_ACACA/297-555	L8GMP4.1 PF07714.15	40.00	0.00
TRINITY_I0YRL6_9CHLO/75-235	I0YRL6.1 PF01554.16	40.00	0.00
TRINITY_V6M7F5_9EUKA/23-107	V6M7F5.1 PF16561.3;	40.00	0.00
TRINITY_A0A015N2M8_9GLOM/217-520	A0A015N2M8.1 PF0597	40.00	0.00
TRINITY_H3NW39_9GAMM/139-517	H3NW39.1 PF09423.8;	40.00	0.00
TRINITY_A8I4J0_CHLRE/1046-1288	A8I4J0.1 PF00664.21	40.00	0.00
TRINITY_A0A0A1U731_ENTIV/754-1013	A0A0A1U731.1 PF0771	40.00	0.00
TRINITY_N4W161_COLOR/21-375	N4W161.1 PF01212.19	40.00	0.00
TRINITY_A0A066X8U2_COLSU/106-230	A0A066X8U2.1 PF0018	40.00	0.00
TRINITY_A0A060SSE0_PYCCI/993-1239	A0A060SSE0.1 PF0772	40.00	0.00
TRINITY_A0A0G2HZ11_9PEZI/139-381	A0A0G2HZ11.1 PF1690	40.00	0.00
TRINITY_D3FF62_CONWI/28-99	D3FF62.1 PF00240.21	40.00	0.00
TRINITY_A0A077ZSB1_STYLE/58-301	A0A077ZSB1.1 PF0052	40.00	0.00
TRINITY_F0Z8D6_DICPU/32-210	F0Z8D6.1 PF00621.18	39.90	0.00
TRINITY_Q248F0_TETTS/19-502	Q248F0.2 PF04916.11	39.90	0.00
TRINITY_A0BHK3_PARTE/94-275	A0BHK3.1 PF03357.19	39.90	0.00
TRINITY_J9EZ03_9SPIT/89-238	J9EZ03.1 PF01694.20	39.90	0.00
TRINITY_A0A078B349_STYLE/444-804	A0A078B349.1 PF0070	39.90	0.00
TRINITY_A0A078A7I2_STYLE/51-320	A0A078A7I2.1 PF0300	39.90	0.00
TRINITY_Q23YB5_TETTS/2-179	Q23YB5.2 PF06017.11	39.90	0.00
TRINITY_I1FE73_AMPQE/40-233	I1FE73.1 PF00149.26	39.90	0.00
TRINITY_I7LX20_TETTS/11-287	I7LX20.2 PF00069.23	39.90	0.00
TRINITY_F4PV70_DICFS/126-308	F4PV70.1 PF05527.9;	39.90	0.00
TRINITY_F4QDL1_DICFS/652-846	F4QDL1.1 PF00350.21	39.90	0.00
TRINITY_T1EMF6_HELRO/7-174	T1EMF6.1 PF00071.20	39.90	0.00
TRINITY_L8HHT7_ACACA/150-448	L8HHT7.1 PF00561.18	39.90	0.00

TRINITY_A0CMN6_PARTE/121-399	A0CMN6.1 PF01457.14	39.90	0.00
TRINITY_A8HXX5_CHLRE/644-876	A8HXX5.1 PF00476.18	39.90	0.00
TRINITY_A0A090M5K8_OSTTA/301-459	A0A090M5K8.1 PF0181	39.90	0.00
TRINITY_D8TCX8_SELML/26-308	D8TCX8.1 PF12931.5;	39.90	0.00
TRINITY_D8TJM5_VOLCA/3-174	D8TJM5.1 PF12999.5;	39.90	0.00
TRINITY_C3Z4J5_BRAFL/1102-1273	C3Z4J5.1 PF05729.10	39.90	0.00
TRINITY_D3ED10_GEOS4/20-227	D3ED10.1 PF01261.22	39.90	0.00
TRINITY_A8J6A0_CHLRE/15-174	A8J6A0.1 PF00071.20	39.90	0.00
TRINITY_A0A0E3ZJB7_9BACT/31-235	A0A0E3ZJB7.1 PF0440	39.90	0.00
TRINITY_A8HPD6_CHLRE/16-276	A8HPD6.1 PF00520.29	39.90	0.00
TRINITY_F0ZDP3_DICPU/30-614	F0ZDP3.1 PF04113.12	39.90	0.00
TRINITY_B9ID98_POPTR/45-361	B9ID98.1 PF04258.11	39.90	0.00
TRINITY_A0A0N4T787_BRUPA/6-154	A0A0N4T787.1 PF0997	39.90	0.00
TRINITY_L8GQC0_ACACA/227-408	L8GQC0.1 PF00621.18	39.90	0.00
TRINITY_K6Z0X8_9ALTE/377-558	K6Z0X8.1 PF02868.13	39.90	0.00
TRINITY_A0A0D2ZUC0_BRAOL/383-663	A0A0D2ZUC0.1 PF1618	39.90	0.00
TRINITY_A0A059LS32_9CHLO/49-519	A0A059LS32.1 PF0050	39.90	0.00
TRINITY_G0R1M3_ICHMG/6-229	G0R1M3.1 PF00566.16	39.90	0.00
TRINITY_D8TL04_VOLCA/85-342	D8TL04.1 PF00069.23	39.90	0.00
TRINITY_L8GIR9_ACACA/27-209	L8GIR9.1 PF13462.4;	39.90	0.00
TRINITY_I0Z2U0_9CHLO/1-234	I0Z2U0.1 PF09468.8;	39.90	0.00
TRINITY_H2Z6S3_CIOSA/41-215	H2Z6S3.1 PF13847.4;	39.90	0.00
TRINITY_J9IQR1_9SPIT/1456-1607	J9IQR1.1 PF05708.10	39.80	0.00
TRINITY_A0BV56_PARTE/20-148	A0BV56.1 PF09229.9;	39.80	0.00
TRINITY_A0A0A1NUK7_9FUNG/102-350	A0A0A1NUK7.1 PF0056	39.80	0.00
TRINITY_Q22D68_TETTS/29-277	Q22D68.3 PF03803.13	39.80	0.00
TRINITY_A0DV77_PARTE/1-119	A0DV77.1 PF01920.18	39.80	0.00
TRINITY_A0A086YPT4_9FIRM/3-287	A0A086YPT4.1 PF0366	39.80	0.00
TRINITY_K3Y8V3_SETIT/56-173	K3Y8V3.1 PF09745.7;	39.80	0.00
TRINITY_J9IG79_9SPIT/139-226	J9IG79.1 PF04969.14	39.80	0.00
TRINITY_K9V5U7_9CYAN/5-202	K9V5U7.1 PF00300.20	39.80	0.00
TRINITY_K9X3A0_9NOST/6-204	K9X3A0.1 PF00106.23	39.80	0.00
TRINITY_B8C3S2_THAPS/695-800	B8C3S2.1 PF00651.29	39.80	0.00
TRINITY_Q239J8_TETTS/159-436	Q239J8.2 PF00069.23	39.80	0.00
TRINITY_X6MTW8_RETFI/11-288	X6MTW8.1 PF07065.12	39.80	0.00
TRINITY_G0QR55_ICHMG/103-222	G0QR55.1 PF02518.24	39.80	0.00
TRINITY_K1QVS3_CRAGI/167-617	K1QVS3.1 PF01432.18	39.80	0.00
TRINITY_D3BD33_POLPA/218-338	D3BD33.1 PF10409.7;	39.80	0.00
TRINITY_A0C5Z6_PARTE/21-244	A0C5Z6.1 PF07722.11	39.80	0.00
TRINITY_A0A078AWD3_STYLE/86-173	A0A078AWD3.1 PF0095	39.80	0.00
TRINITY_A0A0A7KLL9_9DEIO/38-159	A0A0A7KLL9.1 PF0246	39.80	0.00
TRINITY_A0A0G4IZH9_PLABS/340-428	A0A0G4IZH9.1 PF0138	39.80	0.00
TRINITY_Q22CF9_TETTS/26-290	Q22CF9.2 PF06098.9;	39.80	0.00
TRINITY_A0A0N1H5B2_9EURO/227-347	A0A0N1H5B2.1 PF0062	39.80	0.00
TRINITY_R7YPX5_CONA1/736-837	R7YPX5.1 PF04815.13	39.80	0.00
TRINITY_A7SGM4_NEMVE/6-131	A7SGM4.1 PF00188.24	39.80	0.00
TRINITY_L8H5E2_ACACA/175-397	L8H5E2.1 PF10469.7;	39.80	0.00
TRINITY_W5L4N5_ASTMX/13-269	W5L4N5.1 PF01073.17	39.80	0.00
TRINITY_D8U6Z8_VOLCA/20-99	D8U6Z8.1 PF01722.16	39.80	0.00
TRINITY_I0Z8I5_9CHLO/7-129	I0Z8I5.1 PF04145.13	39.80	0.00
TRINITY_A0A0M9YGY2_9ACTN/37-482	A0A0M9YGY2.1 PF1352	39.80	0.00
TRINITY_A8HSL4_CHLRE/1329-1518	A8HSL4.1 PF15785.3;	39.80	0.00
TRINITY_Q54EI7_DICDI/72-491	Q54EI7.1 PF01490.16	39.80	0.00
TRINITY_A0A024URS6_9STRA/382-491	A0A024URS6.1 PF0065	39.80	0.00
TRINITY_A0A0G4IP69_PLABS/26-227	A0A0G4IP69.1 PF0691	39.80	0.00
TRINITY_L8HK60_ACACA/154-481	L8HK60.1 PF07690.14	39.80	0.00
TRINITY_A0A077ZRC9_STYLE/288-425	A0A077ZRC9.1 PF0066	39.80	0.00
TRINITY_A0A022S059_ERYGU/909-1026	A0A022S059.1 PF0058	39.80	0.00
TRINITY_F0ZYQ9_DICPU/7-431	F0ZYQ9.1 PF04791.14	39.80	0.00
TRINITY_I7LWU7_TETTS/6-212	I7LWU7.1 PF03731.13	39.80	0.00
TRINITY_W4FRA5_9STRA/170-345	W4FRA5.1 PF01612.18	39.80	0.00

TRINITY_L8GFH7_ACACA/620-829	L8GFH7.1 PF00616.17	39.80	0.00
TRINITY_L8GI83_ACACA/89-355	L8GI83.1 PF03062.17	39.80	0.00
TRINITY_U9SP08_RHIID/2-187	U9SP08.1 PF04081.11	39.80	0.00
TRINITY_A0A0N0D609_9DELT/671-792	A0A0N0D609.1 PF0007	39.80	0.00
TRINITY_A0D7E3_PARTE/51-173	A0D7E3.1 PF00134.21	39.80	0.00
TRINITY_L8GFZ5_ACACA/382-614	L8GFZ5.1 PF11831.6;	39.80	0.00
TRINITY_H8MU17_CORCM/22-473	H8MU17.1 PF00501.26	39.80	0.00
TRINITY_A8HVJ9_CHLRE/1-298	A8HVJ9.1 PF14870.4;	39.80	0.00
TRINITY_Q245G5_TETTS/3-166	Q245G5.2 PF08613.9;	39.80	0.00
TRINITY_A0A077AWJ9_9RICK/2-198	A0A077AWJ9.1 PF0173	39.80	0.00
TRINITY_J9F4Y5_9SPIT/78-412	J9F4Y5.1 PF01237.16	39.80	0.00
TRINITY_Q2H981_CHAGB/449-680	Q2H981.1 PF00005.25	39.80	0.00
TRINITY_D8TIN5_VOLCA/42-343	D8TIN5.1 PF07082.9;	39.80	0.00
TRINITY_D8TLR9_VOLCA/72-156	D8TLR9.1 PF16561.3;	39.80	0.00
TRINITY_A0A090M3H0_OSTTA/16-175	A0A090M3H0.1 PF0007	39.80	0.00
TRINITY_H1Y6N7_9SPHI/116-335	H1Y6N7.1 PF09992.7;	39.80	0.00
TRINITY_B6IUG7_RHOCS/23-174	B6IUG7.1 PF11911.6;	39.80	0.00
TRINITY_K9SZG2_9CYAN/110-382	K9SZG2.1 PF01636.21	39.80	0.00
TRINITY_G3PTT1_GASAC/1137-1233	G3PTT1.1 PF12755.5;	39.80	0.00
TRINITY_I3BZQ3_9GAMM/37-447	I3BZQ3.1 PF01593.22	39.80	0.00
TRINITY_B8BSZ0_THAPS/4-370	B8BSZ0.1 PF07690.14	39.80	0.00
TRINITY_D8QVL3_SELML/68-528	D8QVL3.1 PF00176.21	39.80	0.00
TRINITY_G0QR68_ICHMG/177-395	G0QR68.1 PF04811.13	39.80	0.00
TRINITY_A0A0F9WG10_9MICR/1882-2185	A0A0F9WG10.1 PF0063	39.70	0.00
TRINITY_Q22NF5_TETTS/2-130	Q22NF5.2 PF15305.4;	39.70	0.00
TRINITY_A0A078A8V4_STYLE/38-150	A0A078A8V4.1 PF0314	39.70	0.00
TRINITY_Q246C6_TETTS/4-223	Q246C6.1 PF00300.20	39.70	0.00
TRINITY_A0ZEC3_NODSP/1242-1563	A0ZEC3.1 PF00698.19	39.70	0.00
TRINITY_A0A0D2U6S3_CAPO3/3-121	A0A0D2U6S3.1 PF0892	39.70	0.00
TRINITY_A0A0J8EQB6_BETVU/47-500	A0A0J8EQB6.1 PF0008	39.70	0.00
TRINITY_A0A0N5B4U9_STREA/94-236	A0A0N5B4U9.1 PF0176	39.70	0.00
TRINITY_Q54U78_DICDI/386-507	Q54U78.1 PF13671.4;	39.70	0.00
TRINITY_F0ZDR8_DICPU/7-189	F0ZDR8.1 PF00025.19	39.70	0.00
TRINITY_L8HE22_ACACA/223-472	L8HE22.1 PF07714.15	39.70	0.00
TRINITY_U5QH35_9CYAN/12-126	U5QH35.1 PF00903.23	39.70	0.00
TRINITY_C3Y0H0_BRAFL/1362-1490	C3Y0H0.1 PF00004.27	39.70	0.00
TRINITY_A0A077ZV54_STYLE/34-212	A0A077ZV54.1 PF0173	39.70	0.00
TRINITY_A7TRV1_VANPO/21-89	A7TRV1.1 PF00505.17	39.70	0.00
TRINITY_L8H8I9_ACACA/41-375	L8H8I9.1 PF04143.12	39.70	0.00
TRINITY_G0QX56_ICHMG/29-236	G0QX56.1 PF01027.18	39.70	0.00
TRINITY_A0CWZ6_PARTE/592-712	A0CWZ6.1 PF00072.22	39.70	0.00
TRINITY_L8GII0_ACACA/41-782	L8GII0.1 PF05786.12	39.70	0.00
TRINITY_Q23Q43_TETTS/594-871	Q23Q43.2 PF00580.19	39.70	0.00
TRINITY_T0RMX0_9STRA/713-836	T0RMX0.1 PF00622.26	39.70	0.00
TRINITY_F6UG60_ORNAN/264-513	F6UG60.2 PF07714.15	39.70	0.00
TRINITY_L8GIA0_ACACA/63-297	L8GIA0.1 PF02230.14	39.70	0.00
TRINITY_D8U323_VOLCA/113-253	D8U323.1 PF12400.6;	39.70	0.00
TRINITY_A0A087SEF3_AUXPR/73-194	A0A087SEF3.1 PF0828	39.70	0.00
TRINITY_D8UCH0_VOLCA/1993-2176	D8UCH0.1 PF08146.10	39.70	0.00
TRINITY_A0A0K9NSC8_ZOSMR/431-668	A0A0K9NSC8.1 PF0481	39.70	0.00
TRINITY_A0A0D2WSN5_CAPO3/1169-1441	A0A0D2WSN5.1 PF0066	39.70	0.00
TRINITY_I7MI37_TETTS/11-167	I7MI37.1 PF00179.24	39.70	0.00
TRINITY_G3HFL8_CRIGR/98-346	G3HFL8.1 PF00069.23	39.70	0.00
TRINITY_A9T7I3_PHYPA/39-263	A9T7I3.1 PF01370.19	39.70	0.00
TRINITY_A9SX47_PHYPA/216-299	A9SX47.1 PF00011.19	39.70	0.00
TRINITY_J9I2H5_9SPIT/998-1122	J9I2H5.1 PF07744.11	39.70	0.00
TRINITY_C1FIB9_MICSR/337-470	C1FIB9.1 PF01728.17	39.70	0.00
TRINITY_I7M6D7_TETTS/137-346	I7M6D7.1 PF03798.14	39.70	0.00
TRINITY_K3X0F0_PYTUL/81-259	K3X0F0.1 PF00092.26	39.70	0.00
TRINITY_Q22D68_TETTS/29-277	Q22D68.3 PF03803.13	39.70	0.00
TRINITY_I0YYN4_9CHLO/278-509	I0YYN4.1 PF07228.10	39.70	0.00

TRINITY_K1VQN0_TRIAC/270-425	K1VQN0.1 PF00069.23	39.70	0.00
TRINITY_W4XFF3_STRPU/197-480	W4XFF3.1 PF01457.14	39.70	0.00
TRINITY_G0R3R5_ICHMG/1-259	G0R3R5.1 PF00069.23	39.70	0.00
TRINITY_Q9TXH9_CAEEL/217-297	Q9TXH9.1 PF00789.18	39.70	0.00
TRINITY_F2U8H2_SALR5/43-141	F2U8H2.1 PF08022.10	39.70	0.00
TRINITY_A0A0G4FKZ5_9ALVE/823-1093	A0A0G4FKZ5.1 PF0066	39.70	0.00
TRINITY_L1JES3_GUITH/22-118	L1JES3.1 PF06058.11	39.70	0.00
TRINITY_A7RIH2_NEMVE/41-162	A7RIH2.1 PF07890.10	39.70	0.00
TRINITY_I7M0W4_TETTS/279-641	I7M0W4.2 PF06602.12	39.70	0.00
TRINITY_Q3M0Y3_PARTE/32-235	Q3M0Y3.1 PF00804.23	39.70	0.00
TRINITY_J9IGS5_9SPIT/95-325	J9IGS5.1 PF07228.10	39.70	0.00
TRINITY_A0CXP9_PARTE/92-386	A0CXP9.1 PF01457.14	39.70	0.00
TRINITY_D8TKD3_VOLCA/49-222	D8TKD3.1 PF04832.10	39.70	0.00
TRINITY_W5P2G8_SHEEP/497-904	W5P2G8.1 PF03372.21	39.70	0.00
TRINITY_K4C5G6_SOLLC/328-493	K4C5G6.1 PF03219.12	39.70	0.00
TRINITY_A0A087SK67_AUXPR/319-613	A0A087SK67.1 PF1618	39.70	0.00
TRINITY_L8H823_ACACA/184-369	L8H823.1 PF07065.12	39.70	0.00
TRINITY_I7MDQ7_TETTS/112-319	I7MDQ7.1 PF01027.18	39.70	0.00
TRINITY_A0A096PK38_MAIZE/7-105	A0A096PK38.1 PF0016	39.70	0.00
TRINITY_D8TTM6_VOLCA/6-138	D8TTM6.1 PF13676.4;	39.70	0.00
TRINITY_D8RM07_SELML/139-188	D8RM07.1 PF00628.27	39.60	0.00
TRINITY_C3YXK2_BRAFL/8-169	C3YXK2.1 PF00071.20	39.60	0.00
TRINITY_A0A078ACA2_STYLE/7-156	A0A078ACA2.1 PF1458	39.60	0.00
TRINITY_A0A067NQ02_PLEOS/5-180	A0A067NQ02.1 PF0002	39.60	0.00
TRINITY_C1N3X3_MICPC/1-109	C1N3X3.1 PF04430.12	39.60	0.00
TRINITY_F2G9F8_ALTMD/624-740	F2G9F8.1 PF02518.24	39.60	0.00
TRINITY_I7M910_TETTS/11-285	I7M910.1 PF00069.23	39.60	0.00
TRINITY_A9TXU9_PHYPA/274-593	A9TXU9.1 PF16529.3;	39.60	0.00
TRINITY_L8GMG4_ACACA/32-285	L8GMG4.1 PF02265.14	39.60	0.00
TRINITY_T2KKS9_9FLAO/14-108	T2KKS9.1 PF02142.20	39.60	0.00
TRINITY_D8TTE6_VOLCA/333-484	D8TTE6.1 PF12499.6;	39.60	0.00
TRINITY_D2VWH0_NAEGR/2-208	D2VWH0.1 PF07722.11	39.60	0.00
TRINITY_A9RMR0_PHYPA/29-269	A9RMR0.1 PF00481.19	39.60	0.00
TRINITY_M2XBK0_GALSU/9-199	M2XBK0.1 PF01612.18	39.60	0.00
TRINITY_C0ZF04_BREBN/23-129	C0ZF04.1 PF13649.4;	39.60	0.00
TRINITY_L8H5S9_ACACA/282-451	L8H5S9.1 PF00621.18	39.60	0.00
TRINITY_K1R3Q5_CRAGI/124-520	K1R3Q5.1 PF05185.14	39.60	0.00
TRINITY_I1CFA0_RHIO9/11-133	I1CFA0.1 PF00241.18	39.60	0.00
TRINITY_G7WK76_METH6/8-255	G7WK76.1 PF01226.15	39.60	0.00
TRINITY_L8HAB9_ACACA/836-943	L8HAB9.1 PF00651.29	39.60	0.00
TRINITY_A0C1Y5_PARTE/3-156	A0C1Y5.1 PF06127.9;	39.60	0.00
TRINITY_J9IGC9_9SPIT/43-342	J9IGC9.1 PF00648.19	39.60	0.00
TRINITY_D8UJS3_VOLCA/84-263	D8UJS3.1 PF02330.14	39.60	0.00
TRINITY_D8TY16_VOLCA/120-295	D8TY16.1 PF13419.4;	39.60	0.00
TRINITY_K4DH13_SOLLC/300-549	K4DH13.1 PF07714.15	39.60	0.00
TRINITY_D8UK64_VOLCA/8-471	D8UK64.1 PF13520.4;	39.60	0.00
TRINITY_U4LEH5_PYROM/784-918	U4LEH5.1 PF04146.13	39.60	0.00
TRINITY_A0A0D1BUD1_USTMA/372-515	A0A0D1BUD1.1 PF0007	39.60	0.00
TRINITY_A0A087SE95_AUXPR/53-291	A0A087SE95.1 PF0014	39.60	0.00
TRINITY_F0ZF55_DICPU/747-981	F0ZF55.1 PF00566.16	39.60	0.00
TRINITY_Q231C5_TETTS/8-146	Q231C5.2 PF10358.7;	39.60	0.00
TRINITY_Q54JI4_DICDI/315-541	Q54JI4.1 PF13236.4;	39.60	0.00
TRINITY_A0A0D2WXG5_CAPO3/1256-1682	A0A0D2WXG5.1 PF1223	39.60	0.00
TRINITY_I0YJI3_9CHLO/128-497	I0YJI3.1 PF07690.14	39.60	0.00
TRINITY_D3AZL5_POLPA/665-855	D3AZL5.1 PF12816.5;	39.60	0.00
TRINITY_A8I5H6_CHLRE/37-316	A8I5H6.1 PF01209.16	39.60	0.00
TRINITY_D4AIZ9_ARTBC/17-274	D4AIZ9.1 PF00248.19	39.60	0.00
TRINITY_A0A078AX44_STYLE/604-698	A0A078AX44.1 PF1349	39.60	0.00
TRINITY_E1ZK66_CHLVA/11-289	E1ZK66.1 PF00856.26	39.60	0.00
TRINITY_D2VE99_NAEGR/20-378	D2VE99.1 PF00266.17	39.50	0.00
TRINITY_F6UJ27_MONDO/44-243	F6UJ27.2 PF01965.22	39.50	0.00

TRINITY_Q3SE55_PARTE/446-749	Q3SE55.1 PF02171.15	39.50	0.00
TRINITY_G0QR05_ICHMG/472-730	G0QR05.1 PF01504.16	39.50	0.00
TRINITY_C4M519_ENTHI/1324-1583	C4M519.1 PF00069.23	39.50	0.00
TRINITY_Q23AB0_TETTS/39-265	Q23AB0.1 PF03372.21	39.50	0.00
TRINITY_I7M6F7_TETTS/1178-1445	I7M6F7.2 PF01504.16	39.50	0.00
TRINITY_C1E7I7_MICSR/92-269	C1E7I7.1 PF00719.17	39.50	0.00
TRINITY_F4PQY7_DICFS/25-186	F4PQY7.1 PF00071.20	39.50	0.00
TRINITY_D8U871_VOLCA/37-497	D8U871.1 PF09751.7;	39.50	0.00
TRINITY_E3T5H3_CROVB/671-790	E3T5H3.1 PF00271.29	39.50	0.00
TRINITY_L8GF10_ACACA/1-543	L8GF10.1 PF04858.11	39.50	0.00
TRINITY_L8GEX5_ACACA/864-1097	L8GEX5.1 PF07714.15	39.50	0.00
TRINITY_Q23QF2_TETTS/248-571	Q23QF2.2 PF03133.13	39.50	0.00
TRINITY_A0A015L3Q8_9GLOM/160-272	A0A015L3Q8.1 PF0266	39.50	0.00
TRINITY_M4AR75_XIPMA/3-277	M4AR75.1 PF06218.9;	39.50	0.00
TRINITY_A0A0G4EWK7_9ALVE/51-336	A0A0G4EWK7.1 PF0006	39.50	0.00
TRINITY_G0R3R5_ICHMG/1-259	G0R3R5.1 PF00069.23	39.50	0.00
TRINITY_L8GXF3_ACACA/15-455	L8GXF3.1 PF03747.12	39.50	0.00
TRINITY_Q86B07_DICDI/215-531	Q86B07.1 PF00657.20	39.50	0.00
TRINITY_L8H0X8_ACACA/537-801	L8H0X8.1 PF07714.15	39.50	0.00
TRINITY_Q22CP8_TETTS/1349-1720	Q22CP8.2 PF10595.7;	39.50	0.00
TRINITY_D3B1F7_POLPA/40-410	D3B1F7.1 PF05625.9;	39.50	0.00
TRINITY_F4PTN1_DICFS/42-388	F4PTN1.1 PF07690.14	39.50	0.00
TRINITY_B0W6G8_CULQU/79-384	B0W6G8.1 PF00561.18	39.50	0.00
TRINITY_A9UQJ2_MONBE/1-91	A9UQJ2.1 PF04178.10	39.50	0.00
TRINITY_L8GZS1_ACACA/293-532	L8GZS1.1 PF07817.11	39.50	0.00
TRINITY_L8GL94_ACACA/389-614	L8GL94.1 PF13672.4;	39.50	0.00
TRINITY_D5XA33_THEPJ/1-342	D5XA33.1 PF00224.19	39.50	0.00
TRINITY_A0A067N0H1_9HOMO/425-1015	A0A067N0H1.1 PF0518	39.50	0.00
TRINITY_B7FVV5_PHATC/1-82	B7FVV5.1 PF00439.23	39.50	0.00
TRINITY_Q67ZB6_ARATH/19-275	Q67ZB6.1 PF13621.4;	39.50	0.00
TRINITY_E9QFH2_DANRE/10-278	E9QFH2.2 PF00069.23	39.50	0.00
TRINITY_I0Z1Q9_9CHLO/19-207	I0Z1Q9.1 PF03962.13	39.50	0.00
TRINITY_A4VCU8_TETTS/13-98	A4VCU8.1 PF15104.4;	39.50	0.00
TRINITY_D8U938_VOLCA/38-185	D8U938.1 PF03652.13	39.50	0.00
TRINITY_D8UJ84_VOLCA/1008-1082	D8UJ84.1 PF04640.12	39.50	0.00
TRINITY_D8THD3_VOLCA/212-384	D8THD3.1 PF00685.25	39.50	0.00
TRINITY_A8JJJ7_CHLRE/93-269	A8JJJ7.1 PF13604.4;	39.50	0.00
TRINITY_W7U749_9STRA/103-319	W7U749.1 PF01925.17	39.50	0.00
TRINITY_D8UJL9_VOLCA/131-436	D8UJL9.1 PF00331.18	39.50	0.00
TRINITY_Q54B73_DICDI/144-412	Q54B73.1 PF02811.17	39.50	0.00
TRINITY_D8ULP0_VOLCA/41-375	D8ULP0.1 PF00194.19	39.50	0.00
TRINITY_K7VKG7_MAIZE/394-579	K7VKG7.1 PF04548.14	39.50	0.00
TRINITY_L8GJU3_ACACA/32-554	L8GJU3.1 PF00135.26	39.50	0.00
TRINITY_A0A067CIC8_SAPPC/301-462	A0A067CIC8.1 PF1029	39.50	0.00
TRINITY_D8UD03_VOLCA/114-402	D8UD03.1 PF00155.19	39.50	0.00
TRINITY_W7XCE4_TETTS/43-439	W7XCE4.1 PF02487.15	39.50	0.00
TRINITY_B3SEY3_TRIAD/1-106	B3SEY3.1 PF00837.15	39.50	0.00
TRINITY_D0NFV6_PHYIT/75-196	D0NFV6.1 PF00043.23	39.50	0.00
TRINITY_A0A0D2X0V9_CAPO3/147-522	A0A0D2X0V9.1 PF0149	39.40	0.00
TRINITY_T1N7W5_TRIUA/11-109	T1N7W5.1 PF04749.15	39.40	0.00
TRINITY_W4KFV5_9HOMO/102-172	W4KFV5.1 PF00076.20	39.40	0.00
TRINITY_A0A077ZQ47_STYLE/65-625	A0A077ZQ47.1 PF0099	39.40	0.00
TRINITY_A0CNQ5_PARTE/260-688	A0CNQ5.1 PF03200.14	39.40	0.00
TRINITY_U9T196_RHIID/51-246	U9T196.1 PF00882.16	39.40	0.00
TRINITY_A0A068SHP7_9FUNG/758-911	A0A068SHP7.1 PF0062	39.40	0.00
TRINITY_F0ZAK5_DICPU/72-456	F0ZAK5.1 PF00854.19	39.40	0.00
TRINITY_A0A074RK62_9HOMO/1-233	A0A074RK62.1 PF0772	39.40	0.00
TRINITY_Q54X10_DICDI/97-238	Q54X10.1 PF01553.19	39.40	0.00
TRINITY_A0A0D2WIP8_CAPO3/213-474	A0A0D2WIP8.1 PF0014	39.40	0.00
TRINITY_I7LV11_TETTS/352-566	I7LV11.1 PF00149.26	39.40	0.00
TRINITY_F0Y7Z4_AURAN/206-283	F0Y7Z4.1 PF03061.20	39.40	0.00

TRINITY_Q3M0Z2_PARTE/41-243	Q3M0Z2.1 PF00804.23	39.40	0.00
TRINITY_F6T310_CIOIN/24-445	F6T310.2 PF04587.13	39.40	0.00
TRINITY_A0A024TG81_9STRA/67-137	A0A024TG81.1 PF0007	39.40	0.00
TRINITY_U5H2U4_USTV1/101-329	U5H2U4.1 PF05721.11	39.40	0.00
TRINITY_W5JCJ5_ANODA/342-485	W5JCJ5.1 PF00388.17	39.40	0.00
TRINITY_D8TIW8_VOLCA/156-378	D8TIW8.1 PF02450.13	39.40	0.00
TRINITY_I7LY38_TETTS/121-191	I7LY38.1 PF13499.4;	39.40	0.00
TRINITY_F2TZK1_SALR5/589-912	F2TZK1.1 PF00176.21	39.40	0.00
TRINITY_I7MER8_TETTS/31-519	I7MER8.2 PF00083.22	39.40	0.00
TRINITY_A4SBF0_OSTLU/16-246	A4SBF0.1 PF04452.12	39.40	0.00
TRINITY_A0A0L0CSS3_LUCCU/14-525	A0A0L0CSS3.1 PF0509	39.40	0.00
TRINITY_Q0C7B1_AEDAE/426-599	Q0C7B1.1 PF00616.17	39.40	0.00
TRINITY_U6JTR5_9EIME/4-219	U6JTR5.1 PF06244.10	39.40	0.00
TRINITY_J9KA86_ACYPI/512-664	J9KA86.2 PF00620.25	39.40	0.00
TRINITY_A8HNM5_CHLRE/2576-2866	A8HNM5.1 PF06011.10	39.40	0.00
TRINITY_A0A078B677_STYLE/33-135	A0A078B677.1 PF0222	39.40	0.00
TRINITY_A0A0M2PJR3_9BACI/20-336	A0A0M2PJR3.1 PF0025	39.40	0.00
TRINITY_J9IQ73_9SPIT/439-537	J9IQ73.1 PF12796.5;	39.40	0.00
TRINITY_I7M2D0_TETTS/65-476	I7M2D0.2 PF00155.19	39.40	0.00
TRINITY_D3AYH4_POLPA/12-342	D3AYH4.1 PF00503.18	39.40	0.00
TRINITY_I7M2G7_TETTS/73-181	I7M2G7.1 PF00787.22	39.40	0.00
TRINITY_J9IME5_9SPIT/326-434	J9IME5.1 PF00651.29	39.40	0.00
TRINITY_S2J1T0_MUCC1/216-489	S2J1T0.1 PF13632.4;	39.40	0.00
TRINITY_A0C8J4_PARTE/154-269	A0C8J4.1 PF15261.4;	39.40	0.00

TRINITY_A0CSW7_PARTE/37-297	A0CSW7.1 PF01715.15	39.40	0.00
TRINITY_W5QAB8_SHEEP/79-201	W5QAB8.1 PF08292.10	39.40	0.00
TRINITY_D2VNG5_NAEGR/186-510	D2VNG5.1 PF00728.20	39.40	0.00
TRINITY_I7M7L8_TETTS/294-508	I7M7L8.2 PF00609.17	39.40	0.00
TRINITY_A0A0A1NE54_9FUNG/345-473	A0A0A1NE54.1 PF0866	39.40	0.00
TRINITY_R1B9I6_EMIHU/246-393	R1B9I6.1 PF07534.14	39.40	0.00
TRINITY_A0D362_PARTE/61-535	A0D362.1 PF00501.26	39.40	0.00
TRINITY_A0A078B349_STYLE/444-804	A0A078B349.1 PF0070	39.40	0.00
TRINITY_Q234Z9_TETTS/1982-2340	Q234Z9.2 PF04054.13	39.40	0.00
TRINITY_A8J9C5_CHLRE/1150-1292	A8J9C5.1 PF03114.16	39.40	0.00
TRINITY_A0A0M0JCK3_9EUKA/69-195	A0A0M0JCK3.1 PF0013	39.40	0.00
TRINITY_A0A061F4R3_THECC/18-215	A0A061F4R3.1 PF0030	39.40	0.00
TRINITY_D1CD72_THET1/15-283	D1CD72.1 PF00580.19	39.40	0.00
TRINITY_A8PYP1_MALGO/3-74	A8PYP1.1 PF00240.21	39.40	0.00
TRINITY_A0A078AB07_STYLE/2-128	A0A078AB07.1 PF0409	39.30	0.00
TRINITY_D7FZS6_ECTSI/5-275	D7FZS6.1 PF00009.25	39.30	0.00
TRINITY_A0A0G4J631_PLABS/71-229	A0A0G4J631.1 PF0489	39.30	0.00
TRINITY_L1JHU7_GUITH/1-193	L1JHU7.1 PF08450.10	39.30	0.00
TRINITY_H3B7B0_LATCH/9-95	H3B7B0.1 PF02970.14	39.30	0.00
TRINITY_I0Z727_9CHLO/78-581	I0Z727.1 PF02990.14	39.30	0.00
TRINITY_A8JIQ5_CHLRE/292-449	A8JIQ5.1 PF00795.20	39.30	0.00
TRINITY_G3HI91_CRIGR/279-409	G3HI91.1 PF00782.18	39.30	0.00
TRINITY_A0DRG1_PARTE/852-1181	A0DRG1.1 PF01237.16	39.30	0.00
TRINITY_Q6BFR2_PARTE/27-205	Q6BFR2.1 PF13462.4;	39.30	0.00
TRINITY_B7FNV6_PHATC/49-254	B7FNV6.1 PF01323.18	39.30	0.00
TRINITY_F0Z9F9_DICPU/146-396	F0Z9F9.1 PF00069.23	39.30	0.00
TRINITY_L8GMR3_ACACA/270-526	L8GMR3.1 PF07714.15	39.30	0.00
TRINITY_A0DMK4_PARTE/24-339	A0DMK4.1 PF03372.21	39.30	0.00
TRINITY_EIF2A_DICDI/257-447	Q54PV7.1 PF08662.9;	39.30	0.00
TRINITY_A8JJQ5_CHLRE/193-340	A8JJQ5.1 PF01764.23	39.30	0.00
TRINITY_Q23BP9_TETTS/506-1002	Q23BP9.2 PF07748.11	39.30	0.00
TRINITY_H2W6J2_CAEJA/21-126	H2W6J2.2 PF00856.26	39.30	0.00
TRINITY_E9QCK7_DANRE/639-797	E9QCK7.1 PF05572.11	39.30	0.00
TRINITY_I7LU61_TETTS/57-381	I7LU61.1 PF00026.21	39.30	0.00
TRINITY_H9G6H0_ANOCA/32-235	H9G6H0.2 PF01852.17	39.30	0.00
TRINITY_Q24C23_TETTS/91-326	Q24C23.3 PF00566.16	39.30	0.00
TRINITY_A0A059C6R8_EUCGR/126-340	A0A059C6R8.1 PF0011	39.30	0.00
TRINITY_Q81N74_BACAN/29-285	Q81N74.1 PF00561.18	39.30	0.00
TRINITY_R4G4L9_RHOPR/45-106	R4G4L9.1 PF01424.20	39.30	0.00
TRINITY_A0A078BA31_STYLE/82-334	A0A078BA31.1 PF0104	39.30	0.00
TRINITY_D8TS85_VOLCA/84-353	D8TS85.1 PF13896.4;	39.30	0.00
TRINITY_F4PTP4_DICFS/618-739	F4PTP4.1 PF01067.20	39.30	0.00
TRINITY_A0CKU6_PARTE/2-124	A0CKU6.1 PF05762.12	39.30	0.00
TRINITY_A0A022RSE6_ERYGU/184-532	A0A022RSE6.1 PF0044	39.30	0.00
TRINITY_I7MMP5_TETTS/4189-4485	I7MMP5.2 PF00632.23	39.30	0.00
TRINITY_D0MW28_PHYIT/147-399	D0MW28.1 PF01399.25	39.30	0.00
TRINITY_J3L0Z6_ORYBR/725-1439	J3L0Z6.1 PF12234.6;	39.30	0.00
TRINITY_A0A0R4IG88_DANRE/151-358	A0A0R4IG88.1 PF0102	39.30	0.00
TRINITY_M1C579_SOLTU/116-321	M1C579.1 PF04616.12	39.30	0.00
TRINITY_U9UD21_RHIID/386-466	U9UD21.1 PF13517.4;	39.30	0.00
TRINITY_A0D2Z5_PARTE/151-399	A0D2Z5.1 PF01457.14	39.30	0.00
TRINITY_A0BU97_PARTE/108-301	A0BU97.1 PF13901.4;	39.30	0.00
TRINITY_A0A078AII6_STYLE/45-323	A0A078AII6.1 PF0026	39.30	0.00
TRINITY_F6HVQ6_VITVI/89-407	F6HVQ6.1 PF13593.4;	39.30	0.00
TRINITY_A0DZ84_PARTE/87-248	A0DZ84.1 PF01746.19	39.30	0.00
TRINITY_A0D2Z5_PARTE/151-399	A0D2Z5.1 PF01457.14	39.30	0.00
TRINITY_F4PTD3_DICFS/26-214	F4PTD3.1 PF01183.18	39.30	0.00
TRINITY_E2ATT4_CAMFO/86-503	E2ATT4.1 PF06602.12	39.30	0.00
TRINITY_D8TTU9_VOLCA/16-183	D8TTU9.1 PF09174.8;	39.30	0.00
TRINITY_D8TV38_VOLCA/14-212	D8TV38.1 PF08638.9;	39.30	0.00
TRINITY_R9AXQ1_WALI9/19-279	R9AXQ1.1 PF00069.23	39.30	0.00

TRINITY_A0A077ZTG6_STYLE/22-262	A0A077ZTG6.1 PF0019	39.30	0.00
TRINITY_F0ZGP7_DICPU/754-1122	F0ZGP7.1 PF02181.21	39.30	0.00
TRINITY_L8GP69_ACACA/67-530	L8GP69.1 PF00443.27	39.30	0.00
TRINITY_D8TV72_VOLCA/776-947	D8TV72.1 PF14288.4;	39.30	0.00
TRINITY_I0YPJ5_9CHLO/9-156	I0YPJ5.1 PF00849.20	39.30	0.00
TRINITY_I0YKY3_9CHLO/1342-1430	I0YKY3.1 PF09324.8;	39.30	0.00
TRINITY_D8U7Q2_VOLCA/436-900	D8U7Q2.1 PF01504.16	39.30	0.00
TRINITY_L8H9F6_ACACA/1730-1984	L8H9F6.1 PF07714.15	39.30	0.00
TRINITY_I7LT28_TETTS/74-187	I7LT28.2 PF06320.11	39.30	0.00
TRINITY_A0A078AYH6_STYLE/8-293	A0A078AYH6.1 PF0006	39.20	0.00
TRINITY_A0A078AUR0_STYLE/268-478	A0A078AUR0.1 PF0377	39.20	0.00
TRINITY_J9ISS6_9SPIT/789-920	J9ISS6.1 PF00072.22	39.20	0.00
TRINITY_D8S7B4_SELML/1034-1394	D8S7B4.1 PF02259.21	39.20	0.00
TRINITY_A0DI54_PARTE/15-331	A0DI54.1 PF00899.19	39.20	0.00
TRINITY_I0GJ09_CALEA/2-167	I0GJ09.1 PF00857.18	39.20	0.00
TRINITY_M5W3M6_PRUPE/244-328	M5W3M6.1 PF16135.3;	39.20	0.00
TRINITY_A0A060SSC6_PYCCI/215-333	A0A060SSC6.1 PF0851	39.20	0.00
TRINITY_T0S798_9STRA/35-224	T0S798.1 PF08648.10	39.20	0.00
TRINITY_Q22PF6_TETTS/62-283	Q22PF6.1 PF12348.6;	39.20	0.00
TRINITY_F4PX77_DICFS/710-859	F4PX77.1 PF00620.25	39.20	0.00
TRINITY_I7LTH0_TETTS/231-521	I7LTH0.1 PF01031.18	39.20	0.00
TRINITY_I7M9Y4_TETTS/83-364	I7M9Y4.1 PF01266.22	39.20	0.00
TRINITY_S2JQ56_MUCC1/127-289	S2JQ56.1 PF00650.18	39.20	0.00
TRINITY_L8GXS7_ACACA/913-1039	L8GXS7.1 PF00241.18	39.20	0.00
TRINITY_D8TKX7_VOLCA/49-142	D8TKX7.1 PF00686.17	39.20	0.00
TRINITY_A0ZJC5_NODSP/73-197	A0ZJC5.1 PF02469.20	39.20	0.00
TRINITY_I7MB87_TETTS/63-523	I7MB87.2 PF00067.20	39.20	0.00
TRINITY_A0DMK4_PARTE/24-339	A0DMK4.1 PF03372.21	39.20	0.00
TRINITY_I7M6D7_TETTS/137-346	I7M6D7.1 PF03798.14	39.20	0.00
TRINITY_W5KK03_ASTMX/493-657	W5KK03.1 PF05572.11	39.20	0.00
TRINITY_D8TTH8_VOLCA/123-760	D8TTH8.1 PF14222.4;	39.20	0.00
TRINITY_R7EL07_9BACE/88-363	R7EL07.1 PF00078.25	39.20	0.00
TRINITY_A9RQK9_PHYPA/52-149	A9RQK9.1 PF04900.10	39.20	0.00
TRINITY_A0EFB4_PARTE/198-687	A0EFB4.1 PF01055.24	39.20	0.00
TRINITY_D3B3I8_POLPA/16-119	D3B3I8.1 PF02893.18	39.20	0.00
TRINITY_W5K1B6_ASTMX/73-584	W5K1B6.1 PF00135.26	39.20	0.00
TRINITY_A9SDG3_PHYPA/268-370	A9SDG3.1 PF01399.25	39.20	0.00
TRINITY_A0YCL9_9GAMM/7-109	A0YCL9.1 PF03807.15	39.20	0.00
TRINITY_A0A0D2QF67_GOSRA/145-287	A0A0D2QF67.1 PF1060	39.20	0.00
TRINITY_R4X8T7_TAPDE/1-82	R4X8T7.1 PF12763.5;	39.20	0.00
TRINITY_L8H1R3_ACACA/495-744	L8H1R3.1 PF07714.15	39.20	0.00
TRINITY_A0A0P7VH63_9TELE/454-527	A0A0P7VH63.1 PF0057	39.20	0.00
TRINITY_Q1K3C1_DESAC/6-561	Q1K3C1.1 PF01411.17	39.20	0.00
TRINITY_C1E9J9_MICSR/312-418	C1E9J9.1 PF04777.11	39.20	0.00
TRINITY_A0D2Z5_PARTE/151-399	A0D2Z5.1 PF01457.14	39.20	0.00
TRINITY_F1A441_DICPU/81-384	F1A441.1 PF01544.16	39.20	0.00
TRINITY_A0A088ATE6_APIME/29-139	A0A088ATE6.1 PF0065	39.20	0.00
TRINITY_B9RRB9_RICCO/502-679	B9RRB9.1 PF02141.19	39.20	0.00
TRINITY_H3ELF8_PRIPA/22-138	H3ELF8.1 PF08477.11	39.20	0.00
TRINITY_D8TWK8_VOLCA/102-558	D8TWK8.1 PF01150.15	39.20	0.00
TRINITY_W5MU09_LEPOC/18-248	W5MU09.1 PF01168.18	39.20	0.00
TRINITY_H2Z453_CIOSA/5-80	H2Z453.1 PF02798.18	39.20	0.00
TRINITY_A8IHE7_CHLRE/70-516	A8IHE7.1 PF13520.4;	39.20	0.00
TRINITY_A8IRB8_CHLRE/110-310	A8IRB8.1 PF06011.10	39.20	0.00
TRINITY_A9TUH0_PHYPA/288-386	A9TUH0.1 PF13499.4;	39.20	0.00
TRINITY_I7LUD7_TETTS/7-211	I7LUD7.1 PF01557.16	39.10	0.00
TRINITY_G0QT60_ICHMG/17-86	G0QT60.1 PF13499.4;	39.10	0.00
TRINITY_L8H3W5_ACACA/971-1093	L8H3W5.1 PF00271.29	39.10	0.00
TRINITY_F0Z6A8_DICPU/12-319	F0Z6A8.1 PF14647.4;	39.10	0.00
TRINITY_A0A0J8BV09_BETVU/20-273	A0A0J8BV09.1 PF0006	39.10	0.00
TRINITY_L0AVA8_THEEQ/18-382	L0AVA8.1 PF00022.17	39.10	0.00

TRINITY_A0A0R4J5L8_SOYBN/6-205	A0A0R4J5L8.1 PF0065	39.10	0.00
TRINITY_F0ZHA2_DICPU/18-202	F0ZHA2.1 PF01209.16	39.10	0.00
TRINITY_A0A084W9Q9_ANOSI/75-143	A0A084W9Q9.1 PF0050	39.10	0.00
TRINITY_Q22Z31_TETTS/156-426	Q22Z31.4 PF00520.29	39.10	0.00
TRINITY_A0DD58_PARTE/2-166	A0DD58.1 PF01086.15	39.10	0.00
TRINITY_A0D7P8_PARTE/1766-1840	A0D7P8.1 PF16561.3;	39.10	0.00
TRINITY_A0A022QCI0_ERYGU/43-203	A0A022QCI0.1 PF0155	39.10	0.00
TRINITY_Q23QF2_TETTS/248-571	Q23QF2.2 PF03133.13	39.10	0.00
TRINITY_PAN3_DICDI/385-541	P0CD65.1 PF00069.23	39.10	0.00
TRINITY_L8GL12_ACACA/119-330	L8GL12.1 PF00112.21	39.10	0.00
TRINITY_F4PPV5_DICFS/1840-2098	F4PPV5.1 PF00481.19	39.10	0.00
TRINITY_W5FLH4_WHEAT/9-228	W5FLH4.1 PF05004.11	39.10	0.00
TRINITY_A0A067NNT9_PLEOS/46-439	A0A067NNT9.1 PF0099	39.10	0.00
TRINITY_E1BWZ1_CHICK/86-384	E1BWZ1.1 PF00561.18	39.10	0.00
TRINITY_H2WNI4_CAEJA/124-192	H2WNI4.2 PF00505.17	39.10	0.00
TRINITY_A8HPS0_CHLRE/20-643	A8HPS0.1 PF04130.11	39.10	0.00
TRINITY_A0A0G4EKP2_9ALVE/14-362	A0A0G4EKP2.1 PF0492	39.10	0.00
TRINITY_E1ZM78_CHLVA/583-838	E1ZM78.1 PF00122.18	39.10	0.00
TRINITY_I1PRK3_ORYGL/102-381	I1PRK3.1 PF00122.18	39.10	0.00
TRINITY_L8GRB2_ACACA/60-255	L8GRB2.1 PF09511.8;	39.10	0.00
TRINITY_H2XVE9_CIOIN/15-255	H2XVE9.1 PF13621.4;	39.10	0.00
TRINITY_A0A015JJN4_9GLOM/42-256	A0A015JJN4.1 PF0035	39.10	0.00
TRINITY_D8UGK8_VOLCA/146-384	D8UGK8.1 PF01547.23	39.10	0.00
TRINITY_I7MI70_TETTS/452-568	I7MI70.1 PF13893.4;	39.10	0.00
TRINITY_D8U1Y3_VOLCA/10-144	D8U1Y3.1 PF03133.13	39.10	0.00
TRINITY_T1FPG7_HELRO/60-154	T1FPG7.1 PF00855.15	39.10	0.00
TRINITY_A0A0D2ULF9_CAPO3/34-349	A0A0D2ULF9.1 PF0190	39.10	0.00
TRINITY_W7X3N9_TETTS/98-166	W7X3N9.1 PF10601.7;	39.10	0.00
TRINITY_A0A0D2UMU4_CAPO3/32-518	A0A0D2UMU4.1 PF1403	39.10	0.00
TRINITY_Q8YQN5_NOSS1/391-516	Q8YQN5.1 PF02517.14	39.10	0.00
TRINITY_M0S0K0_MUSAM/132-223	M0S0K0.1 PF02536.12	39.10	0.00
TRINITY_A0BQL3_PARTE/117-192	A0BQL3.1 PF07258.12	39.10	0.00
TRINITY_X6MTS8_RETFI/89-269	X6MTS8.1 PF01467.24	39.10	0.00
TRINITY_I7MB24_TETTS/12-253	I7MB24.2 PF01370.19	39.10	0.00
TRINITY_L8GVR8_ACACA/47-315	L8GVR8.1 PF03372.21	39.10	0.00
TRINITY_L1IIV3_GUITH/101-232	L1IIV3.1 PF04784.12	39.10	0.00
TRINITY_B9IQV9_POPTR/61-193	B9IQV9.1 PF12894.5;	39.10	0.00
TRINITY_A0A078AGZ3_STYLE/3903-4621	A0A078AGZ3.1 PF0302	39.10	0.00
TRINITY_W1PYV6_AMBTC/587-841	W1PYV6.1 PF07714.15	39.10	0.00
TRINITY_A8J8L4_CHLRE/265-514	A8J8L4.1 PF07714.15	39.10	0.00
TRINITY_G0QVG7_ICHMG/239-716	G0QVG7.1 PF04791.14	39.10	0.00
TRINITY_E4WX43_OIKDI/40-324	E4WX43.1 PF00069.23	39.10	0.00
TRINITY_L8GFV5_ACACA/41-155	L8GFV5.1 PF13669.4;	39.10	0.00
TRINITY_F6S0A5_XENTR/49-219	F6S0A5.1 PF10294.7;	39.10	0.00
TRINITY_I0Z6C3_9CHLO/515-852	I0Z6C3.1 PF04840.10	39.10	0.00
TRINITY_J9I7Z3_9SPIT/528-596	J9I7Z3.1 PF02187.15	39.10	0.00
TRINITY_S7QP61_GLOTA/46-257	S7QP61.1 PF12706.5;	39.10	0.00
TRINITY_D7CQK2_TRURR/26-262	D7CQK2.1 PF12146.6;	39.10	0.00
TRINITY_A8ICQ7_CHLRE/117-203	A8ICQ7.1 PF01985.19	39.10	0.00
TRINITY_I0Z6U9_9CHLO/337-575	I0Z6U9.1 PF07690.14	39.10	0.00
TRINITY_D8TIR1_VOLCA/82-185	D8TIR1.1 PF00085.18	39.10	0.00
TRINITY_I0YIN0_9CHLO/121-407	I0YIN0.1 PF03982.11	39.10	0.00
TRINITY_G0R041_ICHMG/38-325	G0R041.1 PF00069.23	39.10	0.00
TRINITY_A0A0D2X1C4_CAPO3/53-347	A0A0D2X1C4.1 PF0337	39.10	0.00
TRINITY_D3AVM6_POLPA/321-522	D3AVM6.1 PF00753.25	39.10	0.00
TRINITY_A8IDQ0_CHLRE/3389-3498	A8IDQ0.1 PF01477.21	39.10	0.00
TRINITY_C1E3Y1_MICSR/27-178	C1E3Y1.1 PF04051.14	39.00	0.00
TRINITY_Q24CM8_TETTS/58-516	Q24CM8.2 PF00067.20	39.00	0.00
TRINITY_D3B0U2_POLPA/899-1144	D3B0U2.1 PF13086.4;	39.00	0.00
TRINITY_J9I3A6_9SPIT/38-197	J9I3A6.1 PF08534.8;	39.00	0.00
TRINITY_M2X6B6_GALSU/34-151	M2X6B6.1 PF04263.14	39.00	0.00

TRINITY_A0C2Y4_PARTE/17-363	A0C2Y4.1 PF10498.7;	39.00	0.00
TRINITY_C3Y0L7_BRAFL/2-249	C3Y0L7.1 PF10036.7;	39.00	0.00
TRINITY_A0A0M0J5K3_9EUKA/130-214	A0A0M0J5K3.1 PF0183	39.00	0.00
TRINITY_I7MG63_TETTS/20-399	I7MG63.1 PF00022.17	39.00	0.00
TRINITY_D5BZD9_NITHN/8-98	D5BZD9.1 PF02922.16	39.00	0.00
TRINITY_A0A015L086_9GLOM/56-510	A0A015L086.1 PF0390	39.00	0.00
TRINITY_G4UGJ9_NEUT9/259-384	G4UGJ9.1 PF00622.26	39.00	0.00
TRINITY_A9UZH0_MONBE/972-1039	A9UZH0.1 PF08583.8;	39.00	0.00
TRINITY_E1Z963_CHLVA/513-734	E1Z963.1 PF00326.19	39.00	0.00
TRINITY_I7LV11_TETTS/352-566	I7LV11.1 PF00149.26	39.00	0.00
TRINITY_A0A067P386_PLEOS/551-825	A0A067P386.1 PF1323	39.00	0.00
TRINITY_A0DJB3_PARTE/1058-1652	A0DJB3.1 PF04998.15	39.00	0.00
TRINITY_F4PZH3_DICFS/203-744	F4PZH3.1 PF04114.12	39.00	0.00
TRINITY_Q5B9E2_EMENI/63-187	Q5B9E2.1 PF00561.18	39.00	0.00
TRINITY_A0A075AWU8_9FUNG/39-183	A0A075AWU8.1 PF0010	39.00	0.00
TRINITY_G0QWU2_ICHMG/2144-2433	G0QWU2.1 PF00520.29	39.00	0.00
TRINITY_L1J5U3_GUITH/89-197	L1J5U3.1 PF00651.29	39.00	0.00
TRINITY_A0DMK4_PARTE/24-339	A0DMK4.1 PF03372.21	39.00	0.00
TRINITY_G3NMH4_GASAC/16-143	G3NMH4.1 PF00293.26	39.00	0.00
TRINITY_A7RGK5_NEMVE/7-160	A7RGK5.1 PF00106.23	39.00	0.00
TRINITY_K0SXE8_THAOC/71-304	K0SXE8.1 PF07847.10	39.00	0.00
TRINITY_A0A0B4GTV5_9HYPO/34-146	A0A0B4GTV5.1 PF0018	39.00	0.00
TRINITY_X6LHD5_RETFI/4-179	X6LHD5.1 PF00069.23	39.00	0.00
TRINITY_K7IQV8_NASVI/239-599	K7IQV8.1 PF03074.14	39.00	0.00
TRINITY_D8RYT4_SELML/4-165	D8RYT4.1 PF04573.10	39.00	0.00
TRINITY_A0A068RH27_9FUNG/967-1587	A0A068RH27.1 PF0499	39.00	0.00
TRINITY_E0VVX6_PEDHC/30-170	E0VVX6.1 PF05832.10	39.00	0.00
TRINITY_M7B141_CHEMY/446-578	M7B141.1 PF00782.18	39.00	0.00
TRINITY_Q54DW7_DICDI/35-213	Q54DW7.1 PF00621.18	39.00	0.00
TRINITY_E1ZS74_CHLVA/84-367	E1ZS74.1 PF01715.15	39.00	0.00
TRINITY_Y9955_DICDI/662-912	Q55GU0.1 PF07714.15	39.00	0.00
TRINITY_APC8_SCHPO/22-154	O94556.2 PF04049.11	39.00	0.00
TRINITY_A0A0F5YFM2_9CYAN/22-172	A0A0F5YFM2.1 PF0019	39.00	0.00
TRINITY_L8H653_ACACA/846-1065	L8H653.1 PF02854.17	39.00	0.00
TRINITY_C1E6Y9_MICSR/46-332	C1E6Y9.1 PF01256.15	39.00	0.00
TRINITY_A0BD19_PARTE/2961-3202	A0BD19.1 PF04811.13	39.00	0.00
TRINITY_F8W2M8_DANRE/214-518	F8W2M8.1 PF00082.20	39.00	0.00
TRINITY_Q247V8_TETTS/66-225	Q247V8.2 PF01343.16	39.00	0.00
TRINITY_A0A072VMN4_MEDTR/101-212	A0A072VMN4.1 PF0473	39.00	0.00
TRINITY_E4XWM2_OIKDI/171-301	E4XWM2.1 PF02984.17	39.00	0.00
TRINITY_D8U8H2_VOLCA/795-1749	D8U8H2.1 PF02514.14	39.00	0.00
TRINITY_L7U4U2_MYXSD/19-246	L7U4U2.1 PF02230.14	39.00	0.00
TRINITY_G0R3I1_ICHMG/75-256	G0R3I1.1 PF04893.15	39.00	0.00
TRINITY_I7M1F1_TETTS/187-504	I7M1F1.1 PF00728.20	39.00	0.00
TRINITY_A0A0A1U9M9_ENTIV/5-141	A0A0A1U9M9.1 PF0250	39.00	0.00
TRINITY_A0A0A7EJI4_9GAMM/455-573	A0A0A7EJI4.1 PF0007	38.90	0.00
TRINITY_G0R1G3_ICHMG/80-301	G0R1G3.1 PF00566.16	38.90	0.00
TRINITY_A0DMM7_PARTE/3-395	A0DMM7.1 PF03223.13	38.90	0.00
TRINITY_A0A024TCN9_9STRA/58-352	A0A024TCN9.1 PF1034	38.90	0.00
TRINITY_M7BJX8_CHEMY/357-605	M7BJX8.1 PF09822.7;	38.90	0.00
TRINITY_B8P0A1_POSPM/256-355	B8P0A1.1 PF01974.15	38.90	0.00
TRINITY_L8GEX5_ACACA/864-1097	L8GEX5.1 PF07714.15	38.90	0.00
TRINITY_A0A0A0KIZ4_CUCSA/52-272	A0A0A0KIZ4.1 PF0412	38.90	0.00
TRINITY_Q6C321_YARLI/324-454	Q6C321.1 PF05132.12	38.90	0.00
TRINITY_G0R6G3_ICHMG/54-245	G0R6G3.1 PF01145.23	38.90	0.00
TRINITY_W2QTX1_PHYPN/177-320	W2QTX1.1 PF06544.10	38.90	0.00
TRINITY_J9IDD8_9SPIT/348-639	J9IDD8.1 PF03372.21	38.90	0.00
TRINITY_D6WN98_TRICA/134-244	D6WN98.1 PF03556.13	38.90	0.00
TRINITY_W4FX08_9STRA/724-847	W4FX08.1 PF00622.26	38.90	0.00
TRINITY_C3Y9Y1_BRAFL/347-461	C3Y9Y1.1 PF09070.9;	38.90	0.00
TRINITY_I7M6W7_TETTS/15-180	I7M6W7.2 PF14580.4;	38.90	0.00

TRINITY_G0QWI2_ICHMG/263-462	G0QWI2.1 PF13886.4;	38.90	0.00
TRINITY_E1ZQY6_CHLVA/20-215	E1ZQY6.1 PF05891.10	38.90	0.00
TRINITY_W5PED5_SHEEP/51-519	W5PED5.1 PF00067.20	38.90	0.00
TRINITY_L8GGI0_ACACA/67-210	L8GGI0.1 PF13862.4;	38.90	0.00
TRINITY_A8J7M9_CHLRE/742-1038	A8J7M9.1 PF00069.23	38.90	0.00
TRINITY_A0A0J7L3Z3_LASNI/6-218	A0A0J7L3Z3.1 PF0137	38.90	0.00
TRINITY_A8JG37_CHLRE/15-124	A8JG37.1 PF14886.4;	38.90	0.00
TRINITY_A0A0A0KY84_CUCSA/543-894	A0A0A0KY84.1 PF0070	38.90	0.00
TRINITY_D2VCP2_NAEGR/25-231	D2VCP2.1 PF00149.26	38.90	0.00
TRINITY_D8U818_VOLCA/1231-1397	D8U818.1 PF00069.23	38.90	0.00
TRINITY_J3M370_ORYBR/218-403	J3M370.1 PF14580.4;	38.90	0.00
TRINITY_A8HSK5_CHLRE/66-269	A8HSK5.1 PF01596.15	38.90	0.00
TRINITY_A0A087SMX3_AUXPR/167-722	A0A087SMX3.1 PF0491	38.90	0.00
TRINITY_A0A078AXN5_STYLE/270-479	A0A078AXN5.1 PF0481	38.90	0.00
TRINITY_A0A0D2UKQ8_CAPO3/188-572	A0A0D2UKQ8.1 PF0065	38.90	0.00
TRINITY_L8GWI2_ACACA/20-150	L8GWI2.1 PF00782.18	38.90	0.00
TRINITY_A8II37_CHLRE/690-902	A8II37.1 PF10474.7;	38.90	0.00
TRINITY_A0A024U6H0_9STRA/657-862	A0A024U6H0.1 PF1270	38.90	0.00
TRINITY_W7X8T2_TETTS/26-295	W7X8T2.1 PF01267.15	38.90	0.00
TRINITY_PAPP1_HUMAN/499-660	Q13219.3 PF05572.11	38.90	0.00
TRINITY_Q24BY2_TETTS/111-218	Q24BY2.4 PF12066.6;	38.90	0.00
TRINITY_D3B2S6_POLPA/24-418	D3B2S6.1 PF12698.5;	38.90	0.00
TRINITY_C1MN46_MICPC/146-311	C1MN46.1 PF04832.10	38.90	0.00
TRINITY_H3CTV3_TETNG/6-190	H3CTV3.1 PF00106.23	38.90	0.00
TRINITY_F4PQZ7_DICFS/15-97	F4PQZ7.1 PF00300.20	38.80	0.00
TRINITY_A0A0D2X3I2_CAPO3/177-319	A0A0D2X3I2.1 PF1060	38.80	0.00
TRINITY_A0D9S3_PARTE/655-861	A0D9S3.1 PF00566.16	38.80	0.00
TRINITY_I7M4C2_TETTS/42-276	I7M4C2.1 PF00149.26	38.80	0.00
TRINITY_A9RLK7_PHYPA/51-149	A9RLK7.1 PF08551.8;	38.80	0.00
TRINITY_Q22CM5_TETTS/61-238	Q22CM5.2 PF09439.8;	38.80	0.00
TRINITY_A0A0K9PCK3_ZOSMR/121-256	A0A0K9PCK3.1 PF0411	38.80	0.00
TRINITY_Q22X64_TETTS/2-598	Q22X64.2 PF05804.10	38.80	0.00
TRINITY_Q24E70_TETTS/215-694	Q24E70.1 PF04547.10	38.80	0.00
TRINITY_A0A060SQP0_PYCCI/16-139	A0A060SQP0.1 PF0024	38.80	0.00
TRINITY_A0A090M924_OSTTA/112-305	A0A090M924.1 PF1353	38.80	0.00
TRINITY_A0A085N3S5_9BILA/268-600	A0A085N3S5.1 PF0245	38.80	0.00
TRINITY_A0A061FDQ8_THECC/291-540	A0A061FDQ8.1 PF0771	38.80	0.00
TRINITY_I7LZJ1_TETTS/122-329	I7LZJ1.1 PF01027.18	38.80	0.00
TRINITY_D8SBH1_SELML/307-564	D8SBH1.1 PF12697.5;	38.80	0.00
TRINITY_G0QY83_ICHMG/6-220	G0QY83.1 PF08190.10	38.80	0.00
TRINITY_W1NZC1_AMBTC/2-545	W1NZC1.1 PF05786.12	38.80	0.00
TRINITY_G3AUB2_SPAPN/40-172	G3AUB2.1 PF12894.5;	38.80	0.00
TRINITY_A0A0D2UKQ8_CAPO3/188-572	A0A0D2UKQ8.1 PF0065	38.80	0.00
TRINITY_F4NW06_BATDJ/30-213	F4NW06.1 PF00117.26	38.80	0.00
TRINITY_A0A0G4FFD9_9ALVE/90-555	A0A0G4FFD9.1 PF0006	38.80	0.00
TRINITY_H2Z8R3_CIOSA/5-208	H2Z8R3.1 PF05721.11	38.80	0.00
TRINITY_L8HJN0_ACACA/233-963	L8HJN0.1 PF00443.27	38.80	0.00
TRINITY_W9RE27_9ROSA/647-899	W9RE27.1 PF07714.15	38.80	0.00
TRINITY_L8GY59_ACACA/206-368	L8GY59.1 PF01544.16	38.80	0.00
TRINITY_K9RAZ6_9CYAN/9-199	K9RAZ6.1 PF04755.10	38.80	0.00
TRINITY_E5T1Z4_TRISP/103-346	E5T1Z4.1 PF07727.12	38.80	0.00
TRINITY_EF1A_CENSY/8-291	A0RUM4.1 PF00009.25	38.80	0.00
TRINITY_L8HFX4_ACACA/12-318	L8HFX4.1 PF00443.27	38.80	0.00
TRINITY_H3GM81_PHYRM/324-430	H3GM81.1 PF00651.29	38.80	0.00
TRINITY_Q01CK6_OSTTA/516-865	Q01CK6.1 PF05185.14	38.80	0.00
TRINITY_I0YJT0_9CHLO/4-342	I0YJT0.1 PF01266.22	38.80	0.00
TRINITY_L8H4W6_ACACA/18-130	L8H4W6.1 PF07574.11	38.80	0.00
TRINITY_Q23UC9_TETTS/106-388	Q23UC9.2 PF00122.18	38.80	0.00
TRINITY_A0C500_PARTE/33-127	A0C500.1 PF01363.19	38.80	0.00
TRINITY_U6KJ95_EIMTE/1-239	U6KJ95.1 PF08648.10	38.80	0.00
TRINITY_A7RW26_NEMVE/1-259	A7RW26.1 PF00144.22	38.80	0.00

TRINITY_I0Z3X6_9CHLO/64-566	I0Z3X6.1 PF01019.19	38.80	0.00
TRINITY_F4QAY9_DICFS/7-518	F4QAY9.1 PF00899.19	38.80	0.00
TRINITY_D3B2S6_POLPA/24-418	D3B2S6.1 PF12698.5;	38.80	0.00
TRINITY_V7B7B8_PHAVU/1191-1413	V7B7B8.1 PF02854.17	38.80	0.00
TRINITY_D8TST9_VOLCA/621-848	D8TST9.1 PF00211.18	38.80	0.00
TRINITY_J9EFQ5_9SPIT/709-775	J9EFQ5.1 PF13499.4;	38.80	0.00
TRINITY_S8BSW8_9LAMI/2-340	S8BSW8.1 PF05028.12	38.80	0.00
TRINITY_Q17N45_AEDAE/51-246	Q17N45.1 PF00106.23	38.80	0.00
TRINITY_A0A0E9NK18_9ASCO/898-1021	A0A0E9NK18.1 PF0007	38.70	0.00
TRINITY_Q22TC2_TETTS/3-97	Q22TC2.2 PF00686.17	38.70	0.00
TRINITY_A0A078AX59_STYLE/713-871	A0A078AX59.1 PF0303	38.70	0.00
TRINITY_U9UI46_RHIID/30-185	U9UI46.1 PF00810.16	38.70	0.00
TRINITY_E7EN44_HUMAN/12-323	E7EN44.1 PF02190.14	38.70	0.00
TRINITY_R5AGF9_9FIRM/151-583	R5AGF9.1 PF01131.18	38.70	0.00
TRINITY_A0BQW0_PARTE/27-209	A0BQW0.1 PF03357.19	38.70	0.00
TRINITY_F1A037_DICPU/19-205	F1A037.1 PF00300.20	38.70	0.00
TRINITY_I3SRI9_MEDTR/7-144	I3SRI9.1 PF03870.13	38.70	0.00
TRINITY_A0A0R4ICG1_DANRE/101-195	A0A0R4ICG1.1 PF0015	38.70	0.00
TRINITY_I7M0J5_TETTS/133-598	I7M0J5.1 PF01142.16	38.70	0.00
TRINITY_A0A078AJQ0_STYLE/388-594	A0A078AJQ0.1 PF0481	38.70	0.00
TRINITY_A0CDC5_PARTE/838-1107	A0CDC5.1 PF16212.3;	38.70	0.00
TRINITY_I2GIA8_9BACT/29-270	I2GIA8.1 PF03372.21	38.70	0.00
TRINITY_I0YYG8_9CHLO/12-184	I0YYG8.1 PF05063.12	38.70	0.00
TRINITY_C3ZFB5_BRAFL/367-474	C3ZFB5.1 PF00651.29	38.70	0.00
TRINITY_A0CBB5_PARTE/242-397	A0CBB5.1 PF04280.13	38.70	0.00
TRINITY_A0A0M9E7Z9_9DELT/1-155	A0A0M9E7Z9.1 PF0015	38.70	0.00
TRINITY_B7PDP2_IXOSC/71-243	B7PDP2.1 PF13532.4;	38.70	0.00
TRINITY_F0ZN35_DICPU/390-679	F0ZN35.1 PF01504.16	38.70	0.00
TRINITY_D8UDK3_VOLCA/12-513	D8UDK3.1 PF03155.13	38.70	0.00
TRINITY_E1ZL08_CHLVA/10-95	E1ZL08.1 PF13499.4;	38.70	0.00
TRINITY_M4CNA4_BRARP/221-405	M4CNA4.1 PF03031.16	38.70	0.00
TRINITY_L8GQZ4_ACACA/23-685	L8GQZ4.1 PF07093.9;	38.70	0.00
TRINITY_G0S059_CHATD/1296-1468	G0S059.1 PF00616.17	38.70	0.00
TRINITY_D8TTK7_VOLCA/78-378	D8TTK7.1 PF13896.4;	38.70	0.00
TRINITY_A9SWK6_PHYPA/87-335	A9SWK6.1 PF01963.15	38.70	0.00
TRINITY_A0A0B0N5I3_GOSAR/116-417	A0A0B0N5I3.1 PF0044	38.70	0.00
TRINITY_A0A059A8Z1_EUCGR/59-253	A0A059A8Z1.1 PF0728	38.70	0.00
TRINITY_T1G1M0_HELRO/271-426	T1G1M0.1 PF13768.4;	38.70	0.00
TRINITY_F4QE60_DICFS/76-502	F4QE60.1 PF00067.20	38.70	0.00
TRINITY_Q23GZ0_TETTS/399-557	Q23GZ0.1 PF00609.17	38.70	0.00
TRINITY_C3YIS6_BRAFL/616-912	C3YIS6.1 PF00664.21	38.70	0.00
TRINITY_M2W641_GALSU/7-150	M2W641.1 PF00106.23	38.70	0.00
TRINITY_L8GMB8_ACACA/479-700	L8GMB8.1 PF04959.11	38.70	0.00
TRINITY_A0A067CKU8_SAPPC/62-152	A0A067CKU8.1 PF1496	38.70	0.00
TRINITY_I7MB87_TETTS/63-523	I7MB87.2 PF00067.20	38.70	0.00
TRINITY_T1JK96_STRMM/331-541	T1JK96.1 PF07910.11	38.70	0.00
TRINITY_A0A0M2PU93_PROHO/94-505	A0A0M2PU93.1 PF0001	38.70	0.00
TRINITY_L8GVQ1_ACACA/16-224	L8GVQ1.1 PF01991.16	38.70	0.00
TRINITY_A0A0M0BJJ4_9ARCH/6-328	A0A0M0BJJ4.1 PF0000	38.70	0.00
TRINITY_D8TJW1_VOLCA/6-189	D8TJW1.1 PF10225.7;	38.70	0.00
TRINITY_B0D488_LACBS/608-916	B0D488.1 PF02171.15	38.70	0.00
TRINITY_U9T3K7_RHIID/196-498	U9T3K7.1 PF03151.14	38.70	0.00
TRINITY_A9UUA4_MONBE/112-221	A9UUA4.1 PF02996.15	38.70	0.00
TRINITY_G7WPF0_METH6/63-184	G7WPF0.1 PF03351.15	38.70	0.00
TRINITY_A8HS43_CHLRE/13-337	A8HS43.1 PF00225.21	38.70	0.00
TRINITY_K7J987_NASVI/460-578	K7J987.1 PF00665.24	38.70	0.00
TRINITY_D3BI36_POLPA/1029-1279	D3BI36.1 PF07714.15	38.70	0.00
TRINITY_A0E7T1_PARTE/778-901	A0E7T1.1 PF00072.22	38.60	0.00
TRINITY_E9J5D4_SOLIN/482-768	E9J5D4.1 PF00702.24	38.60	0.00
TRINITY_A0A0N4SZ21_BRUPA/274-490	A0A0N4SZ21.1 PF1348	38.60	0.00
TRINITY_J9I3R9_9SPIT/1-81	J9I3R9.1 PF08293.9;	38.60	0.00

TRINITY_I7MAD4_TETTS/869-1238	I7MAD4.2 PF12698.5;	38.60	0.00
TRINITY_L8H9F6_ACACA/1730-1984	L8H9F6.1 PF07714.15	38.60	0.00
TRINITY_V4AA81_LOTGI/7-311	V4AA81.1 PF07714.15	38.60	0.00
TRINITY_A0A0D2WN96_CAPO3/65-326	A0A0D2WN96.1 PF0769	38.60	0.00
TRINITY_A0D109_PARTE/22-300	A0D109.1 PF00069.23	38.60	0.00
TRINITY_Q23RJ8_TETTS/803-963	Q23RJ8.1 PF01979.18	38.60	0.00
TRINITY_H2Z6S3_CIOSA/41-215	H2Z6S3.1 PF13847.4;	38.60	0.00
TRINITY_F0ZWF3_DICPU/198-398	F0ZWF3.1 PF08737.8;	38.60	0.00
TRINITY_A0A0D2UT14_GOSRA/531-649	A0A0D2UT14.1 PF0174	38.60	0.00
TRINITY_A0A0F2QFA2_9RHOB/400-611	A0A0F2QFA2.1 PF1393	38.60	0.00
TRINITY_D3AYP0_POLPA/50-329	D3AYP0.1 PF07992.12	38.60	0.00
TRINITY_L1JQS5_GUITH/295-547	L1JQS5.1 PF00069.23	38.60	0.00
TRINITY_F4PYI7_DICFS/50-458	F4PYI7.1 PF00083.22	38.60	0.00
TRINITY_D3B570_POLPA/109-243	D3B570.1 PF01764.23	38.60	0.00
TRINITY_D3BF56_POLPA/97-304	D3BF56.1 PF06003.10	38.60	0.00
TRINITY_I1FA66_AMPQE/22-157	I1FA66.1 PF01565.21	38.60	0.00
TRINITY_L8GQK2_ACACA/79-161	L8GQK2.1 PF01868.14	38.60	0.00
TRINITY_R7HCF5_9FIRM/44-149	R7HCF5.1 PF11380.6;	38.60	0.00
TRINITY_A0A078BAU4_STYLE/309-610	A0A078BAU4.1 PF0313	38.60	0.00
TRINITY_A0A078AJQ0_STYLE/388-594	A0A078AJQ0.1 PF0481	38.60	0.00
TRINITY_A8TKX4_9PROT/22-377	A8TKX4.1 PF00266.17	38.60	0.00
TRINITY_A8J9L4_CHLRE/43-170	A8J9L4.1 PF10374.7;	38.60	0.00
TRINITY_U9TNM8_RHIID/101-284	U9TNM8.1 PF13472.4;	38.60	0.00
TRINITY_F4NZF5_BATDJ/355-436	F4NZF5.1 PF00789.18	38.60	0.00
TRINITY_L8H9F6_ACACA/1730-1984	L8H9F6.1 PF07714.15	38.60	0.00
TRINITY_Q0DSR4_ORYSJ/108-227	Q0DSR4.1 PF11969.6;	38.60	0.00
TRINITY_K3W780_PYTUL/38-193	K3W780.1 PF01553.19	38.60	0.00
TRINITY_K3WE87_PYTUL/612-725	K3WE87.1 PF09404.8;	38.60	0.00
TRINITY_A0A0D2WMX1_CAPO3/403-580	A0A0D2WMX1.1 PF0061	38.60	0.00
TRINITY_F4PI07_DICFS/810-1059	F4PI07.1 PF07714.15	38.60	0.00
TRINITY_W5MSB0_LEPOC/103-461	W5MSB0.1 PF00176.21	38.60	0.00
TRINITY_D3AYL5_POLPA/432-874	D3AYL5.1 PF06963.10	38.60	0.00
TRINITY_D3B0C1_POLPA/232-428	D3B0C1.1 PF07690.14	38.60	0.00
TRINITY_I1HUS8_BRADI/81-234	I1HUS8.1 PF00650.18	38.60	0.00
TRINITY_F0ZID8_DICPU/269-482	F0ZID8.1 PF02902.17	38.60	0.00
TRINITY_B7GJA4_ANOFW/6-252	B7GJA4.1 PF12705.5;	38.60	0.00
TRINITY_C1MYF3_MICPC/90-234	C1MYF3.1 PF07386.9;	38.60	0.00
TRINITY_F4PS49_DICFS/142-327	F4PS49.1 PF03407.14	38.60	0.00
TRINITY_A8J7I7_CHLRE/57-230	A8J7I7.1 PF13515.4;	38.60	0.00
TRINITY_M0U0Y8_MUSAM/35-510	M0U0Y8.1 PF00067.20	38.60	0.00
TRINITY_B3RN30_TRIAD/6-199	B3RN30.1 PF04072.12	38.60	0.00
TRINITY_U3K3Z9_FICAL/62-517	U3K3Z9.1 PF00067.20	38.60	0.00
TRINITY_A8J6K0_CHLRE/25-233	A8J6K0.1 PF06775.12	38.60	0.00
TRINITY_L8GPA8_ACACA/831-904	L8GPA8.1 PF00240.21	38.60	0.00
TRINITY_Q4DNT3_TRYCC/4-178	Q4DNT3.1 PF02580.14	38.60	0.00
TRINITY_M1P701_DESSD/110-357	M1P701.1 PF07787.10	38.60	0.00
TRINITY_K1QZF2_CRAGI/1141-1565	K1QZF2.1 PF00759.17	38.60	0.00
TRINITY_W5J671_ANODA/34-169	W5J671.1 PF01161.18	38.60	0.00
TRINITY_U3K3Z9_FICAL/62-517	U3K3Z9.1 PF00067.20	38.60	0.00
TRINITY_D8TLX0_VOLCA/633-924	D8TLX0.1 PF03914.15	38.60	0.00
TRINITY_I0YVX3_9CHLO/22-409	I0YVX3.1 PF05282.9;	38.60	0.00
TRINITY_D8U927_VOLCA/125-458	D8U927.1 PF08190.10	38.60	0.00
TRINITY_I0YMF5_9CHLO/166-565	I0YMF5.1 PF05192.16	38.60	0.00
TRINITY_M4BQH7_HYAAE/473-545	M4BQH7.1 PF04433.15	38.60	0.00
TRINITY_L1JID5_GUITH/21-111	L1JID5.1 PF12894.5;	38.60	0.00
TRINITY_Q23FY7_TETTS/410-514	Q23FY7.1 PF04003.10	38.50	0.00
TRINITY_I7MLS9_TETTS/119-280	I7MLS9.1 PF01694.20	38.50	0.00
TRINITY_L8HDM9_ACACA/41-282	L8HDM9.1 PF00149.26	38.50	0.00
TRINITY_M1VHH2_CYAME/294-665	M1VHH2.1 PF02274.15	38.50	0.00
TRINITY_C4LXW0_ENTHI/785-1043	C4LXW0.1 PF07714.15	38.50	0.00
TRINITY_L0DET2_SINAD/319-434	L0DET2.1 PF00578.19	38.50	0.00

TRINITY_A0A0M3K3E5_ANISI/4-332	A0A0M3K3E5.1 PF0856	38.50	0.00
TRINITY_A0DIV5_PARTE/61-259	A0DIV5.1 PF00106.23	38.50	0.00
TRINITY_K1Q3U3_CRAGI/22-247	K1Q3U3.1 PF03388.11	38.50	0.00
TRINITY_A0CE21_PARTE/56-517	A0CE21.1 PF12698.5;	38.50	0.00
TRINITY_A8JFV5_CHLRE/17-152	A8JFV5.1 PF00892.18	38.50	0.00
TRINITY_A8JCL2_CHLRE/11-215	A8JCL2.1 PF07855.10	38.50	0.00
TRINITY_HGH1_DICDI/92-270	Q76NW7.1 PF04063.12	38.50	0.00
TRINITY_I7M8S5_TETTS/121-433	I7M8S5.2 PF01546.26	38.50	0.00
TRINITY_A0A059CCB1_EUCGR/104-189	A0A059CCB1.1 PF0043	38.50	0.00
TRINITY_G4VGM3_SCHMA/71-229	G4VGM3.1 PF08662.9;	38.50	0.00
TRINITY_F1A4V0_DICPU/398-674	F1A4V0.1 PF10186.7;	38.50	0.00
TRINITY_A0A087YCV1_POEFO/340-537	A0A087YCV1.2 PF0285	38.50	0.00
TRINITY_I7LTZ4_TETTS/63-290	I7LTZ4.1 PF02104.13	38.50	0.00
TRINITY_H2MSJ7_ORYLA/5-176	H2MSJ7.1 PF00025.19	38.50	0.00
TRINITY_A4C9D1_9GAMM/22-252	A4C9D1.1 PF00520.29	38.50	0.00
TRINITY_A0A078AX44_STYLE/604-698	A0A078AX44.1 PF1349	38.50	0.00
TRINITY_M0SD20_MUSAM/22-396	M0SD20.1 PF01425.19	38.50	0.00
TRINITY_A0DXX4_PARTE/105-473	A0DXX4.1 PF01457.14	38.50	0.00
TRINITY_Q24CW0_TETTS/47-201	Q24CW0.2 PF01925.17	38.50	0.00
TRINITY_A0A0A0K8Q2_CUCSA/107-523	A0A0A0K8Q2.1 PF0086	38.50	0.00
TRINITY_L8GMR3_ACACA/270-526	L8GMR3.1 PF07714.15	38.50	0.00
TRINITY_C4LYN8_ENTHI/147-276	C4LYN8.1 PF00004.27	38.50	0.00
TRINITY_B3RIX4_TRIAD/81-576	B3RIX4.1 PF00501.26	38.50	0.00
TRINITY_A0A059LQT9_9CHLO/36-318	A0A059LQT9.1 PF0006	38.50	0.00
TRINITY_I7MIQ1_TETTS/357-687	I7MIQ1.2 PF12463.6;	38.50	0.00
TRINITY_A0A024TU53_9STRA/9-168	A0A024TU53.1 PF0007	38.50	0.00
TRINITY_U6NMJ5_HAECO/119-238	U6NMJ5.1 PF00622.26	38.50	0.00
TRINITY_F4Q0Y2_DICFS/53-303	F4Q0Y2.1 PF01151.16	38.50	0.00
TRINITY_D8TTH8_VOLCA/123-760	D8TTH8.1 PF14222.4;	38.50	0.00
TRINITY_A0A0P7THD5_9TELE/11-249	A0A0P7THD5.1 PF0688	38.50	0.00
TRINITY_L8GMP4_ACACA/297-555	L8GMP4.1 PF07714.15	38.50	0.00
TRINITY_A7S1L5_NEMVE/71-266	A7S1L5.1 PF01171.18	38.50	0.00
TRINITY_D8U978_VOLCA/2-203	D8U978.1 PF07714.15	38.50	0.00
TRINITY_A8IY93_CHLRE/47-378	A8IY93.1 PF09728.7;	38.50	0.00
TRINITY_A8IQ59_CHLRE/1465-1608	A8IQ59.1 PF00520.29	38.50	0.00
TRINITY_E3PWL6_CLOSD/1-109	E3PWL6.1 PF07343.9;	38.40	0.00
TRINITY_Q22DT5_TETTS/36-180	Q22DT5.4 PF00106.23	38.40	0.00
TRINITY_A0A0D2UP61_CAPO3/584-737	A0A0D2UP61.1 PF0060	38.40	0.00
TRINITY_G0QW77_ICHMG/43-324	G0QW77.1 PF03619.14	38.40	0.00
TRINITY_A0A0E9NFI9_9ASCO/26-160	A0A0E9NFI9.1 PF1289	38.40	0.00
TRINITY_A9T4V6_PHYPA/145-269	A9T4V6.1 PF01529.18	38.40	0.00
TRINITY_F0ZC32_DICPU/152-235	F0ZC32.1 PF06916.11	38.40	0.00
TRINITY_G7JHQ5_MEDTR/166-270	G7JHQ5.1 PF03171.18	38.40	0.00
TRINITY_I7MAS1_TETTS/403-633	I7MAS1.2 PF00609.17	38.40	0.00
TRINITY_G0R4U6_ICHMG/19-318	G0R4U6.1 PF00266.17	38.40	0.00
TRINITY_I7MIN0_TETTS/9-308	I7MIN0.1 PF08449.9;	38.40	0.00
TRINITY_E7FA74_DANRE/1687-1780	E7FA74.1 PF16770.3;	38.40	0.00
TRINITY_M0S8A8_MUSAM/20-134	M0S8A8.1 PF06246.10	38.40	0.00
TRINITY_D8TU60_VOLCA/39-696	D8TU60.1 PF04916.11	38.40	0.00
TRINITY_SPKA_DICDI/351-612	Q86AT8.1 PF07714.15	38.40	0.00
TRINITY_G7JN28_MEDTR/76-215	G7JN28.1 PF10262.7;	38.40	0.00
TRINITY_C1DYB7_MICSR/138-297	C1DYB7.1 PF01765.17	38.40	0.00
TRINITY_B9RVW1_RICCO/357-463	B9RVW1.1 PF12214.6;	38.40	0.00
TRINITY_A9SWK6_PHYPA/87-335	A9SWK6.1 PF01963.15	38.40	0.00
TRINITY_D0ME80_RHOM4/39-358	D0ME80.1 PF01663.20	38.40	0.00
TRINITY_A9U4C0_PHYPA/2-190	A9U4C0.1 PF14724.4;	38.40	0.00
TRINITY_C4LTX2_ENTHI/61-275	C4LTX2.1 PF00149.26	38.40	0.00
TRINITY_A0CVA1_PARTE/625-865	A0CVA1.1 PF00566.16	38.40	0.00
TRINITY_J9I249_9SPIT/72-231	J9I249.1 PF01343.16	38.40	0.00
TRINITY_L1JM38_GUITH/149-313	L1JM38.1 PF13535.4;	38.40	0.00
TRINITY_A0A067JL15_JATCU/434-599	A0A067JL15.1 PF0007	38.40	0.00

TRINITY_A8IR23_CHLRE/1-184	A8IR23.1 PF13419.4;	38.40	0.00
TRINITY_A0A0A1TCV8_9HYPO/142-382	A0A0A1TCV8.1 PF1319	38.40	0.00
TRINITY_L8GLH0_ACACA/96-270	L8GLH0.1 PF05843.12	38.40	0.00
TRINITY_E1Z4H3_CHLVA/1661-1859	E1Z4H3.1 PF13378.4;	38.40	0.00
TRINITY_G0QLT3_ICHMG/99-440	G0QLT3.1 PF01237.16	38.40	0.00
TRINITY_A0A0D9YAK9_9ORYZ/10-152	A0A0D9YAK9.1 PF0002	38.30	0.00
TRINITY_A0A0G2ZPA9_9DELT/216-336	A0A0G2ZPA9.1 PF0018	38.30	0.00
TRINITY_A0A0D2UJE3_CAPO3/82-201	A0A0D2UJE3.1 PF0004	38.30	0.00
TRINITY_A0A087SHI5_AUXPR/5-193	A0A087SHI5.1 PF1621	38.30	0.00
TRINITY_A0A0N5A8P3_9BILA/8-221	A0A0N5A8P3.1 PF0113	38.30	0.00
TRINITY_A0A0P7UKM0_9TELE/46-174	A0A0P7UKM0.1 PF0478	38.30	0.00
TRINITY_ABCCF_DICDI/875-1144	Q54VC1.1 PF00664.21	38.30	0.00
TRINITY_X6MTW8_RETFI/11-288	X6MTW8.1 PF07065.12	38.30	0.00
TRINITY_L8GPJ8_ACACA/129-640	L8GPJ8.1 PF07690.14	38.30	0.00
TRINITY_E1Z6B0_CHLVA/24-120	E1Z6B0.1 PF10537.7;	38.30	0.00
TRINITY_L8H271_ACACA/165-267	L8H271.1 PF00168.28	38.30	0.00
TRINITY_D3B9W6_POLPA/171-597	D3B9W6.1 PF00501.26	38.30	0.00
TRINITY_D8U150_VOLCA/26-330	D8U150.1 PF04756.11	38.30	0.00
TRINITY_G0QU62_ICHMG/2185-2520	G0QU62.1 PF12777.5;	38.30	0.00
TRINITY_B0WST7_CULQU/148-481	B0WST7.1 PF05028.12	38.30	0.00
TRINITY_J9FJH5_9SPIT/99-246	J9FJH5.1 PF07534.14	38.30	0.00
TRINITY_A0A010QCS2_9PEZI/87-211	A0A010QCS2.1 PF0018	38.30	0.00
TRINITY_T1FWZ5_HELRO/13-142	T1FWZ5.1 PF00782.18	38.30	0.00
TRINITY_A8IY98_CHLRE/366-502	A8IY98.1 PF03188.14	38.30	0.00
TRINITY_D8QRE9_SELML/4-615	D8QRE9.1 PF04130.11	38.30	0.00
TRINITY_I7M6D7_TETTS/137-346	I7M6D7.1 PF03798.14	38.30	0.00
TRINITY_A0A024UBD2_9STRA/265-453	A0A024UBD2.1 PF0223	38.30	0.00
TRINITY_N4W161_COLOR/21-375	N4W161.1 PF01212.19	38.30	0.00
TRINITY_A0A078AGW3_STYLE/63-550	A0A078AGW3.1 PF0050	38.30	0.00
TRINITY_D3B338_POLPA/992-1287	D3B338.1 PF02171.15	38.30	0.00
TRINITY_A0A0N4W0J1_HAEPC/654-749	A0A0N4W0J1.1 PF0222	38.30	0.00
TRINITY_E1ZDV4_CHLVA/257-841	E1ZDV4.1 PF10433.7;	38.30	0.00
TRINITY_B9ILR4_POPTR/776-1025	B9ILR4.2 PF00069.23	38.30	0.00
TRINITY_D3BJN3_POLPA/68-802	D3BJN3.1 PF05879.10	38.30	0.00
TRINITY_I1LKX3_SOYBN/6-130	I1LKX3.1 PF16100.3;	38.30	0.00
TRINITY_J9IYY2_9SPIT/103-419	J9IYY2.1 PF01546.26	38.30	0.00
TRINITY_A0A0A0KAZ4_CUCSA/6-197	A0A0A0KAZ4.1 PF0783	38.30	0.00
TRINITY_D8TQ76_VOLCA/46-459	D8TQ76.1 PF06870.10	38.30	0.00
TRINITY_Q23FG2_TETTS/1702-2050	Q23FG2.2 PF00443.27	38.30	0.00
TRINITY_L8GZN8_ACACA/219-408	L8GZN8.1 PF00617.17	38.30	0.00
TRINITY_F1QXK7_DANRE/170-431	F1QXK7.1 PF00069.23	38.30	0.00
TRINITY_U5D935_AMBTC/366-517	U5D935.1 PF04937.13	38.30	0.00
TRINITY_D8TN95_VOLCA/102-337	D8TN95.1 PF00118.22	38.30	0.00
TRINITY_A8IK98_CHLRE/74-458	A8IK98.1 PF00176.21	38.30	0.00
TRINITY_L8GJ41_ACACA/177-473	L8GJ41.1 PF09423.8;	38.30	0.00
TRINITY_A8J7M9_CHLRE/742-1038	A8J7M9.1 PF00069.23	38.30	0.00
TRINITY_A0A087SFL1_AUXPR/342-523	A0A087SFL1.1 PF0185	38.30	0.00
TRINITY_Q2IQ35_ANADE/179-509	Q2IQ35.1 PF00171.20	38.30	0.00
TRINITY_F4P4T9_BATDJ/43-311	F4P4T9.1 PF00350.21	38.30	0.00
TRINITY_I7MFL4_TETTS/618-718	I7MFL4.2 PF12796.5;	38.20	0.00
TRINITY_R0HMD9_9BRAS/130-349	R0HMD9.1 PF00112.21	38.20	0.00
TRINITY_I7M249_TETTS/257-369	I7M249.1 PF08695.8;	38.20	0.00
TRINITY_G0R560_ICHMG/1-229	G0R560.1 PF00067.20	38.20	0.00
TRINITY_G0QPQ7_ICHMG/153-915	G0QPQ7.1 PF04762.10	38.20	0.00
TRINITY_A2EQY4_TRIVA/71-171	A2EQY4.1 PF08241.10	38.20	0.00
TRINITY_VPS10_PICPG/729-1193	C4R192.1 PF15902.3;	38.20	0.00
TRINITY_B3RUZ1_TRIAD/35-154	B3RUZ1.1 PF03351.15	38.20	0.00
TRINITY_J1FDE1_9BACT/5-271	J1FDE1.1 PF01869.18	38.20	0.00
TRINITY_L8H9P5_ACACA/1162-1679	L8H9P5.1 PF06920.11	38.20	0.00
TRINITY_L8HAW3_ACACA/947-1064	L8HAW3.1 PF12807.5;	38.20	0.00
TRINITY_F8KX77_PARAV/15-456	F8KX77.1 PF01593.22	38.20	0.00

TRINITY_F0Z850_DICPU/211-305	F0Z850.1 PF00153.25	38.20	0.00
TRINITY_I1IR77_BRADI/131-226	I1IR77.1 PF00651.29	38.20	0.00
TRINITY_A0A0D9WVV7_9ORYZ/262-443	A0A0D9WVV7.1 PF0094	38.20	0.00
TRINITY_A0A078B9A8_STYLE/22-221	A0A078B9A8.1 PF0114	38.20	0.00
TRINITY_H0V5S4_CAVPO/197-298	H0V5S4.1 PF00153.25	38.20	0.00
TRINITY_J9HWC2_9SPIT/321-529	J9HWC2.1 PF03770.14	38.20	0.00
TRINITY_E1ZDV4_CHLVA/1238-1591	E1ZDV4.1 PF03178.13	38.20	0.00
TRINITY_D2VCX0_NAEGR/106-317	D2VCX0.1 PF02750.12	38.20	0.00
TRINITY_A0C866_PARTE/289-608	A0C866.1 PF04515.10	38.20	0.00
TRINITY_A0A015M7K9_9GLOM/318-474	A0A015M7K9.1 PF0006	38.20	0.00
TRINITY_D3BCH3_POLPA/1901-2160	D3BCH3.1 PF00481.19	38.20	0.00
TRINITY_C1MGF8_MICPC/33-222	C1MGF8.1 PF13579.4;	38.20	0.00
TRINITY_A0A0K9PV01_ZOSMR/294-407	A0A0K9PV01.1 PF0292	38.20	0.00
TRINITY_Q2HHN2_CHAGB/234-468	Q2HHN2.1 PF00856.26	38.20	0.00
TRINITY_W6G5N1_NODSP/97-330	W6G5N1.1 PF00891.16	38.20	0.00
TRINITY_M4BET0_HYAAE/13-75	M4BET0.1 PF00226.29	38.20	0.00
TRINITY_L8H3W5_ACACA/497-720	L8H3W5.1 PF00176.21	38.20	0.00
TRINITY_K5XK64_AGABU/93-379	K5XK64.1 PF00069.23	38.20	0.00
TRINITY_L8GSQ7_ACACA/503-844	L8GSQ7.1 PF00443.27	38.20	0.00
TRINITY_F0ZW58_DICPU/38-145	F0ZW58.1 PF00307.29	38.20	0.00
TRINITY_D8UGK7_VOLCA/91-281	D8UGK7.1 PF03798.14	38.20	0.00
TRINITY_I0YLE6_9CHLO/727-871	I0YLE6.1 PF13515.4;	38.20	0.00
TRINITY_A0A067C4C7_SAPPC/21-269	A0A067C4C7.1 PF1477	38.20	0.00
TRINITY_A8IPI0_CHLRE/175-335	A8IPI0.1 PF05063.12	38.20	0.00
TRINITY_E1ZLI8_CHLVA/13-184	E1ZLI8.1 PF03152.12	38.20	0.00
TRINITY_A8IWZ7_CHLRE/941-1752	A8IWZ7.1 PF02364.13	38.20	0.00
TRINITY_B3RVL5_TRIAD/13-116	B3RVL5.1 PF02991.14	38.20	0.00
TRINITY_I7M612_TETTS/2-121	I7M612.2 PF08923.8;	38.10	0.00
TRINITY_U5GN11_POPTR/596-830	U5GN11.1 PF02358.14	38.10	0.00
TRINITY_M2TUY0_COCH5/323-879	M2TUY0.1 PF01055.24	38.10	0.00
TRINITY_T0QCC9_9STRA/12-266	T0QCC9.1 PF07690.14	38.10	0.00
TRINITY_Q248B0_TETTS/147-404	Q248B0.3 PF00069.23	38.10	0.00
TRINITY_A0A0F5K5I6_9BURK/5-211	A0A0F5K5I6.1 PF0137	38.10	0.00
TRINITY_K4BMJ3_SOLLC/197-324	K4BMJ3.1 PF00134.21	38.10	0.00
TRINITY_A0BSB9_PARTE/396-700	A0BSB9.1 PF11904.6;	38.10	0.00
TRINITY_L8GIF3_ACACA/110-341	L8GIF3.1 PF07065.12	38.10	0.00
TRINITY_I4Y6B8_WALMC/11-127	I4Y6B8.1 PF00383.21	38.10	0.00
TRINITY_X6MIZ1_RETFI/93-249	X6MIZ1.1 PF02544.14	38.10	0.00
TRINITY_M4XFV7_PSEDE/2-246	M4XFV7.1 PF00795.20	38.10	0.00
TRINITY_D8T6F9_SELML/113-250	D8T6F9.1 PF08652.9;	38.10	0.00
TRINITY_I7M136_TETTS/2403-2719	I7M136.2 PF00520.29	38.10	0.00
TRINITY_A0BPT3_PARTE/448-553	A0BPT3.1 PF11987.6;	38.10	0.00
TRINITY_Q24FP1_TETTS/133-395	Q24FP1.1 PF00069.23	38.10	0.00
TRINITY_J9JVU9_ACYPI/15-416	J9JVU9.1 PF03676.12	38.10	0.00
TRINITY_L8GMR3_ACACA/270-526	L8GMR3.1 PF07714.15	38.10	0.00
TRINITY_Q23QF2_TETTS/248-571	Q23QF2.2 PF03133.13	38.10	0.00
TRINITY_L8GLW0_ACACA/708-815	L8GLW0.1 PF00651.29	38.10	0.00
TRINITY_A9V590_MONBE/142-406	A9V590.1 PF13621.4;	38.10	0.00
TRINITY_A0A075B0K6_9FUNG/81-189	A0A075B0K6.1 PF1179	38.10	0.00
TRINITY_A0DYX2_PARTE/54-188	A0DYX2.1 PF04146.13	38.10	0.00
TRINITY_A0D0L1_PARTE/45-155	A0D0L1.1 PF08240.10	38.10	0.00
TRINITY_Q54PU3_DICDI/548-698	Q54PU3.1 PF08598.9;	38.10	0.00
TRINITY_D8TRL6_VOLCA/310-457	D8TRL6.1 PF12499.6;	38.10	0.00
TRINITY_L8GLF0_ACACA/19-262	L8GLF0.1 PF03896.14	38.10	0.00
TRINITY_Q54HR9_DICDI/14-448	Q54HR9.1 PF01593.22	38.10	0.00
TRINITY_F6YR48_XENTR/198-298	F6YR48.1 PF00168.28	38.10	0.00
TRINITY_D8TY51_VOLCA/72-295	D8TY51.1 PF05721.11	38.10	0.00
TRINITY_Q23AF4_TETTS/189-364	Q23AF4.1 PF13768.4;	38.10	0.00
TRINITY_F6HWI4_VITVI/37-492	F6HWI4.1 PF00450.20	38.10	0.00
TRINITY_D6WDQ2_TRICA/3-115	D6WDQ2.1 PF11969.6;	38.10	0.00
TRINITY_M0ZFR1_HORVD/9-153	M0ZFR1.1 PF01694.20	38.10	0.00

TRINITY_A0A0N0DC34_9HYPO/36-314	A0A0N0DC34.1 PF0026	38.10	0.00
TRINITY_U4U7P0_DENPD/53-185	U4U7P0.1 PF00293.26	38.10	0.00
TRINITY_A0A077C3V9_9RICK/1-137	A0A077C3V9.1 PF0181	38.10	0.00
TRINITY_A0A087SKA1_AUXPR/26-751	A0A087SKA1.1 PF0001	38.10	0.00
TRINITY_S2KEJ0_MUCC1/6-507	S2KEJ0.1 PF05833.9;	38.10	0.00
TRINITY_A0A0J8D1Y5_BETVU/470-757	A0A0J8D1Y5.1 PF1641	38.10	0.00
TRINITY_A0A0E0AI18_9ORYZ/52-271	A0A0E0AI18.1 PF0014	38.10	0.00
TRINITY_A0A061DH64_THECC/47-236	A0A061DH64.1 PF1029	38.10	0.00
TRINITY_J9IR36_9SPIT/409-563	J9IR36.1 PF04280.13	38.10	0.00
TRINITY_F0ZJW7_DICPU/982-1087	F0ZJW7.1 PF01843.17	38.10	0.00
TRINITY_W5LA77_ASTMX/24-315	W5LA77.1 PF09734.7;	38.10	0.00
TRINITY_A0BQJ6_PARTE/96-443	A0BQJ6.1 PF00026.21	38.10	0.00
TRINITY_Q23T76_TETTS/135-542	Q23T76.2 PF10255.7;	38.10	0.00
TRINITY_A0A0J7K9E1_LASNI/612-853	A0A0J7K9E1.1 PF0772	38.10	0.00
TRINITY_D8TW66_VOLCA/1237-1337	D8TW66.1 PF00431.18	38.10	0.00
TRINITY_J9J1N0_9SPIT/5-189	J9J1N0.1 PF00025.19	38.10	0.00
TRINITY_W4GXX2_9STRA/31-251	W4GXX2.1 PF07264.9;	38.10	0.00
TRINITY_A0A0L0NFJ1_9HYPO/1219-1391	A0A0L0NFJ1.1 PF0061	38.10	0.00
TRINITY_A0BKI4_PARTE/27-441	A0BKI4.1 PF07690.14	38.00	0.00
TRINITY_G4MLN4_MAGO7/55-239	G4MLN4.1 PF00625.19	38.00	0.00
TRINITY_C0BL88_9BACT/129-324	C0BL88.1 PF13547.4;	38.00	0.00
TRINITY_A0A078B894_STYLE/114-360	A0A078B894.1 PF1356	38.00	0.00
TRINITY_W7TH15_9STRA/133-315	W7TH15.1 PF03232.11	38.00	0.00
TRINITY_M0TAD0_MUSAM/278-427	M0TAD0.1 PF00005.25	38.00	0.00
TRINITY_L8HBL0_ACACA/285-445	L8HBL0.1 PF02450.13	38.00	0.00
TRINITY_L8GLP7_ACACA/917-1126	L8GLP7.1 PF16095.3;	38.00	0.00
TRINITY_A0DFC5_PARTE/13-272	A0DFC5.1 PF00069.23	38.00	0.00
TRINITY_Q7YT74_CIOIN/5-178	Q7YT74.1 PF00071.20	38.00	0.00
TRINITY_A2EWX3_TRIVA/24-280	A2EWX3.1 PF00069.23	38.00	0.00
TRINITY_A0A0A0LZ06_CUCSA/34-112	A0A0A0LZ06.1 PF0701	38.00	0.00
TRINITY_A0A024U8P3_9STRA/129-293	A0A024U8P3.1 PF0085	38.00	0.00
TRINITY_H3HED1_PHYRM/3-314	H3HED1.1 PF00155.19	38.00	0.00
TRINITY_L8H823_ACACA/184-369	L8H823.1 PF07065.12	38.00	0.00
TRINITY_A0CMN6_PARTE/121-399	A0CMN6.1 PF01457.14	38.00	0.00
TRINITY_L8GTK3_ACACA/373-483	L8GTK3.1 PF00989.23	38.00	0.00
TRINITY_A0A078AI46_STYLE/536-661	A0A078AI46.1 PF0007	38.00	0.00
TRINITY_L8GVF6_ACACA/53-250	L8GVF6.1 PF09414.8;	38.00	0.00
TRINITY_A0A0D5EF57_9BURK/47-125	A0A0D5EF57.1 PF0306	38.00	0.00
TRINITY_A0A0N4WF97_HAEPC/180-314	A0A0N4WF97.1 PF0139	38.00	0.00
TRINITY_E1ZGC8_CHLVA/516-908	E1ZGC8.1 PF04357.11	38.00	0.00
TRINITY_A0A0K0ELA3_STRER/230-425	A0A0K0ELA3.1 PF0173	38.00	0.00
TRINITY_I1CST3_RHIO9/36-202	I1CST3.1 PF08423.9;	38.00	0.00
TRINITY_G0QPA4_ICHMG/1-528	G0QPA4.1 PF16969.3;	38.00	0.00
TRINITY_D2TT12_CITRI/1-140	D2TT12.1 PF13302.5;	38.00	0.00
TRINITY_A0CH91_PARTE/89-233	A0CH91.1 PF01694.20	38.00	0.00
TRINITY_L8GQC0_ACACA/227-408	L8GQC0.1 PF00621.18	38.00	0.00
TRINITY_Q54HM5_DICDI/67-270	Q54HM5.1 PF00804.23	38.00	0.00
TRINITY_A0A087SBT1_AUXPR/20-121	A0A087SBT1.1 PF1605	38.00	0.00
TRINITY_R1BPU4_EMIHU/38-122	R1BPU4.1 PF16561.3;	38.00	0.00
TRINITY_A0BGJ9_PARTE/392-849	A0BGJ9.1 PF06046.11	38.00	0.00
TRINITY_D8TGX6_VOLCA/877-1013	D8TGX6.1 PF01925.17	38.00	0.00
TRINITY_M5VWD4_PRUPE/293-542	M5VWD4.1 PF07714.15	38.00	0.00
TRINITY_D8UCH5_VOLCA/671-922	D8UCH5.1 PF13365.4;	38.00	0.00
TRINITY_J9HY65_9SPIT/97-190	J9HY65.1 PF12894.5;	38.00	0.00
TRINITY_W9S288_9ROSA/14-232	W9S288.1 PF00244.18	38.00	0.00
TRINITY_C5ZVK3_9HELI/37-432	C5ZVK3.1 PF00266.17	38.00	0.00
TRINITY_M1C9K8_SOLTU/8-346	M1C9K8.1 PF07767.9;	38.00	0.00
TRINITY_A0DJB8_PARTE/29-287	A0DJB8.1 PF00194.19	38.00	0.00
TRINITY_A0A087SL48_AUXPR/2-291	A0A087SL48.1 PF0315	38.00	0.00
TRINITY_R0F7Q0_9BRAS/19-271	R0F7Q0.1 PF00069.23	38.00	0.00
TRINITY_A0A0G4IS83_PLABS/350-703	A0A0G4IS83.1 PF0149	38.00	0.00

TRINITY_W5MAW9_LEPOC/320-410	W5MAW9.1 PF12796.5;	38.00	0.00
TRINITY_D0MZH6_PHYIT/325-545	D0MZH6.1 PF00149.26	38.00	0.00
TRINITY_A0A087SK80_AUXPR/36-236	A0A087SK80.1 PF0085	38.00	0.00
TRINITY_L8HIM5_ACACA/67-355	L8HIM5.1 PF00128.22	38.00	0.00
TRINITY_A7SRR7_NEMVE/155-278	A7SRR7.1 PF14641.4;	38.00	0.00
TRINITY_A0A0G4ED92_9ALVE/328-436	A0A0G4ED92.1 PF0065	38.00	0.00
TRINITY_E1Z7R8_CHLVA/67-445	E1Z7R8.1 PF01494.17	38.00	0.00
TRINITY_C1E286_MICSR/50-280	C1E286.1 PF12146.6;	38.00	0.00
TRINITY_D8TYV0_VOLCA/326-491	D8TYV0.1 PF10173.7;	38.00	0.00
TRINITY_C1EBP3_MICSR/11-234	C1EBP3.1 PF01738.16	38.00	0.00
TRINITY_A8IB76_CHLRE/818-1020	A8IB76.1 PF00069.23	38.00	0.00
TRINITY_A0A078AD61_STYLE/66-145	A0A078AD61.1 PF0701	38.00	0.00
TRINITY_I7LZD9_TETTS/6-127	I7LZD9.2 PF01392.20	37.90	0.00
TRINITY_D8M178_BLAHO/1-209	D8M178.1 PF00149.26	37.90	0.00
TRINITY_D3B1J8_POLPA/1226-1403	D3B1J8.1 PF00617.17	37.90	0.00
TRINITY_H2Z3Z7_CIOSA/317-419	H2Z3Z7.1 PF03126.16	37.90	0.00
TRINITY_W5MD04_LEPOC/29-262	W5MD04.1 PF10223.7;	37.90	0.00
TRINITY_J9EQ41_9SPIT/87-364	J9EQ41.1 PF05890.10	37.90	0.00
TRINITY_A1ZJG8_9BACT/275-345	A1ZJG8.1 PF13424.4;	37.90	0.00
TRINITY_I0YSI7_9CHLO/199-342	I0YSI7.1 PF14617.4;	37.90	0.00
TRINITY_Q24CH5_TETTS/1154-1338	Q24CH5.2 PF05708.10	37.90	0.00
TRINITY_D8TTV8_VOLCA/81-324	D8TTV8.1 PF00233.17	37.90	0.00
TRINITY_Q54LK9_DICDI/7-142	Q54LK9.1 PF15982.3;	37.90	0.00
TRINITY_A0A088A958_APIME/17-236	A0A088A958.1 PF0010	37.90	0.00
TRINITY_A9G0Q5_SORC5/53-346	A9G0Q5.1 PF14388.4;	37.90	0.00
TRINITY_A0A088A2A6_APIME/145-298	A0A088A2A6.1 PF0169	37.90	0.00
TRINITY_A0A078AJQ0_STYLE/388-594	A0A078AJQ0.1 PF0481	37.90	0.00
TRINITY_Q28DS2_XENTR/329-467	Q28DS2.1 PF13874.4;	37.90	0.00
TRINITY_PNPP_SCHPO/18-258	Q00472.2 PF00702.24	37.90	0.00
TRINITY_W4Z2A0_STRPU/116-413	W4Z2A0.1 PF00566.16	37.90	0.00
TRINITY_A0A078ALC0_STYLE/35-303	A0A078ALC0.1 PF0170	37.90	0.00
TRINITY_S9UWS8_9TRYP/44-243	S9UWS8.1 PF01027.18	37.90	0.00
TRINITY_Q3M0Z1_PARTE/41-243	Q3M0Z1.1 PF00804.23	37.90	0.00
TRINITY_A0A077ZPS4_STYLE/243-438	A0A077ZPS4.1 PF0011	37.90	0.00
TRINITY_L8GLJ5_ACACA/787-1048	L8GLJ5.1 PF07714.15	37.90	0.00
TRINITY_M3ZGA0_XIPMA/50-521	M3ZGA0.1 PF00083.22	37.90	0.00
TRINITY_A0D801_PARTE/617-862	A0D801.1 PF02714.13	37.90	0.00
TRINITY_C3Z471_BRAFL/355-540	C3Z471.1 PF00616.17	37.90	0.00
TRINITY_H1XPJ5_9BACT/483-700	H1XPJ5.1 PF00326.19	37.90	0.00
TRINITY_F2UIN7_SALR5/19-256	F2UIN7.1 PF03222.11	37.90	0.00
TRINITY_L8HFG4_ACACA/7-102	L8HFG4.1 PF00338.20	37.90	0.00
TRINITY_R4X8L7_TAPDE/1-113	R4X8L7.1 PF08576.8;	37.90	0.00
TRINITY_G0R238_ICHMG/12-332	G0R238.1 PF00162.17	37.90	0.00
TRINITY_A0A077ZQS0_STYLE/68-262	A0A077ZQS0.1 PF1214	37.90	0.00
TRINITY_J9IZJ8_9SPIT/1-251	J9IZJ8.1 PF00069.23	37.90	0.00
TRINITY_B4VYT7_9CYAN/209-366	B4VYT7.1 PF05128.10	37.90	0.00
TRINITY_L8GWK5_ACACA/67-244	L8GWK5.1 PF00621.18	37.90	0.00
TRINITY_I0Z7B1_9CHLO/1-152	I0Z7B1.1 PF05529.10	37.90	0.00
TRINITY_L8GUK1_ACACA/237-488	L8GUK1.1 PF07714.15	37.90	0.00
TRINITY_Q22AB0_TETTS/141-425	Q22AB0.2 PF00566.16	37.90	0.00
TRINITY_M5EIW5_MALS4/63-528	M5EIW5.1 PF00450.20	37.90	0.00
TRINITY_I0YSZ2_9CHLO/11-177	I0YSZ2.1 PF03398.12	37.90	0.00
TRINITY_A0BLH9_PARTE/7-108	A0BLH9.1 PF00168.28	37.90	0.00
TRINITY_D8TTE6_VOLCA/811-970	D8TTE6.1 PF12499.6;	37.90	0.00
TRINITY_A4SBG1_OSTLU/9-269	A4SBG1.1 PF07714.15	37.90	0.00
TRINITY_A0A0J8EFZ9_BETVU/16-511	A0A0J8EFZ9.1 PF0821	37.90	0.00
TRINITY_I7LXB9_TETTS/65-363	I7LXB9.1 PF00996.16	37.90	0.00
TRINITY_Q24CE7_TETTS/28-191	Q24CE7.2 PF09768.7;	37.90	0.00
TRINITY_A0BCK0_PARTE/76-191	A0BCK0.1 PF01569.19	37.90	0.00
TRINITY_F4Q5E9_DICFS/471-609	F4Q5E9.1 PF07534.14	37.90	0.00
TRINITY_F1QJ75_DANRE/180-288	F1QJ75.1 PF00175.19	37.80	0.00

TRINITY_E4X5G9_OIKDI/157-407	E4X5G9.1 PF00069.23	37.80	0.00
TRINITY_G0QNL8_ICHMG/17-231	G0QNL8.1 PF02230.14	37.80	0.00
TRINITY_A0A0L0CLN7_LUCCU/45-413	A0A0L0CLN7.1 PF0251	37.80	0.00
TRINITY_K9RD56_9CYAN/12-439	K9RD56.1 PF00067.20	37.80	0.00
TRINITY_C6W4V6_DYAFD/4-148	C6W4V6.1 PF09424.8;	37.80	0.00
TRINITY_F2UPC9_SALR5/196-493	F2UPC9.1 PF03372.21	37.80	0.00
TRINITY_J0S039_9EURY/955-1172	J0S039.1 PF13087.4;	37.80	0.00
TRINITY_X5DER9_9BACT/3-119	X5DER9.1 PF00072.22	37.80	0.00
TRINITY_A0A0L6UEM4_9BASI/111-332	A0A0L6UEM4.1 PF0155	37.80	0.00
TRINITY_A0ECP3_PARTE/199-326	A0ECP3.1 PF10409.7;	37.80	0.00
TRINITY_A0DKA0_PARTE/20-488	A0DKA0.1 PF12698.5;	37.80	0.00
TRINITY_A0A090M924_OSTTA/112-305	A0A090M924.1 PF1353	37.80	0.00
TRINITY_Q5CR89_CRYPI/181-313	Q5CR89.1 PF10104.7;	37.80	0.00
TRINITY_L8HFX4_ACACA/12-318	L8HFX4.1 PF00443.27	37.80	0.00
TRINITY_A0DYR3_PARTE/708-848	A0DYR3.1 PF01909.21	37.80	0.00
TRINITY_A8IRB8_CHLRE/110-310	A8IRB8.1 PF06011.10	37.80	0.00
TRINITY_Q24DF0_TETTS/748-1037	Q24DF0.2 PF00648.19	37.80	0.00
TRINITY_A0CIS4_PARTE/8-260	A0CIS4.1 PF00069.23	37.80	0.00
TRINITY_A8IVC2_CHLRE/79-199	A8IVC2.1 PF08292.10	37.80	0.00
TRINITY_S2JB42_MUCC1/120-512	S2JB42.1 PF09423.8;	37.80	0.00
TRINITY_L8HIN8_ACACA/120-272	L8HIN8.1 PF01926.21	37.80	0.00
TRINITY_C1N472_MICPC/2-228	C1N472.1 PF04188.11	37.80	0.00
TRINITY_G0QIZ2_ICHMG/16-177	G0QIZ2.1 PF00071.20	37.80	0.00
TRINITY_A0A0D9LKZ3_9EURO/36-303	A0A0D9LKZ3.1 PF0015	37.80	0.00
TRINITY_S2JPC7_MUCC1/1801-1889	S2JPC7.1 PF11919.6;	37.80	0.00
TRINITY_C1N0A5_MICPC/948-1102	C1N0A5.1 PF01927.14	37.80	0.00
TRINITY_LYSG4_DICDI/23-204	Q54BQ8.1 PF01183.18	37.80	0.00
TRINITY_D8U784_VOLCA/435-630	D8U784.1 PF16016.3;	37.80	0.00
TRINITY_A9SRQ1_PHYPA/50-142	A9SRQ1.1 PF00254.26	37.80	0.00
TRINITY_E1ZDU3_CHLVA/68-210	E1ZDU3.1 PF14108.4;	37.80	0.00
TRINITY_H2Q6I7_PANTR/434-570	H2Q6I7.1 PF01529.18	37.80	0.00
TRINITY_A0A078ACA2_STYLE/7-156	A0A078ACA2.1 PF1458	37.80	0.00
TRINITY_Q231F3_TETTS/82-315	Q231F3.1 PF03009.15	37.80	0.00
TRINITY_I0Z6C3_9CHLO/4-420	I0Z6C3.1 PF04841.11	37.80	0.00
TRINITY_D8TVZ9_VOLCA/7-333	D8TVZ9.1 PF00150.16	37.80	0.00
TRINITY_F4QAS0_DICFS/1-239	F4QAS0.1 PF00149.26	37.80	0.00
TRINITY_W3X045_9PEZI/544-639	W3X045.1 PF12796.5;	37.80	0.00
TRINITY_D8TGX3_VOLCA/5-377	D8TGX3.1 PF03054.14	37.80	0.00
TRINITY_L8GTK1_ACACA/146-395	L8GTK1.1 PF07714.15	37.80	0.00
TRINITY_F4QE29_DICFS/40-243	F4QE29.1 PF05057.12	37.80	0.00
TRINITY_U4UTU6_DENPD/81-343	U4UTU6.1 PF00664.21	37.80	0.00
TRINITY_Q23YD4_TETTS/903-1281	Q23YD4.2 PF12698.5;	37.80	0.00
TRINITY_G8BYN1_TETPH/144-326	G8BYN1.1 PF01435.16	37.80	0.00
TRINITY_Q4T616_TETNG/38-496	Q4T616.1 PF00067.20	37.80	0.00
TRINITY_D8RJK1_SELML/4-111	D8RJK1.1 PF00635.24	37.80	0.00
TRINITY_C5KD40_PERM5/279-352	C5KD40.1 PF00076.20	37.80	0.00
TRINITY_A7RH45_NEMVE/1-375	A7RH45.1 PF01979.18	37.70	0.00
TRINITY_I7MHX9_TETTS/57-385	I7MHX9.2 PF00026.21	37.70	0.00
TRINITY_C3ZNS5_BRAFL/55-408	C3ZNS5.1 PF05292.9;	37.70	0.00
TRINITY_C3YB33_BRAFL/139-397	C3YB33.1 PF00939.17	37.70	0.00
TRINITY_A0A078B2E5_STYLE/525-896	A0A078B2E5.1 PF1059	37.70	0.00
TRINITY_M2XYD7_GALSU/14-176	M2XYD7.1 PF00071.20	37.70	0.00
TRINITY_A6BZ09_9PLAN/29-165	A6BZ09.1 PF01878.16	37.70	0.00
TRINITY_A0A068X5Z4_HYMMI/58-189	A0A068X5Z4.1 PF0078	37.70	0.00
TRINITY_A0A058Z129_9EUKA/4-357	A0A058Z129.1 PF0006	37.70	0.00
TRINITY_L8HI30_ACACA/810-1004	L8HI30.1 PF13901.4;	37.70	0.00
TRINITY_H2XTA5_CIOIN/343-503	H2XTA5.1 PF03712.13	37.70	0.00
TRINITY_L8GNS3_ACACA/13-225	L8GNS3.1 PF03357.19	37.70	0.00
TRINITY_E1ZFP4_CHLVA/23-228	E1ZFP4.1 PF05700.9;	37.70	0.00
TRINITY_A0DLL4_PARTE/80-373	A0DLL4.1 PF00648.19	37.70	0.00
TRINITY_M4BSL2_HYAAE/596-661	M4BSL2.1 PF13499.4;	37.70	0.00

TRINITY_H8MJA6_CORCM/946-1175	H8MJA6.1 PF07678.12	37.70	0.00
TRINITY_U9SX29_RHIID/400-662	U9SX29.1 PF00069.23	37.70	0.00
TRINITY_M2Y691_GALSU/133-356	M2Y691.1 PF01556.16	37.70	0.00
TRINITY_A0A077LKW5_9PSED/24-168	A0A077LKW5.1 PF0018	37.70	0.00
TRINITY_B3S5V5_TRIAD/38-487	B3S5V5.1 PF00067.20	37.70	0.00
TRINITY_A0A078B9W1_STYLE/27-329	A0A078B9W1.1 PF0056	37.70	0.00
TRINITY_IKSA_DICDI/261-568	Q54RJ4.1 PF00069.23	37.70	0.00
TRINITY_L8GKH0_ACACA/249-578	L8GKH0.1 PF13598.4;	37.70	0.00
TRINITY_K0R4W7_THAOC/3-179	K0R4W7.1 PF00025.19	37.70	0.00
TRINITY_A0A078BAY4_STYLE/8-267	A0A078BAY4.1 PF0502	37.70	0.00
TRINITY_A0C287_PARTE/52-458	A0C287.1 PF00450.20	37.70	0.00
TRINITY_I7LVA0_TETTS/7-130	I7LVA0.1 PF05762.12	37.70	0.00
TRINITY_A0A059A6B9_EUCGR/506-663	A0A059A6B9.1 PF0060	37.70	0.00
TRINITY_I0Z5N1_9CHLO/328-807	I0Z5N1.1 PF04762.10	37.70	0.00
TRINITY_D6Y1U4_THEBD/46-533	D6Y1U4.1 PF07969.9;	37.70	0.00
TRINITY_F4QC97_DICFS/129-359	F4QC97.1 PF04499.13	37.70	0.00
TRINITY_M7XLX7_RHOT1/1-286	M7XLX7.1 PF13230.4;	37.70	0.00
TRINITY_F4L116_HALH1/265-487	F4L116.1 PF13229.4;	37.70	0.00
TRINITY_D3B9X7_POLPA/156-322	D3B9X7.1 PF10294.7;	37.70	0.00
TRINITY_W7XGH0_TETTS/105-241	W7XGH0.1 PF15294.4;	37.70	0.00
TRINITY_F6WG44_XENTR/1-949	F6WG44.1 PF04762.10	37.70	0.00
TRINITY_F1A080_DICPU/140-306	F1A080.1 PF02544.14	37.70	0.00
TRINITY_Q23G44_TETTS/233-493	Q23G44.2 PF00069.23	37.70	0.00
TRINITY_E4TUQ3_MARTH/24-193	E4TUQ3.1 PF01612.18	37.70	0.00
TRINITY_L8GJ41_ACACA/177-473	L8GJ41.1 PF09423.8;	37.70	0.00
TRINITY_A0A0D2W0W3_CAPO3/475-686	A0A0D2W0W3.1 PF0106	37.70	0.00
TRINITY_G0R402_ICHMG/82-193	G0R402.1 PF00043.23	37.70	0.00
TRINITY_F4PQK5_DICFS/825-1095	F4PQK5.1 PF00664.21	37.70	0.00
TRINITY_F4Q6Y0_DICFS/140-587	F4Q6Y0.1 PF00501.26	37.70	0.00
TRINITY_A0BFR4_PARTE/111-265	A0BFR4.1 PF00650.18	37.70	0.00
TRINITY_A8JGR4_CHLRE/173-480	A8JGR4.1 PF10250.7;	37.70	0.00
TRINITY_I0YSD2_9CHLO/11-512	I0YSD2.1 PF13001.5;	37.70	0.00
TRINITY_I0YKK0_9CHLO/8-180	I0YKK0.1 PF01145.23	37.70	0.00
TRINITY_A0A0D2WTW2_CAPO3/209-314	A0A0D2WTW2.1 PF0780	37.70	0.00
TRINITY_L8GXI7_ACACA/95-287	L8GXI7.1 PF02141.19	37.70	0.00
TRINITY_D8TTE4_VOLCA/34-261	D8TTE4.1 PF00487.22	37.70	0.00
TRINITY_L8GFK9_ACACA/424-687	L8GFK9.1 PF07714.15	37.70	0.00
TRINITY_H2P144_PONAB/1-315	H2P144.1 PF05158.10	37.70	0.00
TRINITY_I0Z379_9CHLO/120-500	I0Z379.1 PF00176.21	37.70	0.00
TRINITY_Q22R41_TETTS/22-209	Q22R41.1 PF01183.18	37.70	0.00
TRINITY_A0A084W9Q9_ANOSI/75-143	A0A084W9Q9.1 PF0050	37.70	0.00
TRINITY_A0CS29_PARTE/2561-2834	A0CS29.1 PF00520.29	37.60	0.00
TRINITY_A0A078AB64_STYLE/56-359	A0A078AB64.1 PF0056	37.60	0.00
TRINITY_A0A024TNB1_9STRA/30-324	A0A024TNB1.1 PF0024	37.60	0.00
TRINITY_Q6CIF8_KLULA/233-352	Q6CIF8.1 PF00581.18	37.60	0.00
TRINITY_A0A067CLJ2_SAPPC/343-839	A0A067CLJ2.1 PF0105	37.60	0.00
TRINITY_I0JIX4_HALH3/7-199	I0JIX4.1 PF13460.4;	37.60	0.00
TRINITY_I2B5C5_SHIBC/31-233	I2B5C5.1 PF04402.12	37.60	0.00
TRINITY_C6Y250_PEDHD/31-271	C6Y250.1 PF03372.21	37.60	0.00
TRINITY_W4XPG3_STRPU/702-984	W4XPG3.1 PF00664.21	37.60	0.00
TRINITY_G0R2W5_ICHMG/613-901	G0R2W5.1 PF00069.23	37.60	0.00
TRINITY_A7SAH3_NEMVE/6-671	A7SAH3.1 PF10168.7;	37.60	0.00
TRINITY_W1PQR7_AMBTC/10-182	W1PQR7.1 PF07855.10	37.60	0.00
TRINITY_K3WRP8_PYTUL/356-499	K3WRP8.1 PF09384.8;	37.60	0.00
TRINITY_L8H8T0_ACACA/97-293	L8H8T0.1 PF00837.15	37.60	0.00
TRINITY_F4P6A0_BATDJ/37-264	F4P6A0.1 PF01872.15	37.60	0.00
TRINITY_D6TVW2_9CHLR/181-385	D6TVW2.1 PF13530.4;	37.60	0.00
TRINITY_A0BG18_PARTE/74-327	A0BG18.1 PF00102.25	37.60	0.00
TRINITY_A0A0L0BTR7_LUCCU/41-339	A0A0L0BTR7.1 PF0056	37.60	0.00
TRINITY_L8GH73_ACACA/285-622	L8GH73.1 PF03124.12	37.60	0.00
TRINITY_W7ARN9_PLAVN/3-118	W7ARN9.1 PF00635.24	37.60	0.00

TRINITY_L8H8C6_ACACA/11-258	L8H8C6.1 PF07714.15	37.60	0.00
TRINITY_F0ZKQ6_DICPU/12-173	F0ZKQ6.1 PF00071.20	37.60	0.00
TRINITY_Q24C23_TETTS/91-326	Q24C23.3 PF00566.16	37.60	0.00
TRINITY_A8J2N5_CHLRE/22-193	A8J2N5.1 PF00535.24	37.60	0.00
TRINITY_D3AXW2_POLPA/67-354	D3AXW2.1 PF06472.13	37.60	0.00
TRINITY_A0A0G4GZH6_9ALVE/419-599	A0A0G4GZH6.1 PF0281	37.60	0.00
TRINITY_A8JDD4_CHLRE/51-266	A8JDD4.1 PF04755.10	37.60	0.00
TRINITY_H6SSM3_RHOPH/62-180	H6SSM3.1 PF07542.9;	37.60	0.00
TRINITY_A0A078DVH1_BRANA/779-996	A0A078DVH1.1 PF0777	37.60	0.00
TRINITY_U4TS54_DENPD/22-129	U4TS54.1 PF00651.29	37.60	0.00
TRINITY_F5RBA9_METUF/25-199	F5RBA9.1 PF00270.27	37.60	0.00
TRINITY_K9RFK2_9CYAN/34-290	K9RFK2.1 PF00069.23	37.60	0.00
TRINITY_I1F267_AMPQE/206-369	I1F267.1 PF13359.4;	37.60	0.00
TRINITY_T0Q5K0_9STRA/19-535	T0Q5K0.1 PF01457.14	37.60	0.00
TRINITY_I0Z5M5_9CHLO/93-249	I0Z5M5.1 PF00650.18	37.60	0.00
TRINITY_G0QK85_ICHMG/890-1299	G0QK85.1 PF02463.17	37.60	0.00
TRINITY_G9NCI0_HYPVG/1-89	G9NCI0.1 PF12796.5;	37.60	0.00
TRINITY_H3FV29_PRIPA/87-225	H3FV29.1 PF01764.23	37.60	0.00
TRINITY_Q23QF2_TETTS/248-571	Q23QF2.2 PF03133.13	37.60	0.00
TRINITY_A0BZF6_PARTE/22-205	A0BZF6.1 PF03357.19	37.60	0.00
TRINITY_E9FXX8_DAPPU/553-865	E9FXX8.1 PF02171.15	37.60	0.00
TRINITY_A0A0D9VS64_9ORYZ/632-730	A0A0D9VS64.1 PF1275	37.60	0.00
TRINITY_K1ZBQ2_9BACT/157-316	K1ZBQ2.1 PF13521.4;	37.60	0.00
TRINITY_E1ZJL7_CHLVA/684-852	E1ZJL7.1 PF00270.27	37.60	0.00
TRINITY_E1ZCW8_CHLVA/18-161	E1ZCW8.1 PF02537.13	37.60	0.00
TRINITY_A0A067TJX2_9AGAR/192-468	A0A067TJX2.1 PF0066	37.50	0.00
TRINITY_K5XB69_AGABU/4-165	K5XB69.1 PF00071.20	37.50	0.00
TRINITY_L0A9E0_DEIPD/46-181	L0A9E0.1 PF09346.8;	37.50	0.00
TRINITY_I7LX20_TETTS/11-287	I7LX20.2 PF00069.23	37.50	0.00
TRINITY_G4TC67_PIRID/198-529	G4TC67.1 PF03062.17	37.50	0.00
TRINITY_F4PYT2_DICFS/180-296	F4PYT2.1 PF07534.14	37.50	0.00
TRINITY_L8H639_ACACA/7-94	L8H639.1 PF16672.3;	37.50	0.00
TRINITY_B8CDM4_THAPS/12-219	B8CDM4.1 PF13532.4;	37.50	0.00
TRINITY_E9H1Y4_DAPPU/4-193	E9H1Y4.1 PF03665.11	37.50	0.00
TRINITY_A0A0F2C634_9MICO/31-148	A0A0F2C634.1 PF0824	37.50	0.00
TRINITY_V8PIQ3_OPHHA/387-668	V8PIQ3.1 PF16187.3;	37.50	0.00
TRINITY_A0A078B0K7_STYLE/11-329	A0A078B0K7.1 PF0769	37.50	0.00
TRINITY_L8GKF2_ACACA/262-608	L8GKF2.1 PF02535.20	37.50	0.00
TRINITY_A9G0Q5_SORC5/53-346	A9G0Q5.1 PF14388.4;	37.50	0.00
TRINITY_H2KUF9_CLOSI/87-186	H2KUF9.1 PF13499.4;	37.50	0.00
TRINITY_A0E8S5_PARTE/21-142	A0E8S5.1 PF02466.17	37.50	0.00
TRINITY_D9SRV2_CLOC7/256-555	D9SRV2.1 PF02836.15	37.50	0.00
TRINITY_D8TS78_VOLCA/34-111	D8TS78.1 PF01253.20	37.50	0.00
TRINITY_D8TRS3_VOLCA/737-937	D8TRS3.1 PF02453.15	37.50	0.00
TRINITY_W7XFP4_TETTS/74-196	W7XFP4.1 PF01597.17	37.50	0.00
TRINITY_A0A0D2WWI6_CAPO3/463-780	A0A0D2WWI6.1 PF0070	37.50	0.00
TRINITY_U9U8A3_RHIID/119-187	U9U8A3.1 PF10601.7;	37.50	0.00
TRINITY_D3B3D2_POLPA/38-262	D3B3D2.1 PF07786.10	37.50	0.00
TRINITY_D8THB4_VOLCA/1-130	D8THB4.1 PF03364.18	37.50	0.00
TRINITY_A0EFC7_PARTE/160-290	A0EFC7.1 PF00782.18	37.50	0.00
TRINITY_D7LDM4_ARALL/9-117	D7LDM4.1 PF00255.17	37.50	0.00
TRINITY_K7TQJ3_MAIZE/33-636	K7TQJ3.1 PF00888.20	37.50	0.00
TRINITY_F4PTD9_DICFS/71-639	F4PTD9.1 PF01457.14	37.50	0.00
TRINITY_E1ZLU2_CHLVA/174-268	E1ZLU2.1 PF00564.22	37.50	0.00
TRINITY_B8P4X6_POSPM/8-248	B8P4X6.1 PF01370.19	37.50	0.00
TRINITY_I0YSS2_9CHLO/125-258	I0YSS2.1 PF01590.24	37.50	0.00
TRINITY_B3S6U8_TRIAD/16-404	B3S6U8.1 PF07690.14	37.50	0.00
TRINITY_A0A0D3FBQ7_9ORYZ/41-191	A0A0D3FBQ7.1 PF0411	37.50	0.00
TRINITY_A0A067BWY7_SAPPC/22-533	A0A067BWY7.1 PF0145	37.50	0.00
TRINITY_Q23ZH8_TETTS/79-176	Q23ZH8.1 PF14769.4;	37.50	0.00
TRINITY_D8U322_VOLCA/1-272	D8U322.1 PF01494.17	37.50	0.00

TRINITY_J9EJ94_9SPIT/12-113	J9EJ94.1 PF00168.28	37.50	0.00
TRINITY_LIPG_RAT/77-377	P04634.1 PF00561.18	37.50	0.00
TRINITY_I0YTX5_9CHLO/28-423	I0YTX5.1 PF00999.19	37.50	0.00
TRINITY_I7MGM3_TETTS/23-732	I7MGM3.2 PF00012.18	37.50	0.00
TRINITY_K7H5G4_CAEJA/381-536	K7H5G4.1 PF03712.13	37.50	0.00
TRINITY_Q22X36_TETTS/3-313	Q22X36.1 PF05462.9;	37.50	0.00
TRINITY_S2JB42_MUCC1/120-512	S2JB42.1 PF09423.8;	37.50	0.00
TRINITY_W7XDL4_TETTS/43-187	W7XDL4.1 PF04099.10	37.50	0.00
TRINITY_F4QDF0_DICFS/315-485	F4QDF0.1 PF09736.7;	37.50	0.00
TRINITY_I0YUA2_9CHLO/24-102	I0YUA2.1 PF07019.10	37.50	0.00
TRINITY_A0A072VJQ0_MEDTR/7-552	A0A072VJQ0.1 PF0560	37.50	0.00
TRINITY_A0A0K9NQX2_ZOSMR/67-408	A0A0K9NQX2.1 PF0002	37.50	0.00
TRINITY_K2C0P0_9BACT/168-244	K2C0P0.1 PF13517.4;	37.50	0.00
TRINITY_I7M6I9_TETTS/8-269	I7M6I9.2 PF14808.4;	37.50	0.00
TRINITY_D8LB49_ECTSI/1135-1295	D8LB49.1 PF00005.25	37.50	0.00
TRINITY_I0YLE6_9CHLO/727-871	I0YLE6.1 PF13515.4;	37.50	0.00
TRINITY_I7M3M5_TETTS/574-892	I7M3M5.1 PF11838.6;	37.40	0.00
TRINITY_I7MFP6_TETTS/20-150	I7MFP6.2 PF14886.4;	37.40	0.00
TRINITY_F4Q1M5_DICFS/126-588	F4Q1M5.1 PF04114.12	37.40	0.00
TRINITY_A0A017SB10_9EURO/268-395	A0A017SB10.1 PF0866	37.40	0.00
TRINITY_Q248F0_TETTS/19-502	Q248F0.2 PF04916.11	37.40	0.00
TRINITY_A0D7M8_PARTE/251-571	A0D7M8.1 PF04515.10	37.40	0.00
TRINITY_F0XWX9_AURAN/3-277	F0XWX9.1 PF00350.21	37.40	0.00
TRINITY_A0A077ZTY2_STYLE/13-164	A0A077ZTY2.1 PF0112	37.40	0.00
TRINITY_V9F6Q9_PHYPR/19-260	V9F6Q9.1 PF05875.10	37.40	0.00
TRINITY_D4JTC6_9FIRM/188-305	D4JTC6.1 PF00072.22	37.40	0.00
TRINITY_A0A067CQV9_SAPPC/27-209	A0A067CQV9.1 PF1029	37.40	0.00
TRINITY_A0A091DGN3_FUKDA/74-189	A0A091DGN3.1 PF0061	37.40	0.00
TRINITY_M7YJF8_TRIUA/295-535	M7YJF8.1 PF00069.23	37.40	0.00
TRINITY_F4Q9I7_DICFS/37-203	F4Q9I7.1 PF10294.7;	37.40	0.00
TRINITY_I7LX20_TETTS/11-287	I7LX20.2 PF00069.23	37.40	0.00
TRINITY_A9G0Q5_SORC5/53-346	A9G0Q5.1 PF14388.4;	37.40	0.00
TRINITY_A0A0F0H7L4_NOCAE/1-259	A0A0F0H7L4.1 PF0056	37.40	0.00
TRINITY_A0A077ZPS4_STYLE/243-438	A0A077ZPS4.1 PF0011	37.40	0.00
TRINITY_A0A087SH04_AUXPR/607-740	A0A087SH04.1 PF0078	37.40	0.00
TRINITY_D3B3V7_POLPA/4-288	D3B3V7.1 PF11945.6;	37.40	0.00
TRINITY_F0ZIE2_DICPU/38-392	F0ZIE2.1 PF10510.7;	37.40	0.00
TRINITY_A8HSJ3_CHLRE/66-324	A8HSJ3.1 PF00069.23	37.40	0.00
TRINITY_M7PL76_PNEMU/444-589	M7PL76.1 PF08646.8;	37.40	0.00
TRINITY_D8LW92_BLAHO/1-121	D8LW92.1 PF05477.9;	37.40	0.00
TRINITY_W1P048_AMBTC/29-693	W1P048.1 PF06419.9;	37.40	0.00
TRINITY_H3GTG5_PHYRM/57-476	H3GTG5.1 PF00450.20	37.40	0.00
TRINITY_A0A0D2QTE0_GOSRA/31-120	A0A0D2QTE0.1 PF0029	37.40	0.00
TRINITY_B2AE59_PODAN/97-307	B2AE59.1 PF00561.18	37.40	0.00
TRINITY_D8UJ57_VOLCA/338-496	D8UJ57.1 PF04937.13	37.40	0.00
TRINITY_D8SXV8_SELML/278-496	D8SXV8.1 PF12348.6;	37.40	0.00
TRINITY_Q55F12_DICDI/374-640	Q55F12.1 PF00149.26	37.40	0.00
TRINITY_A0A068XUL1_ECHMU/28-314	A0A068XUL1.1 PF0006	37.40	0.00
TRINITY_J9ISJ7_9SPIT/109-411	J9ISJ7.1 PF00648.19	37.40	0.00
TRINITY_I7M6D7_TETTS/137-346	I7M6D7.1 PF03798.14	37.40	0.00
TRINITY_F4Q3G0_DICFS/8-596	F4Q3G0.1 PF04916.11	37.40	0.00
TRINITY_Q3SEP6_PARTE/37-615	Q3SEP6.1 PF00995.21	37.40	0.00
TRINITY_W4GQ58_9STRA/105-353	W4GQ58.1 PF00120.22	37.40	0.00
TRINITY_E1Z600_CHLVA/438-727	E1Z600.1 PF01636.21	37.40	0.00
TRINITY_Q54SV5_DICDI/129-469	Q54SV5.2 PF03062.17	37.40	0.00
TRINITY_D8UG16_VOLCA/66-170	D8UG16.1 PF12680.5;	37.40	0.00
TRINITY_A8J7M9_CHLRE/742-1038	A8J7M9.1 PF00069.23	37.40	0.00
TRINITY_I7MEJ1_TETTS/13-399	I7MEJ1.1 PF07690.14	37.40	0.00
TRINITY_A0DI05_PARTE/319-612	A0DI05.1 PF04547.10	37.40	0.00
TRINITY_A8J0W8_CHLRE/37-466	A8J0W8.1 PF01490.16	37.40	0.00
TRINITY_A0A0G4ED92_9ALVE/328-436	A0A0G4ED92.1 PF0065	37.40	0.00

TRINITY_L8H163_ACACA/112-375	L8H163.1 PF00069.23	37.40	0.00
TRINITY_D8THA8_VOLCA/22-139	D8THA8.1 PF01152.19	37.40	0.00
TRINITY_F7FTV2_MONDO/265-614	F7FTV2.1 PF03124.12	37.40	0.00
TRINITY_F4QF50_DICFS/266-485	F4QF50.1 PF04935.10	37.40	0.00
TRINITY_W4YX46_STRPU/98-275	W4YX46.1 PF00644.18	37.40	0.00
TRINITY_A0BN06_PARTE/37-301	A0BN06.1 PF01715.15	37.40	0.00
TRINITY_K9RPD9_9CYAN/8-448	K9RPD9.1 PF00067.20	37.40	0.00
TRINITY_Q22AQ4_TETTS/550-689	Q22AQ4.1 PF01529.18	37.40	0.00
TRINITY_D8T6F9_SELML/113-250	D8T6F9.1 PF08652.9;	37.40	0.00
TRINITY_A0A0G3BJH5_9BURK/5692-5798	A0A0G3BJH5.1 PF1618	37.40	0.00
TRINITY_E3FU34_STIAD/40-322	E3FU34.1 PF01636.21	37.40	0.00
TRINITY_Q23YD6_TETTS/29-282	Q23YD6.1 PF00069.23	37.30	0.00
TRINITY_A0A078BB69_STYLE/58-324	A0A078BB69.1 PF0338	37.30	0.00
TRINITY_V4AT78_LOTGI/35-620	V4AT78.1 PF09739.7;	37.30	0.00
TRINITY_A4Z166_BRASO/3-129	A4Z166.1 PF01124.16	37.30	0.00
TRINITY_M0RKQ4_MUSAM/131-385	M0RKQ4.1 PF07714.15	37.30	0.00
TRINITY_Q54CM5_DICDI/15-438	Q54CM5.1 PF00083.22	37.30	0.00
TRINITY_L8GG36_ACACA/72-255	L8GG36.1 PF02145.13	37.30	0.00
TRINITY_F6RSI8_XENTR/103-443	F6RSI8.1 PF00176.21	37.30	0.00
TRINITY_G0R101_ICHMG/312-470	G0R101.1 PF02872.16	37.30	0.00
TRINITY_Q4YF61_PLABA/106-236	Q4YF61.1 PF08662.9;	37.30	0.00
TRINITY_A0A022QPU9_ERYGU/9-201	A0A022QPU9.1 PF1301	37.30	0.00
TRINITY_S2JQ56_MUCC1/127-289	S2JQ56.1 PF00650.18	37.30	0.00
TRINITY_A8X5X4_CAEBR/298-570	A8X5X4.2 PF00664.21	37.30	0.00

TRINITY_Q54CX3_DICDI/75-194	Q54CX3.1 PF02466.17	37.30	0.00
TRINITY_C0QRB5_PERMH/22-243	C0QRB5.1 PF00194.19	37.30	0.00
TRINITY_Q2J943_FRASC/218-410	Q2J943.1 PF06414.10	37.30	0.00
TRINITY_A0A0D2WGB0_CAPO3/21-228	A0A0D2WGB0.1 PF0681	37.30	0.00
TRINITY_A0A0P7XLZ5_9TELE/343-443	A0A0P7XLZ5.1 PF1615	37.30	0.00
TRINITY_A0CN16_PARTE/18-182	A0CN16.1 PF03357.19	37.30	0.00
TRINITY_A8IPH0_CHLRE/96-246	A8IPH0.1 PF02469.20	37.30	0.00
TRINITY_A8J4Z5_CHLRE/23-117	A8J4Z5.1 PF00686.17	37.30	0.00
TRINITY_I0Z5M5_9CHLO/93-249	I0Z5M5.1 PF00650.18	37.30	0.00
TRINITY_A8J5J8_CHLRE/194-755	A8J5J8.1 PF04130.11	37.30	0.00
TRINITY_A7RNL3_NEMVE/2777-2864	A7RNL3.1 PF00630.17	37.30	0.00
TRINITY_I7LX20_TETTS/11-287	I7LX20.2 PF00069.23	37.30	0.00
TRINITY_A0C424_PARTE/63-322	A0C424.1 PF09511.8;	37.30	0.00
TRINITY_V3ZHK1_LOTGI/146-320	V3ZHK1.1 PF08892.9;	37.30	0.00
TRINITY_G0QR98_ICHMG/111-321	G0QR98.1 PF00481.19	37.30	0.00
TRINITY_L8GW16_ACACA/17-115	L8GW16.1 PF12796.5;	37.30	0.00
TRINITY_G4VDA7_SCHMA/1969-2145	G4VDA7.1 PF02145.13	37.30	0.00
TRINITY_L1IXT3_GUITH/1-178	L1IXT3.1 PF16909.3;	37.30	0.00
TRINITY_B8A5M6_DANRE/403-508	B8A5M6.1 PF00085.18	37.30	0.00
TRINITY_I7MDF9_TETTS/292-610	I7MDF9.1 PF04515.10	37.30	0.00
TRINITY_X6NRX3_RETFI/364-465	X6NRX3.1 PF00085.18	37.30	0.00
TRINITY_A8J465_CHLRE/78-330	A8J465.1 PF09353.8;	37.30	0.00
TRINITY_T0QA30_9STRA/1198-1298	T0QA30.1 PF00637.18	37.30	0.00
TRINITY_A0A067R0B3_ZOONE/86-346	A0A067R0B3.1 PF0006	37.30	0.00
TRINITY_A0CYR0_PARTE/338-447	A0CYR0.1 PF03015.17	37.30	0.00
TRINITY_A0A0J7MWC3_LASNI/91-250	A0A0J7MWC3.1 PF1335	37.30	0.00
TRINITY_J9F4Y5_9SPIT/78-412	J9F4Y5.1 PF01237.16	37.30	0.00
TRINITY_F4NSG0_BATDJ/183-312	F4NSG0.1 PF03188.14	37.30	0.00
TRINITY_F4PJQ6_DICFS/104-474	F4PJQ6.1 PF07690.14	37.30	0.00
TRINITY_D8TP08_VOLCA/394-549	D8TP08.1 PF05843.12	37.30	0.00
TRINITY_D2V884_NAEGR/23-411	D2V884.1 PF02274.15	37.30	0.00
TRINITY_A8HPD4_CHLRE/451-711	A8HPD4.1 PF00520.29	37.30	0.00
TRINITY_A0DXA9_PARTE/13-322	A0DXA9.1 PF03820.15	37.30	0.00
TRINITY_L8HIE7_ACACA/13-192	L8HIE7.1 PF00929.22	37.20	0.00
TRINITY_H3A991_LATCH/268-561	H3A991.1 PF01490.16	37.20	0.00
TRINITY_Q22D95_TETTS/115-205	Q22D95.2 PF00153.25	37.20	0.00
TRINITY_L8HCK5_ACACA/530-801	L8HCK5.1 PF07714.15	37.20	0.00
TRINITY_L8HGY6_ACACA/4-352	L8HGY6.1 PF11397.6;	37.20	0.00
TRINITY_G0QLI8_ICHMG/177-424	G0QLI8.1 PF08367.9;	37.20	0.00
TRINITY_I7MEI2_TETTS/79-268	I7MEI2.2 PF01145.23	37.20	0.00
TRINITY_A0A0A7PE86_9SPHN/62-197	A0A0A7PE86.1 PF0246	37.20	0.00
TRINITY_A7S3C1_NEMVE/118-465	A7S3C1.1 PF14782.4;	37.20	0.00
TRINITY_J9IDJ2_9SPIT/37-276	J9IDJ2.1 PF00194.19	37.20	0.00
TRINITY_A9G0Q5_SORC5/53-346	A9G0Q5.1 PF14388.4;	37.20	0.00
TRINITY_I7M0U2_TETTS/23-1098	I7M0U2.2 PF10266.7;	37.20	0.00
TRINITY_R7EQV0_9BACE/30-279	R7EQV0.1 PF03372.21	37.20	0.00
TRINITY_C1E237_MICSR/66-285	C1E237.1 PF16690.3;	37.20	0.00
TRINITY_B0WXE5_CULQU/211-324	B0WXE5.1 PF00373.16	37.20	0.00
TRINITY_G8BSZ9_TETPH/231-743	G8BSZ9.1 PF03935.13	37.20	0.00
TRINITY_Q54E05_DICDI/198-431	Q54E05.1 PF06027.10	37.20	0.00
TRINITY_A0A0F2NFD9_9DELT/33-203	A0A0F2NFD9.1 PF0018	37.20	0.00
TRINITY_Q22X60_TETTS/11-285	Q22X60.1 PF00069.23	37.20	0.00
TRINITY_Q23QF2_TETTS/248-571	Q23QF2.2 PF03133.13	37.20	0.00
TRINITY_A0A0B1P8K8_UNCNE/869-982	A0A0B1P8K8.1 PF0078	37.20	0.00
TRINITY_Q3SEP6_PARTE/37-615	Q3SEP6.1 PF00995.21	37.20	0.00
TRINITY_H3DYY8_PRIPA/312-477	H3DYY8.1 PF04851.13	37.20	0.00
TRINITY_V7BC73_PHAVU/227-421	V7BC73.1 PF01416.18	37.20	0.00
TRINITY_A0A0D2WGB0_CAPO3/21-228	A0A0D2WGB0.1 PF0681	37.20	0.00
TRINITY_A0A078ABB5_STYLE/6-311	A0A078ABB5.1 PF0799	37.20	0.00
TRINITY_A0C424_PARTE/63-322	A0C424.1 PF09511.8;	37.20	0.00
TRINITY_A0A0G4EHB4_9ALVE/748-1297	A0A0G4EHB4.1 PF0273	37.20	0.00

TRINITY_A9TCF0_PHYPA/176-670	A9TCF0.1 PF02696.12	37.20	0.00
TRINITY_I0Z064_9CHLO/340-703	I0Z064.1 PF10168.7;	37.20	0.00
TRINITY_A0CMN6_PARTE/121-399	A0CMN6.1 PF01457.14	37.20	0.00
TRINITY_A0A075LPQ6_9BACI/7-106	A0A075LPQ6.1 PF0141	37.20	0.00
TRINITY_D8UKQ9_VOLCA/248-630	D8UKQ9.1 PF00150.16	37.20	0.00
TRINITY_L8GV23_ACACA/25-354	L8GV23.1 PF03265.13	37.20	0.00
TRINITY_A0A0J7KGR9_LASNI/8-199	A0A0J7KGR9.1 PF0007	37.20	0.00
TRINITY_L8H5E2_ACACA/175-397	L8H5E2.1 PF10469.7;	37.20	0.00
TRINITY_H2SI60_TAKRU/31-123	H2SI60.1 PF06337.10	37.20	0.00
TRINITY_E1ZTK5_CHLVA/222-684	E1ZTK5.1 PF05292.9;	37.20	0.00
TRINITY_L8GTK1_ACACA/146-395	L8GTK1.1 PF07714.15	37.20	0.00
TRINITY_D8TU13_VOLCA/649-861	D8TU13.1 PF04192.10	37.20	0.00
TRINITY_W7X5Q7_TETTS/17-155	W7X5Q7.1 PF00520.29	37.20	0.00
TRINITY_B8CG90_THAPS/533-811	B8CG90.1 PF00664.21	37.20	0.00
TRINITY_U1GKZ4_ENDPU/432-474	U1GKZ4.1 PF13639.4;	37.20	0.00
TRINITY_W7WZB3_TETTS/5-151	W7WZB3.1 PF07933.12	37.20	0.00
TRINITY_A0D670_PARTE/33-111	A0D670.1 PF07019.10	37.20	0.00
TRINITY_G0QPH0_ICHMG/1-390	G0QPH0.1 PF03917.15	37.10	0.00
TRINITY_Q4G2D6_TRIVA/11-172	Q4G2D6.1 PF00071.20	37.10	0.00
TRINITY_Q5KJW8_CRYNJ/25-95	Q5KJW8.1 PF00076.20	37.10	0.00
TRINITY_W7ANE2_PLAVN/103-241	W7ANE2.1 PF01529.18	37.10	0.00
TRINITY_I7MIN0_TETTS/9-308	I7MIN0.1 PF08449.9;	37.10	0.00
TRINITY_A0A0M8VC58_9ACTN/4-209	A0A0M8VC58.1 PF1341	37.10	0.00
TRINITY_I7LV11_TETTS/352-566	I7LV11.1 PF00149.26	37.10	0.00
TRINITY_W9SM68_9ROSA/116-324	W9SM68.1 PF00566.16	37.10	0.00
TRINITY_N6TSD4_DENPD/146-208	N6TSD4.1 PF10180.7;	37.10	0.00
TRINITY_L8GKH0_ACACA/249-578	L8GKH0.1 PF13598.4;	37.10	0.00
TRINITY_D8S383_SELML/101-256	D8S383.1 PF02270.13	37.10	0.00
TRINITY_A0A0F6W6A6_9DELT/19-223	A0A0F6W6A6.1 PF0501	37.10	0.00
TRINITY_A0EB86_PARTE/167-498	A0EB86.1 PF03133.13	37.10	0.00
TRINITY_L8H1E1_ACACA/5-221	L8H1E1.1 PF01370.19	37.10	0.00
TRINITY_J9EG13_9SPIT/584-905	J9EG13.1 PF05192.16	37.10	0.00
TRINITY_A0CF81_PARTE/292-769	A0CF81.1 PF00443.27	37.10	0.00
TRINITY_S2K416_MUCC1/38-400	S2K416.1 PF00155.19	37.10	0.00
TRINITY_D2W2M6_NAEGR/67-466	D2W2M6.1 PF00450.20	37.10	0.00
TRINITY_A8HTX4_CHLRE/6-135	A8HTX4.1 PF04446.10	37.10	0.00
TRINITY_K1L3B5_9BACT/13-214	K1L3B5.1 PF01596.15	37.10	0.00
TRINITY_D6TFG4_9CHLR/9-160	D6TFG4.1 PF04851.13	37.10	0.00
TRINITY_W7WZC0_TETTS/479-551	W7WZC0.1 PF15016.4;	37.10	0.00
TRINITY_D3BEU5_POLPA/43-608	D3BEU5.1 PF14745.4;	37.10	0.00
TRINITY_T1FN62_HELRO/6-132	T1FN62.1 PF08662.9;	37.10	0.00
TRINITY_C1N2Y5_MICPC/184-742	C1N2Y5.1 PF10433.7;	37.10	0.00
TRINITY_K9WF91_9CYAN/6-199	K9WF91.1 PF00106.23	37.10	0.00
TRINITY_L8H0B7_ACACA/3-150	L8H0B7.1 PF00293.26	37.10	0.00
TRINITY_B8CD21_THAPS/43-201	B8CD21.1 PF01434.16	37.10	0.00
TRINITY_A8JGR9_CHLRE/78-301	A8JGR9.1 PF07859.11	37.10	0.00
TRINITY_J9ICF8_9SPIT/1-131	J9ICF8.1 PF00582.24	37.10	0.00
TRINITY_Q7QIH6_ANOGA/17-126	Q7QIH6.3 PF00651.29	37.10	0.00
TRINITY_F0Z6A8_DICPU/419-855	F0Z6A8.1 PF14648.4;	37.10	0.00
TRINITY_H3GM81_PHYRM/324-430	H3GM81.1 PF00651.29	37.10	0.00
TRINITY_Q81HV9_BACCR/16-265	Q81HV9.1 PF01168.18	37.10	0.00
TRINITY_F4Q584_DICFS/157-485	F4Q584.1 PF03372.21	37.10	0.00
TRINITY_V2YMJ2_MONRO/325-569	V2YMJ2.1 PF13086.4;	37.10	0.00
TRINITY_L8HHX4_ACACA/703-1129	L8HHX4.1 PF00476.18	37.10	0.00
TRINITY_X6NZG4_RETFI/21-287	X6NZG4.1 PF01504.16	37.10	0.00
TRINITY_F6SY88_MONDO/3-177	F6SY88.1 PF00025.19	37.10	0.00
TRINITY_A8IF44_CHLRE/5-261	A8IF44.1 PF16092.3;	37.10	0.00
TRINITY_D8U9S9_VOLCA/599-793	D8U9S9.1 PF00211.18	37.10	0.00
TRINITY_A0A0J7K6W1_LASNI/485-603	A0A0J7K6W1.1 PF0066	37.10	0.00
TRINITY_D8UIZ7_VOLCA/369-849	D8UIZ7.1 PF00759.17	37.10	0.00
TRINITY_I0Z879_9CHLO/15-480	I0Z879.1 PF03155.13	37.10	0.00

TRINITY_A8HPE7_CHLRE/242-337	A8HPE7.1 PF13242.4;	37.10	0.00
TRINITY_A0A074TTD6_HAMHA/22-118	A0A074TTD6.1 PF0364	37.10	0.00
TRINITY_D2VP40_NAEGR/75-303	D2VP40.1 PF07065.12	37.00	0.00
TRINITY_A0A0M0JZK3_9EUKA/232-431	A0A0M0JZK3.1 PF1385	37.00	0.00
TRINITY_C5DH94_LACTC/97-183	C5DH94.1 PF12796.5;	37.00	0.00
TRINITY_G0R1G3_ICHMG/80-301	G0R1G3.1 PF00566.16	37.00	0.00
TRINITY_D7TLT1_VITVI/4-110	D7TLT1.1 PF00168.28	37.00	0.00
TRINITY_C1E5U8_MICSR/55-254	C1E5U8.1 PF13360.4;	37.00	0.00
TRINITY_A7RJR6_NEMVE/10-191	A7RJR6.1 PF13472.4;	37.00	0.00
TRINITY_I0YPY0_9CHLO/587-636	I0YPY0.1 PF00628.27	37.00	0.00
TRINITY_A0DLI9_PARTE/13-277	A0DLI9.1 PF00069.23	37.00	0.00
TRINITY_H3GGZ2_PHYRM/50-226	H3GGZ2.1 PF01569.19	37.00	0.00
TRINITY_G4VRU3_SCHMA/82-202	G4VRU3.1 PF00622.26	37.00	0.00
TRINITY_A4VDT5_TETTS/546-675	A4VDT5.2 PF04499.13	37.00	0.00
TRINITY_Q23RB4_TETTS/70-227	Q23RB4.2 PF01343.16	37.00	0.00
TRINITY_F4WZI8_ACREC/22-270	F4WZI8.1 PF07714.15	37.00	0.00
TRINITY_I7M2X3_TETTS/28-255	I7M2X3.1 PF00149.26	37.00	0.00
TRINITY_Q7RIF5_PLAYO/7-169	Q7RIF5.1 PF00160.19	37.00	0.00
TRINITY_A0A078AF49_STYLE/79-487	A0A078AF49.1 PF1334	37.00	0.00
TRINITY_Q237H9_TETTS/734-967	Q237H9.2 PF12698.5;	37.00	0.00
TRINITY_K7E1N3_MONDO/159-270	K7E1N3.1 PF00651.29	37.00	0.00
TRINITY_D8TTP0_VOLCA/1124-1422	D8TTP0.1 PF00069.23	37.00	0.00
TRINITY_A0A0D1DY50_USTMA/875-1030	A0A0D1DY50.1 PF1271	37.00	0.00
TRINITY_B2IDJ8_BEII9/72-226	B2IDJ8.1 PF10067.7;	37.00	0.00
TRINITY_L8H973_ACACA/26-179	L8H973.1 PF00795.20	37.00	0.00
TRINITY_S8ALH8_DACHA/280-587	S8ALH8.1 PF09423.8;	37.00	0.00
TRINITY_A8HNZ5_CHLRE/73-227	A8HNZ5.1 PF00177.19	37.00	0.00
TRINITY_D8TZI2_VOLCA/63-251	D8TZI2.1 PF01025.17	37.00	0.00
TRINITY_I1GGB4_AMPQE/46-226	I1GGB4.1 PF00092.26	37.00	0.00
TRINITY_A0A0N5B837_STREA/222-429	A0A0N5B837.1 PF0866	37.00	0.00
TRINITY_A0A0D2WK61_CAPO3/512-847	A0A0D2WK61.1 PF0312	37.00	0.00
TRINITY_A0A090LXH8_OSTTA/514-699	A0A090LXH8.1 PF1029	37.00	0.00
TRINITY_L1JGQ3_GUITH/3-193	L1JGQ3.1 PF13419.4;	37.00	0.00
TRINITY_S8EE57_FOMPI/1-174	S8EE57.1 PF05620.9;	37.00	0.00
TRINITY_A0A0J8D429_BETVU/420-1005	A0A0J8D429.1 PF0518	37.00	0.00
TRINITY_R6PMU9_9FIRM/4-154	R6PMU9.1 PF12847.5;	37.00	0.00
TRINITY_D8TTP0_VOLCA/1124-1422	D8TTP0.1 PF00069.23	37.00	0.00
TRINITY_A0A0G4G478_9ALVE/26-579	A0A0G4G478.1 PF0160	37.00	0.00
TRINITY_A8J4Y5_CHLRE/1-425	A8J4Y5.1 PF00450.20	37.00	0.00
TRINITY_A8IEX1_CHLRE/352-562	A8IEX1.1 PF01061.22	37.00	0.00
TRINITY_D8TTP0_VOLCA/1124-1422	D8TTP0.1 PF00069.23	37.00	0.00
TRINITY_D8TZU5_VOLCA/290-568	D8TZU5.1 PF12018.6;	37.00	0.00
TRINITY_A7H7K4_ANADF/264-412	A7H7K4.1 PF00201.16	37.00	0.00
TRINITY_A0A067NVA7_PLEOS/32-317	A0A067NVA7.1 PF0006	37.00	0.00
TRINITY_U2E9J7_9BACT/28-286	U2E9J7.1 PF00561.18	36.90	0.00
TRINITY_F0YGV4_AURAN/12-147	F0YGV4.1 PF00892.18	36.90	0.00
TRINITY_F4PTN1_DICFS/42-388	F4PTN1.1 PF07690.14	36.90	0.00
TRINITY_Q22PK5_TETTS/101-587	Q22PK5.1 PF01425.19	36.90	0.00
TRINITY_D8M5A1_BLAHO/1-212	D8M5A1.1 PF00149.26	36.90	0.00
TRINITY_B8HYX6_CYAP4/26-200	B8HYX6.1 PF13532.4;	36.90	0.00
TRINITY_F4Q1S9_DICFS/33-286	F4Q1S9.1 PF00561.18	36.90	0.00
TRINITY_L8H1C1_ACACA/491-555	L8H1C1.1 PF13432.4;	36.90	0.00
TRINITY_G0QLJ6_ICHMG/4-189	G0QLJ6.1 PF00113.20	36.90	0.00
TRINITY_L8H4K4_ACACA/104-337	L8H4K4.1 PF04445.11	36.90	0.00
TRINITY_F2UAW3_SALR5/119-262	F2UAW3.1 PF03981.10	36.90	0.00
TRINITY_D3HQ64_LEGLN/458-569	D3HQ64.1 PF00072.22	36.90	0.00
TRINITY_I4YMJ8_9RHIZ/5-185	I4YMJ8.1 PF00300.20	36.90	0.00
TRINITY_D8UG85_VOLCA/40-517	D8UG85.1 PF01532.18	36.90	0.00
TRINITY_A0A078AR51_STYLE/8-287	A0A078AR51.1 PF0006	36.90	0.00
TRINITY_A0A087STF8_AUXPR/1-249	A0A087STF8.1 PF0627	36.90	0.00
TRINITY_Q22PH2_TETTS/13-431	Q22PH2.2 PF01137.19	36.90	0.00

TRINITY_F2U800_SALR5/185-462	F2U800.1 PF01602.18	36.90	0.00
TRINITY_C1MVU4_MICPC/988-1289	C1MVU4.1 PF11262.6;	36.90	0.00
TRINITY_A0A0G4IGI5_PLABS/360-474	A0A0G4IGI5.1 PF0338	36.90	0.00
TRINITY_Q236M1_TETTS/41-327	Q236M1.2 PF01025.17	36.90	0.00
TRINITY_SEY11_PARTE/49-739	A0BKG2.1 PF05879.10	36.90	0.00
TRINITY_A8J2X9_CHLRE/125-257	A8J2X9.1 PF00504.19	36.90	0.00
TRINITY_K7JBH5_NASVI/607-815	K7JBH5.1 PF07727.12	36.90	0.00
TRINITY_A0A0A0LDM0_CUCSA/81-222	A0A0A0LDM0.1 PF0159	36.90	0.00
TRINITY_I1FGX7_AMPQE/637-816	I1FGX7.1 PF00644.18	36.90	0.00
TRINITY_A0A0J8FSK4_BETVU/3618-4509	A0A0J8FSK4.1 PF1376	36.90	0.00
TRINITY_A0CCS6_PARTE/5-130	A0CCS6.1 PF01778.15	36.90	0.00
TRINITY_S3D856_OPHP1/4-177	S3D856.1 PF00025.19	36.90	0.00
TRINITY_I0Z118_9CHLO/375-636	I0Z118.1 PF06209.11	36.90	0.00
TRINITY_X6PDX4_RETFI/1195-1371	X6PDX4.1 PF16909.3;	36.90	0.00
TRINITY_S2J1H8_MUCC1/361-991	S2J1H8.1 PF10408.7;	36.90	0.00
TRINITY_Q2JKG0_SYNJB/9-302	Q2JKG0.1 PF00266.17	36.90	0.00
TRINITY_A0A0G4GLJ7_9ALVE/54-165	A0A0G4GLJ7.1 PF0058	36.90	0.00
TRINITY_A0A087SA60_AUXPR/19-283	A0A087SA60.1 PF0006	36.90	0.00
TRINITY_E1Z399_CHLVA/8-173	E1Z399.1 PF11744.6;	36.90	0.00
TRINITY_B3SCI9_TRIAD/108-377	B3SCI9.1 PF00664.21	36.90	0.00
TRINITY_G4V9L8_SCHMA/203-365	G4V9L8.1 PF04004.11	36.90	0.00
TRINITY_D5X9L3_THEPJ/23-309	D5X9L3.1 PF02541.14	36.90	0.00
TRINITY_A0A096LSW9_POEFO/58-245	A0A096LSW9.1 PF0080	36.90	0.00
TRINITY_A0BGJ6_PARTE/13-125	A0BGJ6.1 PF01920.18	36.90	0.00
TRINITY_E1ZPU4_CHLVA/5-453	E1ZPU4.1 PF05327.9;	36.90	0.00
TRINITY_G6FZL1_9CYAN/27-497	G6FZL1.1 PF03055.13	36.90	0.00
TRINITY_G7IA71_MEDTR/108-554	G7IA71.1 PF02133.13	36.80	0.00
TRINITY_A0A0M9GA61_9TRYP/46-331	A0A0M9GA61.1 PF0006	36.80	0.00
TRINITY_I4AKQ4_FLELS/53-342	I4AKQ4.1 PF14388.4;	36.80	0.00
TRINITY_Q244Z6_TETTS/77-284	Q244Z6.1 PF01027.18	36.80	0.00
TRINITY_A0A067EW61_CITSI/22-161	A0A067EW61.1 PF0000	36.80	0.00
TRINITY_A0CY07_PARTE/21-127	A0CY07.1 PF09799.7;	36.80	0.00
TRINITY_W7XDU4_TETTS/448-616	W7XDU4.1 PF01699.22	36.80	0.00
TRINITY_M4ZZ09_9ACTN/56-294	M4ZZ09.1 PF14378.4;	36.80	0.00
TRINITY_A0EA64_PARTE/1-184	A0EA64.1 PF00069.23	36.80	0.00
TRINITY_H2XTA5_CIOIN/343-503	H2XTA5.1 PF03712.13	36.80	0.00
TRINITY_K1XZE6_MARBU/307-454	K1XZE6.1 PF03031.16	36.80	0.00
TRINITY_Q22Z31_TETTS/156-426	Q22Z31.4 PF00520.29	36.80	0.00
TRINITY_A0C7V9_PARTE/171-347	A0C7V9.1 PF06911.10	36.80	0.00
TRINITY_A9TZU1_PHYPA/16-235	A9TZU1.1 PF00687.19	36.80	0.00
TRINITY_K2EBL4_9BACT/19-146	K2EBL4.1 PF03099.17	36.80	0.00
TRINITY_L8GYH6_ACACA/1-275	L8GYH6.1 PF03159.16	36.80	0.00
TRINITY_A0A0L7L536_9NEOP/436-516	A0A0L7L536.1 PF0183	36.80	0.00
TRINITY_G3NPH7_GASAC/10-123	G3NPH7.1 PF10256.7;	36.80	0.00
TRINITY_I1BZM4_RHIO9/2-295	I1BZM4.1 PF07992.12	36.80	0.00
TRINITY_W9WL24_9EURO/493-585	W9WL24.1 PF12796.5;	36.80	0.00
TRINITY_A0A068XUV2_HYMMI/126-436	A0A068XUV2.1 PF0426	36.80	0.00
TRINITY_D8TN28_VOLCA/673-769	D8TN28.1 PF07885.14	36.80	0.00
TRINITY_A0BLH9_PARTE/7-108	A0BLH9.1 PF00168.28	36.80	0.00
TRINITY_A0BLH9_PARTE/7-108	A0BLH9.1 PF00168.28	36.80	0.00
TRINITY_A0A075AYI6_9FUNG/177-491	A0A075AYI6.1 PF0154	36.80	0.00
TRINITY_K9SCW4_9CYAN/247-433	K9SCW4.1 PF02163.20	36.80	0.00
TRINITY_D2W423_NAEGR/65-239	D2W423.1 PF01273.23	36.80	0.00
TRINITY_I7M7F9_TETTS/162-552	I7M7F9.1 PF01619.16	36.80	0.00
TRINITY_I3KIP7_ORENI/454-610	I3KIP7.1 PF05572.11	36.80	0.00
TRINITY_F0YC13_AURAN/81-304	F0YC13.1 PF08450.10	36.80	0.00
TRINITY_A8JDA0_CHLRE/443-662	A8JDA0.1 PF00481.19	36.80	0.00
TRINITY_A0DDF0_PARTE/853-1143	A0DDF0.1 PF00689.19	36.80	0.00
TRINITY_X1X2A4_ACYPI/26-255	X1X2A4.1 PF12697.5;	36.80	0.00
TRINITY_A8J9Y6_CHLRE/15-130	A8J9Y6.1 PF00787.22	36.80	0.00
TRINITY_L8GKH1_ACACA/97-304	L8GKH1.1 PF00856.26	36.80	0.00

TRINITY_H8KM12_SOLCM/12-444	H8KM12.1 PF01593.22	36.80	0.00
TRINITY_I1H6T4_BRADI/588-973	I1H6T4.1 PF00145.15	36.80	0.00
TRINITY_K9Y0F8_STAC7/144-341	K9Y0F8.1 PF09414.8;	36.80	0.00
TRINITY_G0R4M5_ICHMG/51-307	G0R4M5.1 PF00481.19	36.80	0.00
TRINITY_A0A067K1E4_JATCU/180-331	A0A067K1E4.1 PF0493	36.80	0.00
TRINITY_A0A087SBA3_AUXPR/263-464	A0A087SBA3.1 PF0407	36.80	0.00
TRINITY_E1Z8M8_CHLVA/12-352	E1Z8M8.1 PF04177.10	36.80	0.00
TRINITY_A0A0D9VLX2_9ORYZ/403-572	A0A0D9VLX2.1 PF0176	36.80	0.00
TRINITY_A8J0I1_CHLRE/188-606	A8J0I1.1 PF00067.20	36.80	0.00
TRINITY_D8TW39_VOLCA/7-197	D8TW39.1 PF05958.9;	36.80	0.00
TRINITY_L1IL48_GUITH/13-186	L1IL48.1 PF05706.10	36.80	0.00
TRINITY_J9J5M4_9SPIT/47-304	J9J5M4.1 PF00069.23	36.80	0.00
TRINITY_A5FFM2_FLAJ1/36-211	A5FFM2.1 PF13365.4;	36.80	0.00
TRINITY_E1Z251_CHLVA/143-289	E1Z251.1 PF04072.12	36.80	0.00
TRINITY_I7MGW2_TETTS/6-163	I7MGW2.1 PF00106.23	36.80	0.00
TRINITY_A8I3B9_CHLRE/91-352	A8I3B9.1 PF07714.15	36.80	0.00
TRINITY_E7EYU7_DANRE/215-381	E7EYU7.2 PF13359.4;	36.80	0.00
TRINITY_A8JAT8_CHLRE/131-260	A8JAT8.1 PF06463.11	36.80	0.00
TRINITY_D8UJ89_VOLCA/98-1079	D8UJ89.1 PF04931.11	36.80	0.00
TRINITY_C5X8G4_SORBI/415-543	C5X8G4.1 PF02170.20	36.80	0.00
TRINITY_A0A015IWR6_9GLOM/146-259	A0A015IWR6.1 PF1351	36.80	0.00
TRINITY_D8UGJ8_VOLCA/13-286	D8UGJ8.1 PF00069.23	36.80	0.00
TRINITY_Q248E1_TETTS/241-551	Q248E1.1 PF03133.13	36.80	0.00
TRINITY_Q23KE2_TETTS/302-431	Q23KE2.2 PF07534.14	36.80	0.00
TRINITY_W4K3K9_9HOMO/77-277	W4K3K9.1 PF00566.16	36.70	0.00
TRINITY_A0A0A1MXW2_9FUNG/2-121	A0A0A1MXW2.1 PF0156	36.70	0.00
TRINITY_A0A0D2X579_CAPO3/862-1042	A0A0D2X579.1 PF0061	36.70	0.00
TRINITY_I7LZR7_TETTS/58-421	I7LZR7.1 PF03062.17	36.70	0.00
TRINITY_I0HJJ9_ACTM4/22-399	I0HJJ9.1 PF00144.22	36.70	0.00
TRINITY_B3RIS0_TRIAD/58-253	B3RIS0.1 PF00804.23	36.70	0.00
TRINITY_U3I342_ANAPL/203-382	U3I342.1 PF05193.19	36.70	0.00
TRINITY_L8H823_ACACA/184-369	L8H823.1 PF07065.12	36.70	0.00
TRINITY_A0DXK0_PARTE/649-996	A0DXK0.1 PF14744.4;	36.70	0.00
TRINITY_D3B4A4_POLPA/7-179	D3B4A4.1 PF05255.9;	36.70	0.00
TRINITY_D8UJ89_VOLCA/98-1079	D8UJ89.1 PF04931.11	36.70	0.00
TRINITY_A0DLQ7_PARTE/160-514	A0DLQ7.1 PF03133.13	36.70	0.00
TRINITY_I7MDP9_TETTS/397-645	I7MDP9.2 PF00520.29	36.70	0.00
TRINITY_L8H9P5_ACACA/1162-1679	L8H9P5.1 PF06920.11	36.70	0.00
TRINITY_B9XJ07_PEDPL/9-283	B9XJ07.1 PF00749.19	36.70	0.00
TRINITY_A0A0D3DME0_BRAOL/74-311	A0A0D3DME0.1 PF1214	36.70	0.00
TRINITY_S2IYB4_MUCC1/3-369	S2IYB4.1 PF04857.18	36.70	0.00
TRINITY_J9J9X0_9SPIT/234-491	J9J9X0.1 PF00069.23	36.70	0.00
TRINITY_G0R5F7_ICHMG/6-279	G0R5F7.1 PF01457.14	36.70	0.00
TRINITY_D3B6M3_POLPA/229-533	D3B6M3.1 PF00082.20	36.70	0.00
TRINITY_A0A078A6W1_STYLE/181-518	A0A078A6W1.1 PF0072	36.70	0.00
TRINITY_A0EAD7_PARTE/23-277	A0EAD7.1 PF00956.16	36.70	0.00
TRINITY_A8J7M9_CHLRE/742-1038	A8J7M9.1 PF00069.23	36.70	0.00
TRINITY_L8H9P3_ACACA/258-435	L8H9P3.1 PF02145.13	36.70	0.00
TRINITY_A7SGT0_NEMVE/9-138	A7SGT0.1 PF10175.7;	36.70	0.00
TRINITY_A8IR48_CHLRE/1154-1335	A8IR48.1 PF00069.23	36.70	0.00
TRINITY_F6Z5D5_XENTR/729-1003	F6Z5D5.1 PF00664.21	36.70	0.00
TRINITY_Q241A5_TETTS/15-618	Q241A5.1 PF04006.10	36.70	0.00
TRINITY_B1LWC8_METRJ/35-279	B1LWC8.1 PF00561.18	36.70	0.00
TRINITY_D8UCR5_VOLCA/106-316	D8UCR5.1 PF07393.9;	36.70	0.00
TRINITY_E1Z243_CHLVA/12-318	E1Z243.1 PF05625.9;	36.70	0.00
TRINITY_A0A024TE61_9STRA/302-568	A0A024TE61.1 PF0771	36.70	0.00
TRINITY_G0QJG8_ICHMG/371-552	G0QJG8.1 PF01756.17	36.70	0.00
TRINITY_A0A0M0J6D3_9EUKA/396-638	A0A0M0J6D3.1 PF0511	36.70	0.00
TRINITY_W5N0L4_LEPOC/250-397	W5N0L4.1 PF02735.14	36.70	0.00
TRINITY_B8BR90_THAPS/164-450	B8BR90.1 PF09423.8;	36.70	0.00
TRINITY_A8J3K1_CHLRE/1842-2127	A8J3K1.1 PF07714.15	36.70	0.00

TRINITY_A0A078AWY2_STYLE/4041-4144	A0A078AWY2.1 PF0761	36.70	0.00
TRINITY_G0QJM3_ICHMG/11-497	G0QJM3.1 PF01425.19	36.70	0.00
TRINITY_A0A015I1J8_9GLOM/321-462	A0A015I1J8.1 PF0753	36.70	0.00
TRINITY_D8R8Q7_SELML/359-695	D8R8Q7.1 PF04869.12	36.70	0.00
TRINITY_B4J5T1_DROGR/70-196	B4J5T1.1 PF00043.23	36.60	0.00
TRINITY_A0CZW4_PARTE/34-236	A0CZW4.1 PF07162.9;	36.60	0.00
TRINITY_Q23EX0_TETTS/35-292	Q23EX0.1 PF00069.23	36.60	0.00
TRINITY_A0A087ZP88_APIME/3-509	A0A087ZP88.1 PF0560	36.60	0.00
TRINITY_Q23RV5_TETTS/815-1053	Q23RV5.2 PF10474.7;	36.60	0.00
TRINITY_A0A0F8BVN1_CERFI/139-380	A0A0F8BVN1.1 PF1690	36.60	0.00
TRINITY_F4P898_BATDJ/374-522	F4P898.1 PF08030.10	36.60	0.00
TRINITY_A4RRF2_OSTLU/865-1000	A4RRF2.1 PF04096.12	36.60	0.00
TRINITY_L8HCK7_ACACA/3-298	L8HCK7.1 PF04733.12	36.60	0.00
TRINITY_F4PJT8_DICFS/4-196	F4PJT8.1 PF08626.9;	36.60	0.00
TRINITY_I7MB89_TETTS/43-816	I7MB89.2 PF01804.16	36.60	0.00
TRINITY_I7LV11_TETTS/352-566	I7LV11.1 PF00149.26	36.60	0.00
TRINITY_A9G0Q5_SORC5/53-346	A9G0Q5.1 PF14388.4;	36.60	0.00
TRINITY_GSTT2_MOUSE/5-86	Q61133.4 PF13417.4;	36.60	0.00
TRINITY_G6DGH6_DANPL/73-234	G6DGH6.1 PF04727.11	36.60	0.00
TRINITY_A0A0D3C7G0_BRAOL/99-334	A0A0D3C7G0.1 PF0584	36.60	0.00
TRINITY_W1NQP2_AMBTC/158-836	W1NQP2.1 PF05664.9;	36.60	0.00
TRINITY_D8UEC7_VOLCA/2-394	D8UEC7.1 PF03348.13	36.60	0.00
TRINITY_Q22WR6_TETTS/11-159	Q22WR6.2 PF14825.4;	36.60	0.00
TRINITY_A9T695_PHYPA/214-281	A9T695.1 PF14295.4;	36.60	0.00
TRINITY_K3YRE9_SETIT/74-164	K3YRE9.1 PF02225.20	36.60	0.00
TRINITY_F1A057_DICPU/303-489	F1A057.1 PF01331.17	36.60	0.00
TRINITY_AFG2_SCHPO/316-446	O60058.1 PF00004.27	36.60	0.00
TRINITY_D2S257_HALTV/3-143	D2S257.1 PF02837.16	36.60	0.00
TRINITY_A0DCM6_PARTE/47-264	A0DCM6.1 PF01633.18	36.60	0.00
TRINITY_A1S2J4_SHEAM/7-204	A1S2J4.1 PF00106.23	36.60	0.00
TRINITY_W7X474_TETTS/3-234	W7X474.1 PF01876.14	36.60	0.00
TRINITY_A8JF90_CHLRE/27-338	A8JF90.1 PF00520.29	36.60	0.00
TRINITY_I0Z6M2_9CHLO/10-284	I0Z6M2.1 PF03850.12	36.60	0.00
TRINITY_L8H6Q8_ACACA/2-499	L8H6Q8.1 PF04791.14	36.60	0.00
TRINITY_G4ZAC7_PHYSP/224-477	G4ZAC7.1 PF02353.18	36.60	0.00
TRINITY_A8J4M1_CHLRE/402-558	A8J4M1.1 PF12499.6;	36.60	0.00
TRINITY_A0A077ZPR9_STYLE/1002-1272	A0A077ZPR9.1 PF0006	36.60	0.00
TRINITY_D8R3N2_SELML/416-565	D8R3N2.1 PF13638.4;	36.60	0.00
TRINITY_A9SHP2_PHYPA/381-733	A9SHP2.1 PF10373.7;	36.60	0.00
TRINITY_A0A059LS30_9CHLO/1-369	A0A059LS30.1 PF0769	36.60	0.00
TRINITY_A0A0A1U0D6_ENTIV/153-373	A0A0A1U0D6.1 PF0706	36.60	0.00
TRINITY_E1ZF83_CHLVA/18-386	E1ZF83.1 PF00199.17	36.60	0.00
TRINITY_A9UU07_MONBE/109-353	A9UU07.1 PF00487.22	36.60	0.00
TRINITY_I7M791_TETTS/73-229	I7M791.1 PF01343.16	36.60	0.00
TRINITY_G4V6L5_SCHMA/11-298	G4V6L5.1 PF00069.23	36.50	0.00
TRINITY_A9TZG1_PHYPA/59-379	A9TZG1.1 PF14388.4;	36.50	0.00
TRINITY_A0A078ALS5_STYLE/6-235	A0A078ALS5.1 PF1489	36.50	0.00
TRINITY_M5G864_DACSP/168-312	M5G864.1 PF04377.13	36.50	0.00
TRINITY_D3BGC8_POLPA/1-436	D3BGC8.1 PF00022.17	36.50	0.00
TRINITY_D2H277_AILME/182-518	D2H277.1 PF05277.10	36.50	0.00
TRINITY_L8H850_ACACA/5-173	L8H850.1 PF00071.20	36.50	0.00
TRINITY_F4Q789_DICFS/9-129	F4Q789.1 PF00241.18	36.50	0.00
TRINITY_A0A061G2L0_THECC/31-207	A0A061G2L0.1 PF0019	36.50	0.00
TRINITY_I7LTU9_TETTS/76-336	I7LTU9.2 PF00069.23	36.50	0.00
TRINITY_A0A094EAX5_9PEZI/2-375	A0A094EAX5.1 PF0002	36.50	0.00
TRINITY_A8J814_CHLRE/14-119	A8J814.1 PF01920.18	36.50	0.00
TRINITY_Q245W3_TETTS/758-987	Q245W3.2 PF07774.11	36.50	0.00
TRINITY_R6E536_9BACT/23-342	R6E536.1 PF01676.16	36.50	0.00
TRINITY_T0QCM1_9STRA/21-176	T0QCM1.1 PF08617.8;	36.50	0.00
TRINITY_M5Y326_PRUPE/354-929	M5Y326.1 PF05183.10	36.50	0.00
TRINITY_G1KTB3_ANOCA/56-306	G1KTB3.2 PF00378.18	36.50	0.00

TRINITY_W9RER8_9ROSA/123-429	W9RER8.1 PF07993.10	36.50	0.00
TRINITY_F0ZPQ3_DICPU/2-1142	F0ZPQ3.1 PF09735.7;	36.50	0.00
TRINITY_B3S546_TRIAD/200-377	B3S546.1 PF13369.4;	36.50	0.00
TRINITY_L8GMP4_ACACA/297-555	L8GMP4.1 PF07714.15	36.50	0.00
TRINITY_L8H0X8_ACACA/537-801	L8H0X8.1 PF07714.15	36.50	0.00
TRINITY_G0QVW8_ICHMG/43-249	G0QVW8.1 PF03878.13	36.50	0.00
TRINITY_V4MEZ3_EUTSA/141-347	V4MEZ3.1 PF06941.10	36.50	0.00
TRINITY_L8HG29_ACACA/51-299	L8HG29.1 PF00102.25	36.50	0.00
TRINITY_A0CVI3_PARTE/151-343	A0CVI3.1 PF09414.8;	36.50	0.00
TRINITY_W2QJW7_PHYPN/1-171	W2QJW7.1 PF15469.4;	36.50	0.00
TRINITY_A0A0A0LQP7_CUCSA/9-368	A0A0A0LQP7.1 PF1018	36.50	0.00
TRINITY_A8HWI9_CHLRE/348-571	A8HWI9.1 PF07714.15	36.50	0.00
TRINITY_F6S925_CIOIN/133-326	F6S925.2 PF03798.14	36.50	0.00
TRINITY_I7M7H8_TETTS/53-447	I7M7H8.2 PF01490.16	36.50	0.00
TRINITY_D8U2F9_VOLCA/29-477	D8U2F9.1 PF01490.16	36.50	0.00
TRINITY_D3BBV0_POLPA/592-771	D3BBV0.1 PF04678.11	36.50	0.00
TRINITY_F0ZEQ0_DICPU/6-295	F0ZEQ0.1 PF07103.9;	36.50	0.00
TRINITY_Q237B2_TETTS/149-364	Q237B2.1 PF04678.11	36.50	0.00
TRINITY_I0Z8X9_9CHLO/75-263	I0Z8X9.1 PF17047.3;	36.50	0.00
TRINITY_R4XE61_TAPDE/7-107	R4XE61.1 PF00153.25	36.50	0.00
TRINITY_I1FMP4_AMPQE/22-289	I1FMP4.1 PF00701.20	36.50	0.00
TRINITY_Q386Y5_TRYB2/2-167	Q386Y5.1 PF05241.10	36.50	0.00
TRINITY_A0A0D2UAR8_CAPO3/95-270	A0A0D2UAR8.1 PF0062	36.50	0.00
TRINITY_L8HG64_ACACA/2211-2633	L8HG64.1 PF12166.6;	36.50	0.00
TRINITY_A8IBW2_CHLRE/139-344	A8IBW2.1 PF00211.18	36.50	0.00
TRINITY_A0YQS8_LYNSP/82-436	A0YQS8.1 PF13416.4;	36.50	0.00
TRINITY_F6U8N7_XENTR/103-278	F6U8N7.1 PF14580.4;	36.40	0.00
TRINITY_G0R0N7_ICHMG/649-793	G0R0N7.1 PF08752.8;	36.40	0.00
TRINITY_Q22P24_TETTS/166-362	Q22P24.2 PF00149.26	36.40	0.00
TRINITY_F4PKG4_DICFS/116-392	F4PKG4.1 PF00664.21	36.40	0.00
TRINITY_V4AJM9_LOTGI/9-211	V4AJM9.1 PF13419.4;	36.40	0.00
TRINITY_A0DCM6_PARTE/47-264	A0DCM6.1 PF01633.18	36.40	0.00
TRINITY_I7LV51_TETTS/18-193	I7LV51.1 PF13532.4;	36.40	0.00
TRINITY_J9EKQ4_9SPIT/53-232	J9EKQ4.1 PF10294.7;	36.40	0.00
TRINITY_A0A0M0J5U1_9EUKA/369-459	A0A0M0J5U1.1 PF0002	36.40	0.00
TRINITY_G5BU61_HETGA/5-166	G5BU61.1 PF00071.20	36.40	0.00
TRINITY_A0A0A1TVT3_ENTIV/54-407	A0A0A1TVT3.1 PF0115	36.40	0.00
TRINITY_SNX12_SCHPO/701-811	Q9USN1.1 PF00787.22	36.40	0.00
TRINITY_F0ZVQ5_DICPU/78-431	F0ZVQ5.1 PF01925.17	36.40	0.00
TRINITY_A0A078ACH3_STYLE/1513-1666	A0A078ACH3.1 PF1284	36.40	0.00
TRINITY_L8GJ23_ACACA/18-272	L8GJ23.1 PF02900.16	36.40	0.00
TRINITY_J9HLY7_9SPIT/86-256	J9HLY7.1 PF15160.4;	36.40	0.00
TRINITY_G0QJM1_ICHMG/22-245	G0QJM1.1 PF07722.11	36.40	0.00
TRINITY_B1XNR2_SYNP2/58-163	B1XNR2.1 PF13673.5;	36.40	0.00
TRINITY_A0E8S5_PARTE/21-142	A0E8S5.1 PF02466.17	36.40	0.00
TRINITY_A3ZS56_9PLAN/704-819	A3ZS56.1 PF00072.22	36.40	0.00
TRINITY_T1IQI5_STRMM/46-177	T1IQI5.1 PF00782.18	36.40	0.00
TRINITY_G0QXK0_ICHMG/11-169	G0QXK0.1 PF00339.27	36.40	0.00
TRINITY_A0A0H5CAV3_CYBJA/28-146	A0A0H5CAV3.1 PF1015	36.40	0.00
TRINITY_K1QSB7_CRAGI/753-899	K1QSB7.1 PF00339.27	36.40	0.00
TRINITY_I1FWT4_AMPQE/4-97	I1FWT4.1 PF16589.3;	36.40	0.00
TRINITY_T1K7W1_TETUR/55-558	T1K7W1.1 PF12070.6;	36.40	0.00
TRINITY_R0G930_9BRAS/52-456	R0G930.1 PF01494.17	36.40	0.00
TRINITY_Q23YQ8_TETTS/139-489	Q23YQ8.3 PF13868.4;	36.40	0.00
TRINITY_D8UER9_VOLCA/17-281	D8UER9.1 PF00069.23	36.40	0.00
TRINITY_E9G5F2_DAPPU/68-300	E9G5F2.1 PF07808.11	36.40	0.00
TRINITY_A0A068XN59_HYMMI/328-774	A0A068XN59.1 PF0105	36.40	0.00
TRINITY_F2UB11_SALR5/54-261	F2UB11.1 PF01025.17	36.40	0.00
TRINITY_D8U2H6_VOLCA/1-176	D8U2H6.1 PF05603.10	36.40	0.00
TRINITY_J9EPZ7_9SPIT/375-485	J9EPZ7.1 PF00651.29	36.40	0.00
TRINITY_Q23TE6_TETTS/25-352	Q23TE6.2 PF04177.10	36.40	0.00

TRINITY_D8TTP0_VOLCA/1124-1422	D8TTP0.1 PF00069.23	36.40	0.00
TRINITY_Q8CX67_OCEIH/6-370	Q8CX67.1 PF00079.18	36.40	0.00
TRINITY_A8JIE4_CHLRE/4-187	A8JIE4.1 PF10173.7;	36.40	0.00
TRINITY_D8UJ15_VOLCA/1079-1295	D8UJ15.1 PF14633.4;	36.40	0.00
TRINITY_F2ULJ4_SALR5/197-326	F2ULJ4.1 PF00782.18	36.40	0.00
TRINITY_A0A0M2CS90_9MICC/118-335	A0A0M2CS90.1 PF0155	36.40	0.00
TRINITY_I7MLB9_TETTS/842-958	I7MLB9.1 PF00271.29	36.40	0.00
TRINITY_W1QLP0_OGAPD/4-610	W1QLP0.1 PF00012.18	36.40	0.00
TRINITY_D8TTW0_VOLCA/122-380	D8TTW0.1 PF00487.22	36.40	0.00
TRINITY_E2Q1E2_STRC2/205-431	E2Q1E2.1 PF13360.4;	36.40	0.00
TRINITY_A8UVG6_9AQUI/23-374	A8UVG6.1 PF13458.4;	36.40	0.00
TRINITY_G0QUX8_ICHMG/2-192	G0QUX8.1 PF06017.11	36.40	0.00
TRINITY_E1ZN98_CHLVA/91-319	E1ZN98.1 PF00149.26	36.40	0.00
TRINITY_Q23QF2_TETTS/248-571	Q23QF2.2 PF03133.13	36.40	0.00
TRINITY_A0A075ANP7_9FUNG/8-224	A0A075ANP7.1 PF0729	36.40	0.00
TRINITY_D8TM88_VOLCA/93-341	D8TM88.1 PF00348.15	36.40	0.00
TRINITY_I1GE80_AMPQE/220-564	I1GE80.1 PF13598.4;	36.40	0.00
TRINITY_Q22W07_TETTS/20-325	Q22W07.2 PF03224.12	36.40	0.00
TRINITY_F0ZSP4_DICPU/121-459	F0ZSP4.1 PF01619.16	36.40	0.00
TRINITY_K3WBK2_PYTUL/30-635	K3WBK2.1 PF09739.7;	36.40	0.00
TRINITY_F1A4L7_DICPU/143-232	F1A4L7.1 PF02984.17	36.40	0.00
TRINITY_Q86IC6_DICDI/983-1088	Q86IC6.1 PF01843.17	36.40	0.00
TRINITY_A8J752_CHLRE/2729-2884	A8J752.1 PF08016.10	36.40	0.00
TRINITY_I7LXY2_TETTS/864-1187	I7LXY2.2 PF11904.6;	36.30	0.00
TRINITY_A0A0D2WXD3_CAPO3/14-111	A0A0D2WXD3.1 PF0016	36.30	0.00
TRINITY_L8H3W5_ACACA/497-720	L8H3W5.1 PF00176.21	36.30	0.00
TRINITY_F4PH72_DICFS/866-1045	F4PH72.1 PF10443.7;	36.30	0.00
TRINITY_A0DLE6_PARTE/17-195	A0DLE6.1 PF03357.19	36.30	0.00
TRINITY_G3XX32_ASPNA/97-234	G3XX32.1 PF13091.4;	36.30	0.00
TRINITY_L8GNR9_ACACA/6-112	L8GNR9.1 PF00169.27	36.30	0.00
TRINITY_E1Z5D7_CHLVA/48-399	E1Z5D7.1 PF00069.23	36.30	0.00
TRINITY_A0A094GX46_9PEZI/7-210	A0A094GX46.1 PF0546	36.30	0.00
TRINITY_Q23QF2_TETTS/248-571	Q23QF2.2 PF03133.13	36.30	0.00
TRINITY_K2MIF5_TRYCR/448-615	K2MIF5.1 PF00856.26	36.30	0.00
TRINITY_A0A087SSL8_AUXPR/215-510	A0A087SSL8.1 PF0006	36.30	0.00
TRINITY_D8U5L5_VOLCA/20-315	D8U5L5.1 PF13896.4;	36.30	0.00
TRINITY_I7MEK3_TETTS/463-598	I7MEK3.2 PF03914.15	36.30	0.00
TRINITY_I0I669_CALAS/1-240	I0I669.1 PF03577.13	36.30	0.00
TRINITY_A0BFQ3_PARTE/18-326	A0BFQ3.1 PF00069.23	36.30	0.00
TRINITY_A0A059LRX5_9CHLO/1-207	A0A059LRX5.1 PF0097	36.30	0.00
TRINITY_Q22P29_TETTS/54-305	Q22P29.1 PF12697.5;	36.30	0.00
TRINITY_J3LUF2_ORYBR/19-117	J3LUF2.1 PF04749.15	36.30	0.00
TRINITY_D8TJ93_VOLCA/2-159	D8TJ93.1 PF13883.4;	36.30	0.00
TRINITY_K3WHW0_PYTUL/22-237	K3WHW0.1 PF05057.12	36.30	0.00
TRINITY_D8TSN5_VOLCA/497-764	D8TSN5.1 PF07714.15	36.30	0.00
TRINITY_G0QNS7_ICHMG/5-165	G0QNS7.1 PF08613.9;	36.30	0.00
TRINITY_A4S8L4_OSTLU/133-461	A4S8L4.1 PF02535.20	36.30	0.00
TRINITY_F4Q6G4_DICFS/350-494	F4Q6G4.1 PF13015.4;	36.30	0.00
TRINITY_U3JLE8_FICAL/62-177	U3JLE8.1 PF00615.17	36.30	0.00
TRINITY_D8TQB6_VOLCA/2446-2678	D8TQB6.1 PF00069.23	36.30	0.00
TRINITY_A0A078AWE8_STYLE/363-509	A0A078AWE8.1 PF0753	36.20	0.00
TRINITY_W7XA27_TETTS/236-568	W7XA27.1 PF12309.6;	36.20	0.00
TRINITY_W6NXM7_HAECO/3-176	W6NXM7.1 PF00149.26	36.20	0.00
TRINITY_G0R0R4_ICHMG/6-220	G0R0R4.1 PF02114.14	36.20	0.00
TRINITY_U4LTM0_PYROM/303-794	U4LTM0.1 PF00443.27	36.20	0.00
TRINITY_L8GMR3_ACACA/270-526	L8GMR3.1 PF07714.15	36.20	0.00
TRINITY_D3BLY0_POLPA/116-243	D3BLY0.1 PF00004.27	36.20	0.00
TRINITY_A0A0F4NSC0_PSEO7/394-550	A0A0F4NSC0.1 PF0519	36.20	0.00
TRINITY_A0A0H4KTY1_9RHOB/13-238	A0A0H4KTY1.1 PF0056	36.20	0.00
TRINITY_C3YUZ4_BRAFL/44-374	C3YUZ4.1 PF02450.13	36.20	0.00
TRINITY_A0A0D2VQX5_CAPO3/824-1006	A0A0D2VQX5.1 PF0062	36.20	0.00

TRINITY_B4CXZ2_9BACT/104-404	B4CXZ2.1 PF14388.4;	36.20	0.00
TRINITY_D8TTP0_VOLCA/1124-1422	D8TTP0.1 PF00069.23	36.20	0.00
TRINITY_K7M3L4_SOYBN/201-462	K7M3L4.1 PF00069.23	36.20	0.00
TRINITY_L8H3W5_ACACA/497-720	L8H3W5.1 PF00176.21	36.20	0.00
TRINITY_D3B0Y6_POLPA/12-458	D3B0Y6.1 PF05783.9;	36.20	0.00
TRINITY_A0A0C2IY93_THEKT/10-178	A0A0C2IY93.1 PF0483	36.20	0.00
TRINITY_I1FNA5_AMPQE/68-202	I1FNA5.1 PF00043.23	36.20	0.00
TRINITY_I0Z4U3_9CHLO/1399-1742	I0Z4U3.1 PF07714.15	36.20	0.00
TRINITY_U1N7Y3_ASCSU/265-452	U1N7Y3.1 PF00211.18	36.20	0.00
TRINITY_L8GF75_ACACA/5-335	L8GF75.1 PF09786.7;	36.20	0.00
TRINITY_D8U5L5_VOLCA/20-315	D8U5L5.1 PF13896.4;	36.20	0.00
TRINITY_Y1141_DICDI/405-641	Q54F34.1 PF01545.19	36.20	0.00
TRINITY_W1NX46_AMBTC/209-702	W1NX46.1 PF01055.24	36.20	0.00
TRINITY_Q230X1_TETTS/62-338	Q230X1.1 PF00561.18	36.20	0.00
TRINITY_A0A0D2WLC0_CAPO3/58-346	A0A0D2WLC0.1 PF0238	36.20	0.00
TRINITY_A0A077ZSN8_STYLE/26-129	A0A077ZSN8.1 PF1049	36.20	0.00
TRINITY_D8T984_SELML/233-611	D8T984.1 PF00128.22	36.20	0.00
TRINITY_I2GEF6_9BACT/158-355	I2GEF6.1 PF13547.4;	36.20	0.00
TRINITY_I7MI70_TETTS/14-137	I7MI70.1 PF13893.4;	36.20	0.00
TRINITY_A0A0G3XN11_9SPHN/9-200	A0A0G3XN11.1 PF0029	36.20	0.00
TRINITY_M7NVK0_PNEMU/43-199	M7NVK0.1 PF03367.11	36.20	0.00
TRINITY_F0Z8D9_DICPU/33-186	F0Z8D9.1 PF00620.25	36.20	0.00
TRINITY_A0A077ZU40_STYLE/70-398	A0A077ZU40.1 PF0123	36.20	0.00
TRINITY_F4QA18_DICFS/63-236	F4QA18.1 PF00071.20	36.20	0.00
TRINITY_L8GMP4_ACACA/297-555	L8GMP4.1 PF07714.15	36.20	0.00
TRINITY_G6DCU0_DANPL/431-760	G6DCU0.1 PF00702.24	36.20	0.00
TRINITY_F4QCT6_DICFS/87-521	F4QCT6.1 PF12698.5;	36.20	0.00
TRINITY_D3BC27_POLPA/319-631	D3BC27.1 PF05770.9;	36.20	0.00
TRINITY_Q3M0Z1_PARTE/41-243	Q3M0Z1.1 PF00804.23	36.10	0.00
TRINITY_G0QVL4_ICHMG/212-348	G0QVL4.1 PF00153.25	36.10	0.00
TRINITY_Q3M0Y0_PARTE/5-394	Q3M0Y0.1 PF00022.17	36.10	0.00
TRINITY_H7FU47_9FLAO/29-192	H7FU47.1 PF04832.10	36.10	0.00
TRINITY_Q230X4_TETTS/30-358	Q230X4.2 PF16113.3;	36.10	0.00
TRINITY_I7M1G9_TETTS/131-202	I7M1G9.1 PF07258.12	36.10	0.00
TRINITY_A6G6X7_9DELT/30-241	A6G6X7.1 PF03437.13	36.10	0.00
TRINITY_ATG9_DICDI/189-546	Q54NA3.1 PF04109.14	36.10	0.00
TRINITY_F0Z7I9_DICPU/15-122	F0Z7I9.1 PF00168.28	36.10	0.00
TRINITY_Q238Z5_TETTS/22-242	Q238Z5.3 PF07722.11	36.10	0.00
TRINITY_A9V1M8_MONBE/390-579	A9V1M8.1 PF00069.23	36.10	0.00
TRINITY_A0A0D2WV55_CAPO3/693-875	A0A0D2WV55.1 PF0062	36.10	0.00
TRINITY_Q23RD3_TETTS/954-1026	Q23RD3.2 PF13499.4;	36.10	0.00
TRINITY_G0QNB6_ICHMG/7-248	G0QNB6.1 PF00069.23	36.10	0.00
TRINITY_A0DTT7_PARTE/327-627	A0DTT7.1 PF00069.23	36.10	0.00
TRINITY_D8THH2_VOLCA/16-239	D8THH2.1 PF01494.17	36.10	0.00
TRINITY_A0A0G0P0M6_9BACT/2-140	A0A0G0P0M6.1 PF1348	36.10	0.00
TRINITY_LYSG3_DICDI/24-209	Q8T2I5.1 PF01183.18	36.10	0.00
TRINITY_A0A078AJQ0_STYLE/388-594	A0A078AJQ0.1 PF0481	36.10	0.00
TRINITY_I7M910_TETTS/11-285	I7M910.1 PF00069.23	36.10	0.00
TRINITY_A8J0T6_CHLRE/1-157	A8J0T6.1 PF00071.20	36.10	0.00
TRINITY_Q4Q2F7_LEIMA/2-211	Q4Q2F7.1 PF00230.18	36.10	0.00
TRINITY_F6WTJ7_HORSE/297-446	F6WTJ7.1 PF02880.14	36.10	0.00
TRINITY_L8H4A1_ACACA/20-132	L8H4A1.1 PF01398.19	36.10	0.00
TRINITY_W8F274_9BACT/83-211	W8F274.1 PF02469.20	36.10	0.00
TRINITY_S8C7T4_DACHA/264-674	S8C7T4.1 PF00012.18	36.10	0.00
TRINITY_A0A085N9K0_9BILA/353-472	A0A085N9K0.1 PF0066	36.10	0.00
TRINITY_B9HN43_POPTR/31-148	B9HN43.2 PF00188.24	36.10	0.00
TRINITY_A0A059LCS5_9CHLO/41-188	A0A059LCS5.1 PF0006	36.10	0.00
TRINITY_K9SNU5_9CYAN/240-360	K9SNU5.1 PF00182.17	36.10	0.00
TRINITY_A8HPT8_CHLRE/78-408	A8HPT8.1 PF00206.18	36.10	0.00
TRINITY_A0A067Q137_9HOMO/7-249	A0A067Q137.1 PF0536	36.10	0.00
TRINITY_A9V3Z9_MONBE/132-291	A9V3Z9.1 PF08603.9;	36.10	0.00

TRINITY_K9WH26_9CYAN/899-1136	K9WH26.1 PF07993.10	36.10	0.00
TRINITY_B6AF10_CRYMR/13-179	B6AF10.1 PF00071.20	36.10	0.00
TRINITY_L8HAD7_ACACA/61-159	L8HAD7.1 PF00169.27	36.10	0.00
TRINITY_W4YII5_STRPU/107-224	W4YII5.1 PF03283.11	36.10	0.00
TRINITY_I0YUB5_9CHLO/55-241	I0YUB5.1 PF13385.4;	36.10	0.00
TRINITY_C3YB25_BRAFL/63-267	C3YB25.1 PF00149.26	36.10	0.00
TRINITY_A0A0L0CMA9_LUCCU/49-176	A0A0L0CMA9.1 PF0078	36.10	0.00
TRINITY_J9FYL2_9SPIT/398-662	J9FYL2.1 PF00069.23	36.10	0.00
TRINITY_A8J192_CHLRE/156-505	A8J192.1 PF04515.10	36.10	0.00
TRINITY_D8TTP0_VOLCA/1124-1422	D8TTP0.1 PF00069.23	36.10	0.00
TRINITY_I7LSY6_TETTS/44-139	I7LSY6.1 PF13774.4;	36.10	0.00
TRINITY_D2UX37_NAEGR/136-313	D2UX37.1 PF00929.22	36.10	0.00
TRINITY_F9DUI1_9BACL/71-321	F9DUI1.1 PF01636.21	36.10	0.00
TRINITY_C1FGT1_MICSR/532-744	C1FGT1.1 PF12348.6;	36.10	0.00
TRINITY_A8IRM8_CHLRE/54-347	A8IRM8.1 PF00856.26	36.10	0.00
TRINITY_A8IBI1_CHLRE/10-148	A8IBI1.1 PF00179.24	36.10	0.00
TRINITY_A0A0N5DEW8_TRIMR/117-367	A0A0N5DEW8.1 PF0006	36.10	0.00
TRINITY_I7M884_TETTS/59-160	I7M884.2 PF01363.19	36.00	0.00
TRINITY_A0A022QZA6_ERYGU/10-187	A0A022QZA6.1 PF0192	36.00	0.00
TRINITY_A0A0G4IGY6_PLABS/86-378	A0A0G4IGY6.1 PF0118	36.00	0.00
TRINITY_Q22EG9_TETTS/11-197	Q22EG9.1 PF13460.4;	36.00	0.00
TRINITY_H3ALH2_LATCH/392-661	H3ALH2.1 PF07842.10	36.00	0.00
TRINITY_U3IPM0_ANAPL/94-411	U3IPM0.1 PF00026.21	36.00	0.00
TRINITY_D6X1Y5_TRICA/9-227	D6X1Y5.1 PF00335.18	36.00	0.00
TRINITY_D8TLE6_VOLCA/14-651	D8TLE6.1 PF00888.20	36.00	0.00
TRINITY_W4FVI6_9STRA/31-262	W4FVI6.1 PF01063.17	36.00	0.00
TRINITY_A0CLK7_PARTE/302-529	A0CLK7.1 PF00149.26	36.00	0.00
TRINITY_A0A0G4ILS6_PLABS/173-377	A0A0G4ILS6.1 PF0154	36.00	0.00
TRINITY_Q54WT3_DICDI/164-524	Q54WT3.1 PF00026.21	36.00	0.00
TRINITY_W7XHQ2_TETTS/623-861	W7XHQ2.1 PF02714.13	36.00	0.00
TRINITY_W5KWU8_ASTMX/41-151	W5KWU8.1 PF00615.17	36.00	0.00
TRINITY_A0A066V2Y0_9BASI/498-1230	A0A066V2Y0.1 PF0044	36.00	0.00
TRINITY_A0CLK7_PARTE/302-529	A0CLK7.1 PF00149.26	36.00	0.00
TRINITY_L8GIA8_ACACA/26-458	L8GIA8.1 PF00450.20	36.00	0.00
TRINITY_A0A0D2VVY5_CAPO3/15-153	A0A0D2VVY5.1 PF1352	36.00	0.00
TRINITY_Q234K7_TETTS/37-180	Q234K7.2 PF00179.24	36.00	0.00
TRINITY_U4KU25_PYROM/171-293	U4KU25.1 PF00622.26	36.00	0.00
TRINITY_A0A0B0MM04_GOSAR/744-831	A0A0B0MM04.1 PF1279	36.00	0.00
TRINITY_C3Y7X6_BRAFL/35-230	C3Y7X6.1 PF01762.19	36.00	0.00
TRINITY_V7D0Q5_PHAVU/32-200	V7D0Q5.1 PF11715.6;	36.00	0.00
TRINITY_T1IQ34_STRMM/22-421	T1IQ34.1 PF06602.12	36.00	0.00
TRINITY_A0A087SP74_AUXPR/320-455	A0A087SP74.1 PF0176	36.00	0.00
TRINITY_Q10WE1_TRIEI/63-224	Q10WE1.1 PF01434.16	36.00	0.00
TRINITY_F1NN82_CHICK/492-836	F1NN82.2 PF01237.16	36.00	0.00
TRINITY_C1E3A7_MICSR/962-1127	C1E3A7.1 PF13890.4;	36.00	0.00
TRINITY_D8TP23_VOLCA/10-139	D8TP23.1 PF12047.6;	36.00	0.00
TRINITY_V4AHX0_LOTGI/87-328	V4AHX0.1 PF08627.8;	36.00	0.00
TRINITY_A0A0J8C0D6_BETVU/141-301	A0A0J8C0D6.1 PF0155	36.00	0.00
TRINITY_D8SJF1_SELML/791-1057	D8SJF1.1 PF12697.5;	36.00	0.00
TRINITY_I0Z2Y4_9CHLO/747-1368	I0Z2Y4.1 PF12157.6;	36.00	0.00
TRINITY_A8IFL6_CHLRE/1462-1762	A8IFL6.1 PF00069.23	36.00	0.00
TRINITY_K6YJ79_9ALTE/12-122	K6YJ79.1 PF00583.23	36.00	0.00
TRINITY_I0YZB1_9CHLO/66-171	I0YZB1.1 PF00085.18	36.00	0.00
TRINITY_D8TKK7_VOLCA/1-188	D8TKK7.1 PF07714.15	36.00	0.00
TRINITY_U5H7L5_USTV1/264-393	U5H7L5.1 PF03953.15	36.00	0.00
TRINITY_I1F2E6_AMPQE/14-159	I1F2E6.1 PF00112.21	36.00	0.00
TRINITY_A0A059LNH5_9CHLO/51-376	A0A059LNH5.1 PF0149	36.00	0.00
TRINITY_A0A0J8D429_BETVU/420-1005	A0A0J8D429.1 PF0518	36.00	0.00
TRINITY_Q1LQ74_CUPMC/3-125	Q1LQ74.1 PF01124.16	35.90	0.00
TRINITY_A0A0D2W1A4_CAPO3/47-268	A0A0D2W1A4.1 PF0014	35.90	0.00
TRINITY_A0A059LEK0_9CHLO/8-297	A0A059LEK0.1 PF0000	35.90	0.00

TRINITY_A0BZR0_PARTE/192-423	A0BZR0.1 PF00149.26	35.90	0.00
TRINITY_Q76P10_DICDI/22-141	Q76P10.1 PF05005.13	35.90	0.00
TRINITY_I7M9L3_TETTS/26-279	I7M9L3.2 PF02263.17	35.90	0.00
TRINITY_G0QXV2_ICHMG/241-324	G0QXV2.1 PF14759.4;	35.90	0.00
TRINITY_I7M9S7_TETTS/86-331	I7M9S7.2 PF00481.19	35.90	0.00
TRINITY_D3BTZ7_POLPA/115-265	D3BTZ7.1 PF00620.25	35.90	0.00
TRINITY_W0RBU1_9BACT/3-152	W0RBU1.1 PF04525.10	35.90	0.00
TRINITY_T1FNE1_HELRO/126-439	T1FNE1.1 PF01103.21	35.90	0.00
TRINITY_Q0ARS9_MARMM/272-388	Q0ARS9.1 PF02518.24	35.90	0.00
TRINITY_L8GQR1_ACACA/657-1024	L8GQR1.1 PF00176.21	35.90	0.00
TRINITY_A9G0Q5_SORC5/53-346	A9G0Q5.1 PF14388.4;	35.90	0.00
TRINITY_W9XV54_9EURO/15-176	W9XV54.1 PF00071.20	35.90	0.00
TRINITY_D8UJA8_VOLCA/964-1092	D8UJA8.1 PF14443.4;	35.90	0.00
TRINITY_A0CQI4_PARTE/709-830	A0CQI4.1 PF00072.22	35.90	0.00
TRINITY_A0A0L9SQM7_9HYPO/4-181	A0A0L9SQM7.1 PF0015	35.90	0.00
TRINITY_D8UKH2_VOLCA/264-655	D8UKH2.1 PF04857.18	35.90	0.00
TRINITY_A0A067BWY7_SAPPC/22-533	A0A067BWY7.1 PF0145	35.90	0.00
TRINITY_W5KMY9_ASTMX/70-182	W5KMY9.1 PF09430.8;	35.90	0.00
TRINITY_X6NSF5_RETFI/88-345	X6NSF5.1 PF07714.15	35.90	0.00
TRINITY_A0A072TX86_MEDTR/276-421	A0A072TX86.1 PF0517	35.90	0.00
TRINITY_I0Z3H3_9CHLO/15-170	I0Z3H3.1 PF00929.22	35.90	0.00
TRINITY_D5GMB3_TUBMM/25-132	D5GMB3.1 PF06148.9;	35.90	0.00
TRINITY_L5JN97_PTEAL/29-462	L5JN97.1 PF00067.20	35.90	0.00
TRINITY_I7M7B7_TETTS/109-362	I7M7B7.2 PF00069.23	35.90	0.00
TRINITY_A7S4P7_NEMVE/630-748	A7S4P7.1 PF00168.28	35.90	0.00
TRINITY_D8TIB1_VOLCA/100-431	D8TIB1.1 PF07887.9;	35.90	0.00
TRINITY_A0BLH9_PARTE/7-108	A0BLH9.1 PF00168.28	35.90	0.00
TRINITY_A0A078AR51_STYLE/8-287	A0A078AR51.1 PF0006	35.90	0.00
TRINITY_A0A067N7A8_9HOMO/28-183	A0A067N7A8.1 PF0156	35.90	0.00
TRINITY_A2ENP4_TRIVA/27-279	A2ENP4.1 PF00069.23	35.90	0.00
TRINITY_A0A0D2WR04_CAPO3/45-470	A0A0D2WR04.1 PF0075	35.90	0.00
TRINITY_D8TST9_VOLCA/621-848	D8TST9.1 PF00211.18	35.90	0.00
TRINITY_A0A0C2JY18_THEKT/17-771	A0A0C2JY18.1 PF1639	35.80	0.00
TRINITY_W7XIE4_TETTS/21-297	W7XIE4.1 PF01430.17	35.80	0.00
TRINITY_A0A067QH62_ZOONE/133-216	A0A067QH62.1 PF1389	35.80	0.00
TRINITY_S8EMD3_FOMPI/471-598	S8EMD3.1 PF16901.3;	35.80	0.00
TRINITY_H7F3W4_9LIST/162-306	H7F3W4.1 PF01926.21	35.80	0.00
TRINITY_A0EAP9_PARTE/41-162	A0EAP9.1 PF03351.15	35.80	0.00
TRINITY_A0CC42_PARTE/16-287	A0CC42.1 PF00069.23	35.80	0.00
TRINITY_C3XYB9_BRAFL/69-302	C3XYB9.1 PF08627.8;	35.80	0.00
TRINITY_E2BY81_HARSA/5-166	E2BY81.1 PF00071.20	35.80	0.00
TRINITY_G0R352_ICHMG/26-211	G0R352.1 PF13462.4;	35.80	0.00
TRINITY_Q6BFR1_PARTE/25-209	Q6BFR1.1 PF13462.4;	35.80	0.00
TRINITY_A0A0F0HL75_9PSEU/28-183	A0A0F0HL75.1 PF0196	35.80	0.00
TRINITY_H2CIM4_9LEPT/257-517	H2CIM4.1 PF01636.21	35.80	0.00
TRINITY_I7M6Y1_TETTS/72-199	I7M6Y1.1 PF00134.21	35.80	0.00
TRINITY_W4XSR8_STRPU/64-282	W4XSR8.1 PF14675.4;	35.80	0.00
TRINITY_L8HK25_ACACA/40-285	L8HK25.1 PF01637.16	35.80	0.00
TRINITY_A8JGR4_CHLRE/173-480	A8JGR4.1 PF10250.7;	35.80	0.00
TRINITY_I7M910_TETTS/11-285	I7M910.1 PF00069.23	35.80	0.00
TRINITY_A0CXP9_PARTE/92-386	A0CXP9.1 PF01457.14	35.80	0.00
TRINITY_F1QUE8_DANRE/77-293	F1QUE8.1 PF02104.13	35.80	0.00
TRINITY_G0QLS4_ICHMG/523-772	G0QLS4.1 PF07714.15	35.80	0.00
TRINITY_Q23QF2_TETTS/248-571	Q23QF2.2 PF03133.13	35.80	0.00
TRINITY_E1Z6P1_CHLVA/201-305	E1Z6P1.1 PF00168.28	35.80	0.00
TRINITY_I0Z9R3_9CHLO/1171-1635	I0Z9R3.1 PF12698.5;	35.80	0.00
TRINITY_D3BRF9_POLPA/687-869	D3BRF9.1 PF14429.4;	35.80	0.00
TRINITY_L1JXL1_GUITH/9-407	L1JXL1.1 PF04841.11	35.80	0.00
TRINITY_A0A078AX44_STYLE/604-698	A0A078AX44.1 PF1349	35.80	0.00
TRINITY_A0A0D2VP60_CAPO3/1054-1165	A0A0D2VP60.1 PF0220	35.80	0.00
TRINITY_A0A067BWY7_SAPPC/22-533	A0A067BWY7.1 PF0145	35.80	0.00

TRINITY_A8J0X4_CHLRE/280-402	A8J0X4.1 PF02803.16	35.80	0.00
TRINITY_I0ZAU1_9CHLO/192-343	I0ZAU1.1 PF00069.23	35.80	0.00
TRINITY_A0A061DY99_THECC/68-203	A0A061DY99.1 PF0004	35.80	0.00
TRINITY_L8GIZ0_ACACA/22-206	L8GIZ0.1 PF00621.18	35.80	0.00
TRINITY_D3B3M0_POLPA/345-611	D3B3M0.1 PF11817.6;	35.80	0.00
TRINITY_E1Z450_CHLVA/129-264	E1Z450.1 PF07890.10	35.80	0.00
TRINITY_M5ED10_MALS4/335-489	M5ED10.1 PF00620.25	35.80	0.00
TRINITY_A9S9U0_PHYPA/6-313	A9S9U0.1 PF03372.21	35.80	0.00
TRINITY_G0R402_ICHMG/82-193	G0R402.1 PF00043.23	35.80	0.00
TRINITY_F0ZM59_DICPU/346-477	F0ZM59.1 PF04499.13	35.80	0.00
TRINITY_D8TNM8_VOLCA/146-321	D8TNM8.1 PF09750.7;	35.80	0.00
TRINITY_A0A067JAX2_JATCU/600-1007	A0A067JAX2.1 PF0017	35.80	0.00
TRINITY_K1QRM1_CRAGI/216-808	K1QRM1.1 PF04097.12	35.80	0.00
TRINITY_A0BWC2_PARTE/204-435	A0BWC2.1 PF10243.7;	35.80	0.00
TRINITY_A8HP62_CHLRE/179-430	A8HP62.1 PF05548.9;	35.80	0.00
TRINITY_E1Z963_CHLVA/1-418	E1Z963.1 PF02897.13	35.80	0.00
TRINITY_Q23PV4_TETTS/274-435	Q23PV4.2 PF00441.22	35.80	0.00
TRINITY_C5KGV9_PERM5/124-230	C5KGV9.1 PF13646.4;	35.80	0.00
TRINITY_I1FAE8_AMPQE/829-998	I1FAE8.1 PF01734.20	35.80	0.00
TRINITY_C3Z276_BRAFL/163-315	C3Z276.1 PF01936.16	35.80	0.00
TRINITY_A0A0M2HAK2_9MICO/33-201	A0A0M2HAK2.1 PF1348	35.80	0.00
TRINITY_G0R3S9_ICHMG/1-224	G0R3S9.1 PF00155.19	35.80	0.00
TRINITY_A0C099_PARTE/280-614	A0C099.1 PF03133.13	35.70	0.00
TRINITY_A0A078A5X2_STYLE/11-211	A0A078A5X2.1 PF0090	35.70	0.00
TRINITY_F6S9N9_HORSE/80-380	F6S9N9.1 PF00561.18	35.70	0.00
TRINITY_R0HUJ6_9BRAS/114-232	R0HUJ6.1 PF13911.4;	35.70	0.00
TRINITY_A0A0D2WQY8_CAPO3/79-304	A0A0D2WQY8.1 PF0410	35.70	0.00
TRINITY_Q3SDS6_PARTE/10-241	Q3SDS6.1 PF04670.10	35.70	0.00
TRINITY_A0A078B9W1_STYLE/27-329	A0A078B9W1.1 PF0056	35.70	0.00
TRINITY_E8MY14_ANATU/101-205	E8MY14.1 PF16158.3;	35.70	0.00
TRINITY_Q22YH7_TETTS/342-611	Q22YH7.2 PF07714.15	35.70	0.00
TRINITY_A0A0L9SWE8_9HYPO/257-633	A0A0L9SWE8.1 PF0017	35.70	0.00
TRINITY_A4VEV9_TETTS/181-339	A4VEV9.2 PF02518.24	35.70	0.00
TRINITY_G0R3Y2_ICHMG/15-274	G0R3Y2.1 PF00069.23	35.70	0.00
TRINITY_F0ZJS9_DICPU/436-674	F0ZJS9.1 PF00003.20	35.70	0.00
TRINITY_Q231C9_TETTS/30-286	Q231C9.2 PF00069.23	35.70	0.00
TRINITY_F1MJ49_BOVIN/70-386	F1MJ49.2 PF00026.21	35.70	0.00
TRINITY_A0A067GVP2_CITSI/67-435	A0A067GVP2.1 PF1171	35.70	0.00
TRINITY_E2AS81_CAMFO/17-236	E2AS81.1 PF00106.23	35.70	0.00
TRINITY_E1ZHQ5_CHLVA/1633-1861	E1ZHQ5.1 PF02485.19	35.70	0.00
TRINITY_D8UJG8_VOLCA/25-428	D8UJG8.1 PF09724.7;	35.70	0.00
TRINITY_M0XSI6_HORVD/108-224	M0XSI6.1 PF02450.13	35.70	0.00
TRINITY_A0A0M9G675_9TRYP/57-165	A0A0M9G675.1 PF0709	35.70	0.00
TRINITY_A8IKQ0_CHLRE/79-278	A8IKQ0.1 PF00316.18	35.70	0.00
TRINITY_A8J093_CHLRE/30-290	A8J093.1 PF00856.26	35.70	0.00
TRINITY_Q22BA6_TETTS/149-399	Q22BA6.2 PF10192.7;	35.70	0.00
TRINITY_E9GQH9_DAPPU/1-102	E9GQH9.1 PF00651.29	35.70	0.00
TRINITY_FORA_DICDI/352-549	Q54WH2.1 PF06367.14	35.70	0.00
TRINITY_F4Q5N9_DICFS/37-136	F4Q5N9.1 PF06179.10	35.70	0.00
TRINITY_A8IKT9_CHLRE/529-630	A8IKT9.1 PF00134.21	35.70	0.00
TRINITY_EF1A_CENSY/8-291	A0RUM4.1 PF00009.25	35.70	0.00
TRINITY_A0CEF3_PARTE/266-572	A0CEF3.1 PF00443.27	35.70	0.00
TRINITY_A0D5J3_PARTE/683-779	A0D5J3.1 PF00679.22	35.60	0.00
TRINITY_E4XE42_OIKDI/68-172	E4XE42.1 PF00168.28	35.60	0.00
TRINITY_M5G2M9_DACSP/146-250	M5G2M9.1 PF01762.19	35.60	0.00
TRINITY_A0A078B0U0_STYLE/21-275	A0A078B0U0.1 PF0226	35.60	0.00
TRINITY_A0A068S4Y8_9FUNG/2-103	A0A068S4Y8.1 PF1085	35.60	0.00
TRINITY_A0A078ASU1_STYLE/162-297	A0A078ASU1.1 PF1602	35.60	0.00
TRINITY_RUB1_YEAST/3-74	Q03919.1 PF00240.21	35.60	0.00
TRINITY_D8TUC0_VOLCA/1104-1208	D8TUC0.1 PF02656.13	35.60	0.00
TRINITY_L7N2K8_XENTR/57-480	L7N2K8.1 PF05577.10	35.60	0.00

TRINITY_A0A0J7L068_LASNI/157-267	A0A0J7L068.1 PF0007	35.60	0.00
TRINITY_A0A0D2VKV0_CAPO3/160-381	A0A0D2VKV0.1 PF0797	35.60	0.00
TRINITY_K7JAP6_NASVI/824-987	K7JAP6.1 PF00145.15	35.60	0.00
TRINITY_F7BXP1_XENTR/49-181	F7BXP1.1 PF00782.18	35.60	0.00
TRINITY_D3AWF6_POLPA/1305-1480	D3AWF6.1 PF00617.17	35.60	0.00
TRINITY_A8D8P8_BOMMO/315-448	A8D8P8.1 PF02170.20	35.60	0.00
TRINITY_A0C9H6_PARTE/29-196	A0C9H6.1 PF08718.9;	35.60	0.00
TRINITY_F4PHW0_DICFS/359-537	F4PHW0.1 PF01963.15	35.60	0.00
TRINITY_D8TR12_VOLCA/14-230	D8TR12.1 PF00856.26	35.60	0.00
TRINITY_J9JUC1_ACYPI/764-916	J9JUC1.2 PF00620.25	35.60	0.00
TRINITY_A0A078ANW9_STYLE/8-254	A0A078ANW9.1 PF0137	35.60	0.00
TRINITY_I0YLJ5_9CHLO/1438-1705	I0YLJ5.1 PF14228.4;	35.60	0.00
TRINITY_C1EDN7_MICSR/1-299	C1EDN7.1 PF04111.10	35.60	0.00
TRINITY_Q23RP1_TETTS/81-493	Q23RP1.2 PF01457.14	35.60	0.00
TRINITY_A0A067L3G3_JATCU/289-537	A0A067L3G3.1 PF0771	35.60	0.00
TRINITY_L8H6E1_ACACA/61-265	L8H6E1.1 PF00092.26	35.60	0.00
TRINITY_A0A0D9W1M2_9ORYZ/247-310	A0A0D9W1M2.1 PF0040	35.60	0.00
TRINITY_A0A061ETD8_THECC/53-369	A0A061ETD8.1 PF0025	35.60	0.00
TRINITY_T1IQP2_STRMM/60-199	T1IQP2.1 PF10075.7;	35.60	0.00
TRINITY_K1QCA5_CRAGI/20-403	K1QCA5.1 PF01041.15	35.60	0.00
TRINITY_D8TQJ2_VOLCA/22-290	D8TQJ2.1 PF05817.12	35.60	0.00
TRINITY_G0QV43_ICHMG/249-568	G0QV43.1 PF04515.10	35.60	0.00
TRINITY_D8TSG5_VOLCA/121-242	D8TSG5.1 PF08512.10	35.60	0.00
TRINITY_Q22A65_TETTS/524-741	Q22A65.2 PF01612.18	35.60	0.00
TRINITY_D3B0L0_POLPA/251-374	D3B0L0.1 PF00293.26	35.60	0.00
TRINITY_Q231F3_TETTS/82-315	Q231F3.1 PF03009.15	35.60	0.00
TRINITY_D3BKE1_POLPA/249-636	D3BKE1.1 PF01757.20	35.60	0.00
TRINITY_A8J151_CHLRE/549-830	A8J151.1 PF00069.23	35.60	0.00
TRINITY_A0EAD7_PARTE/23-277	A0EAD7.1 PF00956.16	35.50	0.00
TRINITY_W3X1V6_9PEZI/165-391	W3X1V6.1 PF04855.10	35.50	0.00
TRINITY_A0A0G4EJZ4_9ALVE/53-190	A0A0G4EJZ4.1 PF0582	35.50	0.00
TRINITY_A0CJW5_PARTE/64-366	A0CJW5.1 PF00995.21	35.50	0.00
TRINITY_U6NG28_HAECO/90-229	U6NG28.1 PF01764.23	35.50	0.00
TRINITY_A0A0D2X3B4_CAPO3/50-343	A0A0D2X3B4.1 PF0844	35.50	0.00
TRINITY_H2MBE7_ORYLA/98-174	H2MBE7.1 PF13499.4;	35.50	0.00
TRINITY_L8HI32_ACACA/42-157	L8HI32.1 PF00169.27	35.50	0.00
TRINITY_F7BX82_XENTR/13-137	F7BX82.1 PF04488.13	35.50	0.00
TRINITY_F9XH71_ZYMTI/10-116	F9XH71.1 PF00307.29	35.50	0.00
TRINITY_A0A0D2WPQ9_CAPO3/136-387	A0A0D2WPQ9.1 PF1362	35.50	0.00
TRINITY_U6NG28_HAECO/90-229	U6NG28.1 PF01764.23	35.50	0.00
TRINITY_A8IYX5_CHLRE/35-184	A8IYX5.1 PF01694.20	35.50	0.00
TRINITY_C5CDW1_KOSOT/131-317	C5CDW1.1 PF16912.3;	35.50	0.00
TRINITY_G0R6A2_ICHMG/511-619	G0R6A2.1 PF10367.7;	35.50	0.00
TRINITY_A0DDI6_PARTE/14-246	A0DDI6.1 PF16414.3;	35.50	0.00
TRINITY_A2FTW9_TRIVA/10-171	A2FTW9.1 PF00071.20	35.50	0.00
TRINITY_M4BR64_HYAAE/345-692	M4BR64.1 PF00750.17	35.50	0.00
TRINITY_K7TNN3_MAIZE/440-597	K7TNN3.1 PF00004.27	35.50	0.00
TRINITY_A0A077ZSV4_STYLE/29-267	A0A077ZSV4.1 PF0019	35.50	0.00
TRINITY_A0CRH3_PARTE/53-407	A0CRH3.1 PF00155.19	35.50	0.00
TRINITY_D3B059_POLPA/358-943	D3B059.1 PF05667.9;	35.50	0.00
TRINITY_D3B9F0_POLPA/11-162	D3B9F0.1 PF00293.26	35.50	0.00
TRINITY_A0A0M0JZK3_9EUKA/232-431	A0A0M0JZK3.1 PF1385	35.50	0.00
TRINITY_A0DIS6_PARTE/91-235	A0DIS6.1 PF01529.18	35.50	0.00
TRINITY_A0A059AFF4_EUCGR/353-948	A0A059AFF4.1 PF0032	35.50	0.00
TRINITY_L8GMR3_ACACA/270-526	L8GMR3.1 PF07714.15	35.50	0.00
TRINITY_A8ICH8_CHLRE/72-332	A8ICH8.1 PF12146.6;	35.50	0.00
TRINITY_D3BC10_POLPA/164-322	D3BC10.1 PF00620.25	35.50	0.00
TRINITY_A0A0J7MWC3_LASNI/91-250	A0A0J7MWC3.1 PF1335	35.50	0.00
TRINITY_Q23DC1_TETTS/461-1153	Q23DC1.2 PF00443.27	35.50	0.00
TRINITY_A9T8M6_PHYPA/35-493	A9T8M6.1 PF03215.13	35.50	0.00
TRINITY_E1Z2I7_CHLVA/181-844	E1Z2I7.1 PF04858.11	35.50	0.00

TRINITY_W4K730_9HOMO/58-291	W4K730.1 PF02940.13	35.50	0.00
TRINITY_R4XIZ5_TAPDE/64-197	R4XIZ5.1 PF00043.23	35.50	0.00
TRINITY_F4PBS1_BATDJ/109-267	F4PBS1.1 PF01764.23	35.50	0.00
TRINITY_G0QU50_ICHMG/9-210	G0QU50.1 PF00069.23	35.40	0.00
TRINITY_A0A078ACB5_STYLE/210-312	A0A078ACB5.1 PF1349	35.40	0.00
TRINITY_B7G054_PHATC/72-147	B7G054.1 PF03134.17	35.40	0.00
TRINITY_T1K202_TETUR/21-103	T1K202.1 PF12895.5;	35.40	0.00
TRINITY_A0A0J8BUF2_BETVU/178-389	A0A0J8BUF2.1 PF0315	35.40	0.00
TRINITY_A0E6K3_PARTE/11-167	A0E6K3.1 PF00339.27	35.40	0.00
TRINITY_L8HI30_ACACA/54-174	L8HI30.1 PF02759.17	35.40	0.00
TRINITY_C3ZCV4_BRAFL/185-607	C3ZCV4.1 PF00246.22	35.40	0.00
TRINITY_Q22CY1_TETTS/113-205	Q22CY1.1 PF00153.25	35.40	0.00
TRINITY_A0BF36_PARTE/51-151	A0BF36.1 PF10256.7;	35.40	0.00
TRINITY_E0VXM7_PEDHC/29-108	E0VXM7.1 PF13774.4;	35.40	0.00
TRINITY_A0A0F7KJC3_9PROT/7-186	A0A0F7KJC3.1 PF0173	35.40	0.00
TRINITY_Q54GF5_DICDI/555-753	Q54GF5.1 PF02141.19	35.40	0.00
TRINITY_A0A015N049_9GLOM/29-227	A0A015N049.1 PF0136	35.40	0.00
TRINITY_L8GMP4_ACACA/297-555	L8GMP4.1 PF07714.15	35.40	0.00
TRINITY_A0A0G4EYN0_9ALVE/80-431	A0A0G4EYN0.1 PF0862	35.40	0.00
TRINITY_G0PB55_CAEBE/48-208	G0PB55.1 PF00071.20	35.40	0.00
TRINITY_C3ZPP7_BRAFL/1129-1309	C3ZPP7.1 PF00350.21	35.40	0.00
TRINITY_F4Q324_DICFS/3-225	F4Q324.1 PF08709.9;	35.40	0.00
TRINITY_C0GCP5_9FIRM/4-224	C0GCP5.1 PF02517.14	35.40	0.00
TRINITY_A0DS64_PARTE/289-550	A0DS64.1 PF07690.14	35.40	0.00
TRINITY_A8JGC0_CHLRE/1-221	A8JGC0.1 PF04889.10	35.40	0.00
TRINITY_F6ZGD4_CIOIN/495-670	F6ZGD4.2 PF00637.18	35.40	0.00
TRINITY_I0Z2P8_9CHLO/693-909	I0Z2P8.1 PF08172.10	35.40	0.00
TRINITY_C9Z291_STRSW/47-284	C9Z291.1 PF12706.5;	35.40	0.00
TRINITY_M4VDU0_9DELT/480-595	M4VDU0.1 PF00072.22	35.40	0.00
TRINITY_A0A0M2U2L9_9FIRM/82-308	A0A0M2U2L9.1 PF0163	35.40	0.00
TRINITY_A0A078B6G9_STYLE/51-363	A0A078B6G9.1 PF0107	35.40	0.00
TRINITY_A8ITR3_CHLRE/39-175	A8ITR3.1 PF00067.20	35.40	0.00
TRINITY_A9TCE6_PHYPA/157-467	A9TCE6.1 PF08506.8;	35.40	0.00
TRINITY_A9TYN8_PHYPA/180-297	A9TYN8.1 PF04488.13	35.40	0.00
TRINITY_A0A084VME3_ANOSI/413-680	A0A084VME3.1 PF0784	35.40	0.00
TRINITY_H3GRQ7_PHYRM/100-340	H3GRQ7.1 PF13360.4;	35.40	0.00
TRINITY_U5D935_AMBTC/366-517	U5D935.1 PF04937.13	35.40	0.00
TRINITY_I0Z0R8_9CHLO/11-128	I0Z0R8.1 PF00651.29	35.40	0.00
TRINITY_D3BVF7_POLPA/2-430	D3BVF7.1 PF00083.22	35.40	0.00
TRINITY_M2W1N1_GALSU/52-163	M2W1N1.1 PF13911.4;	35.40	0.00
TRINITY_L8GSH4_ACACA/37-217	L8GSH4.1 PF00621.18	35.40	0.00
TRINITY_D8TTC2_VOLCA/10-183	D8TTC2.1 PF07714.15	35.40	0.00
TRINITY_A0A077ZYF9_STYLE/402-524	A0A077ZYF9.1 PF1389	35.40	0.00
TRINITY_T1I4K7_RHOPR/121-187	T1I4K7.1 PF12796.5;	35.40	0.00
TRINITY_D3B803_POLPA/36-206	D3B803.1 PF00445.16	35.30	0.00
TRINITY_A8HPQ8_CHLRE/224-321	A8HPQ8.1 PF01342.19	35.30	0.00
TRINITY_GEFI_DICDI/429-530	Q8IS15.1 PF00618.18	35.30	0.00
TRINITY_I1FBX1_AMPQE/7-152	I1FBX1.1 PF13238.4;	35.30	0.00
TRINITY_A8IGR4_CHLRE/166-1418	A8IGR4.1 PF03813.12	35.30	0.00
TRINITY_A0A0G4EIW3_9ALVE/50-219	A0A0G4EIW3.1 PF0414	35.30	0.00
TRINITY_F1A5P9_DICPU/70-248	F1A5P9.1 PF00621.18	35.30	0.00
TRINITY_A2D9F5_TRIVA/531-673	A2D9F5.1 PF07534.14	35.30	0.00
TRINITY_L8GI47_ACACA/42-316	L8GI47.1 PF09192.8;	35.30	0.00
TRINITY_A8J8W4_CHLRE/13-111	A8J8W4.1 PF13233.4;	35.30	0.00
TRINITY_H2YUM9_CIOSA/1337-1489	H2YUM9.1 PF00620.25	35.30	0.00
TRINITY_A0DMA3_PARTE/467-923	A0DMA3.1 PF04547.10	35.30	0.00
TRINITY_A0A0R3NSP3_DROPS/219-488	A0A0R3NSP3.1 PF0006	35.30	0.00
TRINITY_A0A0D2X2X8_CAPO3/2-320	A0A0D2X2X8.1 PF0126	35.30	0.00
TRINITY_A0DEV0_PARTE/62-177	A0DEV0.1 PF00069.23	35.30	0.00
TRINITY_E1ZH04_CHLVA/10-112	E1ZH04.1 PF01922.15	35.30	0.00
TRINITY_D2VIY6_NAEGR/222-505	D2VIY6.1 PF00657.20	35.30	0.00

TRINITY_M4CMD5_BRARP/71-470	M4CMD5.1 PF01704.16	35.30	0.00
TRINITY_L8GQN2_ACACA/447-624	L8GQN2.1 PF00617.17	35.30	0.00
TRINITY_A8IC13_CHLRE/326-455	A8IC13.1 PF00067.20	35.30	0.00
TRINITY_I7MKV0_TETTS/148-266	I7MKV0.2 PF13863.4;	35.30	0.00
TRINITY_Q23NK4_TETTS/23-187	Q23NK4.2 PF06703.9;	35.30	0.00
TRINITY_F6TP15_ORNAN/12-228	F6TP15.2 PF00817.18	35.30	0.00
TRINITY_A0A0D2VHU9_CAPO3/41-166	A0A0D2VHU9.1 PF0406	35.30	0.00
TRINITY_F1QWL8_DANRE/161-312	F1QWL8.1 PF00620.25	35.30	0.00
TRINITY_I1LEV7_SOYBN/78-478	I1LEV7.1 PF03062.17	35.30	0.00
TRINITY_A0D1C3_PARTE/64-223	A0D1C3.1 PF01343.16	35.30	0.00
TRINITY_A0A078IH43_BRANA/588-899	A0A078IH43.1 PF0225	35.30	0.00
TRINITY_H3FEW2_PRIPA/81-220	H3FEW2.1 PF01764.23	35.30	0.00
TRINITY_L8GMR3_ACACA/270-526	L8GMR3.1 PF07714.15	35.30	0.00
TRINITY_I7M9Y4_TETTS/83-364	I7M9Y4.1 PF01266.22	35.30	0.00
TRINITY_A0BQY4_PARTE/98-531	A0BQY4.1 PF00443.27	35.30	0.00
TRINITY_L8GZD6_ACACA/42-266	L8GZD6.1 PF04938.10	35.30	0.00
TRINITY_D8TTP0_VOLCA/1124-1422	D8TTP0.1 PF00069.23	35.30	0.00
TRINITY_I0ZAB5_9CHLO/587-893	I0ZAB5.1 PF00069.23	35.30	0.00
TRINITY_A0E0G6_PARTE/229-512	A0E0G6.1 PF06664.10	35.30	0.00
TRINITY_Q24FQ7_TETTS/18-275	Q24FQ7.2 PF00069.23	35.30	0.00
TRINITY_A8HSY2_CHLRE/37-412	A8HSY2.1 PF02535.20	35.30	0.00
TRINITY_A0A0M3JZP1_ANISI/75-430	A0A0M3JZP1.1 PF0008	35.30	0.00
TRINITY_I7MH73_TETTS/135-287	I7MH73.1 PF12400.6;	35.20	0.00
TRINITY_A0A067MI27_9HOMO/37-680	A0A067MI27.1 PF0399	35.20	0.00
TRINITY_V7B654_PHAVU/4-119	V7B654.1 PF00168.28	35.20	0.00
TRINITY_F8EER8_RUNSL/34-238	F8EER8.1 PF01734.20	35.20	0.00
TRINITY_T0KSM0_COLGC/10-235	T0KSM0.1 PF05721.11	35.20	0.00
TRINITY_J9IX71_9SPIT/65-299	J9IX71.1 PF00566.16	35.20	0.00
TRINITY_F0ZBZ4_DICPU/39-436	F0ZBZ4.1 PF04801.11	35.20	0.00
TRINITY_Q231F3_TETTS/82-315	Q231F3.1 PF03009.15	35.20	0.00
TRINITY_V6LEA6_9EUKA/78-294	V6LEA6.1 PF02146.15	35.20	0.00
TRINITY_M2XXG5_GALSU/261-390	M2XXG5.1 PF04784.12	35.20	0.00
TRINITY_D3B258_POLPA/403-555	D3B258.1 PF13359.4;	35.20	0.00
TRINITY_A9SAY2_PHYPA/2-220	A9SAY2.1 PF10494.7;	35.20	0.00
TRINITY_Q6BFR2_PARTE/27-205	Q6BFR2.1 PF13462.4;	35.20	0.00
TRINITY_L8HD88_ACACA/139-450	L8HD88.1 PF01266.22	35.20	0.00
TRINITY_D8UJ89_VOLCA/98-1079	D8UJ89.1 PF04931.11	35.20	0.00
TRINITY_F6PSQ8_XENTR/91-562	F6PSQ8.1 PF01425.19	35.20	0.00
TRINITY_Q17I41_AEDAE/77-320	Q17I41.1 PF01663.20	35.20	0.00
TRINITY_B3RMZ8_TRIAD/474-733	B3RMZ8.1 PF00003.20	35.20	0.00
TRINITY_A0A078AR51_STYLE/8-287	A0A078AR51.1 PF0006	35.20	0.00
TRINITY_L2GYX5_VAVCU/24-270	L2GYX5.1 PF00069.23	35.20	0.00
TRINITY_A7SGS9_NEMVE/93-215	A7SGS9.1 PF09335.9;	35.20	0.00
TRINITY_Q54F56_DICDI/852-999	Q54F56.1 PF13926.4;	35.20	0.00
TRINITY_S9W6U9_SCHCR/739-844	S9W6U9.1 PF04003.10	35.20	0.00
TRINITY_A0BXD2_PARTE/279-685	A0BXD2.1 PF01757.20	35.20	0.00
TRINITY_I7M910_TETTS/11-285	I7M910.1 PF00069.23	35.20	0.00
TRINITY_A0D3Z4_PARTE/49-222	A0D3Z4.1 PF01105.22	35.20	0.00
TRINITY_U4TSW9_DENPD/48-334	U4TSW9.1 PF03096.12	35.20	0.00
TRINITY_W7XDG7_TETTS/12-258	W7XDG7.1 PF04252.11	35.20	0.00
TRINITY_D7FWV2_ECTSI/872-1054	D7FWV2.1 PF00092.26	35.20	0.00
TRINITY_F4PMA7_DICFS/179-351	F4PMA7.1 PF00350.21	35.20	0.00
TRINITY_C3XRI3_BRAFL/1097-1484	C3XRI3.1 PF00580.19	35.20	0.00
TRINITY_I7LUW8_TETTS/182-376	I7LUW8.2 PF13886.4;	35.20	0.00
TRINITY_U9SUK0_RHIID/4-94	U9SUK0.1 PF00168.28	35.20	0.00
TRINITY_I7LXL4_TETTS/220-317	I7LXL4.1 PF00153.25	35.20	0.00
TRINITY_W4YIE0_STRPU/7-149	W4YIE0.1 PF00071.20	35.20	0.00
TRINITY_A2E9I9_TRIVA/12-171	A2E9I9.1 PF00071.20	35.20	0.00
TRINITY_A0A078AZ81_STYLE/9-188	A0A078AZ81.1 PF0530	35.20	0.00
TRINITY_A0A072UXG8_MEDTR/130-293	A0A072UXG8.1 PF0316	35.20	0.00
TRINITY_K7IT28_NASVI/57-405	K7IT28.1 PF05147.11	35.20	0.00

TRINITY_A0A091DJ51_FUKDA/231-463	A0A091DJ51.1 PF0115	35.20	0.00
TRINITY_G7I9B4_MEDTR/38-219	G7I9B4.1 PF04413.14	35.20	0.00
TRINITY_G0QIX1_ICHMG/10-323	G0QIX1.1 PF03630.12	35.20	0.00
TRINITY_H3G8Q8_PHYRM/28-251	H3G8Q8.1 PF01738.16	35.10	0.00
TRINITY_Q54VB1_DICDI/23-175	Q54VB1.1 PF14580.4;	35.10	0.00
TRINITY_L1LDB8_THEEQ/9-171	L1LDB8.1 PF00071.20	35.10	0.00
TRINITY_I7M4C2_TETTS/42-276	I7M4C2.1 PF00149.26	35.10	0.00
TRINITY_A0EGI8_PARTE/27-187	A0EGI8.1 PF10294.7;	35.10	0.00
TRINITY_L8GJD9_ACACA/29-126	L8GJD9.1 PF01133.15	35.10	0.00
TRINITY_I7M3M5_TETTS/574-892	I7M3M5.1 PF11838.6;	35.10	0.00
TRINITY_K9VIV2_9CYAN/51-244	K9VIV2.1 PF09511.8;	35.10	0.00
TRINITY_A0A0G4G478_9ALVE/26-579	A0A0G4G478.1 PF0160	35.10	0.00
TRINITY_J9IDK5_9SPIT/476-700	J9IDK5.1 PF12348.6;	35.10	0.00
TRINITY_A0C7I1_PARTE/42-301	A0C7I1.1 PF00069.23	35.10	0.00
TRINITY_M2Y691_GALSU/133-356	M2Y691.1 PF01556.16	35.10	0.00
TRINITY_D3BE12_POLPA/38-242	D3BE12.1 PF00856.26	35.10	0.00
TRINITY_L8H0Q4_ACACA/22-456	L8H0Q4.1 PF00067.20	35.10	0.00
TRINITY_A9U470_PHYPA/136-516	A9U470.1 PF00012.18	35.10	0.00
TRINITY_B0CCN0_ACAM1/40-193	B0CCN0.1 PF13369.4;	35.10	0.00
TRINITY_D8TTA9_VOLCA/13-247	D8TTA9.1 PF01026.19	35.10	0.00
TRINITY_G0QJM1_ICHMG/22-245	G0QJM1.1 PF07722.11	35.10	0.00
TRINITY_A0A0L1IPV0_ASPNO/121-344	A0A0L1IPV0.1 PF0155	35.10	0.00
TRINITY_A0DI05_PARTE/319-612	A0DI05.1 PF04547.10	35.10	0.00
TRINITY_Q23YD6_TETTS/29-282	Q23YD6.1 PF00069.23	35.10	0.00
TRINITY_D8TZX8_VOLCA/219-341	D8TZX8.1 PF01794.17	35.10	0.00
TRINITY_A0A074Y688_9PEZI/256-375	A0A074Y688.1 PF0283	35.10	0.00
TRINITY_A8IXM4_CHLRE/1640-1791	A8IXM4.1 PF03712.13	35.10	0.00
TRINITY_R0GH80_9BRAS/178-465	R0GH80.1 PF06814.11	35.10	0.00
TRINITY_Q22X60_TETTS/11-285	Q22X60.1 PF00069.23	35.10	0.00
TRINITY_K4ABQ6_SETIT/140-318	K4ABQ6.1 PF10294.7;	35.10	0.00
TRINITY_H0HU12_9RHIZ/41-294	H0HU12.1 PF04230.11	35.10	0.00
TRINITY_D2V048_NAEGR/86-253	D2V048.1 PF00092.26	35.10	0.00
TRINITY_Q7NK64_GLOVI/264-552	Q7NK64.1 PF00773.17	35.10	0.00
TRINITY_D8LU94_ECTSI/82-277	D8LU94.1 PF11715.6;	35.10	0.00
TRINITY_K6XP07_9ALTE/44-253	K6XP07.1 PF00149.26	35.10	0.00
TRINITY_I1F267_AMPQE/206-369	I1F267.1 PF13359.4;	35.10	0.00
TRINITY_D8THJ4_VOLCA/30-238	D8THJ4.1 PF01027.18	35.10	0.00
TRINITY_V3ZT54_LOTGI/1-173	V3ZT54.1 PF00025.19	35.10	0.00
TRINITY_A0A0A1NYH9_9FUNG/37-257	A0A0A1NYH9.1 PF0085	35.10	0.00
TRINITY_A0A067SRG2_9AGAR/160-271	A0A067SRG2.1 PF0237	35.10	0.00
TRINITY_A0A090N355_OSTTA/10-180	A0A090N355.1 PF0007	35.10	0.00
TRINITY_E1X1E3_HALMS/17-738	E1X1E3.1 PF01804.16	35.10	0.00
TRINITY_I0I884_CALAS/53-389	I0I884.1 PF01547.23	35.10	0.00
TRINITY_F6XLZ1_XENTR/6-391	F6XLZ1.1 PF04857.18	35.00	0.00
TRINITY_Q54JW6_DICDI/25-310	Q54JW6.1 PF00856.26	35.00	0.00
TRINITY_L8HBK7_ACACA/26-465	L8HBK7.1 PF00067.20	35.00	0.00
TRINITY_B8GKY5_METPE/683-820	B8GKY5.1 PF03422.13	35.00	0.00
TRINITY_E7FFS8_DANRE/98-290	E7FFS8.2 PF07998.9;	35.00	0.00
TRINITY_A8J7M9_CHLRE/742-1038	A8J7M9.1 PF00069.23	35.00	0.00
TRINITY_A0A0N5ART5_9BILA/657-796	A0A0N5ART5.1 PF0176	35.00	0.00
TRINITY_G1KIY9_ANOCA/132-245	G1KIY9.1 PF12796.5;	35.00	0.00
TRINITY_A0A0G4EFZ7_9ALVE/89-487	A0A0G4EFZ7.1 PF0090	35.00	0.00
TRINITY_L8GRV0_ACACA/57-333	L8GRV0.1 PF01490.16	35.00	0.00
TRINITY_D6GSF2_FILAD/8-144	D6GSF2.1 PF02681.12	35.00	0.00
TRINITY_M5WU18_PRUPE/10-171	M5WU18.1 PF00071.20	35.00	0.00
TRINITY_A0DCM6_PARTE/47-264	A0DCM6.1 PF01633.18	35.00	0.00
TRINITY_I7M198_TETTS/52-316	I7M198.2 PF00069.23	35.00	0.00
TRINITY_G0QSH9_ICHMG/120-525	G0QSH9.1 PF01619.16	35.00	0.00
TRINITY_H0VJ55_CAVPO/29-263	H0VJ55.1 PF10223.7;	35.00	0.00
TRINITY_A0A0N0SEV8_9ACTN/25-510	A0A0N0SEV8.1 PF0013	35.00	0.00
TRINITY_D8TU64_VOLCA/75-249	D8TU64.1 PF00089.24	35.00	0.00

TRINITY_A0C319_PARTE/249-448	A0C319.1 PF02735.14	35.00	0.00
TRINITY_L8GKH0_ACACA/249-578	L8GKH0.1 PF13598.4;	35.00	0.00
TRINITY_A0A0E3ZCV1_9BACT/53-433	A0A0E3ZCV1.1 PF0564	35.00	0.00
TRINITY_E1ZSN2_CHLVA/96-517	E1ZSN2.1 PF11957.6;	35.00	0.00
TRINITY_G1PYV1_MYOLU/109-255	G1PYV1.1 PF08662.9;	35.00	0.00
TRINITY_H2SUS1_TAKRU/37-837	H2SUS1.1 PF04563.13	35.00	0.00
TRINITY_A0CC42_PARTE/16-287	A0CC42.1 PF00069.23	35.00	0.00
TRINITY_C6WUF6_METML/905-1020	C6WUF6.1 PF00072.22	35.00	0.00
TRINITY_E1ZMM8_CHLVA/643-864	E1ZMM8.1 PF05548.9;	35.00	0.00
TRINITY_D8TVU5_VOLCA/641-919	D8TVU5.1 PF00233.17	35.00	0.00
TRINITY_L8GPJ3_ACACA/267-618	L8GPJ3.1 PF02386.14	35.00	0.00
TRINITY_A0A0G4J2B1_PLABS/87-313	A0A0G4J2B1.1 PF1485	35.00	0.00
TRINITY_A8J5J3_CHLRE/34-413	A8J5J3.1 PF00266.17	35.00	0.00
TRINITY_A0CS29_PARTE/2561-2834	A0CS29.1 PF00520.29	35.00	0.00
TRINITY_A9T564_PHYPA/113-202	A9T564.1 PF02362.19	35.00	0.00
TRINITY_A8ISQ5_CHLRE/527-928	A8ISQ5.1 PF13086.4;	35.00	0.00
TRINITY_B7P8R1_IXOSC/21-152	B7P8R1.1 PF01138.19	34.90	0.00
TRINITY_W5A4N3_WHEAT/11-265	W5A4N3.1 PF00069.23	34.90	0.00
TRINITY_I7MD42_TETTS/162-405	I7MD42.2 PF00481.19	34.90	0.00
TRINITY_A0A068S7T1_9FUNG/14-119	A0A068S7T1.1 PF0192	34.90	0.00
TRINITY_L8H1U3_ACACA/10-161	L8H1U3.1 PF12239.6;	34.90	0.00
TRINITY_C3YNL0_BRAFL/11-197	C3YNL0.1 PF09808.7;	34.90	0.00
TRINITY_K7IM30_NASVI/14-130	K7IM30.1 PF02893.18	34.90	0.00
TRINITY_K7JWY9_NASVI/466-724	K7JWY9.1 PF00078.25	34.90	0.00

TRINITY_V3Z3J5_LOTGI/39-269	V3Z3J5.1 PF02114.14	34.90	0.00
TRINITY_Q22Y38_TETTS/894-1231	Q22Y38.2 PF01237.16	34.90	0.00
TRINITY_A9VAN1_MONBE/20-210	A9VAN1.1 PF01183.18	34.90	0.00
TRINITY_A8J1A8_CHLRE/209-484	A8J1A8.1 PF04577.12	34.90	0.00
TRINITY_A8IXP6_CHLRE/82-406	A8IXP6.1 PF00704.26	34.90	0.00
TRINITY_A0DFS2_PARTE/641-821	A0DFS2.1 PF05193.19	34.90	0.00
TRINITY_L8GSU3_ACACA/224-441	L8GSU3.1 PF12697.5;	34.90	0.00
TRINITY_L8GIC7_ACACA/9-353	L8GIC7.1 PF02535.20	34.90	0.00
TRINITY_K9RIF6_9CYAN/7-198	K9RIF6.1 PF13460.4;	34.90	0.00
TRINITY_A0A068SB73_9FUNG/109-537	A0A068SB73.1 PF0006	34.90	0.00
TRINITY_A0A087G948_ARAAL/94-480	A0A087G948.1 PF0322	34.90	0.00
TRINITY_T1IVW8_STRMM/219-312	T1IVW8.1 PF00153.25	34.90	0.00
TRINITY_L8HFI1_ACACA/52-233	L8HFI1.1 PF00617.17	34.90	0.00
TRINITY_A0DN68_PARTE/31-272	A0DN68.1 PF00956.16	34.90	0.00
TRINITY_D7U8S3_VITVI/608-818	D7U8S3.1 PF08626.9;	34.90	0.00
TRINITY_D4W7N2_9FIRM/76-204	D4W7N2.1 PF12740.5;	34.90	0.00
TRINITY_B9M8B0_GEODF/64-383	B9M8B0.1 PF01636.21	34.90	0.00
TRINITY_D8QTH1_SELML/234-600	D8QTH1.1 PF09423.8;	34.90	0.00
TRINITY_D3BT39_POLPA/767-918	D3BT39.1 PF01608.15	34.90	0.00
TRINITY_V9F1F2_PHYPR/428-714	V9F1F2.1 PF00481.19	34.90	0.00
TRINITY_A0CBG9_PARTE/12-286	A0CBG9.1 PF00069.23	34.90	0.00
TRINITY_G2MLV0_9ARCH/26-345	G2MLV0.1 PF11199.6;	34.90	0.00
TRINITY_M0T6L8_MUSAM/123-378	M0T6L8.1 PF04811.13	34.90	0.00
TRINITY_A0BV64_PARTE/89-916	A0BV64.1 PF09773.7;	34.90	0.00
TRINITY_D2VET8_NAEGR/14-126	D2VET8.1 PF00307.29	34.90	0.00
TRINITY_A0A067D823_SAPPC/140-257	A0A067D823.1 PF0190	34.90	0.00
TRINITY_D8U479_VOLCA/954-1143	D8U479.1 PF12612.6;	34.90	0.00
TRINITY_L8GMR3_ACACA/270-526	L8GMR3.1 PF07714.15	34.90	0.00
TRINITY_B7QDX1_IXOSC/40-182	B7QDX1.1 PF01699.22	34.90	0.00
TRINITY_J9HX68_9SPIT/57-359	J9HX68.1 PF00648.19	34.90	0.00
TRINITY_I1N4B2_SOYBN/79-285	I1N4B2.1 PF07847.10	34.90	0.00
TRINITY_J9IR36_9SPIT/409-563	J9IR36.1 PF04280.13	34.90	0.00
TRINITY_A0A059LHI8_9CHLO/11-343	A0A059LHI8.1 PF0006	34.90	0.00
TRINITY_A0DI70_PARTE/340-617	A0DI70.1 PF04091.10	34.80	0.00
TRINITY_F9X7C5_ZYMTI/8-244	F9X7C5.1 PF04157.14	34.80	0.00
TRINITY_B9LQN8_HALLT/7-271	B9LQN8.1 PF03372.21	34.80	0.00
TRINITY_Q3SD60_PARTE/9-170	Q3SD60.1 PF00071.20	34.80	0.00
TRINITY_J9JBA7_9SPIT/17-165	J9JBA7.1 PF05188.15	34.80	0.00
TRINITY_L8H2L4_ACACA/85-275	L8H2L4.1 PF05502.11	34.80	0.00
TRINITY_L8H176_ACACA/28-188	L8H176.1 PF06703.9;	34.80	0.00
TRINITY_A8Q838_MALGO/105-416	A8Q838.1 PF00026.21	34.80	0.00
TRINITY_F1NC47_CHICK/135-226	F1NC47.2 PF03908.11	34.80	0.00
TRINITY_L8HCN3_ACACA/51-286	L8HCN3.1 PF04757.12	34.80	0.00
TRINITY_A0A078AR51_STYLE/8-287	A0A078AR51.1 PF0006	34.80	0.00
TRINITY_A0A0G0BAU5_9BACT/23-162	A0A0G0BAU5.1 PF0213	34.80	0.00
TRINITY_D8U8G6_VOLCA/124-246	D8U8G6.1 PF00828.17	34.80	0.00
TRINITY_U3J5E7_ANAPL/1978-2462	U3J5E7.1 PF00476.18	34.80	0.00
TRINITY_I0RDF4_MYCXE/8-426	I0RDF4.1 PF02897.13	34.80	0.00
TRINITY_A0A0D2VQW7_CAPO3/308-459	A0A0D2VQW7.1 PF0441	34.80	0.00
TRINITY_J9IDS2_9SPIT/168-249	J9IDS2.1 PF01485.19	34.80	0.00
TRINITY_H3AZR7_LATCH/38-401	H3AZR7.1 PF07000.9;	34.80	0.00
TRINITY_A0A0D2TLL4_GOSRA/110-403	A0A0D2TLL4.1 PF0972	34.80	0.00
TRINITY_A0A098VN20_9MICR/5-175	A0A098VN20.1 PF0007	34.80	0.00
TRINITY_F0R0T9_BACSH/759-1033	F0R0T9.1 PF02929.15	34.80	0.00
TRINITY_D4M743_9BACT/24-179	D4M743.1 PF13563.4;	34.80	0.00
TRINITY_J9LBD5_ACYPI/71-378	J9LBD5.2 PF00561.18	34.80	0.00
TRINITY_T1EZU6_HELRO/132-220	T1EZU6.1 PF13649.4;	34.80	0.00
TRINITY_L1IVY4_GUITH/10-126	L1IVY4.1 PF10639.7;	34.80	0.00
TRINITY_A0CMN6_PARTE/121-399	A0CMN6.1 PF01457.14	34.80	0.00
TRINITY_Q092W7_STIAD/118-356	Q092W7.1 PF06101.9;	34.80	0.00
TRINITY_F4PR64_DICFS/4-120	F4PR64.1 PF00615.17	34.80	0.00

TRINITY_I0Z7L9_9CHLO/7-158	I0Z7L9.1 PF00179.24	34.80	0.00
TRINITY_M4BT05_HYAAE/2-408	M4BT05.1 PF04188.11	34.80	0.00
TRINITY_K9SZG2_9CYAN/110-382	K9SZG2.1 PF01636.21	34.80	0.00
TRINITY_L8H2X5_ACACA/755-874	L8H2X5.1 PF01067.20	34.80	0.00
TRINITY_A0A075AZS5_9FUNG/23-114	A0A075AZS5.1 PF0468	34.80	0.00
TRINITY_A8IKT9_CHLRE/529-630	A8IKT9.1 PF00134.21	34.80	0.00
TRINITY_A0A0G4IQ86_PLABS/632-902	A0A0G4IQ86.1 PF0218	34.80	0.00
TRINITY_Q54F78_DICDI/37-512	Q54F78.2 PF16134.3;	34.80	0.00
TRINITY_F4PVD5_DICFS/35-289	F4PVD5.1 PF07651.14	34.80	0.00
TRINITY_A0A078B2E5_STYLE/525-896	A0A078B2E5.1 PF1059	34.80	0.00
TRINITY_A8JH49_CHLRE/24-120	A8JH49.1 PF01196.17	34.80	0.00
TRINITY_A0A068Y580_ECHMU/47-420	A0A068Y580.1 PF0341	34.80	0.00
TRINITY_A0A068S0W9_9FUNG/53-352	A0A068S0W9.1 PF0238	34.80	0.00
TRINITY_F5RD44_METUF/90-251	F5RD44.1 PF02955.14	34.80	0.00
TRINITY_A7SJV6_NEMVE/45-509	A7SJV6.1 PF00067.20	34.80	0.00
TRINITY_I0Z4X8_9CHLO/223-518	I0Z4X8.1 PF08540.8;	34.80	0.00
TRINITY_I0YU33_9CHLO/43-470	I0YU33.1 PF02450.13	34.80	0.00
TRINITY_A8J3P1_CHLRE/954-1161	A8J3P1.1 PF00211.18	34.80	0.00
TRINITY_B6K5J7_SCHJY/463-767	B6K5J7.1 PF00632.23	34.70	0.00
TRINITY_A8Q838_MALGO/105-416	A8Q838.1 PF00026.21	34.70	0.00
TRINITY_A0BLH9_PARTE/7-108	A0BLH9.1 PF00168.28	34.70	0.00
TRINITY_G0QNA5_ICHMG/159-338	G0QNA5.1 PF00443.27	34.70	0.00
TRINITY_F0ZDB7_DICPU/280-899	F0ZDB7.1 PF04130.11	34.70	0.00
TRINITY_V6LL14_9EUKA/10-174	V6LL14.1 PF00071.20	34.70	0.00
TRINITY_F4P7C6_BATDJ/5-124	F4P7C6.1 PF08146.10	34.70	0.00
TRINITY_B3KZ91_PLAKH/192-310	B3KZ91.1 PF01569.19	34.70	0.00
TRINITY_J9ING1_9SPIT/21-645	J9ING1.1 PF00888.20	34.70	0.00
TRINITY_W5YNX9_9ALTE/6-200	W5YNX9.1 PF00106.23	34.70	0.00
TRINITY_D0MDL5_RHOM4/19-357	D0MDL5.1 PF05977.11	34.70	0.00
TRINITY_A0A0B2V7Q4_TOXCA/40-174	A0A0B2V7Q4.1 PF0043	34.70	0.00
TRINITY_A0A0D2WPQ9_CAPO3/136-387	A0A0D2WPQ9.1 PF1362	34.70	0.00
TRINITY_A0A078AR51_STYLE/8-287	A0A078AR51.1 PF0006	34.70	0.00
TRINITY_D3BBU1_POLPA/14-124	D3BBU1.1 PF00169.27	34.70	0.00
TRINITY_A0A087H8X7_ARAAL/21-346	A0A087H8X7.1 PF0975	34.70	0.00
TRINITY_Q1AUI2_RUBXD/25-266	Q1AUI2.1 PF03009.15	34.70	0.00
TRINITY_A0A015K3E4_9GLOM/5-105	A0A015K3E4.1 PF0016	34.70	0.00
TRINITY_Q6BJF3_DEBHA/225-415	Q6BJF3.1 PF11715.6;	34.70	0.00
TRINITY_A0A0G4IVB0_PLABS/36-285	A0A0G4IVB0.1 PF1362	34.70	0.00
TRINITY_U1MKK4_ASCSU/61-304	U1MKK4.1 PF13360.4;	34.70	0.00
TRINITY_G1N844_MELGA/89-387	G1N844.1 PF00561.18	34.70	0.00
TRINITY_L8GSM7_ACACA/2-268	L8GSM7.1 PF03372.21	34.70	0.00
TRINITY_Q244W6_TETTS/9-150	Q244W6.2 PF02453.15	34.70	0.00
TRINITY_A0A078ATL6_STYLE/22-309	A0A078ATL6.1 PF1523	34.70	0.00
TRINITY_A8IXZ2_CHLRE/6-158	A8IXZ2.1 PF00582.24	34.70	0.00
TRINITY_F0ZE88_DICPU/11-451	F0ZE88.1 PF08217.9;	34.70	0.00
TRINITY_W7XJ96_TETTS/33-488	W7XJ96.1 PF00067.20	34.70	0.00
TRINITY_H3BZH9_TETNG/49-233	H3BZH9.1 PF01762.19	34.70	0.00
TRINITY_Q23K45_TETTS/268-368	Q23K45.1 PF00085.18	34.70	0.00
TRINITY_A0C868_PARTE/287-682	A0C868.1 PF01757.20	34.70	0.00
TRINITY_K7ITH4_NASVI/179-284	K7ITH4.1 PF00651.29	34.70	0.00
TRINITY_A0A0G0QXC1_9BACT/13-174	A0A0G0QXC1.1 PF0316	34.70	0.00
TRINITY_A0A061D898_BABBI/410-659	A0A061D898.1 PF0150	34.70	0.00
TRINITY_W5JU90_ANODA/491-629	W5JU90.1 PF13716.4;	34.70	0.00
TRINITY_I7LWU9_TETTS/187-305	I7LWU9.2 PF13863.4;	34.70	0.00
TRINITY_A0A078AYH2_STYLE/42-314	A0A078AYH2.1 PF0374	34.70	0.00
TRINITY_C5Z3M4_SORBI/88-327	C5Z3M4.1 PF00481.19	34.70	0.00
TRINITY_I7M7H8_TETTS/53-447	I7M7H8.2 PF01490.16	34.70	0.00
TRINITY_D8TH55_VOLCA/45-544	D8TH55.1 PF03999.10	34.70	0.00
TRINITY_G0PIW0_CAEBE/54-179	G0PIW0.1 PF00651.29	34.70	0.00
TRINITY_V8NLQ1_OPHHA/53-205	V8NLQ1.1 PF00620.25	34.70	0.00
TRINITY_A8J7D3_CHLRE/129-245	A8J7D3.1 PF13640.4;	34.70	0.00

TRINITY_A8IS63_CHLRE/199-437	A8IS63.1 PF00667.18	34.70	0.00
TRINITY_I7LZY1_TETTS/2-293	I7LZY1.2 PF03643.13	34.70	0.00
TRINITY_S2J6X3_MUCC1/13-174	S2J6X3.1 PF00071.20	34.70	0.00
TRINITY_F0ZAI9_DICPU/651-1021	F0ZAI9.1 PF02181.21	34.70	0.00
TRINITY_T0S211_9STRA/307-915	T0S211.1 PF10408.7;	34.70	0.00
TRINITY_A0A075AP74_9FUNG/1-126	A0A075AP74.1 PF0023	34.70	0.00
TRINITY_A0C9E3_PARTE/20-157	A0C9E3.1 PF00179.24	34.70	0.00
TRINITY_Q2SHR5_HAHCH/127-277	Q2SHR5.1 PF06445.13	34.70	0.00
TRINITY_F7AJ67_XENTR/46-153	F7AJ67.1 PF00651.29	34.60	0.00
TRINITY_A0A022RVM8_ERYGU/30-427	A0A022RVM8.1 PF0045	34.60	0.00
TRINITY_K0KRE4_WICCF/375-544	K0KRE4.1 PF02902.17	34.60	0.00
TRINITY_Q09E43_STIAD/320-840	Q09E43.1 PF02128.13	34.60	0.00
TRINITY_Q23MA7_TETTS/15-440	Q23MA7.1 PF01593.22	34.60	0.00
TRINITY_F6YM31_CIOIN/45-180	F6YM31.2 PF00782.18	34.60	0.00
TRINITY_L8GET8_ACACA/107-282	L8GET8.1 PF09637.8;	34.60	0.00
TRINITY_PSMG2_NEMVE/19-240	A7SGU6.1 PF09754.7;	34.60	0.00
TRINITY_Q22MA8_TETTS/122-276	Q22MA8.2 PF00650.18	34.60	0.00
TRINITY_G0RZP4_CHATD/19-209	G0RZP4.1 PF13472.4;	34.60	0.00
TRINITY_A0A084W2F1_ANOSI/532-718	A0A084W2F1.1 PF0161	34.60	0.00
TRINITY_B4N666_DROWI/255-374	B4N666.2 PF00622.26	34.60	0.00
TRINITY_I7MI06_TETTS/309-416	I7MI06.2 PF00085.18	34.60	0.00
TRINITY_A4VDN1_TETTS/4-430	A4VDN1.1 PF00067.20	34.60	0.00
TRINITY_I0ZAV6_9CHLO/30-82	I0ZAV6.1 PF07491.9;	34.60	0.00
TRINITY_A8J9J5_CHLRE/40-249	A8J9J5.1 PF00856.26	34.60	0.00
TRINITY_A0A0L0C5U9_LUCCU/529-741	A0A0L0C5U9.1 PF1270	34.60	0.00
TRINITY_A8JFG7_CHLRE/20-187	A8JFG7.1 PF16016.3;	34.60	0.00
TRINITY_F0ZNA5_DICPU/102-307	F0ZNA5.1 PF06999.10	34.60	0.00
TRINITY_C3Z9X5_BRAFL/97-1081	C3Z9X5.1 PF03813.12	34.60	0.00
TRINITY_A9V0E1_MONBE/11-355	A9V0E1.1 PF03283.11	34.60	0.00
TRINITY_A7APB0_BABBO/869-1003	A7APB0.1 PF04096.12	34.60	0.00
TRINITY_G0QXK0_ICHMG/11-169	G0QXK0.1 PF00339.27	34.60	0.00
TRINITY_A0A078AI13_STYLE/16-119	A0A078AI13.1 PF0030	34.60	0.00
TRINITY_Q55F12_DICDI/374-640	Q55F12.1 PF00149.26	34.60	0.00
TRINITY_E9DJ49_COCPS/180-469	E9DJ49.1 PF08569.9;	34.60	0.00
TRINITY_J9IEP0_9SPIT/637-1073	J9IEP0.1 PF04547.10	34.60	0.00
TRINITY_A0A0R4ICI6_DANRE/29-485	A0A0R4ICI6.1 PF0578	34.60	0.00
TRINITY_D8FV64_9CYAN/82-418	D8FV64.1 PF01547.23	34.60	0.00
TRINITY_A0A024TME2_9STRA/61-491	A0A024TME2.1 PF0006	34.60	0.00
TRINITY_A0A078AEZ9_STYLE/129-236	A0A078AEZ9.1 PF0078	34.60	0.00
TRINITY_M3AUV3_PSEFD/1096-1257	M3AUV3.1 PF04408.21	34.60	0.00
TRINITY_K7L1S8_SOYBN/87-177	K7L1S8.1 PF00686.17	34.60	0.00
TRINITY_L8HB44_ACACA/7-110	L8HB44.1 PF05773.20	34.60	0.00
TRINITY_K9TNS6_9CYAN/49-502	K9TNS6.1 PF02113.13	34.60	0.00
TRINITY_L8H8Z5_ACACA/146-294	L8H8Z5.1 PF13358.4;	34.60	0.00
TRINITY_G0QRX1_ICHMG/11-199	G0QRX1.1 PF04511.13	34.60	0.00
TRINITY_L8H0F7_ACACA/10-170	L8H0F7.1 PF07074.10	34.60	0.00
TRINITY_A0CN02_PARTE/64-622	A0CN02.1 PF00995.21	34.60	0.00
TRINITY_A8J1Y1_CHLRE/568-1125	A8J1Y1.1 PF07969.9;	34.60	0.00
TRINITY_I0YKD6_9CHLO/181-440	I0YKD6.1 PF00520.29	34.60	0.00
TRINITY_D8U5L5_VOLCA/20-315	D8U5L5.1 PF13896.4;	34.60	0.00
TRINITY_F4PUC9_DICFS/84-409	F4PUC9.1 PF00328.20	34.60	0.00
TRINITY_A0A0D3CKW4_BRAOL/732-862	A0A0D3CKW4.1 PF0185	34.60	0.00
TRINITY_D2VMI5_NAEGR/22-391	D2VMI5.1 PF07065.12	34.60	0.00
TRINITY_L1IYY6_GUITH/308-768	L1IYY6.1 PF00082.20	34.60	0.00
TRINITY_Q2UT62_ASPOR/16-158	Q2UT62.1 PF12009.6;	34.50	0.00
TRINITY_D6U5T4_9CHLR/11-461	D6U5T4.1 PF00067.20	34.50	0.00
TRINITY_A0A0K9NNN9_ZOSMR/54-398	A0A0K9NNN9.1 PF0245	34.50	0.00
TRINITY_A0CTF8_PARTE/99-592	A0CTF8.1 PF01425.19	34.50	0.00
TRINITY_G6FNB2_9CYAN/63-231	G6FNB2.1 PF00188.24	34.50	0.00
TRINITY_Q22RP1_TETTS/123-413	Q22RP1.2 PF01513.19	34.50	0.00
TRINITY_D3B9I0_POLPA/31-228	D3B9I0.1 PF00106.23	34.50	0.00

TRINITY_A0A0E3UX38_9BACT/5-226	A0A0E3UX38.1 PF0536	34.50	0.00
TRINITY_V4BAJ3_LOTGI/22-109	V4BAJ3.1 PF14712.4;	34.50	0.00
TRINITY_G0QQC4_ICHMG/15-404	G0QQC4.1 PF07690.14	34.50	0.00
TRINITY_F4PKR3_DICFS/803-1003	F4PKR3.1 PF09184.9;	34.50	0.00
TRINITY_L8H0A3_ACACA/24-404	L8H0A3.1 PF04912.12	34.50	0.00
TRINITY_D8U571_VOLCA/783-1138	D8U571.1 PF00069.23	34.50	0.00
TRINITY_L8GFU2_ACACA/577-721	L8GFU2.1 PF00644.18	34.50	0.00
TRINITY_E1BUG3_CHICK/247-395	E1BUG3.2 PF01794.17	34.50	0.00
TRINITY_A0A0D2UKQ8_CAPO3/188-572	A0A0D2UKQ8.1 PF0065	34.50	0.00
TRINITY_D8SE62_SELML/1-97	D8SE62.1 PF13499.4;	34.50	0.00
TRINITY_A7SN67_NEMVE/6-350	A7SN67.1 PF00443.27	34.50	0.00
TRINITY_F1RS99_PIG/625-793	F1RS99.1 PF00092.26	34.50	0.00
TRINITY_A0A087ST69_AUXPR/27-271	A0A087ST69.1 PF1214	34.50	0.00
TRINITY_F0ZRV9_DICPU/391-664	F0ZRV9.1 PF02714.13	34.50	0.00
TRINITY_J9I240_9SPIT/208-361	J9I240.1 PF05708.10	34.50	0.00
TRINITY_C3Z429_BRAFL/78-495	C3Z429.1 PF00962.20	34.50	0.00
TRINITY_F0ZMQ8_DICPU/111-220	F0ZMQ8.1 PF14593.4;	34.50	0.00
TRINITY_G0R3L9_ICHMG/178-343	G0R3L9.1 PF11721.6;	34.50	0.00
TRINITY_MYLKB_DICDI/20-278	Q86AD7.1 PF00069.23	34.50	0.00
TRINITY_GPA1_DICDI/15-344	P16894.1 PF00503.18	34.50	0.00
TRINITY_A9TF31_PHYPA/14-140	A9TF31.1 PF00241.18	34.50	0.00
TRINITY_V4LC58_9GAMM/34-514	V4LC58.1 PF00135.26	34.50	0.00
TRINITY_C4LUJ0_ENTHI/260-381	C4LUJ0.1 PF03953.15	34.50	0.00
TRINITY_J9HYJ6_9SPIT/30-171	J9HYJ6.1 PF00179.24	34.50	0.00
TRINITY_D8UIJ1_VOLCA/13-334	D8UIJ1.1 PF01266.22	34.50	0.00
TRINITY_Q22P24_TETTS/166-362	Q22P24.2 PF00149.26	34.50	0.00
TRINITY_D8U0C5_VOLCA/2-156	D8U0C5.1 PF00929.22	34.50	0.00
TRINITY_A0BU11_PARTE/3-311	A0BU11.1 PF00459.23	34.50	0.00
TRINITY_PNPP_SCHPO/18-258	Q00472.2 PF00702.24	34.50	0.00
TRINITY_D8TPN9_VOLCA/226-466	D8TPN9.1 PF05548.9;	34.50	0.00
TRINITY_F4PW45_DICFS/16-125	F4PW45.1 PF02991.14	34.50	0.00
TRINITY_A0BXG1_PARTE/29-97	A0BXG1.1 PF10601.7;	34.40	0.00
TRINITY_A0D054_PARTE/220-345	A0D054.1 PF12796.5;	34.40	0.00
TRINITY_W4Y077_STRPU/146-350	W4Y077.1 PF00278.20	34.40	0.00
TRINITY_A0A0K0FFA1_9BILA/12-173	A0A0K0FFA1.1 PF0007	34.40	0.00
TRINITY_F4PXL5_DICFS/1-231	F4PXL5.1 PF10574.7;	34.40	0.00
TRINITY_A0A078B2J0_STYLE/41-343	A0A078B2J0.1 PF0064	34.40	0.00
TRINITY_A0A0G4IYC6_PLABS/25-294	A0A0G4IYC6.1 PF0120	34.40	0.00
TRINITY_C3YV41_BRAFL/27-165	C3YV41.1 PF03188.14	34.40	0.00
TRINITY_A9G0Q5_SORC5/53-346	A9G0Q5.1 PF14388.4;	34.40	0.00
TRINITY_F4PX08_DICFS/1189-1360	F4PX08.1 PF00616.17	34.40	0.00
TRINITY_L8HJL6_ACACA/18-211	L8HJL6.1 PF01195.17	34.40	0.00
TRINITY_A2F029_TRIVA/16-268	A2F029.1 PF00069.23	34.40	0.00
TRINITY_A0CKF8_PARTE/784-1056	A0CKF8.1 PF00664.21	34.40	0.00
TRINITY_L8H7T2_ACACA/124-454	L8H7T2.1 PF16113.3;	34.40	0.00
TRINITY_R7YL61_CONA1/217-308	R7YL61.1 PF00153.25	34.40	0.00
TRINITY_Q232X8_TETTS/36-488	Q232X8.1 PF00067.20	34.40	0.00
TRINITY_B4VYJ3_9CYAN/988-1225	B4VYJ3.1 PF07993.10	34.40	0.00
TRINITY_A0A084WMK6_ANOSI/27-268	A0A084WMK6.1 PF0115	34.40	0.00
TRINITY_A0DN68_PARTE/31-272	A0DN68.1 PF00956.16	34.40	0.00
TRINITY_A0A078AJI8_STYLE/155-505	A0A078AJI8.1 PF1386	34.40	0.00
TRINITY_T1KU00_TETUR/83-406	T1KU00.1 PF00561.18	34.40	0.00
TRINITY_I0I884_CALAS/53-389	I0I884.1 PF01547.23	34.40	0.00
TRINITY_Q54B73_DICDI/144-412	Q54B73.1 PF02811.17	34.40	0.00
TRINITY_D2V9V6_NAEGR/44-290	D2V9V6.1 PF03372.21	34.40	0.00
TRINITY_V4TAC2_9ROSI/92-279	V4TAC2.1 PF04893.15	34.40	0.00
TRINITY_G5HA38_9BACT/48-249	G5HA38.1 PF00106.23	34.40	0.00
TRINITY_A8IPD2_CHLRE/123-314	A8IPD2.1 PF00743.17	34.40	0.00
TRINITY_A0A0G0WSL3_9BACT/52-473	A0A0G0WSL3.1 PF0050	34.40	0.00
TRINITY_A0DKC8_PARTE/3-166	A0DKC8.1 PF14580.4;	34.40	0.00
TRINITY_G0R1P1_ICHMG/34-476	G0R1P1.1 PF13347.4;	34.40	0.00

TRINITY_U4L3F9_PYROM/2305-2422	U4L3F9.1 PF00622.26	34.40	0.00
TRINITY_A0A068RPF7_9FUNG/254-500	A0A068RPF7.1 PF0056	34.40	0.00
TRINITY_T1L2L1_TETUR/51-149	T1L2L1.1 PF04900.10	34.40	0.00
TRINITY_A0A0D2WLJ2_CAPO3/18-142	A0A0D2WLJ2.1 PF0370	34.40	0.00
TRINITY_J9JR19_ACYPI/16-143	J9JR19.2 PF00293.26	34.40	0.00
TRINITY_A0A010REE5_9PEZI/155-273	A0A010REE5.1 PF0246	34.40	0.00
TRINITY_A0A0G4INY1_PLABS/49-372	A0A0G4INY1.1 PF0777	34.40	0.00
TRINITY_A0A0G4EVI0_9ALVE/37-191	A0A0G4EVI0.1 PF0928	34.40	0.00
TRINITY_A0A0M2PU93_PROHO/94-505	A0A0M2PU93.1 PF0001	34.40	0.00
TRINITY_M7BTK2_CHEMY/270-501	M7BTK2.1 PF00233.17	34.40	0.00
TRINITY_D8TGX7_VOLCA/102-547	D8TGX7.1 PF05833.9;	34.40	0.00
TRINITY_X1X2G5_ACYPI/171-335	X1X2G5.1 PF13359.4;	34.40	0.00
TRINITY_D8UBR9_VOLCA/5-112	D8UBR9.1 PF00635.24	34.40	0.00
TRINITY_F0Y6L3_AURAN/5-178	F0Y6L3.1 PF00071.20	34.30	0.00
TRINITY_A0BFQ7_PARTE/4-110	A0BFQ7.1 PF02991.14	34.30	0.00
TRINITY_A0CBG9_PARTE/12-286	A0CBG9.1 PF00069.23	34.30	0.00
TRINITY_F0XQS0_GROCL/534-680	F0XQS0.1 PF01699.22	34.30	0.00
TRINITY_E4XHQ3_OIKDI/2-154	E4XHQ3.1 PF00106.23	34.30	0.00
TRINITY_Q24G73_TETTS/4-188	Q24G73.2 PF04573.10	34.30	0.00
TRINITY_D3BAH8_POLPA/9-101	D3BAH8.1 PF00153.25	34.30	0.00
TRINITY_D3B3S3_POLPA/33-359	D3B3S3.1 PF00328.20	34.30	0.00
TRINITY_K3YS89_SETIT/12-266	K3YS89.1 PF00069.23	34.30	0.00
TRINITY_A0A0A8K1R1_9RHIZ/337-455	A0A0A8K1R1.1 PF0251	34.30	0.00
TRINITY_A0A023XZV5_9GAMM/5-303	A0A023XZV5.1 PF0115	34.30	0.00
TRINITY_W4FHE6_9STRA/391-642	W4FHE6.1 PF07714.15	34.30	0.00
TRINITY_D2VLS7_NAEGR/1151-1256	D2VLS7.1 PF02204.16	34.30	0.00
TRINITY_D2V0G2_NAEGR/7-240	D2V0G2.1 PF06626.10	34.30	0.00
TRINITY_E1ZKH6_CHLVA/2-115	E1ZKH6.1 PF01187.16	34.30	0.00
TRINITY_A0A0F4QY77_9GAMM/47-455	A0A0F4QY77.1 PF0159	34.30	0.00
TRINITY_D3B9C0_POLPA/41-264	D3B9C0.1 PF10607.7;	34.30	0.00
TRINITY_I7M602_TETTS/109-470	I7M602.2 PF07690.14	34.30	0.00
TRINITY_C1ABJ6_GEMAT/11-217	C1ABJ6.1 PF02492.17	34.30	0.00
TRINITY_L8H4W5_ACACA/93-449	L8H4W5.1 PF03372.21	34.30	0.00
TRINITY_A0A0A0LB60_CUCSA/496-822	A0A0A0LB60.1 PF0150	34.30	0.00
TRINITY_A0A0G4H885_9ALVE/380-485	A0A0G4H885.1 PF0008	34.30	0.00
TRINITY_Q8XYB7_RALSO/12-284	Q8XYB7.1 PF12697.5;	34.30	0.00
TRINITY_H6NNK5_9BACL/10-266	H6NNK5.1 PF01026.19	34.30	0.00
TRINITY_A0A075AYI6_9FUNG/177-491	A0A075AYI6.1 PF0154	34.30	0.00
TRINITY_A0A098D2C1_GIBZA/476-613	A0A098D2C1.1 PF1200	34.30	0.00
TRINITY_M3XL57_LATCH/3-188	M3XL57.1 PF00078.25	34.30	0.00
TRINITY_T1L0P2_TETUR/70-215	T1L0P2.1 PF08212.10	34.30	0.00
TRINITY_A0A078A6G9_STYLE/73-422	A0A078A6G9.1 PF0268	34.30	0.00
TRINITY_K7IW89_NASVI/186-293	K7IW89.1 PF00651.29	34.30	0.00
TRINITY_F1QNZ4_DANRE/117-321	F1QNZ4.1 PF05557.11	34.30	0.00
TRINITY_M4F625_BRARP/60-189	M4F625.1 PF04494.13	34.30	0.00
TRINITY_L8H9V4_ACACA/887-1150	L8H9V4.1 PF12931.5;	34.30	0.00
TRINITY_Q22C93_TETTS/132-403	Q22C93.2 PF00561.18	34.30	0.00
TRINITY_D8TX27_VOLCA/36-313	D8TX27.1 PF00481.19	34.30	0.00
TRINITY_E6ZR41_SPORE/93-296	E6ZR41.1 PF01145.23	34.30	0.00
TRINITY_Q23DC1_TETTS/461-1153	Q23DC1.2 PF00443.27	34.30	0.00
TRINITY_A0A086SW02_ACRCH/105-245	A0A086SW02.1 PF0091	34.30	0.00
TRINITY_F9GA28_FUSOF/1-104	F9GA28.1 PF08284.9;	34.30	0.00
TRINITY_D8LGZ7_ECTSI/34-842	D8LGZ7.1 PF04563.13	34.30	0.00
TRINITY_A0CY54_PARTE/132-237	A0CY54.1 PF02517.14	34.30	0.00
TRINITY_Q22WB2_TETTS/1109-1395	Q22WB2.1 PF10300.7;	34.30	0.00
TRINITY_K1RDQ2_CRAGI/2-361	K1RDQ2.1 PF05427.9;	34.20	0.00
TRINITY_I7LX27_TETTS/15-441	I7LX27.1 PF01593.22	34.20	0.00
TRINITY_Q232X8_TETTS/36-488	Q232X8.1 PF00067.20	34.20	0.00
TRINITY_I7LX20_TETTS/11-287	I7LX20.2 PF00069.23	34.20	0.00
TRINITY_G0SXU6_RHOG2/122-373	G0SXU6.1 PF12146.6;	34.20	0.00
TRINITY_D8TTE6_VOLCA/333-484	D8TTE6.1 PF12499.6;	34.20	0.00

TRINITY_D2UXM3_NAEGR/141-412	D2UXM3.1 PF07787.10	34.20	0.00
TRINITY_K3ZS47_SETIT/263-367	K3ZS47.1 PF00168.28	34.20	0.00
TRINITY_W6QM71_PENRO/100-197	W6QM71.1 PF13646.4;	34.20	0.00
TRINITY_A0A074T7I0_HAMHA/599-667	A0A074T7I0.1 PF0220	34.20	0.00
TRINITY_A0A0G2ZUN3_9DELT/30-172	A0A0G2ZUN3.1 PF1358	34.20	0.00
TRINITY_L8HBK7_ACACA/26-465	L8HBK7.1 PF00067.20	34.20	0.00
TRINITY_F2UPY7_SALR5/97-341	F2UPY7.1 PF02263.17	34.20	0.00
TRINITY_V5F2V9_PSEBG/234-429	V5F2V9.1 PF06102.10	34.20	0.00
TRINITY_H1A365_TAEGU/168-504	H1A365.1 PF05277.10	34.20	0.00
TRINITY_Q22U62_TETTS/7-459	Q22U62.1 PF03348.13	34.20	0.00
TRINITY_R5PHN4_9BACT/43-255	R5PHN4.1 PF00149.26	34.20	0.00
TRINITY_A0A0G3A2V9_9DELT/7-233	A0A0G3A2V9.1 PF0536	34.20	0.00
TRINITY_E9DHS1_COCPS/860-1228	E9DHS1.1 PF01237.16	34.20	0.00
TRINITY_W5JHU3_ANODA/948-1221	W5JHU3.1 PF00664.21	34.20	0.00
TRINITY_G1NLD9_MELGA/40-541	G1NLD9.2 PF00443.27	34.20	0.00
TRINITY_D3CUD7_9ACTN/45-303	D3CUD7.1 PF00069.23	34.20	0.00
TRINITY_Q245D9_TETTS/55-158	Q245D9.2 PF08241.10	34.10	0.00
TRINITY_T1JE67_STRMM/3-361	T1JE67.1 PF05427.9;	34.10	0.00
TRINITY_A0E368_PARTE/250-369	A0E368.1 PF14536.4;	34.10	0.00
TRINITY_A0A0D2WWW9_CAPO3/664-1017	A0A0D2WWW9.1 PF0218	34.10	0.00
TRINITY_E1ZDN5_CHLVA/279-378	E1ZDN5.1 PF00153.25	34.10	0.00
TRINITY_A9B4J4_HERA2/210-387	A9B4J4.1 PF13229.4;	34.10	0.00
TRINITY_Q232X8_TETTS/36-488	Q232X8.1 PF00067.20	34.10	0.00
TRINITY_A0CXP9_PARTE/92-386	A0CXP9.1 PF01457.14	34.10	0.00
TRINITY_K1QS27_CRAGI/169-254	K1QS27.1 PF09409.8;	34.10	0.00
TRINITY_A0A078AR51_STYLE/8-287	A0A078AR51.1 PF0006	34.10	0.00
TRINITY_A0A0D2VK03_CAPO3/191-608	A0A0D2VK03.1 PF0316	34.10	0.00
TRINITY_A0A067D6M2_SAPPC/26-209	A0A067D6M2.1 PF1346	34.10	0.00
TRINITY_A0A015KSL8_9GLOM/311-525	A0A015KSL8.1 PF0083	34.10	0.00
TRINITY_A0A0B0PQN3_GOSAR/128-371	A0A0B0PQN3.1 PF0056	34.10	0.00
TRINITY_L2GVH5_VAVCU/142-220	L2GVH5.1 PF14360.4;	34.10	0.00
TRINITY_XPO6_DANRE/103-276	Q8QHJ8.1 PF08389.10	34.10	0.00
TRINITY_A0DT76_PARTE/26-439	A0DT76.1 PF03164.12	34.10	0.00
TRINITY_M7BVW1_CHEMY/82-218	M7BVW1.1 PF07534.14	34.10	0.00
TRINITY_D8THE2_VOLCA/31-230	D8THE2.1 PF00227.24	34.10	0.00
TRINITY_B9SH40_RICCO/305-574	B9SH40.1 PF00069.23	34.10	0.00
TRINITY_D3AWF6_POLPA/1305-1480	D3AWF6.1 PF00617.17	34.10	0.00
TRINITY_A0A024UEU9_9STRA/9-262	A0A024UEU9.1 PF0385	34.10	0.00
TRINITY_A0A068S5U1_9FUNG/59-521	A0A068S5U1.1 PF0006	34.10	0.00
TRINITY_A0A0A1NH50_9FUNG/11-444	A0A0A1NH50.1 PF0360	34.10	0.00
TRINITY_L1LA63_THEEQ/31-247	L1LA63.1 PF00566.16	34.10	0.00
TRINITY_H0Q4S0_9RHOO/6-227	H0Q4S0.1 PF05368.11	34.10	0.00
TRINITY_W8F5N9_9BACT/247-782	W8F5N9.1 PF02128.13	34.10	0.00
TRINITY_E1ZMD3_CHLVA/4-184	E1ZMD3.1 PF01467.24	34.10	0.00
TRINITY_D8TKX7_VOLCA/49-142	D8TKX7.1 PF00686.17	34.10	0.00
TRINITY_I0YKD0_9CHLO/7-215	I0YKD0.1 PF03959.11	34.10	0.00
TRINITY_F4PXG7_DICFS/6-148	F4PXG7.1 PF10358.7;	34.10	0.00
TRINITY_D6TJN6_9CHLR/46-800	D6TJN6.1 PF01804.16	34.10	0.00
TRINITY_X6LE16_RETFI/317-409	X6LE16.1 PF00169.27	34.10	0.00
TRINITY_W7WVY8_TETTS/68-336	W7WVY8.1 PF00069.23	34.10	0.00
TRINITY_W5KRG5_ASTMX/4-257	W5KRG5.1 PF00069.23	34.10	0.00
TRINITY_M2M5B8_BAUCO/1-288	M2M5B8.1 PF04724.11	34.10	0.00
TRINITY_A0A078J9P7_BRANA/13-184	A0A078J9P7.1 PF1265	34.10	0.00
TRINITY_A0A024TMK6_9STRA/125-290	A0A024TMK6.1 PF1029	34.10	0.00
TRINITY_W9RCB9_9ROSA/47-245	W9RCB9.1 PF00106.23	34.10	0.00
TRINITY_L8HBZ7_ACACA/45-347	L8HBZ7.1 PF01148.18	34.10	0.00
TRINITY_A0A087SEB0_AUXPR/837-1291	A0A087SEB0.1 PF0075	34.10	0.00
TRINITY_A8J7J9_CHLRE/33-196	A8J7J9.1 PF12499.6;	34.10	0.00
TRINITY_A0A024UBD2_9STRA/265-453	A0A024UBD2.1 PF0223	34.10	0.00
TRINITY_A0A0J7MYI6_LASNI/104-263	A0A0J7MYI6.1 PF1335	34.10	0.00
TRINITY_Q23YE6_TETTS/45-820	Q23YE6.1 PF01804.16	34.00	0.00

TRINITY_G0QUT0_ICHMG/10-300	G0QUT0.1 PF00069.23	34.00	0.00
TRINITY_L0PEF3_PNEJ8/829-1015	L0PEF3.1 PF00617.17	34.00	0.00
TRINITY_A0A0F5YFY0_9CYAN/7-191	A0A0F5YFY0.1 PF1346	34.00	0.00
TRINITY_A0A0M0K1H4_9EUKA/547-653	A0A0M0K1H4.1 PF0220	34.00	0.00
TRINITY_B7PRH5_IXOSC/38-506	B7PRH5.1 PF00118.22	34.00	0.00
TRINITY_A0CXP9_PARTE/92-386	A0CXP9.1 PF01457.14	34.00	0.00
TRINITY_RTK1_YEAST/303-575	Q12100.1 PF00069.23	34.00	0.00
TRINITY_A0A0A1P2K3_9FUNG/88-539	A0A0A1P2K3.1 PF0006	34.00	0.00
TRINITY_A8J4B3_CHLRE/54-397	A8J4B3.1 PF10250.7;	34.00	0.00
TRINITY_A0A059LJV4_9CHLO/37-203	A0A059LJV4.1 PF0303	34.00	0.00
TRINITY_L8H0Q4_ACACA/22-456	L8H0Q4.1 PF00067.20	34.00	0.00
TRINITY_M2XUX5_GALSU/12-230	M2XUX5.1 PF13489.4;	34.00	0.00
TRINITY_I7MD42_TETTS/162-405	I7MD42.2 PF00481.19	34.00	0.00
TRINITY_D8TIQ2_VOLCA/20-127	D8TIQ2.1 PF12353.6;	34.00	0.00
TRINITY_J3K977_COCIM/635-811	J3K977.2 PF03914.15	34.00	0.00
TRINITY_B0T3U6_CAUSK/89-275	B0T3U6.1 PF07859.11	34.00	0.00
TRINITY_W4GTJ7_9STRA/26-526	W4GTJ7.1 PF01457.14	34.00	0.00
TRINITY_A7S7T2_NEMVE/1-225	A7S7T2.1 PF08423.9;	34.00	0.00
TRINITY_A0A067L538_JATCU/1-438	A0A067L538.1 PF1205	34.00	0.00
TRINITY_A9UU07_MONBE/109-353	A9UU07.1 PF00487.22	34.00	0.00
TRINITY_K1VK27_TRIAC/159-712	K1VK27.1 PF08553.8;	34.00	0.00
TRINITY_V8PGZ0_OPHHA/116-621	V8PGZ0.1 PF16969.3;	34.00	0.00
TRINITY_A8JF93_CHLRE/567-861	A8JF93.1 PF00069.23	34.00	0.00
TRINITY_A8I5V9_CHLRE/18-421	A8I5V9.1 PF07065.12	34.00	0.00
TRINITY_L8H0Q4_ACACA/22-456	L8H0Q4.1 PF00067.20	34.00	0.00
TRINITY_Q22C93_TETTS/132-403	Q22C93.2 PF00561.18	34.00	0.00
TRINITY_F0ZC04_DICPU/25-339	F0ZC04.1 PF09724.7;	34.00	0.00
TRINITY_I1IH10_BRADI/24-280	I1IH10.1 PF00069.23	34.00	0.00
TRINITY_E1Z6J1_CHLVA/60-213	E1Z6J1.1 PF12708.5;	34.00	0.00
TRINITY_E1Z3N0_CHLVA/28-361	E1Z3N0.1 PF00295.15	34.00	0.00
TRINITY_A8I9N4_CHLRE/13-171	A8I9N4.1 PF12499.6;	34.00	0.00
TRINITY_A7T3M9_NEMVE/41-280	A7T3M9.1 PF12146.6;	34.00	0.00
TRINITY_L8H9N6_ACACA/19-590	L8H9N6.1 PF05600.10	34.00	0.00
TRINITY_L8H5A9_ACACA/74-237	L8H5A9.1 PF04387.12	34.00	0.00
TRINITY_D8TS85_VOLCA/84-353	D8TS85.1 PF13896.4;	34.00	0.00
TRINITY_A0A087SMS4_AUXPR/403-512	A0A087SMS4.1 PF1364	34.00	0.00
TRINITY_A8IUH2_CHLRE/119-324	A8IUH2.1 PF07714.15	34.00	0.00
TRINITY_I0YU90_9CHLO/9-650	I0YU90.1 PF04506.11	34.00	0.00
TRINITY_A8JDQ6_CHLRE/523-667	A8JDQ6.1 PF08016.10	34.00	0.00
TRINITY_D8U6K8_VOLCA/164-377	D8U6K8.1 PF03924.11	34.00	0.00
TRINITY_A0A075B447_9FUNG/3-1130	A0A075B447.1 PF0246	33.90	0.00
TRINITY_Q6BJF3_DEBHA/225-415	Q6BJF3.1 PF11715.6;	33.90	0.00
TRINITY_I7MKL5_TETTS/83-500	I7MKL5.2 PF00067.20	33.90	0.00
TRINITY_I7M910_TETTS/11-285	I7M910.1 PF00069.23	33.90	0.00
TRINITY_G1KK61_ANOCA/29-257	G1KK61.1 PF10223.7;	33.90	0.00
TRINITY_A0A0D2VZL3_CAPO3/294-541	A0A0D2VZL3.1 PF0052	33.90	0.00
TRINITY_U6NMJ5_HAECO/119-238	U6NMJ5.1 PF00622.26	33.90	0.00
TRINITY_A0CRH3_PARTE/53-407	A0CRH3.1 PF00155.19	33.90	0.00
TRINITY_A0EBH2_PARTE/32-359	A0EBH2.1 PF00328.20	33.90	0.00
TRINITY_A0A010QE82_9PEZI/10-235	A0A010QE82.1 PF0572	33.90	0.00
TRINITY_C3ZVI6_BRAFL/29-413	C3ZVI6.1 PF04922.10	33.90	0.00
TRINITY_V3ZTL6_LOTGI/3-368	V3ZTL6.1 PF05761.12	33.90	0.00
TRINITY_T0KSM0_COLGC/10-235	T0KSM0.1 PF05721.11	33.90	0.00
TRINITY_F4PJ73_DICFS/7-1148	F4PJ73.1 PF09735.7;	33.90	0.00
TRINITY_A4VDT5_TETTS/546-675	A4VDT5.2 PF04499.13	33.90	0.00
TRINITY_S8B4G2_PENO1/609-970	S8B4G2.1 PF00702.24	33.90	0.00
TRINITY_D2QN84_SPILD/3-301	D2QN84.1 PF01156.17	33.90	0.00
TRINITY_I7M9S7_TETTS/86-331	I7M9S7.2 PF00481.19	33.90	0.00
TRINITY_A4VDN1_TETTS/4-430	A4VDN1.1 PF00067.20	33.90	0.00
TRINITY_E1Z5C6_CHLVA/16-469	E1Z5C6.1 PF03935.13	33.90	0.00
TRINITY_B9I4K9_POPTR/49-179	B9I4K9.1 PF03517.11	33.90	0.00

TRINITY_D2W423_NAEGR/65-239	D2W423.1 PF01273.23	33.90	0.00
TRINITY_A8J514_CHLRE/16-293	A8J514.1 PF00069.23	33.90	0.00
TRINITY_G7CB30_MYCTH/15-324	G7CB30.1 PF01326.17	33.90	0.00
TRINITY_T1GBN4_MEGSC/141-290	T1GBN4.1 PF00620.25	33.90	0.00
TRINITY_D8U200_VOLCA/57-377	D8U200.1 PF07466.9;	33.90	0.00
TRINITY_S3CQ68_OPHP1/12-322	S3CQ68.1 PF00069.23	33.90	0.00
TRINITY_A0A0K2YE41_9NOCA/25-335	A0A0K2YE41.1 PF0132	33.90	0.00
TRINITY_D8U381_VOLCA/40-148	D8U381.1 PF02694.13	33.90	0.00
TRINITY_A0A0N1NYH5_9EURO/40-184	A0A0N1NYH5.1 PF1367	33.90	0.00
TRINITY_T0RK07_9STRA/785-1026	T0RK07.1 PF02714.13	33.90	0.00
TRINITY_D8TH77_VOLCA/22-210	D8TH77.1 PF00069.23	33.90	0.00
TRINITY_D8UAW6_VOLCA/1386-1537	D8UAW6.1 PF00069.23	33.90	0.00
TRINITY_A9RBI6_PHYPA/54-465	A9RBI6.1 PF02897.13	33.80	0.00
TRINITY_G4VH12_SCHMA/450-920	G4VH12.1 PF00899.19	33.80	0.00
TRINITY_A0A087HR20_ARAAL/15-237	A0A087HR20.1 PF0024	33.80	0.00
TRINITY_F0ZI60_DICPU/25-436	F0ZI60.1 PF12698.5;	33.80	0.00
TRINITY_L8H446_ACACA/214-362	L8H446.1 PF00179.24	33.80	0.00
TRINITY_I0Z4A4_9CHLO/51-323	I0Z4A4.1 PF00231.17	33.80	0.00
TRINITY_I7M3L2_TETTS/5-159	I7M3L2.2 PF00790.17	33.80	0.00
TRINITY_I7MJR6_TETTS/77-350	I7MJR6.2 PF00561.18	33.80	0.00
TRINITY_L8HD92_ACACA/1814-2187	L8HD92.1 PF02889.14	33.80	0.00
TRINITY_L8H748_ACACA/27-260	L8H748.1 PF02450.13	33.80	0.00
TRINITY_F1A4E2_DICPU/12-173	F1A4E2.1 PF00071.20	33.80	0.00
TRINITY_I1GE80_AMPQE/220-564	I1GE80.1 PF13598.4;	33.80	0.00
TRINITY_L8GXD6_ACACA/112-229	L8GXD6.1 PF01529.18	33.80	0.00
TRINITY_D3BDY5_POLPA/440-647	D3BDY5.1 PF00924.16	33.80	0.00
TRINITY_D8QSJ9_SELML/486-830	D8QSJ9.1 PF11865.6;	33.80	0.00
TRINITY_D8TRL6_VOLCA/310-457	D8TRL6.1 PF12499.6;	33.80	0.00
TRINITY_D8UJN4_VOLCA/97-364	D8UJN4.1 PF13862.4;	33.80	0.00
TRINITY_W5M6W3_LEPOC/35-350	W5M6W3.1 PF00328.20	33.80	0.00
TRINITY_G3AUB2_SPAPN/40-172	G3AUB2.1 PF12894.5;	33.80	0.00
TRINITY_A0A077ZVR2_STYLE/565-708	A0A077ZVR2.1 PF1526	33.80	0.00
TRINITY_G3P6H1_GASAC/86-386	G3P6H1.1 PF00561.18	33.80	0.00
TRINITY_A0A0D2WSZ7_CAPO3/158-315	A0A0D2WSZ7.1 PF0062	33.80	0.00
TRINITY_A0A078ACA2_STYLE/7-156	A0A078ACA2.1 PF1458	33.80	0.00
TRINITY_A9V2G9_MONBE/59-462	A9V2G9.1 PF00450.20	33.80	0.00
TRINITY_G0R1G3_ICHMG/80-301	G0R1G3.1 PF00566.16	33.80	0.00
TRINITY_Q22E24_TETTS/241-462	Q22E24.1 PF02684.13	33.80	0.00
TRINITY_Q9RZM6_DEIRA/82-311	Q9RZM6.1 PF12697.5;	33.80	0.00
TRINITY_E1ZCR5_CHLVA/245-673	E1ZCR5.1 PF01142.16	33.80	0.00
TRINITY_A0A015JNE6_9GLOM/190-476	A0A015JNE6.1 PF0154	33.80	0.00
TRINITY_K4BM04_SOLLC/882-1171	K4BM04.1 PF00069.23	33.80	0.00
TRINITY_A0A0E0KTV1_ORYPU/5-189	A0A0E0KTV1.1 PF0006	33.80	0.00
TRINITY_D8UD46_VOLCA/316-464	D8UD46.1 PF13671.4;	33.80	0.00
TRINITY_A0A015KAA8_9GLOM/374-518	A0A015KAA8.1 PF0006	33.80	0.00
TRINITY_A0A078AND5_STYLE/23-125	A0A078AND5.1 PF0789	33.70	0.00
TRINITY_S2JNV9_MUCC1/8-108	S2JNV9.1 PF00168.28	33.70	0.00
TRINITY_C2W9U2_BACCE/34-290	C2W9U2.1 PF00561.18	33.70	0.00
TRINITY_A4RXB0_OSTLU/28-319	A4RXB0.1 PF03151.14	33.70	0.00
TRINITY_F4PIF8_DICFS/13-419	F4PIF8.1 PF00083.22	33.70	0.00
TRINITY_B3RRX2_TRIAD/1-238	B3RRX2.1 PF00069.23	33.70	0.00
TRINITY_J9F1T2_9SPIT/1-126	J9F1T2.1 PF14825.4;	33.70	0.00
TRINITY_G0R492_ICHMG/45-429	G0R492.1 PF00026.21	33.70	0.00
TRINITY_L8H4S6_ACACA/29-449	L8H4S6.1 PF12832.5;	33.70	0.00
TRINITY_I1FIZ8_AMPQE/217-575	I1FIZ8.1 PF13598.4;	33.70	0.00
TRINITY_L8H258_ACACA/21-136	L8H258.1 PF00787.22	33.70	0.00
TRINITY_A8HN94_CHLRE/105-201	A8HN94.1 PF01329.17	33.70	0.00
TRINITY_D8RIB2_SELML/16-455	D8RIB2.1 PF06087.10	33.70	0.00
TRINITY_F7FXQ5_ORNAN/179-496	F7FXQ5.2 PF00728.20	33.70	0.00
TRINITY_A0A0F8VF03_9ARCH/29-236	A0A0F8VF03.1 PF0281	33.70	0.00
TRINITY_J9IEP0_9SPIT/637-1073	J9IEP0.1 PF04547.10	33.70	0.00

TRINITY_A0A068SCY1_9FUNG/173-379	A0A068SCY1.1 PF0014	33.70	0.00
TRINITY_B0WT67_CULQU/71-475	B0WT67.1 PF00450.20	33.70	0.00
TRINITY_Q238R6_TETTS/1-312	Q238R6.3 PF10149.7;	33.70	0.00
TRINITY_D8TJN6_VOLCA/1-492	D8TJN6.1 PF07058.9;	33.70	0.00
TRINITY_D8FV64_9CYAN/82-418	D8FV64.1 PF01547.23	33.70	0.00
TRINITY_F0ZHA2_DICPU/18-202	F0ZHA2.1 PF01209.16	33.70	0.00
TRINITY_H2ZXA1_LATCH/316-440	H2ZXA1.1 PF09112.8;	33.70	0.00
TRINITY_X6NEQ3_RETFI/648-804	X6NEQ3.1 PF00644.18	33.70	0.00
TRINITY_A0A090M529_OSTTA/82-337	A0A090M529.1 PF0058	33.70	0.00
TRINITY_W4XPH5_STRPU/280-621	W4XPH5.1 PF14782.4;	33.70	0.00
TRINITY_F4PU75_DICFS/62-443	F4PU75.1 PF02274.15	33.70	0.00
TRINITY_I7ME59_TETTS/50-504	I7ME59.2 PF00067.20	33.60	0.00
TRINITY_Q09AJ8_STIAD/9-122	Q09AJ8.1 PF12680.5;	33.60	0.00
TRINITY_X6M5L4_RETFI/1-369	X6M5L4.1 PF07690.14	33.60	0.00
TRINITY_A0A0M8MRT5_9BASI/14-177	A0A0M8MRT5.1 PF0007	33.60	0.00
TRINITY_W4Y1D7_STRPU/18-141	W4Y1D7.1 PF00168.28	33.60	0.00
TRINITY_Q7S7R7_NEUCR/65-196	Q7S7R7.1 PF00043.23	33.60	0.00
TRINITY_B8C8E8_THAPS/382-562	B8C8E8.1 PF07534.14	33.60	0.00
TRINITY_Q3SE16_PARTE/50-248	Q3SE16.1 PF06705.9;	33.60	0.00
TRINITY_K1VXN6_TRIAC/658-948	K1VXN6.1 PF01504.16	33.60	0.00
TRINITY_Q232X8_TETTS/36-488	Q232X8.1 PF00067.20	33.60	0.00
TRINITY_T1G8Z2_HELRO/76-328	T1G8Z2.1 PF00069.23	33.60	0.00
TRINITY_A0C5A5_PARTE/11-426	A0C5A5.1 PF03348.13	33.60	0.00
TRINITY_A0A087XPQ7_POEFO/32-173	A0A087XPQ7.2 PF0928	33.60	0.00
TRINITY_R6V291_9FIRM/722-839	R6V291.1 PF00072.22	33.60	0.00
TRINITY_J9ITG9_9SPIT/43-340	J9ITG9.1 PF00648.19	33.60	0.00
TRINITY_I7MD42_TETTS/162-405	I7MD42.2 PF00481.19	33.60	0.00
TRINITY_I7M910_TETTS/11-285	I7M910.1 PF00069.23	33.60	0.00
TRINITY_A0E3H2_PARTE/82-288	A0E3H2.1 PF07651.14	33.60	0.00
TRINITY_Q6CEL9_YARLI/113-499	Q6CEL9.2 PF01433.18	33.60	0.00
TRINITY_J9IFM7_9SPIT/442-784	J9IFM7.1 PF05285.10	33.60	0.00
TRINITY_F0Z817_DICPU/213-362	F0Z817.1 PF04088.11	33.60	0.00
TRINITY_A0CAB3_PARTE/485-621	A0CAB3.1 PF00175.19	33.60	0.00
TRINITY_D8TMI5_VOLCA/21-118	D8TMI5.1 PF03645.11	33.60	0.00
TRINITY_E1Z4L1_CHLVA/93-319	E1Z4L1.1 PF13489.4;	33.60	0.00
TRINITY_A0A024UDE2_9STRA/138-385	A0A024UDE2.1 PF0942	33.60	0.00
TRINITY_F8PPC4_SERL3/1288-1661	F8PPC4.1 PF02181.21	33.60	0.00
TRINITY_L8GH56_ACACA/26-138	L8GH56.1 PF08487.8;	33.60	0.00
TRINITY_A8IVV8_CHLRE/112-217	A8IVV8.1 PF04359.12	33.60	0.00
TRINITY_I7IFD4_BABMI/10-147	I7IFD4.1 PF00179.24	33.60	0.00
TRINITY_L8GF85_ACACA/2138-2306	L8GF85.1 PF00520.29	33.60	0.00
TRINITY_A7RPJ7_NEMVE/5-386	A7RPJ7.1 PF00083.22	33.60	0.00
TRINITY_D8SC35_SELML/433-611	D8SC35.1 PF00924.16	33.60	0.00
TRINITY_B7PNE9_IXOSC/191-298	B7PNE9.1 PF00651.29	33.60	0.00
TRINITY_D8U4S8_VOLCA/6-142	D8U4S8.1 PF00582.24	33.60	0.00
TRINITY_G0R1G3_ICHMG/80-301	G0R1G3.1 PF00566.16	33.60	0.00
TRINITY_D8TZV8_VOLCA/186-592	D8TZV8.1 PF14646.4;	33.60	0.00
TRINITY_K0KR26_WICCF/154-404	K0KR26.1 PF00069.23	33.60	0.00
TRINITY_D8TRI1_VOLCA/20-204	D8TRI1.1 PF04720.10	33.60	0.00
TRINITY_I1BR12_RHIO9/37-261	I1BR12.1 PF07819.11	33.60	0.00
TRINITY_A0D933_PARTE/16-192	A0D933.1 PF01105.22	33.50	0.00
TRINITY_M7AQJ1_CHEMY/15-987	M7AQJ1.1 PF11894.6;	33.50	0.00
TRINITY_M2W7G2_GALSU/46-292	M2W7G2.1 PF00294.22	33.50	0.00
TRINITY_Q22X26_TETTS/84-364	Q22X26.2 PF01266.22	33.50	0.00
TRINITY_I4BY26_ANAMD/508-730	I4BY26.1 PF02358.14	33.50	0.00
TRINITY_A0A0C4DL18_MAGP6/21-224	A0A0C4DL18.1 PF0642	33.50	0.00
TRINITY_A0A098EX32_9BACI/31-267	A0A098EX32.1 PF0785	33.50	0.00
TRINITY_Q23LQ9_TETTS/93-385	Q23LQ9.2 PF01457.14	33.50	0.00
TRINITY_G0R625_ICHMG/7-381	G0R625.1 PF05761.12	33.50	0.00
TRINITY_A0DL82_PARTE/243-453	A0DL82.1 PF03403.11	33.50	0.00
TRINITY_G0SZ38_RHOG2/34-256	G0SZ38.1 PF05721.11	33.50	0.00

TRINITY_A0DTM2_PARTE/98-446	A0DTM2.1 PF13868.4;	33.50	0.00
TRINITY_A0A0G4IY77_PLABS/137-395	A0A0G4IY77.1 PF0006	33.50	0.00
TRINITY_H0XA36_OTOGA/16-175	H0XA36.1 PF12706.5;	33.50	0.00
TRINITY_A8J8C1_CHLRE/99-326	A8J8C1.1 PF00557.22	33.50	0.00
TRINITY_D2V5Q4_NAEGR/10-261	D2V5Q4.1 PF00069.23	33.50	0.00
TRINITY_D8TY58_VOLCA/27-330	D8TY58.1 PF09741.7;	33.50	0.00
TRINITY_I0ZAF3_9CHLO/1573-1786	I0ZAF3.1 PF10441.7;	33.50	0.00
TRINITY_W5N1X5_LEPOC/377-632	W5N1X5.1 PF00069.23	33.50	0.00
TRINITY_D3BGC6_POLPA/239-445	D3BGC6.1 PF00616.17	33.50	0.00
TRINITY_T1JUM0_TETUR/213-387	T1JUM0.1 PF00617.17	33.50	0.00
TRINITY_G4TUB8_PIRID/5-178	G4TUB8.1 PF00025.19	33.50	0.00
TRINITY_A0A078AR51_STYLE/8-287	A0A078AR51.1 PF0006	33.40	0.00
TRINITY_L8GPJ8_ACACA/129-640	L8GPJ8.1 PF07690.14	33.40	0.00
TRINITY_SCY1_DICDI/31-371	Q55GS2.1 PF00069.23	33.40	0.00
TRINITY_L8GGA7_ACACA/516-881	L8GGA7.1 PF03378.13	33.40	0.00
TRINITY_PNPP_SCHPO/18-258	Q00472.2 PF00702.24	33.30	0.00
TRINITY_S7Q277_GLOTA/5-167	S7Q277.1 PF00071.20	33.30	0.00
TRINITY_A8JFK1_CHLRE/14-207	A8JFK1.1 PF13589.4;	33.30	0.00
TRINITY_A0CTE6_PARTE/63-497	A0CTE6.1 PF01490.16	33.30	0.00
TRINITY_W4Y9I1_STRPU/534-669	W4Y9I1.1 PF14815.4;	33.30	0.00
TRINITY_I7LXC7_TETTS/17-452	I7LXC7.1 PF00083.22	33.30	0.00
TRINITY_F0ZZN9_DICPU/600-775	F0ZZN9.1 PF00621.18	33.30	0.00
TRINITY_A0BZ71_PARTE/677-791	A0BZ71.1 PF00072.22	33.30	0.00
TRINITY_A0BXG1_PARTE/29-97	A0BXG1.1 PF10601.7;	33.30	0.00
TRINITY_A0BXG1_PARTE/29-97	A0BXG1.1 PF10601.7;	33.30	0.00
TRINITY_V8N4Z4_OPHHA/1-303	V8N4Z4.1 PF00026.21	33.30	0.00
TRINITY_T1H989_RHOPR/142-532	T1H989.1 PF00930.19	33.30	0.00
TRINITY_A0A015M0H2_9GLOM/8-143	A0A015M0H2.1 PF0112	33.30	0.00
TRINITY_L8GT75_ACACA/62-194	L8GT75.1 PF00583.23	33.30	0.00
TRINITY_A0CWW0_PARTE/35-576	A0CWW0.1 PF00995.21	33.30	0.00
TRINITY_C1N6C7_MICPC/171-454	C1N6C7.1 PF00928.19	33.30	0.00
TRINITY_F0ZU75_DICPU/166-360	F0ZU75.1 PF11715.6;	33.30	0.00
TRINITY_V4U0D3_9ROSI/12-641	V4U0D3.1 PF00888.20	33.30	0.00
TRINITY_C4L908_TOLAT/21-158	C4L908.1 PF09588.8;	33.30	0.00
TRINITY_Q5W9T4_CHLRE/42-167	Q5W9T4.1 PF00504.19	33.30	0.00
TRINITY_A0BLH9_PARTE/7-108	A0BLH9.1 PF00168.28	33.30	0.00
TRINITY_D2VMC5_NAEGR/6-132	D2VMC5.1 PF15749.3;	33.30	0.00
TRINITY_U4LLW0_PYROM/192-508	U4LLW0.1 PF00962.20	33.30	0.00
TRINITY_W5LBU9_ASTMX/23-1186	W5LBU9.1 PF06427.9;	33.30	0.00
TRINITY_Q232X8_TETTS/36-488	Q232X8.1 PF00067.20	33.30	0.00
TRINITY_L8HBA4_ACACA/57-414	L8HBA4.1 PF07767.9;	33.30	0.00
TRINITY_GACY_DICDI/544-696	Q54TH9.1 PF00620.25	33.30	0.00
TRINITY_M1W5R4_CLAP2/71-196	M1W5R4.1 PF00043.23	33.30	0.00
TRINITY_Q23AR4_TETTS/1-276	Q23AR4.2 PF04614.10	33.30	0.00
TRINITY_C3YX54_BRAFL/13-254	C3YX54.1 PF13561.4;	33.30	0.00
TRINITY_T1KEW3_TETUR/87-320	T1KEW3.1 PF02902.17	33.30	0.00
TRINITY_W0PGH6_9BURK/176-348	W0PGH6.1 PF05175.12	33.30	0.00
TRINITY_L8GUT4_ACACA/40-399	L8GUT4.1 PF00155.19	33.30	0.00
TRINITY_F0VAK5_NEOCL/36-164	F0VAK5.1 PF04053.12	33.30	0.00
TRINITY_I1GGB4_AMPQE/46-226	I1GGB4.1 PF00092.26	33.30	0.00
TRINITY_A0A099NXC9_PICKU/834-994	A0A099NXC9.1 PF0062	33.30	0.00
TRINITY_A0A0K9NVM7_ZOSMR/73-176	A0A0K9NVM7.1 PF0222	33.30	0.00
TRINITY_A0A0J8F894_BETVU/311-429	A0A0J8F894.1 PF0448	33.30	0.00
TRINITY_A0A078F7K0_BRANA/30-159	A0A078F7K0.1 PF0091	33.30	0.00
TRINITY_K9UAR6_9CHRO/5-101	K9UAR6.1 PF05724.9;	33.30	0.00
TRINITY_W7U3X1_9STRA/182-521	W7U3X1.1 PF10250.7;	33.30	0.00
TRINITY_F0ZW58_DICPU/689-833	F0ZW58.1 PF12335.6;	33.30	0.00
TRINITY_L8HFG7_ACACA/132-452	L8HFG7.1 PF04129.10	33.30	0.00
TRINITY_I3J1D5_ORENI/161-282	I3J1D5.1 PF12755.5;	33.30	0.00
TRINITY_L8GHU6_ACACA/376-526	L8GHU6.1 PF00620.25	33.30	0.00
TRINITY_A0DS68_PARTE/1-166	A0DS68.1 PF06017.11	33.30	0.00

TRINITY_I7LX20_TETTS/11-287	I7LX20.2 PF00069.23	33.30	0.00
TRINITY_A0A084VMY7_ANOSI/56-477	A0A084VMY7.1 PF0213	33.30	0.00
TRINITY_Q55F12_DICDI/374-640	Q55F12.1 PF00149.26	33.30	0.00
TRINITY_A8ISU6_CHLRE/593-795	A8ISU6.1 PF07059.10	33.30	0.00
TRINITY_V3ZMI5_LOTGI/10-184	V3ZMI5.1 PF00071.20	33.30	0.00
TRINITY_F0ZNT4_DICPU/393-543	F0ZNT4.1 PF00620.25	33.30	0.00
TRINITY_V4AJ35_LOTGI/5-108	V4AJ35.1 PF05773.20	33.30	0.00
TRINITY_A0A0R4IAQ8_DANRE/1333-1627	A0A0R4IAQ8.1 PF1211	33.30	0.00
TRINITY_G5IEU5_9CLOT/47-330	G5IEU5.1 PF00664.21	33.30	0.00
TRINITY_W1PHB5_AMBTC/30-149	W1PHB5.1 PF02996.15	33.30	0.00
TRINITY_B8E367_DICTD/33-400	B8E367.1 PF00079.18	33.30	0.00
TRINITY_F0ZVH2_DICPU/16-315	F0ZVH2.1 PF03224.12	33.30	0.00
TRINITY_A0DMA3_PARTE/467-923	A0DMA3.1 PF04547.10	33.30	0.00
TRINITY_W7UA30_9STRA/2-282	W7UA30.1 PF02574.14	33.30	0.00
TRINITY_B3KZ91_PLAKH/192-310	B3KZ91.1 PF01569.19	33.30	0.00
TRINITY_GEFQ_DICDI/967-1144	Q86G47.1 PF00617.17	33.30	0.00
TRINITY_A8J7C9_CHLRE/636-795	A8J7C9.1 PF13925.4;	33.30	0.00
TRINITY_D8TV06_VOLCA/25-163	D8TV06.1 PF08718.9;	33.30	0.00
TRINITY_I0Z854_9CHLO/68-247	I0Z854.1 PF01048.18	33.30	0.00
TRINITY_F1PNA2_CANLF/715-994	F1PNA2.2 PF00664.21	33.30	0.00
TRINITY_A0A075AT68_9FUNG/266-440	A0A075AT68.1 PF0192	33.30	0.00
TRINITY_D8TP82_VOLCA/367-1080	D8TP82.1 PF10408.7;	33.30	0.00
TRINITY_T1IJ26_STRMM/44-286	T1IJ26.1 PF01370.19	33.30	0.00
TRINITY_I0Z4V6_9CHLO/26-104	I0Z4V6.1 PF08142.10	33.30	0.00
TRINITY_A0A098VT88_9MICR/400-812	A0A098VT88.1 PF0808	33.30	0.00
TRINITY_B3S1M2_TRIAD/43-432	B3S1M2.1 PF04100.10	33.30	0.00
TRINITY_A0A0D2WTN7_CAPO3/64-537	A0A0D2WTN7.1 PF0238	33.30	0.00
TRINITY_G0R0X8_ICHMG/6-109	G0R0X8.1 PF03179.13	33.30	0.00
TRINITY_L8GF85_ACACA/2138-2306	L8GF85.1 PF00520.29	33.30	0.00
TRINITY_A8IXF8_CHLRE/79-438	A8IXF8.1 PF03009.15	33.30	0.00
TRINITY_W4Y895_STRPU/330-534	W4Y895.1 PF01858.15	33.30	0.00
TRINITY_I0YSZ2_9CHLO/11-177	I0YSZ2.1 PF03398.12	33.30	0.00
TRINITY_A0BRA8_PARTE/7-108	A0BRA8.1 PF00168.28	33.30	0.00
TRINITY_I3DJE9_9PAST/33-323	I3DJE9.1 PF06472.13	33.30	0.00
TRINITY_A0A0D3G1G3_9ORYZ/41-258	A0A0D3G1G3.1 PF0223	33.30	0.00
TRINITY_I7LX20_TETTS/11-287	I7LX20.2 PF00069.23	33.20	0.00
TRINITY_B9RNK4_RICCO/465-730	B9RNK4.1 PF04437.11	33.20	0.00
TRINITY_A0A0A1NRN8_9FUNG/44-266	A0A0A1NRN8.1 PF0414	33.20	0.00
TRINITY_D8RIB2_SELML/16-455	D8RIB2.1 PF06087.10	33.20	0.00
TRINITY_A0CK34_PARTE/1-235	A0CK34.1 PF02586.12	33.20	0.00
TRINITY_B3ESW8_AMOA5/41-239	B3ESW8.1 PF01734.20	33.20	0.00
TRINITY_I0HYT0_CALAS/7-421	I0HYT0.1 PF05977.11	33.20	0.00
TRINITY_GRLE_DICDI/440-680	Q54ET0.2 PF00003.20	33.20	0.00
TRINITY_Q4N417_THEPA/77-520	Q4N417.1 PF00443.27	33.20	0.00
TRINITY_D8TT61_VOLCA/156-506	D8TT61.1 PF11817.6;	33.20	0.00
TRINITY_E1Z3S2_CHLVA/29-254	E1Z3S2.1 PF02146.15	33.20	0.00
TRINITY_G0S3W8_CHATD/20-666	G0S3W8.1 PF00888.20	33.20	0.00
TRINITY_L8HCX4_ACACA/24-277	L8HCX4.1 PF01026.19	33.20	0.00
TRINITY_A0A078AWW8_STYLE/247-440	A0A078AWW8.1 PF0011	33.20	0.00
TRINITY_K9SZG2_9CYAN/110-382	K9SZG2.1 PF01636.21	33.20	0.00
TRINITY_A0CYW6_PARTE/28-289	A0CYW6.1 PF00069.23	33.20	0.00
TRINITY_I0Z807_9CHLO/145-411	I0Z807.1 PF05116.11	33.20	0.00
TRINITY_B7P719_IXOSC/905-1083	B7P719.1 PF00617.17	33.20	0.00
TRINITY_E2B1J0_CAMFO/1190-1419	E2B1J0.1 PF12348.6;	33.20	0.00
TRINITY_W4GKY8_9STRA/7-270	W4GKY8.1 PF03643.13	33.10	0.00
TRINITY_C3ZGX7_BRAFL/7-151	C3ZGX7.1 PF10513.7;	33.10	0.00
TRINITY_R7J8S3_9PORP/73-374	R7J8S3.1 PF01619.16	33.10	0.00
TRINITY_F8SXZ9_WHEAT/32-152	F8SXZ9.1 PF00188.24	33.10	0.00
TRINITY_I7M9S7_TETTS/86-331	I7M9S7.2 PF00481.19	33.10	0.00
TRINITY_A0EIC2_PARTE/22-327	A0EIC2.1 PF07946.12	33.10	0.00
TRINITY_R4X876_TAPDE/1-135	R4X876.1 PF00125.22	33.10	0.00

TRINITY_Q22C93_TETTS/132-403	Q22C93.2 PF00561.18	33.10	0.00
TRINITY_A0A078A4C1_STYLE/982-1110	A0A078A4C1.1 PF0409	33.10	0.00
TRINITY_Q4FLK1_PELUB/10-176	Q4FLK1.1 PF00994.22	33.10	0.00
TRINITY_A0A077ZQS0_STYLE/68-262	A0A077ZQS0.1 PF1214	33.10	0.00
TRINITY_T1J8W5_STRMM/615-893	T1J8W5.1 PF08925.9;	33.10	0.00
TRINITY_Q24HL5_TETTS/807-923	Q24HL5.2 PF00072.22	33.10	0.00
TRINITY_G0QPS2_ICHMG/376-660	G0QPS2.1 PF00648.19	33.10	0.00
TRINITY_Q22HD0_TETTS/59-363	Q22HD0.1 PF00566.16	33.10	0.00
TRINITY_A0A087SP41_AUXPR/27-351	A0A087SP41.1 PF0398	33.10	0.00
TRINITY_F0ZGP7_DICPU/754-1122	F0ZGP7.1 PF02181.21	33.10	0.00
TRINITY_E1ZSN3_CHLVA/103-252	E1ZSN3.1 PF03114.16	33.10	0.00
TRINITY_D8QTH1_SELML/234-600	D8QTH1.1 PF09423.8;	33.10	0.00
TRINITY_A0A0J7MYI6_LASNI/104-263	A0A0J7MYI6.1 PF1335	33.10	0.00
TRINITY_T1FN62_HELRO/6-132	T1FN62.1 PF08662.9;	33.10	0.00
TRINITY_E9HM72_DAPPU/23-156	E9HM72.1 PF04145.13	33.10	0.00
TRINITY_I0YLE6_9CHLO/727-871	I0YLE6.1 PF13515.4;	33.10	0.00
TRINITY_C4LWM6_ENTHI/12-518	C4LWM6.1 PF04916.11	33.10	0.00
TRINITY_A0A067TKF0_9AGAR/58-206	A0A067TKF0.1 PF0449	33.10	0.00
TRINITY_R5EMZ2_9FIRM/22-195	R5EMZ2.1 PF02146.15	33.10	0.00
TRINITY_A8HUY7_CHLRE/42-247	A8HUY7.1 PF00804.23	33.10	0.00
TRINITY_A0C868_PARTE/287-682	A0C868.1 PF01757.20	33.10	0.00
TRINITY_J9ILZ0_9SPIT/61-293	J9ILZ0.1 PF06011.10	33.10	0.00
TRINITY_A9TI54_PHYPA/6-171	A9TI54.1 PF01956.14	33.10	0.00
TRINITY_Q1ZXE9_DICDI/805-957	Q1ZXE9.1 PF01608.15	33.10	0.00
TRINITY_A8J2X9_CHLRE/125-257	A8J2X9.1 PF00504.19	33.10	0.00
TRINITY_A0A085LV11_9BILA/13-136	A0A085LV11.1 PF0024	33.10	0.00
TRINITY_A7SUH9_NEMVE/38-412	A7SUH9.1 PF00155.19	33.10	0.00
TRINITY_A7HQR5_PARL1/40-217	A7HQR5.1 PF04413.14	33.10	0.00
TRINITY_A0A024UJV3_9STRA/11-461	A0A024UJV3.1 PF0769	33.00	0.00
TRINITY_E1ZEZ8_CHLVA/496-844	E1ZEZ8.1 PF05192.16	33.00	0.00
TRINITY_A0EGI5_PARTE/166-524	A0EGI5.1 PF06602.12	33.00	0.00
TRINITY_V9E5L3_PHYPR/324-430	V9E5L3.1 PF00651.29	33.00	0.00
TRINITY_H2AUK8_KAZAF/18-285	H2AUK8.1 PF00069.23	33.00	0.00
TRINITY_F0ZZN9_DICPU/600-775	F0ZZN9.1 PF00621.18	33.00	0.00
TRINITY_C3Y9V6_BRAFL/10-502	C3Y9V6.1 PF13001.5;	33.00	0.00
TRINITY_V4CF92_LOTGI/13-162	V4CF92.1 PF10572.7;	33.00	0.00
TRINITY_W6KV94_9TRYP/2-375	W6KV94.1 PF00022.17	33.00	0.00
TRINITY_W4HBP1_9STRA/40-313	W4HBP1.1 PF05721.11	33.00	0.00
TRINITY_D8U6K8_VOLCA/164-377	D8U6K8.1 PF03924.11	33.00	0.00
TRINITY_A0A0G2ZKP7_9DELT/512-677	A0A0G2ZKP7.1 PF0357	33.00	0.00
TRINITY_H3B9U4_LATCH/199-406	H3B9U4.1 PF03770.14	33.00	0.00
TRINITY_A0A0K0FG82_9BILA/181-288	A0A0K0FG82.1 PF0065	33.00	0.00
TRINITY_D8U6F6_VOLCA/129-404	D8U6F6.1 PF00211.18	33.00	0.00
TRINITY_ZDHC7_DICDI/244-370	Q552M6.1 PF01529.18	33.00	0.00
TRINITY_E3QHP5_COLGM/37-417	E3QHP5.1 PF01266.22	33.00	0.00
TRINITY_H6NFX2_9BACL/48-284	H6NFX2.1 PF03059.14	33.00	0.00
TRINITY_I4YY54_9RHIZ/5-243	I4YY54.1 PF02353.18	33.00	0.00
TRINITY_E9EJQ9_METRA/376-492	E9EJQ9.2 PF08284.9;	33.00	0.00
TRINITY_I7MA04_TETTS/95-310	I7MA04.1 PF04194.11	33.00	0.00
TRINITY_A7HFL1_ANADF/119-220	A7HFL1.1 PF00011.19	33.00	0.00
TRINITY_D2UXU4_NAEGR/968-1064	D2UXU4.1 PF00169.27	33.00	0.00
TRINITY_D8UFU6_VOLCA/12-507	D8UFU6.1 PF07690.14	33.00	0.00
TRINITY_L1ILW8_GUITH/2-98	L1ILW8.1 PF11715.6;	33.00	0.00
TRINITY_R1BXT9_EMIHU/51-230	R1BXT9.1 PF00350.21	33.00	0.00
TRINITY_L8H1G2_ACACA/704-935	L8H1G2.1 PF10474.7;	33.00	0.00
TRINITY_G1NH86_MELGA/954-1446	G1NH86.2 PF00580.19	33.00	0.00
TRINITY_F1SF79_PIG/221-355	F1SF79.2 PF04326.12	33.00	0.00
TRINITY_F2UN08_SALR5/810-1018	F2UN08.1 PF08767.9;	33.00	0.00
TRINITY_A0A096P7D5_OSTTA/585-791	A0A096P7D5.1 PF1308	33.00	0.00
TRINITY_W9S2R2_9ROSA/1-228	W9S2R2.1 PF02586.12	33.00	0.00
TRINITY_A0BXD2_PARTE/279-685	A0BXD2.1 PF01757.20	32.90	0.00

TRINITY_F1RYU7_PIG/130-359	F1RYU7.1 PF02275.16	32.90	0.00
TRINITY_Q3SDQ2_PARTE/13-174	Q3SDQ2.1 PF00071.20	32.90	0.00
TRINITY_A0BUW7_PARTE/301-621	A0BUW7.1 PF04515.10	32.90	0.00
TRINITY_A0DYR3_PARTE/708-848	A0DYR3.1 PF01909.21	32.90	0.00
TRINITY_G0QVB2_ICHMG/37-311	G0QVB2.1 PF07714.15	32.90	0.00
TRINITY_A0D1D5_PARTE/180-443	A0D1D5.1 PF00069.23	32.90	0.00
TRINITY_F0ZFB3_DICPU/264-425	F0ZFB3.1 PF01554.16	32.90	0.00
TRINITY_B5JEZ5_9BACT/54-327	B5JEZ5.1 PF02353.18	32.90	0.00
TRINITY_I7M6K2_TETTS/65-258	I7M6K2.1 PF01145.23	32.90	0.00
TRINITY_B8HT58_CYAP4/8-334	B8HT58.1 PF13434.4;	32.90	0.00
TRINITY_D3BE55_POLPA/173-325	D3BE55.1 PF04004.11	32.90	0.00
TRINITY_I4YJX6_WALMC/23-406	I4YJX6.1 PF09794.7;	32.90	0.00
TRINITY_J9HRZ5_9SPIT/1043-1207	J9HRZ5.1 PF08623.8;	32.90	0.00
TRINITY_W5WAY5_9PSEU/143-346	W5WAY5.1 PF07859.11	32.90	0.00
TRINITY_A0A0D2WTG5_CAPO3/124-328	A0A0D2WTG5.1 PF0014	32.90	0.00
TRINITY_I0ZAT9_9CHLO/267-451	I0ZAT9.1 PF15469.4;	32.90	0.00
TRINITY_A0CW99_PARTE/11-187	A0CW99.1 PF06017.11	32.90	0.00
TRINITY_W7TVL8_9STRA/1078-1273	W7TVL8.1 PF01734.20	32.90	0.00
TRINITY_A0A024UCN6_9STRA/19-525	A0A024UCN6.1 PF0145	32.90	0.00
TRINITY_D8TQJ4_VOLCA/139-477	D8TQJ4.1 PF05686.10	32.90	0.00
TRINITY_W4Y340_STRPU/212-375	W4Y340.1 PF13359.4;	32.90	0.00
TRINITY_Q7UYG2_RHOBA/201-690	Q7UYG2.1 PF03098.13	32.90	0.00
TRINITY_Q23Q43_TETTS/594-871	Q23Q43.2 PF00580.19	32.90	0.00
TRINITY_I1MDL7_SOYBN/41-338	I1MDL7.2 PF08423.9;	32.90	0.00
TRINITY_D2VJE0_NAEGR/547-1107	D2VJE0.1 PF01457.14	32.90	0.00
TRINITY_G3VBJ7_SARHA/16-176	G3VBJ7.1 PF00071.20	32.90	0.00
TRINITY_A0BLH9_PARTE/7-108	A0BLH9.1 PF00168.28	32.90	0.00
TRINITY_G3PIU7_GASAC/42-204	G3PIU7.1 PF00071.20	32.90	0.00
TRINITY_I1GK72_AMPQE/21-200	I1GK72.1 PF00483.21	32.90	0.00
TRINITY_A0A059DIJ1_EUCGR/3-158	A0A059DIJ1.1 PF0598	32.90	0.00
TRINITY_R1CGH4_EMIHU/60-184	R1CGH4.1 PF00004.27	32.90	0.00
TRINITY_A0A090LZV0_OSTTA/5-163	A0A090LZV0.1 PF0838	32.90	0.00
TRINITY_A0A015JYP1_9GLOM/195-349	A0A015JYP1.1 PF1335	32.90	0.00
TRINITY_A0A078ASZ8_STYLE/2378-2684	A0A078ASZ8.1 PF0052	32.90	0.00
TRINITY_A8JHV6_CHLRE/431-702	A8JHV6.1 PF00069.23	32.90	0.00
TRINITY_A0A078ACE3_STYLE/158-461	A0A078ACE3.1 PF0064	32.90	0.00
TRINITY_G0QNB6_ICHMG/7-248	G0QNB6.1 PF00069.23	32.80	0.00
TRINITY_A0A077ZXG3_STYLE/206-409	A0A077ZXG3.1 PF1388	32.80	0.00
TRINITY_A0A097AZT6_9VIBR/154-276	A0A097AZT6.1 PF0010	32.80	0.00
TRINITY_J0LG47_9BACT/30-232	J0LG47.1 PF04402.12	32.80	0.00
TRINITY_Q10JL7_ORYSJ/13-121	Q10JL7.1 PF02466.17	32.80	0.00
TRINITY_G0R0W9_ICHMG/11-427	G0R0W9.1 PF03348.13	32.80	0.00
TRINITY_J9HNI4_9SPIT/1-275	J9HNI4.1 PF00069.23	32.80	0.00
TRINITY_A0A0A1U484_ENTIV/114-280	A0A0A1U484.1 PF0007	32.80	0.00
TRINITY_Q22HC3_TETTS/1-722	Q22HC3.1 PF04615.11	32.80	0.00
TRINITY_A0A0A1PI03_9FUNG/94-267	A0A0A1PI03.1 PF0065	32.80	0.00
TRINITY_I7LX20_TETTS/11-287	I7LX20.2 PF00069.23	32.80	0.00
TRINITY_T0Q5K0_9STRA/19-535	T0Q5K0.1 PF01457.14	32.80	0.00
TRINITY_A0C424_PARTE/63-322	A0C424.1 PF09511.8;	32.80	0.00
TRINITY_A0A0A1MRK0_9FUNG/22-286	A0A0A1MRK0.1 PF0095	32.80	0.00
TRINITY_A6TPT5_ALKMQ/3-218	A6TPT5.1 PF05368.11	32.80	0.00
TRINITY_W7XDQ2_TETTS/43-354	W7XDQ2.1 PF04111.10	32.80	0.00
TRINITY_A0A0G4EJL3_9ALVE/19-357	A0A0G4EJL3.1 PF0104	32.80	0.00
TRINITY_A0A060LT03_9BACI/620-863	A0A060LT03.1 PF0799	32.80	0.00
TRINITY_B8EP67_METSB/47-569	B8EP67.1 PF00135.26	32.80	0.00
TRINITY_A0A0A1MUZ7_9FUNG/164-334	A0A0A1MUZ7.1 PF0027	32.80	0.00
TRINITY_M2XB22_GALSU/54-251	M2XB22.1 PF05721.11	32.80	0.00
TRINITY_V4ADM5_LOTGI/6-138	V4ADM5.1 PF05615.11	32.80	0.00
TRINITY_D2VR12_NAEGR/55-299	D2VR12.1 PF09511.8;	32.80	0.00
TRINITY_I0YTU9_9CHLO/143-490	I0YTU9.1 PF04515.10	32.80	0.00
TRINITY_A0A0A0LSU3_CUCSA/10-294	A0A0A0LSU3.1 PF0799	32.80	0.00

TRINITY_A0A068S7F9_9FUNG/184-383	A0A068S7F9.1 PF1028	32.80	0.00
TRINITY_W1PKP1_AMBTC/12-420	W1PKP1.1 PF00501.26	32.80	0.00
TRINITY_A9TUK6_PHYPA/186-324	A9TUK6.1 PF02219.15	32.80	0.00
TRINITY_D8U671_VOLCA/42-290	D8U671.1 PF10609.7;	32.80	0.00
TRINITY_A0A067BHM6_SAPPC/10-171	A0A067BHM6.1 PF0007	32.80	0.00
TRINITY_G0QK29_ICHMG/70-205	G0QK29.1 PF02100.15	32.80	0.00
TRINITY_M9X2Z3_MEIRD/146-562	M9X2Z3.1 PF00082.20	32.80	0.00
TRINITY_E9H4L7_DAPPU/18-118	E9H4L7.1 PF14737.4;	32.80	0.00
TRINITY_A0DX79_PARTE/251-413	A0DX79.1 PF01554.16	32.70	0.00
TRINITY_I7M6C4_TETTS/263-473	I7M6C4.1 PF00743.17	32.70	0.00
TRINITY_D0NV41_PHYIT/12-174	D0NV41.1 PF00071.20	32.70	0.00
TRINITY_W7XLQ4_TETTS/8-169	W7XLQ4.1 PF00134.21	32.70	0.00
TRINITY_Q22X60_TETTS/11-285	Q22X60.1 PF00069.23	32.70	0.00
TRINITY_D6TIG8_9CHLR/37-376	D6TIG8.1 PF00144.22	32.70	0.00
TRINITY_A0E0N4_PARTE/14-469	A0E0N4.1 PF00083.22	32.70	0.00
TRINITY_U4UBN8_DENPD/65-319	U4UBN8.1 PF00664.21	32.70	0.00
TRINITY_A0DVZ3_PARTE/7-172	A0DVZ3.1 PF06017.11	32.70	0.00
TRINITY_I7M298_TETTS/2730-2998	I7M298.2 PF00520.29	32.70	0.00
TRINITY_A0A096P8A0_OSTTA/70-215	A0A096P8A0.1 PF1342	32.70	0.00
TRINITY_A9TYN8_PHYPA/180-297	A9TYN8.1 PF04488.13	32.70	0.00
TRINITY_D8TYT5_VOLCA/383-955	D8TYT5.1 PF05664.9;	32.70	0.00
TRINITY_A0CUC4_PARTE/218-328	A0CUC4.1 PF00027.27	32.70	0.00
TRINITY_A2X165_ORYSI/12-266	A2X165.1 PF00069.23	32.70	0.00
TRINITY_A0A087G2T2_ARAAL/107-400	A0A087G2T2.1 PF0315	32.70	0.00
TRINITY_A0A078AN29_STYLE/103-278	A0A078AN29.1 PF0471	32.70	0.00
TRINITY_L8HCA6_ACACA/2-636	L8HCA6.1 PF10191.7;	32.70	0.00
TRINITY_Q22X60_TETTS/11-285	Q22X60.1 PF00069.23	32.70	0.00
TRINITY_A7SE78_NEMVE/44-348	A7SE78.1 PF00648.19	32.70	0.00
TRINITY_L8GY71_ACACA/636-844	L8GY71.1 PF08626.9;	32.70	0.00
TRINITY_F4P722_BATDJ/2-204	F4P722.1 PF12698.5;	32.70	0.00
TRINITY_Q96ZQ4_SULTO/40-200	Q96ZQ4.1 PF07929.9;	32.70	0.00
TRINITY_F9GA28_FUSOF/1-104	F9GA28.1 PF08284.9;	32.70	0.00
TRINITY_A0BRP6_PARTE/103-318	A0BRP6.1 PF04678.11	32.70	0.00
TRINITY_A0A024UUM3_9STRA/144-390	A0A024UUM3.1 PF0052	32.70	0.00
TRINITY_I5C187_9BACT/39-230	I5C187.1 PF07947.12	32.70	0.00
TRINITY_A0A059DBX9_EUCGR/3-141	A0A059DBX9.1 PF0079	32.70	0.00
TRINITY_D8TJ82_VOLCA/9-667	D8TJ82.1 PF04113.12	32.70	0.00
TRINITY_I1HEE0_BRADI/103-213	I1HEE0.1 PF13369.4;	32.70	0.00
TRINITY_E1ZCG6_CHLVA/36-348	E1ZCG6.1 PF00856.26	32.70	0.00
TRINITY_A8J0G9_CHLRE/37-593	A8J0G9.1 PF00067.20	32.70	0.00
TRINITY_W5JMC6_ANODA/81-193	W5JMC6.1 PF04178.10	32.60	0.00
TRINITY_I7M910_TETTS/11-285	I7M910.1 PF00069.23	32.60	0.00
TRINITY_A0A0E0JYX9_ORYPU/177-296	A0A0E0JYX9.1 PF0065	32.60	0.00
TRINITY_C1E040_MICSR/23-99	C1E040.1 PF06093.11	32.60	0.00
TRINITY_K0KX19_WICCF/124-576	K0KX19.1 PF00443.27	32.60	0.00
TRINITY_J9ITG9_9SPIT/43-340	J9ITG9.1 PF00648.19	32.60	0.00
TRINITY_A0A077ZN40_STYLE/4-266	A0A077ZN40.1 PF0006	32.60	0.00
TRINITY_F0ZTC2_DICPU/29-258	F0ZTC2.1 PF16746.3;	32.60	0.00
TRINITY_Q23QF2_TETTS/248-571	Q23QF2.2 PF03133.13	32.60	0.00
TRINITY_D2V7J3_NAEGR/307-411	D2V7J3.1 PF16770.3;	32.60	0.00
TRINITY_L8GI47_ACACA/42-316	L8GI47.1 PF09192.8;	32.60	0.00
TRINITY_I7M7H8_TETTS/53-447	I7M7H8.2 PF01490.16	32.60	0.00
TRINITY_I2GZY6_TETBL/58-201	I2GZY6.1 PF08662.9;	32.60	0.00
TRINITY_A7SFW1_NEMVE/731-908	A7SFW1.1 PF00617.17	32.60	0.00
TRINITY_W4GZP1_9STRA/12-153	W4GZP1.1 PF00179.24	32.60	0.00
TRINITY_A0A0K9PEF3_ZOSMR/44-232	A0A0K9PEF3.1 PF0784	32.60	0.00
TRINITY_A0A0B0PXH5_GOSAR/521-730	A0A0B0PXH5.1 PF0173	32.60	0.00
TRINITY_S8C7T4_DACHA/264-674	S8C7T4.1 PF00012.18	32.60	0.00
TRINITY_E1ZLX0_CHLVA/1313-1748	E1ZLX0.1 PF00176.21	32.60	0.00
TRINITY_A0A0L0C140_LUCCU/786-957	A0A0L0C140.1 PF0061	32.60	0.00
TRINITY_C1E1G3_MICSR/11-130	C1E1G3.1 PF00754.23	32.60	0.00

TRINITY_L0AEB6_NATGS/8-186	L0AEB6.1 PF00406.20	32.60	0.00
TRINITY_V4ABG2_LOTGI/25-214	V4ABG2.1 PF01852.17	32.60	0.00
TRINITY_I7MLJ7_TETTS/146-230	I7MLJ7.1 PF00957.19	32.50	0.00
TRINITY_D2VJG7_NAEGR/71-462	D2VJG7.1 PF00450.20	32.50	0.00
TRINITY_H3GEI2_PHYRM/34-165	H3GEI2.1 PF00782.18	32.50	0.00
TRINITY_M7AJ12_CHEMY/1363-1466	M7AJ12.1 PF02204.16	32.50	0.00
TRINITY_U9T2P2_RHIID/1611-1744	U9T2P2.1 PF13716.4;	32.50	0.00
TRINITY_Q22UG5_TETTS/658-774	Q22UG5.2 PF05786.12	32.50	0.00
TRINITY_A0CS93_PARTE/11-301	A0CS93.1 PF00069.23	32.50	0.00
TRINITY_D2UYS2_NAEGR/34-187	D2UYS2.1 PF07534.14	32.50	0.00
TRINITY_D0N3V3_PHYIT/209-435	D0N3V3.1 PF12348.6;	32.50	0.00
TRINITY_G0QK40_ICHMG/243-478	G0QK40.1 PF07690.14	32.50	0.00
TRINITY_F1QS97_DANRE/63-212	F1QS97.1 PF00520.29	32.50	0.00
TRINITY_A0A0G4J440_PLABS/4-216	A0A0G4J440.1 PF0009	32.50	0.00
TRINITY_A4VDS8_TETTS/15-599	A4VDS8.2 PF05622.10	32.50	0.00
TRINITY_F0ZCL0_DICPU/204-565	F0ZCL0.1 PF00026.21	32.50	0.00
TRINITY_B8E367_DICTD/33-400	B8E367.1 PF00079.18	32.50	0.00
TRINITY_A0A0A1TWN2_ENTIV/81-310	A0A0A1TWN2.1 PF1367	32.50	0.00
TRINITY_D3BTD5_POLPA/174-320	D3BTD5.1 PF07534.14	32.50	0.00
TRINITY_K9WC83_9CYAN/22-380	K9WC83.1 PF06772.9;	32.50	0.00
TRINITY_A8J999_CHLRE/299-570	A8J999.1 PF05206.12	32.50	0.00
TRINITY_D3AWD8_POLPA/21-216	D3AWD8.1 PF01105.22	32.50	0.00
TRINITY_U7PN91_SPOS1/32-123	U7PN91.1 PF00153.25	32.50	0.00
TRINITY_A0A078AR51_STYLE/8-287	A0A078AR51.1 PF0006	32.50	0.00
TRINITY_S9VCG6_9TRYP/10-260	S9VCG6.1 PF00069.23	32.50	0.00
TRINITY_E1ZAT0_CHLVA/107-372	E1ZAT0.1 PF05548.9;	32.50	0.00
TRINITY_I1MS39_SOYBN/1-143	I1MS39.1 PF10075.7;	32.50	0.00
TRINITY_C1MP19_MICPC/54-367	C1MP19.1 PF13350.4;	32.50	0.00
TRINITY_A0A067KTU8_JATCU/670-1158	A0A067KTU8.1 PF1040	32.50	0.00
TRINITY_PK1IP_DANRE/175-262	Q6TNS2.1 PF12894.5;	32.50	0.00
TRINITY_A0A0G0E1A9_9BACT/18-182	A0A0G0E1A9.1 PF1348	32.50	0.00
TRINITY_A0A0D2PI00_GOSRA/591-752	A0A0D2PI00.1 PF1181	32.50	0.00
TRINITY_L8GF17_ACACA/1522-1699	L8GF17.1 PF00617.17	32.50	0.00
TRINITY_I0YJR7_9CHLO/443-593	I0YJR7.1 PF01925.17	32.50	0.00
TRINITY_A0CXP9_PARTE/92-386	A0CXP9.1 PF01457.14	32.50	0.00
TRINITY_H0X419_OTOGA/19-841	H0X419.1 PF04147.10	32.50	0.00
TRINITY_E1ZG88_CHLVA/29-399	E1ZG88.1 PF16670.3;	32.50	0.00
TRINITY_E1Z3D0_CHLVA/45-252	E1Z3D0.1 PF10168.7;	32.50	0.00
TRINITY_A0A078AGI8_STYLE/1904-2163	A0A078AGI8.1 PF0052	32.50	0.00
TRINITY_A0A094F7Z5_9PEZI/1-245	A0A094F7Z5.1 PF0056	32.50	0.00
TRINITY_G2DKS0_9NEIS/32-178	G2DKS0.1 PF08212.10	32.40	0.00
TRINITY_I7MEJ1_TETTS/13-399	I7MEJ1.1 PF07690.14	32.40	0.00
TRINITY_Q23BN3_TETTS/516-1017	Q23BN3.2 PF07748.11	32.40	0.00
TRINITY_A0A078A6W1_STYLE/181-518	A0A078A6W1.1 PF0072	32.40	0.00
TRINITY_A0A078B1L0_STYLE/110-306	A0A078B1L0.1 PF0133	32.40	0.00
TRINITY_A0BND2_PARTE/11-264	A0BND2.1 PF00069.23	32.40	0.00
TRINITY_I1GJ78_AMPQE/13-214	I1GJ78.1 PF01852.17	32.40	0.00
TRINITY_M0U346_MUSAM/95-360	M0U346.1 PF00561.18	32.40	0.00
TRINITY_J9ILZ0_9SPIT/61-293	J9ILZ0.1 PF06011.10	32.40	0.00
TRINITY_F4PL61_DICFS/169-481	F4PL61.1 PF05090.12	32.40	0.00
TRINITY_D3B2M9_POLPA/852-1009	D3B2M9.1 PF12807.5;	32.40	0.00
TRINITY_B8HTJ8_CYAP4/10-264	B8HTJ8.1 PF00069.23	32.40	0.00
TRINITY_L8GXJ8_ACACA/333-582	L8GXJ8.1 PF07065.12	32.40	0.00
TRINITY_A0A0D9WRN5_9ORYZ/29-278	A0A0D9WRN5.1 PF0842	32.40	0.00
TRINITY_L2GMY2_VITCO/126-234	L2GMY2.1 PF00307.29	32.40	0.00
TRINITY_I0YQ81_9CHLO/480-583	I0YQ81.1 PF08244.10	32.40	0.00
TRINITY_X1WBQ3_DANRE/31-235	X1WBQ3.2 PF04548.14	32.40	0.00
TRINITY_A8J0G9_CHLRE/37-593	A8J0G9.1 PF00067.20	32.40	0.00
TRINITY_M5W591_PRUPE/36-647	M5W591.1 PF05470.10	32.40	0.00
TRINITY_A0A0A0LZV6_CUCSA/210-411	A0A0A0LZV6.1 PF0419	32.40	0.00
TRINITY_D7FWV2_ECTSI/872-1054	D7FWV2.1 PF00092.26	32.40	0.00

TRINITY_L8H8T0_ACACA/97-293	L8H8T0.1 PF00837.15	32.40	0.00
TRINITY_F6ZY58_CIOIN/196-409	F6ZY58.2 PF04389.15	32.40	0.00
TRINITY_I1G263_AMPQE/158-459	I1G263.1 PF03133.13	32.40	0.00
TRINITY_D6TJN6_9CHLR/46-800	D6TJN6.1 PF01804.16	32.40	0.00
TRINITY_A0A0N4ZAC6_PARTI/94-236	A0A0N4ZAC6.1 PF0176	32.40	0.00
TRINITY_A0A0D2WV93_CAPO3/26-209	A0A0D2WV93.1 PF0118	32.40	0.00
TRINITY_W5LLC3_ASTMX/8-124	W5LLC3.1 PF01412.16	32.40	0.00
TRINITY_A8HPN3_CHLRE/286-388	A8HPN3.1 PF00254.26	32.40	0.00
TRINITY_A0A0L0BUP4_LUCCU/23-227	A0A0L0BUP4.1 PF0395	32.40	0.00
TRINITY_E1Z656_CHLVA/394-584	E1Z656.1 PF01733.16	32.40	0.00
TRINITY_A0A087SGZ0_AUXPR/671-773	A0A087SGZ0.1 PF0016	32.40	0.00
TRINITY_E1ZNH0_CHLVA/167-474	E1ZNH0.1 PF05548.9;	32.40	0.00
TRINITY_Q24JG1_TETTS/16-326	Q24JG1.1 PF07992.12	32.30	0.00
TRINITY_A0A0M2U2L9_9FIRM/82-308	A0A0M2U2L9.1 PF0163	32.30	0.00
TRINITY_Q23D35_TETTS/322-668	Q23D35.2 PF00616.17	32.30	0.00
TRINITY_A0A078B5Q4_STYLE/58-219	A0A078B5Q4.1 PF0007	32.30	0.00
TRINITY_W4Z6Q3_STRPU/1-667	W4Z6Q3.1 PF09742.7;	32.30	0.00
TRINITY_A9UV73_MONBE/12-344	A9UV73.1 PF00728.20	32.30	0.00
TRINITY_T0Q5K0_9STRA/19-535	T0Q5K0.1 PF01457.14	32.30	0.00
TRINITY_Q54BT0_DICDI/147-432	Q54BT0.1 PF06664.10	32.30	0.00
TRINITY_I4YGY6_WALMC/53-237	I4YGY6.1 PF03798.14	32.30	0.00
TRINITY_F4NZ57_BATDJ/6-142	F4NZ57.1 PF10568.7;	32.30	0.00
TRINITY_A0A078AR51_STYLE/8-287	A0A078AR51.1 PF0006	32.30	0.00
TRINITY_A7RHU2_NEMVE/28-290	A7RHU2.1 PF13621.4;	32.30	0.00
TRINITY_I3KY69_ORENI/162-314	I3KY69.1 PF13358.4;	32.30	0.00
TRINITY_G0QWT0_ICHMG/7-314	G0QWT0.1 PF01457.14	32.30	0.00
TRINITY_U4LD78_PYROM/10-379	U4LD78.1 PF06728.11	32.30	0.00
TRINITY_C3YDJ8_BRAFL/4-449	C3YDJ8.1 PF00067.20	32.30	0.00
TRINITY_D3BDW1_POLPA/33-391	D3BDW1.1 PF01237.16	32.30	0.00
TRINITY_G0QYR4_ICHMG/1316-1557	G0QYR4.1 PF06127.9;	32.30	0.00
TRINITY_E4XXW7_OIKDI/2-170	E4XXW7.1 PF00025.19	32.30	0.00
TRINITY_V3ZIL1_LOTGI/954-1214	V3ZIL1.1 PF02460.16	32.30	0.00
TRINITY_Q38BL0_TRYB2/23-290	Q38BL0.1 PF00069.23	32.30	0.00
TRINITY_I0Z1N8_9CHLO/15-437	I0Z1N8.1 PF00067.20	32.30	0.00
TRINITY_J9IIW2_9SPIT/167-1076	J9IIW2.1 PF09773.7;	32.30	0.00
TRINITY_A0CMN6_PARTE/121-399	A0CMN6.1 PF01457.14	32.30	0.00
TRINITY_H3GC33_PHYRM/138-297	H3GC33.1 PF03959.11	32.30	0.00
TRINITY_A0A024UE41_9STRA/37-929	A0A024UE41.1 PF0246	32.30	0.00
TRINITY_A0A0D2X3B4_CAPO3/50-343	A0A0D2X3B4.1 PF0844	32.30	0.00
TRINITY_L1K102_GUITH/308-439	L1K102.1 PF04499.13	32.30	0.00
TRINITY_A8HPV8_CHLRE/7-364	A8HPV8.1 PF05958.9;	32.30	0.00
TRINITY_V8NB12_OPHHA/389-555	V8NB12.1 PF00092.26	32.30	0.00
TRINITY_F9GA28_FUSOF/1-104	F9GA28.1 PF08284.9;	32.30	0.00
TRINITY_L8GFH4_ACACA/289-545	L8GFH4.1 PF00078.25	32.30	0.00
TRINITY_G0QYY5_ICHMG/38-207	G0QYY5.1 PF13847.4;	32.30	0.00
TRINITY_A0A0J7NHJ8_LASNI/817-1064	A0A0J7NHJ8.1 PF0772	32.30	0.00
TRINITY_D8TP92_VOLCA/138-295	D8TP92.1 PF01556.16	32.30	0.00
TRINITY_L8H4Q7_ACACA/36-291	L8H4Q7.1 PF14494.4;	32.20	0.00
TRINITY_G3HNE2_CRIGR/35-214	G3HNE2.1 PF05241.10	32.20	0.00
TRINITY_V6EY73_9PROT/98-241	V6EY73.1 PF00588.17	32.20	0.00
TRINITY_Q24IK5_TETTS/682-969	Q24IK5.2 PF12719.5;	32.20	0.00
TRINITY_I7M8T5_TETTS/507-687	I7M8T5.2 PF01756.17	32.20	0.00
TRINITY_I7LXH5_TETTS/33-492	I7LXH5.2 PF00067.20	32.20	0.00
TRINITY_A0A0C1GFD0_9FLAO/321-626	A0A0C1GFD0.1 PF0132	32.20	0.00
TRINITY_I3YRY2_AEQSU/34-217	I3YRY2.1 PF13563.4;	32.20	0.00
TRINITY_D2VYX4_NAEGR/317-462	D2VYX4.1 PF01743.18	32.20	0.00
TRINITY_A0EBB5_PARTE/44-269	A0EBB5.1 PF03372.21	32.20	0.00
TRINITY_Q23FZ2_TETTS/149-410	Q23FZ2.1 PF00069.23	32.20	0.00
TRINITY_I7M627_TETTS/31-676	I7M627.2 PF05667.9;	32.20	0.00
TRINITY_D8FWV8_9CYAN/51-244	D8FWV8.1 PF09511.8;	32.20	0.00
TRINITY_M7BHH1_CHEMY/6-378	M7BHH1.1 PF00079.18	32.20	0.00

TRINITY_A0A060SGI4_PYCCI/20-181	A0A060SGI4.1 PF0007	32.20	0.00
TRINITY_CCR4_CANAL/459-769	Q5A761.1 PF03372.21	32.20	0.00
TRINITY_A0DXS7_PARTE/271-599	A0DXS7.1 PF04515.10	32.20	0.00
TRINITY_A0A078AAY6_STYLE/18-304	A0A078AAY6.1 PF0406	32.20	0.00
TRINITY_W3WJ77_9PEZI/10-458	W3WJ77.1 PF00067.20	32.20	0.00
TRINITY_H3FZ36_PRIPA/212-545	H3FZ36.1 PF13598.4;	32.20	0.00
TRINITY_G3W7V8_SARHA/18-241	G3W7V8.1 PF10277.7;	32.20	0.00
TRINITY_A0A078AR51_STYLE/8-287	A0A078AR51.1 PF0006	32.20	0.00
TRINITY_I0YS24_9CHLO/211-868	I0YS24.1 PF10408.7;	32.20	0.00
TRINITY_W4XK16_STRPU/116-662	W4XK16.1 PF00305.17	32.20	0.00
TRINITY_A0EC62_PARTE/59-204	A0EC62.1 PF01699.22	32.20	0.00
TRINITY_OSB7_DICDI/30-402	Q54RM1.1 PF01237.16	32.20	0.00
TRINITY_F9UG22_9GAMM/29-326	F9UG22.1 PF12849.5;	32.20	0.00
TRINITY_G0QK40_ICHMG/243-478	G0QK40.1 PF07690.14	32.10	0.00
TRINITY_K3X309_PYTUL/752-1014	K3X309.1 PF12705.5;	32.10	0.00
TRINITY_Q54I67_DICDI/38-141	Q54I67.1 PF05071.14	32.10	0.00
TRINITY_W7TTJ9_9STRA/6-219	W7TTJ9.1 PF00856.26	32.10	0.00
TRINITY_A0A078A8F8_STYLE/36-193	A0A078A8F8.1 PF0302	32.10	0.00
TRINITY_M7NM14_PNEMU/158-286	M7NM14.1 PF01909.21	32.10	0.00
TRINITY_L8HGT4_ACACA/942-1115	L8HGT4.1 PF14763.4;	32.10	0.00
TRINITY_A0A096N103_PAPAN/23-200	A0A096N103.1 PF0976	32.10	0.00
TRINITY_A0EDW6_PARTE/192-355	A0EDW6.1 PF06757.11	32.10	0.00
TRINITY_K3WX66_PYTUL/31-302	K3WX66.1 PF05721.11	32.10	0.00
TRINITY_C3XRN9_BRAFL/514-649	C3XRN9.1 PF01967.19	32.10	0.00

TRINITY_F0YQR7_AURAN/221-357	F0YQR7.1 PF02518.24	32.10	0.00
TRINITY_K7SIM7_PROA4/3-228	K7SIM7.1 PF00300.20	32.10	0.00
TRINITY_G0QWP9_ICHMG/11-475	G0QWP9.1 PF05783.9;	32.10	0.00
TRINITY_F7BQA9_CIOIN/357-673	F7BQA9.2 PF00995.21	32.10	0.00
TRINITY_A0A0D2PTQ9_GOSRA/835-1324	A0A0D2PTQ9.1 PF0317	32.10	0.00
TRINITY_E1Z463_CHLVA/298-760	E1Z463.1 PF01266.22	32.10	0.00
TRINITY_R0I4R3_9BRAS/1-222	R0I4R3.1 PF00230.18	32.10	0.00
TRINITY_Q55F12_DICDI/374-640	Q55F12.1 PF00149.26	32.10	0.00
TRINITY_Q245X9_TETTS/19-134	Q245X9.1 PF13774.4;	32.10	0.00
TRINITY_B0DCE7_LACBS/69-227	B0DCE7.1 PF04548.14	32.10	0.00
TRINITY_K7JX42_NASVI/338-596	K7JX42.1 PF00078.25	32.10	0.00
TRINITY_A0A087XNQ9_POEFO/109-366	A0A087XNQ9.2 PF0006	32.10	0.00
TRINITY_A0A096PB78_OSTTA/853-1050	A0A096PB78.1 PF0876	32.10	0.00
TRINITY_TRHBN_SYNY3/3-118	P73925.1 PF01152.19	32.10	0.00
TRINITY_Q8F2B5_LEPIN/60-299	Q8F2B5.1 PF00795.20	32.10	0.00
TRINITY_M0SCR3_MUSAM/61-750	M0SCR3.1 PF00063.19	32.00	0.00
TRINITY_F6VUA6_MONDO/10-171	F6VUA6.1 PF00071.20	32.00	0.00
TRINITY_Q16LW8_AEDAE/141-279	Q16LW8.1 PF03188.14	32.00	0.00
TRINITY_A0A0E9NHM9_9ASCO/1410-1710	A0A0E9NHM9.1 PF1211	32.00	0.00
TRINITY_A0A075WQU3_9BACT/7-161	A0A075WQU3.1 PF0015	32.00	0.00
TRINITY_Q234L4_TETTS/1203-1401	Q234L4.2 PF12348.6;	32.00	0.00
TRINITY_Q23WL9_TETTS/100-567	Q23WL9.2 PF14643.4;	32.00	0.00
TRINITY_F4PQD8_DICFS/713-935	F4PQD8.1 PF13236.4;	32.00	0.00
TRINITY_I7MH68_TETTS/52-312	I7MH68.2 PF04045.12	32.00	0.00
TRINITY_M1C2B1_SOLTU/26-431	M1C2B1.1 PF00450.20	32.00	0.00
TRINITY_A0A0G2ZGB8_9DELT/122-254	A0A0G2ZGB8.1 PF0411	32.00	0.00
TRINITY_S2JJ35_MUCC1/555-762	S2JJ35.1 PF00924.16	32.00	0.00
TRINITY_W7X656_TETTS/412-792	W7X656.1 PF12463.6;	32.00	0.00
TRINITY_I1BVZ2_RHIO9/77-520	I1BVZ2.1 PF00067.20	32.00	0.00
TRINITY_F6GXS3_VITVI/23-471	F6GXS3.1 PF06963.10	32.00	0.00
TRINITY_Y5795_DICDI/113-220	Q1ZXL0.1 PF14593.4;	32.00	0.00
TRINITY_A8HZE3_CHLRE/2069-2245	A8HZE3.1 PF00211.18	32.00	0.00
TRINITY_D8UJ57_VOLCA/338-496	D8UJ57.1 PF04937.13	32.00	0.00
TRINITY_I7M8B5_TETTS/65-198	I7M8B5.2 PF01569.19	32.00	0.00
TRINITY_T0RQJ8_9STRA/33-274	T0RQJ8.1 PF01636.21	32.00	0.00
TRINITY_A1SG60_NOCSJ/59-419	A1SG60.1 PF03415.12	32.00	0.00
TRINITY_L8HIN8_ACACA/120-272	L8HIN8.1 PF01926.21	32.00	0.00
TRINITY_D8THR7_VOLCA/300-536	D8THR7.1 PF12697.5;	32.00	0.00
TRINITY_A0D279_PARTE/21-391	A0D279.1 PF01490.16	32.00	0.00
TRINITY_A0A0M0J556_9EUKA/51-512	A0A0M0J556.1 PF1590	32.00	0.00
TRINITY_A9STI9_PHYPA/993-1309	A9STI9.1 PF10350.7;	32.00	0.00
TRINITY_VCP_APIME/62-464	C9WMM5.1 PF00450.20	32.00	0.00
TRINITY_A0A0J7ZH40_STRVR/39-482	A0A0J7ZH40.1 PF1352	32.00	0.00
TRINITY_Y9955_DICDI/662-912	Q55GU0.1 PF07714.15	32.00	0.00
TRINITY_Q23QF2_TETTS/248-571	Q23QF2.2 PF03133.13	32.00	0.00
TRINITY_A8J7W4_CHLRE/77-369	A8J7W4.1 PF04577.12	32.00	0.00
TRINITY_E1Z5D7_CHLVA/48-399	E1Z5D7.1 PF00069.23	32.00	0.00
TRINITY_U5D935_AMBTC/366-517	U5D935.1 PF04937.13	32.00	0.00
TRINITY_Q23TF2_TETTS/1-251	Q23TF2.1 PF03839.14	32.00	0.00
TRINITY_J9IWX7_9SPIT/138-349	J9IWX7.1 PF00069.23	31.90	0.00
TRINITY_Q23G19_TETTS/51-248	Q23G19.1 PF02330.14	31.90	0.00
TRINITY_W7I8A1_9PEZI/104-253	W7I8A1.1 PF02926.15	31.90	0.00
TRINITY_A0A0D2TR40_GOSRA/23-277	A0A0D2TR40.1 PF0006	31.90	0.00
TRINITY_L8HMT0_ACACA/129-213	L8HMT0.1 PF02761.12	31.90	0.00
TRINITY_I7LX20_TETTS/11-287	I7LX20.2 PF00069.23	31.90	0.00
TRINITY_A0A059LS85_9CHLO/131-341	A0A059LS85.1 PF0505	31.90	0.00
TRINITY_F0Z9K8_DICPU/44-444	F0Z9K8.1 PF06728.11	31.90	0.00
TRINITY_E3NHQ1_CAERE/106-152	E3NHQ1.1 PF13639.4;	31.90	0.00
TRINITY_Q22C93_TETTS/132-403	Q22C93.2 PF00561.18	31.90	0.00
TRINITY_F4PB48_BATDJ/1-814	F4PB48.1 PF08553.8;	31.90	0.00
TRINITY_J9F1G3_9SPIT/7-107	J9F1G3.1 PF00168.28	31.90	0.00

TRINITY_G0QJN5_ICHMG/3-324	G0QJN5.1 PF07690.14	31.90	0.00
TRINITY_A0BE52_PARTE/149-264	A0BE52.1 PF03351.15	31.90	0.00
TRINITY_Q23CV6_TETTS/277-487	Q23CV6.1 PF00743.17	31.90	0.00
TRINITY_R6XZG7_9FIRM/10-475	R6XZG7.1 PF00478.23	31.90	0.00
TRINITY_A0A077ZV54_STYLE/34-212	A0A077ZV54.1 PF0173	31.90	0.00
TRINITY_I7M729_TETTS/286-590	I7M729.1 PF02841.12	31.90	0.00
TRINITY_A9V3Z9_MONBE/132-291	A9V3Z9.1 PF08603.9;	31.90	0.00
TRINITY_A0C868_PARTE/287-682	A0C868.1 PF01757.20	31.90	0.00
TRINITY_F0ZYY8_DICPU/171-319	F0ZYY8.1 PF00620.25	31.90	0.00
TRINITY_A0A0D2X5C8_CAPO3/1-688	A0A0D2X5C8.1 PF0578	31.90	0.00
TRINITY_W7WVY8_TETTS/68-336	W7WVY8.1 PF00069.23	31.90	0.00
TRINITY_A0BXD2_PARTE/279-685	A0BXD2.1 PF01757.20	31.90	0.00
TRINITY_I1C8Z7_RHIO9/84-365	I1C8Z7.1 PF00664.21	31.90	0.00
TRINITY_I0YWA5_9CHLO/375-695	I0YWA5.1 PF00069.23	31.90	0.00
TRINITY_A6EKX1_9SPHI/50-266	A6EKX1.1 PF00149.26	31.90	0.00
TRINITY_R4FMH5_RHOPR/43-172	R4FMH5.1 PF04145.13	31.80	0.00
TRINITY_F2SE28_TRIRC/346-532	F2SE28.2 PF05024.13	31.80	0.00
TRINITY_Q81N74_BACAN/29-285	Q81N74.1 PF00561.18	31.80	0.00
TRINITY_F4PW45_DICFS/16-125	F4PW45.1 PF02991.14	31.80	0.00
TRINITY_K2BC96_9BACT/41-359	K2BC96.1 PF07065.12	31.80	0.00
TRINITY_G4V8Q5_SCHMA/60-392	G4V8Q5.1 PF00022.17	31.80	0.00
TRINITY_Q23YD6_TETTS/29-282	Q23YD6.1 PF00069.23	31.80	0.00
TRINITY_M4BKM0_HYAAE/12-184	M4BKM0.1 PF00025.19	31.80	0.00
TRINITY_R0H6R2_9BRAS/49-460	R0H6R2.1 PF13520.4;	31.80	0.00
TRINITY_H3A005_LATCH/325-822	H3A005.1 PF07748.11	31.80	0.00
TRINITY_Q16ZJ5_AEDAE/5-150	Q16ZJ5.1 PF00339.27	31.80	0.00
TRINITY_T0RQK7_9STRA/29-520	T0RQK7.1 PF00118.22	31.80	0.00
TRINITY_C1EE69_MICSR/50-518	C1EE69.1 PF00082.20	31.80	0.00
TRINITY_V4MB11_EUTSA/137-325	V4MB11.1 PF16575.3;	31.80	0.00
TRINITY_F8PU64_SERL3/1255-1441	F8PU64.1 PF00689.19	31.80	0.00
TRINITY_A0CNI6_PARTE/314-523	A0CNI6.1 PF07002.14	31.80	0.00
TRINITY_H1YBJ8_9SPHI/88-371	H1YBJ8.1 PF14388.4;	31.80	0.00
TRINITY_L8GF07_ACACA/1-313	L8GF07.1 PF09766.7;	31.80	0.00
TRINITY_B7KBN2_CYAP7/8-202	B7KBN2.1 PF01734.20	31.80	0.00
TRINITY_I0YX07_9CHLO/5-130	I0YX07.1 PF01124.16	31.80	0.00
TRINITY_I1CH54_RHIO9/77-484	I1CH54.1 PF00067.20	31.80	0.00
TRINITY_A0CXP9_PARTE/92-386	A0CXP9.1 PF01457.14	31.80	0.00
TRINITY_I1G1S2_AMPQE/24-275	I1G1S2.1 PF00069.23	31.80	0.00
TRINITY_K3WUP1_PYTUL/141-255	K3WUP1.1 PF00787.22	31.80	0.00
TRINITY_L8GJ19_ACACA/375-576	L8GJ19.1 PF08320.10	31.80	0.00
TRINITY_D8UFM3_VOLCA/169-291	D8UFM3.1 PF03947.16	31.80	0.00
TRINITY_D8TKE9_VOLCA/222-608	D8TKE9.1 PF00155.19	31.80	0.00
TRINITY_A0A0J8CJD4_BETVU/183-371	A0A0J8CJD4.1 PF1270	31.80	0.00
TRINITY_Q23FP4_TETTS/119-546	Q23FP4.1 PF07690.14	31.80	0.00
TRINITY_A0A0M2PU93_PROHO/94-505	A0A0M2PU93.1 PF0001	31.80	0.00
TRINITY_L8GL94_ACACA/389-614	L8GL94.1 PF13672.4;	31.80	0.00
TRINITY_S8EIK6_FOMPI/624-791	S8EIK6.1 PF00535.24	31.80	0.00
TRINITY_F4PW74_DICFS/177-322	F4PW74.1 PF07534.14	31.80	0.00
TRINITY_I7M1V6_TETTS/314-533	I7M1V6.1 PF07002.14	31.70	0.00
TRINITY_A0A0G0Q6U1_9BACT/6-128	A0A0G0Q6U1.1 PF0857	31.70	0.00
TRINITY_A0BKX3_PARTE/30-221	A0BKX3.1 PF00106.23	31.70	0.00
TRINITY_H2UPR9_TAKRU/31-290	H2UPR9.1 PF00069.23	31.70	0.00
TRINITY_A0A0D9X8Z8_9ORYZ/45-252	A0A0D9X8Z8.1 PF0784	31.70	0.00
TRINITY_F4Q5A9_DICFS/154-532	F4Q5A9.1 PF04547.10	31.70	0.00
TRINITY_A0E368_PARTE/250-369	A0E368.1 PF14536.4;	31.70	0.00
TRINITY_Q1ZXE9_DICDI/3-266	Q1ZXE9.1 PF07651.14	31.70	0.00
TRINITY_I7MMH2_TETTS/14-276	I7MMH2.2 PF00069.23	31.70	0.00
TRINITY_A0C020_PARTE/368-472	A0C020.1 PF00651.29	31.70	0.00
TRINITY_U6B5F2_9RHIZ/125-419	U6B5F2.1 PF00361.18	31.70	0.00
TRINITY_A8HUT6_CHLRE/23-167	A8HUT6.1 PF10487.7;	31.70	0.00
TRINITY_F4Q7N0_DICFS/96-215	F4Q7N0.1 PF00622.26	31.70	0.00

TRINITY_C6RET9_9PROT/18-412	C6RET9.1 PF00266.17	31.70	0.00
TRINITY_J9HNI4_9SPIT/1-275	J9HNI4.1 PF00069.23	31.70	0.00
TRINITY_C5M030_PERM5/217-379	C5M030.1 PF00153.25	31.70	0.00
TRINITY_W5P6H3_SHEEP/32-289	W5P6H3.1 PF00328.20	31.70	0.00
TRINITY_B8HNX9_CYAP4/38-272	B8HNX9.1 PF13472.4;	31.70	0.00
TRINITY_F6UVL5_XENTR/181-312	F6UVL5.1 PF00782.18	31.70	0.00
TRINITY_A0DZ12_PARTE/10-257	A0DZ12.1 PF00069.23	31.70	0.00
TRINITY_B4FTY7_MAIZE/39-199	B4FTY7.1 PF01554.16	31.70	0.00
TRINITY_G8RWJ6_MYCRN/52-447	G8RWJ6.1 PF01593.22	31.70	0.00
TRINITY_A0A067P330_PLEOS/146-376	A0A067P330.1 PF1690	31.70	0.00
TRINITY_Q22B72_TETTS/137-570	Q22B72.1 PF02450.13	31.70	0.00
TRINITY_I1C8D0_RHIO9/8-172	I1C8D0.1 PF00071.20	31.70	0.00
TRINITY_G0QYJ1_ICHMG/448-655	G0QYJ1.1 PF12706.5;	31.70	0.00
TRINITY_G0QX11_ICHMG/67-192	G0QX11.1 PF00043.23	31.70	0.00
TRINITY_Q6CD03_YARLI/252-467	Q6CD03.1 PF01803.14	31.70	0.00
TRINITY_I0Z076_9CHLO/1-257	I0Z076.1 PF01963.15	31.70	0.00
TRINITY_U3IAN3_ANAPL/11-275	U3IAN3.1 PF08423.9;	31.70	0.00
TRINITY_D7DNX3_METV0/558-820	D7DNX3.1 PF04230.11	31.70	0.00
TRINITY_G0QWI2_ICHMG/263-462	G0QWI2.1 PF13886.4;	31.70	0.00
TRINITY_A0A067CTU0_SAPPC/6-130	A0A067CTU0.1 PF0139	31.70	0.00
TRINITY_A0BKH8_PARTE/3-103	A0BKH8.1 PF00168.28	31.70	0.00
TRINITY_A0A0G4J5G9_PLABS/30-194	A0A0G4J5G9.1 PF0033	31.60	0.00
TRINITY_A8WZF7_CAEBR/8-152	A8WZF7.1 PF00179.24	31.60	0.00
TRINITY_I1G8G1_AMPQE/1-365	I1G8G1.1 PF00022.17	31.60	0.00
TRINITY_A0A0Q9WVY2_DROVI/37-349	A0A0Q9WVY2.1 PF0466	31.60	0.00
TRINITY_A0A0M9DTB1_9BACT/125-419	A0A0M9DTB1.1 PF1438	31.60	0.00
TRINITY_B4VJL9_9CYAN/30-242	B4VJL9.1 PF05023.12	31.60	0.00
TRINITY_C5MDZ8_CANTT/1-439	C5MDZ8.1 PF10243.7;	31.60	0.00
TRINITY_E8MZB3_ANATU/34-184	E8MZB3.1 PF01509.16	31.60	0.00
TRINITY_V6CJX7_CAEEL/1231-1361	V6CJX7.1 PF07738.11	31.60	0.00
TRINITY_A8IPQ9_CHLRE/171-429	A8IPQ9.1 PF00069.23	31.60	0.00
TRINITY_G5B4A8_HETGA/3-408	G5B4A8.1 PF00067.20	31.60	0.00
TRINITY_F0ZA31_DICPU/69-581	F0ZA31.1 PF01457.14	31.60	0.00
TRINITY_GGAP2_ARATH/207-382	Q9FLP9.1 PF16269.3;	31.60	0.00
TRINITY_G0QPU9_ICHMG/12-338	G0QPU9.1 PF04547.10	31.60	0.00
TRINITY_M2R6G2_CERS8/26-258	M2R6G2.1 PF01738.16	31.60	0.00
TRINITY_A2ERI4_TRIVA/14-265	A2ERI4.1 PF00069.23	31.60	0.00
TRINITY_I7M910_TETTS/11-285	I7M910.1 PF00069.23	31.60	0.00
TRINITY_G6CWQ7_DANPL/2750-3100	G6CWQ7.1 PF12777.5;	31.60	0.00
TRINITY_A0A0M0JVP8_9EUKA/5-445	A0A0M0JVP8.1 PF0017	31.60	0.00
TRINITY_E9GLS7_DAPPU/17-157	E9GLS7.1 PF07884.12	31.60	0.00
TRINITY_A0A0J7NMJ7_LASNI/194-349	A0A0J7NMJ7.1 PF1335	31.60	0.00
TRINITY_A8IVR0_CHLRE/37-267	A8IVR0.1 PF01547.23	31.60	0.00
TRINITY_B9IHY7_POPTR/46-324	B9IHY7.2 PF00481.19	31.60	0.00
TRINITY_G0QP24_ICHMG/87-456	G0QP24.1 PF03006.18	31.60	0.00
TRINITY_A0CNS7_PARTE/69-227	A0CNS7.1 PF01343.16	31.50	0.00
TRINITY_A0BL69_PARTE/22-130	A0BL69.1 PF00085.18	31.50	0.00
TRINITY_S8BZ88_9LAMI/110-271	S8BZ88.1 PF02518.24	31.50	0.00
TRINITY_R0G3G4_9BRAS/551-816	R0G3G4.1 PF08324.9;	31.50	0.00
TRINITY_F4PJQ6_DICFS/104-474	F4PJQ6.1 PF07690.14	31.50	0.00
TRINITY_A7SCI8_NEMVE/85-243	A7SCI8.1 PF04098.13	31.50	0.00
TRINITY_J9FEL5_9SPIT/980-1214	J9FEL5.1 PF00520.29	31.50	0.00
TRINITY_Q232X8_TETTS/36-488	Q232X8.1 PF00067.20	31.50	0.00
TRINITY_L8H0Q4_ACACA/22-456	L8H0Q4.1 PF00067.20	31.50	0.00
TRINITY_Q245X9_TETTS/19-134	Q245X9.1 PF13774.4;	31.50	0.00
TRINITY_M1VJ71_CYAME/102-468	M1VJ71.1 PF00026.21	31.50	0.00
TRINITY_F6TXH8_CIOIN/149-273	F6TXH8.2 PF04116.11	31.50	0.00
TRINITY_A0A0G4EC88_9ALVE/28-554	A0A0G4EC88.1 PF0145	31.50	0.00
TRINITY_I7LU66_TETTS/331-735	I7LU66.2 PF03133.13	31.50	0.00
TRINITY_D8UGK8_VOLCA/146-384	D8UGK8.1 PF01547.23	31.50	0.00
TRINITY_A0A0H5C7P9_CYBJA/8-351	A0A0H5C7P9.1 PF0492	31.50	0.00

TRINITY_A0A0J8C356_BETVU/154-410	A0A0J8C356.1 PF0006	31.50	0.00
TRINITY_J9IK03_9SPIT/2-253	J9IK03.1 PF00069.23	31.50	0.00
TRINITY_B9IGR6_POPTR/19-179	B9IGR6.2 PF01554.16	31.50	0.00
TRINITY_S2J025_MUCC1/31-192	S2J025.1 PF03031.16	31.50	0.00
TRINITY_I1F8I9_AMPQE/100-276	I1F8I9.1 PF00078.25	31.50	0.00
TRINITY_Q09CN1_STIAD/65-325	Q09CN1.1 PF12146.6;	31.50	0.00
TRINITY_A0CUN4_PARTE/19-217	A0CUN4.1 PF05997.10	31.50	0.00
TRINITY_A0A0N5DGI9_TRIMR/281-442	A0A0N5DGI9.1 PF0438	31.40	0.00
TRINITY_F0ZNA5_DICPU/102-307	F0ZNA5.1 PF06999.10	31.40	0.00
TRINITY_A0EHV9_PARTE/4-278	A0EHV9.1 PF03643.13	31.40	0.00
TRINITY_G0R4Q7_ICHMG/23-419	G0R4Q7.1 PF03062.17	31.40	0.00
TRINITY_A0A0L0CLW7_LUCCU/8-187	A0A0L0CLW7.1 PF0575	31.40	0.00
TRINITY_M7C1Y2_CHEMY/665-827	M7C1Y2.1 PF05236.12	31.40	0.00
TRINITY_F1A450_DICPU/64-267	F1A450.1 PF00804.23	31.40	0.00
TRINITY_U4KVC8_PYROM/118-410	U4KVC8.1 PF00069.23	31.40	0.00
TRINITY_F0ZW42_DICPU/356-671	F0ZW42.1 PF05346.9;	31.40	0.00
TRINITY_H2ZGR1_CIOSA/60-513	H2ZGR1.1 PF00067.20	31.40	0.00
TRINITY_F1A5L9_DICPU/108-371	F1A5L9.1 PF10672.7;	31.40	0.00
TRINITY_I1F4T3_AMPQE/46-289	I1F4T3.1 PF00328.20	31.40	0.00
TRINITY_W4XKP3_STRPU/695-863	W4XKP3.1 PF12348.6;	31.40	0.00
TRINITY_L8H1G1_ACACA/50-875	L8H1G1.1 PF04931.11	31.40	0.00
TRINITY_A8J3P1_CHLRE/954-1161	A8J3P1.1 PF00211.18	31.40	0.00
TRINITY_A8J3P1_CHLRE/954-1161	A8J3P1.1 PF00211.18	31.40	0.00
TRINITY_D5E138_BACMQ/142-922	D5E138.1 PF00311.15	31.40	0.00
TRINITY_A8ZYB6_DESOH/9-118	A8ZYB6.1 PF13911.4;	31.40	0.00
TRINITY_A8IGG5_CHLRE/567-774	A8IGG5.1 PF07714.15	31.40	0.00
TRINITY_H2LC57_ORYLA/21-126	H2LC57.1 PF02991.14	31.40	0.00
TRINITY_A0A0J6VSR9_9MYCO/122-353	A0A0J6VSR9.1 PF0048	31.40	0.00
TRINITY_A0A059CCP9_EUCGR/878-1687	A0A059CCP9.1 PF0236	31.40	0.00
TRINITY_I0Z7N3_9CHLO/842-1054	I0Z7N3.1 PF14533.4;	31.40	0.00
TRINITY_Q54SQ0_DICDI/128-347	Q54SQ0.1 PF01556.16	31.40	0.00
TRINITY_W5Q128_SHEEP/162-441	W5Q128.1 PF00664.21	31.30	0.00
TRINITY_I7M910_TETTS/11-285	I7M910.1 PF00069.23	31.30	0.00
TRINITY_Q9VYJ5_DROME/395-629	Q9VYJ5.3 PF02902.17	31.30	0.00
TRINITY_K1VH25_TRIAC/83-385	K1VH25.1 PF04261.10	31.30	0.00
TRINITY_H0X7C3_OTOGA/6-377	H0X7C3.1 PF00022.17	31.30	0.00
TRINITY_A0A078A4C1_STYLE/982-1110	A0A078A4C1.1 PF0409	31.30	0.00
TRINITY_A0A087SIU5_AUXPR/572-700	A0A087SIU5.1 PF1034	31.30	0.00
TRINITY_A0BYU8_PARTE/71-258	A0BYU8.1 PF00069.23	31.30	0.00
TRINITY_A4S1H5_OSTLU/24-260	A4S1H5.1 PF02353.18	31.30	0.00
TRINITY_A0A059BFS0_EUCGR/89-295	A0A059BFS0.1 PF0085	31.30	0.00
TRINITY_T0PRM4_9STRA/349-595	T0PRM4.1 PF03572.16	31.30	0.00
TRINITY_F4QFK5_DICFS/780-958	F4QFK5.1 PF00617.17	31.30	0.00
TRINITY_D2V9N7_NAEGR/46-382	D2V9N7.1 PF00732.17	31.30	0.00
TRINITY_E1ZNI1_CHLVA/11-818	E1ZNI1.1 PF12832.5;	31.30	0.00
TRINITY_W4G1Y7_9STRA/82-245	W4G1Y7.1 PF00856.26	31.30	0.00
TRINITY_A4S0K0_OSTLU/472-701	A4S0K0.1 PF00702.24	31.30	0.00
TRINITY_A8JF01_CHLRE/2490-2965	A8JF01.1 PF10351.7;	31.30	0.00
TRINITY_L1IYK4_GUITH/62-339	L1IYK4.1 PF05783.9;	31.30	0.00
TRINITY_A0A0G4EFF6_9ALVE/55-556	A0A0G4EFF6.1 PF0299	31.30	0.00
TRINITY_G0R4J9_ICHMG/260-436	G0R4J9.1 PF00406.20	31.20	0.00
TRINITY_V7C012_PHAVU/23-196	V7C012.1 PF00445.16	31.20	0.00
TRINITY_I7LUF5_TETTS/7-205	I7LUF5.1 PF03665.11	31.20	0.00
TRINITY_F4PLR5_DICFS/3-269	F4PLR5.1 PF06454.9;	31.20	0.00
TRINITY_B4L339_DROMO/278-472	B4L339.1 PF10312.7;	31.20	0.00
TRINITY_A0A0D9VST4_9ORYZ/139-314	A0A0D9VST4.1 PF0084	31.20	0.00
TRINITY_U4UBW7_DENPD/35-225	U4UBW7.1 PF00445.16	31.20	0.00
TRINITY_D3BB02_POLPA/74-277	D3BB02.1 PF00804.23	31.20	0.00
TRINITY_I7M910_TETTS/11-285	I7M910.1 PF00069.23	31.20	0.00
TRINITY_K2S878_MACPH/13-120	K2S878.1 PF01624.18	31.20	0.00
TRINITY_K3WZ12_PYTUL/20-301	K3WZ12.1 PF03747.12	31.20	0.00

TRINITY_A0A022RRH3_ERYGU/105-334	A0A022RRH3.1 PF0196	31.20	0.00
TRINITY_E1ZRN8_CHLVA/26-275	E1ZRN8.1 PF04230.11	31.20	0.00
TRINITY_A0A0D9VKR6_9ORYZ/242-451	A0A0D9VKR6.1 PF0545	31.20	0.00
TRINITY_Q24DE8_TETTS/129-738	Q24DE8.1 PF00443.27	31.20	0.00
TRINITY_K7IX85_NASVI/35-141	K7IX85.1 PF00307.29	31.20	0.00
TRINITY_I7MDW0_TETTS/6-395	I7MDW0.2 PF04118.12	31.20	0.00
TRINITY_F4Q2T9_DICFS/499-1098	F4Q2T9.1 PF07748.11	31.20	0.00
TRINITY_C1FEP4_MICSR/76-482	C1FEP4.1 PF13476.4;	31.20	0.00
TRINITY_A0A074T8W2_HAMHA/433-572	A0A074T8W2.1 PF0065	31.20	0.00
TRINITY_A0DPV5_PARTE/314-534	A0DPV5.1 PF07002.14	31.20	0.00
TRINITY_D8UBI0_VOLCA/14-440	D8UBI0.1 PF00067.20	31.20	0.00
TRINITY_Q8RC82_CALS4/24-286	Q8RC82.1 PF12146.6;	31.10	0.00
TRINITY_A0A044QZF2_ONCVO/14-262	A0A044QZF2.1 PF0052	31.10	0.00
TRINITY_S7QGE9_GLOTA/49-890	S7QGE9.1 PF04147.10	31.10	0.00
TRINITY_H2YYQ5_CIOSA/106-224	H2YYQ5.1 PF00615.17	31.10	0.00
TRINITY_A0A074S487_9HOMO/54-500	A0A074S487.1 PF0006	31.10	0.00
TRINITY_I0Z743_9CHLO/13-111	I0Z743.1 PF05328.10	31.10	0.00
TRINITY_A0A078AFR0_STYLE/378-507	A0A078AFR0.1 PF1320	31.10	0.00
TRINITY_F6HEG9_VITVI/51-442	F6HEG9.1 PF00145.15	31.10	0.00
TRINITY_Q23EX8_TETTS/145-491	Q23EX8.2 PF13868.4;	31.10	0.00
TRINITY_E1Z3T1_CHLVA/282-447	E1Z3T1.1 PF06423.10	31.10	0.00
TRINITY_W2RHQ2_PHYPN/15-169	W2RHQ2.1 PF04937.13	31.10	0.00
TRINITY_J9K5F3_ACYPI/607-900	J9K5F3.1 PF02171.15	31.10	0.00
TRINITY_R1EAE4_EMIHU/15-190	R1EAE4.1 PF13360.4;	31.10	0.00
TRINITY_C3ZJ54_BRAFL/64-290	C3ZJ54.1 PF06159.11	31.10	0.00
TRINITY_Q22M35_TETTS/45-209	Q22M35.1 PF03357.19	31.10	0.00
TRINITY_W2RLI5_9EURO/53-181	W2RLI5.1 PF01569.19	31.10	0.00
TRINITY_T0Q9N3_9STRA/459-671	T0Q9N3.1 PF03372.21	31.10	0.00
TRINITY_CRLA_DICDI/62-322	Q54U75.1 PF05462.9;	31.10	0.00
TRINITY_D8M8H6_BLAHO/8-166	D8M8H6.1 PF00071.20	31.10	0.00
TRINITY_D0MW28_PHYIT/147-399	D0MW28.1 PF01399.25	31.10	0.00
TRINITY_I0ZAP1_9CHLO/34-522	I0ZAP1.1 PF03372.21	31.10	0.00
TRINITY_I0YMT6_9CHLO/42-205	I0YMT6.1 PF01569.19	31.10	0.00
TRINITY_F4Q8J5_DICFS/10-193	F4Q8J5.1 PF06371.11	31.10	0.00
TRINITY_Q22AM1_TETTS/51-391	Q22AM1.3 PF00328.20	31.10	0.00
TRINITY_J3M3N4_ORYBR/29-470	J3M3N4.1 PF00759.17	31.10	0.00
TRINITY_A8J3P1_CHLRE/954-1161	A8J3P1.1 PF00211.18	31.10	0.00
TRINITY_V7B6D9_PHAVU/51-187	V7B6D9.1 PF14223.4;	31.10	0.00
TRINITY_I7MMA1_TETTS/931-1194	I7MMA1.2 PF00664.21	31.00	0.00
TRINITY_Q3M0Z2_PARTE/41-243	Q3M0Z2.1 PF00804.23	31.00	0.00
TRINITY_A0D9H0_PARTE/30-354	A0D9H0.1 PF07690.14	31.00	0.00
TRINITY_I7M910_TETTS/11-285	I7M910.1 PF00069.23	31.00	0.00
TRINITY_G3UQI6_MELGA/66-211	G3UQI6.1 PF14996.4;	31.00	0.00
TRINITY_F0Z9S7_DICPU/344-682	F0Z9S7.1 PF04869.12	31.00	0.00
TRINITY_Q22A60_TETTS/15-411	Q22A60.1 PF05761.12	31.00	0.00
TRINITY_Q23YB5_TETTS/2-179	Q23YB5.2 PF06017.11	31.00	0.00
TRINITY_S0E838_GIBF5/48-357	S0E838.1 PF04724.11	31.00	0.00
TRINITY_A0CXP9_PARTE/92-386	A0CXP9.1 PF01457.14	31.00	0.00
TRINITY_Q54PA3_DICDI/95-276	Q54PA3.1 PF01454.17	31.00	0.00
TRINITY_R5Q7T6_9FIRM/2-136	R5Q7T6.1 PF00892.18	31.00	0.00
TRINITY_H2V6H4_TAKRU/7-186	H2V6H4.1 PF00875.16	31.00	0.00
TRINITY_D8U5W0_VOLCA/88-236	D8U5W0.1 PF08546.9;	31.00	0.00
TRINITY_Y1782_THEKO/61-423	Q5JJ64.1 PF00079.18	31.00	0.00
TRINITY_J7LB74_NOCAA/62-403	J7LB74.1 PF00144.22	31.00	0.00
TRINITY_L8HK27_ACACA/473-724	L8HK27.1 PF00069.23	31.00	0.00
TRINITY_A9TYN8_PHYPA/180-297	A9TYN8.1 PF04488.13	31.00	0.00
TRINITY_D8LR70_ECTSI/20-279	D8LR70.1 PF05004.11	31.00	0.00
TRINITY_K1R1V6_CRAGI/17-321	K1R1V6.1 PF05185.14	31.00	0.00
TRINITY_A0A0M0JZK3_9EUKA/232-431	A0A0M0JZK3.1 PF1385	31.00	0.00
TRINITY_W9XUW2_9EURO/422-927	W9XUW2.1 PF00899.19	31.00	0.00
TRINITY_A0A074YH30_9PEZI/92-400	A0A074YH30.1 PF0002	31.00	0.00

TRINITY_A8I0H8_CHLRE/70-331	A8I0H8.1 PF00561.18	31.00	0.00
TRINITY_D8TSN5_VOLCA/497-764	D8TSN5.1 PF07714.15	31.00	0.00
TRINITY_W5KIR2_ASTMX/102-735	W5KIR2.1 PF03343.11	31.00	0.00
TRINITY_K1QYG2_CRAGI/568-1085	K1QYG2.1 PF03177.12	31.00	0.00
TRINITY_A0A0G2LAQ9_DANRE/514-761	A0A0G2LAQ9.1 PF0007	31.00	0.00
TRINITY_F0ZHZ6_DICPU/27-241	F0ZHZ6.1 PF07722.11	31.00	0.00
TRINITY_I1QCG5_ORYGL/32-212	I1QCG5.1 PF00445.16	30.90	0.00
TRINITY_A0A0A1H016_9LACO/29-371	A0A0A1H016.1 PF0029	30.90	0.00
TRINITY_L8GLD9_ACACA/51-301	L8GLD9.1 PF00561.18	30.90	0.00
TRINITY_A0A0F2IYK8_9BACT/61-163	A0A0F2IYK8.1 PF0001	30.90	0.00
TRINITY_C1E094_MICSR/52-268	C1E094.1 PF01988.17	30.90	0.00
TRINITY_B4LQ14_DROVI/5-462	B4LQ14.1 PF00996.16	30.90	0.00
TRINITY_A0A0R4IVS5_DANRE/650-1146	A0A0R4IVS5.1 PF0774	30.90	0.00
TRINITY_F8PWC3_SERL3/70-596	F8PWC3.1 PF08118.9;	30.90	0.00
TRINITY_S2J038_MUCC1/49-202	S2J038.1 PF04112.11	30.90	0.00
TRINITY_GRLE_DICDI/440-680	Q54ET0.2 PF00003.20	30.90	0.00
TRINITY_R5Y700_9CLOT/174-367	R5Y700.1 PF00092.26	30.90	0.00
TRINITY_D0MI23_RHOM4/25-436	D0MI23.1 PF00245.18	30.90	0.00
TRINITY_Q22AM1_TETTS/51-391	Q22AM1.3 PF00328.20	30.90	0.00
TRINITY_Q22Y11_TETTS/1-289	Q22Y11.2 PF05742.10	30.90	0.00
TRINITY_Q22WK2_TETTS/10-120	Q22WK2.1 PF05773.20	30.90	0.00
TRINITY_C5KP64_PERM5/54-554	C5KP64.1 PF15902.3;	30.90	0.00
TRINITY_Q7RW81_NEUCR/971-1135	Q7RW81.3 PF00616.17	30.90	0.00
TRINITY_A8IPG1_CHLRE/11-278	A8IPG1.1 PF08433.8;	30.90	0.00
TRINITY_I0Z419_9CHLO/222-627	I0Z419.1 PF08424.8;	30.90	0.00
TRINITY_I0Z3D8_9CHLO/42-416	I0Z3D8.1 PF00999.19	30.90	0.00
TRINITY_D2VTB2_NAEGR/349-710	D2VTB2.1 PF09423.8;	30.80	0.00
TRINITY_A0CMN6_PARTE/121-399	A0CMN6.1 PF01457.14	30.80	0.00
TRINITY_A0A0K9PG53_ZOSMR/12-168	A0A0K9PG53.1 PF0442	30.80	0.00
TRINITY_Q23CV6_TETTS/29-283	Q23CV6.1 PF00743.17	30.80	0.00
TRINITY_Q24DC8_TETTS/44-337	Q24DC8.2 PF03372.21	30.80	0.00
TRINITY_E2BLD4_HARSA/3131-3319	E2BLD4.1 PF00092.26	30.80	0.00
TRINITY_K2B505_9BACT/11-278	K2B505.1 PF07690.14	30.80	0.00
TRINITY_A0CXP9_PARTE/92-386	A0CXP9.1 PF01457.14	30.80	0.00
TRINITY_A0E0X6_PARTE/134-309	A0E0X6.1 PF00621.18	30.80	0.00
TRINITY_A0A0F3FXH6_9LACO/613-761	A0A0F3FXH6.1 PF0826	30.80	0.00
TRINITY_I7MN29_TETTS/205-557	I7MN29.2 PF03416.17	30.80	0.00
TRINITY_M1VJ71_CYAME/102-468	M1VJ71.1 PF00026.21	30.80	0.00
TRINITY_J9IHM6_9SPIT/49-263	J9IHM6.1 PF02146.15	30.80	0.00
TRINITY_C1MJ95_MICPC/141-430	C1MJ95.1 PF00856.26	30.80	0.00
TRINITY_A8ZW01_DESOH/30-205	A8ZW01.1 PF13365.4;	30.80	0.00
TRINITY_E0VAN1_PEDHC/151-362	E0VAN1.1 PF03770.14	30.80	0.00
TRINITY_F2U7K1_SALR5/94-298	F2U7K1.1 PF01189.15	30.80	0.00
TRINITY_F2UIN7_SALR5/19-256	F2UIN7.1 PF03222.11	30.80	0.00
TRINITY_A0A0D8MM79_PHOLE/324-435	A0A0D8MM79.1 PF0251	30.80	0.00
TRINITY_R1CP07_EMIHU/54-292	R1CP07.1 PF04045.12	30.80	0.00
TRINITY_X6NJ86_RETFI/467-597	X6NJ86.1 PF04499.13	30.80	0.00
TRINITY_E9G7P2_DAPPU/109-254	E9G7P2.1 PF08389.10	30.80	0.00
TRINITY_W7XHQ2_TETTS/623-861	W7XHQ2.1 PF02714.13	30.80	0.00
TRINITY_A0A0J8BKW5_BETVU/1193-1331	A0A0J8BKW5.1 PF1620	30.80	0.00
TRINITY_A0A059LKE0_9CHLO/105-282	A0A059LKE0.1 PF1026	30.80	0.00
TRINITY_D8S9X8_SELML/4-433	D8S9X8.1 PF03372.21	30.80	0.00
TRINITY_A8I935_CHLRE/132-414	A8I935.1 PF13416.4;	30.80	0.00
TRINITY_D6WV93_TRICA/56-185	D6WV93.1 PF00782.18	30.70	0.00
TRINITY_A0A098EX32_9BACI/31-267	A0A098EX32.1 PF0785	30.70	0.00
TRINITY_F4PI23_DICFS/5-167	F4PI23.1 PF00071.20	30.70	0.00
TRINITY_Q232X8_TETTS/36-488	Q232X8.1 PF00067.20	30.70	0.00
TRINITY_Q24C23_TETTS/91-326	Q24C23.3 PF00566.16	30.70	0.00
TRINITY_A0A0M0KA26_9EUKA/367-475	A0A0M0KA26.1 PF0065	30.70	0.00
TRINITY_M2XZ77_GALSU/2-376	M2XZ77.1 PF00022.17	30.70	0.00
TRINITY_D8U6H8_VOLCA/913-1028	D8U6H8.1 PF04091.10	30.70	0.00

TRINITY_A0A0G4GWL3_9ALVE/43-348	A0A0G4GWL3.1 PF1137	30.70	0.00
TRINITY_A1AUW3_PELPD/72-274	A1AUW3.1 PF07859.11	30.70	0.00
TRINITY_A0A0G4EN89_9ALVE/143-459	A0A0G4EN89.1 PF0002	30.70	0.00
TRINITY_K3WFF2_PYTUL/109-395	K3WFF2.1 PF10236.7;	30.70	0.00
TRINITY_F4PBJ7_BATDJ/3-370	F4PBJ7.1 PF04857.18	30.70	0.00
TRINITY_A0A096SRJ4_MAIZE/405-614	A0A096SRJ4.1 PF1336	30.70	0.00
TRINITY_A0CMN6_PARTE/121-399	A0CMN6.1 PF01457.14	30.70	0.00
TRINITY_D8UGU8_VOLCA/204-452	D8UGU8.1 PF03629.16	30.70	0.00
TRINITY_A0A066WPY2_9BASI/380-661	A0A066WPY2.1 PF0008	30.70	0.00
TRINITY_A0A0J8B5S6_BETVU/861-1668	A0A0J8B5S6.1 PF0236	30.70	0.00
TRINITY_T1HCN7_RHOPR/582-767	T1HCN7.1 PF00621.18	30.60	0.00
TRINITY_W7XH58_TETTS/4-284	W7XH58.1 PF04791.14	30.60	0.00
TRINITY_D7MQ99_ARALL/14-316	D7MQ99.1 PF00899.19	30.60	0.00
TRINITY_A0A0A0LN37_CUCSA/5-163	A0A0A0LN37.1 PF0590	30.60	0.00
TRINITY_J9IAM0_9SPIT/410-562	J9IAM0.1 PF10607.7;	30.60	0.00
TRINITY_K3WZW5_PYTUL/199-366	K3WZW5.1 PF02582.12	30.60	0.00
TRINITY_L8HCC5_ACACA/166-433	L8HCC5.1 PF11904.6;	30.60	0.00
TRINITY_V4KH20_EUTSA/12-310	V4KH20.1 PF07992.12	30.60	0.00
TRINITY_I0YQX5_9CHLO/37-339	I0YQX5.1 PF00067.20	30.60	0.00
TRINITY_E1ZRN8_CHLVA/26-275	E1ZRN8.1 PF04230.11	30.60	0.00
TRINITY_A0A075R0X3_BRELA/5-237	A0A075R0X3.1 PF0120	30.60	0.00
TRINITY_E1Z549_CHLVA/41-381	E1Z549.1 PF00497.18	30.60	0.00
TRINITY_T0R5R8_9STRA/279-528	T0R5R8.1 PF00928.19	30.60	0.00
TRINITY_C5MDZ8_CANTT/1-439	C5MDZ8.1 PF10243.7;	30.60	0.00
TRINITY_Q22X60_TETTS/11-285	Q22X60.1 PF00069.23	30.60	0.00
TRINITY_A0A0G4E9R1_9ALVE/130-242	A0A0G4E9R1.1 PF0417	30.60	0.00
TRINITY_C5LAE7_PERM5/461-585	C5LAE7.1 PF08578.8;	30.60	0.00
TRINITY_A0A0M0JWR2_9EUKA/316-647	A0A0M0JWR2.1 PF0179	30.60	0.00
TRINITY_A0DMZ1_PARTE/10-174	A0DMZ1.1 PF03986.11	30.60	0.00
TRINITY_A9SGC0_PHYPA/143-537	A9SGC0.1 PF03969.14	30.60	0.00
TRINITY_W6RZ62_9CLOT/2-307	W6RZ62.1 PF00009.25	30.50	0.00
TRINITY_S9WE56_9CETA/6-377	S9WE56.1 PF00022.17	30.50	0.00
TRINITY_K1QAL0_CRAGI/111-580	K1QAL0.1 PF00012.18	30.50	0.00
TRINITY_I7MCY4_TETTS/74-434	I7MCY4.1 PF06870.10	30.50	0.00
TRINITY_Q22SJ7_TETTS/199-371	Q22SJ7.1 PF00092.26	30.50	0.00
TRINITY_EI24_DICDI/36-246	Q54PW9.1 PF07264.9;	30.50	0.00
TRINITY_I1CVD4_RHIO9/7-523	I1CVD4.1 PF01231.16	30.50	0.00
TRINITY_A0A075B500_9FUNG/145-362	A0A075B500.1 PF0006	30.50	0.00
TRINITY_A0CXP9_PARTE/92-386	A0CXP9.1 PF01457.14	30.50	0.00
TRINITY_A0CRJ5_PARTE/9-482	A0CRJ5.1 PF05833.9;	30.50	0.00
TRINITY_I7MEB9_TETTS/447-795	I7MEB9.2 PF03635.15	30.50	0.00
TRINITY_A0BZG6_PARTE/9-282	A0BZG6.1 PF00069.23	30.50	0.00
TRINITY_I0YQJ0_9CHLO/284-656	I0YQJ0.1 PF03081.13	30.50	0.00
TRINITY_W0DWQ5_9GAMM/10-205	W0DWQ5.1 PF13419.4;	30.50	0.00
TRINITY_W7XA88_TETTS/420-618	W7XA88.1 PF01925.17	30.50	0.00
TRINITY_D8TST9_VOLCA/621-848	D8TST9.1 PF00211.18	30.50	0.00
TRINITY_A0BS05_PARTE/16-359	A0BS05.1 PF00328.20	30.50	0.00
TRINITY_F0Z7H3_DICPU/30-593	F0Z7H3.1 PF00995.21	30.50	0.00
TRINITY_F1A451_DICPU/346-524	F1A451.1 PF05224.10	30.50	0.00
TRINITY_D8SI89_SELML/53-311	D8SI89.1 PF00069.23	30.50	0.00
TRINITY_A6P0S8_9FIRM/99-314	A6P0S8.1 PF00089.24	30.50	0.00
TRINITY_A0A067QPV9_ZOONE/31-282	A0A067QPV9.1 PF0006	30.50	0.00
TRINITY_A0A0D2X509_CAPO3/313-530	A0A0D2X509.1 PF0410	30.50	0.00
TRINITY_D8TI53_VOLCA/419-573	D8TI53.1 PF12499.6;	30.50	0.00
TRINITY_V7B654_PHAVU/4-119	V7B654.1 PF00168.28	30.40	0.00
TRINITY_Q75JR6_DICDI/1186-1338	Q75JR6.1 PF12807.5;	30.40	0.00
TRINITY_Q2IQF1_ANADE/11-98	Q2IQF1.1 PF00027.27	30.40	0.00
TRINITY_A0A0J8C4W3_BETVU/25-204	A0A0J8C4W3.1 PF0044	30.40	0.00
TRINITY_G3PLJ6_GASAC/705-996	G3PLJ6.1 PF00664.21	30.40	0.00
TRINITY_Q9UYG1_PYRAB/4-237	Q9UYG1.1 PF13660.4;	30.40	0.00
TRINITY_E1Z4Y5_CHLVA/59-192	E1Z4Y5.1 PF10033.7;	30.40	0.00

TRINITY_I7LX20_TETTS/11-287	I7LX20.2 PF00069.23	30.40	0.00
TRINITY_G6DNV4_DANPL/1240-1359	G6DNV4.1 PF00638.16	30.40	0.00
TRINITY_A0A075AQC1_9FUNG/976-1266	A0A075AQC1.1 PF0248	30.40	0.00
TRINITY_A0A074X9L6_AURPU/172-439	A0A074X9L6.1 PF0154	30.40	0.00
TRINITY_F0ZFR5_DICPU/149-409	F0ZFR5.1 PF13489.4;	30.40	0.00
TRINITY_Q1H0A7_METFK/148-459	Q1H0A7.1 PF07992.12	30.40	0.00
TRINITY_H2TVA2_TAKRU/382-663	H2TVA2.1 PF16187.3;	30.40	0.00
TRINITY_F4PLG0_DICFS/246-639	F4PLG0.1 PF07690.14	30.40	0.00
TRINITY_L8HCY8_ACACA/6-260	L8HCY8.1 PF06454.9;	30.40	0.00
TRINITY_L8H515_ACACA/218-351	L8H515.1 PF13499.4;	30.40	0.00
TRINITY_A0CXP9_PARTE/92-386	A0CXP9.1 PF01457.14	30.40	0.00
TRINITY_A0CRQ7_PARTE/61-392	A0CRQ7.1 PF02628.13	30.40	0.00
TRINITY_C5MDZ8_CANTT/1-439	C5MDZ8.1 PF10243.7;	30.40	0.00
TRINITY_A0A068S5U1_9FUNG/59-521	A0A068S5U1.1 PF0006	30.40	0.00
TRINITY_L1J437_GUITH/102-337	L1J437.1 PF09423.8;	30.40	0.00
TRINITY_D8TID6_VOLCA/27-604	D8TID6.1 PF02724.12	30.40	0.00
TRINITY_I7M7R3_TETTS/69-230	I7M7R3.1 PF01554.16	30.40	0.00
TRINITY_A8JDG8_CHLRE/162-330	A8JDG8.1 PF03567.12	30.40	0.00
TRINITY_A0A0D2VFF2_CAPO3/256-566	A0A0D2VFF2.1 PF0715	30.40	0.00
TRINITY_D8TZV5_VOLCA/70-558	D8TZV5.1 PF00026.21	30.40	0.00
TRINITY_L8HBK7_ACACA/26-465	L8HBK7.1 PF00067.20	30.40	0.00
TRINITY_A0A0F0KQS0_9MICO/39-242	A0A0F0KQS0.1 PF0023	30.30	0.00
TRINITY_A9TG91_PHYPA/214-888	A9TG91.1 PF10408.7;	30.30	0.00
TRINITY_A0CMN6_PARTE/121-399	A0CMN6.1 PF01457.14	30.30	0.00
TRINITY_F4PK46_DICFS/106-320	F4PK46.1 PF05721.11	30.30	0.00
TRINITY_L1JLX7_GUITH/145-290	L1JLX7.1 PF01764.23	30.30	0.00
TRINITY_J9IFP1_9SPIT/145-520	J9IFP1.1 PF00654.18	30.30	0.00
TRINITY_A0A068SEW5_9FUNG/53-524	A0A068SEW5.1 PF0006	30.30	0.00
TRINITY_Q54YI0_DICDI/175-456	Q54YI0.1 PF06814.11	30.30	0.00
TRINITY_T23O2_RALSO/1-346	Q8XRY7.1 PF03301.11	30.30	0.00
TRINITY_A0A084WNK7_ANOSI/5-261	A0A084WNK7.1 PF0090	30.30	0.00
TRINITY_A0A060SRV2_PYCCI/40-663	A0A060SRV2.1 PF0973	30.30	0.00
TRINITY_H3D3Q8_TETNG/14-393	H3D3Q8.1 PF07690.14	30.30	0.00
TRINITY_A0A099P333_PICKU/90-251	A0A099P333.1 PF0155	30.30	0.00
TRINITY_A0A078BA76_STYLE/103-480	A0A078BA76.1 PF0340	30.30	0.00
TRINITY_W1NQV9_AMBTC/1-195	W1NQV9.1 PF03959.11	30.30	0.00
TRINITY_A0A067CDE6_SAPPC/94-446	A0A067CDE6.1 PF0340	30.30	0.00
TRINITY_GEFQ_DICDI/967-1144	Q86G47.1 PF00617.17	30.30	0.00
TRINITY_A0DEF1_PARTE/574-728	A0DEF1.1 PF11618.6;	30.30	0.00
TRINITY_A9V028_MONBE/91-577	A9V028.1 PF00135.26	30.30	0.00
TRINITY_E1Z4R2_CHLVA/538-725	E1Z4R2.1 PF13385.4;	30.30	0.00
TRINITY_GEFQ_DICDI/967-1144	Q86G47.1 PF00617.17	30.30	0.00
TRINITY_A8JCH8_CHLRE/152-639	A8JCH8.1 PF01532.18	30.30	0.00
TRINITY_C2W3H1_BACCE/1-249	C2W3H1.1 PF10127.7;	30.30	0.00
TRINITY_A0E368_PARTE/250-369	A0E368.1 PF14536.4;	30.20	0.00
TRINITY_Q22RK7_TETTS/62-182	Q22RK7.2 PF16863.3;	30.20	0.00
TRINITY_A0A078AX44_STYLE/604-698	A0A078AX44.1 PF1349	30.20	0.00
TRINITY_W7XJ96_TETTS/33-488	W7XJ96.1 PF00067.20	30.20	0.00
TRINITY_A0DEE9_PARTE/43-499	A0DEE9.1 PF00067.20	30.20	0.00
TRINITY_D3BTH8_POLPA/29-261	D3BTH8.1 PF00149.26	30.20	0.00
TRINITY_L8GQR1_ACACA/657-1024	L8GQR1.1 PF00176.21	30.20	0.00
TRINITY_D3B861_POLPA/591-756	D3B861.1 PF02390.15	30.20	0.00
TRINITY_W4Y5G5_STRPU/17-160	W4Y5G5.1 PF03208.17	30.20	0.00
TRINITY_I0YMH8_9CHLO/78-426	I0YMH8.1 PF00150.16	30.20	0.00
TRINITY_A0A078A5B4_STYLE/158-540	A0A078A5B4.1 PF0850	30.20	0.00
TRINITY_A8JFR2_CHLRE/336-752	A8JFR2.1 PF09797.7;	30.20	0.00
TRINITY_H3GXF1_PHYRM/63-450	H3GXF1.1 PF01490.16	30.20	0.00
TRINITY_V4MAU0_EUTSA/3515-3770	V4MAU0.1 PF00454.25	30.20	0.00
TRINITY_U9SJP4_RHIID/6-321	U9SJP4.1 PF04177.10	30.20	0.00
TRINITY_D3B1K4_POLPA/254-456	D3B1K4.1 PF02886.15	30.20	0.00
TRINITY_A0A088AFF7_APIME/6-240	A0A088AFF7.1 PF0074	30.20	0.00

TRINITY_G0R5R6_ICHMG/21-294	G0R5R6.1 PF00069.23	30.20	0.00
TRINITY_I7MG82_TETTS/90-413	I7MG82.2 PF00225.21	30.20	0.00
TRINITY_G0R213_ICHMG/572-890	G0R213.1 PF11838.6;	30.20	0.00
TRINITY_F9DUI1_9BACL/71-321	F9DUI1.1 PF01636.21	30.20	0.00
TRINITY_V3ZIL1_LOTGI/308-673	V3ZIL1.1 PF02460.16	30.20	0.00
TRINITY_K4BC78_SOLLC/4174-5079	K4BC78.1 PF13764.4;	30.20	0.00
TRINITY_T1IDV6_RHOPR/992-1104	T1IDV6.1 PF12894.5;	30.20	0.00
TRINITY_A5BG02_VITVI/36-278	A5BG02.1 PF09749.7;	30.20	0.00
TRINITY_F0ZWB4_DICPU/26-725	F0ZWB4.1 PF00012.18	30.20	0.00
TRINITY_I0YYH3_9CHLO/2-128	I0YYH3.1 PF01553.19	30.20	0.00
TRINITY_A8HNP6_CHLRE/11-260	A8HNP6.1 PF07727.12	30.20	0.00
TRINITY_A0DWU6_PARTE/3-366	A0DWU6.1 PF04857.18	30.20	0.00
TRINITY_K2BC96_9BACT/41-359	K2BC96.1 PF07065.12	30.20	0.00
TRINITY_G0GCI4_SPITZ/27-140	G0GCI4.1 PF00072.22	30.10	0.00
TRINITY_W7X4A5_TETTS/149-399	W7X4A5.1 PF10192.7;	30.10	0.00
TRINITY_A0A078A0B3_STYLE/47-204	A0A078A0B3.1 PF0152	30.10	0.00
TRINITY_E1ZRN8_CHLVA/26-275	E1ZRN8.1 PF04230.11	30.10	0.00
TRINITY_A0CK29_PARTE/31-245	A0CK29.1 PF01027.18	30.10	0.00
TRINITY_B3S4F9_TRIAD/25-206	B3S4F9.1 PF13462.4;	30.10	0.00
TRINITY_M7NSX7_PNEMU/974-1154	M7NSX7.1 PF00617.17	30.10	0.00
TRINITY_B9NNU9_9RHOB/25-246	B9NNU9.1 PF00149.26	30.10	0.00
TRINITY_I7LXH5_TETTS/33-492	I7LXH5.2 PF00067.20	30.10	0.00
TRINITY_F8WWR8_9PORP/81-208	F8WWR8.1 PF01553.19	30.10	0.00
TRINITY_F0ZKQ6_DICPU/12-173	F0ZKQ6.1 PF00071.20	30.10	0.00
TRINITY_L8GVE8_ACACA/36-197	L8GVE8.1 PF08547.10	30.10	0.00
TRINITY_D3B266_POLPA/273-653	D3B266.1 PF03416.17	30.10	0.00
TRINITY_I0YLB1_9CHLO/37-547	I0YLB1.1 PF00067.20	30.10	0.00
TRINITY_W4YKQ1_STRPU/5-378	W4YKQ1.1 PF00022.17	30.10	0.00
TRINITY_Q22BY1_TETTS/300-566	Q22BY1.1 PF07714.15	30.00	0.00
TRINITY_K1RE36_CRAGI/123-527	K1RE36.1 PF00930.19	30.00	0.00
TRINITY_L8GSU8_ACACA/6-295	L8GSU8.1 PF02535.20	30.00	0.00
TRINITY_C1MQD5_MICPC/1-127	C1MQD5.1 PF00235.17	30.00	0.00
TRINITY_A0CXP9_PARTE/92-386	A0CXP9.1 PF01457.14	30.00	0.00
TRINITY_A0CXP9_PARTE/92-386	A0CXP9.1 PF01457.14	30.00	0.00
TRINITY_A9VAN1_MONBE/20-210	A9VAN1.1 PF01183.18	30.00	0.00
TRINITY_A7RZ78_NEMVE/50-270	A7RZ78.1 PF01633.18	30.00	0.00
TRINITY_D8UC13_VOLCA/1-301	D8UC13.1 PF01795.17	30.00	0.00
TRINITY_Q5CWY7_CRYPI/10-171	Q5CWY7.1 PF00071.20	30.00	0.00
TRINITY_F0ZJS9_DICPU/436-674	F0ZJS9.1 PF00003.20	30.00	0.00
TRINITY_G3AXK5_CANTC/6-172	G3AXK5.1 PF00156.25	30.00	0.00
TRINITY_A9ST26_PHYPA/26-530	A9ST26.1 PF04129.10	30.00	0.00
TRINITY_W7TJL5_9STRA/73-422	W7TJL5.1 PF05686.10	30.00	0.00
TRINITY_Q54FE3_DICDI/69-1056	Q54FE3.1 PF02463.17	30.00	0.00
TRINITY_I4EDR2_9CHLR/14-139	I4EDR2.1 PF07366.10	30.00	0.00
TRINITY_A0A078A5B4_STYLE/158-540	A0A078A5B4.1 PF0850	30.00	0.00
TRINITY_I7LUY7_TETTS/523-836	I7LUY7.2 PF00443.27	30.00	0.00
TRINITY_E9FB92_METRA/40-513	E9FB92.2 PF00067.20	30.00	0.00
TRINITY_A0A059DIR9_EUCGR/255-587	A0A059DIR9.1 PF0337	29.90	0.00
TRINITY_A0CW85_PARTE/39-324	A0CW85.1 PF07766.11	29.90	0.00
TRINITY_A0BFW2_PARTE/49-173	A0BFW2.1 PF00782.18	29.90	0.00
TRINITY_H1LBP6_9LACO/49-210	H1LBP6.1 PF01697.25	29.90	0.00
TRINITY_V4I992_9ACTN/9-199	V4I992.1 PF00106.23	29.90	0.00
TRINITY_I1FF60_AMPQE/550-779	I1FF60.1 PF13621.4;	29.90	0.00
TRINITY_A0A077ZPU9_STYLE/22-310	A0A077ZPU9.1 PF1523	29.90	0.00
TRINITY_F6QNQ3_XENTR/164-1130	F6QNQ3.1 PF03813.12	29.90	0.00
TRINITY_A0A073CK63_PLAAG/22-454	A0A073CK63.1 PF1334	29.90	0.00
TRINITY_V9EVG4_PHYPR/9-198	V9EVG4.1 PF13460.4;	29.90	0.00
TRINITY_V4AP75_LOTGI/63-280	V4AP75.1 PF01633.18	29.90	0.00
TRINITY_N1Q6P2_PSEFD/238-470	N1Q6P2.1 PF02735.14	29.90	0.00
TRINITY_K9SJ15_9CYAN/5-177	K9SJ15.1 PF02739.14	29.90	0.00
TRINITY_H2BYW0_9FLAO/184-344	H2BYW0.1 PF00406.20	29.90	0.00

TRINITY_A0A0R4ICI5_DANRE/15-550	A0A0R4ICI5.1 PF0532	29.90	0.00
TRINITY_K3W895_PYTUL/13-597	K3W895.1 PF16134.3;	29.90	0.00
TRINITY_I0YNC8_9CHLO/238-469	I0YNC8.1 PF00089.24	29.90	0.00
TRINITY_A0A0P7VCG3_9TELE/297-487	A0A0P7VCG3.1 PF0061	29.90	0.00
TRINITY_D3B978_POLPA/402-647	D3B978.1 PF00003.20	29.90	0.00
TRINITY_K9X2I1_9NOST/29-219	K9X2I1.1 PF13532.4;	29.90	0.00
TRINITY_F0ZV95_DICPU/182-735	F0ZV95.1 PF06046.11	29.90	0.00
TRINITY_A8HPW8_CHLRE/149-319	A8HPW8.1 PF00270.27	29.90	0.00
TRINITY_M4E635_BRARP/205-381	M4E635.1 PF04925.13	29.90	0.00
TRINITY_F5Y2K1_RAMTT/298-678	F5Y2K1.1 PF13361.4;	29.90	0.00
TRINITY_Q22CE3_TETTS/270-384	Q22CE3.2 PF01152.19	29.80	0.00
TRINITY_A8AVD3_STRGC/4-144	A8AVD3.1 PF00582.24	29.80	0.00
TRINITY_G4Z177_PHYSP/802-920	G4Z177.1 PF12814.5;	29.80	0.00
TRINITY_G0R213_ICHMG/572-890	G0R213.1 PF11838.6;	29.80	0.00
TRINITY_S9Y9N0_9CETA/22-442	S9Y9N0.1 PF07690.14	29.80	0.00
TRINITY_L8HI91_ACACA/91-321	L8HI91.1 PF05721.11	29.80	0.00
TRINITY_F4PNA6_DICFS/447-711	F4PNA6.1 PF00069.23	29.80	0.00
TRINITY_Q755N2_ASHGO/568-1065	Q755N2.2 PF02386.14	29.80	0.00
TRINITY_A9RDU7_PHYPA/693-887	A9RDU7.1 PF02816.16	29.80	0.00
TRINITY_L8HDY1_ACACA/30-199	L8HDY1.1 PF08603.9;	29.80	0.00
TRINITY_A0A024U0B1_9STRA/44-328	A0A024U0B1.1 PF0006	29.80	0.00
TRINITY_E1Z8T8_CHLVA/250-379	E1Z8T8.1 PF01687.15	29.80	0.00
TRINITY_A0A067GV33_CITSI/9-131	A0A067GV33.1 PF0611	29.80	0.00
TRINITY_I7MEF8_TETTS/134-646	I7MEF8.2 PF12698.5;	29.80	0.00
TRINITY_Q24CL6_TETTS/184-479	Q24CL6.2 PF03715.11	29.80	0.00
TRINITY_F0ZZ45_DICPU/6-254	F0ZZ45.1 PF00069.23	29.80	0.00
TRINITY_G0QVG7_ICHMG/239-716	G0QVG7.1 PF04791.14	29.80	0.00
TRINITY_A0A0B5HNB6_9ARCH/9-134	A0A0B5HNB6.1 PF0857	29.80	0.00
TRINITY_Q24FC1_TETTS/222-393	Q24FC1.1 PF00006.23	29.80	0.00
TRINITY_F4H4R3_CELFA/13-165	F4H4R3.1 PF06445.13	29.80	0.00
TRINITY_A0A0N5D2M8_THECL/28-250	A0A0N5D2M8.1 PF0061	29.80	0.00
TRINITY_B7FVV2_PHATC/256-443	B7FVV2.1 PF02141.19	29.80	0.00
TRINITY_A0A0G3BS48_9BURK/50-535	A0A0G3BS48.1 PF0013	29.80	0.00
TRINITY_W4Y135_STRPU/308-511	W4Y135.1 PF12348.6;	29.80	0.00
TRINITY_A0A067T719_9AGAR/3-173	A0A067T719.1 PF0255	29.80	0.00
TRINITY_D8TPB1_VOLCA/1-169	D8TPB1.1 PF01612.18	29.80	0.00
TRINITY_I1G020_AMPQE/71-386	I1G020.1 PF00026.21	29.80	0.00
TRINITY_I7M910_TETTS/11-285	I7M910.1 PF00069.23	29.80	0.00
TRINITY_J9HYX5_9SPIT/46-224	J9HYX5.1 PF00644.18	29.80	0.00
TRINITY_L8HCD7_ACACA/180-492	L8HCD7.1 PF16529.3;	29.80	0.00
TRINITY_U4L4I7_PYROM/527-843	U4L4I7.1 PF11838.6;	29.70	0.00
TRINITY_D8TZB4_VOLCA/16-197	D8TZB4.1 PF10294.7;	29.70	0.00
TRINITY_V4CK06_LOTGI/5-165	V4CK06.1 PF00071.20	29.70	0.00
TRINITY_A0A0D2UKA6_CAPO3/690-929	A0A0D2UKA6.1 PF0000	29.70	0.00
TRINITY_Q5CXE1_CRYPI/74-329	Q5CXE1.1 PF02434.14	29.70	0.00
TRINITY_A8HPW8_CHLRE/149-319	A8HPW8.1 PF00270.27	29.70	0.00
TRINITY_A0A078AR51_STYLE/8-287	A0A078AR51.1 PF0006	29.70	0.00
TRINITY_V3YZP5_LOTGI/460-707	V3YZP5.1 PF00003.20	29.70	0.00
TRINITY_F0ZSA1_DICPU/430-767	F0ZSA1.1 PF00069.23	29.70	0.00
TRINITY_R9AHK0_WALI9/322-547	R9AHK0.1 PF13086.4;	29.70	0.00
TRINITY_L1I3Z7_GUITH/1-215	L1I3Z7.1 PF00149.26	29.70	0.00
TRINITY_GRLE_DICDI/440-680	Q54ET0.2 PF00003.20	29.70	0.00
TRINITY_V3YZP5_LOTGI/460-707	V3YZP5.1 PF00003.20	29.70	0.00
TRINITY_J9EF32_WUCBA/17-207	J9EF32.1 PF01105.22	29.70	0.00
TRINITY_J9JAY6_9SPIT/8-292	J9JAY6.1 PF00069.23	29.60	0.00
TRINITY_G0QVE8_ICHMG/4-164	G0QVE8.1 PF06017.11	29.60	0.00
TRINITY_Q23EX0_TETTS/35-292	Q23EX0.1 PF00069.23	29.60	0.00
TRINITY_D5GKX1_TUBMM/1-302	D5GKX1.1 PF05158.10	29.60	0.00
TRINITY_W5K0F9_ASTMX/17-1659	W5K0F9.1 PF11894.6;	29.60	0.00
TRINITY_J9I9S9_9SPIT/262-576	J9I9S9.1 PF02841.12	29.60	0.00
TRINITY_F0Z7Y1_DICPU/1320-1613	F0Z7Y1.1 PF06454.9;	29.60	0.00

TRINITY_A0A068RYF4_9FUNG/6-110	A0A068RYF4.1 PF0577	29.60	0.00
TRINITY_D3AVZ9_POLPA/101-404	D3AVZ9.1 PF07946.12	29.60	0.00
TRINITY_D8TRQ9_VOLCA/4-179	D8TRQ9.1 PF01697.25	29.60	0.00
TRINITY_V4BB13_LOTGI/15-188	V4BB13.1 PF00071.20	29.60	0.00
TRINITY_A8J1H4_CHLRE/6324-6636	A8J1H4.1 PF08016.10	29.60	0.00
TRINITY_H2AQB0_KAZAF/11-237	H2AQB0.1 PF00244.18	29.60	0.00
TRINITY_W4XHU3_STRPU/28-549	W4XHU3.1 PF00135.26	29.60	0.00
TRINITY_D8TVP3_VOLCA/12-119	D8TVP3.1 PF00636.24	29.60	0.00
TRINITY_A8J658_CHLRE/123-291	A8J658.1 PF00092.26	29.60	0.00
TRINITY_W4GHI4_9STRA/158-604	W4GHI4.1 PF01457.14	29.60	0.00
TRINITY_I0YKN2_9CHLO/520-718	I0YKN2.1 PF13385.4;	29.60	0.00
TRINITY_A0A0D2WTN7_CAPO3/64-537	A0A0D2WTN7.1 PF0238	29.60	0.00
TRINITY_D6WQX6_TRICA/52-199	D6WQX6.1 PF04495.12	29.60	0.00
TRINITY_D8U6K8_VOLCA/164-377	D8U6K8.1 PF03924.11	29.60	0.00
TRINITY_D8U5B5_VOLCA/230-615	D8U5B5.1 PF09359.8;	29.60	0.00
TRINITY_F7A1M4_XENTR/359-614	F7A1M4.1 PF00078.25	29.60	0.00
TRINITY_E2BLD4_HARSA/3131-3319	E2BLD4.1 PF00092.26	29.60	0.00
TRINITY_I7LU14_TETTS/258-503	I7LU14.1 PF00083.22	29.50	0.00
TRINITY_G7J8G7_MEDTR/22-251	G7J8G7.1 PF01048.18	29.50	0.00
TRINITY_Q22AM1_TETTS/51-391	Q22AM1.3 PF00328.20	29.50	0.00
TRINITY_I7MIS1_TETTS/143-563	I7MIS1.2 PF02450.13	29.50	0.00
TRINITY_G0QVG7_ICHMG/239-716	G0QVG7.1 PF04791.14	29.50	0.00
TRINITY_A0A0L0NFJ1_9HYPO/1219-1391	A0A0L0NFJ1.1 PF0061	29.50	0.00
TRINITY_A0D7F5_PARTE/13-259	A0D7F5.1 PF00069.23	29.50	0.00
TRINITY_D8U7P3_VOLCA/11-149	D8U7P3.1 PF04652.14	29.50	0.00
TRINITY_L8GP97_ACACA/29-332	L8GP97.1 PF03747.12	29.50	0.00
TRINITY_A9ECT1_9FLAO/25-266	A9ECT1.1 PF03372.21	29.50	0.00
TRINITY_G4LXL2_SCHMA/225-332	G4LXL2.1 PF00787.22	29.50	0.00
TRINITY_H2Z7E9_CIOSA/675-974	H2Z7E9.1 PF11262.6;	29.50	0.00
TRINITY_E5S2M9_TRISP/506-757	E5S2M9.1 PF00003.20	29.50	0.00
TRINITY_A8HYT4_CHLRE/20-244	A8HYT4.1 PF05216.11	29.50	0.00
TRINITY_A0A078AGI8_STYLE/1904-2163	A0A078AGI8.1 PF0052	29.50	0.00
TRINITY_A0CXP9_PARTE/92-386	A0CXP9.1 PF01457.14	29.50	0.00
TRINITY_F0Z9N8_DICPU/23-227	F0Z9N8.1 PF05057.12	29.50	0.00
TRINITY_U3IBN3_ANAPL/4-95	U3IBN3.1 PF16589.3;	29.50	0.00
TRINITY_A0BKH8_PARTE/3-103	A0BKH8.1 PF00168.28	29.40	0.00
TRINITY_G0QW56_ICHMG/25-375	G0QW56.1 PF07690.14	29.40	0.00
TRINITY_A0A078AMC9_STYLE/9-109	A0A078AMC9.1 PF0016	29.40	0.00
TRINITY_L5MGU3_MYODS/75-400	L5MGU3.1 PF13347.4;	29.40	0.00
TRINITY_A6GCY6_9DELT/8-435	A6GCY6.1 PF00067.20	29.40	0.00
TRINITY_S0F075_CHTCT/22-224	S0F075.1 PF05721.11	29.40	0.00
TRINITY_A0DMA3_PARTE/467-923	A0DMA3.1 PF04547.10	29.40	0.00
TRINITY_A0CXP9_PARTE/92-386	A0CXP9.1 PF01457.14	29.40	0.00
TRINITY_D8U6K8_VOLCA/164-377	D8U6K8.1 PF03924.11	29.40	0.00
TRINITY_L8HM05_ACACA/513-681	L8HM05.1 PF05729.10	29.40	0.00
TRINITY_A0CTN6_PARTE/65-241	A0CTN6.1 PF00856.26	29.40	0.00
TRINITY_D3B1Z5_POLPA/10-409	D3B1Z5.1 PF10300.7;	29.40	0.00
TRINITY_A0A0D2W0P5_CAPO3/273-680	A0A0D2W0P5.1 PF0149	29.40	0.00
TRINITY_G0QK39_ICHMG/46-377	G0QK39.1 PF00328.20	29.40	0.00
TRINITY_A0BN74_PARTE/148-410	A0BN74.1 PF00481.19	29.40	0.00
TRINITY_K1PIJ8_CRAGI/20-308	K1PIJ8.1 PF13621.4;	29.40	0.00
TRINITY_A0DS64_PARTE/289-550	A0DS64.1 PF07690.14	29.40	0.00
TRINITY_G4TPK8_PIRID/7-365	G4TPK8.1 PF00503.18	29.30	0.00
TRINITY_J9EUT0_9SPIT/265-521	J9EUT0.1 PF02518.24	29.30	0.00
TRINITY_I7M2E4_TETTS/2083-2267	I7M2E4.1 PF08146.10	29.30	0.00
TRINITY_W5N530_LEPOC/336-607	W5N530.1 PF00664.21	29.30	0.00
TRINITY_M5WTD1_PRUPE/231-411	M5WTD1.1 PF00092.26	29.30	0.00
TRINITY_D2UXM9_NAEGR/475-623	D2UXM9.1 PF17177.2;	29.30	0.00
TRINITY_J9I443_9SPIT/34-311	J9I443.1 PF00069.23	29.30	0.00
TRINITY_F4PQC0_DICFS/53-391	F4PQC0.1 PF00328.20	29.30	0.00
TRINITY_W4YHX3_STRPU/44-311	W4YHX3.1 PF04882.10	29.30	0.00

TRINITY_Q556Z2_DICDI/209-508	Q556Z2.1 PF00069.23	29.30	0.00
TRINITY_W7X9E9_TETTS/12-119	W7X9E9.1 PF01398.19	29.30	0.00
TRINITY_I1CCX6_RHIO9/2-444	I1CCX6.1 PF00067.20	29.30	0.00
TRINITY_A0A078AUG4_STYLE/11-173	A0A078AUG4.1 PF0007	29.30	0.00
TRINITY_A0CXP9_PARTE/92-386	A0CXP9.1 PF01457.14	29.30	0.00
TRINITY_A0A077ZUU3_STYLE/1372-1637	A0A077ZUU3.1 PF0052	29.30	0.00
TRINITY_A8IWP6_CHLRE/152-313	A8IWP6.1 PF01734.20	29.30	0.00
TRINITY_D3BU27_POLPA/534-737	D3BU27.1 PF00617.17	29.30	0.00
TRINITY_H2XZL5_CIOIN/130-293	H2XZL5.1 PF10421.7;	29.30	0.00
TRINITY_A8HSY2_CHLRE/37-412	A8HSY2.1 PF02535.20	29.30	0.00
TRINITY_D2V8H7_NAEGR/10-643	D2V8H7.1 PF00888.20	29.30	0.00
TRINITY_A0A024P5U1_9BACI/153-314	A0A024P5U1.1 PF1457	29.20	0.00
TRINITY_Q3A4V9_PELCD/29-209	Q3A4V9.1 PF04832.10	29.20	0.00
TRINITY_Q54CA6_DICDI/121-289	Q54CA6.1 PF02453.15	29.20	0.00
TRINITY_A0CXP9_PARTE/92-386	A0CXP9.1 PF01457.14	29.20	0.00
TRINITY_D8R2B4_SELML/111-306	D8R2B4.1 PF04389.15	29.20	0.00
TRINITY_L8H154_ACACA/1-343	L8H154.1 PF16050.3;	29.20	0.00
TRINITY_A0A0A0LT21_CUCSA/9-195	A0A0A0LT21.1 PF0465	29.20	0.00
TRINITY_W5AE58_WHEAT/91-307	W5AE58.1 PF07228.10	29.20	0.00
TRINITY_F0ZUE1_DICPU/13-577	F0ZUE1.1 PF01457.14	29.20	0.00
TRINITY_I7M910_TETTS/11-285	I7M910.1 PF00069.23	29.20	0.00
TRINITY_A5VCB3_SPHWW/32-371	A5VCB3.1 PF01266.22	29.20	0.00
TRINITY_Q22WX4_TETTS/617-1013	Q22WX4.2 PF12698.5;	29.20	0.00
TRINITY_A0A0G4ISF8_PLABS/111-501	A0A0G4ISF8.1 PF0149	29.20	0.00
TRINITY_E1ZTQ0_CHLVA/88-260	E1ZTQ0.1 PF12239.6;	29.20	0.00
TRINITY_M5X1K9_PRUPE/1-408	M5X1K9.1 PF00067.20	29.20	0.00
TRINITY_S2JWW9_MUCC1/44-618	S2JWW9.1 PF05327.9;	29.20	0.00
TRINITY_Q4G295_TRIVA/9-169	Q4G295.1 PF00071.20	29.20	0.00
TRINITY_W4YU07_STRPU/149-349	W4YU07.1 PF00149.26	29.20	0.00
TRINITY_Q23NK6_TETTS/59-360	Q23NK6.2 PF07690.14	29.20	0.00
TRINITY_D8U4A7_VOLCA/26-266	D8U4A7.1 PF00481.19	29.20	0.00
TRINITY_A0A067MAJ1_9HOMO/103-411	A0A067MAJ1.1 PF0002	29.20	0.00
TRINITY_A8HQD7_CHLRE/69-360	A8HQD7.1 PF00351.19	29.20	0.00
TRINITY_W5QAV6_SHEEP/566-861	W5QAV6.1 PF02171.15	29.20	0.00
TRINITY_Q22U03_TETTS/42-234	Q22U03.2 PF00445.16	29.20	0.00
TRINITY_G0QV22_ICHMG/24-163	G0QV22.1 PF12409.6;	29.20	0.00
TRINITY_A8J5L0_CHLRE/107-1192	A8J5L0.1 PF02463.17	29.20	0.00
TRINITY_E1Z549_CHLVA/41-381	E1Z549.1 PF00497.18	29.20	0.00
TRINITY_B7GBW1_PHATC/8-213	B7GBW1.1 PF01135.17	29.20	0.00
TRINITY_X6M0W0_RETFI/58-401	X6M0W0.1 PF00503.18	29.10	0.00
TRINITY_A0A072TRJ4_MEDTR/55-217	A0A072TRJ4.1 PF0438	29.10	0.00
TRINITY_T1JHP0_STRMM/2-159	T1JHP0.1 PF09767.7;	29.10	0.00
TRINITY_I7LZW4_TETTS/23-359	I7LZW4.2 PF08569.9;	29.10	0.00
TRINITY_Q22SJ7_TETTS/199-371	Q22SJ7.1 PF00092.26	29.10	0.00
TRINITY_K3WIP2_PYTUL/367-652	K3WIP2.1 PF02958.18	29.10	0.00
TRINITY_K7L895_SOYBN/177-790	K7L895.1 PF03343.11	29.10	0.00
TRINITY_A0CXP9_PARTE/92-386	A0CXP9.1 PF01457.14	29.10	0.00
TRINITY_Q233H2_TETTS/5-219	Q233H2.1 PF04750.12	29.10	0.00
TRINITY_M4XLP1_PSEDE/29-263	M4XLP1.1 PF00797.15	29.10	0.00
TRINITY_C5CV60_VARPS/33-328	C5CV60.1 PF02608.12	29.10	0.00
TRINITY_A0A0G4J4N1_PLABS/554-806	A0A0G4J4N1.1 PF0000	29.10	0.00
TRINITY_W7XJ96_TETTS/33-488	W7XJ96.1 PF00067.20	29.10	0.00
TRINITY_C9JUS5_HUMAN/5-152	C9JUS5.2 PF00780.20	29.10	0.00
TRINITY_S2KKM8_MUCC1/1-129	S2KKM8.1 PF11221.6;	29.00	0.00
TRINITY_A3N1G4_ACTP2/5-295	A3N1G4.1 PF04261.10	29.00	0.00
TRINITY_Q22BF9_TETTS/121-285	Q22BF9.1 PF01554.16	29.00	0.00
TRINITY_M2W791_GALSU/1135-1354	M2W791.1 PF14678.4;	29.00	0.00
TRINITY_R1DL91_EMIHU/55-398	R1DL91.1 PF00026.21	29.00	0.00
TRINITY_LYSG4_DICDI/23-204	Q54BQ8.1 PF01183.18	29.00	0.00
TRINITY_A0A0N4VDF3_ENTVE/24-205	A0A0N4VDF3.1 PF0118	29.00	0.00
TRINITY_I7MKH3_TETTS/98-490	I7MKH3.1 PF01457.14	29.00	0.00

TRINITY_A0CXP9_PARTE/92-386	A0CXP9.1 PF01457.14	29.00	0.00
TRINITY_L8GVC5_ACACA/535-841	L8GVC5.1 PF16542.3;	29.00	0.00
TRINITY_I1BNC1_RHIO9/65-389	I1BNC1.1 PF03372.21	29.00	0.00
TRINITY_A0CXP9_PARTE/92-386	A0CXP9.1 PF01457.14	29.00	0.00
TRINITY_K2E113_9BACT/8-206	K2E113.1 PF01734.20	29.00	0.00
TRINITY_A0A078AX44_STYLE/604-698	A0A078AX44.1 PF1349	29.00	0.00
TRINITY_F4NZL9_BATDJ/700-875	F4NZL9.1 PF00617.17	29.00	0.00
TRINITY_F6I1G5_VITVI/22-572	F6I1G5.1 PF12070.6;	29.00	0.00
TRINITY_A9U470_PHYPA/136-516	A9U470.1 PF00012.18	29.00	0.00
TRINITY_A0A078A494_STYLE/100-311	A0A078A494.1 PF0379	28.90	0.00
TRINITY_L8GSS6_ACACA/162-385	L8GSS6.1 PF10271.7;	28.90	0.00
TRINITY_D3BIA5_POLPA/85-280	D3BIA5.1 PF01852.17	28.90	0.00
TRINITY_A0DWP8_PARTE/26-304	A0DWP8.1 PF00069.23	28.90	0.00
TRINITY_A0A0D2U1B4_CAPO3/19-346	A0A0D2U1B4.1 PF0326	28.90	0.00
TRINITY_H1W4V5_COLHI/5-267	H1W4V5.1 PF02460.16	28.90	0.00
TRINITY_D3BAL6_POLPA/2-396	D3BAL6.1 PF01457.14	28.90	0.00
TRINITY_A0DFI8_PARTE/389-577	A0DFI8.1 PF01564.15	28.90	0.00
TRINITY_A0A0G3A2L3_9DELT/83-295	A0A0G3A2L3.1 PF0014	28.90	0.00
TRINITY_A0BN67_PARTE/37-406	A0BN67.1 PF07690.14	28.90	0.00
TRINITY_T1F1D5_HELRO/57-243	T1F1D5.1 PF01762.19	28.90	0.00
TRINITY_B1WBJ2_XENTR/49-505	B1WBJ2.1 PF00067.20	28.90	0.00
TRINITY_Q22U03_TETTS/42-234	Q22U03.2 PF00445.16	28.90	0.00
TRINITY_C5MDZ8_CANTT/1-439	C5MDZ8.1 PF10243.7;	28.90	0.00
TRINITY_E4XHV3_OIKDI/440-695	E4XHV3.1 PF00520.29	28.90	0.00
TRINITY_T1NRF9_TRIUA/62-190	T1NRF9.1 PF01529.18	28.90	0.00
TRINITY_C1E0S8_MICSR/118-273	C1E0S8.1 PF07173.10	28.90	0.00
TRINITY_S6A5L0_9CREN/5-316	S6A5L0.1 PF00009.25	28.90	0.00
TRINITY_A0E5J8_PARTE/12-183	A0E5J8.1 PF01105.22	28.80	0.00
TRINITY_M4BWV5_HYAAE/298-449	M4BWV5.1 PF14580.4;	28.80	0.00
TRINITY_L8GQR1_ACACA/657-1024	L8GQR1.1 PF00176.21	28.80	0.00
TRINITY_A0E368_PARTE/250-369	A0E368.1 PF14536.4;	28.80	0.00
TRINITY_K1Q6I4_CRAGI/226-394	K1Q6I4.1 PF01926.21	28.80	0.00
TRINITY_V3ZQE4_LOTGI/6-334	V3ZQE4.1 PF10239.7;	28.80	0.00
TRINITY_F4PTD9_DICFS/71-639	F4PTD9.1 PF01457.14	28.80	0.00
TRINITY_A9U470_PHYPA/136-516	A9U470.1 PF00012.18	28.80	0.00
TRINITY_G7KQK6_MEDTR/246-556	G7KQK6.2 PF00201.16	28.80	0.00
TRINITY_F4Y1I9_9CYAN/49-252	F4Y1I9.1 PF05050.10	28.80	0.00
TRINITY_D8TJ10_VOLCA/96-269	D8TJ10.1 PF04727.11	28.80	0.00
TRINITY_A3HTV5_9BACT/2-439	A3HTV5.1 PF00171.20	28.80	0.00
TRINITY_A0A0A2EQ48_PORCN/6-187	A0A0A2EQ48.1 PF0496	28.80	0.00
TRINITY_I1FTQ4_AMPQE/27-195	I1FTQ4.1 PF00071.20	28.80	0.00
TRINITY_A0BCK7_PARTE/17-268	A0BCK7.1 PF00069.23	28.80	0.00
TRINITY_A0A0A1N2H7_9FUNG/248-498	A0A0A1N2H7.1 PF0006	28.80	0.00
TRINITY_G2LD78_CHLTF/15-456	G2LD78.1 PF00067.20	28.80	0.00
TRINITY_D2VEC5_NAEGR/29-270	D2VEC5.1 PF12146.6;	28.80	0.00
TRINITY_A0A0B0PRQ4_GOSAR/144-336	A0A0B0PRQ4.1 PF0410	28.80	0.00
TRINITY_A0A0E3UVI8_9BACT/37-178	A0A0E3UVI8.1 PF0821	28.70	0.00
TRINITY_A0A0K0GDU6_9FIRM/47-265	A0A0K0GDU6.1 PF0049	28.70	0.00
TRINITY_A0A067CFY4_SAPPC/57-315	A0A067CFY4.1 PF0771	28.70	0.00
TRINITY_L5K193_PTEAL/37-209	L5K193.1 PF00213.16	28.70	0.00
TRINITY_F6YM31_CIOIN/45-180	F6YM31.2 PF00782.18	28.70	0.00
TRINITY_I7MKW6_TETTS/46-643	I7MKW6.2 PF14745.4;	28.70	0.00
TRINITY_I0Z971_9CHLO/85-456	I0Z971.1 PF01494.17	28.70	0.00
TRINITY_A0A067PK03_9HOMO/425-702	A0A067PK03.1 PF0568	28.70	0.00
TRINITY_Q54NV0_DICDI/913-1146	Q54NV0.1 PF10474.7;	28.70	0.00
TRINITY_A0A0N4ZI70_PARTI/264-369	A0A0N4ZI70.1 PF0016	28.70	0.00
TRINITY_A0A0D2X3D6_CAPO3/12-202	A0A0D2X3D6.1 PF0033	28.70	0.00
TRINITY_T0S962_9STRA/354-560	T0S962.1 PF03572.16	28.70	0.00
TRINITY_C1EJH9_MICSR/428-775	C1EJH9.1 PF13086.4;	28.70	0.00
TRINITY_I0Z1V8_9CHLO/69-348	I0Z1V8.1 PF00069.23	28.70	0.00
TRINITY_A9SFH0_PHYPA/66-379	A9SFH0.1 PF15967.3;	28.70	0.00

TRINITY_A0A091DGF6_FUKDA/4-187	A0A091DGF6.1 PF0002	28.70	0.00
TRINITY_D3BPY7_POLPA/494-667	D3BPY7.1 PF14429.4;	28.60	0.00
TRINITY_H0UZU7_CAVPO/8-169	H0UZU7.1 PF00071.20	28.60	0.00
TRINITY_Q245H7_TETTS/90-237	Q245H7.1 PF11559.6;	28.60	0.00
TRINITY_H2MVG9_ORYLA/11-336	H2MVG9.1 PF06113.10	28.60	0.00
TRINITY_A0A0N4ZLN1_PARTI/5-256	A0A0N4ZLN1.1 PF0243	28.60	0.00
TRINITY_A0A075AQC1_9FUNG/976-1266	A0A075AQC1.1 PF0248	28.60	0.00
TRINITY_H3CAR2_TETNG/6-305	H3CAR2.1 PF00561.18	28.60	0.00
TRINITY_D6WFN7_TRICA/13-460	D6WFN7.1 PF00067.20	28.60	0.00
TRINITY_A0DI05_PARTE/319-612	A0DI05.1 PF04547.10	28.60	0.00
TRINITY_A1ZGJ1_9BACT/4-211	A1ZGJ1.1 PF00106.23	28.60	0.00
TRINITY_I7MKN2_TETTS/45-746	I7MKN2.2 PF05879.10	28.60	0.00
TRINITY_A0A0J7L7U6_LASNI/36-303	A0A0J7L7U6.1 PF0032	28.60	0.00
TRINITY_I1CDS8_RHIO9/36-274	I1CDS8.1 PF01063.17	28.60	0.00
TRINITY_E1ZB98_CHLVA/23-373	E1ZB98.1 PF00497.18	28.60	0.00
TRINITY_X6NPF9_RETFI/5-279	X6NPF9.1 PF07690.14	28.60	0.00
TRINITY_A0A0G4H7S9_9ALVE/200-585	A0A0G4H7S9.1 PF0149	28.60	0.00
TRINITY_U3IS56_ANAPL/520-987	U3IS56.1 PF00580.19	28.60	0.00
TRINITY_Q3SEP6_PARTE/37-615	Q3SEP6.1 PF00995.21	28.50	0.00
TRINITY_Q55E33_DICDI/111-498	Q55E33.1 PF03985.11	28.50	0.00
TRINITY_L5LR78_MYODS/36-348	L5LR78.1 PF00026.21	28.50	0.00
TRINITY_A0A0N4UP19_DRAME/266-429	A0A0N4UP19.1 PF0062	28.50	0.00
TRINITY_H8MPI2_CORCM/14-451	H8MPI2.1 PF00067.20	28.50	0.00
TRINITY_W9QEG3_9ROSA/63-317	W9QEG3.1 PF02854.17	28.50	0.00
TRINITY_W7XJ96_TETTS/33-488	W7XJ96.1 PF00067.20	28.50	0.00
TRINITY_D2HKU2_AILME/39-351	D2HKU2.1 PF05185.14	28.50	0.00
TRINITY_STRAP_DICDI/58-221	Q54LT8.1 PF08662.9;	28.50	0.00
TRINITY_F0ZVQ5_DICPU/78-431	F0ZVQ5.1 PF01925.17	28.40	0.00
TRINITY_J1FAL0_9HELI/98-304	J1FAL0.1 PF01266.22	28.40	0.00
TRINITY_E2BIG5_HARSA/41-541	E2BIG5.1 PF10154.7;	28.40	0.00
TRINITY_D8U6K8_VOLCA/164-377	D8U6K8.1 PF03924.11	28.40	0.00
TRINITY_A0A072NTN1_9EURO/87-314	A0A072NTN1.1 PF0410	28.40	0.00
TRINITY_W5LED3_ASTMX/66-181	W5LED3.1 PF00615.17	28.40	0.00
TRINITY_D3BQD7_POLPA/353-579	D3BQD7.1 PF11744.6;	28.40	0.00
TRINITY_K7M8N3_SOYBN/1351-1725	K7M8N3.1 PF13086.4;	28.40	0.00
TRINITY_B3QUP1_CHLT3/17-307	B3QUP1.1 PF02541.14	28.40	0.00
TRINITY_E1ZSN3_CHLVA/103-252	E1ZSN3.1 PF03114.16	28.40	0.00
TRINITY_A0EHH7_PARTE/8-264	A0EHH7.1 PF02535.20	28.40	0.00
TRINITY_W7X656_TETTS/412-792	W7X656.1 PF12463.6;	28.30	0.00
TRINITY_W7XDU3_TETTS/50-395	W7XDU3.1 PF10974.6;	28.30	0.00
TRINITY_A2BGQ6_DANRE/72-583	A2BGQ6.1 PF03215.13	28.30	0.00
TRINITY_A0E368_PARTE/250-369	A0E368.1 PF14536.4;	28.30	0.00
TRINITY_I1FF60_AMPQE/550-779	I1FF60.1 PF13621.4;	28.30	0.00
TRINITY_A8IX96_CHLRE/150-433	A8IX96.1 PF00069.23	28.30	0.00
TRINITY_L8GVE4_ACACA/1000-1189	L8GVE4.1 PF00621.18	28.30	0.00
TRINITY_J9K7F8_ACYPI/748-1036	J9K7F8.2 PF00664.21	28.30	0.00
TRINITY_F0ZVV4_DICPU/345-650	F0ZVV4.1 PF09770.7;	28.30	0.00
TRINITY_I7M136_TETTS/2403-2719	I7M136.2 PF00520.29	28.30	0.00
TRINITY_L8GZY0_ACACA/7-180	L8GZY0.1 PF00071.20	28.20	0.00
TRINITY_L8GPX0_ACACA/178-559	L8GPX0.1 PF00856.26	28.20	0.00
TRINITY_A0A075AQC1_9FUNG/976-1266	A0A075AQC1.1 PF0248	28.20	0.00
TRINITY_A0E717_PARTE/286-554	A0E717.1 PF02854.17	28.20	0.00
TRINITY_Q23DL4_TETTS/944-1129	Q23DL4.3 PF08625.9;	28.20	0.00
TRINITY_A0A067REZ3_ZOONE/23-292	A0A067REZ3.1 PF0006	28.20	0.00
TRINITY_D3BEG9_POLPA/129-339	D3BEG9.1 PF06814.11	28.20	0.00
TRINITY_A0A067BIS1_SAPPC/108-212	A0A067BIS1.1 PF0400	28.20	0.00
TRINITY_V4U3M1_9ROSI/105-251	V4U3M1.1 PF01699.22	28.20	0.00
TRINITY_B8DM82_DESVM/76-244	B8DM82.1 PF01501.18	28.20	0.00
TRINITY_W4Z461_STRPU/10-245	W4Z461.1 PF13621.4;	28.20	0.00
TRINITY_D2A1W4_TRICA/604-875	D2A1W4.1 PF01757.20	28.20	0.00
TRINITY_A0BE76_PARTE/38-361	A0BE76.1 PF00328.20	28.20	0.00

TRINITY_G7KQK8_MEDTR/195-644	G7KQK8.1 PF00067.20	28.20	0.00
TRINITY_Q22TZ7_TETTS/70-310	Q22TZ7.2 PF15239.4;	28.10	0.00
TRINITY_F4P471_BATDJ/263-623	F4P471.1 PF03081.13	28.10	0.00
TRINITY_W4FXX8_9STRA/2490-2794	W4FXX8.1 PF00520.29	28.10	0.00
TRINITY_H2LYD2_ORYLA/180-491	H2LYD2.1 PF08318.10	28.10	0.00
TRINITY_A0A0D2WIN0_CAPO3/195-762	A0A0D2WIN0.1 PF1596	28.10	0.00
TRINITY_C3XWC4_BRAFL/4-263	C3XWC4.1 PF00069.23	28.10	0.00
TRINITY_A1DC12_NEOFI/39-303	A1DC12.1 PF07859.11	28.10	0.00
TRINITY_L0D867_SINAD/130-368	L0D867.1 PF13621.4;	28.10	0.00
TRINITY_F0ZRG8_DICPU/3225-3518	F0ZRG8.1 PF06650.10	28.10	0.00
TRINITY_D3CRA7_9ACTN/68-327	D3CRA7.1 PF02958.18	28.10	0.00
TRINITY_A0A067QZ59_ZOONE/34-492	A0A067QZ59.1 PF0006	28.10	0.00
TRINITY_D8TLY8_VOLCA/327-524	D8TLY8.1 PF03184.17	28.10	0.00
TRINITY_CHMP7_DICDI/249-433	Q86K93.1 PF03357.19	28.00	0.00
TRINITY_I1C6S2_RHIO9/7-261	I1C6S2.1 PF14938.4;	28.00	0.00
TRINITY_C5DNJ3_LACTC/13-310	C5DNJ3.1 PF00069.23	28.00	0.00
TRINITY_K1QK98_CRAGI/12-176	K1QK98.1 PF03398.12	28.00	0.00
TRINITY_C1FHW0_MICSR/545-914	C1FHW0.1 PF01494.17	28.00	0.00
TRINITY_I7MKL5_TETTS/83-500	I7MKL5.2 PF00067.20	28.00	0.00
TRINITY_F4WZV1_ACREC/185-681	F4WZV1.1 PF00888.20	28.00	0.00
TRINITY_A0A094BAN5_9PEZI/96-296	A0A094BAN5.1 PF0075	28.00	0.00
TRINITY_V8P3G6_OPHHA/26-192	V8P3G6.1 PF10294.7;	28.00	0.00
TRINITY_A0A078AT97_STYLE/378-634	A0A078AT97.1 PF0079	27.90	0.00
TRINITY_J9IHC9_9SPIT/7-346	J9IHC9.1 PF00459.23	27.90	0.00
TRINITY_A0A0P7VEE5_9TELE/81-349	A0A0P7VEE5.1 PF0337	27.90	0.00
TRINITY_Q233I3_TETTS/33-226	Q233I3.1 PF01652.16	27.90	0.00
TRINITY_I1BJN6_RHIO9/14-344	I1BJN6.1 PF00503.18	27.90	0.00
TRINITY_E4RRN3_LEAB4/56-282	E4RRN3.1 PF07859.11	27.90	0.00
TRINITY_E1ZBE8_CHLVA/325-1282	E1ZBE8.1 PF04130.11	27.90	0.00
TRINITY_A0A078A3Q8_STYLE/31-324	A0A078A3Q8.1 PF0064	27.90	0.00
TRINITY_A8J7X1_CHLRE/1-183	A8J7X1.1 PF13419.4;	27.90	0.00
TRINITY_K9RZN8_SYNP3/37-248	K9RZN8.1 PF05023.12	27.90	0.00
TRINITY_A0A0F2QT83_9DELT/39-781	A0A0F2QT83.1 PF0180	27.90	0.00
TRINITY_D5X9C5_THEPJ/8-202	D5X9C5.1 PF01734.20	27.90	0.00
TRINITY_A0A0F0HYQ4_ASPPA/18-273	A0A0F0HYQ4.1 PF0006	27.90	0.00
TRINITY_A0A0K9PP21_ZOSMR/13-263	A0A0K9PP21.1 PF0214	27.90	0.00
TRINITY_A0A099P227_PICKU/5-188	A0A099P227.1 PF0002	27.90	0.00
TRINITY_G3UW78_MOUSE/52-515	G3UW78.1 PF00067.20	27.90	0.00
TRINITY_A0A068XHG0_HYMMI/22-199	A0A068XHG0.1 PF0110	27.80	0.00
TRINITY_A8J3U9_CHLRE/57-225	A8J3U9.1 PF00213.16	27.80	0.00
TRINITY_J9IL07_9SPIT/4-217	J9IL07.1 PF09420.8;	27.80	0.00
TRINITY_A8J7Q4_CHLRE/193-341	A8J7Q4.1 PF01416.18	27.80	0.00
TRINITY_D8TTE6_VOLCA/333-484	D8TTE6.1 PF12499.6;	27.80	0.00
TRINITY_M3XKX3_LATCH/164-471	M3XKX3.1 PF05804.10	27.80	0.00
TRINITY_C5MDZ8_CANTT/1-439	C5MDZ8.1 PF10243.7;	27.80	0.00
TRINITY_F4QAF1_DICFS/89-487	F4QAF1.1 PF00450.20	27.80	0.00
TRINITY_M4AM32_XIPMA/3-276	M4AM32.1 PF01459.20	27.80	0.00
TRINITY_Q4WBN3_ASPFU/39-303	Q4WBN3.1 PF07859.11	27.80	0.00
TRINITY_D8SJK6_SELML/4-253	D8SJK6.1 PF00069.23	27.80	0.00
TRINITY_E0VD49_PEDHC/23-372	E0VD49.1 PF03265.13	27.80	0.00
TRINITY_A9T4L0_PHYPA/60-255	A9T4L0.1 PF05843.12	27.70	0.00
TRINITY_E0W1B6_PEDHC/698-1148	E0W1B6.1 PF00145.15	27.70	0.00
TRINITY_EXOC5_DICDI/344-668	B0G163.1 PF07393.9;	27.70	0.00
TRINITY_A9VAN1_MONBE/20-210	A9VAN1.1 PF01183.18	27.70	0.00
TRINITY_A0A0G4J0Z5_PLABS/30-217	A0A0G4J0Z5.1 PF0394	27.70	0.00
TRINITY_F2TNT7_AJEDA/10-387	F2TNT7.1 PF03849.12	27.70	0.00
TRINITY_A2DY61_TRIVA/6-259	A2DY61.1 PF00069.23	27.70	0.00
TRINITY_F4PGI0_DICFS/385-623	F4PGI0.1 PF00003.20	27.70	0.00
TRINITY_A0A073CX82_PLAAG/32-238	A0A073CX82.1 PF0052	27.70	0.00
TRINITY_A0BKH8_PARTE/3-103	A0BKH8.1 PF00168.28	27.70	0.00
TRINITY_T1HL42_RHOPR/4-488	T1HL42.1 PF00067.20	27.70	0.00

TRINITY_D3BRK2_POLPA/40-468	D3BRK2.1 PF12832.5;	27.70	0.00
TRINITY_F4Q3P7_DICFS/653-804	F4Q3P7.1 PF00620.25	27.70	0.00
TRINITY_C1EJH9_MICSR/428-775	C1EJH9.1 PF13086.4;	27.70	0.00
TRINITY_Q22BS7_TETTS/1-260	Q22BS7.2 PF14938.4;	27.70	0.00
TRINITY_L7FM96_ENTIV/2-126	L7FM96.1 PF00648.19	27.70	0.00
TRINITY_A0A015JEP9_9GLOM/354-682	A0A015JEP9.1 PF1282	27.70	0.00
TRINITY_A0A024TQL6_9STRA/2-322	A0A024TQL6.1 PF0126	27.70	0.00
TRINITY_Q248J3_TETTS/29-415	Q248J3.2 PF07690.14	27.60	0.00
TRINITY_A0A0D2SJZ9_GOSRA/3-647	A0A0D2SJZ9.1 PF0581	27.60	0.00
TRINITY_A0A0G4IQ11_PLABS/110-443	A0A0G4IQ11.1 PF0159	27.60	0.00
TRINITY_I1BRE7_RHIO9/136-327	I1BRE7.1 PF04855.10	27.60	0.00
TRINITY_F4Q385_DICFS/15-119	F4Q385.1 PF02991.14	27.60	0.00
TRINITY_Q23AY7_TETTS/21-318	Q23AY7.2 PF00069.23	27.60	0.00
TRINITY_Q22YP6_TETTS/2455-2737	Q22YP6.2 PF06650.10	27.60	0.00
TRINITY_A5UM62_METS3/3-239	A5UM62.1 PF00230.18	27.60	0.00
TRINITY_A4C850_9GAMM/1-341	A4C850.1 PF07690.14	27.60	0.00
TRINITY_A4VED3_TETTS/46-415	A4VED3.2 PF07690.14	27.60	0.00
TRINITY_A8I935_CHLRE/132-414	A8I935.1 PF13416.4;	27.60	0.00
TRINITY_A6CCI4_9PLAN/65-304	A6CCI4.1 PF07859.11	27.60	0.00
TRINITY_Q769F7_DICDI/20-209	Q769F7.1 PF01105.22	27.50	0.00
TRINITY_M7NM13_PNEMU/359-608	M7NM13.1 PF00009.25	27.50	0.00
TRINITY_D8UC96_VOLCA/50-243	D8UC96.1 PF01547.23	27.50	0.00
TRINITY_G1MLM6_AILME/32-361	G1MLM6.1 PF03414.11	27.50	0.00
TRINITY_I7LT45_TETTS/177-429	I7LT45.2 PF00069.23	27.50	0.00
TRINITY_C5DRW1_ZYGRC/591-883	C5DRW1.1 PF05192.16	27.50	0.00
TRINITY_Q5CXE1_CRYPI/74-329	Q5CXE1.1 PF02434.14	27.50	0.00
TRINITY_V6TYM4_GIAIN/9-206	V6TYM4.1 PF01712.17	27.50	0.00
TRINITY_L8HDK0_ACACA/42-278	L8HDK0.1 PF06454.9;	27.50	0.00
TRINITY_T1L3P6_TETUR/129-463	T1L3P6.1 PF02163.20	27.50	0.00
TRINITY_R7EN63_9FIRM/5-186	R7EN63.1 PF00406.20	27.40	0.00
TRINITY_F0ZHF6_DICPU/91-299	F0ZHF6.1 PF01852.17	27.40	0.00
TRINITY_A0A077ZRI5_STYLE/1-278	A0A077ZRI5.1 PF1014	27.40	0.00
TRINITY_H2YHY8_CIOSA/2-150	H2YHY8.1 PF01762.19	27.40	0.00
TRINITY_A0A068RTV3_9FUNG/3638-3898	A0A068RTV3.1 PF0045	27.40	0.00
TRINITY_A0A0N8JZG7_9TELE/65-179	A0A0N8JZG7.1 PF0061	27.40	0.00
TRINITY_C4R802_PICPG/40-216	C4R802.1 PF00270.27	27.40	0.00
TRINITY_B9RR79_RICCO/3-376	B9RR79.1 PF00022.17	27.40	0.00
TRINITY_H9GJU0_ANOCA/1-314	H9GJU0.2 PF04762.10	27.40	0.00
TRINITY_A6C0H5_9PLAN/160-369	A6C0H5.1 PF03417.14	27.40	0.00
TRINITY_L8GQR1_ACACA/657-1024	L8GQR1.1 PF00176.21	27.40	0.00
TRINITY_B7GD43_PHATC/36-494	B7GD43.1 PF00375.16	27.40	0.00
TRINITY_Q26FF3_FLABB/84-425	Q26FF3.1 PF01494.17	27.40	0.00
TRINITY_D8U8T2_VOLCA/33-264	D8U8T2.1 PF06977.9;	27.40	0.00
TRINITY_I0Z4T6_9CHLO/1812-1957	I0Z4T6.1 PF10075.7;	27.40	0.00
TRINITY_G2XS07_BOTF4/169-858	G2XS07.1 PF04388.10	27.40	0.00
TRINITY_H2WIU9_CAEJA/187-437	H2WIU9.2 PF00069.23	27.40	0.00
TRINITY_P91469_CAEEL/7-159	P91469.1 PF00339.27	27.40	0.00
TRINITY_S2JIZ6_MUCC1/9-545	S2JIZ6.1 PF05091.10	27.40	0.00
TRINITY_L8HCD9_ACACA/473-634	L8HCD9.1 PF12807.5;	27.30	0.00
TRINITY_A0DB40_PARTE/29-327	A0DB40.1 PF00069.23	27.30	0.00
TRINITY_D2A4J0_TRICA/44-278	D2A4J0.1 PF05705.12	27.30	0.00
TRINITY_L8H2E4_ACACA/24-502	L8H2E4.1 PF10508.7;	27.30	0.00
TRINITY_E1Z549_CHLVA/41-381	E1Z549.1 PF00497.18	27.30	0.00
TRINITY_A0A0G2I322_9EURO/41-465	A0A0G2I322.1 PF0122	27.30	0.00
TRINITY_I3TW76_TISMK/34-323	I3TW76.1 PF02608.12	27.30	0.00
TRINITY_A0CKP6_PARTE/17-271	A0CKP6.1 PF00069.23	27.30	0.00
TRINITY_G8BND5_TETPH/198-485	G8BND5.1 PF10296.7;	27.30	0.00
TRINITY_G6DL71_DANPL/3-259	G6DL71.1 PF01633.18	27.30	0.00
TRINITY_A0A099P4Q6_PICKU/55-460	A0A099P4Q6.1 PF0149	27.30	0.00
TRINITY_I7M2I3_TETTS/112-416	I7M2I3.1 PF01733.16	27.30	0.00
TRINITY_A0A067BWY7_SAPPC/22-533	A0A067BWY7.1 PF0145	27.30	0.00

TRINITY_I0YQ78_9CHLO/69-481	I0YQ78.1 PF00474.15	27.30	0.00
TRINITY_B3S6U8_TRIAD/16-404	B3S6U8.1 PF07690.14	27.20	0.00
TRINITY_Q23RZ9_TETTS/247-486	Q23RZ9.3 PF04499.13	27.20	0.00
TRINITY_W4JWJ2_9HOMO/386-617	W4JWJ2.1 PF03572.16	27.20	0.00
TRINITY_D8LU94_ECTSI/82-277	D8LU94.1 PF11715.6;	27.20	0.00
TRINITY_T1JWP1_TETUR/23-284	T1JWP1.1 PF00069.23	27.20	0.00
TRINITY_A0A068XKR2_HYMMI/14-215	A0A068XKR2.1 PF0185	27.20	0.00
TRINITY_K9RNQ6_9CYAN/67-354	K9RNQ6.1 PF14388.4;	27.20	0.00
TRINITY_A0A0E9LX87_9BACT/1-406	A0A0E9LX87.1 PF0008	27.10	0.00
TRINITY_Q22AM1_TETTS/51-391	Q22AM1.3 PF00328.20	27.10	0.00
TRINITY_A0CW99_PARTE/11-187	A0CW99.1 PF06017.11	27.10	0.00
TRINITY_D2VRQ2_NAEGR/487-679	D2VRQ2.1 PF12146.6;	27.10	0.00
TRINITY_F6GU12_VITVI/354-490	F6GU12.1 PF04499.13	27.10	0.00
TRINITY_A0E1I0_PARTE/22-292	A0E1I0.1 PF00069.23	27.10	0.00
TRINITY_A0A068XKR2_HYMMI/14-215	A0A068XKR2.1 PF0185	27.10	0.00
TRINITY_Q28F16_XENTR/33-192	Q28F16.1 PF01652.16	27.10	0.00
TRINITY_D8UJH0_VOLCA/14-270	D8UJH0.1 PF00069.23	27.10	0.00
TRINITY_A8HQJ0_CHLRE/22-173	A8HQJ0.1 PF08617.8;	27.10	0.00
TRINITY_Q248J3_TETTS/29-415	Q248J3.2 PF07690.14	27.00	0.00
TRINITY_I7I8D3_BABMI/250-575	I7I8D3.1 PF02889.14	27.00	0.00
TRINITY_A0A077ZDI3_TRITR/112-709	A0A077ZDI3.1 PF0088	27.00	0.00
TRINITY_K9VNS4_9CYAN/55-223	K9VNS4.1 PF00350.21	27.00	0.00
TRINITY_C5LHN9_PERM5/21-253	C5LHN9.1 PF03661.11	27.00	0.00
TRINITY_F4KFJ2_ARATH/43-179	F4KFJ2.1 PF00790.17	27.00	0.00

TRINITY_U9UHH6_RHIID/72-387	U9UHH6.1 PF00443.27	27.00	0.00
TRINITY_J3N0P0_ORYBR/18-275	J3N0P0.1 PF00069.23	27.00	0.00
TRINITY_I7MKI8_TETTS/449-740	I7MKI8.1 PF13949.4;	27.00	0.00
TRINITY_A0A0G4FFD9_9ALVE/90-555	A0A0G4FFD9.1 PF0006	27.00	0.00
TRINITY_D8TTM6_VOLCA/6-138	D8TTM6.1 PF13676.4;	27.00	0.00
TRINITY_B3RIJ3_TRIAD/63-288	B3RIJ3.1 PF14802.4;	26.90	0.00
TRINITY_L8GYA8_ACACA/88-255	L8GYA8.1 PF07724.12	26.90	0.00
TRINITY_MAEL_XENTR/141-344	Q08D62.1 PF13017.4;	26.90	0.00
TRINITY_D2A1W4_TRICA/604-875	D2A1W4.1 PF01757.20	26.90	0.00
TRINITY_H2W7H8_CAEJA/9-362	H2W7H8.2 PF13598.4;	26.90	0.00
TRINITY_Q011X9_OSTTA/28-214	Q011X9.1 PF00069.23	26.90	0.00
TRINITY_B9GI62_POPTR/146-709	B9GI62.1 PF04114.12	26.90	0.00
TRINITY_H0X825_OTOGA/15-401	H0X825.1 PF01231.16	26.90	0.00
TRINITY_F2UB45_SALR5/592-851	F2UB45.1 PF00481.19	26.90	0.00
TRINITY_A0D550_PARTE/11-336	A0D550.1 PF11945.6;	26.90	0.00
TRINITY_E0VF17_PEDHC/143-369	E0VF17.1 PF10469.7;	26.80	0.00
TRINITY_J9IVC4_9SPIT/69-432	J9IVC4.1 PF01490.16	26.80	0.00
TRINITY_D3B9G1_POLPA/32-541	D3B9G1.1 PF01457.14	26.80	0.00
TRINITY_A0E368_PARTE/250-369	A0E368.1 PF14536.4;	26.80	0.00
TRINITY_A0EBH2_PARTE/32-359	A0EBH2.1 PF00328.20	26.80	0.00
TRINITY_Q10Z33_TRIEI/33-230	Q10Z33.1 PF08495.8;	26.80	0.00
TRINITY_L8GQR1_ACACA/657-1024	L8GQR1.1 PF00176.21	26.80	0.00
TRINITY_A0A067CTW3_SAPPC/789-1030	A0A067CTW3.1 PF0271	26.80	0.00
TRINITY_PEPA_PIG/72-384	P00791.3 PF00026.21	26.80	0.00
TRINITY_D8UKQ9_VOLCA/248-630	D8UKQ9.1 PF00150.16	26.80	0.00
TRINITY_G1QVM3_NOMLE/20-291	G1QVM3.1 PF00780.20	26.70	0.00
TRINITY_D3BQD7_POLPA/353-579	D3BQD7.1 PF11744.6;	26.70	0.00
TRINITY_L8GQR1_ACACA/657-1024	L8GQR1.1 PF00176.21	26.70	0.00
TRINITY_H3AN64_LATCH/64-359	H3AN64.2 PF03253.12	26.70	0.00
TRINITY_A0A024TT64_9STRA/7-312	A0A024TT64.1 PF0253	26.60	0.00
TRINITY_G7E8G4_MIXOS/1907-2076	G7E8G4.1 PF12348.6;	26.60	0.00
TRINITY_W2QLV0_PHYPN/258-463	W2QLV0.1 PF01925.17	26.60	0.00
TRINITY_D2V0I7_NAEGR/175-372	D2V0I7.1 PF01925.17	26.60	0.00
TRINITY_N1RGN0_FUSC4/740-1179	N1RGN0.1 PF02383.16	26.60	0.00
TRINITY_S2JC17_MUCC1/140-436	S2JC17.1 PF06814.11	26.60	0.00
TRINITY_L8GQR1_ACACA/657-1024	L8GQR1.1 PF00176.21	26.60	0.00
TRINITY_K9V140_9CYAN/5-345	K9V140.1 PF01494.17	26.60	0.00
TRINITY_H9GL38_ANOCA/201-374	H9GL38.1 PF03184.17	26.50	0.00
TRINITY_G1KIX3_ANOCA/64-493	G1KIX3.2 PF01490.16	26.50	0.00
TRINITY_J9IBH8_9SPIT/23-371	J9IBH8.1 PF00026.21	26.50	0.00
TRINITY_Q6TU47_DICDI/97-653	Q6TU47.1 PF01457.14	26.50	0.00
TRINITY_A0A0J7KU38_LASNI/286-544	A0A0J7KU38.1 PF0248	26.50	0.00
TRINITY_A0A087GA27_ARAAL/299-431	A0A087GA27.1 PF1598	26.50	0.00
TRINITY_A0A0M8XQT7_9ACTN/42-311	A0A0M8XQT7.1 PF0406	26.50	0.00
TRINITY_F4W7P3_ACREC/12-395	F4W7P3.1 PF00817.18	26.50	0.00
TRINITY_Q46I05_PROMT/21-172	Q46I05.1 PF10503.7;	26.50	0.00
TRINITY_L8GPR5_ACACA/62-354	L8GPR5.1 PF01459.20	26.40	0.00
TRINITY_D2UYT3_NAEGR/492-1002	D2UYT3.1 PF07393.9;	26.40	0.00
TRINITY_A0A0D5LN73_9RHIZ/22-484	A0A0D5LN73.1 PF0129	26.40	0.00
TRINITY_B7FUT8_PHATC/27-372	B7FUT8.1 PF14027.4;	26.40	0.00
TRINITY_J9JL56_ACYPI/38-521	J9JL56.1 PF00201.16	26.40	0.00
TRINITY_A1ZHW6_9BACT/26-221	A1ZHW6.1 PF02146.15	26.40	0.00
TRINITY_H3A969_LATCH/11-441	H3A969.1 PF04180.12	26.40	0.00
TRINITY_M0TY52_MUSAM/34-360	M0TY52.1 PF00150.16	26.40	0.00
TRINITY_U9SVQ2_RHIID/376-642	U9SVQ2.1 PF02714.13	26.40	0.00
TRINITY_D8U7H0_VOLCA/1412-1663	D8U7H0.1 PF00520.29	26.40	0.00
TRINITY_F0ZHF6_DICPU/91-299	F0ZHF6.1 PF01852.17	26.40	0.00
TRINITY_A8IIJ6_CHLRE/68-1078	A8IIJ6.1 PF02463.17	26.40	0.00
TRINITY_A0A077ZPB7_STYLE/91-323	A0A077ZPB7.1 PF0011	26.30	0.00
TRINITY_A0A0M0BJJ4_9ARCH/6-328	A0A0M0BJJ4.1 PF0000	26.30	0.00
TRINITY_F5J2H0_9PORP/1-341	F5J2H0.1 PF06379.10	26.30	0.00

TRINITY_I7LXB6_TETTS/114-386	I7LXB6.2 PF00664.21	26.30	0.00
TRINITY_A0A017SDD2_9EURO/32-296	A0A017SDD2.1 PF0785	26.30	0.00
TRINITY_A8JBD1_CHLRE/17-133	A8JBD1.1 PF01426.16	26.30	0.00
TRINITY_Q55BT0_DICDI/10-412	Q55BT0.1 PF10300.7;	26.30	0.00
TRINITY_A0A0J7L3D4_LASNI/214-502	A0A0J7L3D4.1 PF0032	26.30	0.00
TRINITY_A8HWH9_CHLRE/30-320	A8HWH9.1 PF00069.23	26.30	0.00
TRINITY_R0HFG8_9BRAS/33-499	R0HFG8.1 PF00067.20	26.20	0.00
TRINITY_G3UUP4_MELGA/1-283	G3UUP4.1 PF16094.3;	26.20	0.00
TRINITY_A0A0A1NCR6_9FUNG/111-293	A0A0A1NCR6.1 PF0974	26.20	0.00
TRINITY_A0A075AQC1_9FUNG/976-1266	A0A075AQC1.1 PF0248	26.20	0.00
TRINITY_M0TSF6_MUSAM/147-407	M0TSF6.1 PF00561.18	26.20	0.00
TRINITY_F0ZVI8_DICPU/284-479	F0ZVI8.1 PF01044.17	26.20	0.00
TRINITY_A0A0G4FFD9_9ALVE/90-555	A0A0G4FFD9.1 PF0006	26.20	0.00
TRINITY_J3M769_ORYBR/39-201	J3M769.1 PF04937.13	26.20	0.00
TRINITY_A0CPS5_PARTE/123-450	A0CPS5.1 PF01733.16	26.20	0.00
TRINITY_Q55BN5_DICDI/1-224	Q55BN5.1 PF15001.4;	26.10	0.00
TRINITY_K2RTG0_MACPH/12-173	K2RTG0.1 PF07855.10	26.10	0.00
TRINITY_A0BKU7_PARTE/45-349	A0BKU7.1 PF03416.17	26.10	0.00
TRINITY_E1ZRN8_CHLVA/26-275	E1ZRN8.1 PF04230.11	26.10	0.00
TRINITY_F6RW87_XENTR/18-525	F6RW87.1 PF04129.10	26.10	0.00
TRINITY_Q7V8V6_PROMM/26-254	Q7V8V6.1 PF00520.29	26.10	0.00
TRINITY_A0A059DCB7_EUCGR/572-938	A0A059DCB7.1 PF0337	26.10	0.00
TRINITY_X6NJD7_RETFI/5-321	X6NJD7.1 PF00176.21	26.00	0.00
TRINITY_A0A0G0UKN4_9BACT/1-268	A0A0G0UKN4.1 PF0058	26.00	0.00
TRINITY_A0A091D3J9_FUKDA/201-662	A0A091D3J9.1 PF0412	26.00	0.00
TRINITY_C5E336_LACTC/15-398	C5E336.1 PF03547.16	26.00	0.00
TRINITY_F4Q1V6_DICFS/12-291	F4Q1V6.1 PF10127.7;	26.00	0.00
TRINITY_H3CAL8_TETNG/125-431	H3CAL8.1 PF01733.16	26.00	0.00
TRINITY_A0A067LZ84_9HOMO/33-449	A0A067LZ84.1 PF0123	26.00	0.00
TRINITY_A0DD82_PARTE/42-362	A0DD82.1 PF00328.20	26.00	0.00
TRINITY_E9GZ71_DAPPU/13-257	E9GZ71.1 PF10259.7;	25.90	0.00
TRINITY_A9U470_PHYPA/136-516	A9U470.1 PF00012.18	25.90	0.00
TRINITY_A0A0L0BPK1_LUCCU/166-764	A0A0L0BPK1.1 PF0088	25.90	0.00
TRINITY_W9WWV3_9EURO/2-200	W9WWV3.1 PF05462.9;	25.90	0.00
TRINITY_L7LLY3_9ACTN/5-221	L7LLY3.1 PF00743.17	25.80	0.00
TRINITY_H2VN59_CAEJA/69-458	H2VN59.2 PF08569.9;	25.80	0.00
TRINITY_L8GQR1_ACACA/657-1024	L8GQR1.1 PF00176.21	25.80	0.00
TRINITY_A0A024U517_9STRA/51-338	A0A024U517.1 PF0337	25.80	0.00
TRINITY_F4NT21_BATDJ/3-340	F4NT21.1 PF08610.8;	25.80	0.00
TRINITY_F7DUR0_XENTR/2-304	F7DUR0.1 PF07773.9;	25.80	0.00
TRINITY_A9SW20_PHYPA/5-384	A9SW20.1 PF03348.13	25.80	0.00
TRINITY_A9TJ39_PHYPA/59-364	A9TJ39.1 PF14388.4;	25.80	0.00
TRINITY_K9U7Q7_9CYAN/36-286	K9U7Q7.1 PF00657.20	25.80	0.00
TRINITY_A0A059DF10_EUCGR/35-240	A0A059DF10.1 PF0085	25.80	0.00
TRINITY_D6Y6C0_THEBD/15-172	D6Y6C0.1 PF00583.23	25.80	0.00
TRINITY_F4PGI0_DICFS/385-623	F4PGI0.1 PF00003.20	25.80	0.00
TRINITY_D8LU94_ECTSI/82-277	D8LU94.1 PF11715.6;	25.80	0.00
TRINITY_M3XYU9_MUSPF/194-414	M3XYU9.1 PF01823.17	25.80	0.00
TRINITY_J9HSH9_9SPIT/67-426	J9HSH9.1 PF07690.14	25.70	0.00
TRINITY_A0EEJ2_PARTE/289-396	A0EEJ2.1 PF02204.16	25.70	0.00
TRINITY_E0W2A8_PEDHC/8-1115	E0W2A8.1 PF09735.7;	25.70	0.00
TRINITY_Q17BE1_AEDAE/229-598	Q17BE1.1 PF00443.27	25.70	0.00
TRINITY_F0ZKL8_DICPU/17-302	F0ZKL8.1 PF00856.26	25.70	0.00
TRINITY_G4YY98_PHYSP/92-498	G4YY98.1 PF00561.18	25.70	0.00
TRINITY_G0R5R6_ICHMG/21-294	G0R5R6.1 PF00069.23	25.70	0.00
TRINITY_D8U0A8_VOLCA/366-760	D8U0A8.1 PF04515.10	25.70	0.00
TRINITY_G7J672_MEDTR/109-580	G7J672.2 PF00759.17	25.70	0.00
TRINITY_D2VDC1_NAEGR/86-317	D2VDC1.1 PF01852.17	25.70	0.00
TRINITY_A0A0P7V422_9TELE/1-297	A0A0P7V422.1 PF0032	25.60	0.00
TRINITY_K3W5F5_PYTUL/17-331	K3W5F5.1 PF03198.12	25.60	0.00
TRINITY_D3B2V1_POLPA/16-226	D3B2V1.1 PF08397.9;	25.60	0.00

TRINITY_A0A0D3BPN9_BRAOL/155-477	A0A0D3BPN9.1 PF0577	25.60	0.00
TRINITY_A4S4Y8_OSTLU/1-230	A4S4Y8.1 PF00149.26	25.60	0.00
TRINITY_V7CKY5_PHAVU/9-410	V7CKY5.1 PF03547.16	25.60	0.00
TRINITY_L8HMQ5_ACACA/43-216	L8HMQ5.1 PF00617.17	25.60	0.00
TRINITY_F1SUG6_PIG/247-614	F1SUG6.1 PF12309.6;	25.60	0.00
TRINITY_B5YK45_THEYD/26-379	B5YK45.1 PF13458.4;	25.60	0.00
TRINITY_N1Q1D2_DOTSN/217-616	N1Q1D2.1 PF06772.9;	25.50	0.00
TRINITY_B0C0P2_ACAM1/24-383	B0C0P2.1 PF01494.17	25.50	0.00
TRINITY_J9GCG0_9SPIT/180-340	J9GCG0.1 PF02752.20	25.50	0.00
TRINITY_A0A099NX72_PICKU/5-308	A0A099NX72.1 PF0364	25.50	0.00
TRINITY_D1CCQ7_THET1/1-527	D1CCQ7.1 PF02463.17	25.50	0.00
TRINITY_A0A067L8S5_JATCU/93-350	A0A067L8S5.1 PF0645	25.50	0.00
TRINITY_I1HVD3_BRADI/420-613	I1HVD3.1 PF08161.10	25.50	0.00
TRINITY_A0A0A1N7Q0_9FUNG/347-539	A0A0A1N7Q0.1 PF0357	25.50	0.00
TRINITY_I7ML21_TETTS/173-309	I7ML21.1 PF00892.18	25.40	0.00
TRINITY_A0A067RC97_ZOONE/52-483	A0A067RC97.1 PF0008	25.40	0.00
TRINITY_D8R6G9_SELML/22-403	D8R6G9.1 PF00856.26	25.40	0.00
TRINITY_A0BXC8_PARTE/163-515	A0BXC8.1 PF00450.20	25.40	0.00
TRINITY_I7M0E7_TETTS/70-526	I7M0E7.2 PF07690.14	25.40	0.00
TRINITY_A0A0L0CK75_LUCCU/34-311	A0A0L0CK75.1 PF1362	25.30	0.00
TRINITY_M7PBH8_PNEMU/26-318	M7PBH8.1 PF04756.11	25.30	0.00
TRINITY_E1ZDL1_CHLVA/76-432	E1ZDL1.1 PF00497.18	25.30	0.00
TRINITY_D3B105_POLPA/2231-2476	D3B105.1 PF06650.10	25.30	0.00
TRINITY_G0R306_ICHMG/5-418	G0R306.1 PF01490.16	25.20	0.00
TRINITY_A0A0D2UPS1_CAPO3/347-570	A0A0D2UPS1.1 PF0155	25.20	0.00
TRINITY_E1ZDL1_CHLVA/76-432	E1ZDL1.1 PF00497.18	25.20	0.00
TRINITY_F0ZVQ5_DICPU/78-431	F0ZVQ5.1 PF01925.17	25.20	0.00
TRINITY_A0E368_PARTE/250-369	A0E368.1 PF14536.4;	25.20	0.00
TRINITY_W1NU56_AMBTC/41-438	W1NU56.1 PF07690.14	25.20	0.00
TRINITY_A0E717_PARTE/286-554	A0E717.1 PF02854.17	25.20	0.00
TRINITY_A0A074RN87_9HOMO/392-604	A0A074RN87.1 PF0357	25.20	0.00
TRINITY_A8HNM5_CHLRE/2576-2866	A8HNM5.1 PF06011.10	25.20	0.00
TRINITY_A0A0B5HNS2_9ARCH/5-183	A0A0B5HNS2.1 PF0267	25.10	0.00
TRINITY_A9RVM5_PHYPA/43-322	A9RVM5.1 PF04190.11	25.10	0.00
TRINITY_B6H109_PENRW/42-511	B6H109.1 PF00067.20	25.10	0.00
TRINITY_F4PR02_DICFS/48-357	F4PR02.1 PF10483.7;	25.10	0.00
TRINITY_A0A072TU45_MEDTR/175-442	A0A072TU45.1 PF0776	25.00	0.00
TRINITY_B2HLW3_MYCMM/76-337	B2HLW3.1 PF01636.21	25.00	0.00
TRINITY_D8R6G9_SELML/22-403	D8R6G9.1 PF00856.26	25.00	0.00
TRINITY_A0A0D2W070_CAPO3/571-770	A0A0D2W070.1 PF0501	25.00	0.00
TRINITY_D7FSJ0_ECTSI/914-1098	D7FSJ0.1 PF01852.17	25.00	0.00
TRINITY_W5JZT8_ASTMX/35-207	W5JZT8.1 PF00092.26	25.00	0.00
TRINITY_A7S3U3_NEMVE/57-252	A7S3U3.1 PF00804.23	25.00	0.00
TRINITY_A0A022QP77_ERYGU/8-482	A0A022QP77.1 PF0309	25.00	0.00
TRINITY_A0A090AIL9_9GAMM/348-519	A0A090AIL9.1 PF0009	25.00	0.00
TRINITY_A0A0K6G2C5_9HOMO/50-492	A0A0K6G2C5.1 PF0006	25.00	0.00
TRINITY_G0AAC0_COLFT/211-469	G0AAC0.1 PF00657.20	25.00	0.00
TRINITY_E1ZE38_CHLVA/277-473	E1ZE38.1 PF00534.18	25.00	0.00
TRINITY_A0A061GGV8_THECC/1447-1826	A0A061GGV8.1 PF1308	24.90	0.00
TRINITY_A0A0D2TW14_GOSRA/21-293	A0A0D2TW14.1 PF0645	24.90	0.00
TRINITY_E4XV66_OIKDI/98-386	E4XV66.1 PF13896.4;	24.90	0.00
TRINITY_I2FY45_USTH4/422-750	I2FY45.1 PF03372.21	24.90	0.00
TRINITY_G0R5R6_ICHMG/21-294	G0R5R6.1 PF00069.23	24.90	0.00
TRINITY_A0A0D2WI78_CAPO3/11-639	A0A0D2WI78.1 PF0624	24.90	0.00
TRINITY_G0N5I2_CAEBE/22-342	G0N5I2.1 PF03265.13	24.90	0.00
TRINITY_W7X7K1_TETTS/28-293	W7X7K1.1 PF00069.23	24.80	0.00
TRINITY_G3P833_GASAC/106-328	G3P833.1 PF01556.16	24.80	0.00
TRINITY_T1IUF6_STRMM/243-766	T1IUF6.1 PF04437.11	24.80	0.00
TRINITY_L8HBK7_ACACA/26-465	L8HBK7.1 PF00067.20	24.80	0.00
TRINITY_F1NQI9_CHICK/37-387	F1NQI9.2 PF15053.4;	24.70	0.00
TRINITY_G0QVB2_ICHMG/37-311	G0QVB2.1 PF07714.15	24.70	0.00

TRINITY_G3WPU3_SARHA/33-293	G3WPU3.1 PF00069.23	24.70	0.00
TRINITY_A7URR6_ANOGA/222-617	A7URR6.1 PF01757.20	24.70	0.00
TRINITY_A0A044S4P6_ONCVO/3-385	A0A044S4P6.1 PF0340	24.60	0.00
TRINITY_A3TYU7_OCEBH/4-327	A3TYU7.1 PF03372.21	24.60	0.00
TRINITY_M7NLW5_PNEMU/586-822	M7NLW5.1 PF16910.3;	24.60	0.00
TRINITY_A7RYH4_NEMVE/4-454	A7RYH4.1 PF07778.9;	24.60	0.00
TRINITY_L8H9T2_ACACA/313-518	L8H9T2.1 PF08506.8;	24.50	0.00
TRINITY_W4GAR7_9STRA/101-444	W4GAR7.1 PF03403.11	24.50	0.00
TRINITY_M3VX21_FELCA/61-1205	M3VX21.1 PF15489.4;	24.50	0.00
TRINITY_A0A058Z138_9EUKA/111-404	A0A058Z138.1 PF0085	24.40	0.00
TRINITY_A0A067N0I9_9HOMO/43-515	A0A067N0I9.1 PF0006	24.40	0.00
TRINITY_F4PHT7_DICFS/77-434	F4PHT7.1 PF10165.7;	24.40	0.00
TRINITY_G8BK67_CANPC/204-911	G8BK67.1 PF04615.11	24.30	0.00
TRINITY_I0Z6V9_9CHLO/48-349	I0Z6V9.1 PF13896.4;	24.30	0.00
TRINITY_F2LTV5_HIPMA/22-456	F2LTV5.1 PF00501.26	24.20	0.00
TRINITY_F0ZHM0_DICPU/4-663	F0ZHM0.1 PF05817.12	24.20	0.00
TRINITY_A0A0M9UCN0_9CHLR/210-505	A0A0M9UCN0.1 PF1359	24.20	0.00
TRINITY_A0A087SC64_AUXPR/53-414	A0A087SC64.1 PF0413	24.20	0.00
TRINITY_A0A0D9VCY5_9ORYZ/145-503	A0A0D9VCY5.1 PF0410	24.10	0.00
TRINITY_A0A078DIS1_BRANA/648-890	A0A078DIS1.1 PF0163	24.10	0.00
TRINITY_W1NK52_AMBTC/744-1058	W1NK52.1 PF03178.13	24.00	0.00
TRINITY_CRLA_DICDI/62-322	Q54U75.1 PF05462.9;	24.00	0.00
TRINITY_A8I9D0_CHLRE/49-490	A8I9D0.1 PF00067.20	24.00	0.00
TRINITY_U5DEY2_AMBTC/31-314	U5DEY2.1 PF00856.26	24.00	0.00
TRINITY_R0IVF5_SETT2/22-285	R0IVF5.1 PF00332.16	24.00	0.00
TRINITY_A0DJB8_PARTE/29-287	A0DJB8.1 PF00194.19	23.90	0.00
TRINITY_A0A072VG08_MEDTR/42-480	A0A072VG08.1 PF0006	23.90	0.00
TRINITY_F1QCZ3_DANRE/67-348	F1QCZ3.2 PF09728.7;	23.90	0.00
TRINITY_A0A087ZYS2_APIME/516-832	A0A087ZYS2.1 PF0484	23.80	0.00
TRINITY_A0A0K9P2K7_ZOSMR/43-230	A0A0K9P2K7.1 PF0587	23.80	0.00
TRINITY_F7BBV8_CIOIN/27-291	F7BBV8.2 PF00780.20	23.80	0.00
TRINITY_F2UKP2_SALR5/128-526	F2UKP2.1 PF01490.16	23.80	0.00
TRINITY_G0QXK4_ICHMG/207-399	G0QXK4.1 PF14703.4;	23.80	0.00
TRINITY_A0A024UH59_9STRA/294-590	A0A024UH59.1 PF0145	23.70	0.00
TRINITY_M2WYA1_GALSU/23-335	M2WYA1.1 PF00328.20	23.70	0.00
TRINITY_L8H960_ACACA/401-538	L8H960.1 PF12717.5;	23.70	0.00
TRINITY_S0ETQ6_CHTCT/50-379	S0ETQ6.1 PF13810.4;	23.70	0.00
TRINITY_K9YZ90_CYAAP/163-370	K9YZ90.1 PF01170.16	23.70	0.00
TRINITY_A0DD82_PARTE/42-362	A0DD82.1 PF00328.20	23.70	0.00
TRINITY_J9I221_9SPIT/379-690	J9I221.1 PF00069.23	23.70	0.00
TRINITY_A0A0L0ND85_9HYPO/91-319	A0A0L0ND85.1 PF0085	23.70	0.00
TRINITY_K1QZT6_CRAGI/38-276	K1QZT6.1 PF05705.12	23.60	0.00
TRINITY_A0A087X2S5_POEFO/50-490	A0A087X2S5.1 PF0006	23.60	0.00
TRINITY_A5E0P7_LODEL/777-988	A5E0P7.1 PF01061.22	23.40	0.00
TRINITY_M4BJ52_HYAAE/35-422	M4BJ52.1 PF06870.10	23.40	0.00
TRINITY_L9LAV3_TUPCH/5-202	L9LAV3.1 PF09177.9;	23.40	0.00
TRINITY_A0A078A1U0_STYLE/3115-3435	A0A078A1U0.1 PF0052	23.30	0.00
TRINITY_K1R8G7_CRAGI/78-469	K1R8G7.1 PF10257.7;	23.00	0.00
TRINITY_W7U0H7_9STRA/301-575	W7U0H7.1 PF01793.14	22.90	0.00
TRINITY_Q2L018_BORA1/30-384	Q2L018.1 PF13458.4;	22.80	0.00
TRINITY_A0A015I738_9GLOM/31-277	A0A015I738.1 PF1492	22.70	0.00
TRINITY_A0A0F6W3W3_9DELT/26-274	A0A0F6W3W3.1 PF1269	22.60	0.00
TRINITY_L1J8Y9_GUITH/134-479	L1J8Y9.1 PF08016.10	22.60	0.00
TRINITY_A5ABG2_ASPNC/30-462	A5ABG2.1 PF00067.20	22.60	0.00
TRINITY_A0A0A8JMV7_BACSX/24-281	A0A0A8JMV7.1 PF0056	22.50	0.00
TRINITY_A0A078AEU5_STYLE/182-343	A0A078AEU5.1 PF0275	22.20	0.00
TRINITY_G7DX31_MIXOS/31-346	G7DX31.1 PF01793.14	22.20	0.00
TRINITY_A0A0F6W3W3_9DELT/26-274	A0A0F6W3W3.1 PF1269	22.20	0.00
TRINITY_A0A066UXW6_9HOMO/20-360	A0A066UXW6.1 PF0050	22.20	0.00
TRINITY_A0A0A1MNK3_9FUNG/370-781	A0A0A1MNK3.1 PF0677	22.00	0.00
TRINITY_M1AIX2_SOLTU/365-698	M1AIX2.1 PF00497.18	22.00	0.00

TRINITY_D3B253_POLPA/36-375	D3B253.1 PF03265.13	21.50	0.00
TRINITY_W4YU07_STRPU/149-349	W4YU07.1 PF00149.26	21.40	0.00
TRINITY_S2J7G1_MUCC1/659-1202	S2J7G1.1 PF07919.10	20.60	0.00
TRINITY_T1JPF9_STRMM/592-1878	T1JPF9.1 PF14631.4;	20.40	0.00
TRINITY_V6KHC7_STRNV/37-306	V6KHC7.1 PF04069.10	18.90	0.00
