# Supplementary material for: Transcriptome Analysis of the Accumulation of Astaxanthin in Haematococcus pluvialis Treated with White and Blue Lights as well as Salicylic Acid
Source: Biomed Res Int. 2022 Jul 14;2022:4827595. doi: 10.1155/2022/4827595 (PMC9315456; doi:10.1155/2022/4827595)
Supplement: Supplementary 6 — Table S4: enrichment analysis of differentially expressed genes (DEGs) identified in Haematococcus pluvialis treated with blue light, white light, and blue light with salicylic acid based on the Kyoto Encyclopedia of Genes and Genomes (KEGG) database. [file 4827595.f6.pdf]

PathwayEntry	PathwayDefinition	S gene	TS gene	B gene	:TB gene	pvalue
map04141	Protein processing in endoplasmic reticulum	160	5410	497	19648	0.01
map03008	Ribosome biogenesis in eukaryotes	102	5410	283	19648	0.00
map04145	Phagosome	98	5410	281	19648	0.00
map04146	Peroxisome	91	5410	262	19648	0.01
map00010	Glycolysis / Gluconeogenesis	81	5410	231	19648	0.01
map00630	Glyoxylate and dicarboxylate metabolism	84	5410	198	19648	0.00
map00620	Pyruvate metabolism	91	5410	197	19648	0.00
map00500	Starch and sucrose metabolism	65	5410	196	19648	0.05
map00270	Cysteine and methionine metabolism	60	5410	174	19648	0.03
map00710	Carbon fixation in photosynthetic organisms	67	5410	164	19648	0.00
map00020	Citrate cycle (TCA cycle)	76	5410	148	19648	0.00
map00280	Valine, leucine and isoleucine degradation	50	5410	132	19648	0.01
map00260	Glycine, serine and threonine metabolism	46	5410	131	19648	0.03
map00330	Arginine and proline metabolism	48	5410	129	19648	0.01
map00250	Alanine, aspartate and glutamate metabolism	52	5410	125	19648	0.00
map00195	Photosynthesis	46	5410	122	19648	0.01
map01040	Biosynthesis of unsaturated fatty acids	42	5410	104	19648	0.00
map04933	AGE-RAGE signaling pathway in diabetic complicati	35	5410	98	19648	0.05
map00860	Porphyrin and chlorophyll metabolism	52	5410	96	19648	0.00
map00380	Tryptophan metabolism	34	5410	95	19648	0.05
map00030	Pentose phosphate pathway	34	5410	93	19648	0.04
map00640	Propanoate metabolism	45	5410	92	19648	0.00
map00061	Fatty acid biosynthesis	37	5410	81	19648	0.00
map00410	beta-Alanine metabolism	33	5410	78	19648	0.00
map00360	Phenylalanine metabolism	29	5410	77	19648	0.03
map00100	Steroid biosynthesis	41	5410	73	19648	0.00
map00910	Nitrogen metabolism	26	5410	67	19648	0.03
map00196	Photosynthesis - antenna proteins	39	5410	67	19648	0.00
map00220	Arginine biosynthesis	26	5410	66	19648	0.02
map00400	Phenylalanine, tyrosine and tryptophan biosynthes	26	5410	65	19648	0.02
map00906	Carotenoid biosynthesis	28	5410	58	19648	0.00
map00770	Pantothenate and CoA biosynthesis	22	5410	54	19648	0.02
map00062	Fatty acid elongation	21	5410	52	19648	0.03
map00650	Butanoate metabolism	19	5410	44	19648	0.02
map04712	Circadian rhythm - plant	18	5410	43	19648	0.03
map00960	Tropane, piperidine and pyridine alkaloid biosynt	19	5410	43	19648	0.01
map00670	One carbon pool by folate	19	5410	40	19648	0.01
map00780	Biotin metabolism	17	5410	37	19648	0.01
map00333	Prodigiosin biosyntheses	13	5410	24	19648	0.01
map00290	Valine, leucine and isoleucine biosynthesis	10	5410	21	19648	0.04
map00945	Stilbenoid, diarylheptanoid and gingerol biosynth	7	5410	9	19648	0.00
map00909	Sesquiterpenoid and triterpenoid biosynthesis	4	5410	5	19648	0.02
map00471	D-Glutamine and D-glutamate metabolism	5	5410	5	19648	0.00
map00254	Aflatoxin biosynthesis	4	5410	4	19648	0.01

Pathway:	PathwayDefinition	S gene :	TS gene	B gene :	TB gene	pvalue
map0301	Ribosome	327	8723	657	19648	0.00
map0401	MAPK signaling pathway - plant	200	8723	389	19648	0.00
map0062	Pyruvate metabolism	101	8723	197	19648	0.03
map0026	Glycine, serine and threonine metabol	71	8723	131	19648	0.01
map0493	AGE-RAGE signaling pathway in diabeti	54	8723	98	19648	0.02
map0086	Porphyrin and chlorophyll metabolism	60	8723	96	19648	0.00
map0010	Steroid biosynthesis	45	8723	73	19648	0.00
map0019	Photosynthesis - antenna proteins	40	8723	67	19648	0.01
map0006	Fatty acid elongation	33	8723	52	19648	0.00
map0471	Circadian rhythm - plant	30	8723	43	19648	0.00
map0029	Valine, leucine and isoleucine biosyn	14	8723	21	19648	0.03
map0060	Glycosphingolipid biosynthesis - lact	6	8723	7	19648	0.03

Pathway	PathwayDefinition	S gene	TS gene	B gene	TB gene	pvalue
map0301	Ribosome	270	6540	657	19648	0.00
map0401	MAPK signaling pathway -	159	6540	389	19648	0.00
map0300	Ribosome biogenesis in eu	117	6540	283	19648	0.00
map0414	Phagosome	114	6540	281	19648	0.01
map0019	Oxidative phosphorylation	113	6540	271	19648	0.00
map0462	Plant-pathogen interactio	91	6540	225	19648	0.01
map0305	Proteasome	63	6540	135	19648	0.00
map0056	Ether lipid metabolism	24	6540	53	19648	0.05
map0006	Fatty acid elongation	25	6540	52	19648	0.02
map0471	Circadian rhythm - plant	23	6540	43	19648	0.00
map0096	Tropane, piperidine and p	21	6540	43	19648	0.02
map0051	Other types of O-glycan b	15	6540	30	19648	0.04
map0007	Cutin, suberine and wax b	9	6540	13	19648	0.01
map0047	D-Glutamine and D-glutama	4	6540	5	19648	0.05

Pathway	PathwayDefinition	S gene	TS gene	B gene	TB gene	pvalue
map0301	Ribosome	228	5134	657	19648	0.00
map0300	Ribosome biogenesis in eukaryo	91	5134	283	19648	0.01
map0414	Phagosome	95	5134	281	19648	0.00
map0019	Oxidative phosphorylation	101	5134	271	19648	0.00
map0414	Peroxisome	81	5134	262	19648	0.05
map0001	Glycolysis / Gluconeogenesis	82	5134	231	19648	0.00
map0063	Glyoxylate and dicarboxylate r	82	5134	198	19648	0.00
map0062	Pyruvate metabolism	84	5134	197	19648	0.00
map0097	Aminoacyl-tRNA biosynthesis	64	5134	182	19648	0.00
map0071	Carbon fixation in photosynthe	71	5134	164	19648	0.00
map0002	Citrate cycle (TCA cycle)	70	5134	148	19648	0.00
map0028	Valine, leucine and isoleucine	48	5134	132	19648	0.01
map0026	Glycine, serine and threonine	50	5134	131	19648	0.00
map0033	Arginine and proline metabolis	50	5134	129	19648	0.00
map0025	Alanine, aspartate and glutama	46	5134	125	19648	0.01
map0019	Photosynthesis	52	5134	122	19648	0.00
map0104	Biosynthesis of unsaturated fa	38	5134	104	19648	0.01
map0086	Porphyrin and chlorophyll meta	51	5134	96	19648	0.00
map0003	Pentose phosphate pathway	34	5134	93	19648	0.02
map0064	Propanoate metabolism	41	5134	92	19648	0.00
map0006	Fatty acid biosynthesis	33	5134	81	19648	0.00
map0041	beta-Alanine metabolism	33	5134	78	19648	0.00
map0036	Phenylalanine metabolism	29	5134	77	19648	0.02
map0010	Steroid biosynthesis	37	5134	73	19648	0.00
map0019	Photosynthesis - antenna prote	40	5134	67	19648	0.00
map0022	Arginine biosynthesis	27	5134	66	19648	0.01
map0040	Phenylalanine, tyrosine and tr	27	5134	65	19648	0.00
map0090	Carotenoid biosynthesis	23	5134	58	19648	0.02
map0077	Pantothenate and CoA biosynthe	21	5134	54	19648	0.03
map0065	Butanoate metabolism	19	5134	44	19648	0.01
map0096	Tropane, piperidine and pyridi	18	5134	43	19648	0.02
map0471	Circadian rhythm - plant	18	5134	43	19648	0.02
map0067	One carbon pool by folate	21	5134	40	19648	0.00
map0033	Prodigiosin biosyntheses	11	5134	24	19648	0.03
map0029	Valine, leucine and isoleucine	10	5134	21	19648	0.03
map0043	Taurine and hypotaurine metabo	7	5134	14	19648	0.05
map0094	Stilbenoid, diarylheptanoid ar	6	5134	9	19648	0.01
map0090	Sesquiterpenoid and triterpeno	4	5134	5	19648	0.02
map0047	D-Glutamine and D-glutamate me	4	5134	5	19648	0.02

PathwayEntry	PathwayDefinition	S gene	TS gene	B gene	TB gene	pvalue
map03010	Ribosome	327	6984	657	19648	0.00
map04016	MAPK signaling pathway	161	6984	389	19648	0.01
map04145	Phagosome	122	6984	281	19648	0.00
map00190	Oxidative phosphorylati	122	6984	271	19648	0.00
map04626	Plant-pathogen interact	110	6984	225	19648	0.00
map00620	Pyruvate metabolism	91	6984	197	19648	0.00
map00710	Carbon fixation in phot	75	6984	164	19648	0.00
map00020	Citrate cycle (TCA cycl	65	6984	148	19648	0.02
map00195	Photosynthesis	68	6984	122	19648	0.00
map00860	Porphyrin and chlorophy	52	6984	96	19648	0.00
map00061	Fatty acid biosynthesis	41	6984	81	19648	0.00
map00100	Steroid biosynthesis	36	6984	73	19648	0.01
map00196	Photosynthesis - antenn	40	6984	67	19648	0.00
map00910	Nitrogen metabolism	33	6984	67	19648	0.01
map00590	Arachidonic acid metabo	32	6984	67	19648	0.03
map04130	SNARE interactions in v	33	6984	63	19648	0.00
map00062	Fatty acid elongation	26	6984	52	19648	0.02
map00730	Thiamine metabolism	26	6984	51	19648	0.02
map04712	Circadian rhythm - plan	23	6984	43	19648	0.01
map00514	Other types of O-glycan	16	6984	30	19648	0.03
map00254	Aflatoxin biosynthesis	4	6984	4	19648	0.02

Pathway	Pathway	S gene	TS gene	B gene	TB gene	pvalue
map0023	Purine	44	1076	469	19648	0.00
map0401	MAPK si	41	1076	389	19648	0.00
map0201	ABC tra	42	1076	378	19648	0.00
map0300	Ribosom	26	1076	283	19648	0.01
map0407	Plant h	35	1076	244	19648	0.00
map0462	Plant-p	31	1076	225	19648	0.00
map0050	Starch	19	1076	196	19648	0.01
map0019	Photosy	13	1076	122	19648	0.02
map0006	Fatty a	10	1076	81	19648	0.01
map0059	Arachid	10	1076	67	19648	0.00
map0090	Caroten	7	1076	58	19648	0.04
map0006	Fatty a	7	1076	52	19648	0.02
map0051	Other t	5	1076	30	19648	0.02
map0033	Prodigi	4	1076	24	19648	0.04
map0007	Cutin,	5	1076	13	19648	0.00

Pathway	PathwayDefinition	S gene	TS gene	B gene	TB gene	pvalue
map0301	Ribosome	240	3842	657	19648	0
map0414	Endocytosis	120	3842	508	19648	0.01
map0414	Protein processing in end	132	3842	497	19648	0.00
map0414	Phagosome	77	3842	281	19648	0.00
map0019	Oxidative phosphorylation	90	3842	271	19648	0.00
map0462	Plant-pathogen interactic	59	3842	225	19648	0.01
map0063	Glyoxylate and dicarboxyl	60	3842	198	19648	0.00
map0062	Pyruvate metabolism	51	3842	197	19648	0.02
map0050	Starch and sucrose metabc	55	3842	196	19648	0.00
map0097	Aminoacyl-tRNA biosynthes	47	3842	182	19648	0.02
map0027	Cysteine and methionine m	44	3842	174	19648	0.04
map0071	Carbon fixation in photos	57	3842	164	19648	0.00
map0048	Glutathione metabolism	42	3842	161	19648	0.03
map0002	Citrate cycle (TCA cycle)	62	3842	148	19648	0.00
map0305	Proteasome	65	3842	135	19648	0.00
map0026	Glycine, serine and threc	36	3842	131	19648	0.02
map0033	Arginine and proline meta	34	3842	129	19648	0.04
map0025	Alanine, aspartate and gl	37	3842	125	19648	0.00
map0086	Porphyrin and chlorophyll	29	3842	96	19648	0.01
map0003	Pentose phosphate pathway	26	3842	93	19648	0.03
map0064	Propanoate metabolism	26	3842	92	19648	0.03
map0010	Steroid biosynthesis	21	3842	73	19648	0.04
map0091	Nitrogen metabolism	25	3842	67	19648	0.00
map0059	Arachidonic acid metaboli	22	3842	67	19648	0.01
map0022	Arginine biosynthesis	20	3842	66	19648	0.02
map0471	Circadian rhythm - plant	15	3842	43	19648	0.01
map0025	Aflatoxin biosynthesis	4	3842	4	19648	0.00

Pathway	PathwayDefinition	S gene	TS gene	B gene	TB gene	pvalue
map0023	Purine metabolism	115	3638	469	19648	0.00
map0019	Oxidative phosphorylation	74	3638	271	19648	0.00
map0001	Glycolysis / Gluconeogene	59	3638	231	19648	0.00
map0462	Plant-pathogen interactio	52	3638	225	19648	0.05
map0063	Glyoxylate and dicarboxyla	53	3638	198	19648	0.00
map0062	Pyruvate metabolism	63	3638	197	19648	0.00
map0050	Starch and sucrose metabo	55	3638	196	19648	0.00
map0071	Carbon fixation in photos	57	3638	164	19648	0.00
map0025	Alanine, aspartate and gl	33	3638	125	19648	0.02
map0019	Photosynthesis	62	3638	122	19648	0.00
map0005	Fructose and mannose metal	28	3638	100	19648	0.01
map0086	Porphyrin and chlorophyll	42	3638	96	19648	0.00
map0064	Propanoate metabolism	25	3638	92	19648	0.03
map0006	Fatty acid biosynthesis	23	3638	81	19648	0.02
map0005	Ascorbate and aldarate me	21	3638	77	19648	0.04
map0010	Steroid biosynthesis	23	3638	73	19648	0.01
map0019	Photosynthesis - antenna	37	3638	67	19648	0.00
map0059	Arachidonic acid metaboli	26	3638	67	19648	0.00
map0091	Nitrogen metabolism	25	3638	67	19648	0.00
map0090	Carotenoid biosynthesis	18	3638	58	19648	0.01
map0006	Fatty acid elongation	16	3638	52	19648	0.02
map0471	Circadian rhythm - plant	13	3638	43	19648	0.04
map0051	Other types of O-glycan b	13	3638	30	19648	0.00
map0025	Aflatoxin biosynthesis	4	3638	4	19648	0.00

Pathway	Pathway	S gene	TS gene	B gene	TB gene	pvalue
map0301	Ribosom	93	2002	657	19648	0.00
map0023	Purine	75	2002	469	19648	0.00
map0201	ABC tra	51	2002	378	19648	0.02
map0414	Phagoso	39	2002	281	19648	0.03
map0019	Oxidati	38	2002	271	19648	0.03
map0414	Peroxis	37	2002	262	19648	0.03
map0001	Glycoly	35	2002	231	19648	0.01
map0062	Pyruvat	39	2002	197	19648	0.00
map0050	Starch	43	2002	196	19648	0.00
map0002	Citrate	31	2002	148	19648	0.00
map0305	Proteas	23	2002	135	19648	0.01
map0019	Photosy	28	2002	122	19648	0.00
map0341	Base ex	19	2002	116	19648	0.03
map0086	Porphyr	17	2002	96	19648	0.02
map0006	Fatty a	18	2002	81	19648	0.00
map0041	beta-Al	14	2002	78	19648	0.03
map0005	Ascorba	14	2002	77	19648	0.02
map0019	Photosy	24	2002	67	19648	0.00
map0091	Nitroge	15	2002	67	19648	0.00
map0059	Arachid	13	2002	67	19648	0.02
map0090	Caroten	12	2002	58	19648	0.01
map0096	Tropane	9	2002	43	19648	0.03
map0051	Other t	11	2002	30	19648	0.00
map0046	Cyanoam	7	2002	28	19648	0.02
map0007	Cutin,	6	2002	13	19648	0.00
map0047	D-Gluta	3	2002	5	19648	0.01

Pathway	PathwayDefinition	S gene	TS gene	B gene	TB gene	pvalue
map0301	Ribosome	230	4957	657	19648	0.00
map0414	Protein processin	148	4957	497	19648	0.01
map0301	RNA transport	126	4957	434	19648	0.04
map0300	Ribosome biogenes	100	4957	283	19648	0.00
map0414	Phagosome	96	4957	281	19648	0.00
map0019	Oxidative phospho	104	4957	271	19648	0.00
map0001	Glycolysis / Gluc	70	4957	231	19648	0.05
map0462	Plant-pathogen in	69	4957	225	19648	0.04
map0063	Glyoxylate and di	73	4957	198	19648	0.00
map0062	Pyruvate metaboli	70	4957	197	19648	0.00
map0097	Aminoacyl-tRNA bi	57	4957	182	19648	0.04
map0027	Cysteine and meth	55	4957	174	19648	0.03
map0071	Carbon fixation i	58	4957	164	19648	0.00
map0002	Citrate cycle (TC	67	4957	148	19648	0.00
map0305	Proteasome	52	4957	135	19648	0.00
map0028	Valine, leucine a	51	4957	132	19648	0.00
map0026	Glycine, serine a	44	4957	131	19648	0.02
map0007	Fatty acid degrad	43	4957	126	19648	0.02
map0104	Biosynthesis of u	36	4957	104	19648	0.02
map0086	Porphyrin and chl	32	4957	96	19648	0.05
map0038	Tryptophan metabo	34	4957	95	19648	0.01
map0031	Lysine degradatio	32	4957	93	19648	0.03
map0003	Pentose phosphate	31	4957	93	19648	0.05
map0006	Fatty acid biosyn	34	4957	81	19648	0.00
map0041	beta-Alanine meta	28	4957	78	19648	0.02
map0010	Steroid biosynthe	35	4957	73	19648	0.00
map0019	Photosynthesis -	31	4957	67	19648	0.00
map0413	SNARE interaction	25	4957	63	19648	0.01
map0074	Riboflavin metabo	22	4957	62	19648	0.05
map0090	Carotenoid biosyn	22	4957	58	19648	0.02
map0056	Ether lipid metab	20	4957	53	19648	0.03
map0067	One carbon pool b	16	4957	40	19648	0.03
map0078	Biotin metabolism	18	4957	37	19648	0.00
map0046	Cyanoamino acid m	14	4957	28	19648	0.00
map0033	Prodigiosin biosy	13	4957	24	19648	0.00
map0090	Zeatin biosynthes	11	4957	22	19648	0.01
map0059	Linoleic acid met	7	4957	14	19648	0.04
map0094	Stilbenoid, diary	6	4957	9	19648	0.01

Pathway	PathwayDefinition	S gene	TS gene	B gene	TB gene	pvalue
map0414	Endocytosis	177	5127	508	19648	0.00
map0401	MAPK signaling pathway	130	5127	389	19648	0.00
map0300	Ribosome biogenesis ir	88	5127	283	19648	0.03
map0414	Phagosome	106	5127	281	19648	0.00
map0414	Peroxisome	84	5127	262	19648	0.02
map0056	Glycerophospholipid me	60	5127	182	19648	0.02
map0056	Inositol phosphate met	63	5127	171	19648	0.00
map0002	Citrate cycle (TCA cyc	57	5127	148	19648	0.00
map0305	Proteasome	45	5127	135	19648	0.04
map0028	Valine, leucine and is	49	5127	132	19648	0.00
map0104	Biosynthesis of unsatu	36	5127	104	19648	0.03
map0493	AGE-RAGE signaling pat	39	5127	98	19648	0.00
map0038	Tryptophan metabolism	34	5127	95	19648	0.02
map0031	Lysine degradation	33	5127	93	19648	0.03
map0302	Basal transcription fa	29	5127	80	19648	0.03
map0010	Steroid biosynthesis	35	5127	73	19648	0.00
map0094	Phenylpropanoid biosyr	27	5127	68	19648	0.01
map0056	Ether lipid metabolism	25	5127	53	19648	0.00
map0060	Glycosphingolipid bios	13	5127	28	19648	0.02
map0090	Zeatin biosynthesis	10	5127	22	19648	0.04
map0094	Stilbenoid, diarylhept	6	5127	9	19648	0.01
map0060	Glycosphingolipid bios	6	5127	7	19648	0.00

Pathway	Pathway S	gene :TS	gene B	gene :TB	gene	pvalue
map0301	Ribosom	36	61	657	19648	0.00
map0019	Oxidati	14	61	271	19648	0.00

Pathway	PathwayDefinitic	S gene	TS gene	B gene	TB gene	pvalue
map0414	Endocytosis	149	4607	508	19648	0.00
map0401	MAPK signaling p	135	4607	389	19648	0.00
map0300	Ribosome biogene	85	4607	283	19648	0.01
map0414	Phagosome	87	4607	281	19648	0.00
map0414	Peroxisome	74	4607	262	19648	0.04
map0407	Plant hormone si	72	4607	244	19648	0.02
map0462	Plant-pathogen i	73	4607	225	19648	0.00
map0097	Aminoacyl-tRNA b	56	4607	182	19648	0.01
map0056	Inositol phospha	54	4607	171	19648	0.01
map0028	Valine, leucine	42	4607	132	19648	0.02
map0493	AGE-RAGE signali	34	4607	98	19648	0.01
map0302	Basal transcript	29	4607	80	19648	0.01
map0010	Steroid biosynth	33	4607	73	19648	0.00
map0094	Phenylpropanoid	23	4607	68	19648	0.03
map0056	Ether lipid meta	19	4607	53	19648	0.03
map0006	Fatty acid elong	19	4607	52	19648	0.02
map0090	Brassinosteroid	8	4607	16	19648	0.02
map0094	Stilbenoid, diar	5	4607	9	19648	0.04
